# Web-Based COVID-19 Dashboards and Trackers in the United States: Survey Study

**DOI:** 10.2196/43819

**Published:** 2023-03-20

**Authors:** Melissa D Clarkson

**Affiliations:** 1 Division of Biomedical Informatics University of Kentucky Lexington, KY United States

**Keywords:** COVID-19, data visualization, data dashboard, public health reporting, human information interaction, transparency, trust

## Abstract

**Background:**

The SARS-CoV-2 pandemic provided an opportunity to use public-facing web data visualization tools to help citizens understand the evolving status of the outbreak. Given the heterogeneity of data sources, developers, tools, and designs used in this effort, it raised questions about how visualizations were constructed during a time when daily batches of data were available, but issues of data quality and standardization were unresolved.

**Objective:**

This paper surveyed web-based COVID-19 dashboards and trackers that are likely to be used by the residents of the United States to monitor the spread of infection on a local, national, and global scale. This study is intended to provide insights that will help application developers increase the usefulness, transparency, and trustworthiness of dashboards and trackers for public health data in the future.

**Methods:**

Websites of coronavirus dashboards and trackers were identified in August 2020 using the Google search engine. They were examined to determine the data sources used, types of data presented, types of data visualizations, characteristics of the visualizations, and issues with messy data. The websites were surveyed 3 more times for changes in design and data sources with the final survey conducted in June 2022. Themes were developed to highlight the issues concerning challenges in presenting COVID-19 data and techniques of effective visualization.

**Results:**

In total, 111 websites were identified and examined (84 state focused, 11 nationwide, and 16 with global data), and this study found an additional 17 websites providing access to the state vaccination data. This study documents how data aggregators have played a central role in making data accessible to visualization developers. The designs of dashboards and tracker visualizations vary in type and quality, with some well-designed displays supporting the interpretation of the data and others obscuring the meaning of the data and potentially misleading the viewers. Five themes were identified to describe challenges in presenting COVID-19 data and techniques of effective visualization.

**Conclusions:**

This analysis reveals the extent to which dashboards and trackers informing the American public about the COVID-19 pandemic relied on an ad hoc pipeline of data sources and data aggregators. The dashboards and trackers identified in this survey offer an opportunity to compare different approaches for the display of similar data.

## Introduction

### Background

SARS-CoV-2, a novel coronavirus, was first detected in the United States in mid-January 2020 [1,2], and eventually, many states enacted stay-at-home orders in early March. The SARS-CoV-2 pandemic challenged the public health system in the United States in many ways, including a lack of laboratory testing capacity early in the pandemic, evolving data standards for reporting positive test results and deaths owing to COVID-19, and a lack of coordination among state and federal agencies. In addition, approximately half of all nonfederal hospitals lacked the capacity to electronically exchange information with public health agencies at the beginning of the pandemic [3].

The pandemic also presented challenges in communicating with the public the evolving status of the outbreak and the reasoning behind public health measures, such as stay-at-home orders and masking. As the pandemic progressed, waves of infection rose and fell in the regions of the United States at different times owing to the presence of *superspreader events*, differences in public health responses among states, and the rise of variants [4,5].

### Data Visualization

Data visualization has the potential to modify the course of a pandemic by bringing together information about the state of the pandemic, public policy, and individual behavior in ways that are actionable. However, for the visualizations to have this impact, they must be easily accessible, based on accurate and timely data, and carefully developed with an understanding of both the data and the principles of visual design.

Visualizations have been easily accessible during the pandemic owing to the availability of numerous software tools and platforms for creating graphics and mapping data. Because data sets are available to anyone with an internet connection, early in the pandemic, a number of visualization experts wrote about the need to responsibly use tools and data when creating visualizations [6-9]. Misrepresented and misinterpreted COVID-19 visualizations have inspired one study to use them to help students develop statistical literacy [10].

The large number of visualizations developed and deployed rapidly by public health authorities and data analysts during the pandemic is of interest to visualization and communication researchers. They provide insights and lessons about the process of rapidly designing and developing visualizations [11-15]; efforts to curate global data [16]; the types of visualizations created and who they are for [17-19]; conceptual models linking tools, data, visualizations, and users [20]; and what it means for a visualization to be actionable [21]. In addition, studies have used pandemic data to understand how the users perceive the risk and severity of the pandemic [22-24] and their reactions to the designs of dashboards [25].

### Purpose of This Study

This study complements earlier work by taking a US-focused look at COVID-19 dashboards from August 2020 to June 2022. It documents data sources and data aggregation efforts, identifies themes relevant to designing dashboards for outbreaks, and highlights issues with data availability and standardization. The goals of this work were to provide insights that will help application developers increase the usefulness, transparency, and trustworthiness of dashboards and trackers for public health data in the future and to document the variety of dashboards and trackers used by the residents of the United States and the evolution of these tools for approximately 2 years.

This study encompassed the following 2 broad categories of data visualization: dashboards and trackers. The term *dashboard* generally refers to a set of dynamically updated data visualizations placed in proximity to one another and is used to monitor conditions for the purpose of understanding a system or event. Because several COVID-19 data visualizations took other forms, such as visualizations arranged sequentially with accompanying text, I used the term *tracker* to more broadly refer to these types of dynamically updated displays.

## Methods

This survey began in August 2020, which was approximately 5 months after stay-at-home orders generated widespread public interest in the state of the pandemic and all states were providing data on the web about the pandemic for a public audience.

### Identification of Dashboards and Tracker Websites

To identify web-based dashboards and trackers, I performed a web search using Google on August 12, 2020. Searches were formatted as “coronavirus COVID dashboard tracker” combined with either a state name, “United States,” or “global.” The first 15 results for each keyword combination were examined for their relevance. On the basis of test searches, I determined that the relevant search results were generally in the first 10 results, and results ranked lower than the 15th search result were either links to the dashboards and trackers from other web pages or news or commentary about the pandemic. The websites that were determined to be relevant to this study are listed in Multimedia Appendix 1. Dashboards and trackers were categorized as state focused, nationwide, or global. This study focused on visualizations that are the most likely to be viewed by people in the United States to understand the local and regional status of the pandemic, with less emphasis on global visualizations.

By the end of January 2021, many states had incorporated vaccine dashboards into their state dashboards. To locate vaccine dashboards not integrated into state websites, I performed a Google search for “covid vaccine dashboard tracker” and examined the first 20 results for relevance. Multimedia Appendix 2 lists the websites determined to be relevant to this study.

### Inclusion and Exclusion Criteria

Websites were included in this survey if they displayed up-to-date information, appeared to be updated daily (or nearly daily), and relied mainly on graphs or maps (rather than tables or text) to convey information. Websites were excluded if they showed data limited to regions smaller than a state (such as a single county or city), were specific to a type of setting (such as prisons), or displayed only trackers or dashboards that had been embedded from other websites. The District of Columbia was included in this survey, but the US territories were not. This survey included only publicly available websites accessible on a laptop. Apps developed specifically for smartphones were not included.

The focus of this study was the display of information concerning diagnosed cases of COVID-19, deaths attributed to COVID-19, testing for COVID-19, and vaccination. Visualizations of risk levels, hospital bed availability, and hospital admissions were not central to this study, but designs for these types of data may benefit from these findings.

### Methods of Review

All websites were examined on a MacBook Pro laptop (Mac OS version 10.11) using a Firefox web browser. The data sources used by each dashboard or tracker were documented based on statements from the website itself [26], and for one dashboard, the development team was contacted. Some websites included a statement stating that their data sources have changed as the pandemic has developed, suggesting that their list of sources may not be complete or current. The software tool or method used to create visualizations was also determined. If the name of the software brand was not displayed with the visualization, the *Inspector* tool within Firefox was used to examine the webpage’s HTML and determine the tool or method used.

Each website was examined to determine the following:

1. Does the website credit a data source or sources?
  - What sources are credited? How are they credited?
  - When more than one data source is credited, is it clear which measures come from which source?
  - Rationale: Citing data sources increases the trustworthiness of visualizations; however, there is no established best practice for how to do this. Listing the name of an organization that provided the data may not be sufficient if the data set from that organization cannot be identified with certainty. However, members of the public may not expect data sources to be cited.
2. What types of data are presented?
  - What measures are provided? (such as number of cases, number of tests performed, and number of hospitalizations)
  - What is the level of granularity? (county level or state level)
  - Rationale: Many different measurements relating to COVID-19 were collected by different organizations and public health authorities, with new measures introduced and others discontinued. Differences in granularity are important both for describing the pandemic with more precision and for making the data more relevant to viewers (who have an interest in knowing about COVID-19 in their own area).
3. What graphical forms of visualizations are used? (bar charts, line charts, choropleth maps, etc)
  - Rationale: Surveying graphical forms provides information on which forms designers believe are appropriate for public-facing visualizations and the variety of forms available in visualization tools.
4. Do the visualizations clearly display the data? Might any visualizations lead viewers to make inappropriate conclusions?
  - Rationale: Drawing on my experience as an information designer and instructor for a data visualization course, I examined the designs for issues involving color, size, and labeling; misleading use of space or positioning; and mismatches between the type of data and the chosen graphical form. These present opportunities to increase awareness of good design in data visualization.
5. How do the designers deal with *messy* data, such as lags in reporting and discontinuities in definitions of measures?
  - Rationale: Identifying effective methods for accommodating messy data will help establish best practices.

### Capturing Changes in the Design of Visualizations Over Time

To understand how the dashboards and trackers evolved over the course of the pandemic, the survey of websites was repeated 3 more times. This survey spanned from approximately 7 months after the novel coronavirus was first detected in the United States to nearly 2-and-a-half years after detection. The second review of each website was conducted between January 2021 and March 2021. The third review of each website was conducted in either December 2021 or January 2022. The final review was conducted in June 2022. By the end of the survey period, >1 million deaths in the United States were attributed to COVID-19, with deaths decreasing to <400 per day by June 2022.

Multimedia Appendices 1 and 2 list the information for the websites and any changes to the URLs are noted in the appendices. This review covers only the dashboards and trackers identified in August 2020 and vaccine-focused websites identified from January 2021. Therefore, websites launched after that time were not included.

### Developing Themes

On the basis of a review of the websites, 5 sets of themes were developed to highlight issues concerning challenges in presenting COVID-19 data and techniques of effective visualization.

## Results

### Dashboards and Trackers Identified

#### State Focused

A total of 84 dashboards and trackers focusing on COVID-19 cases in a state (or region composed of several states) were identified. These are listed in Multimedia Appendix 1, with each assigned an identifier in the format *S-x*. (This paper will refer to dashboards and trackers using square brackets with the identifier from the appendix, for example, [S-1] for the dashboard from the Alabama Department of Public Health). At least one dashboard or tracker website was provided by the public health authorities in each state and the District of Columbia as of August 2020. The Massachusetts Department of Public Health originally provided only a downloadable PDF document before switching to a dashboard created with Tableau. An additional 20 dashboards and trackers were developed by newspapers and television news organizations. The remaining websites were associated with nonprofit organizations (n=2), web-based media and marketing companies (n=2), individuals (n=2), a university-associated team, and a health care–related trade organization. All state-focused websites identified in this survey provided data at the county or parish level. As of June 2022, a total of 23 of the 84 dashboards and trackers were removed or no longer updated with new data. Of these, the Florida Department of Health discontinued its dashboard but replaced it with weekly reports that could be downloaded as PDF documents.

#### Nationwide Coverage

In total, 11 websites that displayed data for the entire United States were identified. These are listed in Multimedia Appendix 1, with identifiers in the format *N-x*. Of these, 5 websites displayed data at the state level, whereas 6 provided more granular data at the county level. The Centers for Disease Control and Prevention (CDC) provided 2 trackers [N-1 and N-2]. Other websites were provided by news organizations (n=3), university-associated teams (n=3), technology or web companies (n=2), and a nonprofit organization. As of June 2022, a total of 2 of the 11 websites were discontinued.

#### Global Coverage

An additional 16 websites were identified that displayed worldwide COVID-19 data. These are listed in Multimedia Appendix 1, with identifiers in the format *G-x*. These websites were provided by news organizations (n=3), university-associated teams (n=4), nonprofit organizations (n=3), and technology or web-based businesses (n=6). As of June 2022, a total of 4 of the 16 websites were removed or no longer updated.

#### Vaccine Distribution

Multimedia Appendix 2 lists the additional dashboards and trackers for vaccine distribution. This survey identified 17 state-focused sites with county-level data, 4 with nationwide coverage at the state level, and 3 with global coverage.

### Visualization Tools and Methods

The most popular software platforms used for state-focused dashboards and trackers, particularly among public health authorities, were Tableau, ArcGIS, and Microsoft Power BI. Some dashboards presented all the information in a single page, but it was common for dashboards to have multiple pages to accommodate maps and new types of data that became available during the pandemic. News organizations were more likely to provide trackers arranged as a series of data visualizations with textual explanations and use scalable vector graphics embedded in their web pages. See Multimedia Appendix 1 for information on the visualization tools or methods used for each dashboard and tracker.

### Data Sources and Data Aggregators

#### Overview

As with all data visualizations, it is important for the viewers of COVID-19 dashboards and trackers to know the data sources. A visualization could display data collected by the organization that created the visualization (in the case of public health authorities), data obtained directly from one or more public health authorities, or data from a data aggregator service. Multimedia Appendix 1 documents the data sources stated on the websites. Multimedia Appendix 3 provides a list of data aggregators and prominent dashboard developers with URLs for details on their methodologies and data sources.

#### State Focused

None of the websites provided by state-level public health authorities provided details about data sources or methodology, but it is likely that the data were submitted by local public health departments that received reports from diagnostic laboratories, health clinics, and hospitals. Of the nongovernmental state-focused websites, most stated that the data were from the state public health authority (or, in some cases, a combination of state and local public health authorities), but it is unclear whether these websites were drawing data directly from the public health authorities they credited or if they used a data stream from a data aggregator service. Two nongovernmental websites did not state a source of data or removed the statement [S-30] and [S-54]. One website’s data source [S-77] was credited to a data aggregator.

#### Nationwide Coverage

**Figure 1:**
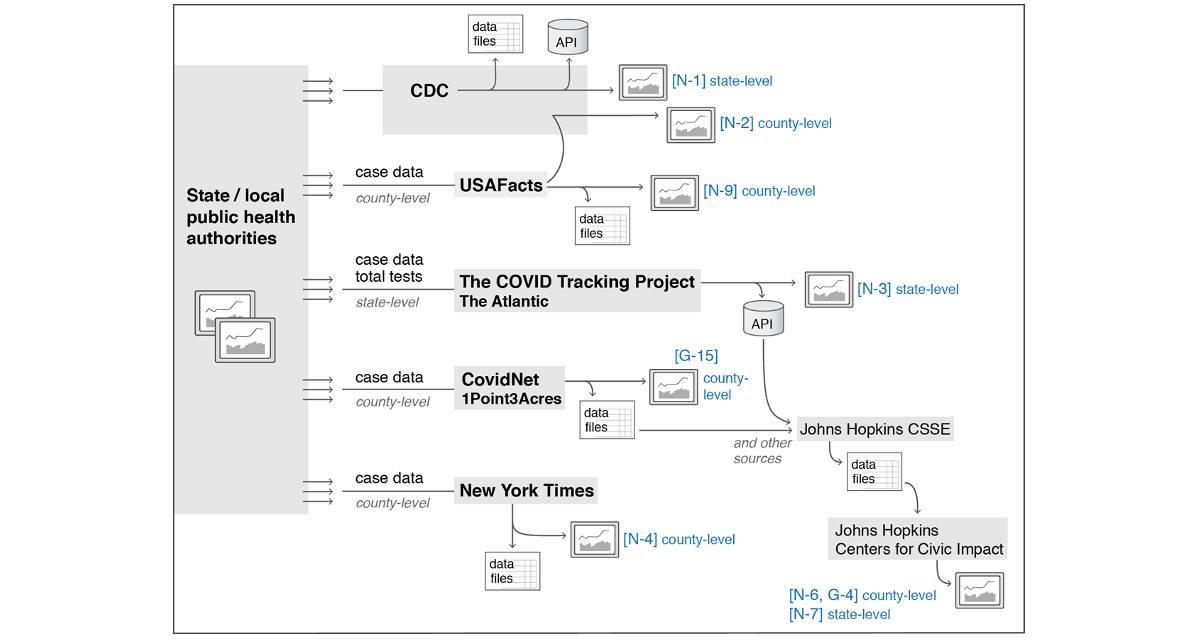
Major data aggregation pathways for the United States’ cases and testing data as of August 2020. References in blue correspond to dashboards and trackers. API: application programming interface; CDC: Centers for Disease Control and Prevention; CSSE: Center for Systems Science and Engineering; G: global; N: nationwide.

Throughout the fall of 2020, the CDC provided only state-level COVID-19 case counts to the public rather than county-level data. Therefore, any website displaying county-level case counts for the United States relied on data aggregated from local and state sources by a nonfederal data aggregator. Figure 1 shows the major data aggregation pathways for case counts and testing data for the United States as of August 2020. It was created by examining data sources and methodology information for the websites and consulting additional reports [26,27]. The following are the 4 major data aggregators used to independently aggregate nationwide data:

- *USAFacts:* A nonprofit civic initiative that gathers government data [28]. County-level data available for download.
- *1Point3Acres (CovidNet):* A volunteer group founded by first-generation Chinese immigrants in the United States [15,29]. County-level data available for download.
- *The New York Times:* County-level data available for download.
- *The COVID Tracking Project:* A volunteer organization launched by *The Atlantic* [30]. State-level data available for download or through an application programming interface (API). It includes data for case counts and total number of tests. This project ended in March 2021, one year after it began.

As shown in Figure 1, in August 2020, only state-level data and not county-level data were available to developers by API. During this survey, several additional resources that claimed to provide APIs for county-level data scraped from the websites of data aggregators were noticed; however, this was in violation of the terms of the service set by those data aggregators.

No nationwide website appeared to use CDC as their only data source. Instead, websites relied on an independent data aggregator or a combination of use of data from the CDC and a data aggregator. Of particular interest is that the county-level tracker provided by the CDC [N-2] credited the USAFacts aggregator as its source of county-level data. In August 2020, a footnote stated “Data courtesy of USAFacts.org downloaded each day at 4:00 pm EST or when earliest update is available” [31]. The web page redesign in November 2020 provided more extensive details on data sources, including the statement “The COVID-19 case and death metrics are generated using data from USAFacts that CDC modifies.” The use of USAFacts was later discontinued and county-level data were obtained directly from the states [32].

#### Global Coverage

The dashboard developed by the Johns Hopkins University (JHU) Centers for Civic Impact displays data from the JHU Center for Systems Science and Engineering (CSSE). JHU CSSE acts as an aggregator of aggregators for worldwide data, relying on a large number of sources, including The COVID Tracking Project and 1Point3Acres for the US data [26]. The complete list of data sources used by the JHU CSSE since January 2020 is provided in their data repository [33].

### Issues in Trust and Transparency

Trust and transparency are emphasized in the guidelines the World Health Organization has assembled for communicating with the public about disease outbreaks [34]. Dashboards and trackers may inform viewers of visualizations about the sources of the data in several ways. The most direct approach is to provide the data source within a caption for each map or graph; however, this may not be feasible for dashboards combining several visualizations. Websites using data aggregators often simply state one or more sources for the entire collection of visualizations. One nationwide dashboard [N-11] was particularly vague about the relationship between the visualizations displayed and the sources of data, crediting *CDC, WHO, The New York Times, JHU, Corona Data Scraper, and official state and county health agencies* without providing further details. When websites list sources in this manner, it raises the following questions:

- Is this website using a data aggregator, but crediting the sources used by the aggregator rather than the aggregator?
- Which measures from which data sources are used in a particular visualization?
- Are all these sources currently used, or is this a list of all sources ever used?
- If only one organization is listed, what is the specific data set from the organization that was used?

Early in the pandemic, data scientists raised concerns about the quality of COVID-19 data [35,36]. The challenges of collecting global data appropriate for display and analysis have led to questions regarding the methodologies and sources used by some aggregators. For example, Worldometer is a private company known for its web counters that estimate world statistics. It became an aggregator of COVID-19 data and provider of popular COVID-19 trackers [G-12] and has been criticized for having an anonymous curation team and opaque methodology [37].

### Visualization Tools and Methods

Multimedia Appendix 1 presents the tools and methods used to construct the visualizations examined in this survey. With the exception of Massachusetts, all state public health authorities provided a web page displaying a dashboard or tracker in August 2020 (with Massachusetts providing PDF downloads). Websites of state public health authorities were often constructed using ArcGIS, whereas state-focused websites from other types of developers relied on a variety of tools (including Tableau, Datawrapper, Infogram, and Microsoft Power BI). Websites providing nationwide coverage tended to be constructed with frameworks using embedded scalable vector graphics. Global dashboards and trackers were created with a variety of methods.

### Critique of Visualizations

#### Overview

Overall, 5 themes were identified from the data visualizations and designs of the dashboards and trackers. Multimedia Appendices 4-18 provide screenshots of the websites taken during the 4 rounds of review. Not every page of the website was captured for multipage websites, but the most relevant visualizations are documented.

#### Theme 1: Data as Imperfect Representation of Reality

**Figure 2:**
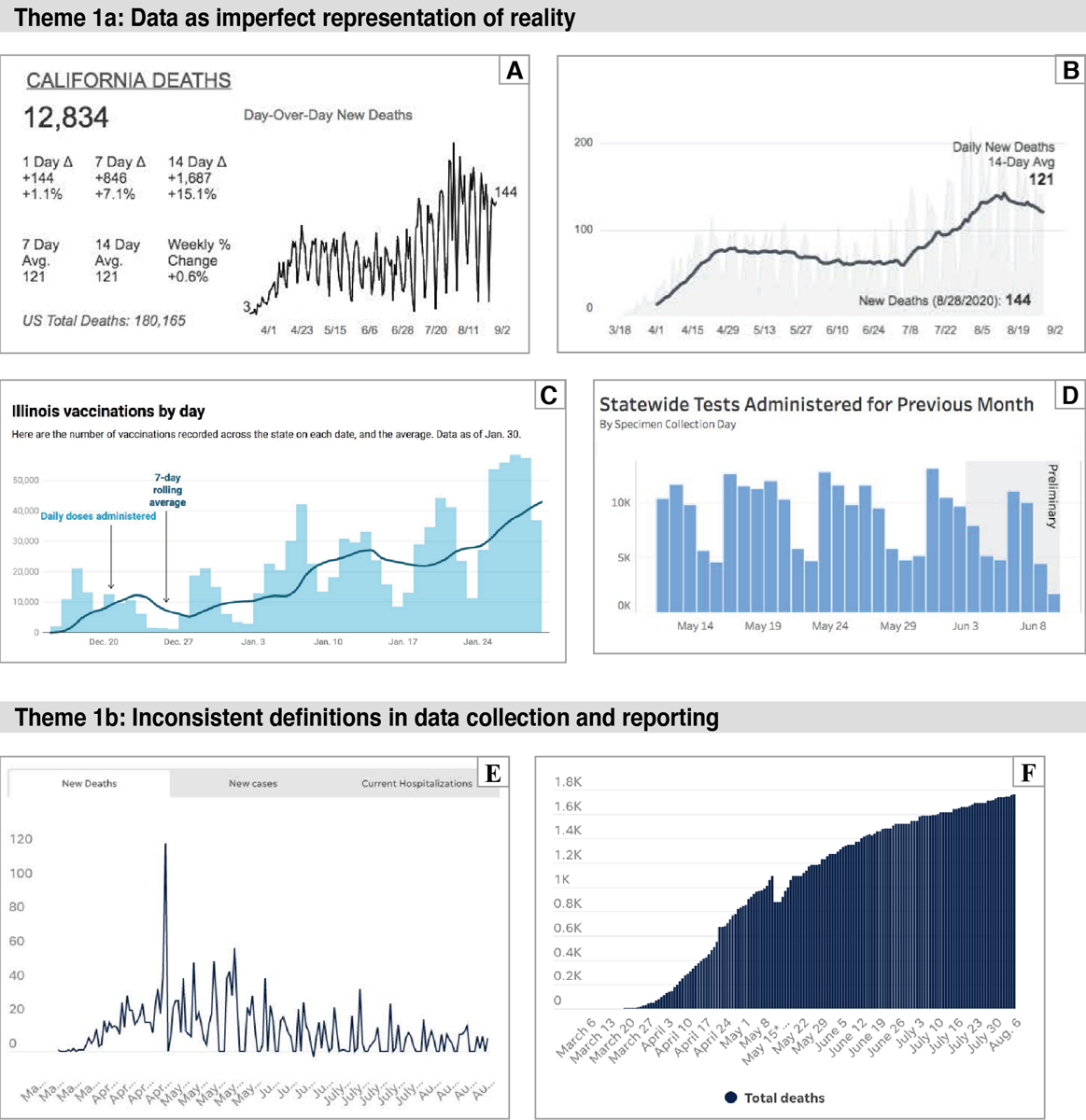
(A and B) Display of daily deaths in California through August 2020 in 2 different dashboards. Notice line for 14-day rolling average in example B. Example A is from [S-9] on August 20, 2020. Example B is from [S-8] on August 29, 2020. (C) Example of 7-day rolling average for daily vaccinations in Illinois. Low values on weekends likely reflect delays in data reporting. From [Svac-6] on January 31, 2021. (D) Tests per day in Indiana. Notice the gray box marking preliminary data. From [S-29] on June 12, 2022. (E) Deaths per day in Colorado, showing a spike on April 24 owing to the inclusion of probable deaths. From [S-13] on August 29, 2020. (F) Cumulative deaths in Colorado, showing a dip on May 15 owing to the change of definition to include only patients who are recorded as dying from COVID-19, rather than testing positive at time of death. From [S-13] on August 14, 2020.

Although the data presented in COVID-19 visualizations are intended to reflect the state of the pandemic, Figure 2 provides examples in which short-term patterns and trends are owing to the methods of data collection and reporting.

##### Theme 1a—Temporal Data Reflect a Combination of Reporting Activity and Public Health Reality

Short-term trends in the data organized by the date of reporting can be misleading. As explained by The COVID Tracking Project:

...this data displays very strong day-of-week effects and is also extremely vulnerable to predictable rise-and-drop artifacts after holidays or other major disruptions, like storms and natural disasters, that affect the ability of counties and states to report their data.

38

To help viewers disregard day-of-the-week variability, most time series graphs include 3-, 7-, or 14-day rolling averages. As time series graphs will have incomplete data for the most recent days (owing to a lag in reporting), the best designs visually indicate the span of incomplete data.

##### Theme 1b—Inconsistent Definitions in Data Collection and Reporting

In the United States, much of the public health infrastructure is regulated and managed at the state and local levels. Therefore, states have different processes for collecting data and use inconsistent definitions. For example, states vary in how they define deaths attributable to COVID-19, whether the number of tests (and positive and negative results) reflects unique people or number of specimens [39], and the diagnosis of asymptomatic cases [40]. In the early months of the pandemic, several states combined the counts of polymerase chain reaction tests (a diagnostic test) and antibody tests (which detect an immune response), leading to distortions in the data on infection rates and testing capacity [41,42]. If data aggregators were unaware of this heterogeneity in state-level data, or unable to correct for known differences, visualizations that provide state-to-state comparisons will be inaccurate. In addition, some states have reported a count of recovered patients with COVID-19. Not only did these states use different definitions for recovered, but referring to patients as recovered when the long-term effects of COVID-19 are not known is misleading [43]. Another potential source of confusion occurred later in the pandemic as people became reinfected, meaning that case counts no longer represented unique individuals if states followed the national case definition [44,45]. The Iowa Department of Public Health noted this change with the following statement:

On September 1, 2021, IDPH adopted the updated 2021 COVID-19 national case definition. As part of this case definition, IDPH began including in its total case counts individuals who were previously reported as a confirmed or probable case, but have become infected again.

S-28

Data regarding vaccinations also had inconsistencies early in 2021. As explained by the *Washington Post* in a footnote below the graphs of state vaccination doses administered by day:

Data before Jan. 12 is inconsistent. On Feb. 19, the CDC altered its reporting of doses administered by federal agencies by adding them to the states where the shots had been given. From Feb. 23 forward, the data reflects doses administered to residents of the states rather than doses administered by the state.

Nvac-3

#### Theme 2: The Importance of Context for Interpretation

**Figure 3:**
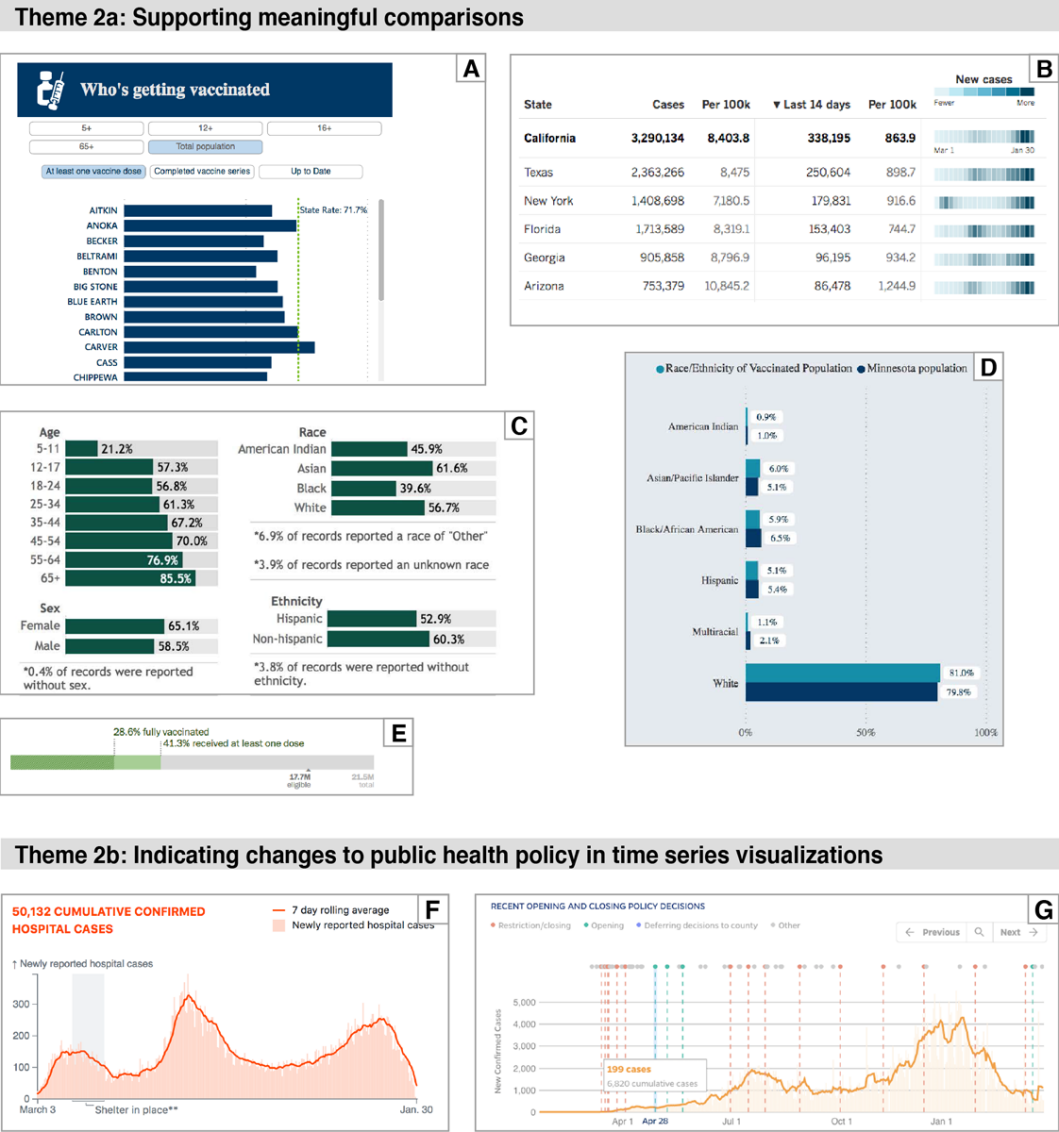
(A) Comparison of vaccination rates in counties of Minnesota against state average. From [Svac-10] on September 25, 2022. (B) Timeline of waves of new cases in California compared with other states. From [S-10] on January 31, 2021. (C) Comparison of vaccination rates in Wisconsin within demographic categories. From [Svac-17] on January 3, 2022. (D) Comparison of vaccination rates in Minnesota within race and ethnicity categories. Graphing on a scale of 100% of the population (rather than proportional to race and ethnicity) makes this design less effective than example C. From [Svac-10] on January 3, 2022. (E) Comparison of percent vaccinated with 1 dose and 2 doses against the eligible population and total population of Florida. From [Nvac-3] on April 30, 2021. (F) Indication of the shelter in place policy as gray band with time series data showing newly reported hospital cases in Georgia. From [S-23] on January 31, 2021. (G) Time points for policy decisions to open or restrict public gathering in Alabama, with time series data showing reported cases. From [N-7] on December 11, 2021.

Data require context for interpretation, and therefore, data visualizations should provide context to help viewers find meaning in a visualization. Figure 3 shows successful and unsuccessful examples of providing context.

##### Theme 2a—Supporting Meaningful Comparisons

Many types of interpretations rely on comparisons. In the context of COVID-19, useful comparisons include differences between regions, differences between demographic groups, differences over time, and differences between vaccinated and unvaccinated populations. It is these comparisons that give meaning to the data.

##### Theme 2b—Indicating Changes to Public Health Policy in Time Series Visualizations

Public health policy affected the trajectory of the pandemic, and policies varied at the state, county, and city levels. Several visualizations superimposed policy changes over time series data.

#### Theme 3: Choosing Values to Display

##### Overview

**Figure 4:**
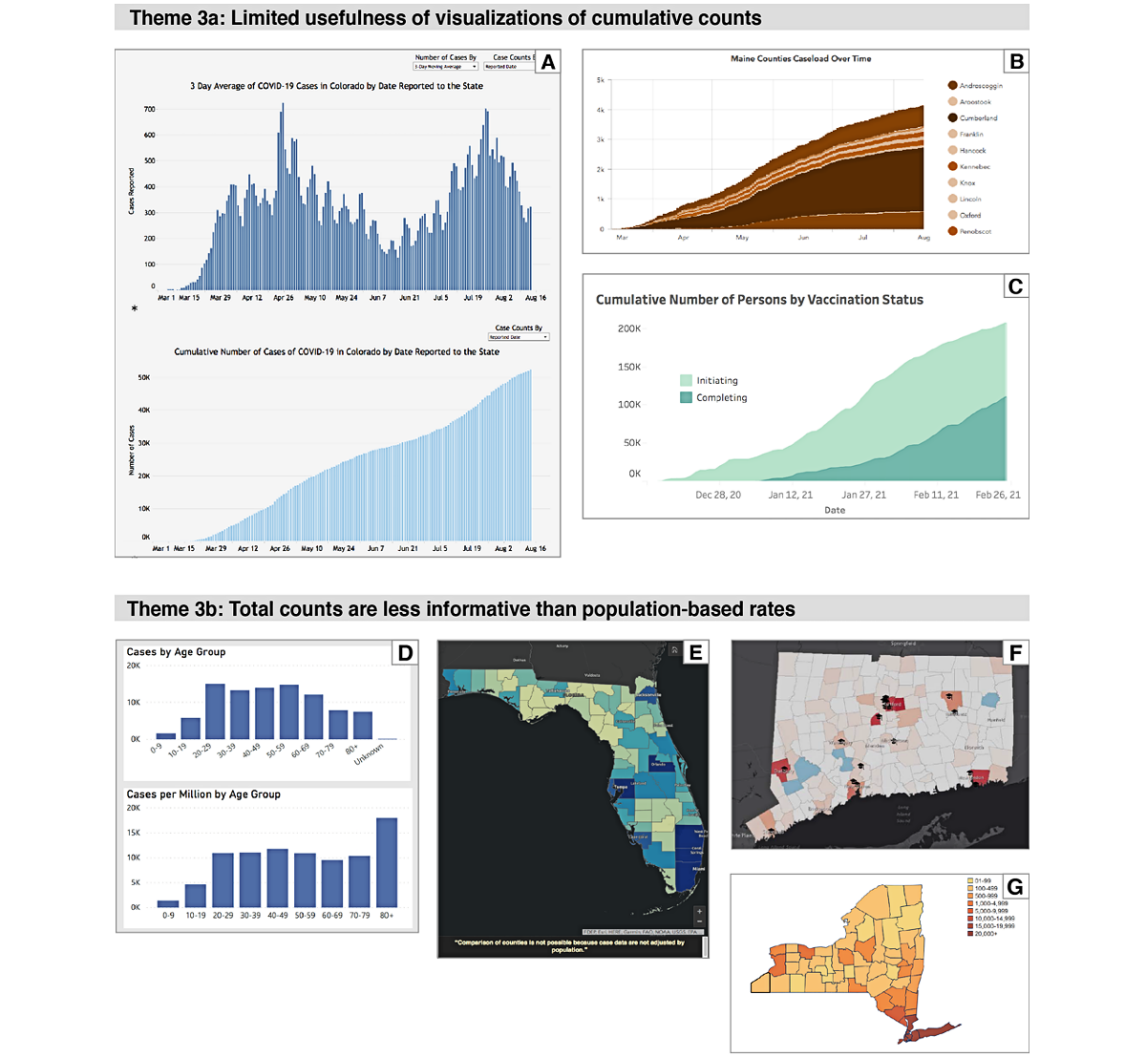
(A) Graphs of the 3-day average of cases (upper graph) and the cumulative number of cases (lower graph) in Colorado. Note that the decrease in new cases in June is difficult to detect in the cumulative graph. From [S-12] on August 15, 2020. (B) Cumulative number of cases in Maine. From [S-37] on August 15, 2020. (C) Cumulative number of persons by vaccination status in Hawaii. The category initiating refers to the first dose, completing indicates receiving both the first and second dose. From [S-25] on February 27, 2021. (D) Case counts by age group (upper graph) and case rates by age group (lower graph) in Michigan. The lower graph shows that patients aged ≥80 years have a higher case rate than the other groups. From [S-40] on August 15, 2020. (E) The home page of the Florida dashboard, with a map showing case counts per county. A note at the bottom says “Comparison of counties is not possible because case data are not adjusted by population.” A color-coding key was not provided. A map displaying the rates by county is available on another tab. From [S-20] on August 15, 2020. (F) Map showing case counts by county. A color-coding key was not provided, but the intensity of red reflects areas of higher population density (with the location of universities indicated). From [S-17] on August 20, 2020. (G) Case counts per county for New York City Long Island has the highest number of cases but also the highest population density.

The COVID-19 pandemic has provided web application developers with access to data and public interest in the visualizations of these data. However, creating useful visualizations often requires more than graphing the raw numbers supplied in a data stream. The examples in Figure 4 demonstrate why it is important to consider whether it is most useful to display the data directly as obtained, a transformation of the data, or cumulative values.

##### Theme 3a—Limited Usefulness of Cumulative Counts

Many dashboards state the total number of COVID-19 cases and deaths, and some also display a time series of cumulative counts. The total number of deaths may be of general interest, but graphs of the cumulative number of cases or deaths are less useful because they show only a rising curve without clearly showing trends during the pandemic. However, it may be that showing a time series of the cumulative number of vaccinated people in a region could help persuade others to become vaccinated.

##### Theme 3b—Total Counts Are Less Informative Than Population-Based Rates

The availability of county-level data helps viewers to understand the geographic distribution of COVID-19 cases and deaths. However, to be more meaningful, data should be displayed as rates (eg, number of cases per 100,000 people) rather than as counts. Visualizing count data on a map is likely to simply show areas with a higher population density and give a misleading impression that COVID-19 has not affected rural areas.

#### Theme 4: Choosing the Graphical Form of the Visualization

**Figure 5:**
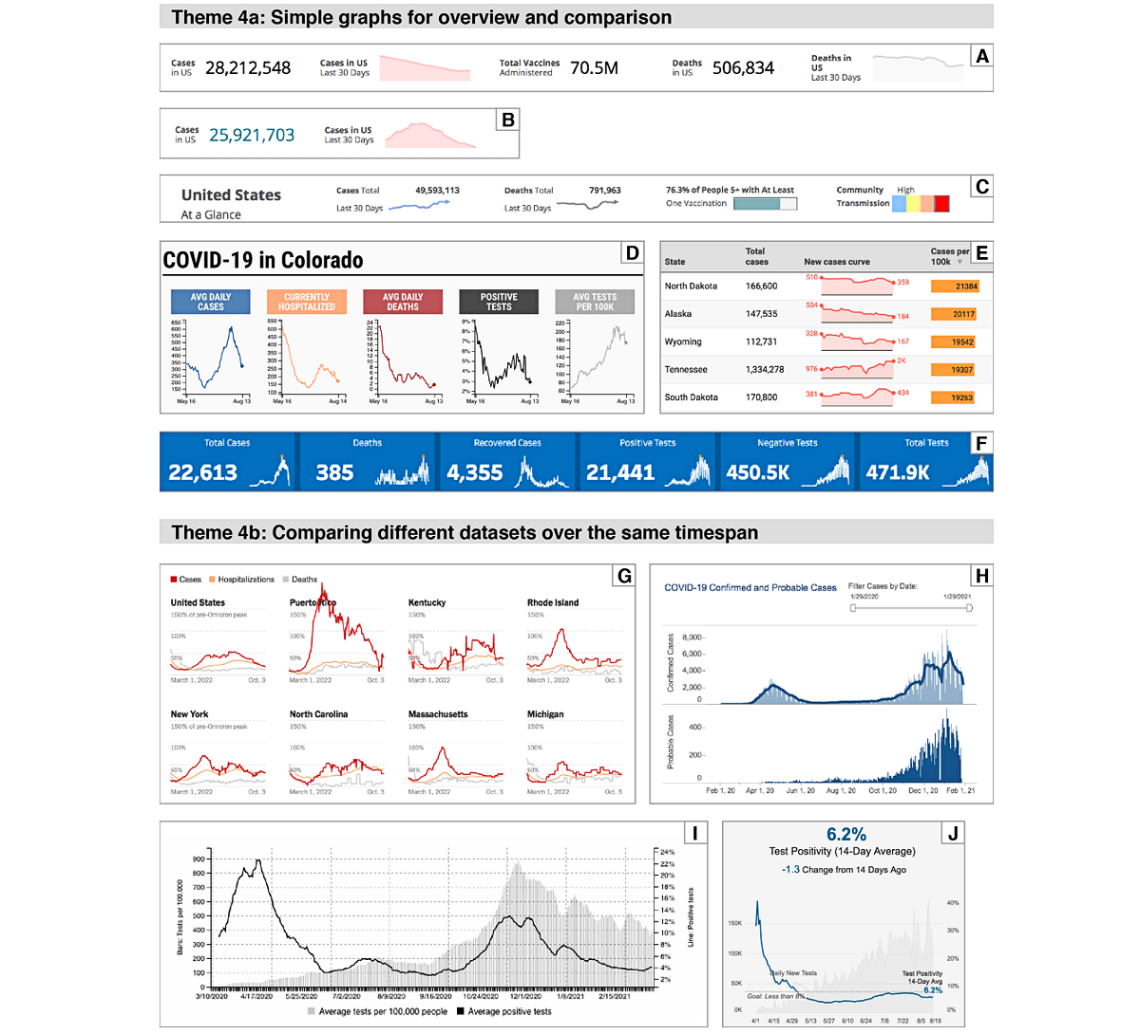
(A-C) Summary visualizations provided by the Ceners for Disease Control and Prevention at the top of their COVID Data Tracker web page [N-2]. Example A was captured on February 1, 2021. This design uses pink shading below the line indicating "Cases in US, last 30 days." This is misleading because the height of the shading does not begin at 0. Example B is the same design captured on February 26, 2021 that deceptively implies that cases have dropped to 0. Example C is the revised design of the summary visualizations captured on December 11, 2021. The shading has been removed and an arrowhead is added. (D) Top-of-page summary provided by the Denver Post [S-14]. The design allows viewers to quickly see and compare trends. Shows data from last 3 months but not from the last 2 days. Captured on August 15, 2020. (E) The first 4 columns and 5 rows of a table comparing each state, ordered by case rates. Sparklines indicate trends over time, but the span of time shown is not defined. Orange bars represent current case rates. From [S-84] on December 11, 2021. (F) Summary for Oregon. This example is less successful in communicating trends because rolling averages are not used. From [S-59] on August 15, 2020. (G) A small portion of a state-by-state comparison provided by the New York Times [N-4] using a small multiples layout. Captured on October 4, 2022. (H) Stacked time series comparing confirmed cases and probable cases in Massachusetts. From [S-39] on January 31, 2021. (I and J) Graphs comparing tests administered and test positivity rate using dual axis graphs. This design is more difficult to interpret than stacked time series. Example I is Colorado data from [S-14] on March 20, 2021. Example J is California data from [S-8] on August 15, 2020.

The graphical forms of the visualizations (including line charts, bar charts, and choropleth maps) and how they were arranged in the dashboards revealed a mixture of effective designs that made good use of perceptual principles as well as less effective designs. Examples are shown in Figure 5.

##### Theme 4a—Simple Graphs for Overview and Comparison

One challenge is to distill the data into simple but meaningful visualizations. Several websites offered simple summary graphics, often in the form of simple time series graphs or sparklines, to communicate the trajectory of the pandemic. However, these simple overviews are only effective if the rolling averages are displayed. Because the pandemic was not uniform across the United States, visualizations also helped people compare the current status and trajectories of different states. However, the key to making these comparisons meaningful is that the underlying data must be comparable, and this relies on the uniformity in data collection or adjustments by data aggregators.

##### Theme 4b—Comparing Different Data Sets Over the Same Timespan

Data displayed as time series are crucial for communicating about the pandemic, and meaning is often derived from comparing different types of data or data from different regions. Small multiples and stacked time series were effective in aiding comparisons. A number of dashboards provided dual axis graphs, often for comparing the numbers of coronavirus tests administered and the positivity rates over time. However, this dual axis design is difficult to interpret, and alternative designs provide better solutions [46,47].

##### Theme 4c—Interactivity of Graphs

Frameworks for developing web visualizations often include functionality for displaying the values of data points when the cursor hovers over points. This method of providing *details-on-demand* is useful for enabling an in-depth exploration of graphs [48] and is often used in time series. Another type of interactivity is to enable a viewer to customize a graph by controlling the data or presentation style through drop-down menus or radio buttons. In this survey, I noted options for choosing between case counts and case rates, setting the length of time for a time series, filtering by demographic group, and switching between a linear or logarithmic scale for case counts. When display options are provided, it is important that a default display is chosen that is suitable for the greatest number of users and minimizes misinterpretation. For example, a linear scale should be the default, but advanced users may choose the option of a logarithmic scale [49]. One particularly useful option for understanding the global spread of the coronavirus is to align outbreaks in different countries based on days since a country’s outbreak reached a particular threshold of cases rather than by date. The former option is the default for a graph provided by Our World in Data [G-10].

#### Theme 5: Pitfalls of Automated Data Display

##### Overview

**Figure 6:**
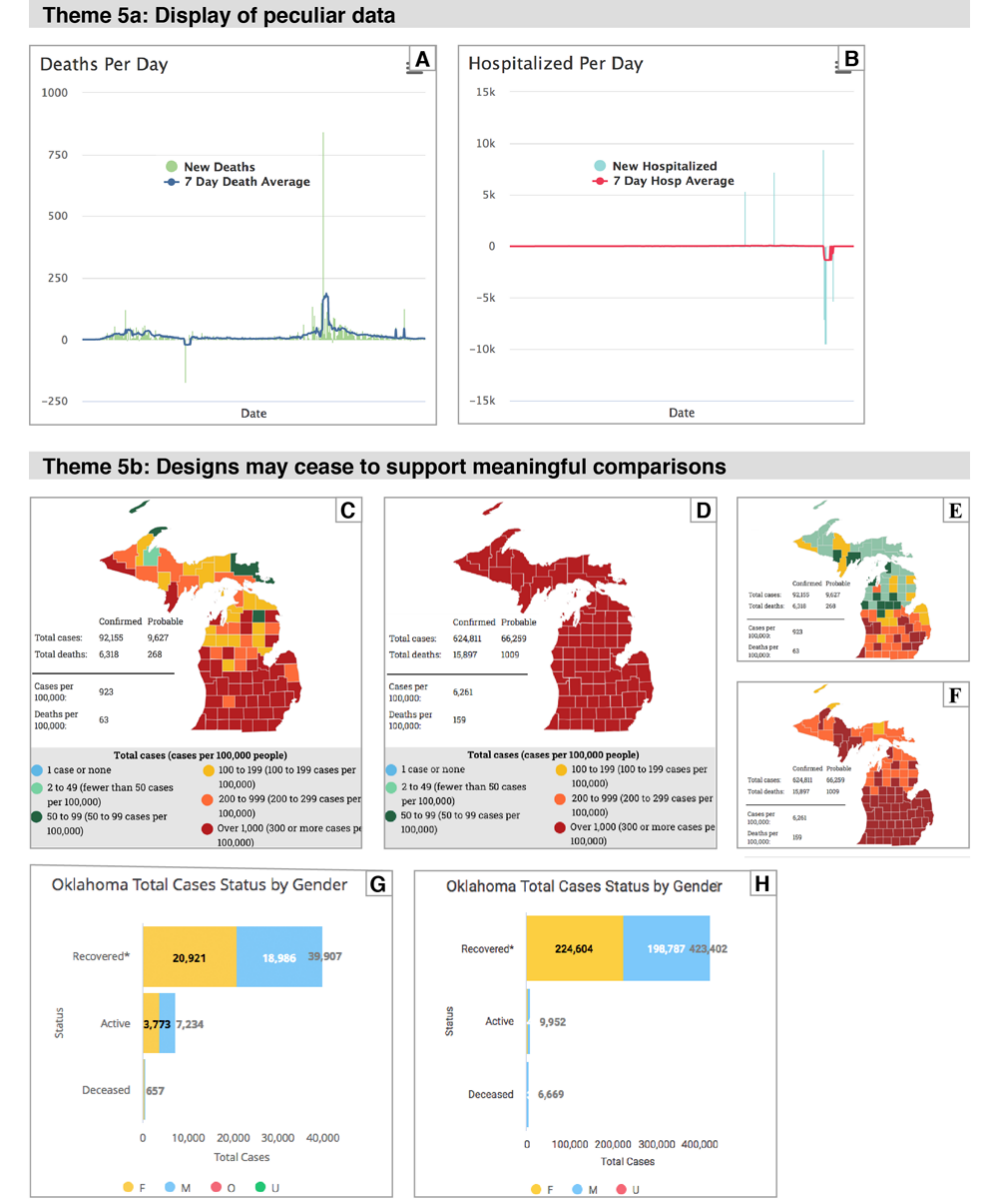
(A) Deaths per day for Colorado, including peaks of −170 and 841. From [N-10] on April 10, 2021. (B) Newly hospitalized patients per day for Kansas, including peaks of 5417, 7257, −9387, and −5290. From [N-10] on April 10, 2021. (C and D) Color coding of counties in Michigan based on case rate. Captured on August 15, 2020, and March 20, 2021. By March 2021 all counties are in the highest bin. From [S-41]. (E and F) Alternate view of Michigan map captured on the same days that display total case counts (rather than rate). Uses same color-coding key as examples C and D. Notice that the data on the August 2020 map spans 5 bins, whereas the March 2021 map uses only 3. (G and H) Patient status in Oklahoma. Captured on August 15, 2020, and April 10, 2021. By April, the number of recovered cases make the length of the active case bar unreadable. From [S-58].

Dashboards and trackers visualize streams of data that are automatically updated. This combination of dynamic data and the lack of human oversight revealed some pitfalls that should be avoided to build more robust systems. These findings also suggest that dashboards need frequent monitoring to detect problems in the design of displays or the handling of data. Figure 6 demonstrates several of the identified problems.

##### Theme 5a—Display of Peculiar Data

Some anomalies in the displayed data cannot be explained by small adjustments to the data or artifacts such as day-of-the-week variations. Extremely high or negative values of counts indicate problems in recording, processing, or transmitting data. The presence of these anomalies should alert developers (and viewers) that the trustworthiness of the entire data set and visualization is questionable.

##### Theme 5b—Designs May Cease to Support Meaningful Comparisons

A design that works well with a particular range of values or size of data set may lose effectiveness as data are dynamically updated. For example, a method of binning data that is effective early in the pandemic will become much less informative if all the data are represented within a single bin later in the pandemic. However, one drawback of adjusting bins over time is that people who periodically view a graph may assume that changes in the distribution of data in bins reflect changes in the data rather than in the definition of the bins.

## Discussion

### Principal Findings

This study identified and examined >100 websites providing COVID-19 dashboards and trackers relevant to the residents of the United States and highlighted the multitude of factors that affect these visualizations. The findings reveal the role data aggregators have played in making data accessible to visualization developers as well as lapses in communicating to viewers the provenance of the data. Decisions by public health experts about data collection and data standards have downstream effects on which data are available to be communicated and compared. In addition, each step of this process is impacted by the evolving nature of the pandemic and political and social systems.

The five themes identified in this work can guide future development of visualizations of public health data for the public: (1) viewers should be made aware that data are an imperfect representation of reality owing to methods of data collection and reporting; (2) viewers need context for interpreting visualizations, such as comparisons with other data or indicators of relevant events on timelines; (3) developers should carefully consider whether plotting a raw data stream, cumulative values, or transformation of values will be the most useful to viewers; (4) the graphical form of a visualization should be chosen to fit the type of data and be designed to make good use of perceptual principles; and (5) visualizations designed to use automated streams of data must be monitored to ensure that the data continue to have reasonable values and that the design of the visualization remains useful with the new data.

### Trust and Transparency Begins With the Data

One of the persistent challenges faced by data aggregators has been managing disparate data sets for analysis and visualization. In the United States, the collection of public health data is governed at the local and state levels [50]. Strategies differ by state, with no central government authority to standardize data collection and reporting. The Council of State and Territorial Epidemiologists published standards for the clinical diagnosis of COVID-19 and data elements to report in April 2020, with updates in August 2020 and August 2021 [40,45,51]. The Council of State and Territorial Epidemiologists also recommended that states enact laws to make cases of COVID-19 reportable to public health authorities. The CDC has no authority to require reporting, stating “COVID-19 case surveillance data are collected by jurisdictions and reported voluntarily to CDC” [52].

Problems with data quality, standards, and availability have been described by dashboard and aggregator teams [53-56] and journalists [57-60]. Problems in data standardization and availability were somewhat alleviated during the first year of the pandemic, but data on case counts became unreliable by early 2022 because of the introduction of rapid at-home test kits [61,62].

Data that are visualized by a person or an organization that did not originally collect the data is an example of data reuse. The movement around Findable, Accessible, Interoperable, Reusable (FAIR) data includes the responsibility of providing appropriate data citations so that the original source and providence of data are discoverable [63]. The disconnect between the vision of the FAIR data and the findings of this survey is important. One challenge is that COVID-19 data are obtained in frequent updates (rather than from archived data sets) and often from data aggregators. This highlights the gap between the real-world need for trustworthy display of data in public health and typical use cases for using FAIR principles.

### Aligning Visualization Goals and Visualized Data

What are the purposes of public-facing visualizations of pandemic data, and what data are needed to achieve those purposes?

Dashboards are often described as tools to support decision-making. Visualizations have played an important role in educating citizens about the pandemic and therefore may encourage changes in behavior to mitigate transmission. However, visualizations are likely to have a constellation of purposes. For example, a visualization could help establish trust between public health authorities and citizens. Further, effectively promoting behavior change may depend on first conveying the magnitude of human suffering caused by the pandemic.

The question of what data are useful for decision-making was addressed early in the pandemic by former CDC Director Dr Tom Frieden. He argued that there is a mismatch between the most commonly available data—counts of cases, hospitalizations, and deaths—and the data that are the most useful for guiding COVID-19 response in communities. He suggested that local decision-making for formulating policies should use data that include the number of unlinked infections, number of health care worker infections, and trends in excess mortality [64].

### Visualizations as Arguments

Data visualizations are often assumed to be neutral and objective mechanisms of communication, but they are not. Designing and developing visualizations require numerous decisions regarding the selection of data and methods of presentation. It has been argued that all visualizations are rhetorical and therefore have the power to influence beliefs and behaviors [65,66].

In the context of the COVID-19 pandemic, public health authorities and government officials have made decisions about what data to collect and what data to *not* collect. These decisions constrain the messages that visualizations can send. In addition, the messages from these visualizations may imply a sense of authority and certainty through their association with organizations that have traditionally been respected (public health agencies, universities, and news organizations) and the “clean lines and structured layouts of traditional visualizations” [65]. This authority and certainty may obscure the extent of human suffering caused by COVID-19, echoing concerns raised by Dragga and Voss [67] in their analysis of graphs depicting fatalities and injuries from causes such as industrial workplaces and baby walkers.

In the United States, the authority of COVID-19 visualizations and the data behind them have been questioned, with various groups asserting that the severity of the pandemic has been either overplayed or downplayed. As many state and local policies for reopening schools and businesses were commonly tied to metrics about the pandemic, such as the test positivity rate or hospitalization rate, people tired of pandemic restrictions have accused COVID-19 data and dashboards of becoming political tools to prevent a return to normal. Other groups adopted a different perspective. For example, in April 2022, a coalition of public health practitioners, scientists, health care workers, educators, and advocates known as The People’s CDC released a statement criticizing the new definitions for categories of community transmission rates. They wrote the following :

The resulting shift from a red map to a green one reflected no real reduction in transmission risk. It was a resort to rhetoric: an effort to craft a success story that would explain away hundreds of thousands of preventable deaths and the continued threat the virus poses.

68

### The Connection Between Data, Usability, and Understandability

Public-facing visualizations of pandemic data are useful only if viewers are able to understand and interpret the data displays they see. Dashboard designers might choose to display large amounts of data with the goal of allowing the viewers to come to their own interpretation of the data without the prescriptive guidance of dashboard designers. However, this effort at transparency can backfire if the viewers are overwhelmed by the complexity or arrive at incorrect conclusions [25,65]. Viewers may assume that websites with more data are more accurate, but the volume of data and visualizations may obscure uncertainties in the data.

Visualization and communication researchers play crucial roles in determining how to better design public-facing dashboards for infectious disease data. Several studies have used COVID-19 data and dashboards in user studies [23-25,69]. Identifying best practices will accelerate the development of effective dashboards and trackers, and the software tools commonly used by public health authorities could incorporate those recommendations into templates. An important area for future investigation is determining if effective design practices for COVID-19 data can be applied to display other types of public health data.

Current research in the field of visualization seeks to develop software tools to assist nonexpert users in choosing effective visualization techniques to support their specific data sets and goals (as demonstrated in studies by Lavalle and Mate [70] and Golfarelli and Lizzi [71]). This aligns with 2 of the themes from this study, choosing the values to display and choosing the graphical form of the visualization. These studies are often based in the domain of business analytics; however, future work could focus on the domain of public health.

### Limitations

This study was limited to dashboards and trackers available to the public as of August 2020 and therefore does not include dashboards used internally by health care and public health organizations. It excludes visualizations produced exclusively for smartphone apps and visualizations that focus on specific populations, such as nursing homes or prisons, or nontraditional data types, such as wastewater sampling.

### Conclusions

This analysis reveals the extent to which dashboards and trackers informing the American public about the COVID-19 pandemic relied on an ad hoc pipeline of data sources and data aggregators. The pandemic has been characterized by disparate and evolving data standards, which has complicated the development of dashboards and trackers that display data over time and across regions. The 128 websites of dashboards and trackers identified in this survey offer an opportunity to compare different approaches to the display of similar data. This work highlights examples that provide clarity in interpreting data, and those that obscure the meaning of the data and may potentially mislead viewers.

## Appendix

Multimedia Appendix 1 State-focused, nationwide, and global dashboards and trackers were examined. Includes the URL for each site, data sources, and type of visualization tool or method used. Government websites are listed in gray shading.

Multimedia Appendix 2 State-focused, nationwide, and global dashboards and trackers for vaccination on websites that are not included in Multimedia Appendix 1. Includes the URL for each site, data sources, and type of visualization tool or method used. Government websites are listed with gray shading.

Multimedia Appendix 3 Web pages for data sources and technical information provided by prominent data aggregators and dashboard developers.

Multimedia Appendix 4 Screenshots for state-focused dashboards and trackers, August 29, 2020.

Multimedia Appendix 5 Screenshots for nationwide dashboards and trackers, August 29, 2020.

Multimedia Appendix 6 Screenshots for global dashboards and trackers, August 29, 2020.

Multimedia Appendix 7 Screenshots for state-focused dashboards and trackers, January 31, 2021.

Multimedia Appendix 8 Screenshots for nationwide dashboards and trackers, February 26, 2021.

Multimedia Appendix 9 Screenshots for global dashboards and trackers, March 1, 2021.

Multimedia Appendix 10 Screenshots for vaccine dashboards and trackers, January 31 or February 27, 2021.

Multimedia Appendix 11 Screenshots for state-focused dashboards and trackers, December 11, 2021.

Multimedia Appendix 12 Screenshots for nationwide dashboards and trackers, December 11, 2021.

Multimedia Appendix 13 Screenshots for global dashboards and trackers, December 11, 2021.

Multimedia Appendix 14 Screenshots for vaccine dashboards and trackers, January 3, 2022.

Multimedia Appendix 15 Screenshots for state-focused dashboards and trackers, June 12, 2022.

Multimedia Appendix 16 Screenshots for nationwide dashboards and trackers, June 16, 2022.

Multimedia Appendix 17 Screenshots for global dashboards and trackers, June 16, 2022.

Multimedia Appendix 18 Screenshots for vaccine dashboards and trackers, June 16, 2022.

## Abbreviations

API: application programming interface
CDC: Centers for Disease Control and Prevention
CSSE: Center for Systems Science and Engineering
FAIR: Findable, Accessible, Interoperable, Reusable
JHU: Johns Hopkins University
