# Supplementary material for: Web-Based COVID-19 Dashboards and Trackers in the United States: Survey Study
Source: JMIR Hum Factors. 2023 Mar 20;10:e43819. doi: 10.2196/43819 (PMC10029858; doi:10.2196/43819)
Supplement: Multimedia Appendix 1 [file humanfactors_v10i1e43819_app1.pdf]

Appendix 1a. Dashboards and trackers for case counts and testing — *State focus, with county-level data*

State public health authorities (or state government) in shaded rows (if no data sources listed, assumed to be direct reporting from counties or districts)

Ref	State	Type*	Host	URL	Data sources**	Vis tool / method***
S-1	AL	govt	Alabama Dept of Public Health	https://alpublichealth.maps.arcgis.com/apps/opsdashboard/index.html -/6d2771faa9da4a2786a509d82c8cf0f7		ArcGIS
S-2	AL	indiv	David Marconnet, CTO of OneTeam.net	https://bamatracker.com/ *(discontinued updates May 2021, still available June 2022)*	state PH, CDC	OneTeam dashboard
S-3	AL	media	Alabama Media Group	https://www.al.com/coronavirus/data/ *(discontinued updates June 2020, still available June 2022)*	state PH	Datawrapper
S-4	AK	govt	Alaska Dept of Health and Social Services	https://coronavirus-response-alaska-dhss.hub.arcgis.com/ *(discontinued)* https://alaska-coronavirus-vaccine-outreach-alaska-dhss.hub.arcgis.com/		ArcGIS
S-5	AK	news	Anchorage Daily News	https://www.adn.com/alaska-news/2020/04/06/covid-19-in-alaska/ *(discontinued updates August 2020, still available June 2022)*	state PH	JPGs
S-6	AZ	govt	Arizona Dept of Health Services	https://www.azdhs.gov/preparedness/epidemiology-disease-control/infectious-disease-epidemiology/covid-19/dashboards/ *(moved to new URL)* https://www.azdhs.gov/covid19/data/index.php		Tableau
S-7	AR	govt	Arkansas Dept of Health	https://experience.arcgis.com/experience/c2ef4a4fcbe5458fbf2e48a21e4fece9 *(moved to new URL)* https://experience.arcgis.com/experience/633006d0782b4544bd5113a314f6268a/		ArcGIS
S-8	CA	govt	California Dept of Public Health	https://update.covid19.ca.gov/		Tableau
S-9	CA	govt	CA Open Data	https://public.tableau.com/profile/ca.open.data#!/vizhome/COVID-19CasesDashboard_15931020425010/Cases *(discontinued)* https://public.tableau.com/app/profile/ca.open.data/viz/COVID-19CasesDashboardv2_0/CaseStatistics	state PH	Tableau
S-10	CA	news	Los Angeles Times	https://www.latimes.com/projects/california-coronavirus-cases-tracking-outbreak/ *(restricted to subscribers only by December 2021)*	state PH	SVGs, Mapbox
S-11	CA	news	CalMatters	https://calmatters.org/health/coronavirus/2020/03/california-coronavirus-by-the-numbers-cases-testing-masks-hospital-beds	state PH, COVID TP^	SVGs
S-12	CO	govt	Colorado Dept of Health & Environment	https://covid19.colorado.gov/data/case-data		Tableau
S-13	CO	news	Coloradoan	https://www.coloradoan.com/story/news/2020/08/01/covid-19-tracker-colorado-cases-deaths-and-hospitalizations-data-coronavirus/5559621002/	state/local PH	Infogram
S-14	CO	news	The Denver Post	https://www.denverpost.com/2020/03/06/coronavirus-map-colorado/	state PH	SVGs, Leaflet
S-15	CT	govt	Connecticut's Official State Website	https://portal.ct.gov/Coronavirus/COVID-19-Data-Tracker		Datawrapper
S-16	CT	npo	CTData Collaborative	https://public.tableau.com/profile/connecticut.state.data.center -!/vizhome/ConnecticutCOVID-19CaseTracking/CTdataCollaborativeCOVID-19	state PH	Tableau
S-17	CT	univ	University of Connecticut Dept of Geography	https://experience.arcgis.com/experience/0d8bc059178d43bdbcfe6406f3dfdc26	state PH	ArcGis
S-18	DC	govt	Government of the District of Columbia	https://coronavirus.dc.gov/page/coronavirus-data		MicroStrategy vis. SDK
S-19	DE	govt	Delaware Environmental Public Health Tracking Network	https://myhealthycommunity.dhss.delaware.gov/locations/state	state PH	SVGs, Leaflet
S-20	FL	govt	Florida Dept of Health	https://experience.arcgis.com/experience/96dd742462124fa0b38ddedb9b25e429 *(site removed, only general COVID information and weekly PDF reports available at https://floridahealthcovid19.gov/)*		ArcGIS
S-21	FL	indiv	Florida COVID Action	https://experience.arcgis.com/experience/d2726d6c01c4486181fec2d4373b01fa *(no longer updated due to lack of state data, still available June 2022)*	state PH	ArcGIS
S-22	GA	govt	Georgia Dept of Public Health	https://dph.georgia.gov/covid-19-daily-status-report *(visualizations discontinued before December 2021)*		SVGs

S-23	GA	news	Atlanta Journal-Constitution	https://www.ajc.com/news/coronavirus-georgia-covid-dashboard/jvoLBozRtBSVSNQDDAuZxH/	state PH	SVGs
S-24	HI	govt	State of Hawaii Dept of Health	https://health.hawaii.gov/coronavirusdisease2019/what-you-should-know/current-situation-in-hawaii/		ArcGIS
S-25	HI	npo	Hawaii Data Collaborative	https://www.hawaiidata.org/covid19<br>*(discontinued December 2021)*	state PH	Tableau
S-26	ID	govt	Idaho Division of Public Health	https://public.tableau.com/profile/idaho.division.of.public.health#!/vizhome/DPHIdahoCOVID-19Dashboard_V2/Story1 *(discontinued)*<br>https://public.tableau.com/profile/idaho.division.of.public.health - !/vizhome/DPHIdahoCOVID-19Dashboard/Home		Tableau
S-27	ID	news	KTVB-7	https://www.ktvb.com/article/news/health/coronavirus/idaho-coronavirus-numbers-map-graph-data-covid-19-timeline-tracking-cases-curve-recoveries-deaths/277-262de30b-ef00-40a2-b40e-43e26f0bb99b<br>*(discontinued updates in August 2021, still available June 2022)*	state/local PH	Flourish
S-28	IL	govt	Illinois Dept of Public Health	https://www.dph.illinois.gov/covid19/covid19-statistics *(discontinued and removed)*<br>https://dph.illinois.gov/covid19/data.html		Plotly
S-29	IN	govt	Indiana State Dept of Health	https://www.coronavirus.in.gov/2393.htm		SVGs
S-30	IN	univ	Polis Center at Indiana University - Purdue University Indianapolis	https://iu.maps.arcgis.com/apps/opsdashboard/index.html - /460c6eb46be7403380091c377e591b0b<br>*(discontinued updates in April, still available June 2022)*	(not stated)	ArcGIS
S-31	IA	govt	Iowa Dept of Public Health	https://coronavirus.iowa.gov/pages/case-counts<br>https://coronavirus.iowa.gov/<br>*(discontinued by June 2022)*		Domo
S-32	KS	govt	Kansas Dept of Health and Environment	https://www.coronavirus.kdheks.gov/160/COVID-19-in-Kansas		Tableau
S-33	KY	govt	Kentucky Dept of Public Health	https://kygeonet.maps.arcgis.com/apps/opsdashboard/index.html - /543ac64bc40445918cf8bc34dc40e334		ArcGIS
S-34	KY	news	WFPL 89.3	https://wfpl.org/looking-for-county-level-data-on-the-coronavirus-heres-our-kentucky-covid-19-tracker/<br>*(discontinued updates in May 2022, still available June 2022)*	NYT	SVGs
S-35	LA	govt	Louisiana Dept of Health	https://ldh.la.gov/coronavirus/		ArcGIS
S-36	ME	govt	Maine Center for Disease Control & Prevention	https://www.maine.gov/dhhs/mecdc/infectious-disease/epi/airborne/coronavirus/data.shtml		Tableau
S-37	ME	govt	Maine Geolibrary	https://geolibrary-maine.opendata.arcgis.com/app/maine-and-covid-19-centralized-dashboard *(discontinued)*<br>https://maine.maps.arcgis.com/apps/MapSeries/index.html?appid=7dcd580d21434c0f8ce74bdb16664b2f<br>*(discontinued updates May 2022, still available June 2022)*		ArcGIS
S-38	MD	govt	Maryland Dept of Health	https://coronavirus.maryland.gov/		ArcGIS
S-39	MA	govt	Massachusetts Dept of Public Health	https://www.mass.gov/info-details/covid-19-response-reporting		*Aug 2020:* PDF<br>*Jan 2021:* Tableau
S-40	MI	govt	Michigan Dept of Health and Human Services	https://www.michigan.gov/coronavirus/0,9753,7-406-98163_98173---,00.html		Microsoft Power BI
S-41	MI	news	Bridge Michigan	https://www.bridgemi.com/michigan-coronavirus-dashboard-cases-deaths-and-maps	state PH	Infogram
S-42	MN	govt	Minnesota Dept of Health	https://www.health.state.mn.us/diseases/coronavirus/situation.html		PNGs
S-43	MS	govt	Mississippi State Dept of Health	https://msdh.ms.gov/msdhsite/_static/14,21882,420,873.html		Microsoft BI
S-44	MO	govt	Missouri Dept of Health and Senior Services	https://missouri-coronavirus-gis-hub-mophep.hub.arcgis.com/app/1aa9c96482d54ccfb309679015313fd2 *(discontinued)*<br>https://showmestrong.mo.gov/data/public-health/		*Aug 2020:* ArcGIS Story Map<br>*Jan 2021:* Tableau
S-45	MT	govt	Montana Dept of Public Health and Human Services	https://dphhs.mt.gov/publichealth/cdepi/diseases/coronavirusmt/demographics<br>*(discontinued embedded PNG images, now provides links to [S-46])*		ArcGIS, PNGs
S-46	MT	govt	Montana State Library	https://montana.maps.arcgis.com/apps/MapSeries/index.html?appid=7c34f3412536439491adcc2103421d4b		ArcGIS

S-47	NC	govt	North Carolina Dept of Health and Human Services	https://covid19.ncdhhs.gov/dashboard		Tableau
S-48	ND	govt	North Dakota Dept of Health	https://www.health.nd.gov/diseases-conditions/coronavirus/north-dakota-coronavirus-cases		Microsoft BI
S-49	NE	govt	Nebraska Dept of Health and Human Services	https://experience.arcgis.com/experience/ece0db09da4d4ca68252c3967aa1e9dd *(removed by December 2021)*		ArcGIS
S-50	NV	govt	Nevada Dept of Health and Human Services	https://nvhealthresponse.nv.gov/		Microsoft Power BI
S-51	NH	govt	New Hampshire Dept of Health and Human Services	https://www.nh.gov/covid19/dashboard/case-summary.htm		Tableau
S-52	NH	news	New Hampshire Public Radio	https://infogram.com/nhpr-covid-19-tracker-1hzj4ozrjnvp2pw?live *(discontinued)*<br>https://www.nhpr.org/post/explore-data-tracking-covid-19-new-hampshire	state PH, COVID TP	*Aug 2020:* Infogram<br>*Jan 2021:*Datawrapper
S-53	NJ	govt	New Jersey Dept of Health	https://www.nj.gov/health/cd/topics/covid2019_dashboard.shtml		ArcGIS Story Map
S-54	NJ	media	NJ Advance Media	https://projects.nj.com/coronavirus-tracker/<br>*(discontinued by June 2022)*	state/local PH (removed source info by Jan 2021)	SVGs, Leaflet
S-55	NM	govt	New Mexico Dept of Health	https://cvprovider.nmhealth.org/public-dashboard.html		SVGs, Leaflet
S-56	NY	govt	New York State Dept of Health	https://covid19tracker.health.ny.gov/views/NYS-COVID19-Tracker/NYSDOHCOVID-19Tracker-Map		Tableau
S-57	OH	govt	Ohio Dept of Health	https://coronavirus.ohio.gov/wps/portal/gov/covid-19/dashboards/overview		Tableau
S-58	OK	govt	Oklahoma State Dept of Health	https://coronavirus.health.ok.gov/ *(redirected to new URL)*<br>https://oklahoma.gov/covid19.html		*Aug 2020, Jan 2021:* Looker<br>*Dec 2021:* Tableau
S-59	OR	govt	Oregon Health Authority	https://public.tableau.com/profile/oregon.health.authority.covid.19 - !/		Tableau
S-60	PA	govt	Pennsylvania Dept of Health	https://www.health.pa.gov/topics/disease/coronavirus/Pages/Cases.aspx		ArcGIS, Microsoft BI
S-61	RI	govt	Rhode Island Dept of Health	https://ri-department-of-health-covid-19-data-rihealth.hub.arcgis.com/		Google Data Studio
S-62	SC	govt	South Carolina Dept of Health and Environment Control	https://www.scdhec.gov/infectious-diseases/viruses/coronavirus-disease-2019-covid-19/sc-testing-data-projections-covid-19 *(discontinued)*<br>https://scdhec.gov/covid19/covid-19-data		ArcGIS<br>*June 2022:* Tableau
S-63	SC	news	WPDE-15	https://wpde.com/news/coronavirus/interactive-watching-the-coronavirus-curve<br>*(discontinued updates in April, still available December 2021)*	state PH	Tableau
S-64	SD	govt	South Dakota Dept of Health	https://doh.sd.gov/news/coronavirus.aspx		Microsoft Power BI
S-65	TN	govt	Tennessee Dept of Health	https://experience.arcgis.com/experience/885e479b688b4750837ba1d291b85aed<br>*(discontinued in January 2022, still available June 2022)*		ArcGIS
S-66	TN	govt	Tennessee Dept of Health	https://www.tn.gov/content/tn/health/cedep/ncov/data.html		Tableau
S-67	TX	govt	Texas Health and Human Services	https://txdshs.maps.arcgis.com/apps/opsdashboard/index.html#/ed483ecd702b4298ab01e8b9cafc8b83 *(discontinued)*		ArcGIS
S-68	TX	news	Houston Chronicle	https://www.houstonchronicle.com/coronavirus/article/covid-interactive-map-houston-texas-us-case-virus-15142609.php	state PH, own reporting	Fluorish
S-69	UT	govt	Utah Dept of Health	https://coronavirus-dashboard.utah.gov/ *(discontinued)*<br>https://coronavirus-dashboard.utah.gov/risk.html		Plotly, Leaflet
S-70	VA	govt	Virginia Dept of Health	https://www.vdh.virginia.gov/coronavirus/covid-19-daily-dashboard/		Tableau
S-71	VT	govt	Vermont Dept of Health	https://www.healthvermont.gov/response/coronavirus-covid-19/current-activity-vermont -dashboard<br>*(discontinued in May 2022)*		ArcGIS
S-72	VT	news	VTDigger	https://vtdigger.org/coronavirus/	state PH	Datawrapper
S-73	WA	govt	Washington State Dept of Health	https://www.doh.wa.gov/Emergencies/NovelCoronavirusOutbreak2020COVID19/DataDashboard		Microsoft BI
S-74	WA	trade	Washington State Hospital Association	https://www.wsha.org/for-patients/coronavirus/coronavirus-tracker/	state PH	Tableau
S-75	WV	govt	West Virginia Dept of Health & Human Resources	https://dhhr.wv.gov/COVID-19/Pages/default.aspx		Microsoft BI

S-76	WI	govt	Wisconsin Dept of Health Services	https://www.dhs.wisconsin.gov/covid-19/county.htm		Tableau
S-77	WI	news	WISN-12	https://www.wisn.com/article/coronavirus-wisconsin-curve-cases-deaths/32110896	JHU	Infogram
S-78	WY	govt	Wyoming Dept of Health	https://health.wyo.gov/publichealth/infectious-disease-epidemiology-unit/disease/novel-coronavirus/covid-19-map-and-statistics/ *(discontinued by December 2021)*		Tableau
S-79	PA NJ NY OH WV MD DE	news	Spotlight PA	https://www.spotlightpa.org/news/2020/03/pa-coronavirus-updates-cases-map-live-tracker/ *(discontinued by June 2022)*	state PH, COVID TP, NYT^	SVGs, Leaflet
S-80	RI MA	news	WPRI-12	https://www.wpri.com/covid-19-tracking-timeline-maps/	state PH	Flourish
S-81	CA US	news	San Francisco Chronicle	https://projects.sfchronicle.com/2020/coronavirus-map/	state PH, JHU, COVID TP, CDC, own reporting	SVGs
S-82	ME US	news	The Maine Monitor	https://www.themainemonitor.org/coronavirus-in-maine-data-dashboard/	state PH, NYT	Tableau
S-83	OR US	news	The Oregonian	https://projects.oregonlive.com/coronavirus/	state/local PH, JHU^, COVID TP^, CDC	SVGs
S-84	AR US global	news	Arkansas Democrat Gazette	https://www.arkansasonline.com/arvirus/ *(discontinued updates May 2022, still available June 2022)*	state PH, JHU, COVID TP, NYT	Datawrapper

Appendix 1b. Dashboards and trackers for case counts and testing — *Nation-wide coverage*

Public health authority in shaded rows

**Ref**	**Granularity**	**Type***	**Host**	**URL**	**Data sources****	**Vis tool / method*****
N-1	state	gov	US Centers for Disease Control and Prevention	https://www.cdc.gov/covid-data-tracker/		SVGs
N-2	county	gov	US Centers for Disease Control and Prevention	https://www.cdc.gov/coronavirus/2019-ncov/cases-updates/county-map.html *(discontinued and combined with [N-1])* https://covid.cdc.gov/covid-data-tracker/ - county-view *(data display not functioning December 2021, working June 2022)*	*Aug 2020:* USAFacts.org *Feb 2021:* CDC	SVGs
N-3	state	news	The COVID Tracking Project / The Atlantic	https://covidtracking.com/ *(project ended March 7, 2021)*	state/local PH	SVGs
N-4	county	news	New York Times	https://www.nytimes.com/interactive/2020/us/coronavirus-us-cases.html	state/local PH, COVID TP	SVGs, Mapbox
N-5	state	news	NPR	https://www.npr.org/sections/health-shots/2020/03/16/816707182/map-tracking-the-spread-of-the-coronavirus-in-the-u-s	JHU	SVGs
N-6	county	univ	Johns Hopkins Centers for Civic Impact	https://coronavirus.jhu.edu/us-map	JHU	ArcGIS
N-7	state	univ	Johns Hopkins Centers for Civic Impact	https://coronavirus.jhu.edu/data/state-timeline	JHU	SVGs
N-8	county	univ	COVID-19 Health Equity Interactive Dashboard / Emory University	https://covid19.emory.edu/	NYT, COVID TP, CDC	SVGs, React Simple Maps
N-9	county	npo	USAFacts	https://usafacts.org/visualizations/coronavirus-covid-19-spread-map/	CDC, state/local PH	SVGs
N-10	state	tech	COVID Mapping Project / Standard Co	https://www.covidmappingproject.com/	COVID TP	SVGs
N-11	county	tech	SAE Industry Technologies Consortia	https://infection2020.com/ *(discontinued updates in October 2021, still available June 2022)*	CDC, WHO, NYT, JHU, Corona Data Scraper, state/local PH	SVGs, Leaflet

Appendix 1c. Dashboards and trackers for case counts and testing — *Global coverage*

Ref	Granularity	Type*	Host	URL	Data sources**	Vis tool / method***
G-1	country	news	New York Times	https://www.nytimes.com/interactive/2020/world/coronavirus-maps.html	JHU, NHCPRC, WHO	SVGs, Mapbox
G-2	country	news	CNN	https://www.cnn.com/interactive/2020/health/coronavirus-maps-and-cases/ *(moved to new URL)* https://www.cnn.com/interactive/2020/health/coronavirus-us-maps-and-cases/	JHU	SVGs
G-3	country	news	Stat / AppliedXL	https://www.statnews.com/feature/coronavirus/covid-19-tracker/	JHU and other global sources listed	chart.js with PNGs
G-4	country, US counties	univ	Johns Hopkins Centers for Civic Impact	https://coronavirus.jhu.edu/map.html *(discontinued updates in March 2022, still available June 2022)*	JHU	ArcGIS
G-5	country, US states	univ	Biocomplexity Institute, University of Virginia	https://nssac.github.io/covid-19/dashboard/	(note stated)	ArcGIS API for JavaScript
G-6	country, US states	univ	HGIS Lab, University of Washington	https://hgis.uw.edu/virus/ *(discontinued updates in June 2021, still available June 2022)*	CDC, WHO, NYT, NBC News, Baidu, Wikipedia, and others as listed	SVGs, CARTO and ESRI map
G-7	by geolocation	univ	HealthMap / various universities	https://www.healthmap.org/covid-19/	Open COVID-19 Data Curation Group	Mapbox
G-8	country	npo	World Health Organization	https://covid19.who.int/	WHO	SVGs
G-9	country	npo	Kaiser Family Foundation	https://www.kff.org/coronavirus-covid-19/fact-sheet/coronavirus-tracker/	JHU, WHO	Tableau
G-10	country	npo	Our World in Data	https://ourworldindata.org/coronavirus	ECDC	Our World in Data Grapher
G-11	country	tech	DXY	https://ncov.dxy.cn/ncovh5/view/en_pneumonia	JHU, WHO, DXY, local media	PNGs
G-12	country, US states	tech	Worldometer	https://www.worldometers.info/coronavirus/	(no list, but references with updates)	SVGs
G-13	country, US states	tech	Acoer	https://www.acoer.com/coronavirus *(discontinued)*	CDC, WHO, IHME, NYT	Acoer HashLog dashboard
G-14	country, some territories	tech	Microsoft	https://www.bing.com/covid	CDC, WHO, other global sources listed	Bing Maps Platform
G-15	country, some territories	tech	1Point3Acres	https://coronavirus.1point3acres.com/en	state/local PH	React with EChart using PNGs, Flourish
G-16	country, some territories	tech	Tableau	https://www.tableau.com/covid-19-coronavirus-data-resources *(discontinued updates April 2022, still available June 2022)*	ECDC, NYT	Tableau

*** Type**

gov: government agency
indiv: individual citizen
media: online media and marketing company
news: news/journalism organization
npo: non-profit organization
trade: trade association
tech: technology or online business
univ: university-associated team

**** Data sources (as stated by site)**

US-based

COVID TP: The COVID Tracking Project
JHU: Johns Hopkins University, Center for Systems Science and Engineering
NYT: The New York Times
own reporting: a news organization's own reporting
state PH: state public health authority
state/local PH: state and county (or district) public health authorities
CDC: Centers for Disease Control and Prevention

Global

ECDC: *European Centre for Disease Prevention and Control*
IHME: Institute for Health Metrics and Evaluation
NHCPRC: National Health Commission of the People's Republic of China
WHO: World Health Organization

Data sources marked with a caret (^) were listed as of August 2020 but not in January/February 2021.

*** **Visualization tool / method**

Tools used to create image of types **SVG** (scalable vector graphics), **PNG** (portable network graphics) and **JPG** could not be identified. Some SVGs are interactive, revealing information when a cursor is over a region. Online
