# Supplementary material for: Web-Based COVID-19 Dashboards and Trackers in the United States: Survey Study
Source: JMIR Hum Factors. 2023 Mar 20;10:e43819. doi: 10.2196/43819 (PMC10029858; doi:10.2196/43819)
Supplement: Multimedia Appendix 2 [file humanfactors_v10i1e43819_app2.pdf]

Appendix 2a. Dashboards and trackers for vaccine distribution (selected examples) — *State focus, with county-level data*

State public health authorities in shaded rows (no data sources listed, assumed to be direct reporting from counties or districts)

Ref	State	Type*	Host	URL	Data sources**	Vis tool / method***
Svac-1	AL	govt	Alabama Dept of Public Health	https://alpublichealth.maps.arcgis.com/apps/opsdashboard/index.html - /e4a232feb1344ce0afd9ac162f3ac4ba		ArcGIS
Svac-2	AL	ind	David Marconnet, CTO of OneTeam.net	https://bamatracker.com/vaccine	state PH	OneTeam dashboard
Svac-3	CA	govt	California Dept of Public Health	https://covid19.ca.gov/vaccines/ - California-vaccines-dashboard *(removed link to Tableau by January 2022)*		Tableau
Svac-4	DC	govt	Government of the District of Columbia	https://coronavirus.dc.gov/data/vaccination		Tableau, Mapbox
Svac-5	CT	news	CT Post	https://www.ctpost.com/projects/coronavirus-vaccine-tracker-connecticut-ct/ *(discontinued updates October 2021, still available June 2022)*	CDC	SVGs, "progress bar" in HTML table
Svac-6	IL	news	Chicago Tribune	https://www.chicagotribune.com/coronavirus/vaccine/ct-viz-illinois-coronavirus-vaccine-tracker-20210113-a6cixchvbrayjhuz7bpwp7wcoy-htmlstory.html	state PH	Datawrapper
Svac-7	IN	govt	Indiana State Dept of Health	https://www.coronavirus.in.gov/vaccine/2680.htm		SVGs
Svac-8	KS	govt	Kansas Dept of Health and Environment	https://www.kansasvaccine.gov/158/Data		Tableau
Svac-9	MI	govt	Michigan Dept of Health and Human Services	https://www.michigan.gov/coronavirus/0,9753,7-406-98178_103214-547150--,00.html *(moved to new URL)* https://www.michigan.gov/coronavirus/resources/covid-19-vaccine/covid-19-dashboard		Microsoft BI
Svac-10	MN	govt	Minnesota Dept of Health	https://mn.gov/covid19/vaccine/data/index.jsp		Microsoft BI
Svac-11	ND	govt	North Dakota Dept of Health	https://www.health.nd.gov/covid19vaccine/dashboard		Microsoft BI
Svac-12	NM	govt	New Mexico Dept of Health	https://cvvaccine.nmhealth.org/public-dashboard.html		SVGs, Leaflet
Svac-13	OH	govt	Ohio Dept of Health	https://coronavirus.ohio.gov/wps/portal/gov/covid-19/dashboards/covid-19-vaccine/covid-19-vaccination-dashboard		Tableau
Svac-14	TN	govt	Tennessee Dept of Health	https://www.tn.gov/health/cedep/ncov/covid-19-vaccine-information.html		Tableau
Svac-15	TX	govt	Texas Health and Human Services	https://tabexternal.dshs.texas.gov/t/THD/views/COVID-19VaccineinTexasDashboard/Summary		Tableau
Svac-16	VT	govt	Vermont Dept of Health	https://www.healthvermont.gov/covid-19/vaccine/covid-19-vaccine-dashboard		Microsoft BI
Svac-17	WI	govt	Wisconsin Dept of Health Services	https://www.dhs.wisconsin.gov/covid-19/vaccine-data.htm		Tableau

Appendix 2b. Dashboards and trackers for vaccine distribution (selected examples) — *Nation-wide coverage*

Ref	Granularity	Type*	Host	URL	Data sources**	Vis tool / method***
Nvac-1	state	news	New York Times	https://www.nytimes.com/interactive/2020/us/covid-19-vaccine-doses.html	CDC	SVGs
Nvac-2	state	news	Springfield News-Leader	https://data.news-leader.com/covid-19-vaccine-tracker	state/local PH, JHU	Mapbox
Nvac-3	state	news	Washington Post	https://www.washingtonpost.com/graphics/2020/health/covid-vaccine-states-distribution-doses/ *(behind paywall as of January 2022)*	state PH, CDC	SVGs
Nvac-4	state	univ	COVID-19 Health Equity Interactive Dashboard / Emory University	https://covid19.emory.edu/Vaccine-Tracker	CDC	SVGs, React Simple Maps

Appendix 2c. Dashboards and trackers for vaccine distribution (selected examples) — *Global coverage*

Ref	Granularity	Type*	Host	URL	Data sources**	Vis tool / method***
Gvac-1	country	npo	Our World in Data	https://ourworldindata.org/covid-vaccinations	gov	Our World in Data Grapher
Gvac-2	country	news	CNN	https://www.cnn.com/interactive/2021/health/global-covid-vaccinations/	OWID	SVGs
Gvac-3	state	univ	Brown School of Public Health	https://globalepidemics.org/vaccine-dashboard/ *(site removed by January 2022)*	OWID, CDC	Microsoft BI

*** Type**

gov: government agency
indiv: individual citizen
news: news/journalism organization
npo: non-profit organization
univ: university-associated team

**** Data sources (as stated by site)**

US-based

JHU: Johns Hopkins University, Center for Systems Science and Engineering
CDC: Centers for Disease Control and Prevention
state PH: state public health authority
state/local PH: state and county (or district) public health authorities

Global

OWID: Our World In Data
gov: governments of various countries

*** **Visualization tool / method**

Tools used to create image of types **SVG** (scalable vector graphics) could not be identified. Some SVGs are interactive, revealing information when a cursor is over a region.
