# Supplementary material for: Web-Based COVID-19 Dashboards and Trackers in the United States: Survey Study
Source: JMIR Hum Factors. 2023 Mar 20;10:e43819. doi: 10.2196/43819 (PMC10029858; doi:10.2196/43819)
Supplement: Multimedia Appendix 3 [file humanfactors_v10i1e43819_app3.pdf]

Appendix 3. Web pages for data sources and technical information provided by prominent dashboard developers and data aggregators (as of August 2020)

Ref	Host	URL	Data sources	Methodology / definitions	Data repository	API info
N-1	CDC	https://data.cdc.gov/Case-Surveillance/COVID-19-Case-Surveillance-Public-Use-Data/vbim-akqf		x	x	x
N-3	The COVID Tracking Project / The Atlantic	https://covidtracking.com/about-data/sources	x			
		https://covidtracking.com/about-data/data-definitions		x		
		https://covidtracking.com/data/api				x
N-4 / G-1	New York Times	https://github.com/nytimes/covid-19-data		x	x	
N-8	COVID-19 Health Equity Interactive Dashboard / Emory University	https://covid19.emory.edu/data-sources		x		
N-9	USAFacts	https://usafacts.org/articles/detailed-methodology-covid-19-data/	x	x		
G-4	Johns Hopkins Centers for Civic Impact	https://coronavirus.jhu.edu/map-faq	x			
G-8	World Health Organization	https://covid19.who.int/info	x		x	
G-10	Our World in Data	https://github.com/owid/covid-19-data/tree/master/public/data	x		x	
G-14	Microsoft	https://help.bing.microsoft.com/#apex/18/en-us/10024	x			
	Center for Systems Science and Engineering at Johns Hopkins University	https://github.com/CSSEGISandData/COVID-19	x		x	
	Open COVID-19 Data Curation Group	https://github.com/beoutbreakprepared/nCoV2019	x	link to publication	x	
