# Supplementary material for: Web-Based COVID-19 Dashboards and Trackers in the United States: Survey Study
Source: JMIR Hum Factors. 2023 Mar 20;10:e43819. doi: 10.2196/43819 (PMC10029858; doi:10.2196/43819)
Supplement: Multimedia Appendix 16 [file humanfactors_v10i1e43819_app16.pdf]

Appendix 16. Screenshots from dashboards and trackers — *Nation-wide coverage* *(captured June 16, 2022)*

Page	Ref	Host
2–5	N-1	US Centers for Disease Control and Prevention
6	N-4	New York Times
7	N-5	NPR
8	N-6	Johns Hopkins Centers for Civic Impact
9	N-7	Johns Hopkins Centers for Civic Impact
10	N-8	COVID-19 Health Equity Interactive Dashboard / Emory University
11	N-9	USAFacts
12	N-10	COVID Mapping Project / Standard Co

COVID Data Tracker

Maps, charts, and data provided by CDC, updates daily by 8 pm ET

COVID Data Tracker will not update on Saturday, June 18, 2022, Sunday, June 19, 2022, and Monday, June 20, 2022. Updates will resume on Tuesday, June 21, 2022.

CDC recommends use of COVID-19 Community Levels to determine the impact of COVID-19 on communities and take action. Community Transmission levels are provided for healthcare facility use only.

United States
At a Glance

**Cases** Total / Case Trends	**85,825,048**	**Deaths** Total / Death Trends	**1,007,964**
Current **Hosp.** / Admission Trends	**24,618**	**83.0% of People 5+ with At Least** One Vaccination	

Data Tracker Home
Cases, Deaths, & Testing
Case & Death Demographic Trends
Vaccination Distribution & Coverage
Vaccine Effectiveness & Breakthrough Surveillance
Health Equity
Pediatric
Pregnancy
People at Increased Risk
Wastewater Surveillance
Health Care Settings
Social Impact & Prevention
Variants & Genomic Surveillance
Antibody Seroprevalence
Other COVID-19 Data
Communications Resources
COVID-19 Home

Get Email Updates

Sign up to receive the COVID Data Tracker Weekly Review.

Email Address:
Email Address

What's this? Submit

Beginning June 13, 2022, instead of daily, jurisdictions and other partners will report vaccine administration and delivery data to CDC weekly on Wednesdays by 4 AM ET. As a result, instead of daily, the following COVID Data Tracker tabs will be refreshed weekly on Thursday by 8:00 PM EST: Vaccinations in the United States, Vaccinations by County, Vaccination Trends, Vaccine Demographics, Vaccination Demographic Trends, Vaccination Equity, Vaccinations and Case Trends, Vaccinations and Other Outcomes.

< Back to Cases, Deaths, & Testing

COVID-19 Integrated County View

Maps, charts, and data provided by CDC, updates daily by 8 pm ET†

This site provides an integrated, county view of key data for monitoring the COVID-19 pandemic in the United States. It allows for the exploration of standardized data across the country.§ The footnotes describe each data source and the methods used for calculating the metrics. For the most complete and up-to-date data for any particular county or state, visit the relevant health department website. Additional data and features are forthcoming.

§County level data are not available for territories. Territory level data are available under the **Cases, Deaths, and Testing tab**. Data presented here for District of Columbia may differ from those presented on the **Cases, Deaths, and Testing tab** due to reporting differences for each tab. For CDC's most up to date data for District of Columbia, select District of Columbia in the dropdown on this tab or see the map below.

The percent of the population coverage metrics are capped at 95%. Learn how CDC estimates vaccination coverage.

View Footnotes and Additional Information

How to Find a COVID-19 Vaccine | Who is Recommended to Receive Booster Doses?

State or territory: Select a State
County or metro area: Select County
Reset Selections

Use the options above or the map below to select a state and county. CDC recommends use of COVID-19 Community Levels to determine the impact of COVID-19 on communities and take action. Community Transmission levels are provided for healthcare facility use only.

Data Type: Cases
Map Metric: Reported cases
All Counties
Download Image

+ -

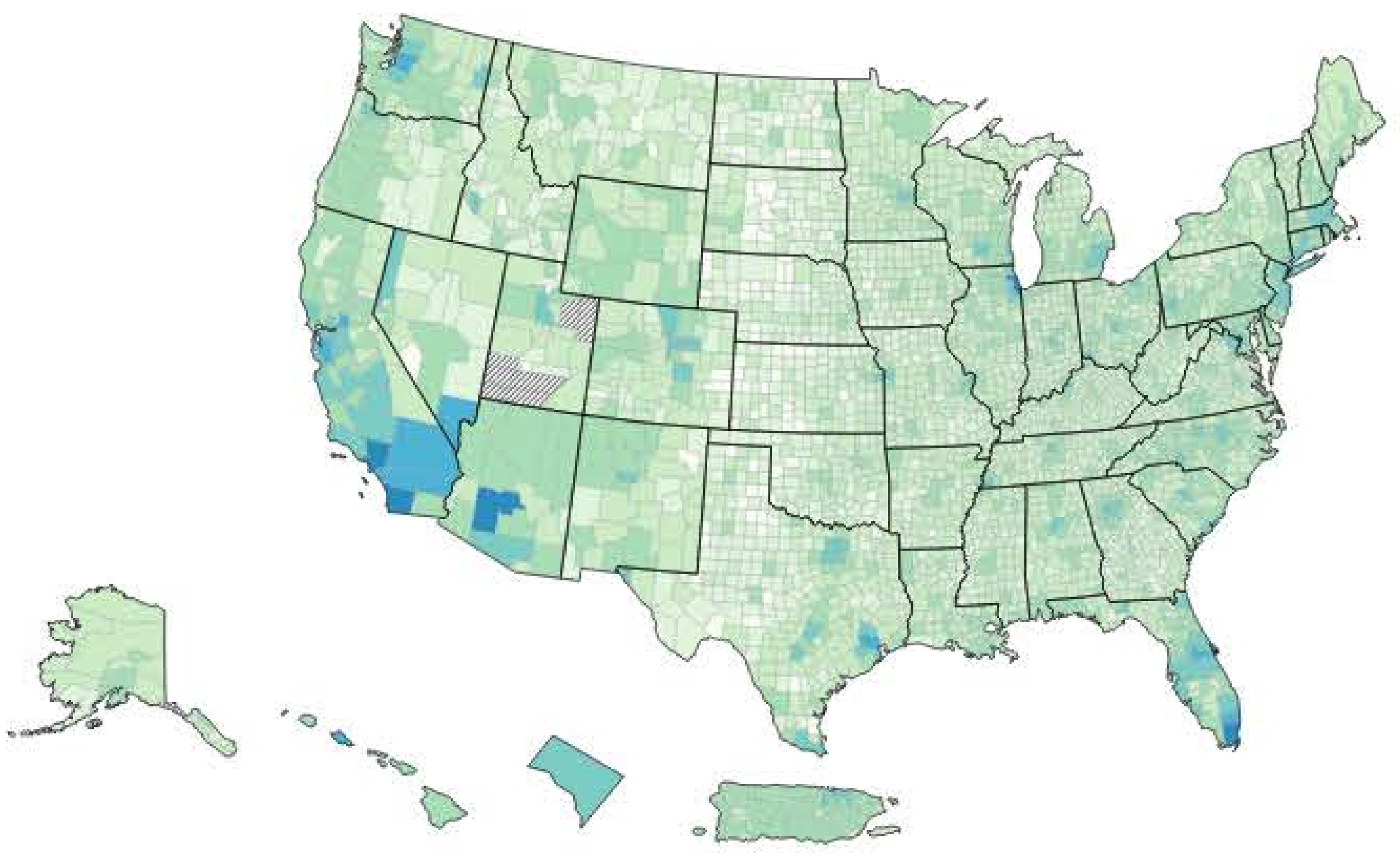

0 | 1 to 9 | 10 to 99 | 100 to 999 | 1,000 to 4,999 | 5,000 to 9,999 | 10,000+ | No Data

Time Period: Thu Jun 09 2022 - Wed Jun 15 2022

Cases in the United States

Reported Cases	# of U.S. Counties at this Level	% of U.S. Counties at this Level	% Point Difference of Counties at this Level Since 7 Days Ago
0	96	3%	– 1%
1 to 9	581	18%	– 3%
10 to 99	1,651	51%	5%
100 to 999	734	23%	0%
1,000 to 4,999	131	4%	– 1%
5,000 to 9,999	14	0%	0%
10,000+	5	0%	0%

Data Downloads and Footnotes

Expand each accordion to view data table and download data

Footnotes and Additional Information

Expand each accordion to view footnotes

View COVID-19 Community Level Data | View Historic Vaccination Data | View County-level Data Sources

Top of Page

Footnotes +

COVID Data Tracker will not update on Saturday, June 18, 2022, Sunday, June 19, 2022, and Monday, June 20, 2022. Updates will resume on Tuesday, June 21, 2022.

CDC recommends use of COVID-19 Community Levels to determine the impact of COVID-19 on communities and take action. Community Transmission levels are provided for healthcare facility use only.

United States
At a Glance

**Cases** Total	85,825,048	Case Trends	
**Deaths** Total	1,007,964	Death Trends	
Current **Hosp.**	24,618	Admission Trends	
**83.0% of People 5+ with At Least** One Vaccination			

Data Tracker Home
Cases, Deaths, & Testing
Case & Death Demographic Trends
Vaccination Distribution & Coverage
Vaccine Effectiveness & Breakthrough Surveillance
Health Equity
Pediatric
Pregnancy
People at Increased Risk
Wastewater Surveillance
Health Care Settings
Social Impact & Prevention
Variants & Genomic Surveillance
Antibody Seroprevalence
Other COVID-19 Data
Communications Resources
COVID-19 Home

Get Email Updates

Sign up to receive the COVID Data Tracker Weekly Review.

Email Address:
Email Address

What's this? Submit

Beginning June 13, 2022, instead of daily, jurisdictions and other partners will report vaccine administration and delivery data to CDC weekly on Wednesdays by 4 AM ET. As a result, instead of daily, the following COVID Data Tracker tabs will be refreshed weekly on Thursday by 8:00 PM EST: Vaccinations in the United States, Vaccinations by County, Vaccination Trends, Vaccination Demographics, Vaccination Demographic Trends, Vaccination Equity, Vaccinations and Case Trends, Vaccinations and Other Outcomes.

< Back to Cases, Deaths, & Testing

COVID-19 Integrated County View

Maps, charts, and data provided by CDC, updates daily by 8 pm ET†

This site provides an integrated, county view of key data for monitoring the COVID-19 pandemic in the United States. It allows for the exploration of standardized data across the country.§ The footnotes describe each data source and the methods used for calculating the metrics. For the most complete and up-to-date data for any particular county or state, visit the relevant health department website. Additional data and features are forthcoming.

§County level data are not available for territories. Territory level data are available under the **Cases, Deaths, and Testing tab**. Data presented here for District of Columbia may differ from those presented on the **Cases, Deaths, and Testing tab** due to reporting differences for each tab. For CDC's most up to date data for District of Columbia, select District of Columbia in the dropdown on this tab or see the map below.

The percent of the population coverage metrics are capped at 95%. Learn how CDC estimates vaccination coverage.

View Footnotes and Additional Information

How to Find a COVID-19 Vaccine | Who is Recommended to Receive Booster Doses?

State or territory: Select a State
County or metro area: Select County
Reset Selections

Use the options above or the map below to select a state and county. CDC recommends use of COVID-19 Community Levels to determine the impact of COVID-19 on communities and take action. Community Transmission levels are provided for healthcare facility use only.

Data Type: Cases
Map Metric: Reported cases per 100,000 population
All Counties
Download Image

\+ -

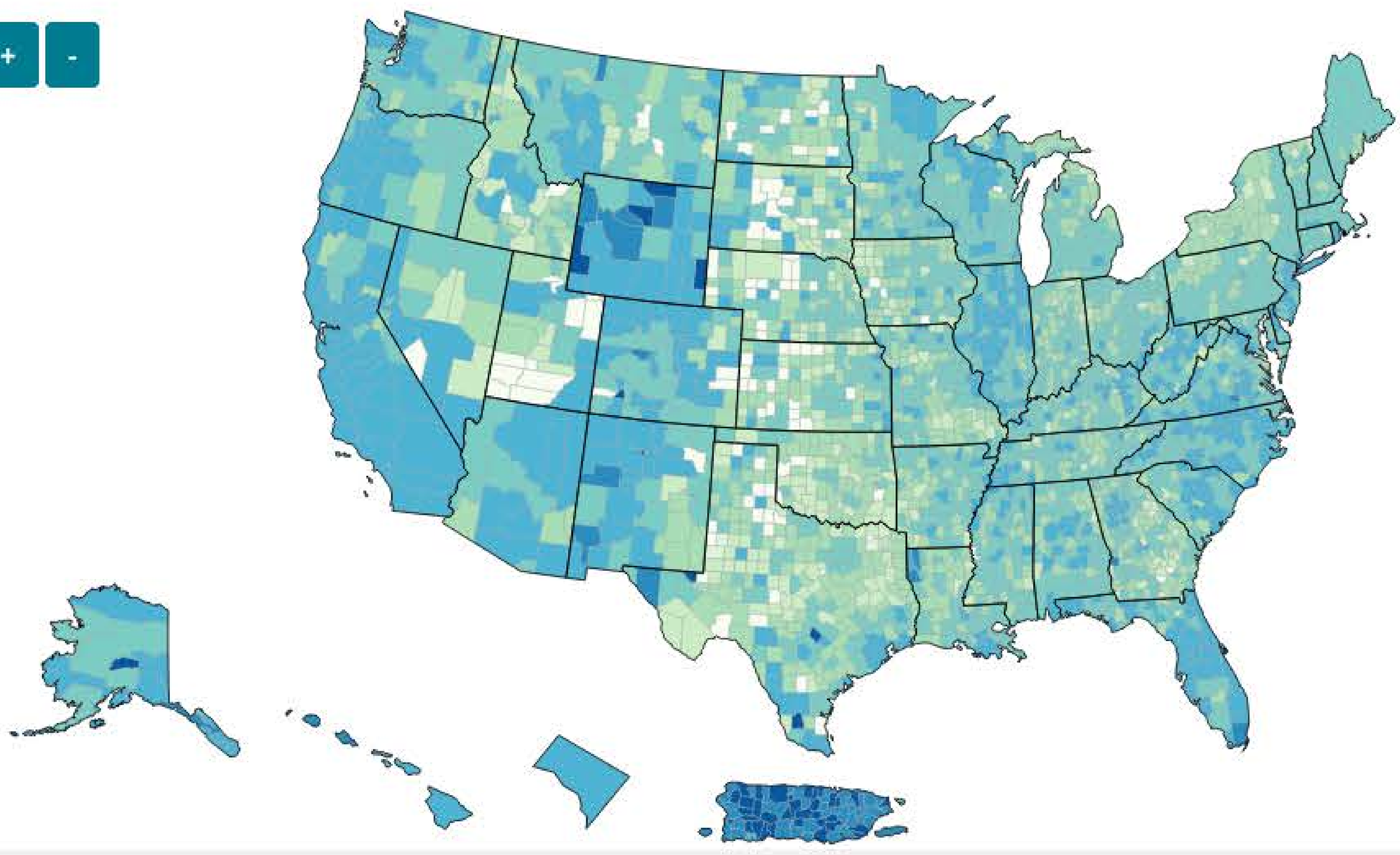

0 | 0.01 to 10 | 10.01 to 50 | 50.01 to 100 | 100.01 to 200 | 200.01 to 500 | 500.01 to 749.99 | 750+ | No Data

Time Period: Thu Jun 09 2022 - Wed Jun 15 2022

Cases in the United States

Reported cases per 100,000 population	# of U.S. Counties at this Level	% of U.S. Counties at this Level	% Point Difference of Counties at this Level Since 7 Days Ago
0	104	3%	– 1%
0.01 to 10	4	0%	0%
10.01 to 50	233	7%	– 4%
50.01 to 100	690	21%	0%
100.01 to 200	1,414	44%	7%
200.01 to 500	670	21%	– 1%
500.01 to 749.99	59	2%	0%
750+	48	1%	0%

Data Downloads and Footnotes

Expand each accordion to view data table and download data

Footnotes and Additional Information

Expand each accordion to view footnotes

View COVID-19 Community Level Data | View Historic Vaccination Data | View County-level Data Sources

Top of Page

Footnotes +

COVID Data Tracker will not update on Saturday, June 18, 2022, Sunday, June 19, 2022, and Monday, June 20, 2022. Updates will resume on Tuesday, June 21, 2022.

CDC recommends use of COVID-19 Community Levels to determine the impact of COVID-19 on communities and take action. Community Transmission levels are provided for healthcare facility use only.

United States At a Glance

**Cases** Total	85,825,048	Case Trends
**Deaths** Total	1,007,964	Death Trends
Current **Hosp.**	24,618	Admission Trends
**83.0% of People 5+ with At Least** One Vaccination		

Data Tracker Home
Cases, Deaths, & Testing
Case & Death Demographic Trends
Vaccination Distribution & Coverage
Vaccine Effectiveness & Breakthrough Surveillance
Health Equity
Pediatric
Pregnancy
People at Increased Risk
Wastewater Surveillance
Health Care Settings
Social Impact & Prevention
Variants & Genomic Surveillance
Antibody Seroprevalence
Other COVID-19 Data
Communications Resources
COVID-19 Home

Beginning June 13, 2022, instead of daily, jurisdictions and other partners will report vaccine administration and delivery data to CDC weekly on Wednesdays by 4 AM ET. As a result, instead of daily, the following COVID Data Tracker tabs will be refreshed weekly on Thursday by 8:00 PM EST: Vaccinations in the United States, Vaccinations by County, Vaccination Trends, Vaccination Demographics, Vaccination Demographic Trends, Vaccination Equity, Vaccinations and Case Trends, Vaccinations and Other Outcomes.

< Back to Vaccination Distribution & Coverage

Trends in Number of COVID-19 Vaccinations in the US

Maps, charts, and data provided by CDC, updates weekly on Thursday by 8pm ET[†]

The percent of the population coverage metrics are capped at 95%. Learn how CDC estimates vaccination coverage.

Select a Location: United States

Select a Metric: Total Doses Administered

View:
- Daily Count
- Cumulative
- Daily Change Reported

Show:
- 7-Day moving average

Bars shown in the darker blue shade represent the most recent six days of reporting where the number of vaccine administrations might be impacted the most due to delays in reporting. All reported numbers might change over time as historical data are reported to CDC.

About These Data | View Footnotes and Download Data

CDC | Data as of: June 16, 2022 6:00am ET. Posted: Thursday, June 16, 2022 2:35 PM ET

Daily Count of Doses by Date of Vaccine Administration, United States

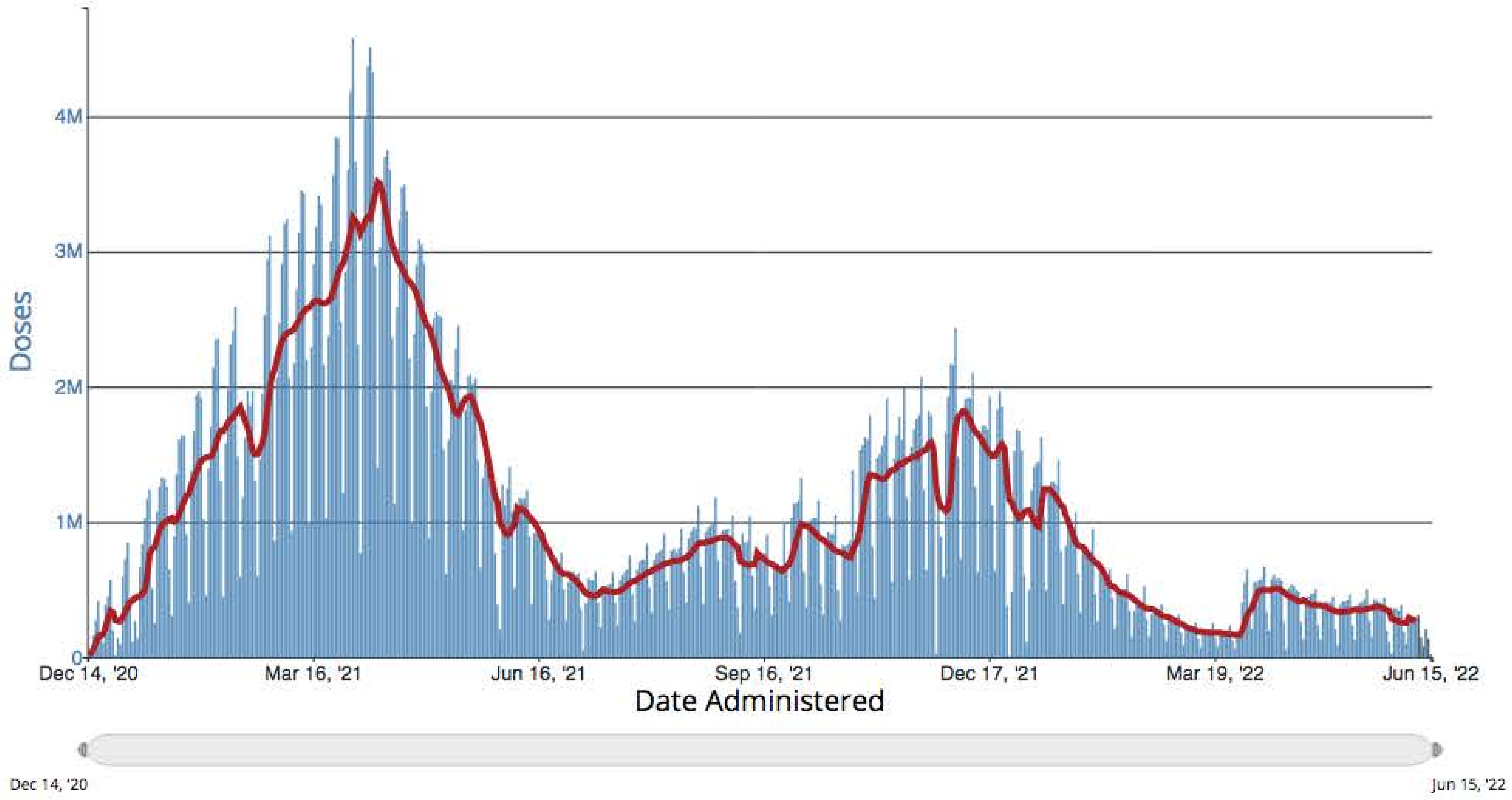

Download Chart

Data Downloads and Footnotes

Expand each accordion to view data table and download data

View Historic Vaccination Data

Data Table for Trends in Number of COVID-19 Vaccinations in the US +

Footnotes +

Get Email Updates

Sign up to receive the COVID Data Tracker Weekly Review.

Email Address:

Email Address

What's this? Submit

COVID Data Tracker will not update on Saturday, June 18, 2022, Sunday, June 19, 2022, and Monday, June 20, 2022. Updates will resume on Tuesday, June 21, 2022.

CDC recommends use of COVID-19 Community Levels to determine the impact of COVID-19 on communities and take action. Community Transmission levels are provided for healthcare facility use only.

United States
At a Glance

**Cases** Total	85,825,048	Case Trends	
**Deaths** Total	1,007,964	Death Trends	
Current **Hosp.**	24,618	Admission Trends	
**83.0% of People 5+ with At Least** One Vaccination			

Data Tracker Home
Cases, Deaths, & Testing
Case & Death Demographic Trends
Vaccination Distribution & Coverage
Vaccine Effectiveness & Breakthrough Surveillance
Health Equity
Pediatric
Pregnancy
People at Increased Risk
Wastewater Surveillance
Health Care Settings
Social Impact & Prevention
Variants & Genomic Surveillance
Antibody Seroprevalence
Other COVID-19 Data
Communications Resources
COVID-19 Home

Get Email Updates

Sign up to receive the COVID Data Tracker Weekly Review.

Email Address:
Email Address

What's this? Submit

Beginning June 13, 2022, instead of daily, jurisdictions and other partners will report vaccine administration and delivery data to CDC weekly on Wednesdays by 4 AM ET. As a result, instead of daily, the following COVID Data Tracker tabs will be refreshed weekly on Thursday by 8:00 PM EST: Vaccinations in the United States, Vaccinations by County, Vaccination Trends, Vaccination Demographics, Vaccination Demographic Trends, Vaccination Equity, Vaccinations and Case Trends, Vaccinations and Other Outcomes.

< Back to Cases, Deaths, & Testing

COVID-19 Integrated County View

Maps, charts, and data provided by CDC, updates daily by 8 pm ET†

This site provides an integrated, county view of key data for monitoring the COVID-19 pandemic in the United States. It allows for the exploration of standardized data across the country.§ The footnotes describe each data source and the methods used for calculating the metrics. For the most complete and up-to-date data for any particular county or state, visit the relevant health department website. Additional data and features are forthcoming.

§County level data are not available for territories. Territory level data are available under the **Cases, Deaths, and Testing tab**. Data presented here for District of Columbia may differ from those presented on the **Cases, Deaths, and Testing tab** due to reporting differences for each tab. For CDC's most up to date data for District of Columbia, select District of Columbia in the dropdown on this tab or see the map below.

The percent of the population coverage metrics are capped at 95%. Learn how CDC estimates vaccination coverage.

View Footnotes and Additional Information

How to Find a COVID-19 Vaccine | Who is Recommended to Receive Booster Doses?

State or territory: Select a State
County or metro area: Select County
Reset Selections

Use the options above or the map below to select a state and county. CDC recommends use of COVID-19 Community Levels to determine the impact of COVID-19 on communities and take action. Community Transmission levels are provided for healthcare facility use only.

Data Type: COVID-19 Community Levels
Map Metric: COVID-19 Community Levels
All Counties
Download Image

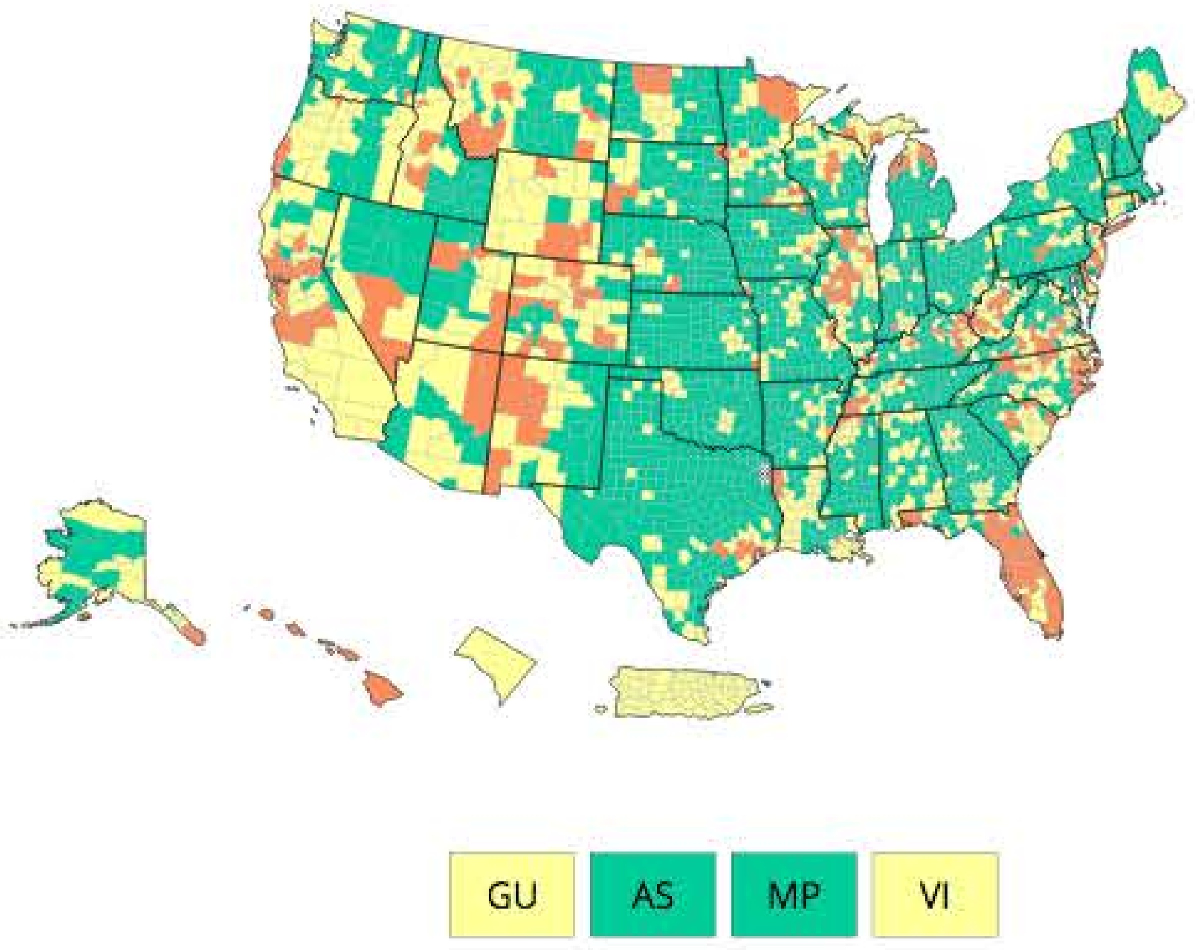

COVID-19 Community Levels in US by County

	Total	Percent	% Change
High	329	10.21%	0.47%
Medium	946	29.37%	– 3.38%
Low	1946	60.42%	2.91%

How are COVID-19 Community Levels calculated?

Low Medium High No Data

Time Period: COVID-19 Community Levels were calculated on Thu Jun 16 2022. New COVID-19 cases per 100,000 population (7-day total) are calculated using data from Thu Jun 09 2022 - Wed Jun 15 2022. New COVID-19 admissions per 100,000 population (7-day total) and Percent of inpatient beds occupied by COVID-19 patients (7-day average) are calculated using data from Wed Jun 08 2022 - Tue Jun 14 2022.

Coronavirus in the U.S.: Latest Map and Case Count

Updated June 16, 2022

New reported cases

All time | Last 90 days

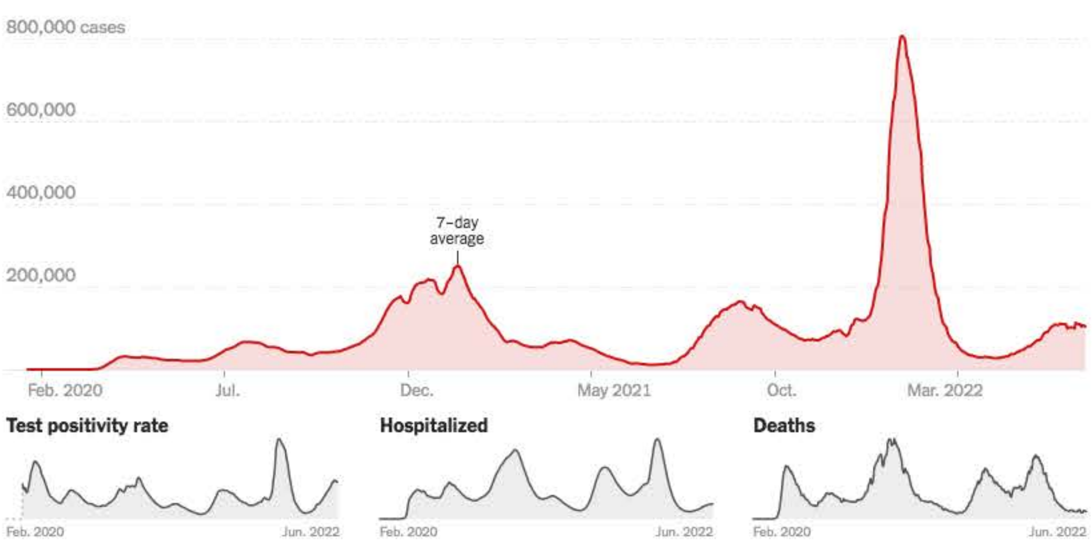

	DAILY AVG. ON JUN. 15	14-DAY CHANGE	TOTAL REPORTED
Cases	104,153	+3%	85,871,798
Test positivity	14%	—	—
Hospitalized	29,858	+8%	—
In I.C.U.s	3,303	+12%	—
Deaths	319	+14%	1,009,151

About this data

Vaccinations

	AT LEAST ONE DOSE	FULLY VACCINATED
All ages	78%	67%
5 and up	83%	71%
65 and up	95%	91%

See more details ›

About this data

State of the virus

Update for June 14

- More than 100,000 cases are currently announced each day in the U.S., a figure that has stayed roughly flat for the month of June.
- Cases are declining in roughly half the states, particularly in the Northeast and Midwest. In the past two weeks, parts of New England have seen cases fall by 50 percent or more.
- In the South and West, however, cases and hospitalizations are increasing substantially. Daily new infections have more than doubled this month in Wyoming, and in Mississippi cases and hospitalizations have both increased by more than 70 percent.
- More than 29,000 people are hospitalized nationwide with the coronavirus, a modest increase from the start of the month. Hospitalizations are decreasing in more than a dozen states, but increases in such populous states as California and Florida have led to continued growth at the national level.
- Fatality data has been volatile in recent weeks because of delays in reporting after Memorial Day. Still, reports of new deaths remain low. Fewer than 350 deaths are being reported each day, down from more than 2,600 a day at the height of the Omicron surge.

Cases by region

This chart shows how average daily cases per 100,000 people have changed in different parts of the country. The state with the highest recent average cases per 100,000 people is shown.

All time | Last 90 days

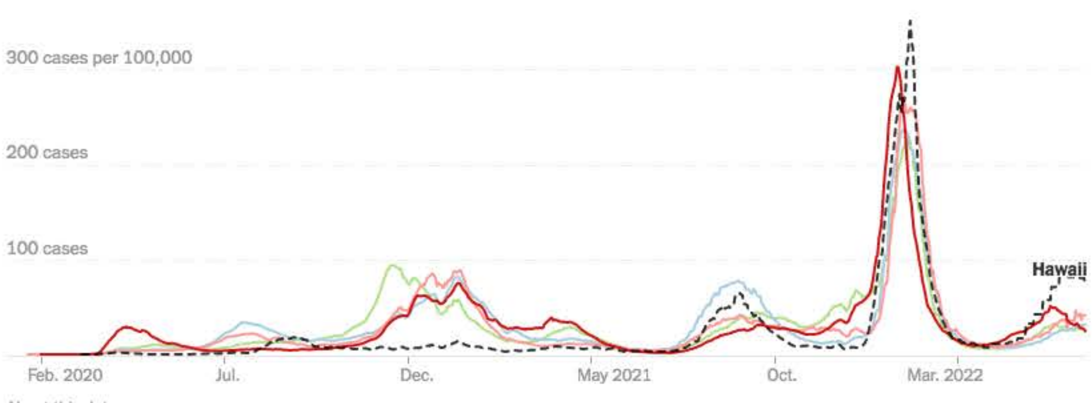

About this data

Hot spots

AVERAGE DAILY CASES PER 100,000 PEOPLE IN PAST WEEK

10 | 30 | 50 | 70 | 100 | 250 | FEW OR NO CASES

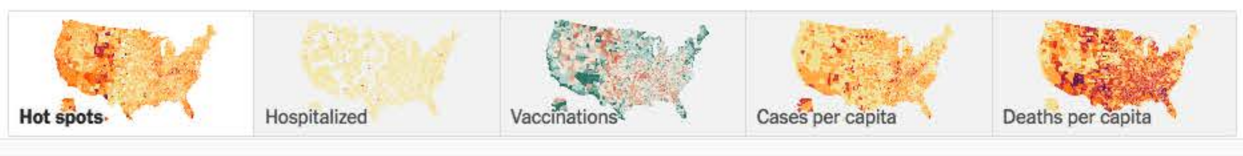

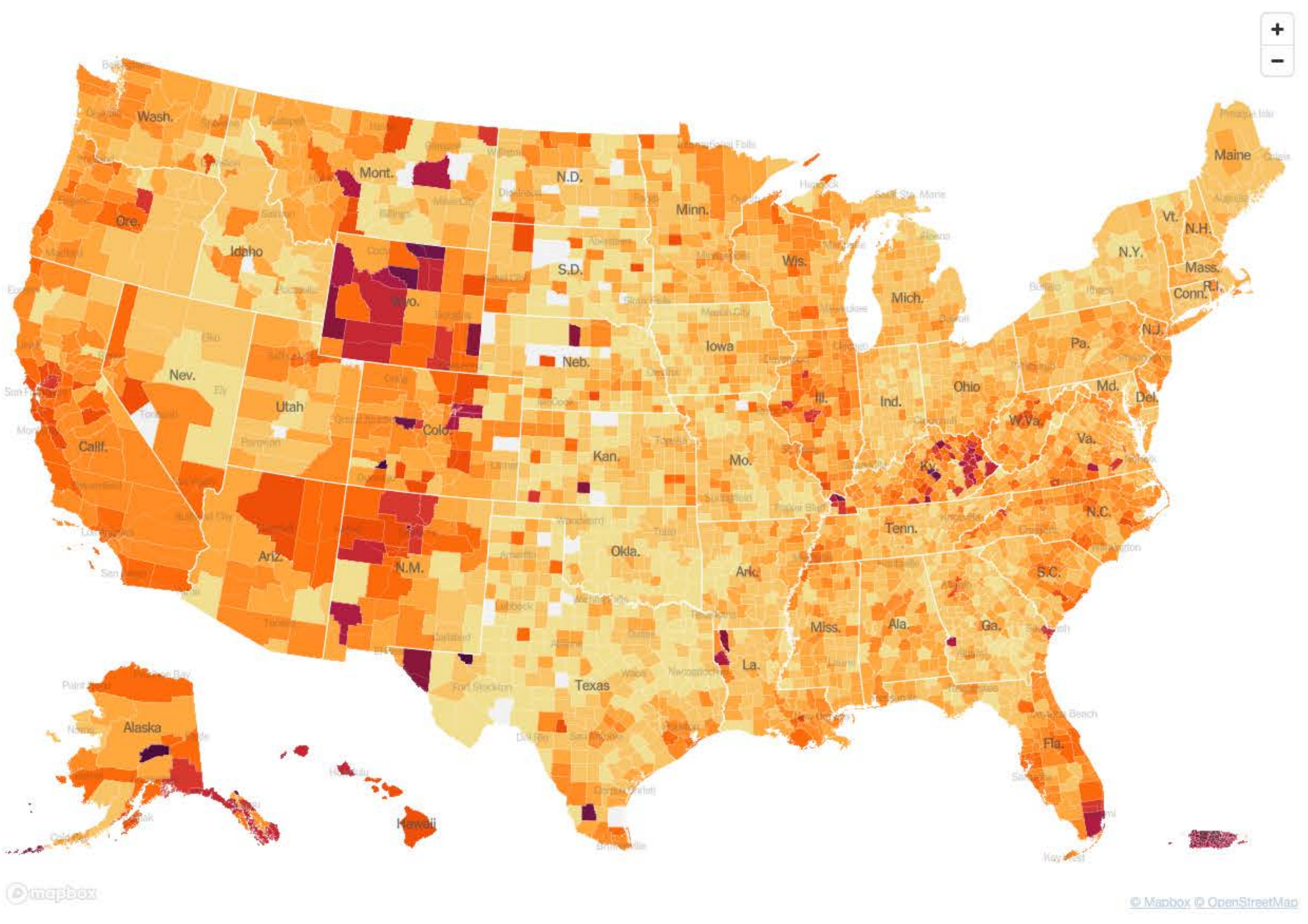

About this data

State trends

This table is sorted by places with the most cases per 100,000 residents in the last seven days. Charts show change in daily averages and are each on their own scale. Select a table header to sort by another metric.

Recent trends | All time | Search states

	CASES DAILY AVG.	PER 100,000	14-DAY CHANGE	TEST POSITIVITY	HOSPITALIZED DAILY AVG.	PER 100,000	14-DAY CHANGE	DEATHS DAILY AVG.	PER 100,000	FULLY VACCINATED
United States	104,153	31	+3%	14%	29,858	9	+8%	318.9	0.10	67%
Puerto Rico ›	3,466	102	−6%	35%	405	12	+6%	5.7	0.17	79%
Hawaii ›	1,011	71	−12%	19%	227	16	+16%	1.3	0.09	79%
Wyoming ›	350	61	+301%	13%	26	5	+67%	0.6	0.10	51%
Guam ›	92	55	+124%	—	—	—	—	0.6	0.34	83%
Colorado ›	2,938	51	+28%	12%	368	6	+48%	7.6	0.13	71%
Florida ›	10,523	49	+10%	19%	3,257	15	+27%	36.7	0.17	68%
Alaska ›	349	48	+9%	14%	54	7	+28%	0	—	63%
Nevada ›	1,365	44	+114%	0%	434	14	+46%	4.6	0.15	61%
New Mexico ›	929	44	+50%	14%	175	8	+19%	4.3	0.20	72%
California ›	17,242	44	+11%	76%	3,087	8	+20%	29.4	0.07	73%

Show all | About this data

How cases, hospitalizations and deaths are trending

Each chart shows how these three metrics compare to the corresponding peak level reached nationwide before Omicron became the dominant variant. For example, a state's case line exceeds 100 percent on the chart when its number of cases per capita exceeds the highest number of U.S. cases per capita reached in January 2021.

Sort by cases | Sort by hospitalizations

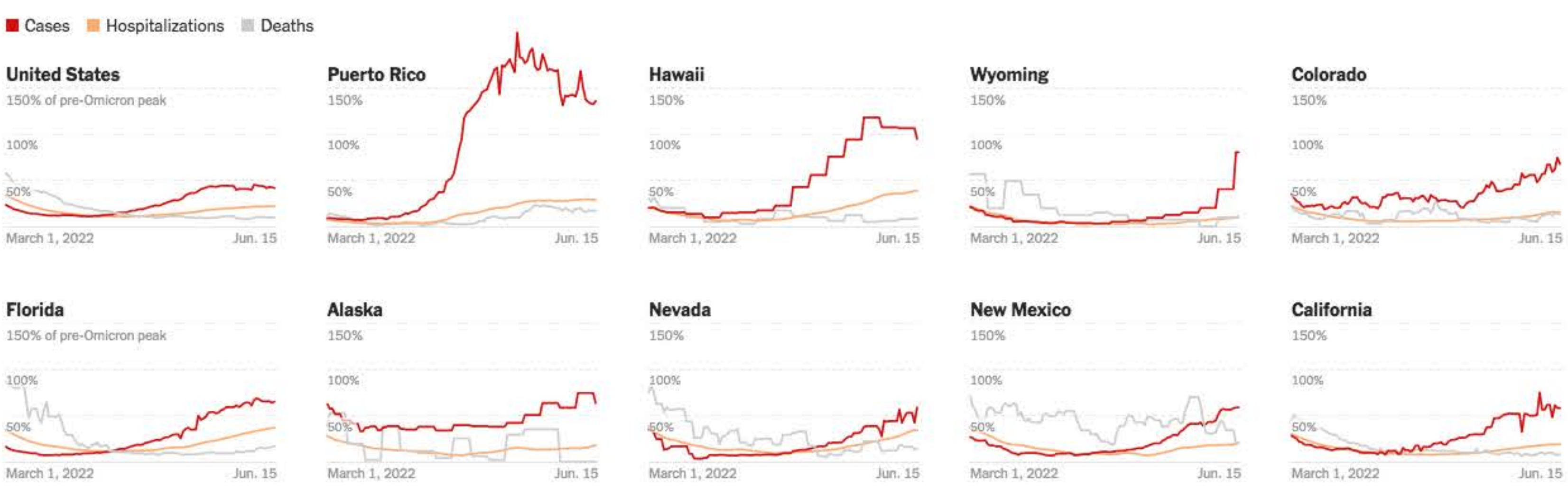

Show all | About this data

Rates for vaccinated and unvaccinated

Data from the Centers for Disease Control and Prevention shows that people who are unvaccinated are at a much greater risk than those who are fully vaccinated to die from Covid-19. These charts compare age-adjusted average daily case and death rates for vaccinated and unvaccinated people in the states and cities that provide this data.

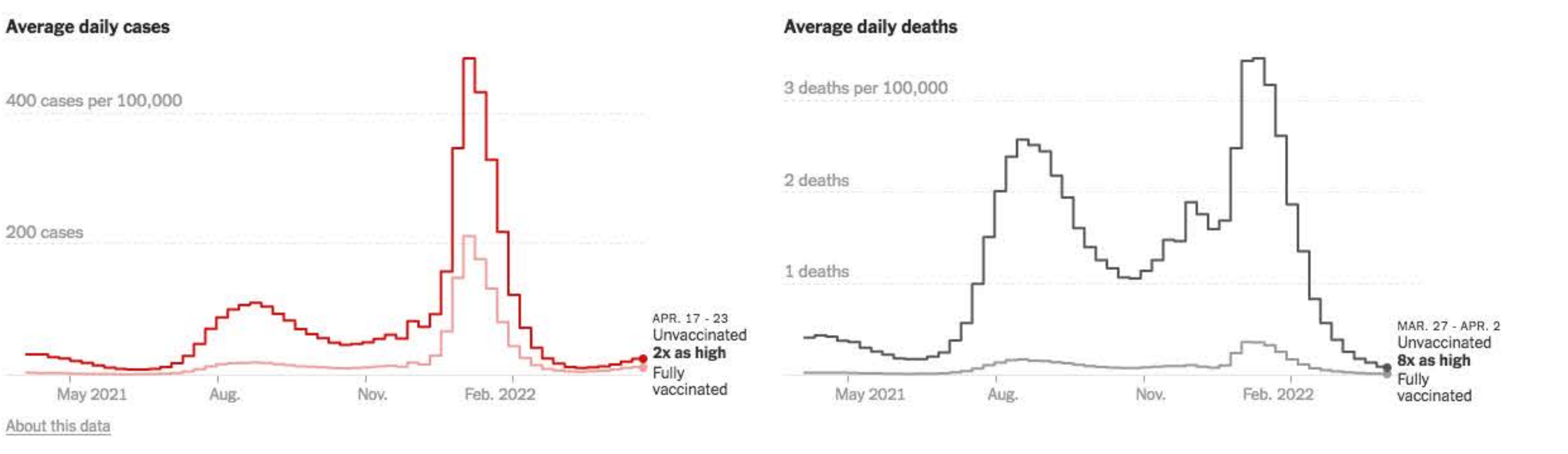

About this data

Daily new hospital admissions by age

This chart shows for each age group the number of people per 100,000 that were newly admitted to a hospital with Covid-19 each day, according to data reported by hospitals to the U.S. Department of Health and Human Services.

All time | Last 90 days

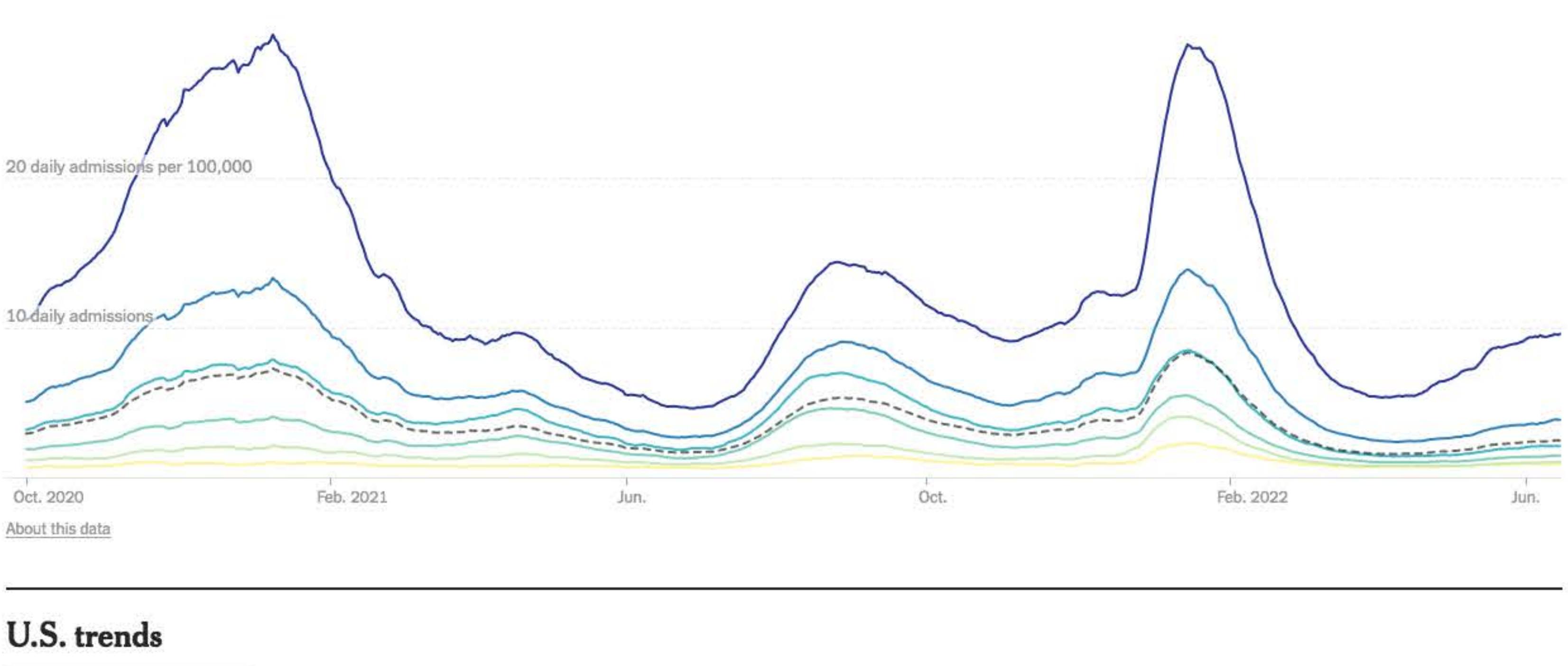

About this data

U.S. trends

All time | Last 90 days

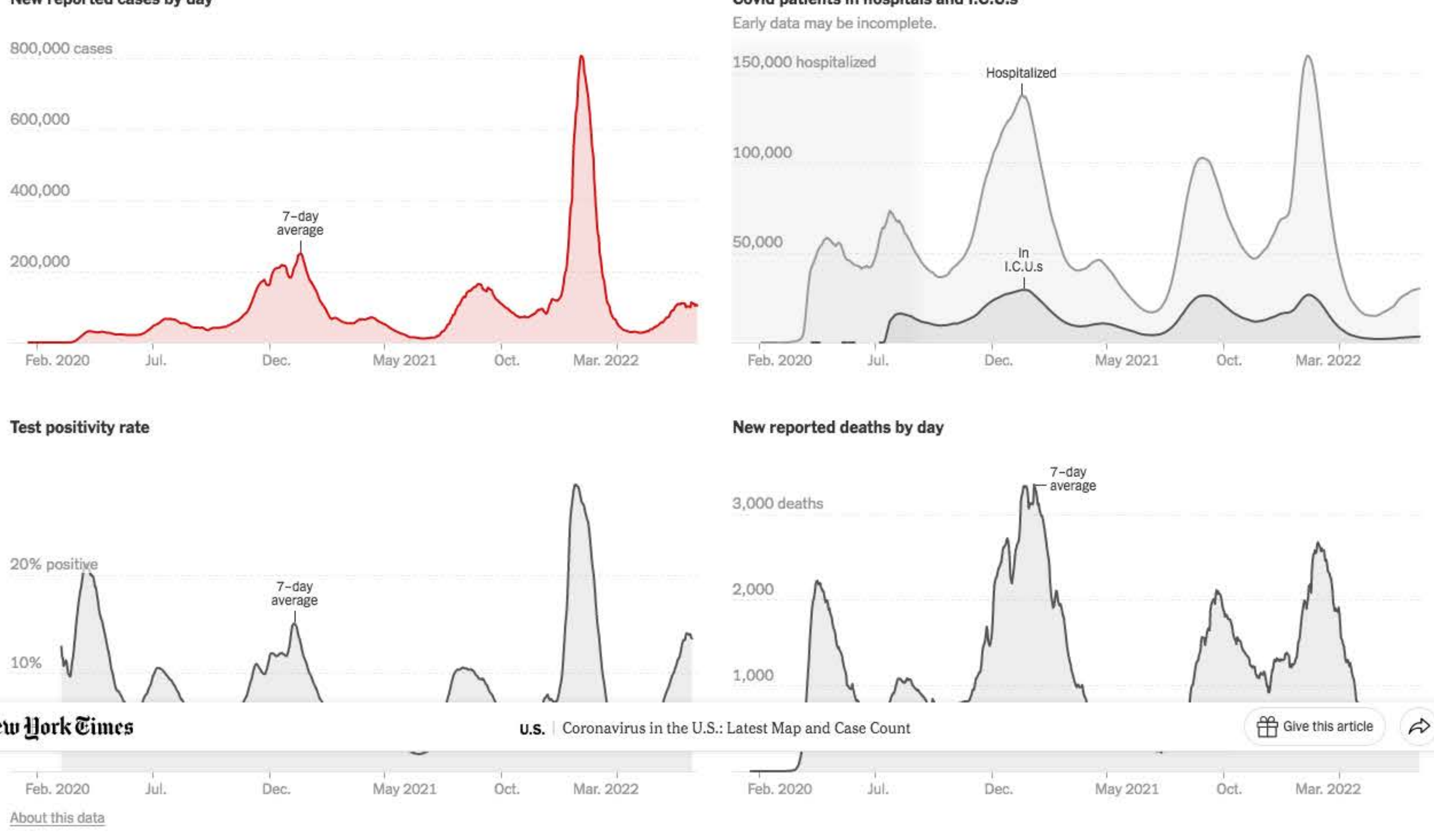

The New York Times | U.S. | Coronavirus in the U.S.: Latest Map and Case Count | Give this article

About this data

County trends

This table is sorted by places with the most cases per 100,000 residents in the last seven days. Charts show change in daily averages and are each on their own scale. Select a table header to sort by another metric.

Recent trends | All time | Search counties and states

	CASES DAILY AVG.	PER 100,000	14-DAY CHANGE	TEST POSITIVITY	HOSPITALIZED AVG. PER 100,000	14-DAY CHANGE	DEATHS DAILY AVG.	PER 100,000	FULLY VACCINATED
McCracken, Ky. ›	147	225	+341%	19%	19	+110%	0.1	0.22	56%
Lincoln, Ky. ›	47	193	+1,046%	11%	5	+87%	0	—	41%
Pitkin, Colo. ›	29	164	+871%	20%	3	—	0	—	84%
Boyle, Ky. ›	49	163	+1,161%	6%	7	+18%	0.1	0.48	57%
Villalba, P.R.	33	153	−1%	36%	—	—	—	—	91%
Sheridan, Wyo. ›	46	151	+1,246%	6%	4	+112%	0	—	54%
Lares, P.R.	36	148	−18%	44%	—	—	—	—	95%
Yauco, P.R.	50	147	+1%	32%	—	—	—	—	88%
Cayey, P.R.	62	147	+12%	42%	—	—	—	—	85%
Peñuelas, P.R.	28	145	+6%	60%	—	—	—	—	85%

Show more | About this data

CORONAVIRUS BY THE NUMBERS

Tracking the coronavirus around the U.S.: See how your state is doing

Updated June 16, 2022 · 5:43 PM ET

STEPHANIE ADELINE | CONNIE HANZHANG JIN | ALYSON HURT | THOMAS WILBURN | DANIEL WOOD | RUTH TALBOT

TRACKING THE CORONAVIRUS
U.S. cases | Vaccines | Hospitals | World cases

Data as of 7:20 a.m. ET on June 16

U.S. CASES

TOTAL	DAILY AVERAGE	2-WEEK TREND
85,935,434	+102,448	-1.7%

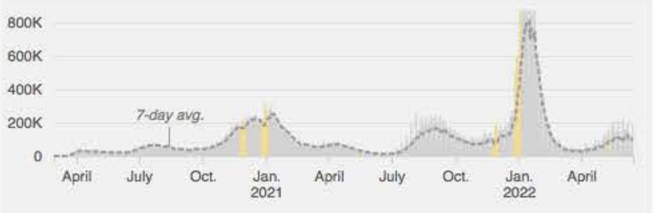

U.S. DEATHS

TOTAL	DAILY AVERAGE	2-WEEK TREND
1,012,573	+293	+6.7%

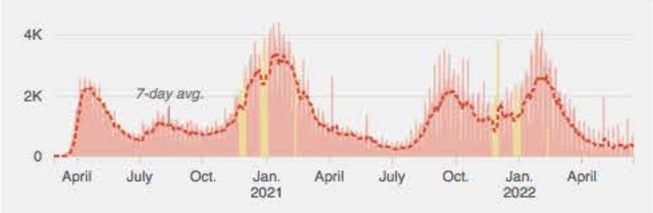

Data irregularities: Data reporting is often delayed on weekends and around major U.S. holidays. Additionally, some states have scaled back how often they report new data, creating more spikes and dips in the daily data.

The daily averages are 7-day averages of new cases and deaths for the week ending June 15. The 2-week trend refers to the percent change in the 7-day average vs. 2 weeks prior.

Source: Center for Systems Science and Engineering at Johns Hopkins University

This page is updated on Mondays and Thursdays.

More than 85 million people in the U.S. have had confirmed coronavirus infections and more than 1 million have died of COVID-19. In the graphics below, explore the trends in your state.

View the data via state-by-state charts (immediately below), a **heat map** that shows state risk levels, a **table** of trends in new infections over four weeks, and a **map** of case and death totals.

Are new cases growing in your state?

7-day average of new cases, per 100,000 people, as of June 15. States are ordered from highest new daily cases to lowest.

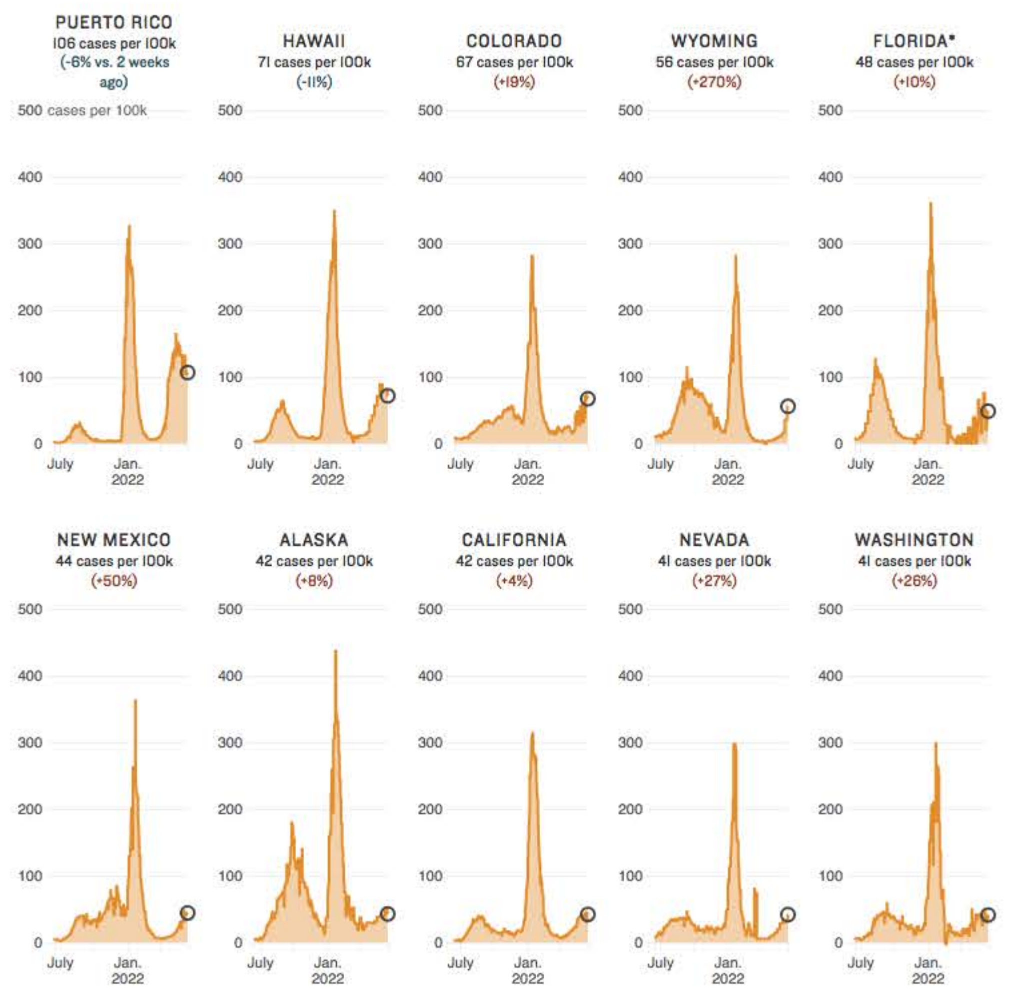

SHOW MORE ▼

Notes

*Data anomalies: In late March, Arizona and Georgia reported backlogged data with their new counts. Florida has shifted to reporting case and death numbers every other week. Johns Hopkins University relies largely on state dashboards for its data. This graphic excludes places where JHU reports no new data in the past week.

Source: Center for Systems Science and Engineering at Johns Hopkins University

The above charts show average new cases per 100,000 people for each state over the last year. In most cases, three waves are visible in these charts: last winter's surge, the delta wave of late summer, and the ongoing omicron wave. In many places the omicron wave has already passed the peak of either previous wave.

34 places are at the highest COVID-19 risk level

Data as of June 15

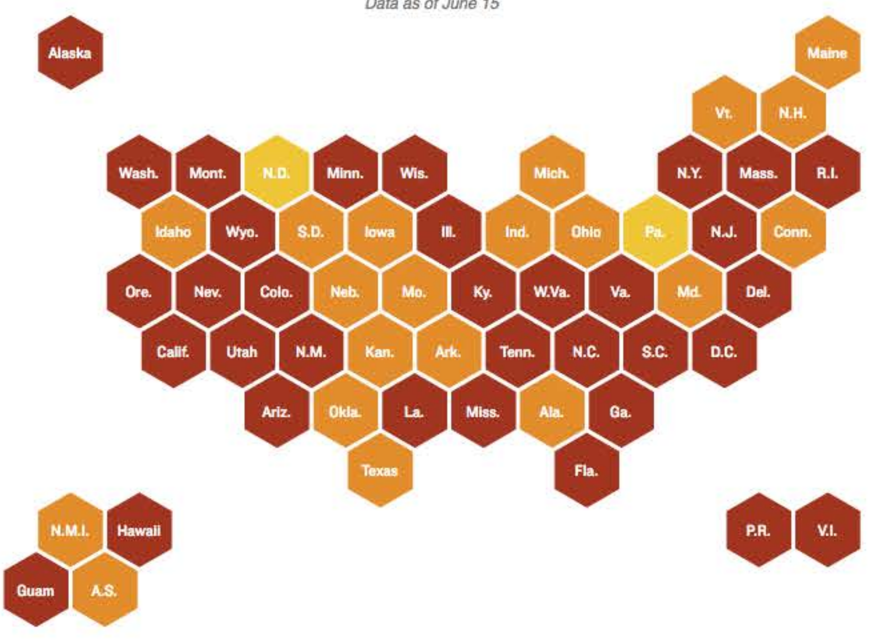

RED	ORANGE	YELLOW	GREEN
**Threshold:** 25+ daily new cases per 100,000 people	**Threshold:** 10-24 daily new cases per 100,000 people	**Threshold:** 1-9 daily new cases per 100,000 people	**Threshold:** <1 daily new case per 100,000 people
**Indicates:** unchecked community spread	**Indicates:** escalating community spread	**Indicates:** potential community spread	**Indicates:** close to containment

Notes

— Daily cases are a 7-day average to smooth out day-to-day variations in the data. Risk levels are based on a scale developed by the Harvard Global Health Institute and a collaboration of top scientists at institutions around the country.

Source: Center for Systems Science and Engineering at Johns Hopkins University, Census Bureau 2021 population estimates, 2020 Census (U.S. territories)

The map above shows the risk of infection in each state based on new daily cases per capita. The consortium of researchers and public health experts who developed these risk levels advises states in the red and orange categories to issue stay-home orders and/or implement rigorous testing and contact tracing. Yellow states should keep up testing and contact tracing as well as social distancing and mask usage.

Total cases and deaths, state by state

Data as of 7:20 a.m. ET on June 16

CASES | CASES PER 100,000 | DEATHS | DEATHS PER 100,000

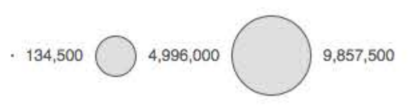

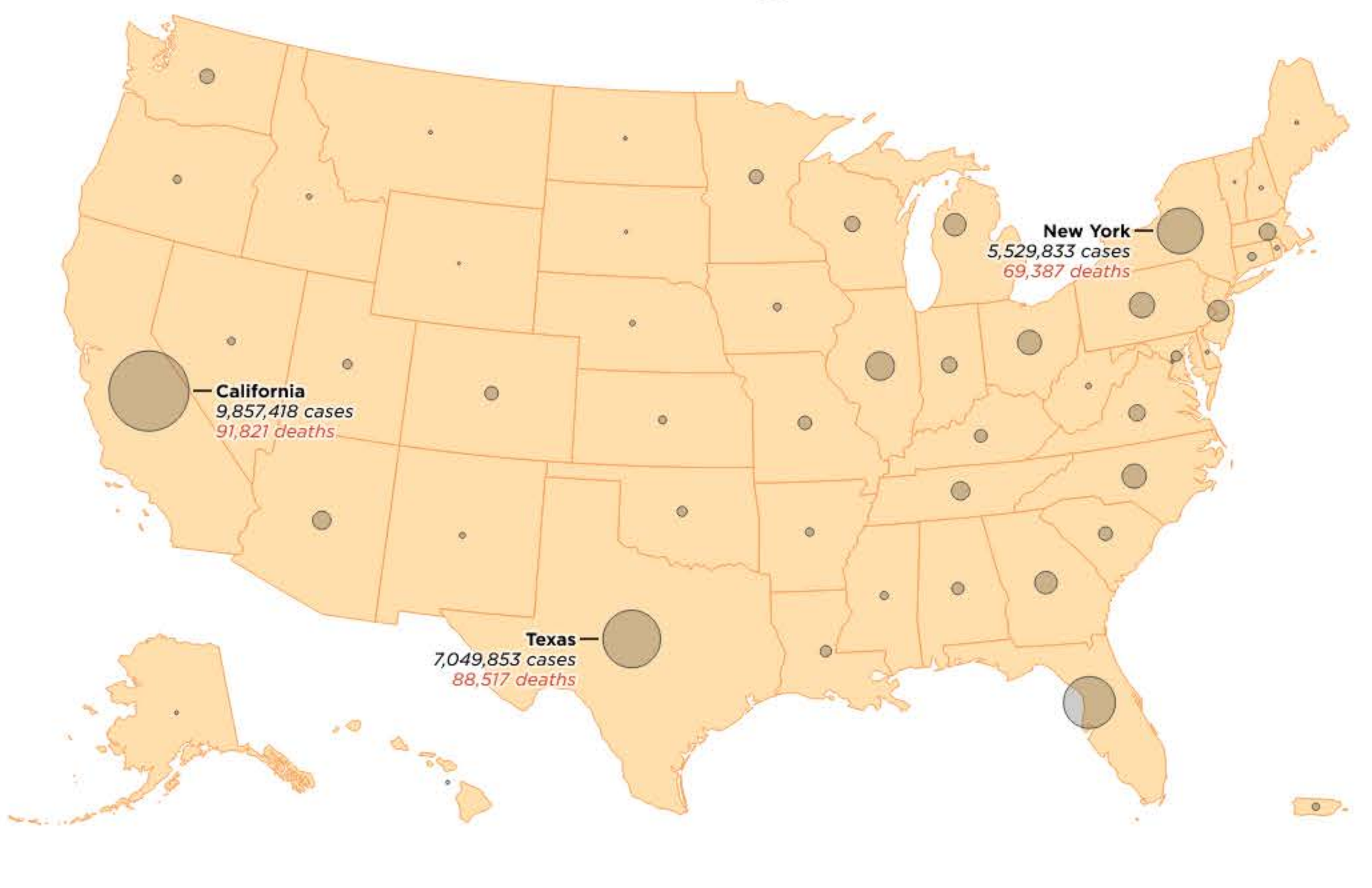

Source: Center for Systems Science and Engineering at Johns Hopkins University

Explore the map above to see totals and per capita figures around the country for both new confirmed cases and reported deaths from COVID-19. New York was the original epicenter of the pandemic in the U.S. As of June 2021, California, Florida and Texas surpass New York for total cases to date.

Click here to see a global map of confirmed cases and deaths.

To show trends, the table below shows the change in average new cases per day in each state, week over week for the last 28 days. States marked in shades of red have growing outbreaks; those in shades of green, are declining.

Total cases and weekly trends, by state and territory

Totals as of 7:20 a.m. ET on June 16. Daily averages as of June 15.

PERCENT CHANGE VS. PREVIOUS WEEK
-100% -50% -5% +5% +50% +100%

STATE	AVG. NEW CASES/DAY: 3 weeks ago	2 weeks ago	Last week	This week	TOTALS SINCE JAN. 2020: CASES	DEATHS
**United States**	109,459	104,233	109,086	102,448	85,941,704	1,012,604
California	15,136	15,802	16,237	16,389	9,857,418	91,821
Texas	4,571	5,236	6,781	7,165	7,049,853	88,517
Florida*	9,020	9,579	11,080	10,523	6,320,890	75,023
New York	8,755	7,622	6,388	5,238	5,529,833	69,387
Illinois	5,296	4,628	5,111	4,103	3,371,262	38,368
Pennsylvania	4,238	3,552	3,223	230	2,956,361	45,413
North Carolina	4,162	3,971	3,826	3,495	2,823,979	25,140
Ohio	2,791	2,792	2,504	2,607	2,798,900	38,657
Georgia*	2,389	2,151	2,529	2,767	2,608,172	38,360
Michigan	3,710	2,791	2,636	2,225	2,581,397	36,675
New Jersey	5,036	3,950	3,778	2,872	2,444,707	33,896
Arizona*	1,643	1,863	2,097	2,333	2,093,680	30,400

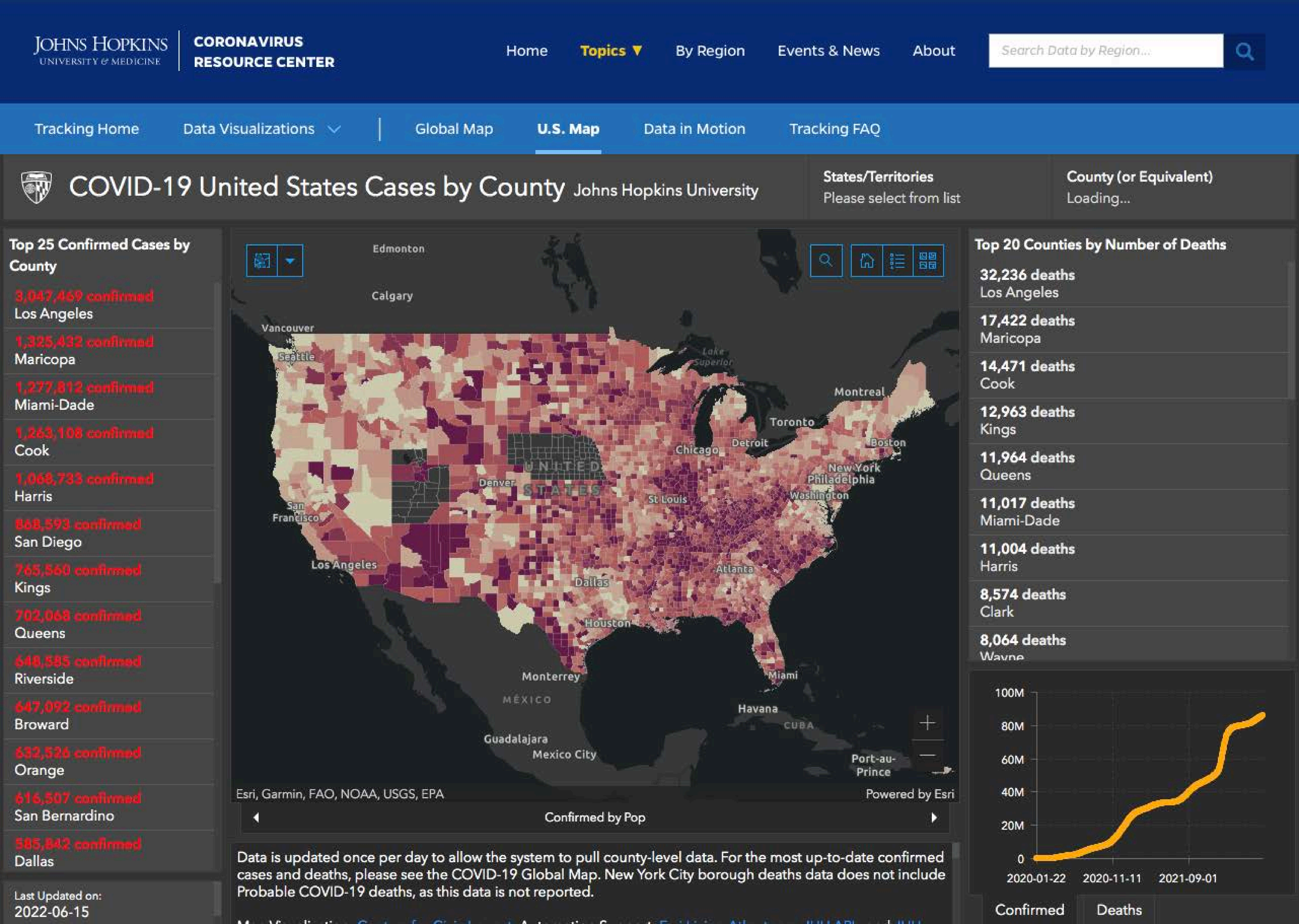
JOHNS HOPKINS UNIVERSITY & MEDICINE
CORONAVIRUS RESOURCE CENTER
Home
Topics
By Region
Events & News
About
Search Data by Region...
Tracking Home
Data Visualizations
Global Map
U.S. Map
Data in Motion
Tracking FAQ
COVID-19 United States Cases by County Johns Hopkins University
States/Territories
Please select from list
County (or Equivalent)
Loading...
Top 25 Confirmed Cases by County
3,047,469 confirmed
Los Angeles
1,325,432 confirmed
Maricopa
1,277,812 confirmed
Miami-Dade
1,263,108 confirmed
Cook
1,068,733 confirmed
Harris
868,593 confirmed
San Diego
765,560 confirmed
Kings
702,068 confirmed
Queens
648,585 confirmed
Riverside
647,092 confirmed
Broward
632,526 confirmed
Orange
616,507 confirmed
San Bernardino
585,842 confirmed
Dallas
Last Updated on:
2022-06-15
Edmonton
Calgary
Vancouver
Seattle
Lake Superior
Montreal
Toronto
Detroit
Chicago
Boston
New York
Philadelphia
Washington
St Louis
UNITED STATES
Denver
San Francisco
Los Angeles
Dallas
Atlanta
Houston
Miami
Monterrey
MÉXICO
Havana
CUBA
Guadalajara
Mexico City
Port-au-Prince
Esri, Garmin, FAO, NOAA, USGS, EPA
Powered by Esri
Confirmed by Pop
Data is updated once per day to allow the system to pull county-level data. For the most up-to-date confirmed cases and deaths, please see the COVID-19 Global Map. New York City borough deaths data does not include Probable COVID-19 deaths, as this data is not reported.
Top 20 Counties by Number of Deaths
32,236 deaths
Los Angeles
17,422 deaths
Maricopa
14,471 deaths
Cook
12,963 deaths
Kings
11,964 deaths
Queens
11,017 deaths
Miami-Dade
11,004 deaths
Harris
8,574 deaths
Clark
8,064 deaths
100M
80M
60M
40M
20M
0
2020-01-22
2020-11-11
2021-09-01
Confirmed
Deaths

MAPS & TRENDS

IMPACT OF OPENING AND CLOSING DECISIONS BY STATE

A look at how social distancing measures may have influenced trends in COVID-19 cases and deaths

SHARE:

ALABAMA

Select another state

Alabama

How to read this graphic

New Confirmed Cases | New Deaths

RECENT OPENING AND CLOSING POLICY DECISIONS

• Restriction/closing • Opening • Deferring decisions to county • Other

← Previous | Next →

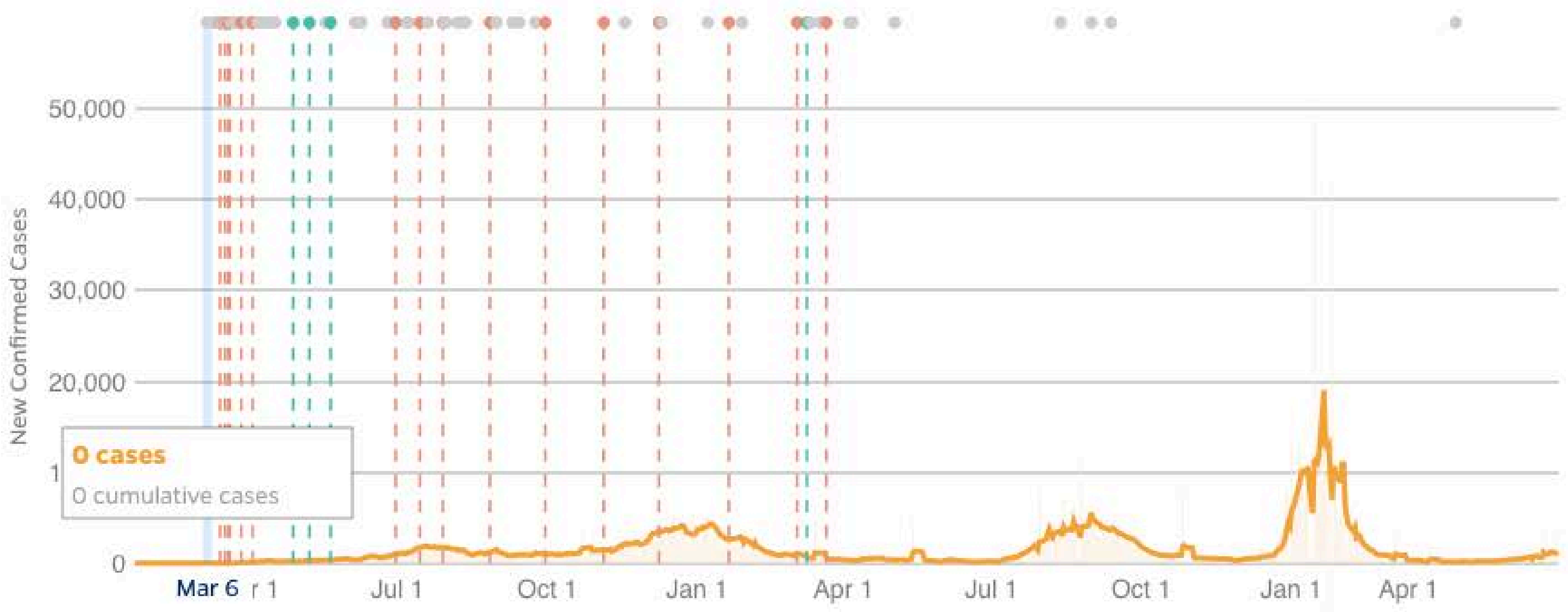

Mar 06, 2020 — 0 CUMULATIVE CASES | 0 CUMULATIVE DEATHS

- Governor Ivey announced the formation of a Coronavirus (COVID-19) Task Force of cabinet and state agency officials, as well as disease specialists in an abundance of caution.

Data Sources: Cases and deaths data from JHU CSSE. As of August 1, 2020, policy events are drawn from from various state-specific sources. Prior to that, the data source for policy events was the National Governor's Association.

ABOUT THIS PAGE:

This page was last updated on Thursday, June 16, 2022 at 6:02 AM EDT.

This timeline shows the major infection control measures and re-openings undertaken by states after the first COVID-19 cases appeared in the United States, alongside the number of new cases and deaths in each state over the same time period.

Dots on the timeline indicate key events in each state – closings (in red), openings (in green) and other policy changes and events (in grey). Clicking on a dot will display event details below the chart. Additionally, the PREVIOUS and NEXT buttons move users through key events by day, allowing them to be viewed in chronological order. The timeline can be viewed against either new cases or new deaths. To see current cumulative case and death counts and daily counts for days with no policy events, click here.

The Latest on this Dashboard

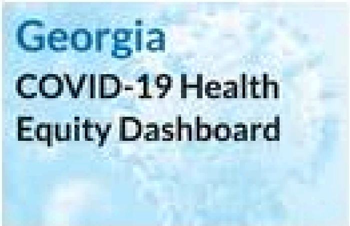

Georgia COVID-19 Health Equity Dashboard
The Georgia COVID-19 Health Equity dashboard is a tool to dynamically track and compare the burden of cases and deaths across counties in Georgia. Click to Access.

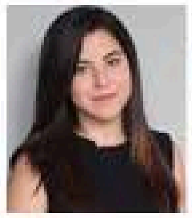

el Nuevo Herald
Miami Herald

Digesting COVID-19 data
Ms. Ana Claudia Chacin talks about the challenges she and other journalists have faced in accessing and reporting accurate COVID-19 data in Florida.for more.

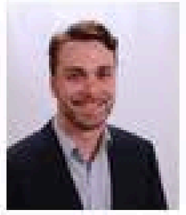

EMORY
ROLLINS SCHOOL OF PUBLIC HEALTH

"Antibodies ar
but it's not th
Dr. David Benl
COVID-19 va
response to th

United States /

See Dashboard Guide (PDF / YouTube)

COVID-19 is affecting every community differently.
Some areas are much harder-hit than others.
What is happening where you live?

Average Daily COVID-19 Cases per 100K

1 11 15 20 25 33 350K
Low High
None Reported

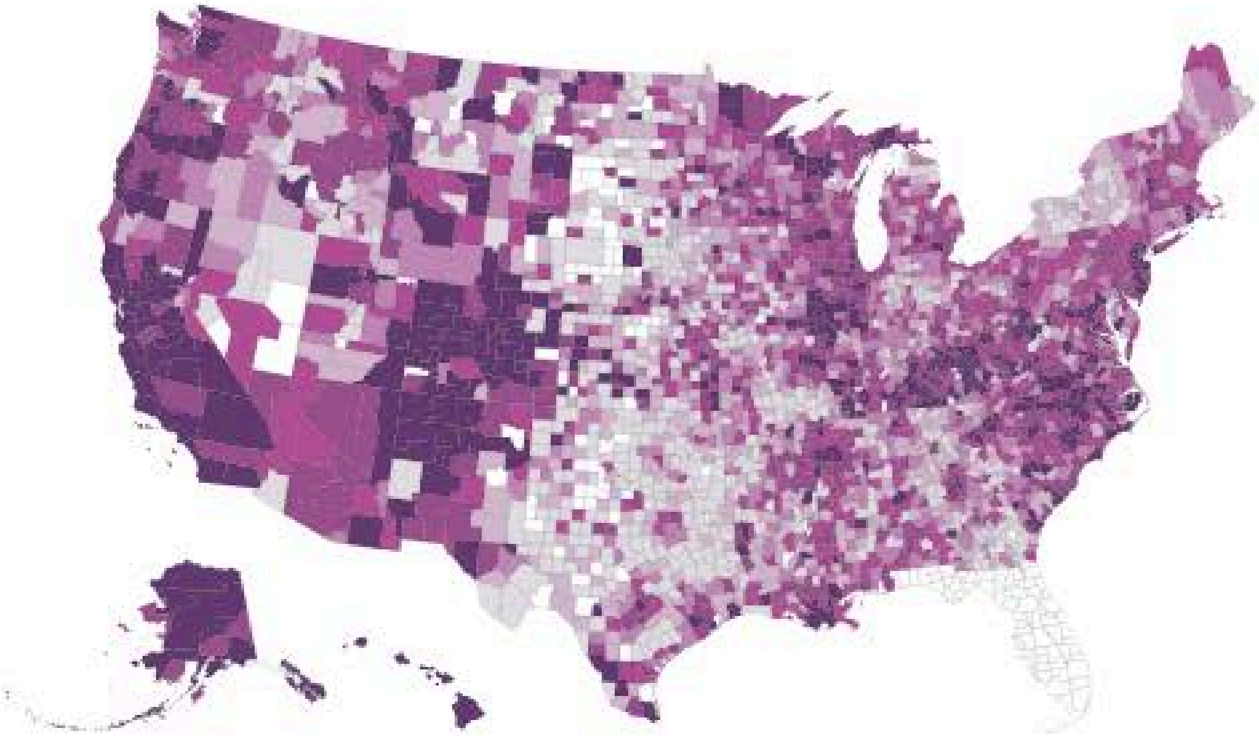

Current Cases and Deaths in The United States

Daily Cases	Daily Deaths
125,403 ↑ 18% 14-day change	271 ↑ 12% 14-day change

*14-day change trends use 7-day averages.

Disparities in COVID-19 Mortality Nation

COVID-19 Death Rates | COVID-19 Death and Population

COVID-19 Death Rate per 100k

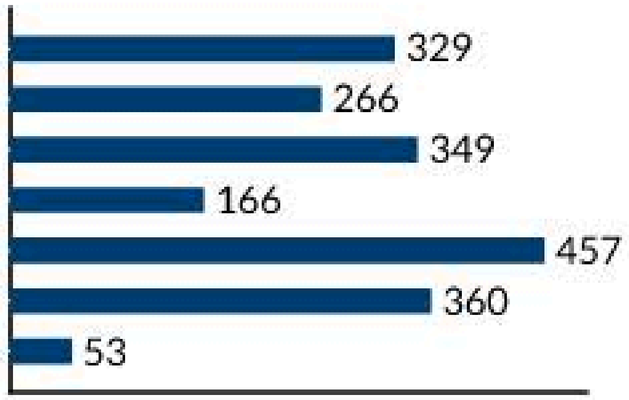

Data as of: 06/13/2022, updated every weekday.

▸ About the data

The chart shows race and ethnicity groups that constitute at least 1% of the state population and have 30 or more deaths. Race and ethnicity data are known for 95% of deaths in the nation. Rates are not reported for race & ethnic groups with < 30 deaths recorded or groups that constitute at least 1% of the state population. NHPI: Native Hawaiians and Pacific Islanders.

Data source: The CDC
Deaths by Race & Ethnicity data as of: 06/02/2022.

US COVID-19 cases and deaths by state

How is the nation faring against COVID-19, both overall and at a state level? Get the answers here, with data on hotspots and infection rates. This map tracks the history of coronavirus cases in America, both recent and all-time.

Data Updated Jun 14 CSV

All states updated case and death data daily early in the pandemic, but many have slowed/stopped. As of spring 2022, dozens of states update daily, others provide data once a week. Nebraska and Missouri have stopped providing regular updates.

	Total Reported	7-Day Average (Jun 14)
**Cases**	83,133,201	79,884
**Deaths**	1,001,988	312

1 Month | 3 Month | 6 Month | All time

NEW CASES PER DAY

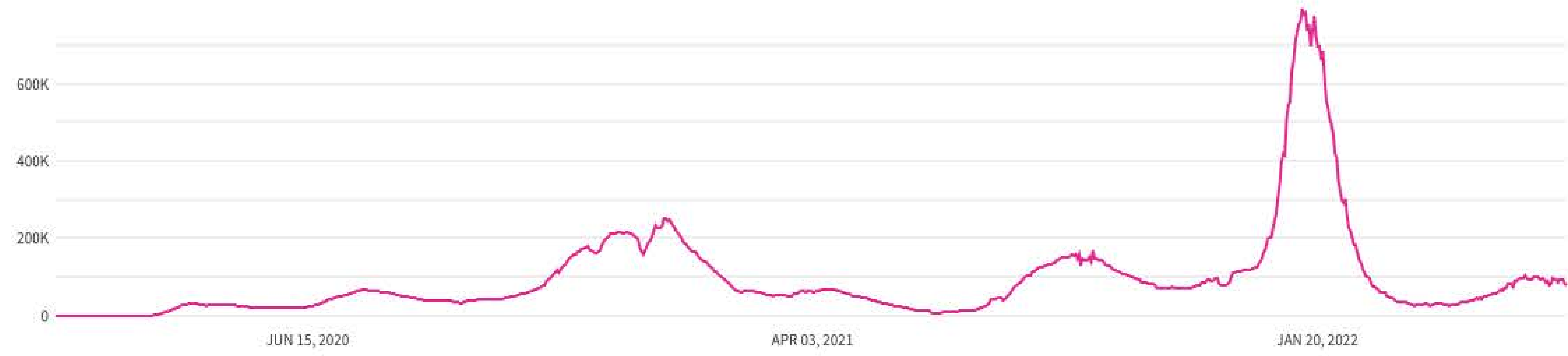

NEW DEATHS PER DAY

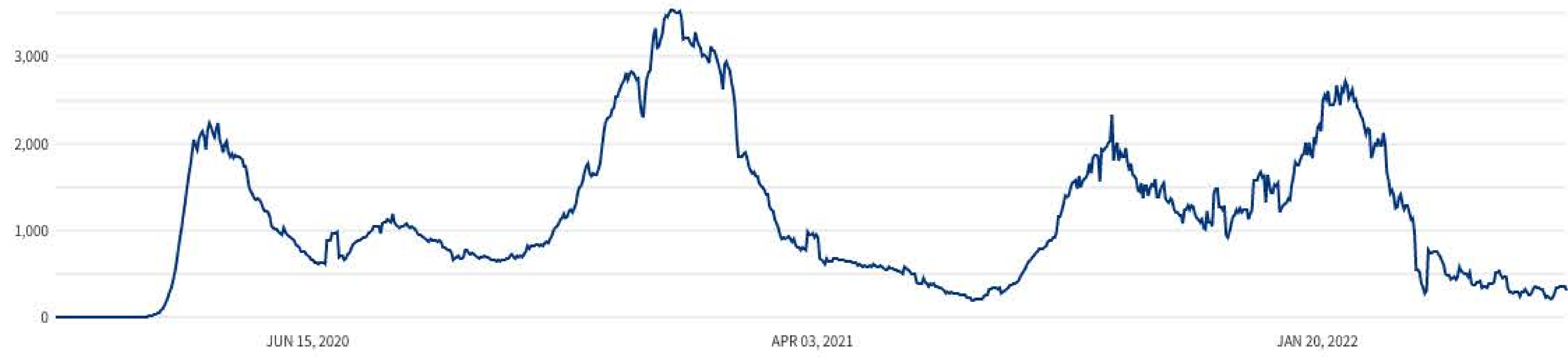

In the United States, there were 49,449 newly reported COVID-19 cases and 159 newly reported COVID-19 deaths on Jun 14, 2022

States sometimes revise case numbers but don't provide historical data, causing our charts to display negative numbers. Learn more in our methodology.

HOT SPOTS PER 100K TOTAL

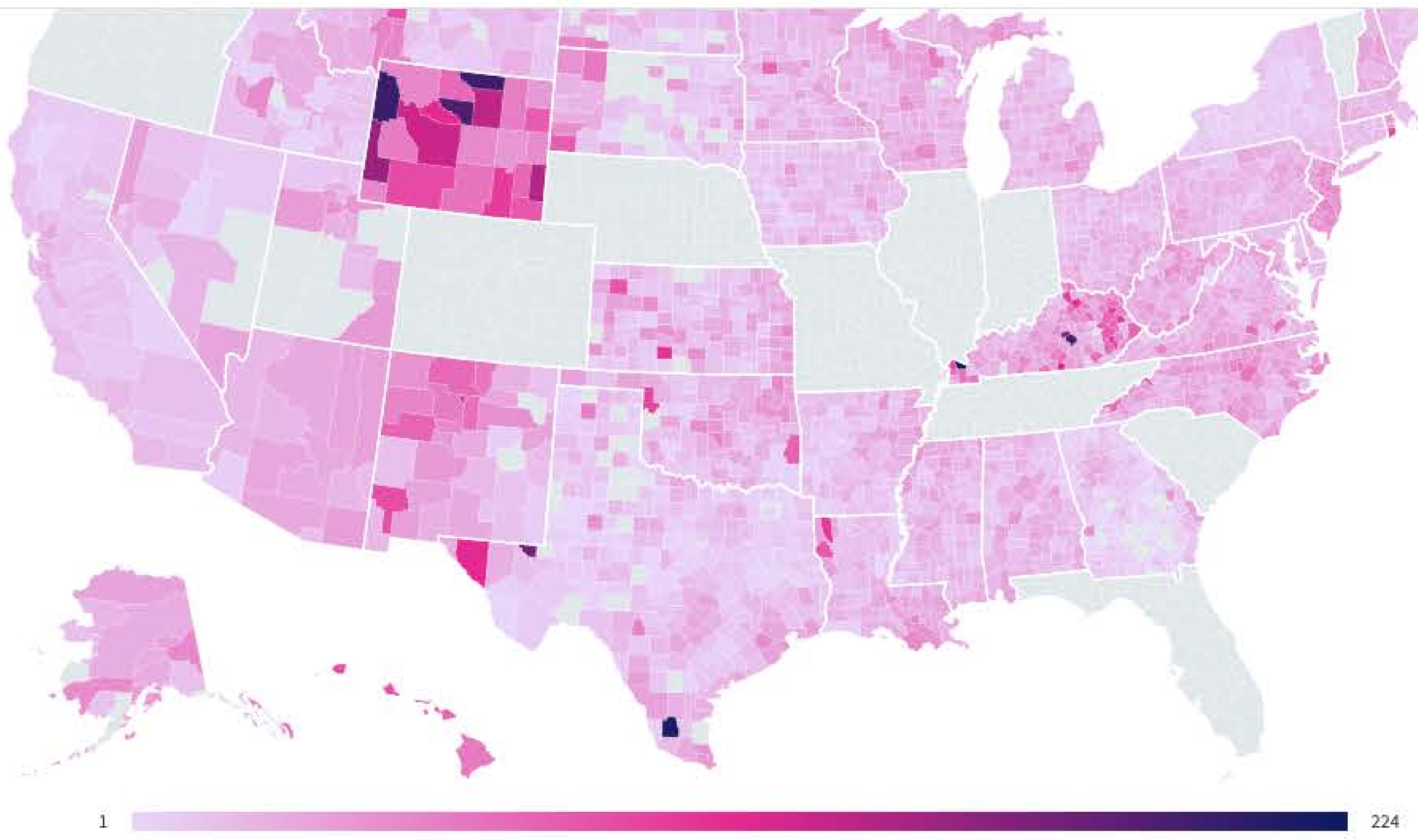

7-DAY AVERAGE CASES PER 100K

From cities to rural towns, stay informed on where COVID-19 is spreading to understand how it could affect families, commerce, and travel. Follow new cases found each day and the number of cases and deaths in the US. The county-level tracker makes it easy to follow COVID-19 cases on a granular level, as does the ability to break down infections per 100,000 people. This county visualization is unique to USAFacts and will be updated with the most recen data as frequently as possible. The underlying data is available for download below the US county map and has helped government agencies like Center for Disease Control and Prevention in its nationwide efforts.

STATE-BY-STATE DATA (TOTALS)

State ↑	7-day avg. cases	7-day avg. deaths	Cases	Deaths	7-day avg. hospitalizations	7-day avg. hospitalizations per 100k
AL	1	2	1,329,827	19,695	68	1
AK	259	0	248,735	1,231	6	0.8
AZ	2	0	2,077,347	29,852	128	1

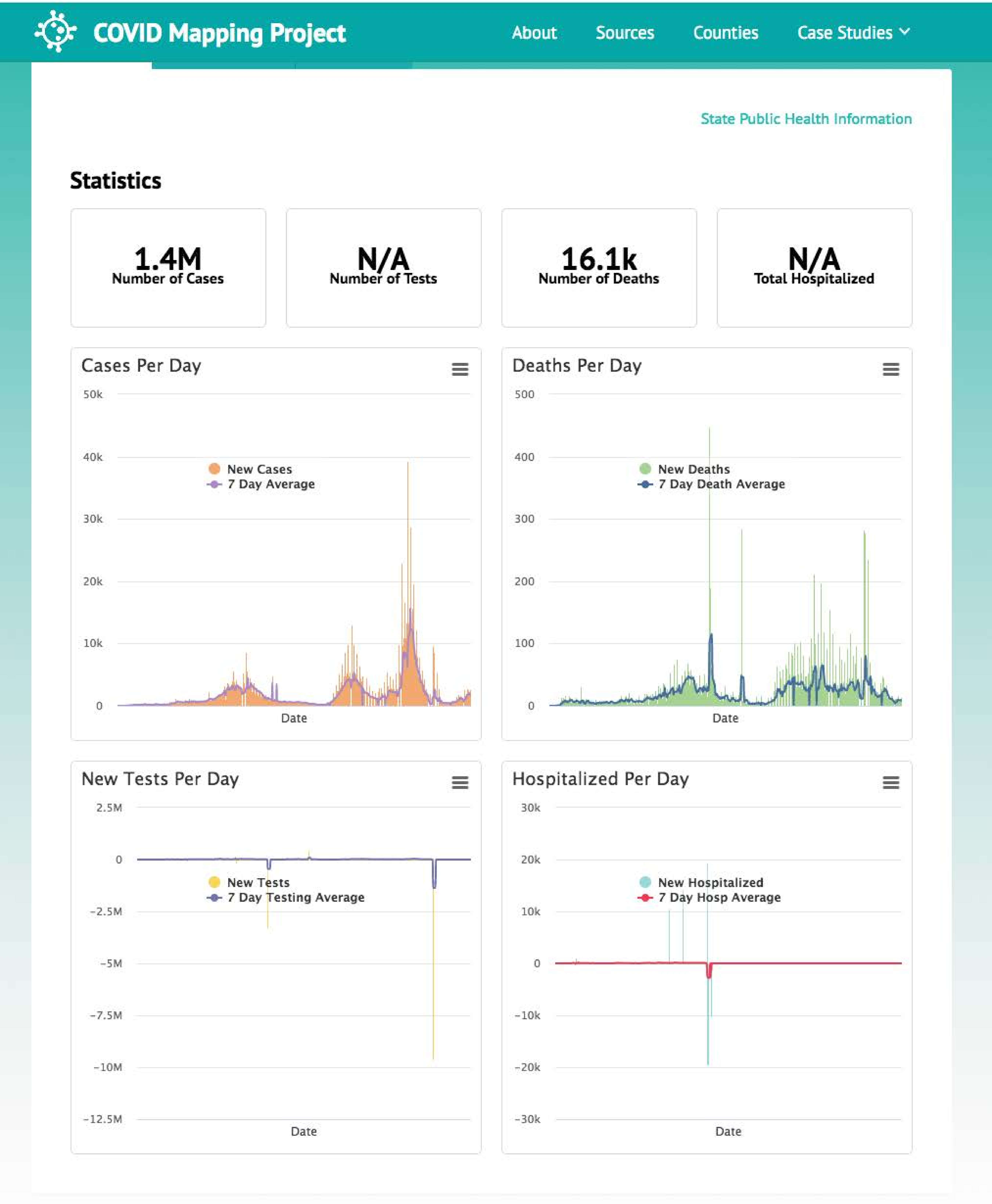
COVID Mapping Project
About
Sources
Counties
Case Studies
State Public Health Information
Statistics
1.4M
Number of Cases
N/A
Number of Tests
16.1k
Number of Deaths
N/A
Total Hospitalized
Cases Per Day
New Cases
7 Day Average
Date
Deaths Per Day
New Deaths
7 Day Death Average
Date
New Tests Per Day
New Tests
7 Day Testing Average
Date
Hospitalized Per Day
New Hospitalized
7 Day Hosp Average
Date
