# Supplementary material for: Web-Based COVID-19 Dashboards and Trackers in the United States: Survey Study
Source: JMIR Hum Factors. 2023 Mar 20;10:e43819. doi: 10.2196/43819 (PMC10029858; doi:10.2196/43819)
Supplement: Multimedia Appendix 17 [file humanfactors_v10i1e43819_app17.pdf]

Appendix 17. Screenshots from dashboards and trackers — *Global coverage* *(captured June 16, 2022)*

**Page**	**Ref**	**Host**
2	G-1	New York Times
3	G-2	CNN
4	G-5	Biocomplexity Institute, University of Virginia
5	G-7	HealthMap / various universities
6	G-8	World Health Organization
7	G-9	Kaiser Family Foundation
8–11	G-10	Our World in Data
12	G-11	DXY
13	G-12	Worldometer
14	G-14	Microsoft
15	G-15	1Point3Acres

Coronavirus World Map: Tracking the Global Outbreak

Updated June 16, 2022

New reported cases

All time | Last 90 days

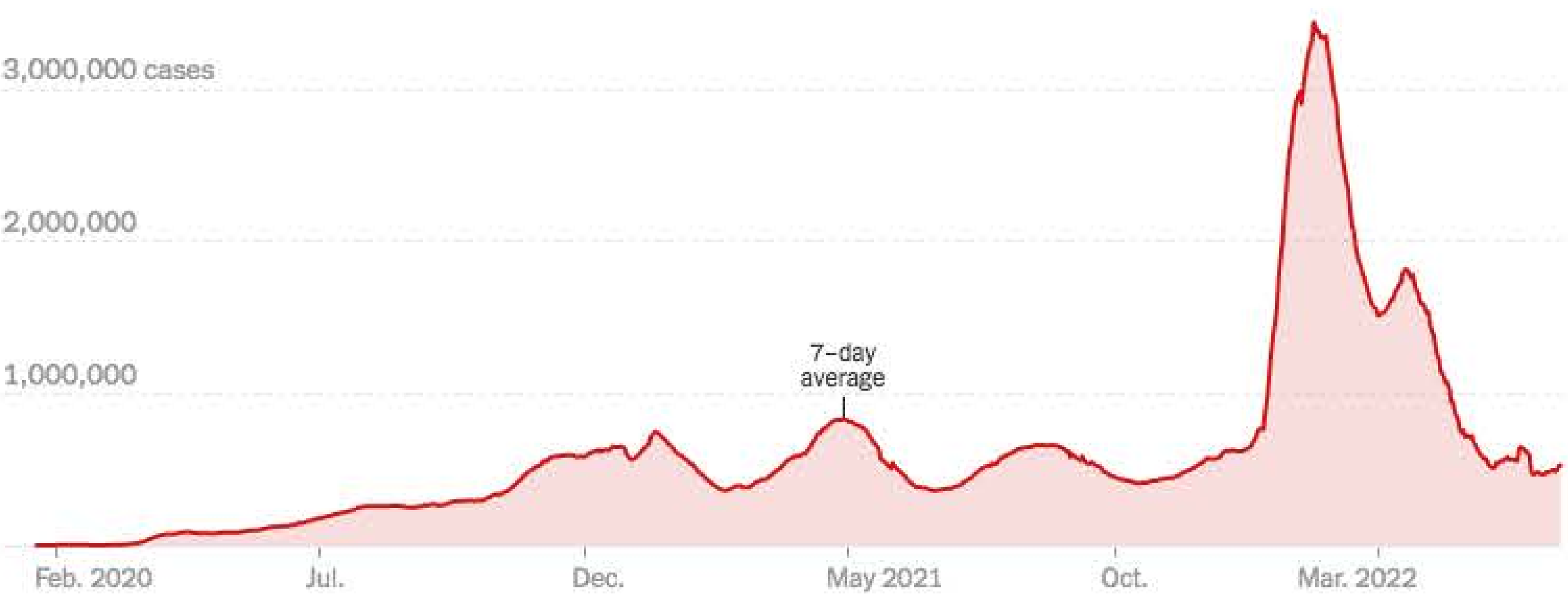

	DAILY AVG. ON JUN. 15	14-DAY CHANGE	TOTAL REPORTED
Cases	527,016	+10%	537,282,756
Deaths	1,373	Flat	6,310,929

About this data

Cases by region

This chart shows how cases per capita have changed in different parts of the world.

All time | Last 90 days

Africa · Asia-Pacific · Europe · Latin America · Middle East · U.S. and Canada

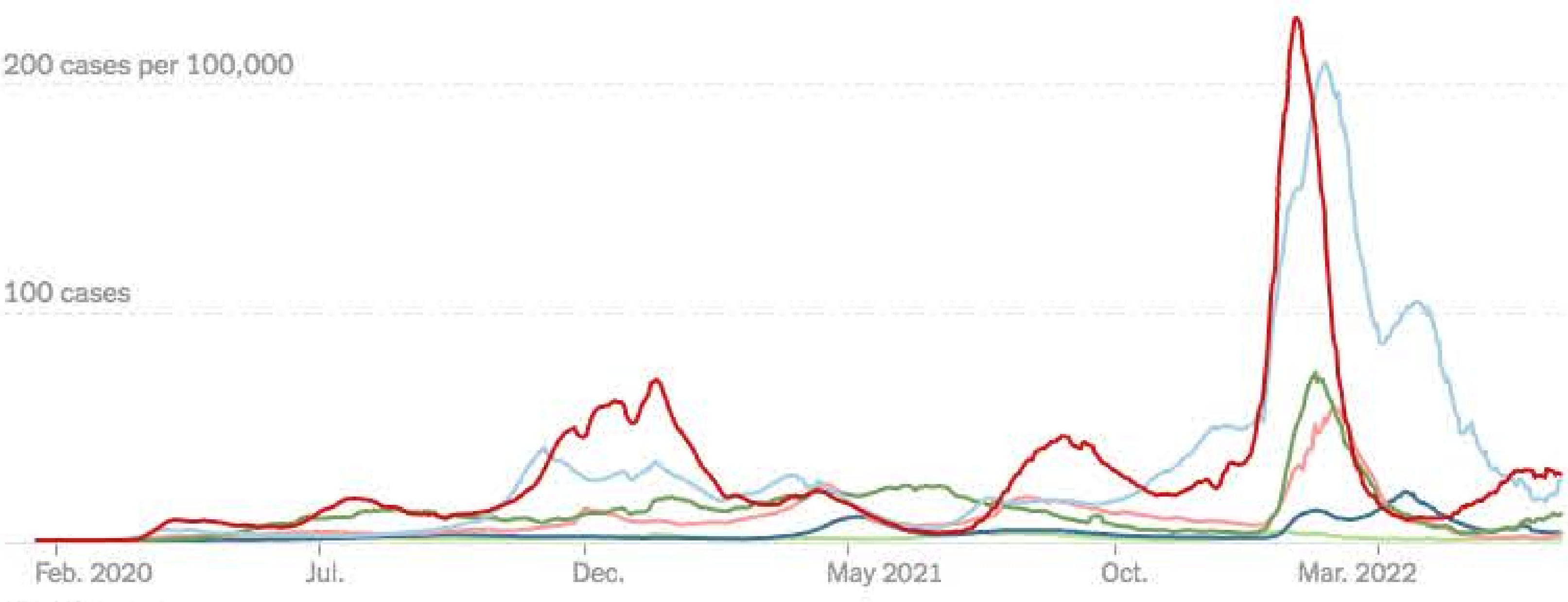

About this data

State of the virus

Update for June 15

- Even as China's outbreaks ease, regular lab testing for Covid-19 has become a feature of daily life. Residents of major cities with no reported cases are being required to present proof of negative P.C.R. tests.
- Canada, continuing to scale back travel restrictions, said it would end a requirement that domestic travelers and federal transport workers show proof of vaccination.
- Executives announced that the first factory in Africa licensed to produce Covid-19 vaccines had not received a single order and could shut down production within weeks.
- Two senior officials in Vietnam were arrested after being accused of involvement in a coronavirus test corruption case that has led to charges against dozens of government officials.
- About 11.2 billion vaccine doses have been administered worldwide, including more than two billion booster or additional doses. Track each country's vaccination progress here.

U.S. Has Far Higher Covid Death Rate Than Other Wealthy Countries

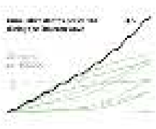

Despite having one of the world's most powerful arsenals of vaccines, the United States has failed to inoculate as much of its population as other wealthy nations.

Hot spots

AVERAGE DAILY CASES PER 100,000 PEOPLE IN PAST WEEK

4 12 20 28 40 68 FEW OR NO CASES MISSING DATA

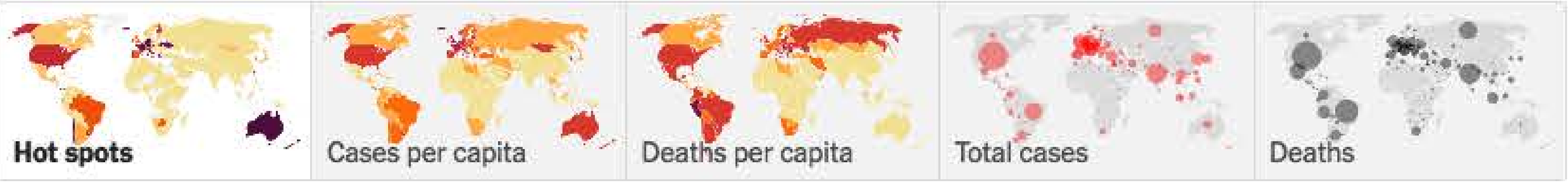

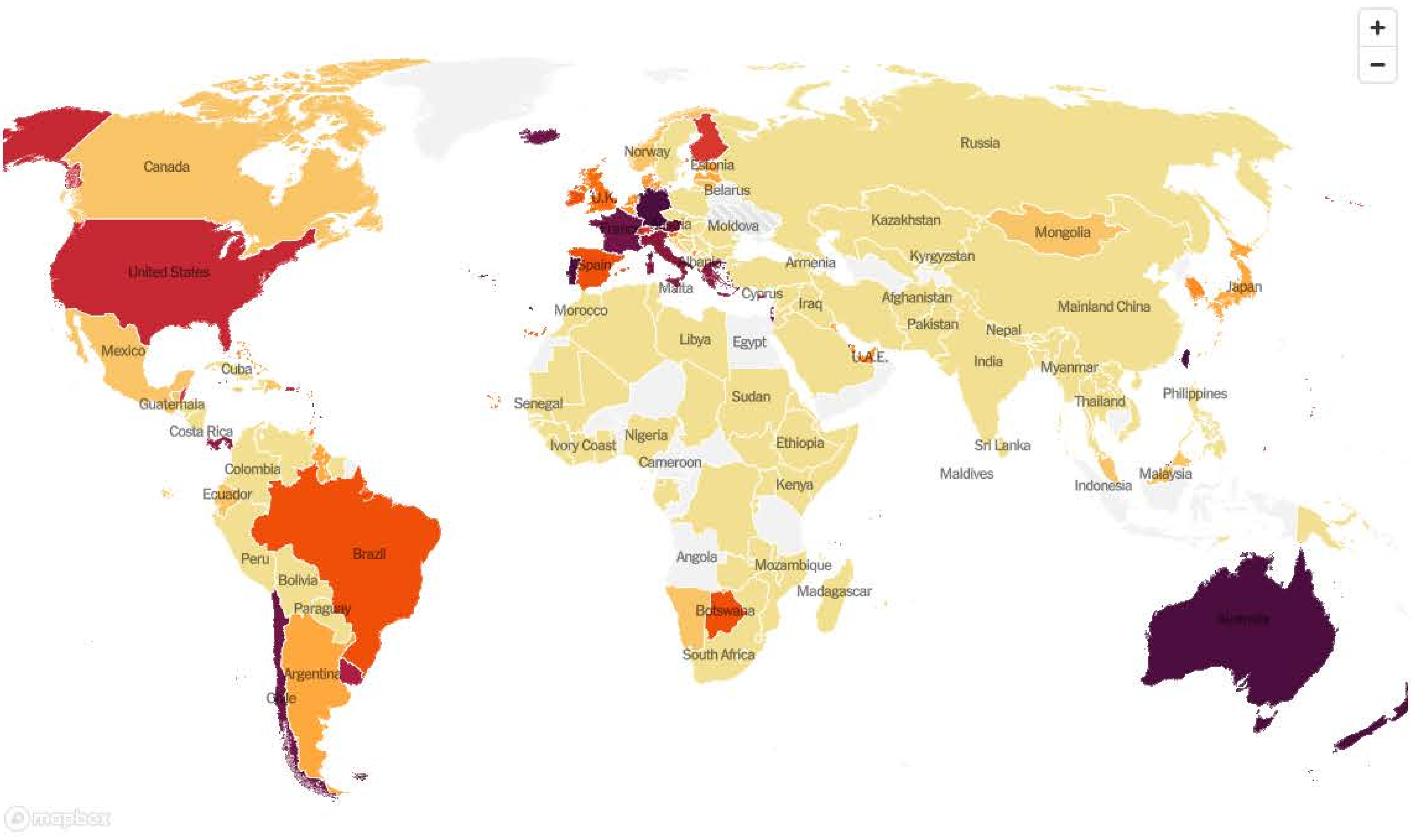

About this data

Reported cases, deaths and vaccinations by country

This table is sorted by places with the most cases per 100,000 residents in the last seven days. Charts show change in daily averages and are each on their own scale.

Recent trends | All time | Search countries

	CASES DAILY AVG.	PER 100,000	14-DAY CHANGE	DEATHS DAILY AVG.	PER 100,000	FULLY VACCINATED
Taiwan	64,499	271	–20%	167.6	0.70	80%
Portugal	15,829	154	–36%	36.9	0.36	86%*
New Zealand	5,531	112	–21%	9.7	0.20	84%
San Marino	36	106	+288%	0	—	70%*
Gibraltar	35	104	+68%	0	—	—
Australia ›	25,993	102	–20%	42.7	0.17	85%
Falkland Islands	3	97	–23%	—	—	—
Cayman Islands	63	97	–48%	0	—	91%
Brunei	368	85	+51%	0.1	0.03	98%

Show all About this data

How global trends have changed

All time | Last 90 days

New reported cases by day

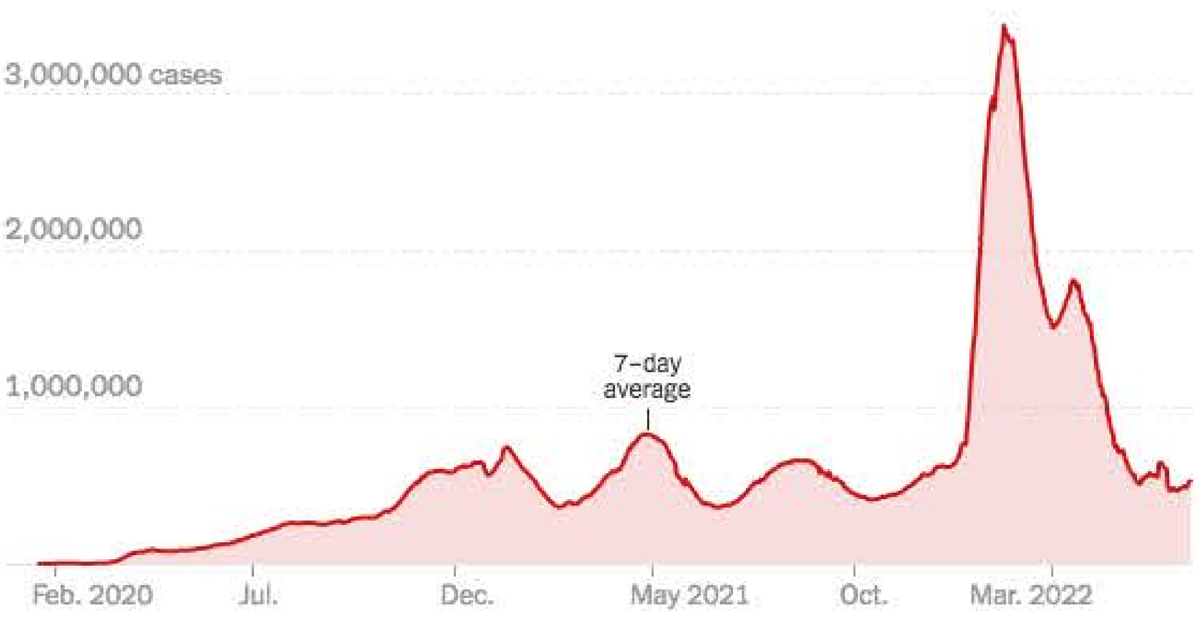

New reported deaths by day

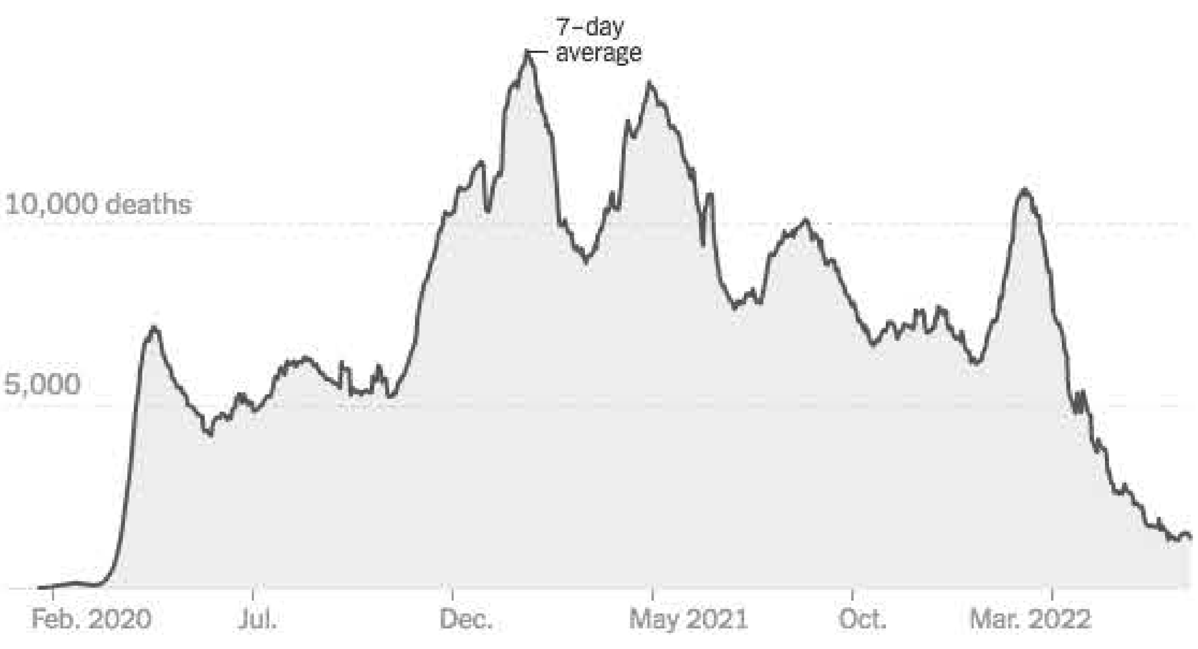

About this data

Tracking Covid-19's global spread

The disease has spread to every continent and case numbers continue to rise

By Henrik Pettersson, Byron Manley and Sergio Hernandez, CNN

Last updated: June 16, 2022 at 7:00 p.m. ET

Authorities in 224 countries and territories have reported about 537.7 million Covid-19 cases and 6.3 million deaths since China reported its first cases to the World Health Organization (WHO) in December 2019.

Cases per 100K | Deaths per 100K | Total cases | Total deaths

0 | 0 to 6,758 | 6,758 to 19,301 | 19,301 to 31,940 | 31,940 to 43,172 | 43,172 to 71,198

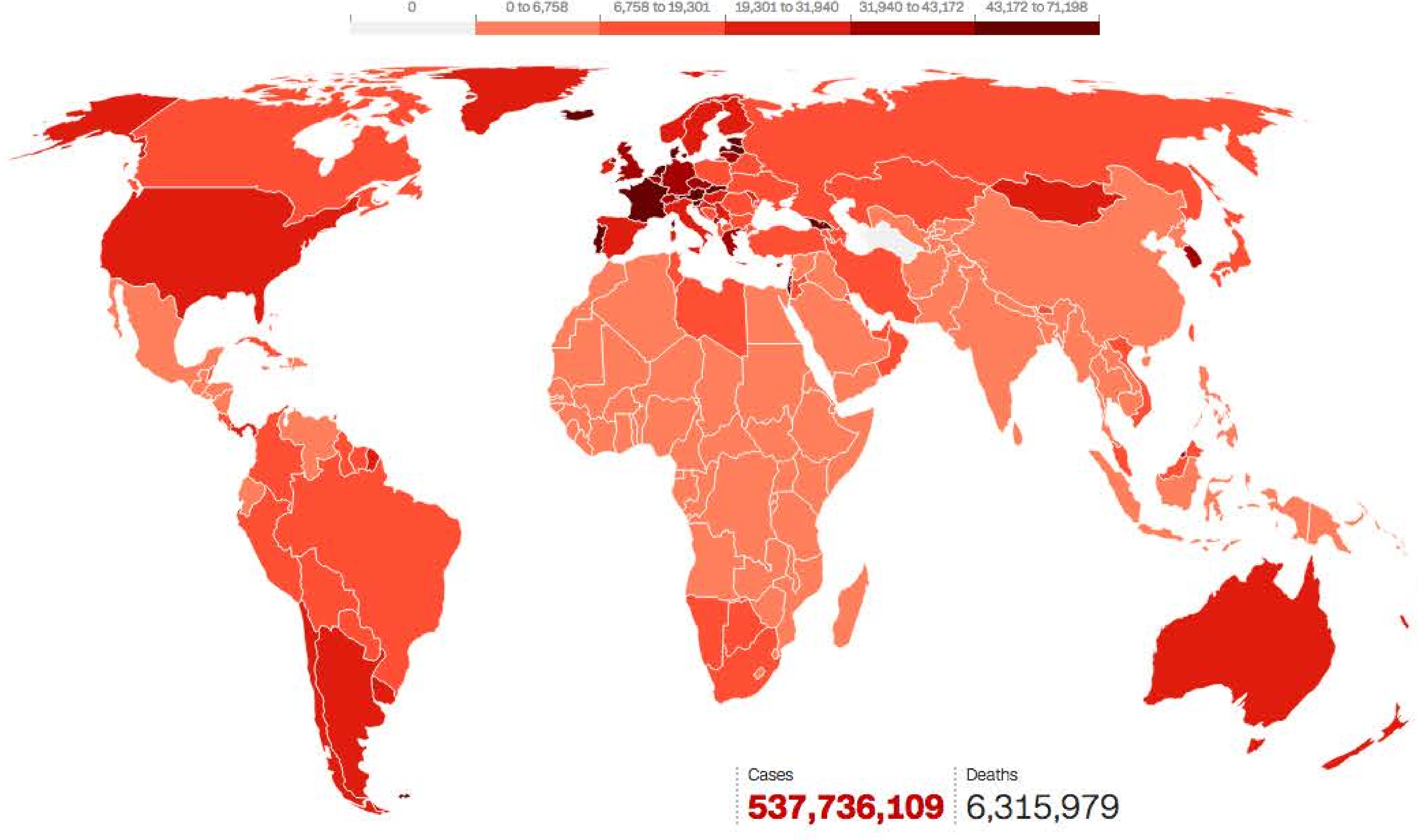

Cases **537,736,109** Deaths 6,315,979

Last updated: June 16, 2022 at 7:00 p.m. ET
Source: Johns Hopkins University Center for Systems Science and Engineering

Reported cases and deaths

The figures below are based on data from the Johns Hopkins University Center for Systems Science and Engineering. These numbers are updated every 15 minutes but may differ from other sources due to differences in reporting times. For up-to-the-minute updates, follow our live coverage.

Location	Cases	...per 100K people	Deaths	...per 100K people
United States	86,050,615	26,216	1,013,006	309
India	43,257,730	3,166	524,803	38
Brazil	31,611,769	14,978	668,693	317
France	29,186,390	43,523	145,711	217
Germany	27,096,571	32,594	140,099	169
United Kingdom	22,537,716	33,722	179,648	269
South Korea	18,256,457	35,306	24,497	47
Russia	18,119,934	12,551	372,579	258
Italy	17,773,764	29,477	167,617	278
Turkey	15,085,742	18,082	98,996	119
Spain	12,515,127	26,585	107,239	228
Vietnam	10,734,925	11,129	43,083	45
Argentina	9,313,453	20,725	128,994	287
Japan	9,094,948	7,203	30,981	25
Netherlands	8,118,312	46,838	22,342	129
Australia	7,724,295	30,453	9,218	36
Iran	7,234,367	8,725	141,360	170
Colombia	6,117,847	12,153	139,894	278
Indonesia	6,064,424	2,241	156,673	58
Poland	6,010,871	15,830	116,390	307

Show more

* Cases identified on cruise ships and at the 2020 Summer Olympics and 2022 Winter Olympics.

Last updated: June 16, 2022 at 7:00 p.m. ET
Sources: Johns Hopkins University Center for Systems Science and Engineering. Population data from World Bank and United Nations.

Since then, cases have been reported on every continent. The vast majority of cases and deaths are now outside mainland China, where the outbreak began.

As the pandemic has spread across the globe, the virus has left a trail of deaths in its wake. Deaths in Europe and North America now outnumber Asia's. In Latin America, South America and the Caribbean, the share of global deaths is still rising.

Daily deaths and cases by region

This chart uses rolling, seven-day averages. This approach makes trends clearer and smooths out anomalies, such as the lack of reporting during the weekend.

Deaths | Cases | Proportional | Actual

Asia • Europe • North America • Latin America and the Caribbean • Africa • Oceania

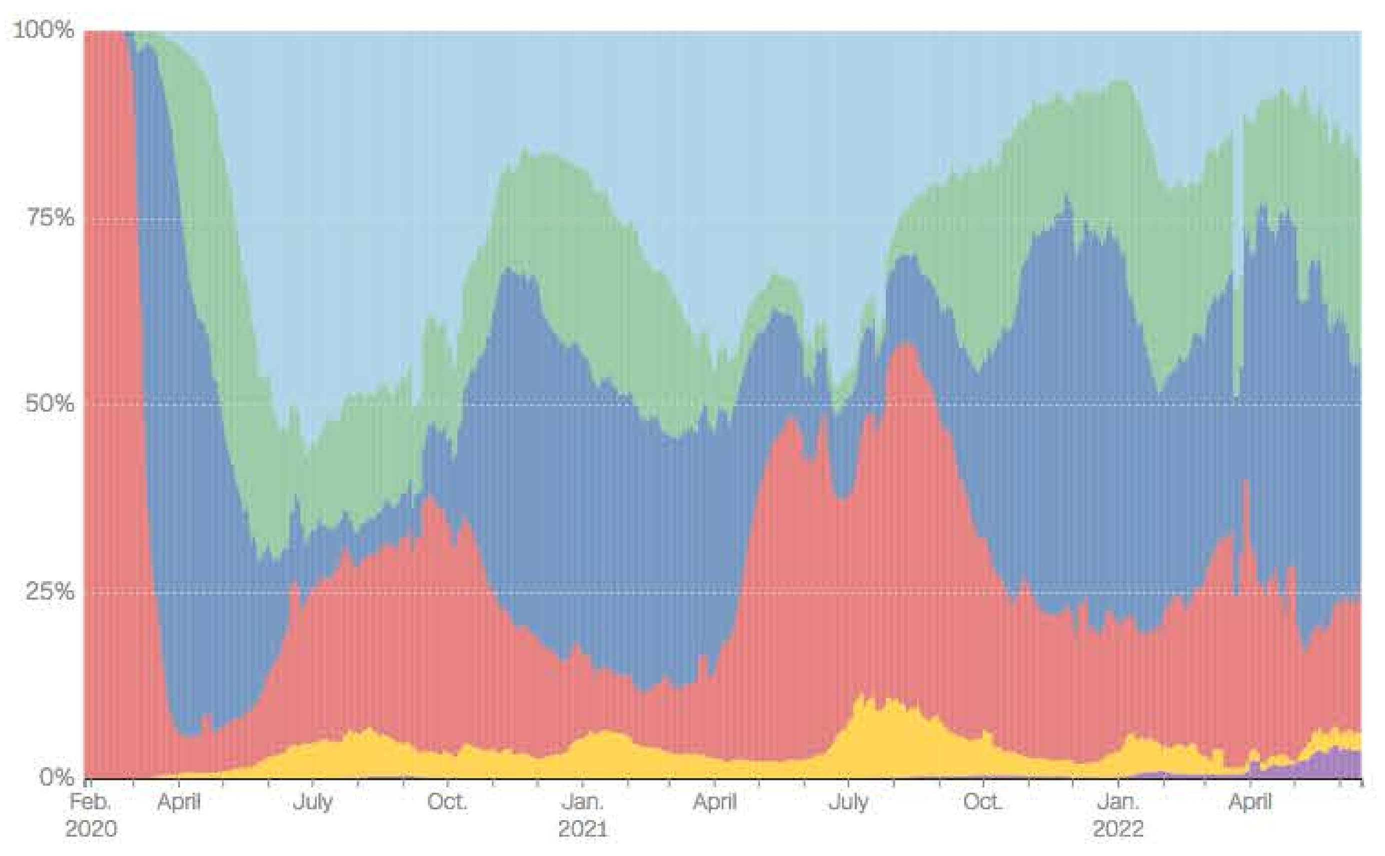

Regions are based on United Nations definitions. Americas have been broken down into subregions (Latin America and the Caribbean and North America).
Last updated: June 16, 2022 at 7:00 p.m. ET
Source: Johns Hopkins University Center for Systems Science and Engineering

Daily reported deaths by country

This timeline of new reported deaths shows how countries and regions have been affected so far.

Jan. 28, 2020 – June 15, 2022

Asia	Total	Latest
India	524,803	11 →
Indonesia	156,670	6 ↑
Iran	141,357	2 →
Turkey	98,996	3 →
Philippines	60,461	1 →

Expand

Europe	Total	Latest
Russia	372,515	65 ↓
United Kingdom	179,587	47 ↓
Italy	167,553	55 ↓
France	145,662	36 ↓
Germany	140,099	68 ↓

Expand

North America	Total	Latest
United States	1,012,607	293 ↓
Canada	41,646	34 ↑
Bermuda	138	0 →
Greenland	21	0 →

Latin America and the Caribbean	Total	Latest
Brazil	668,693	149 ↑
Mexico	325,271	31 ↑
Peru	213,343	8 ↓
Colombia	139,894	4 →
Argentina	128,994	3 →

Expand

Africa	Total	Latest
South Africa	101,576	22 →
Tunisia	28,655	1 →
Egypt	24,720	0 →
Morocco	16,085	0 →
Ethiopia	7,518	0 →

Expand

Oceania	Total	Latest
Australia	9,218	43 ↑
New Zealand	1,308	10 ↓
Fiji	865	0 →
Papua New Guinea	658	0 ↓
French Polynesia	649	0 →

Expand

CNN health

Different countries' epidemics have followed different trajectories. The disease has hit the United States especially hard. About 86.1 million cases have been reported in the country and 1,013,006 patients have died.

Covid-19 case growth rates

The chart below uses a logarithmic scale to show how quickly the number of known Covid-19 cases is growing in each region and territory. Select a location or hover over each line to see how quickly the number of known cases is doubling in that country or territory.

In the United States the number of known cases is doubling about **every two years**.

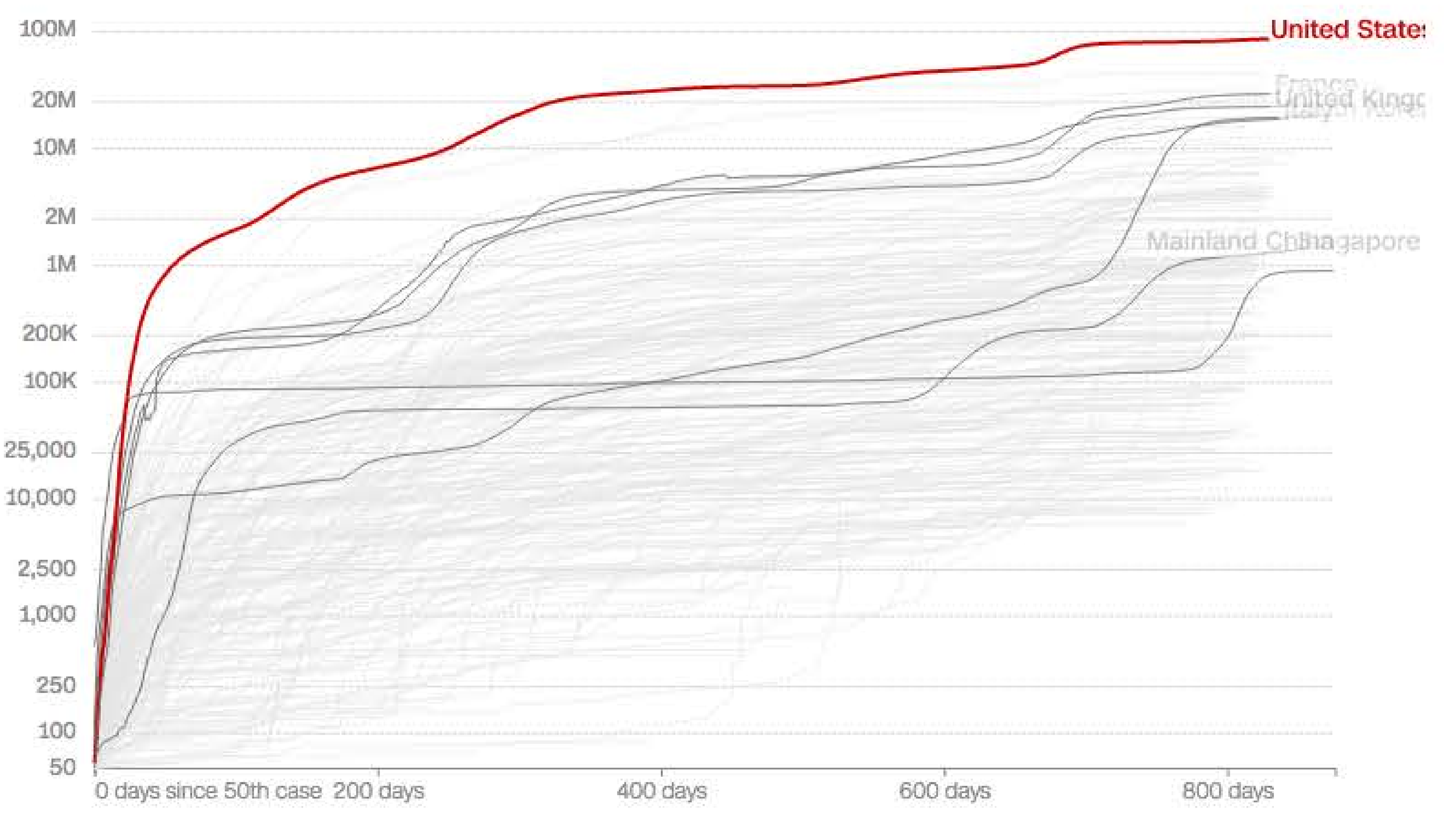

Note: Doubling times are based on the previous week.
Last updated: June 16, 2022 at 7:00 p.m. ET
Source: Johns Hopkins University Center for Systems Science and Engineering

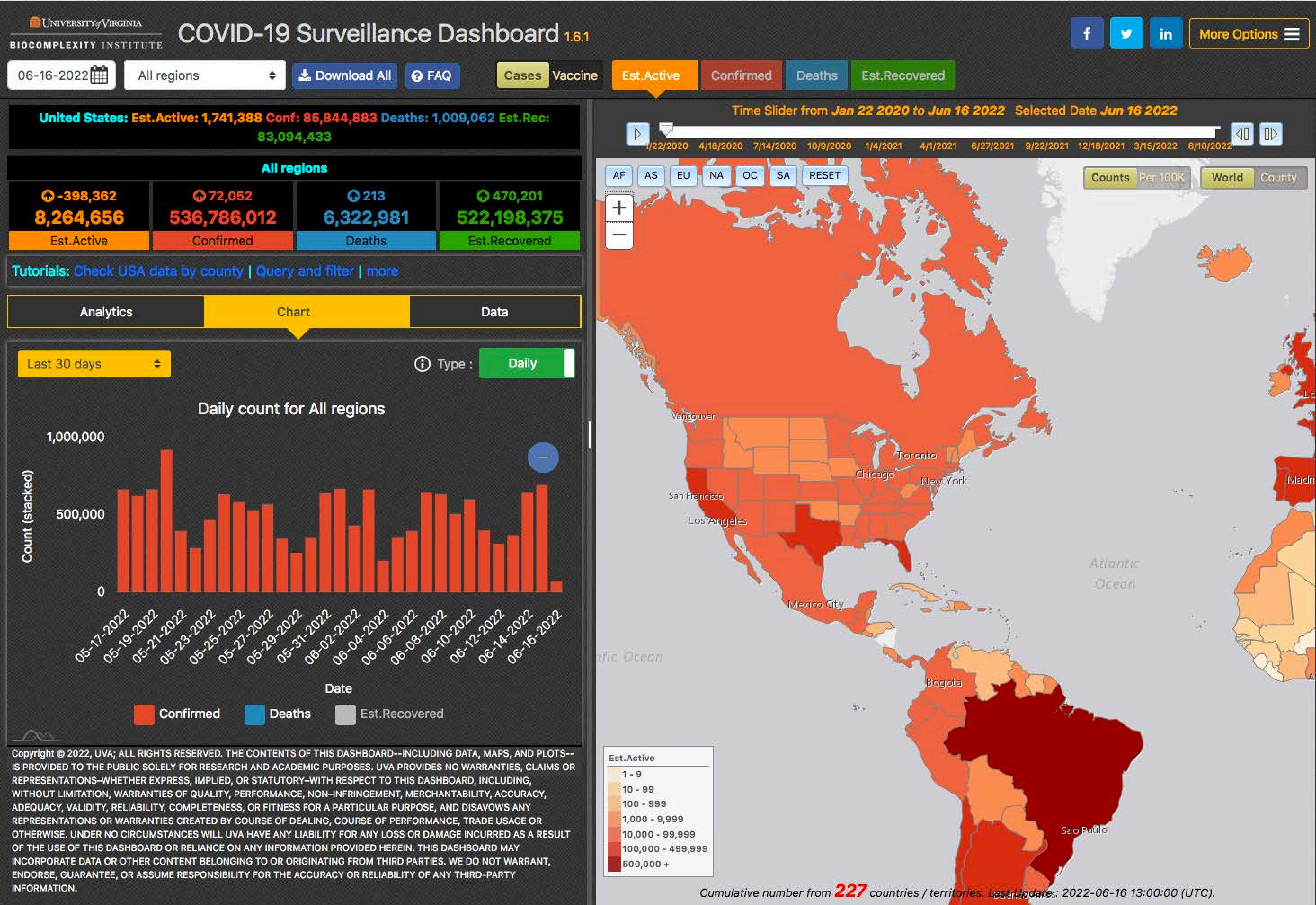
UNIVERSITY of VIRGINIA
BIOCOMPLEXITY INSTITUTE
COVID-19 Surveillance Dashboard 1.6.1
More Options
06-16-2022
All regions
Download All
FAQ
Cases
Vaccine
Est.Active
Confirmed
Deaths
Est.Recovered
United States: Est.Active: 1,741,388 Conf: 85,844,883 Deaths: 1,009,062 Est.Rec: 83,094,433
All regions
-398,362
8,264,656
Est.Active
72,052
536,786,012
Confirmed
213
6,322,981
Deaths
470,201
522,198,375
Est.Recovered
Tutorials: Check USA data by county | Query and filter | more
Analytics
Chart
Data
Last 30 days
Type :
Daily
Daily count for All regions
1,000,000
500,000
0
Count (stacked)
05-17-2022
05-19-2022
05-21-2022
05-23-2022
05-25-2022
05-27-2022
05-29-2022
05-31-2022
06-02-2022
06-04-2022
06-06-2022
06-08-2022
06-10-2022
06-12-2022
06-14-2022
06-16-2022
Date
Confirmed
Deaths
Est.Recovered
Copyright © 2022, UVA; ALL RIGHTS RESERVED. THE CONTENTS OF THIS DASHBOARD--INCLUDING DATA, MAPS, AND PLOTS--IS PROVIDED TO THE PUBLIC SOLELY FOR RESEARCH AND ACADEMIC PURPOSES. UVA PROVIDES NO WARRANTIES, CLAIMS OR REPRESENTATIONS–WHETHER EXPRESS, IMPLIED, OR STATUTORY–WITH RESPECT TO THIS DASHBOARD, INCLUDING, WITHOUT LIMITATION, WARRANTIES OF QUALITY, PERFORMANCE, NON–INFRINGEMENT, MERCHANTABILITY, ACCURACY, ADEQUACY, VALIDITY, RELIABILITY, COMPLETENESS, OR FITNESS FOR A PARTICULAR PURPOSE, AND DISAVOWS ANY REPRESENTATIONS OR WARRANTIES CREATED BY COURSE OF DEALING, COURSE OF PERFORMANCE, TRADE USAGE OR OTHERWISE. UNDER NO CIRCUMSTANCES WILL UVA HAVE ANY LIABILITY FOR ANY LOSS OR DAMAGE INCURRED AS A RESULT OF THE USE OF THIS DASHBOARD OR RELIANCE ON ANY INFORMATION PROVIDED HEREIN. THIS DASHBOARD MAY INCORPORATE DATA OR OTHER CONTENT BELONGING TO OR ORIGINATING FROM THIRD PARTIES. WE DO NOT WARRANT, ENDORSE, GUARANTEE, OR ASSUME RESPONSIBILITY FOR THE ACCURACY OR RELIABILITY OF ANY THIRD-PARTY INFORMATION.
Time Slider from Jan 22 2020 to Jun 16 2022 Selected Date Jun 16 2022
1/22/2020
4/18/2020
7/14/2020
10/9/2020
1/4/2021
4/1/2021
6/27/2021
9/22/2021
12/18/2021
3/15/2022
6/10/2022
AF
AS
EU
NA
OC
SA
RESET
Counts
Per 100K
World
County
Vancouver
Toronto
Chicago
New York
San Francisco
Los Angeles
Atlantic Ocean
Mexico City
Bogota
Sao Paulo
Est.Active
1 - 9
10 - 99
100 - 999
1,000 - 9,999
10,000 - 99,999
100,000 - 499,999
500,000 +
Cumulative number from 227 countries / territories. Last Update: 2022-06-16 13:00:00 (UTC).

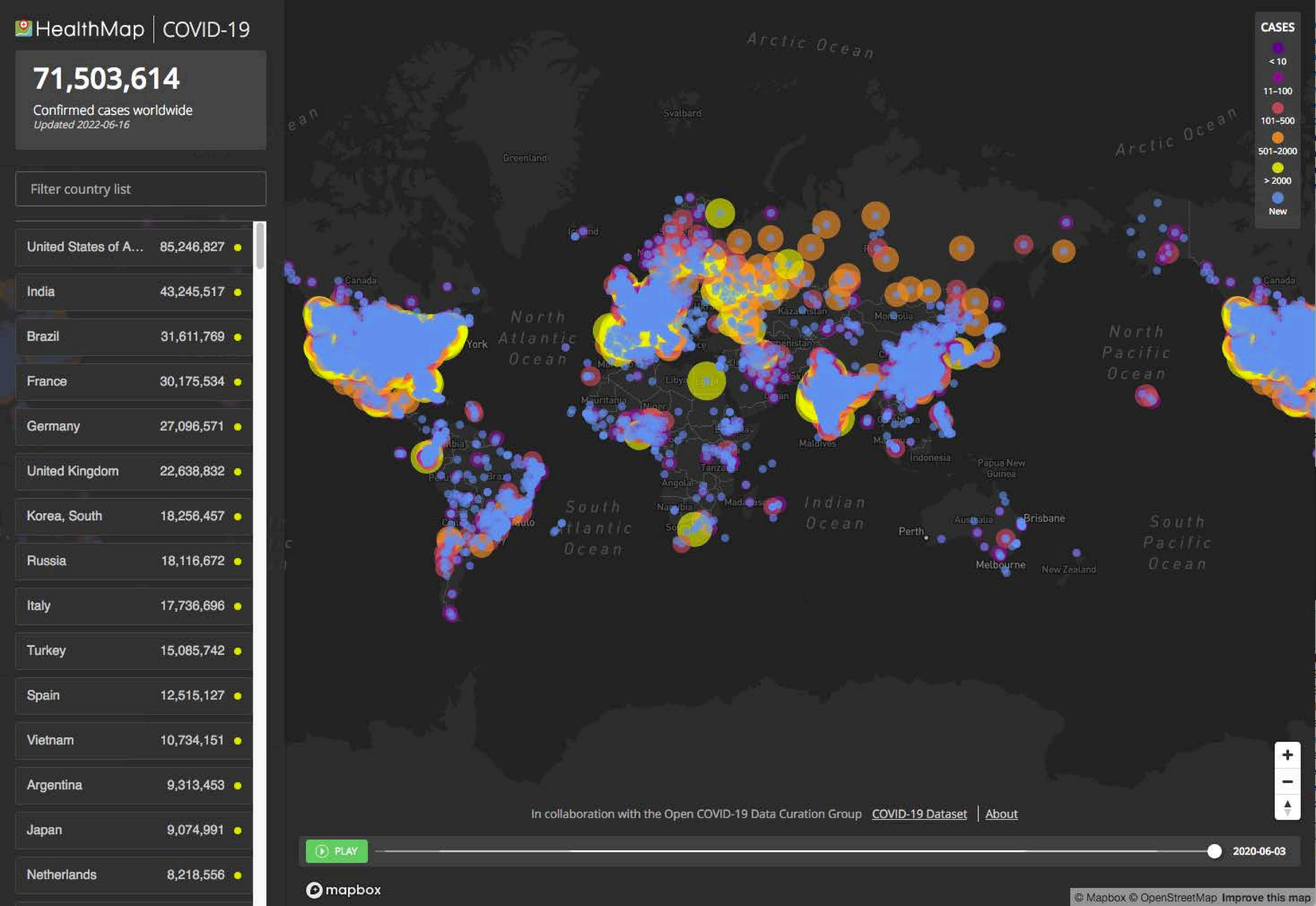
HealthMap | COVID-19
71,503,614
Confirmed cases worldwide
Updated 2022-06-16
Filter country list
United States of A... 85,246,827
India 43,245,517
Brazil 31,611,769
France 30,175,534
Germany 27,096,571
United Kingdom 22,638,832
Korea, South 18,256,457
Russia 18,116,672
Italy 17,736,696
Turkey 15,085,742
Spain 12,515,127
Vietnam 10,734,151
Argentina 9,313,453
Japan 9,074,991
Netherlands 8,218,556
CASES
< 10
11–100
101–500
501–2000
> 2000
New
Arctic Ocean
North Atlantic Ocean
South Atlantic Ocean
Indian Ocean
North Pacific Ocean
South Pacific Ocean
Svalbard
Greenland
Canada
Kazakhstan
Mongolia
Libya
Mauritania
Niger
Angola
Namibia
Maldives
Indonesia
Papua New Guinea
Australia
Perth
Brisbane
Melbourne
New Zealand
In collaboration with the Open COVID-19 Data Curation Group COVID-19 Dataset | About
PLAY
2020-06-03
mapbox
© Mapbox © OpenStreetMap Improve this map

World Health Organization

Search by Country, Territory, or Area

Covid-19 Response Fund

Donate

WHO Coronavirus (COVID-19) Dashboard

Overview | Measures | Table View | Data | More Resources

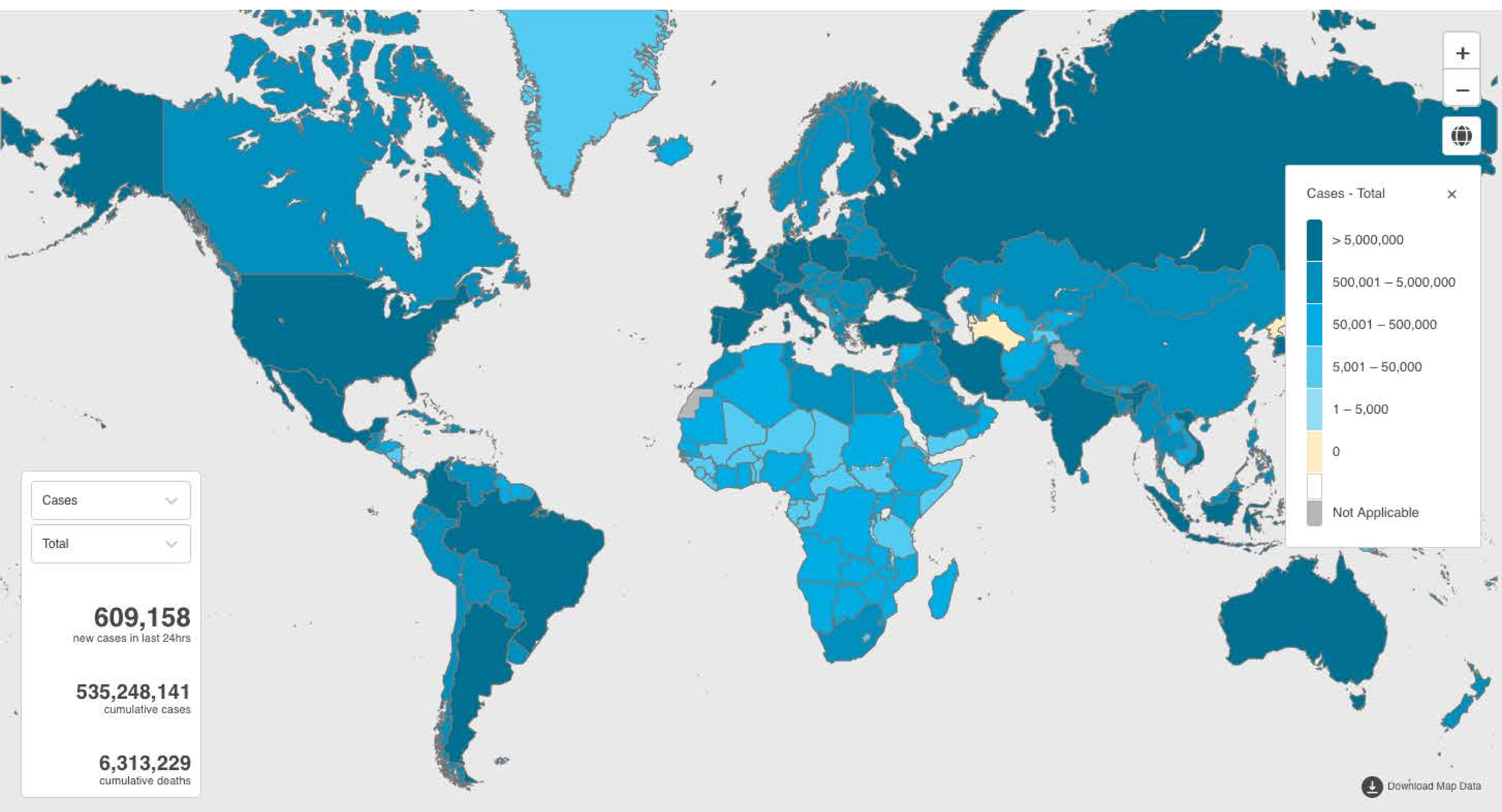

Globally, as of **5:49pm CEST, 16 June 2022**, there have been **535,248,141 confirmed cases** of COVID-19, including **6,313,229 deaths**, reported to WHO. As of **16 June 2022**, a total of **11,902,271,619 vaccine doses** have been administered.

Global Situation

Daily | Weekly

535,248,141
confirmed cases

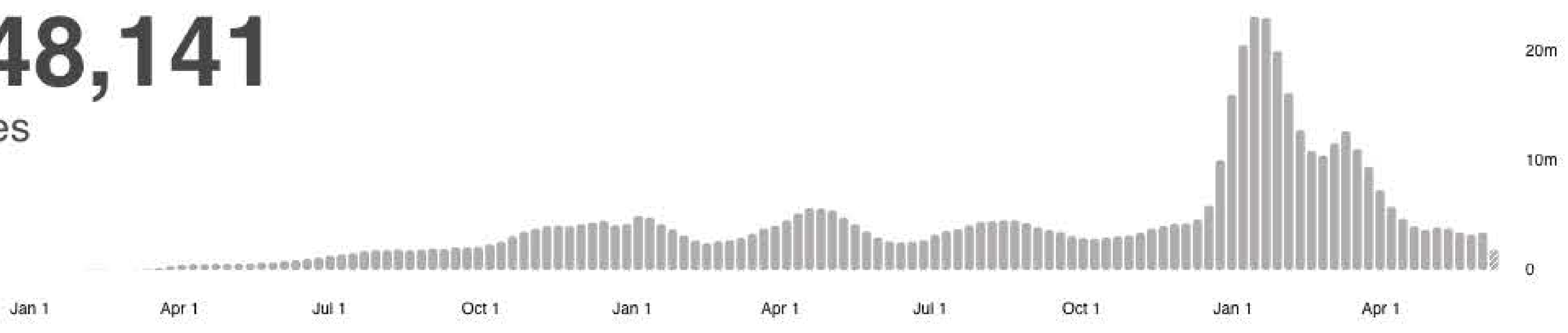

6,313,229
deaths

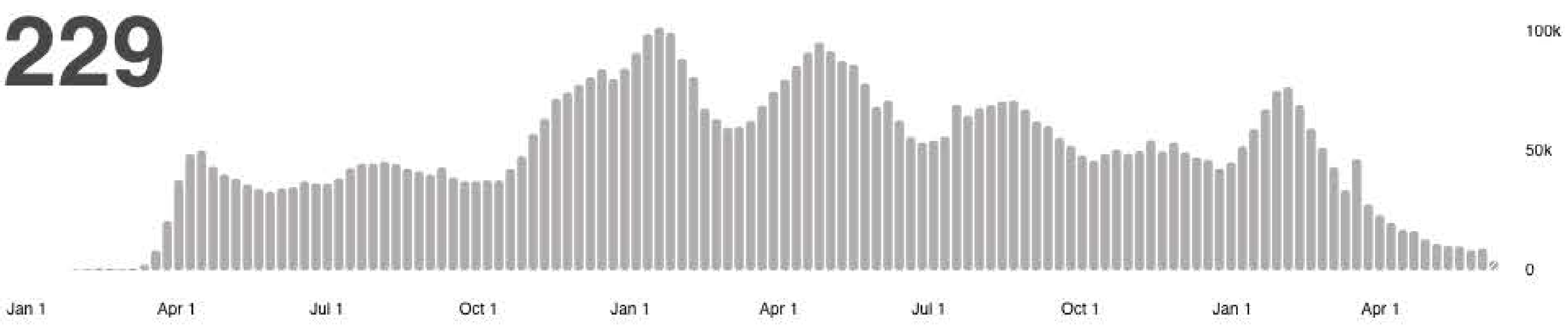

Source: World Health Organization
Data may be incomplete for the current day or week.

Situation by WHO Region

Daily | Weekly | Cases | Deaths | Count

Region	Confirmed
Europe	223,561,062 confirmed
Americas	160,007,700 confirmed
Western Pacific	62,474,187 confirmed
South-East Asia	58,289,012 confirmed
Eastern Mediterranean	21,850,741 confirmed
Africa	9,064,675 confirmed

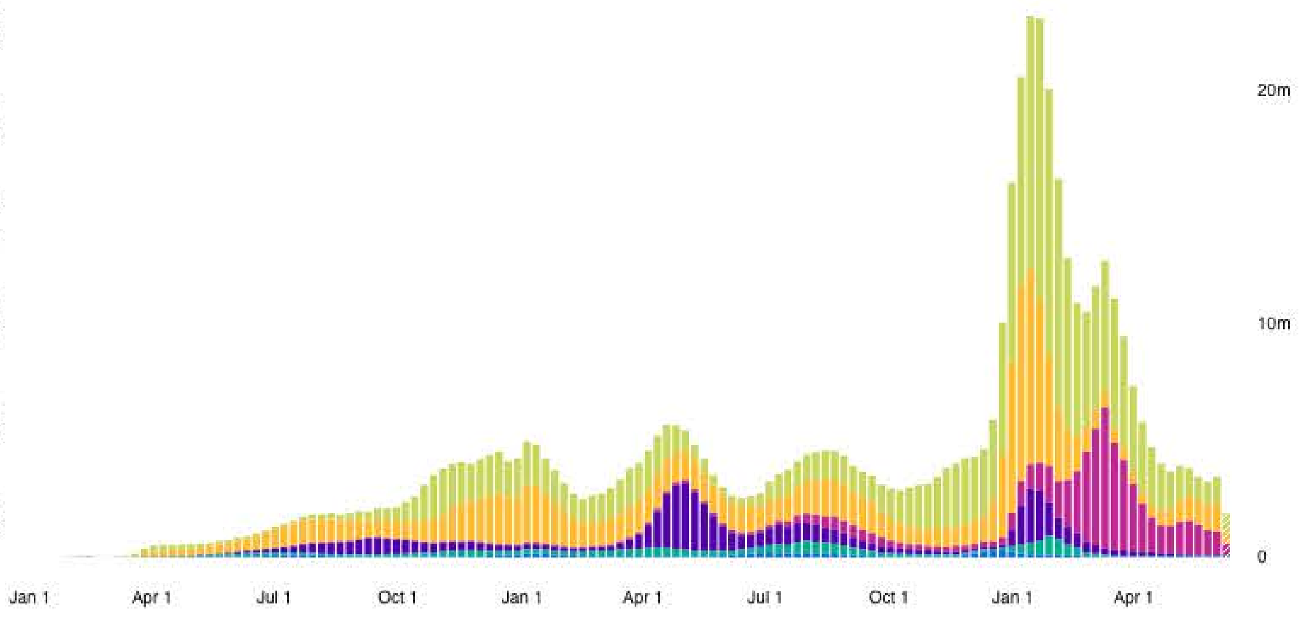

Source: World Health Organization
Data may be incomplete for the current day or week.

Europe
223,561,062
confirmed cases

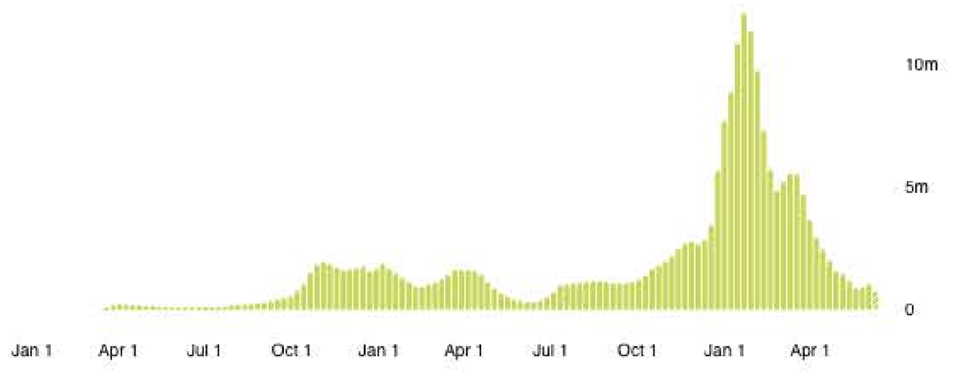

Americas
160,007,700
confirmed cases

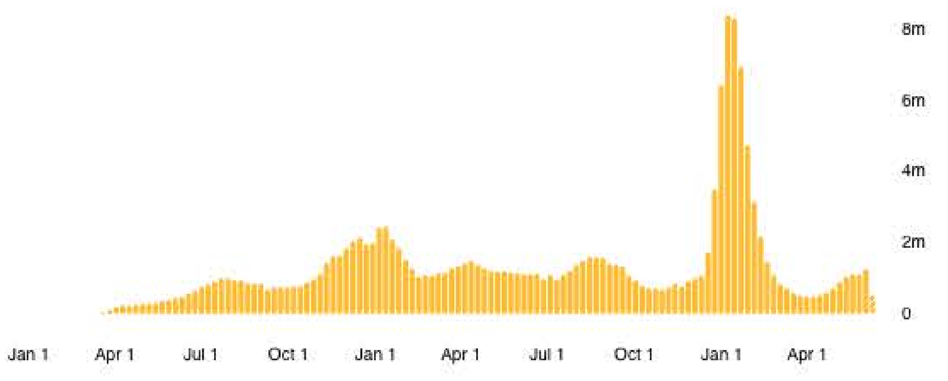

Western Pacific
62,474,187
confirmed cases

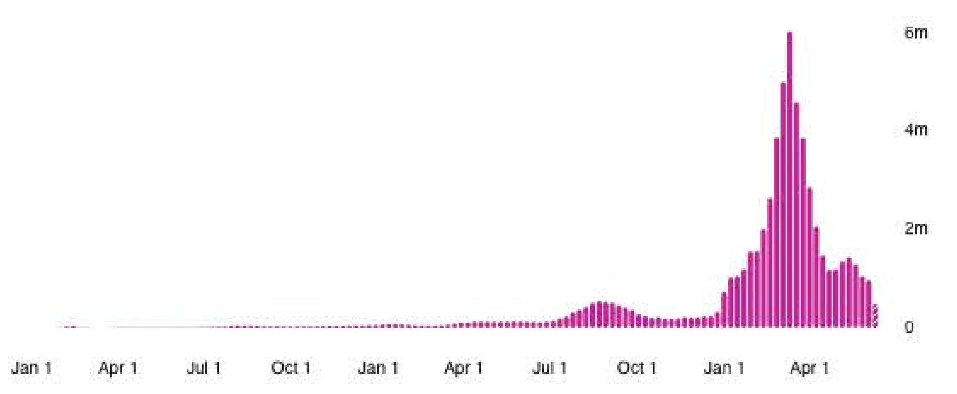

South-East Asia
58,289,012
confirmed cases

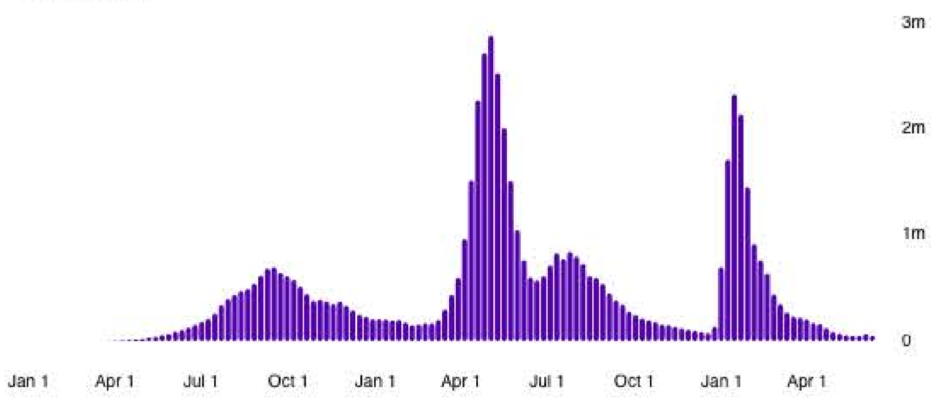

Eastern Mediterranean
21,850,741
confirmed cases

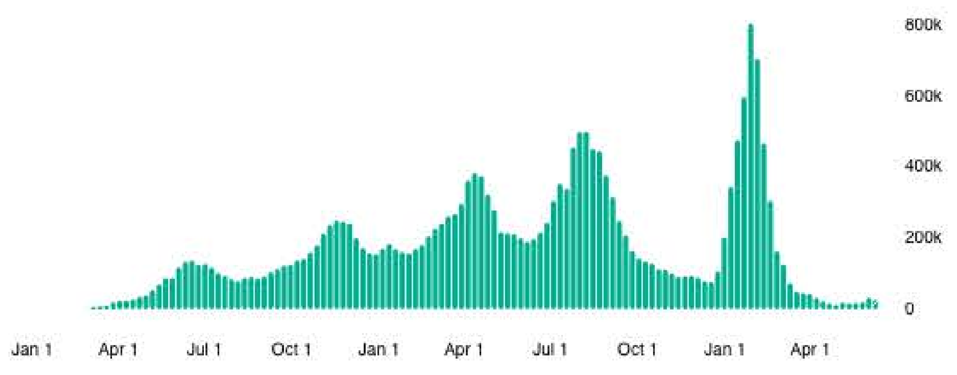

Africa
9,064,675
confirmed cases

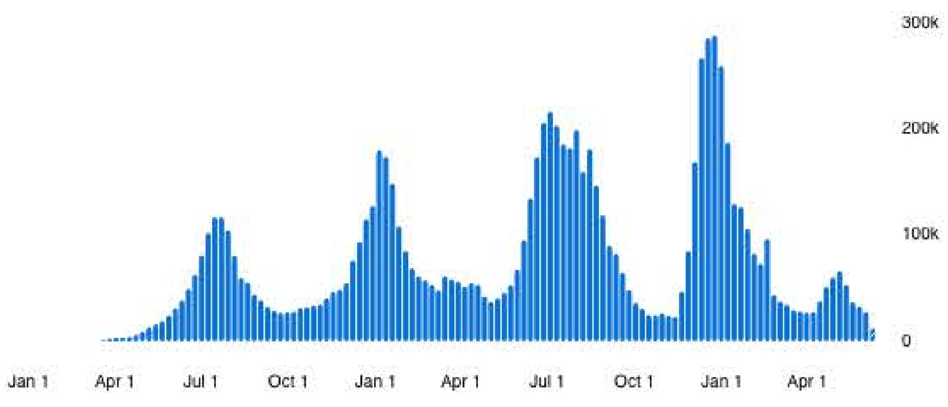

Source: World Health Organization
Data may be incomplete for the current day or week.

Situation by Country, Territory or Area

Cases | Deaths | Total

United States of America
84,868,797
confirmed cases

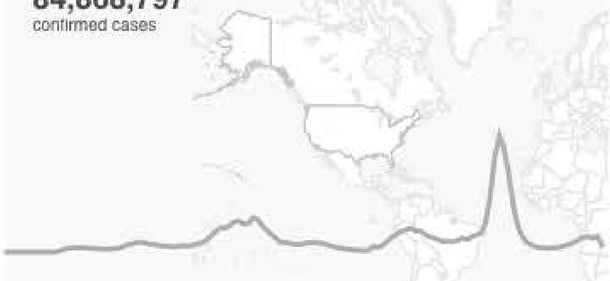

India
43,257,730
confirmed cases

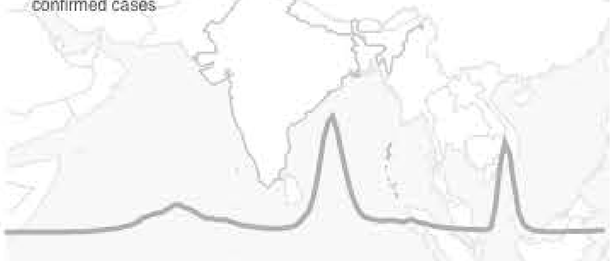

Brazil
31,541,479
confirmed cases

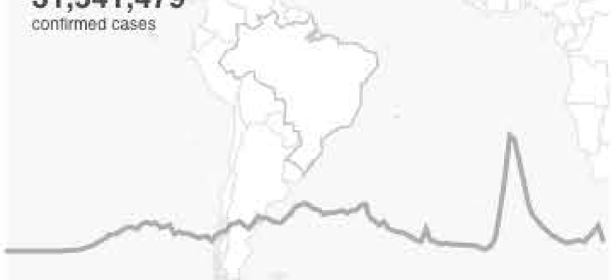

France
29,061,188
confirmed cases

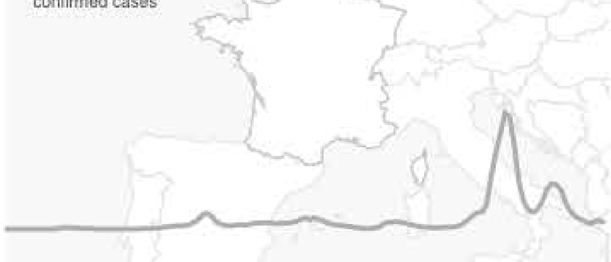

Germany
27,096,571
confirmed cases

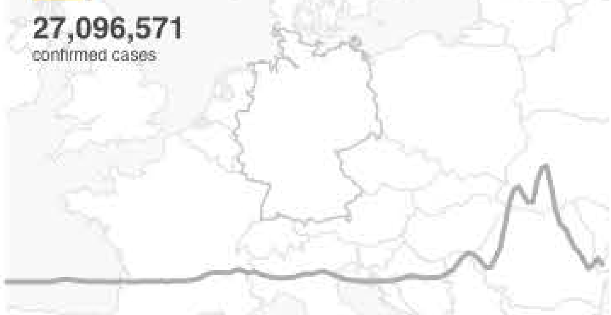

The United Kingdom
22,447,915
confirmed cases

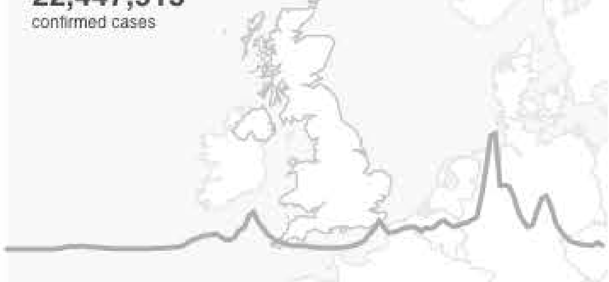

Russian Federation
18,388,424
confirmed cases

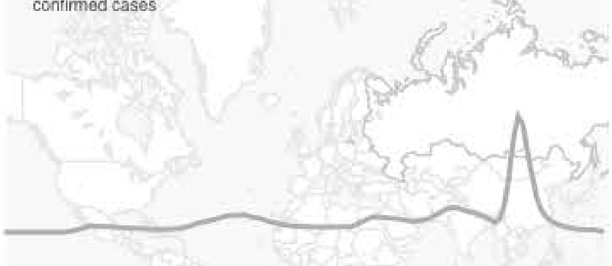

Republic of Korea
18,256,457
confirmed cases

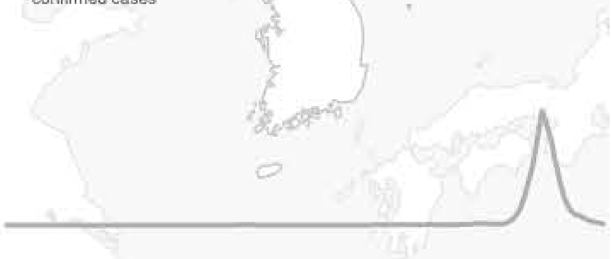

Italy
17,736,696
confirmed cases

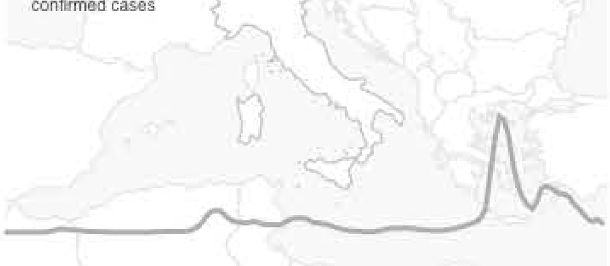

Turkey
15,085,742
confirmed cases

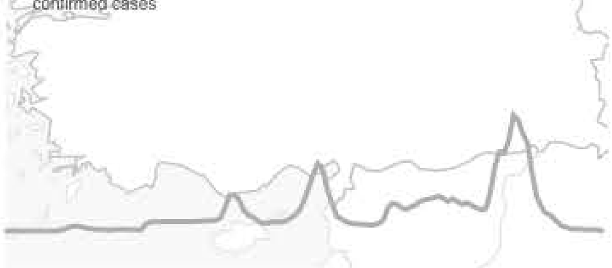

Spain
12,515,127
confirmed cases

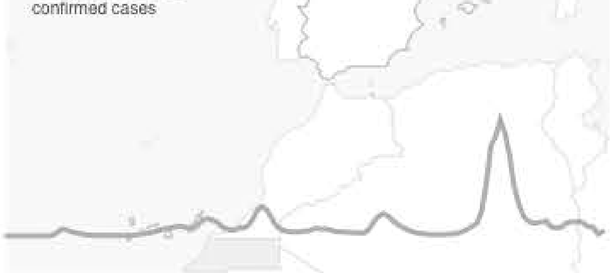

Viet Nam
10,734,151
confirmed cases

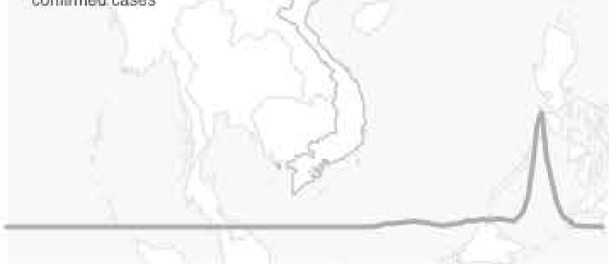

Source: World Health Organization

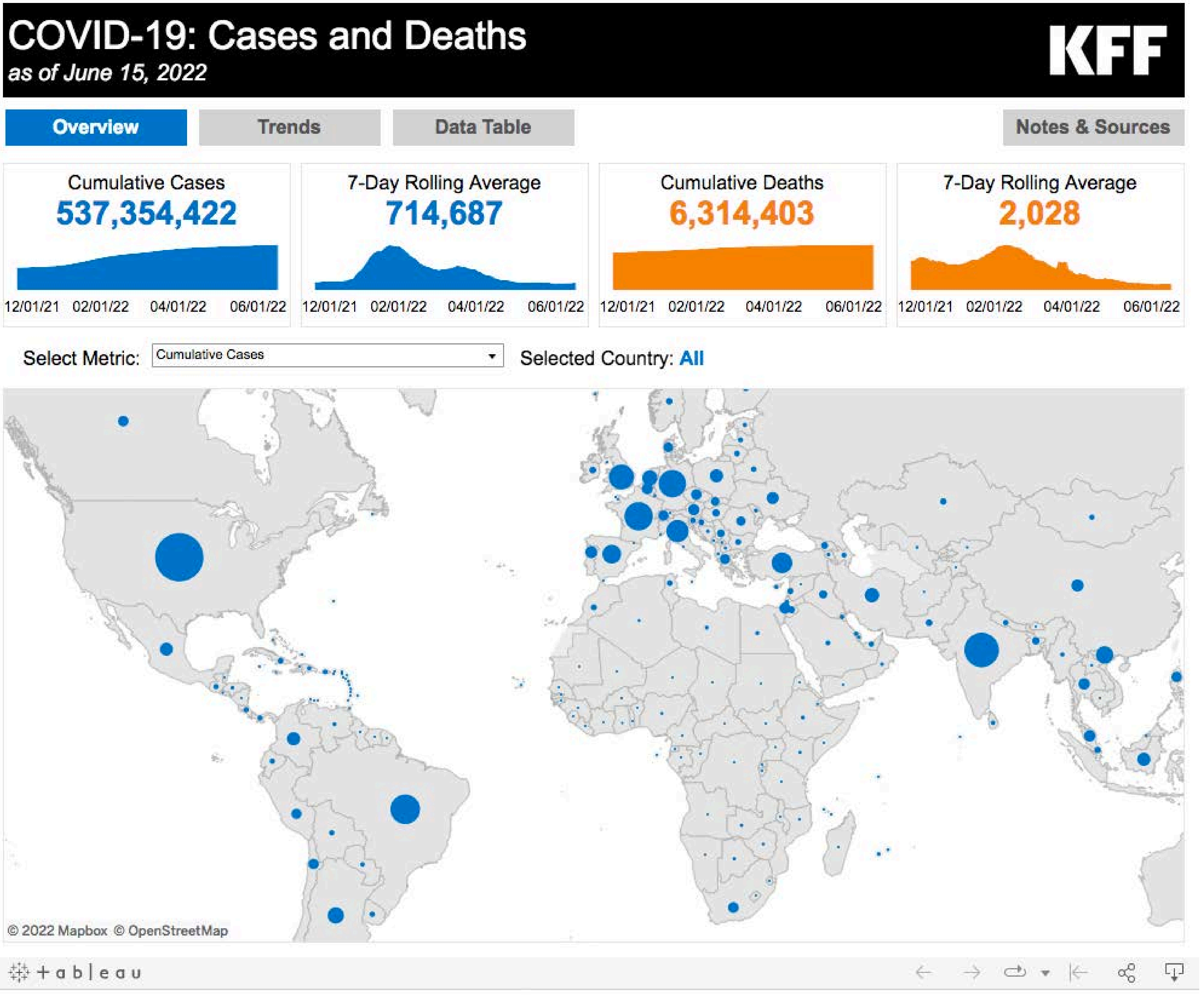
COVID-19: Cases and Deaths
as of June 15, 2022
KFF
Overview
Trends
Data Table
Notes & Sources
Cumulative Cases
537,354,422
7-Day Rolling Average
714,687
Cumulative Deaths
6,314,403
7-Day Rolling Average
2,028
12/01/21 02/01/22 04/01/22 06/01/22
Select Metric:
Cumulative Cases
Selected Country: All
© 2022 Mapbox © OpenStreetMap
+ a b | e a u

Coronavirus Pandemic (COVID-19)

Research and data: Hannah Ritchie, Edouard Mathieu, Lucas Rodés-Guirao, Cameron Appel, Charlie Giattino, Esteban Ortiz-Ospina, Joe Hasell, Bobbie Macdonald, Saloni Dattani and Max Roser

The data on the coronavirus pandemic is updated daily. Last update: **6 hours ago.** Reuse our work freely Cite this research

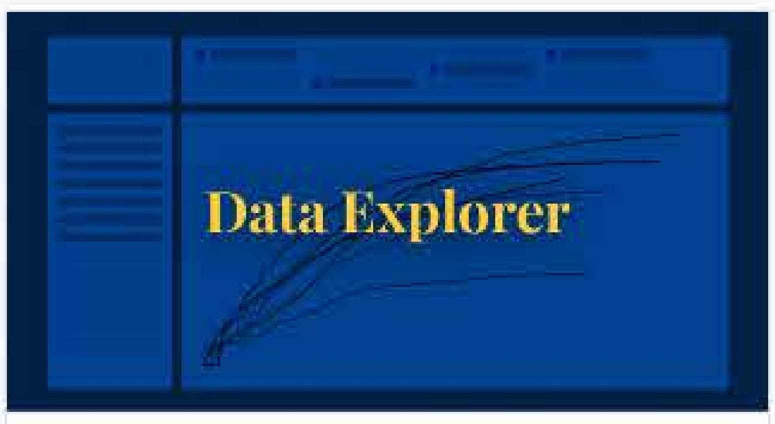

Explore all metrics – including cases, deaths, testing, and vaccinations – in one place.

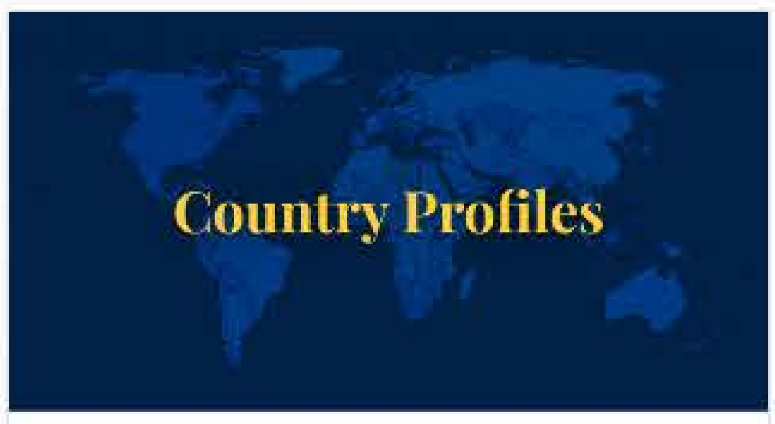

Get an overview of the pandemic for any country on a single page.

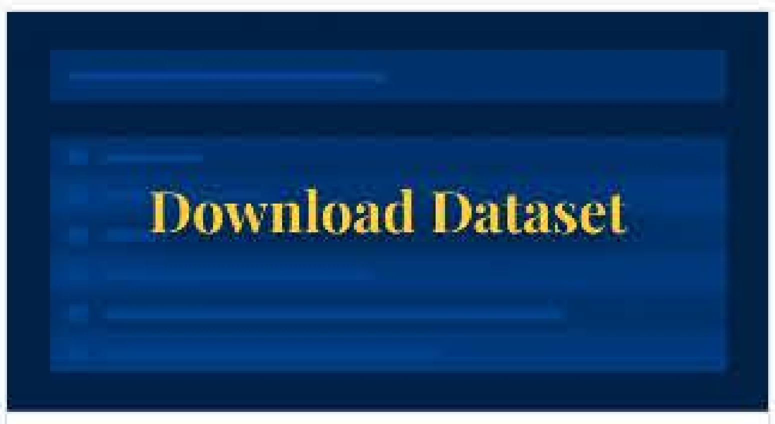

Download our complete dataset of COVID-19 metrics on GitHub. It's open access and free for anyone to use.

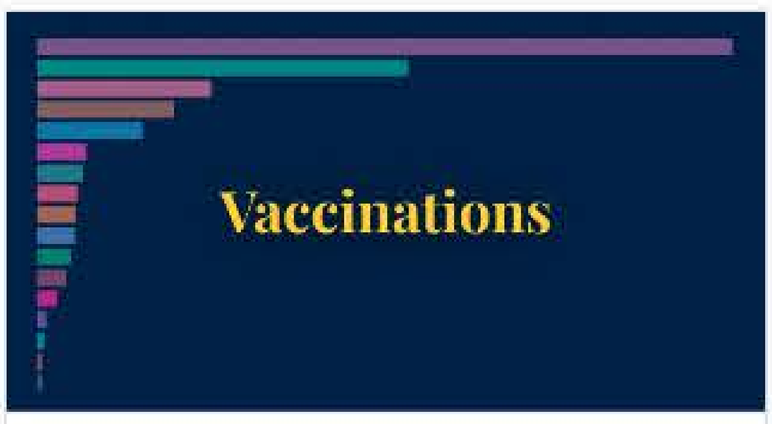

Explore our global dataset on COVID-19 vaccinations.

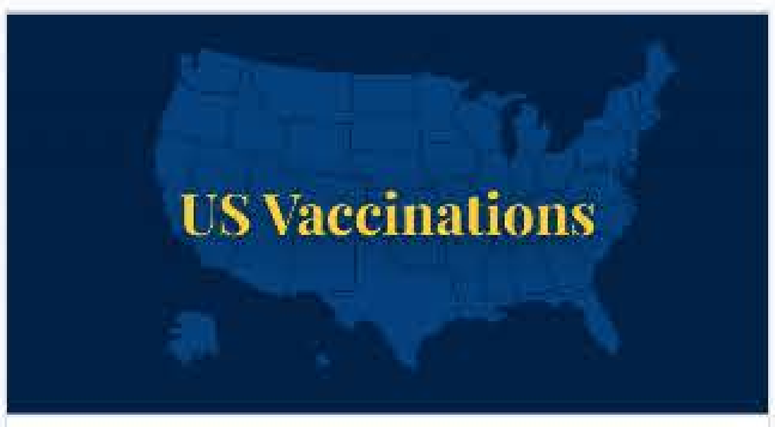

See state-by-state data on vaccinations in the United States.

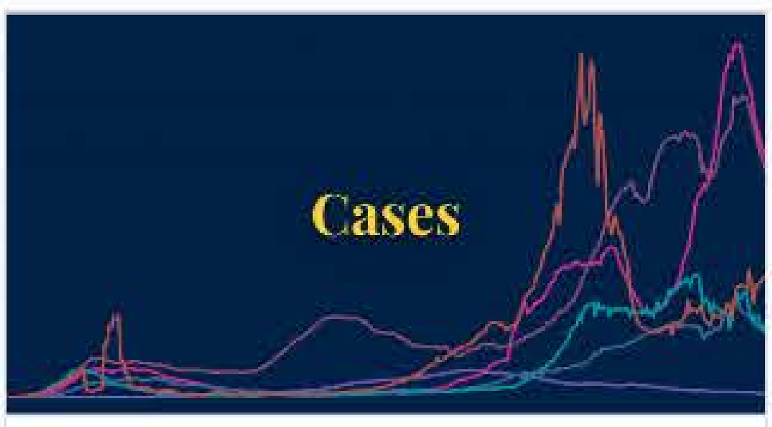

Explore the data on confirmed COVID-19 cases for all countries.

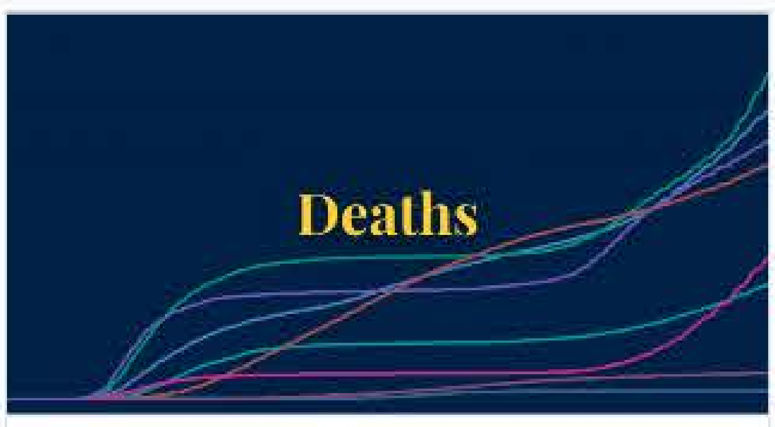

Explore the data on confirmed COVID-19 deaths for all countries.

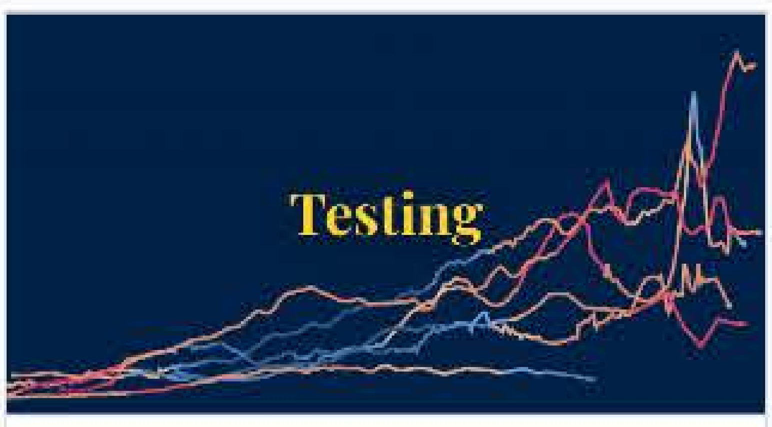

Explore our data on COVID-19 testing to see how confirmed cases compare to actual infections.

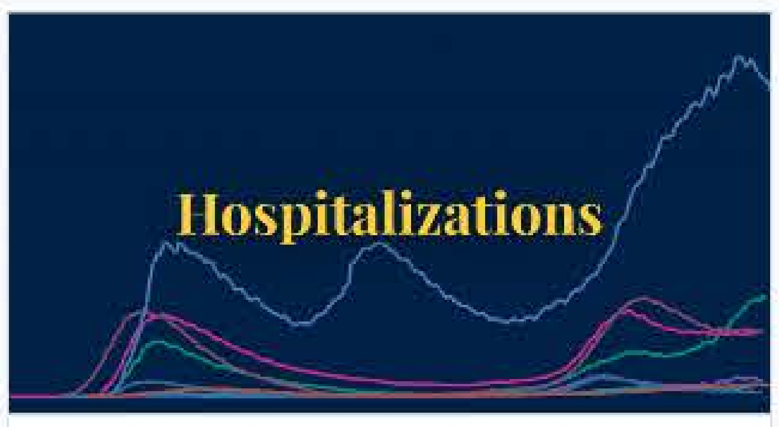

See data on how many people are being hospitalized for COVID-19.

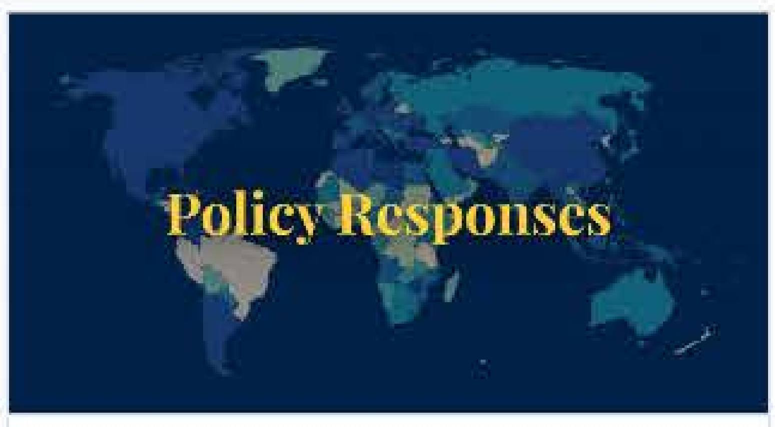

See how government policy responses – on travel, testing, vaccinations, face coverings, and more – vary across the world.

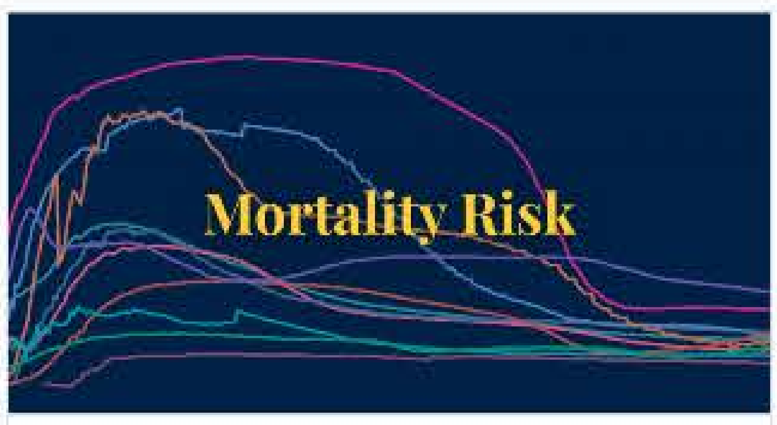

Learn what we know about the mortality risk of COVID-19 and explore the data used to calculate it.

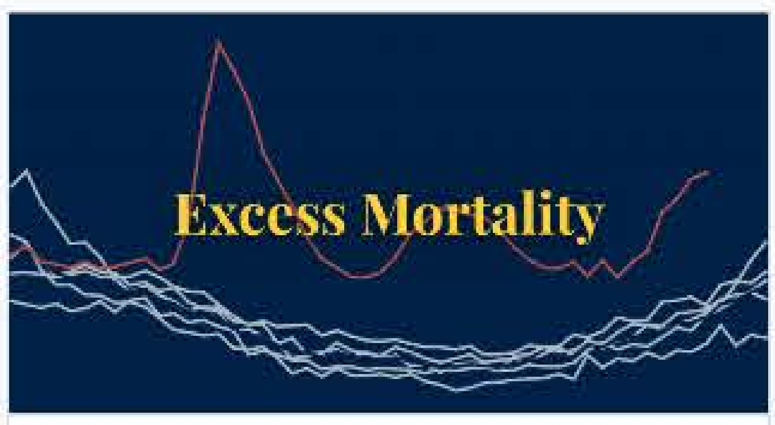

Compare the number of deaths from all causes during COVID-19 to the years before to gauge the total impact of the pandemic on deaths.

Explore the global situation

COVID-19 Data Explorer

Download the complete *Our World in Data* COVID-19 dataset.

METRIC: Confirmed cases
INTERVAL: 7-day rolling average
☑ Relative to Population
☐ Color by test positivity

Type to add a country...

Sort by Select...

Portugal	1.97k
Switzerland	1.91k
Iceland	1.71k
Finland	1.69k
Botswana	1.59k
Ireland	1.56k
British Virgin Islands	1.28k
Australia	1.25k
Brunei	1.18k
New Zealand	1.15k
Curacao	1.13k
Bonaire Sint Eustatius and Saba	1.13k
Luxembourg	984.54
Monaco	910.93
Greece	895.6
Oceania	889.15
Argentina	807.68
Bahrain	784.76

✕ Clear selection

Daily new confirmed COVID-19 cases per million people

7-day rolling average. Due to limited testing, the number of confirmed cases is lower than the true number of infections.

LINEAR LOG

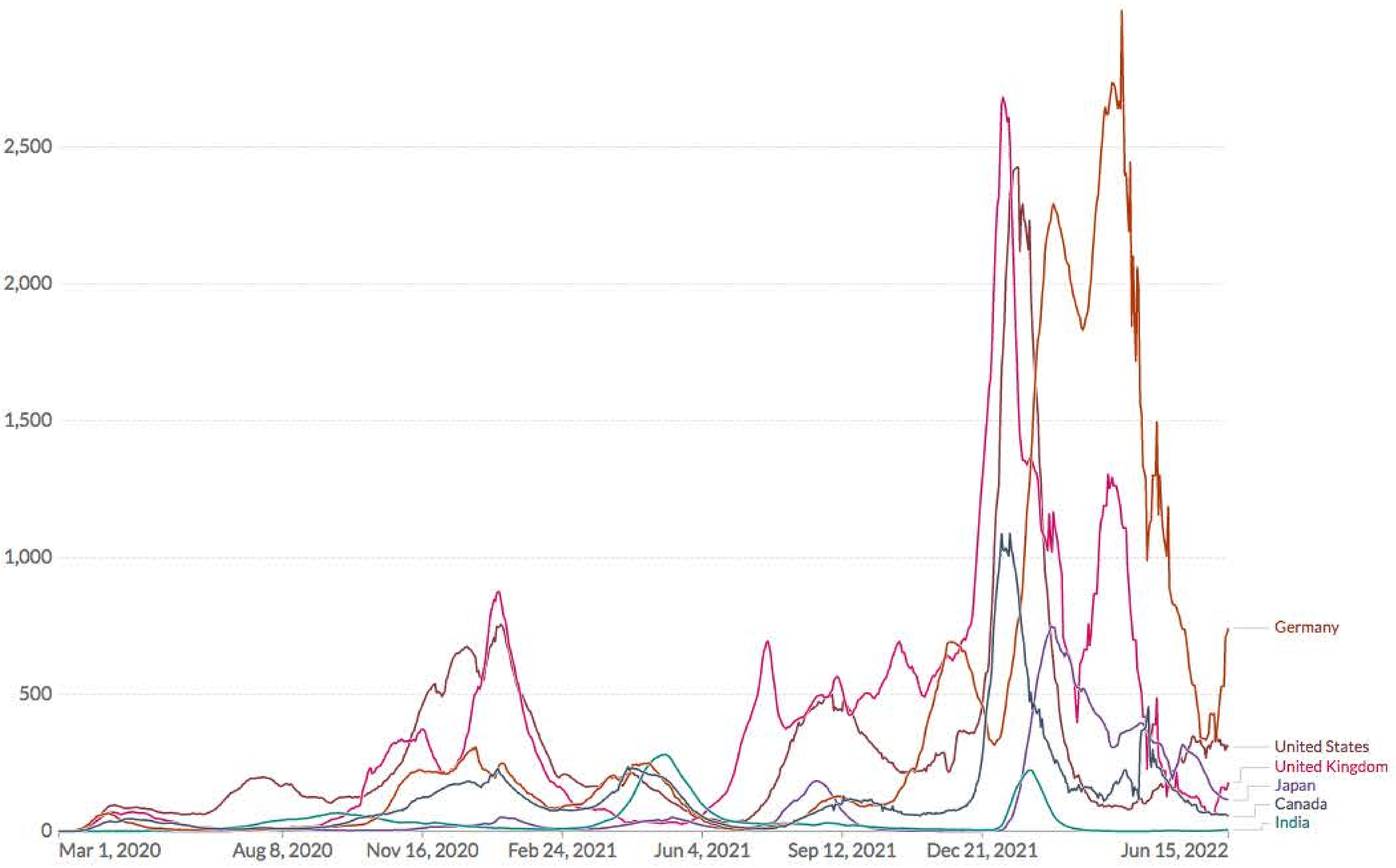

Source: Johns Hopkins University CSSE COVID-19 Data

CC BY

Jan 28, 2020 — Jun 15, 2022

CHART MAP TABLE SOURCES DOWNLOAD

→ Open the Data Explorer in a new tab.

COVID-19 Data Explorer

Download the complete *Our World in Data* COVID-19 dataset.

METRIC: Case fatality rate

INTERVAL: 7-day rolling average

☐ Relative to Population ☐ Color by test positivity

Type to add a country...

Sort by Country name

- ☑ Canada
- ☑ Germany
- ☑ India
- ☑ Italy
- ☑ United Kingdom
- ☑ United States
- ☐ Afghanistan
- ☐ Africa
- ☐ Albania
- ☐ Algeria
- ☐ Andorra
- ☐ Angola
- ☐ Anguilla
- ☐ Antigua and Barbuda
- ☐ Argentina
- ☐ Armenia
- ☐ Aruba

× Clear selection

Moving-average case fatality rate of COVID-19

The case fatality rate (CFR) is the ratio between confirmed deaths and confirmed cases. Our rolling-average CFR is calculated as the ratio between the 7-day average number of deaths and the 7-day average number of cases 10 days earlier.

Our World in Data

LINEAR LOG

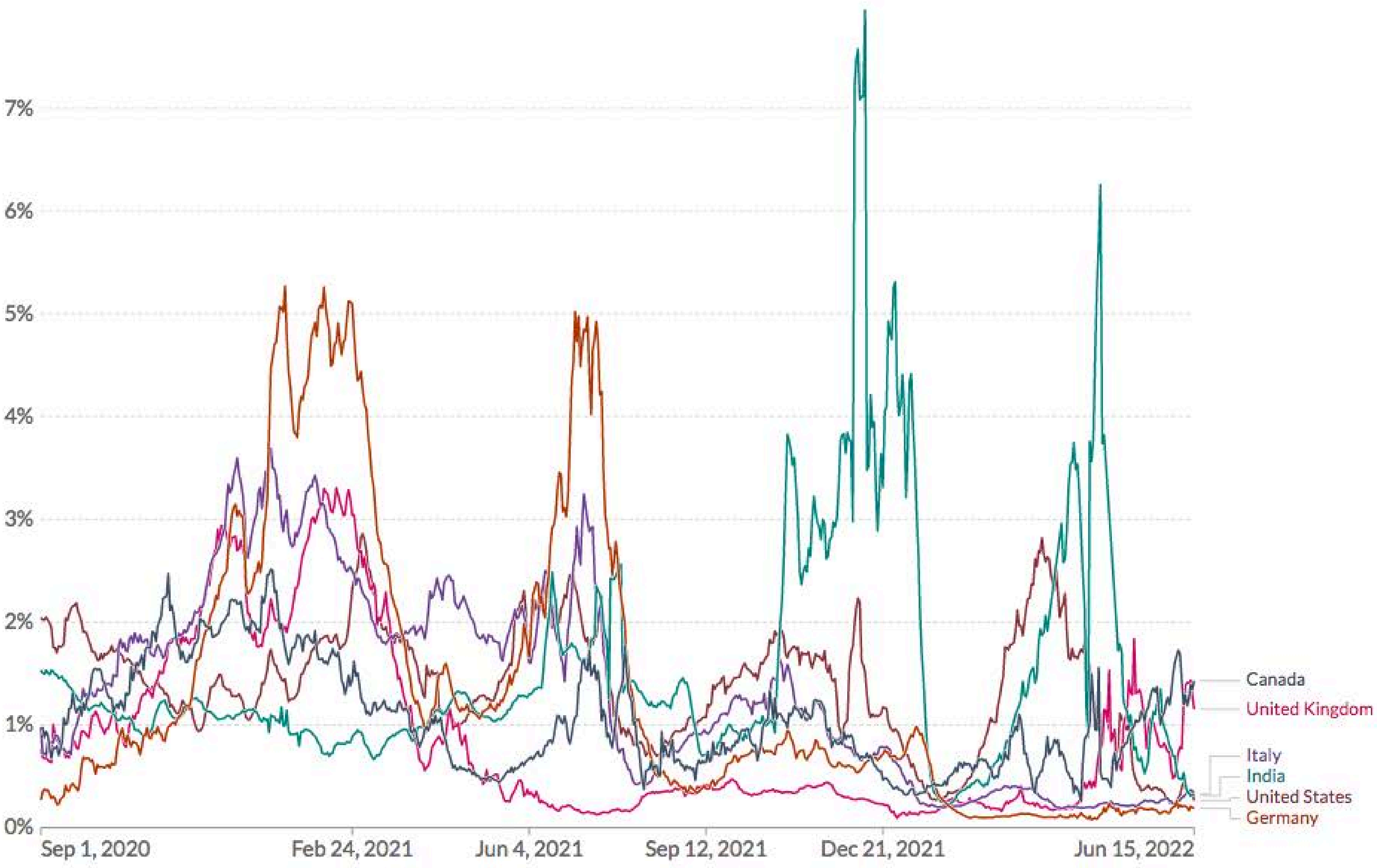

Source: Johns Hopkins University CSSE COVID-19 Data

CC BY

Sep 1, 2020 — Jun 15, 2022

CHART MAP TABLE SOURCES DOWNLOAD

Our World in Data

Articles by topic | Search... | Latest | About | Donate

All charts | Sustainable Development Goals Tracker

OXFORD MARTIN SCHOOL | UNIVERSITY OF OXFORD | GCDL

Coronavirus › By country | Data explorer | Deaths | Cases | Tests | Hospitalizations | Vaccinations | Mortality risk | Excess mortality | Policy responses

COVID-19 Data Explorer

Download the complete *Our World in Data* COVID-19 dataset.

METRIC: Cases, tests, positive and r...

INTERVAL: 7-day rolling average

Relative to Population

Color by test positivity

Type to add a country...

Sort by: Country name

- [x] Canada
- [x] Germany
- [x] India
- [x] Italy
- [x] United Kingdom
- [x] United States
- [ ] Afghanistan
- [ ] Africa
- [ ] Albania
- [ ] Algeria
- [ ] Andorra
- [ ] Angola
- [ ] Anguilla
- [ ] Antigua and Barbuda
- [ ] Argentina
- [ ] Armenia
- [ ] Aruba

× Clear selection

COVID-19 cases, tests, positive rate, and reproduction rate

Our World in Data

7-day rolling average. Due to limited testing, the number of confirmed cases is lower than the true number of infections. Comparisons across countries are affected by differences in testing policies and reporting methods.

LINEAR | LOG | Align axis scales

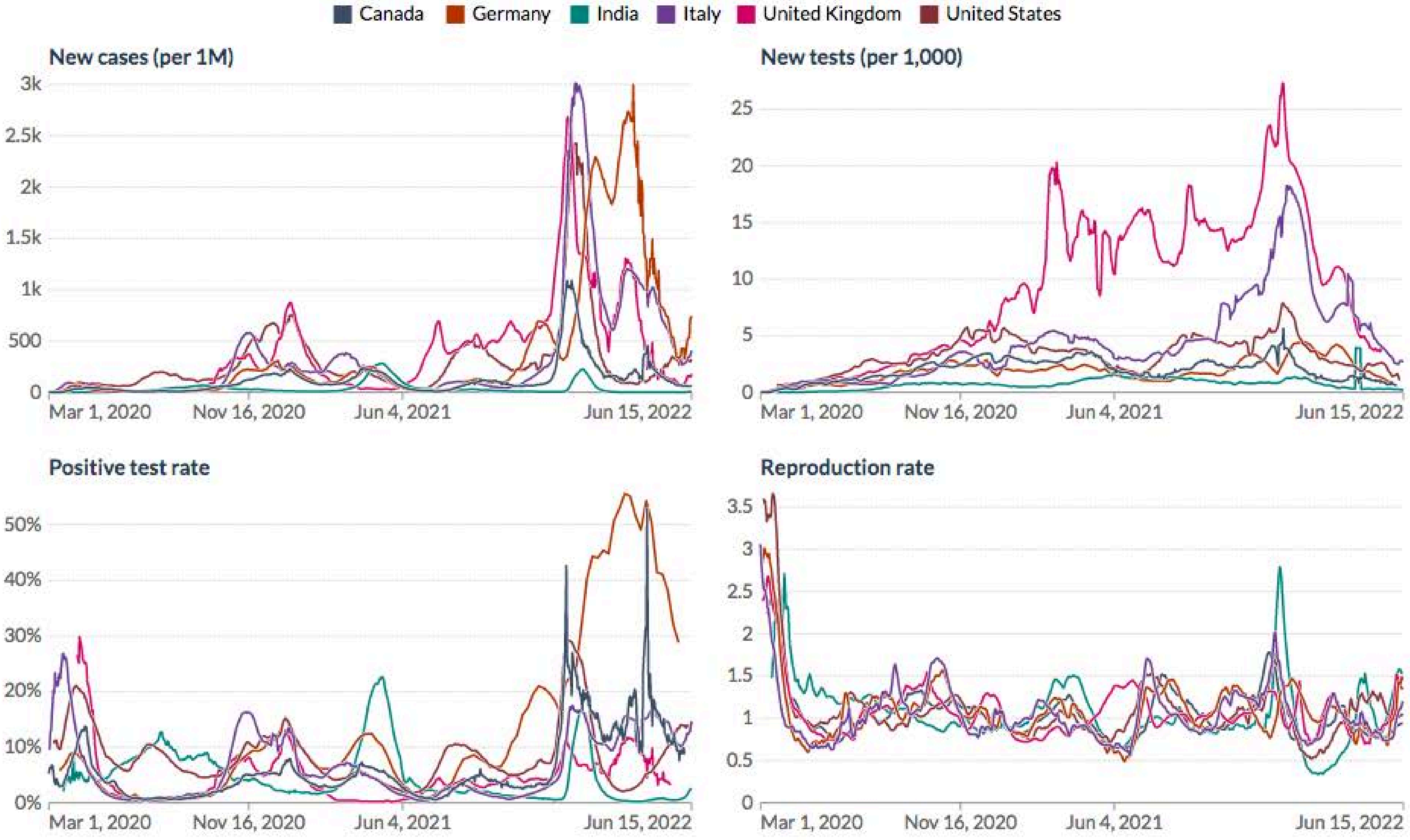

Source: Johns Hopkins University CSSE COVID-19 Data, Official data collated by Our World in Data, Arroyo-Marioli F, Bullano F, Kucinskas S, Rondón-Moreno C (2021) Tracking R of COVID-19: A new real-time estimation using the Kalman filter.

CC BY

Jan 28, 2020 — Jun 15, 2022

CHART | TABLE | SOURCES | DOWNLOAD

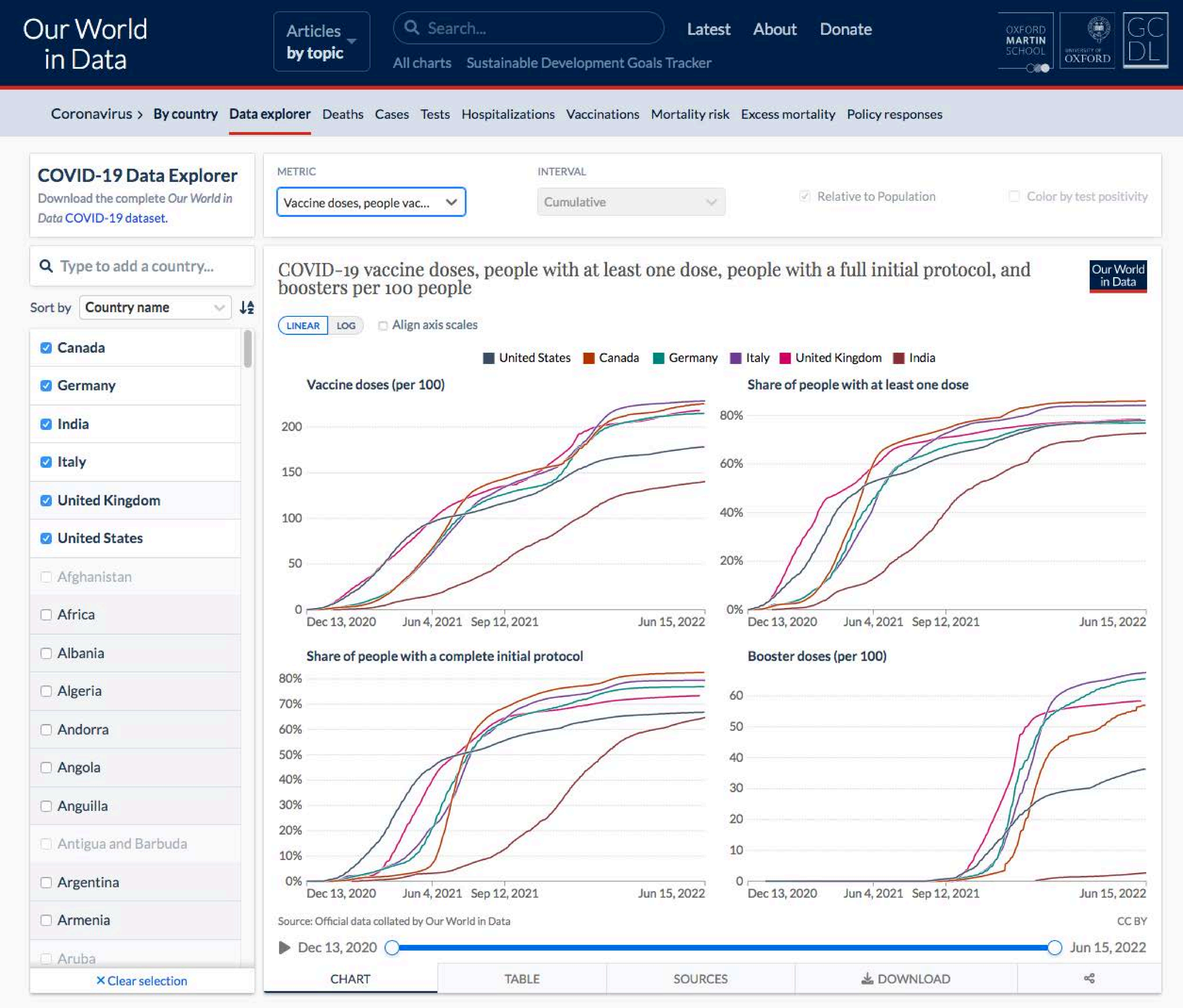
Our World in Data
Articles by topic
Search...
Latest
About
Donate
All charts
Sustainable Development Goals Tracker
OXFORD MARTIN SCHOOL
UNIVERSITY OF OXFORD
GCDL
Coronavirus
By country
Data explorer
Deaths
Cases
Tests
Hospitalizations
Vaccinations
Mortality risk
Excess mortality
Policy responses
COVID-19 Data Explorer
Download the complete Our World in Data COVID-19 dataset.
METRIC
Vaccine doses, people vac...
INTERVAL
Cumulative
Relative to Population
Color by test positivity
Type to add a country...
Sort by
Country name
Canada
Germany
India
Italy
United Kingdom
United States
Afghanistan
Africa
Albania
Algeria
Andorra
Angola
Anguilla
Antigua and Barbuda
Argentina
Armenia
Aruba
Clear selection
COVID-19 vaccine doses, people with at least one dose, people with a full initial protocol, and boosters per 100 people
Our World in Data
LINEAR
LOG
Align axis scales
United States
Canada
Germany
Italy
United Kingdom
India
Vaccine doses (per 100)
Share of people with at least one dose
Share of people with a complete initial protocol
Booster doses (per 100)
Dec 13, 2020
Jun 4, 2021
Sep 12, 2021
Jun 15, 2022
Source: Official data collated by Our World in Data
CC BY
Dec 13, 2020
Jun 15, 2022
CHART
TABLE
SOURCES
DOWNLOAD

中文版

DXY · DX Doctor

COVID-19
Global Pandemic Real-time Report

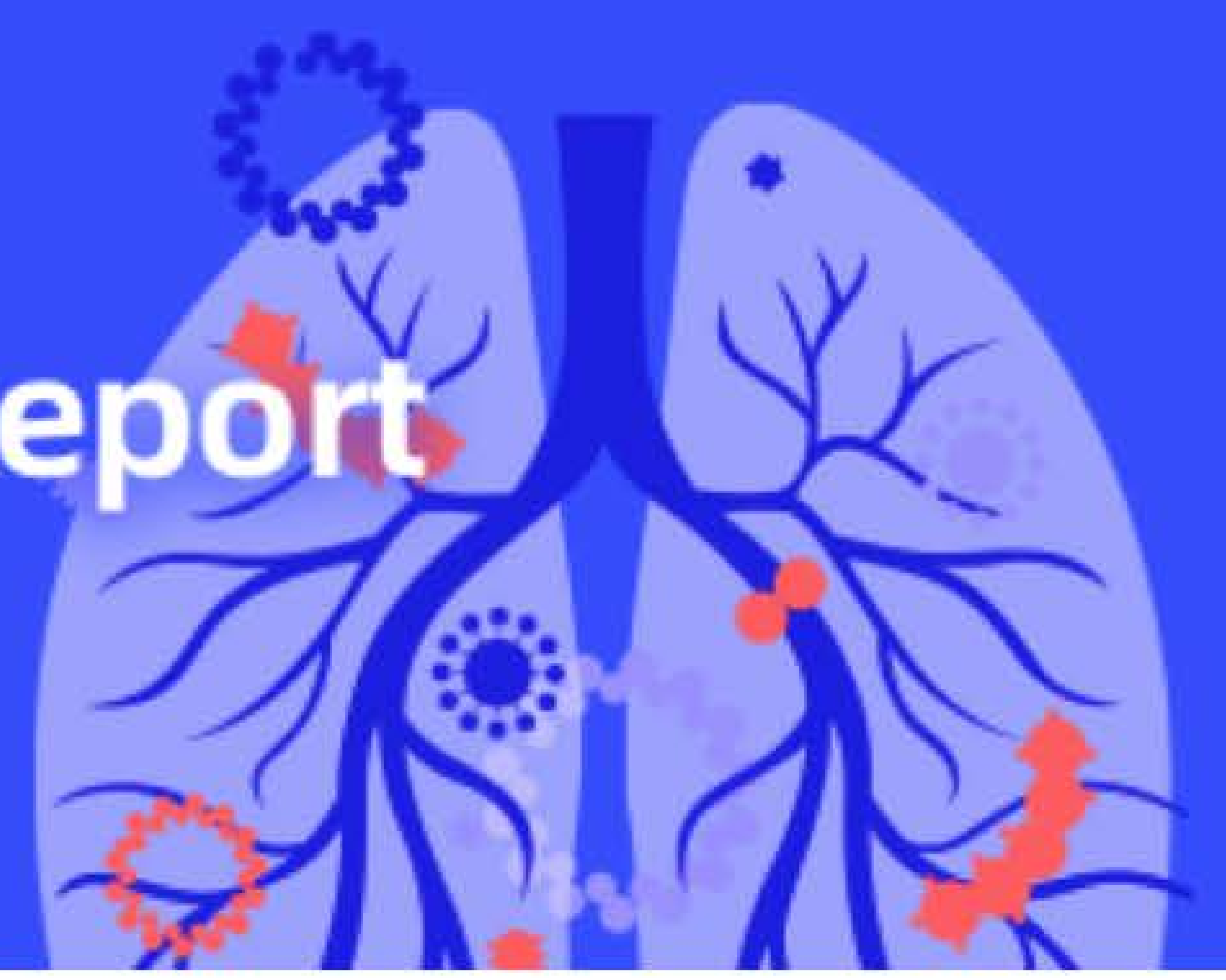

4,703,169,208 Views

Global statistics by UTC+8 2022-06-16 17:18

Data explanations

Active Cases	Total Confirmed	Deaths	Recovered
191,524,690	537,375,315	6,328,566	339,522,059

COVID-19 Global Pandemic Trend

Before Mar. 19, data source: WHO. Time zone: CET 10 AM.
After Mar. 19, data source: WHO, JHU CSSE, Local Media and DXY. Time zone: UTC 10 AM.
Since April 24th, the Spanish Ministry of Health has adjusted the statistical standard of confirmed Covid-19 data, only counting those confirmed by PCR tests.

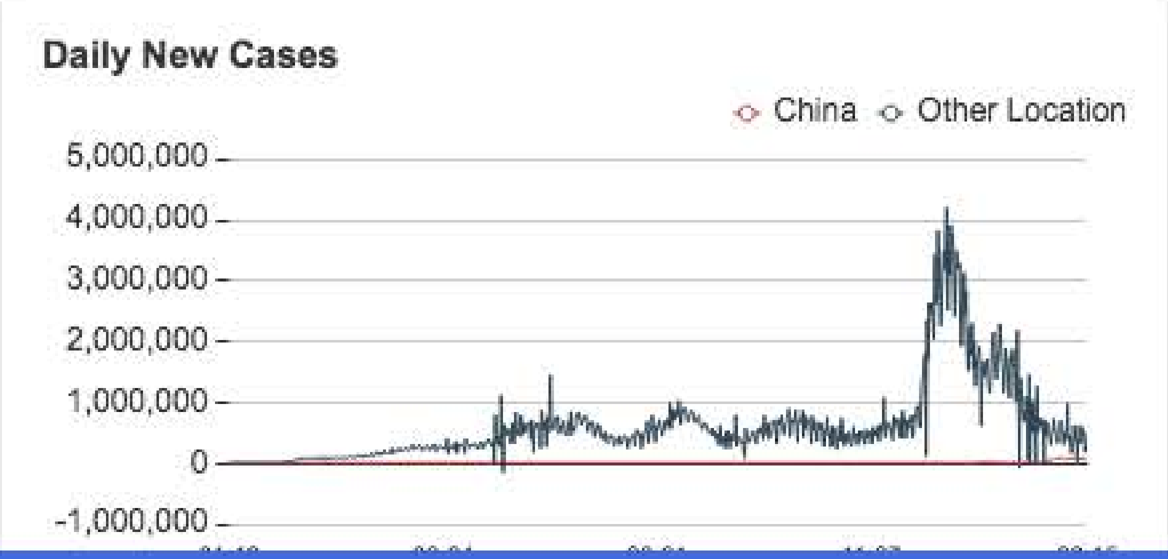

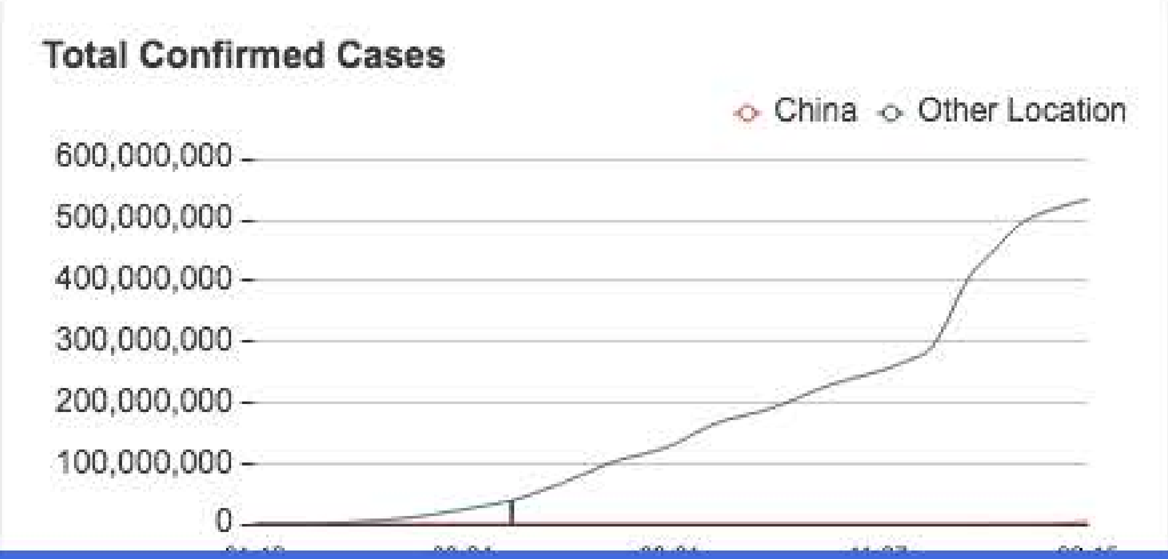

Global Mapping | COVID-19 Knowledge

Daily New Cases | Total Confirmed Cases | Total Deaths Cases

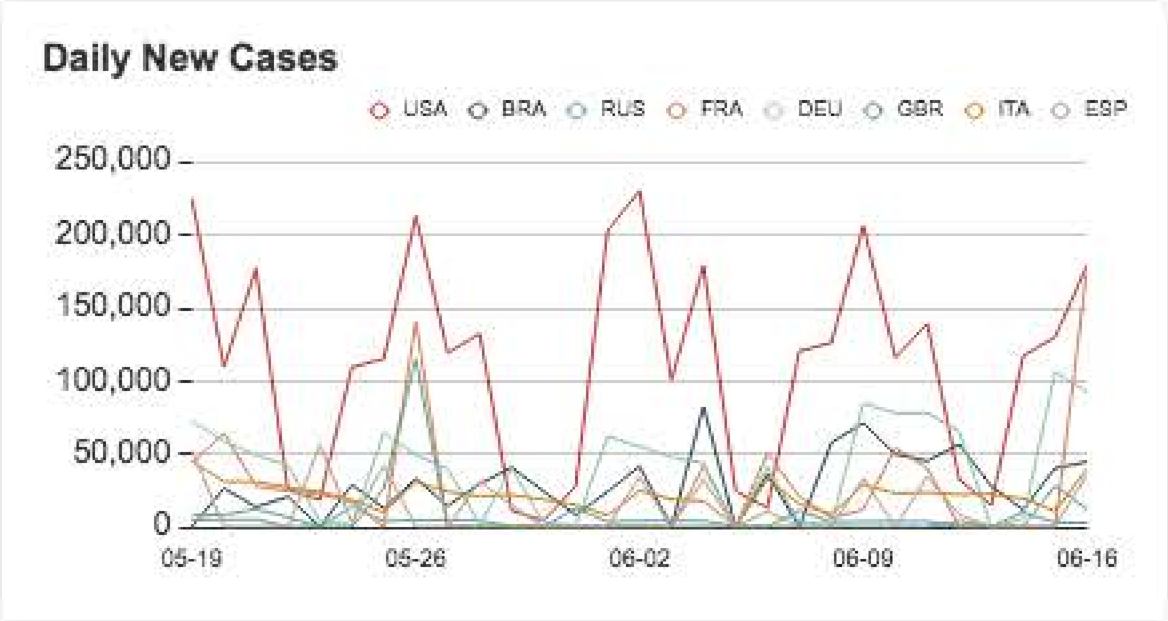

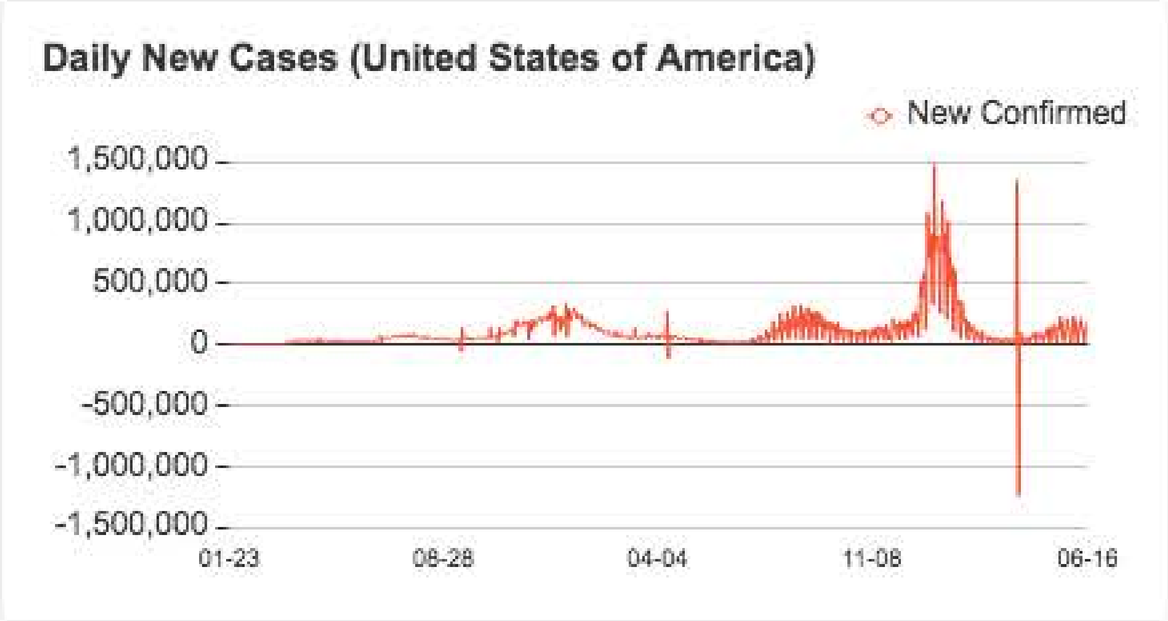

Daily New Cases | United States of America | Brazil | Russian Federation
France | Germany | The United Kingdom | Italy
Spain

Country/Region/ Territory	Active Cases	Total Confirmed	Total Deaths	Total Recovered	More
▼ Europe(49)	140,327,009	200,800,993	1,858,758	58,615,226	
France	29,467,168	29,984,235	149,044	-	More›
Germany	22,539,003	27,007,429	140,026	4,328,400	More›
The United Kingdom	15,829,009	22,499,617	179,539	-	More›
Spain	12,257,512	12,515,127	107,239	150,376	More›
Italy	11,221,938	17,703,887	167,505	6,314,444	More›

Show more ⌄

Country/Region/ Territory	Active Cases	Total Confirmed	Total Deaths	Total Recovered	More
▼ Asia(46)	58,396,512	154,020,050	1,440,055	94,183,483	
Republic of Korea	17,887,532	18,248,479	24,399	336,548	More›
[illegible]	[illegible]	[illegible]	[illegible]	[illegible]	[illegible]

COVID-19 CORONAVIRUS PANDEMIC

Last updated: June 16, 2022, 23:26 GMT

Weekly Trends - Graphs - Countries - News

Coronavirus Cases:

542,975,158

view by country

Deaths:

6,337,630

Recovered:

518,091,297

ACTIVE CASES

CLOSED CASES

daily linear logarithmic

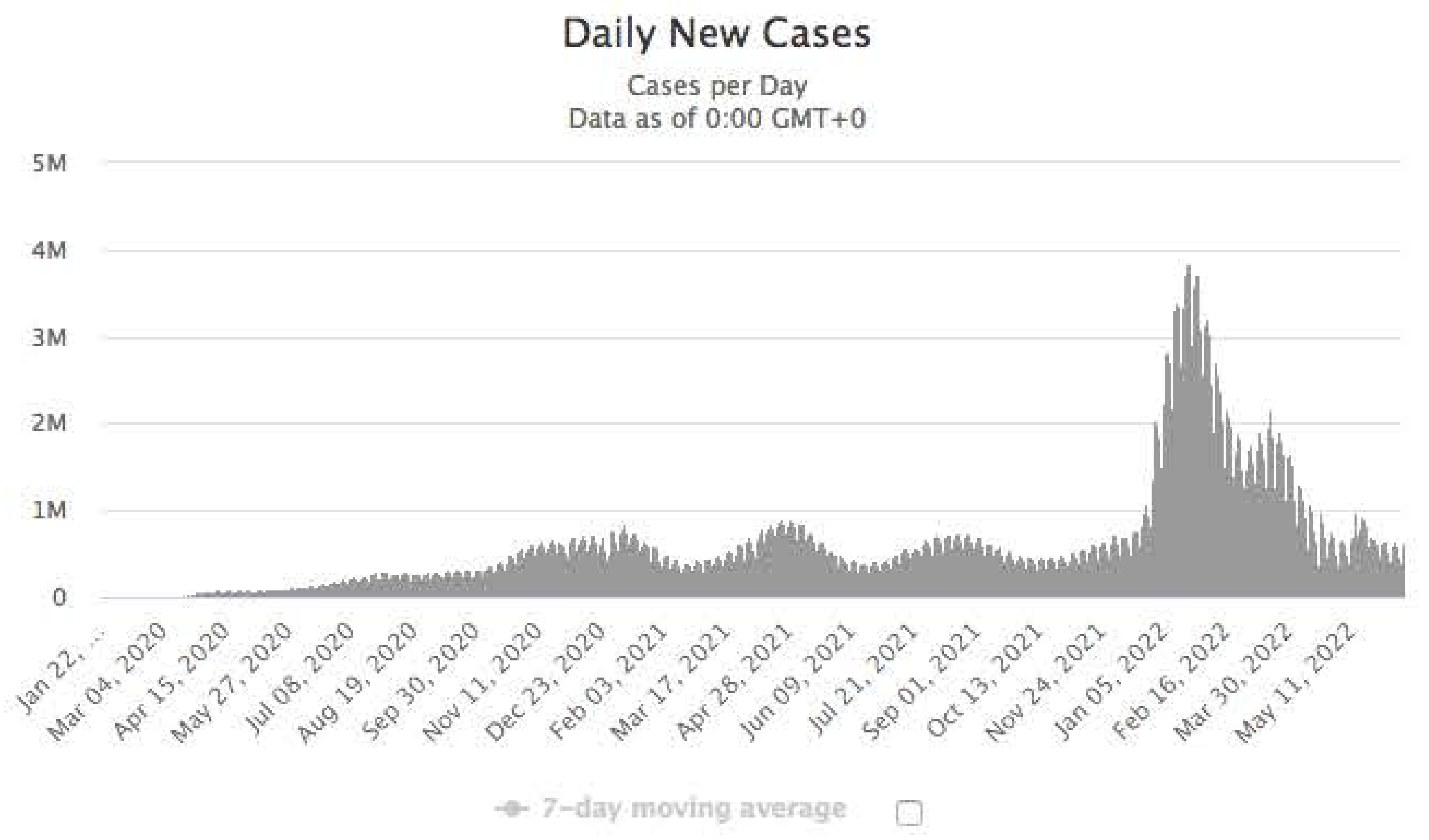

daily linear logarithmic

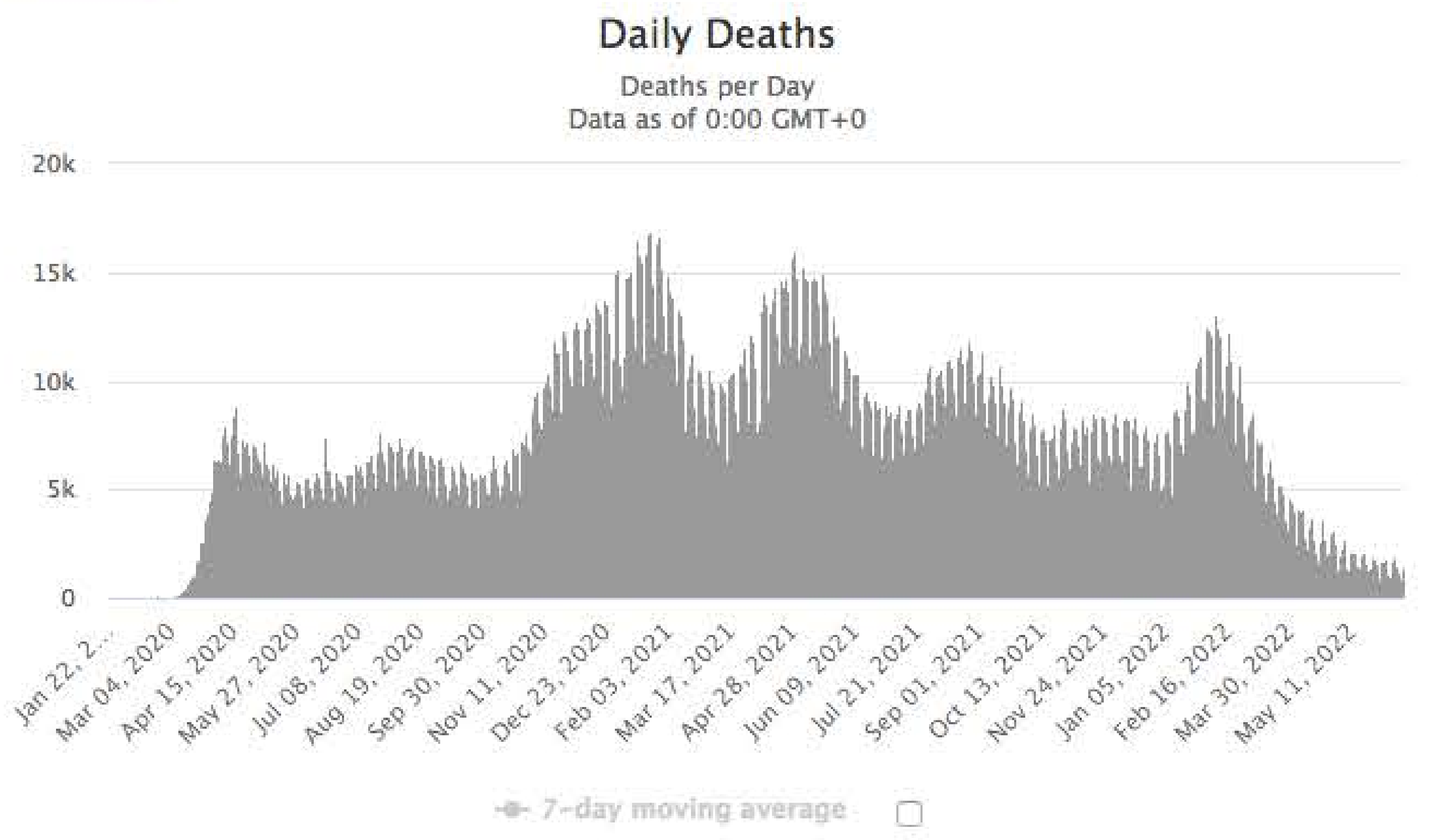

The charts above are updated after the close of the day in GMT+0. See more graphs

Reported Cases and Deaths by Country or Territory

The **coronavirus** COVID-19 is affecting **228 countries and territories. The day is reset after midnight GMT+0.** The list of countries and their regional classification is based on the United Nations Geoscheme. Sources are provided under "Latest News." **Learn more about Worldometer's COVID-19 data**

Report coronavirus cases

MAIN WEEKLY TRENDS

Now Yesterday 2 Days Ago Columns Search:

All Europe North America Asia South America Africa Oceania

#	Country, Other	Total Cases	New Cases	Total Deaths	New Deaths	Total Recovered	New Recovered	Active Cases	Serious, Critical
	World	542,976,728	+529,657	6,337,630	+1,106	518,091,297	+366,560	18,547,801	36,387
1	USA	87,822,126	+49,087	1,037,816	+152	83,569,085	+47,541	3,215,225	3,044

COVID-19 Tracker

Search the web with Bing

Related Searches: coronavirus vaccine registration testing symptoms prevention

Give with Bing

Vaccine doses administered worldwide

11,962,781,283

Confirmed cases worldwide

536,634,986 +883,969

- Active cases *No data*
- Fatal cases 6,312,353 +1,905

Updated 9 min ago

Ready to live a healthier life?

Visit Start Health

Browse Sort

Filter to a location

Location	Cases
Global	536,634,986
United States	85,871,798

Click '+' to drill-down to a state or a city

Location	Cases
+ India	43,257,730
+ Brazil	31,611,769
France	29,984,235
+ Germany	27,096,571
+ United Kingdom	22,499,617
+ Russia	18,388,424
+ South Korea	18,256,457

United States ☆

Cases Vaccines News & Videos Graphs

Overview

Latest Updates

United States

New cases	Deaths	Vaccines
129.5K	382	39,195
-7%	+6%	-15%
14-day change	14-day change	14-day change

Trends

Daily spread trends Expand

Cases Deaths

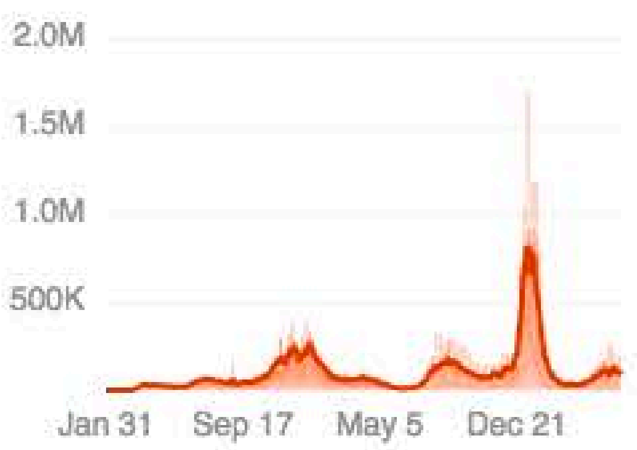

See more charts

Total cases

85.9M

85,871,798

+1.5M -7%

14-day change

Total deaths

1M

+4,820 +6%

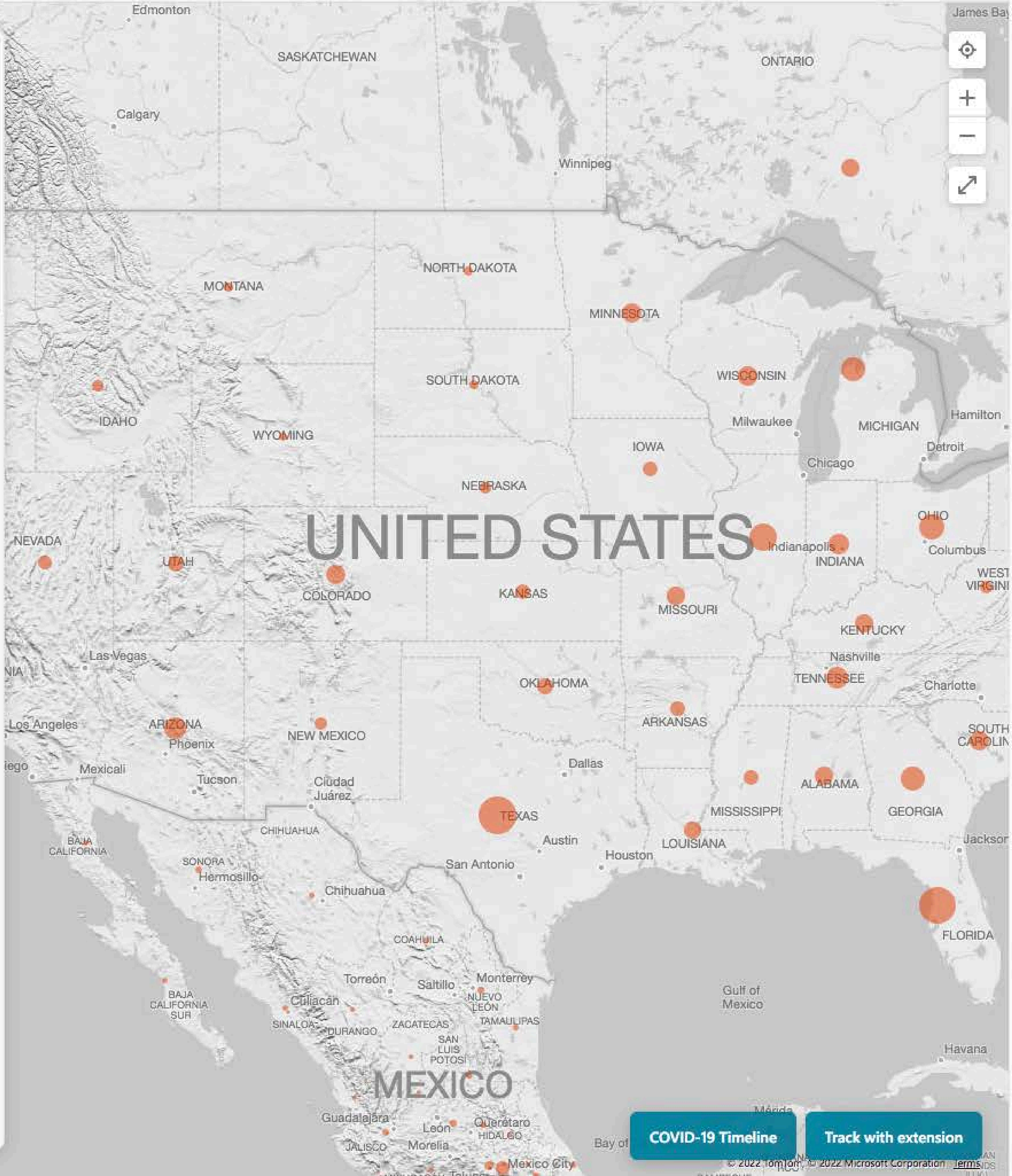

COVID-19 Timeline Track with extension

© 2022 TomTom © 2022 Microsoft Corporation Terms

1Point3Acres

Global COVID-19 Tracker & Interactive Charts

Real Time Updates & Digestable Information for Everyone English 中文版 Français 日本語 español United States

362,033,138 visits Donate

Our paper has been accepted for presentation at BIOKDD 2020 on Aug 24, 2020. Check it here.

Case Summary

Last updated at : 2022-06-16 17:31 EDT

Data source

Location	Cases	Deaths	Hospitalized	Test Pos%
United States	+34,518<br>85,748,398	+210<br>1,025,044	+461<br>30,955	-10.1%<br>4.1%
World	+715,929<br>537,361,665	+2,036<br>6,314,471		

Check out other countries and regions Click to view more

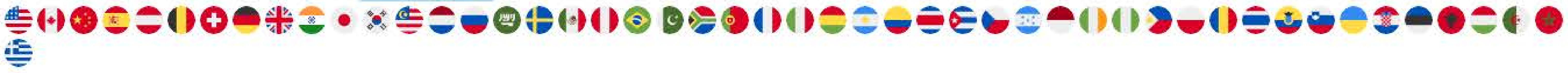

Vaccine Summary

Data source

Location	Vaccine doses administered	First doses administered	Second doses administered
United States	+570,313<br>607,519,950	+99,921<br>265,941,633	+116,389<br>215,896,857
World	+76,801,194<br>5,118,980,464	2,143,228,745	1,884,417,537

Filter the graph by selecting a state from the map below

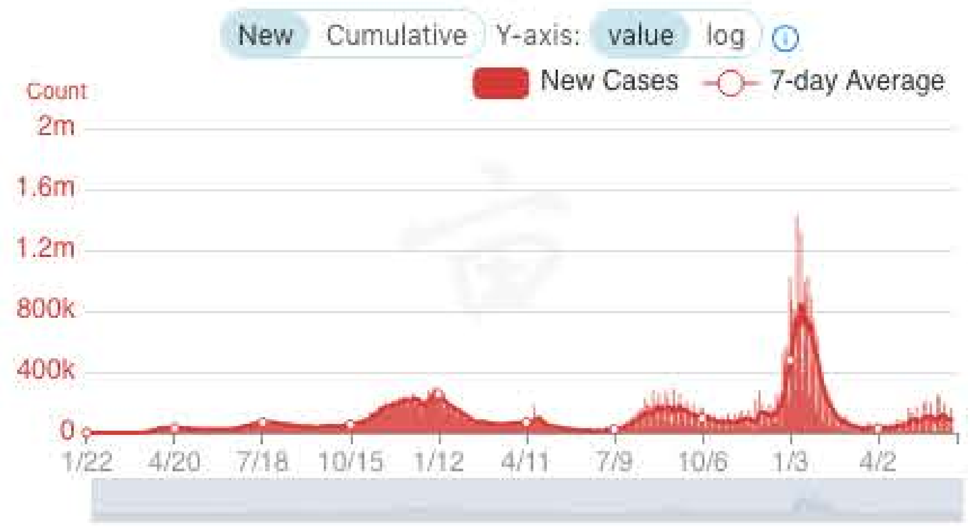

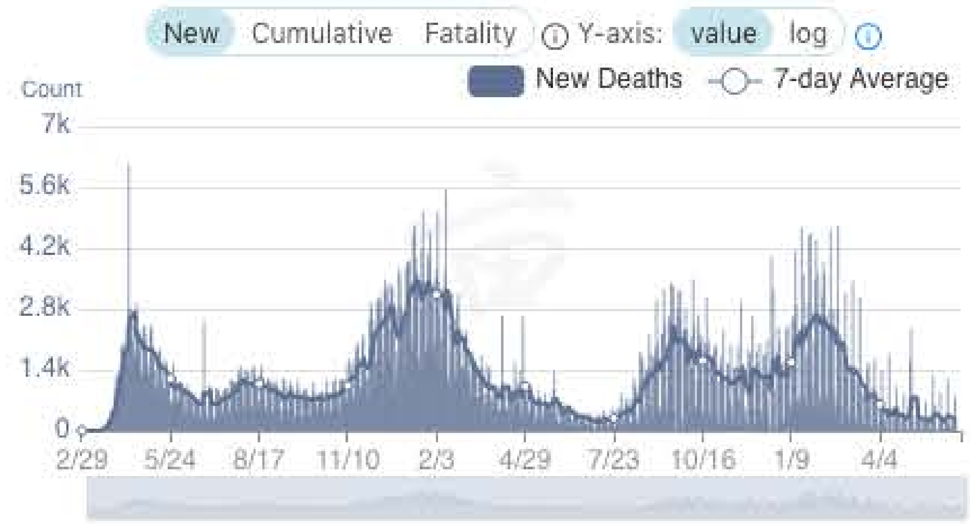

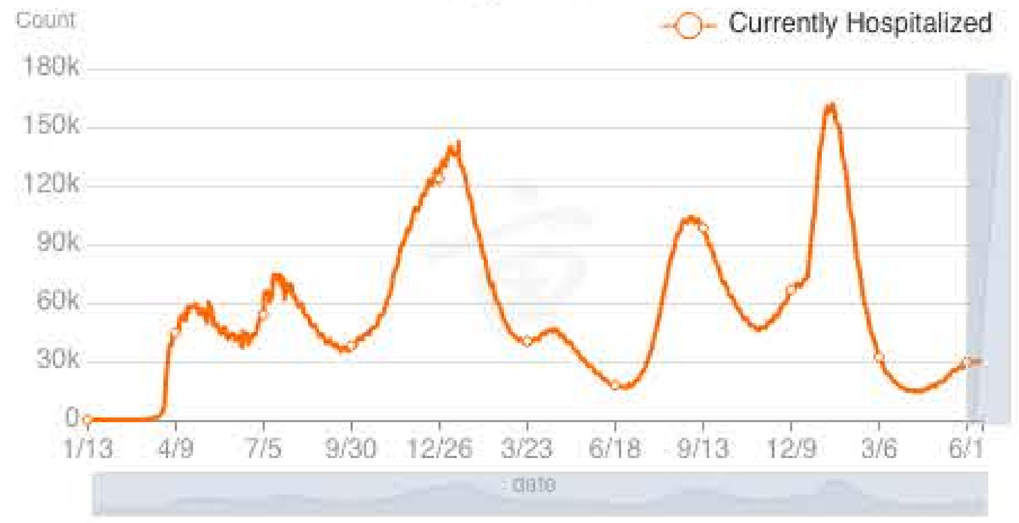

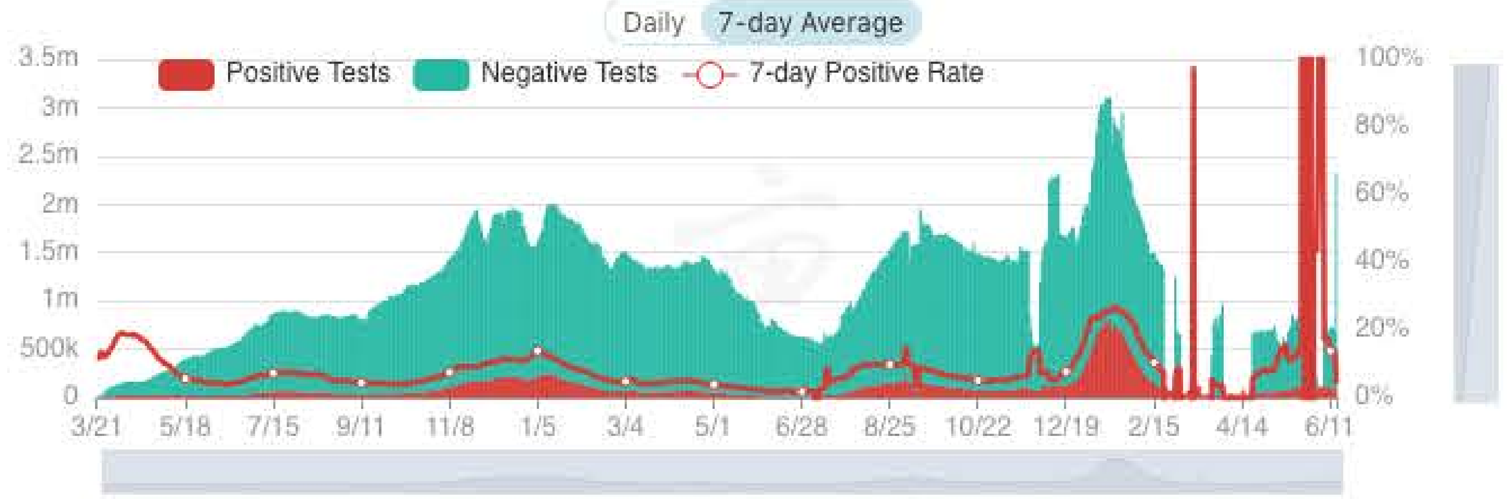

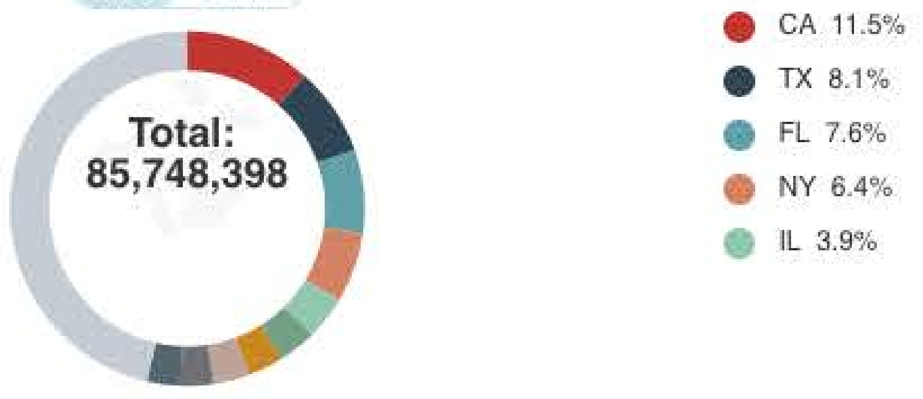

Case Map

Report Error

New Cases Total Cases Vaccination Tests Pop Cases/M Tests/M Deaths/10M

Reset Map

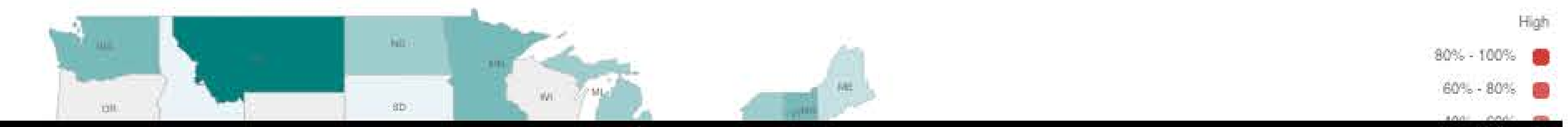

Home Testing World Job Life PPE About Get Data Support Us

Summary Map Cases Supplies Trends

Viewing the data of: United States

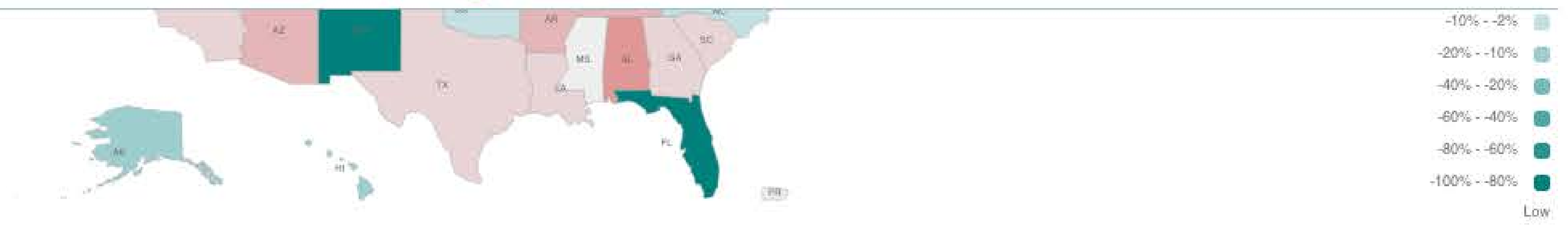

Filter cases by clicking or zooming the map.
Repatriation/Cruise ship cases are separated from TX/CA/NE cases count, consistent with CDC

US Cases

Sort by: Cases

Click here to check state level testing data/location

Location	Cases	Deaths	Fatality	7-Day New Cases	Vaccine doses administered
United States<br>CDC	+34,518<br>85,748,398	+210<br>1,025,044	1.2%	575,176<br>-25.3%	
California	9,856,852	91,973	0.9%	120,683<br>+3%	+41,648<br>77,348,012
Texas	6,976,747	88,715	1.3%	47,789<br>+6%	+24,073<br>47,917,055
Florida	6,512,442	75,175	1.2%	-100%	+57,421<br>38,454,361
New York	5,515,340	69,622	1.3%	36,622<br>-18.3%	+15,644<br>40,135,388
