# Supplementary material for: Web-Based COVID-19 Dashboards and Trackers in the United States: Survey Study
Source: JMIR Hum Factors. 2023 Mar 20;10:e43819. doi: 10.2196/43819 (PMC10029858; doi:10.2196/43819)
Supplement: Multimedia Appendix 18 [file humanfactors_v10i1e43819_app18.pdf]

Appendix 18. Screenshots from selected dashboards and trackers for vaccine distribution — *State, national, and global coverage* *(captured June 16, 2022)*

Page	Ref	Host
2	Svac-1	Alabama Dept of Public Health
3	Svac-2	David Marconnet, CTO of OneTeam.net
4–5	Svac-4	Government of the District of Columbia
6	Svac-6	Chicago Tribune
7	Svac-7	Indiana State Dept of Health
8–11	Svac-8	Kansas Dept of Health and Environment
12	Svac-9	Michigan Dept of Health and Human Services
13–16	Svac-10	Minnesota Dept of Health
17–20	Svac-11	North Dakota Dept of Health
21–22	Svac-12	New Mexico Dept of Health
23	Svac-13	Ohio Dept of Health
24	Svac-14	Tennessee Dept of Health
25–27	Svac-15	Texas Health and Human Services
28–29	Svac-16	Vermont Dept of Health
30	Svac-17	Wisconsin Dept of Health Services
31	Nvac-1	New York Times
32	Nvac-2	Springfield News-Leader
33	Nvac-3	Washington Post
34	Nvac-4	COVID-19 Health Equity Interactive Dashboard / Emory University
35	Gvac-1	Our World in Data
36	Gvac-2	CNN

Alabama's COVID-19 Vaccine Distribution Dashboard

Alabama Department of Public Health | Immunization Division | Updates Tuesday & Thursday by 2:00 p.m.

Select a county
Alabama

Important Notice (05/18/22): The Alabama Department of Public Health (ADPH) recently merged approximately 50,000 COVID-19 vaccine records. Corresponding data on the Vaccine Distribution Dashboard now reflects these changes. The loss of Doses Administered and Number of People Receiving One or More Doses is a result of duplicated doses in the system being merged together. The increase for Number of People Who Have Completed Vaccine Series is due to the merging of data that made previously incomplete records become complete.

Vaccine Doses Administered

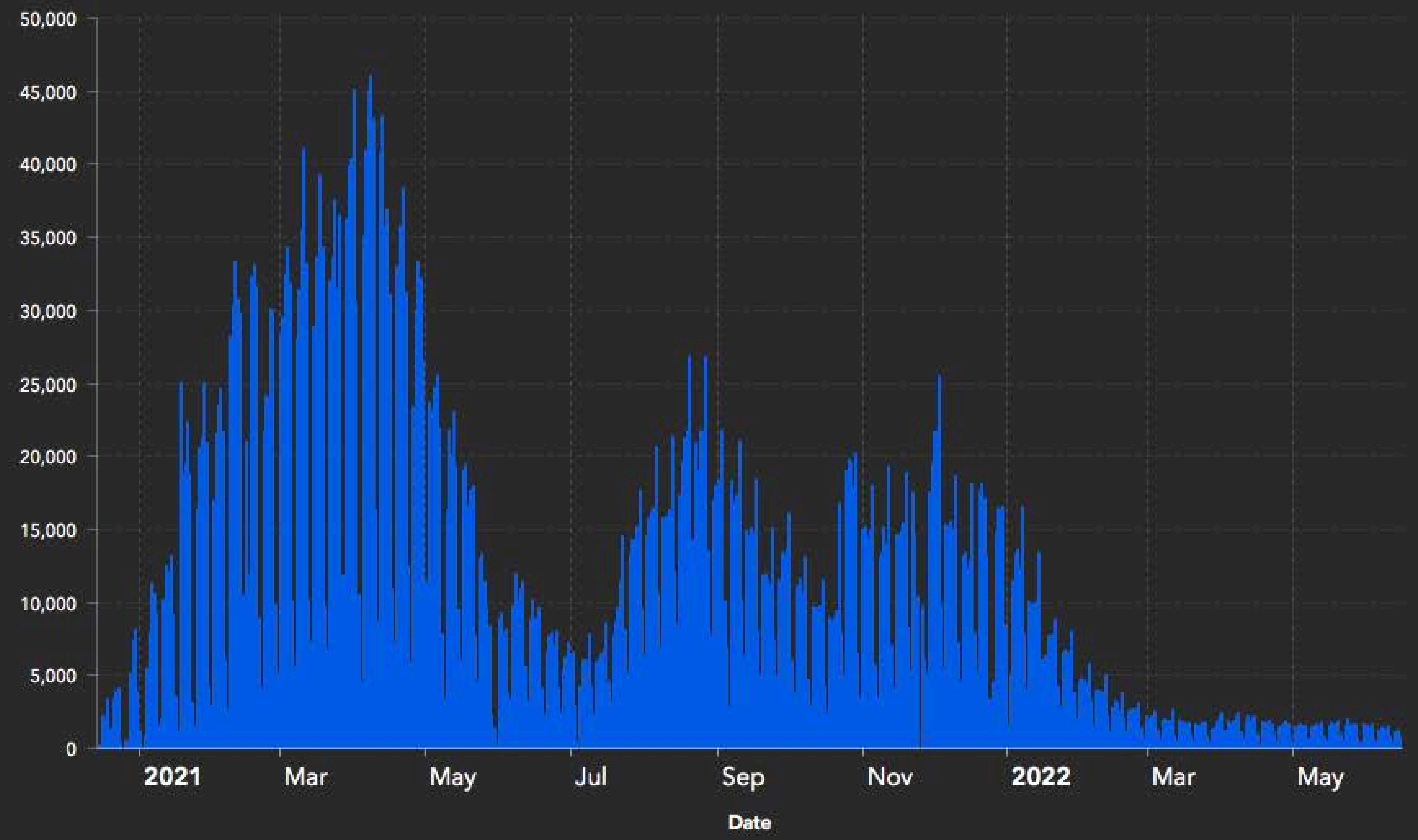

Doses Administered

Doses Administered
6,023,788

Number of people
2,879,968
receiving one or more doses

Number of people
2,380,249
who have completed vaccine series

Additional Doses
842,713
Administered

Doses Delivered to Alabama
9,668,195

Doses Delivered to
4,915,965
Alabama Providers

Doses Delivered to
4,752,230
Federal Programs

Alabama COVID-19 Vaccine Data

As of 5/24/2021, this section is no longer updating. The data shown below is for historical tracking purposes. For the most current data, please check the ADPH Vaccine Distribution Dashboard.

Doses Delivered	Doses Admin.	People Vaccinated	People Vaccinated
4,206,825	2,851,368	1,625,262	1,318,446
3,046,235 AL; 1,181,890 Fed LTC	93.6% of AL delivered	1 or More Doses	Completed Vaccine Series

Administered per Day

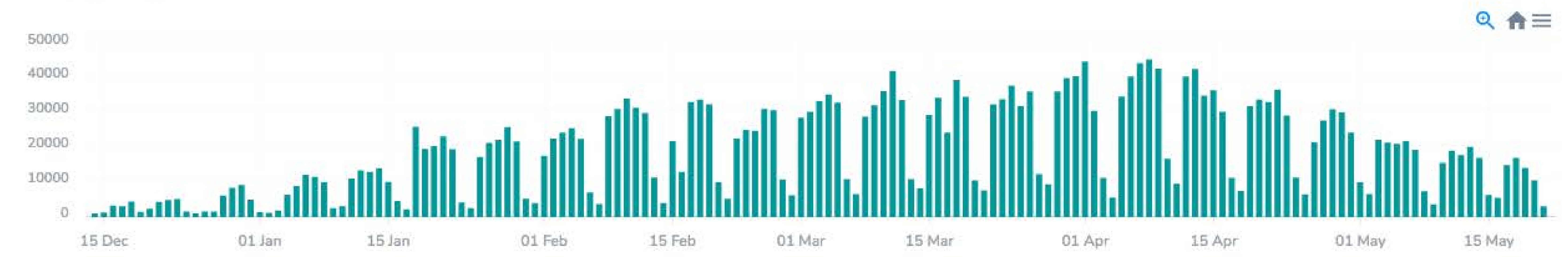

People Vaccinated over time

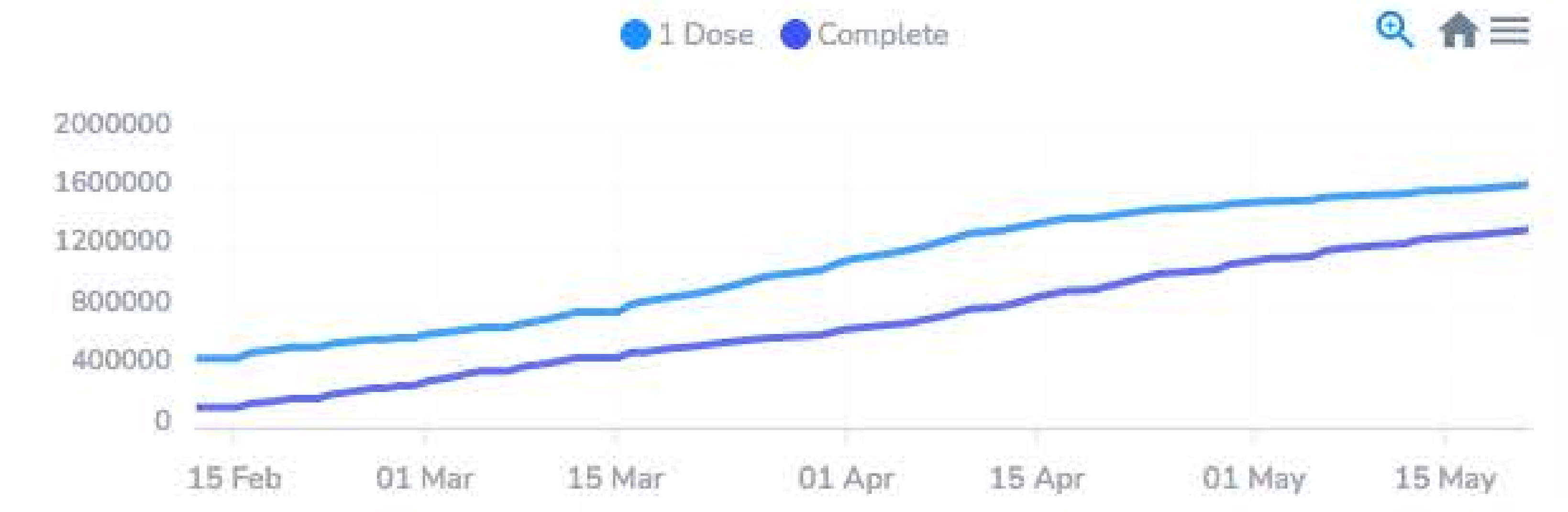

Delivered over time

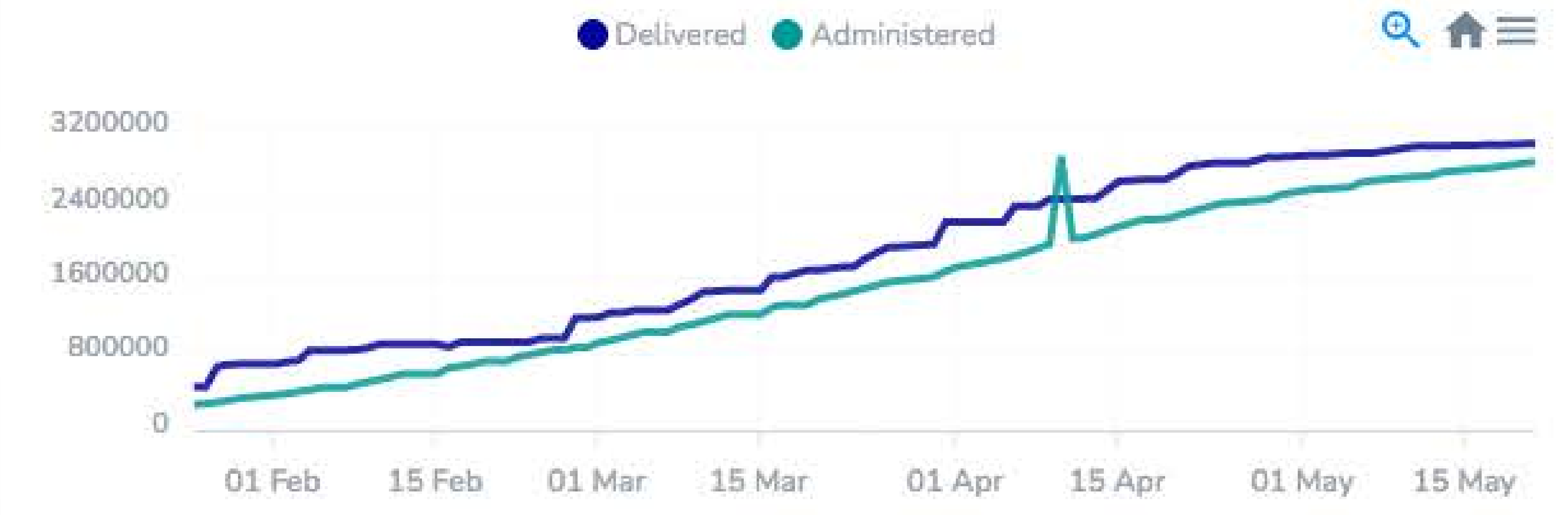

People Complete Change Per Day

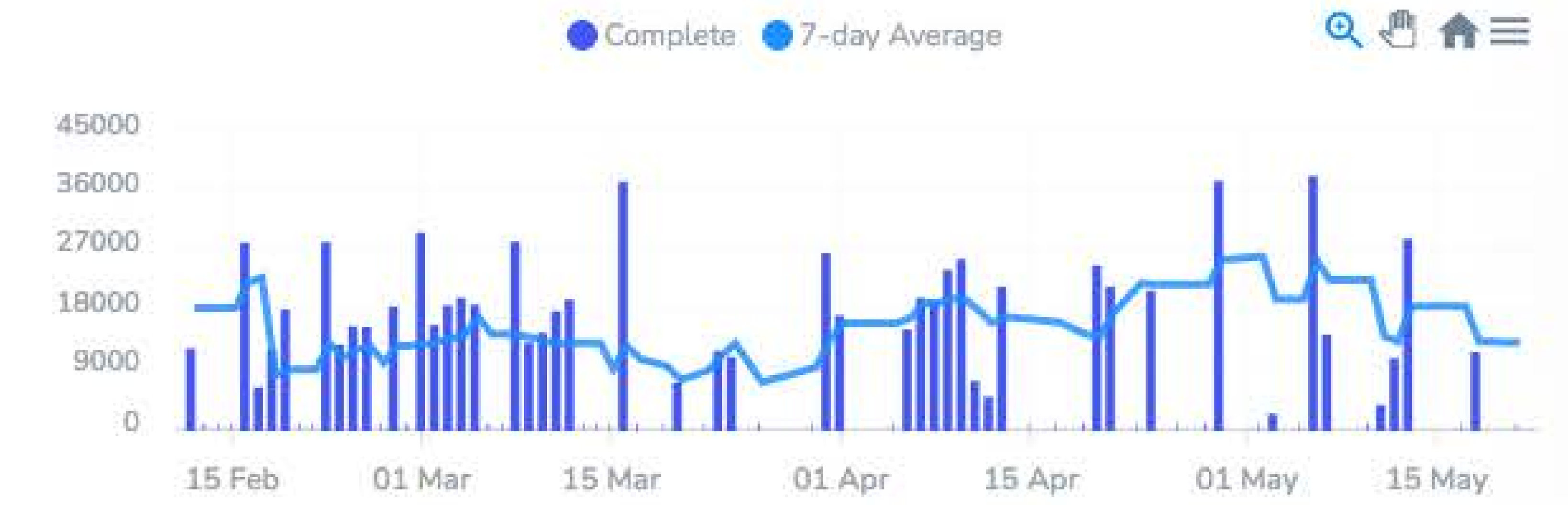

Delivered Change Per Day

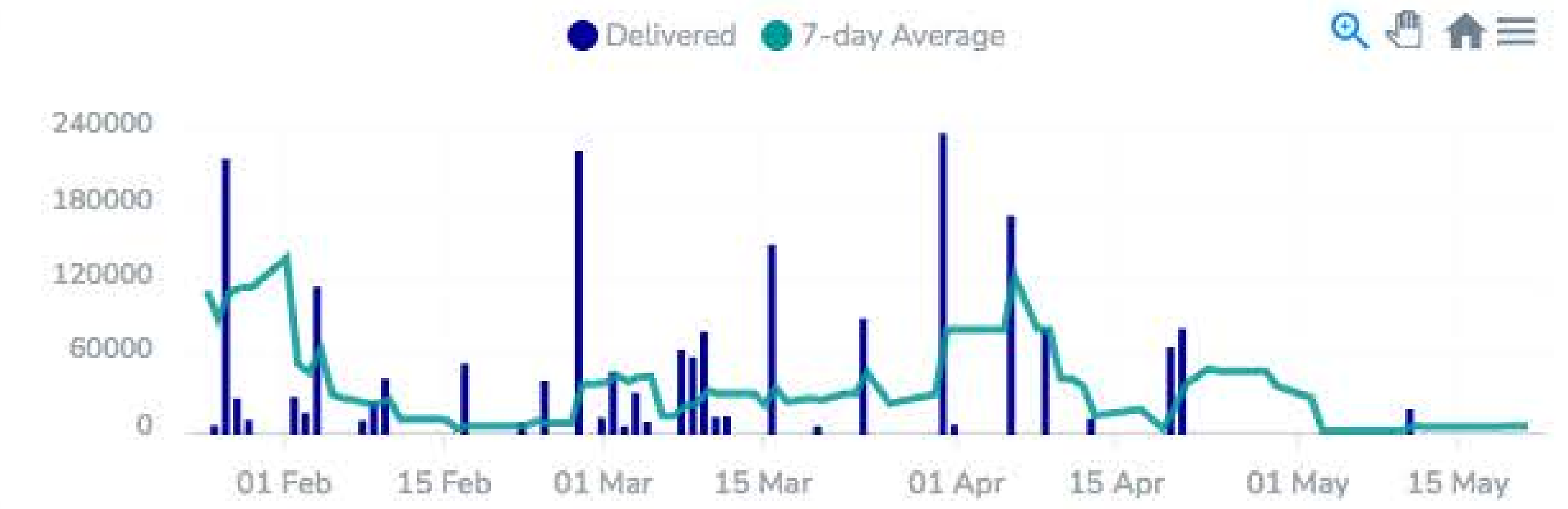

Percent Admin over time (Admin / Total Delivered)

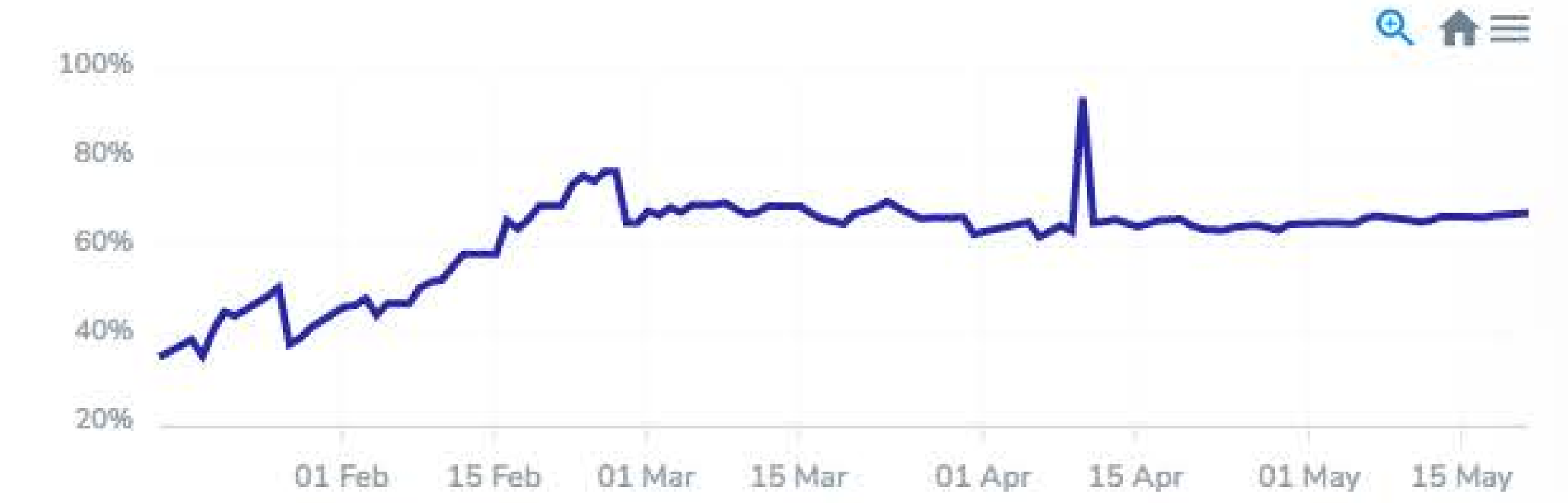

Percent Population over time

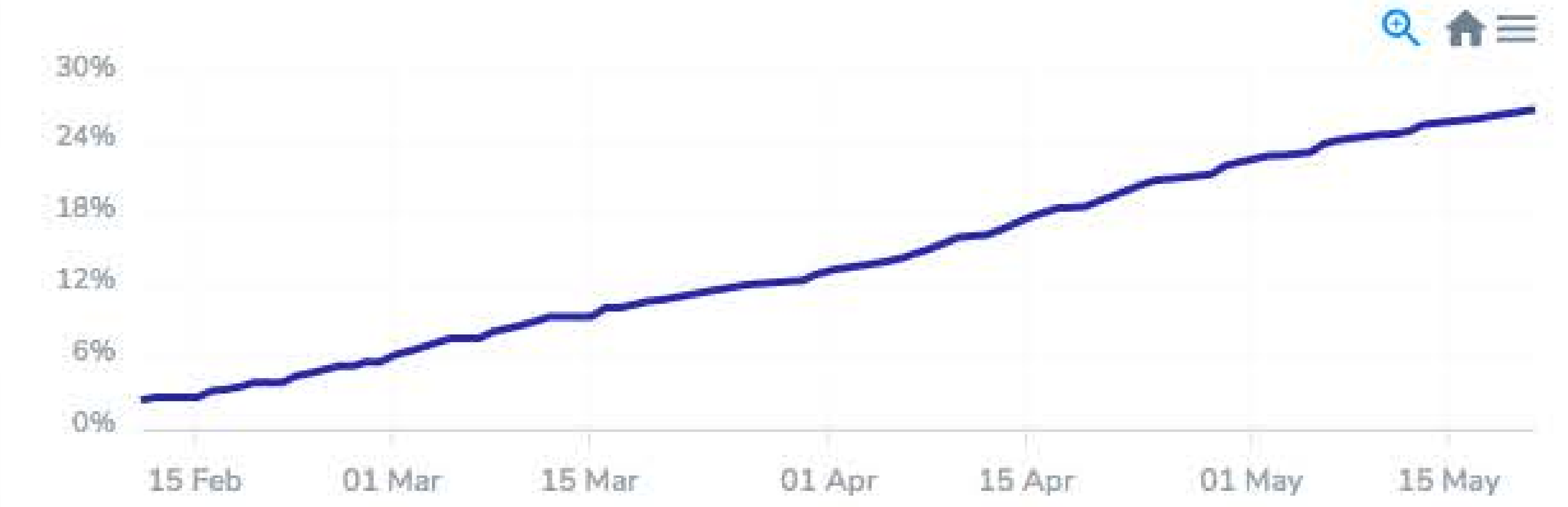

County Details

Show 25 entries Search:

County	People Complete	Percent Complete	1 or More Doses	Admin	7-day Avg Admin	Pop > 16
Jefferson	201,608	30.61%	253,458	440,804	1408.71	658,573
Madison	116,392	31.21%	139,093	245,639	1056.57	372,909
Mobile	104,516	25.29%	122,251	222,947	491.29	413,210
Shelby	66,990	30.77%	82,717	144,507	368.14	217,702

Residents

TOTAL DOSES ADMINISTERED WITHIN DC	ESTIMATED % RESIDENTS WITH AT LEAST ONE DOSE OF PRIMARY SERIES**	ESTIMATED % RESIDENTS COMPLETED PRIMARY SERIES**	ESTIMATED % OF BREAKTHROUGH CASES***
1,590,909	96.8%	76.5%	7.08%

Coverage (%) of Primary Series Completed Residents by Health Planning Neighborhood

Neighborhood | Ward

Count or coverage:

Coverage (%)

- Null
- 1. Primary Series Incomplete
- 2. Primary Series Complete
- 3. Up to Date
- 4. 4th Dose (selected)

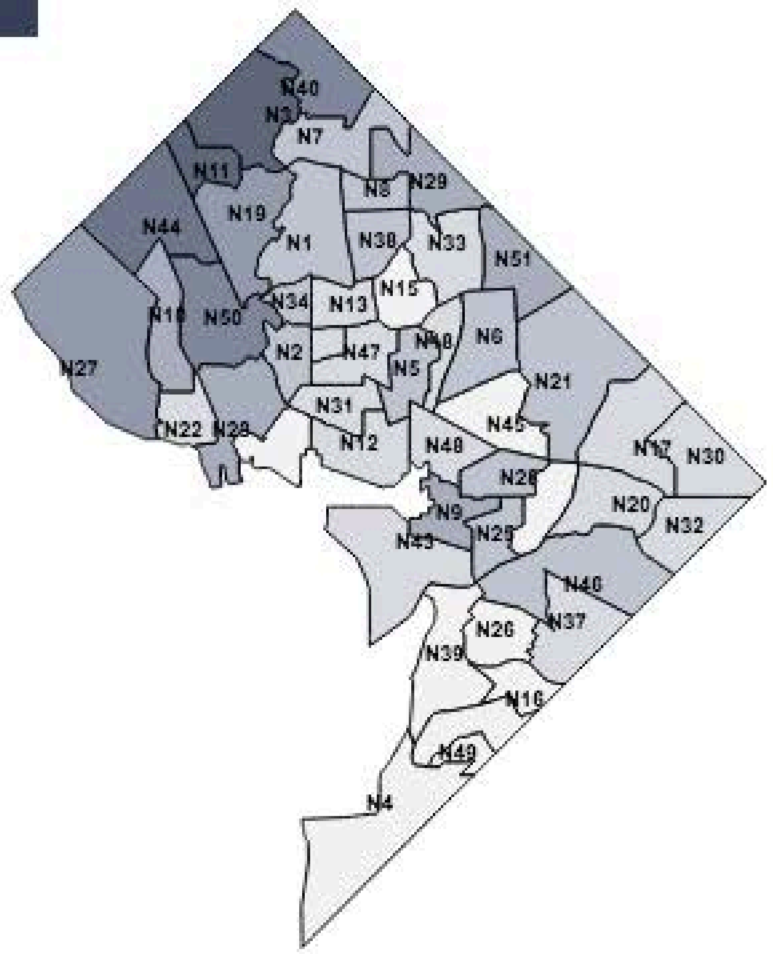

Mapped: 1,125,229

Not Mapped: 108,715

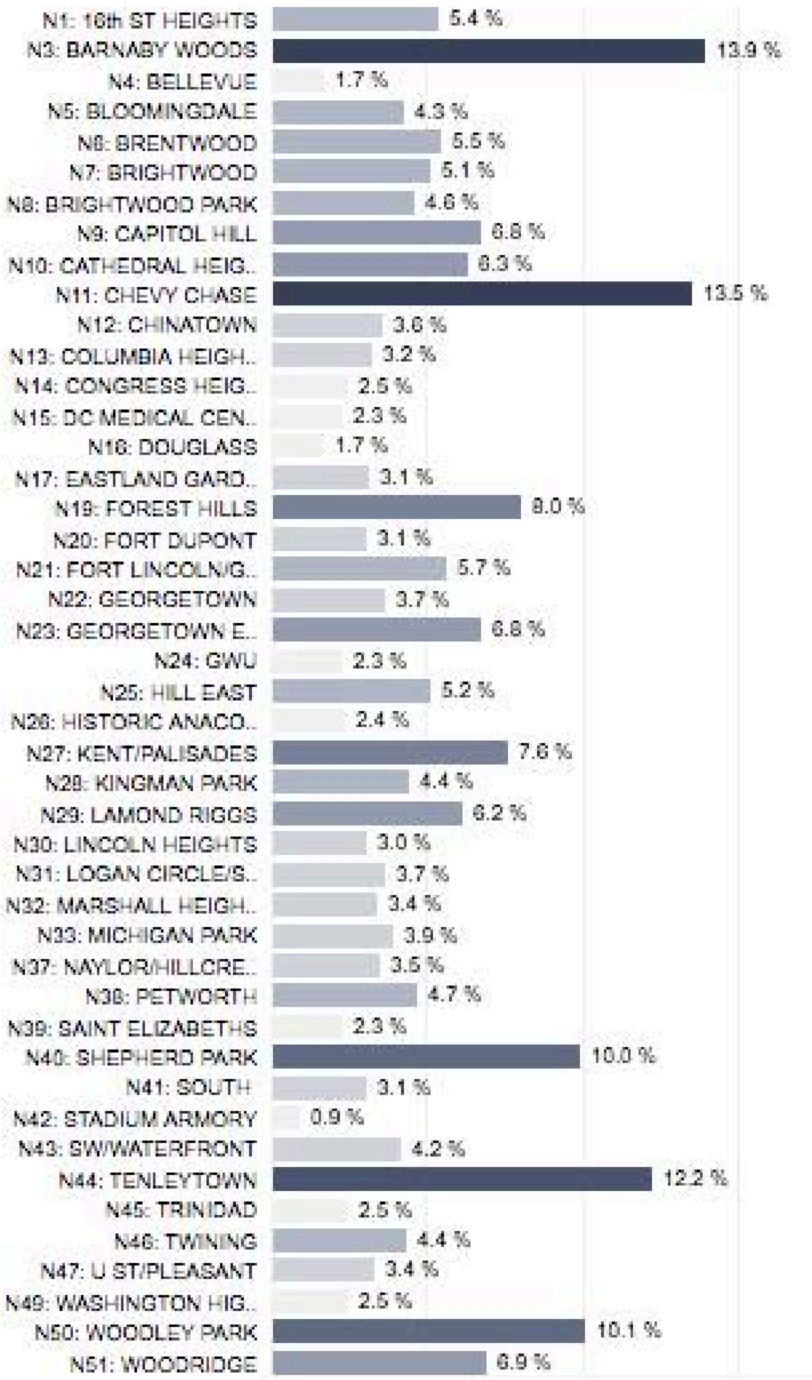

Data Definition

Dashboard Last Refreshed: 6/15/2022

TOTAL DOSES ADMINISTERED WITHIN DC	ESTIMATED % RESIDENTS WITH AT LEAST ONE DOSE OF PRIMARY SERIES**	ESTIMATED % RESIDENTS COMPLETED PRIMARY SERIES**	ESTIMATED % OF BREAKTHROUGH CASES***
**1,590,909**	**96.8%**	**76.5%**	**7.08%**

Total Administrations within DC by Vaccination Date

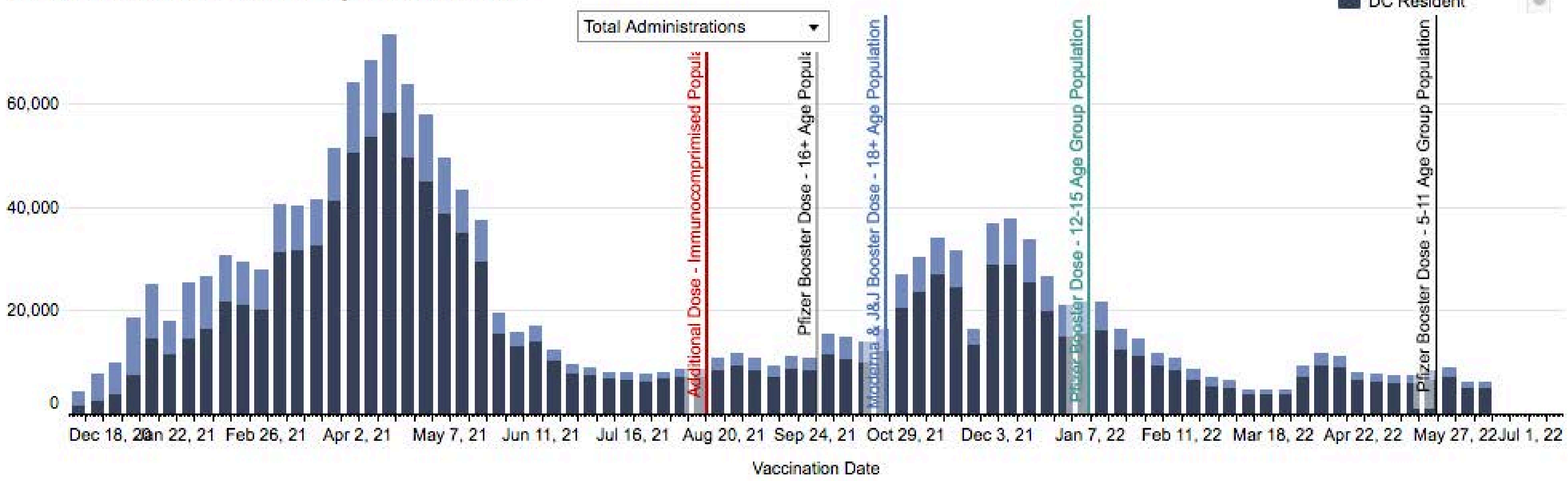

	At Least One Dose	Primary Series	Up to Date	4th Dose	Total Administrations	Dose 7-day Average
DC Resident (within DC)	471,629	407,041	149,481	30,202	1,079,755	621
DC Resident (outside DC)	172,414	96,695	31,066	2,520	291,859	
DC Resident (Federal Entity)	38,784	36,474	8,384	597	84,583	
Total DC Resident	682,827	540,210	188,931	33,319	1,456,197	621
Non DC Resident (within DC)	216,180	134,736	35,809	3,117	386,992	166
Non DC Resident (Federal [illegible]	13,374	14,075	1,638	2,007	39,579	
Total Non DC Resident	229,554	148,811	37,447	5,124	426,571	166

Data Definition

Dashboard Last Refreshed: 6/15/2022

COVID-19 VACCINE

Illinois COVID-19 vaccine tracker: Daily data updates on the state's vaccination effort

By Chicago Tribune staff
Chicago Tribune • Aug 31, 2021 at 4:05 pm

Illinoisans ages 12 and over are now eligible for the coronavirus vaccine. Below are key statistics about the vaccination effort in Illinois.

Here, the Tribune gets your COVID-19 questions answered from experts.

See Coronavirus data on deaths, cases and tests here.

Illinois COVID-19 vaccination rates by county

How Cook County stacks up

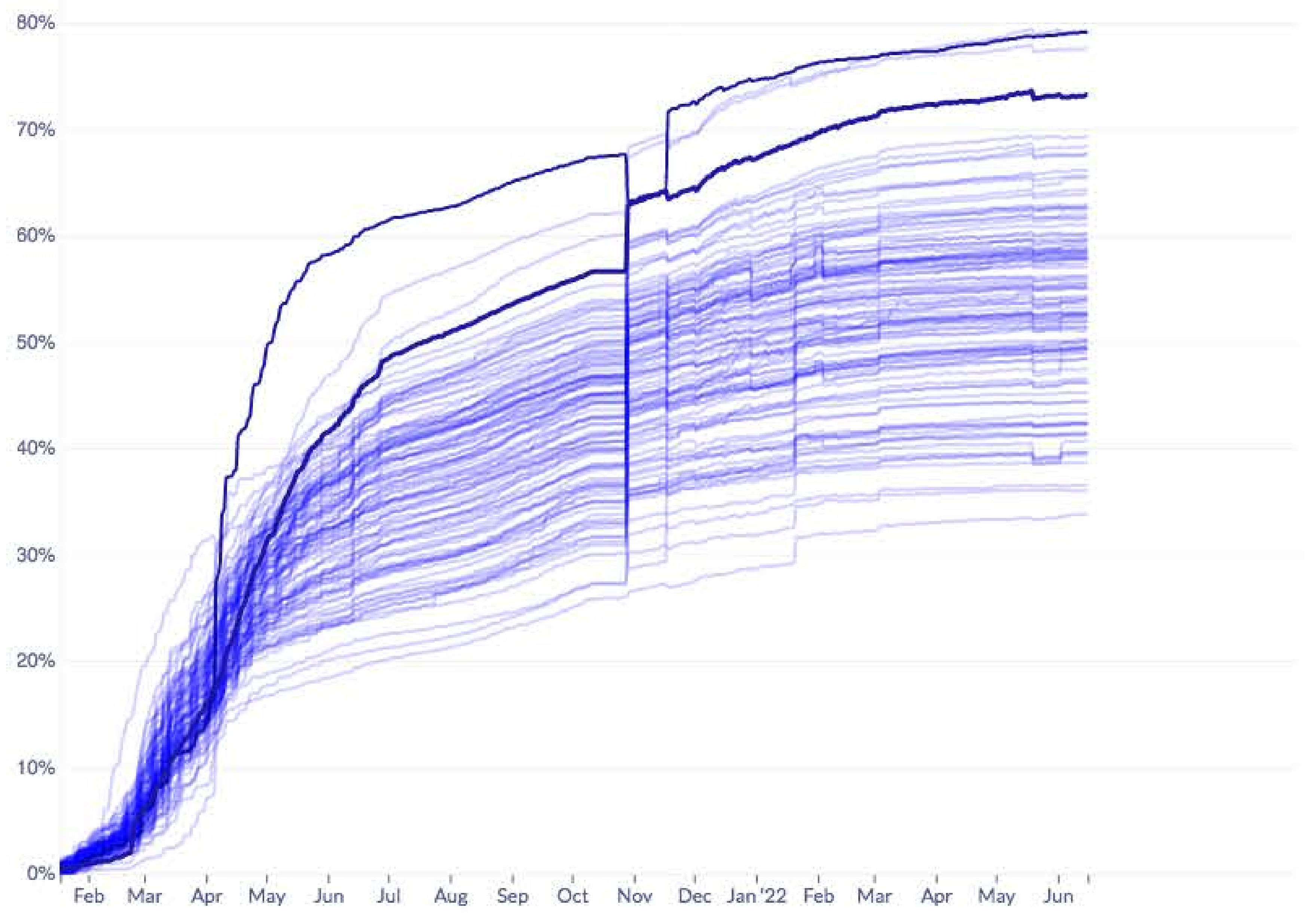

Source: T H

Illinois COVID-19 vaccination rates

Updated June 16, 2022

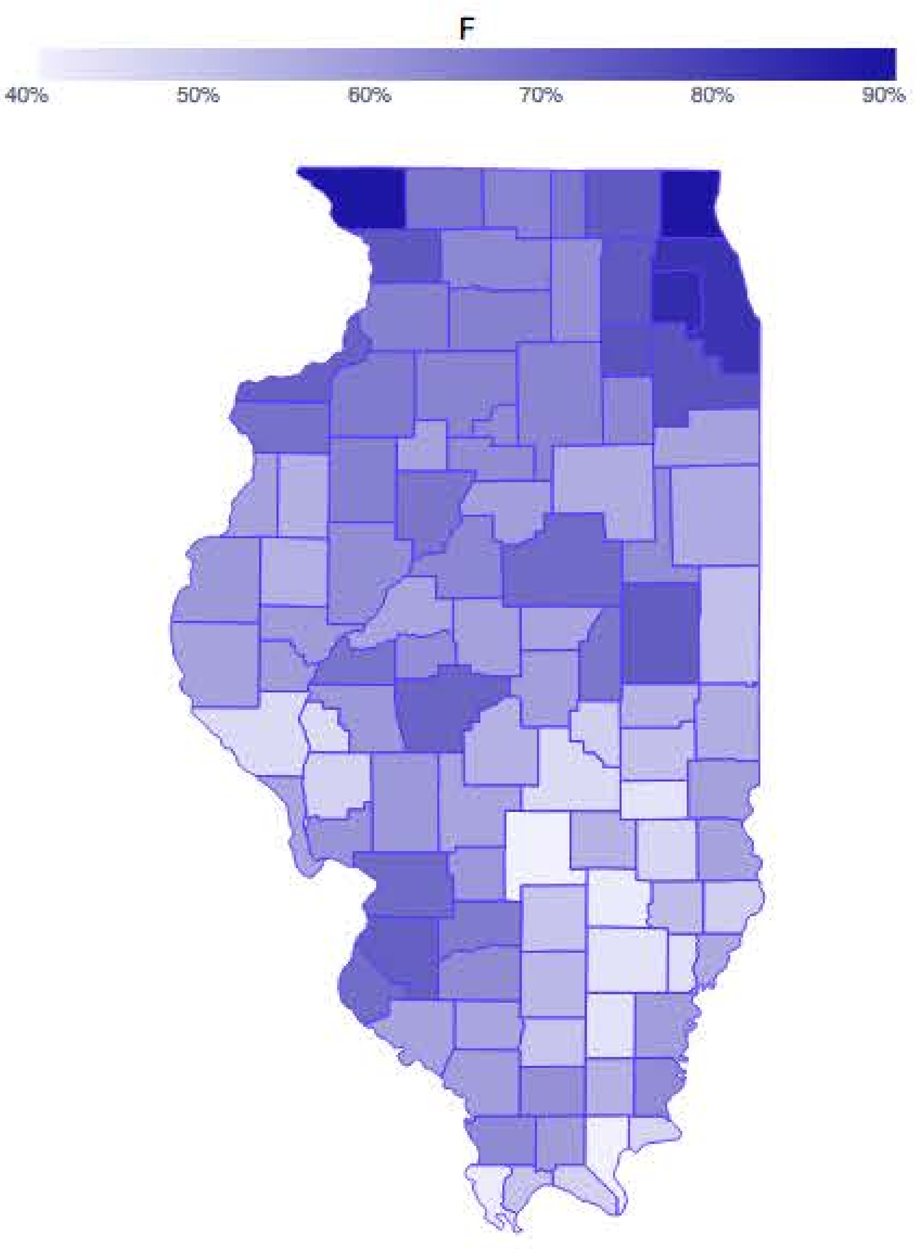

Source: H

COVID-19 vaccination rates for the U.S.

Last Updated on Wednesday 6/15/22

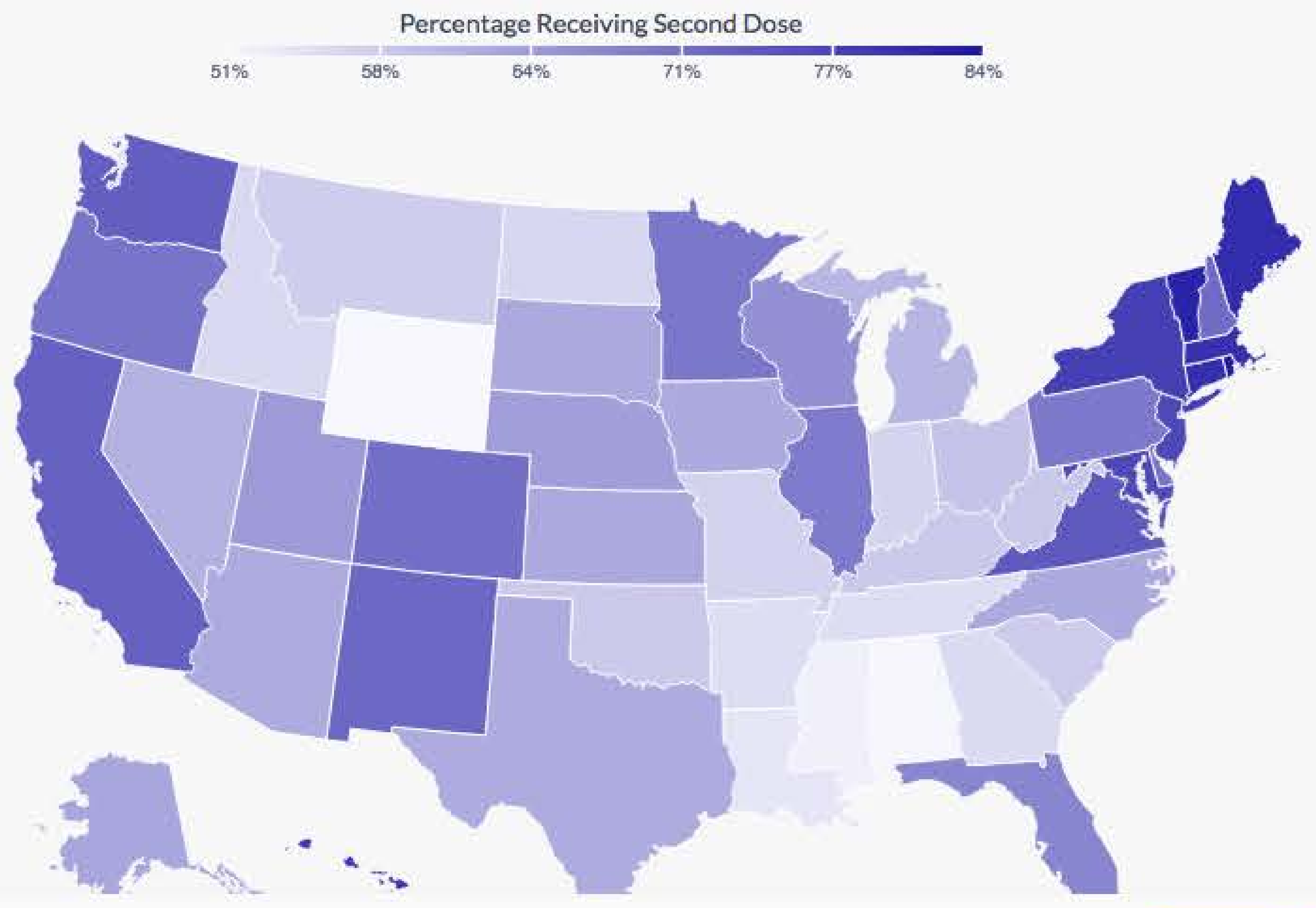

Chicago Tribune
SUBSCRIBE
4 weeks for only 99¢
LOG I

Source: CDC COVID Data Tracker Vaccinations ›

By DATAHERALD
Explore this data

COVID-19 vaccination rates by state

How Illinois stacks up

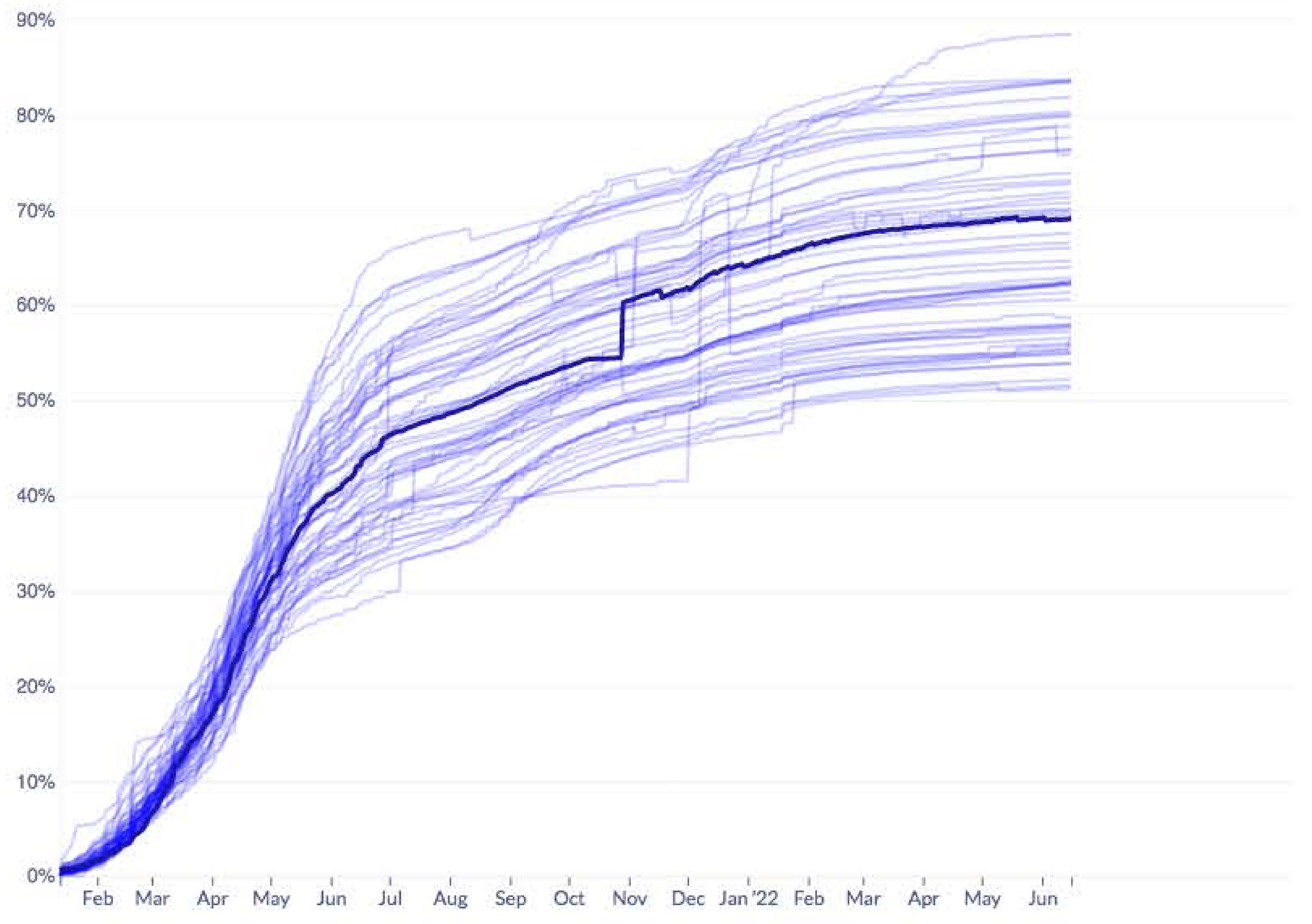

Source: T H

Indiana COVID-19 Vaccination Dashboard

Below results are as of 6/14/2022. Dashboard updates by 5 p.m. every Monday, Wednesday, and Friday.

Return to Landing Page

VACCINE | BREAKTHROUGH

Total Vaccinations

Reported (all-time): 12/14/2020 - 6/14/2022

New
Total

Vaccine Status Series: Fully Vaccinated

Total Doses Administered

Distribution of **9,786,060** total doses administered

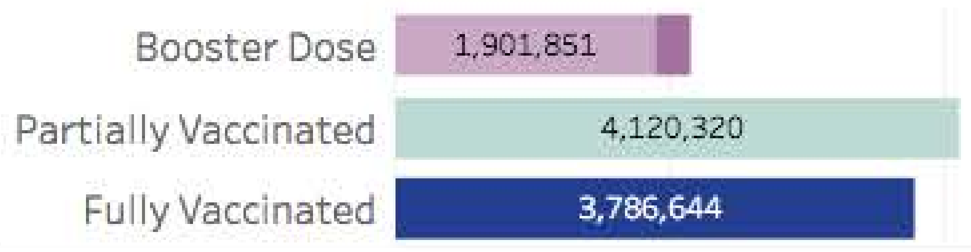

Total Up to Date Individuals

1,901,851

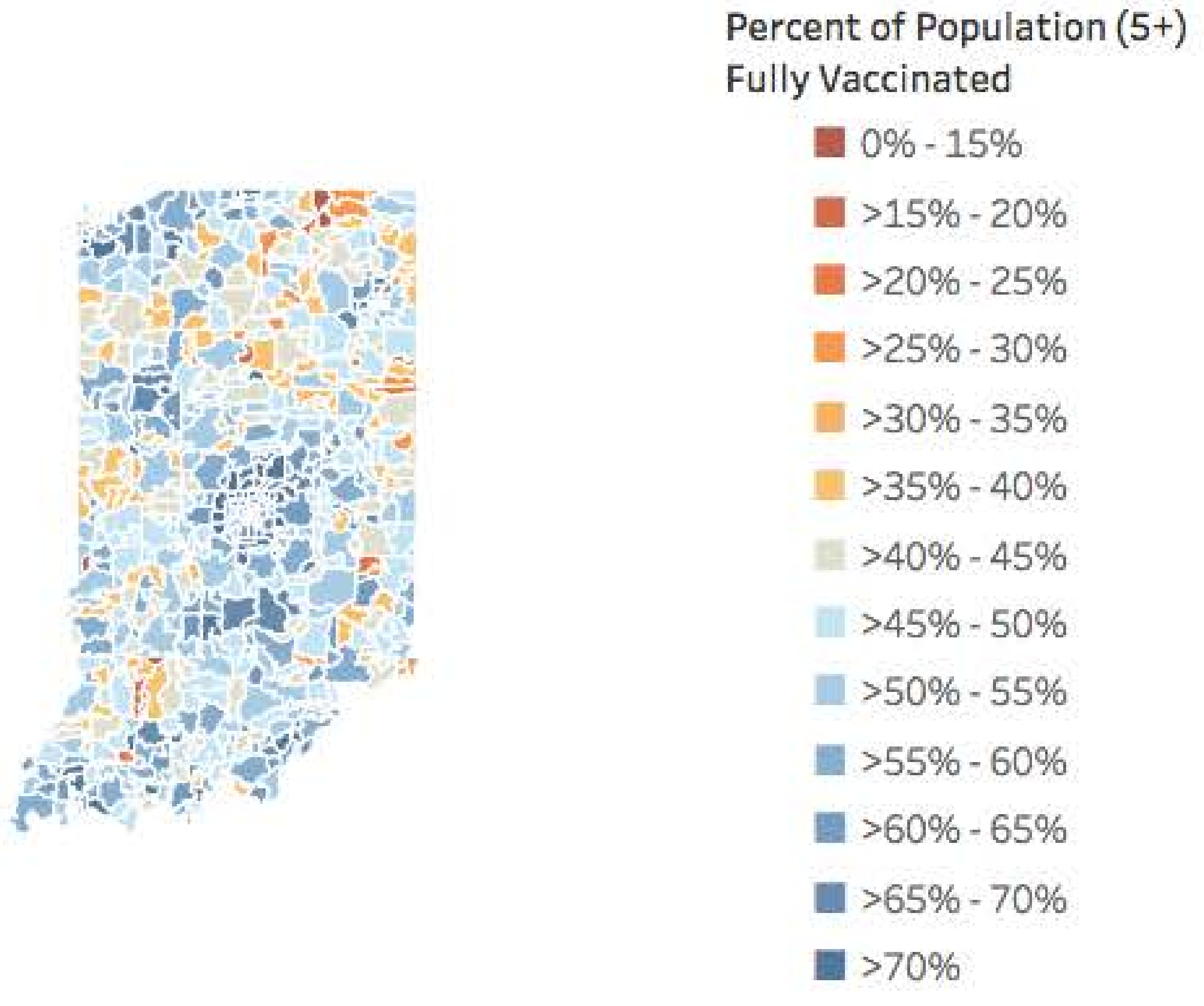

Doses Timeline

Timeframe: All Time

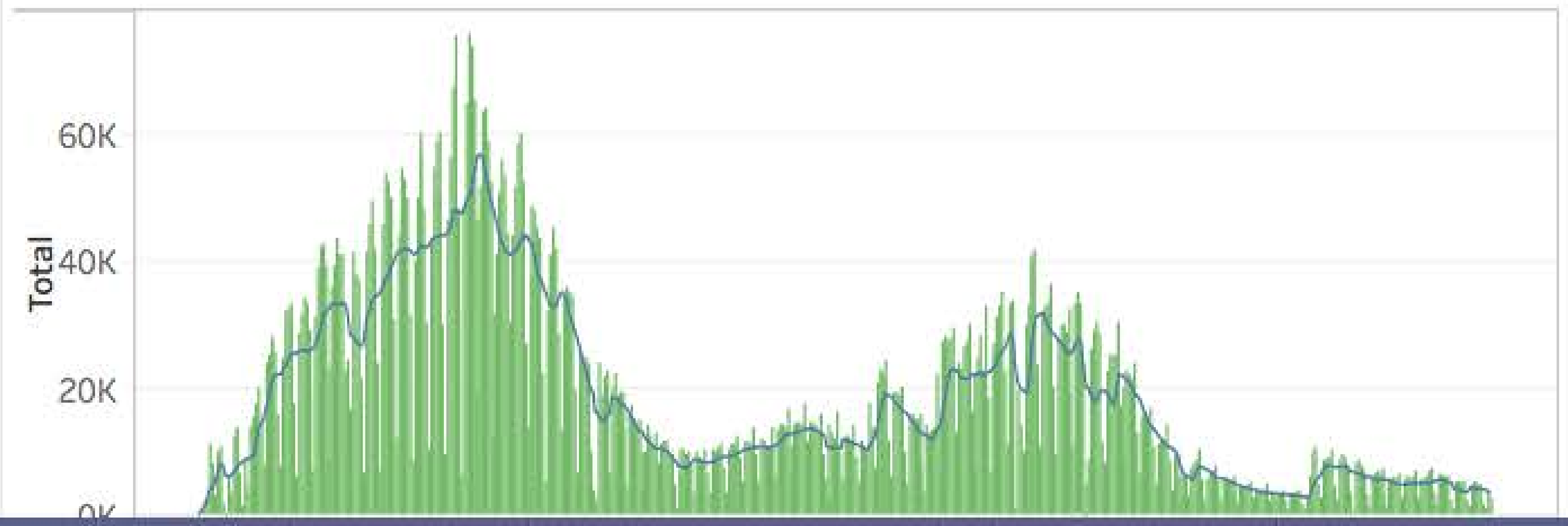

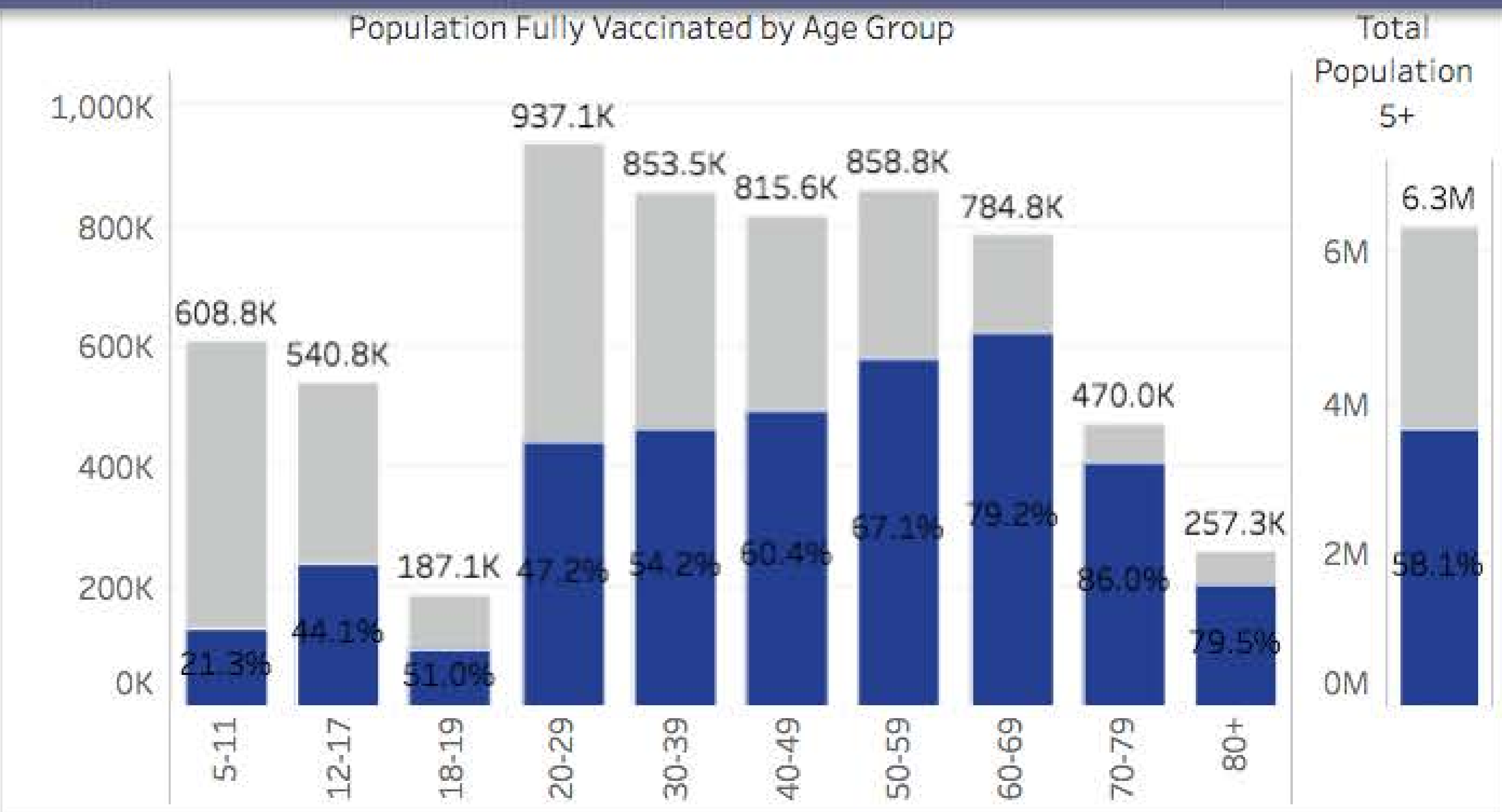

Demographic: Race

Fully Vaccinated

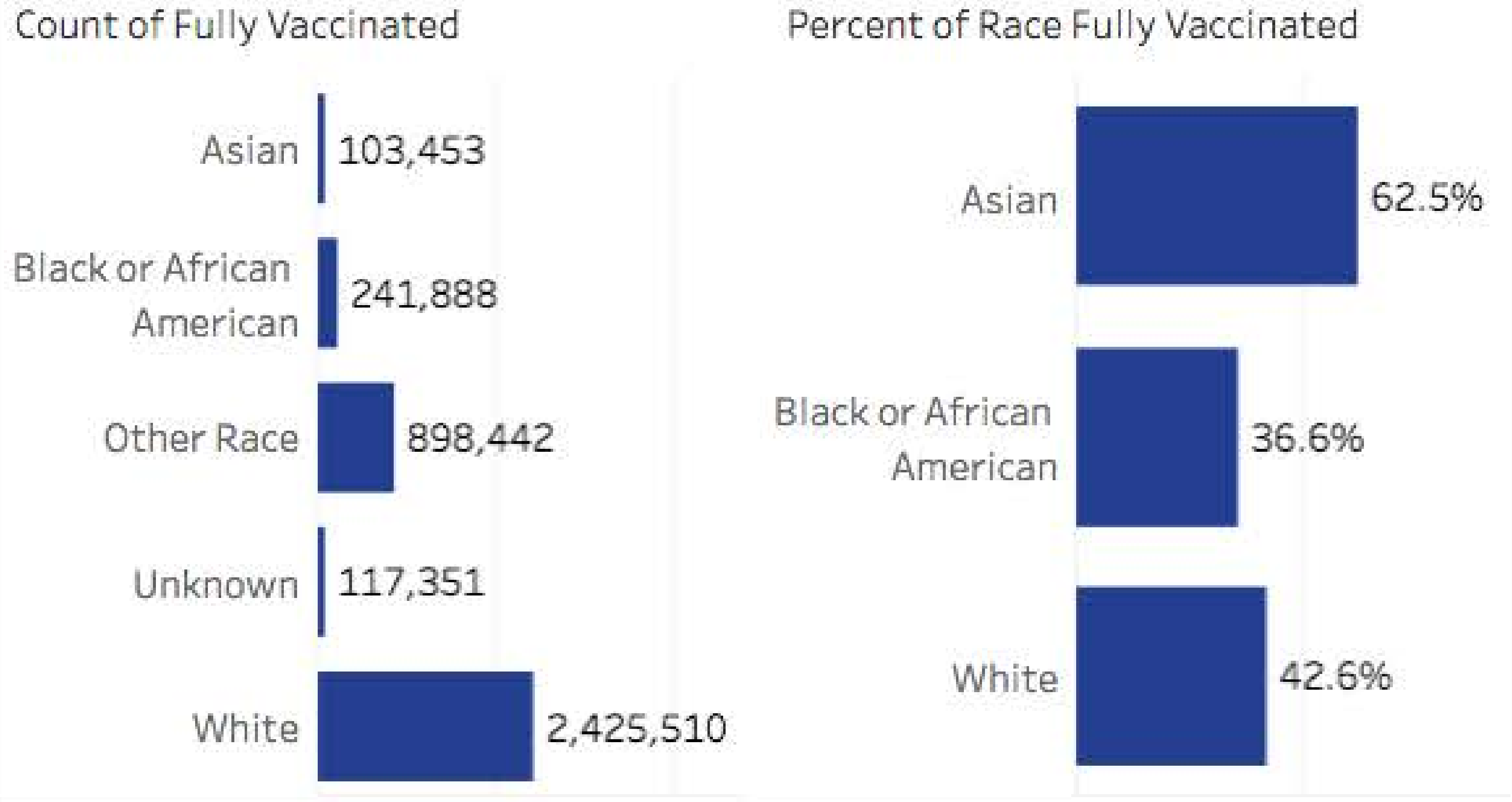

All data displayed is preliminary and subject to change as more information is reported to IDOH. Expect historical data to change as data is reported to IDOH.

Data

Dashboard Information

Beginning May 18, the COVID-19 dashboard will be published on Wednesdays by 12:30 p.m. To maximize your experience, use a device with a larger screen, such as a tablet, laptop or desktop.

Kansas COVID-19
Vaccination Overview

Data are preliminary and subject to quality improvement and quality assurance validation.
Last updated: 6/15/2022
Beginning May 18, the COVID-19 dashboard will be published once a week, Wednesday by 12:30 p.m.
* Third dose includes all instances of 3 doses reported in KSWebIZ. On 8/12/2021, a third dose was approved for those who are severely immunocompromised. On 9/24/2021, booster doses were approved for select populations 6 months following completion of a Pfizer initial series. On 10/21/2021, booster doses for Moderna were approved for select populations 6 months following initial series completion. Johnson and Johnson booster doses were approved for all recipients 2 months after initial series.

1,982,263
People Vaccinated with One Dose

4,476,358
Total Doses Reported as Administered
First Dose: 1,982,263
Second Dose: 1,617,005
Third Dose*: 877,091

5,967,310
Total Doses Distributed
State Distributed: 3,193,400
Federal Pharmacy Program: 2,773,910

63.2%
Percent of Kansans Vaccinated with One Dose

55.3%
Percent of Kansans Completed COVID-19 Vaccine Series

Vaccine Administration

County Vaccination Rates

Understanding the Numbers

Data Source: The data displayed on this dashboard come from the Kansas Immunization Registry (KSWebIZ). As providers in Kansas administer immunizations, including COVID-19 immunizations, they are required by law to submit vaccination information to KSWebIZ. KSWebIZ data are constantly updated and reviewed to ensure data quality and completeness. Vaccine providers outside of Kansas and through federal partnerships do not report to KSWebIZ and are not captured in these data. All states and federal partners report all doses administered to CDC; however, Kansas data used in the CDC COVID-19 Data Tracker has not been updated to reflect removal of duplicates and other updates to data originally submitted. For COVID-19 immunization data that includes federally administered vaccines, please see the CDC COVID-19 Data Tracker https://covid.cdc.gov/covid-data-tracker/#county-view.

Total Doses Administered: Represents the total number of all COVID-19 vaccine doses (e.g. first dose, second dose) that have been given to people in Kansas as reported in KSWebIZ.

Total Doses Distributed: Represents the total number of all COVID-19 vaccine doses that have been delivered to vaccine administration sites throughout Kansas. This includes all first and second doses. Second doses are delivered prior to completion of the appropriate interval.

Population Estimate Totals: U.S. Census Bureau, June 2019 County Characteristics Resident Population Estimates

People Vaccinated: Represents the total number of individuals with a vaccination record reported in the Kansas immunization information system, KSWebIZ. Kansas residents that have been vaccinated in other states or through federal agencies including Veterans Health and Indian Health Services are not currently included in this data.This number may differ from total number of first doses administered if records are complete for second doses but not first.

Percent of Kansans Vaccinated: Represents the number of people with at least one dose of COVID-19 vaccine reported in KSWebIZ as a fraction of the total population of Kansas (2019 Bridged-Race Population Estimates, as reorganized in VSDA Population Master File).

Eligible Population: COVID-19 vaccinations are currently approved under Emergency Use Authorization for individuals 5 years of age and older.

County Vaccination Rate: County vaccination rates are calculated based on vaccine recipient county of residence. KDHE is currently only able to report on doses given in Kansas and reported into the Kansas Immunization Registry (KSWebIZ). Kansas residents that have been vaccinated in surrounding states or through federal agencies are not currently included in this data. Vaccination rates for counties bordering other states or with close ties to federal agencies may have higher vaccination rates than reported using KSWebIZ. Addresses missing zip codes are unable to be associated with a county. Addresses with incomplete or inaccurate zip codes may be associated with an incorrect county. Data cleaning processes are in place to correct these mistakes over time.

Race/Ethnicity Information: Race and ethnicity data is reported into the Kansas Immunization Registry (KSWebIZ) by vaccine providers using patient self-identified race and ethnicity. Multiple races are available for selection with the primary race listed first followed by other selected races. Race is reported into the demographics section of a patient record and can be updated as needed; updated race information will apply to all previous and future vaccine administered to that individual.

Data

Dashboard Information

Beginning May 18, the COVID-19 dashboard will be published on Wednesdays by 12:30 p.m. To maximize your experience, use a device with a larger screen, such as a tablet, laptop or desktop.

Kansas COVID-19
Vaccine Administration

Data are preliminary and subject to quality improvement and quality assurance validation.
Data Source: Kansas Immunization Information System, KSWebIZ

county
(All)

Return to Vaccine Overview

1,840,453 | 67.47%
Eligible Population with at least one dose

1,609,740| 59.01%
Eligible Population Completed COVID-19 Vaccine Series

Eligible Population Vaccinated with One Dose

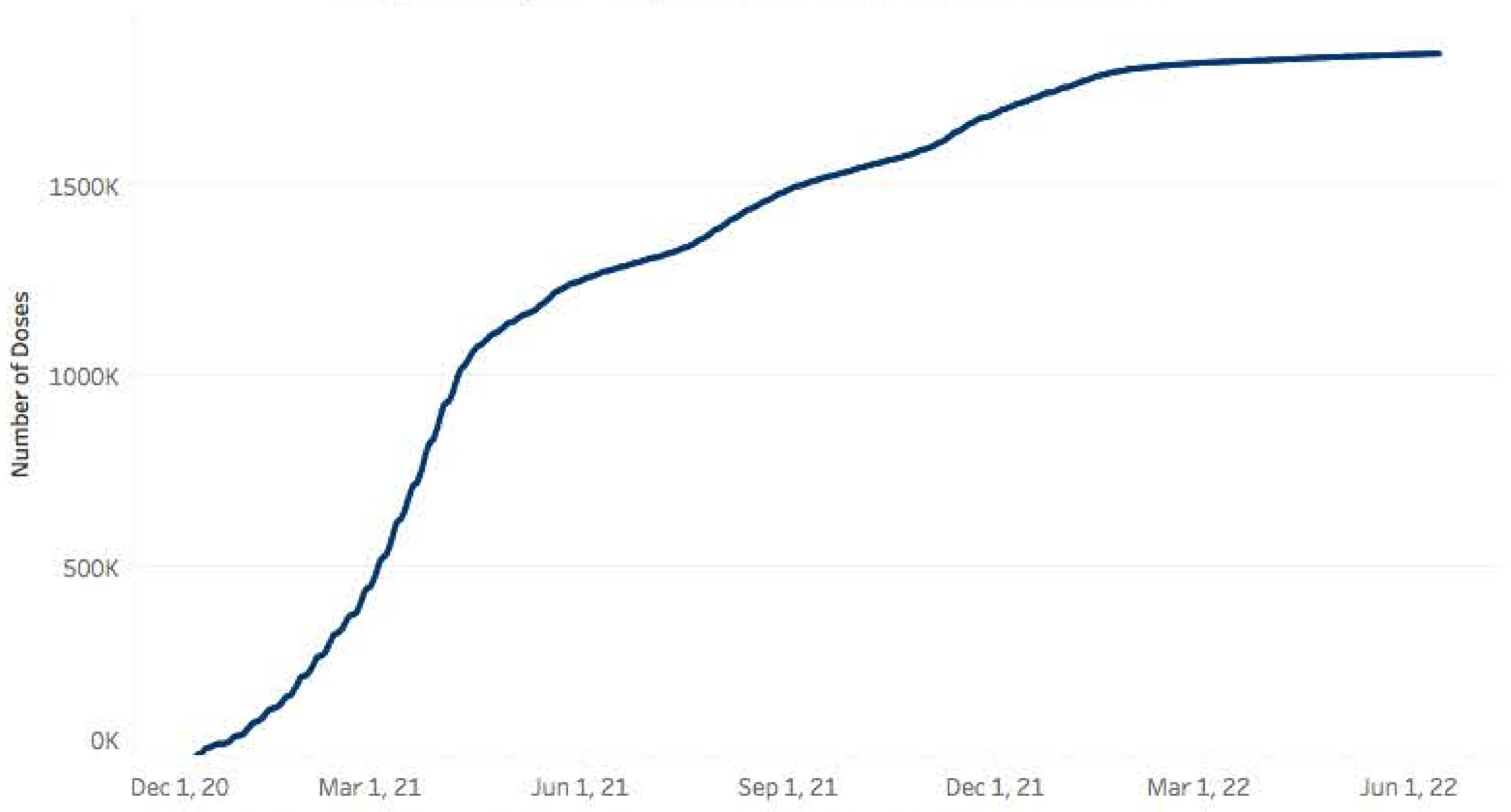

Eligible Population Completed COVID-19 Vaccine Series

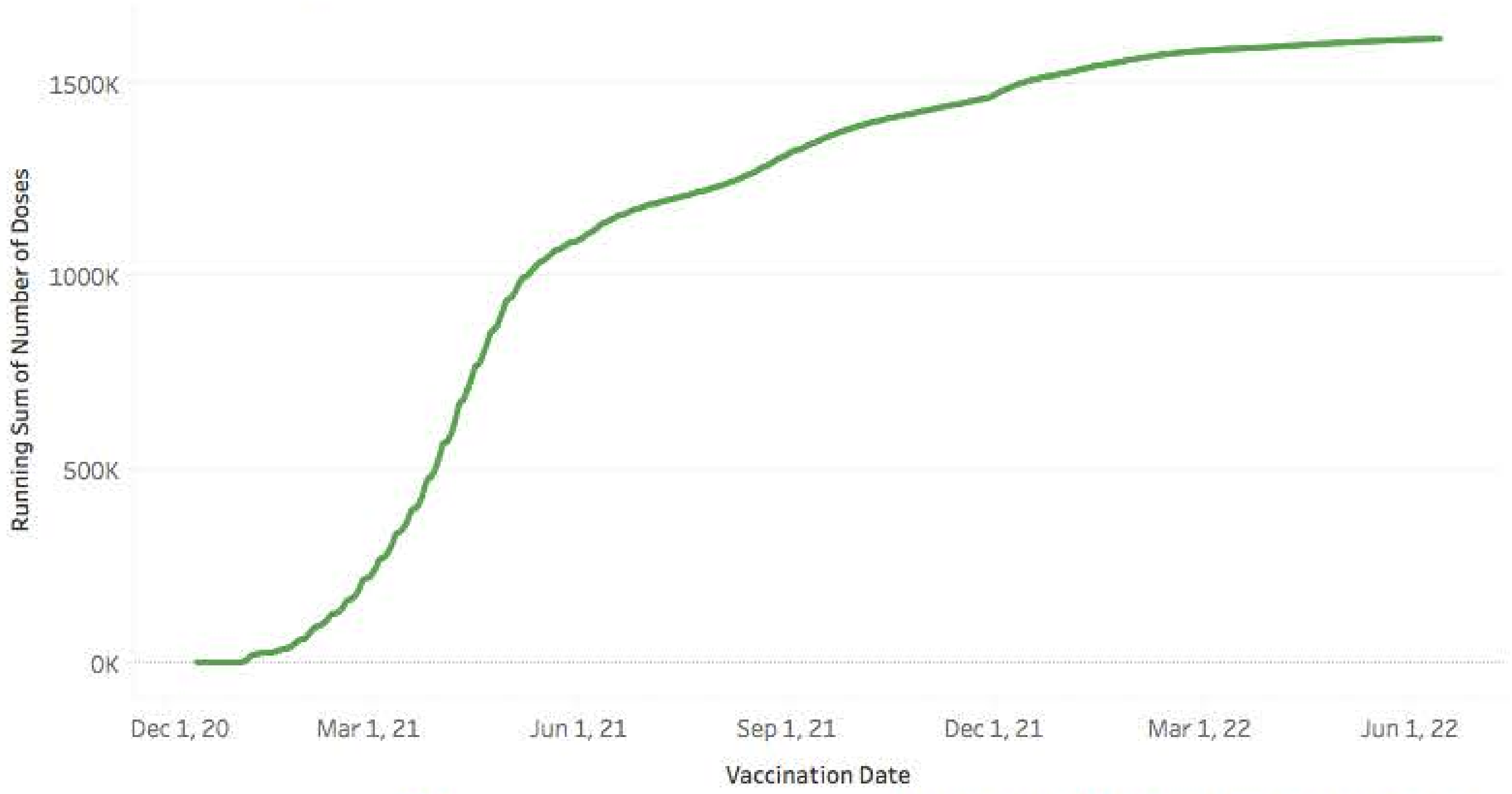

4,476,358

Vaccines Administered by Dose and Manufacturer

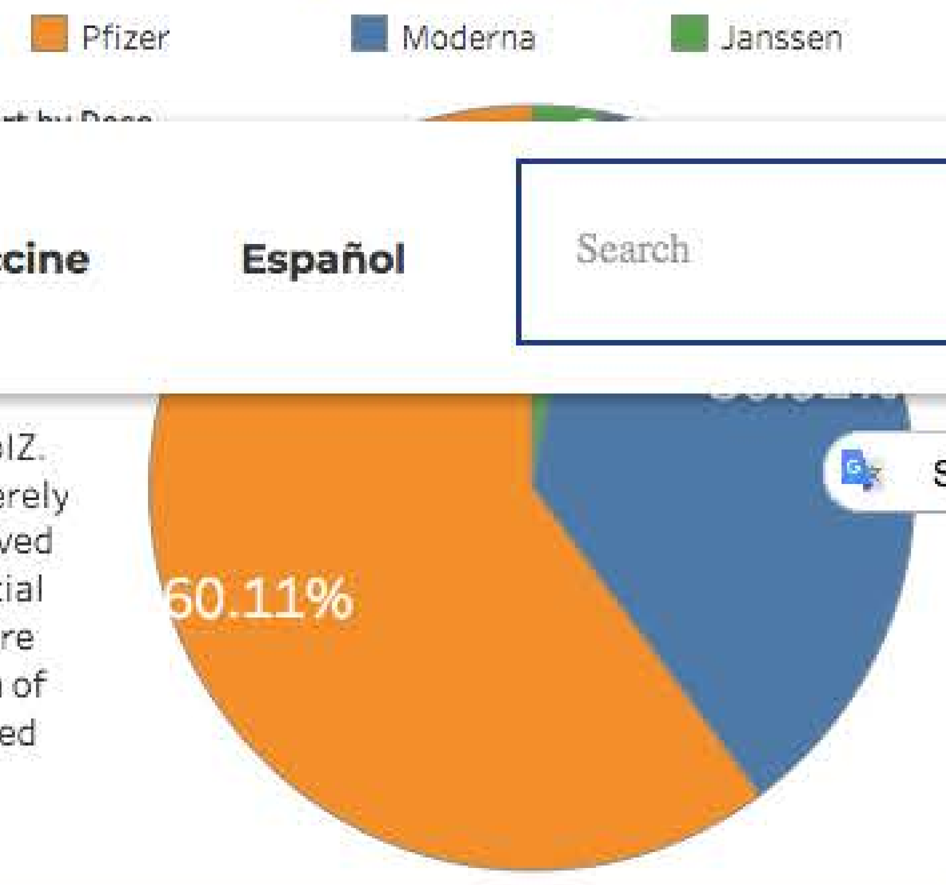

oviders Vaccine Info Data Find My Vaccine Español Search

Select Language

Third Dose : [illegible]

* Third dose includes all instances of 3 doses reported in KSWebIZ. On 8/12/2021, a third dose was approved for those who are severely immunocompromised. On 9/24/2021, a booster dose was approved for select populations 6 months following completion of an initial series of Pfizer. On 10/21/2021, booster doses for Moderna were approved for select populations 6 months following completion of initial series. Johnson and Johnson booster doses were approved for all recipients 2 months after initial series.

COVID-19 Vaccines Administered by Dose Number

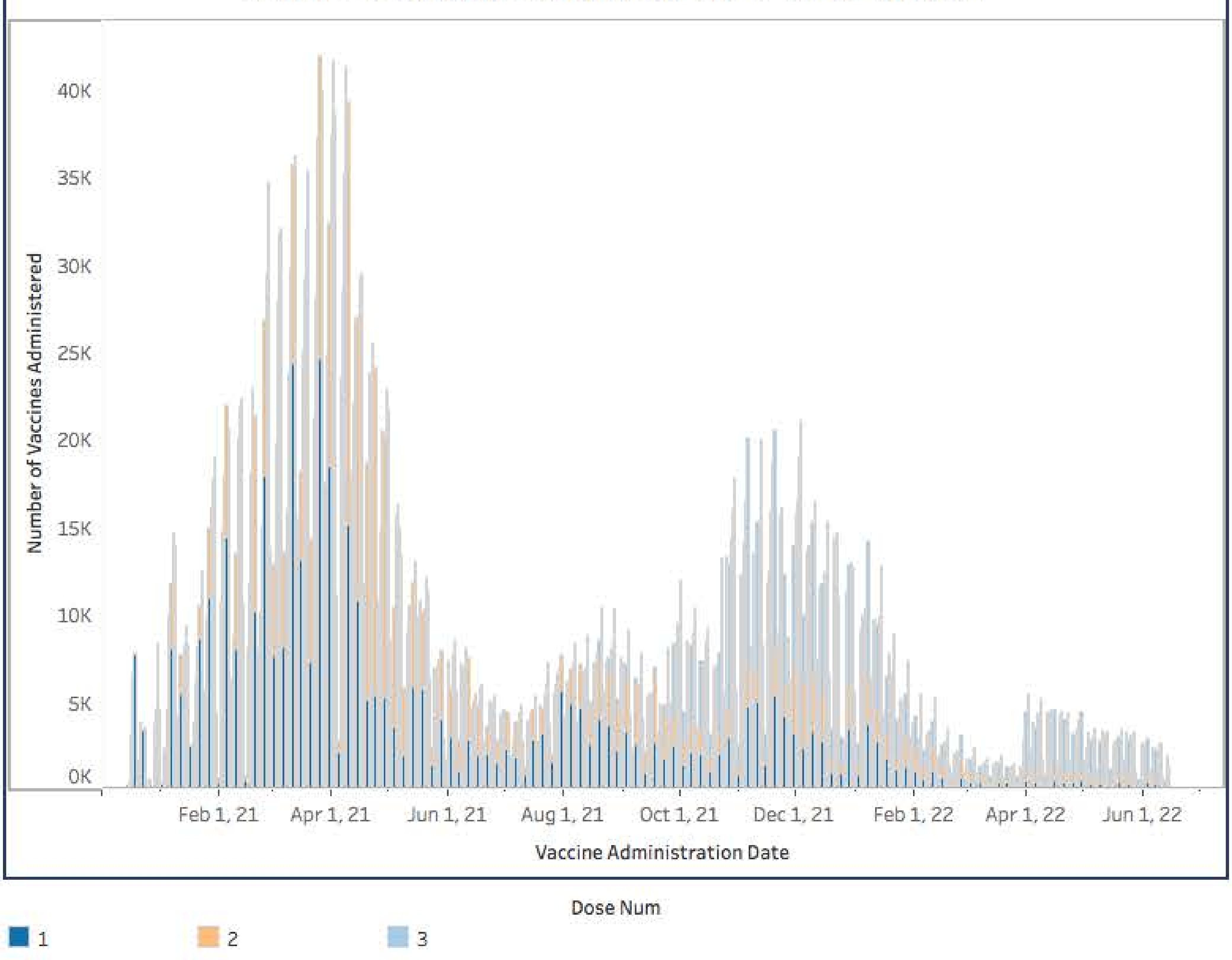

Undo Redo Replay Revert Refresh Pause Share Download Full Screen

Data

Dashboard Information

Beginning May 18, the COVID-19 dashboard will be published on Wednesdays by 12:30 p.m. To maximize your experience, us
device with a larger screen, such as a tablet, laptop or desktop.

Kansas COVID-19
Demographics

Data are preliminary and subject to quality improvement and quality assurance validation. Race and ethnicity data is reported into the Kansas Immunization Registry (KSWebIZ) by vaccine providers using patient self-identified race and ethnicity. Multiple races are available for selection with the primary race listed first followed by other selected races. Race is reported into the demographics section of a patient record and can be updated as needed; updated race information will apply to all previous and future vaccine administered to that individual.

Data Source: Kansas Immunization Information System, KSWebIZ

^Younger than 18 includes all individuals younger than 18 years of age while the vaccine is only approved for persons 5 years of age and older. This rate includes people who are currently ineligible for the vaccine.

Return to Vaccine Overview

People Vaccinated by Sex
(rate per 1,000 population)

	People Vaccinated	Rates per 1,000
Female	976,349	667.99
Male	861,381	593.36
Unknown/Not Reported	2,893	
Total	1,840,623	

People Vaccinated by Age Group
(rate per 1,000 population)

	People Vaccinated	Rate per 1000
5 to 11	76,652	277.44
12 to 17	133,043	557.52
18 to 24	169,692	576.87
25 to 34	236,014	616.35
35 to 44	253,950	703.41
45 to 54	242,494	739.40
55 to 64	292,638	787.71
65 to 74	255,292	937.76
75 to 84	127,024	923.32
85+	53,654	816.92
Grand Total	1,840,453	

People Vaccinated by Race
(rate per 1,000 population)

race	People with at least 1 ..	Rate of at least 1	People Fully Vaccinat..	Rate fully vaccinatd p..
White	1,217,952	484.50	1,137,962	452.68
Black or African Amerian	73,847	413.19	71,296	398.91
American Indian or Alaskan Native	7,022	200.27	6,165	175.83
Asian	52,783	568.40	45,166	486.38
Native Hawaiian or Pacific Islander	1,455	394.63	1,254	340.11
Two or more Races	266,464	2,989.58	252,391	2,831.69
Other	75,124		63,327	
Not Reported/Missing	145,976		121,471	

People Vaccinated by Ethnicity
(rate per 1,000 population)

Ethnicity	People with at least 1	Ethnicity Rate	People Fully Vaccinated	Ethnicity Rate Full
Hispanic or Latino	196,978	553.20	169,313	475.50
Not Hispanic or Latino	1,432,057	560.00	1,353,405	529.24
Unknown or Missing	211,588		176,314	

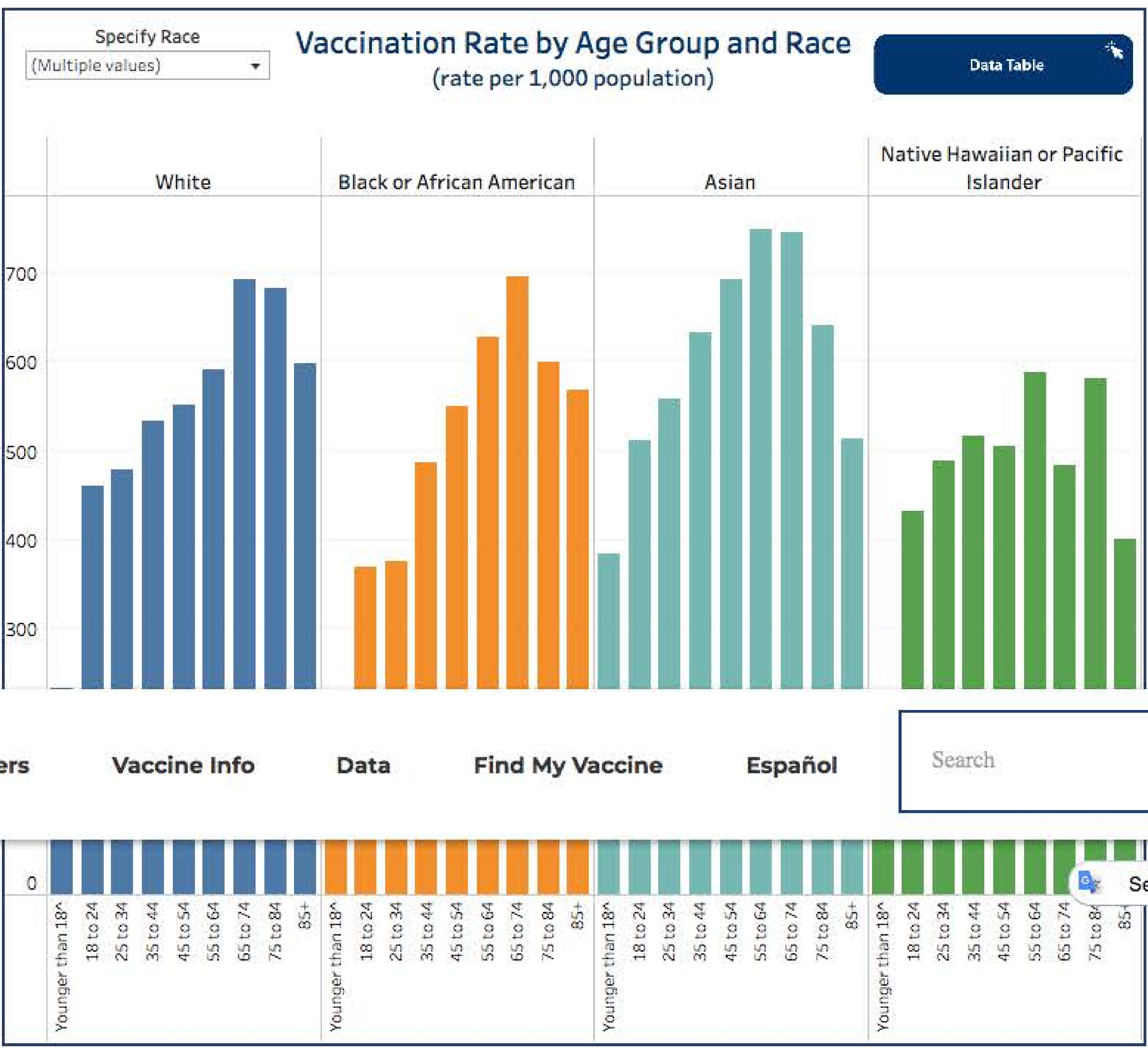

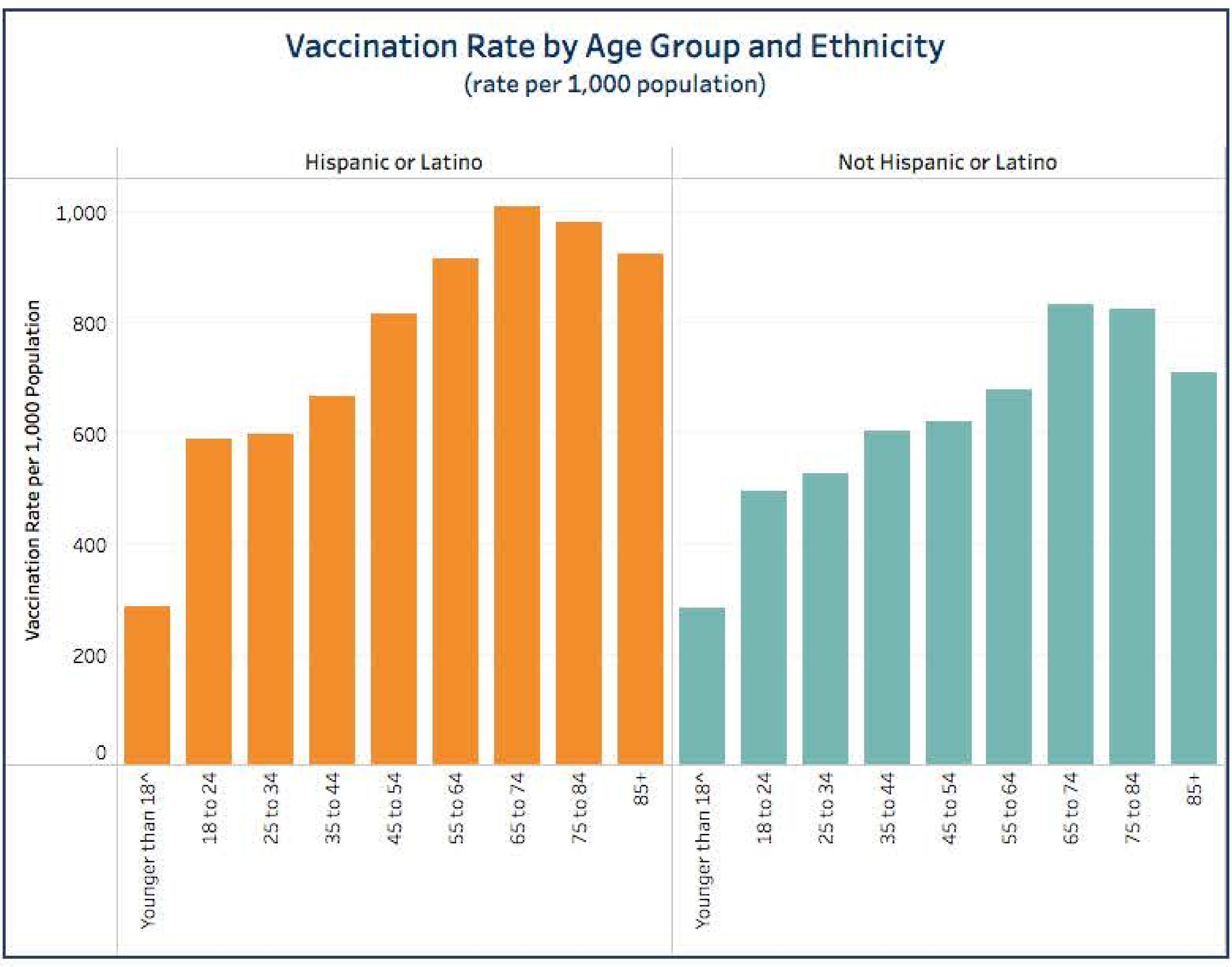

Undo Redo Replay Revert Refresh Pause Share Download Full Screen

Data

Dashboard Information

Beginning May 18, the COVID-19 dashboard will be published on Wednesdays by 12:30 p.m. To maximize your experience, us device with a larger screen, such as a tablet, laptop or desktop.

Kansas COVID-19
Vaccination Rates

Information is provided below on vaccine coverage for people 5 years and older. Information is also provided on vaccine coverage for the total population as a supplement for determining herd immunity.
Vaccine coverage for at least 1 dose includes all individuals who have received their first dose of COVID-19 vaccine. Vaccine coverage for series completion includes all individuals who have completed 2 doses of Pfizer-BioNTech or Moderna vaccine or 1 dose of Johnson and Johnson/Janssen.
Data Source: Kansas Immunization Information System, KSWebIZ

Additional Age Groups | Return to Vaccine Overview

Below Charts Use 5 and Older County Population For Rates

Rate of People ≥ 5 Years of Age Vaccinated with at least 1 Dose
(rate per 1,000 population ≥ 5 years of age; as reported in KSWebIZ)

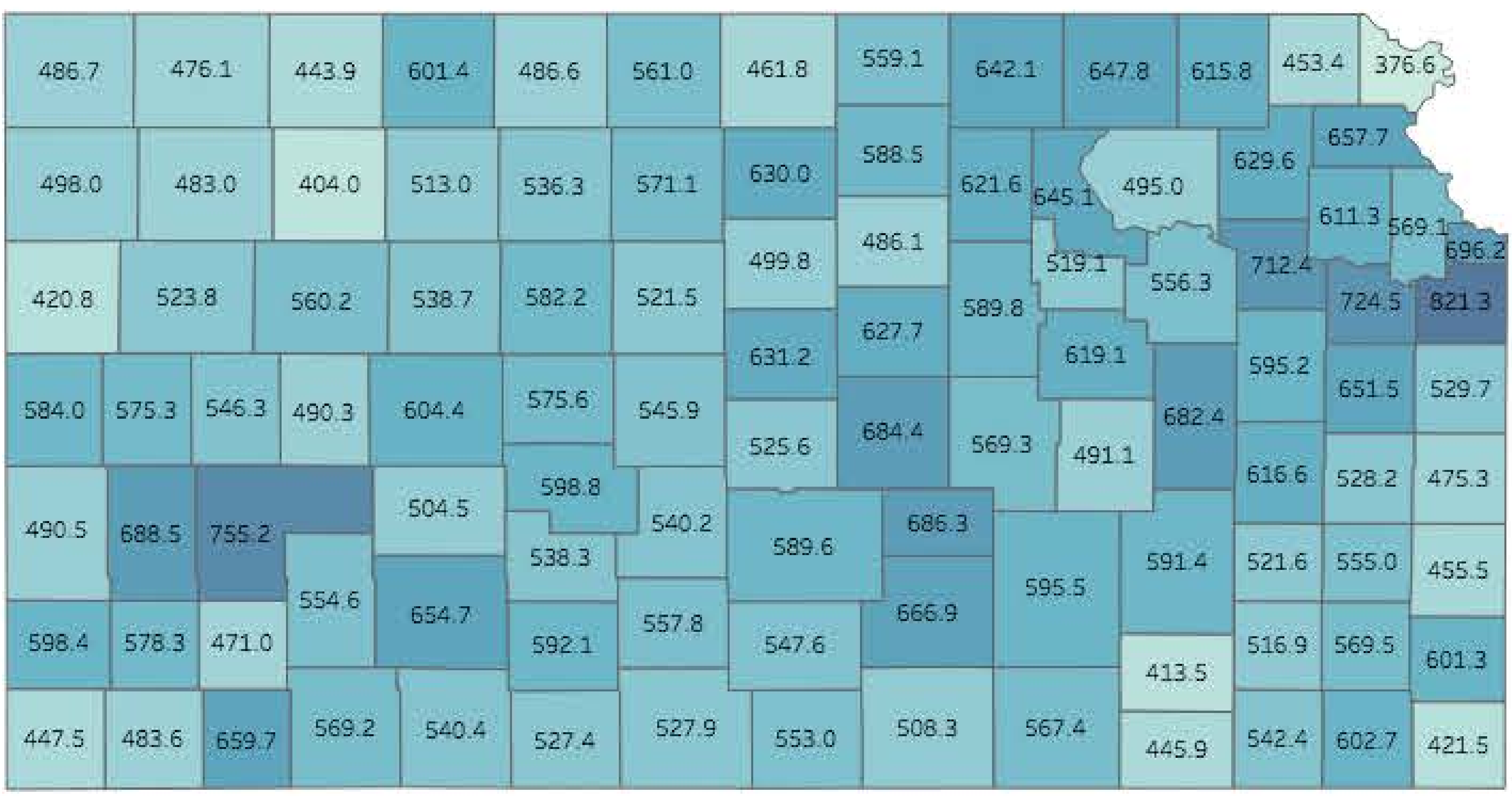

Rate of People ≥ 5 Years of Age with Completed Vaccine Series
(rate per 1,000 population ≥ 5 years; as reported in KSWebIZ)

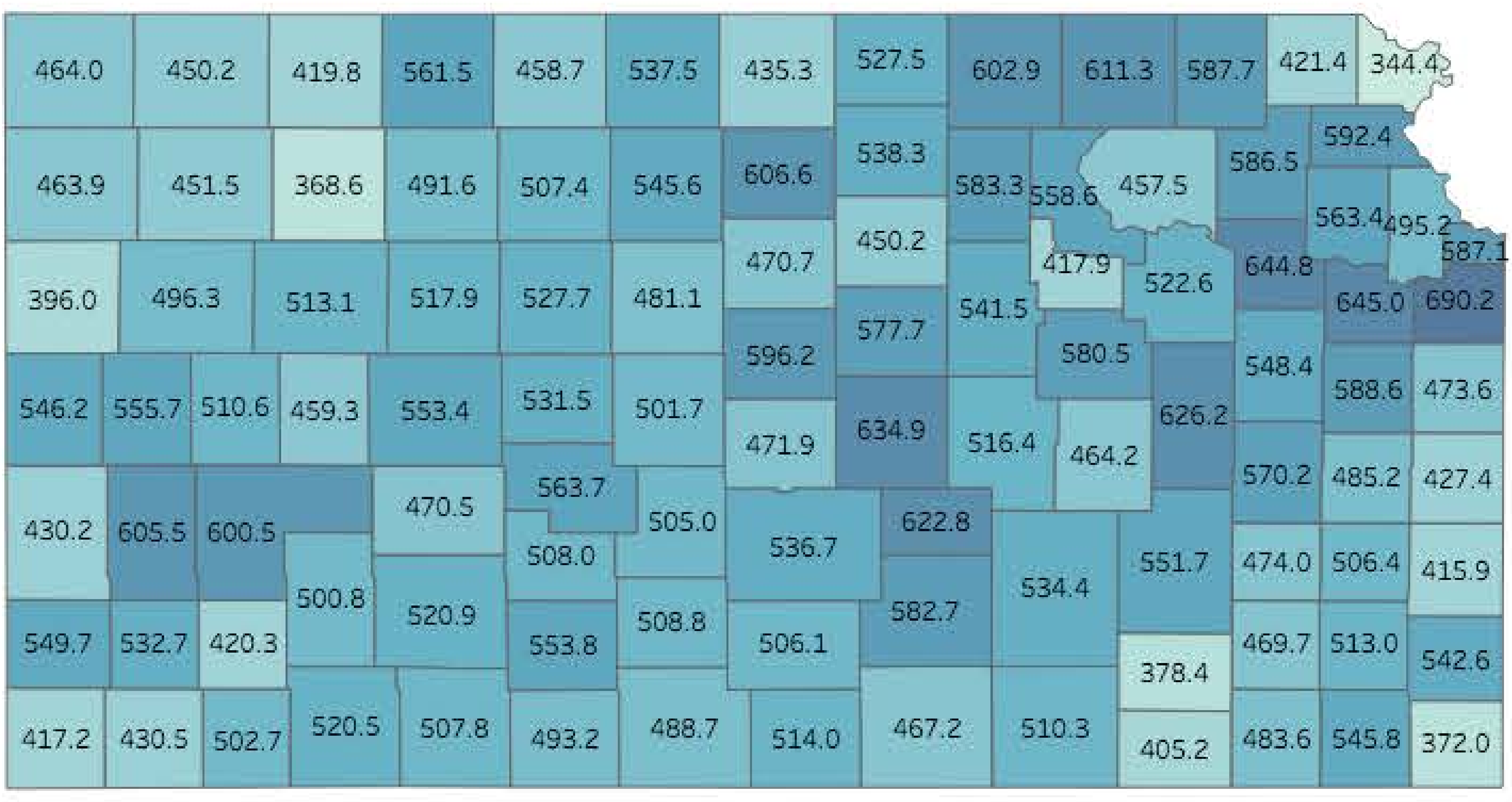

Below Charts Use Total County Population for Rates

Rate of People Vaccinated with at least 1 Dose
(rate per 1,000 total population; as reported in KSWebIZ)

viders Vaccine Info Data Find My Vaccine Español Search

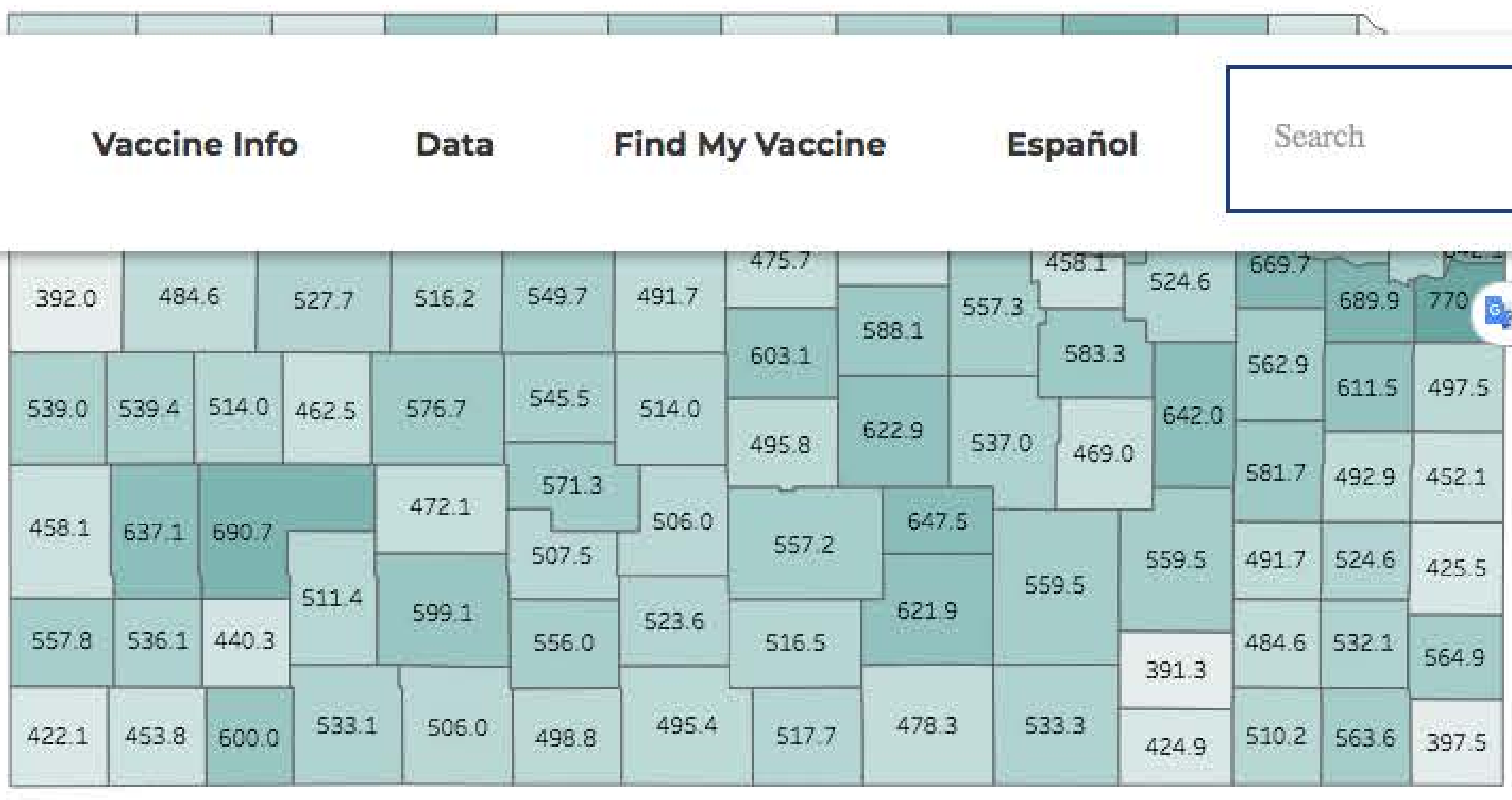

Select

Rate of People with Completed Vaccine Series
(rate per 1,000 total population; as reported in KSWebIZ)

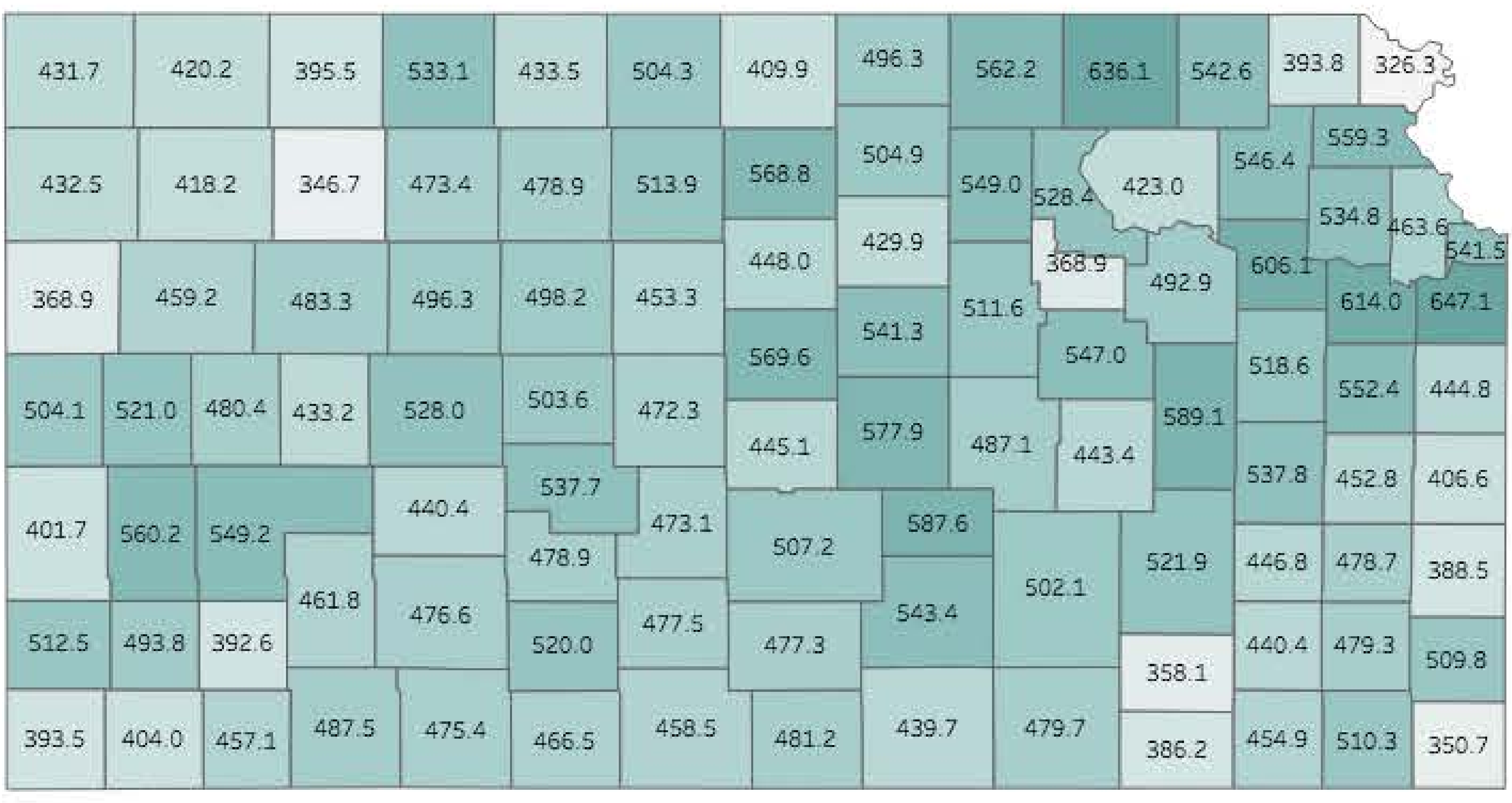

Undo Redo Replay Revert Refresh Pause Share Download Full Screen

Michigan First-Dose Tracker

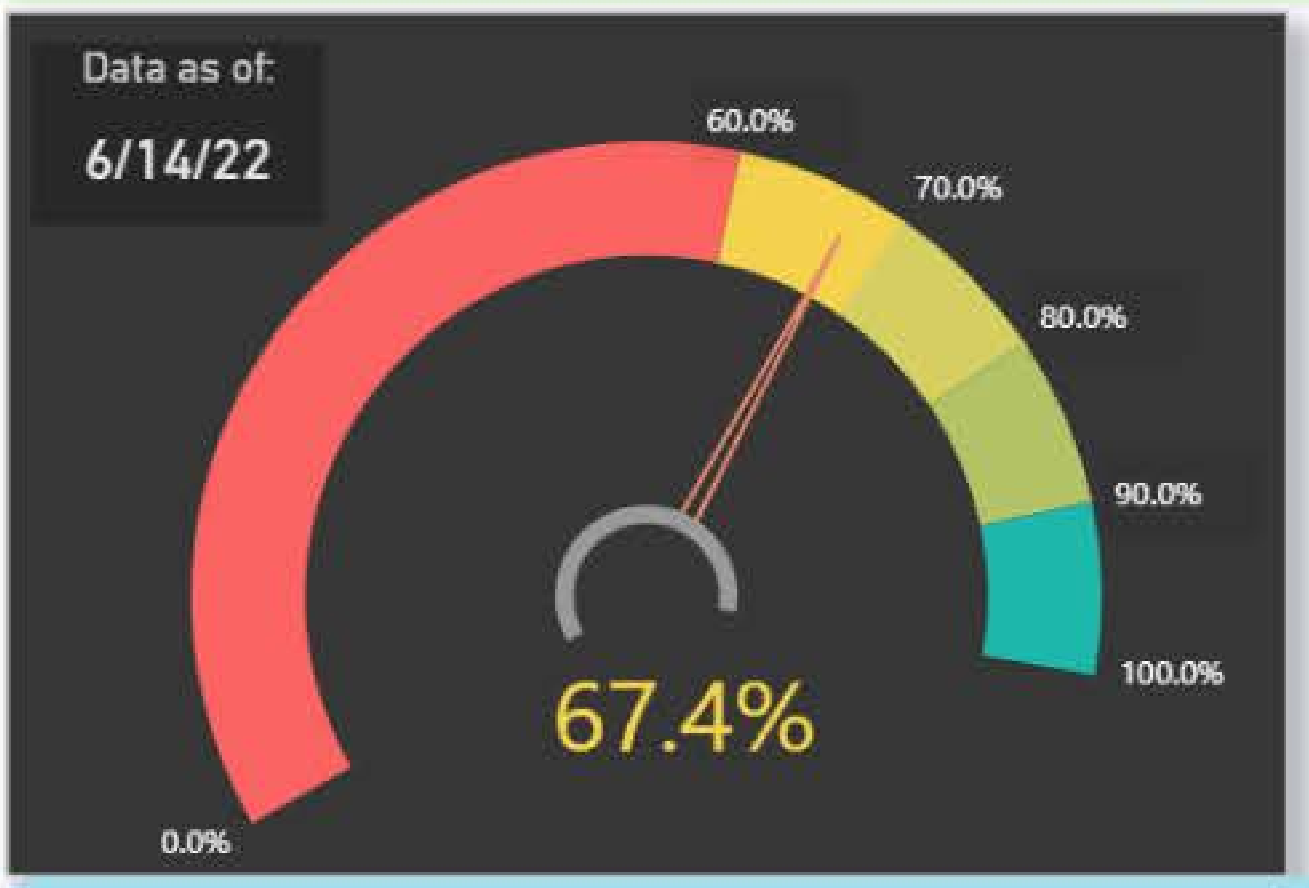

6,726,316 MI Residents with 1+ dose

÷

9,984,503 Total MI Population

=

67.4%

For Reference, 70% is equal to 6,989,152 Michigan Residents
80% is equal to 7,987,602 Michigan Residents
90% is equal to 8,986,053 Michigan Residents

Looking for COVID-19 Case Data? Click here.

Progress is based on the CDC data tracker, which includes MI residents vaccinated by providers not currently reporting to the state dashboard below: Veterans Affairs, Department of Defense, Bureau of Prisons, and most out-of-state providers. Data provided in the Michigan COVID-19 Dashboard below slightly undercounts the true number of doses administered to MI residents. See the "Learn More" tab below for details.

Starting the week of April 4: The first dose tracker, public use files and data reported in the dashboard below will be updated once per week on Wednesdays.

First-dose tracker above is for entire Michigan Population. Coverage maps below are for age 5+.

Michigan's COVID-19 vaccine Public Dashboard now includes Booster Coverage by County under Additional/Booster tab

We have updated the Additional/Booster Doses Administered, this page previously counted the number of third doses administered to individuals who had completed an mRNA series, or second doses administered to individuals who had completed a Janssen primary series. The update counts doses administered after completion of a primary series to account for the fact that multiple additional and/or booster doses may be administered to certain populations

Primary Series Doses Administered | Primary Series Doses by Vaccine | Additional / Booster | Coverage | Age/Sex | Race/Ethnicity | Other | Enrolled Providers | Learn More

COVID Vaccination Coverage - Initiation

Dashboard Updated: June 15, 2022. "Completion" is the percentage of Michigan residents receiving 2 doses of Pfizer or Moderna or 1 dose of J&J. "Initiation" is the percentage who have received either 1 or more doses of ANY vaccine. See the "Learn More" page to learn how percentages are calculated.

Michigan

Preparedness Region: All
Local Health Dept. Jurisdiction: All
County: All

Initiation | Completion | Coverage Slicers | Booster Coverage

COVID Vaccine Coverage by County

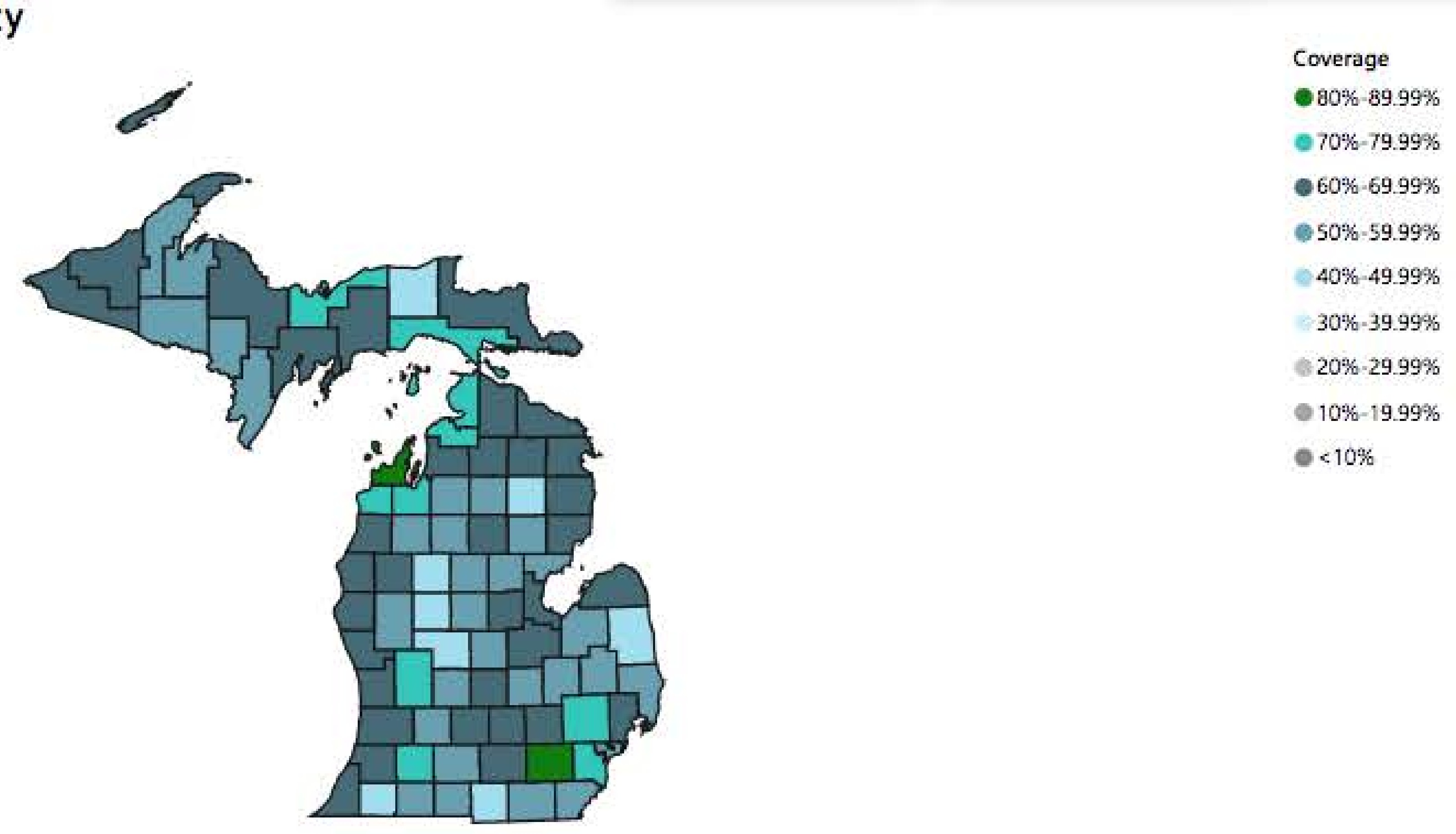

Week Ending Date
12/19/2020 6/18/2022

Residents Vaccinated
6,248,688

Coverage (% of Residents Vaccinated)
66.3%

MI Population*
9,420,414

*2019 US Census estimates for persons 5 years of age and older

85%

Microsoft Power BI

9 of 15

Vaccine Data

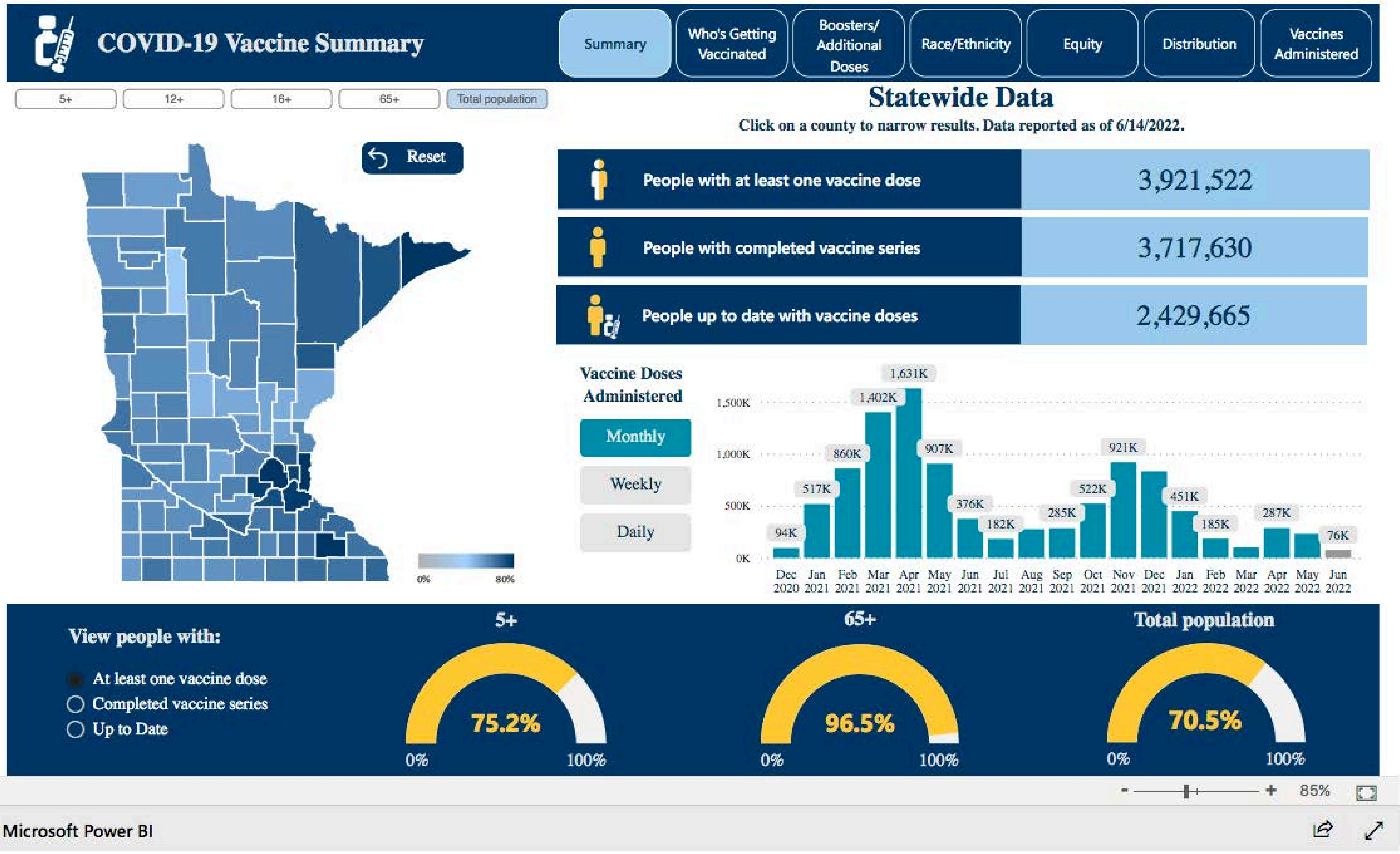

Vaccine Data

Who's getting vaccinated

Summary | Who's Getting Vaccinated | Boosters/ Additional Doses | Race/Ethnicity | Equity | Distribution | Vaccines Administered

5+ | 12+ | 16+ | 65+ | Total population

At least one vaccine dose | Completed vaccine series | Up to Date

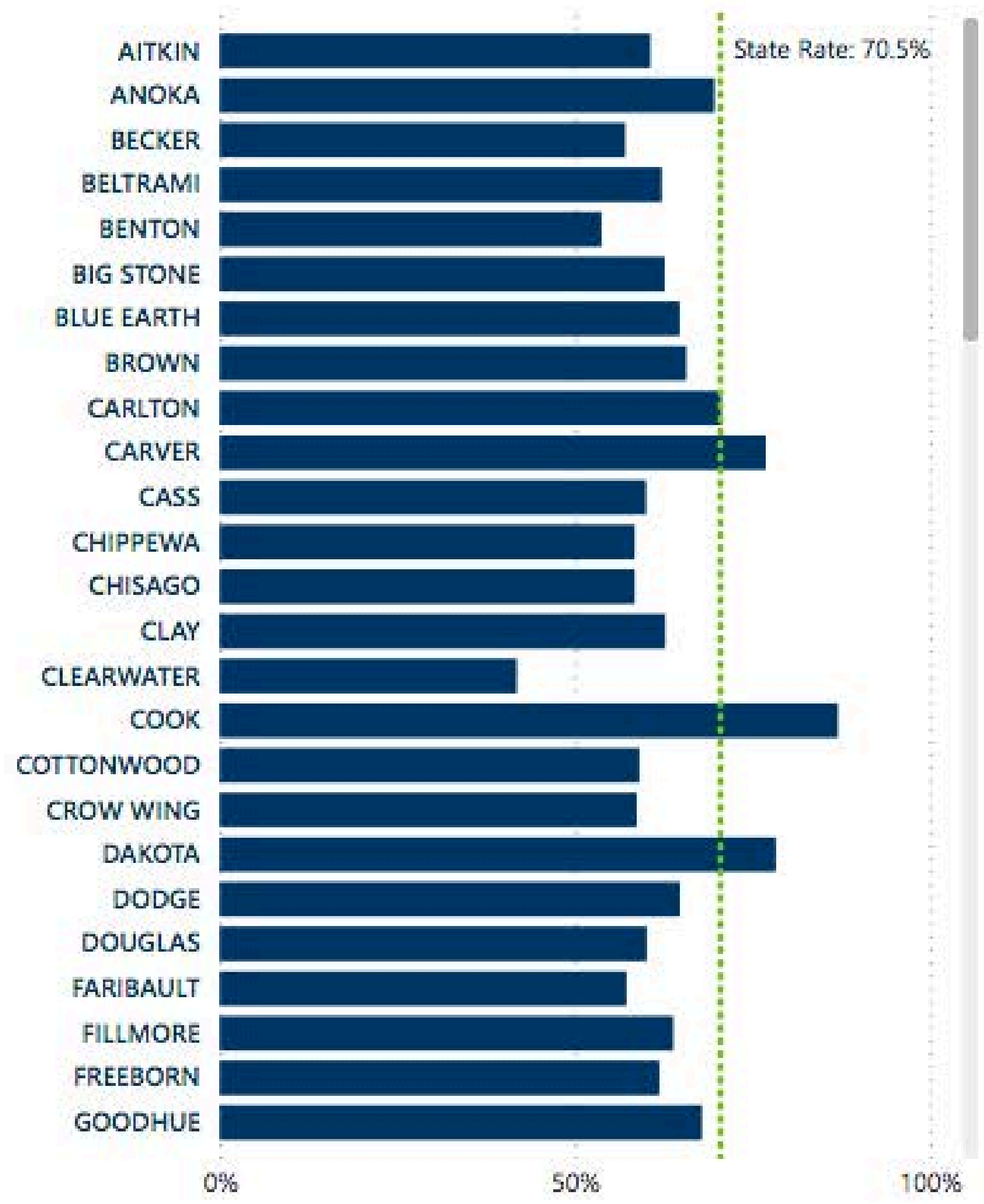

Statewide Data

Click on a county to narrow results. Data reported as of 6/14/2022.

People with at least one vaccine dose	3,921,522	70.5%
People with completed vaccine series	3,717,630	66.8%
People up to date with vaccine doses	2,429,665	43.7%

Age group | Gender

Age group	People with at least one vaccine dose	People with completed vaccine series	People up to date
5-11	215,876	197,010	64,320
12-15	193,730	182,020	70,281
16-17	99,967	94,398	44,505
18-49	1,693,764	1,582,774	902,382
50-64	889,243	859,569	626,031
65+	828,249	801,817	722,140
Unknown/missing	693	42	6
**Total**	**3,921,522**	**3,717,630**	**2,429,665**

Proportion of People Vaccinated by Age Group Population

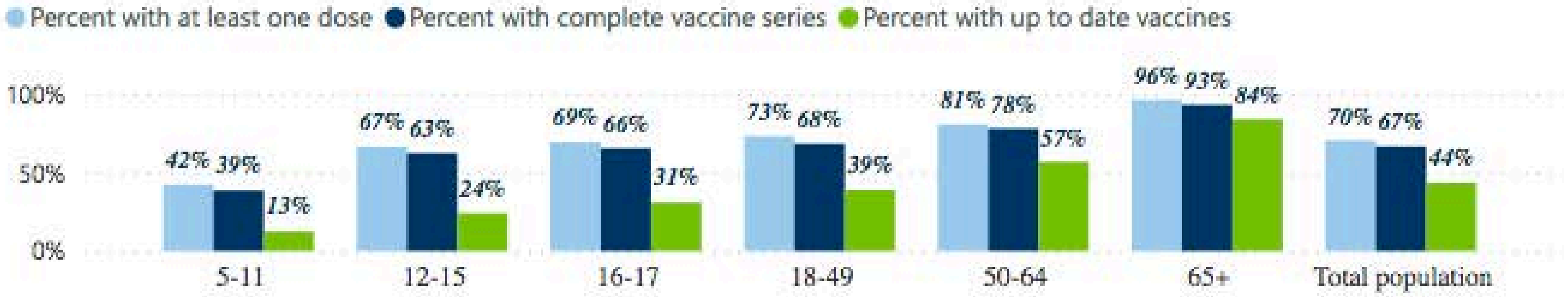

85%

Microsoft Power BI

Pages

Vaccine Data

Race and Ethnicity

Summary | Who's Getting Vaccinated | Boosters/ Additional Doses | Race/Ethnicity | Equity | Distribution | Vaccines Administered

Reset filter

View by people with:

- (●) At least one vaccine dose
- ( ) Completed vaccine series
- ( ) Up to date vaccine doses

View by age group:

- (●) 5+
- ( ) 5-11
- ( ) 12+
- ( ) 12-18
- ( ) 19-44
- ( ) 45-64
- ( ) 65+

The race/ethnicity data provided on this dashboard accounts for 90% of the people who have a COVID-19 immunization record with MDH. It also includes immunizations from VA records which are not included in immunization records with MDH. For the remaining records, race/ethnicity data is not available, unknown, or missing. This percentage may vary slightly for each age demographic.

Data reported as of: **5/28/2022**
Updated bi-weekly on Friday.

Progress with Vaccination by Race and Ethnicity, Age 5+

Totals To Date | Weekly Progress

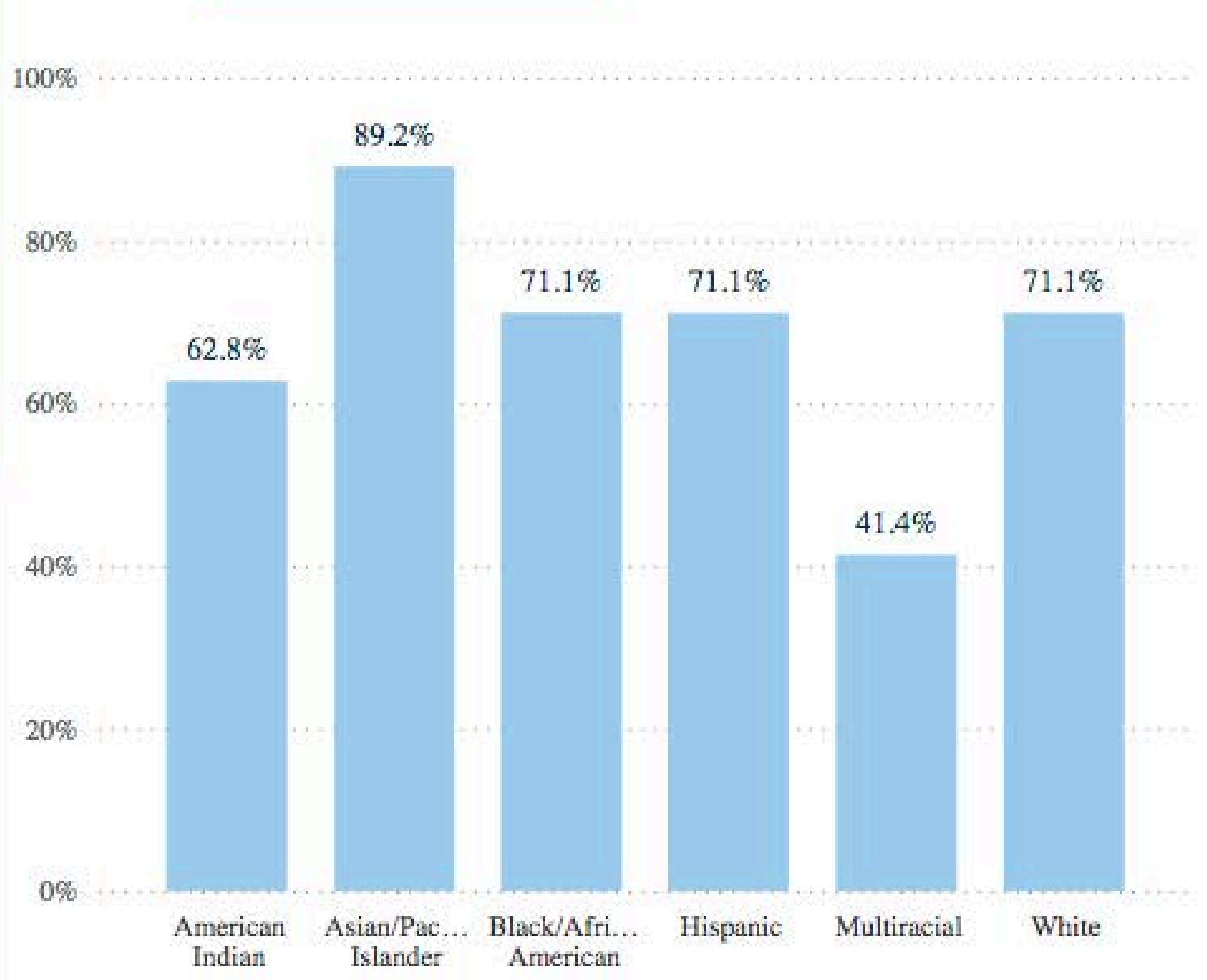

Vaccination Progress by Race and Ethnicity, Age 5+

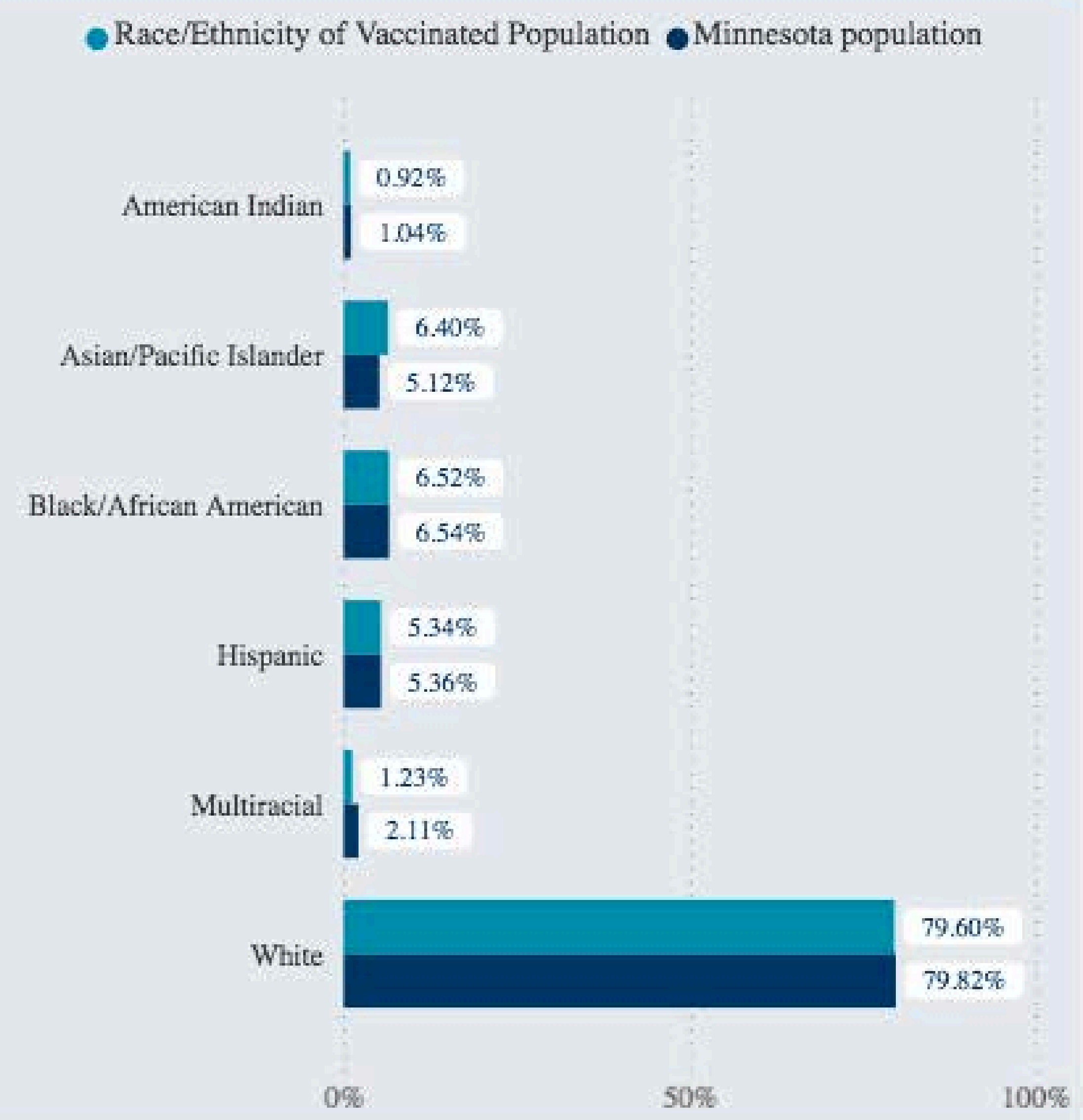

/iew additional information about COVID-19 cases by Race/Ethnicity

85%

Microsoft Power BI

Pages

Vaccine Data

Vaccines administered

Summary | Who's Getting Vaccinated | Boosters/ Additional Doses | Race/Ethnicity | Equity | Distribution | Vaccines Administered

Total vaccine doses administered

10,137,841

Product	Percent	Doses Administered
Pfizer	60.5%	6,135,819
Moderna	36.1%	3,656,271
Johnson & Johnson	3.4%	342,017
Unknown/missing	0.0%	3,734
**Total**	**100.0%**	**10,137,841**

Booster/Additional doses administered

2,289,207

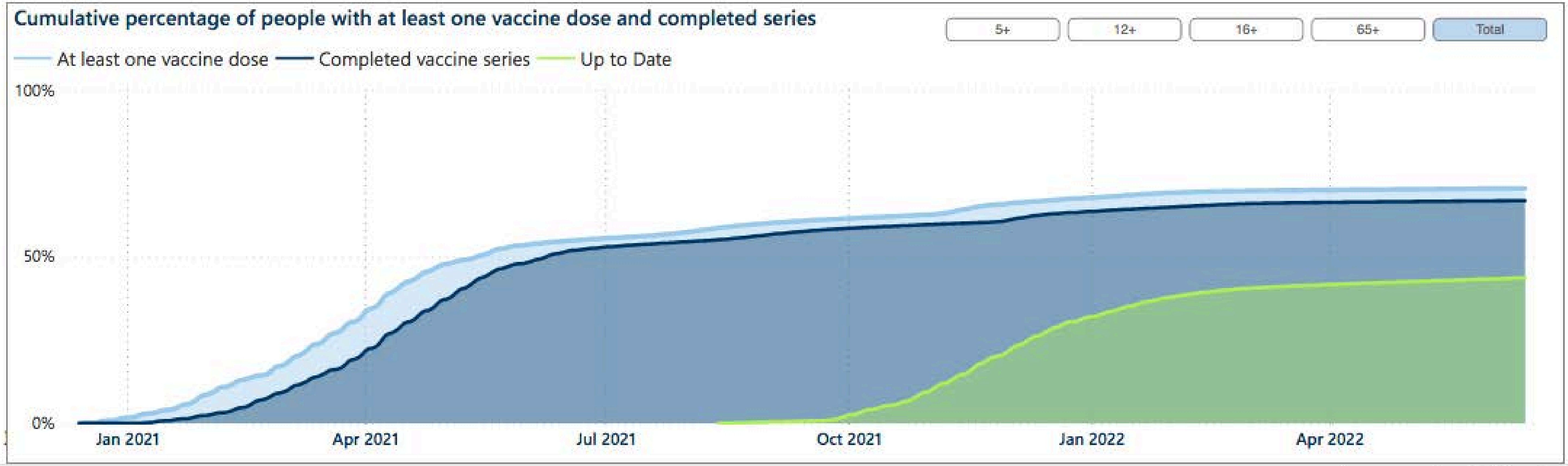

85%

Microsoft Power BI

Pages

Dashboard Last Updated Thursday, 6/16/2022

Data as of Thursday, 6/9/2022

Coverage Rates | Doses Administered | Demographics | County Level | Enrolled Providers

COVID-19 Vaccine Coverage Rates

Select filters to view time or county specific data.

	At least one dose	Primary series complete	Booster dose	Second booster dose
5 and older	58.7%<br>425,692 ND residents	54.6%<br>396,209 ND residents	52.5%<br>208,092 ND residents	
12 and older	58.9%<br>408,525 ND residents	63.1%<br>381,114 ND residents	54.5%<br>207,691 ND residents	
18 and older	65.2%<br>382,601 ND residents	60.8%<br>356,986 ND residents	56.3%<br>200,895ND residents	
65 and older	86.0%<br>103,354 ND residents	81.5%<br>97,839 ND residents	77.2%<br>75,511 ND residents	30.0%<br>22,658 ND residents

Month/ Year: All | Age Group: All | County: All

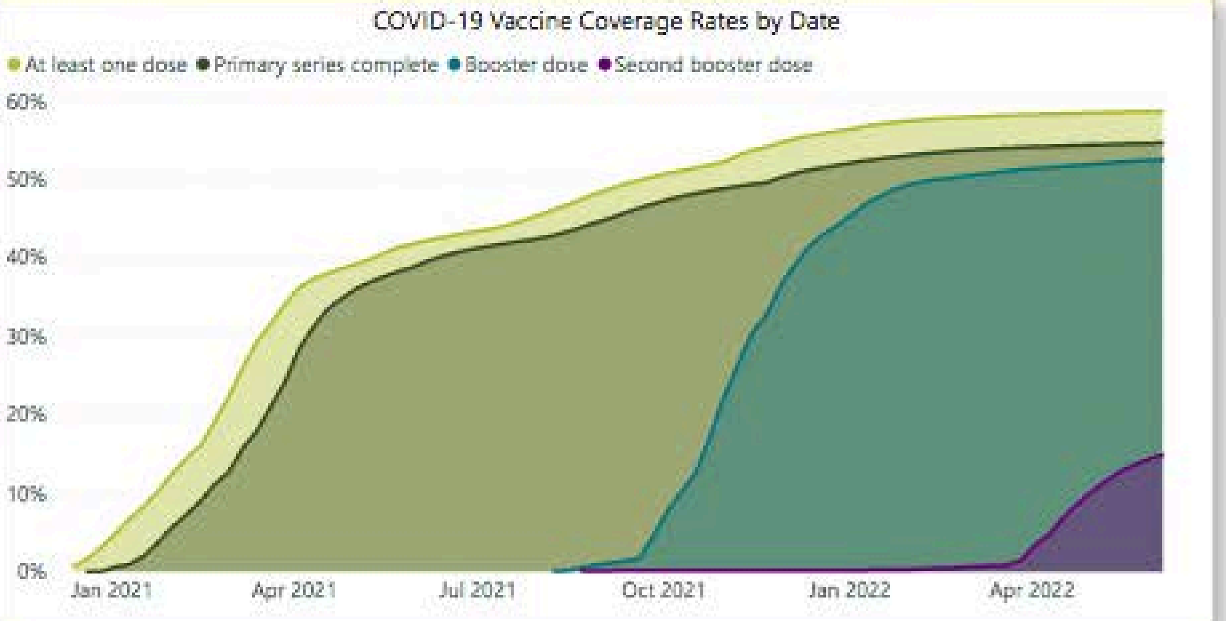

Select View: At least one dose | Primary series comple... | Booster dose | Second booster dose

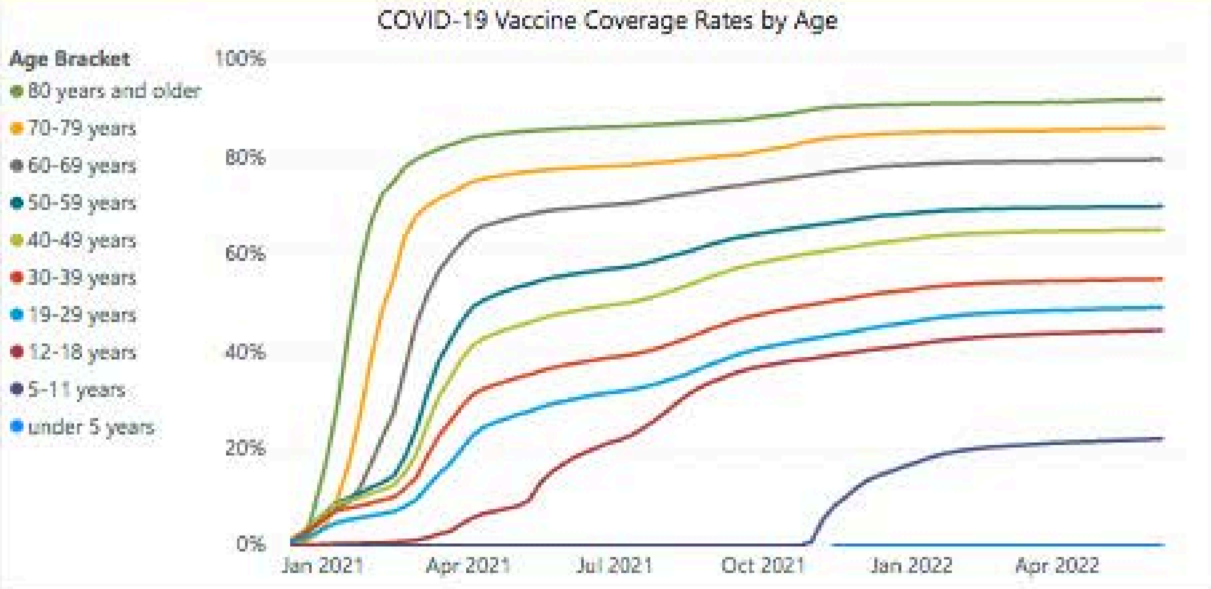

- 56%

Microsoft Power BI

COVID-19 Vaccine Doses Administered

Select Year: All | Select County: All

1.10M
Total Doses Administered

633,436
Pfizer 12+ Doses

35,959
Pfizer 5-11 Doses

389,390
Moderna Doses

38,952
Janssen Doses

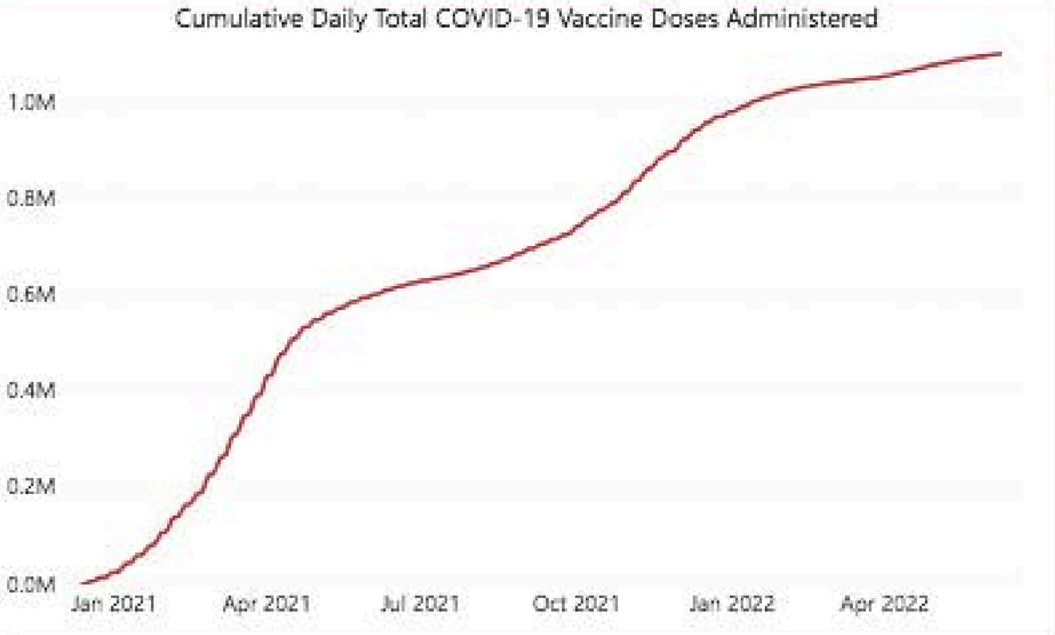

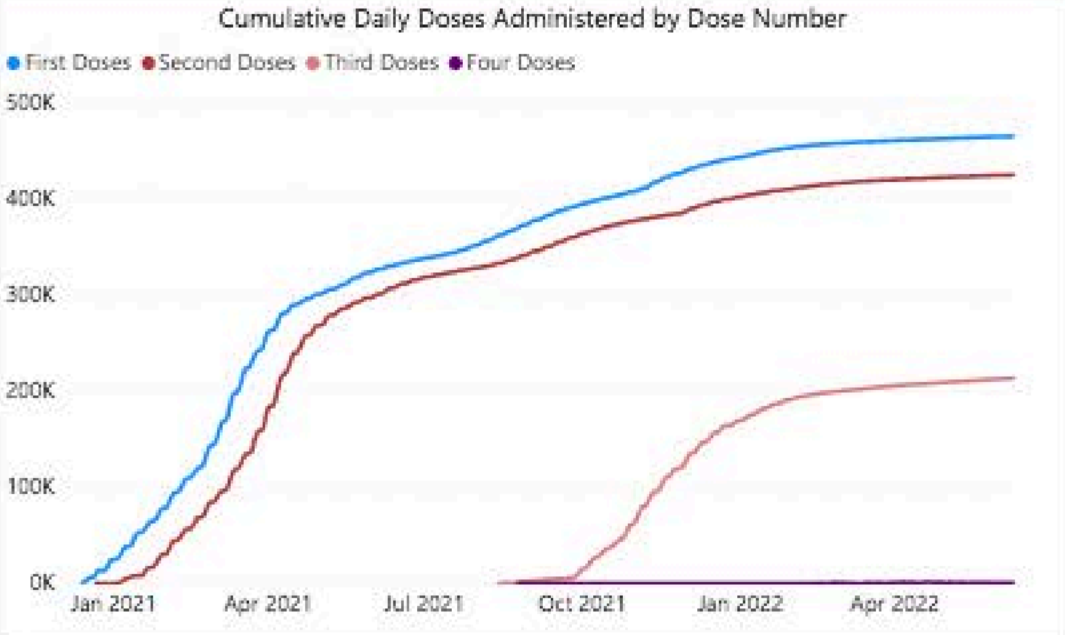

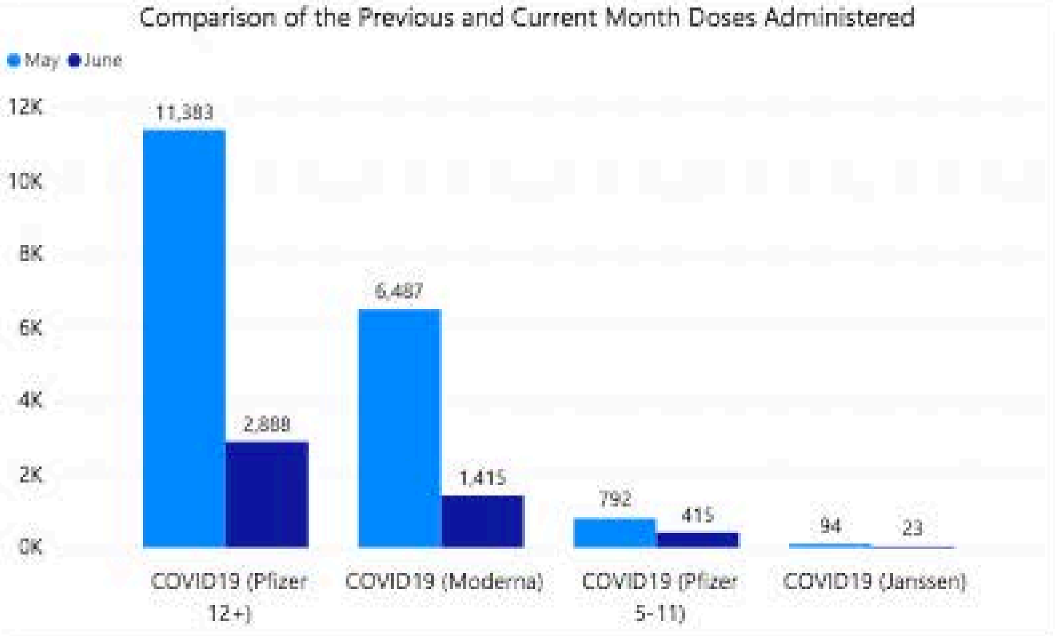

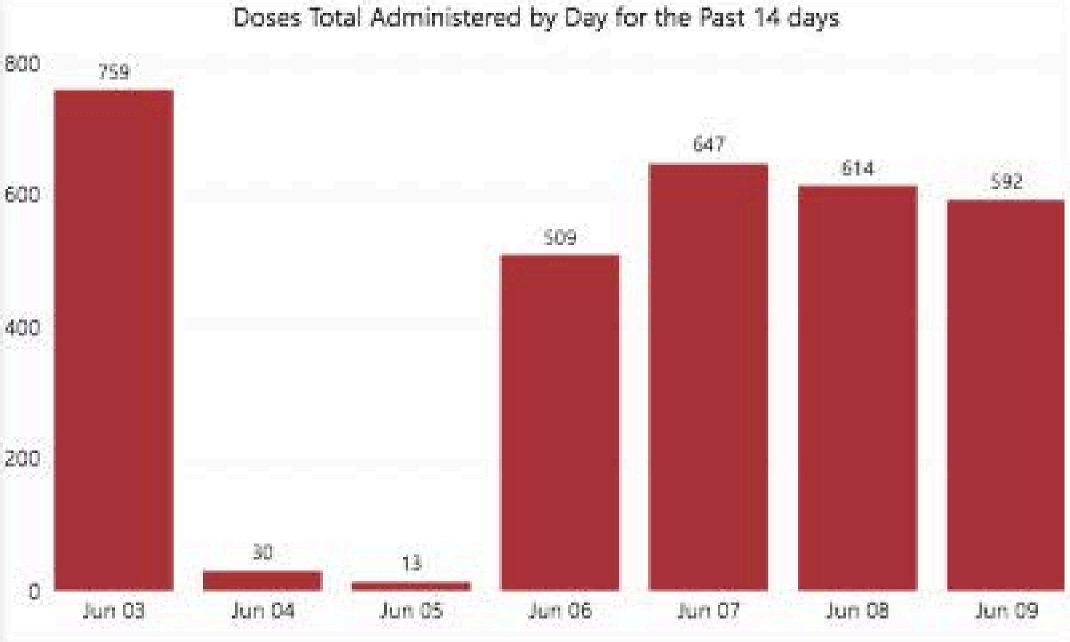

Dashboard Last Updated Thursday, 6/16/2022

Data as of Thursday, 6/9/2022

Coverage Rates | Doses Administered | Demographics | County Level | Enrolled Providers

COVID-19 Vaccine Demographics

Select County: All

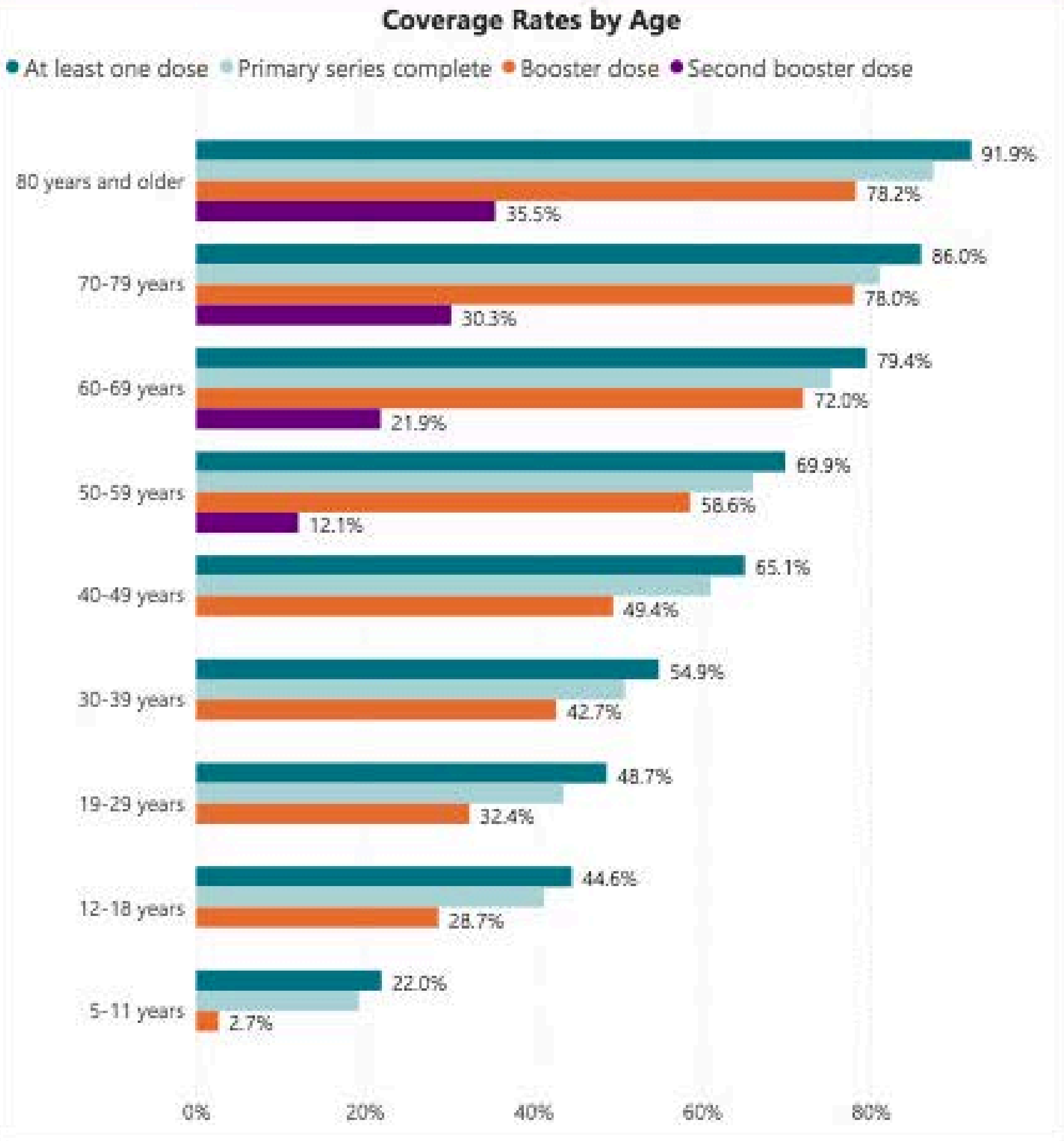

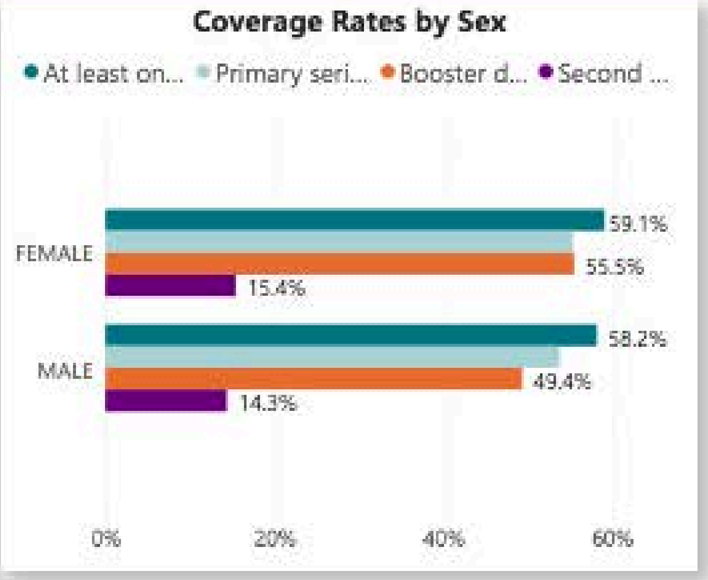

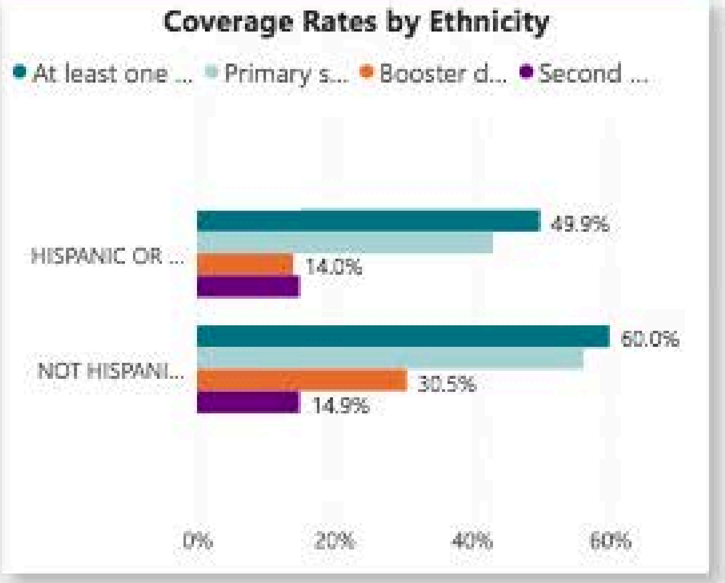

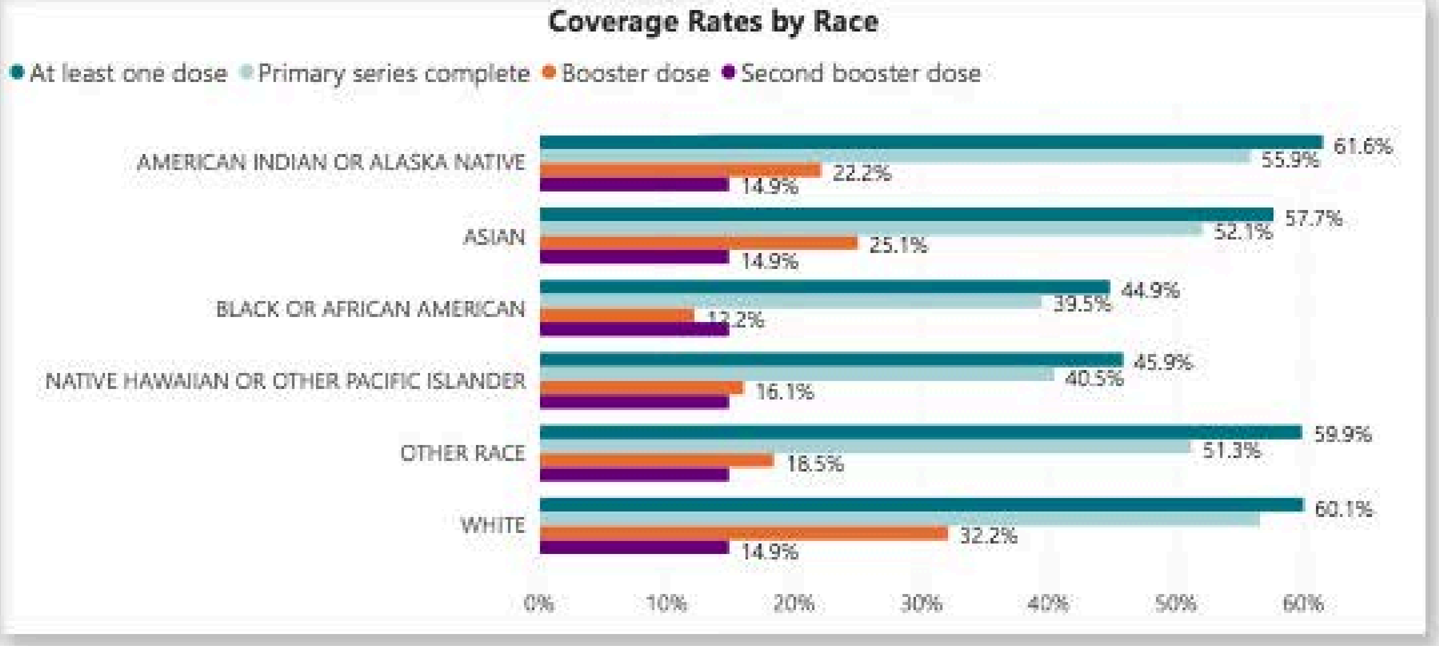

Dashboard Last Updated Thursday, 6/16/2022

Data as of Thursday, 6/9/2022

Coverage Rates | Doses Administered | Demographics | County Level | Enrolled Providers

COVID-19 Vaccine County Level Coverage

1.10M
Total Doses Administered

633,436
Pfizer 12+ Doses

35,959
Pfizer 5-11 Doses

389,390
Moderna Doses

38,952
Janssen Doses

Select Year: All

Select Month: All

Hover over county to view Total COVID-19 Vaccine Doses Administered

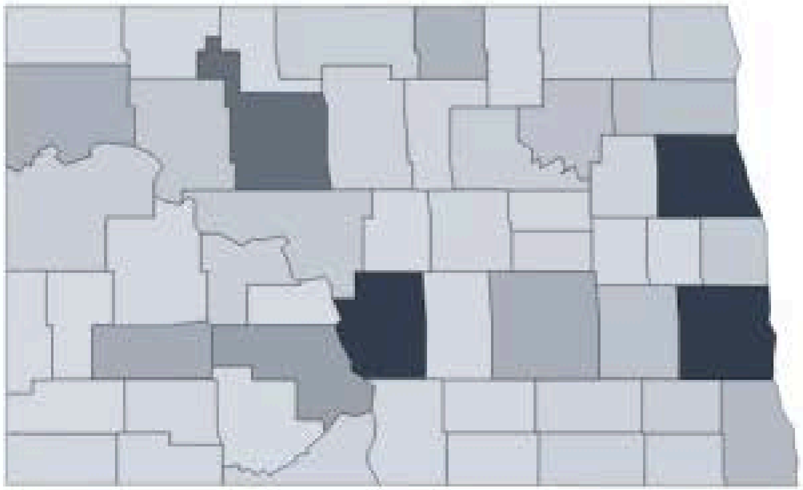

Scroll down to view Total COVID-19 Vaccine Doses Administered

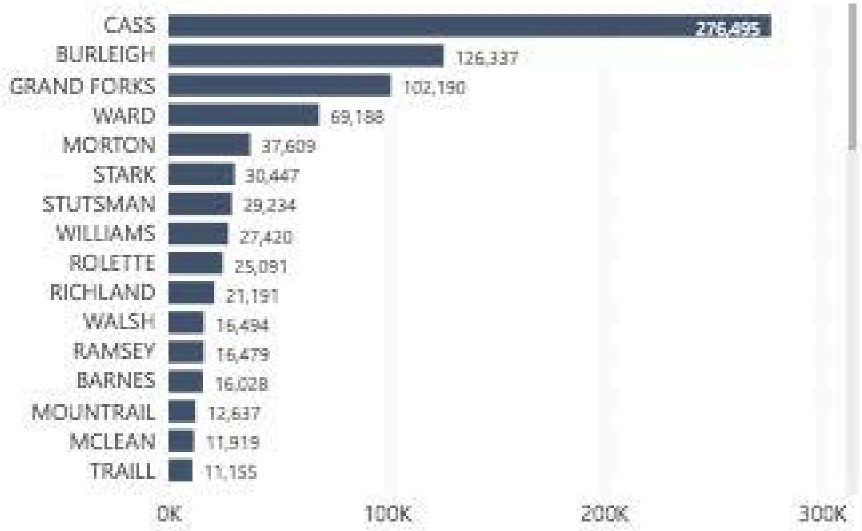

Select At least one dose or Primary series complete

At least one dose | Primary series complete | Booster dose | Second booster dose

5 and older
58.7%
425,692 ND residents

12 and older
63.1%
408,525 ND residents

18 and older
65.2%
382,601 ND residents

65 and older
86.0%
103,354 ND residents

Blank cards appear when there is no data reported.

Hover over county to view COVID-19 Vaccine At least one dose

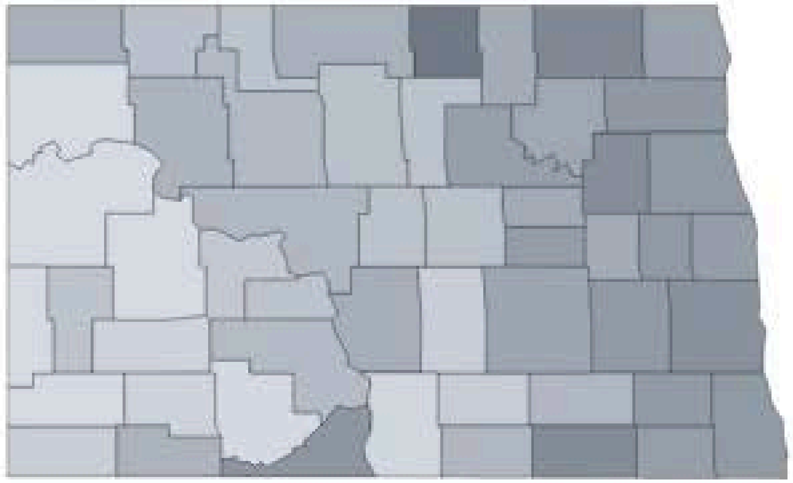

Select Age Group: All

Scroll down to view COVID-19 Vaccine Coverage Rates

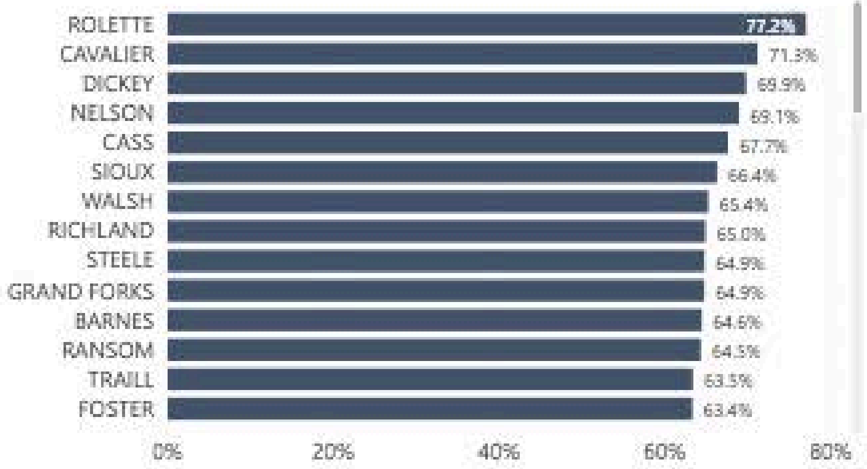

NMDOH

New Mexico Department of Health

COVID-19 Vaccine Dashboard

The New Mexico Department of Health (NMDOH) is leading the State of New Mexico's COVID-19 Vaccination Preparedness Planning in close collaboration with other state agencies, public, private and tribal partners throughout the state.

Learn More

Interested in Phase-Specific Vaccine Data?

If you are interested in information on New Mexico's progress through the phase-based distribution plan, please click here.

COVID-19 Vaccine Dashboard

Last Updated: 6/7/2022

Includes state data (NMSIIS) and Federal data (Tiberius). Data includes doses from the Indian Health Service, Veterans Administration, Department of Defense, Bureau of Prisons, and doses from other states administered to New Mexico residents.

Percentages for "At Least One Dose" and "Primary Series Completed" are based on the population of New Mexicans in the respective age group. Booster data are based on the population of individuals eligible for a booster dose per CDC guidance.

Overall Totals

New Mexicans 18+ With At Least One Dose

1,503,788

New Mexicans 18+ With Primary Series Complete

1,295,667

New Mexicans 18+ With Booster

784,815

Age Group	At Least One Dose	Primary Series Completed	Primary Series With One Booster Dose	Primary Series With More Than One Booster Dose
5-11	74,696 (39.9%)	60,607 (32.4%)	4,030	6.6%
12-17	118,410 (70.2%)	102,964 (61.1%)	35,155 (34.1%)*	N/A
18+	1,503,788 (91.5%)	1,295,667 (78.8%)	784,815 (60.6%)*	163,229 (12.6%)*
65+	417,396 (99.0%)	370,446 (94.4%)	276,183 (74.6%)*	107,954 (29.1%)*

**Booster percentages are based on the population of individuals eligible for a booster dose per CDC guidance.*

Vaccinated By Age Group (State And Federal Data Combined)*

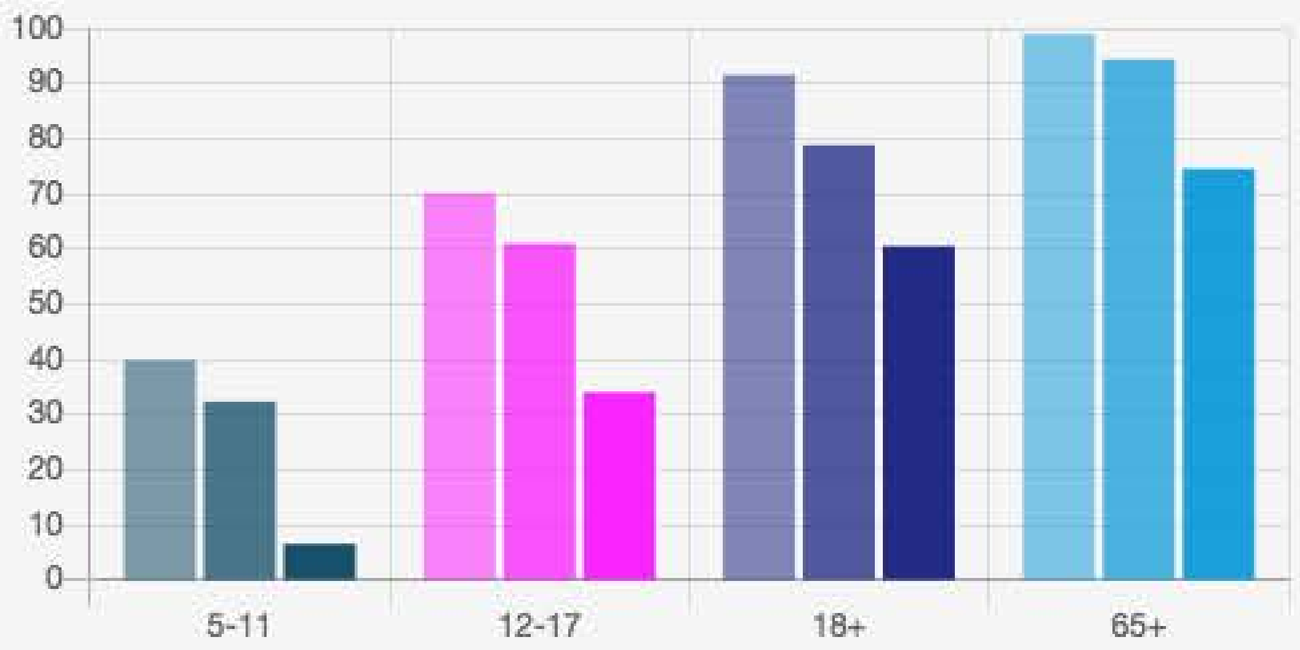

Vaccinated By Race/Ethnicity (State And Federal Data Combined)*

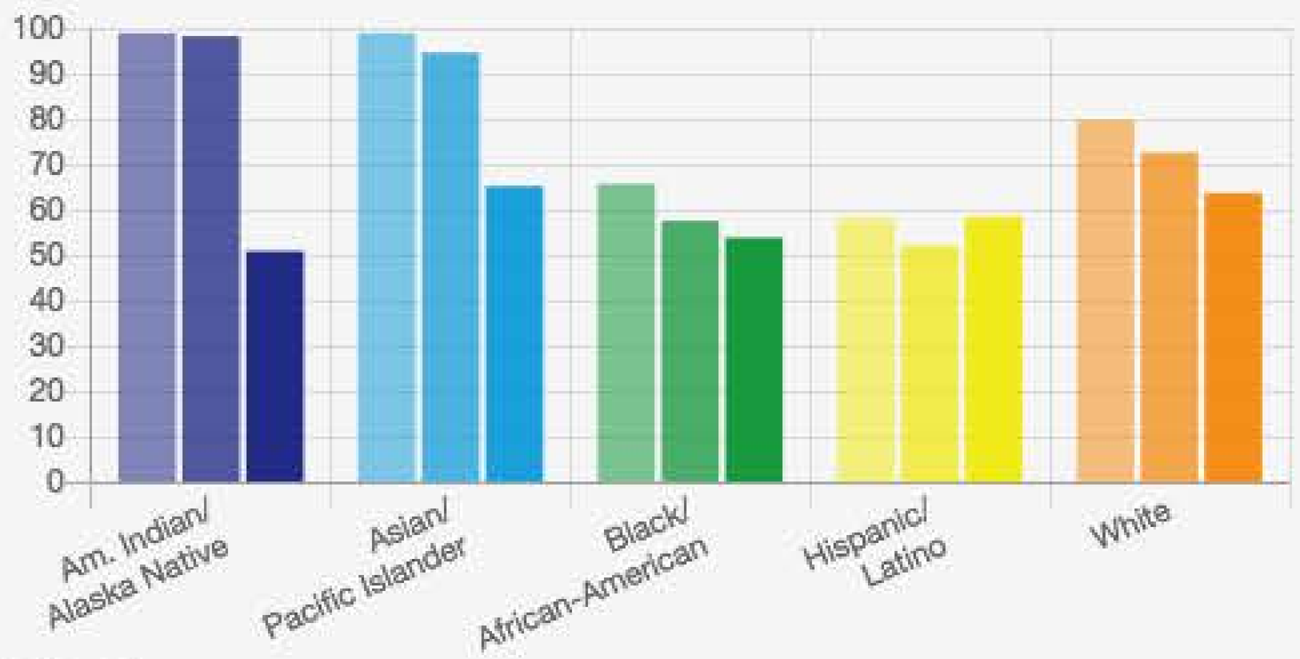

**Booster percentages are based on the population of individuals eligible for a booster dose per CDC guidance.*

NMDOH

New Mexico Department of Health

COVID-19 Vaccine Dashboard

The New Mexico Department of Health (NMDOH) is leading the State of New Mexico's COVID-19 Vaccination Preparedness Planning in close collaboration with other state agencies, public, private and tribal partners throughout the state.

Learn More

Interested in Phase-Specific Vaccine Data?

If you are interested in information on New Mexico's progress through the phase-based distribution plan, please click here.

BASE LAYER

Percent Of Residents With Primary Series Completed

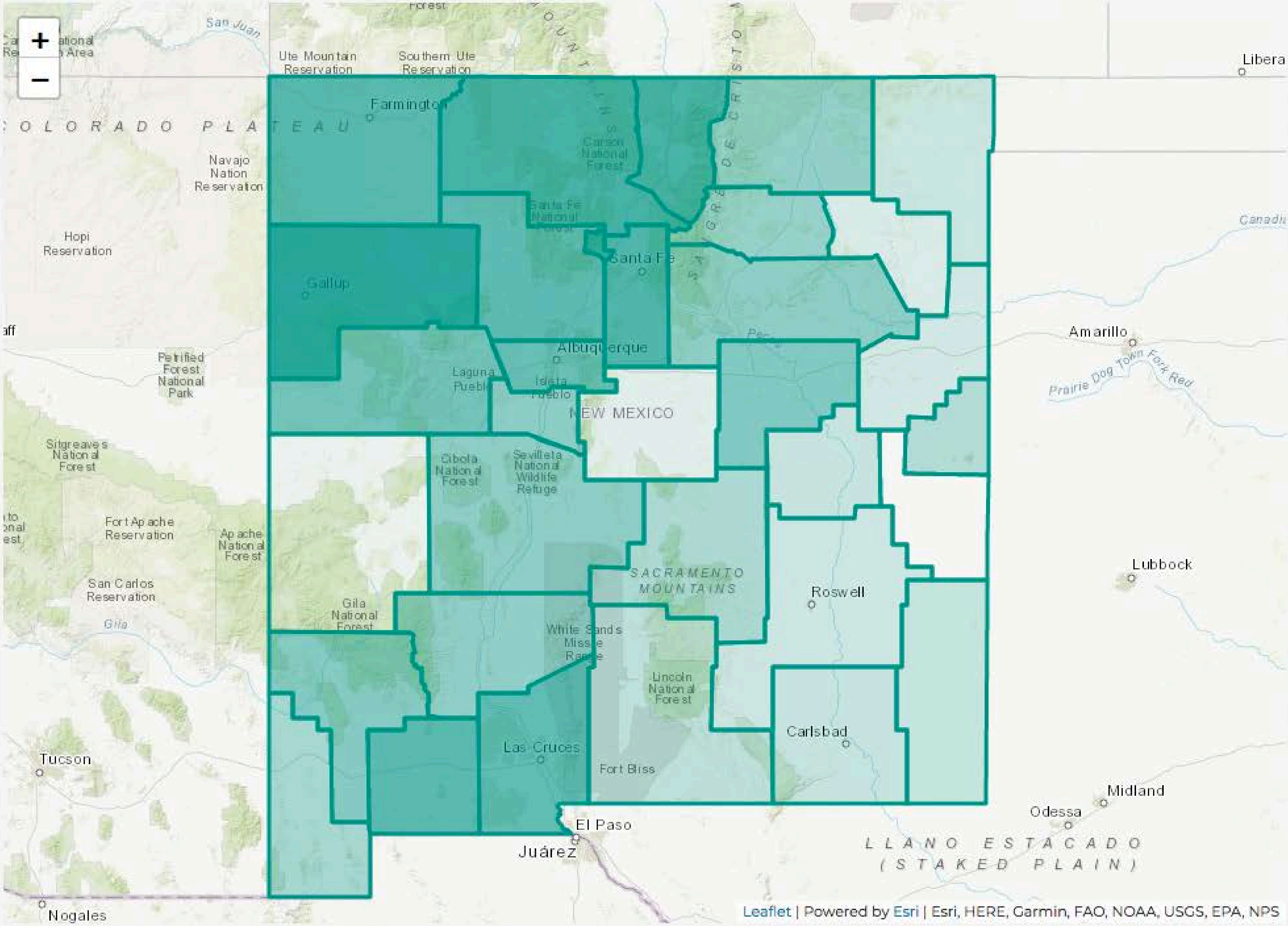

County	Population	Number Of Residents With Primary Series Completed	Percent Of Residents With Primary Series Completed
Bernalillo	534,462	441,360	82.6%
Catron	3,115	1,475	47.4%
Chaves	47,479	26,399	55.6%
Cibola	20,570	16,314	79.3%
Colfax	9,764	6,955	71.2%
Curry	36,809	23,124	62.8%

State of Ohio | COVID-19 Vaccine Dashboard

Last Updated: 06-16-22
Updated Thursdays

CSV

View By
Select to view by Vaccine Started or Completed
Vaccine Started*

Statewide: Vaccine Status
By Total and % of Population

Vaccine Started*, Change from Last Week****	Vaccine Started* By Population	Vaccine Started*, Total
**4,136**	**62.86%**	**7,347,530**

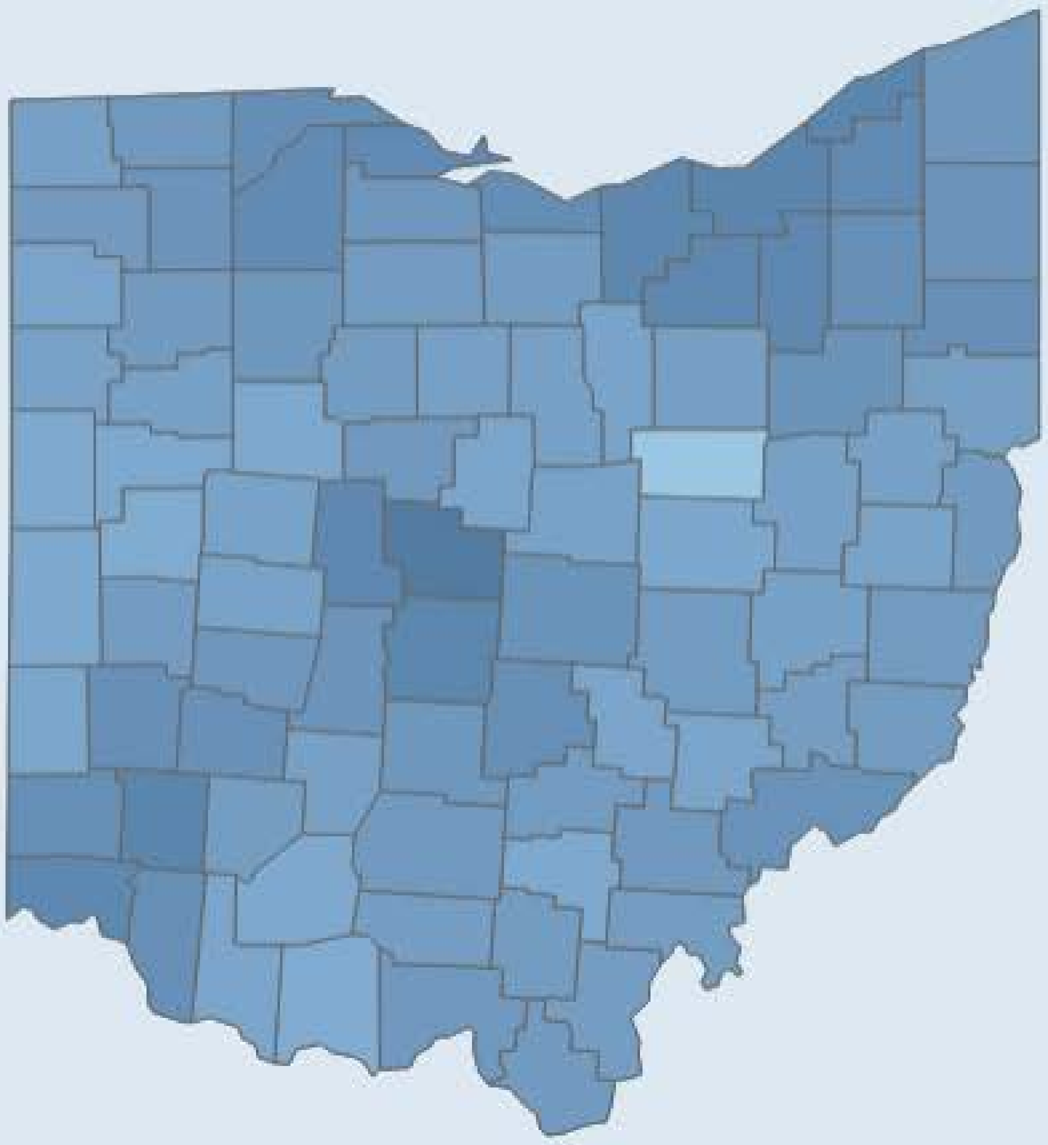

Residency Not Reported
14,664

Residents of Other States - Vaccinated in Ohio
282,698

Key Metrics
Select to view key demographic groups
Age Group

0-19	30.94%	893,519
20-29	58.78%	913,071
30-39	64.45%	952,142
40-49	68.5%	950,221
50-59	74.26%	1,145,356
60-64	82.41%	656,322
65-69	89.40%	596,229
70-74	93.95%	490,821
75-79	89.65%	320,606
80+	85.94%	429,243

View Count By
Select to view counts by daily or cumulative
Daily Total

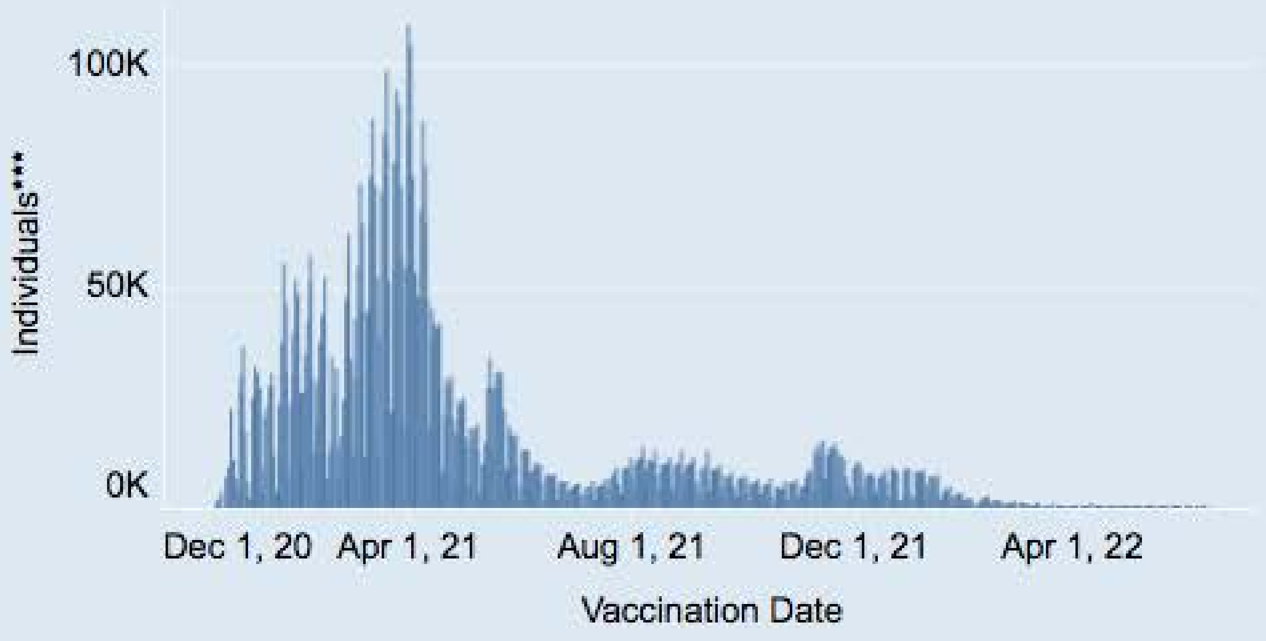

* Indicates that an individual has received at least one reported dose of COVID-19 vaccine.
** Indicates that all recommended COVID-19 vaccine doses have been reported for an individual, and the person is considered fully immunized. Status accounts for the total number of doses needed to be fully immunized only, which is completion of the original vaccine series; other specific requirements (dose spacing, age restrictions) vary depending on COVID-19 vaccine product and the latest CDC/ACIP administration guidance.
*** A person is counted in the "Vaccine Started" category on the day the first COVID-19 vaccination dose was administered. A person is counted in the "Vaccine Completed" category on the day the primary COVID-19 vaccination series was completed.
**** The number of vaccines reported to ODH over the previous week ending at 6 a.m. on Thursdays. Although most vaccines are identified within 24 hours of the vaccine administration date, some may take longer to be reported.

***** The number of people who received a first booster dose includes any person from the population counted as Vaccine Completed who received either an additional dose in their primary series because of an immunocompromising condition or a booster dose of any COVID-19 vaccine on or after August 13, 2021.

****** The county designation is based on the vaccine recipient's address provided for the most recent immunization event. Address changes could cause fluctuations in county vaccination counts and rates.

******* The number of people who received a second booster dose includes any person from the population counted as having received a first booster who also received another booster dose of a COVID-19 mRNA vaccine (Pfizer or Moderna) on or after March 29, 2022.

Population data as reported by the US Census Bureau. There are no population estimates for "Unknown" or "Other" demographic groups, so there is no percentage of population for those groups. Data reported to the Ohio Department of Health. All data displayed are preliminary and subject to change as more information is reported to ODH.

+ t a b l e a u

COVID-19 vaccines are available in all 95 counties. Visit https://www.vaccines.gov/search to locate a facility vaccinating.

TN Department of Health

Tennessee COVID-19 Vaccination Reporting

Last Updated 6/16/2022

Click Here For: Population Metrics

Total Vaccinations Reported	Vaccinations Reported Since 6/9/2022	% of People Statewide With at Least One Dose	% of People Statewide Fully Vaccinated	% of People Statewide With a Booster Dose
9,767,413	23,451	56.8%	50.9%	20.9%

Number of People with:
Series Initiation: 1 dose of Pfizer / Moderna
Series Completion: 2 doses of Pfizer / Moderna OR 1 dose of Janssen
Booster Dose: 3 doses of Pfizer / Moderna

357,117 | 2,070,531 | 1,437,077

Notes:

1. Providers administering COVID-19 vaccines are expected to report vaccine doses to the state immunization information system (TennIIS) within 24 hours of administration and are required to report doses no later than 72 hours after administration.

2. Dashboard does not contain data for COVID-19 vaccinations administered by the Bureau of Prisons (BOP), Department of Defense (DOD), Indian Health Service (IHS) and Veterans Health Administration

3. The number of all vaccine recipients is the sum of Series Initiation, Series Completion, and Booster Dose. The number of fully vaccinated recipients is the sum of Series Completion and Booster Dose.

4. The *Summary Page* includes all vaccinations reported to TennIIS from Tennessee providers, regardless of patient's state of residence. These numbers will differ from the *Population Page*, which contains data on Tennessee residents only.

Age Group

	People Vaccinated	% of People Vaccinated
5-11 years	111,927	2.7
12-15 years	153,387	3.7
16-20 years	226,618	5.4
21-30 years	542,417	13.0
31-40 years	568,943	13.6
41-50 years	585,583	14.0
[illegible]	[illegible]	[illegible]
71-80 years	450,184	10.8
81+ years	190,958	4.6
Pending	256	0.0
Total	4,183,367	100.0

Patient Race

	People Vaccinated	% of People Vaccinated
Asian	76,644	1.8
Black or African American	502,890	11.5
White	2,501,539	59.9
[illegible]	[illegible]	[illegible]
Unknown	143,808	2.6
Total	4,183,367	100.0

TN | Contact Us | Program Areas | News | Health Professionals | Parents | Individuals | Statistical Data | Career Opportunities

Patient Ethnicity

	People Vaccinated	% of People Vaccinated
Hispanic or Latino	251,404	5.4
Not Hispanic or Latino	3,602,028	88.1
Unknown	329,935	6.5
Total	4,183,367	100.0

Patient Sex

	People Vaccinated	% of People Vaccinated
Female	2,259,390	54.0
Male	1,918,130	45.9
Other	1,267	0.0
Unknown	4,387	0.1
Total	4,183,174	100.0

COVID-19 Vaccinations Administered and Reported to TennIIS

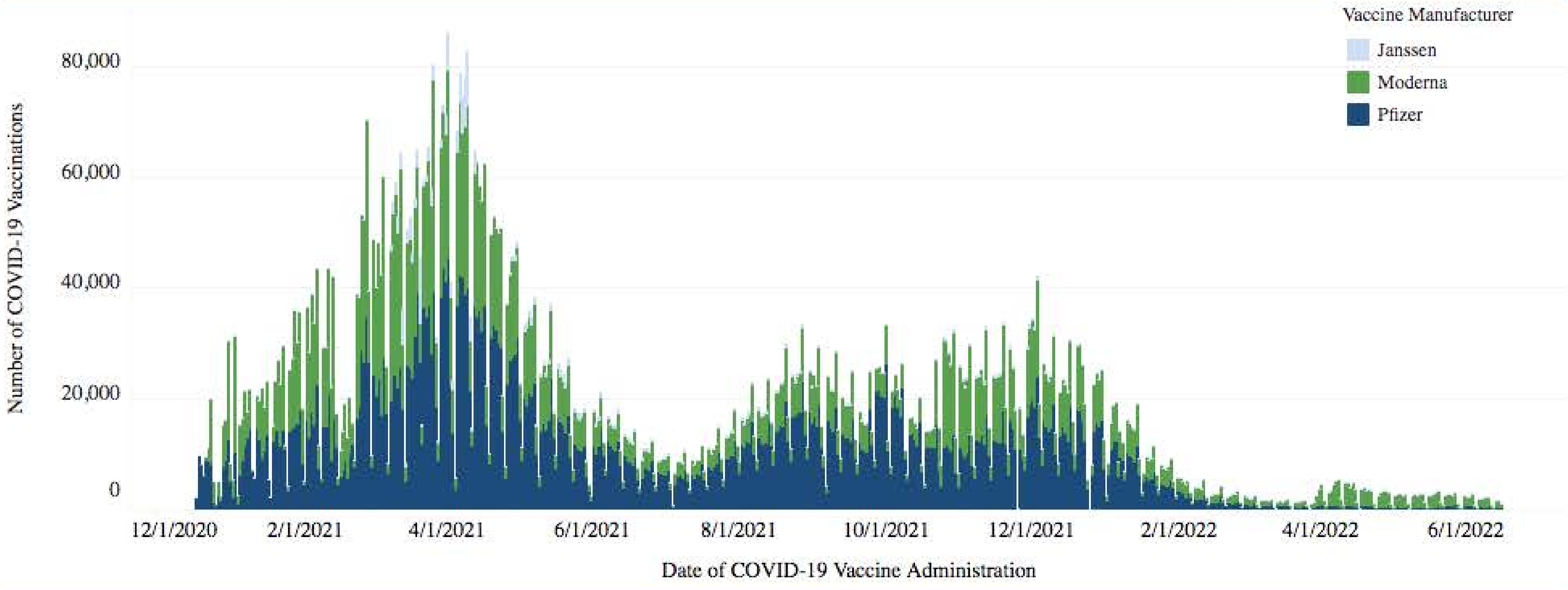

Reported COVID-19 Vaccinations by Patient County of Residence

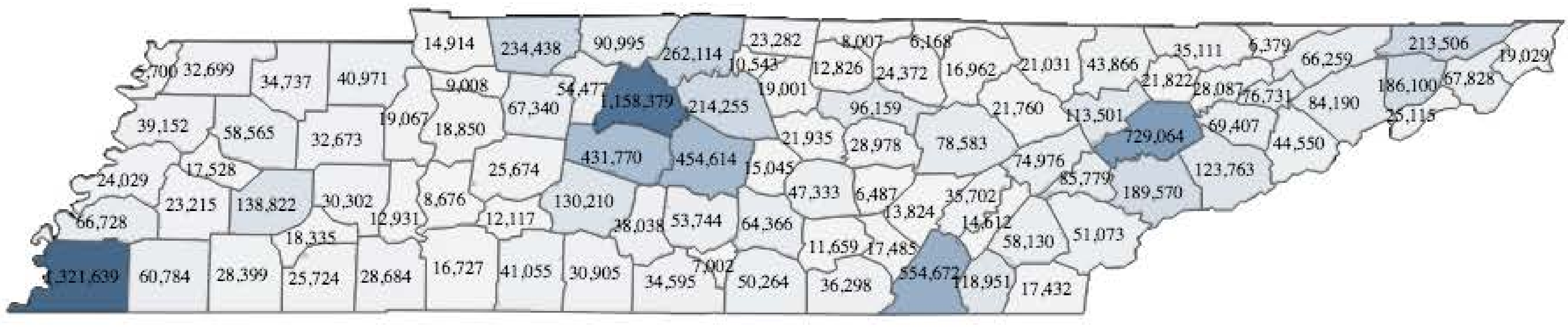

Hover over each county in the map for: county name, number of vaccinations reported, % of county population with at least one dose (any manufacturer), % of county population with series completion (at least 2 doses of Pfizer / Moderna OR 1 dose of Janssen), % of county population with a booster (3 doses of Pfizer / Moderna).

Based on reported doses to the state immunization information system (TennIIS) and 2020 county population data.

Effective 4/1/2022: County assignment of patient residence has been updated from a) the Federal Information Processing Standard (FIPS) national standard of associating zip codes with counties to b) higher precision geocoding by current patient street address and city when a valid address is available. When a valid address is not available (e.g. PO Boxes, missing, etc.), the zip code will be assigned to a Tennessee county per the FIPS standard.

Unexpected response from 'GetSessionInfo' command when fetching online analytics server info.

Summary | Vaccine Doses Administered | People Vaccinated | About the Data

COVID-19 Vaccination in Texas

TEXAS Health and Human Services | Texas Department of State Health Services

DSHS is partnering with vaccine providers across the state to ensure vaccine will be accessible to all Texans at no cost. Visit the DSHS COVID-19 Vaccine Information page for more details on phases and tiers, and other vaccine info here:

Percentage Measure to Show
- (•) Percentage of Population 5+ Vaccinated with At Least One Dose
- ( ) Percentage of Population 5+ Fully Vaccinated
- ( ) Percentage of Population 65+ Vaccinated with At Least One Dose
- ( ) Percentage of Population 65+ Fully Vaccinated

78.40% of Texas State Population Vaccinated

Percentage of County Population Vaccinated

25.99% — 99.99%

This dashboard is updated by 4:00 pm each day with data as of 11:59 pm the previous night. See the About the Data tab for more information on data sources.

Percentage of Population Vaccinated for COVID-19

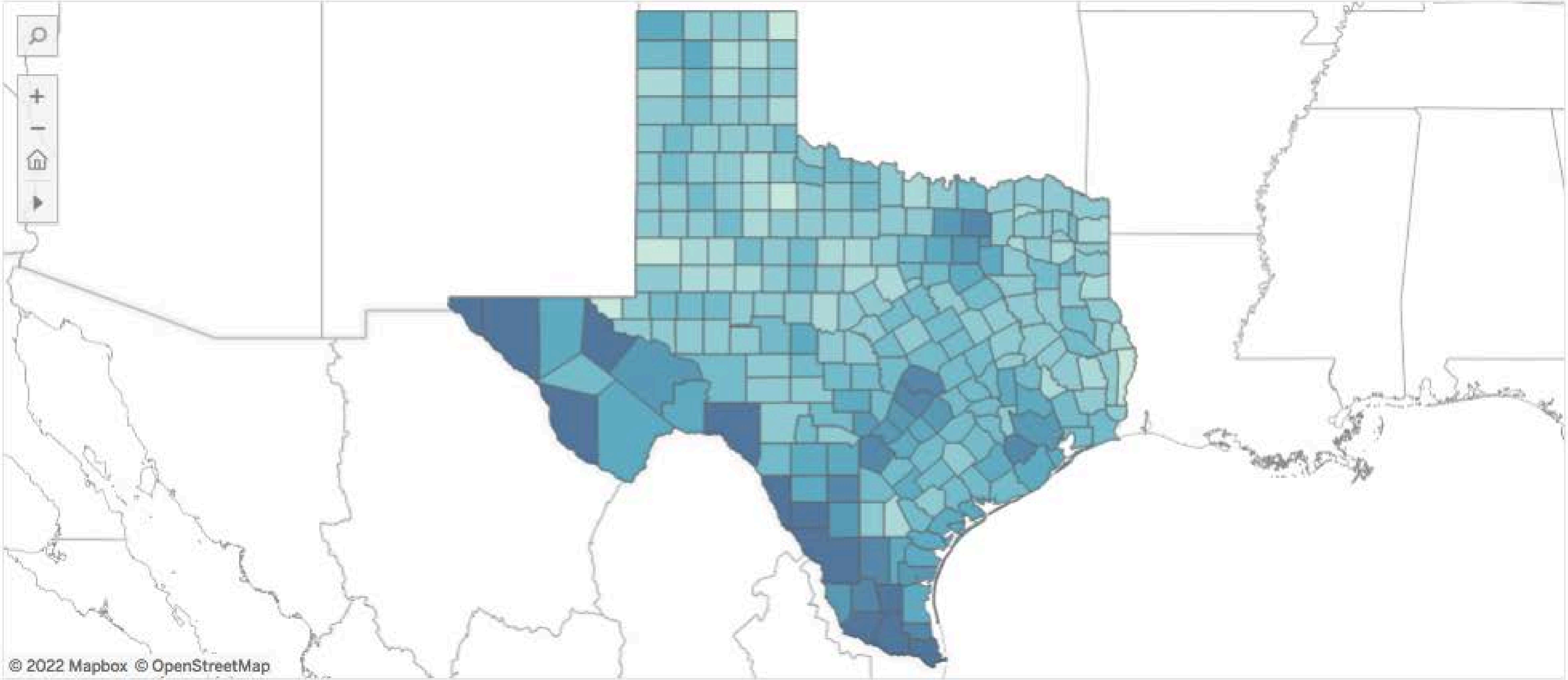

Doses Administered

45,424,231

People Vaccinated

21,143,483

People Fully Vaccinated

17,752,594

People Vaccinated With at Least One Booster

7,017,585

Last Updated:
06/16/2022 12:30 pm

Summary | Vaccine Doses Administered | People Vaccinated | About the Data

TEXAS Health and Human Services | Texas Department of State Health Services

COVID-19 Vaccination in Texas

Doses administered are doses providers have entered into ImmTrac2, the Texas Immunization Registry, by recipient county of residence and date administered.

Use the filter below to select a Texas county and view the number of vaccinations administered to persons living there, by week. Alternatively, click on a county on the map. Click the Revert button at the bottom of the screen to remove filters.

Doses Administered

45,424,231

County Name

(All)

Vaccinations by Week in Texas

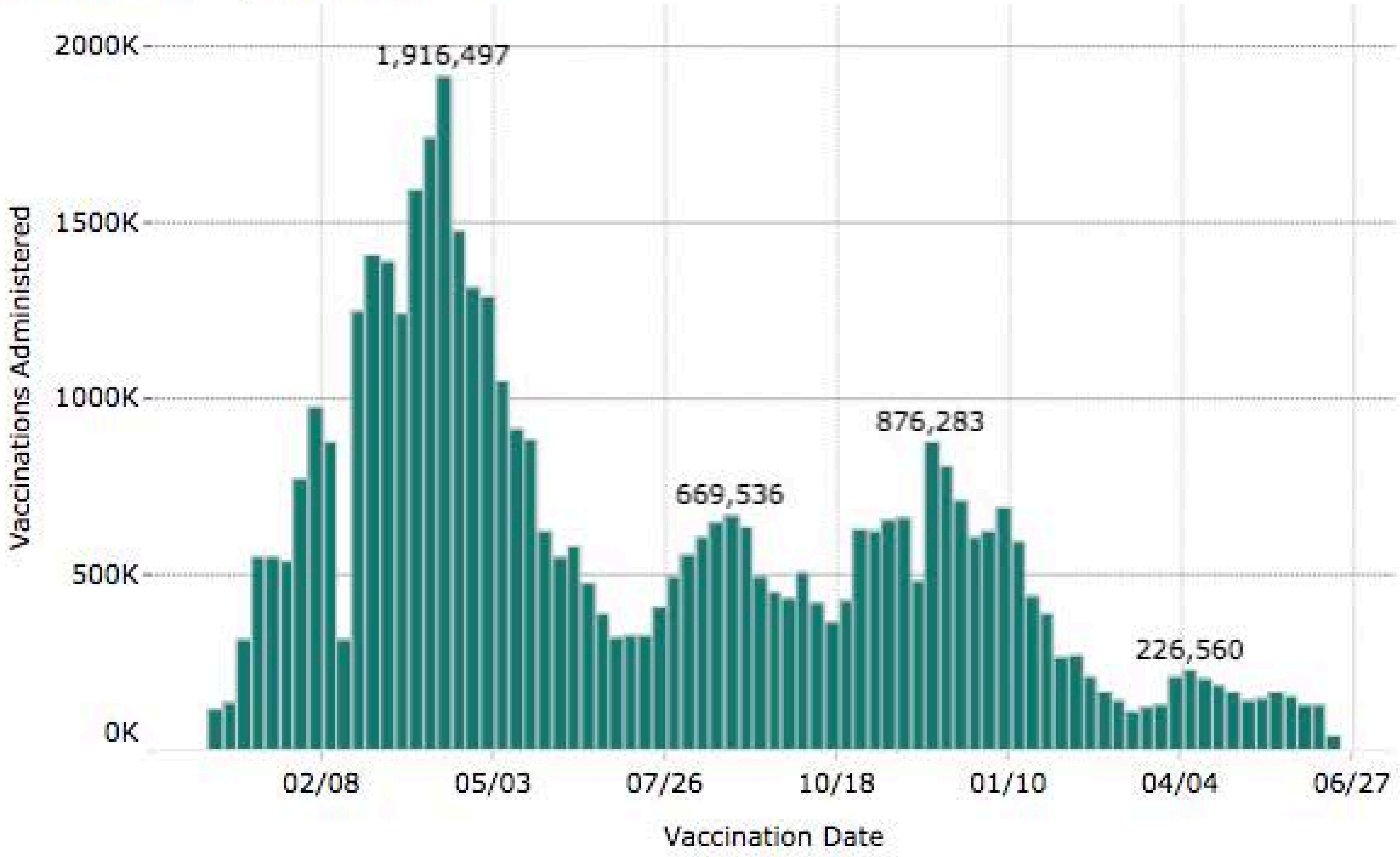

Doses Administered by Organization Type

Emergency Services	1,465,761
Health Center (Community, Federally Qualified, Migrant, Rural)	1,763,515
Hospital	7,560,543
Long-Term Care	127,467
Medical Clinic/Doctor's Office	3,609,955
Public Health/Government	6,097,194
Pharmacy	23,233,337
Other	1,566,459

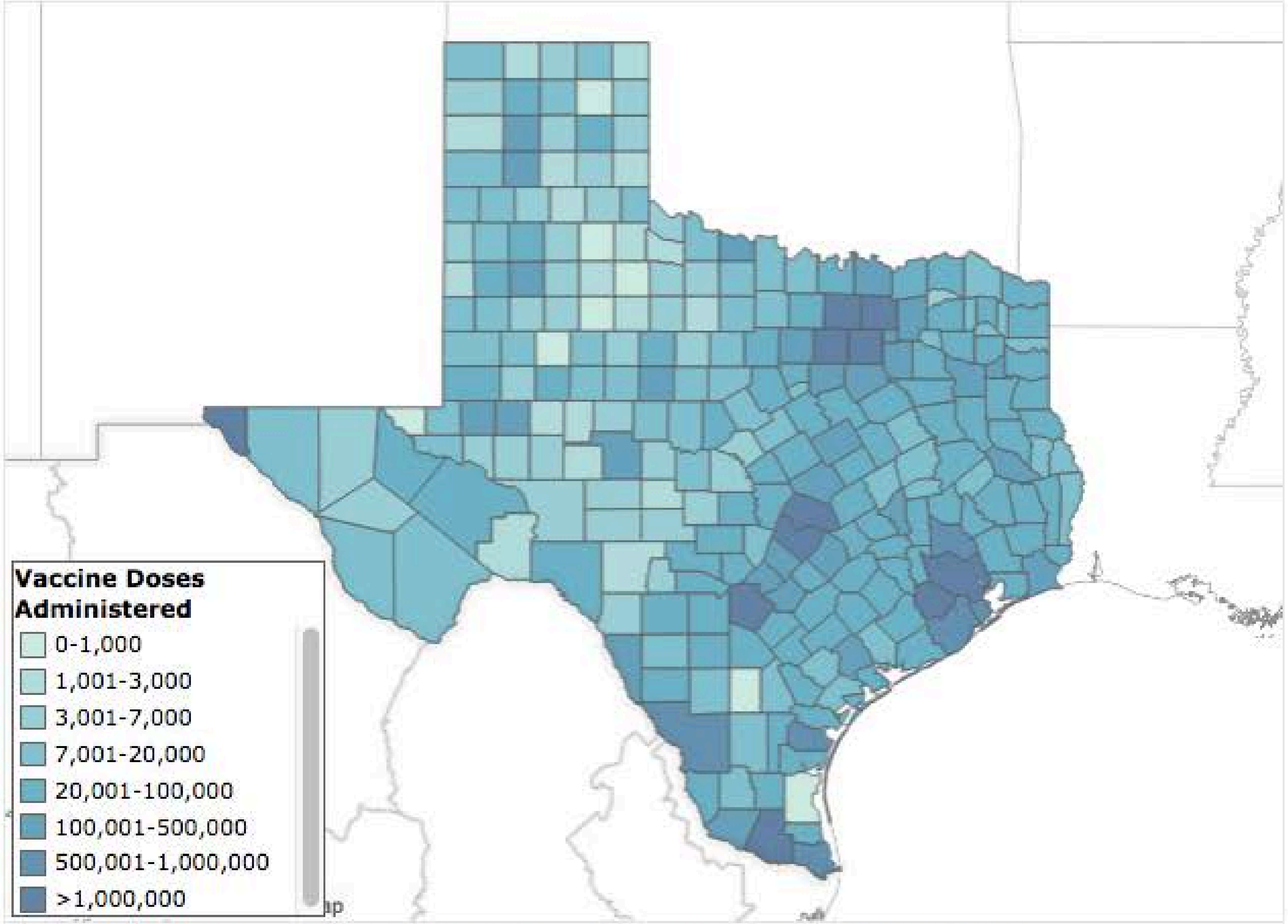

Dashboard is updated daily by 4 p.m. CST with data as of 11:59 the previous night.

For inquiries about the data, email COVIDVaccineQs@dshs.texas.gov.

Summary | Vaccine Doses Administered | People Vaccinated | About the Data

TEXAS Health and Human Services | Texas Department of State Health Services

COVID-19 Vaccination in Texas

Select a Texas county below to view the number people who have received at least one dose of COVID-19 vaccine and those who are fully vaccinated as outlined by the CDC.

Alternatively, click on a county on the map. Click the Revert button at the bottom of the screen to remove filters.

People Vaccinated with at least One Dose	People Fully Vaccinated	People Vaccinated with at least One Booster
**21,143,483**	**17,752,594**	**7,017,585**

Measures to Show
- ○ Vaccinated with at least One Dose
- ○ Fully Vaccinated
- ◉ Vaccinated with at least One Booster

County Name
(All)

Number of People Vaccinated in Texas

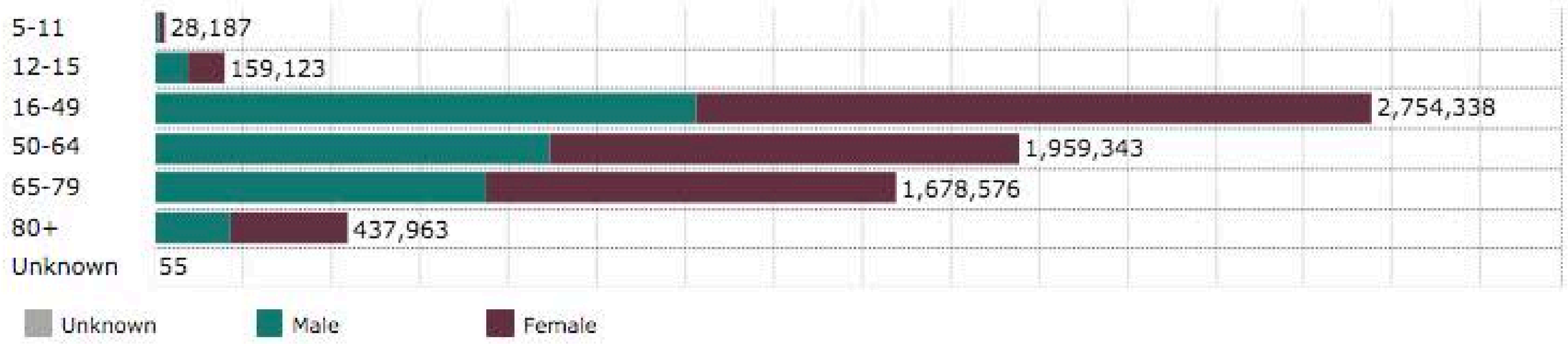

Race/Ethnicity	People Vaccinated	Percent of People Vaccinated
Asian	428,428	6.11%
Black	514,519	7.33%
Hispanic	1,974,707	28.14%
Other	1,580,173	22.52%
Unknown	136,640	1.95%
White	2,383,118	33.96%

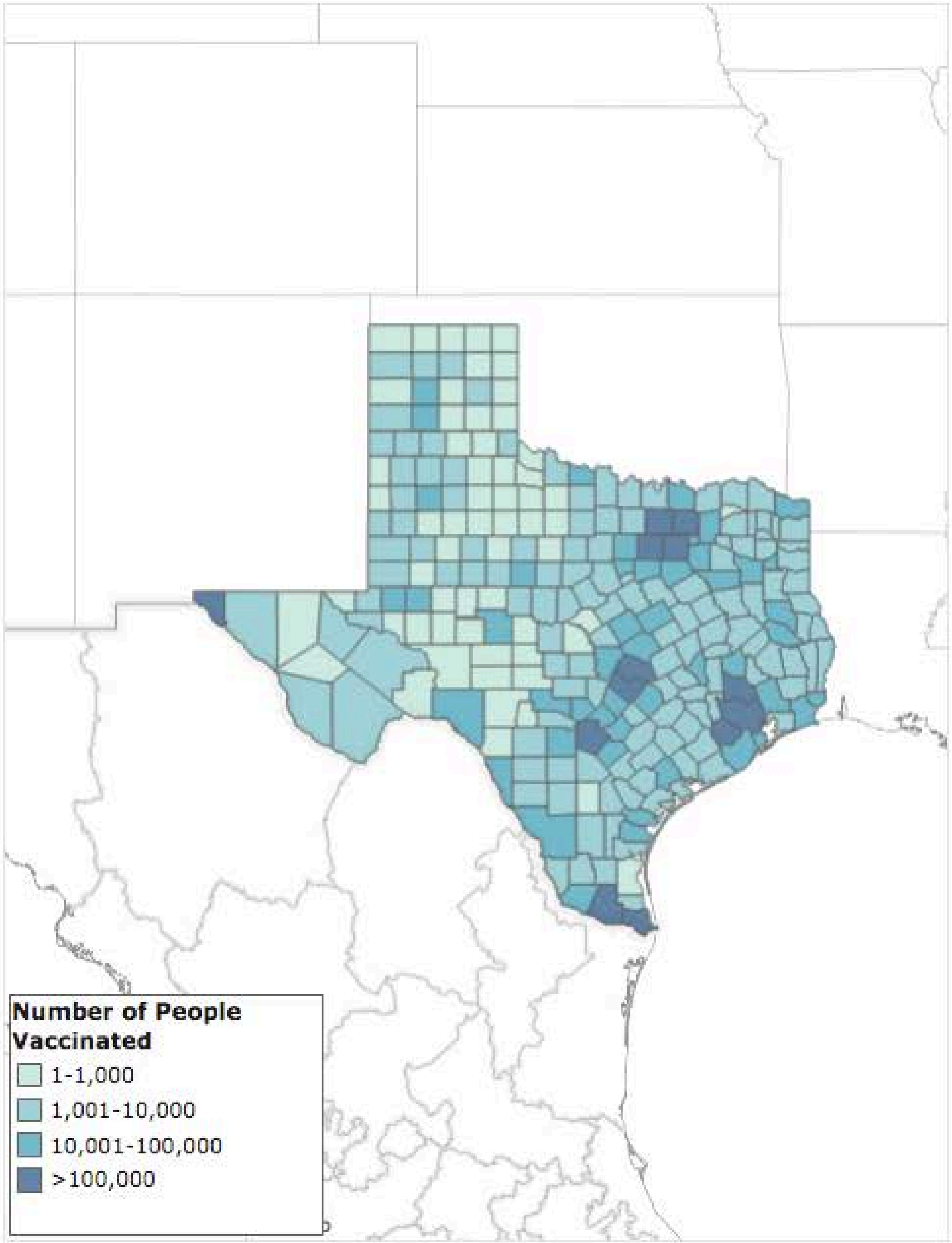

Dashboard is updated daily by 4 p.m. CST with data as of 11:59 the previous night. For inquiries about the data, email COVIDVaccineQs@dshs.texas.gov.

Vermont Vaccination Data

Overall | **Statewide** | By County | By County - Additional

Updated 06/15/2022 12:35 PM

People vaccinated

Up to date | Completed | Additional dose | At least one dose

350.9K
People up-to-date

59%
% of Vermonters age 5+ who have received all recommended vaccines

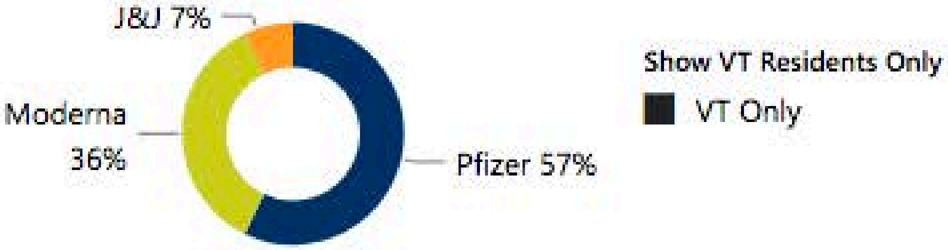

Show VT Residents Only
VT Only

Percent of the statewide population **age 5+ of each subgroup that has received all recommended COVID-19 vaccines, including any booster dose(s) when eligible**

Age	%
5 - 11	56%
12 - 17	42%
18 - 29	33%
30 - 39	51%
40 - 49	56%
50 - 59	60%
60 - 64	71%
65 - 69	83%
70 - 74	92%
[illegible]	[illegible]

Race	%
Asian	52%
Black or African American	42%
Native American, Indigenous, or First Nation	13%
Pacific Islander	15%
Two or more races	63%
White	59%

Ethnicity	%
Hispanic	75%
Not Hispanic	57%

Sex	%
Female	62%
Male	55%

Race/Ethnicity	5-11	12-30	31-64	65+	**Age 5+**
BIPOC	58%	41%	63%	95%	**57%**
Non-Hispanic White	52%	35%	57%	87%	**58%**

Source: Vermont Immunization Registry, VDH Population Estimates (2019)

Data notes

Statewide numbers and percentages are capped at 100%. To protect the identity of individuals, data is suppressed when there are fewer than six people vaccinated in a subgroup.

Race information is not reported for 3% of people vaccinated.

Race/ethnicity information is not reported for 4% of people vaccinated.

Ethnicity information is not reported for 4% of people vaccinated.

BIPOC refers to Black, Indigenous, and people of color.

Sex information is not reported for <0.5% of people vaccinated. The categorization of **male/female** for some people is based on sex assignment at birth, while for others, it is based on gender. This is due to the varying sources and ways the information is reported.
Sex not reported may mean the provider did not collect that information, the patient did not provide it, or the provider or the patient selected a category other than male or female.

96%

Microsoft Power BI

Vermont Vaccination Data

Overall | Statewide | By County | By County - Additional

Updated 06/15/2022 12:35 PM

Data notes

Show rates by: Overall | Race/Ethnicity | Ethnicity | Age | Sex

Select vaccine status: Up to date | Completed | At least one dose

Vaccination rate by county

The percent of the county population age 5+ that has received all recommended COVID-19 vaccines, including any booster dose(s) when eligible by county.

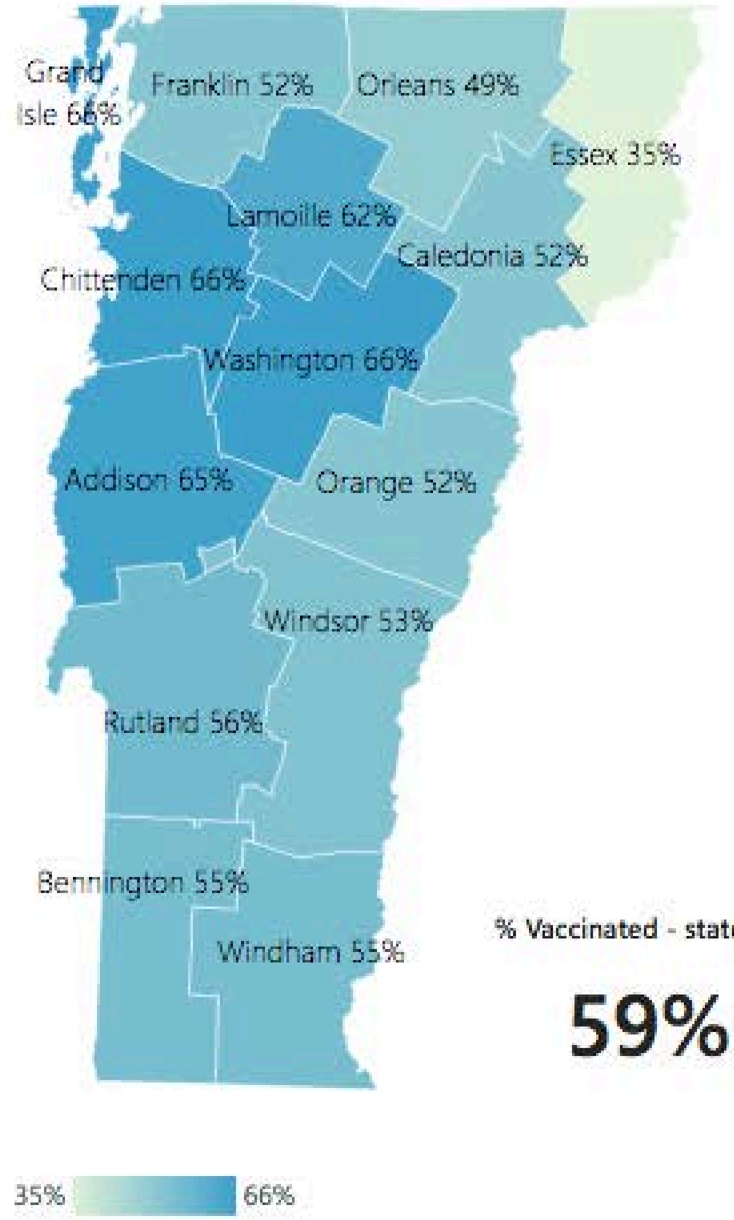

% Vaccinated - statewide

59%

County	Overall progress	5+ pop estimate
Addison	65%	35,214
Bennington	55%	33,828
Caledonia	52%	28,570
Chittenden	66%	156,317
Essex	35%	5,887
Franklin	52%	46,515
Grand Isle	66%	6,900
Lamoille	62%	24,075
Orange	52%	27,542
Orleans	49%	25,742
Rutland	56%	55,626
Washington	66%	55,680
Windham	55%	40,377
Windsor	53%	52,673
**VT**	**59%**	**594,946**

Source: Vermont Immunization Registry, VDH Population Estimates (2019)

96%

Microsoft Power BI

Pages

Total vaccine doses administered
Last updated: 6/16/2022

9,554,059

Johnson & Johnson	Moderna	Pfizer
346,841 doses administered	3,599,106 doses administered	5,608,112 doses administered

Total vaccine doses administered in Wisconsin by day

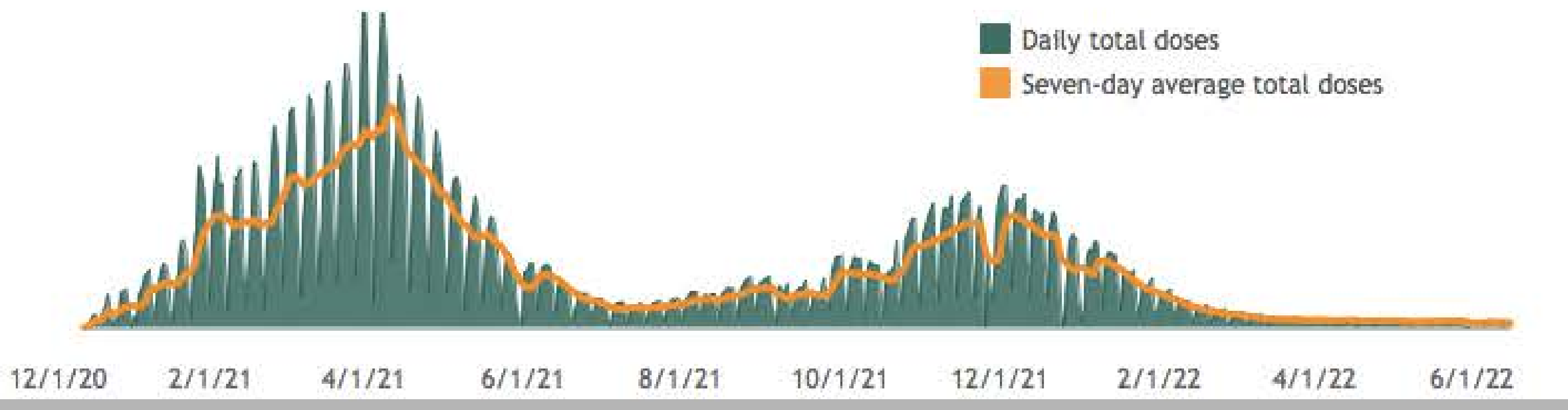

Total additional/booster doses administered

2,052,676

Johnson & Johnson	Moderna	Pfizer
25,632 doses administered	842,197 doses administered	1,184,847 doses administered

Additional/booster vaccine doses administered in Wisconsin by day

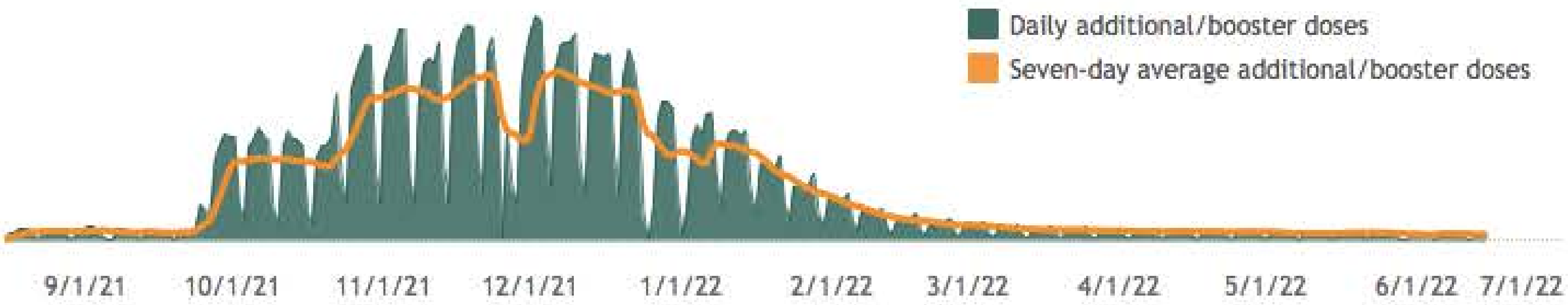

Undo Redo Replay Revert Refresh Pause Share Download Full Screen

Understanding our data: What does this chart mean?

About our data: How do we measure this?

Back to a list of charts on this page.

COVID-19 vaccines for Wisconsin residents
Updated: 6/16/2022

HERC region data

- (●) Total population who have received at least one dose
- ( ) Total population who have completed the series
- ( ) Total population who have received an additional/booster dose

Percent of Wisconsin residents who have received at least one dose by county

Click a county to filter data

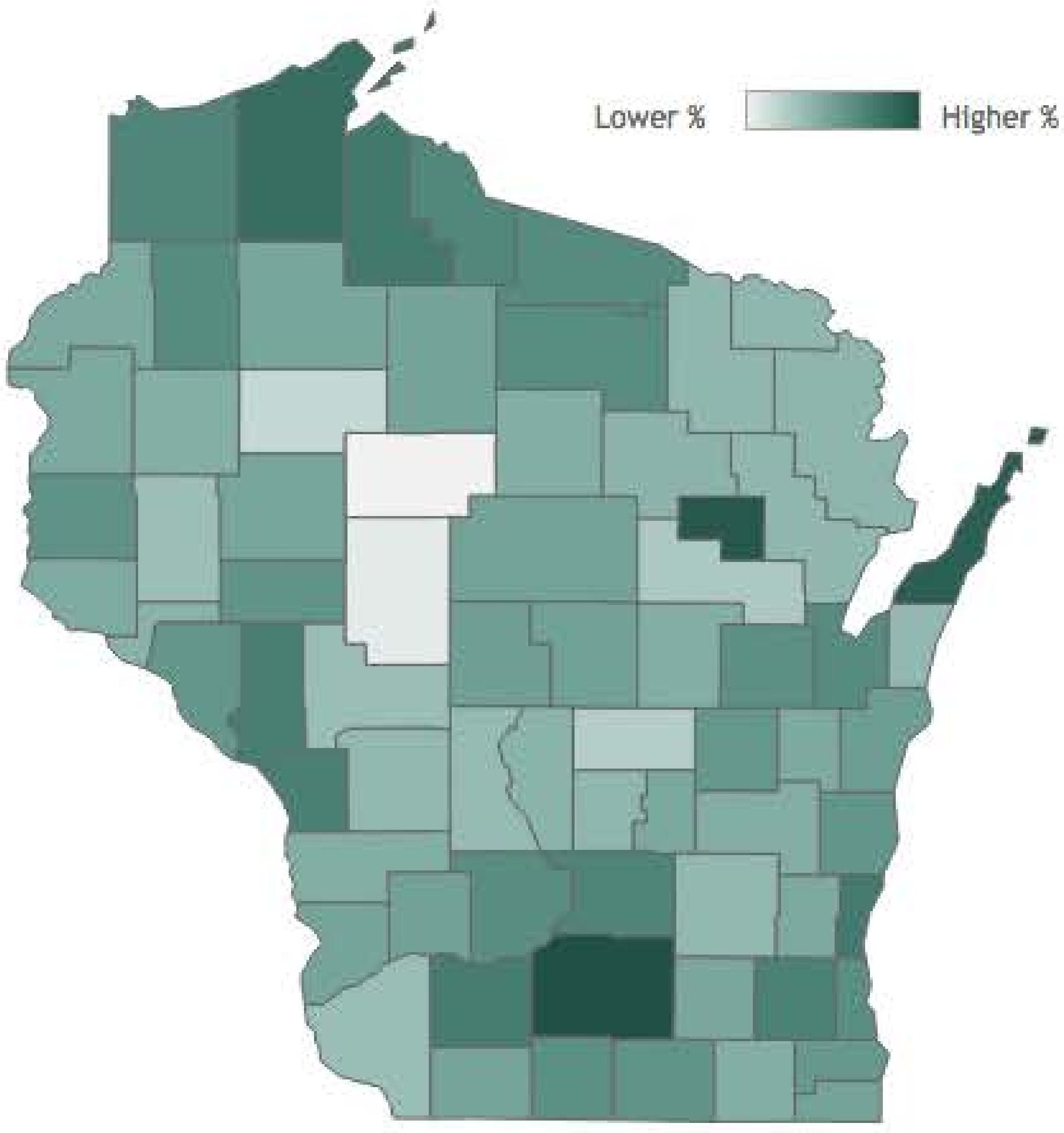

Percent of Wisconsin residents who have received at least one dose

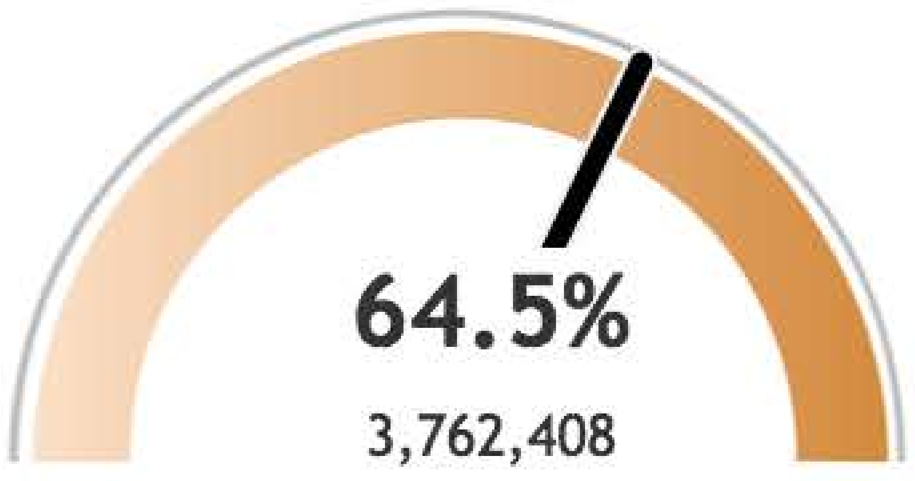

Age	
5-11	28.2%
12-17	62.0%
18-24	60.8%
25-34	64.6%
35-44	69.5%
45-54	71.9%
55-64	78.2%
65+	85.3%

Race	
American Indian	50.0%
Asian	67.8%
Black	44.5%
White	59.4%

*5.8% of records reported a race of "Other"

*3.6% of records reported an unknown race

Sex	
Female	67.4%
Male	61.0%

*0.4% of records were reported without sex.

Ethnicity	
Hispanic	58.6%
Non-hispanic	62.6%

*3.5% of records were reported without ethnicity.

View more data on racial and ethnic disparities in Wisconsin

Vaccine doses for Wisconsin residents by week
(Total: 9,337,074)

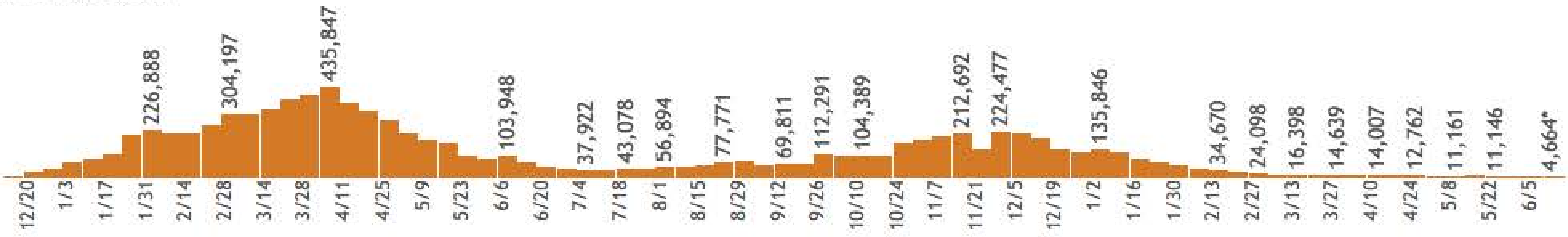

*Current week may be incomplete.

Undo Redo Replay Revert Refresh Pause Share Download Full Screen

See How Vaccinations Are Going in Your County and State

Updated June 15, 2022

Fully vaccinated | Fully vaccinated, 65+ | Boosted

Pct. of residents who are fully vaccinated

30 40 50 60 70% No data

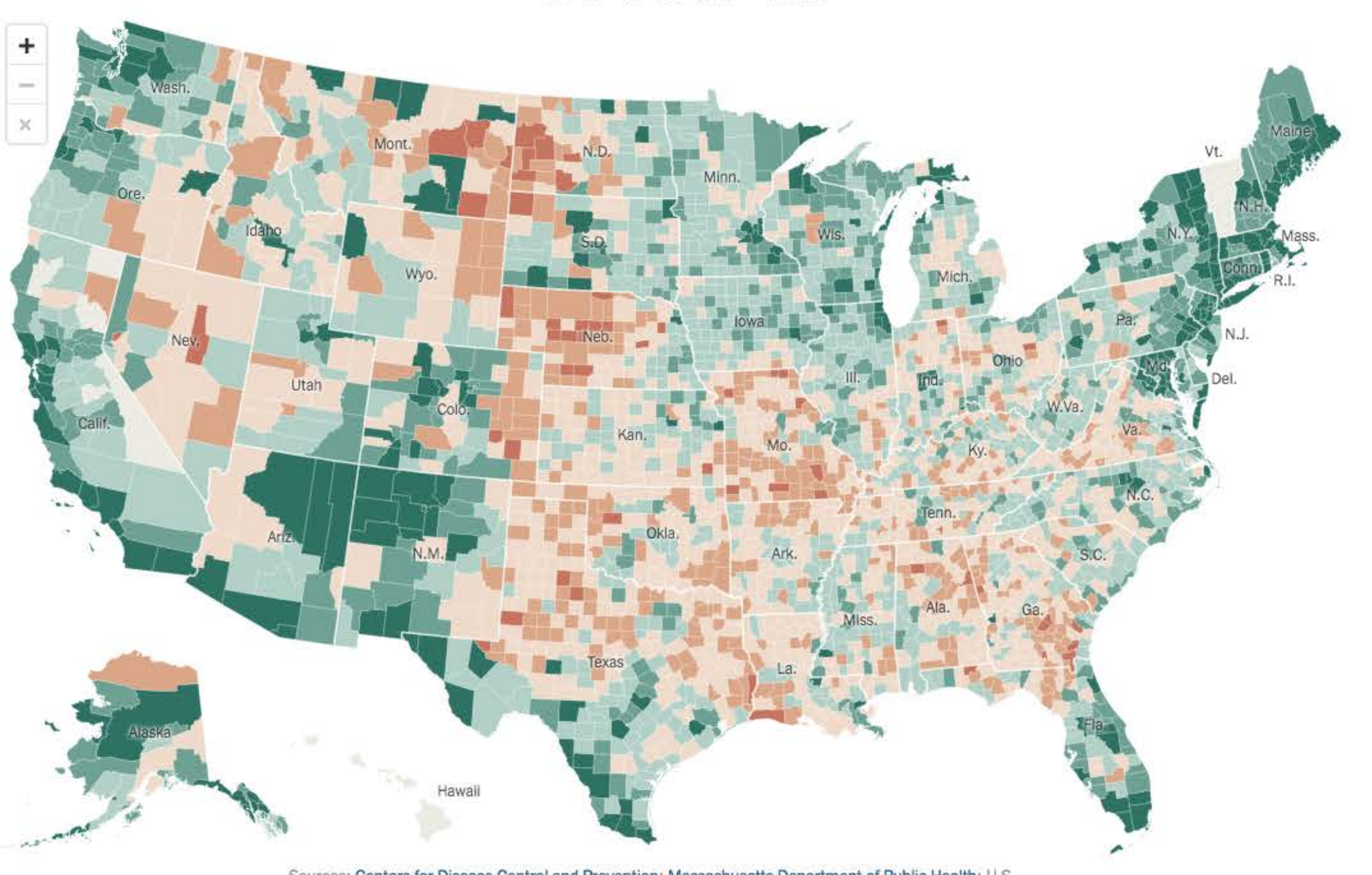

Sources: Centers for Disease Control and Prevention; Massachusetts Department of Public Health; U.S. Census Bureau | Note: No C.D.C. data available for some counties. Vermont was excluded because more than a quarter of data is missing. On Dec. 9, 2021, the C.D.C. capped its vaccination rate figures at 95 percent.

Jump to: Age | New doses | Doses by state | County inequities | Eligibility

The Centers for Disease Control and Prevention said on Wednesday that about 259.1 million people had received at least one dose of a Covid-19 vaccine, including about 221.9 million people who had been fully vaccinated by the Johnson & Johnson single-dose vaccine or the two-dose series made by Pfizer-BioNTech and Moderna.

The C.D.C. also reported that about 104.6 million fully vaccinated people have received an additional vaccine dose or a booster dose, the highest level of protection against the virus.

Share of U.S. population that is...

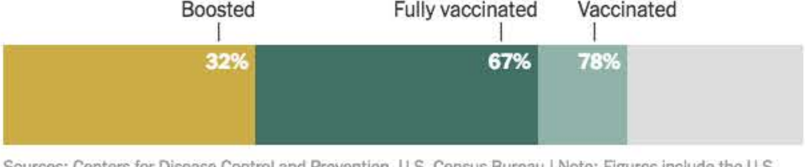

Sources: Centers for Disease Control and Prevention, U.S. Census Bureau | Note: Figures include the U.S. territories and three countries with special agreements. The C.D.C. reported on Nov. 30, 2021 that booster doses are sometimes misclassified as first doses, which may overestimate first dose coverage.

Who Is Vaccinated?

Everyone 5 and older is currently eligible to be fully vaccinated with an initial round or "primary series" of the vaccine. Regulators also recommend a booster dose for those 5 and up who are fully vaccinated and five months past their second shot. Some people 5 and older with weakened immune systems are also eligible for additional shots and they are included in the booster category below.

On March 29, federal regulators authorized second booster shots for those 50 and older, and for individuals with certain immune deficiencies.

Vaccinations by age group

Percentage of fully-vaccinated residents

Name	5 to 11	12 to 17	18 to 64 ▾	65 and up
**U.S. total***	**30%**	**60%**	**73%**	**91%**
Hawaii	43%	84%	91%	93%
Rhode Island	53%	83%	89%	95%
Puerto Rico	65%	90%	88%	90%
New York	38%	74%	86%	93%
Connecticut	44%	78%	86%	95%

Show all

Percentage of residents with a booster

Name	Under 18	18 to 64 ▾	65 and up
**U.S. total***	**6%**	**32%**	**64%**
Puerto Rico	22%	53%	63%
Vermont	16%	50%	85%
Rhode Island	11%	47%	81%
Maine	10%	45%	80%
Hawaii	10%	45%	75%

Show all

*Includes people vaccinated in all 50 states, territories and three countries with special agreements with the United States: Palau, Micronesia and the Marshall Islands.

Sources: Centers for Disease Control and Prevention; Andrew Beveridge, SocialExplorer | Note: Residents under 18 with a booster may include some individuals with unknown age.

Vaccination and booster coverage varies widely by age, in part because older adults became eligible first and some younger children are not yet permitted. In addition, booster eligibility depends on the time since an individual's last shot. Vaccinations also vary among states, and jurisdictions with lower adult vaccination rates also tend to have reduced coverage among children and teens.

How Quickly Are Shots Going in Arms?

Providers are reporting that they have administered about 304,000 doses per day on average, including first, second and additional or booster doses.

New reported doses administered by day

U.S. Total*

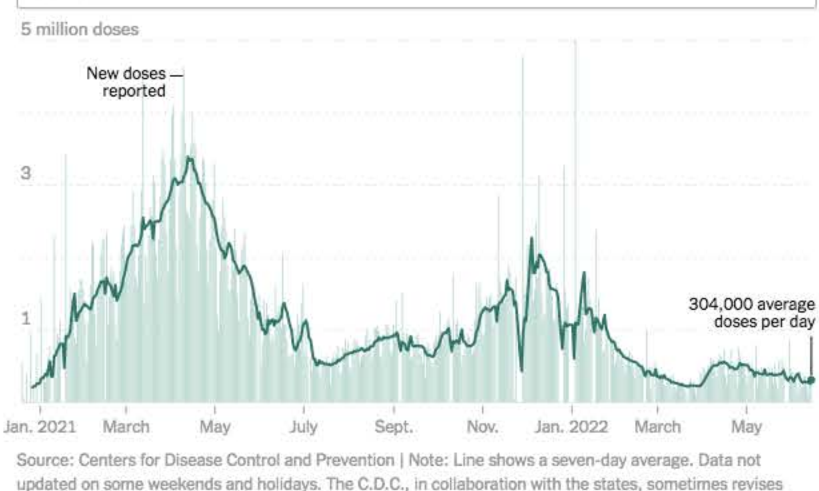

Source: Centers for Disease Control and Prevention | Note: Line shows a seven-day average. Data not updated on some weekends and holidays. The C.D.C., in collaboration with the states, sometimes revises data or reports a single-day large increase in vaccinations from previous dates, which can cause an irregular pattern.

The C.D.C. reported on Nov. 30, 2021, that booster doses are sometimes misclassified as first doses, which may overestimate first dose coverage and underestimate booster coverage.

New reported people vaccinated

Each line shows the seven-day average.

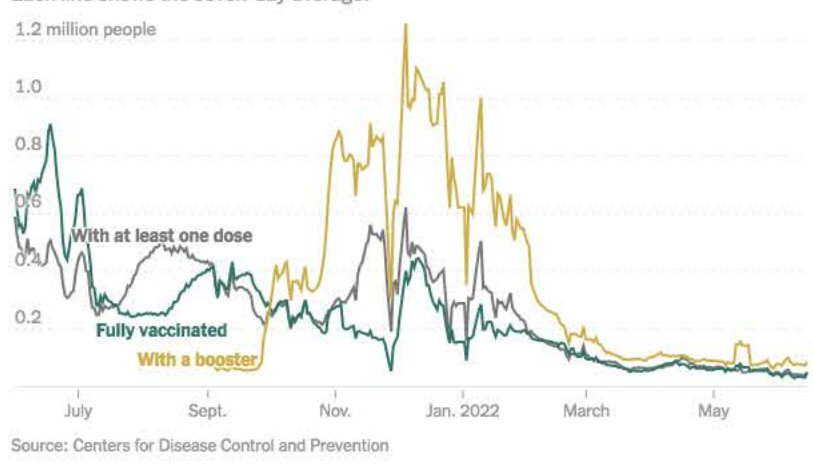

Source: Centers for Disease Control and Prevention

Global vaccinations tracker ›
How many have been vaccinated, by country

YOUR CORONAVIRUS TRACKER: *We'll send you the latest data for places you care about each day.* Sign Up

How Is Each State Doing?

Some jurisdictions have reached a larger share of their population with vaccines and boosters than others.

At least one dose | Fully vaccinated | Boosted

Pct. of residents who are fully vaccinated

55 60 65 70 75%

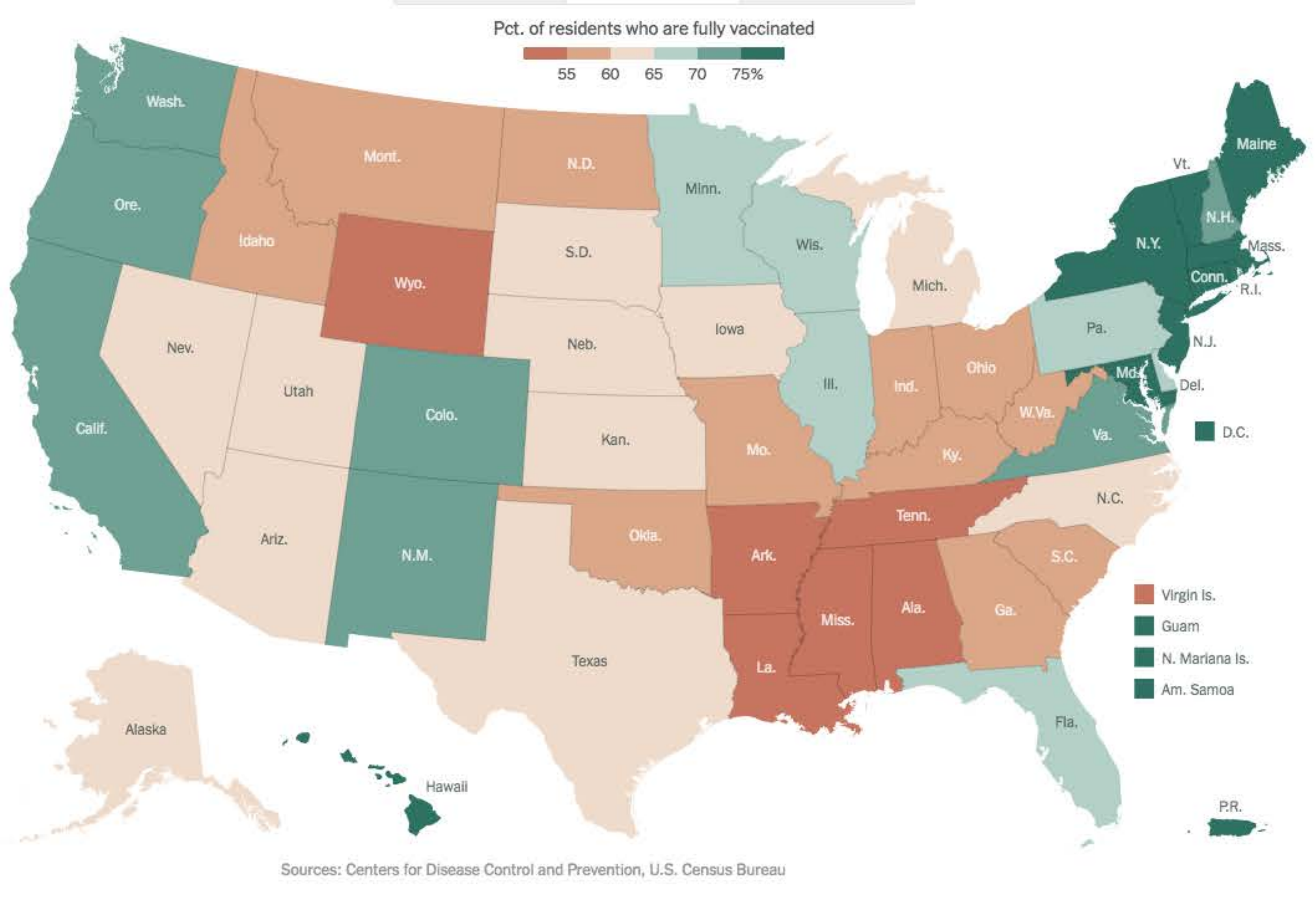

Sources: Centers for Disease Control and Prevention, U.S. Census Bureau

Vaccination rates reported by the C.D.C. can differ from those published by states and territories because of lags and temporary errors in data reporting. Booster doses can also be misclassified as first doses, which may overcount first dose coverage, according to the C.D.C.

The table below includes states, territories, federal agencies and three countries with special agreements with the United States: Palau, Micronesia and the Marshall Islands.

	Pct. of residents			
Name	With at least one dose	Fully ▾ vaccinated	With a booster	Doses administered
**U.S. total***	78%	67%	32%	**591,982,644**
Palau	95%	95%	65%	48,311
American Samoa	92%	85%	48%	111,347
Rhode Island	95%	84%	46%	2,283,681
Guam	92%	83%	38%	360,638
Vermont	94%	82%	51%	1,454,650
Northern Mariana Islands	85%	81%	40%	110,104
Maine	91%	80%	46%	2,952,591
Connecticut	95%	80%	42%	7,826,458
Massachusetts	95%	80%	42%	15,318,166
Hawaii	88%	79%	42%	3,019,831

Show all

*Includes doses provided to Palau, Micronesia and the Marshall Islands.

Sources: Centers for Disease Control and Prevention, U.S. Census Bureau

There are many reasons eligible people are not vaccinated or boosted. Surveys have indicated that some people are adamant in their refusal of the coronavirus vaccines, while others are open to getting a shot but have been putting it off or want to wait and see before making a decision for themselves or for their children.

The first group, surveys have shown, tends to be disproportionately white, rural, evangelical Christian and Republican. The second group tends to be a more diverse and urban group, including many younger people, Black and Latino residents, and Democrats.

Are the Most Vulnerable Counties Being Vaccinated?

Speed hasn't been the only priority for the country's vaccination campaign. The Biden administration has also committed to distributing shots equitably to the communities most affected by the pandemic.

Over a year into the rollout, the most socially vulnerable counties in the U.S. have a lower vaccination rate on average than the nation's least vulnerable. A majority of the most disadvantaged counties with the fewest fully vaccinated people are in the South,

Midwest and the Northeast.

Vaccination rates by county social vulnerability

Percentage of fully-vaccinated residents. Circles sized by county population.

Region ● Midwest ● Northeast ● South ● West

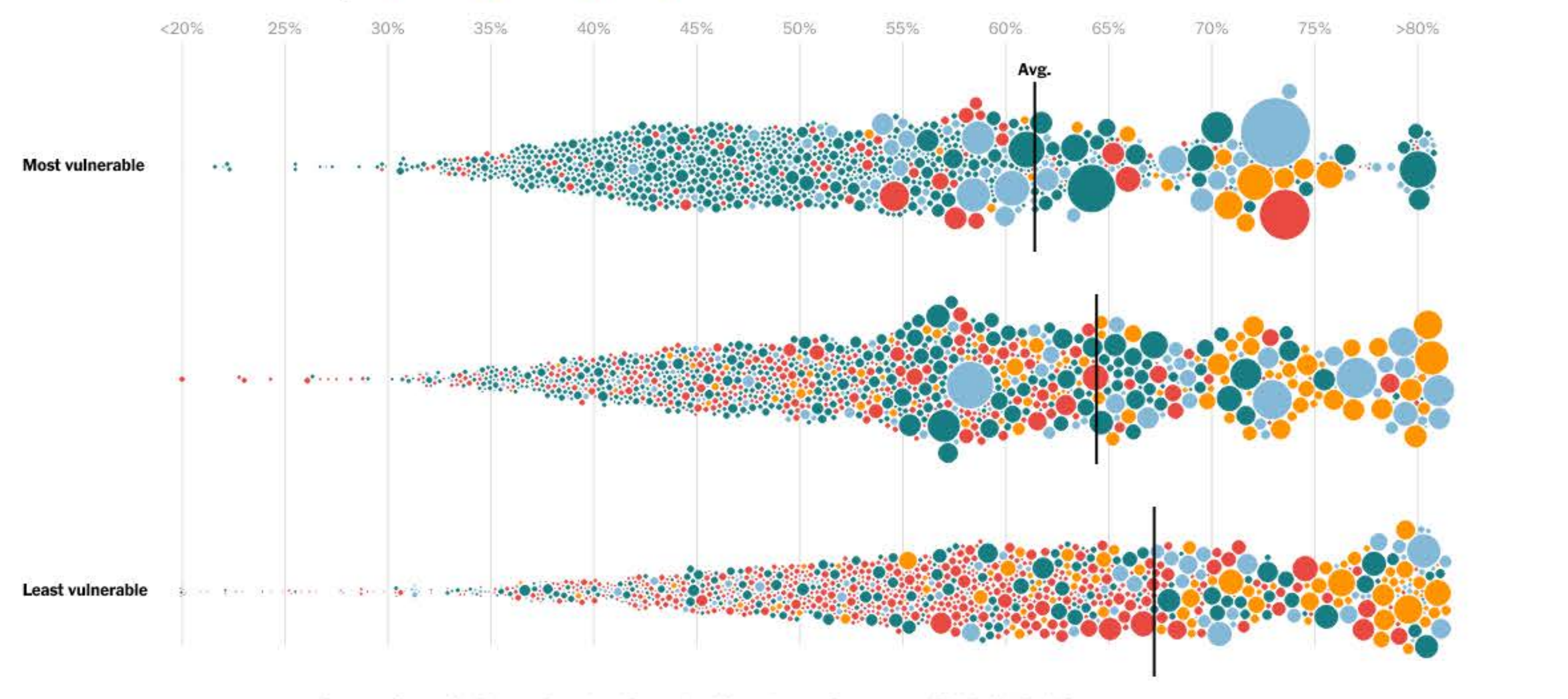

Sources: Centers for Disease Control and Prevention; Massachusetts Department of Public Health; U.S. Census Bureau | Note: No C.D.C. data available for some counties. Vermont was excluded because more than a quarter of data is missing.

Counties are ranked according to the Social Vulnerability Index, a C.D.C. indicator used in public health crises that is based on socioeconomic status, housing, transportation, race, ethnicity and language. Each county's vaccination rate is its share of all residents that have been fully vaccinated, a figure that does not reflect those who have received only one dose of a two-shot vaccine.

Coronavirus (COVID-19)

COVID-19 Vaccine Tracker

This is your state and county equivalent level look at how many have gotten a dose or doses of the COVID-19 vaccine. Click on a state to see how many vaccines have been administered and you can drill down to the county level and see a bar chart that tracks the running total. Data for this interactive map is provided by the Centers for Disease Control and Prevention (CDC) and state health departments and will be updated automatically when states update data.

Map

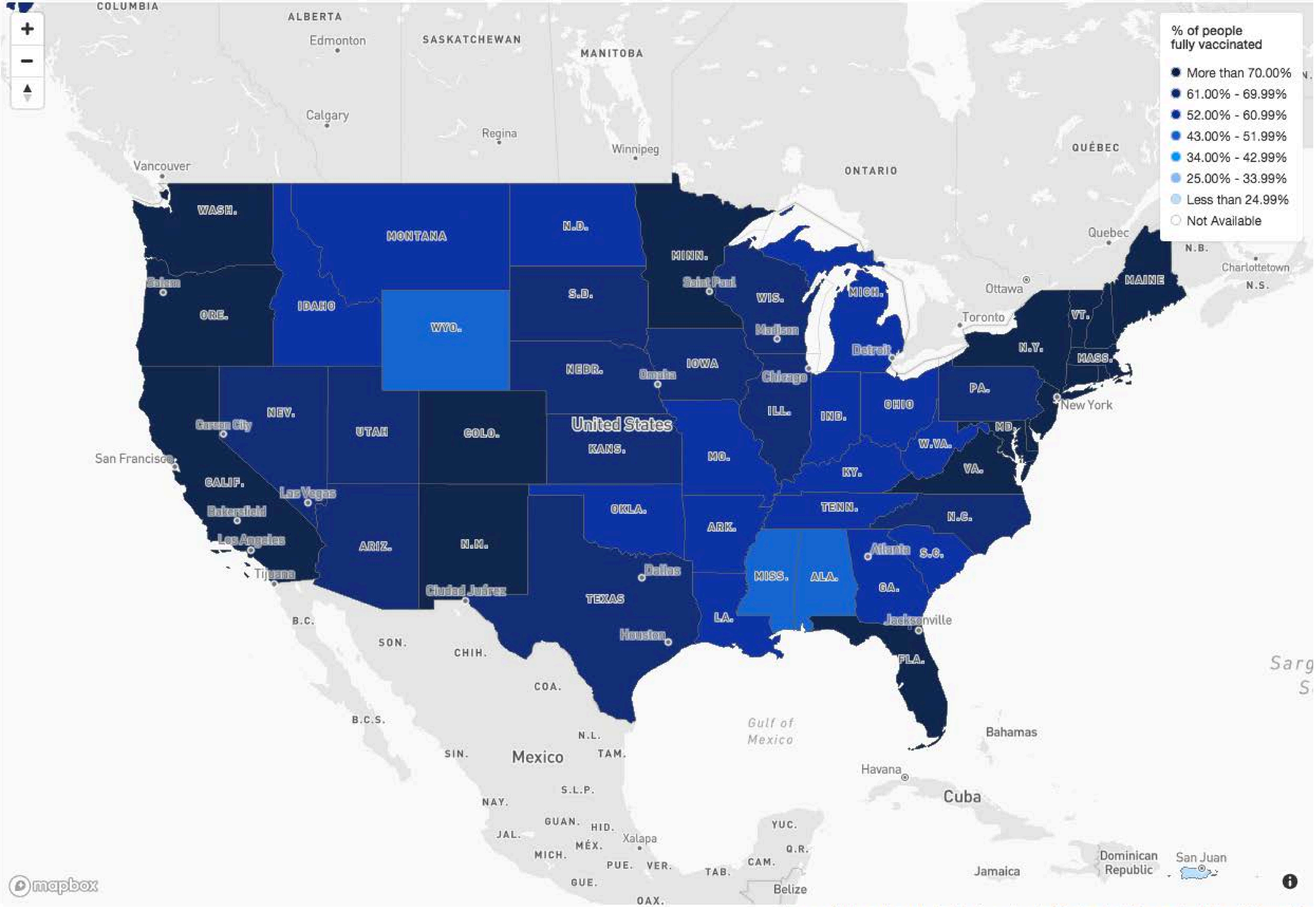

Source: State and county health departments, Centers for Disease Control and Prevention

Health

At least 221.9 million people have been fully vaccinated in the U.S.

This includes more than **104.7 million** people who have received a booster shot.

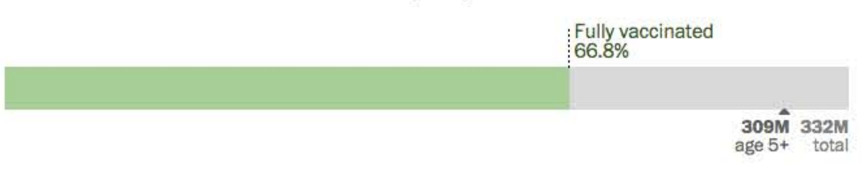

Data as of June 16 at 7:59 p.m.

Home
Share

PLEASE NOTE

The Washington Post is providing this story for free so that all readers have access to this important information about the coronavirus. For more free stories, sign up for our Coronavirus Updates newsletter.

Reported doses administered by day

In the last week, an average of **313.2k** doses per day were administered, a **18%** increase ↑ over the week before.

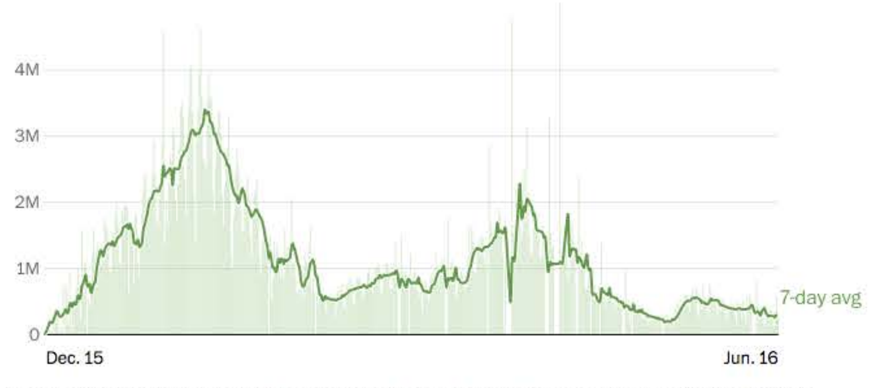

Sources: Centers for Disease Control and Prevention. Note: Due to adjustments in reporting, Pennsylvania removed 1.2 million doses on Nov. 23. On March 13, the CDC reported 4.6m doses due to a data anomaly; in reality, 2.98m fell within a 24-hour period.

Search states

Jump to a state

Select a state

About two-thirds of the country is fully vaccinated in over a year of distributing shots. More than a million doses per day were administered nationwide November through January, but rates have since declined, according to the latest data from the Centers for Disease Control and Prevention, including first doses, second doses and boosters.

Share of population fully vaccinated

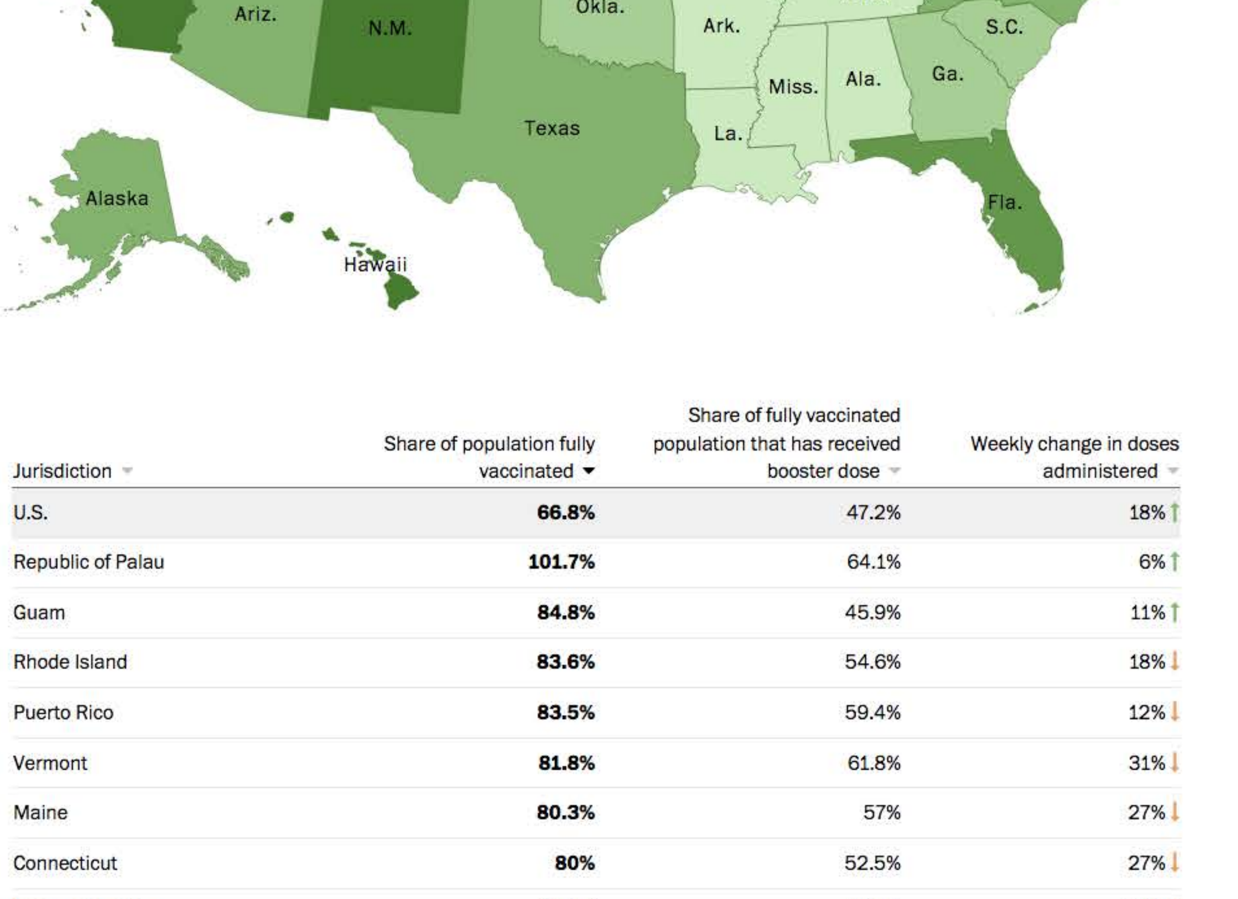

Jurisdiction	Share of population fully vaccinated	Share of fully vaccinated population that has received booster dose	Weekly change in doses administered
U.S.	**66.8%**	47.2%	18% ↑
Republic of Palau	**101.7%**	64.1%	6% ↑
Guam	**84.8%**	45.9%	11% ↑
Rhode Island	**83.6%**	54.6%	18% ↓
Puerto Rico	**83.5%**	59.4%	12% ↓
Vermont	**81.8%**	61.8%	31% ↓
Maine	**80.3%**	57%	27% ↓
Connecticut	**80%**	52.5%	27% ↓
Massachusetts	**79.8%**	52.3%	28% ↓
Hawaii	**78.8%**	52.7%	44% ↓
New York	**77.6%**	46.3%	62% ↑

See all

Note: The U.S. total includes doses provided to Republic of Palau, Federated States of Micronesia and the Marshall Islands, as US-affiliated Pacific Islands. The CDC is underreporting vaccinations in Utah by 100,000 people.

All adults have been eligible for a shot since April. In May, shots were authorized for almost 17 million children between 12 and 15. Another 28.5 million children ages 5 to 11 can now receive vaccinations, making 87 percent of Americans eligible for a coronavirus vaccine.

[For unvaccinated, coronavirus is soaring again]

Public health and government leaders said that racial and ethnic equity would be critical in distributing vaccines, but data collection on the race of recipients has been poor.

These charts show the percent of the population in each racial or ethnic group that has received a vaccine so far. Alaskan Native and Native American populations have a higher rate of vaccination, which tribal leaders have attributed to their sovereignty and emphasis on prioritizing elders and their communities. Because so much race information is missing, the vaccine rate for each group is understated.

Share of each race or ethnicity that has been fully vaccinated

As of June 16, 74.9% of vaccinations include race-ethnicity information

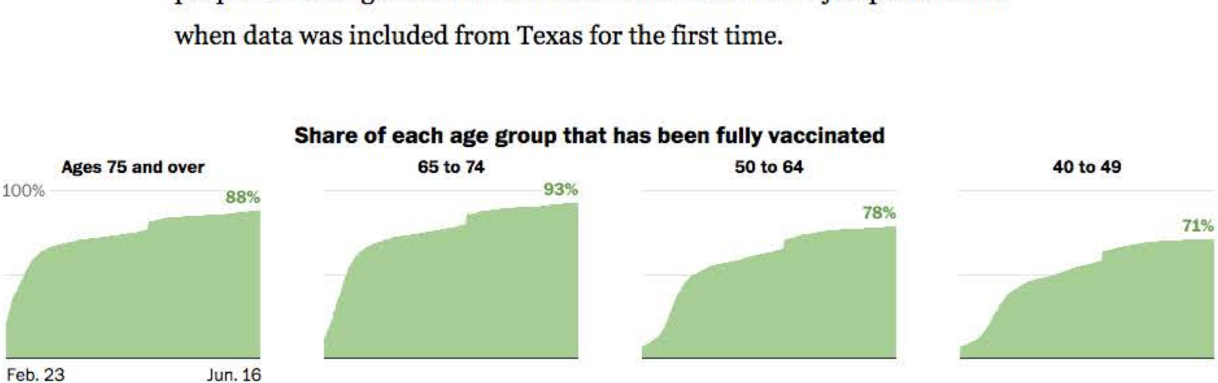

Note: Start date reflects when CDC first made demographic data available.

The risk of hospitalization and death from covid-19 rises with age, and vaccination rates have followed that pattern, with prioritization for people in nursing homes and for older Americans. Rates jumped this fall when data was included from Texas for the first time.

Share of each age group that has been fully vaccinated

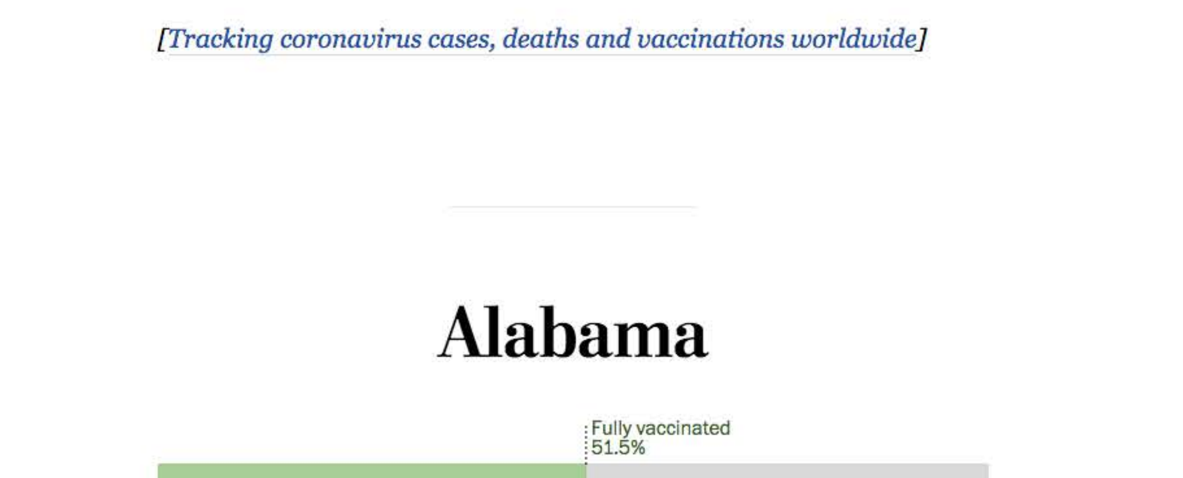

Note: Start date reflects when CDC first made demographic data available. CDC adjusted age calculations on March 12 and May 11.

[Tracking coronavirus cases, deaths and vaccinations worldwide]

Alabama

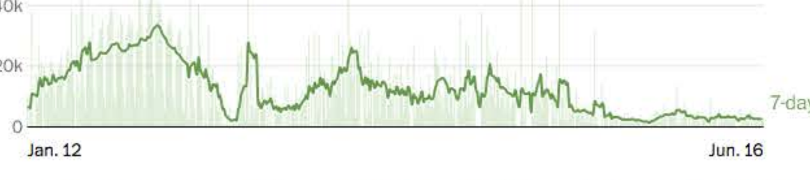

Reported doses administered by day in Alabama

In the last week, an average of **2.3k** doses per day were administered, a **8%** decrease ↓ over the week before.

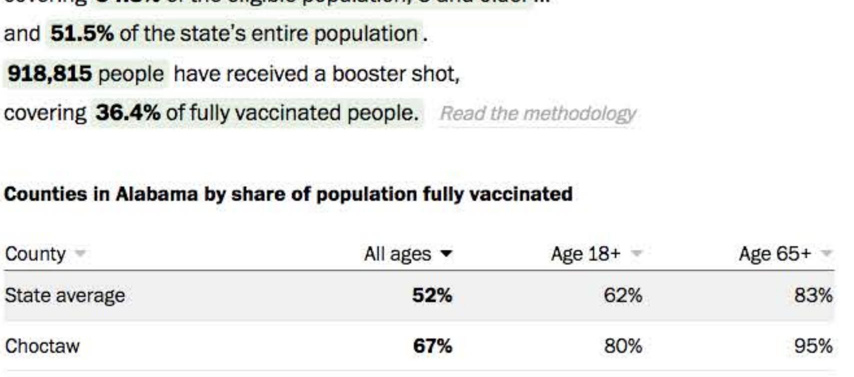

Note: Data before Jan. 12 is inconsistent. On Feb. 19, the CDC added doses administered by federal agencies to the states where the shots had been given. Starting Feb. 23, data reflects doses administered to residents of the state instead of doses administered by the state.

Alabama has fully vaccinated **2,527,041** people,
covering **54.8%** of the eligible population, 5 and older ...
and **51.5%** of the state's entire population.
918,815 people have received a booster shot,
covering **36.4%** of fully vaccinated people. *Read the methodology*

Counties in Alabama by share of population fully vaccinated

County	All ages	Age 18+	Age 65+
State average	**52%**	62%	83%
Choctaw	**67%**	80%	95%
Madison	**63%**	73%	92%
Hale	**62%**	74%	85%
Jefferson	**59%**	70%	92%
Bullock	**55%**	63%	82%

See all

Over the last week, Alabama has seen an average of **1,280** confirmed cases and **2** deaths per day. Alabama has the **28th** most confirmed cases per capita among states and D.C. during the same period.

Alaska

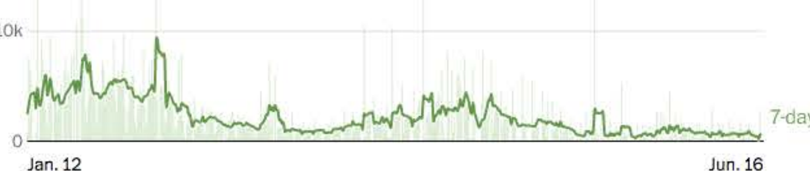

Reported doses administered by day in Alaska

In the last week, an average of **0.5k** doses per day were administered, a **6%** decrease ↓ over the week before.

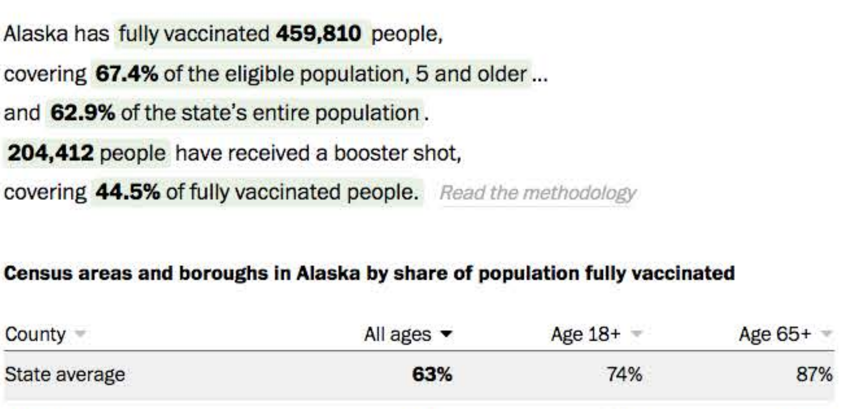

Note: Data before Jan. 12 is inconsistent. On Feb. 19, the CDC added doses administered by federal agencies to the states where the shots had been given. Starting Feb. 23, data reflects doses administered to residents of the state instead of doses administered by the state.

Alaska has fully vaccinated **459,810** people,
covering **67.4%** of the eligible population, 5 and older ...
and **62.9%** of the state's entire population.
204,412 people have received a booster shot,
covering **44.5%** of fully vaccinated people. *Read the methodology*

Census areas and boroughs in Alaska by share of population fully vaccinated

County	All ages	Age 18+	Age 65+
State average	**63%**	74%	87%
Juneau	**78%**	86%	95%
Yukon-Koyukuk	**78%**	90%	93%
Nome	**71%**	86%	93%
Anchorage	**68%**	80%	94%
Bethel	**68%**	81%	86%

See all

Over the last week, Alaska has seen an average of **263** confirmed cases and **0** deaths per day. Alaska has the **12th** most confirmed cases per capita among states and D.C. during the same period.

Arizona

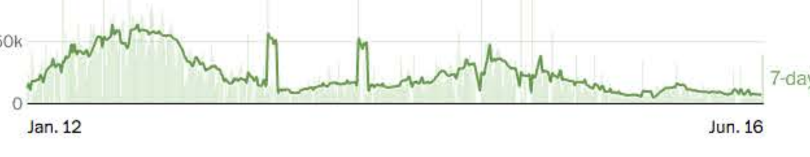

Reported doses administered by day in Arizona

In the last week, an average of **11.2k** doses per day were administered, a **50%** increase ↑ over the week before.

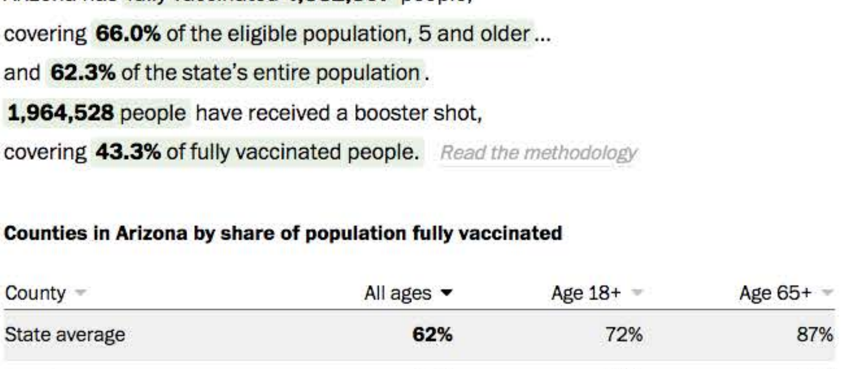

Note: Data before Jan. 12 is inconsistent. On Feb. 19, the CDC added doses administered by federal agencies to the states where the shots had been given. Starting Feb. 23, data reflects doses administered to residents of the state instead of doses administered by the state. On July 1, the CDC reported 258,590 extra doses to account for data reporting issues.

Arizona has fully vaccinated **4,532,807** people,
covering **66.0%** of the eligible population, 5 and older ...
and **62.3%** of the state's entire population.
1,964,528 people have received a booster shot,
covering **43.3%** of fully vaccinated people. *Read the methodology*

Counties in Arizona by share of population fully vaccinated

County	All ages	Age 18+	Age 65+
State average	**62%**	72%	87%
Apache	**95%**	95%	95%
Santa Cruz	**95%**	95%	95%
Navajo	**79%**	87%	95%
Coconino	**78%**	86%	95%

COVID-19 Vaccination Tracker

THE UNITED STATES

Total doses delivered	Total doses administered	Number received at least one dose	Number fully vaccinated
756,780,755	591,406,403	259,048,060	221,768,203

Percent of the U.S. population partially vaccinated
One of two doses of Pfizer or Moderna vaccine received
11.2%

Percent of the U.S. population fully vaccinated
Both doses of Pfizer or Moderna vaccine or one and only dose of Johnson and Johnson received
66.8%

Percent of the U.S. population that received at least one dose
One or more doses of any of the authorized vaccines received
78%

About the data

COVID-19 VACCINATION BY RACE & ETHNICITY

Comparing the racial and ethnic breakdown of all vaccinated individuals, recently vaccinated individuals, and the total US population can help identify groups that require addition efforts to achieve full vaccination coverage.

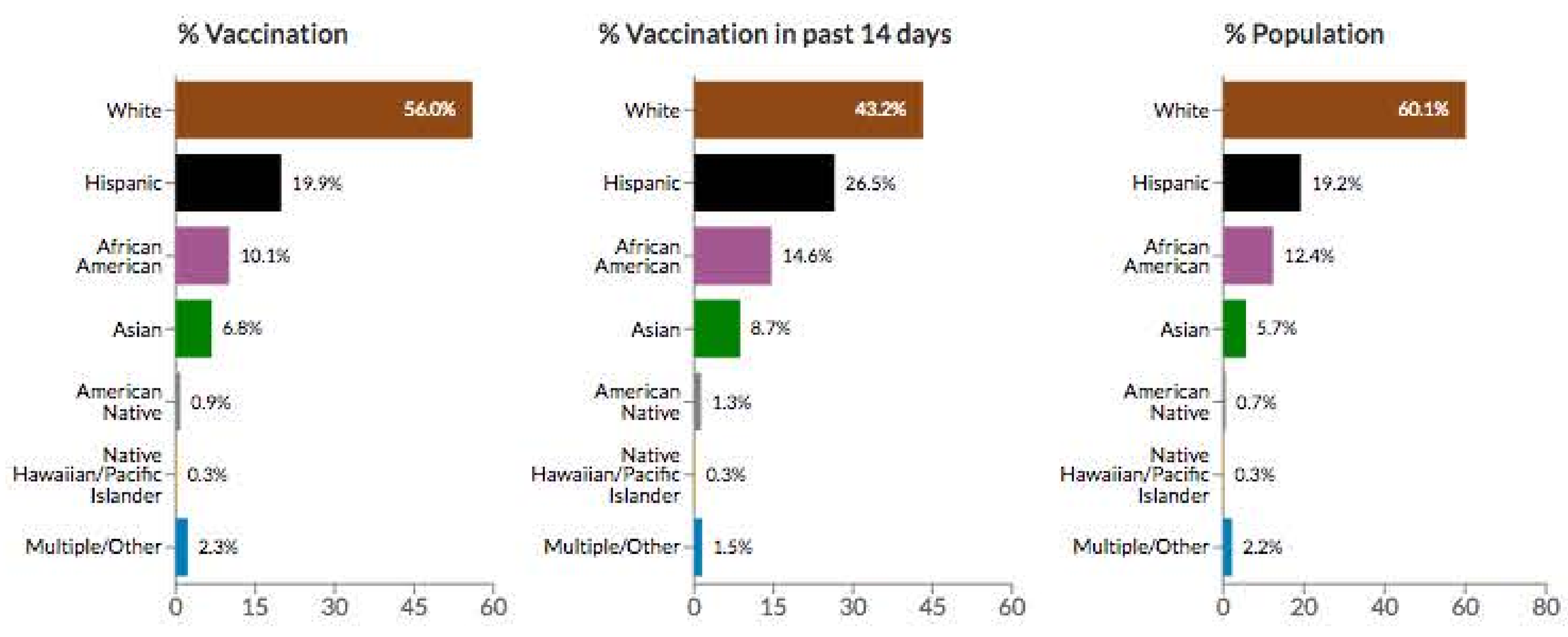

About the data About the data About the data

- White Americans make up 56 % of those fully vaccinated, 43.2 % vaccinated in past 14 days, 59.4 % population.
- Hispanic Americans make up 19.9 % of those fully vaccinated, 26.5 % vaccinated in past 14 days, 19.2 % population.
- African Americans make up 10.1 % of those fully vaccinated, 14.6 % vaccinated in past 14 days, 12.4 % population.
- Asian Americans make up 6.8 % of those fully vaccinated, 14.6 % vaccinated in past 14 days, 5.7 % population.
- Native Americans make up 0.9 % of those fully vaccinated, 1.3 % vaccinated in past 14 days, 0.7 % population.
- Pacific Islanders make up 0.3 % of those fully vaccinated, 0.3 % vaccinated in past 14 days, 0.3 % population.
- Others make up 2.3 % of those fully vaccinated, 1.5 % vaccinated in past 14 days, 2.2 % population.

About the data

COVID-19 VACCINATION BY STATE

State Vaccination Status | State Vaccination Trends

Click on a state.

% of population partially vaccinated (one dose received)

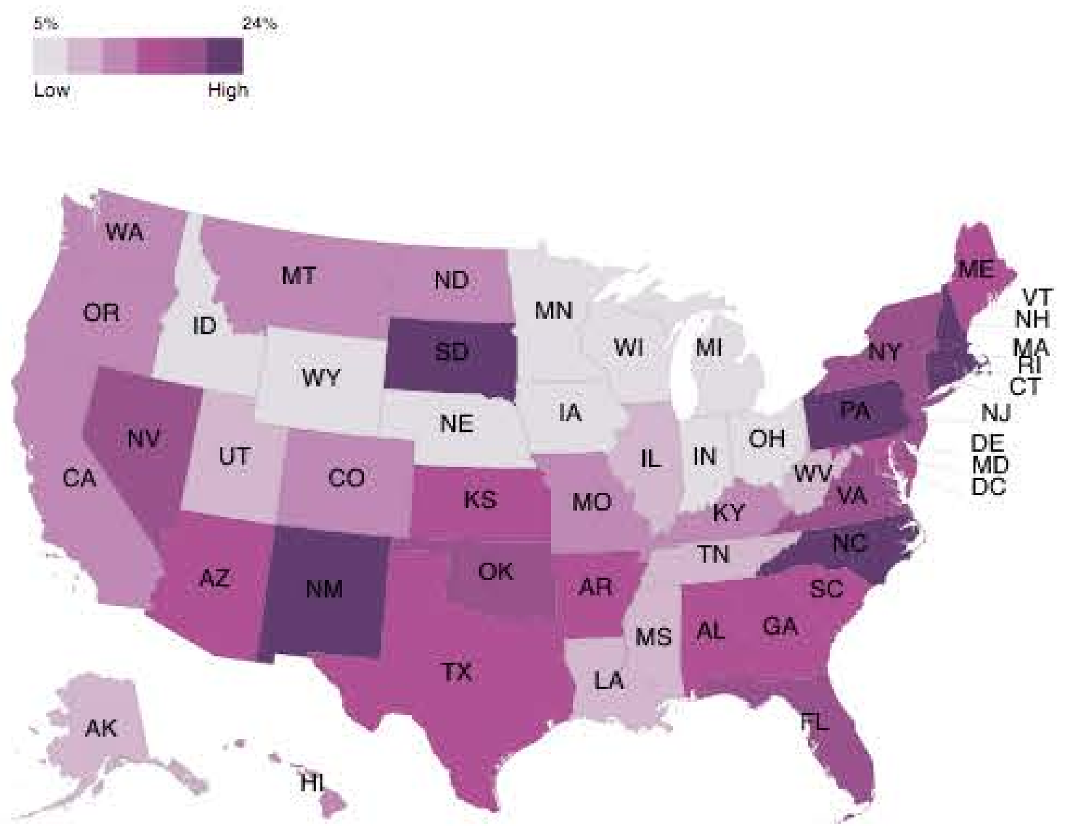

Vaccination Status in
The United States

	Select State	U.S.
Percent fully vaccinated		66.8%
Percent of the U.S population that received at least one dose		78%
Distributed on 06/14/2022		654,400

About the data

COVID-19 VACCINATION BY COUNTY

County Vaccination Trends | County Vaccination Map

African American | Hispanic | Age 65+ | Underlying condition | In poverty | Minority | Native American | Uninsured | Region | Urbanicity | College

Percent vaccinated with at least 1 dose by Community Characteristics

COVID-19 Health Equity Interactive Dashboard | National Report | Vaccination Surveillance | Variant Map | Other Tools | Media Hub | Data Sources & Interpretation | About | EMORY UNIVERSITY

USA Vaccination Tracker
Vaccination by Race & Ethinicity
State Vaccination Tracker
State COVID-19 Burden
COVID-19 Vaccines FAQ
General Information
Vaccine Development
Vaccine Safety
Getting Vaccinated
After You Are Vaccinated

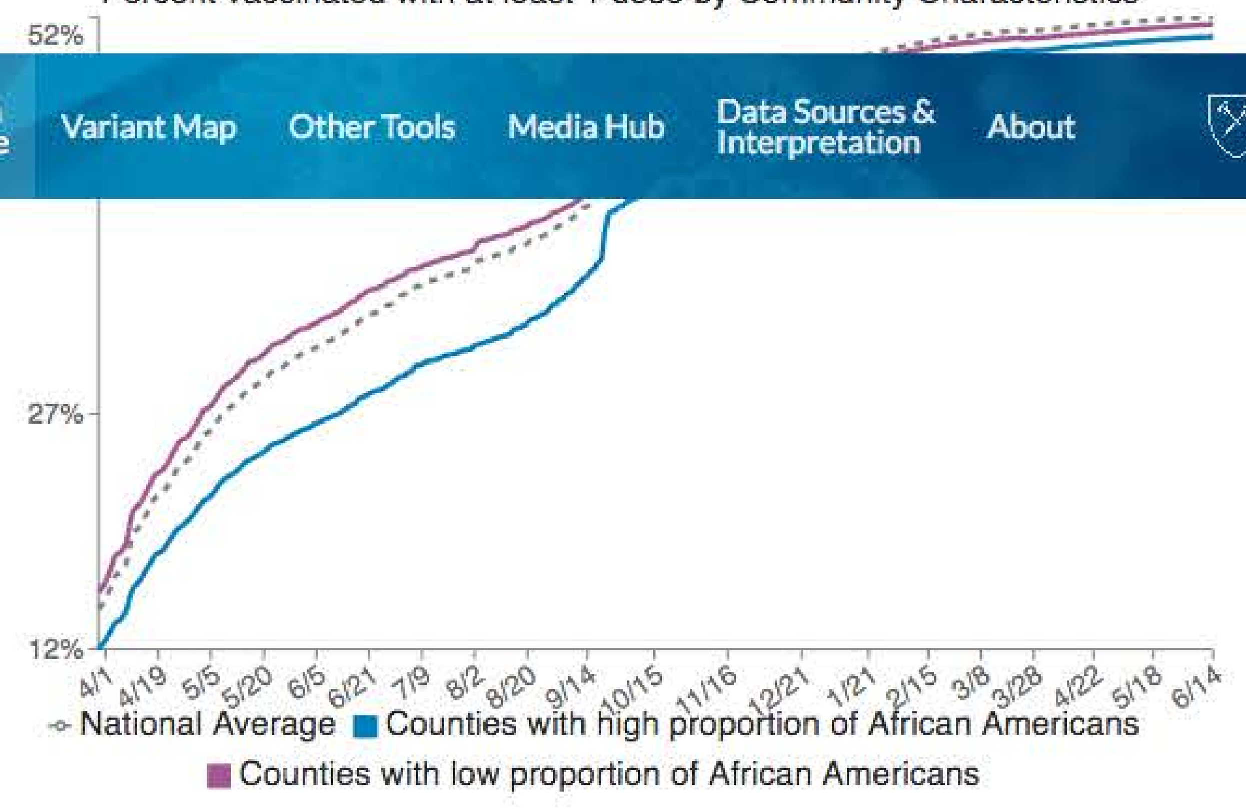

Date Updated: 06/13/2022

About the data

COVID-19 BURDEN IN THE UNITED STATES

Daily Cases
125,403 ↑ 18% 14-day change

Daily Deaths
271 ↑ 12% 14-day change

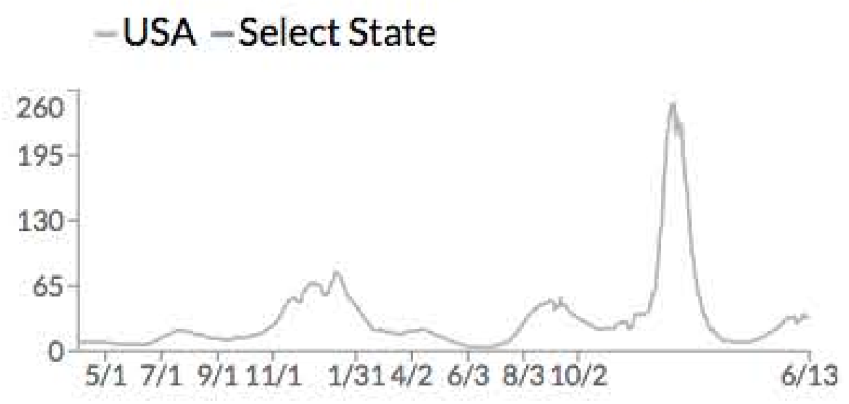

About the data

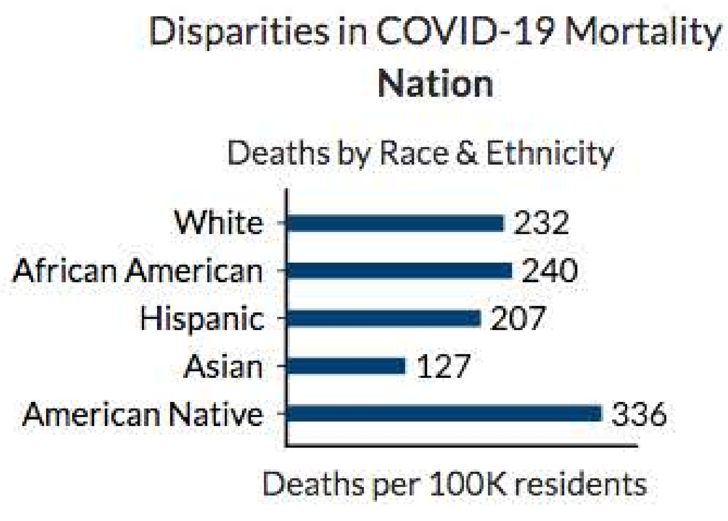

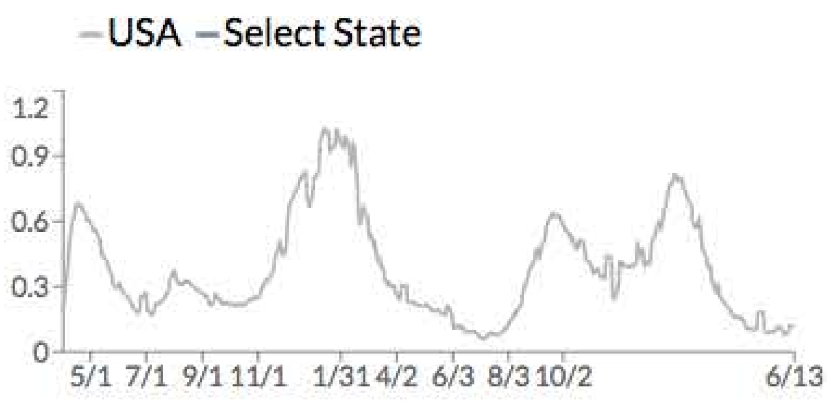

About the data About the data

Coronavirus (COVID-19) Vaccinations

66.2% of the world population has received at least one dose of a COVID-19 vaccine.
11.96 billion doses have been administered globally, and **5.84 million** are now administered each day.
Only **17.7%** of people in low-income countries have received at least one dose.

Our international COVID-19 vaccination dataset is updated each morning (London time), with data up to the previous day. Last update: **17 hours ago.** Reuse our work freely | Cite this research

Our vaccination dataset uses the most recent official numbers from governments and health ministries worldwide. Population estimates for per-capita metrics are based on the United Nations World Population Prospects. Income groups are based on the World Bank classification. A full list of our country-specific sources is available at the bottom of this page, and we also answer frequently-asked questions there.

COVID-19 Data Explorer

Download the complete *Our World in Data* COVID-19 dataset.

METRIC: People vaccinated (by dose)
INTERVAL: Cumulative
☑ Relative to Population
☐ Color by test positivity

☰ Contents | Add a country...

Sort by: Country name

- ☑ Bangladesh
- ☑ Brazil
- ☑ Canada
- ☑ Chile
- ☑ China
- ☑ Cuba
- ☑ Egypt
- ☑ Ethiopia
- ☑ France
- ☑ Germany
- ☑ India
- ☑ Indonesia
- ☑ Iran
- ☑ Italy
- ☑ Japan
- ☑ Mexico
- ☑ Nigeria
- ☑ Pakistan

× Clear selection

Share of people vaccinated against COVID-19, Jun 15, 2022

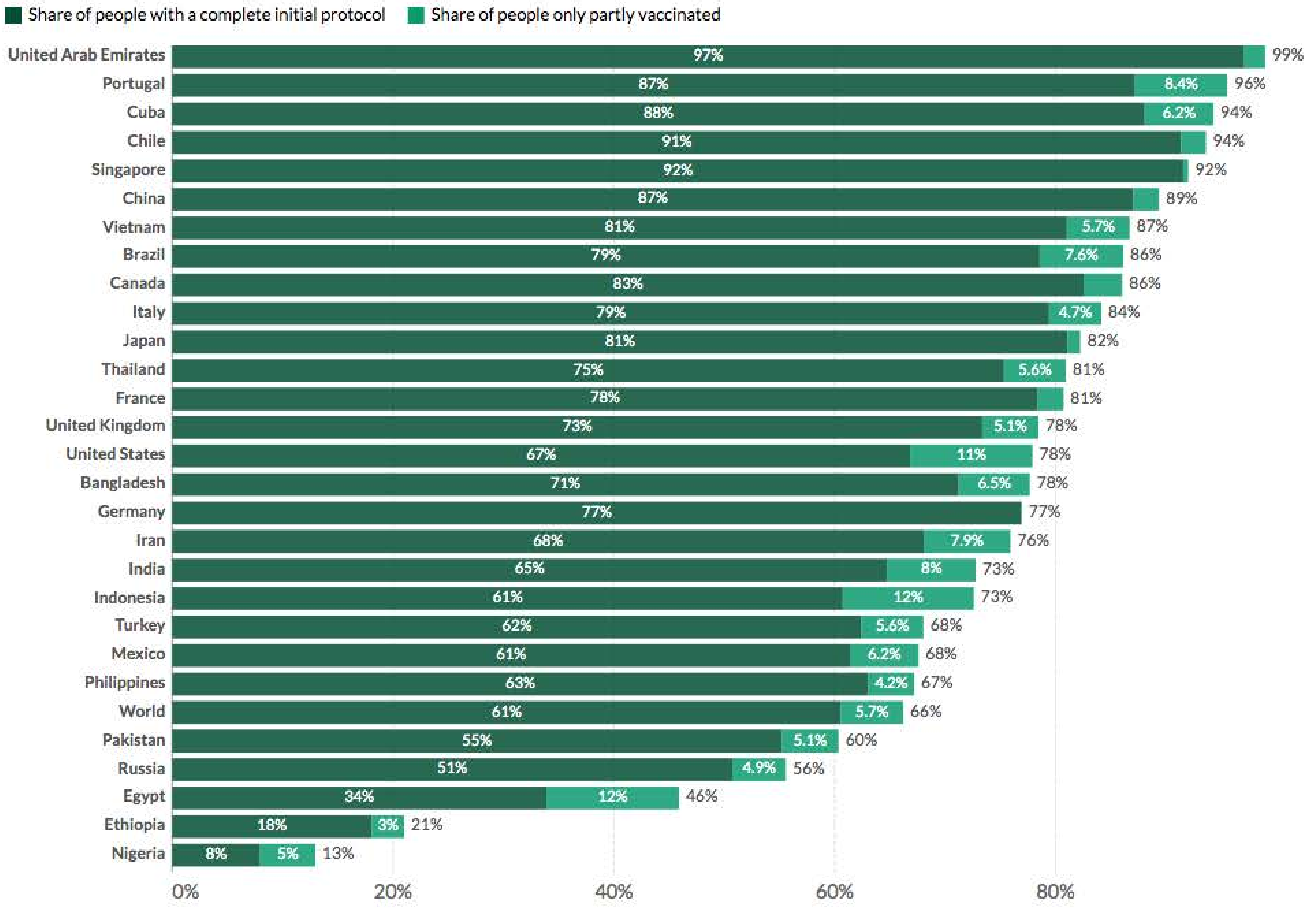

Source: Official data collated by Our World in Data
Note: Alternative definitions of a full vaccination, e.g. having been infected with SARS-CoV-2 and having 1 dose of a 2-dose protocol, are ignored to maximize comparability between countries.

CC BY

Dec 13, 2020 — Jun 15, 2022

CHART | TABLE | SOURCES | DOWNLOAD

Tracking Covid-19 vaccinations worldwide

By Henrik Pettersson, Byron Manley, Sergio Hernandez, Deidre McPhillips and Tatiana Arias, CNN

Last updated: June 16, 2022 at 7:51 p.m. ET

At least 218 countries and territories have administered more than 11 billion doses of a Covid-19 vaccine, since the first Covid-19 case was reported in China in late 2019. Several different vaccines have been developed at record speed, in large part due to years of research on related viruses and billions of dollars in investment.

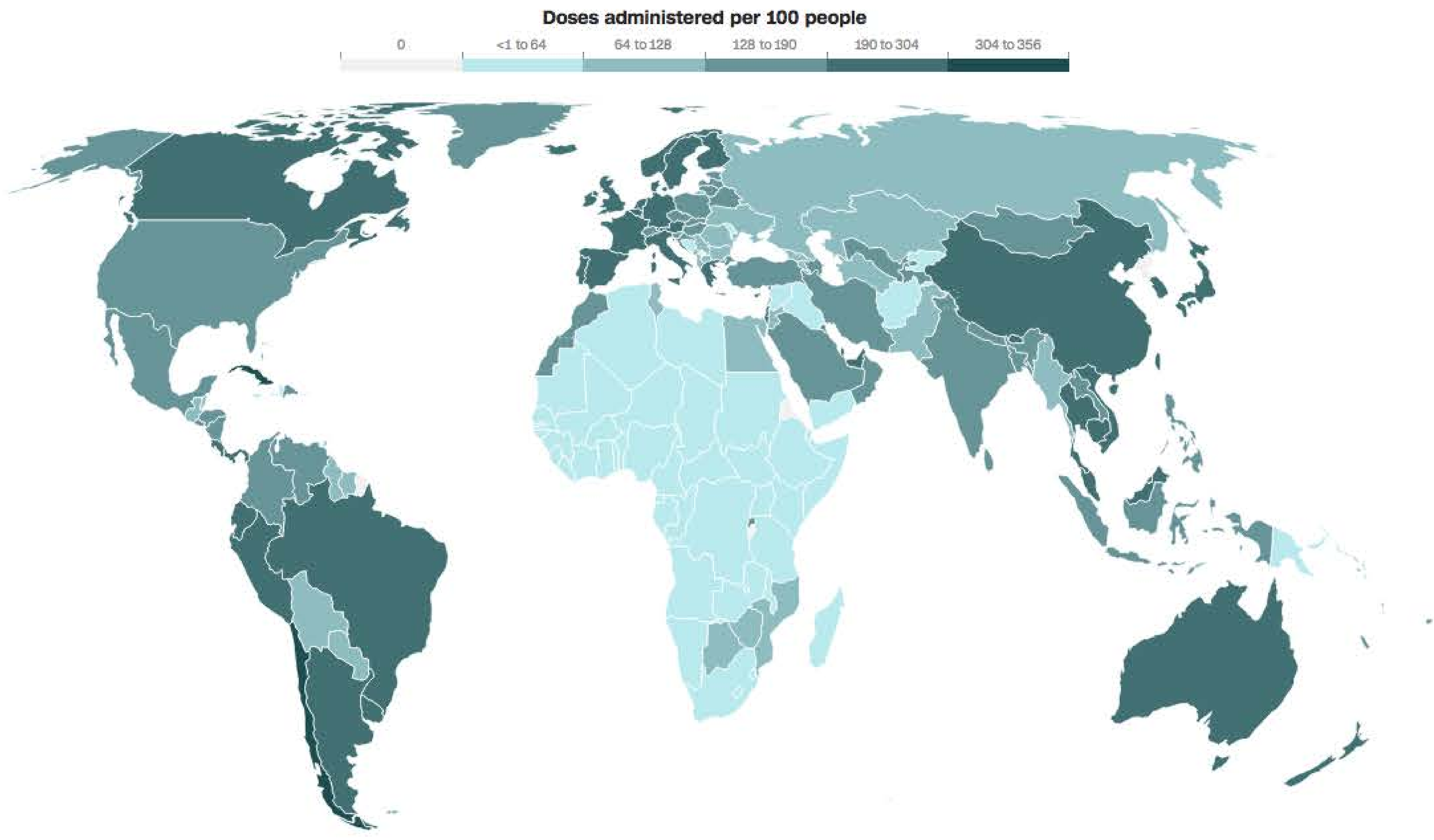

Last updated: June 16, 2022 at 7:51 p.m. ET
Source: Our World in Data

In December 2020, the first dose of a fully tested vaccine — manufactured by Pfizer/BioNTech — was administered in the United Kingdom. Now, multiple vaccines have been authorized for use around the world and dozens of countries and territories have joined the race to vaccinate their residents.

Rate of global vaccination campaign

Currently, the global 7-day moving average of administered doses is about per day.

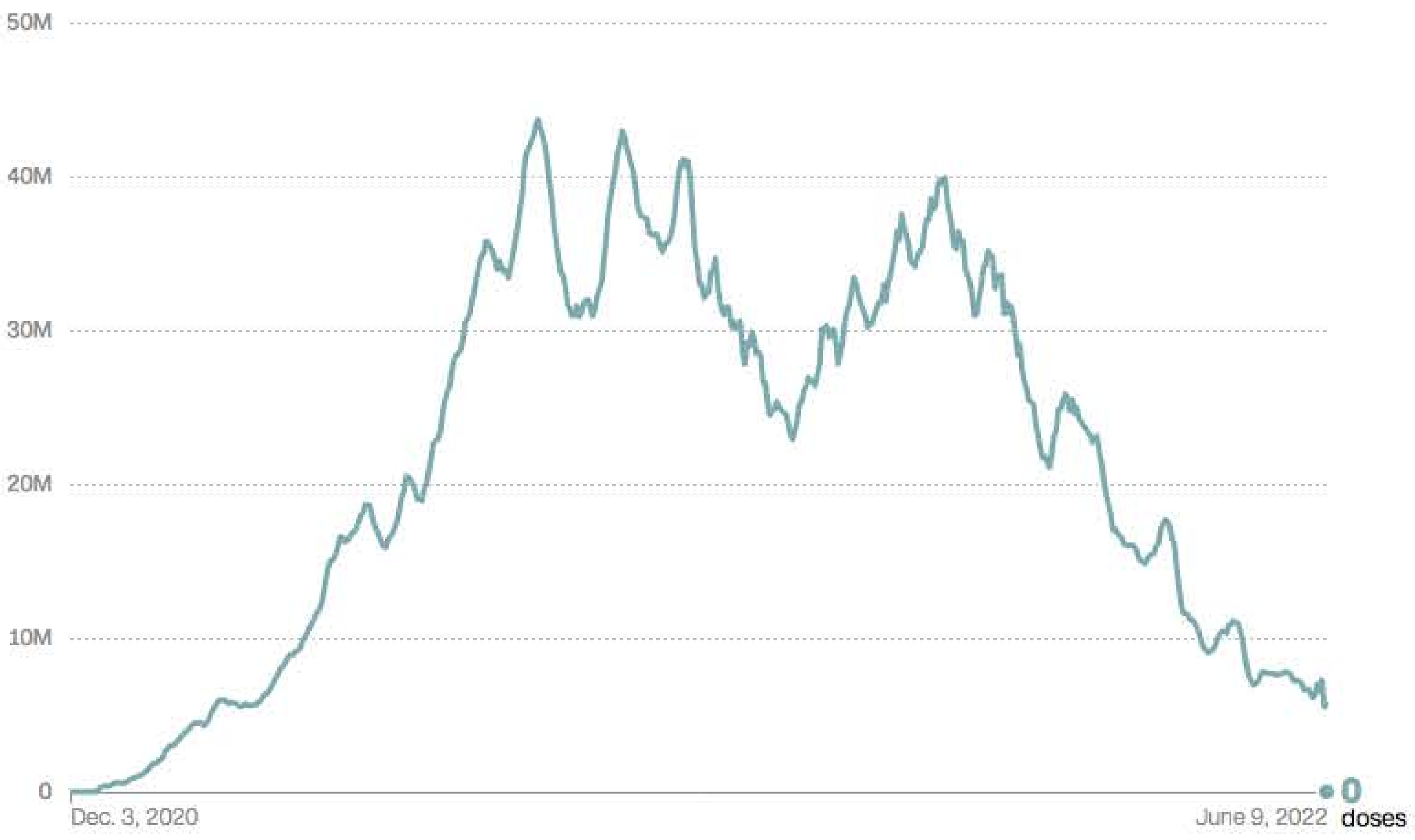

Last updated: June 16, 2022 at 7:51 p.m. ET
Source: Our World in Data

The vaccine rollout strategy varies from country to country. Some have favored vaccinating as many people as possible as quickly as possible, while others have tried to prioritize vaccinating specific vulnerable groups.

Leaders at the World Health Organization have emphasized the need for international cooperation in vaccination campaigns, noting that a global pandemic requires global efforts to end it.

Comparing vaccination rates

At least 218 countries and territories have started their vaccination rollouts. Different countries have different processes for approving vaccines, and not all countries have deals to obtain doses from manufacturers yet. As a result, countries and territories are at different stages of their vaccination campaigns.

Location	Total doses	Doses administered per 100 people	Days since first dose
Gibraltar	119,855	356	522
Cuba	37,823,143	334	402
[illegible]	[illegible]	[illegible]	[illegible]
Singapore	14,171,209	260	533
Niue	4,161	258	378
Malta	1,308,973	254	536
United Arab Emirates	24,880,528	249	532
Samoa	494,684	247	424
Guernsey	156,415	247	546
Uruguay	8,561,243	246	472
South Korea	125,795,763	245	475
Bhutan	1,909,762	245	446
Portugal	24,104,902	237	536
Cambodia	40,160,051	237	491
Taiwan	56,501,765	237	451
Qatar	6,914,015	236	540
Mainland China	3,391,088,000	235	548
Jersey	234,978	232	550
Hong Kong	17,398,698	230	475

Show more

Last updated: June 16, 2022 at 7:51 p.m. ET
Source: Our World in Data, CNN research

Measuring countries' progress is challenging because many are using vaccines that require two doses. Inconsistent data makes it hard to know how many people have been fully or only partially immunized.

The race to get fully vaccinated

Most countries and territories are using vaccines that require two doses to provide the greatest protection. Measuring countries' progress is difficult, because reported data varies from country to country. Many report only the number of doses administered, but at least 206 break down how many people have received one or two doses.

Pitcairn
100.0% fully vaccinated
0% partially vaccinated

Samoa
99.0%
15.6%

United Arab Emirates
97.1%
1.9%

Brunei
96.7%
4.0%

Singapore
91.6%
<1%

Chile
91.4%
2.2%

Tonga
91.0%
10.4%

Malta
91.0%
1.4%

Cayman Islands
89.2%
2.8%

Macao
88.6%
4.3%

Cuba
88.1%
6.2%

Niue
87.8%
14.4%
