# Supplementary material for: Web-Based COVID-19 Dashboards and Trackers in the United States: Survey Study
Source: JMIR Hum Factors. 2023 Mar 20;10:e43819. doi: 10.2196/43819 (PMC10029858; doi:10.2196/43819)
Supplement: Multimedia Appendix 4 [file humanfactors_v10i1e43819_app4.pdf]

Appendix 4. Screenshots from dashboards and trackers — *State-focused coverage* *(captured August 29, 2020)*

Page	Ref	Host
2–4	S-1	Alabama Dept of Public Health
5	S-2	David Marconnet, CTO of OneTeam.net
6	S-3	Alabama Media Group
7–8	S-4	Alaska Dept of Health and Social Services
9	S-5	Anchorage Daily News
10–12	S-6	Arizona Dept of Health Services
13–15	S-7	Arkansas Dept of Health
16	S-8	California Dept of Public Health
17	S-9	CA Open Data
18	S-10	Los Angeles Times
19	S-11	CalMatters
20–22	S-12	Colorado Dept of Health & Environment
23	S-13	Coloradoan
24	S-14	The Denver Post
25	S-15	Connecticut's Official State Website
26–28	S-16	CTData Collaborative
29	S-17	University of Connecticut Dept of Geography
30	S-18	Government of the District of Columbia
31	S-19	Delaware Environmental Public Health Tracking Network
32–34	S-20	Florida Dept of Health
35	S-21	Florida COVID Action
36	S-22	Georgia Dept of Public Health
37	S-23	Atlanta Journal-Constitution
38	S-24	State of Hawaii Dept of Health
39	S-25	Hawaii Data Collaborative
40	S-26	Idaho Division of Public Health
41	S-27	KTVB-7
42	S-28	Illinois Dept of Public Health
43	S-29	Indiana State Dept of Health
44	S-30	Polis Center at Indiana University - Purdue University Indianapolis
45	S-31	Iowa Dept of Public Health
46	S-32	Kansas Dept of Health and Environment
47	S-33	Kentucky Dept of Public Health
48	S-34	WFPL 89.3
49	S-35	Louisiana Dept of Health
50	S-36	Maine Center for Disease Control & Prevention
51–52	S-37	Maine Geolibrary
53	S-38	Maryland Dept of Health
54–55	S-39	Massachusetts Dept of Public Health
56	S-40	Michigan Dept of Health and Human Services
57	S-41	Bridge Michigan
58	S-42	Minnesota Dept of Health

Page	Ref	Host
59	S-43	Mississippi State Dept of Health
60–62	S-44	Missouri Dept of Health and Senior Services
63	S-45	Montana Dept of Public Health and Human Services
64	S-46	Montana State Library
65–66	S-47	North Carolina Dept of Health and Human Services
67	S-48	North Dakota Dept of Health
68	S-49	Nebraska Dept of Health and Human Services
69–71	S-50	Nevada Dept of Health and Human Services
72	S-51	New Hampshire Dept of Health and Human Services
73	S-52	New Hampshire Public Radio
74	S-53	New Jersey Dept of Health
75	S-54	NJ Advance Media
76	S-55	New Mexico Dept of Health
77	S-56	New York State Dept of Health
78	S-57	Ohio Dept of Health
79–81	S-58	Oklahoma State Dept of Health
82–84	S-59	Oregon Health Authority
85–87	S-60	Pennsylvania Dept of Health
88	S-61	Rhode Island Dept of Health
89	S-62	South Carolina Dept of Health and Environment Control
90	S-63	WPDE-15
91–92	S-64	South Dakota Dept of Health
93	S-65	Tennessee Dept of Health
94	S-66	Tennessee Dept of Health
95–96	S-67	Texas Health and Human Services
97	S-68	Houston Chronicle
98	S-69	Utah Dept of Health
99–101	S-70	Virginia Dept of Health
102	S-71	Vermont Dept of Health
103	S-72	VTDigger
104–106	S-73	Washington State Dept of Health
107	S-74	Washington State Hospital Association
108	S-75	West Virginia Dept of Health & Human Resources
109	S-76	Wisconsin Dept of Health Services
110	S-77	WISN-12
111	S-78	Wyoming Dept of Health
112	S-79	Spotlight PA
113	S-80	WPRI-12
114	S-81	San Francisco Chronicle
115	S-82	The Maine Monitor
116	S-83	The Oregonian
117	S-84	Arkansas Democrat Gazette

Alabama's COVID-19 Data and Surveillance Dashboard

ALABAMA PUBLIC HEALTH

Alabama Department of Public Health | Division of Infectious Diseases & Outbreaks | Updates Daily at 10:00 a.m.

Select a County --> None

COVID-19 Confirmed Rate Per 100,000 Person in the Last 14 Days

Rate

- > 100 - 2,000
- > 50 - 100
- > 10 - 50
- 0 - 10

CONFIRMED RATE IN THE LAST 14 DAYS

(Click a county to see the rate.)

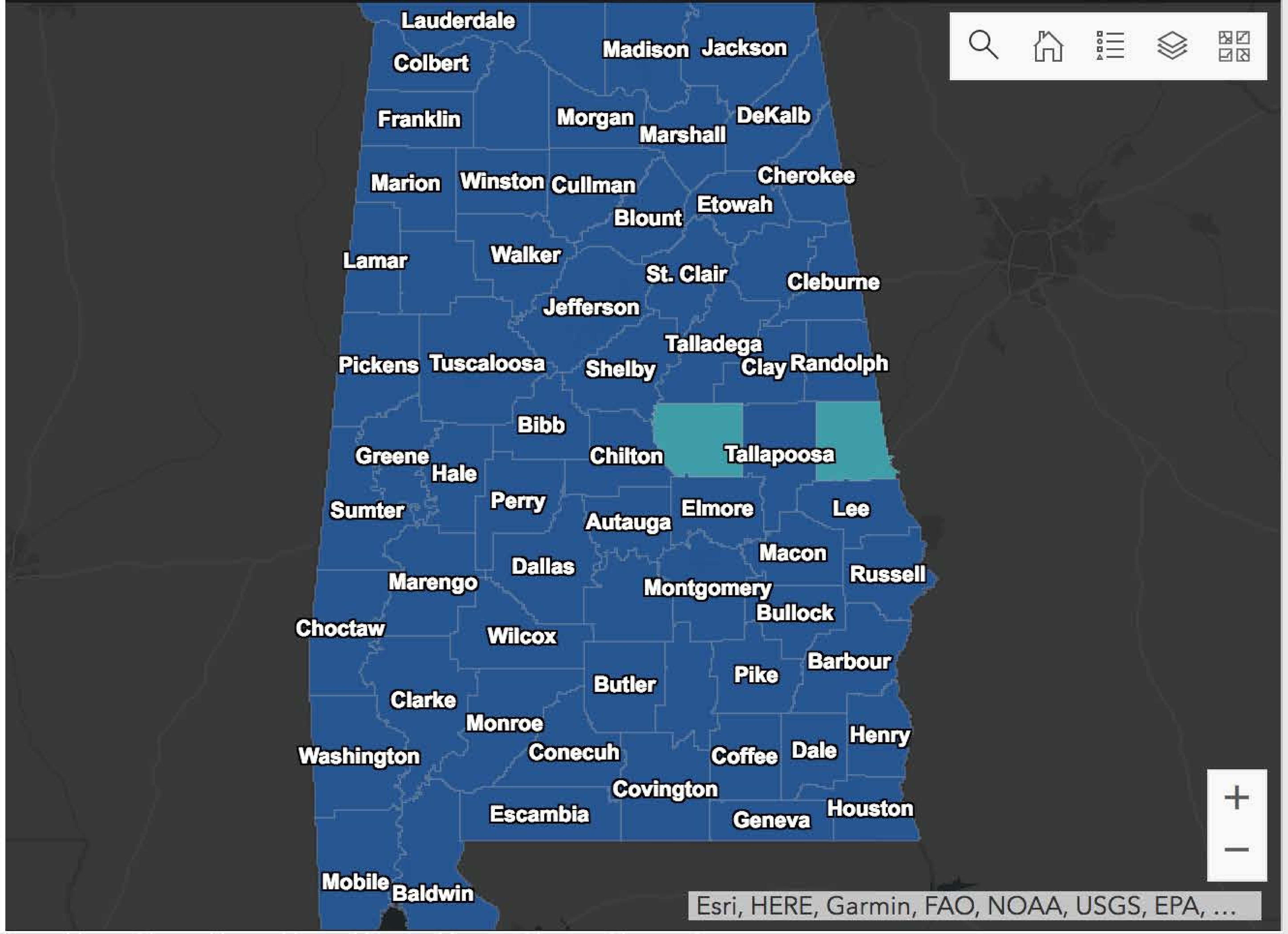

1 2 3 4 5 6 7 8 9 10 11 12 13

CASES	
113,723	8,462
CONFIRMED	PROBABLE

DEATHS	
2,017	90
CONFIRMED	PROBABLE

LAST 14 DAYS	
11,559	198,766
CASES	TESTED

***TOTAL HOSPITALIZATIONS To Date Updates M-F at 3 p.m.**

14,267

STATEWIDE

TOTAL TESTED

956,401

STATEWIDE

PRESUMED RECOVERIES Updated Weekly

48,028

STATEWIDE

About This Dashboard

Data Source: Alabama Department of Public Health (ADPH) **Infectious Diseases & Outbreaks Division.**

This dashboard provides data and surveillance information about Coronavirus Disease 2019 (COVID-19) in Alabama. Data used to produce this dashboard is updated daily from the Alabama NEDSS Base [illegible]

Alabama's COVID-19 Data and Surveillance Dashboard

Alabama Department of Public Health | Division of Infectious Diseases & Outbreaks | Updates Daily at 10:00 a.m.

Select a County --> None

DAILY CASE COUNTS AND MOVING AVERAGES
Statewide

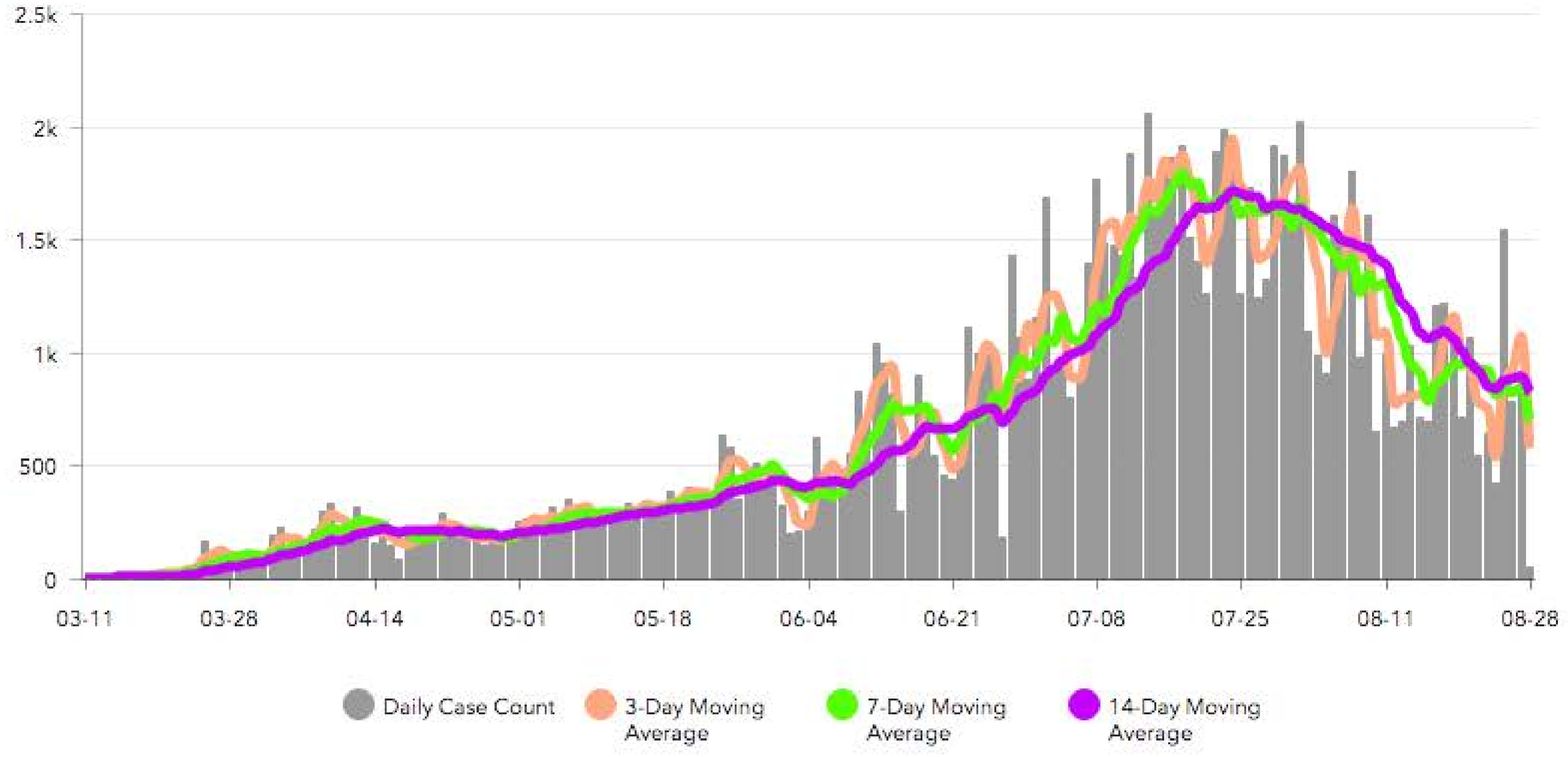

During the week of June 1, an increase in volume of COVID-19 laboratory results being processed by laboratories and sent to state health departments caused delays which affected the number of cases and tested reported. The backlog was resolved by June 5. On June 27, the automatic feeds from laboratories did not process. Therefore the June 28 totals include lab results from June 27 and 28.

1 2 3 4 5 6 7 8 9 10 11 12 13

CASES	
113,723	8,462
CONFIRMED	PROBABLE

DEATHS	
2,017	90
CONFIRMED	PROBABLE

LAST 14 DAYS	
11,559	198,766
CASES	TESTED

***TOTAL HOSPITALIZATIONS**
To Date
Updates M-F at 3 p.m.

14,267

STATEWIDE

TOTAL TESTED

956,401

STATEWIDE

PRESUMED RECOVERIES
Updated Weekly

48,028

STATEWIDE

About This Dashboard

Data Source: Alabama Department of Public Health (ADPH) **Infectious Diseases & Outbreaks Division.**

This dashboard provides data and surveillance information about Coronavirus Disease 2019 (COVID-19) in Alabama. Data used to produce this dashboard is updated daily from the Alabama NEDSS Base

Alabama's COVID-19 Data and Surveillance Dashboard

Alabama Department of Public Health | Division of Infectious Diseases & Outbreaks | Updates Daily at 10:00 a.m.

Select a County --> None

PERCENTAGE OF POSITIVE TESTS

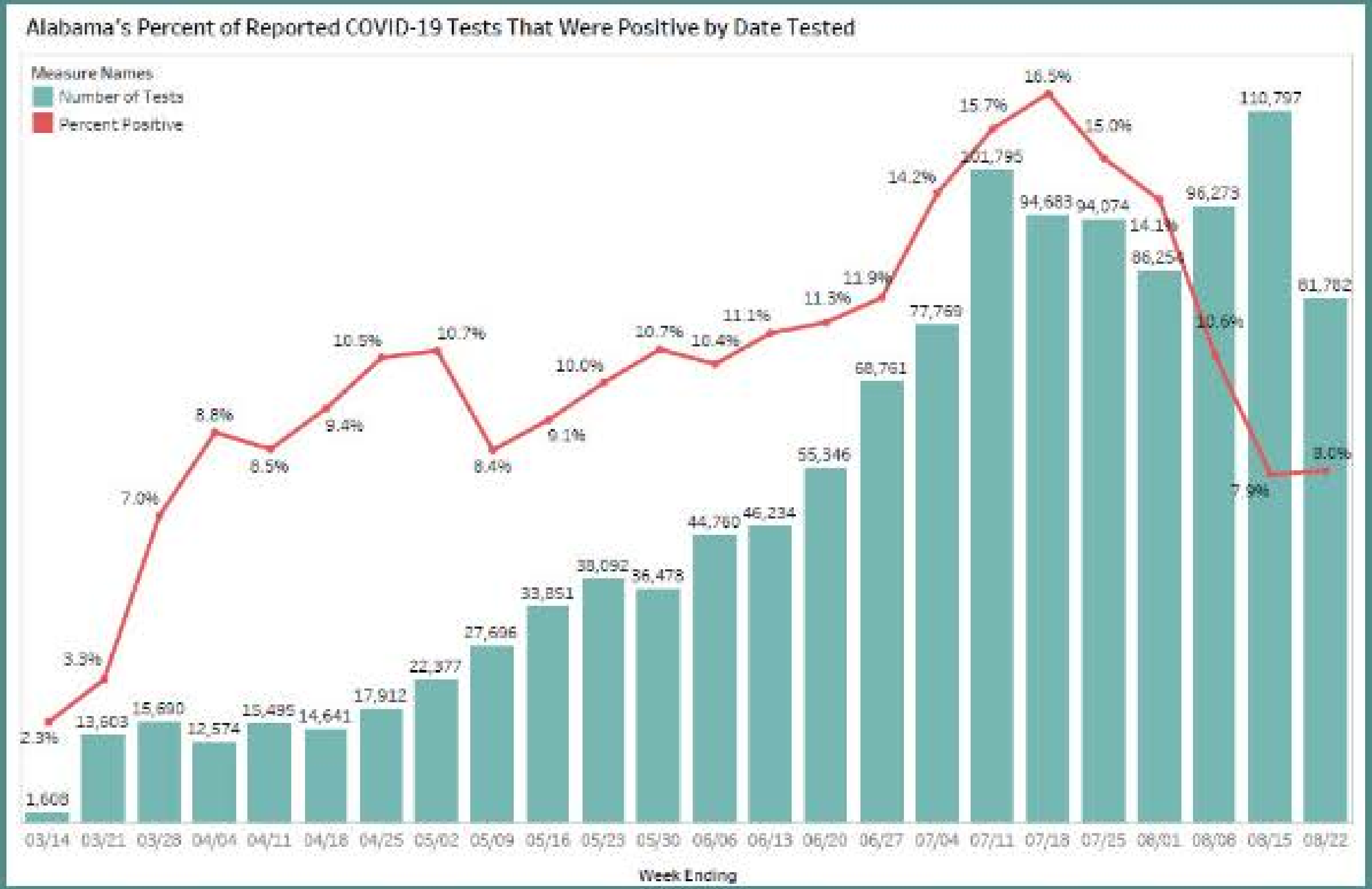

Laboratories are required by law to report both positive and negative COVID-19 test results to ADPH. To accurately calculate the percentage of positive test results, both numerator and denominator data are needed; test results from laboratories that do not report negative results (denominator data) are excluded from the calculation and from this graph. The week is assigned by the collection date (i.e., event date) so it represents the percent that were positive during the week listed. Data are preliminary and subject to change.

1 2 3 4 5 6 7 8 9 10 11 12 13

CASES

113,723	8,462
CONFIRMED	PROBABLE

DEATHS

2,017	90
CONFIRMED	PROBABLE

LAST 14 DAYS

11,559	198,766
CASES	TESTED

*TOTAL HOSPITALIZATIONS To Date Updates M-F at 3 p.m.

14,267

STATEWIDE

TOTAL TESTED

956,401

STATEWIDE

PRESUMED RECOVERIES Updated Weekly

48,028

STATEWIDE

About This Dashboard

Data Source: Alabama Department of Public Health (ADPH) **Infectious Diseases & Outbreaks Division.**

This dashboard provides data and surveillance information about Coronavirus Disease 2019 (COVID-19) in Alabama. Data used to produce this dashboard is updated daily from the Alabama NEDSS Base

Alabama COVID-19
Case Tracker

- Current Totals
- Daily Tracking
- County Maps
- County Details
- Other Stats
- Surveys

Bama Tracker is independently run.
Support it here

ONEteam

Alabama COVID-19 Tracking

Follow on Twitter @BamaTracker for updates.

**115,284** 8,605 Probable Total Cases	**2,059** 93 Probable Total Deaths	**+1,561** +143 Probable Cases Yesterday	**967,213** Total **+10,812** Yesterday Tests Reported
**48,028** Recovered	**0.91** R_t	**12,422** Cases **195,620** Tested Last 14 Days	**14,267** Total **1,002** Current Hospitalizations

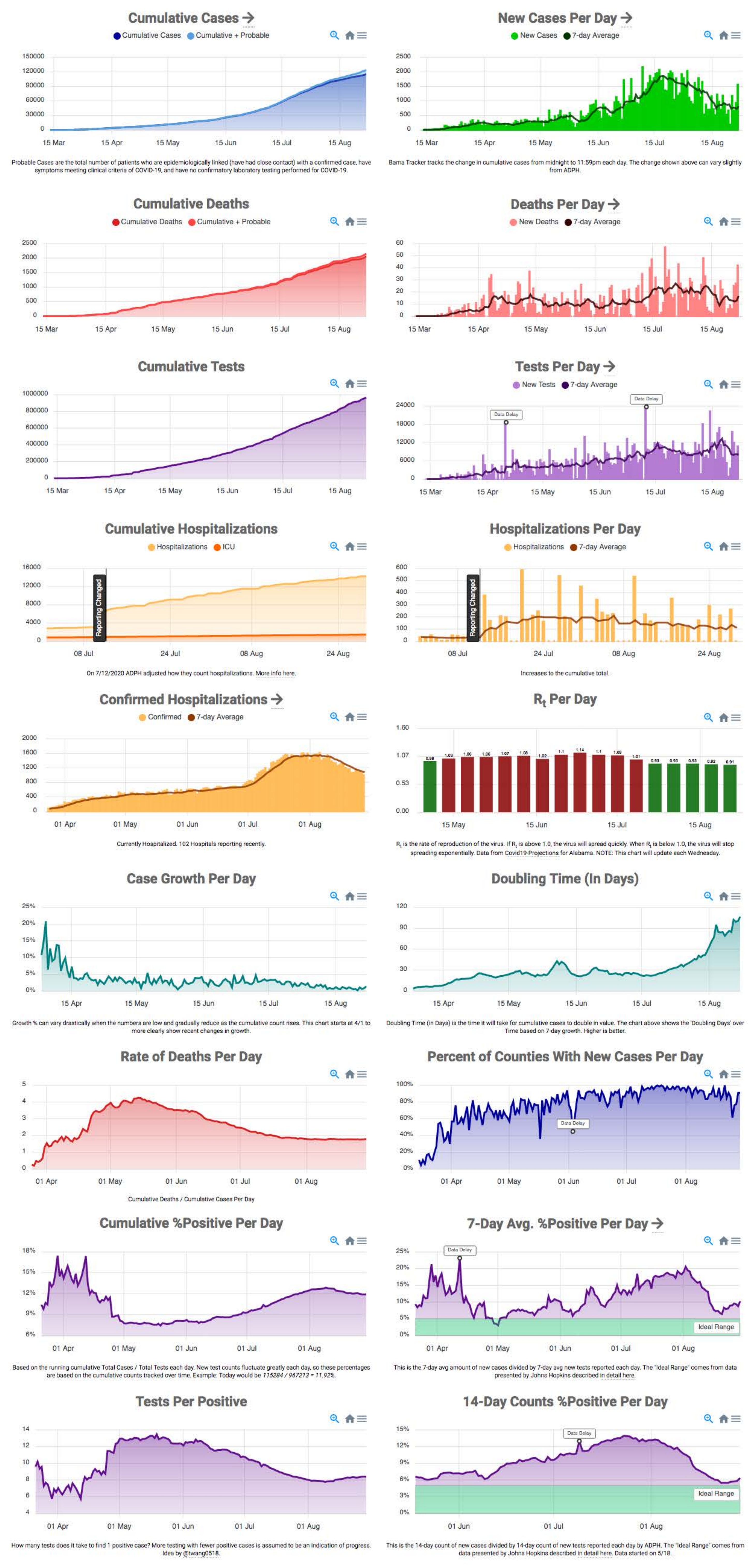

Total Cases By County

7-day Avg Cases By County

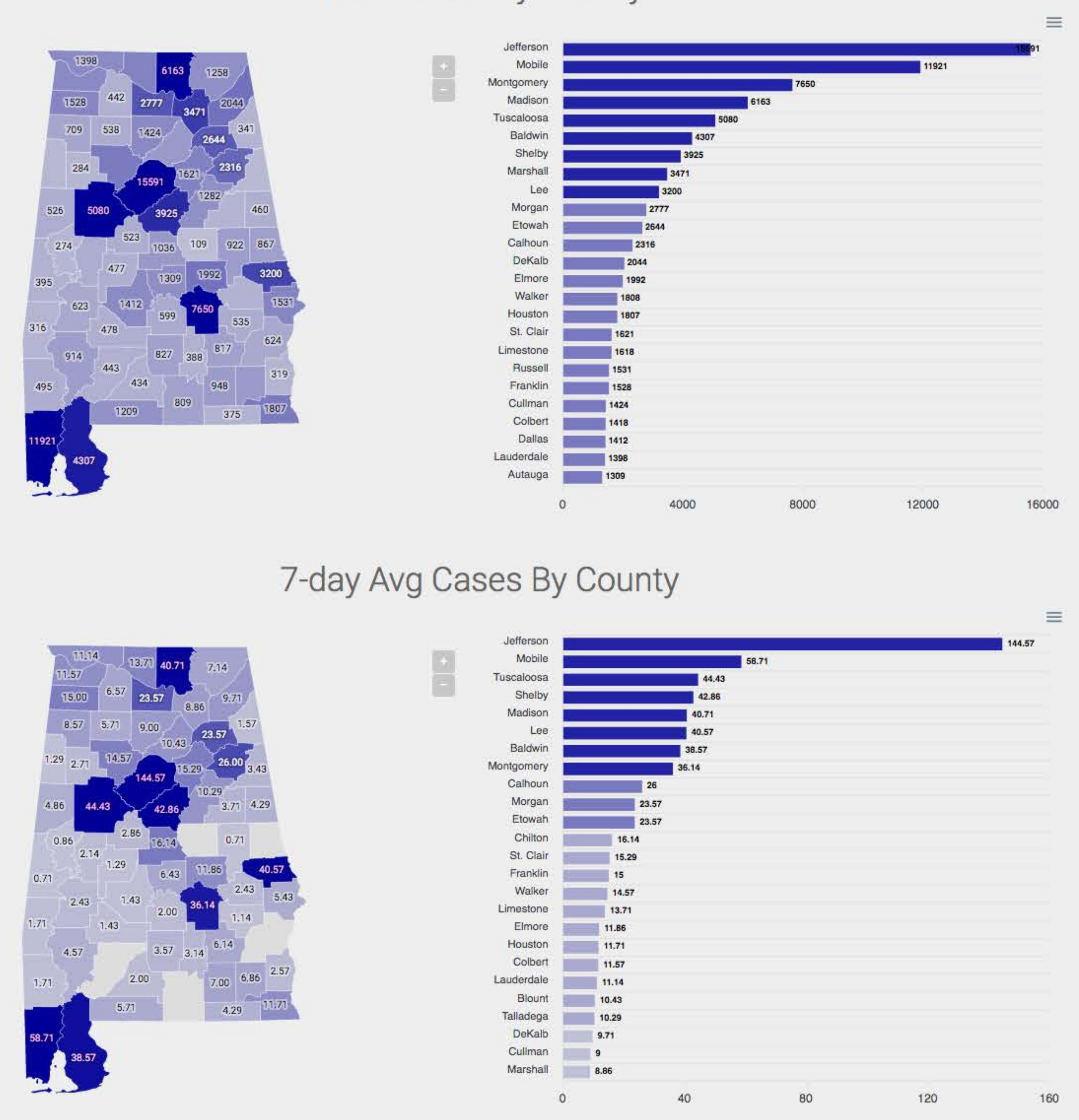

More Alabama COVID-19 Maps

Click on any County in the list below to get to a detailed section of that county's data. You can click/tap on the columns to sort.

Compare Counties | County Sets | Daily History | Data Grid | Bar Chart Race | # Other Stats | Schools | Quick Dashboard

Important: As of 6/3/2020 ADPH began updating their numbers at 10 am once a day. The change in the table below as of 6/3 most likely indicates an increase for the previous day.

County Search

County	Cases	Change	Probable	Change	Deaths	Tests	%Pos (14d)	7day Avg
Jefferson	15,591	+266	542	-3	299	161,932	5.70% ↑	144.57
Mobile	11,921	+91	215	+5	251	84,433	5.93% ↑	58.71
Montgomery	7,650	+68	231	-5	160	45,182	7.21% ↑	36.14
Madison	6,163	+75	203	+2	47	73,559	4.71% ↑	40.71
Tuscaloosa	5,080	+56	694	+53	89	50,159	5.59% ↑	44.43
Baldwin	4,307	+77	117	0	36	37,239	7.09% ↑	38.57
Shelby	3,925	+101	456	+10	42	44,022	5.89% ↑	42.86
Marshall	3,471	+14	134	-1	41	20,746	8.26% ↑	8.86
Lee	3,200	+109	1,183	+44	48	37,530	3.55% ↑	40.57
Morgan	2,777	+35	97	+3	22	21,208	7.05% ↑	23.57
Etowah	2,644	+44	131	+1	39	20,368	11.83% ↑	23.57
Calhoun	2,316	+61	183	-5	29	20,041	9.70% ↑	26.00
DeKalb	2,044	+9	92	-1	14	12,019	8.06% ↑	9.71
Elmore	1,992	+26	167	-1	42	14,946	6.02% ↑	11.86
Walker	1,808	+18	92	-4	72	12,966	7.94% ↑	14.57
Houston	1,807	+22	197	+6	14	19,496	5.57% ↑	11.71
St. Clair	1,621	+17	190	-1	27	16,845	7.78% ↑	15.29
Limestone	1,618	+22	40	-3	15	13,408	6.71% ↑	13.71
Russell	1,531	+19	78	+2	2	9,584	7.52% ↑	5.43
Franklin	1,528	+24	26	-1	27	6,767	14.93% ↑	15.00
Cullman	1,424	+28	188	+1	12	14,152	5.73% ↑	9.00
Colbert	1,418	+25	30	-3	22	9,648	9.09% ↑	11.57
Dallas	1,412	+7	189	+2	25	8,742	5.62% ↑	1.43
Lauderdale	1,398	+14	24	-2	23	15,059	5.34% ↑	11.14
Autauga	1,309	+13	91	-2	22	9,063	7.64% ↑	6.43
Talladega	1,282	+50	451	+4	17	14,284	8.12% ↑	10.29
Jackson	1,258	+11	43	0	7	11,588	6.43% ↑	7.14
Escambia	1,209	+10	187	+4	22	6,921	8.60% ↑	5.71
Chilton	1,036	+29	99	+4	10	6,805	12.69% ↑	16.14
Blount	1,004	+21	244	+8	9	7,876	10.87% ↑	10.43
Dale	1,002	+16	79	+1	37	7,863	6.17% ↑	6.86
Coffee	948	+24	117	+3	5	9,356	5.56% ↑	7.00
Tallapoosa	922	+7	119	0	81	8,955	2.38% ↑	0.71
Clarke	914	+12	66	-3	13	5,385	7.45% ↑	4.57
Chambers	867	+4	157	+3	39	6,187	2.25% ↑	-0.29
Butler	827	+13	28	+2	36	3,720	6.51% ↑	3.57
Pike	817	+3	131	+9	7	7,049	5.29% ↑	6.14
Covington	809	+4	279	+4	24	6,873	4.46% ↑	-1.14
Marion	709	+10	72	-2	28	4,716	10.97% ↑	8.57
Barbour	624	+4	129	+4	7	3,935	5.59% ↓	-0.29
Marengo	623	+3	14	-1	17	4,550	5.65% ↑	2.43
Lowndes	599	+6	18	0	24	2,008	9.24% ↑	2.00
Winston	538	+7	30	+1	11	4,363	7.63% ↓	5.71
Bullock	535	+5	11	0	13	2,165	12.39% ↑	1.14
Hale	527	+5	29	+1	27	3,840	4.03% ↑	2.14
Pickens	526	+11	29	+1	10	3,989	8.56% ↑	4.86
Bibb	523	+6	17	-1	6	4,453	6.80% ↑	2.86
Washington	495	+3	20	0	13	2,937	5.66% ↑	1.71
Wilcox	478	0	35	+2	10	2,349	7.42% ↑	1.43
Perry	477	0	39	-1	5	2,891	6.02% ↓	1.29
Randolph	460	+2	40	+2	11	3,416	6.70% ↓	4.29
Monroe	443	0	98	0	6	3,800	4.87% ↑	-0.43
Lawrence	442	+9	13	-2	5	3,185	10.85% ↑	6.57
Conecuh	434	0	49	0	11	2,025	9.66% ↑	2.00
Sumter	395	0	7	0	19	2,217	5.10% ↓	0.71
Macon	390	+7	39	0	17	3,024	5.65% ↑	2.43
Crenshaw	388	+10	74	+2	26	2,421	6.95% ↑	3.14
Geneva	375	+5	47	0	3	3,982	7.19% ↓	4.29
Clay	342	+4	18	-1	6	2,405	7.00% ↓	3.71
Cherokee	341	+5	57	-1	11	3,080	10.40% ↑	1.57
Henry	319	+5	40	+1	3	2,843	6.04% ↑	2.57
Choctaw	316	+2	10	+1	12	1,351	7.43% ↑	1.71
Fayette	284	+2	27	+2	6	2,615	6.47% ↓	2.71
Greene	274	-1	7	0	15	1,626	7.77% ↓	0.86
Lamar	268	+2	18	-1	2	2,321	4.98% ↑	1.29
Cleburne	200	+3	4	-1	5	1,618	12.38% ↑	3.43
Coosa	109	+1	24	+1	3	1,112	2.95% ↑	-0.43
**Total**	**115,284**	**+1,561**	**8,605**	**+143**	**2059**	**967,213**		58 ↑ 9 ↓

Case & Death Demographics | Alabama Mortality

Other Stats

Nationwide Totals

**6,117,376** US Cases	**+21,141** New Today	**186,382** US Deaths	**+481** New Today

Statewide Percentages

19.73% — **967,213** out of **4,903,000** Population Tested	11.92% — **115,284** out of **967,213** Positive Tests	12.38% — **14,267** out of **115,284** Hospitalized
7.02% — **1,002** Currently Hospitalized	91% — **61** out of **67** Counties with New Cases Today	1.79% — **2059** out of **115,284** Deceased

- **2.351%** of the population of Alabama has tested positive for COVID-19, approximately 2351 in 100,000 people.
- **0.042%** of the population of Alabama have died from COVID-19, approximately 42 in 100,000 people.
- Alabama Cases (115,284) make up **1.88%** of the total US Cases of COVID-19 (6,117,376)

Coronavirus in Alabama: Cases, maps, charts and resources

The Alabama coronavirus charts and map are updated regularly with official numbers as they are released by the Alabama Department of Health, including how many current Alabama coronavirus cases and deaths, statewide and by county. Reports from hospitals, coroners and county departments may show different numbers.

Alabama coronavirus case tracker

Deaths	Tested	Positives	U.S. Deaths	Global Deaths
831	348,687	30,031	120,044	469,122

**The number tested counts only those tested at ADPH's publicly run labs, not for tests done at private, commercial labs*

Table: al.com • Source: Alabama Dept. of Public Health • Get the data • Created with Datawrapper

New Alabama cases (daily)

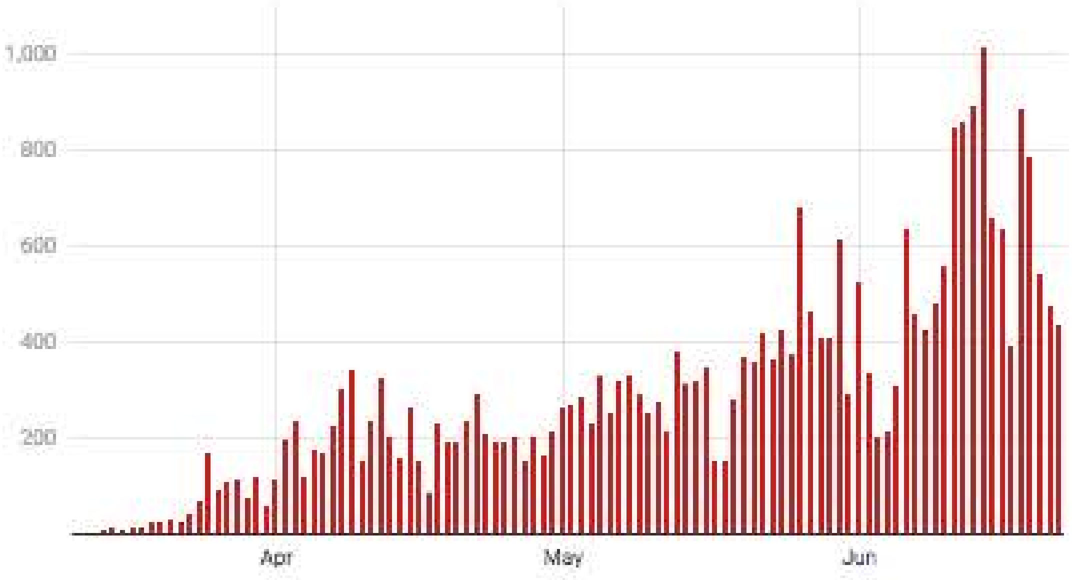

Chart: al.com • Source: Alabama Dept. of Public Health • Get the data • Created with Datawrapper

Total Alabama cases (cumulative)

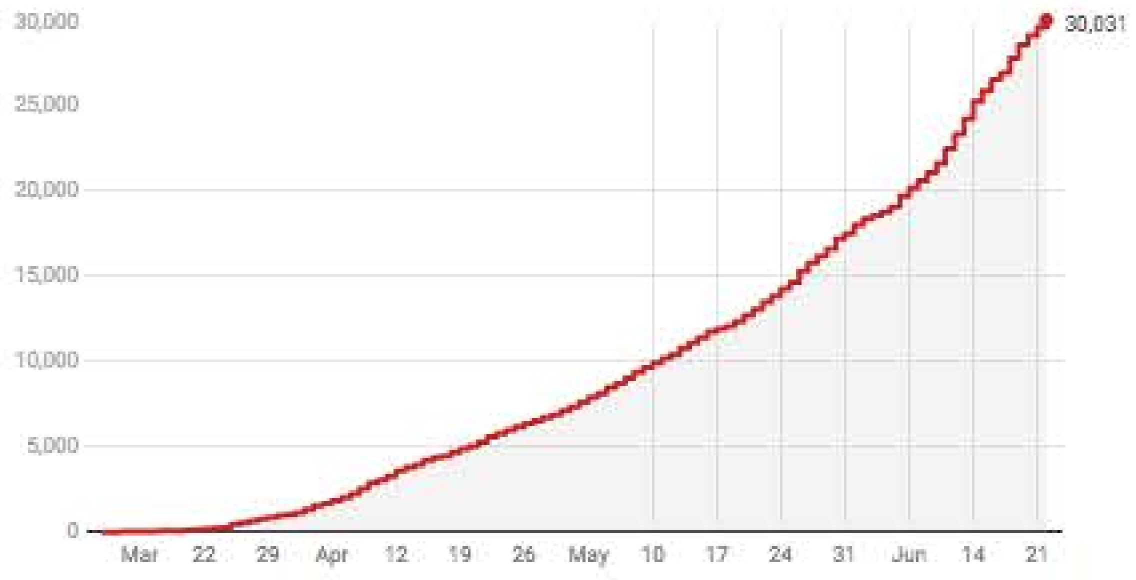

Chart: al.com • Source: Alabama Dept. of Public Health • Get the data • Created with Datawrapper

Page 1 of 3

County	Cases	Confirmed Deaths
Montgomery County	3,254	79
Jefferson County	3,025	125
Mobile County	3,014	132
Tuscaloosa County	1,604	31
Marshall County	1,097	9
Lee County	865	35
Franklin County	779	10
Shelby County	779	22
Morgan County	759	2
Madison County	711	6
Walker County	695	8
Elmore County	677	11
Dallas County	640	6
Butler County	576	26
Chambers County	514	27
Tallapoosa County	513	69
Autauga County	436	8
Baldwin County	422	9
Lowndes County	420	14
Etowah County	415	12
DeKalb County	414	5
Russell County	366	0
Pike County	357	3
Houston County	348	4
Bullock County	325	9
Coffee County	313	1
Cullman County	305	1

**Confirmed deaths includes only deaths reported as official by ADPH*

Table: al.com • Source: Alabama Dept. of Public Health • Get the data • Created with Datawrapper

Alabama coronavirus cases by county

30,031 confirmed cases, 831 deaths (updated 10 a.m. June 22)

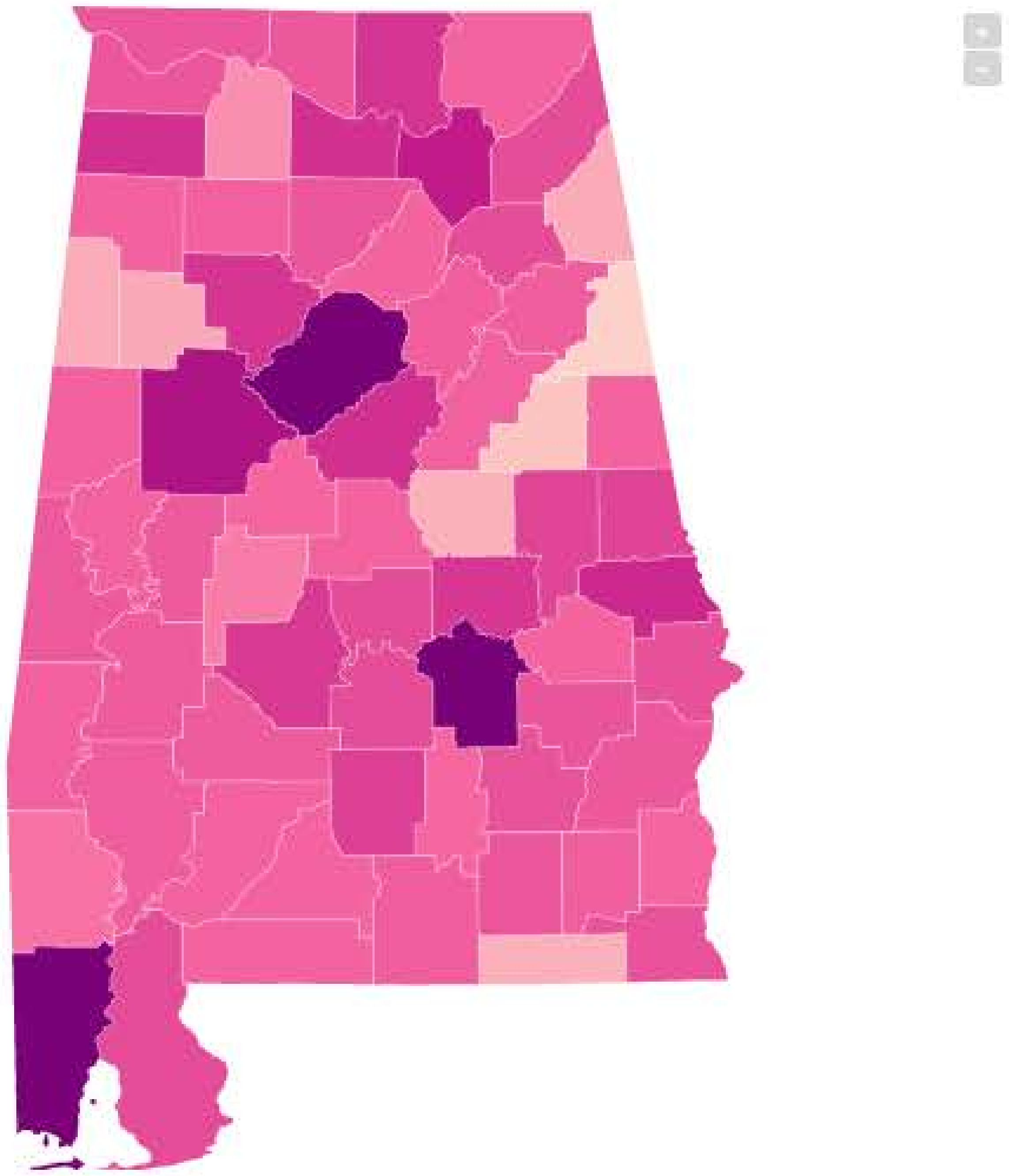

Map: Ramsey Archibald • Source: Alabama Department of Public Health • Get the data • Created with Datawrapper

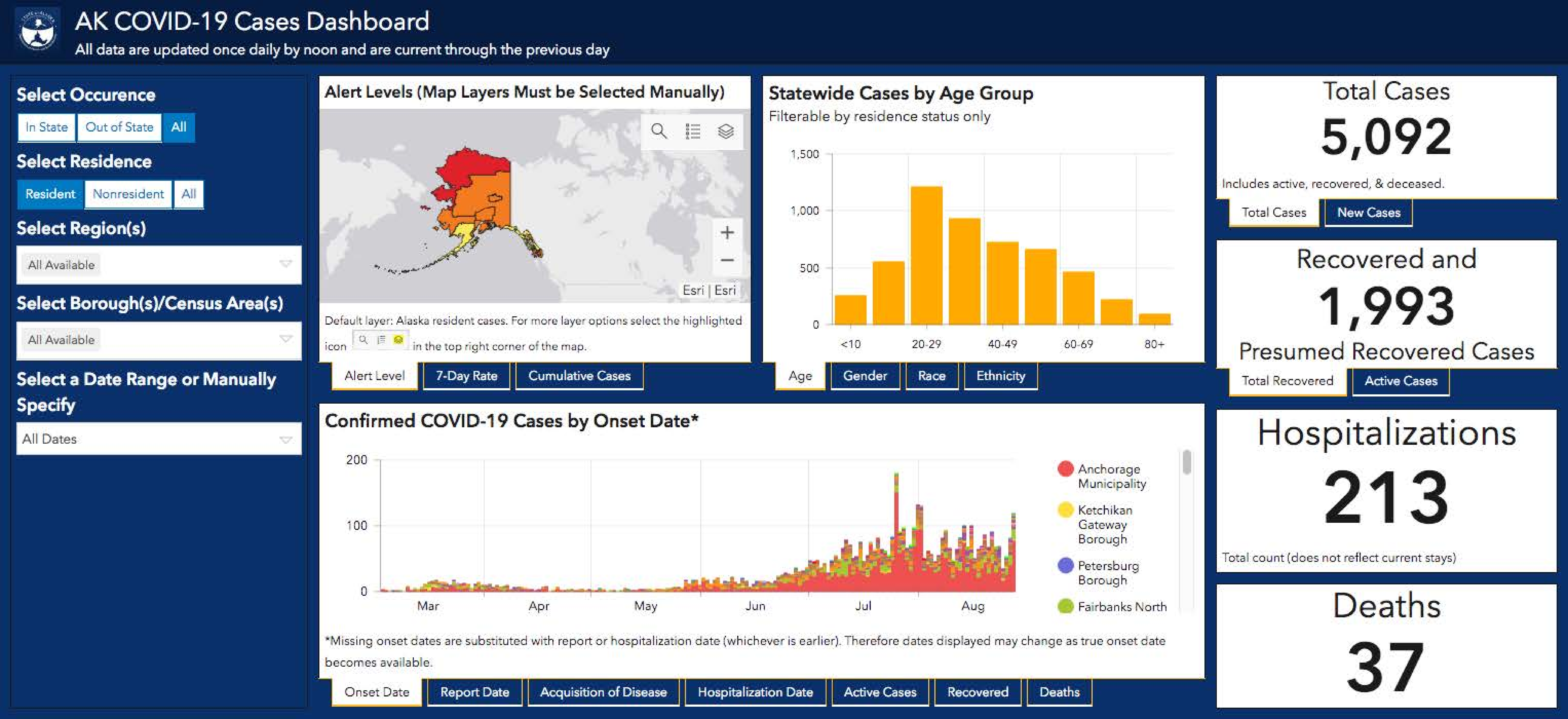
AK COVID-19 Cases Dashboard
All data are updated once daily by noon and are current through the previous day
Select Occurence
In State
Out of State
All
Select Residence
Resident
Nonresident
All
Select Region(s)
All Available
Select Borough(s)/Census Area(s)
All Available
Select a Date Range or Manually Specify
All Dates
Alert Levels (Map Layers Must be Selected Manually)
Esri | Esri
Default layer: Alaska resident cases. For more layer options select the highlighted icon in the top right corner of the map.
Alert Level
7-Day Rate
Cumulative Cases
Statewide Cases by Age Group
Filterable by residence status only
1,500
1,000
500
0
<10
20-29
40-49
60-69
80+
Age
Gender
Race
Ethnicity
Total Cases
5,092
Includes active, recovered, & deceased.
Total Cases
New Cases
Recovered and
1,993
Presumed Recovered Cases
Total Recovered
Active Cases
Confirmed COVID-19 Cases by Onset Date*
200
100
0
Mar
Apr
May
Jun
Jul
Aug
Anchorage Municipality
Ketchikan Gateway Borough
Petersburg Borough
Fairbanks North
*Missing onset dates are substituted with report or hospitalization date (whichever is earlier). Therefore dates displayed may change as true onset date becomes available.
Onset Date
Report Date
Acquisition of Disease
Hospitalization Date
Active Cases
Recovered
Deaths
Hospitalizations
213
Total count (does not reflect current stays)
Deaths
37

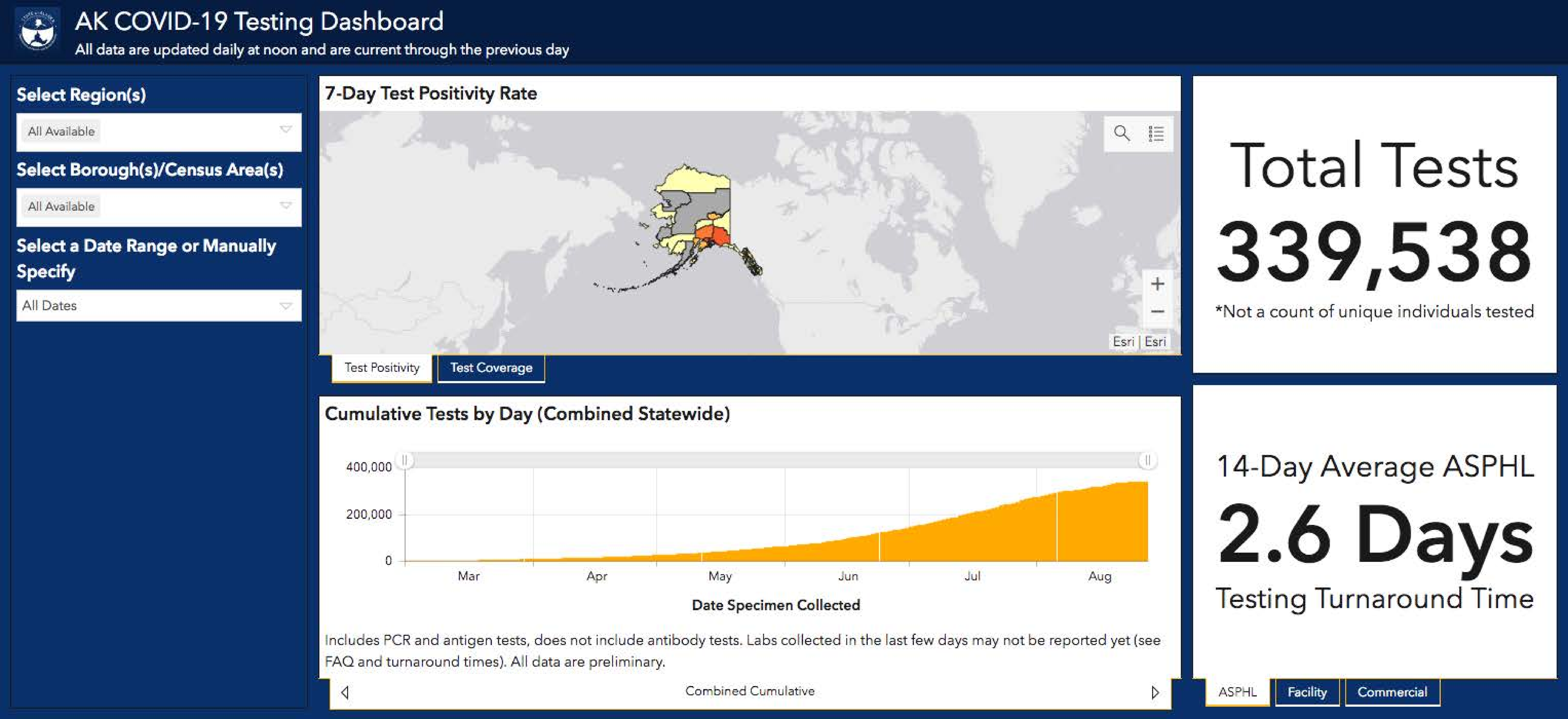
AK COVID-19 Testing Dashboard
All data are updated daily at noon and are current through the previous day
Select Region(s)
All Available
Select Borough(s)/Census Area(s)
All Available
Select a Date Range or Manually Specify
All Dates
7-Day Test Positivity Rate
Esri | Esri
Test Positivity
Test Coverage
Total Tests
339,538
*Not a count of unique individuals tested
Cumulative Tests by Day (Combined Statewide)
400,000
200,000
0
Mar
Apr
May
Jun
Jul
Aug
Date Specimen Collected
Includes PCR and antigen tests, does not include antibody tests. Labs collected in the last few days may not be reported yet (see FAQ and turnaround times). All data are preliminary.
Combined Cumulative
14-Day Average ASPHL
2.6 Days
Testing Turnaround Time
ASPHL
Facility
Commercial

Alaska News

Tracking COVID-19 in Alaska

Author: Anchorage Daily News Updated: August 20 Published April 6

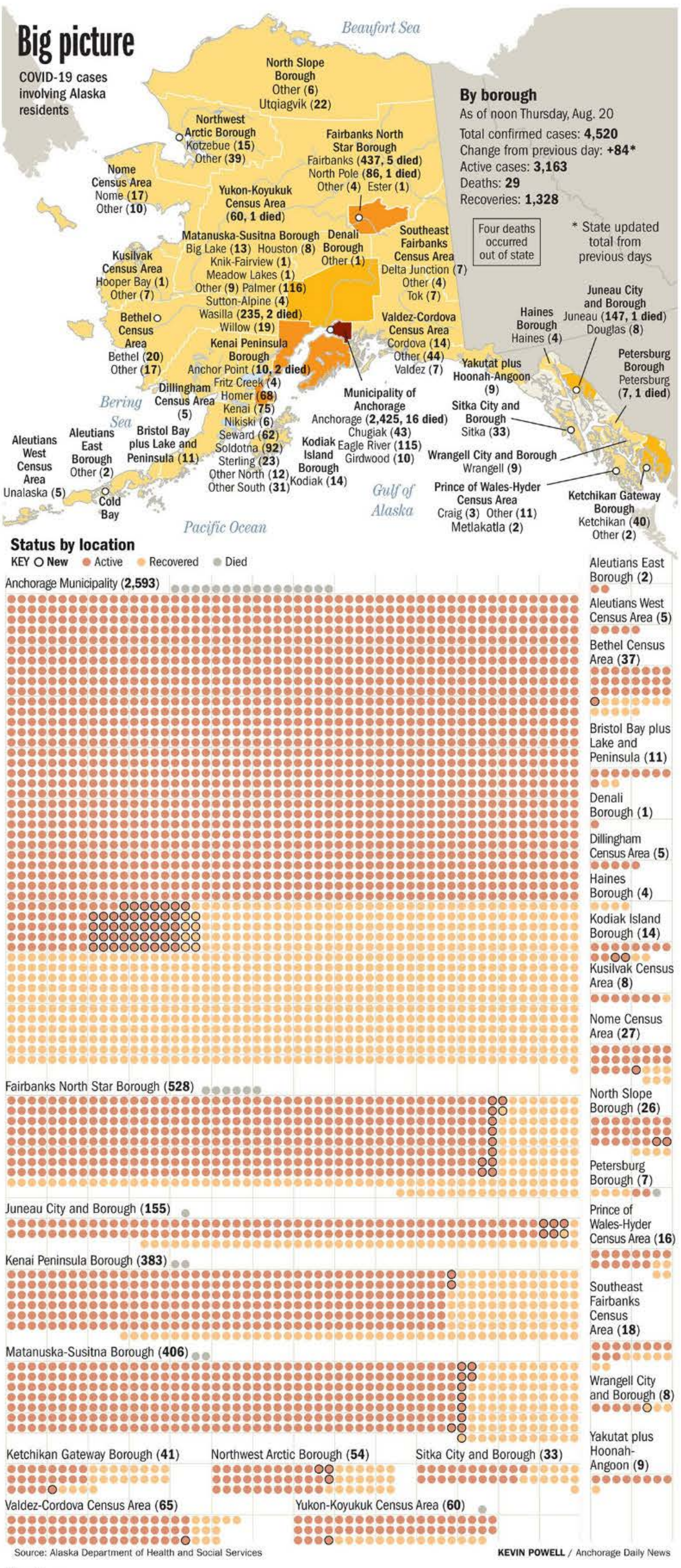

Big picture

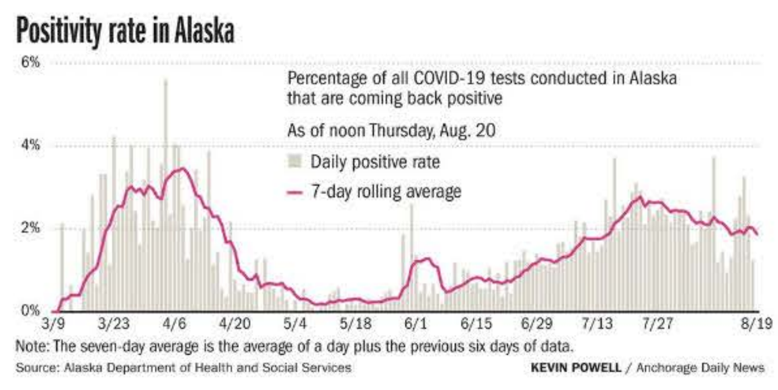

Positivity rate

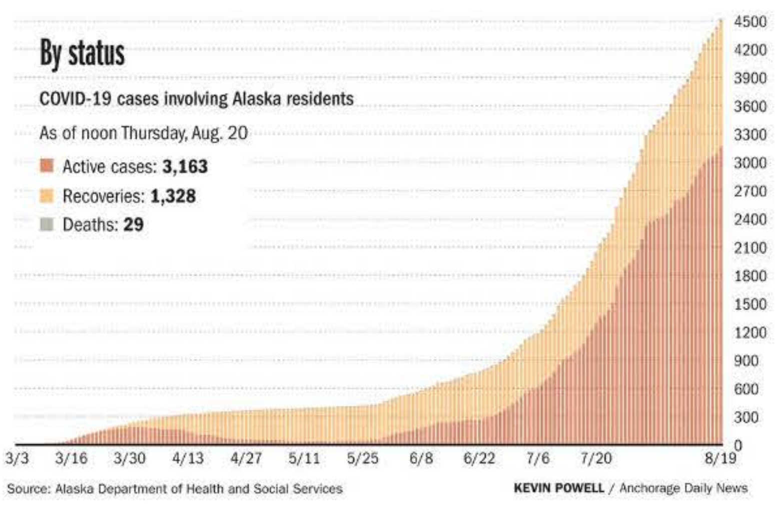

By status

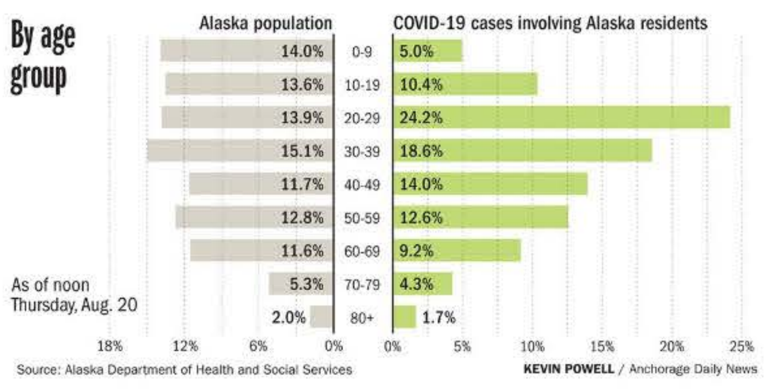

By age group

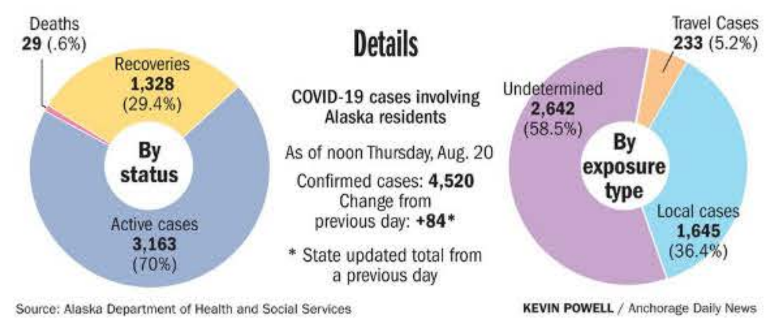

Details

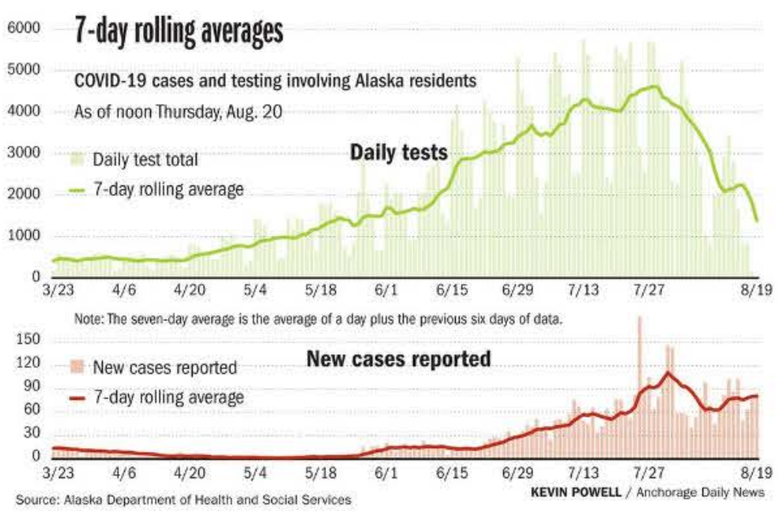

7-day averages

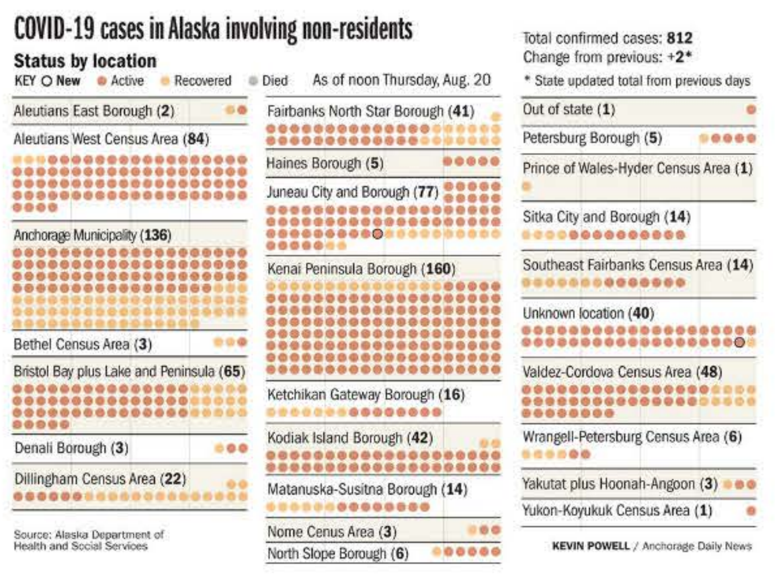

Non-residents

COVID-19 Local News Fund (via Local Media Fou... →
EIN

Anchorage Daily News - COVID-19 Local News Fund

Give to support Anchorage Daily News as we cover the coronavirus crisis in our communities.

$25 $50
$100 $250
$500 $1,000
$ Other

Continue

Powered by Givebutter

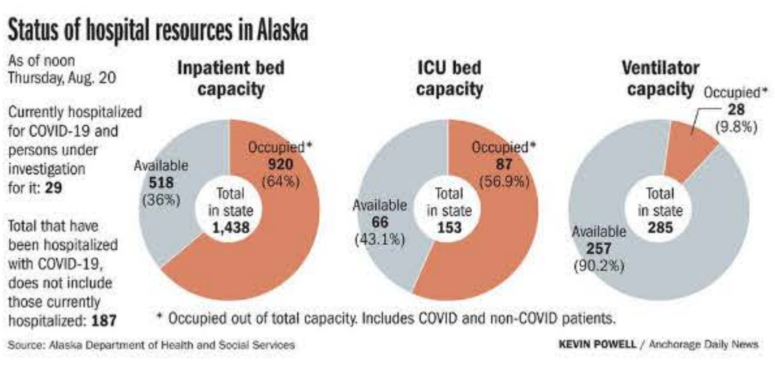

Hospital resources

MYALASKATIX

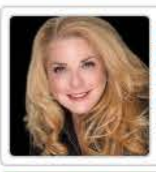
Pivot or Persevere? How to Forge a Strong Personal Brand in Times of Market Disruption
Thursday, September 10, 2020 Get Tickets

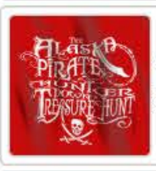
Alaska Pirate Hunker Down Treasure Hunt
Saturday, September 12, 2020 Get Tickets

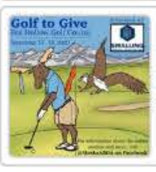
Taps & Apps Golf to Give
Sunday, September 13, 2020 Get Tickets

1 2 3

Most-Read

1 'I really thought I was losing my husband': An Anchorage couple shares their experience with COVID-19
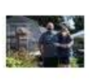

2 Anchorage's bars and restaurants can open to indoor dining Monday at half capacity, with other restrictions
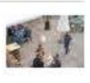

3 A killer whale was headed toward a sea otter in Kachemak Bay. Then the otter hopped on a boat — and stayed there.
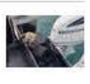

4 Tracking COVID-19 in Alaska: 121 cases reported statewide Friday, no new deaths
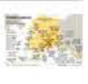

5 Russian navy conducts major maneuvers near Alaska
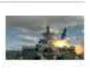

ADN Recommends

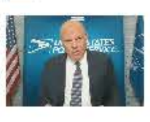
Politics
Alaska's expensive rural mail subsidy is 'on the table,' postmaster general tells Senate committee

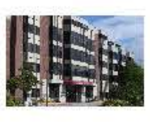
Alaska-news
Anchorage Pioneer Home resident with COVID-19 dies amid cluster of infections in the facility

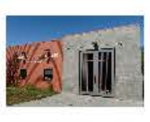
Alaska-news
Anchorage expands virus testing among homeless population after outbreak at Brother Francis Shelter

Data Dashboard

- As our federal partners have updated guidance for identifying COVID-19 deaths, ADHS has enhanced our processes to match. This change may cause the number of new deaths reported on our dashboard to increase more than expected for a few days. Deaths are shown on the bar chart of the COVID-19 deaths section of the dashboard using the date of death, not the day reported. New deaths on the summary section of the dashboard are displayed by the day reported to ADHS.

Summary | Demographics | COVID-19 Deaths | Zip Code
Confirmed COVID-19 Cases by Day | Hospitalization | Hospital COVID-like & Influenza-like illness Surveillance | Laboratory Testing
Congregate Settings with Positive COVID-19 Cases | Hospital Bed Usage & Availability | Ventilator Usage & Availability | Hospital COVID-19 Specific Metrics

Summary

Show case counts or population rates in the map below?
Cases

Select a county to filter the other numbers.
Deaths will not be shown for counties with fewer than three deaths.

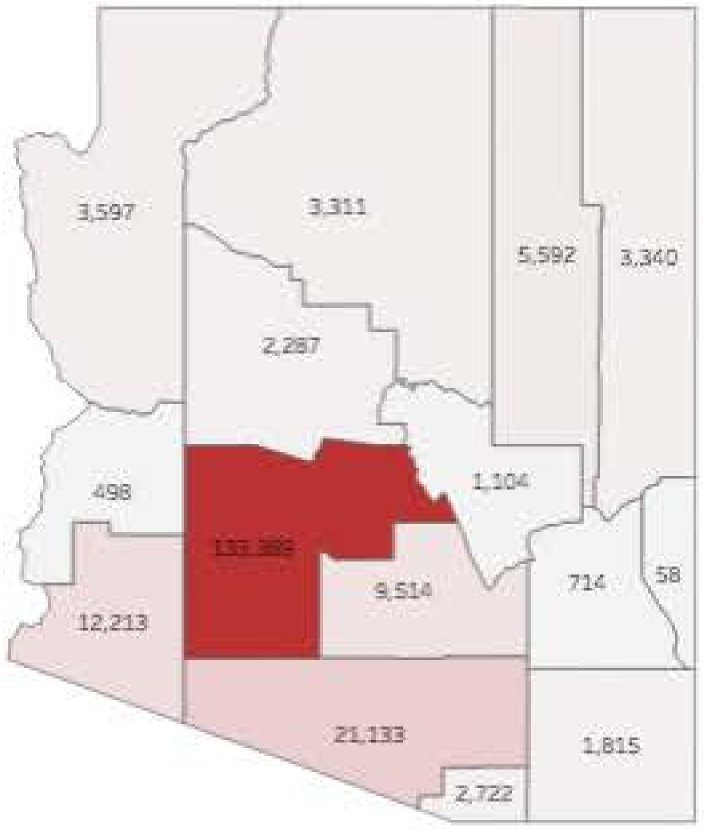

Number of Cases<br>201,287	Number of Deaths<br>5,007	Number of COVID-19 Tests<br>1,456,210
Number of New Cases reported today*<br>629	Number of New Deaths reported today*<br>29	Number of New Tests reported today*<br>9,215
Rate of cases, per 100,000 population<br>2,799.9	Rate of fatalities, per 100,000 population<br>69.65	Total Percent Positive**<br>11.6%
Total COVID-19 PCR Tests<br>1,190,668	New PCR Tests reported today*<br>8,271	PCR Percent Positive**<br>12.8%
Total COVID-19 Serology Tests<br>265,542	New Serology Tests reported today*<br>944	Serology Percent Positive**<br>6.3%

*Counts of new cases, deaths, and numbers tested reflect increases in the total numbers compared to the previous day.

**Percent positive is the number of people with a positive test result, out of all people with COVID-19 testing completed in AZ.

Date Updated: 8/29/2020

Feedback & Support

Data Dashboard

- As our federal partners have updated guidance for identifying COVID-19 deaths, ADHS has enhanced our processes to match. This change may cause the number of new deaths reported on our dashboard to increase more than expected for a few days. Deaths are shown on the bar chart of the COVID-19 deaths section of the dashboard using the date of death, not the day reported. New deaths on the summary section of the dashboard are displayed by the day reported to ADHS.

Summary | Demographics | COVID-19 Deaths | Zip Code

Confirmed COVID-19 Cases by Day | Hospitalization | Hospital COVID-like & Influenza-like illness Surveillance | Laboratory Testing

Congregate Settings with Positive COVID-19 Cases | Hospital Bed Usage & Availability | Ventilator Usage & Availability | Hospital COVID-19 Specific Metrics

COVID-19 Deaths

COVID-19 Deaths (total)	New COVID-19 Deaths Reported Today
5,007	29

Hover over the icon to get more information on the data in this dashboard.

COVID-19 Deaths by County

Data will not be shown for counties with fewer than three deaths.

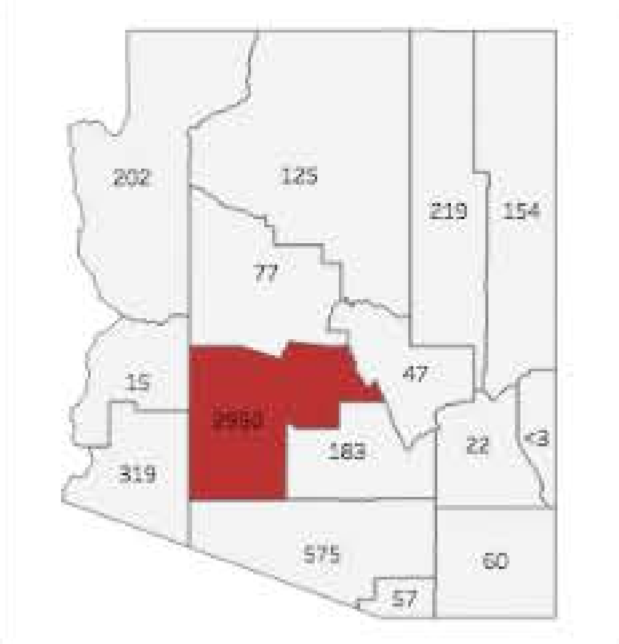

COVID-19 Deaths by Date of Death

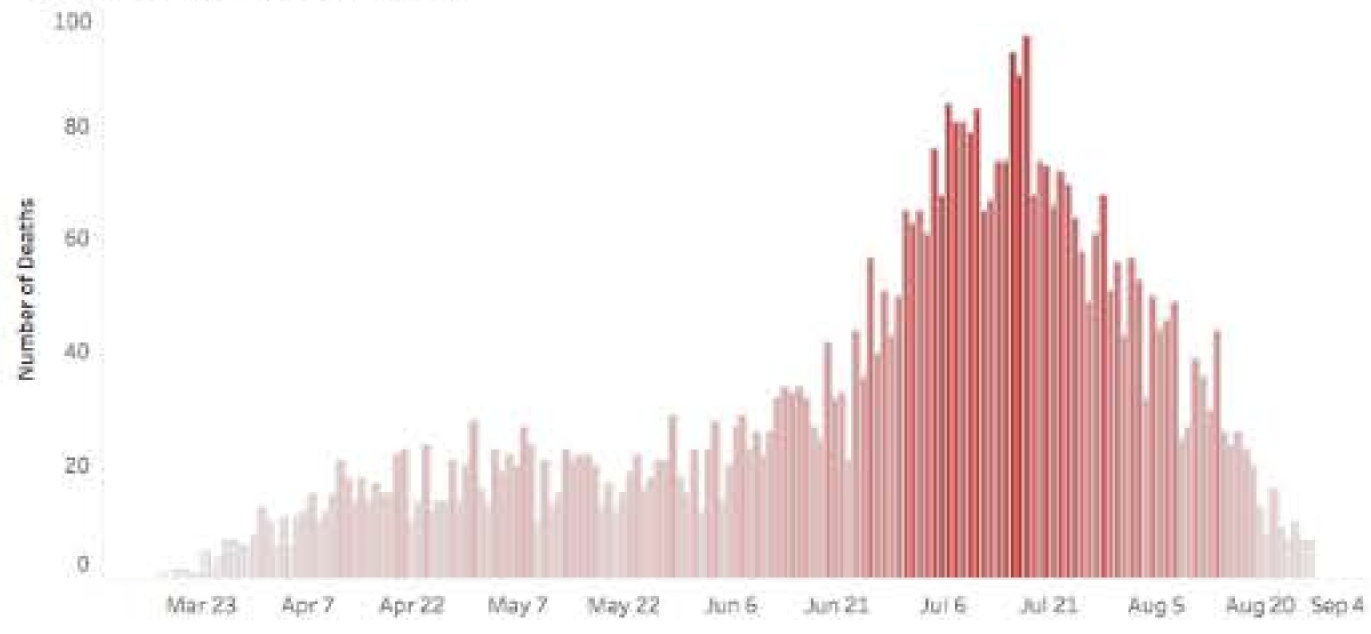

Recent deaths may not be reported yet.

COVID-19 Deaths by Gender

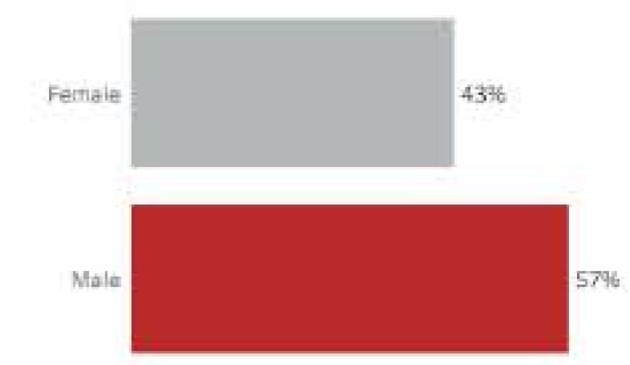

COVID-19 Deaths by Age Group

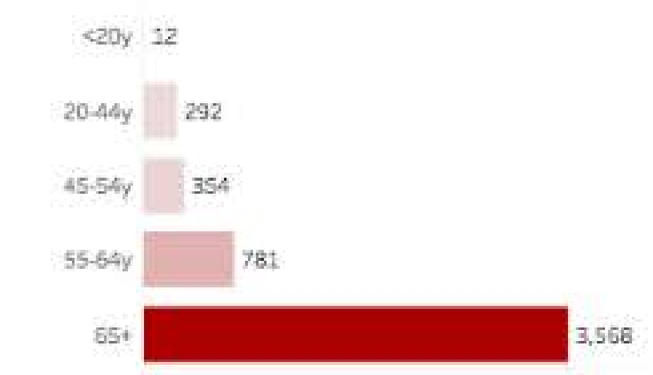

COVID-19 Deaths by Race/Ethnicity

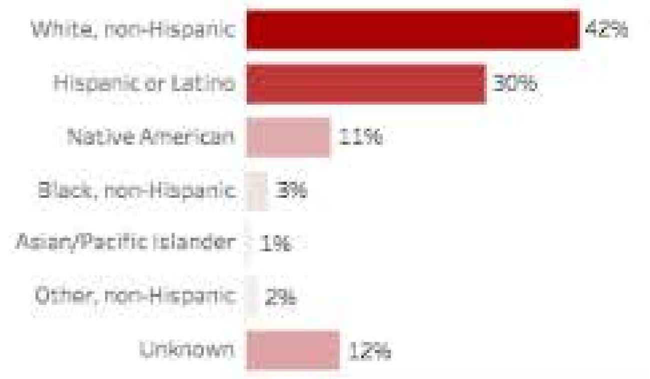

Date Updated: 8/29/2020

Feedback & Support

Data Dashboard

- As our federal partners have updated guidance for identifying COVID-19 deaths, ADHS has enhanced our processes to match. This change may cause the number of new deaths reported on our dashboard to increase more than expected for a few days. Deaths are shown on the bar chart of the COVID-19 deaths section of the dashboard using the date of death, not the day reported. New deaths on the summary section of the dashboard are displayed by the day reported to ADHS.

Summary
Demographics
COVID-19 Deaths
Zip Code
Confirmed COVID-19 Cases by Day
Hospitalization
Hospital COVID-like & Influenza-like illness Surveillance
Laboratory Testing
Congregate Settings with Positive COVID-19 Cases
Hospital Bed Usage & Availability
Ventilator Usage & Availability
Hospital COVID-19 Specific Metrics

Zip Code

COVID-19 Cases by Zip Code

Mobile Version

Case Count
- 0
- 1-10
- 11-100
- 101-500
- 501-1000
- Greater Than 1000
- Data Suppressed

Download Data

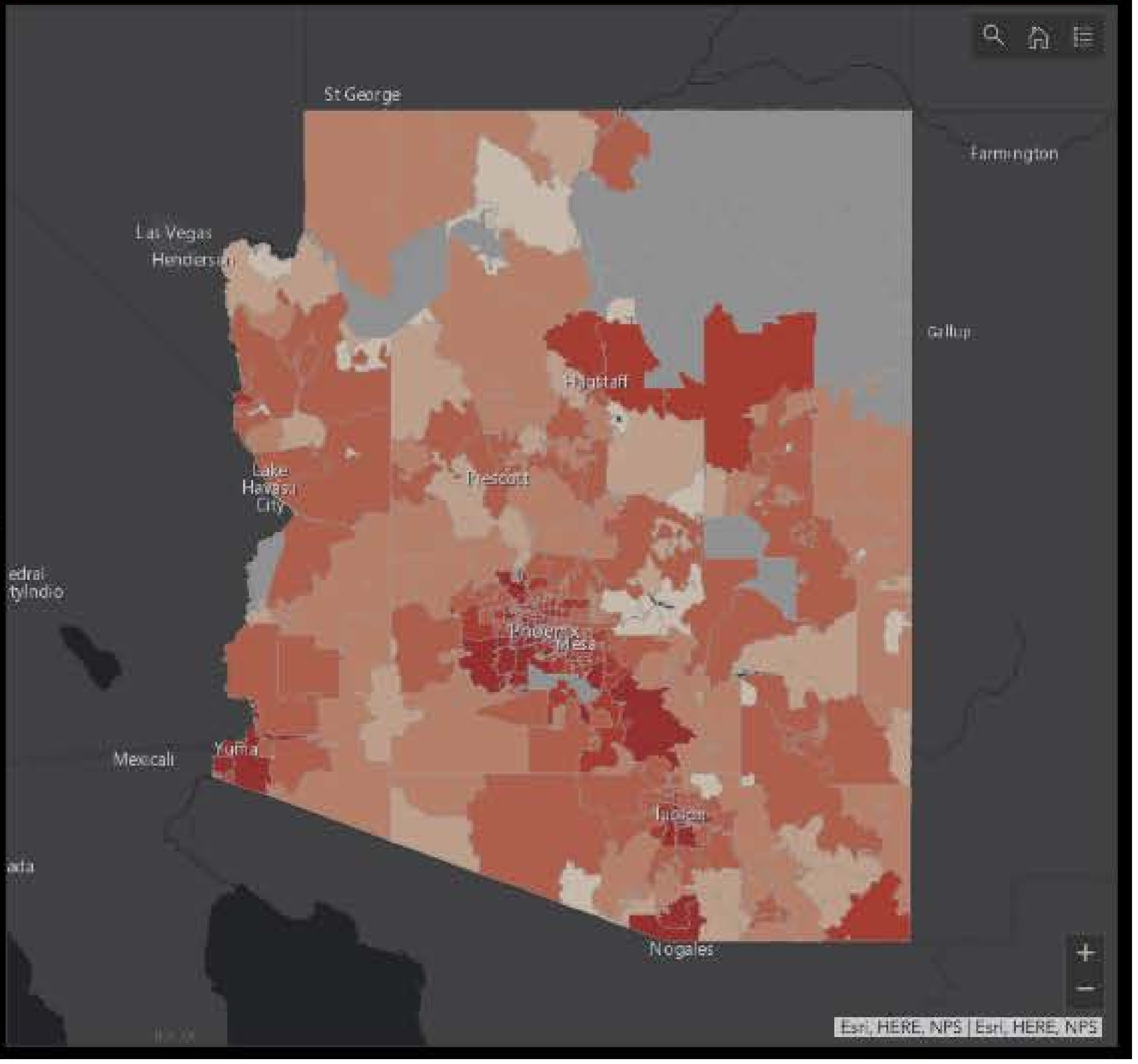

Suppressed data is pending Tribal approval.

Includes cases in MEDSIS, the Arizona surveillance system, with a classification of confirmed or probable.

Over 90% of cases were mapped to the address of the patient's residence. If the patient's address was unknown the case was mapped to the address of the provider followed by the address of the reporting facility.

The zip codes represented on this map are generalized representations of United States Postal Service (USPS) ZIP

Feedback & Support

The Arkansas Department of Health COVID-19 Case Update

Total COVID-19 Cases

59,583

Last update: a few seconds ago

Active Cases

5,496

Last update: a few seconds ago

Recoveries

53,331

Last update: a few seconds ago

Deaths

756

Last update: a few seconds ago

Cumulative Number of Cases by Date

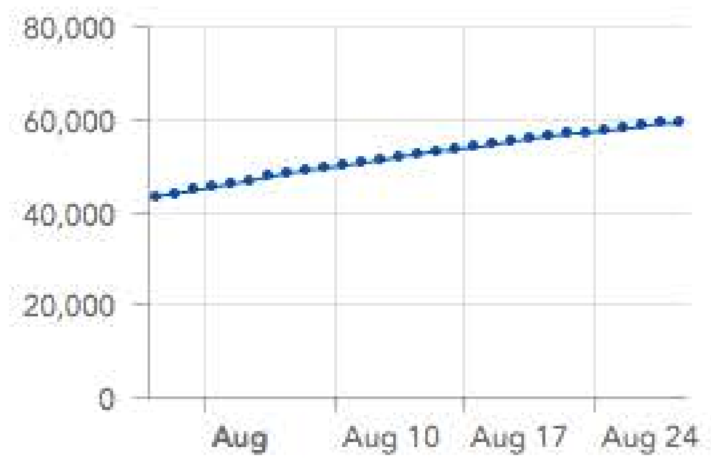

Cases in past 30 days | Cases - All time

Number of COVID-19 Cases by County of Residence

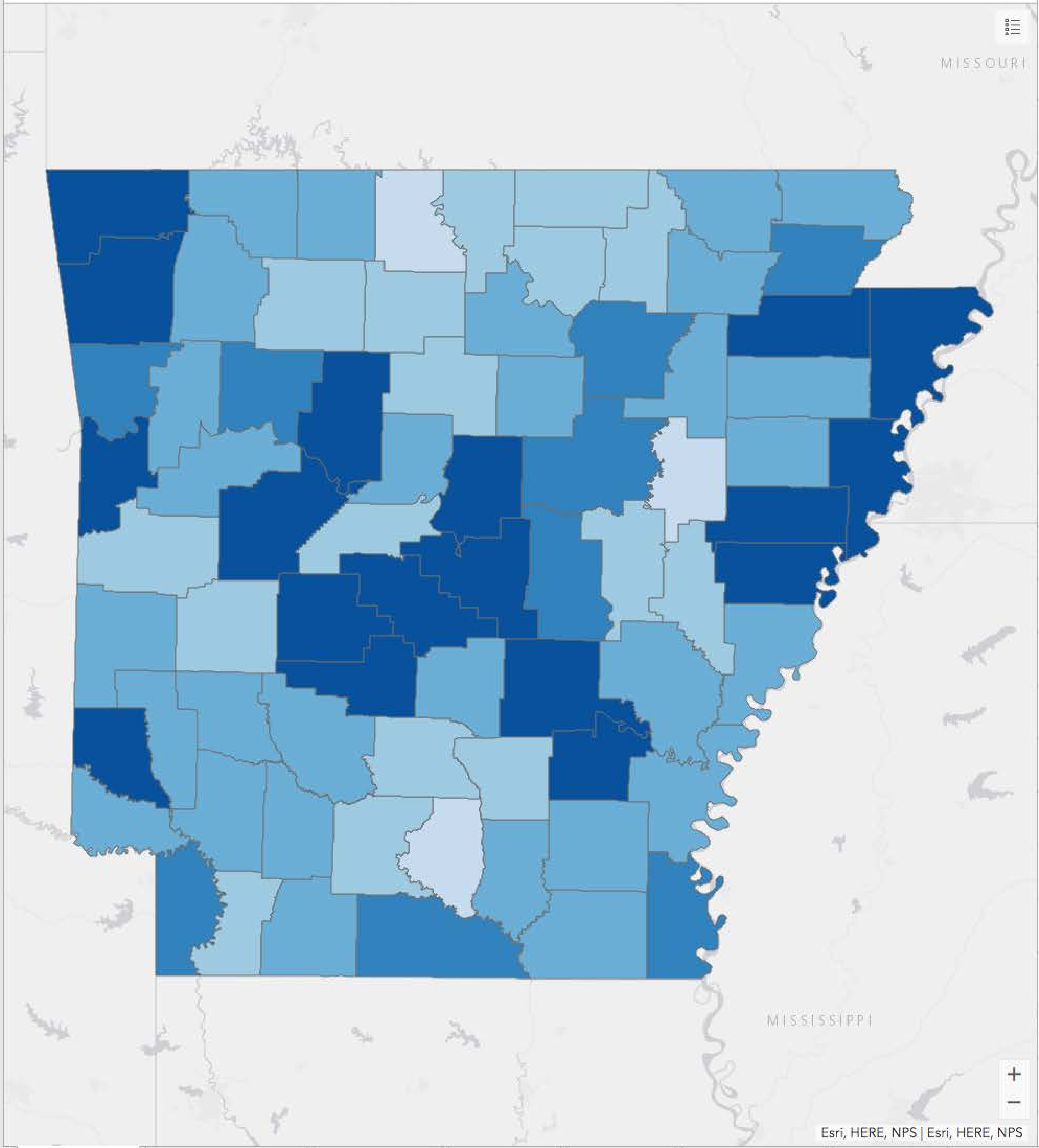

- Arkansas (275 Cases)
- Ashley (387 Cases)
- Baxter (132 Cases)
- Benton (5,238 Cases)
- Boone (309 Cases)
- Bradley (284 Cases)
- Calhoun (23 Cases)
- Carroll (455 Cases)
- Chicot (955 Cases)
- Clark (224 Cases)
- Clay (172 Cases)
- Cleburne (255 Cases)
- Cleveland (135 Cases)
- Columbia (290 Cases)
- Conway (200 Cases)
- Craighead (1,772 Cases)
- Crawford (896 Cases)
- Crittenden (1,624 Cases)
- Cross (261 Cases)
- Dallas (112 Cases)
- Desha (234 Cases)
- Drew (298 Cases)
- Faulkner (1,640 [illegible]

COVID-19 Cases | COVID-19 Active Cases | COVID-19 Deaths | Tests Reported | Health Metrics | Case Demographics | USA and World

The Arkansas Department of Health COVID-19 Case Update

Total COVID-19 Cases

59,583

Last update: a minute ago

Active Cases

5,496

Last update: a minute ago

Recoveries

53,331

Last update: a minute ago

Deaths

756

Last update: a minute ago

Cumulative Number of Cases by Date

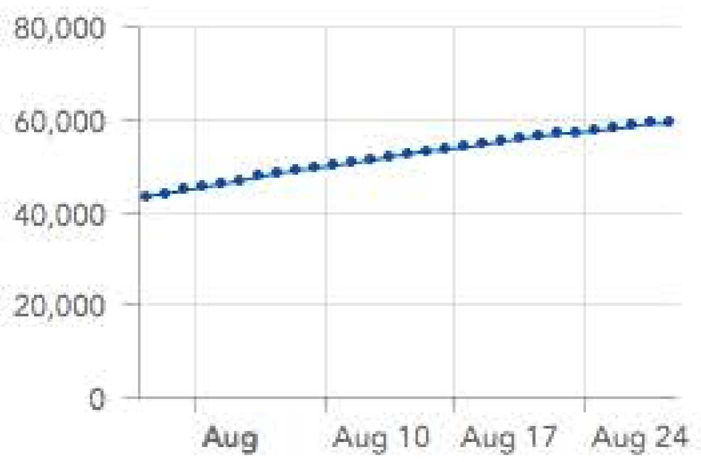

Cases in past 30 days | Cases - All time

COVID-19 Deaths Related to Nursing Homes

262

COVID-19 Deaths Related to Correctional Facilities

37

COVID-19 Deaths by Age Group

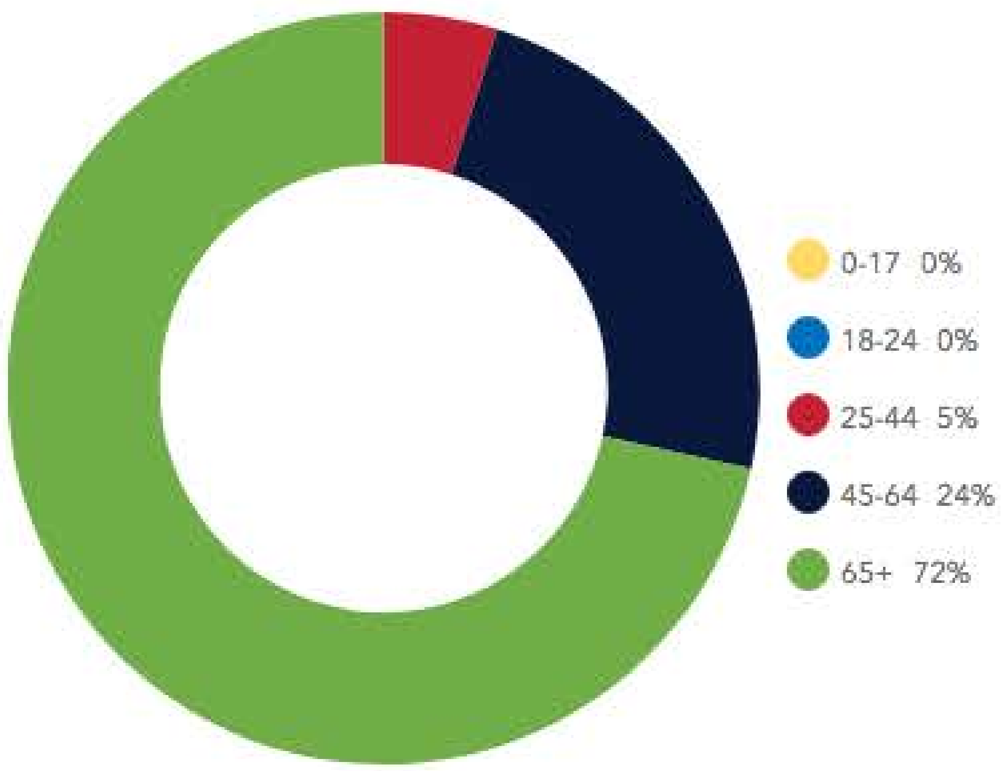

*Statewide

COVID-19 Deaths by Race

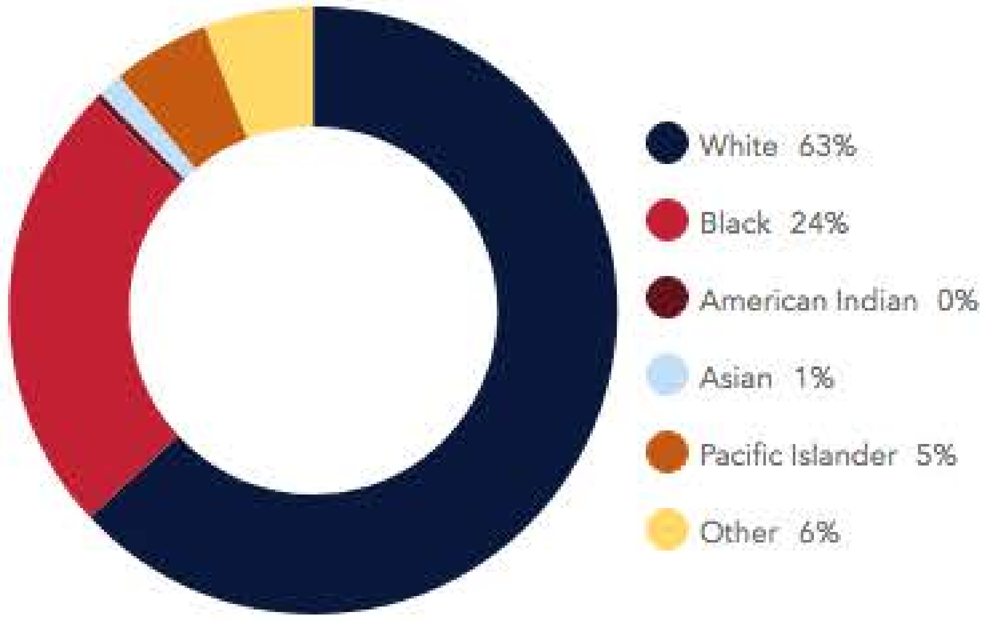

*Statewide

**Percent of deaths of individuals who are Hispanic: 8%

Number of COVID-19 Associated Deaths by County of Residence

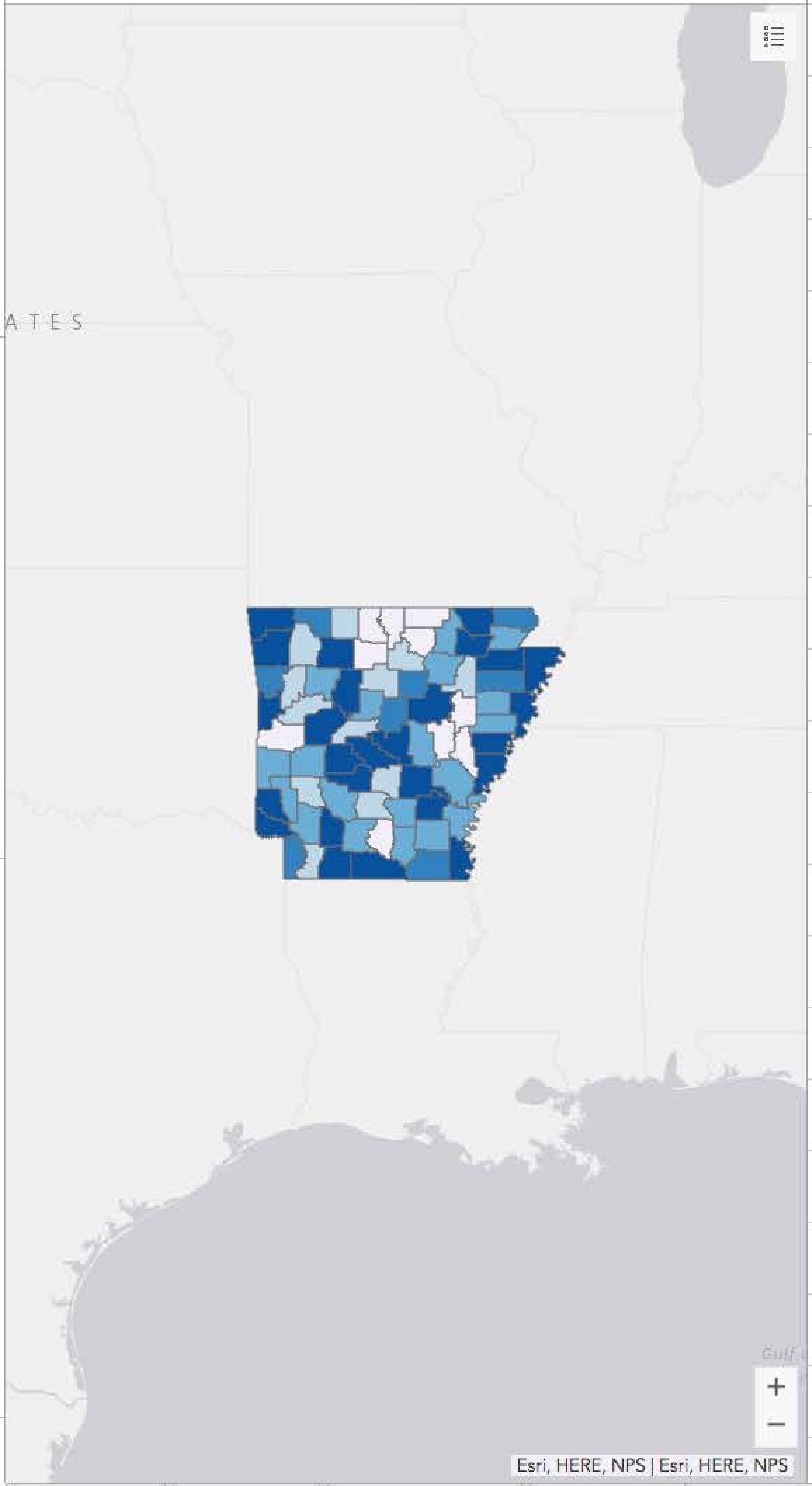

- **Arkansas** (3 Deaths)
- **Ashley** (7 Deaths)
- **Baxter** (0 Deaths)
- **Benton** (48 Deaths)
- **Boone** (2 Deaths)
- **Bradley** (5 Deaths)
- **Calhoun** (0 Deaths)
- **Carroll** (7 Deaths)
- **Chicot** (10 Deaths)
- **Clark** (3 Deaths)
- **Clay** (6 Deaths)
- **Cleburne** (7 Deaths)
- **Cleveland** (3 Deaths)
- **Columbia** (14 Deaths)
- **Conway** (3 Deaths)
- **Craighead** (15 Deaths)
- **Crawford** (6 Deaths)
- **Crittenden** (30 Deaths)
- **Cross** (5 Deaths)
- **Dallas** (2 Deaths)
- **Desha** (5 Deaths)
- **Drew** (4 Deaths)
- **Faulkner** (8 Deaths)

COVID-19 Cases | COVID-19 Active Cases | COVID-19 Deaths | Tests Reported | Health Metrics | Case Demographics | USA and World

The Arkansas Department of Health COVID-19 Case Update

Total COVID-19 Cases

59,583

Last update: a minute ago

Active Cases

5,496

Last update: a minute ago

Recoveries

53,331

Last update: a minute ago

Deaths

756

Last update: a minute ago

Cumulative Number of Cases by Date

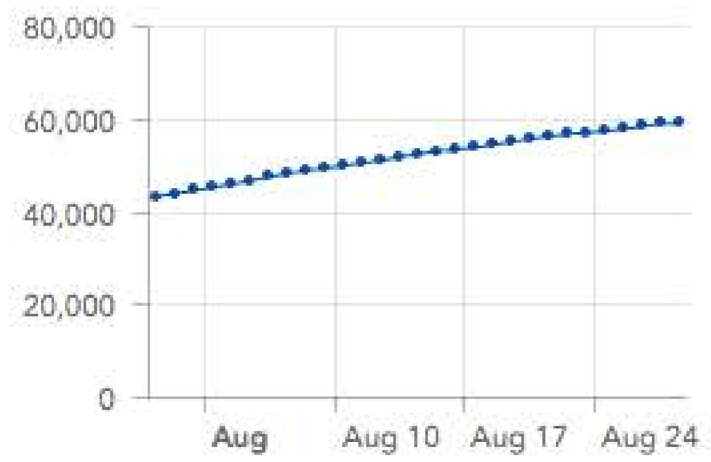

Cases in past 30 days | Cases - All time

COVID-19 Tests Performed per 1,000 Individuals

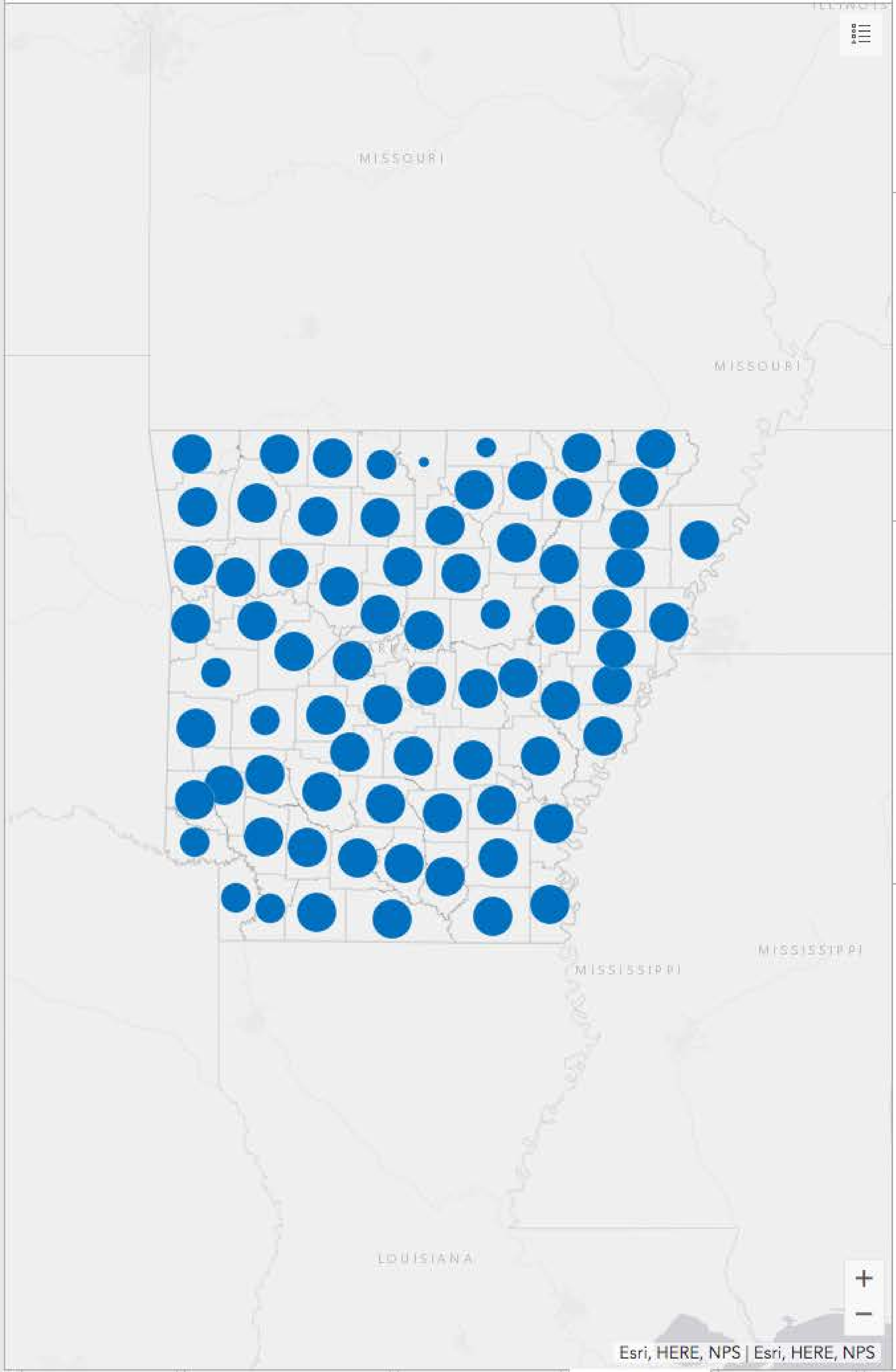

COVID-19 Cases | COVID-19 Active Cases | COVID-19 Deaths | Tests Reported | Health Metrics | Case Demographics | USA and World

Total Positive Cases

59,583

Total Negative Tests Reported

638,472

Total Tests Reported

698,055

Percent Positive Tests

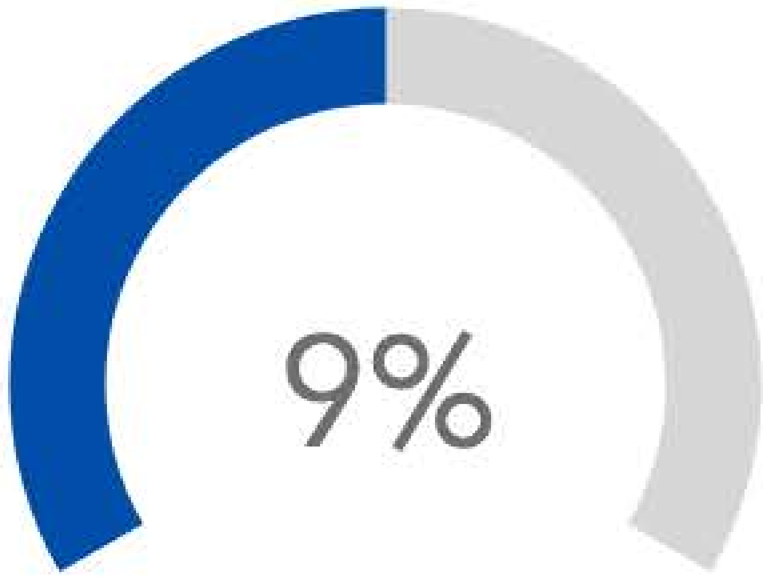

- **Arkansas** (4,193 total tests)
- **Ashley** (4,320 total tests)
- **Baxter** (2,626 total tests)
- **Benton** (59,129 total tests)
- **Boone** (6,252 total tests)
- **Bradley** (3,178 total tests)
- **Calhoun** (1,243 total tests)
- **Carroll** (5,733 total tests)
- **Chicot** (4,955 total tests)
- **Clark** (4,656 total tests)
- **Clay** (3,075 total tests)
- **Cleburne** (4,463 total tests)
- **Cleveland** (1,927 total tests)
- **Columbia** (3,801 total tests)
- **Conway** (4,749 total tests)
- **Craighead** (24,648 total tests)
- **Crawford** (14,010 total tests)
- **Crittenden** (13,120 total tests)
- **Cross** (3,277 total tests)
- **Dallas** (1,513 total tests)
- **Desha** (3,106 total tests)
- **Drew** (4,691 total tests)
- **Faulkner** (27,681 [illegible]

COVID-19 Statewide Update

Update for August 29, 2020

California now has **693,839** confirmed cases of COVID-19, resulting in **12,834** deaths. The number of COVID-related deaths increased by **1.1** percent from Thursday's total of **12,690**. The number of COVID-19 diagnostic test results in California reached a total of **11,109,630**, an increase of **98,993** tests since Thursday. The rate of positive tests over the last 14 days is **5.6** percent.

Updated August 29, 2020 at 11:00 a.m. with data from August 28.

How are COVID-19 cases progressing?

There were 4,981 new confirmed cases Friday. Numbers do not represent true day-over-day change as these results include cases from prior to yesterday. The total number of deaths is 12,834, an increase of 144 from Thursday.

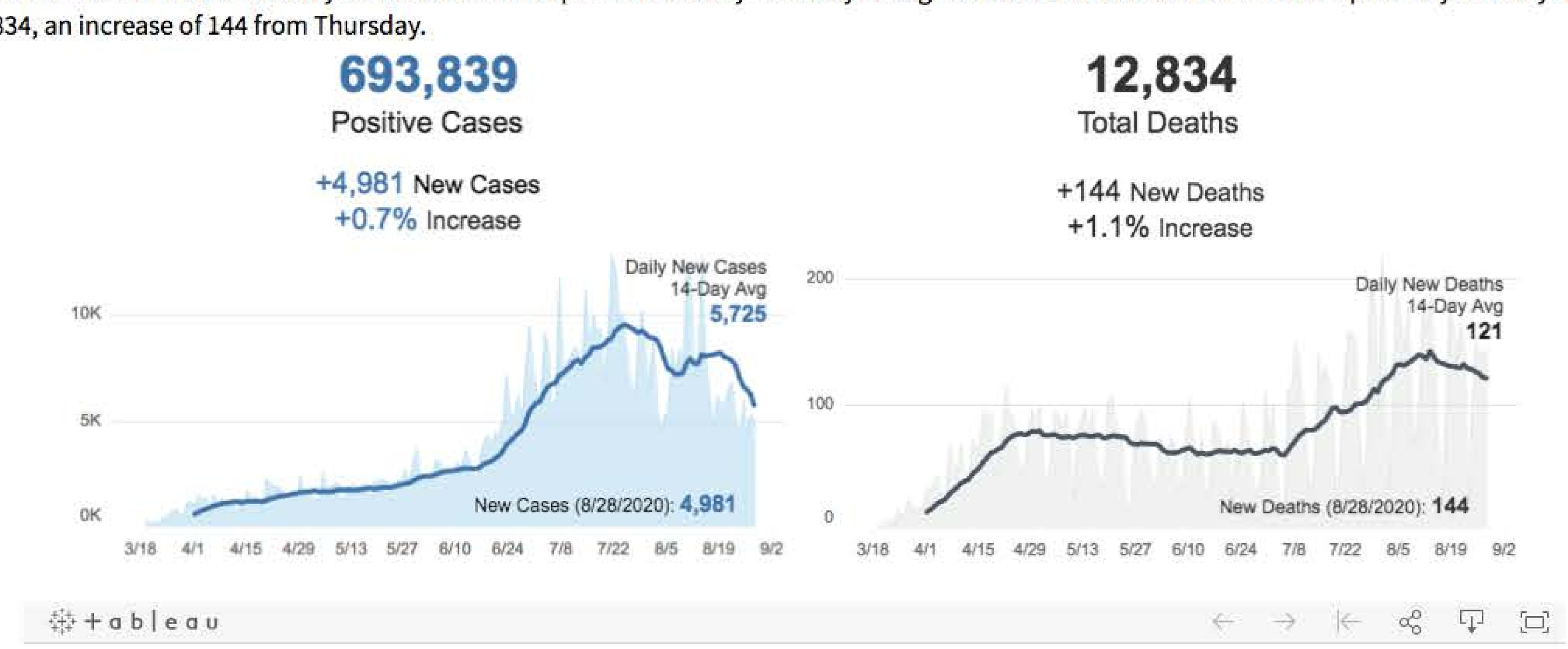

What is the current state of test results reported?

Note: 4/3 and 4/22 testing counts have been adjusted in charts to correct for anomalous reported values.

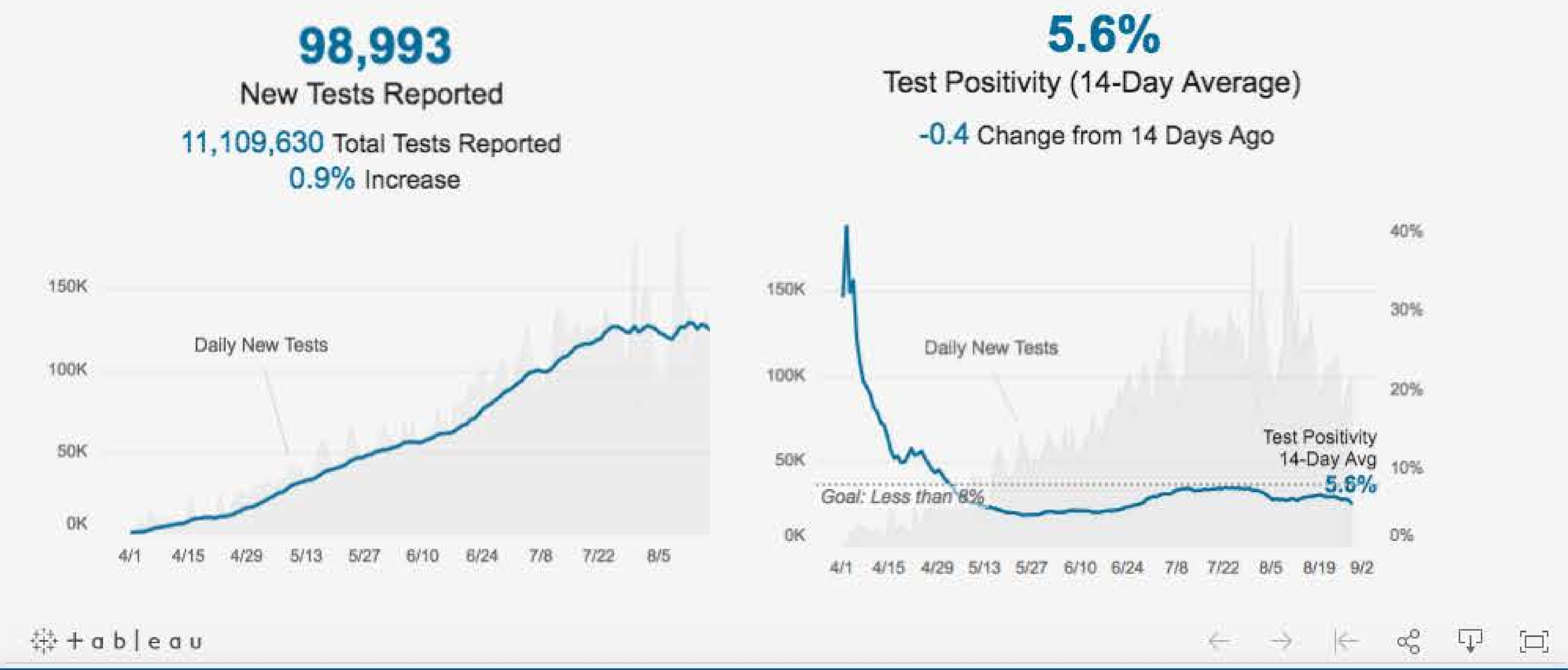

Who is getting infected with COVID-19?

The distribution of confirmed COVID-19 cases reveals significant disparities within California's overall racial and ethnic demographics, with Latino and Native Hawaiian / Pacific Islander groups having a disproportionate proportion of cases relative to their population in the state. Additional disparities are evident in COVID-19 deaths, as seen here. California adults who are Black, Latino, or Native Hawaiian / Pacific Islander have disproportionately more deaths compared with their population in the state. Structural racism, poverty and the increased likelihood of having underlying conditions, such as heart disease and lung disease, are likely to contribute to this disparity. There is a similar proportion of male and female cases in California, but there is a disproportionate proportion of deaths among males than their population.

Note: Percentages may not add up to 100% due to rounding. Ethnicity of deceased is available for a subset of the total number of deaths as reported by law enforcement. In percent of California population, Other appears as zero because it is not used in making population estimates.

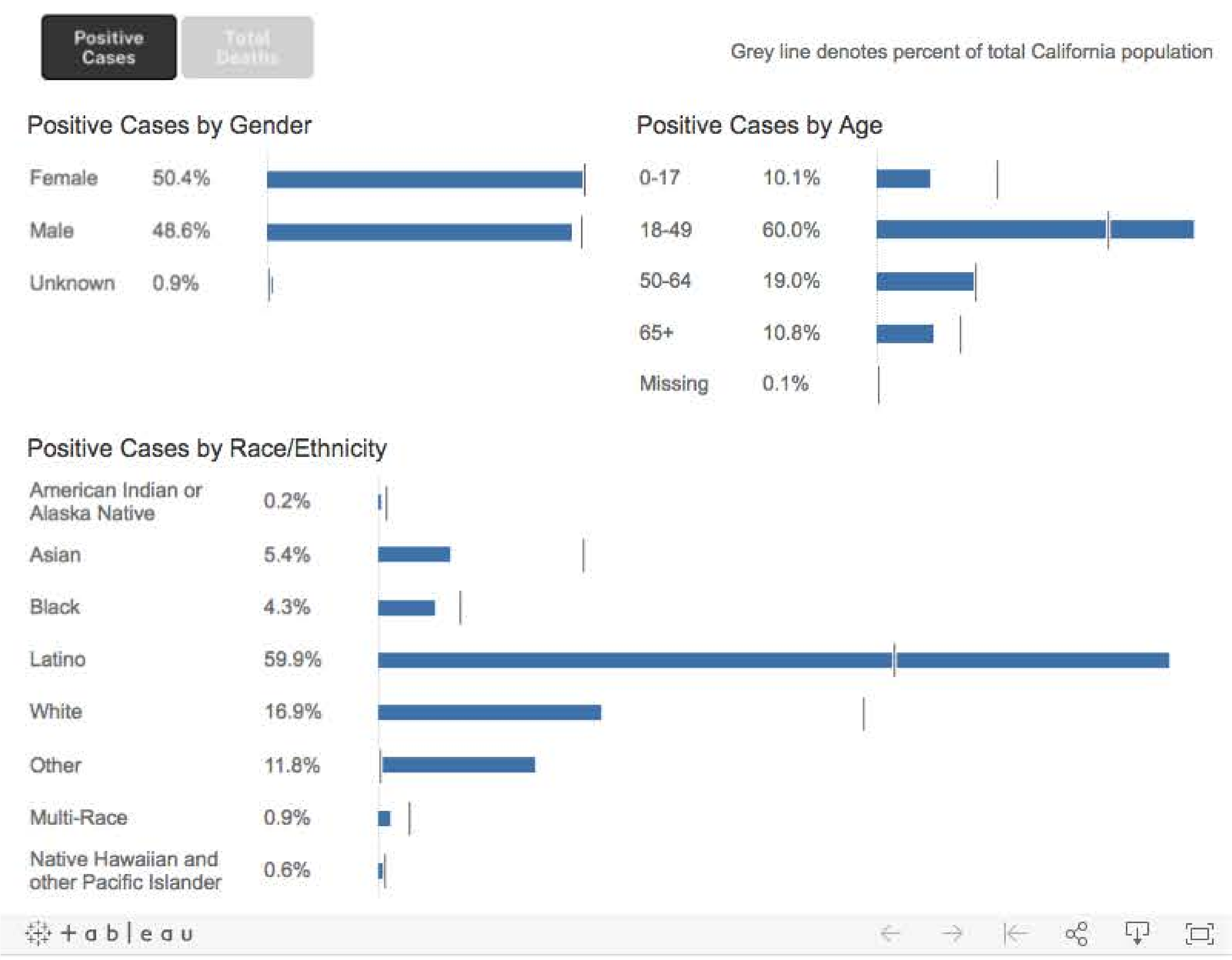

How are COVID-19 cases progressing in each county?

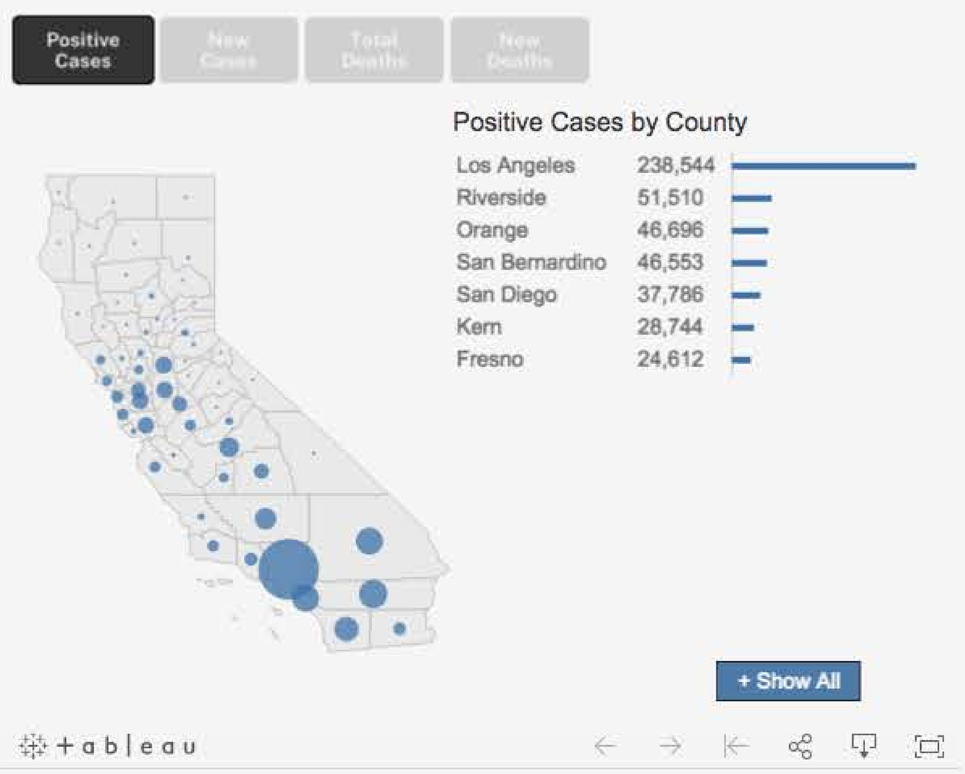

How many hospitalizations are there?

Between Thursday and Friday, there were 133 fewer hospitalizations due to confirmed COVID-19 and 47 fewer cases in intensive care units.

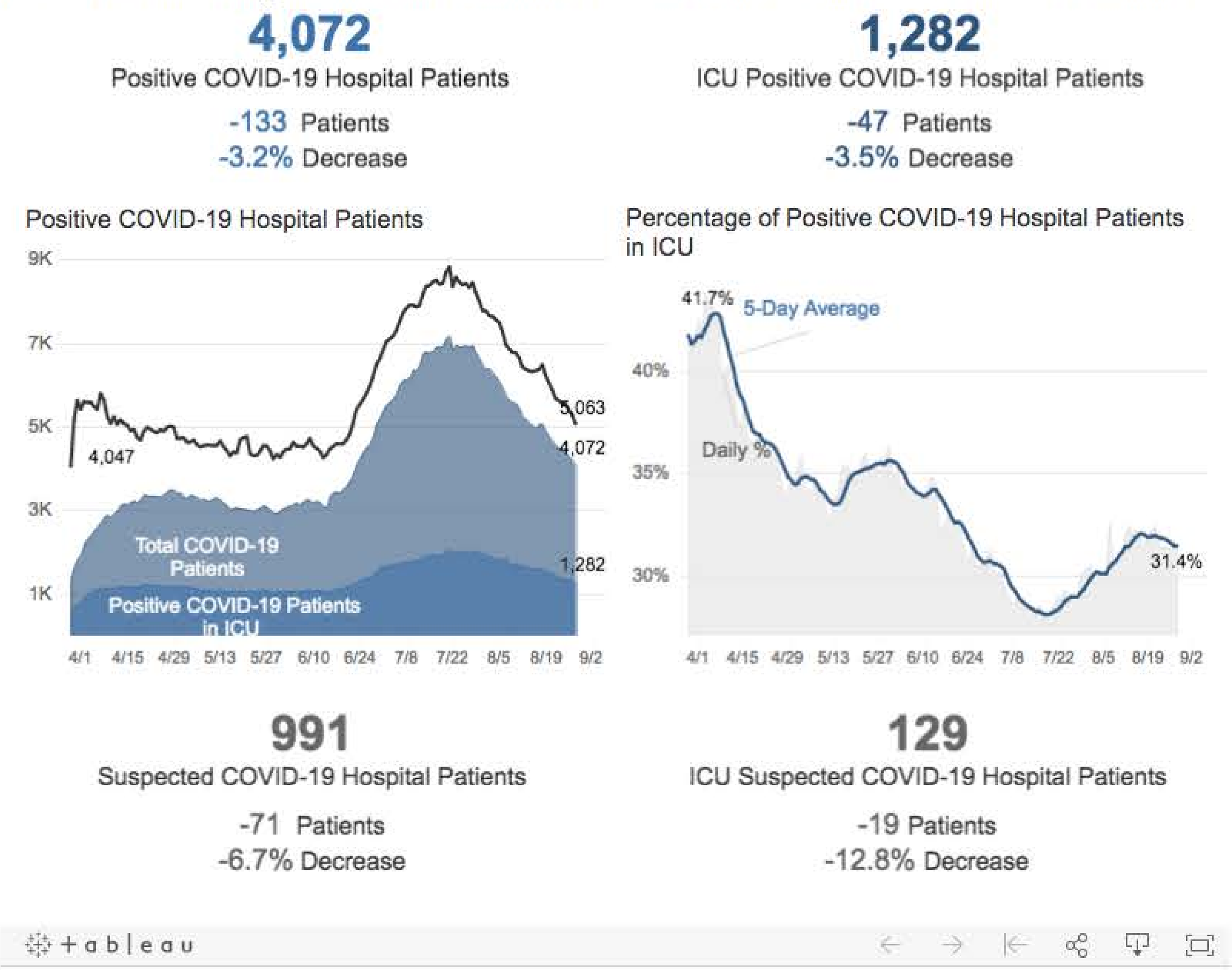

Copyright © 2020 State of California

System time: 8/29/2020 11:45:06 AM (UTC-08:00) Pacific Time (US & Canada) | PST: 8/29/2020 11:45:06 AM

COVID-19: Cases

Sort by
Positive Cases

Note: Numbers do not represent true day-over-day change as these results include cases prior to yesterday.

Positive Cases by County

County	Positive Cases
Los Angeles	238,544
Riverside	51,510
Orange	46,696
San Bernardino	46,553
San Diego	37,786
Kern	28,744
Fresno	24,612
Alameda	17,729
Sacramento	17,119
San Joaquin	17,114
Santa Clara	16,696
Stanislaus	14,176
Tulare	13,776
Contra Costa	13,522
Ventura	10,606
Imperial	9,656
San Francisco	9,135
Santa Barbara	8,001
Merced	7,803
San Mateo	7,783
Monterey	7,591
Marin	5,991
Kings	5,829
Sonoma	5,425
Solano	5,219
Madera	3,552
Placer	2,891
San Luis Obispo	2,849
Yolo	2,323

CALIFORNIA CASES

693,839

1 Day Δ	7 Day Δ	14 Day Δ
+4,981	+36,947	+80,150
+0.7%	+5.6%	+13.1%

7 Day Avg.	14 Day Avg.	Weekly % Change
5,278	5,725	-14.5%

US Total Cases: 5,845,876

Day-Over-Day New Cases

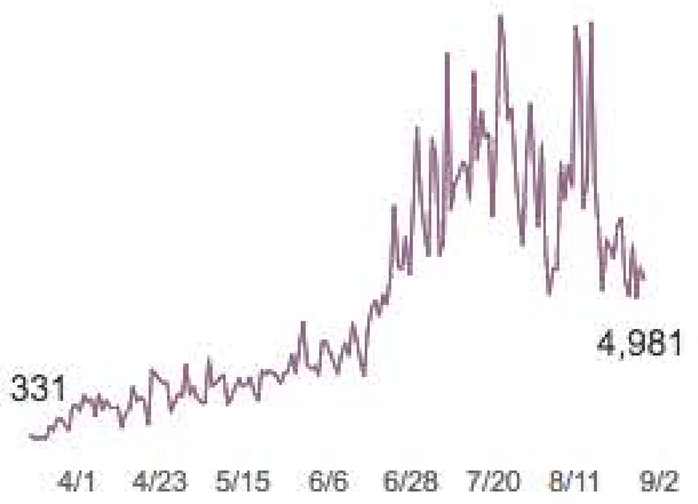

| = California Population %

Gender	%
Female	50%
Male	49%
Unknown	1%

Age	%
0-17	10%
18-49	60%
50-64	19%
65+	11%
Missing	0%

Race/Ethnicity	%
AIAN	0%
Asian	5%
Black	4%
Latino	60%
NHPI	1%
White	17%
Multi-Race	1%
Other	12%

CALIFORNIA TESTING RESULTS

11,109,630

1 Day Δ	14 Day Δ	Positivity:
+98,993	+1,428,519	7 Day: **5.5%**
+0.9%	+14.8%	14 Day: **5.6%**

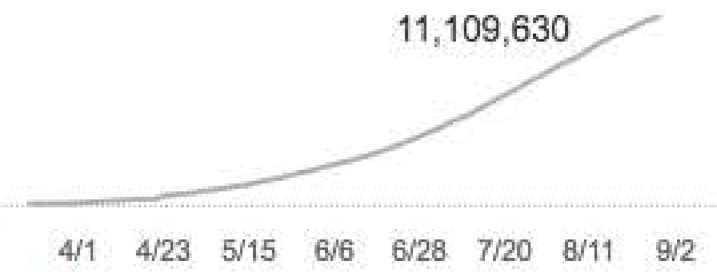

Cumulative Tests Reported by All Labs

CALIFORNIA DEATHS

12,834

1 Day Δ	7 Day Δ	14 Day Δ
+144	+846	+1,687
+1.1%	+7.1%	+15.1%

7 Day Avg.	14 Day Avg.	Weekly % Change
121	121	+0.6%

US Total Deaths: 180,165

Day-Over-Day New Deaths

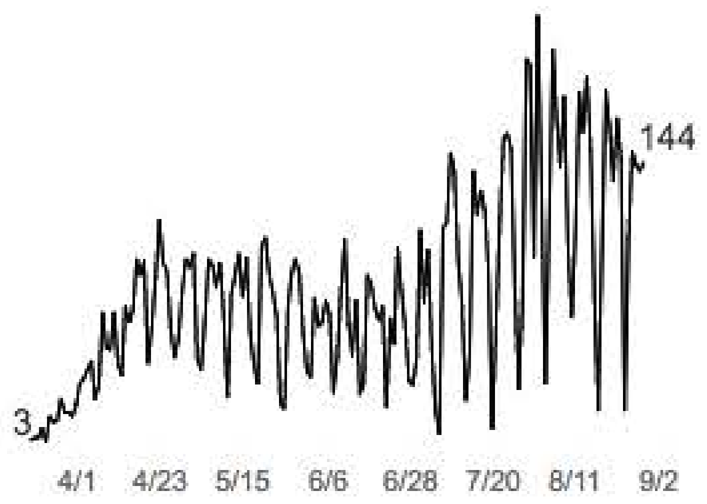

| = California Population %

Gender	%
Female	42%
Male	58%
Unknown	0%

Age	%
0-17	0%
18-49	7%
50-64	18%
65+	74%
Missing	0%

Race/Ethnicity	%
AIAN	0%
Asian	12%
Black	8%
Latino	48%
NHPI	0%
White	30%
Multi-Race	1%
Other	1%

Note: Any instance of a negative number of cases or deaths reflects a correction to previous reporting.

Note: Demographic percentages may not add up to 100% due to rounding. Breakdown of deaths is a subset of total deaths as reported by law enforcement.

Last Updated on 8/28/2020

+ableau

COVID-19 Cases Dashboard

Viz Author: **CA Open Data**

1,659,036 Views ☆ 27

More Detail

Originally Published: Jun 25, 2020

Last Updated: Aug 29, 2020

Workbook Details: 1 Sheet

Metadata: Cases

Tracking the coronavirus in California

By Los Angeles Times Staff
Updated Aug. 29, 8:10 a.m. Pacific

698,458 confirmed cases	12,839 deaths
+230 today	None yet today
+5,425 yesterday	+144 yesterday

The coronavirus pandemic has spread rapidly across California. Experts say the true number of people infected is unknown and likely much higher than official tallies.

To better understand the spread of the virus, The Times is conducting an independent, continual survey of dozens of local health agencies across the state. So far today, two of the 61 agencies have reported new numbers.

What we know

- **The state has changed its reopening plan.** The governor's new system will keep most of the state on lockdown for now. Masks remain required.
- **Officials are pointing to positive signs.** With recent technical bugs sorted out, state and local officials see signs of hope in the recent drops in new cases and hospitalizations.
- **The death toll keeps climbing.** The state has averaged 121.6 deaths per day over the last seven days.
- **Fewer tests are coming back positive.** The statewide positivity rate has decreased to 5.7%.
- **Disparities are wide.** After adjusting for population, Latinos are now 3.4 times more likely to test positive than white people.
- **The highest toll is among seniors.** Roughly 74% of the dead were 65 or older. At least 5,033 were living at a nursing home.

California counties +
Other trackers +
More coverage +

Jump to a section
Trends | Maps | Hospitals | Tests | Demographics | Nursing homes | Reopenings | State rankings

The latest trends

The number of cases in California is now on pace to double every 91.4 days, a number used to measure how quickly the virus is spreading.

Coronavirus can infect people so rapidly that it has continued to spread despite shutdown orders aimed at slowing the growth of new cases and flattening this line.

Cases | Deaths

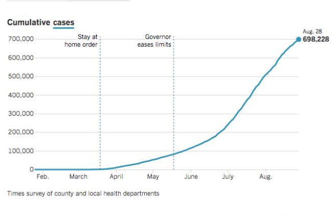

Times survey of county and local health departments

Local governments announce new cases and deaths each day, though bottlenecks in testing and reporting lags can introduce delays. For instance, some agencies do not report new totals on weekends, leading to lower numbers on those days.

State and local officials say they have overcome the technical problems that delayed the tabulation of test results in early August. Tallies dipped, and then surged, due to the bottleneck. Officials say the latest figures are now a more accurate reflection of current trends. The Times aims to rectify past counts as local officials issue corrections.

Over the past week, the state has averaged 5,482 new cases and 121.6 new deaths per day.

The charts here will update when today's Times survey of the state's 61 health agencies is complete.

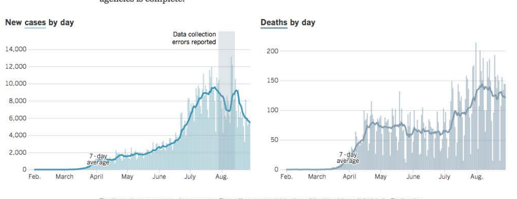

The lines above are seven-day averages. They offer a more stable view of the trend than daily totals. That's why experts wait for lines like these to flatten before they say conditions are improving.

The chart below is adjusted to show how quickly new cases are being confirmed in each county. A good sign is when a line flattens, which indicates that transmission is slowing in that area.

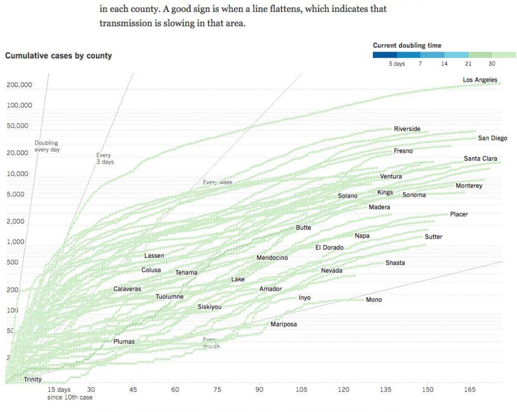

This chart tracks cumulative cases after each county confirmed its 10th case. Case counts are plotted on a logarithmic scale, which makes it easier to see when cases level off. Doubling rate is the estimate of how long it would take the county to double its number of cases, given the trend in the last week. Compare the slope of a county's curve to the slope of the guide lines to estimate its doubling rate at any point.

How COVID-19 crushed California's workforce ↗
The coronavirus outbreak decimated California's economy. The Times is tracking the fallout as businesses begin to reopen.

Cases by county and city

While initial outbreaks were centered in the San Francisco Bay Area, most new cases and deaths are now concentrated in Southern California.

In the last 14 days
Metric: Cases | Deaths
Time frame: Last 14 days | Cumulative
Hover for more information.

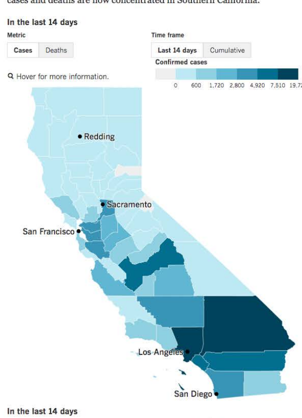

In the last 14 days

County	Cases	▼ Per 100k	Today	Deaths	Per 100k	Today
Fresno »	5,210	532.6	–	60	6.1	–
Kings »	793	528.4	–	8	5.3	–
Madera »	750	483.8	–	7	4.5	–
Stanislaus »	2,304	427.2	–	58	10.8	–
Merced »	1,113	413.6	–	26	9.7	–
Monterey »	1,729	399.1	–	14	3.2	–
Tulare »	1,779	386.3	–	27	5.9	–
San Benito »	227	382.1	–	4	6.7	–
San Bernardino »	7,518	352.1	–	155	7.3	–
Kern »	3,073	348	–	69	7.8	–

Show all

The Times' calculation of per capita rates can vary from what's published elsewhere. To learn more about how and why this count sometimes differs from official figures, consult our FAQ.

Residents of cities, neighborhoods and regions all across the state have contracted the coronavirus. Here are the latest tallies for 1,061 places as released by county health departments.

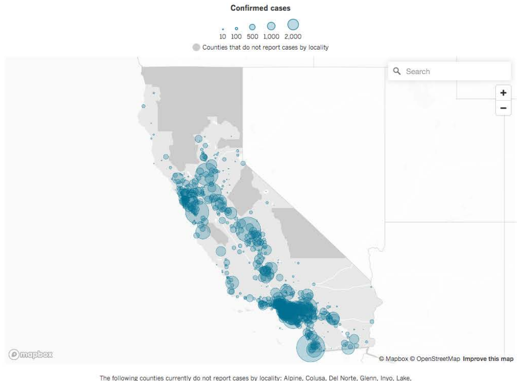

The following counties currently do not report cases by locality: Alpine, Colusa, Del Norte, Glenn, Inyo, Lake, Lassen, Mariposa, Modoc, San Benito, Shasta, Sierra, Siskiyou, Tehama, Trinity and Tuolumne

Filter by county: Los Angeles
Search by name

Area	▼ Confirmed cases
East Los Angeles	5,674
Pomona	4,890
South Gate	4,014
Boyle Heights	3,998
El Monte	3,983
Downey	3,667
Compton	3,621
Palmdale	3,579
North Hollywood	3,261
Unincorporated - Florence-Firestone	3,141

Show all

Hospitals and patients

Lockdown measures aim to slow the virus in hope of preventing hospitals from being overrun. To keep tabs on capacity, officials watch out for rapid increases in the number of patients.

In late July, the Trump administration changed how hospitals must report data. State officials say the transition is complete and that hospital data are now stable.

There are now 4,205 hospital patients statewide with a confirmed case, a change of -19% from two weeks ago.

Confirmed | Suspected | Both

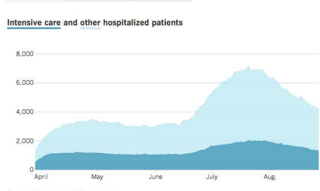

California Department of Public Health

Confirmed patients

County	ICU	Other	Total
San Diego »	79	148	227
Imperial »	12	20	32
Sonoma »	11	29	40
Orange »	98	253	351
Kern »	53	94	147
Sacramento »	55	152	207
San Joaquin »	38	99	137
San Francisco »	36	43	79
Los Angeles »	356	760	1,116
Riverside »	82	141	223

Show all

Officials also closely monitor the number of beds open in intensive-care units. In late July, the state changed its tracking method to exclude beds that are only for infants from the count.

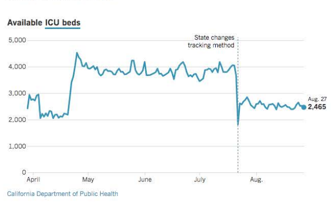

California Department of Public Health

Testing

After a fitful start, California has increased coronavirus testing in the state. Over the last week, an average of 97,324 tests have been conducted each day.

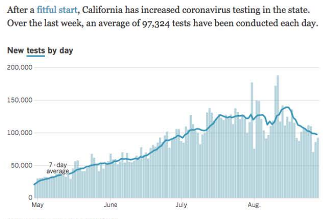

California Department of Public Health

In the last seven days, about 5.7% of the 681,265 tests conducted have returned a positive result.

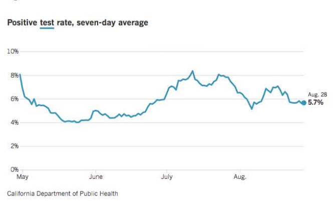

California Department of Public Health

Wide disparities in age and race

While younger adults make up the majority of positive tests, deaths due to the virus have skewed heavily toward the elderly.

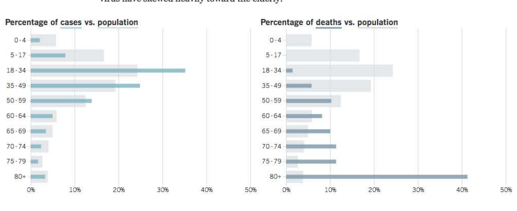

There are 899 cases with an unreported age.
California Department of Public Health

The state has logged the race of the patient in nearly two-thirds of cases.

Latinos and Black people have contracted the virus at a higher rate than white and Asian people. The gap is widening. After adjusting for population, Latinos are now 3.4 times more likely to test positive than white people.

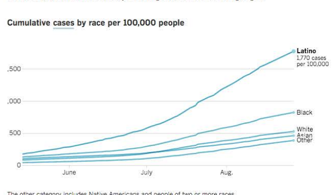

The other category includes Native Americans and people of two or more races.
California Department of Public Health

One outcome is that among most age groups, and especially younger people, Black people and Latinos are dying more often than other races relative to their share of the population.

Percentage of deaths vs. population
Age: 18+ | Cases | Deaths

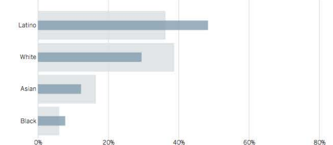

Race	Deaths	Deaths Pct.	Population Pct.
Latino	5,954	48.4%	36.3%
White	3,620	29.5%	38.8%
Asian	1,512	12.3%	16.5%
Black	956	7.8%	6.1%

Note: There are 163 deaths with an unknown race in this age bracket, 1% of the total.

Nursing homes

Nursing homes are a tragic focal point of the coronavirus outbreak. At least 5,033 residents and staff have died from COVID-19, 40% of the statewide total.

Cases | Deaths

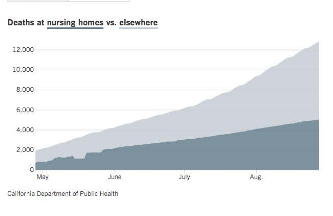

California Department of Public Health

California's Department of Public Health is currently listing 1,121 skilled nursing and 305 assisted-living facilities across the state with COVID-19 outbreaks.

Filter by county: Los Angeles
Search by name

Facility	Residents Cases	Residents Deaths	Staff Cases	Staff Deaths
AFFINITY HEALTHCARE CENTER	36	15	41	-
ALAMEDA CARE CENTER	54	21	37	10 or fewer
ALAMITOS BELMONT REHABILITATION HOSPITAL	-	-	10 or fewer	-
ALCOTT REHABILITATION HOSPITAL	-	-	10 or fewer	10 or fewer
ALDEN TERRACE CONVALESCENT HOSPITAL	107	26	40	-
ALEXANDRIA CARE CENTER	51	24	38	-
ALHAMBRA HEALTHCARE & WELLNESS CENTRE, LP	10 or fewer	10 or fewer	13	-
ALHAMBRA HOSPITAL MEDICAL CENTER D/P SNF	-	-	10 or fewer	-
ALL SAINTS HEALTHCARE	10 or fewer	-	10 or fewer	-
ANGELS NURSING HEALTH CENTER	10 or fewer	-	10 or fewer	-

Show all

The state last updated the list on August 28. Officials have withheld the precise number where there are 10 or fewer cases. The totals are cumulative counts.

Lives lost to COVID-19 ↗
Learn more about those we've lost by reading Times obituaries of Californians who have died from coronavirus.

New guidelines for reopening

Gov. Gavin Newsom unveiled a new plan on Friday to revive the California economy decimated by the COVID-19 pandemic. Counties will be allowed to ease restrictions in four tiers, which will determine when and how businesses reopen.

California's reopening risk tiers

Tier 1	Tier 2	Tier 3	Tier 4
**Widespread**	**Substantial**	**Moderate**	**Minimal**
Most nonessential indoor business operations are closed.	Some nonessential indoor business operations are closed.	Some business operations can open with modifications.	Most business operations can open with modifications

The new groups, known as tiers, are based on a set of metrics developed by state officials. Which tier a county falls in decides what can be reopened.

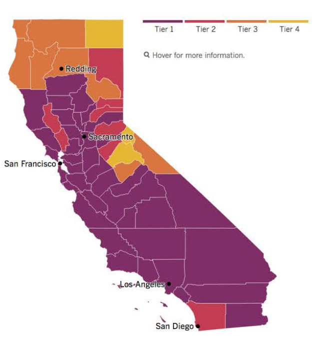

What's open in every county ↗
We're tracking what's open, closed and restricted throughout the state in ten different categories, including parks, retail, restaurants and more. Find out what's open where you live.

Our state in context

To date, the United States has recorded 5,912,216 coronavirus cases and 181,805 deaths. In the last week, the country has averaged 41,528 new cases and 917 deaths per day.

The chart below is adjusted to show how quickly new cases are being confirmed in each state. A good sign is when a line flattens, which indicates that transmission is slowing in that area.

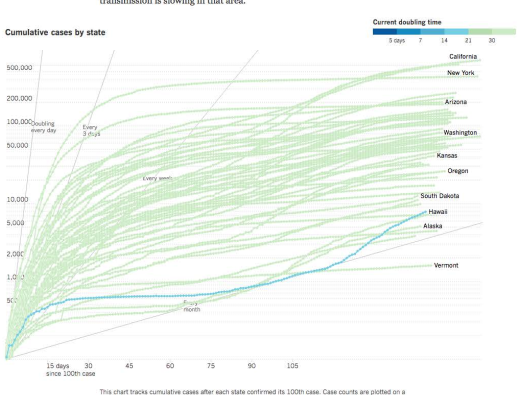

This chart tracks cumulative cases after each state confirmed its 100th case. Case counts are plotted on a logarithmic scale, which makes it easier to see when cases level off. Doubling rate is the estimate of how long it would take the state to double its number of cases, given the trend in the last week. Compare the slope of a state's curve to the slope of the guide lines to estimate its doubling rate at any point.

California, America's most populous state, leads the nation in cases. It ranks much lower after adjusting for population.

The states with the fastest-growing outbreaks over the past two weeks, based on population, are North Dakota, Iowa and Mississippi.

State	▼ Cases	Per 100k	New cases (Fewer–More, Mar 1–Aug 28)
**California**	**698,228**	**1,783.5**	
Texas	619,354	2,221.1	
Florida	615,806	2,989.6	
New York	432,767	2,205.9	
Georgia	265,372	2,577.1	
Illinois	231,186	1,803.1	
Arizona	200,658	2,888.5	
New Jersey	190,971	2,150.1	
North Carolina	162,491	1,600	
Tennessee	150,815	2,267.5	

Show all

No state has had more deaths than New York, though its pace has declined in recent months.

State	▼ Deaths	Per 100k	New deaths (Fewer–More, Mar 1–Aug 28)
New York	32,934	167.9	
New Jersey	15,930	179.4	
**California**	**12,839**	**32.8**	
Texas	12,537	45	
Florida	10,957	53.2	
Massachusetts	9,024	132.1	
Illinois	8,206	64	
Pennsylvania	7,642	59.7	
Michigan	6,712	67.4	
Georgia	5,471	53.1	

Show all

Tracking the coronavirus

California counties
Alameda, Alpine, Amador, Butte, Calaveras, Colusa, Contra Costa, Del Norte, El Dorado, Fresno, Glenn, Humboldt, Imperial, Inyo, Kern, Kings, Lake, Lassen, Los Angeles, Madera, Marin, Mariposa, Mendocino, Merced, Modoc, Mono, Monterey, Napa, Nevada, Orange, Placer, Plumas, Riverside, Sacramento, San Benito, San Bernardino, San Diego, San Francisco, San Joaquin, San Luis Obispo, San Mateo, Santa Barbara, Santa Clara, Santa Cruz, Shasta, Sierra, Siskiyou, Solano, Sonoma, Stanislaus, Sutter, Tehama, Trinity, Tulare, Tuolumne, Ventura, Yolo, Yuba

Other trackers
Lives lost to COVID-19
Unemployment and economic fallout
Housing homeless people
Which counties are open
Which beaches are closed
Frequently asked questions

More coverage
Coronavirus symptoms
How coronavirus spreads
Get our newsletter

About the numbers

This page was created by Swetha Kannan, Casey Miller, Sean Greene, Lorena Iñiguez Elebee, Rong-Gong Lin II, Ryan Murphy, Melody Gutierrez, Priya Krishnakumar, Sandhya Kambhampati, Maloy Moore, Jennifer Lu, Aida Ylanan, Vanessa Martínez, Ryan Menezes, Thomas Suh Lauder, Andrea Roberson, Ben Poston, Nicole Santa Cruz, Iris Lee, Rahul Mukherjee, Jaclyn Cosgrove, Anthony Pesce, Paul Duginski and Phi Do.

State and county totals come from a continual Times survey of California's 58 county health agencies as well as the three run by cities. Those figures are ahead of the totals periodically released by the state's Department of Public Health. State officials acknowledge that their tallies lag behind the updates posted by local agencies throughout the day and do not dispute The Times' method.

Data from other states, Washington, D.C. and Puerto Rico are collected by researchers at the Johns Hopkins University Center for Systems Science and Engineering.

Data on hospitalizations, tests, demographics and nursing homes come from California's Department of Public Health.

Closures and restrictions are drawn from an ongoing Times survey of county governments.

The Times database is available to the public on Github, a popular website for hosting data and computer code. The files will be updated daily at github.com/datadesk/california-coronavirus-data.

Learn more about The Times count by reading this list of frequently asked questions or by reading this interview with members of our team.

CAL MATTERS DONATE SUBSCRIBE ABOUT

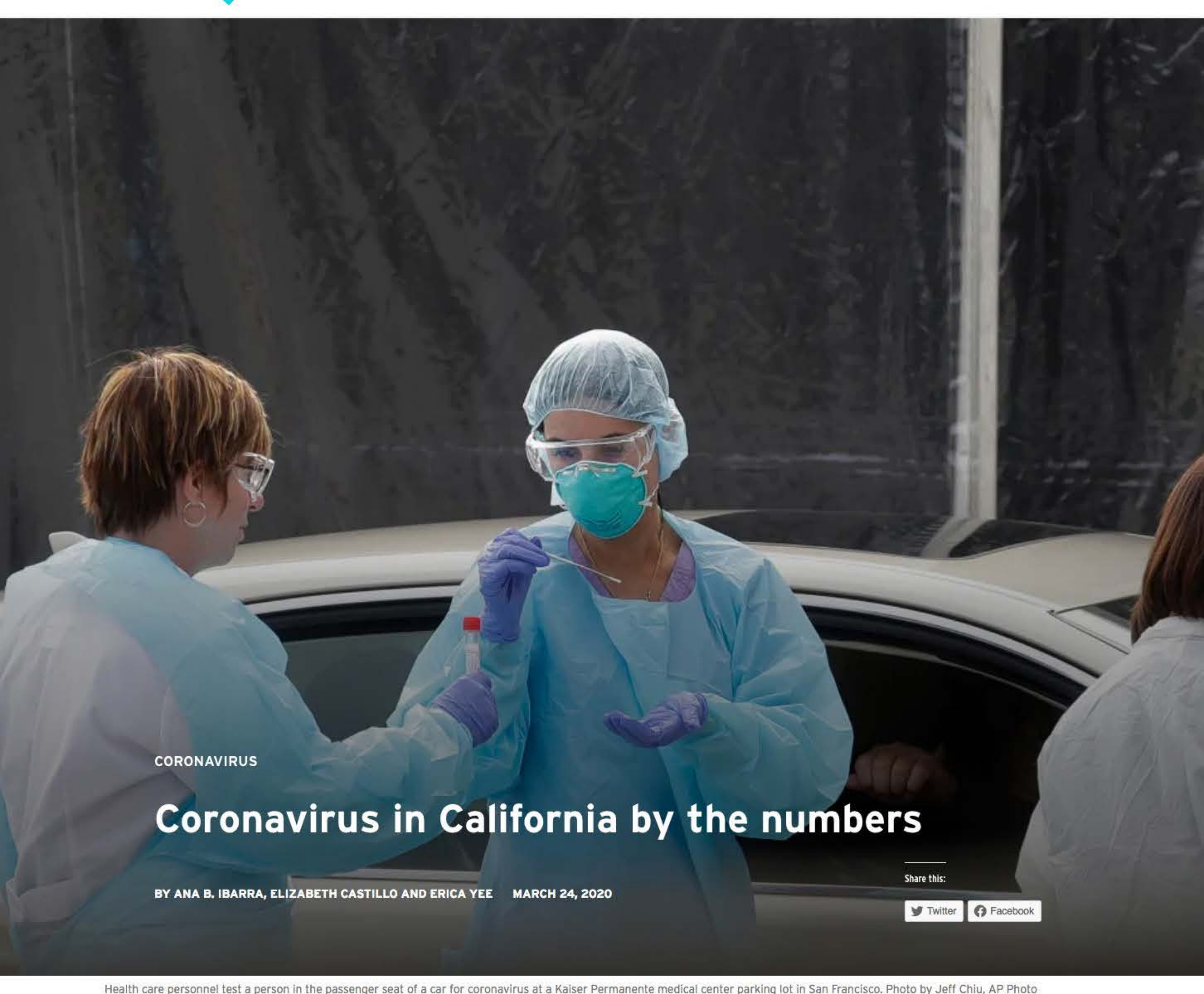

CORONAVIRUS

Coronavirus in California by the numbers

BY ANA B. IBARRA, ELIZABETH CASTILLO AND ERICA YEE MARCH 24, 2020

Share this: Twitter Facebook

Health care personnel test a person in the passenger seat of a car for coronavirus at a Kaiser Permanente medical center parking lot in San Francisco. Photo by Jeff Chiu, AP Photo

IN SUMMARY

Developments in the coronavirus pandemic are changing rapidly. We're working to track the latest California data. Find updates here.

California – having declared a pandemic-induced state of emergency on March 4, followed by a shelter-in-place order two weeks later – is now struggling to figure out what to reopen as coronavirus hospitalizations have plateaued.

A state-forecasted patient surge in mid-May failed to materialize to the degree feared, but Gov. Gavin Newsom continues to warn that the coronavirus threat remains real and dangerous.

The state has made progress on some goals, but lags well behind on others. Testing capacity remains insufficient, contact tracing is not yet up-to-speed throughout the state, and although the state did acquire 15,000 hotel and motel rooms for homeless Californians, thousands remain vacant while tens of thousands remain unsheltered.

Here is a look at what the state has and needs for its continued response, as well as a breakdown of testing, and data of positive cases by age, gender and ethnicity, which shows that the virus has taken a disproportionate toll on communities of color.

This dashboard will be updated regularly with confirmed data from the state's department of public health and the governor's office.

Jump to...

Cases and deaths | By race, age and gender | Testing | Equipment and resources

Also see our hospitalizations tracker

Total cases and deaths

California's public health department announced the first two cases of this new coronavirus in the state on Jan. 26. More than three months later, the state had reported close to 70,000 positive cases and close to 3,000 deaths from the disease the virus causes, COVID-19. The number of infected people, however, is assumed to be much larger because access to testing has been an ongoing challenge.

693,839 cases	12,834 deaths
4,981 on Aug 28	144 on Aug 28
+0.7% from the previous day	+1.1% from the previous day

Retrieved Aug 29, 2020 3:00 PM

Source: California Department of Public Health

Cases and deaths by race, age and gender

Latinos, African Americans and Pacific Islanders are dying from the coronavirus at disproportionately higher levels in California. Pacific Islanders have the highest death rate of any racial group, and the proportion of COVID-19 deaths among African Americans is about twice their population representation. The race/ethnicity is unknown for about 4% of the deaths.

Cases of COVID-19 by race/ethnicity, per 100,000 residents

The race/ethnicity is unknown for 232,632 (33.5%) cases. For 54,595 cases (7.9%), the race/ethnicity is classified as "Other," which is not a census category.

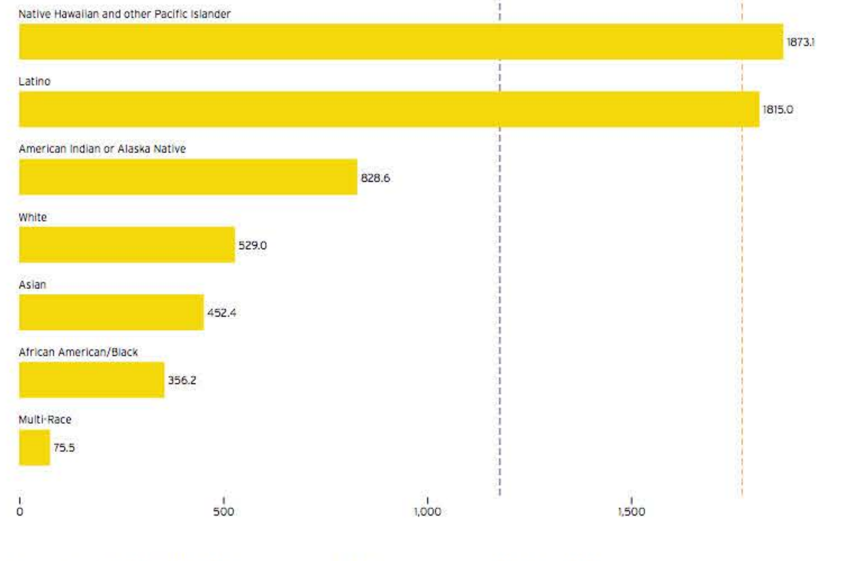

Deaths of COVID-19 by race/ethnicity, per 100,000 residents

The race/ethnicity is unknown for 438 (3.4%) deaths. For 139 deaths (1.1%), the race/ethnicity is classified as "Other," which is not a census category.

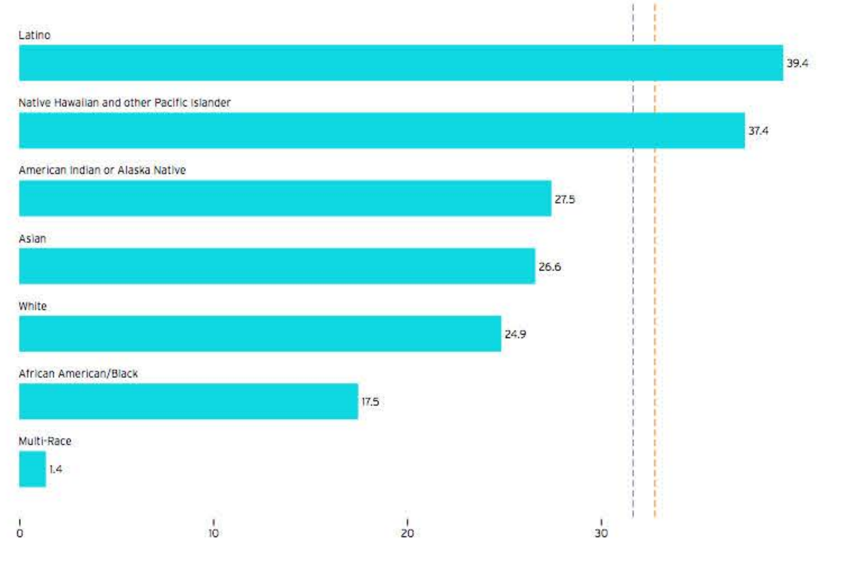

RACE/ETHNICITY	CASES	PERCENT CASES	CASES PER 100K	DEATHS	PERCENT DEATHS	DEATHS PER 100K
Multi-Race	4,171	0.6%	75.5	76	0.6%	1.4
African American/Black	19,680	2.8%	356.2	966	7.5%	17.5
White	77,747	11.2%	529.0	3,655	28.5%	24.9
Asian	24,997	3.6%	452.4	1,471	11.5%	26.6
American Indian or Alaska Native	1,147	0.2%	828.6	38	0.3%	27.5
Native Hawaiian and other Pacific Islander	2,602	0.4%	1,873.1	52	0.4%	37.4
Latino	276,268	39.8%	1,815.0	5,999	46.7%	39.4

Cases of COVID-19 by age, per 100,000 residents

The age is unknown for 0 (0%) cases.

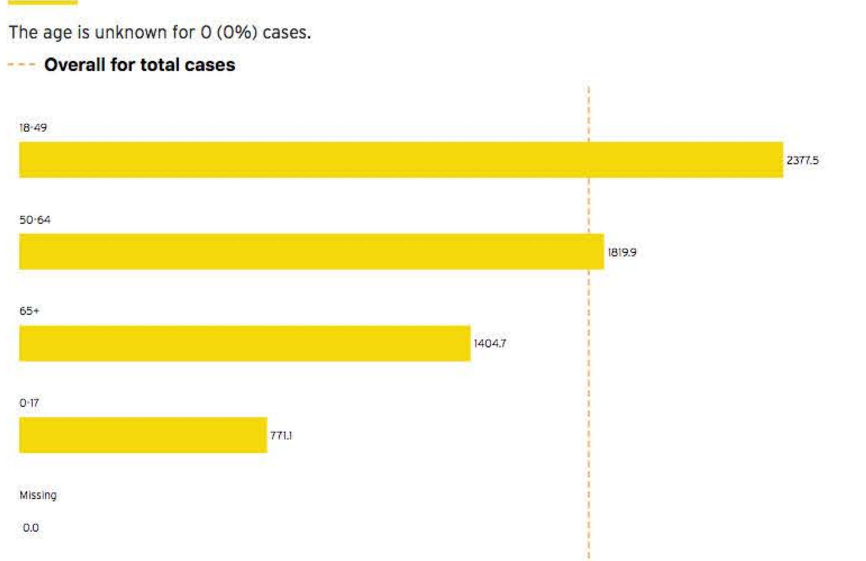

Deaths from COVID-19 by age, per 100,000 residents

The age is unknown for 265 (2.1%) deaths.

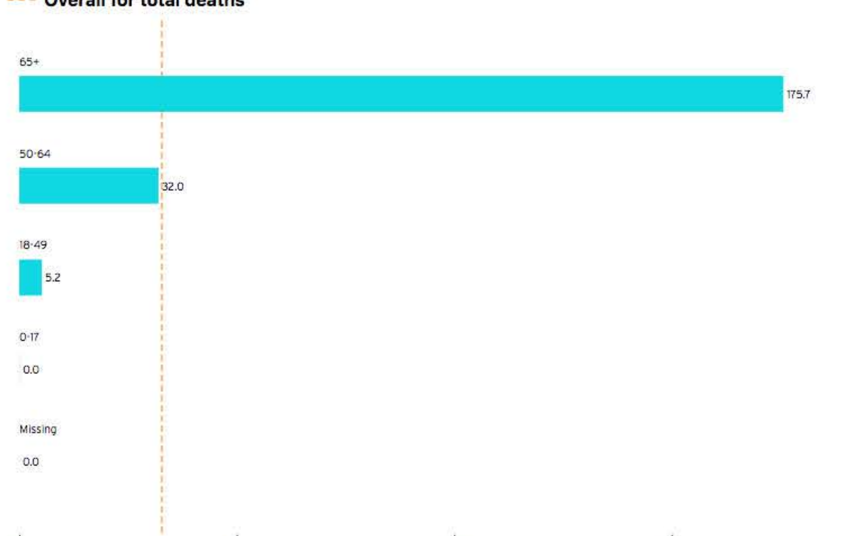

AGE	CASES	PERCENT CASES	CASES PER 100K	DEATHS	PERCENT DEATHS	DEATHS PER 100K
Missing	901	0.1%	0.0	3	0.0%	0.0
0-17	69,964	10.1%	771.1	3	0.0%	0.0
18-49	416,615	60.0%	2,377.5	907	7.1%	5.2
50-64	131,694	19.0%	1,819.9	2,317	18.1%	32.0
65+	74,665	10.8%	1,404.7	9,339	72.8%	175.7

Cases of COVID-19 by gender, per 100,000 residents

The gender is unknown for 6,584 (0.9%) cases.

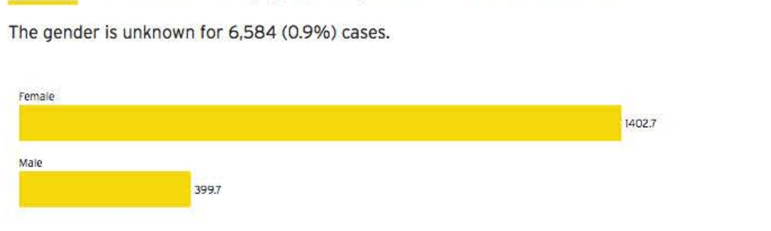

Deaths of COVID-19 by gender, per 100,000 residents

The gender is unknown for 22 (0.2%) deaths.

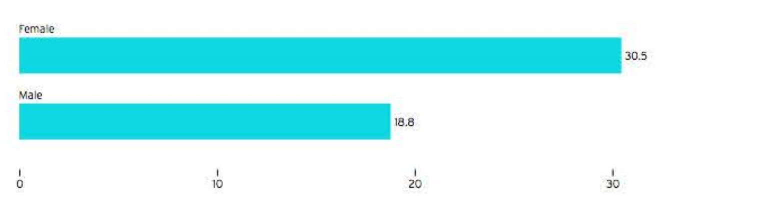

GENDER	CASES	PERCENT CASES	CASES PER 100K	DEATHS	PERCENT DEATHS	DEATHS PER 100K
Male	337,348	48.6%	399.7	7,230	56.3%	18.8
Female	349,907	50.4%	1,402.7	5,317	41.4%	30.5

Updated Aug 29, 2020 3:00 PM

Source: California Department of Public Health

How many tests have been conducted?

Testing nationwide has lagged significantly. Initially labs in California reported they were missing ingredients for extracting genetic material; they also were low on testing supplies such as swabs. For weeks, only those with serious symptoms were being tested. On May 12, the state reported that 1 million Californians had been tested, and that an average of 35,000 tests were being conducted per day. To meet health recommendations for reopening the economy, however, the state needs to almost double that amount of daily tests.

11,010,637

Total tests reported

92,222 on Aug 28

+0.8% from the previous day

Daily tests reported

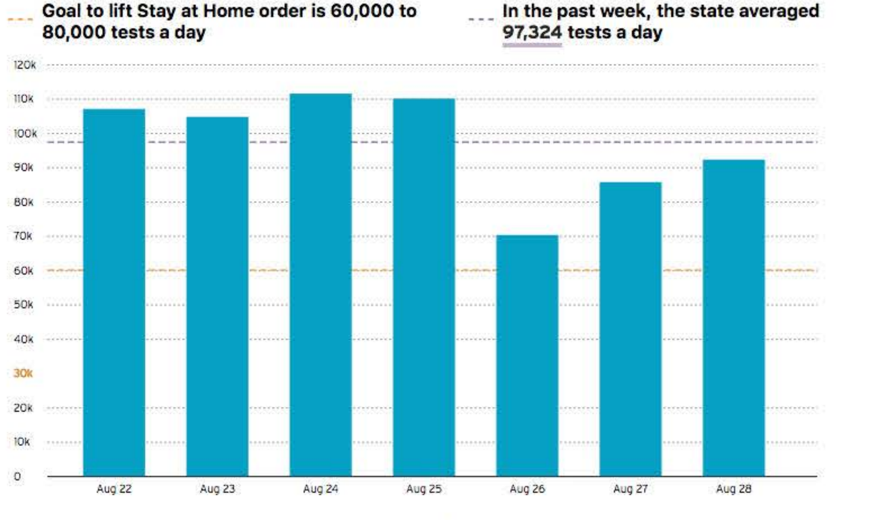

Updated Aug 28

Source: The COVID Tracking Project

What's the status of Project Roomkey?

Project Roomkey is an initative to rent hotels, motels, and trailers to support housing for people experiencing homelessness and other sick or medically vunerable individuals in need of housing.

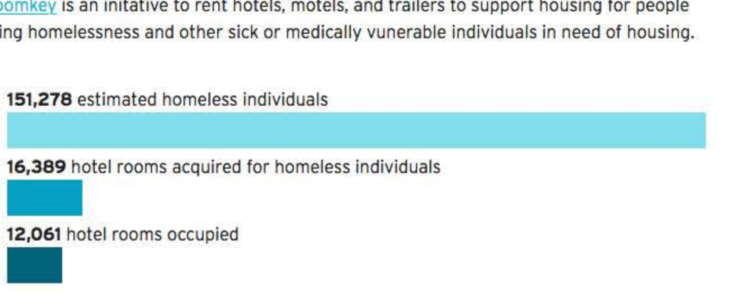

Project Roomkey by county

Hotel rooms | Trailers

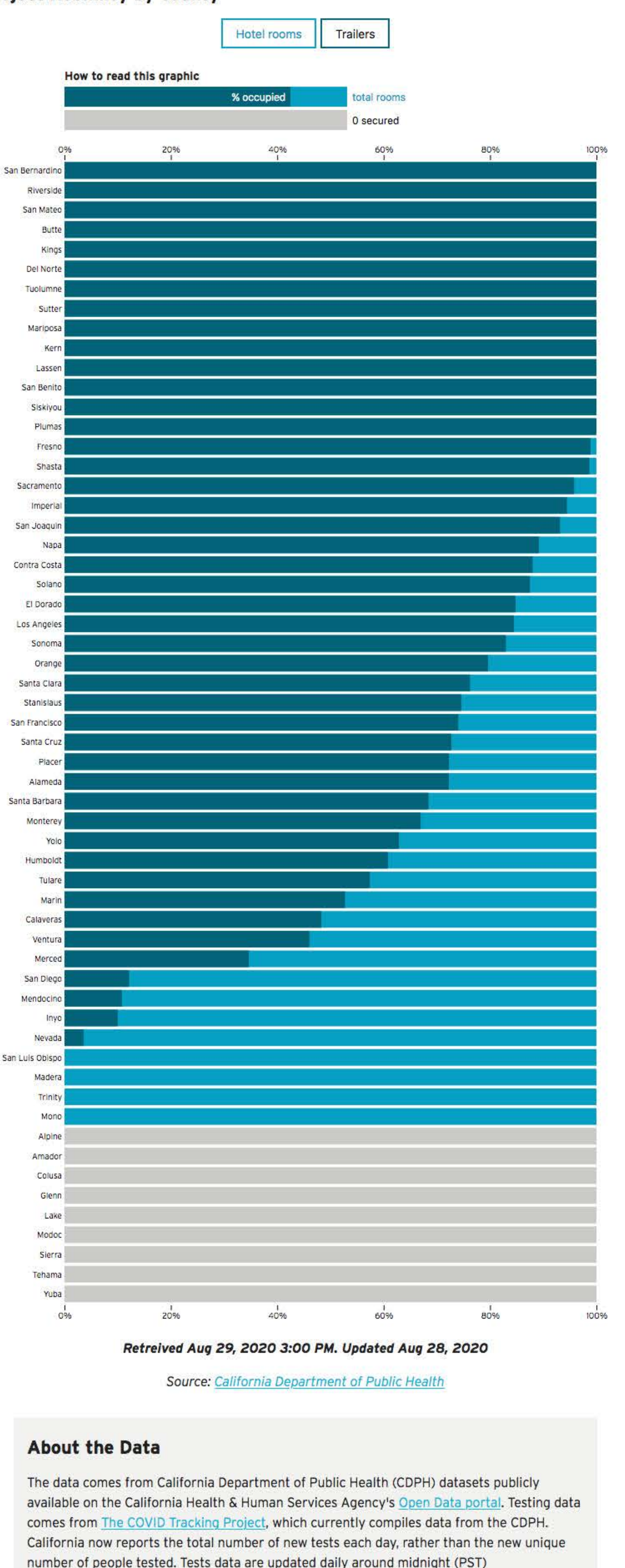

Retreived Aug 29, 2020 3:00 PM. Updated Aug 28, 2020

Source: California Department of Public Health

About the Data

The data comes from California Department of Public Health (CDPH) datasets publicly available on the California Health & Human Services Agency's Open Data portal. Testing data comes from The COVID Tracking Project, which currently compiles data from the CDPH. California now reports the total number of new tests each day, rather than the new unique number of people tested. Tests data are updated daily around midnight (PST)

Case and death numbers are retreived each day around noon. Project Roomkey data is updated irregularly.

Population data is based on the 2018 American Community Survey.

Any questions about the data can be sent to CalMatters health reporter Ana Ibarra.

56.2k Shares

Colorado COVID-19 Data

COLORADO Department of Public Health & Environment

Data is updated daily by about 4 p.m. and includes cases reported through the previous day.
All data, for days past and present, is recalculated daily.

56,773 Cases	6,945 Hospitalized	63 Counties	695,569 People Tested	985,622 Test Encounters	1,937 Deaths Among Cases	1,843 Deaths Due to COVID-19	600 Outbreaks

Case Summary | Demographics | Tests | Hospital Data | Incidence and Epidemic Curve | Syndromic Data | Outbreaks | CDC Provisional Deaths

383 (▲ 50) Cases Reported Today	47 Cases Added with Previous Reported Date	284 (▼ 1) 7-Day Moving Average of Cases Reported	71.1 (▼0.2) 2-Week Cumulative Incidence Per 100K People
14 (▼ 10) New Hospital Admissions	21 (▼ 2) 7-Day Moving Average of New Hospitalizations	29% Adult Critical Care Ventilators in Use	3% Available Hospital Beds Occupied by Confirmed and Suspected COVID Patients
2.63% (▼ 0.51%) State Positivity Rate	2.78% (▼ 0.05%) State 7-Day Positivity Rate	0.98% (▼ 0.11%) Previous Week Emergency Department Syndromic COVID Visits	2.76% (▼ 0.39%) Previous Week Outpatient Syndromic COVID Visits
2.4 (▲ 0.6) Tests Per 1,000 People	1.7 (▲ 0.0) 7-Day Average of Tests Per 1,000 People	13,654 (▲ 3,506) New Individual People Tested	13,648 (▲ 3,412) New Test Encounters

CDPHE

+ a b | e a u

- Case summary snapshot
- Hospital data snapshot
- Cases and deaths by county

CDPHE Open Data Portal

Symptom Support Tool data

Outbreak data

Colorado public survey results

Colorado Department of Corrections COVID-19 Offender Case Dashboard

Additional resources

- Access the data files
- Learn more about the data
- COVID-19 Death Data and Resources
- CDC Provisional Death Counts for Coronavirus Disease (COVID-19)

56.2k Shares

Colorado COVID-19 Data

COLORADO Department of Public Health & Environment

Data is updated daily by about 4 p.m. and includes cases reported through the previous day.
All data, for days past and present, is recalculated daily.

56,773 Cases	6,945 Hospitalized	63 Counties	695,569 People Tested	985,622 Test Encounters	1,937 Deaths Among Cases	1,843 Deaths Due to COVID-19	600 Outbreaks

Case Summary | Demographics | Tests | Hospital Data | Incidence and Epidemic Curve | Syndromic Data | Outbreaks | CDC Provisional Deaths

Cases | Deaths | Maps

Reported Day | Reported Week | Onset Day | Onset Week

Counts | Cumulative | 3-Day Average | 7-Day Average

Cases of COVID-19 in Colorado by Date Reported to the State

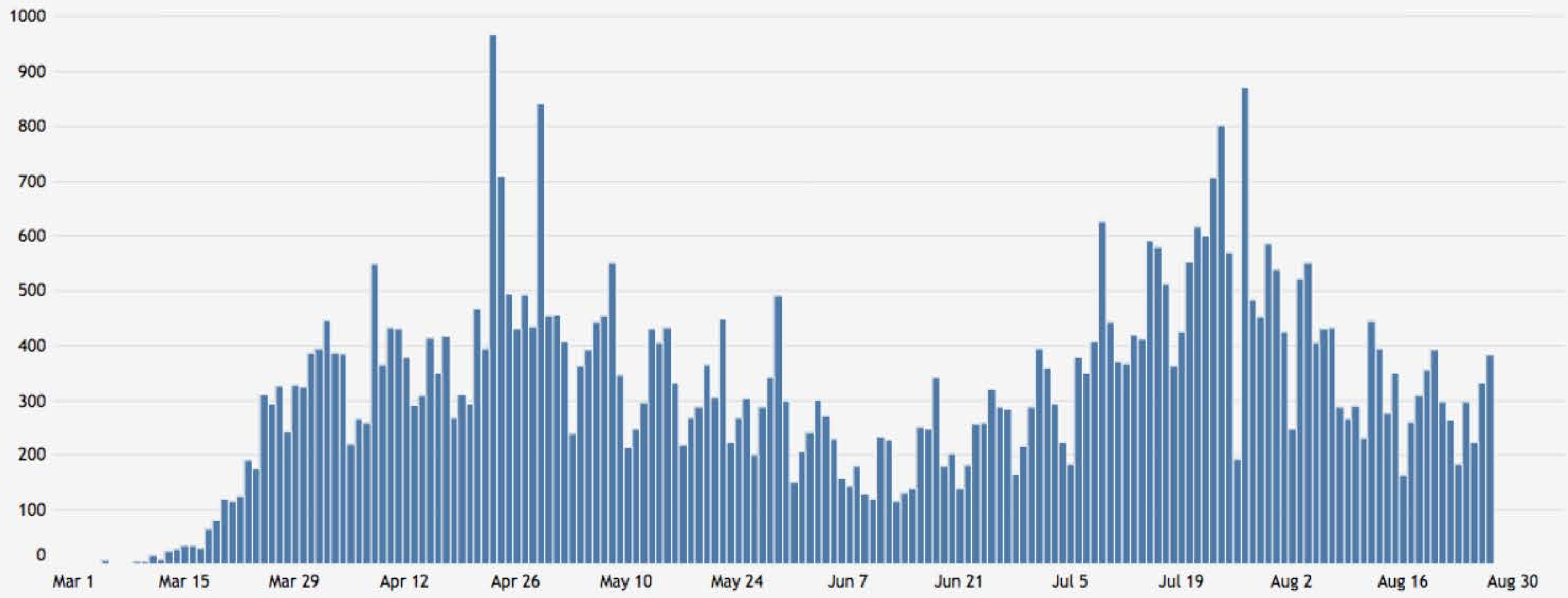

CDPHE

+ableau

Case summary snapshot | Hospital data snapshot | Cases and deaths by county

CDPHE Open Data Portal

Symptom Support Tool data

Outbreak data

Colorado public survey results

Colorado Department of Corrections COVID-19 Offender Case Dashboard

Additional resources

- Access the data files
- Learn more about the data
- COVID-19 Death Data and Resources
- CDC Provisional Death Counts for Coronavirus Disease (COVID-19)

Colorado COVID-19 Data

COLORADO Department of Public Health & Environment

Data is updated daily by about 4 p.m. and includes cases reported through the previous day.
All data, for days past and present, is recalculated daily.

56,773 Cases	6,945 Hospitalized	63 Counties	695,569 People Tested	985,622 Test Encounters	1,937 Deaths Among Cases	1,843 Deaths Due to COVID-19	600 Outbreaks

Case Summary | Demographics | Tests | Hospital Data | Incidence and Epidemic Curve | Syndromic Data | Outbreaks | CDC Provisional Deaths

Cases | Deaths | Maps

Daily | Weekly

Counts | Cumulative | 3-Day Average | 7-Day Average

7-Day Average of Deaths Among COVID-19 Cases in Colorado by Date of Death

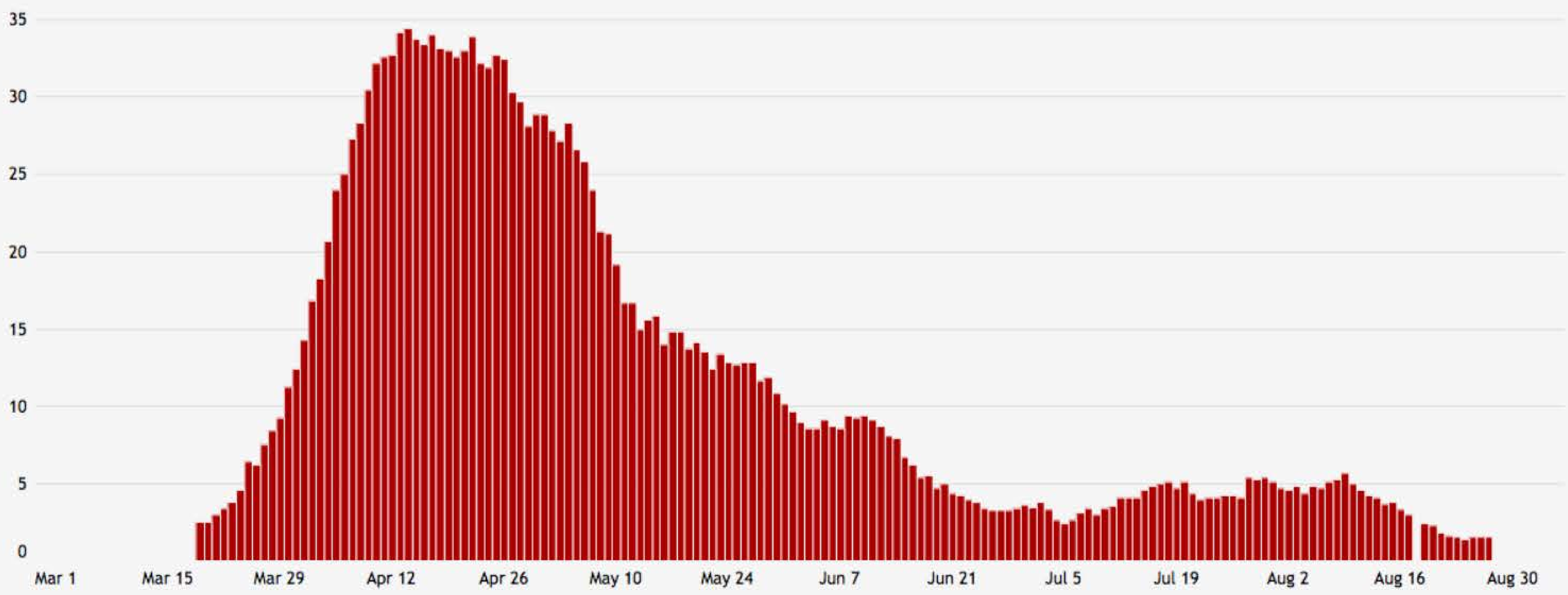

+ a b | e a u

- Case summary snapshot
- Hospital data snapshot
- Cases and deaths by county

CDPHE Open Data Portal

Symptom Support Tool data

Outbreak data

Colorado public survey results

Colorado Department of Corrections COVID-19 Offender Case Dashboard

Additional resources

- Access the data files
- Learn more about the data
- COVID-19 Death Data and Resources
- CDC Provisional Death Counts for Coronavirus Disease (COVID-19)

SUBSCRIBE NOW
$1 for 3 months. Save 97%.

SUBSCRIBE NOW
$1 for 3 months. Save 97%.

NEWS

COVID-19 in Colorado tracker: Larimer and state case, death and hospital data for August

Coloradoan staff

Published 7:56 p.m. MT Aug. 1, 2020 | Updated 4:54 p.m. MT Aug. 28, 2020

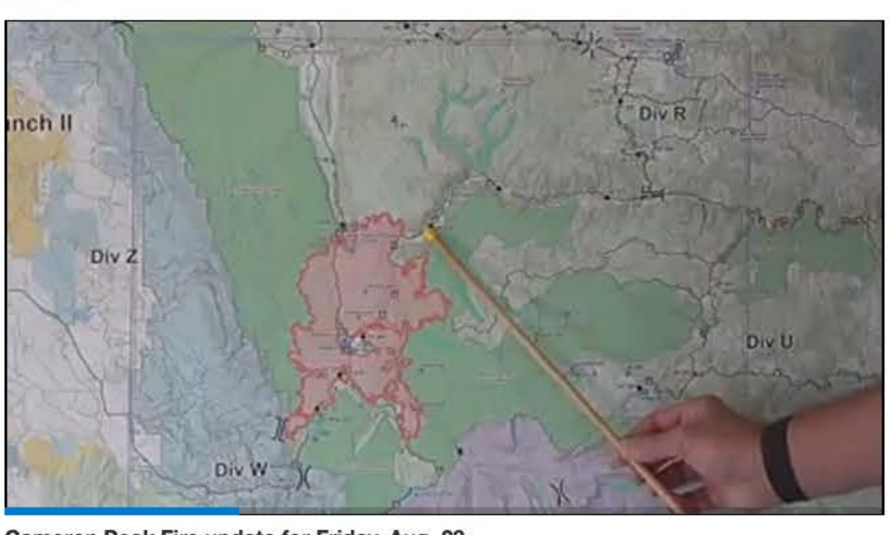

Cameron Peak Fire update for Friday, Aug. 28
Here's what the Centers for Disease Control and Prevention has to say about the use of cloth face coverings in preventing the spread of the coronavirus. *Fort Collins Coloradoan*

Notice our new look? As you get used to things, please let us know what you think!

As the number of confirmed coronavirus cases in Colorado grows, here are the latest updates and information.

Editor's note: *As the coronavirus outbreak continues to evolve, we don't want you to panic. In fact, quite the opposite. That's why the Coloradoan is committed to providing you with accurate, up-to-date information so you can make informed decisions on issues affecting you and the people you love. As such, this story, and many others, are being provided free for all to read. Help us continue this important work by subscribing to the Coloradoan.*

Here are the latest developments on coronavirus in Colorado:

[illegible] in Colorado will be added to the top of [illegible]

4 free articles left.

Note: This tracker includes updates beginning August 1. Looking for past updates?

► A timeline of major updates on coronavirus in Colorado

► Find updates on the spread of coronavirus in Colorado from July

► Find updates on the spread of coronavirus in Colorado from May and June

► Early updates at the beginning of the pandemic in Colorado: March and April

Friday, Aug. 28, 2020

Larimer County cases, deaths:

- New cases reported by county: 23 (Note: This is the number of new confirmed and probable cases reported since The Coloradoan last shared information from the county. This may vary from the "new positive cases" listed on the county's COVID dashboard.)
- New deaths reported by county: 0
- Confirmed cases: 1,814 (positive swab test)
- Probable cases: 53 (people with two of the following: COVID-19 symptoms; epidemiological link to someone who tested positive; positive antibody test)
- Suspect cases: 432 (asymptomatic with positive antibody test)
- Total deaths: 36
- Risk Index in county (based on things such as hospital utilization, new cases, etc.): Medium
- Number of COVID-19 patients in hospitals: 6 (includes Banner and UCHealth)*
- Hospital utilization: 65% (low risk is <70%)*
- ICU utilization: 74% (low risk is <70%)*

Source: Larimer County Department of Health and Environment

Colorado cases, deaths:

- New cases reported by state: 430
- New deaths reported by state (caused by COVID-19): 8
- Current hospitalizations: 134 (84% reporting)
- Hospital transfers or discharges in past 24 hours: 14
- Number of ventilators in use: 29%
- Hospitals anticipating a staff shortage in the next week: 5%
- Facilities anticipating personal protective equipment shortages in the next week: 1%
- Hospitals anticipating ICU bed shortages in the next week: 1%
- Total cases: 56,773
- Total hospitalizations: 6,945
- Total deaths due to coronavirus: 1,843
- Total outbreaks: 600 (reported at residential and nonhospital health care facilities, correctional settings and other facilities in Colorado, such as factories and group homes)

Source: Colorado Department of Public Health & Environment

Coronavirus cases in Larimer County

Confirmed and probable cases | Suspect (asymptomatic antibody) cases

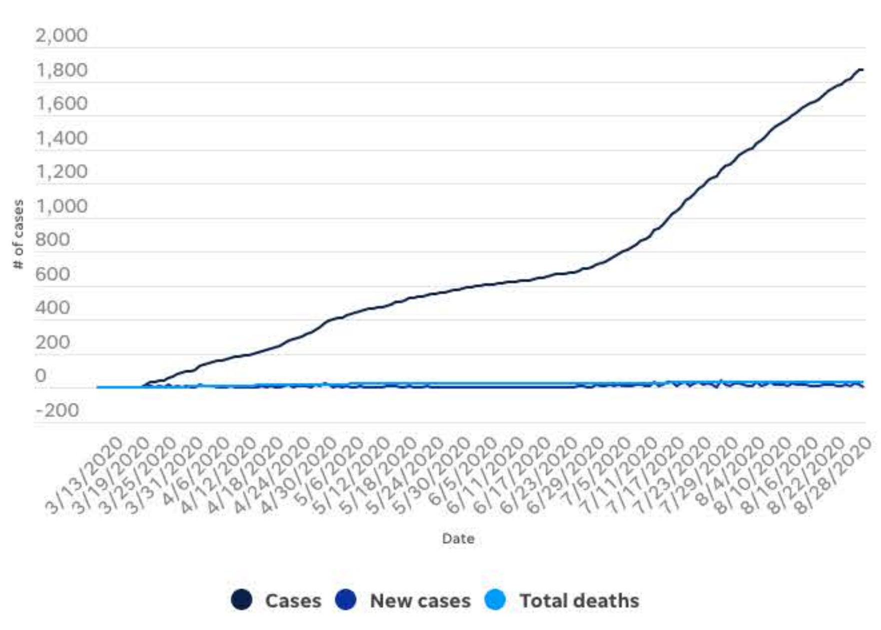

Last updated Aug. 28. Source: Larimer County Department of Health and Environment

The county has three definitions for case types: confirmed, probable and suspect.

- **Confirmed case**: Someone who has tested positive using a molecular amplification detection test (such as a nasal swab tested using PCR testing). This test determines if someone is currently ill and able to spread COVID-19 to others.
- **Probable case**:
  - Has COVID-19 symptoms or clinical characteristics AND epidemiologic evidence with no test confirming the diagnosis
  - Has COVID-19 symptoms or clinical characteristics AND has tested positive using an antigen or serologic (antibody) test
  - Has an epidemiological link based on risk from a known exposure AND has tested positive using an antigen or serologic (antibody) test
  - Has a death certificate that lists COVID-19 disease or SARS-CoV-2 as a cause of death or significant condition contributing to death without positive test results to confirm the diagnosis.
- **Suspect case**: Someone who has tested positive using a serology (antibody) test but who did not have COVID-19 symptoms or clinical characteristics. Antibodies can take several weeks to develop after being exposed so someone who has tested positive for antibodies has likely recovered and is no longer able to spread the virus at the time they are tested for antibodies.

Coronavirus cases by demographic

City | Age | Gender

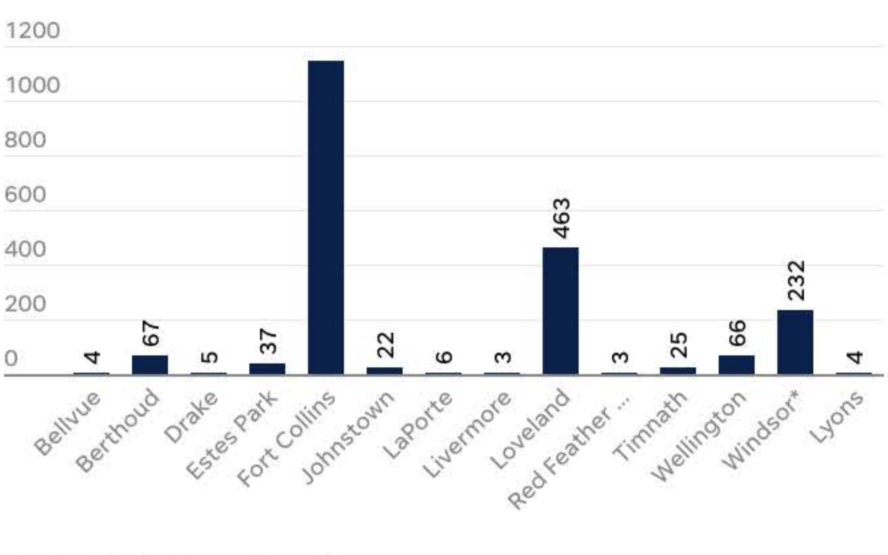

*Data for Windsor includes cases from Weld County.

Demographic data is for Confirmed and Probable cases only, and does not include antibody test results.

Share

Coronavirus cases in Colorado by day

New Deaths | New cases | Current Hospitalizations

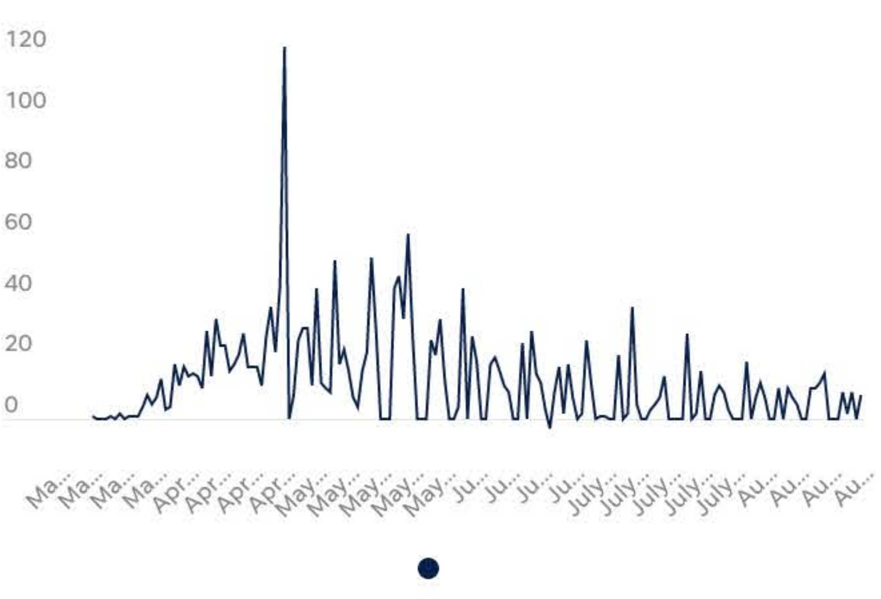

Total Cases | Total Deaths

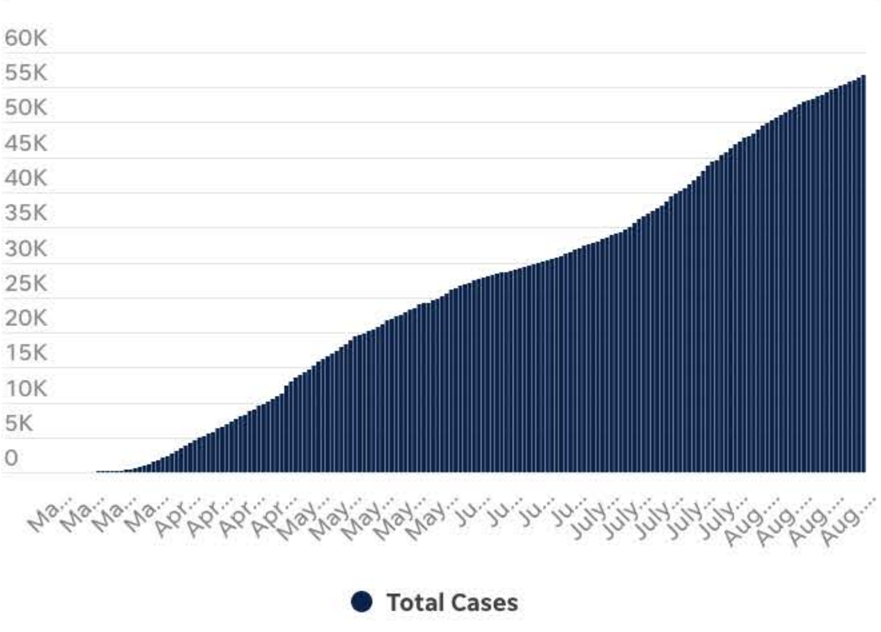

**** Starting with May 15, deaths are defined as people who died as a result of COVID-19. Prior to that date, deaths included anyone who had died and tested positive for coronavirus, regardless of their cause of death. This why you see a change of zero and a dip in total cases on May 15. Unfortunately, this data is not available retroactively.*

*** The April 24/25 reporting figures for deaths may be skewed by retroactive tallying of deaths from coronavirus on previous dates that had not yet been accounted for, Gov. Polis said Friday.*

**The chart here reflects when data was released by the Colorado Department of Health and Environment. On March 20, the health department said it releases data daily at 4 p.m. for the day prior. For example, data on March 20 here represents March 19 cases.*

The state defines cases as "anyone who has tested positive for COVID-19 and anyone who is symptomatic and has had close contact with someone who tested positive."

Hospitalization data is provided to the state of Colorado by hospitals through the Colorado Hospital Association. Not all hospitals provide data, and the percentage of hospitals reporting data each day varies.

Share

Coronavirus cases in Colorado

Hover over the county to see how many confirmed cases there are in that location.

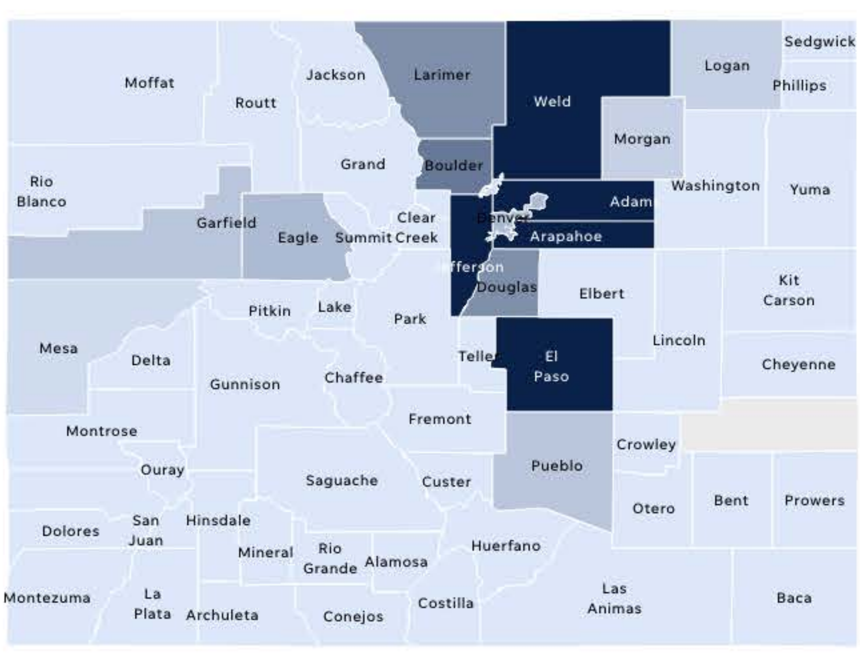

Download data

Last updated 8/28/2020

The health department notes 20 cases as international and therefore not reflected on this map.

Larimer case counts are based on state data, to give an accurate comparison by county.

Death data provided on the county level by the state includes all deaths among COVID-19 cases, regardless of if COVID-19 is listed as a cause of death. The state only provides the number of coronavirus-caused deaths as a total for the state as a whole.

From the health department: Please note initial information may change as more information is collected on each patient.

Share

Thursday, Aug. 27, 2020

Larimer County cases, deaths:

- New cases reported by county: 26 (Note: This is the number of new confirmed and probable cases reported since The Coloradoan last shared information from the county. This may vary from the "new positive cases" listed on the county's COVID dashboard.)
- New deaths reported by county: 0
- Confirmed cases: 1,790 (positive swab test)
- Probable cases: 52 (people with two of the following: COVID-19 symptoms; epidemiological link to someone who tested positive; positive antibody test)
- Suspect cases: 431 (asymptomatic with positive antibody test)
- Total deaths: 36
- Risk Index in county (based on things such as hospital utilization, new cases, etc.): Medium

NEWS › HEALTH · News

Colorado coronavirus tracker: How many new cases are being reported, and where are they?

The charts and maps will be updated daily

By **KEVIN HAMM** | khamm@denverpost.com | The Denver Post
PUBLISHED: March 6, 2020 at 5:41 p.m. | UPDATED: July 28, 2020 at 11:51 a.m.

97

SIGN UP FOR NEWSLETTERS AND ALERTS
SUBMIT YOUR NEWS TIPS OR PHOTOS

MOST POPULAR
1 5 tips for making remote education a success, whether you're a student, parent or teacher
2 The world knows his name now: One year later, how Elijah McClain's death impacted his friends and catalyzed a movement
3 Denver residents to vote on repeal of pit bull ban; Aurora postpones similar decision
4 Kiszla: After loss pushes Nuggets to brink of playoff elimination, what do we say to their championship dreams? Bye, Felicia.
5 Nearly 200 townhomes coming to former CDOT complex in southeast Denver
6 Editorial: Fracas at Denver encampment captures bad behavior by all

COVID-19 in Colorado

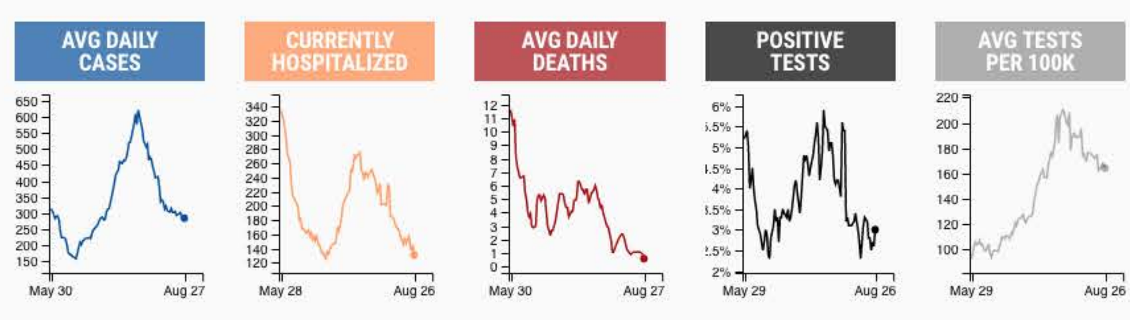

Cases, deaths and hospitalizations

HOW TO READ THE CHARTS

Please note that each chart uses two different data sets: The lines show data by reported date and the bars show data by symptom onset date or date of death.

Lines: *Best for showing trend.* The line charts show when cases, deaths and hospitalizations were reported to the Colorado Department of Public Health and Environment. The lag time between when those occurred and when they were reported to the state can be several days. The chart uses a 7-day moving average to smooth out peaks and valleys in the data due to reporting lag times.

Bars: *Best for showing historical data.* The bar charts show when cases and hospitalizations occurred based on CDPHE's investigation into the onset date of symptoms, and when a death occurred based on the date on the death certificate. **Data for most recent two weeks is not shown because it will be signifcantly revised** with subsequent data releases as CDPHE investigations progress, and is not an accurate representation of recent cases, deaths and hospitalizations.

Totals: The total number of cases, deaths and hospitalizations that have been reported to the CDPHE.

Hover over legend item to highlight, click to add/remove; hover over lines for details
Gray vertical line indicates the end of the stay-at-home order

Charts updated Saturday, August 29

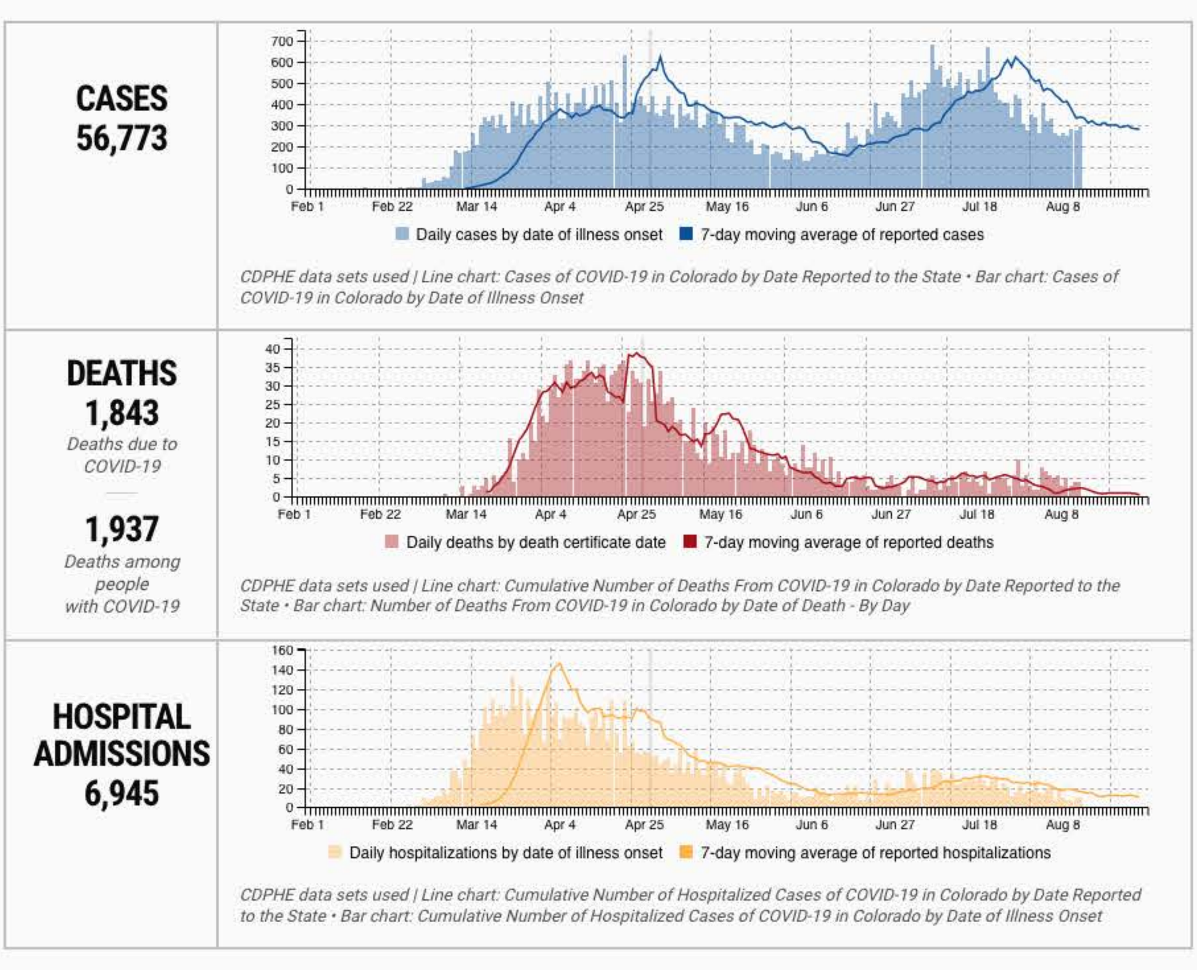

People currently hospitalized

This chart shows the number of people currently hospitalized with either a confirmed or suspected case of COVID-19.

Hover over legend item to highlight, click to add/remove; hover over bars for details

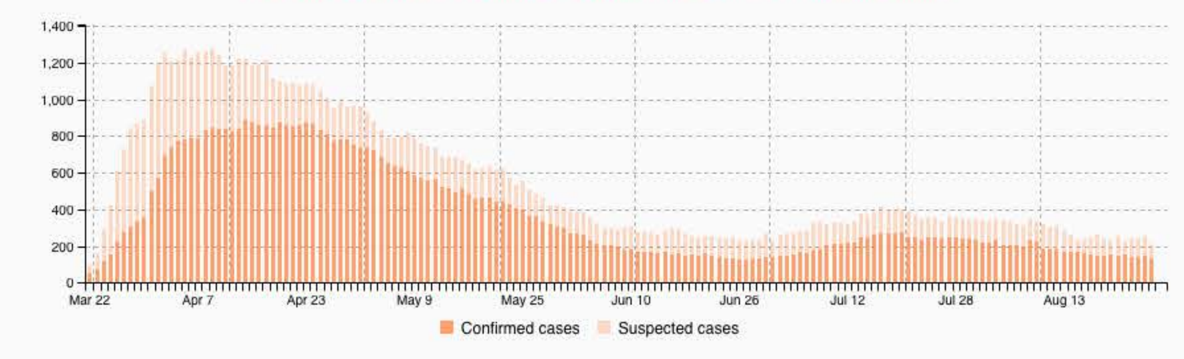

CDPHE data set used | Colorado COVID-19 Hospital Data

Testing rate and results

HOW TO READ THE CHARTS

Bar: The bar chart shows the number of daily tests per 100,000 people, as reported to the CDPHE. Health officials say a testing rate of 152 per 100,000 is necessary to identify the majority of people who are infected.

Line: The line chart shows a 3-day moving average of the percentage of daily tests for COVID-19 that were positive.

Hover over legend item to highlight, click to add/remove; hover over lines for details

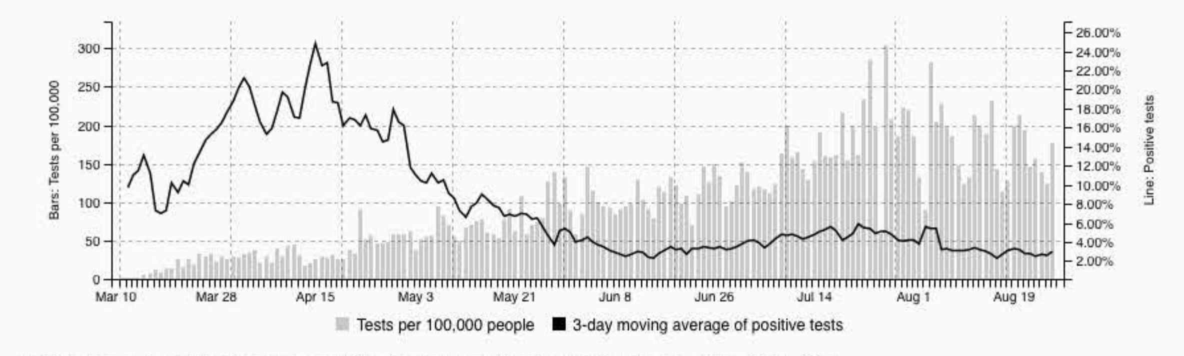

CDPHE data set used | Bar: Total Tests per 100k People | Line: Three-day Moving Average of Percent Positivity

Case and death rates for counties

These charts compare the total case and death rates per 100,000 people for Colorado's 10 most-populous counties using a 7-day moving average of cases and deaths as they are reported to CDPHE. Rates are useful for comparing areas with different populations.

Hover over legend item to highlight, click to add/remove; hover over lines for details
Gray vertical line indicates the end of the stay-at-home order

Cases per 100,000 people

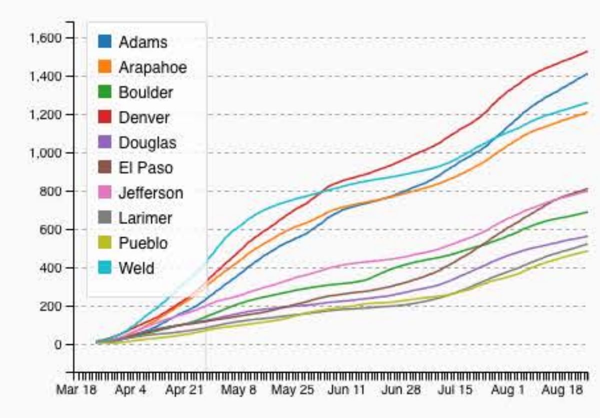

Deaths per 100,000 people

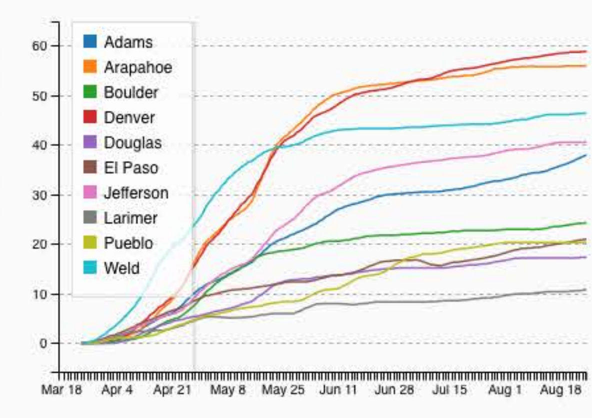

These charts compare total cases and deaths for Colorado's 10 most-populous counties.

Total cases
County (population rank)

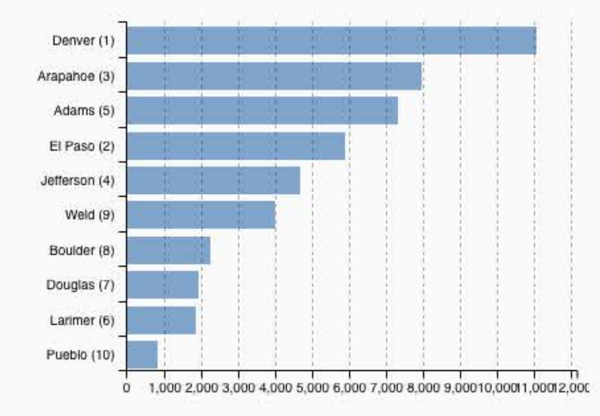

Total deaths
County (population rank)

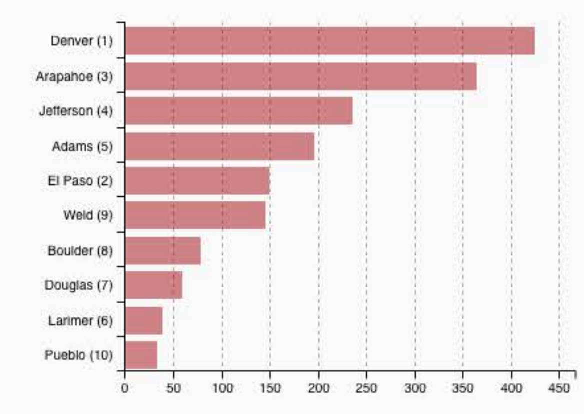

CDPHE data sets used | Colorado Case Counts by County, Number of Deaths by County

The first map shows the total number of cases and deaths by county and the second shows the case rate per 100,000 by county (rate will be slightly different than chart above because it is not a 7-day moving average).

Circles are centered in counties and are not meant to indicate an actual location; click circles for details

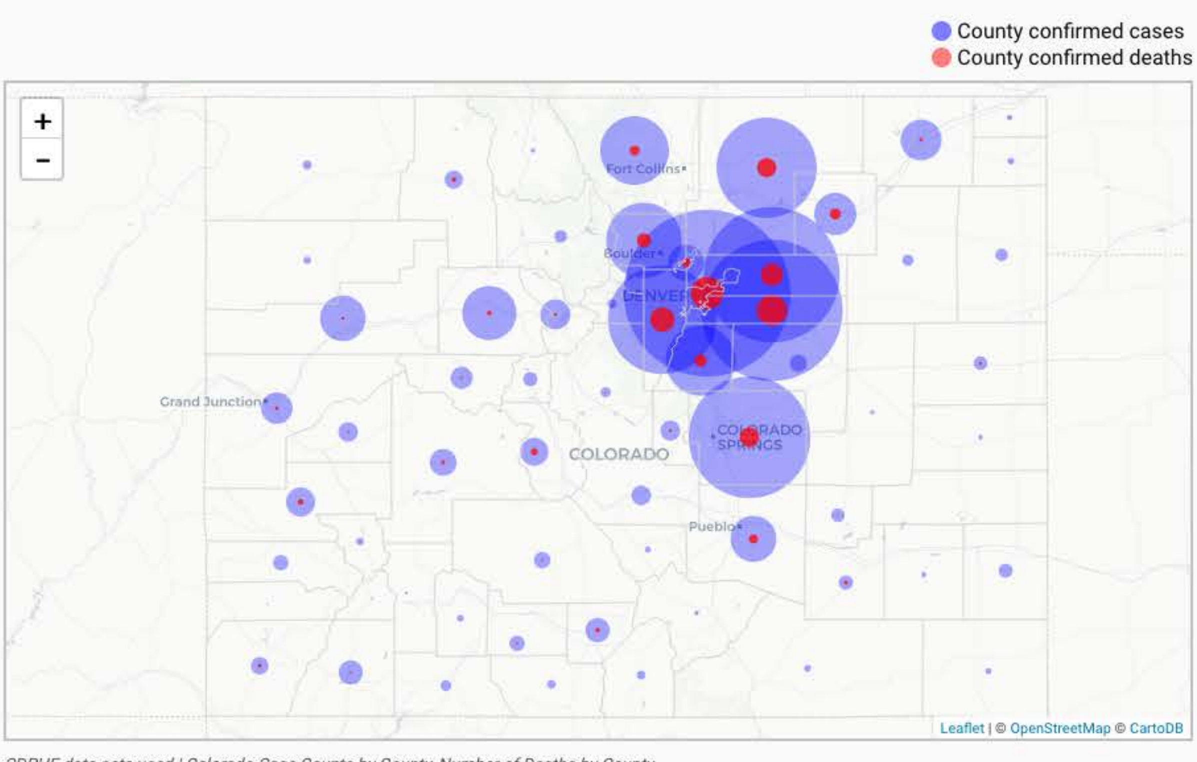

CDPHE data sets used | Colorado Case Counts by County, Number of Deaths by County

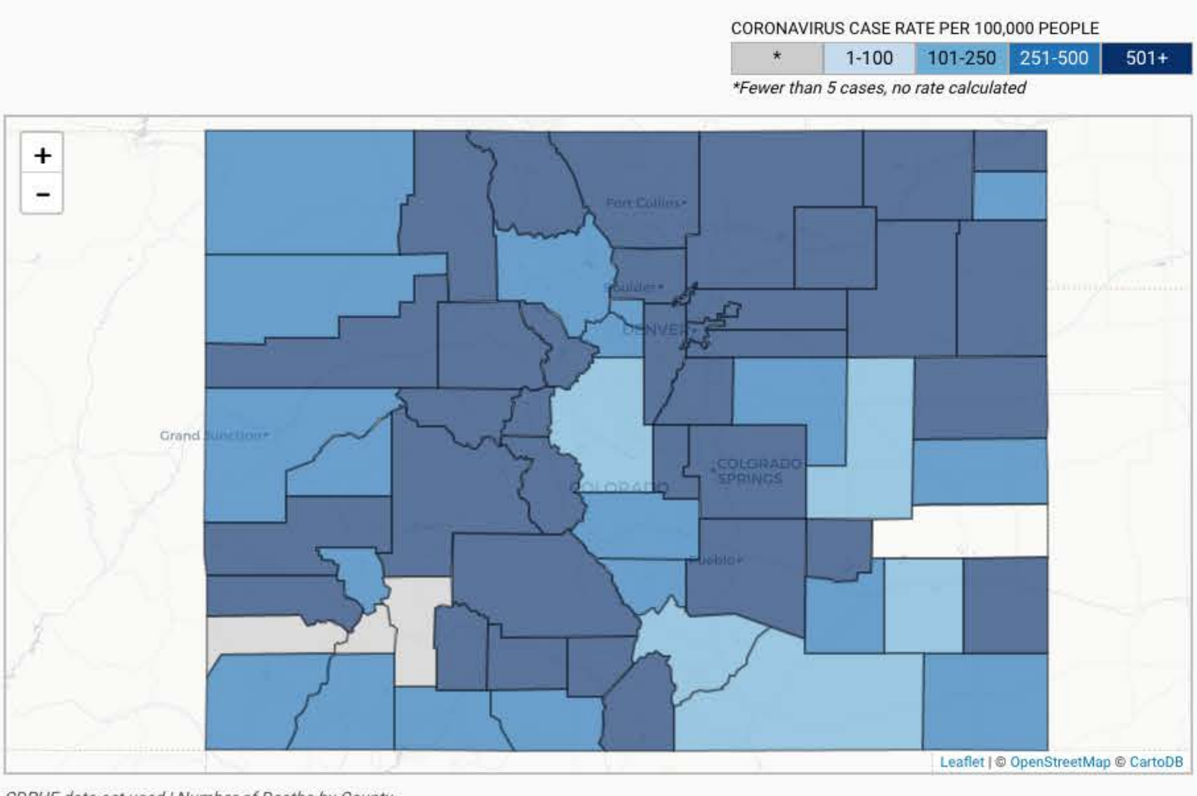

CDPHE data set used | Number of Deaths by County

Cases and deaths by race

These charts show the percentage of statewide cases, deaths and population broken down by race and ethnicity. The dark gray bar shows the percentage of the state's population that race makes up; a cases or deaths bar shorter than the population bar indicates that race is underrepresented, a bar longer than the population bar indicates a race is overrepresented.

Hover over legend item to highlight, click to add/remove; hover over lines for details

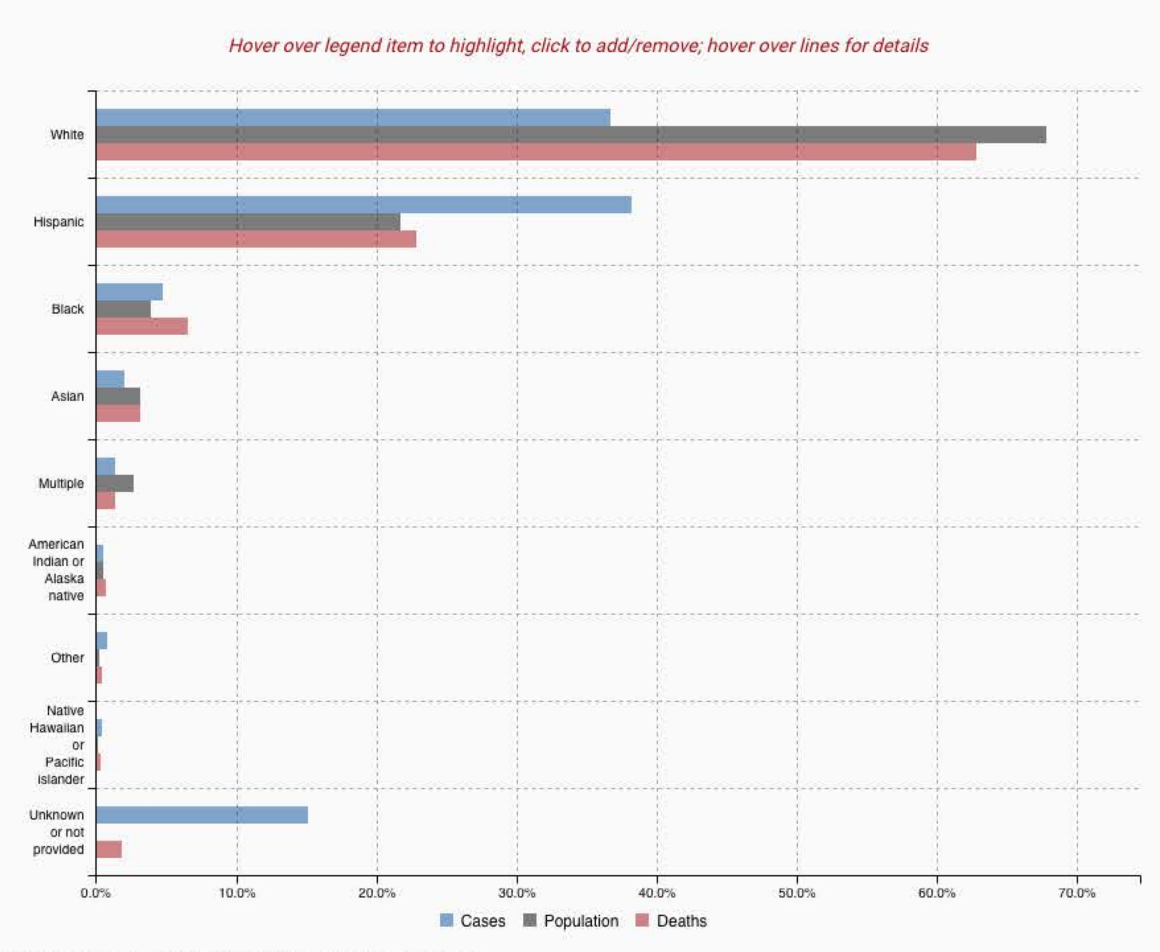

CDPHE data set used | COVID-19 in Colorado by Race & Ethnicity

Cases, hospitalizations and deaths by age

These charts show the total number of cases by age group and breaks them down by the number of people who have been hospitalized, the number who haven't been hospitalized and the number who have died. The second chart shows the same information as percentages of total cases by age group.

Hover over legend item to highlight, click to add/remove; hover over lines for details

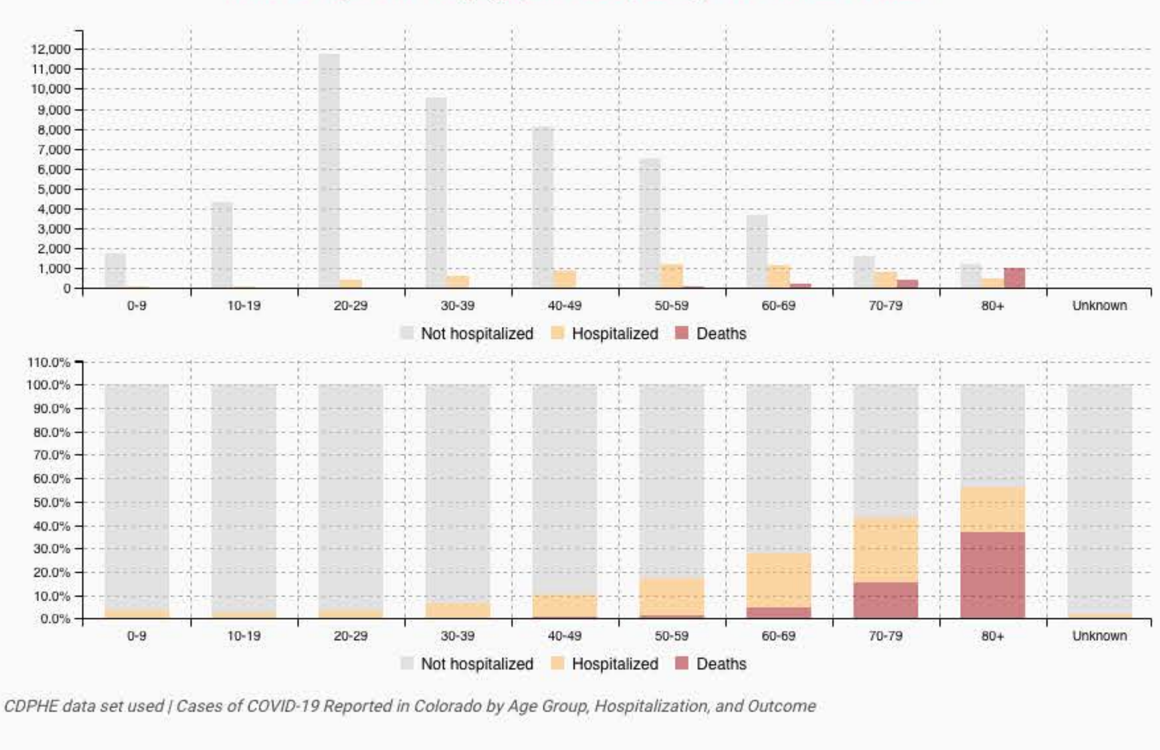

CDPHE data set used | Cases of COVID-19 Reported in Colorado by Age Group, Hospitalization, and Outcome

Stay informed about coronavirus (COVID-19): Connecticut residents are urged to continue taking precautions to prevent the spread of COVID-19. For the latest information, visit **ct.gov/coronavirus**.

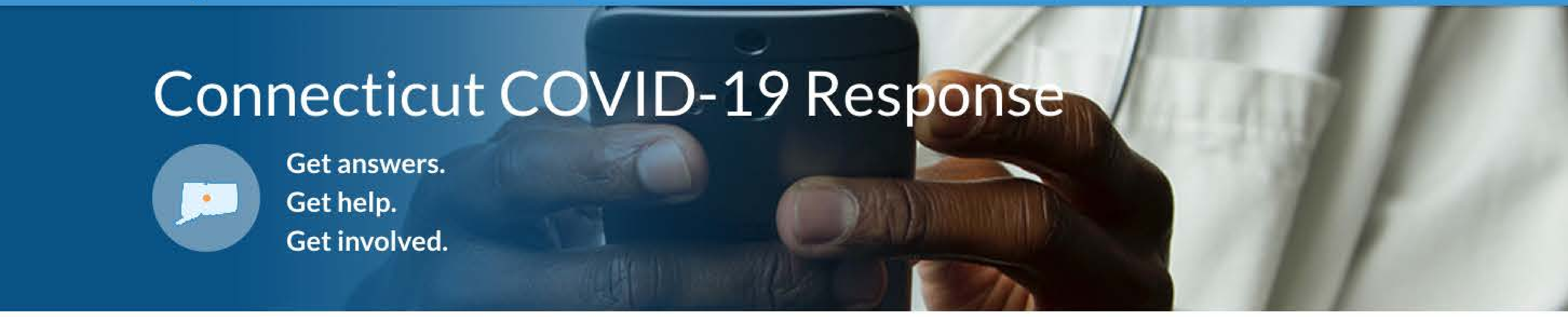

Connecticut COVID-19 Data Tracker

DAILY DATA REPORT
FOR CONNECTICUT
LATEST UPDATE: AUGUST 28, 2020 | 4:00 P.M.

Reporting schedule: The State of Connecticut's COVID-19 metric report is issued once per day, every Monday through Friday. The report that is issued each Monday contains combined data that was collected on Friday, Saturday, and Sunday.

Open data portal: Supplemental data on COVID-19 in Connecticut can be found on the state's open data portal, **data.ct.gov**, in the **COVID-19 data portal**. Data presented on the open data portal is available in its rawest form.

Town Map

Connecticut Towns with Cases of COVID-19

Click town for additional information

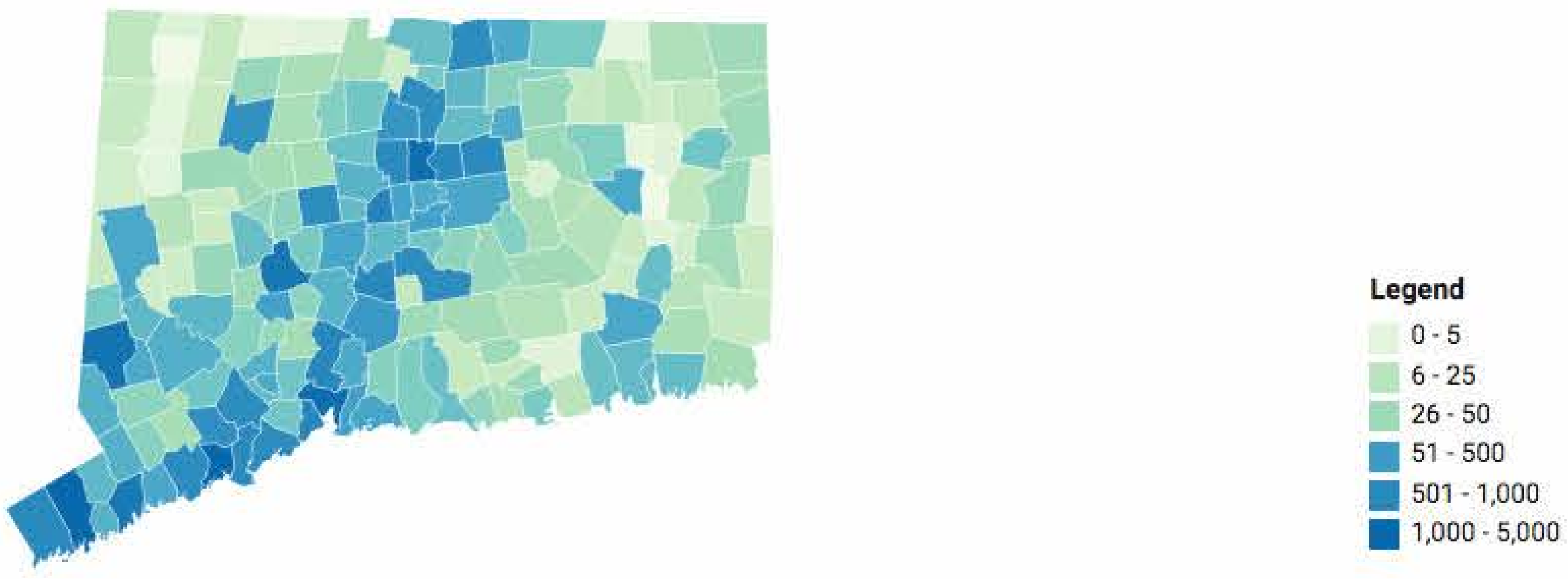

COVID-19 cases and associated deaths include confirmed plus probable.
Source: CT Department of Public Health • Get the data • Created with Datawrapper

State Summary

Connecticut COVID-19 Summary

Summary for the most recent day of reporting. Total COVID-19 Cases and Associated Deaths include confirmed plus probable.

Measure	Total	ChangeDirection	Change
Total COVID-19 Cases	52,495	+	145
Total COVID-19-Associated Deaths	4,465	+	0
Patients Currently Hospitalized with COVID-19	60	+	4
COVID-19 PCR Tests Reported	1,125,398	+	13,997

The CT Summary table represents the date the data were reported to the CT DPH. Cases and deaths are cumulative over time. Hospitalization data are collected by the CT Hospital Association.
Source: CT Department of Public Health • Get the data • Created with Datawrapper

Age Group Chart

Number of COVID-19 Cases, Associated Deaths, and Rates per 100,000 by Age Groups

Total Number of Cases

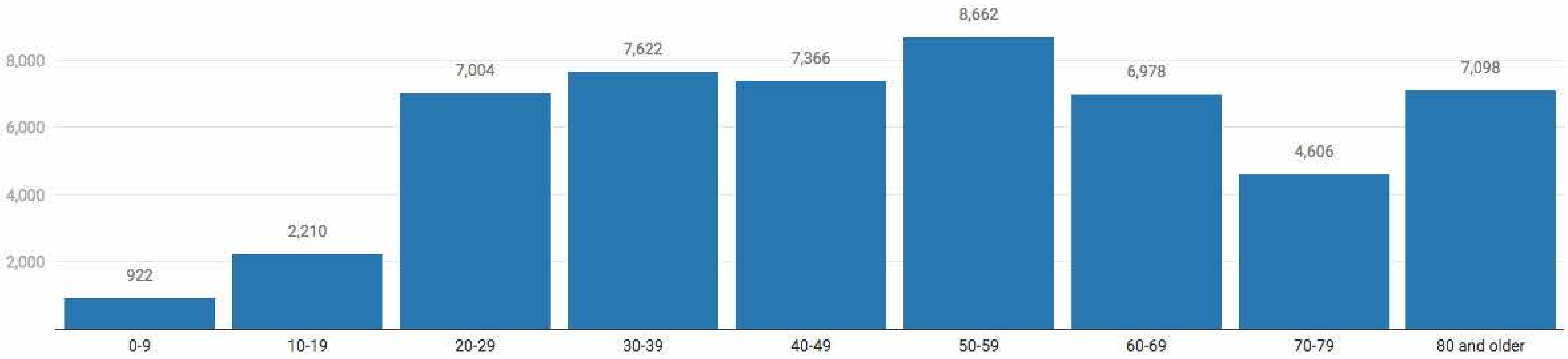

Total COVID-19 cases and associated deaths include confirmed plus probable.
Source: CT Department of Public Health • Get the data • Created with Datawrapper

County Chart

COVID-19 Cases, Rate per 100,000, Patients Currently Hospitalized, and Associated Deaths by County

Cases and deaths are cumulative over time

Number of Total Cases

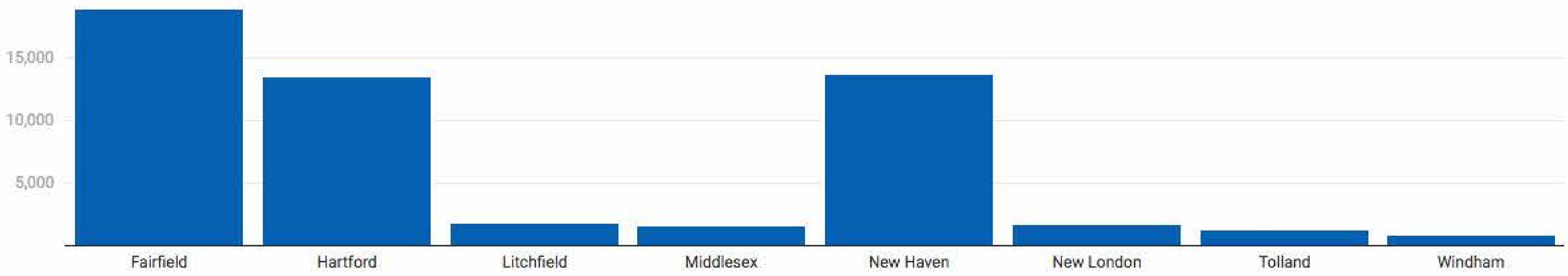

COVID-19 cases, rate per 100,000, and associated deaths are by county of residence. Hospitalization data are collected by CT Hospital Association and represent the number of patients currently hospitalized by location of the hospital and not the patient residence.
Source: CT Department of Public Health • Get the data • Created with Datawrapper

+tableau public GALLERY AUTHORS BLOG RESOURCES ACTIVITY ABOUT SIGN UP SIGN IN

< Connecticut State Data Center - Profile Favorite

CT data collaborative

Connecticut Covid-19 Tracking

This dashboard is updated daily with Covid-19 data from Connecticut Department of Public Health. Data only includes confirmed and reported numbers and are preliminary and subject to change.

As of August 26, 2020, this dashboard has replaced our previous Covid-19 dashboard.

Questions? Comments? Contact Jason Cheung: jc@ctdata.org

Navigate Our Dashboard

Main Dashboard

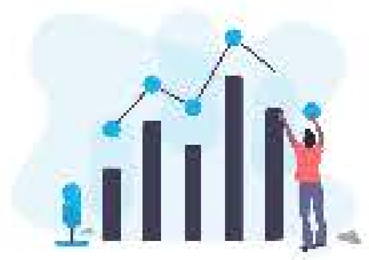

Town and County

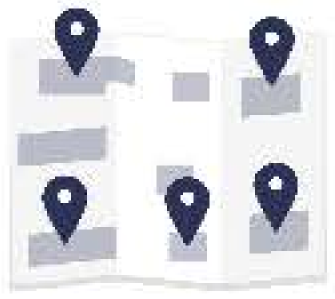

Demographics

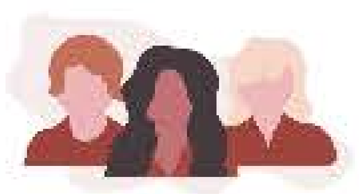

52,350 (+130) CONFIRMED CASES	56 (-1) HOSPITALIZATIONS	4,465 (+2) DEATHS
1,111,401 (+15,452) TESTS COMPLETED	0.25% (-0.10%) POSITIVE TEST RATE	0.96 TRANSMISSION RATE

Select an indicator box above

Daily Confirmed Cases

New laboratory-confirmed and probable Covid-19 cases reported among Connecticut residents, as reported by CT DPH
New cases are calculated by subtracting the previous days data from the most recent day

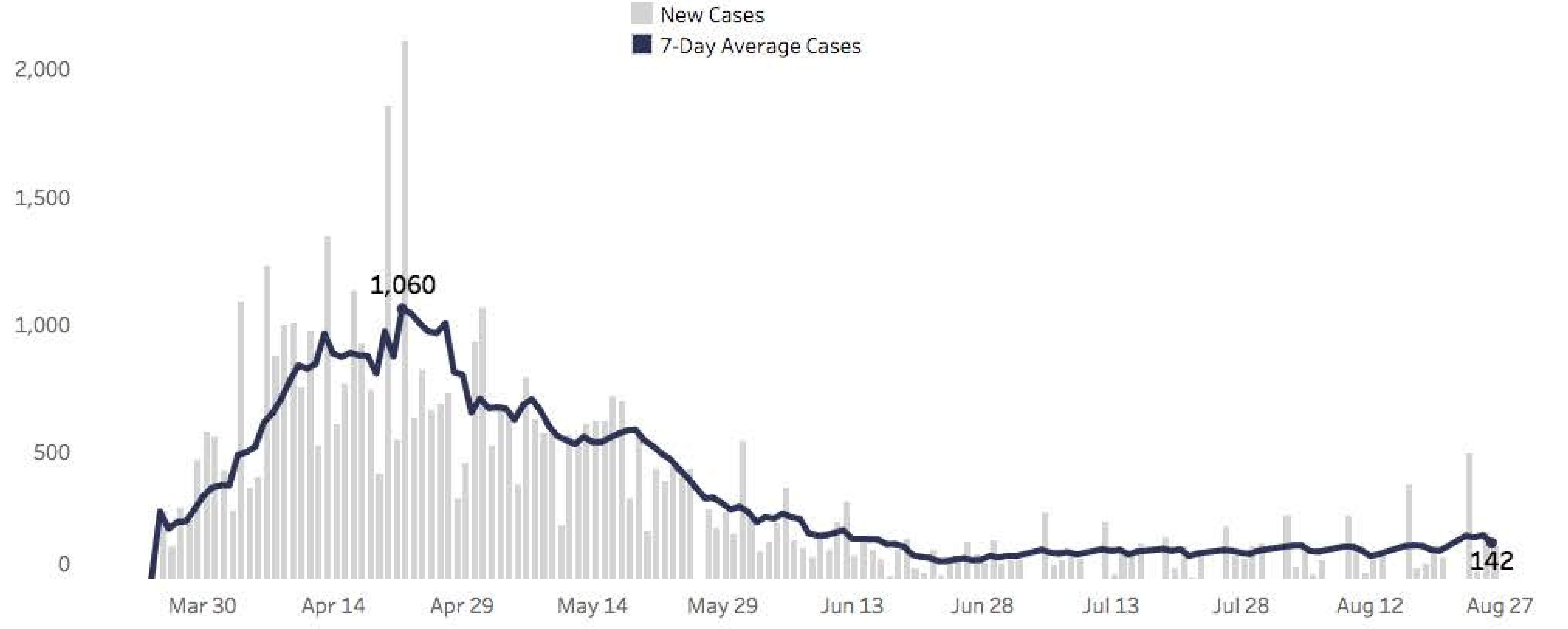

As of July 2020, CT DPH only updates their data on business days, which leads to generally larger increases on Mondays

+tableau

Connecticut Covid-19 Case Tracking

Viz Author: Connecticut State Data Center

45,144 Views ☆16

More Detail

Originally Published: Mar 31, 2020
Last Updated: Aug 28, 2020
Workbook Details: 10 Sheets
Metadata: CTdataCollaborativeCOVID-19
Hosp_Dash
Deaths_Dash
Tests_Dash
PosRate_Dash
Rt_Dash
TownCounty
Demographics_Main
Demographics_RaceEth

+ableau public   GALLERY   AUTHORS   BLOG   RESOURCES   ACTIVITY   ABOUT   SIGN UP   SIGN IN

‹ Connecticut State Data Center - Profile   ☆ Favorite

CT data collaborative

Connecticut Covid-19 Tracking

This dashboard is updated daily with Covid-19 data from Connecticut Department of Public Health. Data only includes confirmed and reported numbers and are preliminary and subject to change.

As of August 26, 2020, this dashboard has replaced our previous Covid-19 dashboard.

Questions? Comments? Contact Jason Cheung: jc@ctdata.org

Navigate Our Dashboard

Main Dashboard

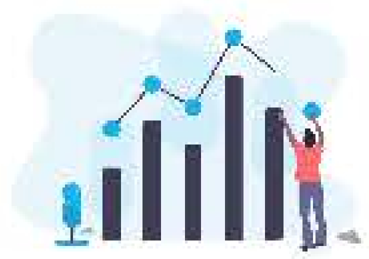

Town and County

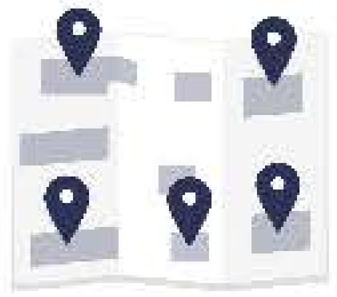

Demographics

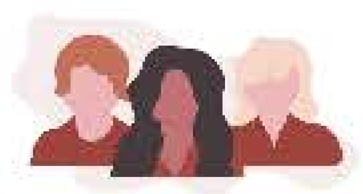

52,350 (+130) CONFIRMED CASES	56 (-1) HOSPITALIZATIONS	4,465 (+2) DEATHS
1,111,401 (+15,452) TESTS COMPLETED	0.25% (-0.10%) POSITIVE TEST RATE	0.96 TRANSMISSION RATE

Select an indicator box above

Daily Deaths

The number of confirmed and probable Covid-19 deaths, as reported to either the Office of the Chief Medical Examiner or CT DPH

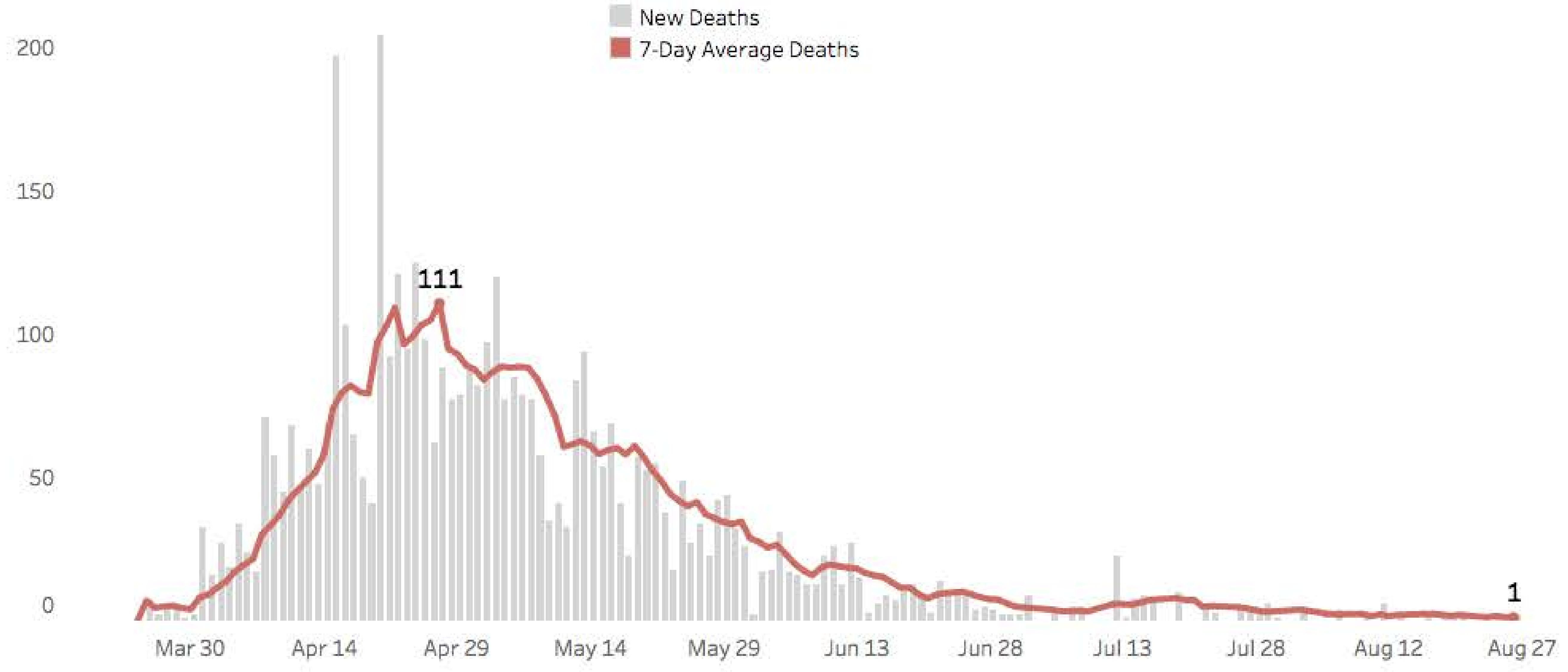

As of July 2020, CT DPH only updates their data on business days, which leads to generally larger increases on Mondays

+ableau

Connecticut Covid-19 Case Tracking

Viz Author: Connecticut State Data Center

45,144 Views ☆ 16

More Detail

Originally Published: Mar 31, 2020

Last Updated: Aug 28, 2020

Workbook Details: 10 Sheets

Metadata: CTdataCollaborativeCOVID-19
Hosp_Dash
Deaths_Dash
Tests_Dash
PosRate_Dash
Rt_Dash
TownCounty
Demographics_Main
Demographics_RaceEth

+ableau public   GALLERY   AUTHORS   BLOG   RESOURCES   ACTIVITY   ABOUT   SIGN UP   SIGN IN

< Connecticut State Data Center - Profile   Favorite

CT data collaborative

Connecticut Covid-19 Tracking

This dashboard is updated daily with Covid-19 data from Connecticut Department of Public Health. Data only includes confirmed and reported numbers and are preliminary and subject to change.

As of August 26, 2020, this dashboard has replaced our previous Covid-19 dashboard.

Questions? Comments? Contact Jason Cheung: jc@ctdata.org

Navigate Our Dashboard

Main Dashboard

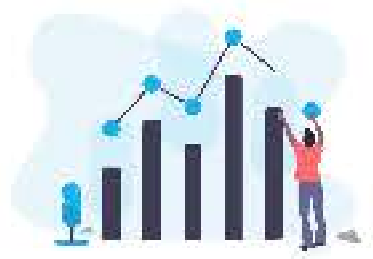

Town and County

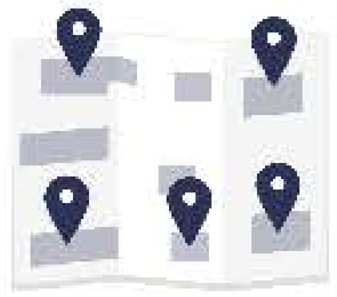

Demographics

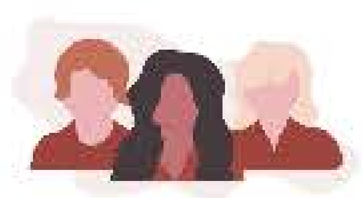

52,350 (+130) CONFIRMED CASES	56 (-1) HOSPITALIZATIONS	4,465 (+2) DEATHS
1,111,401 (+15,452) TESTS COMPLETED	0.25% (-0.10%) POSITIVE TEST RATE	0.96 TRANSMISSION RATE

Select an indicator box above

Daily Tests Completed

The number of new Covid-19 PCR tests completed each day

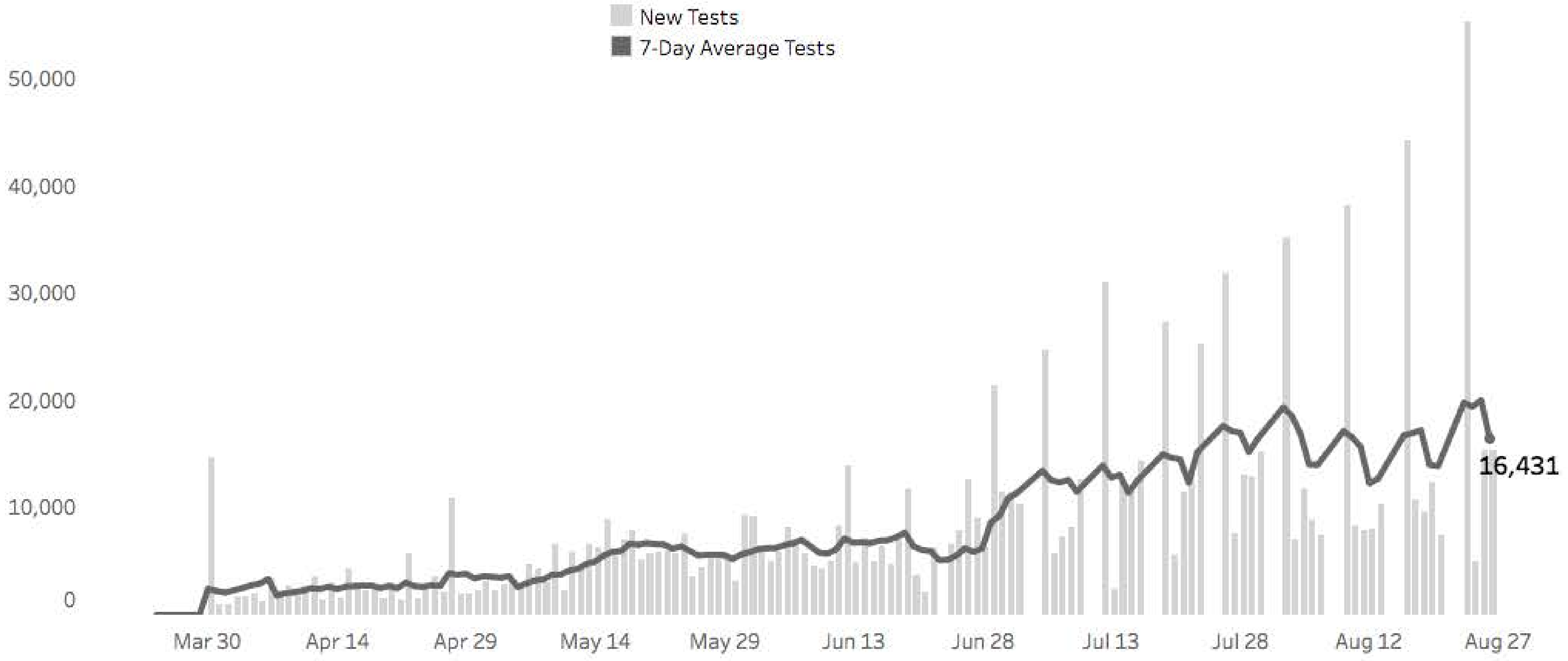

As of July 2020, CT DPH only updates their data on business days, which leads to generally larger increases on Mondays

+ableau

Connecticut Covid-19 Case Tracking

Viz Author: Connecticut State Data Center

45,144 Views ☆16

More Detail

Originally Published: Mar 31, 2020

Last Updated: Aug 28, 2020

Workbook Details: 10 Sheets

Metadata: CTdataCollaborativeCOVID-19
Hosp_Dash
Deaths_Dash
Tests_Dash
PosRate_Dash
Rt_Dash
TownCounty
Demographics_Main
Demographics_RaceEth

Cases of COVID-19 Reported in Connecticut by Town by the Department of Geography at UConn

Last Updated at 8/28/2020, 6:30 PM

Total Confirmed
52,495

Increase Cases
145
as of previous day

Total Deaths
4,465

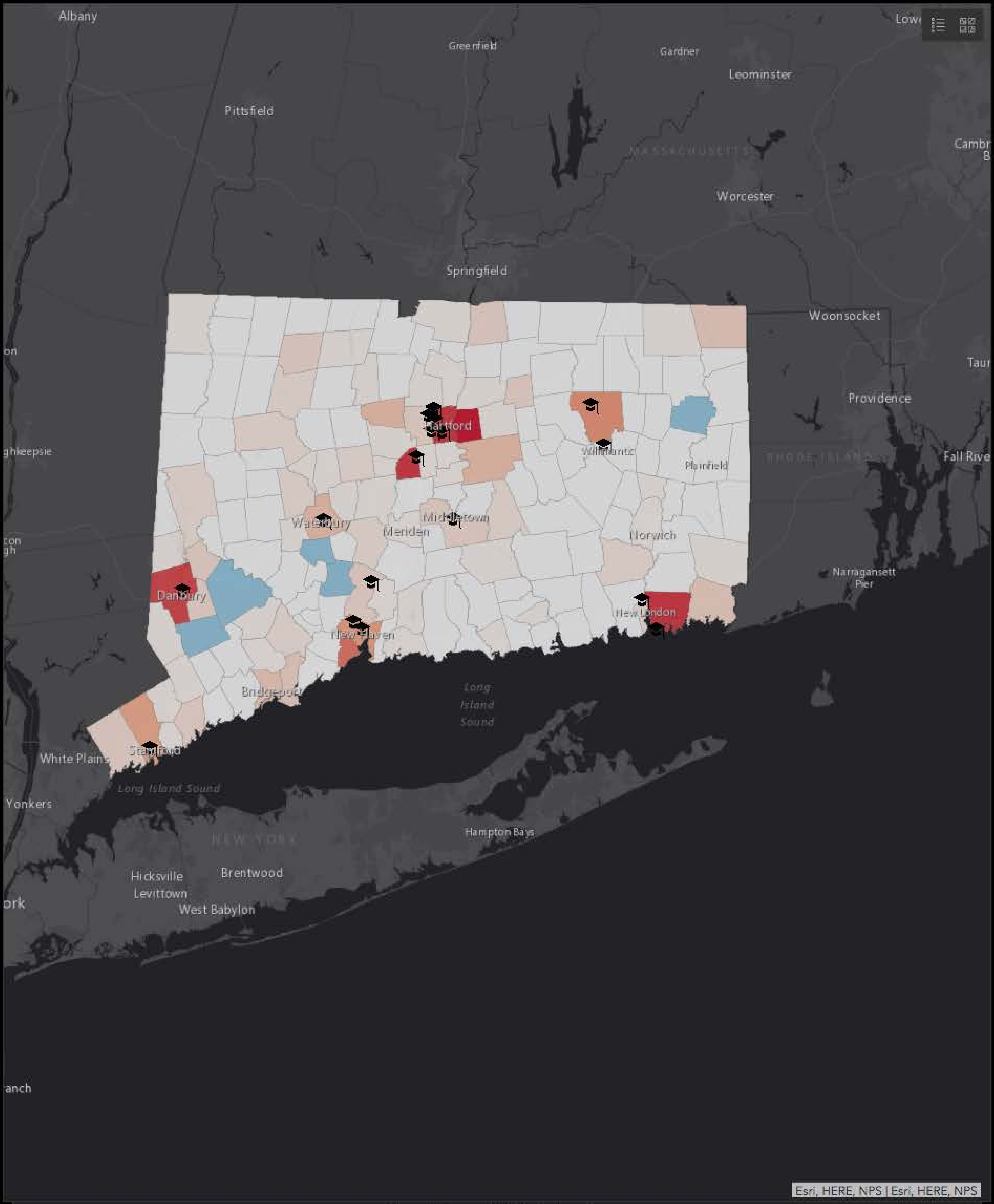

Change in Cases with University Locations

For sources and contact information please click upper right button in this box

Contact
Debarchana (Debs) Ghosh, PhD

Deaths per 100,000 People by Age

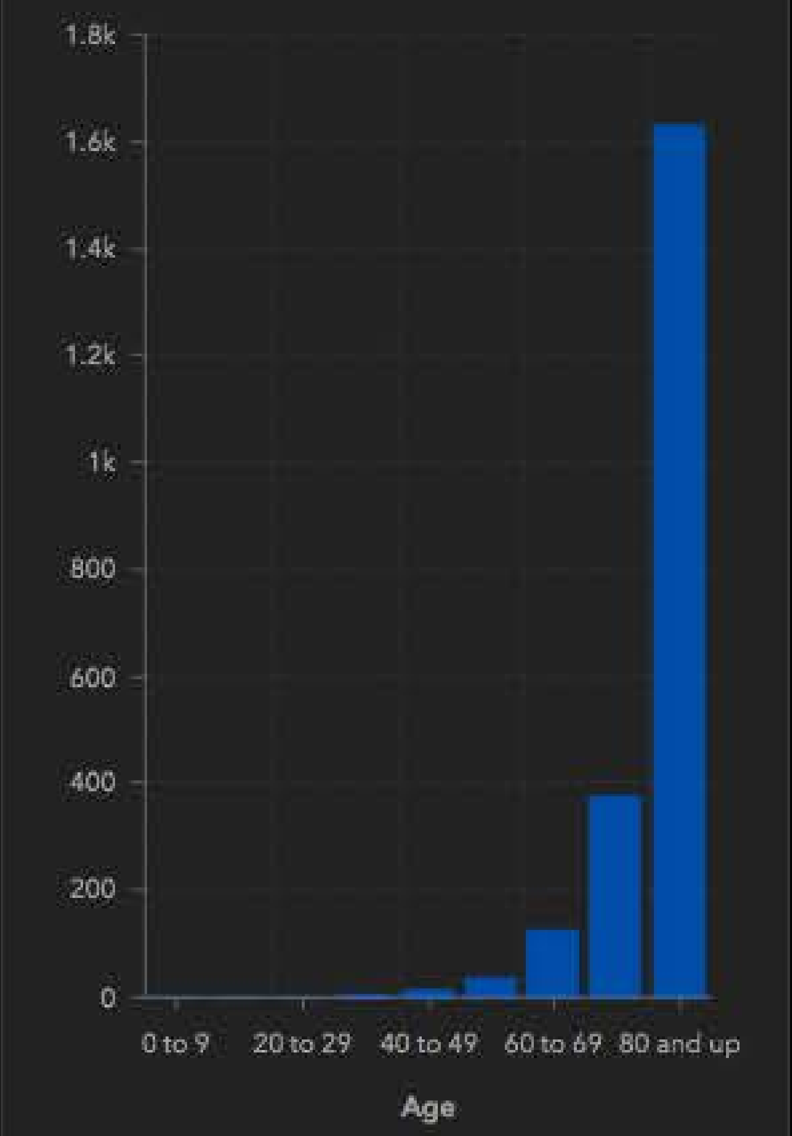

Deaths by Age | Cases by Age

Connecticut Towns

9	Andover
304	Ansonia
23	Ashford
164	Avon
33	Barkhamsted
60	Beacon Falls
192	Berlin
39	Bethany
302	Bethel
13	Bethlehem
557	Bloomfield
24	Bolton
12	Bozrah
369	Branford
4,071	Bridgeport
12	Bridgewater
677	Bristol
201	Brookfield
146	Brooklyn
40	Burlington
20	Canterbury
99	Canton
4	Chaplin
241	Cheshire
[illegible]	Chester

Towns | Counties

Listen

Coronavirus Data

August 28, 2020:

- Total Overall Tested: 285,756*
- Total Number of DC Residents Tested: 177,109*
- Total Positives: 13,925
- Total Lives Lost: 605
- Cleared From Isolation: 11,046

*All data are preliminary and are subject to change based on additional reporting.

- Download copy of DC COVID-19 data

Other Data

- Public Safety Agency Data
- Human Services Agency Data
- DMV Agency Data
- Hospital Status Data

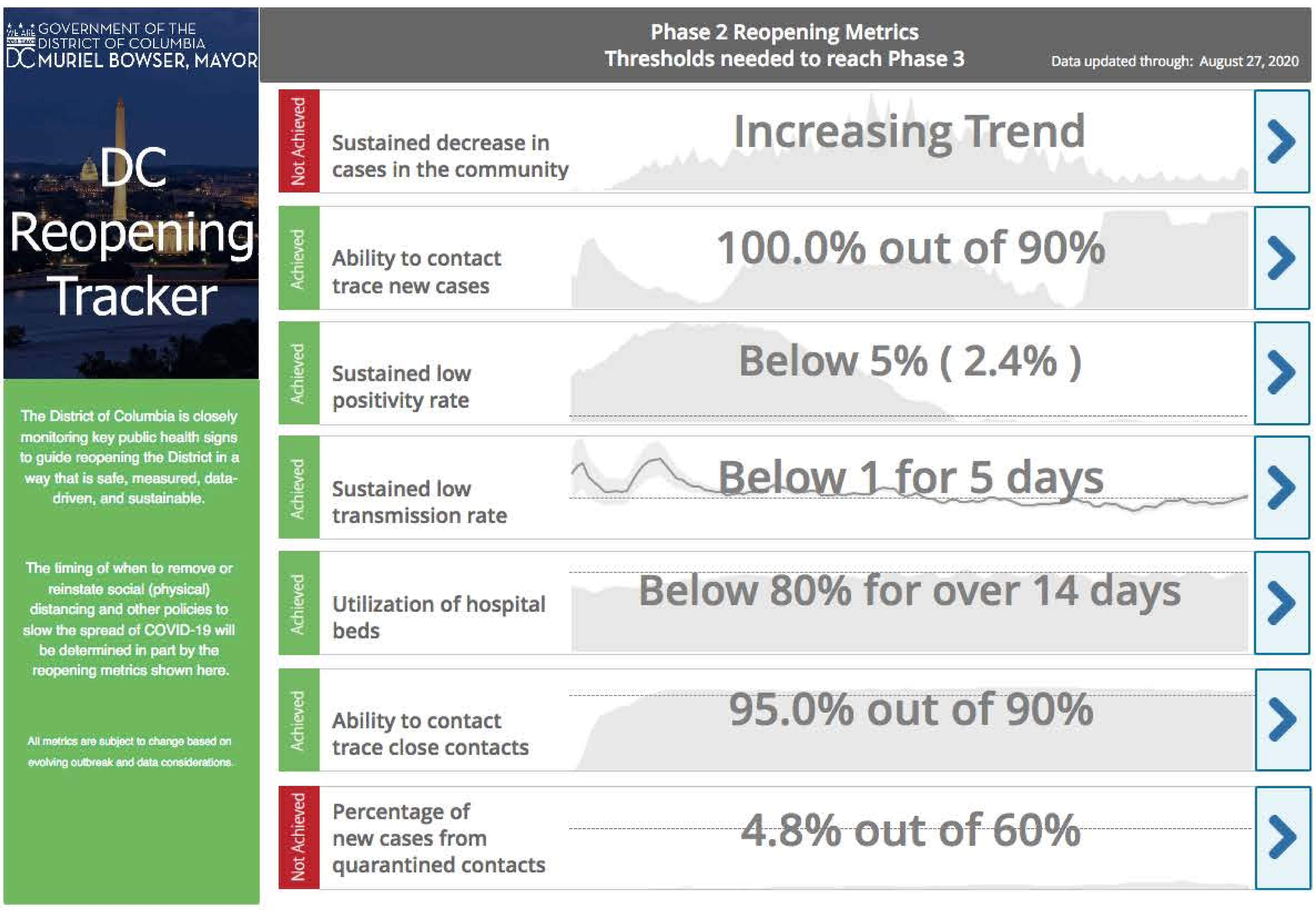

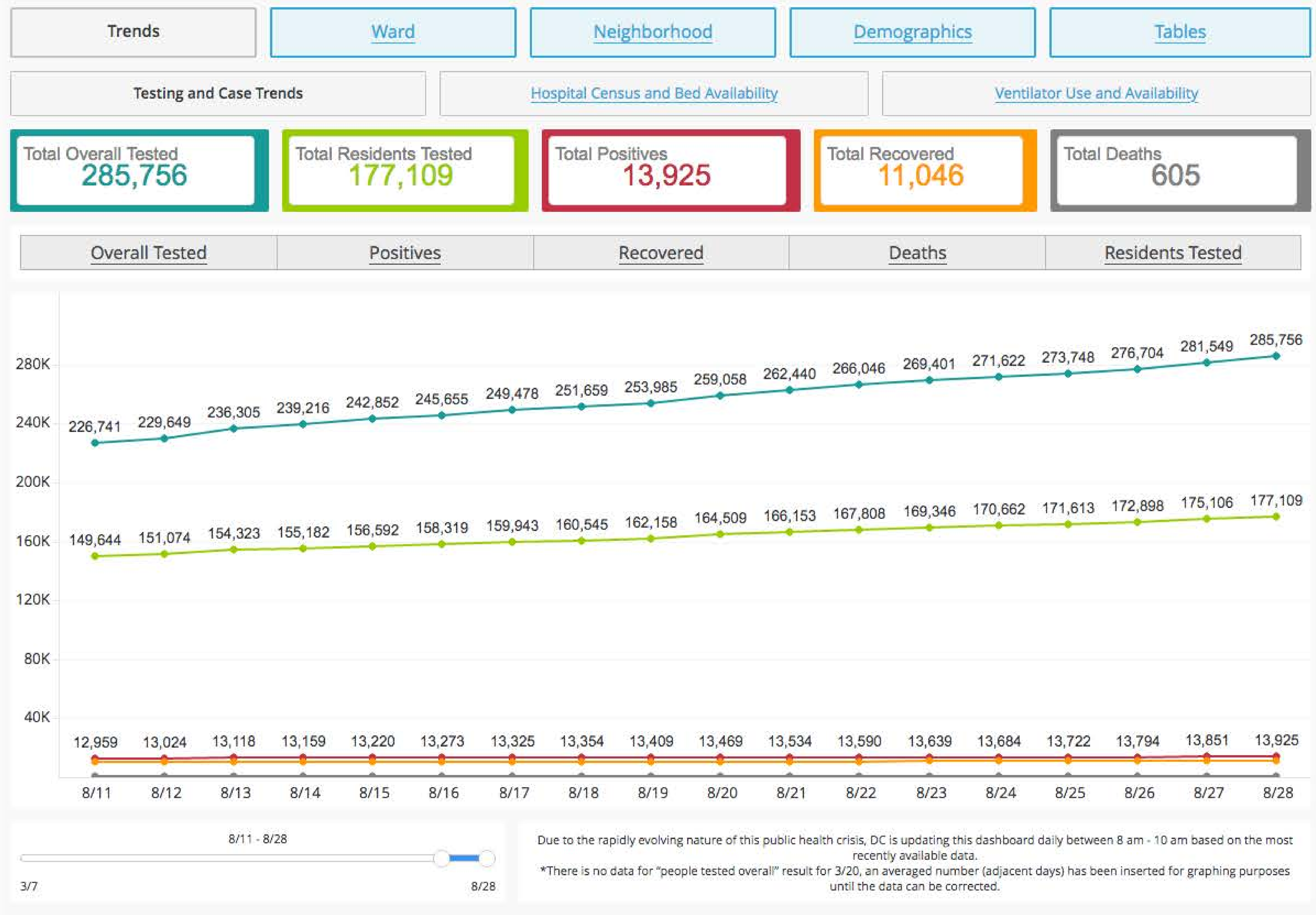

User Guide for Data Visualizations

August 27, 2020:

- Total Overall Tested: 281,549*
- Total Number of DC Residents Tested: 175,106*
- Total Positives: 13,851
- Total Lives Lost: 605
- Cleared From Isolation: 11,010

*All data are preliminary and are subject to change based on additional reporting.

- Download copy of DC COVID-19 data

August 26, 2020:

- Total Overall Tested: 276,704*
- Total Number of DC Residents Tested: 172,898*
- Total Positives: 13,794
- Total Lives Lost: 605
- Cleared From Isolation: 10,986

*All data are preliminary and are subject to change based on additional reporting.

- Download copy of DC COVID-19 data

August 25, 2020:

- Total Overall Tested: 273,748*
- Total Number of DC Residents Tested: 171,613*

Coronavirus (COVID-19) Data Dashboard

State of Delaware

A resource of COVID-19 data and facts for Delaware families and communities.

Delaware's Official Coronavirus Website →
More health data about State of Delaware →

STATE OF DELAWARE
Population: 974,051

Overview | Testing | Cases | Outcomes | Contact Tracing

Overview

About the data | Data download

State of Delaware's Latest Status

Data are current as of 6pm the previous day. Last update: 08/29/2020 11:17 AM.

Current School Planning Scenario
Schools may use a hybrid model of remote learning and in-person learning options with precautions in place.
See More Details →

Contact Tracing
New data are now available about Delaware's contact tracing efforts.
See the Contact Tracing Data →

Rate of COVID cases per 10,000 population

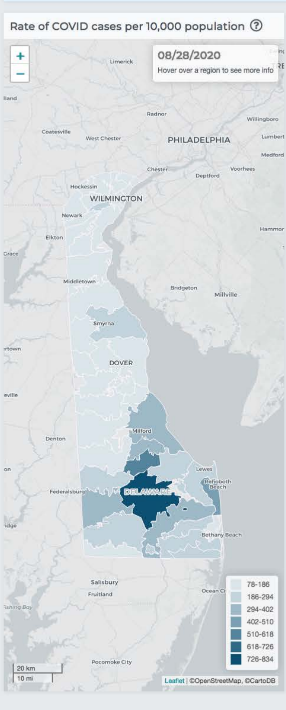

Key Public Health Metrics

215,535
Tested Negative

61
Current Hospitalizations

14
Current Critical Hospitalizations

9,271
Recoveries

See All Tests › | See All Cases › | See All Outcomes ›

Positive Cases in State of Delaware

Total Positive Cases
17,349
183.2 per 10,000 people

Confirmed	Probable	Residents of Long-Term Care Facilities
16,336	1,013	1,243

Positive Cases by County

New Castle County	Kent County	Sussex County	Unknown
8,109	2,612	6,350	278

Deaths in State of Delaware

Total Deaths
604
5.0 per 10,000 people

Confirmed	Probable	Residents of Long-Term Care Facilities
533	71	367

Trends: State of Delaware over the Last 14 Days

14-day charts | 30-day charts | 90-day charts | All available data

Case counts and test data by date of report can vary significantly from day to day for a variety of reasons. In addition to changes due to actual changes in disease incidence, trends are strongly influenced by testing patterns (who gets tested and why), testing availability, lab analysis backlogs, lab reporting delays, new labs joining our electronic laboratory reporting system, mass screenings, etc.

Current Hospitalizations — Updated: 08/29/2020

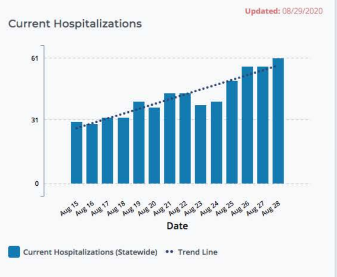

New Hospital Admissions (Confirmed and Suspected with COVID-19) — Updated: 08/29/2020

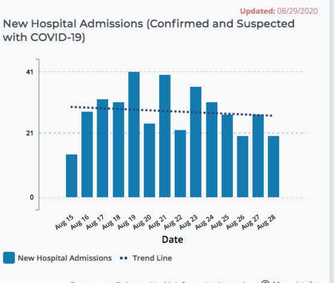

Data source: Delaware Health Information Network | More details

Percentage of Persons Who Tested Positive — Updated: 08/29/2020

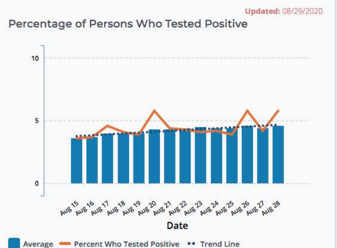

Averaging method: averages are a moving calculation that smooth unstable data. For each data point, seven values are averaged: the data point and the six preceding data points. Toward the beginning of the time series, when there are fewer than six values before a data point, instead of averaging seven values, only the available values are used in the calculation. More details

New Positive Cases — Updated: 08/29/2020

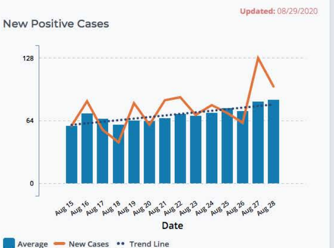

Averaging method: averages are a moving calculation that smooth unstable data. For each data point, seven values are averaged: the data point and the six preceding data points. Toward the beginning of the time series, when there are fewer than six values before a data point, instead of averaging seven values, only the available values are used in the calculation. More details

Percentage of Emergency Department Visits for Influenza-Like Illness (ILI) and COVID-19-Like Illness (CLI) by Week — Updated: 08/29/2020

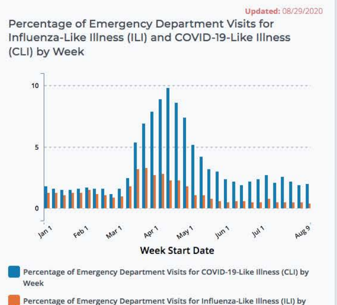

More details

Cumulative Number of Positive Cases — Updated: 08/29/2020

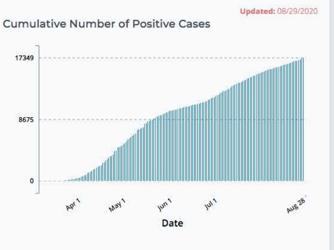

Cases by County (Cumulative)

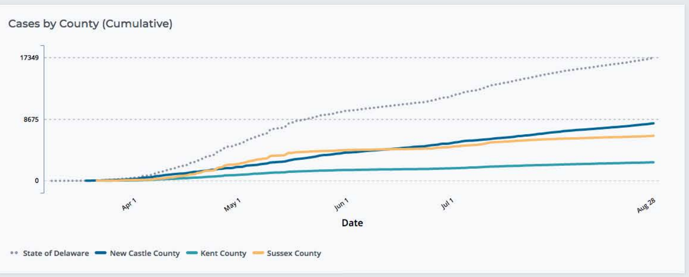

School Reopening Criteria

CURRENT STATUS
*Data are updated every Saturday**

2 Minimal-to-Moderate Community Spread

Schools may use a hybrid model of remote learning and in-person learning options with precautions in place.

*Unlike other COVID data on My Healthy Community, historical data presented on the school reopening dashboard are not updated daily. This is because the reopening dashboard is used to make decisions based on the best available data at the time and are not informed by real-time updates to historical data.

Breakdown of This Week's Status (08/22/2020 - 08/28/2020)

Overall Rating	New Cases (per 100,000 people)	% of Persons Tested Positive	Avg. Daily Hospitalizations (per 100,000 people)
Minimal-to-Moderate — *Schools are partially open*	61.5 — Minimal-to-Moderate, 10 to 100	4.5% — Minimal-to-Moderate, 3 to 10%	5.1 — Minimal, Fewer than 10

Planning Scenarios for Schools	1 Minimal Community Spread	2 Minimal-to-Moderate Community Spread	3 Significant Community Spread
Description	Schools are open for in-person instruction.	Schools may use a hybrid model of remote learning and in-person learning options with precautions in place.	School buildings are closed — remote learning only.
**Criteria**			
New Cases (per 100,000 people)	Fewer than 10	10 to 100	More than 100
% of Persons Tested Positive	Less than 3%	3 to 10%	More than 10%
Avg. Daily Hospitalizations (per 100,000 people)	Fewer than 10	10 to 25	More than 25
Overall Rating*	2+ green	2+ yellow	2+ red

*If there is one of each color, % of Persons Tested Positive determines the overall rating.

Status by Week	Overall Rating	New Cases (per 100,000 people)	% of Persons Tested Positive	Avg. Daily Hospitalizations (per 100,000 people)
08/22/2020 - 08/28/2020	Minimal-to-Moderate	61.5	4.5	5.1
08/15/2020 - 08/21/2020	Minimal-to-Moderate	47.8	4.1	3.6
08/08/2020 - 08/14/2020	Minimal-to-Moderate	50.8	4.0	3.7
08/01/2020 - 08/07/2020	Minimal-to-Moderate	56.5	4.2	4.3
07/25/2020 - 07/31/2020	Minimal-to-Moderate	70.0	4.3	6.0
07/18/2020 - 07/24/2020	Minimal-to-Moderate	70.7	4.2	5.7

Healthcare System Status

Delaware's PPE Inventory Status — Updated: 08/29/2020

- Fluid Resistant Gowns and Isolation Gowns — 2 Weeks+ Left (Stable)
- Face Shields — 2 Weeks+ Left (Stable)
- Glasses and Goggles — 2 Weeks+ Left (Stable)
- Gloves — 2 Weeks+ Left (Stable)
- Isolation and Procedural-Ear Loop Masks — 2 Weeks+ Left (Stable)
- N95 Masks — 2 Weeks+ Left (Stable)
- Surgical Masks — 2 Weeks+ Left (Stable)

Delaware's Healthcare System Status — Updated: 08/29/2020

- ICU Bed Availability — Stable (75% or less occupied)
- Ventilator Availability — Stable (75% or less occupied)
- Hospital ED Wait Times — < 5 hours
- Hospital Census/Capacity — < 98%

Testing

About the data | Data download | How can I get tested? →

Latest Status

Total Persons Tested per 10,000 Population

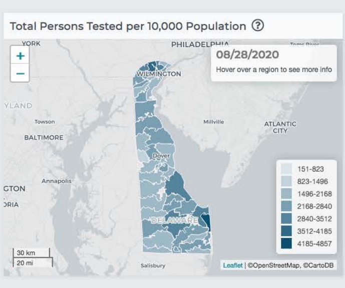

Total Persons Tested in State of Delaware

State of Delaware
232,884
2,452.7 per 10,000 people

Total Persons Tested by County

New Castle County	Kent County	Sussex County	Unknown
129,329	31,446	57,810	14,299

Trends

Total Persons Tested — Updated: 08/29/2020

232,884 — Total count on 08/29/2020
2,452.7 — rate per 10,000 people on 08/29/2020

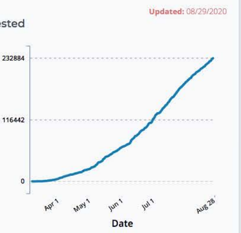

Tested Negative — Updated: 08/29/2020

215,535 — Total count on 08/29/2020
2,270.0 — rate per 10,000 people on 08/29/2020

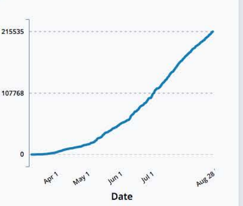

More details

Demographics

Total Persons Tested by Race/Ethnicity

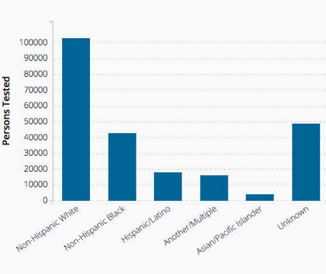

Total Persons Tested by Race/Ethnicity & County — Updated: 08/29/2020

RACE/ETHNICITY	STATE OF DELAWARE	NEW CASTLE	KENT	SUSSEX
Non-Hispanic White	102,983	55,407	11,390	30,570
Non-Hispanic Black	42,804	26,919	6,455	6,396
Hispanic/Latino	17,925	9,907	1,416	5,753
Another/Multiple	16,052	9,338	1,501	3,919
Asian/Pacific Islander	4,284	3,328	402	399
Unknown	48,836	24,430	10,282	10,773

Total Population by Race/Ethnicity for State of Delaware →

Rate of Persons Tested by Race/Ethnicity

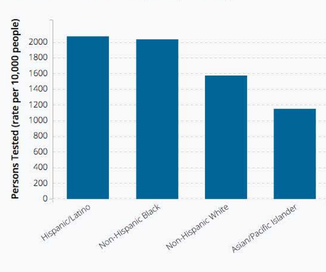

Rate of Persons Tested by Race/Ethnicity & County — Updated: 08/29/2020

RACE/ETHNICITY	STATE OF DELAWARE	NEW CASTLE	KENT	SUSSEX
Hispanic/Latino	2,076.7	1,832.2	1,162.6	2,867.3
Non-Hispanic Black	2,039.3	1,942.1	1,457.4	2,369.6
Non-Hispanic White	1,572.5	1,546.9	982.2	1,691.2
Asian/Pacific Islander	1,151.6	1,081.4	1,126.7	1,396.6

Total Population by Race/Ethnicity for State of Delaware →

Cases (State Of Delaware)

About the data | Data download

Latest Status

Rate of COVID cases per 10,000 population (State of Delaware)

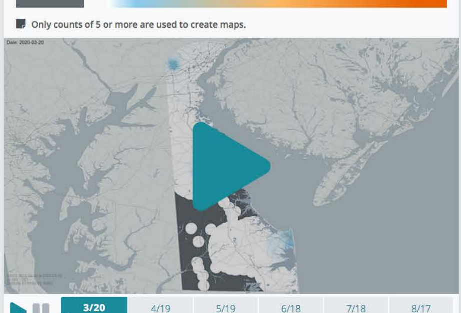

Total Positive Cases in State of Delaware

Total Positive Cases
17,349
183.2 per 10,000 people

Confirmed	Probable	Residents of Long-Term Care Facilities
16,336	1,013	1,243

Positive Cases by County

New Castle County	Kent County	Sussex County	Unknown
8,109	2,612	6,350	278

Trends

Positive Cases — Updated: 08/29/2020

17,349 — Total count on 08/29/2020
183.2 — rate per 10,000 people on 08/29/2020

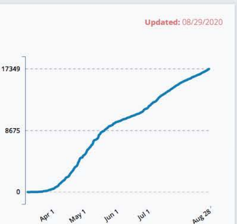

Current Hospitalizations — Updated: 08/29/2020

61 — Total count on 08/29/2020
0.6 — rate per 10,000 people on 08/29/2020

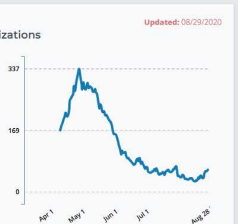

Demographics

Rate charts | Count charts

Positive Cases per 10,000 Population by Age Group

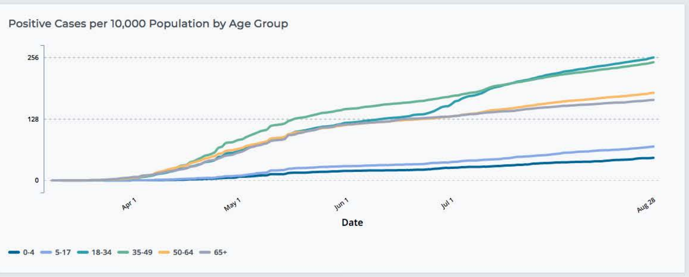

Rate of Cases by Age

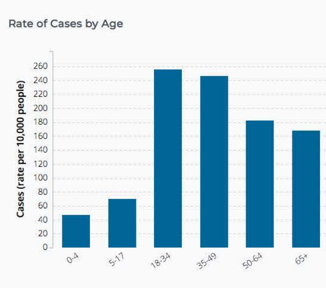

Rate of Cases by Age & County — Updated: 08/29/2020

AGE	STATE OF DELAWARE	NEW CASTLE	KENT	SUSSEX
0-4	47.2	39.3	28.2	86.6
5-17	70.7	54.8	39.8	137.9
18-34	256.4	192.7	177.3	544.1
35-49	246.5	176.6	208.6	481.2
50-64	182.8	151.5	176.4	250.6
65+	168.0	164.3	173.6	164.8

Total Population by Age for State of Delaware →

Rate of Cases by Sex

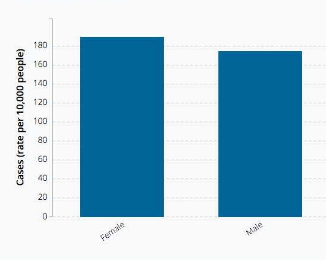

Rate of Cases by Sex & County — Updated: 08/29/2020

SEX	STATE OF DELAWARE	NEW CASTLE	KENT	SUSSEX
Female	189.7	157.1	154.9	287.2
Male	174.7	134.0	143.1	290.5

Rate of Cases by Race/Ethnicity

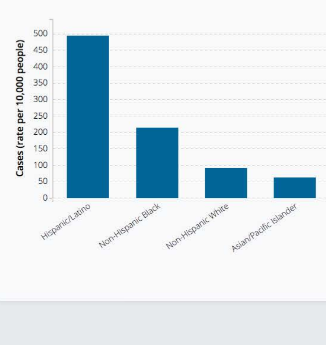

Rate of Cases by Race/Ethnicity & County — Updated: 08/29/2020

RACE/ETHNICITY	STATE OF DELAWARE	NEW CASTLE	KENT	SUSSEX
Hispanic/Latino	495.3	346.6	257.0	1,015.7
Non-Hispanic Black	214.5	177.6	197.1	410.5
Non-Hispanic White	92.7	74.0	89.8	126.0
Asian/Pacific Islander	62.6	45.8	123.3	164.5

Total Population by Race/Ethnicity for State of Delaware →

Outcomes (State Of Delaware)

About the data | Data download

Latest Status

Total Deaths in State of Delaware

Total Deaths
604
5.0 per 10,000 people

Confirmed	Probable	Residents of Long-Term Care Facilities
533	71	367

Deaths by County

New Castle County	Kent County	Sussex County	Unknown
300	109	195	0

Most Recent Deaths (Announced 08/28/2020)

Total Deaths
0

Age Range	Individuals Having Underlying Health Conditions	Residents of Long-Term Care Facilities
N/A	0	0

Deaths by County

New Castle County	Kent County	Sussex County	Unknown
0	0	0	0

Trends

Recoveries — Updated: 08/29/2020

9,271 — Total count on 08/29/2020
99.6 — rate per 10,000 people on 08/29/2020

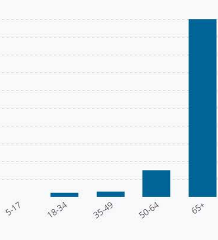

Deaths — Updated: 08/29/2020

604 — Total count on 08/29/2020
5.0 — rate per 10,000 people on 08/29/2020

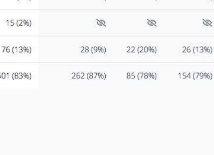

Demographics

Total Deaths by Age

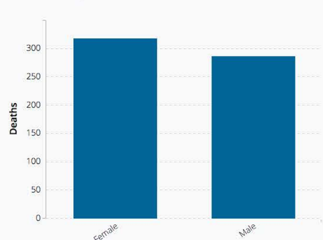

Total Deaths by Age & County — Updated: 08/29/2020

AGE	STATE OF DELAWARE	NEW CASTLE	KENT	SUSSEX
0-4	0 (0%)	0 (0%)	0 (0%)	0 (0%)
5-17	0 (0%)	0 (0%)	0 (0%)	0 (0%)
18-34	12 (2%)			
35-49	15 (2%)			
50-64	76 (13%)	28 (9%)	22 (20%)	26 (13%)
65+	501 (83%)	262 (87%)	85 (78%)	154 (79%)

Total Population by Age for State of Delaware →

Total Deaths by Sex

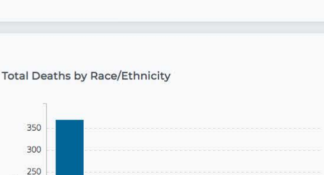

Total Deaths by Sex & County — Updated: 08/29/2020

SEX	STATE OF DELAWARE	NEW CASTLE	KENT	SUSSEX
Female	318 (53%)	156 (52%)	55 (50%)	107 (55%)
Male	286 (47%)	144 (48%)	54 (50%)	88 (45%)

Total Deaths by Race/Ethnicity

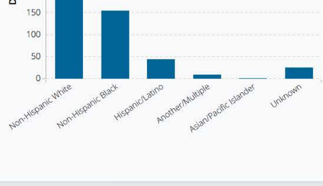

Total Deaths by Race/Ethnicity & County — Updated: 08/29/2020

RACE/ETHNICITY	STATE OF DELAWARE	NEW CASTLE	KENT	SUSSEX
Non-Hispanic White	369 (61%)	185 (62%)	62 (57%)	122 (63%)
Non-Hispanic Black	155 (26%)	73 (24%)	40 (37%)	42 (22%)
Hispanic/Latino	44 (7%)			
Another/Multiple	9 (1%)			
Asian/Pacific Islander	1 (0%)			
Unknown	26 (4%)			

Total Population by Race/Ethnicity for State of Delaware →

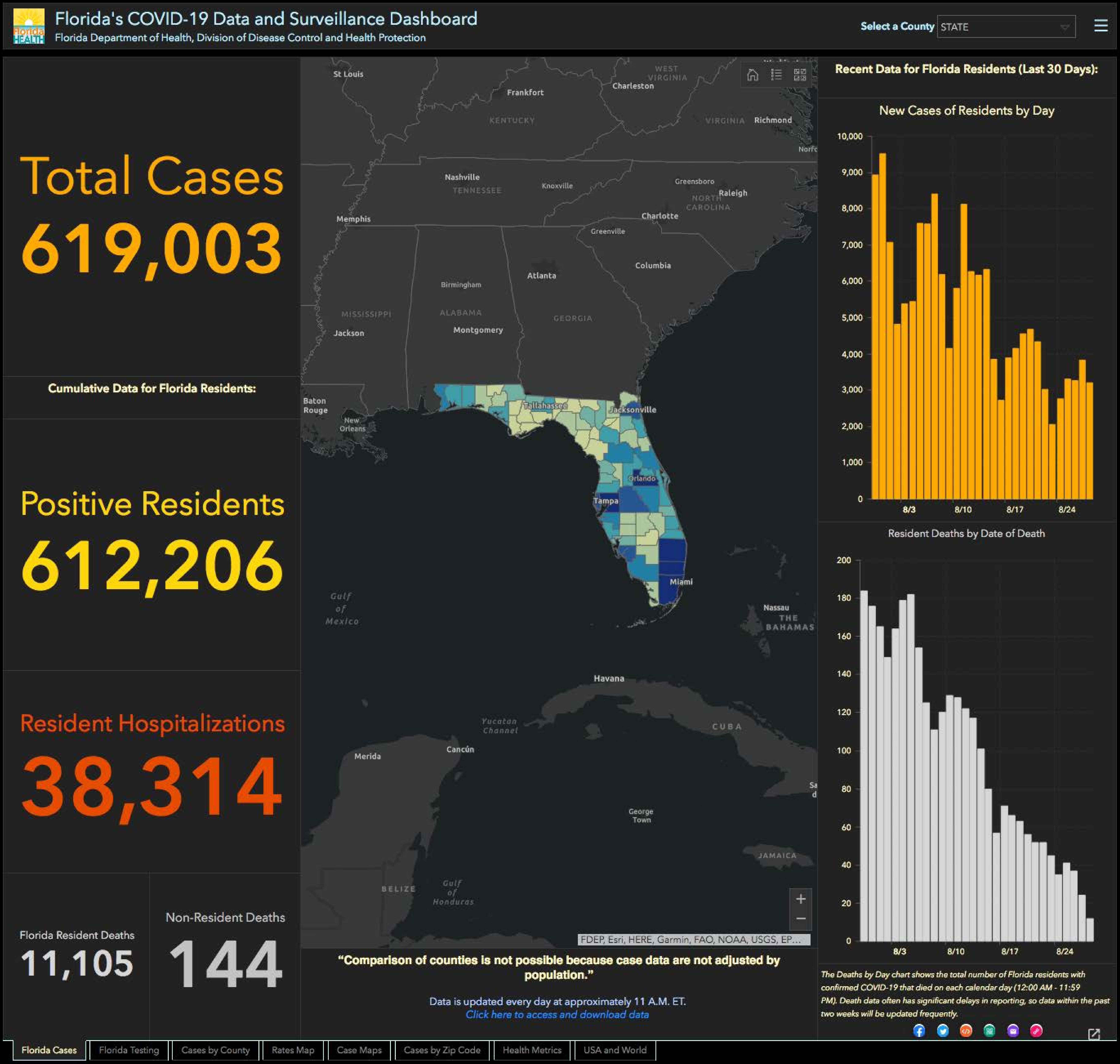
Florida's COVID-19 Data and Surveillance Dashboard
Florida Department of Health, Division of Disease Control and Health Protection
Select a County
STATE
Total Cases
619,003
Cumulative Data for Florida Residents:
Positive Residents
612,206
Resident Hospitalizations
38,314
Florida Resident Deaths
11,105
Non-Resident Deaths
144
St Louis
Frankfort
Charleston
WEST VIRGINIA
KENTUCKY
VIRGINIA
Richmond
Nashville
TENNESSEE
Knoxville
Greensboro
Raleigh
NORTH CAROLINA
Memphis
Charlotte
Greenville
Columbia
Atlanta
Birmingham
MISSISSIPPI
ALABAMA
GEORGIA
Jackson
Montgomery
Baton Rouge
New Orleans
Tallahassee
Jacksonville
Orlando
Tampa
Miami
Gulf of Mexico
Nassau
THE BAHAMAS
Havana
Yucatan Channel
CUBA
Merida
Cancún
George Town
JAMAICA
BELIZE
Gulf of Honduras
FDEP, Esri, HERE, Garmin, FAO, NOAA, USGS, EP...
"Comparison of counties is not possible because case data are not adjusted by population."
Data is updated every day at approximately 11 A.M. ET.
Click here to access and download data
Recent Data for Florida Residents (Last 30 Days):
New Cases of Residents by Day
10,000
9,000
8,000
7,000
6,000
5,000
4,000
3,000
2,000
1,000
0
8/3
8/10
8/17
8/24
Resident Deaths by Date of Death
200
180
160
140
120
100
80
60
40
20
0
8/3
8/10
8/17
8/24
The Deaths by Day chart shows the total number of Florida residents with confirmed COVID-19 that died on each calendar day (12:00 AM - 11:59 PM). Death data often has significant delays in reporting, so data within the past two weeks will be updated frequently.
Florida Cases
Florida Testing
Cases by County
Rates Map
Case Maps
Cases by Zip Code
Health Metrics
USA and World

Florida's COVID-19 Data and Surveillance Dashboard

Florida Department of Health, Division of Disease Control and Health Protection

Select a County: STATE

Data for Florida Residents from Previous Day

Total FL-Residents Tested

64,904

FL-Residents Positive

3,207

FL-Residents Negative

61,697

Previous Day Percent Positive

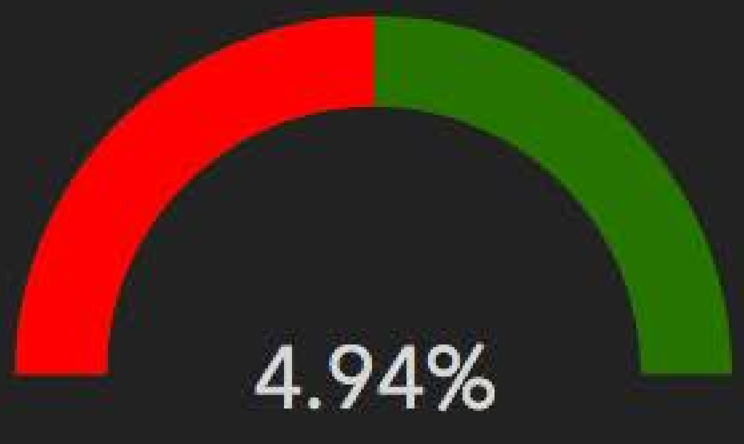

Positivity rate of new cases by week

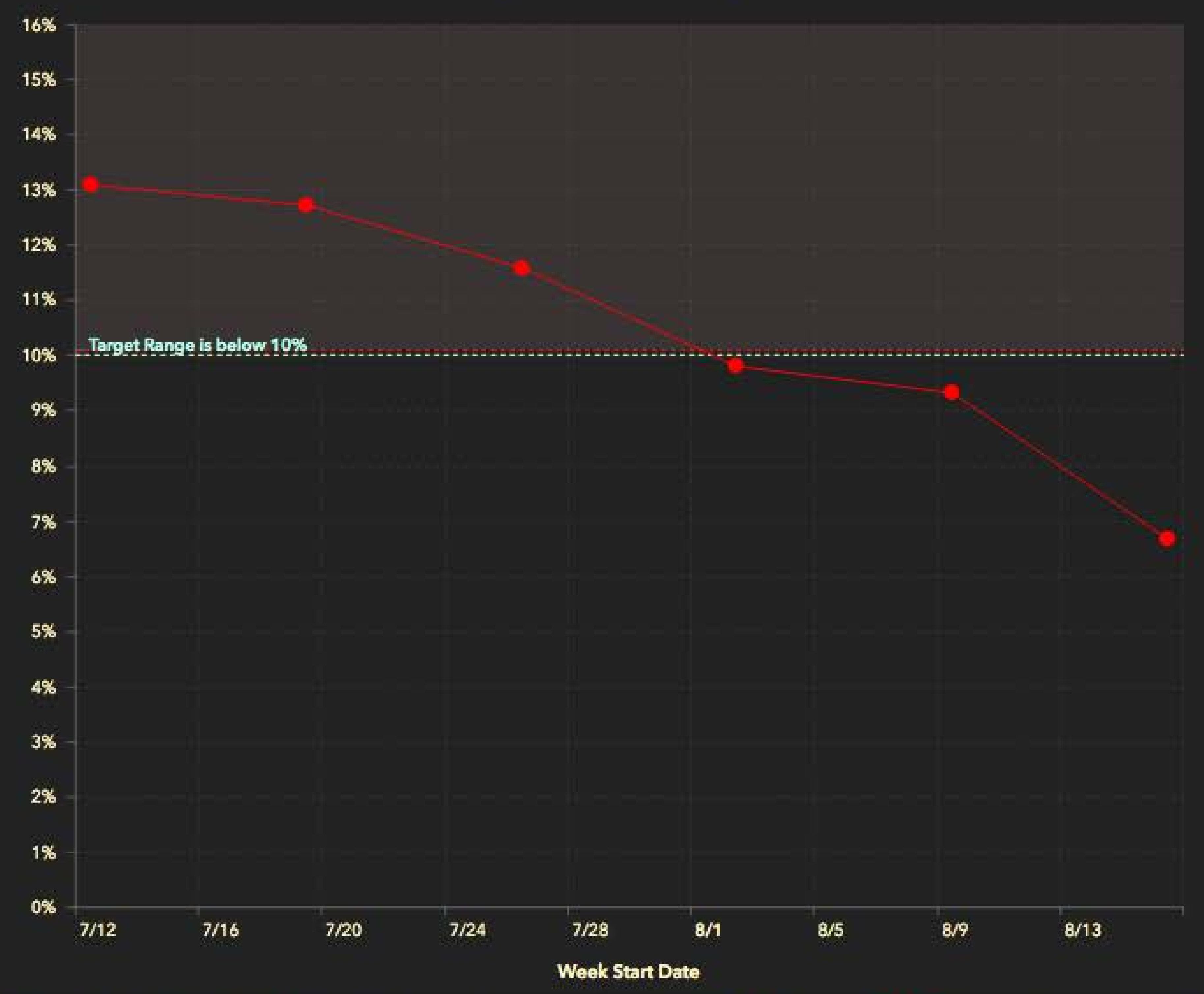

Testing Data for State from previous day

Total FL Residents Tested: 64,904
Positive: 3,207
Negative: 61,697

Percent Positive: 4.94 %

Statewide Negative and Positive Persons Tested by Week

*Date of data shown is the last date of the reporting period and includes the total from the last report date **and** the previous six days.*

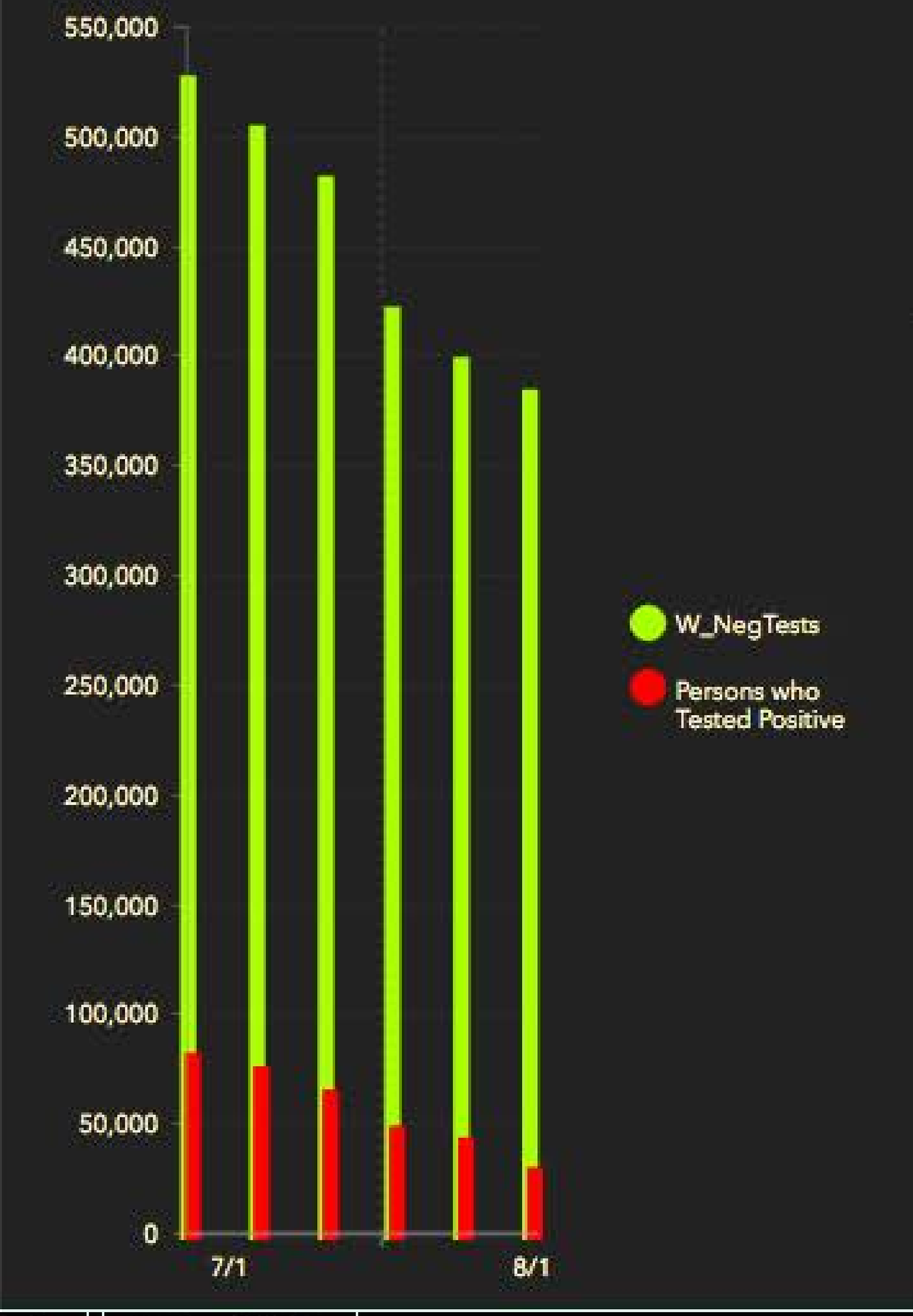

Use the drop-down menu above to select a county.

Cumulative Data for Florida and Non-Florida Residents

Total People Tested

4,582,134

Positive

619,003

Negative

3,956,208

Cumulative percent positive

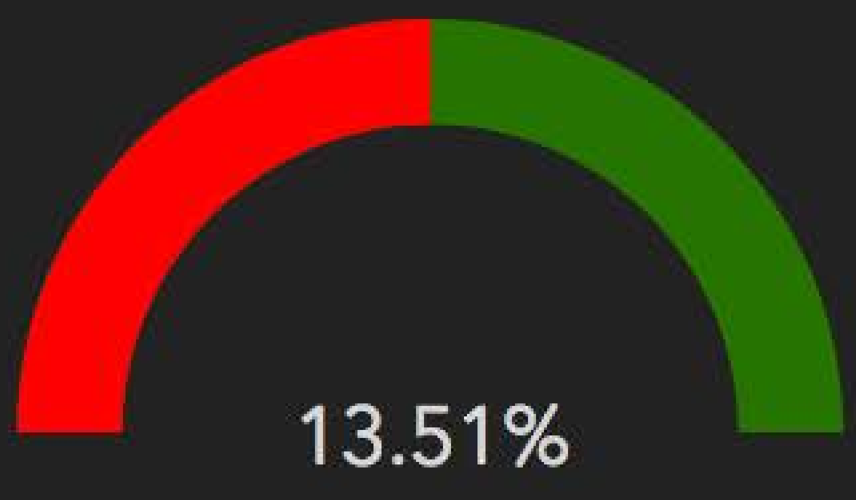

Florida Cases | Florida Testing | Cases by County | Rates Map | Case Maps | Cases by Zip Code | Health Metrics | USA and World

Florida's COVID-19 Data and Surveillance Dashboard

Florida Department of Health, Division of Disease Control and Health Protection

Select a County STATE

Number of Florida resident COVID-19 cases per 100,000 population by county.

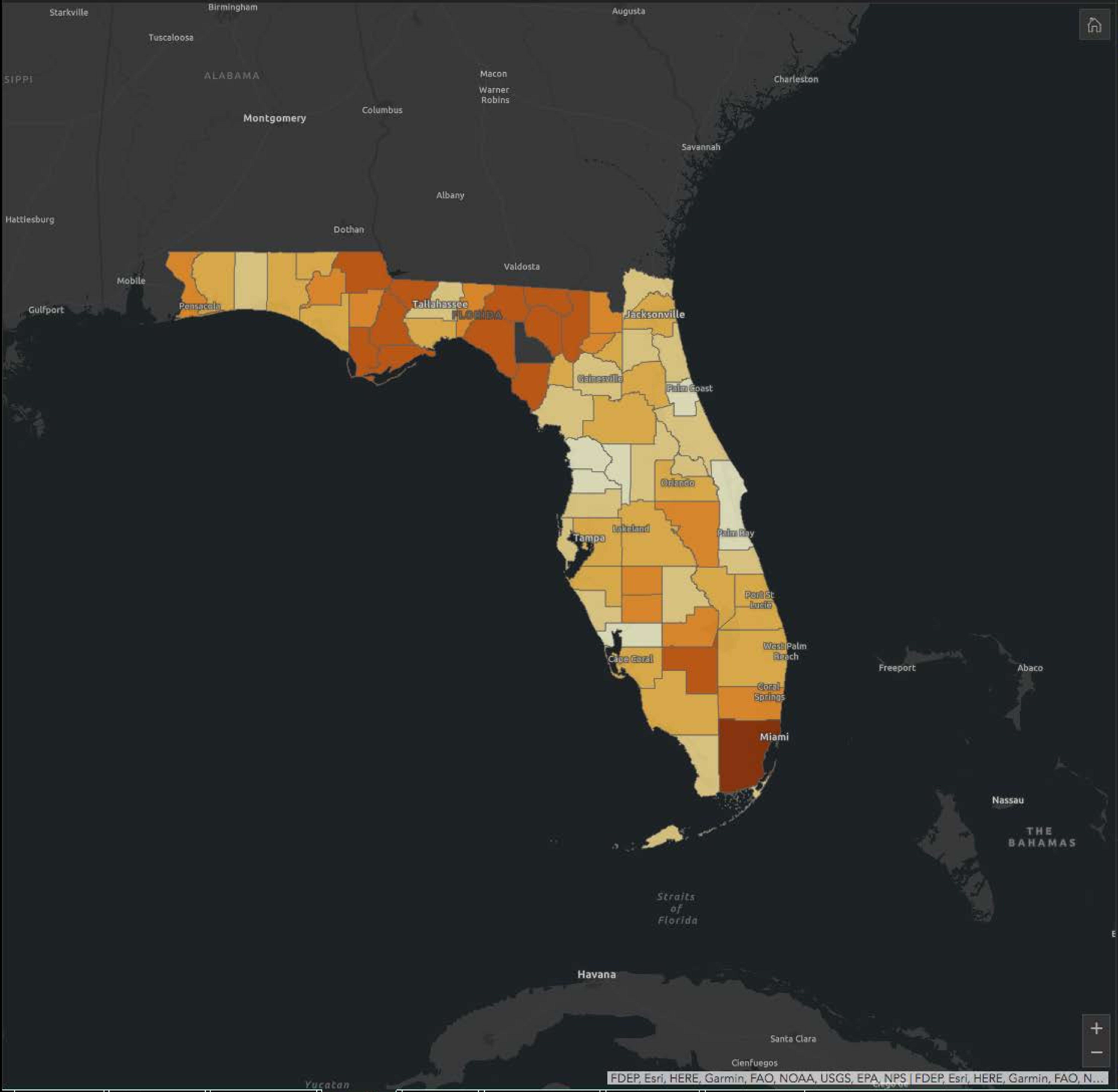

Case Maps for COVID19

CovidRate

- > 5,679 - 13,541
- > 4,234 - 5,679
- > 3,233 - 4,234
- > 2,258 - 3,233
- > 1,547 - 2,258
- 0 - 1,547

Case Rate per 100,000 people

2,972

Florida Cases | Florida Testing | Cases by County | Rates Map | Case Maps | Cases by Zip Code | Health Metrics | USA and World

Florida COVID Action

Get Data. Get Tested. Get Hel

Welcome to our COVID Action site.

Page Menu

NEWS AND UPDATES:

The COVID Monitor now tracks cases in every K-12 school district in the USA.

Visit Website

Data last updated: 8/29/2020 4:44 PM UTC

Total COVID-19 Positive:
666,517
New Cases Today: 3,197
New persons tested today: 27,571

All Florida COVID-19 Deaths:
11,249
New Deaths Reported Today: 150

Our new dashboard experience provides information in an easy-to-navigate format, with much more data and shorter load times. If you prefer to use the old format, click the link below.

Click here to access old dashboard

Where do you want to go?

Home | Summary Info | Local Data | Healthcare and Hospitals | Get Tested | Get Help | #COVIDStorm

Data last updated: 8/29/2020 4:44 PM

Total positive since March 1:
666,517
Includes those who received positive results from PCR, antigen and antibody testing. To learn more about the different types of tests, click here.

Total deaths since March 1:
11,249
New deaths today: 150
Includes only those deaths in which COVID-19 was listed as a primary reason for death, as determined by the Florida Department of Health. Data may differ from the number of deaths reported by each county's Medical Examiner. Florida does not include probable cases and deaths, despite CDC advisement.

Total DOH Cases since March 1:
619,003
New cases today: 3,197
DOH Case data includes only those who tested positive with a molecular (PCR) test and/or antibody test, but excludes antibody positive. DOH includes antigen tests in their calculations of percent positivity, but does not specify how many antigen tests are included in those figures.

Click here to download our county master file.

Select a county
- All -

Long-term care facilities (LTCF):
Cumulative LTCF cases: 39,488
"Active" LTCF Cases: 9,208
LTCF Deaths: 4,525

Prisons:
Prison cases: 20,246
Prison deaths: 91

Pediatrics:
Cases: 50,327
Admitted to an ER: 3,418
In-patient admissions to a hospital: 633
Pediatric Deaths: 8

Cases in county schools:
Last two weeks: 206

Testing stats:

Antibody testing:
Antibody positive: 47,514
Antibody negative: 403,867

PCR (Diagnostic) and Antigen testing:
Total Positive (PCR): 610,689
Total Positive (Antigen): 8,314
Total tests to date: 4,582,134

Statewide Hospital Stats:
To view hospital data by county, visit our "Healthcare and Hospitals" Page.

Current hospitalizations with a primary diagnosis of COVID-19:
4,009

Available beds:
Adult ICU: 1,253
Pediatric ICU: 244

Today's Percent Positive:
Note: Statewide positivity data does not filter by county.

New persons tested:
11.6%

All people tested: 7.13%
Labs processed: 0%

Looking to download or plug into the data you see here?
You can download the Master File for our county data here.
For all of our available datasets and feeds, visit our website's data library or go straight to our GIS Data hub.

Florida COVID Action Data Library

Florida COVID Action GIS Data Hub

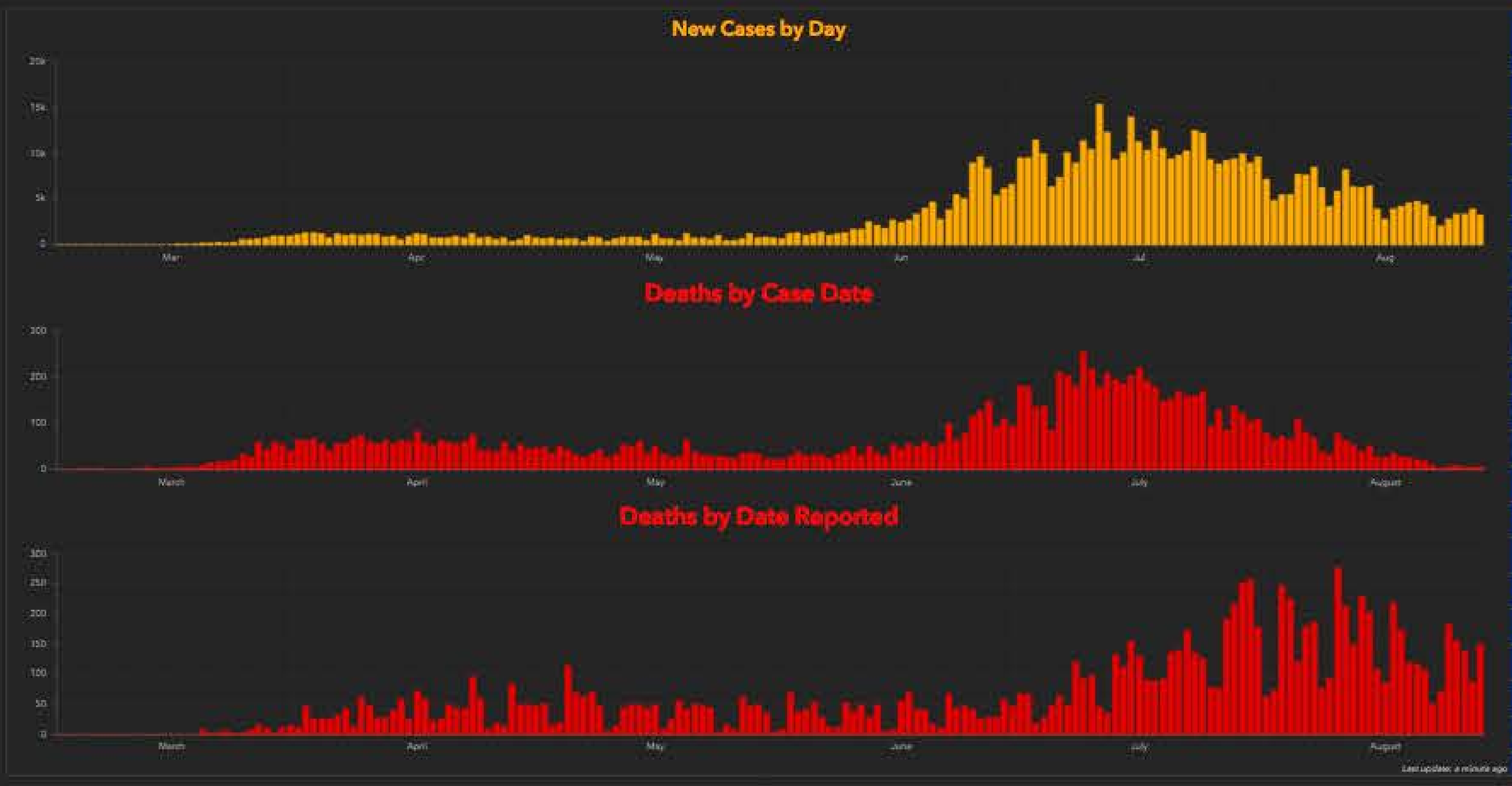

Select a county to filter the charts
Alachua
Baker
Bay
Bradford
Brevard
Broward
Calhoun
Charlotte
Citrus
Clay
Collier
Columbia
Dade
Desoto
Dixie
Duval
Escambia
Flagler
Franklin
Gadsden
Gilchrist

Download the cases by day, deaths by case data, and deaths by date reported data here.
Download DOH's deaths by date of death (residents only for those with a confirmed date of death) here.

Total Positive
666,517

Florida COVID Deaths
11,249

New Cases Today
3,197

New Deaths Reported Today
150
New Deaths this Week
838
New Cases this week
21,802
Total DOH Cases
619,003

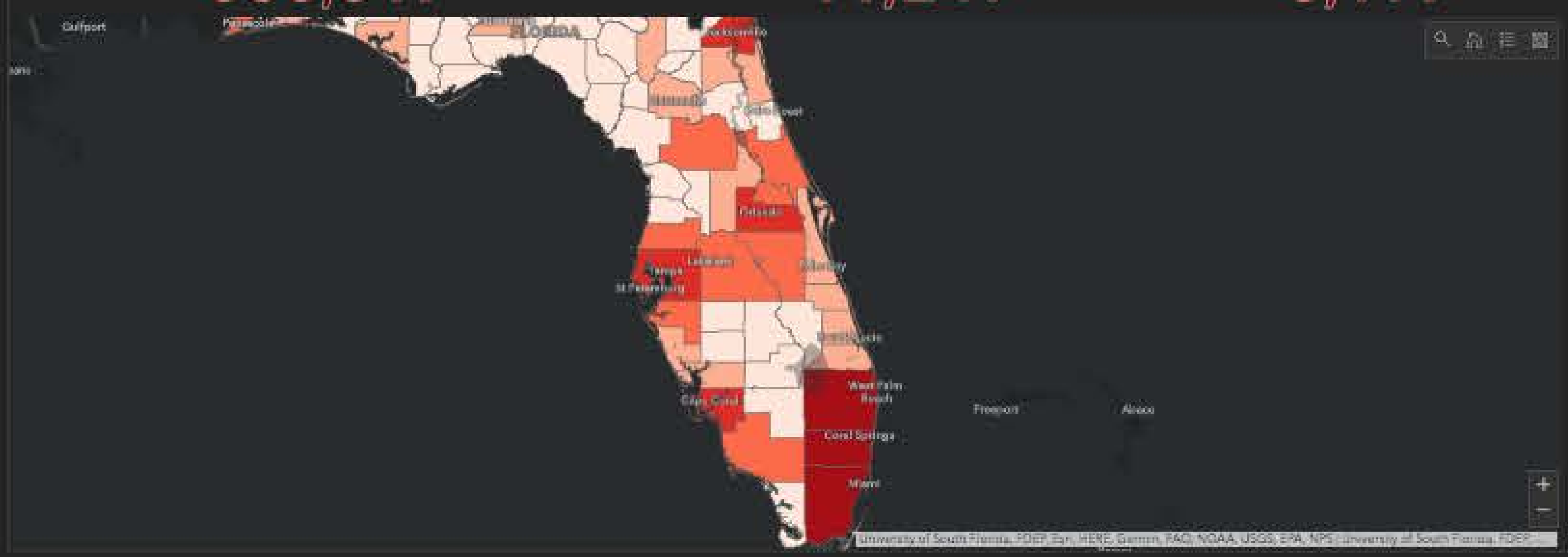

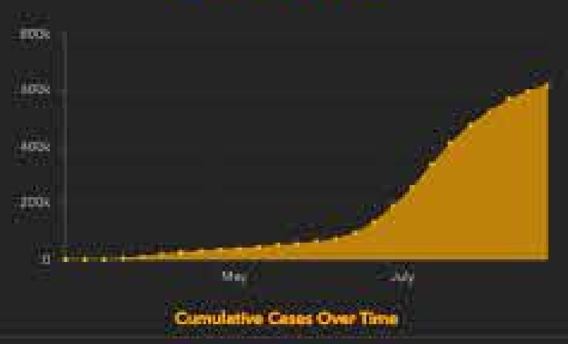

Select a county to filter this page
Alachua
Baker
Bay
Bradford
Brevard
Broward
Calhoun
Charlotte
Citrus
Clay
Collier
Columbia
Dade
Desoto
Dixie
Duval
Escambia
Flagler

Home | Summary Info | Local Data | Healthcare and Hospitals | Get Tested | Get Help | #COVIDStorm

Main Website | Download Data | Support

Facebook Twitter Embed QR code Email Share link

Georgia Department of Public Health Daily Status Report

Note: To ensure the most updated version of this webpage, please refresh your browser.

These data represent confirmed cases of COVID-19 reported to the Georgia Department of Public Health (DPH) as of 8/29/2020, 2:50:08 PM.

The latest: What's new with this report?
Guide: Understand the data (PDF)
Download the data (CSV)
GEMA Daily COVID-19 Information
Georgia DCH Daily COVID-19 Long-Term Care Facility Report
Historical Covid Maps (PDF)
Georgia Hospital Beds and Ventilator Use
Georgia Medical Facility Patient Census

Georgia Overall COVID-19 Status

Below you will find information reported to DPH on the total number of COVID-19 tests, confirmed COVID-19 cases (PCR positive), ICU admissions, hospitalizations, and deaths attributed to COVID-19. These data are based on available information at the time of the report and may not reflect all cases or tests performed in Georgia.

COVID-19 questions?

Confirmed Cases	Deaths	Hospitalizations	ICU Admissions
267,758	5,576	24,533	4,479

Reported Today

Confirmed Cases	Deaths	Hospitalizations
2,428	106	198

COVID-19 Testing

	Total PCR/ Molecular	PCR/Molecular Reported Today	Total Antibody (Serology)
Number of Tests	2,312,179	25,195	275,453
Number of Positive Tests	244,513	2,193	20,980
% Positive	10.6%	8.7%	7.6%

Note: these data only include lab tests reported to DPH by electronic laboratory reporting (ELR) and do not represent all tests performed in Georgia. The number of positive tests in this chart will not match the number of confirmed cases because the case numbers include all reporting sources, not just ELR. People with a positive test often get retested and may test positive multiple times. These repeat positive tests will be counted as new positive tests, but they will not be reported as new cases.

Georgia Cases by County

The maps below show the number and rate of confirmed COVID-19 cases and percent positive PCR tests by county of residence. Choose "Cases", Testing" or "Deaths", then use the drop-down menu to choose cumulative "Cases", "Cases per 100K", "Deaths", "Deaths per 100K", "% Positive", or data for the "Last 2 Weeks". By clicking on the date above the map, you can select a past date to see historic maps, using the current color scale. On the map, you can hover or click to find out additional details such as number of deaths, hospitalizations, case rate, etc. Selecting a county will also update the cases over time charts.

The color scale is based on the distribution of county-level case counts or rates, outlier values are removed from the scale calculation. The scale will change every month to accommodate increasing or decreasing case counts and maintain distinctions between counties, and will be calculated based on the current data at the beginning of each month. DPH will publish historic maps when the scale changes.

COVID-19 By County

Cases | Testing | Deaths

Last 2 weeks ▾ | August 29th

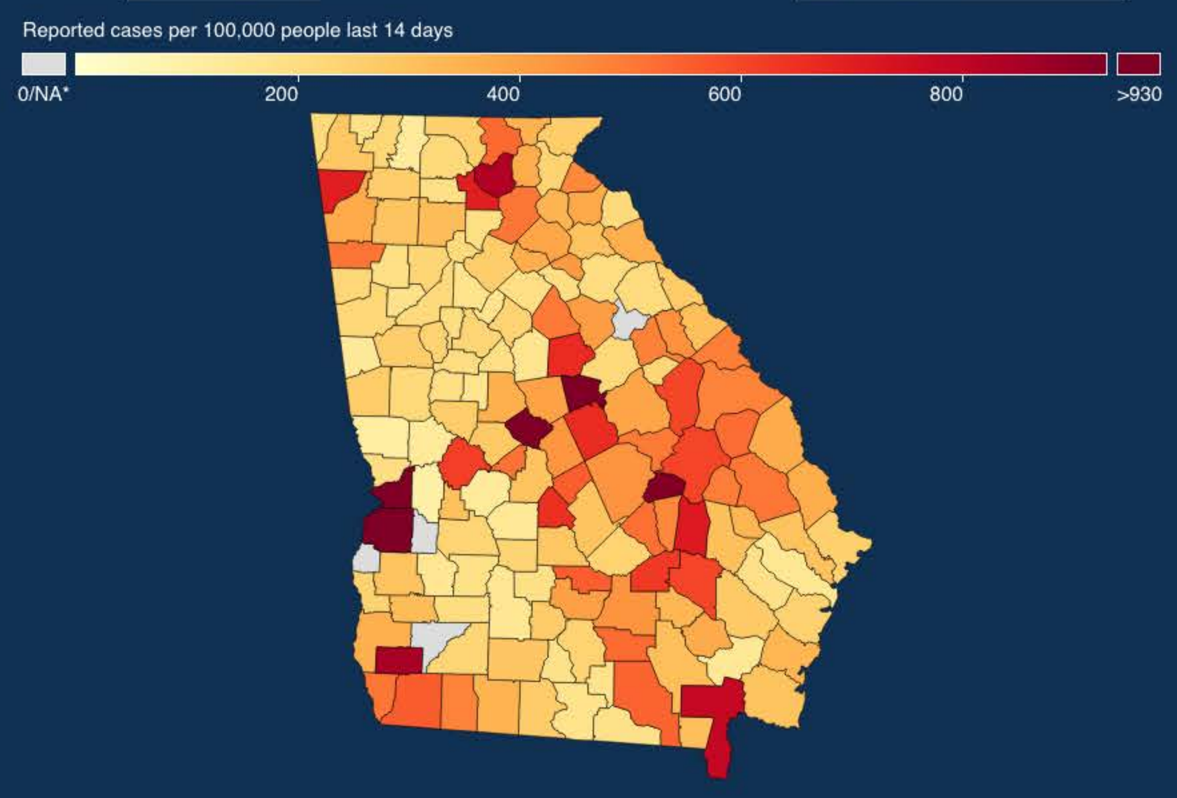

COVID-19 Over Time

Cases | Cumulative Cases | Deaths | Total Tests | Percent Positive

Georgia ▾ | Date of Report ▾

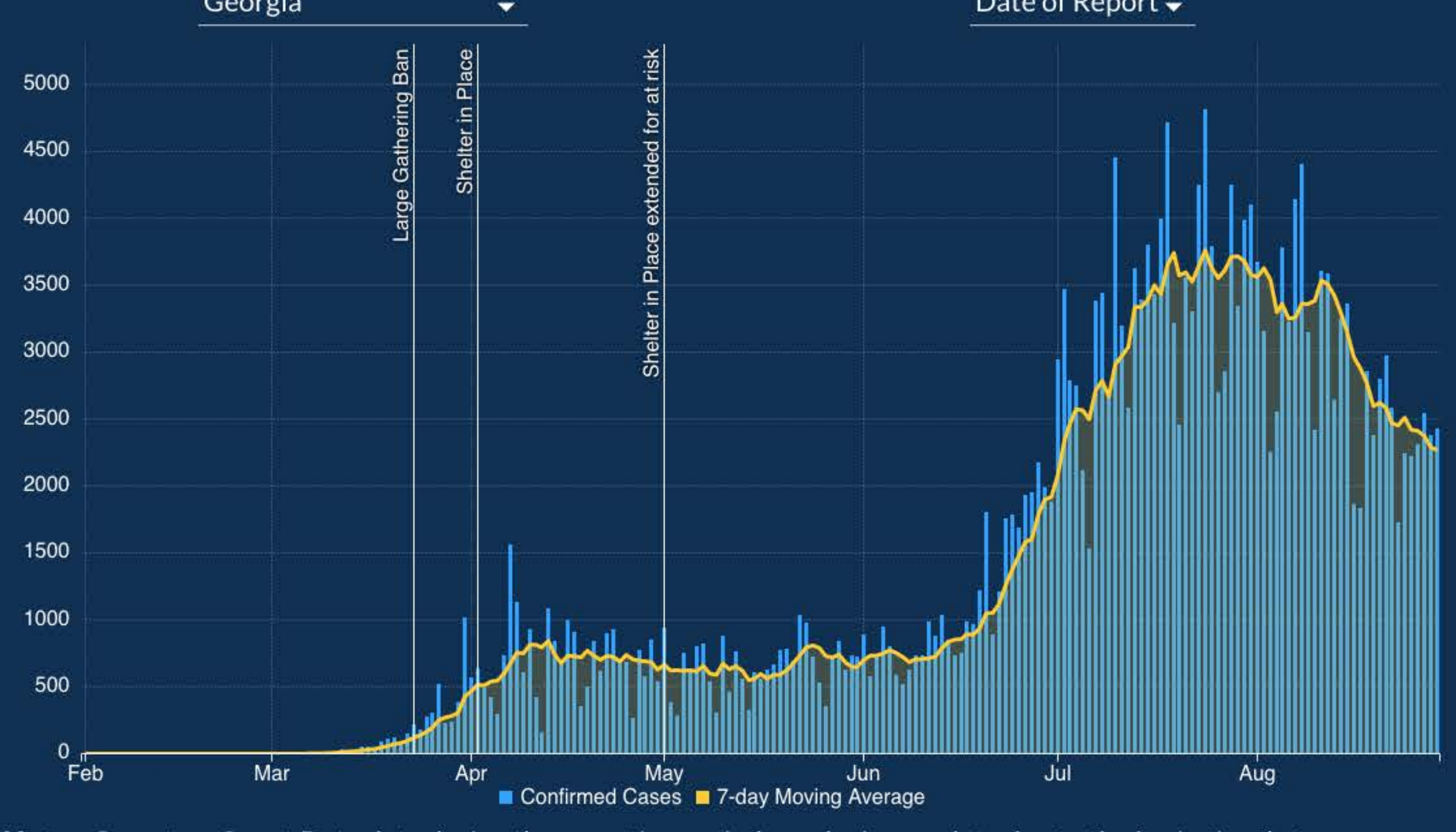

Note – Symptom Onset Date data during the reporting period may be incomplete due to the lag in time between when the case was tested and/or reported and submitted to the Georgia DPH for reporting purposes. This delay can vary depending on the testing facility and/or jurisdiction.
* Rates may not be accurate when case counts are <5 and are not presented. Percent positive may not be accurate when total PCR tests are <10 and are not presented.

Counties | Top Five Counties | Deaths in Georgia | Race and Ethnicity

County ↑	Confirmed Cases	Cases per 100K	Total Deaths	Hospitalizations
Appling	883	4757.29	23	82
Atkinson	403	4837.94	3	49
Bacon	513	4498.42	8	38
Baker	71	2278.56	3	16
Baldwin	1644	3700.37	50	128
Banks	368	1841.66	6	50
Barrow	1717	1987.66	37	212
Bartow	2407	2172.95	70	242
Ben Hill	620	3724.84	12	64

Demographics | Healthcare Workers | Lab Testing | Risks/ Comorbidities

COVID-19 Case Demographics

Confirmed COVID-19 Cases, Hospitalizations, Deaths by Age Group

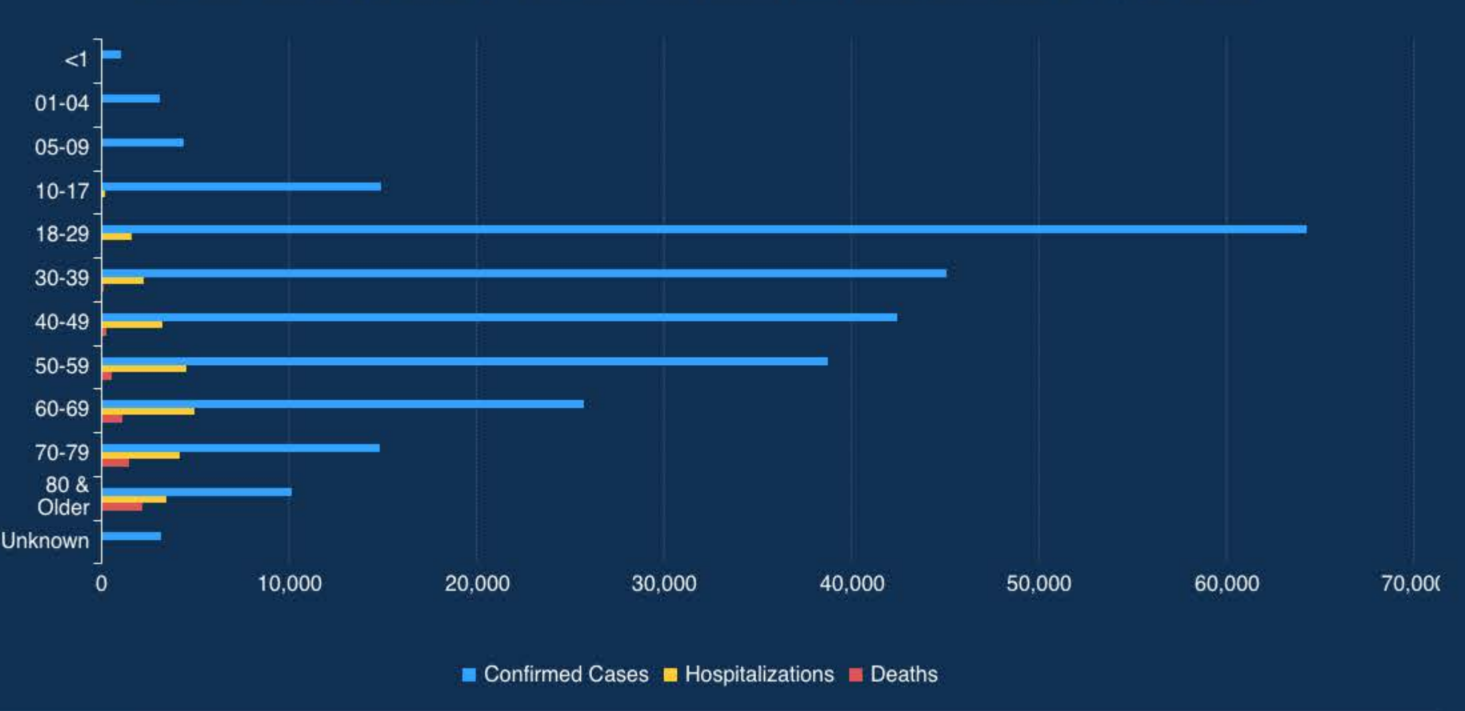

Race and Sex | Race and Ethnicity

Confirmed Cases by Race and Sex

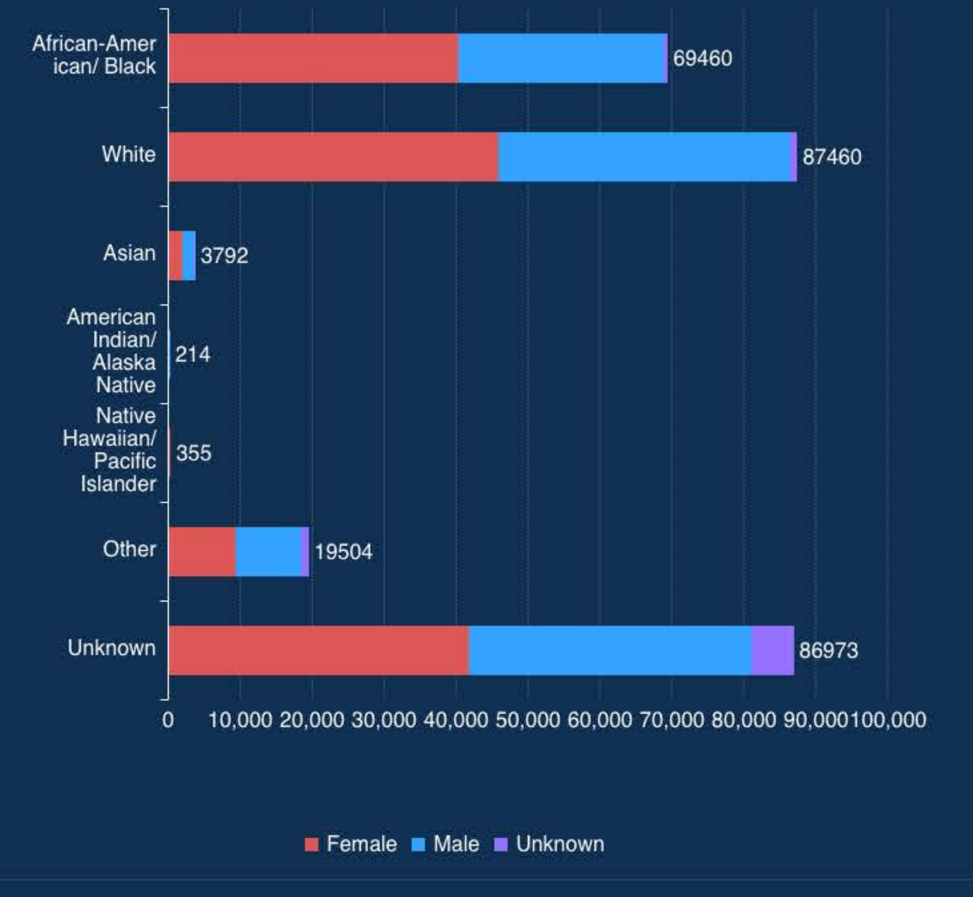

Confirmed COVID-19 Cases by Sex

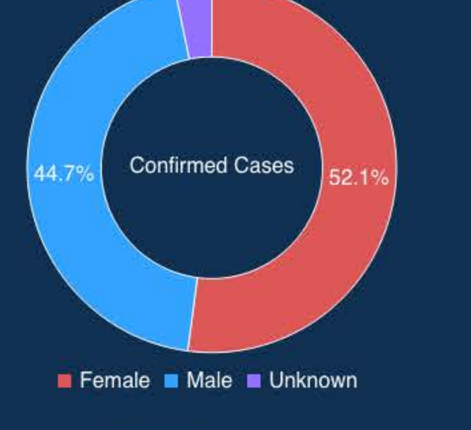

Chart | Table

Confirmed COVID-19 Cases by Race and Sex

Race	↓ Total Cases	Female Cases	Male Cases
White	87460	45782	40728
Unknown	86973	41860	39234
African-American/ Black	69460	40353	28413
Other	19504	9319	9119
Asian	3792	1863	1829
Native Hawaiian/ Pacific Islander	355	189	164
American Indian/ Alaska Native	214	110	100

Coronavirus in Georgia: COVID-19 Dashboard

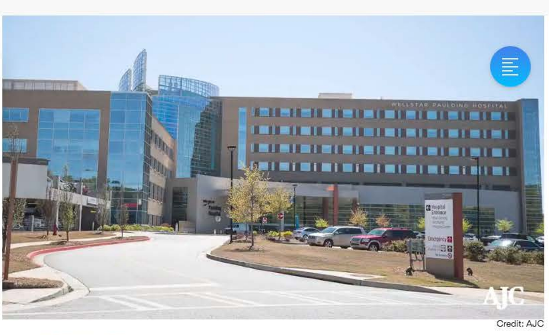
Credit: AJC

CORONAVIRUS | Updated Aug 26, 2020

Latest stats and the news on the coronavirus outbreak

This dashboard gives up-to-date counts for cases, deaths and other key statewide measures for the novel coronavirus. We update it at 3:15 p.m. (Sources: Georgia Department of Public Health, Georgia Emergency Management & Homeland Security Agency, Atlanta Journal-Constitution analysis)

The dashboard reflects the day the state reported confirmed cases and deaths. This means that the new confirmed cases could be reported a day or more after the test results came back, several days after the test was taken, and a week or more after the patient was infected. So new confirmed cases reflect the spread of actual infections from a week or more ago.

Explore AJC's new charts track virus' impact in Georgia

We have added additional charts that present the data based on first reported symptom for confirmed cases or date of death, as reported by the state.

Q: What is the latest on coronavirus cases in Georgia?

Newly reported cases by day in Georgia

Here, new confirmed cases of coronavirus are shown by the date they first appeared on the DPH website. Because of the lag between first symptoms and DPH reporting a positive test result, other charts representing symptom onset can be misleading without proper context, as they tend to show a recent steep decline due to incomplete data. Looking at newly reported cases in this chart adds context by providing a static snapshot of each day's reports.

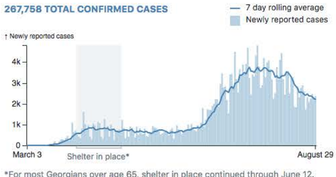

*For most Georgians over age 65, shelter in place continued through June 12. Medically fragile Georgians must continue to shelter in place through at least August 31; residents of long term care facilities are subject to stricter restrictions through at least August 31.
Source: Georgia Department of Public Health
Updated August 29 at 2:50 p.m.

[If you are on mobile and are not seeing our charts, please click here to reload full version of page]

Confirmed cases by date of symptom onset* in Georgia

Here, new confirmed cases of coronavirus are shown by the date of symptom onset* as reported by DPH. But because there is a lag between first symptoms and the date DPH reports a positive test result, more recent data is likely to be adjusted upward in the future.

While DPH may adjust the number of cases from any date, the dashed line and lighter colored bars represent the window when the most adjustments are likely to occur, according to our analysis.

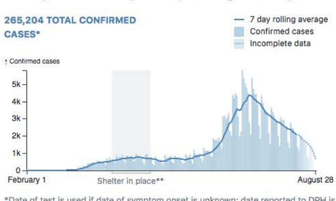

*Date of test is used if date of symptom onset is unknown; date reported to DPH is used if none of the above are known. Cases where DPH is unable to determine a date are not included, which accounts for the discrepancy between the number of confirmed cases on this chart and the newly reported cases chart.
**For most Georgians over age 65, shelter in place continued through June 12. Medically fragile Georgians must continue to shelter in place through at least August 31; residents of long term care facilities are subject to stricter restrictions through at least August 31.
Source: Georgia Department of Public Health
Updated August 28 at 2:50 p.m.

Q: What is the latest on coronavirus deaths in Georgia?

Newly reported deaths by day in Georgia

Here, new deaths from coronavirus are shown by the date they first appeared on the DPH website. Because of the lag between the date of a death and DPH reporting it, other charts representing day of death can be misleading without proper context, as they tend to show a recent steep decline due to incomplete data. Looking at newly reported deaths in this chart adds context by providing a static snapshot of each day's reports.

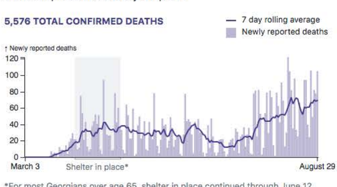

*For most Georgians over age 65, shelter in place continued through June 12. Medically fragile Georgians must continue to shelter in place through at least August 31; residents of long term care facilities are subject to stricter restrictions through at least August 31.
Source: Georgia Department of Public Health
Updated August 29 at 2:50 p.m.

Confirmed deaths by day of death in Georgia

Here, new deaths from coronavirus are shown by the date they occurred, as reported by DPH. But because there is a lag between the date of death and the date DPH reports it, more recent data is likely to be adjusted upward in the future.

While DPH may adjust the number of deaths from any date, the dashed line and lighter colored bars represent the window when the most adjustments are likely to occur, according to our analysis.

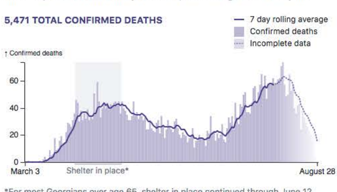

*For most Georgians over age 65, shelter in place continued through June 12. Medically fragile Georgians must continue to shelter in place through at least August 31; residents of long term care facilities are subject to stricter restrictions through at least August 31.
Source: Georgia Department of Public Health
Updated August 28 at 2:50 p.m.

Explore » Follow the full AJC coverage of the coronavirus outbreak

Q: What is the latest on people hospitalized for COVID-19 in Georgia?

Current hospitalizations by day in Georgia

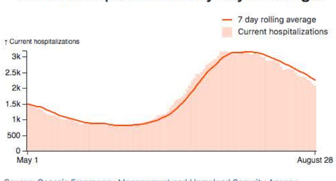

Source: Georgia Emergency Management and Homeland Security Agency
Updated August 28 at 4:05 p.m.

A: This is the number of people confirmed to have COVID-19 who are currently hospitalized, as published by the Georgia Department of Emergency Management & Homeland Security Agency. It began reporting this measure daily on May 2. It does not include those hospitalized who remain under investigation for suspected COVID. This number is updated daily.

Newly reported hospital cases* by day in Georgia

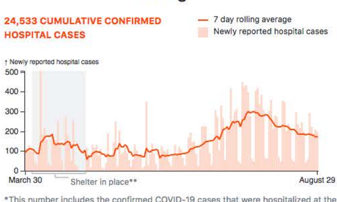

*This number includes the confirmed COVID-19 cases that were hospitalized at the time the case was reported to DPH or when the case was interviewed. This number does not capture hospitalizations that occur after a confirmed case was reported and, as such, is likely an underestimation of actual hospitalizations. It does not represent the number of COVID-19 cases currently hospitalized.
**For most Georgians over age 65, shelter in place continued through June 12. Medically fragile Georgians must continue to shelter in place through at least August 31; residents of long term care facilities are subject to stricter restrictions through at least August 31.
Source: Georgia Department of Public Health
Updated August 29 at 2:50 p.m.

Q: How many Georgians are testing positive for Coronavirus?

Percent testing positive for Coronavirus in Georgia

Each bar in this chart shows the percent testing positive for coronavirus each day as reported to the Georgia Department of Public Health (DPH). The line running through the bars shows the seven-day rolling average of the daily rates. The data does not include serologic tests, which identify if someone was exposed to the virus in the past and developed antibodies. The 5% line highlighted on the graph is the upper threshold identified by the White House Coronavirus Task Force for states to be in the green, or safe, zone for reopening.

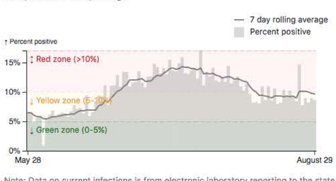

Note: Data on current infections is from electronic laboratory reporting to the state, which does not include all tests performed in Georgia. DPH excludes other testing sources because they do not consistently provide negative results, which must be factored in to arrive at positivity percentage. DPH also notes that, "People with a positive test often get retested and may test positive multiple times. These repeat positive tests will be counted as new positive tests."
Data: Georgia Department of Public Health
Zones: White House Coronavirus Task Force
Updated August 29

Q: Does today's case data show how the virus is spreading right now in Georgia?

A: No. Epidemiologists agree that today's counts are a snapshot of what the virus did roughly two weeks ago. It can take a week or more for a person to become infected, show symptoms, get tested and have their results reported to public health officials. Deaths are generally reported to the state quickly, but it can take weeks for an infected person to succumb to the virus.

Q: What does the 7-day average line indicate?

A: This measure makes it easier to see trends in the spread of the virus that can be obscured by unrelated bumps and dips. For instance, Georgia's daily case counts tend to be lower on the weekends because reporting and testing slows down, not because fewer people are getting sick.

Q: Where is coronavirus spreading now?

Recently reported coronavirus cases in Georgia

Rate of recently reported coronavirus cases in each county by population (cases reported in the last 14 days by the Georgia Department of Public Health based on each patient's reported county of residence, divided by the county's population and then multiplied by 100,000).

Tap each county for details on recently reported cases there.

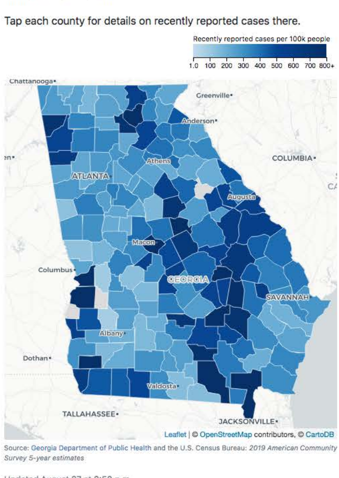

Source: Georgia Department of Public Health and the U.S. Census Bureau: *2019 American Community Survey 5-year estimates*

Updated August 27 at 2:50 p.m.

Q: What areas in metro Atlanta have had the most confirmed cases?

Confirmed coronavirus cases by ZIP code

Cumulative COVID-19 cases per 100,000 residents, plotted by ZIP Code Tabulation Areas (ZCTAs), which are approximations of actual postal ZIP code areas used by the U.S Census Bureau.

Because the Census only reports population estimates for full ZTCAs, we are unable to determine a rate for those which cross county lines into one or more counties for which we did not gather data. Those areas are displayed in gray but you can still find confirmed case totals by hovering over the ZIP code.

Fulton County reported fewer than 10 cases in some ZIP codes; those ZIPs and others for which no data was available are denoted on the map as a white area.

This data is updated weekly. Data is accurate to Aug. 24 for Gwinnett, Newton and Rockdale, Aug. 25 for DeKalb, Aug. 26 for Fulton, Cobb, Douglas and Clayton.

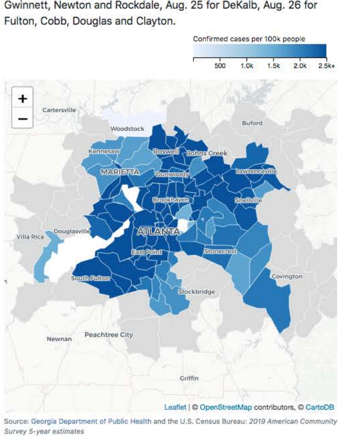

Source: Georgia Department of Public Health and the U.S. Census Bureau: *2019 American Community Survey 5-year estimates*

Q: Are there are other measures that can indicate how the virus is spreading?

A: Yes: the effective reproduction number, or Rt.

Rt is an epidemiological statistic that represents transmissibility, or the number of people a sick person infects at a given stage in the epidemic. If each person gets one person sick, the epidemic neither grows nor shrinks. Less than one means each person infects, on average, fewer than one person, so the epidemic shrinks. If Rt is larger than one, the epidemic grows. As Rt moves farther from 1, the epidemic shrinks or grows more rapidly.

The AJC calculated and compared several independent methods to estimate a value for Rt in Georgia. While the results from all were very similar, we're displaying a method designed by the Imperial College of London that uses epidemic curves to produce Rt estimates. The college's method is unrelated to its COVID-19 estimates.

As with other data presented on this dashboard, Rt values lag because of testing lags.

Transmission estimates in Georgia

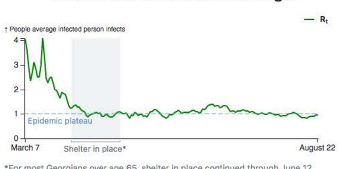

*For most Georgians over age 65, shelter in place continued through June 12. Medically fragile Georgians must continue to shelter in place through at least August 31; residents of long term care facilities are subject to stricter restrictions through at least August 31.
Source: Georgia Department of Public Health
Updated August 26 at 1:59 a.m.

Map | Epi Curve | County Curve | Lab Testing | Age/Gender | Age Trends | Race

Hawaii COVID-19 Cases

Hawaii County

Honolulu County

Kauai

Total

7,830

Released

2,410

Required

485

Deaths

59

Cases with Onset Dates in the Past 28 Days

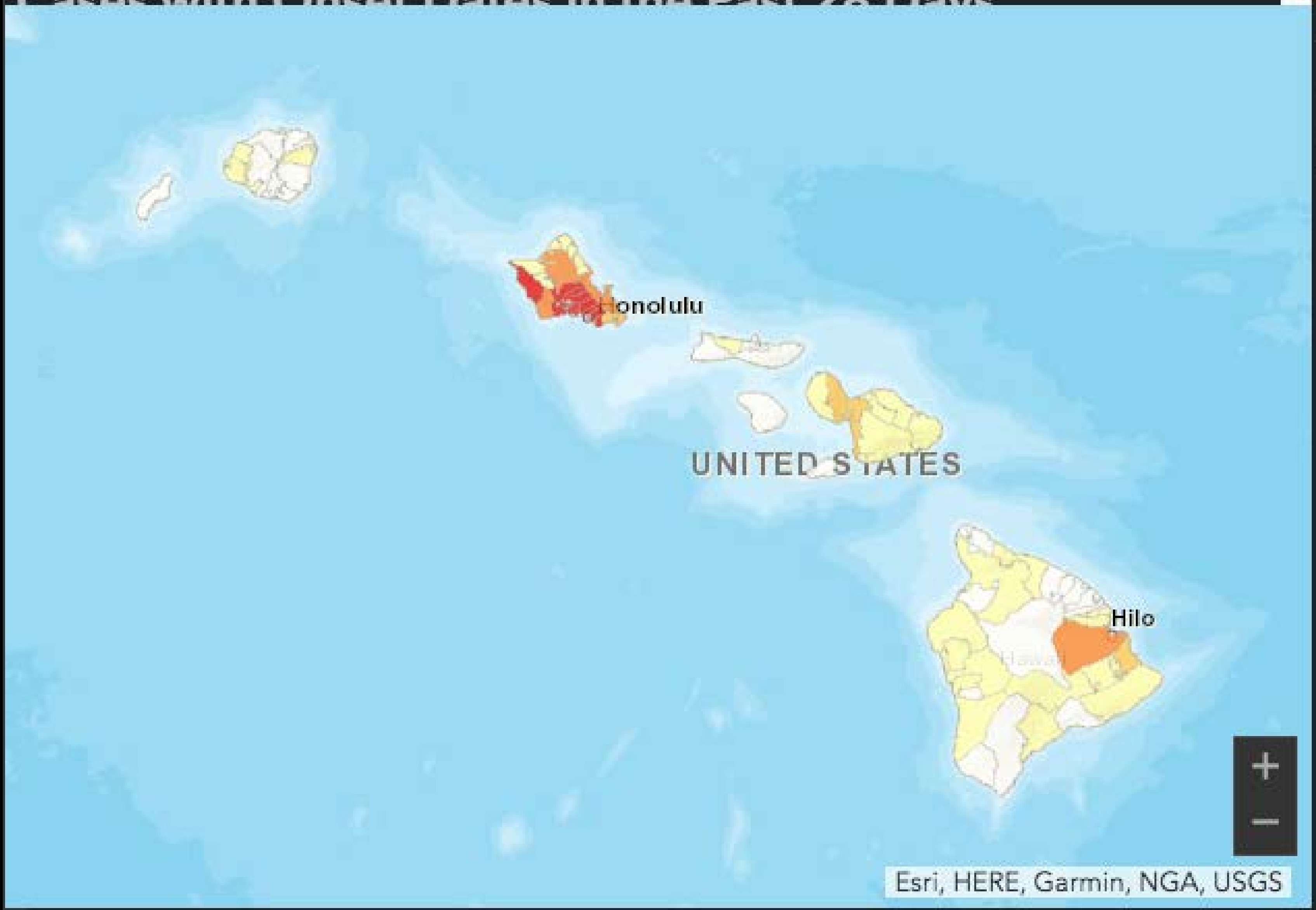

Cases with onset dates in the past 28 days

- 0
- 1-25
- 26-5
- 51-1
- >150

Cases in the Past 28 Days

Data as of August 27, 2020. Data are preliminary and subject to change.

Maps include all cases for which location data are available. Case location based on ZCTA of case residence or location stayed while in Hawaii. The maps do not imply that risk of transmission is isolated to

Tracking COVID-19 In Hawaii

*During these uncertain times, the Hawaii Data Collaborative is **working to unlock the power of publicly available data to support better decision making** for shaping our community's response both during and in the aftermath of the COVID-19 crisis. We are maintaining this easy-to-digest local source for timely information, while working to build collaborations to ensure we have the best possible COVID-19 data for critical decision making going forward.*

*The Hawaii Data Collaborative is not affiliated, associated, or officially connected in any way with the State of Hawaii Department of Health; their COVID-19 webpage can be accessed at the link here.

COVID-19 Hawaii Insights | Visitor & Unemployment Tracking ↓ | Local & National Resources ↓

7-Day Averages of New COVID-19 Cases in Hawaii

Values in parentheses refer to (new cases | total cases | 7-day avg of daily cases/100k)

State

252.0

(265 | 7,830 | 17.7)

14-Day Trend:

(0 cases pending)

Honolulu 229.3 (233 | 7,147 | 23.5)

Hawaii 14.6 (26 | 279 | 7.2)

Maui 7.9 (6 | 324 | 4.7)

Kauai 0.0 (0 | 56 | 0.0)

14-Day Trend:

Additional State-Wide 7-Day Averages:
Hover over each indicator to see county breakdown

Active Cases	Hospitalizations	Deaths
201.0	21.1	2.0
(221 | 5,361 | 14.1)	(23 | 485 | 1.5)	(4 | 59 | 0.1)

Data Format:
7-Day Avg of New Cases
(New Cases | Total Cases | 7-Day Avg of Daily Cases/100k)

Last updated Aug 28

+ a b | e a u

Hawaii COVID-19 Detailed Trends

7-Day Avg of Daily Cases per 100,000 people

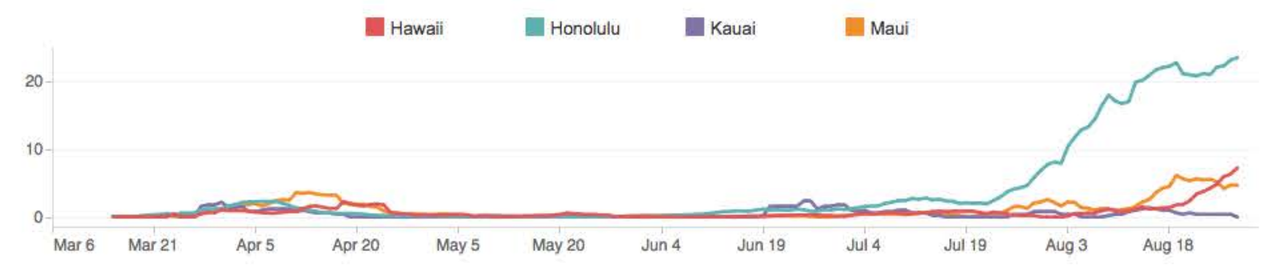

Trends by Metric and Region

Bars represent daily values and line represents 7-day averages

Metric: New Cases Region: State

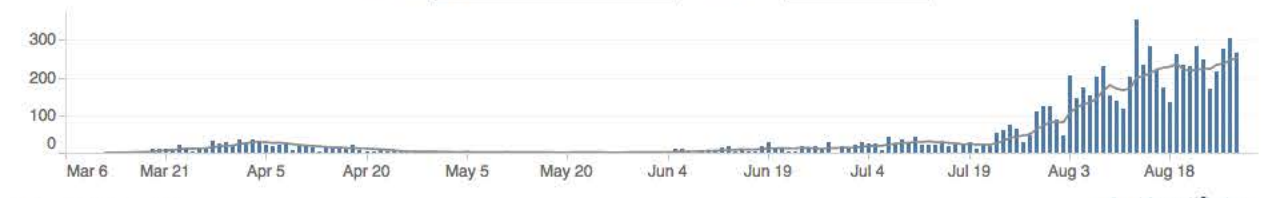

Last updated August 28

+ a b | e a u

Hawaii COVID-19 ICU Hospitalizations

Data for this dashboard currently draws from daily updates provided by Lieutenant Governor Josh Green. At this time, active hospitalization data is not reported by the Department of Health.

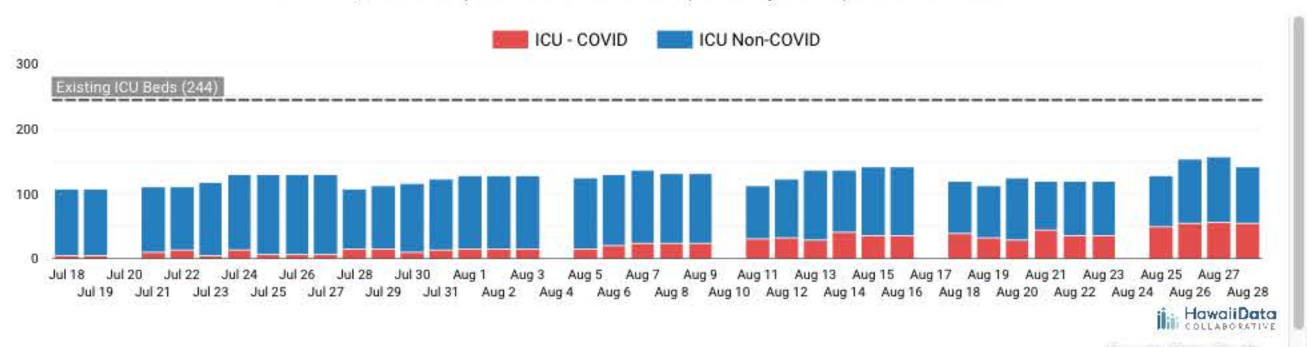

Click here to access the data for the dashboards above

Hawaii COVID-19 Cases by Risk Factor

Note: This dashboard is maintained by the Hawaii Department of Health, and is included here in its original form.

COVID-19 Epidemic Curve, Hawaii 2020 (Updated Aug 28, 2020)

View Trend Line

View as Table

Total Cases: 7,806**

View by Risk Factor (All)

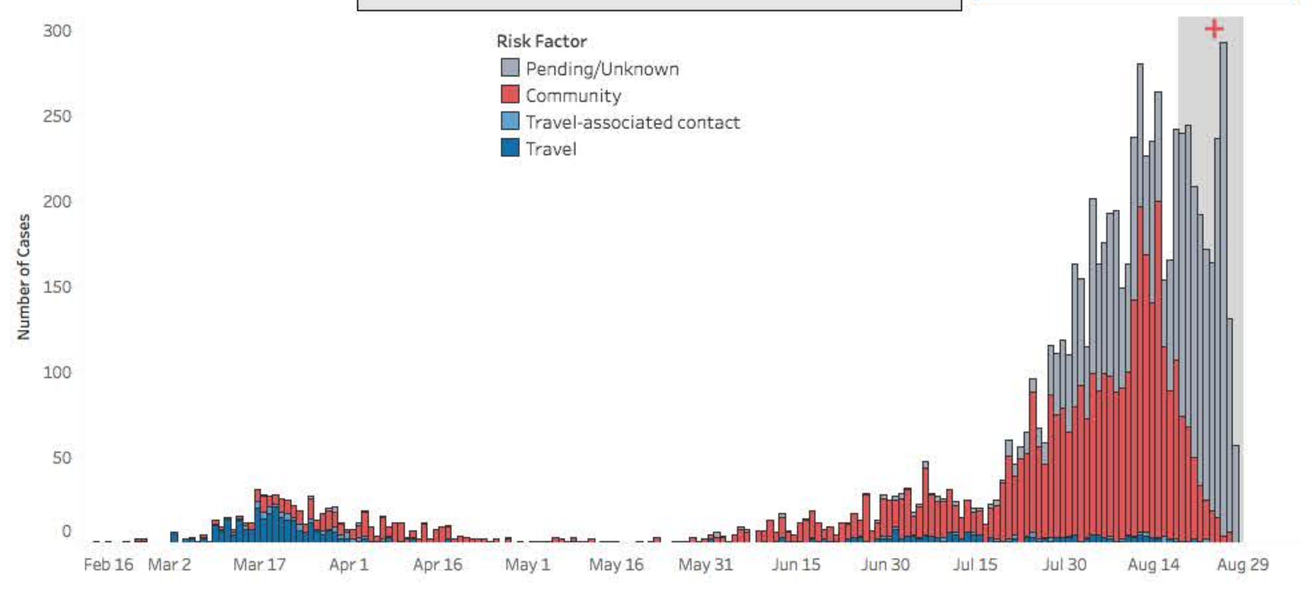

* When symptom onset date is not available, test date is used
+ Illnesses that began in the past 10 days may not yet have been reported

+ a b | e a u

Hawaii COVID-19 Testing

Note: This dashboard is maintained by the Hawaii Department of Health, and is included here in its original form.

COVID-19 Testing* in Hawaii, 2020 Updated Aug 28, 2020

Select County: (Note: y-axis scale changes for each county) (All)

Total Number of Tests Performed:	Percent Positive (past 7 days):
251,710	6.2%

View Turnaround Time

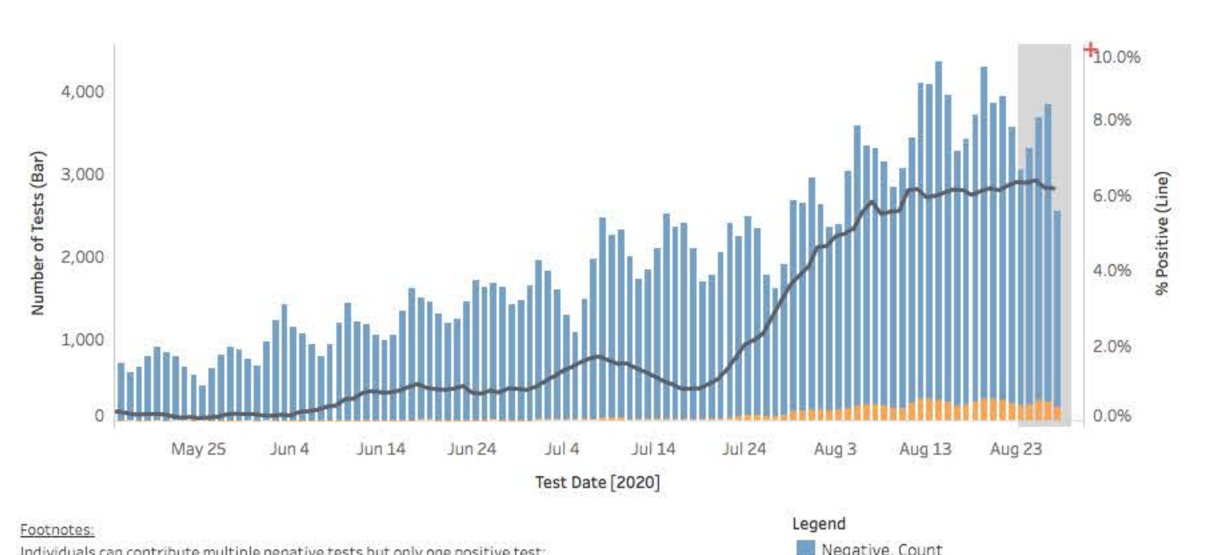

Footnotes:
Individuals can contribute multiple negative tests but only one positive test;
* Based on electronic laboratory reporting of PCR tests; uses a 3-day moving average;
+ Tests performed in the previous 3-5 days may not yet have been reported;

+ a b | e a u

Anonymous Mobility Data

The mobility index map below indicates changes in mobility by county, compared to a predetermined baseline (100). Mobility data is based on anonymized geolocation reports, obtained by Descartes Labs, from smartphones and other mobile devices. This data is statistically aggregated, removing the ability to characterize the behavior of any single device

Numbers above 100 represent increases in community movement, while numbers below 100 represent decreases:

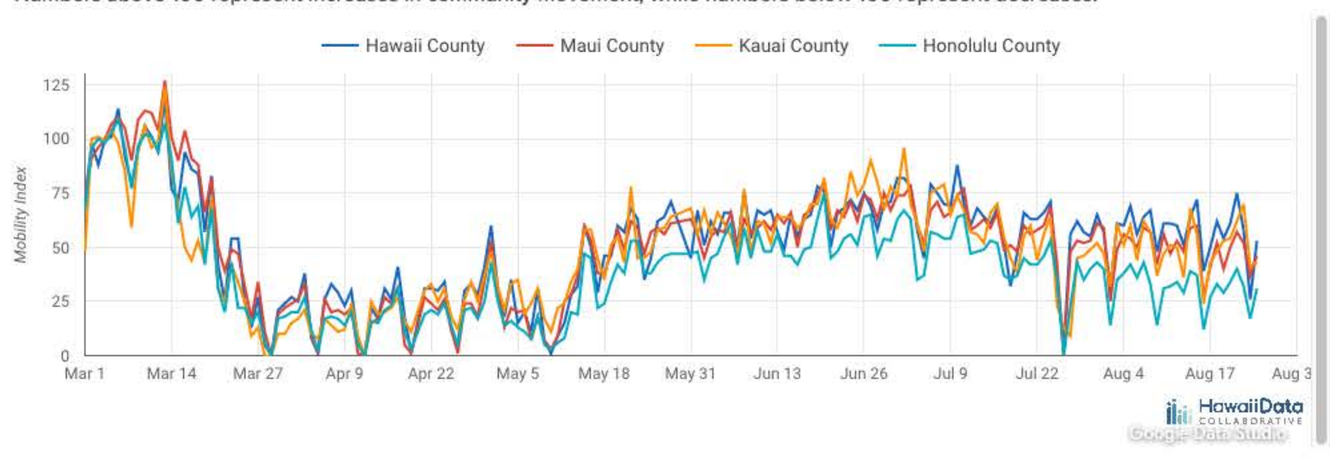

Daily Passenger Arrivals by Air

Region: State Sort by reason for travel: Visitor

Reason for Travel: Visitor Resident Military New Resident Other

Daily Arrivals – State

The average daily number of passangers arriving in Hawaii last year was roughly 30,000

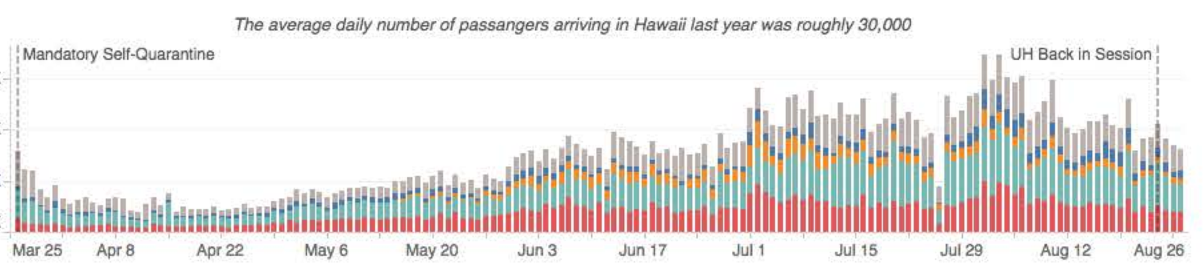

7-day Averages – State

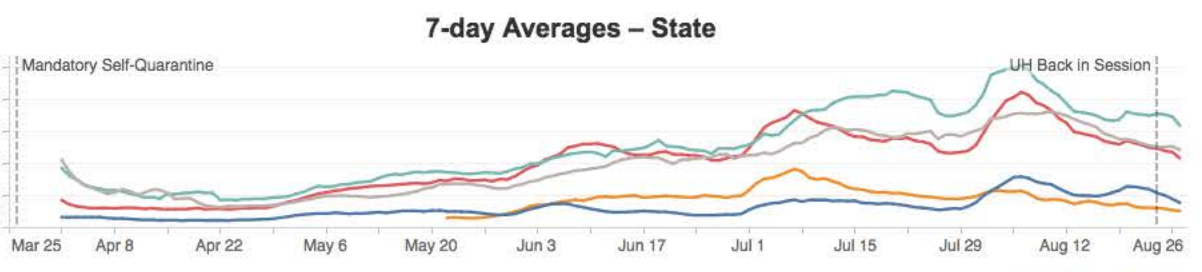

+ a b | e a u

Click here to access the data for passenger arrivals by air

Unemployment Insurance Claims & Claimant Characteristics

Hawaii's Unemployment Insurance Trends in 2020

Area
Hawaii County
Honolulu County
Kauai County
Maui County
State of Hawaii

UI Claims

Select Area to view number of Initial Claims and Weeks Claimed by Week Ending Date

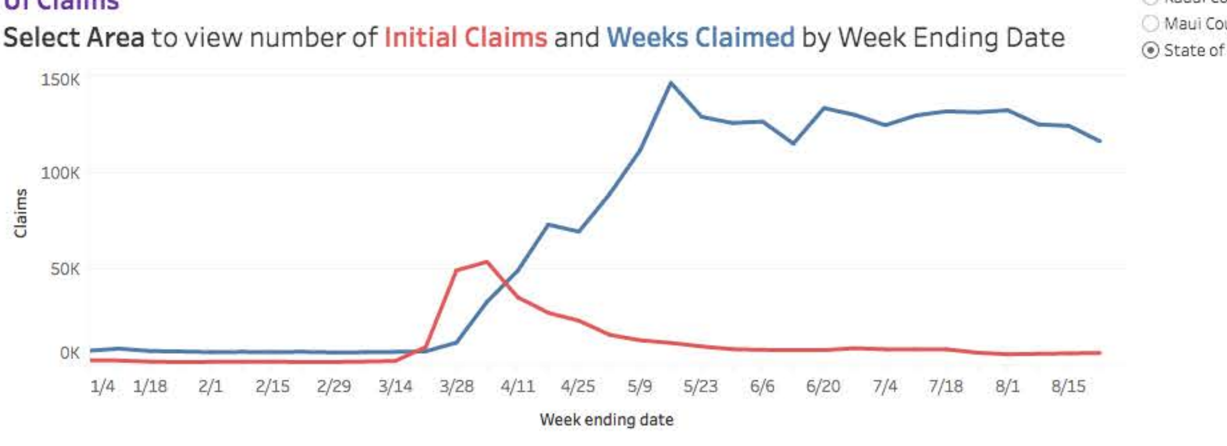

UI Claimant Characteristics, July 2020, State of Hawaii

Select one or more Categories

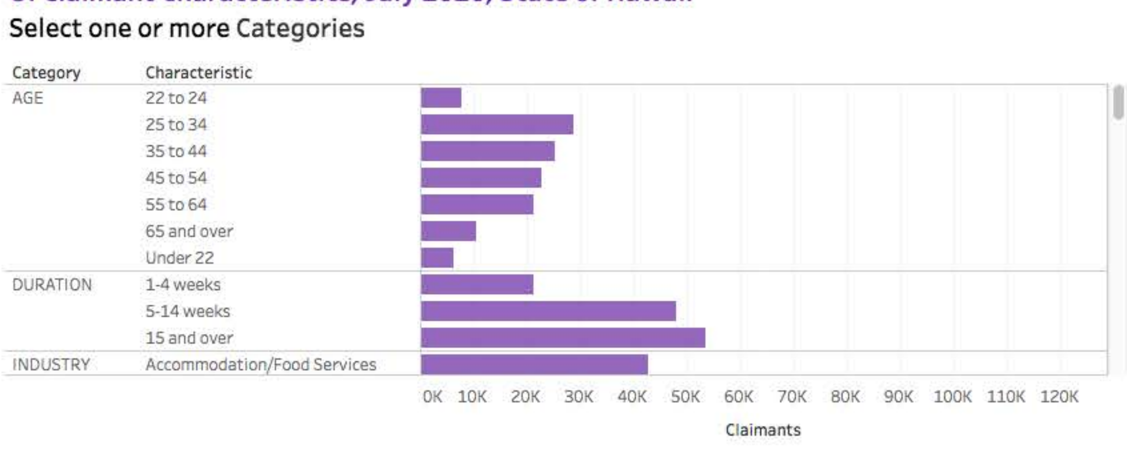

Category
(All)
AGE
DURATION
INDUSTRY
OCCUPATION
RACE
SEX
TOTAL

+ a b | e a u

Note: This dashboard is maintained by the Hawaii Department of Labor and Industrial Relations, and is included here in its original form. More information can be found here.

COVID-19 in Idaho

DIVISION OF PUBLIC HEALTH

31,384 ***(262 New)***

Statewide Cases

Idaho case counts include both probable and confirmed cases, classified based on criteria contained in CSTE position statement 20-ID-02, effective August 3, 2020.

Confirmed: A person is classified as a confirmed case if they tested positive for SARS-CoV-2 infection using a molecular amplification test.

Probable: A person is classified as a probable case if 1) they tested positive for SARS-CoV-2 infection using an antigen test, 2) they have a clinically compatible illness and epidemiologic risk for infection, or 3) their death certificate lists COVID-19 or SARS-CoV-2 as an underlying cause of death or significant condition contributing to death, without confirmation using a molecular amplification test.

Trend *(Number of Cumulative Cases)*

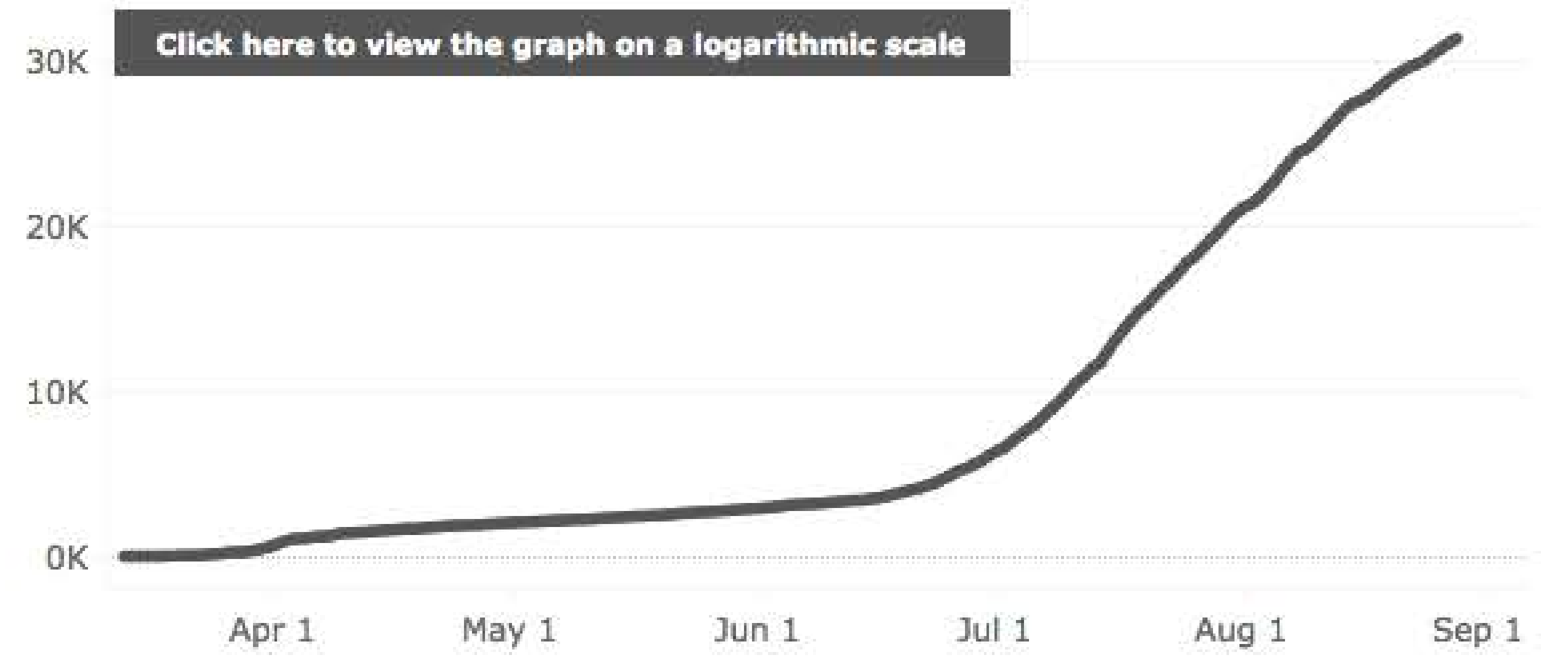

Cases by Date State Notified

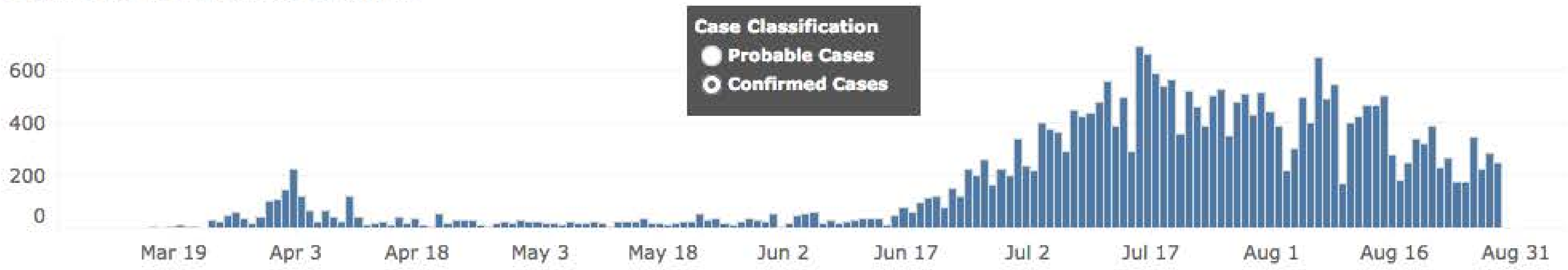

COVID-19 by Date of Onset

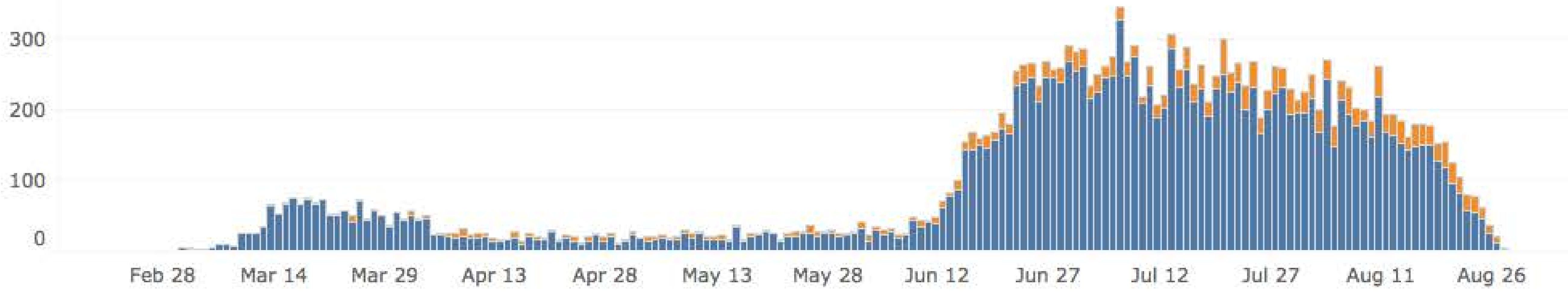

The Department of Health and Welfare will update data daily at coronavirus.idaho.gov at 5 p.m. ***Data are preliminary and subject to change.*** Idaho case counts include both probable and confirmed cases, classified based on criteria contained in CSTE position statement 20-ID-02, effective August 3, 2020.

IDAHO DEPARTMENT OF HEALTH & WELFARE

+ableau

DPH Idaho COVID-19 Dashboard.V2

Viz Author: Idaho Division of Public Health

3,480,019 Views ☆118

More Detail

Originally Published: Apr 2, 2020

Last Updated: Aug 28, 2020

Workbook Details: 1 Sheet

Metadata: Story 1

CORONAVIRUS

Interactive map and timelines tracking coronavirus numbers and trends in Idaho

See the latest COVID-19 data as we track deaths as well as confirmed, probable and recovered coronavirus cases.

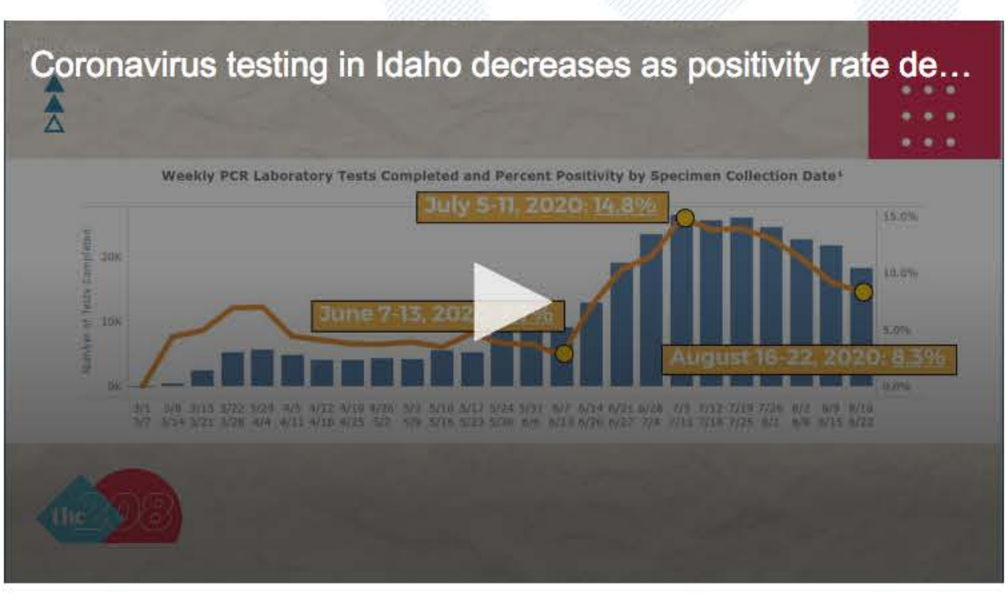

Author: KTVB Staff
Published: 4:13 PM MDT March 13, 2020
Updated: 5:22 PM MDT August 28, 2020

BOISE, Idaho — As Idaho reopens, we're continuing to post live daily updates on the coronavirus situation in Idaho (see the latest real-time updates here).

We're also tracking the coronavirus case numbers and overall trends over time below.

How we track COVID-19 cases:

Every day, KTVB works diligently to ensure we are providing the most accurate, up-to-date information regarding COVID-19 cases in Idaho. We track our numbers the same way the Idaho Dept. of Health and Welfare does. However, we update our total number more frequently, meaning our numbers may not always match with the state.

Local Idaho health districts are releasing confirmed COVID-19 cases before they are submitted to the state and added to the official Idaho state total. We're adding up the state total and showing the latest total count in our real-time updates live blog and below in this article.

The state updates the number of total cases every day around 5 p.m. KTVB updates our running total once the state and local health districts have confirmed their total number of cases.

Regional health districts release updated numbers at different times than the state Department of Health and Welfare, which can sometimes cause a discrepancy in the number reported. We use the latest information reported by the state and the health districts to keep the total as updated as possible.

Some of those numbers local districts release might change if a positive case is determined to be an out of state resident - they will be added to another state's total number of cases instead of Idaho.

It is important to note the difference between "confirmed" and "probable". Confirmed cases refer to a person who was tested for COVID-19 and tested positive. Probable cases refer to a person who is exhibiting symptoms after coming in contact with a person who has a confirmed case. Though a probable case is not confirmed, the state will monitor the person for symptoms.

"Total cases" refers to the number of confirmed and probable cases combined. We will always clarify how many of the total cases are confirmed and how many are probable in our daily updates.

Scroll down to see our multiple graphs and timelines looking at the coronavirus case trends in Idaho, including a new graph showing a 14-day average that the State of Idaho is tracking to determine which phase of reopening certain areas or the entire Gem State is ready for.

Idaho coronavirus latest: Latest news and daily updates | Idaho cities requiring masks | Help nonprofits

Download the KTVB news app to get urgent news alerts.

Latest Idaho coronavirus numbers:

See more and the latest coronavirus updates here.

- 354 deaths
- 29,241 confirmed cases (31,384 confirmed and probable cases)
- 14,175 recovered (estimated)

RELATED: 'It's just the right thing to do': Gov. Little says all Idahoans should be wearing masks in grocery and retail stores

Map of Idaho confirmed coronavirus (COVID-19) cases:

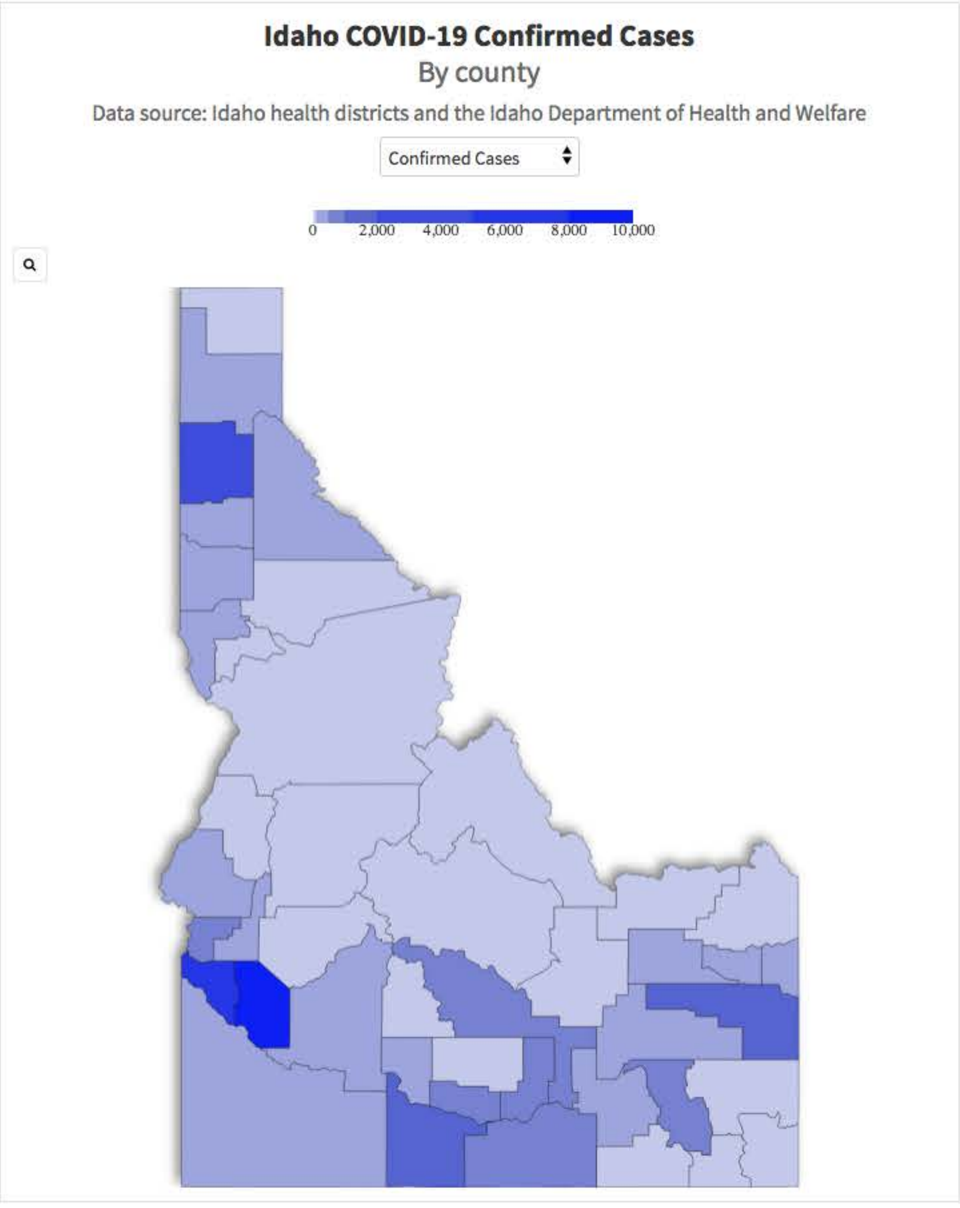

You can hover over each county to see its latest numbers.

Timeline of COVID-19 cases in Idaho:

Mouse over or tap each data point for more details. (Mobile users can tap here for a larger version).

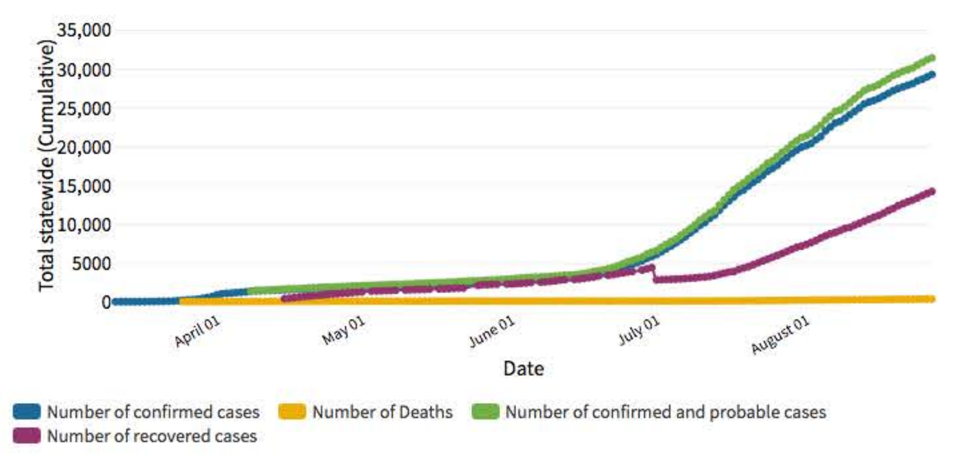

Note on the drop in recovered cases: Idaho Department of Health and Welfare says "an error in auto-calculation was discovered 7/1/2020."

"Probable" cases are people showing symptoms who had close contact with a confirmed coronavirus case, such as a member of the same household.

Timeline of total COVID-19 related deaths in Idaho:

Mouse over or tap each data point for more details. (Mobile users can tap here for a larger version).

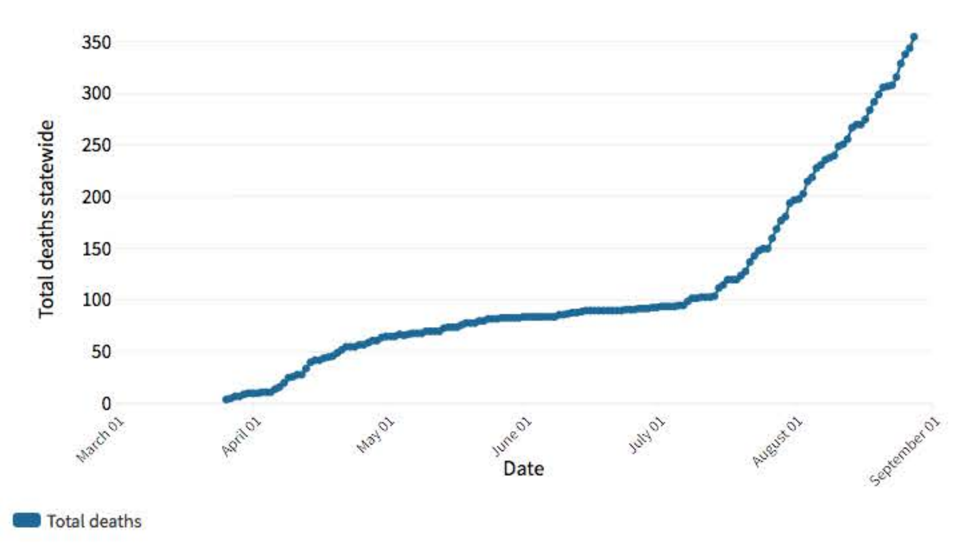

Timeline of total number of COVID-19 cases in Idaho and new cases reported (updated daily):

Mouse over or tap each data point for more details. (Mobile users can tap here for a larger version).

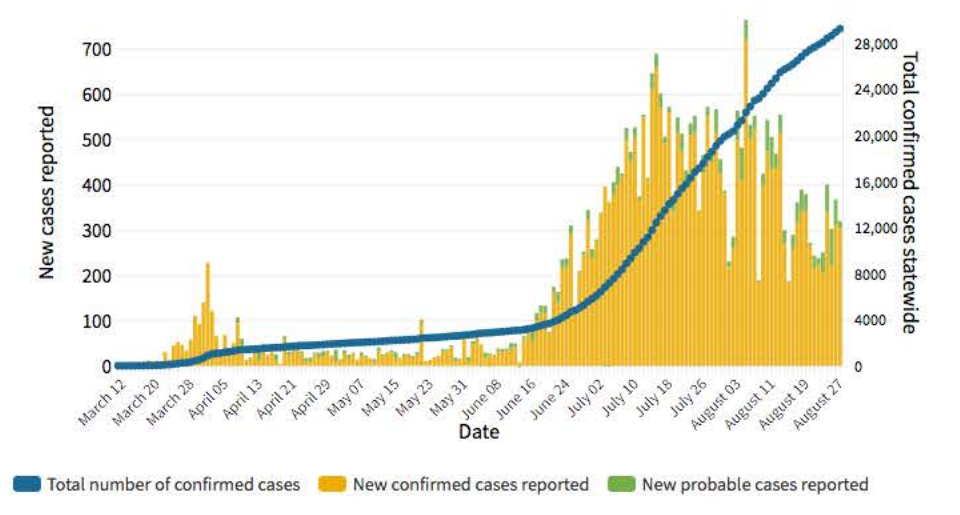

Timeline tracking the trend in newly reported cases over a 14-day period (updated daily):

Mouse over or tap each data point for more details. (Mobile users can tap here for a larger version).

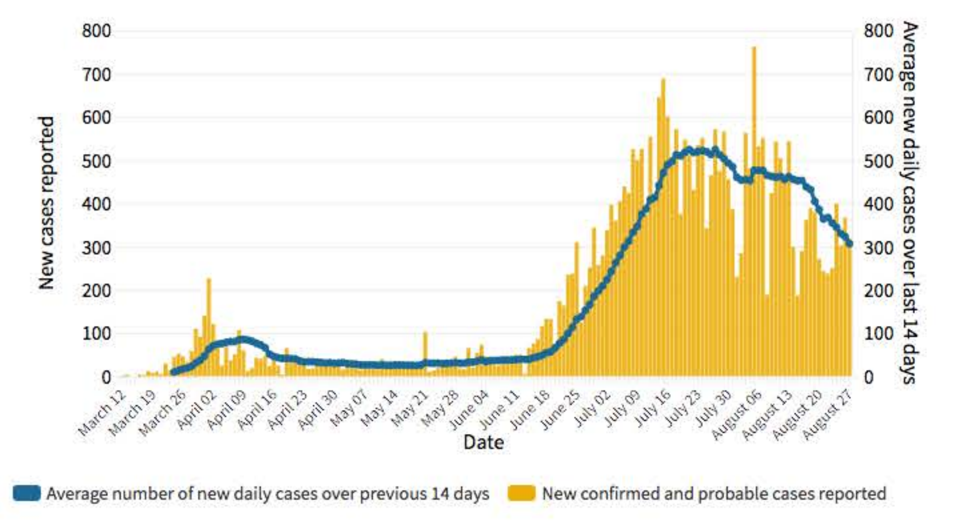

The State of Idaho has been using the 14-day average in its tracking to determine when the state could move to the next stage of reopening.

Timeline tracking the trend in new daily cases being reported over the last two weeks statewide (updated daily):

Mouse over or tap each data point for more details. (Mobile users can tap here for a larger version).

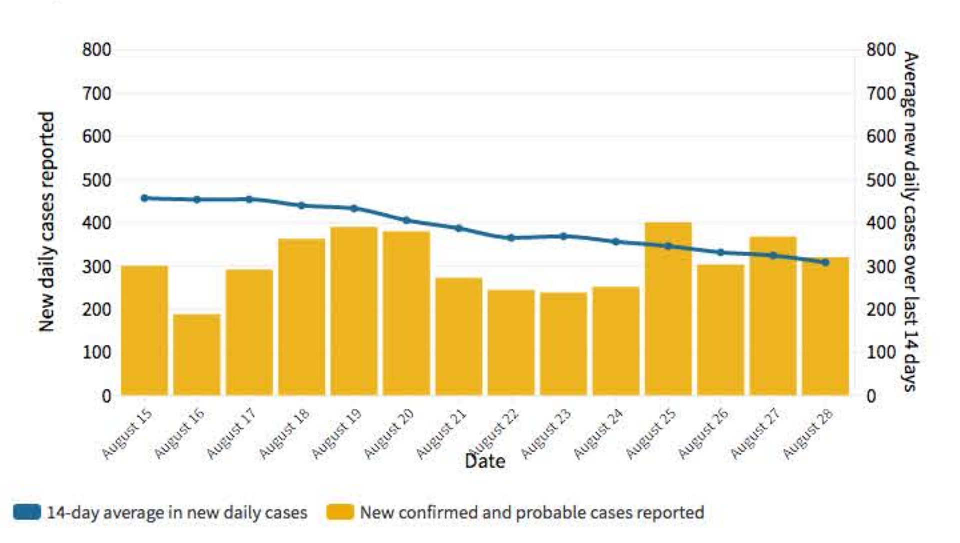

Here are the separate Idaho health districts we are monitoring to get the latest statewide COVID-19 case total:

State of Idaho (this will not immediately include the latest information released by the below lower districts)

Central District Health

South Central Public Health District

Southeastern Idaho Public Health

Eastern Idaho Public Health

Panhandle Health District

Idaho North Central District

Southwest District Health

RELATED: How Idaho counts confirmed COVID-19 cases

COVID-19 Home
Guidance +
Illinois Data +
Testing +
Resources & Information +
Health Care Providers & Facilities +
Media & Publications +

COVID-19 Statistics

Positive Cases (Confirmed)	Deaths	Total Tests Performed*
231,363	8,008	3,973,089

Recovery Rate**
95%

*Total number of tests performed and reported electronically for testing of COVID-19 at IDPH, commercial or hospital laboratories. All numbers displayed are provisional and will change.
** Recovered cases are defined as persons with initial positive specimen collection date > 42 days who have not expired.
Recovery rate is calculated as the recovered cases divided by the sum of recovered cases and deceased cases.
Information regarding the number of persons under investigation updated on 8/29/2020.

By County | By Zip

Select Zip:

Illinois

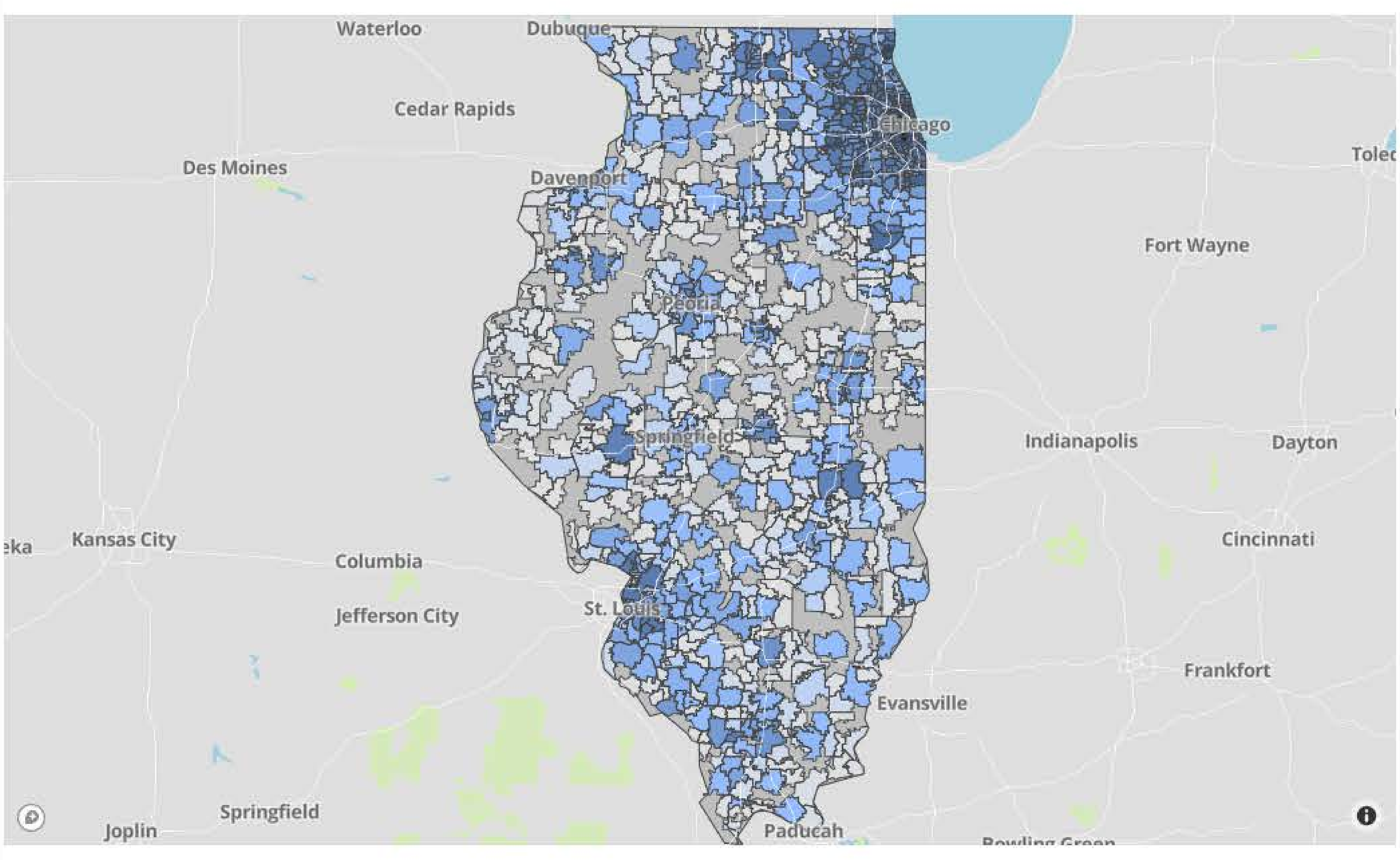

The data on this site is what has been entered into Illinois' National Electronic Disease Surveillance System (I-NEDSS) data system.
The data is constantly being entered and may change as cases are investigated. Therefore, numbers may vary from other sites where data is published.
*Data shown is >5 cases per zip code which is consistent with Collection, Disclosure, and Confidentiality of Health Statistics (77 Ill. Adm. Code 1005).
It should be assumed that COVID-19 exposure can occur in every county in IL.
Zip code is zip code of residence, which may not be location of exposure.

Search

Zip	Tested	Positive Cases	Deaths
60002	3,321	184	N/A
60004	16,554	550	N/A
60005	8,547	364	N/A
60007	7,207	508	N/A
60008	7,415	492	N/A
60010	9,496	412	N/A
60012	1,952	135	N/A
60013	4,175	256	N/A
60014	8,620	564	N/A
60015	6,975	306	N/A

1 2 3 4 5 6 7 8 9 10 11 12 13 14 15 16 17 18 19 20 21 22 23 24 25 26
27 28 29 30 31 32 33 34 35 36 37 38 39 40 41 42 43 44 45 46 47 48 49 50
51 52 53 54 55 56 57 58 59 60 61 62 63 64 65 66 67 68 69 70 71 72 73 74
75 76 77 78 79 80 81 82 83 84 85 86 87 88 89 90 91 92 93 94 95 96 97 All

Age Demographics

Confirmed Cases | Tested | Deaths

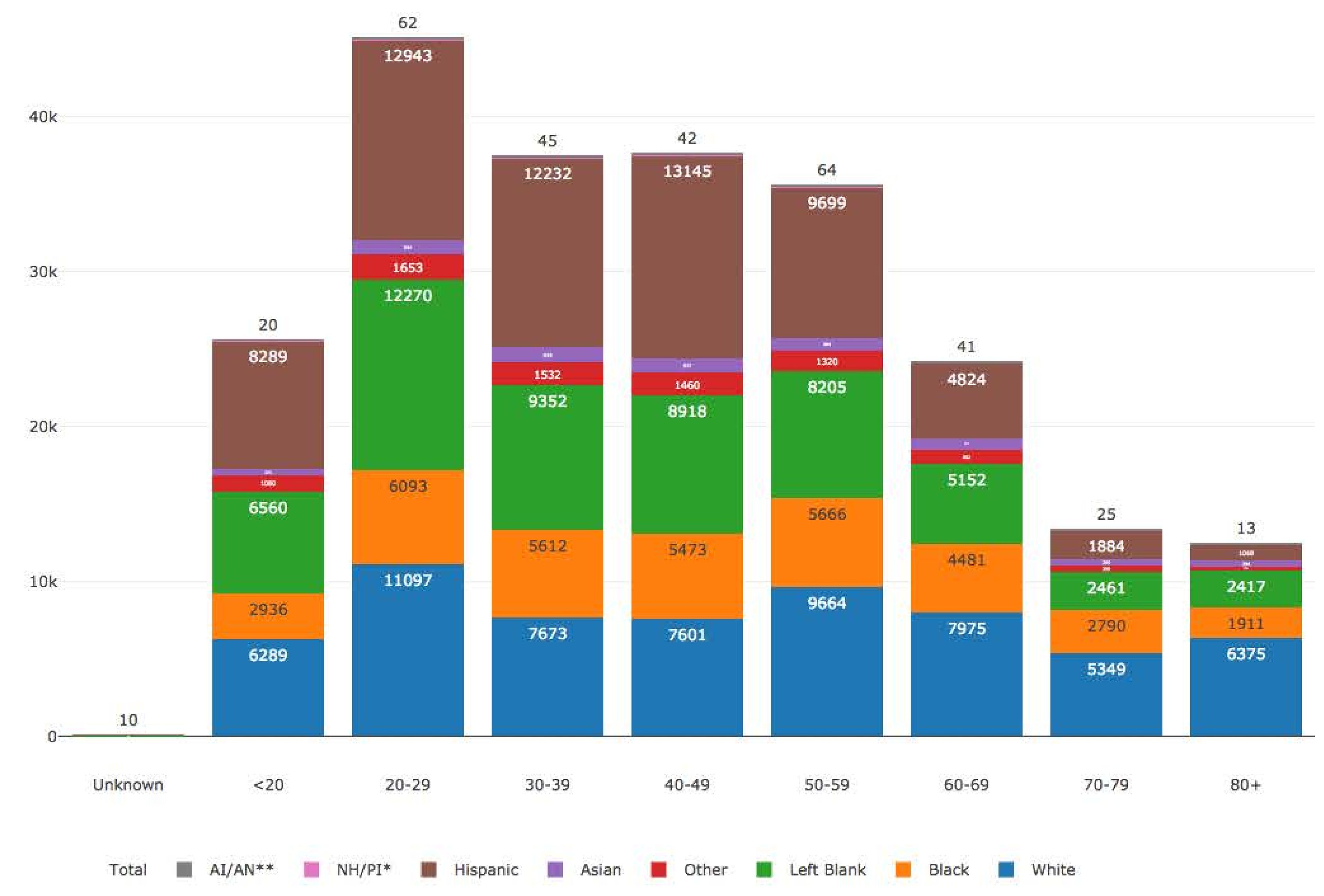

Race Demographics

Confirmed Cases | Tested | Deaths

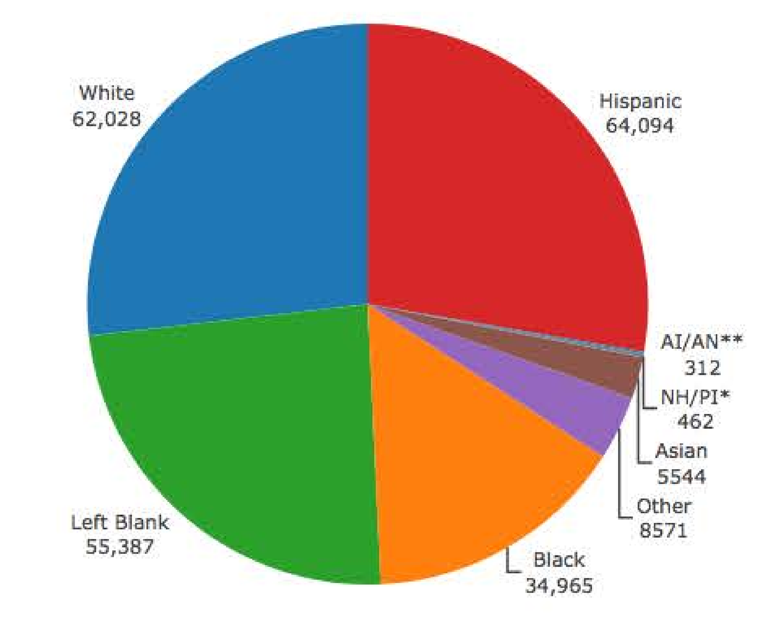

Gender

Confirmed Cases | Tested | Deaths

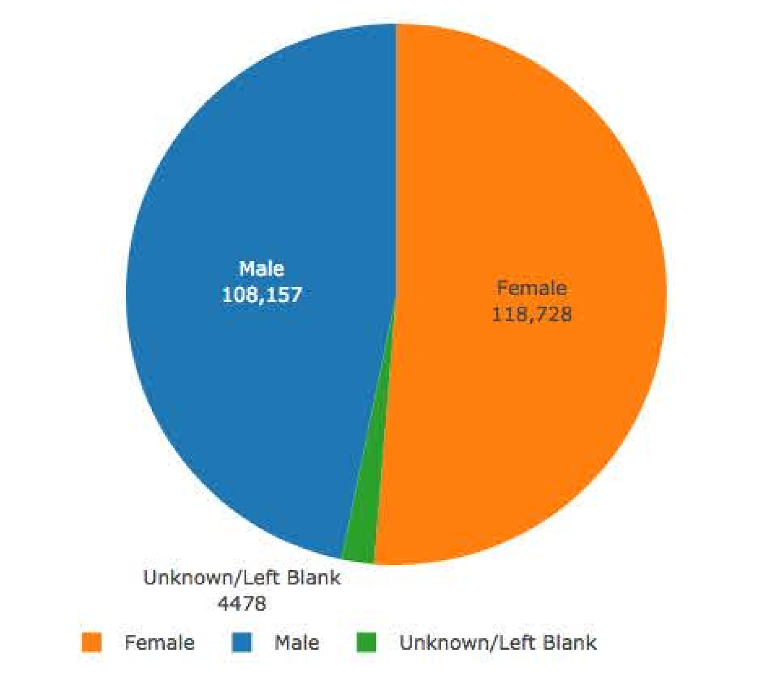

*Native Hawaiian or Other Pacific Islander
**American Indian or Alaskan Native

Tested Results Last 5 days

Tested | Cases | Deaths

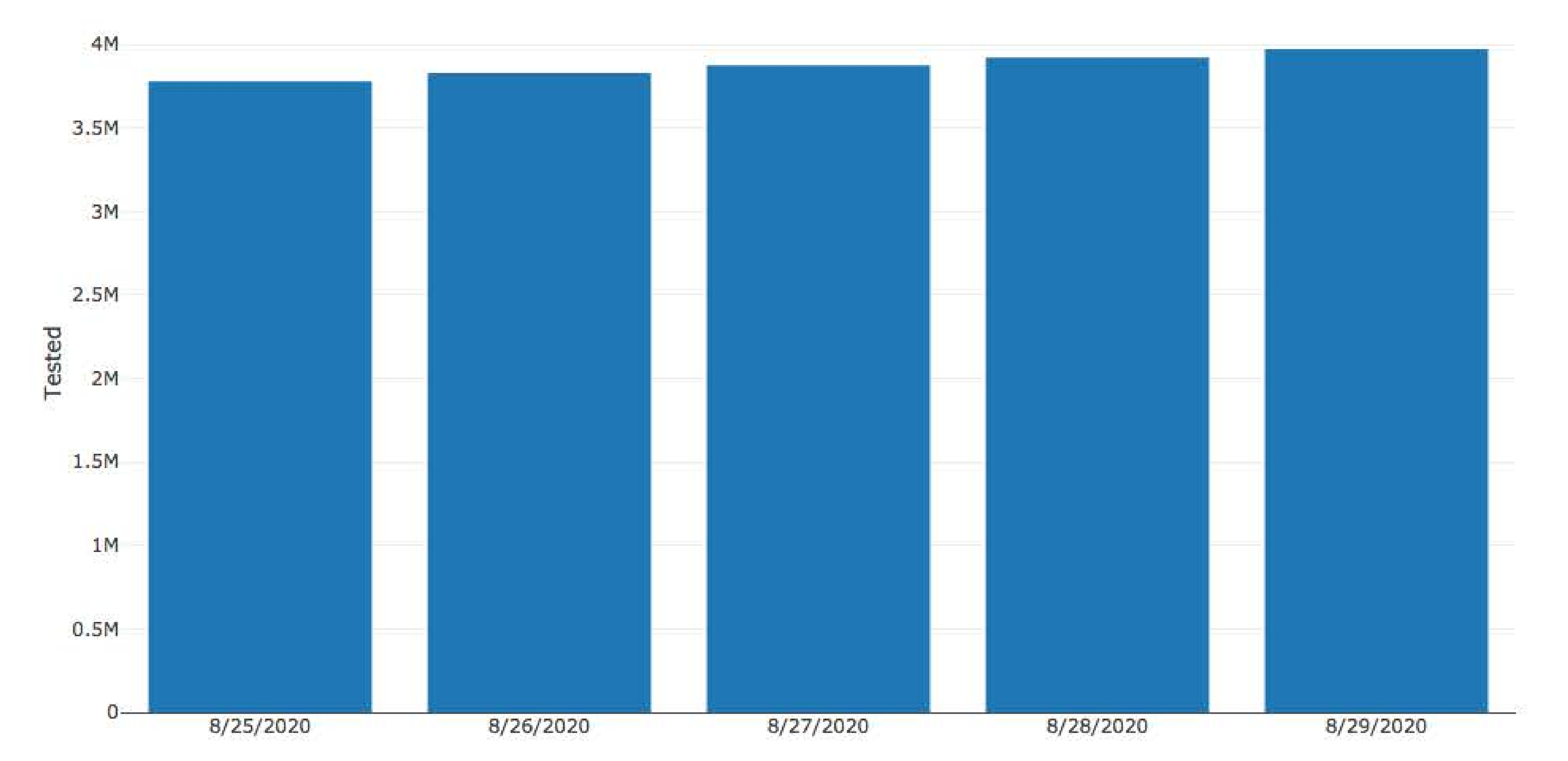

5d | 10d | 30d | All Time

Tested Change Last 5 days

Tested | Cases | Deaths

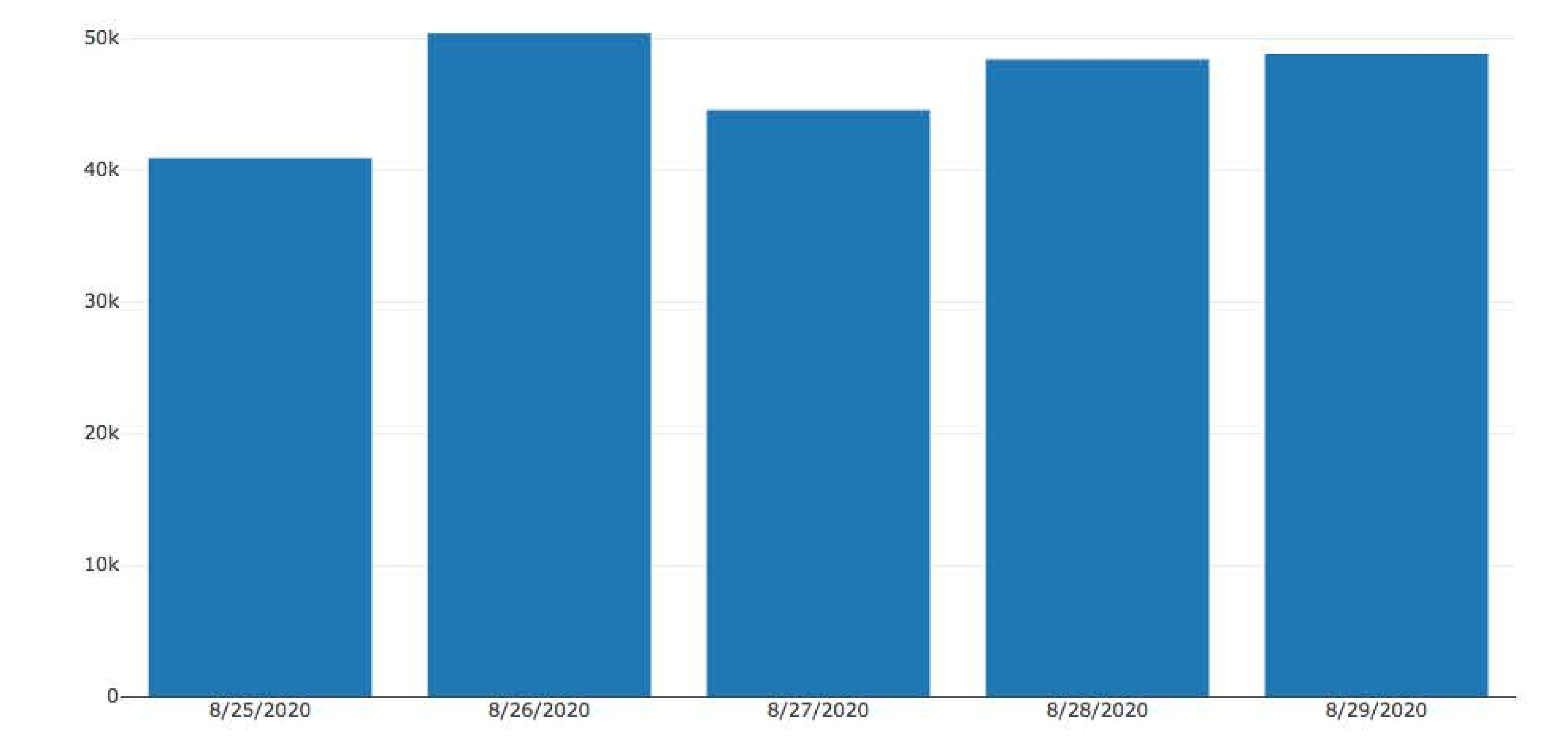

5d | 10d | 30d | All Time

Indiana COVID-19 Dashboard

Indiana COVID-19 Data Report

Additional Resources

Dashboard | LTC

Below results are as of **08/28/2020, 11:59 PM**. Dashboard updated daily at **12:00 PM**.
New positive cases, deaths and tests have occurred over a range of dates but were reported to ISDH in the last 24 hours.

Newly Reported Confirmed COVID-19 Counts

New Individuals Tested
12,033
21,492 New Tests Administered
between 03/27/2020 and 08/28/2020

New Positive Cases
1,140
between 08/12/2020 and 08/28/2020

Positivity - Unique Individuals
6.6 % 7-Day Rate
8.8 % cumulative rate
between 08/15/2020 and 08/22/2020

New Deaths
8
between 08/23/2020 and 08/28/2020

Total Confirmed COVID-19 Counts

Total Individuals Tested
1,055,793
1,396,309 Tests Administered

Total Positive Cases
92,434

Positivity - All Tests
5.3 % 7-Day Rate
6.8 % cumulative rate
between 08/15/2020 and 08/22/2020

Total Deaths
3,066

Ask Barb the Bot

County Distributions

Total Count

Positive Cases | Deaths | Tested | Positivity - All Tests

Select a county below by tap or click — ISDH District: Statewide

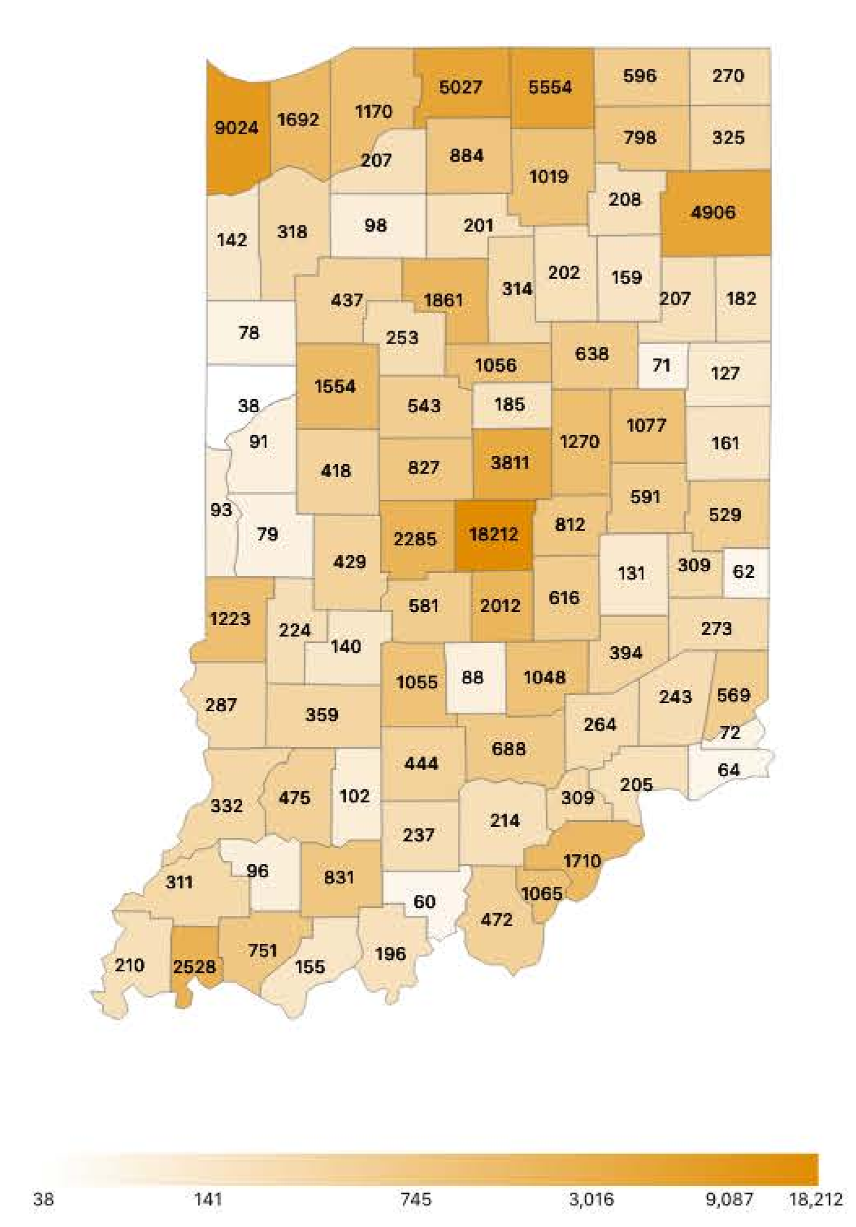

Positive Cases and Tested

Statewide Positive Cases by Day

Showing: All

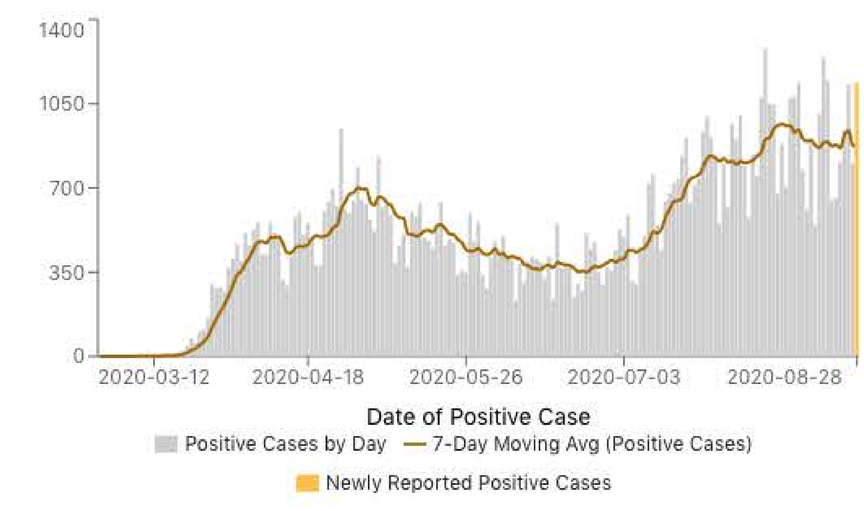

Statewide Tests by Day

Showing: All

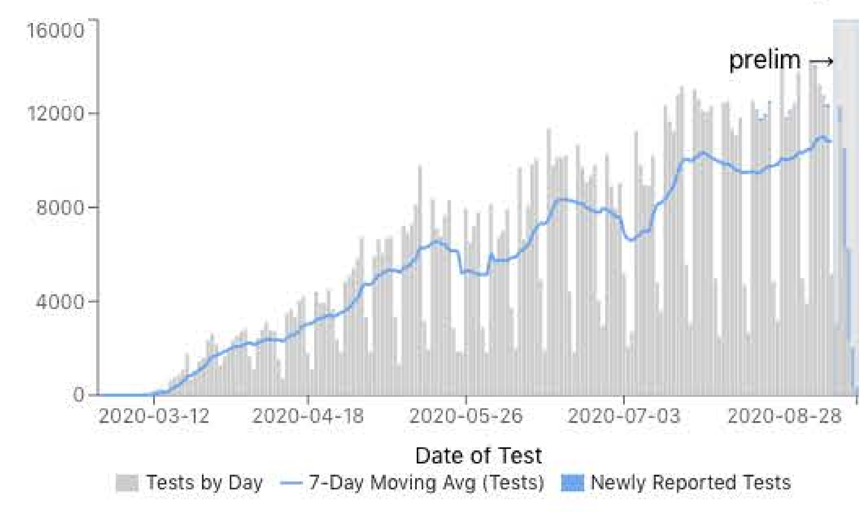

Deaths

Statewide Deaths by Day

Showing: All

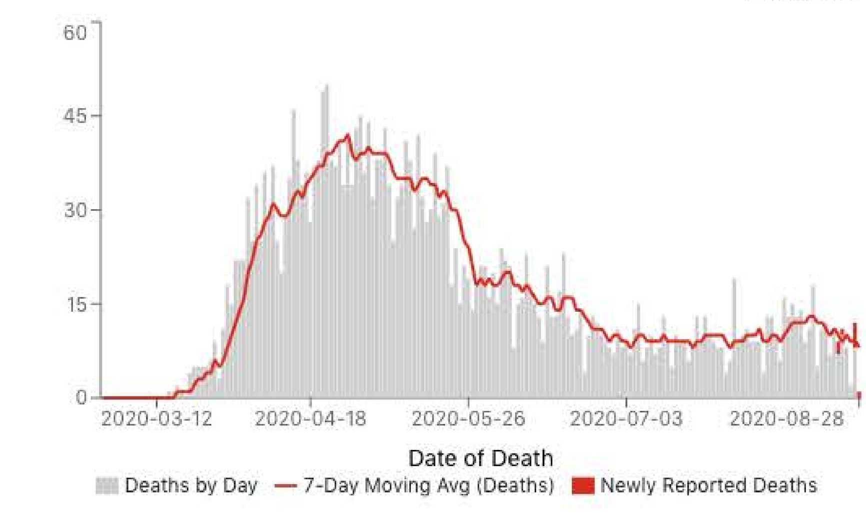

Positivity

Statewide Daily Positive Test Rate

What does prelim mean?

Showing: All Tests

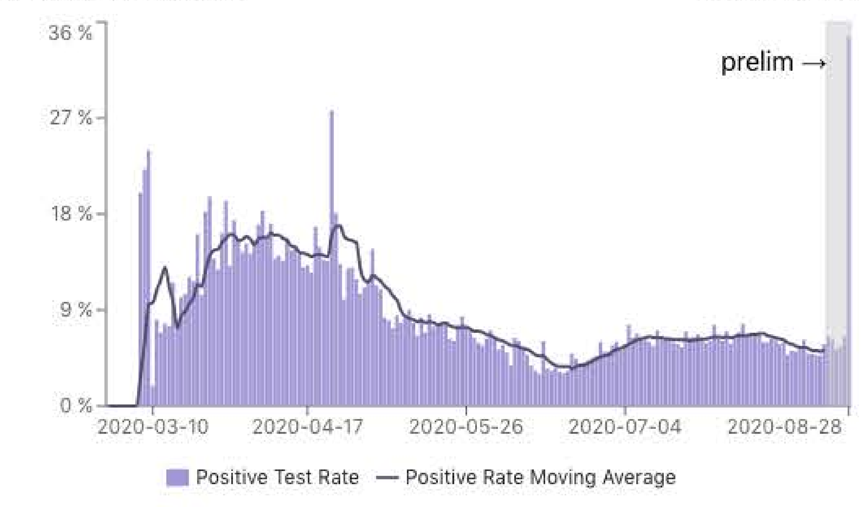

Demographic Distributions

Why do I see "Unknowns"?

Positive Cases | Deaths | Tested

Statewide Demographics for Positive Cases*

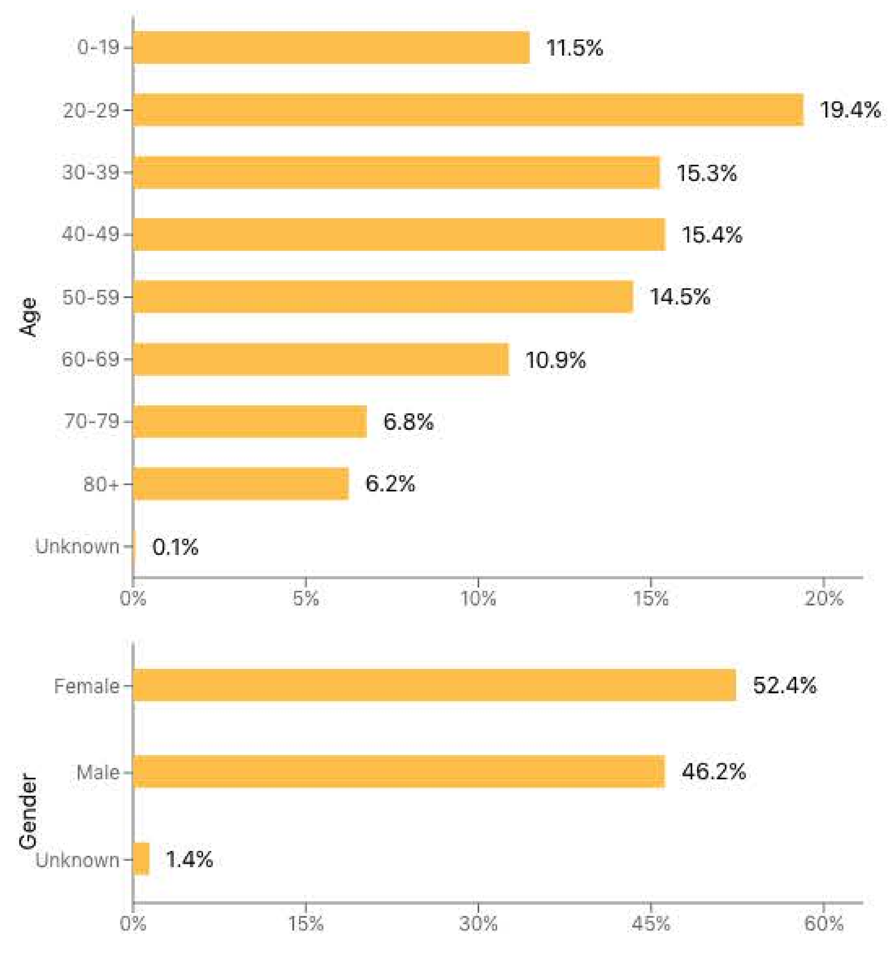

Race	% of Cases	% of Indiana Population
White	50.3 %	85.1 %
Other Race	14.8 %	2.6 %
Black or African American	10.5 %	9.8 %
Asian	1.1 %	2.5 %
Unknown	23.2 %	0 %

Ethnicity	% of Cases	% of Indiana Population
Not Hispanic or Latino	36.5 %	92.9 %
Hispanic or Latino	9.5 %	7.1 %
Unknown	54.1 %	0 %

** Indiana population percentages provided by 2019 U.S. Census Bureau; Population Estimate Program.*

Resource Usage

Today | By Day

Today's Statewide ICU Bed Usage

36.0%
ICU Beds Available

11.9%
ICU Beds in Use - COVID

52.1%
ICU Beds in Use - Non-COVID

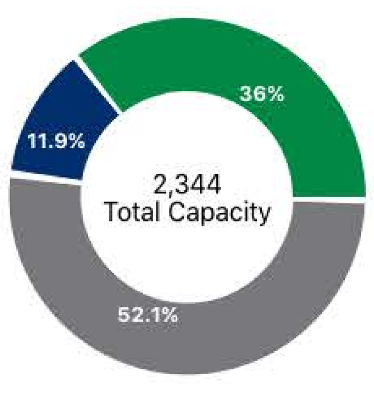

Today's Statewide Ventilator Usage

83.2%
Ventilators Available

2.5%
Ventilators in Use - COVID

14.3%
Ventilators in Use - Non-COVID

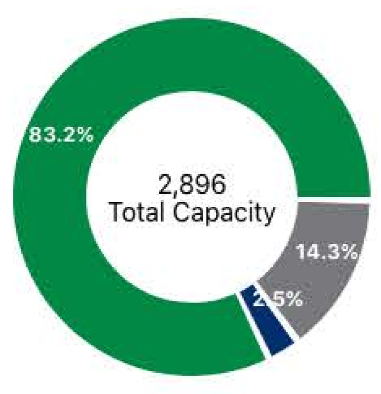

Hospitalizations

Census | Admissions

Statewide COVID-19 Hospital Census

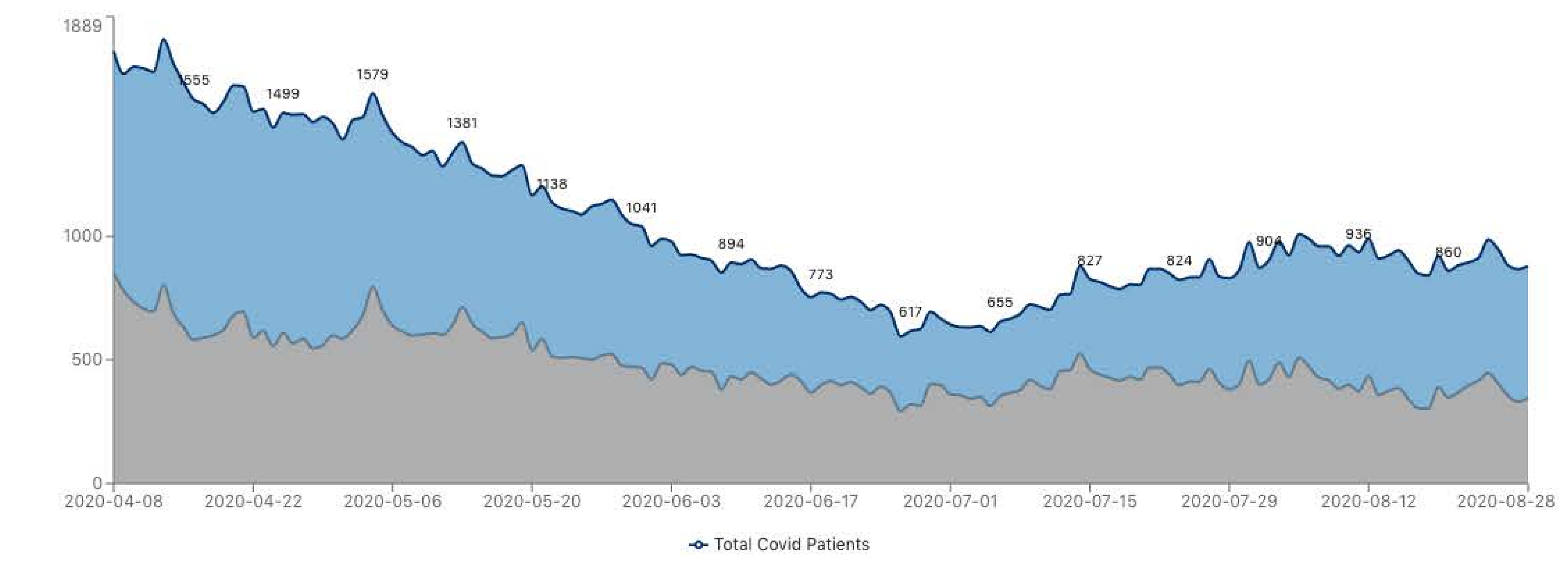

Probable COVID-19 Counts

Total Probable Deaths
219

All data displayed is preliminary and subject to change as more information is reported to ISDH. Tests are displayed by the date the test was performed and deaths are displayed by the date the death occurred.

Expect historical data to change as data is reported to ISDH.

Indiana COVID-19 Cases Tracker

The Polis Center at IUPUI

Adams County

Cases	Tested	Deaths
92,434	1,055,793	3,066
139.262 per 10k	1,590.666 per 10k	4.619 per 10k

Total Cases

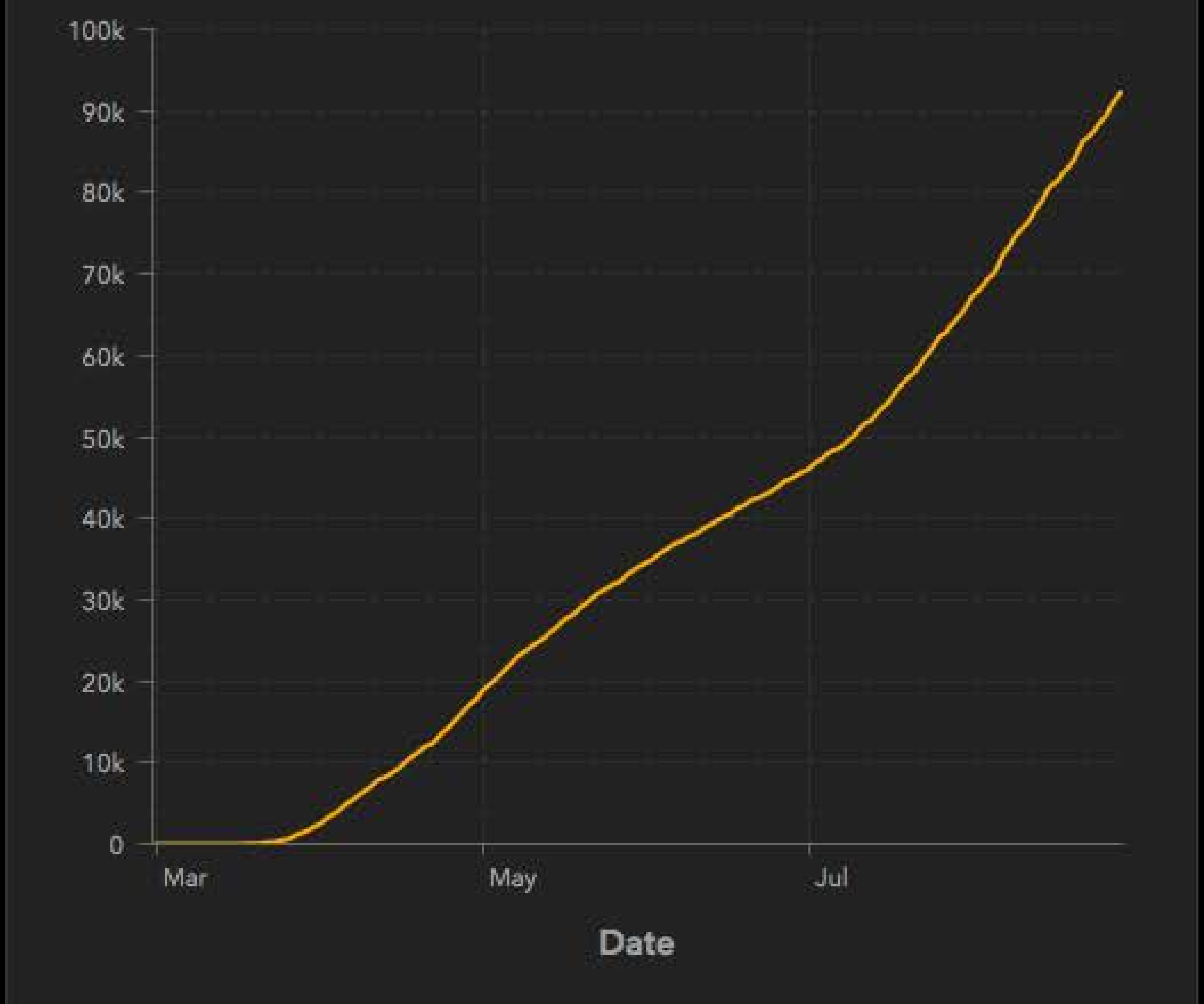

Total Cases | Cases - Log Scale | Total Deaths | Total Tests

Cases by Day

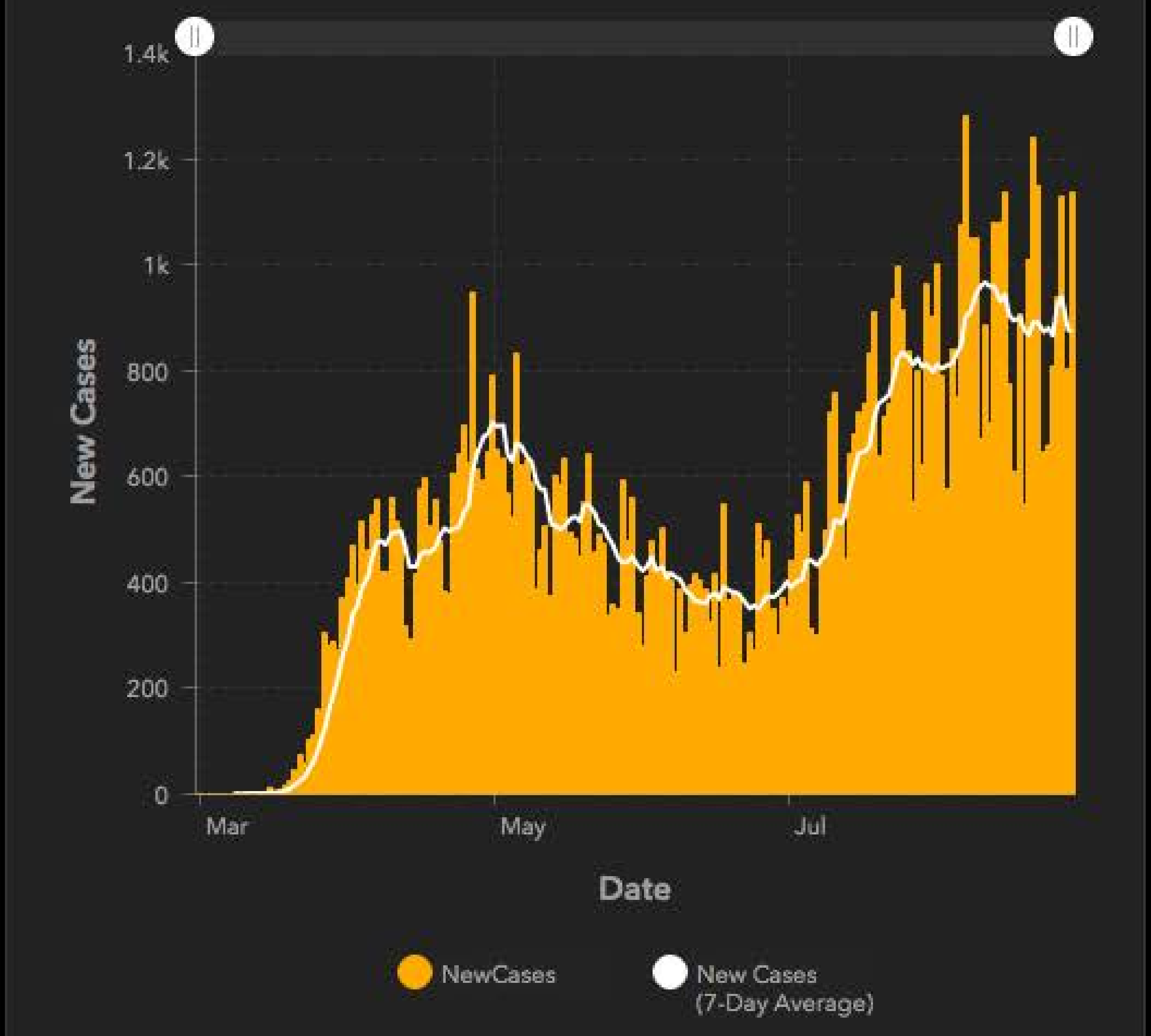

Daily Cases | Daily Deaths | Daily Tests | ICU Beds | Ventilators

Last updated on 8/29/2020

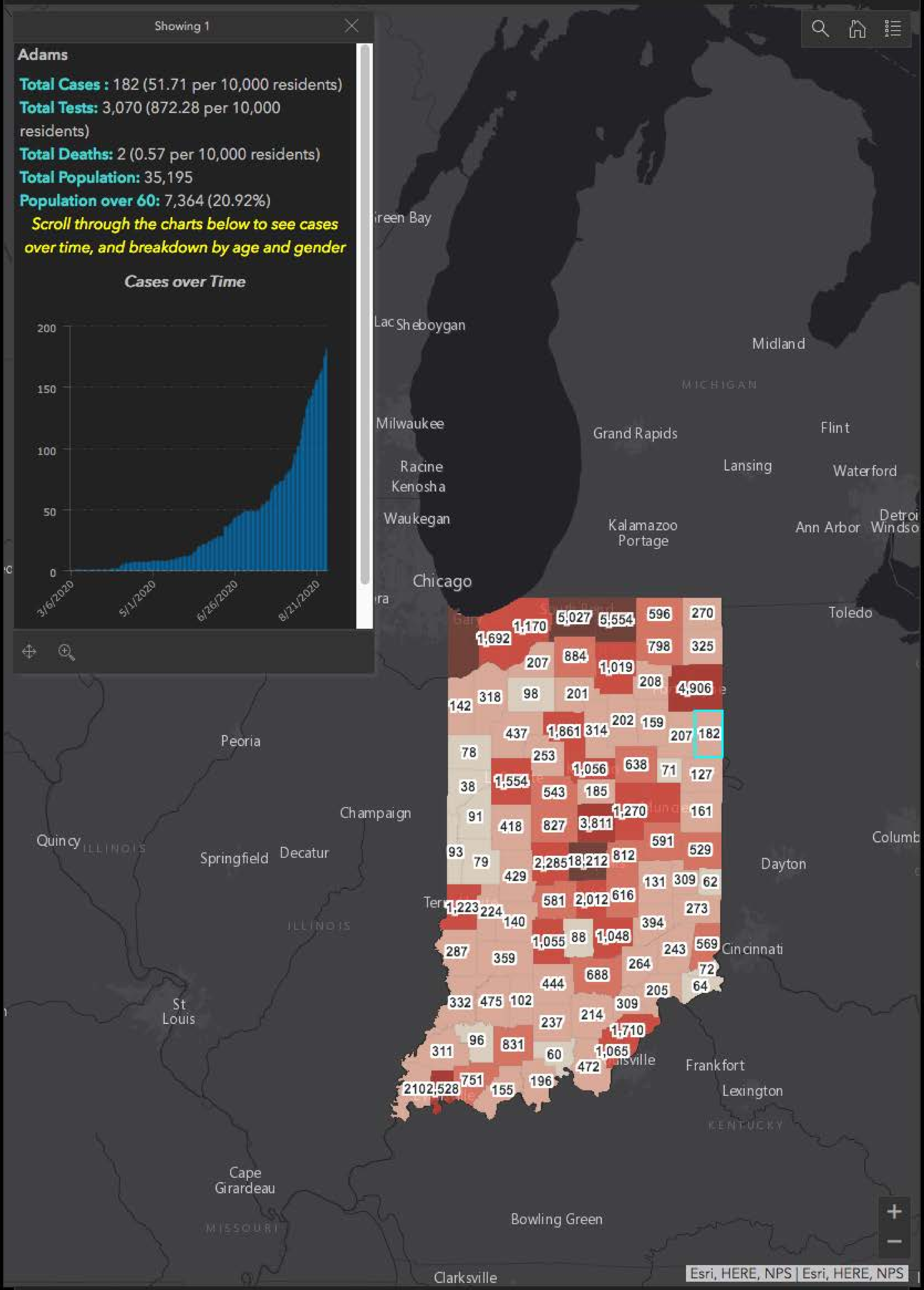

Total Cases

Positive Case Analysis

% Positive Analysis - Public Schools
Dashboard showing percent positive analysis by county and corresponding school districts.
View

Long Term Care
Iowa Long Term Care COVID-19 current outbreak information.
View

RMCC Dashboard
View the most current Hospital data by RMCC Region.
View

Test Iowa Assessments
View summary of Test Iowa assessments that have been completed.
View

Serology Testing
View summary of Test Iowa assessments that have been completed.
View

Outcome Analysis Recovered
View summary dashboard of COVID-19 cases resulting in recovery.
View

Outcome Analysis Deaths
View summary dashboard of COVID-19 cases resulting in death.
View

Positive Case Analysis

Lab Results Received Yesterday

Individuals Reported	Individuals Negative	Individuals Positive
6,798	5,820	927

PCR

Individuals Tested	Individuals Negative	Individuals Positive	% Individuals Positive
614,094	550,830	61,749	10.1%

Antigen

Individuals Tested	Individuals Negative	Individuals Positive	% Individuals Positive
11,775	10,325	1,450	12.3%

Total

Individuals Tested	Individuals Negative	Individuals Positive	% Individuals Positive
625,869	561,155	63,199	10.1%

Individuals Tested

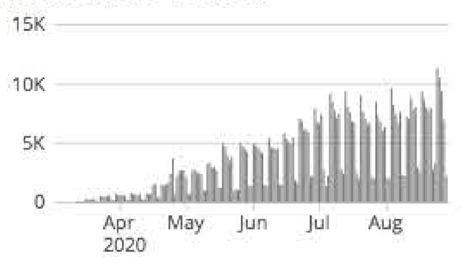

Individuals Negative

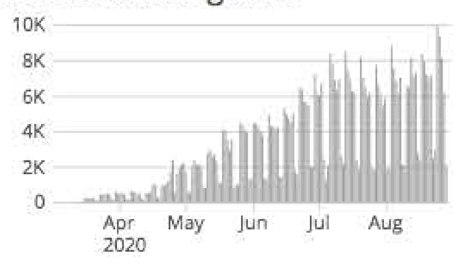

Individuals Positive

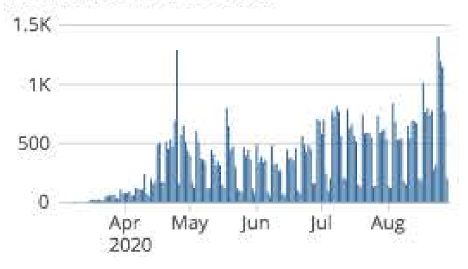

% Individuals Positive

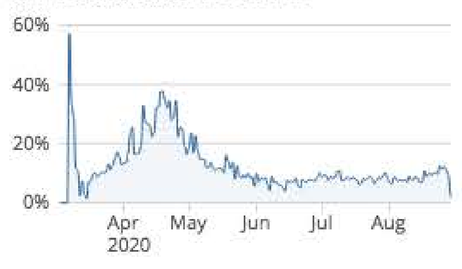

1 of 5	1 of 6	1 of 50	10.4%
Iowans Tested	Iowans Tested Negative	Iowans Tested Positive	% Positive Cases (Past 14 Day Average)

Individual Positive Cases

This interactive dashboard allows you to explore cases by county. Select a county name within the map or table to filter to specific counties.

Positive Cases by County

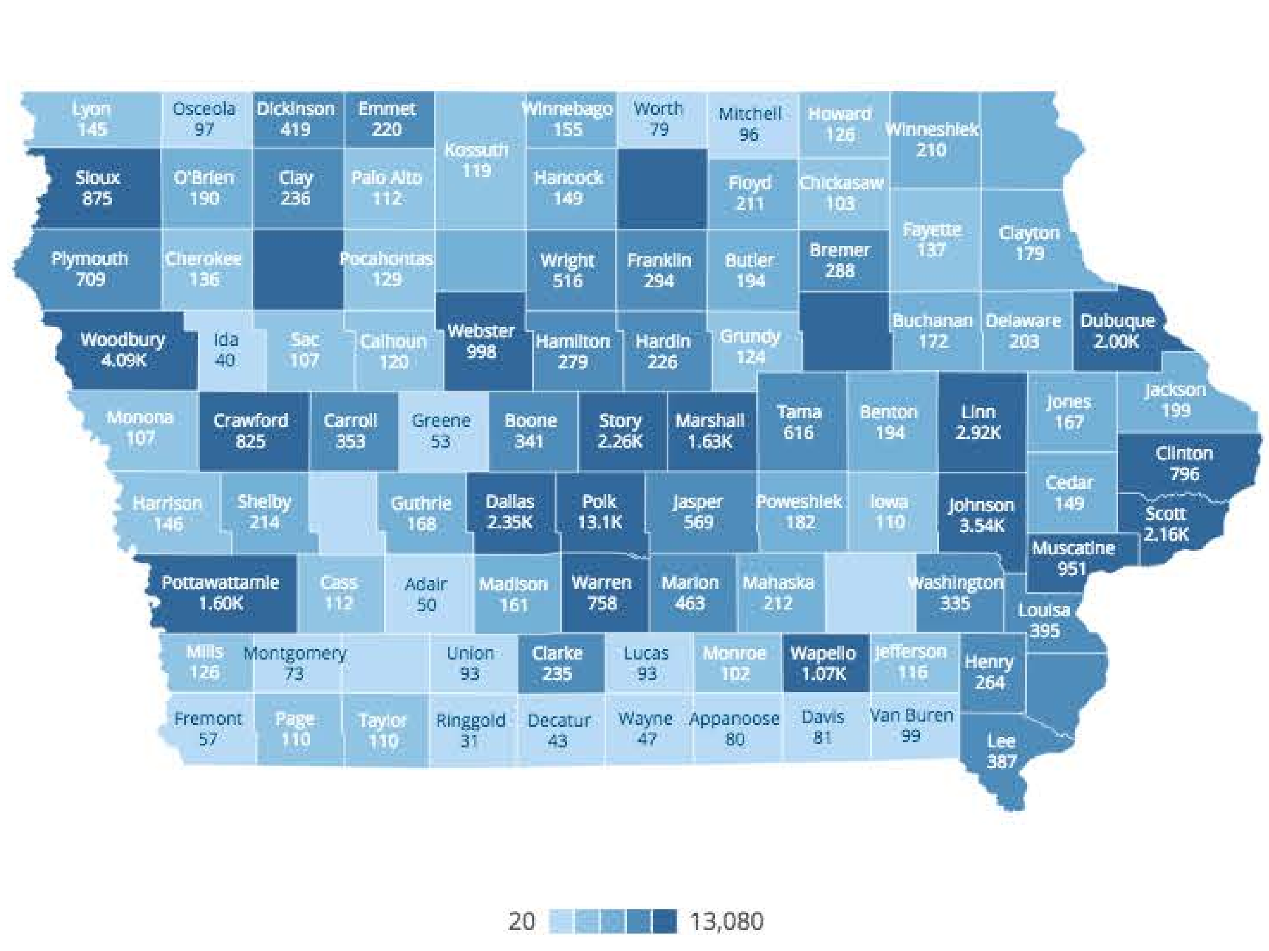

Positive Cases Symptomatic vs. Asympto...

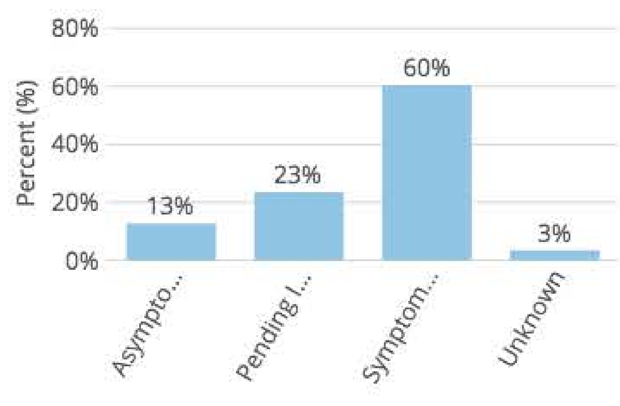

Positive Cases by Age Group

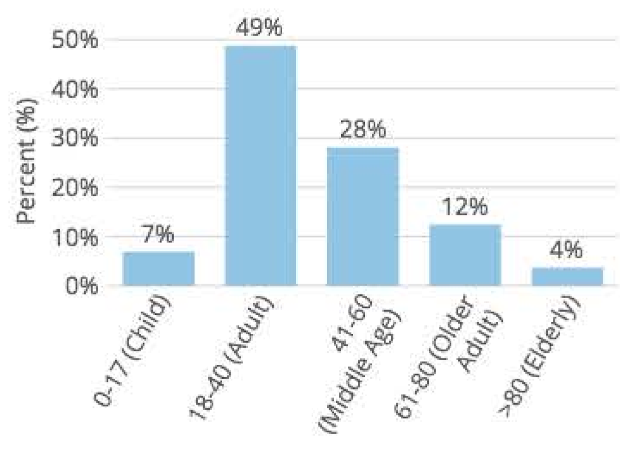

Positive Cases

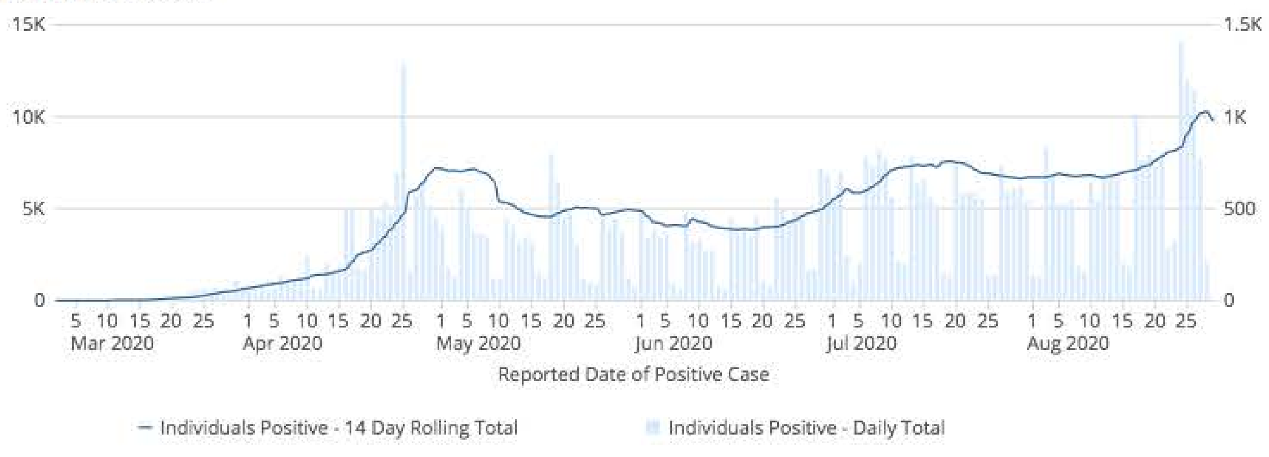

Positive Cases by Sex

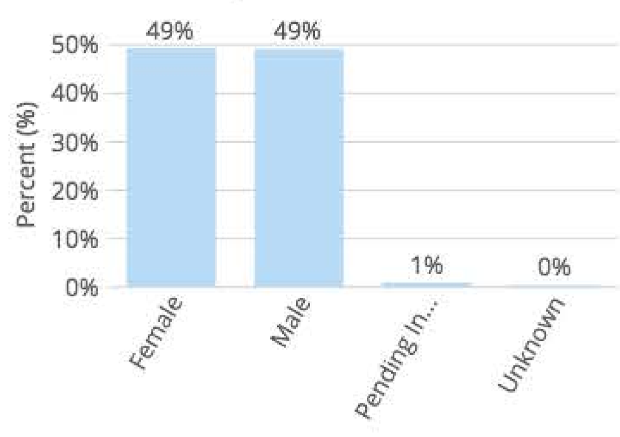

County	Positive Cases	Cases Per 100,000 Population
Polk	13,080	2,685
Woodbury	4,085	3,984
Black Hawk	3,715	2,806
Johnson	3,540	2,340
Linn	2,916	1,291
Dallas	2,345	2,600
Story	2,263	2,307
Scott	2,155	1,244
Dubuque	2,002	2,067
Buena Vista	1,825	9,183
Marshall	1,627	4,069
Pottawattamie	1,601	1,712
Wapello	1,067	3,031
Webster	998	2,751
Muscatine	951	2,215
Sioux	875	2,507
Crawford	825	4,808
Cerro Gordo	806	1,890
Clinton	796	1,711
Warren	758	1,485
Plymouth	709	2,825

Positive Cases by Race

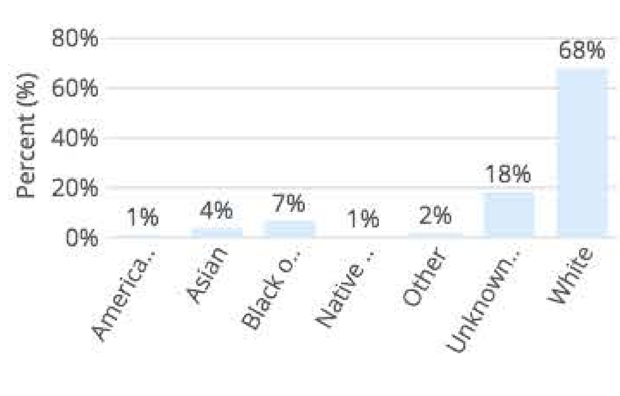

Positive Cases by Ethnicity

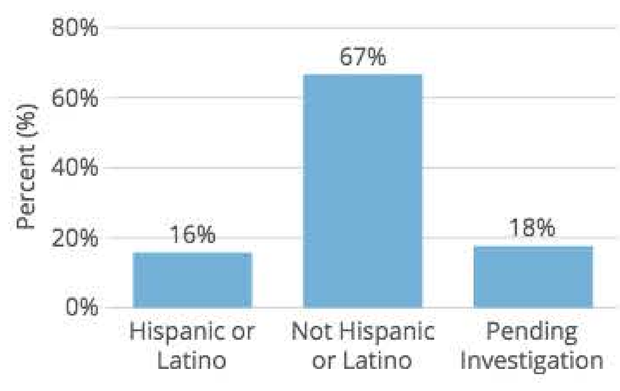

Total Positive Cases by Pre-Existing Condition

Pre-Existing Condition	No Pre-Existing Condition	Pre-Existing Condition Unknown
13,528	32,203	2,465

Positive Cases by Pre-Existing Condition

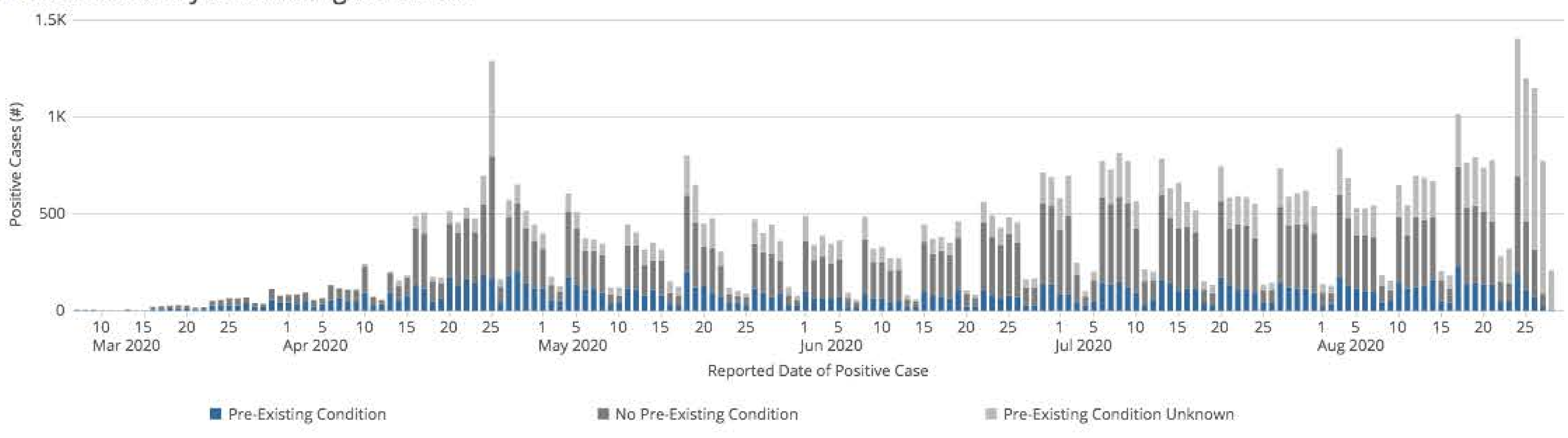

Positive Cases by Pre-Existing Condition

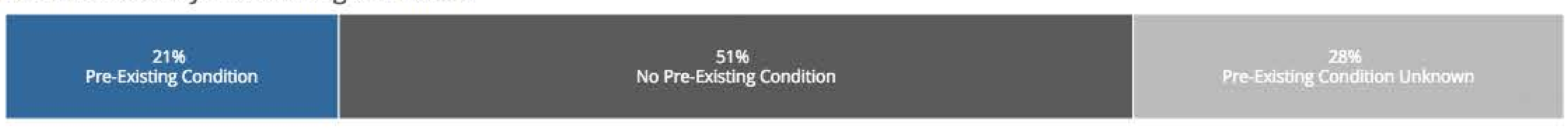

Positive Cases - Pre-Existing Condition by Age Group

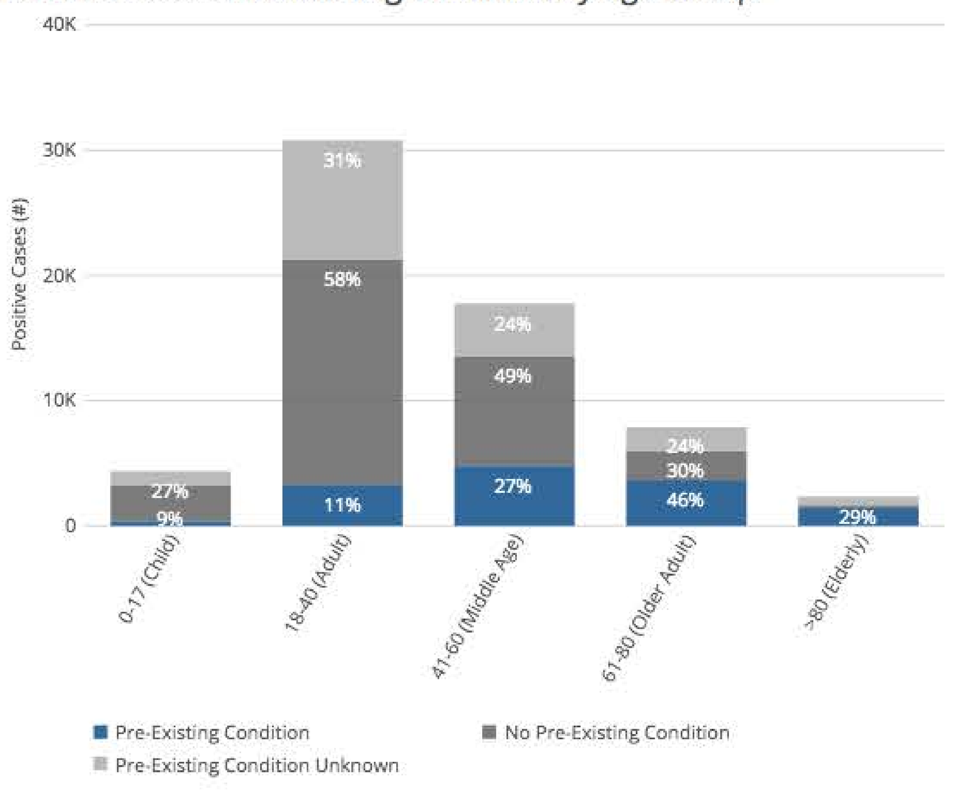

Positive Cases - Pre-Existing Condition by Occupation

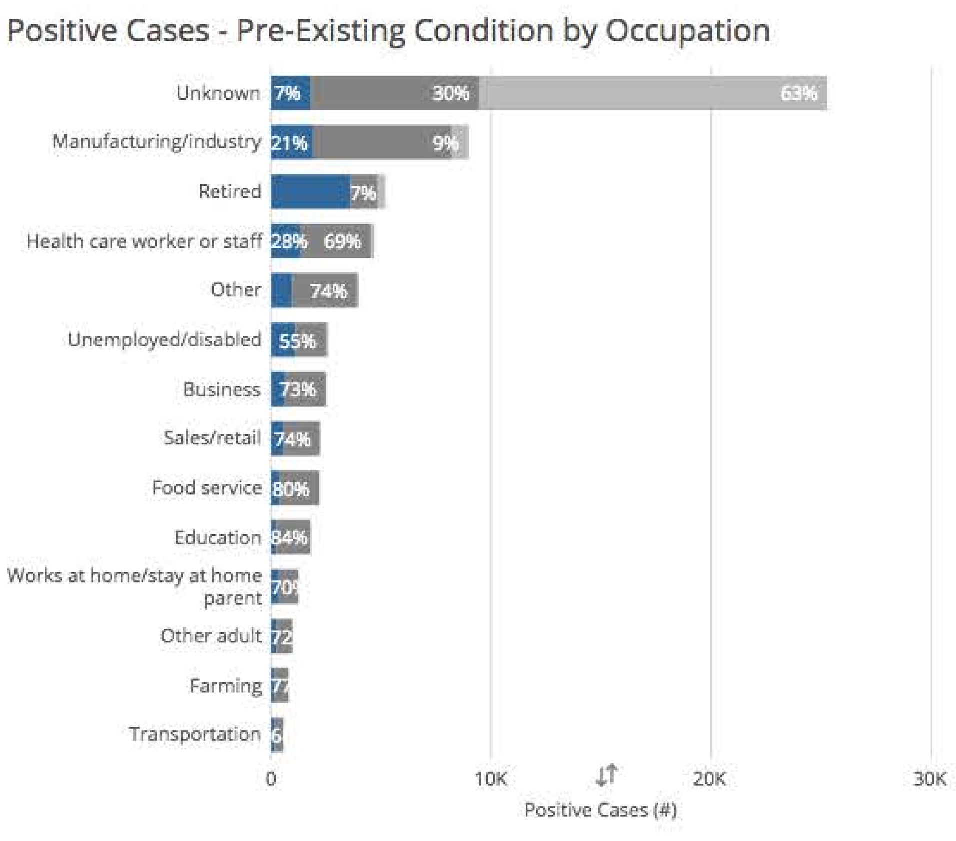

Last updated on: 2020-08-29
COVID-19 data are reported as timely, accurately and completely as we have available. Data are updated as we receive information that is more complete and is subject to change. Positive case count trend line represents 14 day rolling total.

LATEST UPDATE **Watch the COVID-19 Press Conferences** — Want to know the latest news about COVID-19 in Kansas? Watch Governor Laura Kelly and Secretary Lee Norman, M.D.'s press conferences. Learn more...

COVID-19 Cases in Kansas

Map Information

The map is updated Monday, Wednesday and Friday by 12:30 p.m. The map contains a variety of data at the state and county level, including the Reopen Kansas Metrics. Click on the gray buttons to access the data. To maximize your experience, use a device with a larger screen, such as a tablet, laptop or desktop. The Map FAQ Document (PDF) includes detailed instructions on how to use and share the data.

Daily Case Summary & Historical Data

The COVID-19 Summary is published Monday, Wednesday and Friday at 12:30 p.m. and includes historical data. The summary may not be published during holiday observances.

Kansas Coronavirus Disease 2019 (COVID-19): Overview

Cases*	Hospitalizations	Statewide Deaths	Negative Tests
41,048	2,278	443	362,099

*As of May 7, 2020, confirmed and probable cases are included in the case totals. A confirmed case is defined as a person who tested positive by molecular testing for the novel coronavirus (SARS-CoV-2), which causes Coronavirus Disease 2019 (COVID-19). A probable case is defined as a person with: a) presumptive lab evidence (serology) with COVID-19 symptoms or epi-linked to a confirmed case, or b) no lab testing with COVID-19 symptoms and epi-linked to a confirmed case. Cases are based on individual people, not on the number of tests. A person with multiple positive tests is only counted once.

Case counts are preliminary and subject to verification.

Last updated: 08/28/2020 at 9:00 AM. There were 1,111 new cases and 6 new deaths from Wednesday, 8/26/2020.

Select county from map below to update charts to county specific details.

State Map of COVID-19 Cases by County of Residence

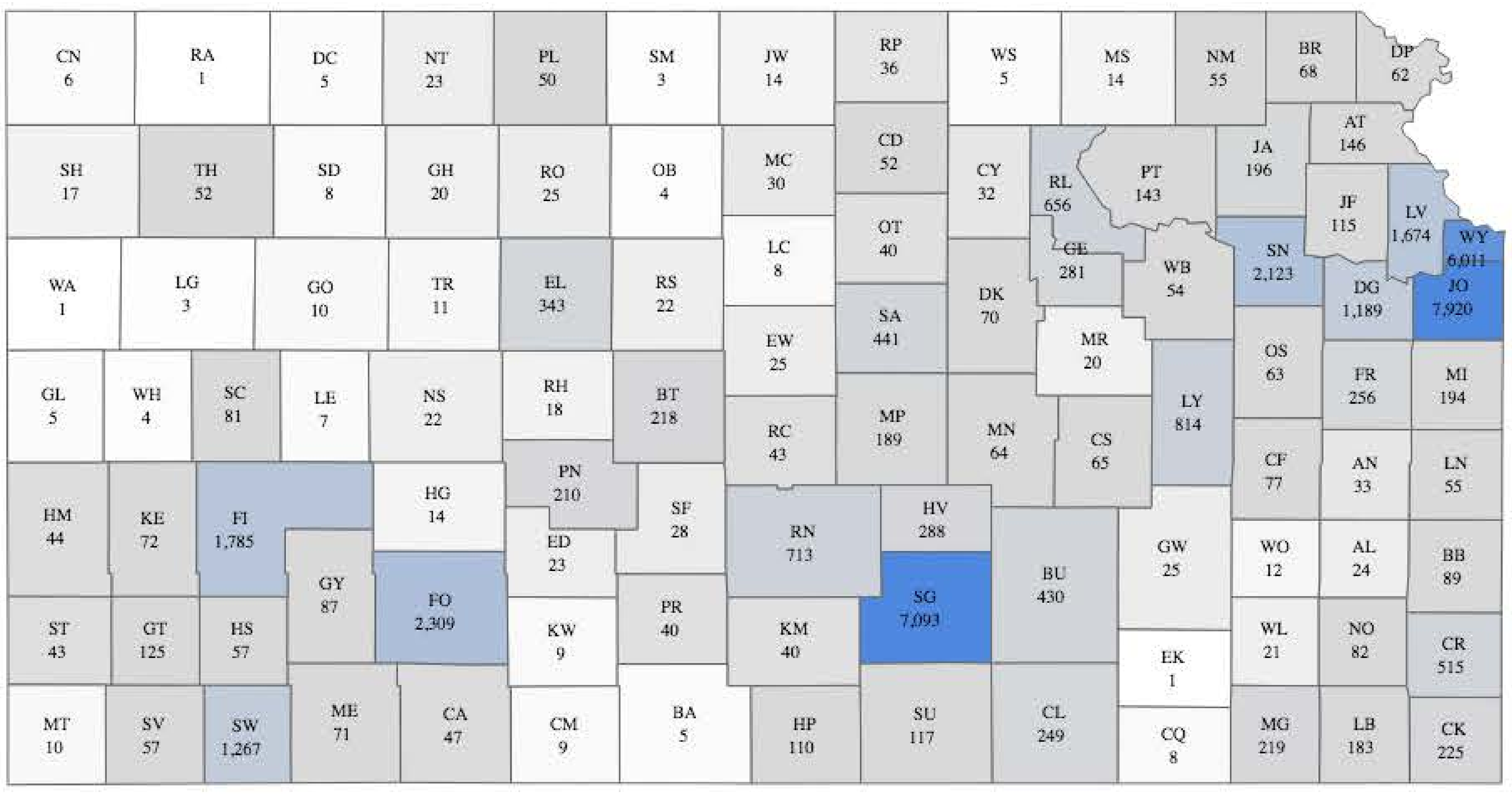

Statewide COVID-19 Cases by Symptom Onset Date

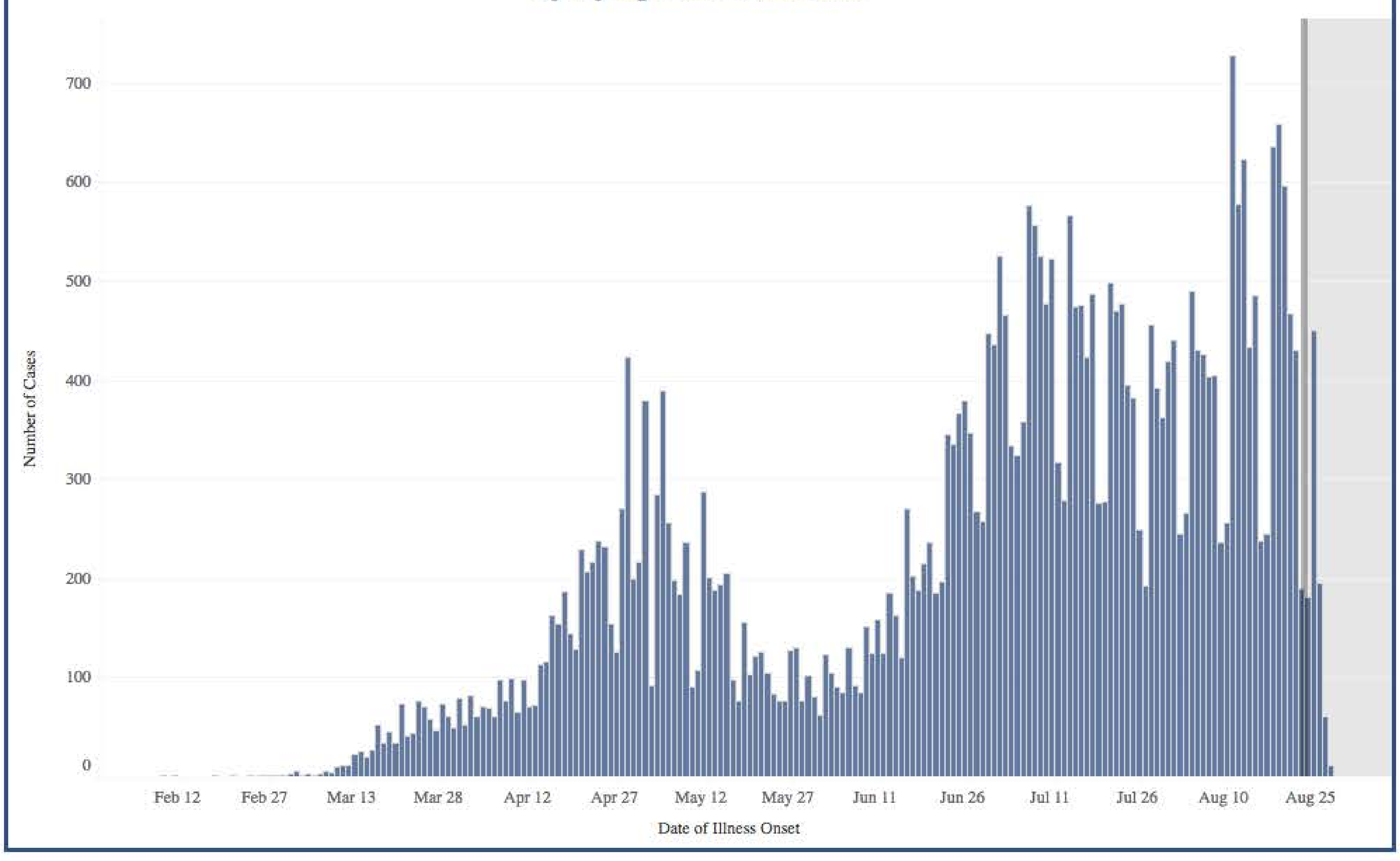

IMPORTANT NOTE: If symptom onset date is missing, the date of specimen collection is used as a proxy for the onset date.

Daily Counts of New Cases and Total Cases Reported by Date Diagnosed

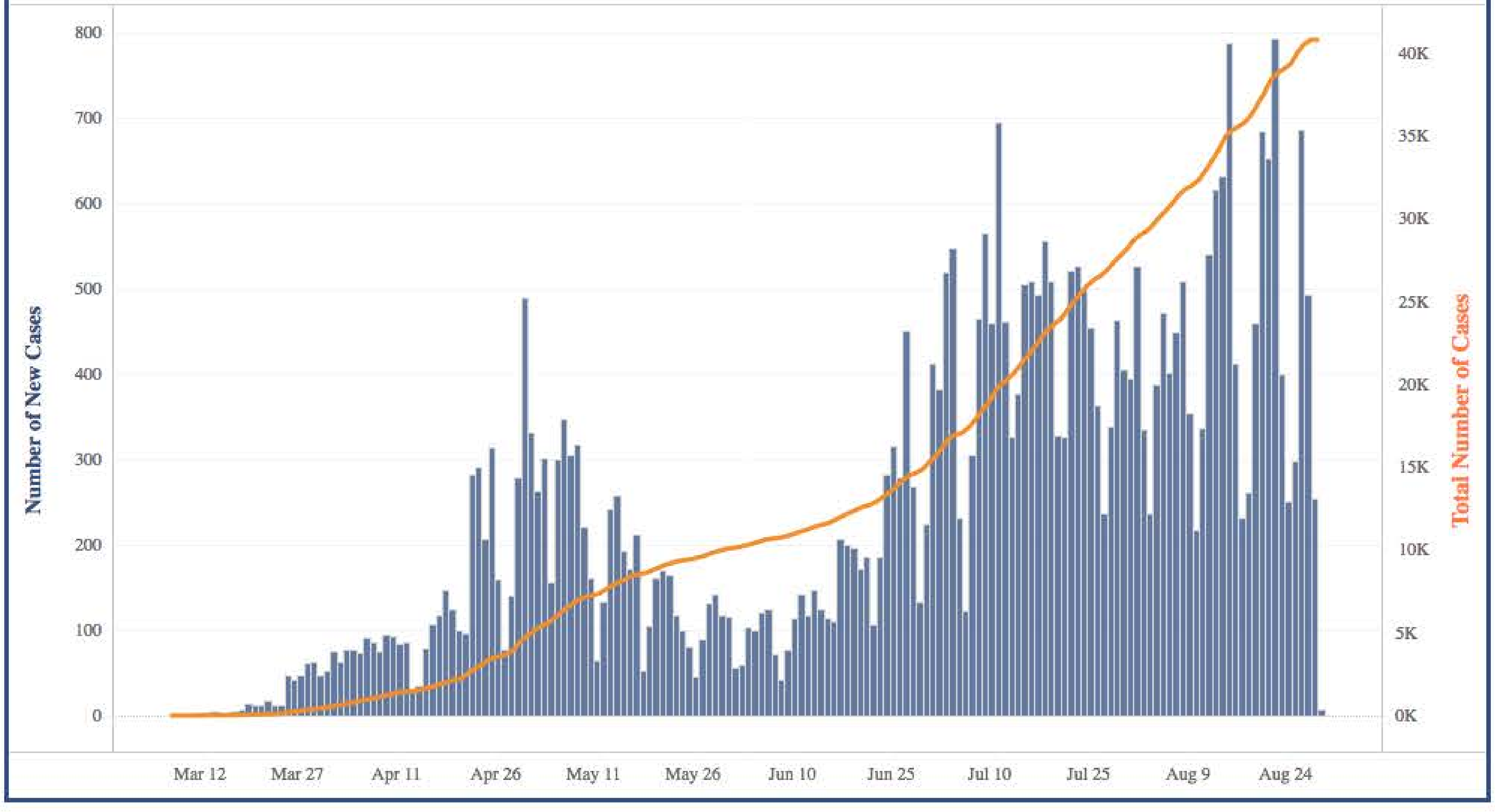

Cases by County

+ a b l e a u

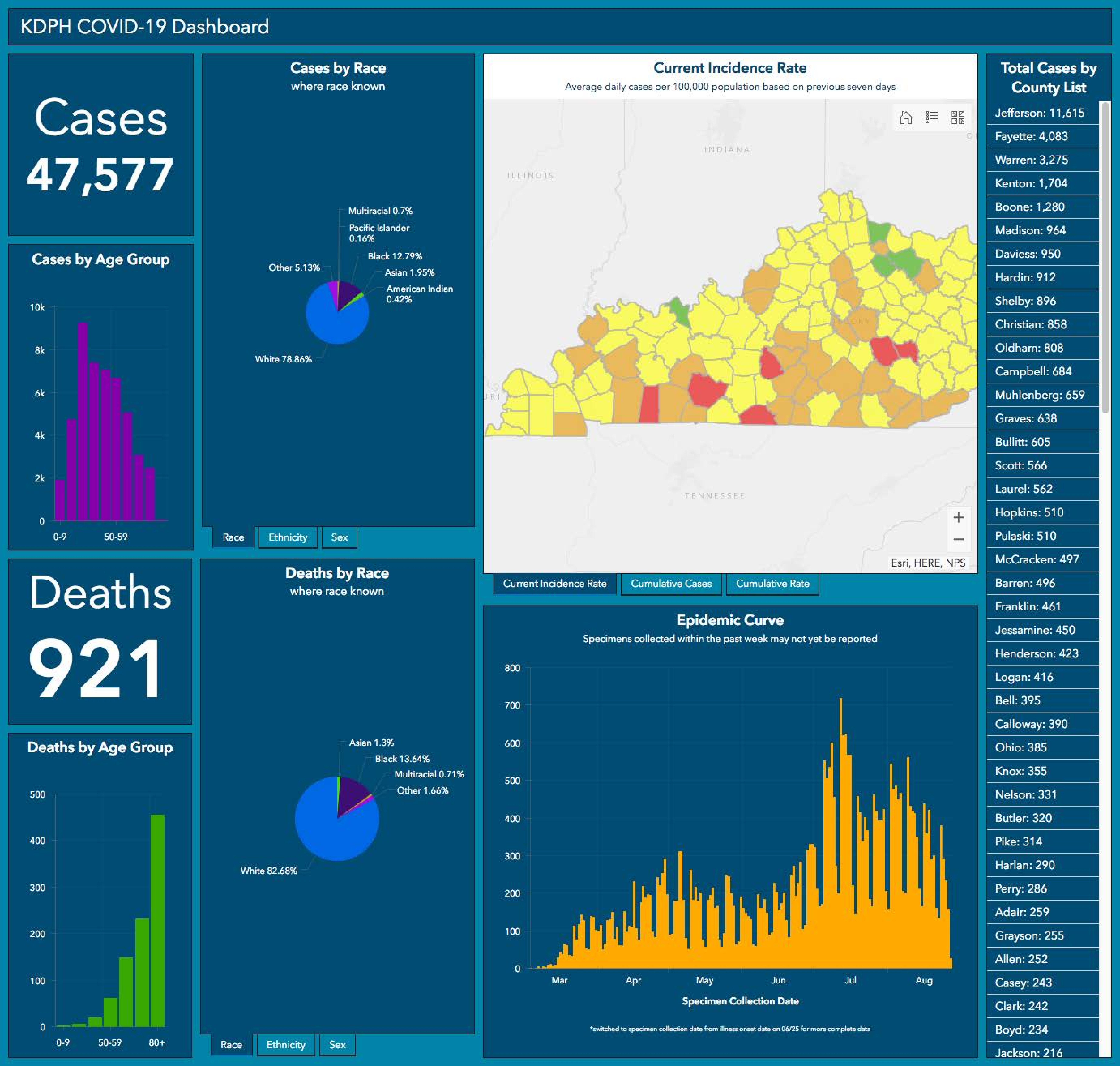
KDPH COVID-19 Dashboard
Cases
47,577
Cases by Age Group
10k
8k
6k
4k
2k
0
0-9
50-59
Cases by Race
where race known
Multiracial 0.7%
Pacific Islander 0.16%
Black 12.79%
Asian 1.95%
American Indian 0.42%
Other 5.13%
White 78.86%
Race
Ethnicity
Sex
Current Incidence Rate
Average daily cases per 100,000 population based on previous seven days
INDIANA
ILLINOIS
TENNESSEE
Esri, HERE, NPS
Current Incidence Rate
Cumulative Cases
Cumulative Rate
Total Cases by County List
Jefferson: 11,615
Fayette: 4,083
Warren: 3,275
Kenton: 1,704
Boone: 1,280
Madison: 964
Daviess: 950
Hardin: 912
Shelby: 896
Christian: 858
Oldham: 808
Campbell: 684
Muhlenberg: 659
Graves: 638
Bullitt: 605
Scott: 566
Laurel: 562
Hopkins: 510
Pulaski: 510
McCracken: 497
Barren: 496
Franklin: 461
Jessamine: 450
Henderson: 423
Logan: 416
Bell: 395
Calloway: 390
Ohio: 385
Knox: 355
Nelson: 331
Butler: 320
Pike: 314
Harlan: 290
Perry: 286
Adair: 259
Grayson: 255
Allen: 252
Casey: 243
Clark: 242
Boyd: 234
Jackson: 216
Deaths
921
Deaths by Age Group
500
400
300
200
100
0
0-9
50-59
80+
Deaths by Race
where race known
Asian 1.3%
Black 13.64%
Multiracial 0.71%
Other 1.66%
White 82.68%
Race
Ethnicity
Sex
Epidemic Curve
Specimens collected within the past week may not yet be reported
800
700
600
500
400
300
200
100
0
Mar
Apr
May
Jun
Jul
Aug
Specimen Collection Date
*switched to specimen collection date from illness onset date on 06/25 for more complete data

Looking For County-Level Data On The Coronavirus? Here's Our Kentucky COVID-19 Tracker

By Alexandra Kanik

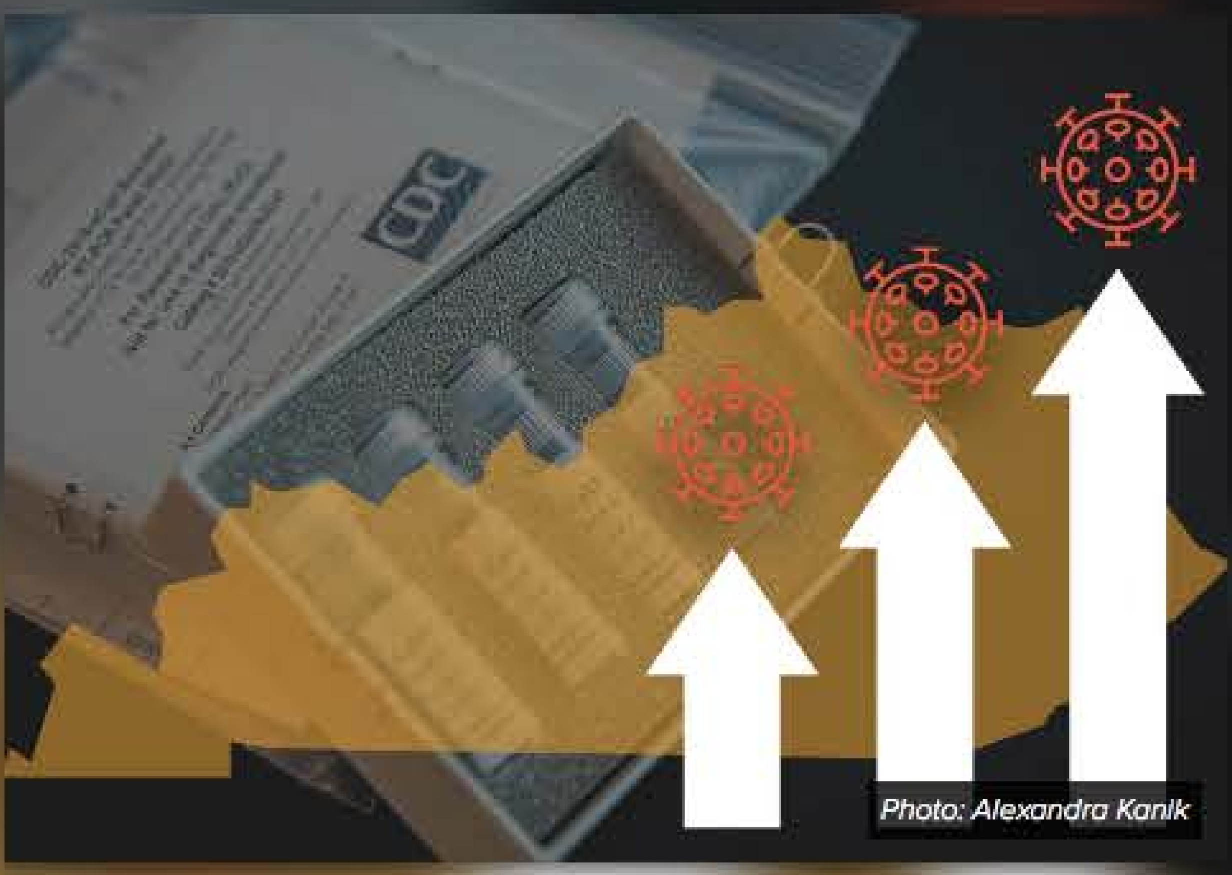

Photo: Alexandra Kanik

HEALTH June 29, 2020

Kentucky COVID-19 Tracker

This dashboard provides a localized look at COVID-19-related cases and deaths in your county, as well as a risk assessment for your county's population.

Data last updated August 28, 2020.

Use the dropdown below to display more information.

Select a county...

Total cases

There have been **48,937 cases** of COVID-19 in Kentucky. The population of this region is 4,440,204. That's 1102 cases per 100,000 people.

Newly reported cases

There were **620 newly reported cases** of COVID-19 on August 28, 2020 in Kentucky. Over time, new cases indicate how fast the virus is spreading in a community.

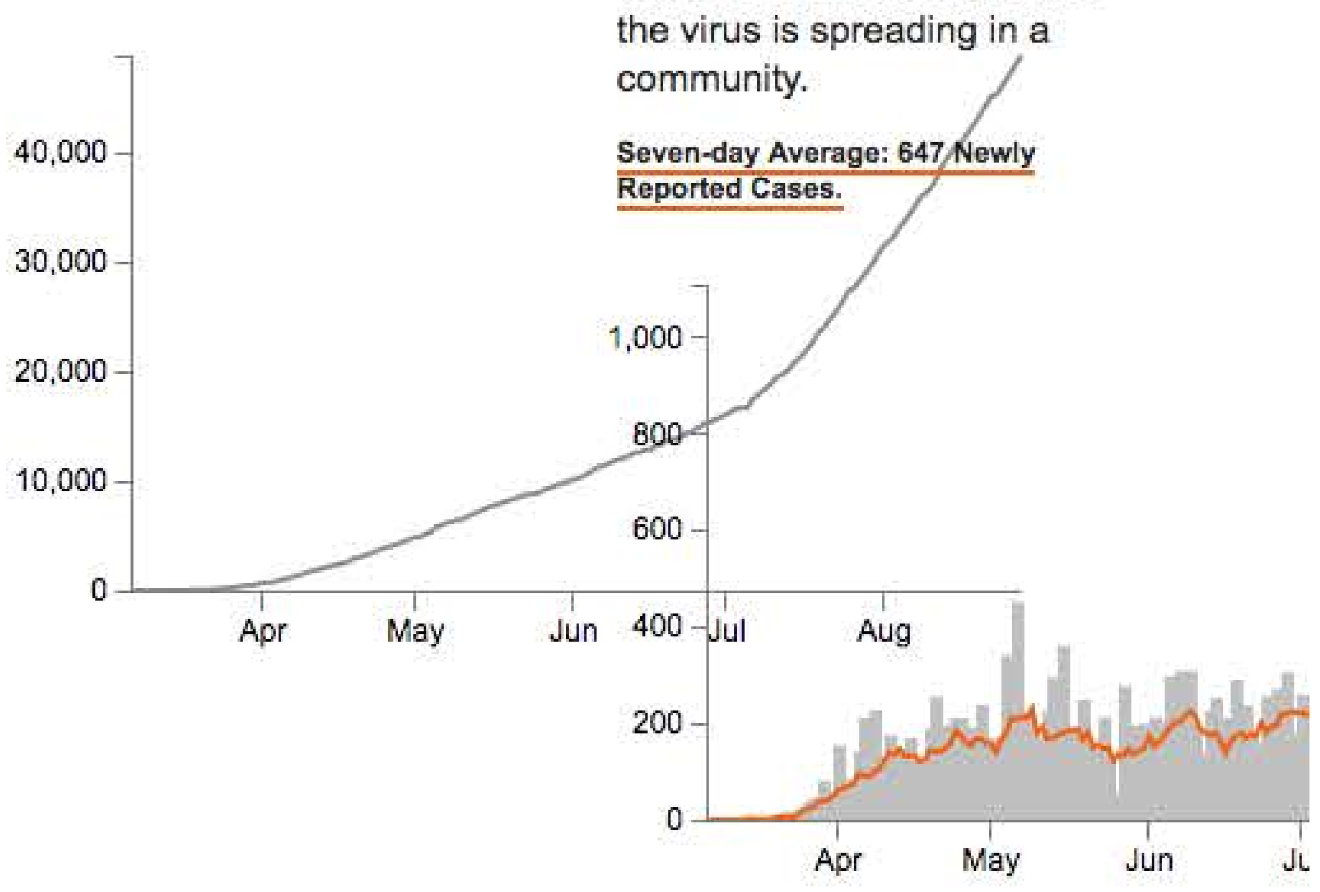

Total deaths

There have been **946 reported deaths** related to COVID-19 in Kentucky.

Newly reported deaths

There were **8 new deaths** related to COVID-19 reported on August 28, 2020 in Kentucky.

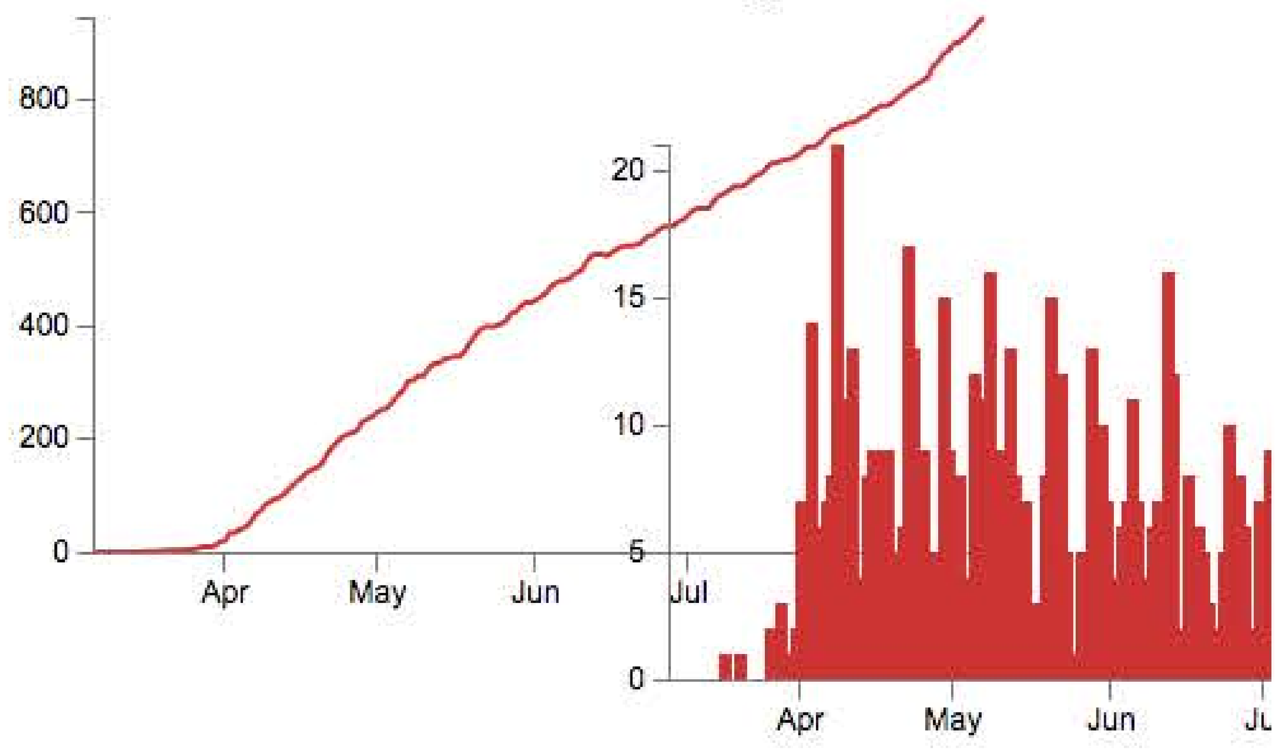

Disclaimer & Data Sources

Age data provided by the 2018 5 year American Community Survey. Disease data are from 2014 and are provided by the Institute for Health Metrics and Evaluation's Global Health Data Exchange

As of April 27, 2020, case and death data for Kentucky counties are provided by the New York Times county dataset. Read more about the data and potential caveats here.

The data presented here only includes cases in which a person has tested positive for COVID-19, but it may be lower than the actual number of infected people in a community due to a lack of testing.

As counties report cases when test results come in, some of the increases in counties' cases in one day could be due to many pending results coming in rather than many people coming down with symptoms in one day.

Credits

This dashboard was created by Allie Kanik at Louisville Public Media, Lisa Pickoff-White at KQED, Emily Zentner at CapRadio, and Dana Amihere at KPCC.

Share on Twitter | Share on Facebook

CORONAVIRUS

LOUISIANA DEPARTMENT OF HEALTH

*Filtered to: Louisiana

Louisiana Coronavirus (COVID-19) Information

627<br>New Cases	30<br>New Deaths	10,275<br>New Tests
146,243<br>Total Cases*	4,741<br>Total Deaths*	1,828,478<br>Total Tests*

Presumed Recovered<br>127,918<br>as of 8/24/2020	Probable Cases<br>701<br>as of 8/26/2020	COVID Patients<br>900<br>in Hospitals	COVID Patients<br>141<br>on Ventilators

Cumulative 2 Week (7/30 to 8/12/2020) Incidence by Parish

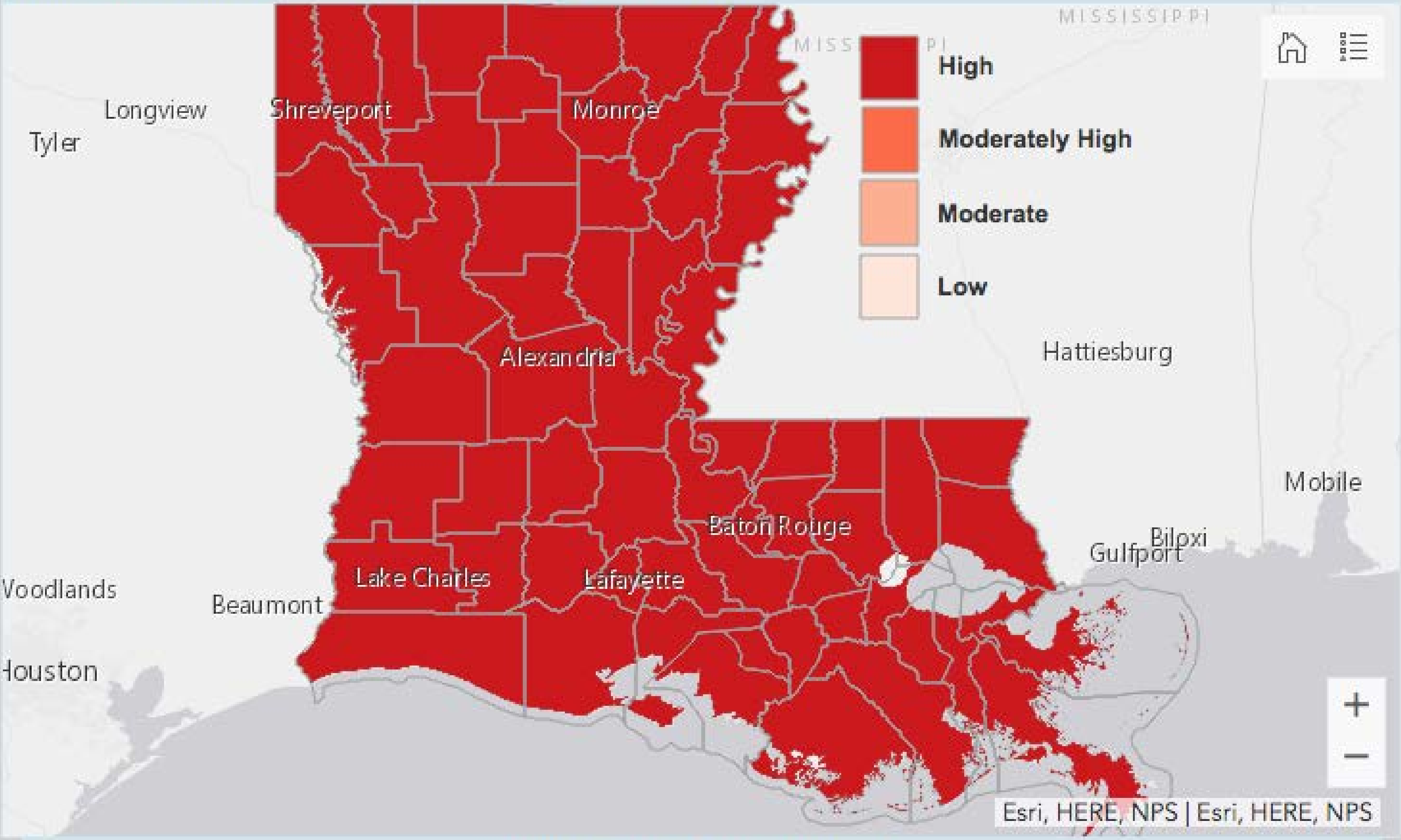

Cumulative 2 Week Incidence by Parish

Click the < > arrows to navigate tabs containing other information and downloadable data
This dashboard is updated daily by noon, except on Saturday.

211 Social services help and information about COVID-19 in Maine, call 211, email info@211maine.org, text your ZIP code to 898-211, or if out-of-state call 1-877-463-6207.

COVID-19: Maine Data

On June 23, 2020, Maine CDC changed how we show the dates of cases, deaths, recoveries, and hospitalizations. We now show events on the day they occurred, instead of the day the event was reported to us. As we investigate cases, we learn things that help our understanding of when events such as hospitalizations and recoveries occurred. For example, if a person reports on Friday that they recovered Wednesday, we now show this recovery on Wednesday. We do this to give the public a better picture of the spread of COVID-19 in the state.

This means updates on June 24 with data as of June 23 at 11:59 PM will be on trend charts on June 23. With this change, you may see that recoveries are higher for previous days. That is because our knowledge of who is released from isolation becomes better every day, as case investigators follow up with people with COVID-19.

Maine CDC updates data daily at 9:30 AM with COVID-19 case investigation data included as of 11:59 PM the previous day. All data are preliminary and may change as Maine CDC investigates cases. For more information about the data, please see the "Read Details About the Data" section below.

Access a Google Sheet with the most recent cumulative case data by county, sex, age, and race.

Cumulative COVID-19 Cases by County

Export table to PDF

Cumulative COVID-19 Cases by County

Table updated: August 29, 2020
Data through: Friday, August 28, 2020 11:59 pm

County	Cases	Confirmed cases	Probable cases	Deaths	Hospitalizations	Recoveries	Case rate (per 10K people)
**Statewide**	**4,489**	**4,032**	**457**	**132**	**418**	**3,899**	**33.5**
Cumberland	2,183	1,948	235	70	197	1,938	74.4
York	822	734	88	15	83	671	39.9
Androscoggin	610	548	62	10	49	546	56.6
Penobscot	228	214	14	6	26	189	15.1
Kennebec	187	172	15	11	28	158	15.3
Waldo	69	65	4	14	5	52	17.4
Sagadahoc	60	59	1	0	7	53	16.8
Oxford	60	48	12	0	1	56	10.4
Somerset	50	43	7	1	2	39	9.9
Franklin	50	48	2	1	3	44	16.7
Hancock	45	40	5	1	3	41	8.2
Aroostook	38	37	1	1	5	34	5.7
Lincoln	35	30	5	1	3	32	10.2
Knox	29	24	5	1	5	26	7.3
Washington	15	15	0	0	0	13	4.8
Piscataquis	8	7	1	0	1	7	4.8

+ableau

Download the most recent Cases by County Table

COVID-19 Case Trends

1. Case trends by county | 2. New cases by date | 3. Cases by age trend | 4. Maine cases v. other states | 5. Case rates by county | 6. COVID-19 race

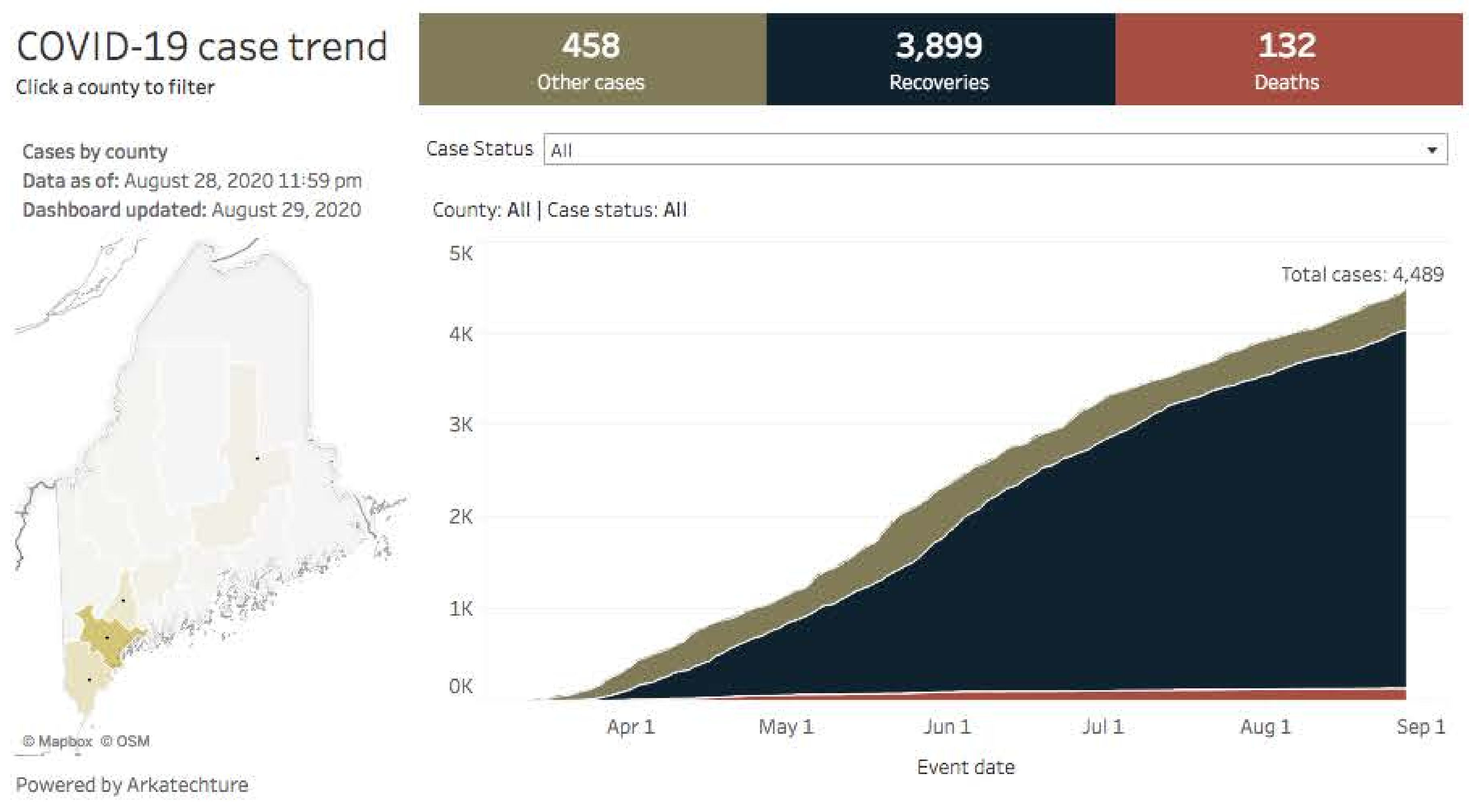

+ableau

Maine COVID-19 Reopening Gating Metrics

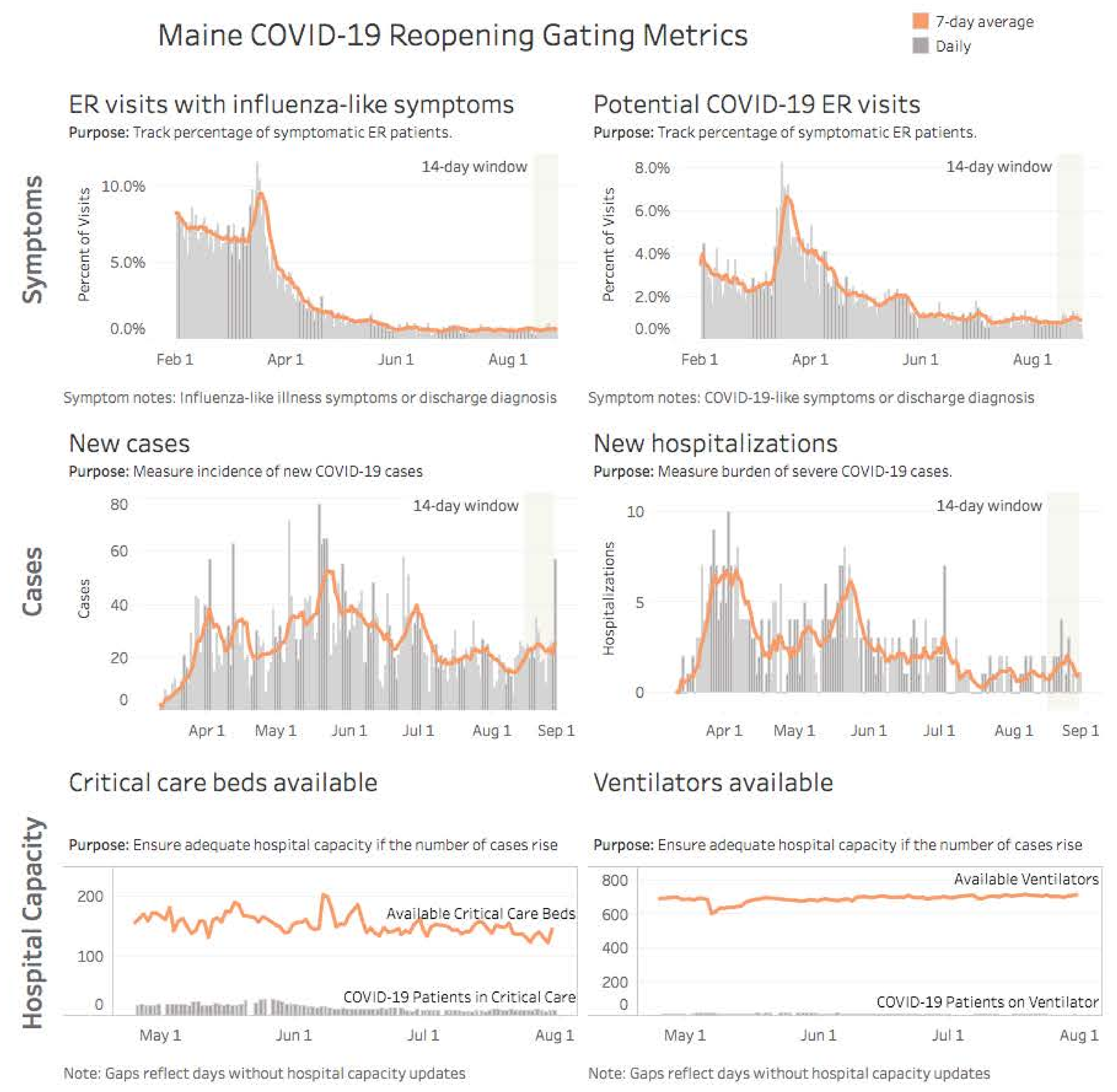

+ableau

Cumulative COVID-19 Cases by ZIP Code

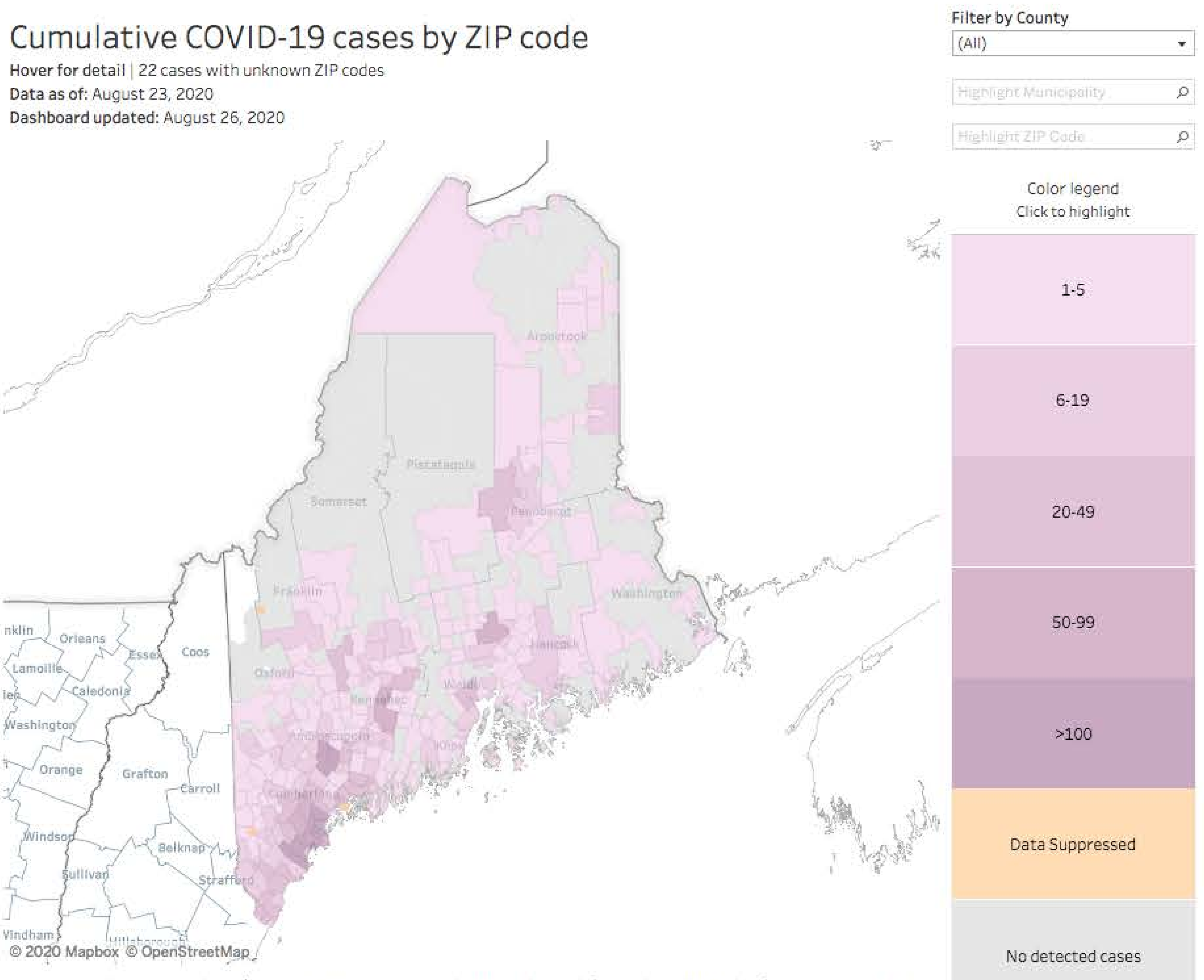

Per Maine CDC's privacy policy, 1) Case counts are suppressed in zip codes with fewer than 50 people. 2) Exact case counts are suppressed in all zip codes with 5 or fewer cases. 3) In counties where total case counts are low enough that zip code data could indirectly reveal the identity of a case, all zip codes in the county are suppressed.

+ableau

Maine and COVID-19

Maine COVID-19 Cumulative County Case Data

Last Updated: Saturday, August 29, 2020 at 12:30PM EDT

Total Cases*	Recovered	Hospitalizations	Deaths
4,489	3,899	418	132

*Total cases includes both confirmed and probable cases.

Cumulative COVID-19 Cases by County

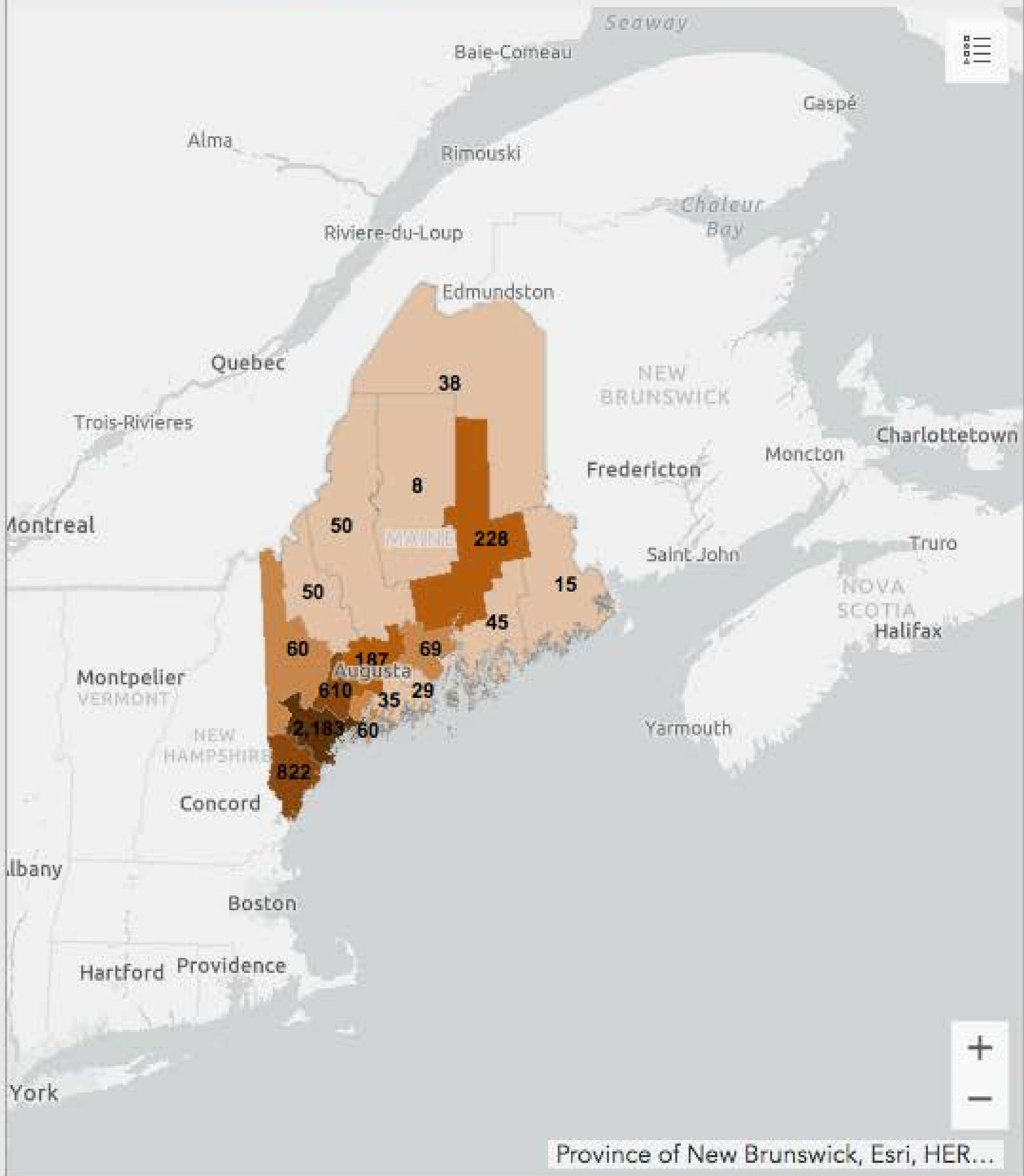

Note: The case distribution displayed on this map is likely an underrepresentation of the true number of cases in Maine since not all individuals are being tested. For individuals not considered to be at high risk, medical providers are currently advised to diagnose COVID-19 based on symptoms.

COVID-19 Progression in Maine

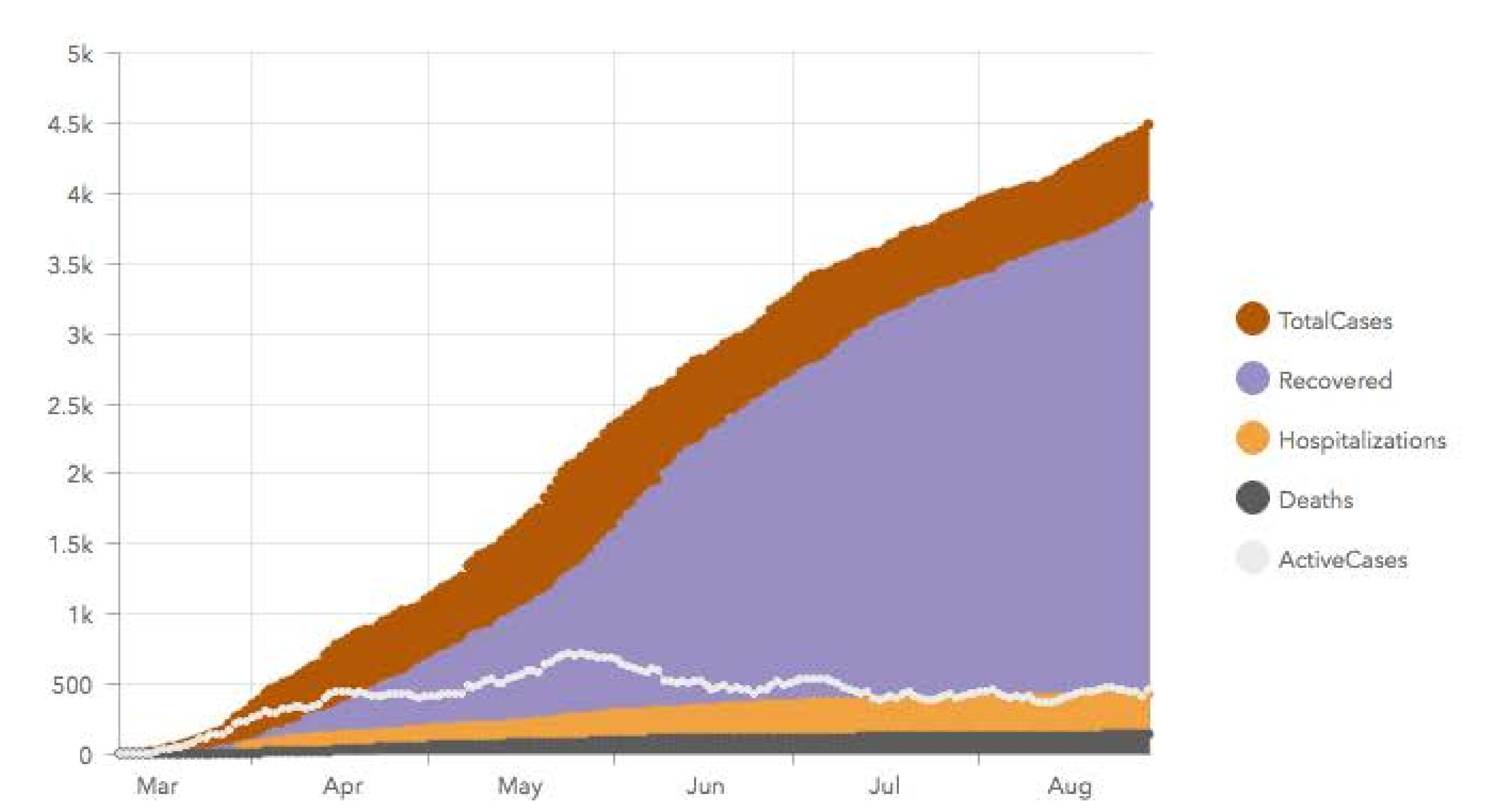

Case data are preliminary due to day-to-day changes in available information reported by the counties.

Maine Counties Caseload Over Time

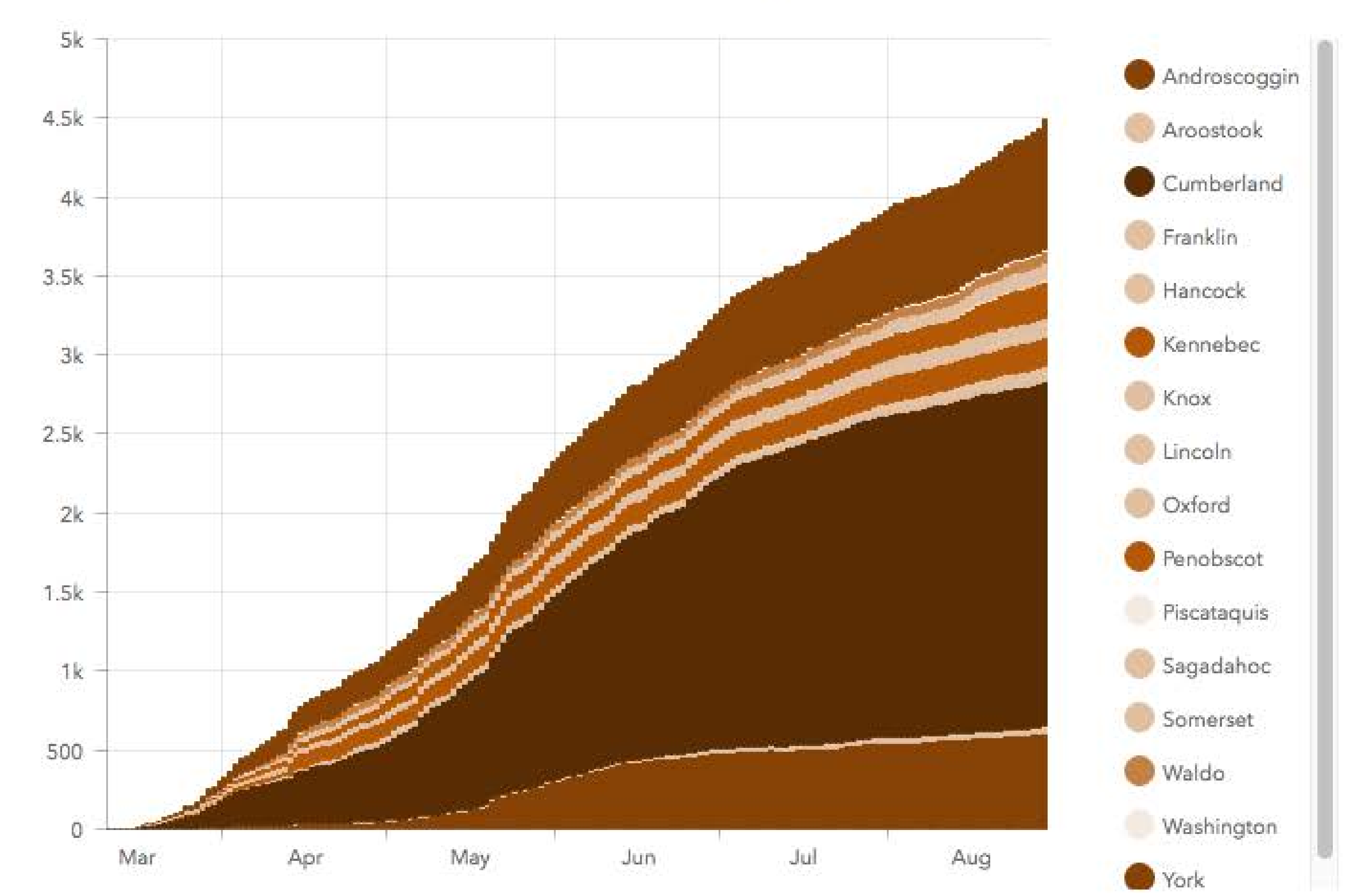

Maine and COVID-19

Maine COVID-19 Population and Case Rates

Last Updated: Saturday, August 29, 2020 at 12:30PM EDT

Total Cumulative Cases	Total Active Cases	Total Tests
4,489	458<br>+9 from yesterday	264,408

Statewide Cumulative Cases by Age

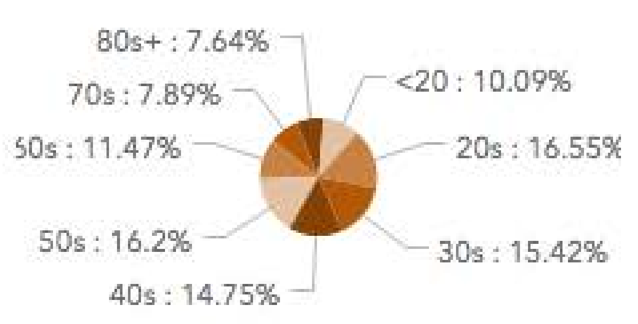

Statewide Cumulative Cases by Sex

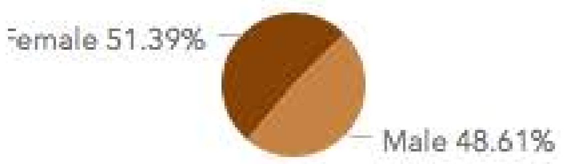

Statewide Cumulative Cases by Race

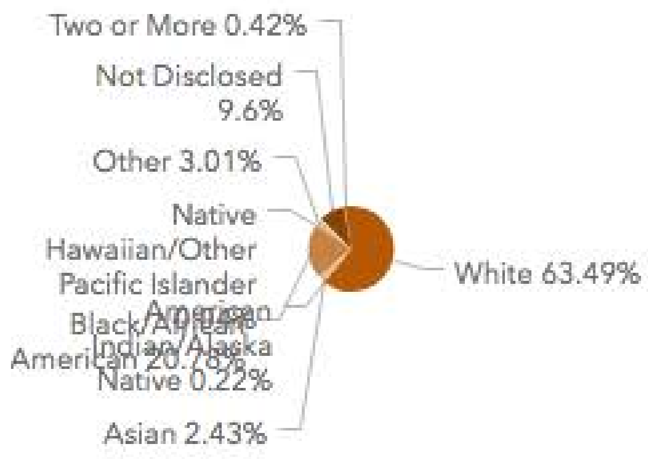

COVID-19 Case Rates by County

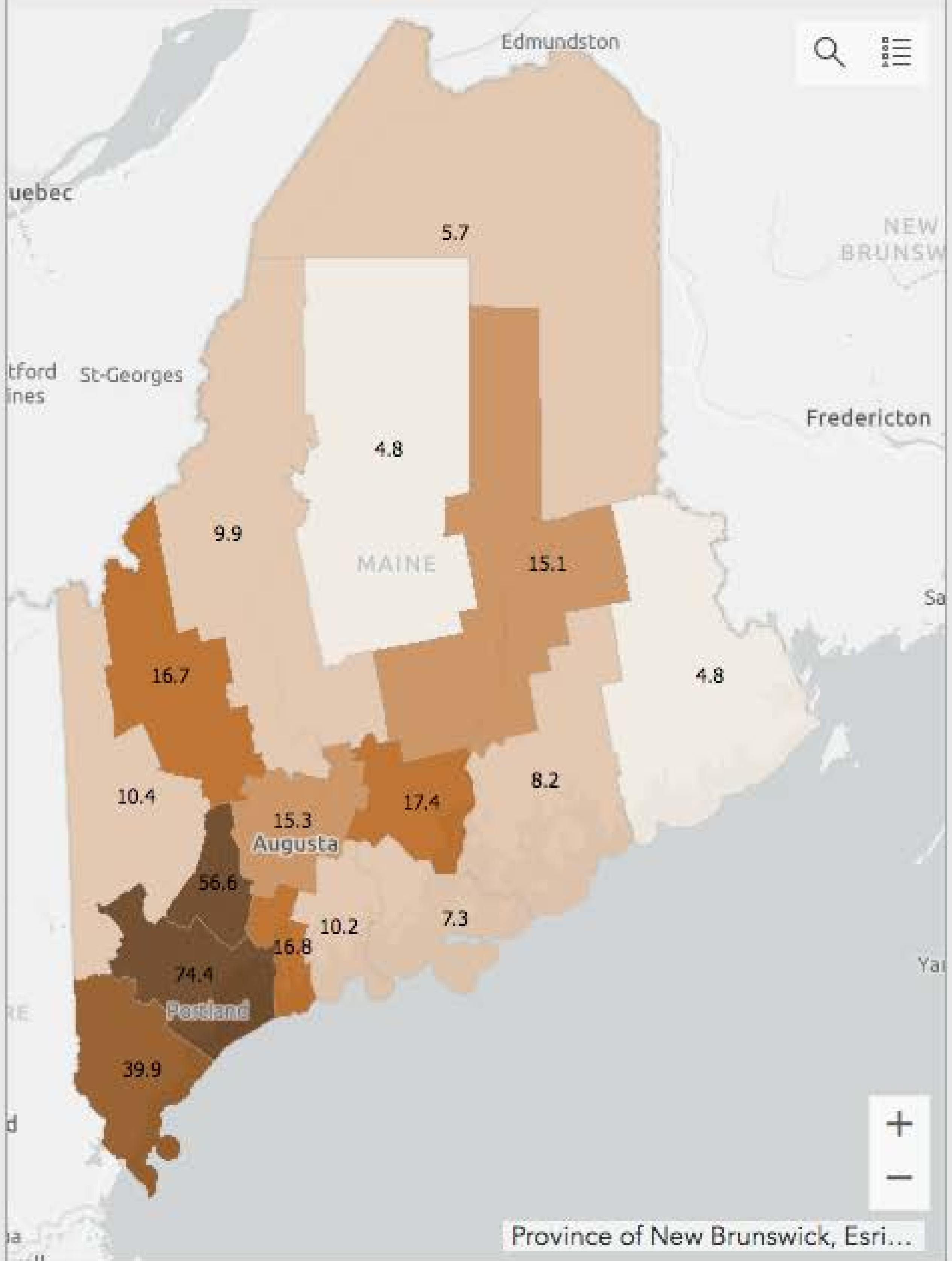

Note: Case rates are calculated by dividing the total cumulative number of confirmed positive cases in a county by the total population of that county multiplied by 10,000. Confirmed data represent only those individuals with positive test results, which likely under-represents the true number of cases in Maine.

Rates (%) of Recovery, Hospitalization, and Deceased by County

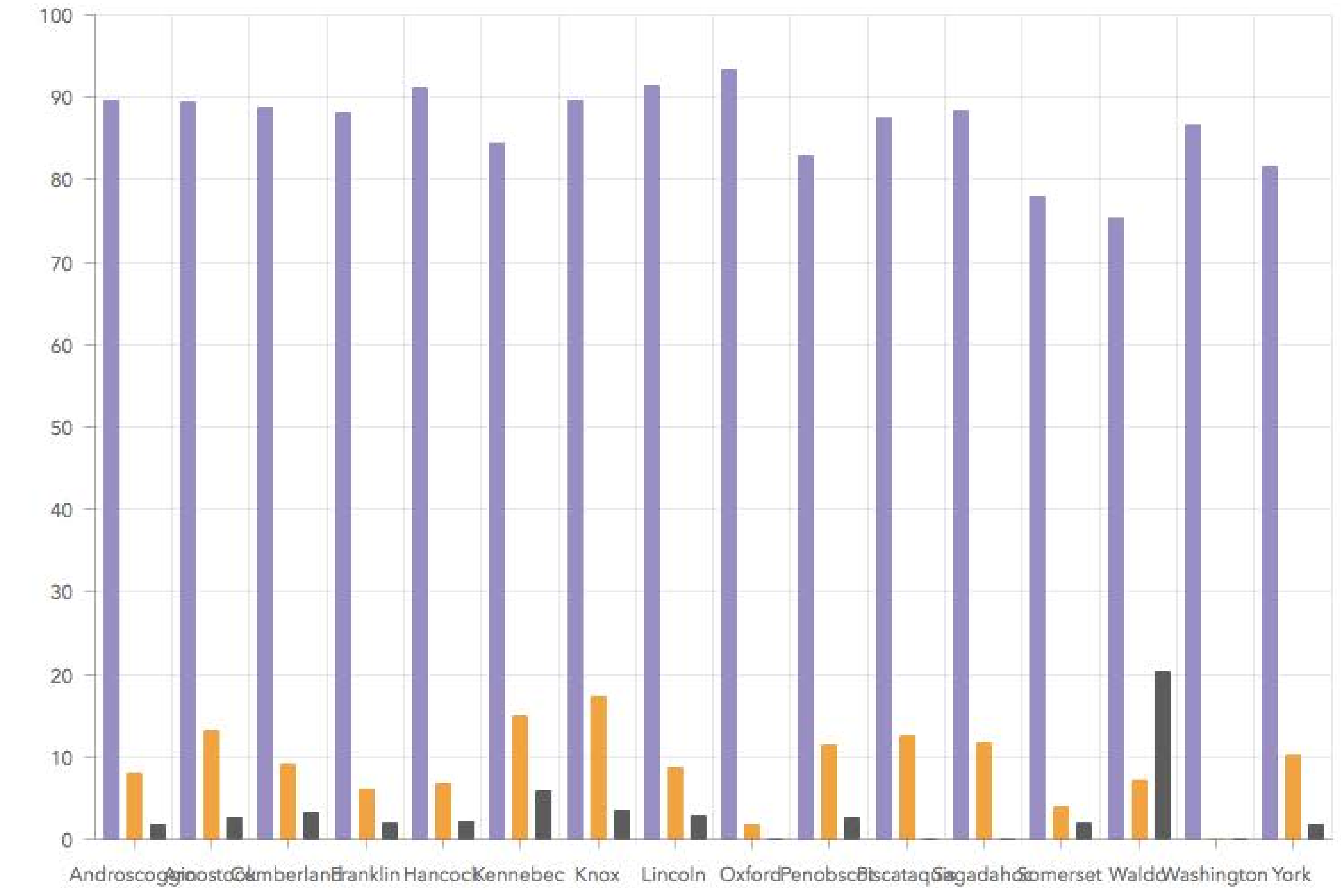

Calculation: (Category divided by total county caseload) multiplied by 100

MARYLAND
DEPARTMENT OF HEALTH

Maryland

Coronavirus Disease 2019 (COVID-19) Outbreak

This is a rapidly evolving situation and the Maryland Department of Health (MDH) will provide updates as they become available.

Notice of Non-Discrimination and Accessibility Statement

Maryland COVID-19 Data Dashboard

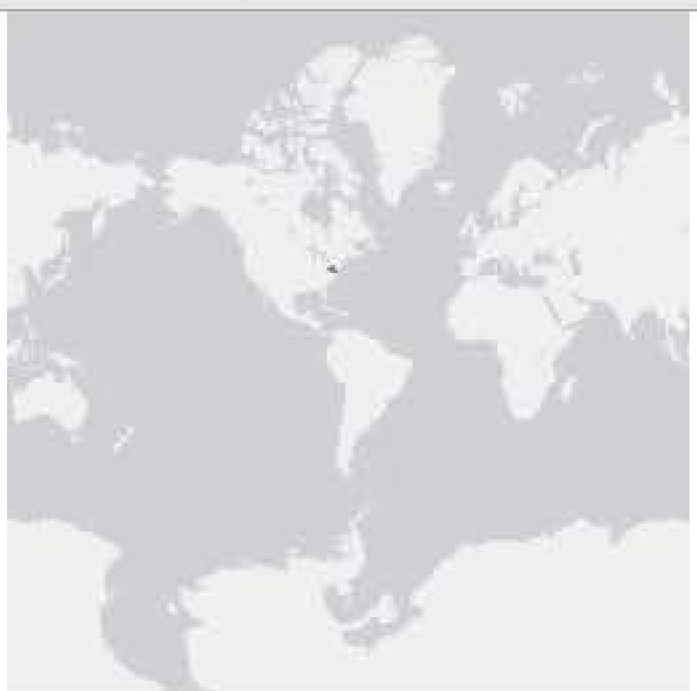

Esri

Affected Counties:

- Prince George's - 26,064
- Montgomery - 19,866
- Baltimore - 15,274
- Baltimore City - 14,383
- Anne Arundel - 8,264
- Howard - 4,354

Cases by County | Cases by ZIP Code | Testing by County

ICU and Acute Hospital Beds for COVID-19, Currently in Use

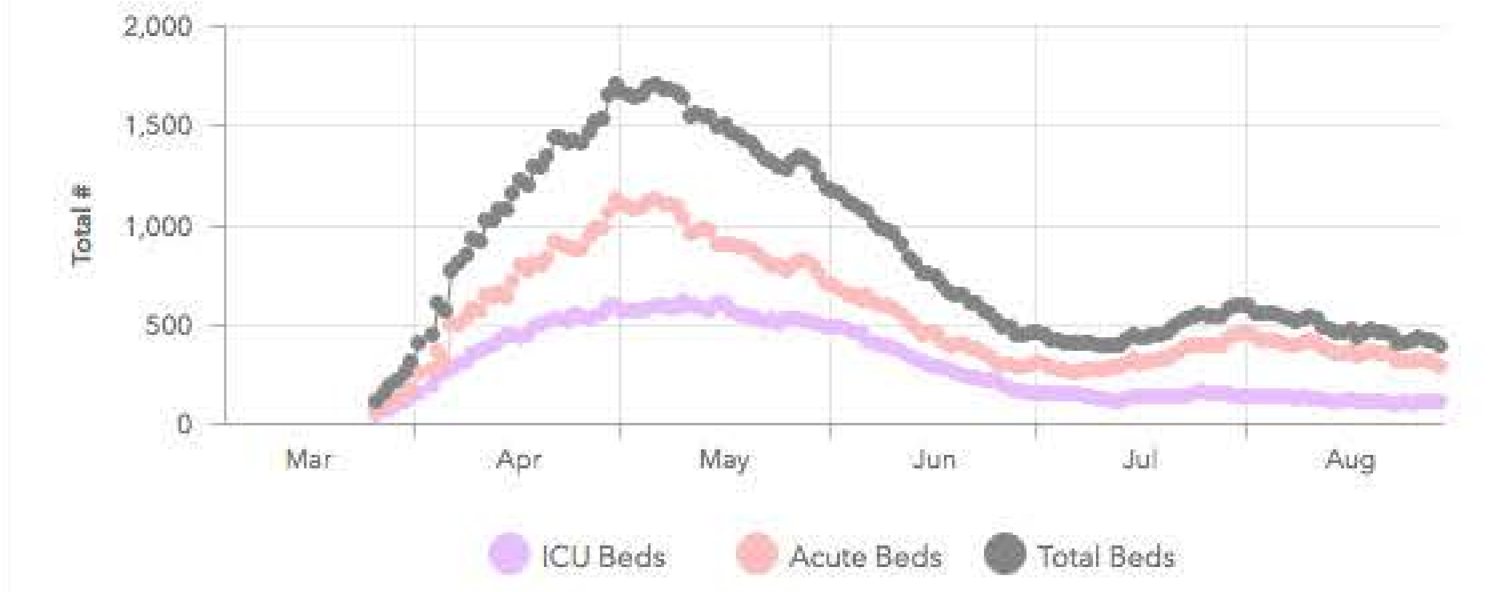

Hospitalizations, Current

Testing Volume, Tests per Day and Percent Positive Rate (7-Day Avg)
- Methodology

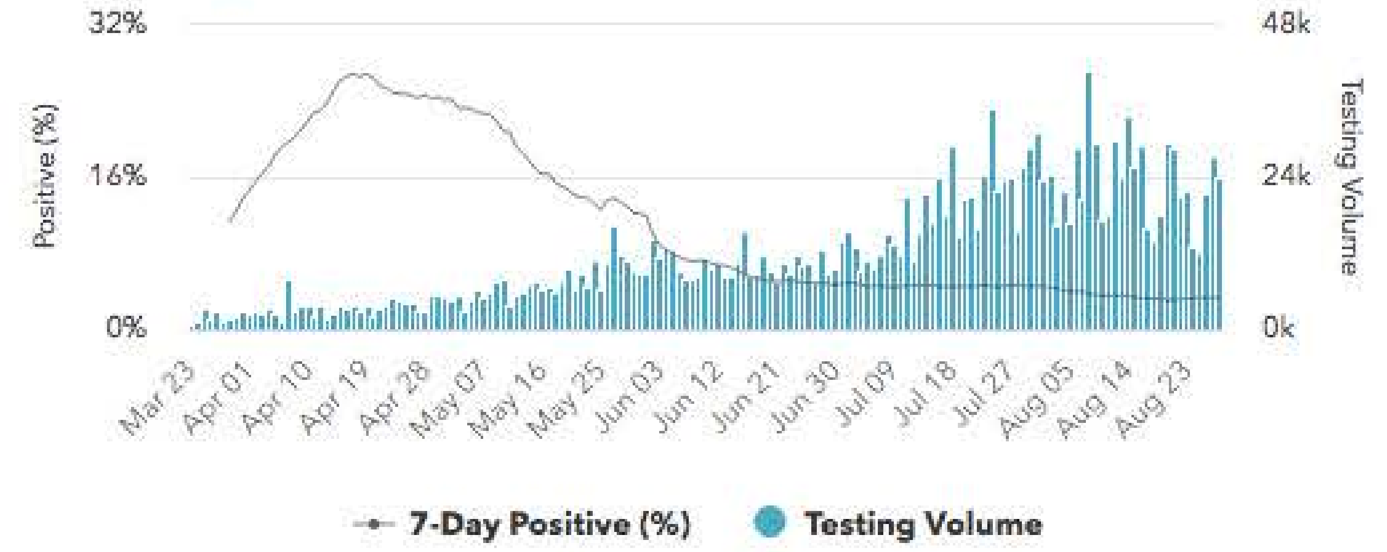

Positivity Rate

Confirmed Cases	Persons Tested Negative	Testing Volume	Testing % Positive	Confirmed Deaths	Currently Hospitalized
107,294	1,197,189	1,894,410	3.37%	3,603	387
24hr Change: +630	24hr Change: +12,583	24hr Change: +25,483	24hr Change: 0.05	24hr Change: +10	24hr Change: -25

Note: The Maryland Department of Health updates these data daily during the 10 a.m. hour. All data are preliminary and subject to change based on additional reporting. Case and death data reflect Maryland residents only. Death data are presented by both date of report and by date of death and are updated as amendments to the death record are received. Data for ZIP codes with 7 or fewer cases are suppressed. Hospitalization data reflect COVID-19 bed occupancy in Maryland hospitals. Testing volume data represent the static daily total of PCR COVID-19 tests electronically reported; this count does not include test results submitted by labs and other clinical facilities through non-electronic means. The percent positive rate is a seven day rolling average of positive results as a percentage of all tests. MDH is continuously evaluating its data and reporting systems and will make updates as more data becomes available.

Massachusetts Department of Public Health COVID-19 Dashboard - Friday, August 28, 2020

Dashboard of Public Health Indicators

Newly Reported Confirmed Cases Today	Total Confirmed Cases	Newly Reported Deaths among Confirmed Today	Total Deaths among Confirmed Cases
438	117,888	16	8,791

New Individuals Tested by Molecular Tests	Total Individuals Tested by Molecular Tests
23,716	1,673,552

Total Molecular Tests Administered
2,290,752

Legend
- Green: Positive trend
- Yellow: In progress
- Red: Negative trend

Below is the current status:

Measure	Status
COVID-19 positive test rate	Green
Number of individuals who died from COVID-19	Yellow
Number of patients with COVID-19 in hospitals	Green
Healthcare system readiness	Yellow
Testing capacity	Green
Contact tracing capabilities	Green

The front page of the dashboard has been reformatted. Antibody tests (individual and total numbers) can be found on page 17.

Massachusetts Department of Public Health COVID-19 Dashboard - Friday, August 28, 2020

Dashboard of Public Health Indicators

Percent or Count of Change Since Lowest Observed Value (LOV)

7 Day Weighted Average of Positive Molecular Test Rate*

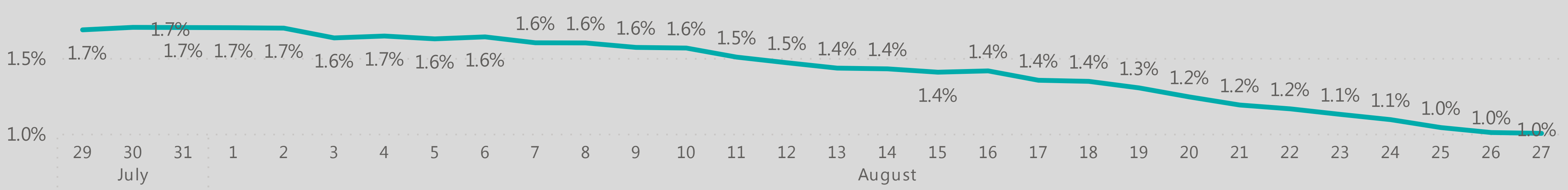

0 %

LOV = 1.0%

3 Day Average of Number of COVID-19 Patients in Hospital**

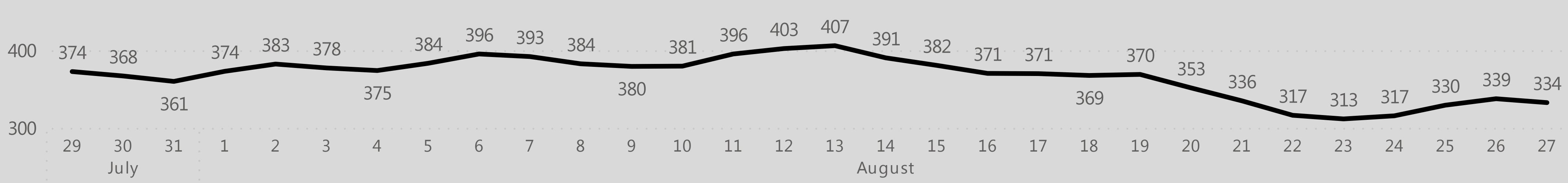

7 %

LOV = 313

Number of Hospitals using Surge Capacity

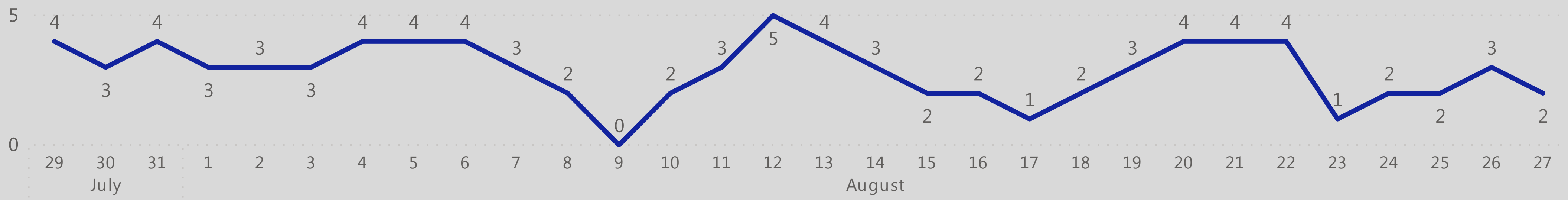

2

LOV = 0

3 Day Average of COVID-19 Deaths***

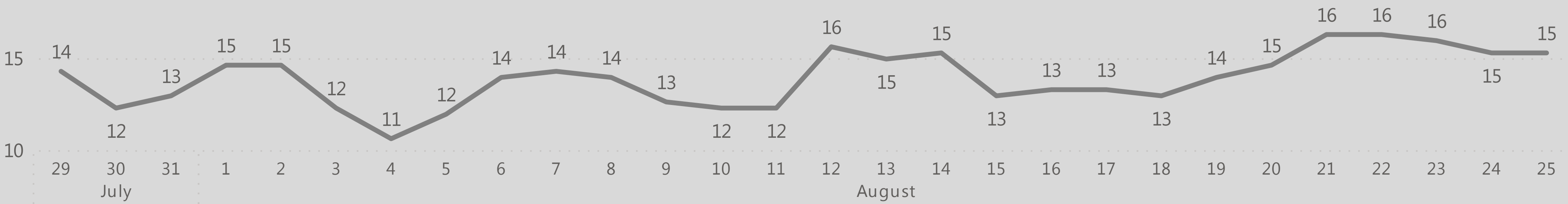

44 %

LOV = 11

*Calculated from total molecular tests; **Includes both confirmed and suspected cases of COVID-19; ***Includes deaths in only confirmed cases of COVID-19

Note: Hospital-reported data included here reflects a transition to new federal reporting standards imposed as of 7/22. The third graph (number of hospitals using surge) does not include data from July 22 due to this transition. As a result, data may not be directly comparable to hospital data previously reported. LOV = Lowest observed value, i.e. the lowest value the public health indicator has been since tracking started.

CURRENT STATUS | DAILY CASES | CUMULATIVE TRENDS | DEMOGRAPHICS | TOTAL TESTING | DIAGNOSTIC TESTING | LEARN MORE

View Cases

View Deaths

View Table

Select Preparedness Region:

All

Select County:

All

This report is provisional and subject to change. City of Detroit and Wayne County are reported separately. MDHHS began tracking probable cases April 5, 2020. FCI indicates federal correctional institute. MDOC indicates Michigan Department of Corrections. Diagnostic tests, including nucleic acid amplification/real-time polymerase chain reaction (NAAT/RT-PCR) and rapid virus antigen detection point of care (POC) tests, look for current infection. Serology (antibody) tests are used to look for the possibility of previous infection.

COVID-19 Cases by County

Confirmed cases include individuals with a positive diagnostic test for COVID-19. Probable cases include individuals with COVID-19 symptoms and an epidemiologic link to confirmed COVID-19, but no diagnostic test.

Cases by County

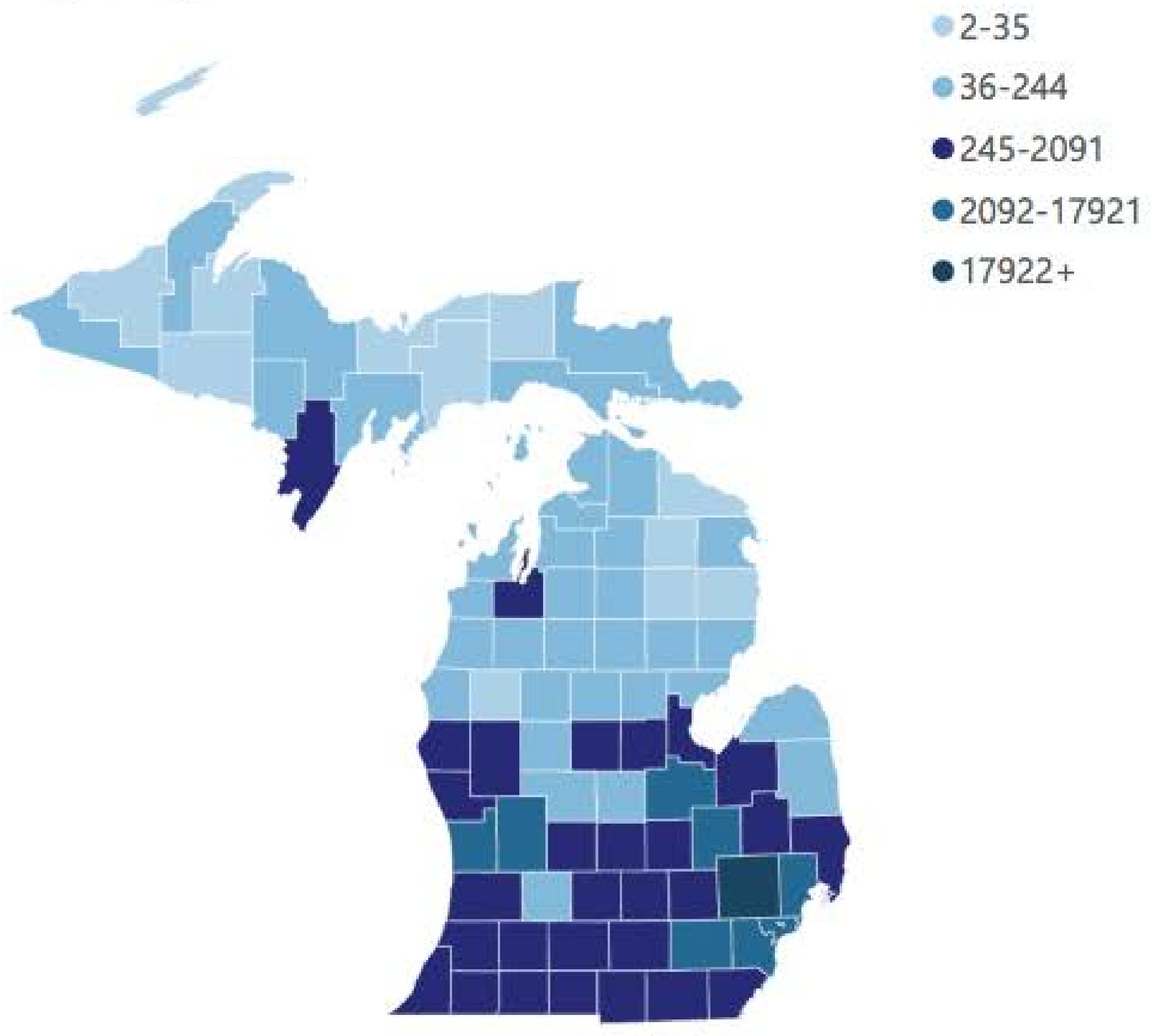

Michigan

Saturday, August 29, 2020

Michigan (Statewide) Case Fatality Rate

6.4%

Current Totals

Case Status	Cases	Deaths
Confirmed	100,699	6,446
Probable	10,437	266
**Total**	**111,136**	**6,712**

Laboratory Testing

Test Type	Tests Performed
Diagnostic	2,715,128
Serology	253,766
**Total**	**2,968,894**

Microsoft Power BI

1 of 7

Bridge Michigan's nonpartisan, nonprofit news source

If you care about Michigan, please support our work. DONATE TODAY

Michigan coronavirus dashboard: cases, deaths and maps

Coronavirus in Michigan

Click the tabs to see total numbers and per-capita rates by county.

Total numbers | Per 100,000

	Confirmed	Probable
Total cases:	101,478	10,437
Total deaths:	6,467	266

Cases per 100,000: 1,017

Deaths per 100,000: 65

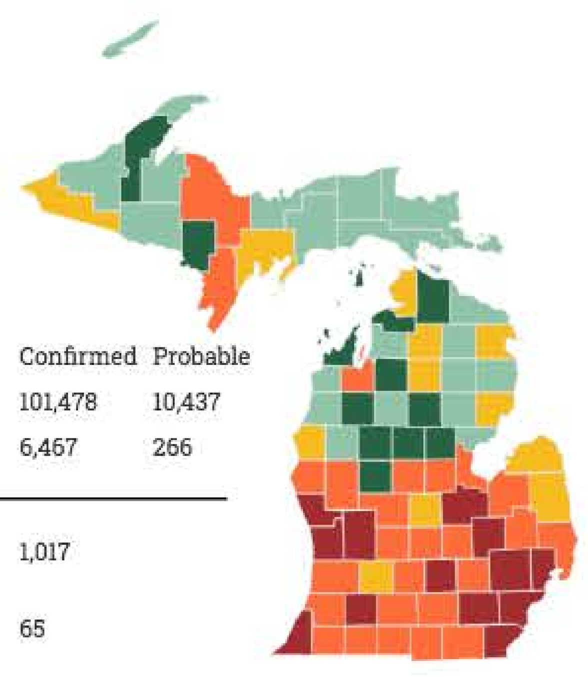

Total cases (cases per 100,000 people)
- 1 case or none
- 2 to 49 (fewer than 50 cases per 100,000)
- 50 to 99 (50 to 99 cases per 100,000)
- 100 to 199 (100 to 199 cases per 100,000)
- 200 to 999 (200 to 299 cases per 100,000)
- Over 1,000 (300 or more cases per 100,000)

Daily cases

New cases each day since March 25

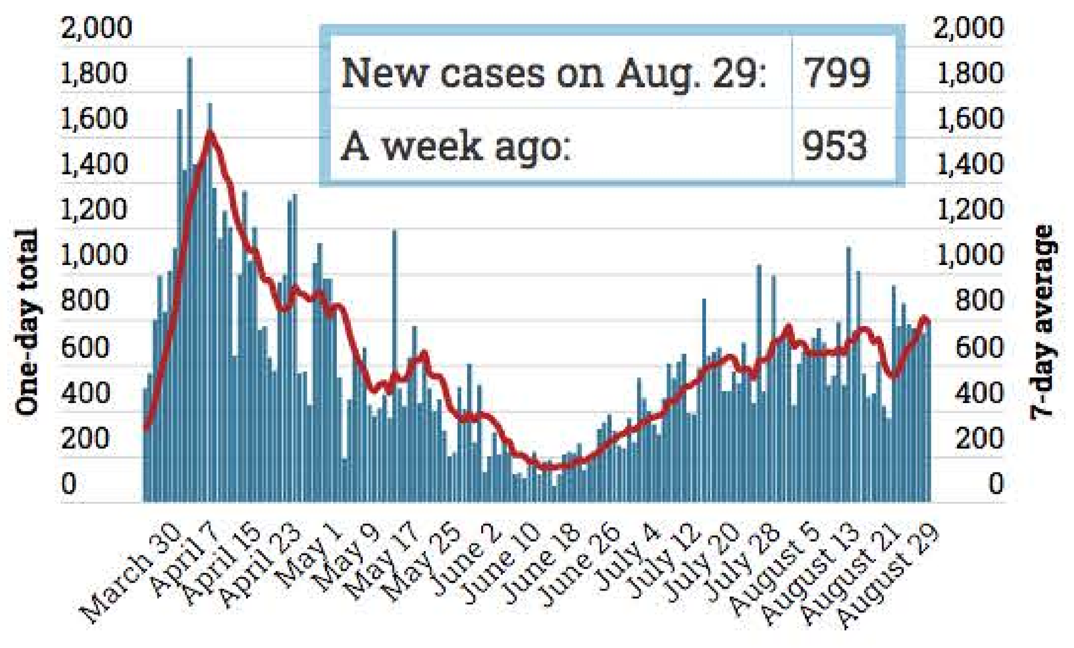

● One-day total ● 7-day average

Daily deaths

Reported deaths by day since March 25

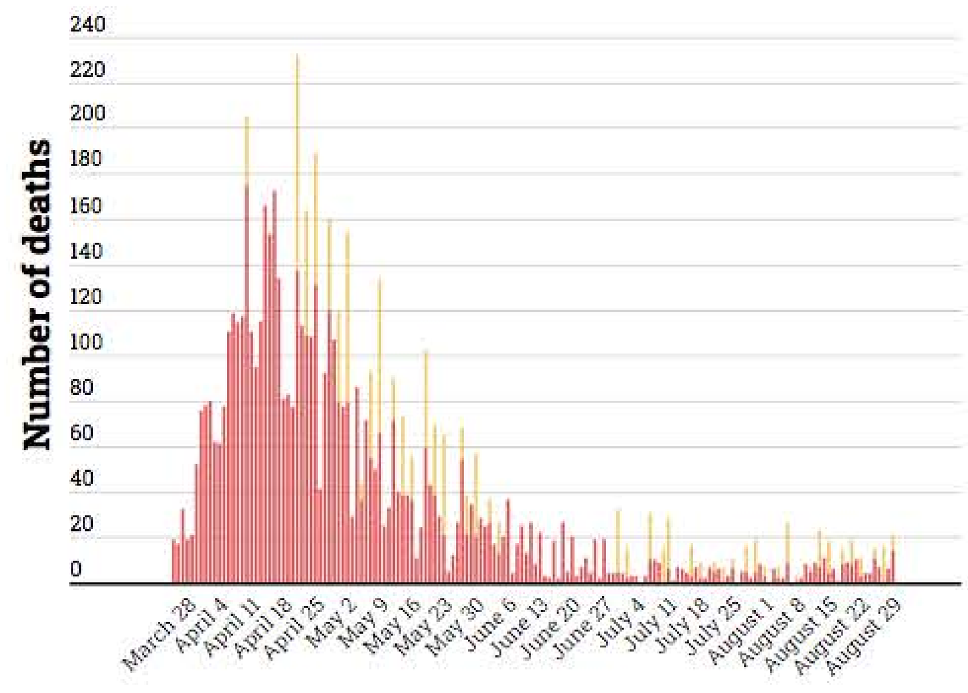

● Deaths ● Added deaths

*Days on which the state has added deaths based on review of earlier death records.

Cases and testing

You can use this new graphic to check daily changes in cases and tests, as well as compare to the previous week.

Cases | Testing

Today: Aug. 29	Cases today	7-day average	Prior week avg.	Cases per 100,000 rate	Prior week rate
Wayne	102	126	91	7	5
Macomb	99	122	98	14	11
Oakland	89	118	96	9	8
Kent	58	60	30	9	5
Isabella	31	27	2	38	3
Saginaw	29	30	18	16	9
Branch	26	9	4	21	9
Ottawa	25	14	12	5	4
Detroit City	22	36	28	5	4
Kalamazoo	22	18	10	7	4
Lenawee	16	7	4	7	4
Luce	16	2	0	32	0
Berrien	15	14	12	9	8
Genesee	15	16	14	4	3
Ingham	14	13	10	4	3

Bridge — *Mike Wilkinson*

Hospitalizations

The state has recently changed how it reports hospitalization data, now including confirmed and suspected cases. Bridge has changed this graphic to match. Data does not exist for every day and it is not updated on weekends.

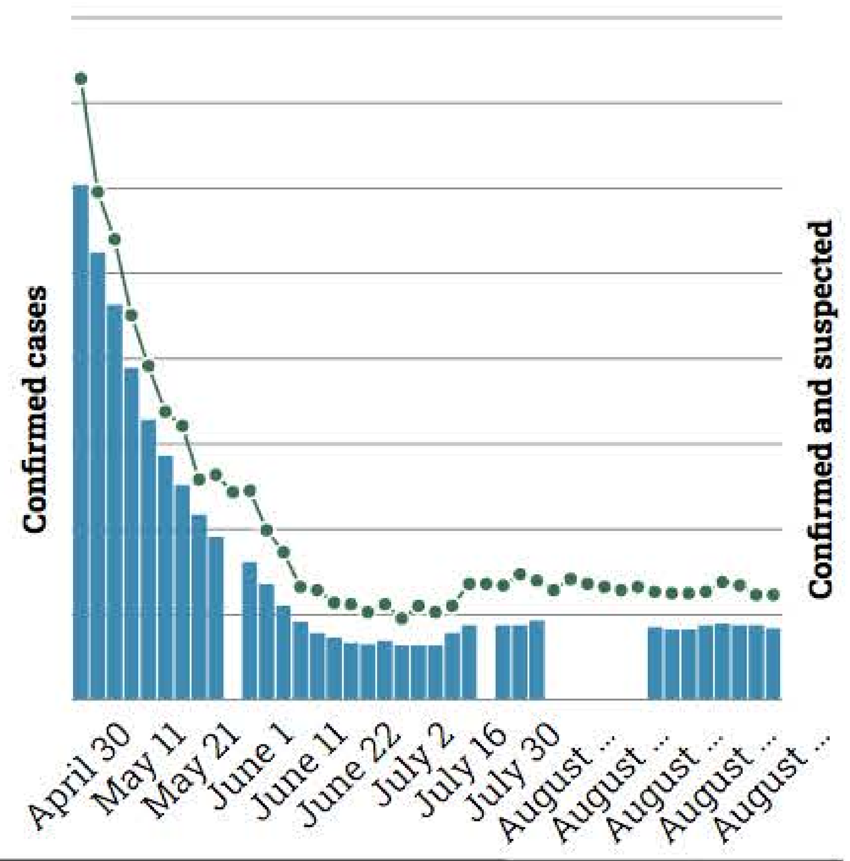

Source: Michigan Department of Health and Human Services and Western Michigan University Libraries.

Bridge — *Mike Wilkinson*

COVID-19 deaths in Michigan

This map shows home residence of those who have died from the coronavirus.

Total deaths: 6,467

- No deaths
- 1 death
- 2 to 49 deaths
- 50 to 99 deaths
- More than 100 deaths

*As of 10 a.m. Aug. 29

Source: Michigan Department of Health & Human Services

Share

Bridge

Age breakdown

The coronavirus has infected and killed people of all ages in Michigan but has hit the elderly particularly hard. Here's the current breakdown.

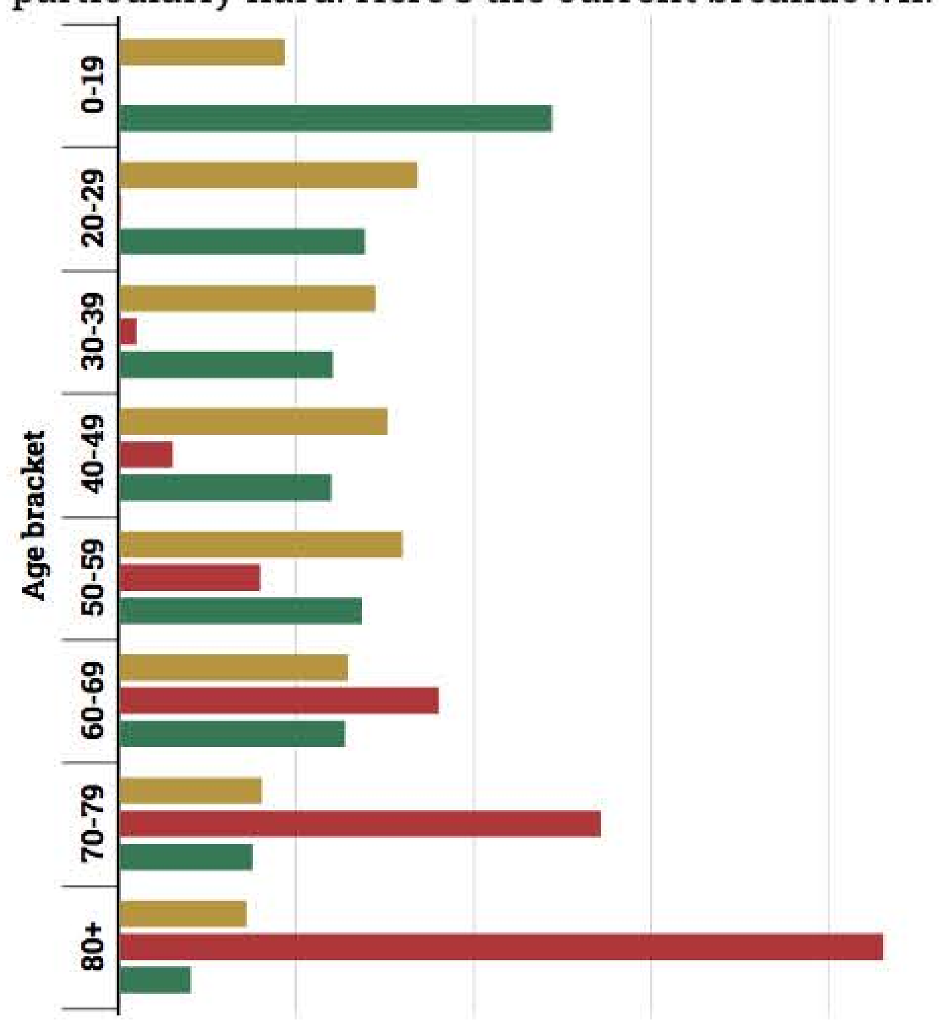

● Percent of cases ● Percent of deaths
● Percent of population

Source: Michigan Department of Health & Human Services

Nursing homes

State data on nursing home coronavirus cases and deaths since March.

Resident Confirmed Cases	8,152
Resident COVID-19 Deaths	2,103
Staff Confirmed Cases	4,324
Staff COVID-19 Deaths	21

Total deaths

Deaths reported going back 7 days

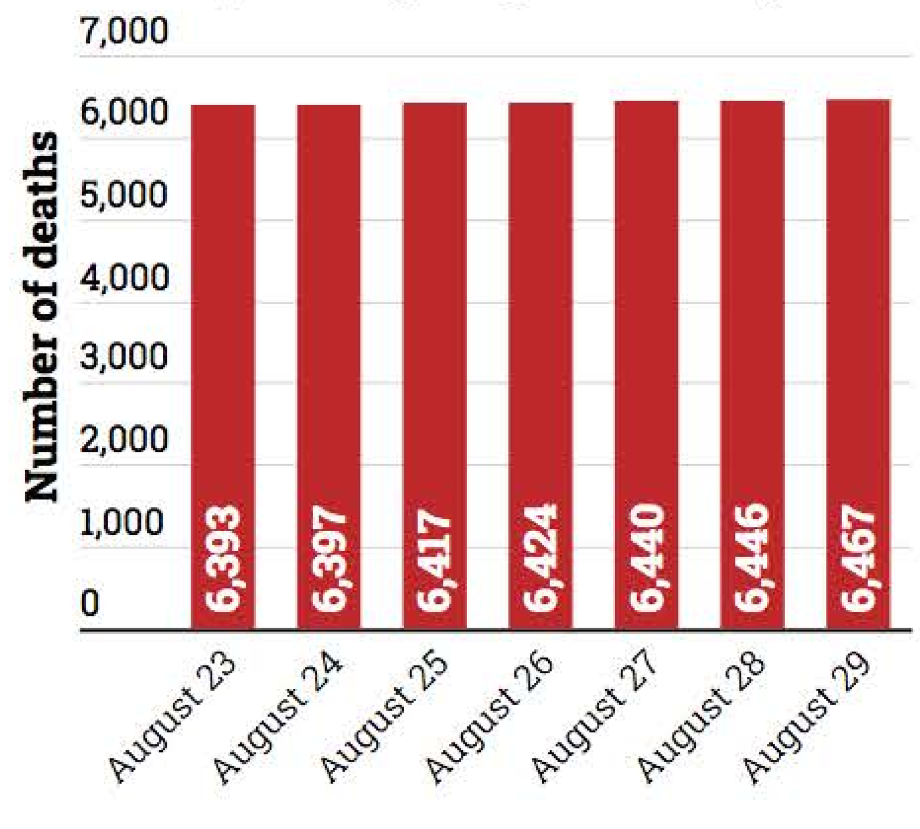

Daily testing results

Michigan health officials say the goal is to keep the positive test rate at 3 percent or lower, where it has hovered for over two months.

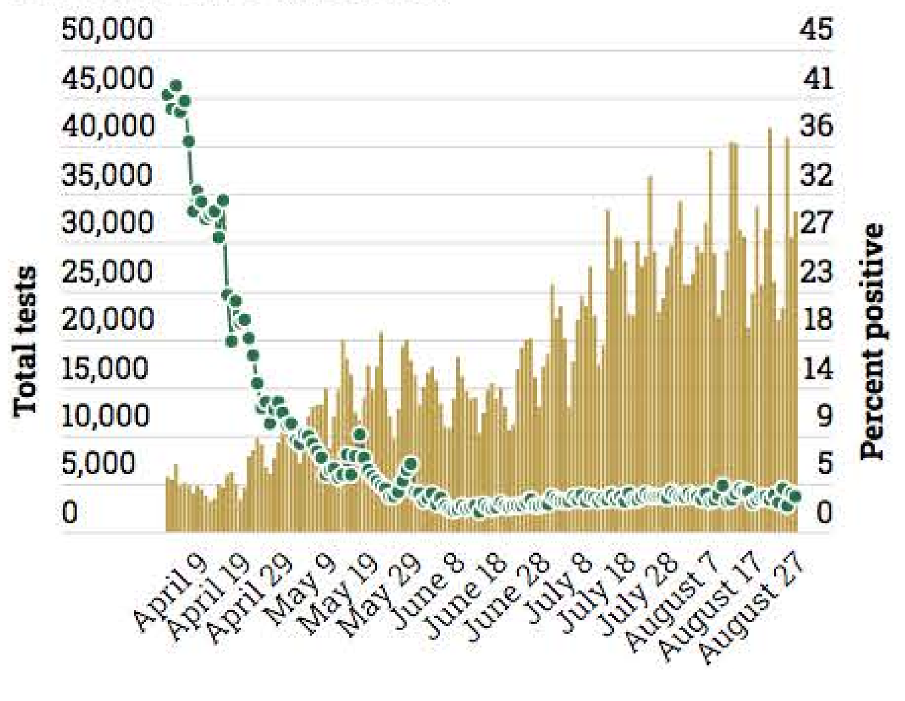

● Total tests ● Percent positive

COVID-19
COVID-19 Home
Situation Update
About COVID-19
Symptoms & Testing
If You Are Sick
Protect Yourself & Others
Daily Life & Coping
Materials and Resources
Guidance Library

Information for:
Health Care
Community Settings
Businesses & Employers
Schools & Child Care
Institutes of Higher Education

Related Topics
COVID-19 Research Studies in Minnesota
COVID-19 Contracts for Diverse Media Messaging and Community Outreach
Disabilities and Unique Health Needs During COVID-19
Infectious Respiratory Illness

Situation Update for COVID-19

Subscribe: COVID-19 updates
Sign up to receive email or mobile alerts when this data is updated, as well as other periodic COVID-19 updates.

Updated August 29, 2020
Updated daily at 11 a.m., with data current as of 4 p.m. the previous day.

Data is for cases that were tested and returned positive. All data is preliminary and may change as cases are investigated. Many data points are collected during case interviews. Data presented below is for all cases, regardless of interview status. Data for cases pending interview may be listed as "unknown/missing."

Not all suspected cases of COVID-19 are tested, so this data is not representative of the total number of people in Minnesota who have or had COVID-19. Cumulative numbers are since Jan. 20, 2020.

On this page:
Daily Update
Testing
Minnesota Case Overview
Hospitalization
Case Demographics
Likely Exposure
Residence
Weekly Report
Care Facilities
Other COVID-19 Data and Statistics

Expand All Contract All

Daily Update:

Because all data is preliminary, the change in number of cumulative positive cases and deaths from one day to the next may not equal the newly reported cases or deaths.

74,257<br>Total positive cases (cumulative)	
1,032<br>Newly reported cases	4<br>Newly reported deaths

Newly reported cases detail
Newly reported deaths detail

Testing

Total approximate number of completed tests: 1,460,381

- Total approximate number of people tested: 1,111,137

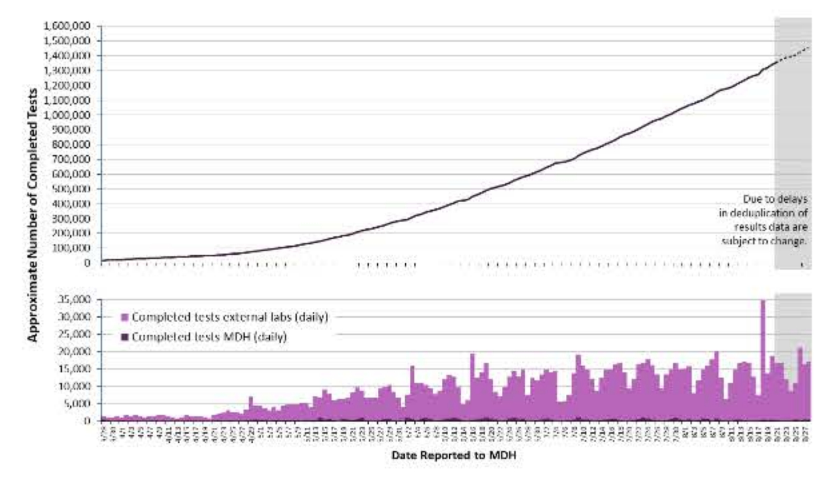

Testing data table
More about testing data

Minnesota Case Overview

- **Total positive cases:** 74,257
  - Number of health care workers: 8,175

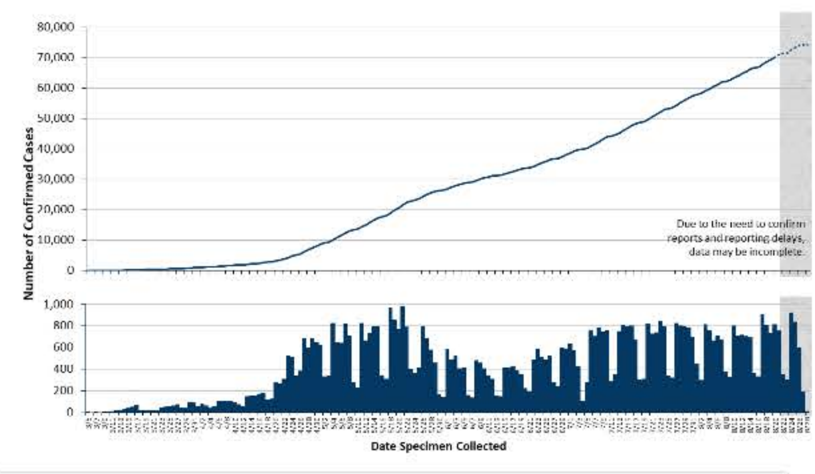

Positive cases by date specimen collected data table
More about positive cases

Patients no longer needing isolation

- **Patients no longer needing isolation:** 66,107

More about patients no longer needing isolation

Deaths

- **Deaths:** 1,814
  - Deaths among cases that resided in long-term care or assisted living facilities: 1,337
- **Probable COVID-19 Deaths*:** 49

* COVID-19 listed on death certificate but a positive test not documented for the person.

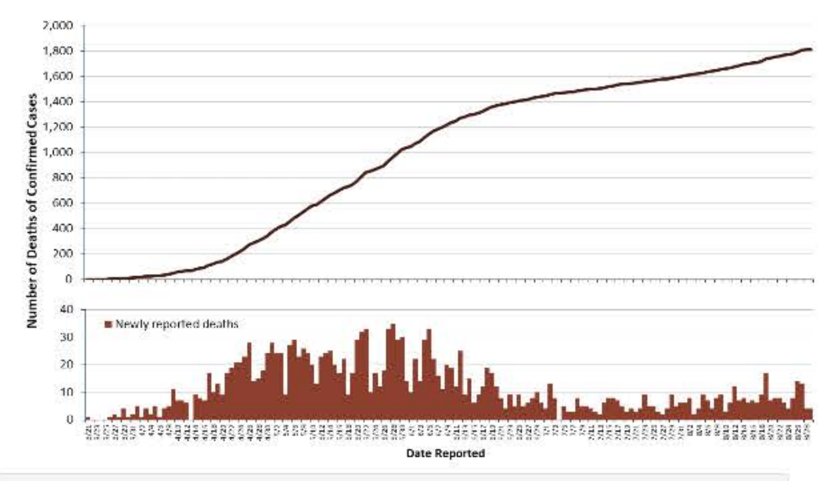

Deaths data table
More about deaths

Hospitalization

Total cases hospitalized: 6,411
- Hospitalized as of today*: 313
  - Hospitalized in ICU as of today*: 134

* Refer to "More about hospitalizations" for notes.

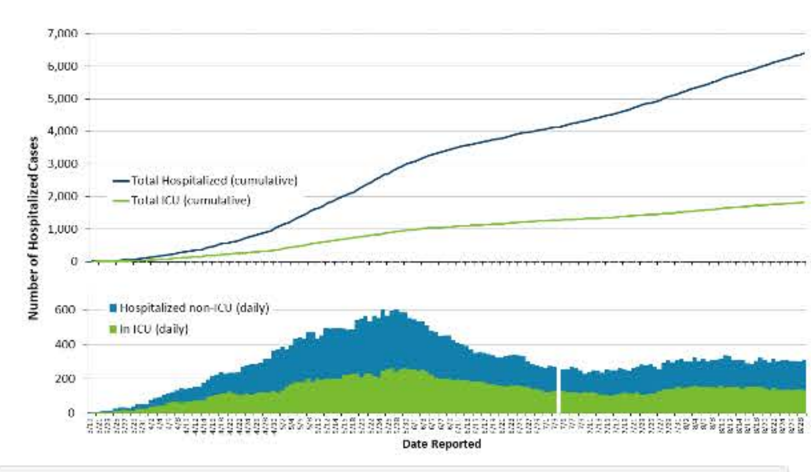

Hospitalization data table
More about hospitalizations

Case Demographics

Age

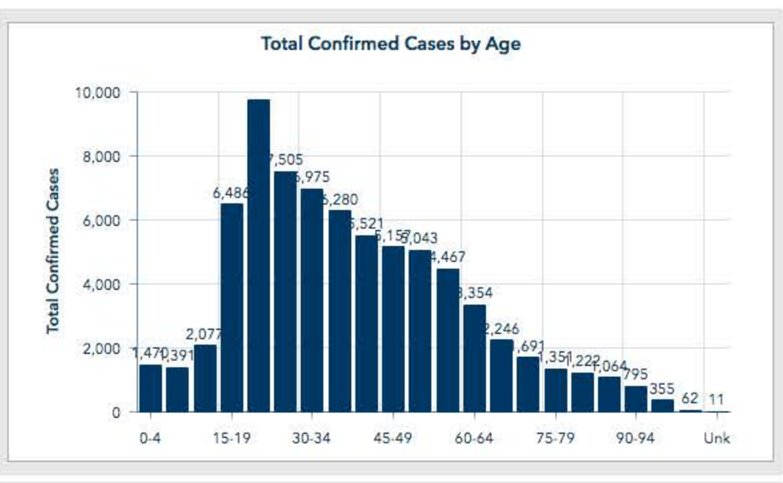

Age group data table:
Including age group of deaths
More about age

Gender

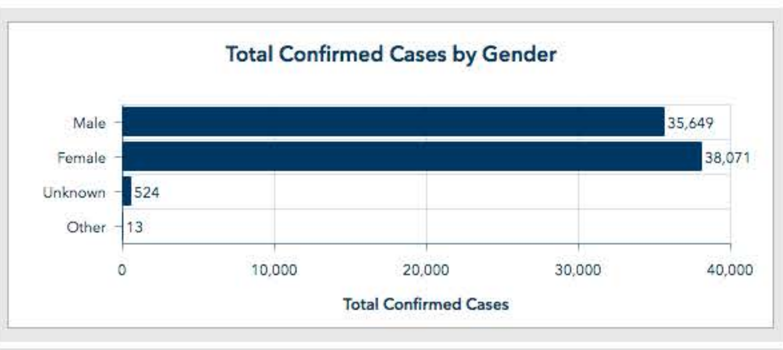

Gender data table
More about gender

Race & Ethnicity

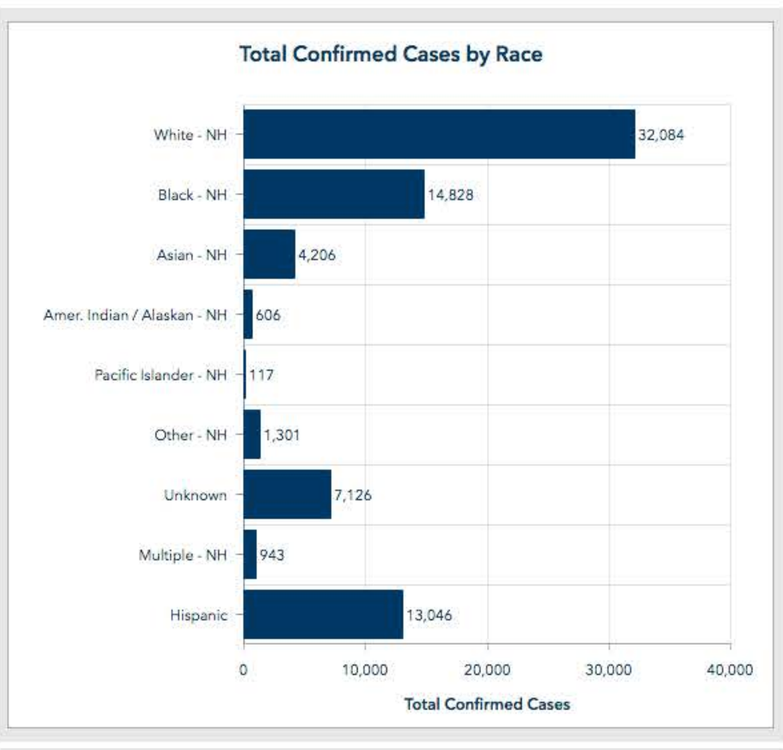

Race and ethnicity data table:
Including race and ethnicity of deaths
More about race and ethnicity

Likely Exposure

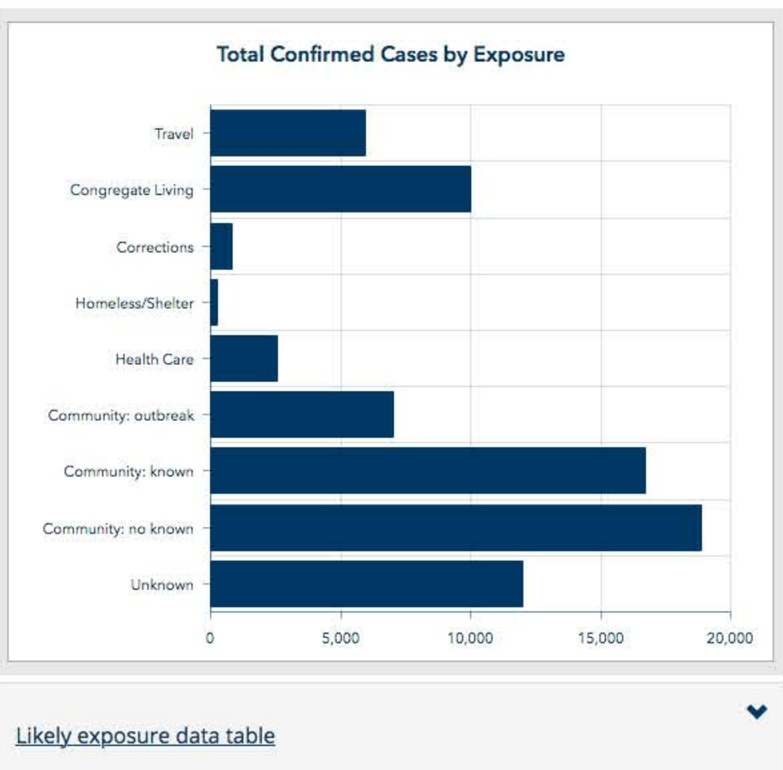

Likely exposure data table
More about likely exposure

Not all cases among health care workers have a likely exposure as health care staff.

Residence

Cases by County of Residence

County of residence is confirmed during the case interview. At the time of this posting not all interviews have been completed.

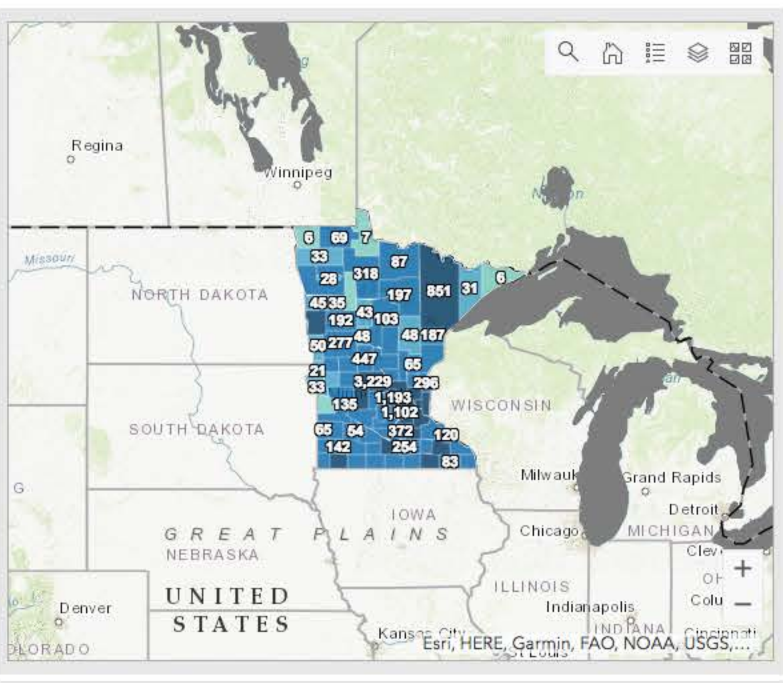

County of residence data table:
Including county of residence of deaths
More about location data

Residence Type

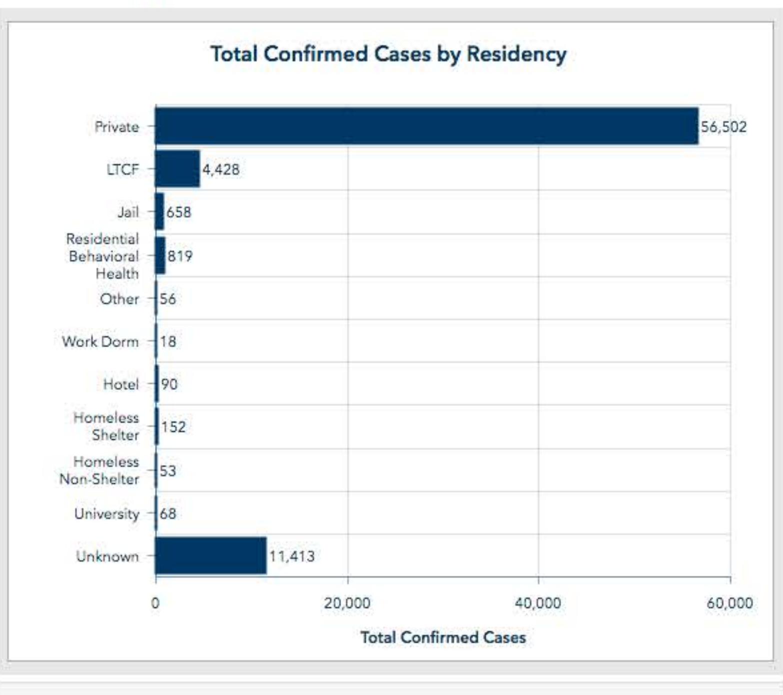

Share This
Subscribe: COVID-19 updates
Planning Guidance and Data for Schools
Masks and Face Coverings
Videos for COVID-19 Response

Minnesota Helpline:
For questions related to the COVID-19 pandemic, call 651-297-1304 or 1-800-657-3504
Mon.-Fri.: 9 a.m. to 4 p.m.

Mental Health Crisis Line - Call: **CRISIS (**274747)

COVID-19 information in other languages:
Kab Mob Khaus Las Nas Vais Lav 2019 (COVID-19) (Hmong)
Cudurka Koronafayras 2019 (COVID-19) (Somali)
Enfermedad del Coronavirus 2019 (COVID-19) (Spanish)
American Sign Language (ASL) Videos

Contact us:
If you have questions or comments about this page, use our IDEPC Comment Form or call 651-201-5414 for the MDH Infectious Disease Epidemiology, Prevention and Control Division.

E-Newsletter
Get disease updates, inspection results, health news and much more with our weekly e-mail newsletter
email address
SUBSCRIBE

Interactive Map: Mississippi COVID-19 Cases

Select Language

▸ More COVID-19 charts and data

Mississippi COVID-19
Cases and Deaths by Race with Ethnicity
as of 6 pm CT, August 28, 2020

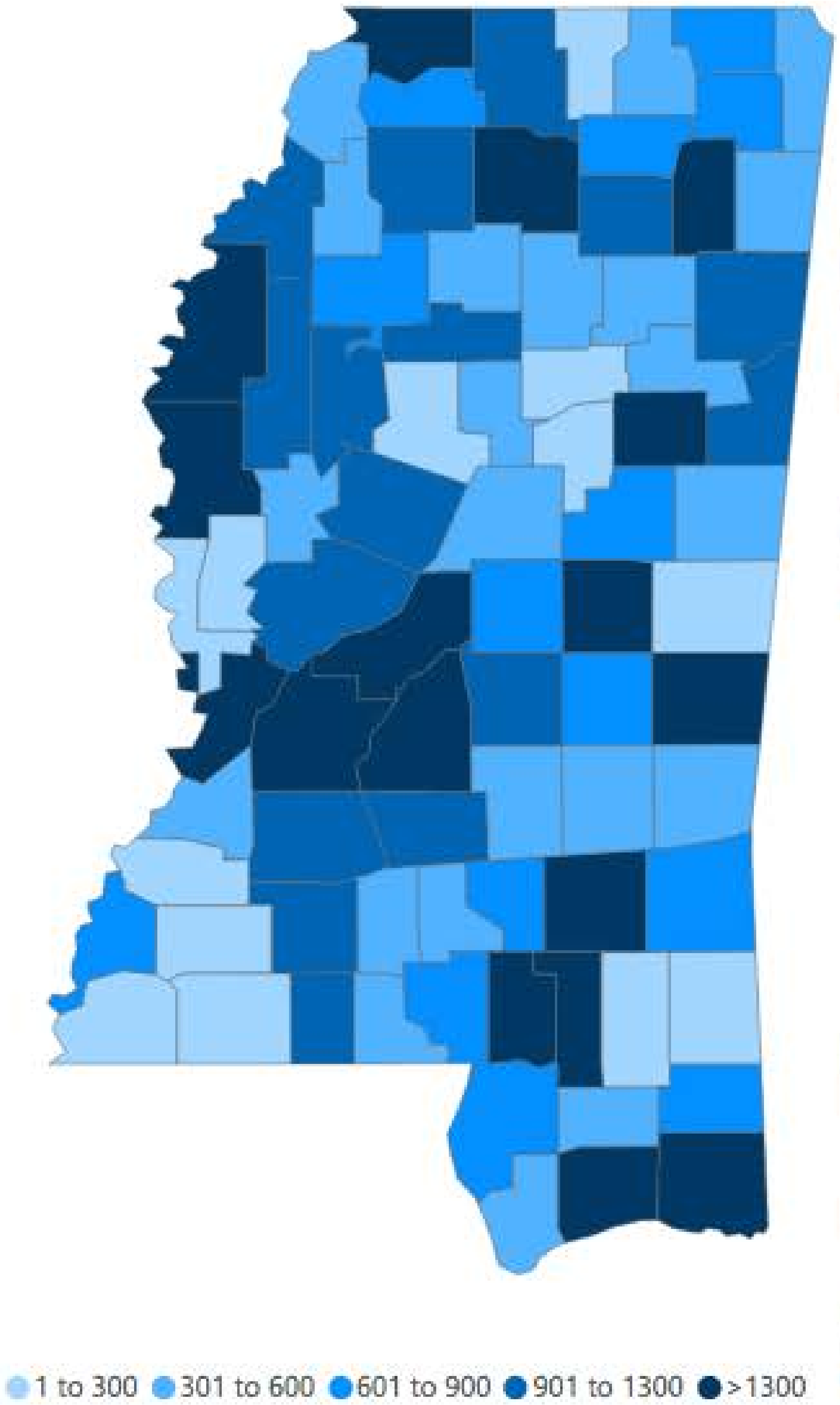

Total Cases
82,029

	American Indian or Alaska Native	Asian	Black	White	Other	Unknown
Non Hispanic	468	217	26,631	21,264	872	414
Hispanic	14	11	103	682	1,545	97
Unknown Ethnicity	779	63	6,731	5,783	4,442	11,913

Total Deaths
2,427

	American Indian or Alaska Native	Asian	Black	White	Other	Unknown
Non Hispanic	64	3	1,041	966	12	2
Hispanic	1	0	3	17	13	1
Unknown Ethnicity	20	1	109	117	22	35

Microsoft Power BI

Missouri Coronavirus GIS Hub For more information on COVID-19, click here.

Missouri COVID-19 Dashboard: The Spread of COVID-19 in Missouri

Show Me Strong RECOVERY PLAN

COVID - Overview | Cases - Demographics | Cases - County | Testing - PCR | Testing - Serology | Deaths | Hospitalizations

THE SPREAD OF COVID-19 IN MISSOURI

See documentation for more information

Mobile Site

(Click to view on mobile devices.)

Due to an increased COVID-19 test volume, DHSS may experience delays in data processing and reporting from laboratories and providers.

Lab Confirmed Cases in Missouri

82,190

Deaths Attributed to COVID-19

1,496

Number of New Cases Added in the Last 24 Hours

1,198

Number of New Deaths Reported in the Last 24 Hours

32

7 Day Percent Positive of PCR Tested Individuals

12.2%

Number of Hospitalizations due to COVID-19

976

*Data reflects 72 hour delay

COVID-19 Cases By Jurisdiction Of Residence

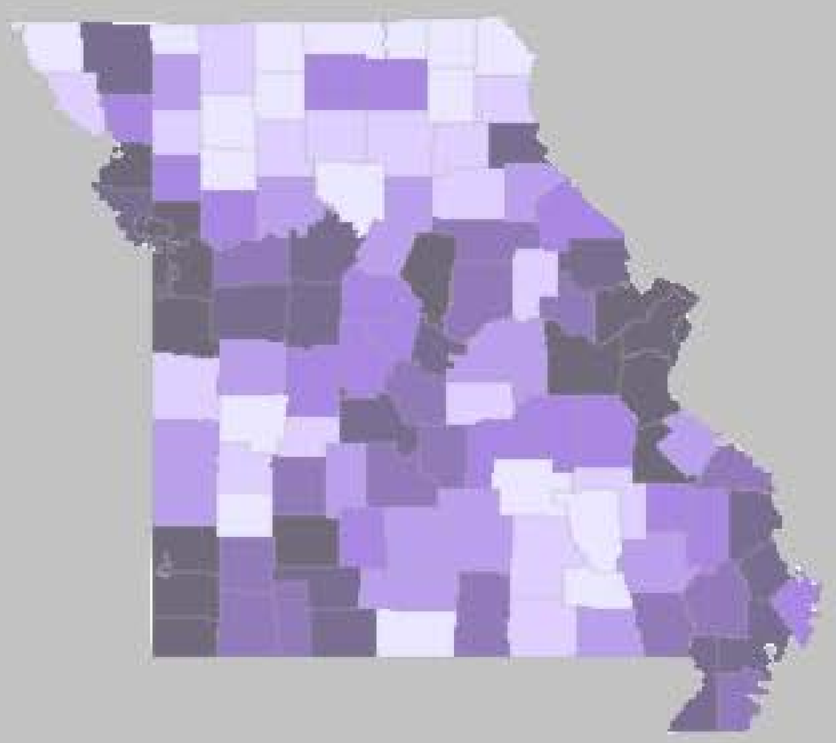

Time-based data, including one- and seven-day percent increase in statewide cases, daily COVID-19 test results, and cases and deaths by reported date, are subject to a 72-hour delay to ensure that the data are accurate and complete. (Example, on 5/15 the dashboard will report data for a period ending on 5/12). These data are provisional and will change daily. Investigations are being done on new cases, so as additional information is determined on these cases, such as county, demographics, and the total counts, will continue to change. Efforts are continually being made to improve data quality so that DHSS is providing the most accurate information possible.

Cases By Jurisdiction

ADAIR	224
ANDREW	127
ATCHISON	28
AUDRAIN	260
BARRY	352
BARTON	102
BATES	70
BENTON	155
BOLLINGER	121
BOONE	2,358
BUCHANAN	1,276
BUTLER	373
CALDWELL	41
CALLAWAY	316
CAMDEN	510
CAPE GIRARDEAU	944
CARROLL	110
CARTER	36
CASS	1,053
CEDAR	48
CHARITON	25
CHRISTIAN	634
CLARK	41
CLAY	1,355
CLINTON	128
COLE	820
COOPER	220
CRAWFORD	178
DADE	26
DALLAS	108
DAVIESS	22
DEKALB	63

See documentation for more information. Page last [illegible]

RECENT UPDATES (8/25/20)

Added a map of Current Consecutive Days of Downward Trajectory by County on the Cases-County tab

Past Updates (7/25/20)

Hospitalization reporting has resumed with data beginning 7/25/2020.
Included a smaller age breakdown of the cases (0-9 and 10-19)

Past Updates (7/24/20)

DHSS has listened to your feedback and has included some additional metrics to the COVID-19 dashboard.

- Restructured front page of dashboard to include new metrics
- Added New Cases Reported in Last 24 Hours
- Added New Deaths Reported in Last 24 Hours
- Added Average Age of COVID-19 patient (cumulative and within last 7 days)
- Added 7 Day Percent Positive of PCR Tested Individuals
- Updated documentation information to include more information about calculations, new metrics and how the data is analyzed. Updated location of documentation can be found here.

Thank you for helping DHSS [illegible]

esri A Story Map

Missouri COVID-19 Dashboard: The Spread of COVID-19 in Missouri

PCR Tested Individuals

965,326

Individuals with Positive PCR Results

81,308

Percent Positive of All Patients Tested by PCR

8.4%

7 Day Percent Positive of PCR Tested Individuals

12.2%

Daily COVID-19 PCR Test Encounter Results

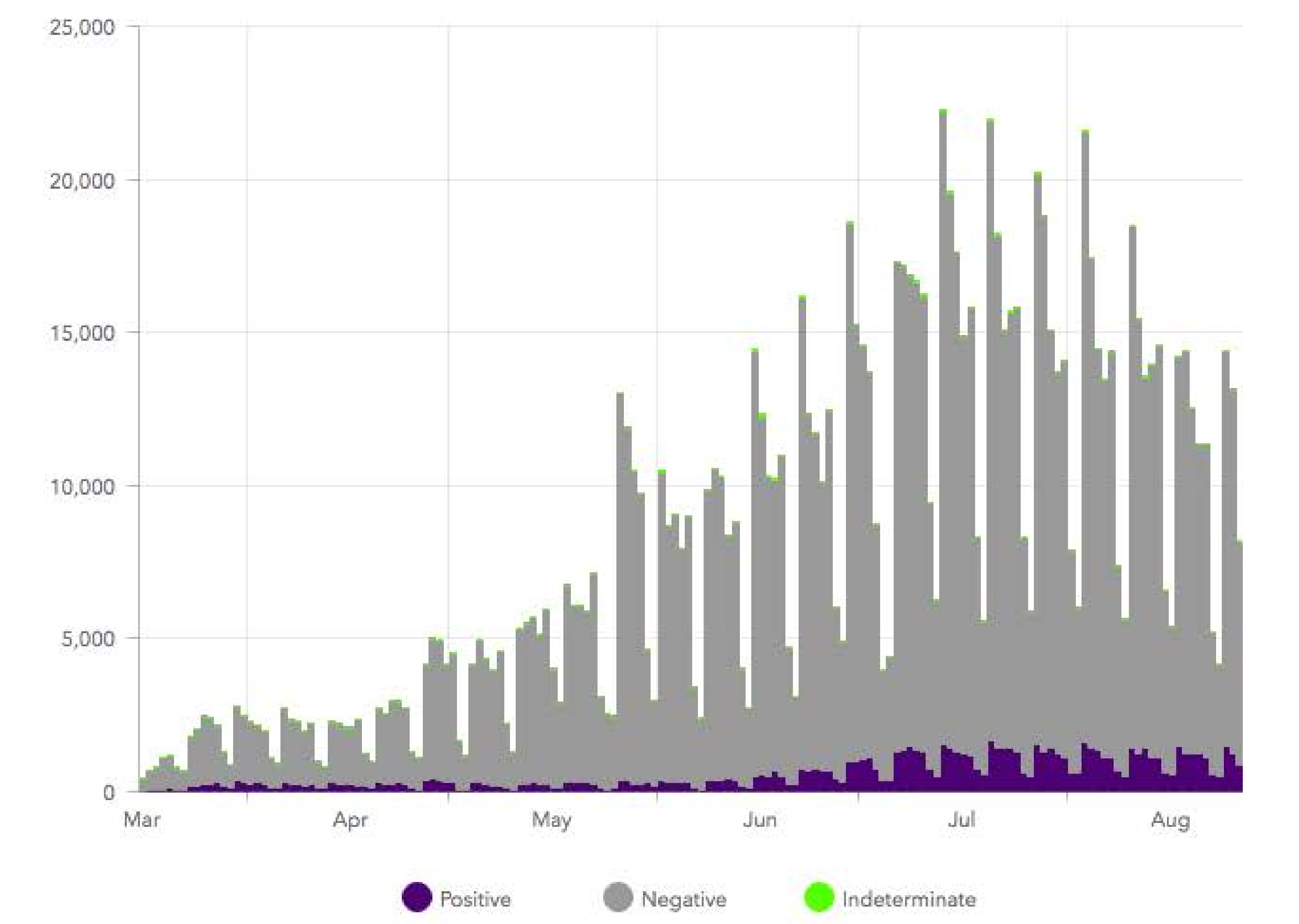

Age Distribution of PCR Tested Patients

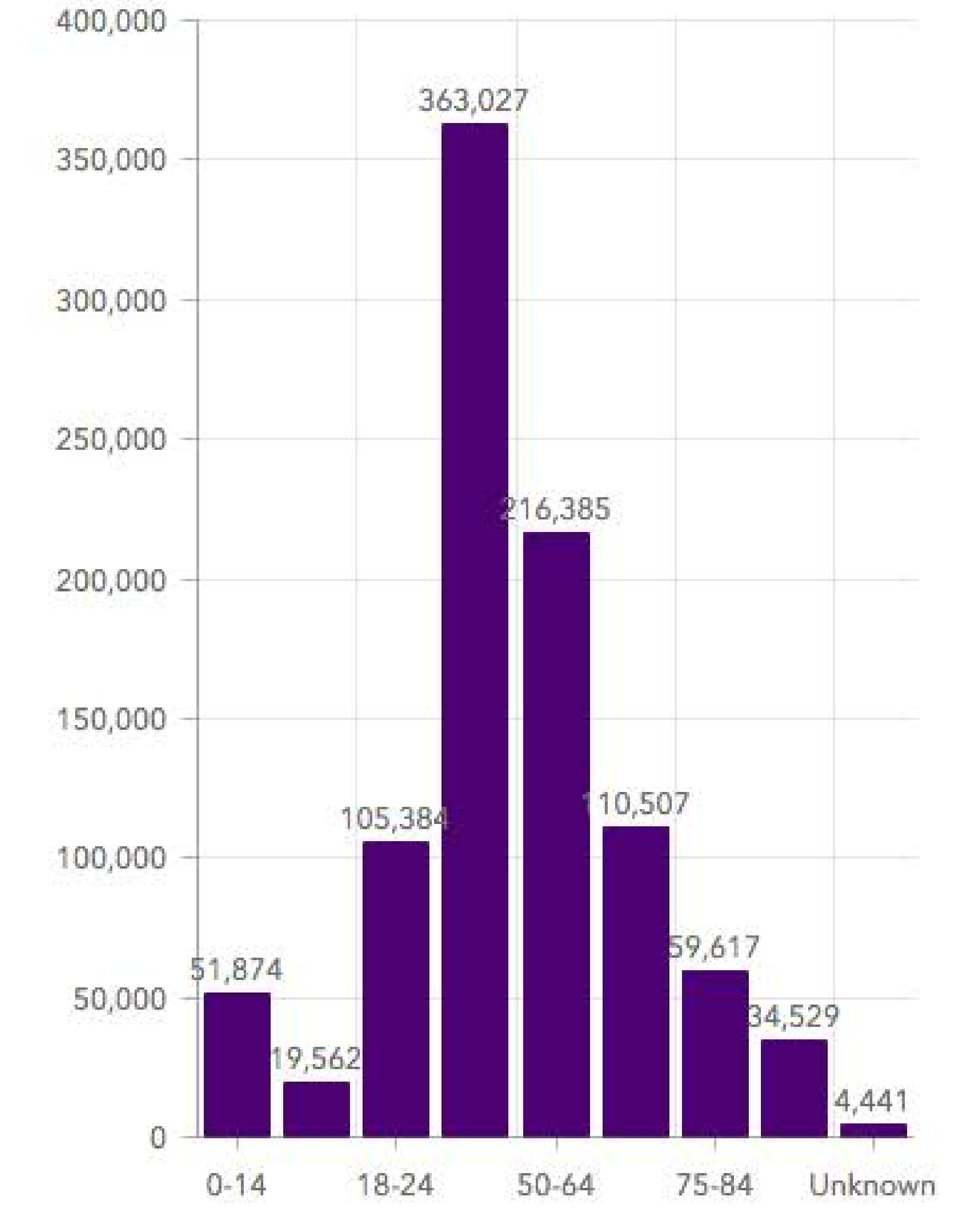

PCR Testing Per 100k Population

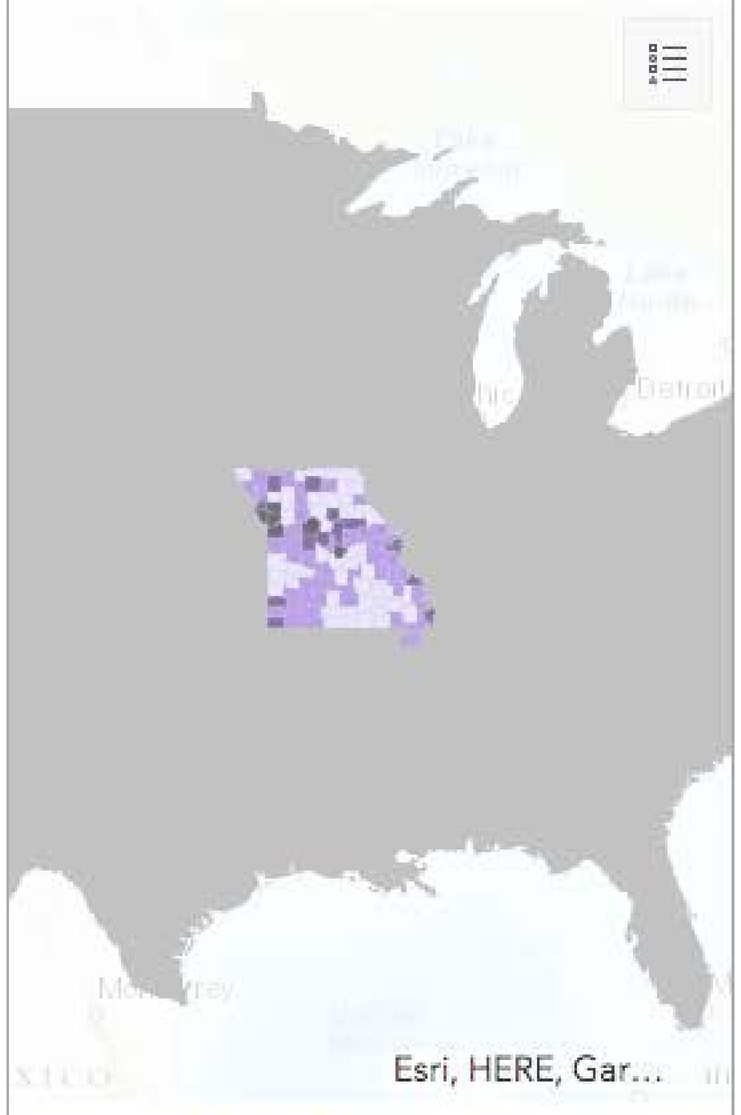

Note: Limited patient County information; where this information is missing (1/3 of test data), it is inferred from health facility or lab location

See documentation for more information.
Page last updated 8/29/2020

About Testing:

A **PCR** test looks for the viral RNA in the nose, throat, or other areas in the respiratory tract to determine if there is an active infection with SARS-CoV-2, the virus that causes COVID-19. A positive PCR test means that the person has an active COVID-19 infection.

A **Serology** test looks for antibodies against SARS-CoV-2 in the blood to determine if there has been an infection in the past. Antibodies are formed by the body to fight off infections. A positive antibody test means that the person was infected with COVID-19 in the past or recently, and that their immune system developed antibodies to try to fight it off.

Test encounters: The number of tests conducted. An individual patient may have multiple test encounters.

Missouri Coronavirus GIS Hub   For more information on COVID-19, click here.

Missouri COVID-19 Dashboard: The Spread of COVID-19 in Missouri

Show Me Strong RECOVERY PLAN

COVID - Overview | Cases - Demographics | Cases - County | Testing - PCR | Testing - Serology | Deaths | Hospitalizations

Deaths Attributed to COVID-19

1,496

New Deaths Reported in the Last 24 Hours

32

Total Deaths By County

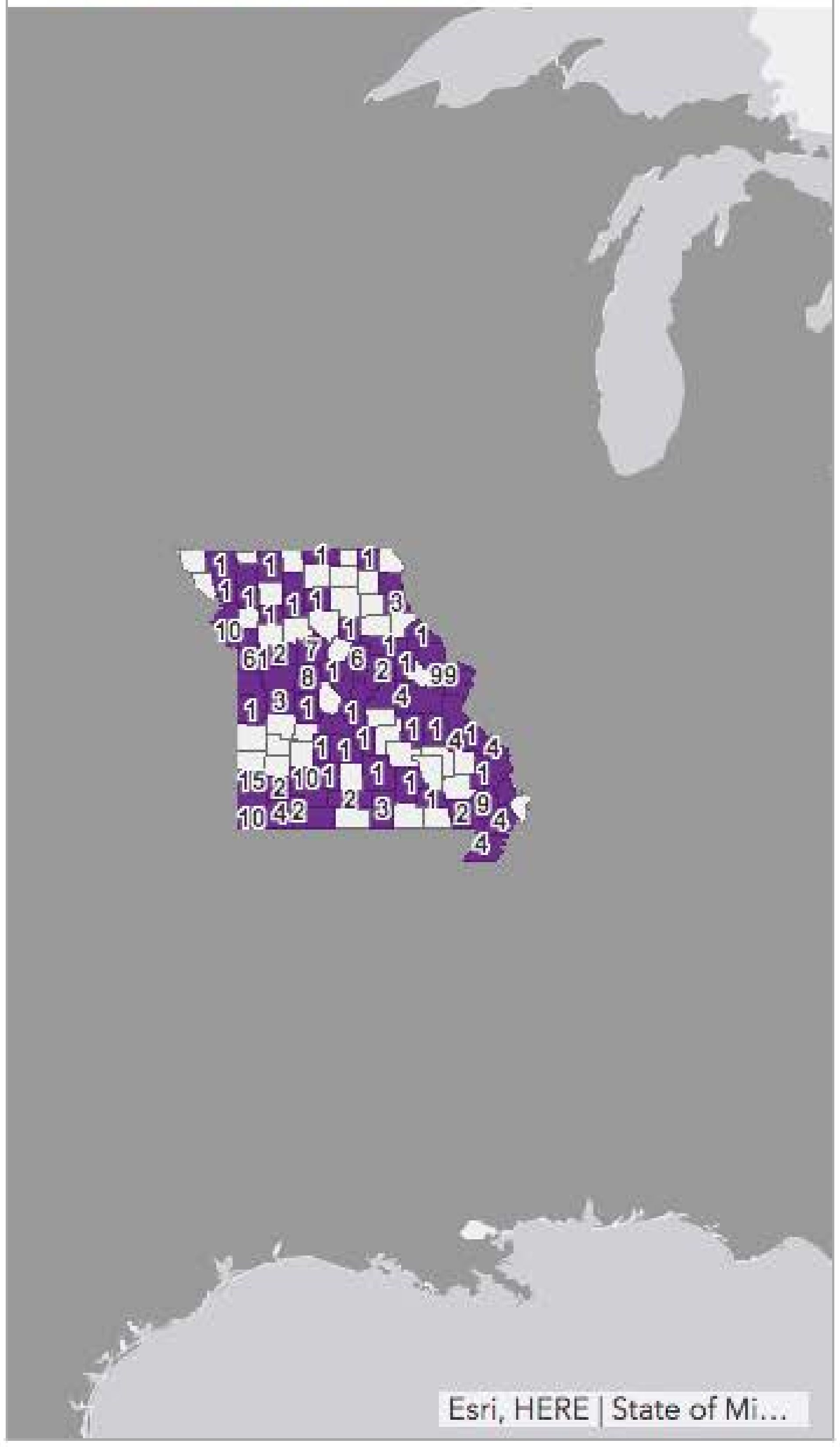

County Deaths

ADAIR: 0
ANDREW: 1
ATCHISON: 0
AUDRAIN: 1
BARRY: 4
BARTON: 0
BATES: 1
BENTON: 1
BOLLINGER: 1
BOONE: 6
BUCHANAN: 10
BUTLER: 2
CALDWELL: 1

Deaths By Biological Sex

Male : 738
Female: 753
Unknown*: 5

See documentation for more information.
Page last updated 8/29/2020

Deaths by Race

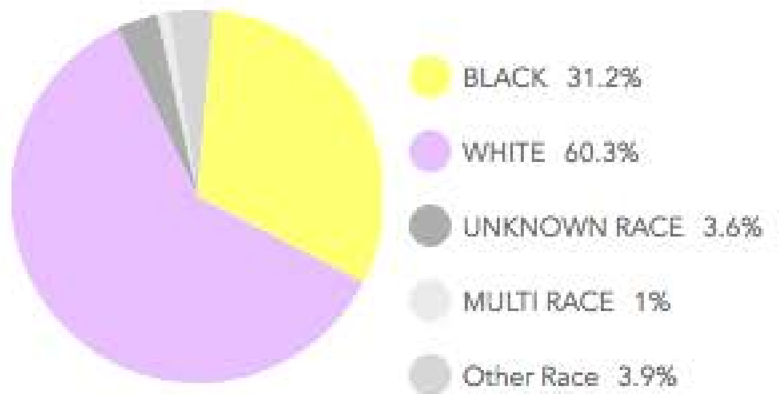

*Other includes: Asian, Indian, Pacific, and Other Races. Each with less than 1% of cases.

Deaths by Ethnicity

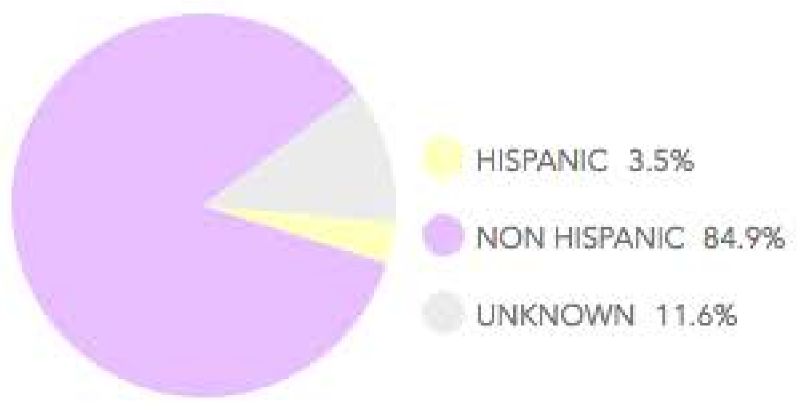

Daily Deaths

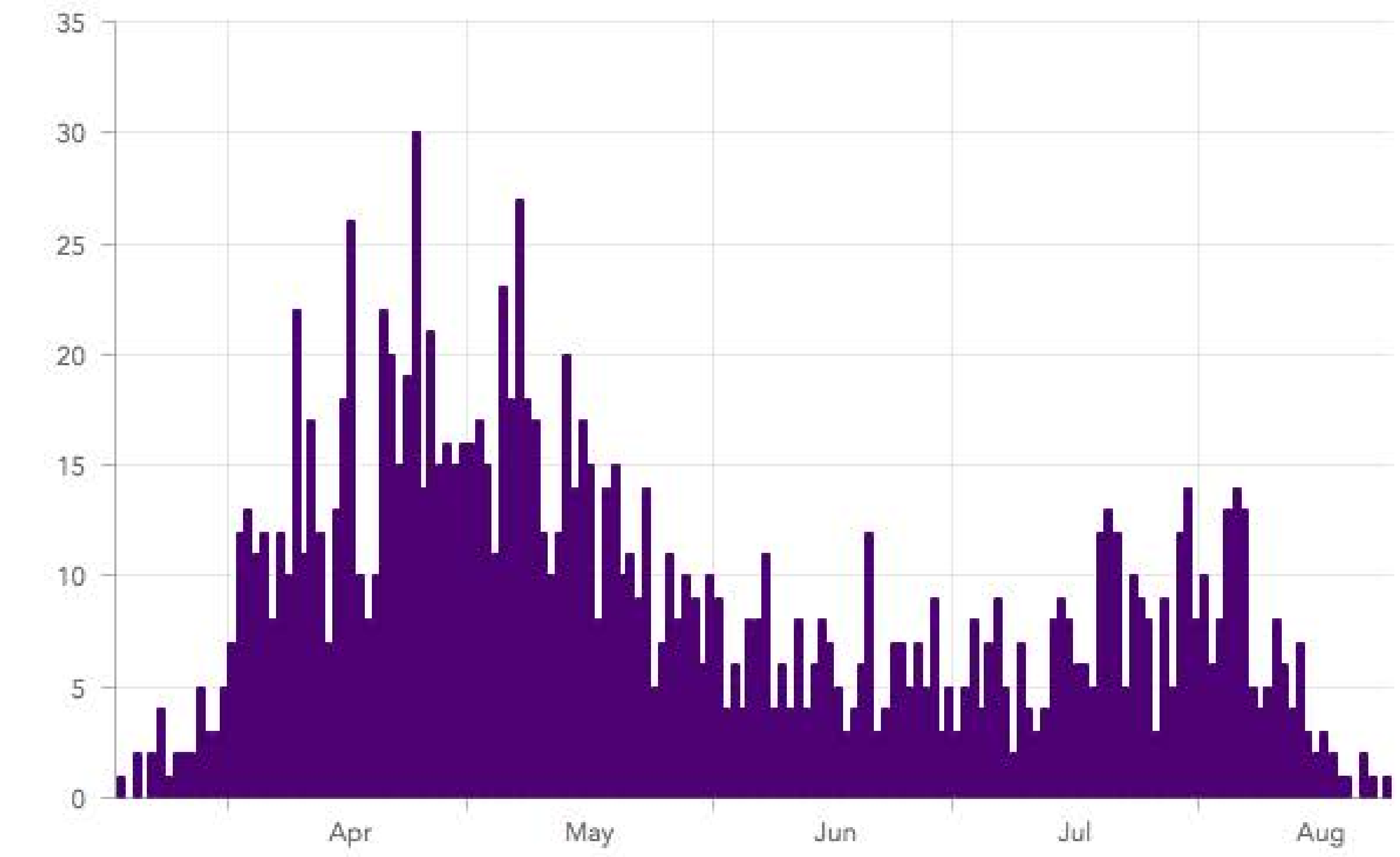

Daily Deaths | Cumulative Total

Deaths by Age Range

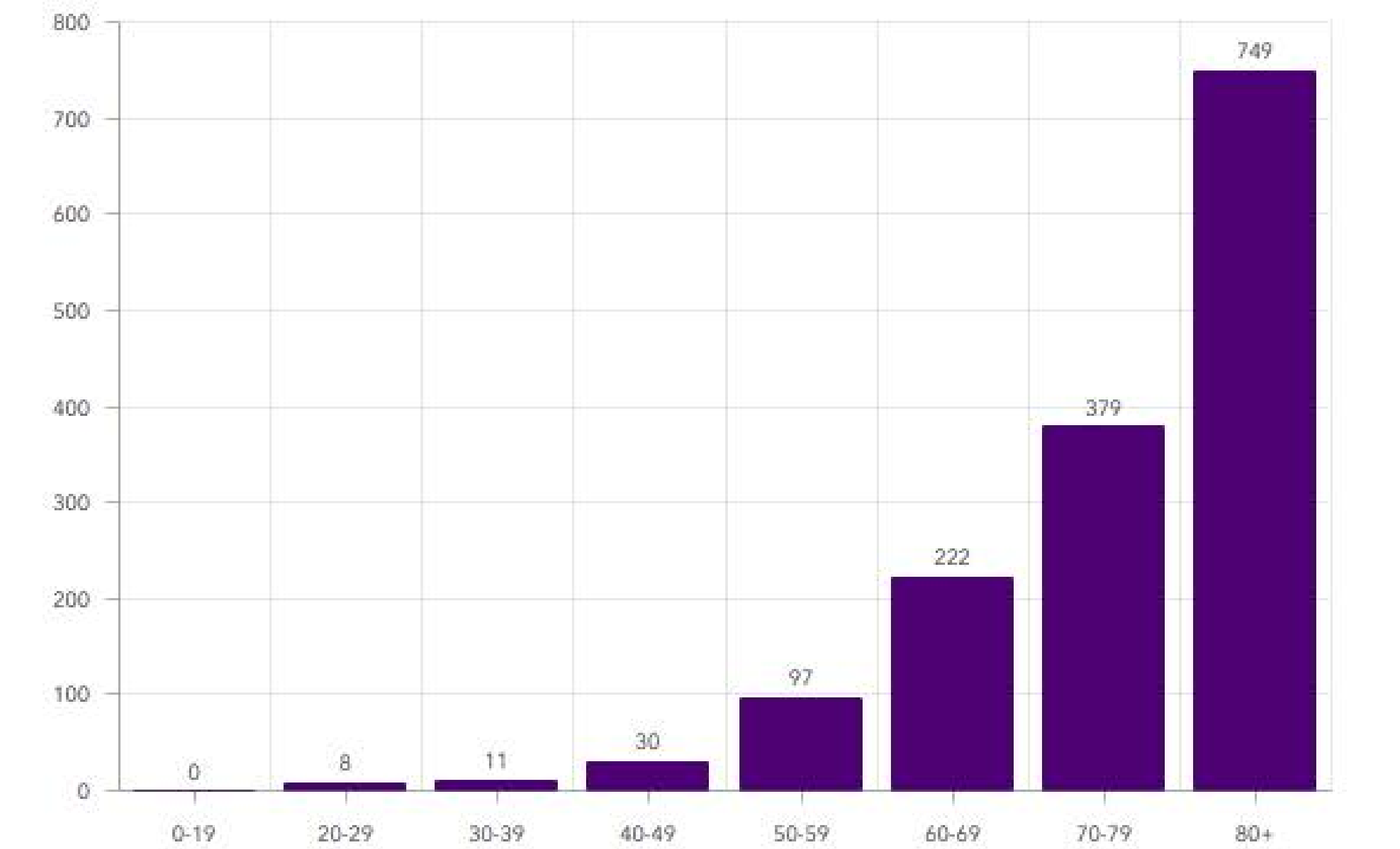

Source: Missouri Department of Health and Senior Services

esri A Story Map

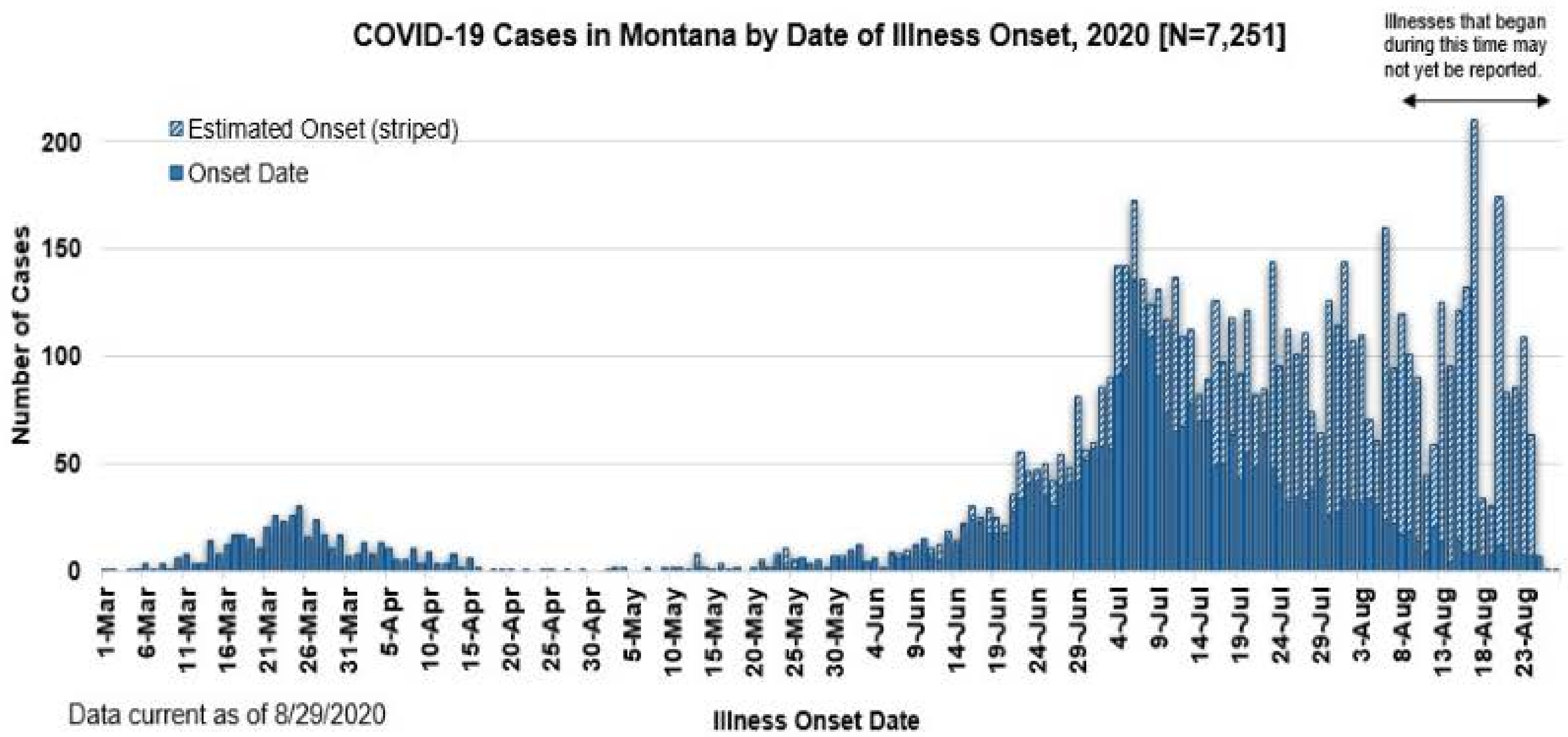

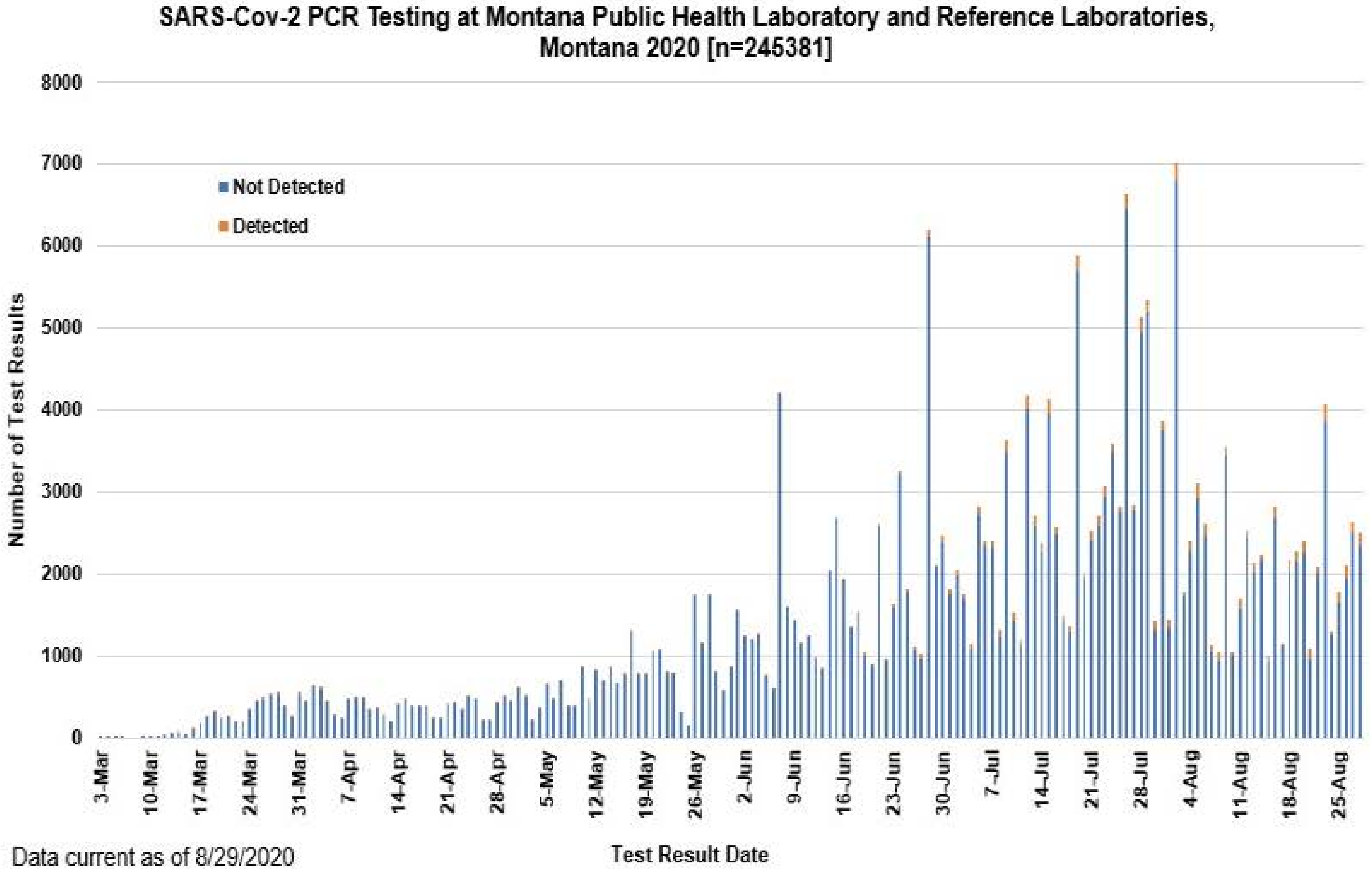

Hospitalization Status	Number of Cases (percent of total)
Ever hospitalized	429 (6%)
Not Hospitalized	6822 (94%)
Total	7251

MONTANA RESPONSE: COVID-19 - Coronavirus - Global, National, and State Information

Montana Maps

MT COVID-19 Cases | Montana Situation Update | Current Information | EOC Status | MT Dept. of Public Health | Global Outbreak Dashboard | CDC | Travel Info | WHO

Total Confirmed Cases
7,251

187 New Cases

Total Confirmed, New Daily, Active Cases by County

Yellowstone County Cases
2,012 Total | **54** New | **908** Active

Flathead County Cases
585 Total | **28** New | **153** Active

Rosebud County Cases
232 Total | **27** New | **206** Active

Big Horn County Cases
652 Total | **22** New | **121** Active

Gallatin County Cases
1,069 Total | **18** New | **52** Active

Cascade County Cases
280 Total | **15** New | **114** Active

Missoula County Cases
431 Total | **4** New | **49** Active

Lake County Cases
202 Total | **3** New | **14** Active

Ravalli County Cases
107 Total | **3** New | **13** Active

Hill County Cases
81 Total | **2** New | **33** Active

McCone County Cases
11 Total | **2** New | **4** Active

Sweet Grass County Cases

Montana Coronavirus Active Cases by County

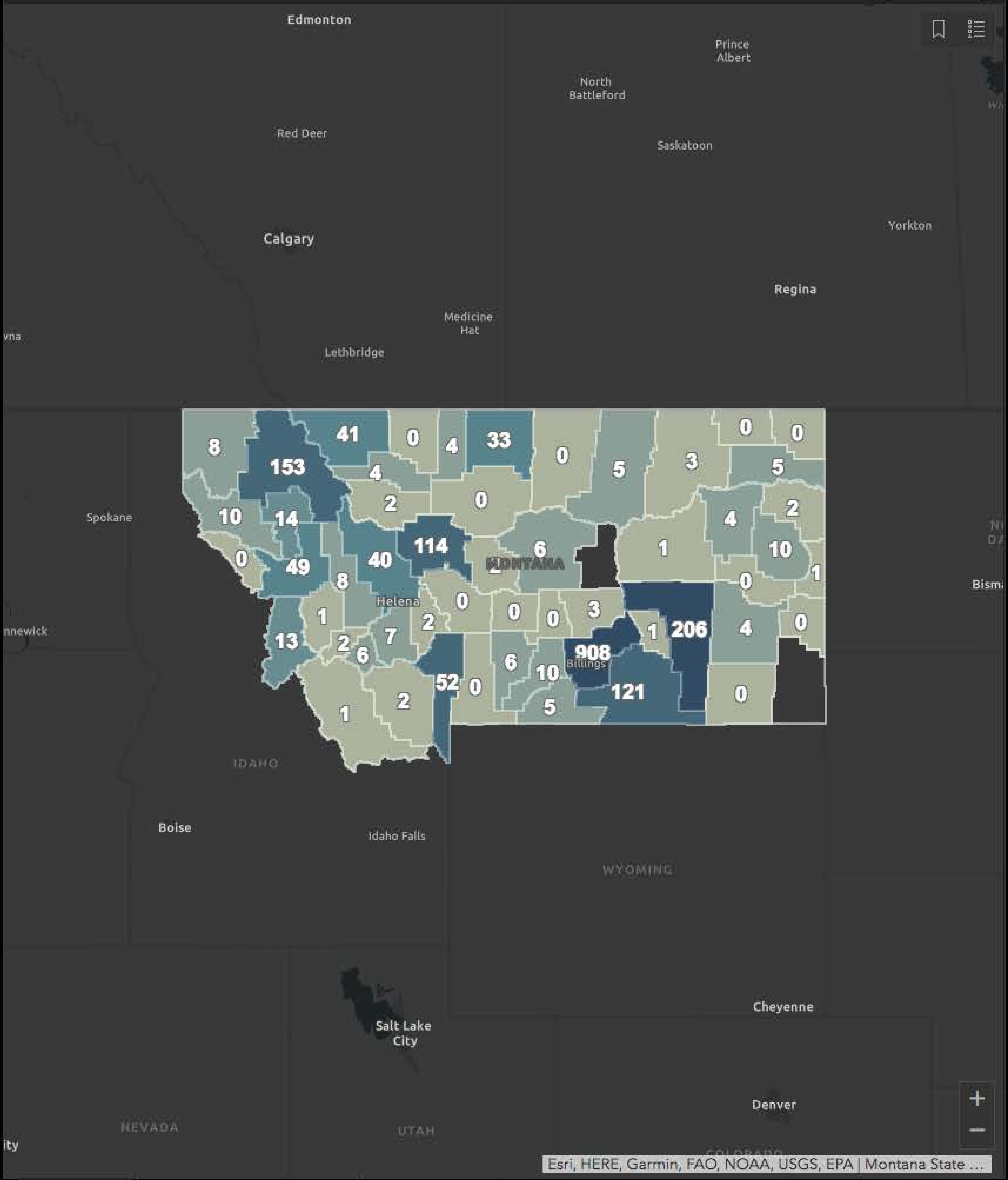

Active Cases | Cumulative Confirmed Cases

Updates are made by 10:00 a.m. daily. Information is reported based on the previous day. It is highly likely that the information on this dashboard and map will change based on further public health investigation. To learn more about the information on this map and dashboard: More Information. For information on demographics and nonresidents, click here. Source Data: Montana Department of Health & Human Services, Montana State Library. Date and time of last update: 8/29/2020, 11:33:22 AM.

Total Deaths
104

Recovered
5,278

Active
1,869

Total Tests
245,381
New Tests: 2,506

Active Hospitalizations
128

Total Hospitalizations
429

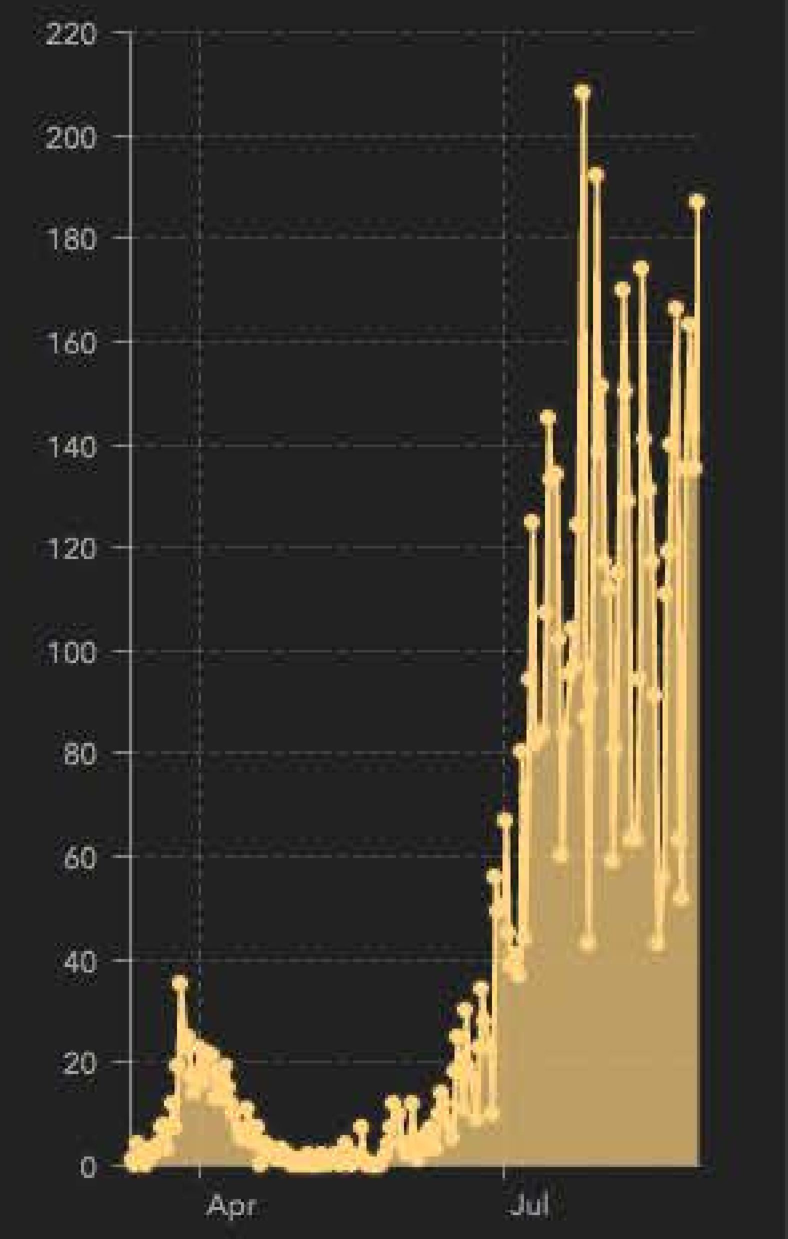

Cases by Day

COVID-19 · Resources, information and assistance from across state government Visit the Information Hub

COVID-19 North Carolina Dashboard

The total new lab-confirmed cases on the North Carolina Department of Health and Human Services (NCDHHS) COVID-19 Data Dashboard for Aug. 29 reflects an increase caused, in part, by approximately 1,000 positive tests from dates in the first half of the month that were reported to NCDHHS by LabCorp in the past 24 hours. The Department is working with LabCorp to understand the cause of the delayed reporting; however, LabCorp confirmed that individuals were not delayed in receiving their results.

Knowing when to dial up or down measures that slow the spread of the virus depends on North Carolina's testing, tracing and trends. This dashboard provides an overview on the metrics and capacities that the state is following.

Updated daily by approximately 12:00 p.m. Last updated August 29, 2020 at 12:35 p.m.

Daily Cases

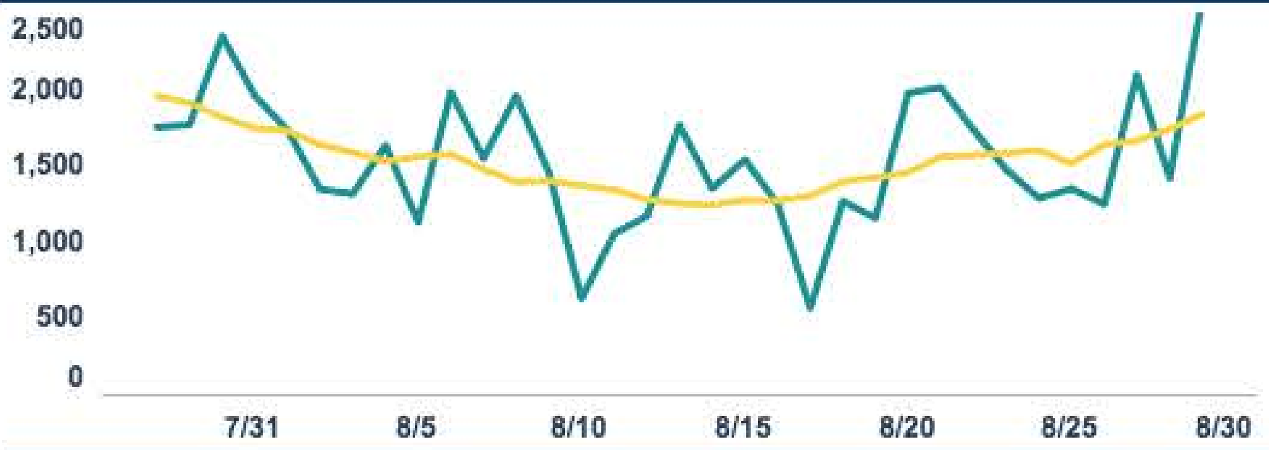

LAB-CONFIRMED CASES

165,076

Daily Tests

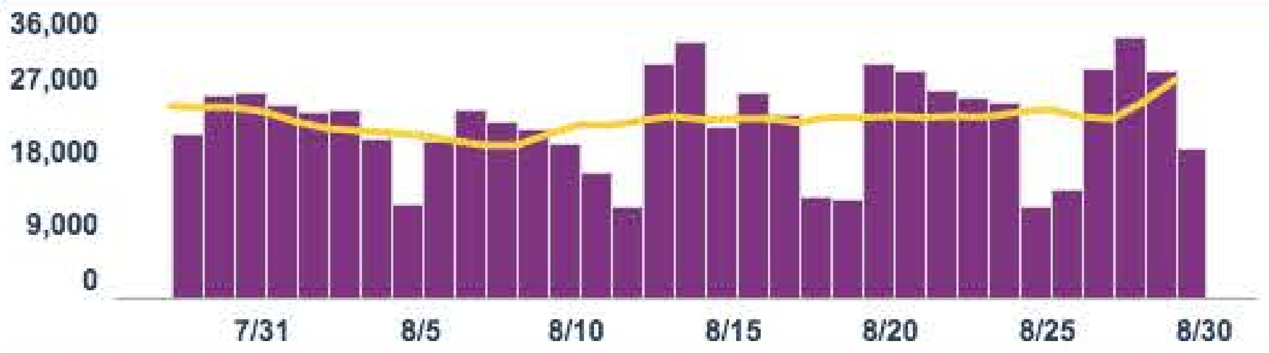

COMPLETED TESTS

2,218,149

Daily Number of People Currently Hospitalized

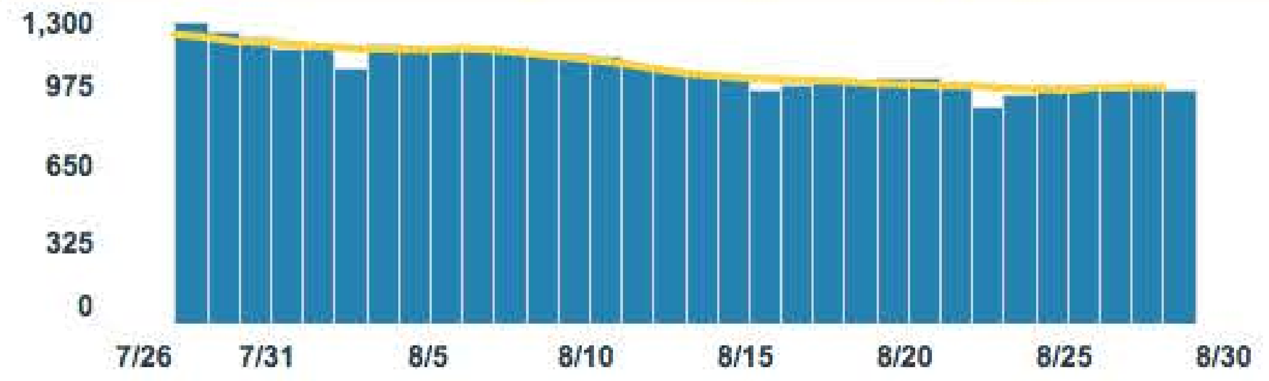

CURRENTLY HOSPITALIZED

965

County Map by Cases

(Click to highlight map, unclick to revert map)

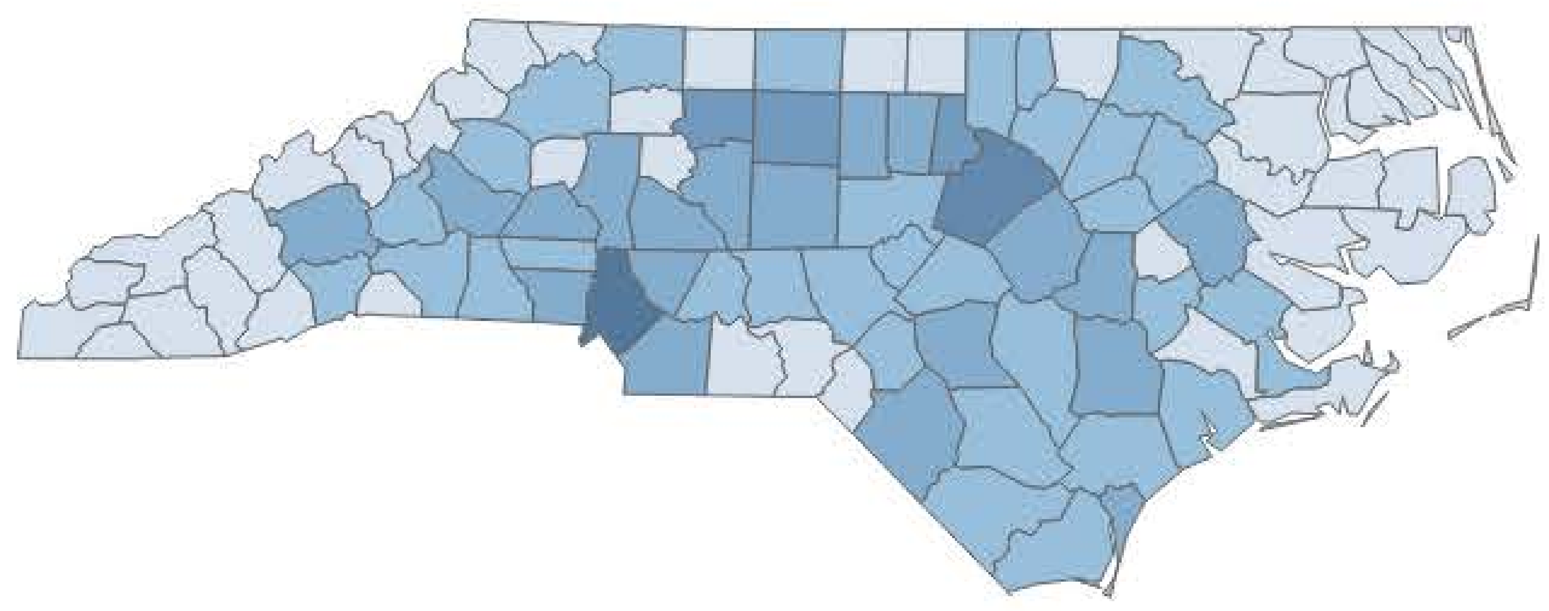

Map Credit: NCDHHS

- 27-700
- 701-1800
- 1801-5000
- 5001-12000
- 12001-21015
- 21015+

Select Geography
- County
- ZIP Code

Search for County

Select Metric
- Cases
- Cases per 10,000 residents

County Cases and Deaths

(Click to highlight map, unclick to revert map)

County	Cases	Deaths
Alamance	3,076	46
Alexander	396	2
Alleghany	203	0
Anson	437	3
Ashe	194	1
Avery	133	0
Beaufort	558	9

August 29, 2020

+ a b l e a u

Dashboard

CLI Surveillance

Cases

Testing

Hospitalizations

Contact Tracing

PPE

Outbreaks and Clusters

Reports

About the Data

Testing

NCDHHS is working to transition away from manual reporting processes by working to onboard all labs into NC EDSS

Positive Tests as a Percent of Total Tests

Is North Carolina seeing a 14-day downward trajectory or sustained leveling of positive tests as a percentage of total tests?

As we ramp up testing, there will be more laboratory-confirmed positive tests. Looking at what percent of total tests are positive helps us understand whether positive tests are increasing in comparison to the total number of tests conducted.

Laboratory results received electronically for previous dates may cause slight variation in day-to-day reporting.

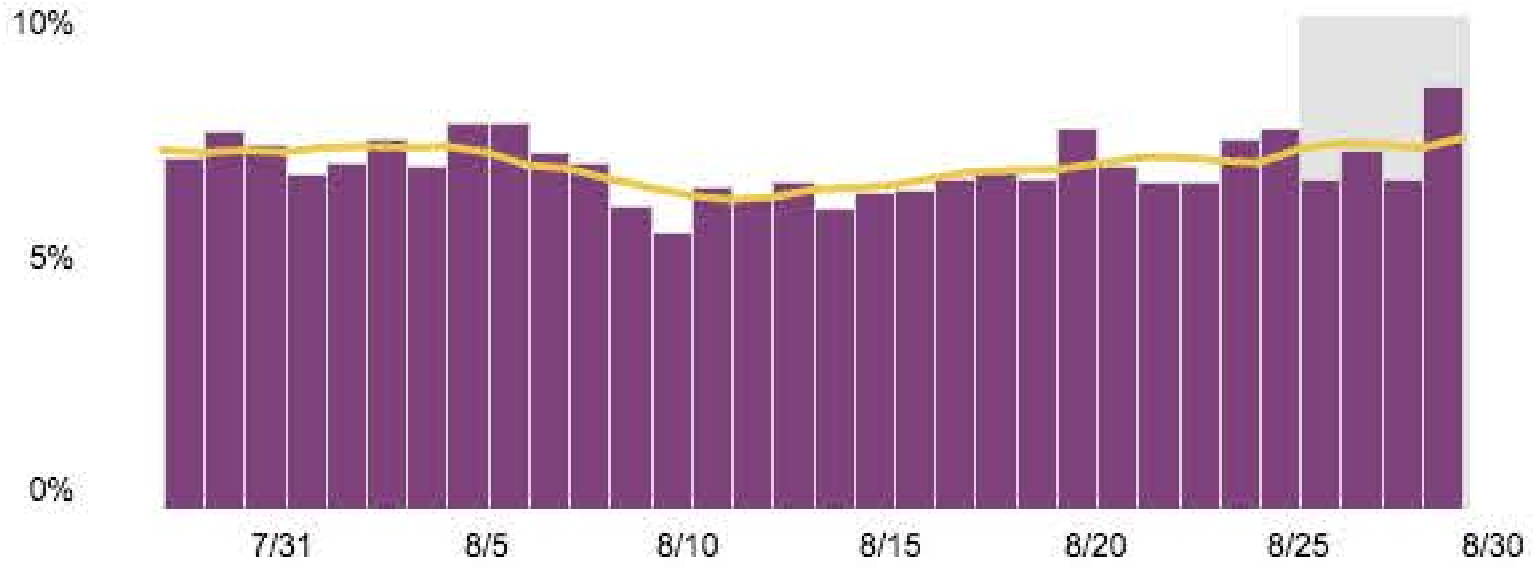

Percent Positive by County

Which counties are seeing a decline or sustained level of percent of tests that are positive?

It is important to recognize that the amount of testing being conducted varies across counties. Because the percent positive is calculated using electronically reported data from NC EDSS only, which is a subset of total tests reported, county-level percent positive rates may be impacted by how many of the total tests for residents in the county were reported electronically through NC EDSS.

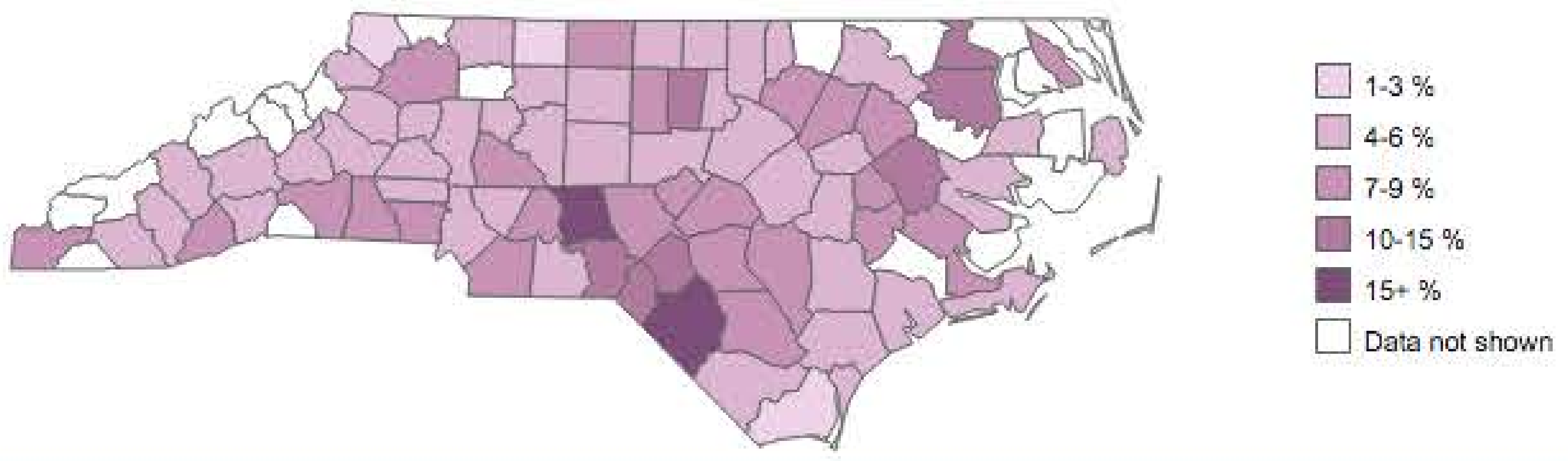

Testing Turnaround Time

There are multiple stages that make up the time it takes from when a person is tested to the time the person receives their results – the testing turnaround time. The first stage is the time between when a specimen is collected to when it is received by a laboratory. Several factors can impact this timing, including how and when the specimen is transported to a laboratory. The second stage is the time between when the specimen is received and when the laboratory has a result. This first and second stage is shown in the dark purple in the graph below. The third stage is the time between when the laboratory determines a result and the laboratory electronically reported to NCDHHS. This third stage is shown in the light purple. The fourth stage, which is not represented in the graph below, is the time between when a laboratory reports a result and the patient is notified of their results. This fourth stage happens between the health care provider and patient and is not reported to NCDHHS. The yellow line shows the 7-day rolling average of the dark purple, when a specimen is collected to when the laboratory has a result.

Please note that the graph shows the average turnaround time across all laboratories reporting electronically to NCDHHS. Individual laboratories may have shorter or longer turnaround times and, therefore people's individual experience will vary.

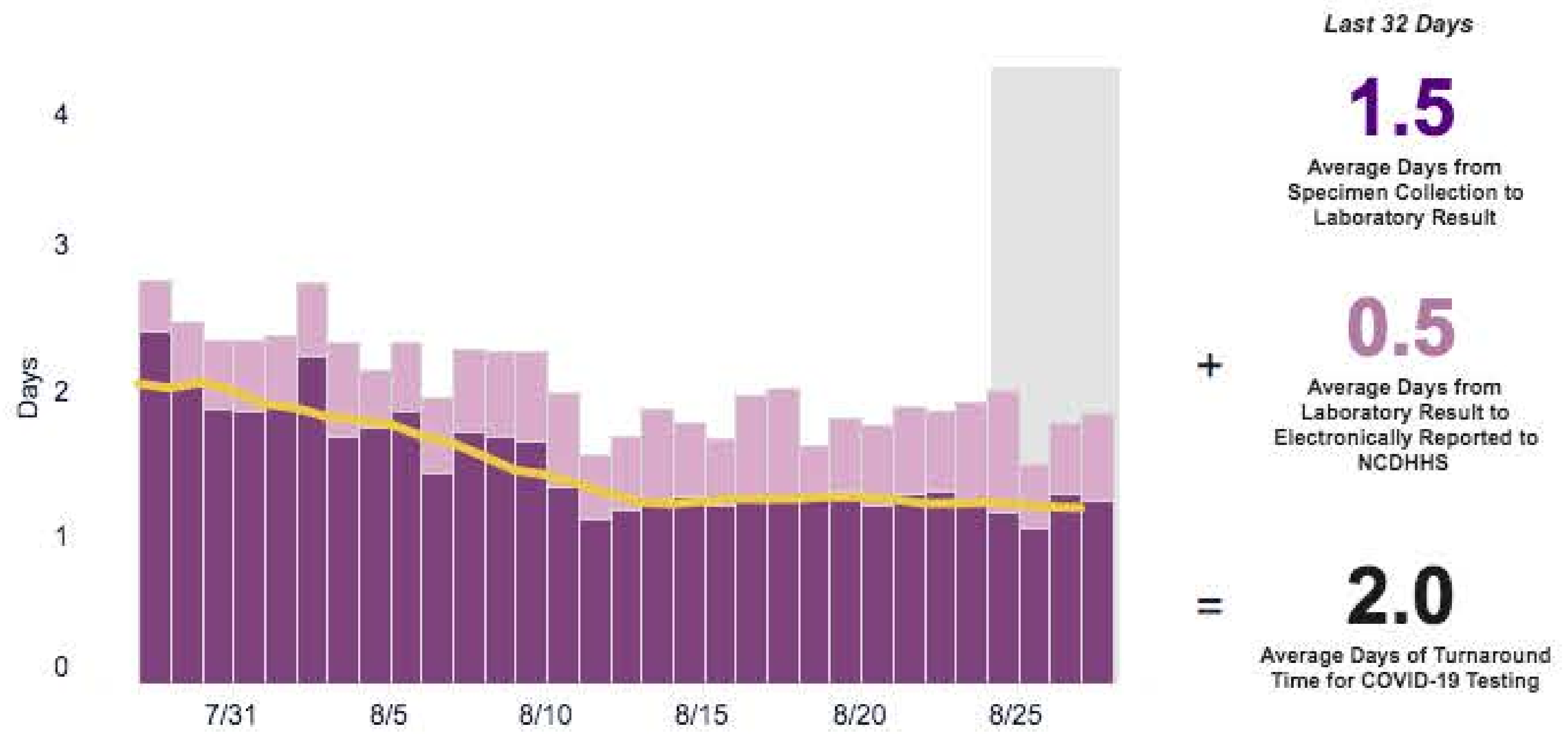

August 29, 2020

tableau

Dashboard

CLI Surveillance

Cases

Testing

Hospitalizations

Contact Tracing

PPE

Outbreaks and Clusters

Reports

About the Data

Coronavirus Cases

WELCOME to the refreshed COVID-19 dashboard. In this revised format, you'll find new county-level breakdowns, as well as additional statewide data. The dashboard is best viewed on a computer, but turning your mobile device to landscape will improve your experience. We welcome your feedback!

Share Your Feedback

The North Dakota Department of Health dashboard is updated daily by 11 am and includes cases reported through the previous day. The investigations are ongoing and information on the website is likely to change as cases are investigated. The information on the dashboard is the most up to date and may be different than previous news releases.

STATE and COUNTY LEVEL INFORMATION

LAST UPDATED
8/29/2020 8:26:12 AM

COVID-19 STATUS REPORT

POPULATION 760,077 | COUNTY All | % of POPULATION TESTED 25.40%

Hover over a value and click the icon for more information

TODAY

NEW TESTS
- TOTAL PROCESSED: 7,898
- FIRST TIME TESTED: 1,751

NEW POSITIVES
- TOTAL: 375
- PREVIOUSLY TESTED NEG. BEFORE POS. TEST: 164

POSITIVITY RATE
- TOTAL: 4.75%

RECOVERED: 210

ACTIVE POSITIVES: 2,325

ACTIVE POSITIVES

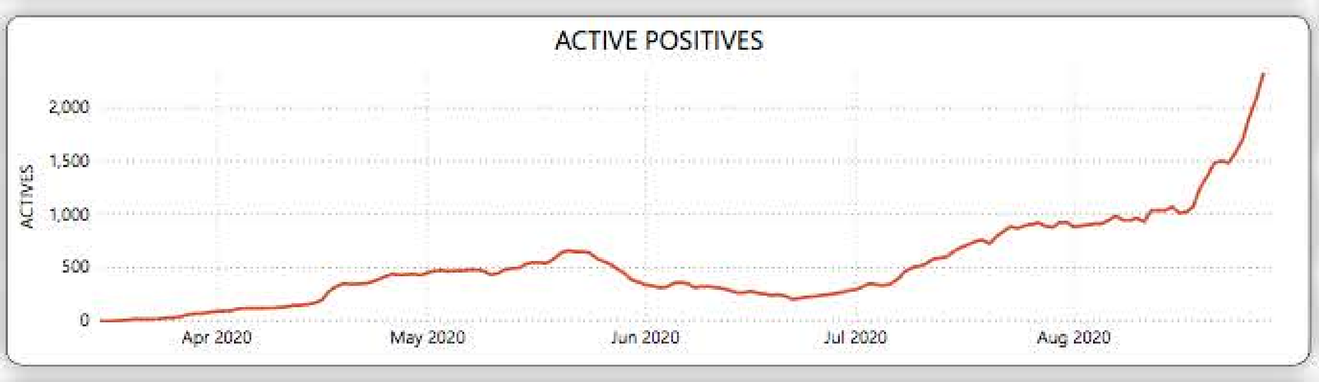

14 DAY ROLLING AVERAGE POSITIVITY RATE (TOTAL TESTS)

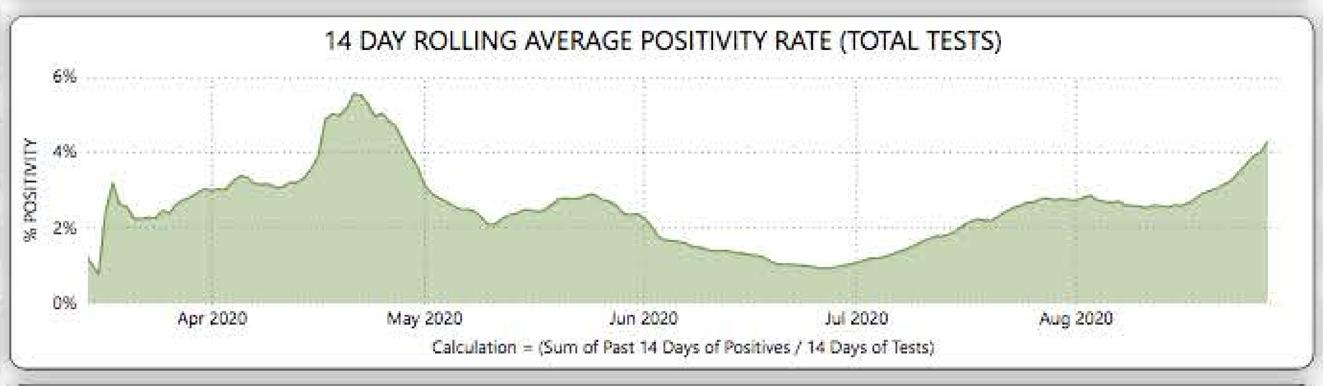

Calculation = (Sum of Past 14 Days of Positives / 14 Days of Tests)

8/28/2020

CUMULATIVE

TESTS
- TOTAL PROCESSED: 465,649
- UNIQUE INDIVIDUALS: 199,747

POSITIVES
- TOTAL: 11,484
- PREVIOUSLY TESTED NEG. BEFORE POS. TEST: 3,404

POSITIVITY RATE
- TOTAL: 5.75%

RECOVERED: 9,018 | DEATHS: 141

Feedback (+)

COVID-19 TRENDS

Hover over a value and click the icon for more information

DAILY NEW POSITIVES

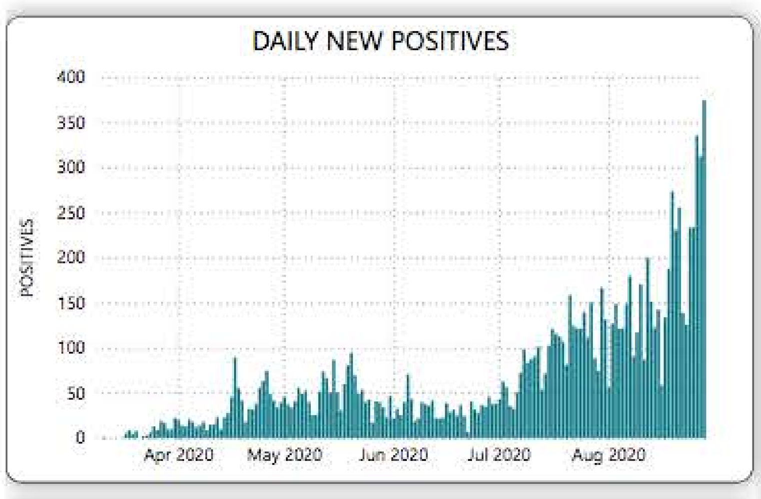

AGE COMPARISON

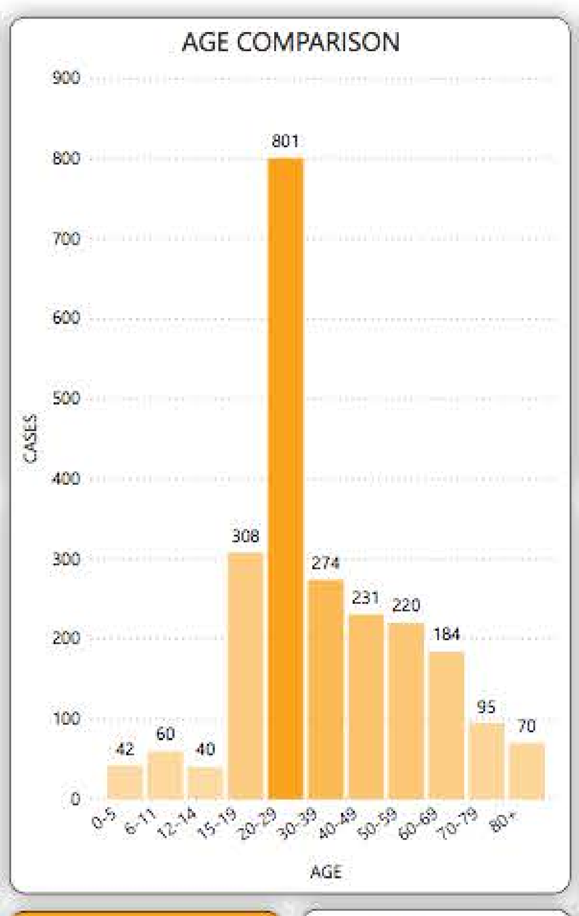

CUMULATIVE POSITIVES AND RECOVERED

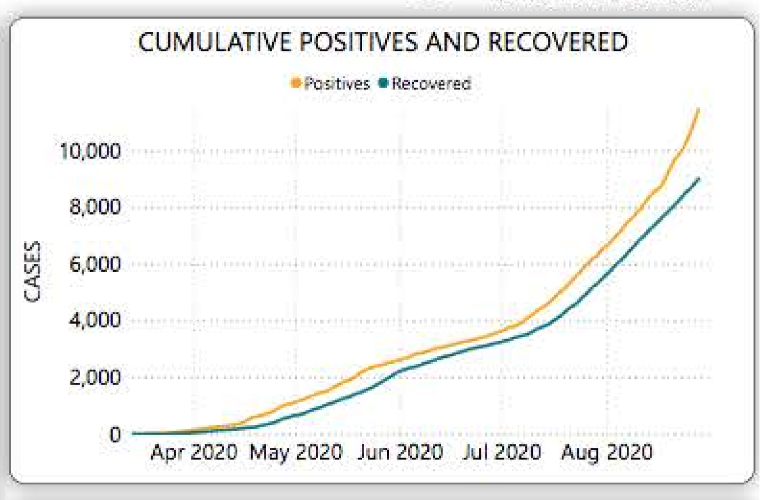

DAILY UNIQUE AND SERIAL TESTS

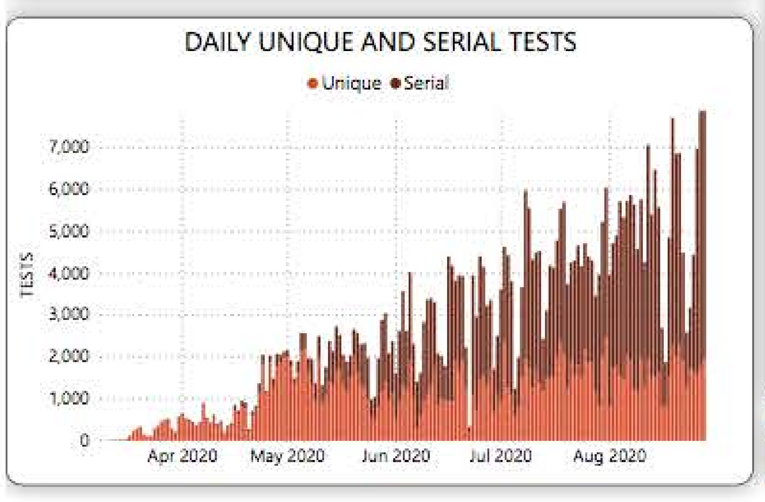

14 DAY ROLLING AVERAGE DAILY TOTAL TESTS

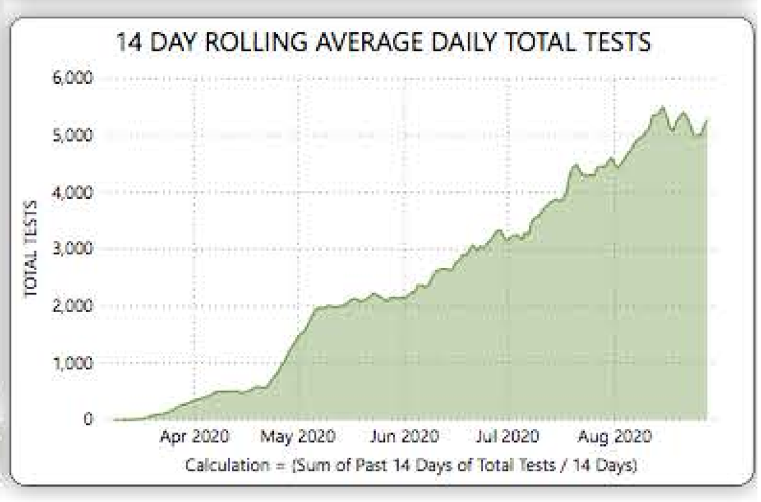

Calculation = (Sum of Past 14 Days of Total Tests / 14 Days)

PRESS FOR ACTIVES | PRESS FOR POSITIVES

STATE LEVEL INFORMATION

COVID-19 DEMOGRAPHICS and SOURCE of EXPOSURE

Hover over a value and click the icon for more information

GENDER

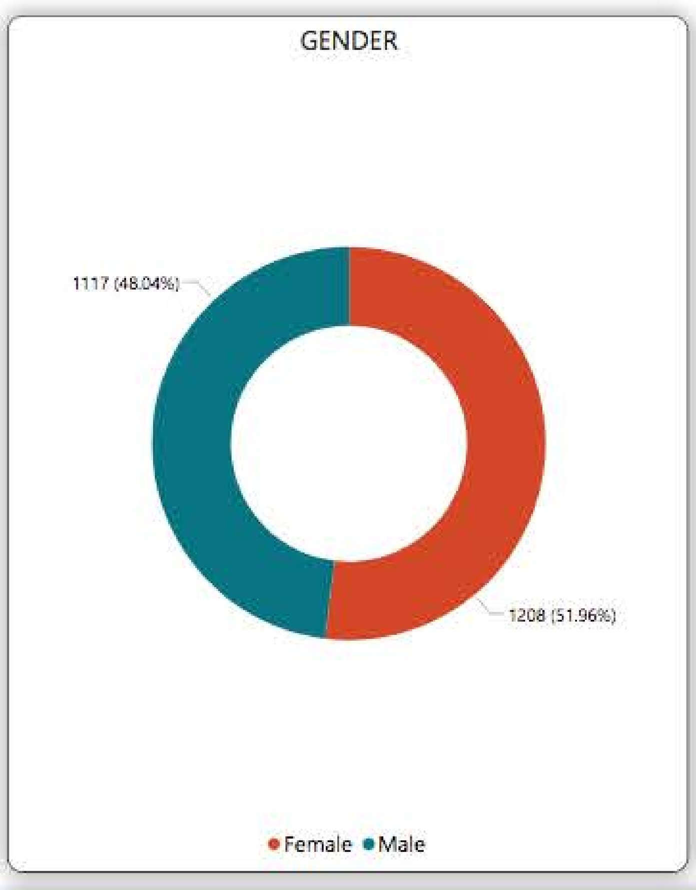

SPREADTYPE

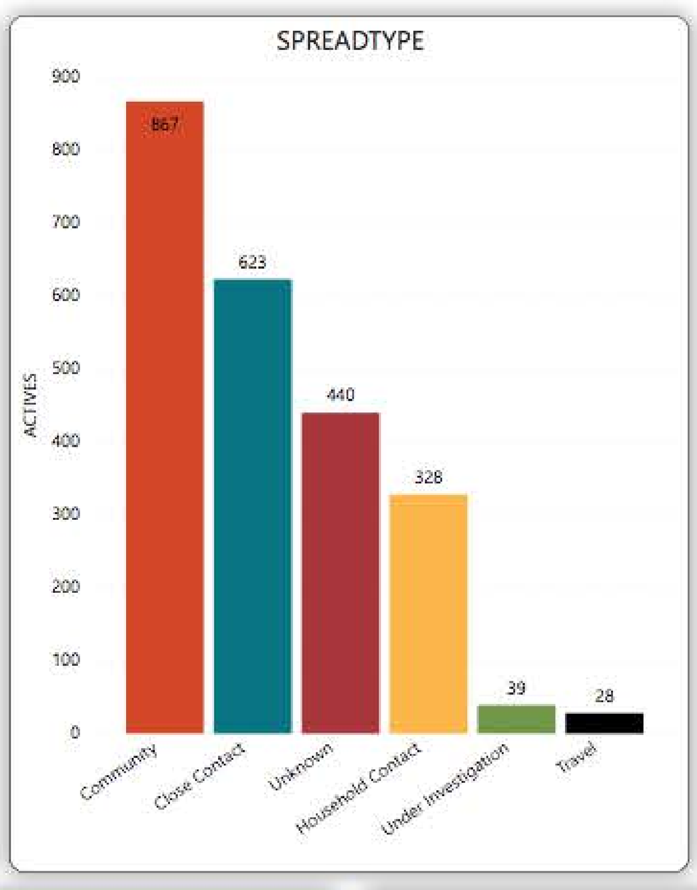

RACE

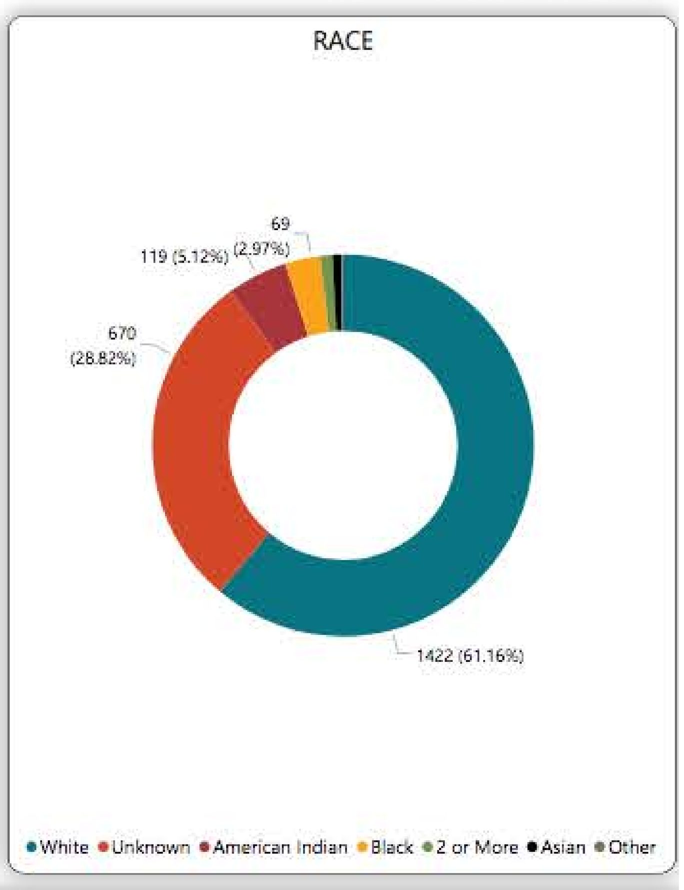

PRESS FOR ACTIVE POSITIVES | PRESS FOR CUMULATIVE POSITIVES

STATE HOSPITALIZATIONS and DEATHS

Hover over a value and click the icon for more information

HOSPITALIZATIONS

ACTIVE HOSPITALIZATIONS

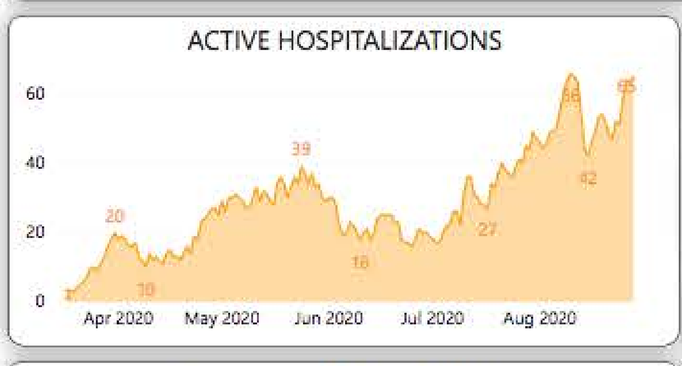

- COVID ACTIVE NON-ICU: 48
- COVID ACTIVE HOSPITALIZATIONS: 65
- COVID ACTIVE ICU: 17
- COVID TOTAL NON-ICU: 404
- COVID TOTAL HOSPITALIZATIONS: 551
- COVID TOTAL ICU: 147

AGE OF TOTAL HOSPITALIZED

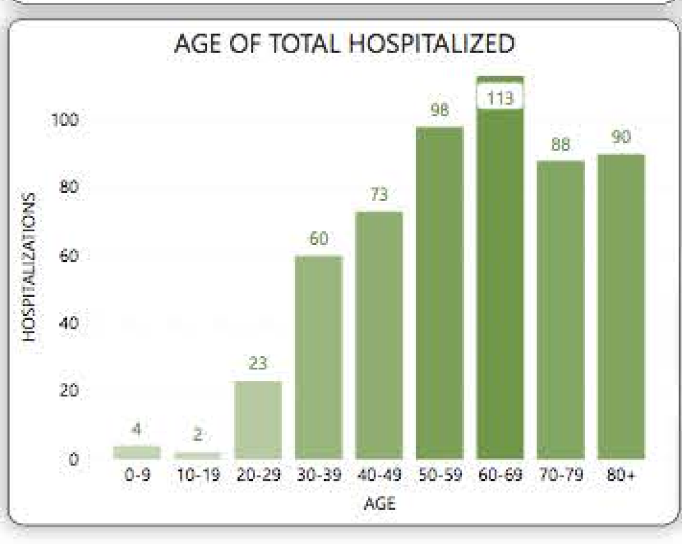

OCCUPIED NON-ICU BEDS

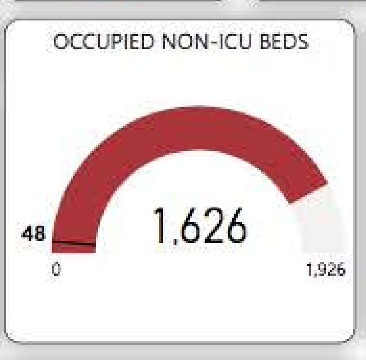

OCCUPIED ICU BEDS

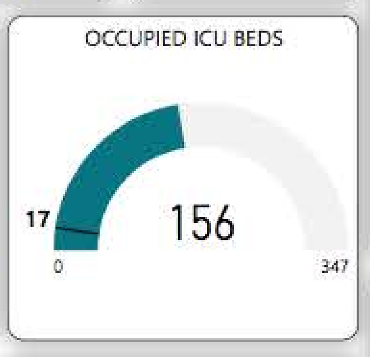

- NON-ICU BEDS OCCUPIED DUE TO COVID: 2.49%
- ICU BEDS OCCUPIED DUE TO COVID: 4.90%
- AVERAGE HOSPITALIZATION LENGTH OF STAY (DAYS): 8.36

DEATHS

CUMULATIVE DEATHS: 141

CUMULATIVE DEATHS BY AGE GROUP

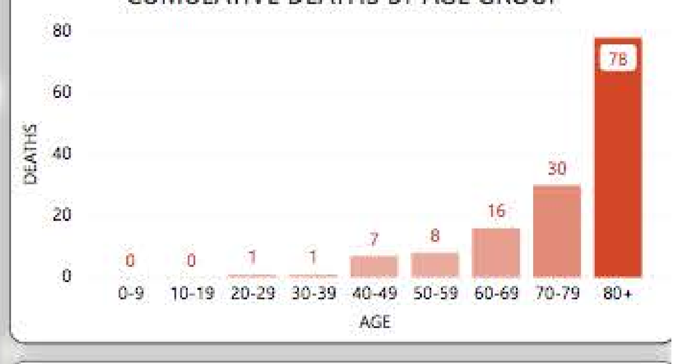

DEATHS BY MONTH

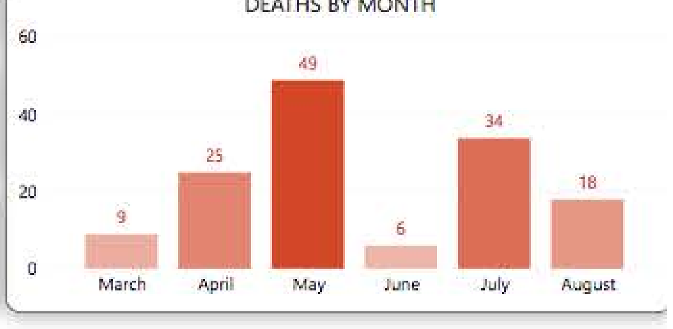

NORTH DAKOTA COUNTY ANALYSIS

Hover over a value and click the icon for more information

PRESS FOR ACTIVE POSITIVES

PRESS FOR DAILY POSITIVES

PRESS FOR CUMULATIVE POSITIVES

PRESS FOR CUMULATIVE DEATHS

PRESS FOR ACTIVE PER 10K

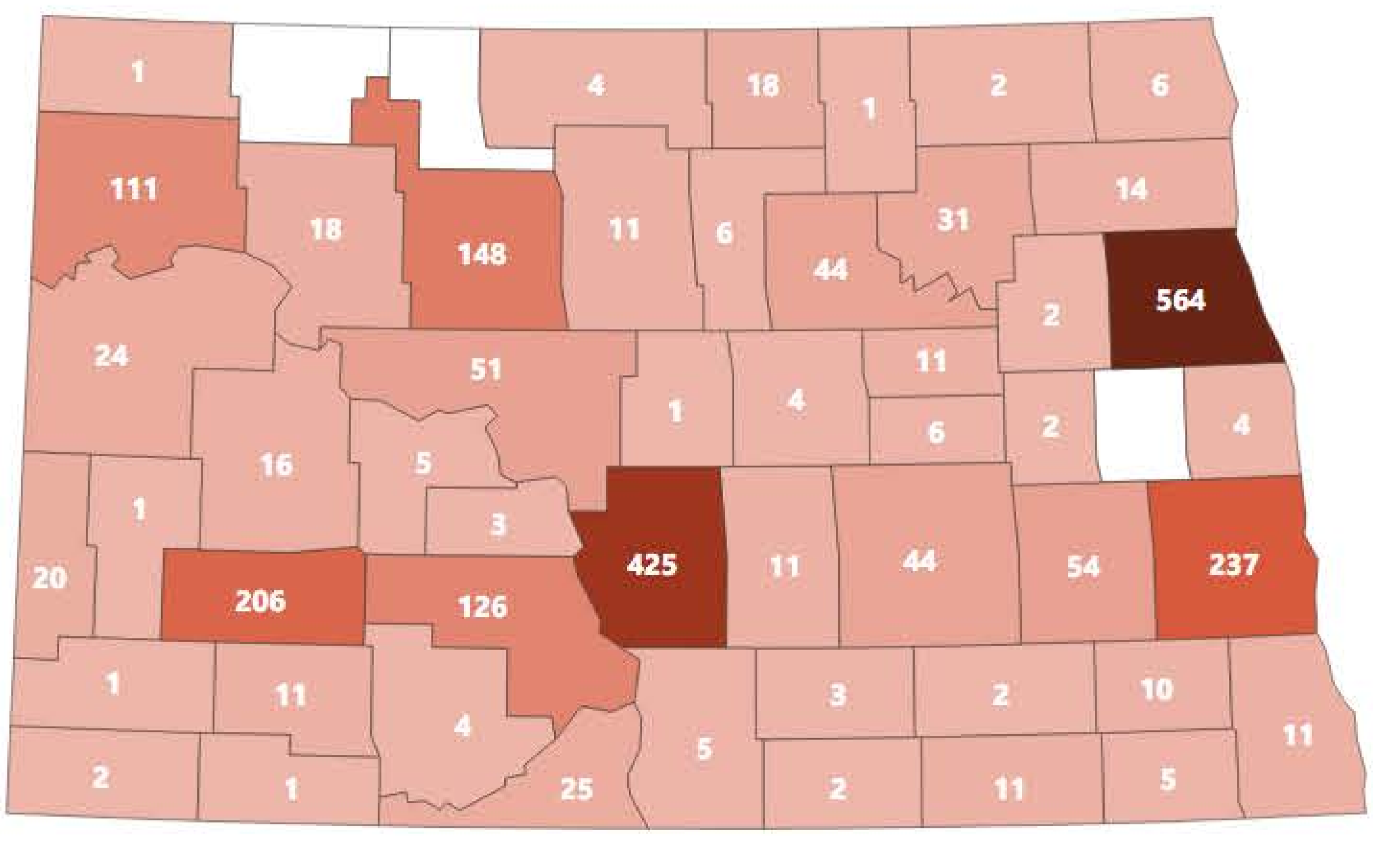

Coronavirus COVID-19 Nebraska Cases by the Nebraska Department of Health and Human Services (DHHS)

Total Positive Cases

33,436

Total Tested	Tested: Not Detected	Deaths
355,731	321,782	392

*Does not include inconclusive results

New positive cases by date results were received

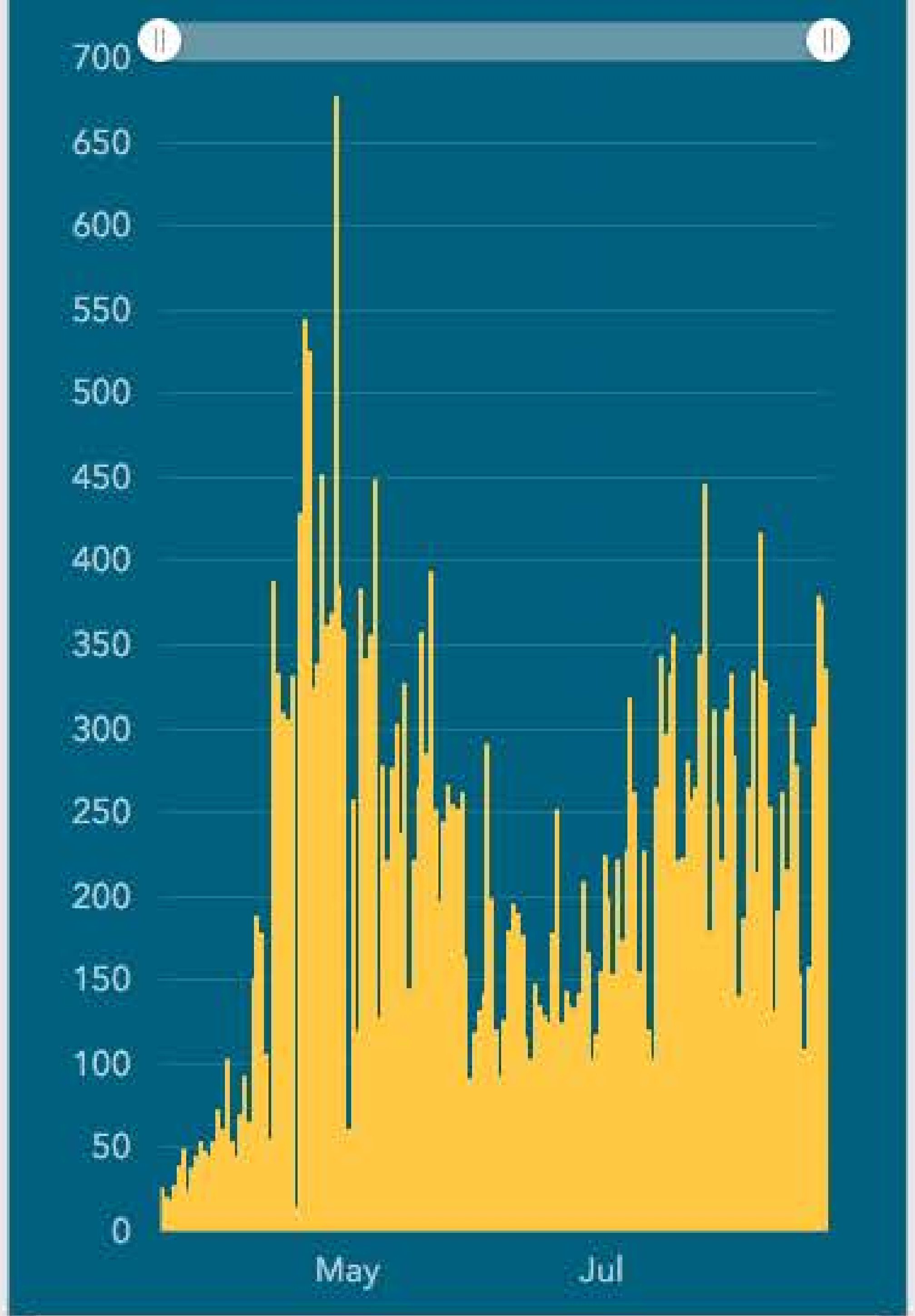

Positives by Date

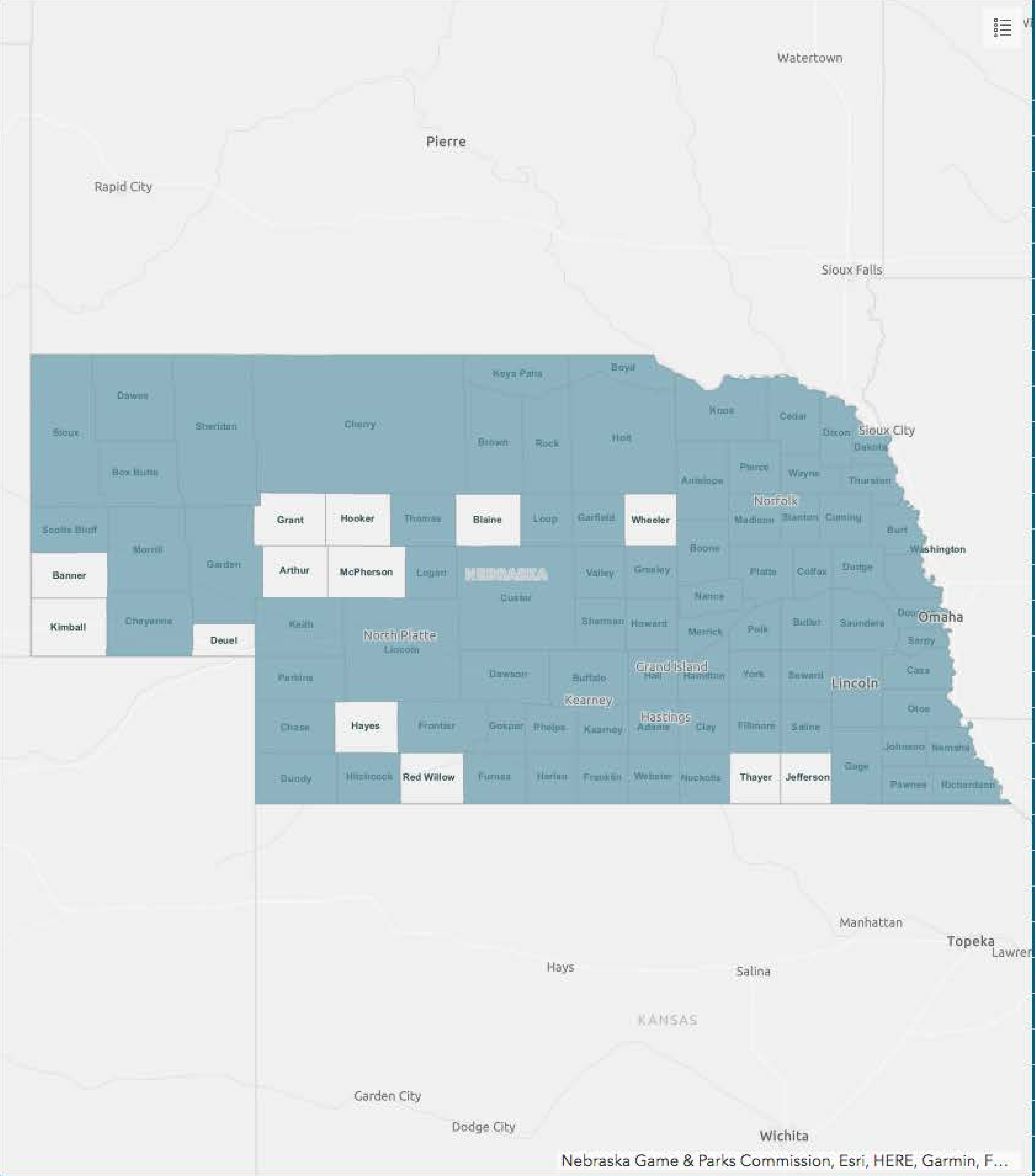

Number of positive tests by county in the last 14 days

Cases	County
1,258	Douglas
455	Lancaster
327	Sarpy
171	Buffalo
101	Lincoln
90	Madison
63	Dodge
59	Hall
56	Dawes
53	Dakota
46	Otoe
43	Scotts Bluff
42	Saunders
36	Washington
35	Cass
33	Platte
32	Seward
31	Adams
31	Saline
30	Gage
28	Nemaha
28	Kearney
27	Dawson
24	Pierce
22	Phelps
21	Knox
19	Cedar
18	York
17	Butler
15	Cherry
15	Custer

Last Updated: 8/28/2020, 7:20 PM CDT

Last 14 Day Positive Cases

Contact Nebraska Department of Health and Human Services (DHHS)
http://dhhs.ne.gov/coronavirus
Information Line: (402) 552-6645
Data are representative of Nebraska residents, are provisional and data reported by the local health department should be considered the most up to date.

NEBRASKA
Good Life. Great Mission.
DEPT. OF HEALTH AND HUMAN SERVICES

COVID-19 (Coronavirus)

State of Nevada
Department of Health
and
Human Services
Office of Analytics

For More Information Please Visit:

Nevada Health Response

Last updated on 08/29/2020 at 8:50 am, with data through the previous day. Please note that updates apply incrementally and may be in process for up to an hour after the update time listed here.

Total Tests (molecular)

844,391
+ 7,334

Confirmed Cases

68,461
+ 609

Deaths

1,302
+15

Tests per 1,000 per week*

13.2

Daily Positivity Rate*

8.2%

Cumulative Positivity Rate*

10.5%

Please reference technical notes page for updated

Current Hospitalizations

Confirmed	Suspected
557	118
-8	-24

Intensive Care Unit (ICU)

182
-15

Hospitalization data are not updated on Sundays.

COVID-19 data are reported as timely, accurately, and completely as we have available. Data are updated as we receive information that is more complete and will change over time as we learn more.
**The daily positivity rate and number of tests per 1,000 per week are reported on a 5-day lag and based on the date the sample was collected (specimen collection date). The cumulative positivity rate is cumulative through the most recent specimen collection date with data available.*

COVID-19 (Coronavirus)

State of Nevada
Department of Health
and
Human Services
Office of Analytics

Cumulative Cases

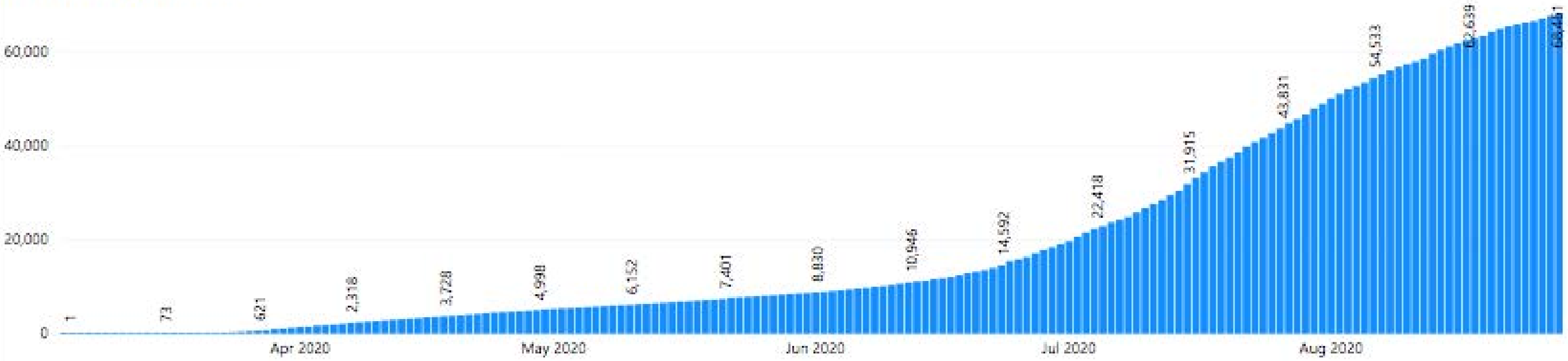

Daily New Cases

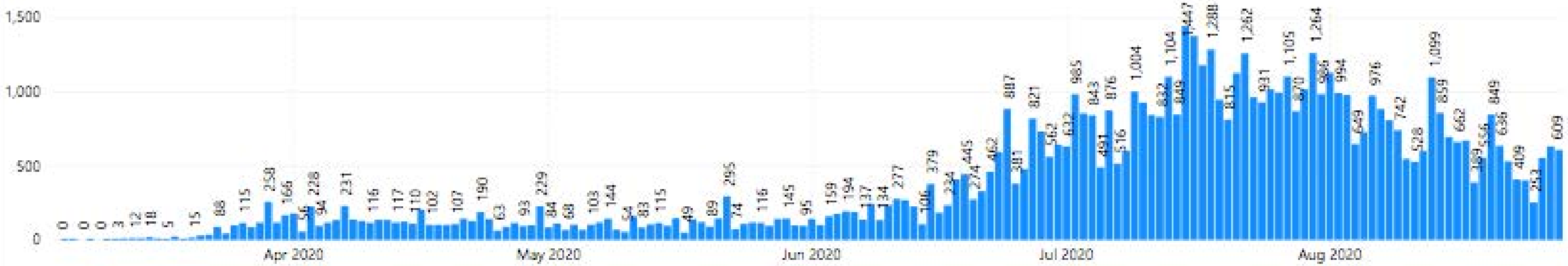

Counts may be artificially low Saturday - Sunday and artificially high on Monday due to limited lab capacity on weekends and limited weekend reporting.

COVID-19 (Coronavirus)

State of Nevada
Department of Health
and
Human Services
Office of Analytics

Total Tests (molecular)
844,391

Total People Tested
592,115

Cumulative Tests Reported

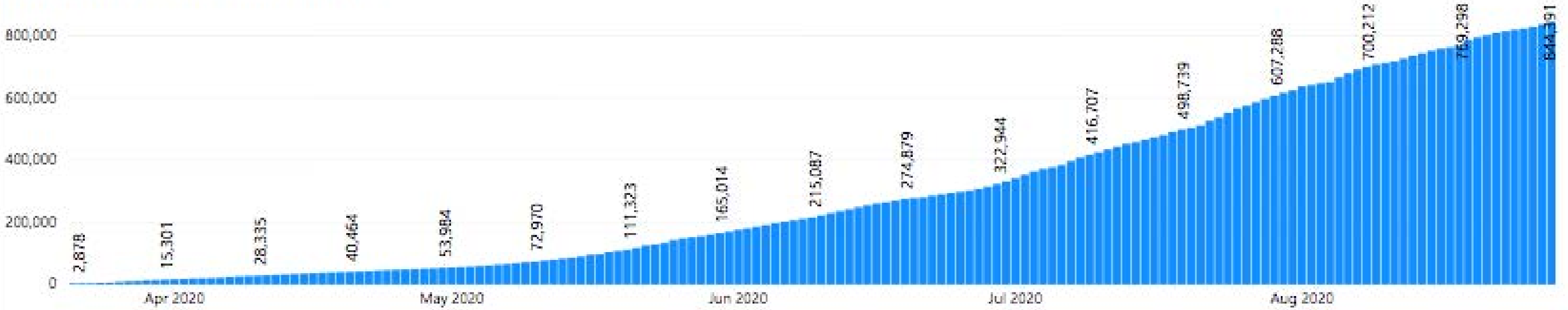

Daily New Tests Reported

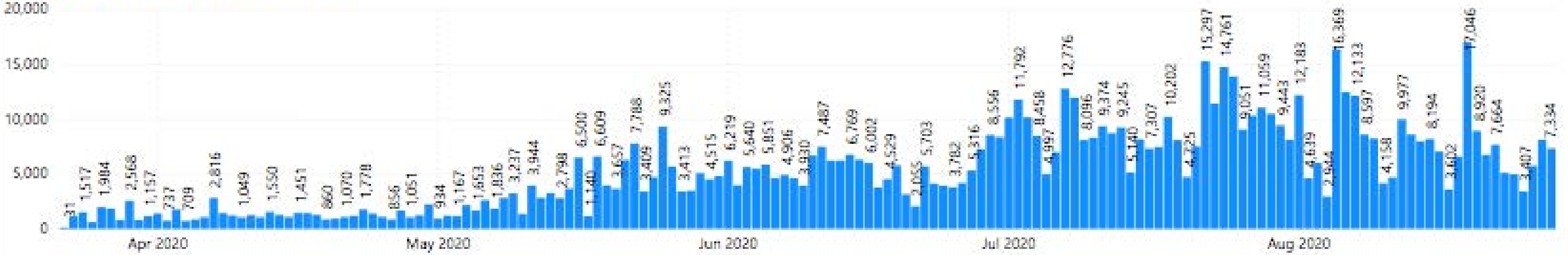

Counts may be artificially low Saturday - Sunday and artificially high on Monday due to limited lab capacity on weekends and limited weekend reporting.

Summary | Risk / Epidemic Curve | Map of Cumulative Positive Ca... | Map of Current Positive Cases

NEW HAMPSHIRE LABORATORY-CONFIRMED COVID-19 CASES

Health Outcomes	Infections		Hospitalizations		Intensive Care Unit		Recovered and Off Isolation	Deaths
	Current	Cumulative	Current	Cumulative	Current	Cumulative		
Persons	243	7,246	7	714	n/a	220	6,571	432

NH DIVISION OF Public Health Services Department of Health and Human Services

Persons with COVID-19	Infections		Hospitalizations		Deaths	
	Persons	% of Total	Persons	% of Total	Persons	% of Total
Healthcare Workers*	1,745	24.1%	56	7.8%	8	1.9%
Long-Term Care Settings**	2,168	29.9%	207	29.0%	351	81.3%
**Sex**						
Female	4,102	56.6%	314	44.0%	221	51.2%
Male	3,141	43.3%	400	56.0%	211	48.8%
Unknown	3	0.0%	0	0.0%	0	0.0%
Grand Total	7,246		714		432	
**Age Group (in Years)**						
0 - 9	162	2.2%	4	0.6%	0	0.0%
10 - 19	386	5.3%	5	0.7%	0	0.0%
20 - 29	1,202	16.6%	17	2.4%	1	0.2%
30 - 39	1,015	14.0%	35	4.9%	0	0.0%
40 - 49	948	13.1%	61	8.5%	7	1.6%
50 - 59	1,142	15.8%	102	14.3%	10	2.3%
60 - 69	908	12.5%	169	23.7%	38	8.8%
70 - 79	578	8.0%	161	22.5%	103	23.8%
80 +	905	12.5%	160	22.4%	273	63.2%
Grand Total	7,246		714		432	

Race / Ethnicity	% of NH Population ****	Infections		Hospitalizations		Deaths	
		Persons	% of Total	Persons	% of Total	Persons	% of Total
White**	90.0%	4,698	73.7%	528	79.3%	396	93.6%
Hispanic or Latino*	3.9%	870	13.7%	69	10.4%	12	2.8%
Black or African American**	1.4%	357	5.6%	32	4.8%	9	2.1%
Other***	1.8%	253	4.0%	15	2.3%	2	0.5%
Asian**	3.0%	193	3.0%	22	3.3%	4	0.9%
Grand Total		6,371		666		423	

Race/Ethnicity is known for 87.9% of COVID-19 infections, 93.3% of hospitalizations, and 97.9% of deaths

COVID-19 CASES BY SEX (n= 7243)

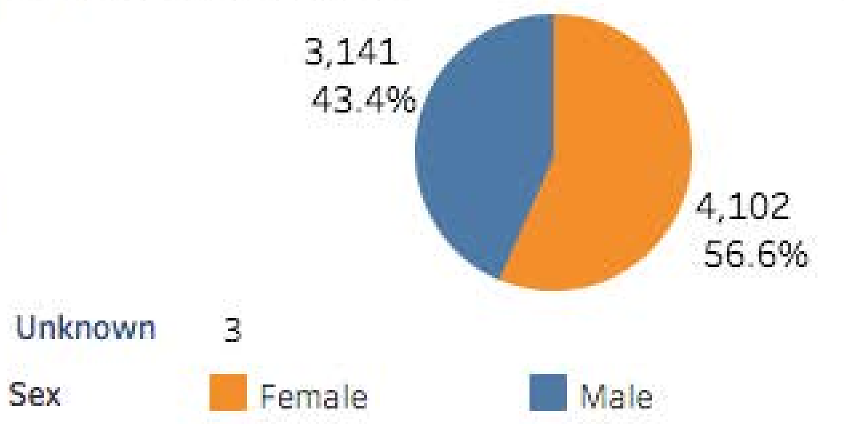

COVID-19 CASES BY AGE GROUP (n= 7246)

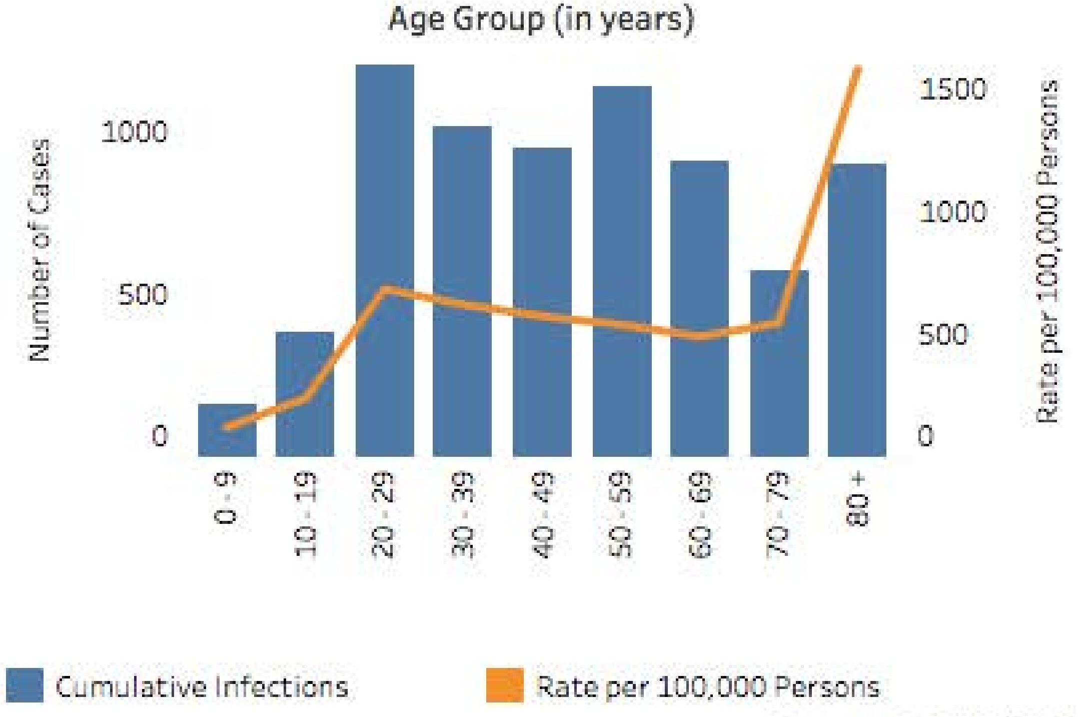

Data as of: 8/29/2020

Undo Redo Revert Refresh Pause Share Download

infogram

Most of the information below comes directly from New Hampshire's ongoing COVID-19 data reports and COVIDTracking.com. If you have questions about the data or think we made a mistake, please email us at coronavirus@nhpr.org.

How to use the charts below: Hovering over individual bars in each chart will let you view the specific number reported for a particular date. Clicking on a category in the legend for each chart will temporarily hide it, allowing you to isolate individual pieces of data. Hovering over individual categories in the legend will display only that category of data.

How Many Cases Are Emerging Each Day?

Here, you can track New Hampshire's COVID-19 "curve" — or the number of new cases the state is reporting each day — as well as the seven-day average. Please keep in mind, the numbers in the chart below sometimes might differ from what you would get if you subtracted the state's total COVID-19 cases from one day to the next. The state says this is because some cases are still under investigation when daily updates are published.

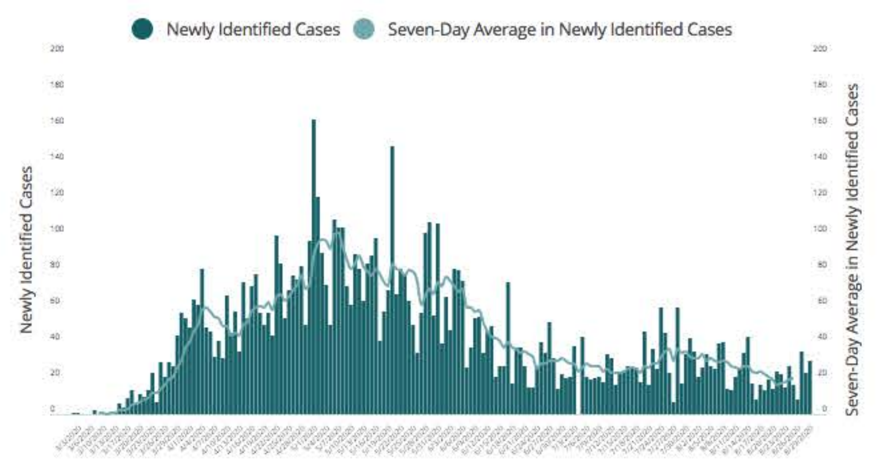

How Many Have Been Identified So Far?

In addition to charting the overall total of COVID-19 cases by date, we also included a rolling 14-day case total because that was one of the criteria the White House advised states to track as they consider reopening.

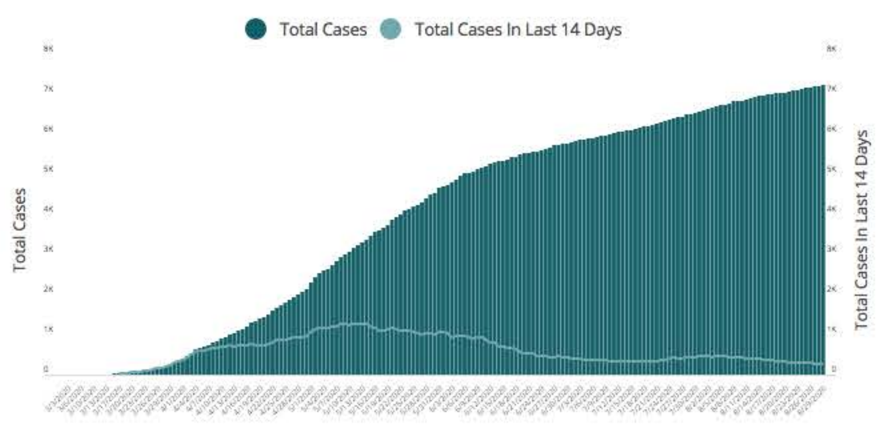

A Closer Look: Who's Been Infected With COVID-19

As reported in New Hampshire's COVID-19 Summary Dashboard (since May 29) and Weekly COVID-19 Summary Reports (prior to May 29). These charts were last updated Aug. 17.

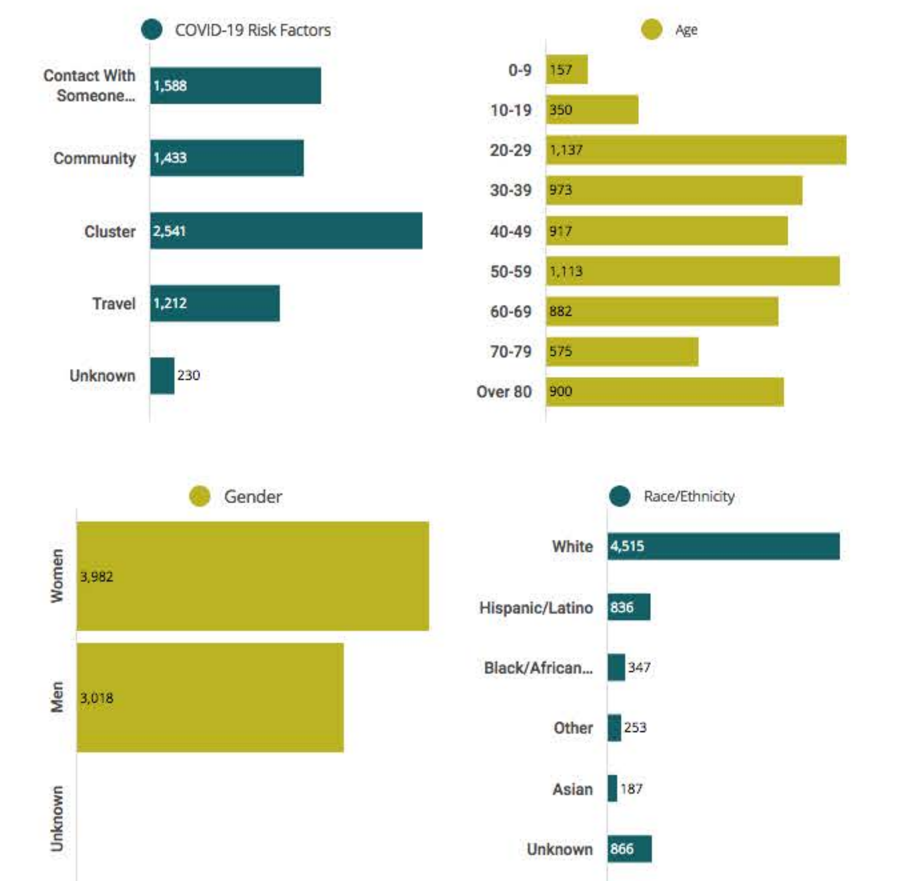

A note about these categories: When tracking COVID-19 Risk Factors, "Community" refers to cases acquired COVID-19 through community transmission. "Cluster" refers to cases where "3 or more cases within a workplace or facility with evidence of transmission occurring within that location." "Travel" refers to cases acquired through either domestic or international travel. When tracking Race/Ethnicity, "Other" includes "non-Hispanic American Indian/Alaska Native, Native Hawaiian/Other Pacific Islander, Other race alone, or two or more races." More information on these definitions can be found in the state's Weekly Summary Reports.

How Many Are Dying From COVID-19?

New Deaths Reported By Day | Total Deaths

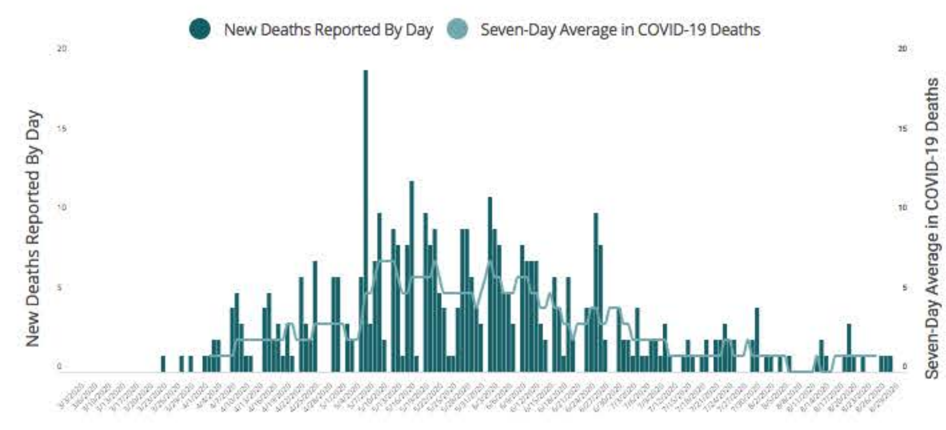

A Closer Look: Who Has Died from COVID-19

As reported in New Hampshire's COVID-19 Summary Dashboard (since May 29) and Weekly COVID-19 Summary Reports (prior to May 29). These charts were last updated Aug. 17.

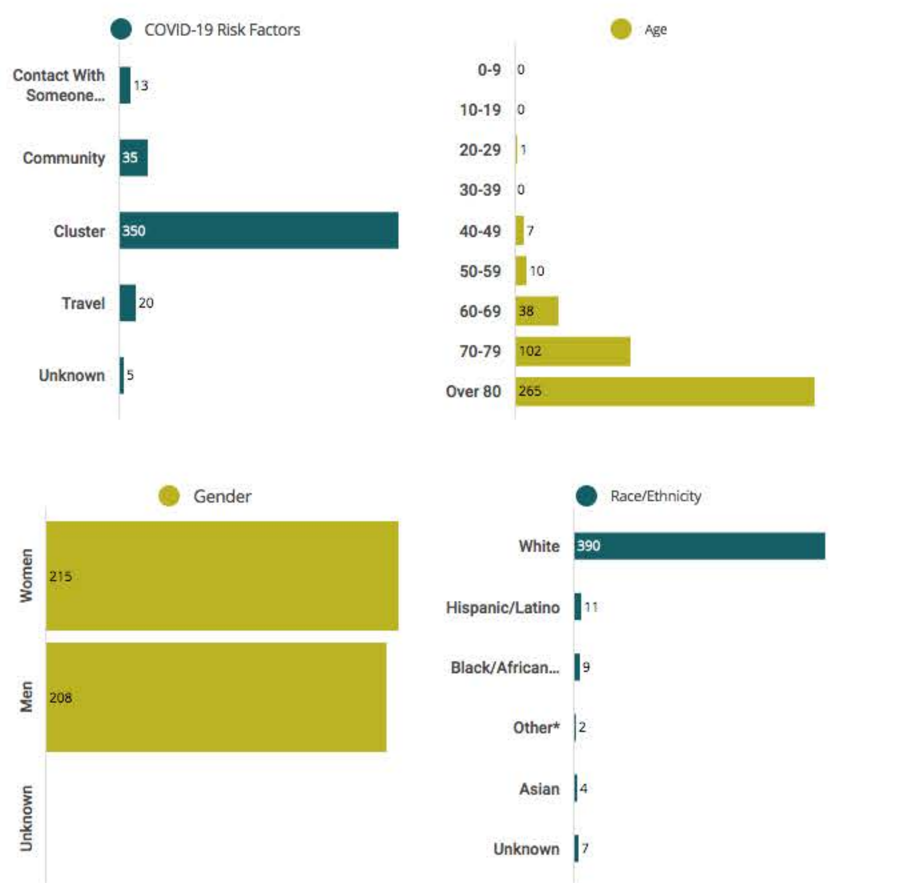

A note about these categories: When tracking COVID-19 Risk Factors, "Community" refers to cases acquired COVID-19 through community transmission. "Cluster" refers to cases where "3 or more cases within a workplace or facility with evidence of transmission occurring within that location." "Travel" refers to cases acquired through either domestic or international travel. When tracking Race/Ethnicity, "Other" includes "non-Hispanic American Indian/Alaska Native, Native Hawaiian/Other Pacific Islander, Other race alone, or two or more races." More information on these definitions can be found in the state's Weekly Summary Reports.

How Many Have Been Hospitalized?

Please note: While New Hampshire reported its first COVID-19 case in early March, cumulative hospitalizations have only been reported since March 23, and daily hospitalizations since April 14. Additionally, on July 14, the state disclosed that they were reporting "2 newly identified hospitalizations and 74 additional hospitalizations identified after further review and quality assurance of previous data." It is not yet clear when those hospitalizations took place.

Current Hospitalizations | Total Hospitalizations

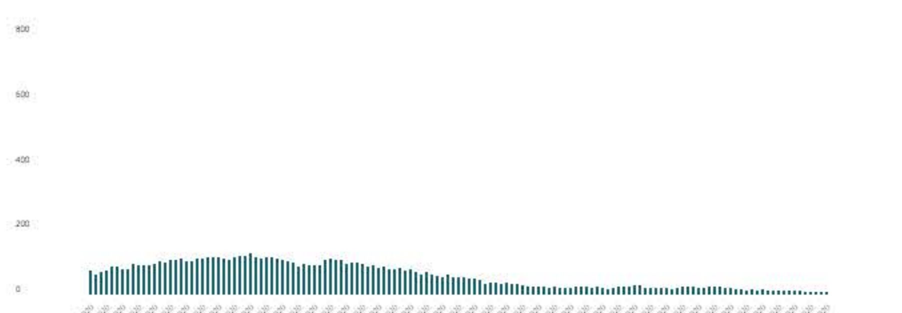

A Closer Look: Who's Been Hospitalized for COVID-19

As reported in New Hampshire's COVID-19 Summary Dashboard (since May 29) and Weekly COVID-19 Summary Reports (prior to May 29). These charts were last updated Aug. 17.

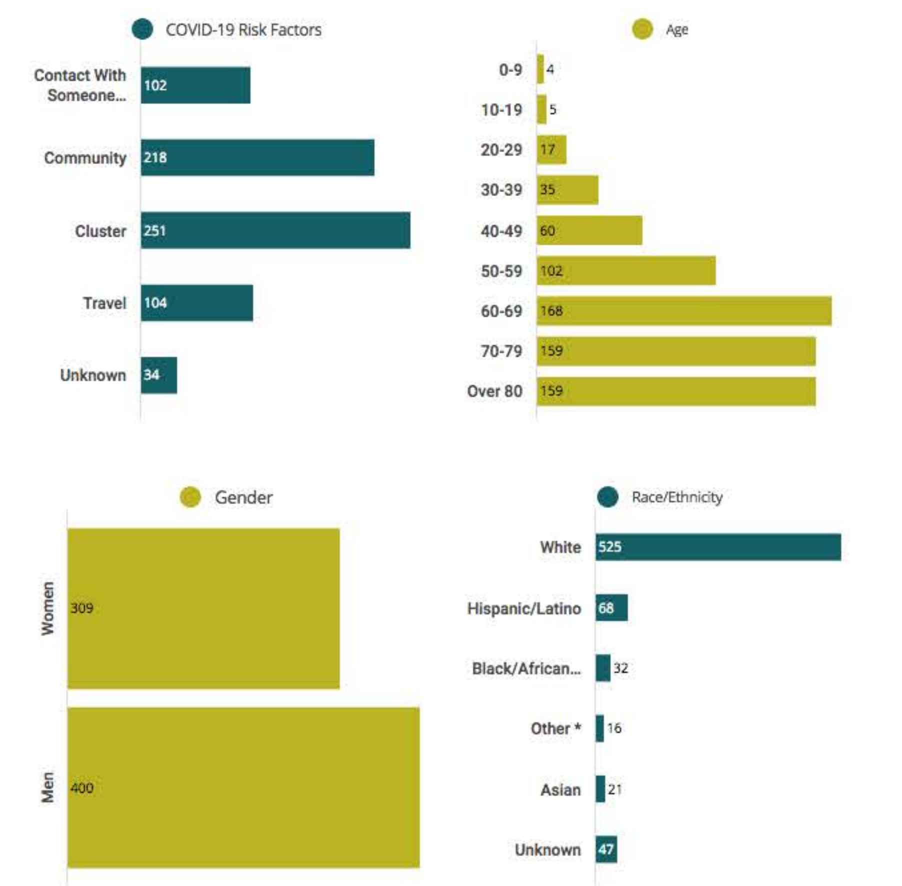

A note about these categories: When tracking COVID-19 Risk Factors, "Community" refers to cases acquired COVID-19 through community transmission. "Cluster" refers to cases where "3 or more cases within a workplace or facility with evidence of transmission occurring within that location." "Travel" refers to cases acquired through either domestic or international travel. When tracking Race/Ethnicity, "Other" includes "non-Hispanic American Indian/Alaska Native, Native Hawaiian/Other Pacific Islander, Other race alone, or two or more races." More information on these definitions can be found in the state's Weekly Summary Reports.

How Much Is N.H. Testing?

New Hampshire's approach to reporting its testing results has varied over time. Since the end of April, the state has reported daily updates on the number of tests reported from individual sources: hospitals, public health laboratories, private companies and more. The state does not provide raw data on the number of positive results from each source.

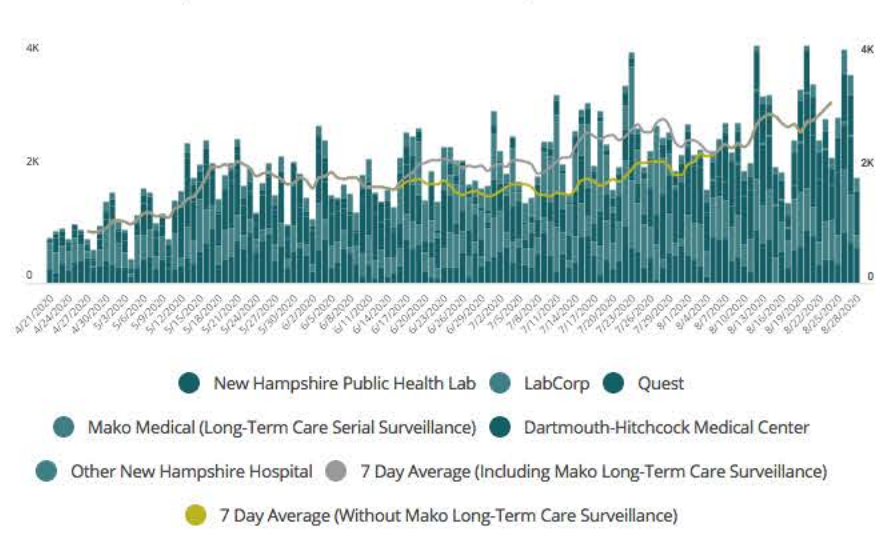

Testing Rates (%) | Testing Results (Absolute)

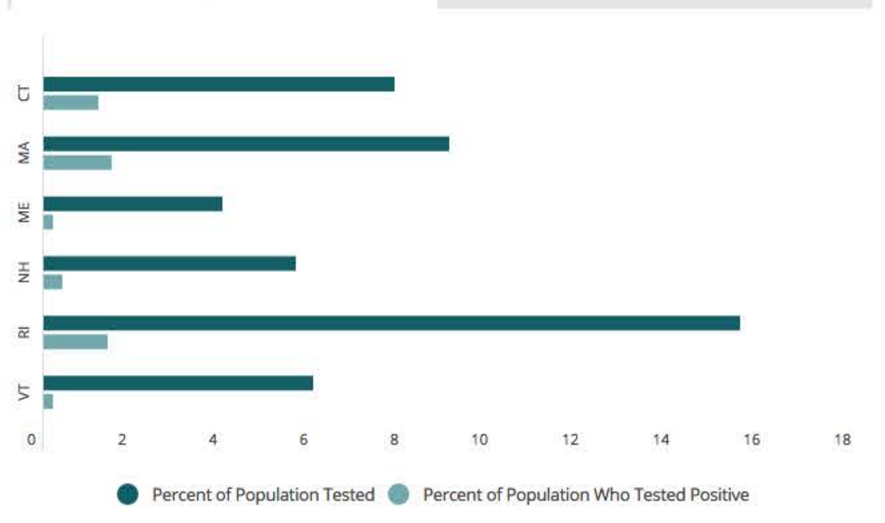

Notes: Testing numbers are collected from The COVID Tracking Project, which draws on data reported by public health agencies in each state. Individual states might report different metrics, so the numbers might not reflect the full scope of testing in each place. In some cases, the daily totals for each state could lag behind due to data entry delays. We used 2018 estimates reported by the U.S. Census Bureau to calculate population testing rates. While New Hampshire reports "pending" test results on a daily basis, those are not included in the above totals.

Source: New Hampshire COVID-19 Data Reports, COVIDTracking.com, Census Data

nhpr

NHPR COVID-19 Tracker

Report

All rights reserved © 2020 Infogram. Terms & Privacy
Infogram and Infogr.am are registered trademarks of Prezi, Inc.

New Jersey COVID-19

NJ COVID-19 Hub

Cases and Trends | Case and Mortality Summaries | Hospital Census | Contact Tracing | Hospital Discharges

COVID-19 Dashboard

Stage 2: Moderate Risk Activities Restarted with

THE ROAD BACK
Restoring Economic Health through Public Health

Updated: 8/29/2020

Comments about this dashboard: Covid.Dashboard@doh.nj.gov

COVID-19 Cases by County

Data is provisional and subject to revision.

Atlantic County

9 New Positives

240 Confirmed Deaths

3,729 Positive Test Results

11 Probable Deaths

Bergen County

49 New Positives

1,790 Confirmed Deaths

21,520 Positive Test Results

242 Probable Deaths

Burlington County

18 New Positives

442 Confirmed Deaths

6,370 Positive Test Results

39 Probable Deaths

Camden County

24 New Positives

538 Confirmed Deaths

9,116 Positive Test

52 Probable Deaths

Cases with Unassigned County
337

Deaths with Unassigned County
0

Cases by County:

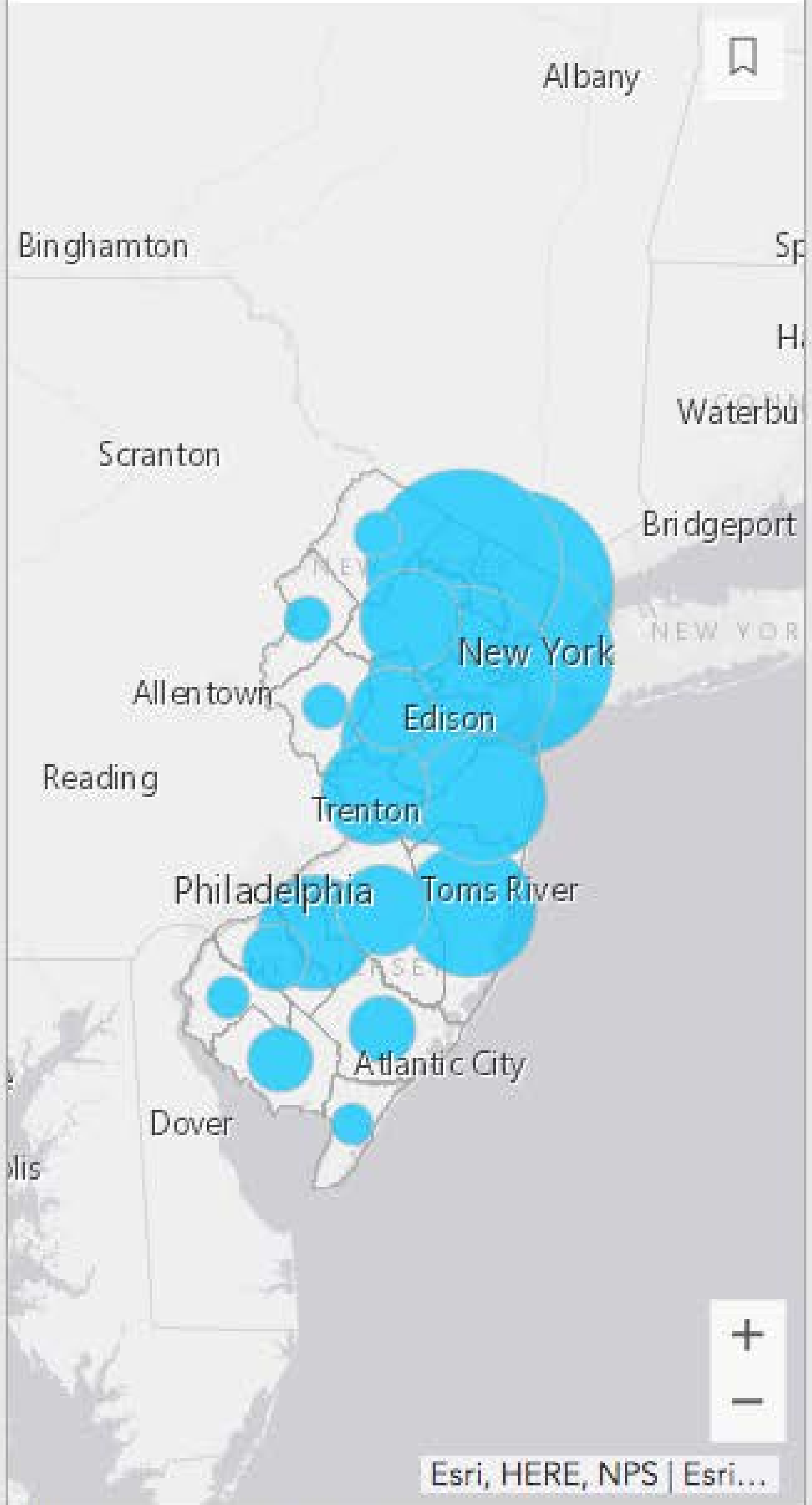

Click county to see details.

Map (scroll for more content)

Total Cases
191,320

Total Confirmed Deaths
14,153

Total Probable Deaths
1,780

New Lab-Confirmed Deaths
4

Source: CDRSS

Hospital-reported Deaths (last 24h)

Hospital-reported Deaths (last 24h)
2

Note: Data as-reported from NJHA facility survey, deaths are

Total PCR Tests Reported
2,807,747

Rate of Transmission (Rt)
0.81

New Cases Over Time

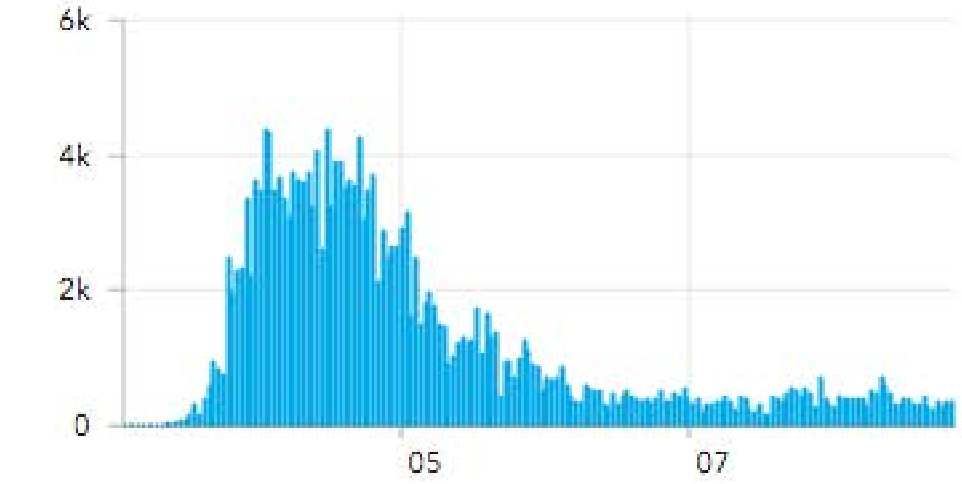

Data are provisional and subject to revision with further investigation.

esri A Story Map

N.J. Coronavirus Tracker

Pick a location Select county...

Confirmed cases in *New Jersey*

Total infected: 190,971

Total deaths: 14,150

Total counts by day in *New Jersey*

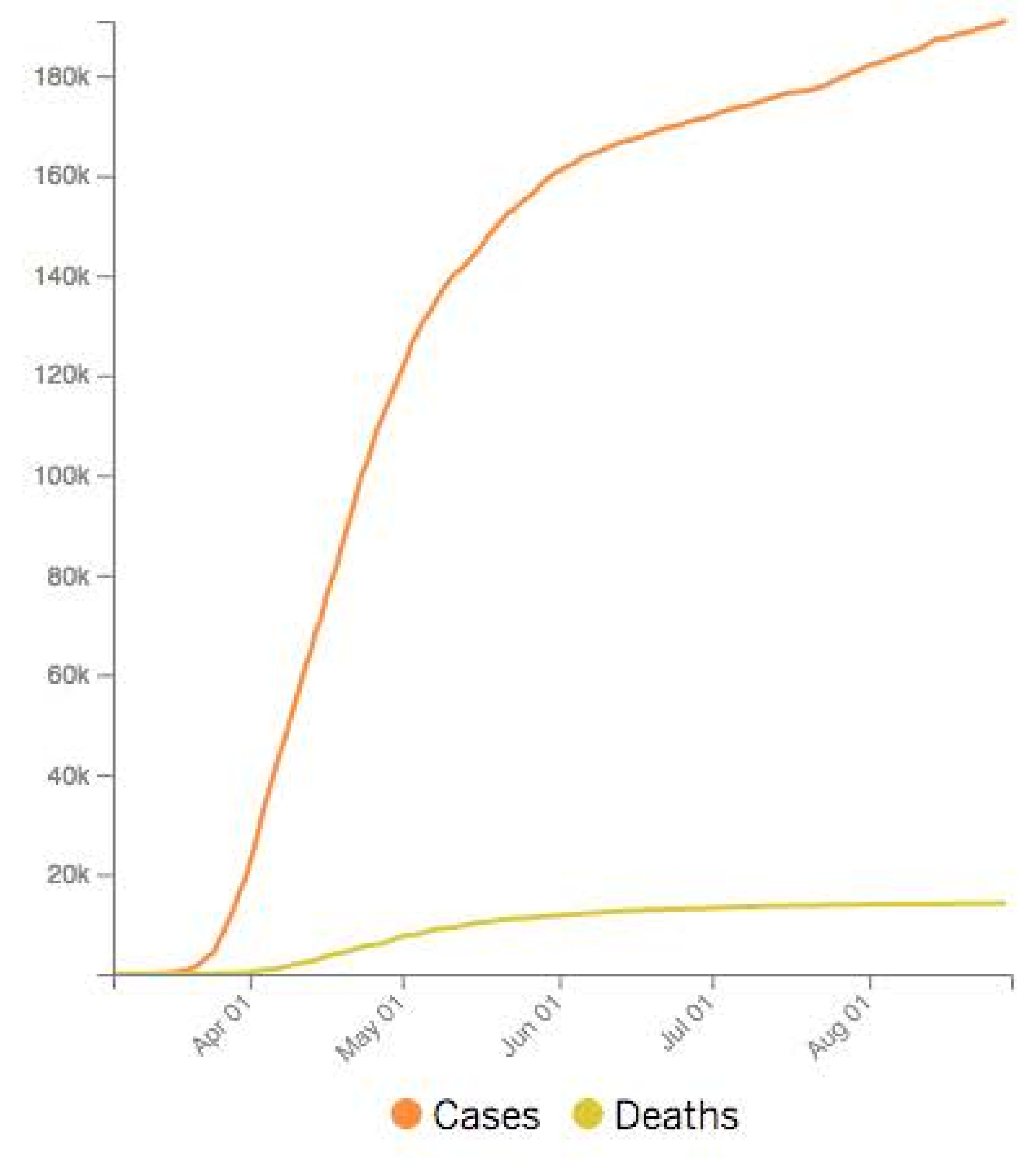

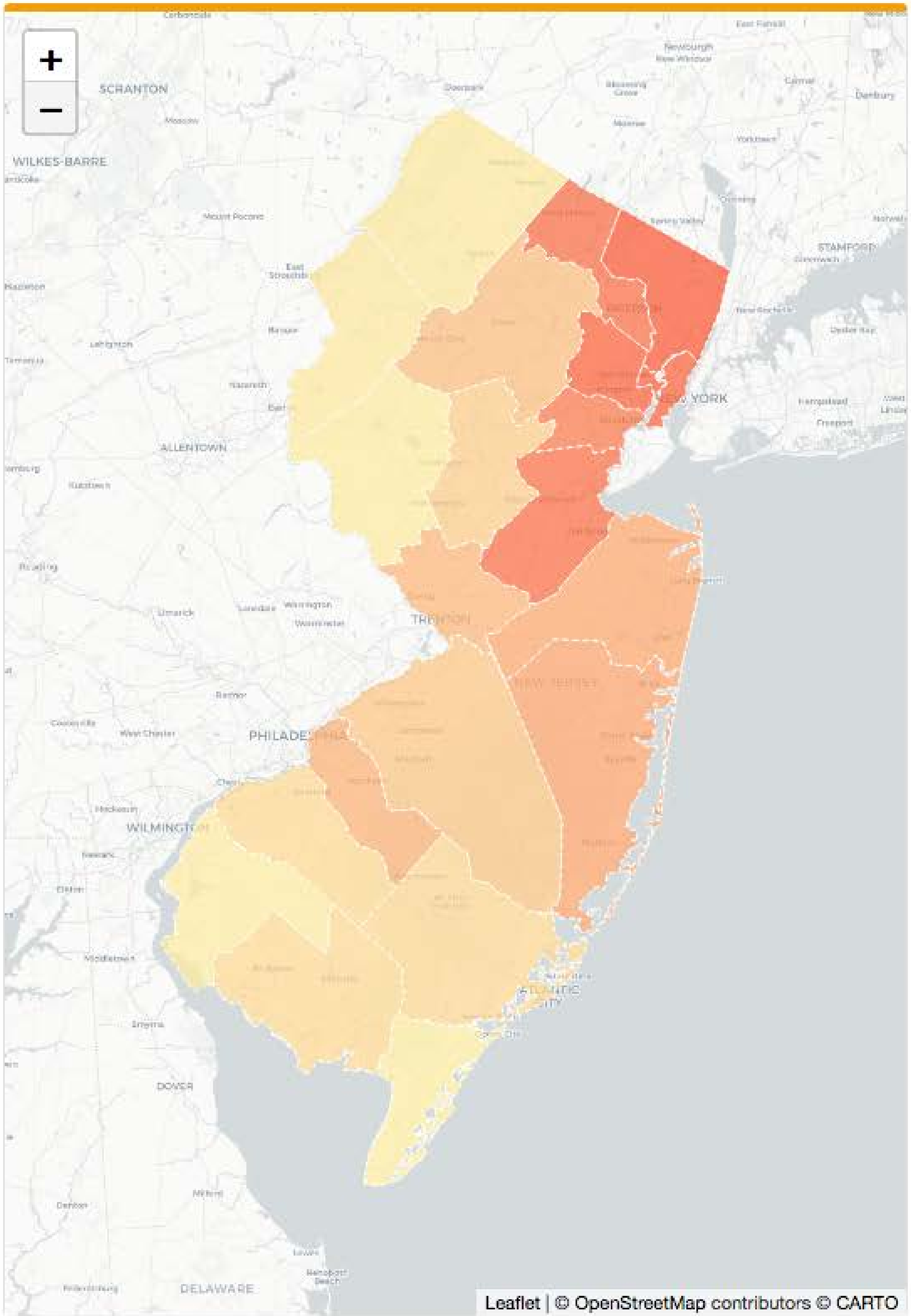

Confirmed U.S. cases

Total infected: 5,884,053

Total deaths: 173,754

Information provided by the COVID Tracking Project.

Coronavirus in N.J.

Journalism like this project needs your support. Subscribe today.

See all N.J. coronavirus news

What's open near you? Check out NJ is Open*

All N.J. numbers are reported by state and county health departments and tracked by NJ Advance Media

Updated 46 minutes ago.

© NJ Advance Media | Development by Arjun Kakkar and Cassidy Grom | Contact

NEW MEXICO DEPARTMENT OF HEALTH

New Mexico Department of Health

COVID-19 in New Mexico

Back to CV.NMHEALTH.ORG

NUMBER OF CASES

25,178

TOTAL # OF TESTS PERFORMED

752,522

TOTAL HOSPITALIZATIONS

3,116

CURRENT HOSPITALIZATIONS

67

TOTAL DEATHS

769

TOTAL RECOVERED

12,820

Show Historical Statewide Data

Last Updated: 8/29/2020, 5:50:04 PM

Information about the Data

View Map by County | View Map by Tests Performed | View Map by Zip Code | View Correctional Facilities | View Cases By County

Map Key: Fewer Cases — More Cases

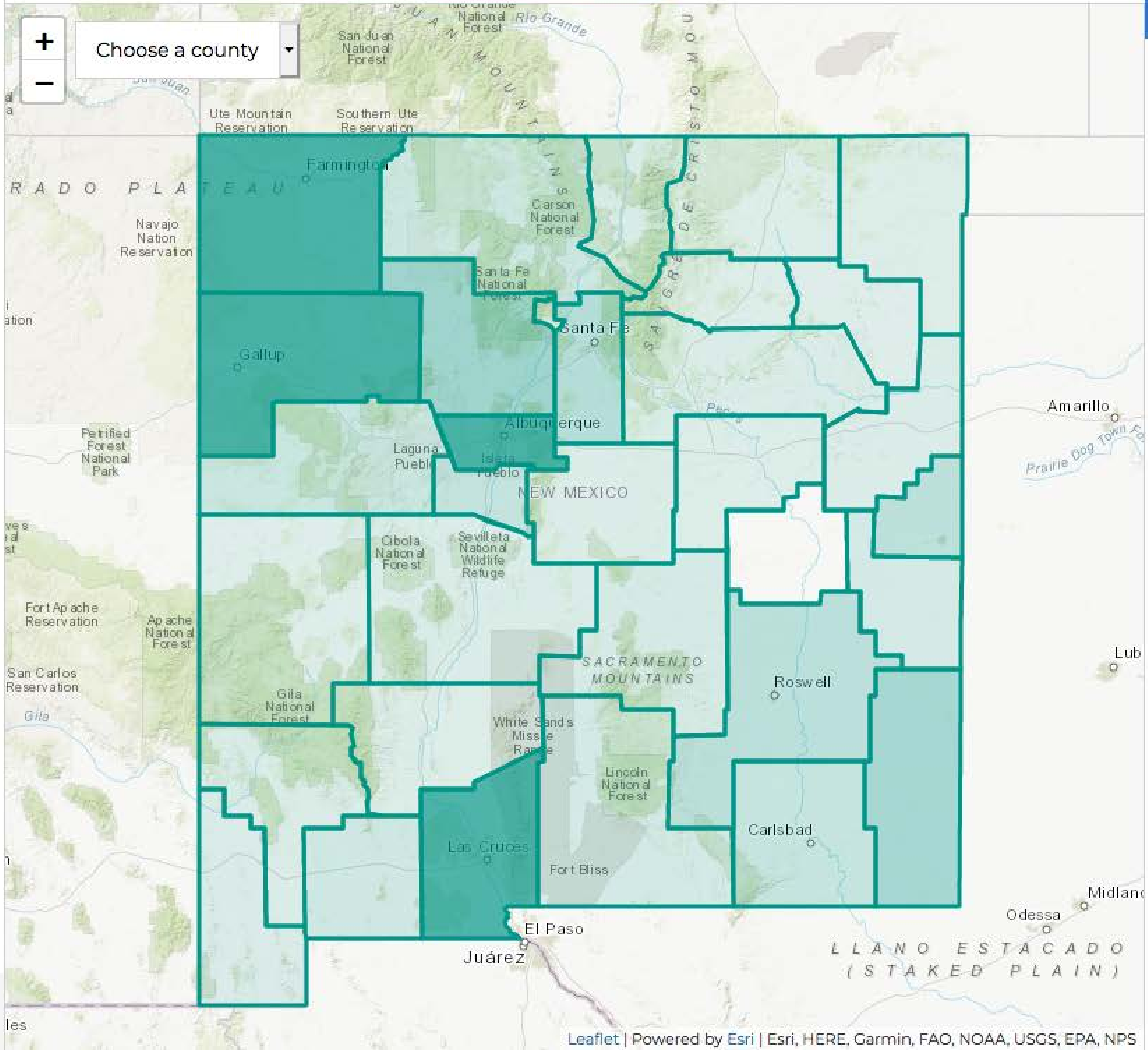

EXPAND DEMOGRAPHICS VIEW

Statewide Gender Breakdown

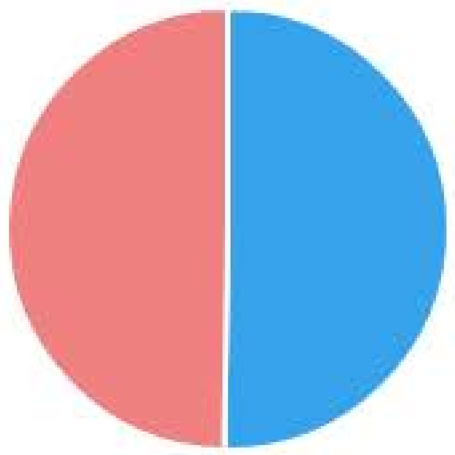

Gender	Cases
Male	50.28%
Female	49.72%

Statewide Age Breakdown

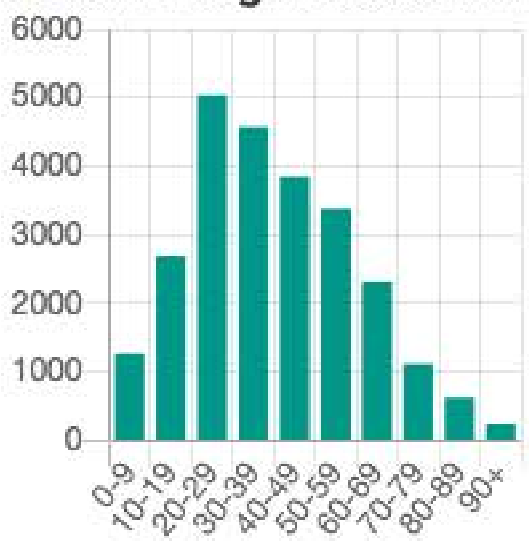

Age	Cases
0-9	1271
10-19	2697
20-29	5044
30-39	4582

New York State Department of Health COVID-19 Tracker

Testing data as of: 8/28/2020 Midnight
Testing data last updated on: 8/29/2020
(Updated daily before 2 PM)

Statewide

Total Persons Tested
8,096,770

Total Tested 8/28
93,873

Total Tested Positive
433,402

Sex Distribution of Positive Cases

Female	Male	Unknown
48.9%	50.3%	0.7%

New Positives 8/28
635

Persons Tested Positive by County

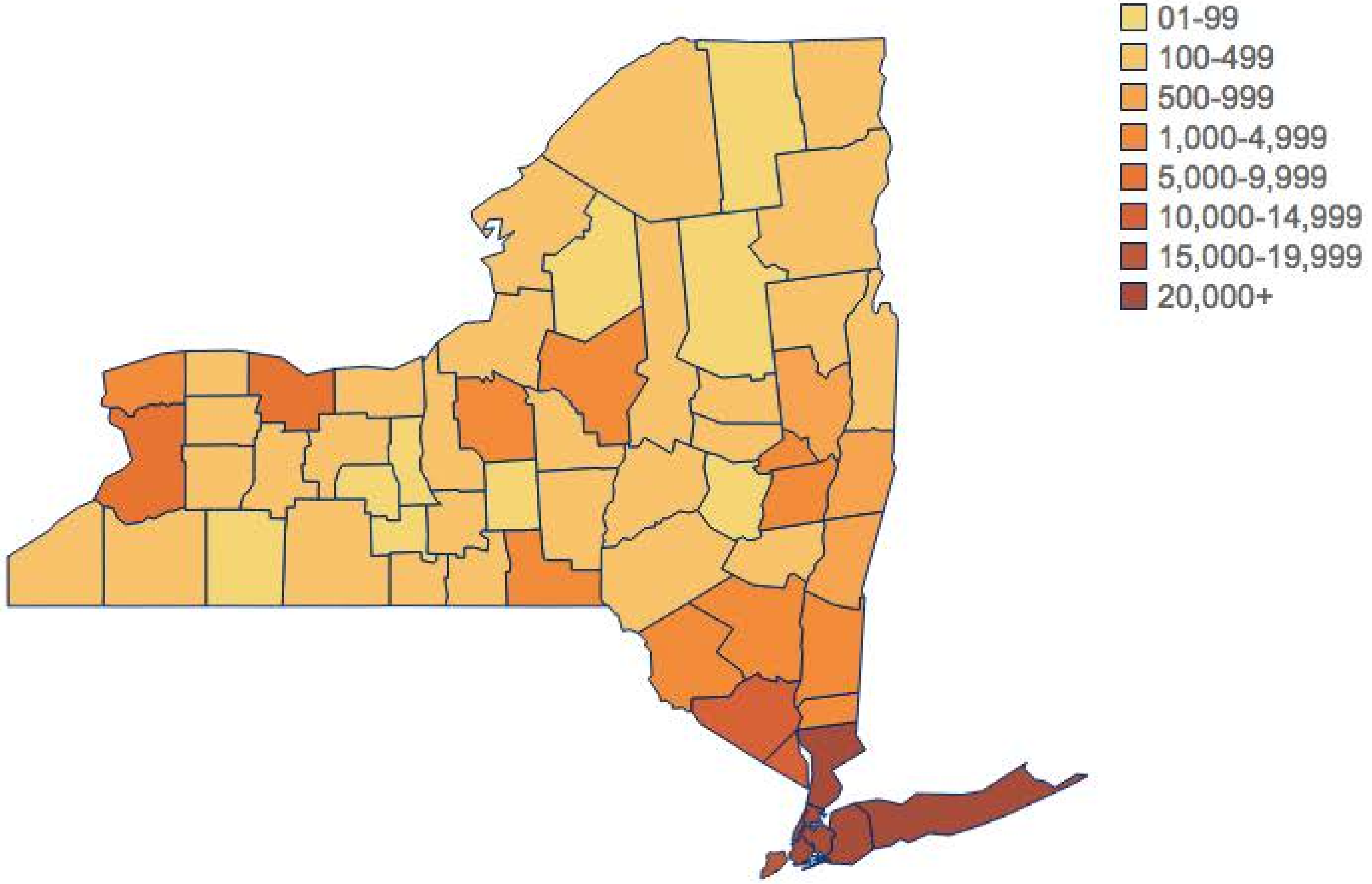

County Stats

County Name	Number of Persons Tested	Tested Positive	% Positive Results	Persons Tested Today	New Positives Today

Click County to See Detail
Click Again for Statewide

Albany	2,754
Allegany	88
Bronx	51,468
Broome	1,286
Cattaraugus	197
Cayuga	177
Chautauqua	373
Chemung	196
Chenango	225
Clinton	150
Columbia	569
Cortland	98
Delaware	112
Dutchess	4,846
Erie	9,741
Essex	126
Franklin	58
Fulton	311
Genesee	293
Greene	307

Click for Daily Trends

Click for Table View

Click for Fatality Data

FAQs & Helpful Links

State of Ohio | COVID - 19 Dashboard

Last Updated: 08-29-20
Updated daily at 2 p.m.

County Map | Cases

Select a County To Filter Dashboard

Case Classification Status
Filter all views by case classification
(All) Total Cases

View By
Pick an option to view in map
Cases

County Search
Search a county to highlight
No items highlighted

Ethnicity:
Select a Ethnicity to filter the dashboard
All

Race
Select a race to filter the dashboard
All

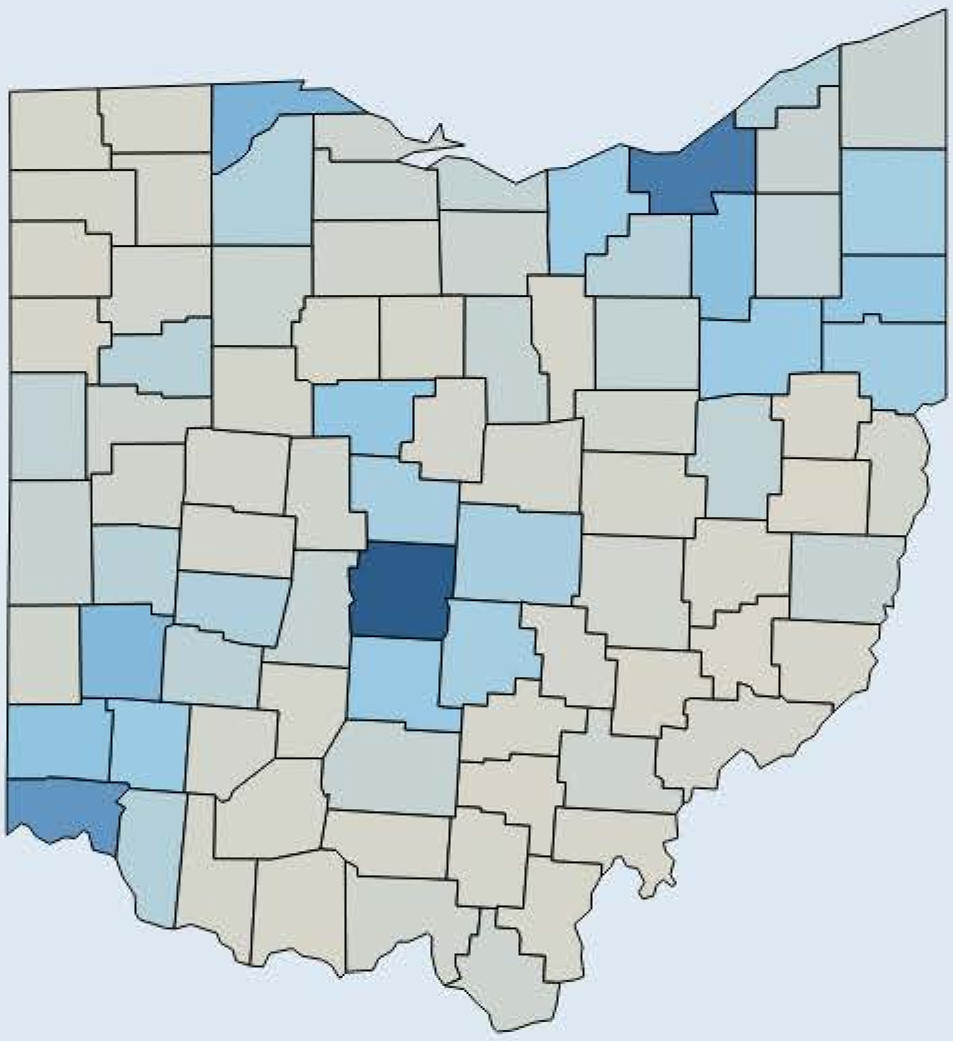

© OpenStreetMap

Metrics | Cumulative and Daily Count

View Count By:
Select to view counts by daily or cumulative
Daily Count

Cases

121,340

* Preliminary

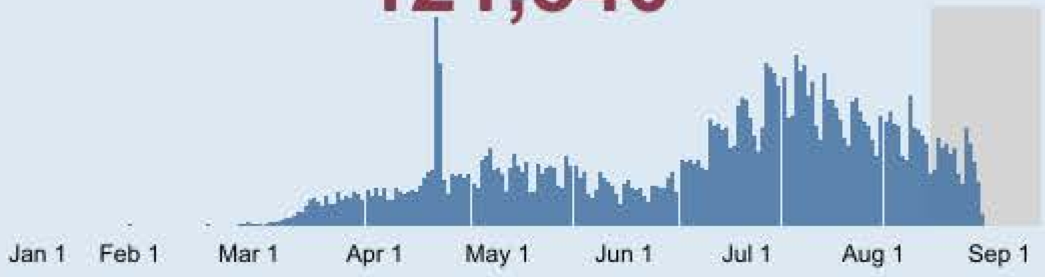

Hospitalizations

13,288

* Preliminary

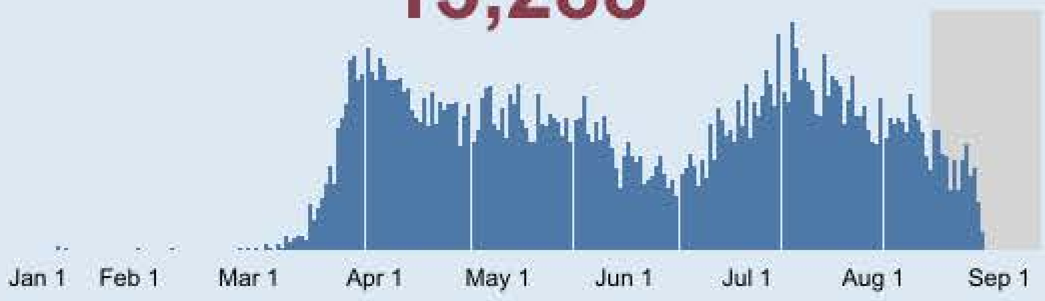

Deaths

4,126

* Preliminary

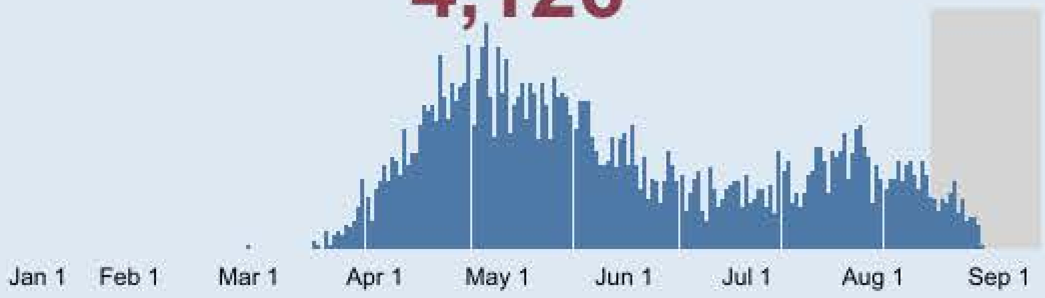

Presumed Recovered ***

101,185

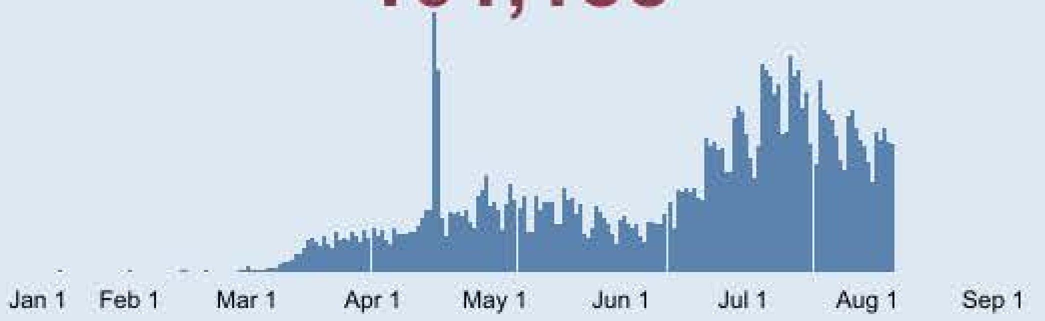

Key Metrics | By County

View By
Pick an options to change the view
County

View By	Case Count	Hospitalization Count	Death Count
Mercer	[illegible]	[illegible]	[illegible]
Erie	769	109	36
Wayne	751	79	61
Richland	695	93	17
Madison	688	42	11
Ross	685	89	10
Belmont	681	49	26
Geauga	611	102	46
Darke	607	66	35
Ashtabula	605	95	46
Hancock	512	44	7
Sandusky	510	74	17
Huron	469	46	6
Ottawa	446	49	29
Lawrence	429	47	14
Auglaize	409	39	8
Athens	385	21	2
Union	358	18	1
Holmes	349	32	7
Shelby	346	32	4

Note: Totals include Confirmed and CDC Expanded Case Definition (Probable)

All data displayed is preliminary and subject to change as more information is reported to ODH. Trends are based on the date of illness onset. If onset date is unknown, the earliest known date associated with the case is used as a substitute for the date of illness onset.

*Preliminary case data in the shaded area is likely under-reported to ODH because of the delay between initial contact with a health care provider, testing, and diagnosis.

**Definitive cause of death can take weeks to determine. Data displayed illustrates current status.

*** Presumed Recovered is defined as cases with a symptom onset date >21 days prior who are not deceased.

Ohio Department of Health

COVID-19 Cases - Main Page

BETA

OK Summary	OK Cases	OK Deaths	OK Active	OK Recovered
Cases Updated As Of:	57,353	797	7,949	48,607
2020-08-29	↑ 1,093 DoD*	↑ 11 DoD*	↑ 237 DoD*	↑ 845 DoD*
Source: OK Government	1.9% DoD*	1.4% DoD*	3.1% DoD*	1.8% DoD*

US Summary	US Cases	US Deaths	US Active	US Recovered
Cases Updated As Of:	5,913,941	181,800	3,613,774	2,118,367
2020-08-28	↑ 46,156 DoD*	↑ 976 DoD*	↑ 28,139 DoD*	↑ 17,041 DoD*
Source: Johns Hopkins	0.8% DoD*	0.5% DoD*	0.8% DoD*	0.8% DoD*

Oklahoma Case Status by Date of Onset

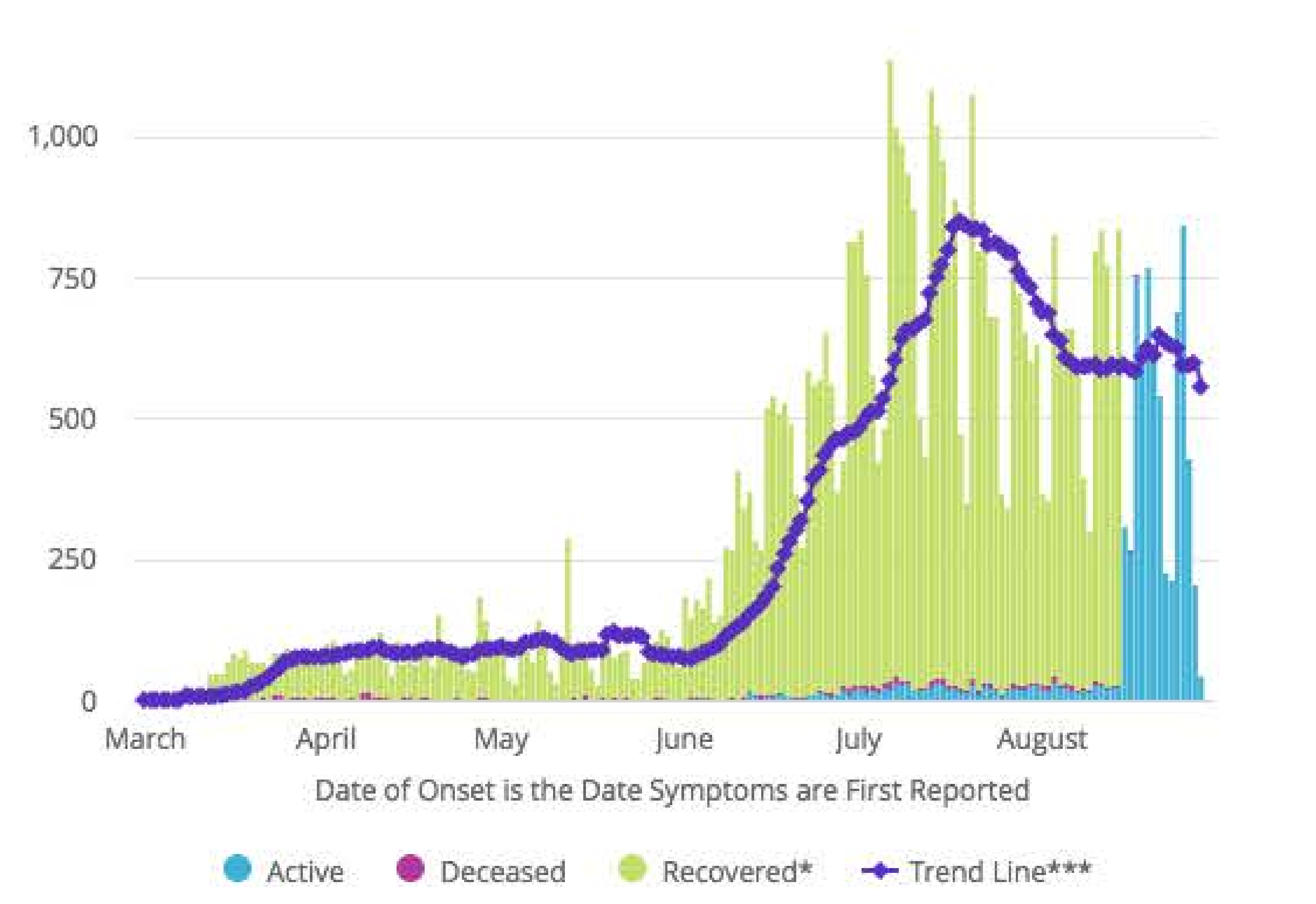

Oklahoma Cumulative Cases by Date Reported

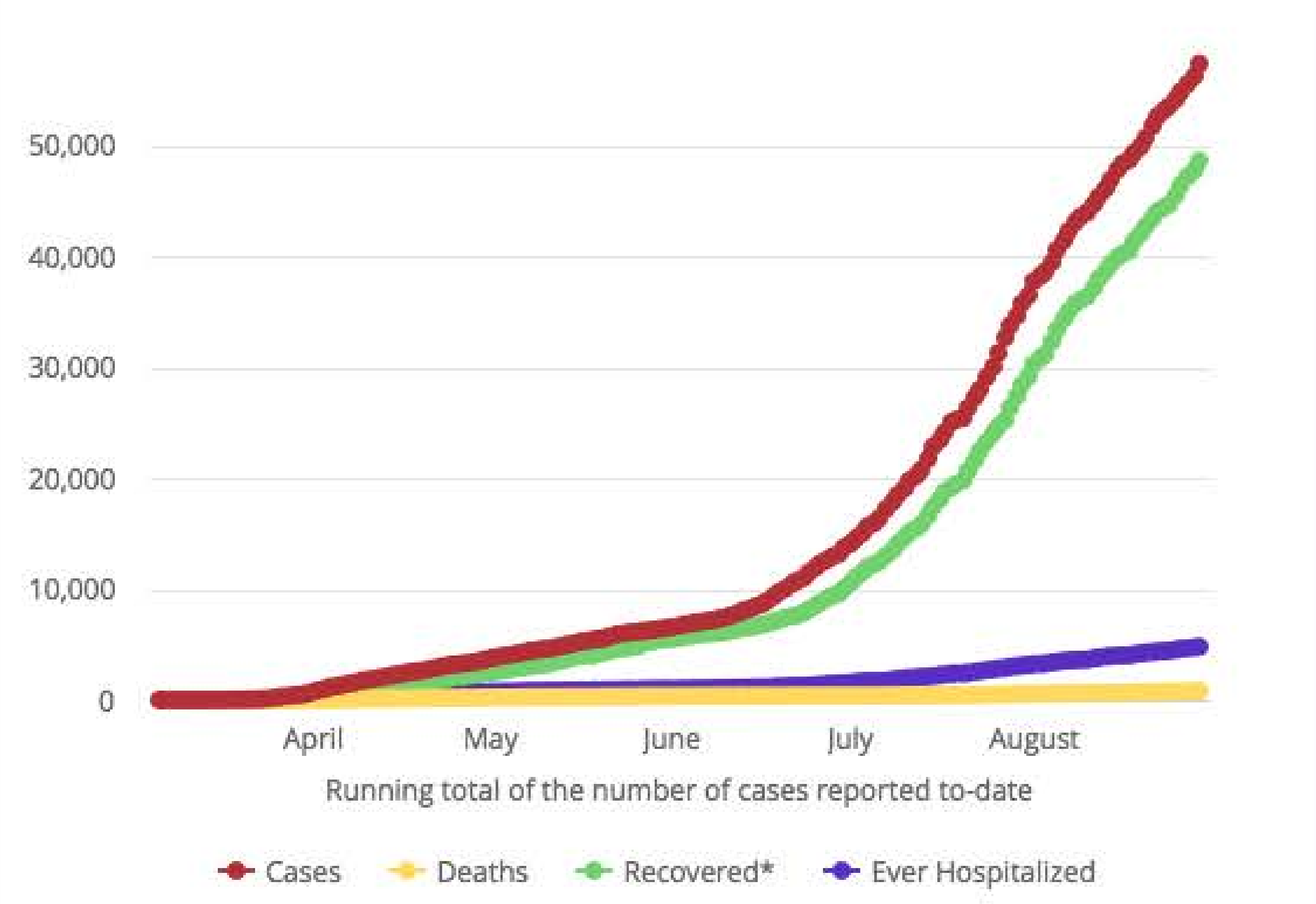

Oklahoma Total Cases by Race

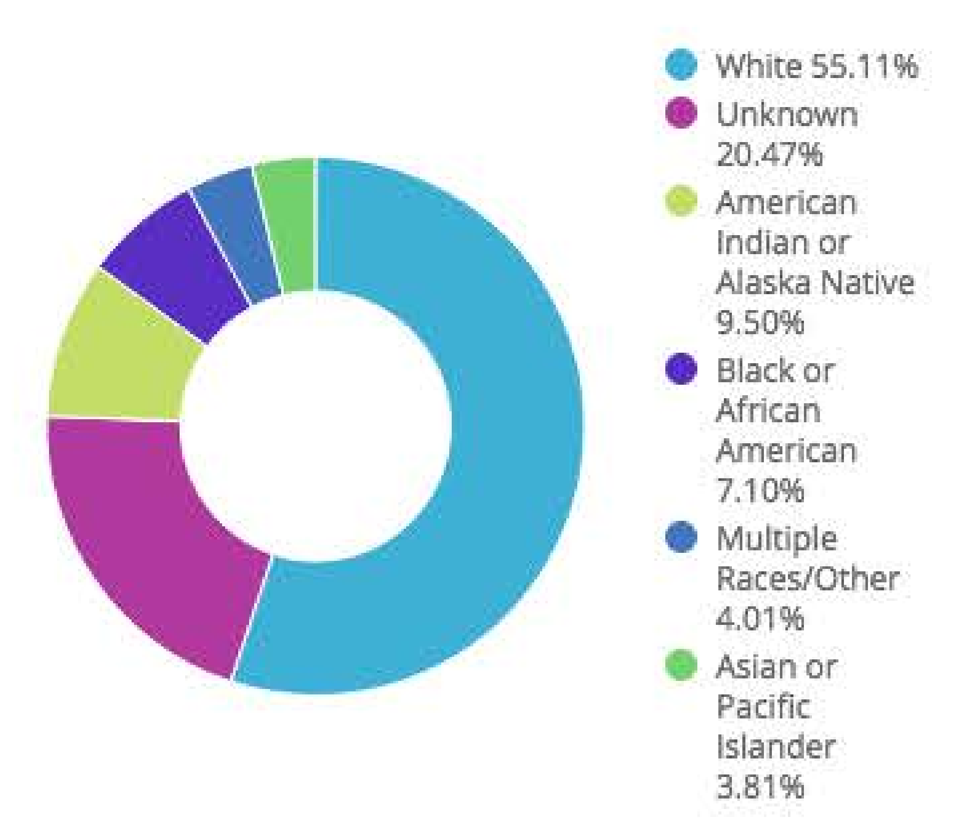

Oklahoma Total Cases by Age Group

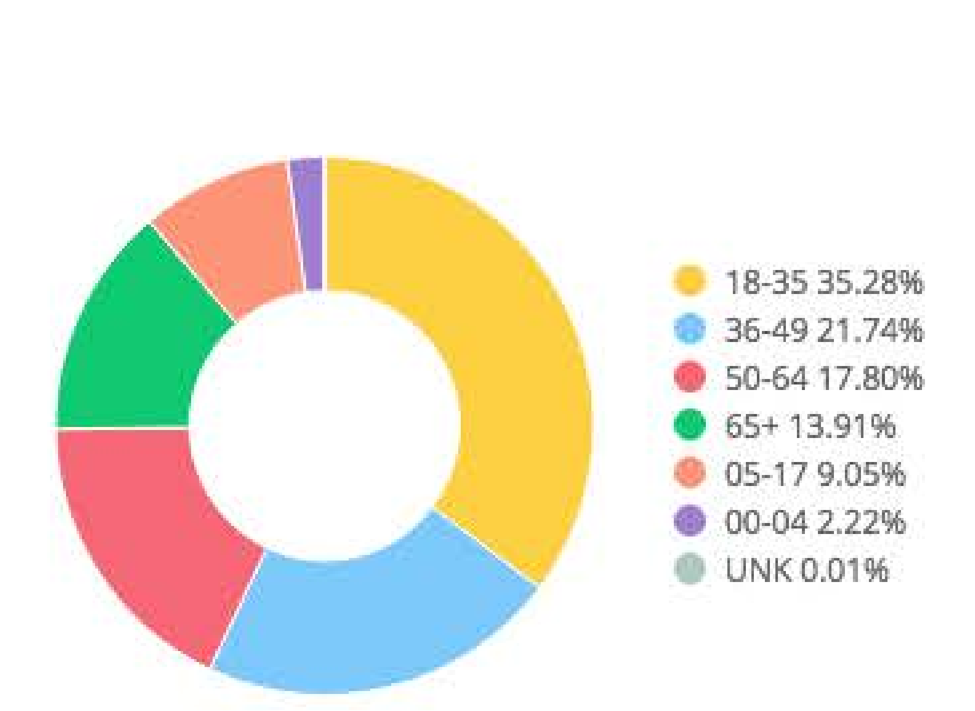

Oklahoma Total Cases Status by Gender

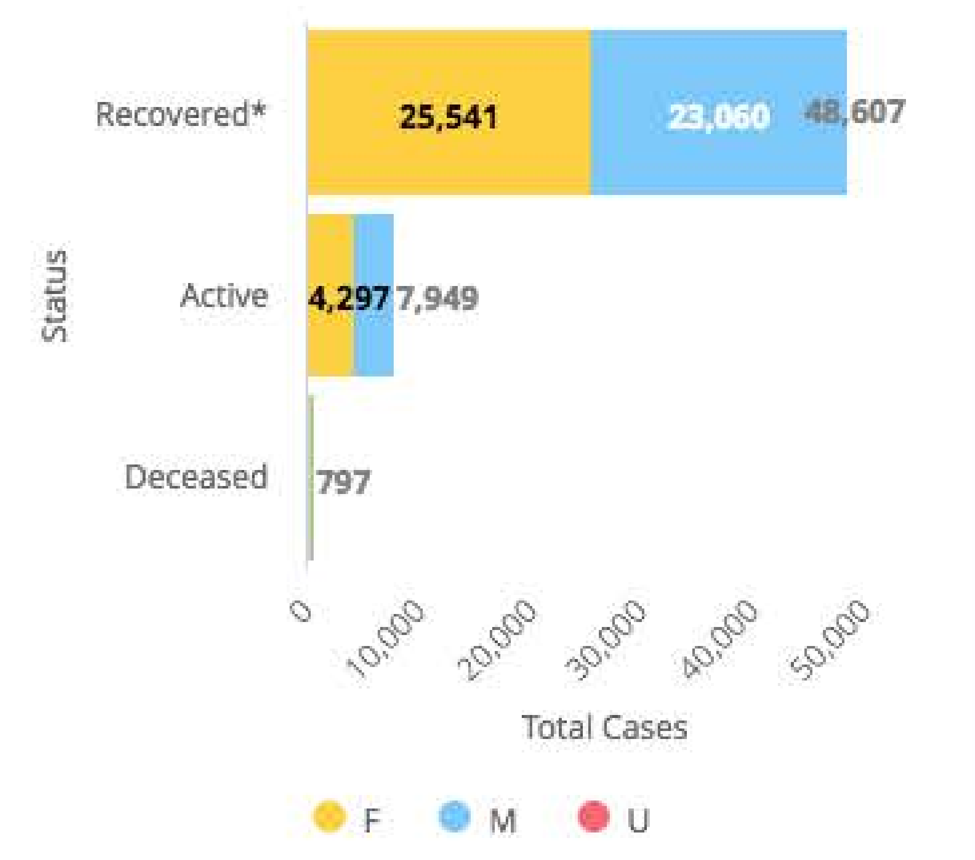

View Data by County

View Data by City

View Data by Zip

View Data for Confirmed Cases

View Data for Deaths

View Data for Recoveries

Symptom Tracker

Personal Protective and Hospital Equipment

OSDH District Breakout

View Data Downloads

COVID-19 Cases by County

View Data
Downloads

Confirmed Cases by County

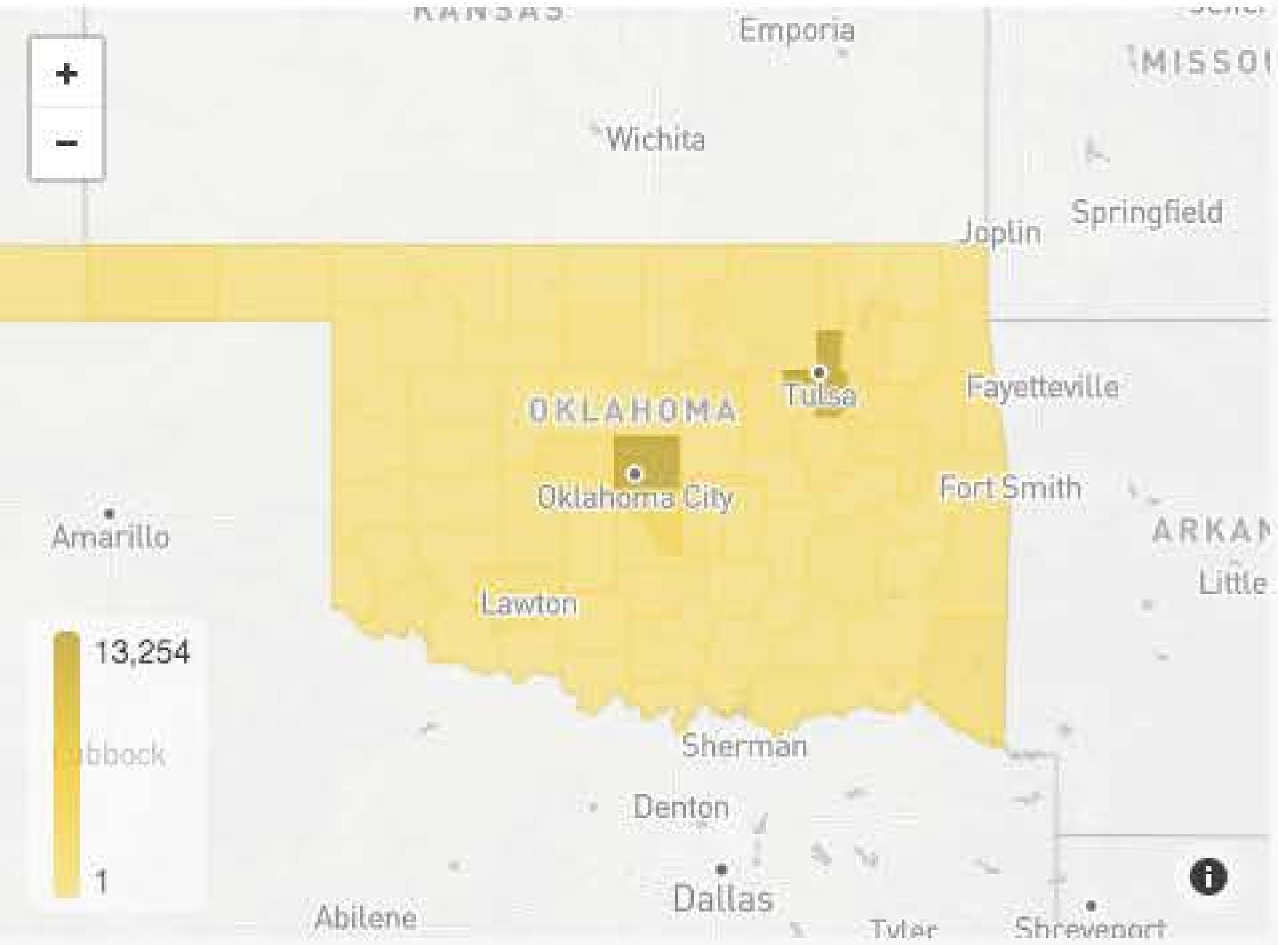

Deaths by County

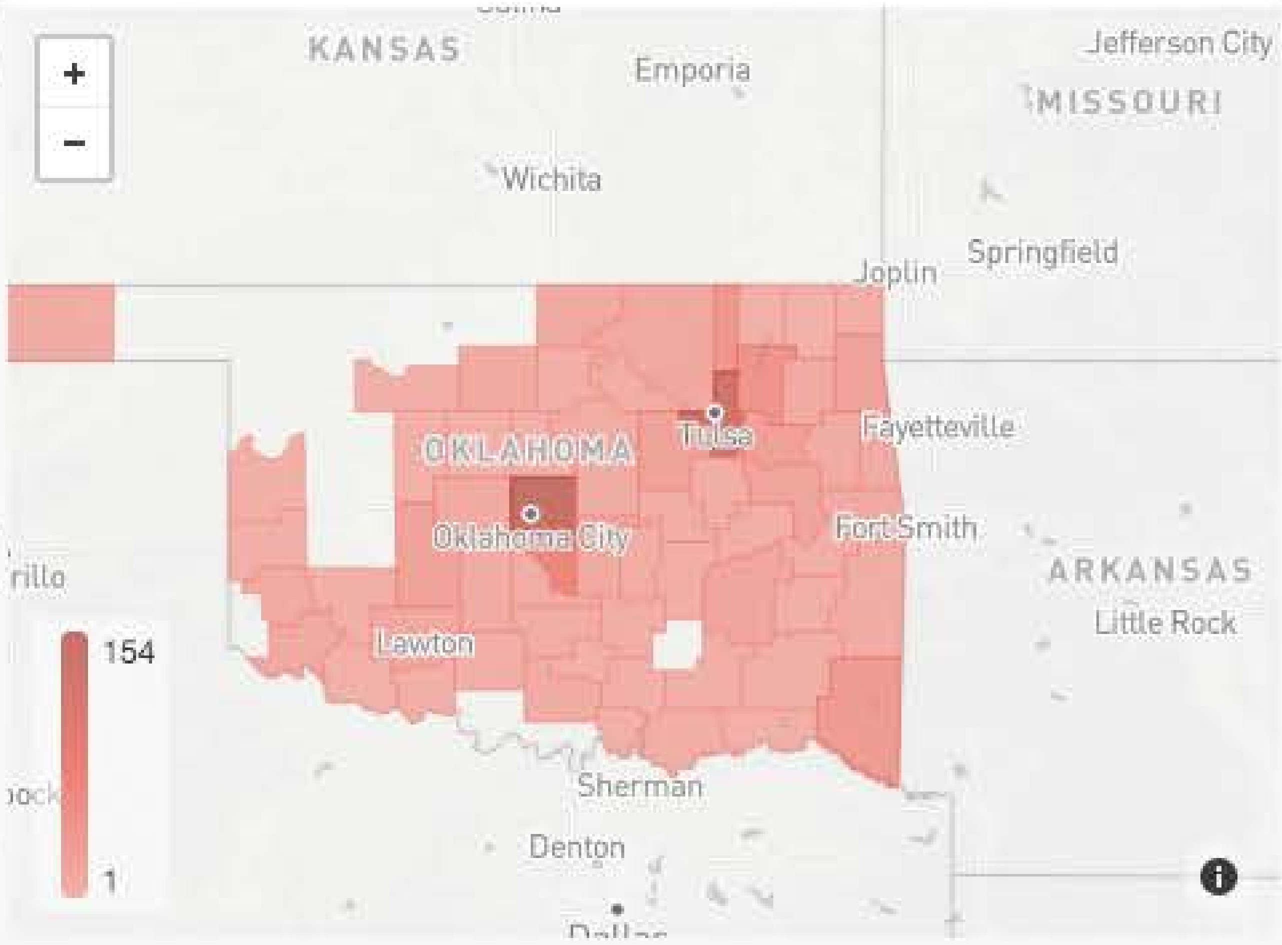

Recovered by County

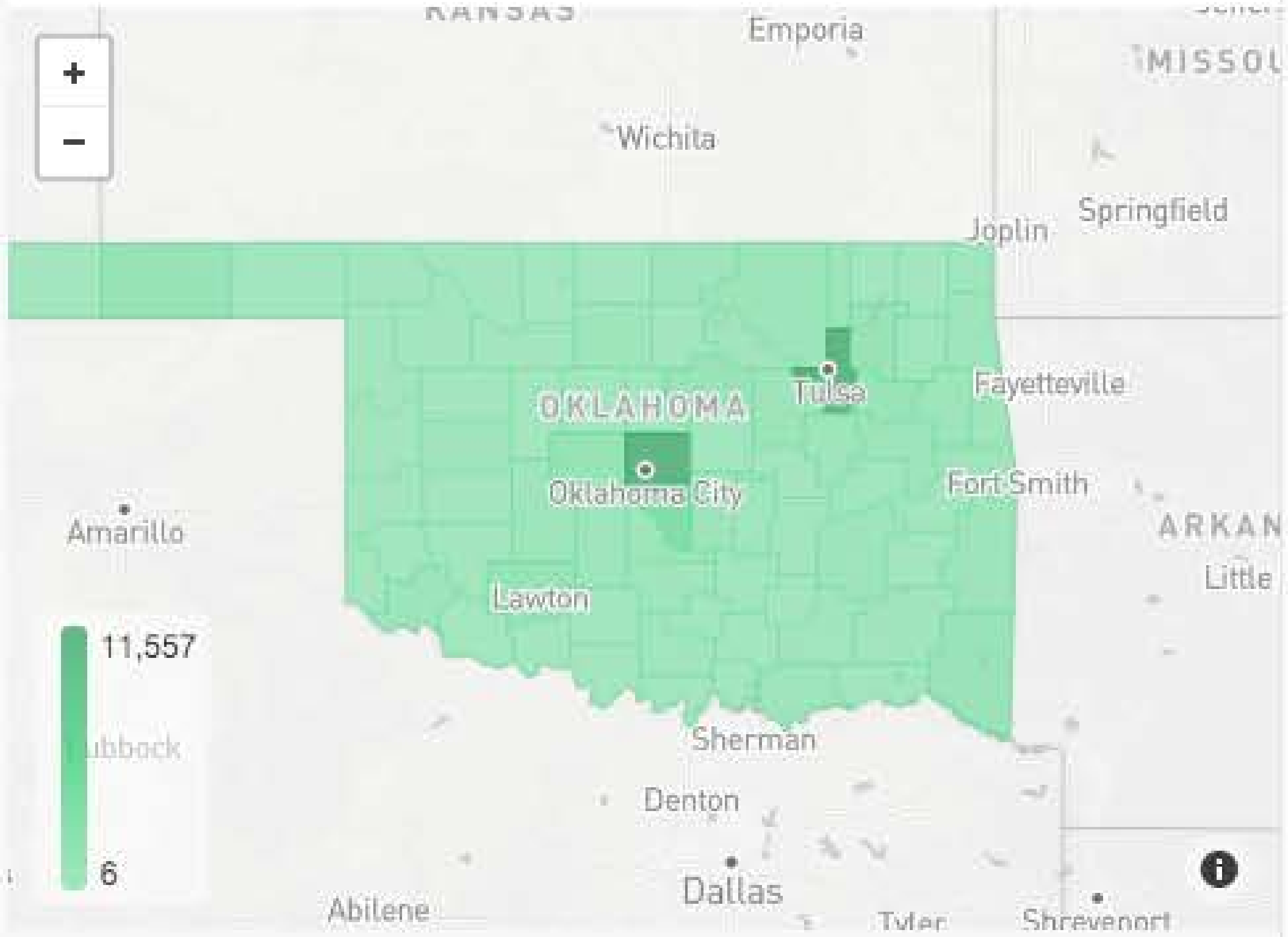

County Summary

Patient County	Total Cases	Total Deaths	Total Recovered
Rogers	1,302	41	1,073
Comanche	1,218	11	1,125
Texas	1,143	7	1,081
Wagoner	1,127	23	971
Payne	1,096	5	831
Mccurtain	1,006	29	825
Muskogee	949	16	550
Garfield	932	12	619
Pottawatomie	829	9	608
Creek	796	21	687
Washington	780	39	672
Cherokee	648	6	490
Osage	629	12	559
Le Flore	620	5	457
Okmulgee	613	4	511
Bryan	592	3	516
Jackson	582	9	545
**Totals**	57,353	797	48,607

County Summary

Patient County	Total Cases	Total Deaths	Total Recovered
Adair	438	8	336
Alfalfa	12	0	6
Atoka	108	1	90
Beaver	44	0	41
Beckham	124	1	106
Blaine	73	1	46
Bryan	592	3	516
Caddo	560	20	455
Canadian	1,509	11	1,301

Powered by looker

COVID-19 Cases - Deaths Summary

just now

View Data Downloads

Oklahoma Total Deaths by Race

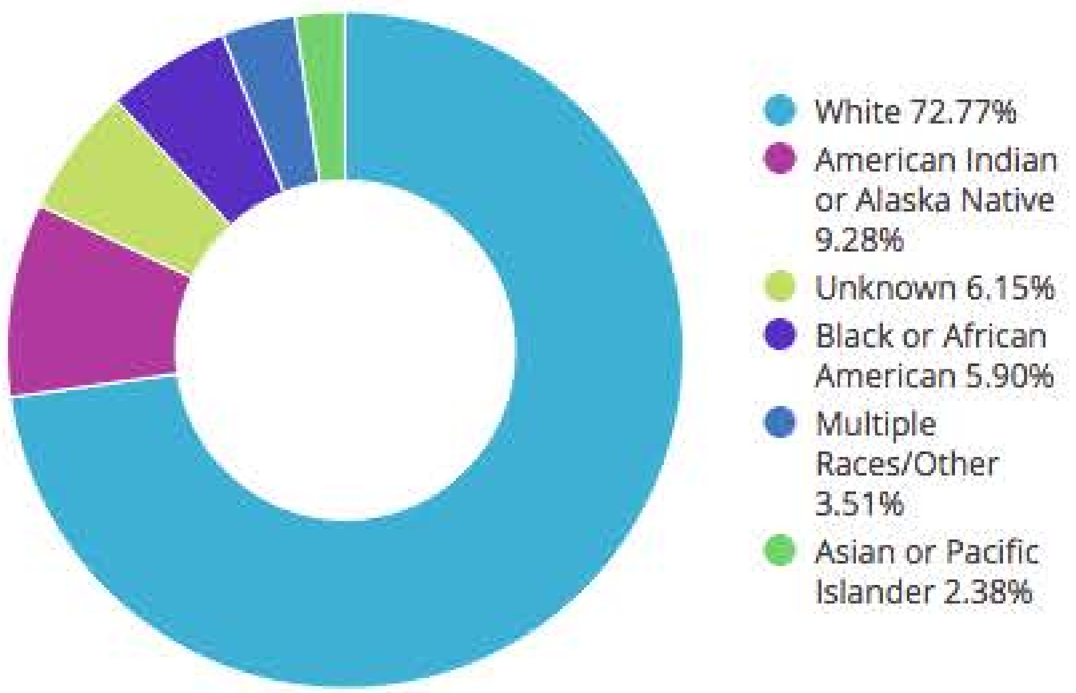

Oklahoma Total Deaths by Age Group

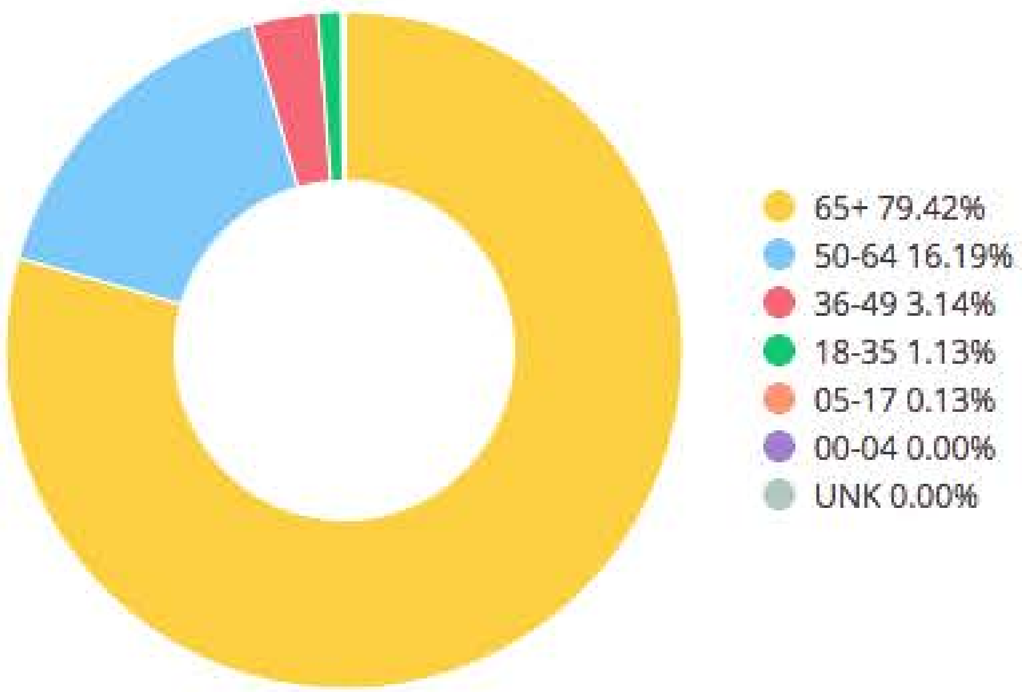

Oklahoma Total Deaths by Gender

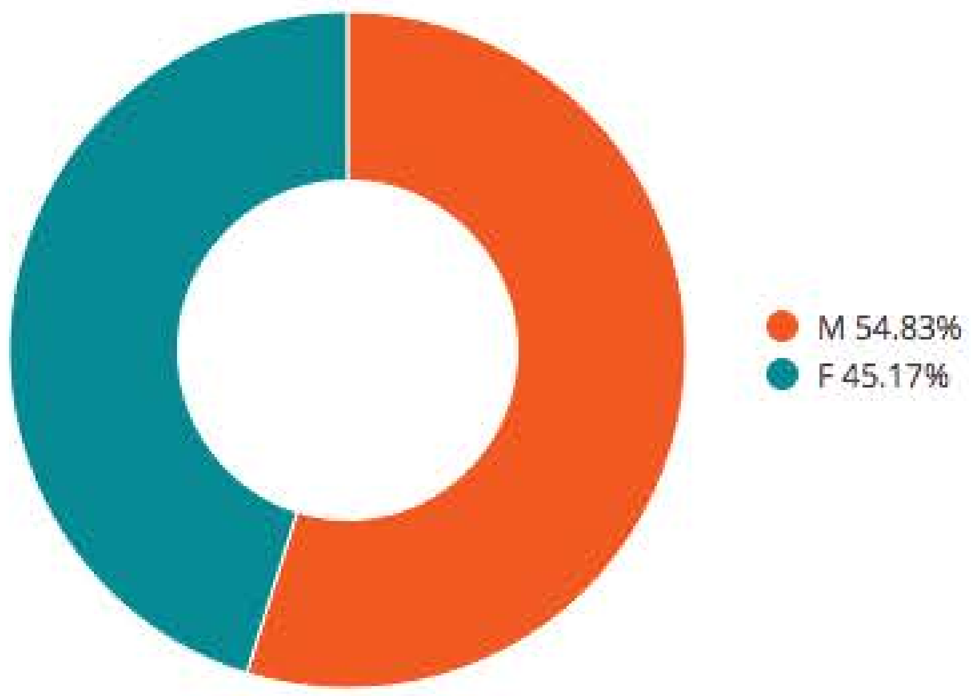

Deaths by County

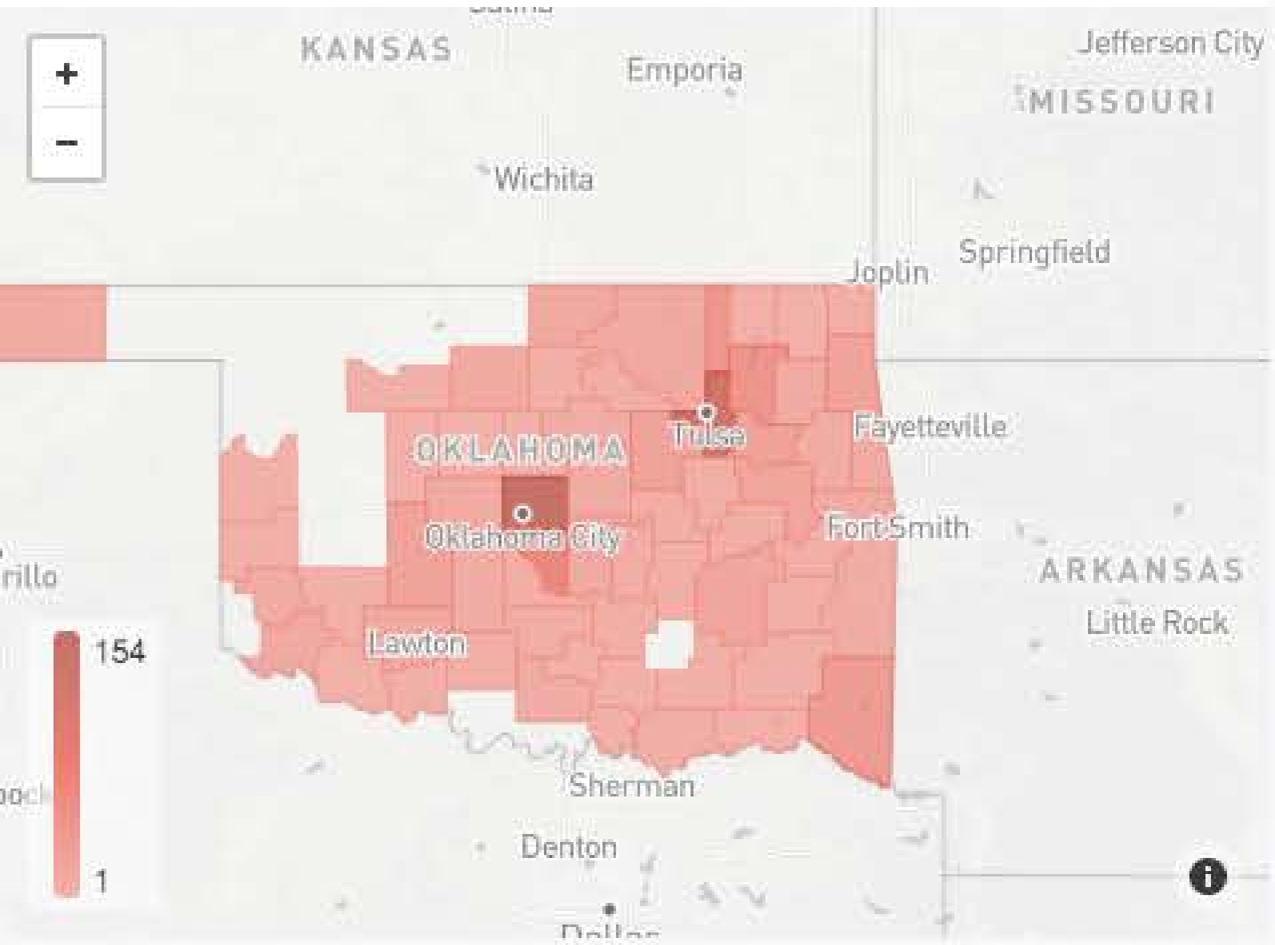

Deaths by City

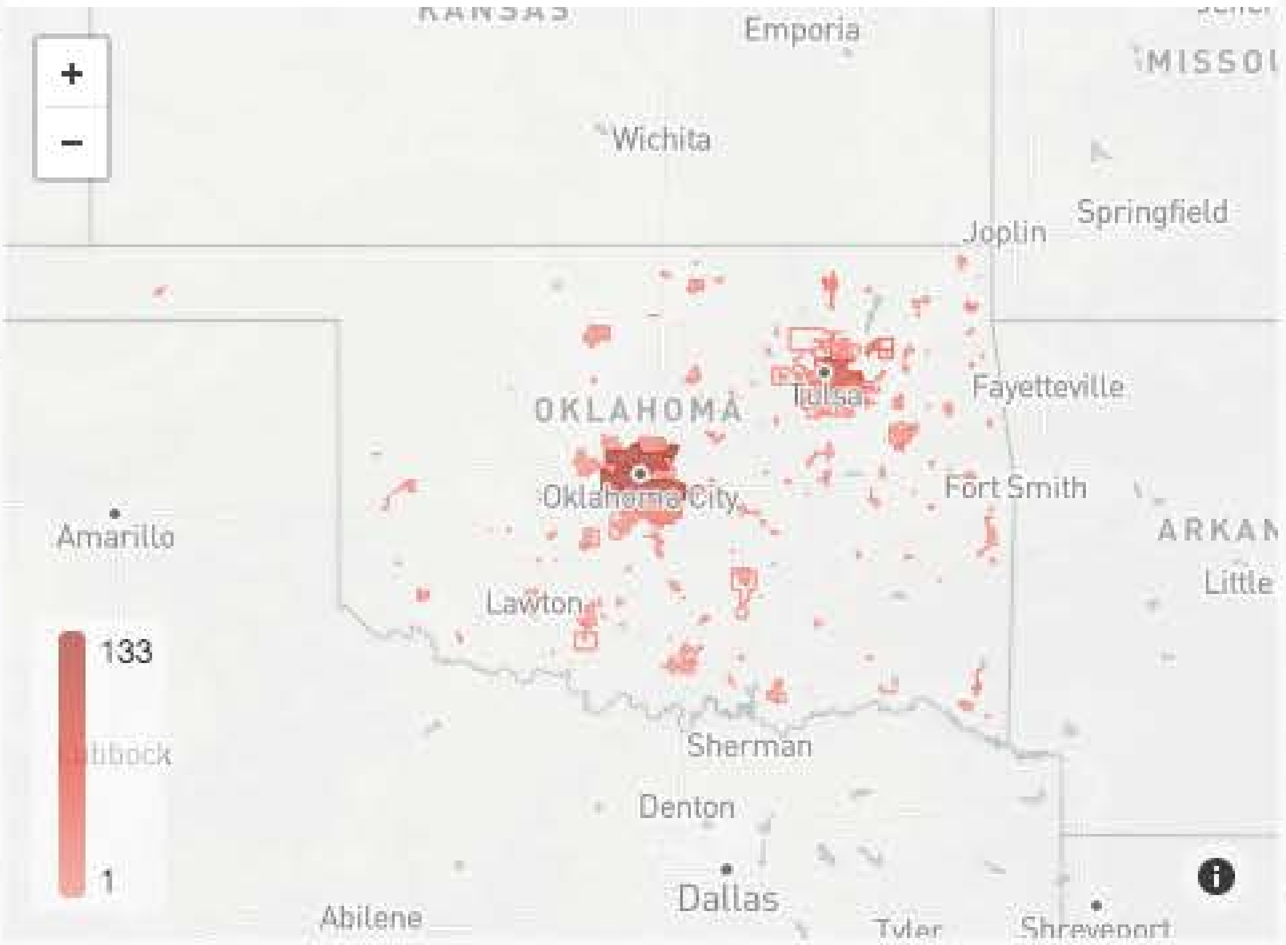

Deaths by Zip

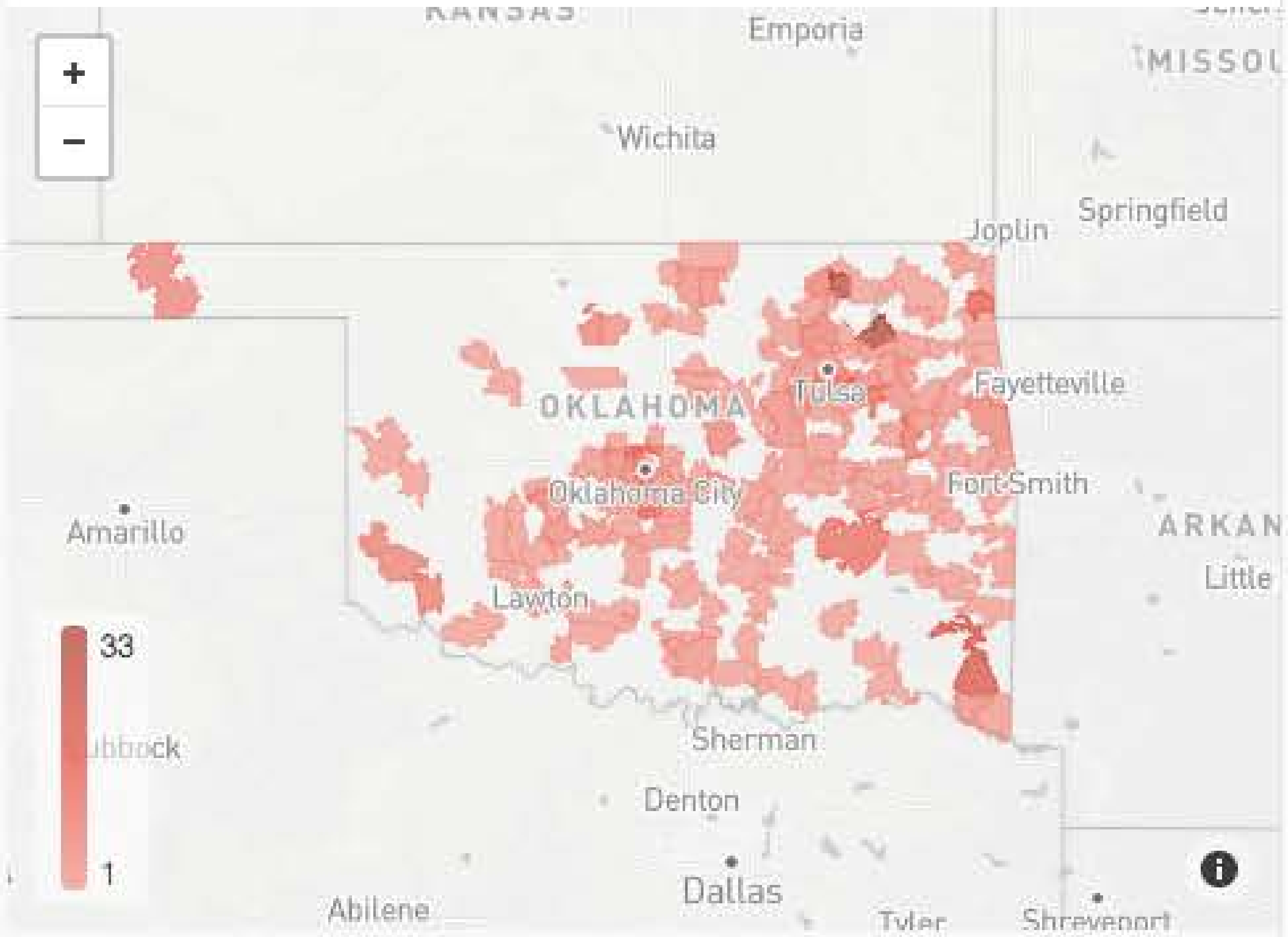

County Summary

Patient Cou...	Total Deaths
Oklahoma	154
Tulsa	132
Cleveland	63
Rogers	41
Washington	39
Mccurtain	29
Wagoner	23
Delaware	21
Creek	21
Caddo	20
Muskogee	16
Pittsburg	15
Garfield	12
Kay	12

City Summary

Patient City	Total Deaths
Oklahoma City	133
Tulsa	94
Claremore	38
Bartlesville	37
Norman	34
Broken Arrow	27
Broken Bow	22
Edmond	20
Grove	17
Mcalester	14
Coweta	13
Moore	13
Enid	12
Muskogee	12

Zip Summary

Patient Zip	Total Deaths
74017	33
74006	29
74728	21
73013	16
74344	16
74012	16
74136	15
73072	14
Other***	14
73120	13
73160	13
74429	13
74501	12
73170	12

Powered by looker

+ableau public GALLERY AUTHORS BLOG RESOURCES ACTIVITY ABOUT SIGN UP SIGN IN

< Oregon Health Authority COVID-19 - Profile ☆ Favorite

Oregon Health Authority

The Oregon Health Authority (OHA) is part of the statewide response working to address and reduce the effects of COVID-19 on the lives of Oregonians. Data presented here are from the OHA Public Health Division.

OHA COVID-19 Website

Contact Us

Oregon's COVID-19 Update

New cases and deaths are reported from the previous day.
NOTE: Monday's report includes weekend data.

Case and Testing | Hospital Capacity | Emergency Department

301 new cases*

9 new deaths

Data current as of 12:01am 8/28/2020

Oregon COVID-19 Testing Results as Reported to OPERA†

Cumulative COVID-19 Testing in Oregon

Positive	24,776
Negative	517,813
Total Tested	542,589
Total Deaths	447

Testing at Oregon State Public Health Lab

Specimens received yesterday	389
Test results released yesterday	27
Specimens pending as of 8:00 PM yesterday	808
Total Tested at OSPHL	21,257

Age, Sex and Hospitalization Status for Confirmed and Presumptive COVID-19 Cases (Cumulative)

Cases by Age Group

0 to 9	1,213 (4.7%)
10 to 19	2,674 (10.3%)
20 to 29	5,635 (21.6%)
30 to 39	4,596 (17.6%)
40 to 49	4,293 (16.5%)
50 to 59	3,320 (12.7%)
60 to 69	2,128 (8.2%)
70 to 79	1,265 (4.9%)
80+	909 (3.5%)
Not available	21 (0.1%)
Total	26,054 (100.0%)

Cases by Sex (as reported to OHA)

Female	13,503 (51.8%)
Male	12,477 (47.9%)
Non-binary	3 (0.0%)
Not available	71 (0.3%)
Total	26,054 (100.0%)

Hospitalized During Illness?

Yes	2,108 (8.1%)
No	21,158 (81.2%)
Not available	2,788 (10.7%)
Total	26,054 (100.0%)

Race, Ethnicity, Select Symptoms and Risk Factors for Confirmed and Presumptive COVID-19 Cases

Cases by Race

White	10,416 (40.0%)
Black	898 (3.4%)
Asian	730 (2.8%)
American Indian/Alaska Native	629 (2.4%)
Pacific Islander	505 (1.9%)
Other	9,115 (35.0%)
Multiracial	494 (1.9%)
Not available	3,267 (12.5%)
Total	26,054 (100.0%)

Health Care Worker?

Yes	2,587 (9.9%)
No	19,653 (75.4%)
Not available	3,814 (14.6%)
Total	26,054 (100.0%)

Congregate Setting‡

Yes	3,371 (12.9%)
No	19,076 (73.2%)
Not available	3,607 (13.8%)
Total	26,054 (100.0%)

Cases by Ethnicity

Hispanic	10,438 (40.1%)
Not Hispanic	12,401 (47.6%)
Not available	3,215 (12.3%)
Total	26,054 (100.0%)

The Oregon COVID-19 epidemiological curve is available on the OHA COVID-19 webpage in the "Case and Testing Counts" Tableau Dashboard. Please click the orange box to the right to be redirected.

Oregon Epi Curve

Notes

Data are provisional and subject to change. *Monday's report includes new cases and deaths reported between 12:01am Friday - 12:01am Monday. Monday's 'Testing at OSPHL' numbers are summarized from the entire weekend.*
*Total cases include both presumptive and confirmed cases. Presumptive cases are people without a positive diagnostic test who have COVID-19-like symptoms and had close contact with a labratory confirmed case.
†OPERA is the state of Oregon's electronic disease surveillance system for COVID-19.
‡Congregate settings include, but are not limited to, long-term care facilities, group homes, shelters, prisons, etc.

+ableau

Oregon COVID-19 Update

Viz Author: Oregon Health Authority COVID-19

Oregon Health Authority's daily update on COVID-19 cases, hospital capacity, and emergency department visits statewide. #Oregon #OHA #COVID19 #coronavirus #PublicHealth

367,293 Views ☆ 9

More Detail

Originally Published:	Jun 29, 2020
Last Updated:	Aug 28, 2020
Workbook Details:	3 Sheets
Metadata:	Case and Testing<br>Hospital Capacity<br>Emergency Department

Oregon Health Authority

The Oregon Health Authority (OHA) is part of the statewide response working to address and reduce the effects of COVID-19 on the lives of Oregonians. Data presented here are from the OHA Public Health Division.

OHA COVID-19 Website

Summary Table

Contact Us

Total Cases	Deaths	Recovered Cases	Positive Tests	Negative Tests	Total Tests
26,054	447	4,884	24,776	517.8K	542.6K

Oregon's Epi Curve | View total persons tested

Data current as of 12:01am 8/28/2020

Oregon's Epi Curve: COVID-19 cases

This chart shows the number of Oregonians who have been identified as COVID-19 cases and whether they were ever hospitalized for their illness.‡

Total Cases	Hospitalized	Not Hospitalized	Hospitalization Status Unknown
26,054	2,108	21,158	2,788

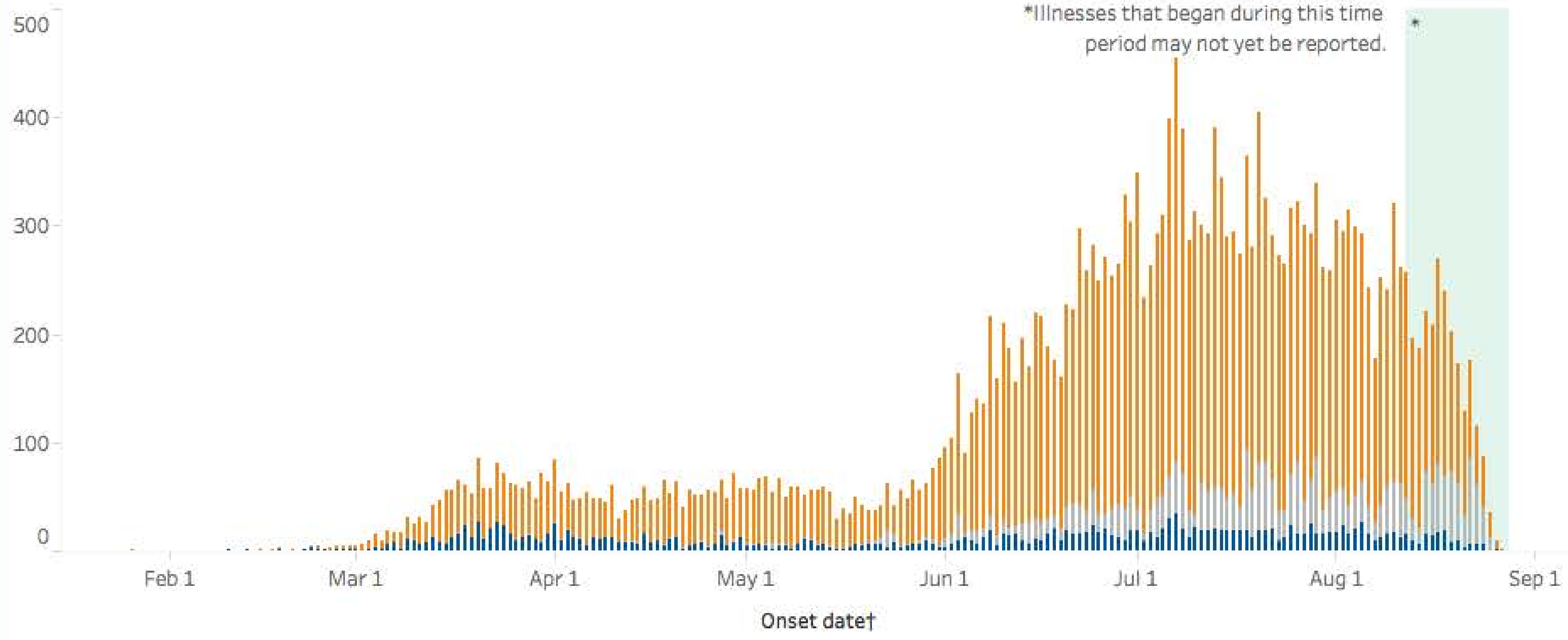

Enter or drag the cursors to change the onset date range.

January 26, 2020 — August 27, 2020

Not Hospitalized | Hospitalized | Hospitalization Status Unknown

When people say we want to "flatten the curve," this is the curve they are talking about.

We want to slow the number of new cases of COVID-19 so our healthcare system isn't overwhelmed and can provide care to everyone who needs it. Staying home except for essential needs and practicing social distancing are important ways we can flatten the curve.

Please note: The light blue shaded area shows the past 17 days, and not all people who got sick during this time period have been reported yet to OHA.

Notes

† Cases are counted by onset date (the date the person started having symptoms). When a person with symptoms did not report their onset date or when a person without symptoms is tested, their onset date is calculated as 2 days before the specimen was collected for testing.

‡ COVID-19 cases include both confirmed and presumptive cases.

COVID-19 data are provisional and subject to change.

+ableau

Oregon COVID-19 Case and Testing Counts Statewide

Viz Author: Oregon Health Authority COVID-19

Oregon Health Authority's dashboard on COVID-19 case and testing counts statewide. #Oregon #OHA #COVID19 #coronavirus #PublicHealth #EpiCurve

952,962 Views ☆ 72

More Detail

Originally Published: Apr 2, 2020

Last Updated: Aug 28, 2020

Workbook Details: 2 Sheets

Metadata: COVID-19 EPI Cases
COVID-19 EPI Tested

Oregon Health Authority

The Oregon Health Authority (OHA) is part of the statewide response working to address and reduce the effects of COVID-19 on the lives of Oregonians. Data presented here are from the OHA Public Health Division.

OHA COVID-19 Website

Summary Table

Contact Us

Total Cases	Deaths	Recovered Cases	Positive Tests	Negative Tests	Total Tests
26,054	447	4,884	24,776	517.8K	542.6K

Testing and Outcomes by County | Trends in Test Positivity

Data current as of 12:01am 8/28/2020

Oregon's COVID-19 Testing and Outcomes by County

This map shows the number of COVID-19 cases per 100,000 people by county in Oregon.* The number of cases in a community depends, in part, on the number of people who live there. Looking at the numbers this way helps us compare counties of different sizes more evenly than total case counts alone. Hover over a county in the map below to see the total number of cases, recovered cases, deaths, and number of people with positive and negative tests in that county. The table summarizes this information as rates and percentages for all counties.

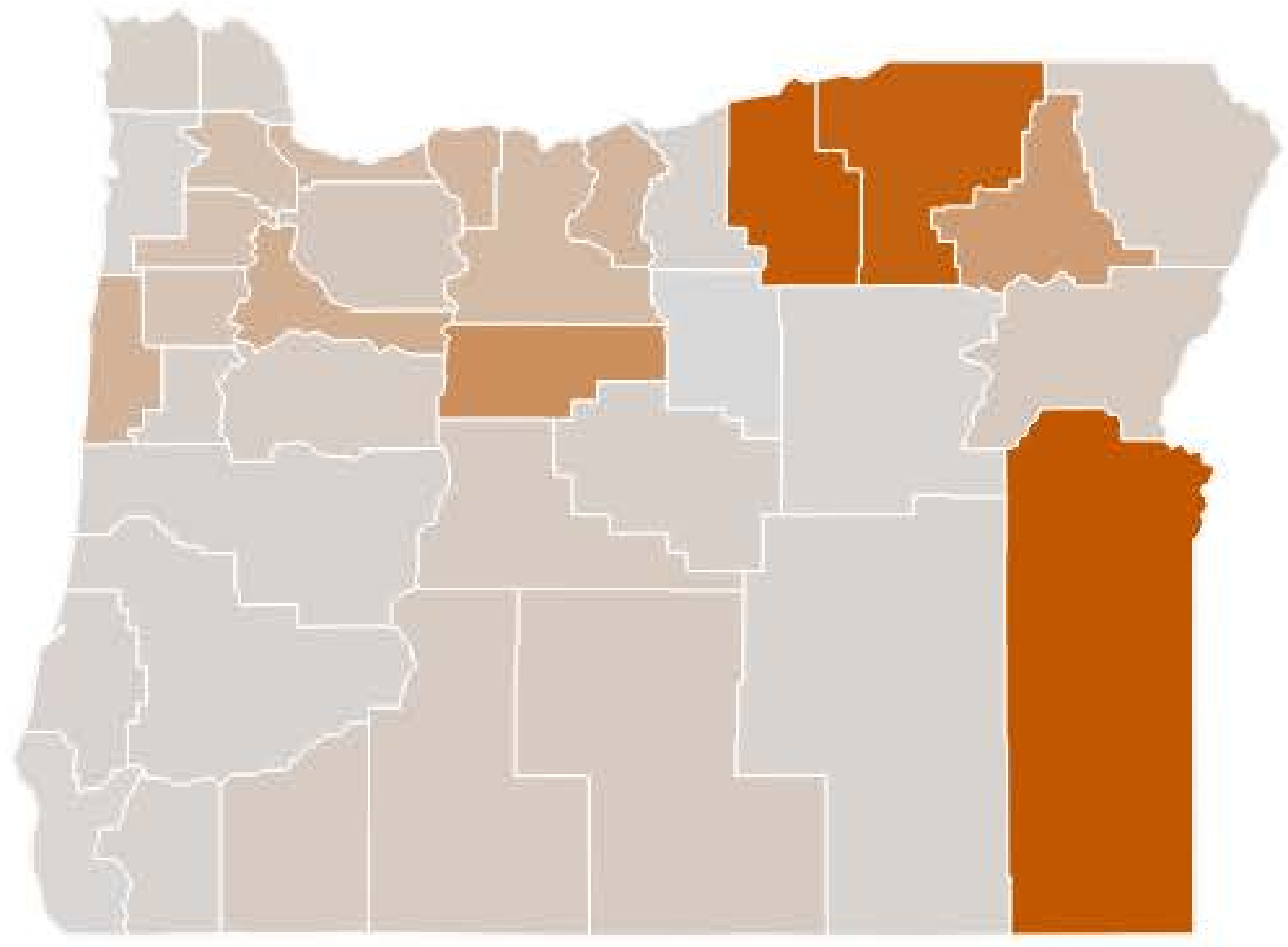

Cases per 100,000 0.0 – 3,502.9

	Case Count per 100,000	Deaths (%)	Recovered Cases (%)	Positive Tests (%)	Negative Tests (%)	Test Count per 100,000
All	615.0	2%	19%	5%	95%	12,807.8
Malheur	3,502.9	2%	6%	21%	79%	16,259.2
Morrow	3,382.5	1%	13%	22%	78%	14,901.8
Umatilla	3,223.3	1%	17%	18%	82%	17,111.9
Jefferson	1,904.2	2%	53%	9%	91%	19,155.3
Union	1,542.4	0%	11%	12%	88%	12,298.3
Marion	1,065.7	2%	15%	8%	92%	12,167.6
Lincoln	959.4	3%	7%	6%	94%	16,686.7
Sherman	902.9	0%	6%	4%	96%	17,833.0
Hood River	875.3	0%	23%	4%	96%	17,945.6
Wasco	756.3	1%	9%	4%	96%	16,290.3
Multnomah	718.2	2%	16%	5%	95%	14,760.4
Washington	602.2	1%	17%	5%	95%	12,874.1
Yamhill	568.2	2%	16%	4%	96%	13,574.7
Polk	491.9	3%	79%	5%	95%	8,865.1
Clackamas	449.7	3%	10%	4%	96%	12,225.3
Baker	404.3	3%	1%	4%	96%	8,395.8
Lake	371.2	0%	10%	3%	97%	8,538.5
Deschutes	356.0	2%	70%	3%	97%	12,552.3
Jackson	350.7	0%	28%	3%	97%	10,956.7
Klamath	343.2	1%	22%	3%	97%	13,093.2
Wallowa	307.6	5%	32%	2%	98%	11,774.6
Linn	297.9	3%	23%	3%	97%	10,871.0
Columbia	242.7	1%	20%	2%	98%	11,064.1
Clatsop	241.5	0%	21%	2%	98%	12,241.9
Crook	230.4	2%	50%	2%	98%	9,602.8

Notes

*Rates shown on this dashboard are calculated using population estimates from the 2019 Annual Population Report from Portland State University's College of Urban & Public Affairs: Population Research Center. For our calculations, we round population numbers to the nearest whole number, resulting in slight variation from the numbers in that report (within +/- 4 persons).

COVID-19 data are provisional and subject to change.

+ a b | e a u

Oregon COVID-19 Testing and Outcomes by County

Viz Author: **Oregon Health Authority COVID-19**

Oregon Health Authority's COVID-19 dashboard on testing and COVID-19 case outcomes by county. #Oregon #OHA #COVID19 #coronavirus #PublicHealth

439,677 Views ☆23

More Detail

Originally Published: May 26, 2020

Last Updated: Aug 28, 2020

Workbook Details: 2 Sheets

Metadata: Oregon's COVID-19 Testing and Outcomes by County
Oregon's COVID-19 Trends in Test Positivity

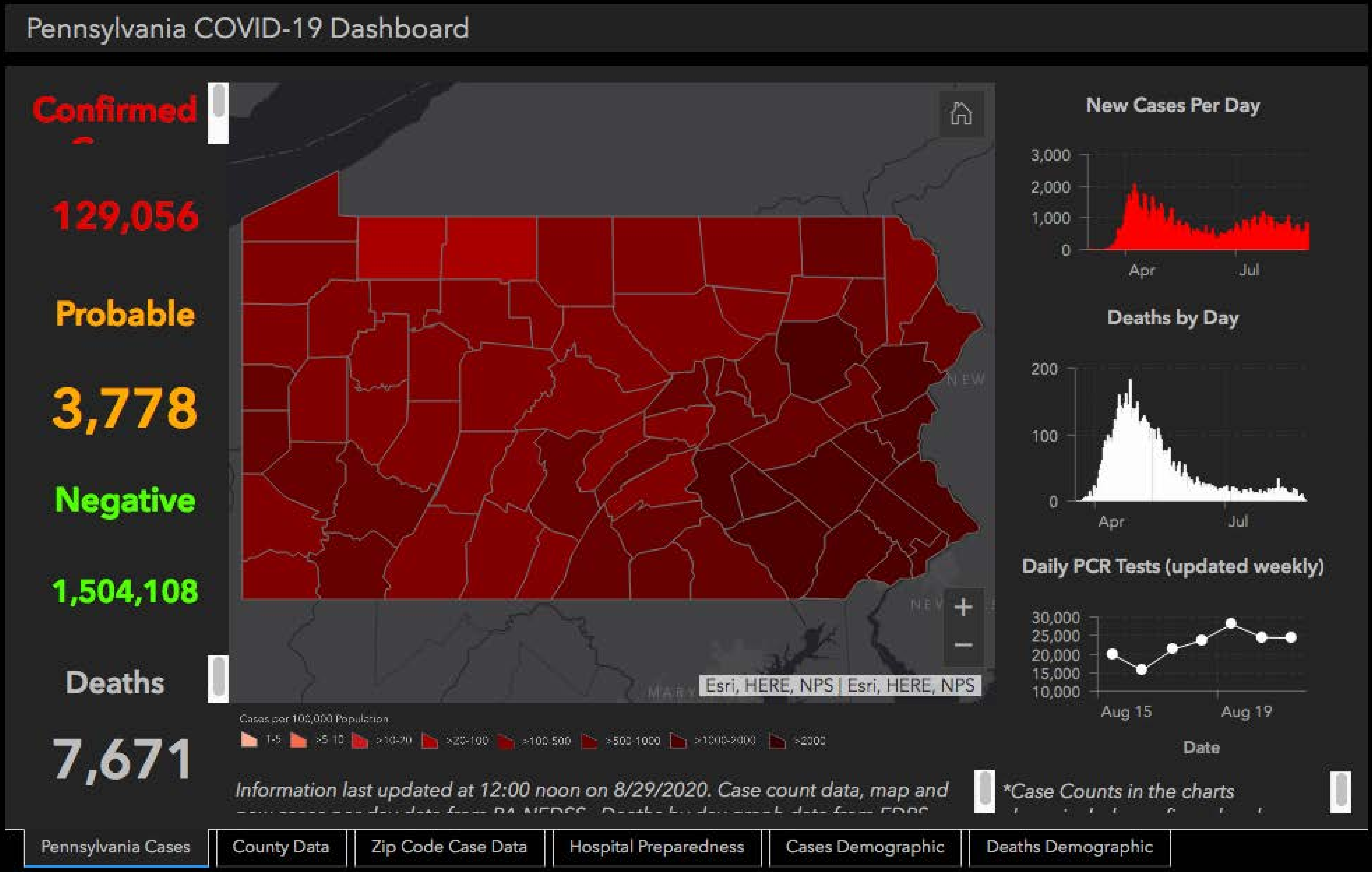

Pennsylvania COVID-19 Dashboard
Confirmed
129,056
Probable
3,778
Negative
1,504,108
Deaths
7,671
NEW
Esri, HERE, NPS | Esri, HERE, NPS
Cases per 100,000 Population
1-5
>5-10
>10-20
>20-100
>100-500
>500-1000
>1000-2000
>2000
Information last updated at 12:00 noon on 8/29/2020. Case count data, map and
New Cases Per Day
3,000
2,000
1,000
0
Apr
Jul
Deaths by Day
200
100
0
Apr
Jul
Daily PCR Tests (updated weekly)
30,000
25,000
20,000
15,000
10,000
Aug 15
Aug 19
Date
*Case Counts in the charts
Pennsylvania Cases
County Data
Zip Code Case Data
Hospital Preparedness
Cases Demographic
Deaths Demographic

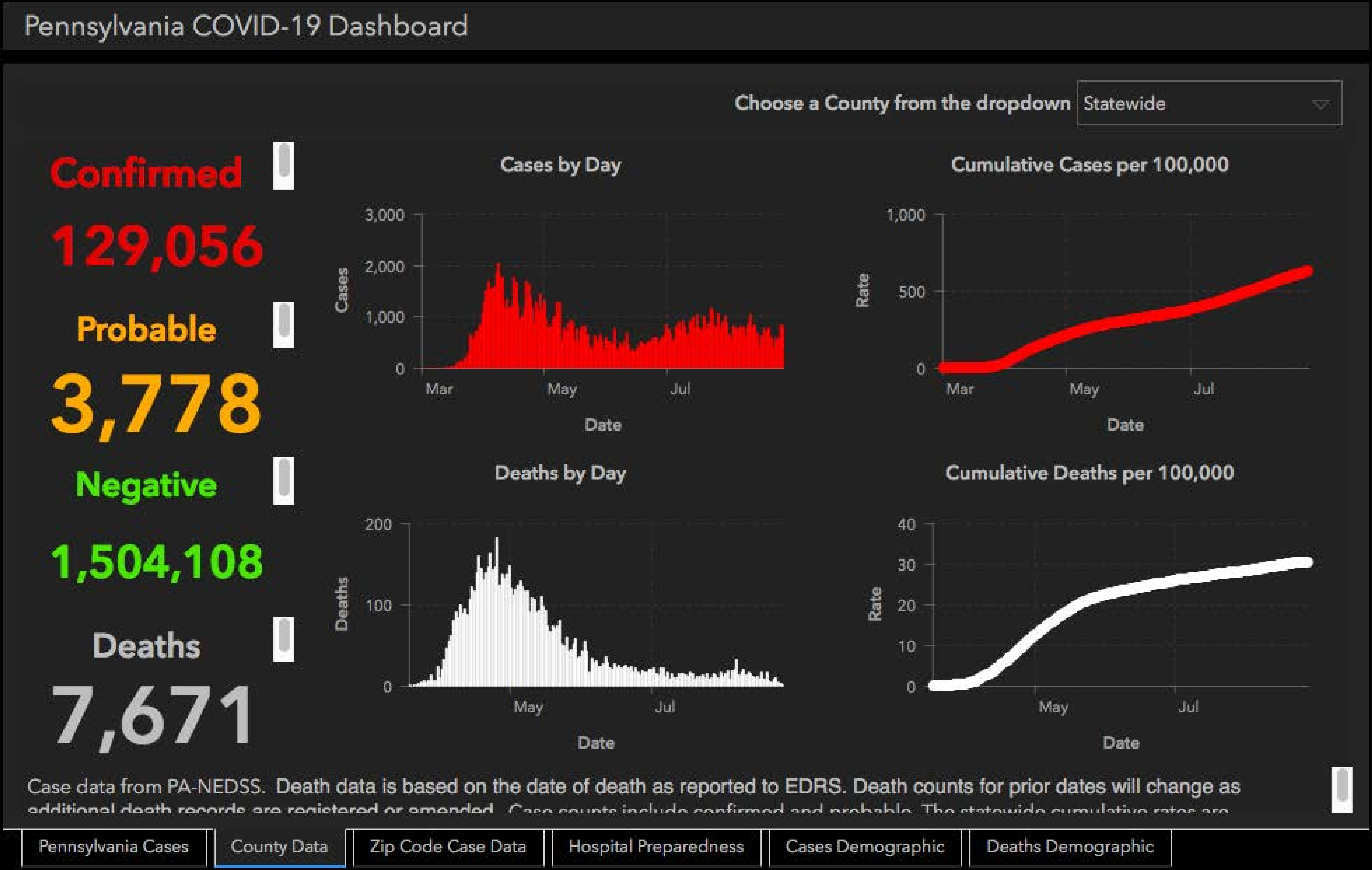
Pennsylvania COVID-19 Dashboard
Choose a County from the dropdown
Statewide
Confirmed
129,056
Probable
3,778
Negative
1,504,108
Deaths
7,671
Cases by Day
3,000
2,000
1,000
0
Cases
Mar
May
Jul
Date
Cumulative Cases per 100,000
1,000
500
0
Rate
Mar
May
Jul
Date
Deaths by Day
200
100
0
Deaths
May
Jul
Date
Cumulative Deaths per 100,000
40
30
20
10
0
Rate
May
Jul
Date
Case data from PA-NEDSS. Death data is based on the date of death as reported to EDRS. Death counts for prior dates will change as
Pennsylvania Cases
County Data
Zip Code Case Data
Hospital Preparedness
Cases Demographic
Deaths Demographic

Pennsylvania COVID-19 Dashboard

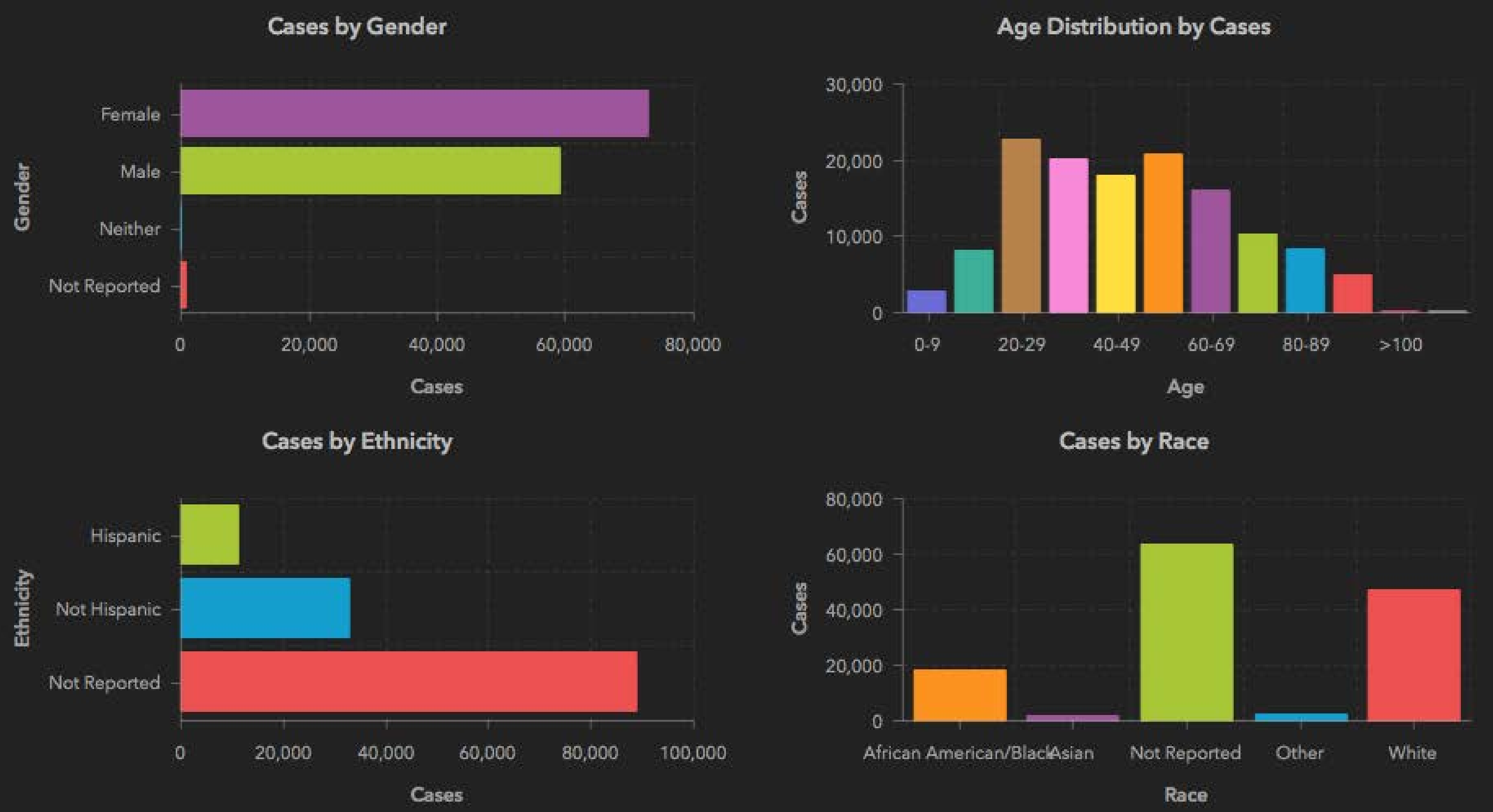

*Case Counts include confirmed and probable cases. All data from PA-NEDSS

Pennsylvania Cases | County Data | Zip Code Case Data | Hospital Preparedness | Cases Demographic | Deaths Demographic

Rhode Island Department of Health COVID-19 Data Tracker by Date

Updated Monday through Friday by 12pm

Aug 28, 2020

Total Tests

504,904

Total Positive Cases

21,683

Tests Prior Day

7,222

New Positive Cases

58

Percent Positive Prior Day

0.8%

Total Fatalities

1,046

New Fatalities

2

New Positive Cases by Date and 3-day Average

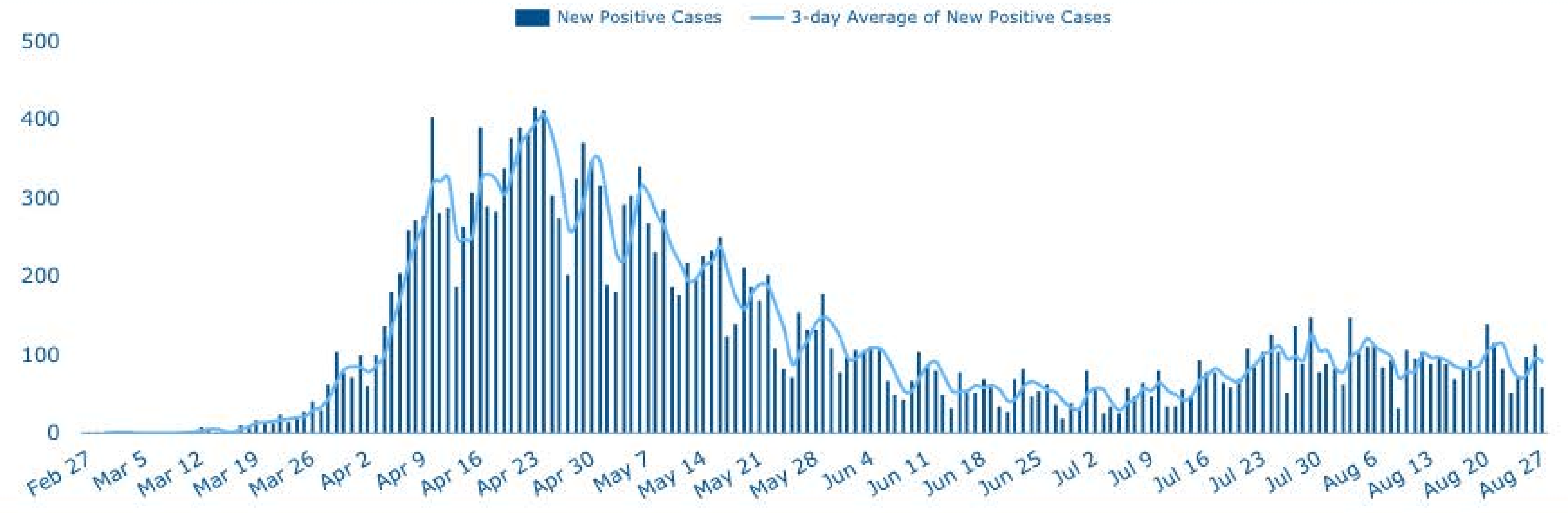

People Hospitalized by Date and 3-day Average

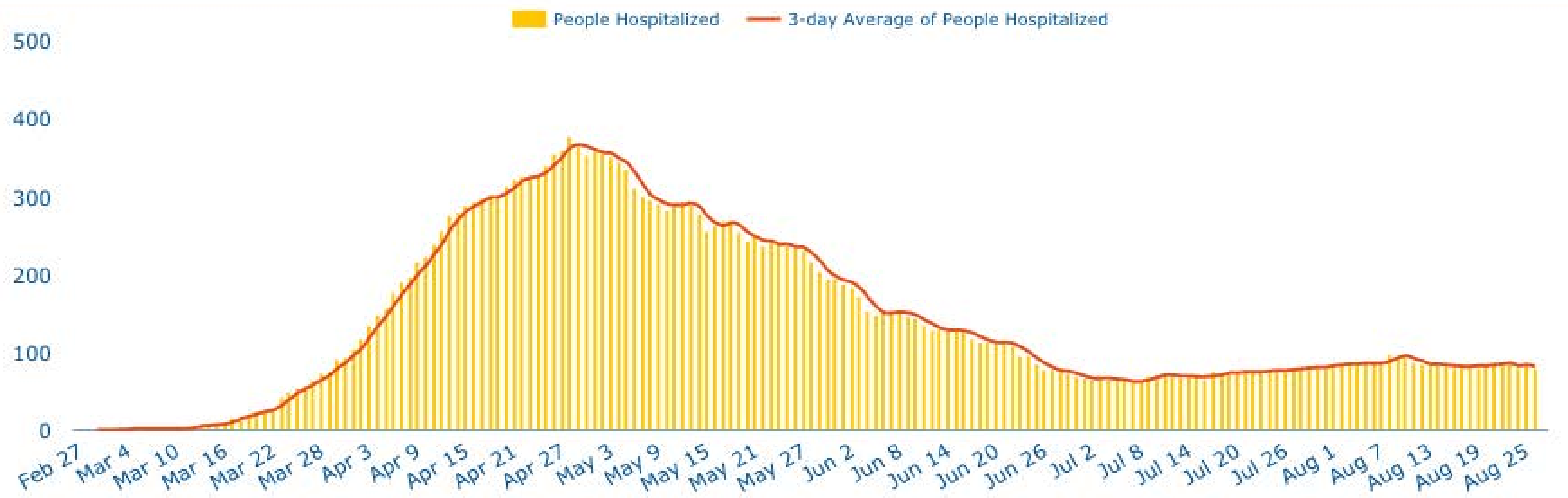

3-day averages are an average of the reported day and the two days prior.

Hospitalization Data

People Hospitalized	People in ICU	People on a Ventilator	3-day Average of People Hospitalized
81	8	4	83

Google Data Studio

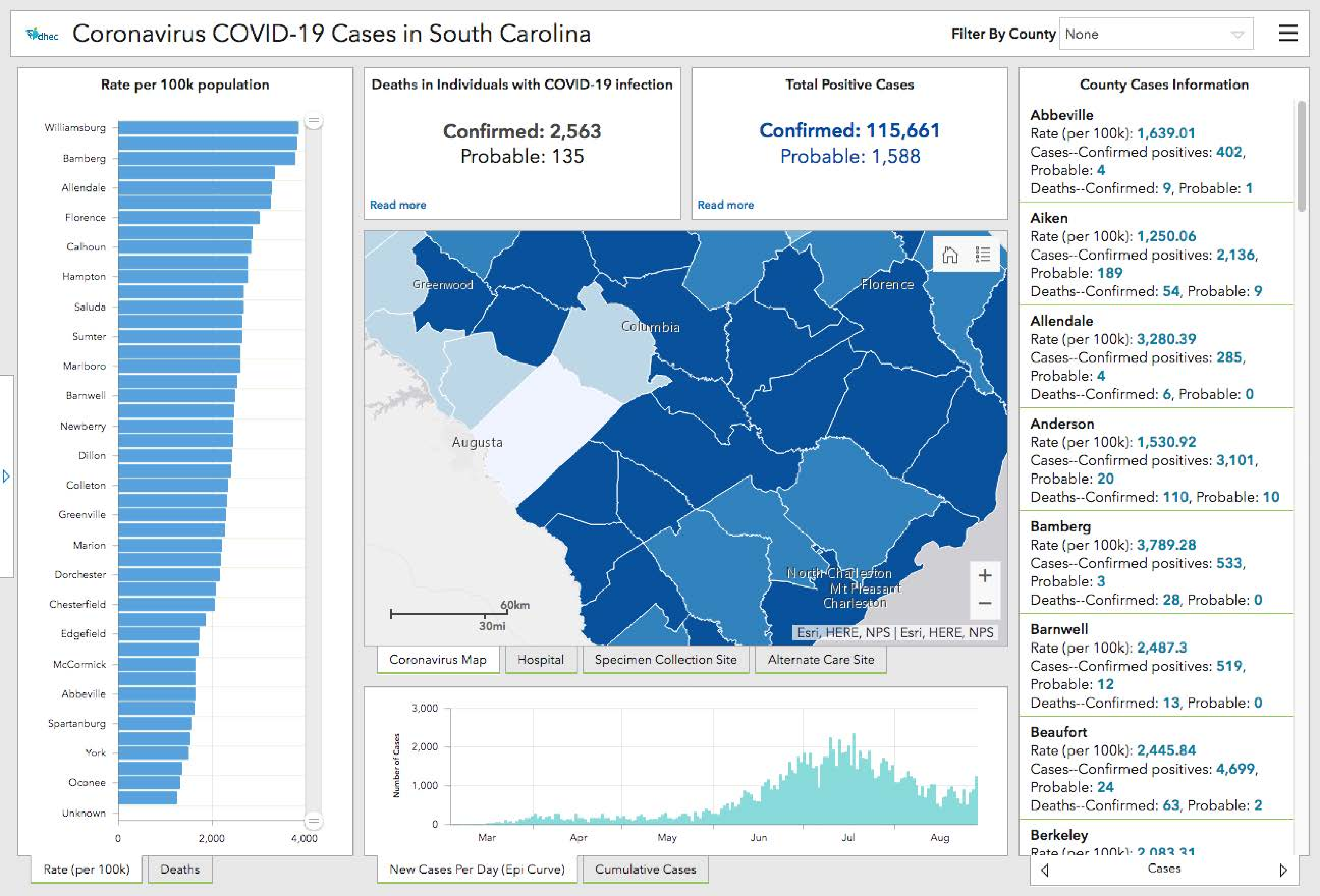

dhec
Coronavirus COVID-19 Cases in South Carolina
Filter By County
None
Rate per 100k population
Williamsburg
Bamberg
Allendale
Florence
Calhoun
Hampton
Saluda
Sumter
Marlboro
Barnwell
Newberry
Dillon
Colleton
Greenville
Marion
Dorchester
Chesterfield
Edgefield
McCormick
Abbeville
Spartanburg
York
Oconee
Unknown
0
2,000
4,000
Rate (per 100k)
Deaths
Deaths in Individuals with COVID-19 infection
Confirmed: 2,563
Probable: 135
Read more
Total Positive Cases
Confirmed: 115,661
Probable: 1,588
Read more
Greenwood
Columbia
Florence
Augusta
North Charleston
Mt Pleasant
Charleston
60km
30mi
Esri, HERE, NPS | Esri, HERE, NPS
Coronavirus Map
Hospital
Specimen Collection Site
Alternate Care Site
3,000
2,000
1,000
0
Number of Cases
Mar
Apr
May
Jun
Jul
Aug
New Cases Per Day (Epi Curve)
Cumulative Cases
County Cases Information
Abbeville
Rate (per 100k): 1,639.01
Cases--Confirmed positives: 402, Probable: 4
Deaths--Confirmed: 9, Probable: 1
Aiken
Rate (per 100k): 1,250.06
Cases--Confirmed positives: 2,136, Probable: 189
Deaths--Confirmed: 54, Probable: 9
Allendale
Rate (per 100k): 3,280.39
Cases--Confirmed positives: 285, Probable: 4
Deaths--Confirmed: 6, Probable: 0
Anderson
Rate (per 100k): 1,530.92
Cases--Confirmed positives: 3,101, Probable: 20
Deaths--Confirmed: 110, Probable: 10
Bamberg
Rate (per 100k): 3,789.28
Cases--Confirmed positives: 533, Probable: 3
Deaths--Confirmed: 28, Probable: 0
Barnwell
Rate (per 100k): 2,487.3
Cases--Confirmed positives: 519, Probable: 12
Deaths--Confirmed: 13, Probable: 0
Beaufort
Rate (per 100k): 2,445.84
Cases--Confirmed positives: 4,699, Probable: 24
Deaths--Confirmed: 63, Probable: 2
Berkeley
Rate (per 100k): 2,083.31
Cases

Interactive: COVID-19 curves for each South Carolina county

by Nicholas Papantonis | Monday, March 16th 2020

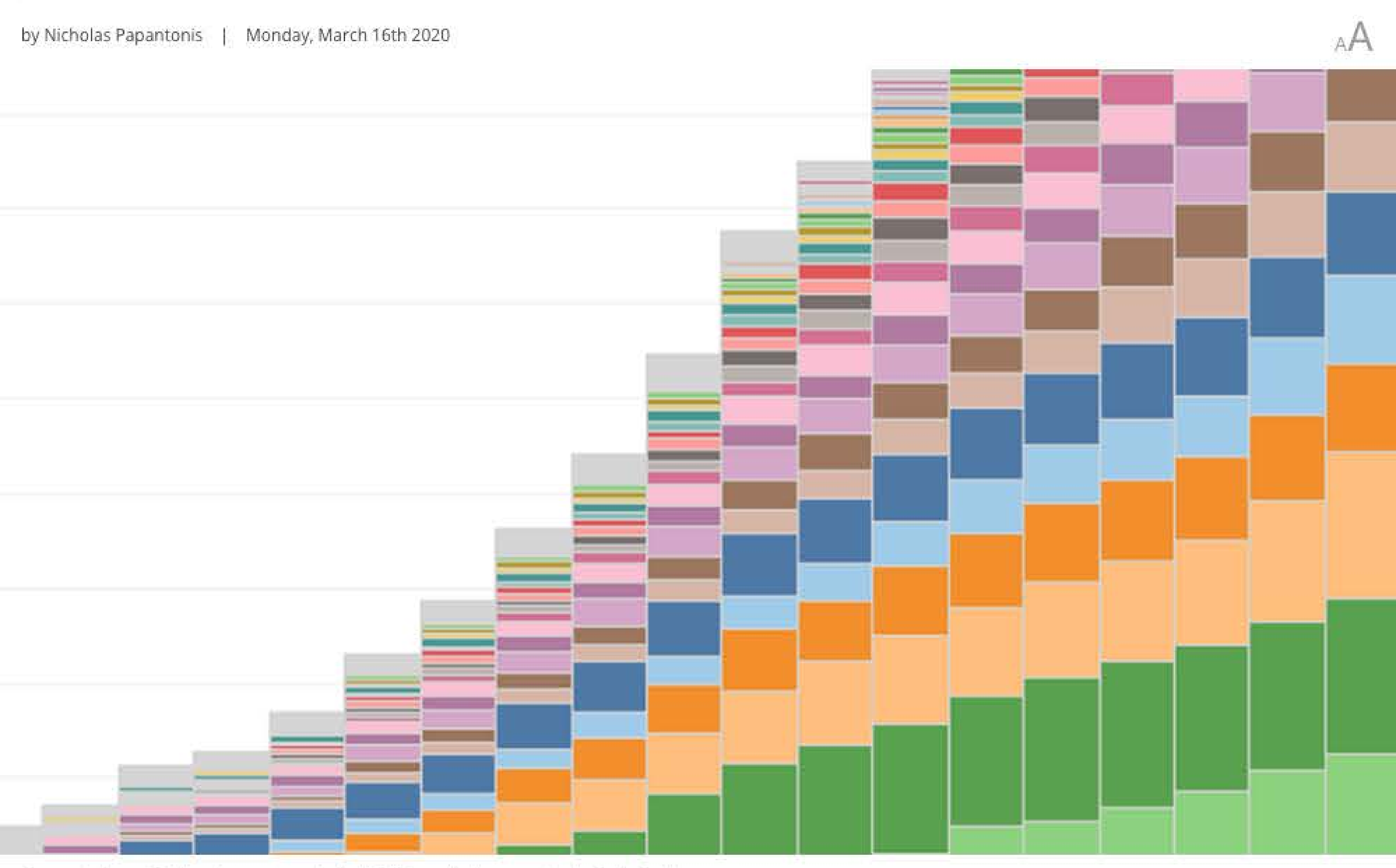

Screen shot from a Tableau chart put together by ABC 15 to track the coronavirus in South Carolina.

CONWAY, S.C. (WPDE) — Welcome to the COVID-19 data center, where you can track and compare efforts to flatten the curve in South Carolina and each of its counties.

A quickly spreading virus can overwhelm the state's health care system, forcing doctors and nurses to choose which patients to care for as resources like beds and ventilators run scarce.

By keeping people relatively isolated from each other, the virus has a more difficult time transmitting from one person to another. The practice and effects were simulated by The Washington Post.

You can check out the current number of cases and deaths in South Carolina (and along the North Carolina border belt) in an interactive map updated with each new case.

DHEC epidemiologists warn the number of confirmed cases is far fewer than the actual number of cases, since so many people are asymptomatic or only experience minor symptoms.

You can see a break down of the daily state tally in this chart below.

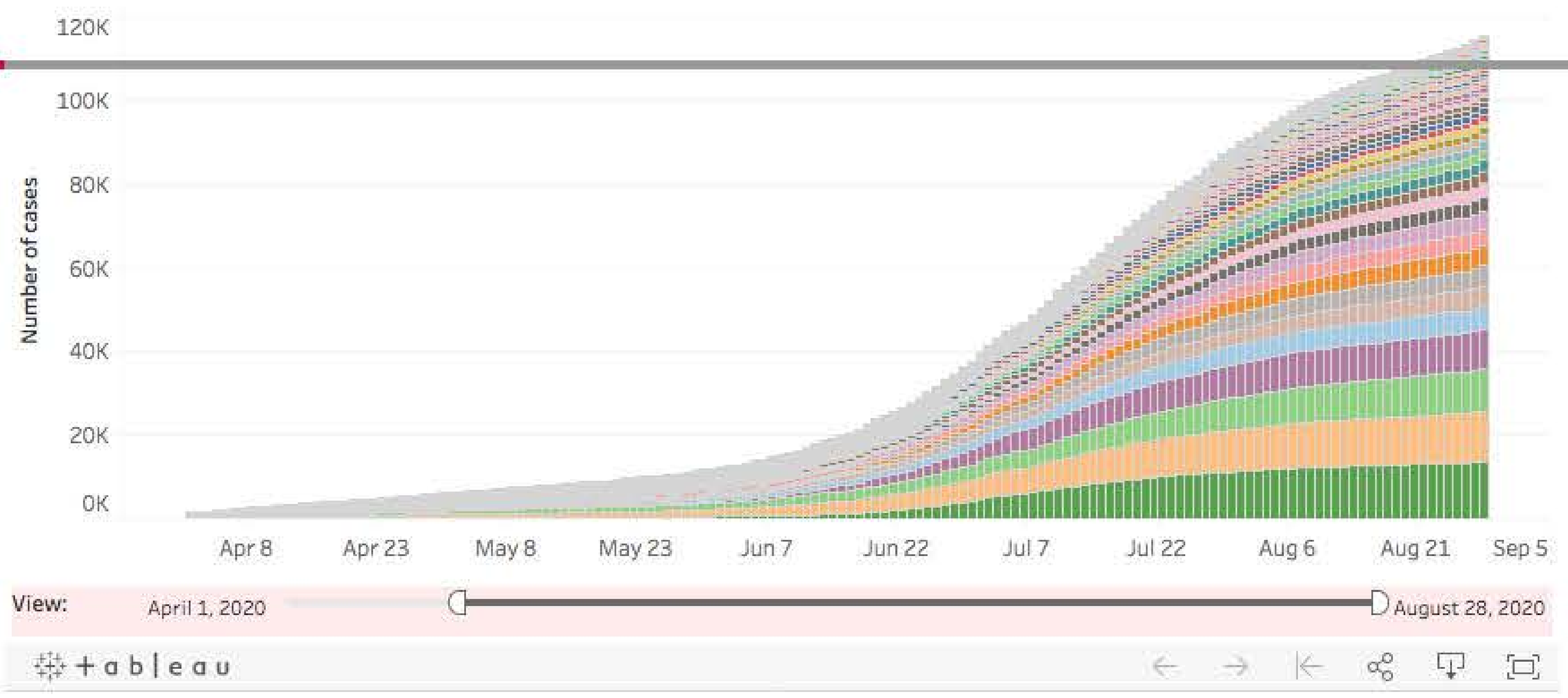

Can't see the chart on mobile? Tap here.

Next, we can see how each county is managing its curve.

Of course, smaller, more rural counties tend to have fewer cases, and gain new cases slower than larger, urban counties. That makes for a flatter curve.

You can view each county's curve over time in the third chart below. While the default view is set to all counties, you can look at each county one at a time, or compare multiple counties using the drop down to the right.

For reference, the following county pairings have similar populations:

- Horry County and Spartanburg County
- Charleston County and Richland County
- Florence County and Dorchester County
- Berkeley County and York County
- Lexington County and Spartanburg County
- Beaufort County and Anderson County
- Sumter County and Lancaster County
- Darlington, Georgetown, Laurens and Kershaw Counties

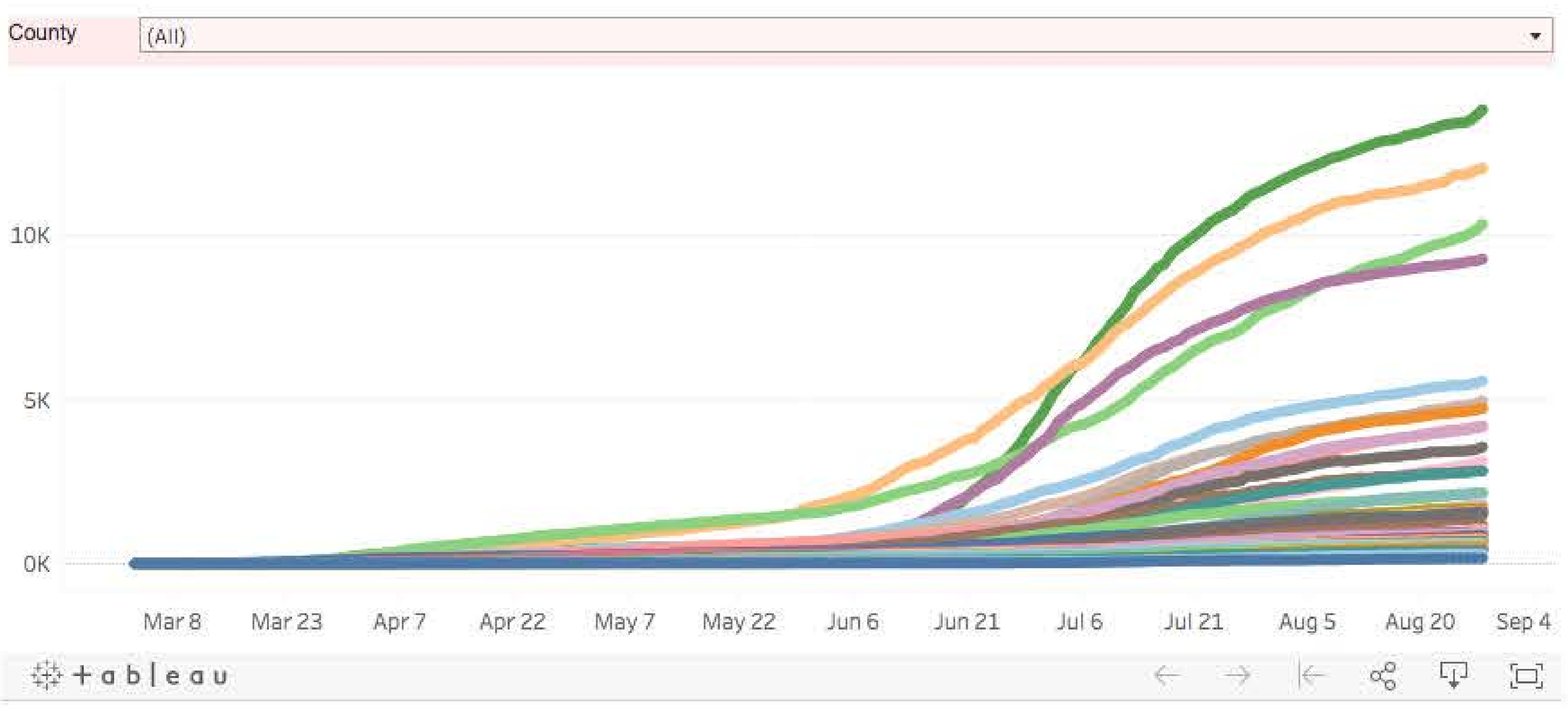

Can't see the chart on mobile? Tap here.

All of these charts show one set of data: the running totals for cases in South Carolina, which can be slightly more difficult to comprehend.

In making a decision to open or close businesses and schools, health experts and government leaders often rely on a "moving average" of the number of new cases, or an average of the past few days to smooth out the wild day-to-day swings in numbers.

The average lags behind the number of new cases, meaning that while cases are on the rise, the average appears lower than the daily case count. When cases are falling, the average appears higher. This helps by building a few days' worth of data into the trend line to prevent any sudden decisions from being made.

You can view the 7-day moving average of South Carolina's new cases, deaths and hospitalizations below:

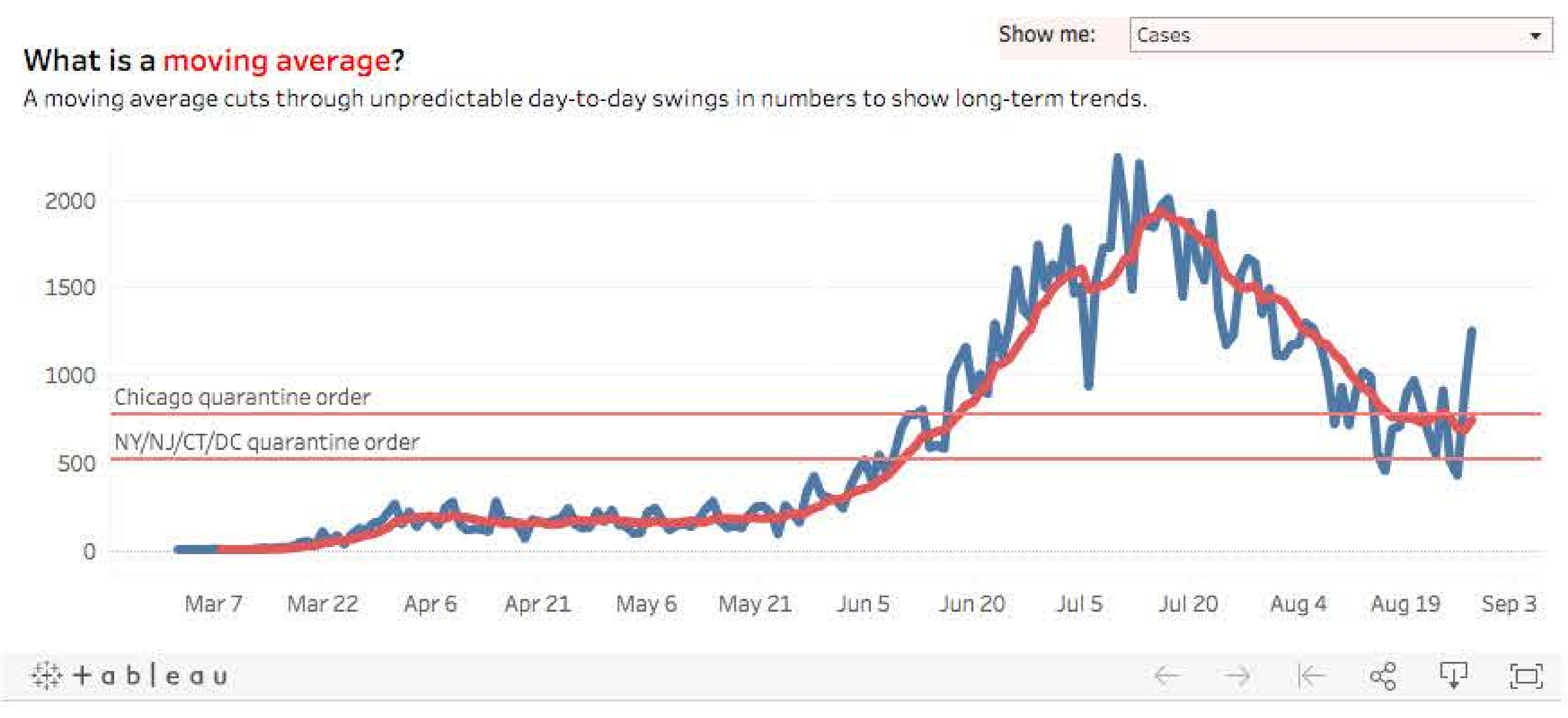

Can't see the chart on mobile? Tap here.

Related: Interactive map: tracking COVID-19 cases by county and zip code

We have also built a chart to track deaths in South Carolina over time. Because most of the counties have relatively few deaths, they appear in clusters and have fairly jagged lines.

You can use the drop down menu to filter counties individually or in small groups.

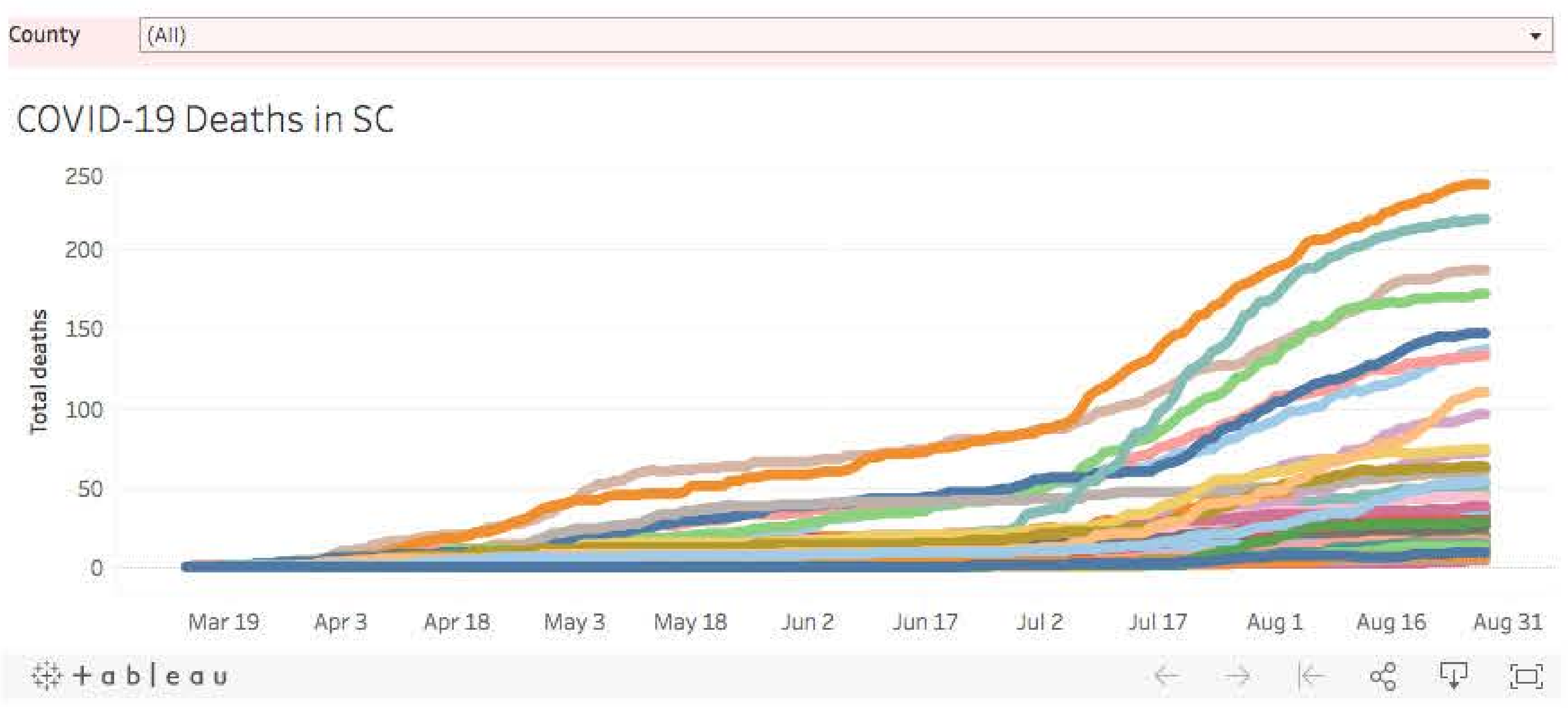

And finally, here's how the state is doing compared to the United States.

In this chart, we've scaled down the US population to 1.6% to match South Carolina's population.

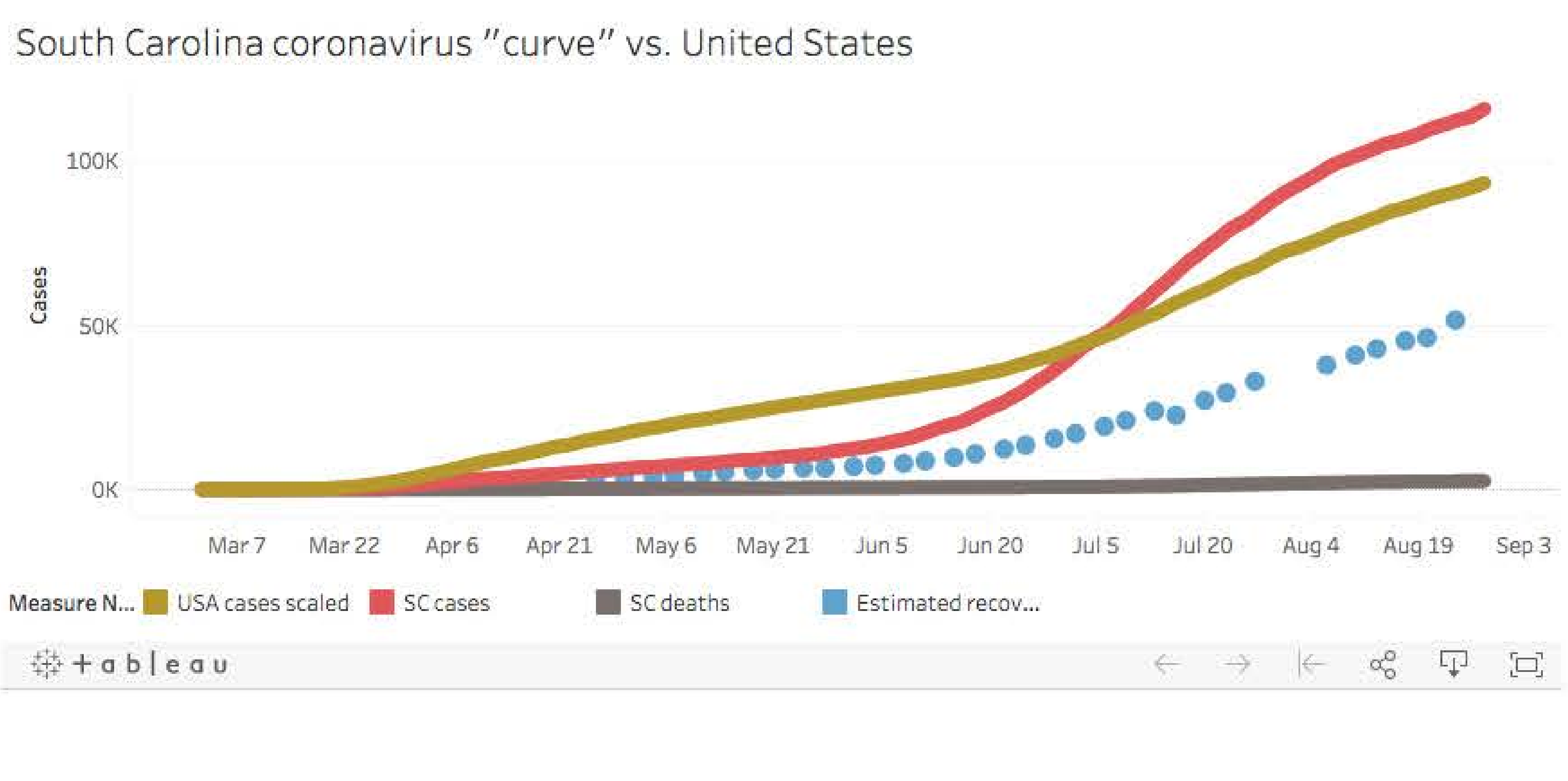

SOUTH DAKOTA DEPARTMENT OF HEALTH covid.sd.gov

NOVEL CORONAVIRUS (COVID-19) UPDATES AND INFORMATION

Updates | COVID-19 in SD
Multisystem Inflammatory Syndrome (MIS-C)
Precautions to Avoid Illness | If You Develop Symptoms
Plasma Donation | Community Guidance
Multiple Language Resources
For Medical Providers | SD Healthcare Provider Guidance
Remdesivir Distribution | SD Registries & Data Collection
CDC Healthcare Provider Guidance | CDC Website

The South Dakota Department of Health (SD-DOH) continues to respond to the emergence of the novel coronavirus. We are working with the CDC and our healthcare partners across the state to protect South Dakotans from COVID-19.

SOUTH DAKOTA
COVID-19 INFORMATION LINE
Questions about COVID-19? We're here to help.
PLEASE CALL **1-800-997-2880**

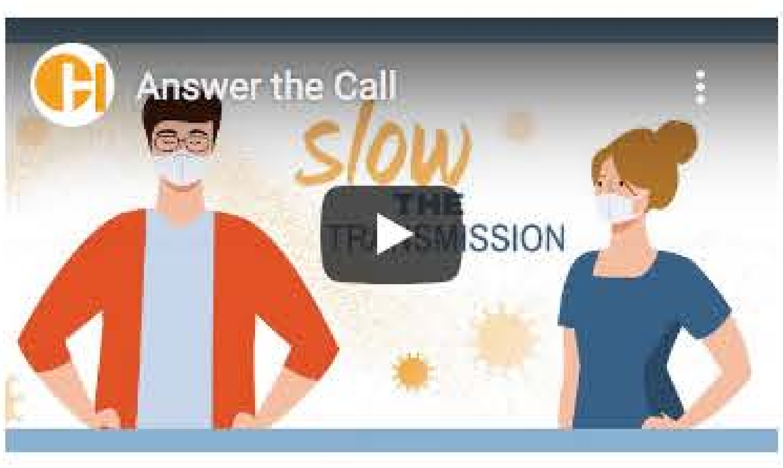

UPDATES

- South Dakota FAQs: Symptoms and potential exposure, socializing risks, food safety, Care19 Diary app, and general resources.
- South Dakota COVID-19 News
- Risks and Benefits: Considerations for Social Activities during COVID-19
- COVID-19 Contact Tracing: What to expect if you've had close contact with a positive case
- Frequently Asked Questions (CDC)
- South Dakota's 'Back to Normal' Plan | Spanish
- Download the Care19 App
- Great Plains Tribal Chairman's Health Board COVID-19 Resources
- Interested in volunteering your services? Join the State Emergency Registry of Volunteers for South Dakota.
- What should I do? Infographic
- COVID-19 Testing Facts
- Guidance on Interpreting COVID-19 Test Results
- COVID-19 Symptoms and Screening App and Website from Apple (in partnership with CDC and the White House).

BACK TO TOP

COVID-19 IN SOUTH DAKOTA

Please wait. The data dashboard below takes a moment to load and is best viewed on desktop or in landscape mode on mobile (i.e. holding the phone sideways).

SD Overview | Demographics | Hospital Capacity | Trends | Tables

New Cases Today	Active Cases	Recovered Cases
425	2,428	10,347
**Currently Hospitalized**	**Ever Hospitalized**	**Deaths**
79	1,006	167
**Total Cases**	**Total Persons Tested**	**Total Tests**
12,942	144,506	189,359
**% Progress (June Goal: 44,233 Tests)**	**% Progress (July Goal: 44,233 Tests)**	**% Progress (August Goal: 44,233 Tests)**
97%	106%	108%

Cases: *Persons who meet confirmed or probable case definition according to the national surveillance case definition for COVID-19. A positive case moves from active to recovered status based on the CDC guidelines for removal from isolation.*

Hospitalized: *Currently Hospitalized numbers include COVID-19 cases and suspects that are in the hospital under transmission-based precautions. The number of persons currently hospitalized may include out-of-state cases and suspects. Data are reported directly by hospitals through the South Dakota HAvBED (Hospital Available Beds for Emergencies and Disaster) system. Ever Hospitalized include COVID-19 cases that were, at some point during their infection, hospitalized under transmission-based precautions. The number of persons ever hospitalized are cumulative and includes only South Dakota resident cases. Data are reported from the Maven electronic disease surveillance system.*

Deaths: *SD-DOH does not report deaths until a certified death record has been filed with COVID-19 as a cause or contributing factor. By law, a death record must be filed within 5 days of the date of death.*

Tests: *% Progress shows the percentage of viral tests completed toward the monthly goal of 44,233. The monthly goal for South Dakota is equal to 5% of the state's population. The federal government established a 2% goal for SARS-CoV-2 testing for each state. Total tests are the number of tests performed, not the number of individuals tested, and include tests conducted by the South Dakota Public Health Laboratory and hospital, commercial, Indian Health Service, Veteran Affairs,*

Case Map by County of Residence

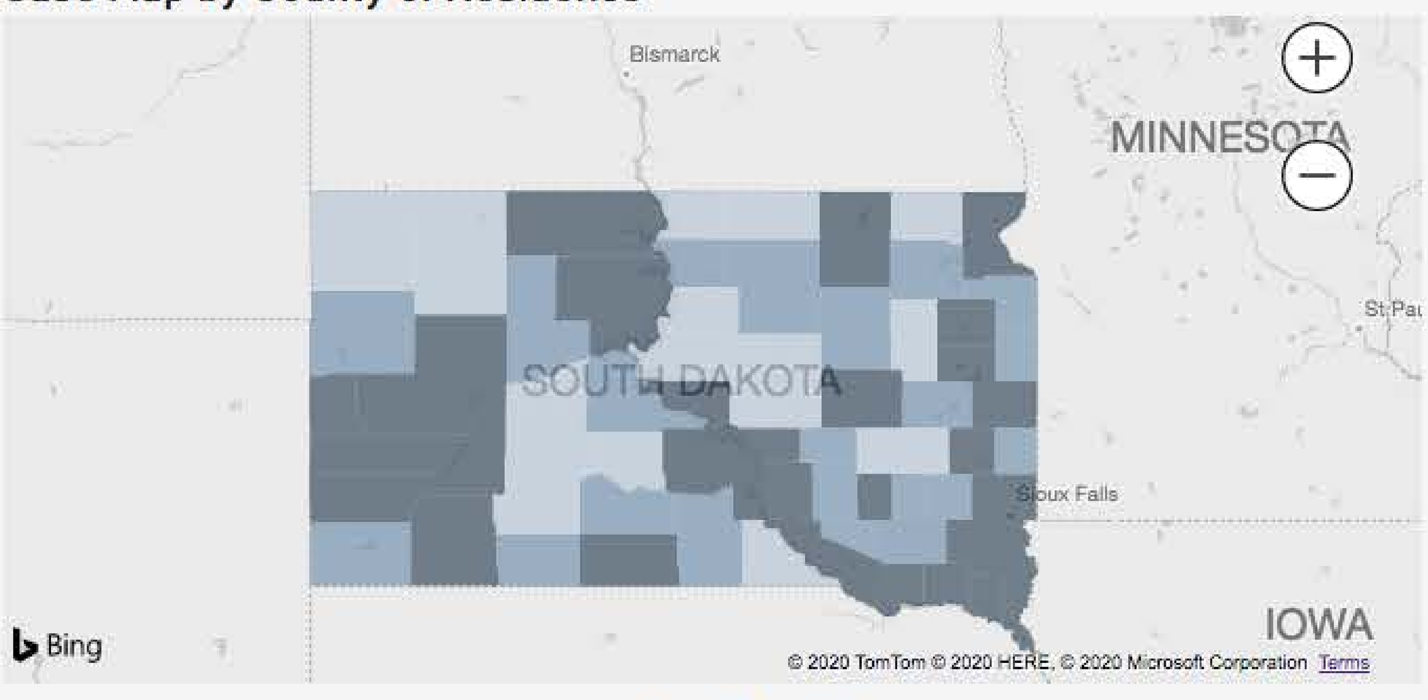

Cases 0 1-19 20-49 50+

Hover over a county to see its details, or click county to update the orange boxes above.

Community spread is defined as:
None: *0 cases per 100,000 OR 0 cases**
Minimal: *<10 cases per 100,000 OR 1-3 cases**
Moderate: *10-100 cases per 100,000 OR 4-9 cases**
Substantial: *>100 cases per 100,000 OR 10+ cases**
**Counties that have a population of < 35,000 will use a minimum number for determination of spread.*
Community spread is updated every Monday based on the previous week's (Sat-Sun) number/rate of new cases.

Cases by County of Residence

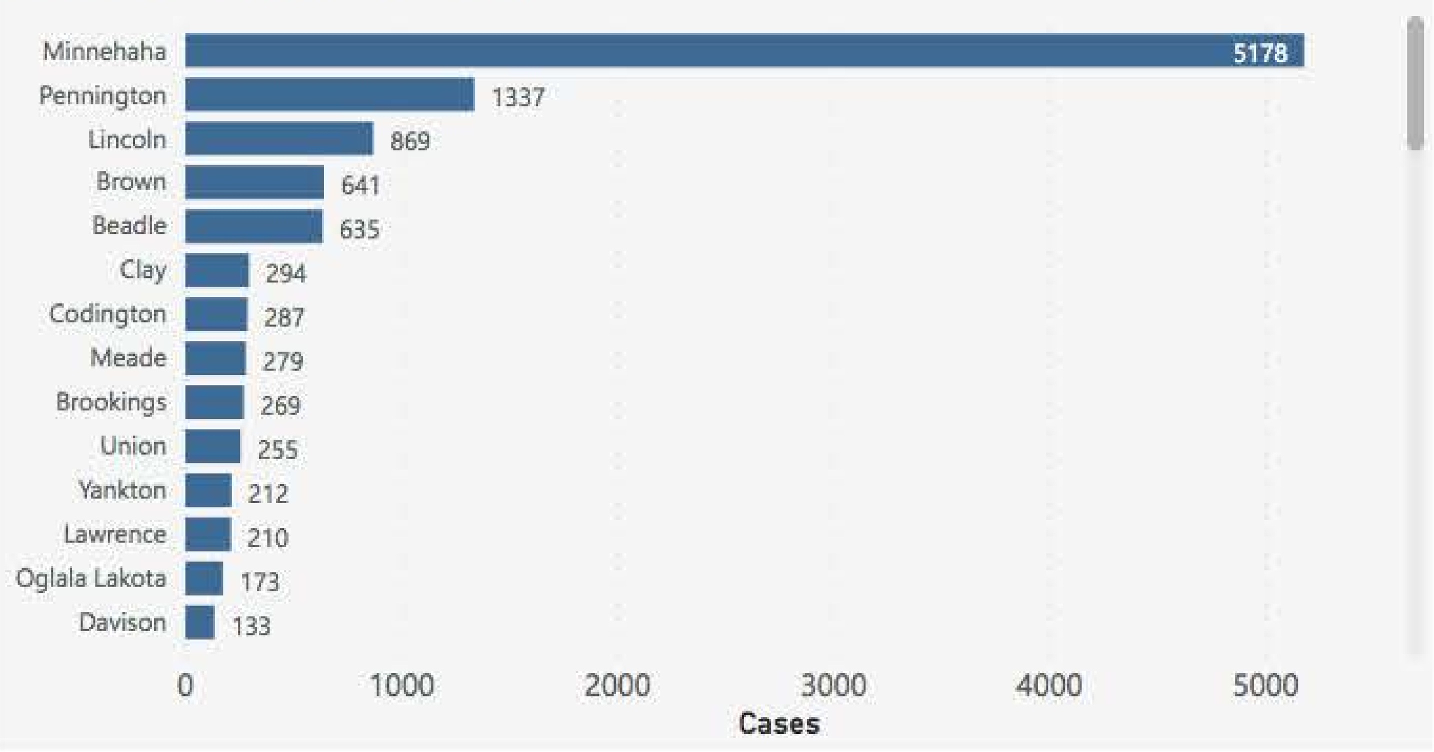

Note: *Data are provisional and subject to change. Data are updated daily by about 12 p.m. and include cases reported through 1 p.m. the previous day.*

LAST UPDATED
8/29/2020 10:38:32 AM

Microsoft Power BI 1 of 5

Note:

- The Community Impact Map uses the **definitions** for none, minimal, moderate, and substantial community transmission. The level of spread is aligned with the **CDC's Community Mitigation Framework**.
- The number of persons positive for COVID-19 and number of tests positive for COVID-19 reflect only South Dakota residents, regardless of where testing was conducted (in-state or out-of-state).
- Investigation of a person positive for COVID-19 is completed by the public health agency in the state where the person resides. A South Dakota resident testing positive in another state is reported to SD-DOH for investigation.
- States notify the public health agency in which close contacts of a positive case reside so notification can be made. A South Dakota resident will be notified if they were a close contact to a confirmed case by the SD-DOH.
- A public notice is issued by SD-DOH when a person positive for COVID-19 is unable to identify all close contacts (spent 15 minutes or more within 6 feet) where there may be risk of exposure so that individuals can monitor themselves for symptoms and seek guidance from their medical provider.
- A person is considered recovered after they have been released from isolation by SD-DOH upon meeting the following: (1) at least 10 days have elapsed after their symptoms began, and (2) at least 24 hours have elapsed after their fever resolved and their other symptoms have improved.
- COVID-19 cases within **South Dakota K-12 Schools and Colleges, Universities, and Technical Schools** will be updated weekly on Monday.

BACK TO TOP

SOUTH DAKOTA DEPARTMENT OF HEALTH covid.sd.gov

NOVEL CORONAVIRUS (COVID-19) UPDATES AND INFORMATION

Updates | COVID-19 in SD
Multisystem Inflammatory Syndrome (MIS-C)
Precautions to Avoid Illness | If You Develop Symptoms
Plasma Donation | Community Guidance
Multiple Language Resources
For Medical Providers | SD Healthcare Provider Guidance
Remdesivir Distribution | SD Registries & Data Collection
CDC Healthcare Provider Guidance | CDC Website

The South Dakota Department of Health (SD-DOH) continues to respond to the emergence of the novel coronavirus. We are working with the CDC and our healthcare partners across the state to protect South Dakotans from COVID-19.

SOUTH DAKOTA
COVID-19 INFORMATION LINE
Questions about COVID-19? We're here to help.
PLEASE CALL **1-800-997-2880**

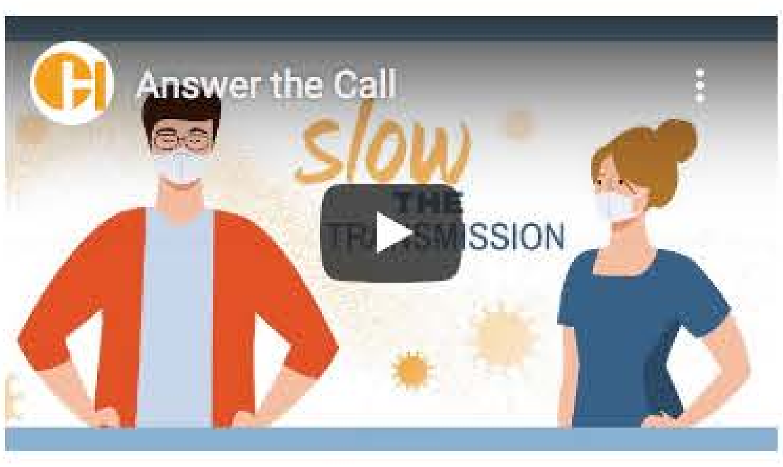

UPDATES

- South Dakota FAQs: Symptoms and potential exposure, socializing risks, food safety, Care19 Diary app, and general resources.
- South Dakota COVID-19 News
- Risks and Benefits: Considerations for Social Activities during COVID-19
- COVID-19 Contact Tracing: What to expect if you've had close contact with a positive case
- Frequently Asked Questions (CDC)
- South Dakota's 'Back to Normal' Plan | Spanish
- Download the Care19 App
- Great Plains Tribal Chairman's Health Board COVID-19 Resources
- Interested in volunteering your services? Join the State Emergency Registry of Volunteers for South Dakota.
- What should I do? Infographic
- COVID-19 Testing Facts
- Guidance on Interpreting COVID-19 Test Results
- COVID-19 Symptoms and Screening App and Website from Apple (in partnership with CDC and the White House).

BACK TO TOP

COVID-19 IN SOUTH DAKOTA

Please wait. The data dashboard below takes a moment to load and is best viewed on desktop or in landscape mode on mobile (i.e. holding the phone sideways).

SD Overview | Demographics | Hospital Capacity | Trends | Tables

County
All

Test Positivity Rate, Last 1 Day	Test Positivity Rate, Last 7 Days	Test Positivity Rate, Last 14 Days	Test Positivity Rate, Cumulative
19.0%	15.4%	12.5%	8.0%

Test Positivity Rate: *This rate is equal to the number of Positive Tests divided by Total Tests, within specified period of time.*

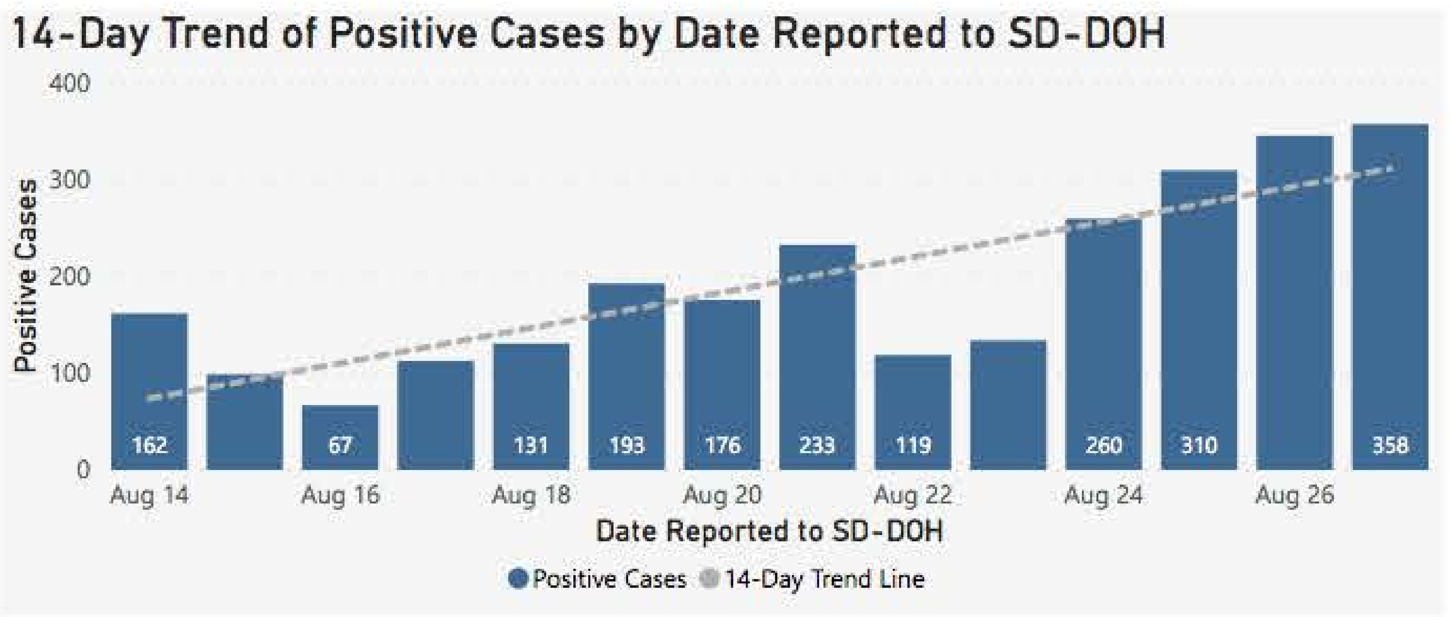

14-Day Trend of Positive Cases by Date Reported to SD-DOH: *This graph shows the trend (increasing or decreasing) at the state or county level.*

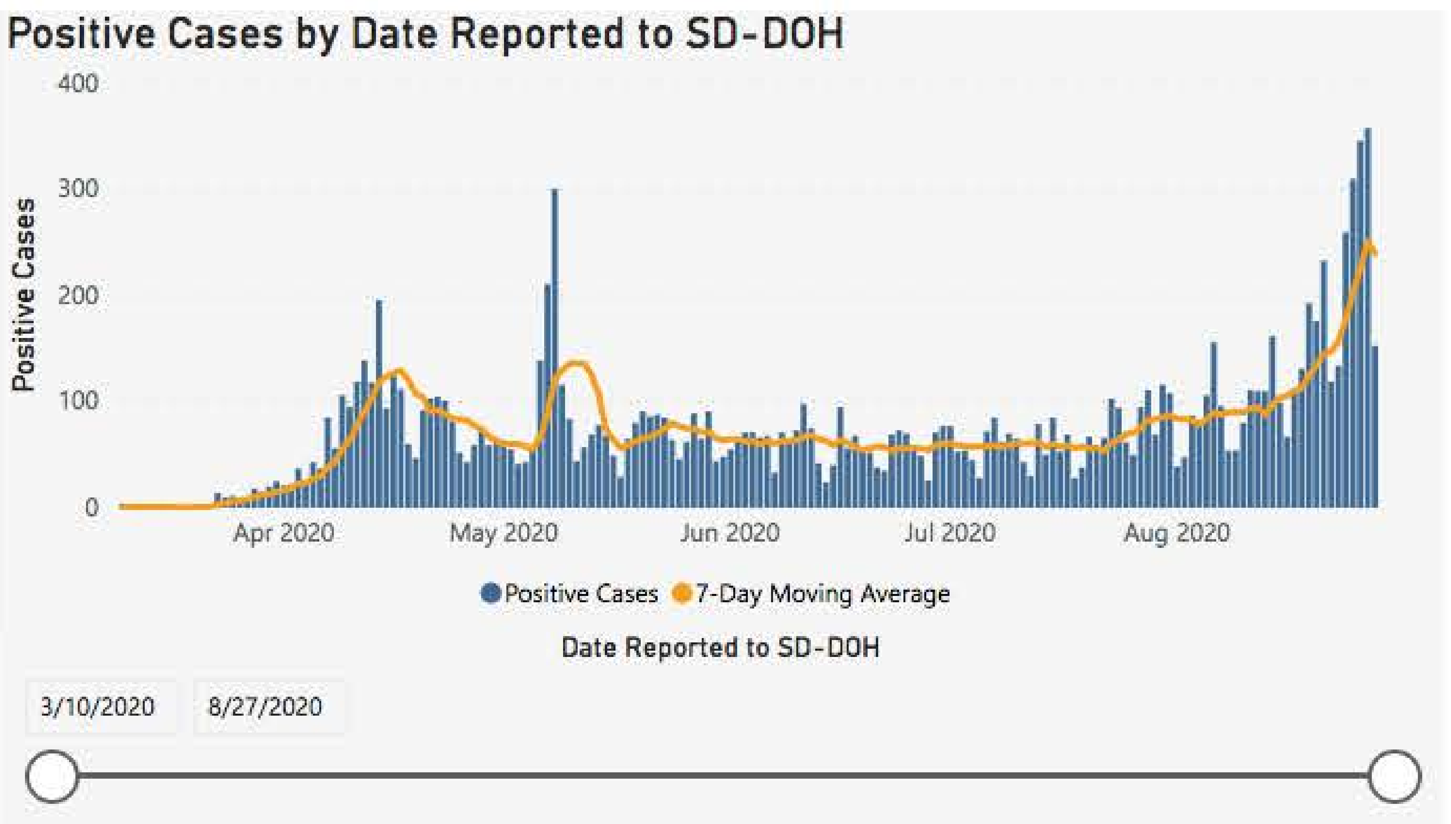

Positive Cases by Date Reported to SD-DOH: *This graph shows positive cases over time includes results received by midnight and may not align with the daily data update at noon.*

Rate of Cases, Last 7 Days	Rate of Cases, Cumulative
191 (per 100,000)	1467 (per 100,000)

Rate of Cases: *Number of cases reported to SD-DOH in the last 7 days (or cumulative) divided by the state or county population multiplied by 100,000. This is done to compare disease burden across geographic areas (eg. state or county).*

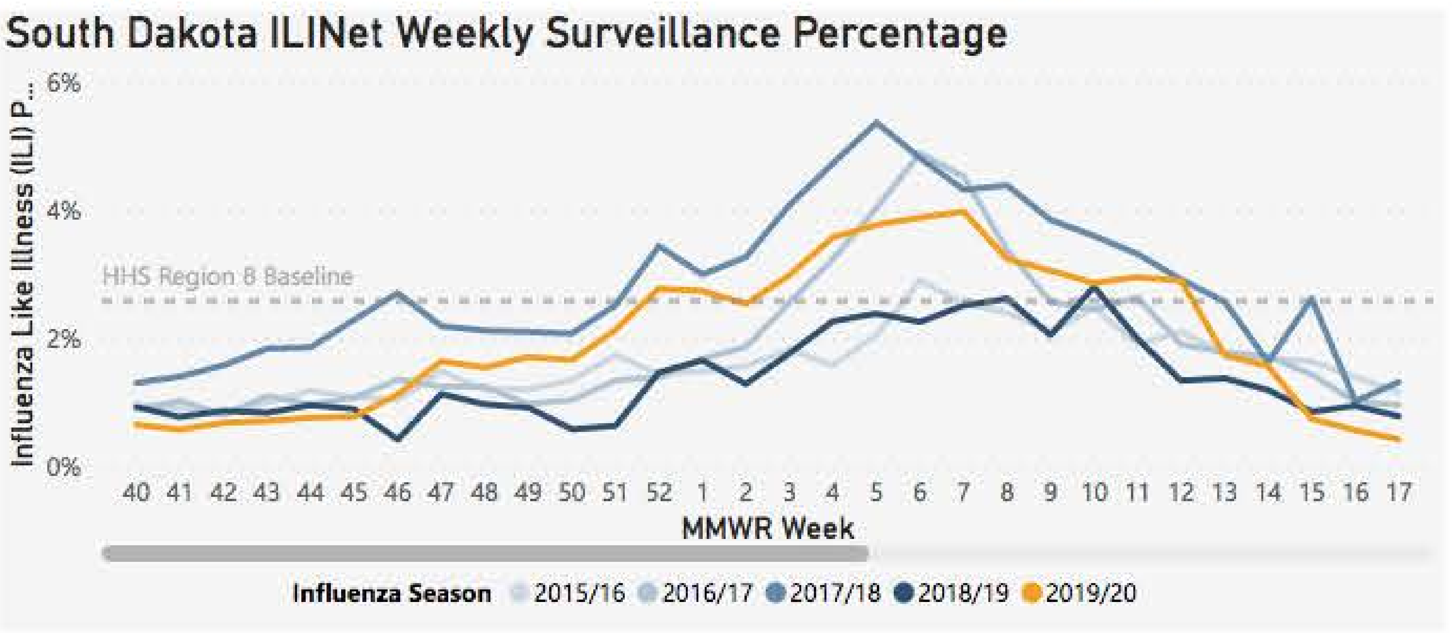

ILINet: *Influenza-like illness US Influenza Surveillance System.This includes any illness that meets influenza symptoms of a fever (100.4+ degrees Fahrenheit), cough, and/or sore throat. Participation includes all 50 states and 3 territories. MMWR Week is a numbered week baseline established by the CDC that defines 52 weeks per year.*

Latest MMWR Week (Week ending 8/22/2020, MMWR week 34)	HHS Region 8 Baseline
0.31%	2.60%

Note: *Data are provisional and subject to change. Data are updated daily by about 12 p.m. Results include tests conducted by the South Dakota Public Health Laboratory and hospital, commercial, Indian Health Service, Veteran Affairs, and other medical laboratories.*

LAST UPDATED
8/29/2020 10:38:32 AM

Microsoft Power BI ‹ 4 of 5 ›

Note:

- The Community Impact Map uses the **definitions** for none, minimal, moderate, and substantial community transmission. The level of spread is aligned with the **CDC's Community Mitigation Framework**.
- The number of persons positive for COVID-19 and number of tests positive for COVID-19 reflect only South Dakota residents, regardless of where testing was conducted (in-state or out-of-state).
- Investigation of a person positive for COVID-19 is completed by the public health agency in the state where the person resides. A South Dakota resident testing positive in another state is reported to SD-DOH for investigation.
- States notify the public health agency in which close contacts of a positive case reside so notification can be made. A South Dakota resident will be notified if they were a close contact to a confirmed case by the SD-DOH.
- A public notice is issued by SD-DOH when a person positive for COVID-19 is unable to identify all close contacts (spent 15 minutes or more within 6 feet) where there may be risk of exposure so that individuals can monitor themselves for symptoms and seek guidance from their medical provider.
- A person is considered recovered after they have been released from isolation by SD-DOH upon meeting the following: (1) at least 10 days have elapsed after their symptoms began, and (2) at least 24 hours have elapsed after their fever resolved and their other symptoms have improved.
- COVID-19 cases within **South Dakota K-12 Schools and Colleges, Universities, and Technical Schools** will be updated weekly on Monday.

BACK TO TOP

TN Novel Coronavirus (COVID-19) Unified Command

Tennessee State Government

Choose date: 8/28/2020

Confirmed Cases

click to toggle visibility

26,839 Shelby
380 deaths, 295,361 negative

23,399 Davidson
255 deaths, 244,244 negative

7,939 Hamilton
73 deaths, 98,688 negative

7,770 Rutherford
68 deaths, 62,709 negative

6,275 Knox
56 deaths, 96,763 negative

4,328 Williamson
27 deaths, 51,662 negative

4,051 Sumner
86 deaths, 33,861 negative

2,791 Wilson
31 deaths, 29,219 negative

2,460 Bradley
16 deaths, 19,591 negative

2,452 Montgomery
28 deaths, 31,320 negative

2,345 Putnam
30 deaths, 22,418 negative

2,219 Sevier
12 deaths, 20,160 negative

1,992 Blount
17 deaths, 20,769 negative

1,869 Robertson
26 deaths, 13,532 negative

1,763 Madison
41 deaths, 20,049 negative

1,725 Maury
14 deaths, 18,655 negative

1,700 Washington
12 deaths, 22,016 negative

TN

95

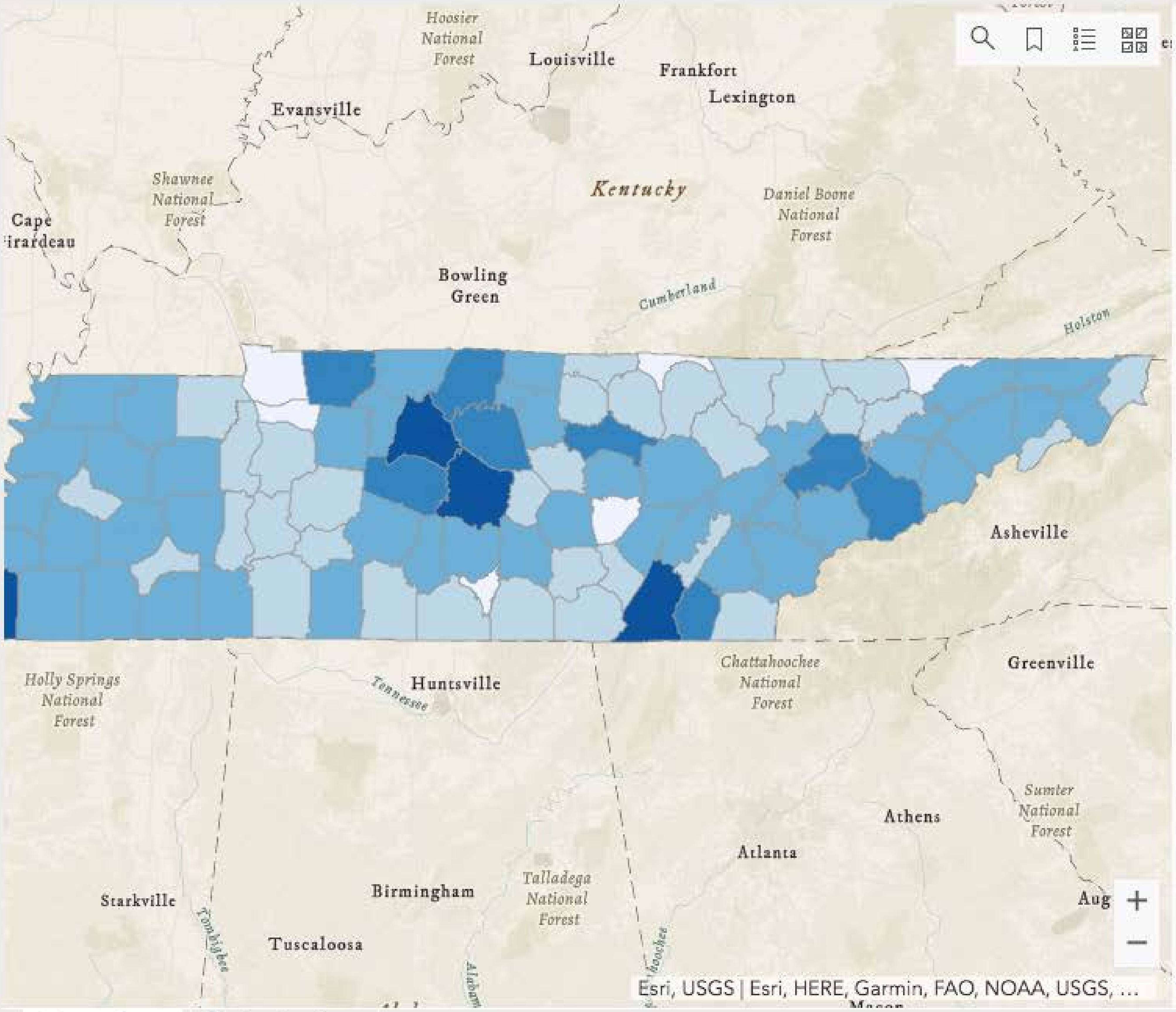

Affected Counties

Total Cases:
- > 7,000 - 27,000
- > 2,000 - 7,000
- > 500 - 2,000
- > 100 - 500
- 1 - 100

County Cases | Testing Locations

Statewide: 8/28/2020

150,815 Confirmed Cases

1,701 Fatalities

6,751 Hospitalizations

113,313 Recovered

Daily: 8/28/2020

1,636 New Cases

28 New Fatalities

74 New Hospitalizations

1,897 New Recovered

Total Confirmed Cases

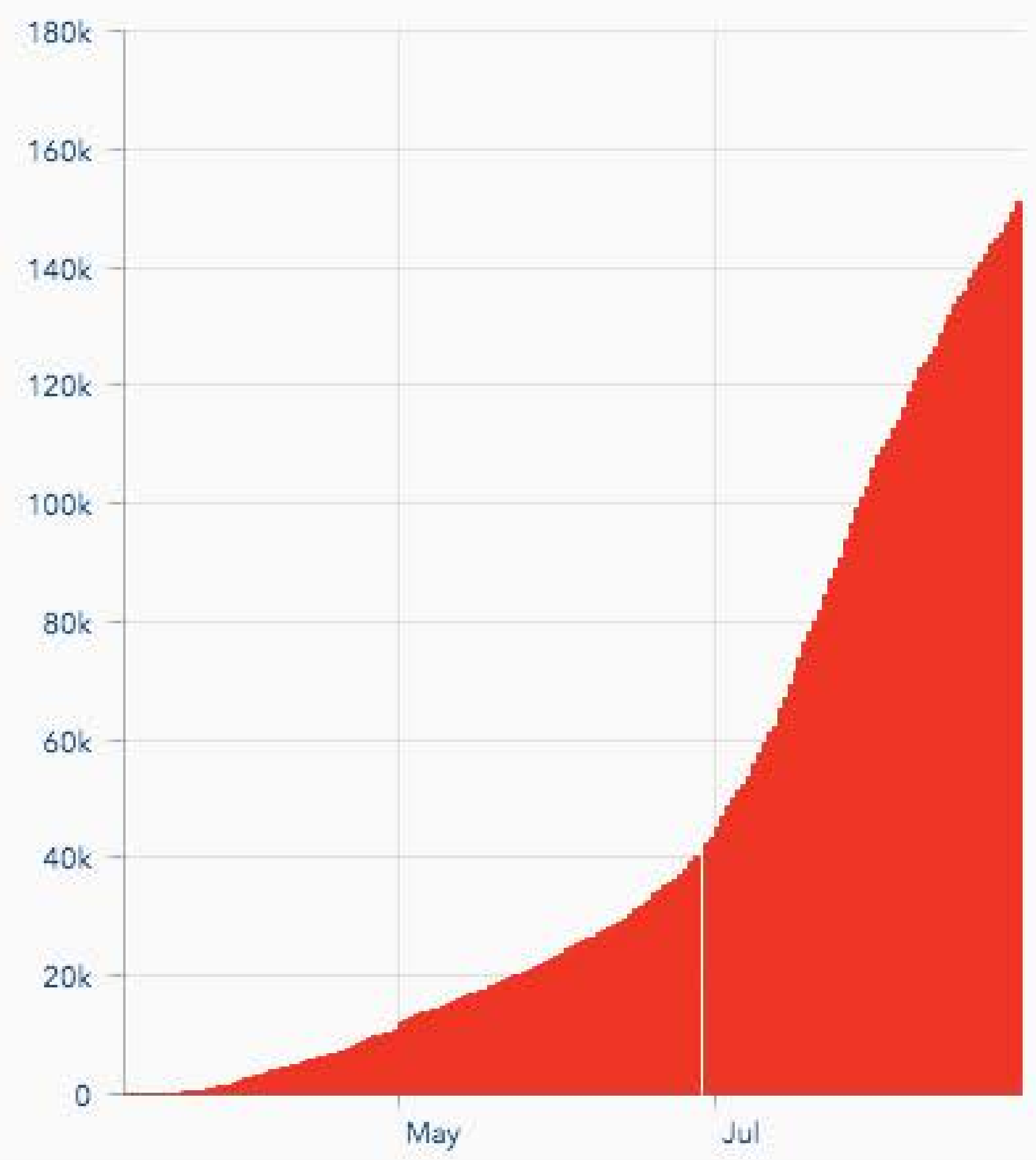

Confirmed Cases By Age

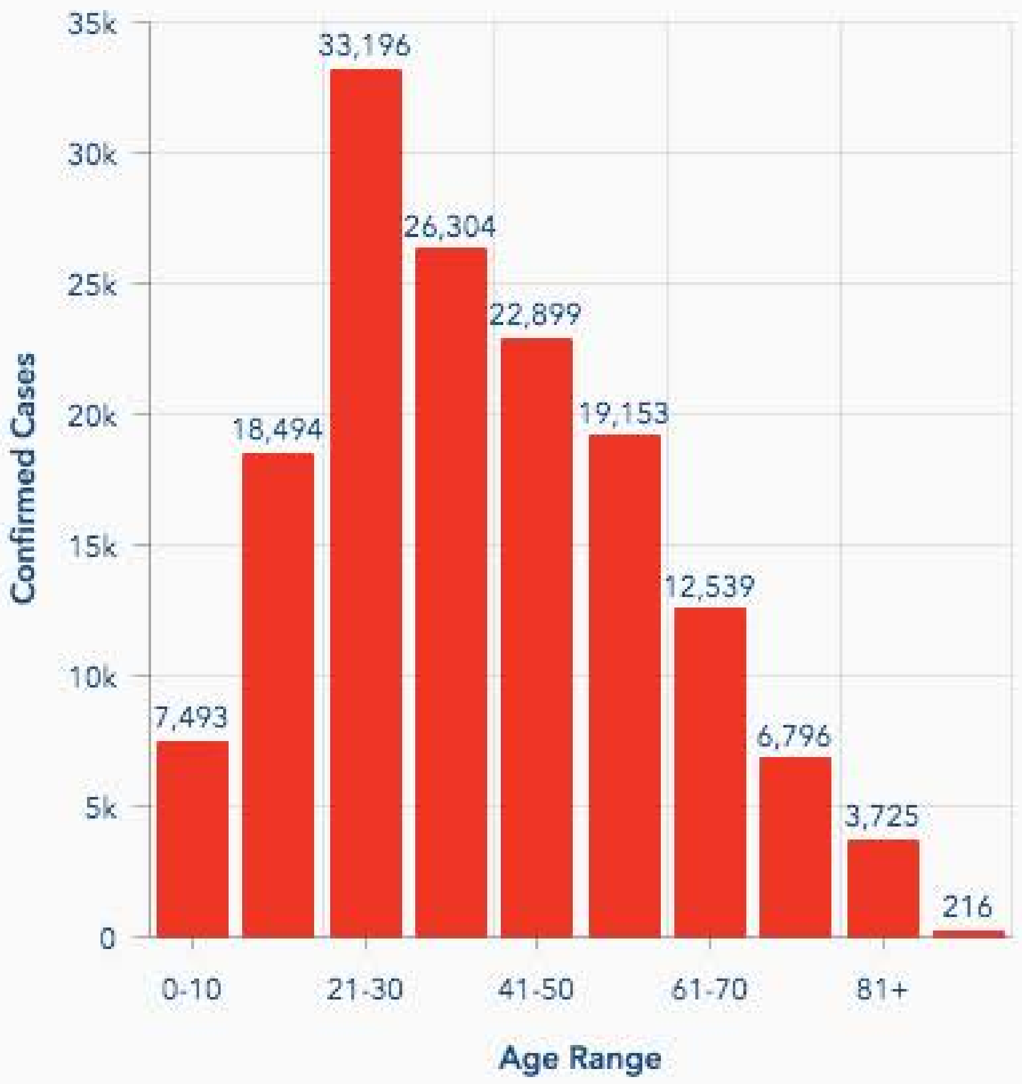

Cases by Age | New Daily Cases by Age

Tennessee COVID-19 - August 29, 2020
Epidemiology and Surveillance Data

TN Department of Health

Total Cases:	152,280	Confirmed Cases:	148,681	Probable Cases:	3,599	Recovered Cases:	114,099
Total Deaths:	1,725	Confirmed Deaths:	1,677	Probable Deaths:	48		

Cases by County

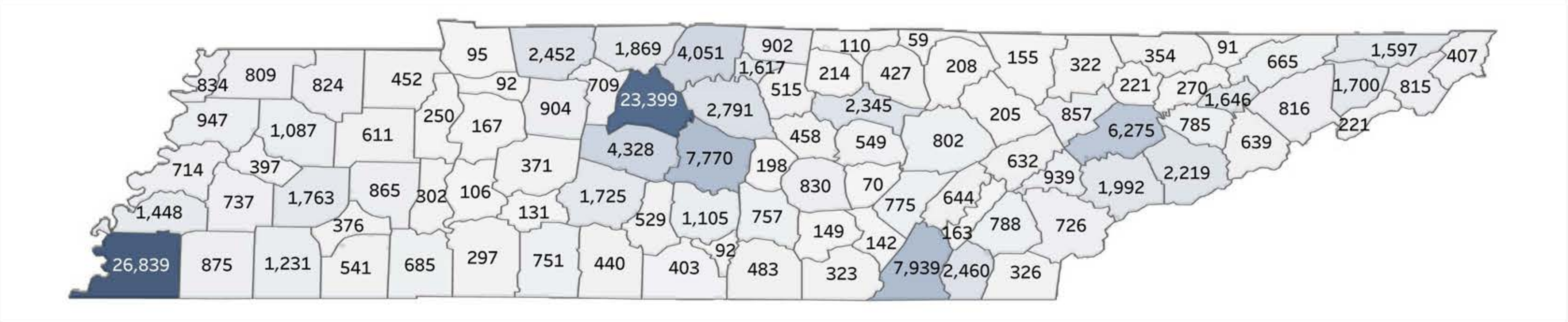

Daily Counts of Cases

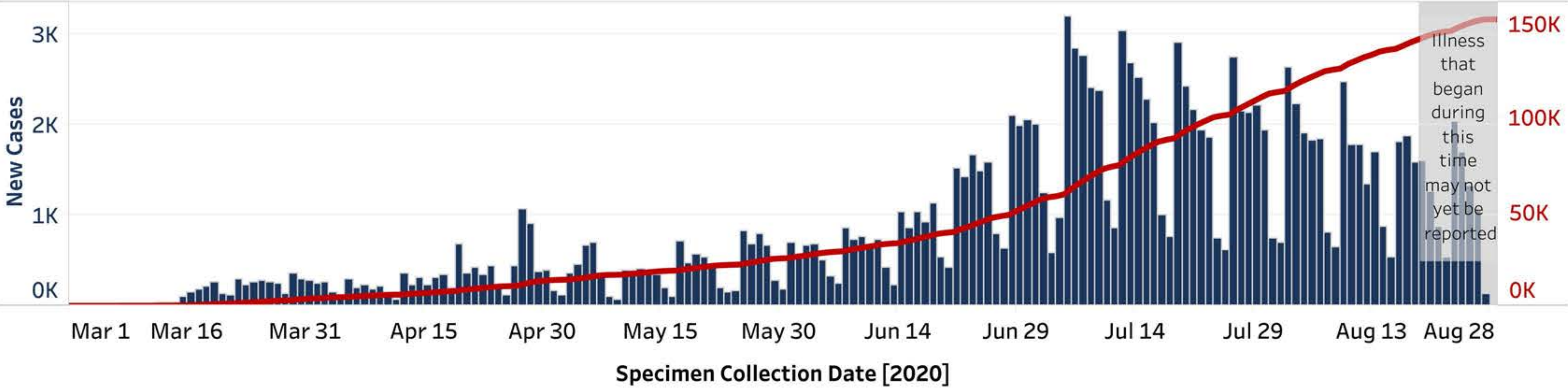

Demographics

Minimum Age	Average Age	Maximum Age
0	39	106

Age (years)	Cases	% of Cases	Deaths	% of Deaths
0-10	7,571	5%	4	0%
11-20	18,735	12%	1	0%
21-30	33,484	22%	16	1%
31-40	26,531	17%	34	2%
41-50	23,094	15%	80	5%
51-60	19,311	13%	176	10%
61-70	12,688	8%	336	19%
71-80	6,895	5%	510	30%
81+	3,762	2%	568	33%
Pending	209	0%	0	0%

Patient Sex	Cases	% of Cases	Deaths	% of Deaths
Female	77,308	51%	740	43%
Male	73,636	48%	984	57%
Pending	1,336	1%	1	0%

Race	Cases	% of Cases	Deaths	% of Deaths
American Indian or Alaska Native	202	0%	4	0%
White	75,948	50%	1,078	62%
Black or African American	29,268	19%	522	30%
Native Hawaiian or Other Pacific Islander	112	0%	2	0%
Asian	1,324	1%	15	1%
Other/Multiracial	19,851	13%	89	5%
Pending	25,575	17%	15	1%

Ethnicity	Cases	% of Cases	Deaths	% of Deaths
Not Hispanic or Latino	94,356	62%	1,591	92%
Hispanic or Latino	22,358	15%	122	7%
Pending	35,566	23%	12	1%

Outcomes

Ever Hospitalized	Cases	% of Cases
Yes	6,817	4%
No	89,893	59%
Pending	55,570	36%

Please note for hospitalizations: Information about hospitalization status is gathered at the time of diagnosis, therefore this information may be incomplete. This number indicates the number of patients that were *ever* hospitalized during their illness, it does not indicate the number of patients currently hospitalized.

Recovered	Cases	% of Cases
Yes	114,099	75%

Please note for recovered: TDH defines "recovered" as people who (1) have been confirmed to be asymptomatic by their local or regional health department and have completed their required isolation period or (2) are at least 21 days beyond the first test confirming their illness.

Deceased	Cases	% of Cases
Yes	1,725	1%
No	150,555	99%

Please note for deaths: Some deaths may be reported by health care providers, hospitals, medical examiners, local health departments, or others before they are included in the statewide count.

Laboratory Testing

Results	Number of Tests
PCR Positive	180,543
PCR Negative	1,992,863
Total	2,173,406

Please note, these data are meant to provide a rough estimate of testing volume. Due to different source data, numbers may differ slightly from day to day. Laboratory reports of positive cases are reported to metro and local health departments as soon as results are available. State numbers are updated at 2pm daily and there may be a lag in the reporting of cumulative numbers at the state level. Please note numbers within tables may not add up to total confirmed number of cases due to reporting delays and/or missing data. Percentages may not add up to 100.0% due to rounding.

Texas Case Counts
COVID-19

Note that a backlog of 18 confirmed cases were included in the statewide total but excluded from statewide and Dallas county new confirmed cases (258). Details can be found here: https://www.dallascounty.org/covid-19/

Also, a backlog of 12 confirmed cases were included in the statewide total but excluded from statewide and Montgomery county new confirmed cases (53). Details can be found here: https://mcphd-tx.org/coronavirus-covid-19/confirmed-cases/

Molecular Tests
4,900,972
as of 8/28/2020

606,530
Confirmed Cases

12,420
Fatalities

Daily New Cases
4,732
8/29/2020

Newly Reported Fatalities
154
8/29/2020

Estimated*
492,921
Recoveries

Estimated*
101,189
Active Cases

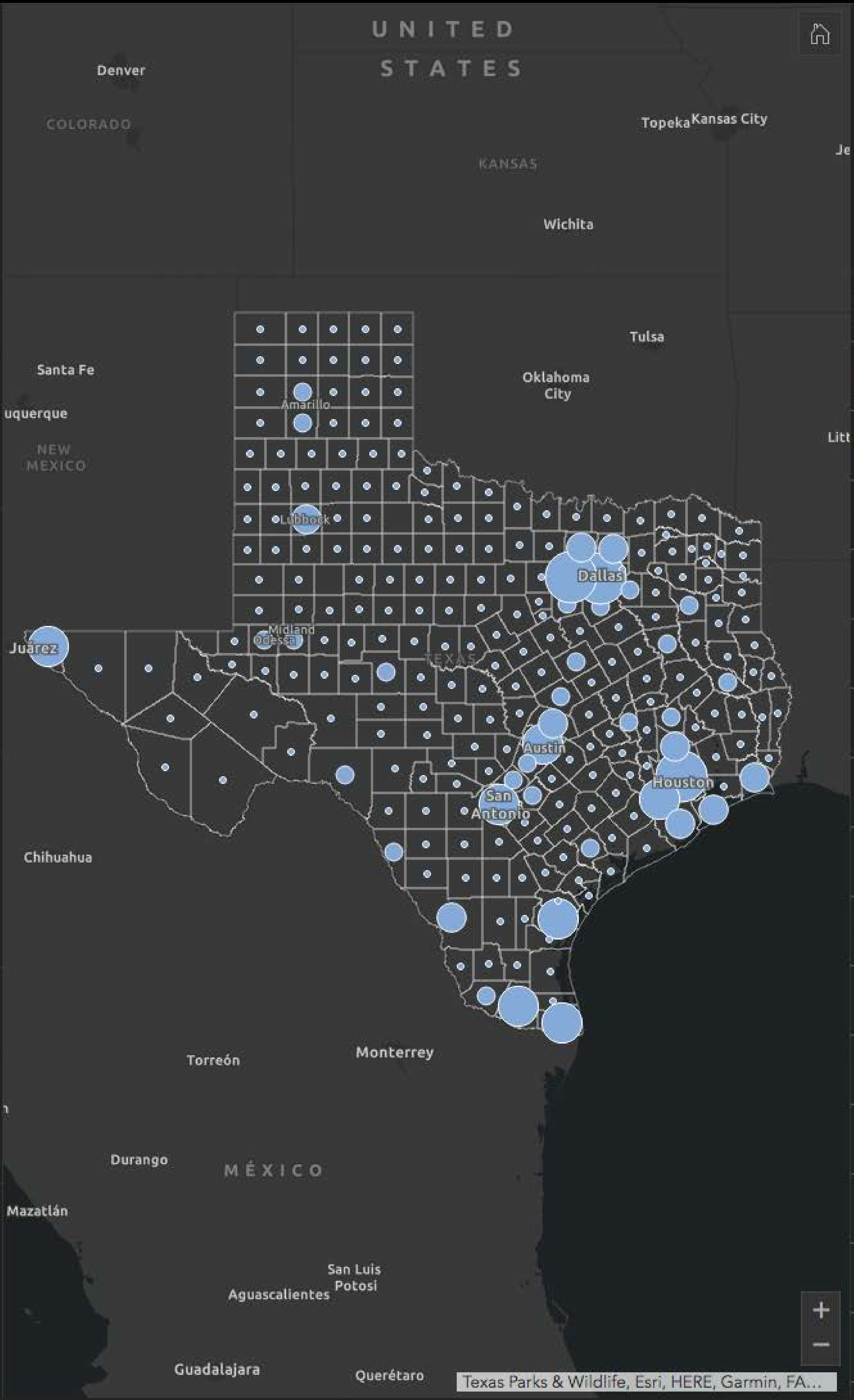

Cases by County

251 of 254
Counties Reporting Cases

County	Cases
Harris County	103,088
Dallas County	70,376
Tarrant County	38,846
Bexar County	37,641
Hidalgo County	27,252
Travis County	26,307
Cameron County	20,853
El Paso County	20,217
Nueces County	14,927
Fort Bend County	14,845
Collin County	10,889
Galveston County	10,509
Webb County	10,482
Denton County	9,875
Brazoria County	9,220
Montgomery County	8,580

Disclaimer: All data are provisional and subject to change.
*Probable cases **are not** included in the total case numbers.*
This dashboard will be updated daily by 4:00PM.

COVID-19 Test and Hospital Data

Last Updated
8/29/2020 3:05PM

TEXAS Health and Human Services | Texas Department of State Health Services

Texas Case Counts COVID-19

CORONAVIRUS DISEASE 2019

Total Tests Reported

5,306,731

as of 8/29/2020

Tests by Type

as of 8/28/2020

Total Molecular Tests

4,900,972

Total Antigen Tests	Positive Antigen Tests
24,176	3,371

Total Antibody Tests	Positive Antibody Tests
356,229	29,635

Total Tests Reported

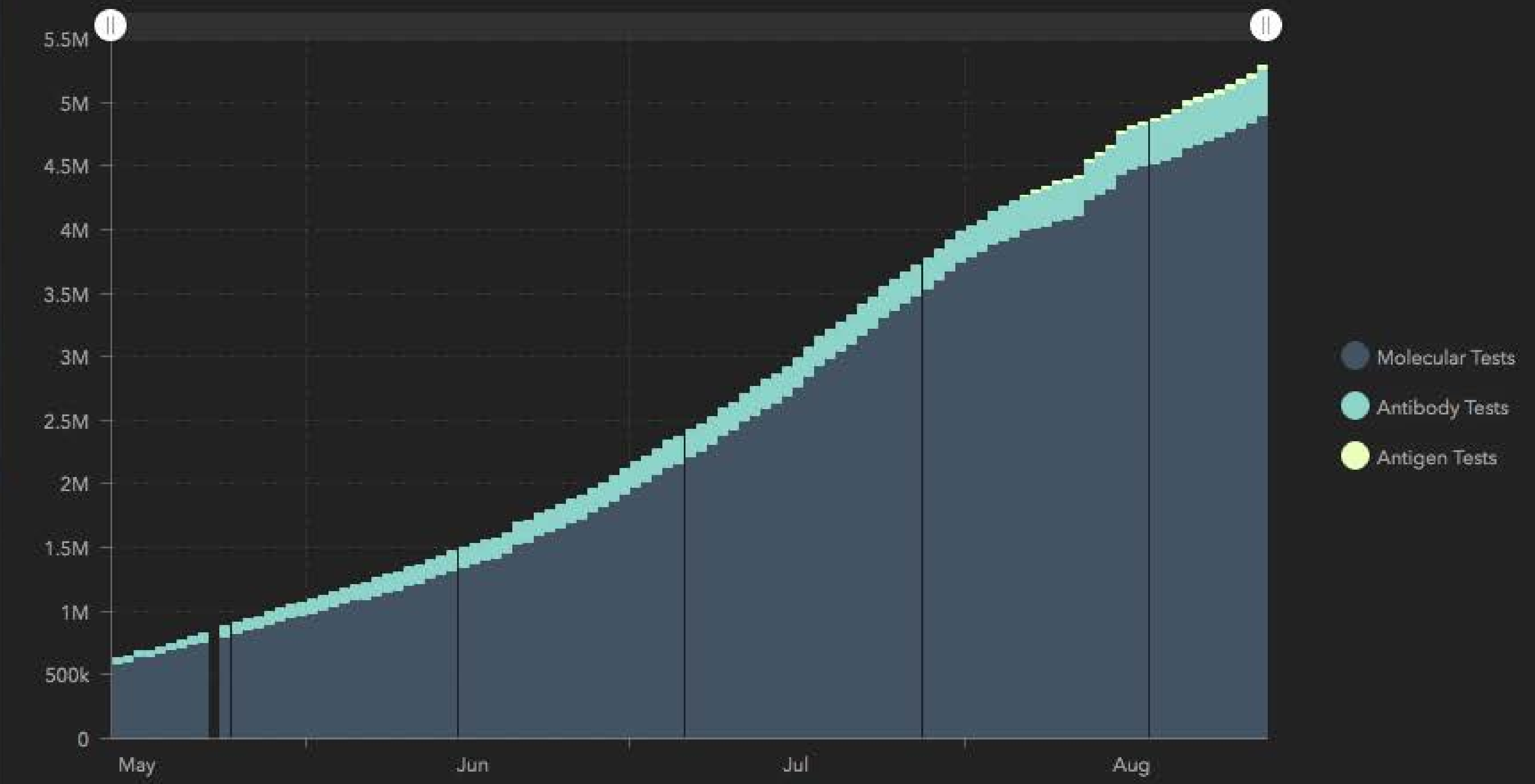

Statewide total tests displayed here and posted on the case counts webpage are raw test counts. Cumulative test totals include tests performed by public labs (Laboratory Response Network) AND non-public labs (commercial labs, hospitals, physician offices, and drive-thru sites) reported electronically and non-electronically. Total test numbers are unable to be de-duplicated for private labs. All data are provisional and subject to change over time.

Daily New Tests (7 day average)

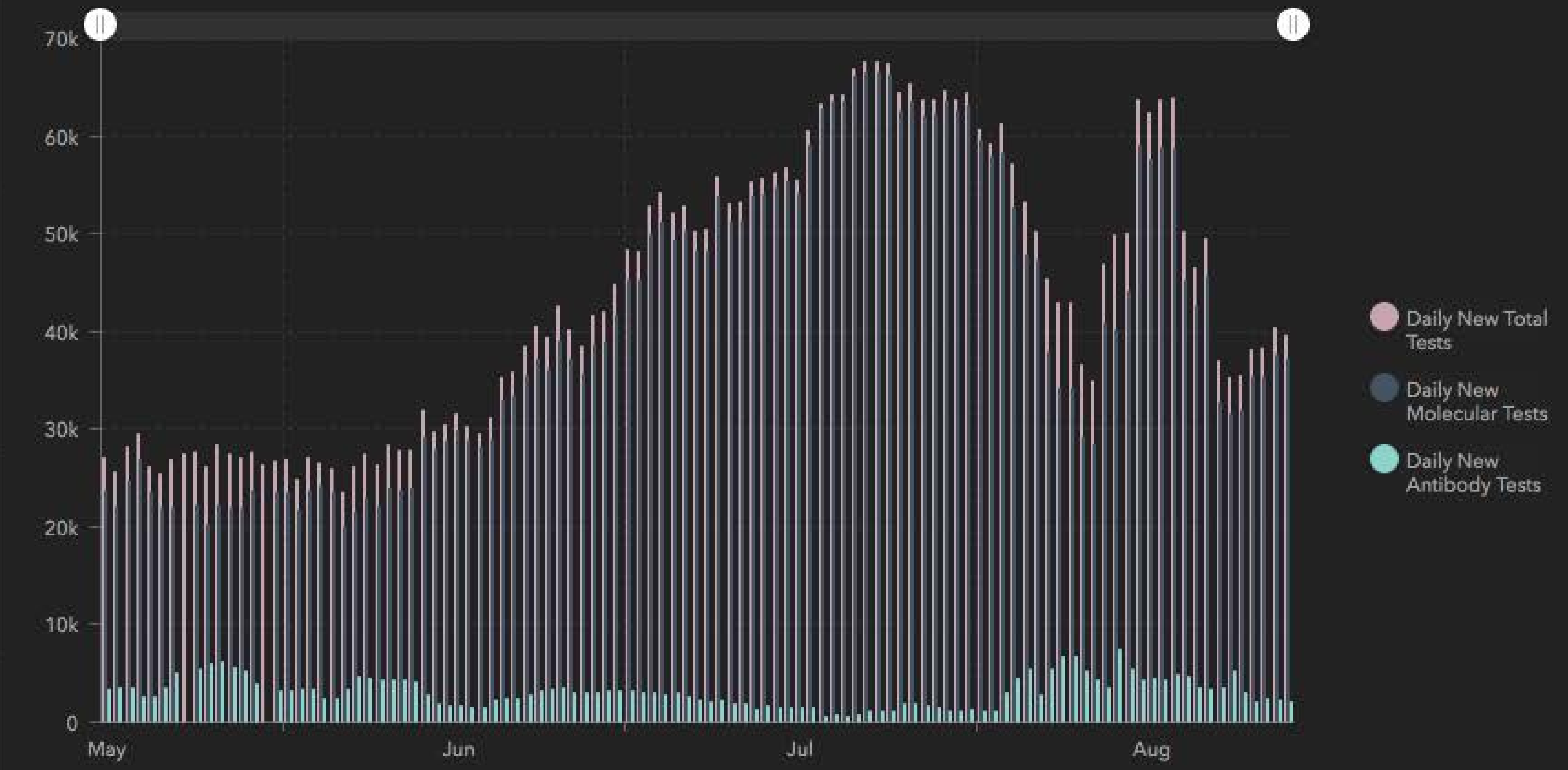

Due to technical issues, data processing could not be completed for viral and antibody lab tests on May 23. Consequently, we are unable to calculate the seven-day average of viral or antibody tests for May 23 and May 30.

Testing Data | Testing Positivity Rate | Total Tests by County | Hospitals - Statewide | Hospitals - Regional | Data Definitions | Data Sources | Additional Data

Disclaimer: All data are provisional and subject to change.
This dashboard will be updated daily by 4:00PM.

COVID-19 Case Data

Last Updated
8/29/2020 3:05PM

CORONAVIRUS

Tracking coronavirus: Interactive maps, charts show spread of COVID-19 across Houston, rest of Texas

Jordan Ray , Jordan Rubio , Matt Dempsey and Stephanie Lamm | Aug. 28, 2020 | Updated: Aug. 28, 2020 1:11 p.m.

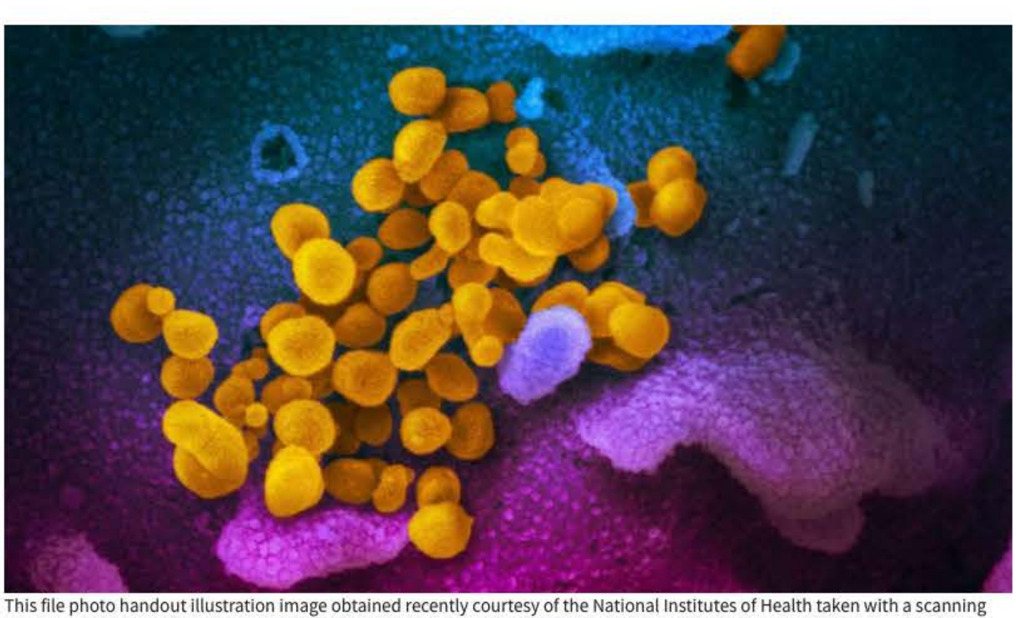

This file photo handout illustration image obtained recently courtesy of the National Institutes of Health taken with a scanning electron microscope shows the virus that causes the disease COVID-19 isolated from a patient in the U.S., emerging from the surface of cells (blue/pink) cultured in the lab.
Photo: HANDOUT / National Institutes of Health / AFP via Getty Images ... more

Most Popular

'Black Panther' star Chadwick Boseman dies of cancer at 43

'Black Panther' star Chadwick Boseman dies of cancer at 43

Door-to-door no more: How Jehovah's Witnesses have adapted in COVID quarantine

Republicans ask Texas Supreme Court to boot Libertarians off ballot

Astros protest racial injustice by sitting out game against A's

Carbon monoxide poisoning from generators poses new threat in areas hit by Hurricane Laura

Alison Cook tried Whataburger's new Pico de Gallo Burger

To keep you up-to-date with the ever-growing number of COVID-19 cases in Houston, Texas and the rest of the world, the Houston Chronicle has come up with a few eye-catching, but easy-to-use interactives, based on our reporting and official data from the Texas Department of State Health Services.

CORONAVIRUS UPDATES: Stay informed with accurate reporting you can trust

Daily COVID-19 case totals for Houston, rest of Texas

DWIGHT SILVERMAN, HOUSTON CHRONICLE
Coronavirus live updates: Texas adds 5,331 cases for Friday

BY JULIAN GILL AND MATT DEMPSEY, STAFF WRITER
Daily coronavirus numbers for Houston, rest of Texas: Aug. 23

HOUSTON
Salvation Army to serve food in Texas communities left without power by...

HOUSTON
Laura evacuees return to devastated terrain

HOUSTON
Three years after Harvey, Houston's home repair program continues to lag....

HOUSTON
Dogs, cats displaced by Hurricane Laura on their way to Houston

If you're mostly concerned with the spread of coronavirus specifically in

Positive test rate

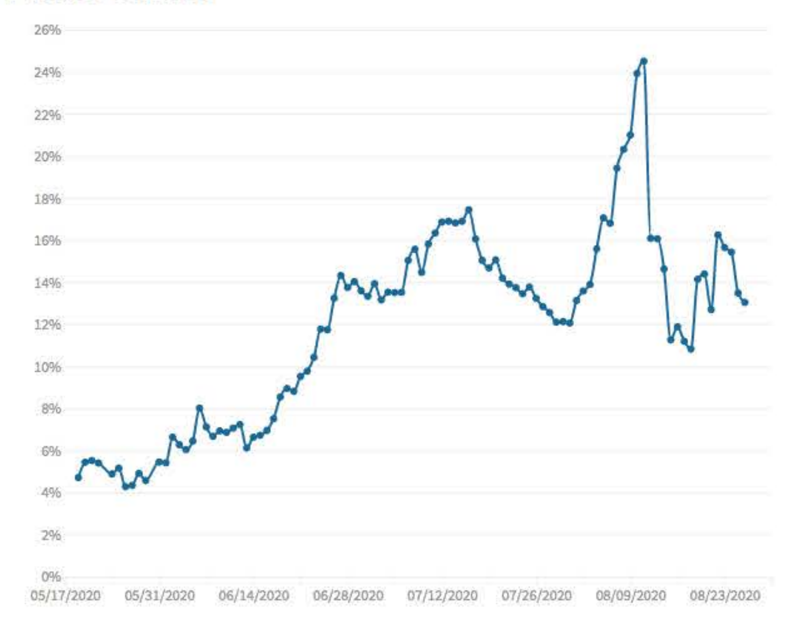

Starting May 19, antibody tests are excluded from the denominator

A Flourish chart

Next, the seven-day rolling average of new cases around the Lone Star State.

7-day rolling average of daily new cases

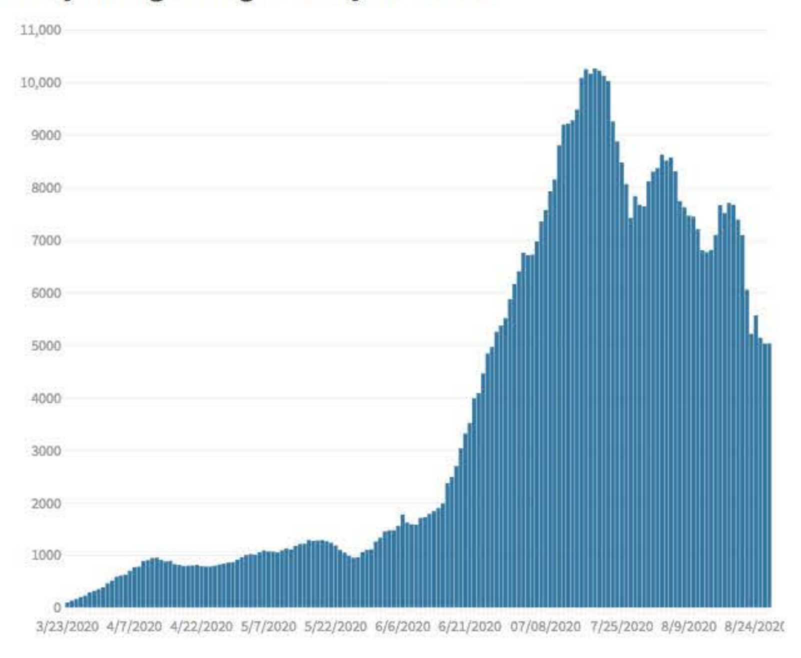

A Flourish chart

The next interactive will help you compare the number of cases in Texas to the number of tests being done.

Rolling average of new tests by day

This shows the seven-day average of new viral tests, by date, in Texas since May 20

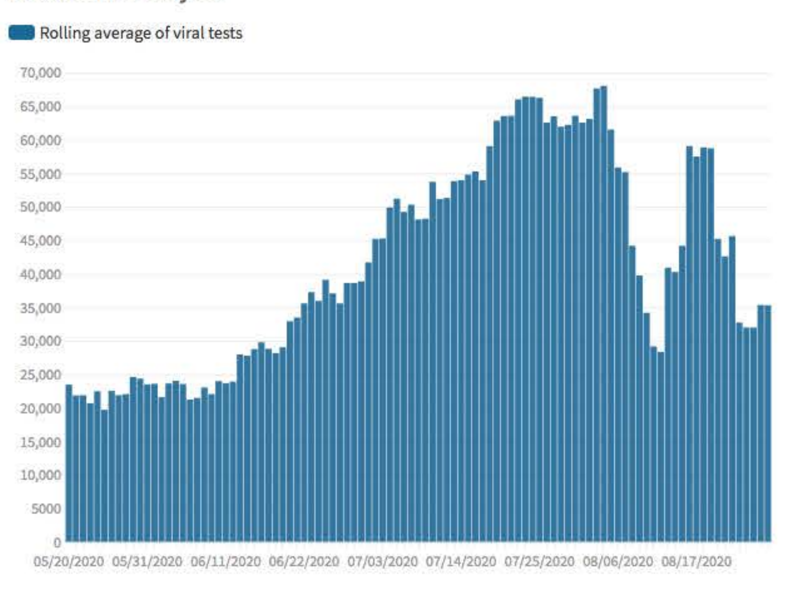

A Flourish chart

For an overview of the Lone Star State, we've got the Texas Coronavirus Map, one of the only comprehensive case trackers for the region. The map includes the number of confirmed cases, deaths and recoveries.

622,496 confirmed coronavirus cases, 12,526 deaths in Texas.

We are tracking cases of coronavirus disease using data collected from the Centers for Disease Control and Prevention and state and local public health departments and our own reporting.

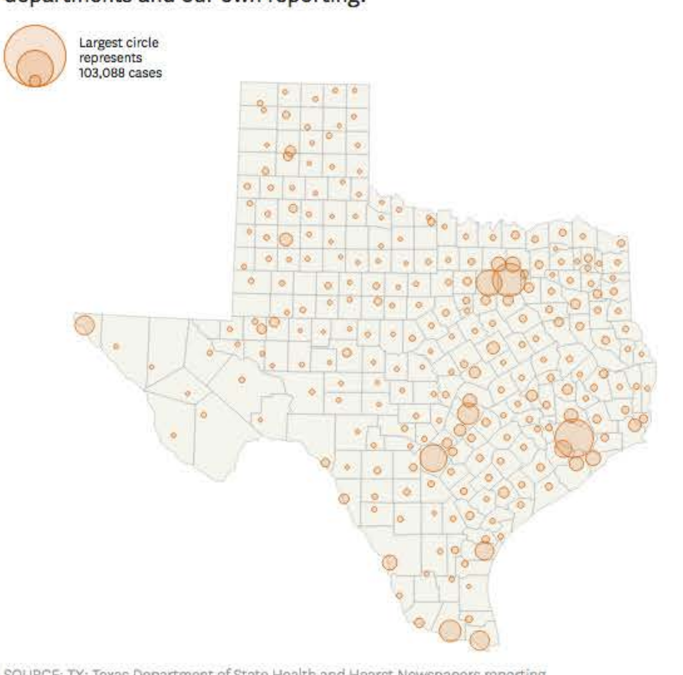

SOURCE: TX: Texas Department of State Health and Hearst Newspapers reporting

You can also get an in-depth look at the cumulative number of deaths in Texas by date below.

Deaths due to the coronavirus in Texas by date

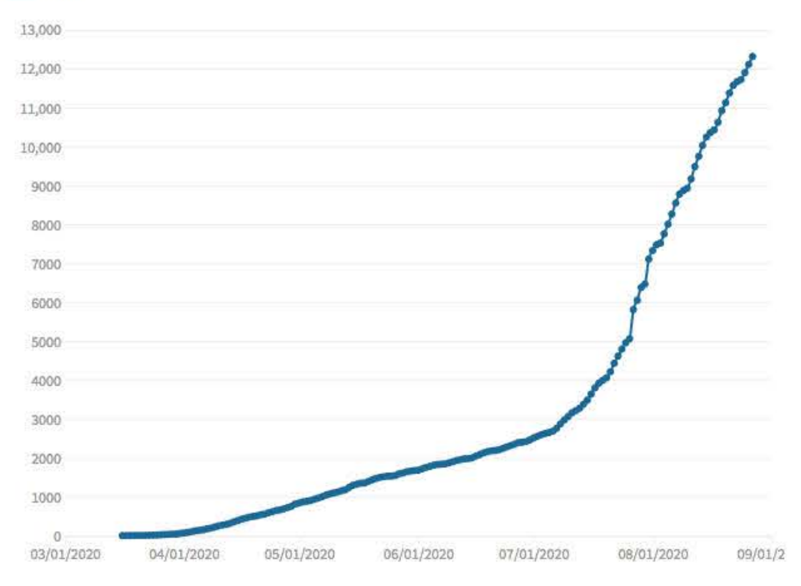

Source: Texas Department of State Health Services, Hearst Newspaper reporting

A Flourish chart

This GIF shows the spread of confirmed and probable COVID cases through Texas.

Confirmed and probable cases per 10,000 people

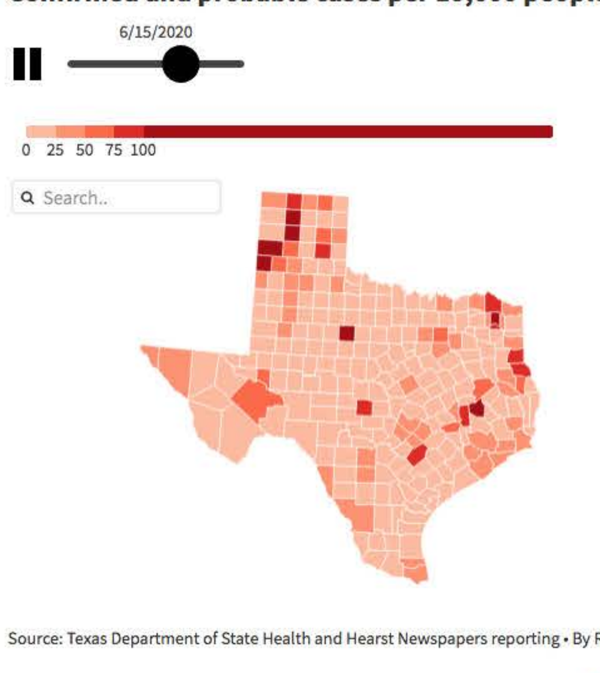

Source: Texas Department of State Health and Hearst Newspapers reporting • By Ryan Serpico

A Flourish data visualization

The next chart breaks down how coronavirus has affected the Houston region, including Harris, Fort Bend, Montgomery, Galveston, Brazoria, Liberty, Chambers and Waller counties.

Seven-day rolling average of new cases for each county in the Houston region

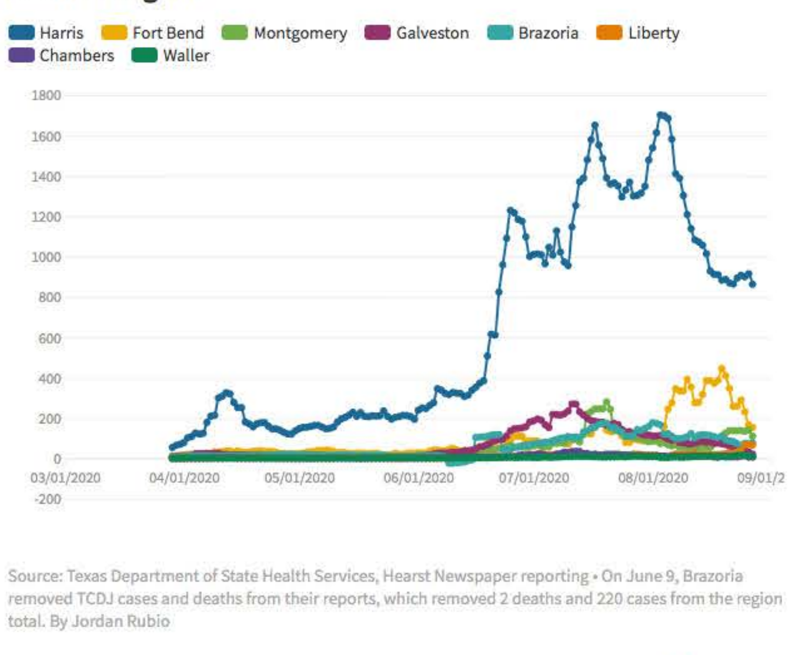

Source: Texas Department of State Health Services, Hearst Newspaper reporting • On June 9, Brazoria removed TCDJ cases and deaths from their reports, which removed 2 deaths and 220 cases from the region total. By Jordan Rubio

A Flourish chart

Pivoting to Houston-area hospitals, you can see the number of confirmed or suspected COVID-19 patients below.

Confirmed or suspected COVID patients in Houston region hospitals

Daily COVID-related patient admissions in Houston-area hospitals

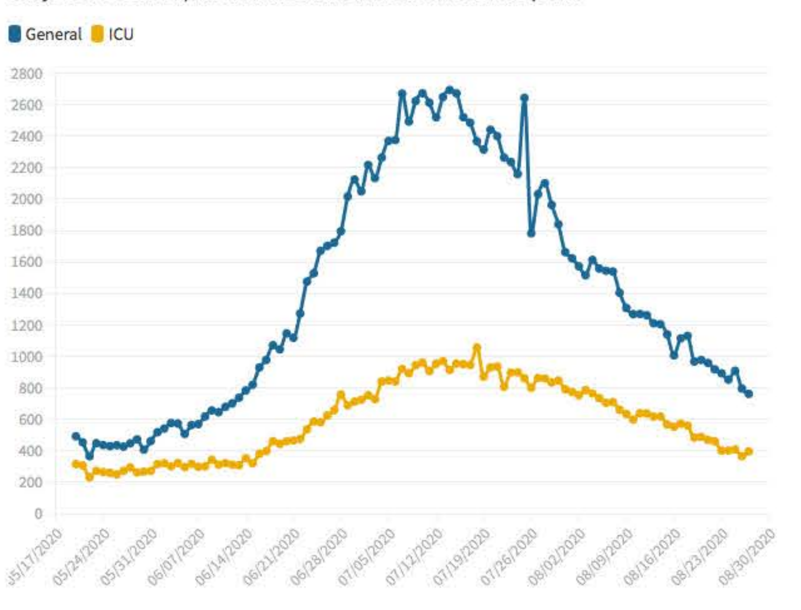

A Flourish chart

The next two charts highlight deaths in the Houston region. The first shows the number of cumulative deaths by date.

Confirmed coronavirus deaths in the Houston region by date

These are all the known deaths in Harris, Fort Bend, Montgomery, Waller, Liberty, Brazoria, Galveston and Chambers counties since April 1.

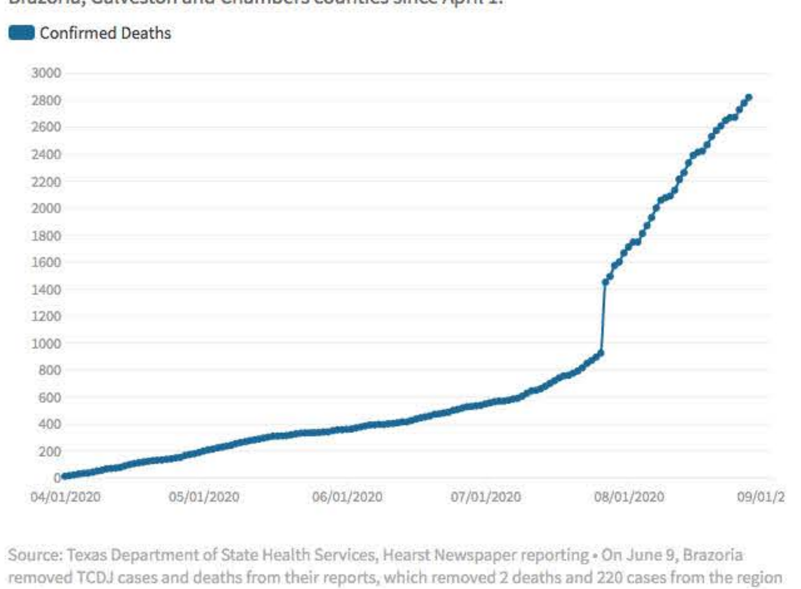

Source: Texas Department of State Health Services, Hearst Newspaper reporting • On June 9, Brazoria removed TCDJ cases and deaths from their reports, which removed 2 deaths and 220 cases from the region total.

A Flourish chart

The second shows the number of new deaths in the region by date.

New coronavirus deaths in the Houston region by date

These are the new deaths, by date, for the eight counties that comprise the Houston region.

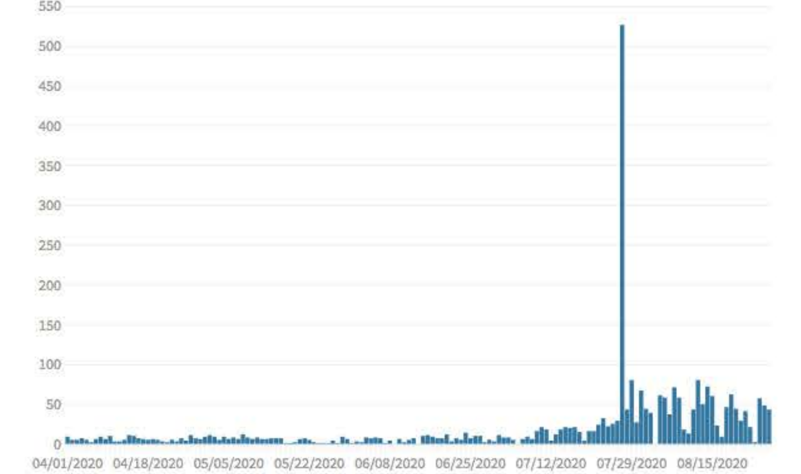

Source: Houston Chronicle reporting, county heath authorities • Created by Jordan Rubio

A Flourish chart

Finally, we've got a breakdown of national coronavirus cases in the style of a U.S. map, helping to show how the numbers in Texas compare to the rest of the country.

5,954,676 confirmed coronavirus cases that we know of, 182,665 deaths

U.S. reports of COVID-19 are tabulated by Johns Hopkins University, which is tracking cases using data collected from the Centers for Disease Control and Prevention and state and local public health departments.

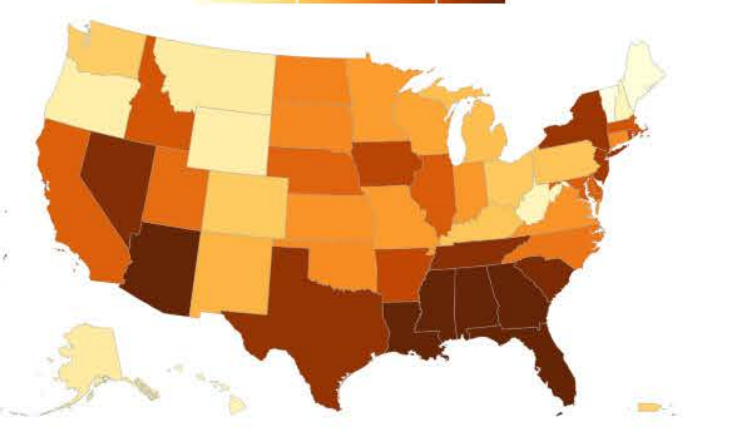

* 152 cases without available location data not mapped. Figures include presumed COVID-19 deaths.

Sources: The Center for Systems Science and Engineering at Johns Hopkins University, U.S. Census Bureau

UTAH DEPARTMENT OF HEALTH COVID-19 Surveillance | Overview | Risk Factors | Hospitalizations & Mortality | Demographics | Trends | Outbreaks

Overview of COVID-19 Surveillance

This report is updated daily at approximately 1:00 p.m. Information on COVID-19 cases changes rapidly, and this report may not reflect updates made after 1:00 p.m. by local health departments or health care systems. Most of the tables, charts, and maps in this report are interactive. Tables can be sorted by clicking on column headings. Maps and charts can be sorted, zoomed, selected, etc. using the mouse cursor and data will appear when hovering or clicking the mouse cursor. Controls will appear at the top right corner of charts when the mouse cursor is placed on the chart.

Report Date: August 29, 2020

51,406	650,560	803,709	3,057	407
Total COVID-19 Cases	Total People Tested	Total Tests Reported	Total COVID-19 Hospitalizations	Total COVID-19 Deaths

Total Number of Lab-Confirmed COVID-19 Cases Living in Utah

Jurisdiction	Cases	Hospitalizations	Deaths
Bear River	2550	130	11
Central Utah	506	29	3
Davis County	3841	215	23
Salt Lake County	23708	1541	230
San Juan	664	89	28
Southeast Utah	141	6	1
Southwest Utah	3565	210	27
Summit County	839	53	1
Tooele County	675	33	0
TriCounty	204	17	1
Utah County	10774	495	46
Wasatch County	629	25	7
Weber-Morgan	3310	214	29
State Total	51406	3057	407

COVID-19 Case Rates per 100,000 Population

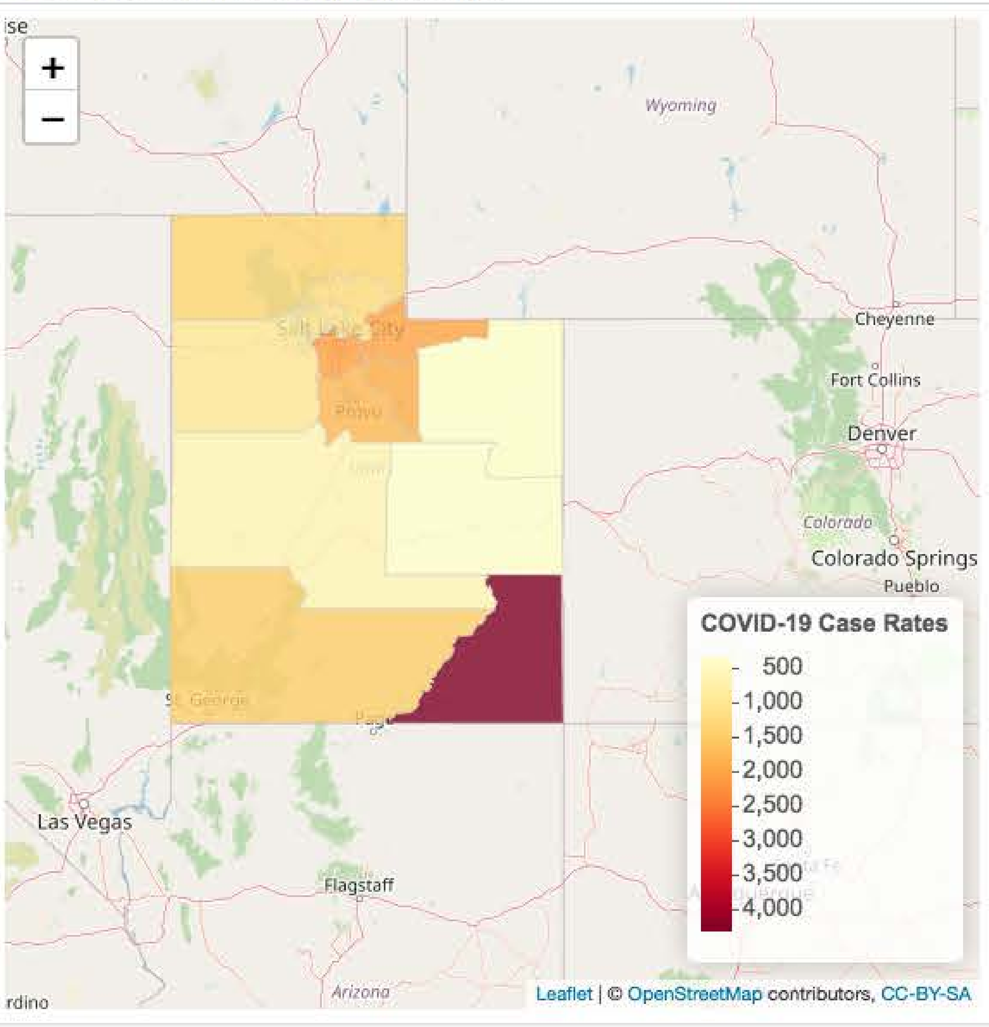

Cases Reported by Indian Health Service and Tribal Nations (these cases are also reflected in case counts by health district)

Reporting Jurisdiction	Cases
Navajo Nation Reservation (Utah) - Utah Navajo Health System	467
Uintah and Ouray Reservation: Uintah & Ouray Indian Health Services (IHS)	16
State Total	51406

COVID-19 Cases by Positive Test Report Date | COVID-19 Cases by Date of Symptom Onset or Diagnosis

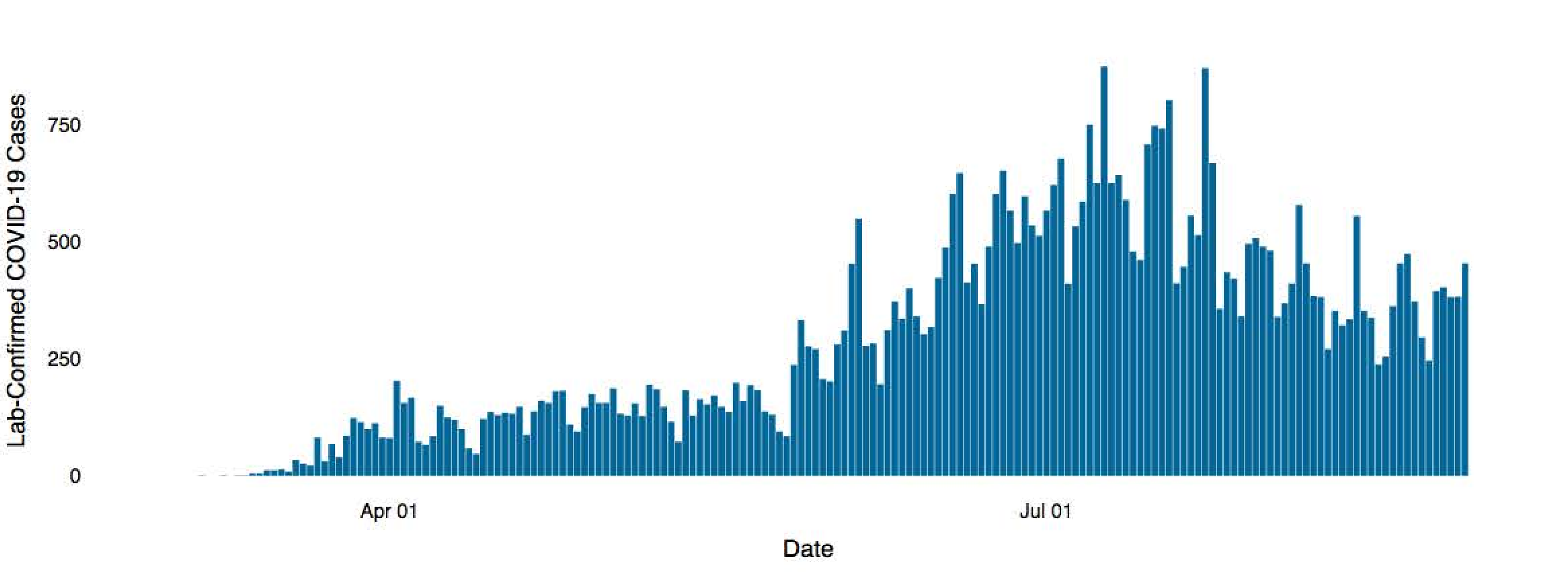

Number of People Tested by Date | Total Tests by Date

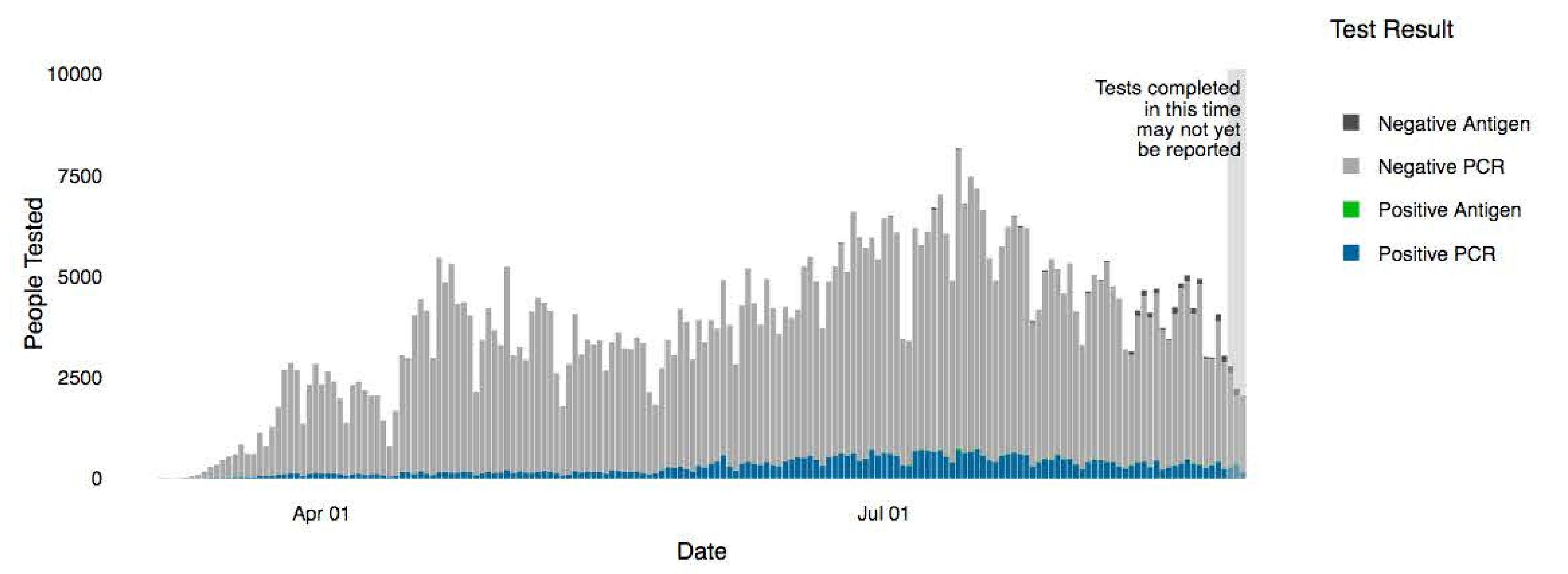

Cumulative COVID-19 Cases with Estimated Recoveries * (does not include the current day)

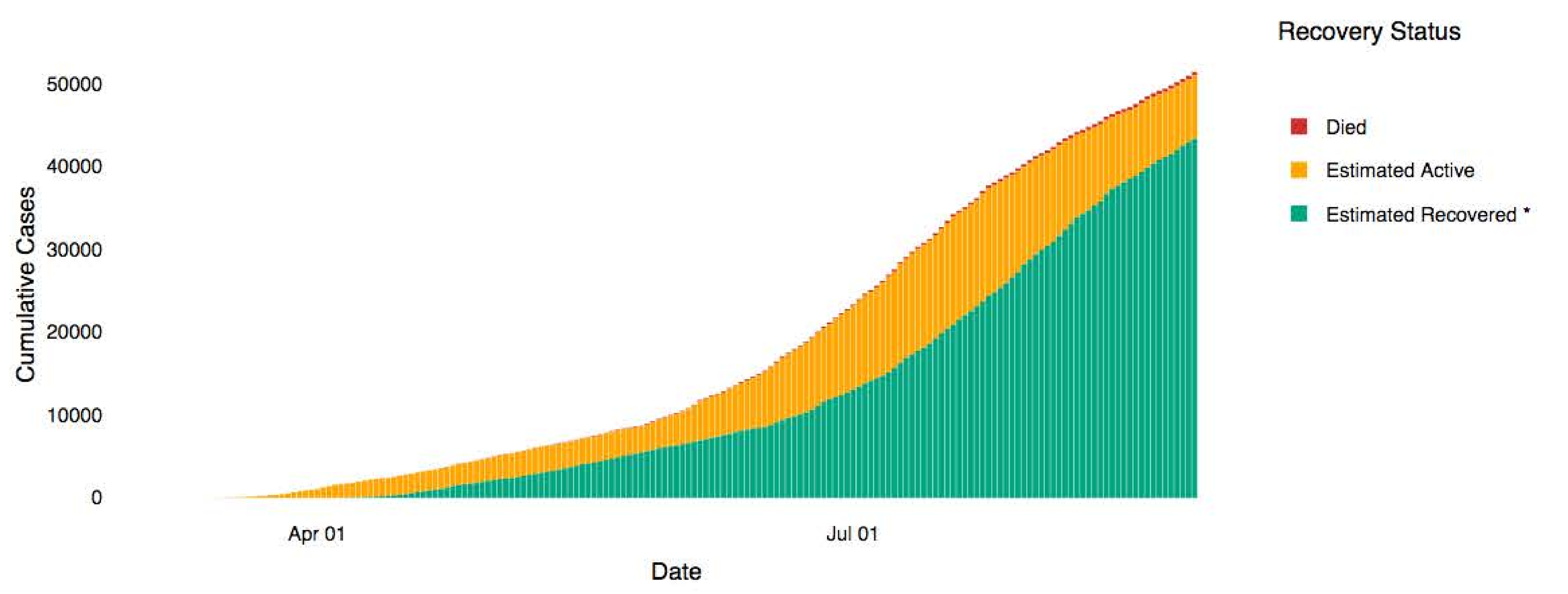

Small Area Case Rates

The below map shows the crude case rate per 100,000 people per Utah Small Area. Crude case rates show the number of COVID-19 cases in an area given its population. Case rates may be higher or lower depending on the age of people living in a small area and how much testing has occurred there. Small areas with low populations may have disproportionately high or low rates depending on few cases and the number of tests performed in an area. A high rate in an area does not necessarily indicate a current outbreak because rates are calculated from the beginning of the pandemic. "Utah Small Areas" are geographic areas with population sizes ranging from about 8,000 to 86,000. Small areas were created by UDOH and local health departments to assess health data and policies. More information on Utah Small Areas can be found at: ibis.health.utah.gov.

Crude Case Rate Per 100,000 People in Utah by Small Area

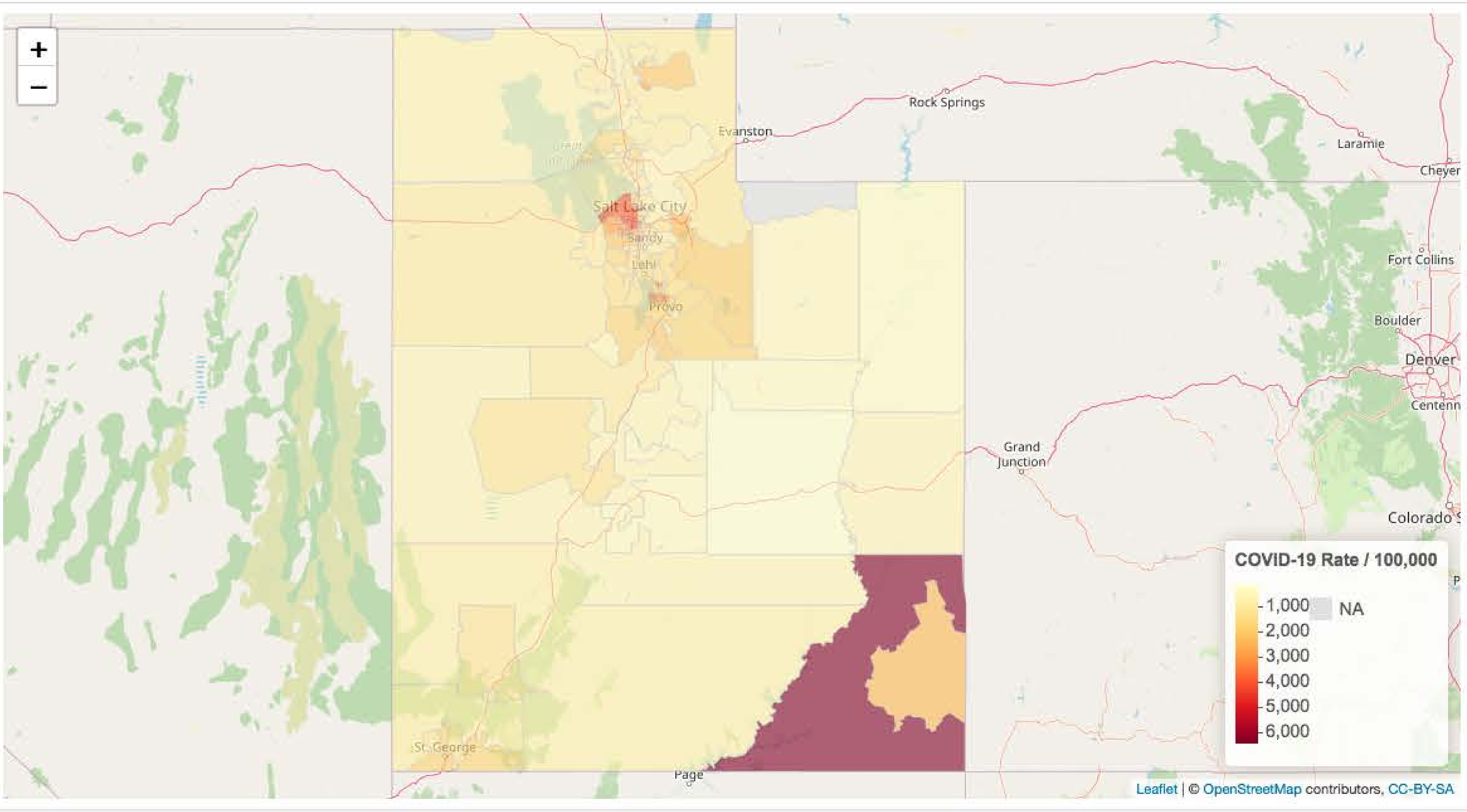

Cases by Local Health Department

Cases counts and total case rates by Local Health Department (LHD) are shown below. Utah's LHDs may also report their own case data. Local reports should be considered the most up to date and accurate information for their local area due to small differences in case classifications at any given time. The "Cumulative Case Rate per 100,000 population by LHD" shows the total number of laboratory-confirmed COVID-19 cases in an area given its population size since the start of the pandemic.

COVID-19 Cases by the Date a Positive Test was Reported to Public Health by LHD | Cumulative Case Rate per 100,000 Population by LHD

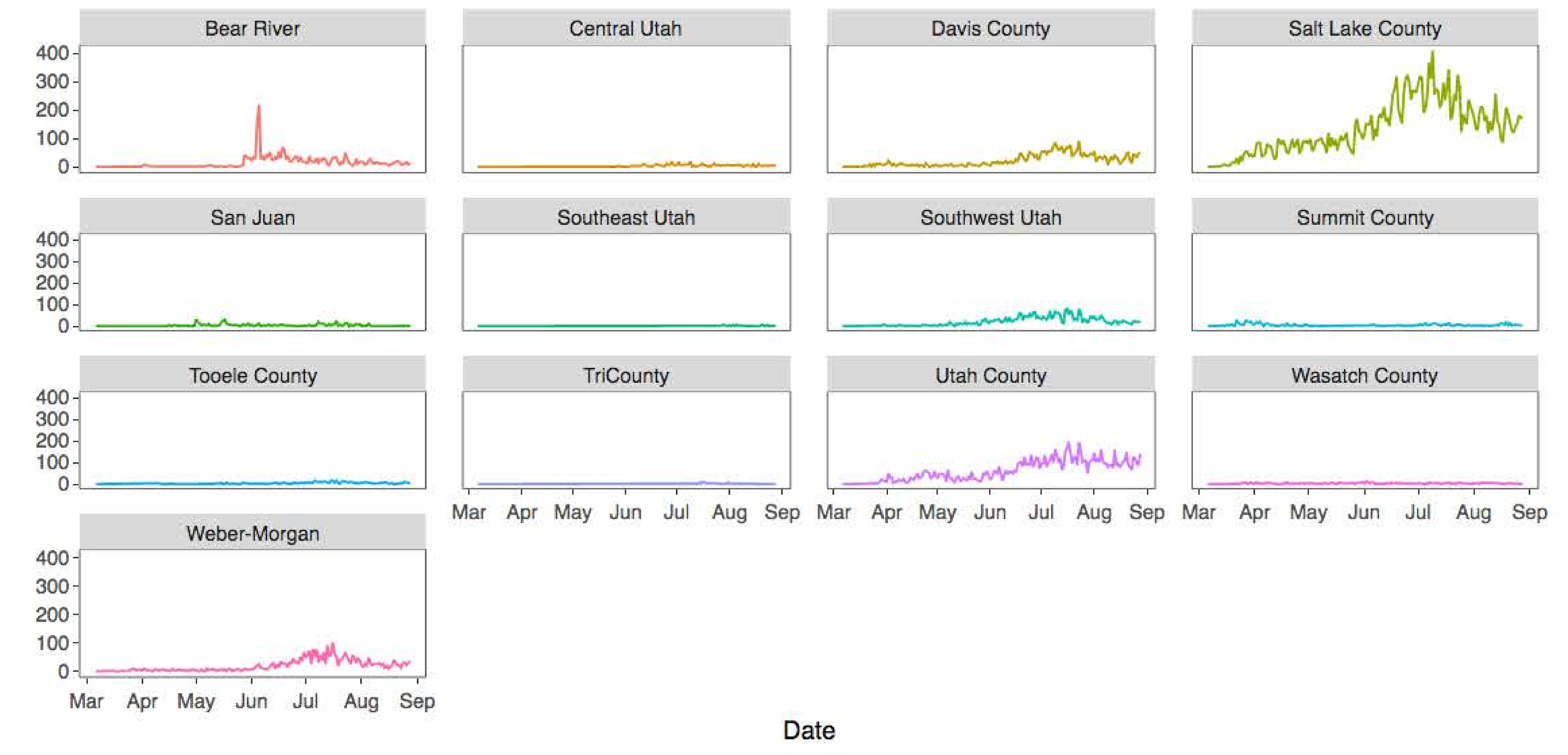

Data Notes:

* The number of recovered persons is estimated by the number of cases whose first positive laboratory test was reported at least 21 days ago, excluding deaths.

Laboratory: The Utah Department of Health (UDOH) is currently reporting PCR and antigen test results and will begin reporting serology results in the coming weeks. Positive test results are reported immediately; negative test results may not be reported for 24-72 hours. Results prior to March 19, 2020 may be under-reported. Laboratory positives may not match confirmed cases due to ongoing investigations and confirmatory testing. When available, laboratory data is shown by the date a sample was tested. If unavailable, it is shown by the date reported to public health. Laboratory data are shown as both the total number of people tested, and as total number of tests performed. For the total number of people tested, one test per person is included by their earliest positive result. If there are no positive tests, their earliest negative result is used.

Case Dates: As of April 15, 2020, case data are displayed by both the date the first positive laboratory result (FPLR) is reported to public health and the self-reported symptom onset date. Previously, this was reported by the first day a person was reported (through laboratory results or contact tracing) to public health. The FPLR date will provide a more stable estimate of the new cases reported to public health; however, there will be small differences in cases by date as public health receives additional information. Onset date is taken preferentially from the following dates when available: 1) self-reported date of first symptoms, 2) date diagnosed by a clinician, 3) date first positive specimen was collected, 4) first report to public health. The actual number of cases in Utah is higher than what is reported due to mild cases not feeling ill enough to seek care, clinician judgment for testing, and expanding laboratory capacity. For more information on how COVID-19 deaths are registered in the state of Utah, visit coronavirus.utah.gov/COVID-19-deaths.

Death Counts: Deaths reported by UDOH include confirmed and probable cases as defined by the Council of State and Territorial Epidemiologists (CSTE) case definition. This includes: 1) confirmed cases with a positive COVID-19 PCR result and no alternative cause of death noted on the death certificate or reported by the Office of the Medical Examiner (OME), 2) probable cases where the death certificate lists COVID-19 disease or SARS-CoV-2 as a cause of death or a significant condition contributing to death and no alternative cause of death reported by the OME, and 3) probable cases with COVID-19 symptoms and close contact to a laboratory confirmed case and no alternative cause of death reported by the OME or the death certificate. Death counts are provisional and subject to change as investigations are completed.

Test Types
PCR Tests: COVID-19 PCR tests detect viral genetic material and are used to diagnose active infections.
Antigen Tests: COVID-19 antigen tests are rapid diagnostic tests that detect specific fragments of the virus. Like PCR tests, they are very specific to the SARS-CoV2 virus and detect active infections. Antigen tests can often be performed at point-of-care facilities. While all test results must be reported, as of July 10, 2020 some facilities had not yet fully implemented reporting for all negative antigen tests.
Antibody Tests: COVID-19 antibody tests, also called serological tests, detect the presence of antibodies to the virus in blood samples. They are typically used to identify people with prior infections. There is a lag between infection and antibody production by the immune system, so antibody tests usually cannot be used to detect active infections.

Case Definitions: UDOH assigns case status following the national case definition, with the exception of considering antigen positive tests as evidence of a confirmed case. A confirmed case is any person with a positive SARS-CoV2 PCR or antigen test.

Data for this report were accessed on August 29, 2020 09:00 AM.

VIRGINIA'S HEALTH IS IN OUR HANDS. Do your part, stop the spread.

COVID-19 in Virginia: [illegible]

VDH VIRGINIA DEPARTMENT OF HEALTH

Dashboard Updated: 8/29/2020

Data entered by 5:00 PM the prior day.

Cases, Hospitalizations and Deaths

Total Cases*		Total Hospitalizations**		Total Deaths	
118,809		9,512		2,568	
(New Cases: 1,217)^					
Confirmed†	Probable†	Confirmed†	Probable†	Confirmed†	Probable†
113,623	5,186	9,448	64	2,435	133

* Includes both people with a positive test (Confirmed), and symptomatic with a known exposure to COVID-19 (Probable).

** Hospitalization status at time case was investigated by VDH. This underrepresents the total number of hospitalizations in Virginia.

^New cases represent the number of confirmed and probable cases reported to VDH in the past 24 hours.

† Probable case status was defined starting April 5 by CSTE, for more information click here: https://cdn.ymaws.com/www.cste.org/resource/resmgr/2020ps/interim-20-id-01_covid-19.pdf

Source: Cases - Virginia Electronic Disease Surveillance System (VEDSS), data entered by 5:00 PM the prior day.

Outbreaks

Total Outbreaks*	Outbreak Associated Cases
832	17,460

* At least two (2) lab confirmed cases are required to classify an outbreak.

For more information about the Long Term Care Facilities, please visit this link: https://www.vdh.virginia.gov/coronavirus/health-professionals/virgini long-term-care-task-force/

Testing (PCR Only)

Testing Encounters PCR Only*	Current 7-Day Positivity Rate PCR Only**
1,555,487	6.9%

* PCR" refers to "Reverse transcriptase polymerase chain reaction laboratory testing."

** Lab reports may not have been received yet. Percent positivity is not calculated for days with incomplete data.

Multisystem Inflammatory Syndrome in Children

Total Cases*	Total Deaths
8	0

*Cases defined by CDC HAN case definition: https://emergency.cdc.gov/han/2020/han00432.asp

Undo Redo Revert Refresh Pause Download Full Screen

COVID-19 in Virginia: Cases

VIRGINIA'S HEALTH IS IN OUR HANDS. Do your part, stop the spread.

VDH VIRGINIA DEPARTMENT OF HEALTH

Dashboard Updated: 8/29/2020
Data entered by 5:00 PM the prior day.

Total Cases*		Total Hospitalizations**		Total Deaths	
118,809		9,512		2,568	
Confirmed†	Probable†	Confirmed†	Probable†	Confirmed†	Probable†
113,623	5,186	9,448	64	2,435	133

Select Measure
(Affects Map and Bar Chart)
- ⦿ Cases
- ○ Hospitalizations
- ○ Deaths

Select Counts or Rates
(Affects Map)
- ⦿ Counts
- ○ Rates per 100,000

Rates that are based on counts less than 15 should be interpreted with caution

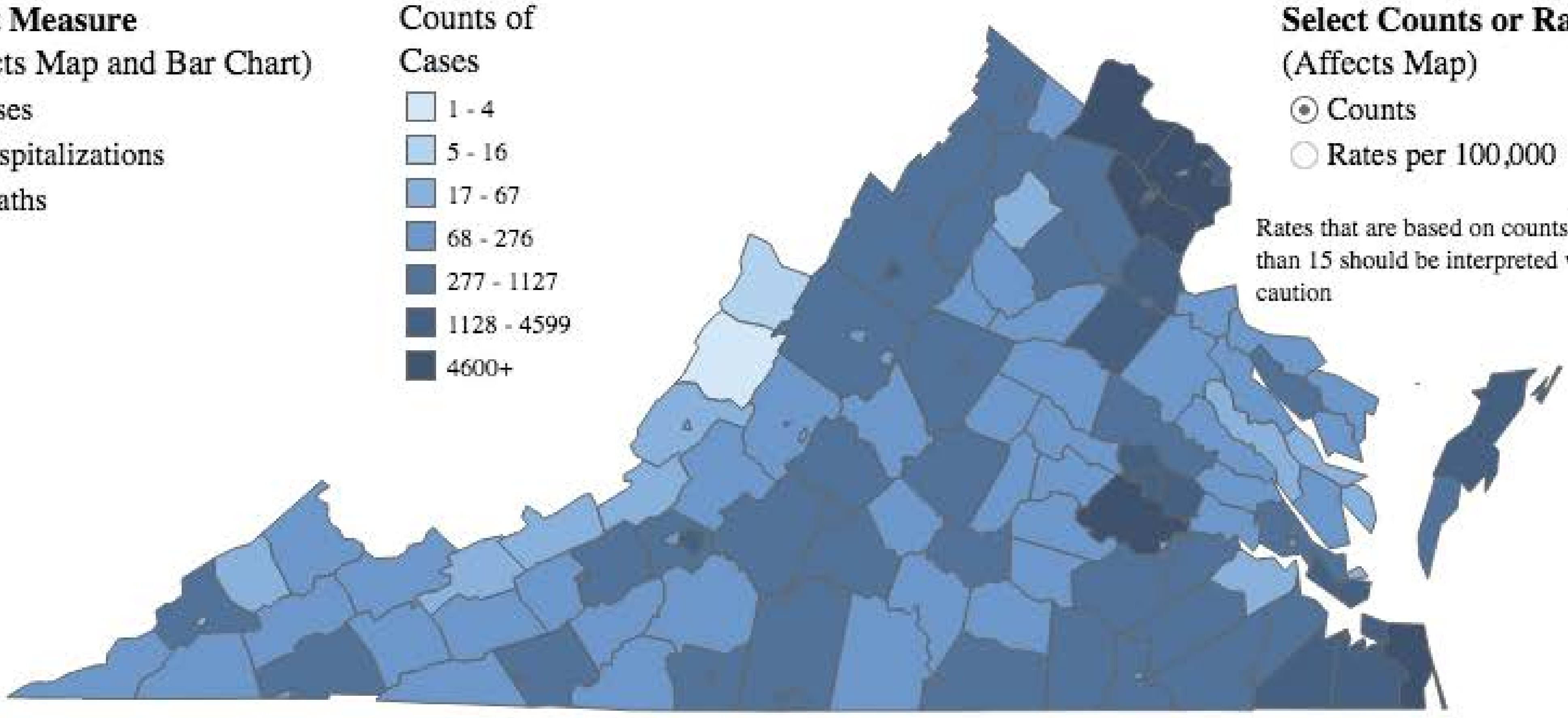

Number of Cases by Date of Symptom Onset

Number of cases by the day closest to when symptoms began.

Select Region
(Affects Bar Chart)
(All)

Illness may not have been reported yet.

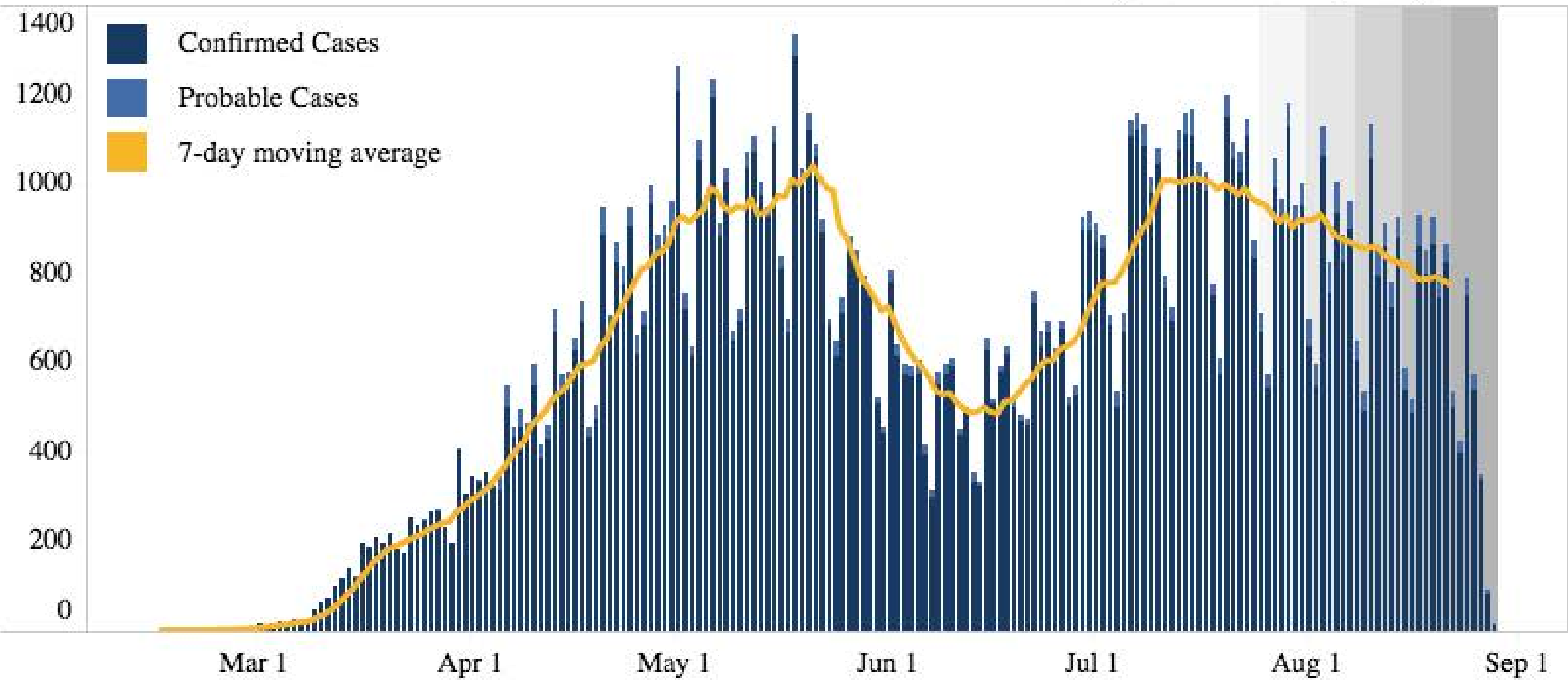

* Includes both people with a positive test (Confirmed), and symptomatic with a known exposure to COVID-19 (Probable).
** Hospitalization status at time case was investigated by VDH. This underrepresents the total number of hospitalizations in Virginia.
† Probable case status was defined starting April 5 by CSTE, for more information click here: https://cdn.ymaws.com/www.cste.org/resource/resmgr/2020ps/interim-20-id-01_covid-19.pdf
Source: Cases - Virginia Electronic Disease Surveillance System (VEDSS), data entered by 5:00 PM the prior day.

Undo Redo Revert Refresh Pause Download Full Screen

COVID-19 in Virginia: Testing

VIRGINIA'S HEALTH IS IN OUR HANDS. Do your part, stop the spread.

VDH VIRGINIA DEPARTMENT OF HEALTH

Dashboard Updated: 8/29/2020
Data entered by 5:00 PM the prior day.

Select Health District from Drop Down Menu
(Affects Number of Testing Encounters, Current Positivity Rate, and Both Bar Charts)
(All)

Select PCR* or Total Tests from Drop Down M
(Affects Table and Both Bar Charts)
PCR Only

All Health Districts
Testing Encounters PCR Only
1,555,487

All Health Districts
Current 7-Day Positivity Rate PCR Only
6.9%

Testing Encounters by Health District, PCR Only

Fairfax	185,764
Prince William	97,212
Loudoun	66,265
Virginia Beach	83,913
Chesterfield	69,847
Henrico	76,998
Rappahannock	50,538
Arlington	43,510
Norfolk	51,284
Lord Fairfax	42,755
Richmond	47,258
Peninsula	58,467
Alexandria	30,773
Thomas Jefferson	47,968
Roanoke	51,282
Chesapeake	46,037
Central Shenandoah	40,721
Rappahannock Rapidan	28,135
Crater	32,843
Central Virginia	41,430
Western Tidewater	33,194
Chickahominy	25,165
Portsmouth	22,534
Three Rivers	23,796
Mount Rogers	24,689
Eastern Shore	11,247
Piedmont	21,023
Hampton	20,224
Alleghany	22,231
New River	31,373
Southside	16,224
Lenowisco	11,304
West Piedmont	15,147
Cumberland Plateau	10,375
Pittsylvania-Danville	16,214
Out of State	49,741
Unknown	8,006

Number of Testing Encounters, Number of Positive Testing Encounters, and Percent Positivity by La Report Date - All Health Districts, PCR Only**

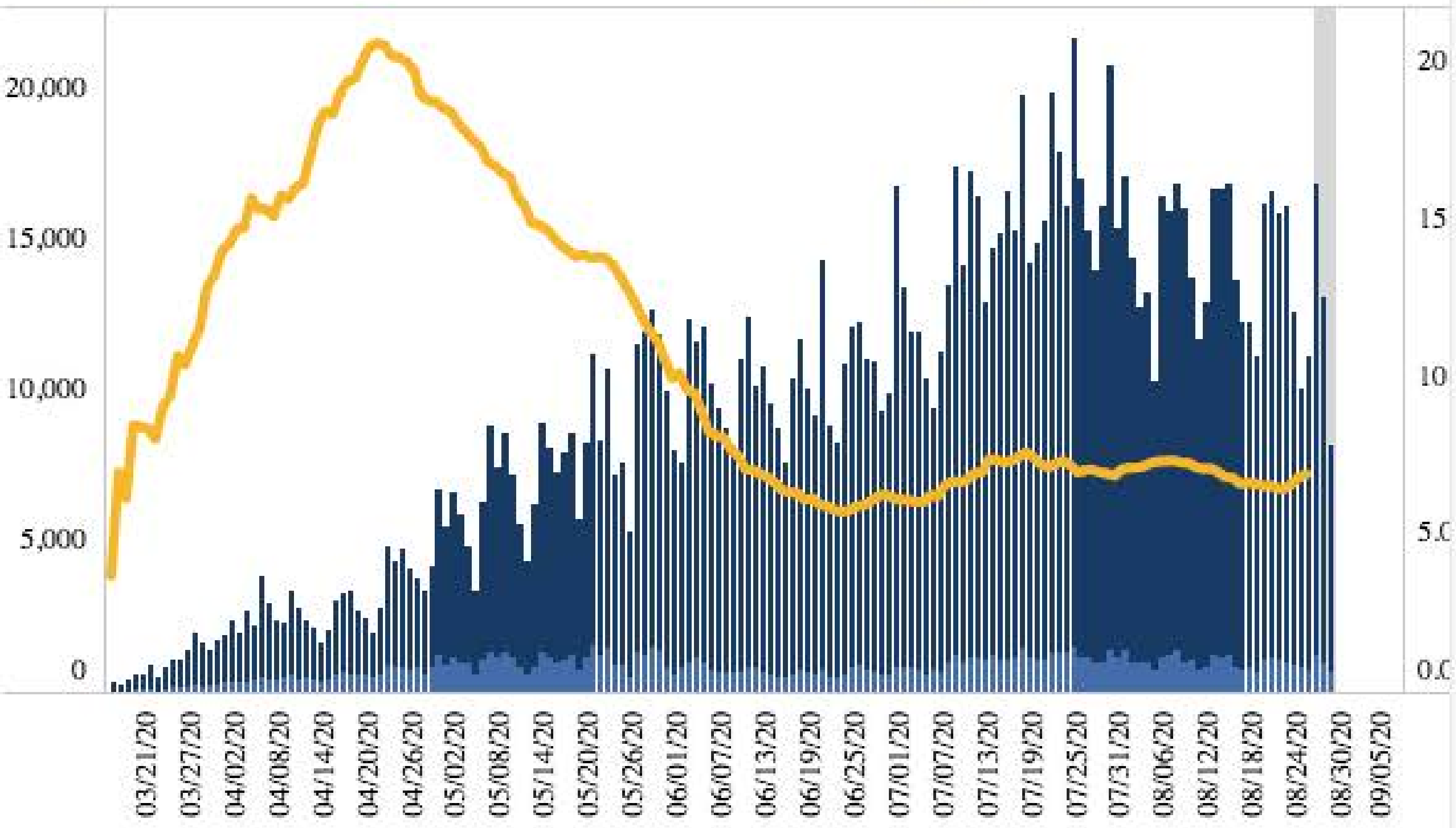

Number of Testing Encounters by Lab Report Date - All Health Districts, PCR Only

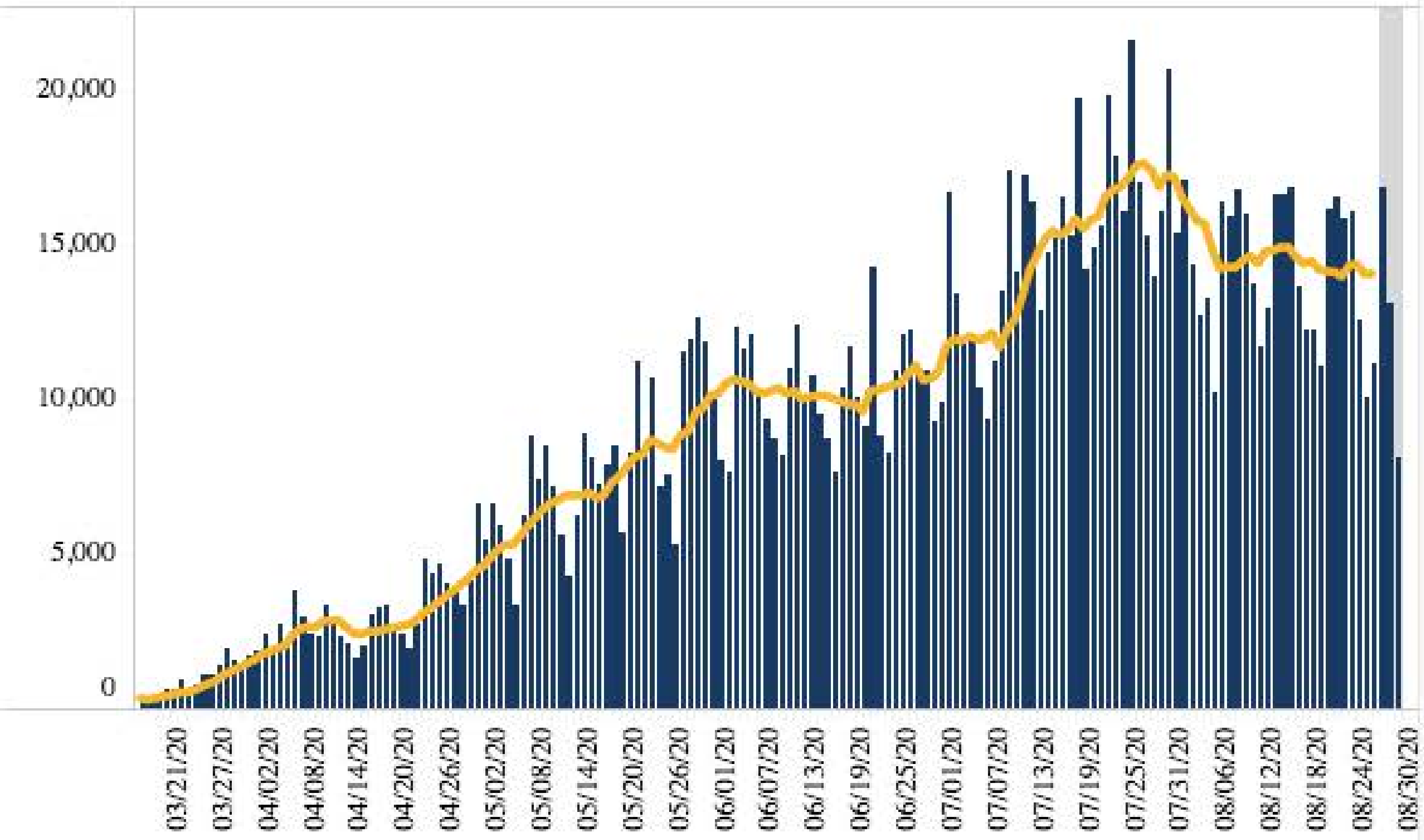

* PCR" refers to "Reverse transcriptase polymerase chain reaction laboratory testing."
** Lab reports may not have been received yet. Percent positivity is not calculated for days with incomplete data.

Undo Redo Revert Refresh Pause Download Full Screen

COVID-19 in Vermont Vermont Department of Health (VDH)

Total Cases
1,605
15 New

Currently Hospitalized
2

Hospitalized Under Investigation
8

Total People Recovered
1,413

Deaths
58

People Tested
130,180

Travelers Monitored
765

Contacts Monitored
68

People Completed Monitoring
7,421

Last Updated: 8/29/2020, 10:40:24 AM

COVID-19 Positive Cases by County

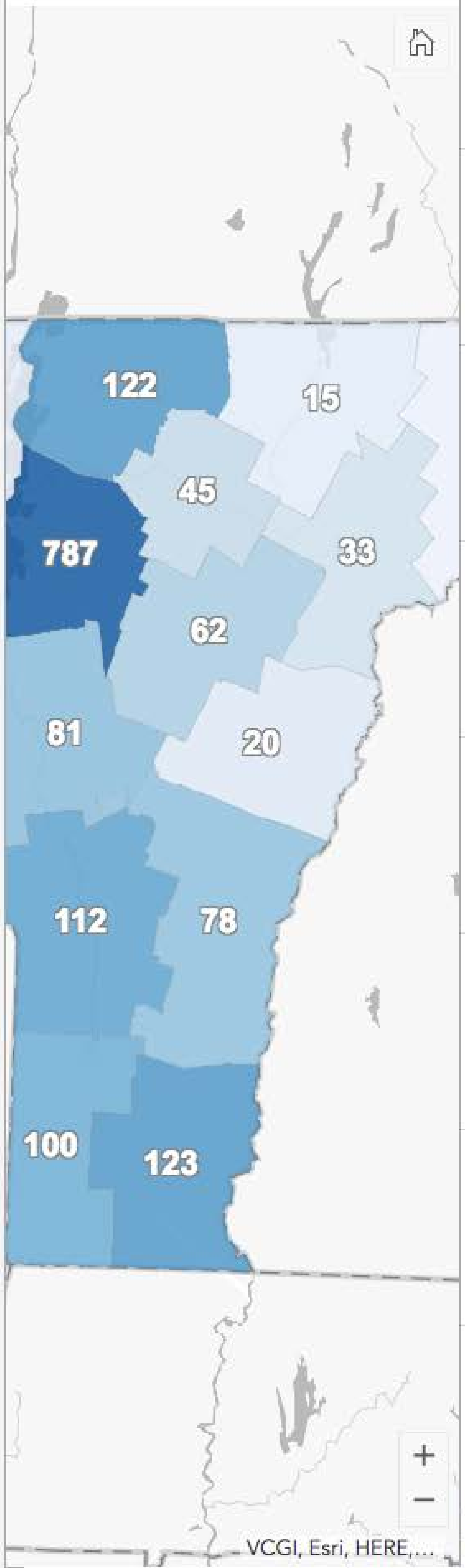

Chittenden County
Total Cases: **787**
New Cases: **8**
Total Deaths: **39**
Cases Per 10,000: **47.82**

Windham County
Total Cases: **123**
New Cases: **2**
Total Deaths: **3**
Cases Per 10,000: **28.77**

Franklin County
Total Cases: **122**
New Cases: **0**
Total Deaths: **7**
Cases Per 10,000: **24.69**

Rutland County
Total Cases: **112**
New Cases: **1**
Total Deaths: **1**
Cases Per 10,000: **19.09**

Bennington County
Total Cases: **100**
New Cases: **0**
Total Deaths: **1**
Cases Per 10,000: **28.07**

Addison County
Total Cases: **81**
New Cases: **2**
Total Deaths: **2**
Cases Per 10,000: **21.91**

Windsor County
Total Cases: **78**
New Cases: **1**
Total Deaths: **2**
Cases Per 10,000: **14.11**

Washington County
Total Cases: **62**
New Cases: **0**
Total Deaths: **2**
Cases Per 10,000:

Click a County to highlight it on the map.

Cases by County

All data are compiled by the Vermont Department of Health, and are preliminary and subject to change. Case information is updated daily by 12:00pm ET, reflecting counts as of end of previous day. For more information, see the full VDH COVID-19 page. Application is dynamically resized if viewing on a mobile device. Click tabs to view other info, use sliders or resize elements if needed by dragging the divider between them, or use the expand button in upper right to enlarge section:

Total Cases includes Vermont residents who tested positive at VDH, commercial, and other public health labs, and non-Vermont

COVID-19 **Cases** and **Deaths** by Age Group

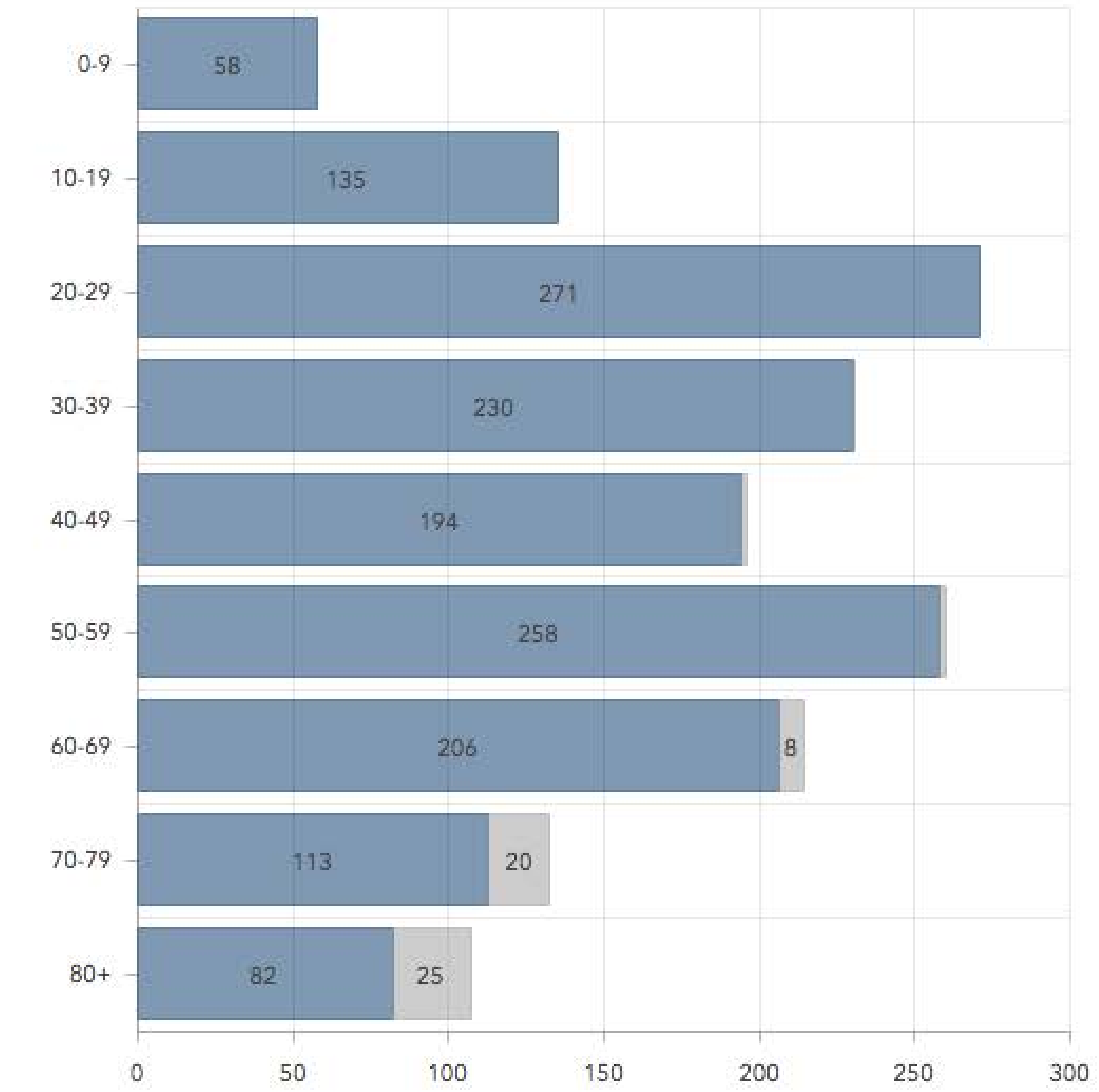

Vermont COVID-19 **New** and **Cumulative** Cases, **Cumulative** Deaths

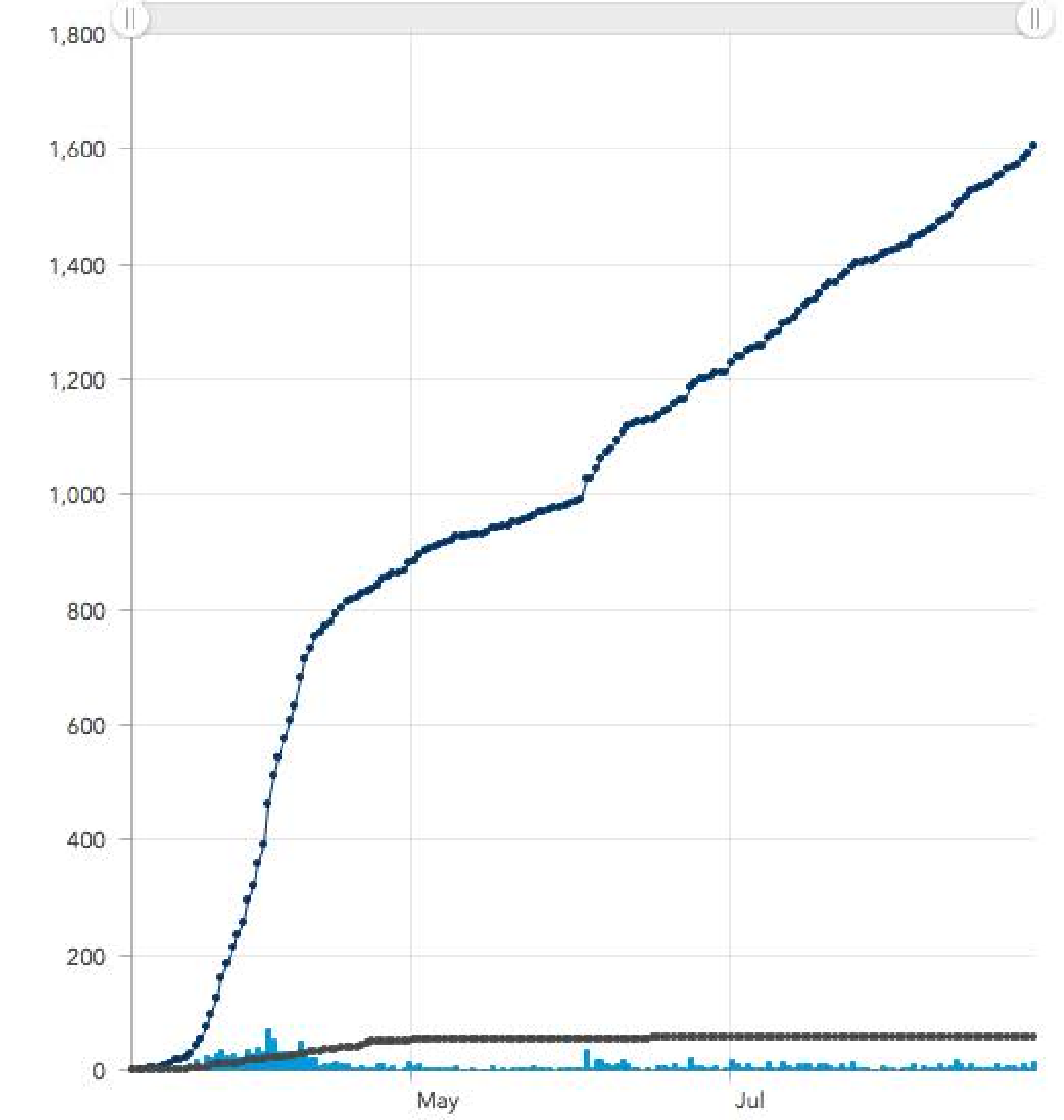

Hover over chart to see values, and drag scrollbar at top of chart to zoom into date range

Cases and Deaths by Date and Age

Support VTDigger
Rigorous nonprofit news for Vermont.
DONATE → Email SIGN UP
VTDIGGER
News in pursuit of truth

The latest numbers in Vermont

Vermont public health labs are currently testing for coronavirus at the order of doctors. Results take 24-48 hours to come back.

As of 8/27/20:	
People tested positive	1586
People hospitalized	4
People who have died	58
People who have recovered	1396
Total people tested	124831
Contacts being monitored	63
People who are hospitalized under investigation	8

[illegible]

The rise of coronavirus in Vermont

Cases from 3/3/2020 to today, according to the Vermont Department of Health.

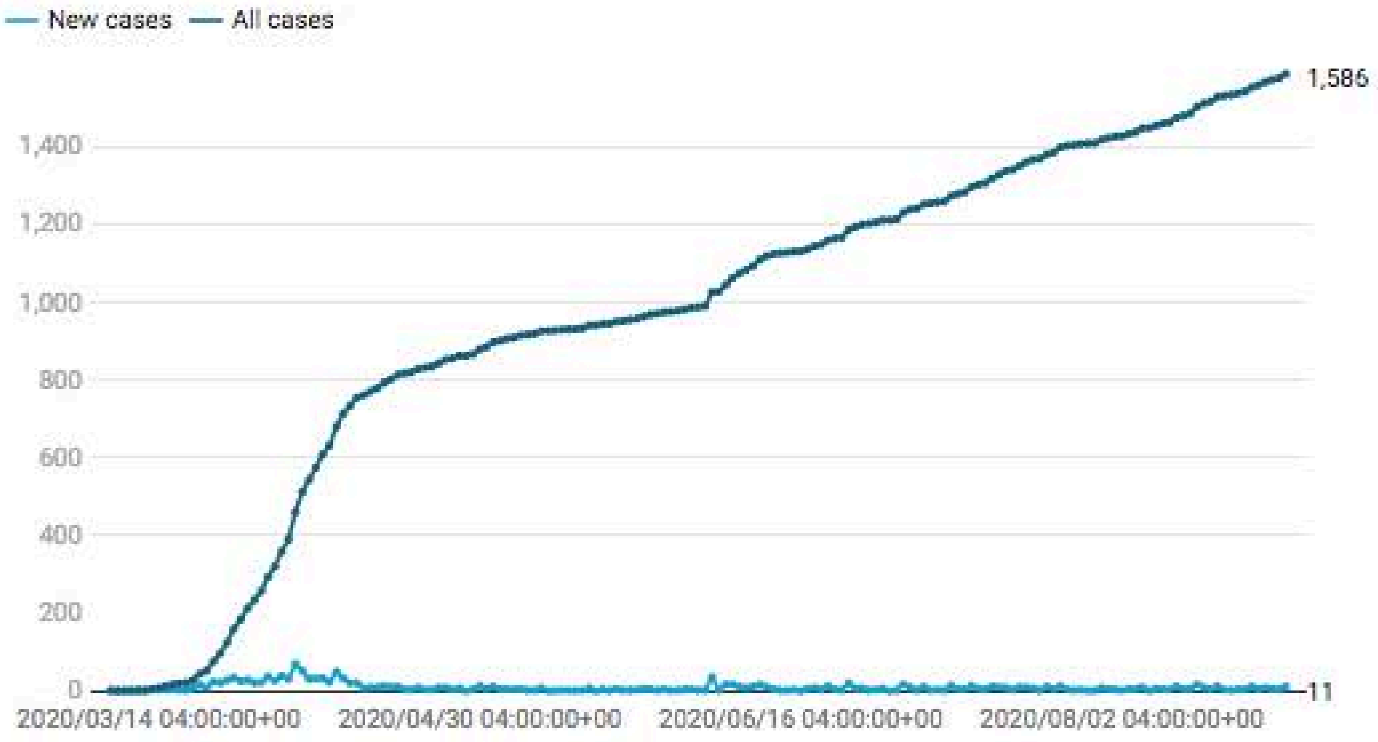

Updated around 1 p.m. each day.

Chart: Erin Petenko • Source: VTDOH via VT Center for Geographic Information • Get the data • Created with Datawrapper

COVID-19 cases, by county

Mouse over or click on a county to view deaths and the rate by population. Updated by the VT Department of Health by 1 p.m. daily.

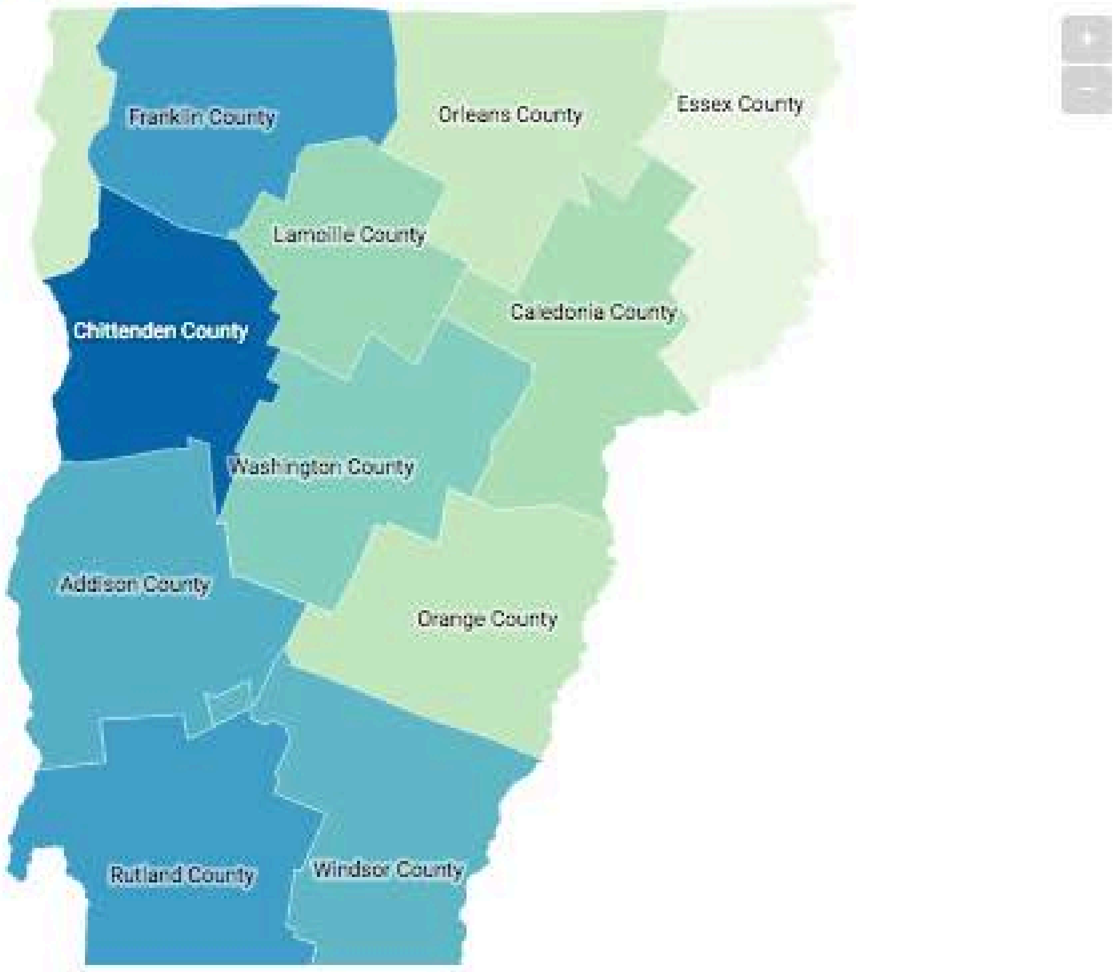

Current Status | Epidemiologic Curves | Cumulative Counts | Demographics | Testing | COVID-like Illness Hospitalizations

CURRENT STATUS

Data as of August 28, 2020 11:59PM PT

Select an Option

Confirmed Cases

Hospitalizations

Deaths

Select County

All

Tabular View

County-level information can be found on Local Health Jurisdiction (LHJ) websites

LHJ websites

COVID-19 in Washington State

Confirmed Cases, Hospitalizations and Deaths by County

COVID-19 has spread throughout most of Washington State. Confirmed cases, hospitalizations and deaths happened to people who have had a positive molecular test for COVID-19.

Learn More

Confirmed Cases	**73,879**
Hospitalizations	**6,723**
Deaths	**1,905**
Percent of Deaths (deaths/confirmed cases)	**2.6%**
Total Tests	**1,447,460**

Please click "Learn More" for more information.

Confirmed Cases by County

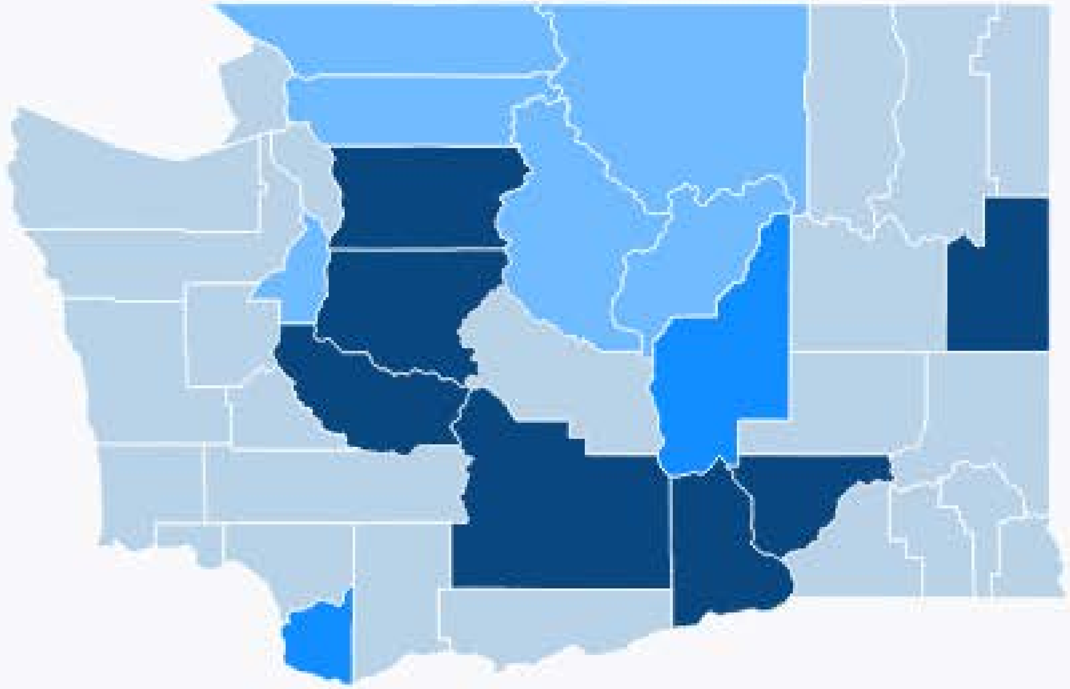

Legend

- 3,880 +
- 2,911 to 3,880
- 1,941 to 2,910
- 971 to 1,940
- 1 to 970
- 0

267 of 73,879 confirmed cases do not have an assigned county

Microsoft Power BI

Current Status | Epidemiologic Curves | Cumulative Counts | Demographics | Testing | COVID-like Illness Hospitalizations

EPIDEMIOLOGIC CURVES

Data as of August 28, 2020 11:59PM PT

Select an Option

Confirmed Cases

Hospitalizations

Deaths

Select County

All

Tabular View

Click on Tabular View to see and download the data.

COVID-19 in Washington State

Confirmed Cases and Deaths by Illness Onset Date, and Hospitalizations by Admission Date

This chart shows the progression of the COVID-19 outbreak in Washington by cases, hospitalizations and deaths over time and is known as an epidemiologic curve. The epidemiologic curve is the curve referred to in the phrase, "flatten the curve."

Learn More

Deaths Counts

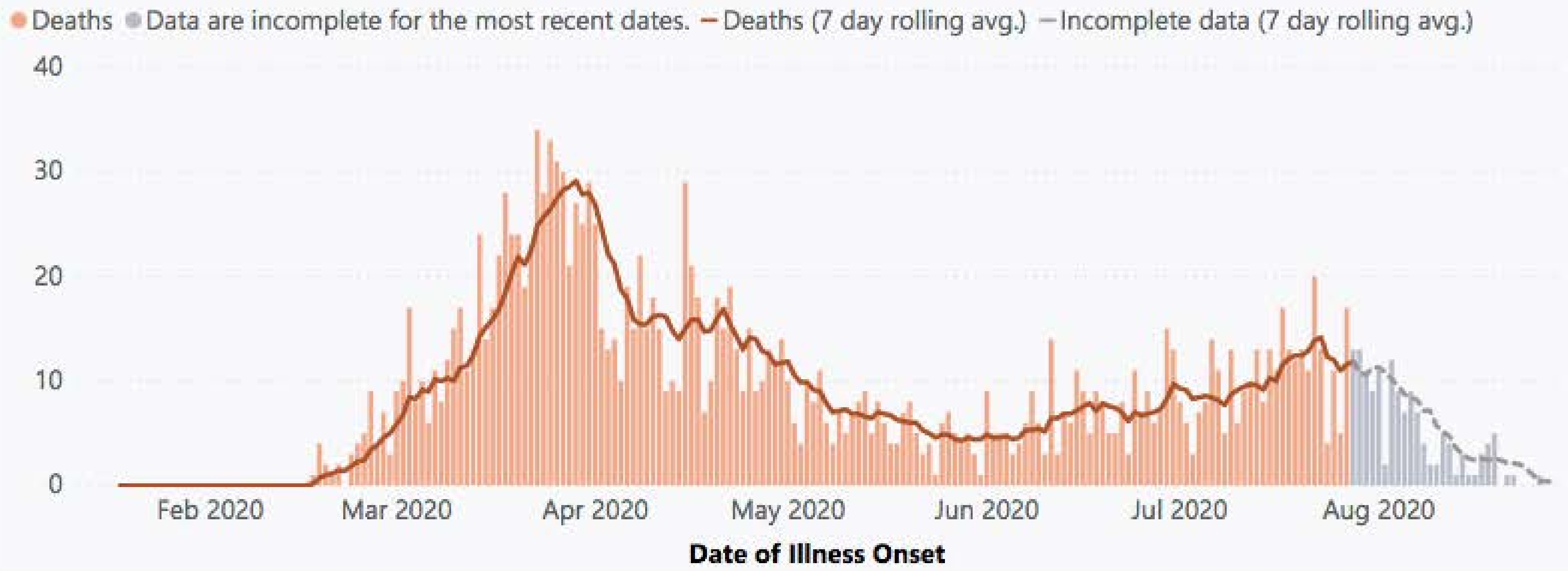

4 of 1,905 deaths do not have an assigned county. Deaths from the last 3-4 weeks may not yet be reported.

Current Status | Epidemiologic Curves | Cumulative Counts | Demographics | Testing | COVID-like Illness Hospitalizations

TESTING

Data as of August 28, 2020 11:59PM PT

Select a County

All

Download

COVID-19 in Washington State

Number of Tests by Specimen Collection Date

Testing was initially limited, but since late June, we have averaged over 10,000 tests per day. We continue to work on increasing test availability and accessibility across Washington.

Learn More

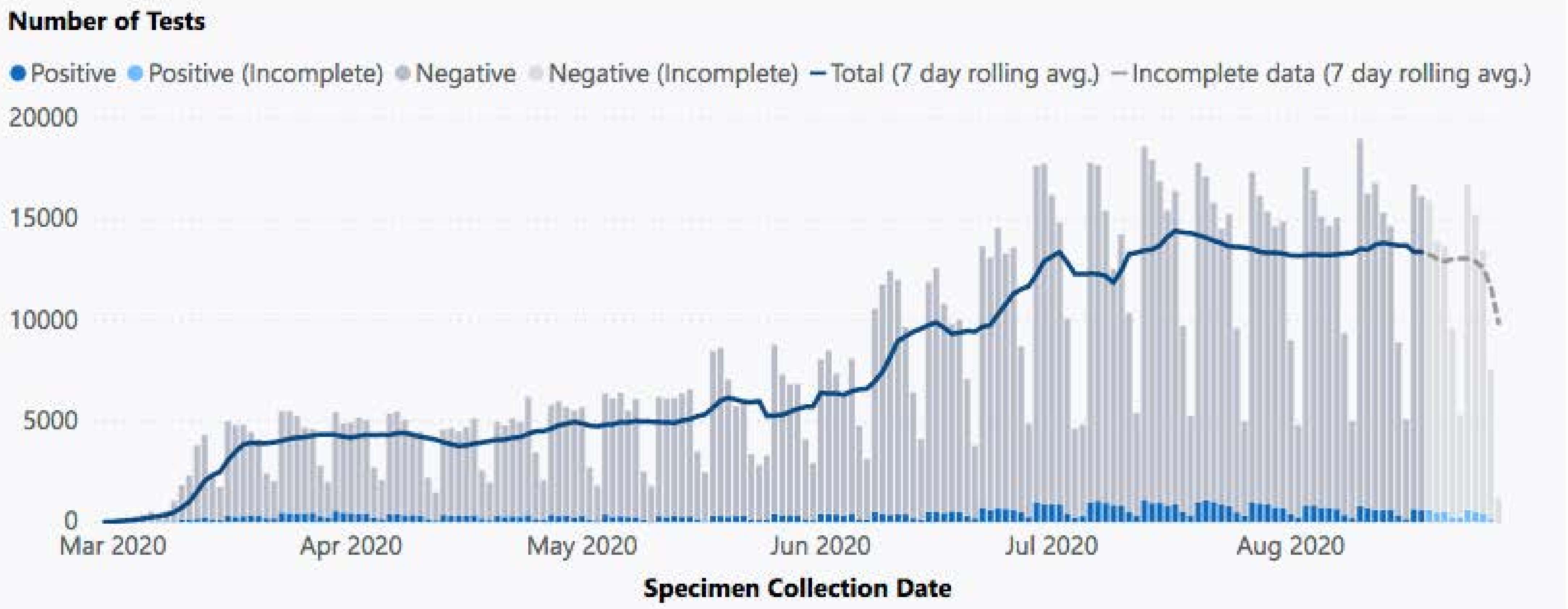

262,080 out of 1,447,460 test results do not have an assigned county.

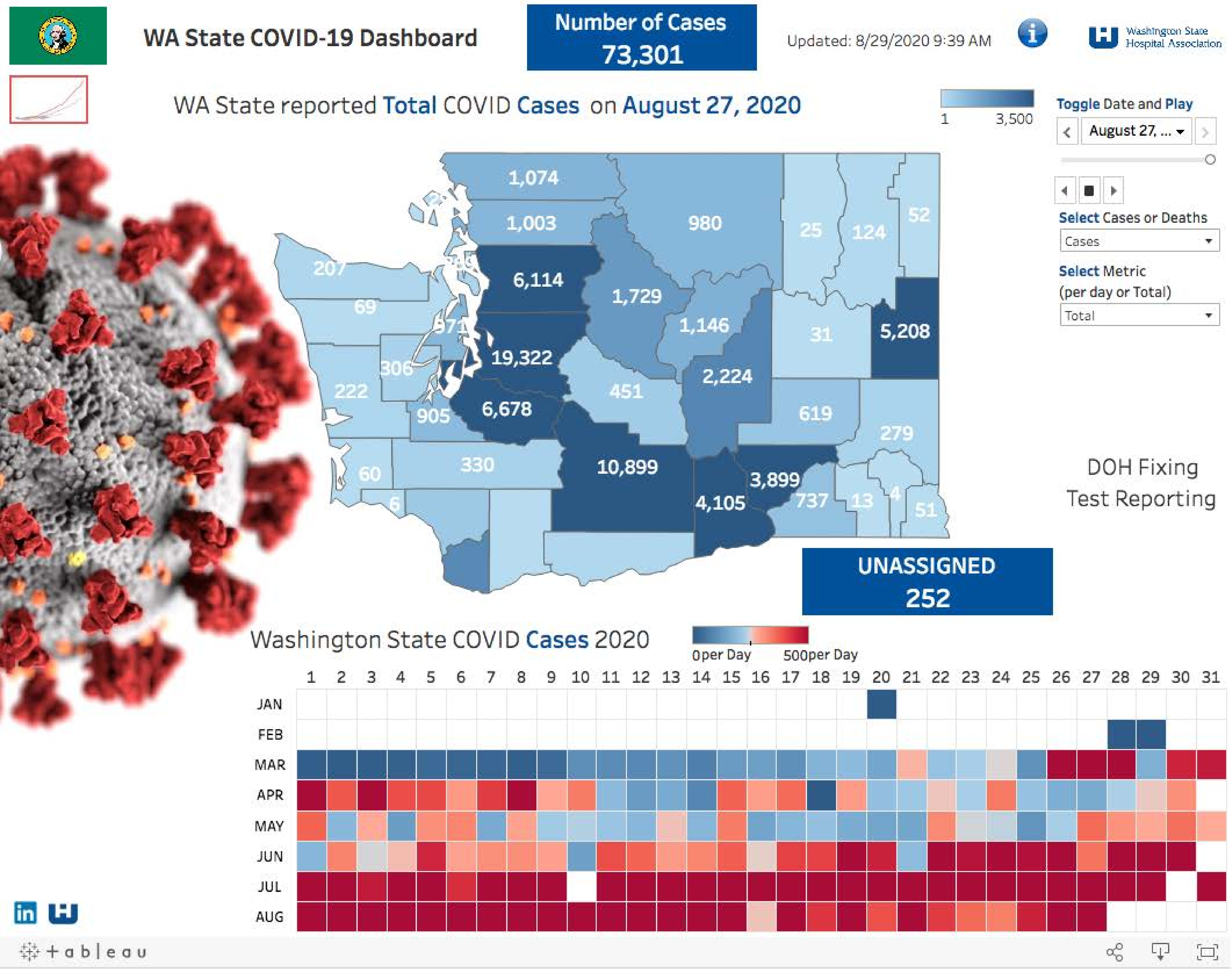
WA State COVID-19 Dashboard
Number of Cases
73,301
Updated: 8/29/2020 9:39 AM
Washington State Hospital Association
WA State reported Total COVID Cases on August 27, 2020
1
3,500
Toggle Date and Play
August 27, ...
Select Cases or Deaths
Cases
Select Metric
(per day or Total)
Total
1,074
1,003
980
25
124
52
207
6,114
1,729
69
1,146
31
5,208
571
19,322
306
2,224
222
451
905
6,678
619
279
330
10,899
60
3,899
4,105
737
13
4
51
6
DOH Fixing
Test Reporting
UNASSIGNED
252
Washington State COVID Cases 2020
0per Day
500per Day
1 2 3 4 5 6 7 8 9 10 11 12 13 14 15 16 17 18 19 20 21 22 23 24 25 26 27 28 29 30 31
JAN
FEB
MAR
APR
MAY
JUN
JUL
AUG
+ a b | e a u

West Virginia COVID-19

WEST VIRGINIA Department of Health & Human Resources BUREAU FOR PUBLIC HEALTH

Overview | County Alert System | 7 Day Trend | Cumulative Summary | Case and Lab Trends | Other Trends | Hospital | Long-Term Care | State Comparison

i

**9,967**<br>Total Number of Cases<br>Includes Probable and Confirmed Cases (Click to View)	**212**<br>Deaths<br>Includes Probable and Confirmed Deaths (Click to View)	**426,074**<br>Confirmatory Lab Test	**2.34%**<br>Cumulative Percent Positive<br>Total Cases / Confirmatory Lab Test
**143**<br>New Cases Received In The Last 24 Hours<br>*Compared to yesterday's data	**1,820**<br>Current Active Cases		**2.47%**<br>Daily Percent Positive<br>Daily Cases / Confirmatory Lab Test

Note: Data posted on dashboard is from 12:00 midnight the previous day. All data are provisional and subject to change based on information obtained during public health investigations.

Click here to view CDC's definition on Confirmed and Probable cases

Updated 10:00 AM 8/29/2020

Microsoft Power BI

COVID-19: County Data

COVID-19: Wisconsin Data
- Summary
- Activity Level by Region and County
- Cases
- Data 101
- Deaths
- Facility-Wide Investigations
- Hospital Capabilities
- COVID-19

About our data

All data are laboratory-confirmed cases of COVID-19 that we extract from our live Wisconsin Electronic Disease Surveillance System (WEDSS) and freeze once a day. These numbers are the official state numbers, though counties may report their own totals independent of DHS. Combining the DHS and local totals may result in inaccurate totals.

Deaths must be reported by health care providers, medical examiners/coroners, and recorded by local health departments in order to be counted.

The number of people with negative test results includes only Wisconsin residents who had negative test results reported **electronically** to DHS or entered manually into the WEDSS electronic laboratory module. As a result, this number underestimates the total number of Wisconsin residents with negative test results.

Data shown below are subject to change. We strive for transparency and accuracy in our data, and as individual cases are investigated by public health, there may be corrections to the status and details of cases that result in changes to this information. Some examples of corrections or updates that may lead to changes to our data, such as case and negative counts and deaths going up or down, include:

- Update or correction of case's address, resulting in a change to their location of residence to another county or state,
- Correction to laboratory result,
- Correction to a case's status from confirmed to unconfirmed (for example, if they were marked as confirmed because a blood test detecting antibodies was positive instead of a test detecting the virus causing COVID-19),
- De-duplication or merging and consolidation of case records,
- Update of case's demographic information from missing or unknown to complete information.

For information on testing, see: COVID-19, testing criteria section.

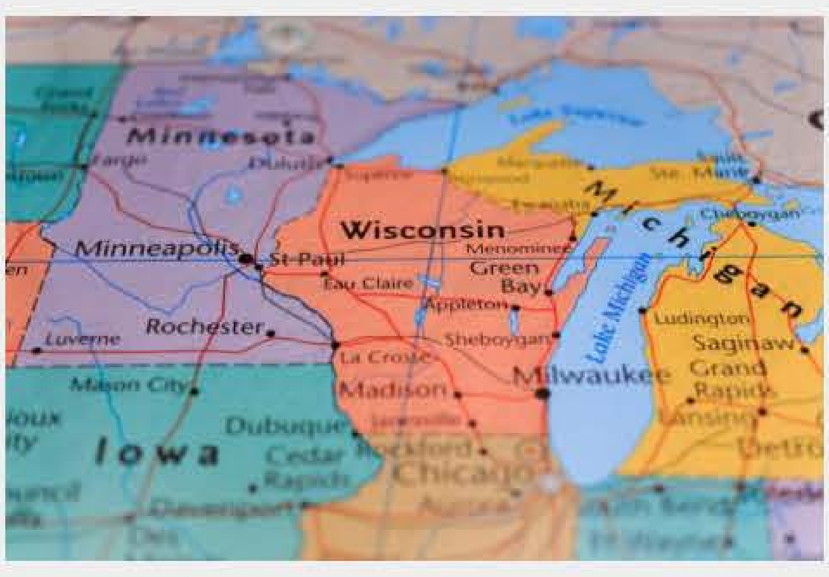

View COVID-19 Maps

- Cases by county and census tract
- Deaths by county and census tract
- Community testing sites

Download the following data:

- State
- County
- Testing sites

Need more information? Look at our Data Dictionary (PDF) or visit our Data 101 section where you can find information on how we calculate and report COVID-19 data.

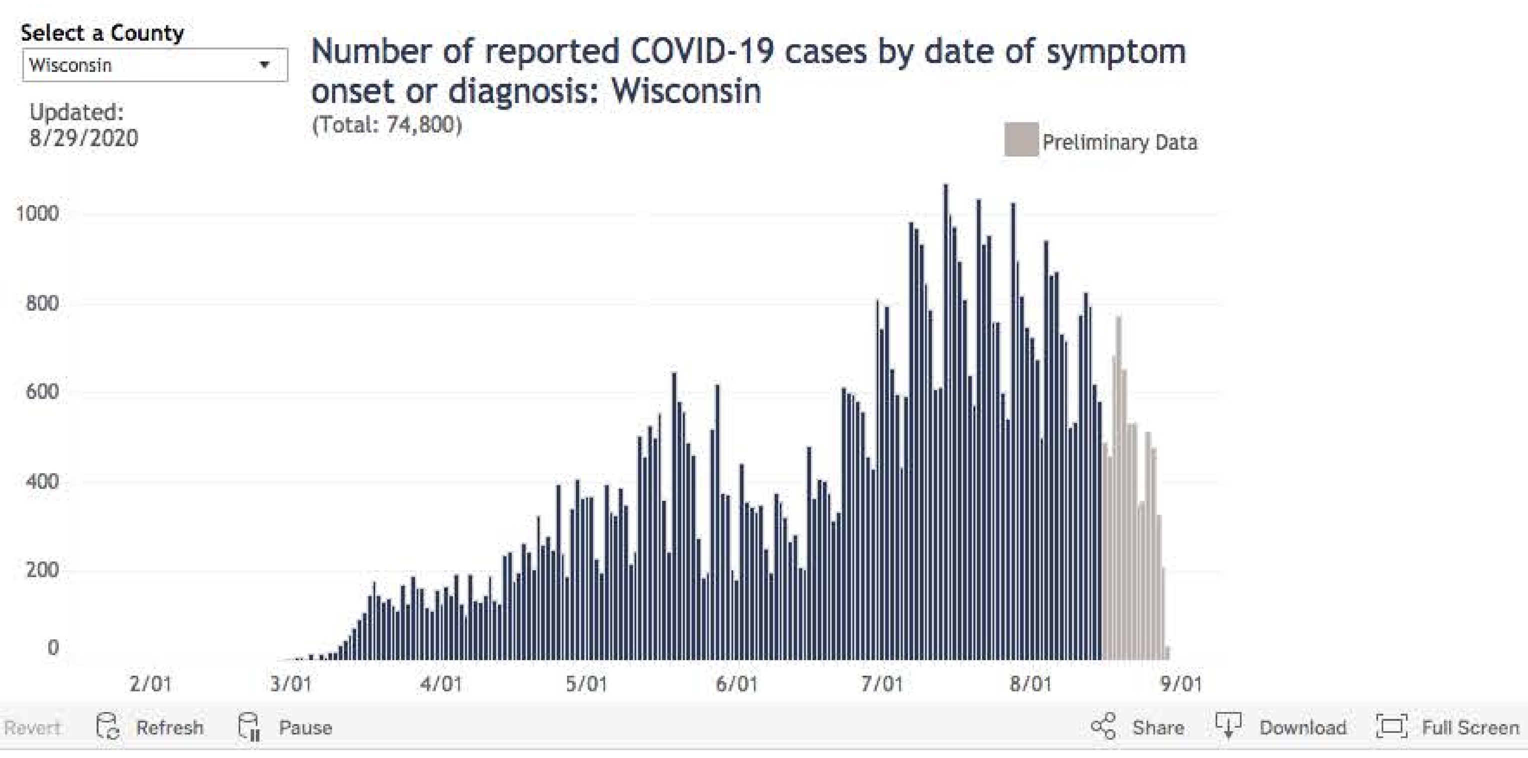

This chart is called an epidemic curve (or "epi curve"). It is used to track the number of illnesses over time and see when peaks of illnesses occur. This figure is showing data by when someone's symptoms began, also called symptom onset date (or by diagnosis date if symptom onset date is missing, or the patient did not have symptoms). Symptom onset date is more meaningful than using the date when the case was reported because it represents when illnesses occurred, instead of when the person was tested and the result was reported to public health.

When using symptom onset date, any downward trends that are seen during the most recent two weeks are usually not true decreases in illness and need to be interpreted with caution. This downward trend usually represents the data lag time; thus, data during the most recent two weeks are highlighted as **preliminary data.** When people have an acute illness, such as COVID-19, it may take several days for them to see a doctor or be tested. It also takes time for the tests to be completed and the results to be sent to public health to be included in case counts.

The figure titled "Cumulative total and newly reported COVID-19 cases by date confirmed" on our COVID-19: Wisconsin Cases webpage does present data by the date a case was reported as being laboratory-confirmed (and not by symptom onset date). These data are also available by county and can be downloaded by clicking the "Download data by county" hyperlink above.

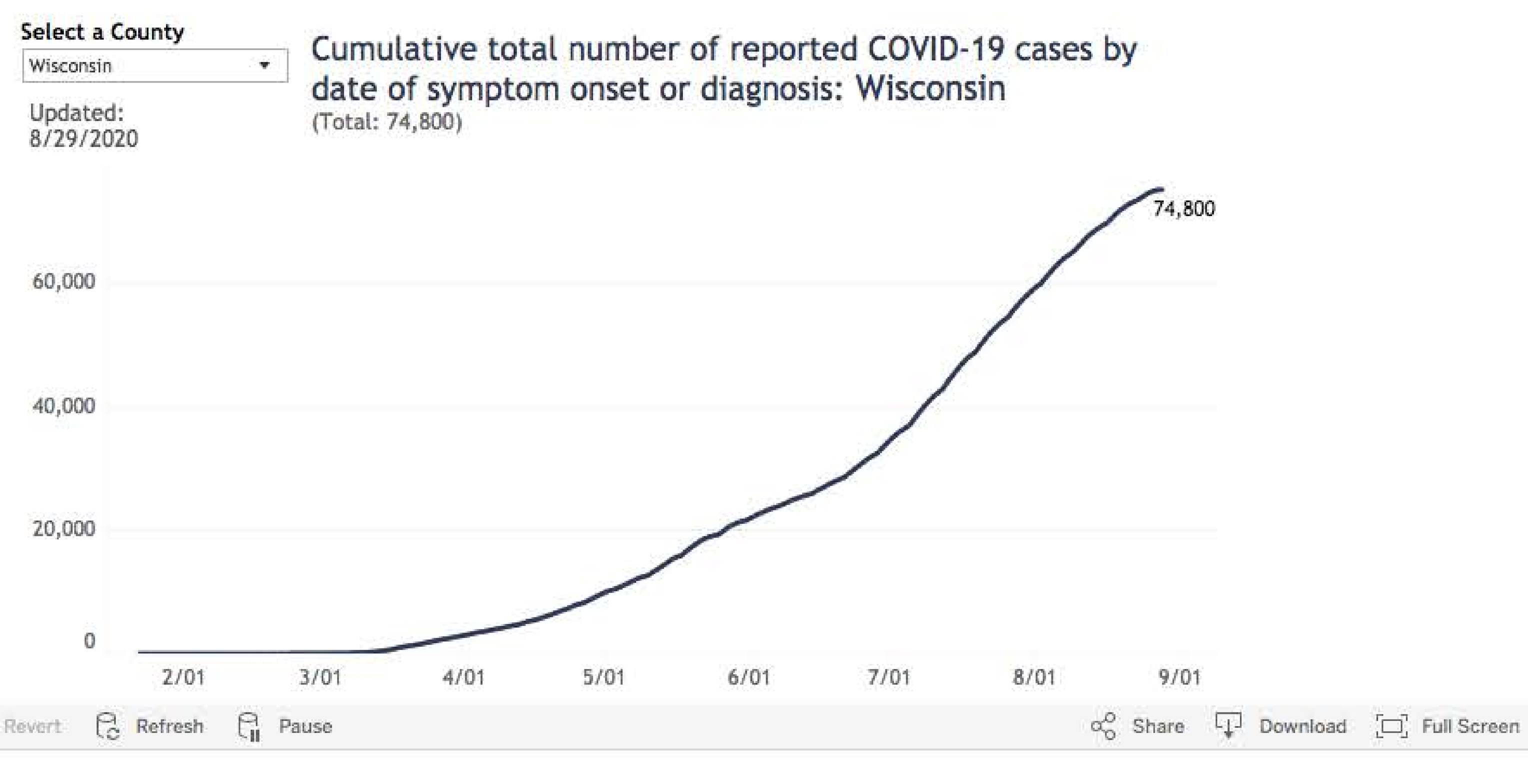

This chart is called a cumulative epidemic curve. It shows the total (cumulative) number of confirmed cases, regardless of whether the cases have recovered. It represents how COVID-19 is spreading and expanding statewide and by county over time. An upward trend in the curve shows a time period where the number of cases are growing. A steeper curve in the chart signals that cases are growing at a higher rate.

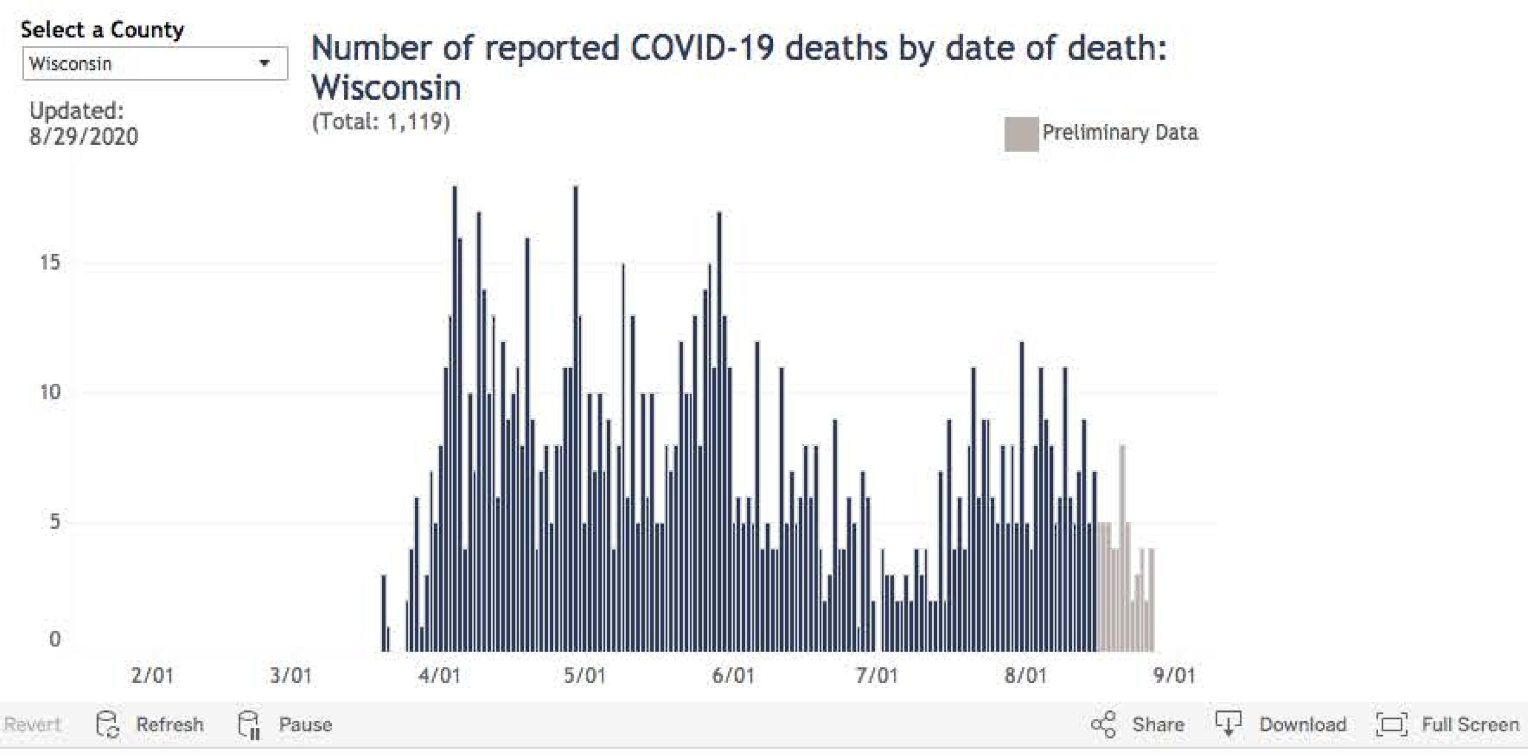

This chart is called a mortality curve. It is used to track the number of deaths over time and see when peaks occur. This figure is showing data by when a person died. Date of death is more meaningful than using the date when the person's death was reported to public health.

When presenting data by the date of death, any downward trends that are seen during the most recent two weeks are usually not true decreases in deaths and need to be interpreted with caution. This downward trend usually represents the data lag time; thus, data during the most recent two weeks are highlighted as **preliminary data**. It takes time for patient deaths to be reported to public health and to be included in death counts.

The figure titled "Cumulative total and newly reported COVID-19 deaths by date reported" on our COVID-19: Wisconsin Deaths webpage does present data by the date a death was reported as being associated with COVID-19 (and not by date of death). These data are also available by county and can be downloaded by clicking the "Download data by county" hyperlink above.

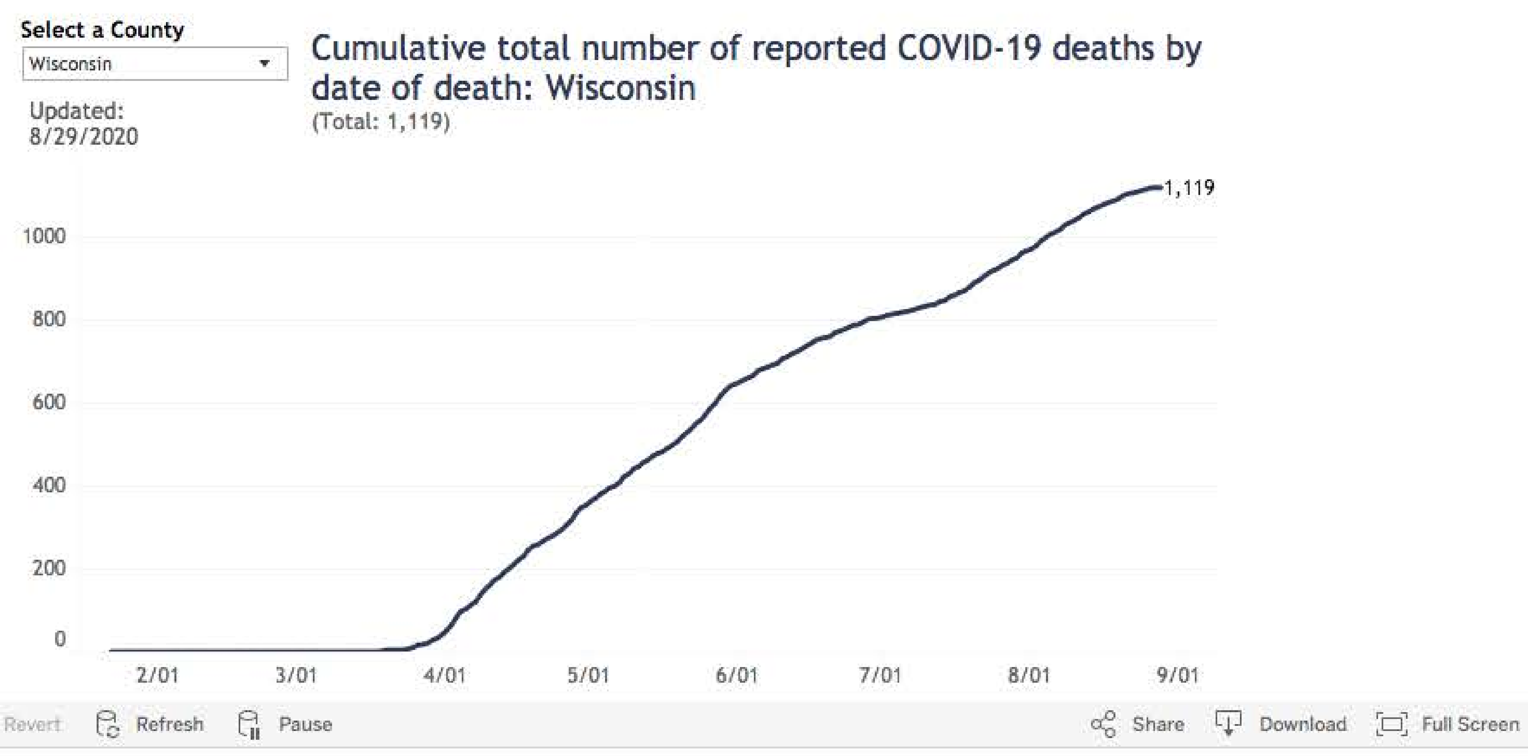

This chart is called a cumulative mortality curve. It shows the total (cumulative) number of deaths among confirmed cases statewide and by county over time. An upward trend in the curve shows a time period where the number of COVID-19-related deaths are increasing. A steeper curve in the chart signals a higher rate of deaths.

Number of positive cases and deaths by county

Wisconsin County	Positive as of 8/29/2020	Negative as of 8/29/2020	Deaths as of 8/29/2020	Rate (positive cases per 100,000 people) as of 8/29/2020	Case fatality percentage (percent of cases who died) as of 8/29/2020
Adams	108	3,125	3	538.0	3%
Ashland	34	2,018	1	216.4	3%
Barron	371	7,376	3	819.9	1%
Bayfield	44	2,445	1	293.5	2%

Coronavirus: Tracking COVID-19 curve of cases, deaths in Wisconsin

We're tracking the curve of coronavirus cases and coronavirus-related deaths that have occurred in Wisconsin.

101
Shares

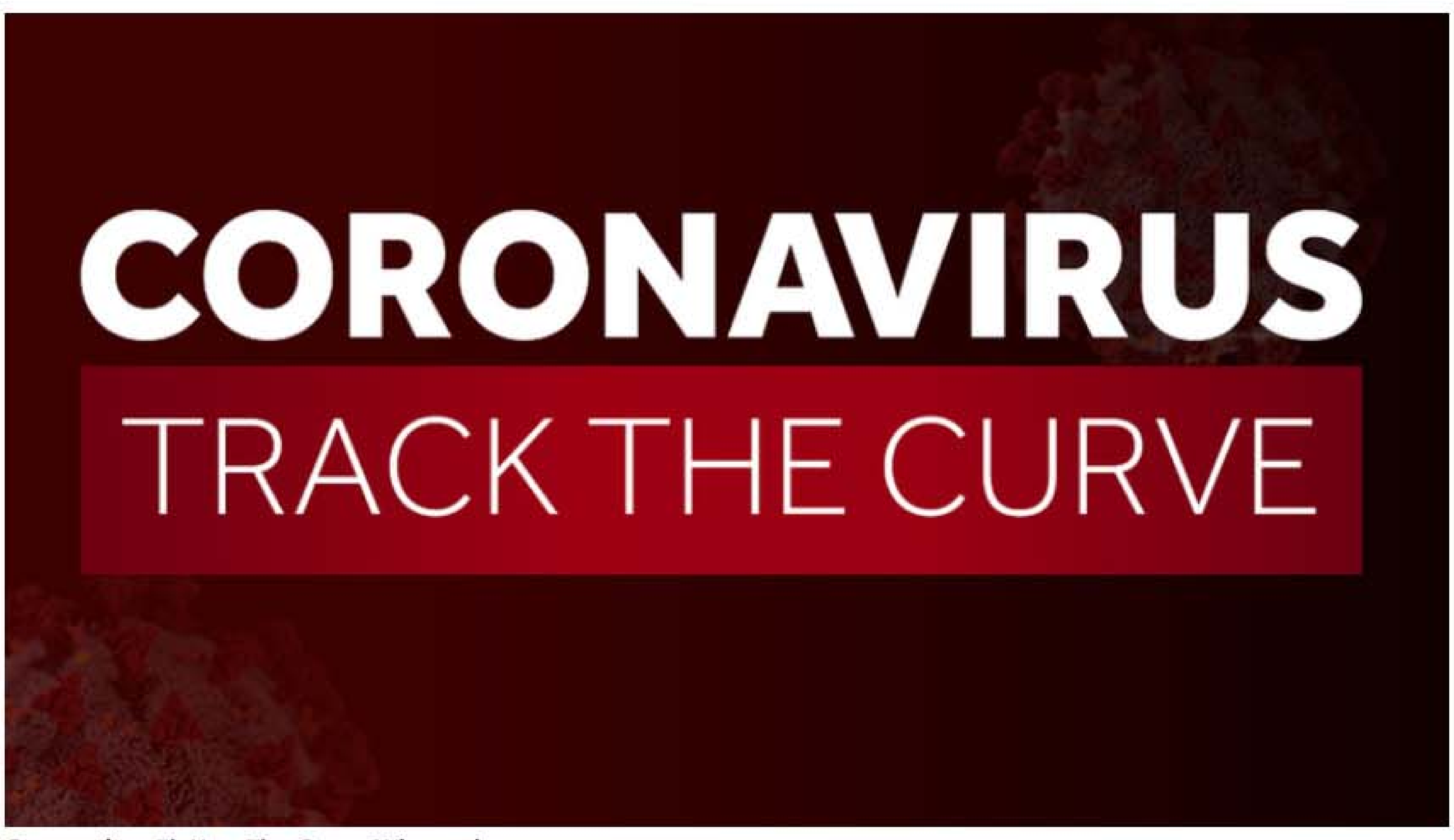

Coronavirus Flatten The Curve Wisconsin

We're tracking the curve of coronavirus cases and coronavirus-related deaths that have occurred in Wisconsin.

Continuing Coverage: Coronavirus in Wisconsin

The data shows the total number of COVID-19 cases along with the number of COVID-19 cases per day.

In addition, it includes the total number of deaths related to COVID-19 complications along with the number of deaths from coronavirus occurring daily.

The coronavirus case numbers and death toll are updated daily.

App users click here

Tracking the COVID-19 curve in Wisconsin

COVID-19 Cases Total

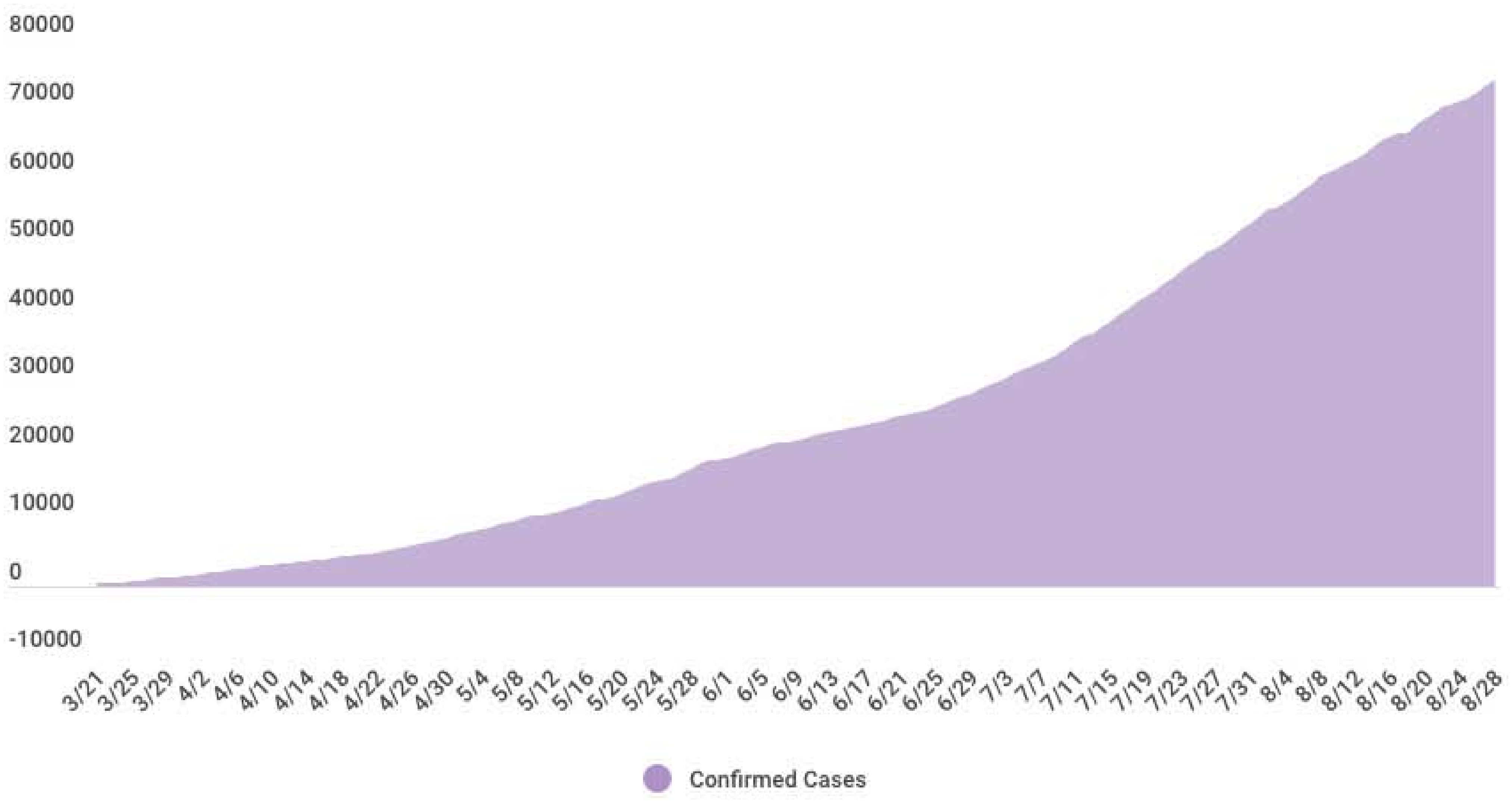

COVID-19 Cases Per Day

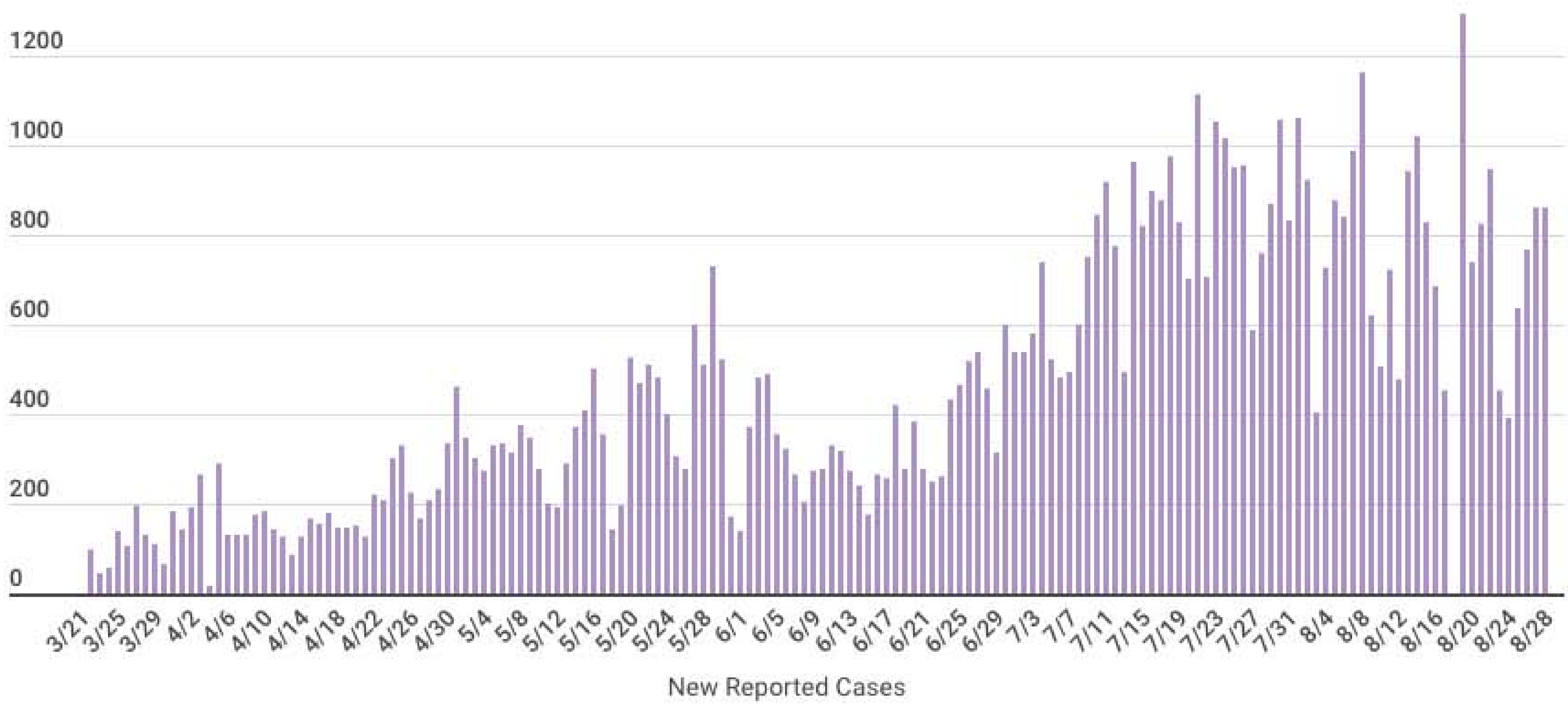

COVID-19 Deaths Total

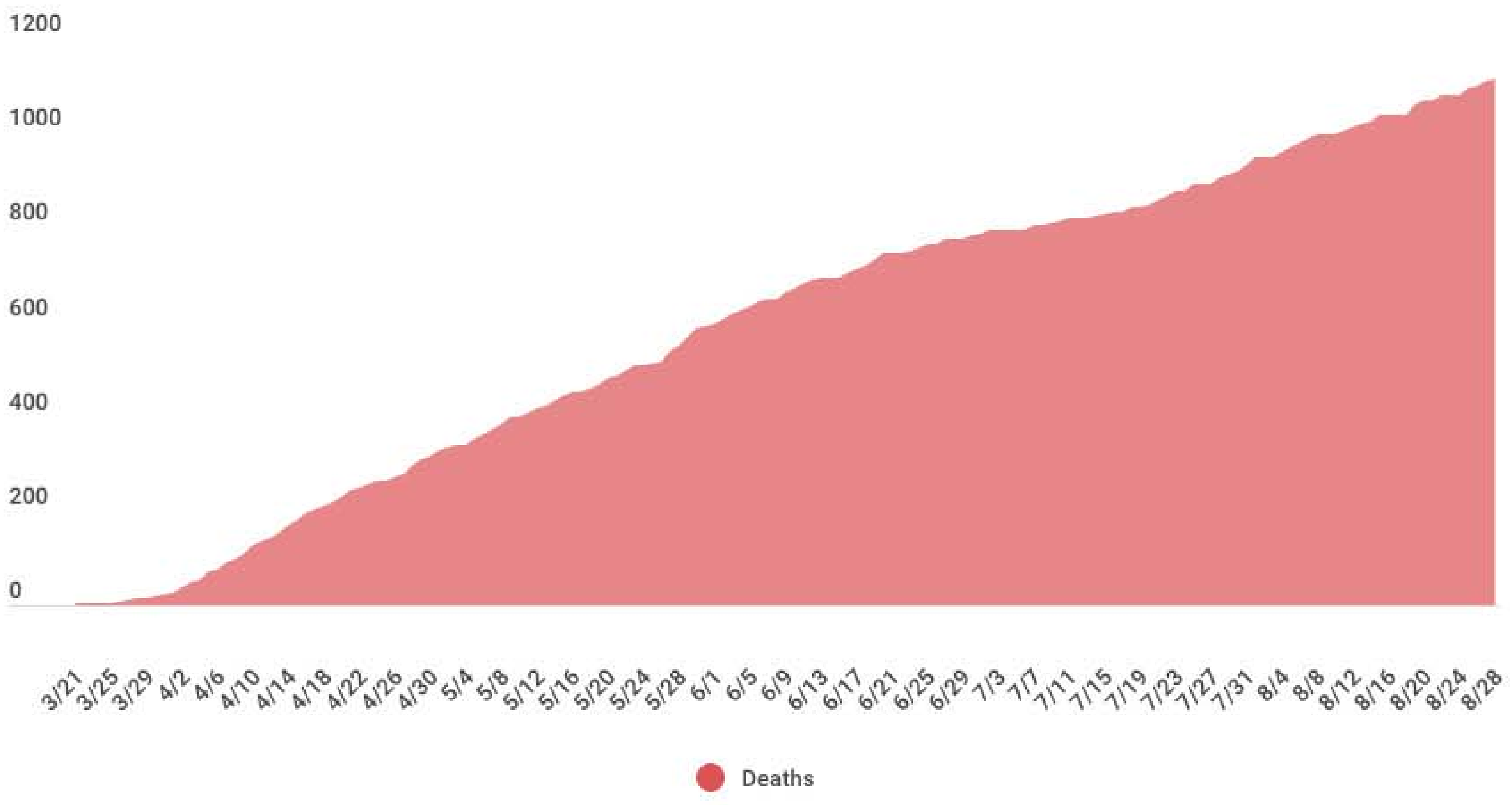

COVID-19 Deaths Per Day

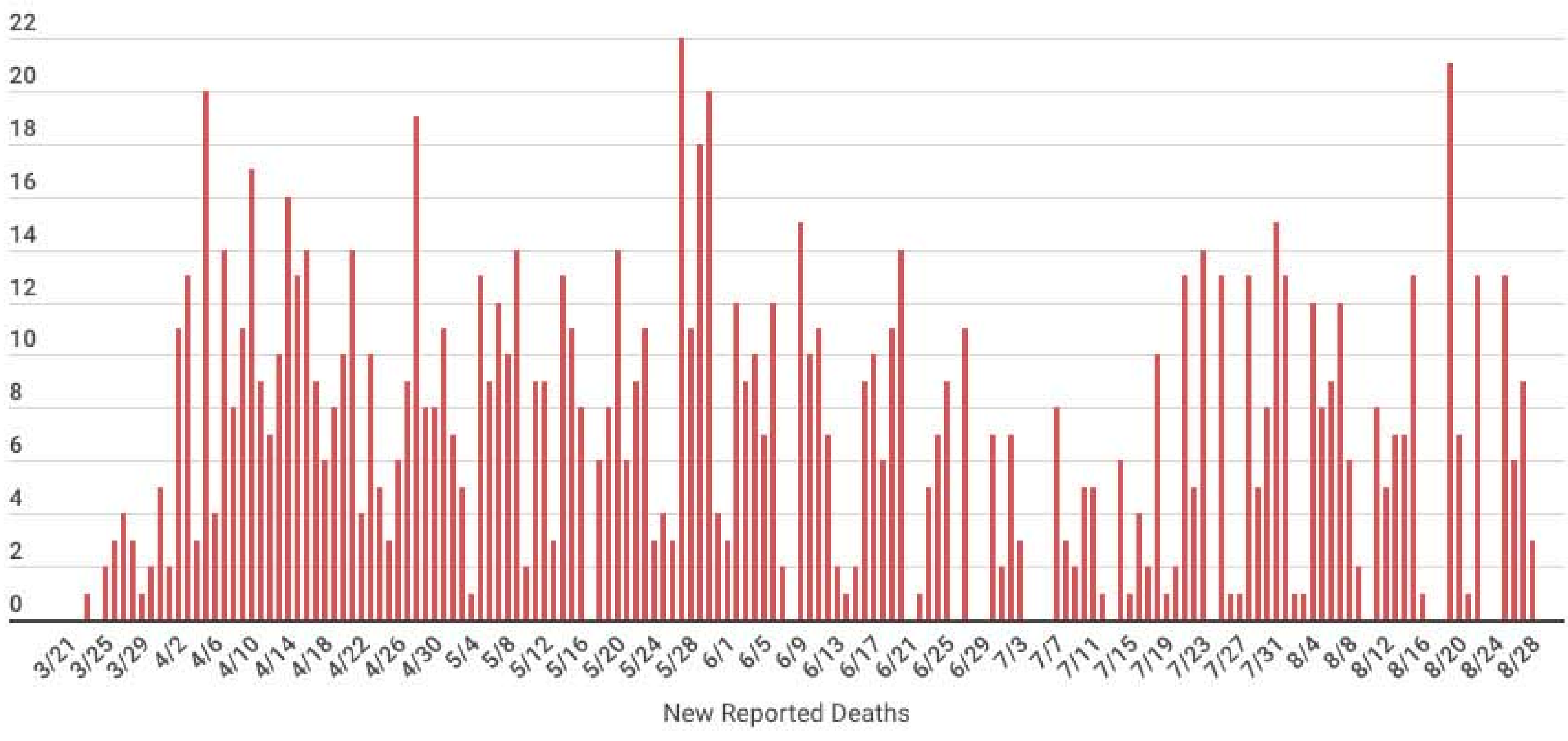

These charts are powered by data from Johns Hopkins University CSSE.
The coronavirus case numbers and death toll are updated daily.
This data may vary from state and local health department tallies.

COVID-19 County by County

HEARST television

Infectious Disease Epidemiology Unit

Conducts surveillance for infectious diseases and investigates clusters and outbreaks

Contact Info:
1-877-996-9000

Home » Public Health Division » Infectious Disease Epidemiology » Disease Information » Coronavirus Disease 2019 (COVID-19) » COVID-19 Map and Statistics

Infectious Disease Epidemiology
- Disease Information
  - Coronavirus Disease 2019 (COVID-19)
    - COVID-19 Information for Healthcare Providers
    - COVID-19 Map and Statistics
    - COVID-19 Orders and Guidance
    - COVID-19 State and County Dashboards
    - COVID-19 Testing Data
    - COVID-19 Media Campaign
  - Carbapenem-Resistant Enterobacteriaceae
  - Prion Diseases
  - Anthrax
  - Botulism
  - Brucellosis
  - Campylobacter
  - Colorado Tick Fever
  - Cryptosporidium
  - Ebola Virus Disease
  - E. Coli
  - Giardia
  - Hepatitis A
  - Influenza
  - Meningitis
  - MRSA
  - Norovirus
  - Ozone
  - Plague
  - Pertussis
  - Rabies
  - Rocky Mountain Spotted Fever
  - Salmonella
  - Tularemia
  - Varicella
  - West Nile Virus
- Meaningful Use
- Reportable Diseases and Conditions
- Reports and Statistics
- Staff
- Case Counts by County
- Foodborne Illness Complaint
- Food Safety
- Heathcare-Associated Infections

COVID-19 Map and Statistics

For an explanation of the graphs shown on this dashboard, please view the Wyoming COVID-19 Map and Statistics Dashboard Interpretation

3,210 Lab Confirmed Cases	574 Probable Cases	37 deaths
2,637 Recovered	479 Recovered	Click here for COVID-19 Related Death Information

For testing data please see our COVID-19 Testing Data page:
https://health.wyo.gov/publichealth/infectious-disease-epidemiology-unit/disease/novel-coronavirus/covid-19-testing-data/

Wyoming COVID-19 Lab Confirmed Cases by County of Residence

Hover over a county name to see county level breakdown | 9:59 AM Saturday, August 29, 2020 |

Click here to view county numbers in a table

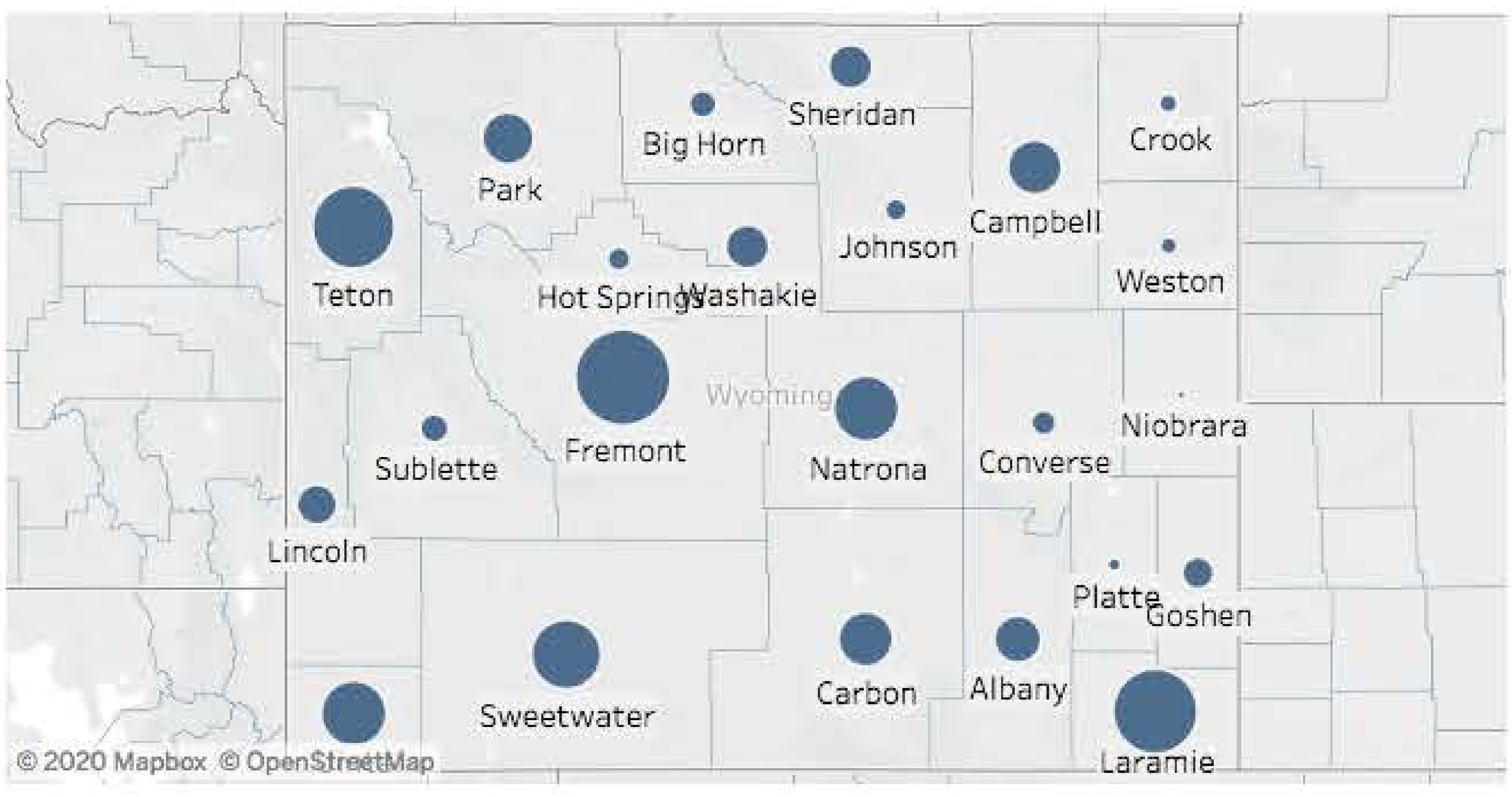

Click here to view cases by City/Town

Select range of dates to view trend graphs:

June 1, 2020 — August 27, 2020

Lab Confirmed Cases by Date of Symptom Onset

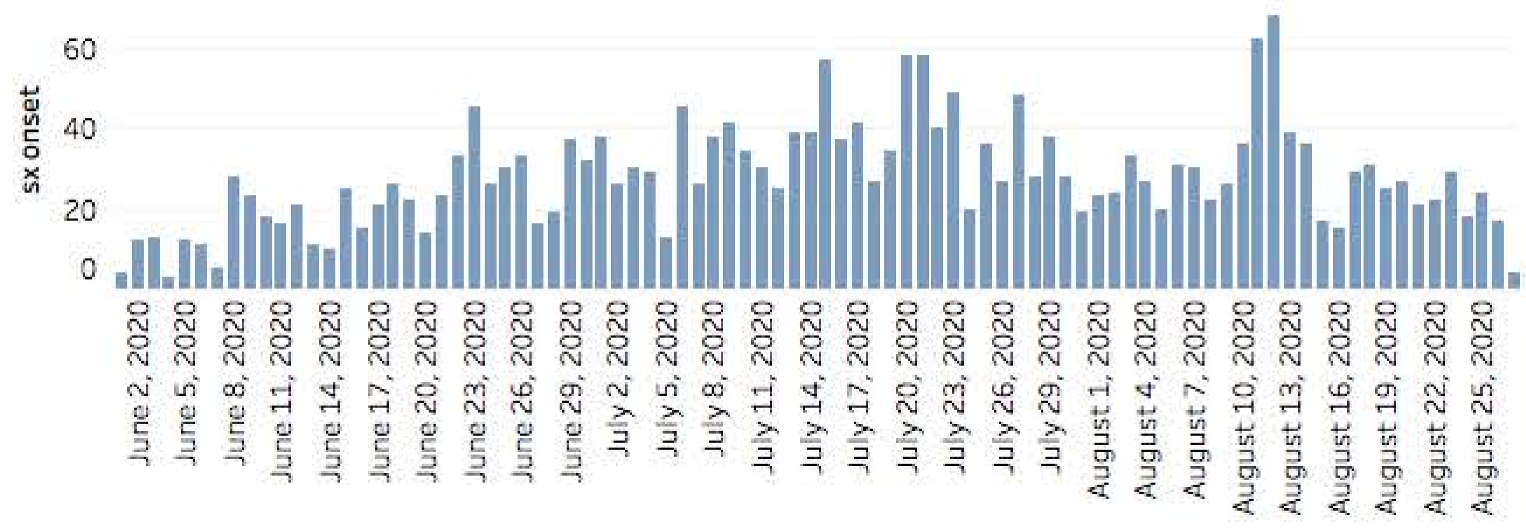

Lab Confirmed Cases by Date of Report, Total Number of Recovered Lab Confirmed Cases; and Percent of Cases with Community Spread or Unknown Exposure

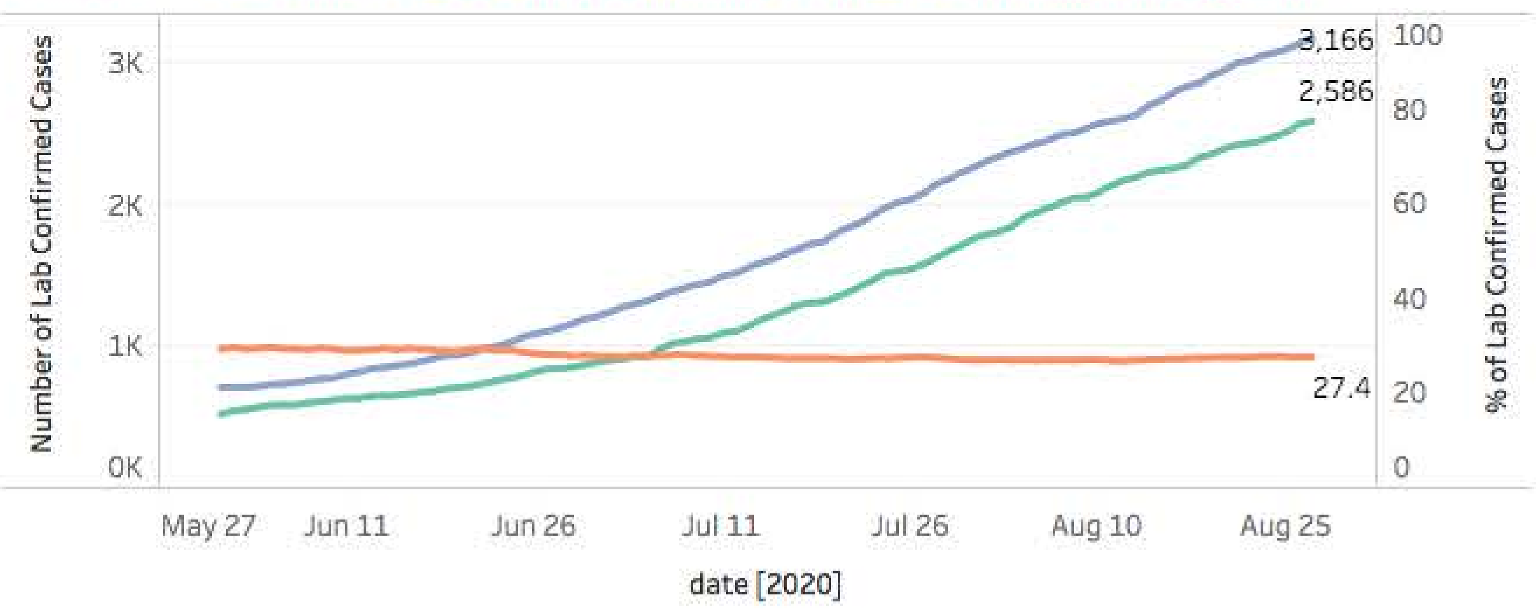

Average Number of Lab Confirmed Cases by Date of Report

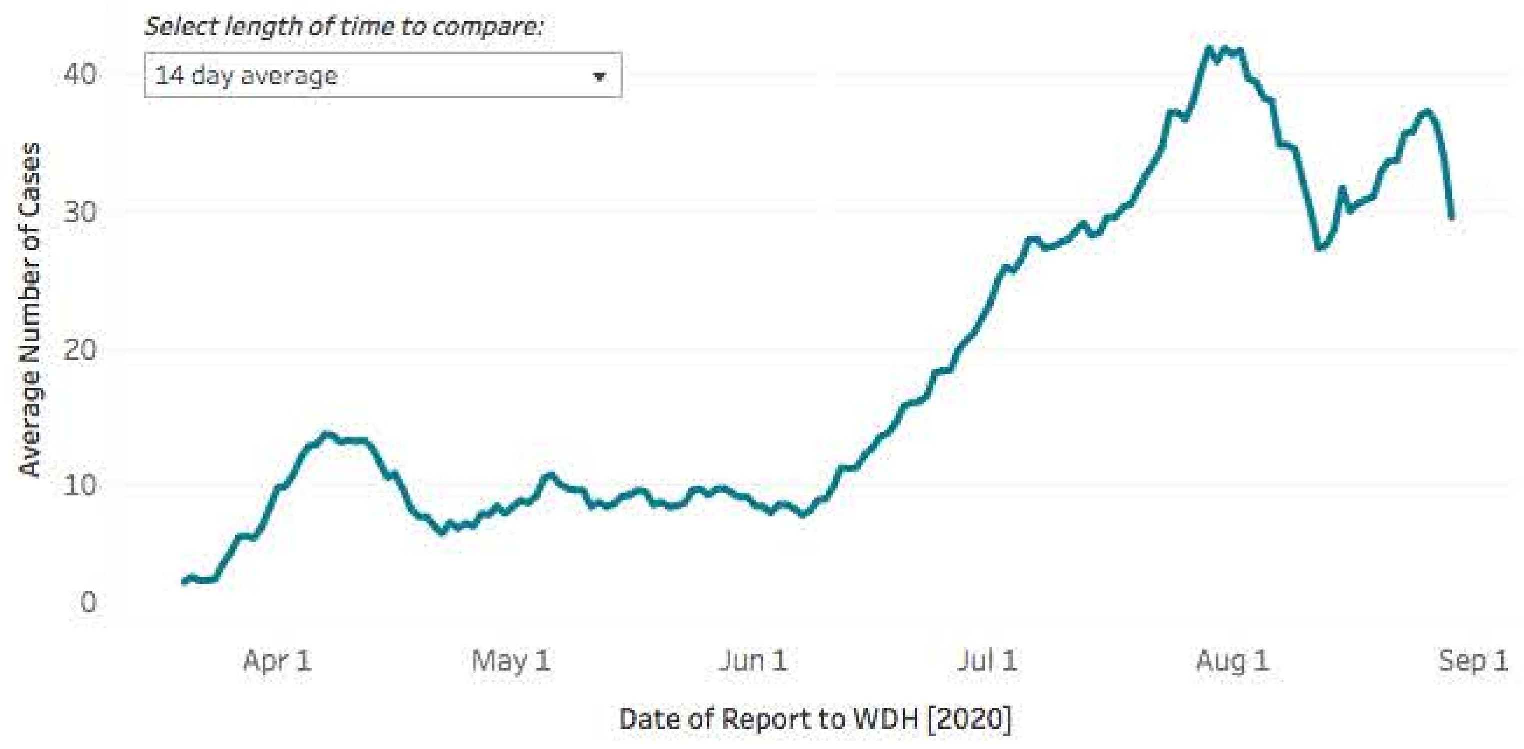

Lab Confirmed Cases by Age Group

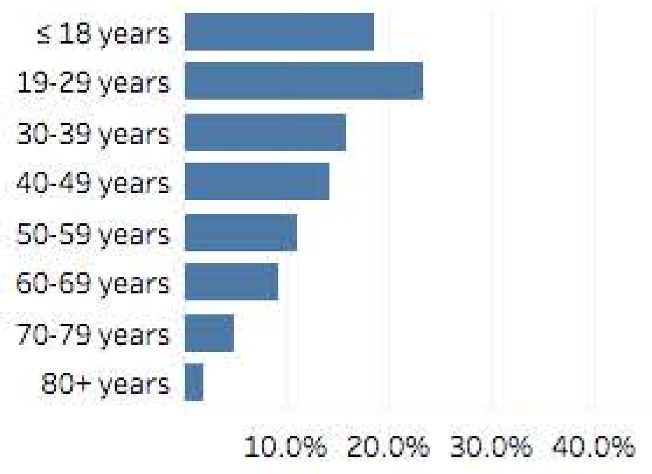

Lab Confirmed Cases by Exposure Risk

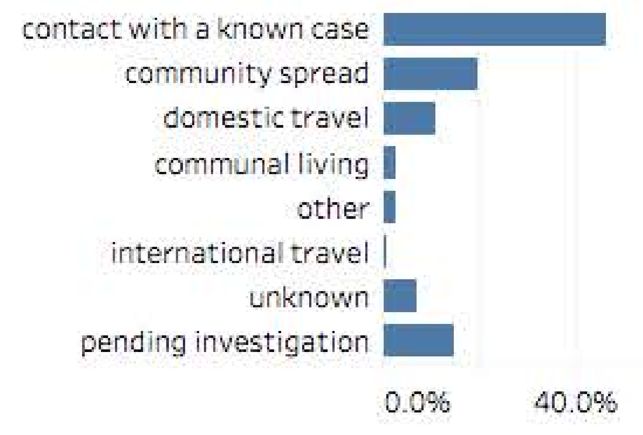

SPOTLIGHT PA

OUR MISSION OUR TEAM SUPPORTERS FAQ SEND A TIP SUPPORT US

COVID-19 ALERTS: Sign up for a free weekly email with the latest data customized to your county.

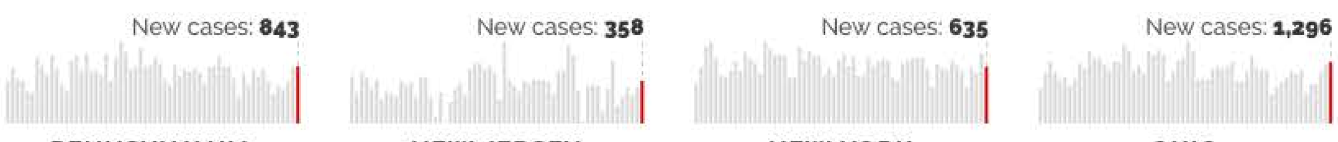

PENNSYLVANIA NEW JERSEY NEW YORK OHIO

Coronavirus: Tracking The Spread

Since the first case of the coronavirus was identified in Pennsylvania on March 6, we've been tracking its spread. These numbers tell only part of the story. Tests are still not widely available, and you can have the virus — and transmit it — without feeling sick. The numbers shown here represent known cases. Because of limited testing, undiagnosed cases are probably widespread. Every day, we are compiling data from multiple sources, including state and county health departments, to provide a more comprehensive picture of the coronavirus in the region.

COVID-19 ALERTS

Sign up for a weekly update on the latest coronavirus data for your county.

Email address

Sign up

Tracking Coronavirus in Pennsylvania

LAST UPDATED: 8/29/2020 • SOURCES: Pa. Department of Health; COVID Tracking Project

TOTAL TESTS
1,633,164 +16,079 from day before

TOTAL CASES
132,834 +843 from day before

TOTAL DEATHS
7,671 +16 from day before

REGIONAL TRENDS

This chart shows a 7-day moving average of new cases added each day per 100,000 people.

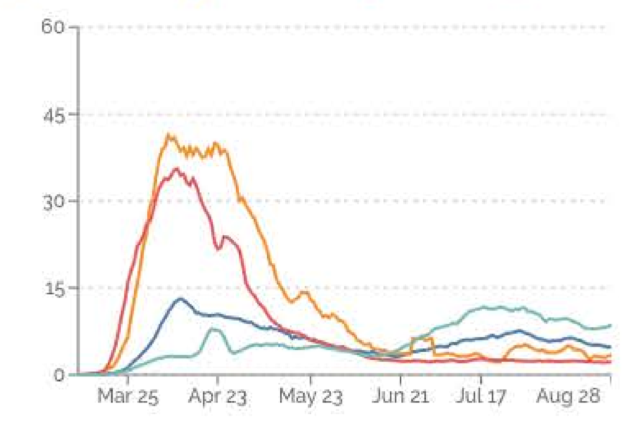

CASES DEATHS

Per capita Total

Total reported cases per 100,000 people over past 14 days

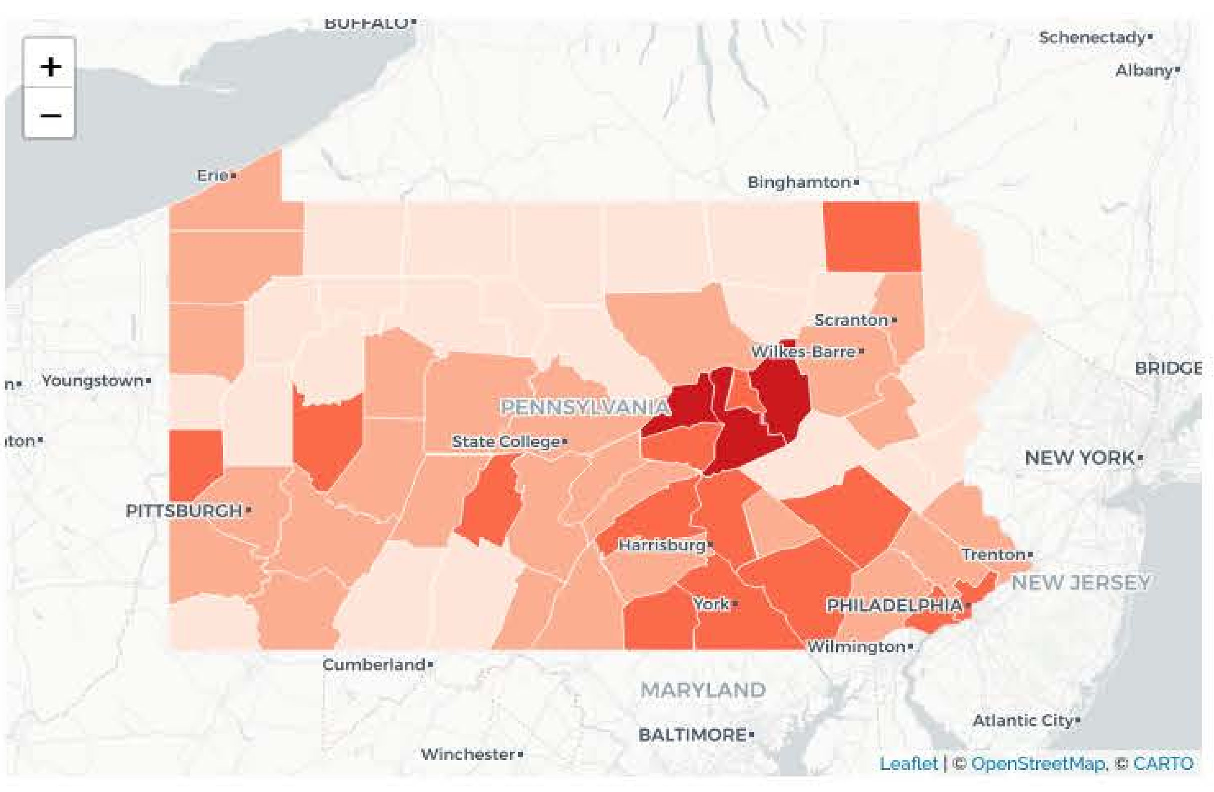

SINCE MARCH PAST 14 DAYS

Cases by county

Over the past two weeks, 10 counties in Pennsylvania saw a rise in new cases, while 26 were falling. 31 were statistically unchanged or had an unclear trend. The table below shows data since **March 5**. To see data for only the past two weeks, use the toggle above.

Search by county

SORT BY: Name 14-day Trend **Cases** Deaths Tests

County	Past 14-day trend*	Cases Total	Cases Per 100k people	Deaths Total	Deaths Per 100k people	Tests Total	Tests Per 100k people
Philadelphia	Falling	28,880	1,823	1,767	112	251,579	15,882
Montgomery	Unclear	10,947	1,317	861	104	129,080	15,535
Delaware	Falling	10,281	1,814	724	128	96,072	16,951
Allegheny	Falling	10,211	840	313	26	155,349	12,775
Bucks	Falling	7,744	1,233	586	93	84,511	13,451
Lancaster	Unclear	6,660	1,220	432	79	68,973	12,639
Berks	Unclear	6,012	1,427	379	90	45,399	10,779
Chester	Falling	5,589	1,065	353	67	69,263	13,193
Lehigh	Falling	5,184	1,404	343	93	54,676	14,805
Northampton	Falling	4,112	1,347	300	98	49,872	16,336

(Each row also shows a DAILY CASES TREND chart, Mar 5 – Aug 29.)

SHOW ALL

NOTE: Past 14-day trend is derived from a linear regression analysis of the 7-day moving average of new cases added each day over the past 14 days. Care should be taken in interpreting 'rising' or 'falling' trends for rural counties with low numbers of new daily cases. Read our FAQ for more information about our methodology.
SOURCES: Pa. Department of Health; Spotlight Pa/Philadelphia Inquirer analysis

DAILY TOTALS CUMULATIVE TOTALS

Cases and deaths in Pennsylvania

There have been an average of 629 new cases and 14 deaths each day over the past week in Pennsylvania. Below are 7-day moving averages since March 5.

CASES

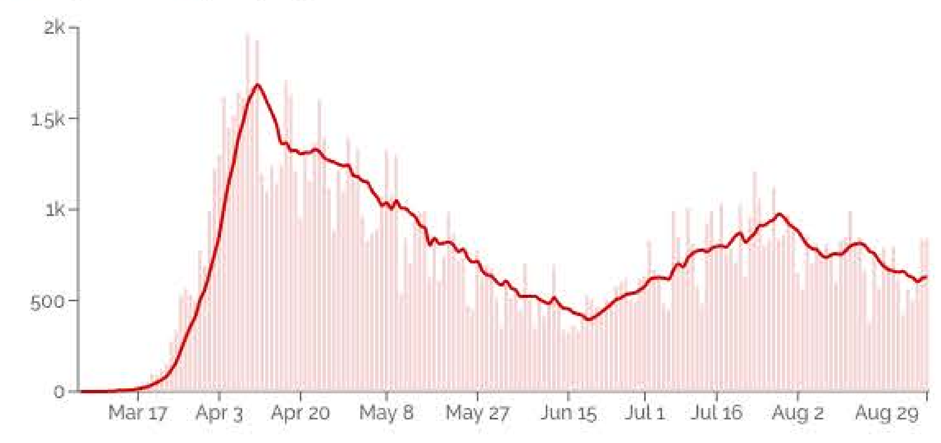

DEATHS

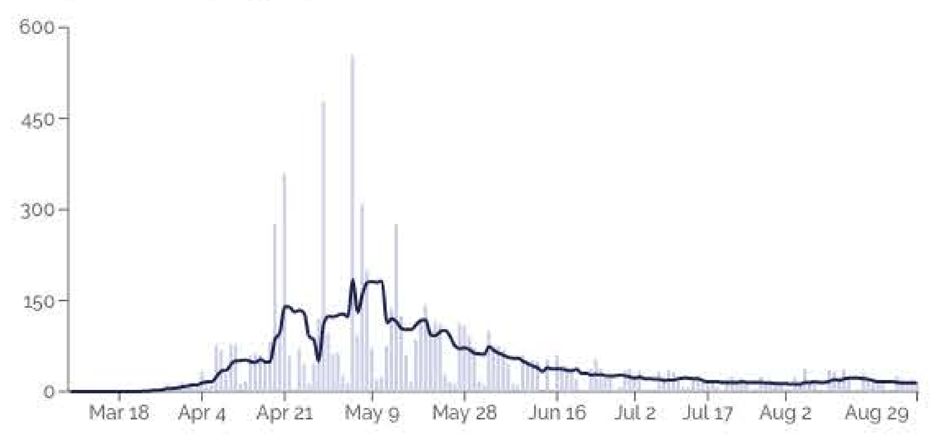

NOTE: Cases include both lab-confirmed positive results and cases deemed probable based on federal criteria. Several large spikes are present in death totals in April and May due to changes the Pa. Department of Health made to how it counts COVID-19 deaths .
SOURCE: Pa. Department of Health

DAILY TOTALS MORE DATA

Testing in Pennsylvania

Pennsylvania reported an average of 13,748 new test results each day over the past week and had a positivity rate of 4.4% . The positivity rate is the average daily number of new positive tests over the past seven days divided by the average daily number of new tests over the same period.

TESTS PER DAY

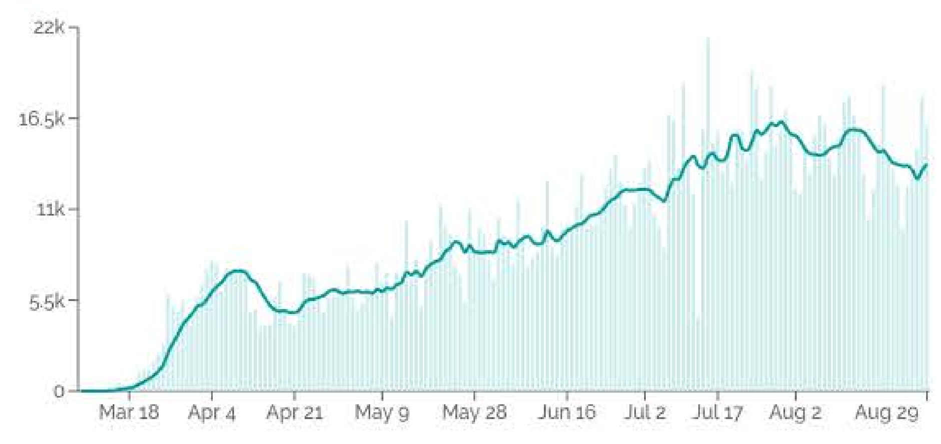

POSITIVITY RATE

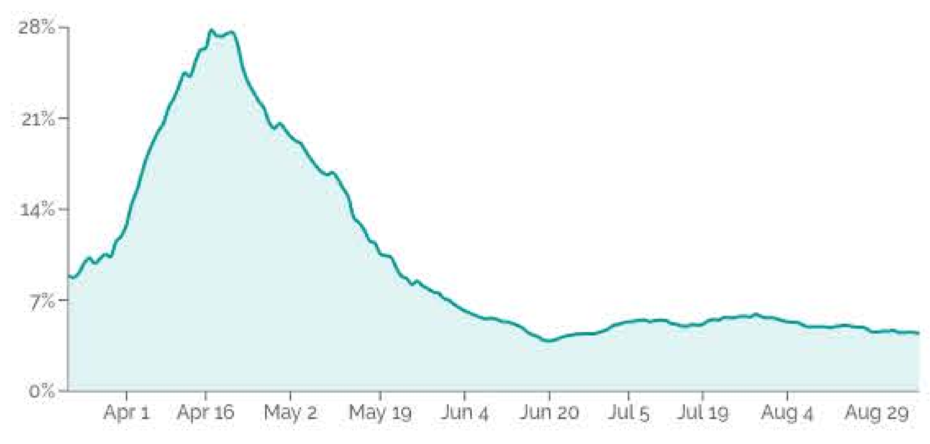

NOTE: Total test numbers are calculated by adding together positive and negative lab results. Note that 'total' and 'positive' numbers prior to July 13 include a small number of probable cases in addition to lab-confirmed results. Probable cases are cases that are determined likely to be COVID-19 based on federal criteria rather than lab results.
SOURCE: Pa. Department of Health

Hospitalizations in Pennsylvania

As of August 29, 509 people in Pennsylvania were in the hospital due to the coronavirus and 78 people were on ventilators. There have been an average of 525 people in the hospital and 78 people on ventilators each day over the past week.

PEOPLE IN HOSPITAL

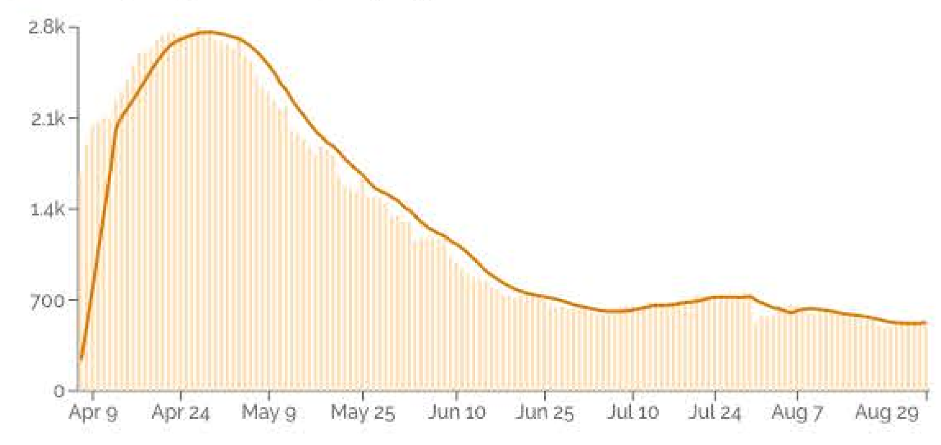

PEOPLE ON VENTILATORS

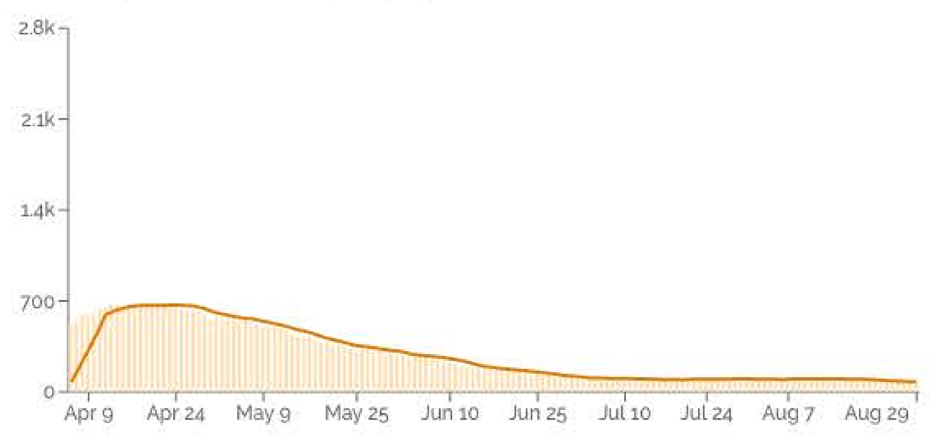

NOTE: Gaps in hospitalization and ventilator numbers may exist for certain periods of time. These gaps may be due to a lack of publicly-available data or because the data was not being compiled by the COVID Tracking Project at that time.
SOURCE: COVID Tracking Project

Cases, deaths, and tests data from the Pa. Department of Health is compiled daily by Spotlight PA/The Philadelphia Inquirer from the department's interactive dashboard. The data in the department's dashboard may not always be consistent with the numbers reported in its press materials or other pages of its website. When major discrepancies exist, Spotlight PA/The Philadelphia Inquirer either defers to the numbers reported in its press materials or contacts the department for clarification. Read our FAQ for more information about our sourcing and methodology.

CREDITS

Graphics: Daniel Simmons-Ritchie, *Spotlight PA* and John Duchneskie, Dylan Purcell and Dominique DeMoe, *Philadelphia Inquirer*.

Developers: Daniel Simmons-Ritchie, *Spotlight PA* and Garland Potts and Dain Saint, *Philadelphia Inquirer*.

Editors: Chris Baxter, *Spotlight PA* and Jessica Parks, *Philadelphia Inquirer*.

75° Providence

WPRI.com Eyewitness News

NEWS | WEATHER | WATCH | TARGET 12 | SPORTS | LOCAL PROGRAMS | THE RHODE SHOW | JOBS | MORE

LIVE NOW / 12 on 12 Digital Original: Distance Learning Divide

COVID-19 TRACKING: Maps, Charts & Interactive Data

The very latest visual data on cases and statistics of Coronavirus in Rhode Island and Massachusetts

NOTE: This tracking page includes the most current data available. The R.I. Department of Health is now only updating data Monday through Friday. Some categories – including geographic and demographic data – are updated intermittently.

COVID-19 in Rhode Island

Total New Cases	Total Deaths	Currently Hospitalized	Currently in ICU	Currently on Ventilator	Total Hospital Discharges
21,683	1,046	81	8	4	2,084

Digital graphic created by Target 12 * Total cases cumulative since March 1, 2020

Interactive content by Flourish

COVID-19 Deaths in Rhode Island
(cumulative total)

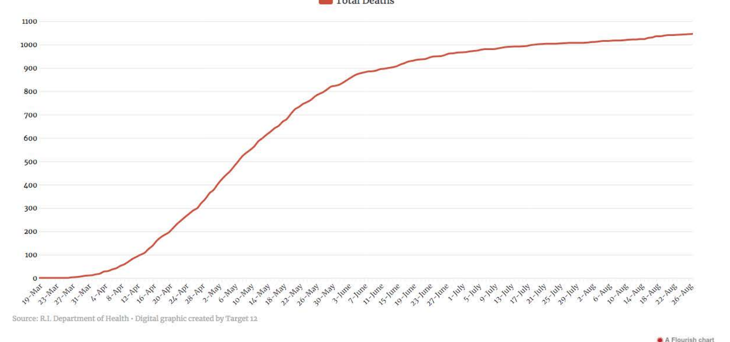

Source: R.I. Department of Health • Digital graphic created by Target 12

A Flourish chart

7-Day Average of Deaths in Rhode Island
(RI death data revised daily due to lag in reporting)

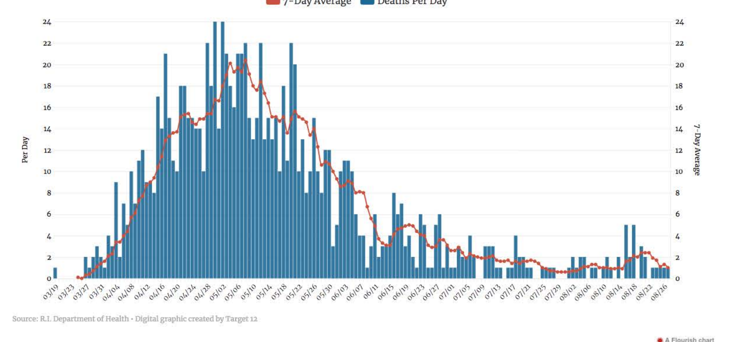

Source: R.I. Department of Health • Digital graphic created by Target 12

A Flourish chart

7-Day Average of New Positive Cases in Rhode Island
RI expanded testing on April 2 to anyone with symptoms

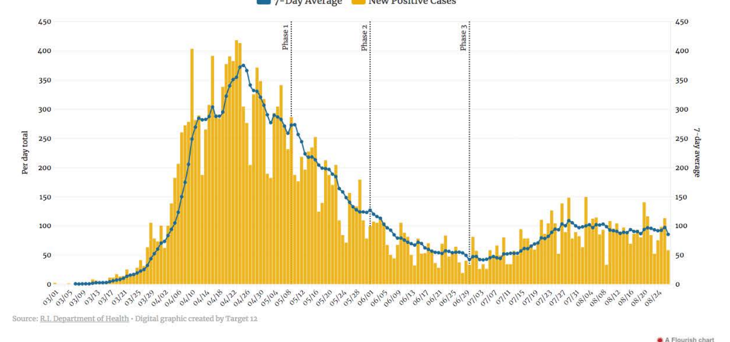

Source: R.I. Department of Health • Digital graphic created by Target 12

A Flourish chart

7-Day Average of All Positive Cases in Rhode Island
RI started releasing repeat positive cases beginning July 8

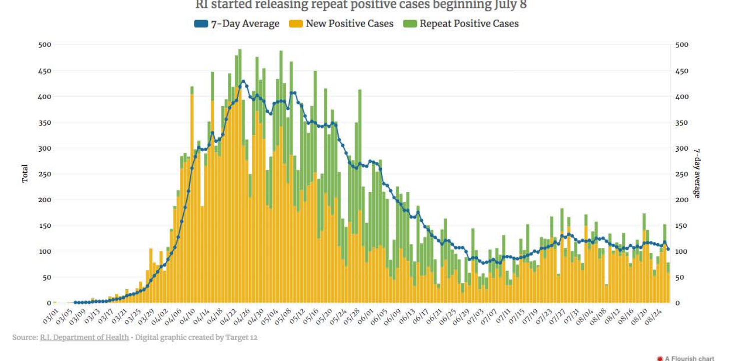

Source: R.I. Department of Health • Digital graphic created by Target 12

A Flourish chart

Rhode Island Positivity Rate

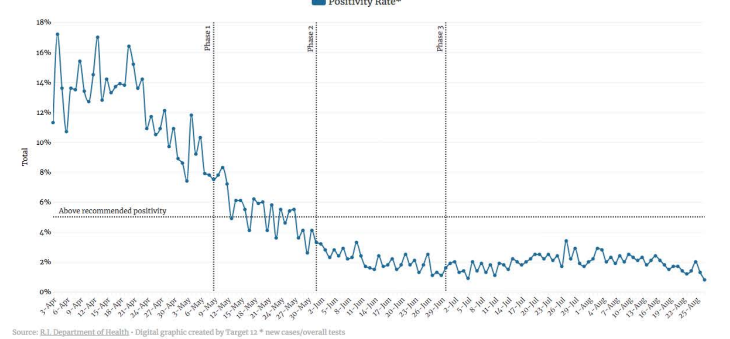

Source: R.I. Department of Health • Digital graphic created by Target 12 * new cases/overall tests

A Flourish chart

7-Day Average of Daily Testing in Rhode Island
Includes all negative and positive tests from each day

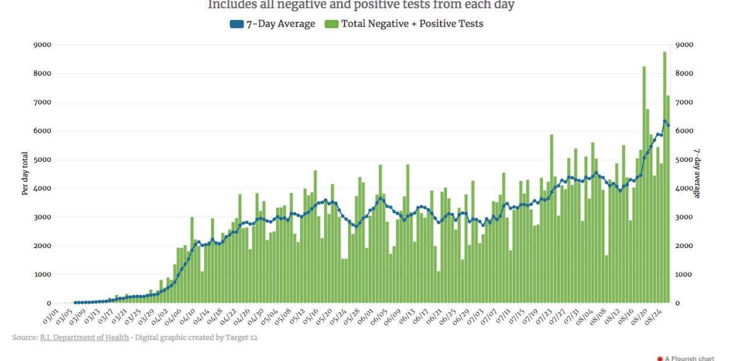

Source: R.I. Department of Health • Digital graphic created by Target 12

A Flourish chart

Total COVID-19 Hospitalizations in Rhode Island
(RI data revised daily with a two-day lag in reporting)

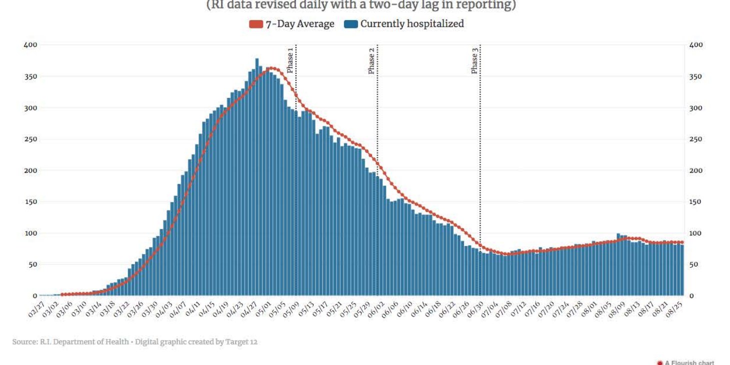

Source: R.I. Department of Health • Digital graphic created by Target 12

A Flourish chart

COVID-19 Intensive Care in Rhode Island
(RI data revised daily with a two-day lag)

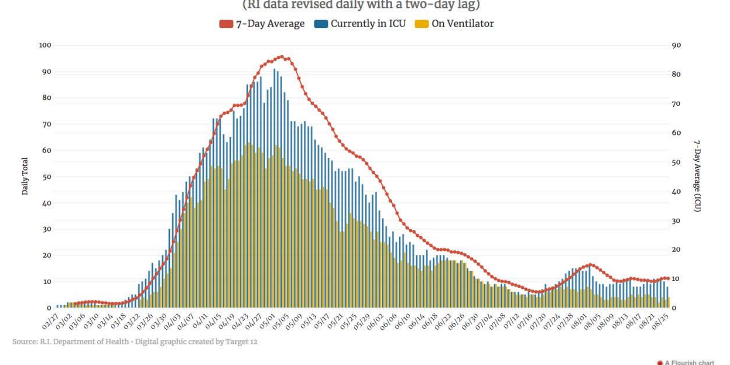

Source: R.I. Department of Health • Digital graphic created by Target 12

A Flourish chart

New Hospital Admissions in Rhode Island
(RI data revised daily with a two-day lag)

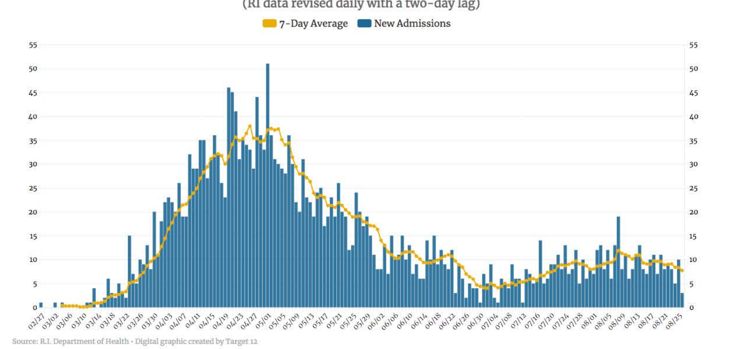

Source: R.I. Department of Health • Digital graphic created by Target 12

A Flourish chart

New Hospital Discharges in Rhode Island
(RI data revised daily with a two-day lag in reporting)

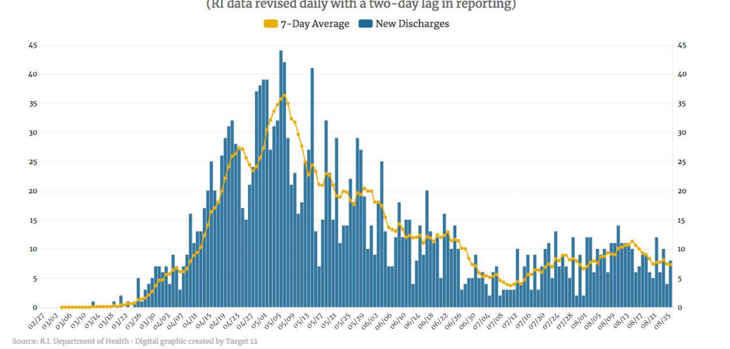

Source: R.I. Department of Health • Digital graphic created by Target 12

A Flourish chart

COVID-19 Cases, Hospitalizations and Deaths by Municipality in Rhode Island

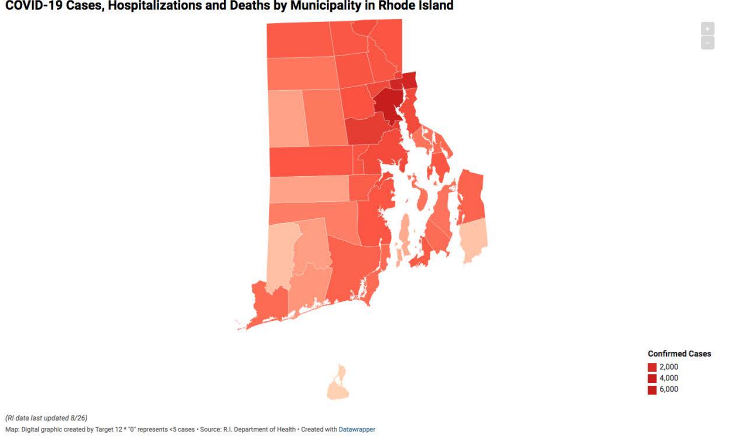

(RI data last updated 8/26)

Map: Digital graphic created by Target 12 * "0" represents <5 cases • Source: R.I. Department of Health • Created with Datawrapper

COVID-19 Cases, Hospitalizations and Deaths Per Capita by Municipality in Rhode Island

n/a = data suppressed

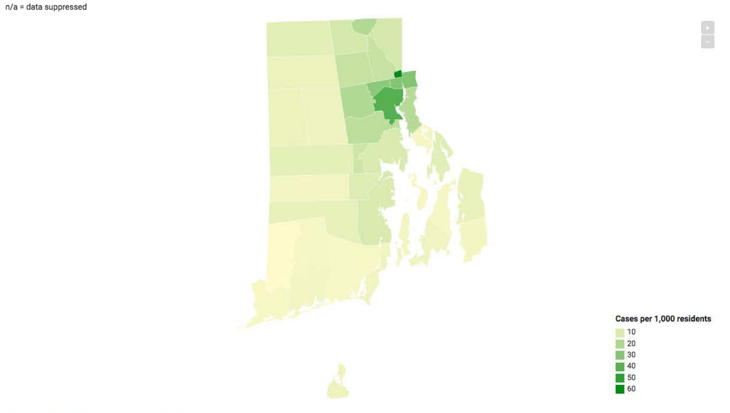

(RI data last updated 8/26) * New Shoreham case data suppressed

Map: Created by Target 12 • Source: R.I. Department of Health; U.S. Census Bureau • Created with Datawrapper

COVID-19 Cases by ZIP in Rhode Island

(NOTE: 0 = 0 or fewer than 5 cases)

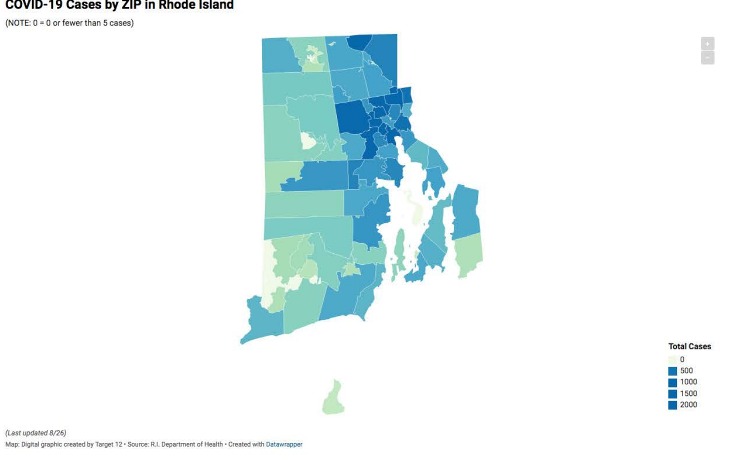

(Last updated 8/26)

Map: Digital graphic created by Target 12 • Source: R.I. Department of Health • Created with Datawrapper

(NOTE: MA only updates municipal data once per week on Wednesdays. Maps will be updated accordingly.)

COVID-19 Cases by Municipality in Massachusetts

(1 = Fewer than 5)

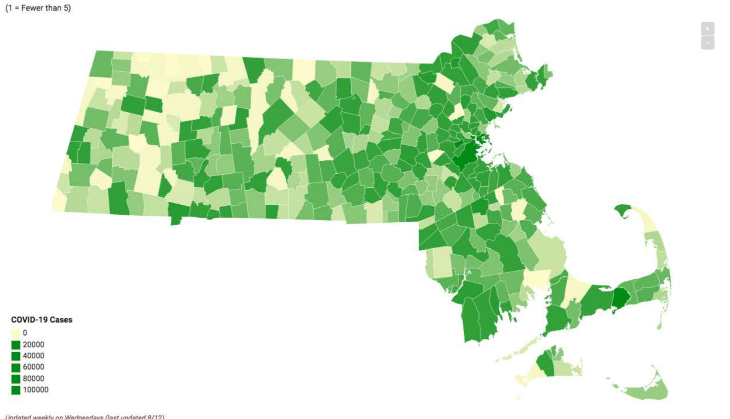

Updated weekly on Wednesdays (last updated 8/12)

Map: Created by Target 12 • Source: Mass. Department of Public Health • Map data: MassGIS • Created with Datawrapper

Total COVID-19 Cases by Age in Rhode Island
(Since March 1)

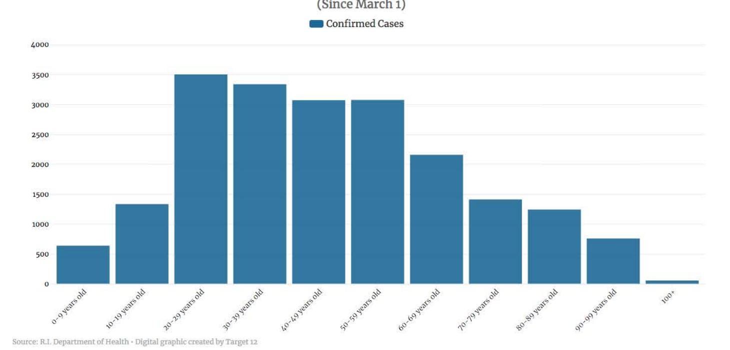

Source: R.I. Department of Health • Digital graphic created by Target 12

A Flourish chart

COVID-19 Hospitalizations & Deaths by Age in RI*
*1 = Fewer than 5

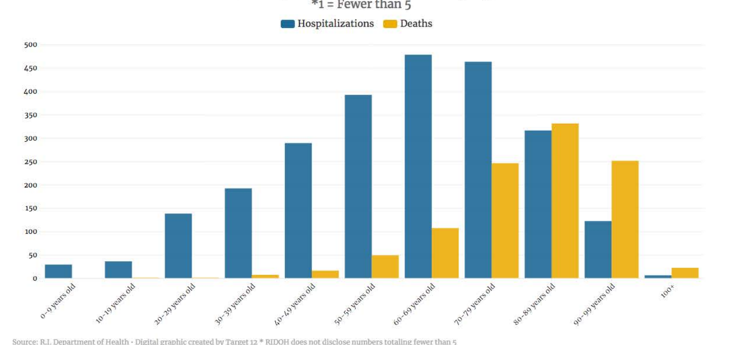

Source: R.I. Department of Health • Digital graphic created by Target 12 * RIDOH does not disclose numbers totaling fewer than 5

A Flourish chart

COVID-19 Cases, Hospitalizations and Deaths by Sex in Rhode Island
(% of total)

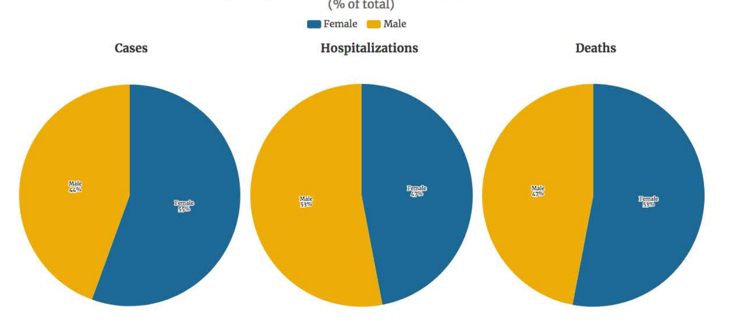

Source: R.I. Department of Health • Digital graphic created by Target 12

A Flourish chart

COVID-19 by Race and Ethnicity in Rhode Island
Percentages based on known cases*

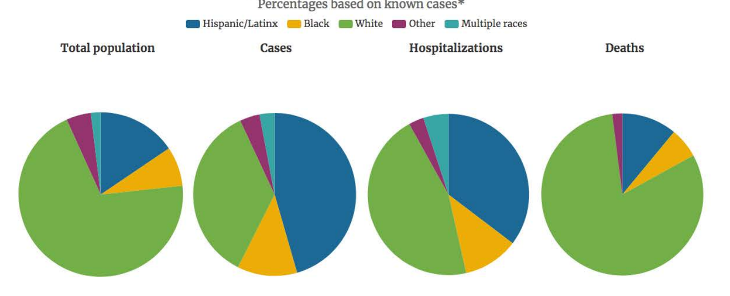

Source: R.I. Department of Health • Digital graphic created by Target 12 * Total may not equal 100% due to rounding

A Flourish chart

Unemployment Claims in Rhode Island

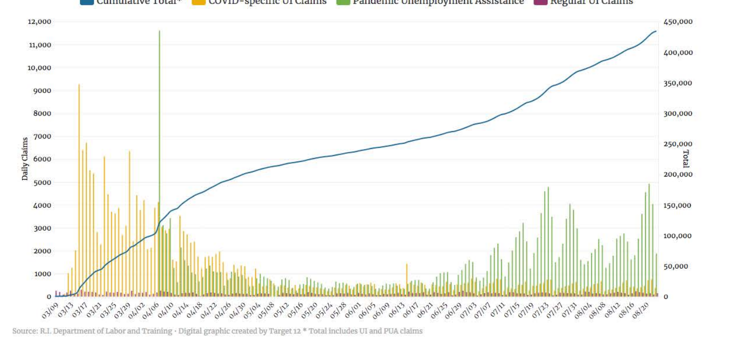

Source: R.I. Department of Labor and Training • Digital graphic created by Target 12 * Total includes UI and PUA claims

A Flourish chart

Temporary Caregiver Insurance Claims in Rhode Island

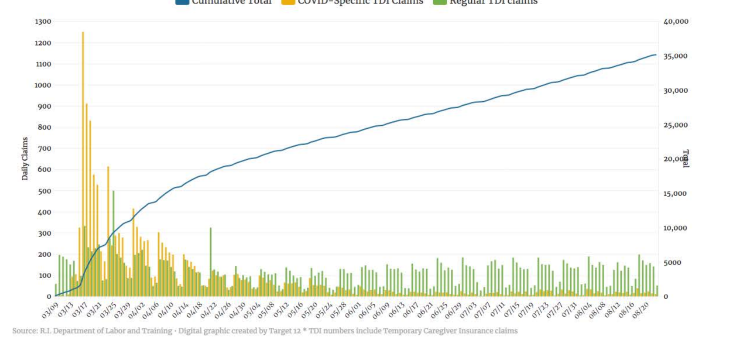

Source: R.I. Department of Labor and Training • Digital graphic created by Target 12 * TDI numbers include Temporary Caregiver Insurance claims

A Flourish chart

Unemployment Rate (RI, MA, USA)
2006-2020 (seasonally adjusted)

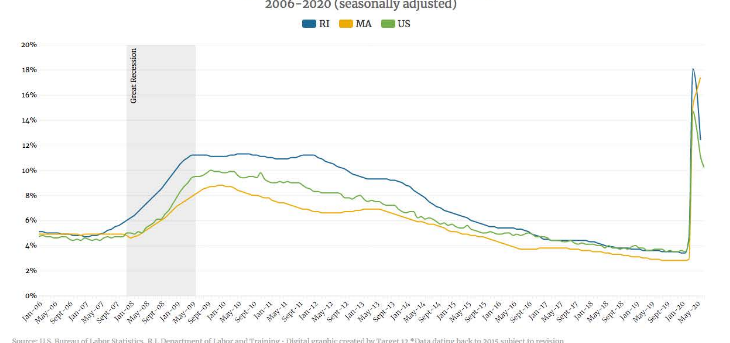

Source: U.S. Bureau of Labor Statistics, R.I. Department of Labor and Training • Digital graphic created by Target 12 *Data dating back to 2015 subject to revision

Bay Area		California		United States	
84,087 cases	1,111 deaths	698,370 cases	12,837 deaths	5,958,486 cases	182,711 deaths
99 on Aug. 29	0 on Aug. 29	99 on Aug. 29	0 on Aug. 29	46,156 on Aug. 28	976 on Aug. 28

Coronavirus Tracker

The Chronicle is mapping every reported coronavirus case in the Bay Area, California and the U.S. We are tallying the number of confirmed cases, deaths, hospitalizations and tests reported across the state by day.

By CHRONICLE DIGITAL TEAM | LAST UPDATED: Aug. 29, 2020 9:58 a.m.

Total cases | New cases

Total confirmed cases as share of the population

1 in 131 | 1 in 70 | 1 in 49 | 1 in 34

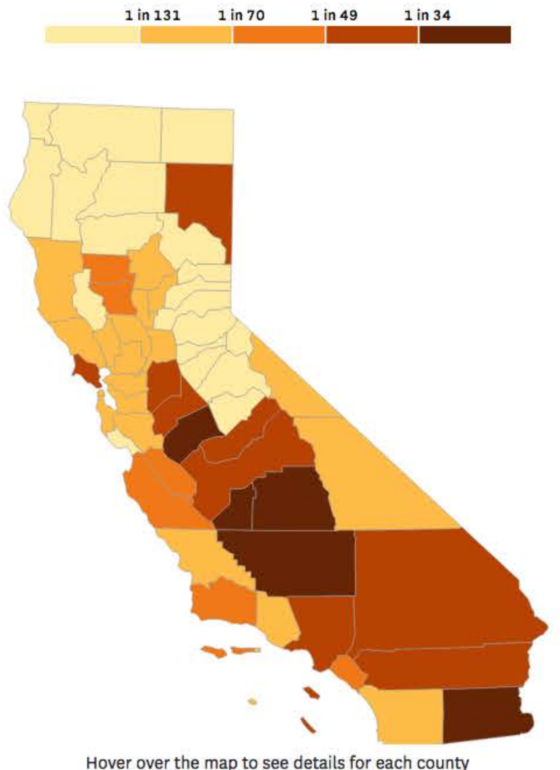

Hover over the map to see details for each county

California cases are organized by reporting county. Cases are based on reports from the U.S. Centers for Disease Control and Prevention, the California Department of Public Health and county public health departments, plus exclusive Chronicle reporting. For more information on the data, please read about our methodology.

What are the latest developments?

California introduced a new reopening plan Friday that will determine how businesses can reopen and when activities can resume based on based on a county's recent data for coronavirus transmissions. Gov. Gavin Newsom said the new plan is simpler and slower than the original plan introduced in the spring. San Francisco and Napa counties are not in the most restricted tier and, in addition to being allowed to open more businesses, could see students back in classrooms in just a few weeks. San Francisco also announced that it will allow outdoor personal care services, including hair and nail salons and gyms, to reopen beginning Tuesday.

For the latest news and developments, read The Chronicle's coronavirus live updates or sign up for our coronavirus newsletter.

New reported cases and deaths in the Bay Area, by day

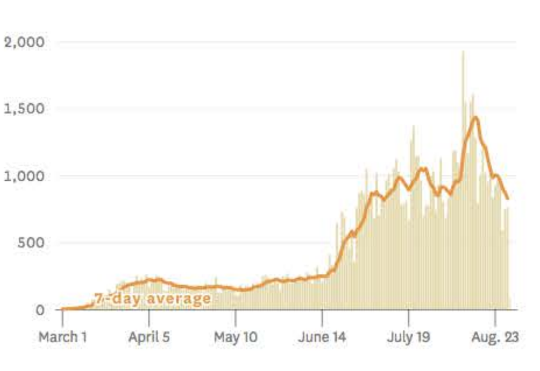

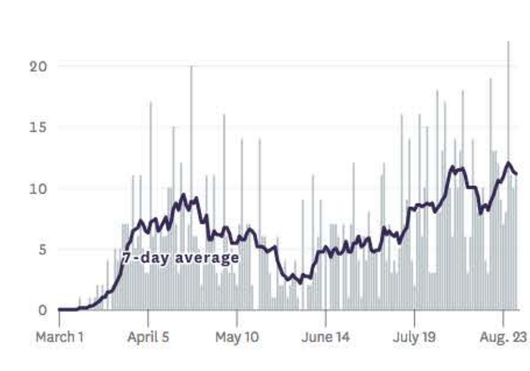

New reported cases and deaths in California, by day

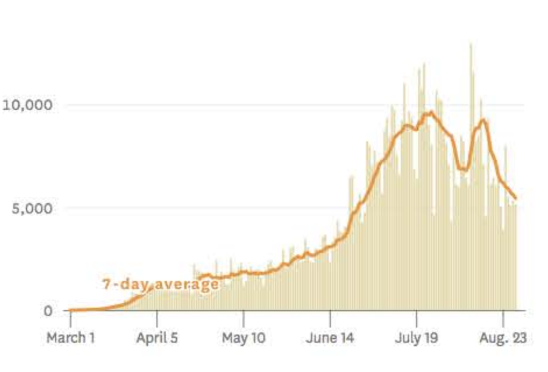

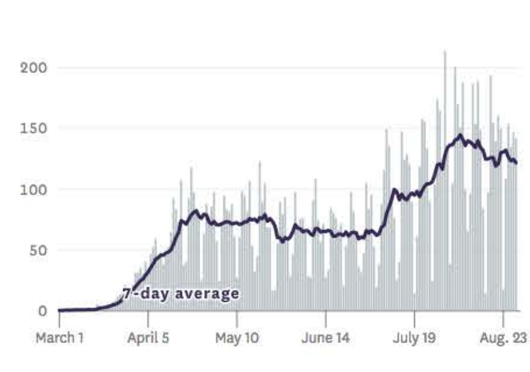

Sources: U.S. Centers for Disease Control and Prevention, California Department of Public Health and county public health departments, Chronicle reporting

The seven-day trailing average represents the average number of cases or deaths reported each day over the weeklong span ending on the given day. It is used to account for daily variances in reporting and large one-off changes in daily counts, such as April 20 when nearly 1,500 cases were reported in L.A. County as the result of a lab clearing a large backlog. Some counties do not provide daily updates which, combined with daily variances in the number of tests given, could result in randomly higher or lower counts for daily reported cases.

Where do Bay Area counties stand in reopening?

The Bay Area's overall reopening has come to a halt as all nine counties are subject to the state's more restrictive orders after being on the state list for not meeting key metrics for three or more consecutive days, forcing them to close or delay opening many indoor businesses and all bars. Napa County came off the watch list on Friday, but what that means in terms of reopening will depend on new guidance from state officials, which is expected soon. Alameda recently announced that outdoor operation of businesses such as hair and nail salons, barbershops, swimming pools, wineries and tasting rooms, could resume on Aug. 28. Check the reopening tracker to see what's open in each county and the progress they're making to meet key indicators.

Find the Bay Area reopening checklist, a detailed list of what's open and what's not for each Bay Area county and the reopening status for each county in California in The Chronicle's reopening tracker

What are the current trends in the Bay Area?

After reporting nearly 10,000 new cases two weeks ago while clearing a backlog of test results created by the recent computer issue with the state reporting agency, the Bay Area reported just more than 6,700 last week. That number is generally in line with data reported the past two months. The nine-county region was averaging around 2,000 per week in early June. Officials remain concerned about how little they've learned about the virus and its spread, citing lack of data as a key factor. Bay Area cases have surpassed 80,000 with more than 1,000 virus-related deaths.

Cases and deaths by Bay Area county

The new case curve represents the 7-day moving average which accounts for variances in daily reporting. The change in new cases compares the number of cases in the past seven days to the previous seven days.

County	Total deaths	Total cases	New cases curve	Weekly change in new cases	
Alameda	247	17,847	178 (March 1 – Aug. 28)	▼286	-19%
Santa Clara	245	16,814	188	▼322	-20%
Contra Costa	173	13,562	167	▼350	-23%
San Francisco*	83	9,349	66	▼113	-20%
San Mateo	131	8,074	71	▼89	-15%
Marin	95	6,068	27	▲17	+10%
Sonoma	78	5,588	92	▼139	-18%
Solano	46	5,388	49	▼112	-25%
Napa	13	1,397	13	▼11	-11%

Sources: U.S. Centers for Disease Control and Prevention, the California Department of Public Health and county public health departments, exclusive Chronicle reporting

* San Francisco reports cases with a three-day lag. The weekly change calculation for San Francisco uses the most recent 14-day period for which there is data.

Note: 159 reported cases at San Quentin state prison were added to the Marin County total on June 20

Bay Area cases by city or region

Some Bay Area county health departments are now providing details on where people who have tested positive reside, either by region, city or jurisdiction. Use the drop down to search counties (more will be added if data becomes available).

Alameda County

Place	Total cases
Alameda	317
Albany	49
Berkeley	526
Castro Valley MAC	464
Dublin	214
Eden Area MAC	1,088

Show more

ADVERTISEMENT

How many people have been hospitalized?

The number of COVID-19 patients in California hospitals continued to drop last week and is at level last seen in late June. The peak was nearly 7,200 patients on July 21 and recently dropped below 4,500. The number of ICU patients across the state has seen a similar decline, from more than 2,000 in late July to fewer than 1,400. The recent computer issue with the state reporting agency was not believed to have altered hospitalization statistics. In the Bay Area, the number of patients has remained elevated for both total patients and ICU patients but is still below peak levels. The ability for hospitals to handle future outbreaks is being closely monitored and will help shape California's attempt to reopen.

Confirmed ICU and non-ICU COVID-19 patients in Bay Area hospitals

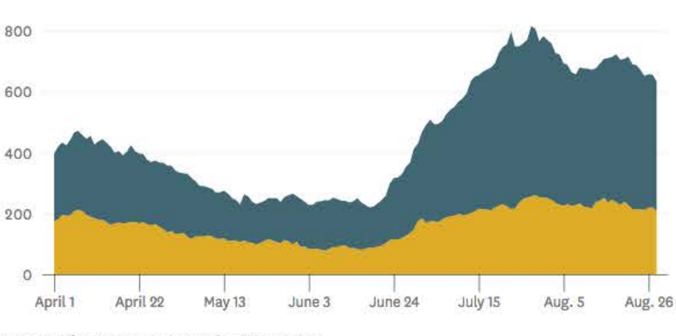

Source: California Department of Public Health

Confirmed ICU and non-ICU COVID-19 patients in California hospitals

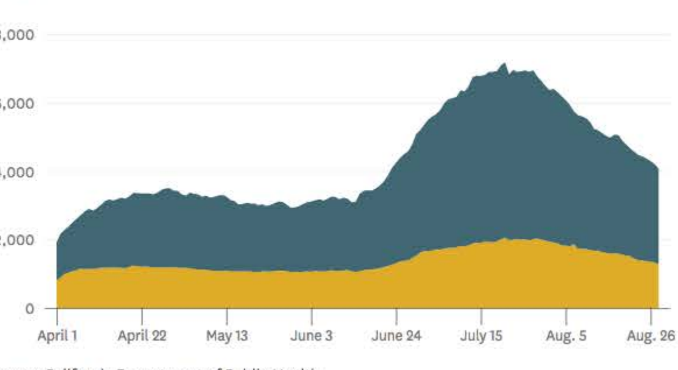

Source: California Department of Public Health

Due to limited testing capacity, not all hospitalized patients with COVID-19 symptoms have been tested. While not all suspected cases will ultimately test positive, including these patients may give a more comprehensive picture of the demands on hospitals. Even with suspected cases included, hospitals have not seen a surge in virus-related patients as feared.

Total confirmed and suspected COVID-19 patients in California hospitals

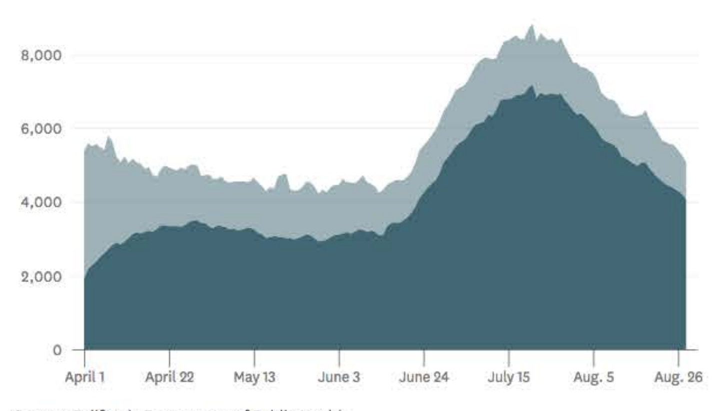

Source: California Department of Public Health

What is the status of testing in California?

The state processed nearly 108,000 test results per day last week with a positivity rate of 5.7%. That number is lower than the 7.0% reported the previous week when the state cleared its backlog and is in line with the 5.6% reported during the glitch. The rate was up to nearly 8% prior to the computer issue. The state uses an 8% or less positive rate over seven days as a standard counties must meet in order to continue moving ahead in reopening. As more people get tested, the positive test rate provides a more clear picture of how many people are carrying the virus, including those without symptoms, and the possibility for a second surge in cases.

Daily test results reported in California

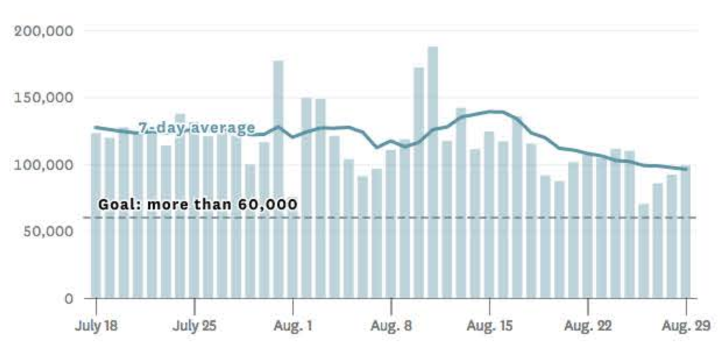

Positive test rate in California

The positive test rate, or positivity rate, is the percentage of total tests that come back positive each day. A lower positivity rate can indicate that enough tests are being conducted. Read more about the positive test rate here.

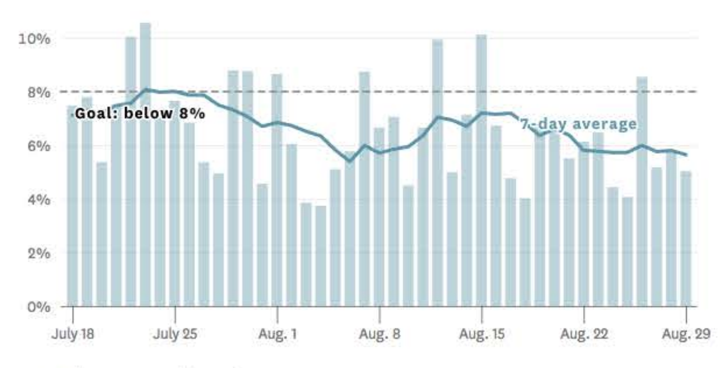

Source: The COVID Tracking Project

Tests are reported by the California Department of Public Health. On April 22, the CDPH began reporting total number of tests conducted, rather than individual people tested, causing test numbers to jump because some individuals are tested more than once.

On July 1, the state reported a backlog of nearly 4,000 positive test results which elevated the positivity rate for that day. This type of variation is accounted for by the trailing seven-day average.

How does California compare to the rest of the nation?

Coronavirus cases in the U.S. are approaching 6 million since the start of the outbreak. The seven-day average for new cases was below 45,000 last week, continuing a downward trend from more than 65,000 per day in late July. For comparison, the U.S. was averaging about 21,000 new cases per day in mid June. After a data correction attributed to the delay in reporting two weeks ago, California averaged fewer than 6,500 cases per day last week. That's on par with late June. The Bay Area saw a smaller decline from highs reported in July with just fewer than 1,000 cases per day last week. Global estimates are that 65% of all patients who tested positive have recovered, but figures for recoveries are not available for the Bay Area or California.

5,958,486 confirmed cases in the U.S.
182,711 deaths

Cases per 100,000 people

250 | 1,000 | 2,000 | 2,500+

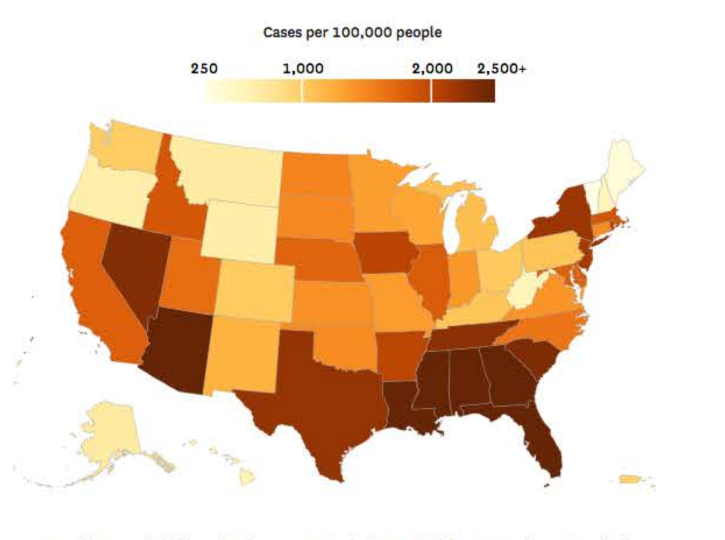

* 152 cases without available location data not mapped. Figures include presumed COVID-19 deaths.

Sources: The Center for Systems Science and Engineering at Johns Hopkins University, U.S. Census Bureau

U.S. reports of COVID-19 are tabulated by Johns Hopkins, which is tracking cases using data collected from the Centers for Disease Control and Prevention and state and local public health departments. Numbers from Johns Hopkins for California may not match the numbers from the top map, which are being compiled by The Chronicle using county reports, county public health departments and Chronicle reporting.

CORONAVIRUS, HEALTH

Coronavirus in Maine: Data Dashboard

Explore trends in Maine's COVID-19 cases by county, age and sex of the afflicted individuals and more in charts updated daily with data from Maine's CDC.

BY DARREN FISHELL | APRIL 2, 2020

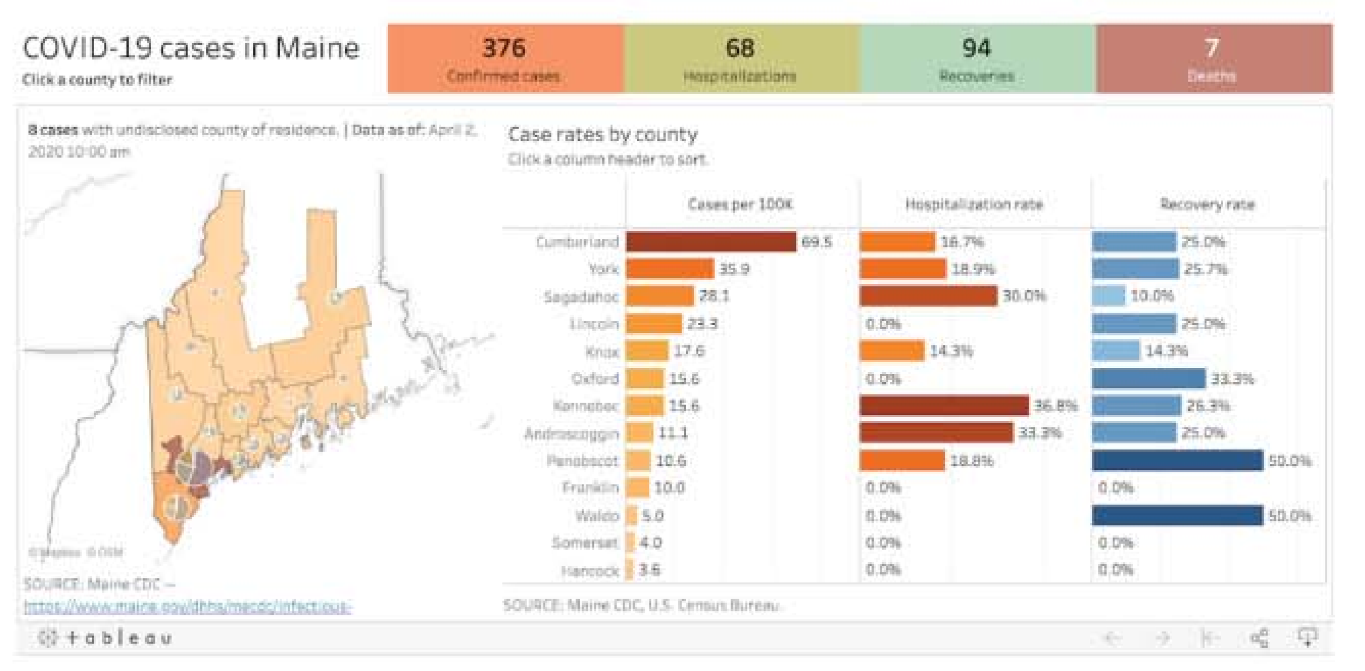

COVID-19, the illness caused by the novel coronavirus, continues to spread throughout Maine.

The Maine Center for Disease Control and Prevention is publishing details about each confirmed case in the state, which are visualized below.

As the CDC publishes these numbers, its director, Dr. Nirav Shah, has noted that it's unclear how much the data lags behind the real spread of the virus.

"What we know about outbreaks is that we are often just detecting the tip of the iceberg," Shah told reporters on March 22.

Total cases

The Maine CDC breaks out cases at the county level by deaths, hospitalizations, recoveries and total confirmed cases. Each of the categories subtracted from total confirmed cases results in the other confirmed cases, shown below.

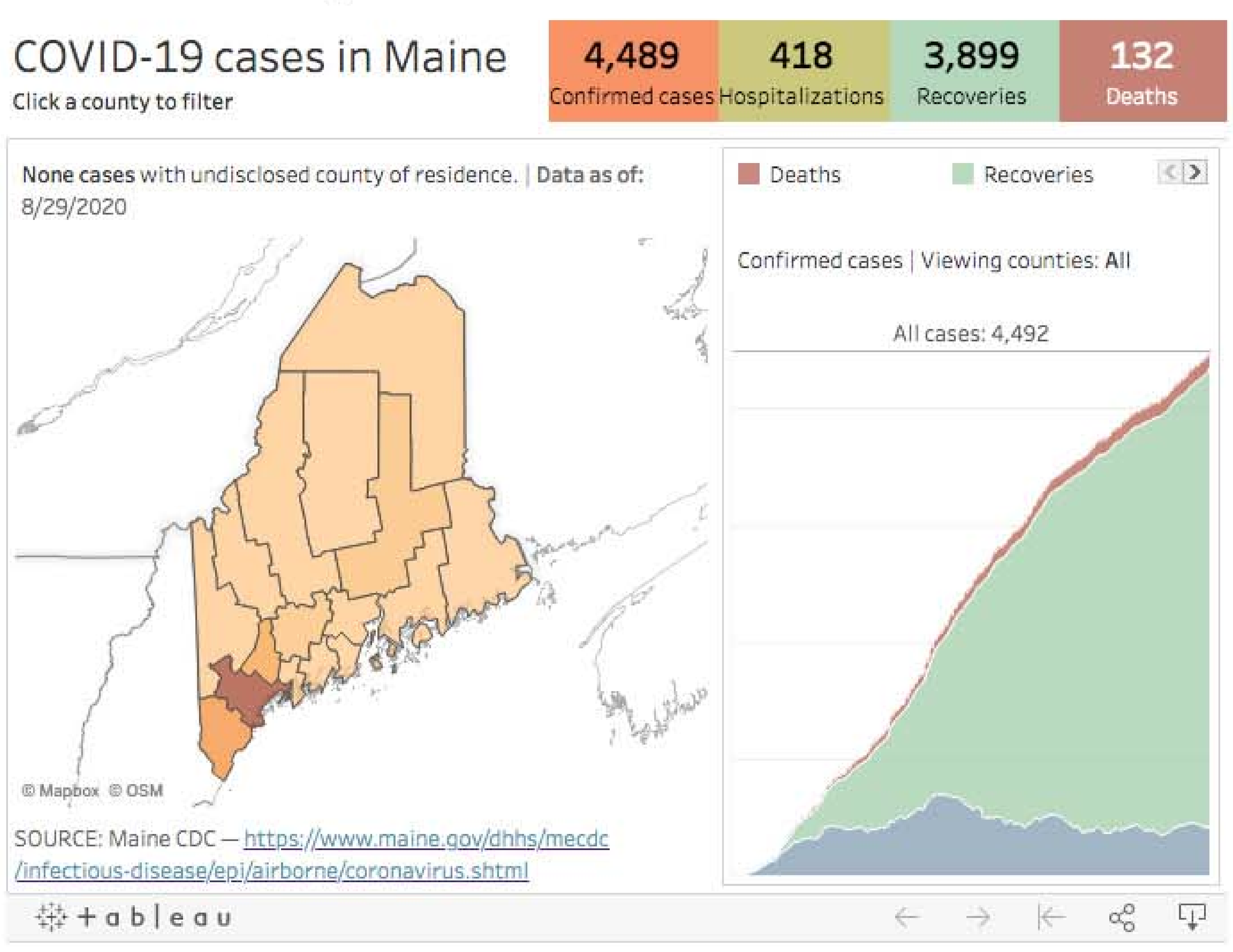

New cases

The rate of growth of cases is important to watch. Social distancing measures intend to slow the spread of the disease so that fewer people are sick all at once, which stands to overwhelm the healthcare system.

Cumberland County has seen the largest spikes in new cases, hitting a high for new cases each day. The early numbers could be impacted by delays in testing, as well.

The chart also illustrates some of the path the disease has taken through Maine counties, starting from March 18. Piscataquis, Aroostook and Washington counties had not reported a case as of April 1.

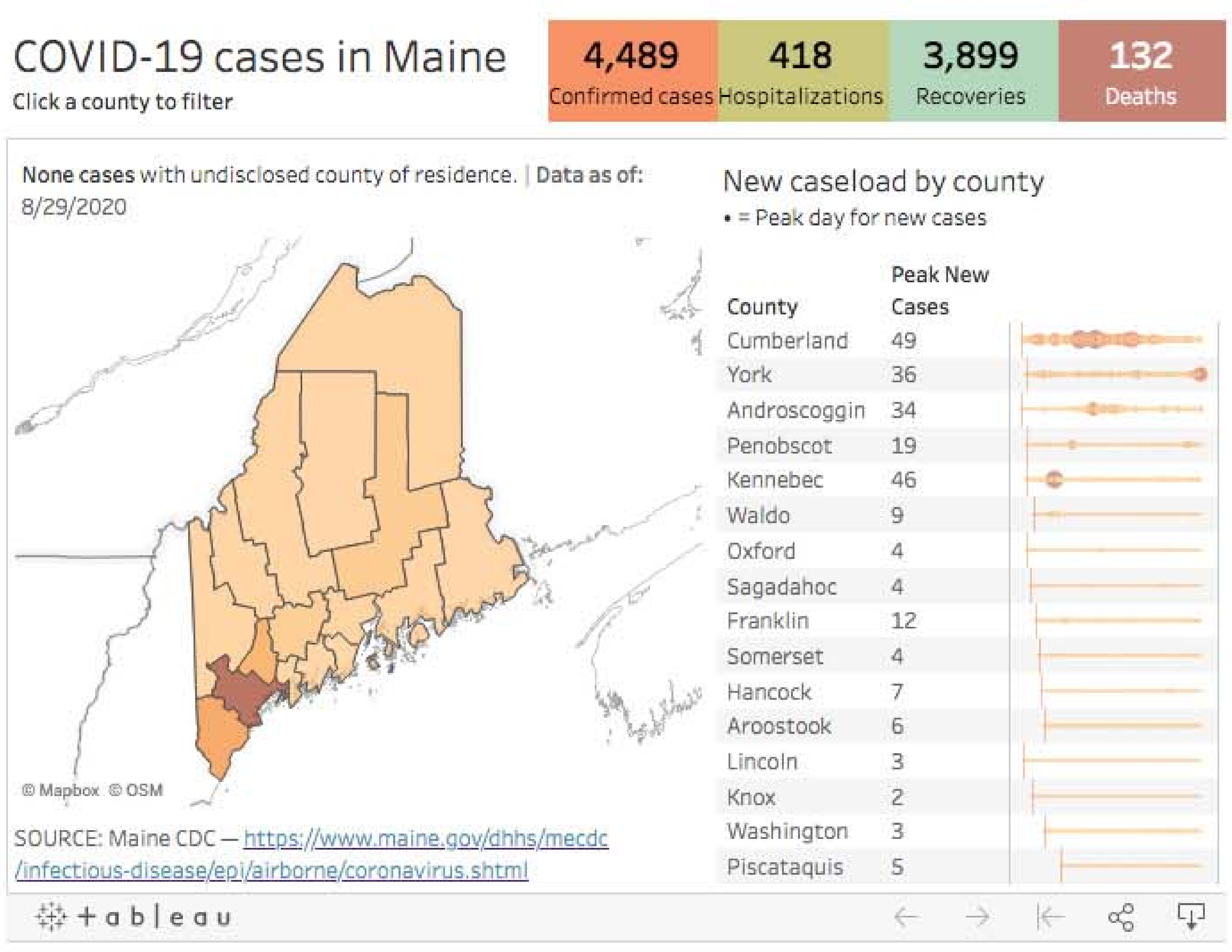

Case rates

For overall metrics, Cumberland and York will have the highest case counts because they have the most people.

The charts below adjust for population, showing the counties that have the highest current rates of confirmed infection.

These are shown alongside the latest calculations for the share of confirmed cases that have resulted in either recovery or hospitalization. These numbers only provide a small snapshot of the current status of testing and the disease in Maine.

In other words, those numbers reflect the situation on the ground in Maine and not necessarily a broader set of insights about the illness, COVID-19.

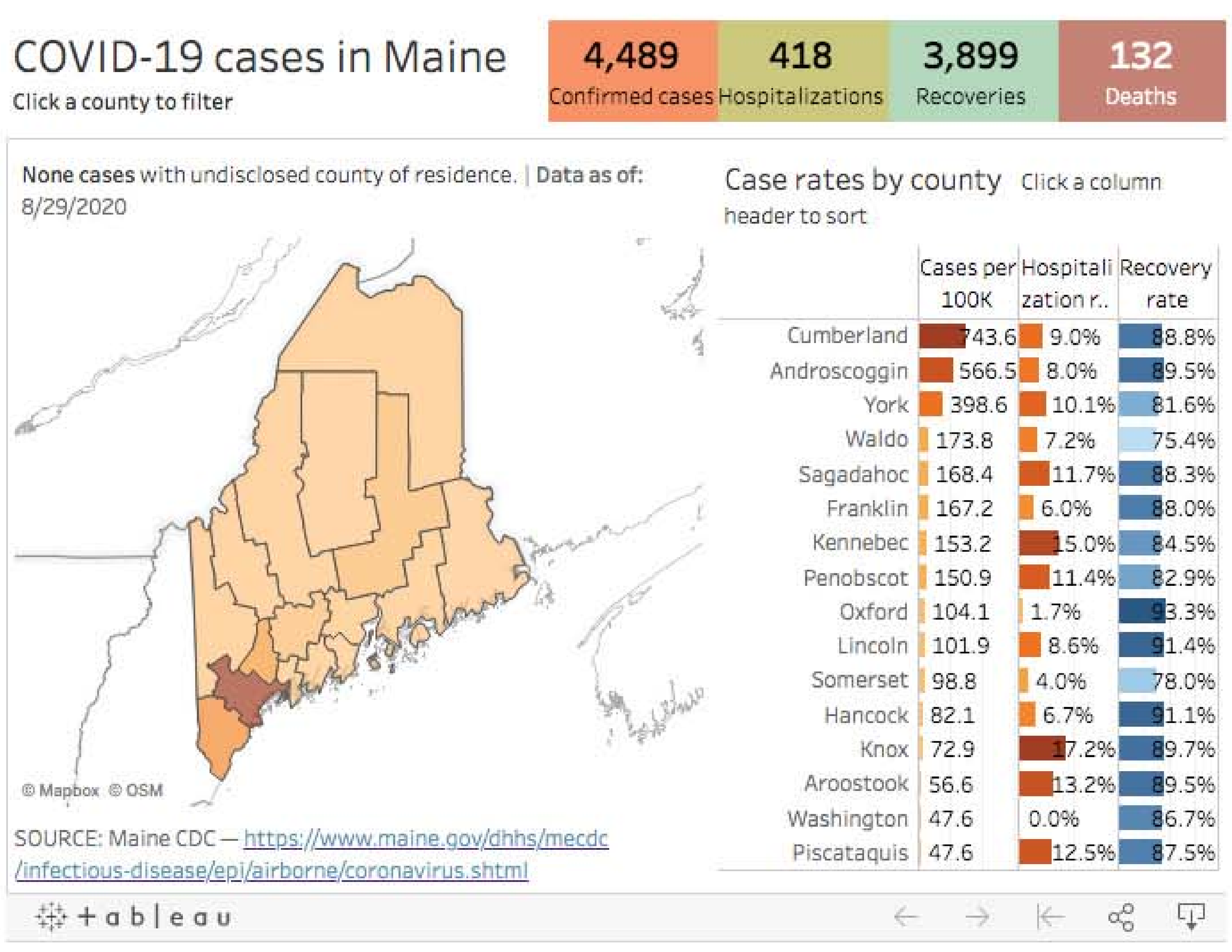

Age trends

The majority of Mainers infected so far have been in their 60s, followed by people in their 50s.

The latest studies of the disease have found that it is more deadly for older people, especially people over 80.

That has not meant that younger people are in the clear — the disease can still be fatal. The line chart shows how cases have trended among Mainers of different ages.

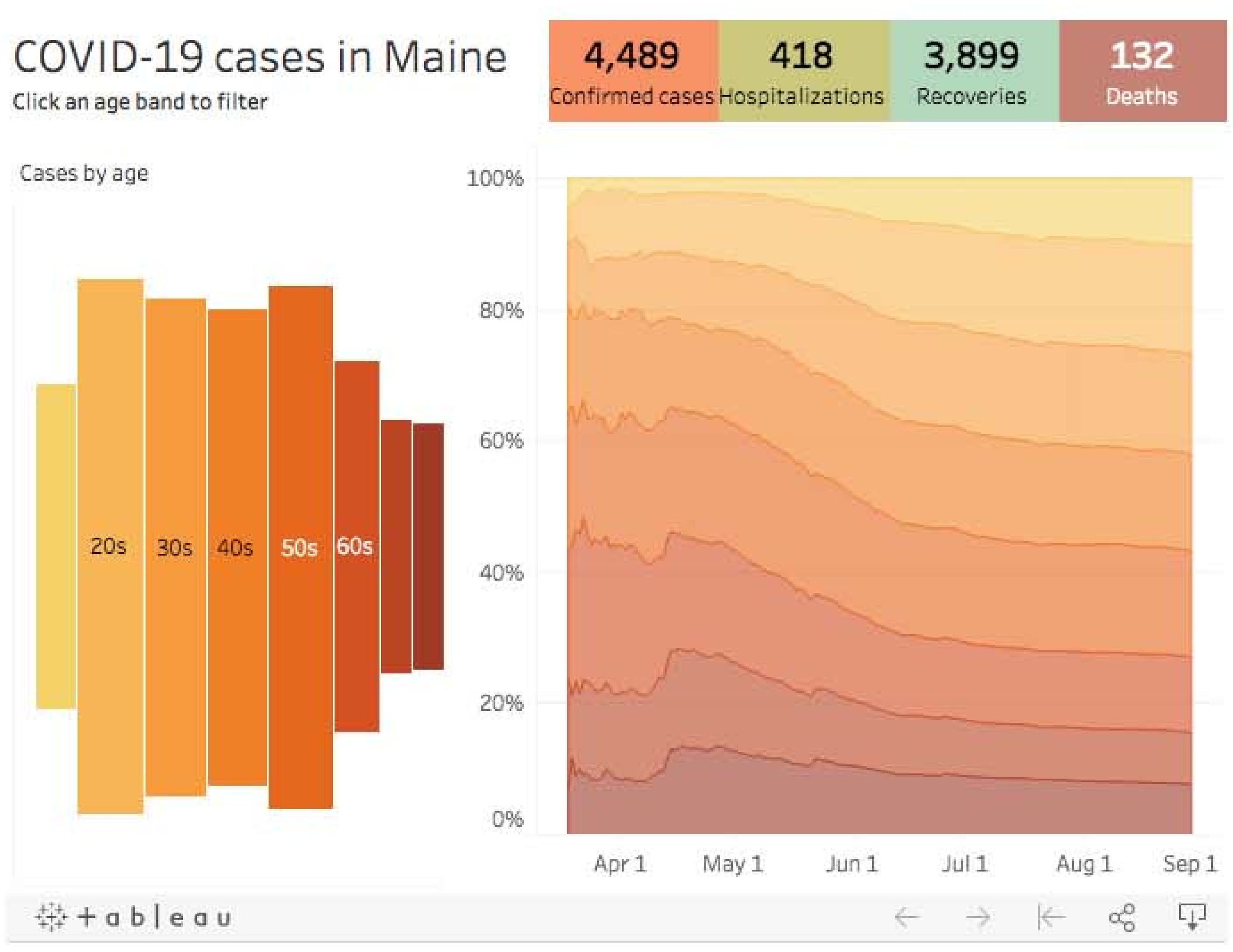

Comparing Maine with other states

The big question is: Can America stop the spread of this virus?

At the state level, this is reflected by looking at the rate of cases day by day. How quickly are they increasing? How many days is it taking for cases to double?

The goal is to slow that growth by: keeping six feet of distance from people you do not live with; washing your hands frequently and thoroughly; and disinfecting regularly touched surfaces.

The chart below uses a logarithmic y-axis, where the values double at every tick on the axis. As The New York Times explained, this makes it easier to tell when case growth begins to slow:

> Unconstrained, the coronavirus spreads exponentially, the caseload doubling at a steady rate. That curve, plotted linearly, is a skyrocketing curve. Plotted logarithmically, however, it transforms into a straight line — which means that deviations from the exponential spread of the virus become much easier to discern.

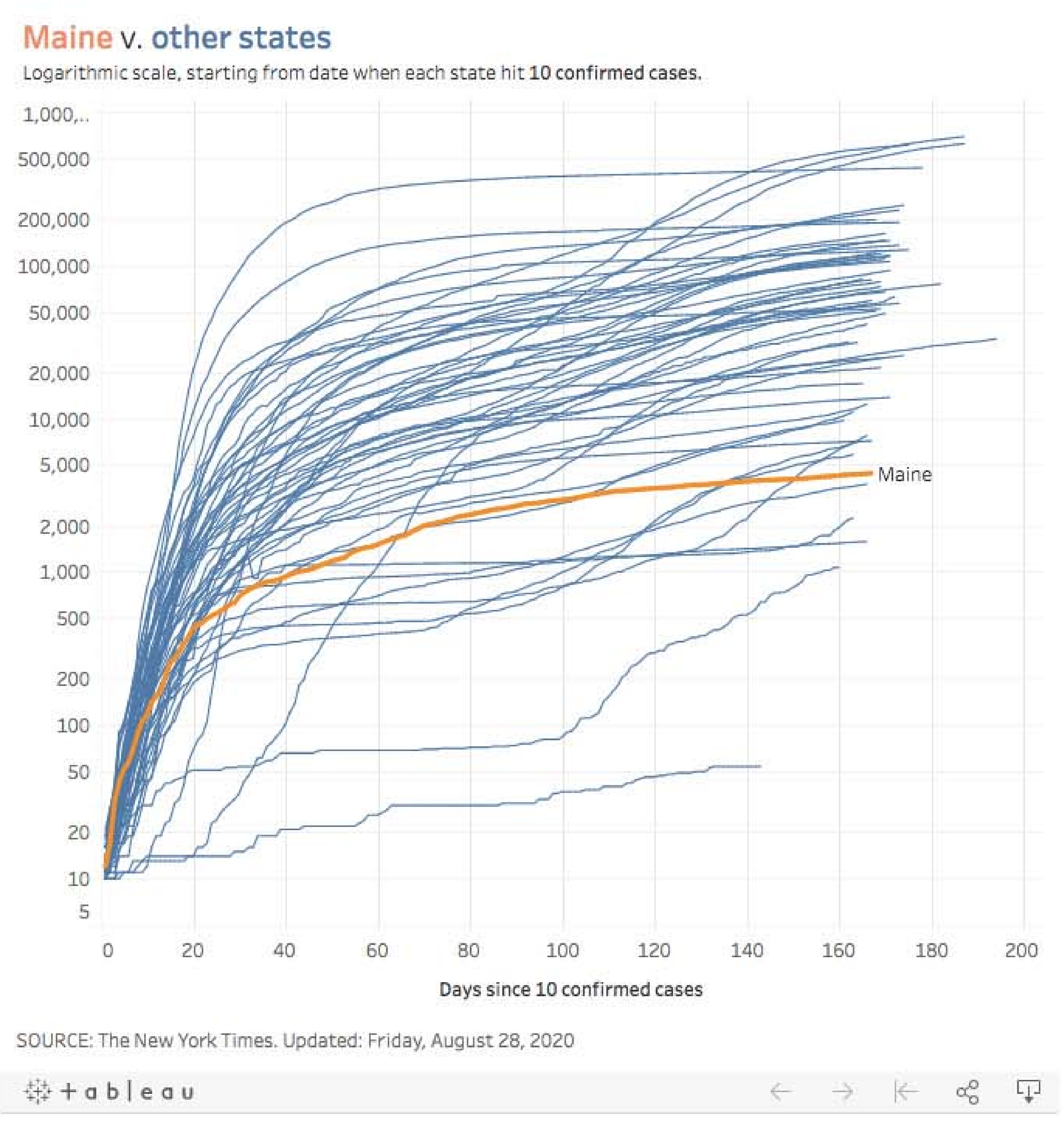

Like 2K Share

Coronavirus at a glance

The latest numbers, maps and charts to keep you up to date.

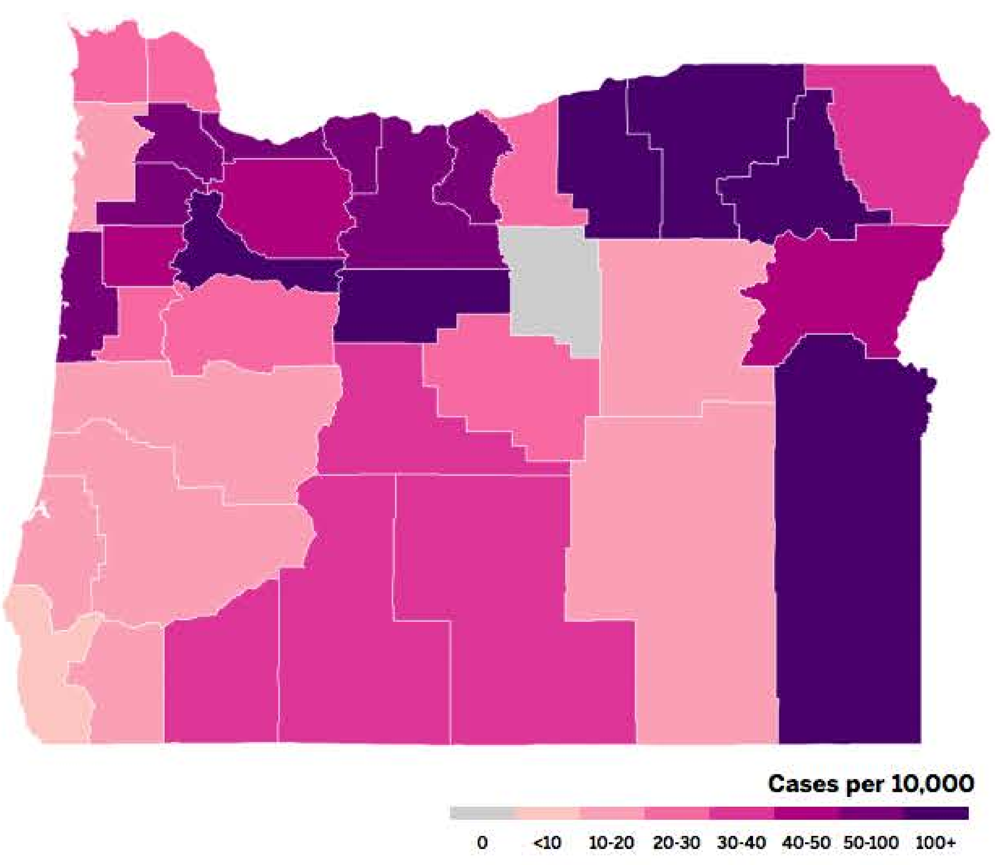

26,054
CASES REPORTED

233
AVG. NEW CASES PER DAY

114
HOSPITALIZATIONS

447
DEATHS

See county-level numbers »

Daily positive test rates

The proportion of daily reported tests in Oregon that have resulted in a confirmed case of coronavirus infection. The Oregon Health Authority does not report complete testing numbers on the weekends.

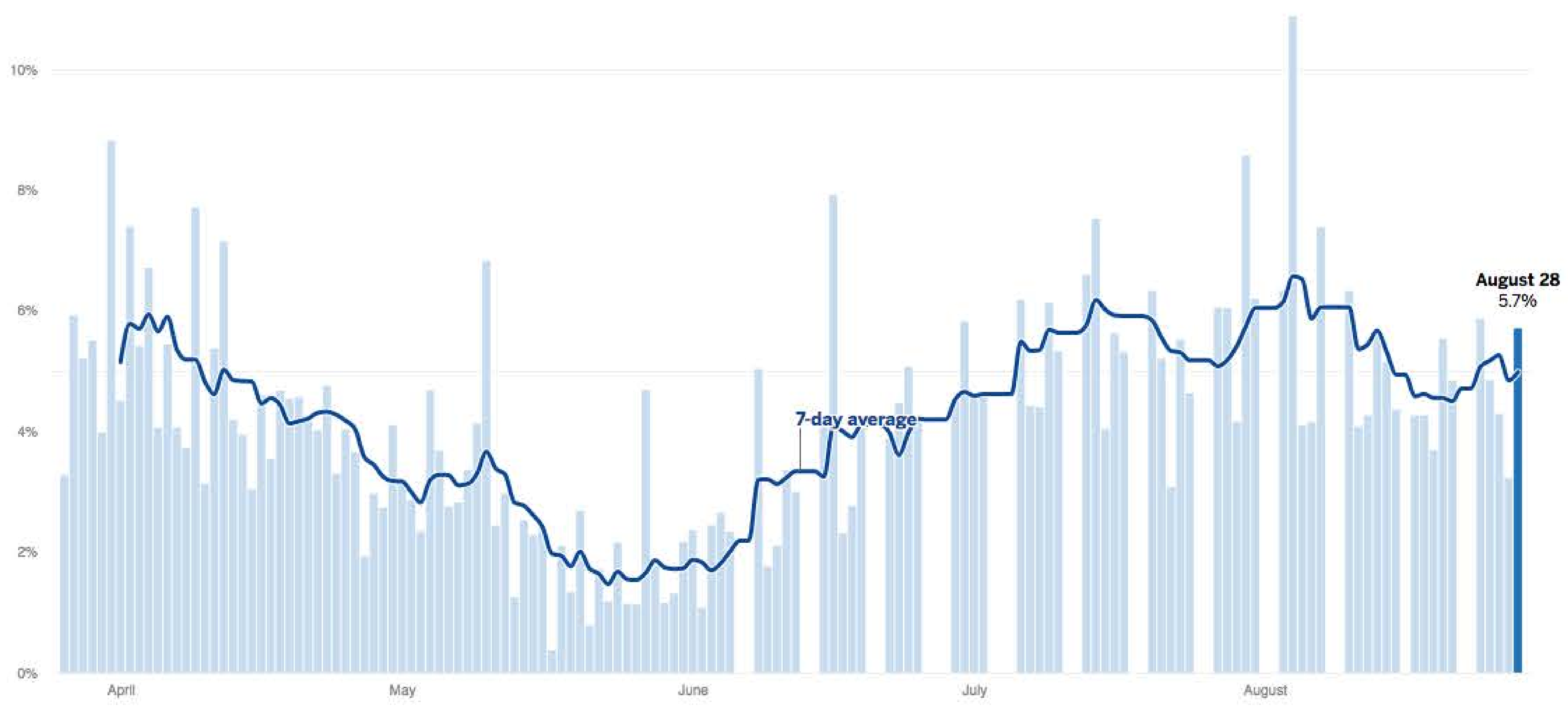

Daily new cases

Daily confirmed and presumptive coronavirus cases reported in Oregon and the 7-day rolling average.

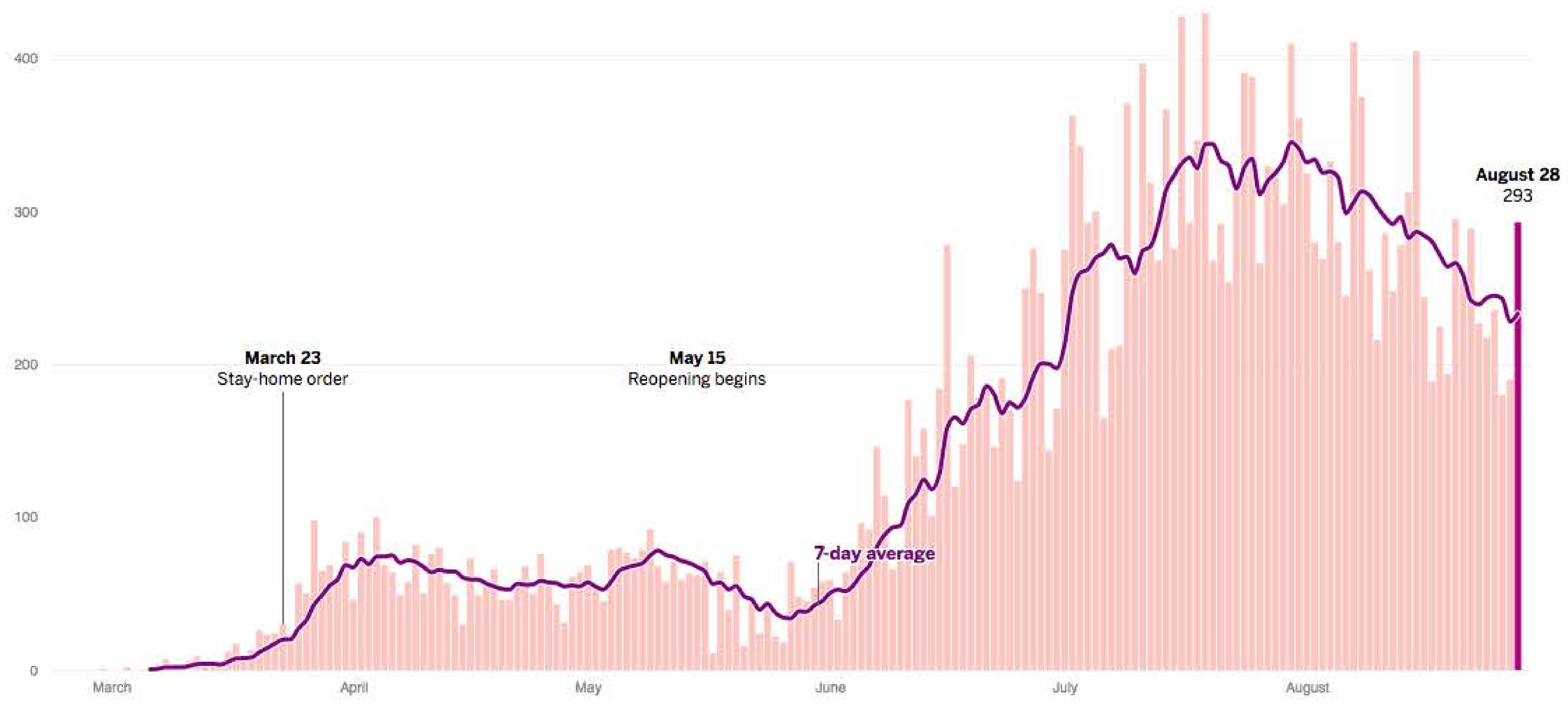

Daily new deaths

Daily coronavirus deaths reported in Oregon and the 7-day rolling average.

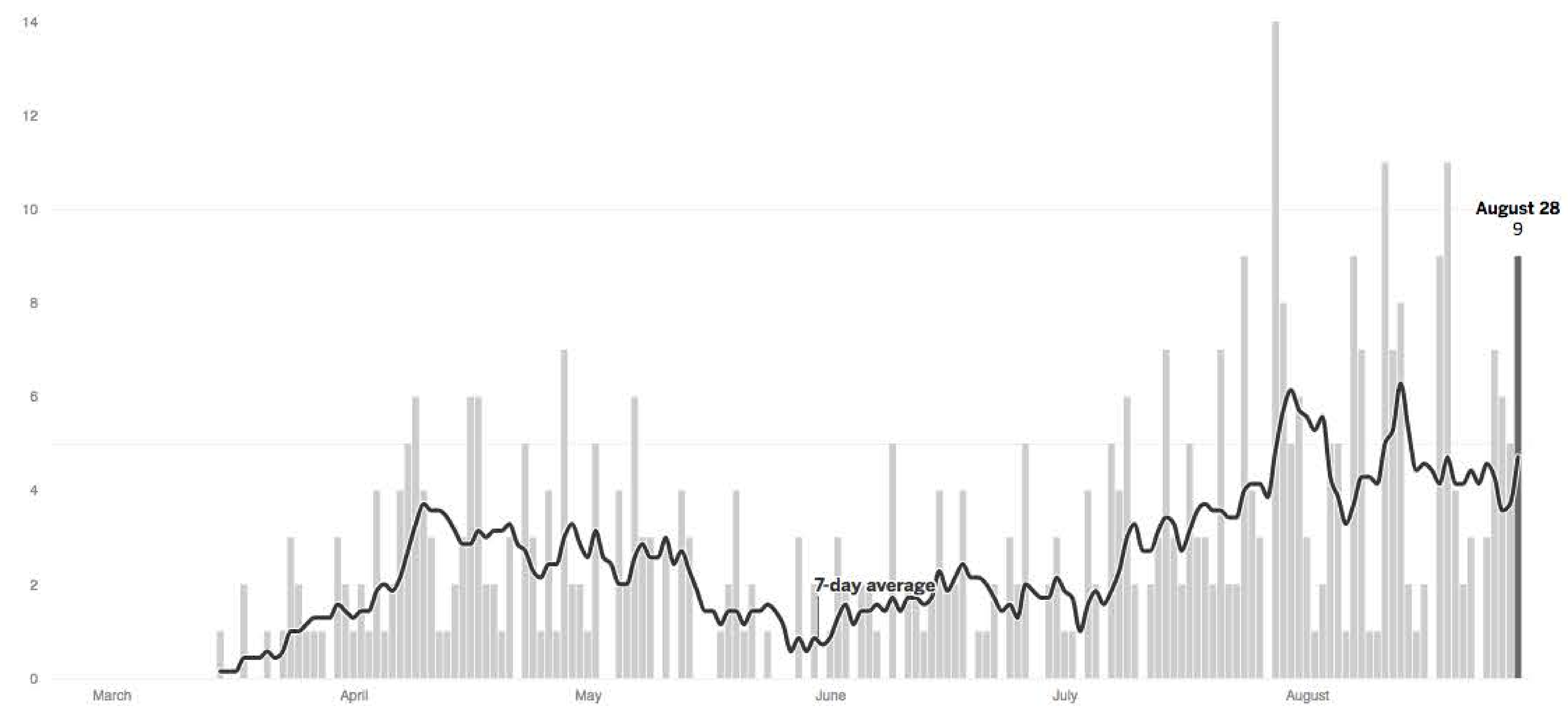

Daily hospitalization status

Number of confirmed coronavirus patients in Oregon who are **hospitalized**, in the **ICU** or on a **ventilator**

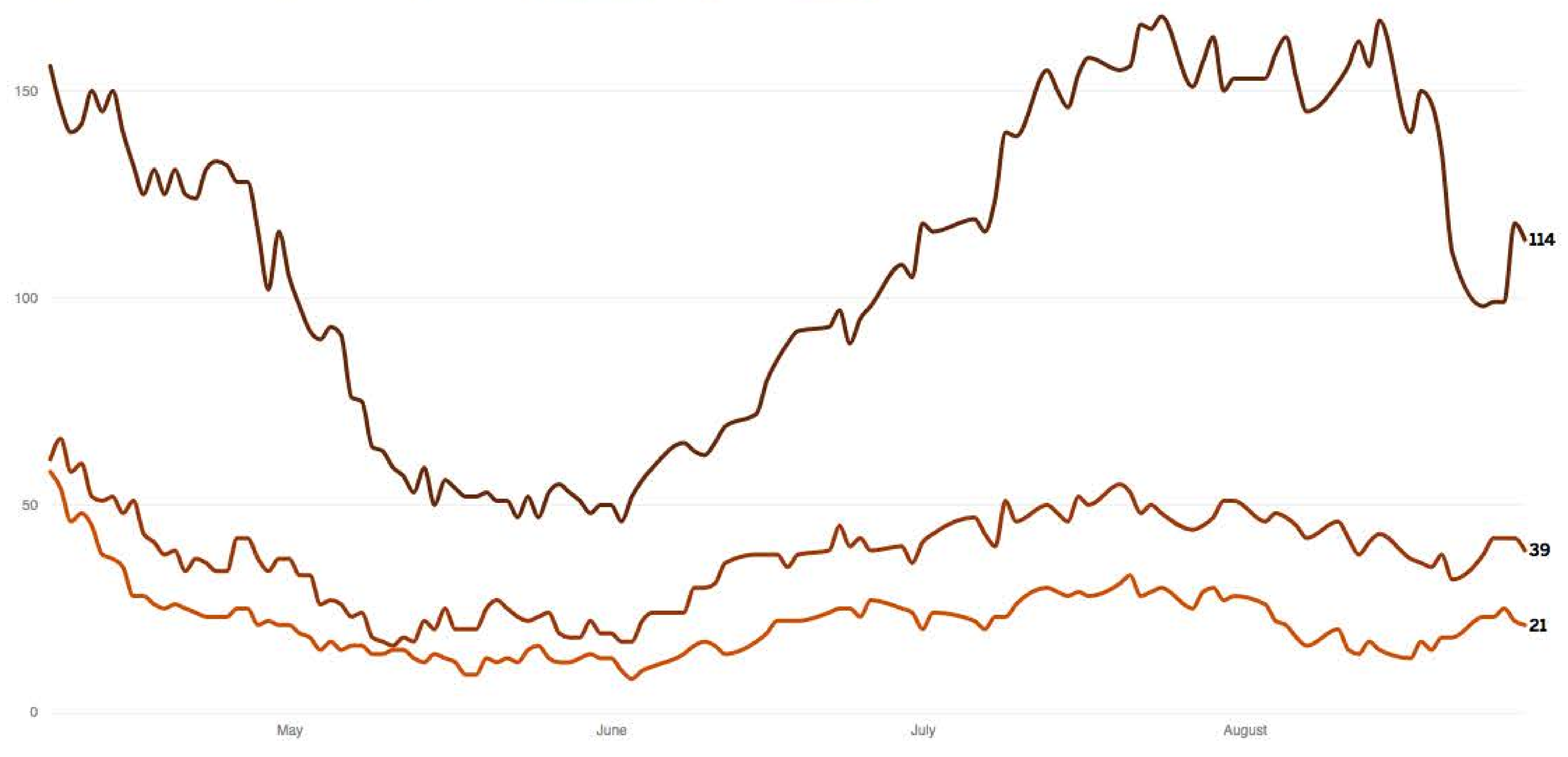

Confirmed U.S. coronavirus cases

5,958,486 cases **182,711** deaths

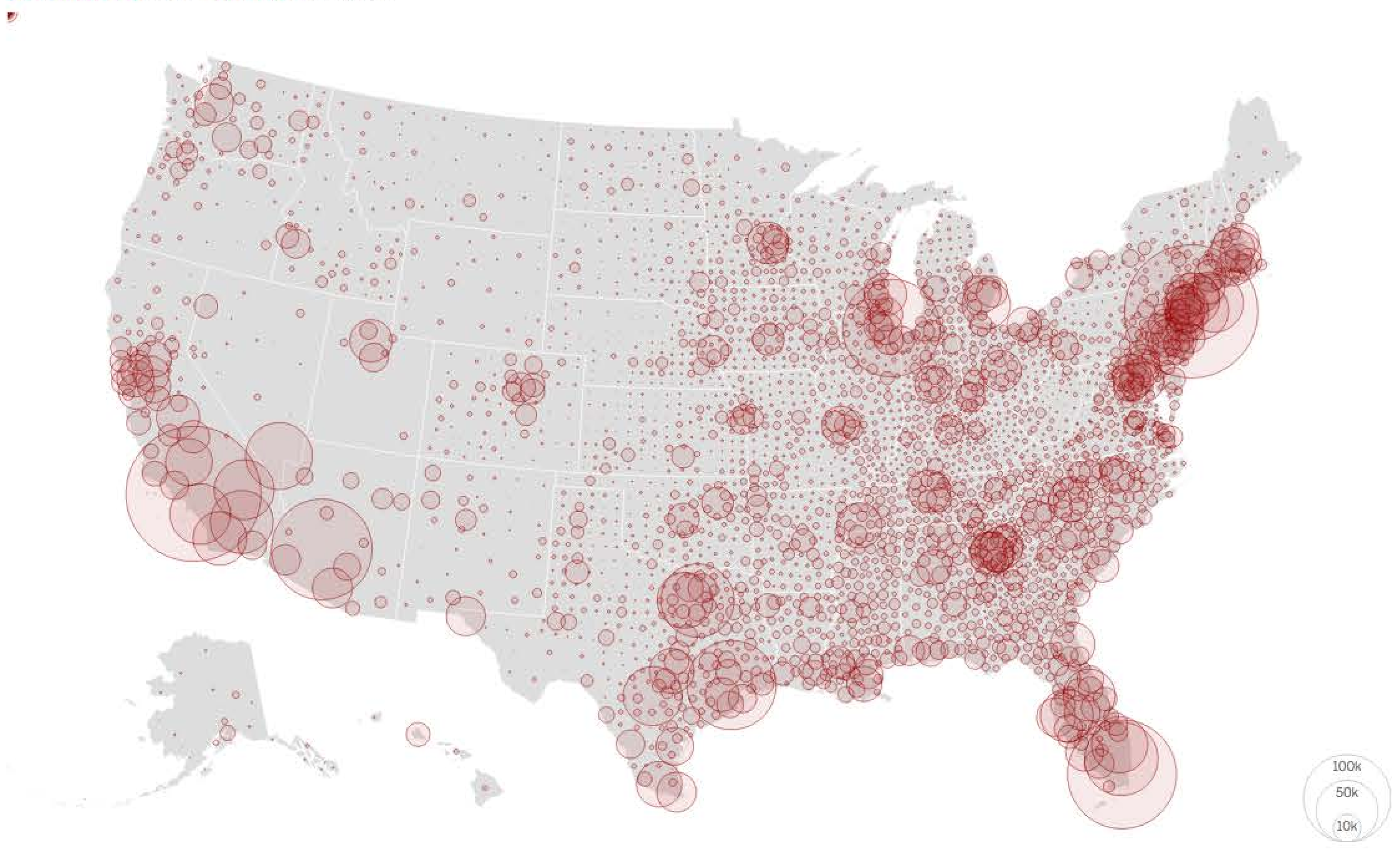

Coronavirus timeline

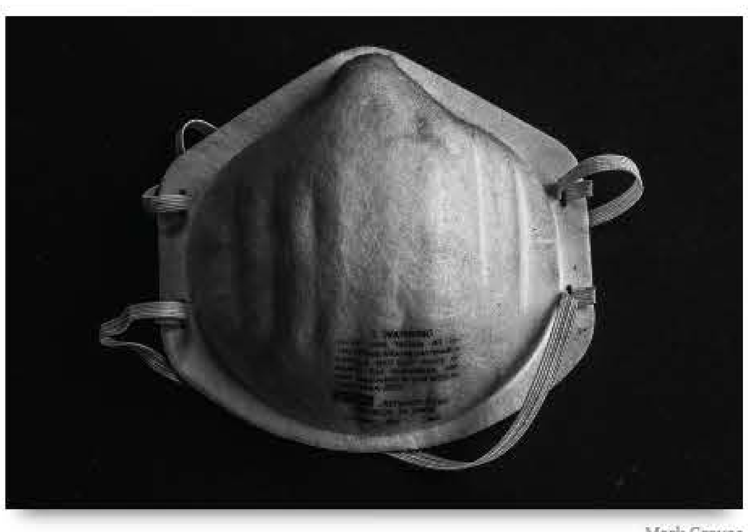

Mark Graves

CORONAVIRUS IN OREGON

It was winter when the novel coronavirus struck Oregon. Now, summer is here and the state is struggling to reopen after Gov. Kate Brown loosened her stay-home restrictions. Still, the virus continues to influence every aspect of our daily lives. Here's a timeline of key events so far.

PATIENT ONE

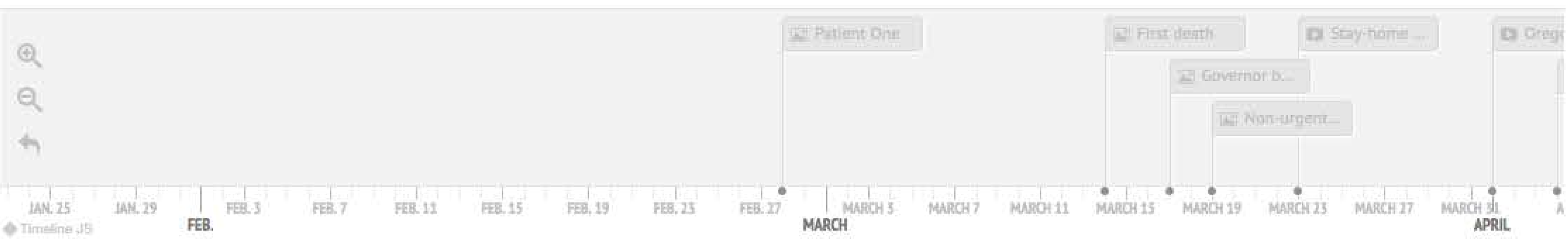

Interactives by Mark Friesen, David Cansler and Mark Graves/staff

Sources: Oregon Health Authority, Oregon Military Department Office of Emergency Management, county health departments, Center for Systems Science and Engineering at Johns Hopkins University, The COVID Tracking Project

Arkansas Democrat-Gazette

SUBSCRIBE

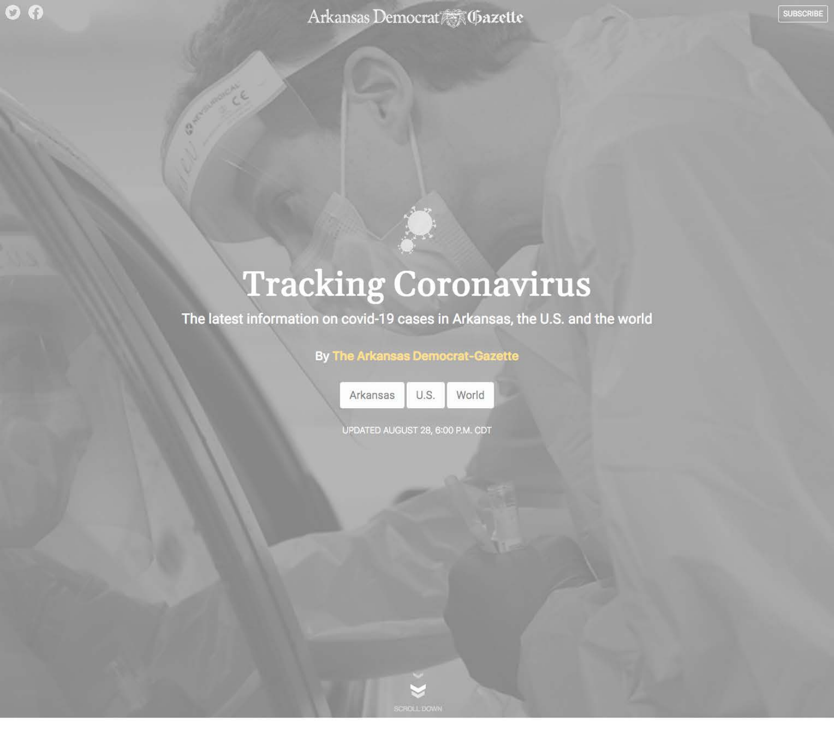

Tracking Coronavirus

The latest information on covid-19 cases in Arkansas, the U.S. and the world

By The Arkansas Democrat-Gazette

Arkansas | U.S. | World

UPDATED AUGUST 28, 6:00 P.M. CDT

SCROLL DOWN

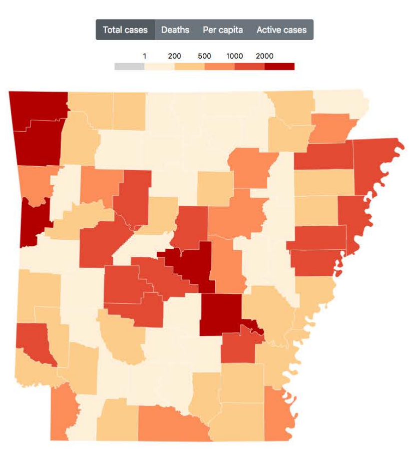

59,583 confirmed cases

5,496 active cases

756 deaths

53,331 recoveries

638,472 negative tests

* 815 cases with missing county information.

Arkansas' first presumptive case of covid-19, the illness caused by the coronavirus, was detected in Pine Bluff on Wednesday, March 11. The patient had an out-of-state travel history.

Also on March 11, the Centers for Disease Control and Prevention announced that Arkansas will receive $6,205,347 to support coronavirus preparedness and response, according to the agency's website.

Symptoms of covid-19, including fever, cough and shortness of breath, have been mild for most people but severe or even deadly for some. The elderly and people with underlying health conditions considered most at risk.

Arkansas covid-19 cases distribution

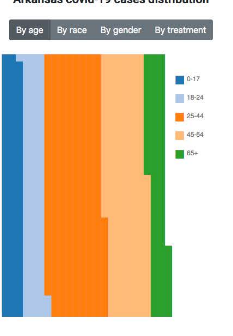

The disease is thought to spread mainly from person-to-person through droplets produced when an infected person coughs or sneezes. Spread generally occurs between people who are in close contact (within about 6 feet).

People can also catch covid-19 by touching a contaminated surface and then touching their face.

There is also evidence suggesting people can spread covid-19 without developing symptoms, although the Centers for Disease Control and Prevention believes that people are most contagious when they have symptoms.

New cases by day

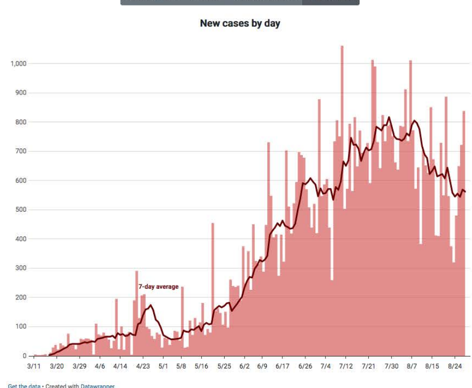

Get the data • Created with Datawrapper

Counties with the most covid-19 cases in Arkansas

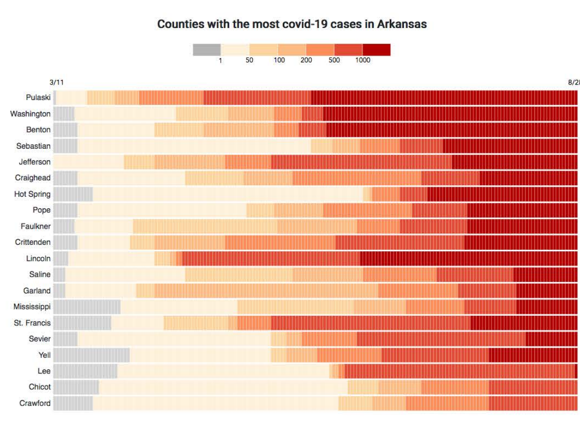

Nursing homes across the state have been in lockdown under federal orders to protect their residents.

Gov. Asa Hutchinson announced plans to test every Arkansas nursing home resident and worker for the coronavirus in June.

Covid-19 cases in Arkansas long-term care facilities

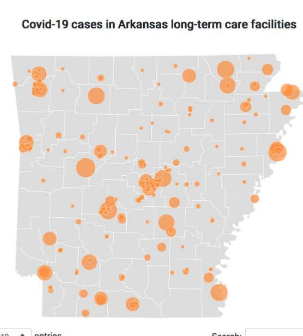

Show 10 entries Search:

Facility	Positive residents	Positive staff members	Total cases	Resident deaths
Advanced Health and Rehab of Union County	0	3	3	0
Allay Health and Rehab	5	6	11	2
Amberwood Health & Rehab	0	4	4	0
Apple Creek Health and Rehabilitation	1	0	1	0
Arbor Oaks Healthcare & Rehab	16	0	16	0
Arkansas Nursing & Rehab	2	3	5	0
Arkansas State Veteran's Home	6	4	10	2
Arkansas State Veterans Home at North Little Rock	0	2	2	0
Ash Flat Healthcare & Rehab	37	18	55	4
Ashley Health & Rehabilitation	3	1	4	0

Showing 1 to 10 of 213 entries

Previous 1 2 3 4 5 ... 22 Next

Coronavirus infections have spread through more than a dozen prisons, jails and juvenile lock-ups in the state.

Officials have struggled to keep the virus contained even when they identified outbreaks, while prisoners and their families have accused the Arkansas Department of Corrections of bungling their response to the virus. Some have complained that prison officials have kept them in the dark about their loved-one's illness.

In April, Gov. Asa Hutchinson ordered a review of nearly 2,000 inmates who are serving time for non-violent and non-sexual offenses to determine which ones can memade eligible for early release. Of those, 730 had been released as of the end of June.

Covid-19 cases in Arkansas correctional facilities

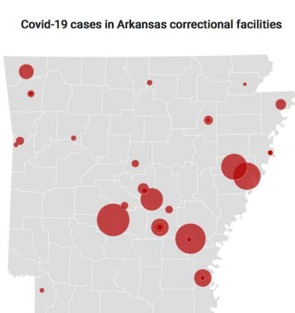

Show 10 entries Search:

Facility	Positive inmate	Positive staff members	Total cases	Inmate/staff member deaths
Barbara Ester Unit	62	4	64	0
Benton County Jail	189	15	204	0
Benton Unit	20	2	22	0
Central Arkansas Community Correction Center	63	27	90	1
Central Office-ERT Parole Transport	0	8	8	0
Crawford County Jail	33	0	33	0
Cummins State Prison	1062	69	1131	11
Delta Corrections Facility	302	5	307	0
Dermott Juvenile Correctional Facility	0	2	2	0
East Arkansas Community Corrections Center	3	2	5	0

Showing 1 to 10 of 32 entries

Previous 1 2 3 4 Next

People who think they may have covid-19 should call their health care providers or schedule a test at a local health unit, according to the state Department of Health.

A health care provider will decide if a test is appropriate based on the symptoms and possible exposure to the coronavirus.

The University of Arkansas for Medical Sciences is also offering free online screenings and over the phone at (800) 632-4502.

For children, Arkansas Children's Hospital is offering drive-thru screenings and phone screenings at (800) 743-3616.

Coronavirus testing and screening locations in Arkansas

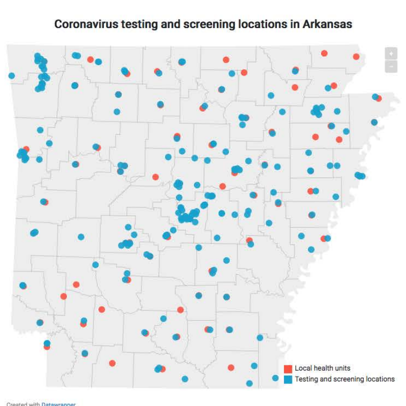

Created with Datawrapper

How Arkansas compares in testing

* States sized by population.

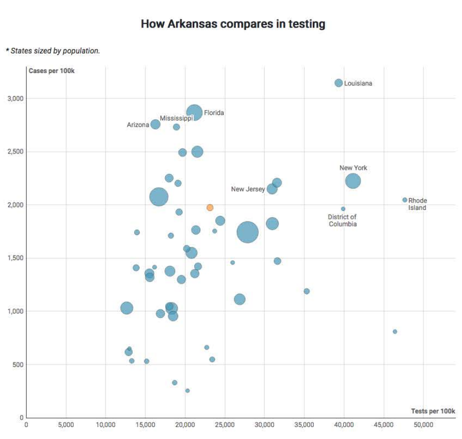

Created with Datawrapper

Gov. Asa Hutchinson signed legislation creating a $173 million rainy-day fund to respond to the coronavirus outbreak on March 28.

On March 30, Hutchinson announced that legislative leaders approved his administration's request to tap $45 million from the fund created by the General Assembly.

Larry Walther, secretary of the state Department of Finance and Administration, said $30 million of the request will be used to purchase personal protective equipment for the state's hospitals, first responders, community health clinics and others whose work is critical to the response to the ongoing crisis.

An additional $13.5 million will be used to purchase more ventilators and associated parts and equipment as well as any shipping or freight charges required to equip the state's hospitals with this life-saving equipment.

Cumulative covid-19 cases in the United States by county

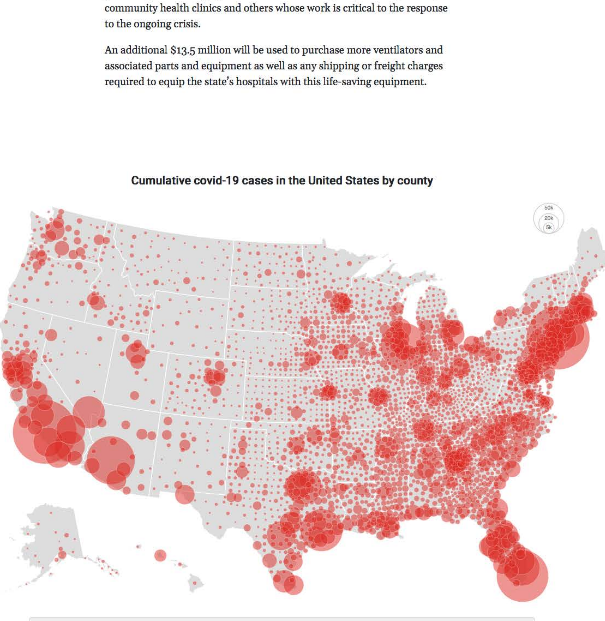

5,912,016 confirmed cases 181,655 deaths 2,118,367 recoveries

Covid-19 cases in the United States by state

* Numbers used for new cases curves are 7-day averages.

Search in table — Page 1 of 4

	State	Confirmed cases	New cases curve	Cases per 100k	Deaths	Total tests	% of positive	Tests per 100k
1	Louisiana	146,243	646	3146	4,904	1,828,478	8.0%	39332
2	Florida	615,806	3k	2867	11,099	4,547,677	13.5%	21174
3	Arizona	200,658	537	2757	4,978	1,184,077	16.9%	16268
4	Mississippi	81,294	710	2732	2,413	562,882	14.4%	18913
5	Georgia	265,372	2k	2499	5,471	2,286,984	11.6%	21540
6	Alabama	122,185	1k	2492	2,107	964,863	12.7%	19678
7	South Carolina	115,951	796	2252	2,655	926,509	12.5%	17995
8	New York	432,767	608	2225	25,312	8,002,897	5.4%	41138
9	Tennessee	150,815	1k	2208	1,701	2,155,504	7.0%	31563
10	Nevada	67,852	488	2203	1,287	588,551	11.5%	19108
11	New Jersey	190,971	308	2150	15,930	2,749,194	6.9%	30952
12	Texas	601,768	5k	2075	12,266	4,842,362	12.4%	16700
13	Rhode Island	21,683	60	2047	1,046	504,904	4.3%	47661
14	Arkansas	59,583	562	1974	756	698,055	8.5%	23131
15	District of Columbia	13,851	55	1963	605	281,549	4.9%	39894

Created with Datawrapper

The outbreak in the United States first emerged in Washington state after a resident tested positive on January 20.

The first coronavirus-related death occurred in Northern California's Santa Clara County on February 6.

Cases have been discovered in all 50 states after West Virginia reported its first on March 17, although testing and reporting discrepancies exist among different states.

Thousands of new cases are now being detected across the country each day.

Cumulative covid-19 cases worldwide

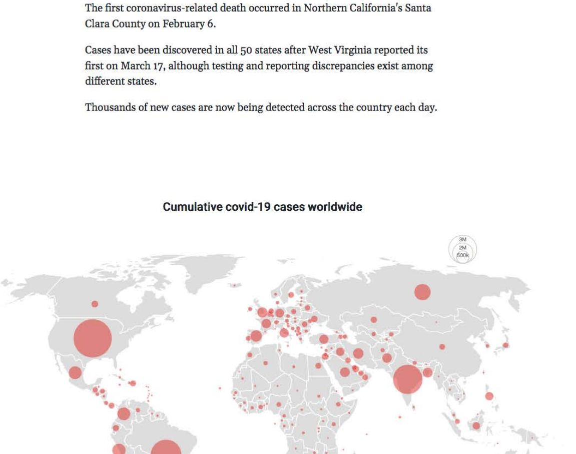

24,578,557 confirmed cases 833,943 deaths 16,074,810 recoveries

Countries with the most covid-19 cases

Countries	Cases: Total (Daily Change)	Cases: Trend (vs. Prev. Week)	Deaths: Total (Daily Change)
U.S.	5,957,687 (+41,640)	↘ -6%	182,474 (+920)
Brasil	3,846,153 (+38,920)	↗ +6%	120,262 (+880)
India	3,463,972 (+69,750)	↗ +9%	62,550 (+970)
Russia	982,573 (+4,720)	→ -2%	16,977 (+100)
Peru	629,961 (+7,850)	↗ +8%	28,471 (+180)
South Africa	622,551 (+2,400)	↘ -31%	13,981 (+130)
Colombia	590,492 (+9,760)	↘ -11%	18,766 (+310)
Mexico	585,738 (+5,140)	↘ -6%	63,146 (+510)
Spain	439,286 (+7,600)	↗ +23%	29,011 (+20)
Chile	408,009 (+1,740)	→ +4%	11,181 (+60)

*Daily Change: 7-day average of new confirmed cases.

Created with Datawrapper

Cumulative covid-19 cases 200 days since 100th case

* This chart is plotted on a logarithmic scale.

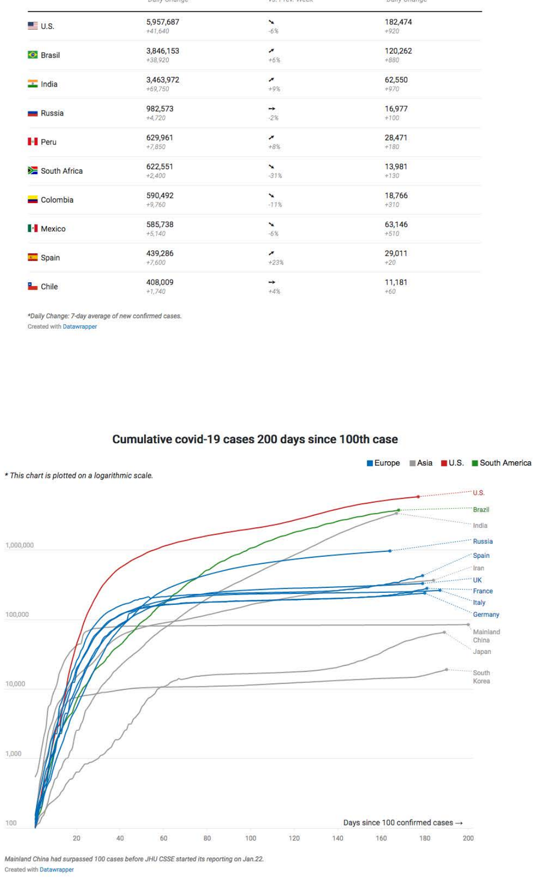

Mainland China had surpassed 100 cases before JHU CSSE started its reporting on Jan.22.

Created with Datawrapper
