# Supplementary material for: Web-Based COVID-19 Dashboards and Trackers in the United States: Survey Study
Source: JMIR Hum Factors. 2023 Mar 20;10:e43819. doi: 10.2196/43819 (PMC10029858; doi:10.2196/43819)
Supplement: Multimedia Appendix 5 [file humanfactors_v10i1e43819_app5.pdf]

Appendix 5. Screenshots from dashboards and trackers — *Nation-wide coverage* *(captured August 29, 2020)*

Page	Ref	Host
2–3	N-1	US Centers for Disease Control and Prevention
4	N-2	US Centers for Disease Control and Prevention
5	N-3	The COVID Tracking Project / The Atlantic
6–7	N-4	New York Times
8	N-5	NPR
9	N-6	Johns Hopkins Centers for Civic Impact
10	N-7	Johns Hopkins Centers for Civic Impact
11	N-8	COVID-19 Health Equity Interactive Dashboard / Emory University
12	N-9	USAFacts
13	N-10	COVID Mapping Project / Standard Co
14	N-11	SAE Industry Technologies Consortia

CDC COVID Data Tracker

Maps, charts, and data provided by the CDC

The CDC COVID-19 Data Tracker has a new look and location to make the data easier to find and use. All previous data remains and will be updated on the same schedule. Please update bookmarks and links.

Also, as of 8/27, the Tracker includes correctional facility information, NIH Vulnerability Index data, and upgraded testing information.

United States COVID-19 Cases and Deaths by State

Reported to the CDC since January 21, 2020

USA
5,890,532
TOTAL CASES
CDC | Updated: Aug 29 2020 12:16PM

USA
181,143
TOTAL DEATHS
CDC | Updated: Aug 29 2020 12:16PM

USA
291,985
Cases in Last 7 Days
CDC | Updated: Aug 29 2020 12:16PM

Total Cases by State/Territory

State/Territory	Total Cases	Confirmed	Probable
California	688,858	N/A	N/A
Florida	609,074	N/A	N/A
Texas	601,768	N/A	N/A
Georgia	265,372	N/A	N/A
New York City*	234,509	229,751	4,758
Illinois	231,185	229,483	1,702
Arizona	200,658	198,978	1,680
New York*	199,357	N/A	N/A
New Jersey	190,971	N/A	N/A
North Carolina	162,491	N/A	N/A
[illegible]	[illegible]	[illegible]	[illegible]

CDC | Updated: Aug 29 2020 12:16PM

Total Cases | Cases in Last 7 Days | Cases per 100,000 | Total Deaths | Deaths per 100,000

Total Number of COVID-19 Cases in the US Reported to the CDC, by State/Territory

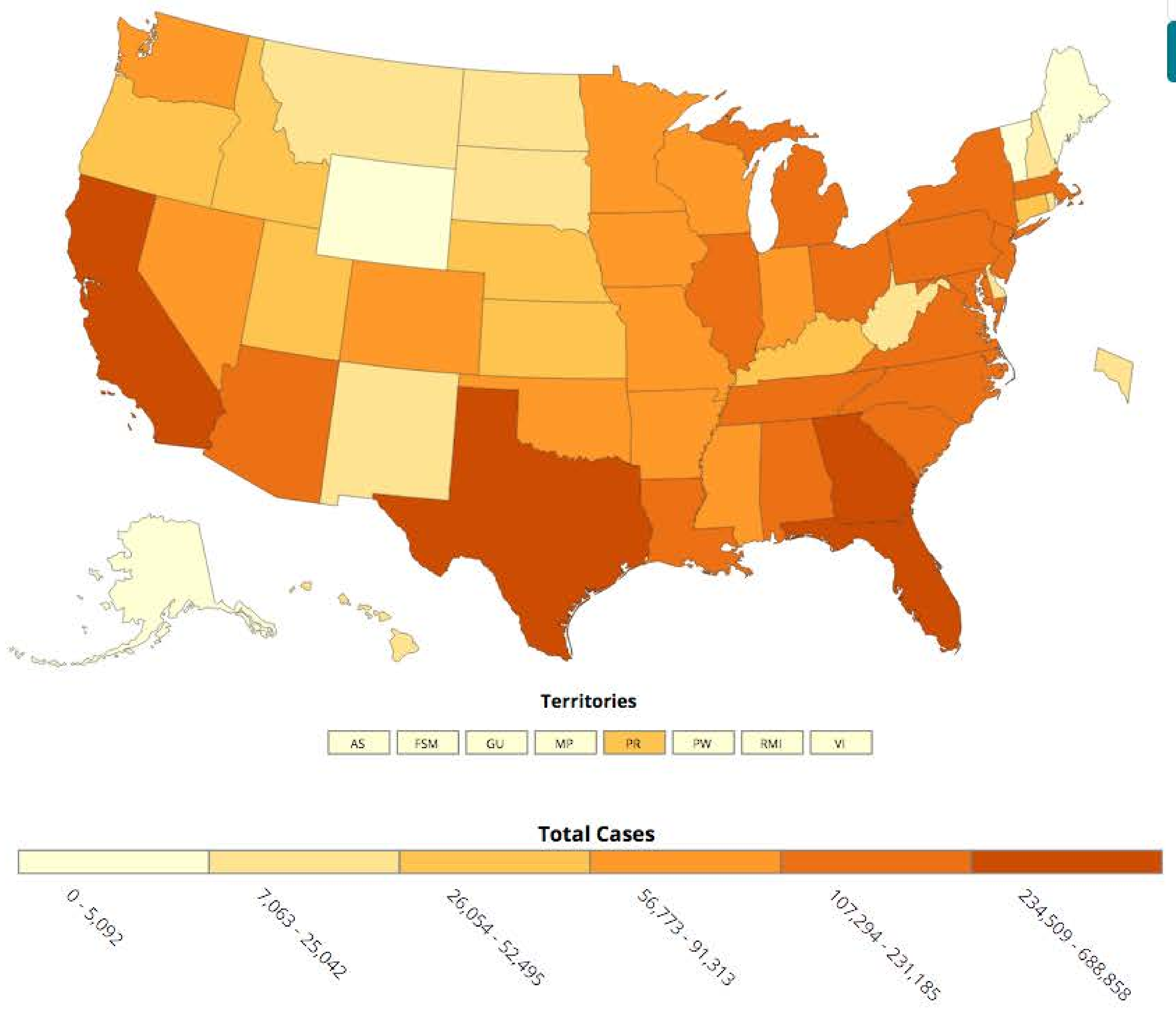

Data Sources, References & Notes: Total cases are based on aggregate counts of COVID-19 cases reported by state and territorial jurisdictions to the Centers for Disease Control and Prevention (CDC) since January 21, 2020, with the exception of persons repatriated to the United States from Wuhan, China, and Japan. The numbers are confirmed and probable COVID-19 cases as reported by U.S. states, U.S. territories, New York City, and the District of Columbia from the previous day. *Counts for New York City and New York state are shown separately; data for New York State show total cases and deaths for the State excluding data for NYC. When not available to CDC this is annotated by N/A. Rates are calculated using U.S. Census Bureau, 2018 American Community Survey 1-year estimates and are shown as cases/100,000 people. The map shows total cases per state, new cases in the last 7 days per state, and the rate (cases/100,000) per state. The 7-day moving average of new cases (current day + 6 preceding days / 7) was calculated to smooth expected variations in daily counts. Demographic data for deaths is based on a subset of cases where case-level data is available. Case numbers reported on other websites may differ from what is posted on CDC's website because CDC's overall case numbers are validated through a confirmation process with each jurisdiction. Differences between reporting jurisdictions and CDC's website may occur due to the timing of reporting and website updates. The process used for finding and confirming cases displayed by other sites may differ.

Coronavirus Disease 2019 (COVID-19)

CDC COVID Data Tracker

Maps, charts, and data provided by the CDC

The CDC COVID-19 Data Tracker has a new look and location to make the data easier to find and use. All previous data remains and will be updated on the same schedule. Please update bookmarks and links.

Also, as of 8/27, the Tracker includes correctional facility information, NIH Vulnerability Index data, and upgraded testing information.

Case Trends ▾ | Laboratory ▾ | Community Impact ▾ | Unique Populations ▾ | COVID-19 Home

Testing | Serology Surveillance

United States Laboratory Testing

USA

82,779,999

TESTS REPORTED

CDC | Updated: Aug 29 2020 12:16PM

USA

7,172,609

POSITIVE TESTS

CDC | Updated: Aug 29 2020 12:16PM

USA

9%

OVERALL % POSITIVE

CDC | Updated: Aug 29 2020 12:16PM

Tests Performed | Percent Positive | Tests per 100,000

COVID-19 Viral (RT-PCR) Laboratory Tests Performed by State/Territory

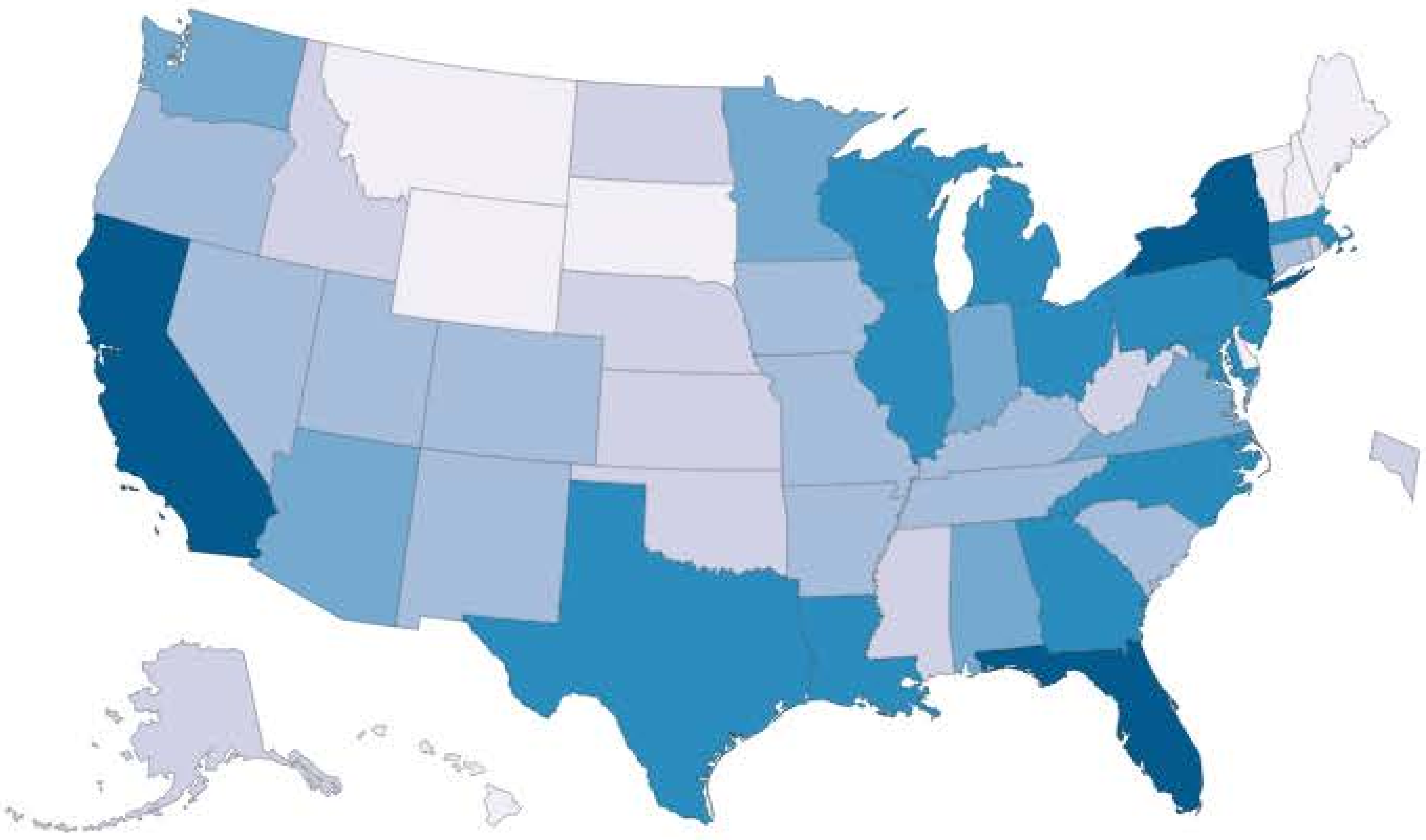

Territories

GU | PR | VI

Cumulative Tests Performed

17,432 - 293,363 | 363,398 - 610,988 | 660,964 - 1,049,031 | 1,233,094 - 1,773,884 | 1,829,023 - 4,917,736 | 7,707,231 - 10,845,124

COVID-19 Viral (RT-PCR) Tests Performed by State/Territory

State	# Tests Performed	% Positive
California	10,845,124	6-10%
New York	8,152,614	6-10%
Florida	7,707,231	11-20%
Texas	4,917,736	11-20%
Illinois	3,611,610	6-10%
Michigan	2,848,730	0-5%
Pennsylvania	2,416,999	6-10%
Massachusetts	2,239,216	6-10%
New Jersey	2,238,111	0-5%
Georgia	2,198,412	11-20%
Ohio	2,087,026	6-10%
North Carolina	2,061,563	6-10%
Maryland	2,024,001	6-10%
Wisconsin	1,849,480	0-5%
Louisiana	1,829,023	11-20%
Indiana	1,773,884	6-10%
Virginia	1,621,389	11-20%
Minnesota	1,559,916	6-10%
Arizona	1,500,252	11-20%
Alabama	1,371,384	11-20%
[illegible]	[illegible]	[illegible]

CDC | Updated: Aug 29 2020 12:16PM

Disclaimer: The data presented above represent viral COVID-19 laboratory test (reverse transcription polymerase chain reaction) results from laboratories in the United States, including commercial and reference laboratories, public health laboratories, hospital laboratories, and other testing locations. The data represent laboratory test totals—not individual people—and exclude antibody and antigen tests. The data are provisional and subject to change. The data presented may not include counts for the most recent 3 days due to the time it takes to report testing information. The data may also not include results from all testing sites within a jurisdiction (e.g., point-of-care test sites) and therefore reflect the majority, but not all, COVID-19 tests in the United States. The data do not include COVID-19 antigen or antibody testing results.

The data are reported to state and jurisdictional health departments in accordance with applicable state or local law and in accordance with the Coronavirus Aid, Relief, and Economic Security (CARES) Act (CARES Act Section 18115). The data for each state are sourced from either data submitted directly by the state health department via COVID-19 electronic laboratory reporting (CELR), or a combination of commercial, public health, and in-house hospital laboratories. CELR data are used for states that either submit line-level data or submit aggregate counts that do not include antigen or antibody tests. The data are available for public use on HealthData.Gov.

Coronavirus Disease 2019 (COVID-19)

CDC COVID Data Tracker

Maps, charts, and data provided by the CDC

The CDC COVID-19 Data Tracker has a new look and location to make the data easier to find and use. All previous data remains and will be updated on the same schedule. Please update bookmarks and links.

Also, as of 8/27, the Tracker includes correctional facility information, NIH Vulnerability Index data, and upgraded testing information.

United States COVID-19 Cases and Deaths by County

State: Select a State   Data: Total Number of Cases

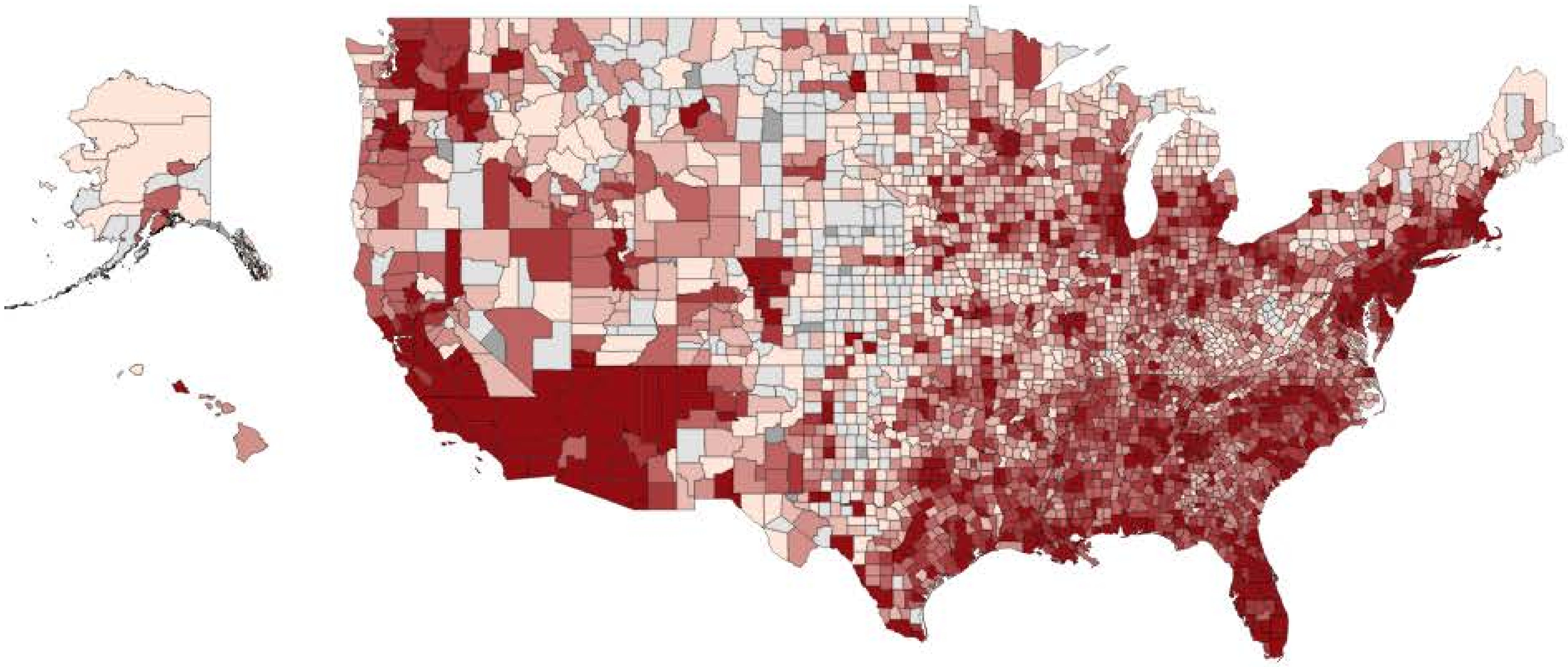

Total Cases

- 0
- <20
- 20 - 75
- 76 - 175
- 176 - 352
- 353 - 727
- 728 - 1,821
- 1,822 - 238,458

Select a county to view data*

*Data courtesy of USAFacts.org downloaded each day at 4:00pm EST or when earliest update is available. Refer to USAFacts.org for data collection and processing methodology. Official verified statistics from CDC are provided on the US Cases page.

Cases By County -

Our Data

We update all our data each day between about 5:30pm and 7pm Eastern Time.

Sign up for our newsletter for the latest news, and to help us gather the best data possible.

Our dataset was last updated at Aug 29 2020

Get the data as: CSV | API | Spreadsheet

Cases (Historical data)

Total cases (Definition)	5,928,381
New cases today (Calculated)	44,328
Change over 7 days (Calculated)	5.1%+

Tests (Historical data)

Total tests (Definition)	76,325,567
New tests today (Calculated)	760,818
Change over 7 days (Calculated)	6.8%+

Hospitalization (Historical data)

Currently hospitalized (Definition)	36,470
Currently in ICU (Definition)	7,426
Currently on ventilator (Definition)	2,060

Outcomes (Historical data)

Recovered (Definition)	2,140,614
Deaths (Definition)	174,768

US states and territories report data in differing units and with varying definitions. Our national summary Cases, Tests, and Outcomes numbers are simple sums of the data states and territories provide, but because of the disparate metrics they include, they should be considered estimates.

Last 90 days | Full range

National 7-day average

New tests (Calculated)
Total test results

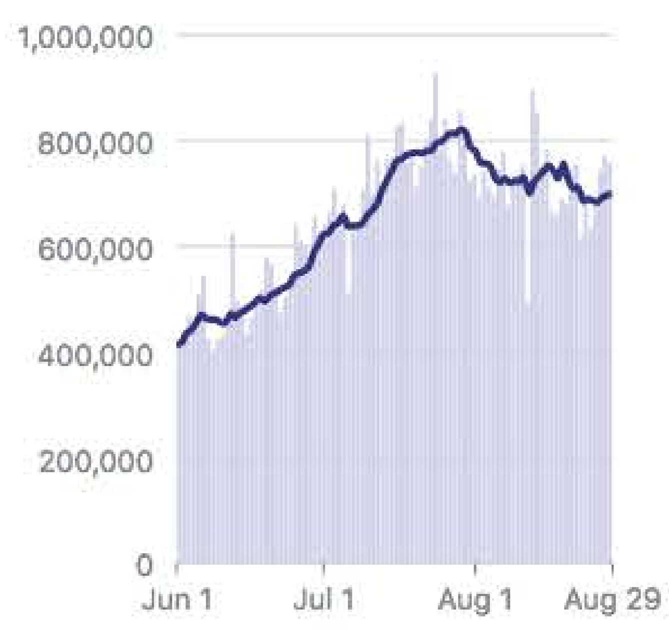

New cases (Calculated)

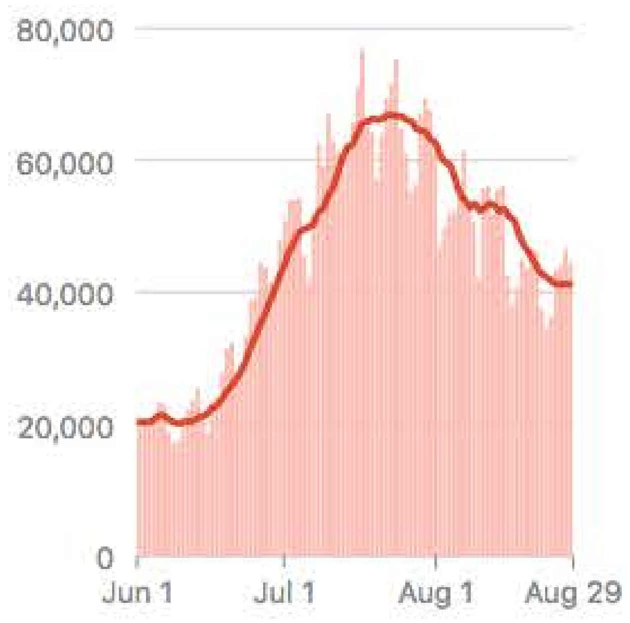

Current hospitalizations (Notes)

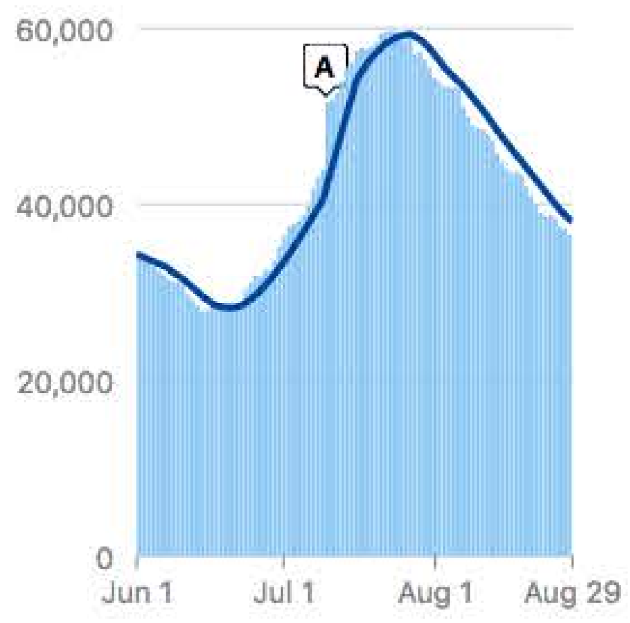

New deaths (Calculated)

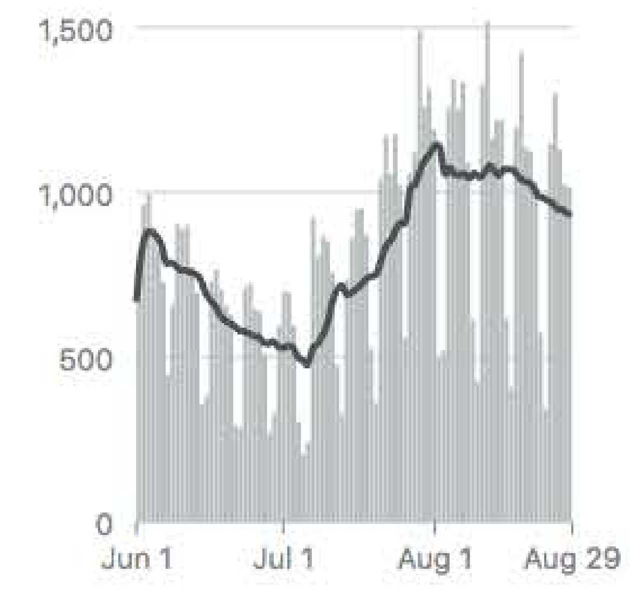

Chart information and data ↓

The COVID Tracking Project

Data by State

Jump to a state

AL	AK	AS	AZ
AR	CA	CO	CT
DE	DC	FL	GA
GU	HI	ID	IL
IN	IA	KS	KY
LA	ME	MD	MA
MI	MN	MS	MO
MT	NE	NV	NH
NJ	NM	NY	NC
ND	MP	OH	OK
OR	PA	PR	RI
SC	SD	TN	TX
VI	UT	VT	VA
WA	WV	WI	WY

Alabama

Current data quality grade: B

Cases (Historical data)

Total cases (Definition)	123,889
New cases today (Calculated)	1,704
Change over 7 days (Calculated)	8.2%+

Viral (PCR) tests (Historical data)

Total tests (in test encounters) (Definition)	Not Reported
Total tests (in specimens) (Definition)	967,213
Total tests (in people) (Definition)	Not Reported

Antibody tests (Historical data)

Total tests (Definition)	Not Reported

Hospitalization (Historical data)

Ever hospitalized (Definition)	14,267
└ Now hospitalized (Definition)	986
Ever in ICU (Definition)	1,459
└ Now in ICU (Definition)	Not Reported
Ever on ventilator (Definition)	801
└ Now on ventilator (Definition)	Not Reported

Outcomes (Historical data)

Recovered (Definition)	48,028
Total deaths (Definition)	2,152
└ Probable deaths (Definition)	93
└ Confirmed deaths (Definition)	2,059

Where this data comes from ↓ State's dataset was last updated at 8/29/20 7:00 am ET

Notes:

On **July 11, 2020**, Alabama began using direct reports from hospitals for cumulative hospitalizations (which we report in our **Ever hospitalized** metric), rather than state case surveillance. Its number of cumulative hospitalizations increased by 3,616 from 3,129 to 6,745 as a result.

On **July 15, 2020**, we changed our data reporting to reflect that Alabama reports test totals in specimens rather than unique people tested, as we had initially believed. All test results were moved into the correct API field and metric.

Negative test results reported in our API and CSVs are calculated by subtracting **Positive PCR tests (people)** from **Total PCR tests (specimens)** in the absence of better data.

Back to top.

Alaska

Current data quality grade: A

Cases (Historical data)

Total cases (Definition)	6,035
New cases today (Calculated)	112
Change over 7 days (Calculated)	9.8%+

Viral (PCR) tests (Historical data)

Total tests (in test encounters) (Definition)	Not Reported
Total tests (in specimens) (Definition)	345,695
Total tests (in people) (Definition)	Not Reported

Antibody tests (Historical data)

Total tests (Definition)	Not Reported

WORLD COUNTRIES U.S.A. STATES TESTING

Coronavirus in the U.S.: Latest Map and Case Count

By The New York Times Updated August 29, 2020, 2:15 P.M. E.T.

Leer en español

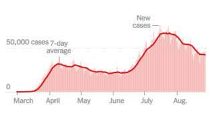

TOTAL CASES
5.9 million+
DEATHS
182,175
Includes confirmed and probable cases where available

Map | Cases by state | Hot spots | Clusters | Latest news »

At least 1,010 new coronavirus deaths and 46,556 new cases were reported in the United States on Aug. 28. Over the past week, there have been an average of 41,980 cases per day, a decrease of 6 percent from the average two weeks earlier.

As of Saturday afternoon, more than 5,952,500 people in the United States have been infected with the coronavirus and at least 182,100 have died, according to a New York Times database.

Hot spots | Total cases | Deaths | Per capita

Average daily cases per 100,000 people in the past week

8 24 40 56 Few or no cases

Double-click to zoom into the map.

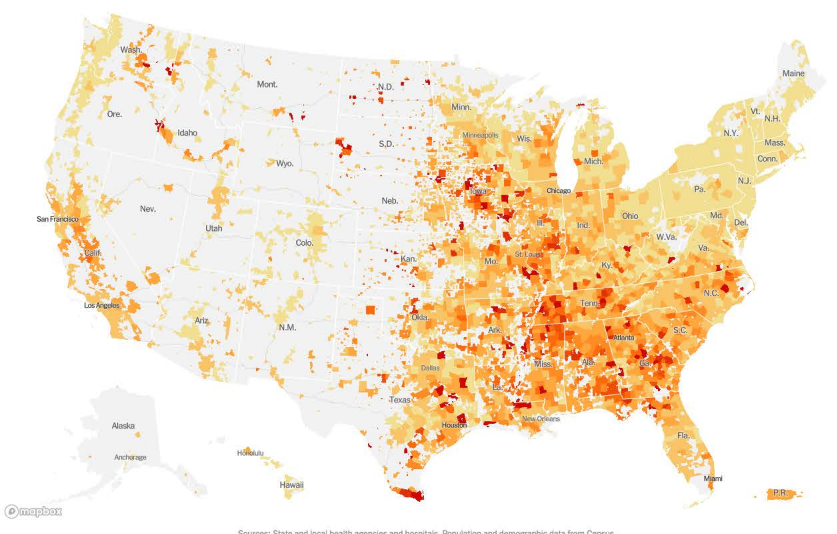

Sources: State and local health agencies and hospitals. Population and demographic data from Census Bureau.
About this data

See our maps tracking the coronavirus outbreak around the world.

Case numbers remain persistently high across much of the country, though reports of new cases have dropped considerably since late July, when the country averaged well over 60,000 per day.

But as much of the South and West saw vast improvement, and as the Northeast kept case reports low, new infections were rising by late summer across parts of the Midwest and on several of America's islands.

Deaths, though still well below their peak spring levels, averaged around 1,000 per day through most of August, more than double the average from early July.

Where new cases are increasing

Cases per capita | Total cases

Charts show daily cases per capita and are on the same scale. States are sorted by cases per capita for the most recent day. Tap a state to see detailed map page.

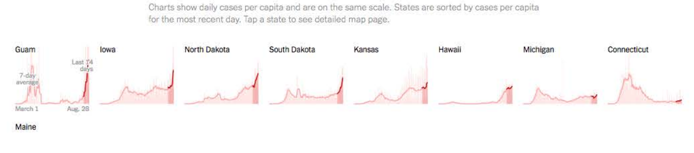

These states have had recent growth in newly reported cases over the last 14 days. The White House released criteria for states to reopen based on a "downward trajectory" of cases over the last 14 days, though it did not define how to measure the trajectory.

Where new cases are mostly the same

Cases per capita | Total cases

Charts show daily cases per capita and are on the same scale. States are sorted by cases per capita for the most recent day. Tap a state to see detailed map page.

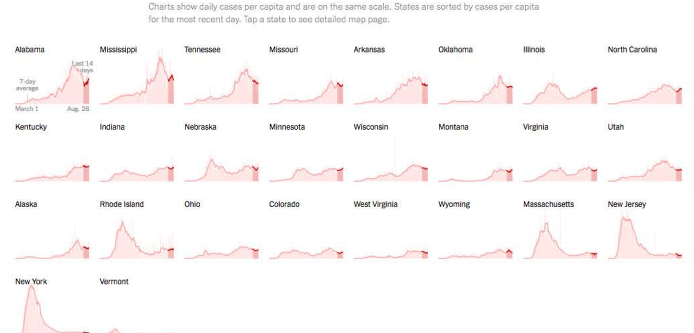

Where new cases are decreasing

Cases per capita | Total cases

Charts show daily cases per capita and are on the same scale. States are sorted by cases per capita for the most recent day. Tap a state to see detailed map page.

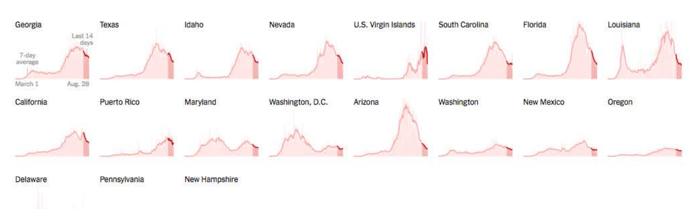

Note: States and territories are grouped according to how the seven-day average of new cases has changed from two weeks ago to today.

Where new deaths are increasing

Deaths per capita | Total deaths

Charts show daily deaths per capita and are on the same scale. States are sorted by deaths per capita for the most recent day. Tap a state to see detailed map page.

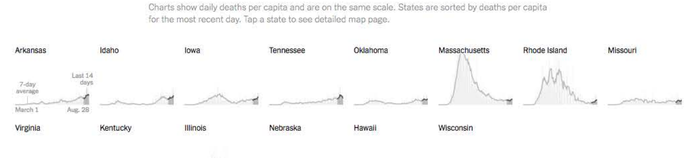

These states have had the highest growth in newly reported deaths over the last 14 days. Deaths tend to rise a few weeks after a rise in infections, as there is typically a delay between when people are infected, when they die and when deaths are reported. Some deaths reported in the last two weeks may have occurred much earlier because of these delays.

A year that started out normal — with packed sports arenas, busy airports and handshake-heavy political campaigning — quickly became defined by the pandemic.

In late February, there were just a few dozen known cases in the United States, most of them linked to travel. But by summer, the virus had torn through every state, infecting more people than the combined populations of Louisiana and Wyoming. The national death toll exceeded 170,000, more than the population of Jackson, Miss. And after glimmers of progress in the late spring, cases surged to new records in July.

Cases and deaths by state and county

This table is sorted by places with the most cases per 100,000 residents in the last seven days. Charts are colored to reveal when outbreaks emerged.

Cases | Deaths | Search counties

	TOTAL CASES	PER 100,000	CASES IN LAST 7 DAYS	▼PER 100,000	WEEKLY CASES PER CAPITA (FEWER – MORE)
Guam	2,256	1,339	520	309	March 1 – Aug. 28
+ Iowa MAP »	63,112	2,000	7,497	238	
+ North Dakota MAP »	11,488	1,507	1,636	215	
+ South Dakota MAP »	12,942	1,463	1,633	185	
+ Alabama MAP »	123,889	2,527	8,553	174	
+ Mississippi MAP »	81,294	2,732	4,971	167	
+ Georgia MAP »	249,317	2,348	16,148	152	
+ Kansas MAP »	41,862	1,437	4,397	151	
+ Tennessee MAP »	147,814	2,164	9,882	145	
+ Missouri MAP »	82,917	1,351	8,184	133	

Show all

About this data

American life has been fundamentally reordered because of the virus. Concerts, parades and baseball games continue to be called off. Countless people have found themselves jobless and struggling to afford housing. Many schools and colleges will hold few or no in-person classes this fall. More than 26,000 cases have been linked to colleges and universities over the course of the pandemic.

New reported cases by day in the United States

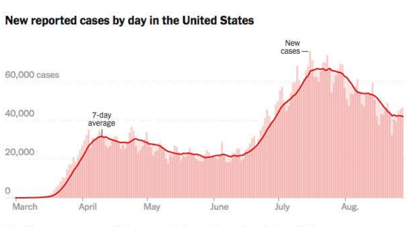

Note: The seven-day average is the average of a day and the previous six days of data.

New reported deaths by day in the United States

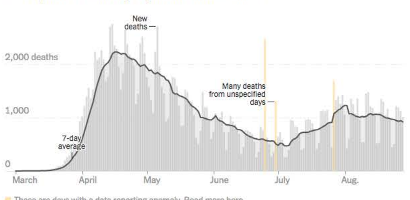

These are days with a data reporting anomaly. Read more here.

The New York Times has found that official tallies in the United States and in more than a dozen other countries have undercounted deaths during the coronavirus outbreak because of limited testing availability.

The New York Times is engaged in a comprehensive effort to track information on every coronavirus case in the United States, collecting information from federal, state and local officials around the clock. The numbers in this article are being updated several times a day based on the latest information our journalists are gathering from around the country. The Times has made that data public in hopes of helping researchers and policymakers as they seek to slow the pandemic and prevent future ones.

The Times's data collection for this page is based on reports from state and local health agencies, a process that is unchanged by the Trump administration's requirement that hospitals bypass the Centers for Disease Control and Prevention and send all patient information to a central database in Washington.

The places hit hardest

The coronavirus has left no state unscathed. But its impact has been wildly uneven.

Officials in California, Florida and Texas, the states with the most known cases, have each identified more than 600,000 cases. In a few less populous states, including Vermont and Wyoming, there are fewer than 5,000 patients. And in a handful of remote counties, there has been not even one positive test.

The nation's most populous places have all suffered tremendously. In Cook County, Ill., which includes Chicago, more than 5,000 people have died. In Los Angeles County, Calif., at least 238,000 people have had the virus, more than in most states. And in New York City, about one of every 360 residents has died.

But unlike in the early days of the pandemic, it is not so simple to say that big cities have been hit hardest. On a per capita basis, many of the places with the most cases have been small and mid-sized metros in the Southwest with large Native American or Hispanic populations. In Yuma County, Ariz., along the country's border with Mexico, about one of every 18 residents is known to have had the virus. In McKinley County, N.M., which includes part of the hard-hit Navajo Nation, one of every 300 residents has died from Covid-19.

Hot spots: Counties with the highest number of recent cases per resident

Search counties

COUNTY	TOTAL CASES	PER 100,000	CASES IN LAST 7 DAYS	PER 100,000	WEEKLY CASES PER CAPITA (FEWER – MORE)
Pawnee, Kan.	210	3,274	131	2,042	March 1 – Aug. 28
East Feliciana, La.	1,054	5,508	346	1,808	
Chattahoochee, Ga.	1,088	9,975	192	1,760	
Luce, Mich.	136	2,183	103	1,654	
Harper, Kan.	110	2,024	68	1,251	
Madison, La.	783	7,150	133	1,215	
Lincoln, Ark.	1,529	11,740	158	1,213	
Maverick, Texas	3,258	5,548	662	1,127	
Radford, Va.	334	1,830	191	1,047	
Madison, Texas	833	5,832	145	1,015	

Show all

Note: Recent cases are from the last seven days.

Hundreds of thousands of cases traced to clusters

Coronavirus outbreaks have been traced to funerals, fast food restaurants, cruise ships and Navy vessels. But most of the biggest known clusters have been in nursing homes, food processing plants and correctional facilities, all places where people are packed in close quarters with little opportunity for social distancing.

***Read more here** about some of the country's less-noticed coronavirus clusters.*

Coronavirus cases have been reported in more than 18,000 nursing homes and other long-term care facilities, according to data collected by The New York Times from states, counties, the federal government and facilities themselves. More than 439,000 residents and employees have been infected in those homes, and more than 72,000 have died. That means about 40 percent of deaths from the virus in the United States have been tied to nursing homes and other long-term care facilities.

We're tracking the devastating effects of the coronavirus in more than 16,000 nursing homes across the country »

"This disease creates the potential for a perfect storm in a long-term care facility — large groups of vulnerable people living together and a highly transmissible virus that may not cause symptoms in those who care for them," said Dr. Daniel Rusyniak, the chief medical officer for Indiana's state social services agency.

In American jails and prisons, more than 176,000 people have been infected and at least 1080 inmates and correctional officers have died. During interviews with dozens of inmates across the country, many said they were frightened and frustrated by what prison officials have acknowledged has been an uneven response to the virus.

"I am very concerned," said Adamu Chan, an inmate at San Quentin State Prison in California, which has become one of the nation's largest coronavirus clusters with more than 2,600 infections and 27 deaths. "There's no way to social distance. We all eat together. We have a communal bathroom. There's no way to address a public health issue in an overcrowded facility."

Search clusters

CASES CONNECTED TO	CASES
San Quentin State Prison — San Quentin, Calif.	2,612
Marion Correctional Institution — Marion, Ohio	2,443
Miami-Dade County Jail — Miami, Fla.	2,099
Avenal State Prison — Avenal, Calif.	1,956
Pickaway Correctional Institution — Scioto Township, Ohio	1,795
Columbia Correctional Institution — Lake City, Fla.	1,425
Seagoville federal prison — Seagoville, Texas	1,391
Trousdale Turner Correctional Center — Hartsville, Tenn.	1,385
North County jail — Castaic, Calif.	1,384
Ouachita River Unit prison — Malvern, Ark.	1,326

Show all

About the data

In data for the United States, The Times uses reports from state, county and regional health departments. Most governments update their data on a daily basis, and report cases and deaths based on an individual's residence.

Not all governments report these the same way. The Times uses the total of confirmed and probable counts when they are available individually or combined. To see whether a state includes probable cases and deaths, visit the individual state pages listed at the bottom of this page.

The Times has identified the following reporting anomalies or methodology changes in the data:

- **June 25:** New Jersey began reporting probable deaths.
- **June 30:** New York City released deaths from earlier periods but did not specify when they were from.
- **July 27:** Texas began reporting deaths based on death certificates, causing a one-day increase.
- To see a detailed list of all reporting anomalies, visit the individual state pages listed at the bottom of this page.

The U.S. data includes cases and deaths that have been identified by public health officials as confirmed coronavirus patients, and also includes probable coronavirus cases and deaths when governments report them. **Confirmed cases and deaths**, which are widely considered to be an undercount of the true toll, are counts of individuals whose coronavirus infections were confirmed by a molecular laboratory test. **Probable cases and deaths** count individuals who meet criteria for other types of testing, symptoms and exposure, as developed by national and local governments.

Governments often revise data or report a single-day large increase in cases or deaths from unspecified days without historical revisions, which can cause an irregular pattern in the daily reported figures. The Times is excluding these anomalies from seven-day averages when possible.

Read more about the methodology and download county-level data for coronavirus cases in the United States from The New York Times on GitHub.

Tracking the Coronavirus

Is Your State Doing Enough Coronavirus Testing?

By Keith Collins Updated August 29, 2020

The number of daily coronavirus tests being conducted in the United States is only **52 percent** of the level considered necessary to mitigate the spread of the virus, as many states struggle to increase testing and catch up to the recent surge in cases.

10 states* meet the testing target | **5** states are near the target | **35** states are far below the target

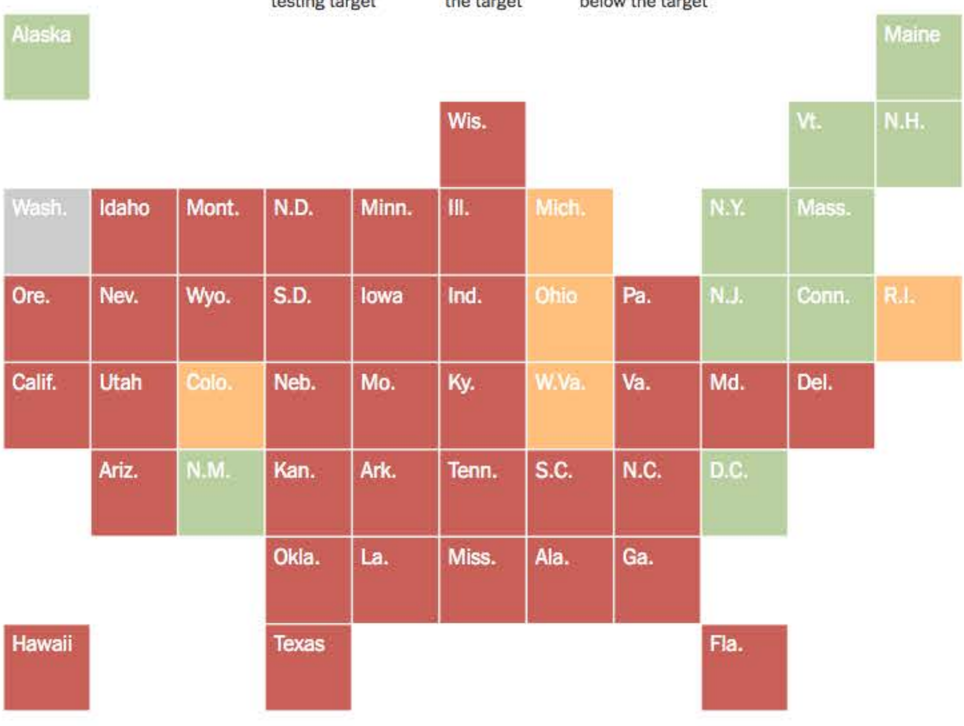

*Includes 9 states and Washington, D.C. States within 20 percent of the testing target are considered "near" the target.

Washington state is currently not reporting testing figures and is excluded from this analysis until it resumes reporting.

An average of **727,000 tests per day** were performed over the past week, according to data collected by the Covid Tracking Project, far below the current nationwide target of **1.4 million daily tests.** The target, which is based on a methodology developed by researchers at the Harvard Global Health Institute, is different for each state and varies over time as infection rates change.

How each state's current testing measures up

Average daily testing and hospitalizations in the last two weeks

	DAILY TESTS PER 100,000	▲ PERCENTAGE OF TESTING TARGET	POSITIVE TEST RATE	HOSPITALIZED PER 100,000
**United States**	222	52	7%	15
Idaho	157	16	16%	11
Mississippi	149	17	18%	38
Nevada	154	26	16%	33
Florida	166	28	17%	31
Alabama	202	28	12%	30
Texas	158	28	16%	25
Iowa	153	29	10%	8
Arkansas	243	30	9%	16
Arizona	131	30	11%	22
North Dakota	203	32	9%	7
Utah	137	32	9%	7
Indiana	148	33	9%	14
Georgia	256	34	11%	27
Nebraska	155	34	9%	8
South Dakota	118	34	9%	6
Tennessee	310	34	8%	19
Oklahoma	212	34	9%	14
Hawaii	175	36	8%	9
Kansas	129	38	12%	10
Minnesota	162	41	7%	6
South Carolina	197	42	10%	26
Missouri	170	43	11%	15
Wyoming	74	43	10%	3
Kentucky	186	44	7%	15
Wisconsin	204	45	7%	6
Montana	169	49	6%	8
Louisiana	431	51	6%	29
North Carolina	214	55	6%	10
Virginia	183	56	7%	15
Pennsylvania	118	58	5%	5
Maryland	233	59	5%	9
California	316	62	6%	17
Illinois	337	64	4%	12
Oregon	126	66	6%	5
Delaware	183	77	6%	4
Colorado	197	81	3%	5
Ohio	191	84	5%	8
West Virginia	262	113	3%	7
Michigan	296	118	3%	7
Rhode Island	371	119	2%	8
Washington, D.C.	474	145	2%	12
New Hampshire	123	173	2%	1
New Mexico	328	175	2%	6
Massachusetts	247	196	2%	6
Alaska	584	222	2%	5
New Jersey	279	286	1%	6
Maine	204	407	1%	1
Vermont	183	469	1%	2
Connecticut	306	523	1%	2
New York	383	670	1%	3

Note: In many states, if a person is tested more than once, each test is counted in data for total tests. Some states, including Nevada, Rhode Island and Wyoming, report the actual number of people tested, which is a more accurate way to measure the share of a population that has been tested, but can result in a lower number of total tests. Positive test rates and percentages of targets are calculated using cumulative figures from the last 14 days. All other values are 14-day rolling averages. Trendlines for daily tests and hospitalizations show averages for the last two weeks.

Testing operations in the United States were delayed at the start of the outbreak after tests developed by the Centers for Disease Control and Prevention were found to be faulty. Since then, testing levels have steadily increased across the country, but many states have struggled to stay ahead of new outbreaks as overwhelmed labs report processing delays and shortages of crucial testing supplies. The resulting backlog can leave sick people undiagnosed, with the potential to further spread the virus as states reopen.

The Harvard researchers say that at minimum there should be enough daily capacity to test anyone who has flu-like symptoms and an additional 10 people for any symptomatic person who tests positive for the virus. That level of testing — which, according to researchers, is the minimum necessary to mitigate the disease — would require roughly double the number of daily tests currently being performed. Their estimates for the testing required to *suppress* the spread of the virus are much higher.

Aside from current testing levels, another important indicator of a state's testing performance is its positive test rate, which is the percent of tests that come back positive. Lower rates suggest that testing is more widespread and that it is not limited to those with severe symptoms. Positive rates should be at or below 5 percent for at least 14 days before a state or country can safely reopen, according to the World Health Organization. In the United States, the current positive rate is **7 percent.**

Where testing is far below recommended levels

Most states remain far from the minimum level of testing needed to reduce the spread of the virus.

Rate of testing compared to target

States are ordered by current testing shortfall

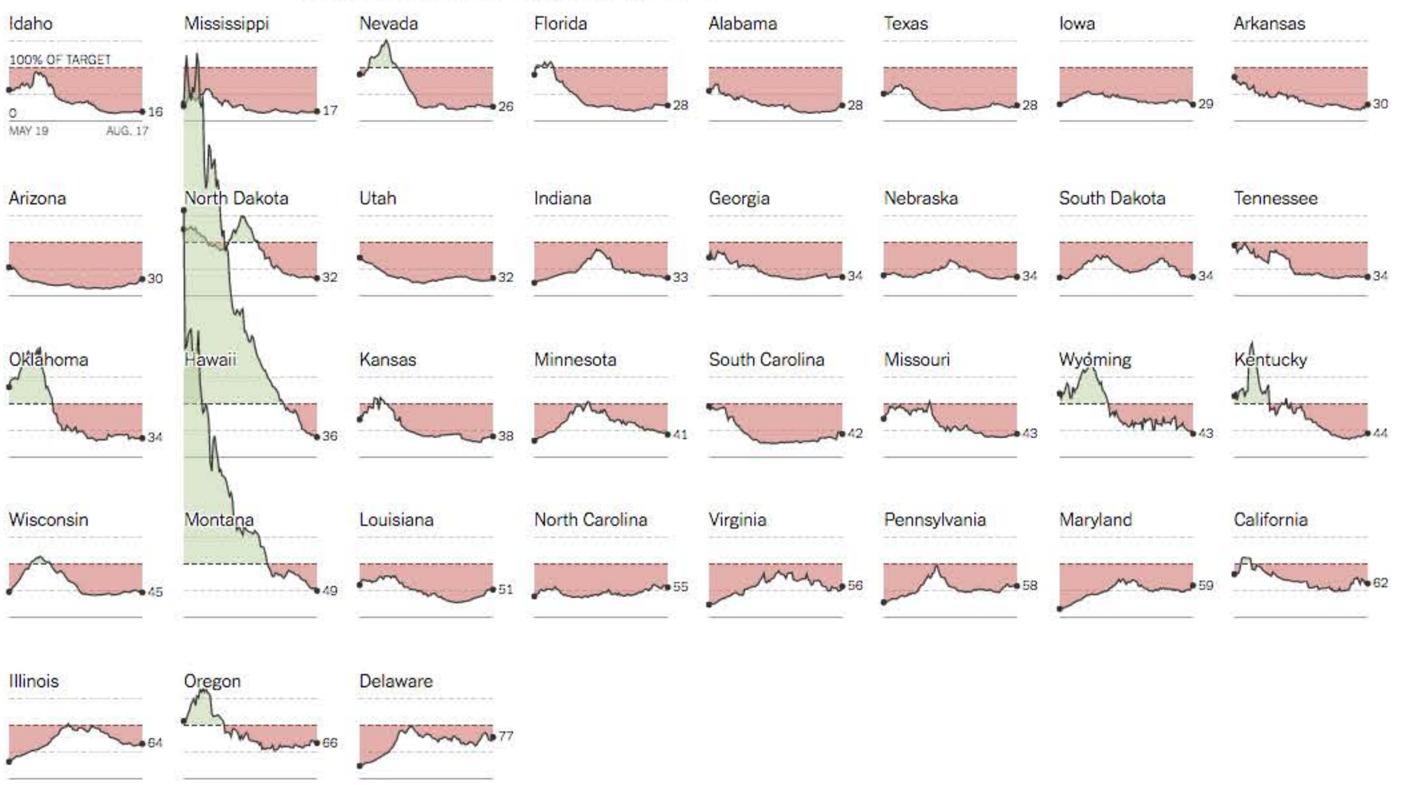

Where testing is above or near recommended levels

Over time, some states have reached the minimum testing target, and a few have exceeded it.

Rate of testing compared to target

States are ordered by current testing levels

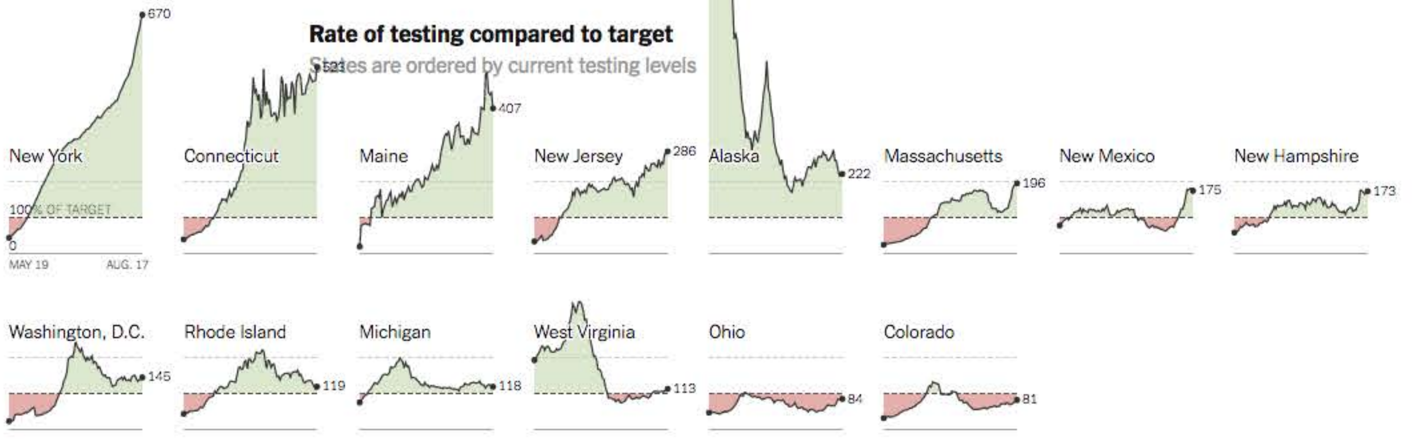

Sources: The Covid Tracking Project; the Harvard Global Health Institute; Youyang Gu's Covid-19 Forecasts; and the Centers for Disease Control and Prevention

Note: Testing targets are calculated daily using the methodology developed by the Harvard Global Health Institute (exact figures may differ because of recent changes in underlying projections). Two-week rolling averages are calculated using the most recent 14 days for which data is available. Some states may include non-diagnostic tests, like antibody tests, in the data they release, which can affect reported testing rates. Some states don't consistently release data on current hospitalizations (see more information about missing data here.) Case data is from the Covid Tracking Project for consistency with daily testing data.

CORONAVIRUS BY THE NUMBERS

Coronavirus Maps: How Severe Is Your State's Outbreak?

August 28, 2020 · 8:20 AM ET

STEPHANIE ADELINE · CONNIE HANZHANG JIN · ALYSON HURT · THOMAS WILBURN · DANIEL WOOD · RUTH TALBOT

COVID-19 In The United States

Data as of 7:28 a.m. ET, Aug. 28

CASES		DEATHS	
TOTAL	AUG. 27	TOTAL	AUG. 27
5,869,540	+45,966	180,854	+1,116
LAST 3 WEEKS: Aug. 7 +58,173; Aug. 27 +45,966 (7-day avg.)		LAST 3 WEEKS: Aug. 7 +1,243; Aug. 27 +1,116 (7-day avg.)	

Source: Center for Systems Science and Engineering at Johns Hopkins University

This page is updated regularly.

More than 5.5 million people in the U.S. have been infected with the coronavirus and more than 180,000 have died. Tens of thousands of new cases are reported daily. Some state outbreaks are worse than others. In the graphics below, explore the trends in your state.

View the data via a **heat map** (immediately below), **curve charts**, a **table** of state-by-state trends over four weeks, or a **map** of total cases and deaths.

Which Places Have The Most New Daily Cases?

Data as of 11:59 p.m. ET, August 27

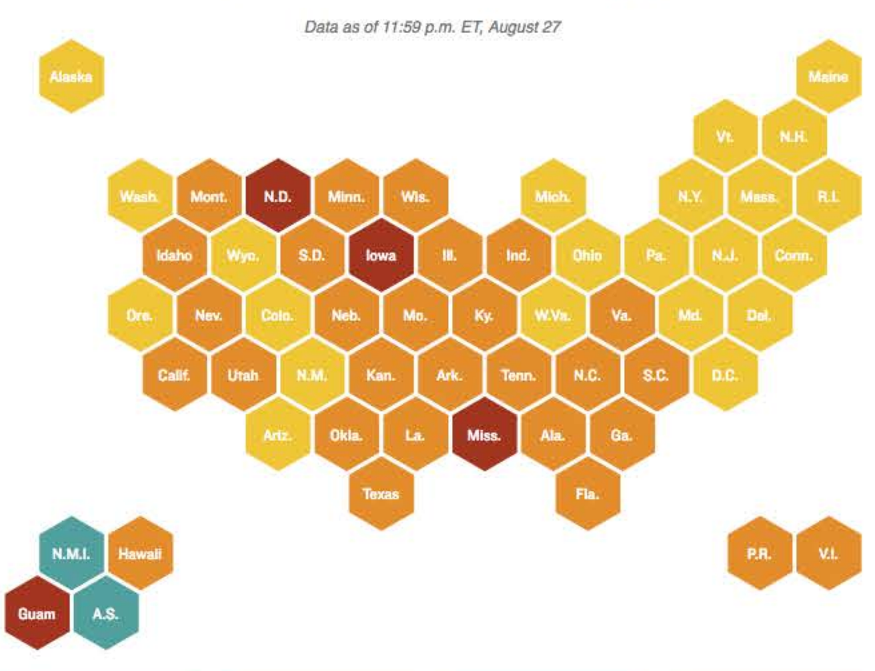

RED	ORANGE	YELLOW	GREEN
**Threshold:** 25+ daily new cases per 100,000 people	**Threshold:** 10-24 daily new cases per 100,000 people	**Threshold:** 1-9 daily new cases per 100,000 people	**Threshold:** <1 daily new case per 100,000 people
**Indicates:** unchecked community spread	**Indicates:** escalating community spread	**Indicates:** potential community spread	**Indicates:** close to containment

THE HIGHEST-RISK PLACES INCLUDE:

STATE	RISK LEVEL	AVG. THIS WEEK	PER 100K	% CHANGE VS. 2 WEEKS AGO
Guam	RED	75 new cases/day	47 per 100K	+733%
Iowa	RED	994 new cases/day	32 per 100K	+147%
North Dakota	RED	223 new cases/day	29 per 100K	+57%
Mississippi	RED	749 new cases/day	25 per 100K	-6%
South Dakota	ORANGE	215 new cases/day	24 per 100K	+142%

Note: Daily cases are a 7-day average to smooth out day-to-day variations in the data. Risk levels are based on a scale developed by the Harvard Global Health Institute and a collaboration of top scientists at institutions around the country.

Source: Center for Systems Science and Engineering at Johns Hopkins University, Census Bureau 2019 population estimates, 2010 Census (U.S. territories)

The map above shows the risk of infection in each state based on new daily cases per capita. These color-coded risk levels were developed by a consortium of researchers and public health experts. The group advises states in the red category to issue stay-home orders. Orange states may need to consider stay-home orders, along with increased testing and contact tracing. Yellow states need to keep up social distancing and mask usage, and both yellow and green states should continue testing and contact tracing.

Are New Cases And Deaths Still Growing In Your State?

Data as of 11:59 p.m. ET, August 27

NEW DAILY CASES | NEW DAILY DEATHS

All States

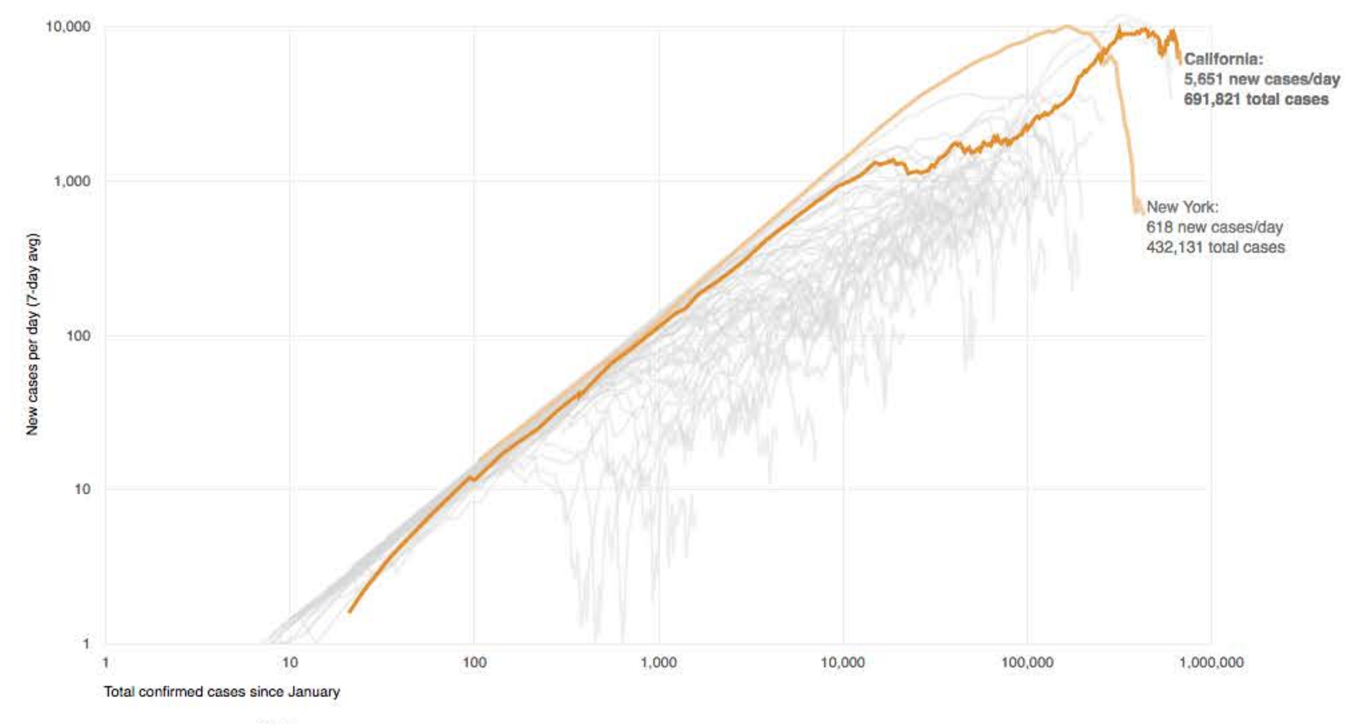

Notes

This chart uses a logarithmic scale, which is meant to emphasize rate of change and allows for the display of a wide range of numbers. Every axis line is 10 times greater than the previous one. The x-axis uses total confirmed cases or deaths on a logarithmic scale rather than date to underscore the overall size of a state's outbreak relative to its daily growth.

Source: Center for Systems Science and Engineering at Johns Hopkins University

To compare state outbreaks, the chart above graphs trend lines for average new daily cases and deaths against each state's totals to date. This type of visualization highlights a state's daily growth or decline relative to the overall size of its outbreak.

When both new and total case and death counts grow quickly, the curves bend upward. As new cases and deaths slow, the curves level or bend down. In New York, the curve rose sharply before reaching over 170,000 total cases in April. Since then, new cases have fallen from about 10,000 per day in mid-April to under 600 per day in mid August.

Total Cases And Deaths, State By State

Data as of 7:28 a.m. on Aug. 29

CASES | CASES PER 100,000 | DEATHS | DEATHS PER 100,000

1,000 · 10,000 · 100,000

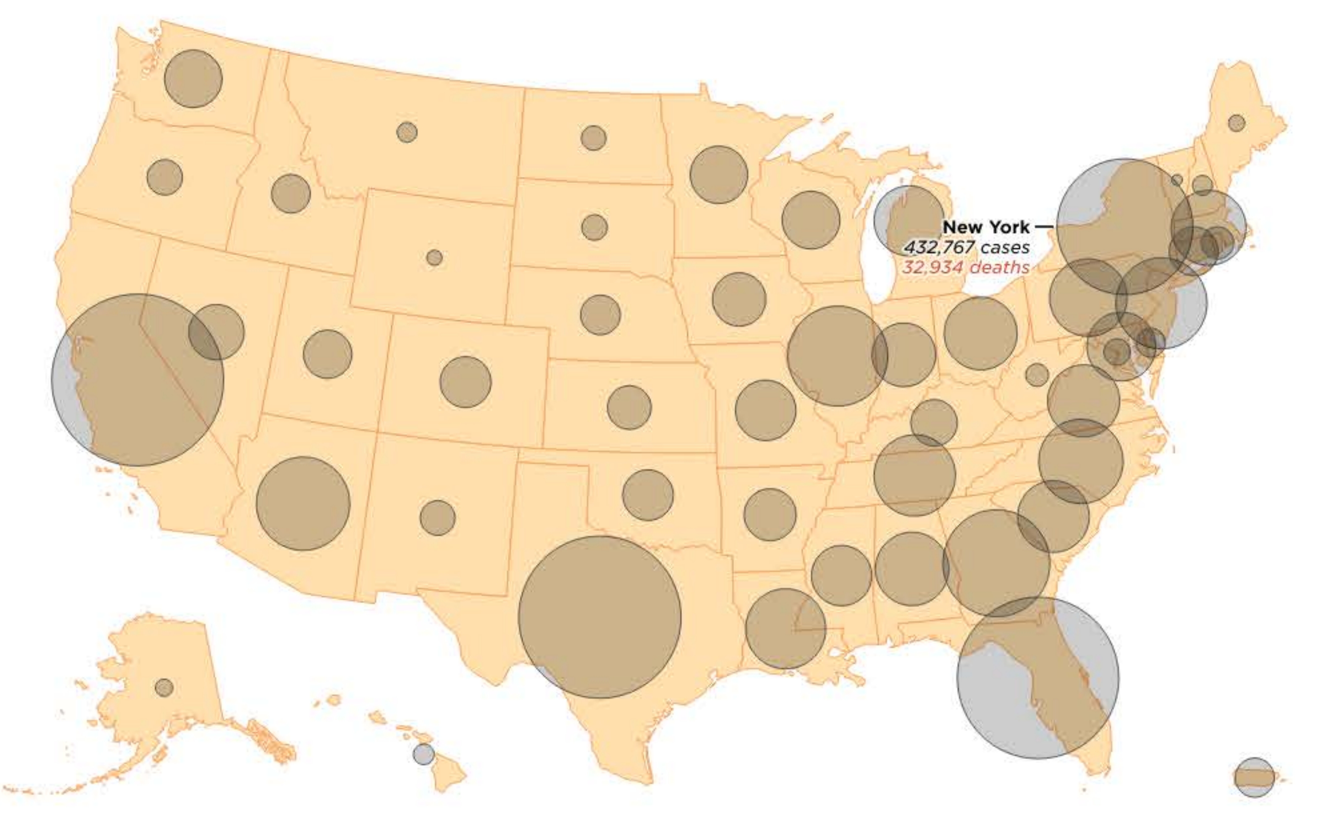

Source: Center for Systems Science and Engineering at Johns Hopkins University

Explore the map above to see totals and per capita figures around the country for both new confirmed cases and reported deaths from COVID-19. New York was the original epicenter of the pandemic in the U.S. As of mid August, California, Florida and Texas have surpassed New York for total cases to date, though New York still has the highest death total.

Click here to see a global map of confirmed cases and deaths.

To show trends state by state, the table below shows the change in average new cases per day in each state, week over week for the last 28 days. States marked in shades of red are showing growth, those in shades of green, are showing declines.

Total Cases And Weekly Trends, By State And Territory

Totals as of 7:28 a.m. on Aug. 29. Daily averages as of 11:59 p.m. ET, August 27.

PERCENT CHANGE VS. PREVIOUS WEEK: -100% -50% -5% +5% +50% +100%

STATE	AVG. NEW CASES/DAY				TOTALS SINCE JANUARY	
	3 weeks ago	2 weeks ago	Last week	This week	CASES	DEATHS
**United States**	55,510	52,197	46,413	41,991	5,918,229	181,776
California	6,915	8,092	7,755	5,651	698,494	12,840
Texas	7,956	6,705	7,005	4,951	621,745	12,475
Florida	7,001	6,678	4,495	3,341	615,806	10,957
New York	651	645	623	618	432,767	32,934
Georgia	3,230	3,396	2,582	2,333	265,372	5,471
Illinois	1,653	1,717	1,904	1,957	231,186	8,206
Arizona	1,837	1,020	784	551	200,658	4,978
New Jersey	390	413	276	298	190,971	15,930
North Carolina	1,610	1,289	1,297	1,596	162,491	2,652
Tennessee	1,926	1,737	1,523	1,429	150,815	1,701
Louisiana	1,824	1,170	769	688	146,243	4,904
Pennsylvania	777	808	684	603	136,781	7,642
Massachusetts	397	364	329	342	127,584	9,024
Alabama	1,547	1,281	985	1,225	122,185	2,107
Ohio	1,121	1,136	940	975	120,124	4,105
Virginia	995	1,108	894	956	117,578	2,550
South Carolina	1,318	1,016	773	754	115,951	2,655
Michigan	696	743	680	818	111,136	6,712
Maryland	833	736	581	548	106,664	3,727
Indiana	823	936	816	1,032	91,313	3,277
Mississippi	974	798	780	749	81,294	2,413
Missouri	1,091	1,094	1,132	1,123	81,672	1,500
Wisconsin	810	775	718	701	73,981	1,113
Washington	731	632	579	473	73,301	1,905
Minnesota	707	622	616	726	73,240	1,859
Nevada	962	728	705	519	67,852	1,287
Iowa	468	402	594	994	62,830	1,108
Arkansas	753	677	428	569	59,583	756
Colorado	462	397	287	302	56,753	1,937
Oklahoma	809	672	652	697	56,260	786
Connecticut	82	77	93	131	52,495	4,465
Utah	460	358	365	368	50,948	407
Kentucky	553	633	563	622	46,757	918
Kansas	390	410	489	636	41,050	445
Nebraska	294	263	241	250	33,436	392
Puerto Rico	480	645	528	463	32,550	428
Idaho	451	461	354	286	31,376	353
Oregon	299	297	266	228	26,054	447
New Mexico	198	173	138	138	25,042	767
Rhode Island	94	90	90	103	21,683	1,046
Delaware	97	86	107	37	17,083	604
District of Columbia	66	72	55	55	13,851	605
South Dakota	84	89	113	215	12,517	165
North Dakota	125	142	153	223	11,484	141
West Virginia	129	126	118	92	9,826	201
Hawaii	132	200	219	246	7,830	59
New Hampshire	28	26	18	21	7,216	432
Montana	113	115	95	122	7,063	100
Alaska	86	69	80	65	5,092	37
Maine	16	13	23	23	4,436	132
Wyoming	38	24	48	39	3,803	37
Vermont	5	6	8	7	1,589	58
Guam	8	9	32	75	1,287	10
Virgin Islands	17	26	27	26	1,075	14
Northern Marianas	1	0	1	0	56	2
American Samoa	0	0	0	0	0	0

Source: Center for Systems Science and Engineering at Johns Hopkins University

Methodology

The graphics on this page pull from data compiled by the Center for Systems Science and Engineering at Johns Hopkins University from several sources, including the Centers for Disease Control and Prevention; the World Health Organization; national, state and local government health departments; 1point3acres; and local media reports.

The JHU team automates its data uploads and regularly checks them for anomalies. This may result in occasional data discrepancies on this page as the JHU team resolves anomalies and updates its feeds. State-by-state recovery data are unavailable at this time. There may be discrepancies between what you see here and what you see on your local health department's website. Figures shown do not include cases on cruise ships.

In early June, Michigan health authorities started including probable cases in its totals. This resulted in a brief spike in the state's daily numbers until the JHU team was able to reconcile the historical data. On June 25, New Jersey included 1,854 previously-unreported probable deaths in its totals, resulting in a spike in the state's daily death numbers.

This story was originally published on March 16, 2020. Elena Renken was a co-author on that version.

Sean McMinn contributed to and Carmel Wroth edited this story.

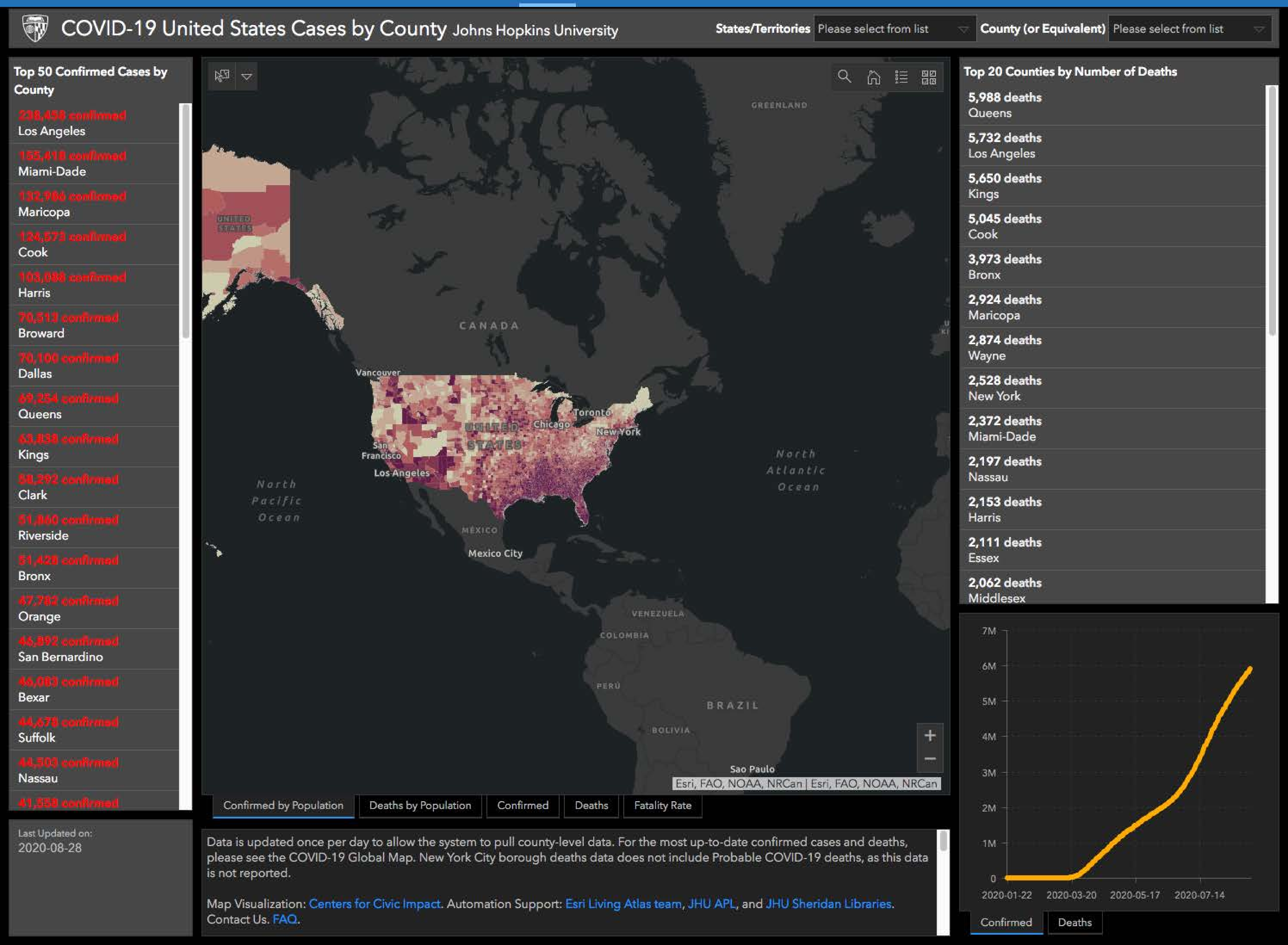
COVID-19 United States Cases by County Johns Hopkins University
States/Territories
Please select from list
County (or Equivalent)
Please select from list
Top 50 Confirmed Cases by County
238,458 confirmed
Los Angeles
155,418 confirmed
Miami-Dade
132,986 confirmed
Maricopa
124,573 confirmed
Cook
103,088 confirmed
Harris
70,513 confirmed
Broward
70,100 confirmed
Dallas
69,254 confirmed
Queens
63,838 confirmed
Kings
58,292 confirmed
Clark
51,860 confirmed
Riverside
51,428 confirmed
Bronx
47,782 confirmed
Orange
46,892 confirmed
San Bernardino
46,083 confirmed
Bexar
44,678 confirmed
Suffolk
44,503 confirmed
Nassau
41,558 confirmed
Last Updated on:
2020-08-28
GREENLAND
CANADA
UNITED STATES
Vancouver
Toronto
Chicago
New York
San Francisco
Los Angeles
North Pacific Ocean
North Atlantic Ocean
MÉXICO
Mexico City
VENEZUELA
COLOMBIA
PERÚ
BRAZIL
BOLIVIA
Sao Paulo
Esri, FAO, NOAA, NRCan | Esri, FAO, NOAA, NRCan
Confirmed by Population
Deaths by Population
Confirmed
Deaths
Fatality Rate
Data is updated once per day to allow the system to pull county-level data. For the most up-to-date confirmed cases and deaths, please see the COVID-19 Global Map. New York City borough deaths data does not include Probable COVID-19 deaths, as this data is not reported.
Map Visualization: Centers for Civic Impact. Automation Support: Esri Living Atlas team, JHU APL, and JHU Sheridan Libraries. Contact Us. FAQ.
Top 20 Counties by Number of Deaths
5,988 deaths
Queens
5,732 deaths
Los Angeles
5,650 deaths
Kings
5,045 deaths
Cook
3,973 deaths
Bronx
2,924 deaths
Maricopa
2,874 deaths
Wayne
2,528 deaths
New York
2,372 deaths
Miami-Dade
2,197 deaths
Nassau
2,153 deaths
Harris
2,111 deaths
Essex
2,062 deaths
Middlesex
7M
6M
5M
4M
3M
2M
1M
0
2020-01-22
2020-03-20
2020-05-17
2020-07-14
Confirmed
Deaths

MAPS & TRENDS

IMPACT OF OPENING AND CLOSING DECISIONS BY STATE

A look at how social distancing measures may have influenced trends in COVID-19 cases and deaths

SHARE:

ALABAMA

Select another state

Alabama

How to read this graphic

New Confirmed Cases | New Deaths

RECENT OPENING AND CLOSING POLICY DECISIONS

Restriction/closing · Opening · Deferring decisions to county · Other

Previous | Next

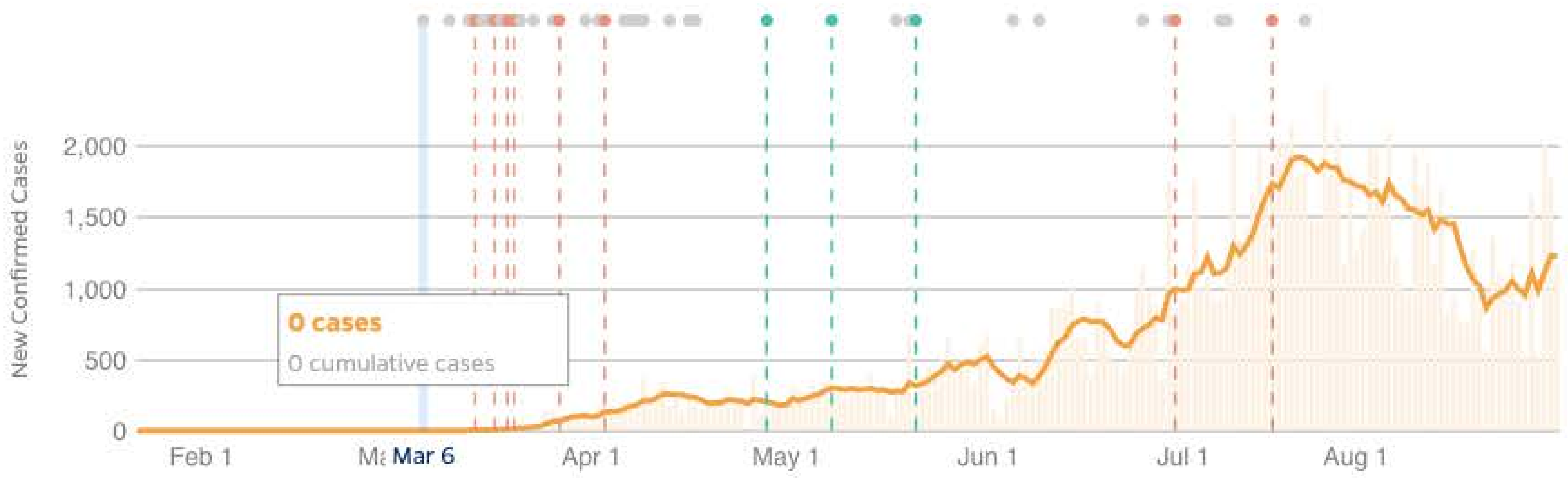

Mar 06, 2020 0 CUMULATIVE CASES | 0 CUMULATIVE DEATHS

- Governor Ivey announced the formation of a Coronavirus (COVID-19) Task Force of cabinet and state agency officials, as well as disease specialists in an abundance of caution.

Data Sources: Cases and deaths data from JHU CSSE. Policy actions from the National Governor's Association, D.C. Policy Center, and Washington, DC's Coronavirus website.

As of July 31, 2020, the data source for the policy events, the National Governor's Association, has not updated this information on their website. We are currently researching alternative data sources. Exception: Washington, D.C. continues to be updated.

ABOUT THIS PAGE:

This page was last updated on Saturday, August 29, 2020 at 3:00 AM EDT.

This timeline shows the major infection control measures and re-openings undertaken by states after the first COVID-19 cases appeared in the United States, alongside the number of new cases and deaths in each state over the same time period.

Dots on the timeline indicate key events in each state – closings (in red), openings (in green) and other policy changes and events (in grey). Clicking on a dot will display event details below the chart. Additionally, the PREVIOUS and NEXT buttons move users through key events by day, allowing them to be viewed in chronological order. The timeline can be viewed against either new cases or new deaths. To see current cumulative case and death counts and daily counts for days with no policy events, click here.

United States /

See Dashboard Guide (PDF / YouTube)

COVID-19 is affecting every community differently.
Some areas are much harder-hit than others.
What is happening where you live?

Average Daily COVID-19 Cases

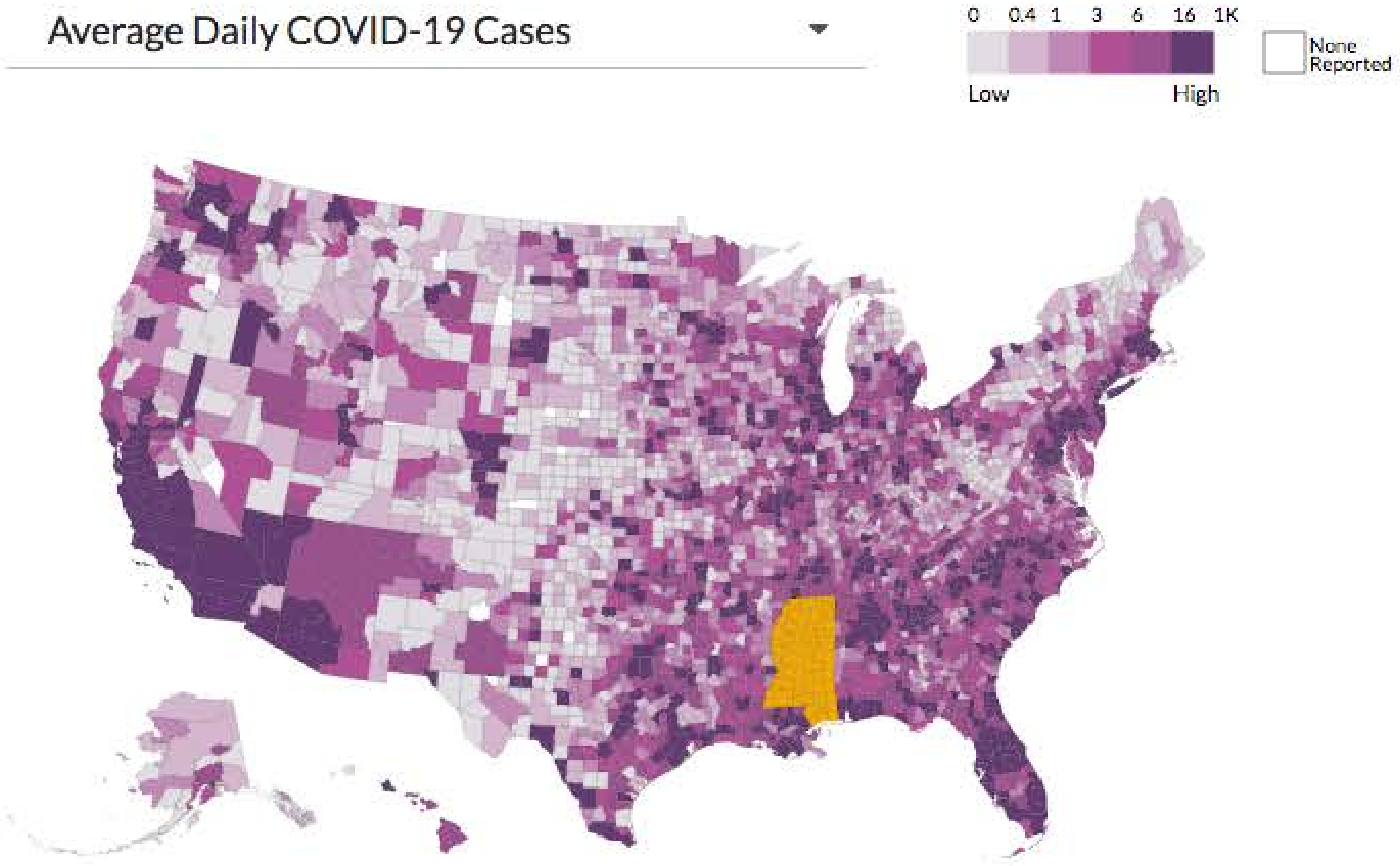

A Snapshot of Health Disparities in **Mississippi**

Counties with higher proportions of African American residents tend to have higher rates of death from COVID-19.
Click on the map to explore your state and county.

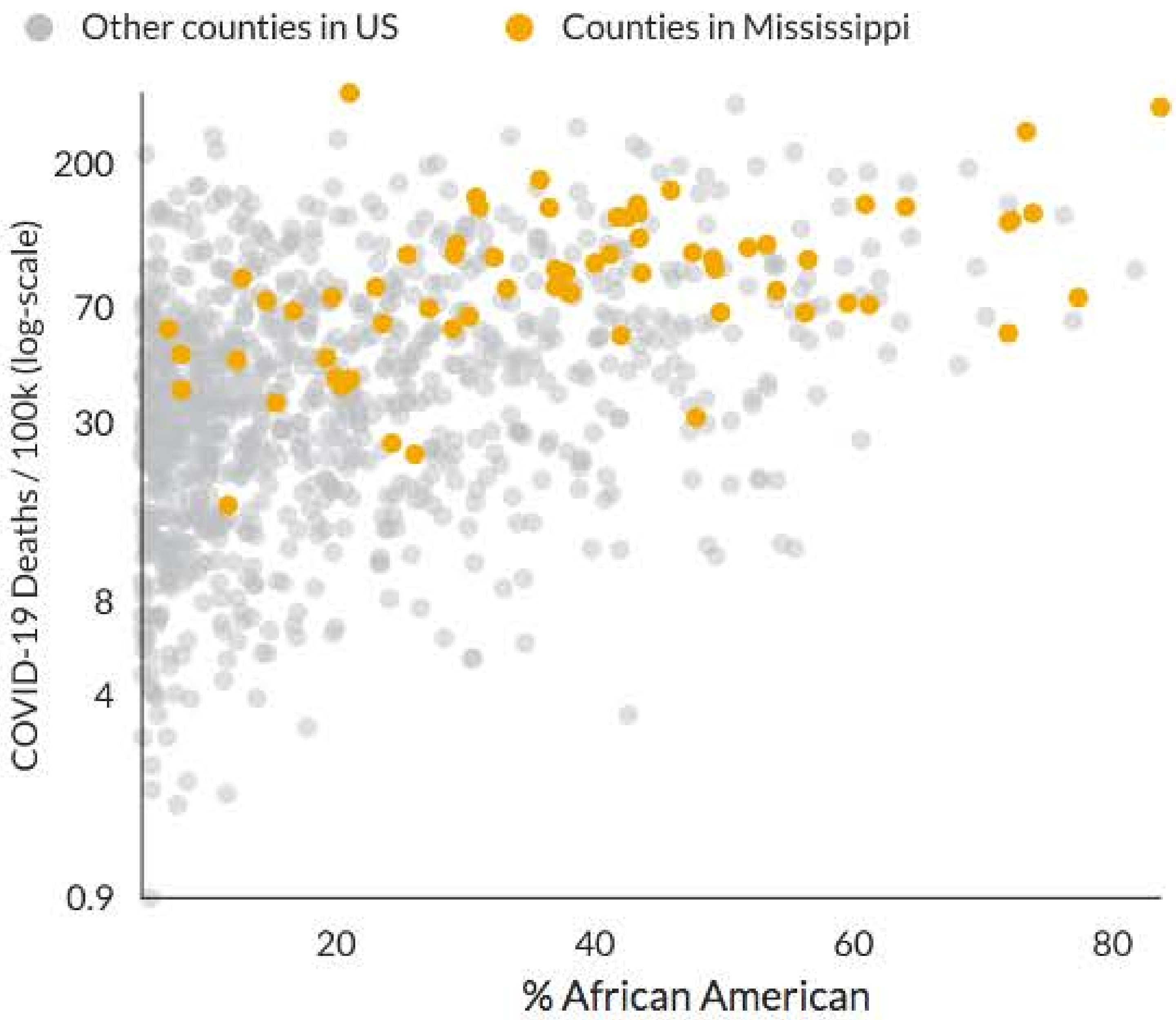

Daily Cases is the average number of new positive cases for COVID-19 infection over the last seven days.
Daily Deaths is the average number of new deaths due to confirmed or presumed COVID-19 infection over the last seven days.
For a complete table of variable definition, click here.

Data last updated: 08/27/2020, updated every weekday.
The chart does not contain those counties with less than 10,000 population and less than 5% African American.

VISUALIZATIONS

US Coronavirus Cases and Deaths

Track COVID-19 data daily by state and county

Data Updated Aug 29, 2020

Coronavirus cases continue to grow in America. From cities to rural towns, stay informed on where COVID-19 is spreading to understand how it could affect families, commerce, and travel. Follow new cases found each day and the total number of cases and deaths in the US. The county-level tracker makes it easy to follow COVID-19 cases on a granular level, as does the ability to break down infections per 100,000 people. This county visualization is unique to USAFacts and will be updated with the most recent data as frequently as possible. The underlying data is available for download below the US county map and has helped government agencies like the Centers for Disease Control and Prevention in its nationwide efforts.

KNOWN CASES	DEATHS
**5,865,041**	**180,329**
(+44,956 reported Aug 28)	(+997 reported Aug 28)

COUNTY MAP

KNOWN CASES | KNOWN CASES (PER 100K) | DEATHS

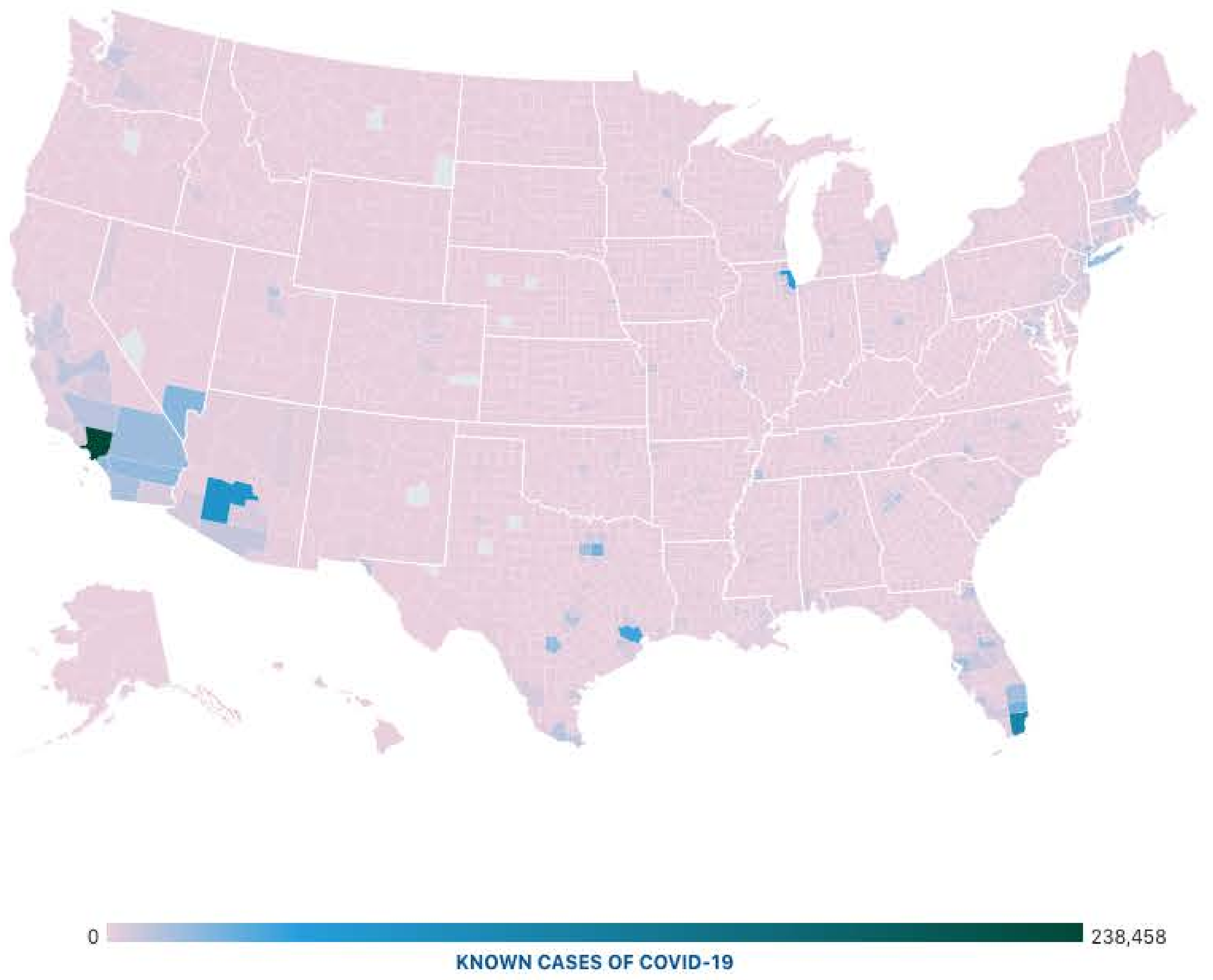

FIND USAFACTS AT

CDC FEEDING AMERICA Google KAISER PERMANENTE MIT The Weather Channel

NEW KNOWN CASES PER DAY

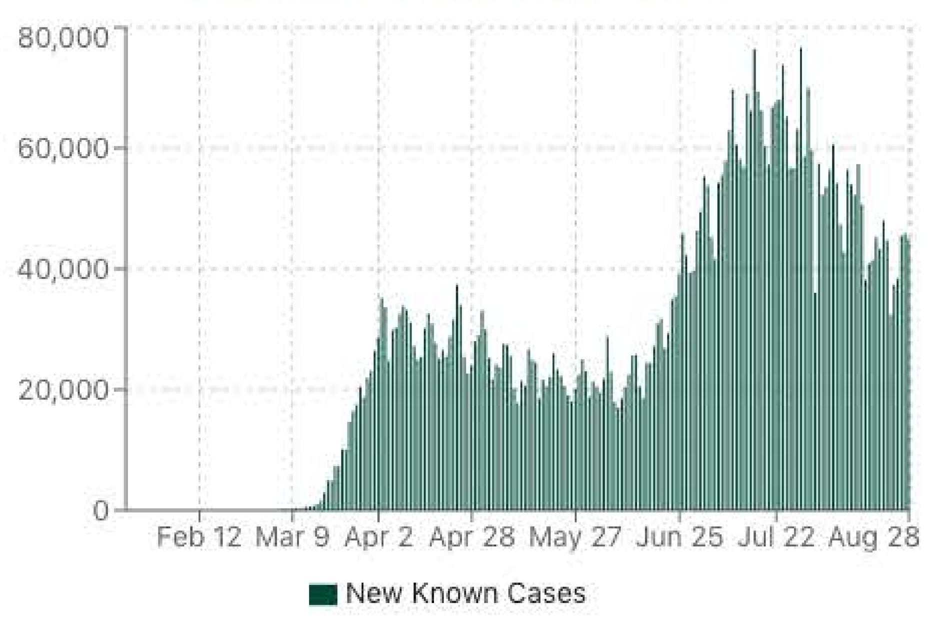

NEW KNOWN DEATHS PER DAY

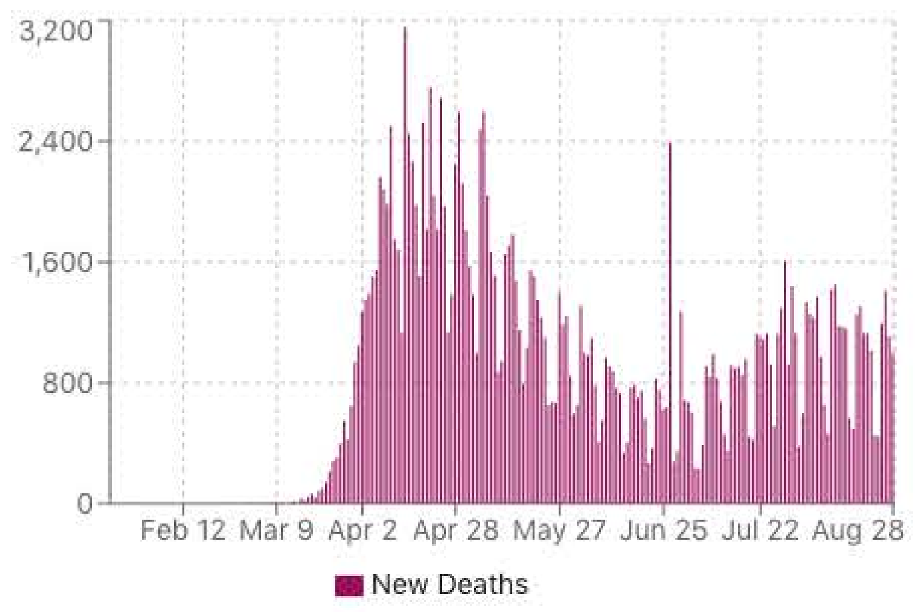

CUMULATIVE CASES

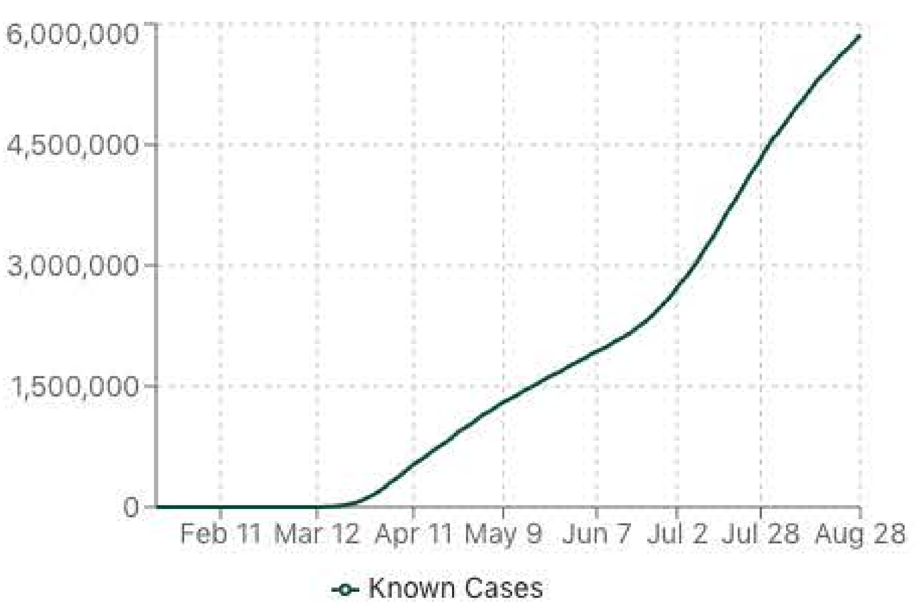

CUMULATIVE DEATHS

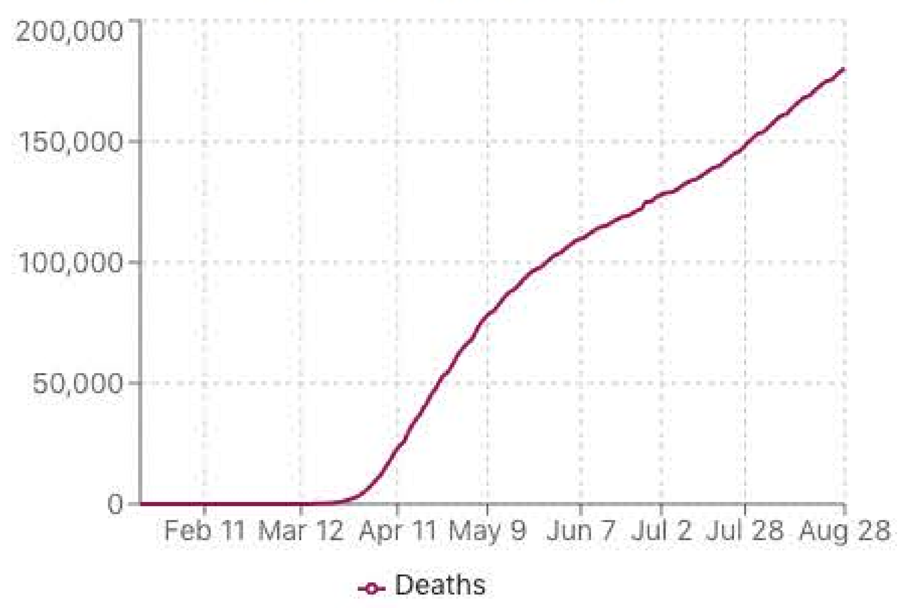

KNOWN CASES | KNOWN CASES (PER 100K) | DEATHS

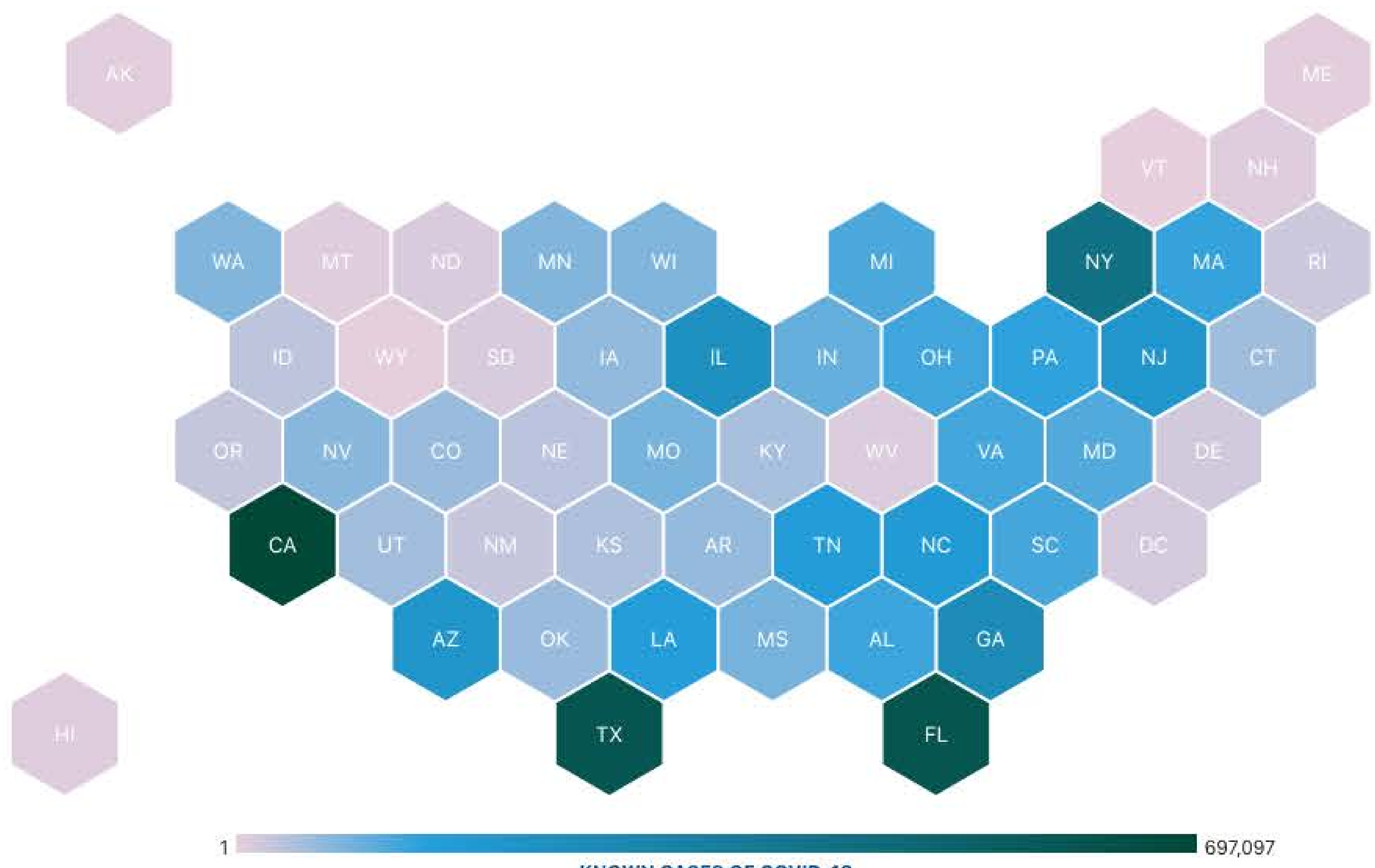

STATE-BY-STATE DATA (TOTALS)

State	Confirmed	Deaths
Alabama	122,185	2,107
Alaska	5,092	37
Arizona	200,658	4,978
Arkansas	59,583	756
California	697,097	12,801
Colorado	56,773	1,937
Connecticut	52,495	4,465
Delaware	17,083	604
District of Columbia	13,851	605

MORE INFO

COVID-19: RECOVERY HUB

COVID-19: STATS & DATA

DOWNLOAD DATA

KNOWN CASES

DEATHS

COUNTY POPULATIONS

RELATED ARTICLES

CORONAVIRUS
Here's the data on five key issues from Biden and Trump's convention speeches

CHILD CARE
How are children going back to school in America's 225 largest public school districts?

CORONAVIRUS
Coronavirus and college football: what might this mean for colleges' bottom lines?

COVID Mapping Project

About | Sources | Counties | Case Studies | Tweet

Kentucky

Overview | Case Studies | Counties

State Public Health Information

State 2.0

State: KY

47,577	815.6k	921	4560
Number of Cases	Number of Tests	Number of Deaths	Total Hospitalized

Deaths Per Day

New Deaths · 7 Day Death Average

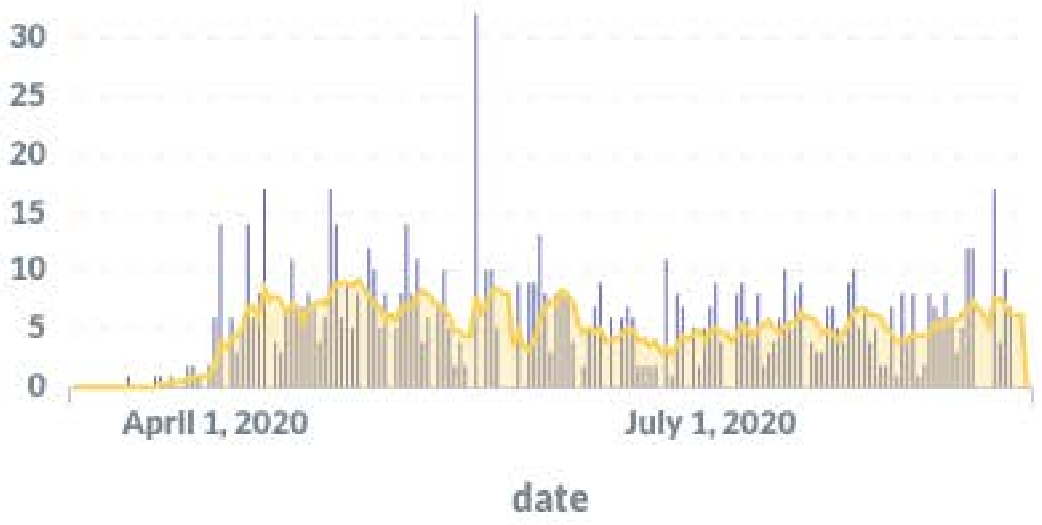

Cases Per Day

7 Day Average · New Cases

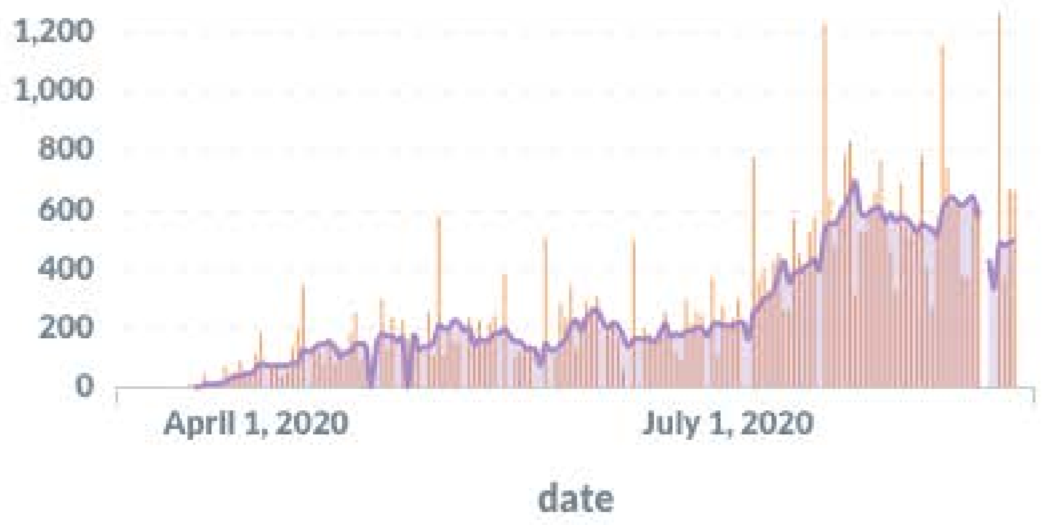

Hospitalized Per Day

New Hospitalized · 7 Day Hosp Average

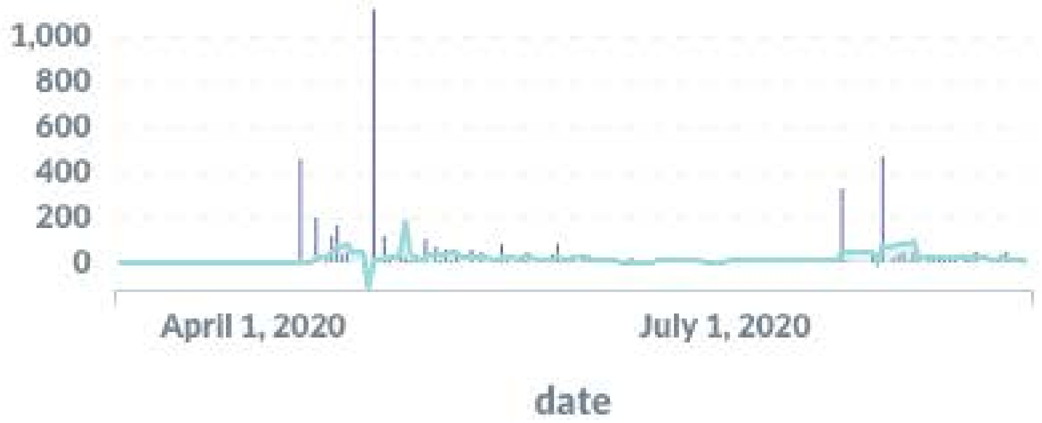

New Tests Per Day

Seven Day Testing Average · New Tests

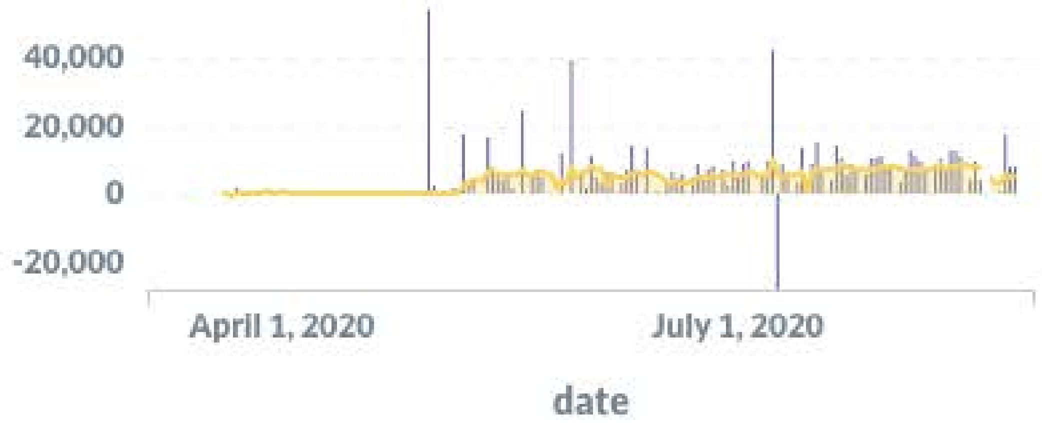

Select Language Powered by Google Translate

DASHBOARD

United States Coronavirus (COVID-19) Tracker

Posted August 2nd

♥ **August is here and we need your help. We are fully committed to maintaining this free resource until the pandemic ends. However, all operational costs are paid for with our own pockets. Your generous support is what keeps us running. Could you chip in today so we can continue providing this service? Thank you.**

Donate

I've Already Donated This Week

Infection Map (hide)

Click to autodetect your location

Inform your friends & family:

Share | Share | Email | Tweet | Share

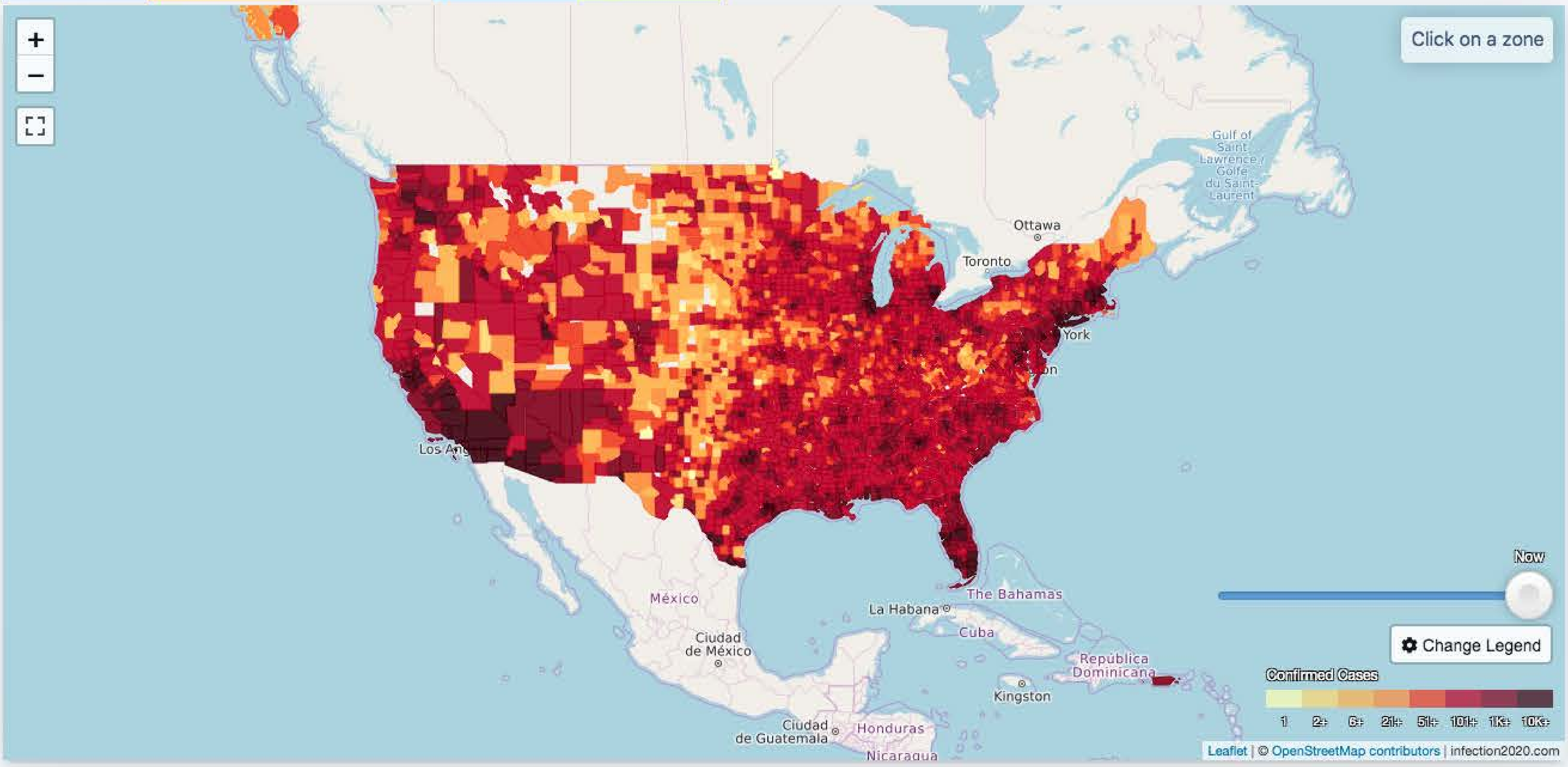

Nationwide Live Data

Note: As of 4/17, the number of cases and deaths now include probable cases to match the new CDC guidelines. This contributed to the spike in new deaths displayed for 4/17, which includes 4,226 probable deaths.

CASES	DEATHS	CASE FATALITY RATE	RECOVERED
5,838,827	182,803	3.13%	3,375,838
↑ 1% (+44,525 past 24hr)	↑ 1% (+1,099 past 24hr)	-	↑ 1% (+27,898 past 24hr)

Cases by County (hide)

Tap a state row to show its counties.
Tap the blue chart icon to see historical data.
Scroll down to see more.

State	Cases	Deaths
CA	692,965	12,698
TX	622,121	12,432
FL	611,073	10,867
NY	436,852	33,300
GA	243,425	5,251
IL	226,317	7,988
AZ	200,173	4,933
NJ	193,200	15,979

Nationwide Cases & Deaths

Cases | Deaths | All | linear | log

Touch the chart to view datapoints.
Tap on legend to hide specific dataset.

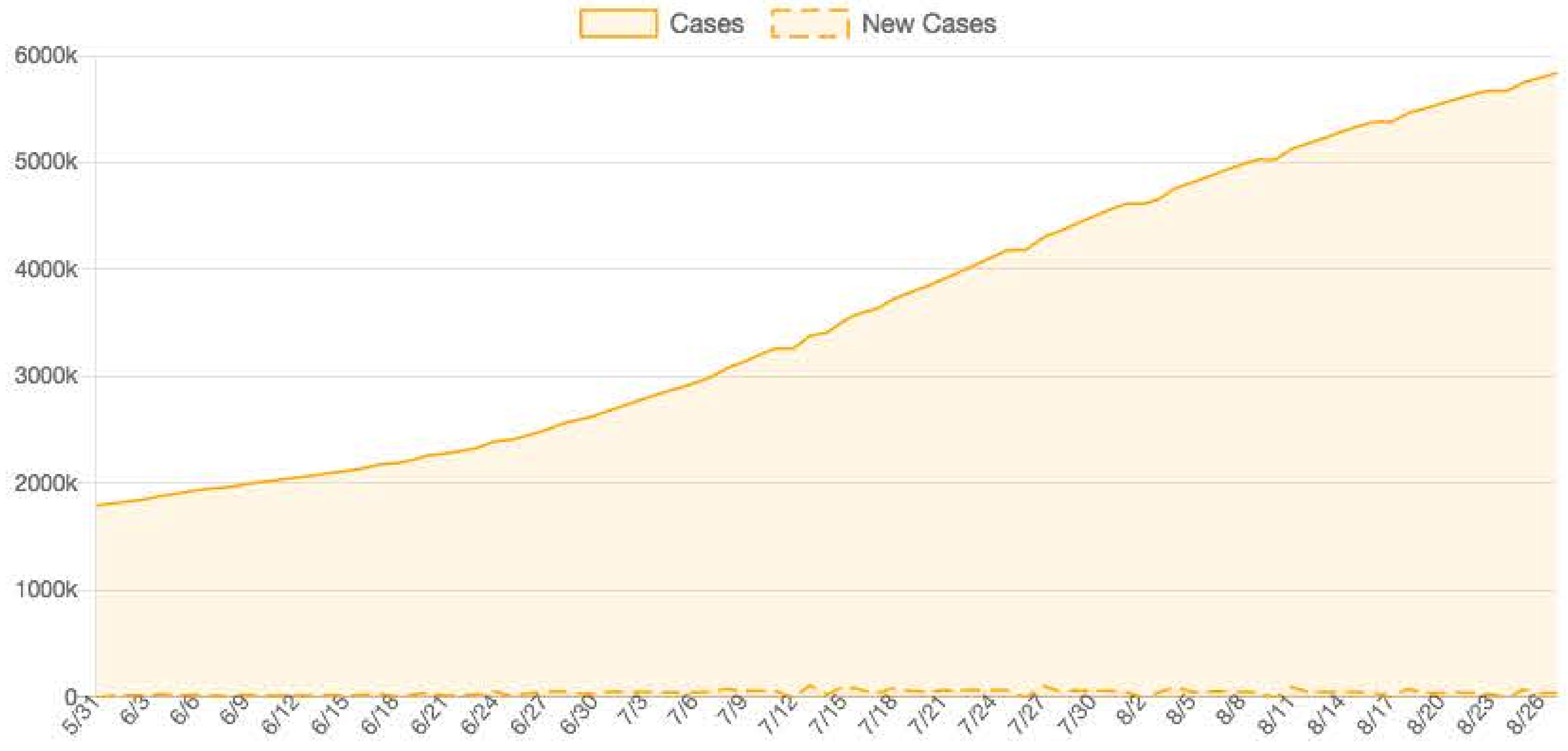

14 days | All days

Data sources: CDC, WHO, The New York Times, JHU, Corona Data Scraper, and official state and county health agencies.
Please contact us if you have any suggestions or feedback.
If you would like to volunteer, please fill out this form.
Big thanks to Netlify and Mapbox for supporting us during this outbreak.

Disclaimer
