# Supplementary material for: Web-Based COVID-19 Dashboards and Trackers in the United States: Survey Study
Source: JMIR Hum Factors. 2023 Mar 20;10:e43819. doi: 10.2196/43819 (PMC10029858; doi:10.2196/43819)

Appendix 6. Screenshots from dashboards and trackers — *Global coverage* *(captured August 29, 2020)*

**Page**	**Ref**	**Host**
2	G-1	New York Times
3	G-2	CNN
4	G-3	Stat / AppliedXL
5	G-4	Johns Hopkins Centers for Civic Impact
6	G-5	Biocomplexity Institute, University of Virginia
7	G-6	HGIS Lab, University of Washington
8	G-7	HealthMap / various universities
9–11	G-8	World Health Organization
12	G-9	Kaiser Family Foundation
13	G-10	Our World in Data
14	G-11	DXY
15	G-12	Worldometer
16	G-13	Acoer
17	G-14	Microsoft
18–19	G-15	1Point3Acres
20–21	G-16	Tableau

Coronavirus Map: Tracking the Global Outbreak

By The New York Times Updated August 29, 2020, 2:15 P.M. E.T.

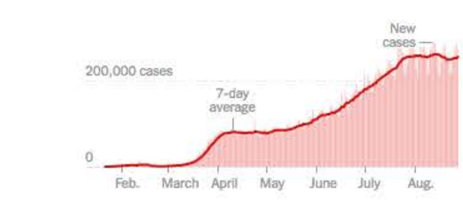

TOTAL CASES
24.7 million+
DEATHS
838,322
Includes confirmed and probable cases where available

Map | Country table | New cases | Tips | Latest news »

The coronavirus pandemic has sickened more than 24,793,400 people, according to official counts. As of Saturday afternoon, at least 838,300 people have died, and the virus has been detected in nearly every country, as these maps show.

Hot spots | Total cases | Deaths | Per capita

Average daily cases per 100,000 people in the past week

2 | 6 | 10 | 14 | Few or no cases

Double-click to zoom into the map.

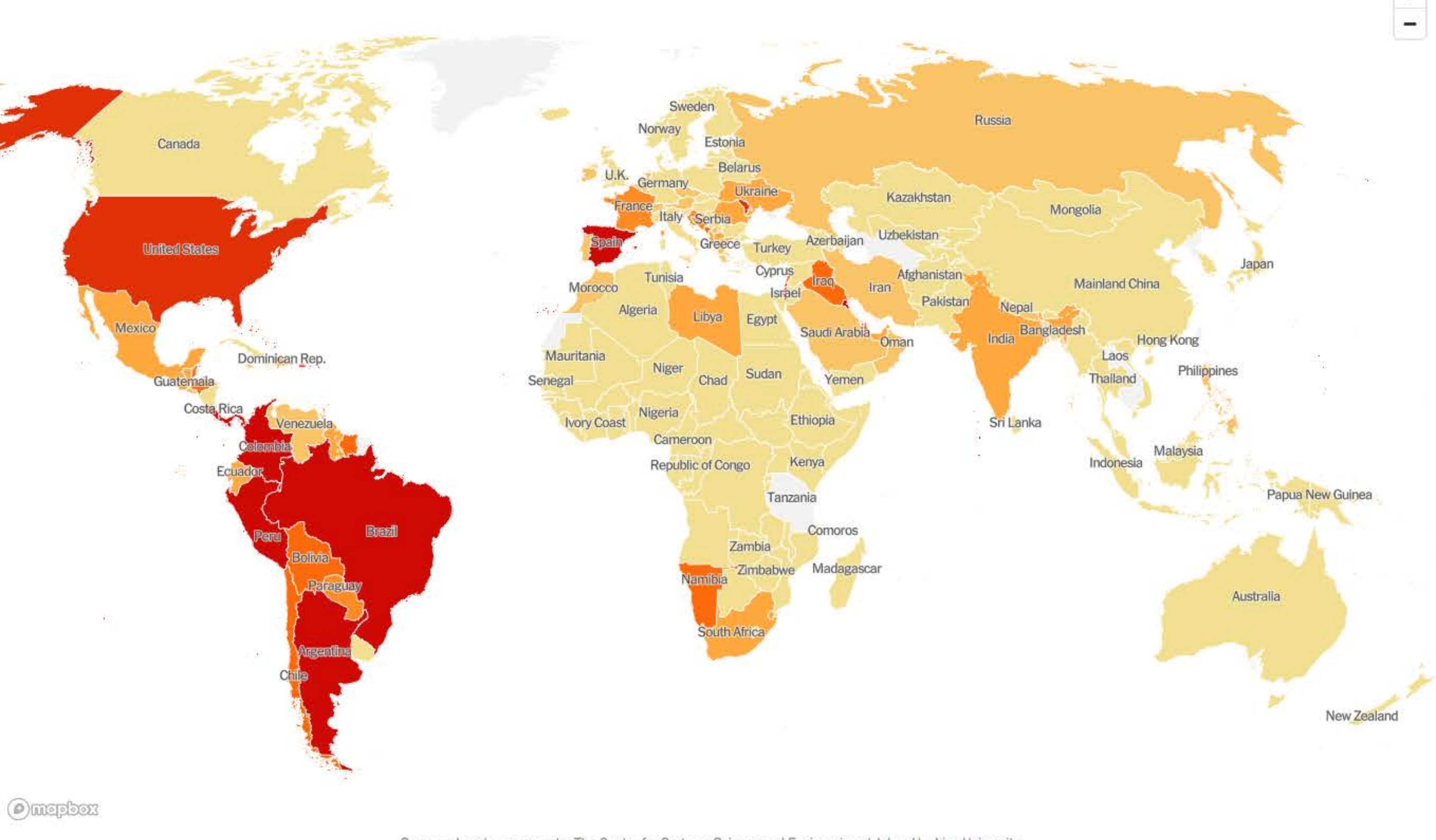

Sources: Local governments; The Center for Systems Science and Engineering at Johns Hopkins University; National Health Commission of the People's Republic of China; World Health Organization.
About this data

The coronavirus pandemic is ebbing in some of the countries that were hit hard early on, but the number of new cases is growing faster than ever worldwide, with more than 200,000 reported each day on average.

Cases | Deaths | Search countries

	TOTAL CASES	PER 100,000	CASES IN LAST 7 DAYS	▼ PER 100,000	WEEKLY CASES PER CAPITA (FEWER – MORE)
Aruba	1,906	1,801	442	418	Jan. 22 – Aug. 28
Turks and Caicos Islands	490	1,301	148	393	
Sint Maarten	444	1,092	91	224	
Maldives	7,469	1,448	905	175	
Peru	629,961	1,969	54,938	172	
Bahrain	51,113	3,257	2,452	156	
Panama	90,624	2,170	6,232	149	
Argentina	392,009	881	62,966	142	
Colombia	590,492	1,189	68,276	138	
Brazil MAP »	3,804,803	1,816	272,473	130	

Show all

Weekly cases per capita shows the share of population with a new reported case for each week. Weeks without a reported case are shaded gray.

The virus continues to affect every region of the world, but some countries are experiencing high rates of infection, while others appear to have mostly controlled the virus.

Where new cases are increasing

Total cases | Cases per capita

Charts show daily cases and are individually scaled to the maximum for each country.

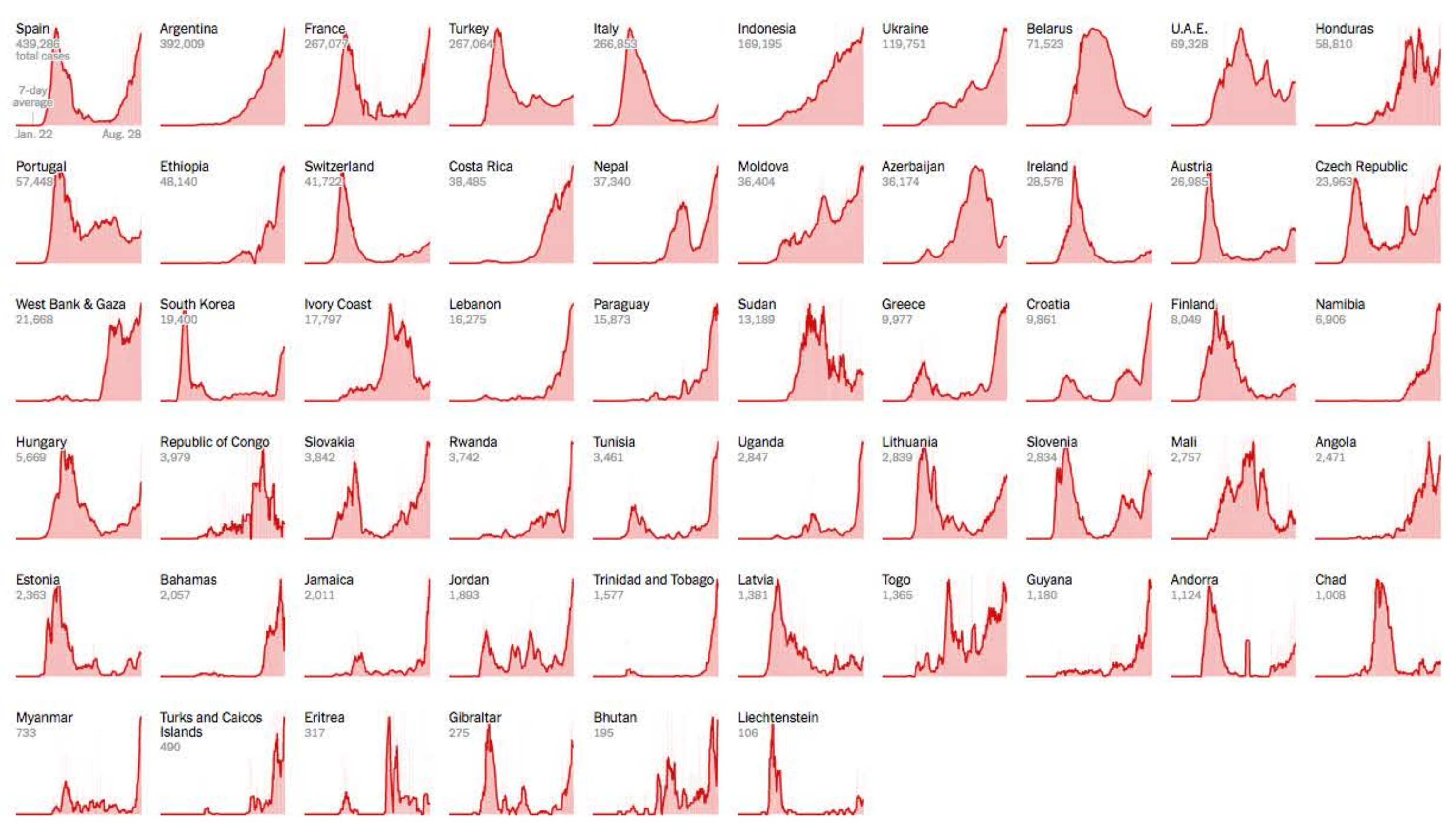

These countries have had growth in newly reported cases in the last 14 days.

Where new cases are mostly the same

Total cases | Cases per capita

Charts show daily cases and are individually scaled to the maximum for each country.

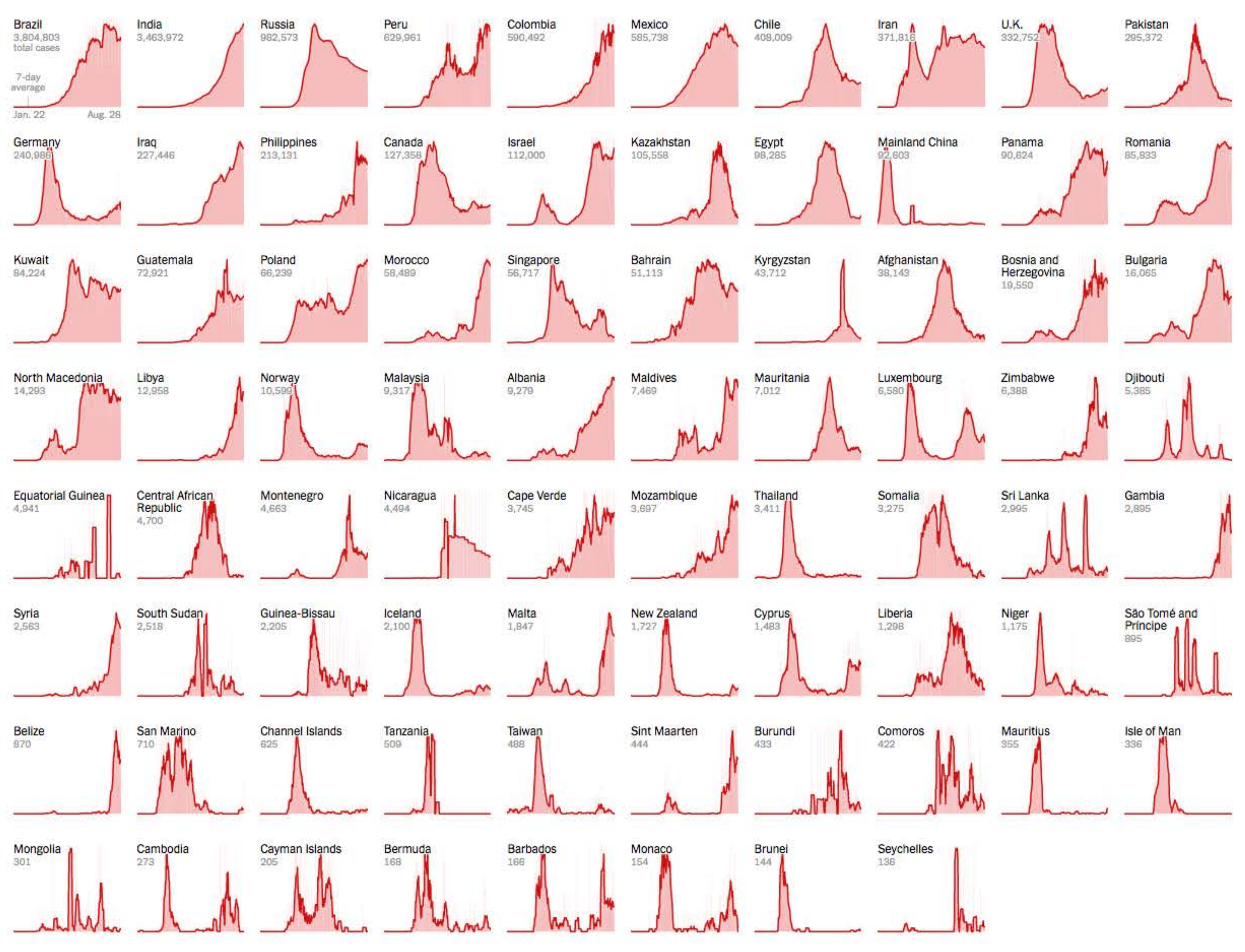

Where new cases are decreasing

Total cases | Cases per capita

Charts show daily cases and are individually scaled to the maximum for each country.

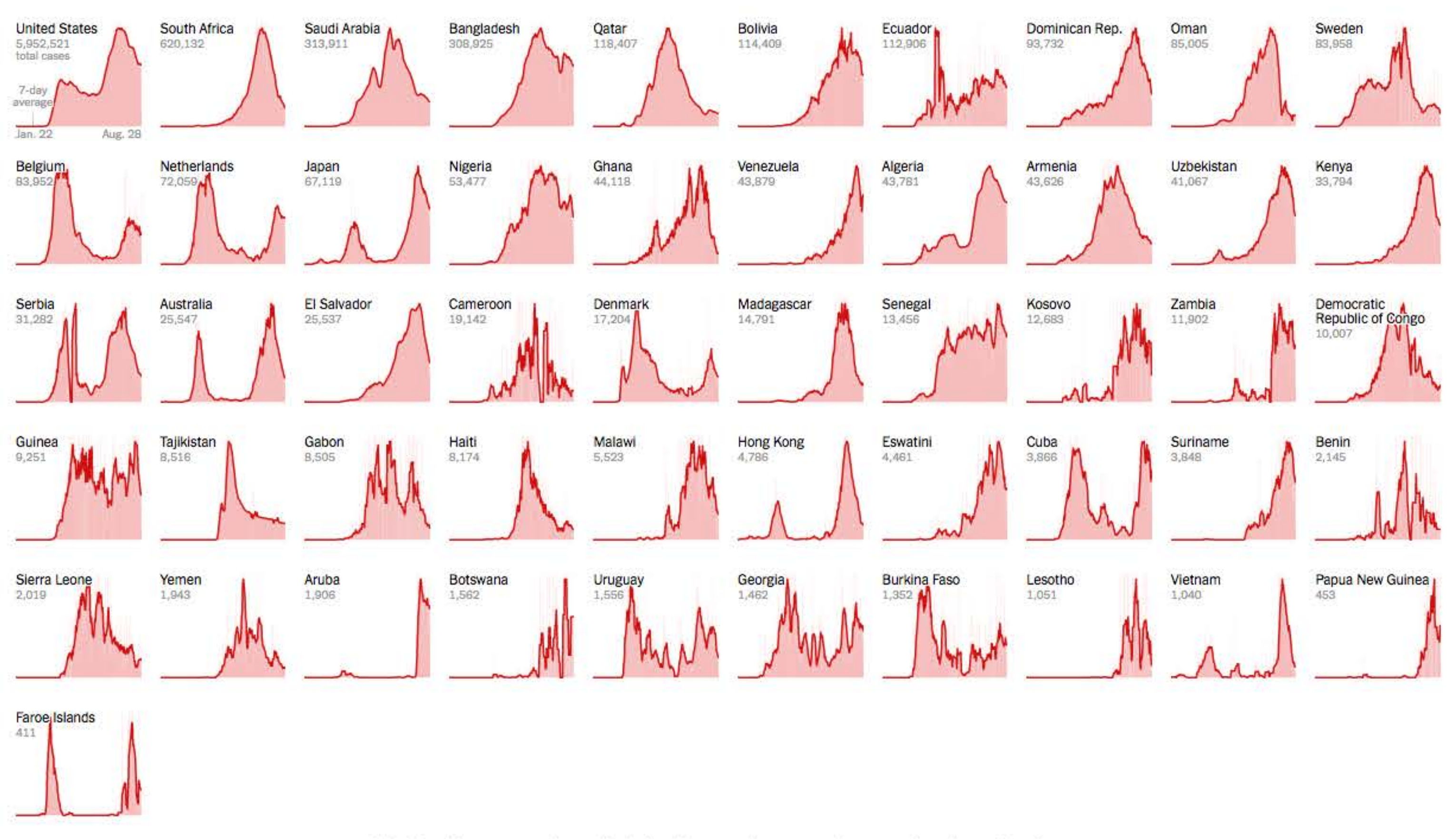

Note: Countries are grouped according to how the seven-day average of new cases has changed from two weeks ago to today. Countries with fewer than 100 cases are not shown.

Where new deaths are increasing

Total deaths | Deaths per capita

Charts show daily deaths and are individually scaled to the maximum for each country.

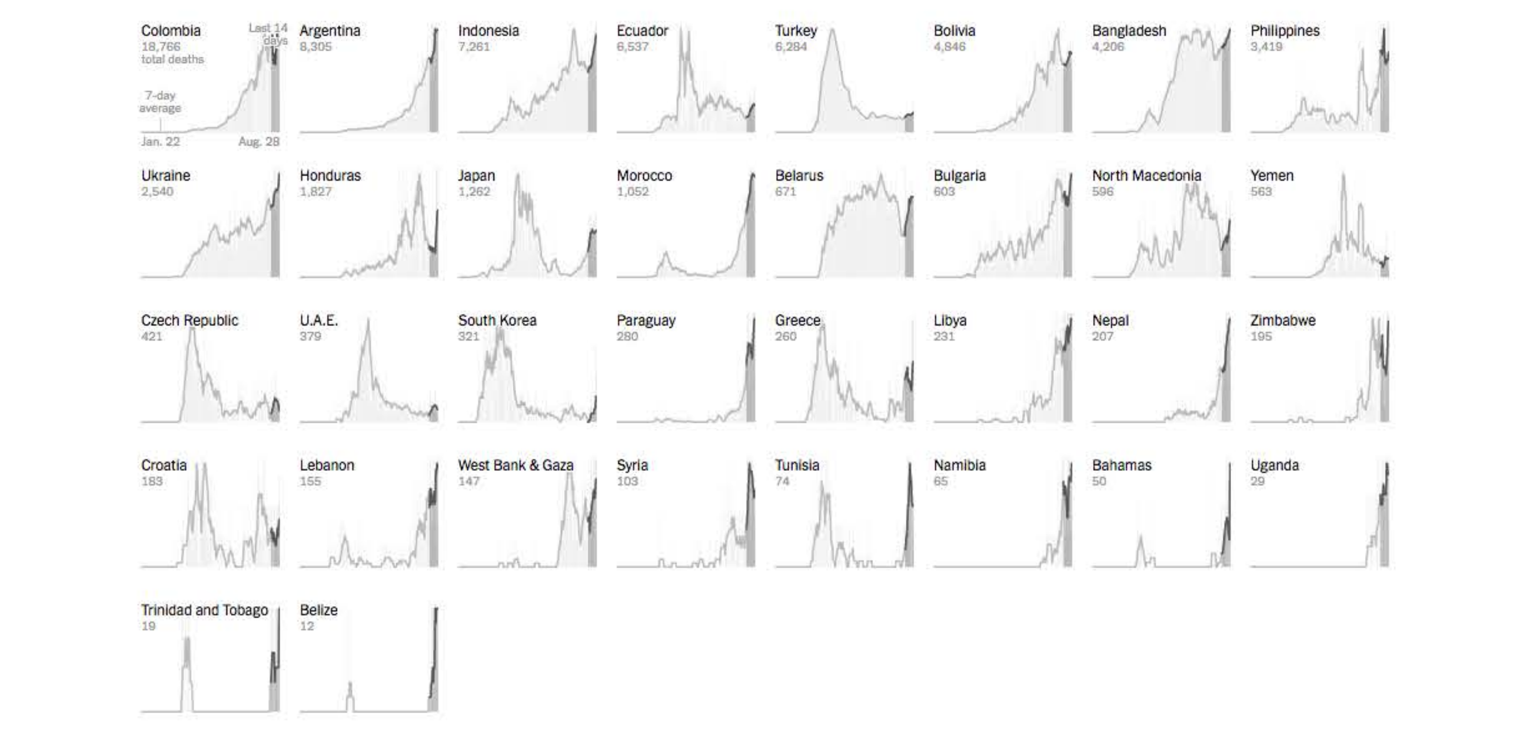

These countries have had the highest growth in newly reported deaths over the last 14 days. Deaths tend to rise a few weeks after a rise in infections, as there is typically a delay between when people are infected, when they die and when deaths are reported. Some deaths reported in the last two weeks may have occurred much earlier because of these delays.

The outbreak was initially defined by a series of shifting epicenters — including Wuhan, China; Iran; northern Italy; Spain; and New York.

Cases worldwide leveled off in April after social distancing measures were put in place in many of the areas with early outbreaks.

But as countries began to reopen in May and June, the United States was unable to contain a resurgence of the disease, making it one of the main drivers of rising case numbers around the world. Many South American countries are also experiencing high rates of infection, with Brazil, Colombia, Peru and Argentina reporting large numbers of cases every day.

New reported cases by day across the world

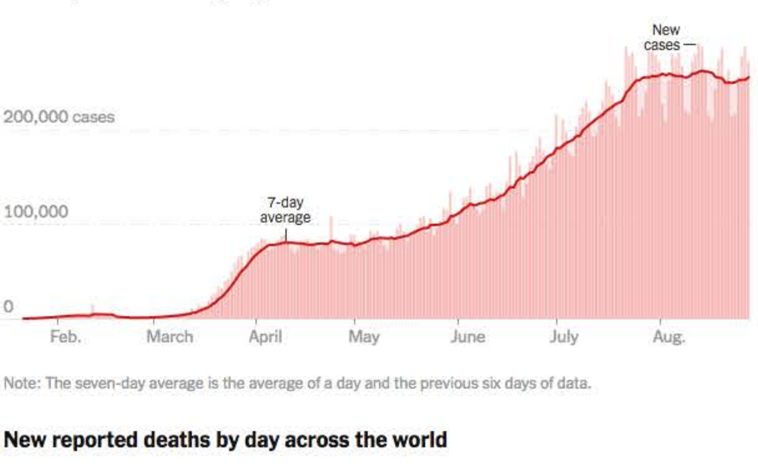

Note: The seven-day average is the average of a day and the previous six days of data.

New reported deaths by day across the world

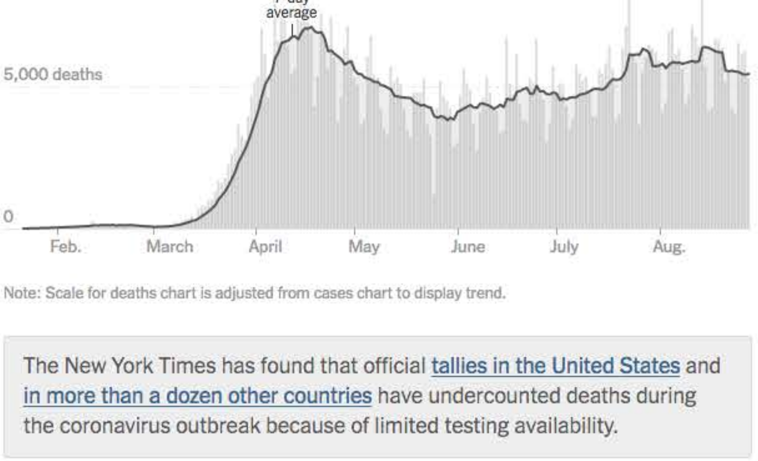

Note: Scale for deaths chart is adjusted from cases chart to display trend.

The New York Times has found that official tallies in the United States and in more than a dozen other countries have undercounted deaths during the coronavirus outbreak because of limited testing availability.

Follow our coverage of the coronavirus pandemic here.

United States

The number of known coronavirus cases in the United States continues to grow. As of Saturday afternoon, at least 5,952,500 people across every state, plus Washington, D.C., and four U.S. territories, have tested positive for the virus, according to a New York Times database, and at least 182,100 patients with the virus have died.

Reported cases in the United States

Average daily cases per 100,000 people in the past week

← Fewer | More →

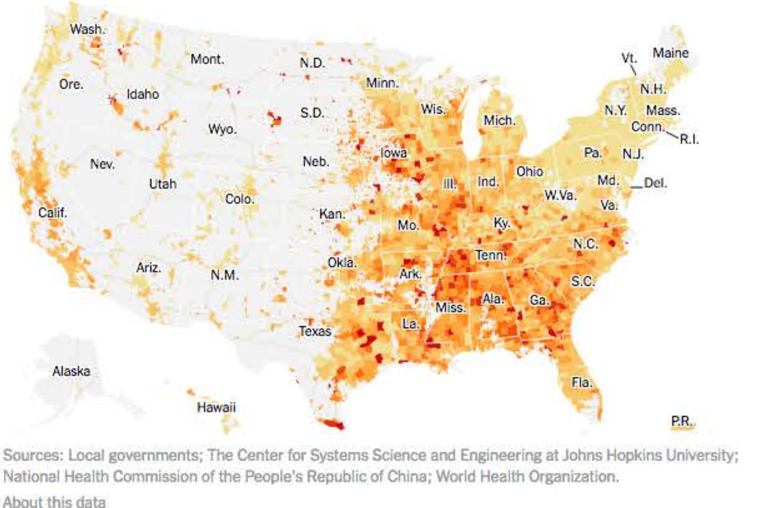

Sources: Local governments; The Center for Systems Science and Engineering at Johns Hopkins University; National Health Commission of the People's Republic of China; World Health Organization.
About this data

See our page of maps, charts and tables tracking every coronavirus case in the U.S.

After case numbers fell steadily in April and May, cases in the United States are growing again at about the same rapid pace as when infections were exploding in New York City in late March. But the hotspots are now mainly spread across the southern and western parts of the country.

The New York Times is engaged in an effort to track the details of every reported case in the United States, collecting information from federal, state and local officials around the clock. The numbers in this article are being updated several times a day based on the latest information our journalists are gathering from around the country. The Times has made that data public in hopes of helping researchers and policymakers as they seek to slow the pandemic and prevent future ones.

Read more about the methodology and download county-level data for coronavirus cases in the United States from The New York Times on GitHub.

About the data

Governments often revise data or report a single-day large increase in cases or deaths from unspecified days without historical revisions, which can cause an irregular pattern in the daily reported figures. The Times is excluding these anomalies from seven-day averages when possible.

CNN health

● Follow CNN's live coverage →

Tracking coronavirus' global spread

Since December, the virus has spread to nearly every continent and case numbers continue to rise

By Henrik Pettersson, Byron Manley and Sergio Hernandez, CNN

Last updated: August 29, 2020 at 7:45 p.m. ET

Authorities in 214 countries and territories have reported about 24.9 million Covid-19 cases and 840,000 deaths since China reported its first cases to the World Health Organization (WHO) in December.

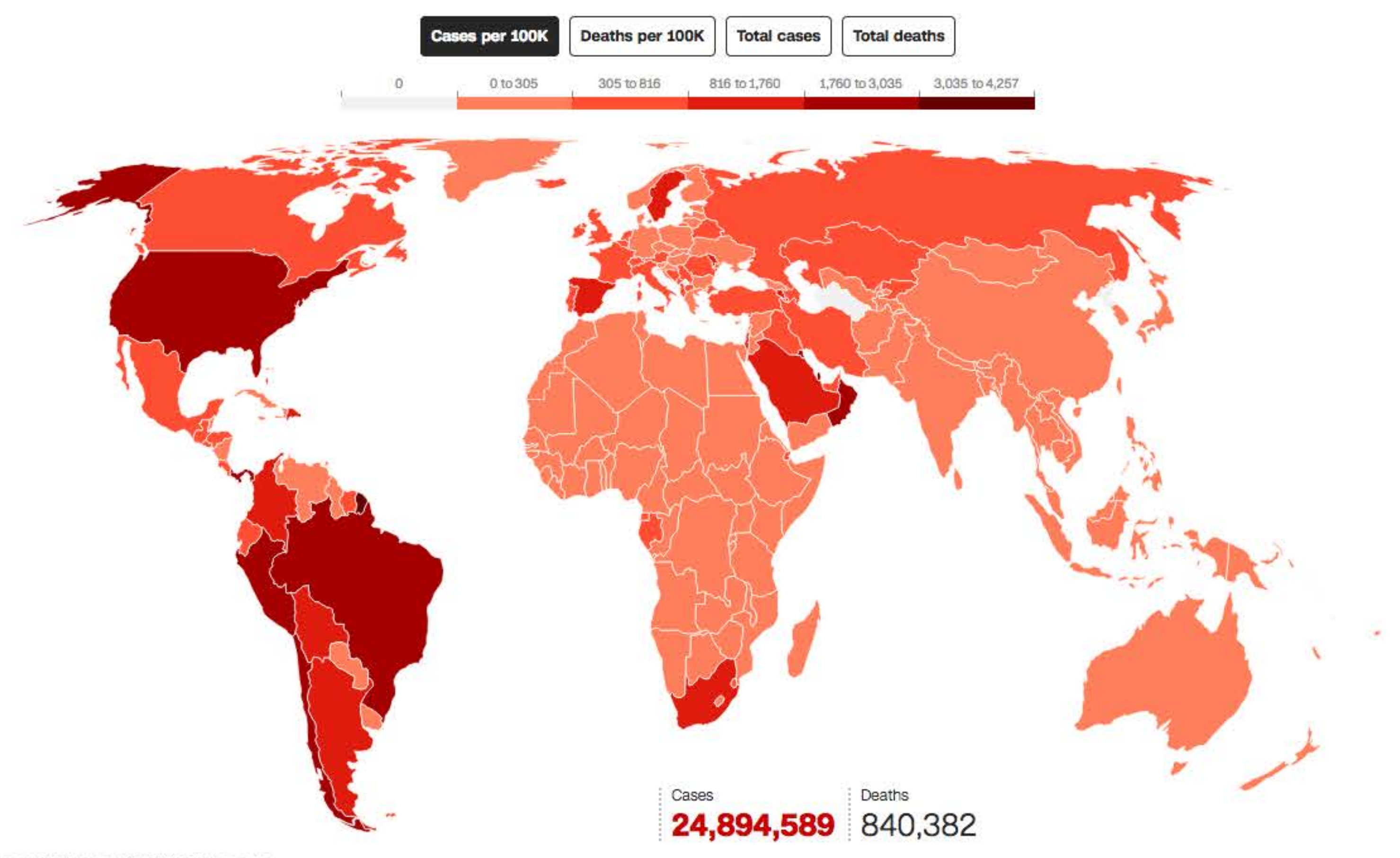

Last updated: August 29, 2020 at 7:45 p.m. ET
Source: Johns Hopkins University Center for Systems Science and Engineering

Reported cases and deaths

The figures below are based on data from the Johns Hopkins University Center for Systems Science and Engineering. These numbers are updated every 15 minutes but may differ from other sources due to differences in reporting times. For up-to-the-minute updates, follow our live coverage.

Location	Cases	...per 100K people	Deaths	...per 100K people
United States	5,958,902	1,821	182,718	56
Brazil	3,846,153	1,836	120,262	57
India	3,463,972	256	62,550	5
Russia	982,573	680	16,977	12
Peru	629,961	1,969	28,471	89
South Africa	622,551	1,077	13,981	24
Colombia	590,492	1,189	18,766	38
Mexico	585,738	464	63,146	50
Spain	439,286	940	29,011	62
Chile	408,009	2,178	11,181	60
Argentina	401,239	902	8,353	19
Iran	371,816	455	21,359	26
United Kingdom	332,752	500	41,498	62
Saudi Arabia	313,911	931	3,840	11
Bangladesh	308,925	191	4,206	3
Pakistan	295,372	139	6,284	3
France	288,531	431	30,460	45
Turkey	267,064	324	6,284	8
Italy	266,853	442	35,473	59
Germany	242,835	293	9,299	11

Show more

* Cases identified on cruise ships.

Last updated: August 29, 2020 at 7:45 p.m. ET
Sources: Johns Hopkins University Center for Systems Science and Engineering. Population data from World Bank and United Nations.

Since then, cases have been reported on every continent except Antarctica. The vast majority of cases and deaths are now outside mainland China, where the outbreak began.

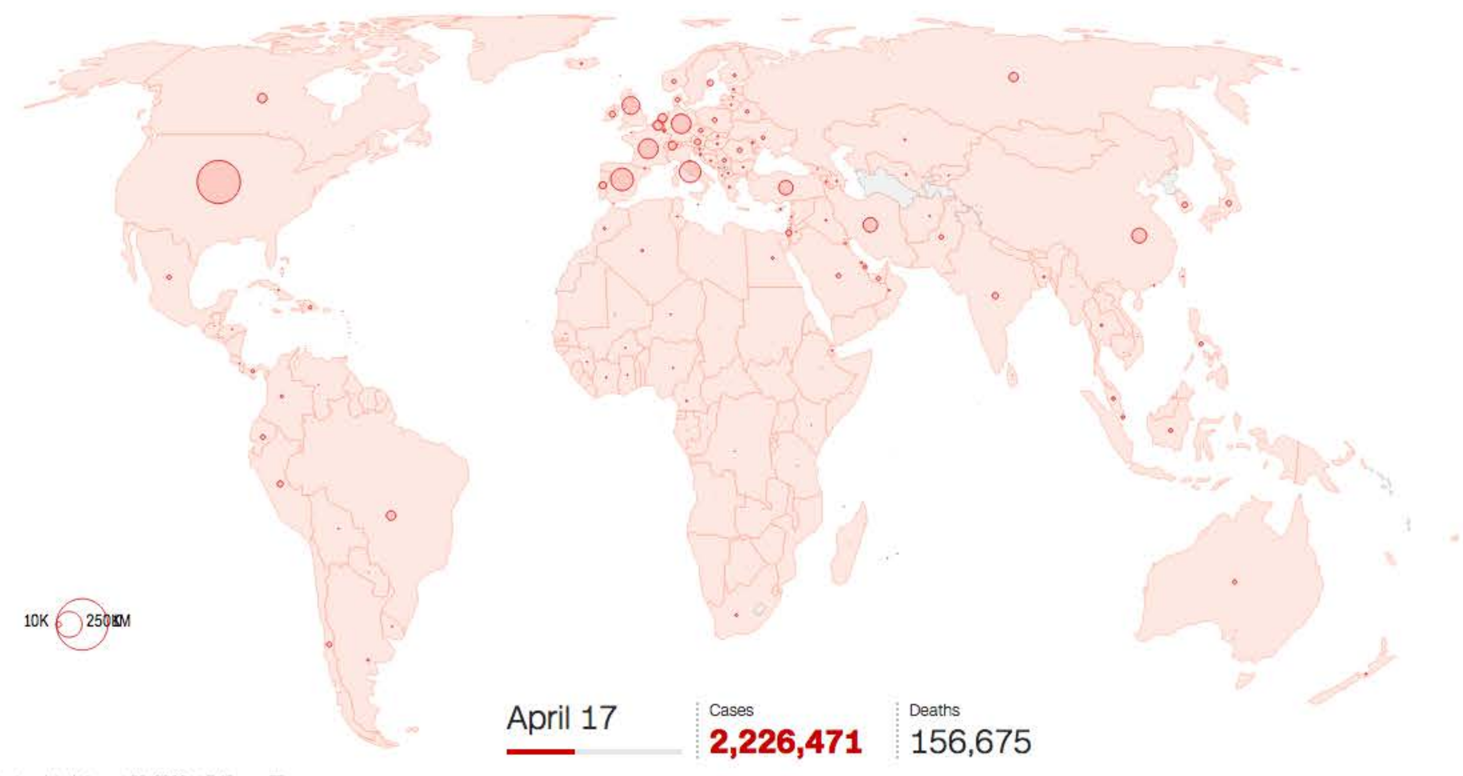

Last updated: August 29, 2020 at 7:45 p.m. ET
Source: Johns Hopkins University Center for Systems Science and Engineering

As the pandemic has spread across the globe, the virus has left a trail of deaths in its wake. Deaths in Europe and North America now outnumber Asia's. In Latin America, South America and the Caribbean, the share of global deaths is still rising.

Daily deaths and cases by region

This chart uses rolling, seven-day averages. This approach makes trends clearer and smooths out anomalies, such as the lack of reporting during the weekend.

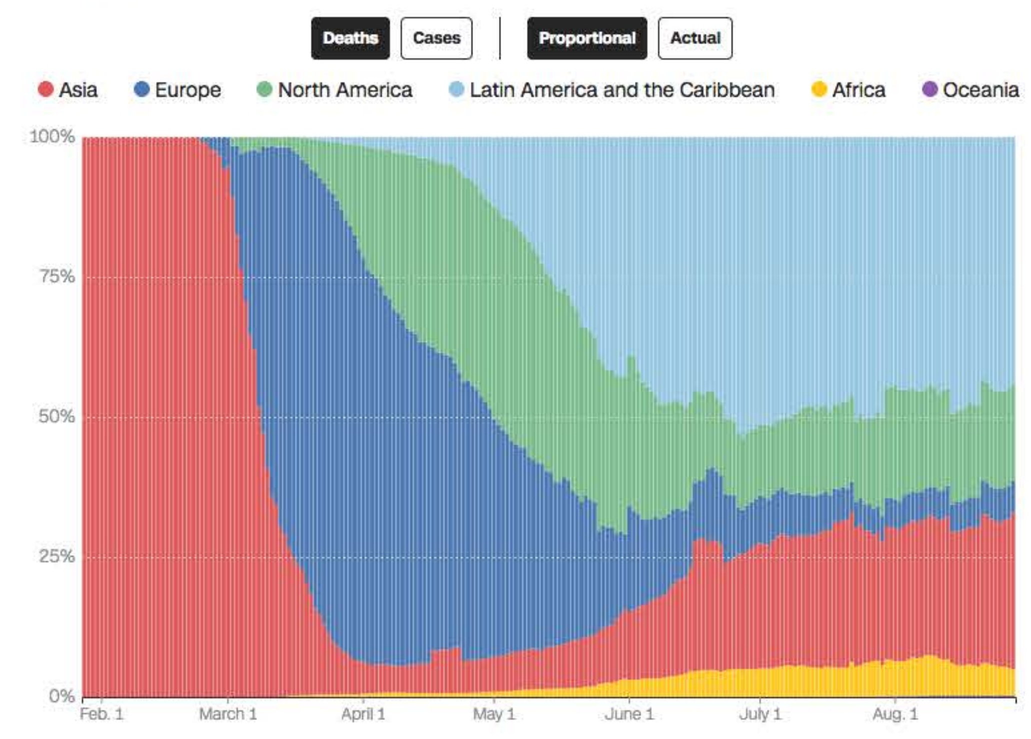

Regions are based on United Nations definitions. Americas have been broken down into subregions (Latin America and the Caribbean and North America).
Last updated: August 29, 2020 at 7:45 p.m. ET
Source: Johns Hopkins University Center for Systems Science and Engineering

Daily reported deaths by country

This timeline of new reported deaths shows how countries and regions have been affected so far.

Based on 7-day rolling averages | ↑↓ Trend in rolling average

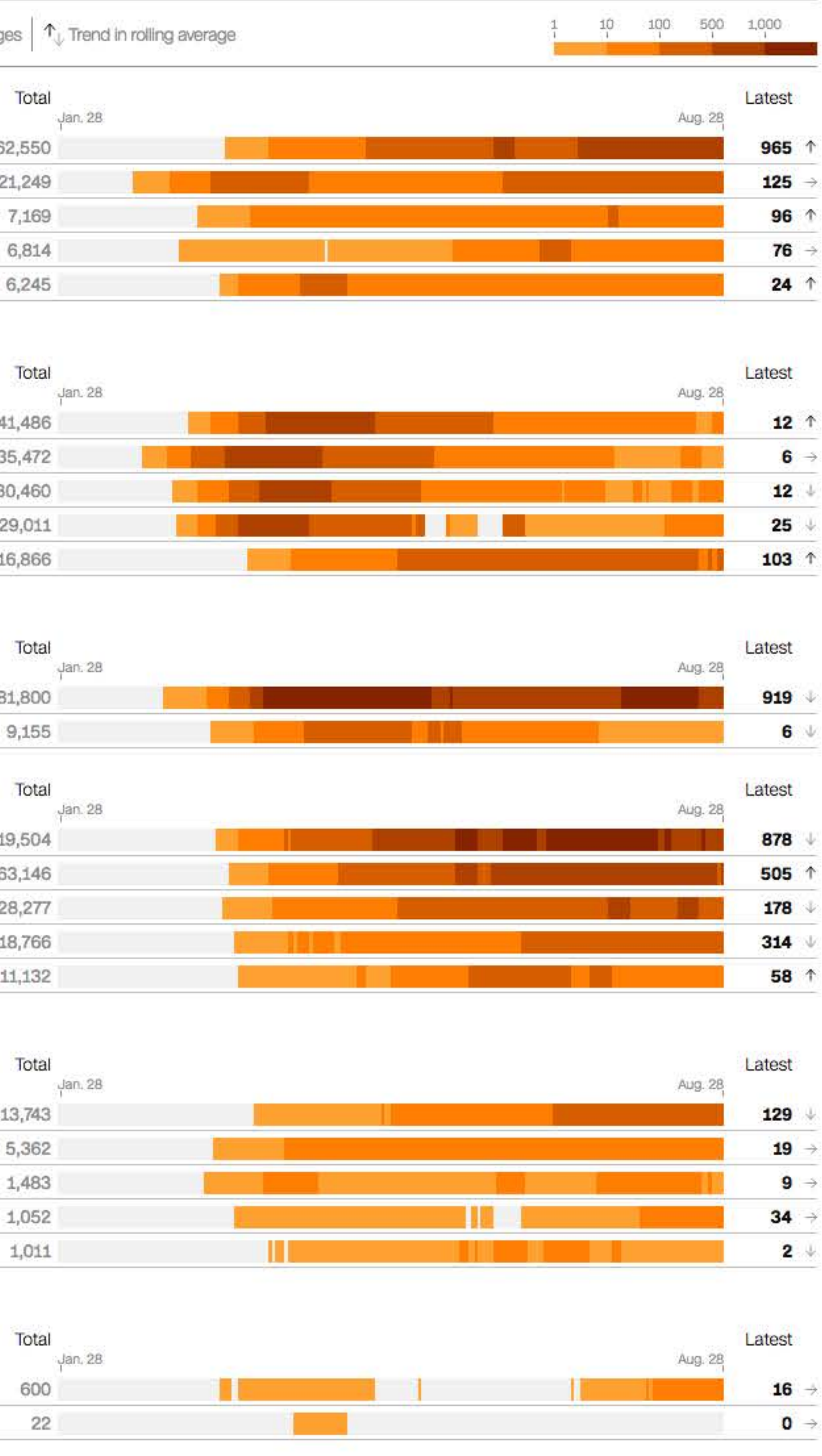

Asia	Total	Latest
India	62,550	965 ↑
Iran	21,249	125 →
Indonesia	7,169	96 ↑
Iraq	6,814	76 →
Turkey	6,245	24 ↑

Expand

Europe	Total	Latest
United Kingdom	41,486	12 ↑
Italy	35,472	6 →
France	30,460	12 ↓
Spain	29,011	25 ↓
Russia	16,866	103 ↑

Expand

North America	Total	Latest
United States	181,800	919 ↓
Canada	9,155	6 ↓

Latin America and the Caribbean	Total	Latest
Brazil	119,504	878 ↓
Mexico	63,146	505 ↑
Peru	28,277	178 ↓
Colombia	18,766	314 ↓
Chile	11,132	58 ↑

Expand

Africa	Total	Latest
South Africa	13,743	129 ↓
Egypt	5,362	19 →
Algeria	1,483	9 →
Morocco	1,052	34 →
Nigeria	1,011	2 ↓

Expand

Oceania	Total	Latest
Australia	600	16 →
New Zealand	22	0 →

Note: The tables above only include countries and territories with 10 or more deaths. Deaths have also been reported in: Cruise ships (15), Bermuda (9), Taiwan (7), Comoros (7), Barbados (7), Réunion (6), Myanmar (6), Papua New Guinea (5), Saint Martin (5), Monaco (4), Turks and Caicos Islands (3), Brunei (3), Botswana (3), Antigua and Barbuda (3), Fiji (2), Western Sahara (1), Montserrat (1), Cayman Islands (1), British Virgin Islands (1), Curaçao (1), Liechtenstein (1), Burundi (1)

Last updated: August 29, 2020 at 7:45 p.m. ET
Source: Johns Hopkins University Center for Systems Science and Engineering

Different countries' epidemics have followed different trajectories. The disease has hit the United States especially hard. About 6 million cases have been reported in the country and 182,718 patients have died.

Covid-19 case growth rates

The chart below uses a logarithmic scale to show how quickly the number of known Covid-19 cases is growing in each region and territory. Select a location or hover over each line to see how quickly the number of known cases is doubling in that country or territory.

In **the United States** ▾ the number of known cases is doubling about **every three months**.

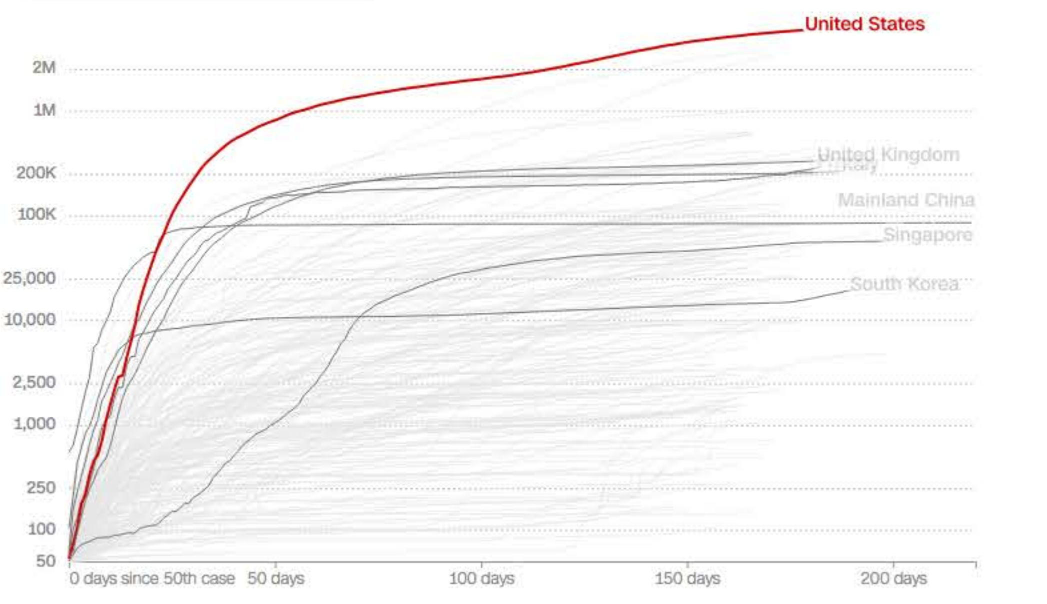

Note: Doubling times are based on the previous week.
Last updated: August 29, 2020 at 7:45 p.m. ET
Source: Johns Hopkins University Center for Systems Science and Engineering

But experts say it's unclear how deadly the virus really is, in part because a large number of cases — including mild or asymptomatic infections — may never be diagnosed. In the United States, New York City has even told doctors not to test most sick people unless they're hospitalized.

The director of the US National Institute of Allergy and Infectious Diseases, Dr. Anthony Fauci, has put the virus's mortality rate at about 2%, while the WHO has estimated 3.4%. ■

Covid-19 Worldwide

Last updated: August 29, 2020 at 8:16 PM EDT

Search by country or region

Total Number of Cases | Cases Per 100k Population

Worldwide	24,806,834
United States	5,958,902
Brazil	3,846,153
India	3,463,972
Russia	982,573
Peru	629,961
South Africa	622,551
Colombia	590,492
Mexico	585,738
Spain	439,286
Chile	408,009

TOTAL CASES	FIRST CASE	RECOVERED	DEATHS
24,806,834	242 DAYS AGO	16,159,958	828,712

2 WEEK AVERAGE CHANGE IN NEW CASES -16.1%

Average New Cases Yesterday	262,573
Average New Cases 2 Weeks Ago	313,111

2 WEEK AVERAGE CHANGE IN DEATHS -36.1%

Average Deaths Yesterday	6,641
Average Deaths 2 Weeks Ago	10,389

2 Week Trend | Cases Over Time

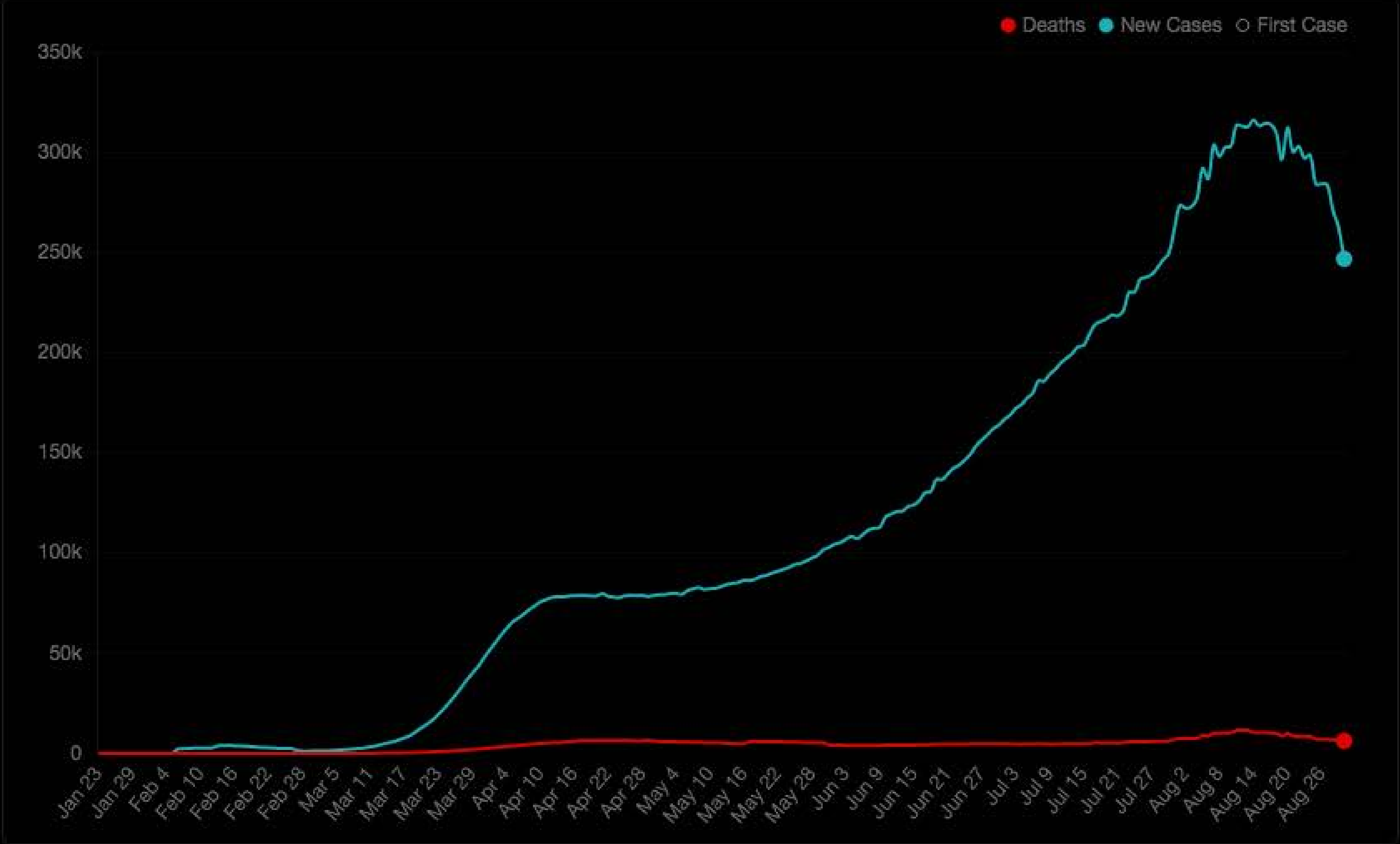

COVID-19 Dashboard by the Center for Systems Science and Engineering (CSSE) at Johns Hopkins University (J...

Global Cases
24,894,589

Cases by Country/Region/Sovereignty

5,958,902	US
3,846,153	Brazil
3,463,972	India
982,573	Russia
629,961	Peru
622,551	South Africa
590,492	Colombia
585,738	Mexico
439,286	Spain
408,009	Chile
401,239	Argentina
371,816	Iran
334,915	United Kingdom
313,911	Saudi Arabia
308,925	Bangladesh
304,947	France
295,372	Pakistan
267,064	Turkey
266,853	Italy
242,835	Germany
[illegible]	Iraq

Admin0

Last Updated at (M/D/YYYY)
8/29/2020, 7:28:21 PM

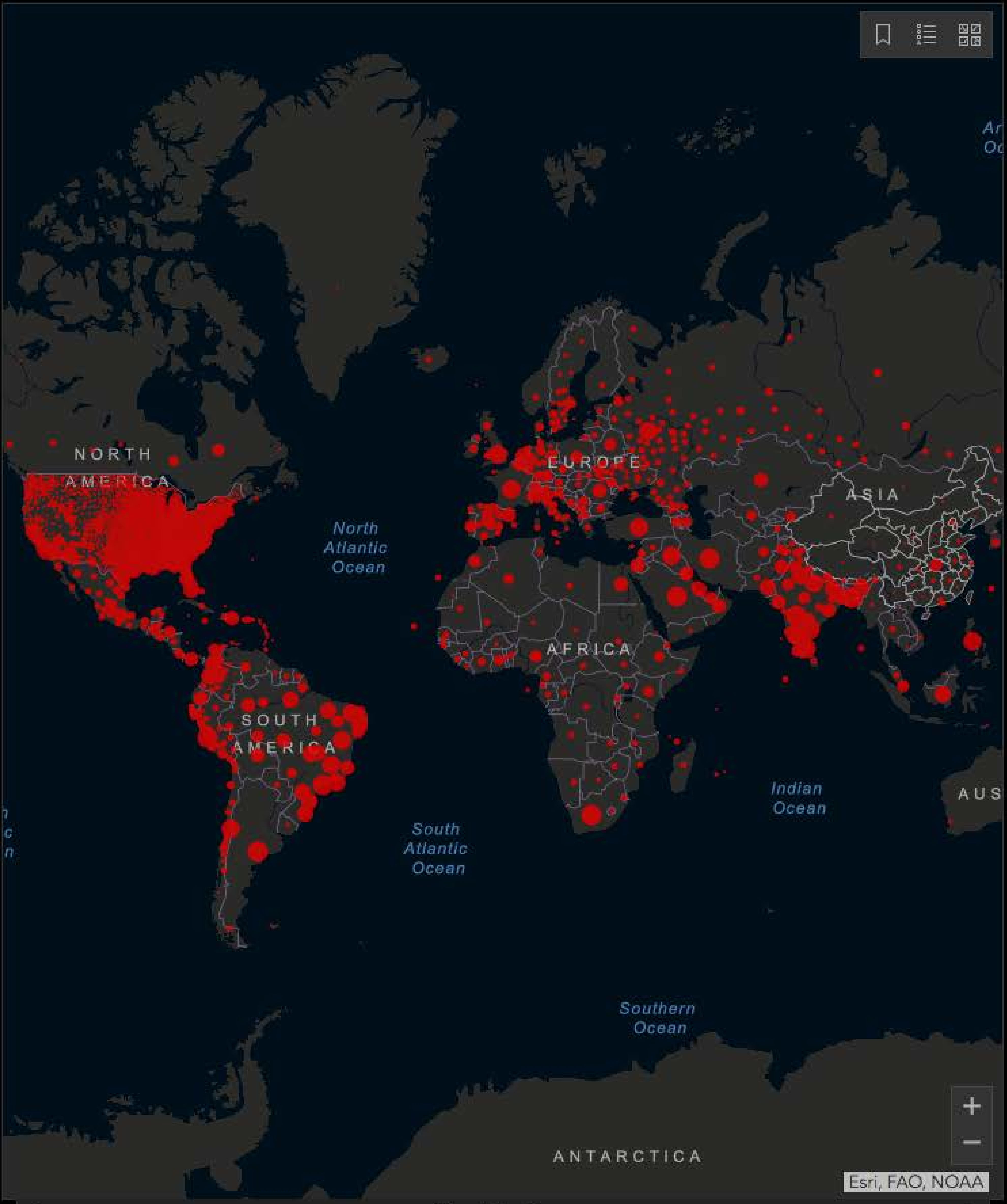

Cumulative Cases

188
countries/regions

Lancet Inf Dis Article: Here. Mobile Version: Here. Data sources: Full list. Downloadable database: GitHub, Feature Layer.
Lead by JHU CSSE. Technical Support: Esri Living Atlas team and JHU APL. Financial Support: JHU, NSF, Bloomberg Philanthropies and Stavros Niarchos Foundation. Resource support: Slack, Github and AWS. Click *here* to **donate** to the CSSE dashboard team, and other JHU COVID-19 Research Efforts. FAQ. Read more in this blog. Contact US.

Global Deaths
840,382

182,718 deaths	US
120,262 deaths	Brazil
63,146 deaths	Mexico
62,550 deaths	India
41,585 deaths	United Kingdom
35,473 deaths	Italy
30,601 deaths	France
29,011 deaths	Spain
28,471 deaths	Peru
21,359 deaths	Iran

Global Deaths

US State Level
Deaths, Recovered

32,937 deaths, 74,990 recovered
New York US

15,933 deaths, 33,827 recovered
New Jersey US

12,889 deaths, recovered
California US

12,594 deaths, 492,921 recovered
Texas US

11,105 deaths, recovered
Florida US

9,036 deaths, 103,920 recovered
Massachusetts US

8,217 deaths, recovered
[illegible]

US Deaths, Re...

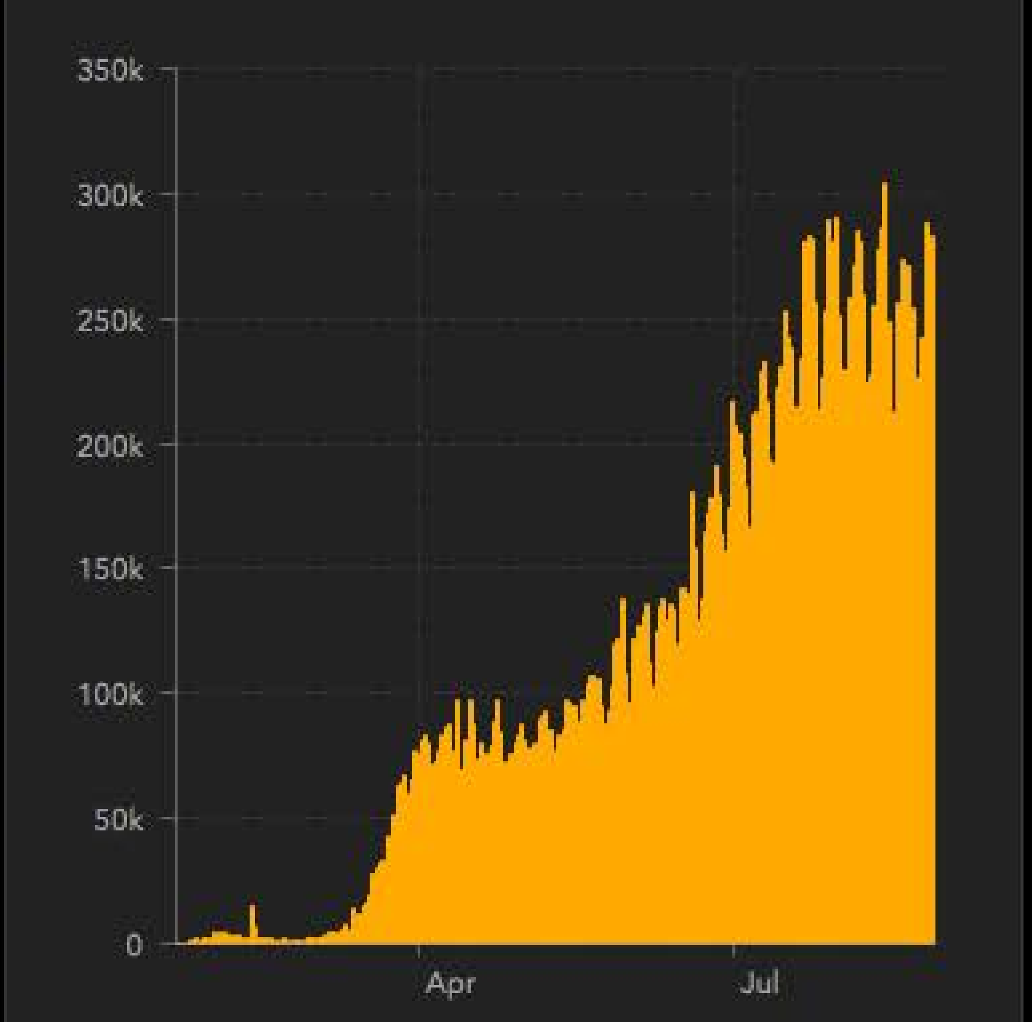

Daily Cases

UNIVERSITY of VIRGINIA
BIOCOMPLEXITY INSTITUTE

COVID-19 Surveillance Dashboard 1.5.0

COVID-19 Medical Resource Demand Dashboard - Virginia

More Options

08-29-2020 | All regions | FAQ

Est.Active | Confirmed | Deaths | Est.Recovered | Cases | Tests

United States: Est.Active: 734,291 Conf: 6,037,667 Deaths: 184,771 Est.Rec: 5,118,605

All regions

-96,903	126,840	1,872	221,871
3,760,058	24,993,796	842,414	20,391,324
Est.Active	Confirmed	Deaths	Est.Recovered

What's estimated recovered? Tutorials: USA testing data & toggles | USA county | more

Chart | Data

World | Last 30 days | Type : Daily

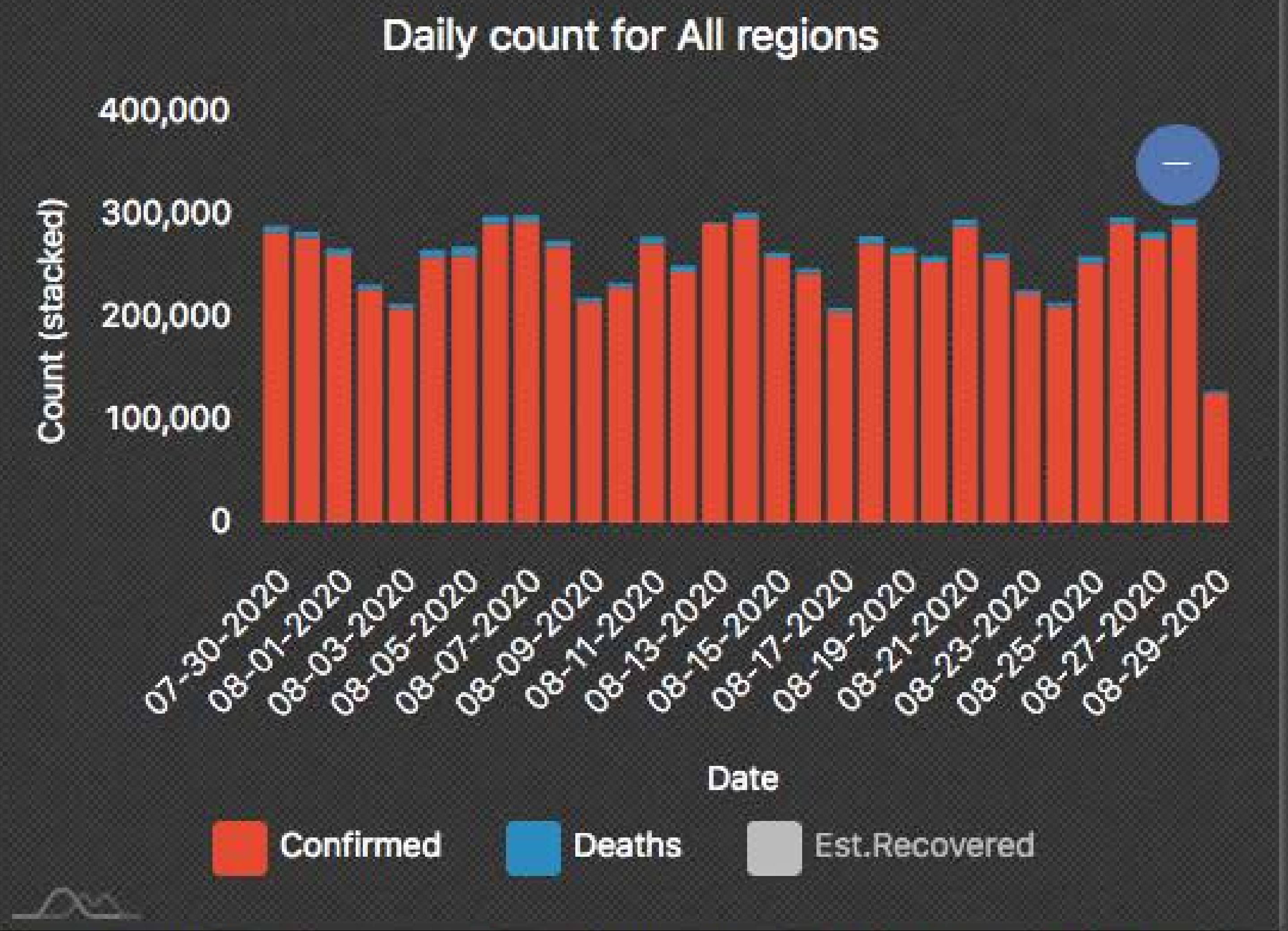

COPYRIGHT © 2020, UVA; ALL RIGHTS RESERVED. THE CONTENTS OF THIS DASHBOARD--INCLUDING DATA, MAPS, AND PLOTS--IS PROVIDED TO THE PUBLIC SOLELY FOR RESEARCH AND ACADEMIC PURPOSES. UVA PROVIDES NO WARRANTIES, CLAIMS OR REPRESENTATIONS–WHETHER EXPRESS, IMPLIED, OR STATUTORY–WITH RESPECT TO THIS DASHBOARD, INCLUDING, WITHOUT LIMITATION, WARRANTIES OF QUALITY, PERFORMANCE, NON-INFRINGEMENT, MERCHANTABILITY, ACCURACY, ADEQUACY, VALIDITY, RELIABILITY, COMPLETENESS, OR FITNESS FOR A PARTICULAR PURPOSE, AND DISAVOWS ANY REPRESENTATIONS OR WARRANTIES CREATED BY COURSE OF DEALING, COURSE OF PERFORMANCE, TRADE USAGE OR OTHERWISE. UNDER NO CIRCUMSTANCES WILL UVA HAVE ANY LIABILITY FOR ANY LOSS OR DAMAGE INCURRED AS A RESULT OF THE USE OF THIS DASHBOARD OR RELIANCE ON ANY INFORMATION PROVIDED HEREIN. THIS DASHBOARD MAY INCORPORATE DATA OR OTHER CONTENT BELONGING TO OR ORIGINATING FROM THIRD PARTIES. WE DO NOT WARRANT, ENDORSE, GUARANTEE, OR ASSUME RESPONSIBILITY FOR THE ACCURACY OR RELIABILITY OF ANY THIRD-PARTY INFORMATION.

Time Slider from *Jan 22 2020 to Aug 29 2020* Selected Date *Aug 29 2020*

1/22/2020 2/13/2020 3/6/2020 3/28/2020 4/19/2020 5/11/2020 6/2/2020 6/24/2020 7/16/2020 8/7/2020 8/29/2020

AF | AS | EU | NA | OC | SA | RESET

Counts | Per 100K | World | County

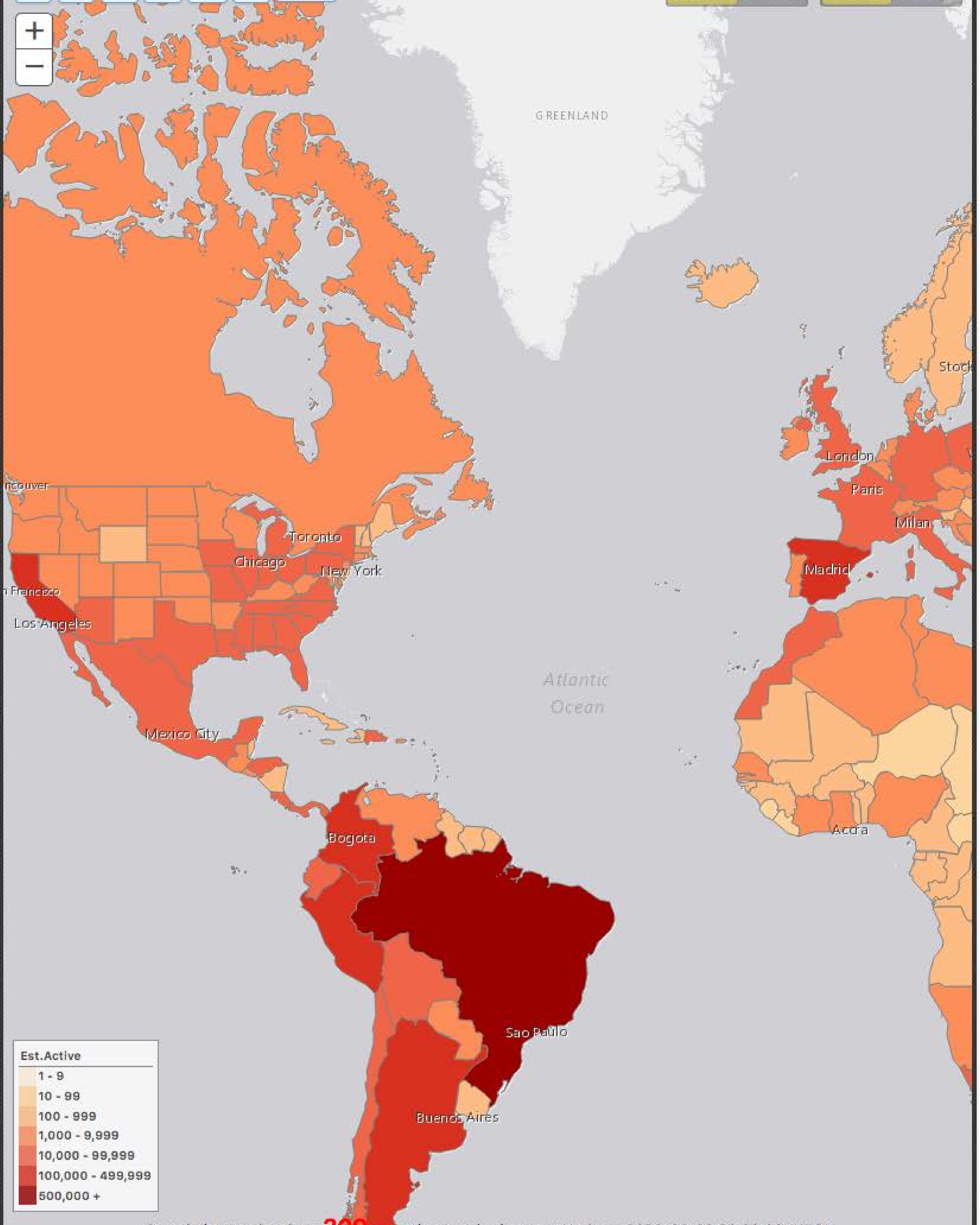

Cumulative number from 209 countries / territories. Last Update : 2020-08-29 20:00:00 (UTC) Esri, HERE | Esri, HERE

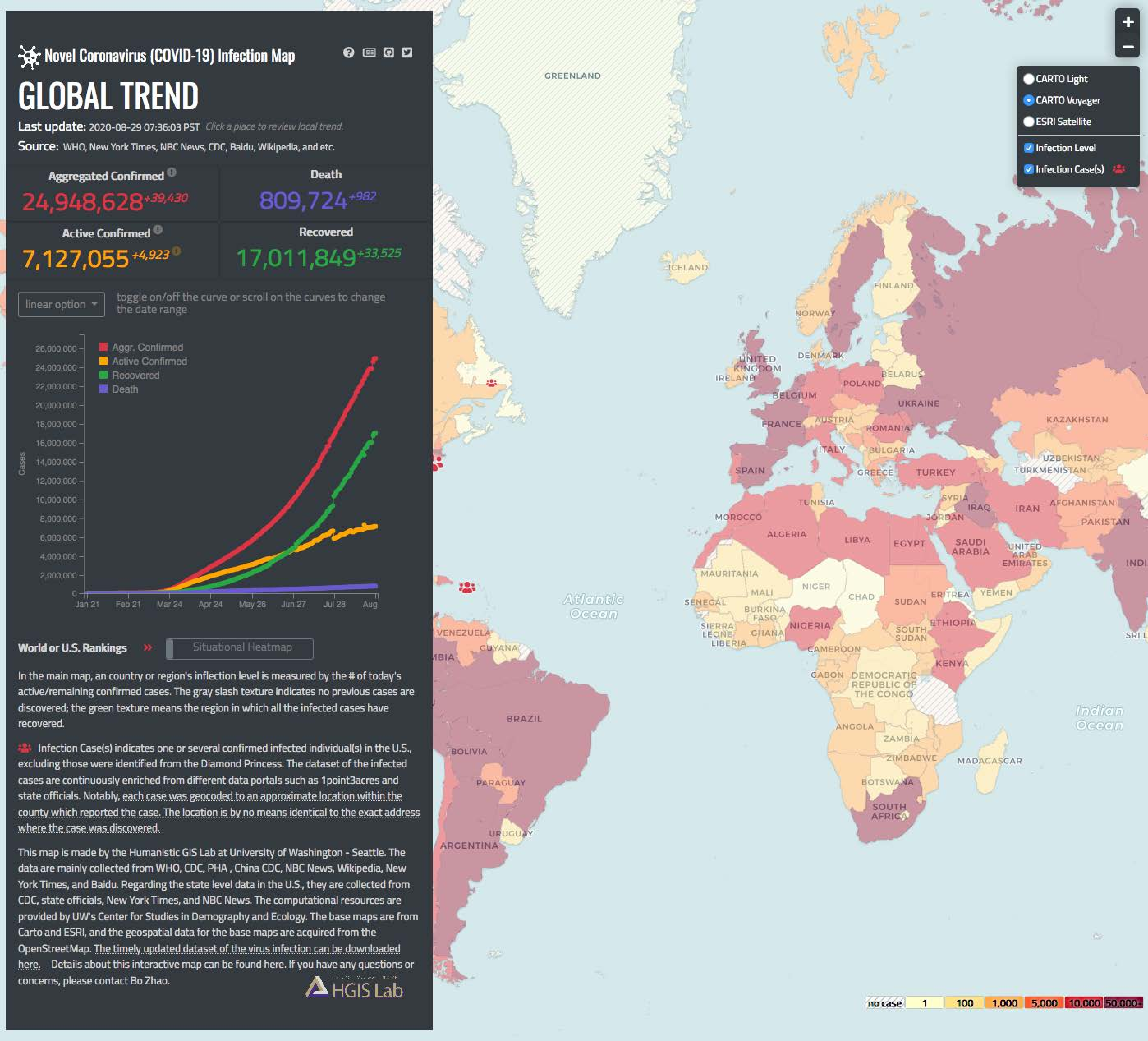
Novel Coronavirus (COVID-19) Infection Map
GLOBAL TREND
Last update: 2020-08-29 07:36:03 PST Click a place to review local trend.
Source: WHO, New York Times, NBC News, CDC, Baidu, Wikipedia, and etc.
Aggregated Confirmed
24,948,628 +39,430
Death
809,724 +982
Active Confirmed
7,127,055 +4,923
Recovered
17,011,849 +33,525
linear option
toggle on/off the curve or scroll on the curves to change the date range
Aggr. Confirmed
Active Confirmed
Recovered
Death
Cases
26,000,000
24,000,000
22,000,000
20,000,000
18,000,000
16,000,000
14,000,000
12,000,000
10,000,000
8,000,000
6,000,000
4,000,000
2,000,000
0
Jan 21
Feb 21
Mar 24
Apr 24
May 26
Jun 27
Jul 28
Aug
World or U.S. Rankings
Situational Heatmap
In the main map, an country or region's inflection level is measured by the # of today's active/remaining confirmed cases. The gray slash texture indicates no previous cases are discovered; the green texture means the region in which all the infected cases have recovered.
Infection Case(s) indicates one or several confirmed infected individual(s) in the U.S., excluding those were identified from the Diamond Princess. The dataset of the infected cases are continuously enriched from different data portals such as 1point3acres and state officials. Notably, each case was geocoded to an approximate location within the county which reported the case. The location is by no means identical to the exact address where the case was discovered.
This map is made by the Humanistic GIS Lab at University of Washington - Seattle. The data are mainly collected from WHO, CDC, PHA , China CDC, NBC News, Wikipedia, New York Times, and Baidu. Regarding the state level data in the U.S., they are collected from CDC, state officials, New York Times, and NBC News. The computational resources are provided by UW's Center for Studies in Demography and Ecology. The base maps are from Carto and ESRI, and the geospatial data for the base maps are acquired from the OpenStreetMap. The timely updated dataset of the virus infection can be downloaded here. Details about this interactive map can be found here. If you have any questions or concerns, please contact Bo Zhao.
HGIS Lab
CARTO Light
CARTO Voyager
ESRI Satellite
Infection Level
Infection Case(s)
GREENLAND
ICELAND
FINLAND
NORWAY
DENMARK
UNITED KINGDOM
IRELAND
BELARUS
POLAND
BELGIUM
UKRAINE
FRANCE
AUSTRIA
ROMANIA
KAZAKHSTAN
ITALY
BULGARIA
UZBEKISTAN
TURKMENISTAN
SPAIN
GREECE
TURKEY
TUNISIA
SYRIA
IRAQ
IRAN
AFGHANISTAN
MOROCCO
JORDAN
PAKISTAN
ALGERIA
LIBYA
EGYPT
SAUDI ARABIA
UNITED ARAB EMIRATES
INDI
MAURITANIA
MALI
NIGER
CHAD
SUDAN
ERITREA
YEMEN
Atlantic Ocean
SENEGAL
BURKINA FASO
SIERRA LEONE
LIBERIA
GHANA
NIGERIA
SOUTH SUDAN
ETHIOPIA
SRI L
VENEZUELA
GUYANA
CAMEROON
KENYA
GABON
DEMOCRATIC REPUBLIC OF THE CONGO
BRAZIL
Indian Ocean
ANGOLA
ZAMBIA
BOLIVIA
ZIMBABWE
MADAGASCAR
PARAGUAY
BOTSWANA
SOUTH AFRICA
URUGUAY
ARGENTINA
no case
1
100
1,000
5,000
10,000
50,000+

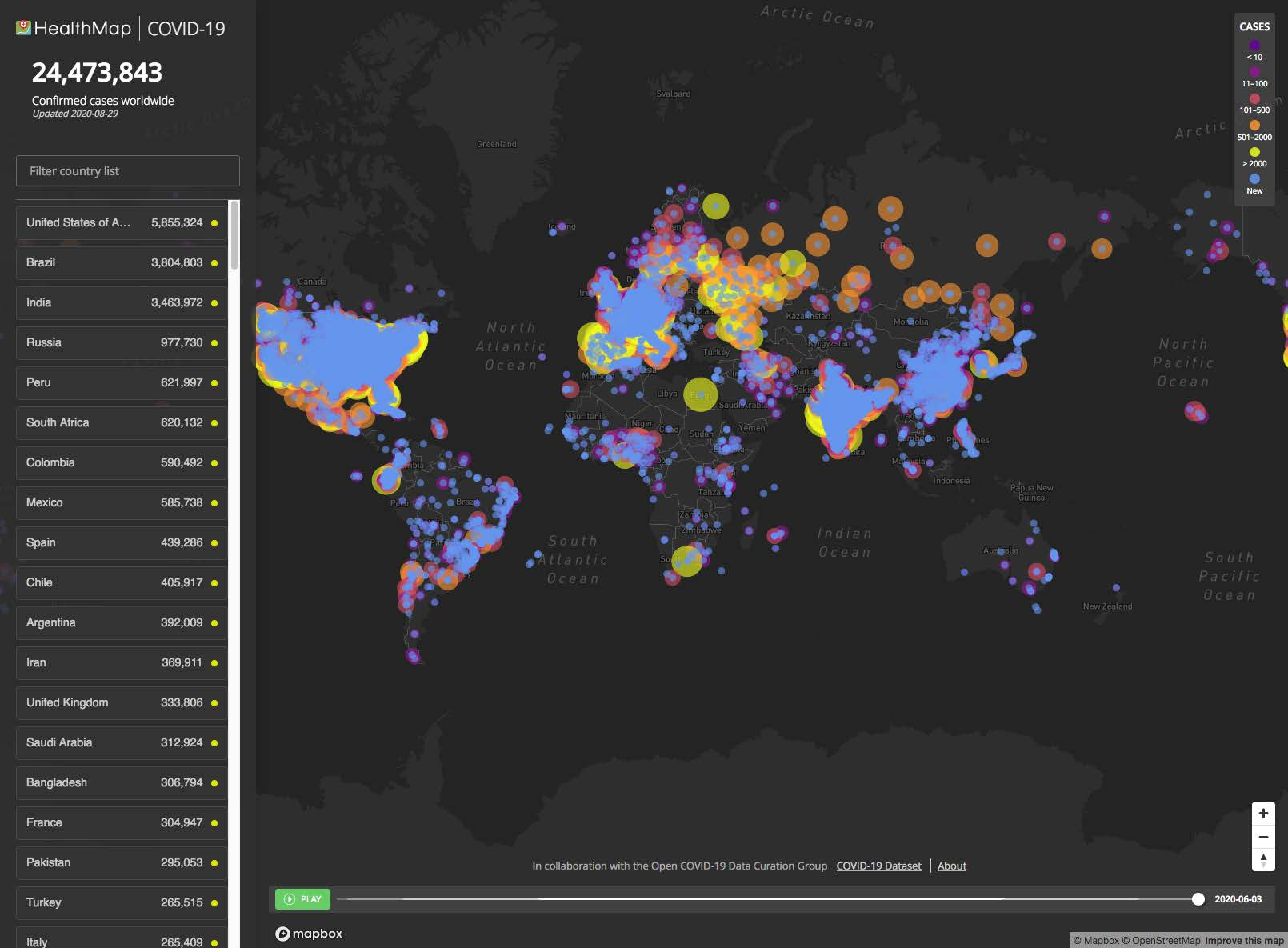
HealthMap | COVID-19
24,473,843
Confirmed cases worldwide
Updated 2020-08-29
Filter country list
United States of A... 5,855,324
Brazil 3,804,803
India 3,463,972
Russia 977,730
Peru 621,997
South Africa 620,132
Colombia 590,492
Mexico 585,738
Spain 439,286
Chile 405,917
Argentina 392,009
Iran 369,911
United Kingdom 333,806
Saudi Arabia 312,924
Bangladesh 306,794
France 304,947
Pakistan 295,053
Turkey 265,515
Italy 265,409
CASES
< 10
11–100
101–500
501–2000
> 2000
New
Arctic Ocean
Svalbard
Greenland
Arctic
Canada
North Atlantic Ocean
North Pacific Ocean
South Atlantic Ocean
Indian Ocean
South Pacific Ocean
Kazakhstan
Mongolia
Kyrgyzstan
Turkey
Libya
Saudi Arabia
Mauritania
Niger
Chad
Sudan
Yemen
Indonesia
Papua New Guinea
Australia
New Zealand
In collaboration with the Open COVID-19 Data Curation Group
COVID-19 Dataset
About
PLAY
2020-06-03
mapbox
© Mapbox © OpenStreetMap Improve this map

World Health Organization

Search by Country, Territory, or Area

Covid-19 Response Fund

Donate

WHO Coronavirus Disease (COVID-19) Dashboard

Data last updated: 2020/8/29, 3:44pm CEST

Overview | Data Table | Explore

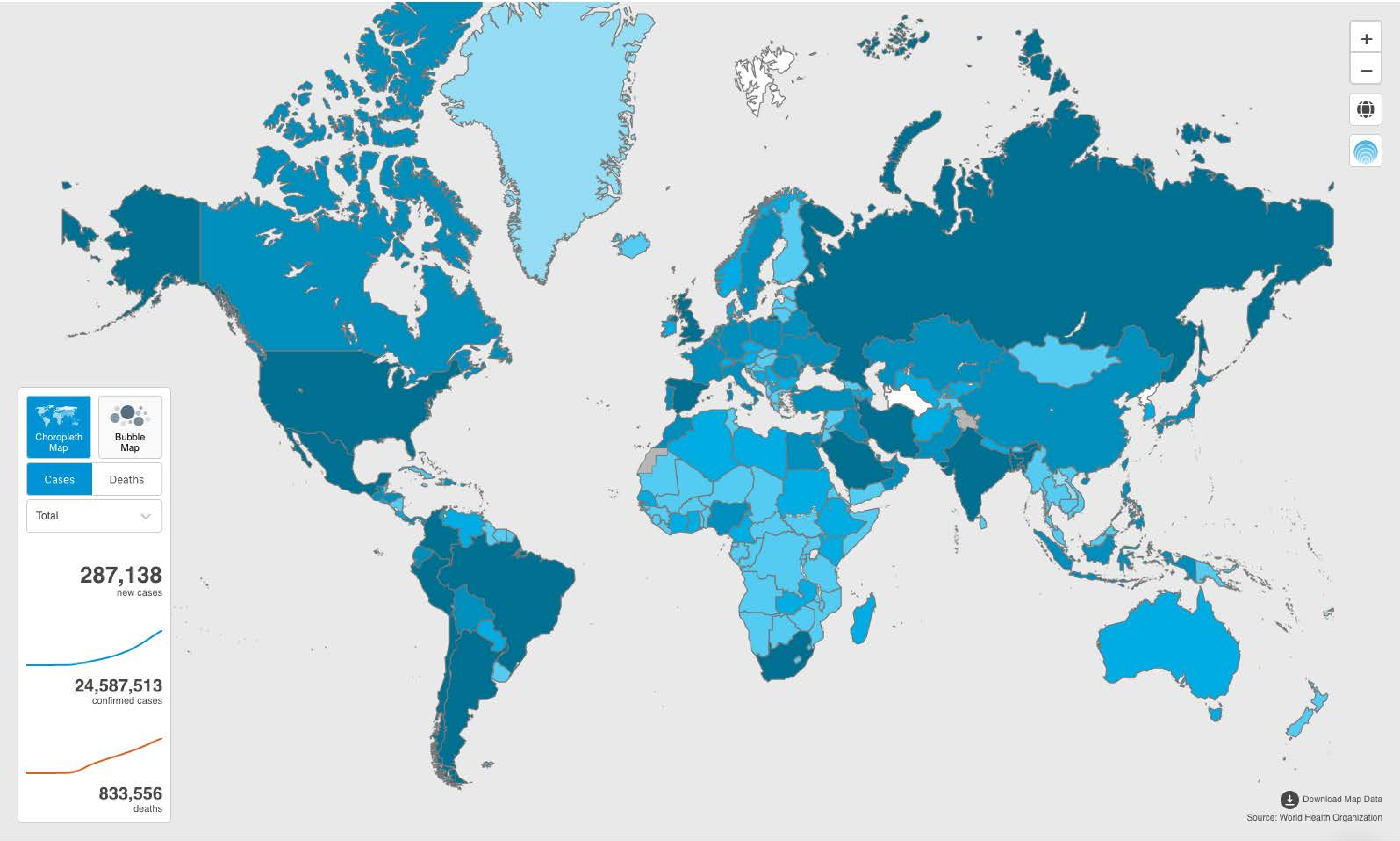

Globally, as of **3:44pm CEST, 29 August 2020**, there have been **24,587,513 confirmed cases** of COVID-19, including **833,556 deaths**, reported to WHO.

World Health Organization

Search by Country, Territory, or Area

Covid-19 Response Fund

Donate

WHO Coronavirus Disease (COVID-19) Dashboard

Data last updated: 2020/8/29, 3:44pm CEST

Overview | Data Table | Explore

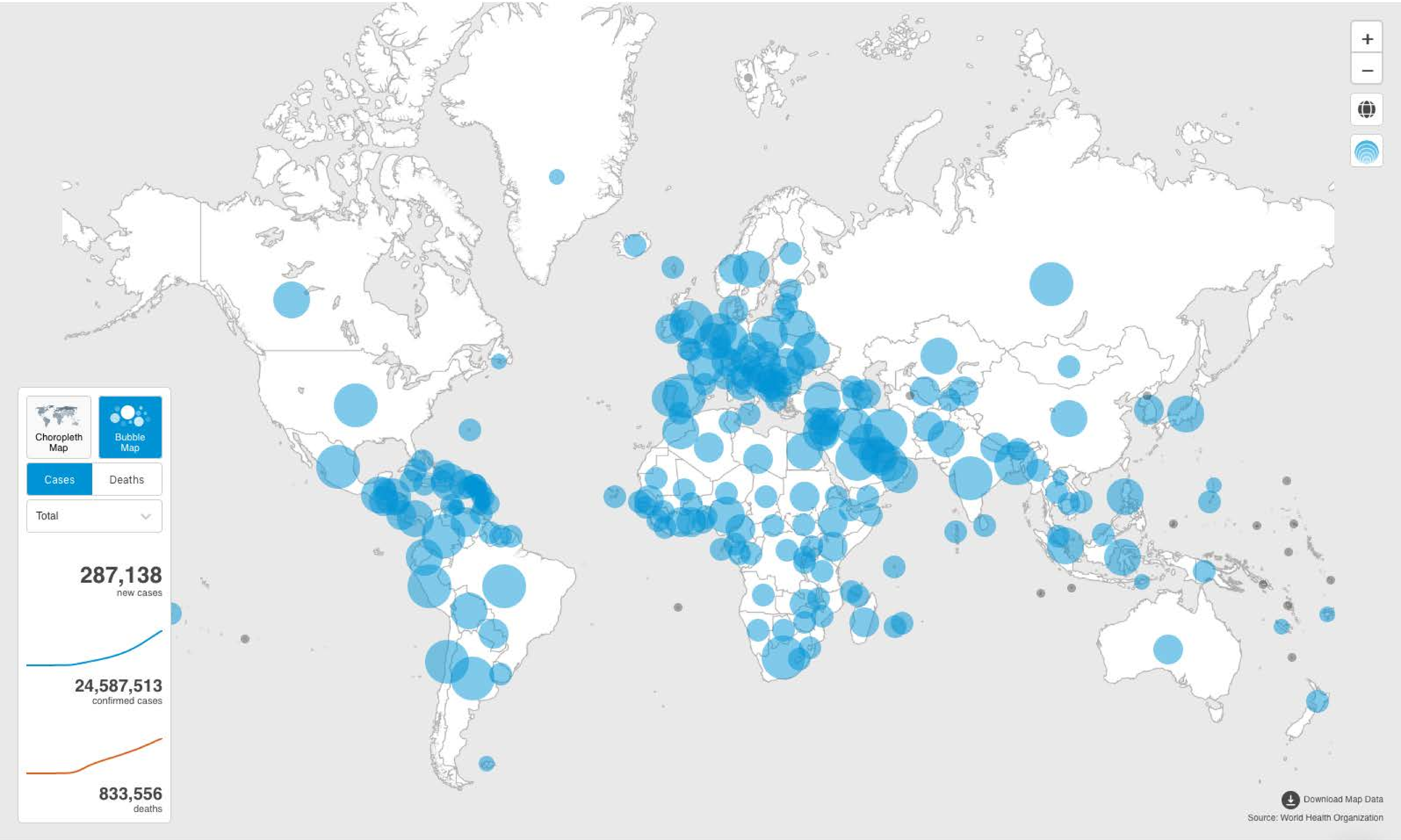

Globally, as of **3:44pm CEST, 29 August 2020**, there have been **24,587,513 confirmed cases** of COVID-19, including **833,556 deaths**, reported to WHO.

World Health Organization

Search by Country, Territory, or Area

Covid-19 Response Fund

Donate

WHO Coronavirus Disease (COVID-19) Dashboard

Data last updated: 2020/8/29, 3:44pm CEST

Overview Data Table Explore

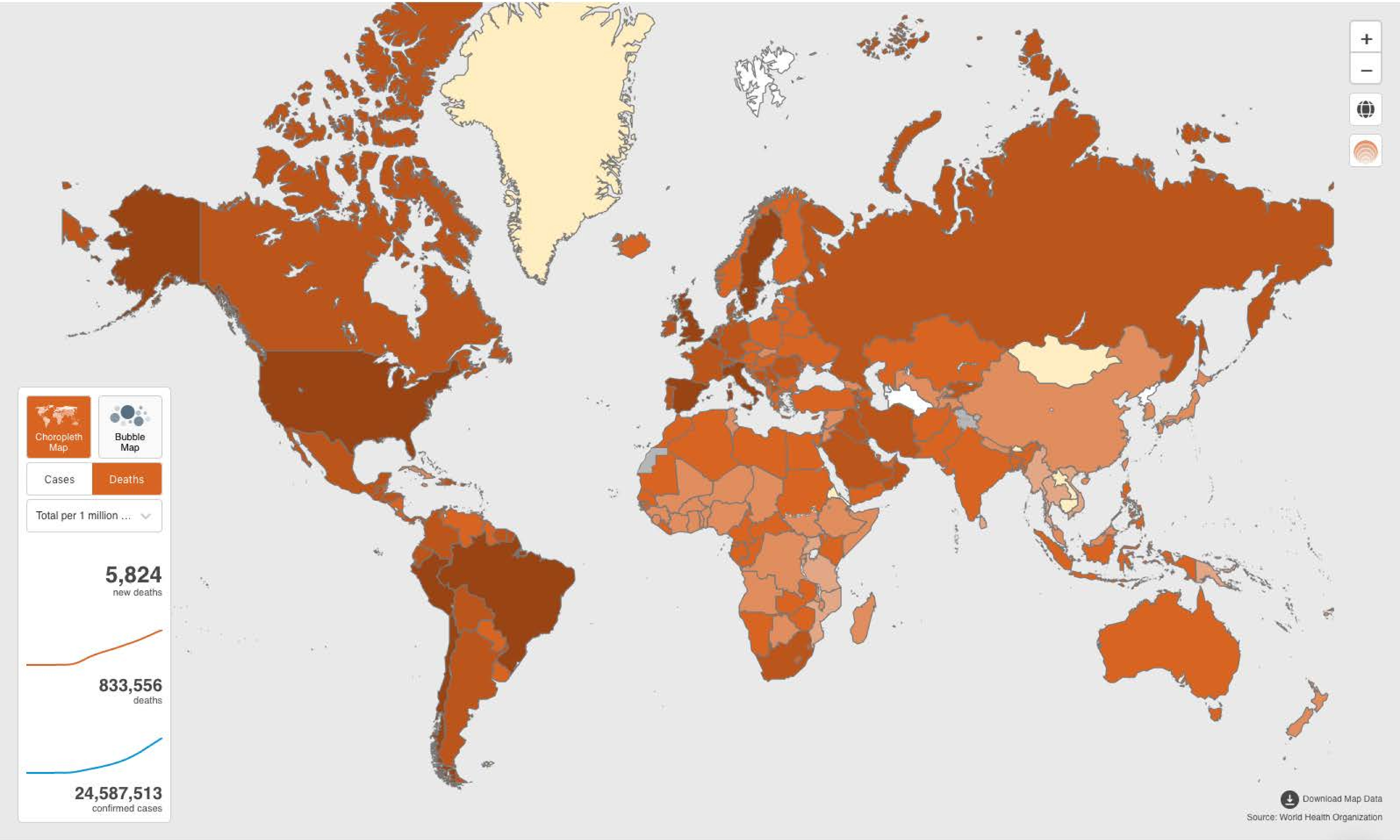

Globally, as of **3:44pm CEST, 29 August 2020**, there have been **24,587,513 confirmed cases** of COVID-19, including **833,556 deaths**, reported to WHO.

COVID-19: Confirmed Cases & Deaths by Country

as of 28 August, 2020

KFF

Overview | Analyze Trends | View Data Table | Government Actions

Total Cases

24,734,439

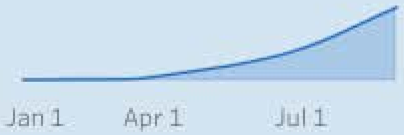

New Cases on 8/28/2020

283,107

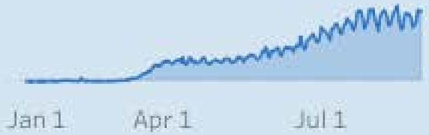

Total Deaths

837,122

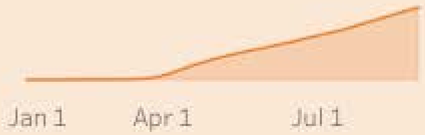

New Deaths on 8/28/2020

5,538

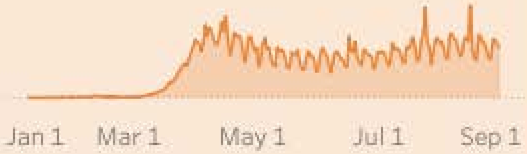

Select Metric Cases

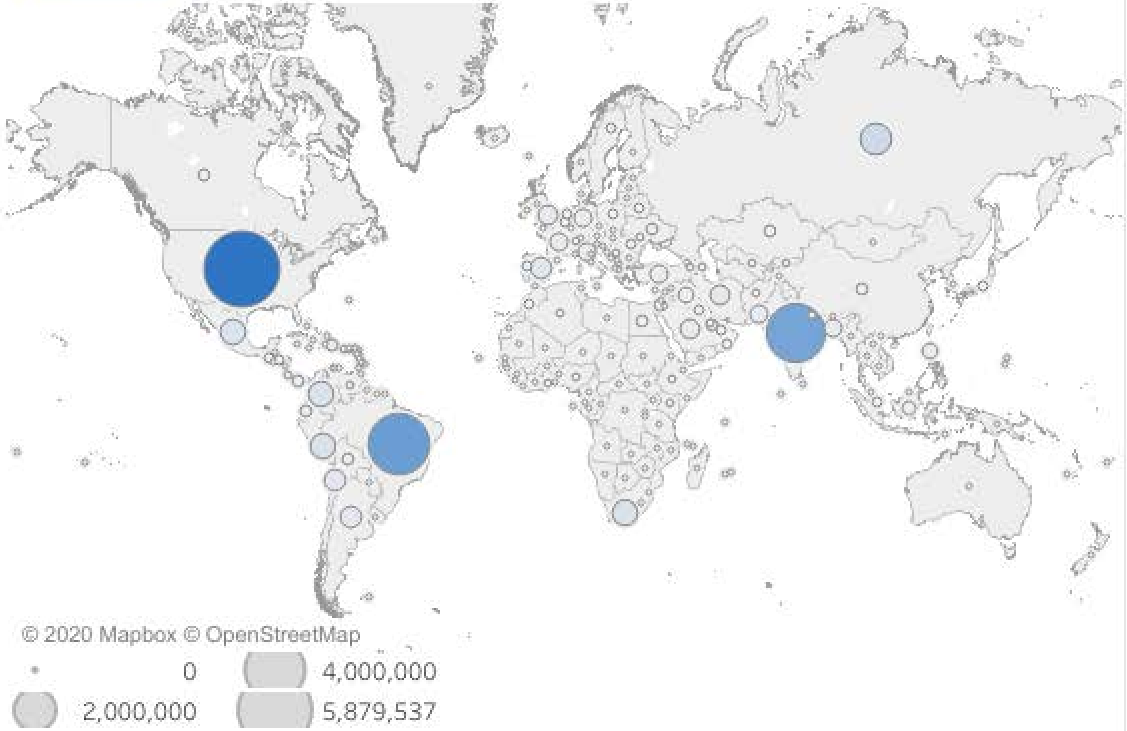

Select a Country to the left to highlight it on the chart below

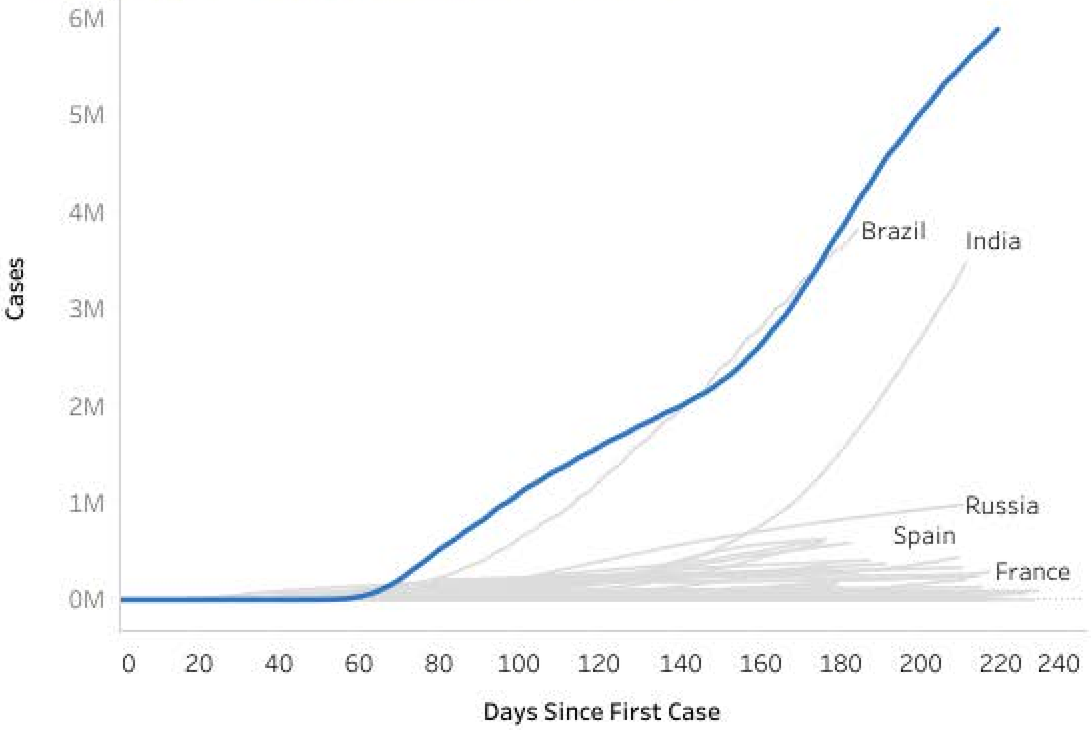

Source: Johns Hopkins University (JHU) Coronavirus Resource Center; last updated with data from 8/28/2020. Data prior to January 22, 2020 are from the World Health Organization's (WHO) Coronavirus disease (COVID-2019) situation reports. Notes: Cumulative case totals include both laboratory confirmed and clinically diagnosed cases; prior to February 14, 2020, totals include only laboratory confirmed cases. Japan's totals include cases that have been identified on the Diamond Princess cruise ship (except in cases that have been re-categorized by a reporting country).

Our World in Data | Articles by topic | Search... | Latest | About | Donate
All charts | Sustainable Development Goals Tracker

Statistics and Research

Coronavirus Pandemic (COVID-19)

Research and data: Hannah Ritchie, Esteban Ortiz-Ospina, Diana Beltekian, Edouard Mathieu, Joe Hasell, Bobbie Macdonald, Charlie Giattino, and Max Roser
Web development: Breck Yunits, Ernst van Woerden, Daniel Gavrilov, Matthieu Bergel, Shahid Ahmad, and Jason Crawford

We are grateful to everyone whose editorial review and expert feedback on this work helps us to continuously improve our work on the pandemic. Thank you. Here you find the acknowledgements.

The data on the coronavirus pandemic is updated daily. Last update: **August 29, 2020** (10:30, London time).

Reuse our work freely | Cite this research

You can **download** our complete – daily updated – Our World in Data COVID-19 database.

What is the global situation now?

Only if we end the pandemic everywhere can we end the pandemic anywhere. The entire world has the same goal: cases of COVID-19 need to go to zero.

The chart below shows which countries are making progress to this goal and which are not.

The trajectories show the daily number of confirmed cases. But the widely available data on confirmed cases only becomes meaningful when it can be interpreted in light of how much a country is testing. This is why Our World in Data built the global database on COVID-19 testing and the line colors in this chart show whether a country is testing adequately or not.

A country is not testing adequately when it is finding a case for every few tests they perform. Here it is likely that the true number of new cases is much higher than the number of cases that were confirmed by tests. When the *positive rate* of tests is high the line is shown in shades of red.

Blue lines mean that a country does many tests for each case it finds; the testing effort in these countries is adequate.

→ We explain this chart and the metrics in much more detail further below in the section 'The chart to monitor the fight against the global pandemic'.

To be safe anywhere, every region in the world needs to make progress against the pandemic – and this means dark blue lines hitting zero.

Progress is possible – some countries bent the curve of new cases and are monitoring the outbreak well. But globally we are very far from the goal and the global number of confirmed cases is rising extremely fast.

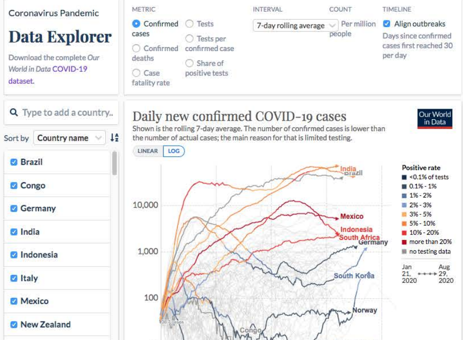

→ Open the Data Explorer in a new tab.

Is it possible to make progress against the pandemic?

IN THIS SECTION
↓ Coronavirus Country Profiles
↓ Deaths due to COVID-19

Two questions guide this daily updated publication on the global COVID-19 pandemic:

- How can we make progress against the pandemic?
- And, are we making progress?

To answer these questions we need data. But data is not enough. This is especially true in this pandemic because even the best available data is far from perfect. Much of our work therefore focuses on explaining what the data can – and can not – tell us about the pandemic.

Our goal is two-fold:

1. To **provide reliable, global and open data and research** on how the COVID-19 pandemic is spreading, what impact the pandemic has, how we can make progress against the pandemic, and whether the measures countries are taking are successful or not;
2. And to **build an infrastructure** that allows research colleagues – and everyone who is interested – to navigate and understand this data and research.

Before we study *how* to make progress we should consider the more basic question: is it possible to do so?

The answer is very clear: While some countries have failed in their response to the pandemic, others met the challenge much more successfully. Perhaps the most important thing to know about the pandemic is that *it is possible to fight the pandemic.*

Responding successfully means two things: limiting the *direct* and the *indirect* impact of the pandemic. Countries that have responded most successfully were able to avoid choosing between the two: they avoided the trade-off between a high mortality and a high socio-economic impact of the pandemic. New Zealand has been able to bring infections down and open up their country internally. Other island nations were also able to almost entirely prevent an outbreak (like Taiwan, Australia, and Iceland). But not only islands were able to bend the curve of infections and prevent large outbreaks – Norway, Uruguay, Switzerland, South Korea, and Germany are examples. These countries suffered a smaller direct impact, but they also limited the indirect impacts because they were able to release lockdown measures earlier.

Together with colleagues at the Robert Koch Institute, the Chan School of Public Health, the UK Public Health Rapid Support Team, the London School of Hygiene and Tropical Medicine and other institutions we study countries that responded most successfully in detail.

The point of this work is to understand those countries so that the rest of the world can learn from them. We have published three country specific studies:

- Emerging COVID-19 success story: **Vietnam**'s commitment to containment
- Emerging COVID-19 success story: **Germany**'s strong enabling environment
- Emerging COVID-19 success story: **South Korea** learned the lessons of MERS

Together with epidemiologists Anna Seale, Dave Kennedy, and Daniel Bausch we wrote this introduction to the Pandemic Exemplar project. We will continue this work over the course of the pandemic so that the world can learn from those countries that are most successful in their fight against the pandemic.

Coronavirus Country Profiles

We built 207 country profiles which allow you to explore **the statistics on the coronavirus pandemic for every country in the world.**

In a fast-evolving pandemic it is not a simple matter to identify the countries that are most successful in making progress against it. Excess mortality and the rate of the confirmed deaths is what we focus on in the sections below, but for a fuller assessment a wider perspective is useful. For this purpose we track the impact of the pandemic across our publication and we built country profiles for 207 countries to study **the statistics on the coronavirus pandemic for every country in the world** in depth.

Each profile includes **interactive visualizations, explanations** of the presented metrics, and the details on the **sources of the data.**

Every country profile is **updated daily.**

Search for a country...

Our 12 most visited country profiles

United States	United Kingdom	Germany	India
Spain	France	Brazil	Canada
Australia	New Zealand	Sweden	Mexico

Every profile includes four sections:

1. **Deaths:** How many deaths from Coronavirus have been reported? Is the number of deaths still increasing? How does the death rate compare to other countries?
2. **Testing:** How much testing for coronavirus do countries conduct? When did they start and how does it compare with other countries?
3. **Cases:** How many cases were confirmed? How many tests did a country do to find one COVID-19 case? And is your country bending the curve?
4. **Government responses:** What measures did countries take in response to the pandemic?

Deaths due to COVID-19

While the Country Profiles provide the in-depth perspective, the chart shown below provides the global, comparative perspective. The trajectories of the death counts make clear just how very differently different countries were impacted.

The *steepness* of the slope shows how rapidly the death count was rising at a particular moment in the outbreak. The *level* shows how many people were confirmed to have died due to COVID-19 in each country.

Among the countries with the highest death toll are some of the most populous countries in the world such as the US, Brazil, and Mexico. If you prefer to adjust for the differences in population size you can switch to per capita statistics by clicking the 'per million people' tickbox.

We can see three different ways in which the pandemic has affected countries:

- Some countries have not been able to contain the pandemic. The death toll there continues to rise quickly week after week.
- Some countries saw large outbreaks, but then 'bent the curve' and brought the number of deaths down again. Italy, Germany, and many European countries followed this trajectory.
- Some were able to prevent a large outbreak altogether. Shown in the chart are South Korea and Norway. These countries had rapid outbreaks, but were then able to reduce the number of deaths very quickly to low numbers.

While some commentaries on the pandemic have the premise that all countries failed to respond well to the pandemic the exact opposite stands out to us: Even at this early stage of the pandemic we see very large differences between countries – as the chart shows. While some suffer terrible outbreaks others have managed to contain rapid outbreaks or even prevented bad outbreaks entirely. It is possible to respond successfully to the pandemic.

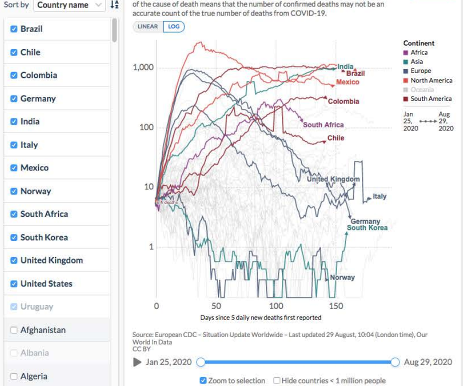

→ Open the Explorer in a new tab.

In-depth: The Data on confirmed deaths

Detailed information about the data sources for these death statistics, the definition of what is counted as a death from COVID-19, limitations of cross-country comparisons, and many additional detailed charts can be found in our page dedicated to deaths from the coronavirus.

How many people have died from the coronavirus disease?
Coronavirus Deaths →

In-depth: The mortality risk from COVID-19

If someone is infected with COVID-19, how likely is that person to die? In our page dedicated to the mortality risk we explain why it is difficult to answer this question and what we do know – we explain the difference between the infection and the case fatality rate, explain common mistakes in interpreting mortality statistics, and we report the case fatality rate for countries around the world – as always we update it daily.

What do we know about the risk of dying from COVID-19? And what is still unknown?
Mortality Risk of the Coronavirus Disease →

In-depth: Excess mortality

Excess mortality refers to the number of deaths *from all causes* above and beyond what we would have expected to see under 'normal' conditions.[1] In this case, we're interested in how deaths during the COVID-19 pandemic compare to the average number of deaths over the same period in previous years.

Looking at excess mortality is helpful for understanding the total impact of the pandemic on deaths – both direct and indirect. It helps us understand the direct impact by capturing deaths caused by COVID-19 that were not correctly diagnosed and reported, for example because no test for the virus was conducted. It helps us understand the indirect mortality impact by capturing the many ways in which the pandemic has affected health systems and living conditions. For example, if the pandemic overwhelms health systems or diverts resources away from other health problems, more people may die from non-COVID causes than we would expect. Excess mortality is thus a more comprehensive measure of the impact of the pandemic on deaths than the confirmed COVID-19 death count alone.

The chart here shows excess mortality during the pandemic as the *percentage difference* between the number of weekly deaths in 2020 and the average number of deaths in the same week over the previous five years (2015–2019) – a measure called the P-score.[2]

Because the P-score measures percentage difference *within* a country, it allows for direct comparisons *across* countries. You can see from the chart that some countries – such as the UK and Spain – suffered high levels of excess mortality, while others – such as Germany and Norway – experienced much more modest increases in mortality.

The P-scores shown here combine all ages and do not account for differences in mortality risk by age and countries' age distributions.

How do excess mortality P-scores compare when **grouping by age**?
Excess mortality during COVID-19, by age →

You can learn in more depth about the P-score and other measures of excess mortality and their comparability across countries in our work with John Muellbauer and Janine Aron.

Our in-depth article on measuring excess mortality:
A pandemic primer on excess mortality statistics and their comparability across countries →

Our main page covering excess mortality:
Excess mortality during the Coronavirus Pandemic →

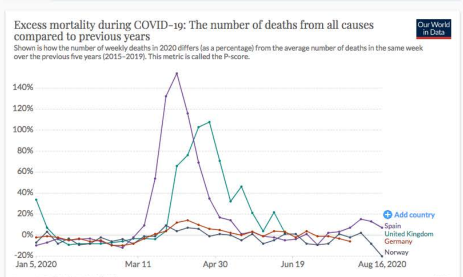

Beyond disease and deaths: the indirect impact of the pandemic

The COVID-19 pandemic could be (hopefully!) one of the most impactful events in our lifetime. That is because the direct and indirect impacts of COVID-19 are so extraordinarily large.

Once a country experiences a rapid outbreak of the disease it can only respond with one of two bad alternatives: leaving the pandemic unchecked would mean that a very large share of the population would get sick and many would die. But the *indirect impacts* that result from the alternative – the containment measures of shutting down public life – also lead to widespread suffering: these include higher unemployment, lower production (and therefore increasing rates of poverty), and likely also higher mortality from other causes.

To properly monitor the impact of the pandemic we would need **metrics that are unfortunately not available:** the total number of deaths due COVID-19 and the metrics that capture the many ways the pandemic indirectly impacts our lives.

The goal of researchers – in our team and elsewhere – is to work towards these ideal measures. In the meantime we have to learn from **the metrics that are available currently:** the number of confirmed deaths due to COVID-19, excess mortality, and the early results of the ongoing research on the pandemic's indirect impacts.

To capture both of these are ongoing efforts of our work. Here at *Our World in Data* we aim to provide a broad perspective on the living conditions of people around the world – from poverty, to education, to human rights, and health more broadly – look at the full list of the topics we work on. In this project, the impact of the pandemic will be one of our focus areas as we move forward.

For example, if a country had a P-score of 100% in a given week in 2020, that would mean the death count for that week was 100% higher than – that is, double – the average death count in the same week over the previous five years.

Which countries are making progress against the pandemic?

To end the Coronavirus pandemic, we have a clear and simple goal: cases need to go to zero everywhere.

Viruses don't respect borders – even the 1918 influenza pandemic reached remote islands within months, and that was long before the days of global air travel. It is therefore *the entire world* that needs to make progress against the virus if we want to prevent a situation where countries either need to lock themselves off from the rest of the world or suffer recurring COVID-19 outbreaks.

Only if we end the pandemic everywhere can the pandemic end anywhere.

The chart to monitor the fight against the global pandemic

If the *total* number of cases was known it would be straightforward to build a good visualization to monitor the pandemic. We would simply report new cases over time – a chart that the literature refers to as the *epidemic curve*, often shortened to *epi curve*.

However, with the COVID-19 pandemic we are in the unfortunate situation that the number of total cases is not known. Only a fraction of total cases – those confirmed by a test – is known.

This means that we need to be able to monitor the number of confirmed cases and the testing that a country does. Because no academically sound organization built a database on testing we here at Our World in Data spend much of our work on building the required international testing database that allows us to make sense of the available counts of confirmed cases.

Below we will explain in more detail what we can learn from this chart, but the quick summary of this chart is that each line in this chart shows you the new confirmed cases over time – that part of the chart is simply the classic epi curve – while the line color shows you the quality of the data at each point in time: the rate of tests that are positive. Darker shades of blue indicate a low positive rate, which means that a country is testing very widely so that the confirmed case count is likely closer to the true total number of cases.

The chart allows everyone to monitor whether the world is successful in the fight against the pandemic: what the world needs to achieve is that all lines turn into dark blue and hit zero.

Related charts:

What does the same chart look like in ***per capita*** terms?
Cases per capita and the positive rate →

When did countries bend the curve?
Daily new cases vs cumulative cases →

When did countries bend the curve?
In *per capita* terms: Daily new cases vs cumulative cases →

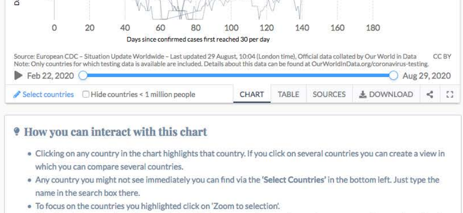

How you can interact with this chart

- Clicking on any country in the chart highlights that country. If you click on several countries you can create a view in which you can compare several countries.
- Any country you might not see immediately you can find via the **'Select Countries'** in the bottom left. Just type the name in the search box there.
- To focus on the countries you highlighted click on 'Zoom to selection'.
- The default view on a logarithmic y-axis is helpful to compare the growth rates between countries: on a logarithmic axis the steepness of the line corresponds to the growth rate. [Here is an explanation for how to read logarithmic axes.]
- But in this chart – as in many of our charts – you can switch to a linear axis – just click on 'Linear' in the top left.

Why are not all countries included in this chart?

- For a country to be included in this chart, we require data on testing for this country (here is our testing database – all sources are documented there and we report for which countries we were not able to find testing data.)
- If you are interested in the number of confirmed cases for a country not shown here, have a look at the Data Explorer, you'll find the data there.

Case counts are only meaningful if we also know how much *testing* a country does

As the chart title says, the epi curve for each country shows the number of *confirmed* cases only: those cases that were confirmed by a *laboratory test.*

Confirmed cases are only a fraction of the number of *total cases.* The question is: how big of a fraction of the total number of cases are confirmed? This is not known, but we can get an indication by looking at the extent of testing.

How big a fraction of total cases get confirmed depends on how much a country actually tests. To understand the spread of the disease we need to interpret the number of cases – the epidemic curve – in light of how much testing for COVID-19 the country actually does.

For this reason we at *Our World in Data* built a global database on testing. We made it available for everyone – epidemiologists, the WHO, the UN, and many governments rely on our database for their daily work. We update it continuously.

When we decided to build the testing database we did so because we wanted to build this chart: one that brings together the epi curve with the relevant information about how much a country actually tests to allow everyone to monitor the quality of the reported data. We can only include countries in this chart for which we have data on testing. You can find the complete overview – including a detailed description of each source – in our testing database here. Our data covers currently 66% of the world population.

The rate of positive tests: the line color indicates the reliability of the case count

Just as it is not informative to look at case counts in isolation it is also not informative to look at the number of tests in isolation. We need to see the number of tests in relation to the size of the outbreak: countries with large outbreaks need to do much more testing to monitor the spread of the pandemic than countries where the disease is under control. See the work of our colleague Joe Hasell who looked at this in detail.

Based on this principle we calculate a quality metric for the case counts that answers the question: what share of tests in a particular country confirm a case of COVID-19?

This metric is referred to as the positive rate or positivity rate.[3]

The world map here shows this data – it is exactly the same data shown as the line color in the chart above.

By moving the time-line below the map you can see how this metric has changed around the world; by clicking on a country you can see how it changed in every country.

The world map shows enormous differences across countries:

- Some countries, like Australia, South Korea and Slovenia do hundreds, or even thousands of tests for each case they find – the positive rate of these countries is therefore below 1% or even 0.1%.
- Others, such as Mexico, Nigeria, and Bangladesh do very few tests – five or fewer – for every confirmed case. Their positive rate is very high.

Countries that do very few tests per confirmed case are unlikely to be testing widely enough to find all cases. The WHO has suggested a positive rate lower than 10% – but better lower than 3% – as a general benchmark of adequate testing.[4]

The countries that have a positive rate below 3% are shown in shades of blue. Those that have a positive rate higher than 5% are shown in shades of red.

In countries that test very little in relation to their outbreak – shown in shades of red in the chart – many cases are likely to go unreported. In these countries, the number of confirmed cases indicated may represent only a fraction of the total number of cases.

Only countries for which we have data on testing are included in this world map (here is our testing database – all sources are documented there and we report for which countries we were not able to find testing data.)

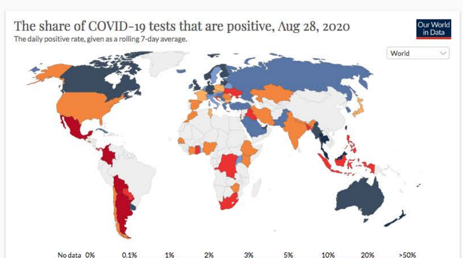

You can also think of this quality metric the other way around:
The number of tests per one confirmed case →

What we learn from this chart: Some countries bent the curve early and monitor the outbreak well, others not

What stands out from this chart is that the differences between countries are indeed very large. In the charts below we highlight countries that make these differences especially clear.

- The data for Slovakia, Thailand, New Zealand, South Korea, and Germany shows that these countries monitored the outbreak well from the start or caught up rapidly after an initial outbreak. Eventually they were able to bend the curve and bring down the number of confirmed cases, while increasing the ratio of tests to confirmed cases. These are not the only countries that achieved this; you can add for example Austria, Iceland, Slovenia, Tunisia, or Latvia to the chart and you will find similar trajectories.
- The data for Brazil, Mexico, the United States, Panama, India, Pakistan, South Africa, and Nigeria shows that these countries test little relative to the size of the outbreak. Additionally these countries report unfortunately still very high daily case counts – their lines are red and far from zero.

In the early phase of the outbreak there was not always a big difference between these two groups, but while the first group of countries managed to catch up with the outbreak, the second one did not achieve this yet.

You can of course select other countries too. By hovering over the epi curves – or finding a country via 'Select countries' in the bottom left – you can see how the testing relative to the size of the outbreak has changed over time for each country.

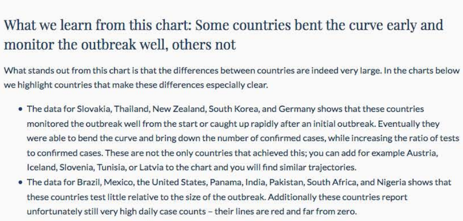

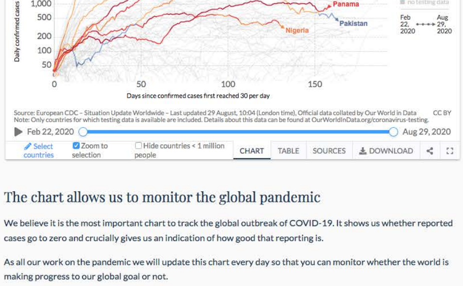

The chart allows us to monitor the global pandemic

We believe it is the most important chart to track the global outbreak of COVID-19. It shows us whether reported cases go to zero and crucially gives us an indication of how good that reporting is.

As all our work on the pandemic we will update this chart every day so that you can monitor whether the world is making progress to our global goal or not.

To be safe anywhere, every region in the world needs to make progress against the pandemic – and this means dark blue lines hitting zero. Currently we are far from it.

But just as the chart before that focused on confirmed deaths, it is clear that a rapid, devastating outbreak is not the fate that countries inevitably face. We need to look at the countries that responded most successfully and learn what allows to make progress against the pandemic.

4,291,751,158 Views

DXY · DX Doctor

COVID-19
Global Pandemic Real-time Report

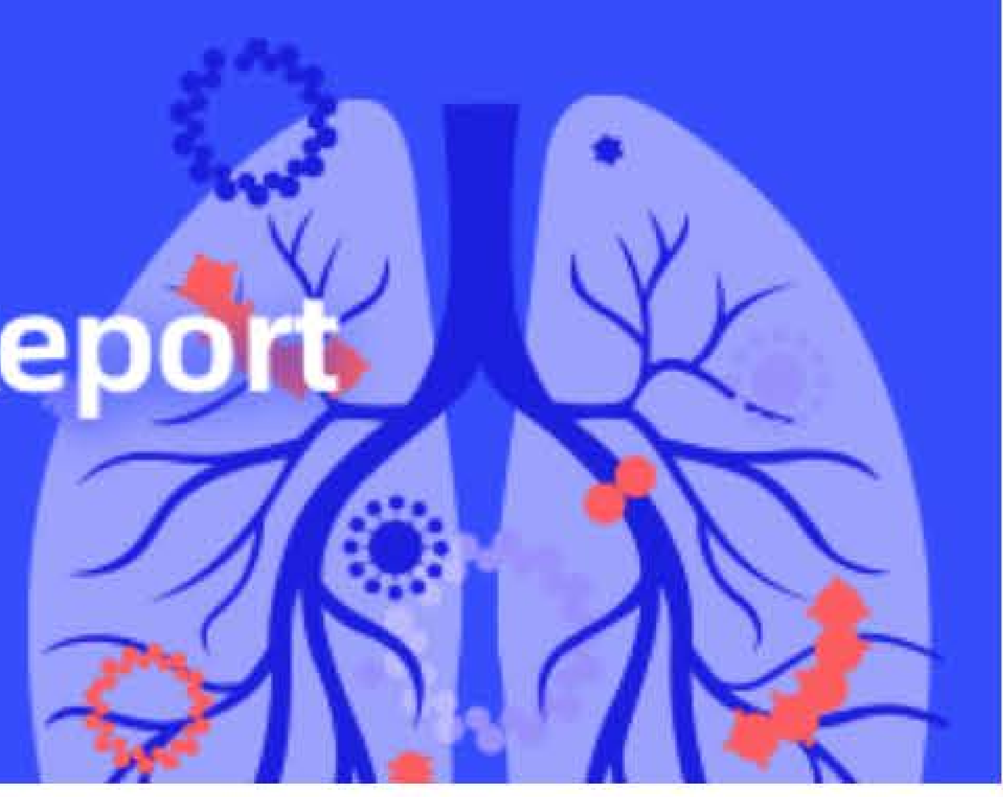

⇄ 切换到中文版

Global Mapping | COVID-19 Knowledge

Global statistics by UTC+8 2020-08-30 08:33

ⓘ Data explanations

8,297,789	24,938,525	841,058	15,799,678
Active Cases	Total Confirmed	Deaths	Recovered

COVID-19 Global Pandemic Trend

Before Mar. 19, data source: WHO. Time zone: CET 10 AM.
After Mar. 19, data source: WHO, JHU CSSE, Local Media and DXY. Time zone: UTC 10 AM.
Since April 24th, the Spanish Ministry of Health has adjusted the statistical standard of confirmed Covid-19 data, only counting those confirmed by PCR tests.

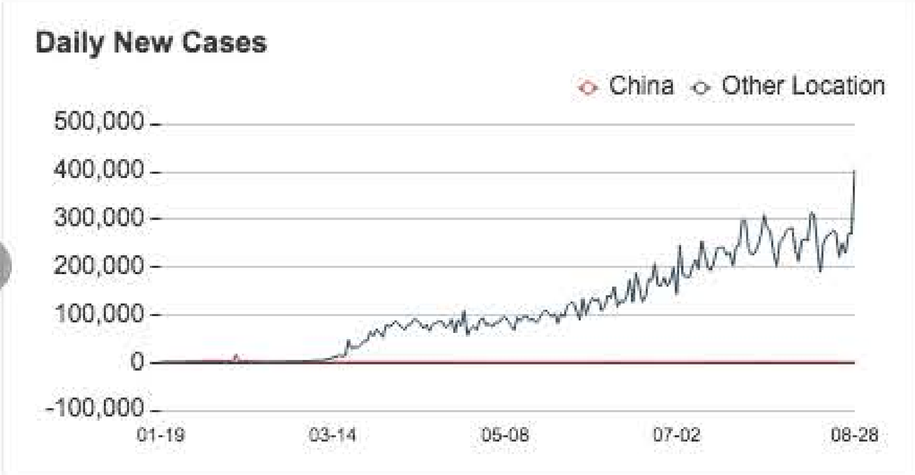

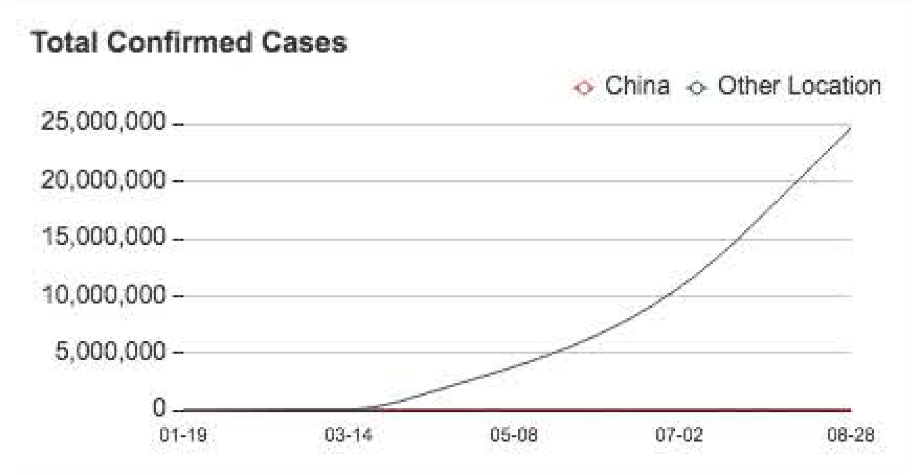

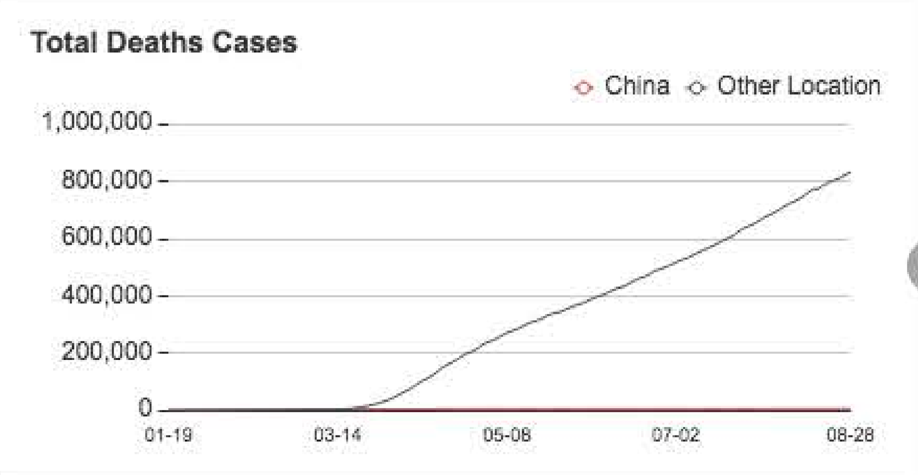

Daily New Cases | Total Confirmed Cases | Total Deaths Cases

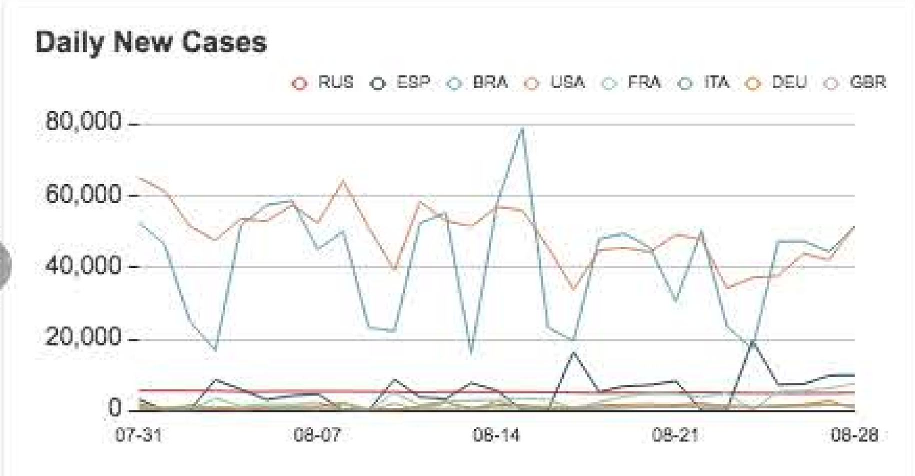

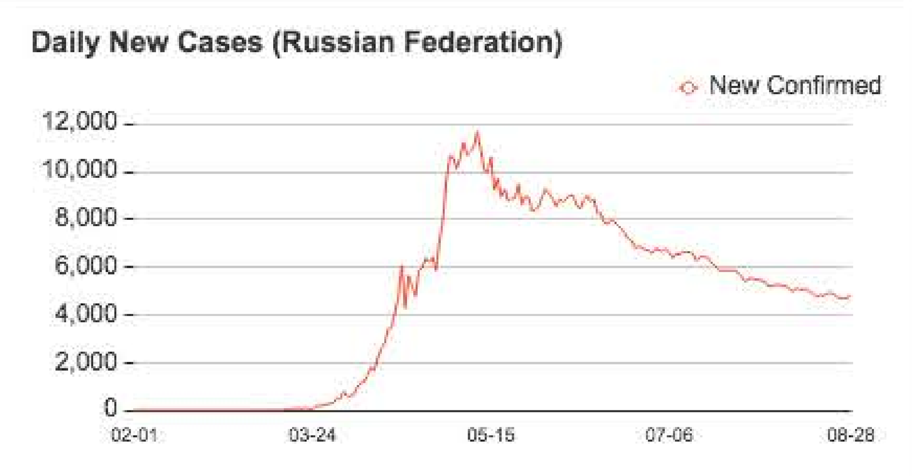

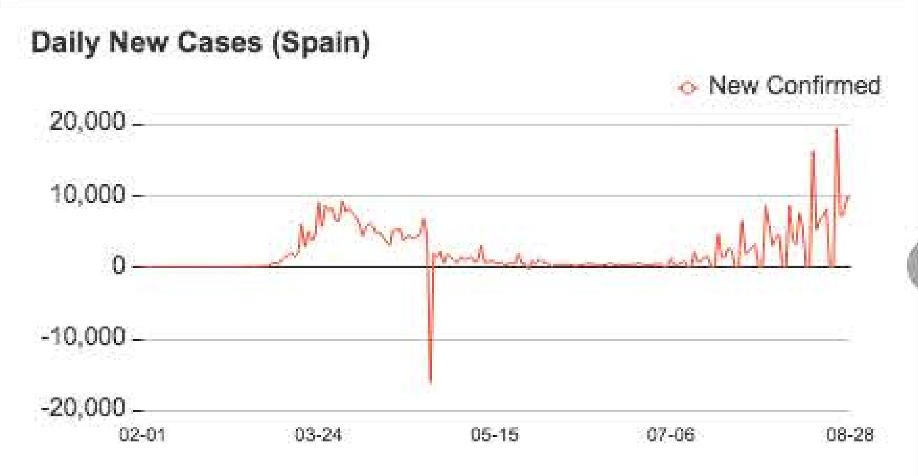

Daily New Cases | Russian Federation | Spain | Brazil
United States of America | France | Italy | Germany
The United Kingdom

Country/Region/ Territory	Active Cases	Total Confirmed	Total Deaths	Total Recovered	More
▼ **North America(40)**	**4,114,279**	**7,127,666**	**265,639**	**2,747,748**	
United States of America	3,635,161	5,958,486	182,711	2,140,614	More ›
Mexico	117,925	585,738	63,146	404,667	More ›
Guatemala	67,089	72,921	2,709	3,123	More ›
Honduras	54,099	57,669	1,803	1,767	More ›
Panama	53,903	89,982	1,948	34,131	More ›
Show more ⌄					
▼ **Europe(49)**	**1,337,481**	**3,530,367**	**207,345**	**1,985,541**	
The United Kingdom	290,715	332,752	41,498	-	More ›
Spain	259,899	439,286	29,011	150,376	More ›
Russian Federation	163,938	985,346	17,025	804,383	More ›
France	155,751	272,530	30,602	86,177	More ›
Sweden	76,280	87,072	5,821	4,971	More ›
Show more ⌄					
▼ **Asia(46)**	**1,284,764**	**6,837,507**	**138,868**	**5,413,875**	
India	752,424	3,463,973	62,550	2,648,999	More ›
Bangladesh	105,856	308,925	4,206	198,863	More ›
Philippines	74,611	213,131	3,419	135,101	More ›
Iraq	51,535	227,446	6,891	169,020	More ›
Indonesia	39,132	169,195	7,261	122,802	More ›
Show more ⌄					
▼ **South America(15)**	**1,279,868**	**6,175,458**	**199,356**	**4,696,234**	
Brazil	718,889	3,846,153	120,452	3,006,812	More ›
Peru	163,473	629,961	28,471	438,017	More ›
Colombia	142,133	590,520	18,767	429,620	More ›
Argentina	112,734	401,239	8,353	280,152	More ›
Bolivia (Plurinational State of)	55,817	113,129	4,791	52,521	More ›
Show more ⌄					
▼ **Africa(56)**	**274,520**	**1,237,643**	**29,213**	**933,910**	
South Africa	72,454	620,132	13,743	533,935	More ›
Ethiopia	30,768	49,654	770	18,116	More ›
Egypt	21,819	98,497	5,376	71,302	More ›
Morocco	14,360	60,056	1,078	44,618	More ›
Kenya	13,632	33,794	572	19,590	More ›
Show more ⌄					
▼ **Oceania(8)**	**6,723**	**29,143**	**624**	**21,796**	
Australia	4,237	25,448	583	20,628	More ›
Guam	1,269	1,279	10	0	More ›
French Polynesia	482	482	0	0	More ›
Papua New Guinea	448	453	5	0	More ›
New Zealand	186	1,376	22	1,168	More ›
Show more ⌄					
▼ **Other(1)**	**154**	**741**	**13**	**574**	
International conveyance (Diamond Princess)	154	741	13	574	More ›

COVID-19 CORONAVIRUS PANDEMIC

Last updated: August 30, 2020, 00:52 GMT

Graphs - Countries - Death Rate - Symptoms - Incubation - Transmission - News

Coronavirus Cases:

25,155,358

view by country

Deaths:

845,955

Recovered:

17,499,497

ACTIVE CASES		CLOSED CASES	
**6,809,906** Currently Infected Patients		**18,345,452** Cases which had an outcome:	
**6,748,666** (99%) in Mild Condition	**61,240** (1%) Serious or Critical	**17,499,497** (95%) Recovered / Discharged	**845,955** (5%) Deaths
Show Graph		Show Graph	

daily linear logarithmic

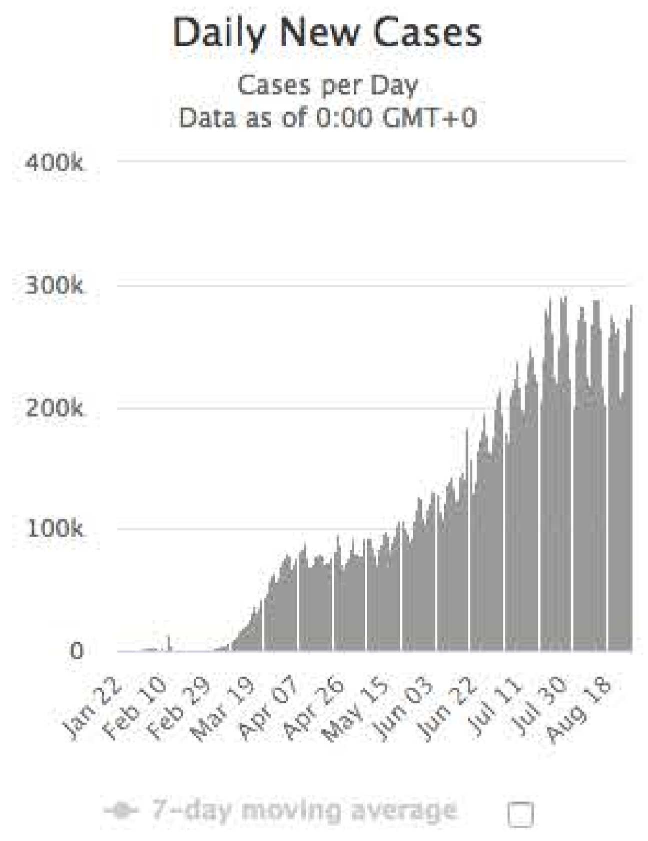

daily linear logarithmic

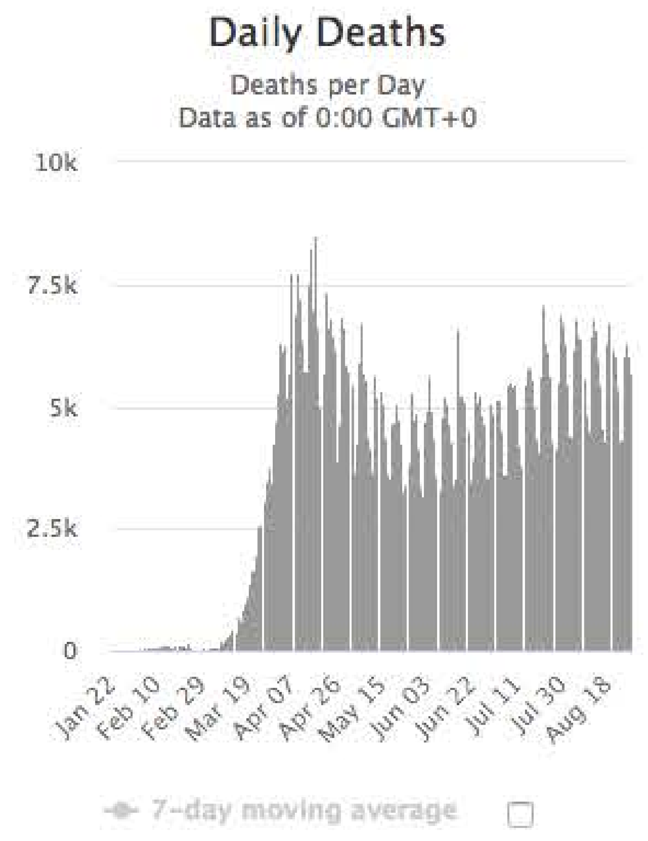

The charts above are updated after the close of the day in GMT+0. See more graphs

Reported Cases and Deaths by Country, Territory, or Conveyance

The **coronavirus** COVID-19 is affecting **213 countries and territories** around the world and 2 international conveyances. **The day is reset after midnight GMT+0**. The list of countries and territories and their continental regional classification is based on the United Nations Geoscheme. Sources are provided under "Latest Updates".

Learn more about Worldometer's COVID-19 data

Report coronavirus cases

Now Yesterday 2 Days Ago Columns ▾ Search:

All Europe North America Asia South America Africa Oceania

#	Country, Other	Total Cases	New Cases	Total Deaths	New Deaths	Total Recovered	Active Cases	Serious, Critical	Tot Cases/ 1M pop	Deaths/ 1M pop	Total Tests	Tests/ 1M pop	Population
	World	25,155,358	+255,763	845,955	+5,278	17,499,497	6,809,906	61,240	3,227	108.5			
1	USA	6,138,748	+42,513	186,855	+954	3,408,726	2,543,167	16,012	18,528	564	81,101,049	244,782	331,319,877
2	Brazil	3,846,965	+34,360	120,498	+904	3,006,812	719,655	8,318	18,077	566	14,352,484	67,444	212,805,498
3	India	3,539,712	+78,472	63,657	+944	2,712,520	763,535	8,944	2,561	46	40,406,609	29,234	1,382,196,924
4	Russia	985,346	+4,941	17,025	+111	804,383	163,938	2,300	6,751	117	36,000,000	246,669	145,944,843
5	Peru	639,435	+9,474	28,607	+136	446,675	164,153	1,524	19,350	866	3,156,679	95,526	33,045,190
6	South Africa	622,551	+2,419	13,981	+238	536,694	71,876	539	10,476	235	3,652,970	61,468	59,428,553
7	Colombia	599,914	+9,394	19,064	+297	440,574	140,276	1,493	11,770	374	2,685,355	52,685	50,970,324
8	Mexico	585,738	+5,824	63,146	+552	404,667	117,925	3,221	4,535	489	1,319,957	10,220	129,151,233
9	Spain	455,621		29,011		N/A	N/A	751	9,744	620	8,517,446	182,161	46,757,783
10	Chile	408,009	+2,037	11,181	+49	381,183	15,645	959	21,314	584	2,365,753	123,584	19,142,814
11	Argentina	401,239	+9,230	8,353	+82	287,220	105,666	2,192	8,865	185	1,222,359	27,006	45,262,921
12	Iran	371,816	+1,905	21,359	+110	319,847	30,610	3,791	4,418	254	3,184,857	37,841	84,165,180
13	UK	332,752	+1,108	41,498	+12	N/A	N/A	60	4,897	611	16,273,209	239,508	67,944,393
14	Saudi Arabia	313,911	+987	3,840	+27	288,441	21,630	1,555	8,995	110	5,026,127	144,015	34,900,013
15	[illegible]	[illegible]	[illegible]	[illegible]	[illegible]	[illegible]	[illegible]	[illegible]	[illegible]	[illegible]	[illegible]	[illegible]	[illegible]

HOME CORONAVIRUS PRODUCTS ABOUT PARTNERS WHAT'S NEW Contact

If you see an authentication issue, please clear your cache.

Covid 19 Tracker

Data as of 27 August 2020

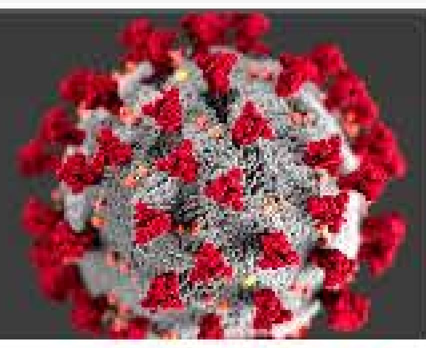

Decentralized on Hedera Hashgraph

Total Confirmed

24,454,076

Total Deaths

831,605

Mortality Rate: 3.4%

COUNTRIES | PROJECTIONS

Case Confirmed by Country

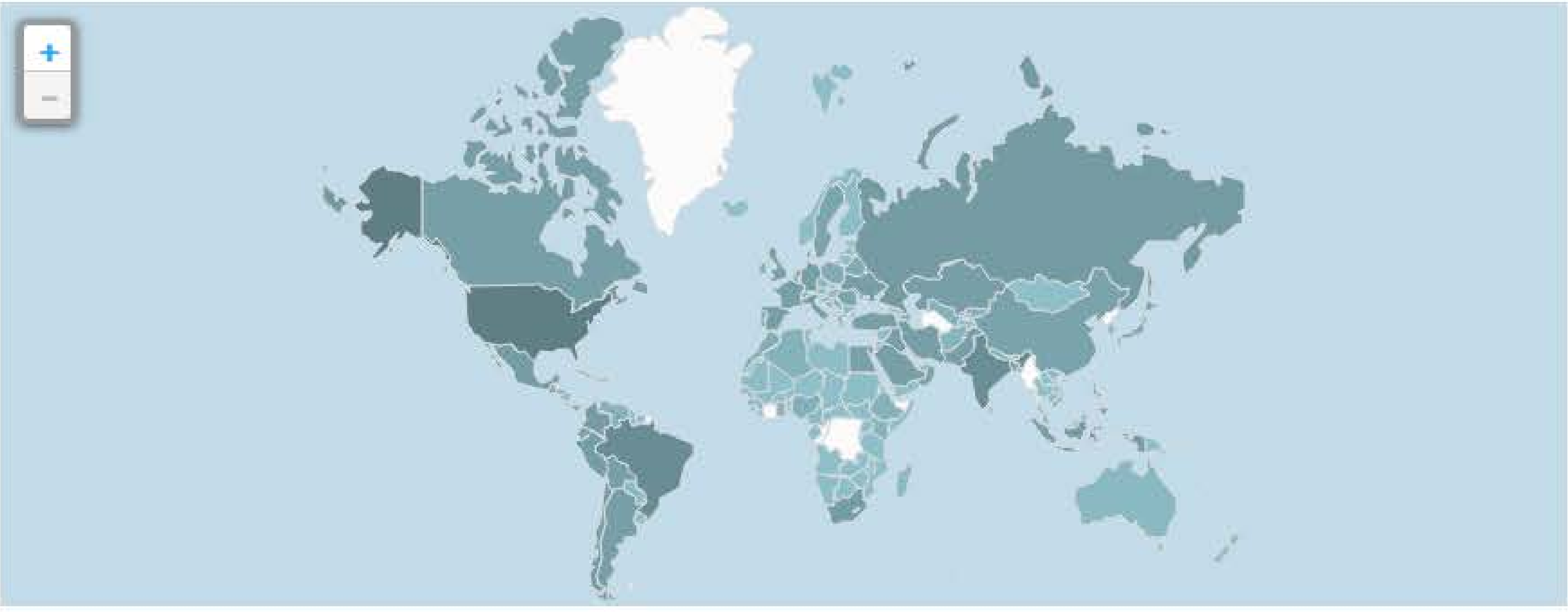

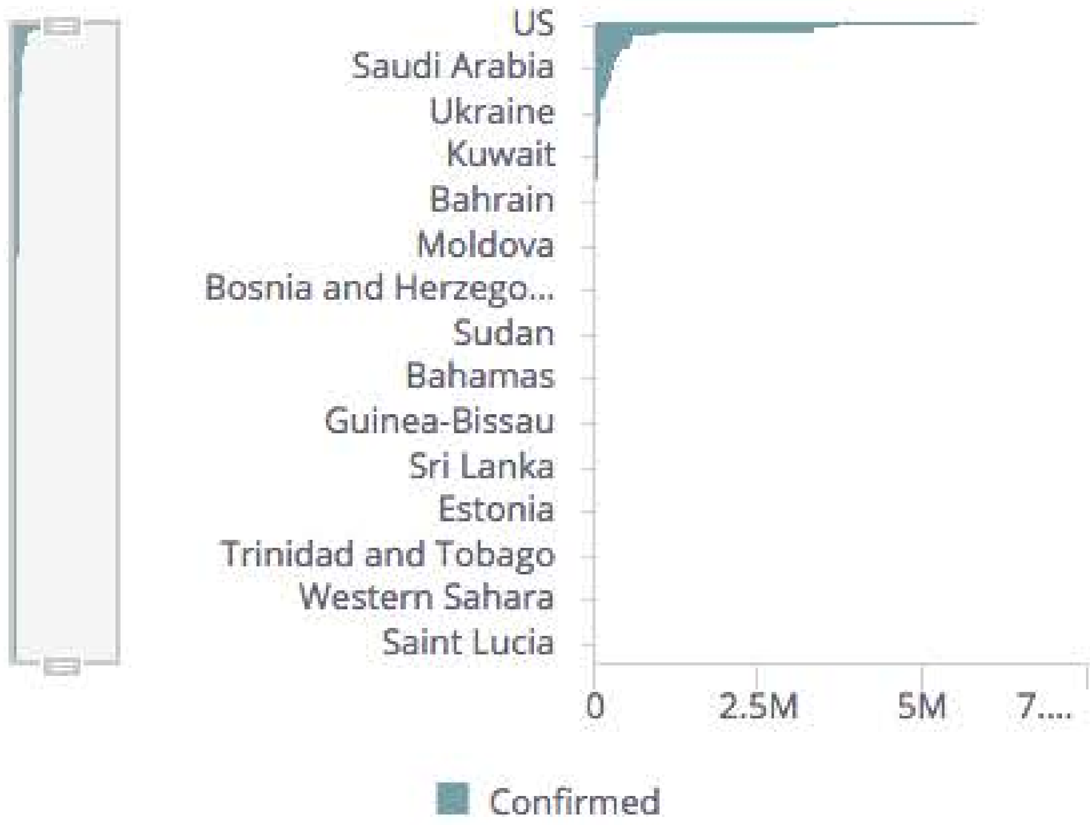

Daily New Cases

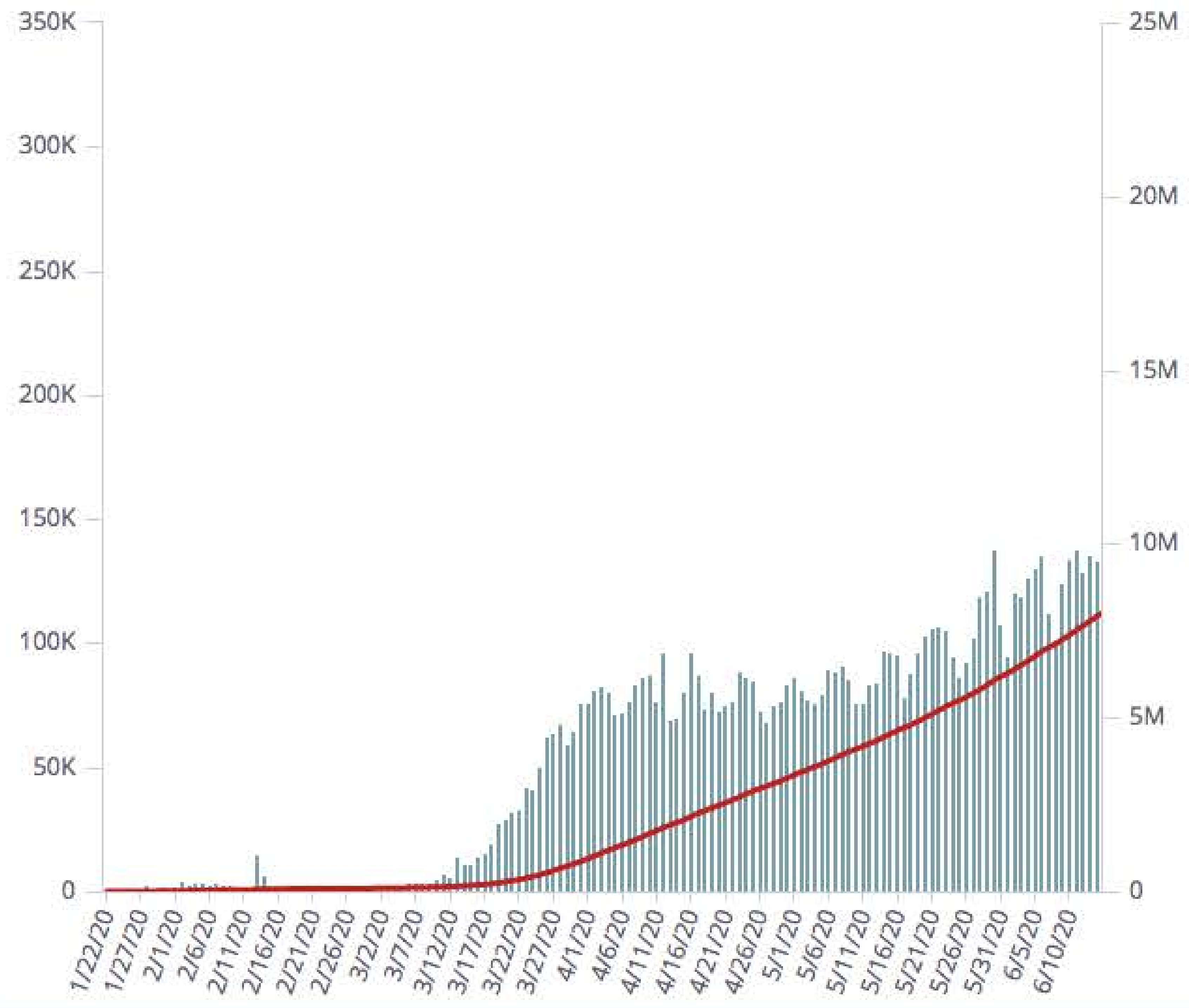

Daily New Deceases

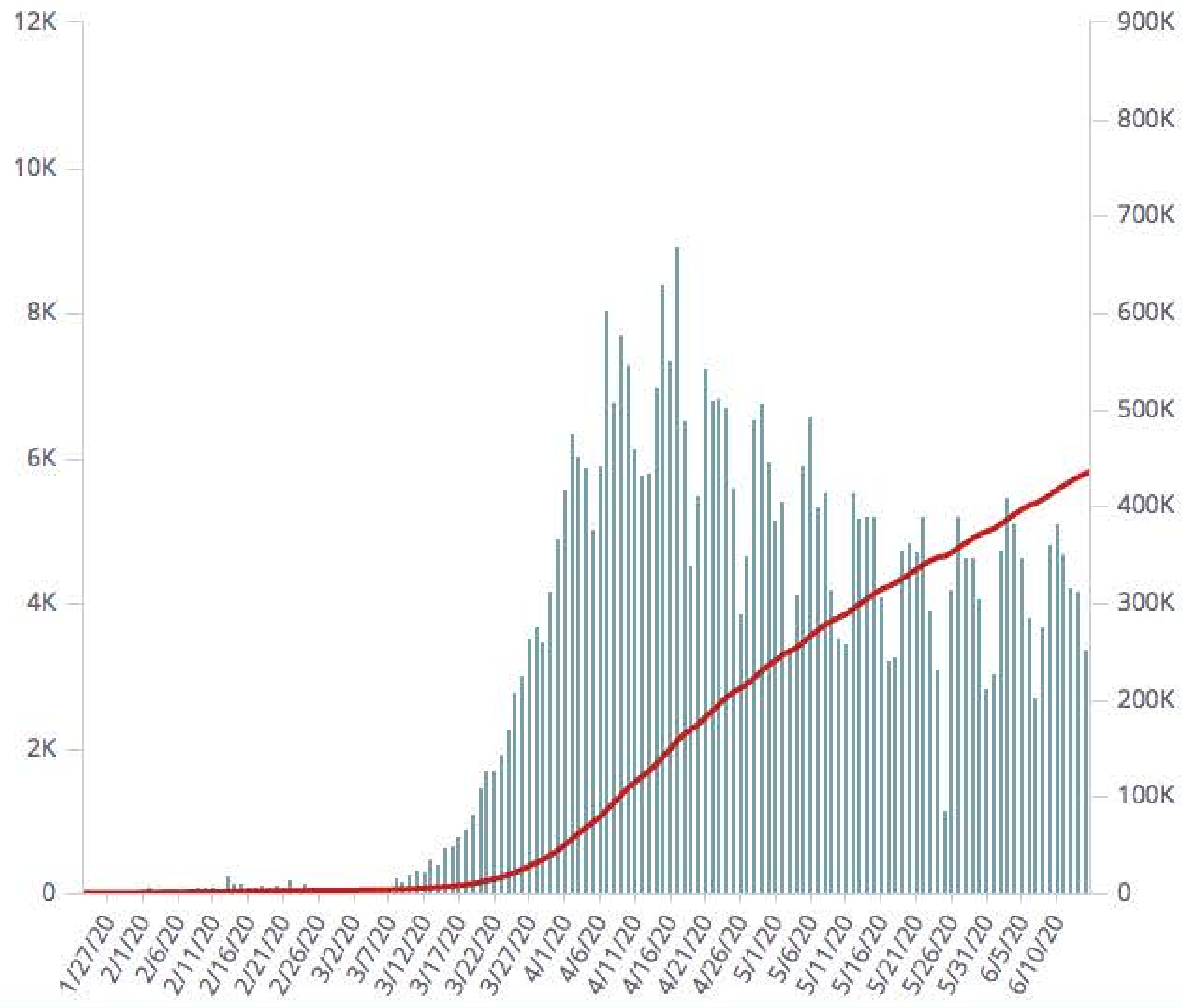

* Coronavirus (COVID-19) data is collected from a wide set of public data, including data from the Center for Disease Control (CDC), the World Health Organization (WHO), the Institute for Health Metrics and Evaluation (IHME) and The New York Times.

** Explicit written permission from Acoer LLC is required for use of Acoer Coronavirus Tracker assets. Contact us for more information or to request permission for use of Acoer assets.

COVID-19 Tracker

Search the web with Bing

Give with Bing

Related Searches: coronavirus testing prevention treatment symptoms work from home jobs

Track 174+ vaccine trials

Total confirmed cases

Updated 17 min ago

24,776,988 +139,444

Active cases	7,711,158	
Recovered cases	16,227,851	+119,992
Fatal cases	837,979	+2,934

Browse Sort

Filter to a location

Global	24,776,988
United States	6,008,588
California	693,839
Florida	619,003
Texas	606,530
New York	433,402
Georgia	267,758
Illinois	231,363
Arizona	201,287
New Jersey	191,320
North Carolina	165,076
Tennessee	152,280
Louisiana	146,243

United States

Save location

Overview News & Videos Graphs Vaccines

Overview

6,008,588 +6,924

Active cases	2,659,859	
Recovered cases	3,164,376	+7,136
Fatal cases	184,353	+162

Trends

New and fatal cases Expand

New Cases

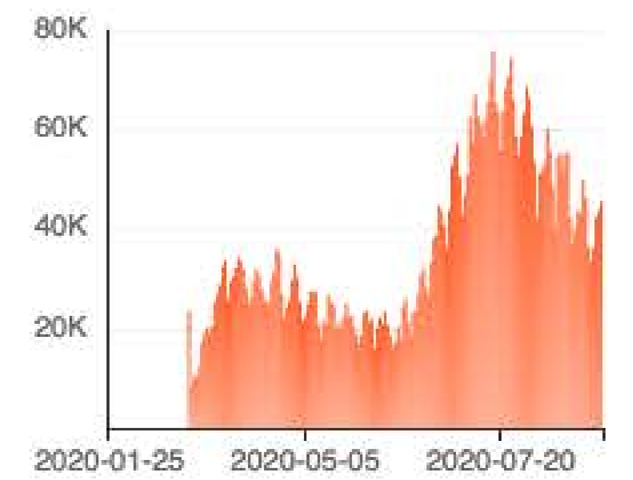

See more charts

News

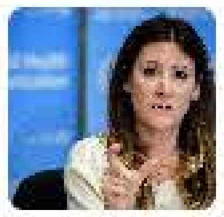

Doctors: 1st Covid reinfection case 'not surprising'

Yahoo! News · 24m

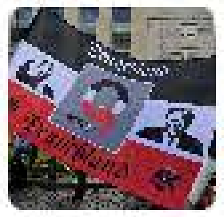

Berlin police break up 'anti-coronavirus' protest of 18,000

CBS News · 52m

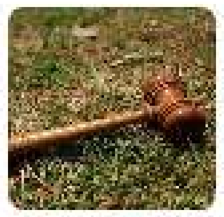

2 Florida men used fake IDs, bank accounts to steal more than $3M

The Hill · 2h

1,200 students test

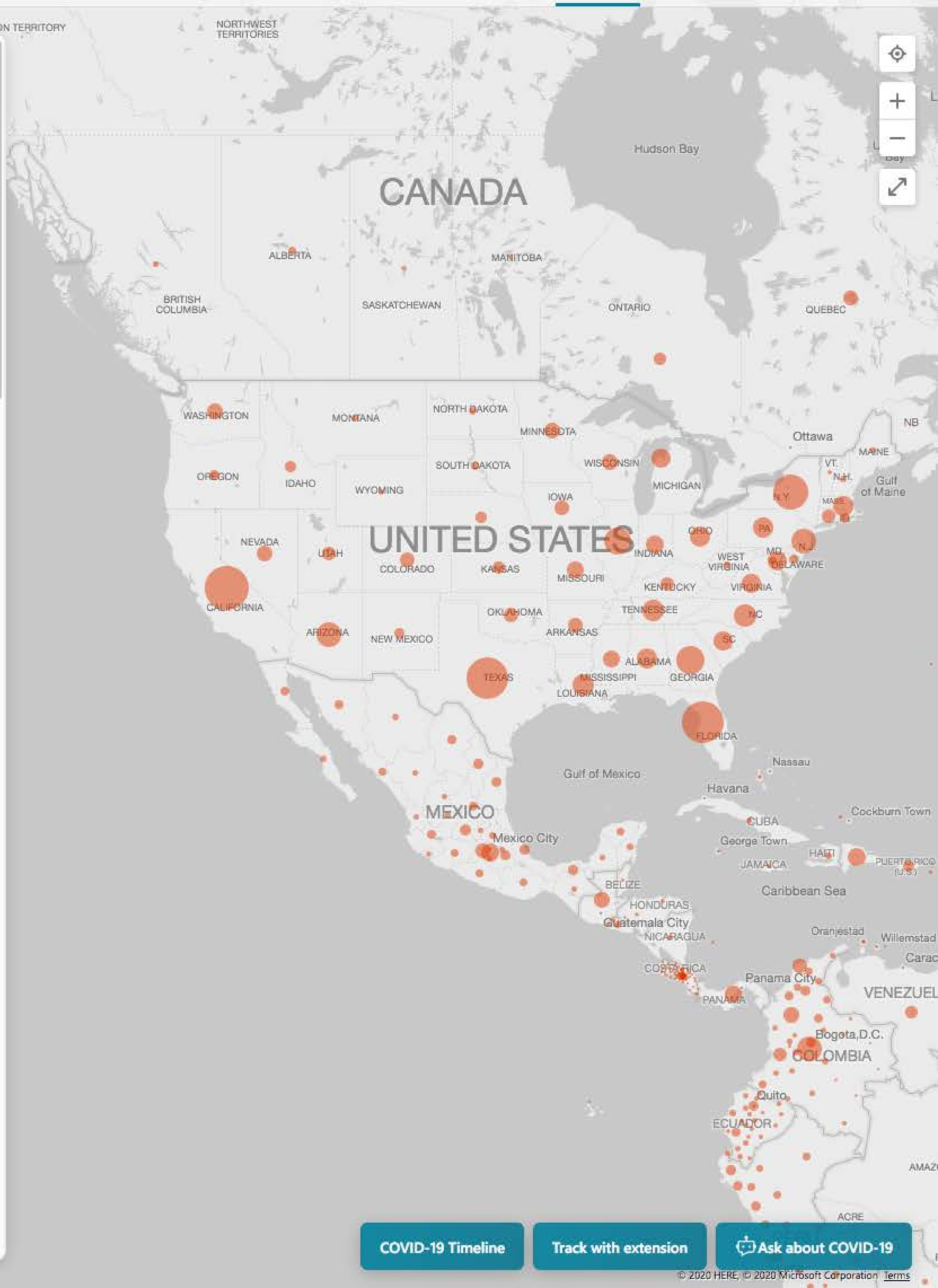

COVID-19 Timeline Track with extension Ask about COVID-19

© 2020 HERE, © 2020 Microsoft Corporation Terms

1Point3Acres

248,686,692 visits

Global COVID-19 Tracker & Interactive Charts

Real Time Updates & Digestable Information for Everyone

English | 中文版 | Français | 日本語 | español | United States

Home Testing World News Job Life PPE About Get Data Support Us

Our reporting is not affected by official COVID reporting moving from CDC to HHS. Read More about Why

Case Summary

Last updated at : 2020-08-29 20:45 EDT

Data source

Location	Cases	Deaths	Recovered	Fatality
United States	+41,249<br>6,086,536	+1,285<br>186,087	+19,058<br>3,184,963	3.1%
World	+281,759<br>24,734,448	+5,538<br>837,124	+200,884<br>16,198,165	3.4%

We just added more countries and regions! Click to view more

Filter the graph by selecting a state from the map below

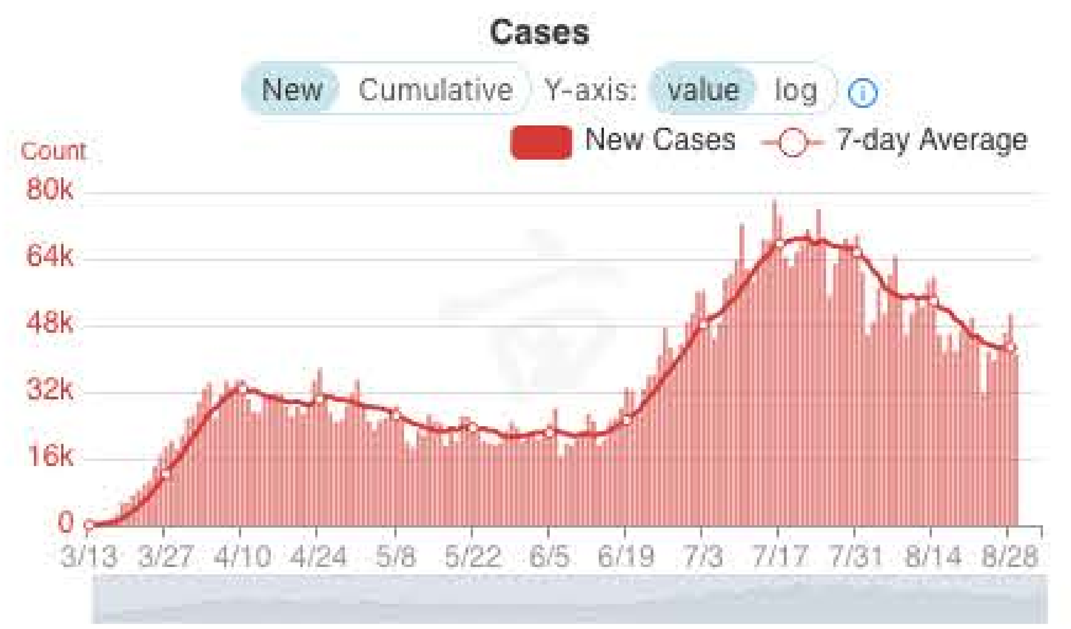

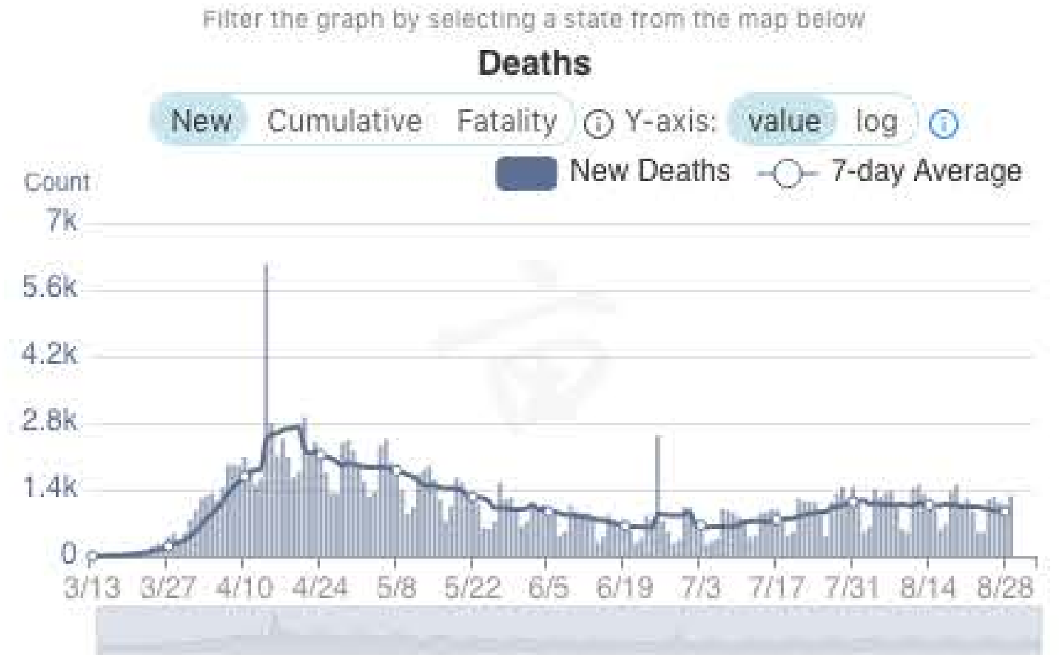

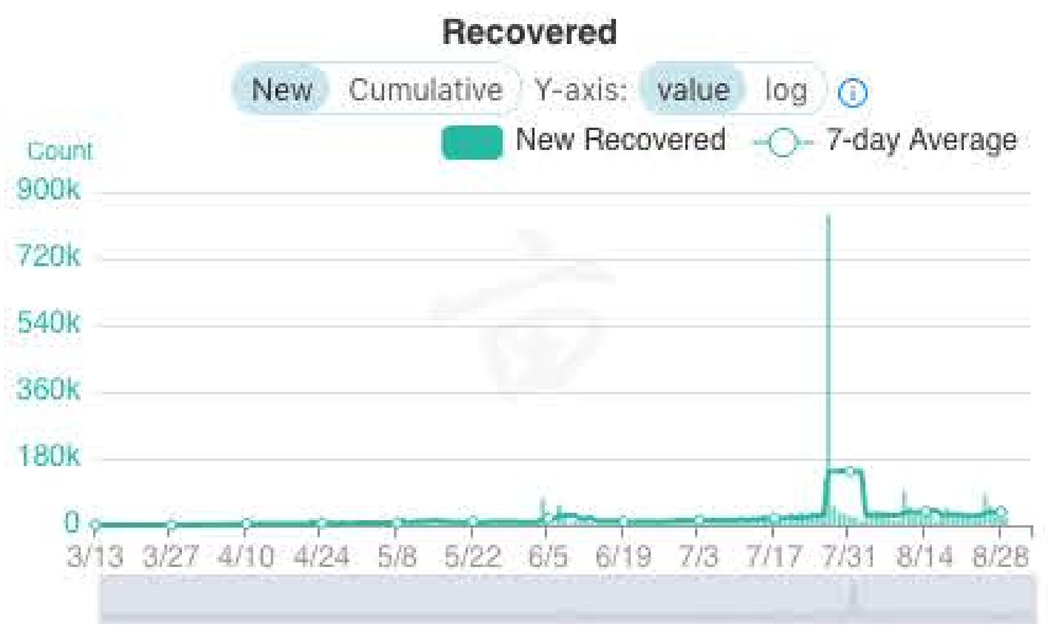

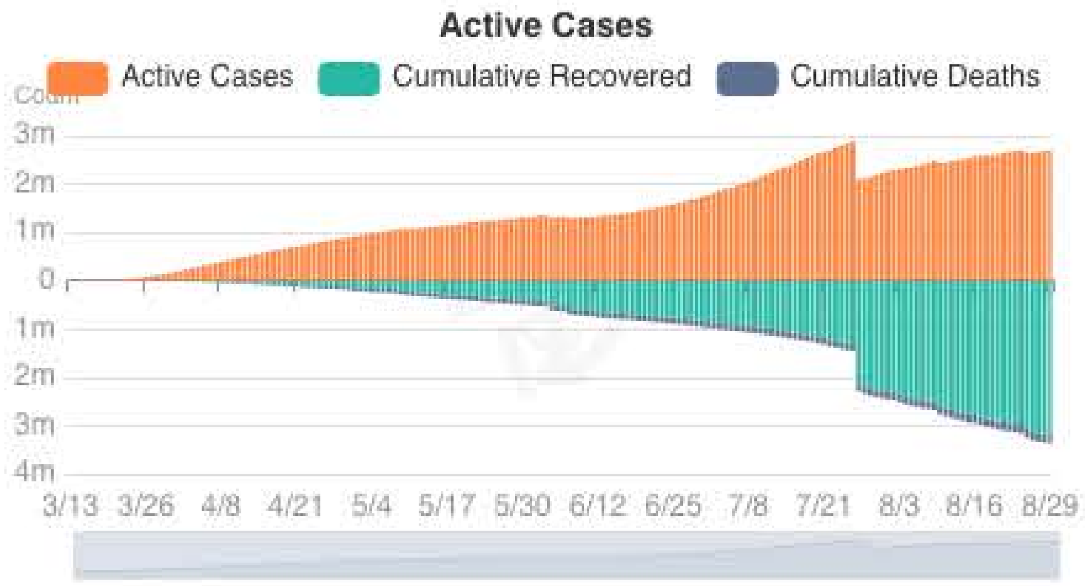

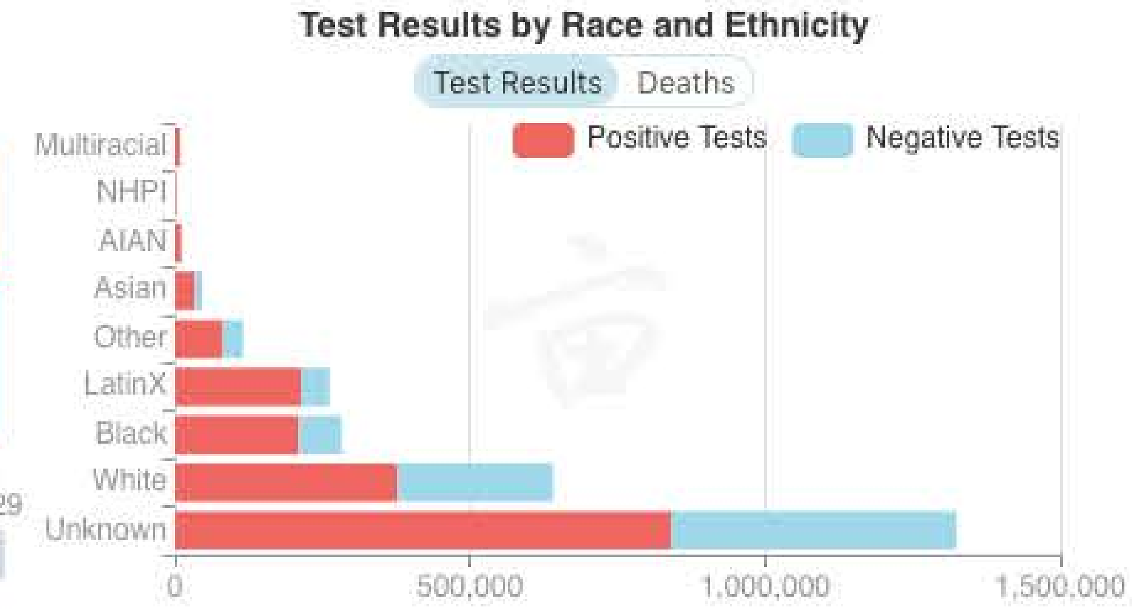

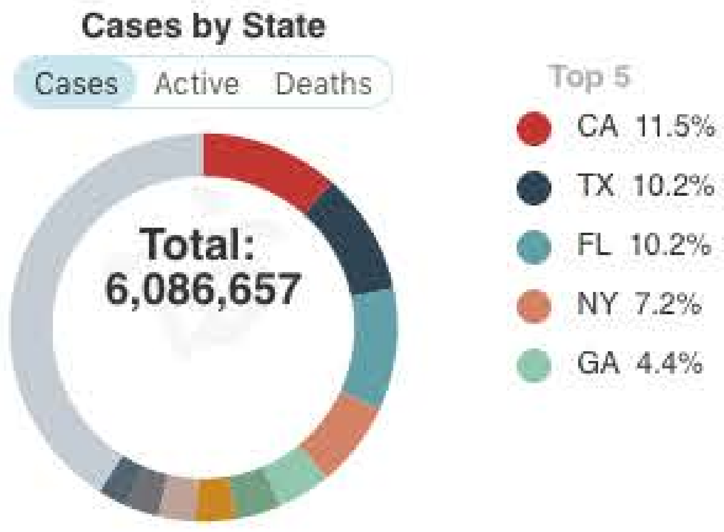

Case Map

Report Error

Cases | Active | New Daily Cases | Tests | Popⁿ | Cases/M | Tests/M | Deaths/10M

Reset Map

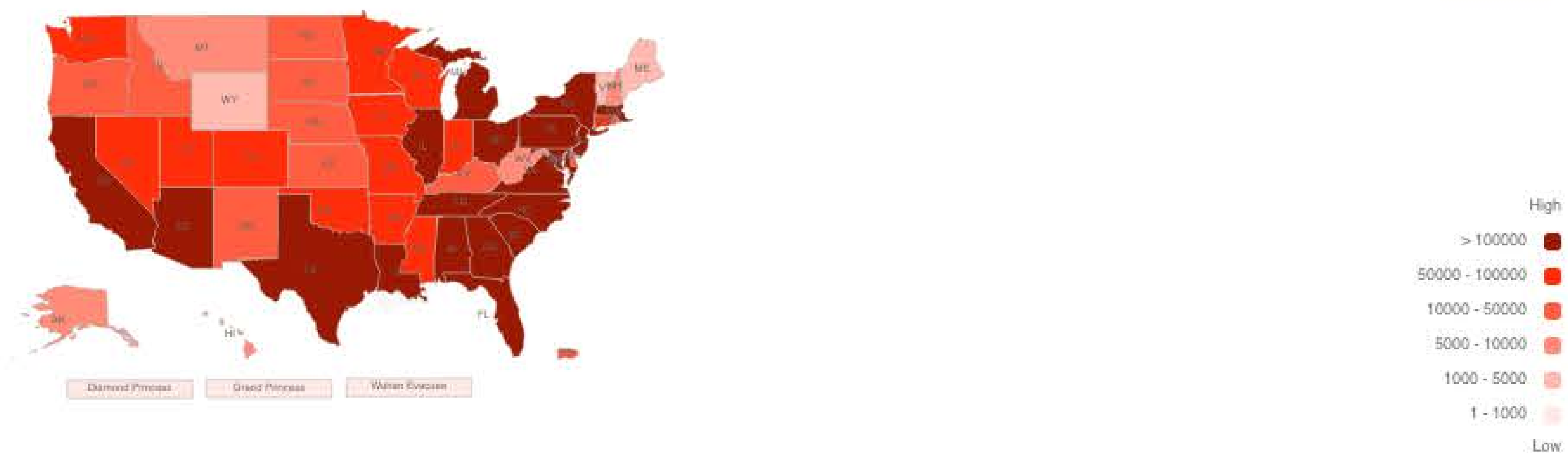

Filter cases by clicking or zooming the map.

Repatriation/Cruise ship cases are separated from TX/CA/NE cases count, consistent with CDC

US Cases

Sort by: Cases

Click here to check state level testing data/location

United States<br>CDC	+41,249<br>6,086,536	+1,285<br>186,087	3.1%	+19,058<br>3,184,963	+11.2%
California	+2,670<br>700,778	+66<br>12,907	1.8%	+2,334<br>294,976	+13.4%
Texas	+4,969<br>622,804	+129<br>12,318	2%	+11,124<br>500,233	+14.3%
Florida	+3,198<br>619,004	+148<br>11,105	1.8%	381	+8.7%
New York	+617<br>438,497	+4<br>32,938	7.5%	64,300	+2%
Georgia	+2,344<br>268,344	+104<br>5,581	2.1%	927	+15.8%
Illinois	+1,880<br>231,362	+10<br>8,007	3.5%	79,803	+13.1%
Arizona	+629<br>201,287	+30<br>5,007	2.5%	174	+4.5%
[illegible]	+318<br>[illegible]	+4<br>[illegible]	[illegible]	[illegible]	[illegible]

Trends

Cases Mobility Employment

Cases Death Active | Total Daily new per million people | US Canada World

Coronavirus Case Trajectories in US

You can Customize/ScreenShot, then share with your friends!

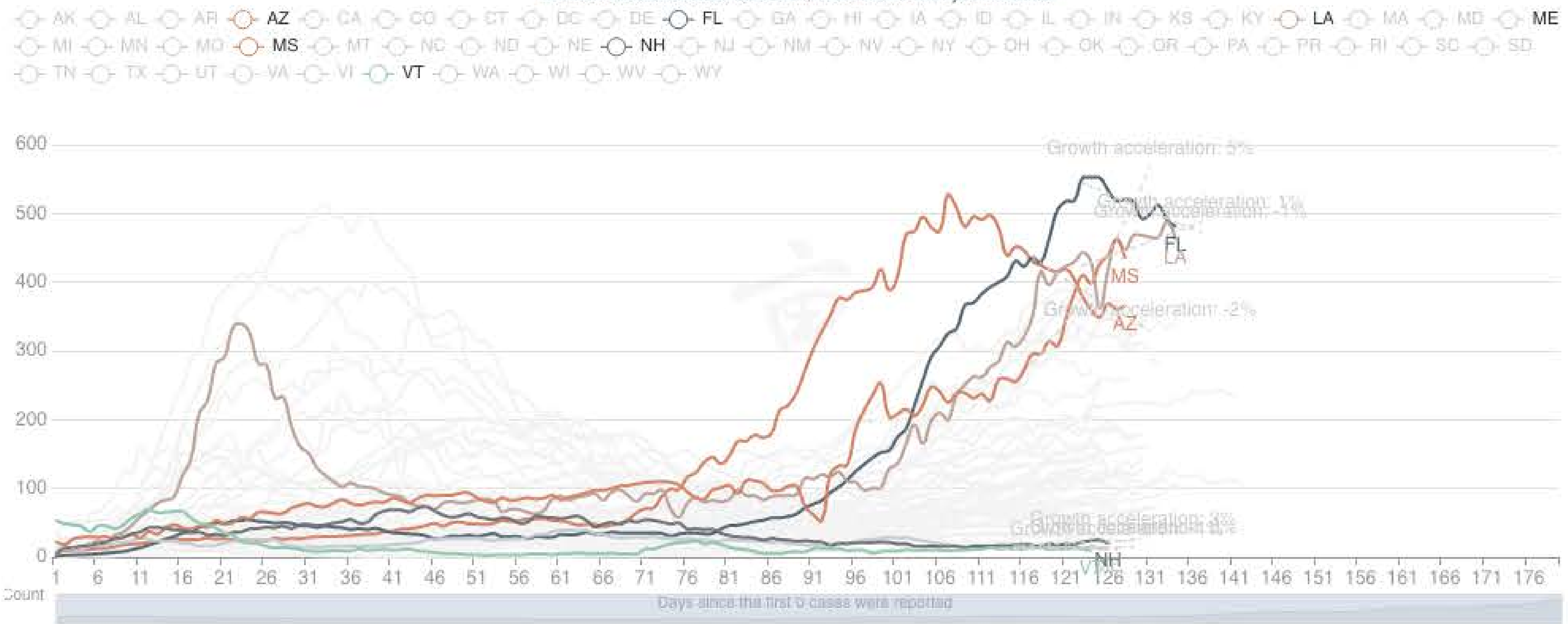

Counting each region from the first day when 100+ cases were reported. China/Hubei started on the 4 days after the first 400-500 cases were reported to align the graph.
Data based on Johns Hopkins University COVID-19 dataset. Inspired by Coronavirus tracked: the latest figures as the pandemic spreads

value log ⓘ New cases based on average of recent 1 day 3 days 5 days 7 days

Coronavirus Case Trajectories in US

You can Customize/ScreenShot, then share with your friends!

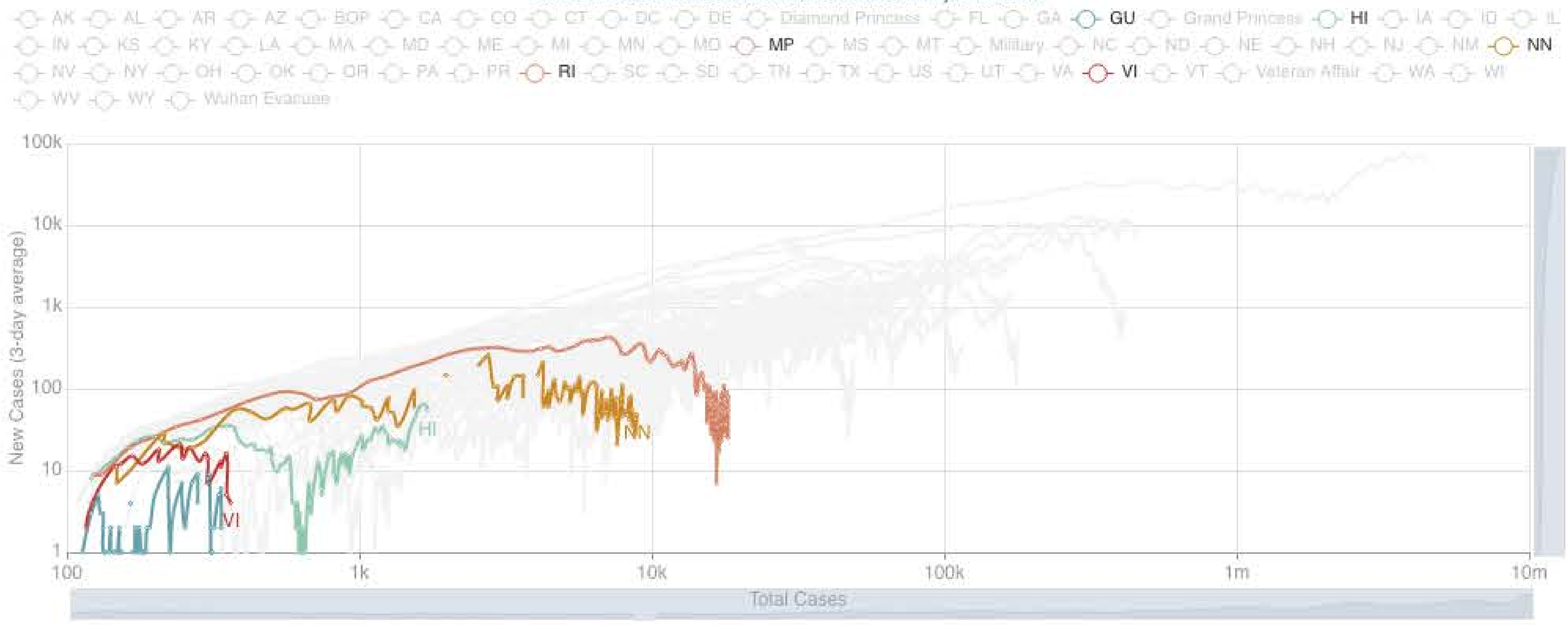

How to read this graph Inspired by https://aatishb.com/covidtrends/

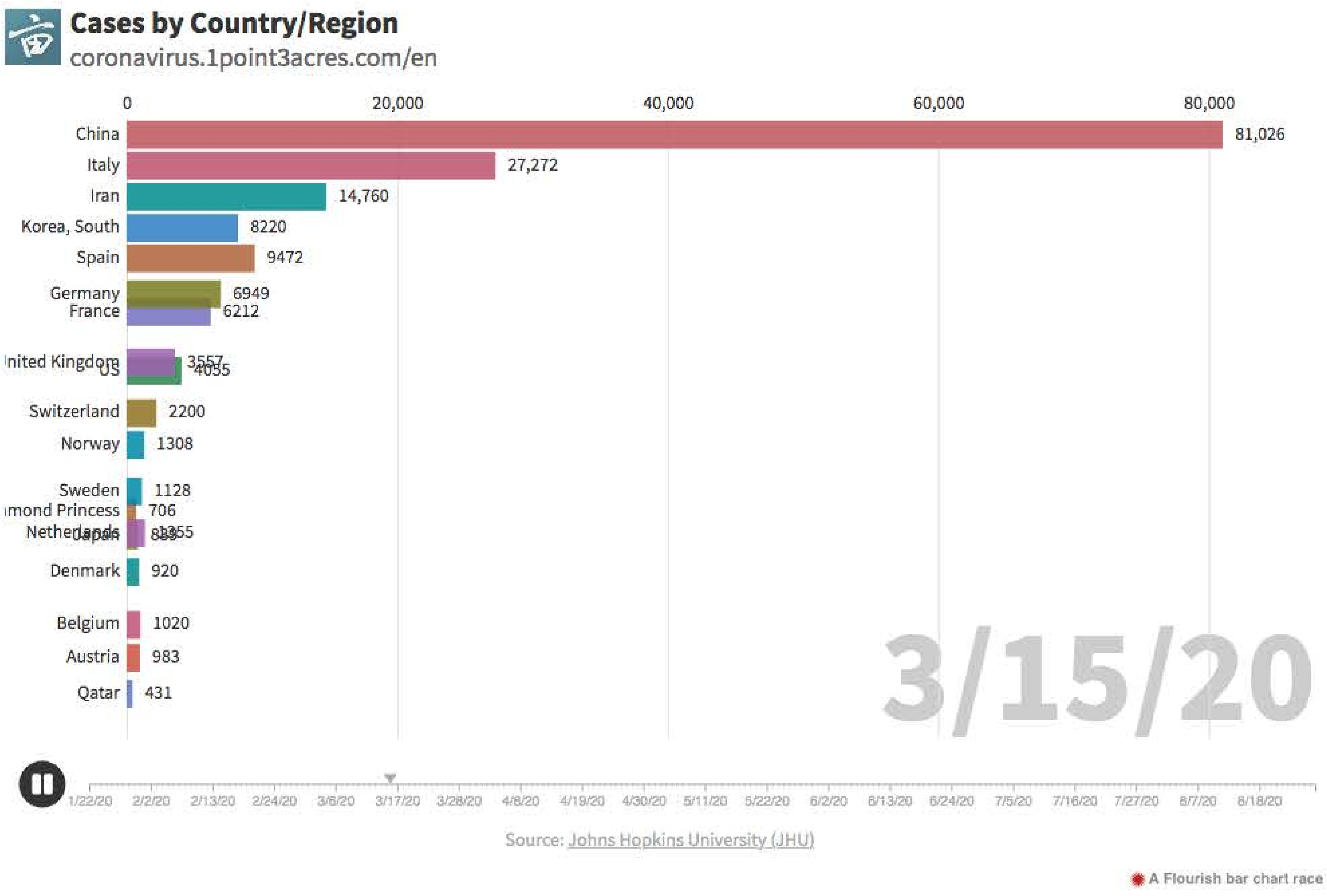

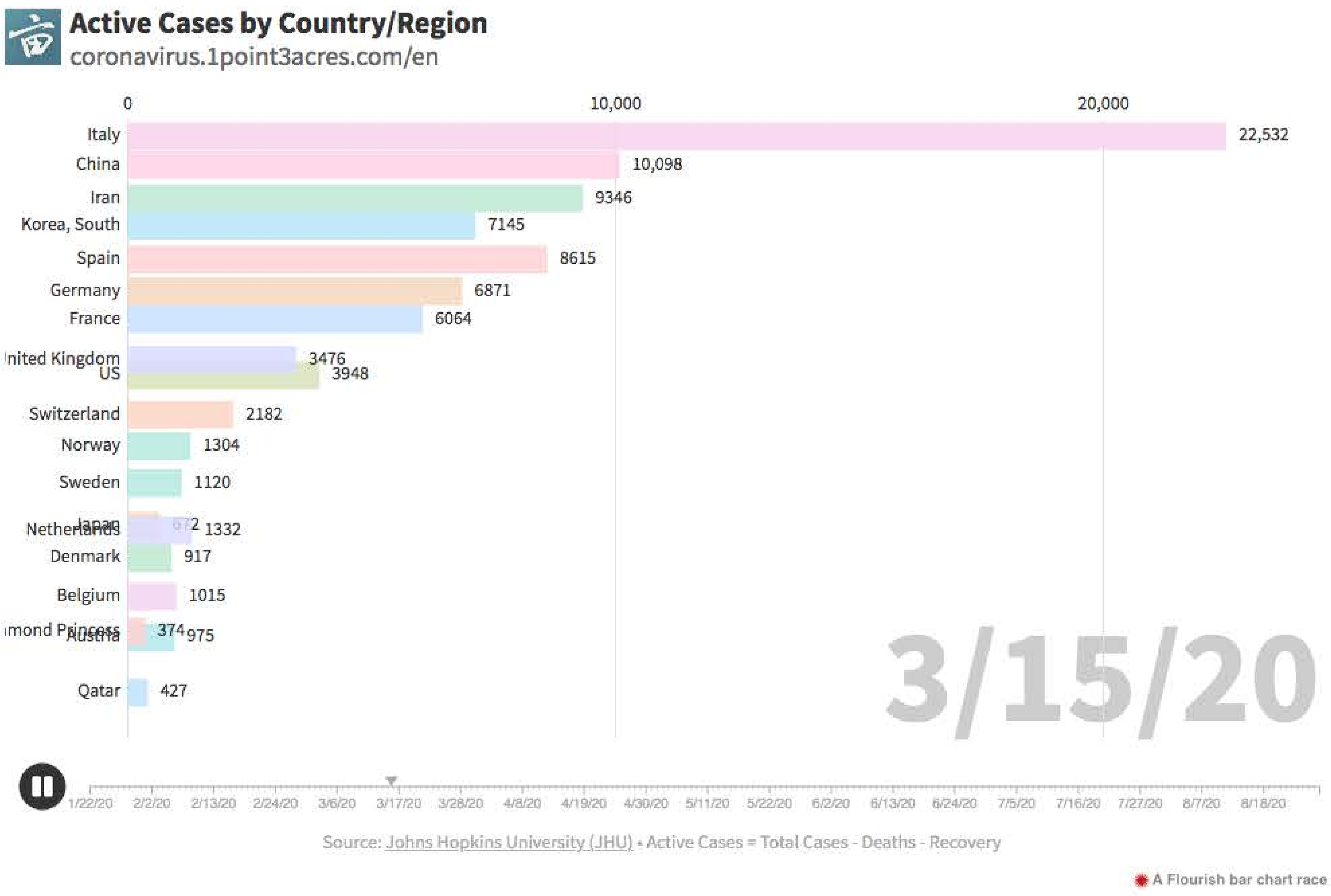

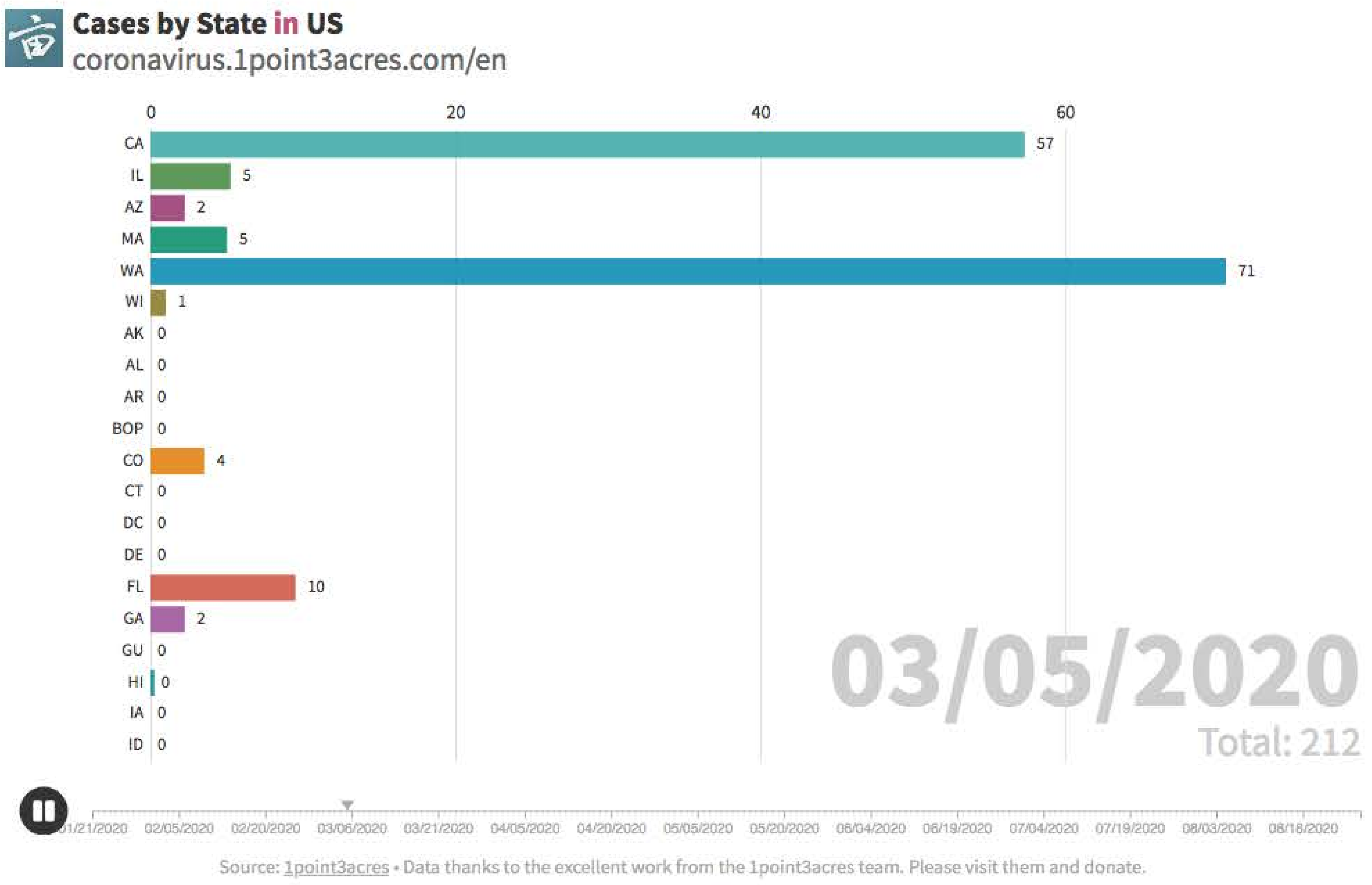

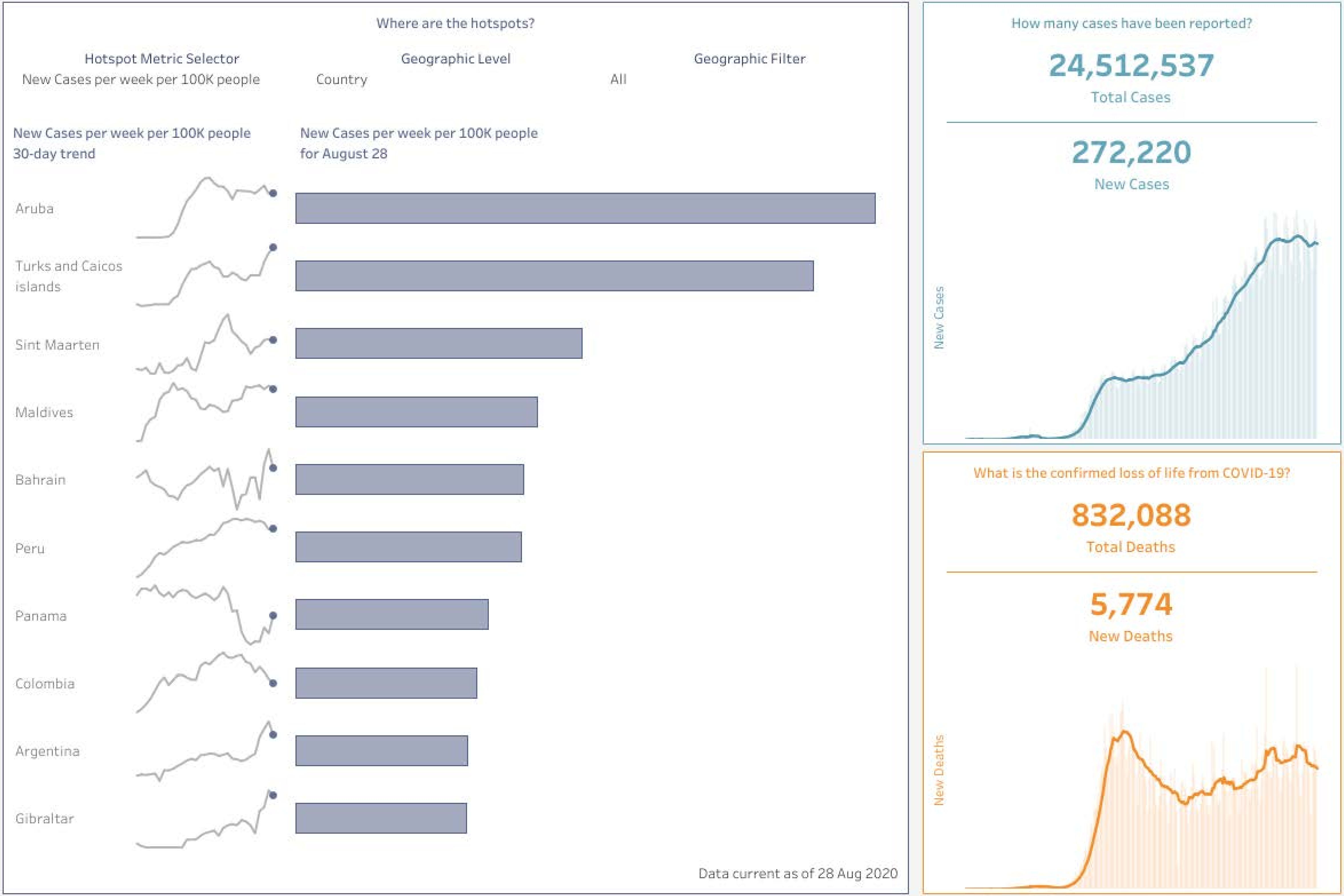
Where are the hotspots?
Hotspot Metric Selector
New Cases per week per 100K people
Geographic Level
Country
Geographic Filter
All
New Cases per week per 100K people 30-day trend
New Cases per week per 100K people for August 28
Aruba
Turks and Caicos islands
Sint Maarten
Maldives
Bahrain
Peru
Panama
Colombia
Argentina
Gibraltar
Data current as of 28 Aug 2020
How many cases have been reported?
24,512,537
Total Cases
272,220
New Cases
New Cases
What is the confirmed loss of life from COVID-19?
832,088
Total Deaths
5,774
New Deaths
New Deaths

Global COVID-19 Tracker

December 31, 2019 - August 28, 2020

Global | United States

Cumulative or New
New

Positive Cases or Deaths
Positive Cases

New Positive Cases

272,220

▼ -3.1% vs previous day
280,883

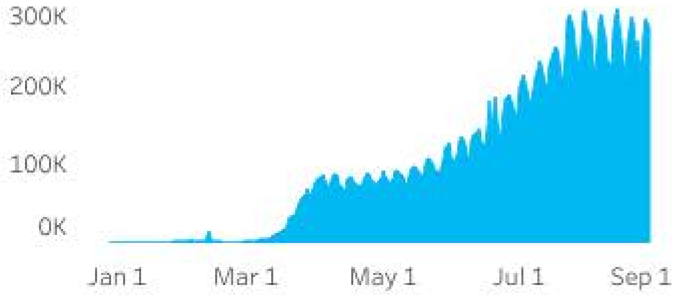

New Deaths

5,774

▼ -6.9% vs previous day
6,200

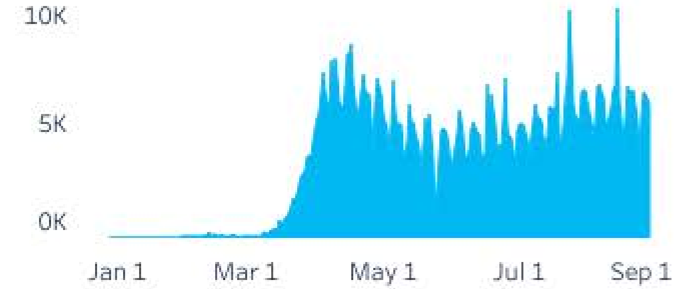

New Positive Cases

Select a Country to see more details

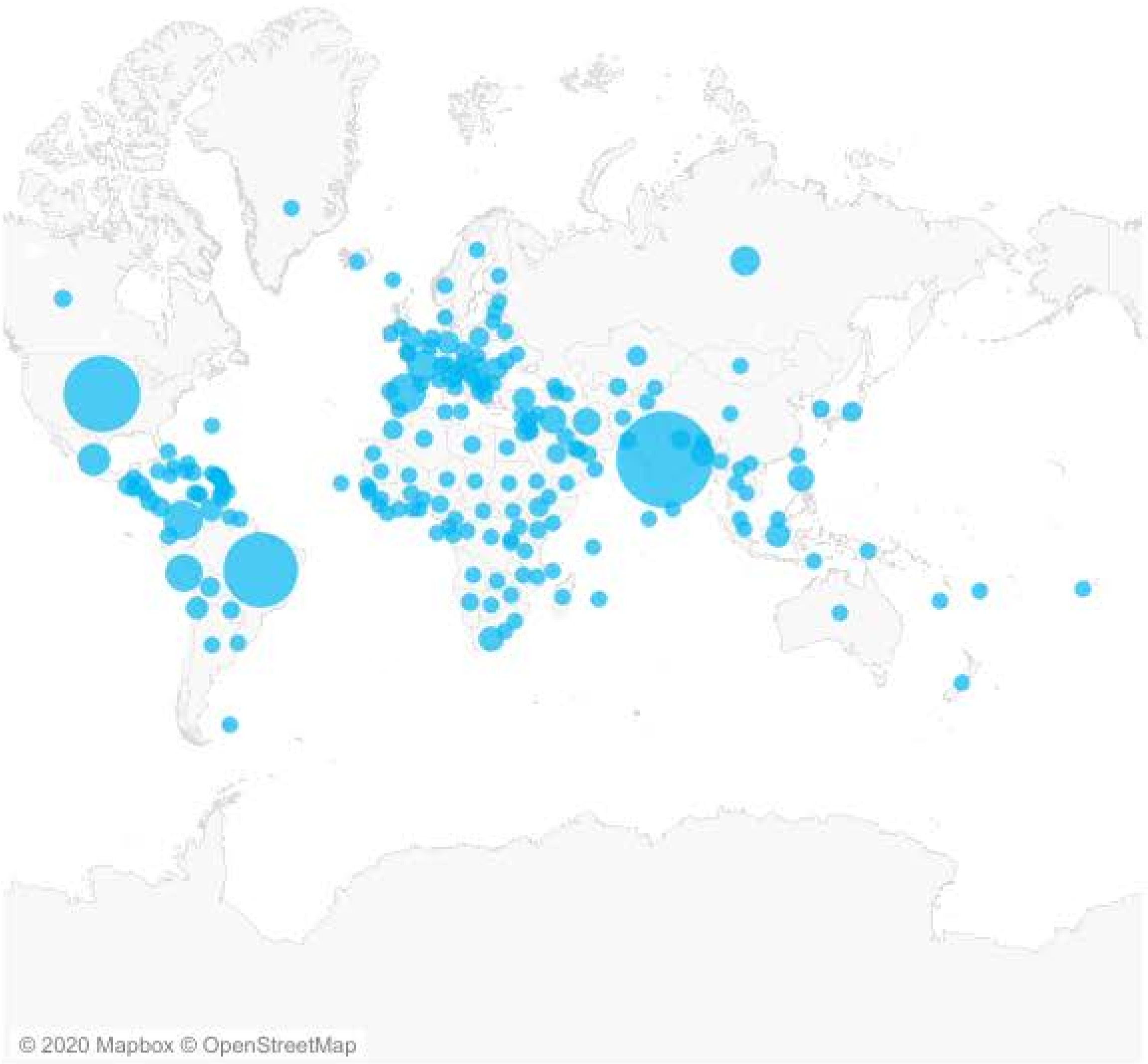

New Positive Cases

Select a Country to see more details

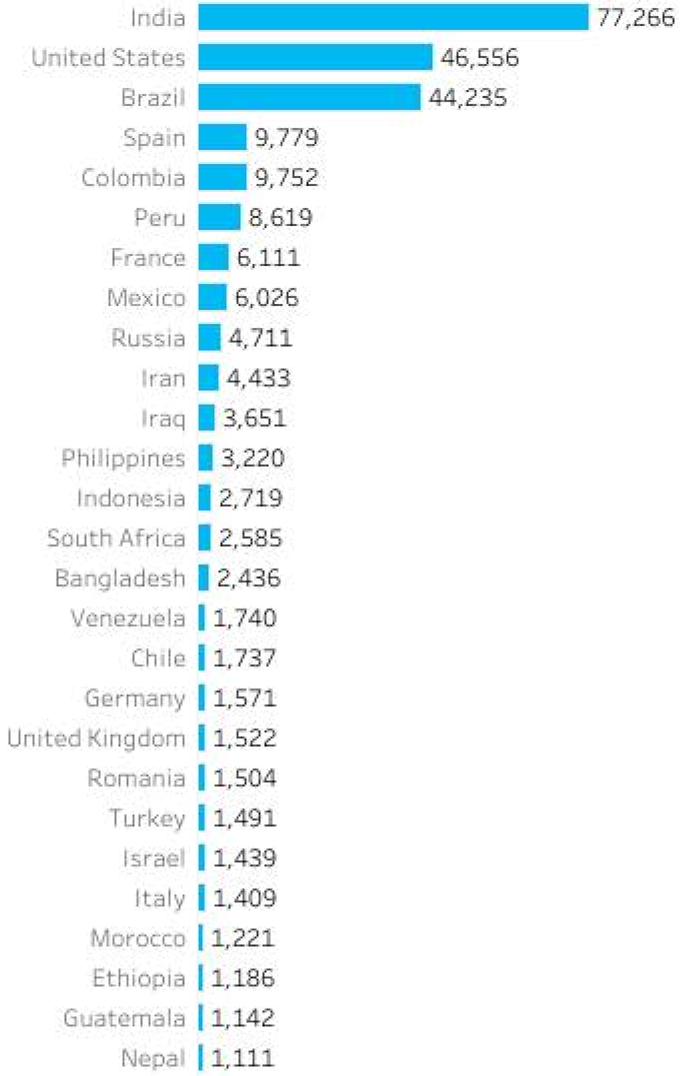

Supplement: Multimedia Appendix 6 [file humanfactors_v10i1e43819_app6.pdf]
