# Supplementary material for: Web-Based COVID-19 Dashboards and Trackers in the United States: Survey Study
Source: JMIR Hum Factors. 2023 Mar 20;10:e43819. doi: 10.2196/43819 (PMC10029858; doi:10.2196/43819)

Appendix 7. Screenshots from dashboards and trackers — *State-focused coverage* *(captured January 31, 2021)*

Page	Ref	Host
2–4	S-1	Alabama Dept of Public Health
5	S-2	David Marconnet, CTO of OneTeam.net
6–7	S-4	Alaska Dept of Health and Social Services
8–11	S-6	Arizona Dept of Health Services
12–14	S-7	Arkansas Dept of Health
15	S-8	California Dept of Public Health
16	S-9	CA Open Data
17	S-10	Los Angeles Times
18	S-11	CalMatters
19–23	S-12	Colorado Dept of Health & Environment
24	S-13	Coloradoan
25	S-14	The Denver Post
26	S-15	Connecticut's Official State Website
27–29	S-16	CTData Collaborative
30	S-17	University of Connecticut Dept of Geography
31	S-18	Government of the District of Columbia
32	S-19	Delaware Environmental Public Health Tracking Network
33–35	S-20	Florida Dept of Health
36	S-21	Florida COVID Action
37	S-22	Georgia Dept of Public Health
38	S-23	Atlanta Journal-Constitution
39	S-24	State of Hawaii Dept of Health
40	S-25	Hawaii Data Collaborative
41–45	S-26	Idaho Division of Public Health
46	S-27	KTVB-7
47	S-28	Illinois Dept of Public Health
48	S-29	Indiana State Dept of Health
49	S-30	Polis Center at Indiana University - Purdue University Indianapolis
50	S-31	Iowa Dept of Public Health
51–52	S-32	Kansas Dept of Health and Environment
53	S-33	Kentucky Dept of Public Health
54	S-34	WFPL 89.3
55	S-35	Louisiana Dept of Health
56	S-36	Maine Center for Disease Control & Prevention
57–58	S-37	Maine Geolibrary
59–60	S-38	Maryland Dept of Health
61–63	S-39	Massachusetts Dept of Public Health
64–66	S-40	Michigan Dept of Health and Human Services
67	S-41	Bridge Michigan
68	S-42	Minnesota Dept of Health
69	S-43	Mississippi State Dept of Health

Page	Ref	Host
70–72	S-44	Missouri Dept of Health and Senior Services
73–74	S-46	Montana State Library
75–77	S-47	North Carolina Dept of Health and Human Services
78–80	S-48	North Dakota Dept of Health
81–82	S-49	Nebraska Dept of Health and Human Services
83–85	S-50	Nevada Dept of Health and Human Services
86	S-51	New Hampshire Dept of Health and Human Services
87	S-52	New Hampshire Public Radio
88–89	S-53	New Jersey Dept of Health
90	S-54	NJ Advance Media
91	S-55	New Mexico Dept of Health
92	S-56	New York State Dept of Health
93	S-57	Ohio Dept of Health
94–96	S-58	Oklahoma State Dept of Health
97–100	S-59	Oregon Health Authority
101–105	S-60	Pennsylvania Dept of Health
106	S-61	Rhode Island Dept of Health
107	S-62	South Carolina Dept of Health and Environment Control
108	S-63	WPDE-15
109–111	S-64	South Dakota Dept of Health
112	S-65	Tennessee Dept of Health
113–114	S-66	Tennessee Dept of Health
115–116	S-67	Texas Health and Human Services
117	S-68	Houston Chronicle
118	S-69	Utah Dept of Health
119–123	S-70	Virginia Dept of Health
124	S-71	Vermont Dept of Health
125	S-72	VTDigger
126–129	S-73	Washington State Dept of Health
130	S-74	Washington State Hospital Association
131	S-75	West Virginia Dept of Health & Human Resources
132	S-76	Wisconsin Dept of Health Services
133	S-77	WISN-12
134	S-78	Wyoming Dept of Health
135	S-79	Spotlight PA
136	S-80	WPRI-12
137	S-81	San Francisco Chronicle
138	S-82	The Maine Monitor
139	S-83	The Oregonian
140	S-84	Arkansas Democrat Gazette

Alabama's COVID-19 Data and Surveillance Dashboard

Alabama Department of Public Health | Division of Infectious Diseases & Outbreaks | Updates Daily at 10 a.m.

Select a County --> None

COVID-19 Cases Rate Per 100,000 Person in the Last 14 Days

Rate

- > 749 - 5,000
- > 499 - 749
- > 199 - 499
- > 100 - 199
- > 50 - 100
- > 9 - 50
- > 4 - 9
- 0 - 4

Rate (per 100,000 persons) of Cases Reported in the Last 14 Days
(Click a county to see the rate.)

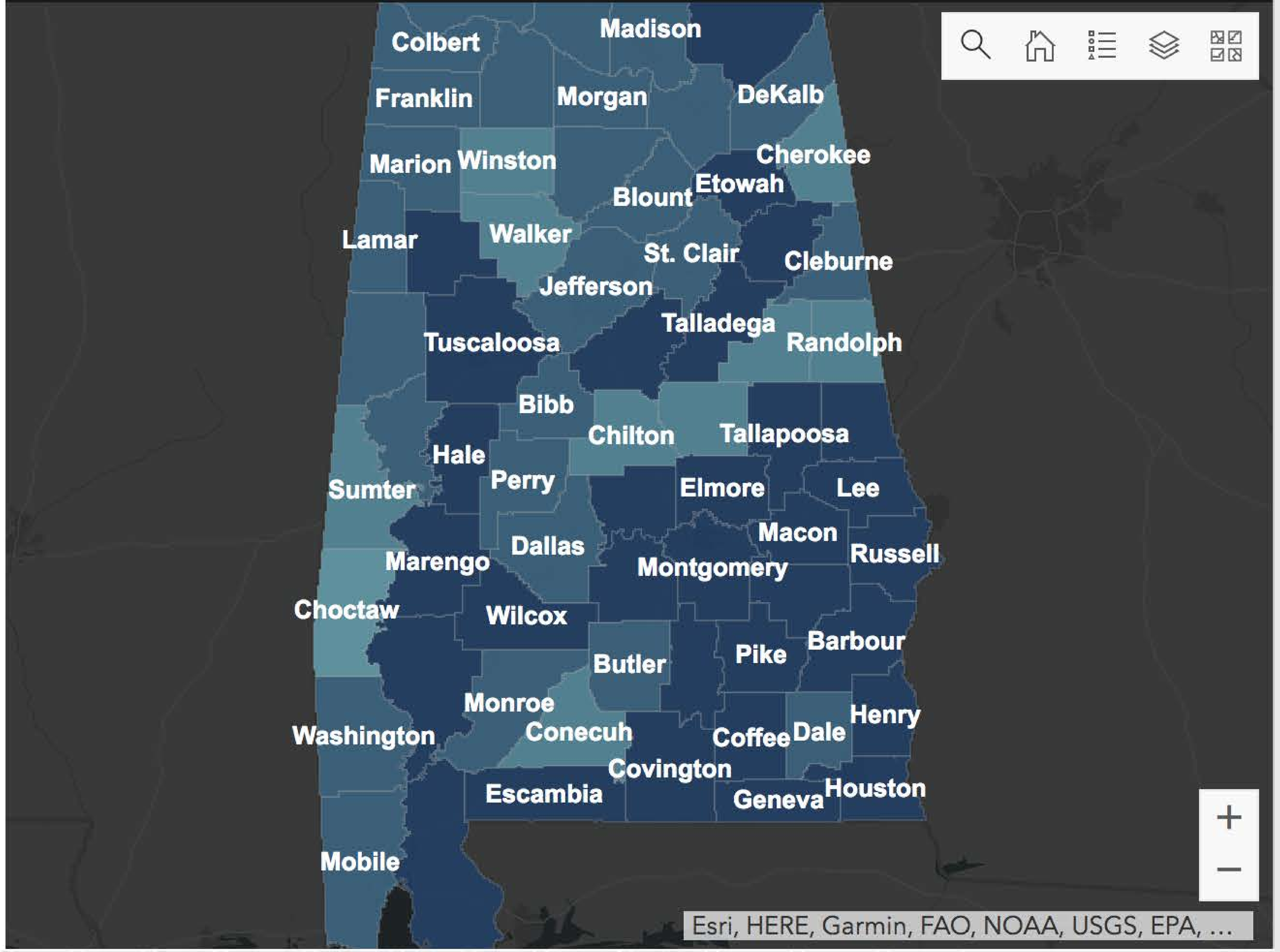

0 | 1 | 2 | 3 | 4 | 5 | 6 | 7A | 7B | 8 | 9 | 10 | 11 | 12 | 13 | 14

CASES	
459,639 TOTAL	
362,319	97,320
CONFIRMED	PROBABLE

DEATHS	
7,688 TOTAL	
6,171	1,517
CONFIRMED	PROBABLE

LAST 14 DAYS	
38,051	238,029
CASES	TESTED (DIAGNOSTIC)

TOTAL TESTED	
2,140,705	105,584
DIAGNOSTIC	ANTIBODY

***TOTAL HOSPITALIZATIONS To Date Updates M-F at 3 p.m.**

41,859

STATEWIDE

PRESUMED RECOVERIES Updated Weekly

242,143

STATEWIDE

About This Dashboard

Data Source: Alabama Department of Public Health (ADPH) **Infectious Diseases & Outbreaks Division.**

This dashboard provides data and surveillance information about Coronavirus Disease 2019 (COVID-19) in Alabama. Data used to produce this dashboard is updated daily from the Alabama NEDSS Base System [illegible]

Alabama's COVID-19 Data and Surveillance Dashboard

Alabama Department of Public Health | Division of Infectious Diseases & Outbreaks | Updates Daily at 10 a.m.

Select a County --> None

Daily Number and Moving Averages of Cases by Date of Report (Statewide)

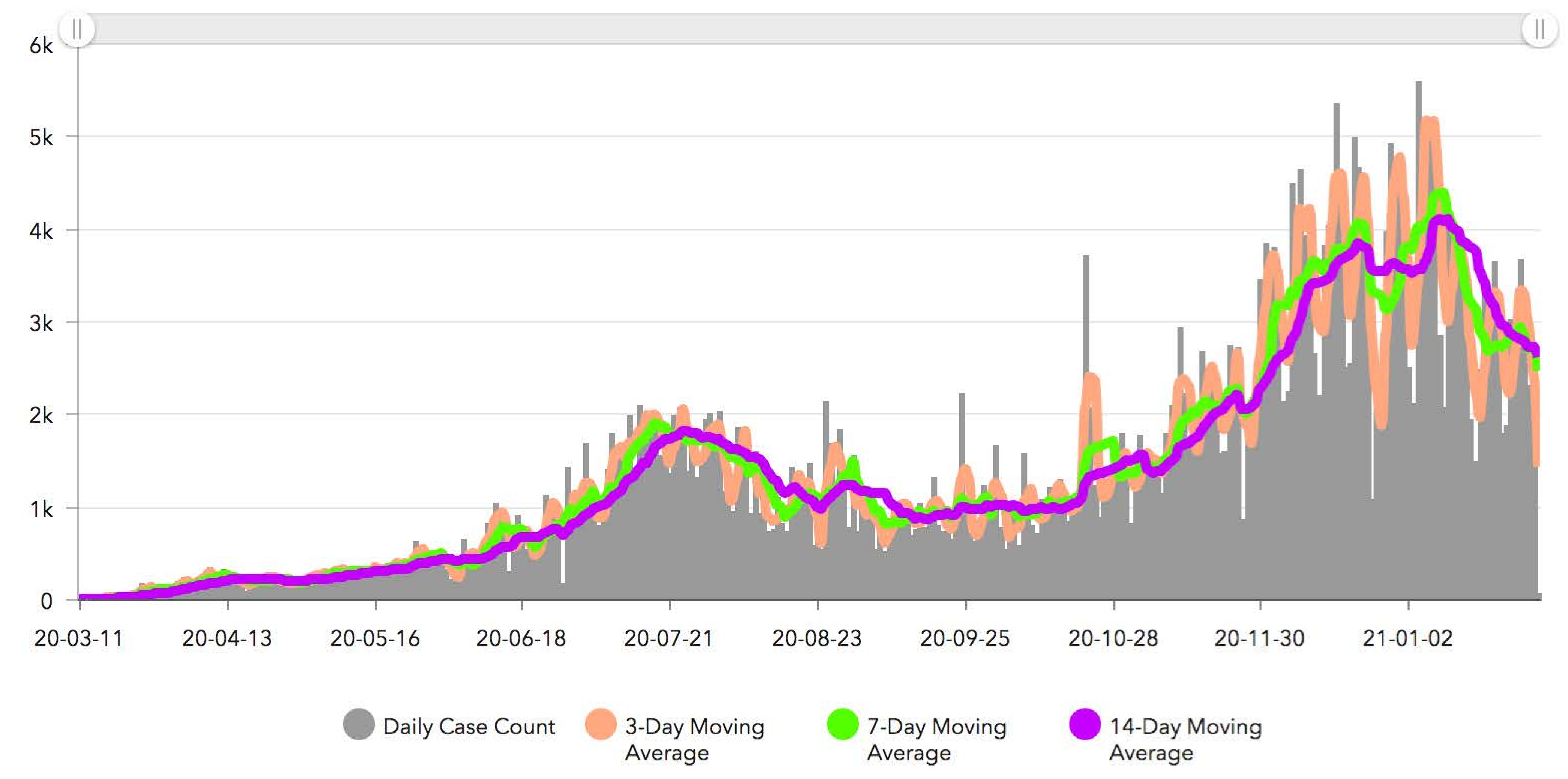

Due to technical issues, laboratory errors, and backlogs from onboarding new laboratories, the following dates: 06/02, 06/28, 08/11, 08/25, 09/24, 09/29, 10/05, 10/22, 10/30, 10/31, 11/13, 11/14, 12/1, 12/08, 12/10, 12/14, and 12/17 saw an inflation in total number of daily cases. All laboratories are required by law to report all results for SARS-CoV-2 to ADPH. Delays in reporting by required reporters is not within control of ADPH.

0 | 1 | 2 | 3 | 4 | 5 | 6 | 7A | 7B | 8 | 9 | 10 | 11 | 12 | 13 | 14

CASES

459,639

TOTAL

362,319	97,320
CONFIRMED	PROBABLE

DEATHS

7,688

TOTAL

6,171	1,517
CONFIRMED	PROBABLE

LAST 14 DAYS

38,051	238,029
CASES	TESTED (DIAGNOSTIC)

TOTAL TESTED

2,140,705	105,584
DIAGNOSTIC	ANTIBODY

***TOTAL HOSPITALIZATIONS**
To Date
Updates M-F at 3 p.m.

41,859

STATEWIDE

PRESUMED RECOVERIES
Updated Weekly

242,143

STATEWIDE

About This Dashboard

Data Source: Alabama Department of Public Health (ADPH) **Infectious Diseases & Outbreaks Division**.

This dashboard provides data and surveillance information about Coronavirus Disease 2019 (COVID-19) in Alabama. Data used to produce this dashboard is updated daily from the Alabama NEDSS Base System [illegible]

Alabama's COVID-19 Data and Surveillance Dashboard

Alabama Department of Public Health | Division of Infectious Diseases & Outbreaks | Updates Daily at 10 a.m.

Select a County --> None

PERCENTAGE OF POSITIVE TESTS

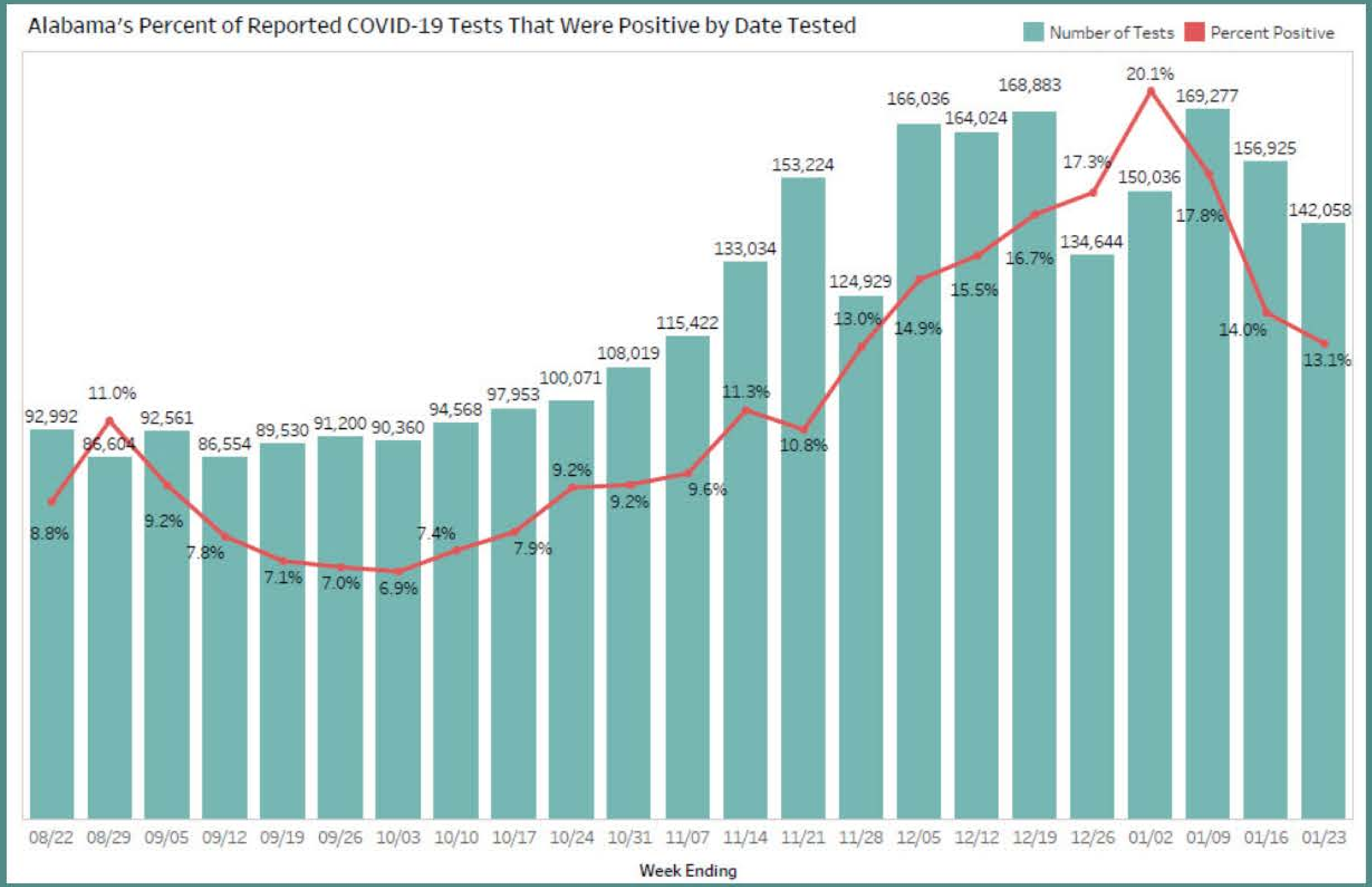

Laboratories are required by law to report both positive and negative COVID-19 test results to ADPH. To accurately calculate the percentage of positive test results, both numerator and denominator data are needed; test results from laboratories that do not report negative results (denominator data) are excluded from the calculation and from this graph. Only nucleic acid amplification and antigen test methods are included in this calculation. The week is assigned by the collection date (i.e., event date) so it represents the percent that were positive during the week listed. Data are preliminary and subject to change.

0 | 1 | 2 | 3 | 4 | 5 | 6 | 7A | 7B | 8 | 9 | 10 | 11 | 12 | 13 | 14

CASES

459,639

TOTAL

362,319	97,320
CONFIRMED	PROBABLE

DEATHS

7,688

TOTAL

6,171	1,517
CONFIRMED	PROBABLE

LAST 14 DAYS

38,051	238,029
CASES	TESTED (DIAGNOSTIC)

TOTAL TESTED

2,140,705	105,584
DIAGNOSTIC	ANTIBODY

*TOTAL HOSPITALIZATIONS To Date Updates M-F at 3 p.m.

41,859

STATEWIDE

PRESUMED RECOVERIES Updated Weekly

242,143

STATEWIDE

About This Dashboard

Data Source: Alabama Department of Public Health (ADPH) **Infectious Diseases & Outbreaks Division**.

This dashboard provides data and surveillance information about Coronavirus Disease 2019 (COVID-19) in Alabama. Data used to produce this dashboard is updated daily from the Alabama NEDSS Base System [illegible]

BAMATRACKER   Health   BECOME A PATRON

COVID-19

- Main Tracker
- Charts
- Counties
- School Data
- Vaccine Tracking
- Maps
- Mortality
- Demographics
- National Totals
- Daily Email
- F.A.Q.

OTHER

- Updates

Alabama COVID-19 Tracking

Total Cases	Total Deaths	New Cases	Recoveries
459,639	7,688	+4,057	242,143
362,319 Conf · 97,320 Prob	6,171 Conf · 1,517 Prob	+2,964 Conf · +1,093 Prob	As of 2021-01-27 · 66.83%

Total Tested	Vaccinations	Last 14 Days	Hospitalized
2,140,705	323,875	38,051	1,879
+13,397 Yesterday	772,275 Delivered · 41.94%	238,029 Tested · 15.99% Pos	41,859 Total

Confirmed Cases Per Day

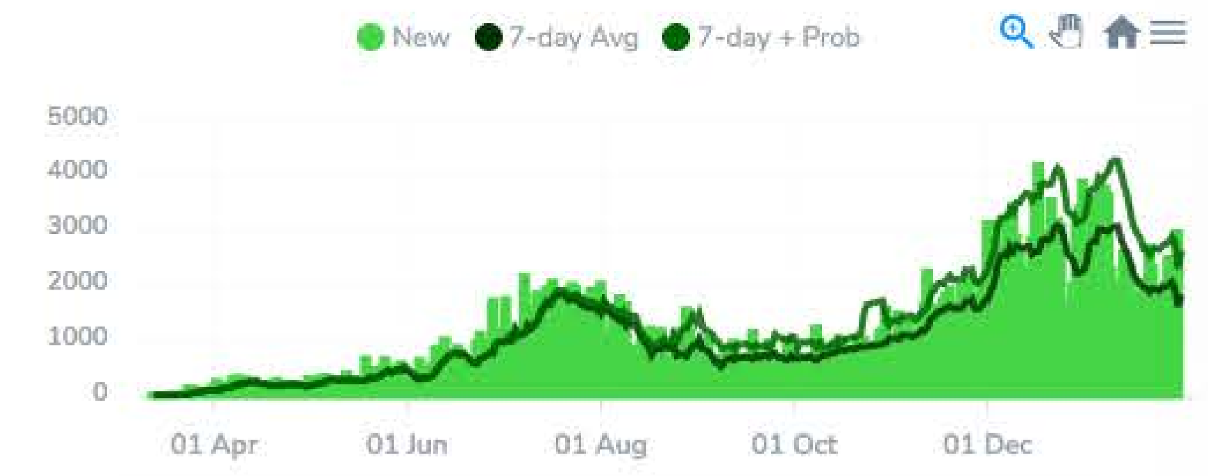

Deaths by Date

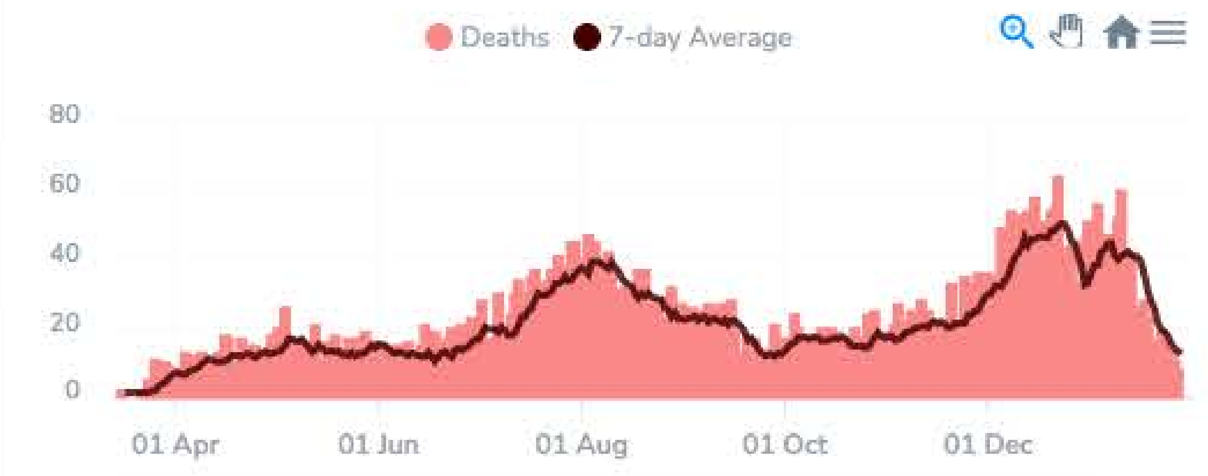

Confirmed Hospitalizations

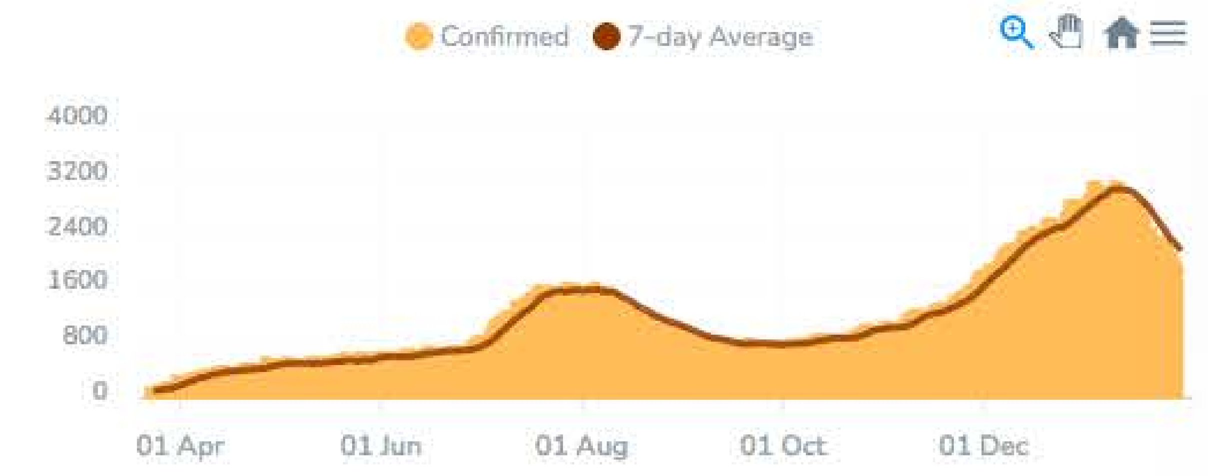

R_t Per Day

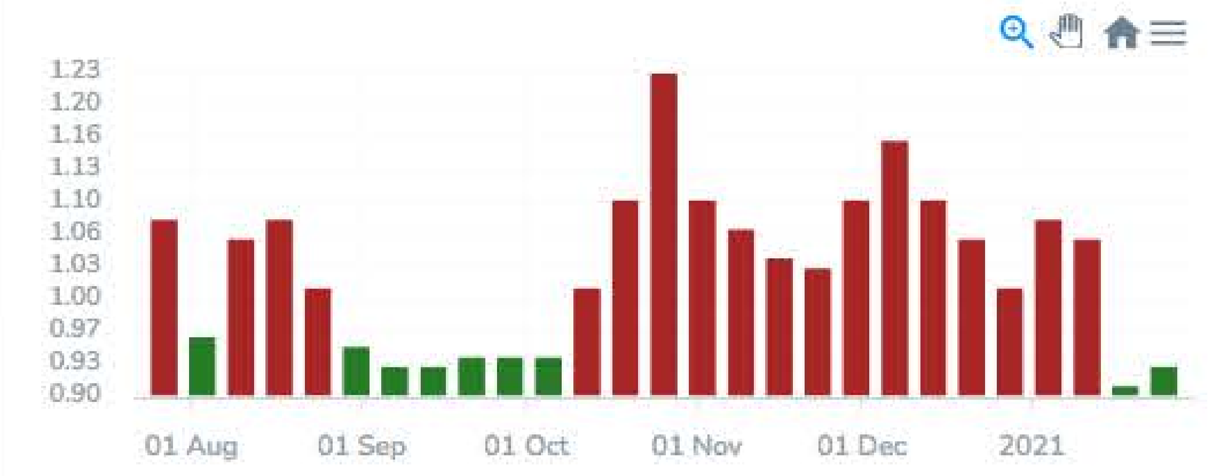

7-Day Avg. %Positive Per Day

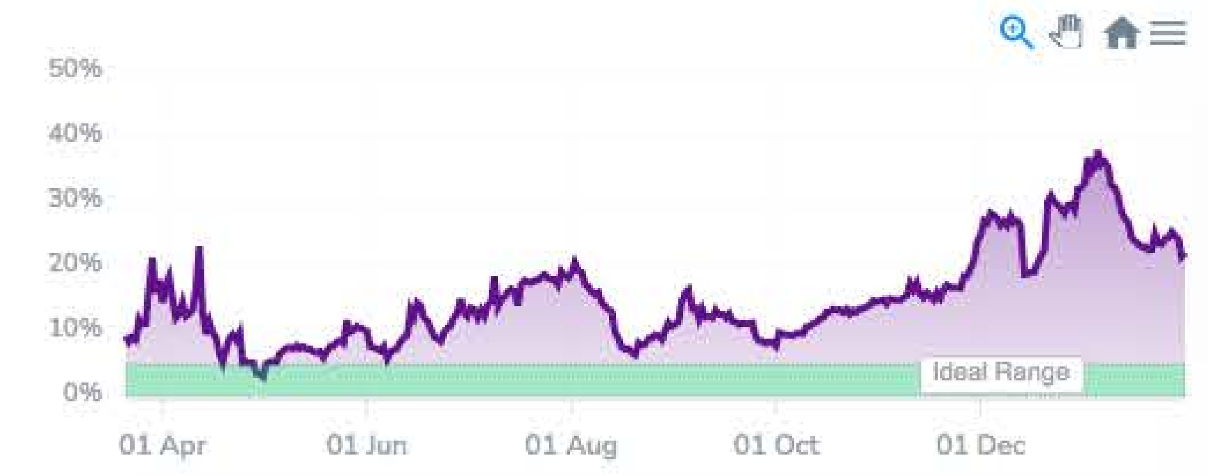

14-Day Counts %Positive Per Day

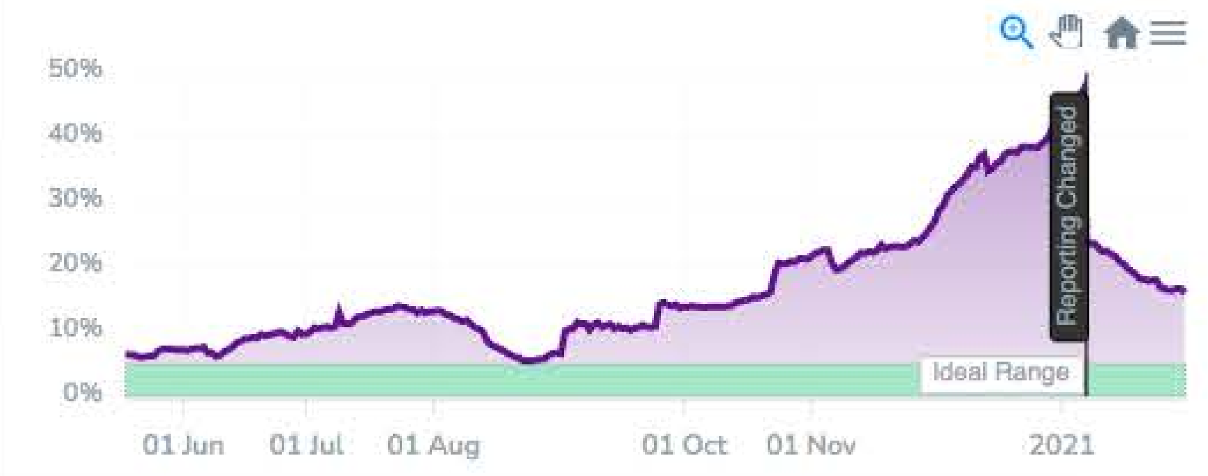

County Details

Show 25 entries   Search:

County	Cases	Change	Probable	Change	Deaths	Change	Tests	%Pos (14d)	7day Avg
Jefferson	60,429	+476	6,455	+85	1128	+16	359,748	12.74% ↑	252.86
Madison	26,147	+282	3,533	+65	287	+13	166,872	15.43% ↑	147.71
Mobile	24,906	+236	8,154	+77	611	+3	164,066	15.51% ↓	151.71
Montgomery	19,033	+209	1,888	+15	370	+7	89,369	17.76% ↓	121.43
Shelby	17,721	+191	2,495	+12	160	+10	98,724	16.50% ↓	112.57
Tuscaloosa	15,334	+95	7,087	+85	302	+3	127,634	12.01% ↑	62.57
Baldwin	12,884	+146	5,242	+58	224	0	88,854	20.01% ↑	73.86
Morgan	10,903	+56	2,098	+9	175	+4	47,122	16.12% ↓	36.71
Etowah	9,830	+56	2,685	+35	246	+6	50,585	14.84% ↓	38.86
Marshall	9,370	+51	1,410	+15	152	+8	39,532	17.56% ↓	34.86
Calhoun	8,987	+54	3,024	+39	232	0	53,082	16.04% ↑	34.57
Lee	7,832	+75	5,997	+75	128	+4	72,722	17.44% ↑	46.86
Limestone	7,467	+52	1,209	+6	106	+1	31,956	19.48% ↓	36.00
DeKalb	7,060	+32	1,073	+12	137	+1	27,028	20.03% ↑	26.71
St. Clair	6,874	+47	1,285	+21	155	+6	36,261	17.77% ↓	32.43
Elmore	6,476	+39	2,126	+29	134	+2	40,050	17.35% ↓	29.43
Cullman	5,838	+22	2,713	+25	149	+2	34,935	14.72% ↓	19.86
Lauderdale	5,352	+26	2,828	+20	156	+5	36,639	15.31% ↑	28.29
Walker	5,329	+26	829	+1	197	+3	24,475	13.97% ↓	16.57
Jackson	5,296	+34	949	+11	70	+1	26,673	20.54% ↑	24.14
Houston	5,035	+45	4,436	+57	209	+9	42,920	20.51% ↑	28.57
Autauga	4,977	+58	692	+15	69	0	22,521	19.06% ↑	33.29
Talladega	4,691	+47	2,146	+28	117	+1	30,232	17.51% ↓	40.14
Blount	4,375	+51	1,338	+7	100	+2	20,329	17.41% ↑	30.43
Colbert	4,100	+10	1,636	+16	104	0	23,272	16.29% ↑	22.14
Total	362,319	2,964	97,320	1,093	7,688	122	2,140,705	40 ↑ 27 ↓	

Showing 1 to 25 of 67 entries

Previous 1 2 3 Next

2020 - 2021 © Magic & Wires LLC. This website is for educational and academic research purposes only. Do not rely upon the content of this website as a basis for making any business, legal or health decisions. COVID-19 Data collected from ADPH, CDC & HHS.

Terms & Conditions

AK COVID-19 Cases Dashboard

All data are updated once daily by noon and are current through the previous day

Select Occurence

In State | Out of State | All

Select Residence

Resident | Nonresident | All

Select Region(s)

All Available

Select Borough(s)/Census Area(s)

All Available

Select a Date Range or Manually Specify

All Dates

Alert Levels (Map Layers Must be Selected Manually)

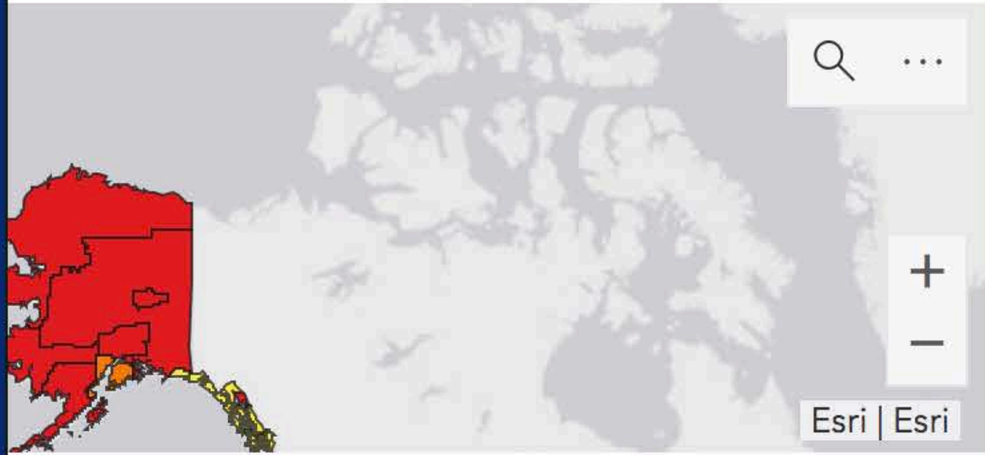

Default layer: Alaska resident cases. For more layer options select the highlighted icon in the top right corner of the map.

Alert Level | 7-Day Rate | Cumulative Cases

Statewide Cases by Age Group

Filterable by residence status only

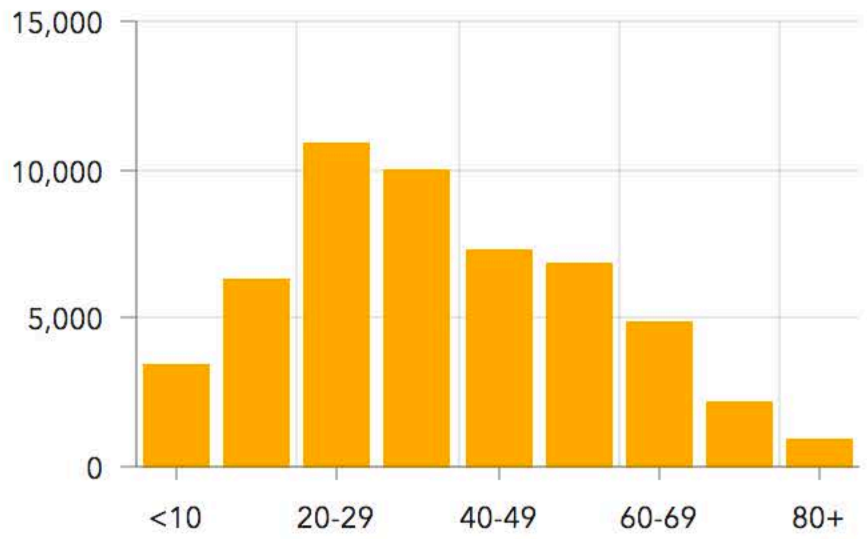

Age | Gender | Race | Ethnicity

Total Cases

52,470

Cases have not **been updated for today.** Please check back later.

Hospitalizations

1,179

Total count (does not reflect current stays)

Deaths

260

COVID-19 Cases by Report Date

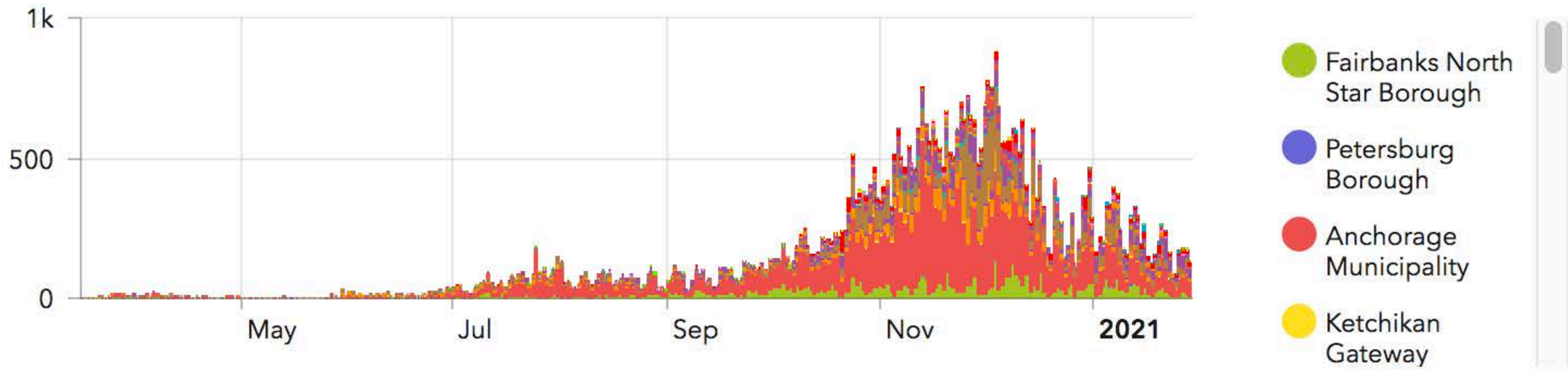

Includes "confirmed" and "probable". Report date reflects the date case was submitted to DHSS, not the onset date of symptoms.

Report Date | Onset Date | Acquisition of Disease | Hospitalization Date | Deaths

AK COVID-19 Cases Dashboard

All data are updated once daily by noon and are current through the previous day

Select Occurence

In State | Out of State | All

Select Residence

Resident | Nonresident | All

Select Region(s)

All Available

Select Borough(s)/Census Area(s)

All Available

Select a Date Range or Manually Specify

All Dates

Average Daily Case Rate in Last 7 Days

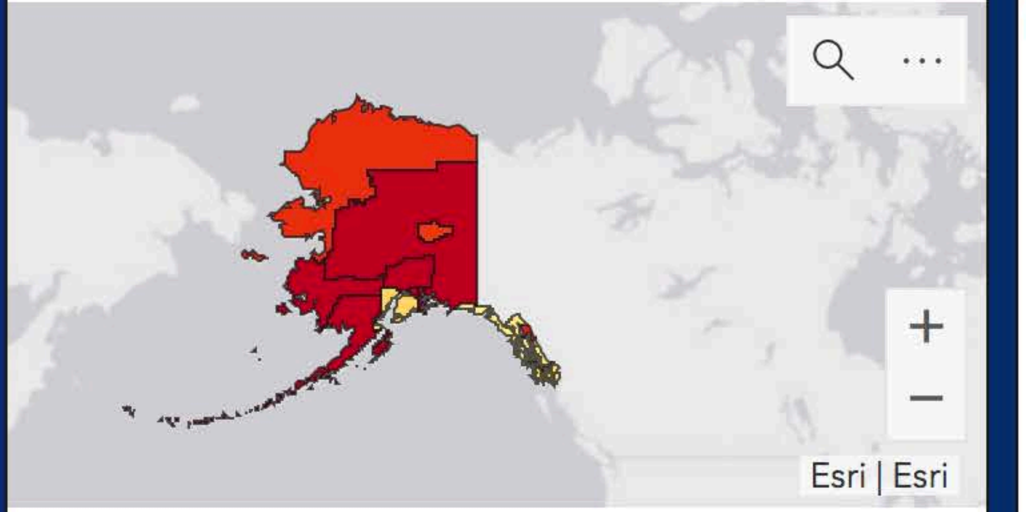

Default layer: Alaska resident cases. For more layer options select the highlighted icon in the top right corner of the map.

Alert Level | 7-Day Rate | Cumulative Cases

Statewide Cases by Race

Filterable by residence status only

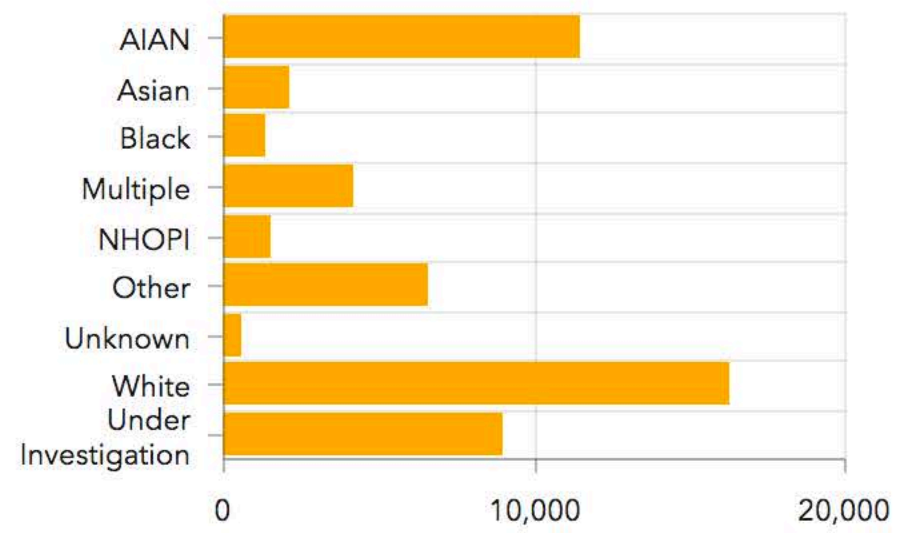

Age | Gender | Race | Ethnicity

Statewide COVID-19 Hospitalizations by Hospitalization Date

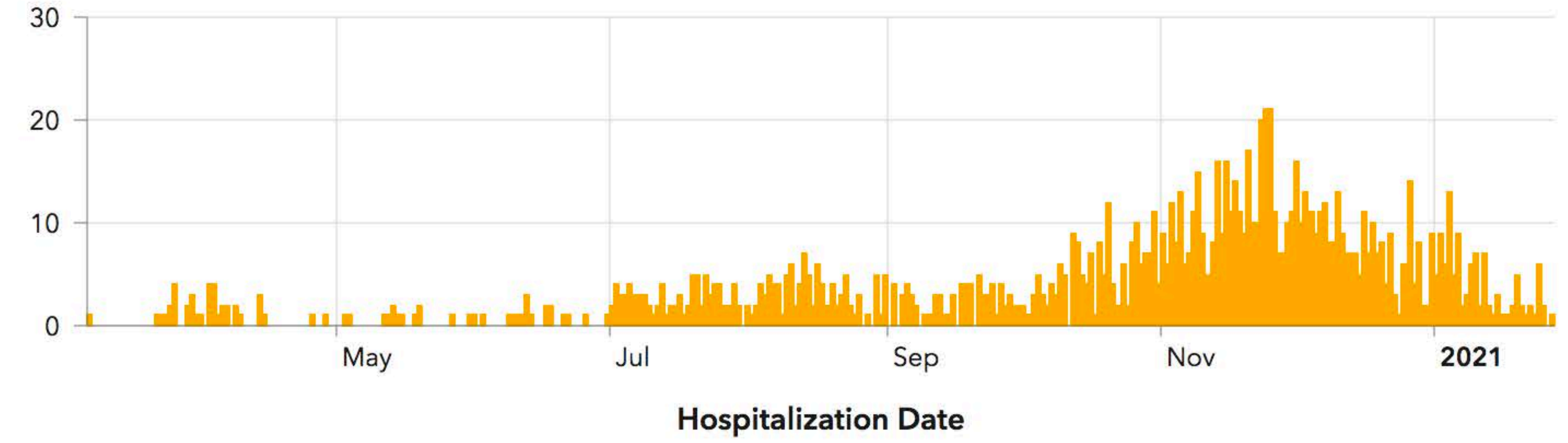

Report Date | Onset Date | Acquisition of Disease | Hospitalization Date | Deaths

Total Cases

52,470

Cases have not **been updated for today.** Please check back later.

Hospitalizations

1,179

Total count (does not reflect current stays)

Deaths

260

Data Dashboard

- As our federal partners have updated guidance for identifying COVID-19 deaths, ADHS has enhanced our processes to match. This change may cause the number of new deaths reported on our dashboard to increase more than expected for a few days. Deaths are shown on the bar chart of the COVID-19 deaths section of the dashboard using the date of death, not the day reported. New deaths on the summary section of the dashboard are displayed by the day reported to ADHS.

Summary
Demographics
COVID-19 Deaths
Zip Code
Confirmed COVID-19 Cases by Day
Hospitalization
Hospital COVID-like & Influenza-like illness Surveillance
Laboratory Testing
Congregate Settings with Positive COVID-19 Cases
Hospital Bed Usage & Availability
Ventilator Usage & Availability
Hospital COVID-19 Specific Metrics

Summary

Show case counts or population rates in the map below?
Cases

Select a county to filter the other numbers.
Deaths will not be shown for counties with fewer than three deaths.

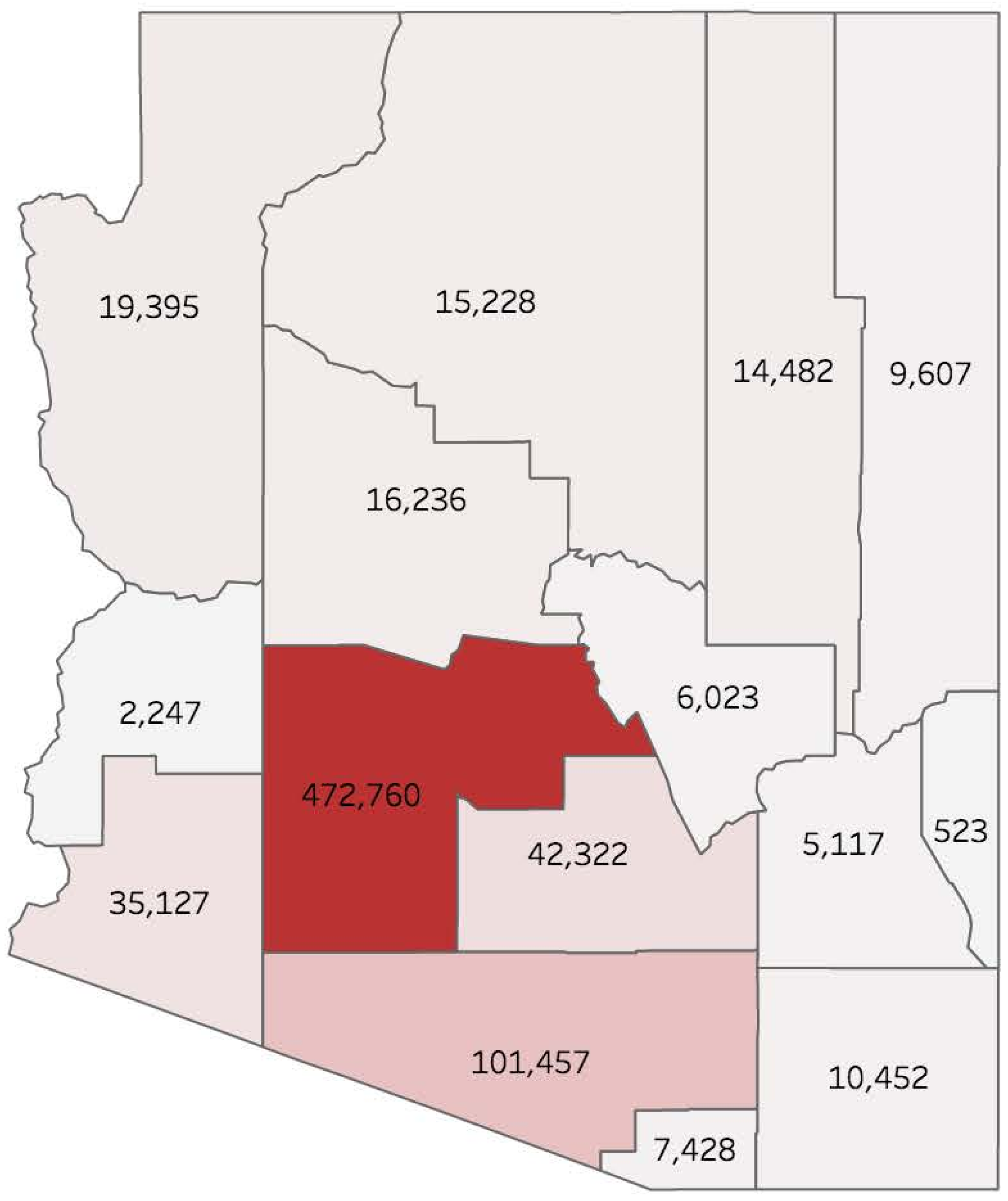

Number of Cases<br>758,404	Number of Deaths<br>13,120	Number of COVID-19 Tests<br>3,875,353
Number of New Cases reported today*<br>5,025	Number of New Deaths reported today*<br>22	Number of New Tests reported today*<br>18,430
Rate of cases, per 100,000 population<br>10,549.5	Rate of fatalities, per 100,000 population<br>182.50	Total Percent Positive**<br>14.3%
Total COVID-19 Diagnostic Tests***<br>3,457,316	New Diagnostic Tests reported today*<br>17,199	Diagnostic Tests Percent Positive**<br>14.8%
Total COVID-19 Serology Tests<br>418,037	New Serology Tests reported today*<br>1,231	Serology Percent Positive**<br>11.3%

*Counts of new cases, deaths, and numbers tested reflect increases in the total numbers compared to the previous day.

**Percent positive is defined as number of people with a positive test result, reported electronically out of all people with COVID-19 testing reported electronically completed in AZ.

***Diagnostic tests include PCR and antigen testing.

Date Updated: 1/31/2021

Feedback & Support

Data Dashboard

- As our federal partners have updated guidance for identifying COVID-19 deaths, ADHS has enhanced our processes to match. This change may cause the number of new deaths reported on our dashboard to increase more than expected for a few days. Deaths are shown on the bar chart of the COVID-19 deaths section of the dashboard using the date of death, not the day reported. New deaths on the summary section of the dashboard are displayed by the day reported to ADHS.

Summary | Demographics | COVID-19 Deaths | Zip Code

Confirmed COVID-19 Cases by Day | Hospitalization | Hospital COVID-like & Influenza-like illness Surveillance | Laboratory Testing

Congregate Settings with Positive COVID-19 Cases | Hospital Bed Usage & Availability | Ventilator Usage & Availability | Hospital COVID-19 Specific Metrics

COVID-19 Deaths

COVID-19 Deaths (total)	New COVID-19 Deaths Reported Today
13,120	22

Hover over the icon to get more information on the data in this dashboard.

COVID-19 Deaths by County

Data will not be shown for counties with fewer than three deaths.

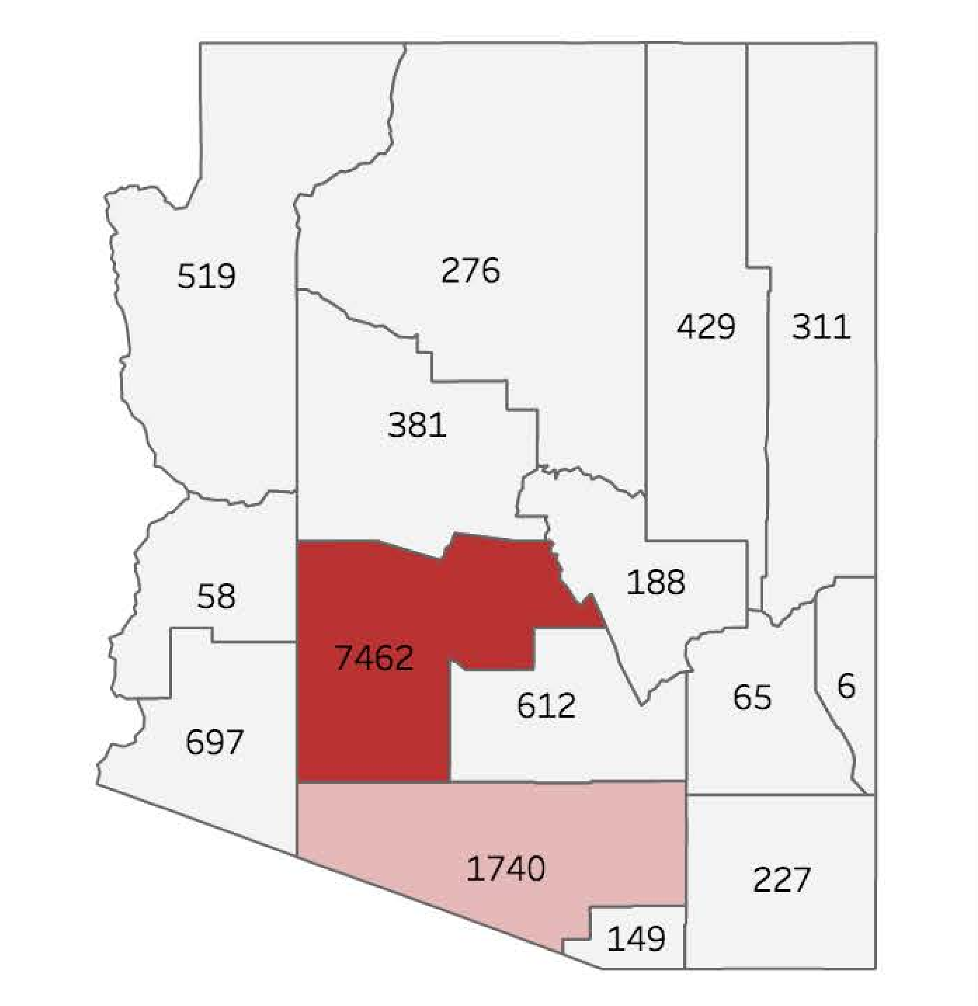

COVID-19 Deaths by Date of Death

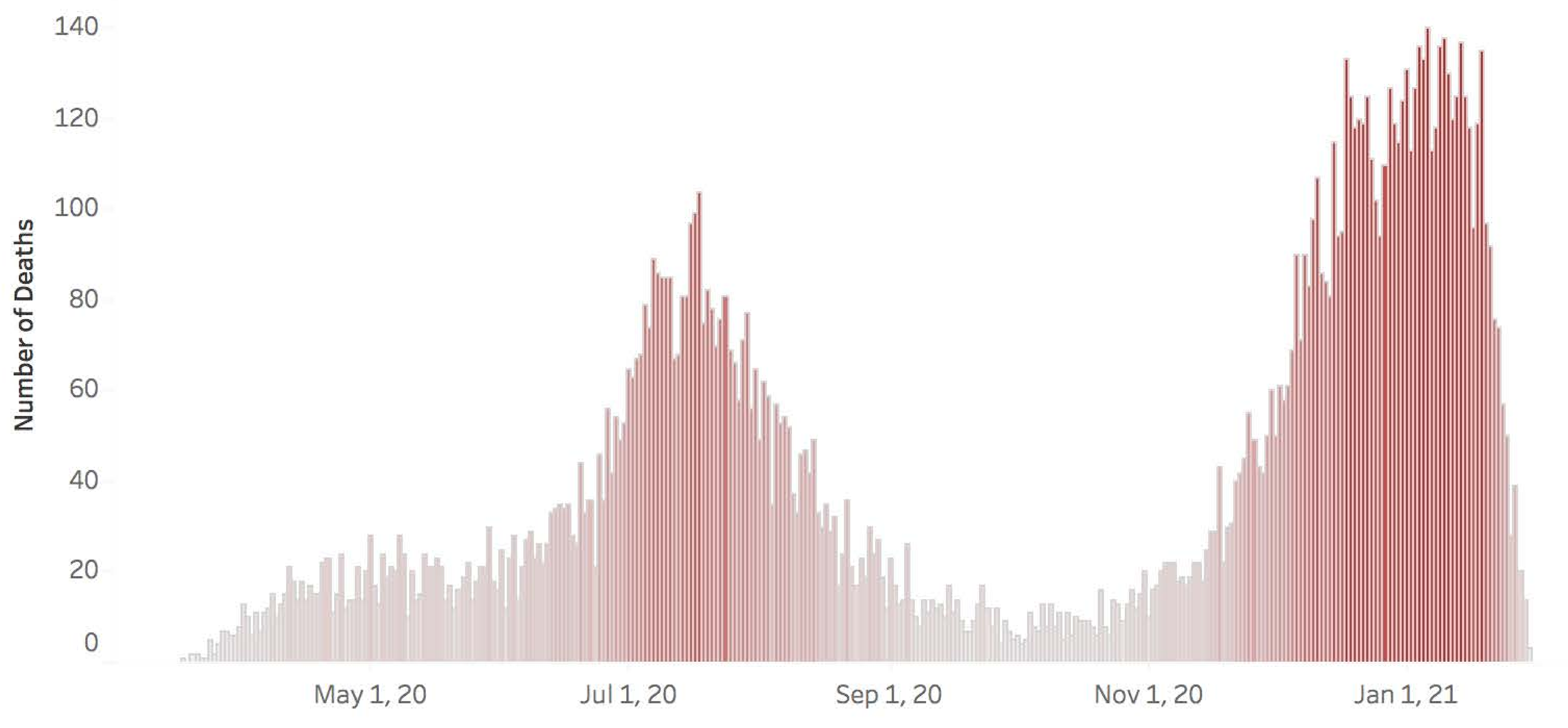

Recent deaths may not be reported yet. Cases missing the date of death are excluded from the graph above, but are included in all other numbers.

COVID-19 Deaths by Gender

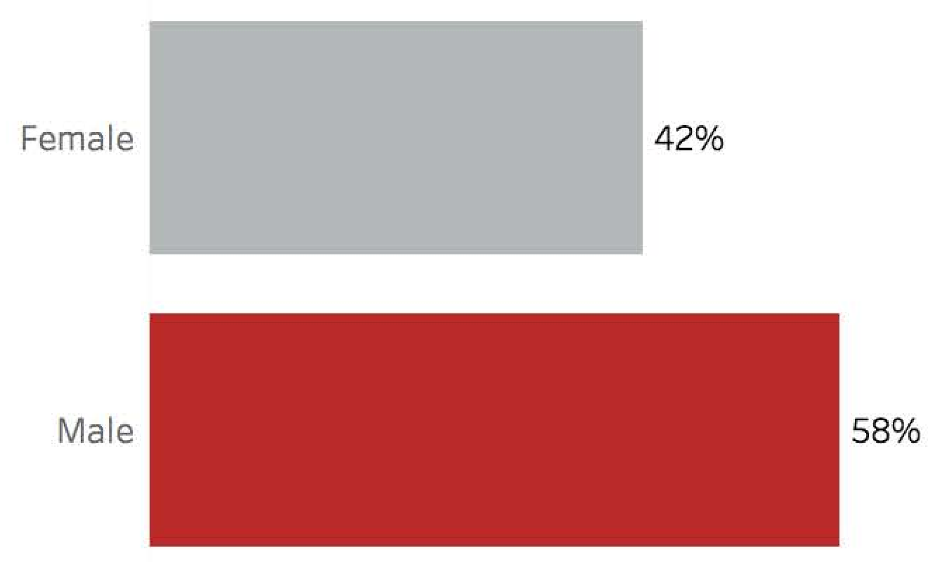

COVID-19 Deaths by Age Group

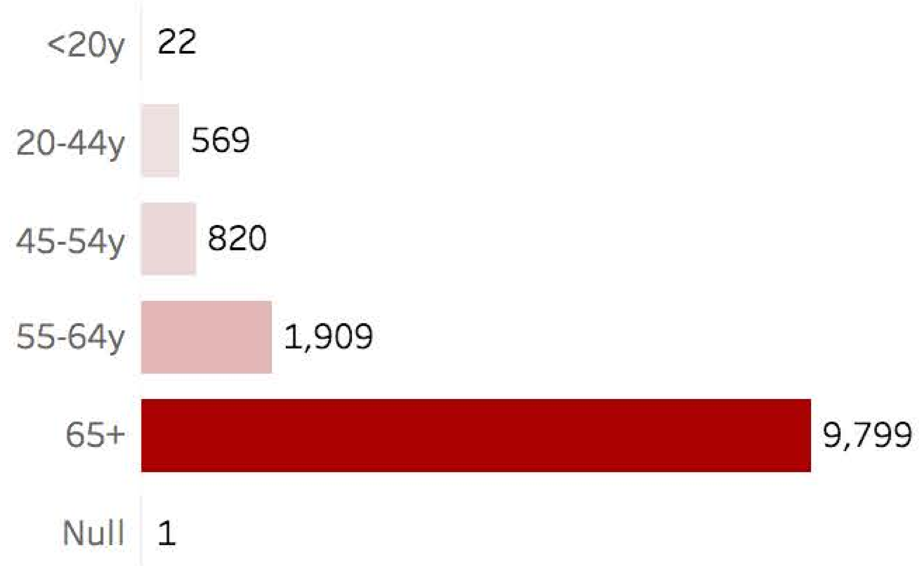

COVID-19 Deaths by Race/Ethnicity

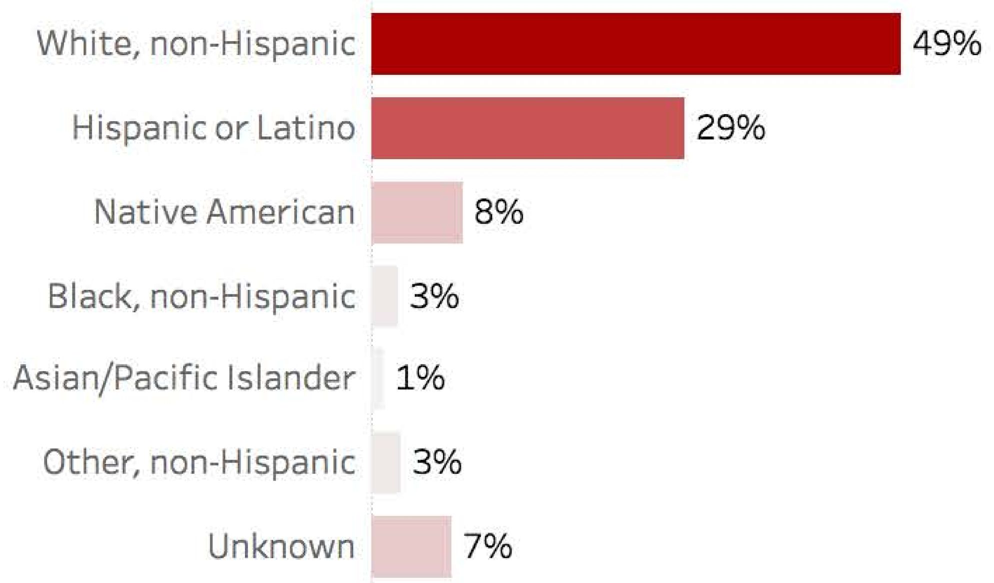

Date Updated: 1/31/2021

Feedback & Support

Data Dashboard

- As our federal partners have updated guidance for identifying COVID-19 deaths, ADHS has enhanced our processes to match. This change may cause the number of new deaths reported on our dashboard to increase more than expected for a few days. Deaths are shown on the bar chart of the COVID-19 deaths section of the dashboard using the date of death, not the day reported. New deaths on the summary section of the dashboard are displayed by the day reported to ADHS.

Summary
Demographics
COVID-19 Deaths
Zip Code
Confirmed COVID-19 Cases by Day
Hospitalization
Hospital COVID-like & Influenza-like illness Surveillance
Laboratory Testing
Congregate Settings with Positive COVID-19 Cases
Hospital Bed Usage & Availability
Ventilator Usage & Availability
Hospital COVID-19 Specific Metrics

Zip Code

COVID-19 Cases by Zip Code

Mobile Version

Case Count

- 0
- 1-10
- 11-100
- 101-500
- 501-1000
- Greater Than 1000
- Data Suppressed

Download Data

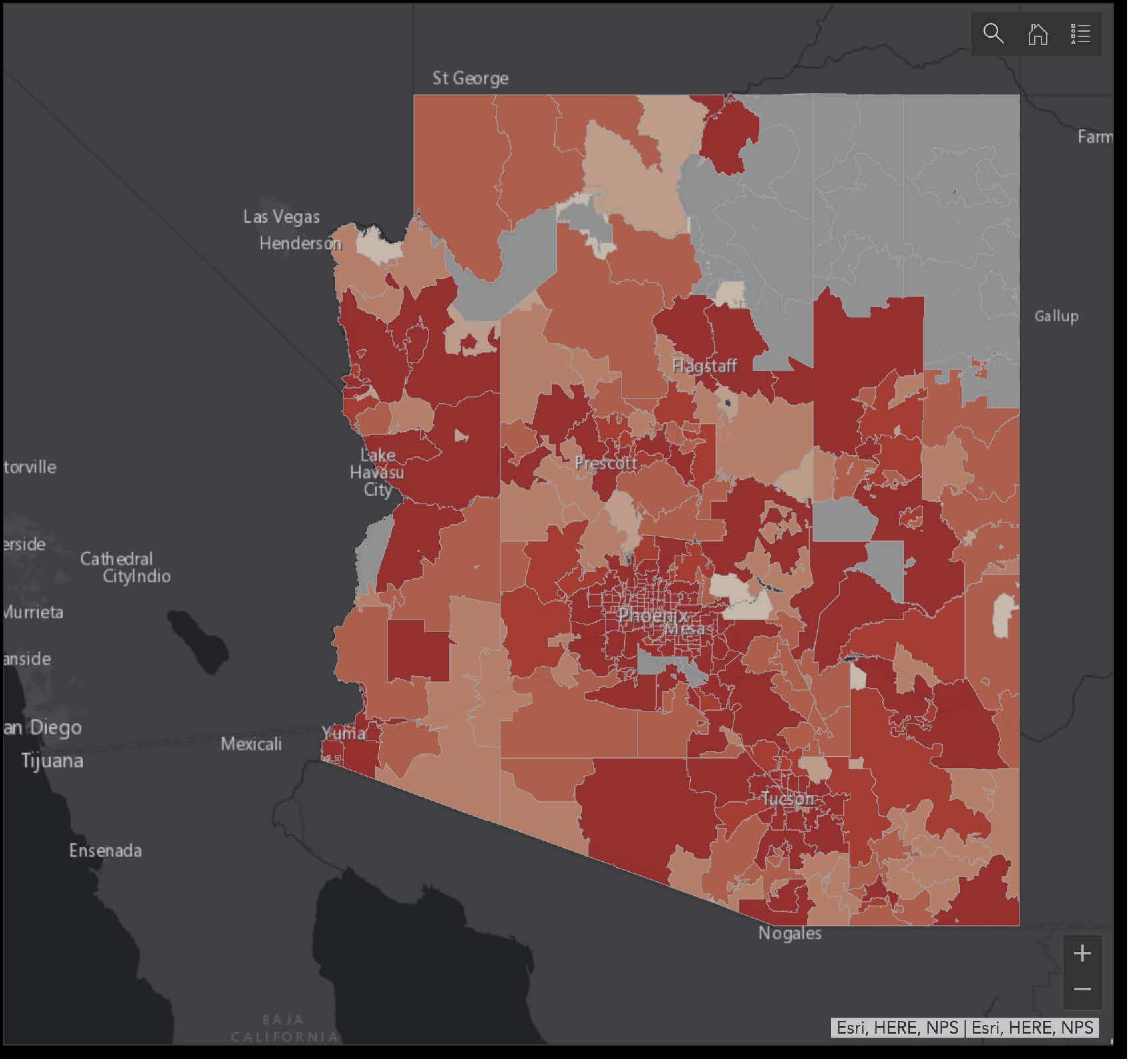

Suppressed data is pending Tribal approval.

Includes cases in MEDSIS, the Arizona surveillance system, with a classification of confirmed or probable.

Over 90% of cases were mapped to the address of the patient's residence. If the patient's address was unknown the case was mapped to the address of the provider followed by the address of the reporting facility.

The zip codes represented on this map are generalized representations of United States Postal Service (USPS) ZIP Code service areas. Any case with a [illegible]

Feedback

Data Dashboard

ADHS Home / Public Health Preparedness / Epidemiology & Disease Control / Infectious Disease Services / Coronavirus Disease 2019 (COVID-19) / Data Dashboard

- As our federal partners have updated guidance for identifying COVID-19 deaths, ADHS has enhanced our processes to match. This change may cause the number of new deaths reported on our dashboard to increase more than expected for a few days. Deaths are shown on the bar chart of the COVID-19 deaths section of the dashboard using the date of death, not the day reported. New deaths on the summary section of the dashboard are displayed by the day reported to ADHS.

Summary | Demographics | COVID-19 Deaths | Zip Code

Confirmed COVID-19 Cases by Day | Hospitalization | Hospital COVID-like & Influenza-like illness Surveillance | Laboratory Testing

Congregate Settings with Positive COVID-19 Cases | Hospital Bed Usage & Availability | Ventilator Usage & Availability | Hospital COVID-19 Specific Metrics

Laboratory Testing

People Tested | Tests Conducted

Choose a test type to filter the data.
All

Number of People Tested
See info button for more information.

Hover over the icon to get more information on the data in this dashboard.

People tested using All tests completed for COVID-19	People tested using All tests reported yesterday in Arizona	Total % Positive COVID-19 All Tests
3,875,353	18,430	14.3%

People tested using All tests by county
Select a county to filter the data.

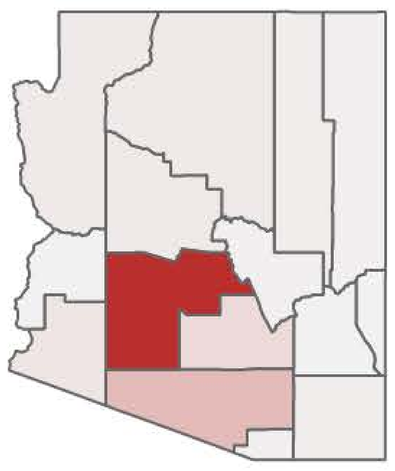

People tested using All tests by date of collection

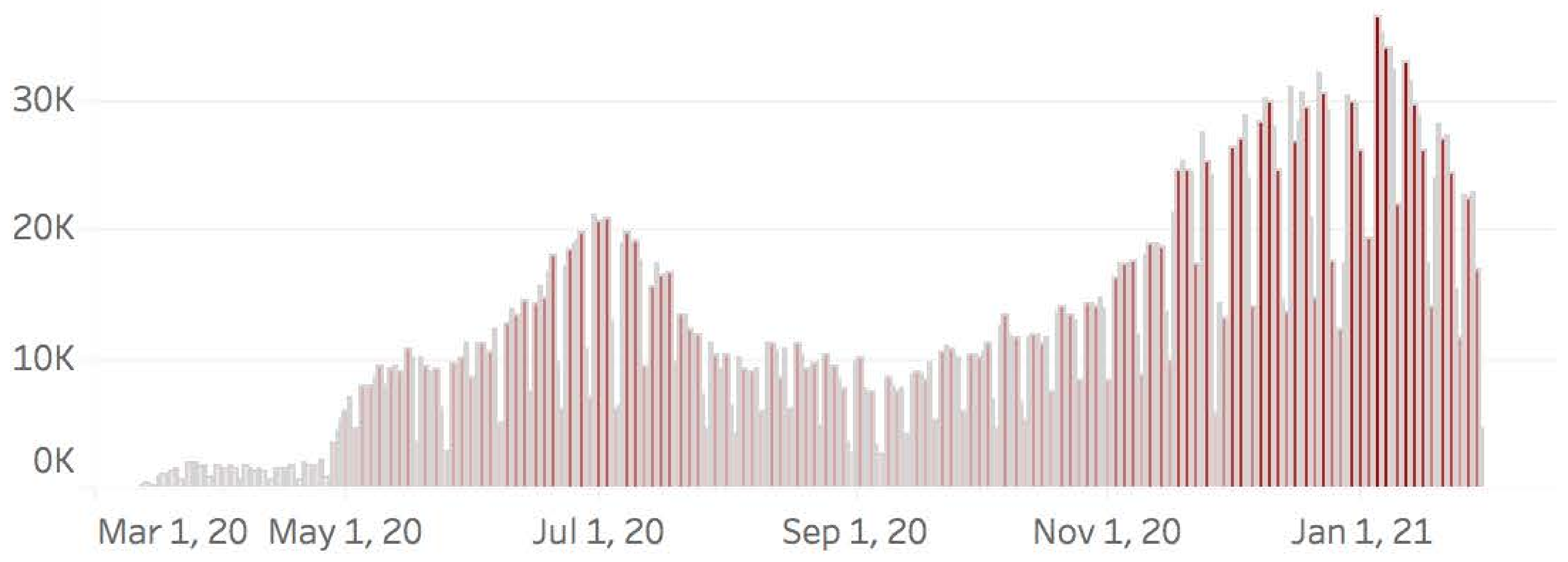

People tested using All Testing by Age Group

Age Group	People tested
Less than 20 years	620,315
20 - 44 years	1,569,343
45 - 54 years	531,032
55 - 64 years	507,046
65 years and older	638,149
Unknown	9,468

People tested for COVID-19 and percent positive by week

Percent positive is the number of people with a positive result in Electronic Laboratory Reporting (ELR), out of all people with COVID-19 testing reported via ELR in AZ. Diagnostic tests include PCR

Total % Positive COVID-19 Diagnostic Tests: **14.8%**

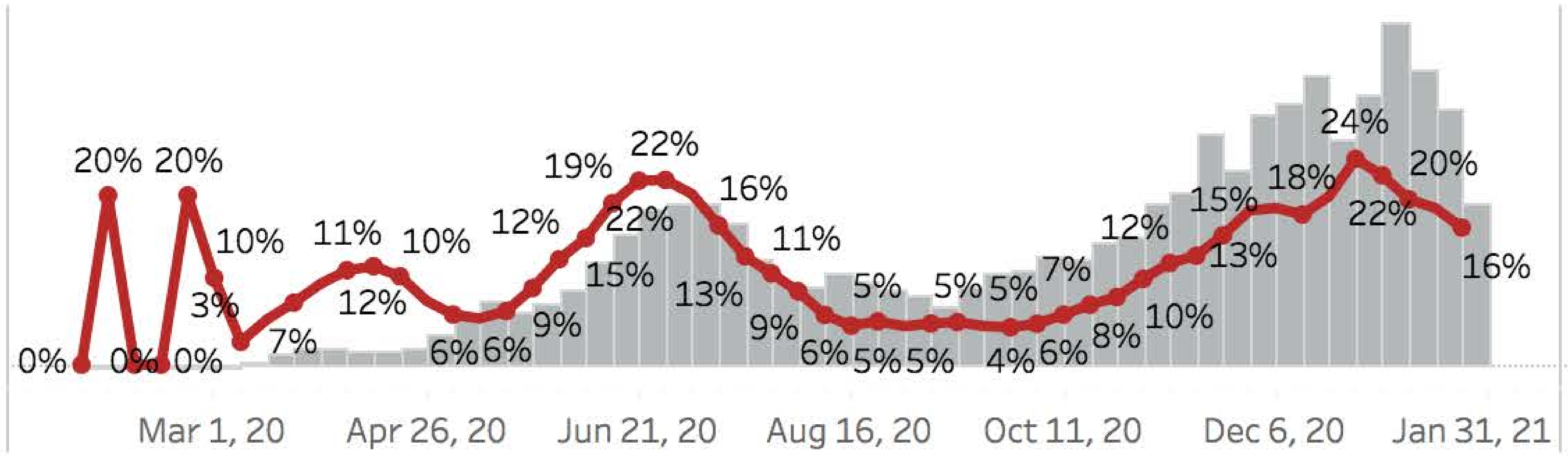

Total % Positive COVID-19 Serology Tests: **10.0%**

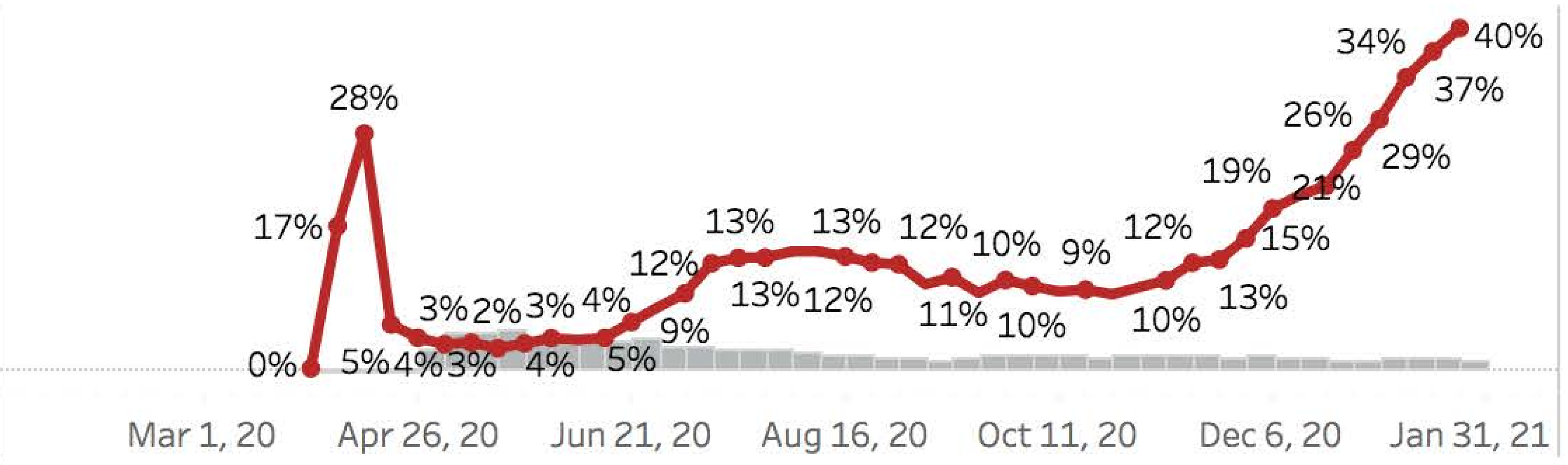

Date Updated: 1/31/2021

*NOTE: Results from the last 4-7 days may not be reported yet.

Feedback

The Arkansas Department of Health COVID-19 Case Update

Total COVID-19 Cases

294,387

Confirmed Cases

234,905

Probable Cases

59,482

Recovered Cases

271,911

Cumulative Number of Cases
(last 14 days)

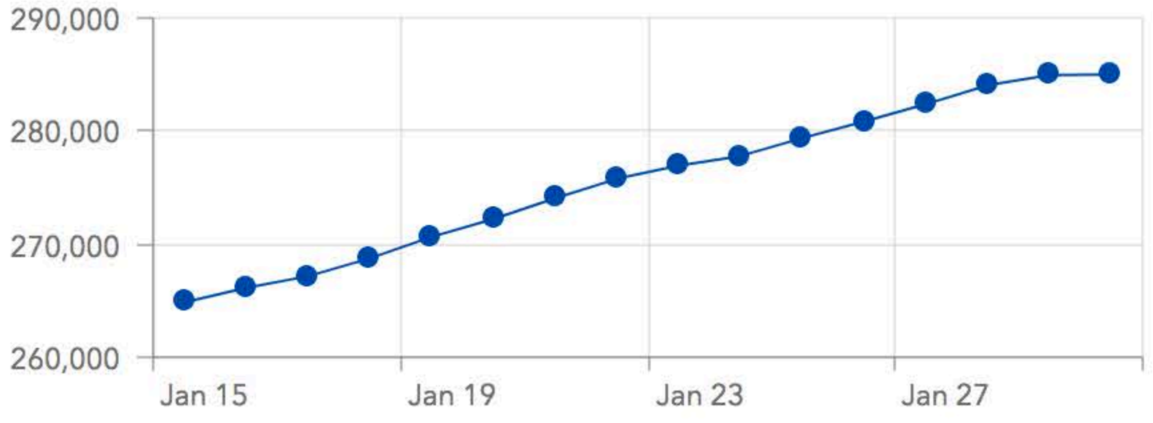

Number of COVID-19 Cases by County of Residence

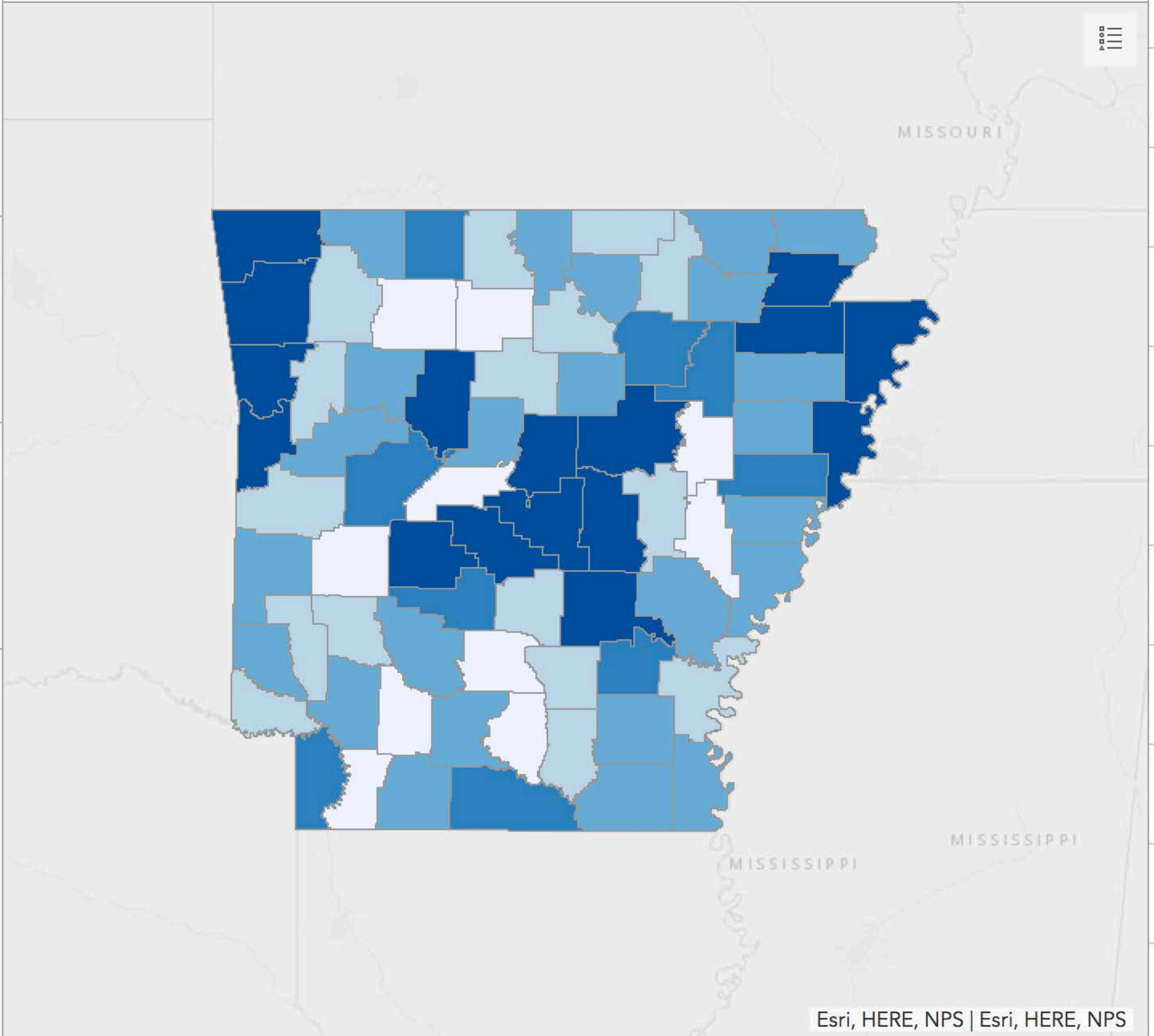

Arkansas (1,770 Total Positive Cases)

Ashley (1,637 Total Positive Cases)

Baxter (2,629 Total Positive Cases)

Benton (25,025 Total Positive Cases)

Boone (3,329 Total Positive Cases)

Bradley (1,247 Total Positive Cases)

Calhoun (344 Total Positive Cases)

Carroll (2,575 Total Positive Cases)

Chicot (1,551 Total Positive Cases)

Clark (1,831 Total Positive Cases)

Clay (1,562 Total Positive Cases)

Total Cases | Active Cases | Deaths | Tests | Case Demographics | Health Metrics | Hospital Information | USA and Global

The Arkansas Department of Health COVID-19 Case Update

Totals Cases by Gender

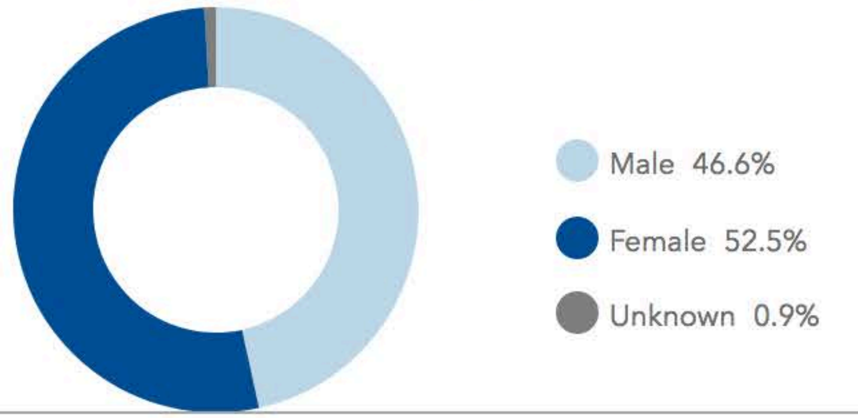

Cases by Race

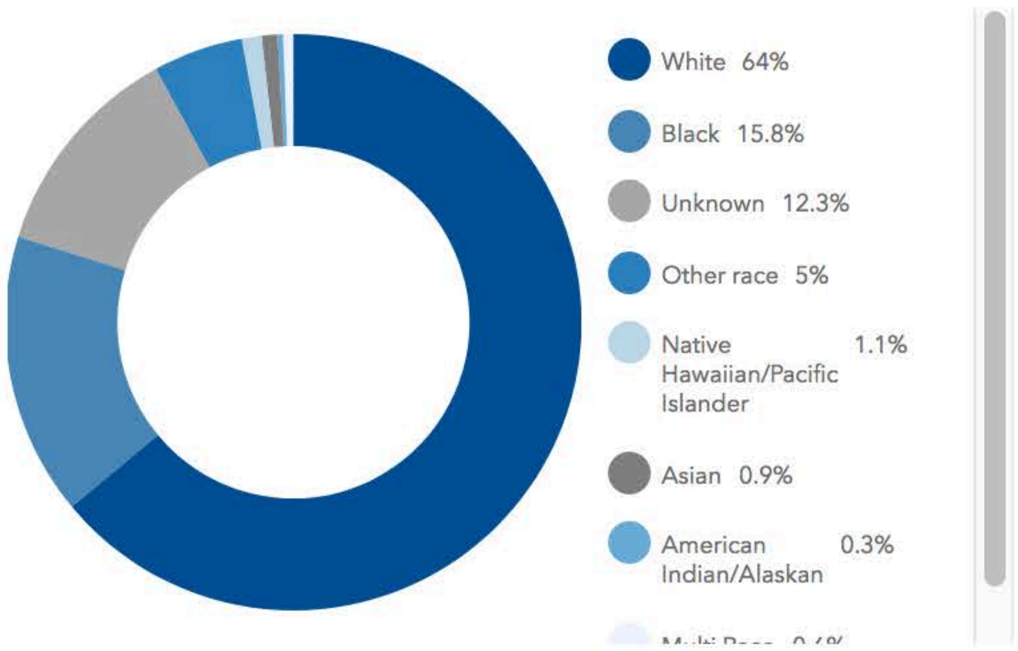

Cases by Ethnicity

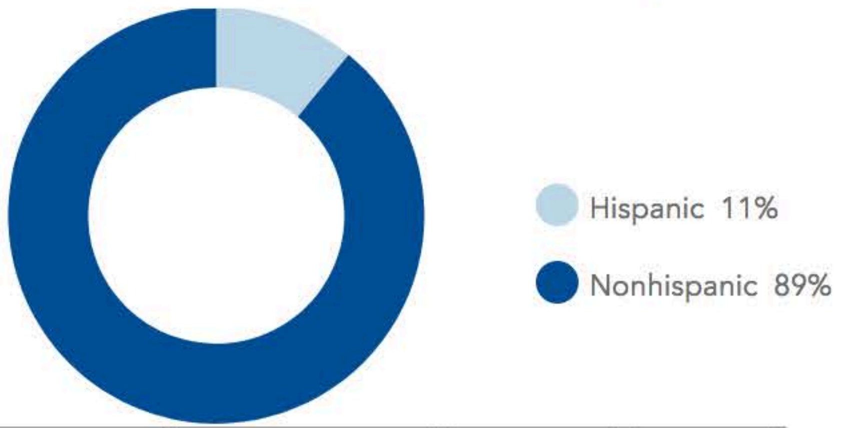

Cases by Age Category

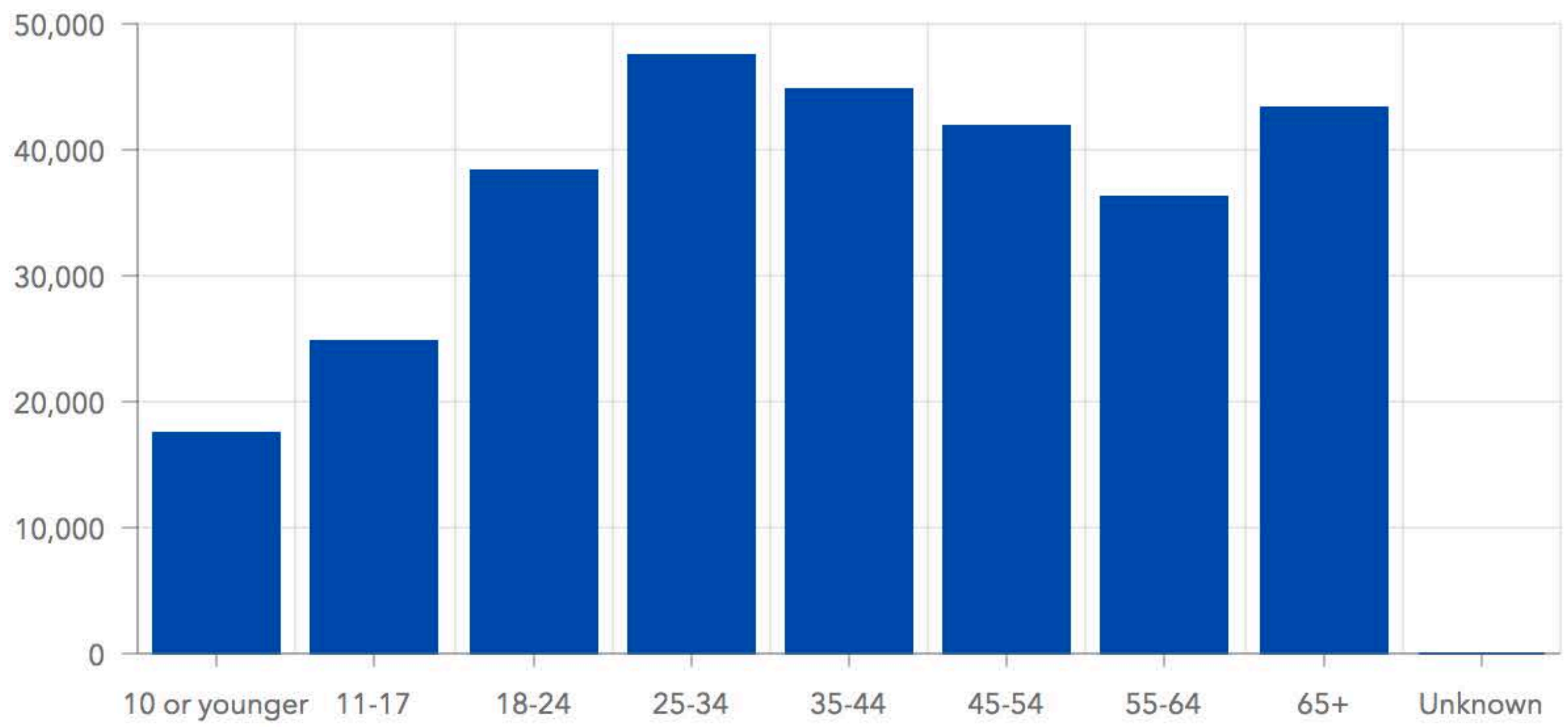

Cases by Underlying Health Concerns

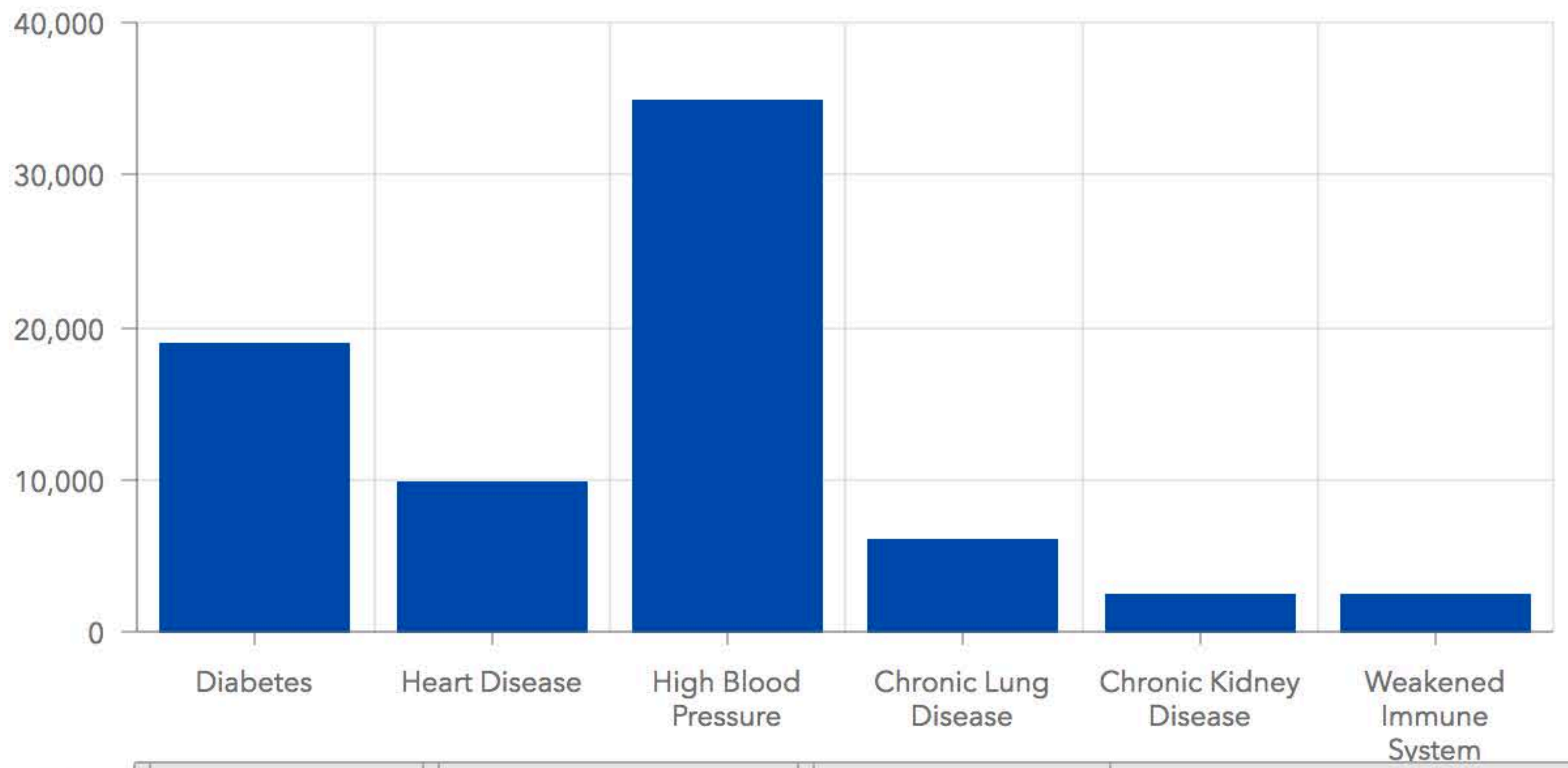

Percent of Cases That Are Healthcare Workers

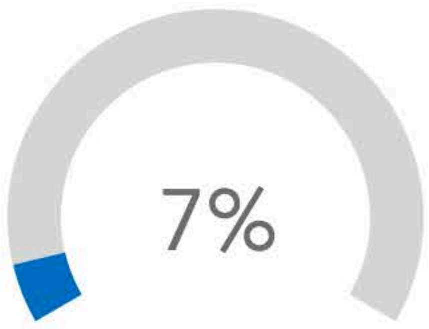

Percent of Cases In Nursing Homes

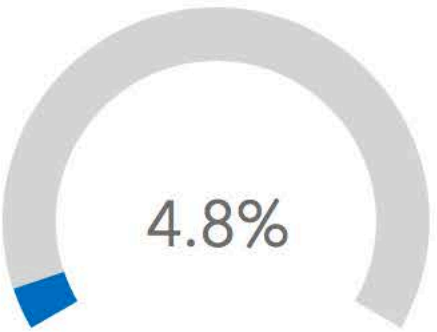

Percent of Cases In Correctional Facilities

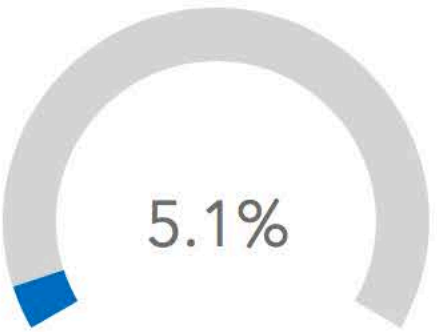

The Arkansas Department of Health COVID-19 Case Update

Total Tests Reported

2,781,116

Percent Positive PCR Tests	Percent Positive Antigen Tests
9.7 %	19.3 %

14 Day Testing Trend - PCR

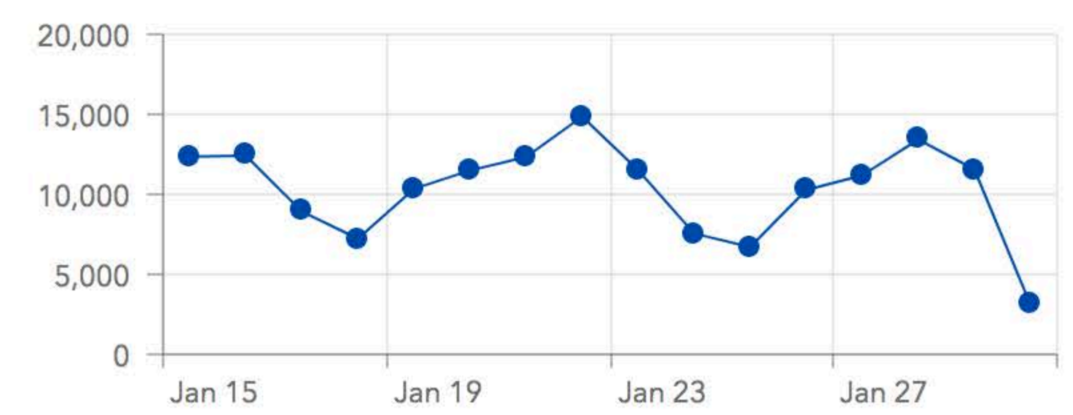

14 Day Testing Trend - Antigen

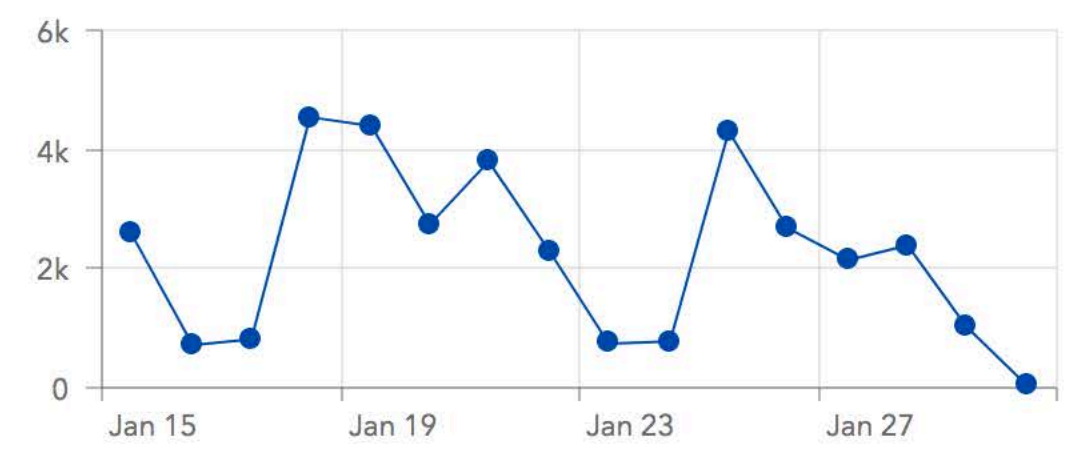

COVID-19 Tests Resulted per 1,000 Individuals

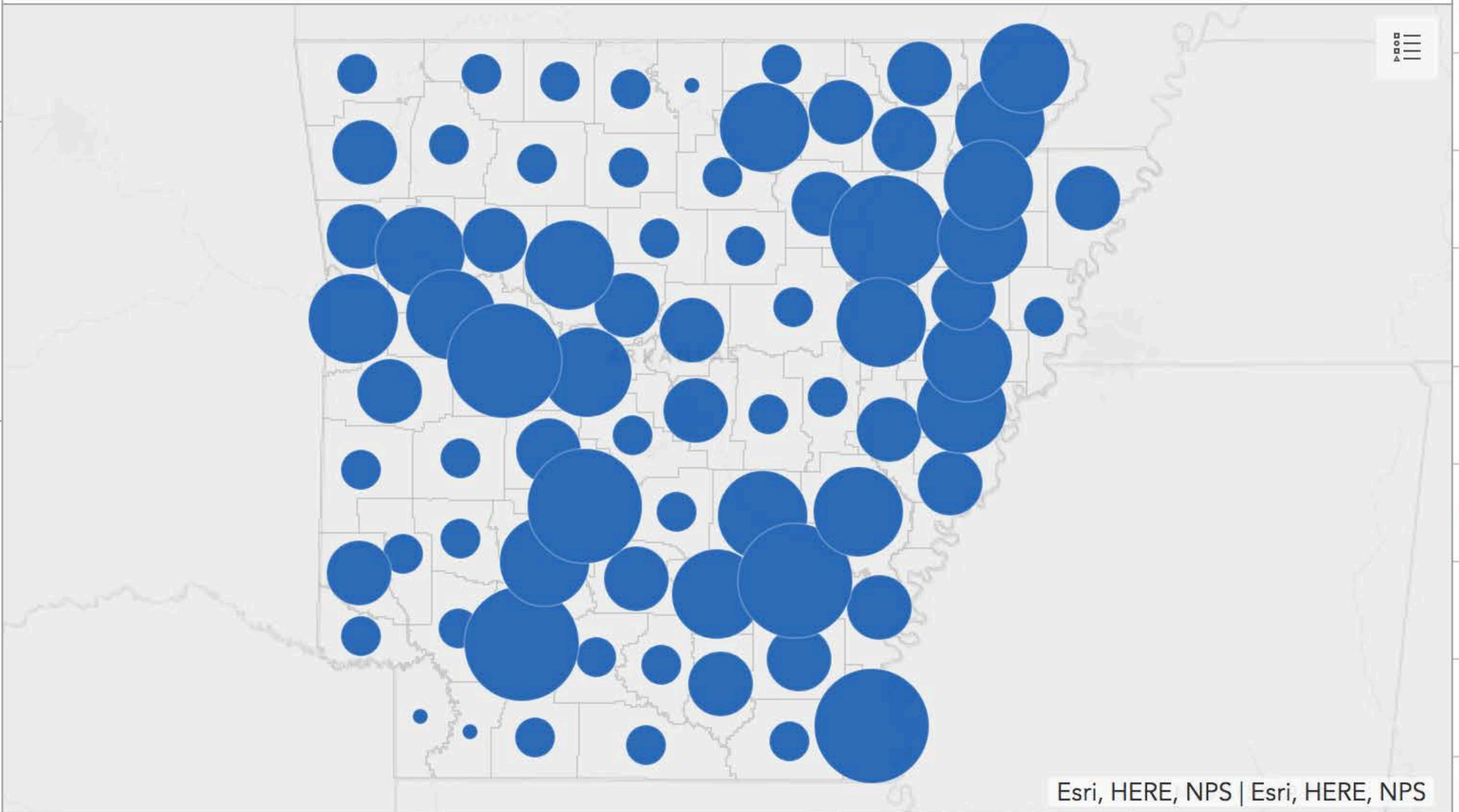

Arkansas (19,627 total tests)
Ashley (14,679 total tests)
Baxter (19,003 total tests)
Benton (195,414 total tests)
Boone (30,697 total tests)
Bradley (10,364 total tests)
Calhoun (3,663 total tests)
Carroll (19,539 total tests)
Chicot (14,117 total tests)
Clark (23,532 total tests)
Clay (16,439 total tests)
Cleburne (20,047 total tests)
Cleveland (8,714 total

COVID-19 Negative Tests		COVID-19 Positive Tests	
2,476,470	PCR Negatives 2,185,289	304,646	PCR 234,905
	Antigen 291,181		Antigen 69,741

Total Cases | Active Cases | Deaths | Tests | Case Demographics | Health Metrics | Hospital Information | USA and Global

Tracking COVID-19 in California

Last updated January 30, 2021 at 11:00 AM

Update for January 30, 2021

As of January 30, California has 3,224,374 confirmed cases of COVID-19, resulting in 40,216 deaths. The number of COVID-related deaths increased by 1.6 percent from the prior day total of 39,578.

Updated January 30, 2021, with data from January 29, 2021.

Note: Numbers do not represent true day-over-day change as these results include cases from prior to yesterday. All-time series data is by reported date (the date information was reported to the California Department of Public Health).

CASES	DEATHS	TESTS	VACCINES ADMINISTERED
**3,224,374** total	**40,216** total	**41,980,456** total	**3,266,914** total
**18,427** today	**638** today	**303,051** today	
0.6% increase from prior day total	1.6% increase from prior day total	0.7% increase from prior day total	

Vaccination data

COVID-19 vaccination is one of the most important tools to end the pandemic. We're tracking the progress of vaccinating Californians across the state.

VIEW VACCINATION DATA

See the data statewide and in each county

Enter a county | Get county data

Daily cases and deaths

California has 3,224,374 confirmed cases of COVID-19, resulting in 40,216 deaths.

Total cases in California

3,224,374 positive cases
18,427 new cases
0.6% increase from prior day total

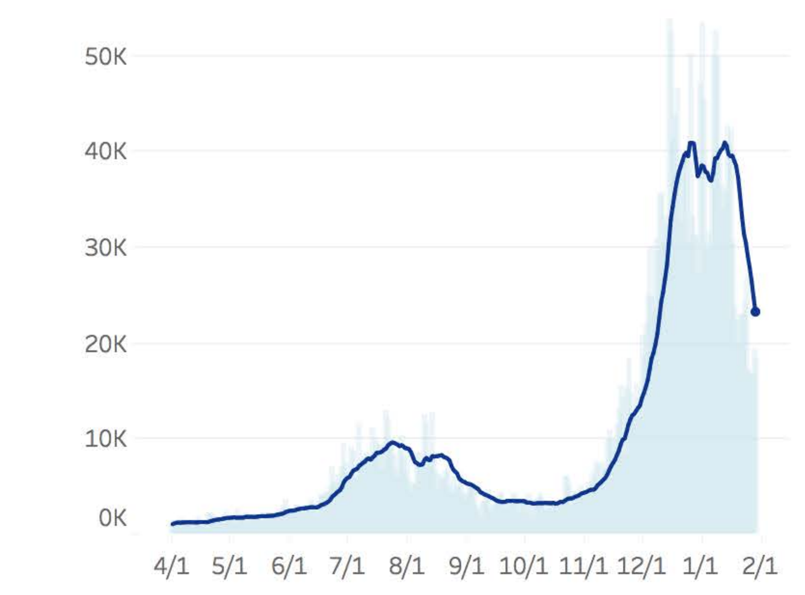

Total deaths in California

40,216 total deaths
638 new deaths
1.6% increase from prior day total

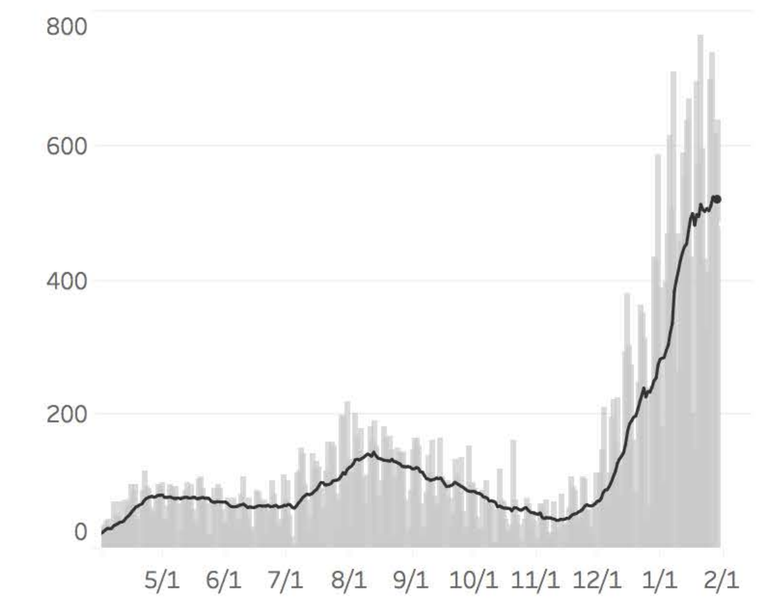

tableau

Note: Numbers do not represent true day-over-day change as these results include cases from prior to yesterday.

Testing for COVID-19

The number of COVID-19 diagnostic test results in California reached a total of 41,980,456, an increase of 303,051 tests from the prior day total. The rate of positive tests over the last 14 days is 7.8 percent.

Total tests reported in California

303,051 new tests reported
41,980,456 total tests reported
0.7% increase from prior day total

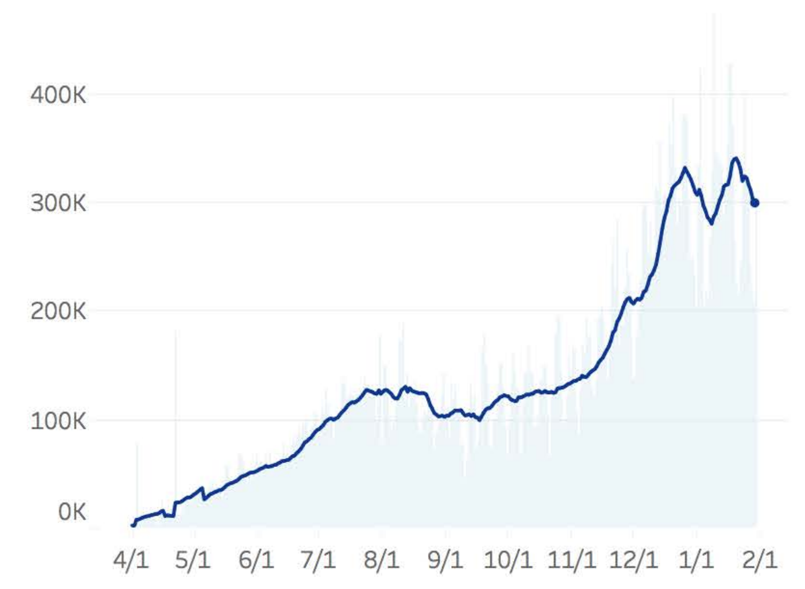

Positivity rate in California

7.8% test positivity (14-day average)
-4.7% increase from 14 days ago

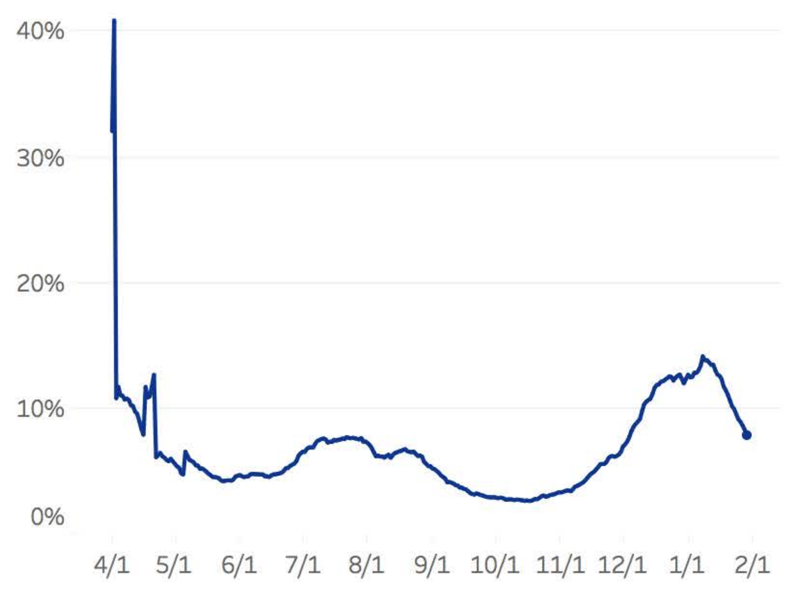

tableau

Impact on hospitals and ICUs

The number of hospitalizations due to confirmed and suspected COVID-19 cases in California reached a total of 15,941, a decrease of 640 from the prior day total. The number of ICU patients due to confirmed and suspected COVID-19 cases in California reached a total of 4,042, a decrease of 190 from the prior day total.

COVID-19 hospitalized patients in California

Hospitalized | ICU

15,941 COVID-19 hospitalized patients
-640 patients
4.0% decrease from prior day

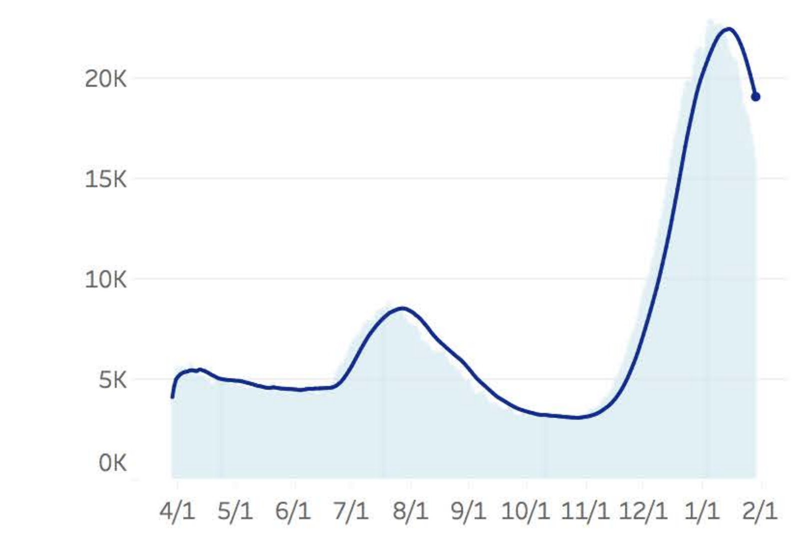

ICU beds in California

1,184 ICU beds available
3 decrease from prior day

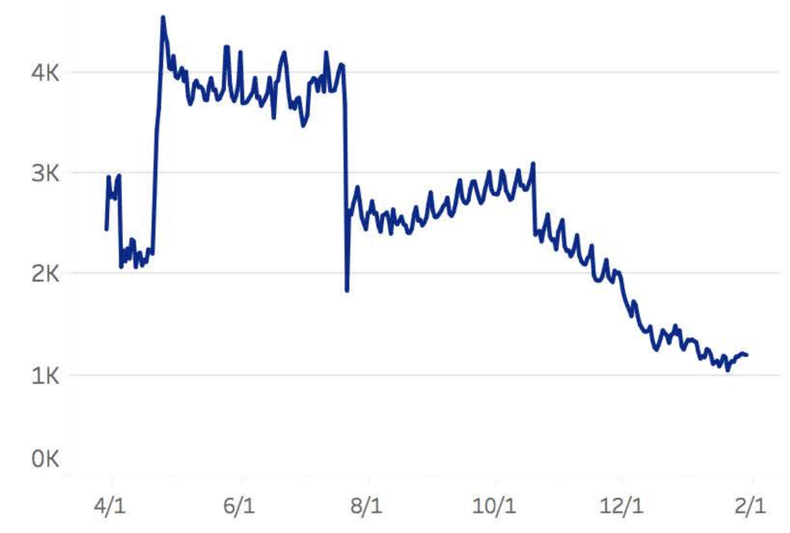

tableau

COVID-19: Cases

Sort by: Positive Cases

Note: Numbers do not represent true day-over-day change as these results include cases prior to yesterday.

Positive Cases by County

Los Angeles	1,078,329
San Bernardino	267,490
Riverside	263,504
San Diego	234,637
Orange	229,917
Santa Clara	101,010
Fresno	87,936
Sacramento	86,320
Kern	85,044
Alameda	73,210
Ventura	69,511
San Joaquin	60,899
Contra Costa	56,318
Stanislaus	45,466
Tulare	43,925
Monterey	38,539
San Mateo	35,223
San Francisco	31,294
Santa Barbara	28,105
Solano	27,352
Imperial	26,045
Merced	25,865
Sonoma	25,710
Kings	20,466
Placer	18,367
[illegible]	[illegible]
Marin	12,145
Yolo	11,612

CALIFORNIA CASES

| = California Population %

3,224,374

1 Day Δ	7 Day Δ	14 Day Δ
+18,427	+139,334	+324,128
+0.6%	+4.5%	+11.2%

7 Day Avg.	14 Day Avg.	Weekly % Change
19,905	23,152	-24.6%

US Total Cases: 25,615,268

Day-Over-Day New Cases

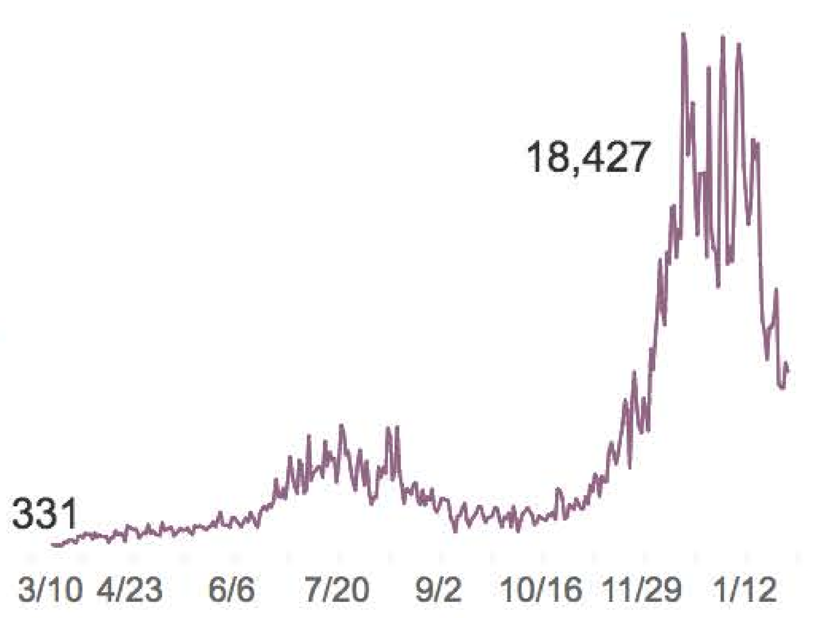

Gender	
Female	51%
Male	48%
Unknown	2%

Age	
0-17	13%
18-49	58%
50-64	19%
65+	11%
Missing	0%

Race/Ethnicity	
AIAN	0%
Asian	7%
Black	4%
Latino	55%
NHPI	1%
White	20%
Multi-Race	1%
Other	12%

CALIFORNIA TESTING RESULTS

41,980,456

1 Day Δ	14 Day Δ	Positivity:
+303,051	+4,178,201	7 Day: **6.9%**
+0.7%	+11.1%	14 Day: **7.8%**

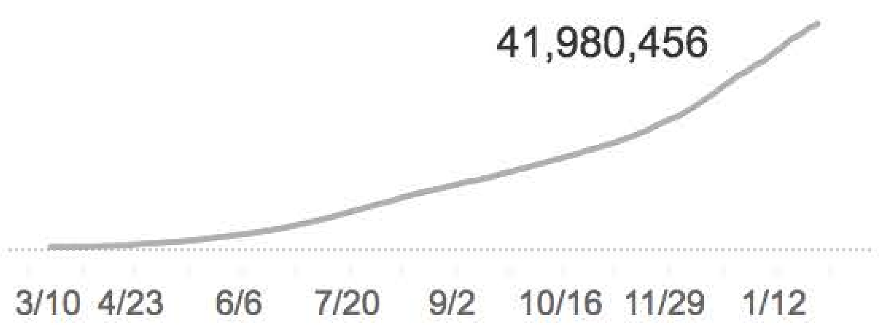

Cumulative Tests Reported by All Labs

CALIFORNIA DEATHS

| = California Population %

40,216

1 Day Δ	7 Day Δ	14 Day Δ
+638	+3,855	+7,256
+1.6%	+10.6%	+22.0%

7 Day | 14 Day | Weekly % [illegible]

US Total Deaths: 431,619

Day-Over-Day New Deaths

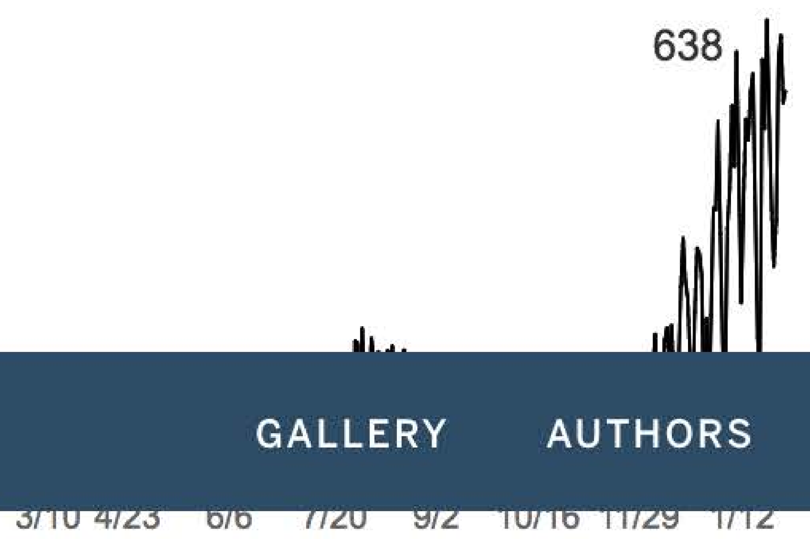

Gender	
Female	42%
Male	58%
Unknown	1%

Age	
0-17	0%
18-49	7%
50-64	19%
[illegible]	[illegible]

Race/Ethnicity	
AIAN	0%
Asian	12%
Black	7%
Latino	46%
NHPI	1%
[illegible]	[illegible]
Other	2%

Note: Negative numbers are data corrections. Please contact the Local Health Department for more information.

Note: Demographic percentages may not add up to 100% due to rounding. Breakdown of deaths is a subset of total deaths as reported by law enforcement.

Last Updated on 1/29/2021

+tableau+public GALLERY AUTHORS BLOG RESOURCES ACTIVITY ABOUT SIGN UP SIGN IN

+ a b | e a u

COVID-19 Cases Dashboard

Viz Author: **CA Open Data**

3,051,144 Views ☆56

More Detail

Originally Published: Jun 25, 2020

Last Updated: Jan 30, 2021

Workbook Details: 1 Sheet

Metadata: Cases

Tracking the coronavirus in California

By Los Angeles Times Staff
Updated Jan. 31 8:38 a.m. Pacific

3,290,134 confirmed cases	40,701 deaths	3,266,914 vaccines administered
+168 today	+1 today	None yet today
+16,290 yesterday	+469 yesterday	+188,664 yesterday

To better understand the COVID-19 pandemic, The Times is conducting an independent, continual survey of dozens of local health agencies across the state. So far today, one of the 61 agencies has reported new numbers.

What we know

- **The state has lifted regional stay-at-home orders.** Though many restrictions are likely to remain, the change could allow restaurants to reopen outdoor dining and some services to resume.
- **Case counts have started to sink.** Over the last seven days, the state has averaged 20,768 cases per day, a 49.6% decrease from two weeks ago. Roughly 6.9% of tests this past week have come back positive.
- **The death toll is near all-time highs.** The state has averaged 551 daily deaths over the last week, an increase of 4.2% from two weeks ago.
- **The hospitalization surge has crested.** Statewide, there are 15,113 people hospitalized with a confirmed case, a 27.1% decrease from two weeks ago.
- **Vaccines are slowly arriving.** 6.5% of the population has received at least one dose. Learn more about the rollout with The Times' new vaccine tracker.
- **Disparities in age and race persist.** Roughly 75% of the dead were 65 or older. After adjusting for population, Latinos are now 2.6 times more likely than whites to test positive.

California counties +
Other trackers +
More coverage +

Jump to a section
Totals Hotspots Maps Hospitals Vaccines Tests Demographics Nursing homes Prisons States

The latest totals

Coronavirus can infect people so rapidly that it has continued to spread despite shutdown orders aimed at slowing the growth of new cases and flattening the line below.

So far, 1 out of every 12 people in the state has tested positive. The number statewide is now on pace to double every 131.5 days, a number used to measure how quickly the virus is spreading.

Cases | Deaths

Cumulative cases

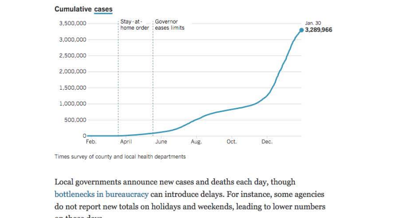

Times survey of county and local health departments

Local governments announce new cases and deaths each day, though bottlenecks in bureaucracy can introduce delays. For instance, some agencies do not report new totals on holidays and weekends, leading to lower numbers on those days.

Over the past week, the state has averaged 20,768 new cases and 551 new deaths per day. Experts say the true number of people infected is unknown and likely much higher than official tallies.

The charts here will update when today's Times survey of the state's 61 health agencies is complete.

New cases by day / **Deaths by day**

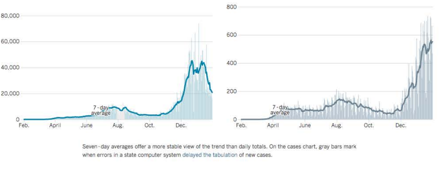

Seven-day averages offer a more stable view of the trend than daily totals. On the cases chart, gray bars mark when errors in a state computer system delayed the tabulation of new cases.

Track hospitals in California ↗
Follow the data and look up the latest patient numbers and beds at hundreds of hospitals across the state.

Where new cases are concentrated

State officials study the latest data and then rate regions and counties to determine when and how businesses reopen.

The government doesn't release enough data to replicate its analysis, but the rate of new cases per 100,000 residents over the last seven days provides insight into where the virus is spreading.

Metric: Cases | Deaths — Method: Per 100k | Totals

Regions ranked by new cases per 100,000 residents

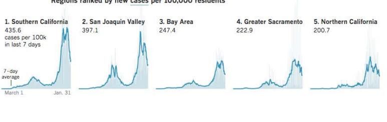

After adjusting for population, the virus is now categorized as widespread in 54 the state's 58 counties, which results in stricter rules being put into place. Together they are home to 99% of California residents.

Metric: Cases | Deaths — Method: Per 100k | Totals

Counties ranked by new cases per 100,000 residents

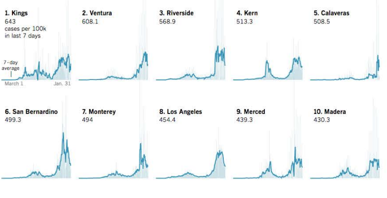

Show all

The Times' calculation of per capita rates can vary from what's published elsewhere. To learn more about how and why this count sometimes differs from official figures, consult our FAQ.

What's open where? ↗
See how the governor has rated all 58 counties, and what that means for reopening, in our county reopening tracker.

Mapping the toll

The coronavirus has been found in all 58 counties, from urban Southern California to the state's rural north.

Cumulative totals

Metric: Cases | Deaths — Method: Per 100k | Totals

Hover for more information.

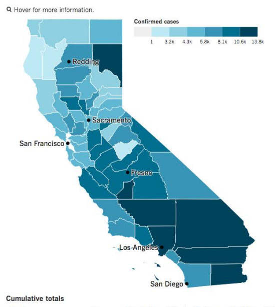

Cumulative totals

County	Cases	▼Per 100k	Today	Deaths	Per 100k	Today
Lassen »	5,393	17,293.6	–	16	51.3	–
Imperial »	25,948	14,398.3	–	532	295.2	–
Kings »	20,668	13,771.8	–	171	113.9	–
San Bernardino »	273,520	12,808.8	–	1,821	85.3	–
Riverside »	271,910	11,409	–	3,091	129.7	–
Los Angeles »	1,111,391	11,006	–	16,652	164.9	–
Kern »	93,628	10,602.8	–	624	70.7	–
Merced »	26,230	9,748.2	–	350	130.1	–
Tulare »	44,518	9,667.8	–	588	127.7	–
Madera »	14,250	9,192.8	–	175	112.9	–

Show all

Residents of cities, neighborhoods and regions all across the state have contracted the coronavirus. Here are the latest tallies for 1,352 places as released by county health departments.

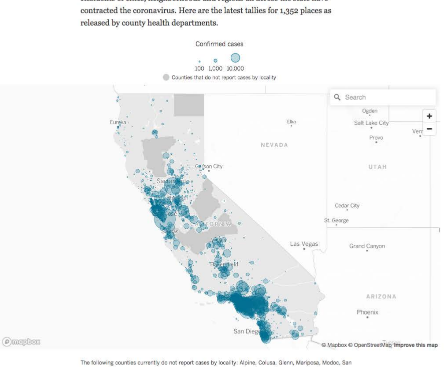

The following counties currently do not report cases by locality: Alpine, Colusa, Glenn, Mariposa, Modoc, San Benito, Tehama and Tuolumne

Filter by county: Los Angeles — Search by name

Area	▼Confirmed cases
East Los Angeles	23,222
Pomona	22,492
Palmdale	22,086
Lancaster	19,422
North Hollywood	18,402
Santa Clarita	17,340
South Gate	17,095
Glendale	16,635
Boyle Heights	16,303
Downey	15,978

Show all

Hospitals and patients

Stay-at-home orders aim to slow the virus in hope of preventing hospitals from being overrun. To keep tabs on available beds, officials watch out for rapid increases in the number of patients.

There are now 15,113 hospital patients statewide with a confirmed case, a change of -27% from two weeks ago.

Confirmed | Suspected | Both

Intensive care and other hospitalized patients

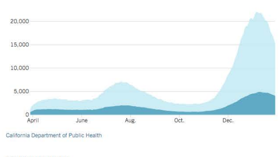

California Department of Public Health

Confirmed patients

County	ICU	Other	▼Total
Los Angeles »	1,418	3,910	5,328
Orange »	394	1,048	1,442
San Diego »	379	938	1,317
Riverside »	295	808	1,103
San Bernardino »	277	823	1,100
Santa Clara »	133	354	487
Fresno »	72	403	475
Ventura »	82	294	376
Sacramento »	107	245	352
Alameda »	106	241	347

Show all

Officials also closely monitor the number of beds open in intensive-care units. The latest data show that there are 1,184 available statewide.

Available ICU beds

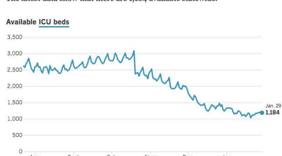

California Department of Public Health

Officials have organized the state's counties into five regions. Previously, if a region's available percentage of intensive-care beds fell below 15%, a stay-at-home order was issued. The numbers are adjusted to account for a variety of factors and are not a precise measurement of availability. After a change on Jan. 25, it's unclear how these ICU figures will be used in the future.

Available ICU beds by region

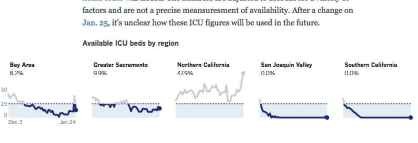

Track hospitals in California ↗
Follow the data and look up the latest patient numbers and beds at hundreds of hospitals across the state.

Vaccines

California's ultimate goal will be to fully vaccinate nearly 40 million people. Two COVID-19 vaccines have received emergency use authorization so far, and both require two doses given a few weeks apart to provide full inoculation.

To date, 3,266,914 shots of have been administered, about 68.6% of the 4.8 million doses the state has received. Over the last seven days, an average of 181,040 doses per day have been given out.

Cumulative vaccine doses administered

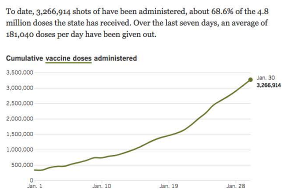

California Department of Public Health

Track coronavirus vaccinations in California ↗
Explore the data tracking the state's coronavirus vaccine supply to see how and where it has been distributed so far.

Testing

After a fitful start, California has increased coronavirus testing in the state. Over the last week, an average of 287,191 tests have been conducted each day.

New tests by day

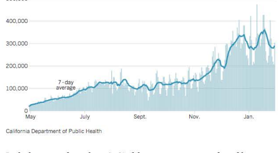

California Department of Public Health

In the last seven days, about 6.9% of the 2,010,337 tests conducted have returned a positive result.

Positive test rate, seven-day average

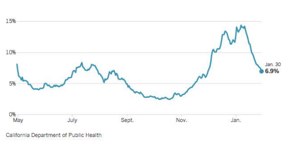

California Department of Public Health

Wide disparities in age and race

While younger adults make up the majority of positive tests, deaths due to the virus have skewed heavily toward the elderly.

Percentage of cases vs. population / **Percentage of deaths vs. population**

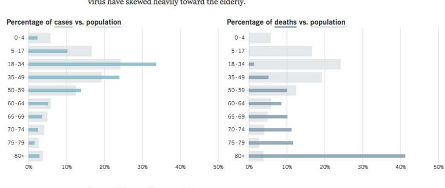

There are 2089 cases with an unreported age.
California Department of Public Health

The state has logged the race of the patient in nearly two-thirds of cases.

Latinos and Black people have contracted the virus at a higher rate than white and Asian people. After adjusting for population, Latinos are now 2.6 times more likely to test positive than white people.

Cumulative cases by race per 100,000 people

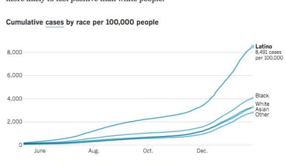

The other category includes Native Americans and people of two or more races.
California Department of Public Health

One outcome is that among most age groups, and especially younger people, Black people and Latinos are dying more often than other races relative to their share of the population.

Percentage of deaths vs. population

Age: 18+ — Cases | Deaths

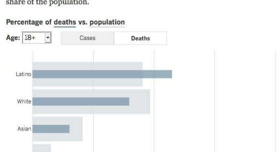

Race	Deaths	Deaths Pct.	Population Pct.
Latino	17,141	46.0%	36.3%
White	11,874	31.9%	38.8%
Asian	4,560	12.2%	16.5%
Black	2,422	6.5%	6.1%

Note: There are 784 deaths with an unknown race in this age bracket, 2% of the total.

Lives lost to COVID-19 ↗
Learn more about those we've lost by reading Times obituaries of Californians who have died from COVID-19.

Nursing homes

Nursing homes are a tragic focal point of the outbreak. Residents and staff have accounted for 5% the state's coronavirus cases, but 30% of its deaths.

Cases | Deaths

Deaths at nursing homes vs. elsewhere

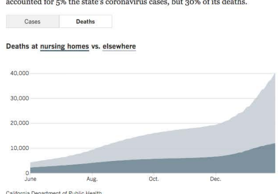

California Department of Public Health

Track outbreaks in California nursing homes ↗
Follow the data and look up the latest tallies at the hundreds of skilled-nursing and assisted-living facilities across the state.

State prisons

The Department of Corrections and Rehabilitation has faced criticism around its handling of recent surge in cases across its 35 facilities in the state.

Cumulative cases | Active cases | Deaths

Cumulative cases

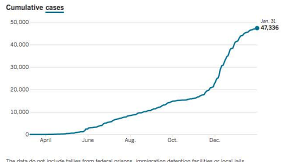

The data do not include tallies from federal prisons, immigration detention facilities or local jails.
California Department of Corrections and Rehabilitation

Tracking outbreaks in California' state prisons ↗
Follow the latest data on cases and deaths in the state prison system.

California in context

To date, the United States has recorded 26,040,296 coronavirus cases and 439,303 deaths. In the last week, the country has averaged 151,012 new cases and 3,141 deaths per day.

California — America's most populous state — has the nation's top case count. Home to 12% of the country's population, thus far it has accounted for roughly 13% of cases.

New cases in California vs. the rest of the country

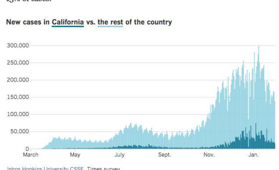

Johns Hopkins University CSSE, Times survey

State	Cases	Per 100k	▼Last 14 days	Per 100k	New cases (Fewer – More; Mar 1 – Jan 30)
California	3,290,134	8,403.8	338,195	863.9	
Texas	2,363,266	8,475	250,604	898.7	
New York	1,408,698	7,180.5	179,831	916.6	

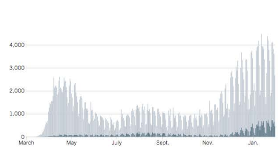

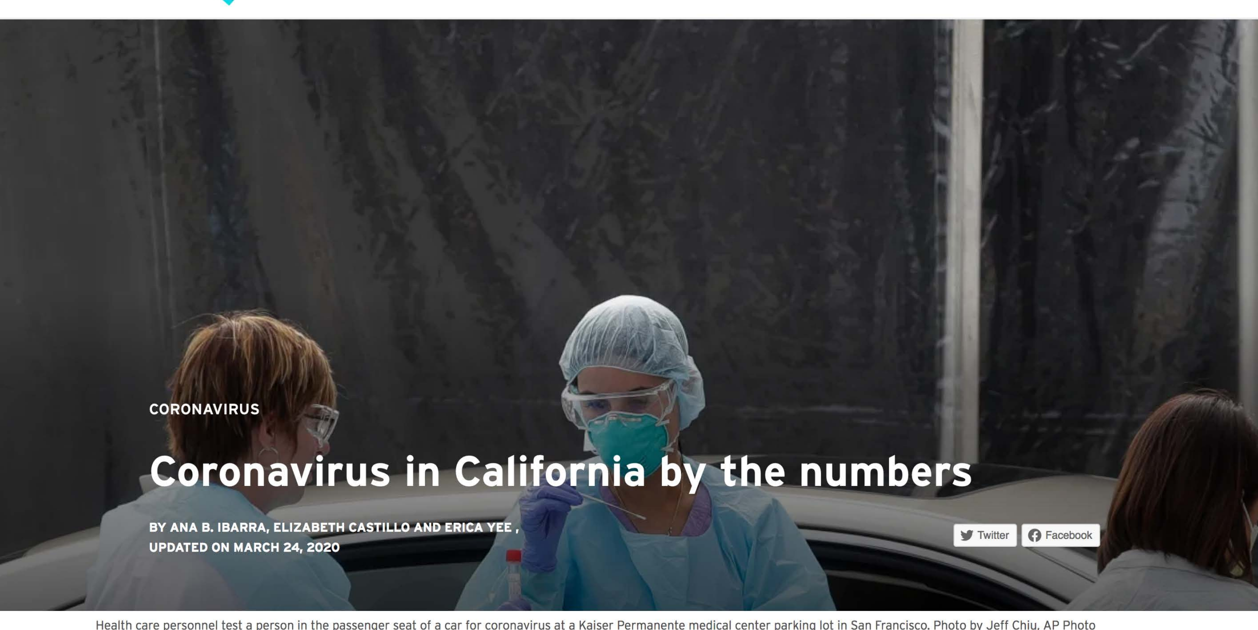

CORONAVIRUS

Coronavirus in California by the numbers

BY ANA B. IBARRA, ELIZABETH CASTILLO AND ERICA YEE ,
UPDATED ON MARCH 24, 2020

Twitter Facebook

Health care personnel test a person in the passenger seat of a car for coronavirus at a Kaiser Permanente medical center parking lot in San Francisco. Photo by Jeff Chiu, AP Photo

IN SUMMARY

Developments in the coronavirus pandemic are changing rapidly. We're working to track the latest California data. Find updates here.

California – having declared a pandemic-induced state of emergency on March 4, followed by a shelter-in-place order two weeks later – is now struggling to figure out what to reopen as coronavirus hospitalizations have plateaued.

Support nonprofit journalism in California

We rely on your generous support to cover the stories that matter most to you. If you find our work valuable in these difficult times, please support our journalism.

ONE-TIME MONTHLY ANNUALLY

$10 $15 $25 Other

Your contribution is appreciated.

DONATE

I'M NOT INTERESTED

A state-forecasted patient surge in mid-May failed to materialize to the degree feared, but Gov. Gavin Newsom continues to warn that the coronavirus threat remains real and dangerous.

The state has made progress on some goals, but lags well behind on others. Testing capacity remains insufficient, contact tracing is not yet up-to-speed throughout the state, and although the state did acquire 15,000 hotel and motel rooms for homeless Californians, thousands remain vacant while tens of thousands remain unsheltered.

Here is a look at what the state has and needs for its continued response, as well as a breakdown of testing, and data of positive cases by age, gender and ethnicity, which shows that the virus has taken a disproportionate toll on communities of color.

Keep tabs on the latest California policy and politics news

Enter your email

SUBSCRIBE

By clicking subscribe, you agree to share your email address with CalMatters to receive marketing, updates, and other emails from the site owner. Use the unsubscribe link in those emails to opt out at any time.

I'M NOT INTERESTED

This dashboard will be updated regularly with confirmed data from the state's department of public health and the governor's office.

Jump to...

Cases and deaths | By race, age and gender | Testing | Equipment and resources

Also see our hospitalizations tracker

Total cases and deaths

California's public health department announced the first two cases of this new coronavirus in the state on Jan. 26. More than three months later, the state had reported close to 70,000 positive cases and close to 3,000 deaths from the disease the virus causes, COVID-19. The number of infected people, however, is assumed to be much larger because access to testing has been an ongoing challenge.

**3,224,374**<br>cases<br>18,427 on Jan 29<br>+0.6% from the previous day	**40,216**<br>deaths<br>638 on Jan 29<br>+1.6% from the previous day

Retreived Jan 30, 2021 3:00 PM

Source: *California Department of Public Health*

Cases and deaths by race, age and gender

Latinos, African Americans and Pacific Islanders are dying from the coronavirus at disproportionately higher levels in California. Pacific Islanders have the highest death rate of any racial group, and the proportion of COVID-19 deaths among African Americans is about twice their population representation. The race/ethnicity is unknown for about 4% of the deaths.

Cases of COVID-19 by race/ethnicity, per 100,000 residents

The race/ethnicity is unknown for 775,978 (24.1%) cases. For 298,712 cases (9.3%), the race/ethnicity is classified as "Other," which is not a census category.

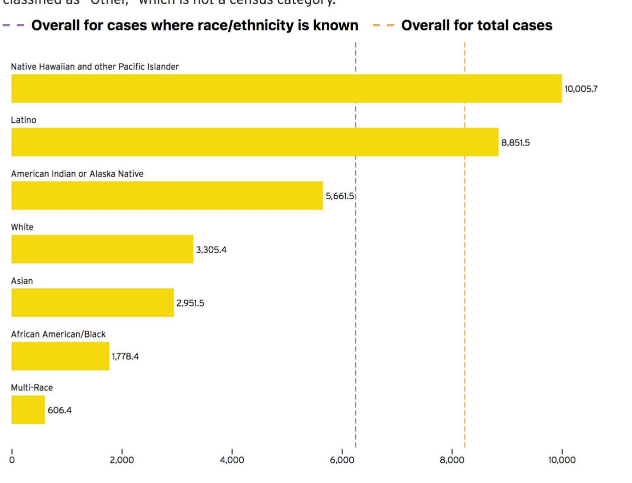

Deaths of COVID-19 by race/ethnicity, per 100,000 residents

The race/ethnicity is unknown for 1,742 (4.3%) deaths. For 776 deaths (1.9%), the race/ethnicity is classified as "Other," which is not a census category.

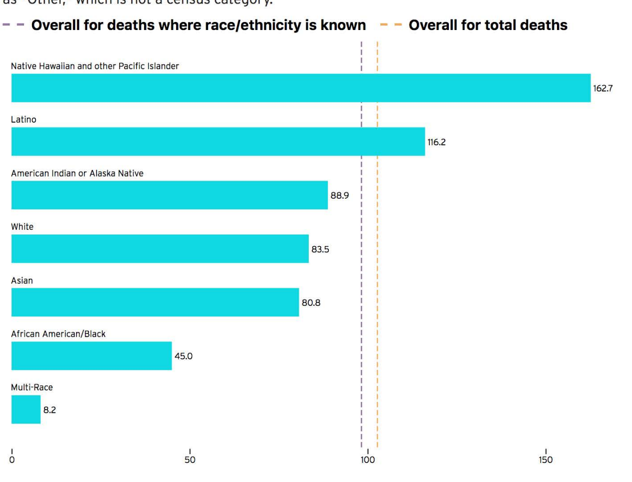

RACE/ETHNICITY	CASES	PERCENT CASES	CASES PER 100K	DEATHS	PERCENT DEATHS	DEATHS PER 100K
Multi-Race	33,506	1.0%	606.4	451	1.1%	8.2
African American/Black	98,263	3.0%	1,778.4	2,488	6.2%	45.0
Asian	163,086	5.1%	2,951.5	4,462	11.1%	80.8
White	485,750	15.1%	3,305.4	12,267	30.5%	83.5
American Indian or Alaska Native	7,837	0.2%	5,661.5	123	0.3%	88.9
Latino	1,347,343	41.8%	8,851.5	17,681	44.0%	116.2
Native Hawaiian and other Pacific Islander	13,899	0.4%	10,005.7	226	0.6%	162.7

Cases of COVID-19 by age, per 100,000 residents

The age is missing for 2,116 (0.1%) cases.

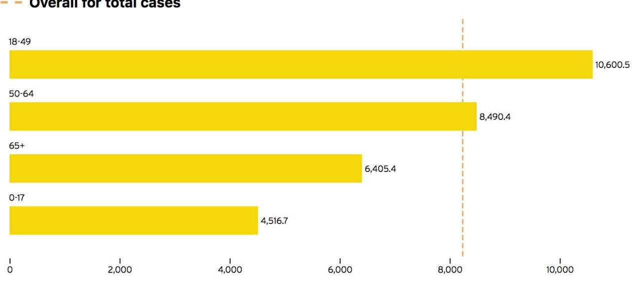

Deaths from COVID-19 by age, per 100,000 residents

The age is missing for 9 (0.0%) deaths.

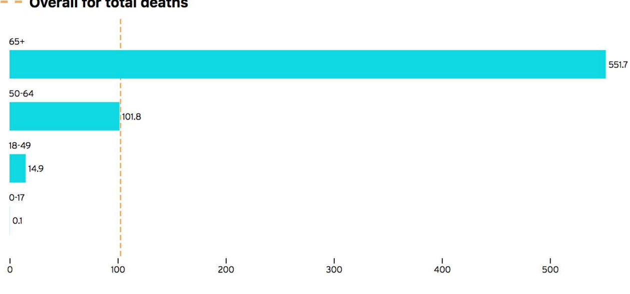

AGE	CASES	PERCENT CASES	CASES PER 100K	DEATHS	PERCENT DEATHS	DEATHS PER 100K
0-17	409,826	12.7%	4,516.7	11	0.0%	0.1
18-49	1,857,546	57.6%	10,600.5	2,616	6.5%	14.9
50-64	614,408	19.1%	8,490.4	7,364	18.3%	101.8
65+	340,478	10.6%	6,405.4	29,323	72.9%	551.7

Cases of COVID-19 by gender, per 100,000 residents

The gender is unknown for 49,912 (1.5%) cases.

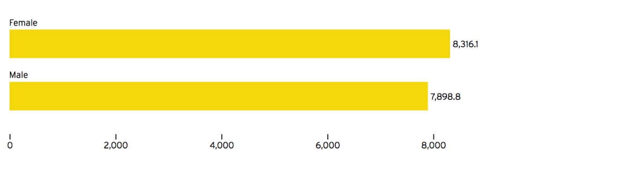

Deaths of COVID-19 by gender, per 100,000 residents

The gender is unknown for 177 (0.4%) deaths.

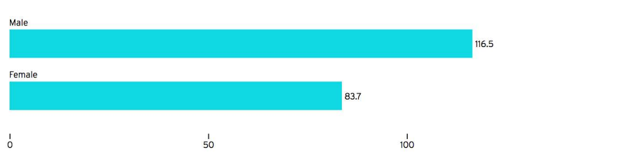

GENDER	CASES	PERCENT CASES	CASES PER 100K	DEATHS	PERCENT DEATHS	DEATHS PER 100K
Female	1,637,851	50.8%	8,316.1	16,477	41.0%	83.7
Male	1,536,611	47.7%	7,898.8	22,669	56.4%	116.5

Updated Jan 30, 2021 3:00 PM

Source: *California Department of Public Health*

How many tests have been conducted?

Testing nationwide has lagged significantly. Initially labs in California reported they were missing ingredients for extracting genetic material; they also were low on testing supplies such as swabs. For weeks, only those with serious symptoms were being tested. On May 12, the state reported that 1 million Californians had been tested, and that an average of 35,000 tests were being conducted per day.

3,224,374
Total tests reported
303,051 on Jan 30
+9.5% from the previous day

Daily tests reported

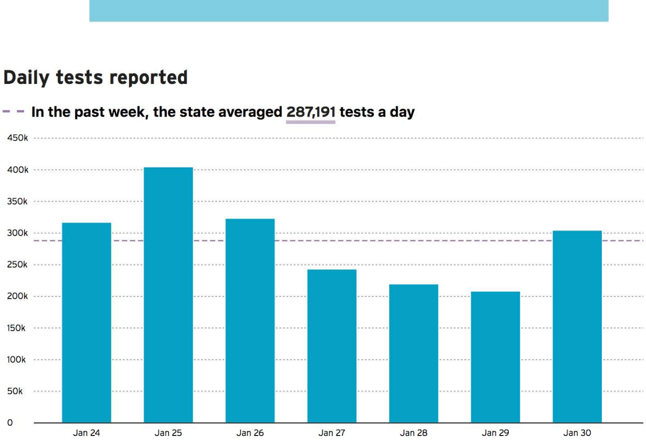

Updated Jan 30

109k Shares

Colorado COVID-19 Data

Click here for a mobile friendly view
Data is updated daily by about 4 p.m. and includes cases reported through the previous day.
All data, for days past and present, is recalculated daily.

COLORADO
Department of Public Health & Environment

395,016 Cases	21,812 Hospitalized	64 Counties	2,393,750 People Tested	5,426,059 Test Encounters	5,616 Deaths Among Cases	4,928 Deaths Due to COVID-19	3,4 Outbr

Case Summary | Demographics | Tests | Hospital Data | Incidence and Epidemic Curve | Syndromic Data | Outbreaks | CDC Provisional D

1,278 (▼ 338) Cases Reported Today	360 Cases Added with Previous Reported Date	1,316 (▼ 88) 7-Day Moving Average of Cases Reported	354.7 (▼ 13.4) 2-Week Cumulative Incidence Per 100K People
27 (▼ 31) New Hospital Admissions	85 (▼ 4) 7-Day Moving Average of New Hospitalizations	29% Adult Critical Care Ventilators in Use	7% Available Hospital Beds Occupied by Confirmed and Suspected COVID Patients
4.47% (▼ 0.00%) State Positivity Rate	5.03% (▼ 0.10%) State 7-Day Positivity Rate	3.05% (▲ 0.29%) Previous Week Emergency Department Syndromic COVID Visits	4.29% (▲ 0.34%) Previous Week Outpatient Syndromic COVID Visits
5.2 (▼ 1.1) Tests Per 1,000 People	4.6 (▼ 0.20) 7-Day Average of Tests Per 1,000 People	7,224 (▼ 1,327) New Individual People Tested	29,924 (▼ 6,248) New Test Encounters

+ableau

Trouble viewing? Access the data from Tableau or view the data tables below.

Case summary snapshot | Hospital data snapshot | Cases and deaths by county

CDPHE Open Data Portal | Symptom Support Tool data | More outbreak data

COVID-19 dial | COVID-19 cases over time | Colorado Department of Corrections COVID-19 Offender Case Dashboard

Additional resources

- Access the data files
- Learn more about the data
- COVID-19 Death Data and Resources
- CDC Provisional Death Counts for Coronavirus Disease (COVID-19)
- Colorado public survey results

Colorado COVID-19 Data

Click here for a mobile friendly view
Data is updated daily by about 4 p.m. and includes cases reported through the previous day.
All data, for days past and present, is recalculated daily.

COLORADO Department of Public Health & Environment

395,016 Cases	21,812 Hospitalized	64 Counties	2,393,750 People Tested	5,426,059 Test Encounters	5,616 Deaths Among Cases	4,928 Deaths Due to COVID-19	3,4 Outbr

Case Summary | Demographics | Tests | Hospital Data | Incidence and Epidemic Curve | Syndromic Data | Outbreaks | CDC Provisional D

Cases | Deaths Among Cases | Maps

Reported Day | Reported Week | Onset Day | Onset Week

Counts | Cumulative | 3-Day Average | 7-Day Average

Cases of COVID-19 in Colorado by Date Reported to the State

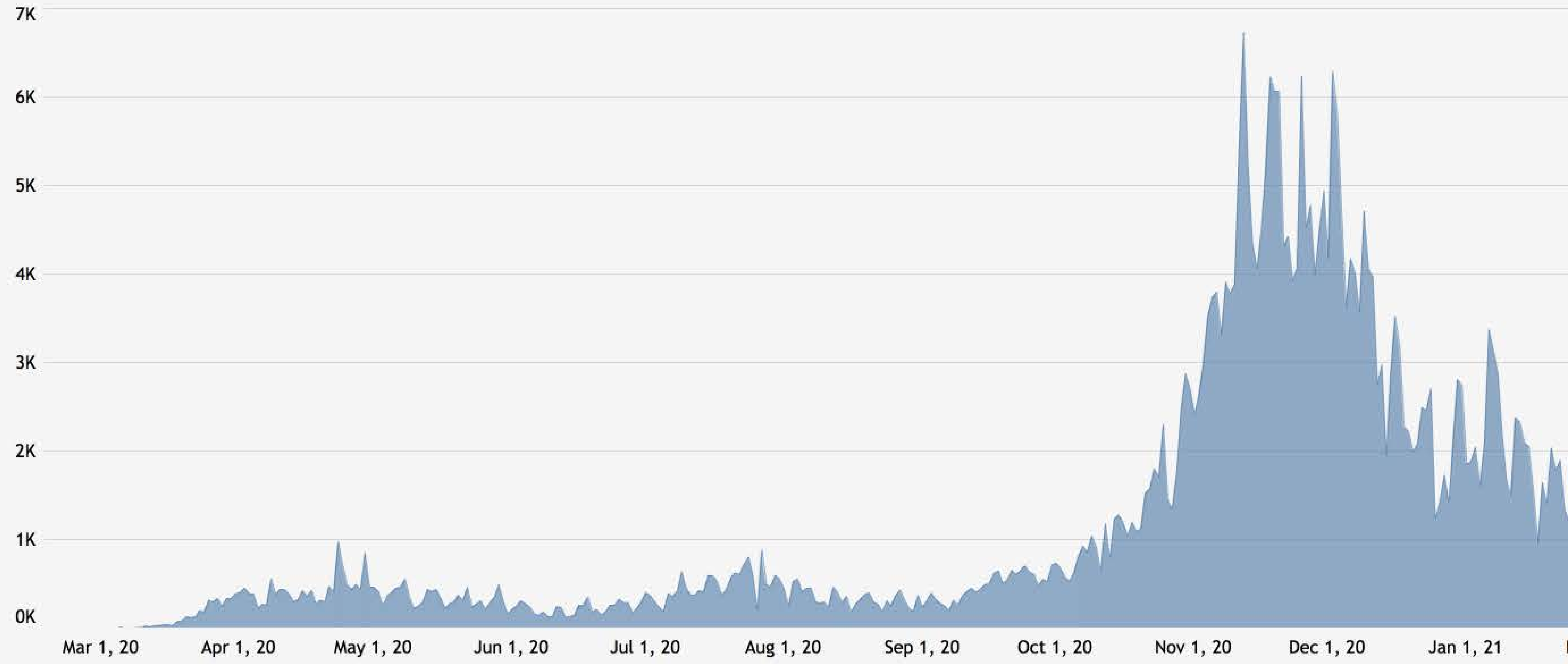

+ableau

Trouble viewing? Access the data from Tableau or view the data tables below.

- Case summary snapshot
- Hospital data snapshot
- Cases and deaths by county

CDPHE Open Data Portal | Symptom Support Tool data | More outbreak data

COVID-19 dial | COVID-19 cases over time | Colorado Department of Corrections COVID-19 Offender Case Dashboard

Additional resources

- Access the data files
- Learn more about the data
- COVID-19 Death Data and Resources
- CDC Provisional Death Counts for Coronavirus Disease (COVID-19)
- Colorado public survey results

Colorado COVID-19 Data

Click here for a mobile friendly view

Data is updated daily by about 4 p.m. and includes cases reported through the previous day.
All data, for days past and present, is recalculated daily.

COLORADO
Department of Public Health & Environment

395,016 Cases	21,812 Hospitalized	64 Counties	2,393,750 People Tested	5,426,059 Test Encounters	5,616 Deaths Among Cases	4,928 Deaths Due to COVID-19	3,4 Outbr

Case Summary | Demographics | Tests | Hospital Data | Incidence and Epidemic Curve | Syndromic Data | Outbreaks | CDC Provisional D

Cases | Deaths Among Cases | Maps

Daily | Weekly

Counts | Cumulative | 3-Day Average | 7-Day Average

7-Day Average of Deaths Among COVID-19 Cases in Colorado by Date of Death

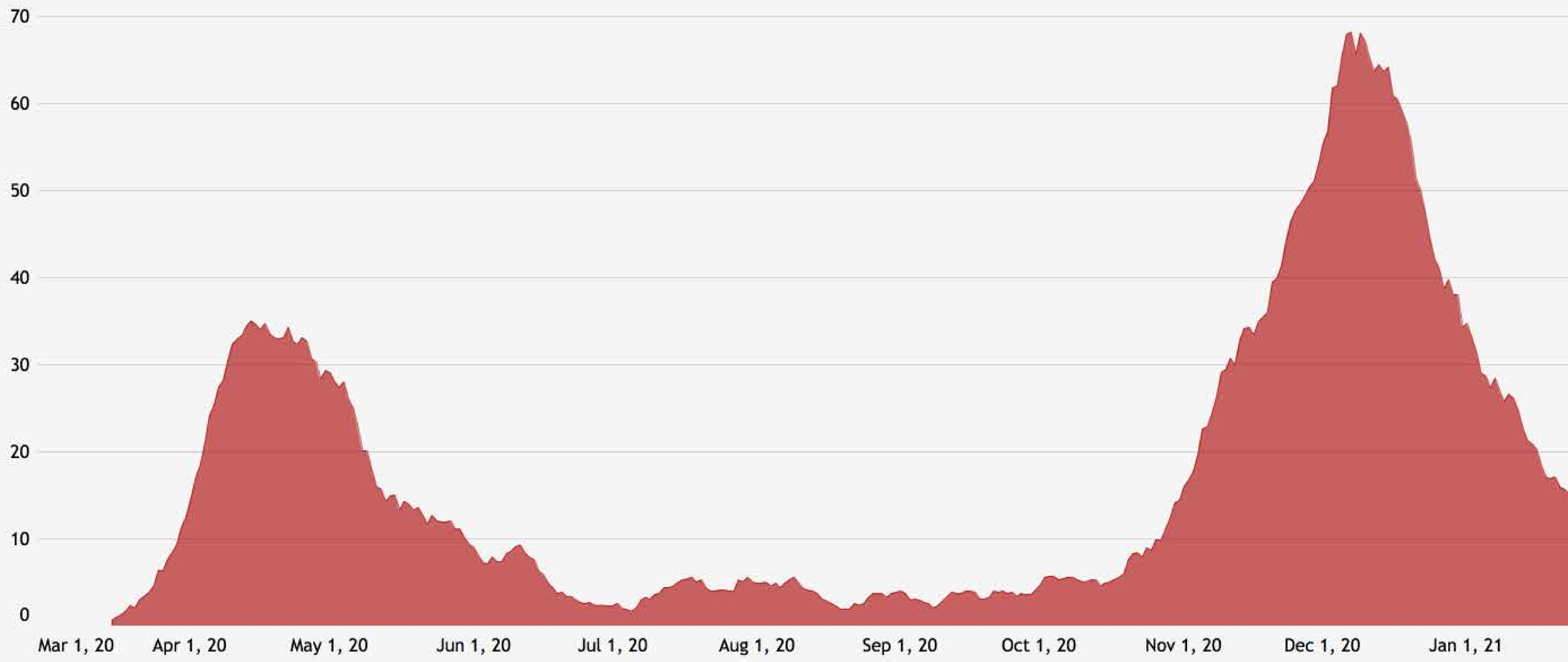

+ableau

Trouble viewing? Access the data from Tableau or view the data tables below.

Case summary snapshot | Hospital data snapshot | Cases and deaths by county

CDPHE Open Data Portal

Symptom Support Tool data

More outbreak data

COVID-19 dial

COVID-19 cases over time

Colorado Department of Corrections COVID-19 Offender Case Dashboard

Additional resources

- Access the data files
- Learn more about the data
- COVID-19 Death Data and Resources
- CDC Provisional Death Counts for Coronavirus Disease (COVID-19)
- Colorado public survey results

© 2021 State of Colorado | Transparency Online | Accessibility | Privacy Statement | Security Statement

COLORADO Official State Web Portal

Colorado COVID-19 Data

Click here for a mobile friendly view
Data is updated daily by about 4 p.m. and includes cases reported through the previous day.
All data, for days past and present, is recalculated daily.

COLORADO
Department of Public Health & Environment

395,016 Cases	21,812 Hospitalized	64 Counties	2,393,750 People Tested	5,426,059 Test Encounters	5,616 Deaths Among Cases	4,928 Deaths Due to COVID-19	3,49 Outbre

Case Summary | Demographics | Tests | Hospital Data | Incidence and Epidemic Curve | Syndromic Data | Outbreaks | CDC Provisional De

Daily | Weekly | Maps

PCR Tests | Antibody Tests | Daily Positivity | 3-Day Avg Positivity | 7-Day Avg Positivity

Daily Tests | Cumulative Tests

Daily COVID-19 PCR Test Data From Clinical Laboratories With 7-Day Average Positivity

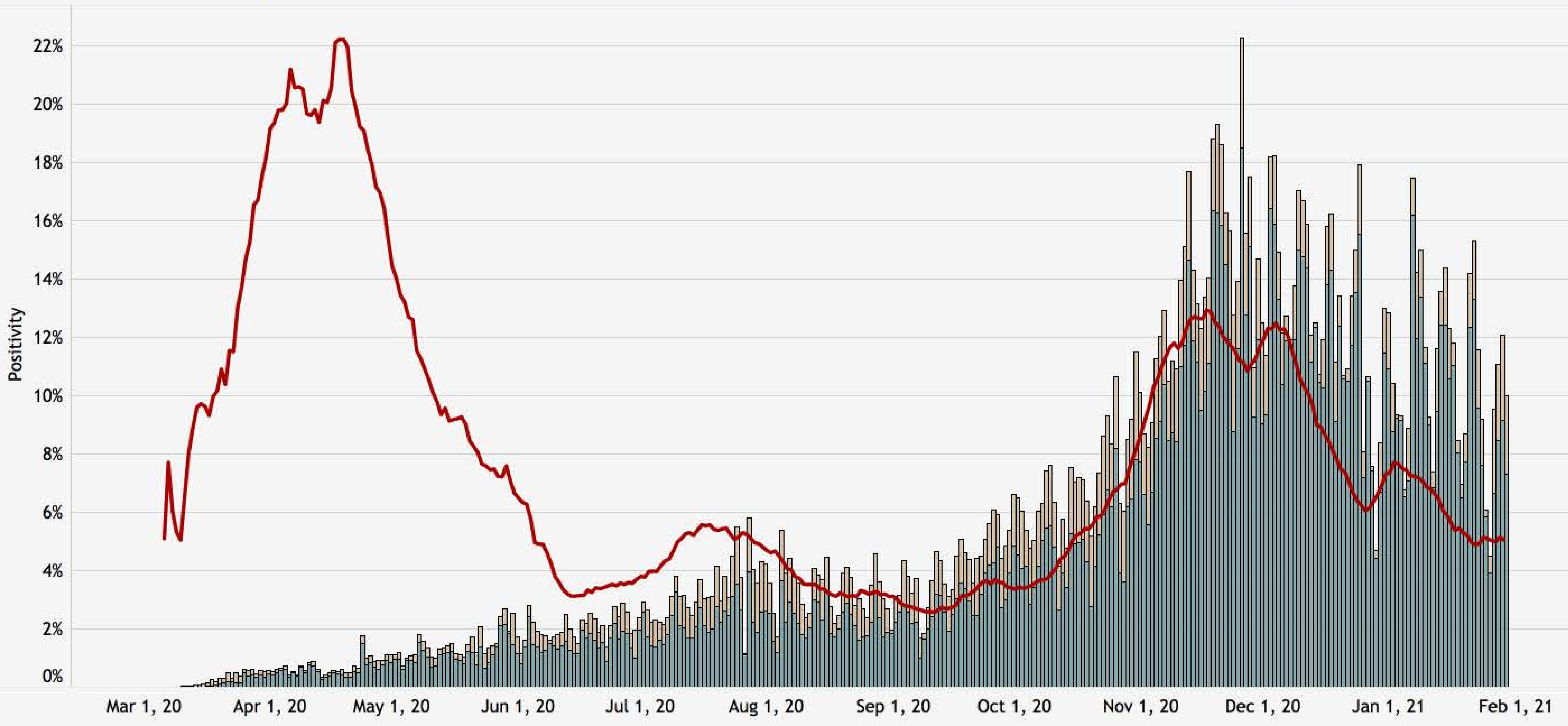

+ableau

Trouble viewing? Access the data from Tableau or view the data tables below.

Case summary snapshot | Hospital data snapshot | Cases and deaths by county

CDPHE Open Data Portal | Symptom Support Tool data | More outbreak data

COVID-19 dial | COVID-19 cases over time | Colorado Department of Corrections COVID-19 Offender Case Dashboard

Additional resources

- Access the data files
- Learn more about the data
- COVID-19 Death Data and Resources
- CDC Provisional Death Counts for Coronavirus Disease (COVID-19)
- Colorado public survey results

Colorado COVID-19 Data

Click here for a mobile friendly view
Data is updated daily by about 4 p.m. and includes cases reported through the previous day.
All data, for days past and present, is recalculated daily.

COLORADO Department of Public Health & Environment

395,016 Cases	21,812 Hospitalized	64 Counties	2,393,750 People Tested	5,426,059 Test Encounters	5,616 Deaths Among Cases	4,928 Deaths Due to COVID-19	3,4 Outbr

Case Summary | Demographics | Tests | Hospital Data | Incidence and Epidemic Curve | Syndromic Data | Outbreaks | CDC Provisional D

Daily | Weekly | Maps

PCR Tests | Antibody Tests | Daily Positivity | 3-Day Avg Positivity | 7-Day Avg Positivity

Daily Tests | Cumulative Tests

Daily COVID-19 Antibody Test Data From Clinical Laboratories With 7-Day Average Positivity

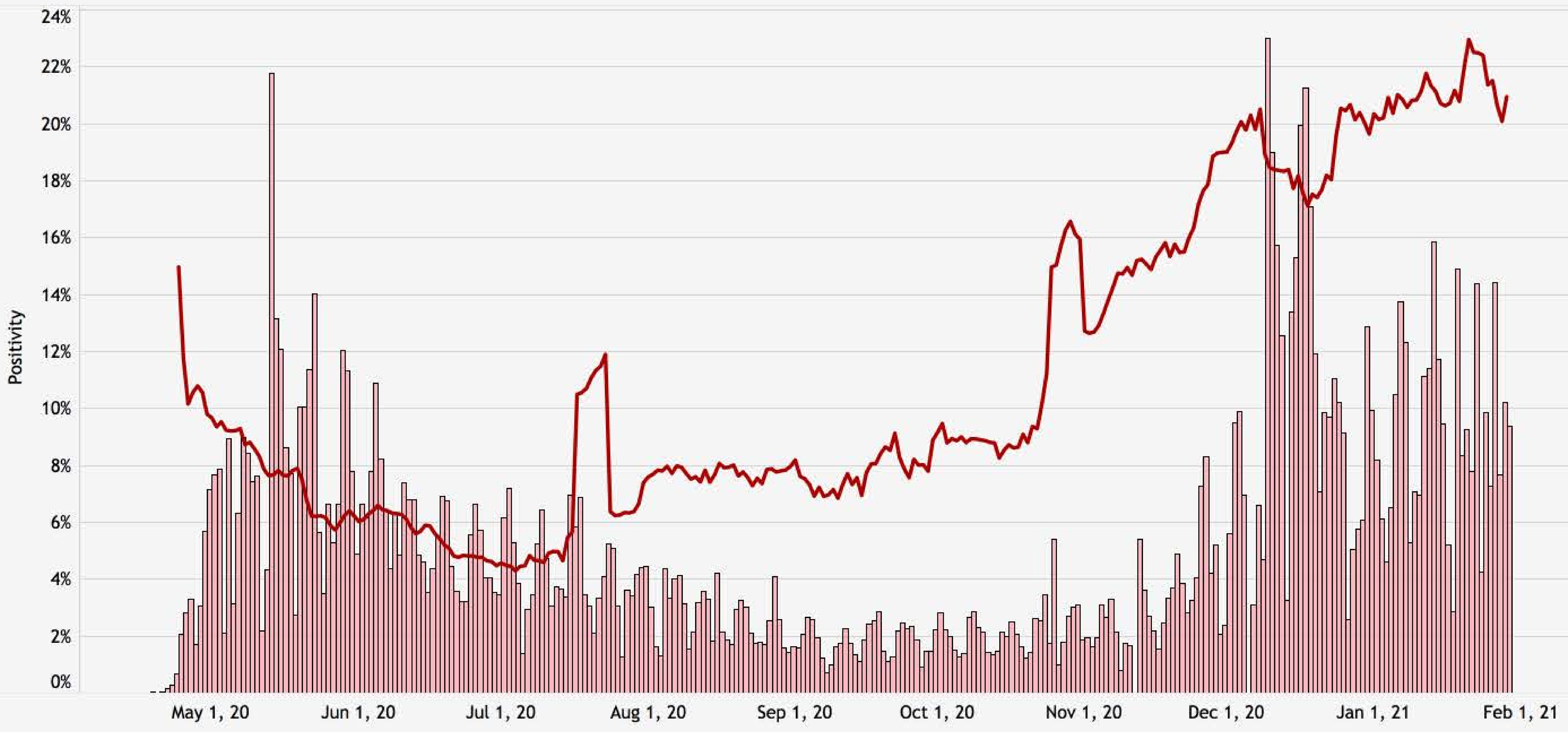

+tableau

Trouble viewing? Access the data from Tableau or view the data tables below.

Case summary snapshot | Hospital data snapshot | Cases and deaths by county

CDPHE Open Data Portal | Symptom Support Tool data | More outbreak data

COVID-19 dial | COVID-19 cases over time | Colorado Department of Corrections COVID-19 Offender Case Dashboard

Additional resources

- Access the data files
- Learn more about the data
- COVID-19 Death Data and Resources
- CDC Provisional Death Counts for Coronavirus Disease (COVID-19)
- Colorado public survey results

COVID-19 in Colorado tracker: Larimer and state case, death and hospital data for August

Coloradoan staff

Published 7:56 p.m. MT Aug. 1, 2020 | Updated 4:35 p.m. MT Sep. 1, 2020

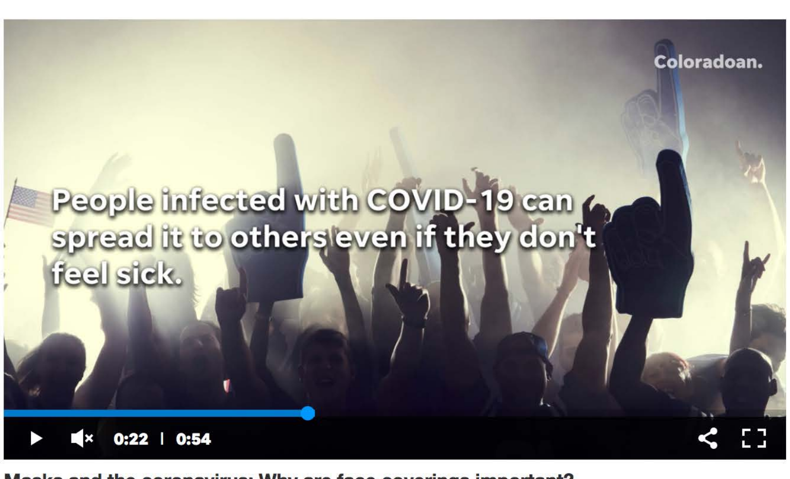

Masks and the coronavirus: Why are face coverings important?
Here's what the Centers for Disease Control and Prevention has to say about the use of cloth face coverings in preventing the spread of the coronavirus. *Holly Engelman/The Coloradoan, Wochit*

Notice our new look? As you get used to things, please let us know what you think!

As the number of confirmed coronavirus cases in Colorado grows, here are the latest updates and information.

Editor's note: *As the coronavirus outbreak continues to evolve, we don't want you to panic. In fact, quite the opposite. That's why the Coloradoan is committed to providing you with accurate, up-to-date information so you can make informed decisions on issues affecting you and the people you love. As such, this story, and many others, are being provided free for all to read. Help us continue this important work by subscribing to the Coloradoan.*

Here are the latest developments on coronavirus in Colorado:

Note: This tracker includes updates from August.

Looking for September updates?

► Find updates on the spread of COVID-19 in Colorado in September

Looking for past updates?

► A timeline of major updates on coronavirus in Colorado

► Find updates on the spread of coronavirus in Colorado from July

► Find updates on the spread of coronavirus in Colorado from May and June

► Early updates at the beginning of the pandemic in Colorado: March and April

Monday, Aug. 31, 2020

Larimer County cases, deaths:

- New cases reported by county: 27 (Note: This is the number of new confirmed and probable cases reported since The Coloradoan last shared information from the county. **In this case, that was Saturday, Aug. 29, 2020.** This may vary from the "new positive cases" listed on the county's COVID dashboard.)
- New deaths reported by county: 0
- Confirmed cases: 1,861 (positive swab test)
- Probable cases: 51 (people with two of the following: COVID-19 symptoms; epidemiological link to someone who tested positive; positive antibody test)
- Suspect cases: 433 (asymptomatic with positive antibody test)
- Total deaths: 36
- Risk Index in county (based on things such as hospital utilization, new cases, etc.): Medium
- Number of COVID-19 patients in hospitals: 5 (includes Banner and UCHealth)*
- Hospital utilization: 62% (low risk is <70%)*
- ICU utilization: 71% (low risk is <70%)*

Source: Larimer County Department of Health and Environment

Colorado cases, deaths:

- New cases reported by state **(since Saturday, Aug. 29)**: 383
- New deaths reported by state (caused by COVID-19; **since Saturday, Aug. 29**): 6
- Current hospitalizations: 148 (75% reporting)
- Hospital transfers or discharges in past 24 hours: 7
- Number of ventilators in use: 27%
- Hospitals anticipating a staff shortage in the next week: 5%
- Facilities anticipating personal protective equipment shortages in the next week: 1%
- Hospitals anticipating ICU bed shortages in the next week: 1%
- Total cases: 57,424
- Total hospitalizations: 7,028
- Total deaths due to coronavirus: 1,849
- Total outbreaks: 604 (reported at residential and nonhospital health care facilities, correctional settings and other facilities in Colorado, such as factories and group homes)

Source: Colorado Department of Public Health & Environment

Coronavirus cases in Larimer County

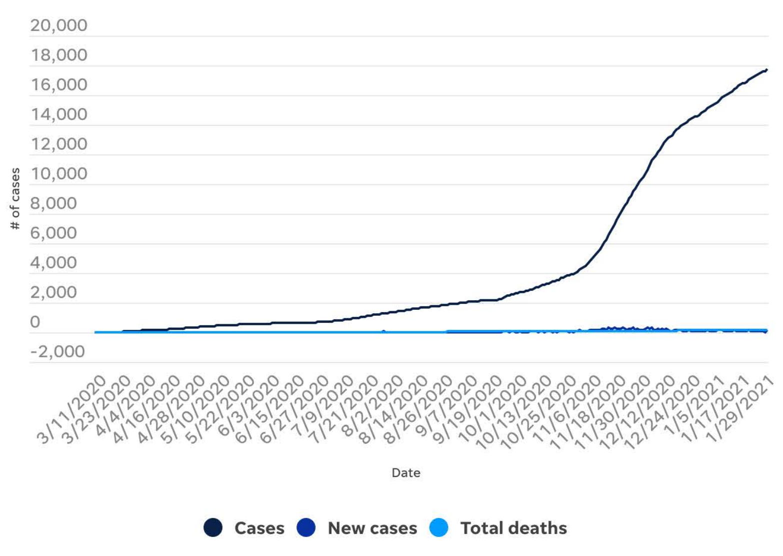

Last updated Jan. 30, 2021. Source: Larimer County Department of Health and Environment

The county has three definitions for case types: confirmed, probable and suspect.
- **Confirmed case**: Someone who has tested positive using a molecular amplification detection test (such as a nasal swab tested using PCR testing). This test determines if someone is currently ill and able to spread COVID-19 to others.
- **Probable case**:
  - Has COVID-19 symptoms or clinical characteristics AND epidemiologic evidence with no test confirming the diagnosis
  - Has COVID-19 symptoms or clinical characteristics AND has tested positive using an antigen or serologic (antibody) test
  - Has an epidemiological link based on risk from a known exposure AND has tested positive using an antigen or serologic (antibody) test
  - Has a death certificate that lists COVID-19 disease or SARS-CoV-2 as a cause of death or significant condition contributing to death without positive test results to confirm the diagnosis.
- **Suspect case**: Someone who has tested positive using a serology (antibody) test but who did not have COVID-19 symptoms or clinical characteristics. Antibodies can take several weeks to develop after being exposed so someone who has tested positive for antibodies has likely recovered and is no longer able to spread the virus at the time they are tested for antibodies.

Coronavirus cases by demographic

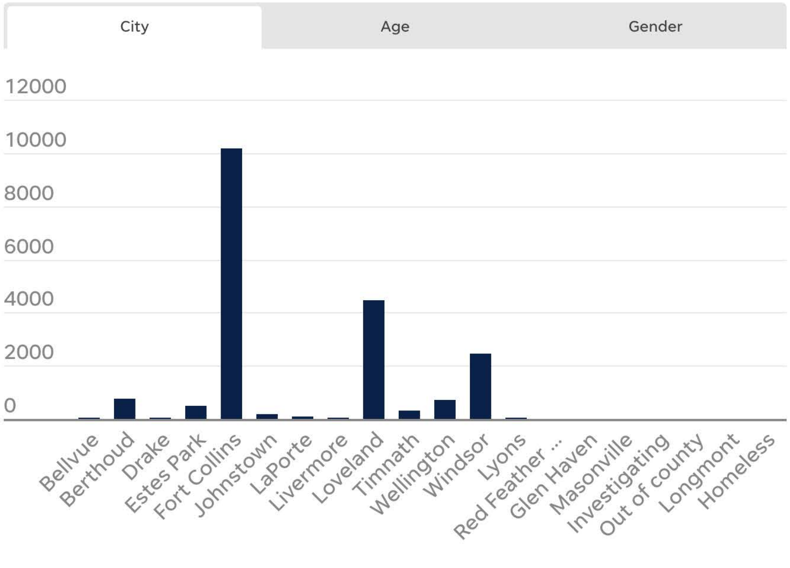

*Data for Windsor does not include cases from Weld County. Locations listed are those included in data from the Larimer County health department. Some locations may be outside of Larimer County.

Demographic data is for Confirmed and Probable cases only, and does not include antibody test results.

Share

Coronavirus cases in Colorado by day

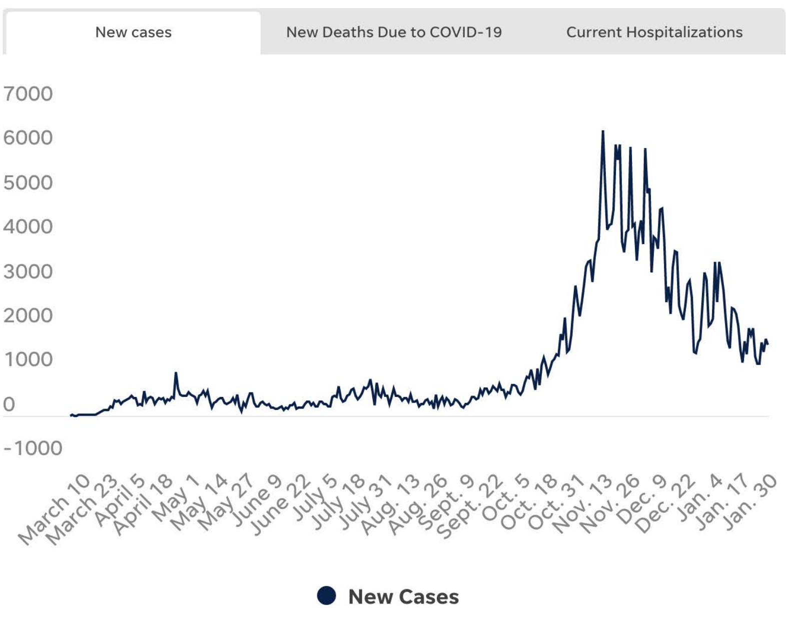

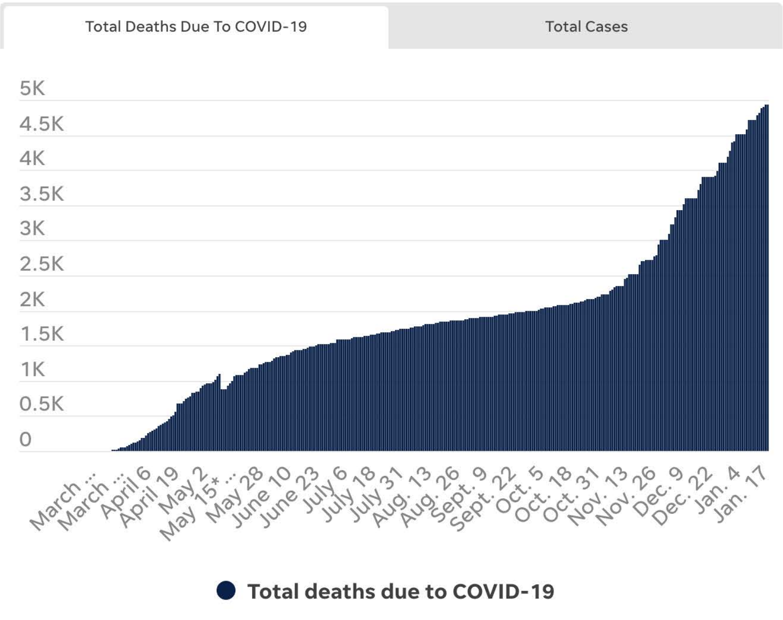

The Colorado health department releases data daily at 4 p.m. These charts reflect the dates cases were first reported by the state, not necessarily the date when cases were determined or first reported to the state.

**The state health department added 462 backdated positive test results on Jan. 29, according to a news release.*

**For Oct. 24 and Oct. 26, the number of new cases reflects the addition of older cases from as early as Oct. 1 that had not previously been included in charts due to formatting issues at the state health department.*

**Starting with May 15, deaths are defined as people who died as a result of COVID-19. Prior to that date, deaths included anyone who had died and tested positive for coronavirus, regardless of their cause of death. This why you see a change of zero and a dip in total cases on May 15. Unfortunately, this data is not available retroactively.*

**The April 24-25 reporting figures for deaths may be skewed by retroactive tallying of deaths from coronavirus on previous dates that had not yet been accounted for.*

The state defines cases as "anyone who has tested positive for COVID-19 and anyone who is symptomatic and has had close contact with someone who tested positive."

Hospitalization data is provided to the state of Colorado by hospitals through the Colorado Hospital Association. Not all hospitals provide data, and the percentage of hospitals reporting data each day varies.

Share

Coronavirus cases in Colorado

Hover over the county to see how many confirmed cases there are in that location.

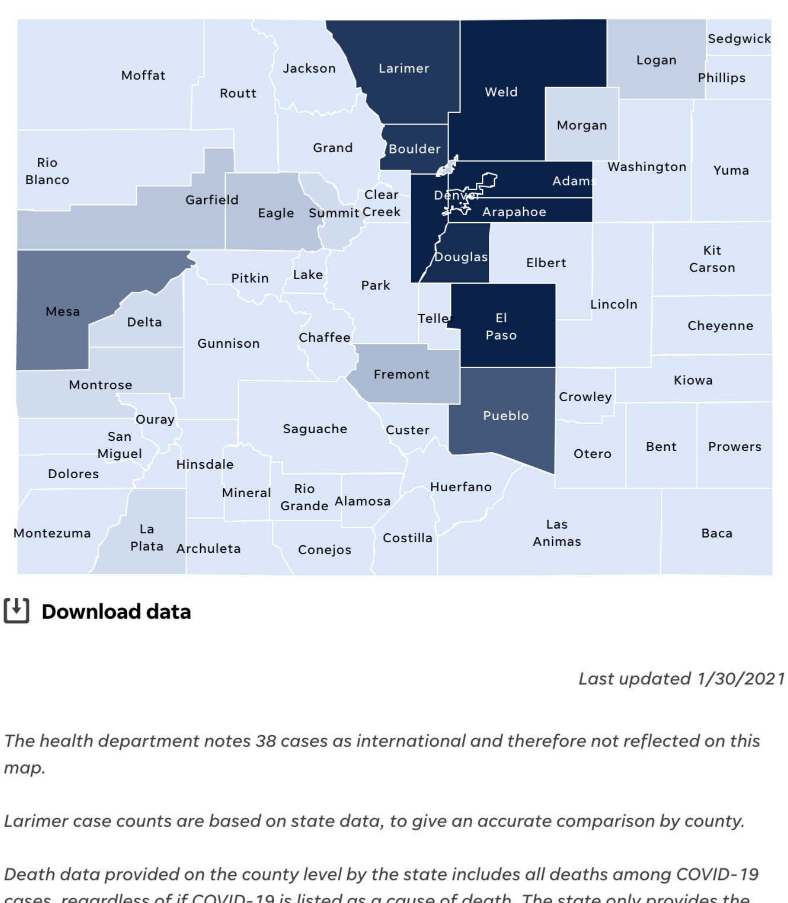

Download data

Last updated 1/30/2021

The health department notes 38 cases as international and therefore not reflected on this map.

Larimer case counts are based on state data, to give an accurate comparison by county.

Death data provided on the county level by the state includes all deaths among COVID-19 cases, regardless of if COVID-19 is listed as a cause of death. The state only provides the number of coronavirus-caused deaths as a total for the state as a whole.

From the health department: Please note initial information may change as more information is collected on each patient.

Share

Saturday, Aug. 29, 2020

Larimer County cases, deaths:

- New cases reported by county: 22 (Note: This is the number of new confirmed and probable cases reported since The Coloradoan last shared information from the county. This may vary from the "new positive cases" listed on the county's COVID dashboard.)
- New deaths reported by county: 0
- Confirmed cases: 1,834 (positive swab test)
- Probable cases: 51 (people with two of the following: COVID-19 symptoms; epidemiological link to someone who tested positive; positive antibody test)
- Suspect cases: 433 (asymptomatic with positive antibody test)
- Total deaths: 36
- Risk Index in county (based on things such as hospital utilization, new cases, etc.): Medium
- Number of COVID-19 patients in hospitals: 6 (includes Banner and UCHealth)*
- Hospital utilization: 65% (low risk is <70%)*
- ICU utilization: 74% (low risk is <70%)*

Source: Larimer County Department of Health and Environment

Colorado cases, deaths:

- New cases reported by state: 268
- New deaths reported by state (caused by COVID-19): 0
- Current hospitalizations: 134 (84% reporting)
- Hospital transfers or discharges in past 24 hours: 14

SUBSCRIBE NOW
$3 for 3 months. Save 90%.
Coloradoan.
SUBSCRIBE NOW
$3 for 3 months. Save 90%.
[News] Sports Business NoCo Life Opinion Obituaries E-Edition Legals 37°F Subscribe Sign In

[illegible] week: 1%
- Hospitals anticipating ICU bed shortages in the next week: 1%
- Total cases: 57,041
- Total hospitalizations: 6,993
- Total deaths due to coronavirus: 1,843
- Total outbreaks: 602 (reported at residential and nonhospital health care facilities, correctional settings and other facilities in Colorado, such as factories and group homes)

Source: Colorado Department of Public Health & Environment

NEWS › HEALTH • News

Colorado coronavirus tracker: How many new cases are being reported, and where are they?

The charts and maps will be updated daily

By **KEVIN HAMM** | khamm@denverpost.com | The Denver Post

PUBLISHED: March 6, 2020 at 5:41 p.m. | UPDATED: January 19, 2021 at 7:08 p.m.

Sign up for Newsletters and Alerts

SUBSCRIBE

Follow Us

98

COVID-19 in Colorado

Charts updated Sunday, January 31

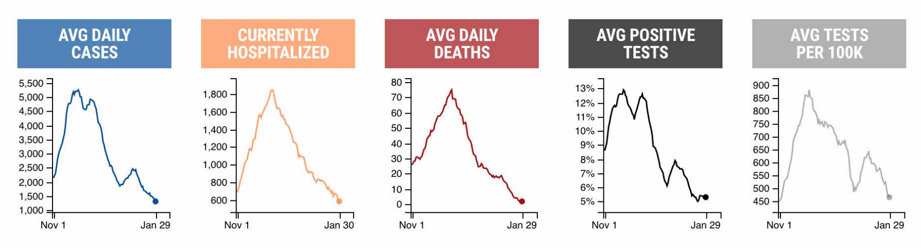

Vaccinations

Colorado population vaccinated through Jan 29

Current vaccine distribution phase: 1A and 1B

ONE DOSE	TWO DOSES
7.6%	2.2%
440,445 people vaccinated	*124,686 people vaccinated*

Vaccines administered

By week; current week will update daily

Hover/click chart for details; click legend item to filter chart

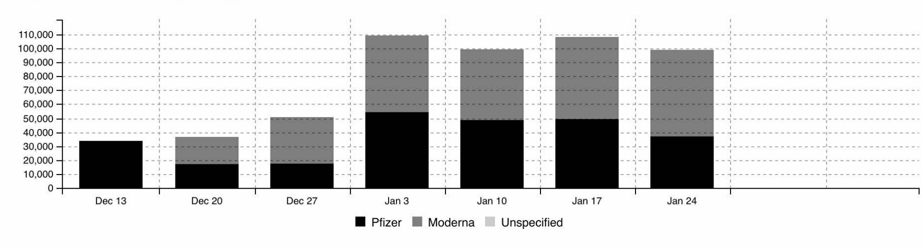

Cases, deaths and hospitalizations

HOW TO READ THE CHARTS

Please note that each chart uses two different data sets: The lines show data by the date it was reported to the Colorado Department of Public Health and Environment, and the bars show data by symptom onset date or date of death.

Lines: *Best for showing trend.* The line charts show when cases, deaths and hospitalizations were reported to the Colorado Department of Public Health and Environment. The lag time between when those occurred and when they were reported to the state can be several days. The chart uses a 7-day moving average to smooth out peaks and valleys in the data due to reporting lag times.

Bars: *Best for showing historical data.* The bar charts show when cases and hospitalizations occurred based on CDPHE's investigation into the onset date of symptoms, and when a death occurred based on the date on the death certificate. **Data for most recent two weeks is not shown because it will be signifcantly revised** with subsequent data releases as CDPHE investigations progress, and is not an accurate representation of recent cases, deaths and hospitalizations.

Totals: The total number of cases, deaths and hospitalizations that have been reported to the CDPHE.

Hover/click chart for details; click legend item to filter chart
Gray area indicates the statewide stay-at-home order

CASES
395,016

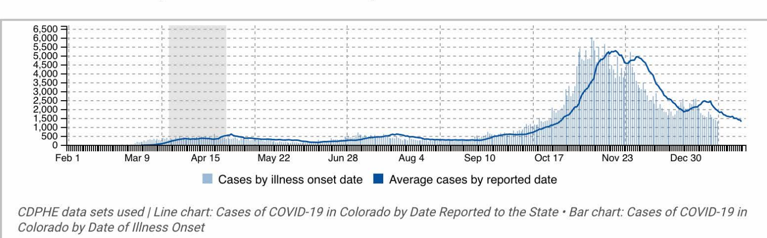

CDPHE data sets used | Line chart: Cases of COVID-19 in Colorado by Date Reported to the State • Bar chart: Cases of COVID-19 in Colorado by Date of Illness Onset

DEATHS
4,928
Deaths due to COVID-19

5,616
Deaths among people with COVID-19

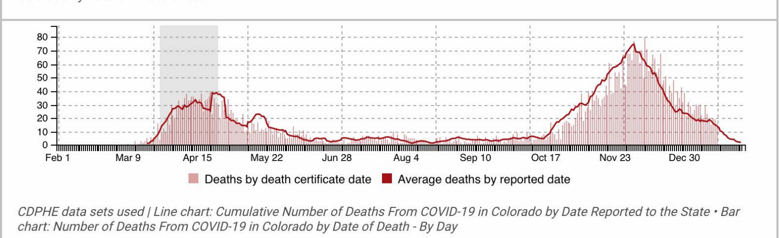

CDPHE data sets used | Line chart: Cumulative Number of Deaths From COVID-19 in Colorado by Date Reported to the State • Bar chart: Number of Deaths From COVID-19 in Colorado by Date of Death - By Day

HOSPITAL ADMISSIONS
21,812

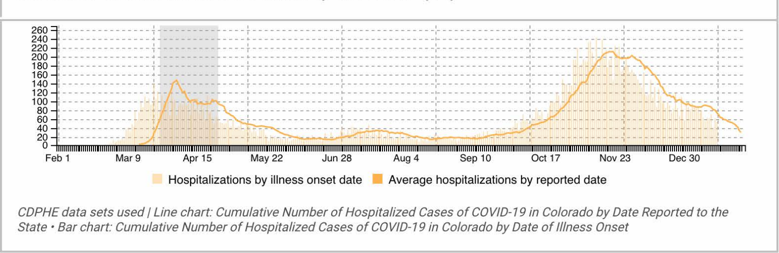

CDPHE data sets used | Line chart: Cumulative Number of Hospitalized Cases of COVID-19 in Colorado by Date Reported to the State • Bar chart: Cumulative Number of Hospitalized Cases of COVID-19 in Colorado by Date of Illness Onset

People currently hospitalized

This chart shows the number of people currently hospitalized with either a confirmed or suspected case of COVID-19.

Hover/click chart for details; click legend item to filter chart

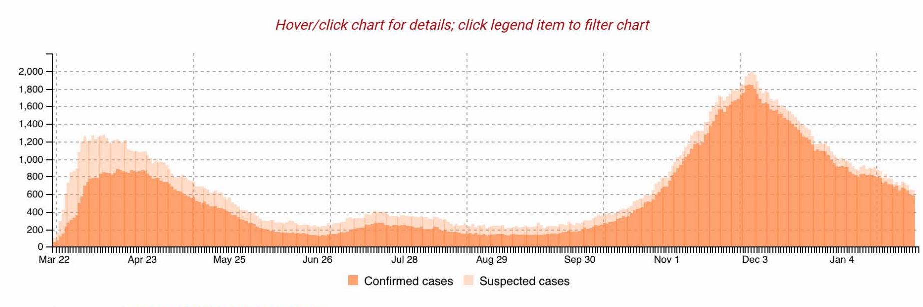

CDPHE data set used | Colorado COVID-19 Hospital Data

Testing rate and results

HOW TO READ THE CHARTS

Bar: The bar chart shows the 7-day moving average of the number of daily tests per 100,000 people, as reported to the CDPHE. Health officials say a testing rate of 152 per 100,000 is necessary to identify the majority of people who are infected.

Line: The line chart shows a 7-day moving average of the percentage of daily tests for COVID-19 that were positive.

Hover/click chart for details; click legend item to filter chart

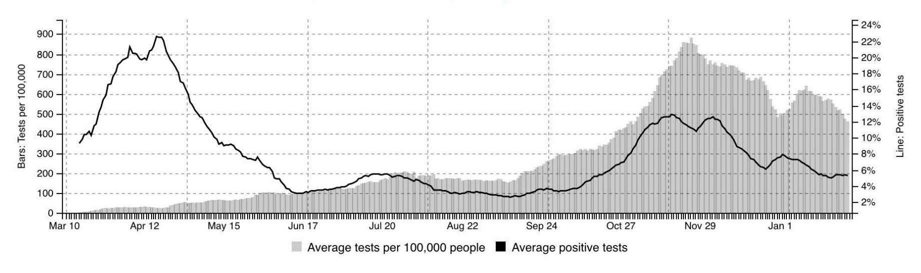

CDPHE data set used | Bar: Total Tests per 100k People | Line: Positivity Data from Clinical Laboratories

Daily cases in Colorado's largest counties

These charts show the daily case count and 7-day average of reported COVID-19 cases for the state's 10 most-populous counties.
Note: Each chart's vertical axis range is unique to its data; change from a week ago is based on averages.

Hover/click chart for details; click legend item to filter chart

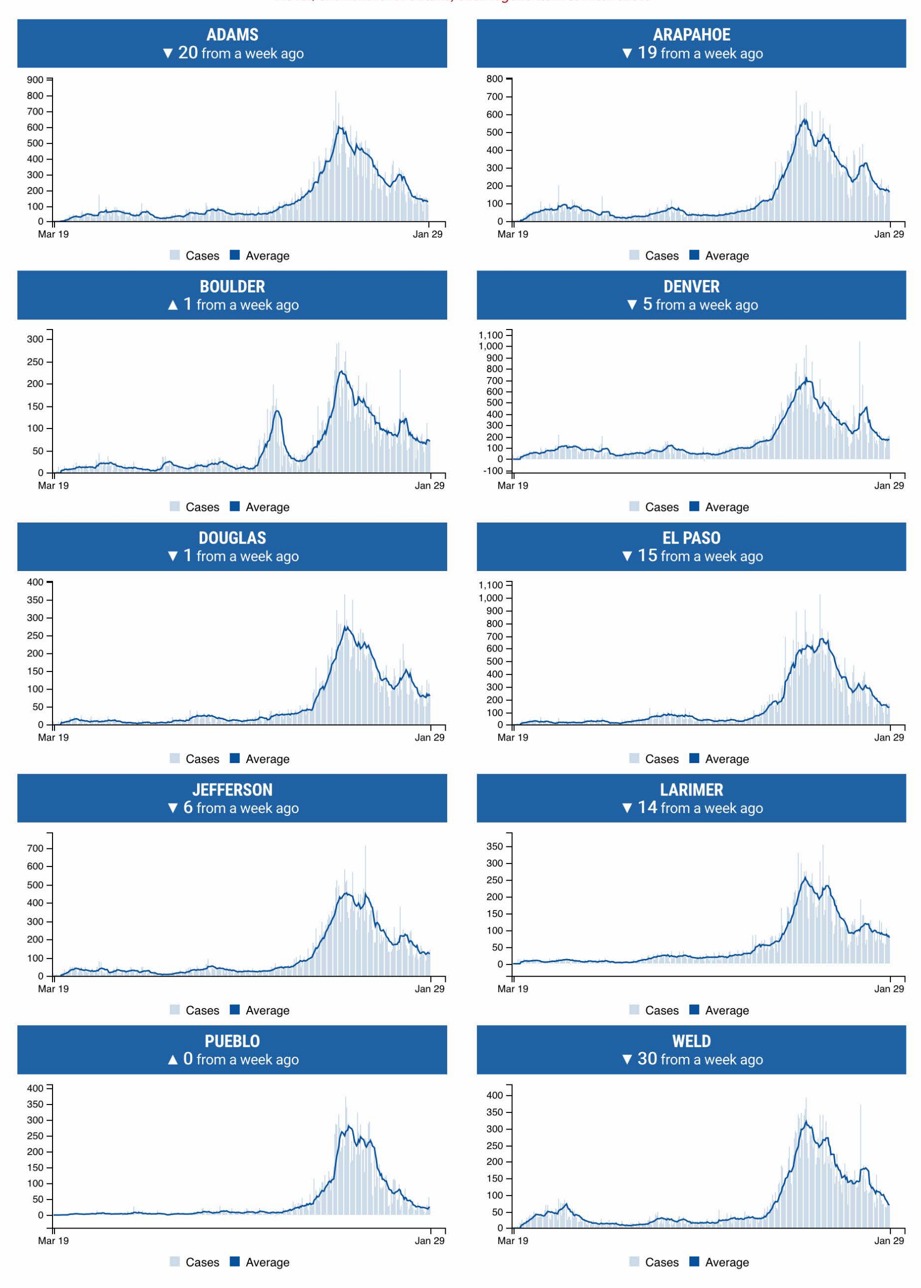

Case and death rates for counties

These charts compare the total case and death rates per 100,000 people for Colorado's 10 most-populous counties using a 7-day moving average of cases and deaths as they are reported to CDPHE. Rates are useful for comparing areas with different populations.

Hover/click chart for details; click legend item to filter chart

Cases per 100,000 people

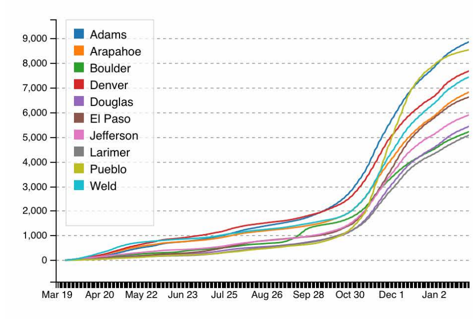

Deaths per 100,000 people

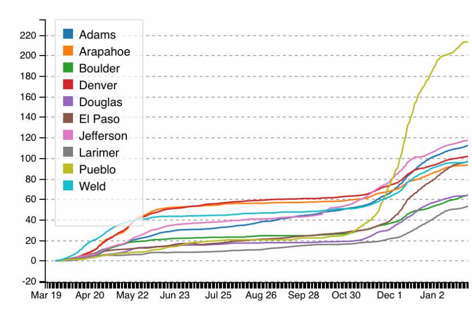

These charts compare total cases and deaths for Colorado's 10 most-populous counties.

Total cases
County (population rank)

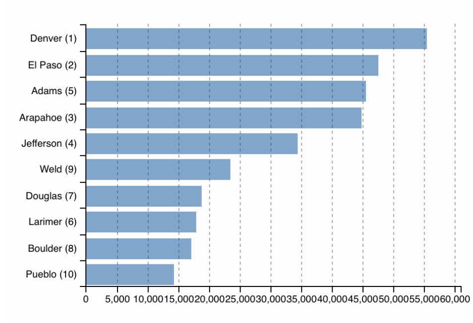

Total deaths
County (population rank)

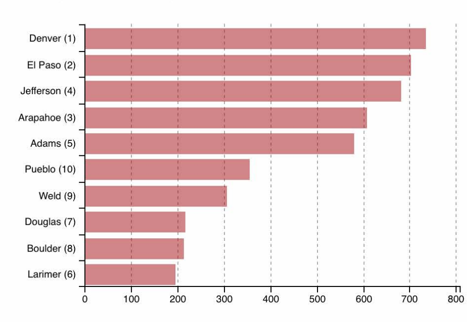

CDPHE data sets used | Colorado Case Counts by County, Number of Deaths by County

Maps: County cases and deaths

The first map shows the total number of cases and deaths by county and the second shows the case rate per 100,000 by county (rate will be slightly different than chart above because it is not a 7-day moving average).

Circles are centered in counties and are not meant to indicate an actual location; click circles for details

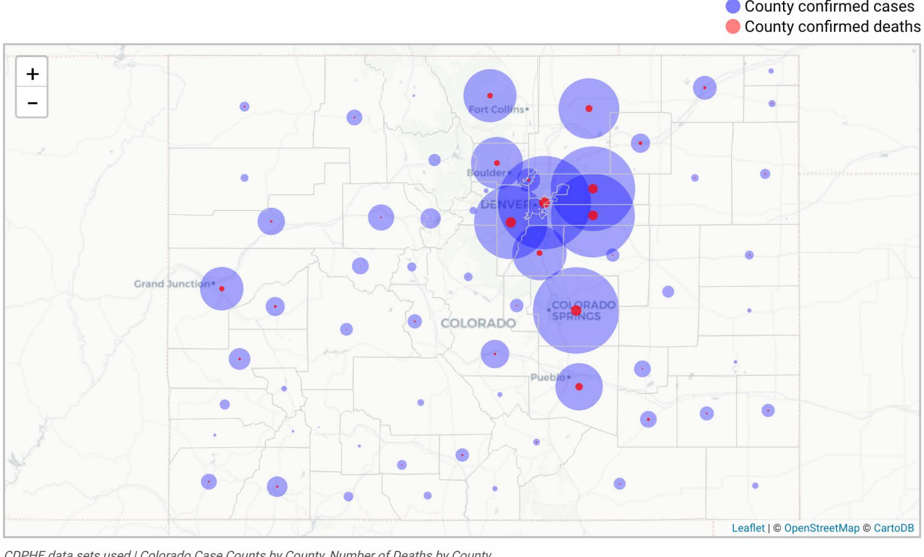

CDPHE data sets used | Colorado Case Counts by County, Number of Deaths by County

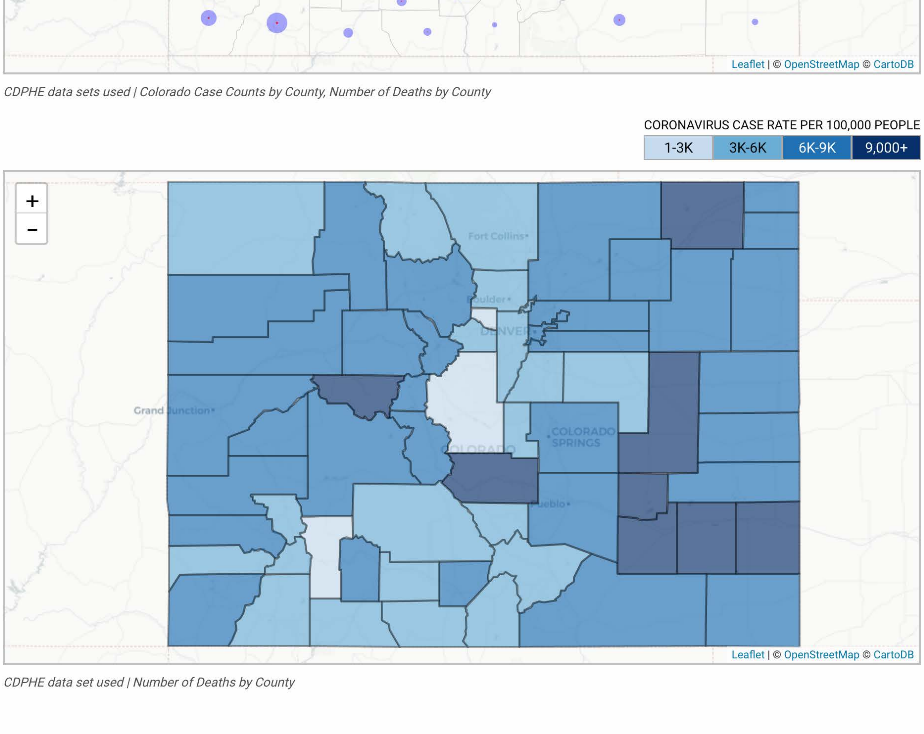

CDPHE data set used | Number of Deaths by County

Cases and deaths by race

These charts show the percentage of statewide cases, deaths and population broken down by race and ethnicity. The dark gray bar shows the percentage of the state's population that race makes up; a cases or deaths bar shorter than the population bar indicates that race is underrepresented, a bar longer than the population bar indicates a race is overrepresented.

Hover/click chart for details; click legend item to filter chart

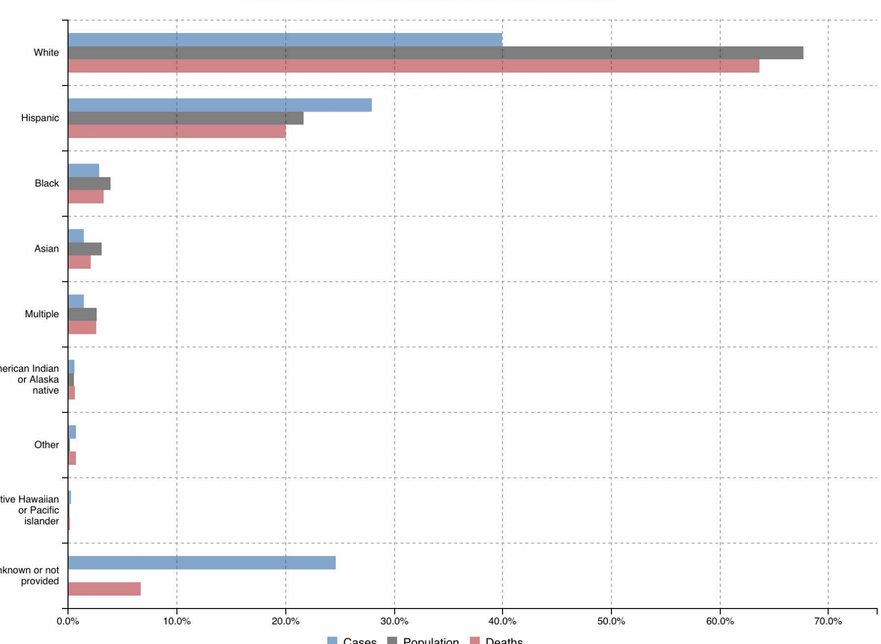

CDPHE data set used | COVID-19 in Colorado by Race & Ethnicity

Cases, hospitalizations and deaths by age

These charts show the total number of cases by age group and breaks them down by the number of people who have been hospitalized, the number who haven't been hospitalized and the number who have died. The second chart shows the same information as percentages of total cases by age group.

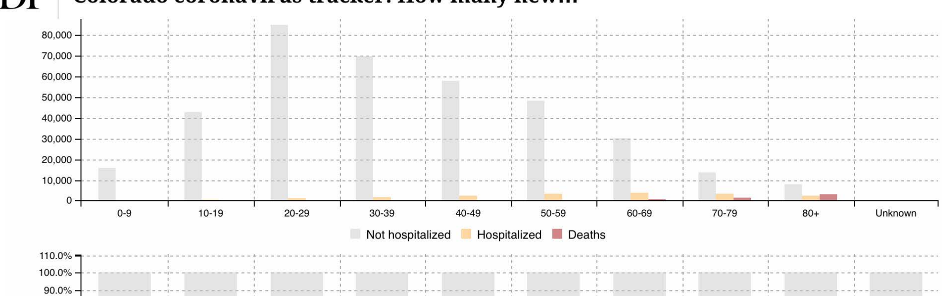

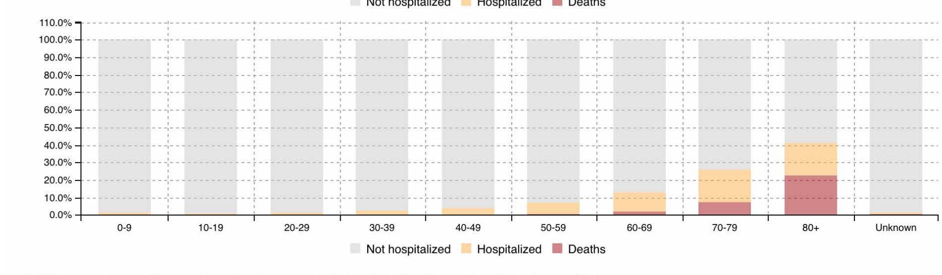

CDPHE data set used | Cases of COVID-19 Reported in Colorado by Age Group, Hospitalization, and Outcome

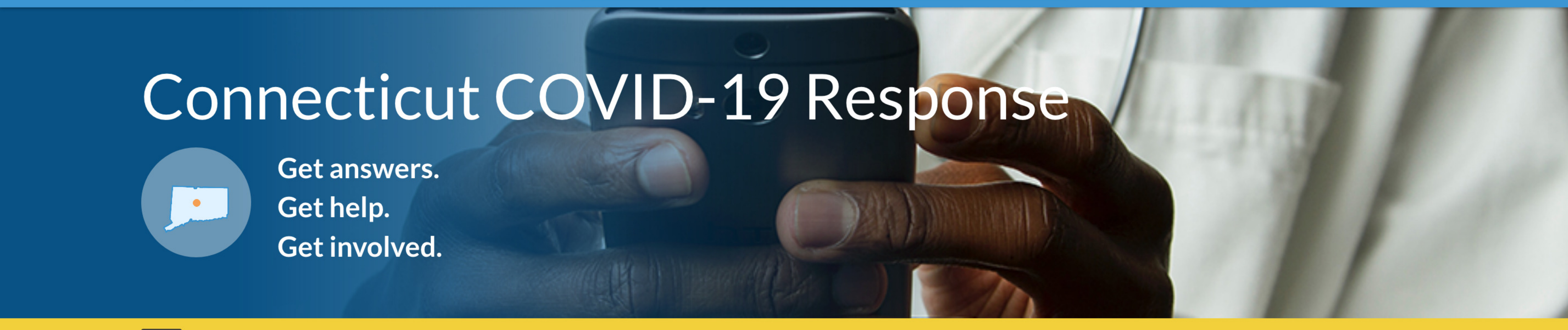

Connecticut COVID-19 Response

Get answers.
Get help.
Get involved.

COVID-19 VACCINES >> Due to high traffic volume at websites and phone lines, eligible residents in Phase 1B may encounter delays scheduling appointments to receive the COVID-19 vaccine. Please try back later. Appointment availability is limited based on the limited supply of vaccine that is currently available, but slots will continue to open up as they become available.

DAILY DATA REPORT
FOR CONNECTICUT

LATEST UPDATE: JANUARY 29, 2021 AT 4:00 P.M.

Reporting schedule: The State of Connecticut's COVID-19 metric report is issued once per day, every Monday through Friday, usually around 4:00 p.m. The report that is issued each Monday contains combined data that was collected on Friday, Saturday, and Sunday.

Open data portal: Supplemental data on COVID-19 in Connecticut can be found on the state's open data portal, data.ct.gov, in the COVID-19 data portal. Data presented on the open data portal is available in its rawest form.

Town Map

Average Daily Rate of COVID-19 Cases Among Persons Living in Community Settings per 100,000 Population By Town

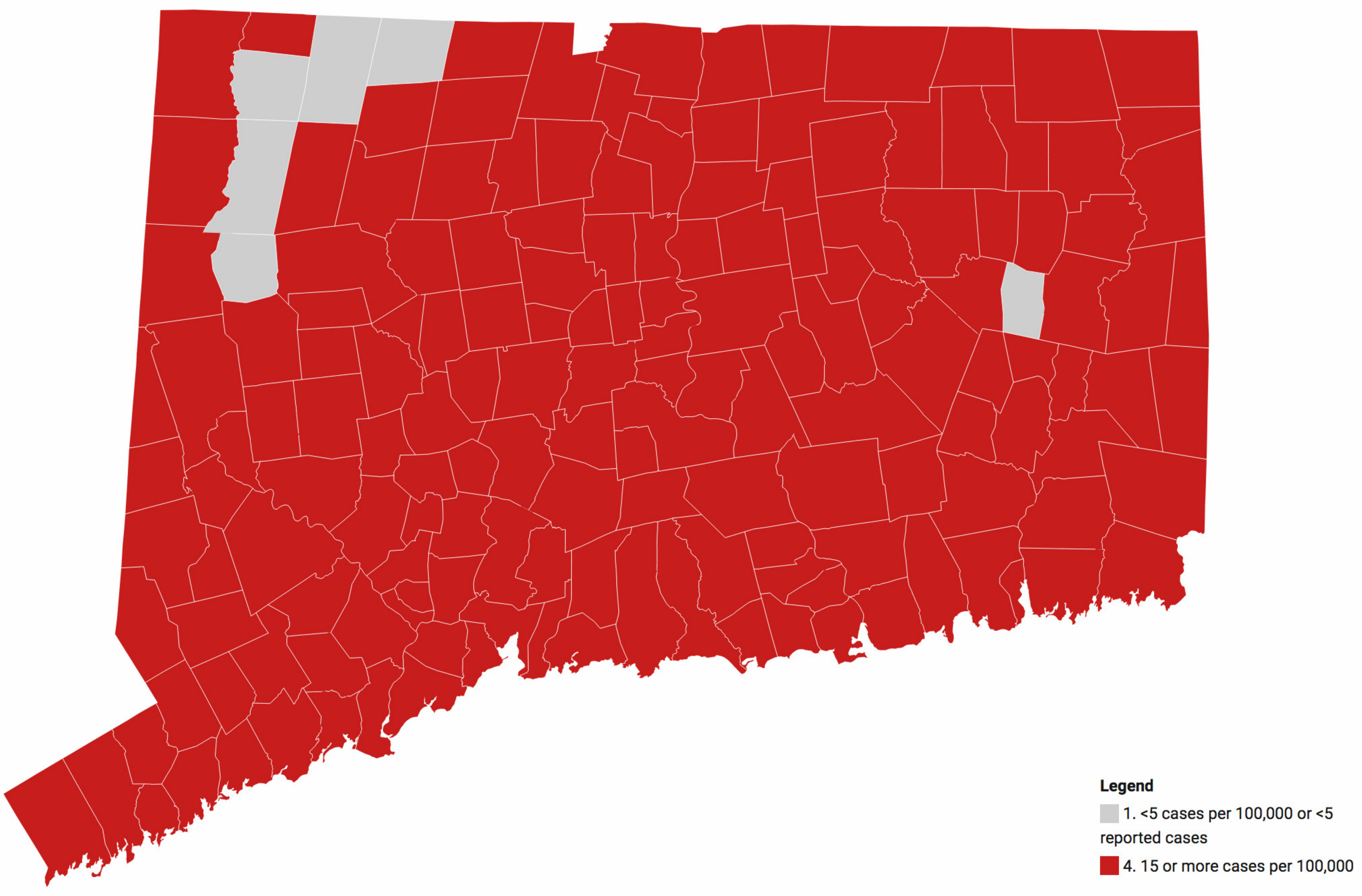

This map shows the average daily rate of new cases of COVID-19 by town during the past two weeks. Only cases among persons living in community settings are included in this map; the map does not include cases among people who reside in nursing home, assisted living, or correctional facilities.

Map: Ver 12.1.2020 • Source: CT Department of Public Health • Get the data • Created with Datawrapper

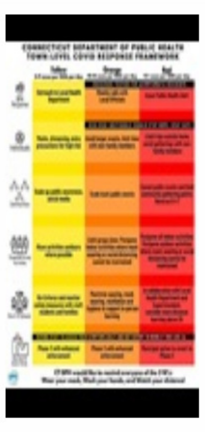

Connecticut Department of Public Health Town-level COVID Response Framework

State Summary

Connecticut COVID-19 Summary

Summary for the most recent day of reporting. Includes confirmed plus probable cases; probable cases include persons with positive antigen results

Measure	Total	ChangeDirection	Change
COVID-19 Cases (confirmed and probable)	250,023	+	1,258
COVID-19 Tests Reported (molecular and antigen)	5,693,083	+	34,577
Daily Test Positivity*			3.64%
Patients Currently Hospitalized with COVID-19	985	-	10
COVID-19-Associated Deaths	7,046	+	26

Daily test positivity is the number of new positive molecular and antigen cases divided by the number of new molecular and antigen tests reported in the past 24 hours.

Table: Ver 12.1.2020 • Source: CT Department of Public Health • Get the data • Created with Datawrapper

Age Group Chart

Number of COVID-19 Cases, Associated Deaths, and Rates per 100,000 by Age Groups

Total Number of Cases

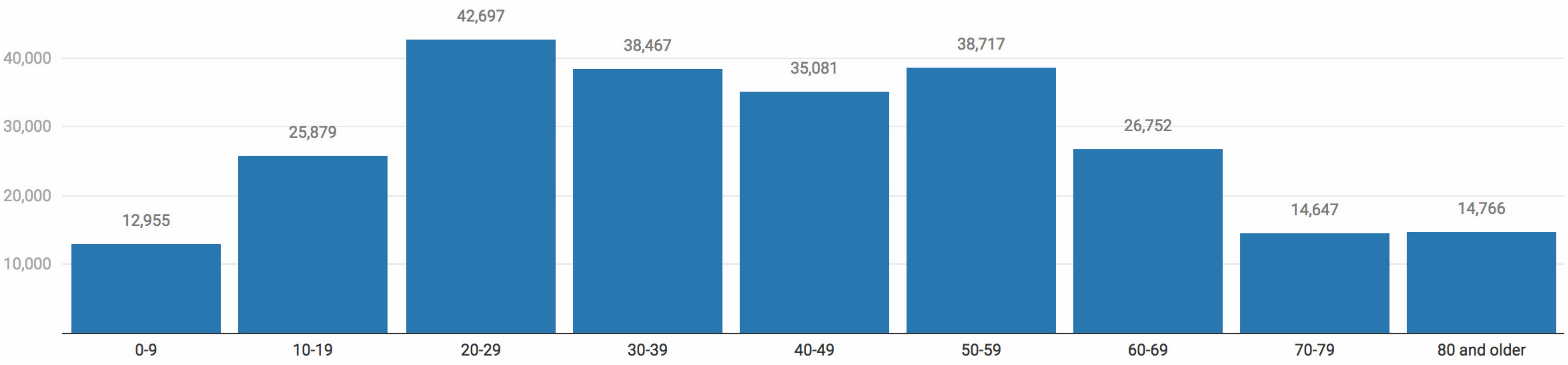

Total COVID-19 cases and associated deaths include confirmed plus probable.

Source: CT Department of Public Health • Get the data • Created with Datawrapper

County Chart

COVID-19 Cases, Rate per 100,000, Patients Currently Hospitalized, and Associated Deaths by County

Cases and deaths are cumulative over time

Number of Total Cases

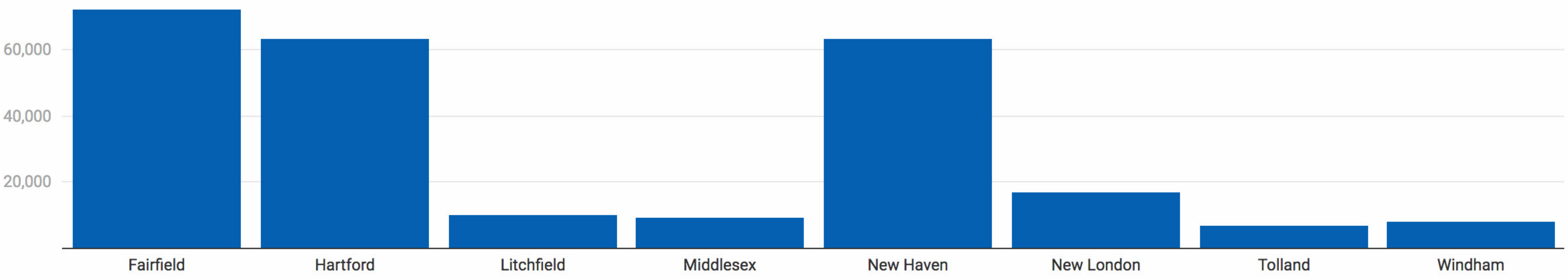

COVID-19 cases, rate per 100,000, and associated deaths are by county of residence. Hospitalization data are collected by CT Hospital Association and represent the number of patients currently hospitalized by location of the hospital and not the patient residence.

Source: CT Department of Public Health • Get the data • Created with Datawrapper

CT data collaborative

247,339 (+2,440) CONFIRMED CASES	1,016 (-52) HOSPITALIZATIONS	6,976 (+42) DEATHS
5,618,321 (+55,474) TESTS COMPLETED	4.40% (+0.73%) POSITIVE TEST RATE	1.00 TRANSMISSION RATE

Select an indicator box above

Connecticut Covid-19 Tracking

This dashboard is updated daily with Covid-19 data from Connecticut Department of Public Health. Data only includes confirmed and reported numbers and are preliminary and subject to change.

As of August 26, 2020, this dashboard has replaced our previous Covid-19 dashboard.

Questions? Comments? Contact Jason Cheung: jc@ctdata.org

Navigate Our Dashboard

Main Dashboard

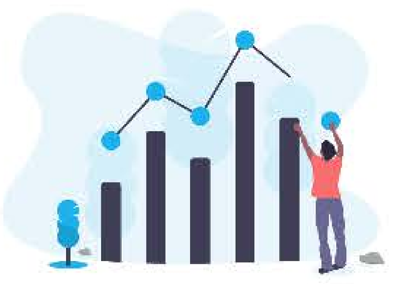

Town and County

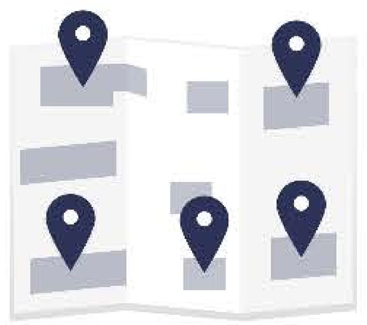

Demographics

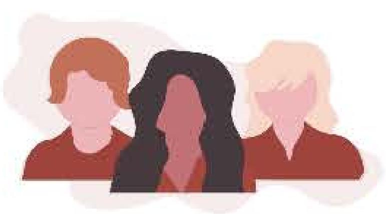

Daily Confirmed and Probable Cases

This chart shows new Covid-19 cases reported by CT DPH. The **blue 7-day moving average line** provides the best insight about state trends since daily counts may vary as a result of various reasons. Please focus on the **7-day moving average** rather than daily increases. Fluctuations in daily increases can be a result of many factors, such as errors in data collection, CT DPH not reporting on weekends as of July 2020 (resulting in larger increases on Mondays), and more.*

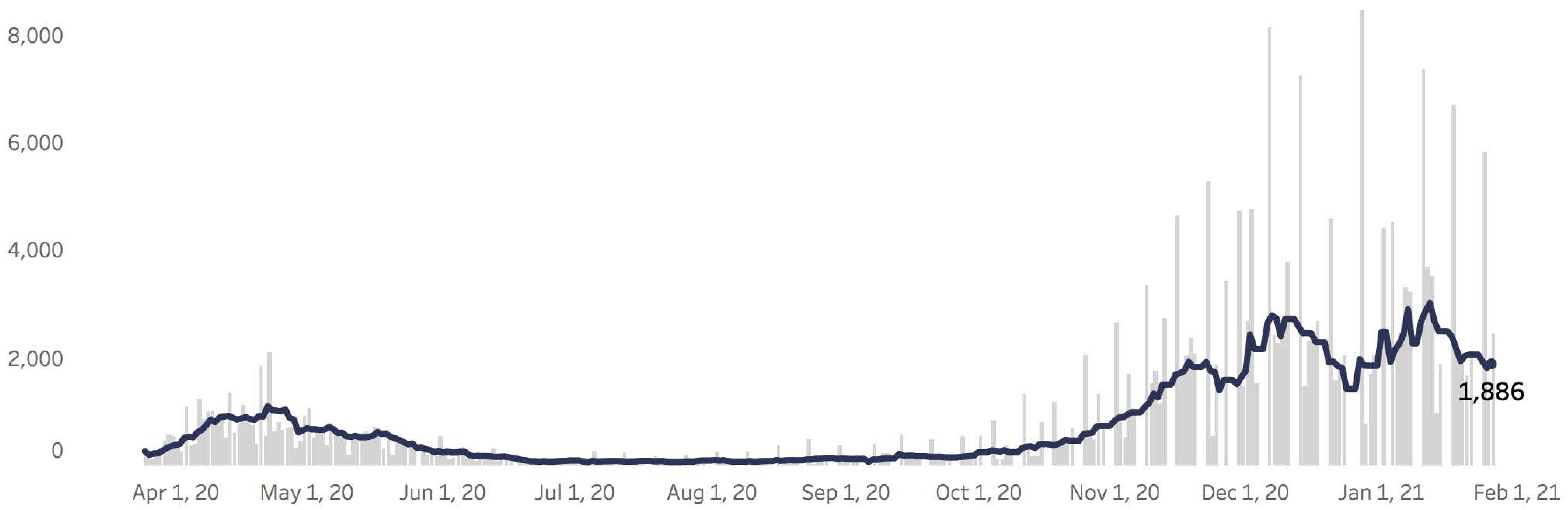

* The Date of Specimen Collection data is recommended by epidemiologists at CT DPH as it provides a more accurate daily increase in Covid-19 cases based on test date. **However, daily increases may be undercounted for days or weeks depending on the time it takes to receive test results.** To provide an accurate and recent snapshot of Covid-19, CTData has decided to use the Daily Report data with a focus on the **7-day moving average**.

+ a b | e a u

Connecticut Covid-19 Case Tracking

Viz Author: **Connecticut State Data Center**

134,290 Views ☆ 34

More Detail

+ab|eau+public GALLERY AUTHORS BLOG RESOURCES ACTIVITY ABOUT SIGN UP SIGN IN

Last Updated: Jan 27, 2021

Workbook Details: 17 Sheets

Metadata: CTdataCollaborativeCOVID-19
Cases_Number
Hosp_Number
Positive_Rate_Number
Tests_Number
Rt_Number
Deaths_Number
Hosp_Dash
Daily Confirmed and Probable Cases This chart shows new Covid-19 cases reported by CT DPH. The blue 7-day moving average line provides the best insight about state trends since daily counts may vary as a result of various reasons. Please focus on the 7-da
Deaths_Dash
Tests_Dash
PosRate_Dash
Rt_Dash
TownCounty
Demographics_Main
Demographics_RaceEth
Demographics_Gender

247,339 (+2,440) CONFIRMED CASES	1,016 (-52) HOSPITALIZATIONS	6,976 (+42) DEATHS
5,618,321 (+55,474) TESTS COMPLETED	4.40% (+0.73%) POSITIVE TEST RATE	1.00 TRANSMISSION RATE

Select an indicator box above

CTdata collaborative

Connecticut Covid-19 Tracking

This dashboard is updated daily with Covid-19 data from Connecticut Department of Public Health. Data only includes confirmed and reported numbers and are preliminary and subject to change.

As of August 26, 2020, this dashboard has replaced our previous Covid-19 dashboard.

Questions? Comments? Contact Jason Cheung: jc@ctdata.org

Navigate Our Dashboard

Main Dashboard

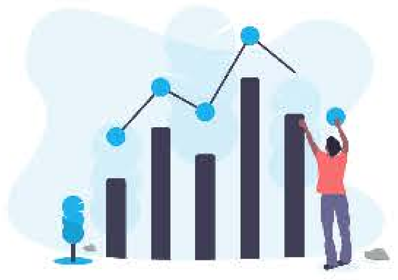

Town and County

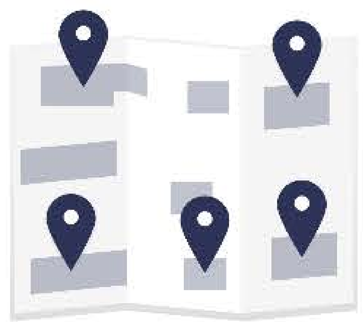

Demographics

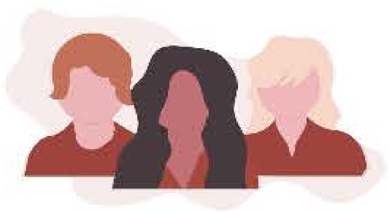

Daily Deaths

The number of confirmed and probable Covid-19 deaths, as reported to either the Office of the Chief Medical Examiner or CT DPH

Please focus on the **7-day moving average** rather than daily increases. Fluctuations in daily increases can be a result of many factors, such as errors in data collection, CT DPH not reporting on weekends as of July 2020 (resulting in larger increases on Mondays), and more.

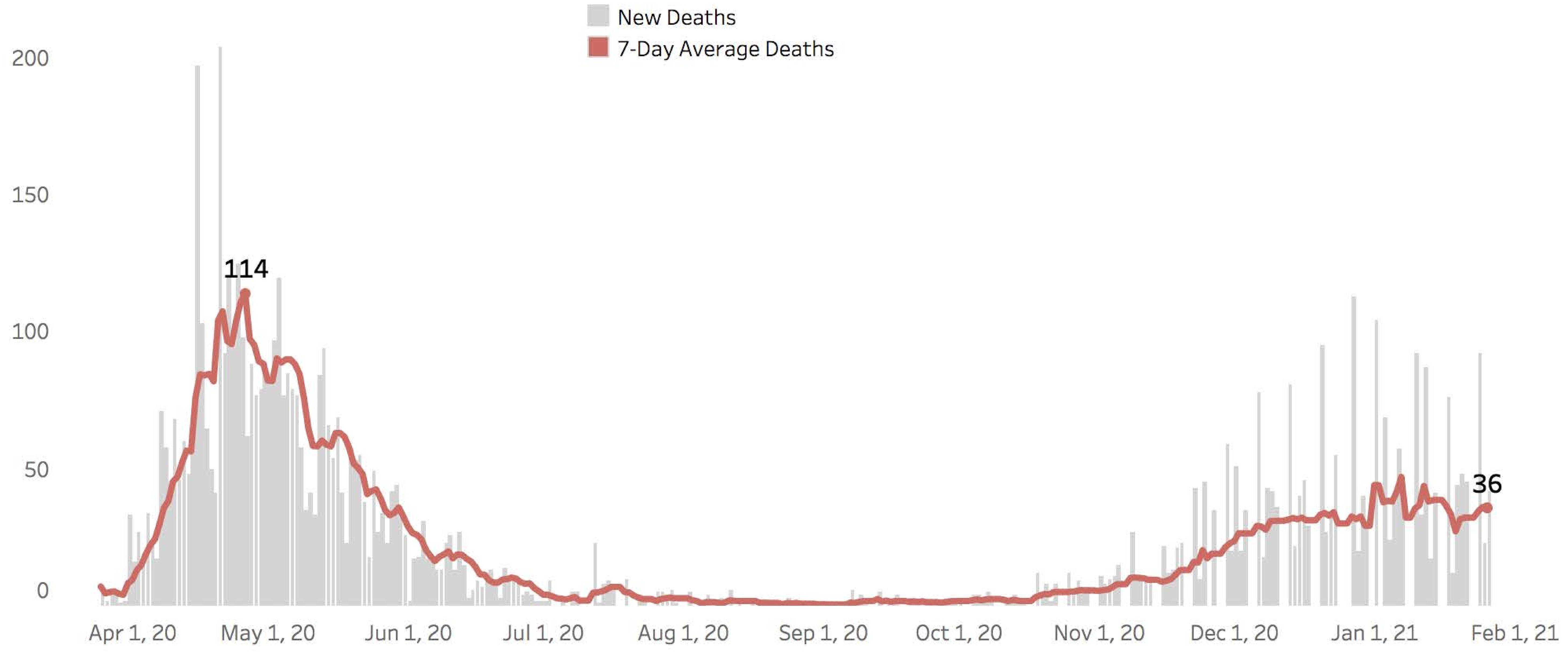

+ableau

Connecticut Covid-19 Case Tracking

Viz Author: **Connecticut State Data Center**

134,290 Views ☆ 34

More Detail

Originally Published: Mar 31, 2020

[illegible]

+ableau public GALLERY AUTHORS BLOG RESOURCES ACTIVITY ABOUT SIGN UP SIGN IN

Metadata: CTdataCollaborativeCOVID-19
Cases_Number
Hosp_Number
Positive_Rate_Number
Tests_Number
Rt_Number
Deaths_Number
Hosp_Dash
Daily Confirmed and Probable Cases This chart shows new Covid-19 cases reported by CT DPH. The blue 7-day moving average line provides the best insight about state trends since daily counts may vary as a result of various reasons. Please focus on the 7-da
Deaths_Dash
Tests_Dash
PosRate_Dash
Rt_Dash
TownCounty
Demographics_Main
Demographics_RaceEth
Demographics_Gender

CT data collaborative

247,339 (+2,440) CONFIRMED CASES

1,016 (-52) HOSPITALIZATIONS

6,976 (+42) DEATHS

5,618,321 (+55,474) TESTS COMPLETED

4.40% (+0.73%) POSITIVE TEST RATE

1.00 TRANSMISSION RATE

Select an indicator box above

Connecticut Covid-19 Tracking

This dashboard is updated daily with Covid-19 data from Connecticut Department of Public Health. Data only includes confirmed and reported numbers and are preliminary and subject to change.

As of August 26, 2020, this dashboard has replaced our previous Covid-19 dashboard.

Questions? Comments? Contact Jason Cheung: jc@ctdata.org

Navigate Our Dashboard

Main Dashboard

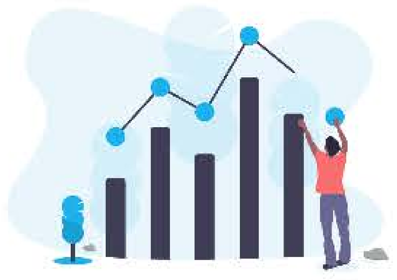

Town and County

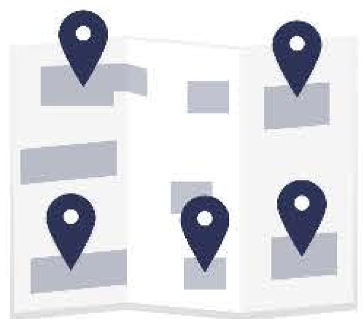

Demographics

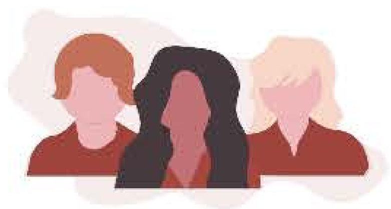

Daily Tests Completed

The number of new Covid-19 PCR tests completed each day

Please focus on the **7-day moving average** rather than daily increases. Fluctuations in daily increases can be a result of many factors, such as errors in data collection, CT DPH not reporting on weekends as of July 2020 (resulting in larger increases on Mondays), and more.

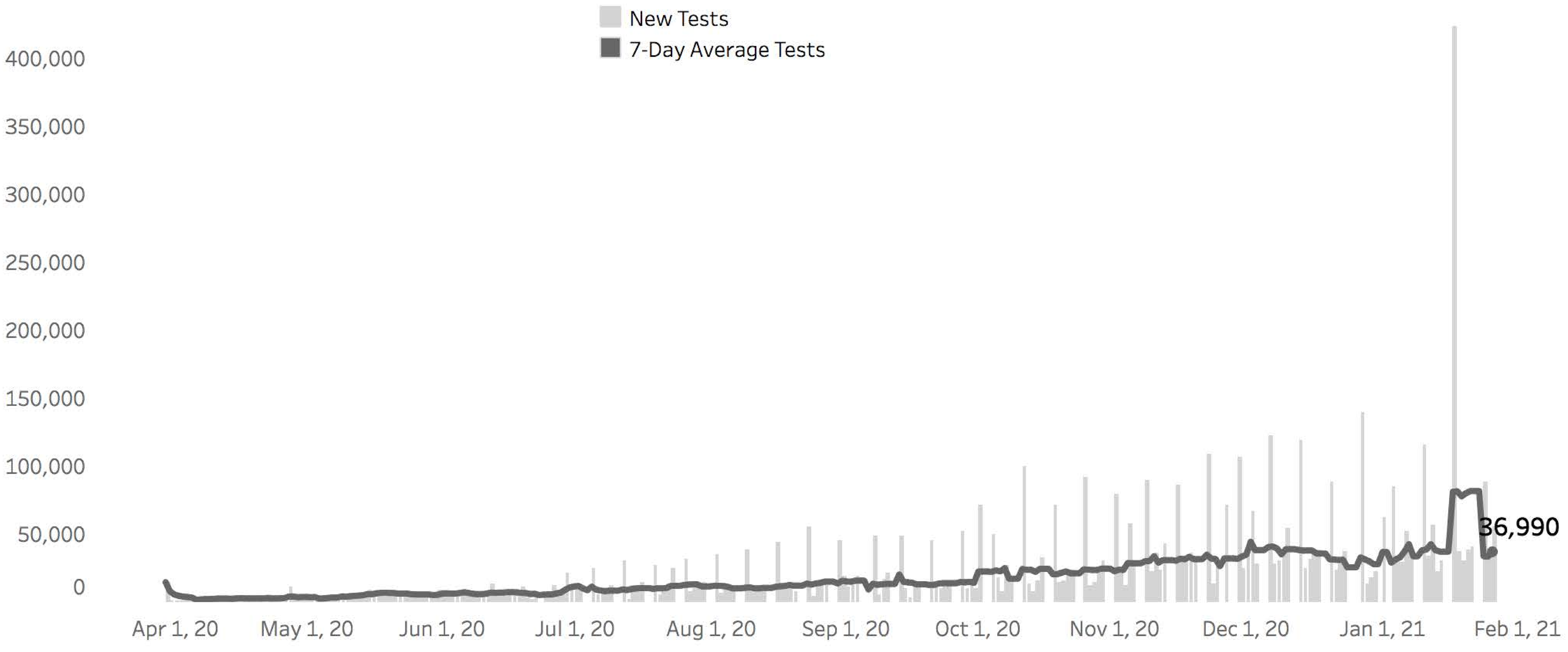

+ a b | e a u

Connecticut Covid-19 Case Tracking

Viz Author: **Connecticut State Data Center**

134,290 Views ☆ 34

More Detail

Originally Published: Mar 31, 2020

Last Updated: Jan 27, 2021

Workbook Details: 17 Sheets

+ab|eau public GALLERY AUTHORS BLOG RESOURCES ACTIVITY ABOUT SIGN UP SIGN IN

[illegible]
Hosp_Number
Positive_Rate_Number
Tests_Number
Rt_Number
Deaths_Number
Hosp_Dash
Daily Confirmed and Probable Cases This chart shows new Covid-19 cases reported by CT DPH. The blue 7-day moving average line provides the best insight about state trends since daily counts may vary as a result of various reasons. Please focus on the 7-da
Deaths_Dash
Tests_Dash
PosRate_Dash
Rt_Dash
TownCounty
Demographics_Main
Demographics_RaceEth
Demographics_Gender

Cases of COVID-19 Reported in Connecticut by Town by the Department of Geography at UConn

Last Updated at
1/29/2021, 6:30 PM

Total Confirmed
250,023

Increase Cases
1,258
as of previous day

Total Deaths
7,046

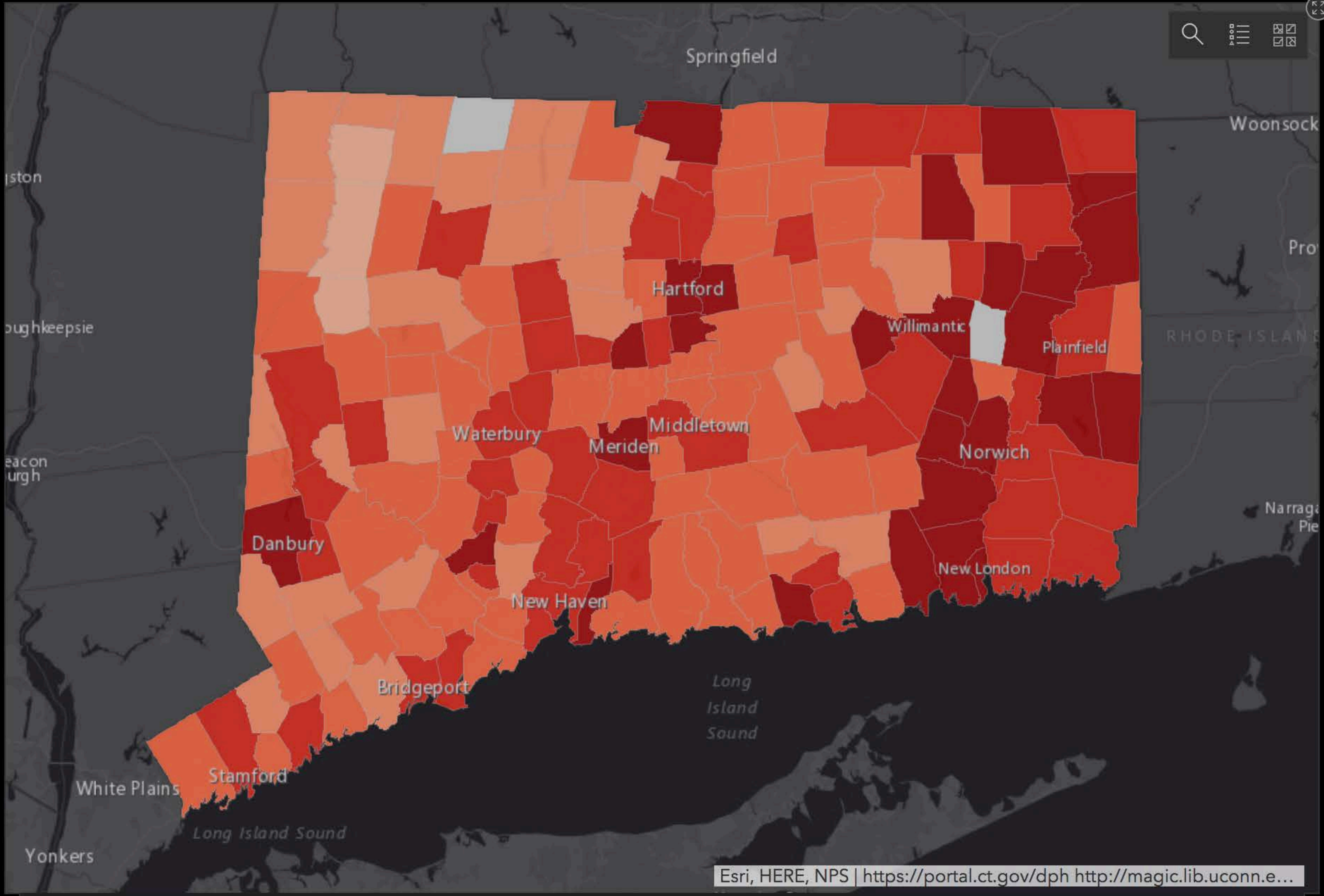

Case Rate per 10,000 People in Past 14-Days

For sources and contact information please click upper right button in this box

Deaths per 100,000 People by Age

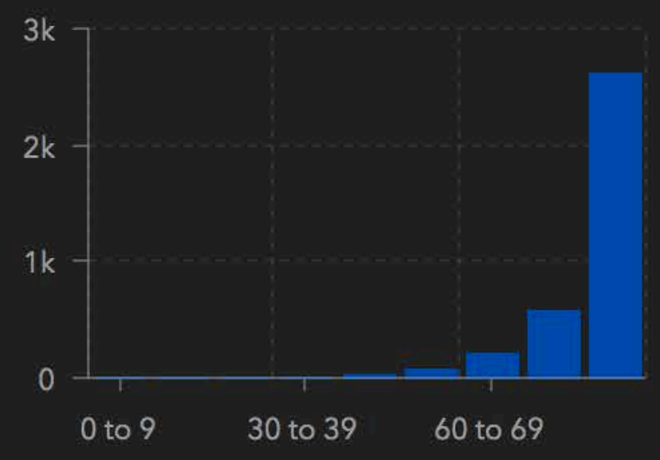

Deaths by Age | Cases by Age

Connecticut Towns

- 128 Andover
- 1,327 Ansonia
- 193 Ashford
- 649 Avon
- 122 Barkhamsted
- 391 Beacon Falls
- 1,177 Berlin
- 290 Bethany
- 1,455 Bethel
- 152 Bethlehem
- 1,571 Bloomfield
- 196 Bolton

Towns | Counties

Data
COVID-19 Surveillance
Reopening Metrics
Exposure Activities
Outbreak Data
Vaccination Data

COVID-19 Surveillance

COVID-19 Surveillance | Reopening Metrics | Outbreak Data
Exposure Activities | Vaccinations

January 30, 2021

- Total Overall Number of Tests: 1,100,466*
- Total Number of DC Residents Tested: 411,140*
- Total Positives: 36,872
- Total Lives Lost: 913
- Cleared from Isolation: 25,810

*All data are preliminary and are subject to change based on additional reporting

Download copy of DC COVID-19 data

Other Data

- Public Safety Agency Data
- Human Services Agency Data
- DMV Agency Data
- Hospital Status Data
- DC Public Schools Data

Trends | Ward | Neighborhood | Demographics | Tables

Testing and Case Trends | Hospital Census and Bed Availability | Ventilator Use and Availability

Total Overall Tested	Total Residents Teste	Total Positives	Total Recovered	Total Lives Lost
1,100,466	411,140	36,872	25,810	913

Overall Tested | Positives | Recovered | Lives Lost | Residents Tested

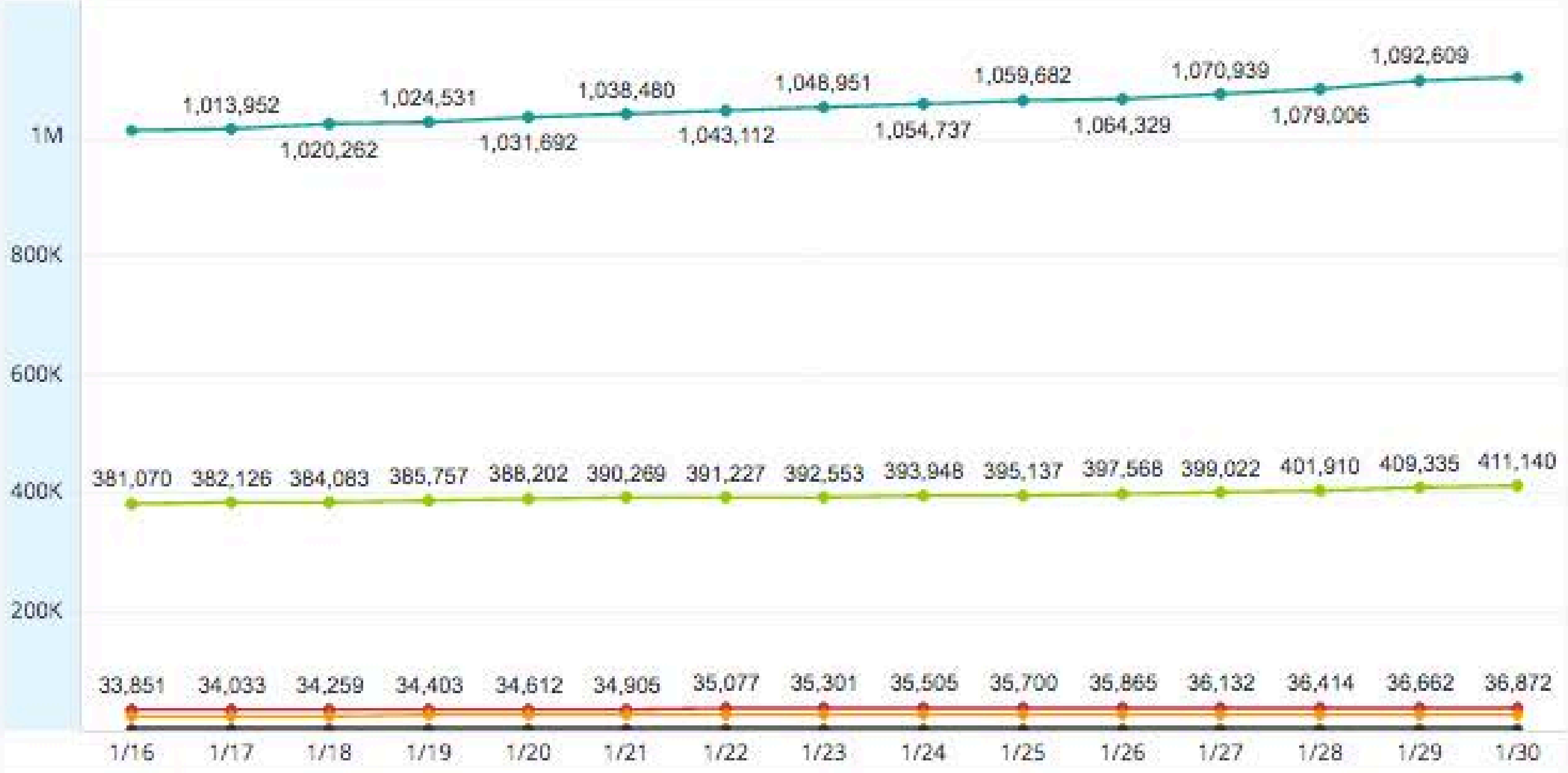

1/16 - 1/30
3/7 1/30

Due to the rapidly evolving nature of this public health crisis, DC is updating this dashboard daily between 8 am - 10 am based on the most recently available data.
*There is no data for "people tested overall" result for 3/20, an averaged number (adjacent days) has been inserted for graphing purposes until the data can be corrected.

Delaware.gov
Agencies News Topics Contact

My Healthy Community
Delaware Environmental Public Health Tracking Network

Search for your community

Home / State of Delaware

Coronavirus (COVID-19) Data Dashboard

Overview
About the data | Data download

State of Delaware's Latest Status

Data are current as of 6pm the previous day. Last update: 01/31/2021 11:52 AM.

In-Person Contagious School Cases Dashboard
This new dashboard contains information about COVID-19 cases in Delaware's schools.
View dashboard →

New Positive Cases per Week (per 100,000 Population)
Date Range: 01/23/2021 - 01/29/2021

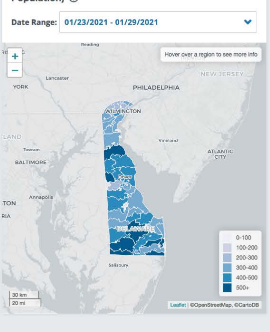

Key Public Health Metrics

507,941 Persons Tested Negative
315 Current Hospitalizations
43 Current Critical Hospitalizations
532 New Positive Cases

More Tests Data > | More Hospital Data > | More Cases Data >

Positive Cases in State of Delaware

Total Positive Cases
78,071
832.6 per 10,000 people

Confirmed 74,279 | Probable 3,792 | Residents of Long-Term Care Facilities 2,130

Positive Cases by County

New Castle County 44,340 | Kent County 12,821 | Sussex County 20,728
Unknown 182

Deaths in State of Delaware

Total Deaths
1,090
9.0 per 10,000 people

Confirmed 982 | Probable 108 | Residents of Long-Term Care Facilities 575

Trends: State of Delaware over the Last 14 Days

14-day charts | 30-day charts | 90-day charts | All available data

Case counts and test data by date of report can vary significantly from day to day for a variety of reasons. In addition to changes due to actual changes in disease incidence, trends are strongly influenced by testing patterns (who gets tested and why), testing availability, lab analysis backlogs, lab reporting delays, new labs joining our electronic laboratory reporting system, mass screenings, etc.

Current Hospitalizations
Updated: 01/31/2021

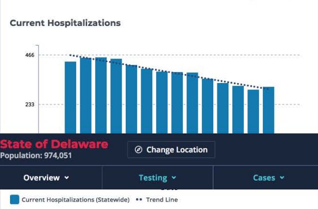

Current Hospitalizations (Statewide) •• Trend Line

New Hospital Admissions (Confirmed and Suspected with COVID-19)
Updated: 01/31/2021

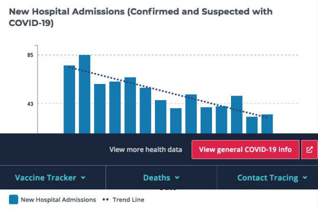

New Hospital Admissions •• Trend Line
Data source: Delaware Health Information Network | More details

State of Delaware
Population: 974,051
Change Location | View more health data | View general COVID-19 info
Overview | Testing | Cases | Vaccine Tracker | Deaths | Contact Tracing

Percent Positive
Updated: 01/31/2021

Percent of persons testing positive, and percent of tests that are positive are helpful indicators in understanding the spread of infection in a community. Percent of persons testing positive indicate one result reported for each individual tested (positive or negative), even if that person is tested more than once with the same result. As a result, it often reflects higher positivity rates.

Percent of tests that are positive indicates the total number of specimens tested, including when an individual is tested more than once, and reflects higher reported test numbers and lower positivity rates. It is also more widely used by other states and the Federal government, and is therefore useful to compare Delaware to other jurisdictions.

Because of the increasing frequency of repeat testing, using person-based percent positives could be misleading on a daily basis. Therefore, using the percent of tests that are positive is a more useful measure to make operational decisions. Data for percent of tests that are positive are based on the testing date, while data for persons testing positive is based on the date results are reported to DPH. There is a two-day lag for presenting data related to percent of tests that are positive to account for the time delay between the date of the test and the date that DPH receives the test result.

Percent of persons testing positive | Percent of tests that are positive

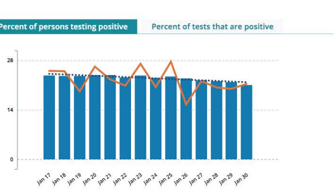

Average — Percent Positive •• Trend Line

Averaging method: averages are a moving calculation that smooth unstable data. For each data point, seven values are averaged: the data point and the six preceding data points. Toward the beginning of the time series, when there are fewer than six values before a data point, instead of averaging seven values, only the available values are used in the calculation. More details

New Positive Cases
Updated: 01/31/2021

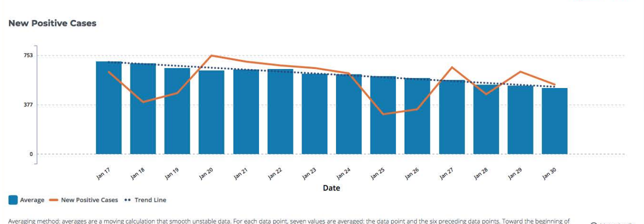

Average — New Positive Cases •• Trend Line

Averaging method: averages are a moving calculation that smooth unstable data. For each data point, seven values are averaged: the data point and the six preceding data points. Toward the beginning of the time series, when there are fewer than six values before a data point, instead of averaging seven values, only the available values are used in the calculation. More details

Percentage of Emergency Department Visits for Influenza-Like Illness (ILI) and COVID-19-Like Illness (CLI) by Week
Updated: 01/31/2021

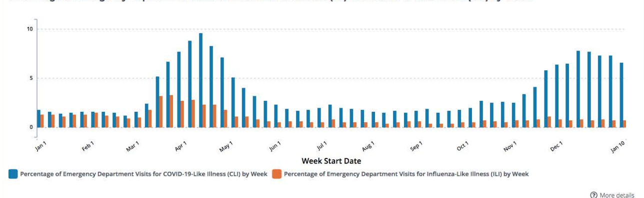

Percentage of Emergency Department Visits for COVID-19-Like Illness (CLI) by Week | Percentage of Emergency Department Visits for Influenza-Like Illness (ILI) by Week
More details

Tests Performed
Updated: 01/31/2021

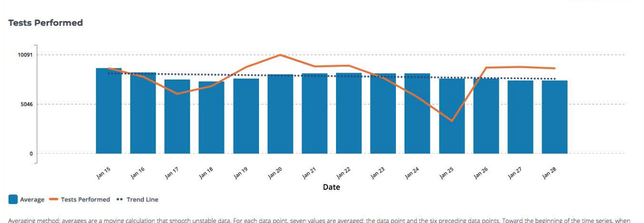

Average — Tests Performed •• Trend Line

Averaging method: averages are a moving calculation that smooth unstable data. For each data point, seven values are averaged: the data point and the six preceding data points. Toward the beginning of the time series, when there are fewer than six values before a data point, instead of averaging seven values, only the available values are used in the calculation.

Cases by County (Cumulative)

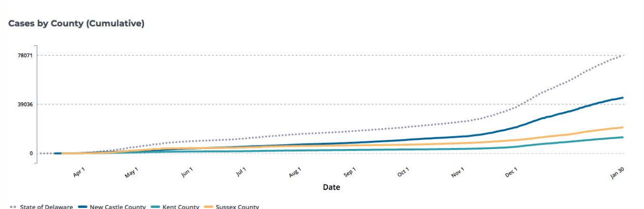

State of Delaware — New Castle County — Kent County — Sussex County

Weekly Community Spread Summary

Note about Updates to This Section
This section has been updated to provide a weekly snapshot measuring the level of COVID-19 community spread in the State of Delaware. The School Reopening dashboard has been replaced with a weekly community spread summary. The previous School Reopening dashboard was based on the State's understanding of COVID-19 at the time it was developed, in August 2020. We have learned a lot since the summer months and no longer believe that the reopening dashboard was an accurate reflection of the conditions in our schools. The Division of Public Health (DPH) will continue to work closely with school leaders to monitor conditions. A new dashboard reflecting data about in-person attendance and cases among students and staff in schools - including district level data - has been added to the My Healthy Community COVID-19 data portal. It can be found here.

Breakdown of This Week's Status
01/16/2021 - 01/22/2021
Updated: Every Monday

New cases rate (per 100,000 people)	Percent of tests that are positive (per 100,000 people)	Avg. daily hospitalizations (per 100,000 people)
471.0	8.6%	44.9

Unlike other COVID data on My Healthy Community, historical data presented on the weekly community spread summary are not updated daily. These data are updated on Mondays. This is to allow for complete reporting of the week's test data.

Status by Week

	New cases rate (per 100,000 people)	Percent of tests that are positive	Avg. daily hospitalizations (per 100,000 people)
01/16/2021 - 01/22/2021	471.0	8.6	44.9
01/09/2021 - 01/15/2021	507.1	8.3	47.7
01/02/2021 - 01/08/2021	579.9	9.8	45.6
12/26/2020 - 01/01/2021	452.9	9.3	42.8
12/19/2020 - 12/25/2020	448.2	8.5	43.2

Show/hide additional weeks ↓

View more detailed data →

Healthcare System Status

Delaware's PPE Inventory Status
Updated: 01/31/2021

- Fluid Resistant Gowns and Isolation Gowns — 2 Weeks+ Left (Stable)
- Face Shields — 2 Weeks+ Left (Stable)
- Glasses and Goggles — 2 Weeks+ Left (Stable)
- Gloves — 2 Weeks+ Left (Stable)
- Isolation and Procedural-Ear Loop Masks — 2 Weeks+ Left (Stable)
- N95 Masks — 2 Weeks+ Left (Stable)
- Surgical Masks — 2 Weeks+ Left (Stable)

Delaware's Healthcare System Status
Updated: 01/31/2021

- ICU Bed Availability — Stable (75% or less occupied)
- Ventilator Availability — Stable (75% or less occupied)
- Hospital ED Wait Times — < 5 hours
- Hospital Census/Capacity — < 98%

Testing
About the data | Data download | How can I get tested?

Percent of persons testing positive, and percent of tests that are positive are helpful indicators in understanding the spread of infection in a community. Percent of persons testing positive indicate one result reported for each individual tested (positive or negative), even if that person is tested more than once with the same result. As a result, it often reflects higher positivity rates.

Percent of tests that are positive indicates the total number of specimens tested, including when an individual is tested more than once, and reflects higher reported test numbers and lower positivity rates. It is also more widely used by other states and the Federal government, and is therefore useful to compare Delaware to other jurisdictions.

Because of the increasing frequency of repeat testing, using person-based percent positives could be misleading on a daily basis. Therefore, using the percent of tests that are positive is a more useful measure to make operational decisions. Data for percent of tests that are positive are based on the testing date, while data for persons testing positive is based on the date results are reported to DPH. There is a two-day lag for presenting data related to percent of tests that are positive to account for the time delay between the date of the test and the date that DPH receives the test result.

Latest Status

Test Rates by Zip Code and Week
Date Range: 01/23/2021 - 01/29/2021
Total Persons Tested | Total Tests

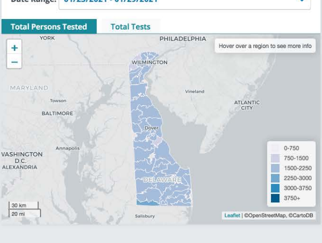

Testing Totals in State of Delaware
Total Persons Tested | Total Tests

State of Delaware
586,012
61,718.3 per 100,000 people

Total Persons Tested by County
New Castle County 343,114 | Kent County 80,314 | Sussex County 132,195
Unknown 30,389
More details

Trends

Testing Totals
Updated: 01/31/2021
Total Persons Tested | Total Tests

586,012 Total count on 01/31/2021
61,718.3 rate per 100,000 people on 01/31/2021

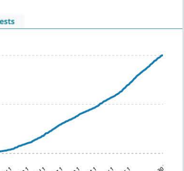

Total Persons Tested
More details

Persons Tested Negative
Updated: 01/31/2021

507,941 Total count on 01/31/2021
5,349.6 rate per 10,000 people on 01/31/2021

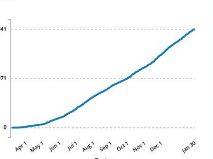

Persons Tested Negative
More details

Demographics

Updated: 01/31/2021

Total Persons Tested by Race/Ethnicity

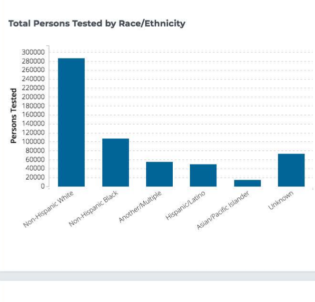

Total Persons Tested by Race/Ethnicity & County

RACE/ETHNICITY	STATE OF DELAWARE	NEW CASTLE	KENT	SUSSEX
Non-Hispanic White	286,430	155,846	37,415	78,758
Non-Hispanic Black	107,099	69,220	18,315	13,179
Another/Multiple	54,934	34,459	5,496	10,790
Hispanic/Latino	49,757	31,704	4,619	11,438
Asian/Pacific Islander	14,466	11,750	1,307	1,021
Unknown	73,326	40,135	13,162	17,009

Total Population by Race/Ethnicity for State of Delaware →

Updated: 01/31/2021

Rate of Persons Tested by Race/Ethnicity

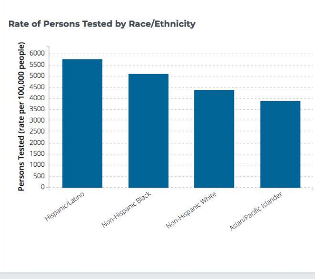

Rate of Persons Tested by Race/Ethnicity & County

RACE/ETHNICITY	STATE OF DELAWARE	NEW CASTLE	KENT	SUSSEX
Hispanic/Latino	5,764.6	5,863.4	3,792.3	5,700.8
Non-Hispanic Black	5,102.6	4,993.9	4,135.1	4,882.6
Non-Hispanic White	4,373.6	4,351.0	3,226.5	4,357.0
Asian/Pacific Islander	3,888.7	3,818.0	3,663.1	3,573.7

Total Population by Race/Ethnicity for State of Delaware →

Cases (State Of Delaware)
About the data | Data download

Latest Status

Movement of the Disease over Time
New Positive Cases | Total Positive Cases (Early Days of the Pandemic)

This animated map shows the rate of new cases of COVID-19 across Delaware as a 7 day average. We can see the period during the summer when the disease was less prevalent, and the areas of the State where people were hit hardest.

COVID-19 New Positive Cases (State of Delaware)
Insufficient data | Rate per 10,000 people 0 – 17.12
Only counts of 5 or more are used to create maps.

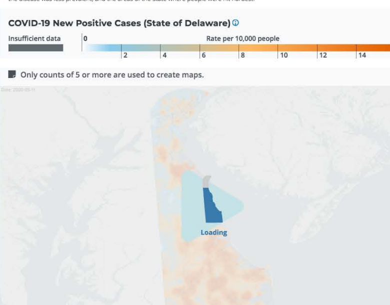

Total Positive Cases in State of Delaware

Total Positive Cases
78,071
832.6 per 10,000 people

Confirmed 74,279 | Probable 3,792 | Residents of Long-Term Care Facilities 2,130

Positive Cases by County
New Castle County 44,340 | Kent County 12,821
Sussex County 20,728 | Unknown 182

Trends

Positive Cases
Updated: 01/31/2021

78,071 Total count on 01/31/2021
832.6 rate per 10,000 people on 01/31/2021

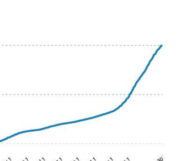

Positive Cases

Percent Positive by Zip Code and Week
Date Range: 01/23/2021 - 01/29/2021
Percent of persons testing positive | Percent of tests that are positive

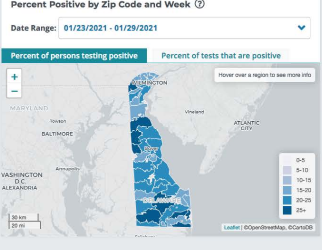

Venues Visited by Cases

Case counts broken down by participation in an event or visitation to an entertainment or recreational venue in the two weeks prior to COVID-19 symptom onset. These counts suggest potential exposure during the event/visit — they do not indicate virus contraction.

Select date range: All Weeks

All Weeks

6,525 (19%) of interviewed cases reported participating in an event or visiting a venue in the two weeks prior to the onset of their symptoms (or if no symptoms, date of their test)
28,136 (81%) of interviewed cases did not report participating in an event or visiting a venue

Top three venues per category

VENUE CATEGORY	CASE COUNT*
**Beaches**	**506**
**Events**	**2,318**
Religious service**	679
House party	442
Other large gathering	423
**Entertainment and Recreational Places**	**3,701**
Restaurant	1,596
Other tourist attraction	549
Gym	472

* Individuals who test positive are interviewed to determine the venues visited in the two weeks prior to the onset of their symptoms (or if no symptoms, date of their test). This information was used to calculate the data above. Counts do not necessarily suggest a degree of risk associated with these venues but may suggest the need for further investigation.

** Includes choir or singing practice.

Cases Who Visited Venues (Reported All Weeks)

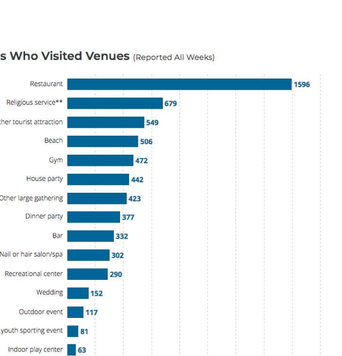

Demographics

Rate charts | Count charts

Positive Cases per 10,000 Population by Age Group

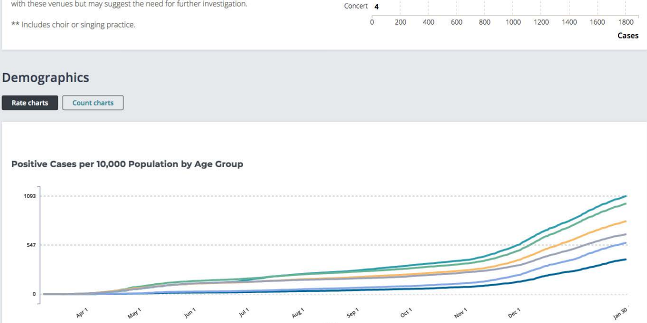

0-4 | 5-17 | 18-34 | 35-49 | 50-64 | 65+

Updated: 01/31/2021

Rate of Cases by Age

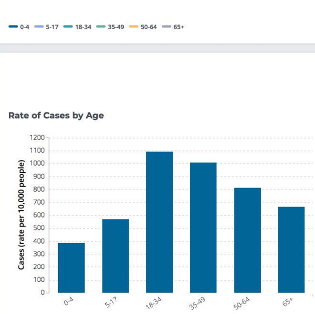

Rate of Cases by Age & County

AGE	STATE OF DELAWARE	NEW CASTLE	KENT	SUSSEX
0-4	386.5	401.3	306.6	417.1
5-17	571.6	587.2	432.5	659.1
18-34	1,092.6	1,051.3	882.8	1,459.6
35-49	1,008.2	940.0	914.1	1,295.3
50-64	812.1	767.1	813.8	907.8
65+	666.7	633.6	702.7	689.7

Total Population by Age for State of Delaware →

Updated: 01/31/2021

Rate of Cases by Sex

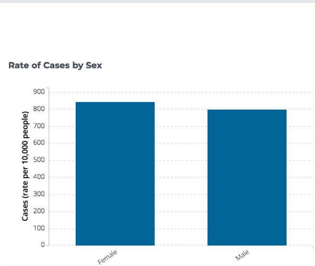

Rate of Cases by Sex & County

SEX	STATE OF DELAWARE	NEW CASTLE	KENT	SUSSEX
Female	841.9	823.9	747.5	954.5
Male	799.1	770.0	716.9	930.4

Updated: 01/31/2021

Rate of Cases by Race/Ethnicity

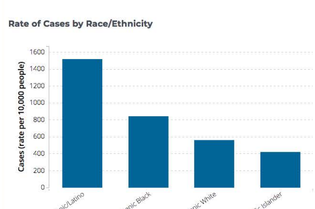

Rate of Cases by Race/Ethnicity & County

RACE/ETHNICITY	STATE OF DELAWARE	NEW CASTLE	KENT	SUSSEX
Hispanic/Latino	1,516.3	1,471.0	1,041.9	1,917.4
Non-Hispanic Black	843.7	809.1	806.9	1,072.2
Non-Hispanic White	564.9	530.5	549.7	640.3
Asian/Pacific Islander	424.7	374.3	703.5	609.0

Total Population by Race/Ethnicity for State of Delaware →

Deaths (State Of Delaware)
About the data | Data download

540

Age Range | Residents of Long-Term Care Facilities

0

Trends

recovered from the disease. Cases considered recovered included only infected persons who reported to DPH that they had reached the end of their isolation period. It does not capture instances such as in long-term care and other congregate settings where patients are cleared by facility medical staff, not DPH. In addition, it does not include those who do not respond to,

Total count on 01/31/2021

Total Deaths by Sex & County

STATE OF DELAWARE

Florida's COVID-19 Data and Surveillance Dashboard

Florida Department of Health, Division of Disease Control and Health Protection

Select a County STATE

Total Cases

1,713,589

Cumulative Data for Florida Residents:

Positive Residents

1,682,096

Resident Hospitalizations

72,135

Florida Resident Deaths

26,360

Non-Resident Deaths

435

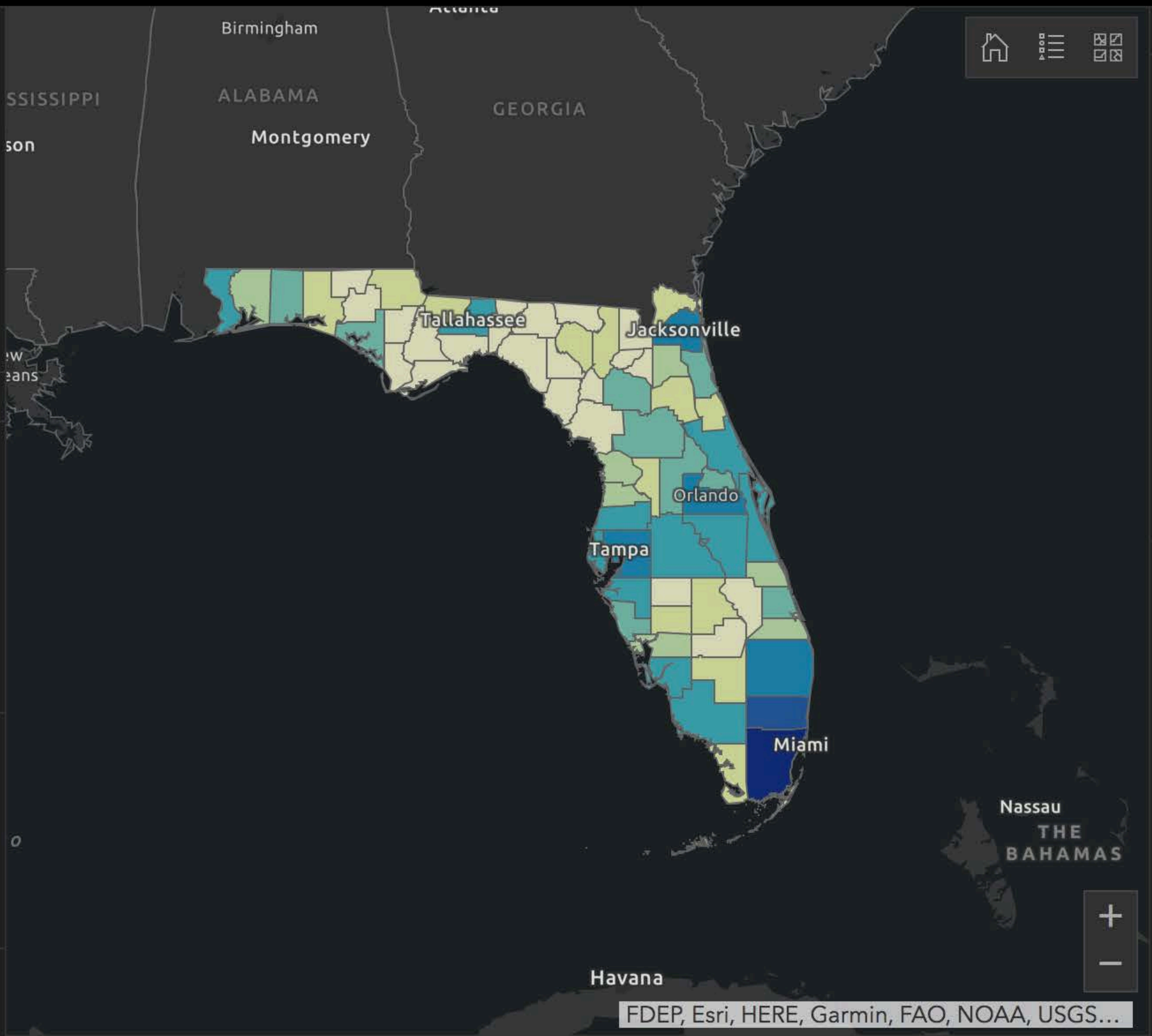

Comparison of counties is not possible because case data are not adjusted by population.

Recent Data for Florida Residents (Last 30 Days):

New Cases of Residents by Day

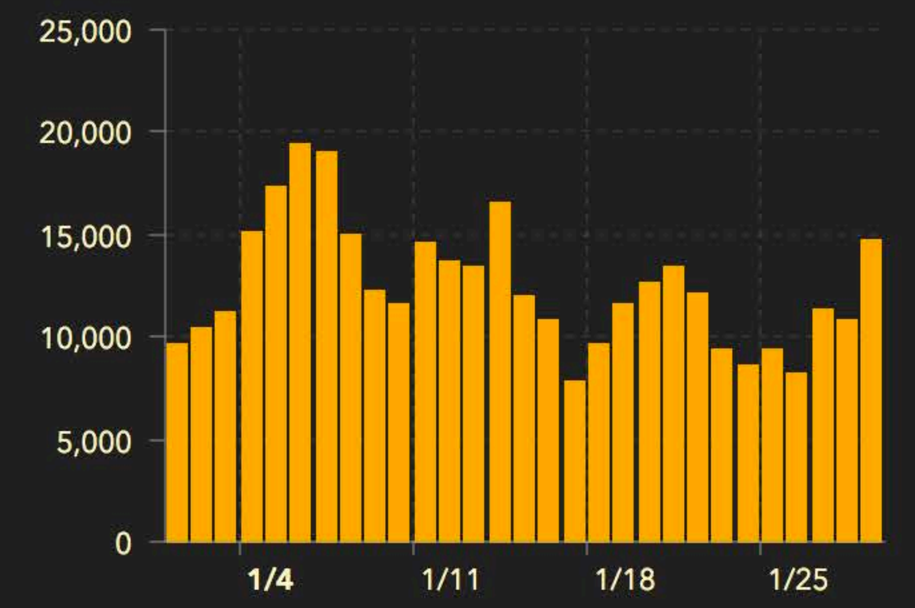

Resident Deaths by Date of Death

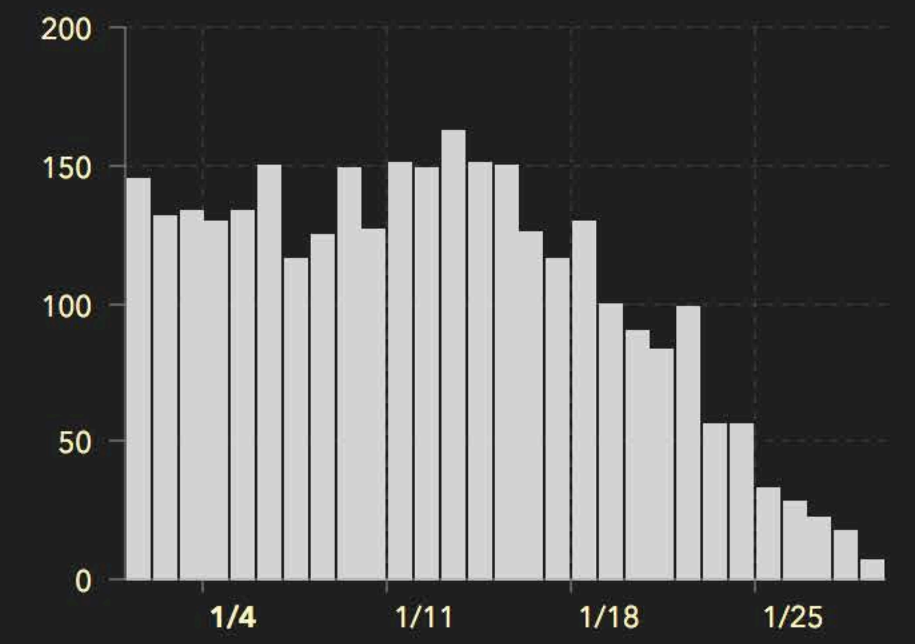

The Deaths by Day chart shows the total number of Florida residents with confirmed COVID-19 that died on each calendar day (12:00 AM - 11:59 PM). Death data often has significant delays in reporting, so

Florida Cases | Florida Testing | Cases by County | Rates Map | Case Maps | Cases by Zip Code | Health Metrics | USA and World

Florida's COVID-19 Data and Surveillance Dashboard

Florida Department of Health, Division of Disease Control and Health Protection

Select a County: STATE

Data for Florida Residents from Previous Day

Total FL Residents Tested

222,516

FL Residents Positive

14,735

FL Residents Negative

207,781

Previous Day Percent Positive

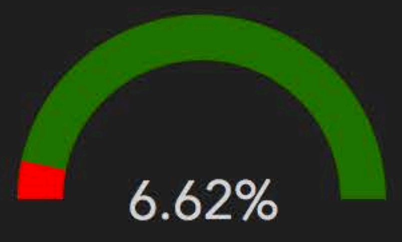

Testing Data for State from previous day

Total FL Residents Tested: 222,516
Positive: 14,735
Negative: 207,781

Percent Positive: 6.62 %

Use the drop-down menu above to select a county.

Statewide Negative and Positive Persons Tested by Week

*Date of data shown is the last date of the reporting period and includes the total from the last report date **and** the previous six days.*

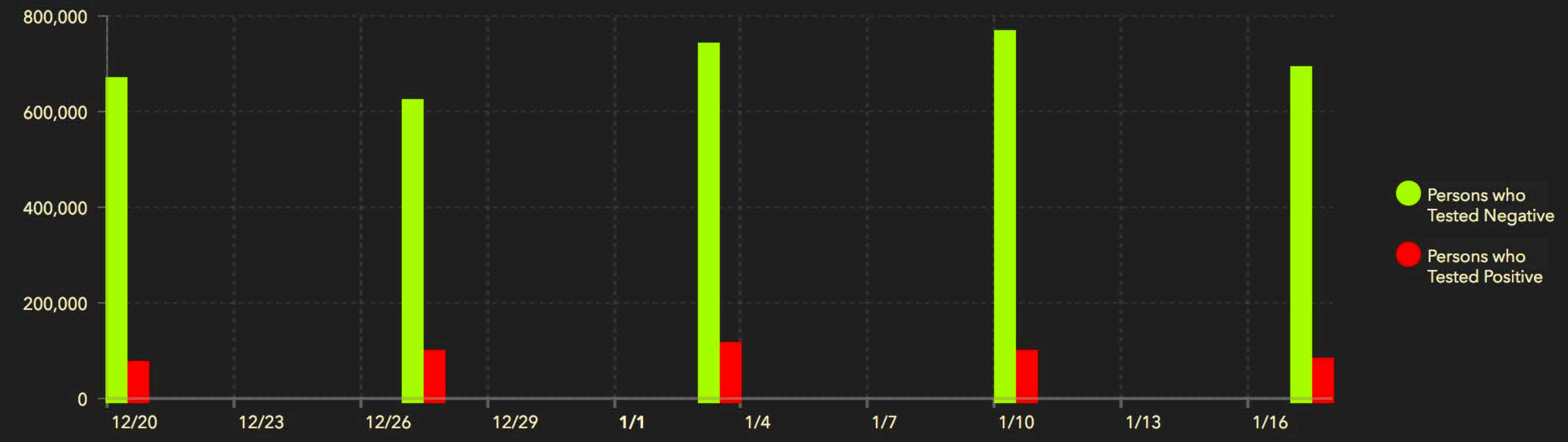

Positivity Rate of New Cases by **Week**

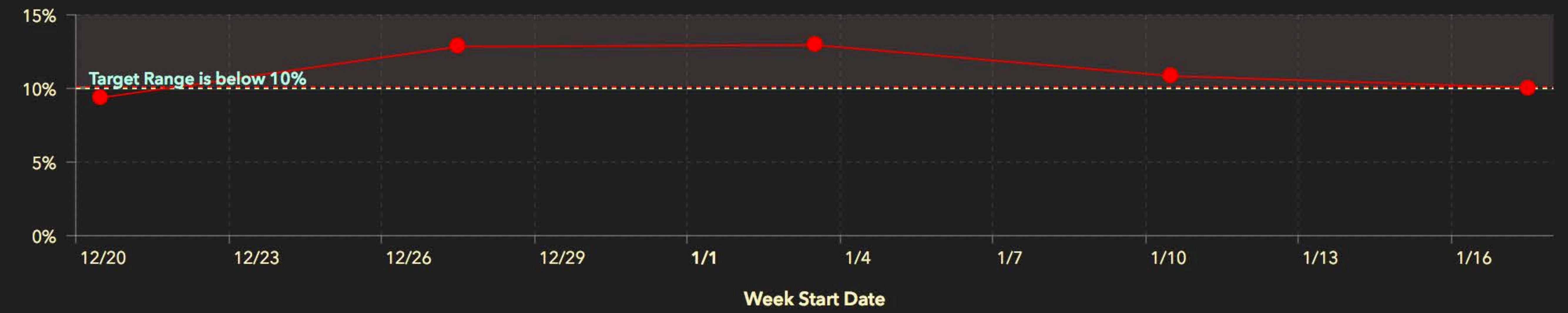

Florida Cases | Florida Testing | Cases by County | Rates Map | Case Maps | Cases by Zip Code | Health Metrics | USA and World

Florida's COVID-19 Data and Surveillance Dashboard

Florida Department of Health, Division of Disease Control and Health Protection

Select a County: STATE

Number of Florida resident COVID-19 cases per 100,000 population by county.

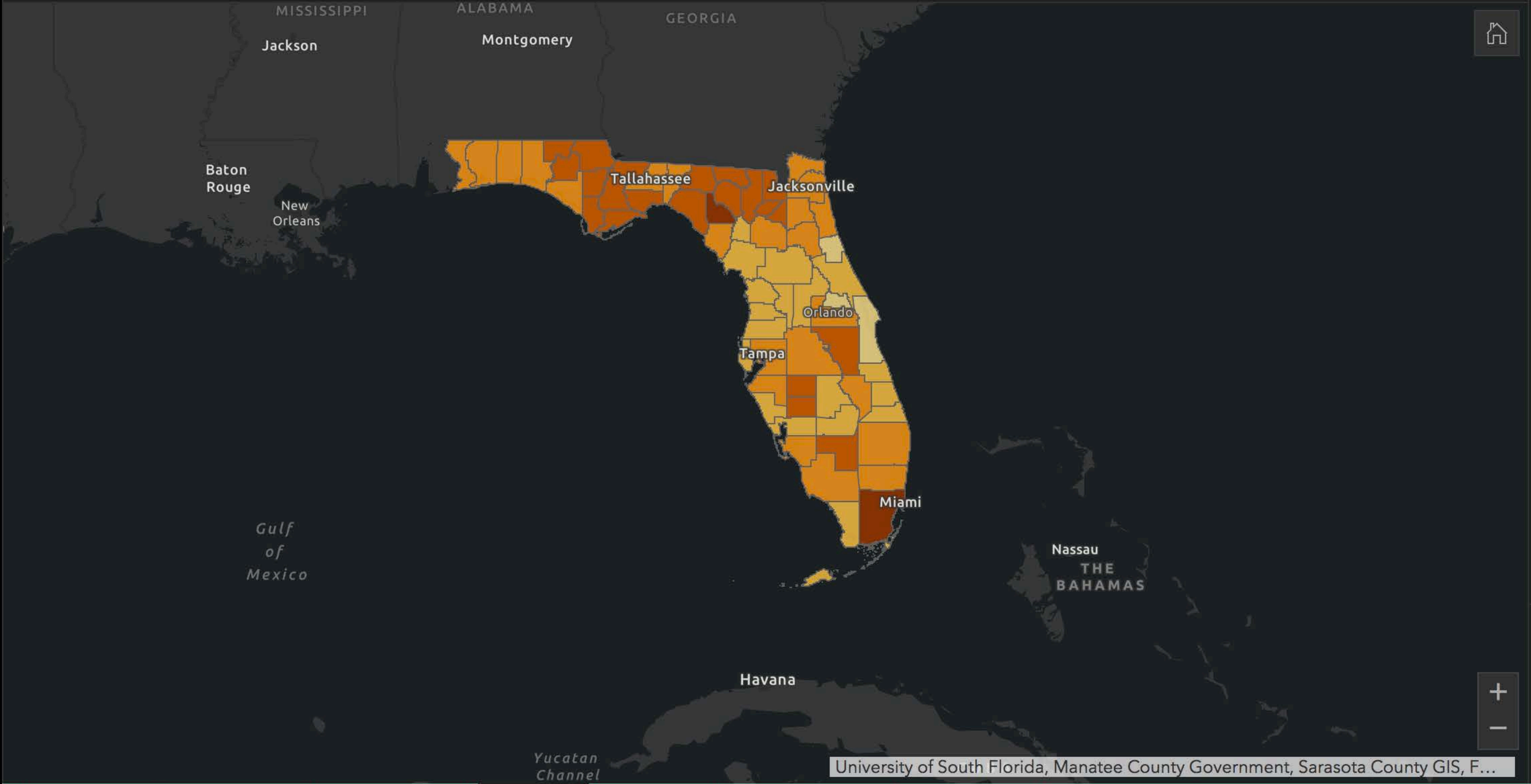

Case Maps for COVID19

CovidRate

- > 11,886 – 20,000
- > 8,982 – 11,886
- > 7,042 – 8,982
- > 5,376 – 7,042
- > 1 – 5,376
- 0 – 1

Case Rate per 100,000 People

8,166

Florida Cases | Florida Testing | Cases by County | Rates Map | Case Maps | Cases by Zip Code | Health Metrics | USA and World

Welcome to our COVID Action site.

Page Menu

NEWS AND UPDATES:

the COVID MONITOR

The COVID Monitor now tracks cases in every K-12 school district in the USA.

Visit Website

IMPORTANT VACCINE INFORMATION: The Pfizer-BioNTech and Moderna vaccines have been authorized for emergency use by the FDA. Read more about this here. The Florida Department of Health website for COVID-19 vaccination information can be accessed here.

Some counties have begun taking appointments for those 65 years and older, healthcare workers, and long-term care facility staff and residents to receive the vaccine. To see what options are available in your county, click here.

Data last updated: 1/30/2021 8:19 PM EDT

Total COVID-19 lab-confirmed infections:
1,808,930

New Cases Today: 15,019
New persons tested today: 78,130

All Florida COVID-19 Deaths:
26,795

New Deaths Reported Today: 110

Our new dashboard experience provides information in an easy-to-navigate format, with much more data and shorter load times. If you prefer to use the old format, click the link below.

Click here to access old dashboard

Where do you want to go?

Home | Summary Info | Local Data | Healthcare and Hospitals | Get Tested | Get Help

Data last updated: 1/30/2021 8:19 PM

Total lab-confirmed infections since March 1:
1,808,930
Includes those who received positive results from PCR, antigen and antibody testing. To learn more about the different types of tests, click here.

Total deaths since March 1:
26,795
New deaths today: 110
Includes only those deaths in which COVID-19 was listed as a primary reason for death, as determined by the Florida Department of Health. Data may differ from the number of deaths reported by each county's Medical Examiner. Florida does not include probable cases and deaths, despite CDC advisement.

Total DOH Cases since March 1:
1,713,589
New cases today: 15,019
DOH Case data includes only those who tested positive with a molecular (PCR) test and/or antigen test, but excludes antibody positive. DOH includes antigen tests in their calculations of percent positivity, but does not specify how many antigen tests are included in those figures.

Total first-dose vaccines given*:
1,651,366
Total complete vaccines given*:
296,782
Total overdue for second shot:
Florida no longer reports this information

**Number of vaccinations reflects state-wide total for persons receiving the first of two shots in a series (top) and total receiving the complete series (bottom), for either the Moderna of Pfizer-BioNTech vaccines. Click here to see DOH's latest vaccination report.*

Select a county
- All -

Testing stats:

Antibody testing:
Antibody positive: 95,341
Antibody negative: 560,932

PCR (Diagnostic) and Antigen testing:
Total Positive (PCR): 1,420,088
Total Positive (Antigen): 293,501
Total tests to date: 10,144,844

Long-term care facilities (LTCF):
Cumulative LTCF cases: 76,233
"Active" LTCF Cases: 7,369
LTCF Deaths: 9,174

Prisons:
Prison cases: 29,195
Prison deaths: 209

Pediatrics:
Cases: 182,774
Admitted to an ER: 6,935
In-patient admissions to a hospital: 1,244
Pediatric Deaths: 9

Cases in K-12 schools*:
Total cases: 48,817
Students: 34,812
Staff: 13,993
*To see more about cases in schools, click here.

Click here to download our county master file (csv, geojson, and other API formats available)

Statewide Hospital Stats:
To view hospital data by county, visit our "Healthcare and Hospitals" Page.

Current hospitalizations with a primary diagnosis of COVID-19:
6,573

Available beds:
Adult ICU: 997

"The COVID-19 "currently hospitalized" data only counts persons who are hospitalized with a "primary diagnosis of COVID-19." This excludes persons with multiple diagnosis in which COVID-19 is a factor but not primary diagnosis, those who were hospitalized at the time they contracted COVID-19, or any case where a doctor doesn't feel COVID-19 is the primary cause of illness. Additionally, VA hospitals do not report to AHCA, as they are not licensed by or under the jurisdiction of the state.

Statewide Average Percent Positive for Today:
Select a county for local positivity:
New persons tested: 21.35%

Statewide data (does not filter by county):
All people tested: 9.58%
Labs processed: 23.814 %

New persons tested positivity equals the number of people testing positive for the first time (new cases) divided by the total number of people tested for the first time (excluding all people who have already been tested.
All people tested positivity equals the total number of people who tested positive divided by the total number of people tested, including duplicate positive and negative persons tested.
Labs processed positivity equals the number of people tested positive divided by all people tested at each laboratory for the first time.

Looking to download or plug into the data you see here?
You can download the Master File for our county data here.
For all of our available datasets and feeds, visit our website's data library or go straight to our GIS Data hub.

Florida COVID Action Data Library

Florida COVID Action GIS Data Hub

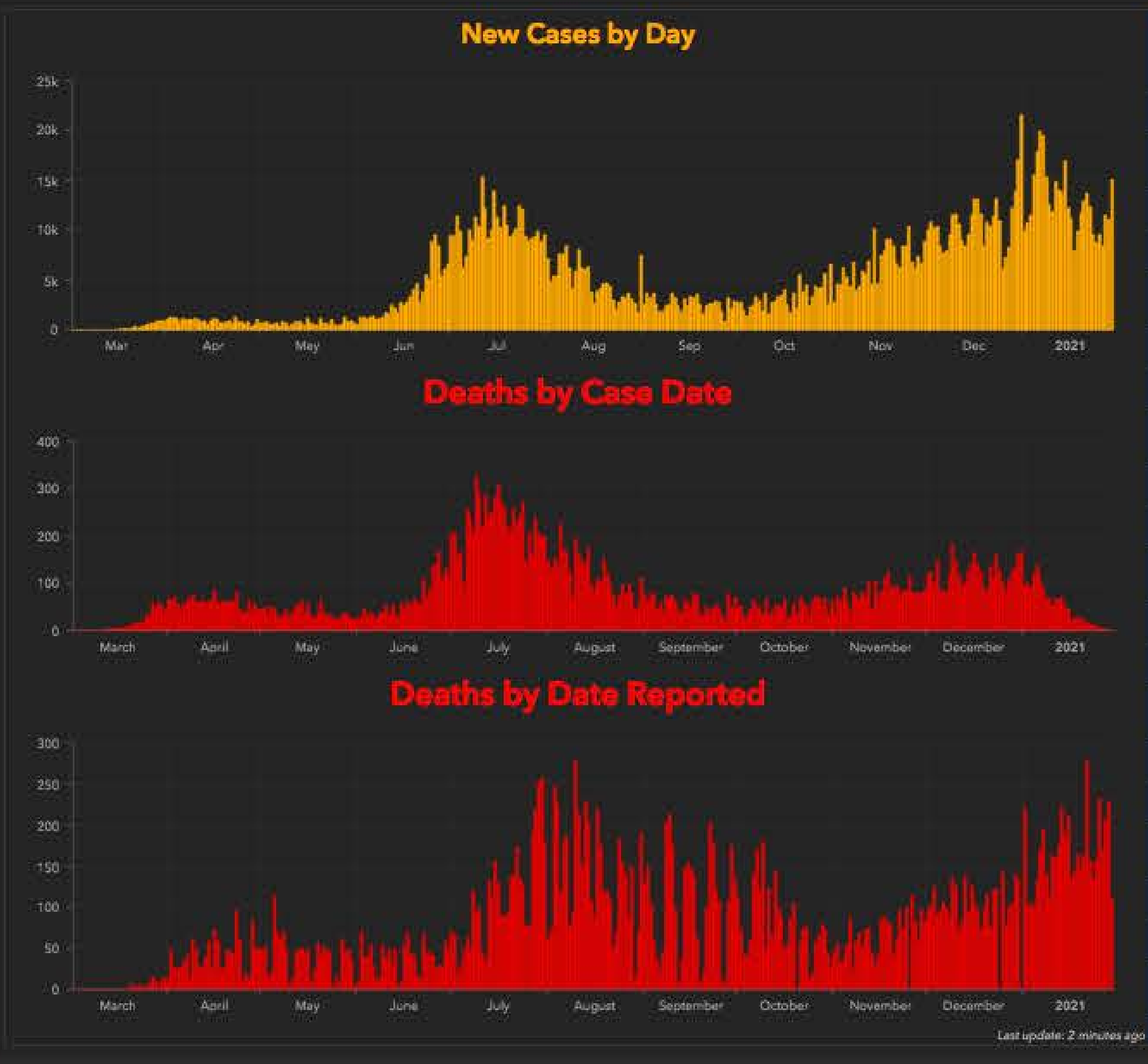

Select a county to filter the charts
Alachua
Baker
Bay
Bradford
Brevard
Broward
Calhoun
Charlotte
Citrus
Clay
Collier
Columbia
Dade
Desoto
Dixie
Duval
Escambia
Flagler
Franklin
Gadsden
Gilchrist
Glades

Download the cases by day, deaths by case data, and deaths by date reported data here.
Download DOH's deaths by date of death (residents only for those with a confirmed date of death) here.

Total Positive
1,808,930

Florida COVID Deaths
26,795

New Cases Today
15,019

New Deaths Reported Today
110

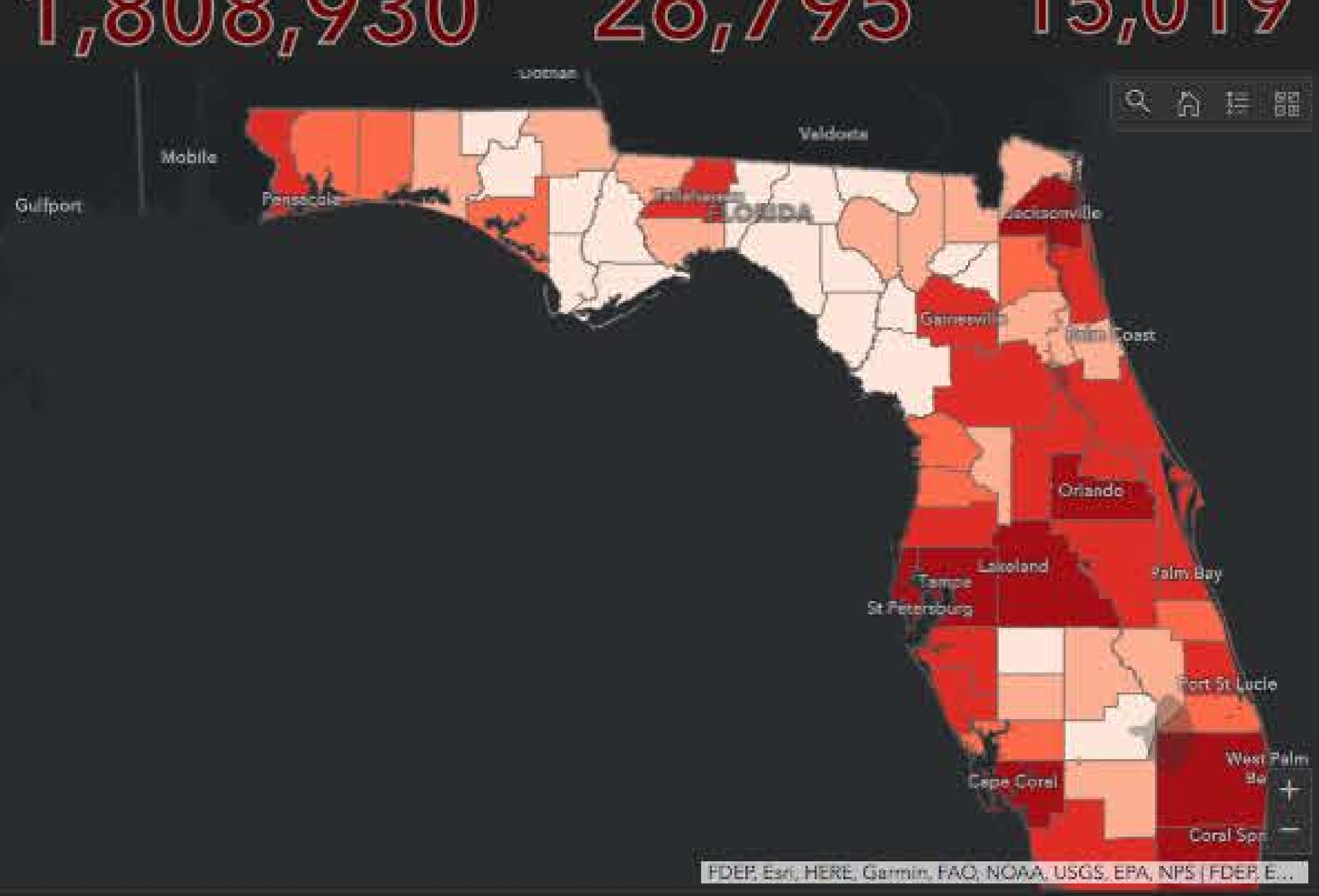

New Deaths this Week
1,234
New Cases this week
74,060
Total DOH Cases
1,713,589

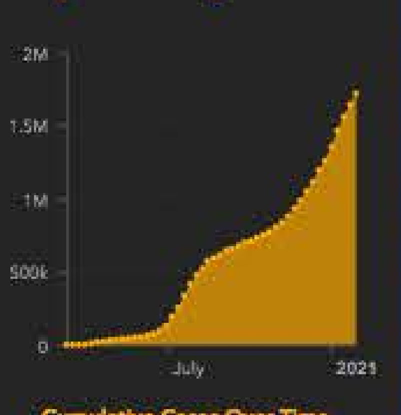

Cumulative Cases Over Time

Select a county to filter this page
Alachua
Baker
Bay
Bradford
Brevard
Broward
Calhoun
Charlotte
Citrus
Clay
Collier
Columbia
Dade
Desoto
Dixie
Duval
Escambia
Flagler
Franklin

Home | Summary Info | Local Data | Healthcare and Hospitals | Get Tested | Get Help

Georgia Department of Public Health Daily Status Report

Note: To ensure the most updated version of this webpage, please refresh your browser.

These data represent confirmed cases of COVID-19 reported to the Georgia Department of Public Health (DPH) as of 1/30/2021, 2:50:12 PM.

- The latest: What's new with this report?
- Guide: Understand the data (PDF)
- Download the data (CSV)
- GEMA Daily COVID-19 Information
- Georgia DCH Daily COVID-19 Long-Term Care Facility Report
- Historical Covid Maps (PDF)
- Georgia Hospital Beds and Ventilator Use
- Georgia Medical Facility Patient Census

Georgia Overall COVID-19 Status

Below you will find information reported to DPH on the total number of COVID-19 tests, confirmed COVID-19 cases (PCR positive), ICU admissions, hospitalizations, and deaths attributed to COVID-19. These data are based on available information at the time of the report and may not reflect all cases or tests performed in Georgia.

Confirmed Cases	Confirmed Deaths	Hospitalizations
746,867	12,568	50,132

ICU Admissions	Antigen Positive Cases	Probable Deaths
8,390	158,991	1,628

Reported Today

Confirmed Cases	Antigen Positive Cases	Confirmed Deaths	Hospitalizations
4,933	1,717	159	263

COVID-19 Testing

	Total PCR/ Molecular	PCR/Molecular Reported Today	Total Antibody (Serology)
Number of Tests	6,425,042	46,500	443,440
Number of Positive Tests	716,476	5,480	60,474
% Positive	11.2%	11.8%	13.6%

Note: these data only include lab tests reported to DPH by electronic laboratory reporting (ELR) and do not represent all tests performed in Georgia. The number of positive tests in this chart will not match the number of confirmed cases because the case numbers include all reporting sources, not just ELR. People with a positive test often get retested and may test positive multiple times. These repeat positive tests will be counted as new positive tests, but they will not be reported as new cases.

Georgia Cases by County

The maps below show the number and rate of confirmed COVID-19 cases and percent positive PCR tests by county of residence. Choose "Cases", Testing" or "Deaths", then use the drop-down menu to choose cumulative "Cases", "Cases per 100K", "Deaths", "Deaths per 100K", "% Positive", or data for the "Last 2 Weeks". By clicking on the date above the map, you can select a past date to see historic maps, using the current color scale. On the map, you can hover or click to find out additional details such as number of deaths, hospitalizations, case rate, etc. Selecting a county will also update the cases over time charts.

The color scale is based on the distribution of county-level case counts or rates, outlier values are removed from the scale calculation. The scale will change as needed to accommodate increasing or decreasing case counts and maintain distinctions between counties, and will be calculated based on the current data. Historic maps can be viewed by changing the date above the map.

COVID-19 By County

Cases | Testing | Deaths

Last 2 weeks | January 30th

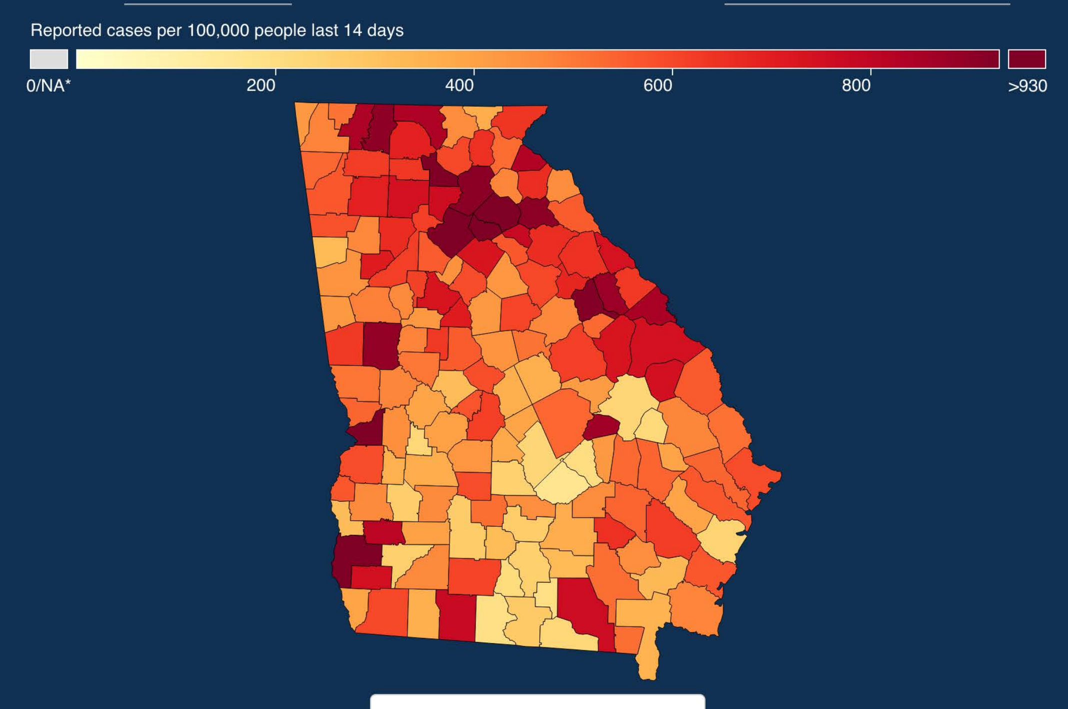

Georgia - January 30
Cases (last 2 weeks): 72,378
Cases per 100k (last 2 weeks): 668
Cases (total): 746,867
Cases per 100k (total): 6894
Population: 10,833,472

COVID-19 Over Time

Cases | Cumulative Cases | Deaths | Total Tests | Percent Positive

Georgia | Date of Report

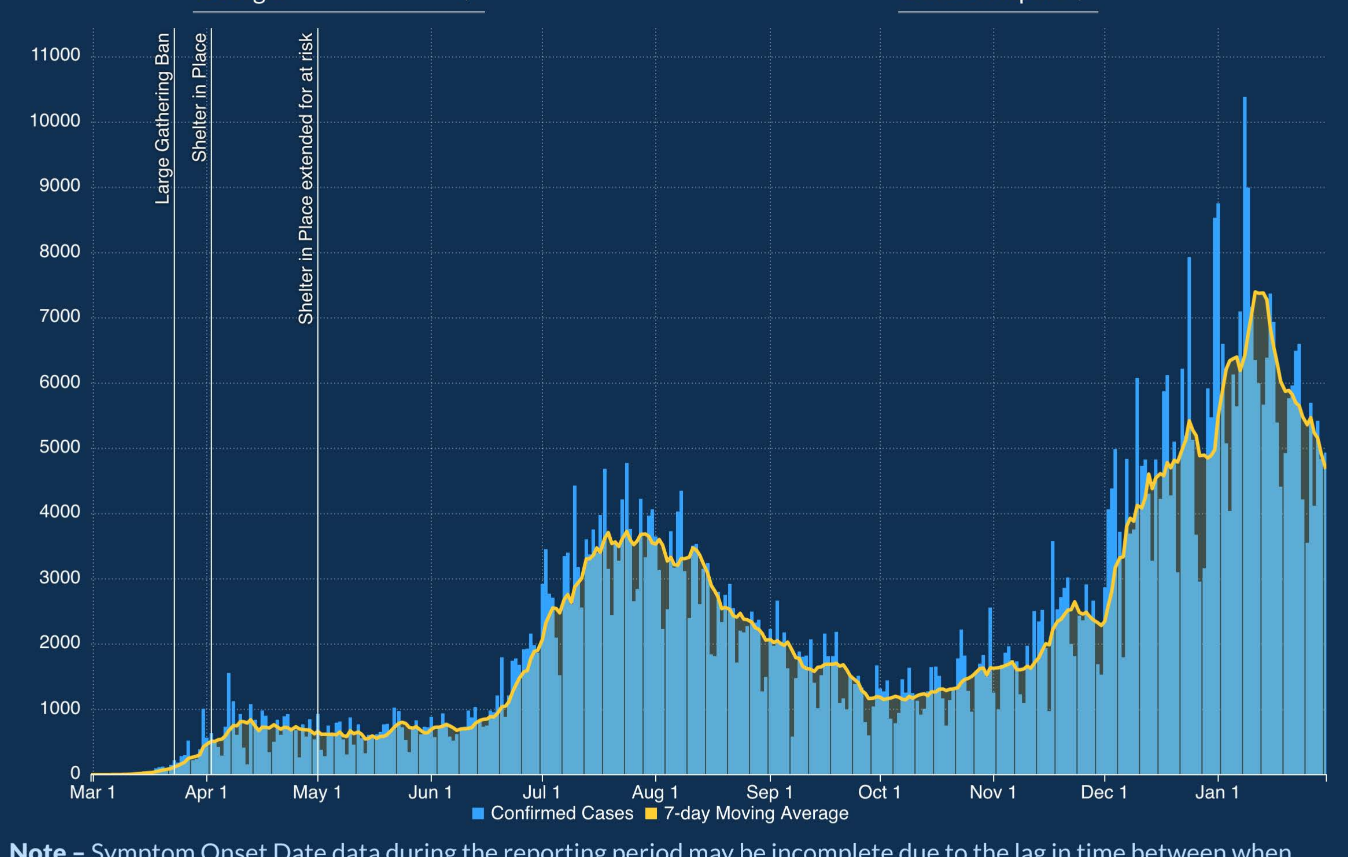

Note – Symptom Onset Date data during the reporting period may be incomplete due to the lag in time between when the case was tested and/or reported and submitted to the Georgia DPH for reporting purposes. This delay can vary depending on the testing facility and/or jurisdiction.
* Rates may not be accurate when case counts are <5 and are not presented. Percent positive may not be accurate when total PCR tests are <10 and are not presented.

Counties | Top Five Counties | Deaths in Georgia | Race and Ethnicity

County ↑	Confirmed Cases	Confirmed Cases per 100K	Antigen Positive Cases	Confirmed Deaths	Probable Deaths	Hospitalizati[illegible]
Appling	1694	9126.66	310	50	3	[illegible]
Atkinson	739	8871.55	198	14	0	[illegible]
Bacon	991	8689.93	318	24	6	[illegible]
Baker	151	4845.96	57	6	0	[illegible]
Baldwin	3464	7796.88	480	91	3	[illegible]
Banks	1437	7191.47	111	27	1	[illegible]
Barrow	7272	8418.32	744	93	1	[illegible]
Bartow	8951	8080.63	2244	155	12	[illegible]

Demographics | Healthcare Workers | Lab Testing | Risks/ Comorbidities

COVID-19 Case Demographics

Confirmed COVID-19 Cases, Hospitalizations, Deaths by Age Group

Age Group | Race | Ethnicity

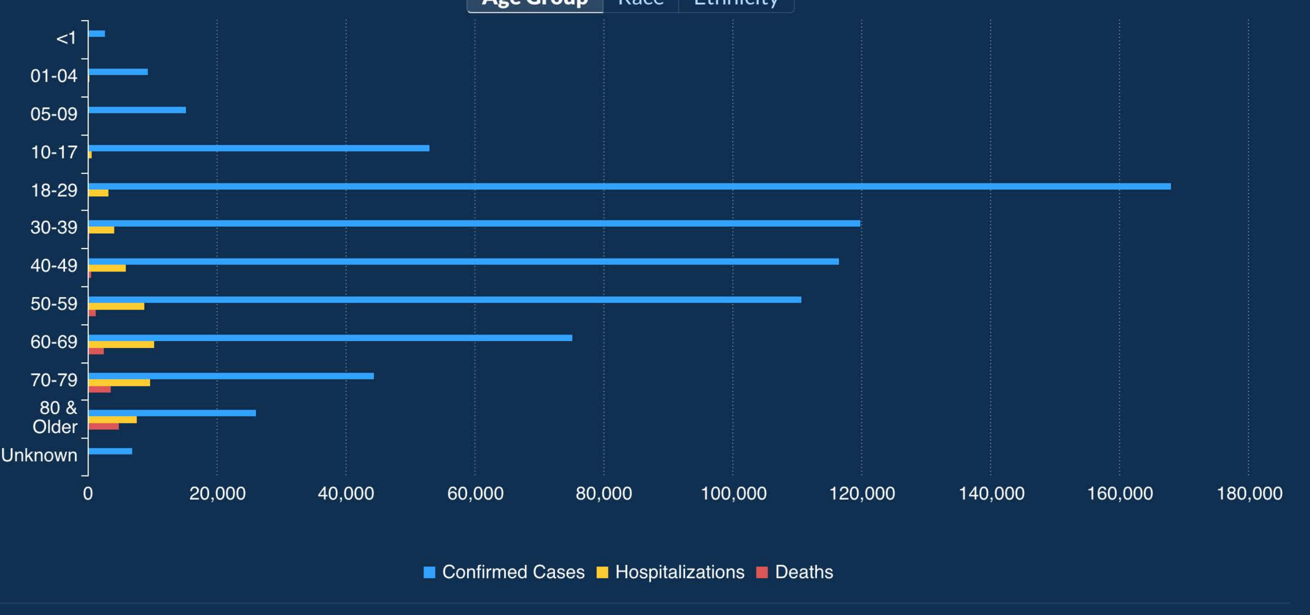

Cases by Age Over Time (7 Day Moving Average)

All Ages | Children Only

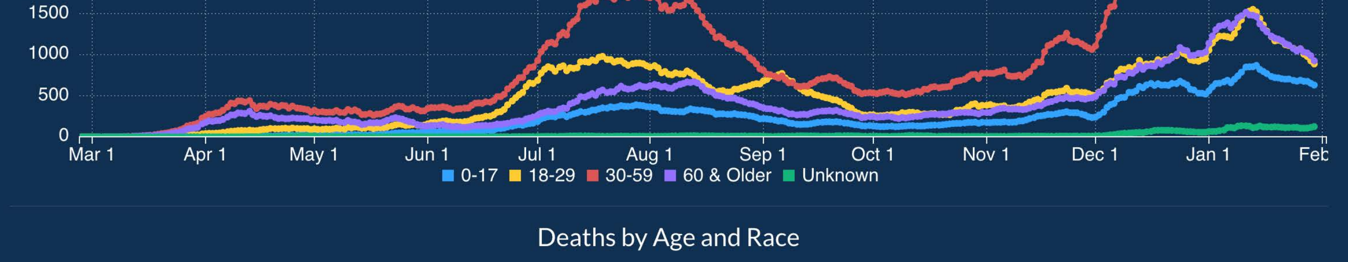

Deaths by Age and Race

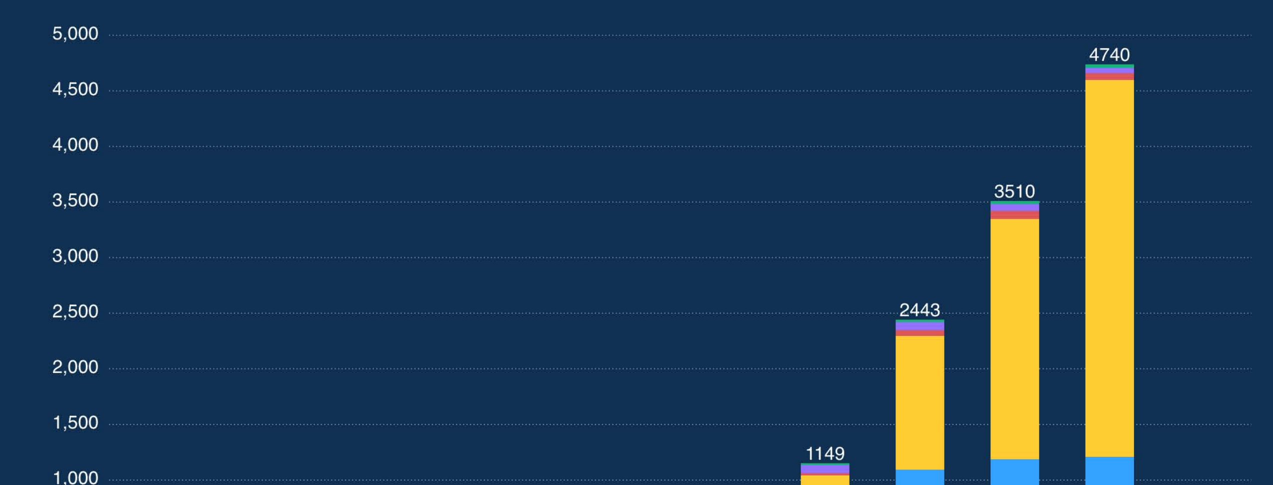

Race and Sex

Confirmed Cases by Race and Sex

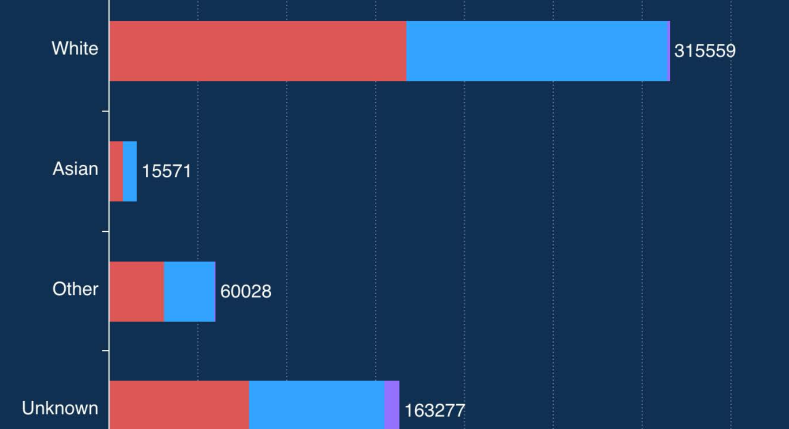

Confirmed COVID-19 Cases by Sex

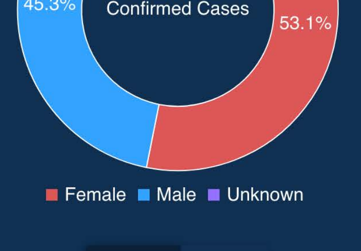

Chart | Table

Confirmed COVID-19 Cases by Race and Sex

Race	↓ Total Cases	Female Cases	Male Cases
White	315559	167312	146842
African-American/ Black	192432	112203	79358
Unknown	163277	78637	76414
Other	58607	30205	27580
Asian	15571	7763	7720
Native Hawaiian/ Pacific Islander	837	420	412

Coronavirus in Georgia: COVID-19 Dashboard

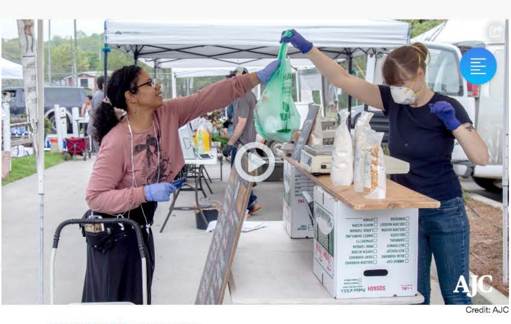

Credit: AJC

CORONAVIRUS | UPDATED DAILY

Latest stats and the news on the coronavirus outbreak

This dashboard gives up-to-date counts for confirmed and probably cases, deaths and other key statewide measures for the novel coronavirus. We update it at 3:15 p.m. (Sources: Georgia Department of Public Health, Georgia Emergency Management & Homeland Security Agency, Atlanta Journal-Constitution analysis)

The dashboard reflects the day the state reported confirmed and probable cases and confirmed deaths. This means that the new confirmed cases could be reported a day or more after the test results came back, several days after the test was taken, and a week or more after the patient was infected. So new confirmed cases reflect the spread of actual infections from a week or more ago.

Georgia DPH tracks confirmed cases (via molecular PCR tests) and probable cases (typically rapid antigen test positive cases), and the AJC has updated its dashboard to reflect both varieties of test.

We have added additional charts that present the data based on first reported symptom for confirmed cases or date of death, as reported by the state. The AJC also tracks hospitalization data, rates of spread by county and computes transmission estimates, which are explained in detail below.

Jump to a specific chart: Cases | Deaths | Hospitalizations | Tests | County Data | Metro Atlanta Map | Transmission Rates | Vaccines

Q: What is the latest on confirmed and probable coronavirus cases in Georgia?

Newly reported cases by day in Georgia

Here, new confirmed* and antigen positive** cases of coronavirus are shown by the date they first appeared on the DPH website. Because of the lag between first symptoms and DPH reporting a positive test result, other charts representing symptom onset can be misleading without proper context, as they tend to show a recent steep decline due to incomplete data. Looking at newly reported cases in this chart adds context by providing a static snapshot of each day's reports.

- Newly reported confirmed* cases
- Confirmed* 7 day rolling average
- Including probable** cases
- Including probable** 7 day rolling average

746,867 TOTAL CONFIRMED* CASES
905,858 TOTAL INCLUDING PROBABLE CASES**

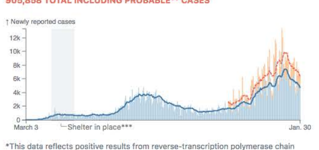

*This data reflects positive results from reverse-transcription polymerase chain reaction, or PCR, tests.
**This data reflects positive results from rapid antigen tests, which are considered probable cases. DPH tracks cases by person, not individual tests. That means DPH moves antigen positive cases to the confirmed category if a person also receives a PCR positive. That change in data is updated daily. DPH first started reporting antigen positive cases on Nov. 4.
***For most Georgians over age 65, shelter in place continued through June 12. Georgians who are residents of long term care facilities or are medically fragile must continue to shelter in place through at least Dec. 31.
Source: Georgia Department of Public Health
Updated January 30 at 3:30 p.m.

[If you are on mobile and are not seeing our charts, please click here to reload full version of page]

Explore See how cases are trending in your county

Confirmed cases by date of symptom onset* in Georgia

Here, new confirmed cases of coronavirus are shown by the date of symptom onset* as reported by DPH. But because there is a lag between first symptoms and the date DPH reports a positive test result, more recent data is likely to be adjusted upward in the future.

While DPH may adjust the number of cases from any date, the dashed line and lighter colored bars represent the window when the most adjustments are likely to occur, according to our analysis.

745,970 TOTAL CONFIRMED CASES*

- 7 day rolling average
- Confirmed cases
- Incomplete data

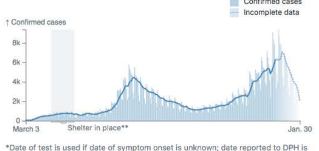

*Date of test is used if date of symptom onset is unknown; date reported to DPH is used if none of the above are known. Cases where DPH is unable to determine a date are not included, which accounts for the discrepancy between the number of confirmed cases on this chart and the newly reported cases chart. This data reflects positive results from reverse-transcription polymerase chain reaction, or PCR, tests. Antigen positive tests are currently not included.
**For most Georgians over age 65, shelter in place continued through June 12. Georgians who are residents of long term care facilities or are medically fragile must continue to shelter in place through at least Nov. 30.
Source: Georgia Department of Public Health
Updated January 30 at 3:31 p.m.

Q: What is the latest on coronavirus deaths in Georgia?

Newly reported deaths by day in Georgia

Here, new deaths from coronavirus are shown by the date they first appeared on the DPH website. Because of the lag between the date of a death and DPH reporting it, other charts representing day of death can be misleading without proper context, as they tend to show a recent steep decline due to incomplete data. Looking at newly reported deaths in this chart adds context by providing a static snapshot of each day's reports.

- Newly reported confirmed* deaths
- Confirmed* 7 day rolling average
- Including probable** deaths
- Including probable** 7 day rolling average

12,568 TOTAL CONFIRMED* DEATHS
14,196 TOTAL INCLUDING PROBABLE DEATHS**

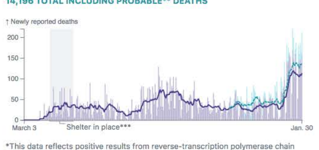

*This data reflects positive results from reverse-transcription polymerase chain reaction, or PCR, tests.
**This data reflects positive results from rapid antigen tests, which are considered probable. DPH first started reporting probable deaths on Nov. 4.
***For most Georgians over age 65, shelter in place continued through June 12. Georgians who are residents of long term care facilities or are medically fragile must continue to shelter in place through at least Dec. 31.
Source: Georgia Department of Public Health
Updated January 30 at 3:30 p.m.

Confirmed deaths by day of death in Georgia

Here, new deaths from coronavirus are shown by the date they occurred, as reported by DPH. But because there is a lag between the date of death and the date DPH reports it, more recent data is likely to be adjusted upward in the future.

While DPH may adjust the number of deaths from any date, the dashed line and lighter colored bars represent the window when the most adjustments are likely to occur, according to our analysis.

12,568 TOTAL CONFIRMED DEATHS

- 7 day rolling average
- Confirmed deaths
- Incomplete data

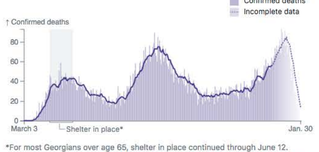

*For most Georgians over age 65, shelter in place continued through June 12. Georgians who are residents of long term care facilities or are medically fragile must continue to shelter in place through at least Nov. 30.
Source: Georgia Department of Public Health
Updated January 30 at 3:31 p.m.

Explore » Follow the full AJC coverage of the coronavirus outbreak

Q: What is the latest on people hospitalized for COVID-19 in Georgia?

Current hospitalizations by day in Georgia

- 7 day rolling average
- Current hospitalizations

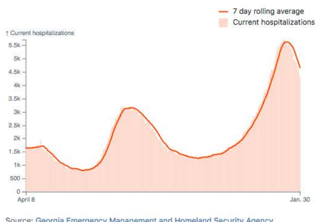

Source: Georgia Emergency Management and Homeland Security Agency
Updated January 30 at 2:30 p.m.

A: This is the number of people confirmed to have COVID-19 who are currently hospitalized, as published by the Georgia Department of Emergency Management & Homeland Security Agency. It began reporting this measure daily on May 2. It does not include those hospitalized who remain under investigation for suspected COVID. This number is updated daily.

Newly reported hospital cases* by day in Georgia

50,132 CUMULATIVE CONFIRMED HOSPITAL CASES

- 7 day rolling average
- Newly reported hospital cases

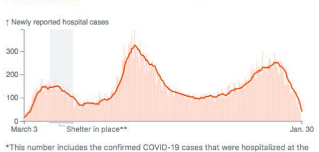

*This number includes the confirmed COVID-19 cases that were hospitalized at the time the case was reported to DPH or when the case was interviewed. This number does not capture hospitalizations that occur after a confirmed case was reported and, as such, is likely an underestimation of actual hospitalizations. It does not represent the number of COVID-19 cases currently hospitalized.
**For most Georgians over age 65, shelter in place continued through June 12. Georgians who are residents of long term care facilities or are medically fragile must continue to shelter in place through at least Nov. 30.
Source: Georgia Department of Public Health
Updated January 30 at 3:30 p.m.

Q: How many Georgians are testing positive for Coronavirus?

Percent testing positive for Coronavirus in Georgia

Each bar in this chart shows the percent testing positive for coronavirus each day as reported to the Georgia Department of Public Health (DPH). The line running through the bars shows the seven-day rolling average of the daily rates. The data does not include serologic tests, which identify if someone was exposed to the virus in the past and developed antibodies. The 5% line highlighted on the graph is the upper threshold identified by the White House Coronavirus Task Force for states to be in the green, or safe, zone for reopening.

- Percent positive
- 7 day rolling average

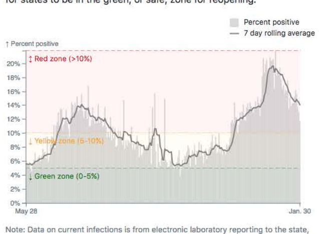

Note: Data on current infections is from electronic laboratory reporting to the state, which does not include all tests performed in Georgia. DPH excludes other testing sources because they do not consistently provide negative results, which must be factored in to arrive at positivity percentage. DPH also notes that, "People with a positive test often get retested and may test positive multiple times. These repeat positive tests will be counted as new positive tests."
Data: Georgia Department of Public Health
Zones: White House Coronavirus Task Force
Updated January 30 at 3:31 p.m.

Q: Does today's case data show how the virus is spreading right now in Georgia?

A: No. Epidemiologists agree that today's counts are a snapshot of what the virus did roughly two weeks ago. It can take a week or more for a person to become infected, show symptoms, get tested and have their results reported to public health officials. Deaths are generally reported to the state quickly, but it can take weeks for an infected person to succumb to the virus.

Q: What does the 7-day average line indicate?

A: This measure makes it easier to see trends in the spread of the virus that can be obscured by unrelated bumps and dips. For instance, Georgia's daily case counts tend to be lower on the weekends because reporting and testing slows down, not because fewer people are getting sick.

Q: Where is coronavirus spreading now?

Recently reported confirmed* coronavirus cases in Georgia

Rate of recently reported confirmed* coronavirus cases in each county by population (confirmed* cases reported in the last 14 days by the Georgia Department of Public Health based on each patient's reported county of residence, divided by the county's population and then multiplied by 100,000).

Tap each county for details on recently reported confirmed* and probable** cases there.

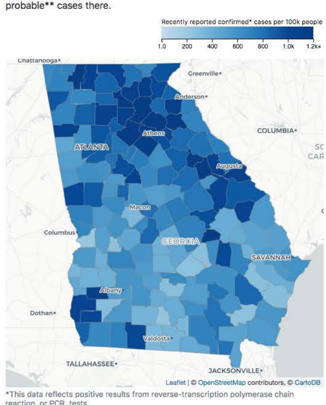

*This data reflects positive results from reverse-transcription polymerase chain reaction, or PCR, tests.
**This data reflects positive results from rapid antigen tests, which are considered probable cases. DPH tracks cases by person, not individual tests. That means DPH moves antigen positive cases to the confirmed category if a person also receives a PCR positive. That change in data is updated daily.
Source: Georgia Department of Public Health and the U.S. Census Bureau: *2019 American Community Survey 5-year estimates*

Updated January 20 at 8:31 p.m.

Explore See how cases are trending in your county

Q: What areas in metro Atlanta have had the most confirmed cases?

Confirmed coronavirus cases by ZIP code

Cumulative COVID-19 cases per 100,000 residents, plotted by ZIP Code Tabulation Areas (ZCTAs), which are approximations of actual postal ZIP code areas used by the U.S Census Bureau.

Because the Census only reports population estimates for full ZTCAs, we are unable to determine a rate for those which cross county lines into one or more counties for which we did not gather data. Those areas are displayed in gray but you can still find confirmed case totals by hovering over the ZIP code.

Fulton County reported fewer than 10 cases in some ZIP codes; those ZIPs and others for which no data was available are denoted on the map as a white area.

This data is updated weekly. Data is accurate to Nov. 11 for Clayton, Nov. 13 for Fulton, Nov. 16 for Gwinnett, Newton and Rockdale, Nov. 17 for DeKalb and Nov. 18 for Cobb and Douglas.

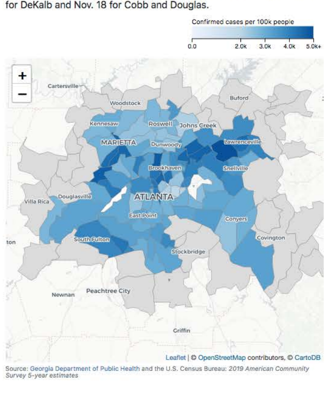

Source: Georgia Department of Public Health and the U.S. Census Bureau: *2019 American Community Survey 5-year estimates*

Q: Are there are other measures that can indicate how the virus is spreading?

A: Yes: the effective reproduction number, or Rt.

Rt is an epidemiological statistic that represents transmissibility, or the number of people a sick person infects at a given stage in the epidemic. If each person gets one person sick, the epidemic neither grows nor shrinks. Less than one means each person infects, on average, fewer than one person, so the epidemic shrinks. If Rt is larger than one, the epidemic grows. As Rt moves farther from 1, the epidemic shrinks or grows more rapidly.

The AJC calculated and compared several independent methods to estimate a value for Rt in Georgia. While the results from all were very similar, we're displaying a method designed by the Imperial College of London that uses epidemic curves to produce Rt estimates. The college's method is unrelated to its COVID-19 estimates.

As with other data presented on this dashboard, Rt values lag because of testing lags.

Transmission estimates in Georgia

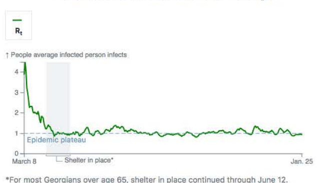

*For most Georgians over age 65, shelter in place continued through June 12. Georgians who are residents of long term care facilities or are medically fragile must continue to shelter in place through at least Dec. 31.
Source: Georgia Department of Public Health
Updated January 29 at 1:48 p.m.

Q: What do we know about the allocation and distribution of vaccines in Georgia?

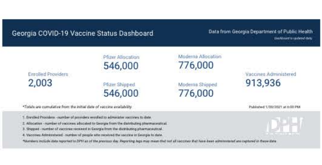

Explore COVID-19 vaccine in metro Atlanta: Everything you need to know

Do you have more questions? Email us at coronavirus@ajc.com.

In Other News

CORONAVIRUS
Coronavirus: How many people have died from the virus in the U.S.?

CORONAVIRUS
COVID-19 can cause fertility problems in men, study finds

CORONAVIRUS
WHO team visits Wuhan hospital that had early COVID patients

CORONAVIRUS
NY's Cuomo admits undercounting COVID nursing home deaths

CORONAVIRUS
South African virus variant found in SC, first U.S. appearance

CORONAVIRUS
Michael Strahan reportedly tests positive for COVID-19

CORONAVIRUS
As COVID-19 variant spreads through Georgia, health officials emphasize need to vaccinate

CORONAVIRUS
Here are the places Dr. Anthony Fauci says you should continue avoiding

AJC
Atlanta. News. Now.
The Atlanta Journal-Constitution

Sign up for our newsletters

About: Help Center | About The Atlanta Journal-Constitution | Newsroom Ethics Code | Careers | Archive
Contact Us: Contact Us | Send a News Tip | Advertise | AJC Newsroom
Subscriptions: Print Subscription | Digital Subscription | Manage Subscription | Your Profile | Why Subscribe? | NIE/Newspapers in Education
Our Products: ePaper | Newsletters | All AJC Podcasts | Download iOS App | Download Android App
Follow

© 2021 The Atlanta Journal-Constitution. All Rights Reserved. By using this website, you accept the terms of our Visitor Agreement and Privacy Policy, and understand your options regarding Ad Choices. Learn about Careers at Cox Enterprises.

State of Hawai'i – Department of Health
Disease Outbreak Control Division | COVID-19

Home | News | What You Should Know | What You Can Do | For Clinicians | Resources | Travel | DOCD Home

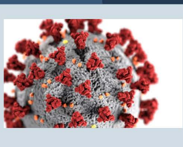

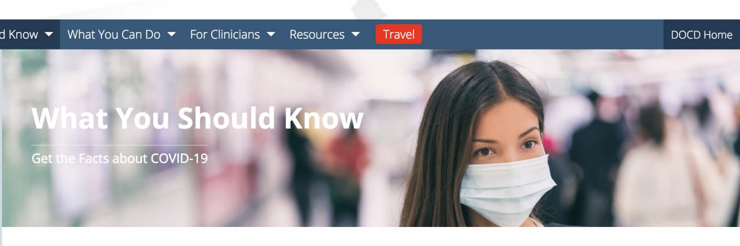

More in this section:

- COVID-19 Vaccine Frequently Asked Questions
- Hawaii COVID-19 Data
- Current Situation in the U.S.
- Background
- How it Spreads
- Symptoms
- FAQs
- Other Resources
- What the Hawaii Department of Health is Doing

Hawaii COVID-19 Data

Summary Metrics | Vaccine | Maps | Epidemic Curve | Travel (new) | County Curve | Lab Testing

Timeliness (new) | Outcomes | Age Trends | Race | Mask Use | Probable Cases | School Reopening Metrics |

Long Term Care Facilities | Cluster Report

There have been 25771 cases of COVID-19 identified in Hawaii. Of those cases, 7% have required hospitalization, and 24036 (93%) were residents.

Hawaii COVID-19 Summary Metrics

Learn more about these metrics, why they are important and how they are calculated.

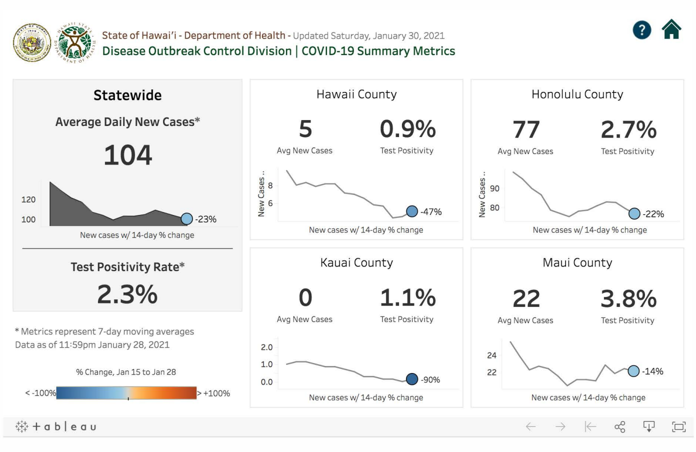

Hawaii COVID-19 Vaccine Administration (back to top)

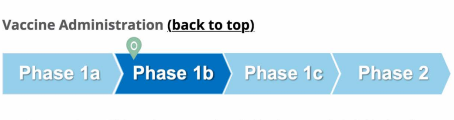

Current Phase *(Click on phases to see the prioritization groups included in that phase)*

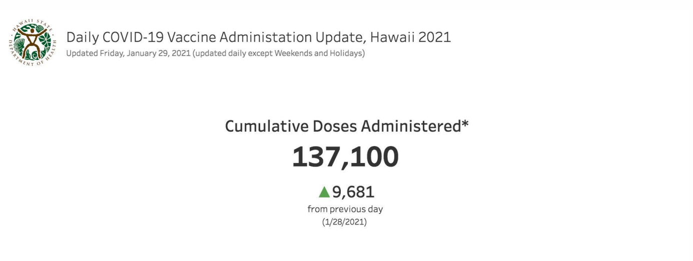

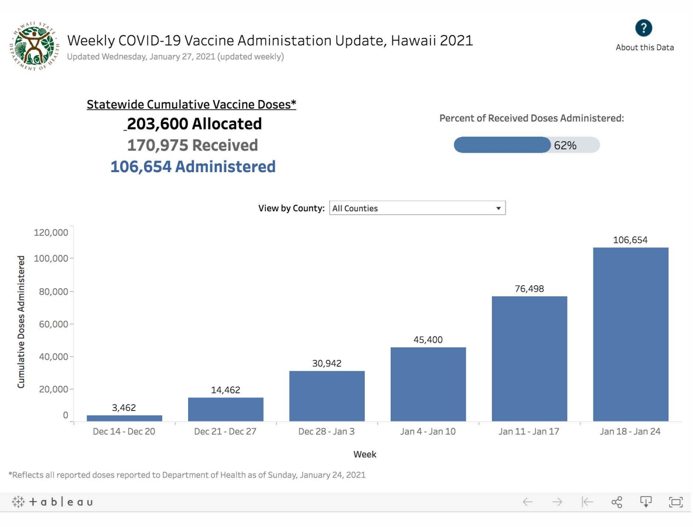

Hawaii COVID-19 Maps (back to top)

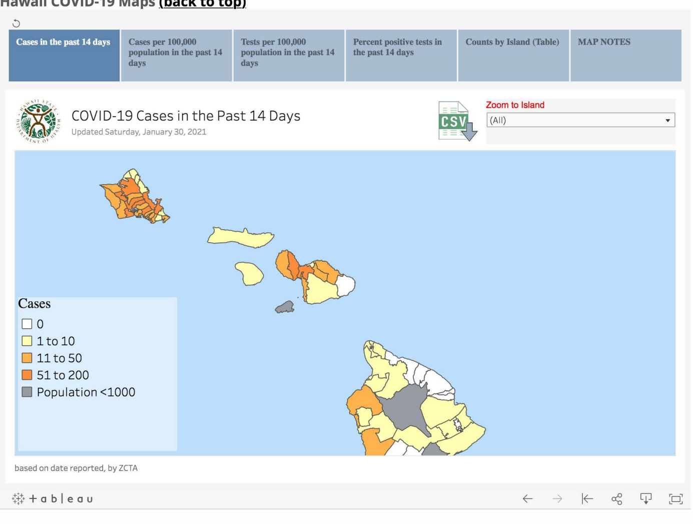

How is Hawaii Doing at Flattening the Epidemic Curve? (back to top)

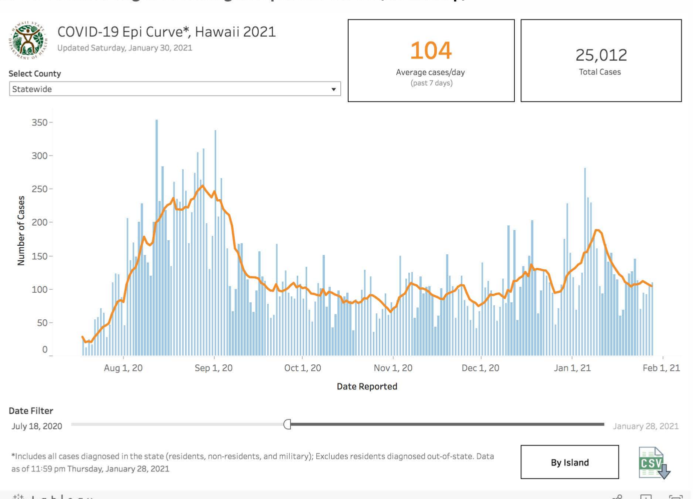

How many of the cases were related to Travel? (back to top)

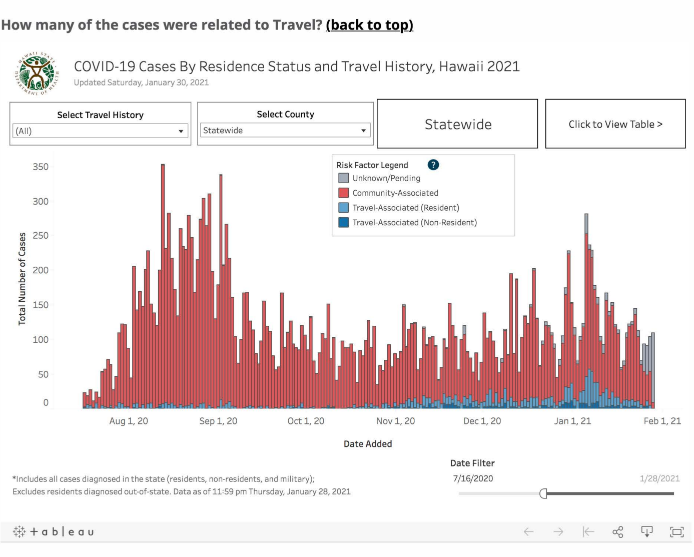

How do Hawaii's Counties Compare to One Another? (back to top)

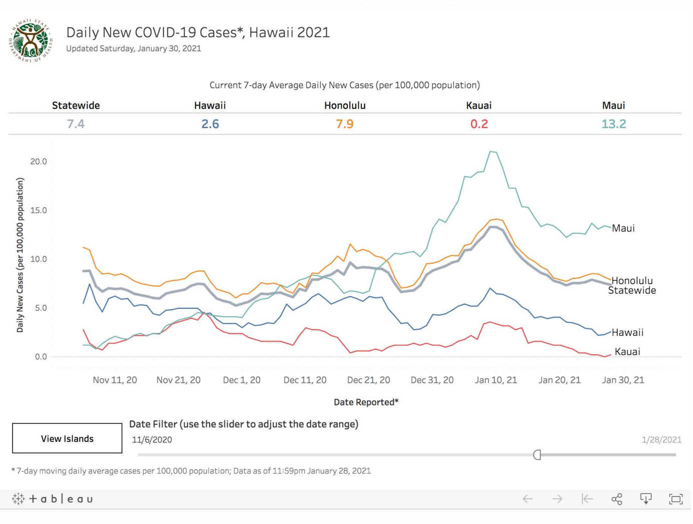

How Many People are Being Tested? (back to top)

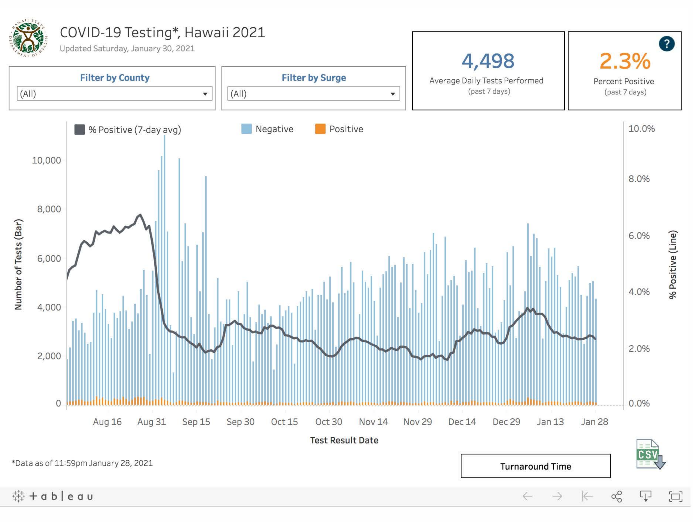

What proportion of COVID-19 Cases are being reached in a timely manner? (back to top)

Learn more about understanding case investigation metrics.

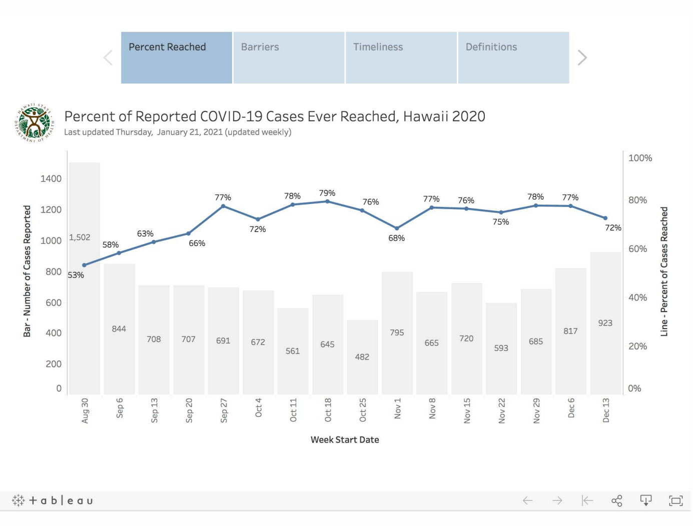

What are the Ages, Gender, and Outcomes of COVID-19 Cases? (back to top)

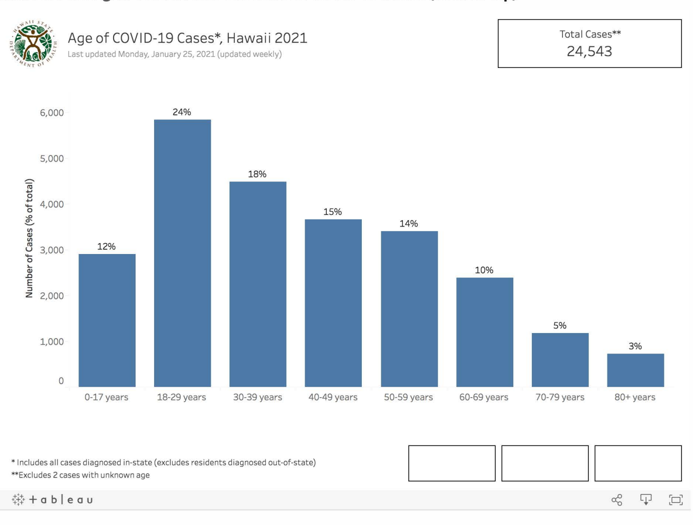

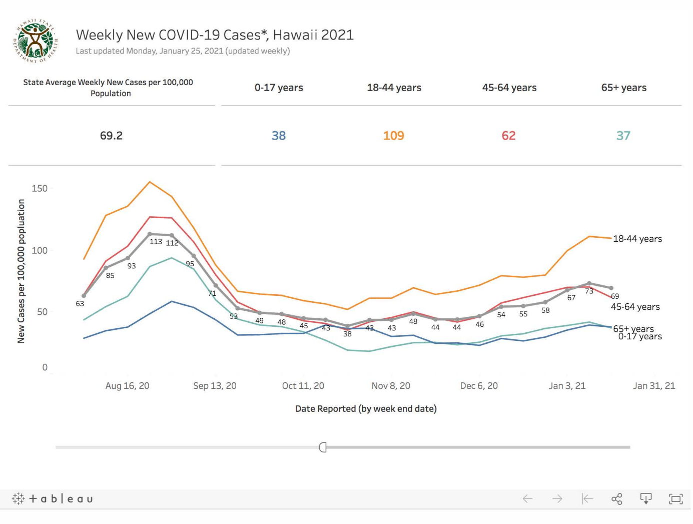

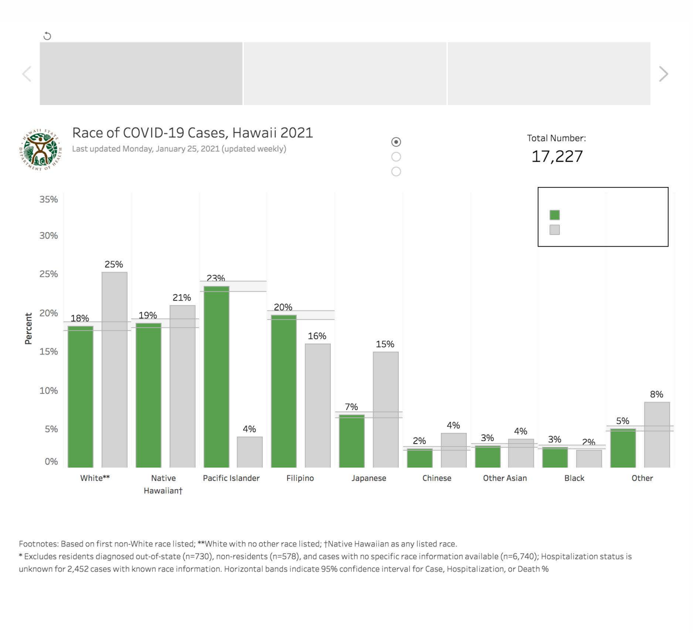

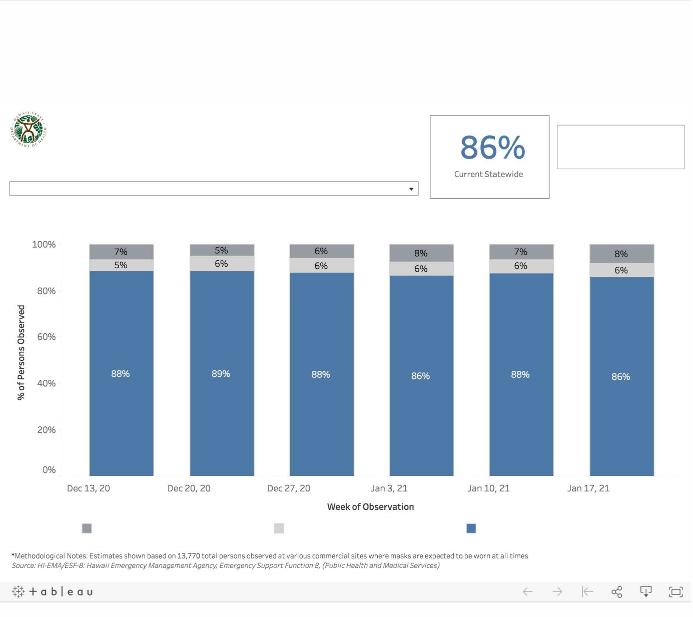

COVID Data Resources

With the onset of COVID in March 2020, we launched this page as a platform for providing consistent and accessible data relevant for responding to the pandemic. Since then, multiple public and private data resources have evolved to meet this need. As such, we have repurposed this page to serve as a directory of these resources while pivoting to support key COVID data partners going forward.

Overviews & Forecasts ↓ | Detailed COVID Data ↓ | Economic Recovery Data ↓

COVID-19 in Hawaii – Current Situation

State

104.1

(116 | 25,771 | 7.8)

14-Day Trend: (0 cases pending)

Additional State-Wide 7-Day Averages:
Hover over each indicator to see county breakdown

Hospitalizations	Deaths
5.4	1.7
(11 | 1,694 | 0.4)	(59 | 407 | 0.1)

Honolulu 76.7 (82 | 20,864 | 7.9) — 14-Day Trend:

Hawaii 5.1 (8 | 2,151 | 2.6)

Maui 22.1 (19 | 1,858 | 13.2)

Kauai 0.1 (1 | 178 | 0.4)

Data Format:
7-Day Avg of New Cases
(New Cases | Total Cases | 7-Day Avg of Daily Cases/100k)

Updated with data as of Jan 29

View Detailed Trends →

+ t a b l e a u

Base data for this dashboard is pulled daily from DOH's Current Situation in Hawaii page.
Click here to access the data for this dashboard.

COVID Overviews & Forecasts

Summary Metrics

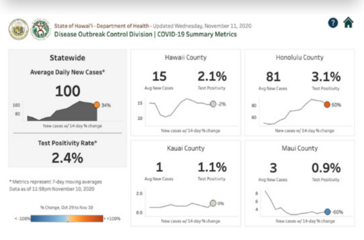

State and County current 7-day average of new cases and test positivity rate.
Source: DOH Disease Outbreak Control Division
Updated daily

Maps

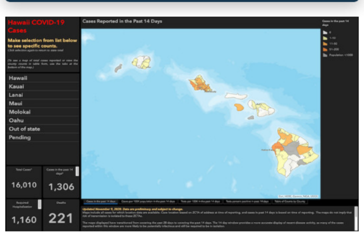

Case count, case rate, testing rate, and test positivity rate in the past 14 days, by zip code.
Source: DOH Disease Outbreak Control Division
Updated daily

HIEMA Dashboard

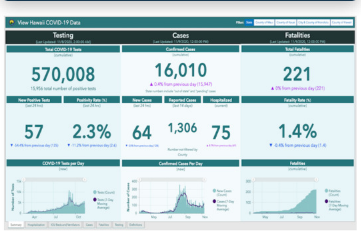

Dashboard of key indicators the State relies on to inform policy decisions.
Source: Hawaii Emergency Management Agency
Updated daily

COVID Pau Key Indicators

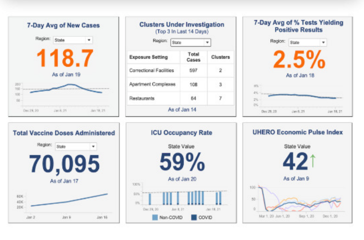

Overview of key indicators and easy-to-digest information and resources.
Source: COVID Pau
Updated daily

HiPAM Forecasts

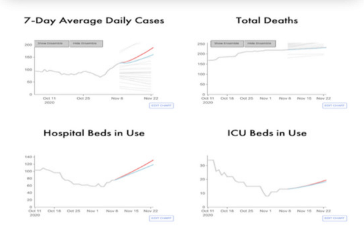

Forecasts of key indicators, based on pessimistic and expected inputs.
Source: Hawaii Pandemic Applied Modeling
Updated daily

CDC COVID Data Tracker

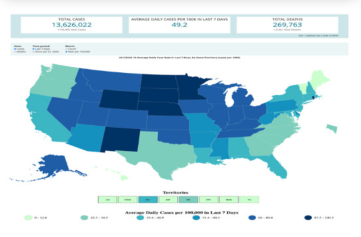

Maps, charts and downloadable data for various COVID indicators, by state.
Source: Centers for Disease Control and Prevention
Updated daily

COVID Tracking Project

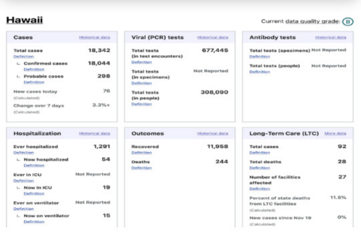

This project compiles and publishes various COVID indicators. Data API available.
Source: The COVID Tracking Project
Updated daily

Rate of Transmission

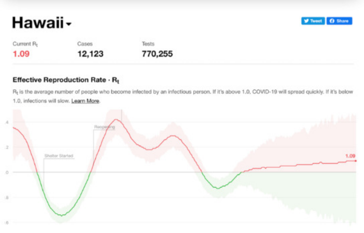

Up-to-date Rt values by state, a key measure of how fast the virus is growing.
Source: Rt Live
Updated daily

Detailed COVID Data

County Curve

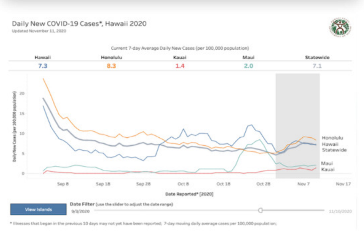

State and county trend of 7-day average of daily new cases per 100,000 population.
Source: DOH Disease Outbreak Control Division
Updated daily

Lab Testing

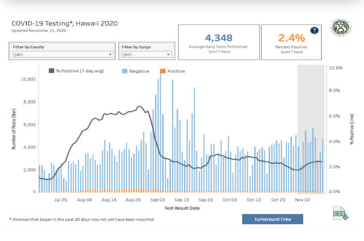

Daily number of tests broken down by test result, and weekly test turnaround time.
Source: DOH Disease Outbreak Control Division
Updated daily

Outcomes

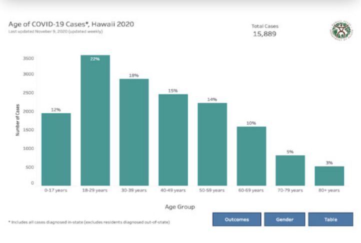

Cases by age group and gender, as well as deaths and hospitalizations by gender.
Source: DOH Disease Outbreak Control Division
Updated weekly

Age Trends

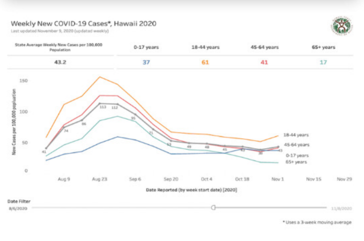

Weekly trends of new cases per 100,000 population by age group.
Source: DOH Disease Outbreak Control Division
Updated weekly

Race Trends

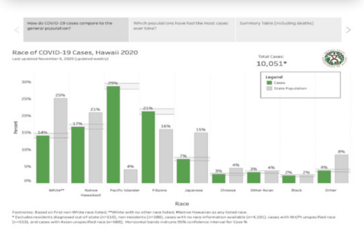

Breakdown of cases by race as it compares to the State's general population.
Source: DOH Disease Outbreak Control Division
Updated weekly

Mask Use

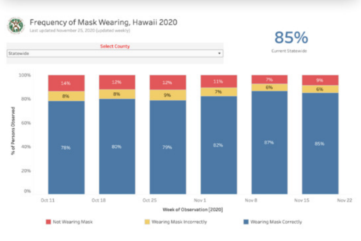

Percentage of people by county observed following mask wearing recommendations.
Source: DOH Disease Outbreak Control Division
Updated weekly

Epi Curve

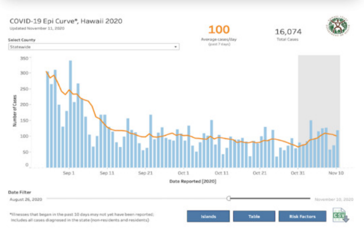

Number of COVID-19 cases over time by risk factor, statewide and by county.
Source: DOH Disease Outbreak Control Division
Updated daily

Contact Tracing

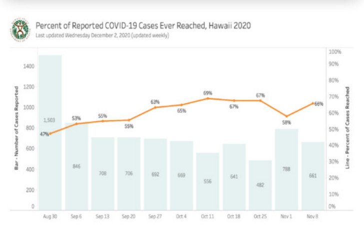

Percent of reported cases ever reached and contact tracing barriers and timeliness.
Source: DOH Disease Outbreak Control Division
Updated weekly

Cluster Reports

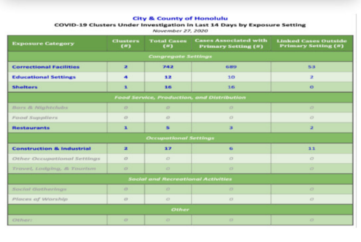

Summary of identified COVID-19 clusters that are actively under investigation.
Source: DOH Disease Outbreak Control Division
Updated weekly

Vaccinations

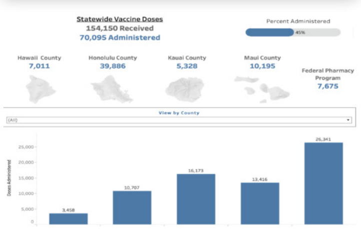

Cumulative and weekly umber of doses administered, statewide and by county.
Source: DOH Behavioral Health & Homelessness Statewide Unified Response Group
Updated monthly

BHHSURG PPE

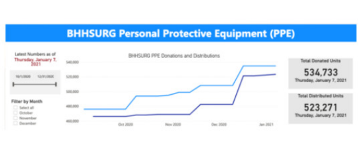

Summary of personal protective equipment donated to BHHSURG and distributed.
Source: DOH Behavioral Health & Homelessness Statewide Unified Response Group
Updated monthly

Isolation & Quarantine

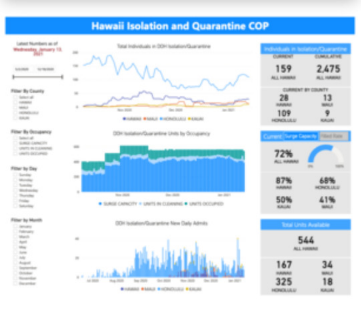

Individuals in DOH Isolation/Quarantine, statewide and by county.
Source: DOH Behavioral Health & Homelessness Statewide Unified Response Group
Updated weekly

Hawaii CARES

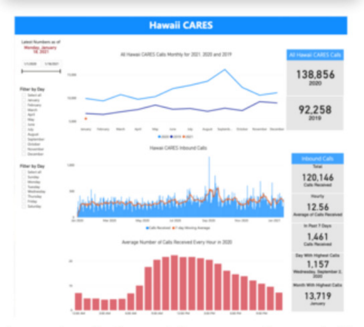

Inbound calls for crisis support, mental health resources, and substance use treatment services.
Source: DOH Behavioral Health & Homelessness Statewide Unified Response Group
Updated weekly

Economic Recovery Data

UHERO Economic Pulse

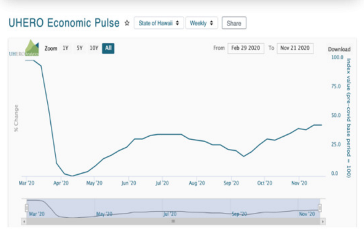

Indicates the extent of recovery from the trough of the COVID-recession.
Source: University of Hawaii Economic Research Organization
Updated weekly

Passenger Arrivals

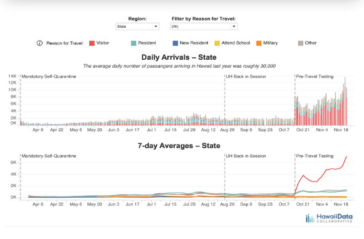

Daily counts of passenger arrivals by air, broken down by reason for travel.
Source: Hawaii Tourism Authority
Updated daily

Unemployment Claims

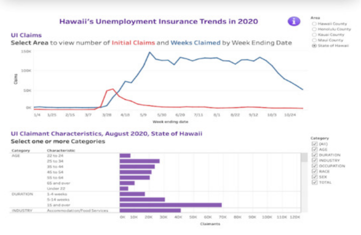

Unemployment insurance initial claims and weeks claimed, and claimant characteristics.
Source: Department of Labor & Industrial Relations
Updated weekly

Community Mobility

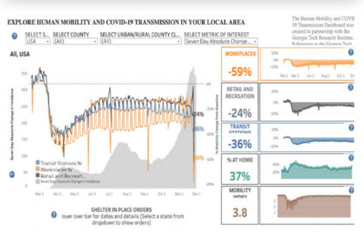

State and county trends of change in visits to different categories of places. Select "HI" from the state drop-down menu.
Source: Dashboard created by CDC, using data from Google's Community Mobility Reports
Updated weekly

Tourism

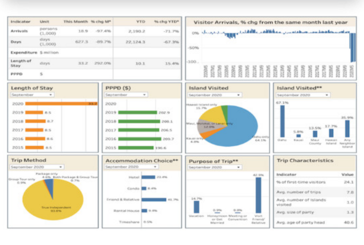

Visitor indicators, including length of stay, spending, and other trip characteristics.
Source: Department of Business, Economic Development & Tourism
Updated monthly

Safe Travels Program

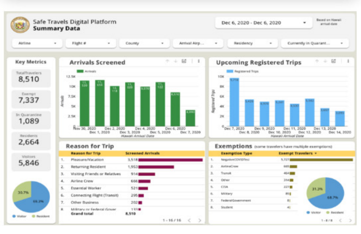

Summary data on incoming travelers collected through the Safe Travels Program.
Source: State of Hawaii
Updated daily

Our Partners

COVID PAU
A PROJECT OF THE HAWAIʻI COVID COLLABORATIVE

HiPAM
Hawaiʻi Pandemic Applied Modeling

Hawaii State Department of Health

< Idaho Division of Public Health - Profile

Statewide Cases

162,683 Total Statewide Cases
(Daily Update: +328)

Trend *(Number of Cumulative Confirmed and Probable Cases)*

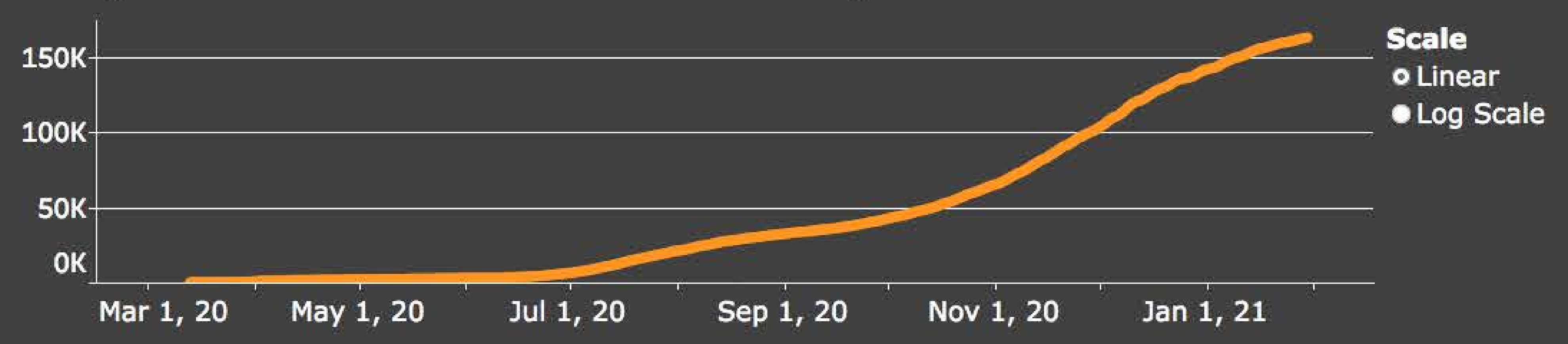

Daily Cases *(by Date of Official State Notification)*

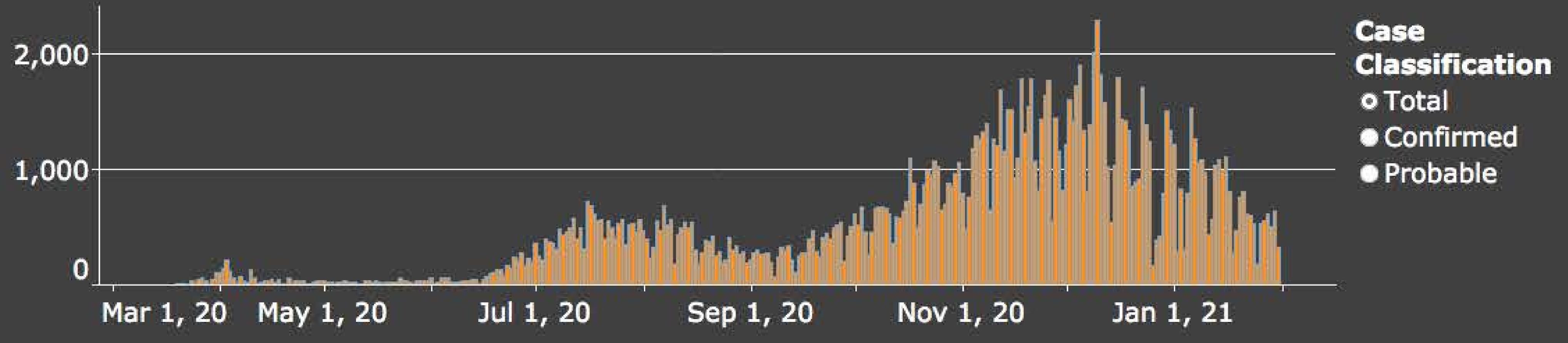

Onset Date of Cases

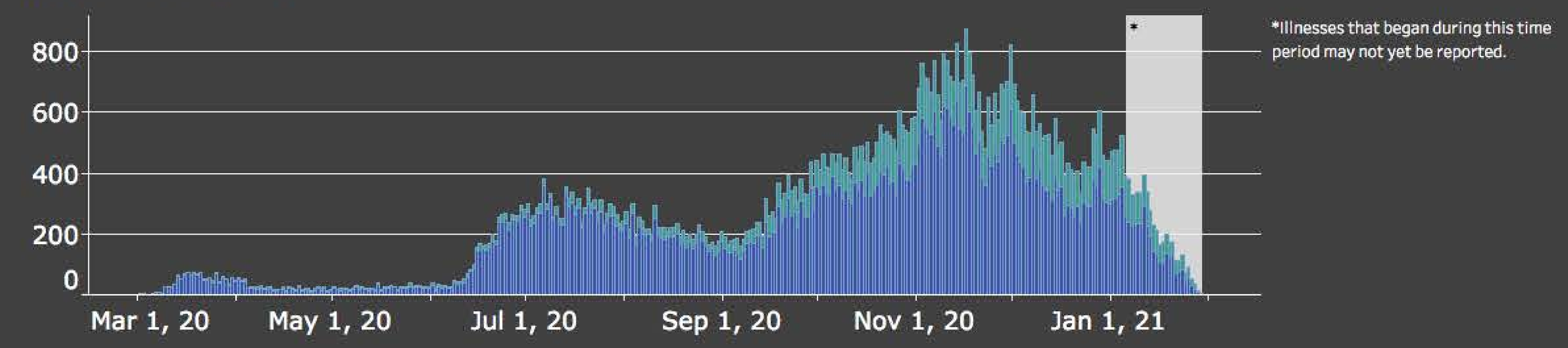

For definitions of confirmed and probable cases, as well as details on the data release schedule, please visit the Notes page at the top right.

IDAHO DEPARTMENT OF HEALTH & WELFARE

+ t a b l e a u

DPH Idaho COVID-19 Dashboard

Viz Author: **Idaho Division of Public Health**

4,083,886 Views ☆17

More Detail

Originally Published: Sep 3, 2020
Last Updated: Jan 30, 2021
Workbook Details: 12 Sheets
Metadata: Home
Statewide Cases
Demographics
County
HotSpots
ER
Testing
Deaths
Death Demographics
Ventilators and ICU Beds
Patients Currently Hospitalized in an Inpatient Bed who have Suspected or Confirmed COVID-10
Notes

© 2021 Tableau Software, LLC, a Salesforce Company.
All Rights Reserved

Cases by County

Moving Average Incidence Rate

Select a county from the dropdown to view a trend of the daily 7-day moving average incidence rate per 100,000 population

Select County

Statewide

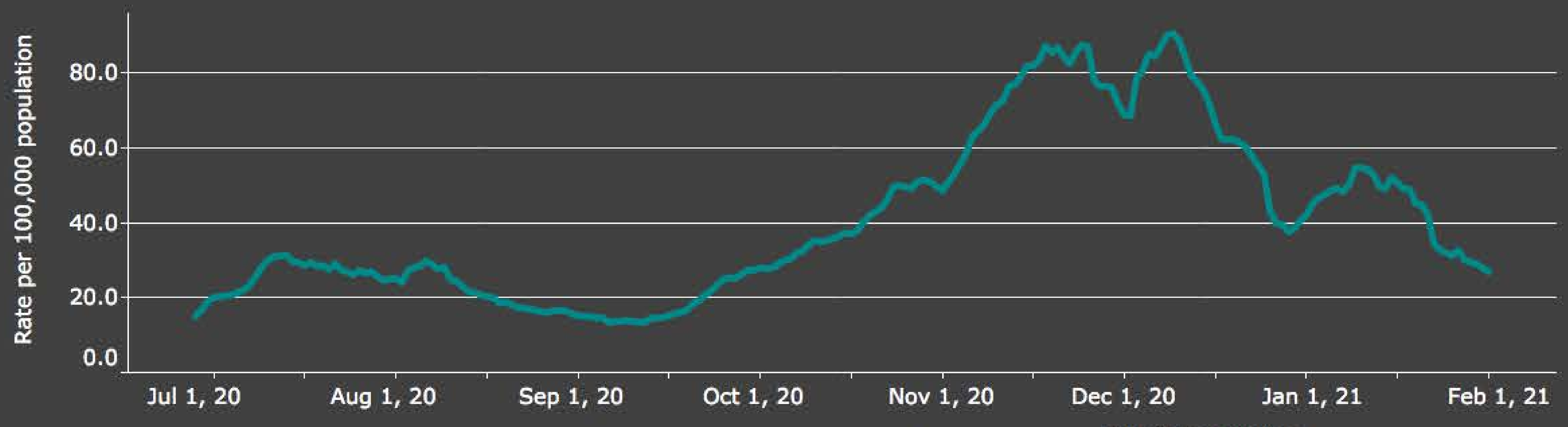

County	Confirmed	Probable	Total Cases	Cumulative Incidence Rate	Daily 7-Day Moving Average Incidence Rate	Deaths
Ada	37,939	6,544	44,483	9,236.8	26.0	409
Adams	237	73	310	7,219.4	0.0	3
Bannock	4,405	2,959	7,364	8,386.5	22.6	84
Bear Lake	235	93	328	5,355.1	14.0	2
Benewah	415	119	534	5,743.2	13.8	6
Bingham	2,476	1,581	4,057	8,666.8	20.4	58
Blaine	1,768	178	1,946	8,453.2	63.3	14
Boise	220	68	288	3,677.7	5.5	2
Bonner	2,185	506	2,691	5,883.4	44.7	27
Bonneville	8,404	3,052	11,456	9,621.9	24.1	136
Boundary	347	382	729	5,953.5	23.3	10
Butte	148	37	185	7,123.6	11.0	1
Camas	49	16	65	5,877.0	12.9	0
Canyon	20,151	3,592	23,743	10,329.8	27.3	256
Caribou	301	309	610	8,525.5	14.0	11
Cassia	2,385	379	2,764	11,502.3	20.2	22
Clark	44	10	54	6,390.5	16.9	0
Clearwater	741	233	974	11,123.8	42.4	12
Custer	175	52	227	5,260.7	6.6	3
Elmore	1,173	338	1,511	5,492.3	13.0	10
Franklin	808	207	1,015	7,314.8	15.4	12
Fremont	842	107	949	7,244.8	14.2	14
Gem	1,288	356	1,644	9,076.9	36.3	27
Gooding	892	346	1,238	8,156.0	14.1	23
Idaho	849	294	1,143	6,857.9	9.4	17
Jefferson	1,682	581	2,263	7,575.9	18.7	20
Jerome	1,904	517	2,421	9,917.3	11.7	18
Kootenai	13,950	1,760	15,710	9,481.2	39.7	168
Latah	2,373	128	2,501	6,235.7	24.2	6
Lemhi	395	80	475	5,917.5	3.6	9
Lewis	274	94	368	9,588.3	22.3	6
Lincoln	367	109	476	8,870.7	29.3	10
Madison	5,134	728	5,862	14,689.2	43.0	21
Minidoka	1,870	359	2,229	10,594.6	19.7	26
Nez Perce	2,995	294	3,289	8,139.5	14.8	45
Oneida	193	118	311	6,863.8	44.1	3
Owyhee	849	123	972	8,221.3	10.9	24
Payette	1,846	468	2,314	9,661.4	15.5	27
Power	443	158	601	7,824.5	33.5	6
Shoshone	908	56	964	7,483.3	22.2	30
Teton	581	416	997	8,211.2	82.4	4
Twin Falls	6,828	1,922	8,750	10,071.6	19.7	119
Valley	574	141	715	6,276.3	51.4	4
Washington	835	322	1,157	11,349.8	35.0	20

Cases by Date of Onset and Public Health District

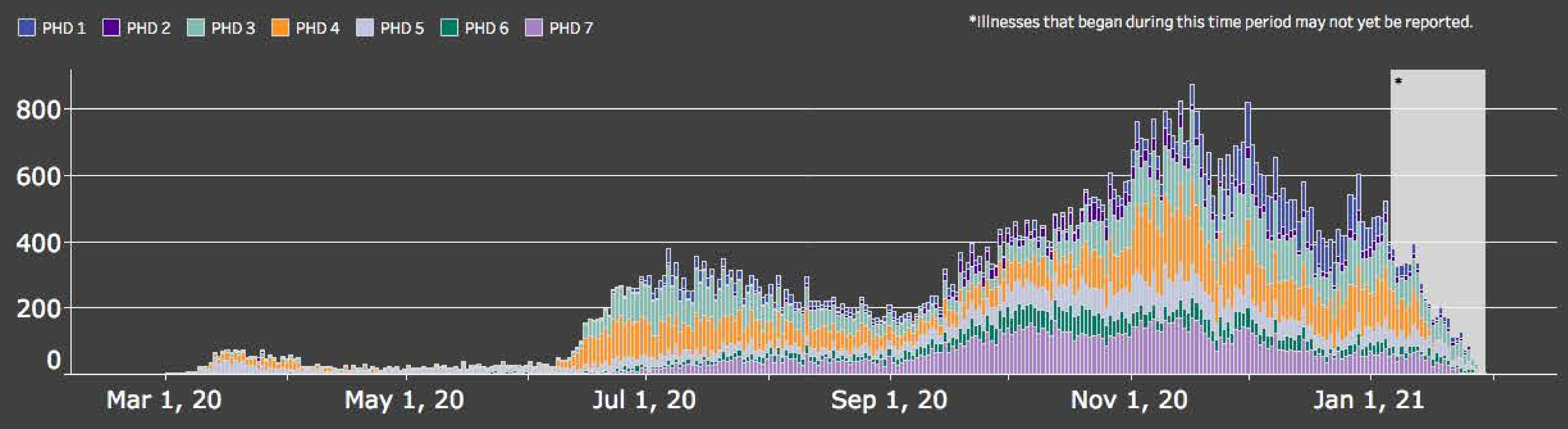

For notes on county cases from the above visualizations, please visit the Notes page at the top right.

IDAHO DEPARTMENT OF HEALTH & WELFARE

+tableau

DPH Idaho COVID-19 Dashboard

Viz Author: Idaho Division of Public Health

4,083,886 Views ☆17

More Detail

Originally Published: Sep 3, 2020
Last Updated: Jan 30, 2021
Workbook Details: 12 Sheets
Metadata: Home
Statewide Cases
Demographics
County
HotSpots
ER
Testing
Deaths
Death Demographics
Ventilators and ICU Beds
Patients Currently Hospitalized in an Inpatient Bed who have Suspected or Confirmed COVID-10
Notes

© 2021 Tableau Software, LLC, a Salesforce Company.
All Rights Reserved

Laboratory Testing for SARS-CoV-2

PCR Tests — **990,444 Tests Performed** / **603,115 Idahoans Tested**

PCR Tests Performed and Percent Positivity by Week of Specimen Collection

Select Jurisdiction State

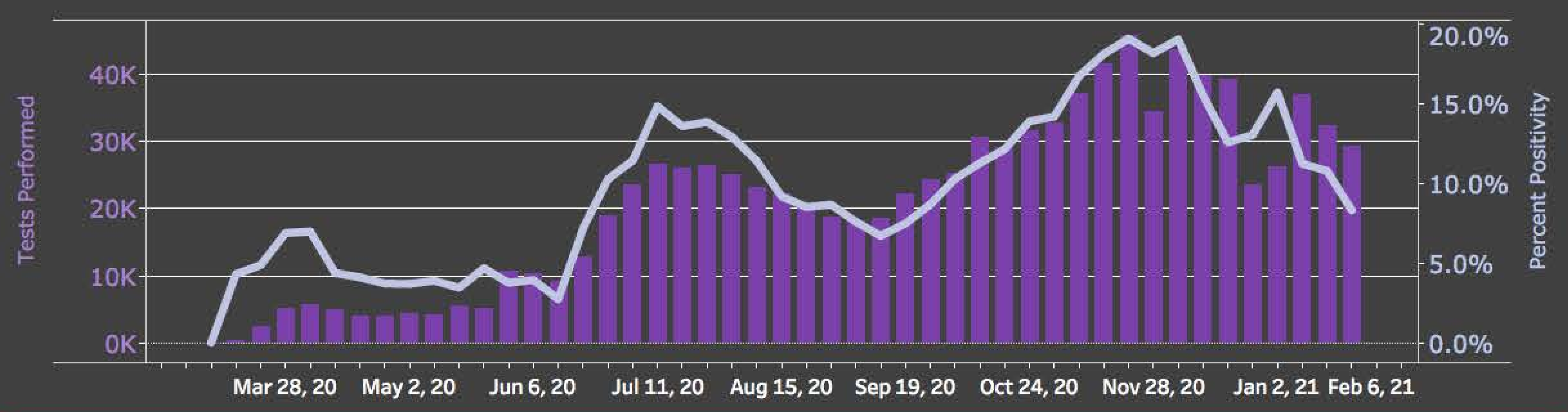

Antigen Tests — **78,973 Tests Performed**

Number of Positive Antigen Tests Reported by Week of Specimen Collection

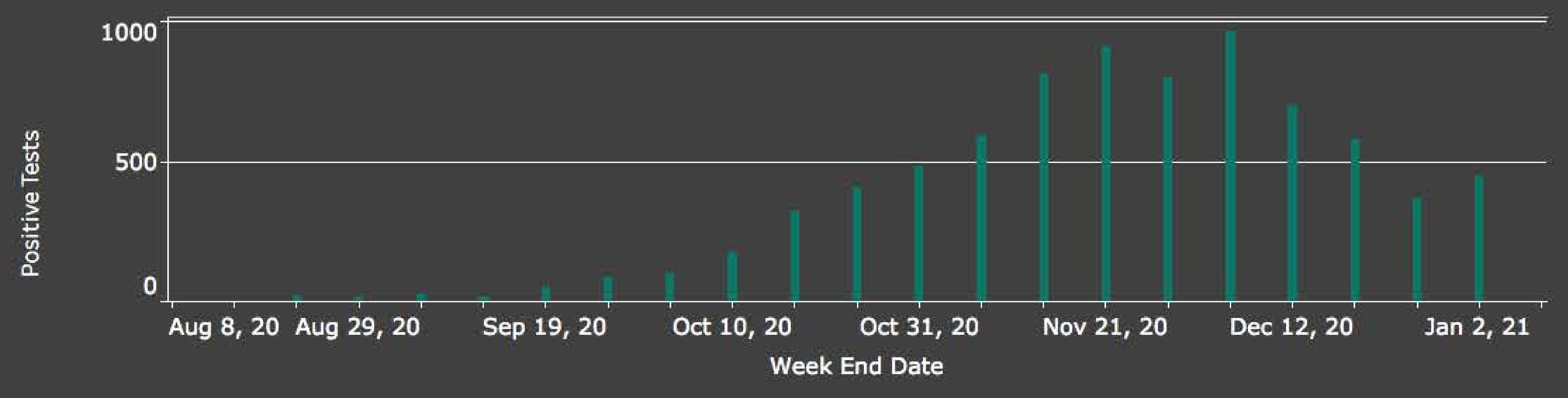

For notes and details on testing calculations, please visit the Notes page at top right.

IDAHO DEPARTMENT OF HEALTH & WELFARE

+ableau

DPH Idaho COVID-19 Dashboard

Viz Author: Idaho Division of Public Health

4,083,886 Views ☆17

More Detail

Originally Published: Sep 3, 2020

Last Updated: Jan 30, 2021

Workbook Details: 12 Sheets

Metadata: Home
Statewide Cases
Demographics
County
HotSpots
ER
Testing
Deaths
Death Demographics
Ventilators and ICU Beds
Patients Currently Hospitalized in an Inpatient Bed who have Suspected or Confirmed COVID-10
Notes

© 2021 Tableau Software, LLC, a Salesforce Company. All Rights Reserved

+ableau public GALLERY AUTHORS BLOG RESOURCES ACTIVITY ABOUT SIGN UP SIGN IN
Idaho Division of Public Health - Profile
Favorite Make a Copy Download

HOME | Statewide Cases | Statewide Demographics | Cases by County | Weekly Hotspots & Local Trends | Emergency Department Visits | Laboratory Testing | COVID-19 Related Deaths | Deaths by Demographics | Ventilators & ICU Beds | Hospitalizations | NOTES

Demographics of COVID-19 Related Deaths

Idaho Resident Deaths: 1,725 *(1,513 Confirmed Cases, 212 Probable Cases)*
96.5 Deaths per 100,000 Population

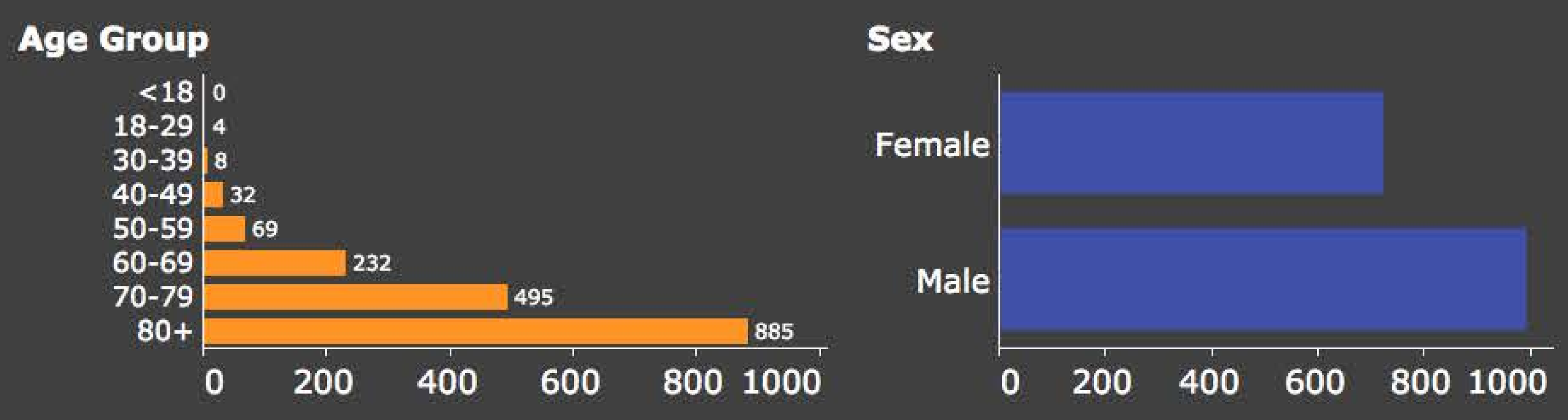

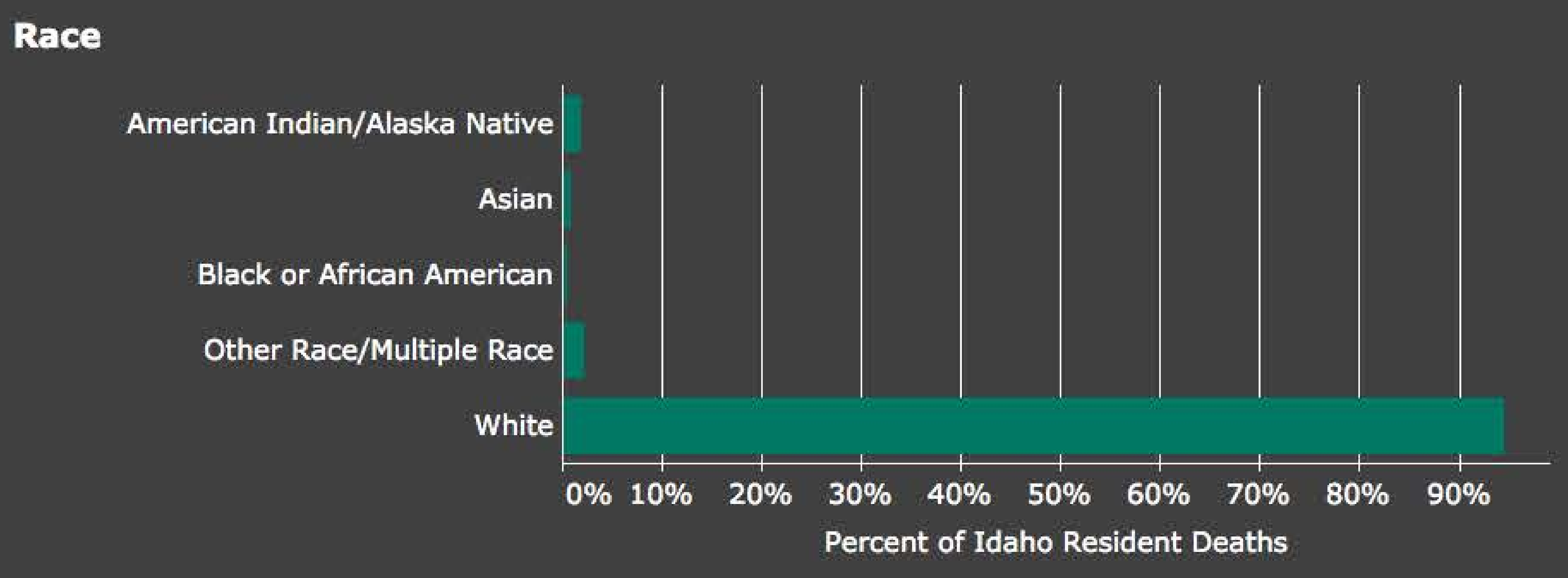

The race category for 15 deaths is pending

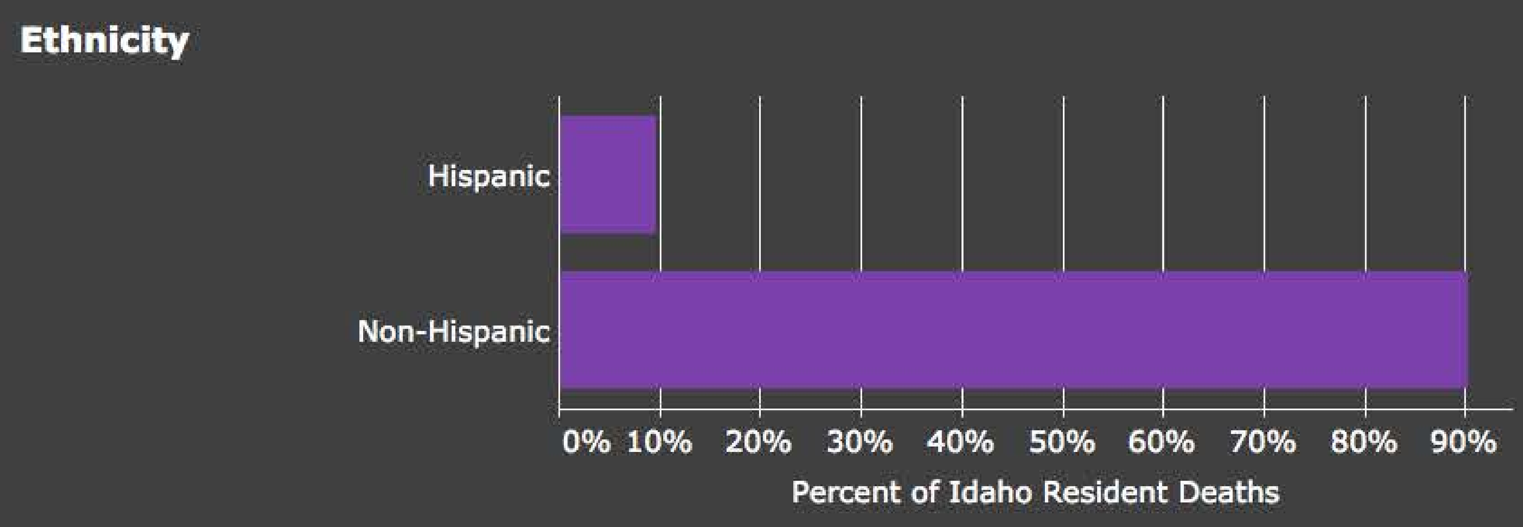

The ethnicity category for 13 deaths is pending

For definitions, please visit the Notes page at the top right.

IDAHO DEPARTMENT OF HEALTH & WELFARE

DPH Idaho COVID-19 Dashboard

Viz Author: Idaho Division of Public Health
4,083,886 Views ☆17

More Detail

Originally Published: Sep 3, 2020
Last Updated: Jan 30, 2021
Workbook Details: 12 Sheets
Metadata: Home
Statewide Cases
Demographics
County
HotSpots
ER
Testing
Deaths
Death Demographics
Ventilators and ICU Beds
Patients Currently Hospitalized in an Inpatient Bed who have Suspected or Confirmed COVID-19
Notes

+ableau
© 2021 Tableau Software, LLC, a Salesforce Company. All Rights Reserved
Products: Tableau Desktop, Tableau Server, Tableau Online, Tableau Prep, Tableau Public Free
Legal: Privacy Policy, Data Policy, Terms of Service, Patents & IP, Do Not Sell My Personal Information
About: What is Tableau Public?, Current Status, FAQ, Careers, Contact Us
English

COVID-19 Related Deaths Among Idaho Residents

Idaho Resident Deaths: 1,725 *(1,513 Confirmed Cases, 212 Probable Cases)*
96.5 Deaths per 100,000 Population

COVID-19 Related Deaths by Public Health District | **COVID-19 Related Deaths by County**

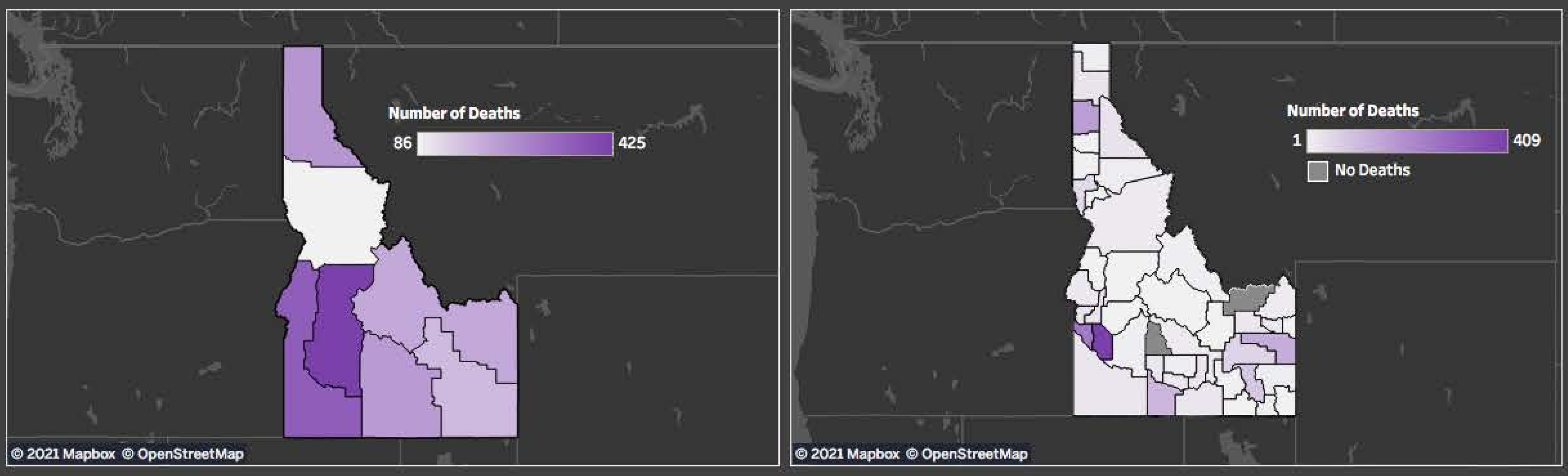

COVID-19-Related Deaths Among Idaho Residents by Date of Death

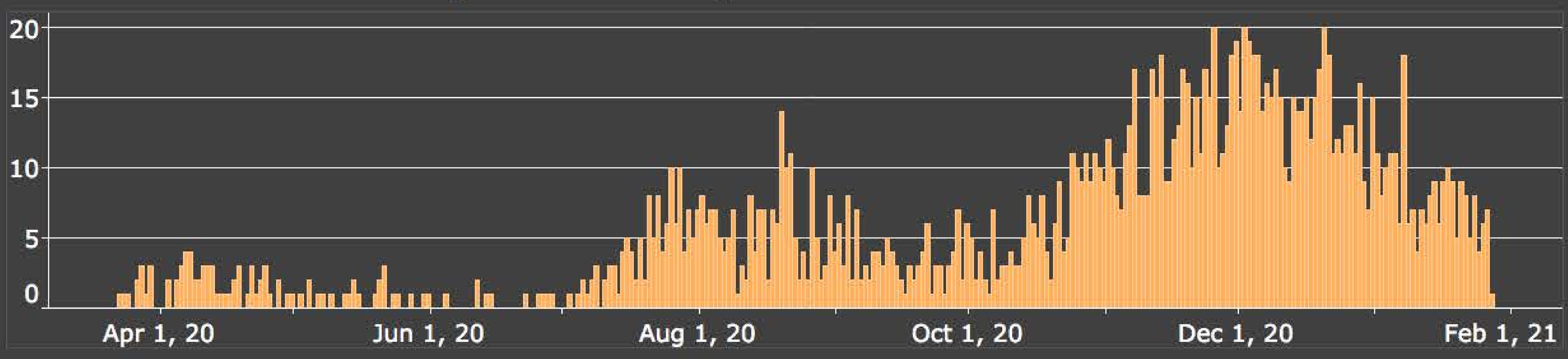

For definitions and notes, please visit the Notes page at the top right.

IDAHO DEPARTMENT OF HEALTH & WELFARE

+tableau

DPH Idaho COVID-19 Dashboard

Viz Author: **Idaho Division of Public Health**
4,083,886 Views ☆17

More Detail

Originally Published: Sep 3, 2020
Last Updated: Jan 30, 2021
Workbook Details: 12 Sheets
Metadata: Home
Statewide Cases
Demographics
County
HotSpots
ER
Testing
Deaths
Death Demographics
Ventilators and ICU Beds
Patients Currently Hospitalized in an Inpatient Bed who have Suspected or Confirmed COVID-10
Notes

+tableau
© 2021 Tableau Software, LLC, a Salesforce Company.
All Rights Reserved

Products
Tableau Desktop
Tableau Server
Tableau Online
Tableau Prep
Tableau Public Free

Legal
Privacy Policy
Data Policy
Terms of Service
Patents & IP
Do Not Sell My Personal Information

About
What is Tableau Public?
Current Status
FAQ
Careers
Contact Us

English

CORONAVIRUS

Interactive map and timelines tracking COVID-19 numbers and trends in Idaho

See the latest COVID-19 data as we track deaths as well as confirmed, probable and recovered coronavirus cases.

Author: **KTVB Staff**
Published: **4:13 PM MDT March 13, 2020**
Updated: **5:33 PM MST January 30, 2021**

BOISE, Idaho — We're continuing to post live daily updates on the coronavirus situation in Idaho while tracking the coronavirus case numbers and overall trends.

Scroll down to see our multiple graphs and timelines looking at the coronavirus case trends in Idaho.

Underneath all of the graphs and timelines, you can read about our methodology for tracking numbers and a terminology breakdown. Scroll all the way to the bottom to see it.

Latest Idaho coronavirus numbers:

See a more detailed daily number breakdown here.

Friday's (01/29/2021) numbers:

- 621 total new cases
- 4 new deaths reported
- See more details here.

Since the start of the pandemic:

- 1,727 deaths
- 132,316 confirmed cases (162,355 confirmed and probable cases)
- 81,034 recovered (estimated)
- 114,372 total COVID-19 vaccine doses administered
- 94,340 people have received the vaccine
- 73,770 people have received only the first dose of the vaccine
- 20,301 people have received both doses of the vaccine

Scroll all the way down to see definitions of the terminology we use for recovered, confirmed, probable, etc, as well as information about how we gather this COVID-19 data.

Download the KTVB news app to get urgent news alerts.

Timeline of COVID-19 in Idaho:

Mouse over or tap each data point for more details. (Mobile users can tap here for a larger version).

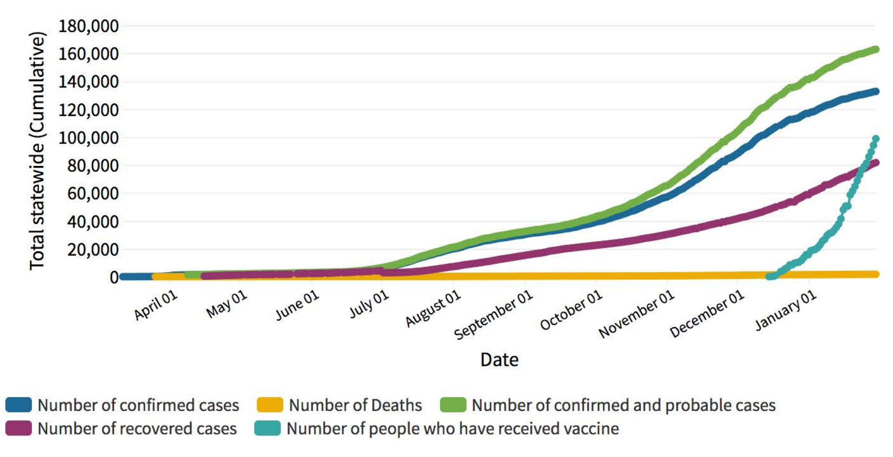

Note on the drop in recovered cases: Idaho Department of Health and Welfare says "an error in auto-calculation was discovered 7/1/2020."

"Probable" cases are people who have received a positive antigen test but not a lab PCR test, or people showing symptoms who had close contact with a confirmed coronavirus case, such as a member of the same household.

Timeline: New Idaho daily cases reported over the last two weeks

Mouse over or tap each data point for more details. (Mobile users can tap here for a larger version).

This graph is updated daily.

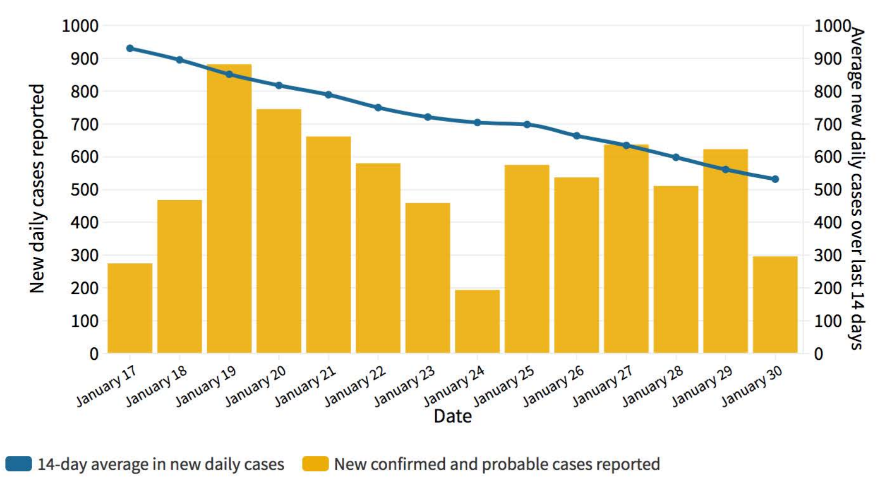

Timeline: Trend in newly reported cases over a 14-day period

Mouse over or tap each data point for more details. (Mobile users can tap here for a larger version).

This graph is updated daily.

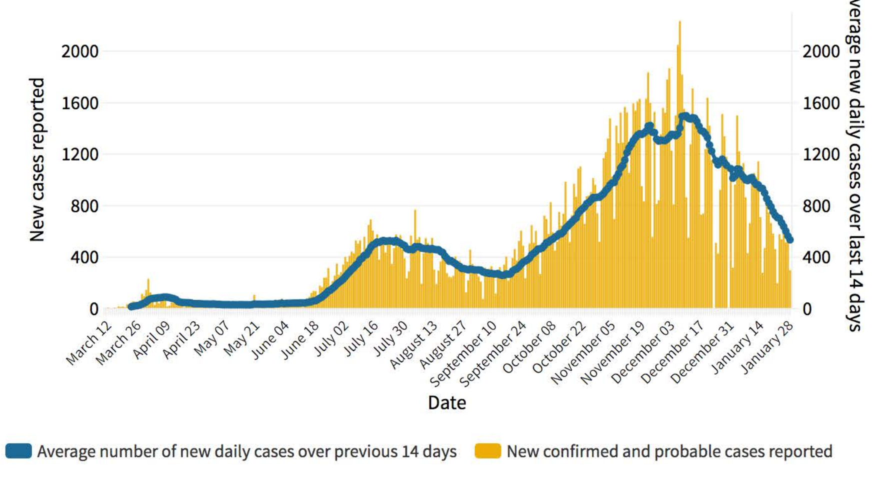

The State of Idaho has been using the 14-day average in its tracking to determine when the state could move to the next stage of reopening.

Timeline of total COVID-19 related deaths in Idaho:

Mouse over or tap each data point for more details. (Mobile users can tap here for a larger version).

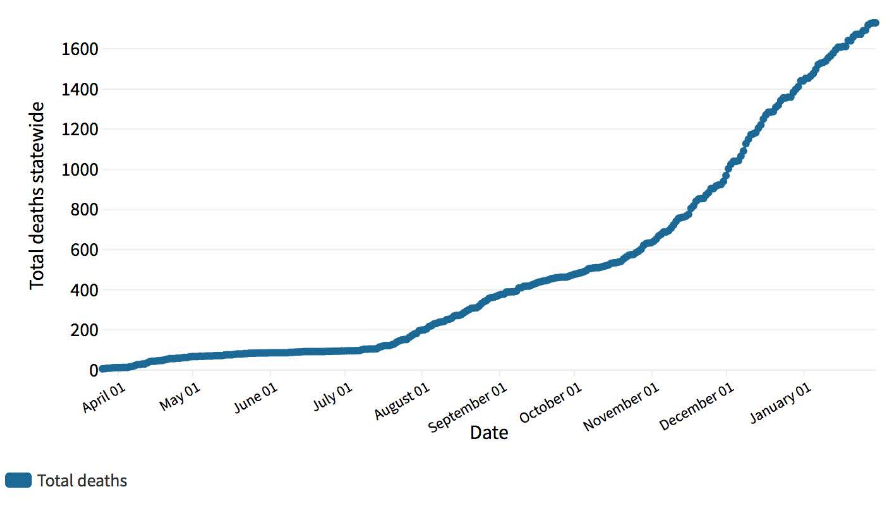

Map of Idaho confirmed coronavirus (COVID-19) cases:

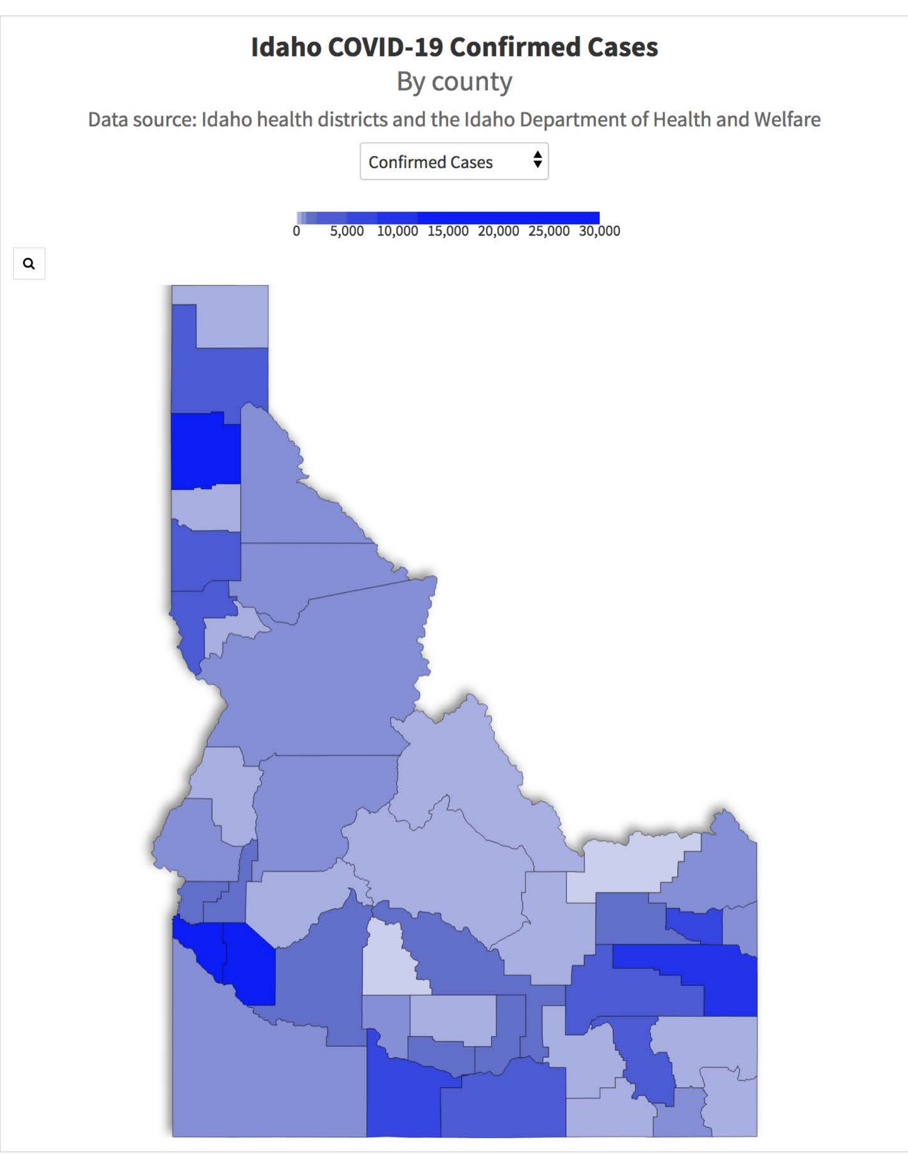

You can hover over each county to see its latest numbers.

Timeline of total number of COVID-19 cases in Idaho and new cases reported (updated daily):

Mouse over or tap each data point for more details. (Mobile users can tap here for a larger version).

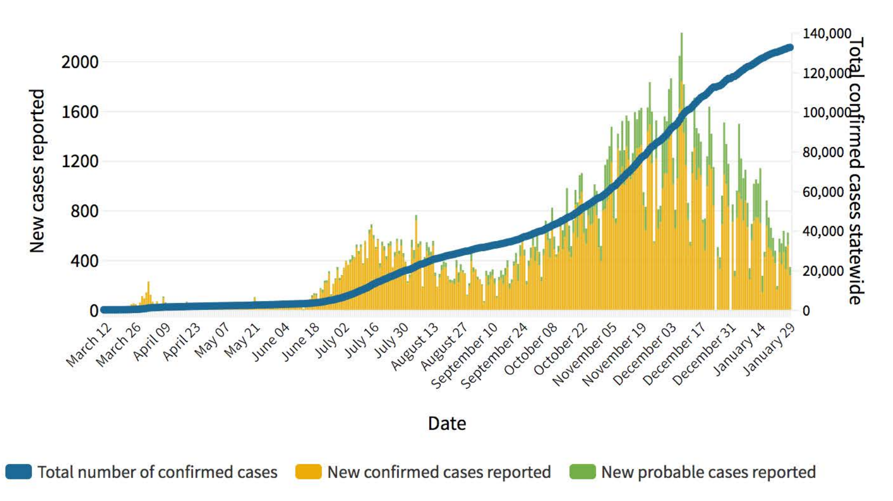

How we track COVID-19 cases:

Every day, KTVB works diligently to ensure we are providing the most accurate, up-to-date information regarding COVID-19 cases in Idaho. We track our numbers the same way the Idaho Dept. of Health and Welfare does. However, we update our total number more frequently, meaning our numbers may not always match with the state.

Local Idaho health districts are releasing confirmed COVID-19 cases before they are submitted to the state and added to the official Idaho state total. We're adding up the state total and showing the latest total count in our real-time updates live blog and below in this article.

The state updates the number of total cases every day around 5 p.m. KTVB updates our running total once the state and local health districts have confirmed their total number of cases.

Regional health districts release updated numbers at different times than the state Department of Health and Welfare, which can sometimes cause a discrepancy in the number reported. We use the latest information reported by the state and the health districts to keep the total as updated as possible.

Some of those numbers local districts release might change if a positive case is determined to be an out of state resident - they will be added to another state's total number of cases instead of Idaho.

Terminology:

- "**Recoveries**" are tracked by the Idaho Department of Health and Welfare. The state says it "Is defined as the number of persons with a confirmed COVID-19 diagnosis who are alive 30 days post-onset (or referral date if onset is not available) based upon total cases, deaths, and any clinical info available."
- **"Confirmed" cases** refer to a person who was tested for COVID-19 and tested positive.
- **"Probable" cases** refer to a person who is exhibiting symptoms after coming in contact with a person who has a confirmed case. Though a probable case is not confirmed, the state will monitor the person for symptoms.
- **"Total cases"** refers to the number of confirmed and probable cases combined. We will always clarify how many of the total cases are confirmed and how many are probable in our daily updates.

KTVB7 News Weather Sports Connect Watch RIGHT NOW Boise, ID » 49°

COVID-19 Statistics

Total Cases	Deaths	Probable Deaths
1,126,301	19,243	2,010

Total Tests Performed*	Recovery Rate**
16,039,292	98%

*Total molecular and antigen tests performed and reported electronically for testing of COVID-19 at IDPH, commercial or hospital laboratories. All numbers displayed are provisional and will change.

** Recovered cases are defined as persons with initial positive specimen collection date > 42 days who have not expired.

Recovery rate is calculated as the recovered cases divided by the sum of recovered cases and deceased cases.

Information regarding the number of persons under investigation updated on 1/31/2021.

By County | By Zip

Select Zip:

Illinois

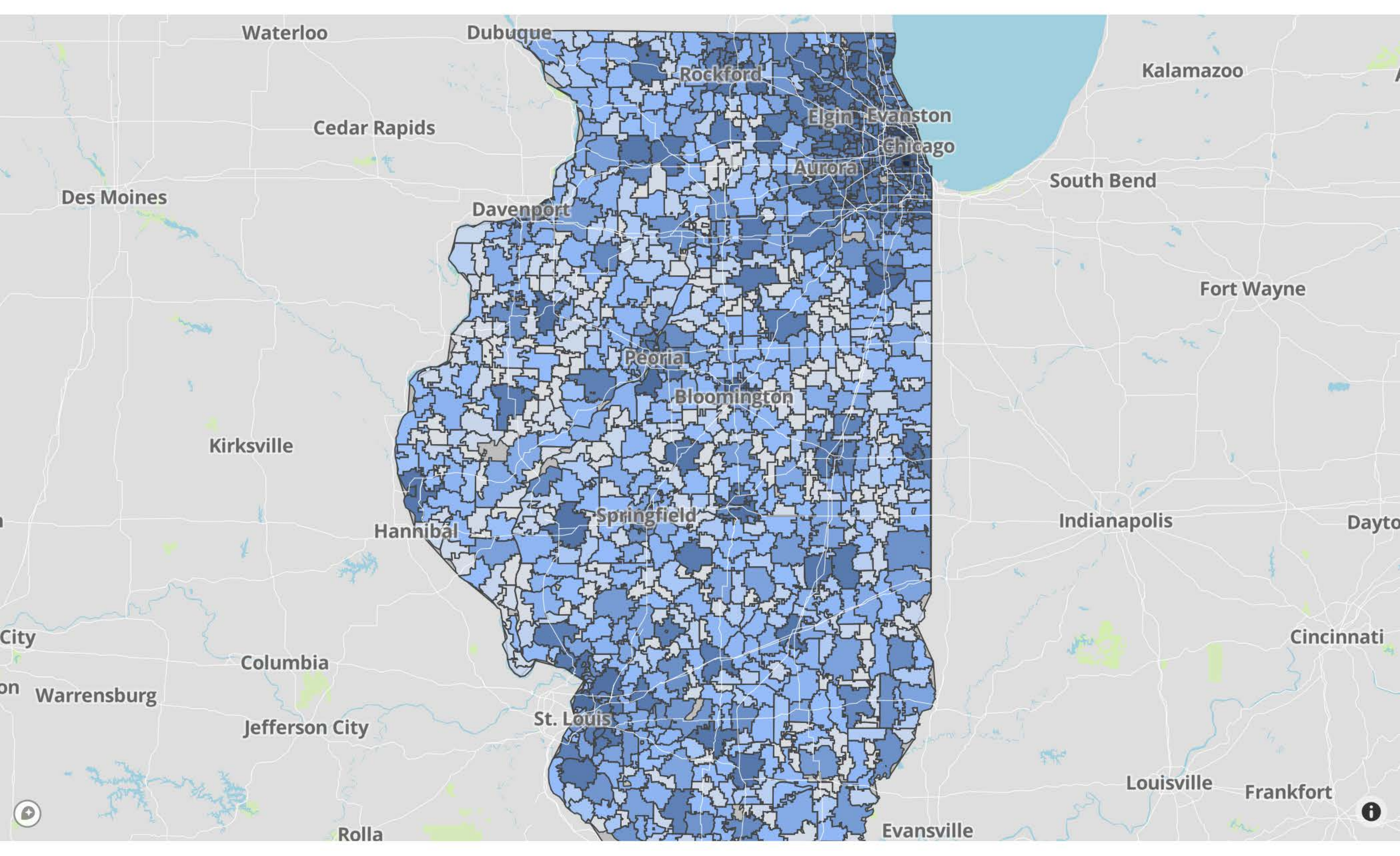

The data on this site is what has been entered into Illinois' National Electronic Disease Surveillance System (I-NEDSS) data system.

The data is constantly being entered and may change as cases are investigated. Therefore, numbers may vary from other sites where data is published.

*Data shown is >5 cases per zip code which is consistent with Collection, Disclosure, and Confidentiality of Health Statistics (77 Ill. Adm. Code 1005).

It should be assumed that COVID-19 exposure can occur in every county in IL.

Zip code is zip code of residence, which may not be location of exposure.

Search

Zip	Tested	Total Cases	Deaths
60000	416	42	N/A
60001	158	6	N/A
60002	13,692	1,573	N/A
60004	51,205	3,282	N/A
60005	31,872	2,065	N/A
60006	210	14	N/A
60007	27,636	2,508	N/A
60008	24,170	2,225	N/A
60009	189	19	N/A
60010	37,273	2,606	N/A
60011	188	8	N/A
60012	11,903	827	N/A
60013	17,222	1,639	N/A

Age Demographics

Total Cases | Tested | Deaths

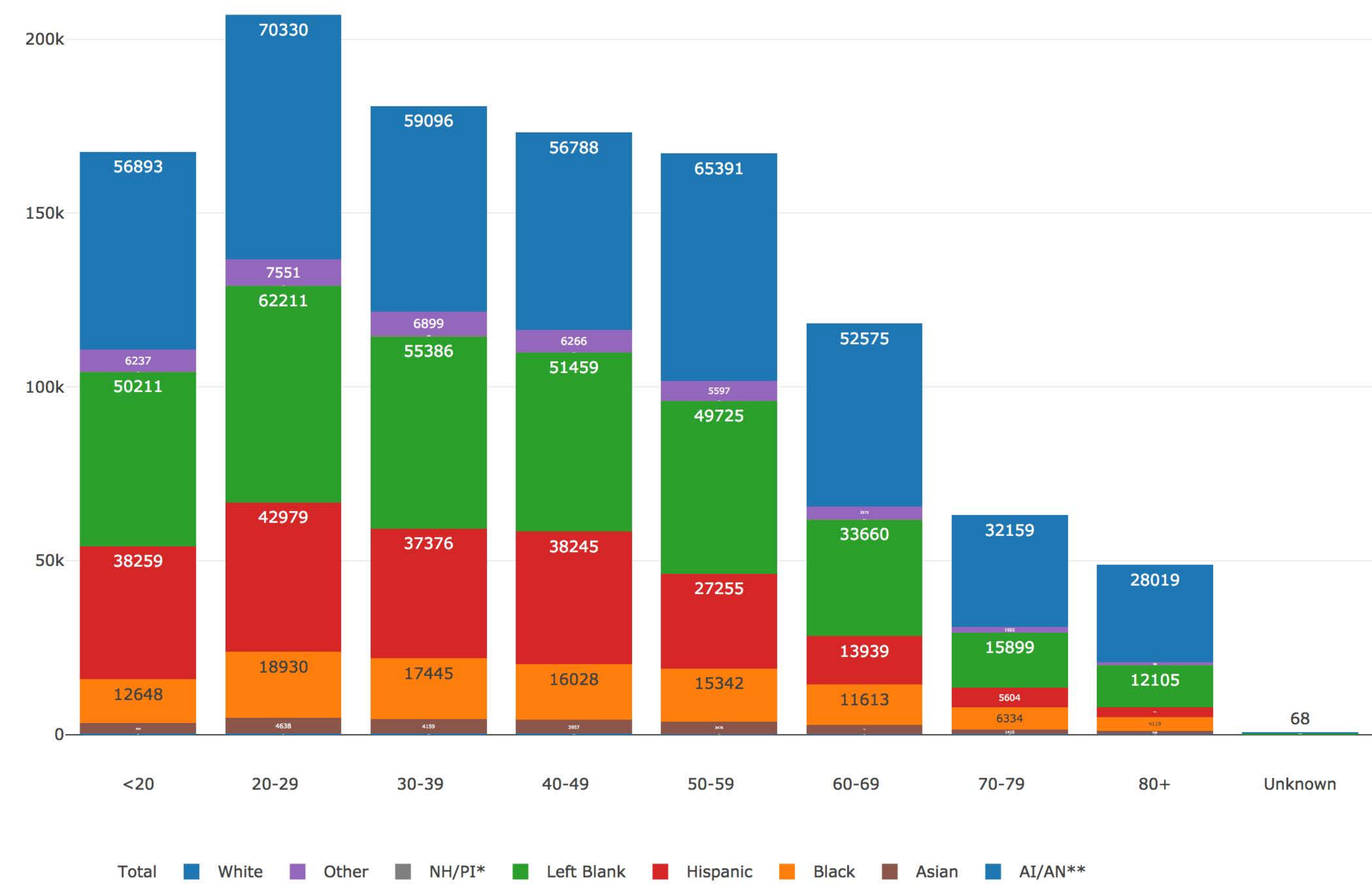

Race Demographics

Total Cases | Tested | Deaths

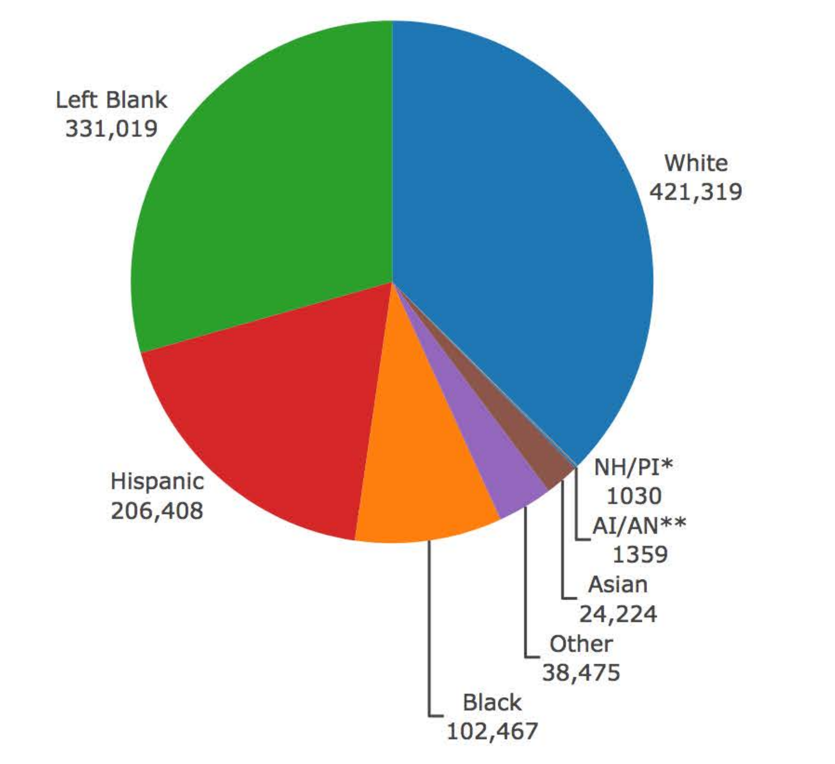

Gender

Total Cases | Tested | Deaths

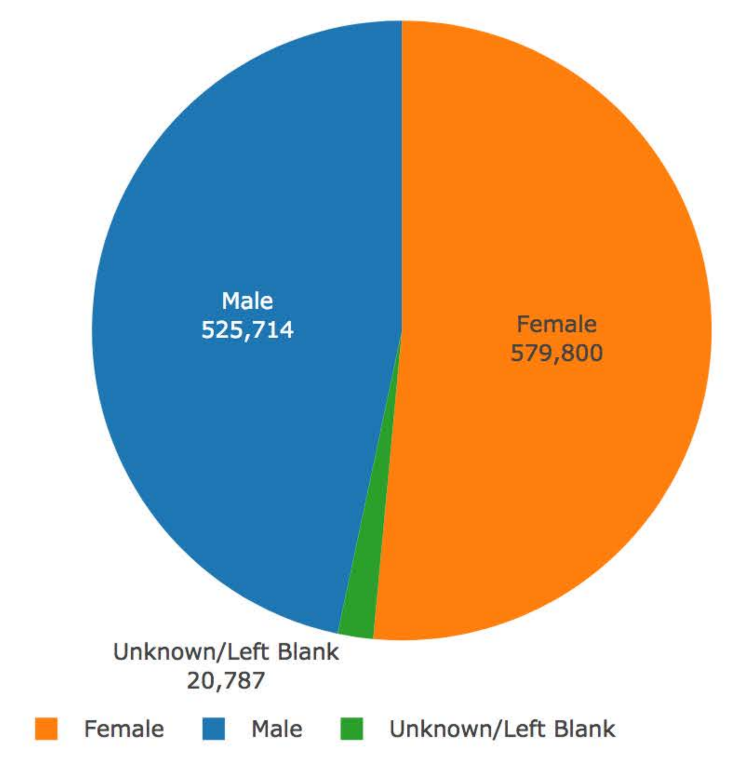

*Native Hawaiian or Other Pacific Islander

**American Indian or Alaskan Native

Tested Results Last 15 days

Tested | Cases | Deaths

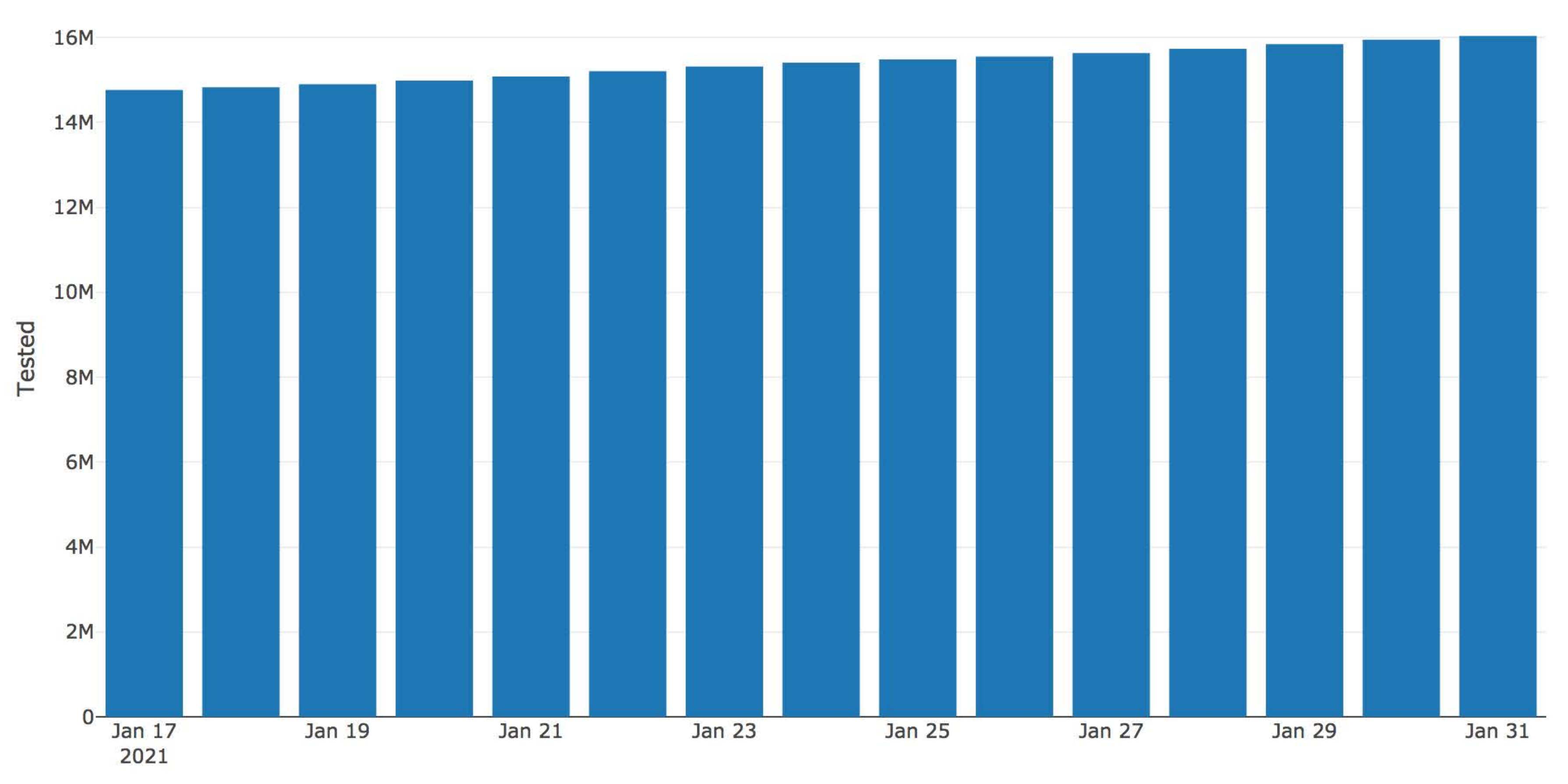

15d | 30d | 90d | All Time

Tested Change Last 15 days

Tested | Cases | Deaths

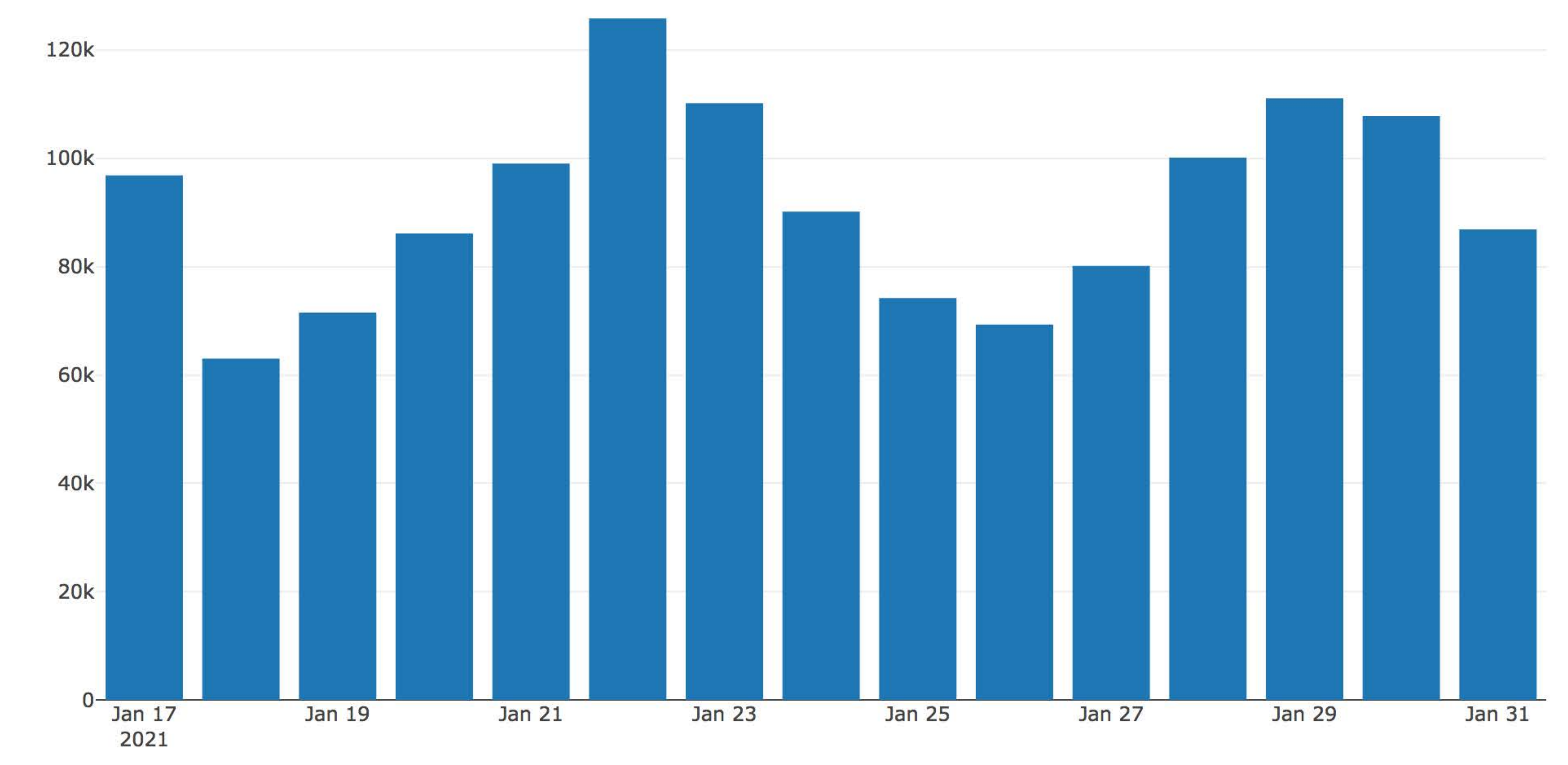

15d | 30d | 90d | All Time

On January 22, 2021, IDPH began including in the total case count all probable deaths reported since the beginning of the pandemic. Due to this change, all probable deaths, 1,903, have been added to the one-day, January 22, 2021, total of new cases.
Beginning November 6, 2020 and going forward, IDPH will report confirmed cases and probable cases combined. Due to this change, all probable cases previously reported separately, 7,673, have been added to the one-day, November 6, 2020 total of new cases

Indiana COVID-19 Dashboard and Map

Indiana COVID-19 Data Report

Additional Resources

Dashboard | LTC | School

Below results are as of **01/30/2021, 11:59 PM**. Dashboard updated daily at **12:00 PM**.
New positive cases, deaths and tests have occurred over a range of dates but were reported to the state Department of Health in the last 24 hours.

Filters

Date Range   Last 30 days   Last 45 days   Last 60 days   Last 90 days

Newly Reported Confirmed COVID-19 Counts

New Tests Administered
38,155
08/25/2020 ... 01/30/2021
8,703 New Individuals Tested

New Positive Cases
1,750
11/25/2020 ... 01/30/2021

Positivity - All Tests
8.5 % 7-Day Rate
01/18/2021 ... 01/24/2021
10.6 % cumulative rate

New Deaths
5
01/29/2021 ... 01/30/2021

Total Confirmed COVID-19 Counts

Total Tests Administered
7,014,523
02/26/2020 ... 01/30/2021
2,957,867 Individuals Tested

Total Positive Cases
626,682
03/06/2020 ... 01/30/2021

Positivity - Unique Individuals
17 % 7-Day Rate
01/18/2021 ... 01/24/2021
21.2 % cumulative rate

Total Deaths
9,598
03/16/2020 ... 01/30/2021

County Distributions

Select a county below by tap or click

Cases | Deaths | Tested | Positivity | County Metrics

Below results are as of **01/25/2021, 11:59 PM**. The county metrics map is updated Wednesdays at **12:00 PM** and reflects data through the previous Sunday.
Click here to learn more about the county metrics map

Map Filter: Weekly 2-Metric Score   ISDH District: Statewide

Advisory Level

Advisory level refers to the current guidelines the county must follow. A county must remain at a lower Weekly Two-Metric Score for two consecutive weeks to move down to a lower advisory level.

Weekly 2-Metric Score

- Blue (0 and .5)
- Yellow (1 and 1.5)
- Orange (2 and 2.5)
- Red (3)

Weekly Cases Per 100,000 Residents

- Less Than 10 new cases(0)
- 10 to 99 new cases(1)
- 100 to 199 new cases(2)
- 200 or more(3)

7-Day All Tests Positivity Rate

- Less than 5% (0)
- 5% to 9.9% (1)
- 10% to 14.9% (2)
- 15% or greater (3)

The ⚠ indicates a disclaimer alert for the county. Hover over the symbol for additional details.

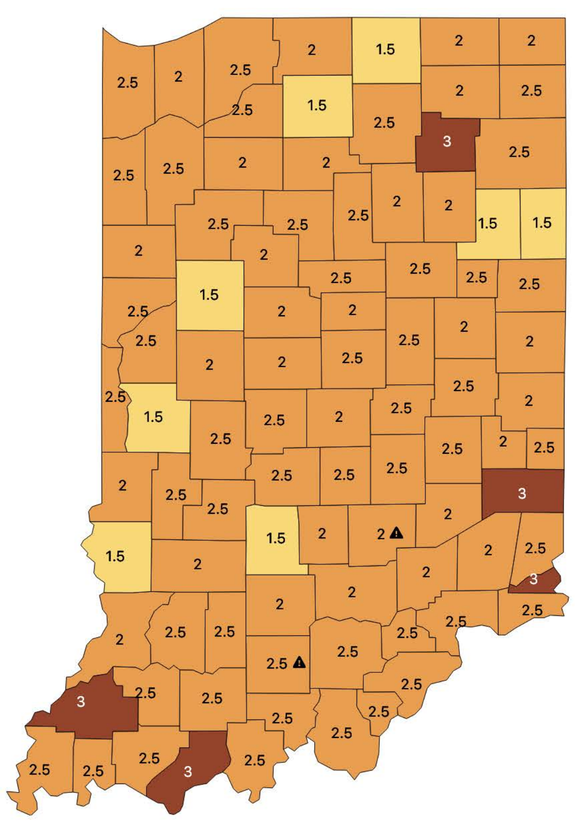

Positive Cases

Statewide Positive Cases by Day

All | Newly Reported

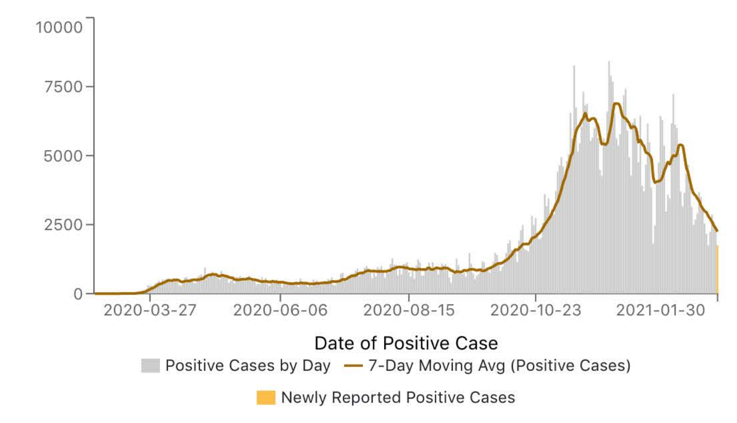

Tests

Statewide Tests by Day

All | Newly Reported
All Tests | Individuals

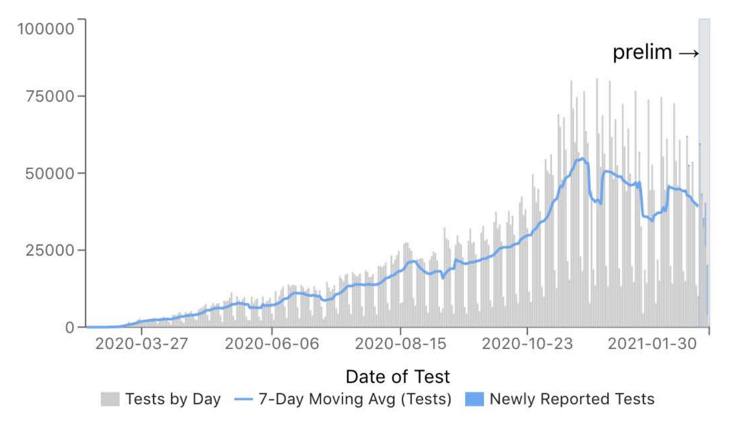

Deaths

Statewide Deaths by Day

All | Newly Reported

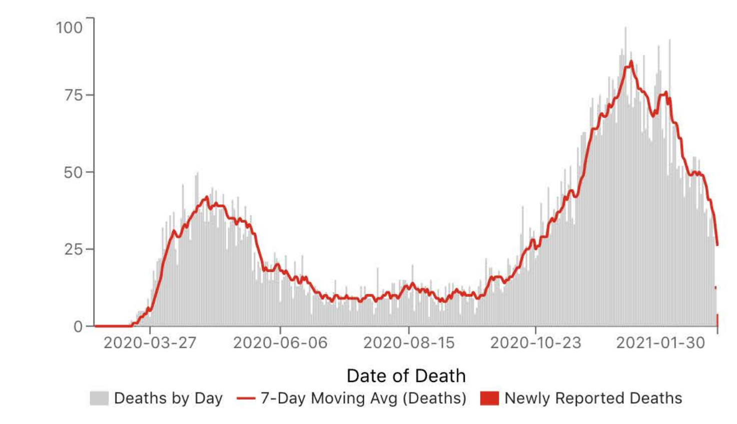

Positivity

Statewide Daily Positive Test Rate

All Tests | Individuals

What does prelim mean?

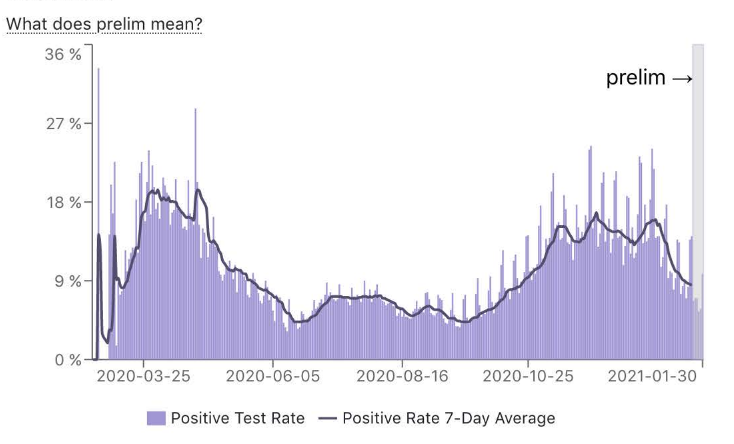

Demographic Distributions

Why do I see "Unknowns"?

Positive Cases | Deaths | Tested Individuals

Statewide Demographics for Positive Cases*

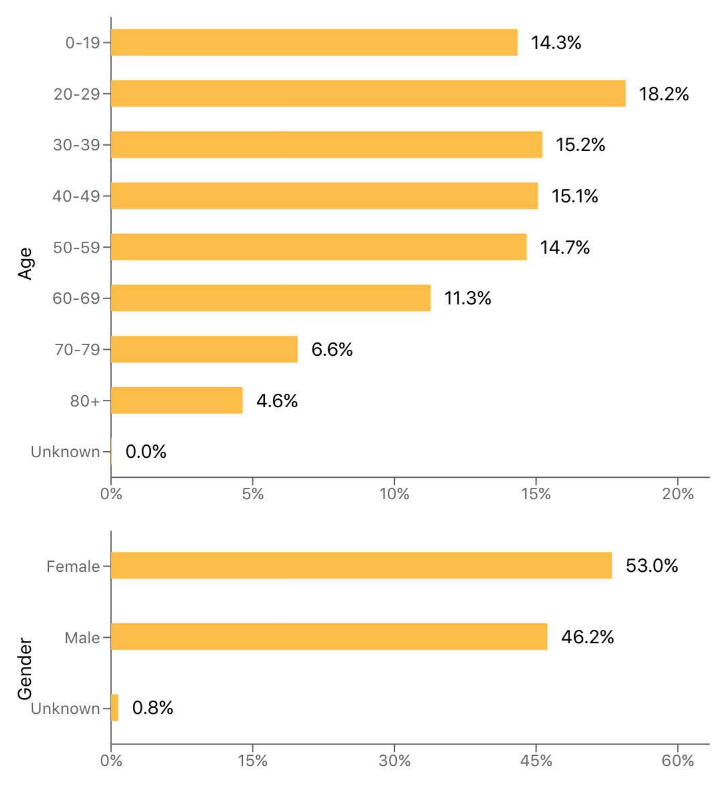

Race	% of Cases	% of Indiana Population
White	66.9 %	85.1 %
Other Race	11.0 %	2.6 %
Black or African American	6.6 %	9.8 %
Asian	1.3 %	2.5 %
Unknown	14.2 %	0 %

Ethnicity	% of Cases	% of Indiana Population
Not Hispanic or Latino	51.5 %	92.9 %
Hispanic or Latino	5.8 %	7.1 %
Unknown	42.7 %	0 %

** Indiana population percentages provided by 2019 U.S. Census Bureau; Population Estimate Program.*

Resource Usage

Today | By Day

Today's Statewide ICU Bed Usage

33.6% ICU Beds Available

17.2% ICU Beds in Use - COVID

49.1% ICU Beds in Use - Non-COVID

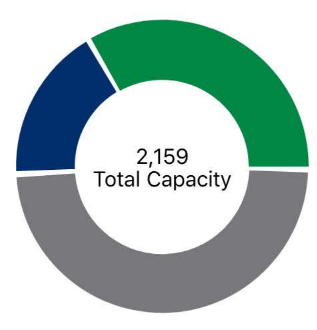

Today's Statewide Ventilator Usage

77.3% Ventilators Available

7.6% Ventilators in Use - COVID

15.1% Ventilators in Use - Non-COVID

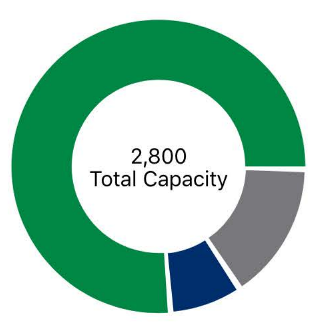

Hospitalizations

Census | Admissions

Statewide COVID-19 Hospital Census

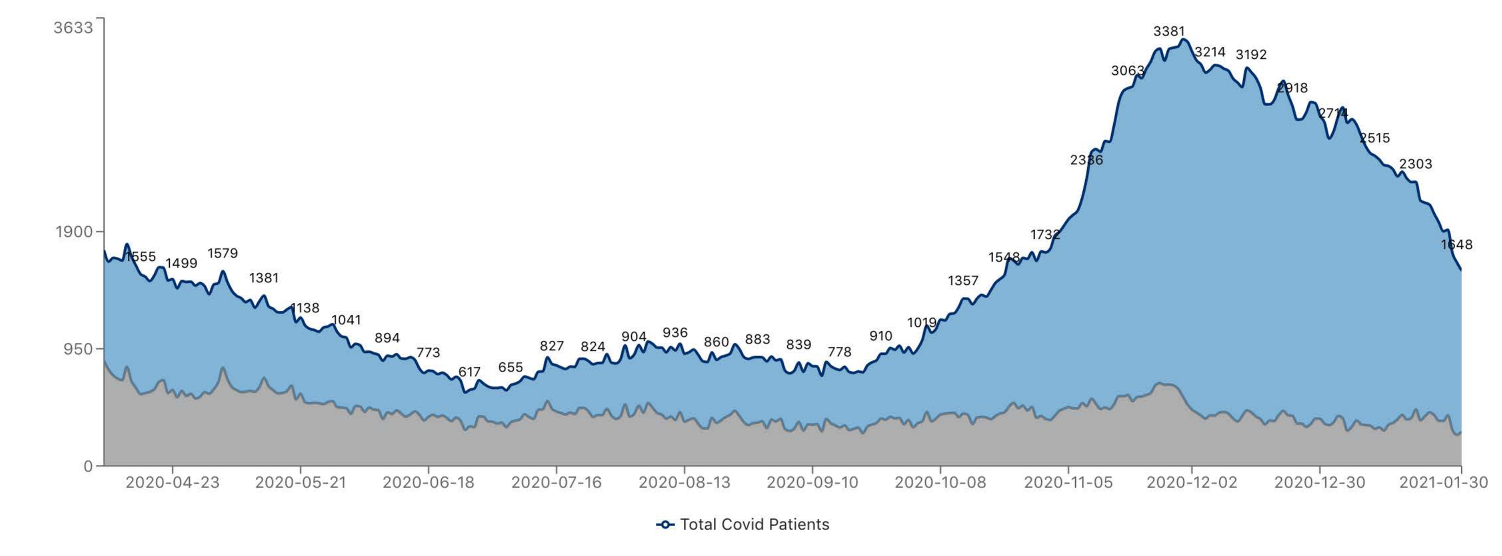

Probable COVID-19 Counts

Total Probable Deaths
376

All data displayed is preliminary and subject to change as more information is reported to ISDH. Tests are displayed by the date the test was performed and deaths are displayed by the date the death occurred.

Expect historical data to change as data is reported to ISDH.

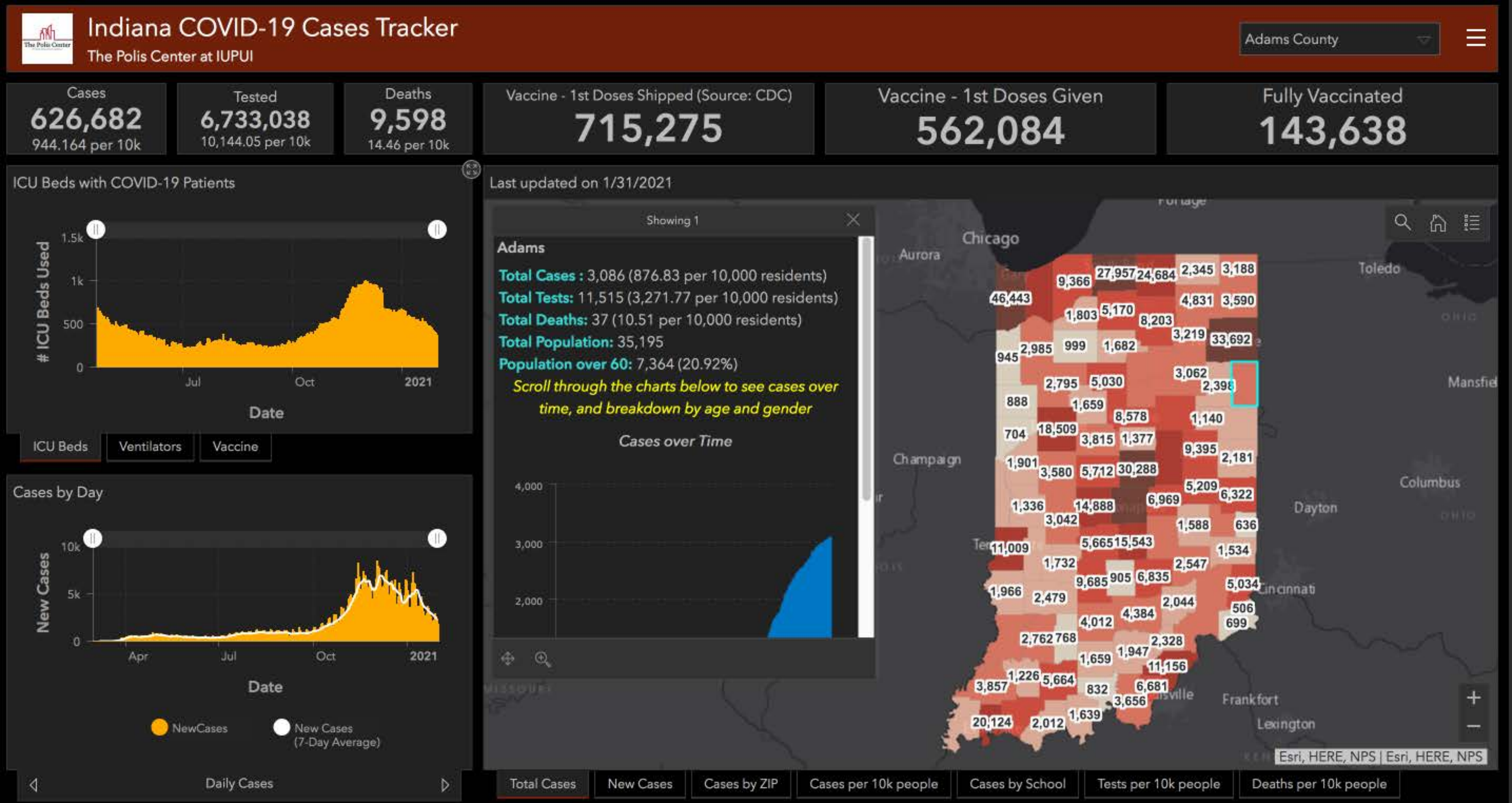
Indiana COVID-19 Cases Tracker
The Polis Center at IUPUI
Adams County
Cases
626,682
944.164 per 10k
Tested
6,733,038
10,144.05 per 10k
Deaths
9,598
14.46 per 10k
Vaccine - 1st Doses Shipped (Source: CDC)
715,275
Vaccine - 1st Doses Given
562,084
Fully Vaccinated
143,638
ICU Beds with COVID-19 Patients
ICU Beds Used
1.5k
1k
500
0
Jul
Oct
2021
Date
ICU Beds
Ventilators
Vaccine
Cases by Day
New Cases
10k
5k
0
Apr
Jul
Oct
2021
Date
NewCases
New Cases (7-Day Average)
Daily Cases
Last updated on 1/31/2021
Showing 1
Adams
Total Cases : 3,086 (876.83 per 10,000 residents)
Total Tests: 11,515 (3,271.77 per 10,000 residents)
Total Deaths: 37 (10.51 per 10,000 residents)
Total Population: 35,195
Population over 60: 7,364 (20.92%)
Scroll through the charts below to see cases over time, and breakdown by age and gender
Cases over Time
4,000
3,000
2,000
Chicago
Aurora
Toledo
Champaign
Columbus
Dayton
Cincinnati
Frankfort
Lexington
Esri, HERE, NPS | Esri, HERE, NPS
Total Cases
New Cases
Cases by ZIP
Cases per 10k people
Cases by School
Tests per 10k people
Deaths per 10k people

Positive Case Analysis

% Positive Analysis - Public Schools

Dashboard showing percent positive analysis by county and corresponding school districts.

View

Long Term Care

Iowa Long Term Care COVID-19 current outbreak information.

View

RMCC Dashboard

View the most current Hospital data by RMCC Region.

View

Test Iowa Assessments

View summary of Test Iowa assessments that have been completed.

View

Serology Testing

View summary of Test Iowa assessments that have been completed.

View

Outcome Analysis Recovered

View summary dashboard of COVID-19 cases resulting in recovery.

View

Outcome Analysis Deaths

View summary dashboard of COVID-19 cases resulting in death.

View

Hospitalization Analysis

Dashboard summary of current hospitalization data.

View

Positive Case Analysis

PCR

Total Tests	Negative Tests	Positive Tests
2,495,359	2,192,505	289,850
**Individuals Tested**	**Individuals Negative**	**Individuals Positive**
1,256,023	986,128	267,557

Antigen

Total Tests	Negative Tests	Positive Tests
1,127,395	1,072,309	54,961
**Individuals Tested**	**Individuals Negative**	**Individuals Positive**
216,688	165,002	51,686

Total

Total Tests	Negative Tests	Positive Tests	% Positive Tests (Past 14 Days)
3,622,754	3,264,814	344,811	6.3%

Total Tests by Day | Negative Tests by Day | Positive Tests | % Positive Tests by Day

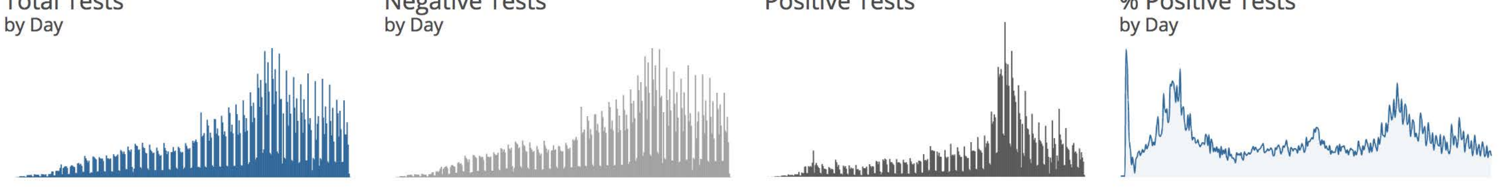

Individuals Tested	Individuals Negative	Individuals Positive	% Indiv. Positive (Past 14 days)
1,472,945	1,151,130	319,243	10.0%

Individuals Tested by Day | Individuals Negative by Day | Individuals Positive by Day | % Individuals Positive by Day

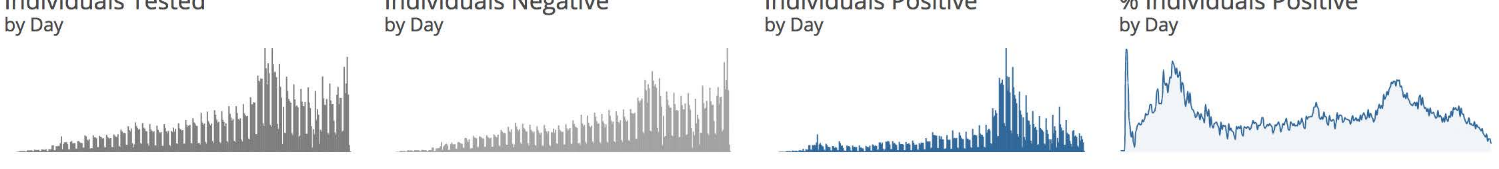

% Positive Cases 14 Day and 7 Day Average

% Positive Cases average is calculated by the sum total of Individuals Positive divided by the sum total of Total Individuals Tested for the past period of days (14 or 7)

Past 14 Day Average	Past 7 Day Average
10.0%	7.6%

Individuals Positive per 100,000 population

% Change in Individual Positive cases per 100,000 population during last 7 days compared to previous 7 days (negative values indicated improving trend)

Individuals Positive per 100,000 population Last 7 Days	% Change last 7 days compared with previous 7 days
184.6	-0.73%

Individual Positive Cases

This interactive dashboard allows you to explore cases by county. Select a county name within the map or table to filter to specific counties.

Positive Cases Last 7 Days

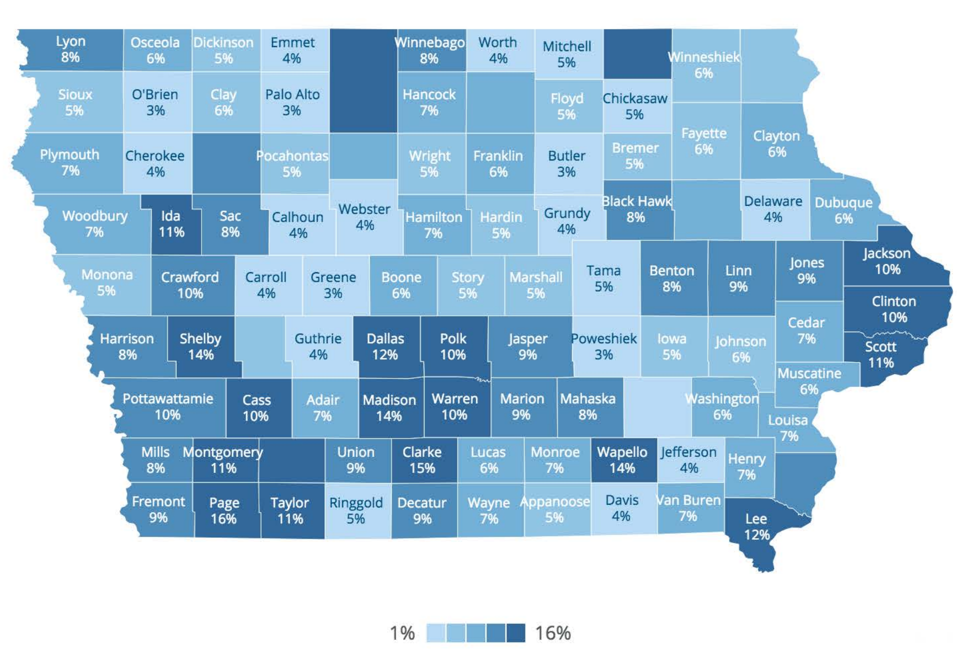

Positive Cases Last 7 days by Age Group

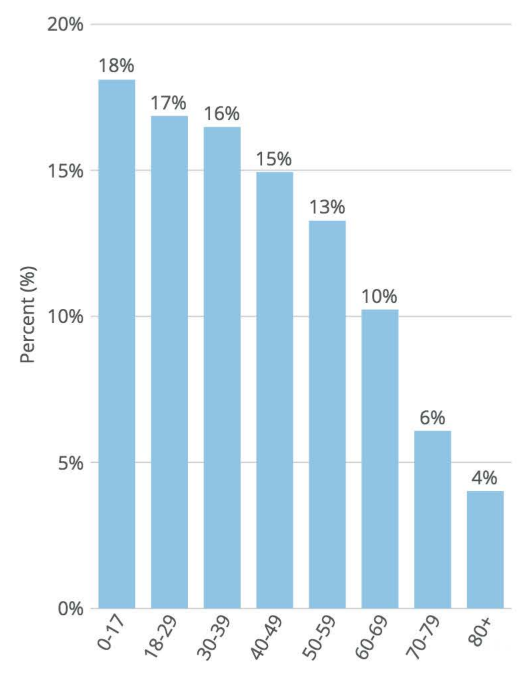

Positive Cases Last 14 Days

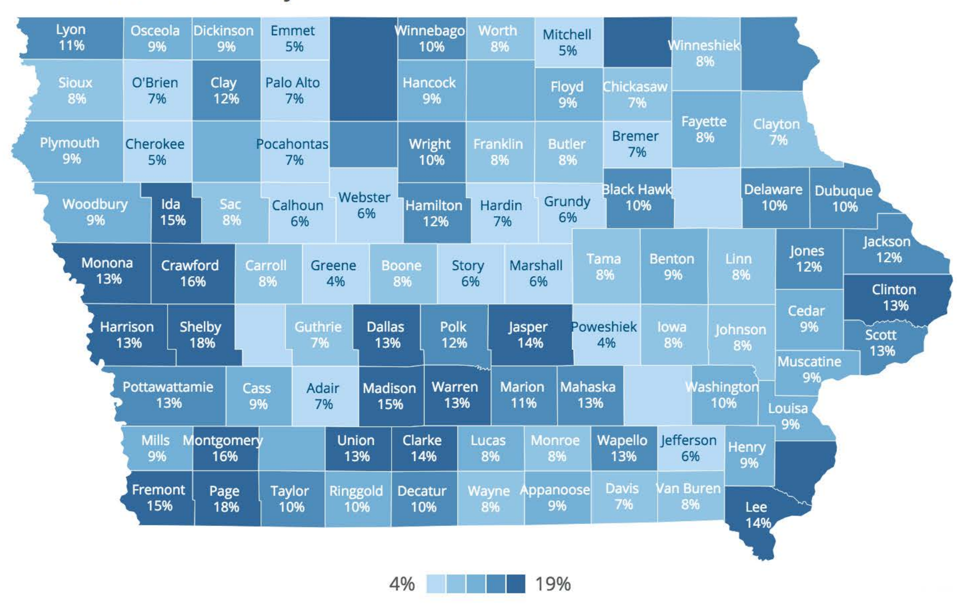

Positive Cases Last 14 days by Age Group

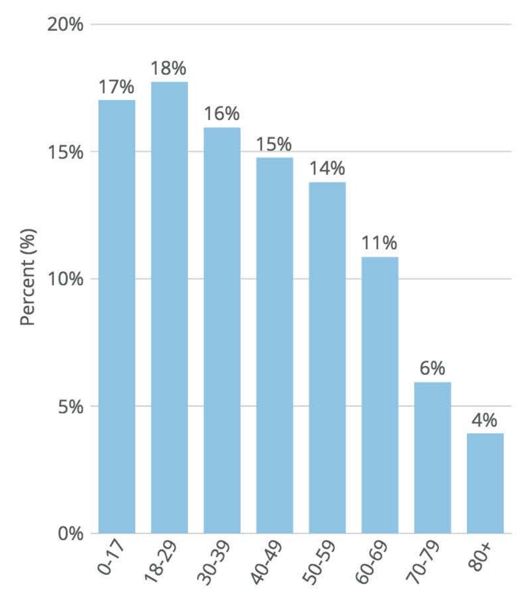

Positive Cases

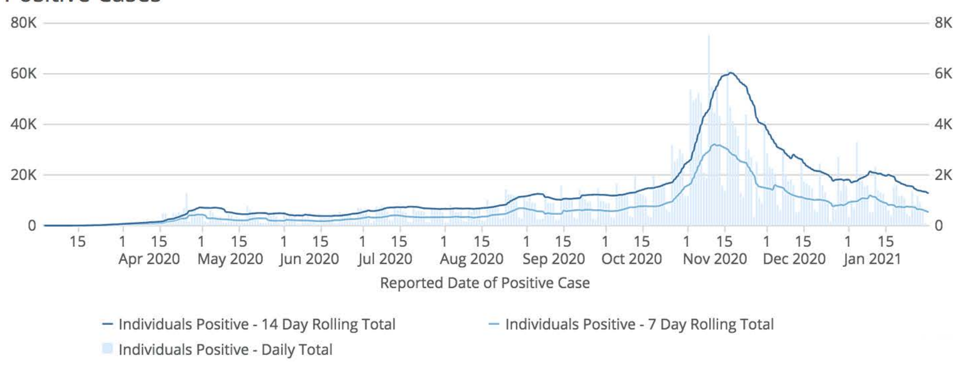

Positive Cases by Sex

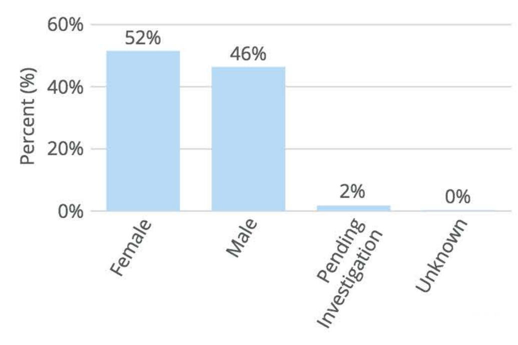

County	Positive Cases	Cases Per 100,000 Population
Polk	47,925	9,837
Linn	18,504	8,191
Scott	16,229	9,366
Black Hawk	14,375	10,857
Woodbury	13,255	12,927
Johnson	12,460	8,237
Dubuque	11,826	12,210
Pottawattamie	9,330	9,975
Dallas	9,326	10,342
Story	9,011	9,185
Cerro Gordo	4,813	11,286
Webster	4,771	13,152
Warren	4,745	9,294
Clinton	4,695	10,093
Sioux	4,628	13,257
Marshall	4,384	10,965
Des Moines	4,041	10,325
Muscatine	4,008	9,336
Buena Vista	4,006	20,157
Wapello	3,589	10,195
[illegible]	[illegible]	[illegible]

Positive Cases by Race

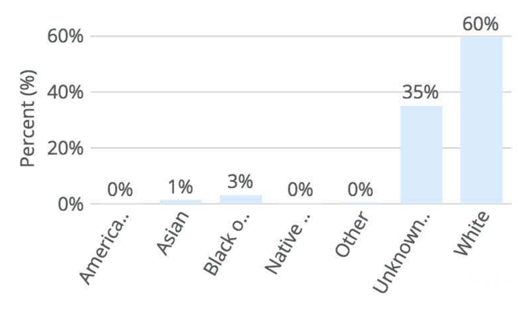

Positive Cases by Ethnicity

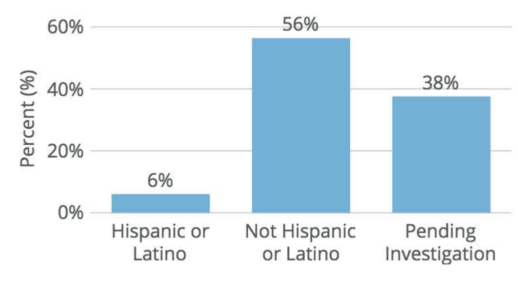

Total Positive Cases by Pre-Existing Condition

Pre-Existing Condition	No Pre-Existing Condition	Pre-Existing Condition Unknown
30,543	71,552	217,148

Positive Cases by Pre-Existing Condition

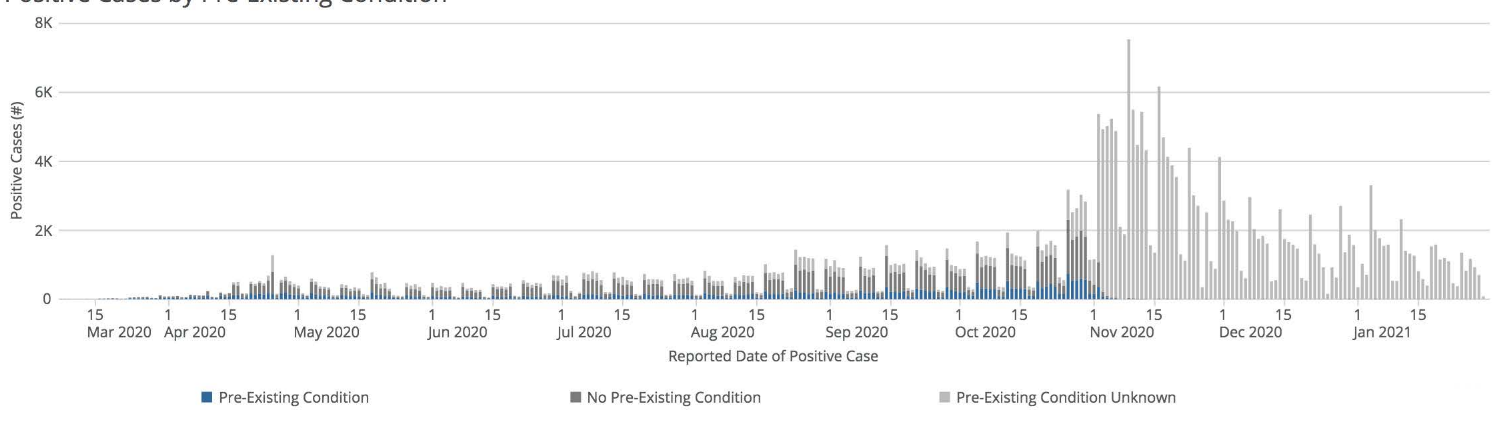

Positive Cases by Pre-Existing Condition

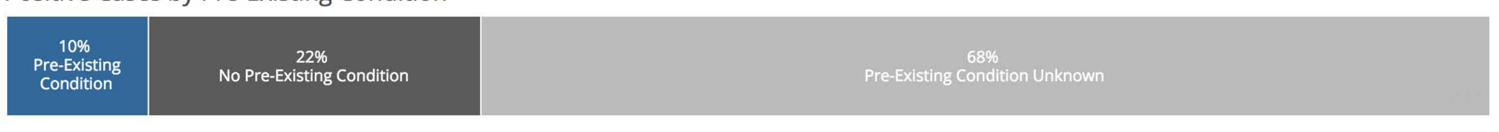

Positive Cases - Pre-Existing Condition by Age Group

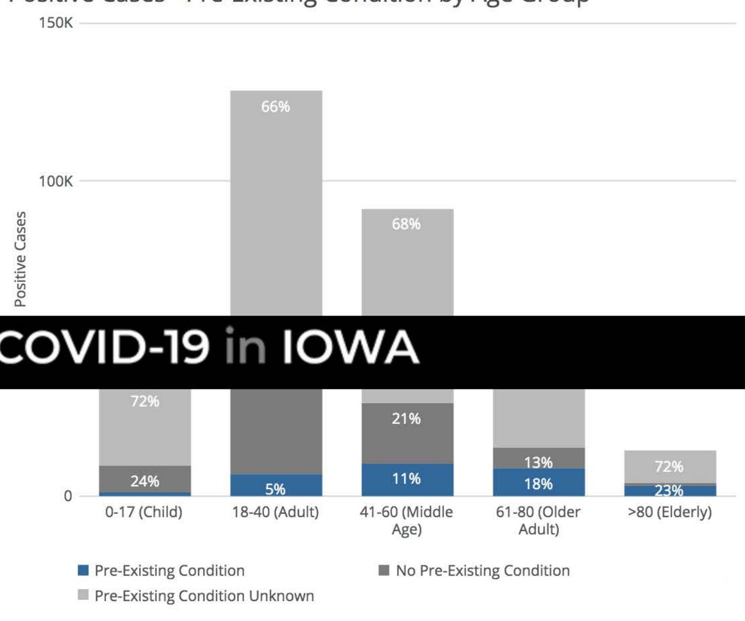

Positive Cases - Pre-Existing Condition by Occupation

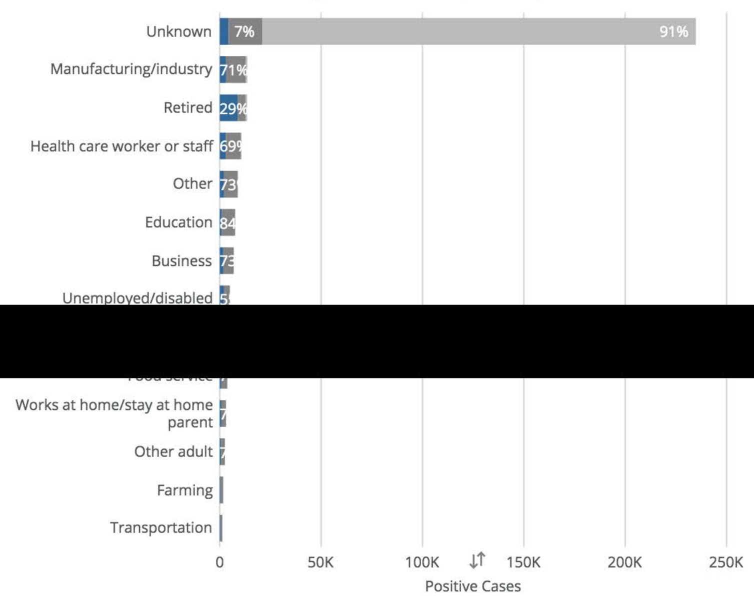

COVID-19 in IOWA

Last updated on: 2021-01-31

COVID-19 data are reported as timely, accurately and completely as we have available. Data are updated as we receive information that is more complete and is subject to change. Positive case count trend line represents 14 day rolling total.

LATEST UPDATE **New Kansas COVID-19 Vaccine Website —** Do you have questions about the COVID-19 vaccine? Find the answers you seek, weekly updates, resources & more on our new website: KansasVaccine.gov

COVID-19 Cases in Kansas

Map Information
The map is updated Monday, Wednesday and Friday by 12:30 p.m. The map contains a variety of data at the state and county level, including the Reopen Kansas Metrics. Click on the gray buttons to access the data. To maximize your experience, use a device with a larger screen, such as a tablet, laptop or desktop. The Map FAQ Document (PDF) includes detailed instructions on how to use and share the data.

Daily Case Summary & Historical Data
The COVID-19 Summary is published Monday, Wednesday and Friday at 12:30 p.m. and includes historical data. The summary may not be published during holiday observances.

COVID-19 Case Definition
As of May 7, 2020, confirmed and probable cases are included in the case totals. From May 7 to August 31, 2020, in accordance with the CDC surveillance case definition at the time, a confirmed case is defined as a person who tested positive by molecular testing for the novel coronavirus (SARS-CoV-2), which causes Coronavirus Disease 2019 (COVID-19). A probable case is defined as a person with: a) presumptive lab evidence (serology) with COVID-19 symptoms or epi-linked to a confirmed case, or b) no lab testing with COVID-19 symptoms and epi-linked to a confirmed case.

As of September 1, 2020, in accordance with an updated CDC surveillance case definition, a confirmed case is defined as a person who tested positive by PCR. A probable case is defined as a person with: a) presumptive lab evidence (antigen) or b) no lab testing but meeting clinical case criteria and is epi-linked (see CDC/CSTE surveillance definition). Cases are based on individual people, not on the number of tests. A person with multiple tests is only counted once.

Location Identification
KDHE will release the names of locations that have five or more cases with symptom-onset dates in the last 14 days every Wednesday. KDHE's policy can be viewed online. Also, please review our Frequently Asked Questions concerning the listing.

COVID-19 Vaccine Data
Data about the COVID-19 vaccine can be found at KansasVaccine.gov.

Kansas Department of Health and Environment

KS COVID-19 Overview | Testing Rates | Hospital Capacity | Cluster Summary
Case Characteristics | Nursing Home Metrics | Hospital Summary | Death Summary
Case Rates | School Gating Metrics | ED Summary | Reopen Kansas Metrics

Kansas Coronavirus Disease 2019 (COVID-19): Overview

COVID-19 Cases	Hospitalizations	Statewide Deaths	Negative Tests
274,685	8,417	3,779	894,038

Data are preliminary and subject to quality improvement and quality assurance validation.

Last updated: 01/29/2021 at 9:00 AM. There were 2,168 new cases, 61 new deaths, and 149 new hospitalizations reported since Wednesday, 01/27/2021.

Select county from map below to update charts to county specific details.

State Map of COVID-19 Cases by County of Residence

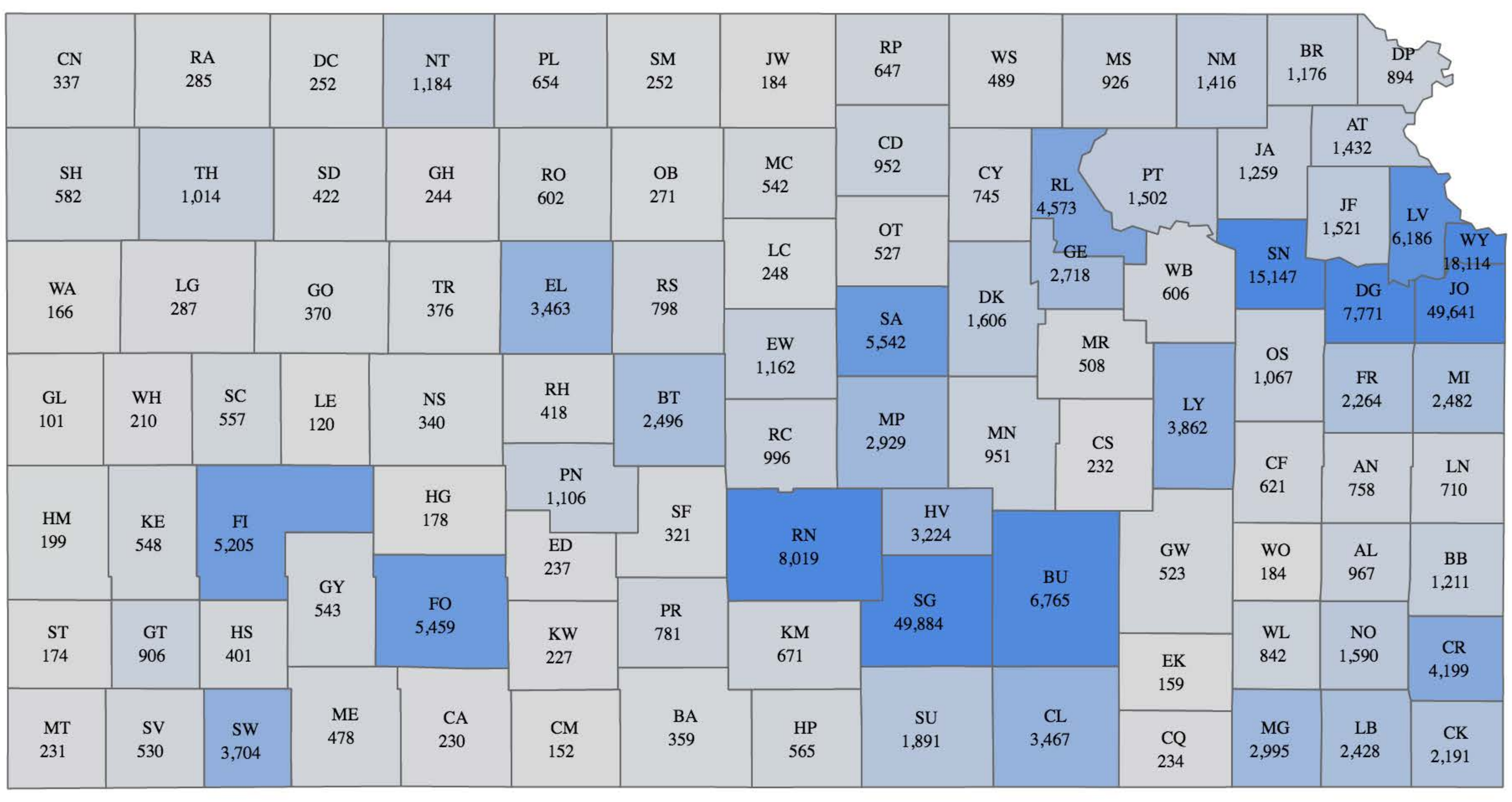

Statewide COVID-19 Cases by Symptom Onset Date

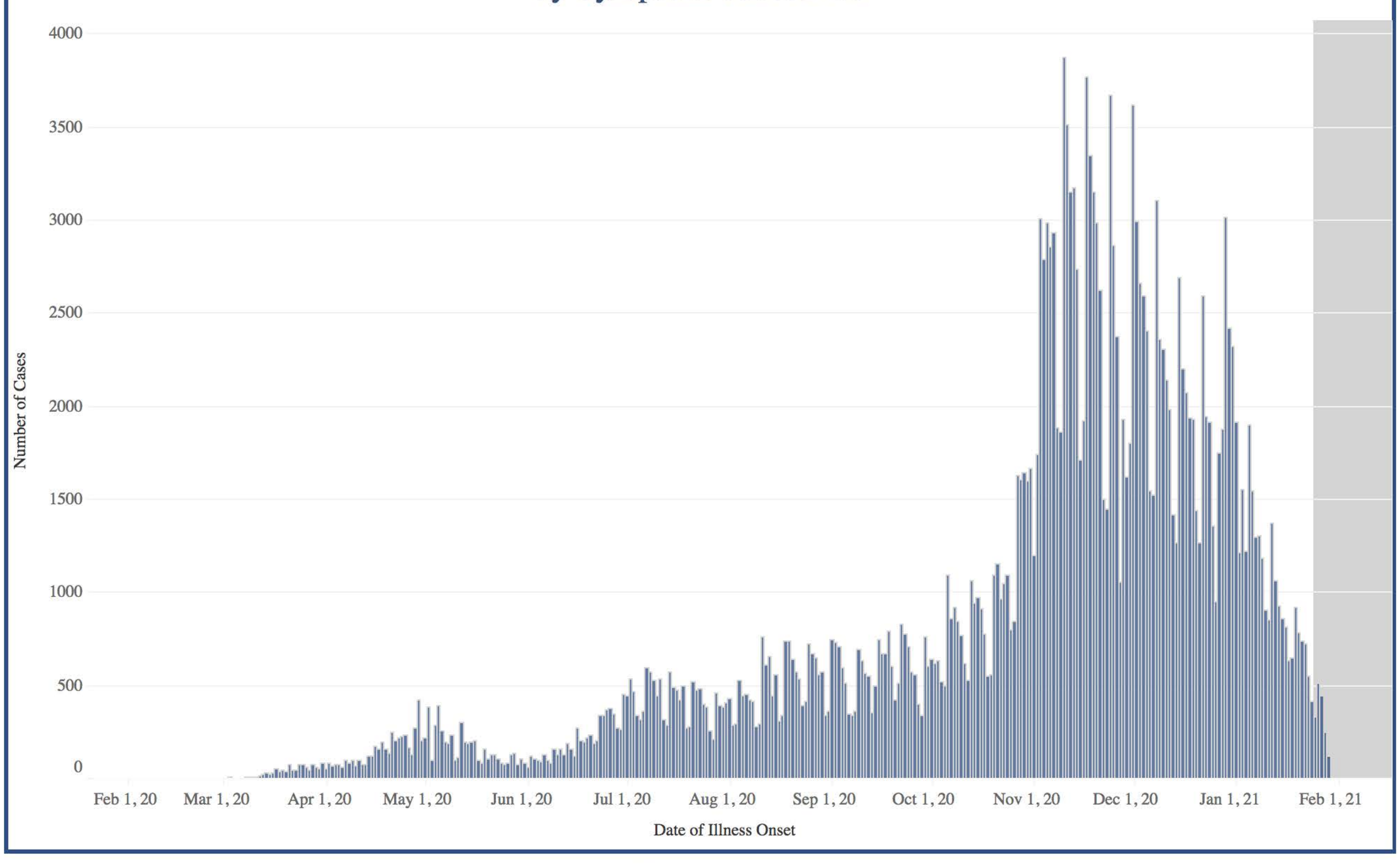

IMPORTANT NOTE: If symptom onset date is missing, the date of specimen collection is used as a proxy for the onset date.

Daily Counts of New Cases and Total Cases Reported by Date Diagnosed

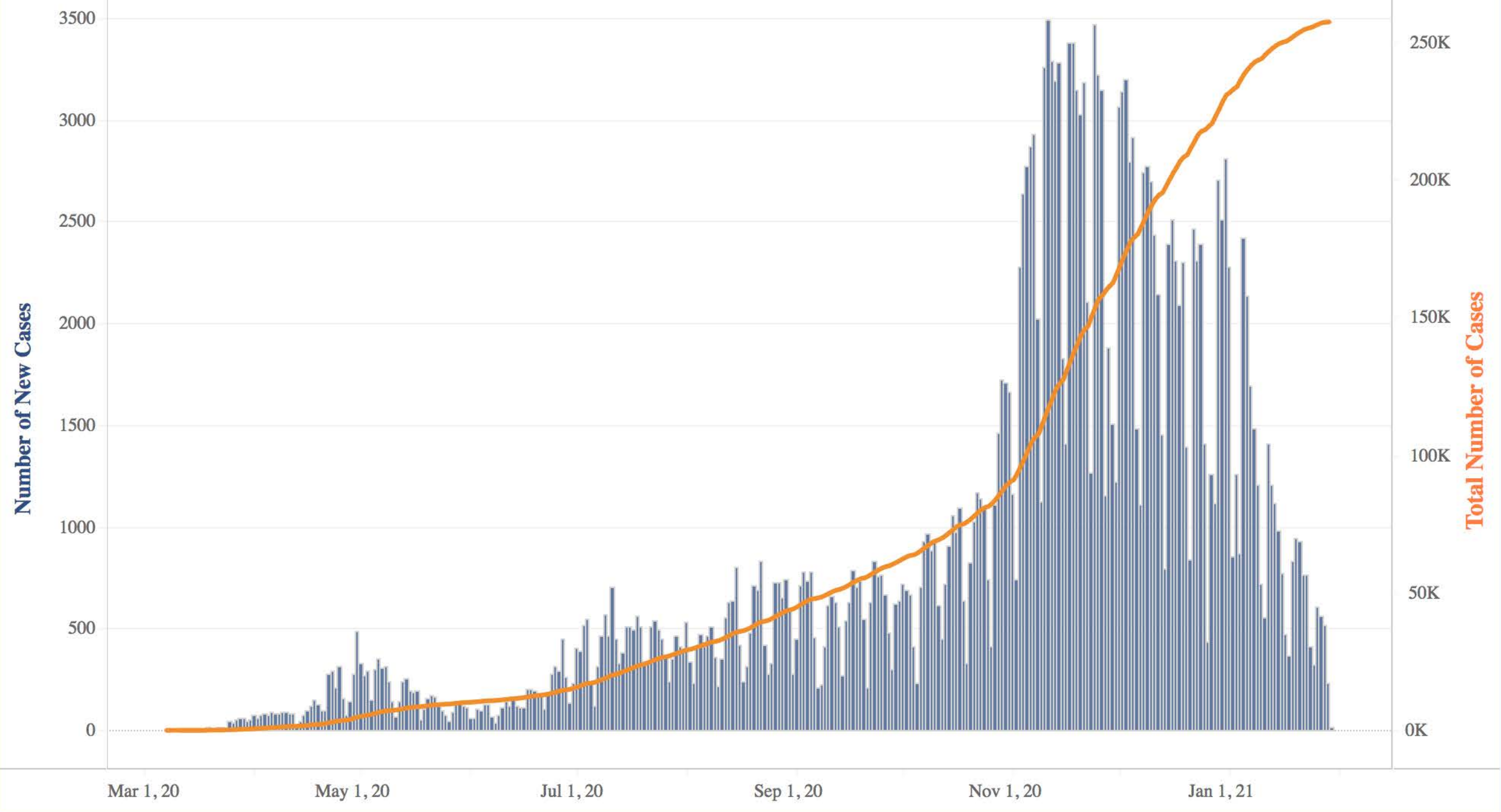

Cases by County

+ a b l e a u

Select Language

Kansas Department of Health and Environment

KS COVID-19 Overview | Testing Rates | Hospital Capacity | Cluster Summary | Case Characteristics | Nursing Home Metrics | Hospital Summary | Death Summary | Case Rates | School Gating Metrics | ED Summary | Reopen Kansas Metrics

Kansas COVID-19: Testing Rates

Overall Testing	Negative Individuals	Monthly Percent Positive	Monthly Testing Rate
1,168,723 People<br>2,194,854 PCR Tests	894,038	8.8%	40,117 per 100,000

IMPORTANT NOTE: The number of people tested represents any Kansas resident that was tested for the SARS-CoV-2 virus by a diagnostic test (e.g., PCR, antigen). Data as reported by laboratories into the KDHE electronic disease surveillance system (EpiTrax). Data are preliminary and subject to quality improvement and quality assurance validation. As of July 27, 2020 the definition of number of people tested was updated to include only diagnostic testing. Monthly testing rate is per 100,000 population.

Positive PCR Tests by Performing Lab

Laboratory	Number of Cases	% of Cases
State Lab (KHEL)	36,902	16.4%
Private Labs	188,618	83.6%
Total	225,520	100.0%

Cases by Lab Type

Test Type	Percent of Cases
PCR	83.2%
Antigen	16.7%
Serology*	0.1%

*Cases with serology results are required to have either an epidemiologic link to a case or signs and symptoms compatible with COVID-19.

Race Testing Rates per 1,000

Race	Persons Tested	Rate per 1,000
White	731,894	287.61
Black or African American	54,976	252.18
Asian	21,857	203.67
American Indian or Alaska Native	7,357	113.53
Other Race	39,322	427.12
Not Reported/Missing	313,317	

Ethnicity Testing Rates per 1,000

Ethnicity	People Tested	Rate per 1,0..
Hispanic or Latino	86,979	249.62
Not Hispanic or Latino	626,445	244.41
Unknown or Missing	455,299	

Weekly Testing Rate by County and Age Group (All PCR Tests) (Per 1,000 Population)

Filter by Age Groups: (All)

Filter by County: All (Kansas)

Week of Lab Test	0-4 years	5-10 years	11-13 years	14-17 years	18-24 years	25-34 years	35-44 years	45-54 years	55-64 years	65-74 years	75-84 years	85+ years
01/17/2021	9.2	12.9	13.8	22.4	48.0	35.8	36.5	33.8	30.4	26.4	31.9	73.7
01/10/2021	9.8	13.9	16.1	26.5	47.8	39.6	39.9	38.8	33.5	29.7	35.3	78.3
01/03/2021	9.8	13.3	16.9	25.7	50.4	44.6	43.8	41.6	38.4	34.3	39.3	81.2
12/27/2020	7.4	8.5	10.0	17.9	35.0	35.6	34.5	32.3	31.8	29.6	36.6	83.8
12/20/2020	8.2	10.4	13.0	19.4	35.0	38.0	38.0	35.2	33.5	30.9	37.0	80.9
12/13/2020	9.8	12.9	14.0	23.3	39.3	40.6	40.2	39.0	36.0	33.8	41.2	97.5
12/06/2020	9.5	11.3	13.4	22.5	39.3	39.6	38.8	37.7	34.5	30.7	39.1	87.4
11/29/2020	9.2	9.9	12.2	20.9	38.2	39.0	38.0	35.7	32.9	29.1	38.2	88.1
11/22/2020	9.2	8.7	9.9	17.9	37.4	36.3	34.9	32.5	29.4	26.4	34.6	77.8
11/15/2020	12.8	13.1	16.2	24.7	48.5	42.9	41.2	38.1	34.0	29.4	35.7	78.5
11/08/2020	10.2	11.3	14.0	22.0	38.5	35.0	34.8	32.7	29.7	26.9	33.0	64.0
11/01/2020	8.2	9.3	11.0	17.4	33.1	29.9	28.1	26.8	25.1	22.8	28.4	56.9
10/25/2020	6.3	6.6	7.7	13.6	27.1	26.3	25.1	23.7	22.5	20.1	24.6	51.9
10/18/2020	6.1	6.3	7.2	12.1	24.8	24.2	22.6	22.0	21.1	19.6	24.7	50.7
10/11/2020	6.5	6.9	7.2	14.3	25.9	24.3	23.1	21.6	20.6	19.7	24.9	50.3
10/04/2020	6.8	7.4	7.6	14.8	27.6	25.6	23.7	22.3	20.9	20.0	27.2	52.0
09/27/2020	5.8	6.7	8.5	14.3	25.5	23.0	22.1	20.8	19.5	18.7	24.2	46.1
09/20/2020	5.9	7.5	8.8	15.4	28.3	24.8	23.4	21.4	20.2	18.5	23.5	43.0
09/13/2020	4.9	5.8	7.6	12.4	27.1	24.1	23.0	20.8	19.5	17.8	22.0	39.3
09/06/2020	3.9	3.9	5.2	9.2	22.9	18.8	17.6	17.1	15.8	14.9	18.3	33.7
08/30/2020	4.2	3.7	4.9	8.2	27.7	18.3	16.0	15.4	14.1	13.6	17.1	30.6
08/23/2020	4.3	3.4	4.2	9.1	33.8	17.6	15.4	15.0	14.0	14.1	16.9	27.2
08/16/2020	4.0	3.2	4.1	8.4	43.4	20.0	17.3	15.8	14.9	14.7	17.6	27.7
08/09/2020	4.3	3.2	3.4	7.5	30.7	17.8	15.8	15.0	13.1	13.9	15.6	23.3
08/02/2020	4.0	3.0	2.9	6.5	18.7	16.1	14.1	13.6	12.9	13.7	15.5	21.4
07/26/2020	4.2	3.1	3.9	7.6	16.0	16.3	14.9	14.7	13.3	14.0	16.6	26.6
[illegible]	[illegible]	[illegible]	[illegible]	[illegible]	[illegible]	[illegible]	[illegible]	[illegible]	[illegible]	[illegible]	[illegible]	[illegible]

Kansas Monthly Testing Rates

	New People Tested	Test Rate per 100,000
February 2020	1	0
March 2020	6,604	227
April 2020	27,742	952
May 2020	64,132	2,201
June 2020	80,686	2,770
July 2020	122,622	4,209
August 2020	121,830	4,182
September 2..	115,184	3,954
October 2020	129,199	4,435
November 20..	185,339	6,362
December 20..	180,956	6,211
January 2021	123,386	4,235

Kansas Monthly Percent Positivity

Month, Year of Lab ..	Percent Positivity
February 2020	0.0%
March 2020	7.3%
April 2020	13.6%
May 2020	6.8%
June 2020	4.9%
July 2020	7.8%
August 2020	7.8%
September 2020	6.7%
October 2020	8.6%
November 2020	16.7%
December 2020	11.5%
January 2021	8.8%

Excluded from the Kansas Monthly Testing Rates table are people with a missing laboratory test date.

Total laboratory tests (the denominator) are the number of PCR tests performed, not the number of individuals tested (i.e. unit of measurement for percent positivity is the test, not a person). For each individual, only the first positive test result is included in the numerator and the denominator (multiple positive tests for a single person will not appear as multiple COVID-19 cases). All negative PCR tests are included in the denominator.

Specify County: All (Kansas)

Percent Positive and Number of Negative and Positive COVID-19 Molecular Assay Testing (e.g. PCR) Performed by Laboratory Test Date

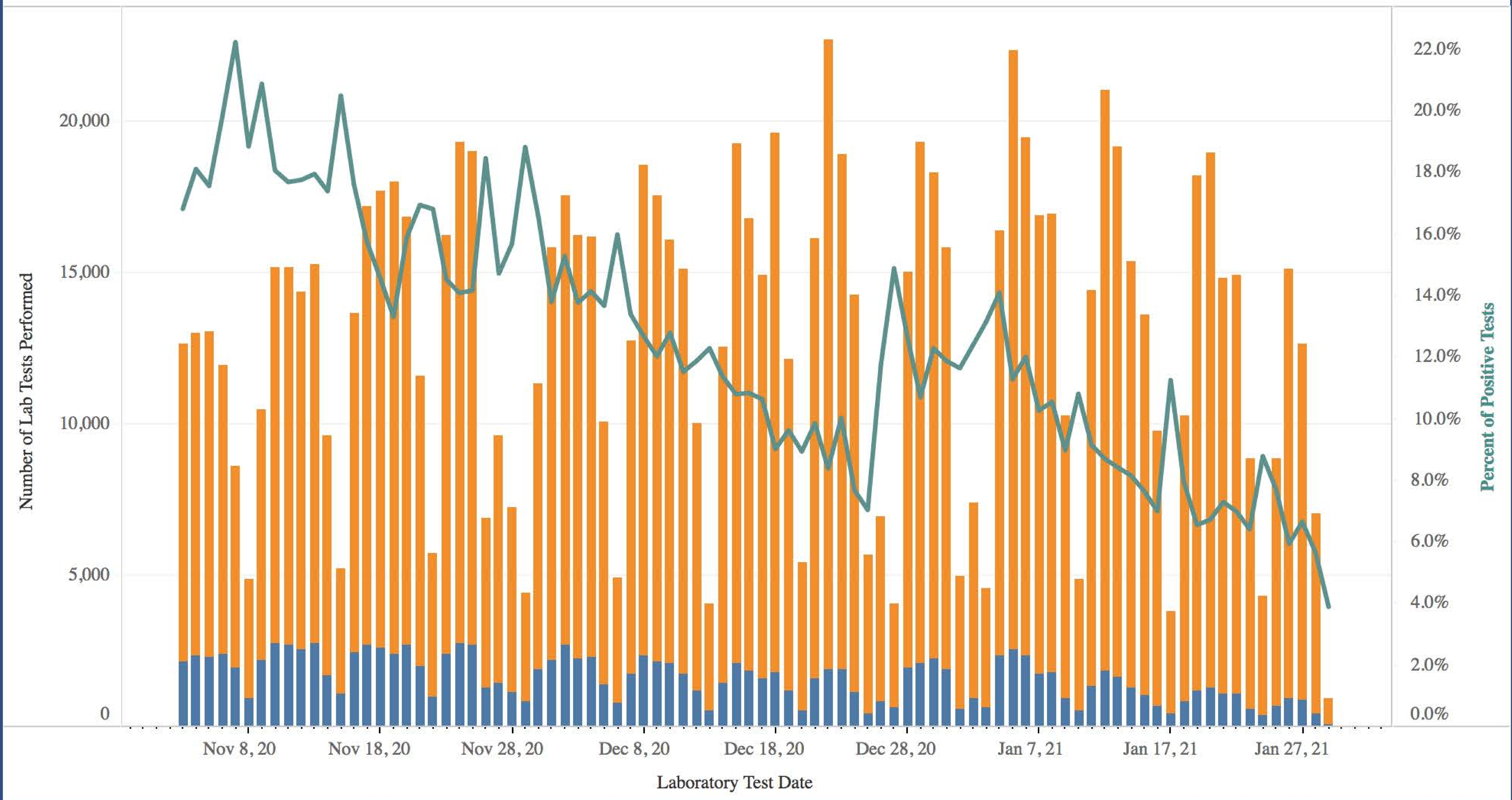

+ableau

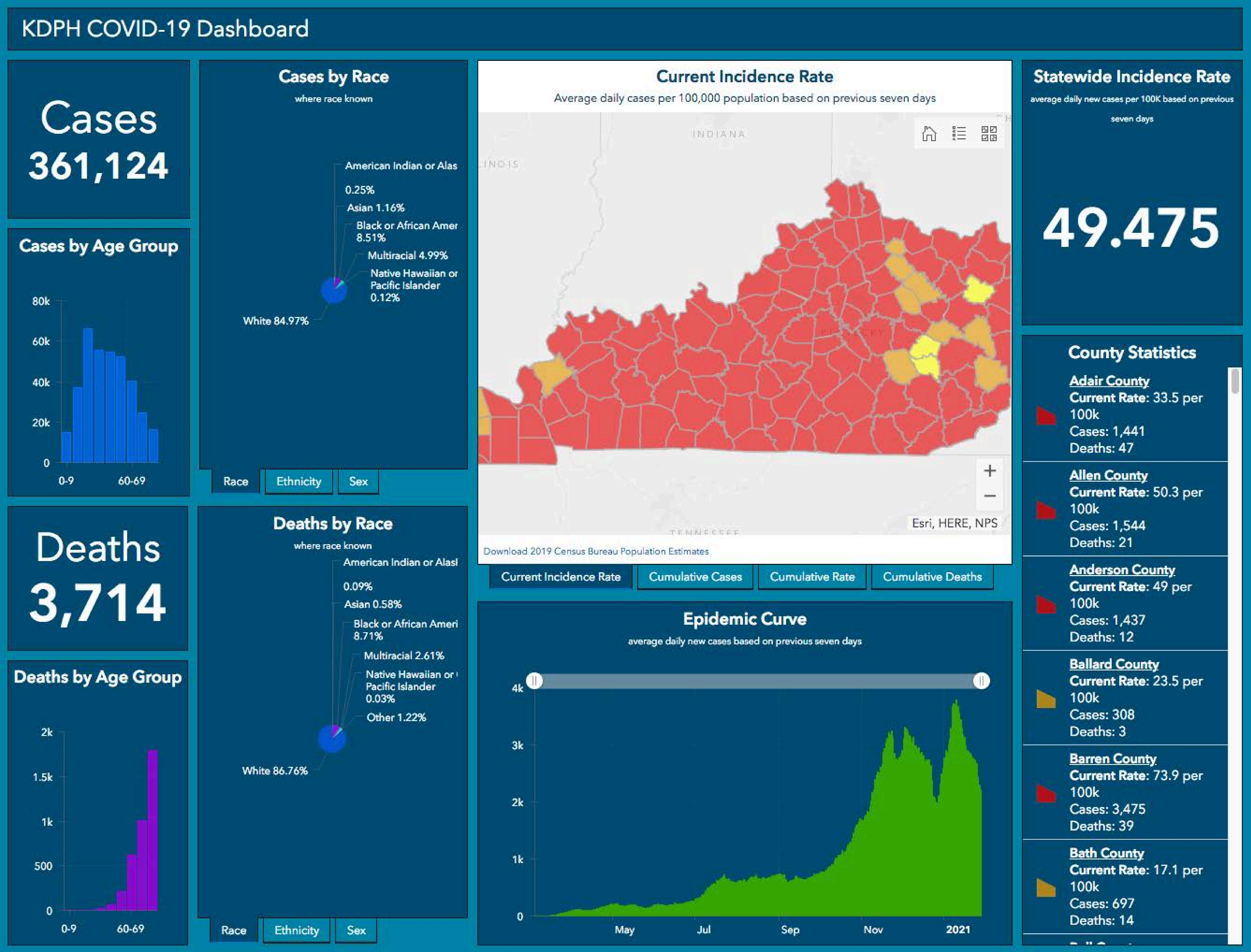
KDPH COVID-19 Dashboard
Cases
361,124
Cases by Age Group
80k
60k
40k
20k
0
0-9
60-69
Deaths
3,714
Deaths by Age Group
2k
1.5k
1k
500
0
0-9
60-69
Cases by Race
where race known
American Indian or Alas
0.25%
Asian 1.16%
Black or African Amer
8.51%
Multiracial 4.99%
Native Hawaiian or
Pacific Islander
0.12%
White 84.97%
Race
Ethnicity
Sex
Deaths by Race
where race known
American Indian or Alasl
0.09%
Asian 0.58%
Black or African Ameri
8.71%
Multiracial 2.61%
Native Hawaiian or
Pacific Islander
0.03%
Other 1.22%
White 86.76%
Race
Ethnicity
Sex
Current Incidence Rate
Average daily cases per 100,000 population based on previous seven days
INDIANA
Esri, HERE, NPS
Download 2019 Census Bureau Population Estimates
Current Incidence Rate
Cumulative Cases
Cumulative Rate
Cumulative Deaths
Epidemic Curve
average daily new cases based on previous seven days
4k
3k
2k
1k
0
May
Jul
Sep
Nov
2021
Statewide Incidence Rate
average daily new cases per 100K based on previous seven days
49.475
County Statistics
Adair County
Current Rate: 33.5 per 100k
Cases: 1,441
Deaths: 47
Allen County
Current Rate: 50.3 per 100k
Cases: 1,544
Deaths: 21
Anderson County
Current Rate: 49 per 100k
Cases: 1,437
Deaths: 12
Ballard County
Current Rate: 23.5 per 100k
Cases: 308
Deaths: 3
Barren County
Current Rate: 73.9 per 100k
Cases: 3,475
Deaths: 39
Bath County
Current Rate: 17.1 per 100k
Cases: 697
Deaths: 14

Looking For County-Level Data On The Coronavirus? Here's Our Kentucky COVID-19 Tracker

By Alexandra Kanik

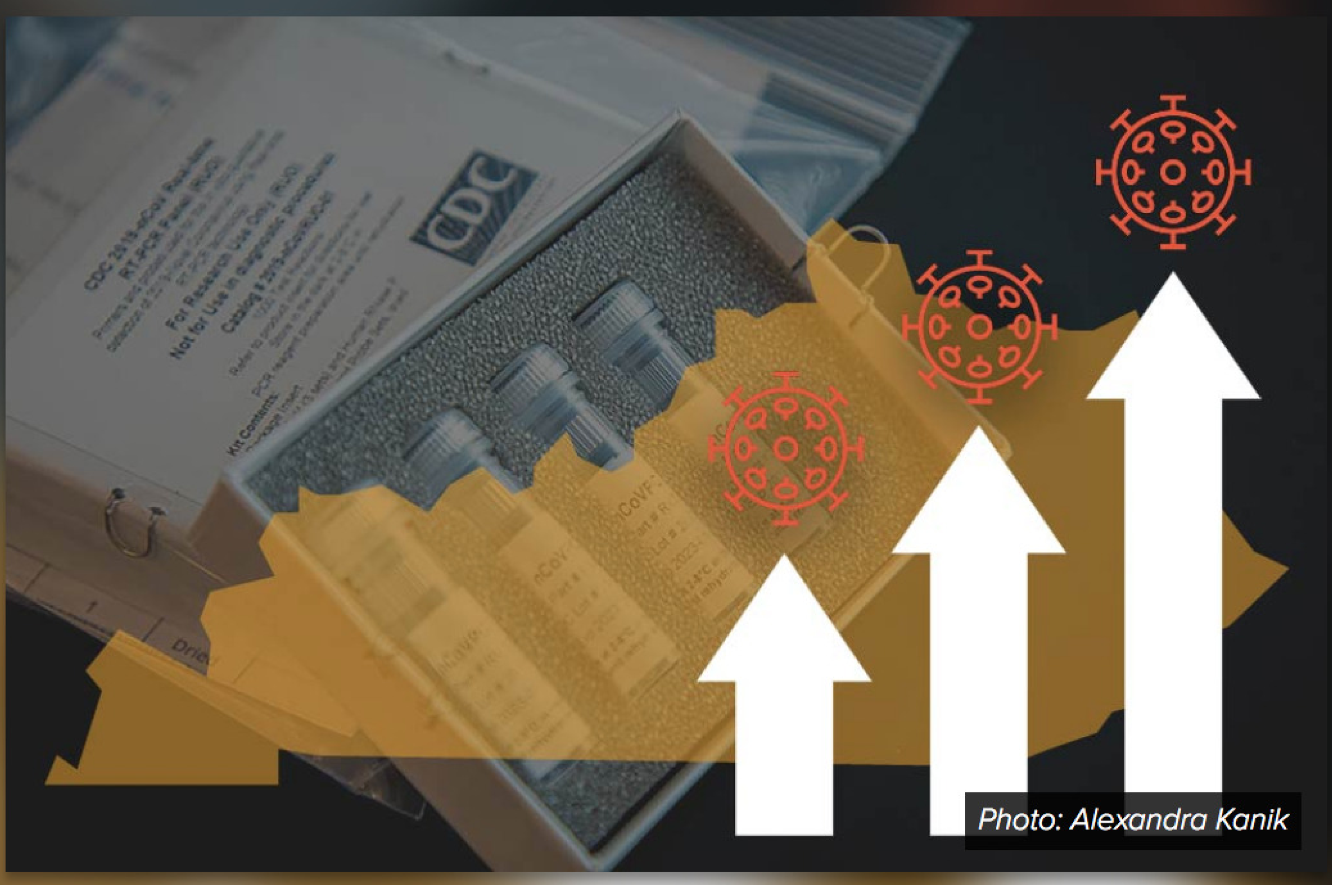

HEALTH June 29, 2020

Kentucky COVID-19 Tracker

This dashboard provides a localized look at COVID-19-related cases and deaths in your county, as well as a risk assessment for your county's population.

Data last updated January 30, 2021.

Use the dropdown below to display more information.

Select a county...

Total cases

There have been **365,016 cases** of COVID-19 in Kentucky. The population of this region is 4,440,204. That's 8221 cases per 100,000 people.

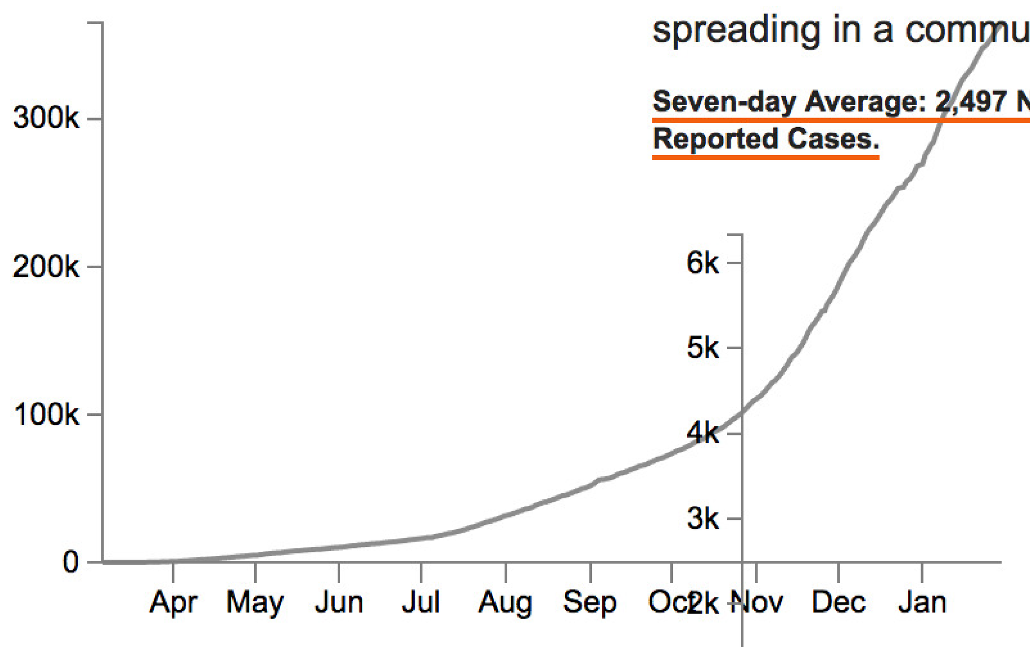

Newly reported cases

There were **2,898 newly reported cases** of COVID-19 on January 30, 2021 in Kentucky. Over time, new cases indicate how fast the virus is spreading in a community.

Seven-day Average: 2,497 Newly Reported Cases.

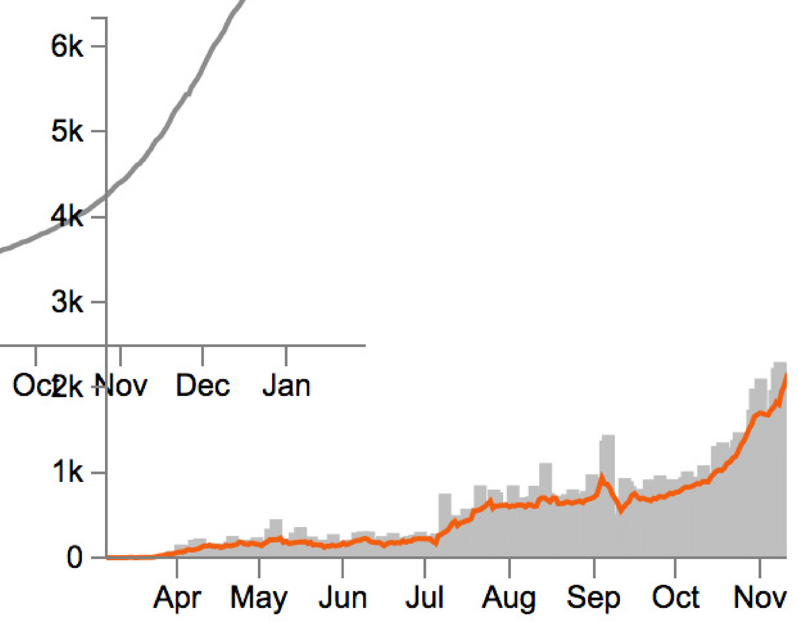

Total deaths

There have been **3,967 reported deaths** related to COVID-19 in Kentucky.

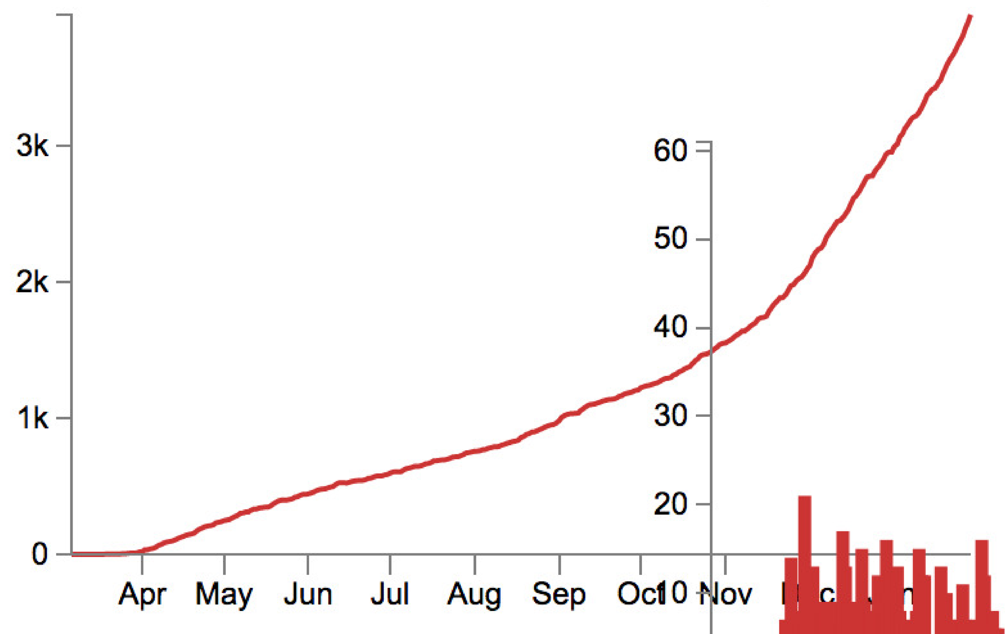

Newly reported deaths

There were **57 new deaths** related to COVID-19 reported on January 30, 2021 in Kentucky.

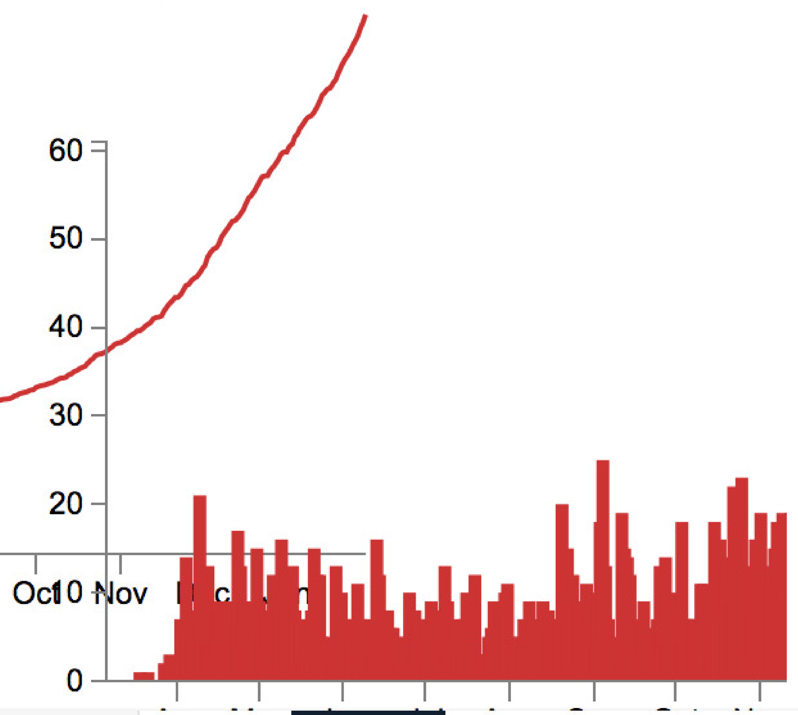

Disclaimer & Data Sources

Age data provided by the 2018 5 year American Community Survey. Disease data are from 2014 and are provided by the Institute for Health Metrics and Evaluation's Global Health Data Exchange

As of April 27, 2020, case and death data for Kentucky counties are provided by the New York Times county dataset. Read more about the data and potential caveats here.

The data presented here only includes cases in which a person has tested positive for COVID-19, but it may be lower than the actual number of infected people in a community due to a lack of testing.

As counties report cases when test results come in, some of the increases in counties' cases in one day could be due to many pending results coming in rather than many people coming down with symptoms in one day.

Credits

This dashboard was created by Allie Kanik at Louisville Public Media, Lisa Pickoff-White at KQED, Emily Zentner at CapRadio, and Dana Amihere at KPCC.

LOUISIANA DEPARTMENT OF HEALTH

Filtered to: Louisiana

Louisiana Coronavirus (COVID-19) Information

New Cases*	New Deaths*	New Tests*
3,355	58	48,745

Total Cases	Total Deaths	Total Tests
400,626	8,859	5,315,681

349,697 Confirmed	50,929 Probable	8,291 Confirmed	568 Probable	4,956,519 Molecular	359,162 Antigen

COVID Patients 1,416 in Hospitals*	COVID Patients 199 on Ventilators*	Presumed Recovered 344,321 as of January 25th*

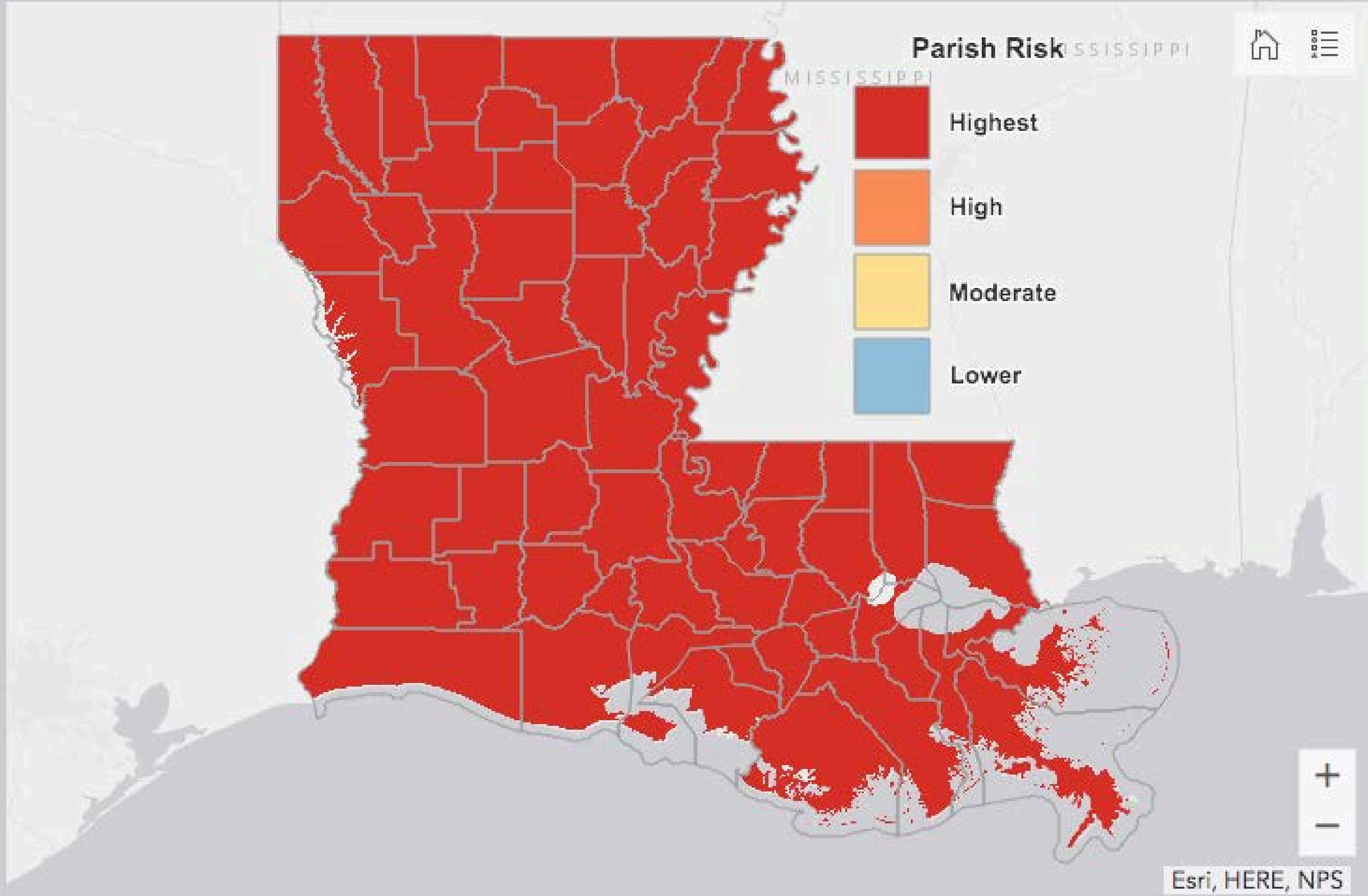

Community Risk by Parish

Click the < > arrows to navigate tabs containing other information and downloadable data

*Statewide only indicator

This dashboard is updated daily by noon, except on Saturday.

Coronavirus Disease 2019 (COVID-19)

- COVID-19 Homepage
- Maine Data
- General Information
- Contact Tracing
- Travelers
- Healthcare Providers
- Long Term Care Facilities and Congregate Living
- Communities, Schools, and Workplaces

EPI Information

- A-Z Index of Epidemiology Diseases
- Contact Us
- Disease Reporting
- Request for Data

211 Get Connected. Get Answers. Social services help and information about COVID-19 in Maine, call 211, email info@211maine.org, text your ZIP code to 898-211, or if out-of-state call 1-877-463-6207.

COVID-19: Maine Data

Maine CDC updates data daily by 9:30 AM with COVID-19 case investigation data included as of 11:59 PM the previous day. All data are preliminary and may change as Maine CDC investigates cases. For more information about the data, please see the "Read Details About the Data" section below.

Prior to November 25th, Maine CDC attempted multiple follow-up calls with every identified case of COVID-19 in Maine in order to assess whether their isolation period was completed. Since that date, Maine CDC has not conducted routine follow-up calls with every identified case, so the count of people who have completed isolations is no longer updated.

Starting December 5th, Maine CDC has focused its efforts to follow up with cases of COVID-19 on cases who are under the age of 19, over the age of 64, or who meet certain other criteria (including being hospitalized, identified as a minority, identified as having a disability, being a health care worker or first responder, living or working in congregate settings, or associated with a school or child care facility). Depending on available resources and case levels, Maine CDC may follow up with other cases who do not meet those criteria.

Download csv files with the most recent cumulative case data by zip code, county, age, sex, race, and ethnicity.

Download a csv file with historical case data by county and date.

Download a csv file with syndromic surveillance gate metrics by date.

View Maine's complete COVID-19 vaccination dashboard.

COVID-19 Case Trends

Daily lab results | New cases by date | Cases by age trend | Case rates by county | COVID-19 data by race | Maine v. other states

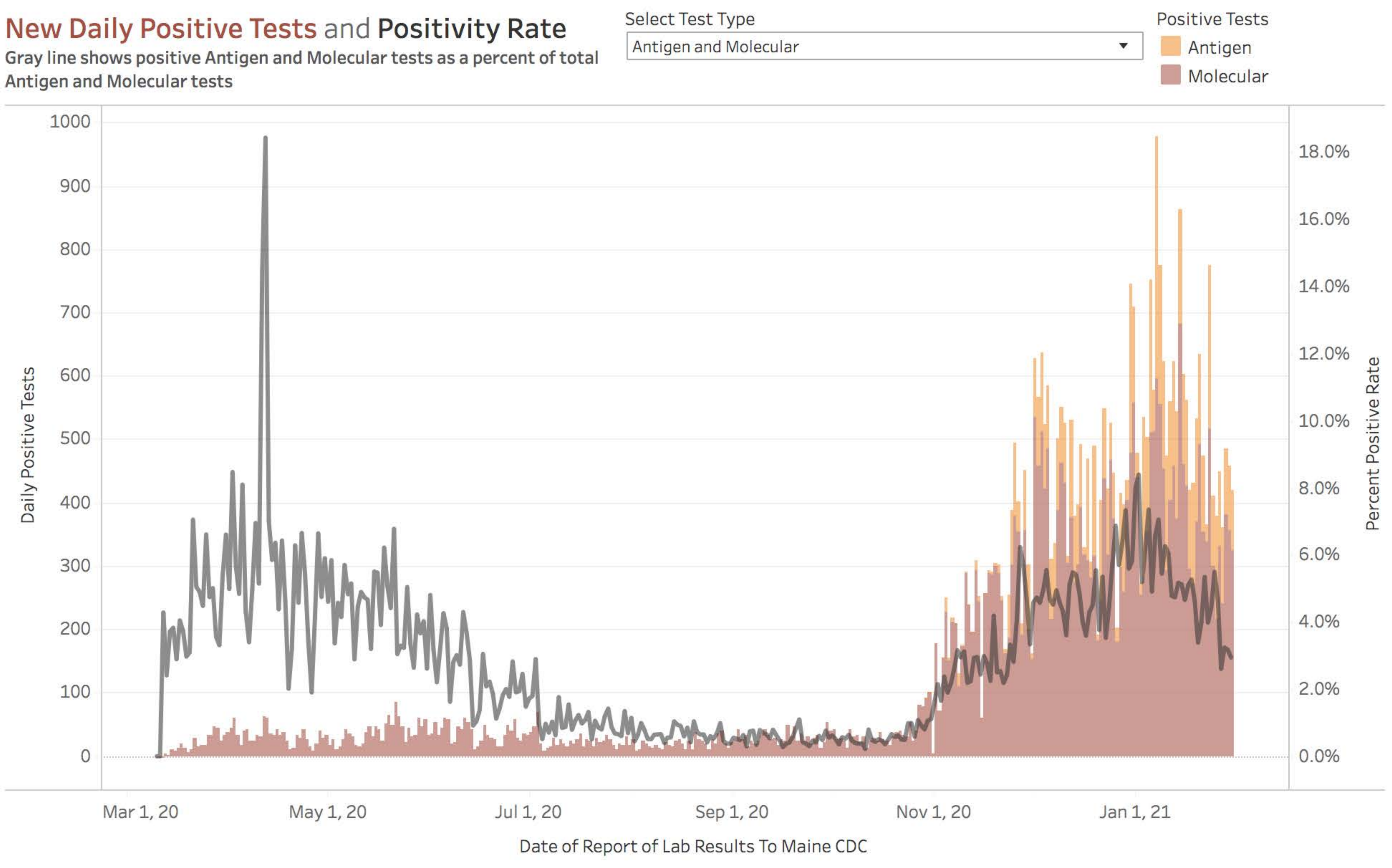

Undo | Redo | Revert | Refresh | Pause | Share | Download | Full Screen

Download csv files with the most recent cumulative case data by zip code, county, age, sex, race, and ethnicity.

Download a csv file with historical case data by county and date.

Cumulative COVID-19 Cases by County

Export table to PDF

Cumulative COVID-19 Cases by County

Table updated: January 31, 2021
Data through: Saturday, January 30, 2021 11:59 pm

Patient County	Cases	Confirmed cases	Probable cases	Deaths	Hospitalizations	Completed Isolations	Case rate (per 10K people)
**Statewide**	**39,324**	**31,689**	**7,635**	**590**	**1,390**	**12,409**	**293.8**
Cumberland	11,197	9,102	2,095	156	372	4,188	381.4
York	8,429	7,138	1,291	100	285	2,561	408.7
Androscoggin	4,313	3,334	979	55	141	1,480	400.5
Penobscot	3,420	2,707	713	63	166	904	226.3
Kennebec	3,113	2,292	821	43	107	880	255.0
Oxford	1,908	1,485	423	35	53	389	331.1
Aroostook	1,141	909	232	39	56	177	170.0
Somerset	1,089	885	204	25	51	415	215.3
Hancock	779	653	126	30	37	224	142.1
Sagadahoc	776	607	169	1	16	176	217.8
Franklin	771	625	146	8	16	203	257.9
Washington	622	399	223	10	23	179	197.5
Knox	578	547	31	4	18	206	145.3
Waldo	512	449	63	18	23	212	129.0
Lincoln	471	415	56	2	15	161	137.1
Piscataquis	203	140	63	1	11	54	120.8
Unknown	2	2	0	0	0	0	

Download a csv file with the most recent cumulative case data by county.

Download a csv file with historical case data by county and date.

Maine COVID-19 Reopening Gating Metrics

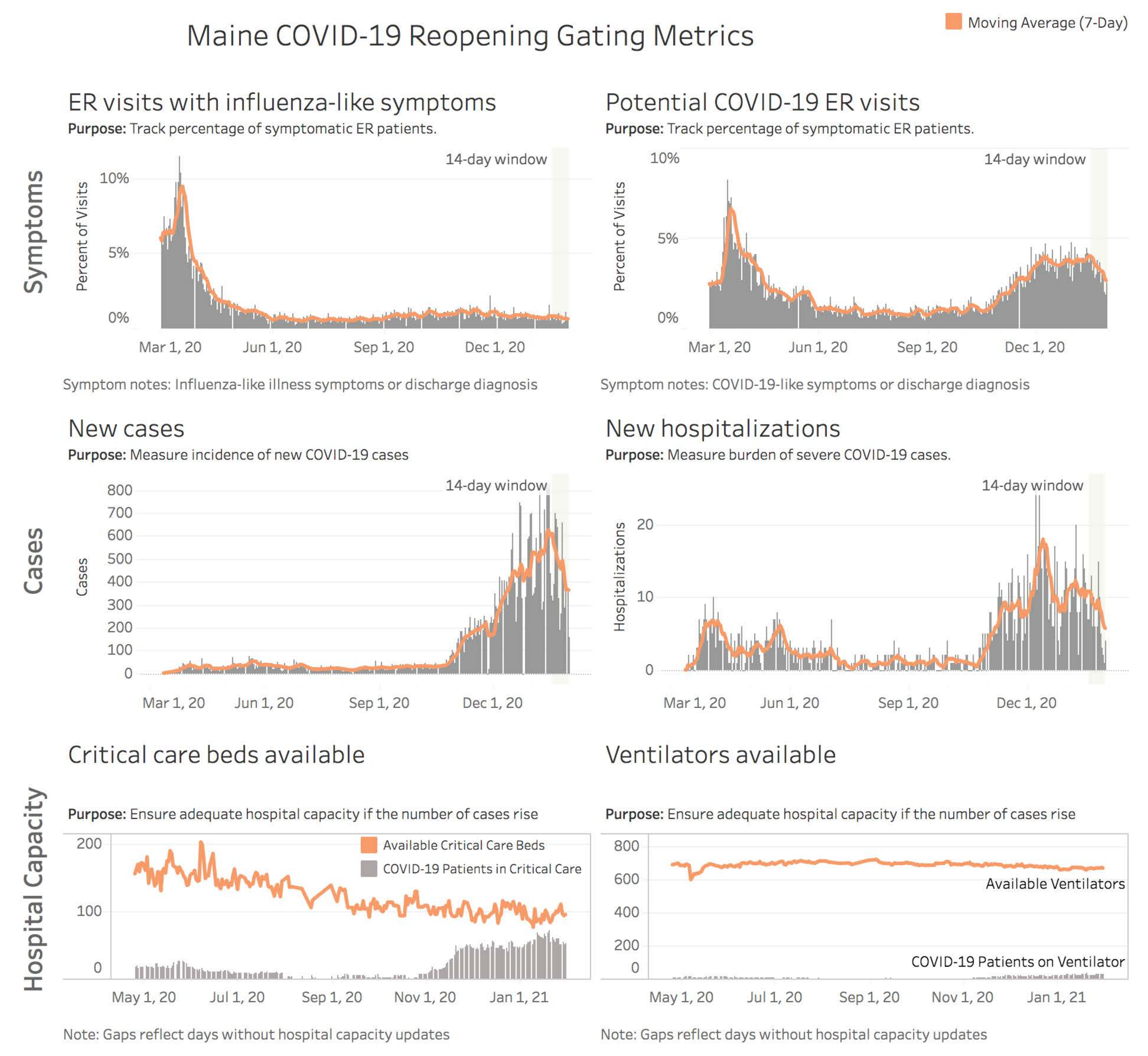

Undo | Redo | Revert | Refresh | Pause | Share | Download | Full Screen

Cumulative COVID-19 Cases by ZIP Code

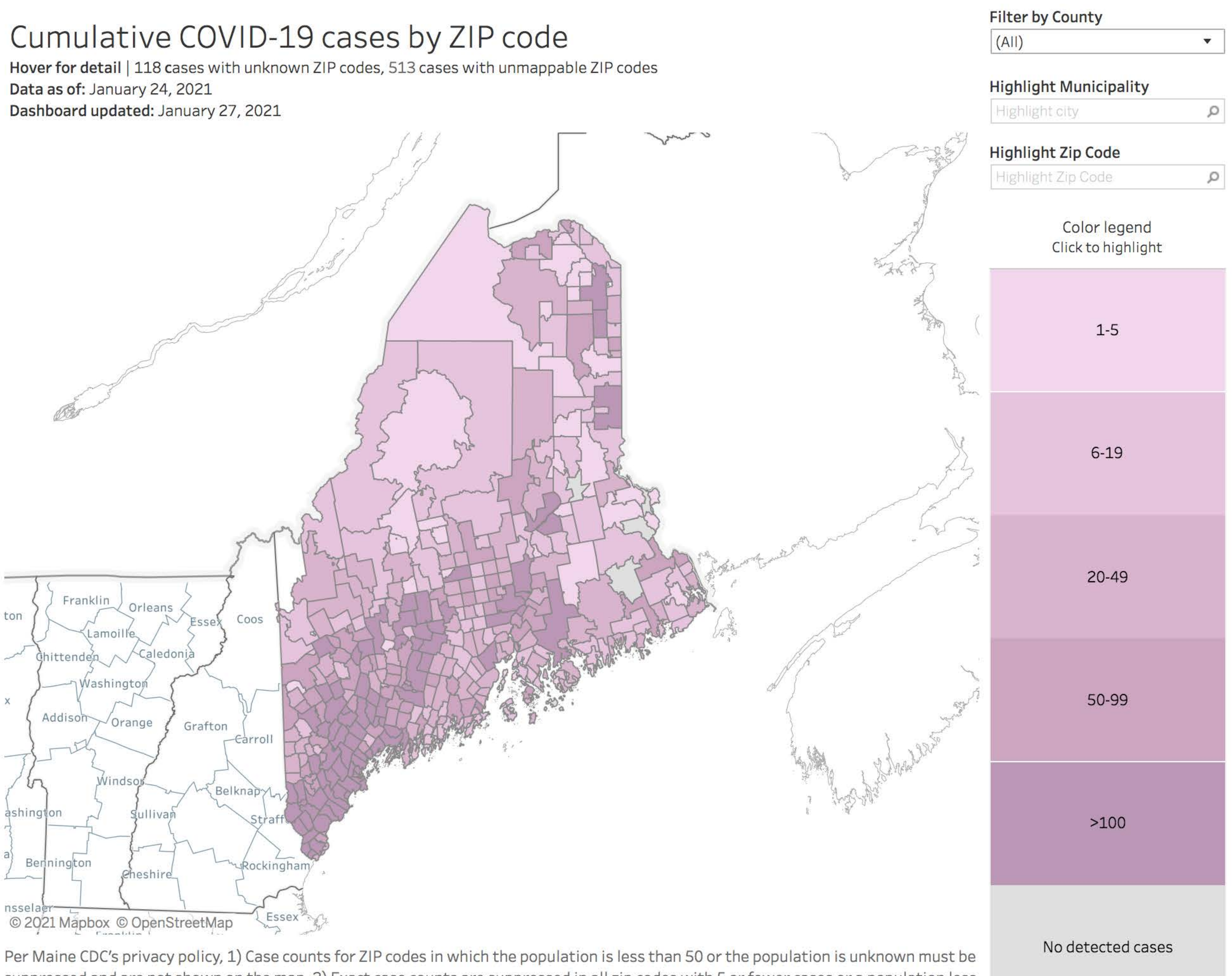

Per Maine CDC's privacy policy, 1) Case counts for ZIP codes in which the population is less than 50 or the population is unknown must be suppressed and are not shown on the map. 2) Exact case counts are suppressed in all zip codes with 5 or fewer cases or a population less than 2,000.

Download a csv file with the most recent cumulative case data by zip code.

Maine and COVID-19

Maine COVID-19 Centralized Dashboard

Maine and COVID-19 | Cumulative Case Data | Hospital Capacity Data | Population and Case Rate Data | 24-Hour Case Changes

Maine COVID-19 Cumulative County Case Data

Last Updated: Sunday, January 31, 2021 at 12:25PM EDT

Total Cases*	Recovered	Hospitalizations	Deaths
39,324	12,409	1,390	590

*Total cases includes both confirmed and probable cases.

Cumulative Total COVID-19 Cases by County

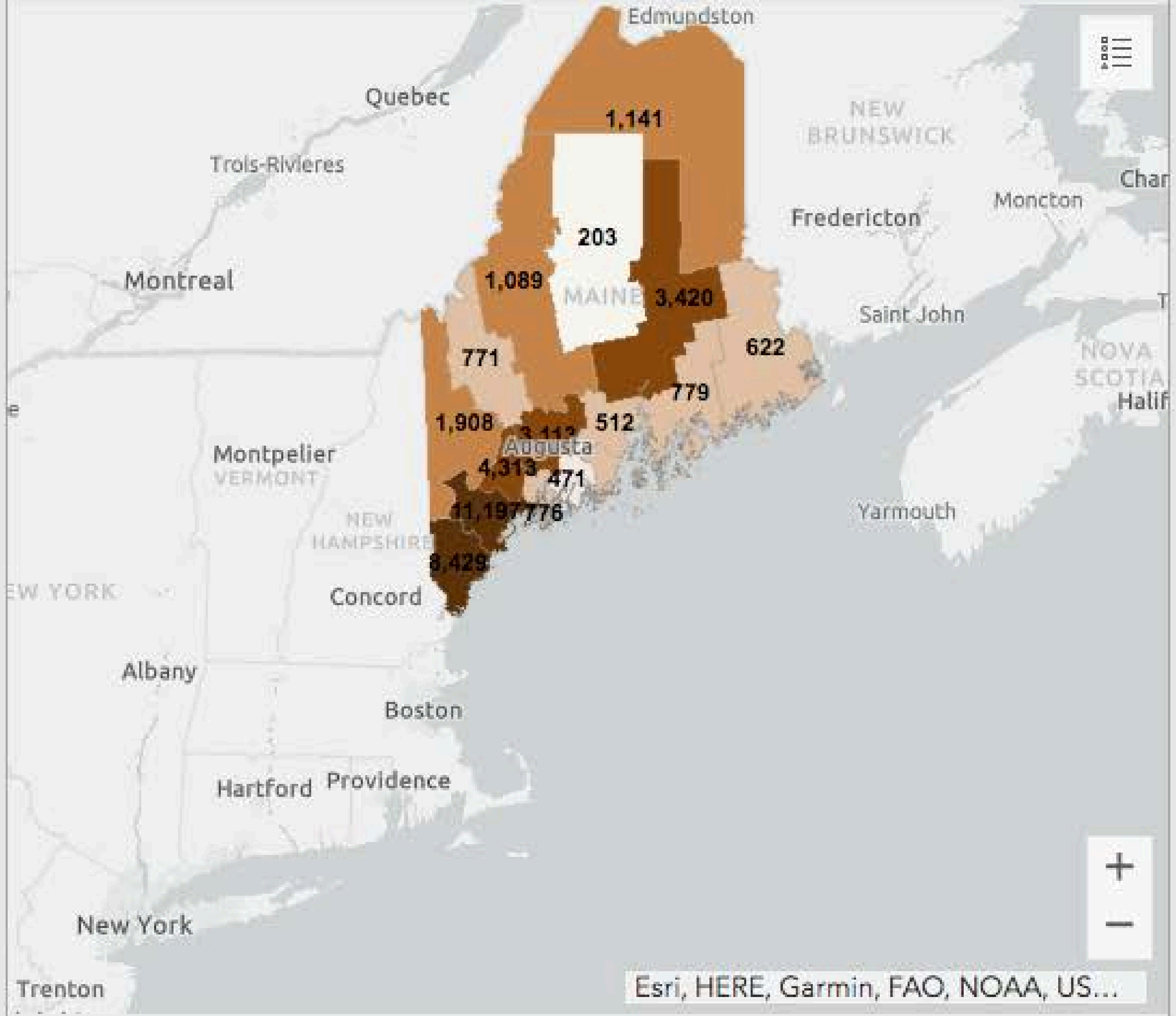

Note: The case distribution displayed on this map is likely an **underrepresentation** of the true number of cases in Maine since not all individuals are being tested. For individuals not considered to be at high risk, medical providers are currently advised to diagnose COVID-19 based on symptoms.

COVID-19 Progression in Maine

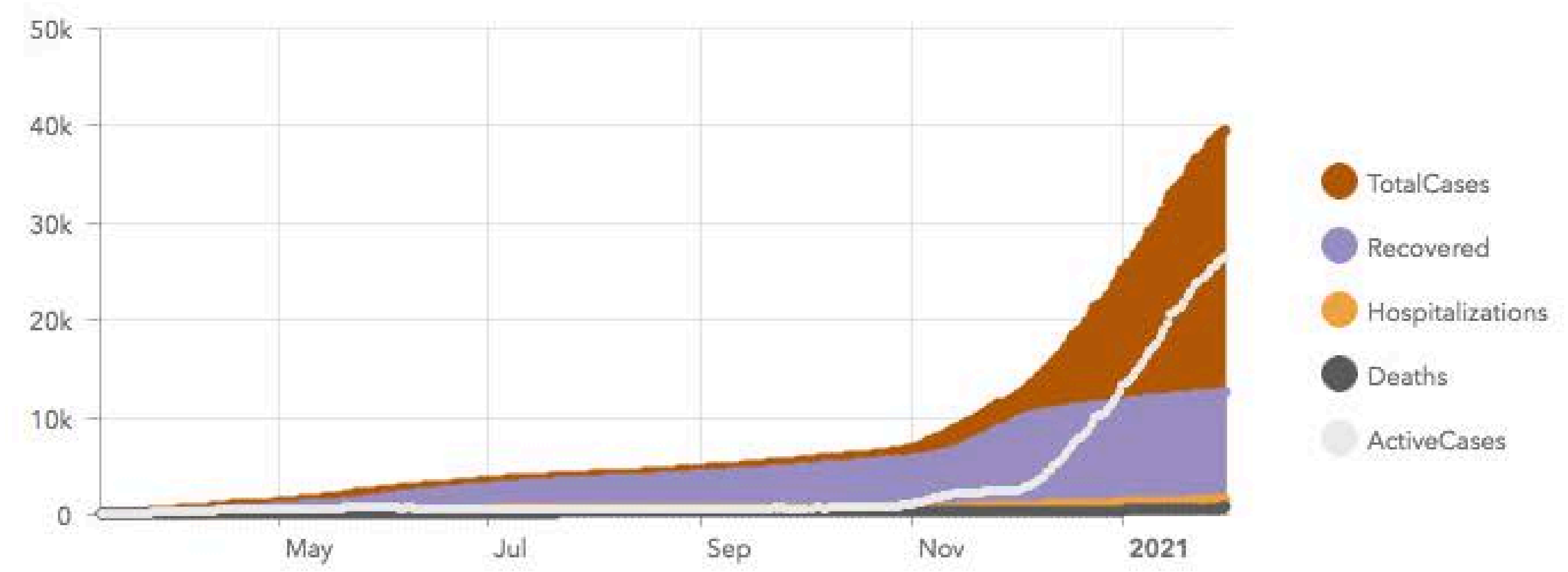

Case data are preliminary due to day-to-day changes in available information reported by the counties.

Note: Data for October 2, 2020 and January 24, 2021 are unavailable due to MECDC technical difficulties.

Maine Counties Caseload Over Time

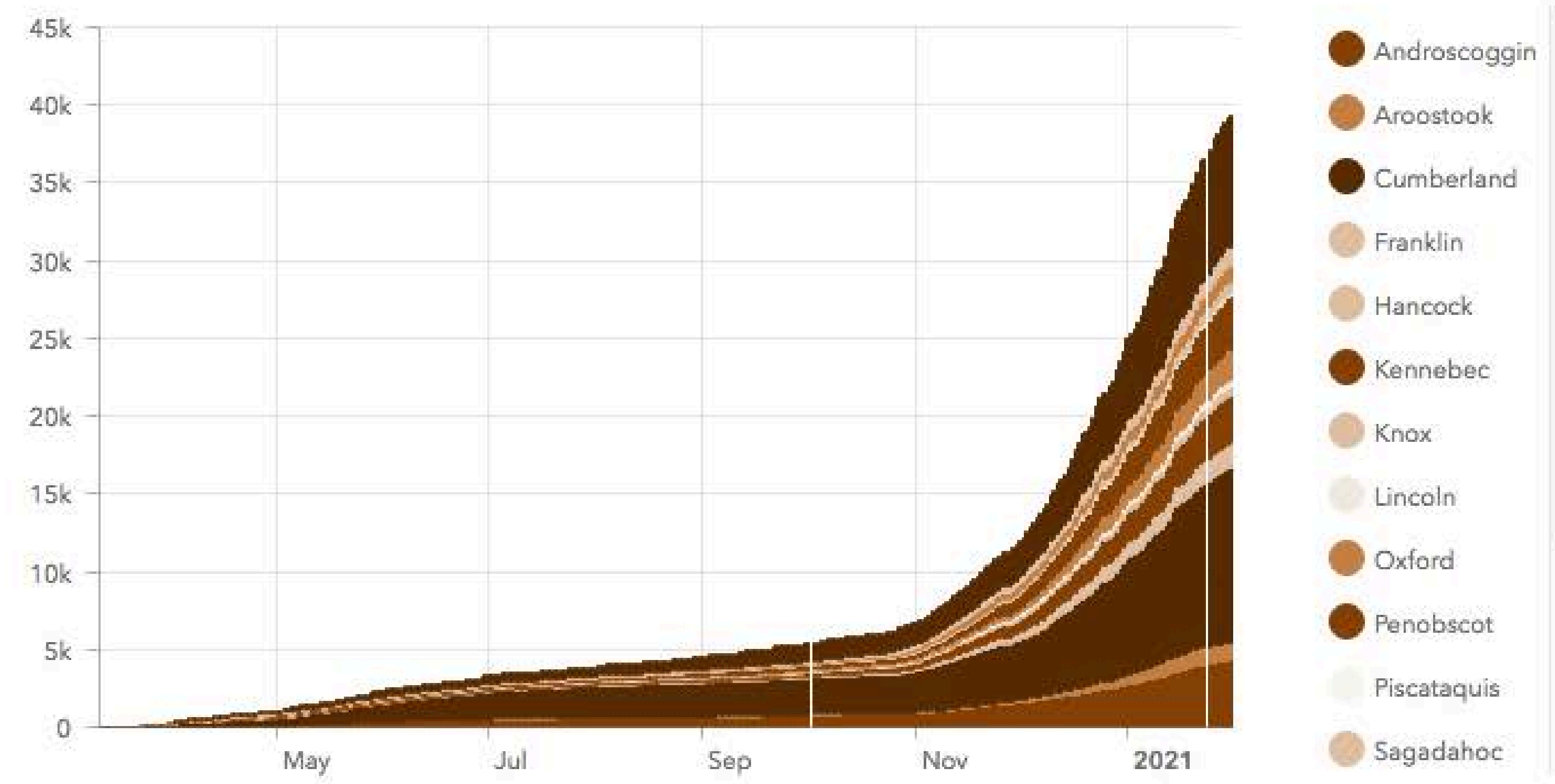

Note: Data for October 2, 2020 and January 24, 2021 are unavailable due to MECDC technical difficulties.

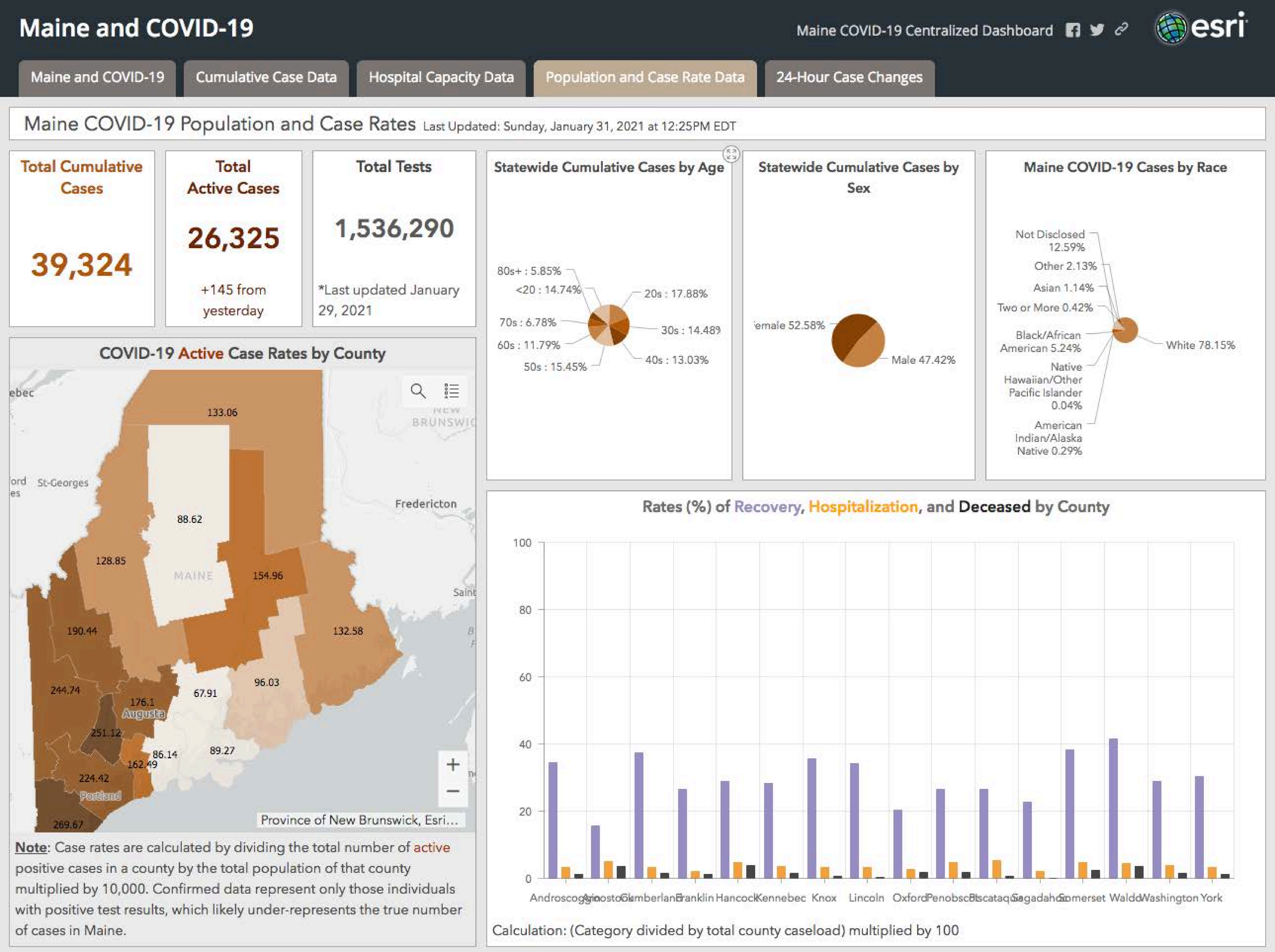
Maine and COVID-19
Maine COVID-19 Centralized Dashboard
esri
Maine and COVID-19
Cumulative Case Data
Hospital Capacity Data
Population and Case Rate Data
24-Hour Case Changes
Maine COVID-19 Population and Case Rates Last Updated: Sunday, January 31, 2021 at 12:25PM EDT
Total Cumulative Cases
39,324
Total Active Cases
26,325
+145 from yesterday
Total Tests
1,536,290
*Last updated January 29, 2021
Statewide Cumulative Cases by Age
80s+ : 5.85%
<20 : 14.74%
20s : 17.88%
70s : 6.78%
30s : 14.489
60s : 11.79%
40s : 13.03%
50s : 15.45%
Statewide Cumulative Cases by Sex
'emale 52.58%
Male 47.42%
Maine COVID-19 Cases by Race
Not Disclosed 12.59%
Other 2.13%
Asian 1.14%
Two or More 0.42%
Black/African American 5.24%
Native Hawaiian/Other Pacific Islander 0.04%
American Indian/Alaska Native 0.29%
White 78.15%
COVID-19 Active Case Rates by County
133.06
88.62
128.85
154.96
190.44
132.58
244.74
176.1
Augusta
67.91
96.03
251.12
86.14
89.27
162.49
224.42
Portland
269.67
MAINE
Fredericton
St-Georges
Province of New Brunswick, Esri...
Note: Case rates are calculated by dividing the total number of active positive cases in a county by the total population of that county multiplied by 10,000. Confirmed data represent only those individuals with positive test results, which likely under-represents the true number of cases in Maine.
Rates (%) of Recovery, Hospitalization, and Deceased by County
100
80
60
40
20
0
Androscoggin
Aroostook
Cumberland
Franklin
Hancock
Kennebec
Knox
Lincoln
Oxford
Penobscot
Piscataquis
Sagadahoc
Somerset
Waldo
Washington
York
Calculation: (Category divided by total county caseload) multiplied by 100

MARYLAND DEPARTMENT OF HEALTH
Maryland

Coronavirus Disease 2019 (COVID-19) Outbreak

This is a rapidly evolving situation and the Maryland Department of Health (MDH) will provide updates as they become available.

Notice of Non-Discrimination and Accessibility Statement

Additional COVID-19 Resources at CovidLINK

Search

Maryland COVID-19 Data Dashboard

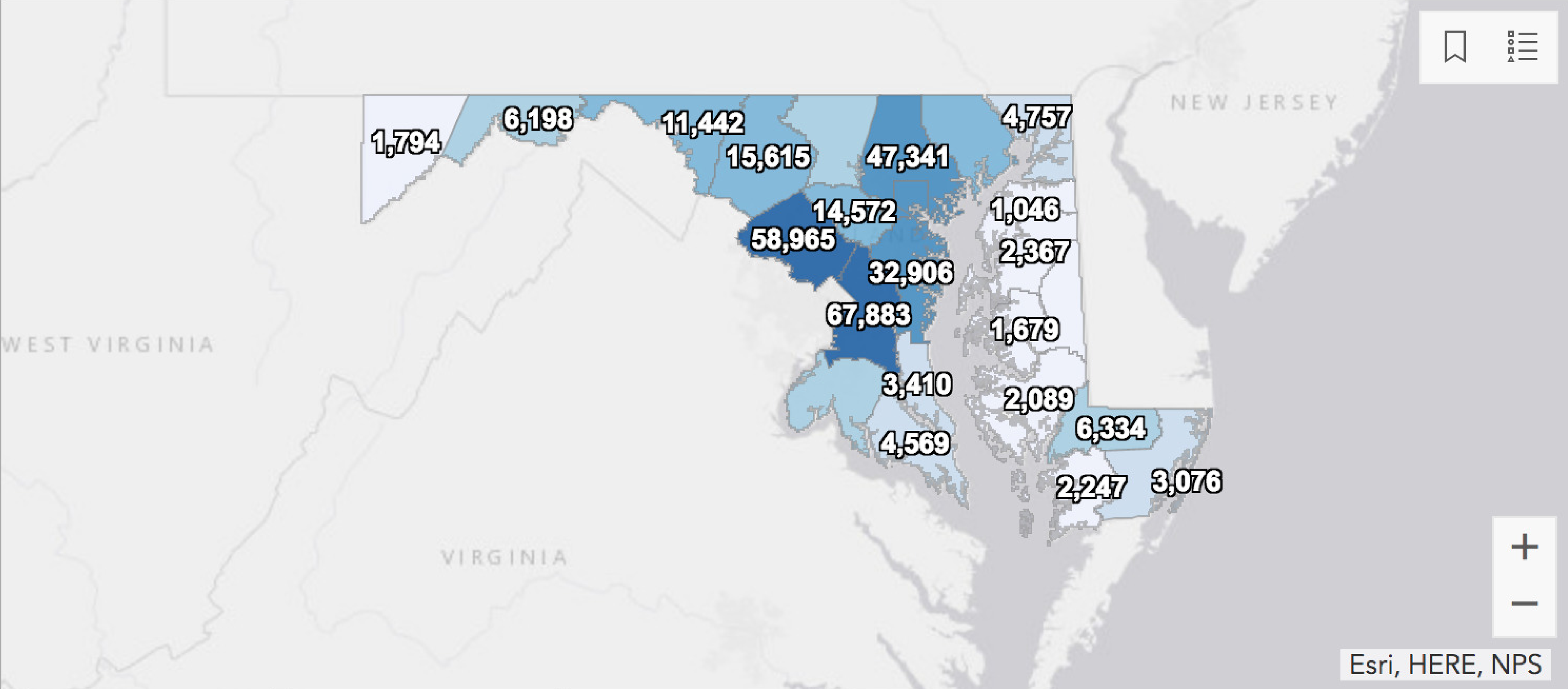

Affected Counties:

- Prince George's - 67,883
- Montgomery - 58,965
- Baltimore - 47,341
- Baltimore City - 37,951
- Anne Arundel - 32,906
- Frederick - 15,615

Cases by County | Cases by ZIP Code | Testing by County

ICU and Acute Hospital Beds for COVID-19, Currently in Use

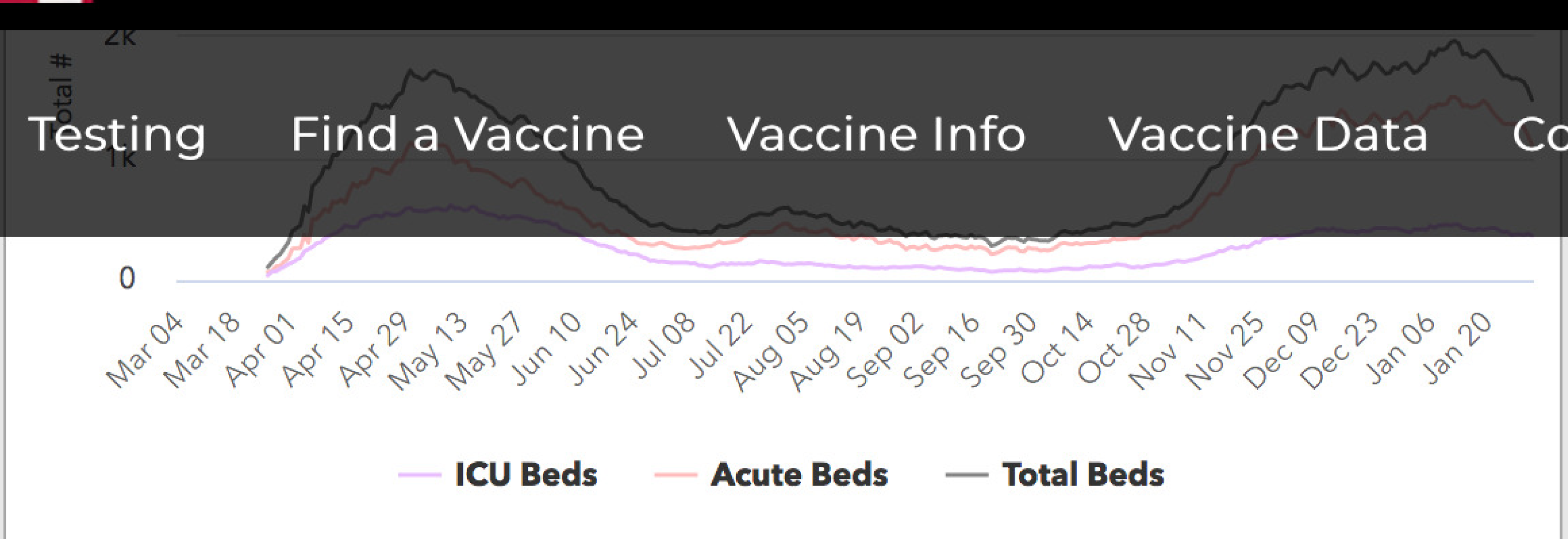

Hospitalizations, Current

Testing Volume, Tests per Day and Percent Positive Rate (7-Day Avg)

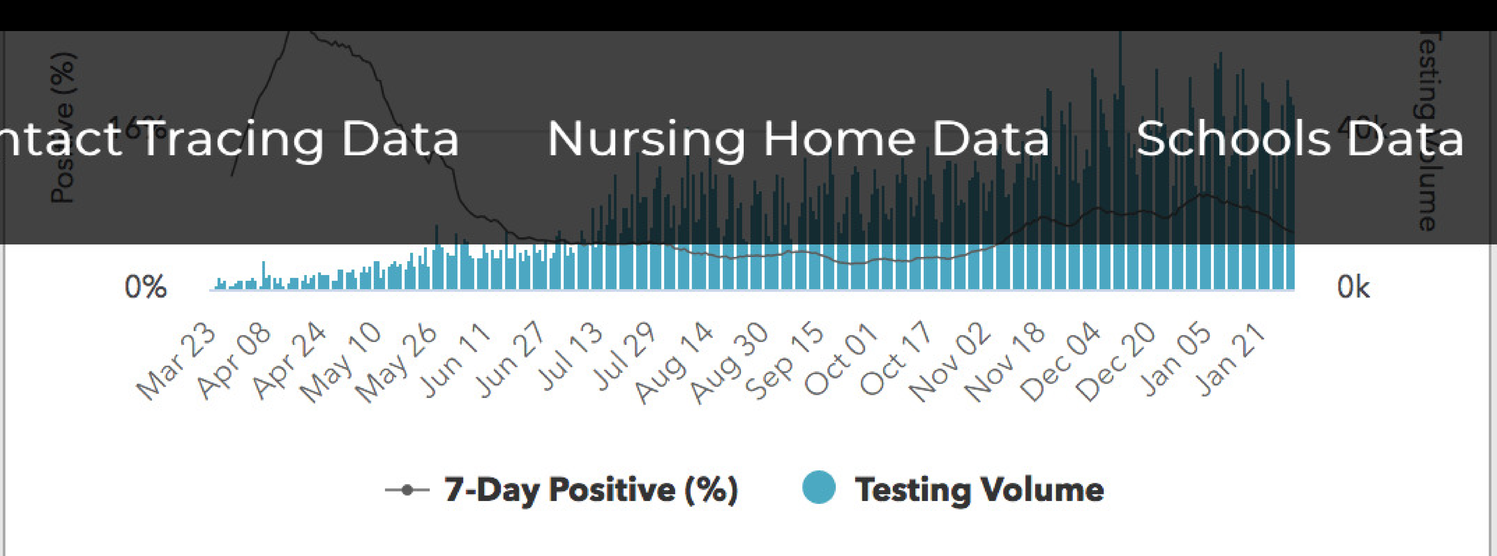

Positivity Rate

Maryland.gov

LANGUAGE ASSISTANCE SERVICES

Testing | Find a Vaccine | Vaccine Info | Vaccine Data | Contact Tracing Data | Nursing Home Data | Schools Data

Confirmed Cases	Persons Tested Negative	Testing Volume	Testing % Positive	Confirmed Deaths	Currently Hospitalized
354,473	2,841,114	7,013,154	5.66%	6,951	1,471
24hr Change: +1,747	24hr Change: +9,301	24hr Change: +46,230	24hr Change: -0.13	24hr Change: +20	24hr Change: -89

Note: The Maryland Department of Health updates these data daily during the 10 a.m. hour. All data are preliminary and subject to change based on additional reporting. Case and death data reflect Maryland residents only. Death data are presented by both date of report and by date of death and are updated as amendments to the death record are received. Data for ZIP codes with 7 or fewer cases are suppressed. Hospitalization data reflect COVID-19 bed occupancy in Maryland hospitals. Testing volume data represent the static daily total of PCR COVID-19 tests electronically reported; this count does not include test results submitted by labs and other clinical facilities through non-electronic means. The percent positive rate is a seven day rolling average of positive results as a percentage of all tests. MDH is continuously evaluating its data and reporting systems and will make updates as more data becomes available. View the Maryland COVID-19 Data Dashboard FAQ.

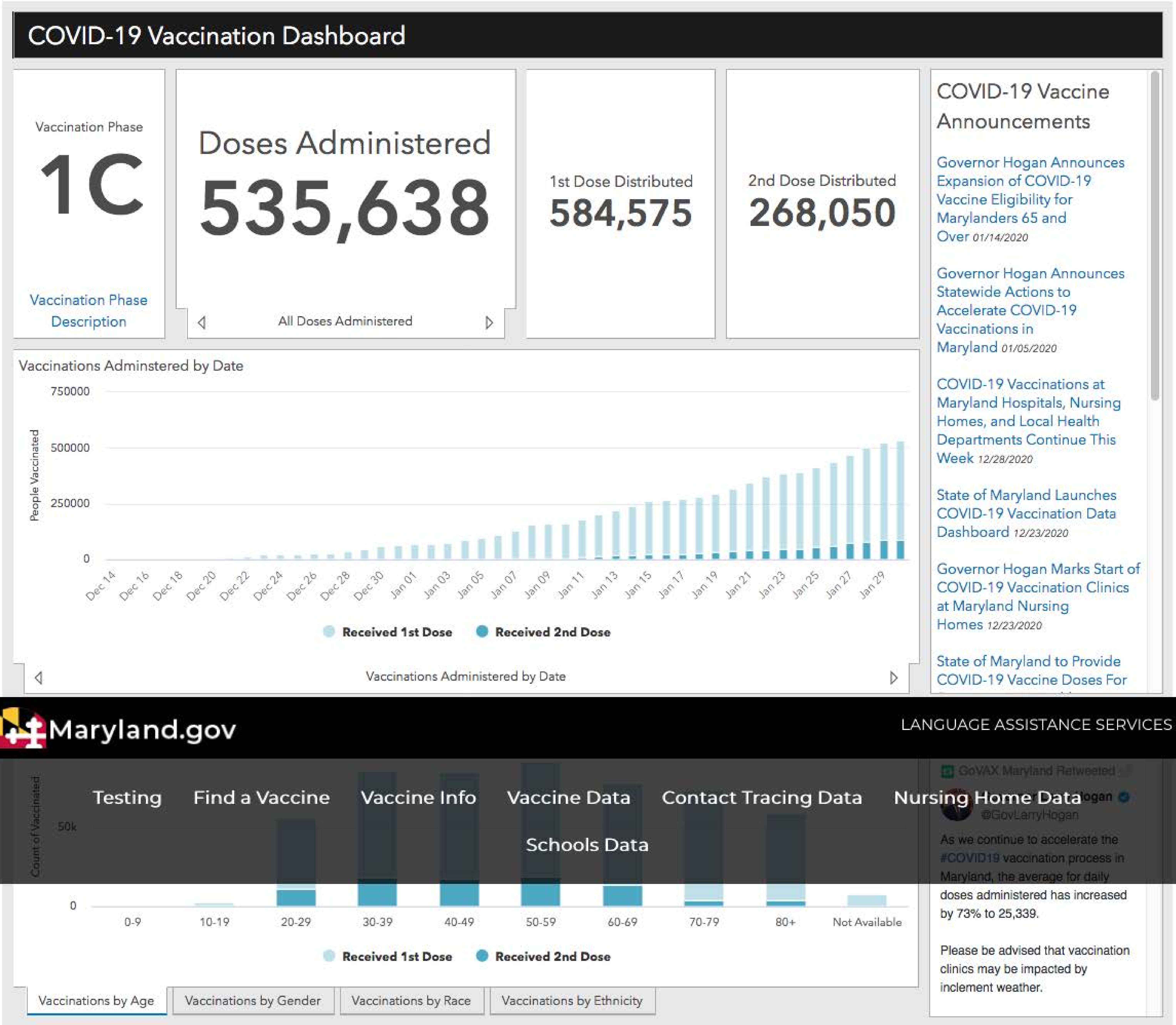

Note: The Maryland Department of Health updates these data daily during the 10 a.m. hour. Data are based on facility reports to MDH, which may be revised if additional information becomes available. Some vaccination data may be unavailable due to the time lag between vaccine administration and data entry. All data are preliminary and subject to change based on additional reporting. Doses distributed and administered both account for the 8,000 doses of vaccine provided to the District of Columbia to vaccinate Maryland residents who work in DC. Percent of population vaccinated is calculated based on total population, not total population eligible to be vaccinated. MDH is continuously evaluating its data and reporting systems and will make updates as more data becomes available.

Massachusetts Department of Public Health | COVID-19 Dashboard

Today at a Glance

Data as of: January 29, 2021
Caution: recent data may be incomplete

Navigation

- Today's Overview
- Overview Trends
- COVID-19 Cases
- COVID-19 Testing
- Hospitalization
- COVID-19 Deaths
- Higher Ed & LTCF
- Patient Breakdown
- City and Town
- Resources
- Data Archive

Cases

Confirmed Cases

Today there were 3,957 new, confirmed cases reported bringing the total to 495,599 total confirmed cases.

Probable Cases

Today, there were 151 new, probable cases reported bringing the total to 25,761 total probable cases.

Estimated Active Cases

There are an estimated 72,965 currently active COVID-19 cases.

Cases by age during the last two weeks

*Data updated weekly

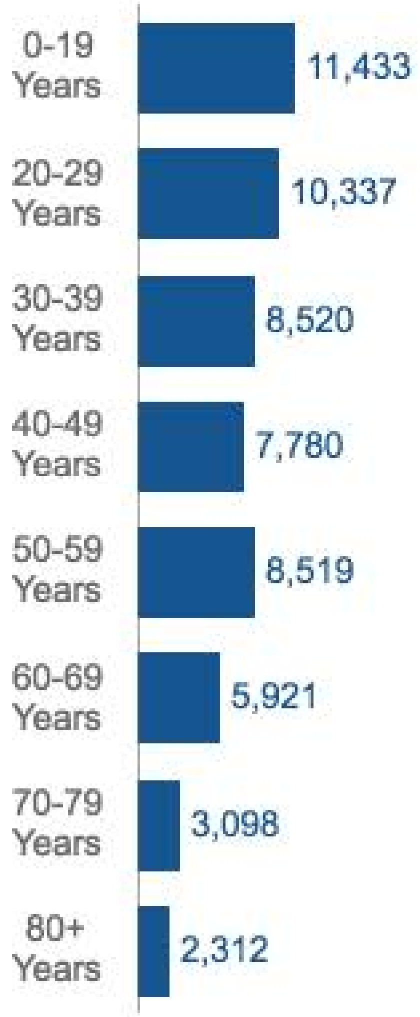

Testing

Tests Reported

There were 143,342 new COVID-19 molecular tests reported, bringing the cumulative total to 13,538,980 tests.

Turn Around Time

The current turn around time for a molecular test is 1.92 days.

Percent Positivity

The 7-day average of percent positivity is 3.89%.

Hospitalizations

Hospitalizations

There are 1,739 patients hospitalized for COVID-19.

ICU & Intubated Patients

There are 393 patients in Intensive Care Units (ICU) and 239 patients are intubated.

Average Age of Patients

The average age of patients who were hospitalized for COVID-19 was 70 years old.

Deaths

Confirmed Deaths

There were 87 new, confirmed deaths reported. There have been 14,241 confirmed deaths in total.

Probable Deaths

There were 0 new, probable deaths reported. There have been 290 probable deaths in total.

For details on the definitions of each indicator hover over the box or graph. All data included in this dashboard are preliminary and subject to change. Data Sources: COVID-19 Data provided by the Bureau of Infectious Disease and Laboratory Sciences and the Registry of Vital Records and Statistics; COVID-19 Hospitalization Data provided by the MDPH survey of hospitals (hospital survey data are self-reported); Created by the Massachusetts Department of Public Health, Bureau of Infectious Disease and Laboratory Sciences, Office of Integrated Surveillance and Informatics Services.

+ a b | e a u

Massachusetts Department of Public Health | COVID-19 Dashboard
Trends: 7-day Averages Over Time

Released on: January 30, 2021
Data as of: January 29, 2021
Caution: recent data may be incomplete

Cases

7-day average of COVID-19 confirmed cases

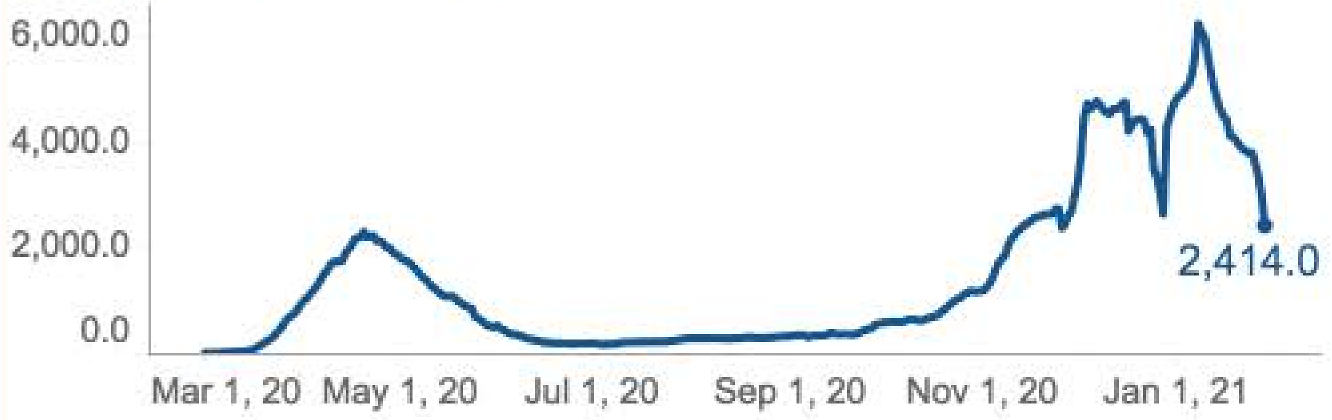

The lowest observed value was 156.7 on 7/4/2020.

Testing

7-day weighted average percent positivity

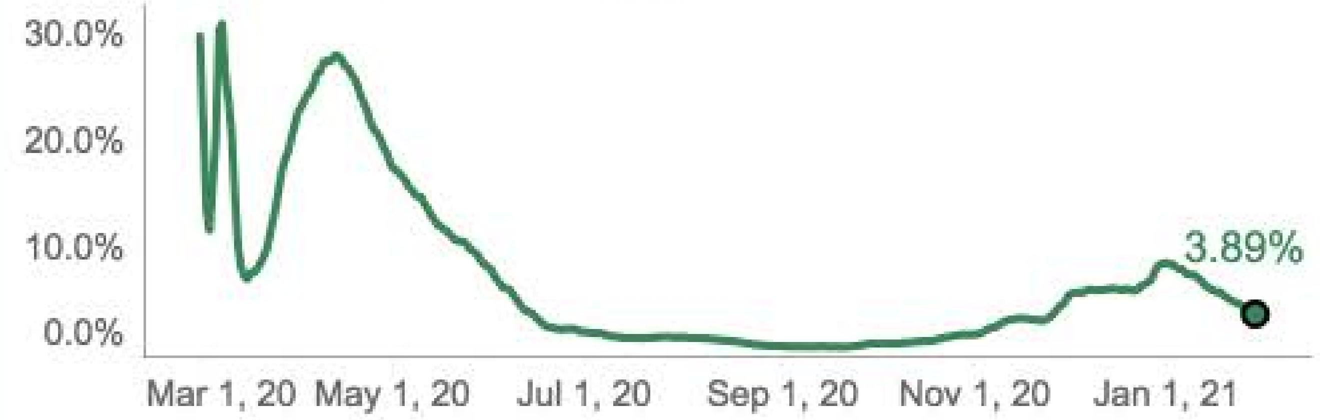

The lowest observed value was 0.8% on 9/21/2020.

Hospitalizations

7-day average of hospitalizations

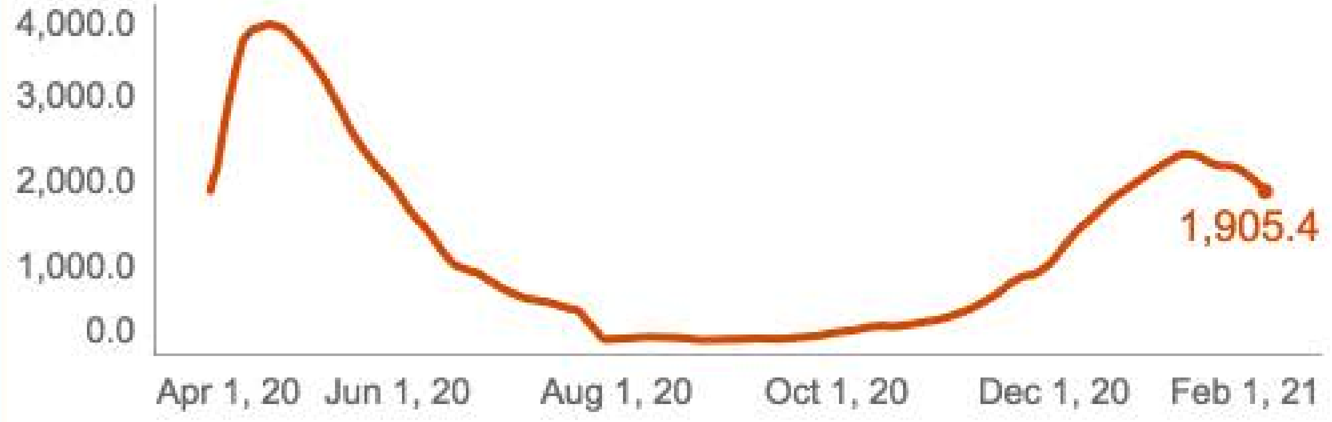

The lowest observed value was 155.3 on 8/26/2020.

Deaths

7-day average of confirmed deaths

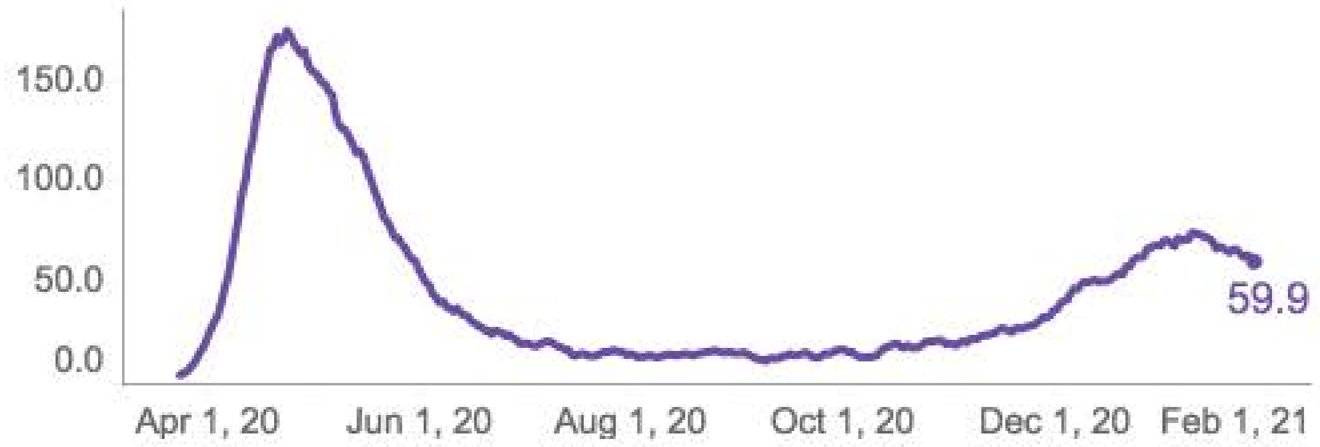

The lowest observed value was 3.7 on 9/9/2020.

Date Filter

3/1/2020 1/29/2021

For details on the definitions of each indicator please see the corresponding tab for that indicator. All data included in this dashboard are preliminary and subject to change. Data Sources: COVID-19 Data provided by the Bureau of Infectious Disease and Laboratory Sciences and the Registry of Vital Records and Statistics; Created by the Massachusetts Department of Public Health, Bureau of Infectious Disease and Laboratory Sciences, Office of Integrated Surveillance and Informatics Services.

+ a b l e a u

Massachusetts Department of Public Health | COVID-19 Dashboard
Cases of COVID-19

Released on: January 30, 2021
Data as of: January 29, 2021
Caution: recent data may be incomplete

Navigation

- Today's Overview
- Overview Trends
- COVID-19 Cases
- COVID-19 Testing
- Hospitalization
- COVID-19 Deaths
- Higher Ed & LTCF
- Patient Breakdown
- City and Town
- Resources
- Data Archive

Confirmed Cases

Today there were 3,957 new, confirmed cases reported bringing the total to 495,599 total confirmed cases.

Probable Cases

Today, there were 151 new, probable cases reported bringing the total to 25,761 total probable cases.

Incidence Rate

The 14-day, statewide incidence rate is 59.40 per 100,000 Massachusetts residents.

Estimated Active Cases

There are an estimated 72,965 currently active COVID-19 cases.

COVID-19 Confirmed and Probable Cases

Filter Cases by Date:
1/29/2020 — 1/29/2021

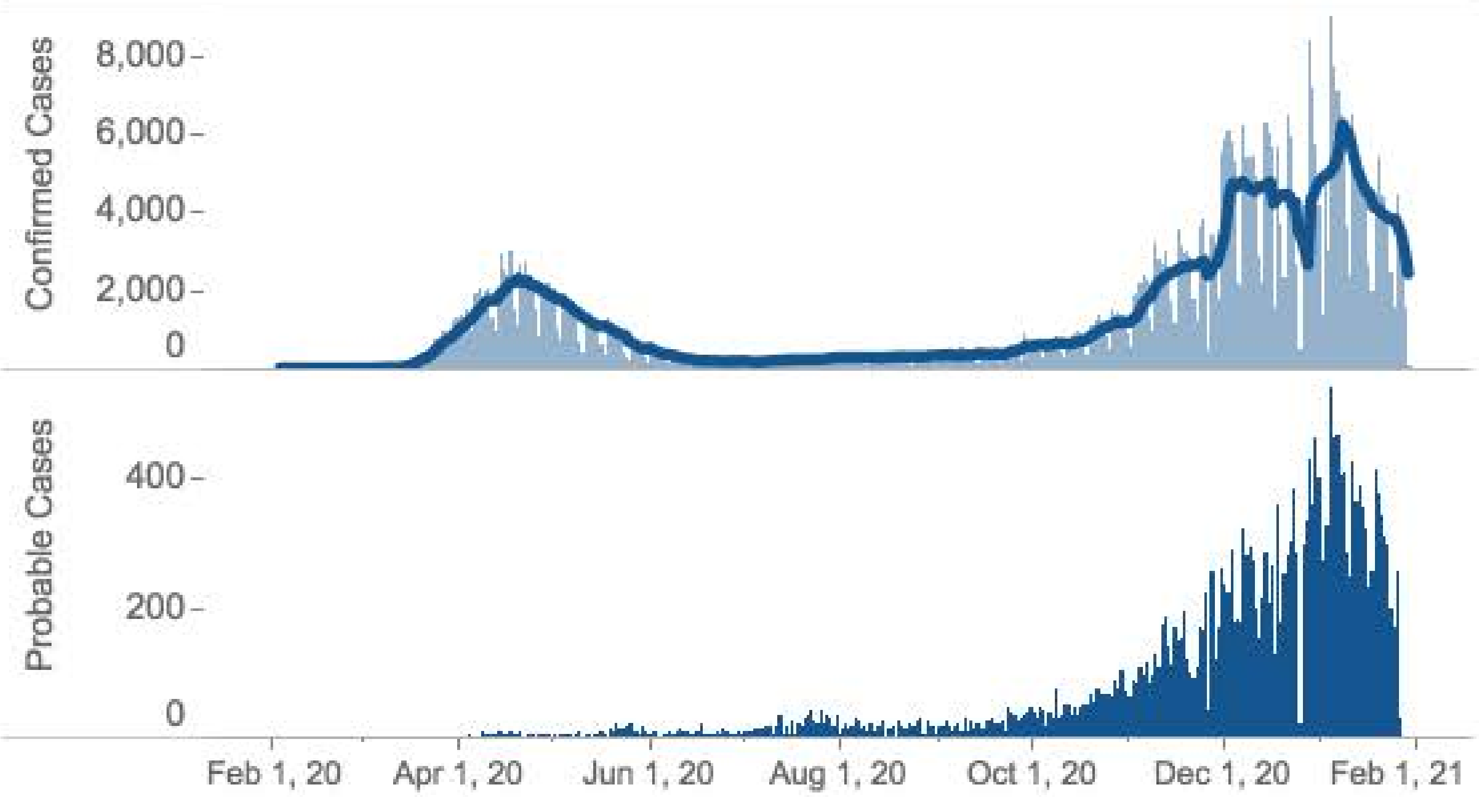

New confirmed cases reported by county

Case Type: New confirmed cases
Select Date: 1/30/2021

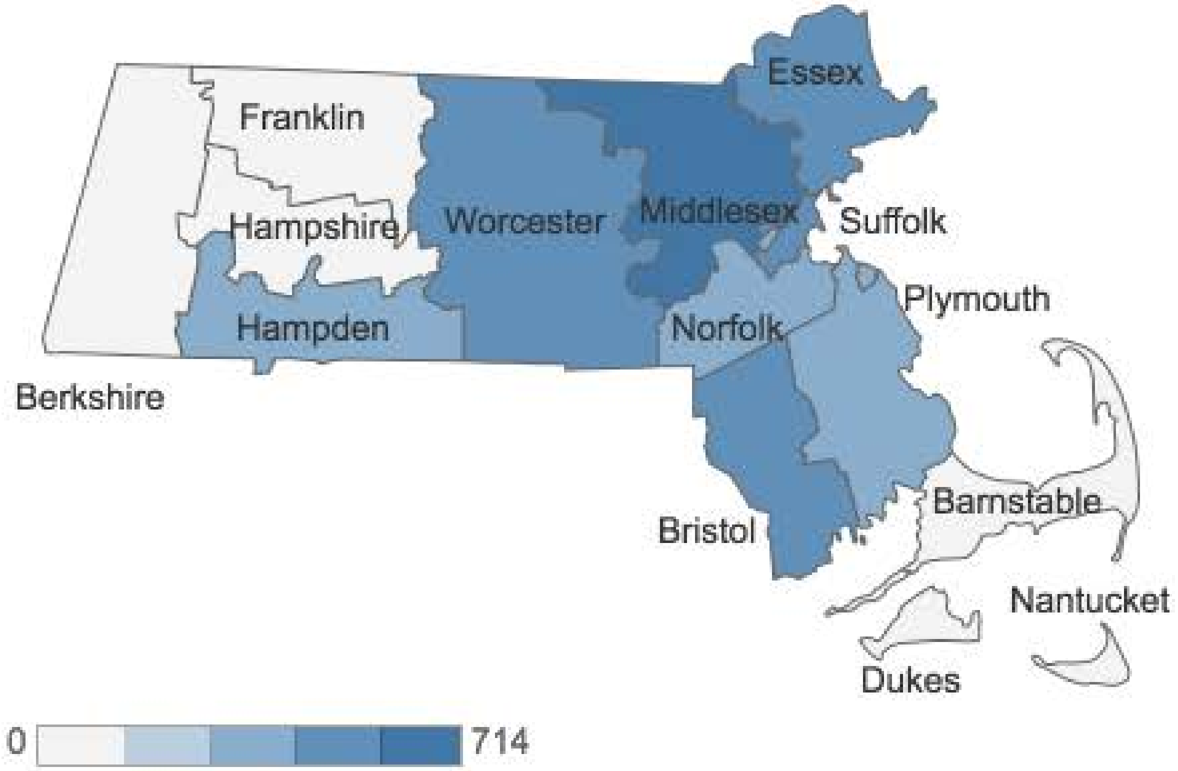

All data included in this dashboard are preliminary and subject to change. Data Sources: COVID-19 Data provided by the Bureau of Infectious Disease and Laboratory Sciences and the Registry of Vital Records and Statistics; Created by the Massachusetts Department of Public Health, Bureau of Infectious Disease and Laboratory Sciences, Office of Integrated Surveillance and Informatics Services.

tableau

CURRENT STATUS | DAILY CASES | CUMULATIVE TRENDS | DEMOGRAPHICS | TOTAL TESTING | DIAGNOSTIC TESTING | LEARN MORE

View Cases

View Deaths

View Table

Select Preparedness Region:

All

Select County:

All

This report is provisional and subject to change. City of Detroit and Wayne County are reported separately. MDHHS began tracking probable cases April 5, 2020. FCI indicates federal correctional institute. MDOC indicates Michigan Department of Corrections. Diagnostic tests, including nucleic acid amplification/real-time polymerase chain reaction (NAAT/RT-PCR) and rapid virus antigen detection point of care (POC) tests, look for current infection. Serology (antibody) tests are used to look for the possibility of previous infection.

COVID-19 Cases by County

Confirmed cases include individuals with a positive diagnostic test for COVID-19. Probable cases include individuals with COVID-19 symptoms and an epidemiologic link to confirmed COVID-19, but no diagnostic test.

Cases by County

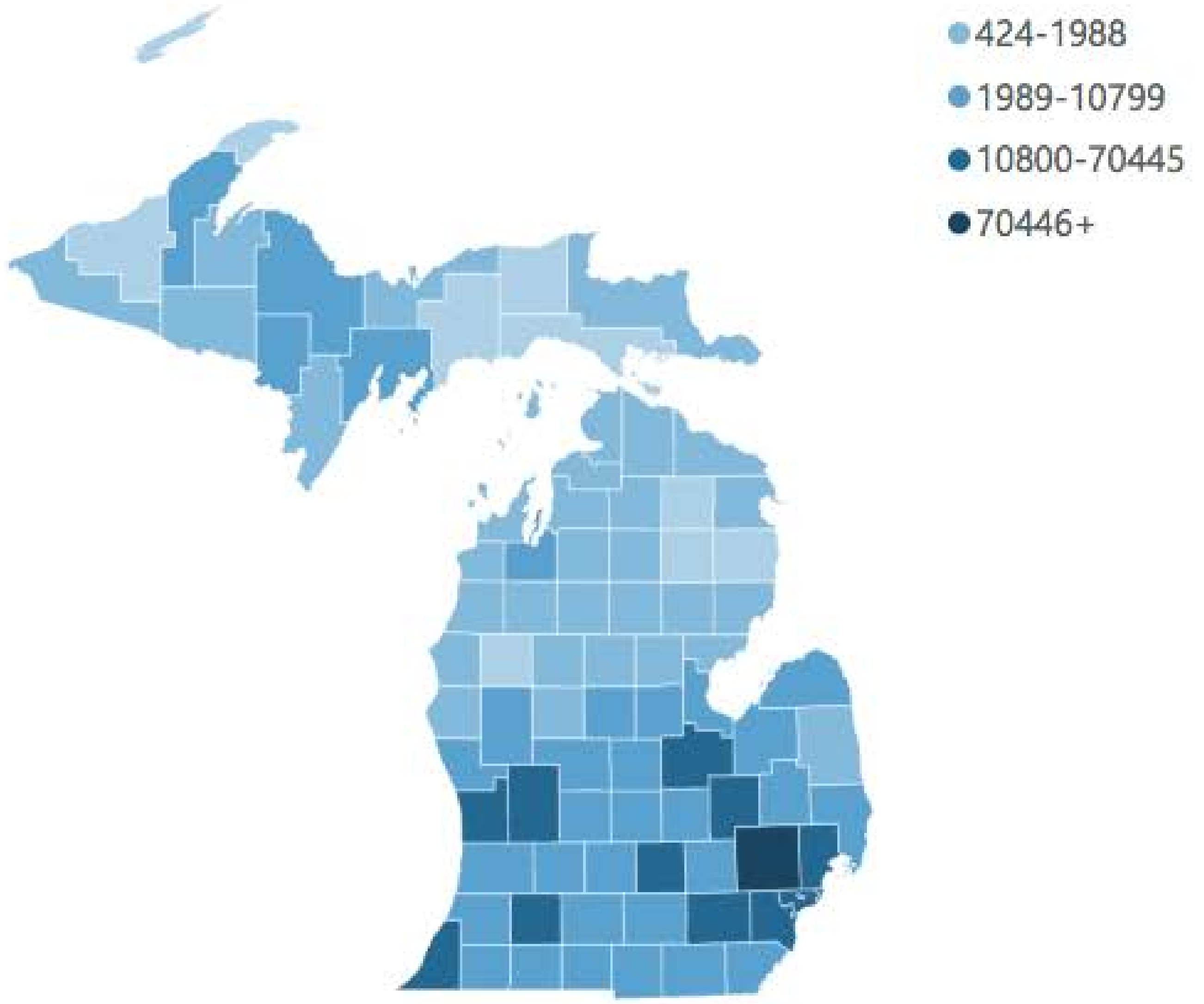

Michigan

Saturday, January 30, 2021

Michigan (Statewide) Case Fatality Rate

2.6%

Current Totals

Case Status	Cases	Deaths
Confirmed	559,241	14,601
Probable	50,897	924
**Total**	**610,138**	**15,525**

Laboratory Testing

Test Type	Tests Performed
Diagnostic	9,465,145
Serology	490,470
**Total**	**9,955,615**

Confirmed Cases

Deaths

View Table

Select Preparedness Region:

All

Select County:

All

Cases are shown by onset date or by lab specimen collection date if onset date was unavailable. Deaths are shown by date of death or date or last status update if date of death was unavailable. Tracking of probable cases and deaths did not begin until April 5, 2020, therefore only probable cases referred for investigation on or after April 5, 2020 are displayed (onset may have been prior).

Notes: FCI indicates federal correctional institute. MDOC indicates Michigan Department of Corrections.

Daily Cases by Status

The number of daily confirmed cases represent the total number of people who had a positive diagnostic test for COVID-19 by the date of the onset of their symptoms.

Filter by Date:

3/1/2020 1/30/2021

Cases by Date of Onset

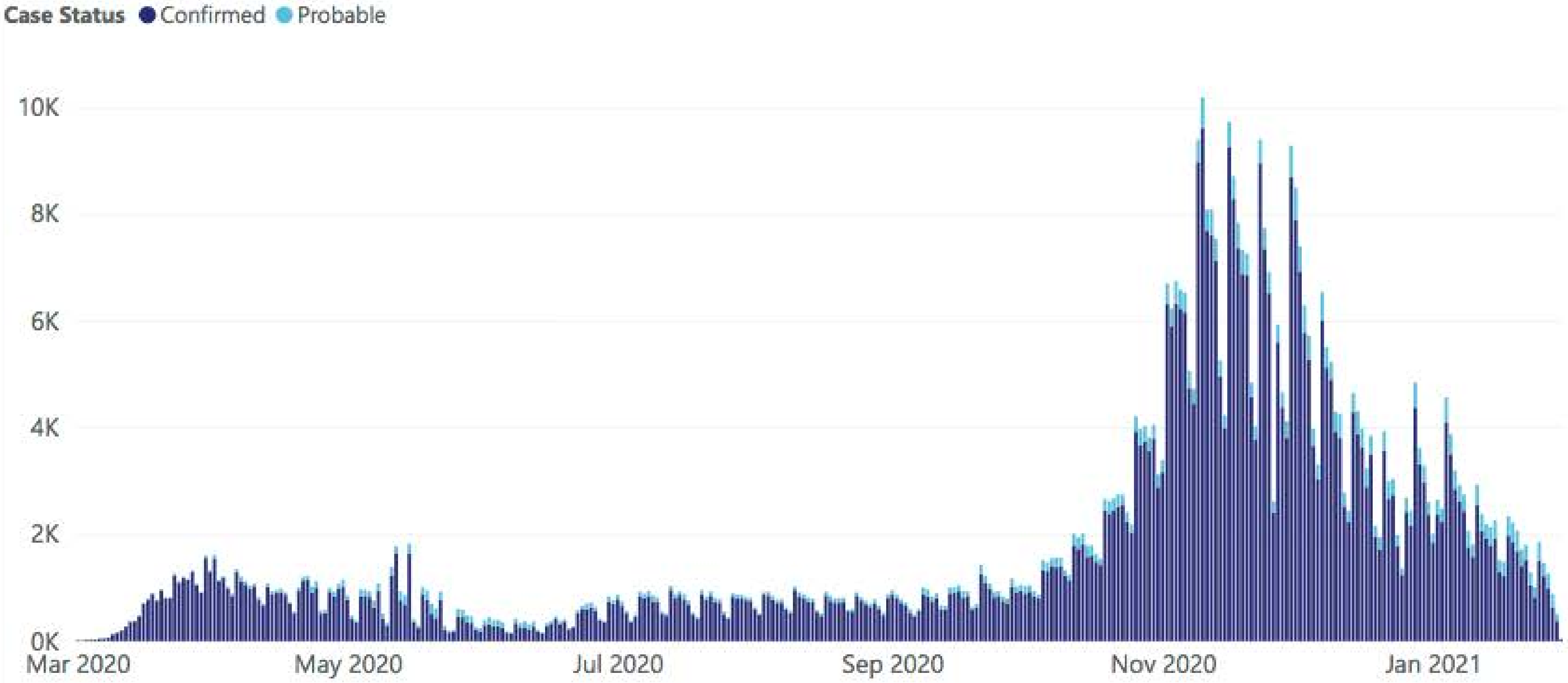

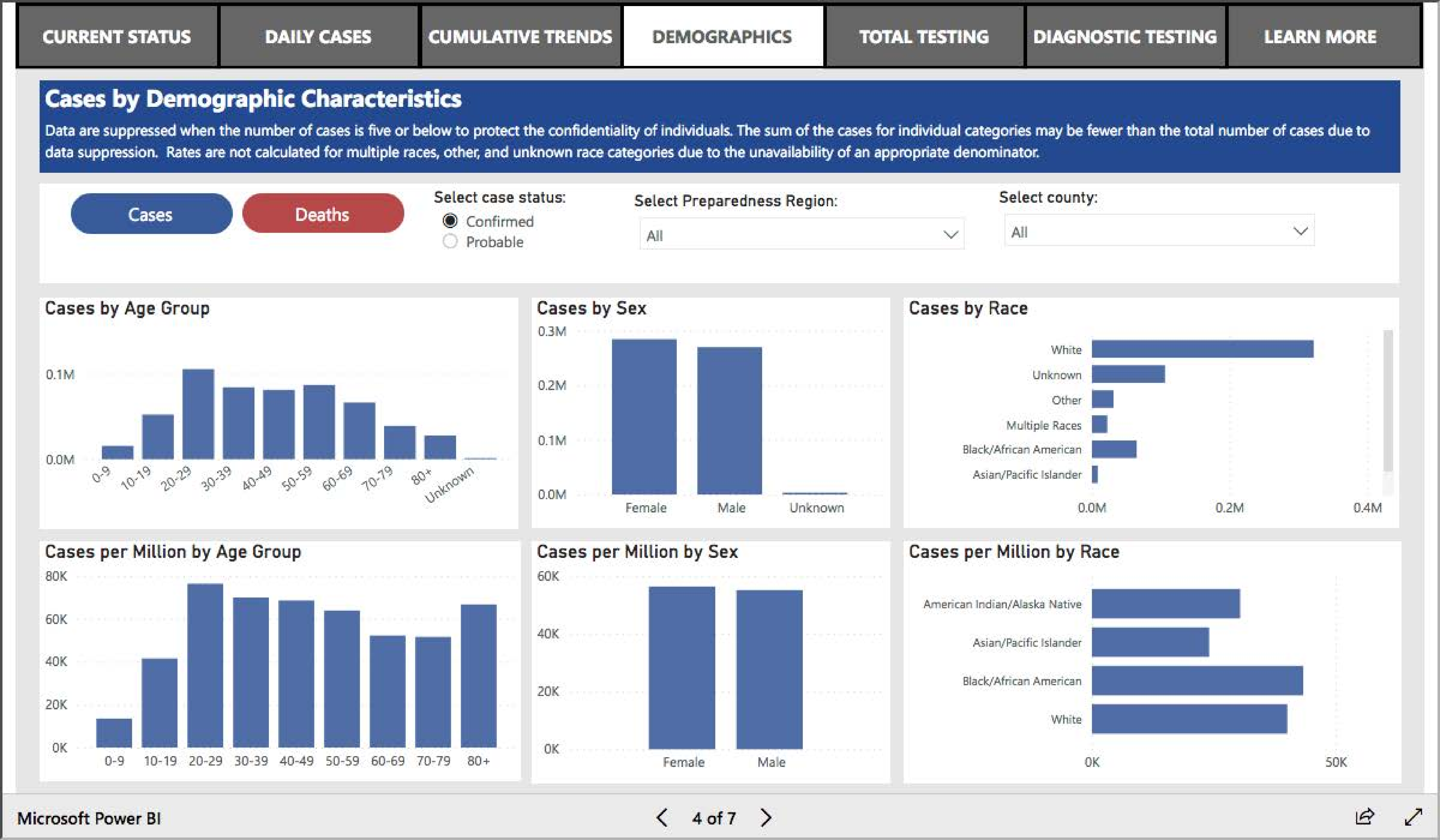
CURRENT STATUS
DAILY CASES
CUMULATIVE TRENDS
DEMOGRAPHICS
TOTAL TESTING
DIAGNOSTIC TESTING
LEARN MORE
Cases by Demographic Characteristics
Data are suppressed when the number of cases is five or below to protect the confidentiality of individuals. The sum of the cases for individual categories may be fewer than the total number of cases due to data suppression. Rates are not calculated for multiple races, other, and unknown race categories due to the unavailability of an appropriate denominator.
Cases
Deaths
Select case status:
Confirmed
Probable
Select Preparedness Region:
All
Select county:
All
Cases by Age Group
0.1M
0.0M
0-9
10-19
20-29
30-39
40-49
50-59
60-69
70-79
80+
Unknown
Cases by Sex
0.3M
0.2M
0.1M
0.0M
Female
Male
Unknown
Cases by Race
White
Unknown
Other
Multiple Races
Black/African American
Asian/Pacific Islander
0.0M
0.2M
0.4M
Cases per Million by Age Group
80K
60K
40K
20K
0K
0-9
10-19
20-29
30-39
40-49
50-59
60-69
70-79
80+
Cases per Million by Sex
60K
40K
20K
0K
Female
Male
Cases per Million by Race
American Indian/Alaska Native
Asian/Pacific Islander
Black/African American
White
0K
50K
Microsoft Power BI
4 of 7

Bridge MICHIGAN | Michigan's nonpartisan, nonprofit news source

If you care about Michigan, please support our work.
DONATE TODAY

Michigan coronavirus dashboard: vaccines, cases, deaths and maps

Michigan does not report new coronavirus data on Sundays. It provides updates Monday–Saturday, with Monday's report covering two days of new data.

Coronavirus in Michigan

Find coronavirus infections rates across much of the state. **Click on a county to see the current day's* cases and the rate per 100,000 over the past week. Note: Scale was changed Nov. 24 to reflect surge in cases.**

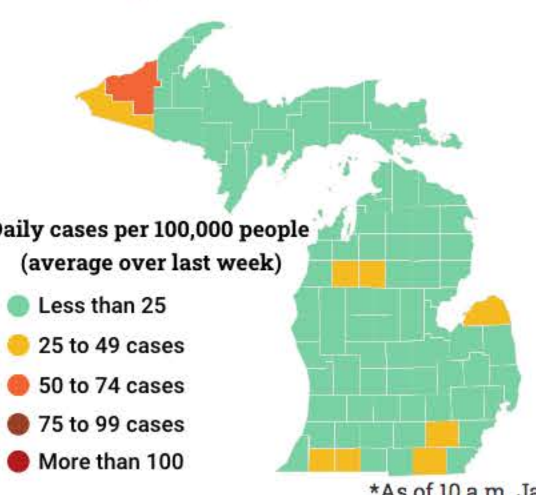

*As of 10 a.m. Jan. 30

	Cases today	Daily cases per 100,000
Michigan	1,358	16
Detroit**	58	12

** Detroit not included in Wayne County's numbers in map

Source: Michigan Department of Health & Human Services

Bridge — *Mike Wilkinson*

Cases and testing

You can use this graphic to check daily changes in cases and tests, as well as compare to the previous week. **NOTE: Monday totals include cases from both Sunday and Monday. The state does not report case information on Sundays.**

Cases | Testing

Today: Jan. 30	Cases today	7-day average	Prior week avg.	Cases per 100,000 rate	Prior week rate
Wayne	139	158	183	15	17
Oakland	134	161	192	13	15
Washtenaw	103	105	66	28	18
Kent	102	126	142	19	22
Macomb	100	117	164	13	19
Ottawa	60	61	76	21	26
Detroit City	58	78	85	12	13
Genesee	43	40	52	10	13
Jackson	40	28	32	18	20

Note: In counties with state prisons, new cases are often first reported within the county. Later, they are subtracted out and identified as having occurred within the Michigan Department of Corrections. This can create days where a county will have a negative number of new cases.

Bridge — *Mike Wilkinson*

Hospitalizations

The second wave of coronavirus cases spurred a rapid increase in the number of people being treated for COVID-19 in Michigan hospitals. But since early December the number has steadily fallen.

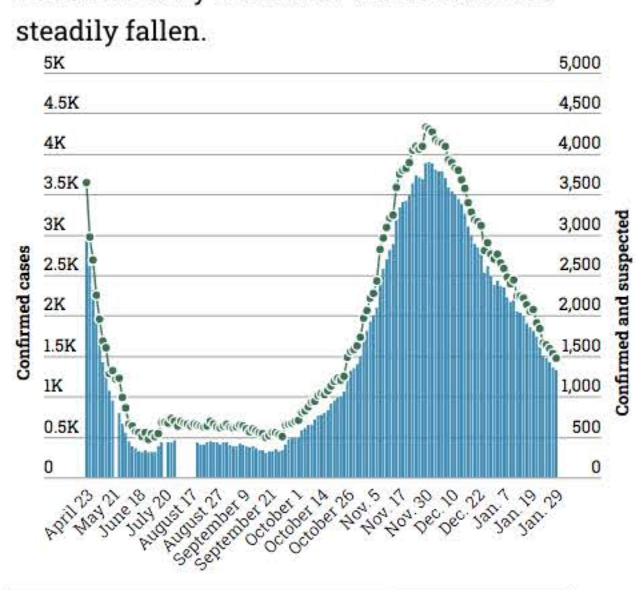

Source: Michigan Department of Health and Human Services and Western Michigan University Libraries.

Bridge — *Mike Wilkinson*

Daily cases

New cases each day since March 25

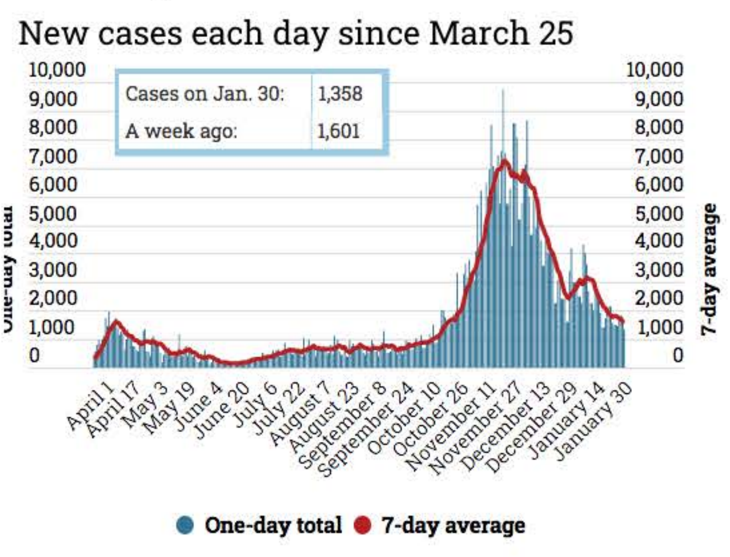

Coronavirus in Michigan

Click the tabs to see total numbers and per-capita rates by county.

	Confirmed	Probable
Total cases:	559,241	50,897
Total deaths:	14,601	924

Cases per 100,000:	5,604
Deaths per 100,000:	146

Total cases (cases per 100,000 people)
- 1 case or none
- 2 to 49 (fewer than 50 cases per 100,000)
- 50 to 99 (50 to 99 cases per 100,000)
- 100 to 199 (100 to 199 cases per 100,000)
- 200 to 999 (200 to 299 cases per 100,000)
- Over 1,000 (300 or more cases per 100,000)

COVID-19 vaccines given, by date

Michigan health officials are trying to vaccinate as many people as possible with the limited quantities of vaccine that they have. Here's how it is going, by date.

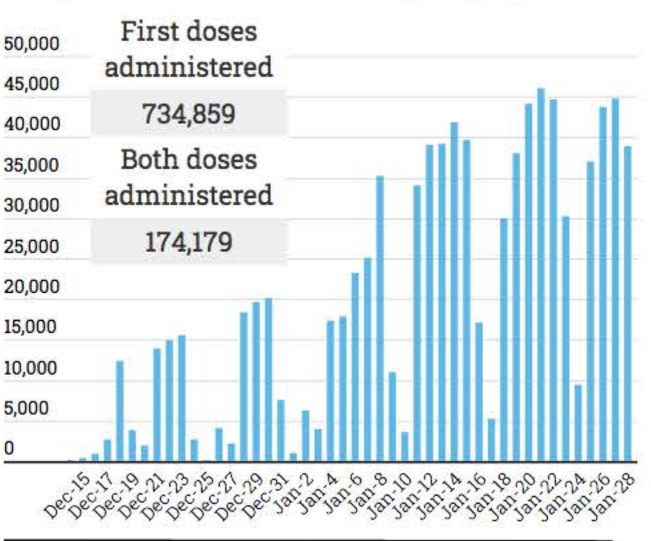

Source: Michigan Department of Health and Human Services

Bridge — *Mike Wilkinson*

Age breakdown

The coronavirus has infected and killed people of all ages in Michigan but has hit the elderly particularly hard. Here's the current breakdown.

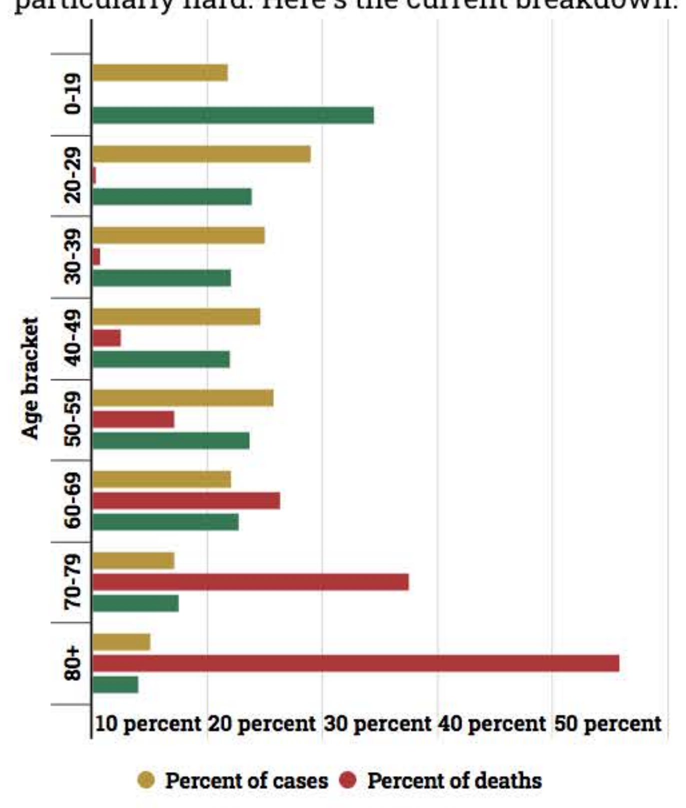

Source: Michigan Department of Health & Human Services

Who's getting the vaccine?

Health care workers in Michigan were first in line for the coronavirus vaccine, which is now being offered to people over 65 and other essential workers. Track where people have gotten at least the first dose below.

First doses administered
734,859
Both doses administered
174,179

County/City	First dose administered	Both doses administered
Alcona	919	105
Alger	1,013	111
Allegan	6,212	1,483
Alpena	3,478	737
Antrim	1,721	244
Arenac	1,096	138
Baraga	1,070	133
Barry	3,210	567
Bay	7,550	2,018

Source: Michigan Department of Health and Human Services

Bridge — *Mike Wilkinson*

Vaccines in Michigan

The first COVID vaccine from Pfizer/BioNTech is being sent and administered across the state. Health care workers are the top priority in the first wave of vaccines. You can track vaccine shipments here by clicking on the counties.

Total doses shipped:
1,479,975

Doses shipped:
- More than 10,000 doses
- 5,000 to 10,000
- 2,500 to 4,999
- Up to 2,500
- No doses yet

Source: Michigan Department of Health & Human Services

Bridge — *Mike Wilkinson*

School outbreaks

Michigan public health officials have begun identifying coronavirus outbreaks at schools, including preschool, K-12 and higher ed institutions. **Report date: Dec. 14, 2020. In ongoing outbreaks, counts with asterisk (*) refer only to last 28 days.**

New outbreaks | Ongoing

County (community)	School	Cases
Allegan (Martin)	Brandon Elementary School	2 staff
Berrien (Saint Joseph)	Brown Elementary School	3 staff and students
Berrien (Niles)	Brandywine Middle/High School	2 staff
Berrien (Stevensville)	Lakeshore High School	2 students
Berrien (Berrien Springs)	Blossomland	4 staff and students
Detroit City (Detroit City)	Mumford High School	4 staff
Detroit City (Detroit City)	Charles R. Drew Transtion Center	2 staff
Genesee (Flint)	Armstrong Middle School	2 staff
Genesee (Flint)	Kearsley High School	3 staff
Livingston (Hartland)	Creekside Elementary School	2 staff and students
Macomb (Macomb)	Little Turtle Early Childhood School	2 students
Macomb	[illegible]	[illegible]

Source: Michigan Department of Health and Human Services

Bridge — *Mike Wilkinson*

Daily testing results

Michigan health officials say the goal is to keep the positive test rate at 3 percent or lower, where it had hovered for months before begining to rise in October, alarming state leaders.

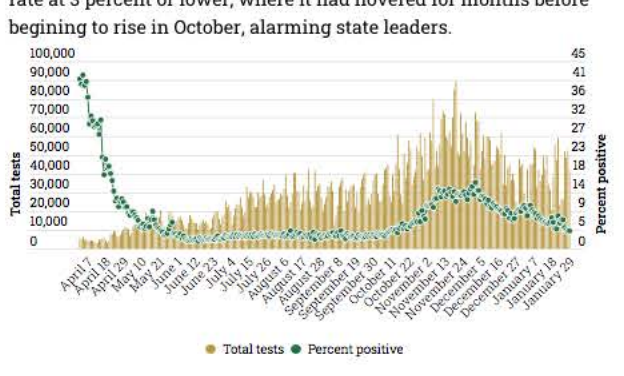

COVID-19 deaths in Michigan

This map shows home residence of those who have died from the coronavirus.

Total deaths: 14,601

- No deaths
- 1 to 25 deaths
- 26 to 49 deaths
- 50 to 99 deaths
- More than 100 deaths

*As of 10 a.m. Jan. 30

Source: Michigan Department of Health & Human Services

Share | Bridge

Daily deaths

Deaths by day reported, since March 25

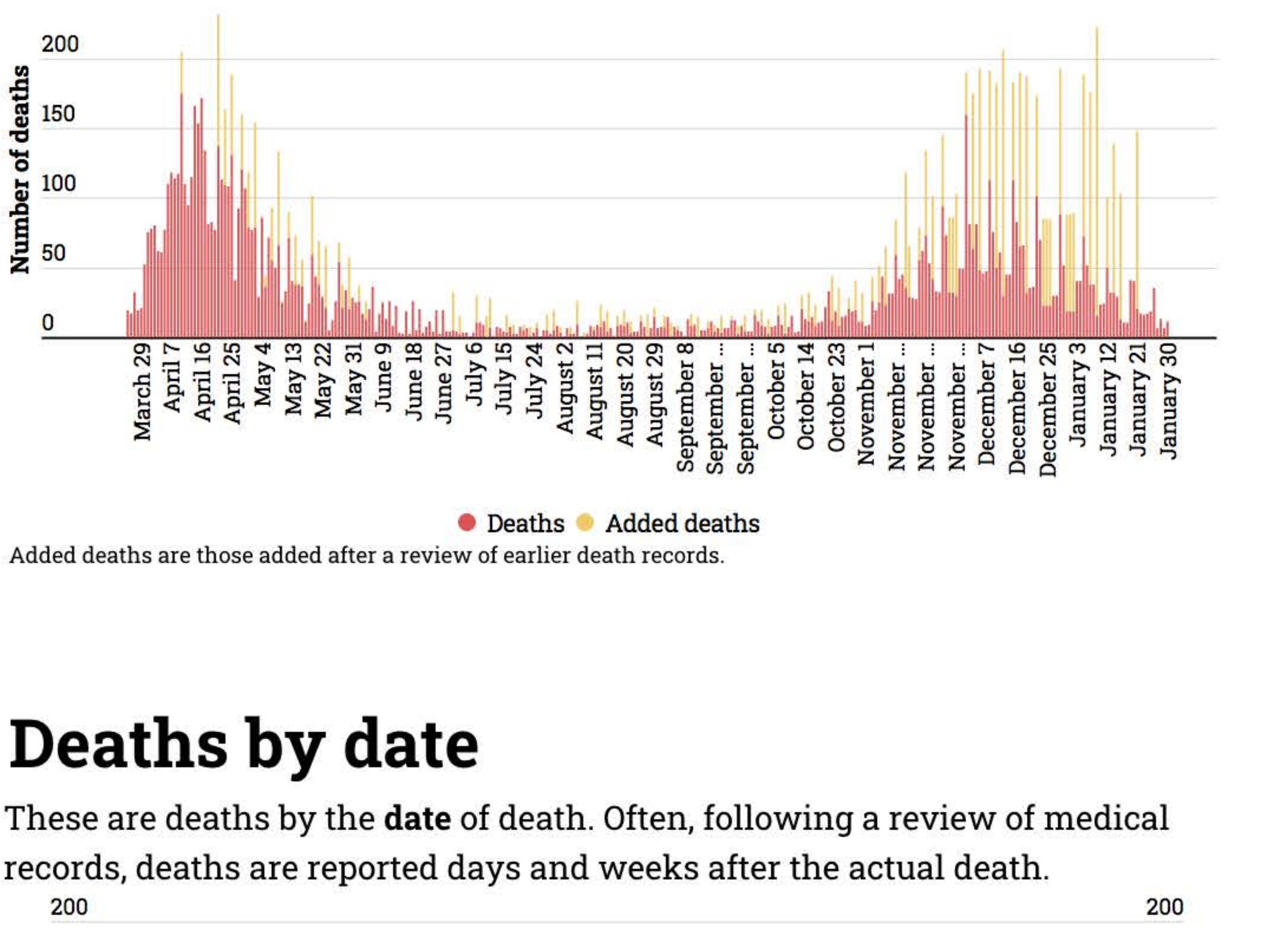

Added deaths are those added after a review of earlier death records.

Deaths by date

These are deaths by the **date** of death. Often, following a review of medical records, deaths are reported days and weeks after the actual death.

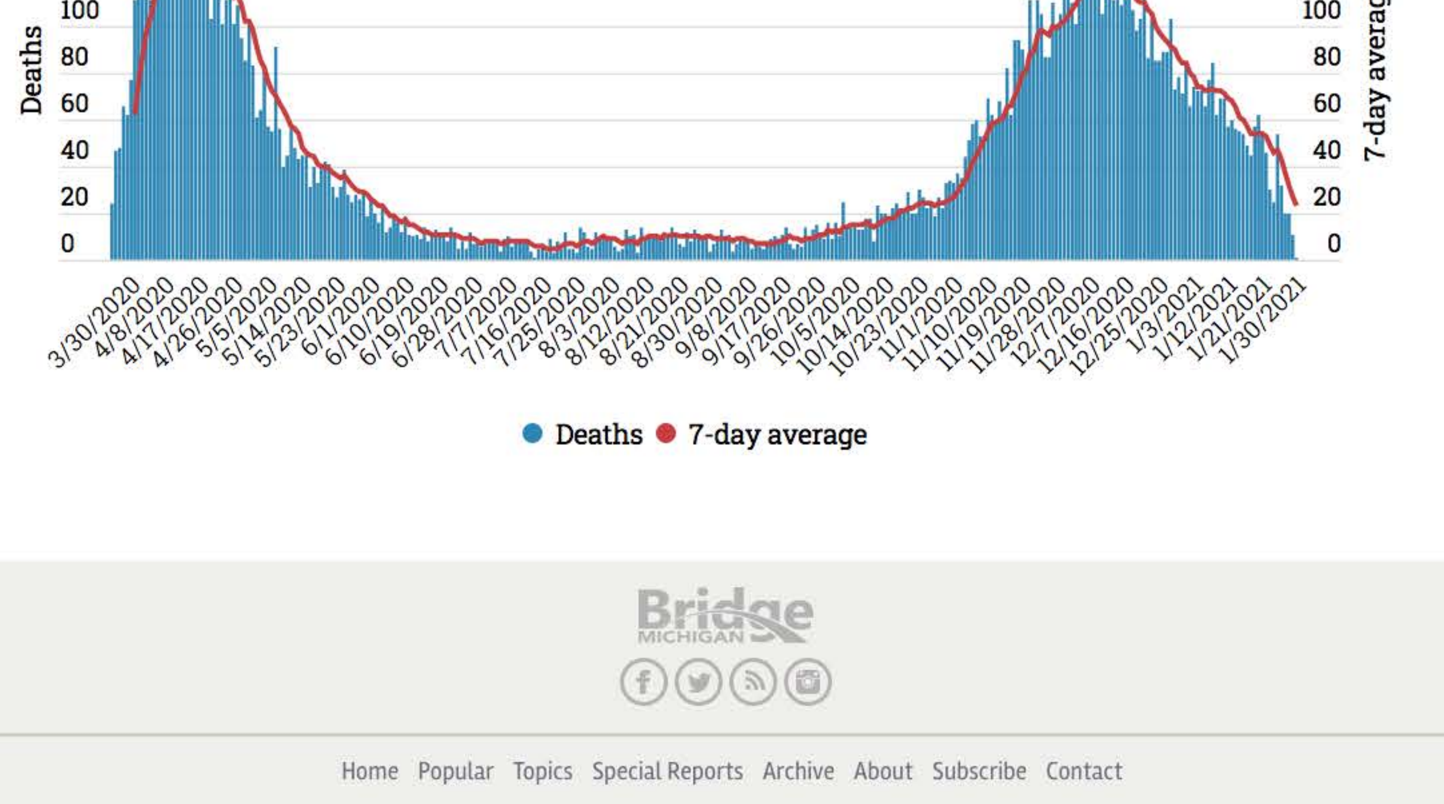

© 2021 Bridge Michigan. All rights reserved.

Website by Gravity Works

COVID-19
- COVID-19 Home
- Situation Update
- About COVID-19
- Testing
- If You Are Sick
- Vaccine
- Close Contacts & Tracing
- Protect Yourself & Others
- Daily Life & Coping
- Materials & Resources
- Guidance Library
- Stories of Community Outreach & Partnership

Information for:
- Health Care
- Community Settings
- Businesses & Employers
- Schools & Child Care
- Institutes of Higher Education

Related Topics
- COVID-19 Research Studies in Minnesota
- COVID-19 Contracts for Diverse Media Messaging & Community Outreach
- Disabilities & Unique Health Needs During COVID-19
- Infectious Respiratory Illness

Situation Update for COVID-19

Subscribe: COVID-19 updates
Sign up to receive email or mobile alerts when this data is updated, as well as other periodic COVID-19 updates.

Updated January 31, 2021
Updated daily at 11 a.m., with data current as of 4 p.m. the previous day.

Data is for cases that were tested and returned positive. All data is preliminary and may change as cases are investigated. Many data points are collected during case interviews. Data presented below is for all cases, regardless of interview status. Data for cases pending interview may be listed as "unknown/missing."

Positive PCR test results are considered confirmed cases. Positive antigen test results are considered probable cases.

All probable cases get the same public health follow up and recommendations as cases confirmed by PCR tests.

On this page:

Daily Update
Testing
Minnesota Case Overview
Case Demographics
Likely Exposure
Residence
Weekly Report
Facilities with Cases & Exposures
Other COVID-19 Data and Statistics

Expand All Contract All

Daily Update:

Because all data is preliminary, the change in number of cumulative positive cases and deaths from one day to the next may not equal the newly reported cases or deaths.

Total positive cases (cumulative)	461,807
Total confirmed cases (PCR positive) (cumulative)	441,778
Total probable cases (Antigen positive) (cumulative)	20,029
Newly reported cases	996
Newly reported confirmed cases	846
Newly reported probable cases	150

Newly reported cases detail

Newly reported deaths	13

Newly reported deaths detail

Testing

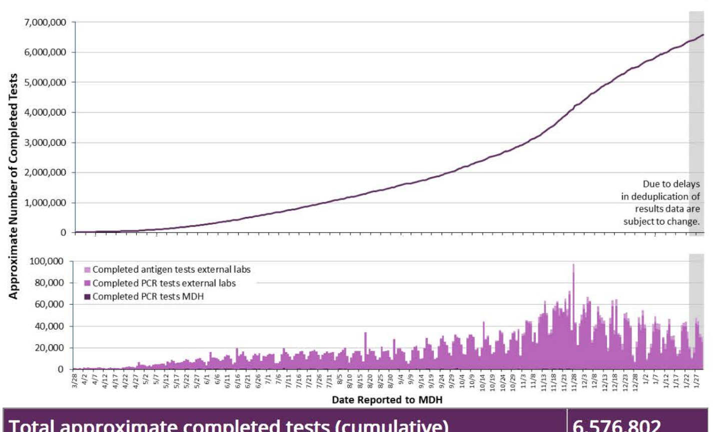

Total approximate completed tests (cumulative)	6,576,802
Total approximate number of completed PCR tests (cumulative)	6,236,640
Total approximate number of completed antigen tests (cumulative)	340,162

Testing data table

Total approximate number of people tested (cumulative)	3,264,300

More about testing data

Minnesota Case Overview

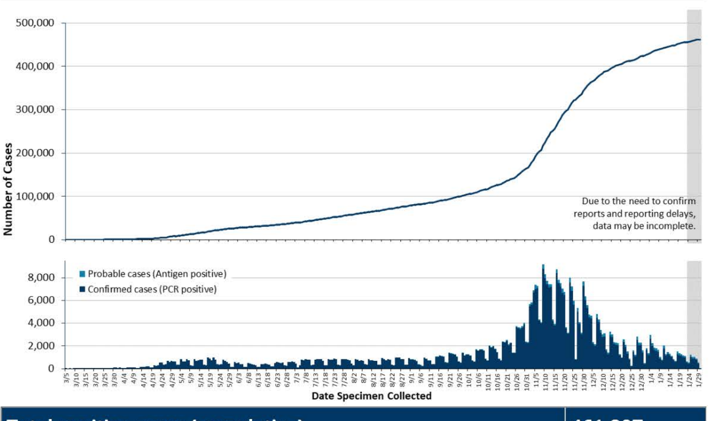

Total positive cases (cumulative)	461,807
Total confirmed cases (PCR positive) (cumulative)	441,778
Total probable cases (Antigen positive) (cumulative)	20,029

Positive cases by date specimen collected data table

Total number of health care workers (cumulative)	36,456

More about positive cases

Patients no longer needing isolation

Patients no longer needing isolation (cumulative)	446,137

More about patients no longer needing isolation

Deaths

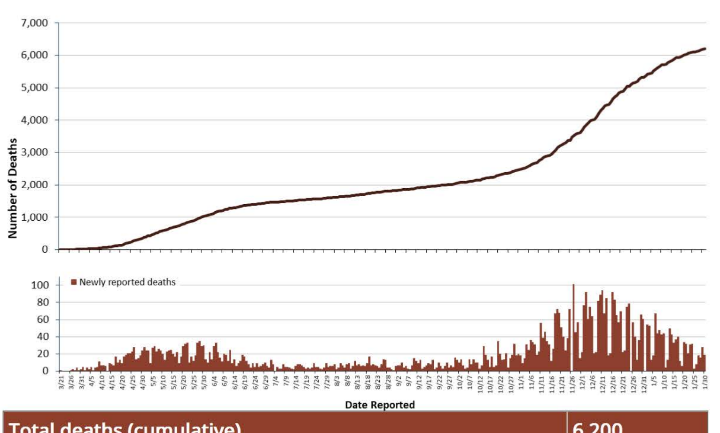

Total deaths (cumulative)	6,200
Deaths from confirmed cases (cumulative)	5,952
Deaths from probable cases (cumulative)	248
Deaths among cases that resided in long-term care or assisted living facilities (cumulative)	3,929

Deaths data table

Total non-laboratory-confirmed COVID-19 deaths (death certificate) (cumulative)	68

More about deaths

Hospitalizations

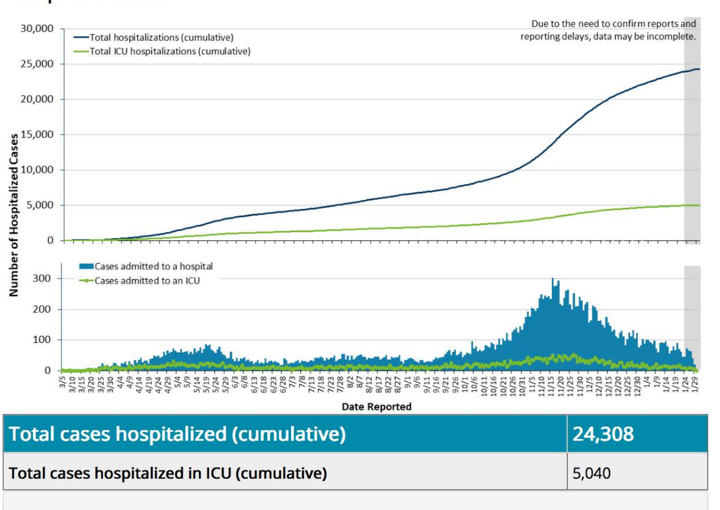

Total cases hospitalized (cumulative)	24,308
Total cases hospitalized in ICU (cumulative)	5,040

Hospitalization data table

More about hospitalizations

Case Demographics

Age

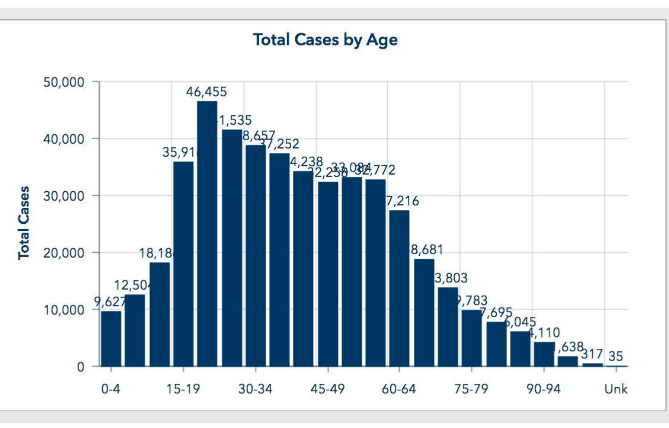

Age group data table:
Including age group of deaths

More about age

Gender

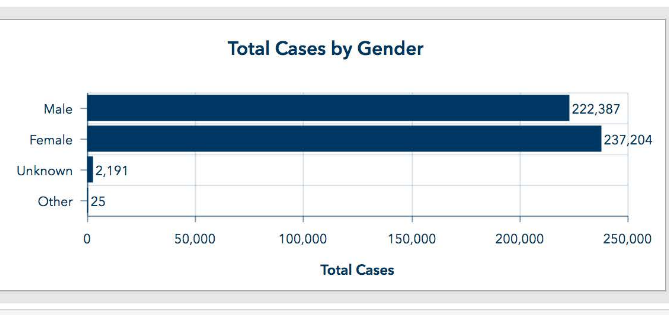

Gender data table

More about gender

Race & Ethnicity

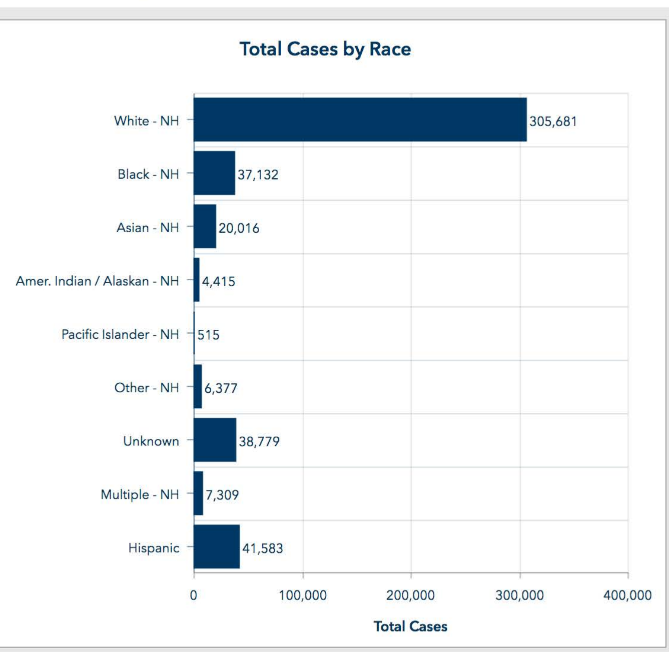

Race and ethnicity data table:
Including race and ethnicity of deaths

More about race and ethnicity

Likely Exposure

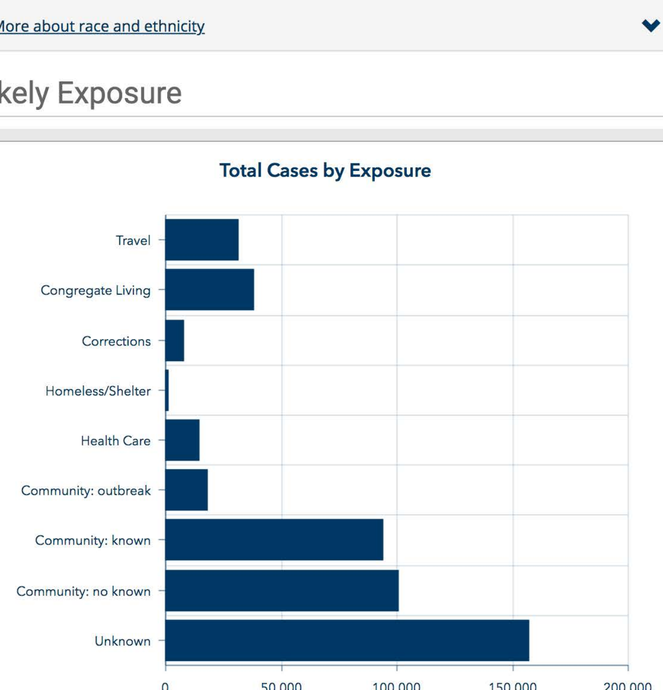

Likely exposure data table

More about likely exposure

Not all cases among health care workers have a likely exposure as health care staff.

Residence

Cases by County of Residence

County of residence is confirmed during the case interview. At the time of this posting not all interviews have been completed.

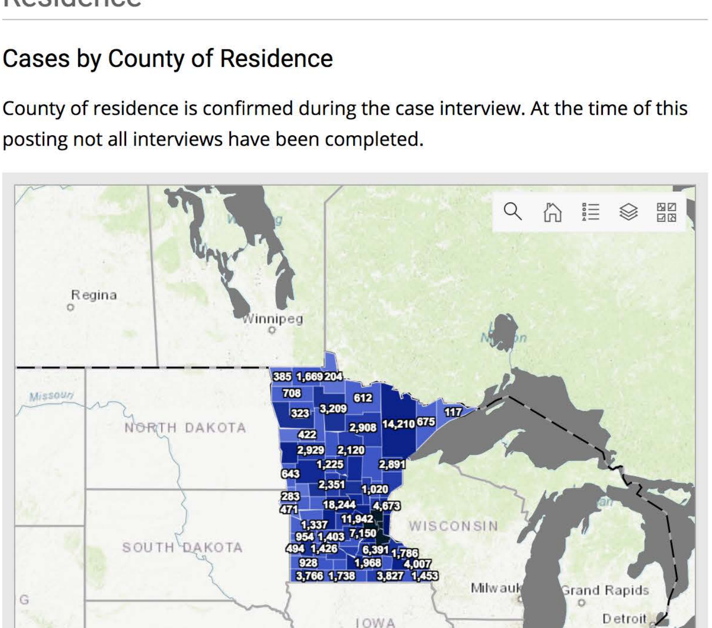

County of residence data table:
Including county of residence of deaths

More about location data

Minnesota Department of Health

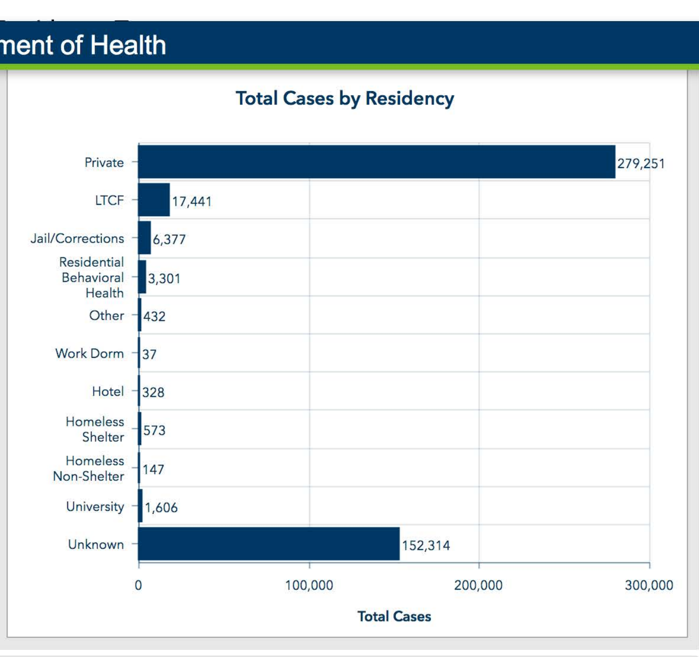

Residence type data table

- MN DOC: COVID-19 Testing of People Incarcerated in MN Prisons

Share This

Subscribe: COVID-19 updates

STAY SAFE MN
Minnesota COVID-19 Response

COVID-19 VACCINE

TESTING FOR COVID-19

SCHOOLS PLANNING & DATA

Minnesota Helpline:
For questions related to the COVID-19 pandemic, call 651-297-1304 or 1-800-657-3504
Mon.-Fri.: 9 a.m. to 4 p.m.

Mental Health Crisis Line - Call: **CRISIS (**274747)

COVID-19 information in other languages:
- Kab Mob Khaus Las Nas Vais Lav 2019 (COVID-19) (Hmong)
- Cudurka Koronafayras 2019 (COVID-19) (Somali)
- Enfermedad del Coronavirus 2019 (COVID-19) (Spanish)
- American Sign Language (ASL) Videos

Contact us:
If you have questions or comments about this page, use our IDEPC Comment Form or call 651-201-5414 for the MDH Infectious Disease Epidemiology, Prevention and Control Division.

E-Newsletter
Get disease updates, inspection results, health news and much more with our weekly e-mail newsletter
email address
SUBSCRIBE

Interactive Maps: Mississippi COVID-19 Cases and Incidence

Select Language
▸ More COVID-19 charts and data

Current COVID-19 Activity: 7-Day Case Incidence

FULL SCREEN

Mississippi COVID-19 Cases Incidence Rate *
as of January 29, 2021

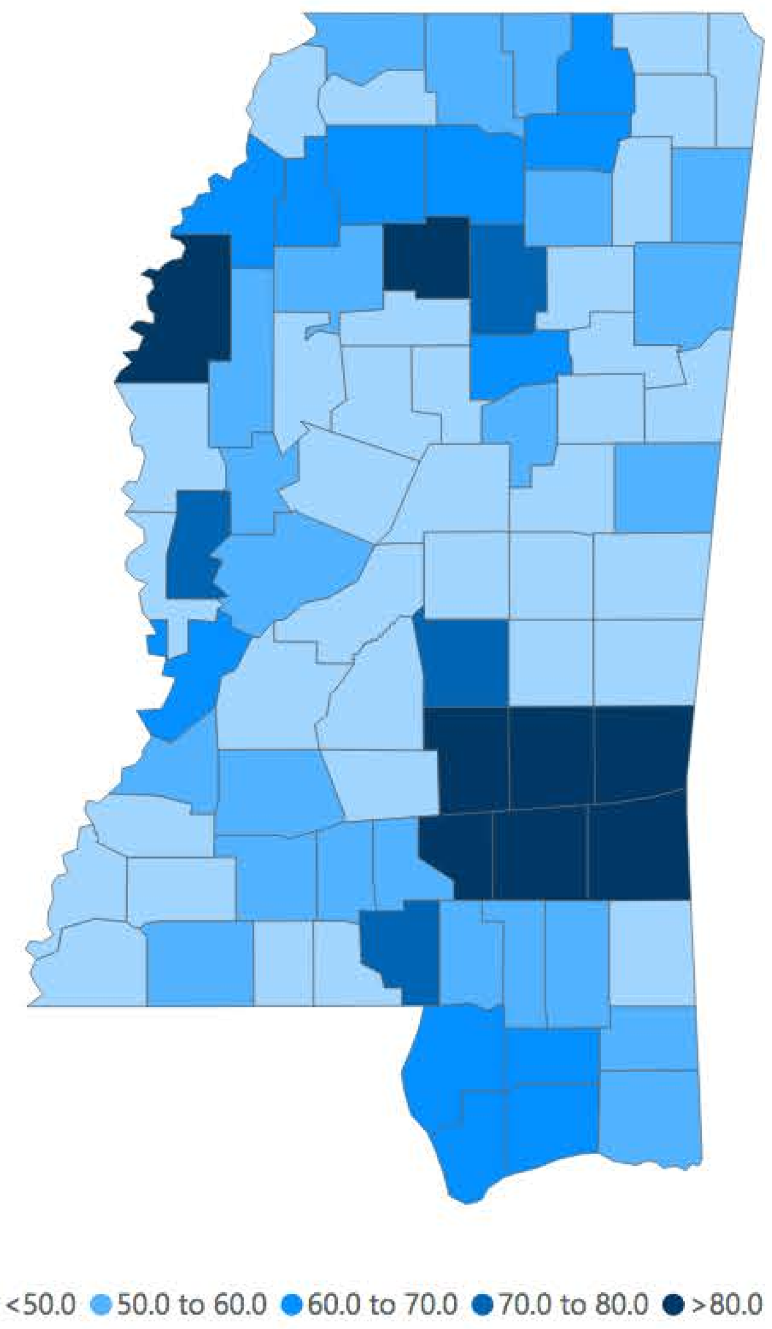

7 Day Average New Cases
1,595.3

7 Day Average Incidence Rate *
54.0

Cumulative Cases
274,190

2019 Estimated Population
2,976,148

County	Incidence Rate	New Cases (7-Day Avg)	2019 Est. Population	Mask Mandate Status	Mask Mandate Started	Mask Mandate Ended
Jasper	113.4	18.6	16,383	None		
Wayne	109.7	22.1	20,183	None		
Jones	104.5	71.1	68,098	Active	11/24/20	
Bolivar	90.5	27.7	30,628	Active	11/24/20	
Yalobusha	89.7	10.9	12,108	Active	11/10/20	
Smith	88.0	14.0	15,916	None		
Covington	83.6	15.6	18,636	Active	11/11/20	
Clarke	81.8	12.7	15,541	None		
Sharkey	79.3	3.4	4,321	None		
Marion	77.3	19.0	24,573	None		

** Based on average daily new cases of past 7 days per 100,000 population.*

Microsoft Power BI

Cumulative COVID-19 Cases and Deaths

FULL SCREEN

Mississippi COVID-19
Cases and Deaths by Race with Ethnicity
as of 6 pm CT, January 29, 2021

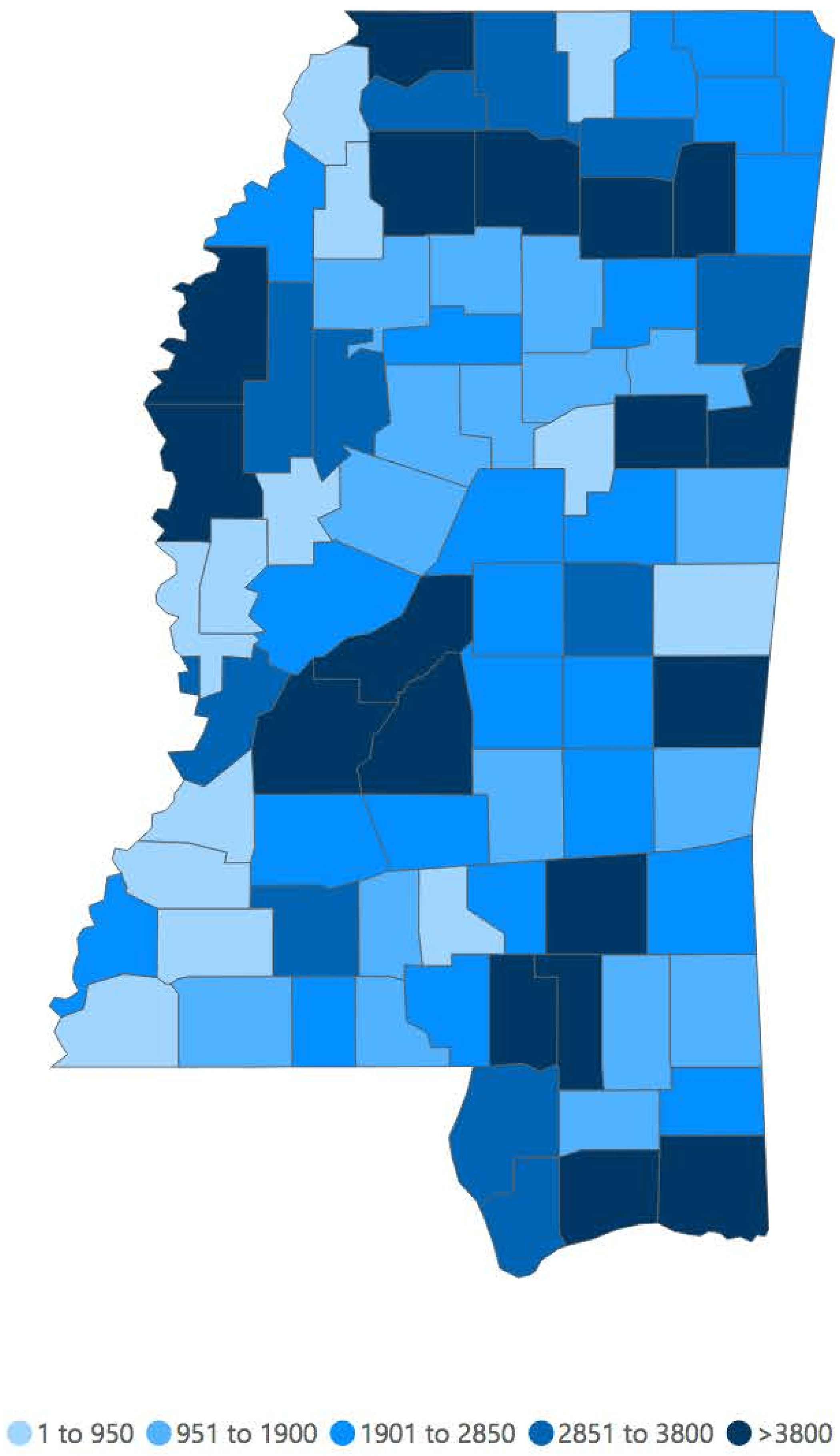

Total Cases
274,190

	American Indian or Alaska Native	Asian	Black	White	Other	Unknown
Non Hispanic	1,083	942	73,811	103,459	2,674	2,229
Hispanic	49	32	358	1,790	3,176	629
Unknown Ethnicity	962	317	16,926	25,511	6,862	33,380

Total Deaths
6,018

	American Indian or Alaska Native	Asian	Black	White	Other	Unknown
Non Hispanic	81	10	2,083	2,710	33	12
Hispanic	1	0	7	28	17	2
Unknown Ethnicity	23	7	313	496	54	141

Microsoft Power BI

STATEWIDE | COUNTY | VACCINE | SCHOOL DISTRICTS | DEMOGRAPHICS | HEALTHCARE SYSTEM | TESTING

COVID-19 in Missouri

NOTE on dates: This dashboard was last updated on the morning of **1/31/2021** and includes data reported through **1/30/2021**. All 7-day metrics are based on the date a test or death *occurred*, and are subject to a 3-day delay to ensure data are accurate and complete. See FAQ for details.
Range for 7-Day metrics: 1/22/2021 through 1/28/2021

Note: Please see our "Details about Metrics" page for a message regarding Pettis County data.

COVID-19 in Missouri to Date

Confirmed Cases to Date
458,819

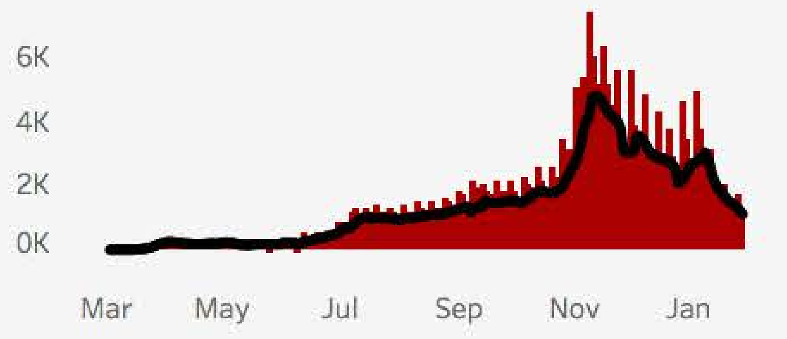

Confirmed Deaths to Date
6,748

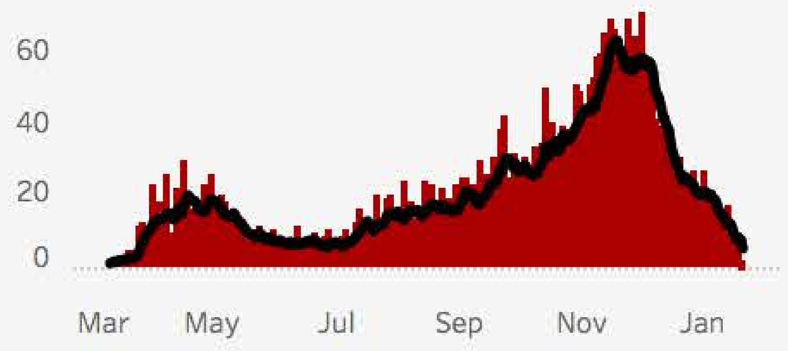

PCR Tests to Date
4,156,792
Antigen Tests: **638,827**

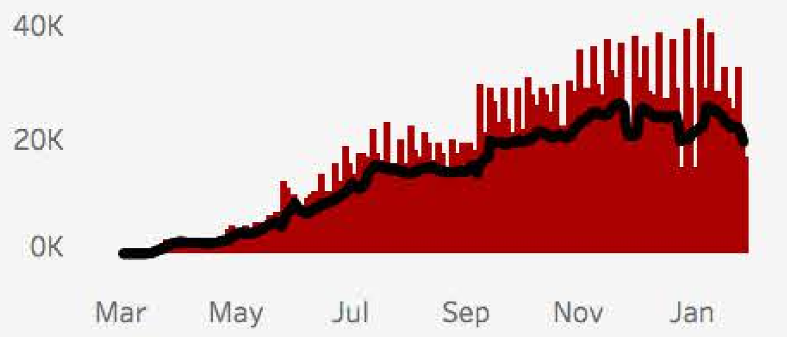

COVID-19 in Missouri - Past 7 Days (based on date of occurrence)

	1/28	1/27	1/26	1/25	1/24	1/23	1/22
Confirmed Cases: Past 7 Days **7,913** (daily avg. of 1,130)	1,041	1,031	1,238	1,797	649	805	1,352
Confirmed Deaths: Past 7 Days **53** (see note)	2	10	8	3	4	9	17
PCR Tests: Past 7 Days **92,743**	11,311	12,144	15,988	23,945	6,492	7,303	15,560
Antigen Tests: **46,893** (total daily avg. of 19,948)	5,989	7,054	7,437	9,653	6,778	3,629	6,353

For the maps and bar charts, select what you'd like to display:
See the FAQs to learn more about what each metric means.

Deaths | Testing | Positivity Rate | Cases | Total Volume | Per 100k

Cases by Jurisdiction to Date
Colored by Per 100k

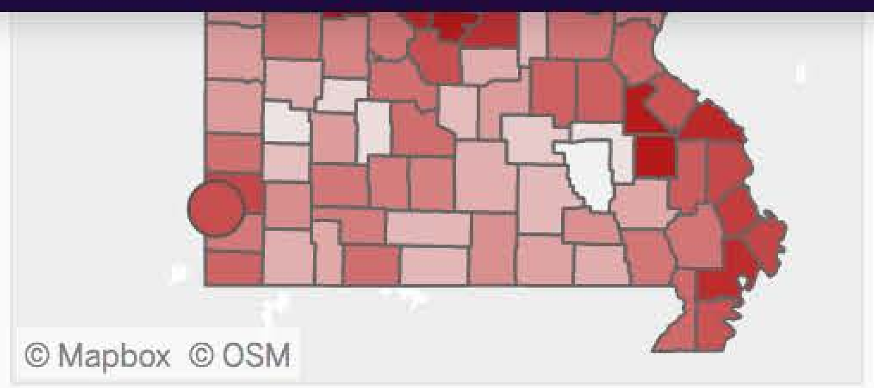

Sorted by Cases (Per 100k)
Colored by Total Volume

[illegible]	[illegible]	10,912
COLE	6	10,887
PERRY	7	10,410
MONITEAU	8	10,259
SALINE	9	10,254
NEW MADRID	10	10,219
OSAGE	11	9,982
[illegible]	12	[illegible]

Past 7 Days: Cases by Juris.
Colored by Per 100k

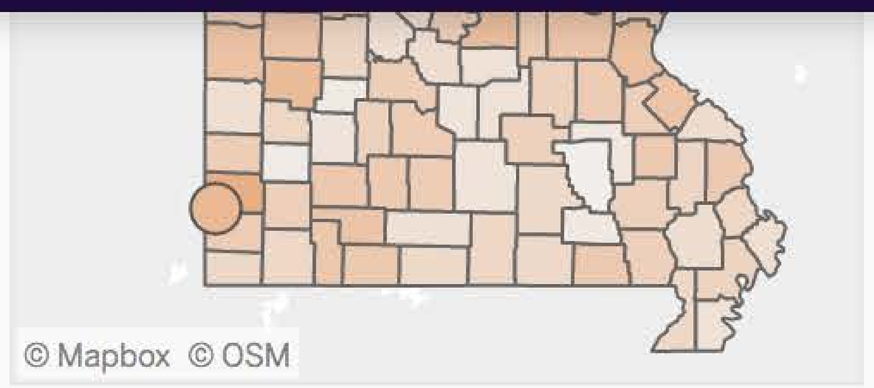

Sorted by Cases (Per 100k)
Colored by Total Volume

[illegible]	5	204.2
ADAIR	6	201.2
LIVINGSTON	7	197.0
ST CLAIR	8	191.6
RAY	9	191.2
OSAGE	10	176.3
JEFFERSON	11	174.6
[illegible]	[illegible]	[illegible]

Show Me Strong RECOVERY PLAN | HOME | BUSINESSES | COMMUNITIES | CITIZENS | DATA | FAQ | HIRING HEALTHCARE HEROES | RESOURCES | Select Language

HOW TO READ THIS CHART

What can I do to help?

- Wash your hands often
- Social distancing (>= 6ft)
- Wear a mask
- Cover coughs & sneezes
- Clean & disinfect frequently
- Monitor your health daily

Source: CDC | Protecting yourself & others

The Status of COVID-19 in Missouri vs. Other States

Based on White House Coronavirus Task Force thresholds (see "How to read this chart" for details)

Severity: Past 7 Days
How large is the problem now?

New Cases	**7,913** (129 per 100k)	#24 (#34)
New Deaths	**53** (0.9 per 100k)	#31 (#39)
7-Day Positivity Rate	**9.4% (CDC method)**	

Trajectory: Past 7 vs. Prior 7
In what direction is the problem moving?

% Change in Cases	**-25.4%**
% Change in Deaths	**-36.1%**
%p Change in Positivity Rate	**-1.7%p**

Response
What is being done now?

New Tests (Past 7 Days)	**92,743** (1,511 per 100k)	#27 (#49)
% Change in Tests (Past 7 vs. Prior 7)	**-11.6%**	
Testing Turnaround (Past 7 Days)	**1.7 days**	

COVID-19 Testing in Missouri

This dashboard was last updated on the morning of **1/31/2021** and includes data reported through **1/30/2021**.

Statewide PCR Positivity Rates Over Time

Calculated two different ways - Hover over the chart and read the tooltip to understand the methodology, and see the FAQ for more detail.

Moving 7-day PCR Positivity Rate over Time - Not Deduplicated to the Individual (CDC method)

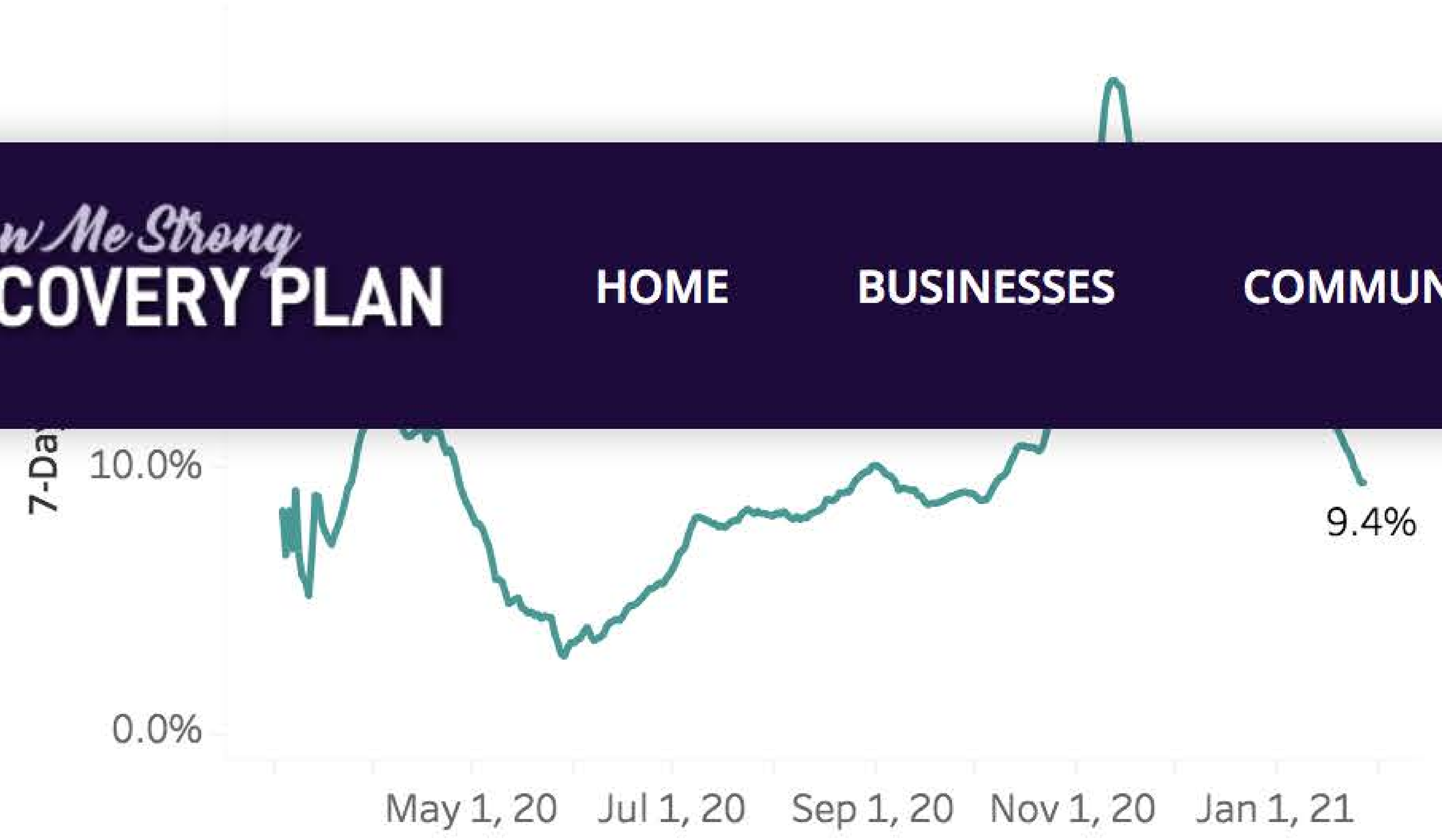

Cumulative PCR Positivity Rate Over Time - Deduplicated to the Individual

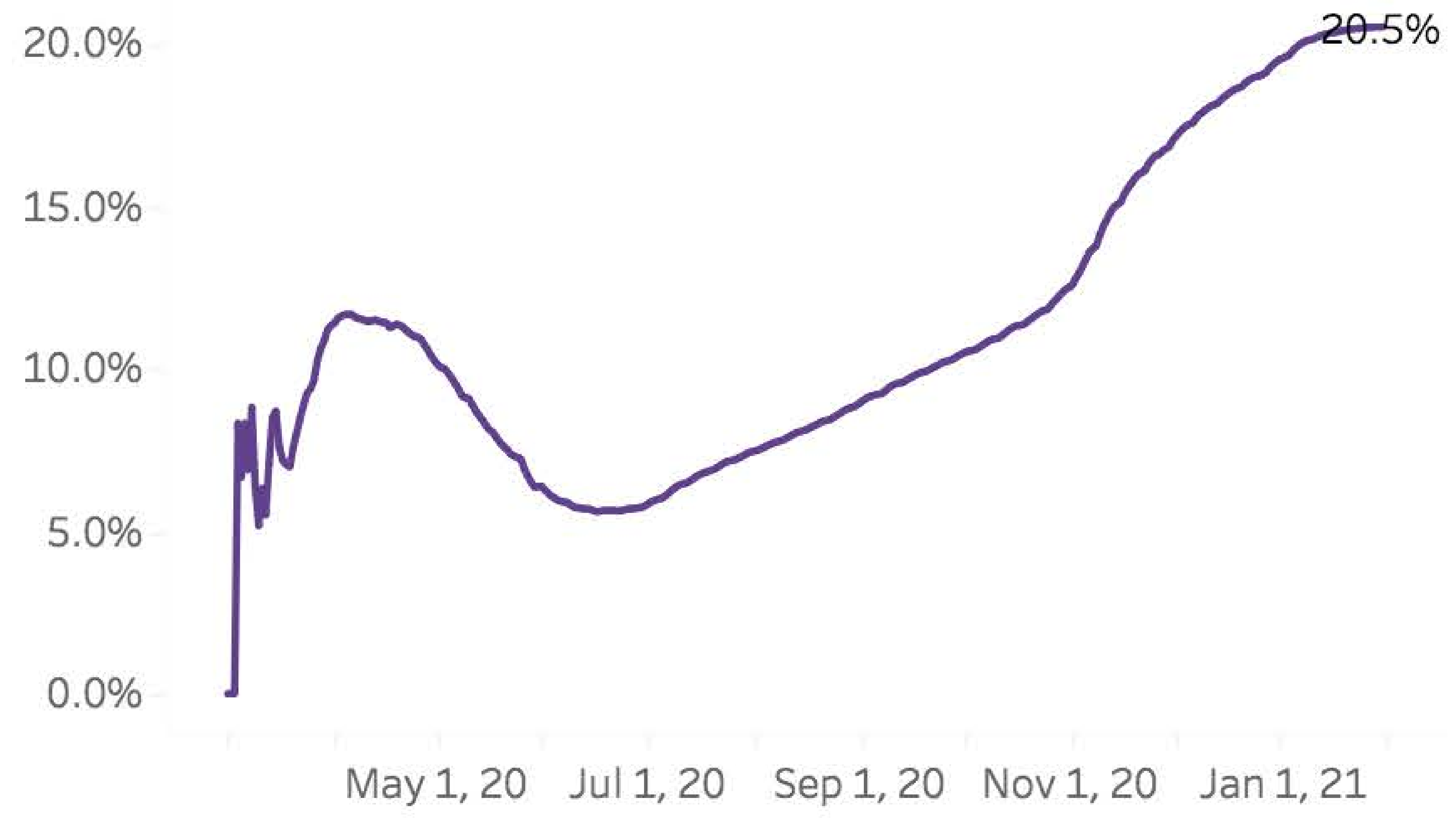

Test Results by Test Type

See the FAQ for more details about types of tests.

Test Type	Total Tests	
PCR	4,156,792	3,641,261 88%
[illegible]	[illegible]	89%

Individuals Tested by Test Type

This metric counts people, instead of tests.

Test Type	Total Individuals Tested
PCR	2,234,208
[illegible]	[illegible]

Result: Positive | Negative | Indeterminate

Weekly Total Testing by Test Type

See the FAQ for more details about types of tests.

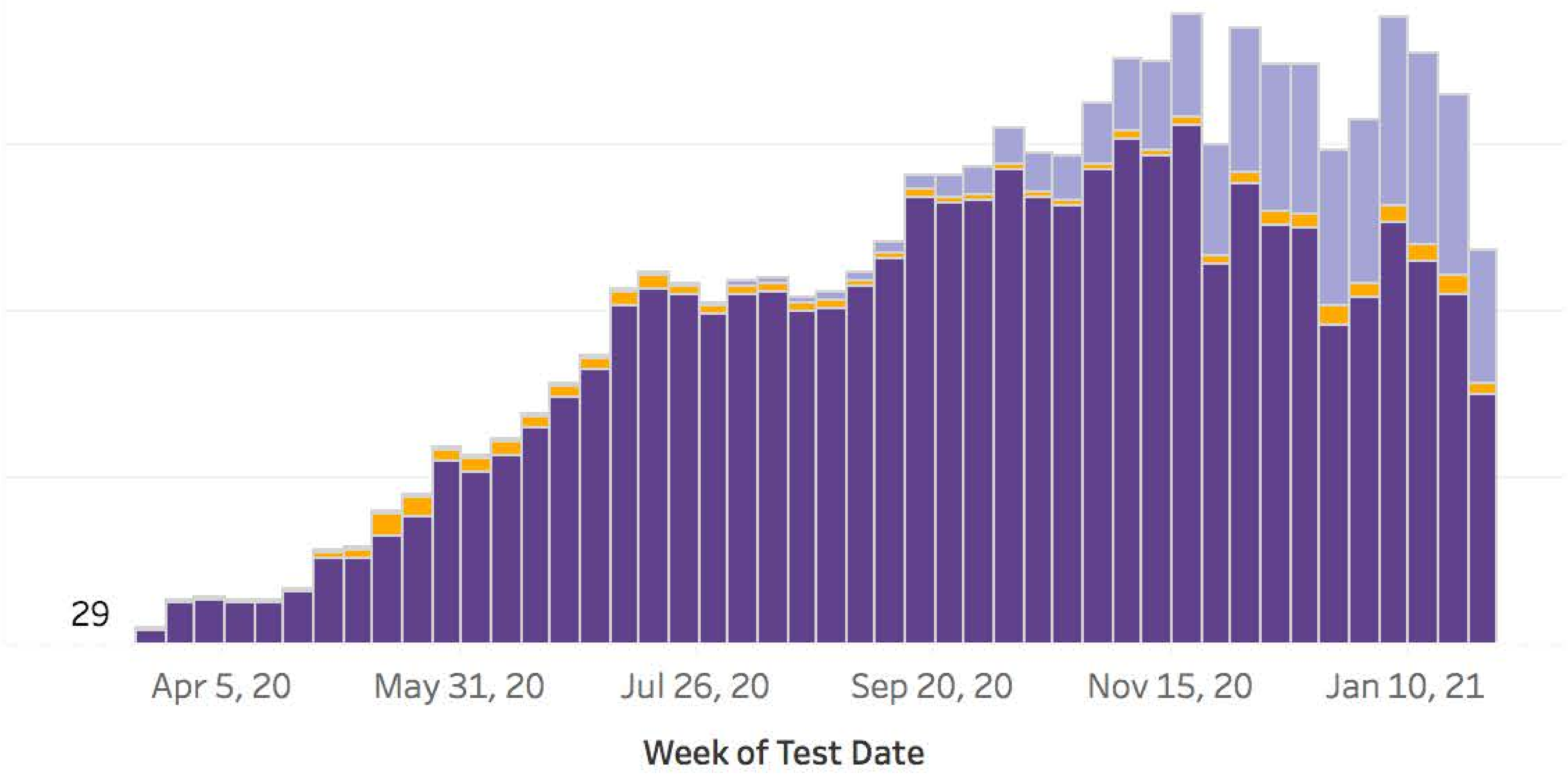

Test Type: ANTIGEN | SEROLOGY | PCR

COVID-19 Vaccinations in Missouri

Overall Vaccinations in Missouri

This dashboard was last updated on **1/31/2021** and contains data on vaccinations adminstered through **1/31/2021**. Historical numbers, especially over the most recent few days, will update as providers report data to the state. Differences between this dashboard's numbers and those on CDC's dashboard are primarily due to timing.

Total doses administered (includes first and second doses)	Number of people who have received at least one dose	Number of people who have received a second dose	Share of population who have received at least one dose	Doses administered in past 7 days (1/23/2021 through 1/29/2021)
**511,657**	**394,065**	**117,592**	**6.4%** (1.9% have received two doses)	**143,512** (average of 20,502 per day)

Doses Administered Over Time

Line represents 7-day average. Note that data from the most recent days may be incomplete because of delays in reporting.

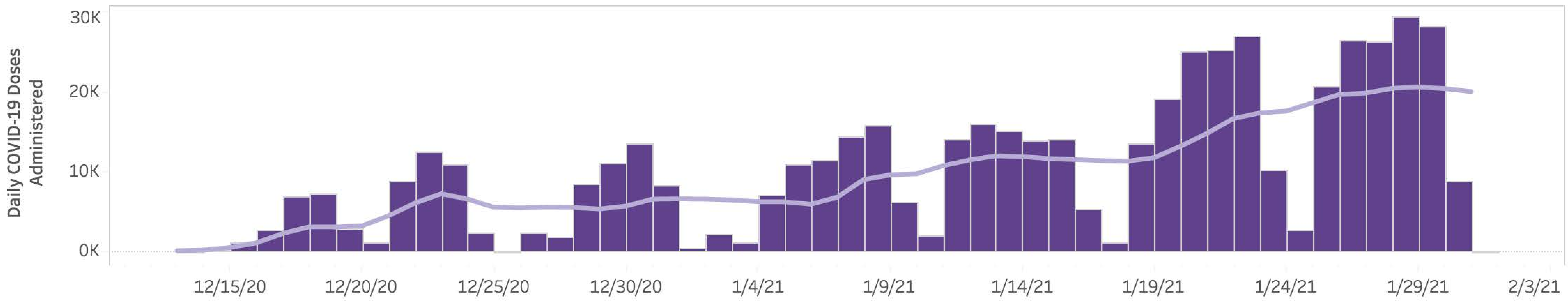

Who Has Been Vaccinated?

These charts show vaccination progress by demographic group. The % rates reflect the share of the population group who has received at least one dose. These rates will increase over time as more people are vaccinated. Hover over the bar chart to see the number of first doses for that population group.

Notes: These rates exclude records for which we have no sex, race, and/or ethnicity on file, so the actual rates may be higher. These rates include persons who list their address as outside of Missouri but received their vaccination in Missouri.

Share of Population Initiating Vaccination by Age Group

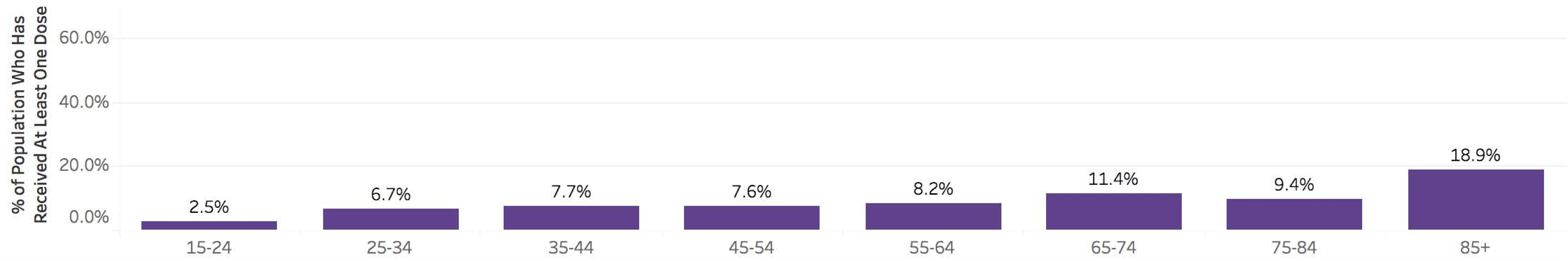

Share of Population Initiating Vaccination by Sex

Share of Population Initiating Vaccination by Race

Share of Population Initiating Vaccination by Ethnicity

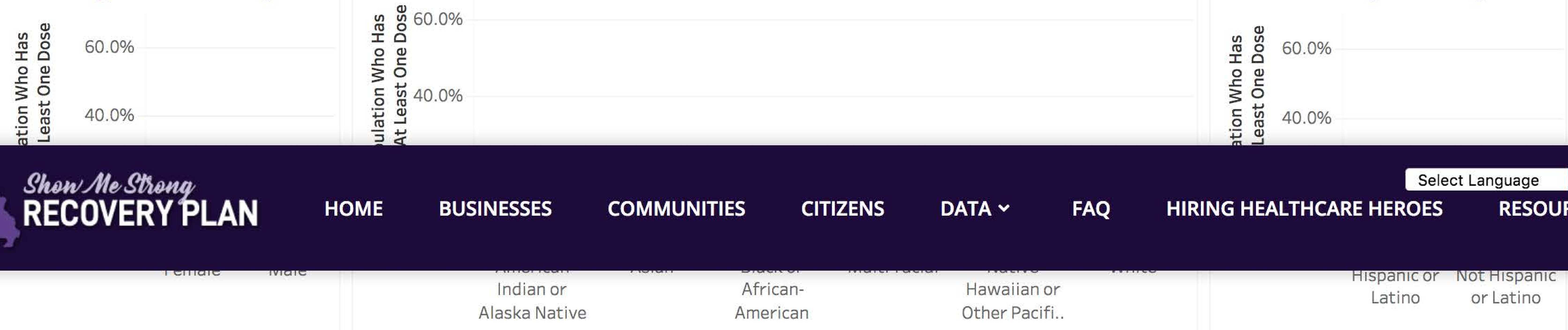

Show Me Strong RECOVERY PLAN
HOME | BUSINESSES | COMMUNITIES | CITIZENS | DATA | FAQ | HIRING HEALTHCARE HEROES | RESOURCES
Select Language

Where Do Vaccinated Residents Live?

Note: Some recipients of Missouri vaccines list their address as in another state. They do not appear in this county data, so the county data will not add to total doses administered.

Share of Population who have received at least first dose

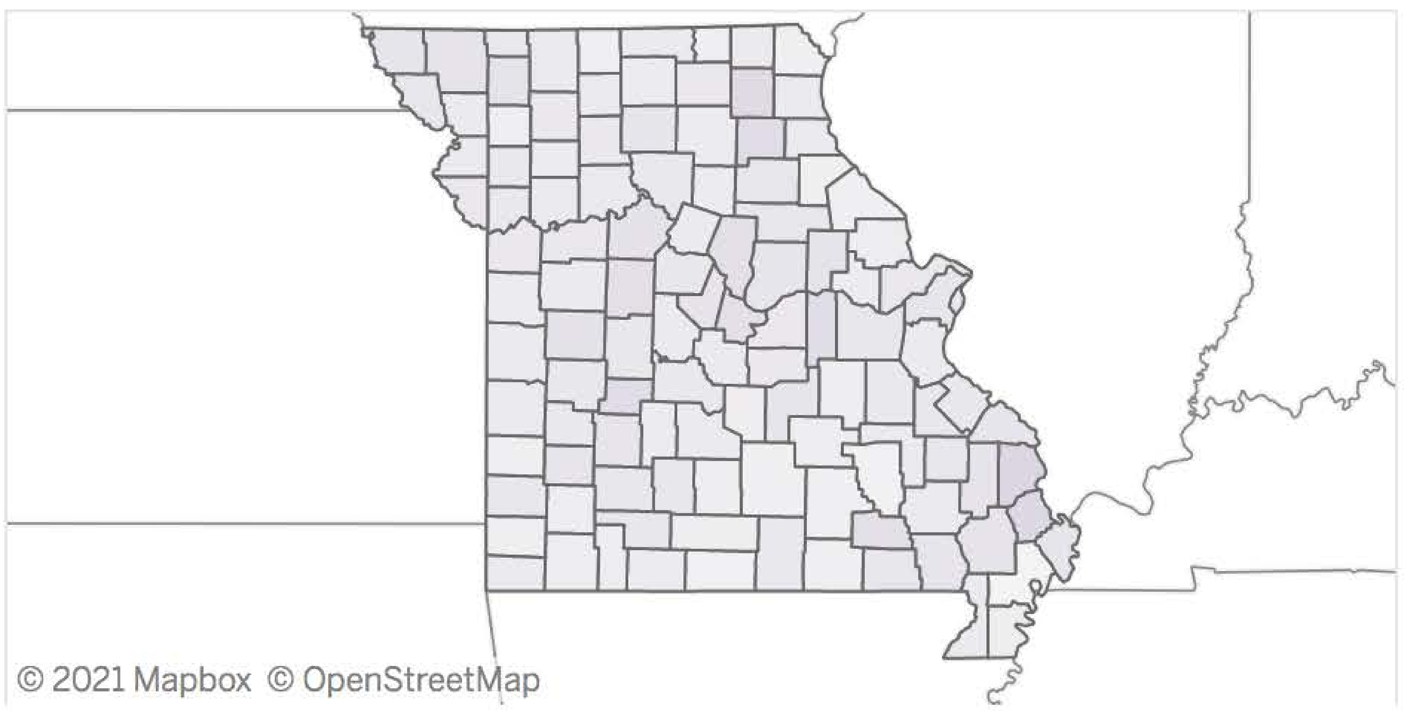

Percent of Population First Dose
0.0% – 100.0%

Detail by County

County Name	Total COVID-19 Doses Administered	First COVID-19 Dose Administered	Second COVID-19 Dose Administered	Percent of Population Who Has Received First Dose	7-Day COVID-19 Doses Administered
Adair	1,840	1,301	537	5.1%	122
Andrew	1,006	741	265	4.2%	244
Atchison	402	321	81	6.2%	161
Audrain	1,752	1,517	235	6.0%	497
Barry	1,391	1,104	280	3.1%	423
Barton	373	289	83	2.5%	89
Bates	901	662	239	4.1%	395
Benton	1,298	1,089	209	5.6%	629
Bollinger	970	852	118	7.0%	331
Boone	22,153	15,755	6,376	8.7%	4,792

MONTANA RESPONSE: COVID-19 - Coronavirus - Global, National, and State Information

Montana Maps | Montana State Library

COVID-19 Vaccine | MT COVID-19 Cases | COVID-19 Demographics | Frequently Asked Questions | MT Dept. of Public Health | Global Outbreak Dashboard | Community Planning Info

Cumulative Cases
93,949

191 New Cases

Cumulative, New Daily, Active Cases by County

Flathead County Cases
10,153 Total | 41 New | 635 Active

Yellowstone County Cases
15,449 Total | 29 New | 798 Active

Missoula County Cases
7,704 Total | 26 New | 263 Active

Gallatin County Cases
11,366 Total | 20 New | 302 Active

Lewis and Clark County Cases
5,895 Total | 11 New | 196 Active

Glacier County Cases
1,465 Total | 8 New

Montana Coronavirus Active Cases by County

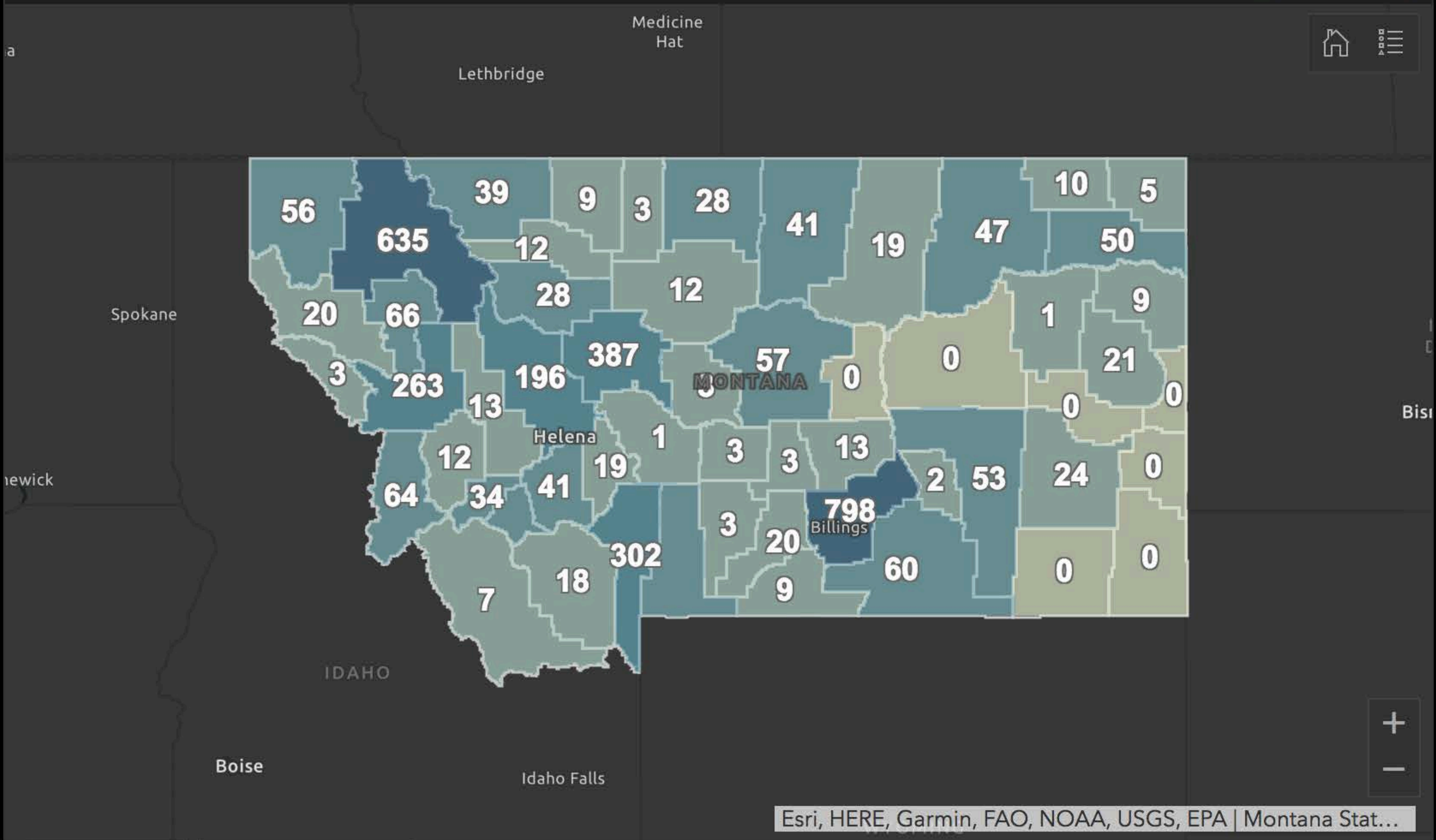

Active Cases | Cumulative Cases

Updates are made between 10:00 and 11:30 a.m. daily. Information is reported based on the previous day. It is highly likely that the information on this dashboard and map will change based on further public health investigation. To learn more about the information on this map and dashboard: More Information. For information on demographics and nonresidents, click here. Source Data: Montana Department of Health & Human Services, Montana State Library. Date and time of last update: [illegible]

Total Deaths
1,233

Recovered
89,073

Active
3,643

Total Tests
954,249
New Tests: 3,506

Active Hospitalizations
101

Total Hospitalizations
4,245

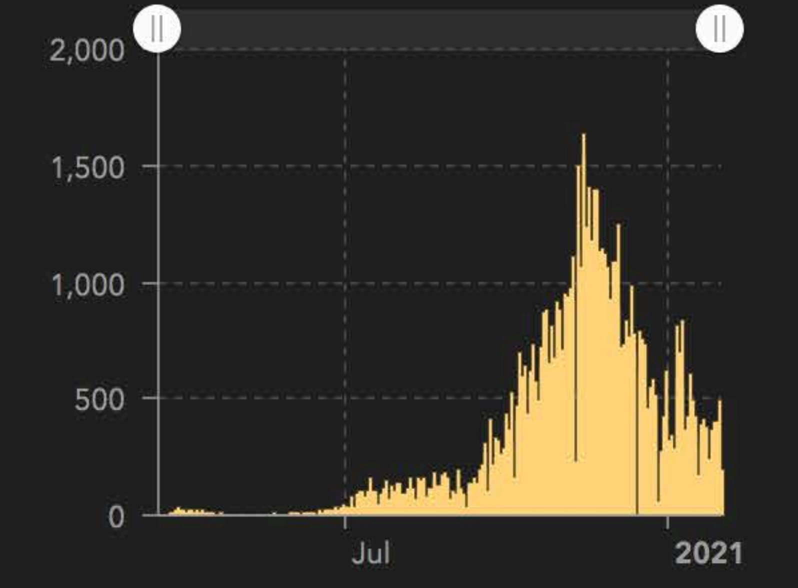

Cases by Day

MONTANA RESPONSE: COVID-19 - Coronavirus - Global, National, and State Information

Montana Maps

COVID-19 Vaccine | MT COVID-19 Cases | COVID-19 Demographics | Frequently Asked Questions | MT Dept. of Public Health | Global Outbreak Dashboard | Community Planning Info

Total Doses Administered

106,121

Total Montanans Fully Immunized

26,388

COVID-19 Vaccinations By County

Beaverhead County Vaccines:
626 Doses Given | **83** Fully Immunized

Big Horn County Vaccines:
1,503 Doses Given | **259** Fully Immunized

Blaine County Vaccines:
1,147 Doses Given | **365** Fully Immunized

Broadwater County Vaccines:
186 Doses Given | **28** Fully Immunized

Carbon County Vaccines:
742 Doses Given | **133** Fully Immunized

Carter County Vaccines:
110 Doses Given | **11** Fully Immunized

Cascade County Vaccines:
5,891 Doses Given | **1,099** Fully Immunized

Chouteau County Vaccines:
422 Doses Given | **12** Fully Immunized

Custer County Vaccines:
820 Doses Given | **107** Fully Immunized

COVID-19 Vaccinations by County of Residence: Administered Doses per 1,000 Eligible People

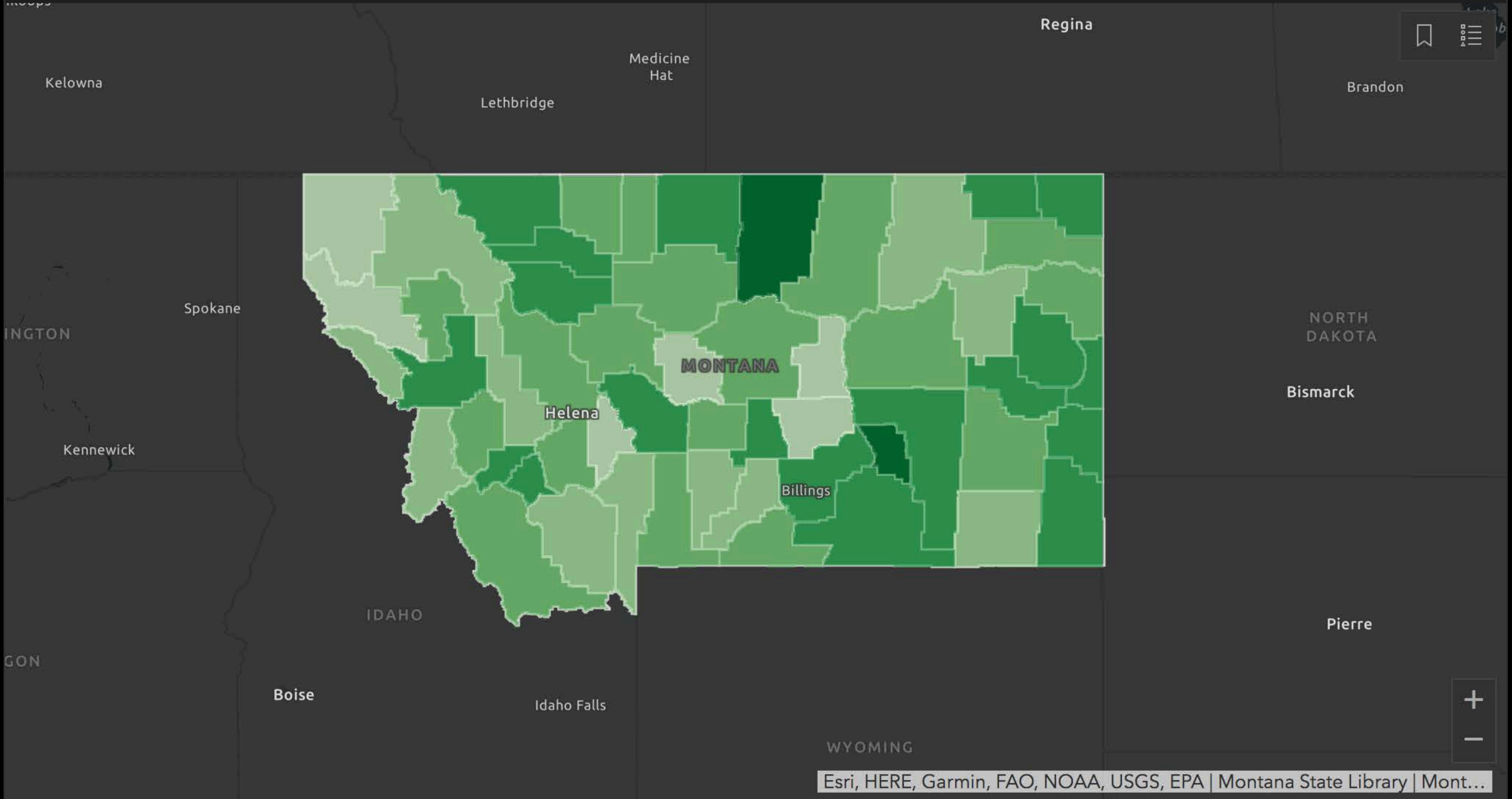

Information is reported based on the previous day/week. Total Doses Administered and Total Montanans Fully Immunized are updated daily between 10:00 and 11:30 a.m. Last Updated: **1/31/2021, 11:23 AM.** County Vaccine Information are updated every Monday, between 10:00 and 11:30 a.m. To learn more about the information on this map and dashboard: More Information. For information on vaccines in Montana click here. For assistance, please e-mail or

COVID-19 · County Alert System: 86 Counties Red and 13 Counties Orange. Read the report

NCDHHS COVID-19 » COVID-19 North Carolina Dashboard

COVID-19 North Carolina Dashboard

Knowing when to dial up or down measures that slow the spread of the virus depends on North Carolina's testing, tracing and trends. This dashboard provides an overview on the metrics and capacities that the state is following.

Updated daily by approximately 12:00 p.m. Last updated January 31, 2021 at 12:45 p.m.

Daily Cases

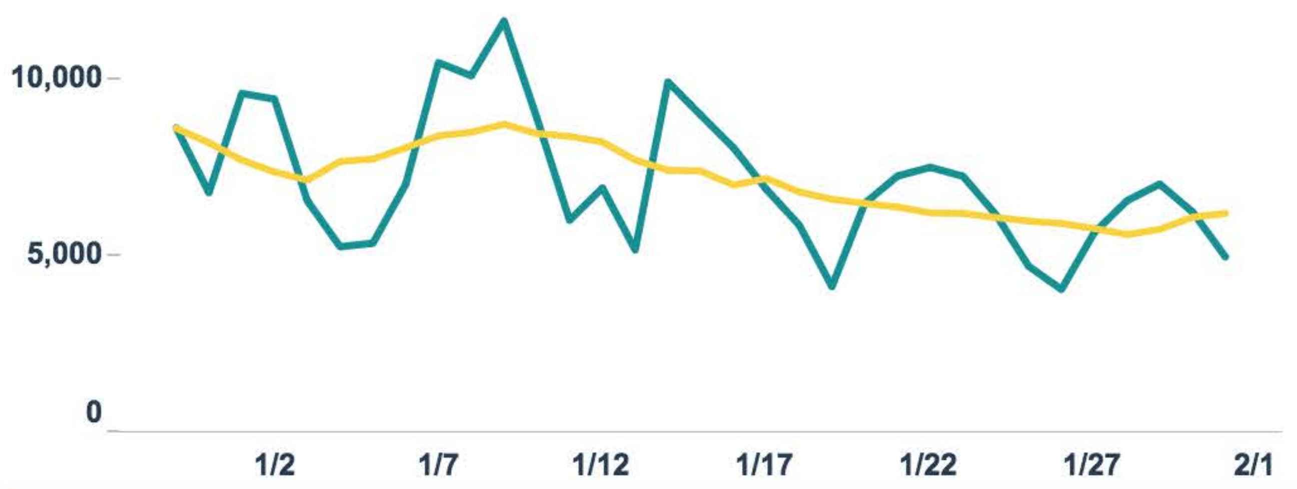

TOTAL CASES

757,526

Molecular (PCR) Positive Cases

676,973

Antigen Positive Cases

80,553

Daily Tests

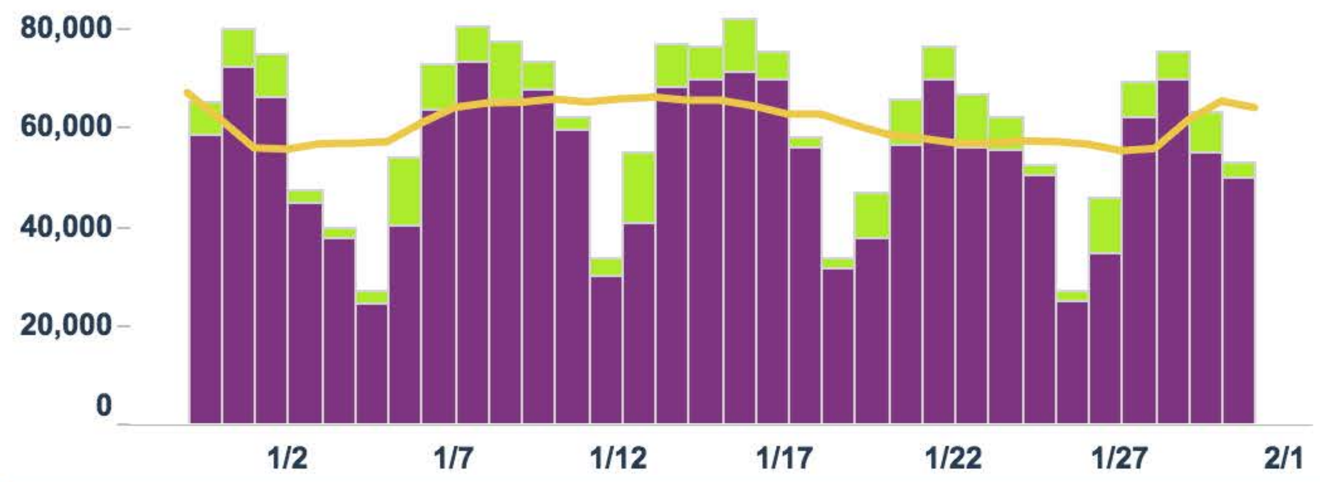

COMPLETED TESTS

8,863,068

Daily Number of People Currently Hospitalized

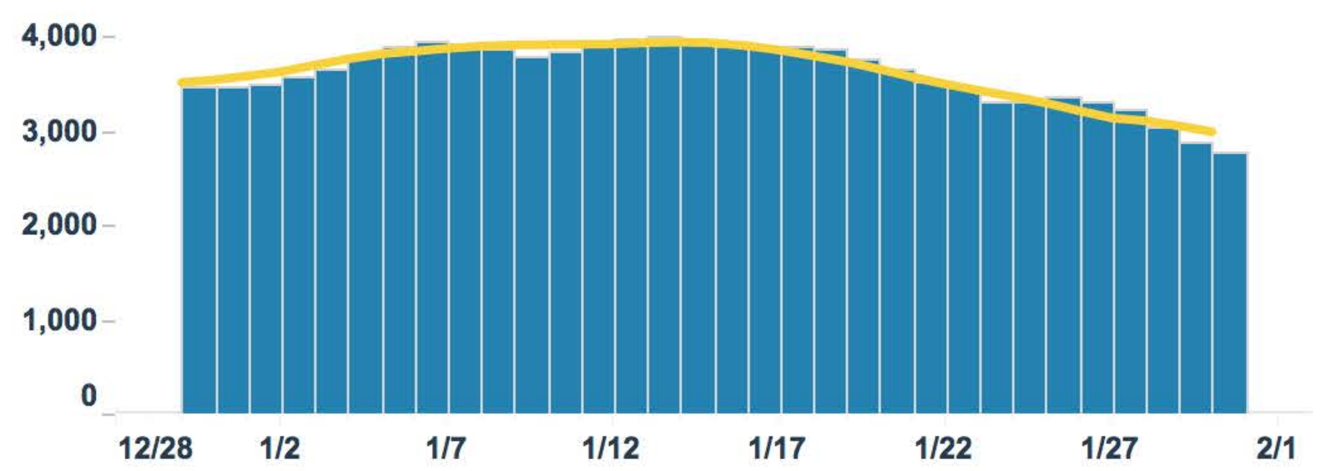

CURRENTLY HOSPITALIZED

2,782

County Map by Cases per 100,000 Residents (Molecular (PCR) and Antigen)
(Click to highlight map, unclick to revert map)

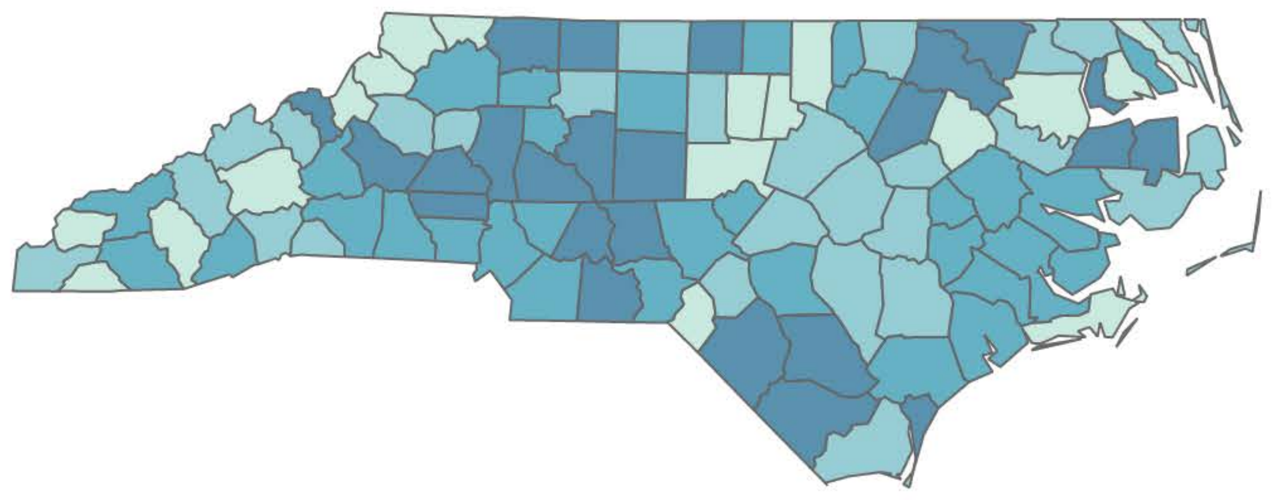

150-400 | 601-750 | 901+
401-600 | 751-900

Dashboard

Summary

County Alert System

Vaccinations

CLI Surveillance

Cases

Cases Demographics

Testing

Hospitalizations

Hospitalization Demographics

Contact Tracing

PPE

Outbreaks and Clusters

Reports

Data Behind the Dashboards

Home | About COVID-19 | Vaccines | Dashboard | Information For | Guidance | Slow The Spread

Cases per 100,000 Residents

Date Range
Last 14 Days

Total County Cases and Deaths (Molecular (PCR) and Antigen)
(Click to highlight map, unclick to revert map)

County	Cases	Deaths
Alamance	13,788	172
Alexander	3,503	59
Alleghany	827	4
Anson	2,031	42
Ashe	1,682	34
Avery	1,683	16
Beaufort	3,788	71
Bertie	1,523	35

Hover over icons for information.

January 31, 2021

+ a b l e a u

Testing

Dashboard

Summary

County Alert System

Vaccinations

CLI Surveillance

Cases

Cases Demographics

Testing

Hospitalizations

Hospitalization Demographics

Contact Tracing

PPE

Outbreaks and Clusters

Reports

Data Behind the Dashboards

Total Tests Reported

Is North Carolina increasing its testing capacity?

Testing is an important tool to help diagnose and isolate people who are infected. Testing is also important to help characterize the prevalence, spread and contagiousness of the disease.

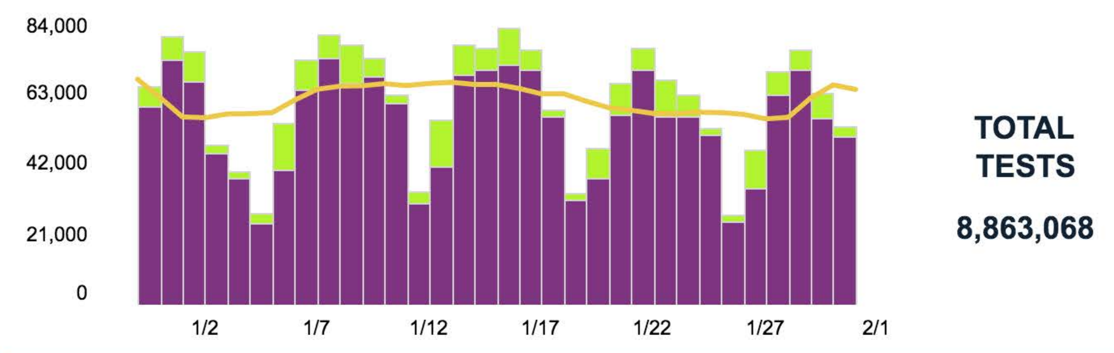

Labs Reported Electronically

Labs submit testing data to the State in two ways: at the patient-level, and in aggregate.

Patient-level testing data (positives and negatives) is submitted either electronically through NC COVID or manually via secure fax or electronic communications. Approximately 80% of total patient-level tests are now submitted electronically through NC COVID. The other 20% that are manually submitted must then be hand-entered into NC COVID.

Aggregate testing data is provided by labs that are not using electronic laboratory reporting. These labs submit the daily number of tests performed to NCDHHS using the electronic COVID-19 Aggregate Test Reporting (eCATR) survey tool.

Test totals are combined from these two sources of testing data to obtain the daily and cumulative report of total tests performed. NCDHHS has been working to improve data reporting processes to minimize redundancy with all labs to move to reporting exclusively through NC COVID to improve data integrity.

NCDHHS is working to transition away from manual reporting processes by working to onboard all labs into NC

Positive Tests as a Percent of Total Tests

Is North Carolina seeing a 14-day downward trajectory or sustained leveling of positive tests as a percentage of total tests?

As we ramp up testing, there will be more positive tests. Looking at what percent of total tests are positive helps us understand whether *positive* Molecular (PCR) tests are increasing in comparison to the total number of PCR tests conducted.

Laboratory results received electronically for previous dates may cause slight variation in day-to-day reporting.

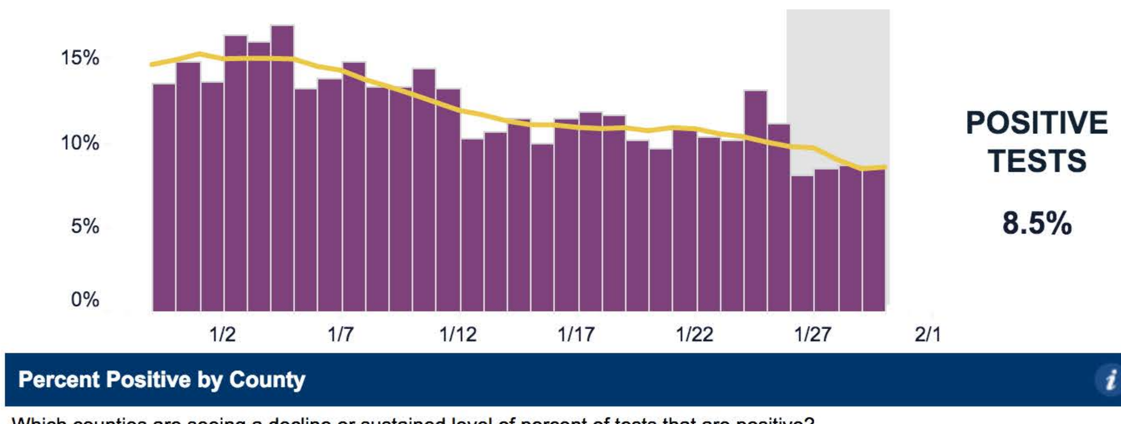

Percent Positive by County

Which counties are seeing a decline or sustained level of percent of tests that are positive?

It is important to recognize that the amount of testing being conducted varies across counties. Because the percent positive is calculated using only molecular (PCR) testing data reported electronically in to NC COVID, which is a subset of total tests reported, county-level percent positive rates may be impacted by how many of the total tests for residents in the county were reported electronically through NC COVID.

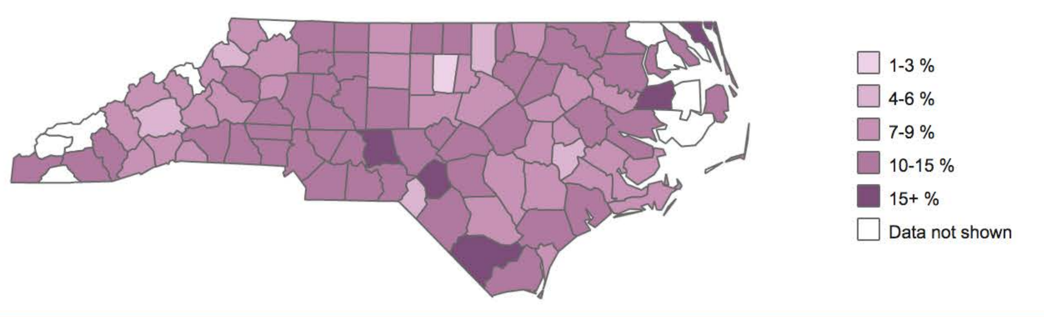

Testing Turnaround Time

There are multiple stages that make up the time it takes from when a person is tested to the time the person receives their results — the testing turnaround time The first stage is the time between when a specimen is collected to when it is received by a laboratory. Several factors can impact this timing, including how and when the specimen is transported to a laboratory. The second stage is the time between when the specimen is received and when the laboratory has a result. This first and second stage is shown in the dark purple in the graph below. The third stage is the time between when the laboratory determines a result and the laboratory electronically reported to NCDHHS. This third stage is shown in the light purple. The fourth stage, which is not represented in the graph below; is the time between when a laboratory reports a result and the patient is notified of their results. This fourth stage happens between the health care provider and patient and is not reported to NCDHHS. The yellow line shows the 7-day rolling average of the dark purple, when a specimen is collected to when the laboratory has a result.

Please note that the graph shows the average turnaround time across all laboratories reporting electronically to NCDHHS. Individual laboratories may have shorter or longer turnaround times and, therefore people's individual experience will vary. Antigen tests are excluded from testing turnaround calculations because antigen tests are designed to be rapid and are typically resulted within an hour or less. Including these tests in testing turnaround time calculations would potentially artificially decrease testing turnaround times for molecular (PCR) test results, which are the most widely used tests today. Laboratory results received electronically for previous dates may cause slight variation in day-to-day reporting.

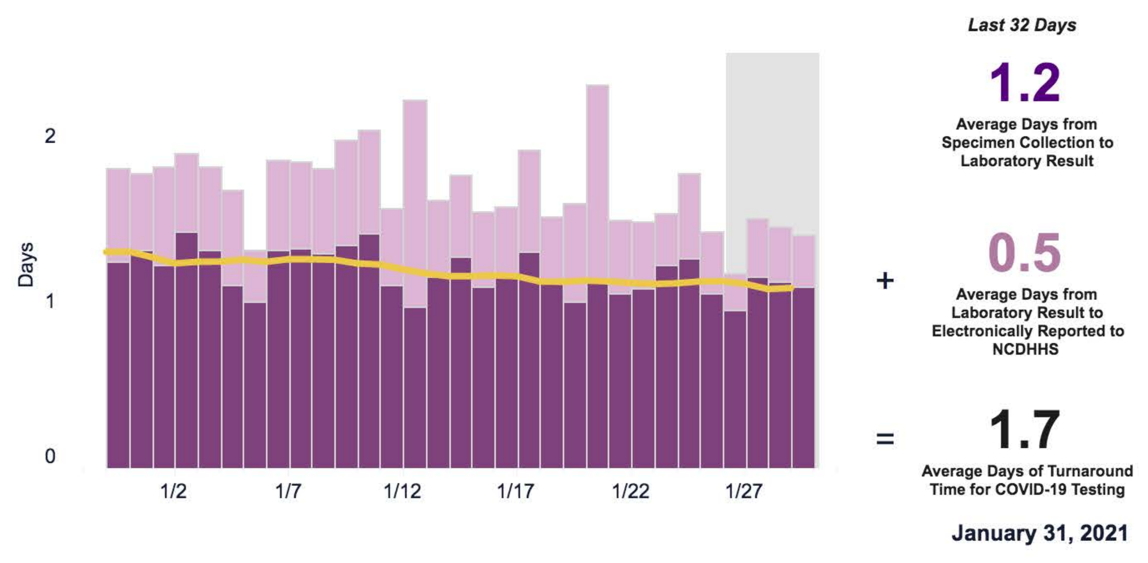

+ableau

COVID-19 Testing

Labs submit testing data to the State in two ways: at the patient-level, and in aggregate.

Patient-level testing data (positives and negatives) is submitted either electronically through NC COVID or manually via secure fax or electronic communications. Approximately 80% of total patient-level tests are now submitted electronically through NC COVID. The other 20% that are manually submitted must then be hand-entered into NC COVID.

Aggregate testing data is provided by labs that don't have electronic laboratory reporting. These labs submit the daily number of tests performed to NCDHHS using the electronic COVID-19 Aggregate Test Reporting (eCATR) survey tool.

Test totals are combined from these two sources of testing data to obtain the daily and cumulative report of total tests performed. NCDHHS has been working to improve data reporting processes to minimize redundancy with all labs to move to reporting exclusively through NC COVID to improve data integrity with test totals from NC COVID to obtain the total number of tests conducted.

Tests can be reported in batches (e.g., a lab reports three days of testing data at once) and tests are not always reported on the day they occurred. When a new lab begins reporting, it may report a backlog of tests from the past days or weeks. When tests are reported in batches or a new laboratory begins reporting, those tests are assigned back to the correct date. Therefore, previous days of testing are updated as more tests are reported. The number of tests completed by day is a dynamic number and is dependent on reporting labs.

Total tests represent the number of tests reported to NCDHHS for that day. This tells us how much daily testing is increasing throughout the state. This includes both total tests that report daily through electronic laboratory reporting, and those that are submitted manually. Because tests can be reported in batches, dates may be backfilled, and data might fluctuate. Tests have been broken out to show molecular (PCR) vs. antigen tests completed starting with data on Sept. 22, 2020 on the "Total Tests Reported" chart. For dates prior to Sept. 22, 2020, test totals cannot be broken out according to test type due to data limitations in the process previously used for laboratory reporting of test totals. However, it is likely that antigen tests were included in aggregate totals submitted by laboratories in daily reports in the past.

Percent positive represents the number of positive molecular (PCR) tests as a percentage of all molecular (PCR) tests reported electronically directly into NC COVID. We know that as we test more, the number of infections detected may go up. This metric gives us a sense of how many positive tests we are seeing in comparison to how much testing is being done. All data are preliminary and may change.

To calculate **positive tests as a percent of total tests** NCDHHS only uses molecular (PCR) test results from laboratories that report both positives and negatives through electronic laboratory reporting in NC COVID. This ensures that the positive and negative tests were from the same day to calculate an accurate daily percent positive. This is because test totals that are not reported electronically can occur in batches, and it cannot be confirmed that the positives and negatives occurred on the same day. Antigen tests are excluded from percent positivity calculations at this time, but NCDHHS will be evaluating including them in calculations in the future.

Average Percent Positive by County

North Carolina uses labs reported electronically to provide county level testing data. Antigen tests are excluded from percent positivity calculations to align with current CDC definitions used to calculate percent positivity.

A limitation of this data is that we know that not all tests are reported electronically, and some counties may be more likely to use a lab that does not report electronically. County data serves as a starting place to help counties gauge their testing volumes.

Percent positive shows the trends in positive tests as a percent of total tests for tests that were reported electronically. Counties may not have any labs reported electronically on a given day. These will show up as no tests, and 0% positive for that day.

The percent positive displayed in the county map is the average percent positive during the last two weeks. If a county has only one week with less than an average of 50 daily tests, then that week will not be shown and not included in the average percent positive. If a county has both weeks with fewer than an average of 50 daily tests, then the graph will not be shown. Counties with fewer than an average of 50 daily tests for the two weeks are not shown because there are not enough electronically reported tests in that county to provide a reliable percent positive calculation. The percent positive may fluctuate substantially as there are a smaller number of tests than the state percent positive. All data are preliminary and subject to change.

Testing Turnaround Time

Testing turnaround time measures the time between when a person is tested, and when the result feeds into public health for tests that are electronically reported. Please note that the testing turnaround time doesn't necessarily include the time between when a person is tested and notified of their results.

There are multiple stages that make up the time it takes from when a person is tested to the time the person receives their results – the testing turnaround time. The first stage is the time between when a specimen is collected to when it is received by a laboratory. Several factors can impact this timing, including how and when the specimen is transported to a laboratory. The

Home About COVID-19 Vaccines Dashboard Information For Guidance Slow The Spread

[illegible] stage, which is not represented in the graph below, is the time between when a laboratory reports a result and the patient is notified of their results. This fourth stage happens between the health care provider and patient and is not reported to NCDHHS. The yellow line shows the 7-day rolling average of the dark purple, when a specimen is collected to when the laboratory has a result.

Individual laboratories may have shorter or longer turnaround times and, therefore people's individual experience will vary. Antigen tests are excluded from testing turnaround calculations because antigen tests are designed to be rapid and are typically resulted within an hour or less. Including these tests in testing turnaround time calculations would potentially artificially decrease testing turnaround times for molecular (PCR) test results, which are the most widely used tests today.

Laboratory results received electronically for previous dates may cause slight variation in day-to-day reporting.

COVID-19 · County Alert System: 86 Counties Red and 13 Counties Orange. Read the report

Vaccinations

NC is currently providing vaccinations to individuals in Group 1: Health care workers & Long-Term Care staff and residents; and Group 2 : Anyone 65 years or older, regardless of health status or living situation.

This dashboard shows the total number of doses that have been administered by NC providers and through the federal program administering long-term care vaccinations.

Current vaccines require two doses. The dashboard shows the total number of doses that have been administered by week, and the number of people by reported county of residence who have received either the first or second dose of the COVID-19 vaccine. It also includes statewide demographic data on people who have received either the first or second dose of the vaccine by race, ethnicity, gender, and age group. Data shown by week, county of residence, and demographics do not include information on doses that have been administered through the federal long-term care (LTC) facilities program. Data shown are preliminary and may change. There can be a 72-hour lag in data reported to NCDHHS.

Vaccinations Data: December 14, 2020 – January 27, 2021 at 11:59 p.m.

NC Providers - Vaccinations Summary

	Allocated by Federal Government	Arrived in North Carolina	Total Doses Administered	Percent of Arrived Doses Administered
FIRST DOSES	942,100	794,525	699,885	88%
SECOND DOSES	479,375	387,650	122,158	32%
TOTAL DOSES	1,421,475	1,182,175	822,043	70%

Federal Long-Term Care Program - Vaccinations Summary

	Allocated by Federal Government	Arrived in North Carolina	Total Doses Administered	Percent of Arrived Doses Administered
FIRST DOSES	165,900	165,900	87,982	53%
SECOND DOSES	165,900	131,400	4,192	3%
TOTAL DOSES	331,800	297,300	92,174	31%

Data on doses administered in long-term care (LTC) facilities through the federal government program with CVS and Walgreens are not included in data for 'Weekly Vaccinations', 'People Vaccinated by Demographics', or 'People Vaccinated by County of Residence'.

Weekly Vaccinations

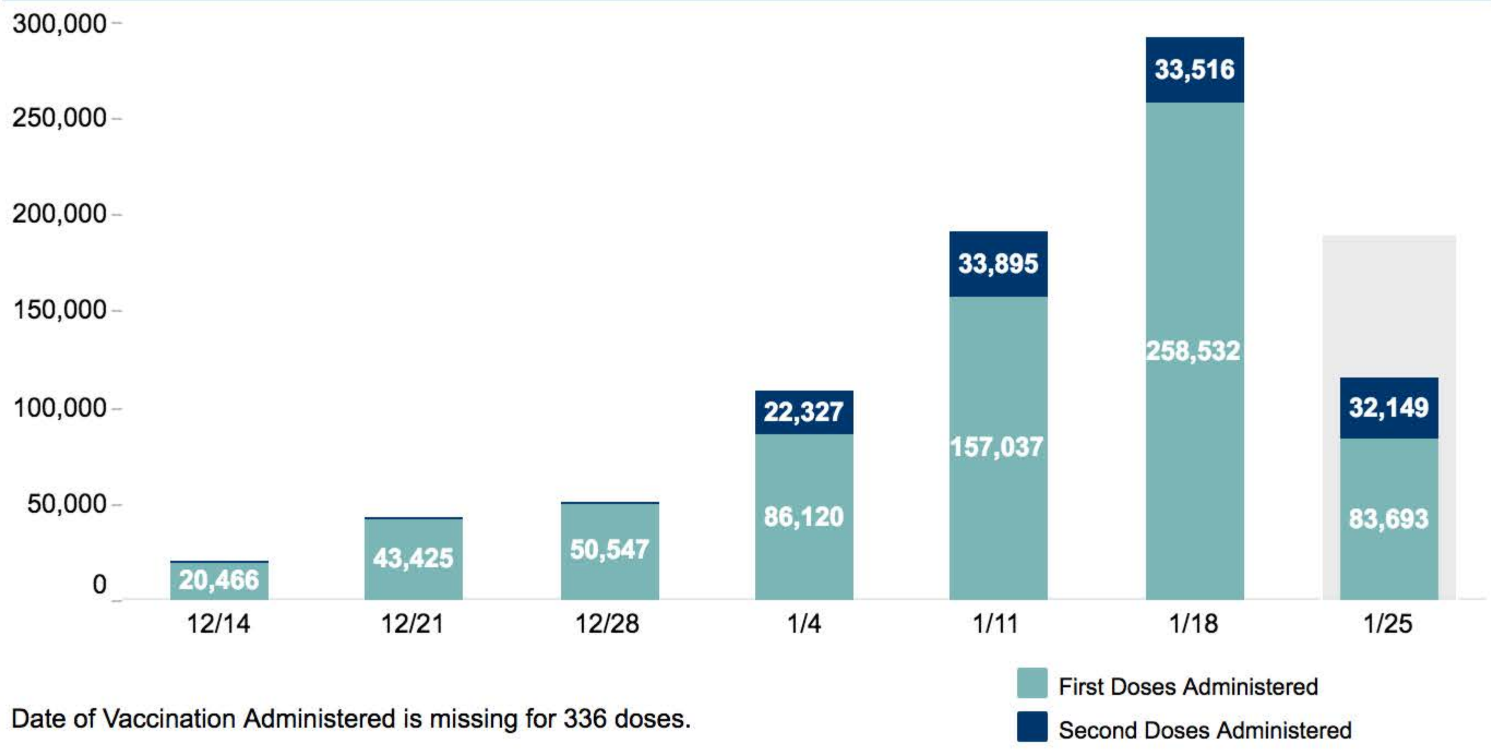

Date of Vaccination Administered is missing for 336 doses.

People Vaccinated by Demographics

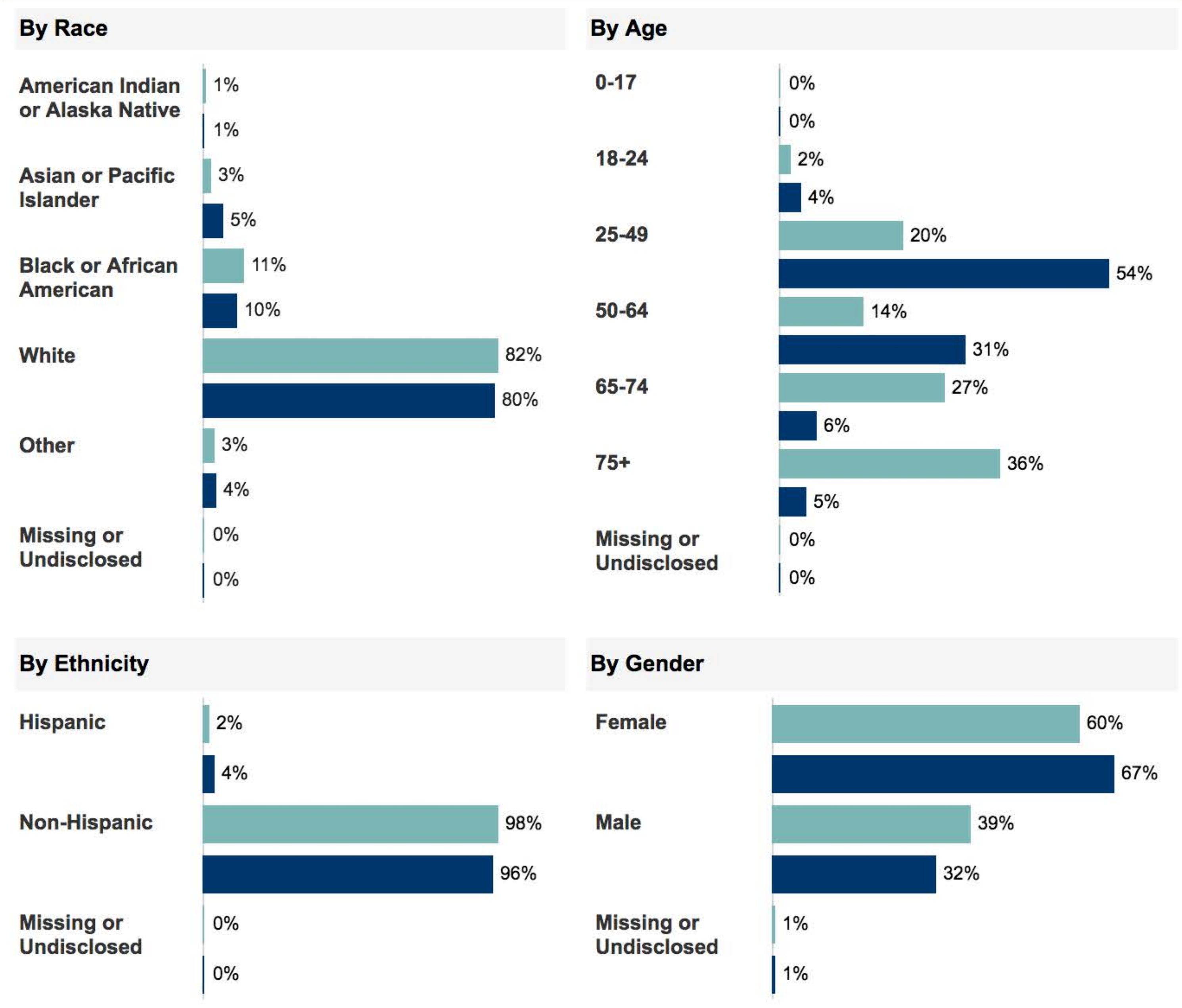

People Vaccinated by County of Residence - First Doses Administered

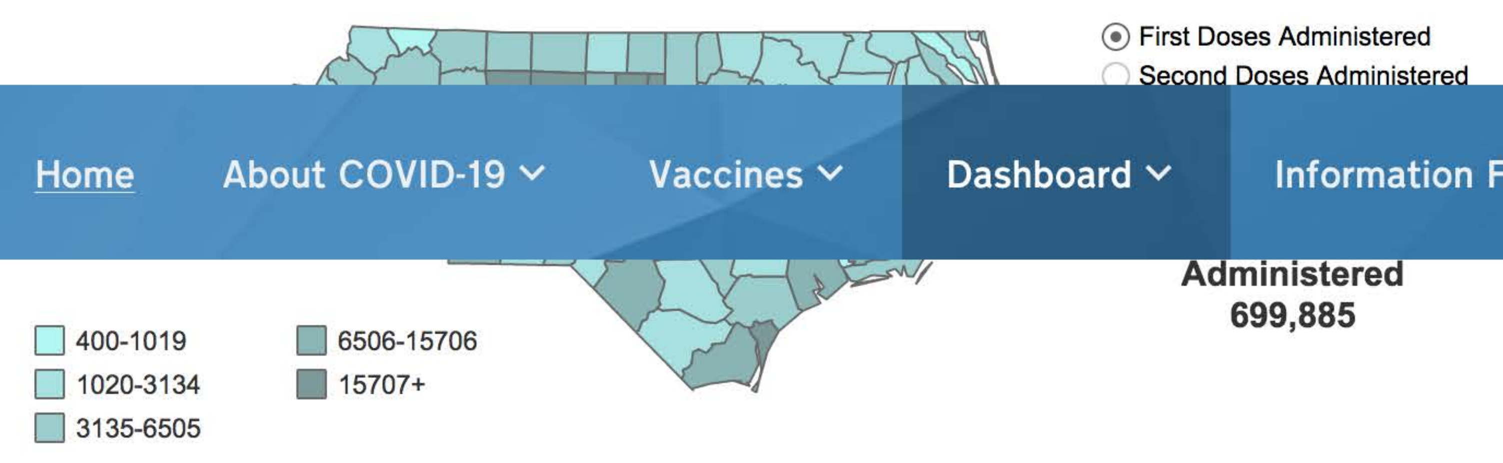

First Doses Administered: 15,862 county of residence missing.

January 28, 2021

+ableau

'Missing or Undisclosed' may include data for people that did not provide demographic information, or where vaccine providers did not submit the demographic information. Numbers may not add up to 100% due to rounding.

Dashboard

Summary
County Alert System
Vaccinations
CLI Surveillance
Cases
Cases Demographics
Testing
Hospitalizations
Hospitalization Demographics
Contact Tracing
PPE
Outbreaks and Clusters
Reports
Data Behind the Dashboards

North Dakota | Health
Be Legendary.

Overview | Trends | Demographics | Hospitalizations | County Analysis

Last Updated: 1/31/2021

POPULATION
762,062

County
All

% of POPULATION TESTED
51.71%

Hover over a value and click the icon for more information

TODAY

POSITIVITY RATE
3.11%

NEW TESTS

TOTAL PROCESSED
2,495

SUSCEPTIBLE ENCOUNTERS
2,152

NEW POSITIVES

TOTAL (PCR AND ANTIGEN TESTS)
67

CONFIRMED (PCR TESTS)
43

PROBABLE (ANTIGEN TESTS)
24

NEW RECOVERED

RECOVERED
128

ACTIVE POSITIVES

981

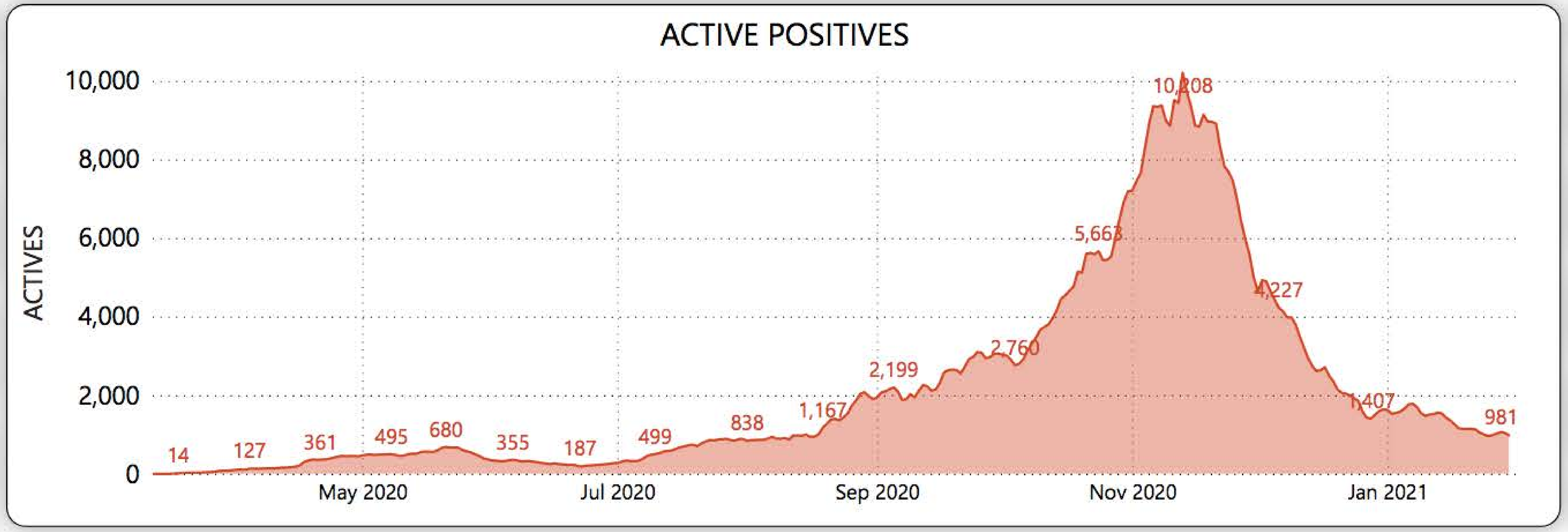

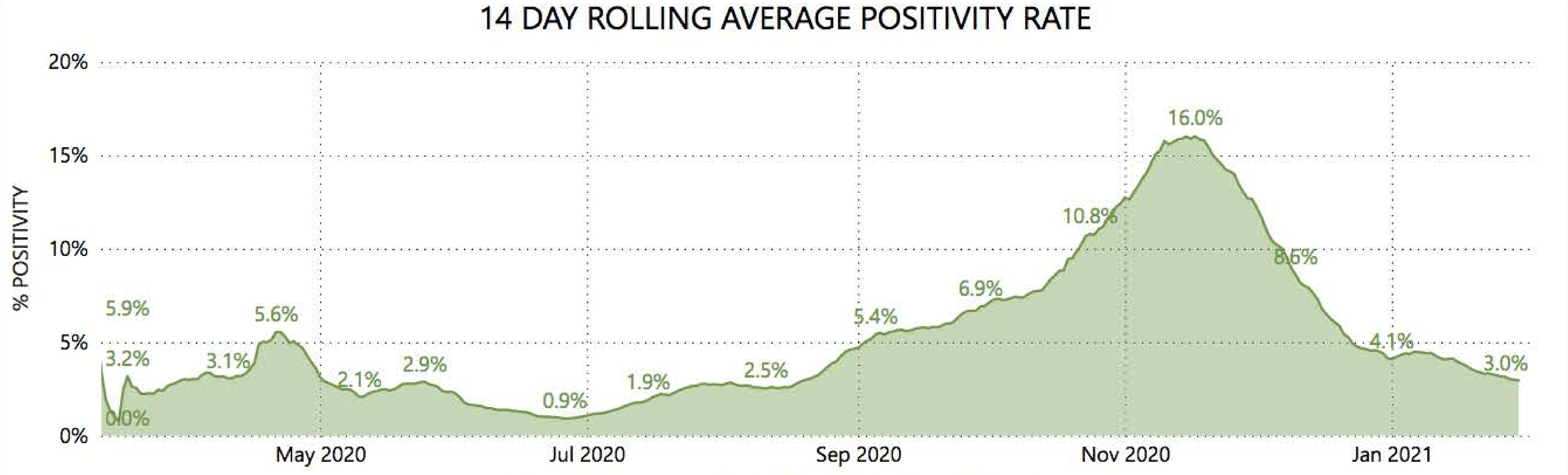

Calculation = (Sum of Past 14 Days of Positives / 14 Days of Tests)

CUMULATIVE

POSITIVITY RATE
24.77%

TESTS

TOTAL PROCESSED
1,524,151

SUSCEPTIBLE ENCOUNTERS
1,425,425

UNIQUE INDIVIDUALS
394,086

POSITIVES

TOTAL (PCR AND ANTIGEN TESTS)
97,630

CONFIRMED (PCR TESTS)
93,293

PROBABLE (ANTIGEN TESTS)
4,337

RECOVERED and DEATHS

RECOVERED	DEATHS
95,227	1,422

Overview | Trends | Demographics | Hospitalizations | County Analysis

Hover over a value and click the icon for more information

DAILY		CUMULATIVE		AGE COMPARISON
CONFIRMED POSITIVES	PROBABLE POSITIVES	CONFIRMED POSITIVES	PROBABLE POSITIVES	ACTIVE POSITIVES
14 DAY AVERAGE TOTAL PROCESSED TESTS		RECOVERED	DEATHS	DAILY POSITIVES
				CUMULATIVE POSITIVES

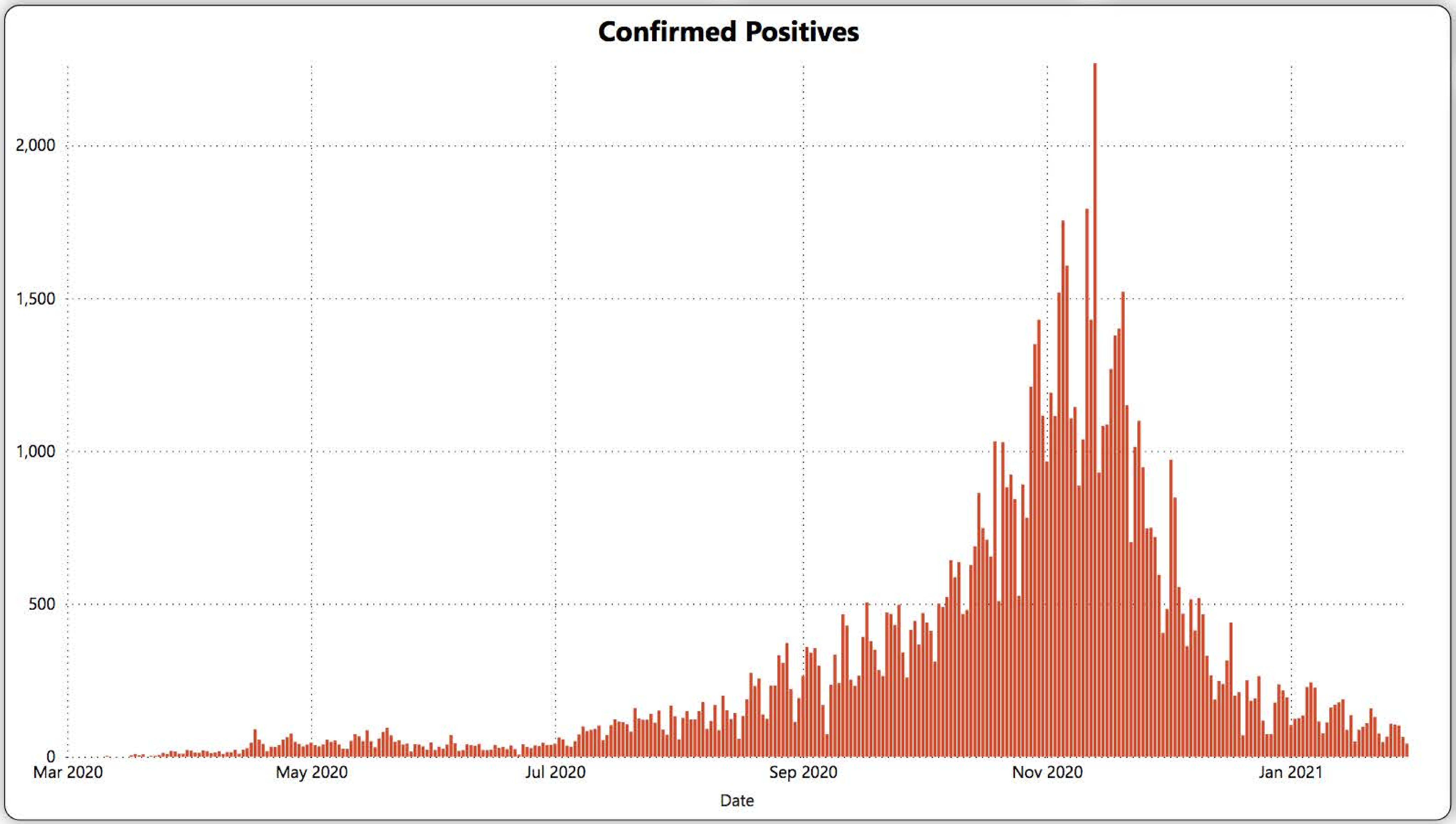

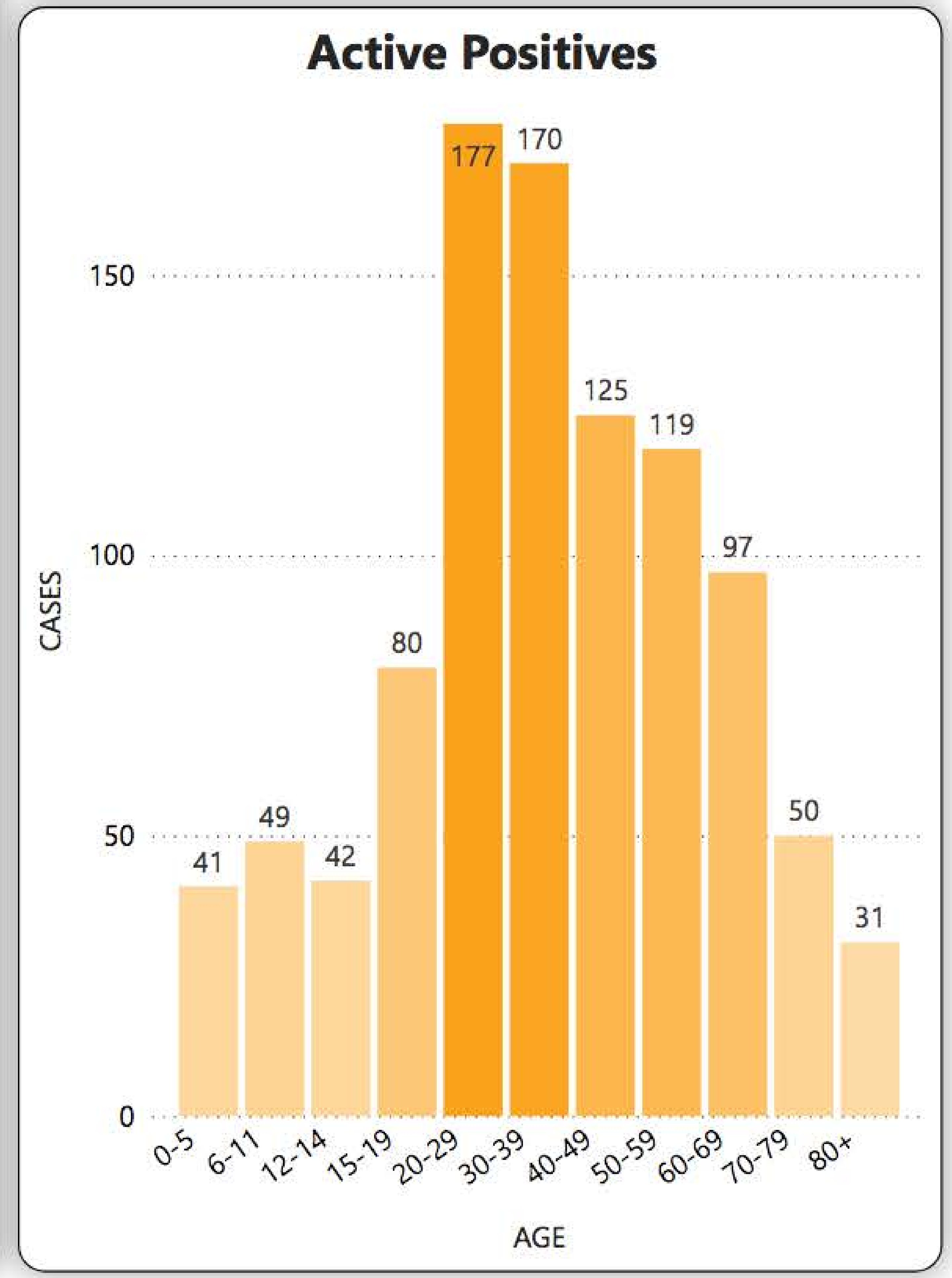

Hover over a value and click the icon for more information

PRESS FOR ACTIVE POSITIVES | PRESS FOR CUMULATIVE POSITIVES

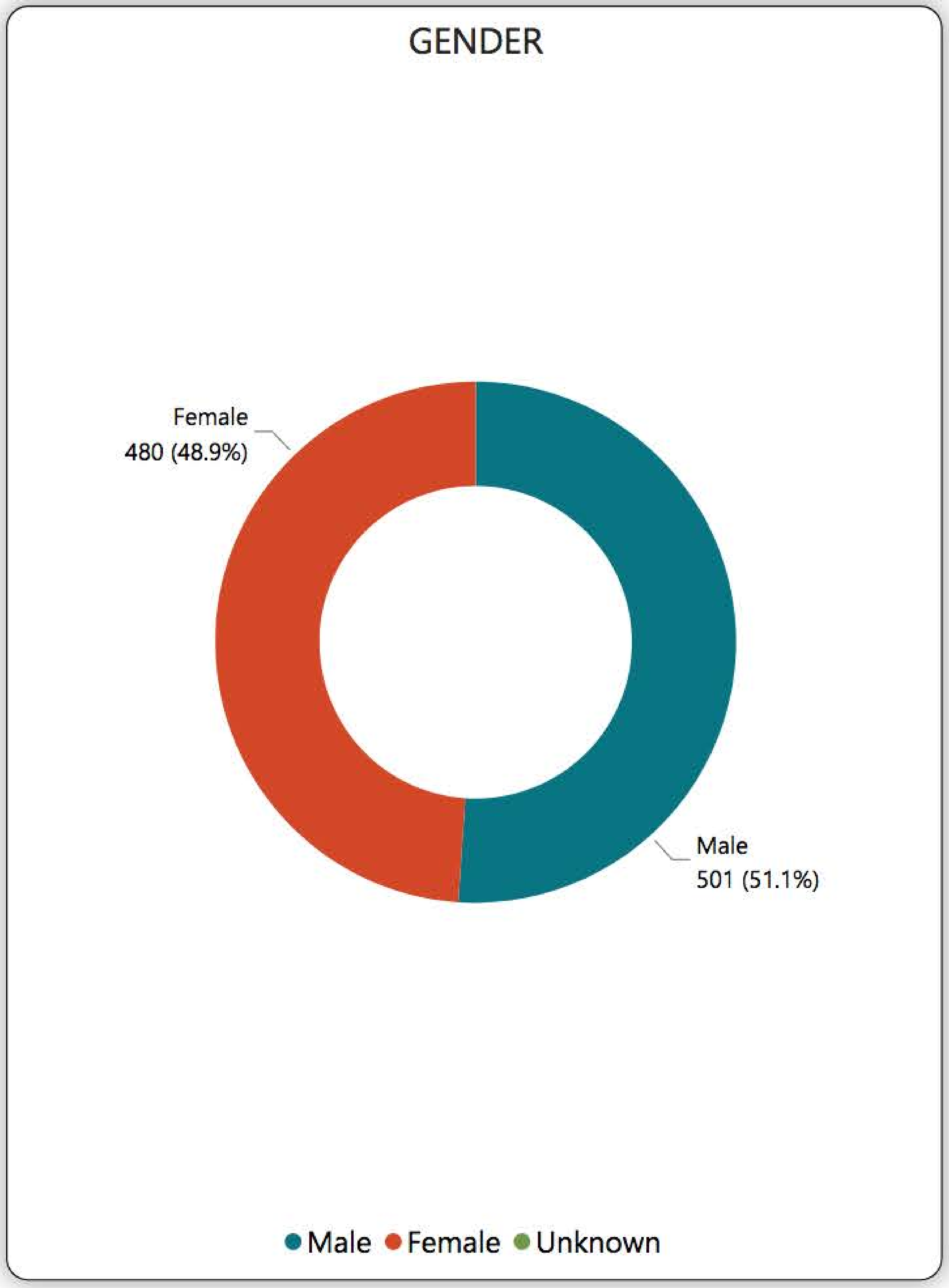

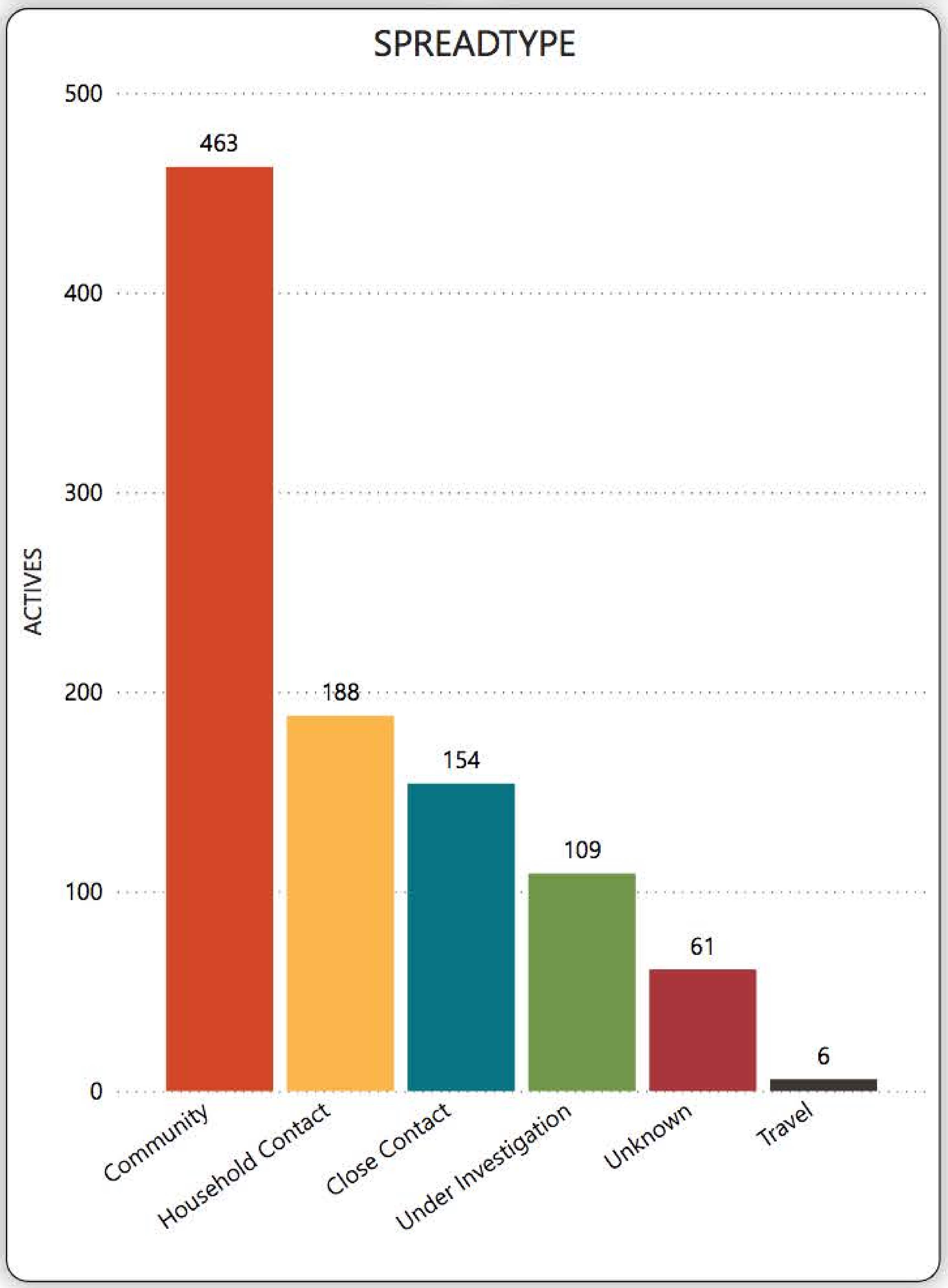

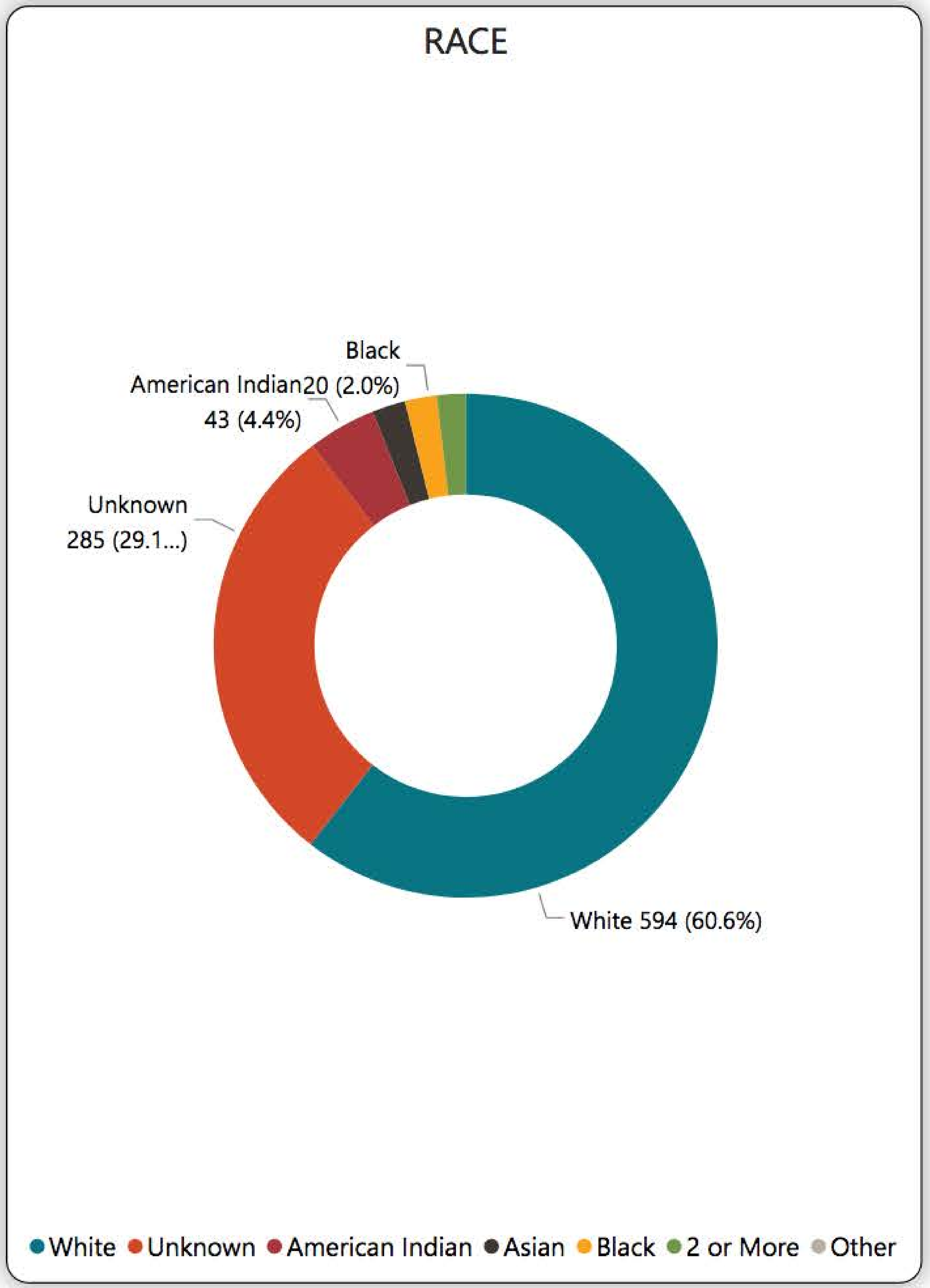

Go to COVID-19 Vaccination Dashboard

Coronavirus COVID-19 Nebraska Cases by the Nebraska Department of Health and Human Services (DHHS)

Total Positive Cases
190,713

People Tested: Not Detected
732,719

*Excludes inconclusives and out of state residents

People Tested
923,914

Total Tests
2,100,123

Active Hospitalizations
320

Total Staffed Beds, 14 Day Avg.
4,066

% Positive by Specimen Collection Date

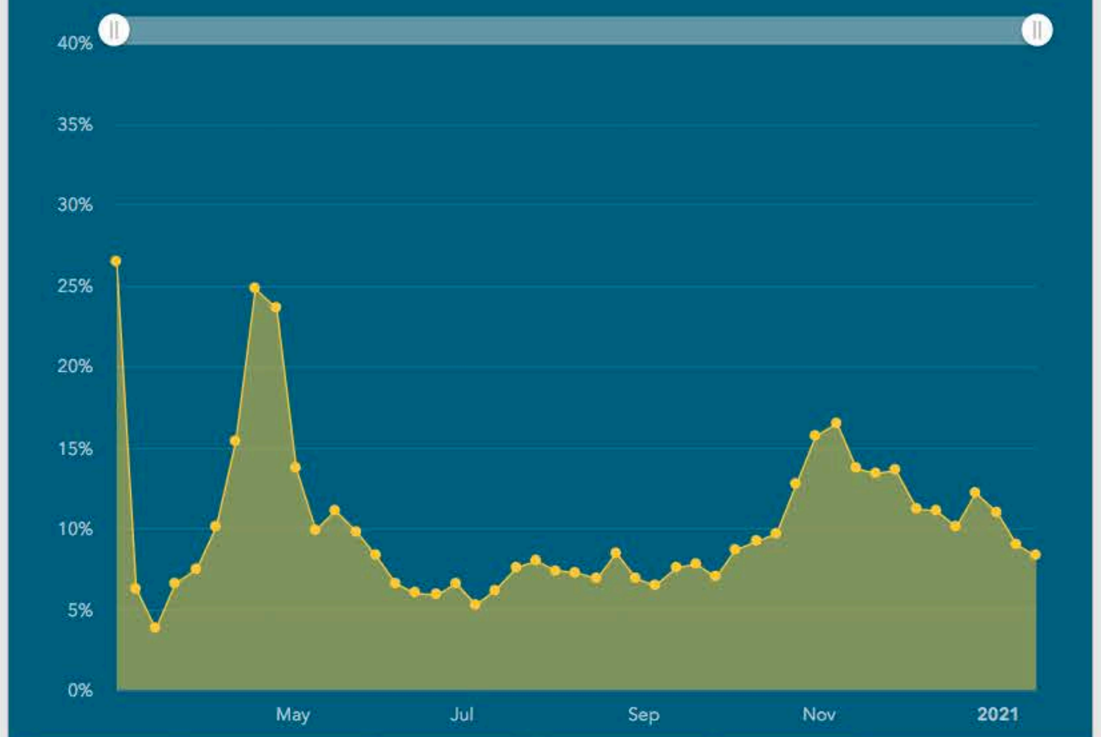

% Positive | Positive Cases | Total Cases | Tests by Date | Total Tests

Daily Active Hospitalizations

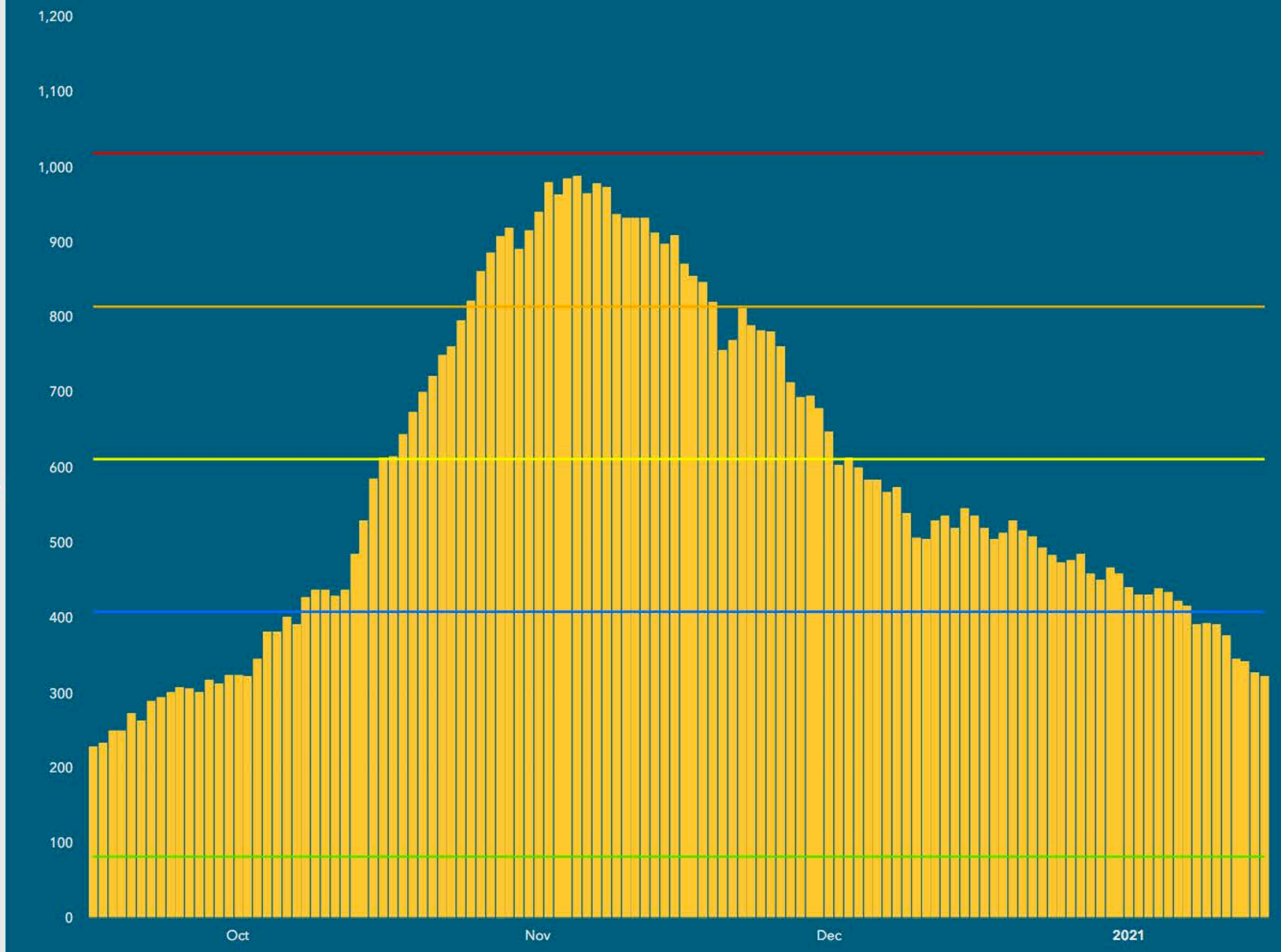

Colored lines represent the corresponding thresholds for changing DHM Color Phases

DHM Thresholds | Last 14 Day Positive Cases | Total Positive Cases | Hospital Capacity | Positive Cases | Hospitalized Cases | Recoveries | Deaths | Race | Ethnicity | Turn Around Time | About the Data

Contact Nebraska Department of Health and Human Services (DHHS)
http://dhhs.ne.gov/coronavirus
Information Line: (402) 552-6645

Data are representative of Nebraska residents, are provisional and data reported by the local health department should be considered the most up to date.

Race and Ethnicity tabs were last updated January 22th, 2021

NEBRASKA
Good Life. Great Mission.
DEPT. OF HEALTH AND HUMAN SERVICES

Go to COVID-19 Case Dashboard

Nebraska COVID-19 Vaccinations | Nebraska DHHS

Total Vaccines Allocated

254,000

Total Vaccinations Administered

180,842

First Dose in Series Received

134,606

Second Dose in Series Received

46,236

Percentage of Population 16 and Older Completing Vaccination

3.12%

of 1.48M People

Daily New Vaccinations Administered

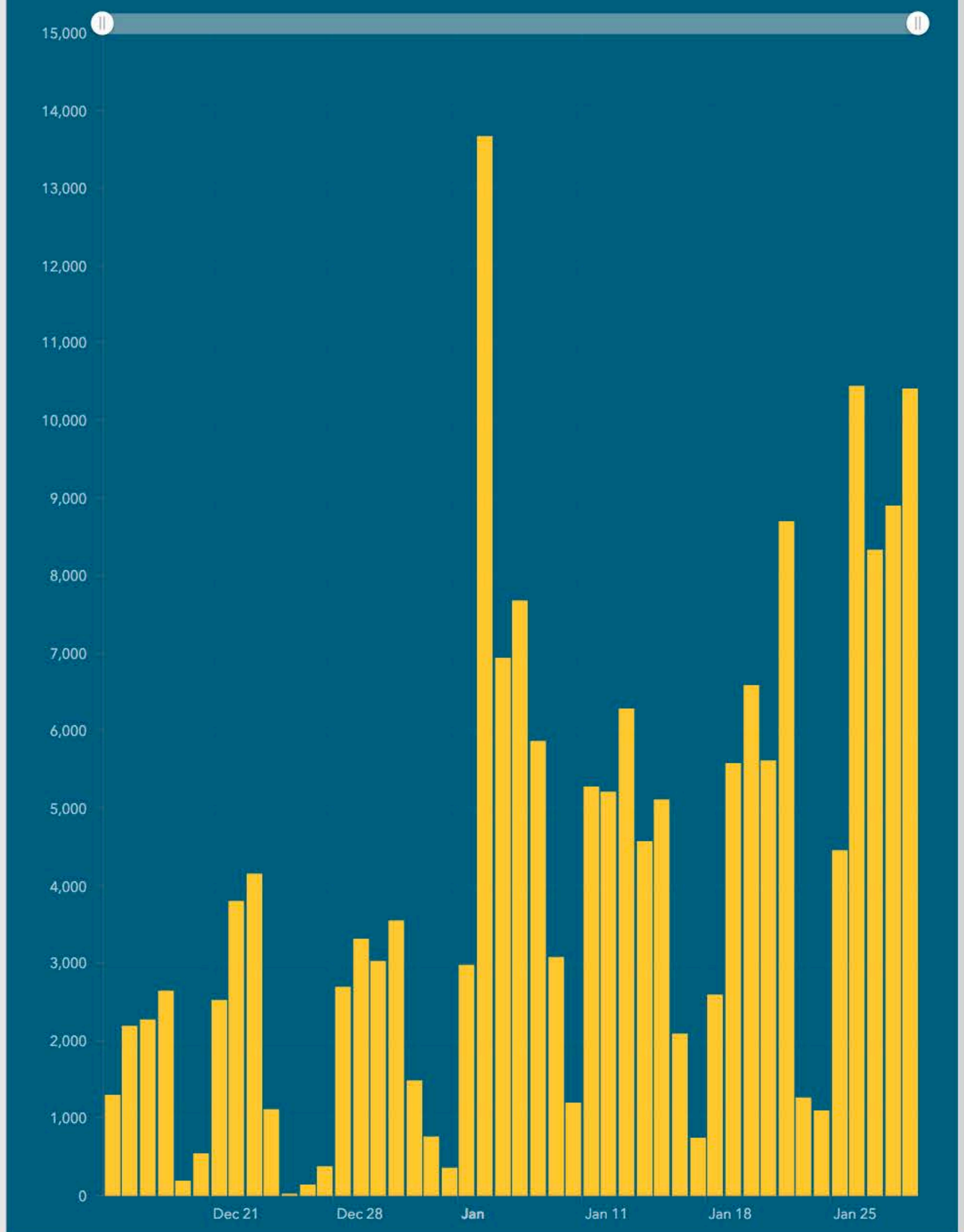

Cumulative Total of Vaccinations Administered

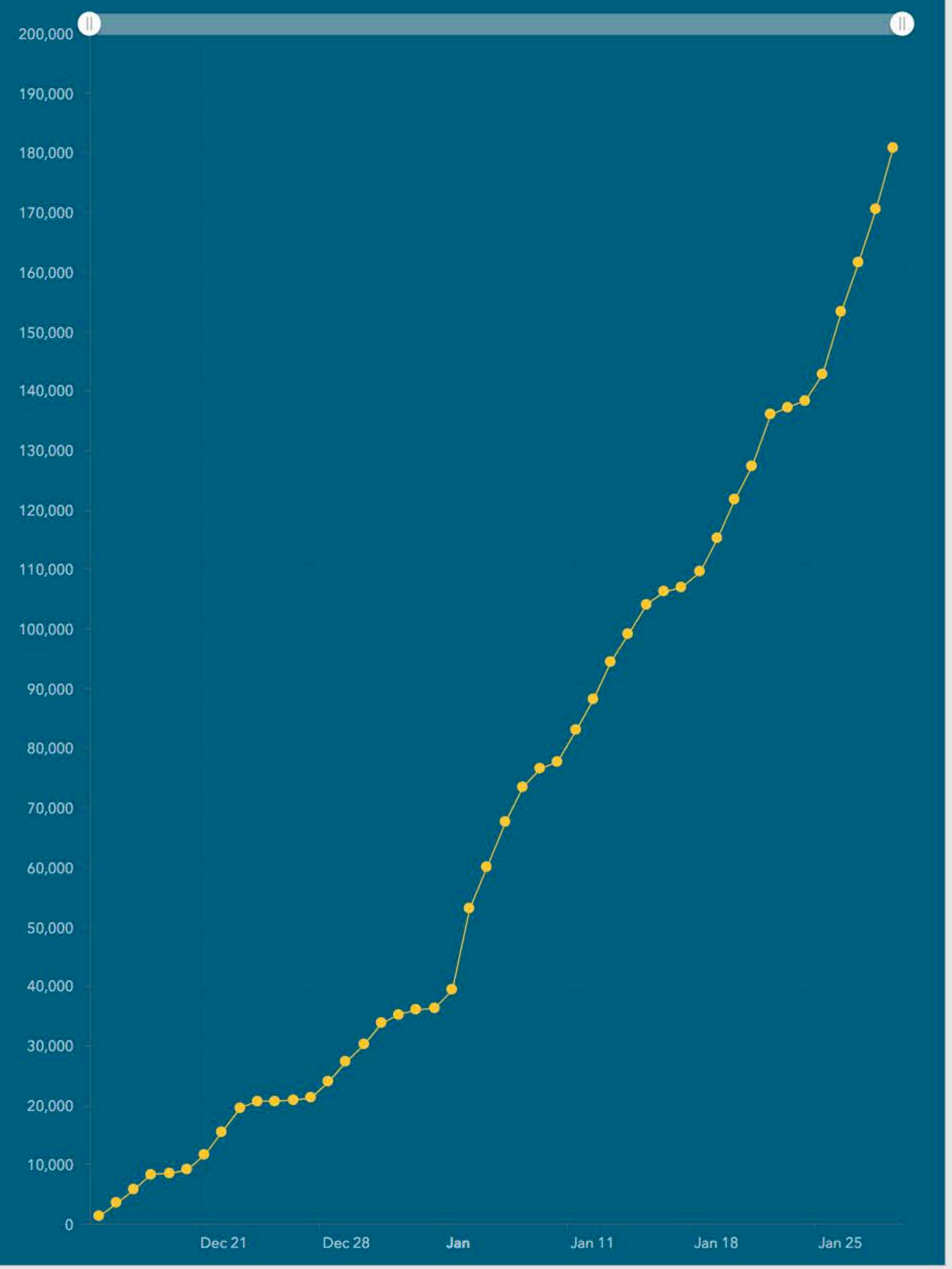

Overview | 1st Dose Demographics | 2nd Dose Demographics | Phases | About the Data | Map

Current Status | Trends | Demographics | County Tracker

Download Data

Confirmed Cases | Testing | Hospitalizations | Deaths

DASHBOARD UPDATED ON 1/31/2021

CURRENT STATUS - CONFIRMED CASES

DATA AS OF 1/30/2021

Confirmed case data are summarized below. Daily new cases are displayed as 14-day moving averages. Cases per 100,000 are displayed for the most recent 30-day period and cumulatively. In the maps below, counties with higher rates are shaded darker. **Learn more**

SELECT A COUNTY

Search

- North
  - Carson City
  - Washoe
- South
  - Clark
- Rural
  - Churchill
  - Douglas
  - Elko
  - Esmeralda
  - Eureka
  - Humboldt
  - Lander
  - Lincoln
  - Lyon
  - Mineral
  - Nye
  - Pershing
  - Storey
  - White Pine

KEY METRICS

1,002

NEW DAILY CASES

14-day moving average

1,409 Cases per 100K

30-day period

278,308 Cumulative cases

8,837 Cumulative cases per 100K

344

NEW DAILY TESTS PER 100K

14-day moving average with 7-day lag

18

CASES PER 100K (30-day period)

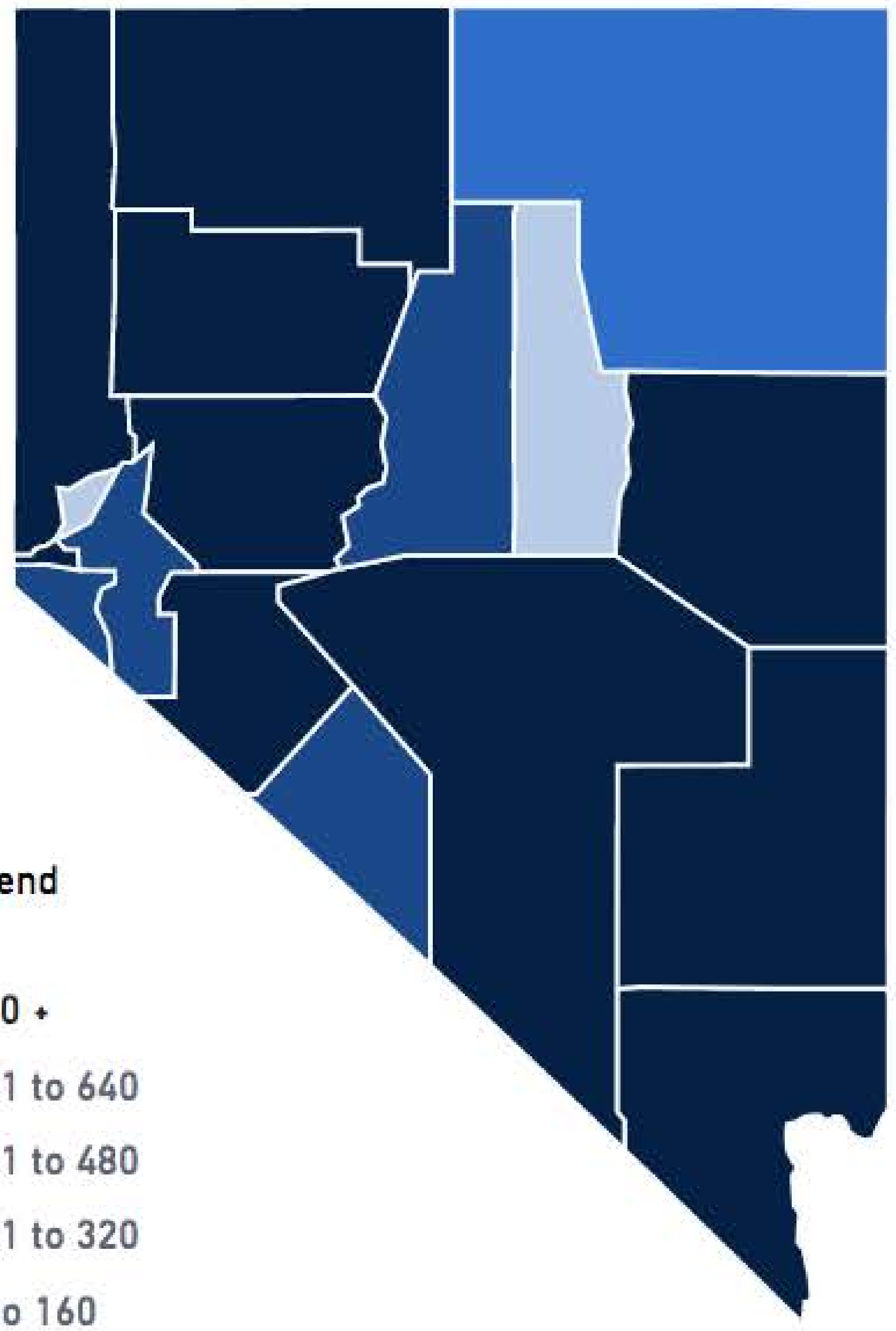

Legend

- 640 +
- 481 to 640
- 321 to 480
- 161 to 320
- 1 to 160
- 0

DAILY NEW CASES (14-day moving avg.)

STATEWIDE ONLY

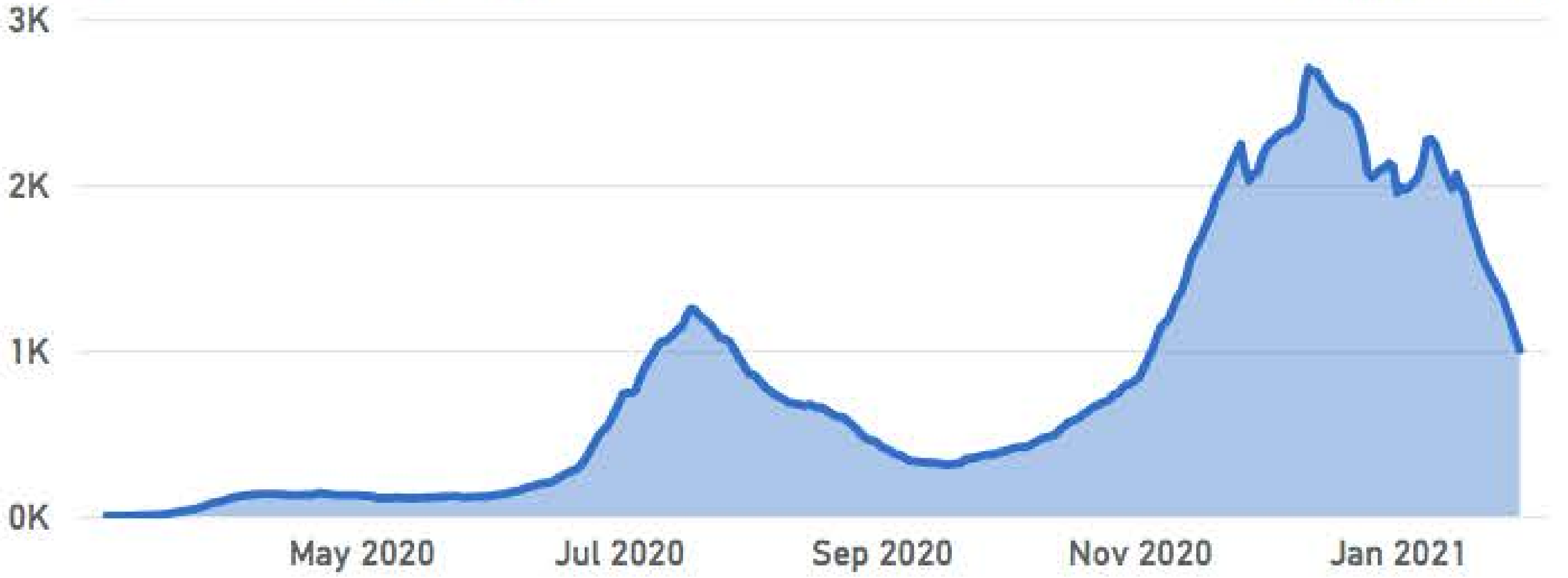

COUNTY	POPULATION	CUMULATIVE CASES	CUMULATIVE CASES PER 100K
Carson City	56,546	5,700	10,080
Churchill	25,876	1,828	7,065
Clark	2,318,174	214,108	9,236
Douglas	49,695	2,380	4,789
Elko	54,985	4,263	7,753
Esmeralda	974	36	3,696
Eureka	1,966	44	2,238
Humboldt	17,062	1,013	5,937
Lander	5,996	433	7,221
**Total**	**3,149,234**	**278,308**	**8,837**

This dashboard is updated daily at approximately 10:00 am. Please note that updates apply incrementally and may be in process for up to an hour. The Nevada DHHS also maintains a facility-specific COVID-19 dashboard, available **Here**

Microsoft Power BI

Current Status | Trends | Demographics | County Tracker

Download Data

Confirmed Cases | Testing | Test Positivity Rate | Hospitalizations | ICU & Ventilator Use | Deaths

DASHBOARD UPDATED ON 1/31/2021

TRENDS - CONFIRMED CASES

DATA AS OF 1/30/2021

Confirmed case data are reported by the regional health authorities and the Office of Public Health Investigations and Epidemiology (OPHIE), within the Division of Public and Behavioral Health (DPBH). The counts of confirmed cases may go down when any case which was previously reported in Nevada was later identified to be from another jurisdiction or out of state. Counts may be artificially low Saturday - Monday due to limited laboratory capacity and less reporting over the weekends.
Learn more

SELECT A COUNTY

Search

- North
  - Carson City
  - Washoe
- South
  - Clark
- Rural
  - Churchill
  - Douglas
  - Elko
  - Esmeralda
  - Eureka
  - Humboldt
  - Lander
  - Lincoln
  - Lyon
  - Mineral
  - Nye
  - Pershing
  - Storey
  - White Pine

KEY METRICS

1,002

DAILY NEW CASES
14-day moving average

1,409 Cases per 100K
30-day period

When people talk about "flattening the curve", this is the curve they are talking about. We want to slow the number of new cases so that our healthcare system isn't overwhelmed and can provide care to everyone who needs it.

Staying home, practicing social distancing, and wearing a face mask are important ways to help flatten the curve.

DAILY NEW CASES, BY SPECIMEN COLLECTION DATE

STATEWIDE ONLY

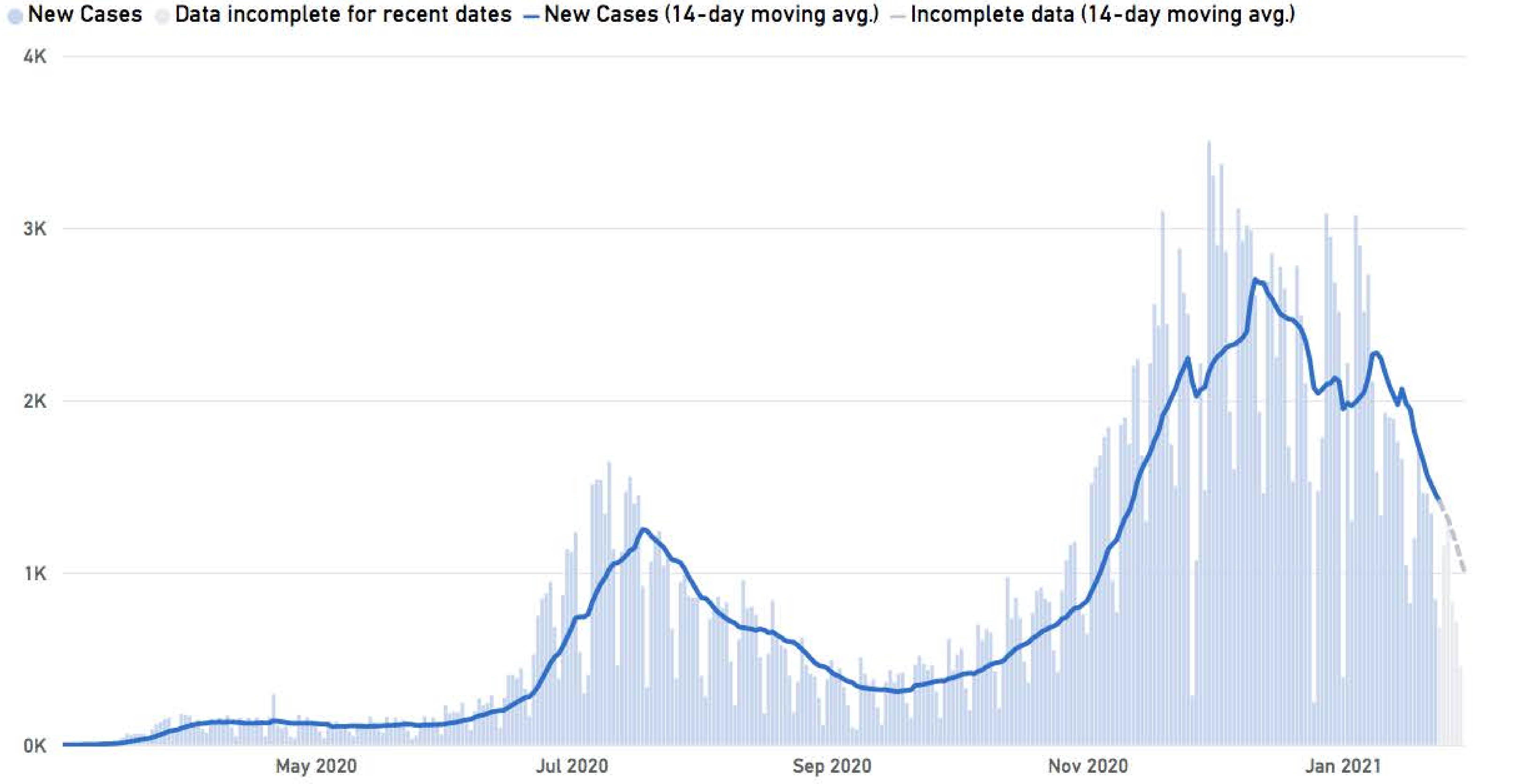

This dashboard is updated daily at approximately 10:00 am. Please note that updates apply incrementally and may be in process for up to an hour. The Nevada DHHS also maintains a facility-specific COVID-19 dashboard, available Here

Microsoft Power BI

Pages

Current Status | Trends | Demographics | County Tracker

Download Data

Confirmed Cases | Tests | Deaths

DASHBOARD UPDATED ON 1/31/2021

DEMOGRAPHICS - CONFIRMED CASES

DATA AS OF 1/30/2021

COVID-19 can affect people of all age groups, including children. However, adults in Nevada are more frequently tested and diagnosed with COVID-19. Children can spread COVID-19 despite the absence of signs or symptoms. Adults aged 60 years or above are more vulnerable to COVID-19-associated mortality as opposed to other age groups. A significant number of laboratory records for people tested and confirmed cases are missing race and ethnicity information. Some laboratory records for people tested and confirmed cases are also missing age and gender information. This is similar to the completeness of these data fields in other states and nationally. **Learn more**

Group	CASES %	POPULATION %	POPULATION
**AGE**	**100.0%**	**100.0%**	**3,149,234**
<10	4.0%	12.4%	389,978
10-19	9.8%	13.6%	429,790
20-29	20.3%	13.9%	437,588
30-39	18.7%	13.5%	423,831
40-49	16.5%	13.1%	414,074
50-59	14.5%	12.7%	398,817
60-69	9.0%	11.0%	345,779
70+	7.3%	9.8%	309,377
**GENDER**	**100.0%**	**100.0%**	**3,149,234**
Female	51.3%	50.0%	1,573,738
Male	48.7%	50.0%	1,575,496
**RACE/ETHNICITY**	**100.0%**	**100.0%**	**3,149,234**
Hispanic	35.0%	30.2%	952,600
Non-Hispanic American Indian or Alaska Native	0.5%	1.1%	35,742
Non-Hispanic Asian/Pacific Islander	7.9%	9.9%	312,158
Non-Hispanic Black	7.5%	8.9%	280,643
Non-Hispanic Other Race	12.6%	0.0%	0

This dashboard is updated daily at approximately 10:00 am. Please note that updates apply incrementally and may be in process for up to an hour. The Nevada DHHS also maintains a facility-specific COVID-19 dashboard, available **Here**

Microsoft Power BI

Pages

NEW HAMPSHIRE LABORATORY-CONFIRMED COVID-19 CASES

Health Outcomes	Infections		Hospitalizations		Intensive Care Unit		Recovered and Off Isolation	Deaths
	Current	Cumulative	Current	Cumulative	Current	Cumulative		
Persons	5,238	65,362	217	1,033	n/a	329	59,082	1,042

NH DIVISION OF Public Health Services Department of Health and Human Services

Persons with COVID-19	Infections		Hospitalizations		Deaths	
	Persons	% of Total	Persons	% of Total	Persons	% of Total
Healthcare Workers*	3,738	5.7%	76	7.4%	11	1.1%
Long-Term Care Settings**	5,763	8.8%	249	24.1%	770	73.9%
**Sex**						
Female	33,231	50.8%	444	43.0%	521	50.0%
Male	30,861	47.2%	585	56.6%	519	49.8%
Unknown	1,270	1.9%	4	0.4%	2	0.2%
Grand Total	**65,362**		**1,033**		**1,042**	
**Age Group (in Years)**						
0 - 9	2,977	4.6%	7	0.7%	0	0.0%
10 - 19	6,758	10.3%	7	0.7%	0	0.0%
20 - 29	12,289	18.8%	24	2.3%	1	0.1%
30 - 39	9,578	14.7%	45	4.4%	2	0.2%
40 - 49	8,798	13.5%	84	8.1%	9	0.9%
50 - 59	10,508	16.1%	131	12.7%	18	1.7%
60 - 69	7,059	10.8%	244	23.6%	82	7.9%
70 - 79	3,795	5.8%	243	23.5%	234	22.5%
80 +	3,510	5.4%	248	24.0%	696	66.8%
Unknown	90	0.1%	0	0.0%	0	0.0%
Grand Total	**65,362**		**1,033**		**1,042**	
**Test Type**						
Antigen Positive	18,585	28.4%	80	7.7%	179	17.2%
PCR Positive	46,777	71.6%	953	92.3%	863	82.8%
Grand Total	**65,362**		**1,033**		**1,042**	

Race / Ethnicity	% of NH Population ****	Infections		Hospitalizations		Deaths	
		Persons	% of Total	Persons	% of Total	Persons	% of Total
White**	90.0%	31,521	80.4%	777	80.8%	803	94.5%
Hispanic or Latino*	3.9%	3,705	9.5%	93	9.7%	20	2.4%
Black or African American**	1.4%	879	2.2%	38	4.0%	12	1.4%
Other***	1.8%	2,229	5.7%	19	2.0%	8	0.9%
Asian**	3.0%	851	2.2%	35	3.6%	7	0.8%
Grand Total		**39,185**		**962**		**850**	

Race/Ethnicity is known for 60.0% of COVID-19 infections, 93.1% of hospitalizations, and 81.6% of deaths

COVID-19 CASES BY SEX (n= 64092)

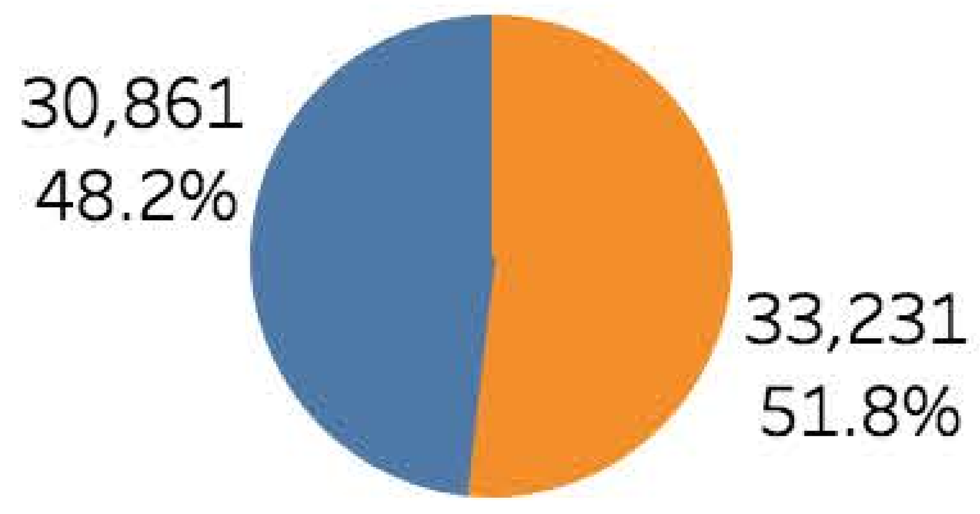

Unknown 1,270

Sex: Female, Male

COVID-19 CASES BY AGE GROUP (n= 65272)

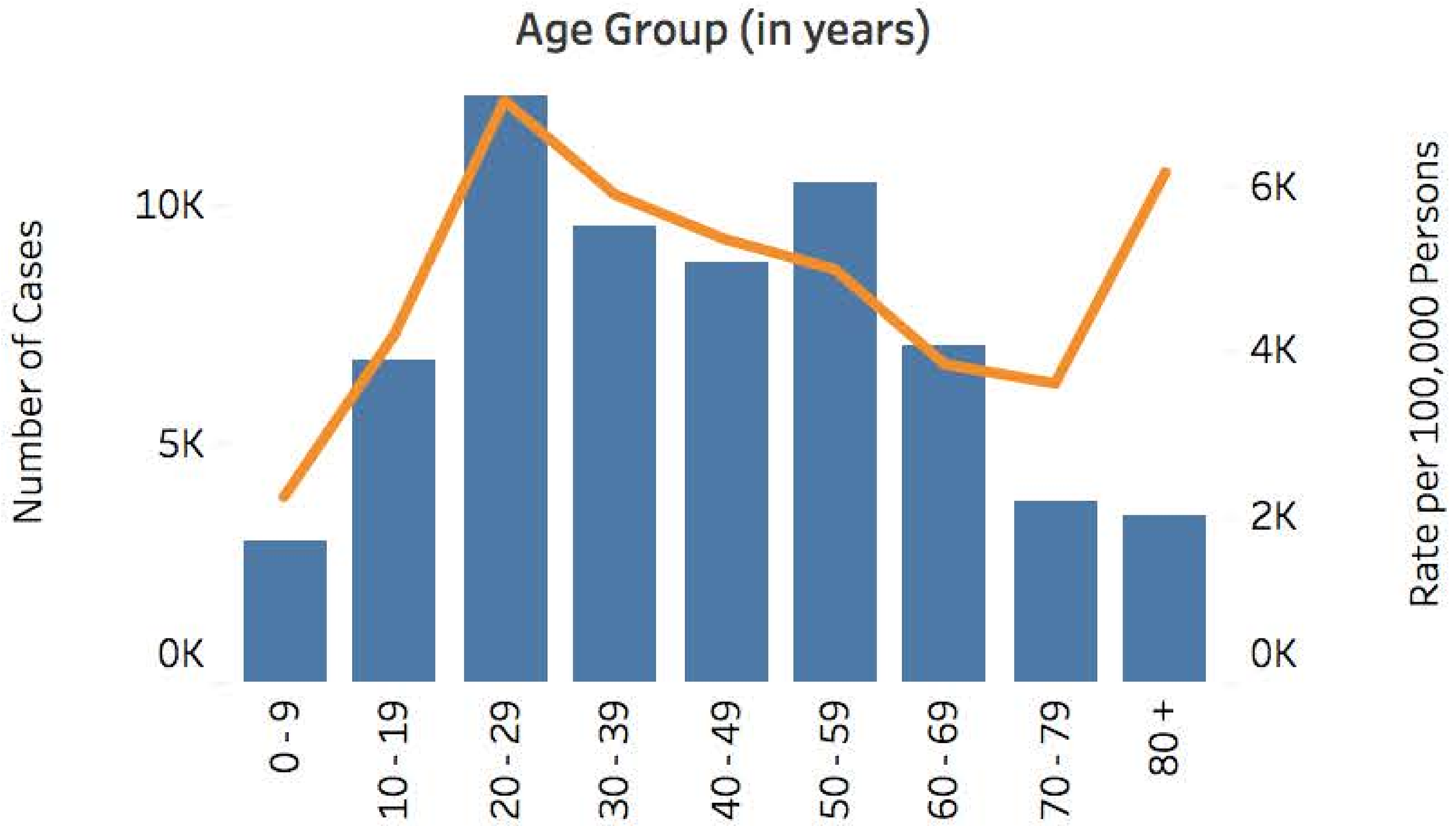

Unknown 90

Cumulative Infections | Rate per 100,000 Persons

Data as of: 1/30/2021

Undo Redo Revert Refresh Pause Share Download Full Screen

Most of the information below comes directly from New Hampshire's COVID-19 data updates and the COVID Tracking Project at The Atlantic. If you have questions or think we made a mistake, please email us at coronavirus@nhpr.org.

Just the basics: Total cases, hospitalizations, deaths and other key metrics for New Hampshire

These numbers above are pulled directly from New Hampshire's COVID-19 website. If you notice a mistake, please email us at **coronavirus@nhpr.org** so that we can correct any mistakes as soon as possible.

65,362	People Have Tested Positive for COVID-19 Since March 2020
59,082	People Have Recovered from COVID-19 Since March 2020 *
1,042	People Have Died from COVID-19
5,238	People Currently Have COVID-19 *
217	People Are Currently Hospitalized With COVID-19
415	People Are Awaiting Pending Test Results From the N.H. Public Health Lab

** The state stopped doing universal contact tracing and case monitoring in November, focusing on "high risk" populations. Cases that aren't being actively monitored "are considered recovered after 10 days from the date the case was reported to us," according to the state health department.*

Table: NHPR • Source: N.H. Department of Health and Human Services • Created with Datawrapper

How many people have been vaccinated?

If you're having trouble viewing this chart on a mobile device, try clicking here to open it in a new window.

These numbers come directly from New Hampshire's Department of Health and Human Services, as of Jan. 21. We'll continue to update this chart as new information is reported.

Click here for more official details on New Hampshire's COVID-19 vaccine plans, or check out NHPR's vaccine explainer here.

Vaccination Site	New Doses Available Since Last Week	New Doses Administered Since Last Week	Of the Available Doses, How Many Have Been Administered to Patients?	Of the Available Doses, How Many Have Been Delivered to Vaccination Sites?	Total Doses Administered to Patients	Fi
Statewide Totals	+32,005	+25,010	56%	79%	85,771	[illegible]
Hospitals	+12,590	+8,567	79%	85%	38,295	[illegible]
Long-Term Care Pharmacy Partnership Program	+8,775	+7,867	35%	68%	20,306	[illegible]
State-Managed Fixed Sites	+6,285	+7,633	64%	94%	24,039	[illegible]
Regional Public Health Network Mobile Sites	+2,900	+761	43%	56%	2,535	
Other	+1,455	+182	21%	24%	596	

Notes: "Doses Available" includes doses that have already arrived in New Hampshire and those at distributors waiting to be shipped, according to DHHS. "Doses Delivered" refers to the number of vaccine doses received at each location. According to state officials, "orders are based on clinic schedules and average uptake of vaccine by participants." More information on the Long-Term Care Pharmacy Partnership Program, overseen by the federal government, can be found at this link. Additionally, the state says "doses administered may exceed the number of doses distributed due to the ability to get extra doses out of a vial, depending on the type of syringe used."

Table: NHPR • Source: N.H. Department of Health and Human Services • Created with Datawrapper

Vaccines By Phase 1A Group (First Dose Only)

New Hampshire is prioritizing health care workers, first responders and people associated with long-term care settings in its first phase of vaccine distribution. This chart shows how many doses have been allocated to each group as of Jan. 14, but not how many people have been vaccinated. The state says it's not able to provide data on the number of doses administered to New Hampshire residents in each group at this time.

We'll continue to update this chart as new information is reported. In the meantime, click here for more details on New Hampshire's COVID-19 vaccine plans.

(Hover over the categories below for more details on the doses set aside for each group.)

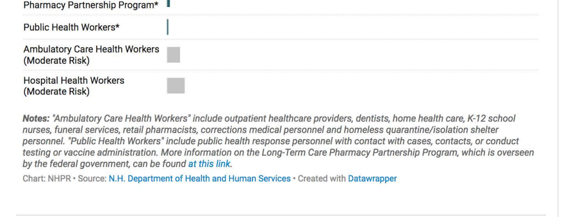

Notes: "Ambulatory Care Health Workers" include outpatient healthcare providers, dentists, home health care, K-12 school nurses, funeral services, retail pharmacists, corrections medical personnel and homeless quarantine/isolation shelter personnel. "Public Health Workers" include public health response personnel with contact with cases, contacts, or conduct testing or vaccine administration. More information on the Long-Term Care Pharmacy Partnership Program, which is overseen by the federal government, can be found at this link.

Chart: NHPR • Source: N.H. Department of Health and Human Services • Created with Datawrapper

How many new cases are emerging each day?

If you're having trouble viewing this chart on a mobile device, click here to open it in a new window.

This shows how many new COVID-19 cases were announced each day by state health officials, and the seven-day average for the same metric. It doesn't reflect the number of new positive tests by date or new infections by illness onset date, because the state updates those figures on an ongoing basis. For the latest testing and illness onset data, check out the state's dashboard.

Note: New Hampshire began including positive antigen tests, in addition to positive PCR tests, in its case totals in early October. The state didn't report any data on Thanksgiving Day (Nov. 26), Christmas Day (Dec. 25) or New Year's Day (Jan. 1), which could make it appear cases decreased and then spiked more than they actually did after those dates.

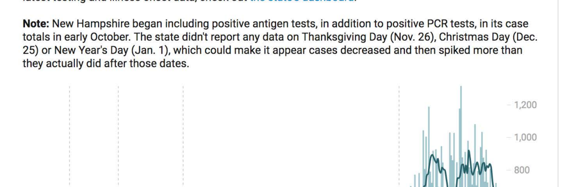

The dotted lines above reflect major policy steps in New Hampshire's response to COVID-19:
'Stay At Home' (March 26) Non-essential businesses closed, residents ordered to remain home
'Stay At Home 2.0' (May 1) Guidelines loosened, non-essential businesses allowed to reopen in phases
'Safer At Home' (June 15) Stay At Home mandate lifted, statewide business guidelines imposed
Statewide Mask Mandate (Nov. 19) Applies to public spaces where distancing can't be achieved

Chart: NHPR • Source: New Hampshire Department of Health and Human Services • Created with Datawrapper

How many people have COVID-19 right now?

If you're having trouble viewing this chart on a mobile device, click here to open it in a new window.

This shows how many people in New Hampshire were infected with COVID-19 on each date, as reported by state health officials, plus the seven-day rolling average for the same metric. The state stopped doing universal contact tracing and case monitoring in November, focusing on "high risk" populations. Cases that aren't being actively monitored "are considered recovered after 10 days from the date the case was reported to us," according to the state health department.

Note: The state didn't report any data on Thanksgiving Day (Nov. 26), Christmas Day (Dec. 25) or New Year's Day (Jan. 1), which could make it appear cases decreased and then spiked more than they actually did after those dates.

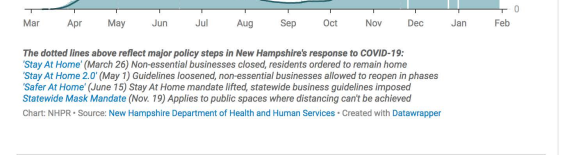

The dotted lines above reflect major policy steps in New Hampshire's response to COVID-19:
'Stay At Home' (March 26) Non-essential businesses closed, residents ordered to remain home
'Stay At Home 2.0' (May 1) Guidelines loosened, non-essential businesses allowed to reopen in phases
'Safer At Home' (June 15) Stay At Home mandate lifted, statewide business guidelines imposed
Statewide Mask Mandate (Nov. 19) Applies to public spaces where distancing can't be achieved

Chart: NHPR • Source: New Hampshire Department of Health and Human Services • Created with Datawrapper

How many people are hospitalized?

If you're having trouble viewing this chart on a mobile device, click here to open it in a new window.

This shows how many people were hospitalized in New Hampshire with confirmed COVID-19 cases on each date, according to state health officials, as well as the rolling-seven day average for the same metric.

Note: New Hampshire didn't start reporting daily hospitalization numbers until mid-April. The state didn't report any data on Thanksgiving Day (Nov. 26), Christmas Day (Dec. 25) and New Year's Day (Jan. 1), which could make it appear cases decreased and then spiked more than they actually did after those dates.

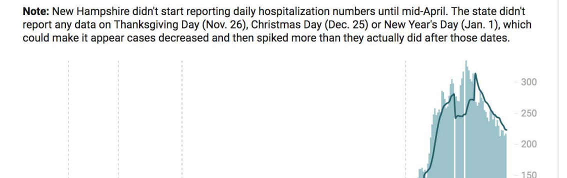

The dotted lines above reflect major policy steps in New Hampshire's response to COVID-19:
'Stay At Home' (March 26) Non-essential businesses closed, residents ordered to remain home
'Stay At Home 2.0' (May 1) Guidelines loosened, non-essential businesses allowed to reopen in phases
'Safer At Home' (June 15) Stay At Home mandate lifted, statewide business guidelines imposed
Statewide Mask Mandate (Nov. 19) Applies to public spaces where distancing can't be achieved

Chart: NHPR • Source: New Hampshire Department of Health and Human Services • Created with Datawrapper

How full are N.H.'s inpatient beds with COVID-19 patients?

If you're having trouble viewing this chart on a mobile device, click here to open it in a new window.

New Hampshire hospitals report data on inpatient bed use and COVID-19 directly to the U.S. Department of Health and Human Services, which provides a window into how the virus is affecting hospital capacity over time. The chart below reflects rolling seven-day averages for each value, meant to adjust for single-day spikes or other gaps in reporting.

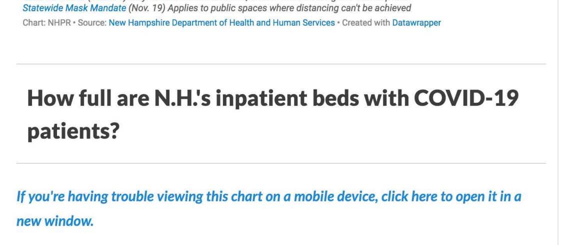

Chart: NHPR • Source: U.S. Department of Health and Human Services • Created with Datawrapper

How full are N.H.'s ICU beds with COVID-19 patients?

If you're having trouble viewing this chart on a mobile device, click here to open it in a new window.

These figures come directly from numbers reported by New Hampshire hospitals to the U.S. Department of Health and Human Services. The chart below reflects rolling seven-day averages, meant to adjust for single-day spikes or other gaps in daily reporting.

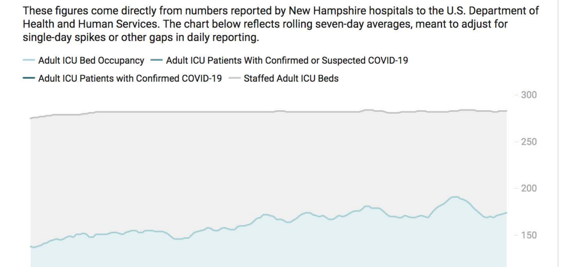

The data above comes from New Hampshire's 26 short-term and critical access hospitals, as well as these rehabilitation and psychiatric institutions: Northeast Rehabilitation Hospital, Encompass Health Rehabilitation Hospital of Concord, New Hampshire Hospital and Hampstead Hospital.

Chart: NHPR • Source: U.S. Department of Health and Human Services • Created with Datawrapper

What's the situation like at your local hospital?

If you're having trouble viewing this chart on a mobile device, click here to open it in a new window.

Federal data gives us a deeper look into the disparate effects of COVID-19 on different hospitals across the state. The figures below are averages for the third week of January.

Why is data missing for some facilities? If a hospital reported four or fewer patients for a particular category, the U.S. Department of Health and Human Services suppressed that data in its public reports. In those cases, the fields without data will be blank in the table below.

Search in table

Page 1 of 6

Facility	How Full Were Inpatient Beds Overall?	How Full Were Staffed Adult ICU Beds?	How Full Were Inpatient Beds With COVID-19 Patients?	How Full Were Staffed ICU Beds With COVID-19 Patients?	Total Adult Inpatien Beds
Valley Regional Hospital (Claremont)	94%		28%		18
New London Hospital (New London)	89%		0%		25
Mary Hitchcock Memorial Hospital (Lebanon)	89%	77%	4%	17%	391
Elliot Hospital (Manchester)	88%	81%	9%		268
Portsmouth Regional Hospital (Portsmouth)	88%	86%	13%		184

These numbers were self-reported from New Hampshire healthcare institutions to the U.S. Department of Health and Human Services, and the criteria used for each category might vary from hospital to hospital. While this is the most recent data available, it is a bit delayed: The values are averages reported for the week of Jan. 15 through Jan. 21, rounded to the nearest whole number.

Table: NHPR • Source: United States Department of Health and Human Services • Created with Datawrapper

How many people are dying from COVID-19?

If you're having trouble viewing this chart on a mobile device, click here to open it in a new window.

This shows how many deaths were reported on each date, plus the rolling-seven day average in the same metric. Please keep in mind that there can be a lag between when someone dies from COVID-19 and when their death is confirmed by state health officials. The charts below only reflect the death totals *reported* on a particular date, not the number of deaths that occurred on each date.

Note: The state didn't report any data on Thanksgiving Day (Nov. 26), Christmas Day (Dec. 25) or New Year's Day (Jan. 1), which could make it appear cases decreased and then spiked more than they actually did after those dates.

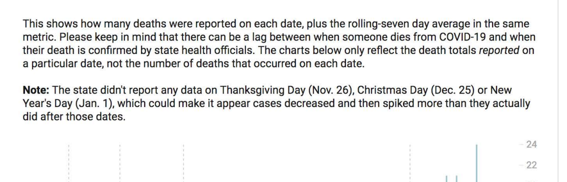

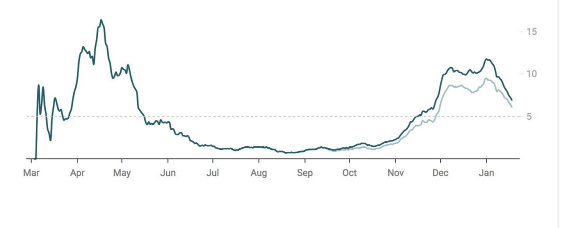

New Jersey COVID-19

NJ COVID-19 Hub

Cases and Trends | Vaccination Overview | Hospital Census | Schools | Case and Mortality Summaries | Contact Tracing

COVID-19 Dashboard

Stage 2: Moderate Risk Activities Restarted with Safeguarding

THE ROAD BACK
Restoring Economic Health through Public Health

Updated: 1/31/2021
Comments about this dashboard: Covid.Dashboard@doh.nj.gov
Please note that due to the high volume of email this mailbox receives, not all messages can be addressed individually. For general

Last update: a few seconds ago

COVID-19 Cases by County

Data is provisional and subject to revision.

"Confirmed" cases are established through PCR testing.

"Probable" cases are established through viral antigen testing.

Bergen County

372 **New Confirmed Cases**
2,206 **Confirmed Deaths**
67 **New Probable Cases**
60,252 **Total Confirmed Cases**
268 **Probable Deaths**
7,001 **Total Probable Cases**

Hudson County

369 **New Confirmed Cases**
1,696 **Confirmed Deaths**
43 **New Probable Cases**

Confirmed Cases with Unassigned County
1,154

Deaths with Unassigned County
0

New Cases by County

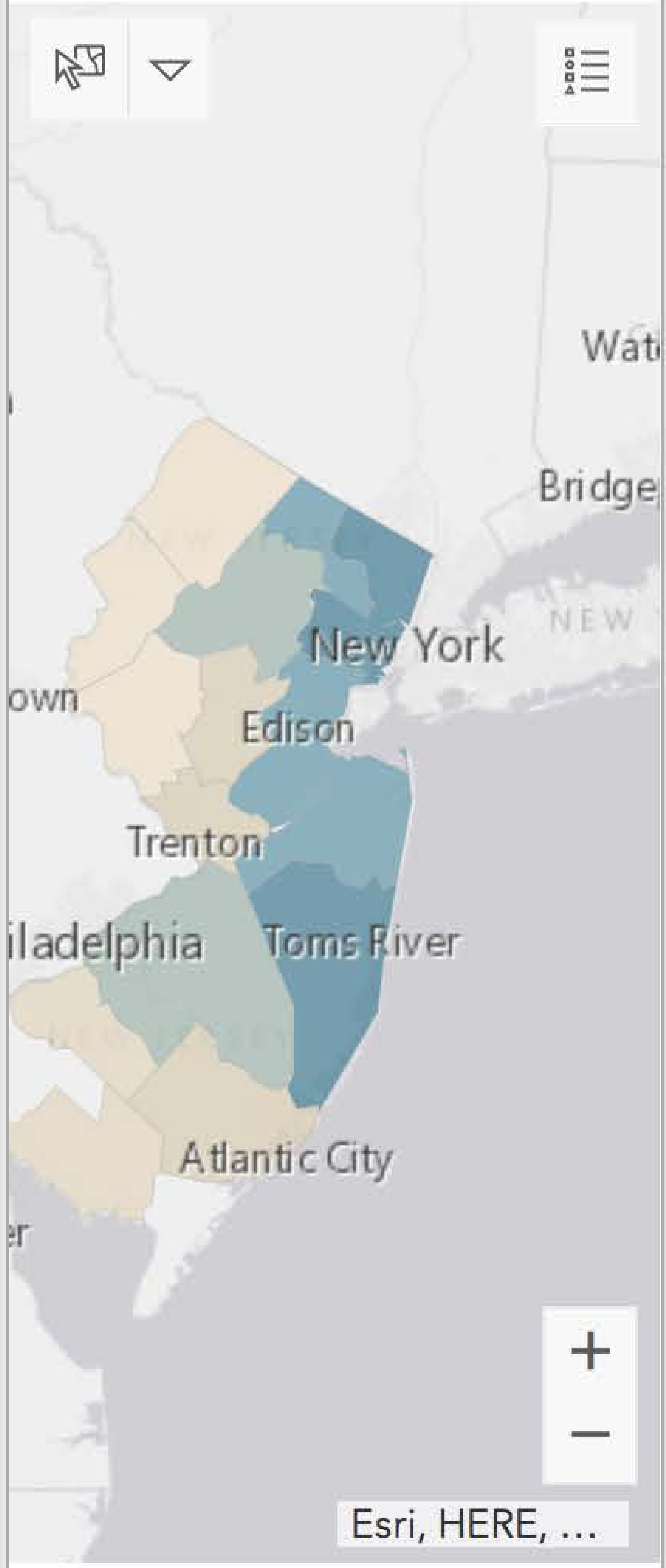

Click county to see details.

Map (scroll for mor...

Total Confirmed* Cases
623,541
*Established through PCR testing

Total Confirmed Deaths
19,355

Total PCR Tests Reported
9,326,347

Probable* Cases
73,288
*Established through viral antigen testing

Total Probable Deaths*
2,129
*Updated Wednesdays

Last update: a few seconds ago

Cumulative | Daily | Notes

New Confirmed* Cases Over Time

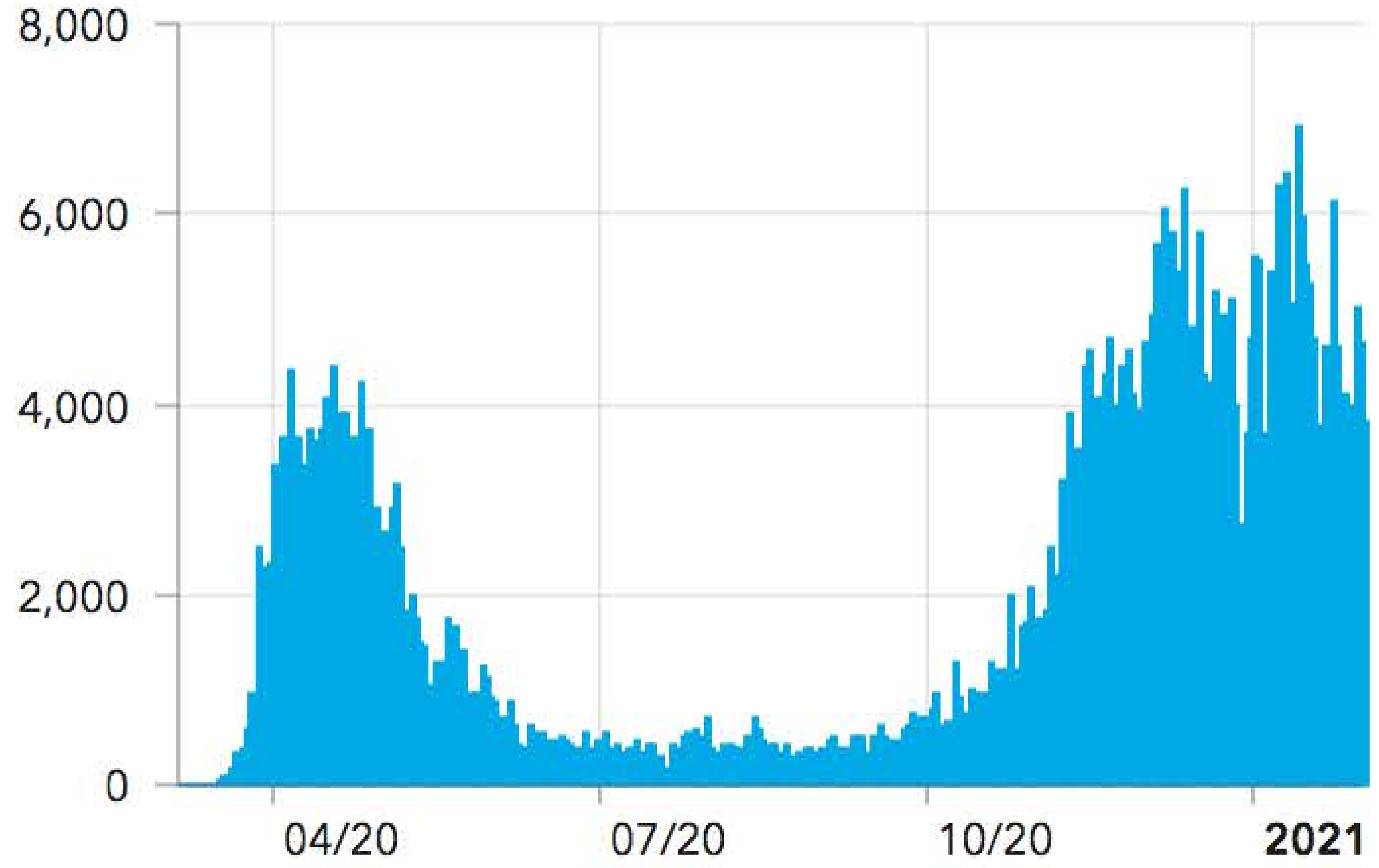

* Established via PCR testing
Data are provisional and subject to revision with further investigation.

esri A Story Map

New Jersey COVID-19

NJ COVID-19 Hub

Cases and Trends | Vaccination Overview | Hospital Census | Schools | Case and Mortality Summaries | Contact Tracing

Total Vaccine Doses Administered*

785,588

Current as of 31-Jan-2021 10:10 ET

First Doses Administered

656,174

Second Doses Administered

124,612

*Total vaccine doses administered includes dose 1, dose 2 and a small number of doses that do not have a dose number yet indicated at the time of data refresh. Therefore, summing across dose 1 and dose 2 will not

By Gender

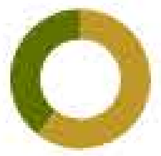

- Female 60%
- Male 40%
- Other 0%

Last update: a few seconds ago

By Race/Ethnicity

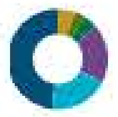

- Asian 6%
- Black 3%
- Hispanic/Latinx 5%
- Other 19%
- Unknown 19%
- White 48%

Last update: a few seconds ago

By Age Group

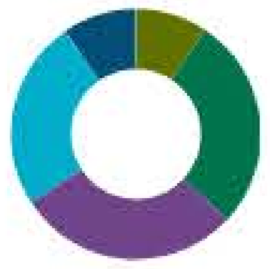

- 16-17 0%
- 18-29 9%
- 30-49 28%
- 50-64 29%
- 65-79 25%
- 80+ 10%
- (Missing) 0%

Last update: a few seconds ago

Vaccine Brand

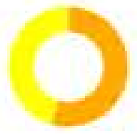

- Moderna 55%
- Pfizer 45%

Last update: a few seconds ago

Statewide data only

Key Vaccination Statistics | Vaccination Doses Over Time | Vaccination Site Map

Select a county or counties to filter the graphs.

ATLANTIC COUNTY
22,813 Doses Administered

BERGEN COUNTY
86,616 Doses Administered

BURLINGTON COUNTY
38,742 Doses Administered

CAMDEN COUNTY
46,359 Doses Administered

CAPE MAY COUNTY
11,358 Doses Administered

CUMBERLAND COUNTY
11,498 Doses Administered

ESSEX COUNTY
62,777 Doses Administered

GLOUCESTER COUNTY
26,024 Doses Administered

HUDSON COUNTY

Last update: a few seconds ago

esri A Story Map

N.J. Coronavirus Tracker

nj.com

subscribe

Select a location: All N.J.

Daily Summary: All N.J.

New Cases	Total Cases
3,809	623,541
**New Deaths**	**Total Deaths**
29	19,355
**New Tests**	**Total Tests**
-	9,326,347
**Today's Positivity**	**Overall Positivity**
-	6.69%

Daily Cases: All N.J.

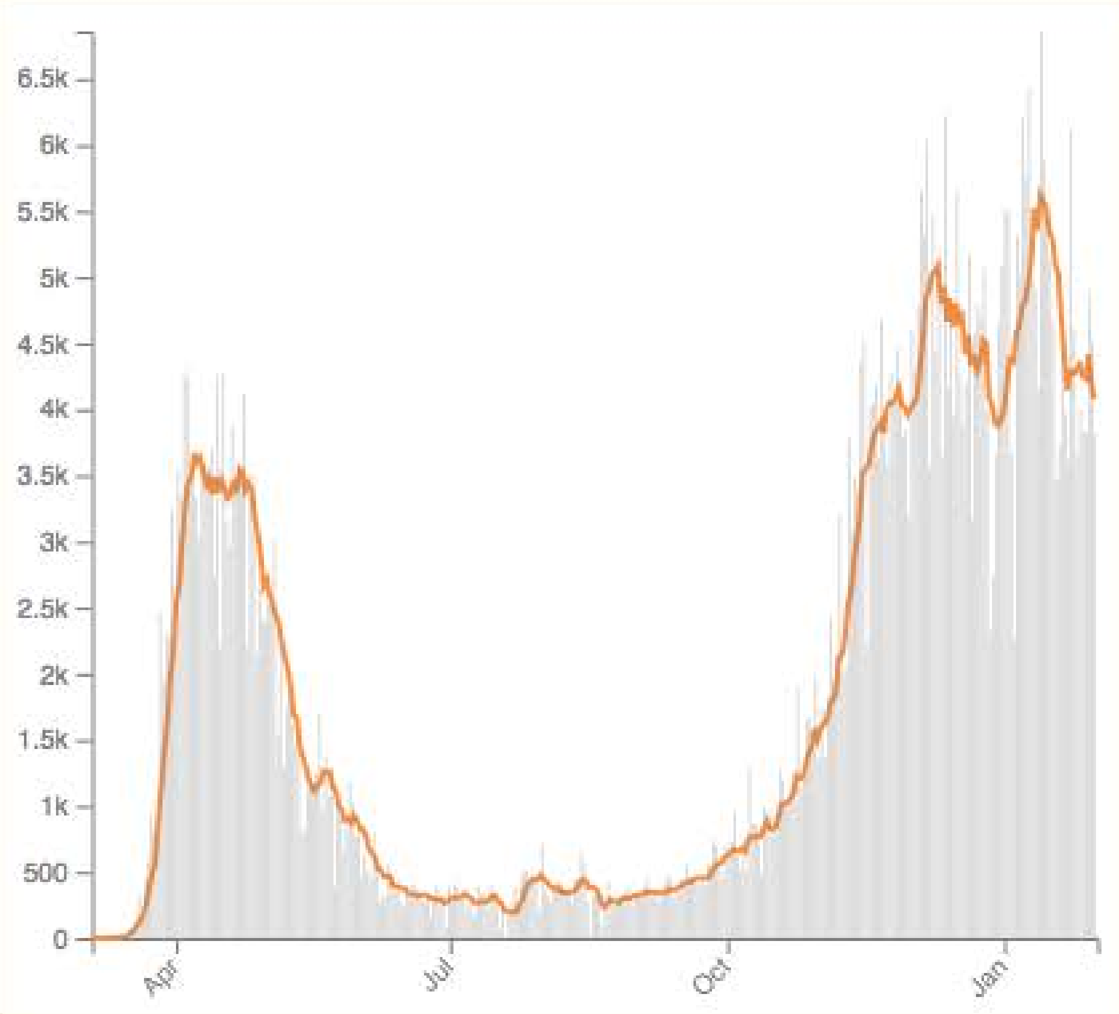

Daily Deaths: All N.J.

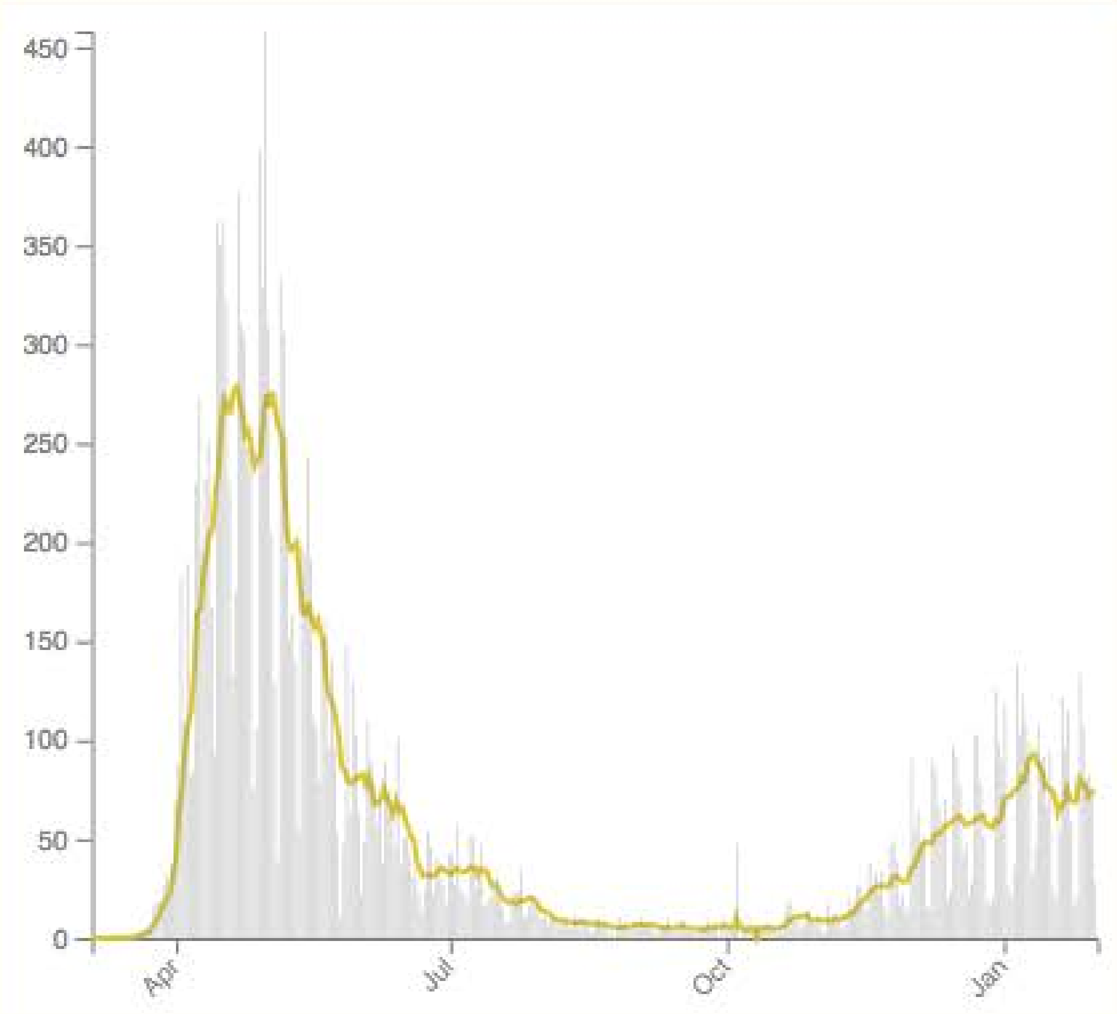

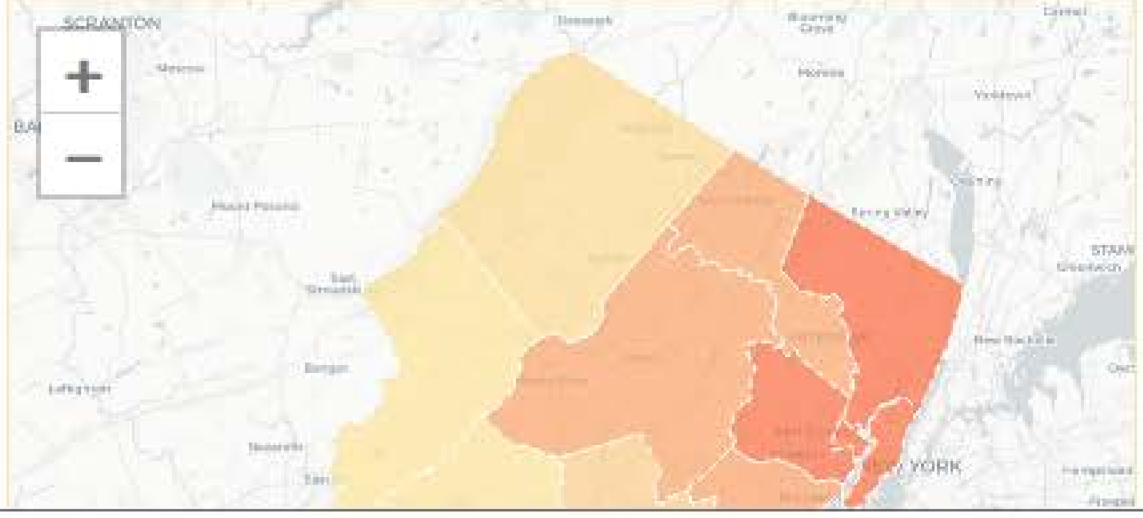

Total Cases & Deaths: All N.J.

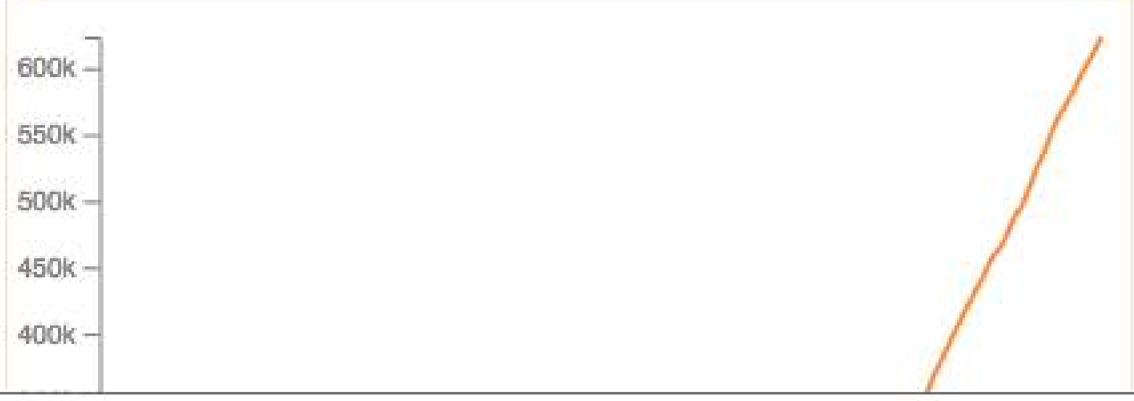

Daily Testing

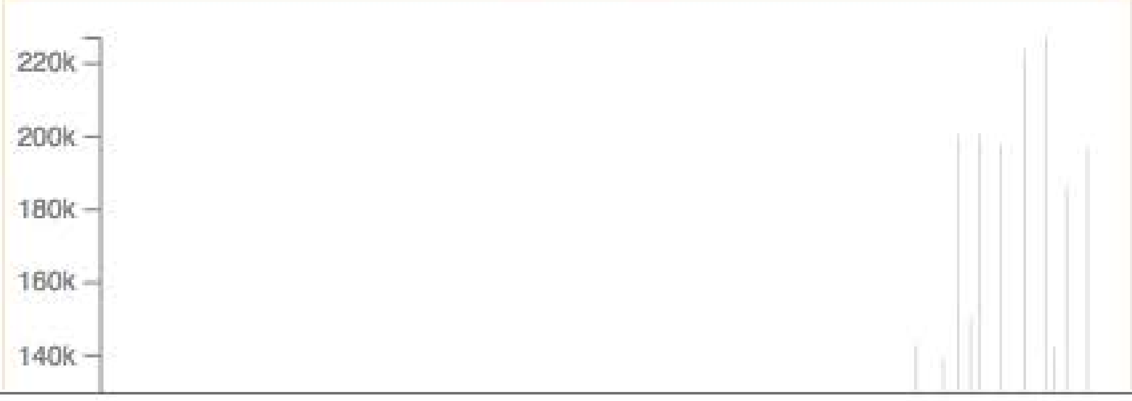

Contact | © NJ Advance Media | Developed by Arjun Kakkar

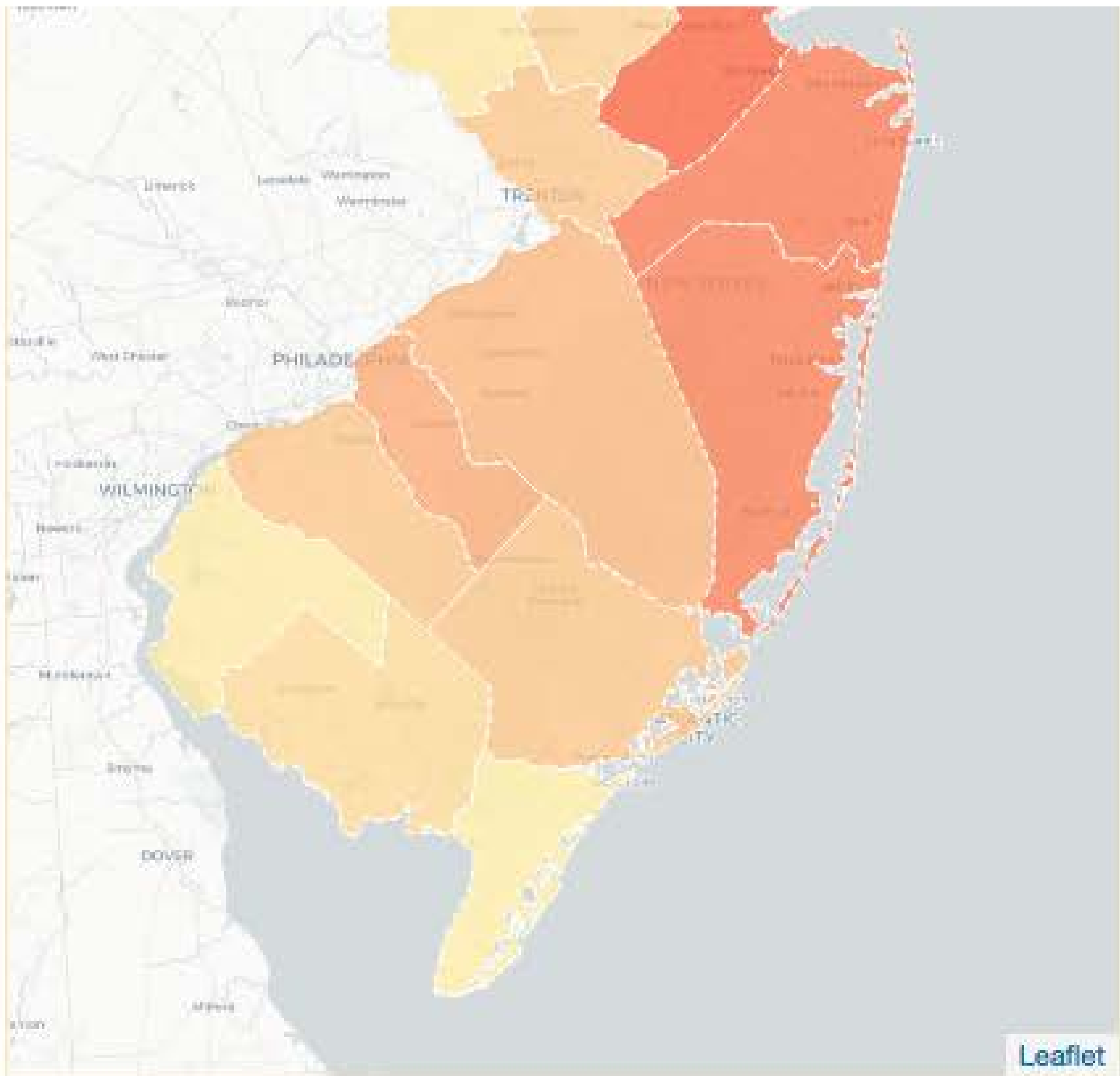

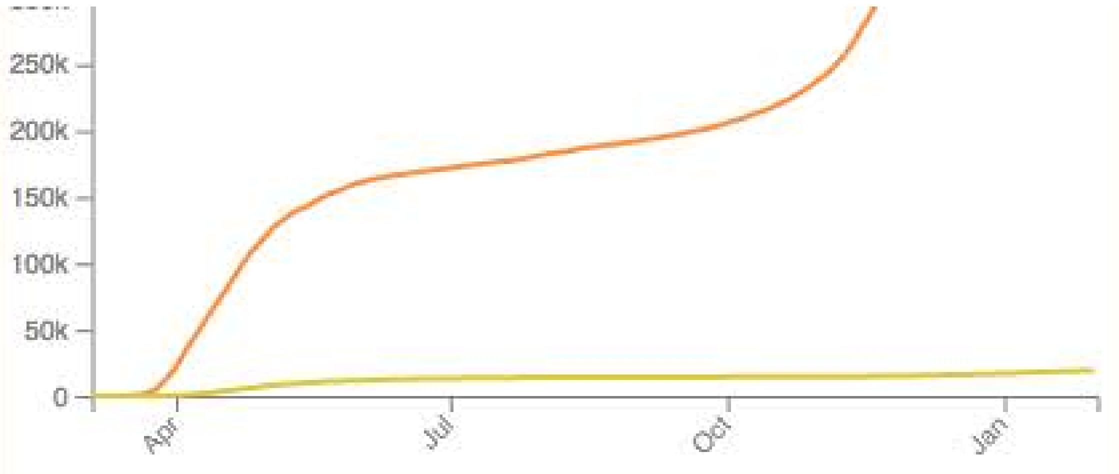

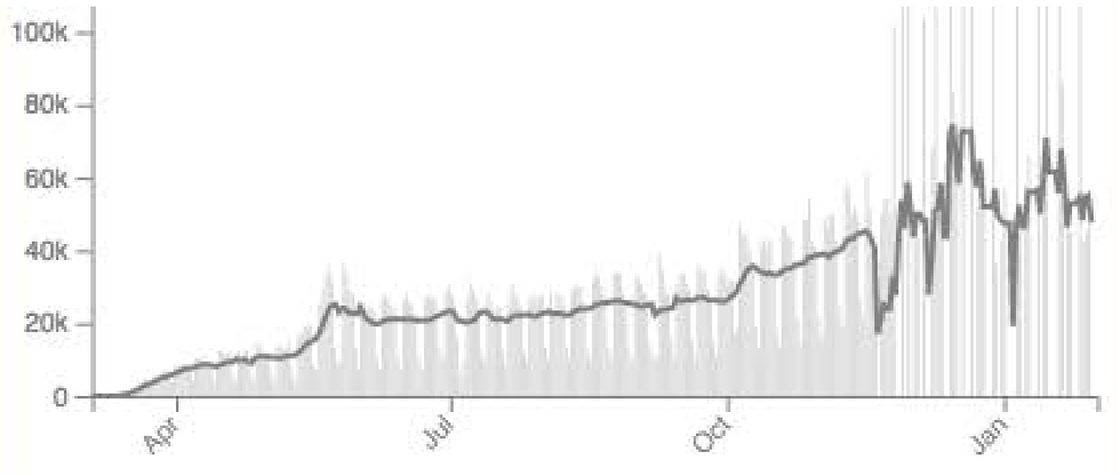

U.S. Summary

Cumulative Cases	Cumulative Deaths
25,697,888	430,120

Resources

- Sign up for coronavirus text alerts, emails and other resources.
- See all N.J. coronavirus news.

New Mexico Department of Health

COVID-19 in New Mexico

Back to CV.NMHEALTH.ORG

NUMBER OF CASES

173,539

TOTAL # OF TESTS PERFORMED

2,328,334

TOTAL HOSPITALIZATIONS

12,047

CURRENT HOSPITALIZATIONS

548

TOTAL DEATHS

3,265

TOTAL RECOVERED

102,298

Show Historical Statewide Data

Last Updated: 1/30/2021, 5:55:35 PM

Information about the Data

Current COVID case outlook 1/12/2021 - 1/25/2021

DATA IS UPDATED EVERY OTHER WEEK. NEXT UPDATE: 2/10/2021

Map Key: Fewer Cases — More Cases View Map Key

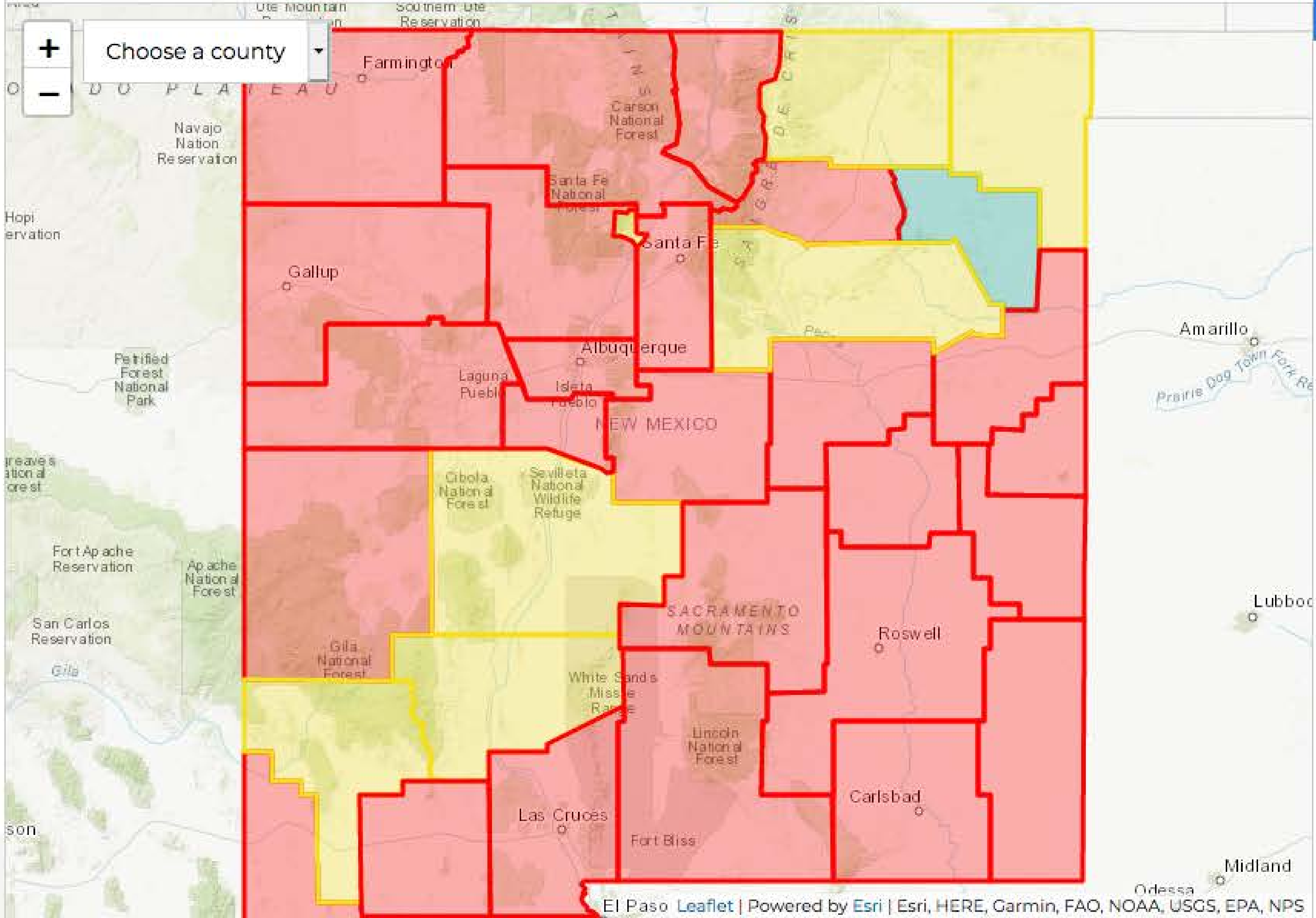

EXPAND DEMOGRAPHICS VIEW

Statewide Gender Breakdown

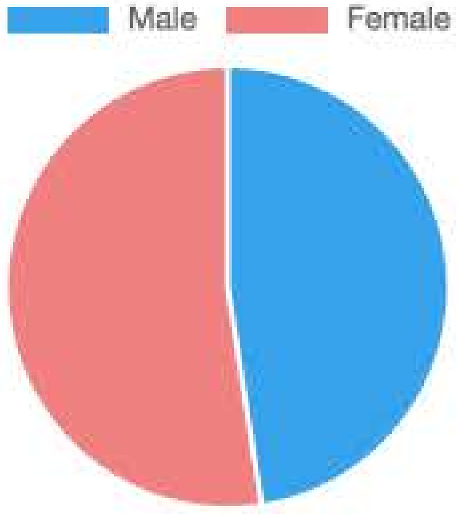

Gender	Cases
Male	47.55%
Female	52.45%

Statewide Age Breakdown

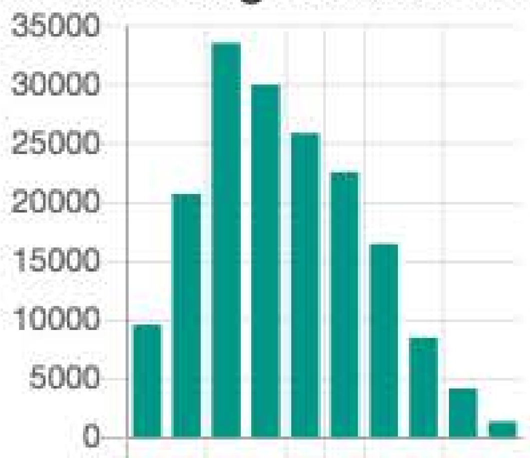

New York State Department of Health

NYSDOH COVID-19 Tracker

Testing data as of: 1/30/2021 2 PM
Testing data last updated on: 1/31/2021

Statewide

Total Persons Tested
32,161,751

Total Tested 1/30
243,066

Total Tested Positive
1,410,656

Sex Distribution of Positive Cases

Female	Male	Unknown
50.5%	48.4%	1.2%

New Positives 1/30
10,793

Persons Tested Positive by County

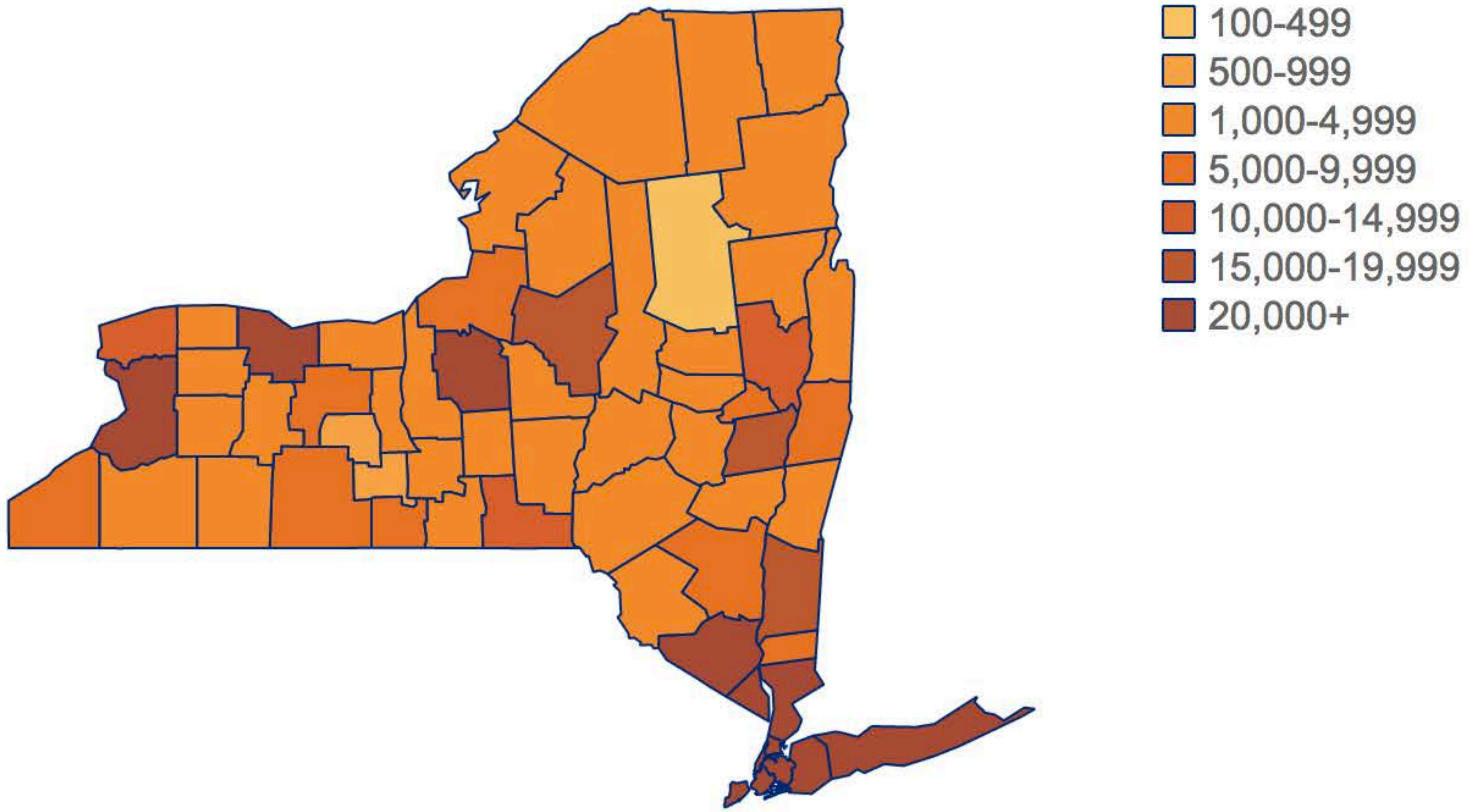

County Stats: None

Click County to See Detail
Click Again for Statewide

County	Positive
Albany	18,701
Allegany	2,578
Bronx	115,427
Broome	12,748
Cattaraugus	3,797
Cayuga	4,903
Chautauqua	6,481
Chemung	6,041
Chenango	2,110
Clinton	2,720
Columbia	2,971
Cortland	2,846
Delaware	1,217
Dutchess	19,123
Erie	57,406
Essex	1,111
Franklin	1,539
Fulton	2,593

Click for Daily Trends

Click for Table View

Click for Fatality Data

FAQs & Helpful Links

State of Ohio | COVID - 19 Dashboard

Last Updated: 01-31-21
Updated daily at 2 p.m.

County Map | Cases
Select a County To Filter Dashboard

Case Classification Status
Filter all views by case classification
(All) Total Cases

View By
Pick an option to view in map
Cases

County Search
Search a county to highlight
Highlight County

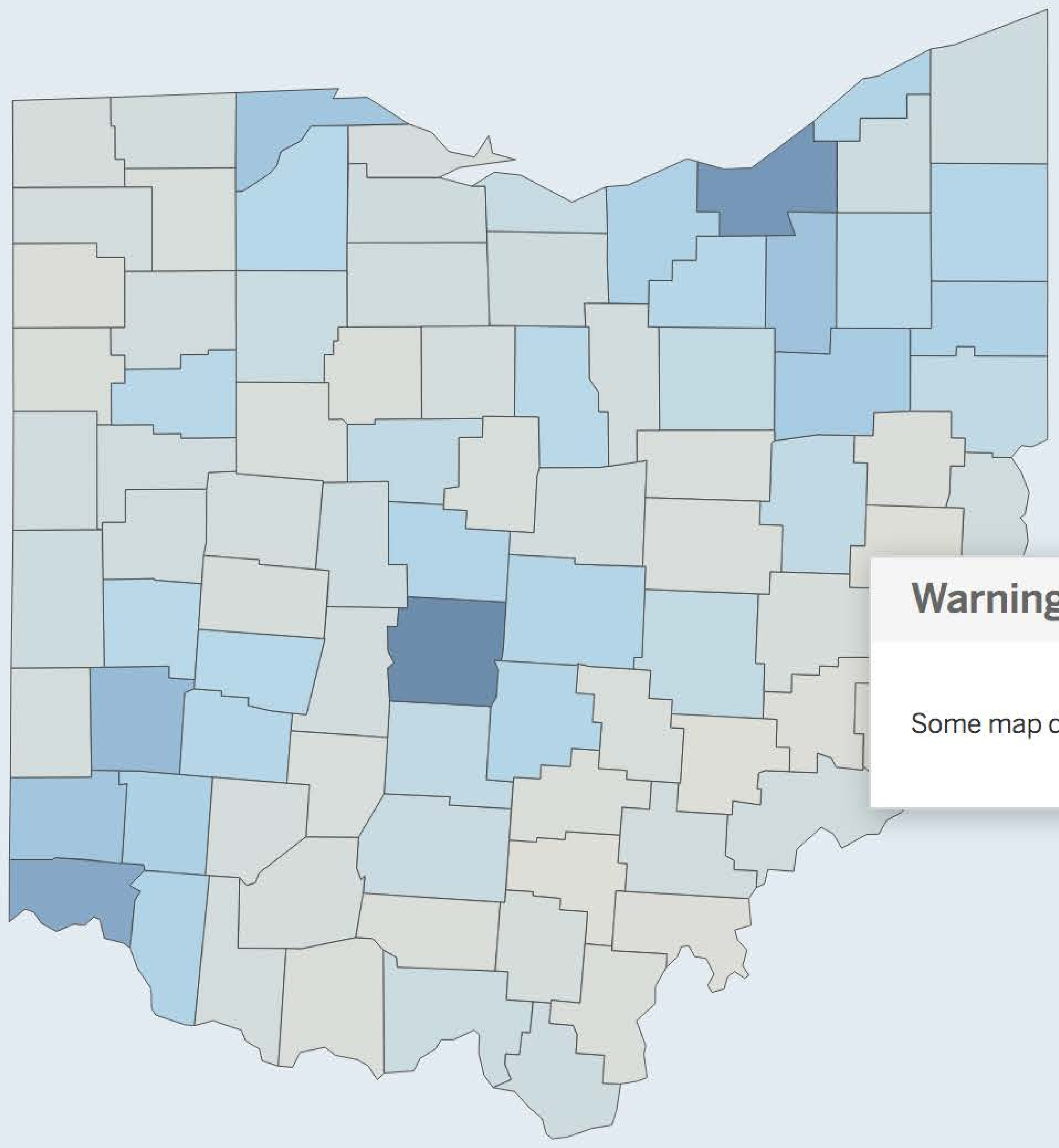

Warning
Some map details are not available.

© OpenStreetMap

Metrics | Cumulative and Daily Count

View Count By:
Select to view counts by daily or cumulative
Daily Count

Cases

[illegible]

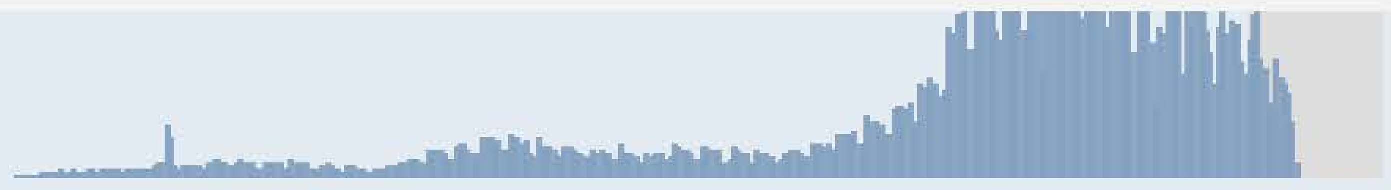

Hospitalizations

46,215

* Preliminary

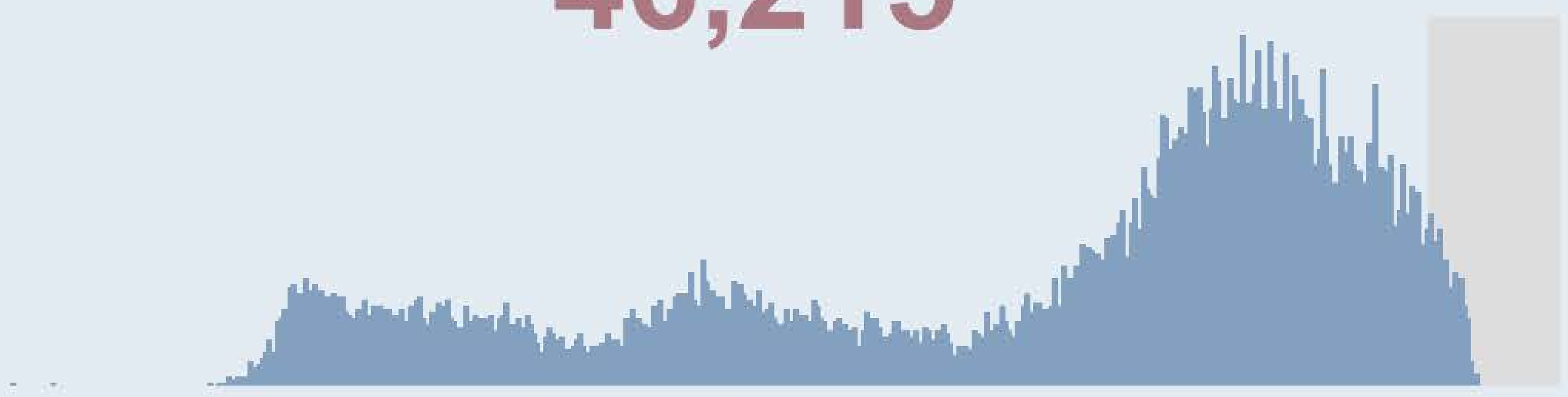

Deaths

11,175

* Preliminary

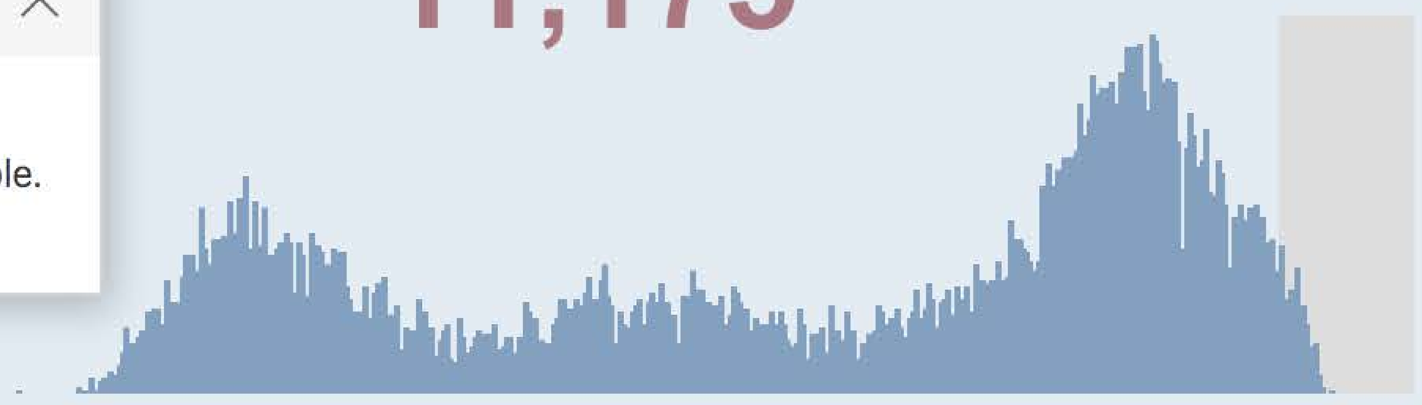

Presumed Recovered ***

779,888

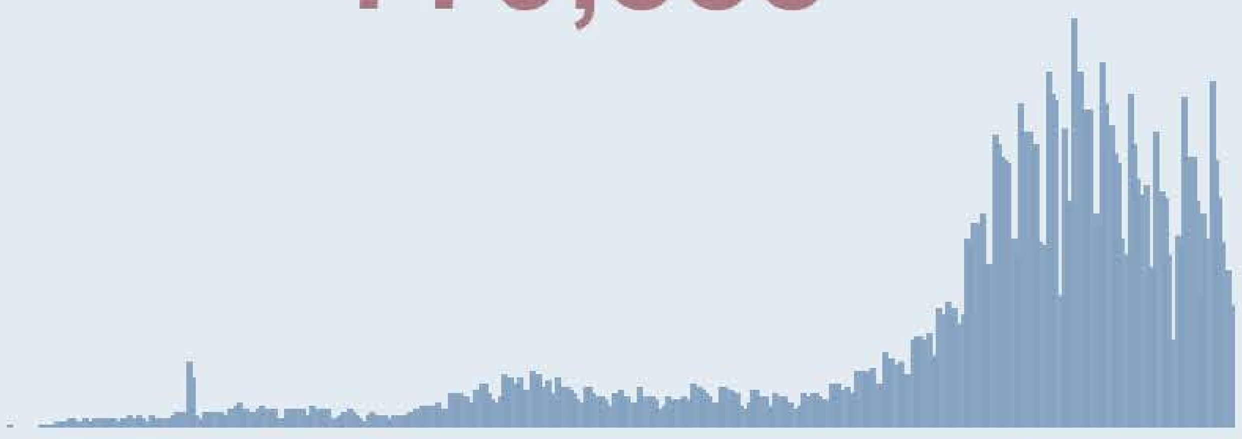

Key Metrics | By County

View By
Pick an option to change the view
County

View By	Case Count	Hospitalization Count	Death Count
Franklin	104,761	3,362	709
Cuyahoga	89,042	5,444	1,203
Hamilton	67,154	2,380	460
Montgom..	44,586	3,868	430
Summit	36,414	3,082	803
Lucas	32,637	1,738	669
Butler	32,348	939	242
Stark	26,971	1,563	466
Warren	20,531	591	149
Lorain	20,012	1,039	237
Medina	[illegible]	[illegible]	[illegible]

Language Translation

An Official Site of Ohio.gov

Ohio Department of Health | Coronavirus (COVID-19) | Search

Ohio Public Health Advisory System | Responsible RestartOhio | COVID-19 Vaccination Program | Families and Individuals | Healthcare Providers and Local Health Districts

Note: Totals include Confirmed and CDC Expanded Case Definition (Probable)

All data displayed is preliminary and subject to change as more information is reported to ODH. Trends are based on the date of illness onset. If onset date is unknown, the earliest known date associated with the case is used as a substitute for the date of illness onset.

*Preliminary case data in the shaded area is likely under-reported to ODH because of the delay between initial contact with a health care provider, testing, and diagnosis.

**Definitive cause of death can take weeks to determine. Data displayed illustrates current status.

*** Presumed Recovered is defined as cases with a symptom onset date >21 days prior who are not deceased.

+ a b l e a u

COVID-19 Cases - Main Page BETA

OK Case Summary

Source: Oklahoma Government

Case Updated As Of: 2021-01-31

OK Cases	OK Deaths	OK Active	OK Recovered
389,472	3,547	29,539	356,386
↑ 2,882 DoD*	↑ 43 DoD*	↑ 676 DoD*	↑ 2,163 DoD*
0.7% DoD*	1.2% DoD*	2.3% DoD*	0.6% DoD*

OK Immunization Summary

Source: Oklahoma Government

Immunization Updated As Of: 2021-01-29

315,034	58,245	373,279
Prime Doses Given	Completed Series	Total Doses Administered
▲ 72705 WOW*		▲ 95128 WOW*

315,600	195,750
Prime Doses Allocated	Boost Doses Allocated

US Summary

Source: Johns Hopkins

Case Updated As Of: 2021-01-30

US Cases	US Deaths
26,074,885	439,530
↑ 142,091 DoD*	↑ 2,731 DoD*
0.5% DoD*	0.6% DoD*

Oklahoma Case Status by Date of Onset

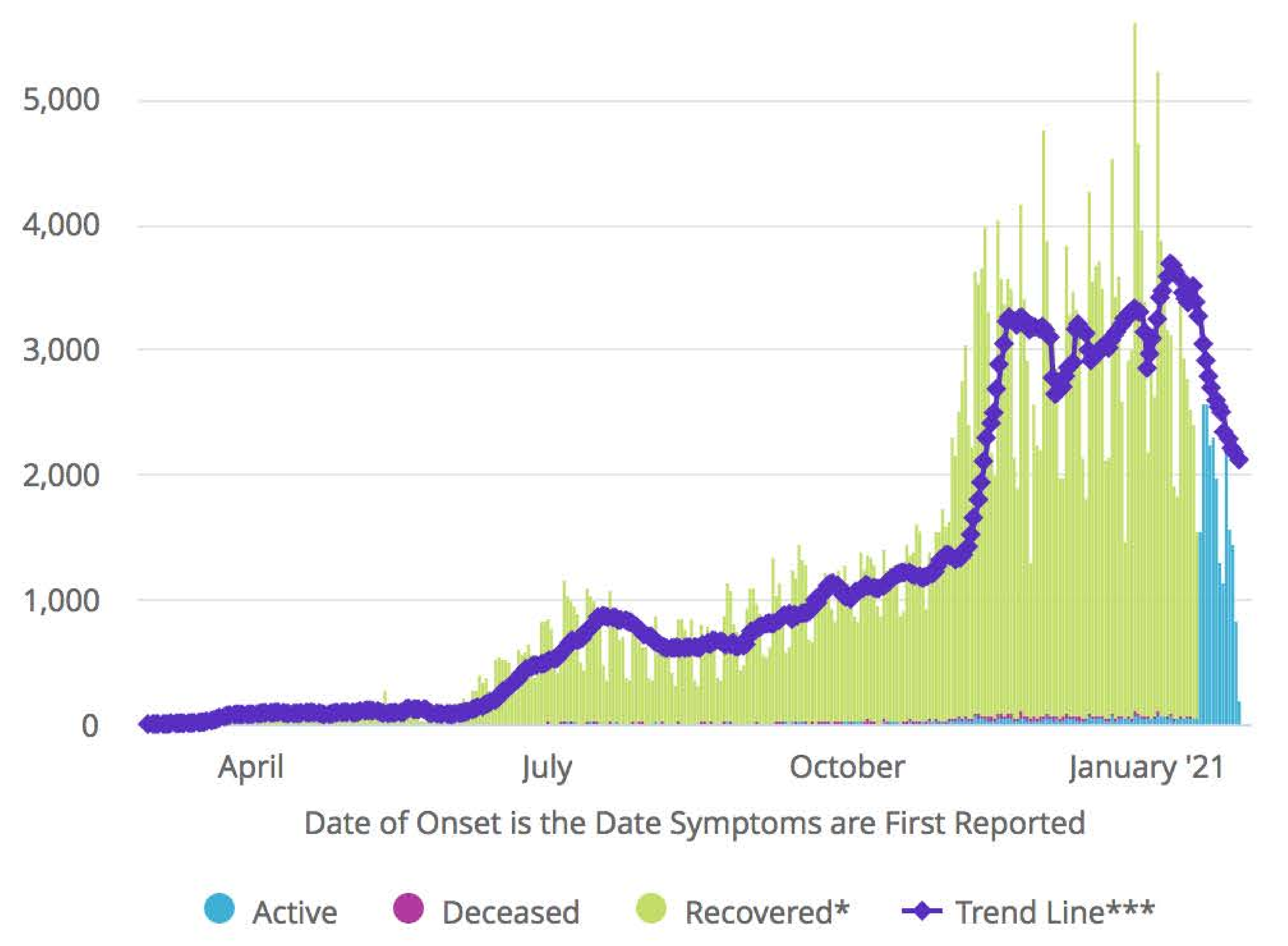

Oklahoma Cumulative Cases by Date Reported

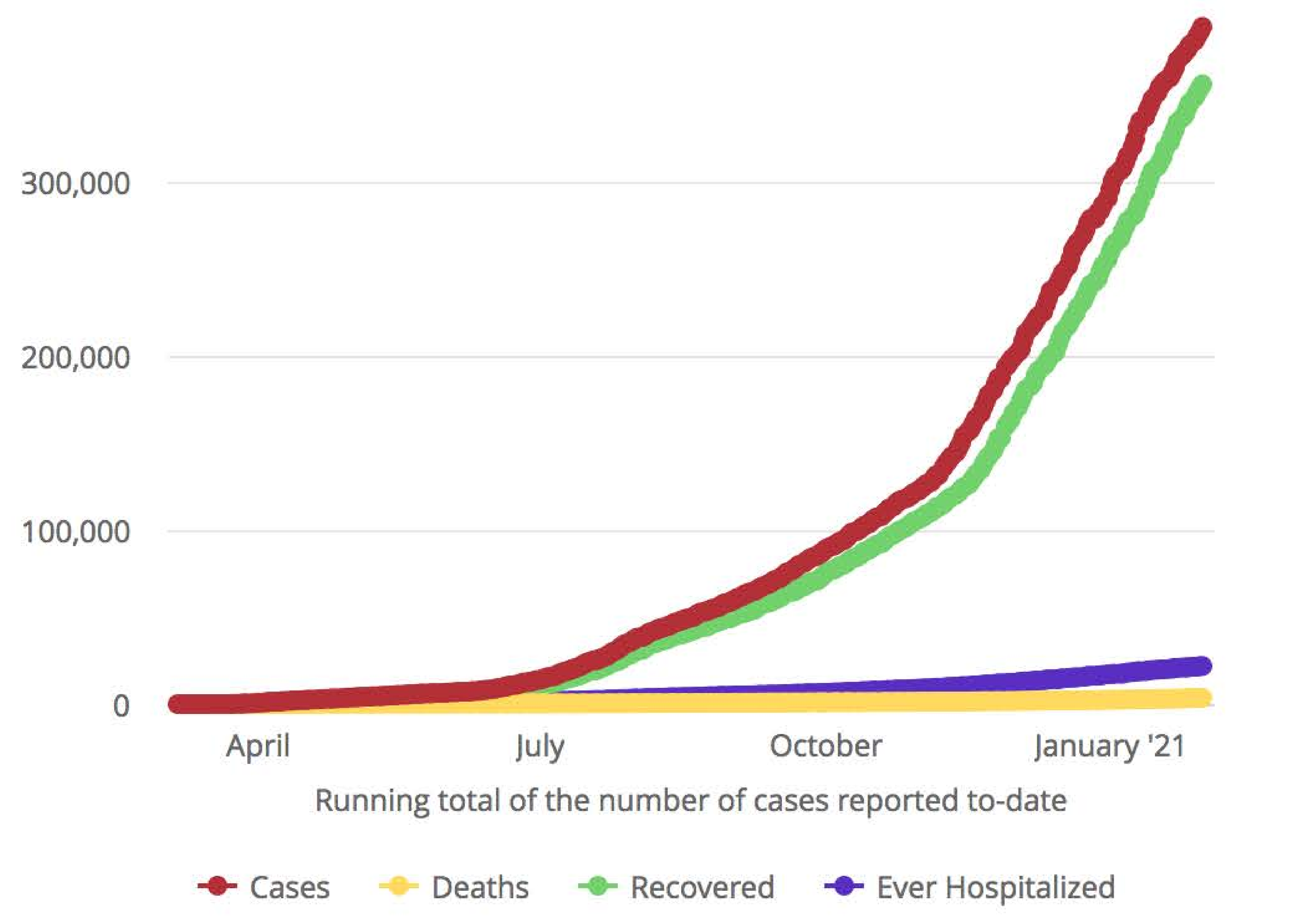

Oklahoma Total Cases by Race

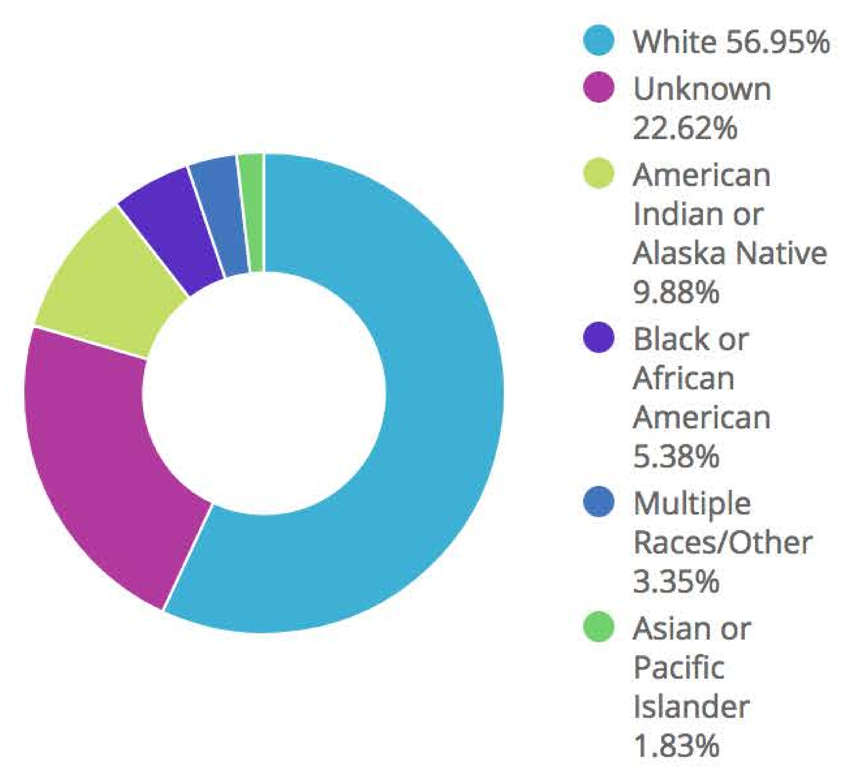

Oklahoma Total Cases by Age Group

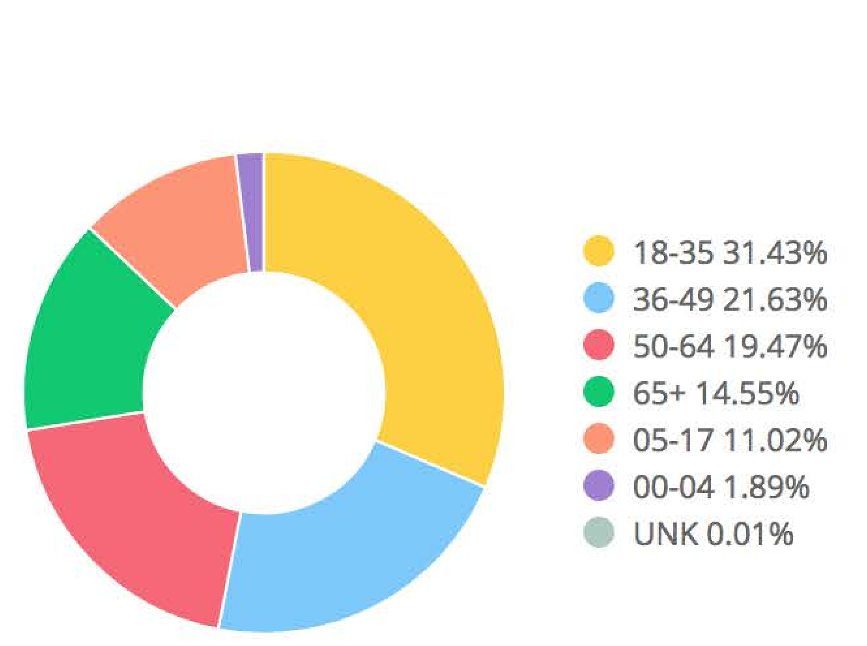

Oklahoma Total Cases Status by Gender

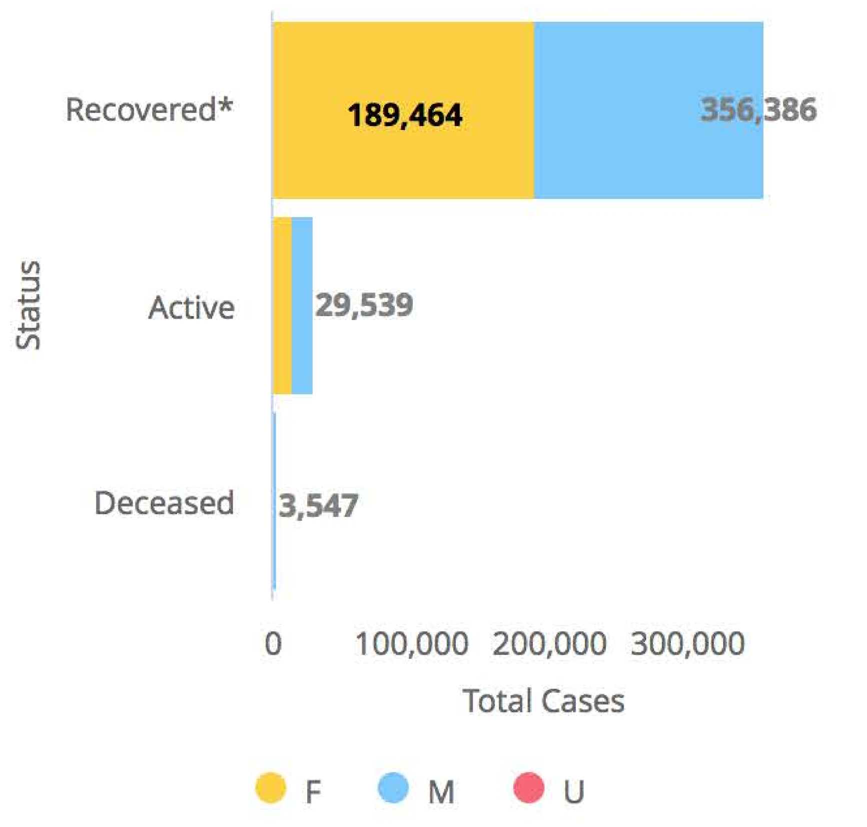

View Data by County | View Data by City | View Data by Zip

View Data for Cases | View Data for Deaths | View Data for Recoveries

Symptom Tracker | Personal Protective and Hospital Equipment | Testing Sites

OSDH District Breakout | View Data by County and Duration Filters | View Data Downloads

COVID-19 Cases by County

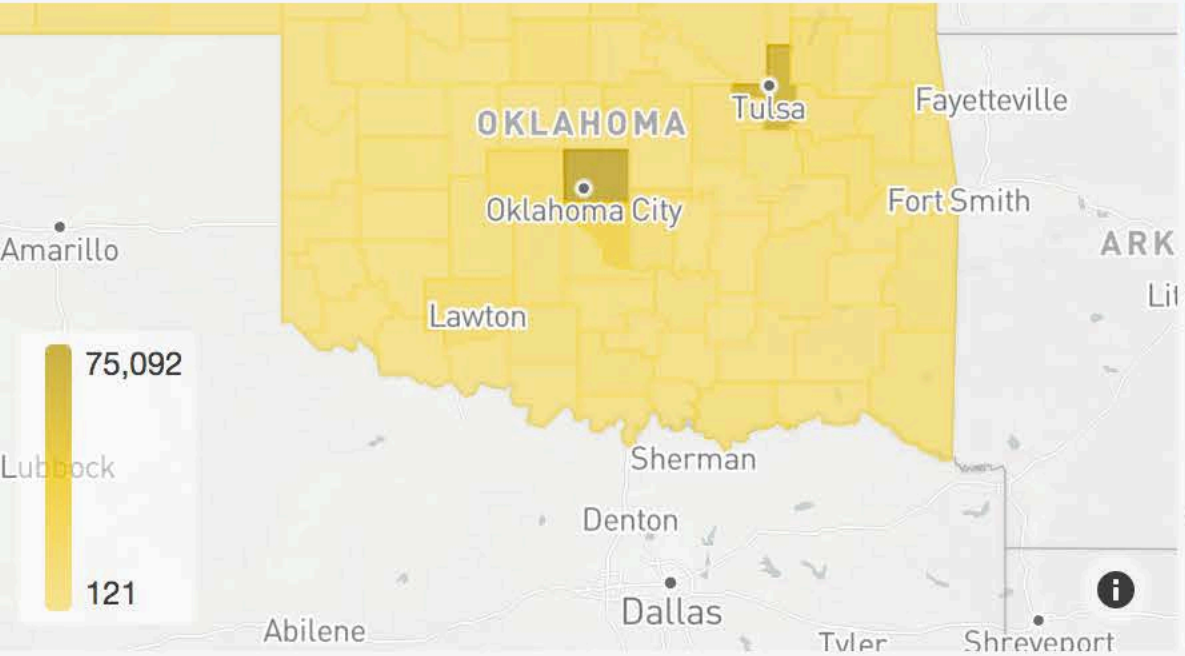

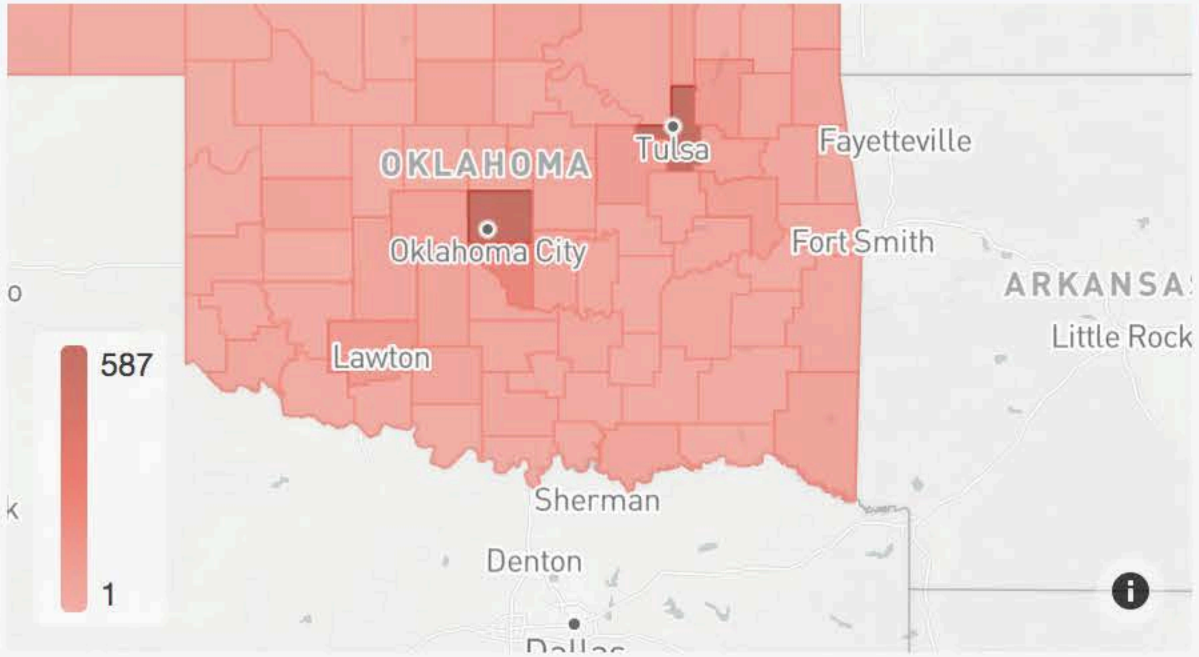

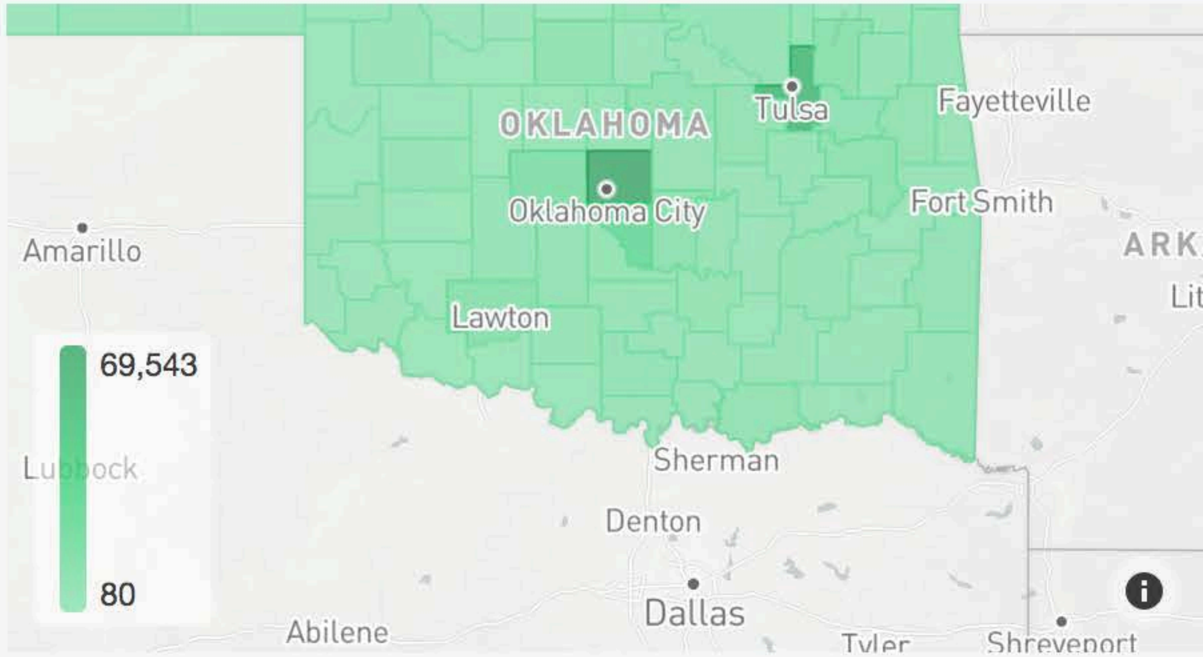

County Summary

Patient County	Total Cases	Total Deaths	Total Recovered
Oklahoma	75,092	577	69,543
Tulsa	64,115	587	58,745
Cleveland	26,314	227	23,941
Canadian	14,290	65	13,397
Comanche	10,251	106	8,953
Rogers	9,151	101	8,289
Muskogee	8,443	78	7,583
Payne	7,851	42	7,230
Pottawatomie	7,183	55	6,668
Garfield	6,979	60	6,504
Wagoner	6,938	69	6,199
Creek	5,807	95	5,208
Bryan	5,315	45	4,783
Grady	5,219	58	4,825
Cherokee	5,044	32	4,573
Carter	5,025	32	4,465
Le Flore	4,919	39	4,551

COVID-19 Cases - Deaths Summary

View Data Downloads

Oklahoma Total Deaths by Race

3m ago

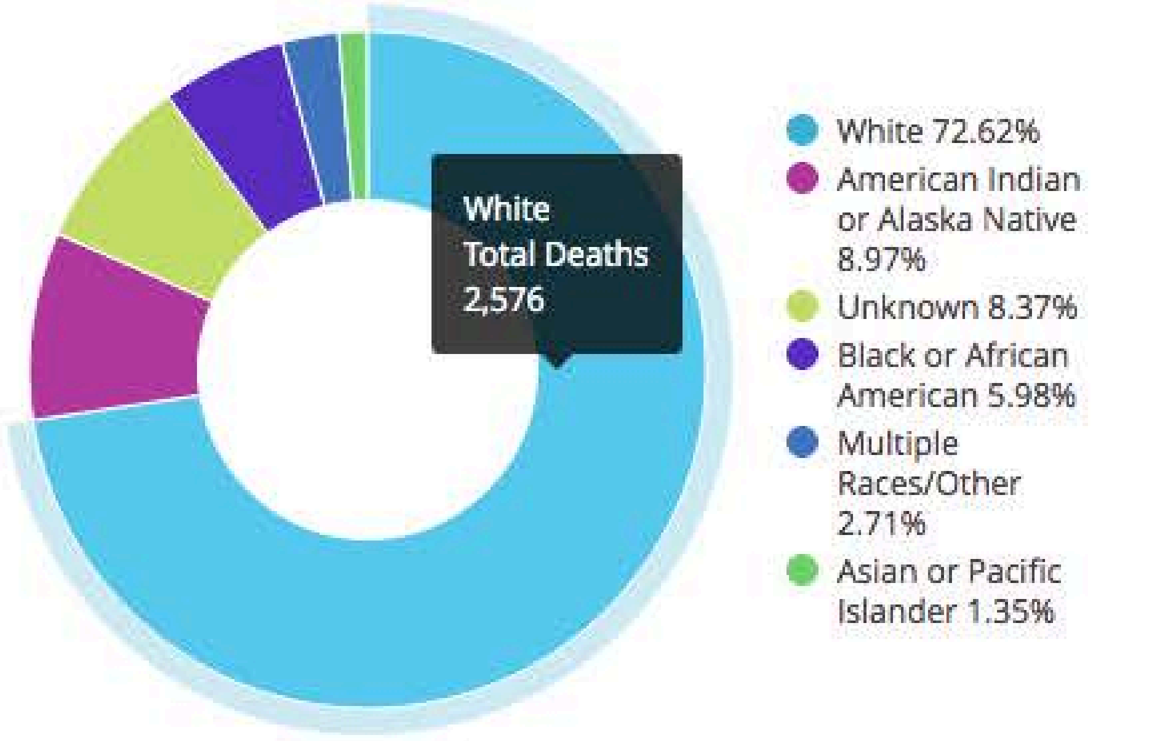

Oklahoma Total Deaths by Age Group

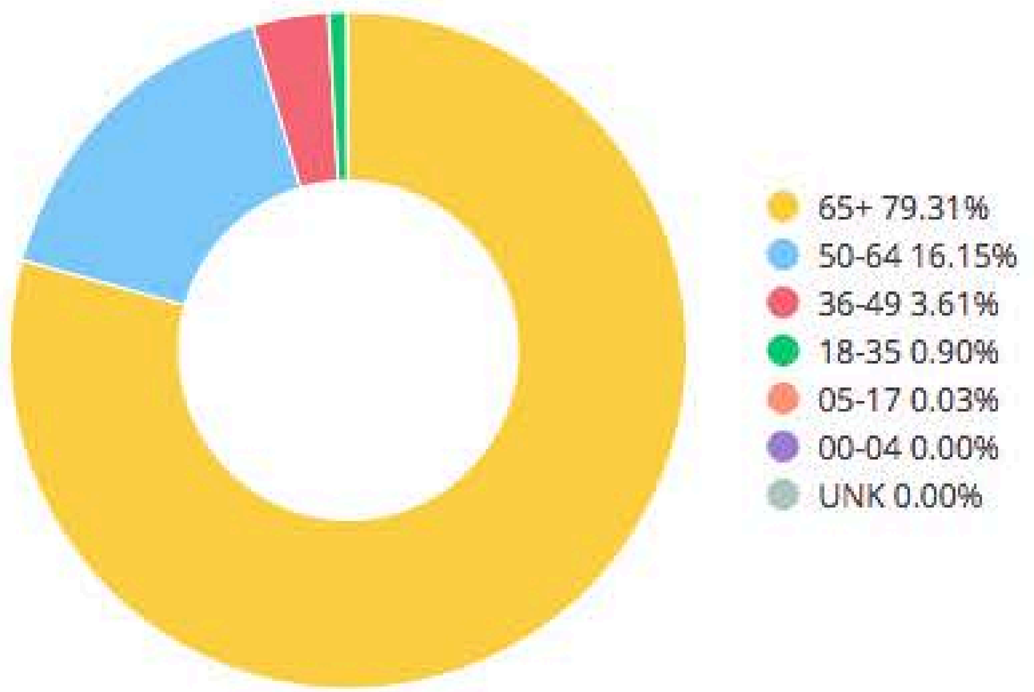

Oklahoma Total Deaths by Gender

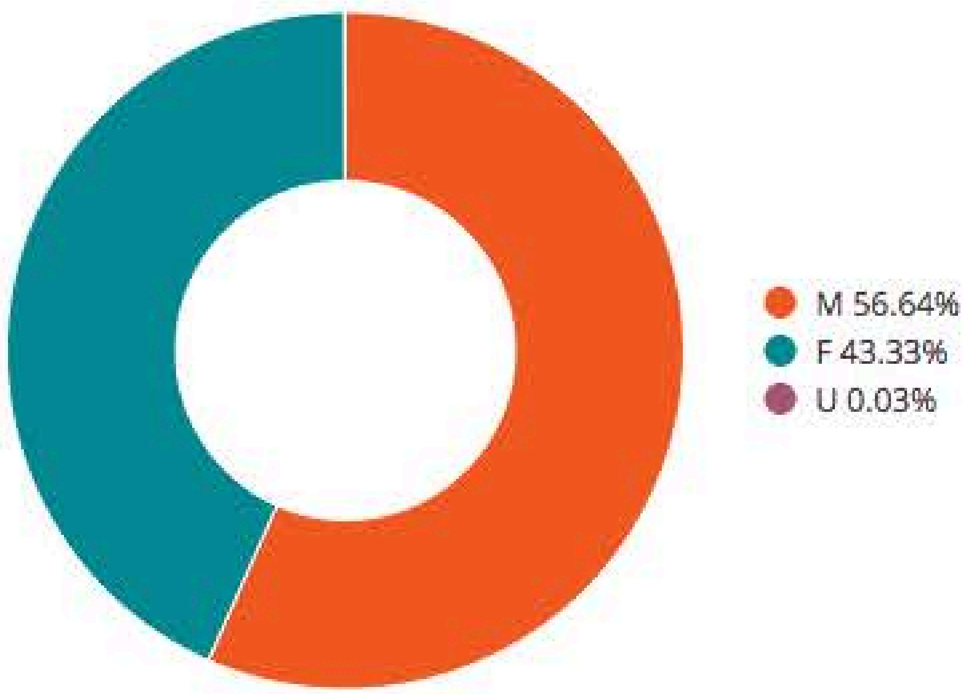

Deaths by County

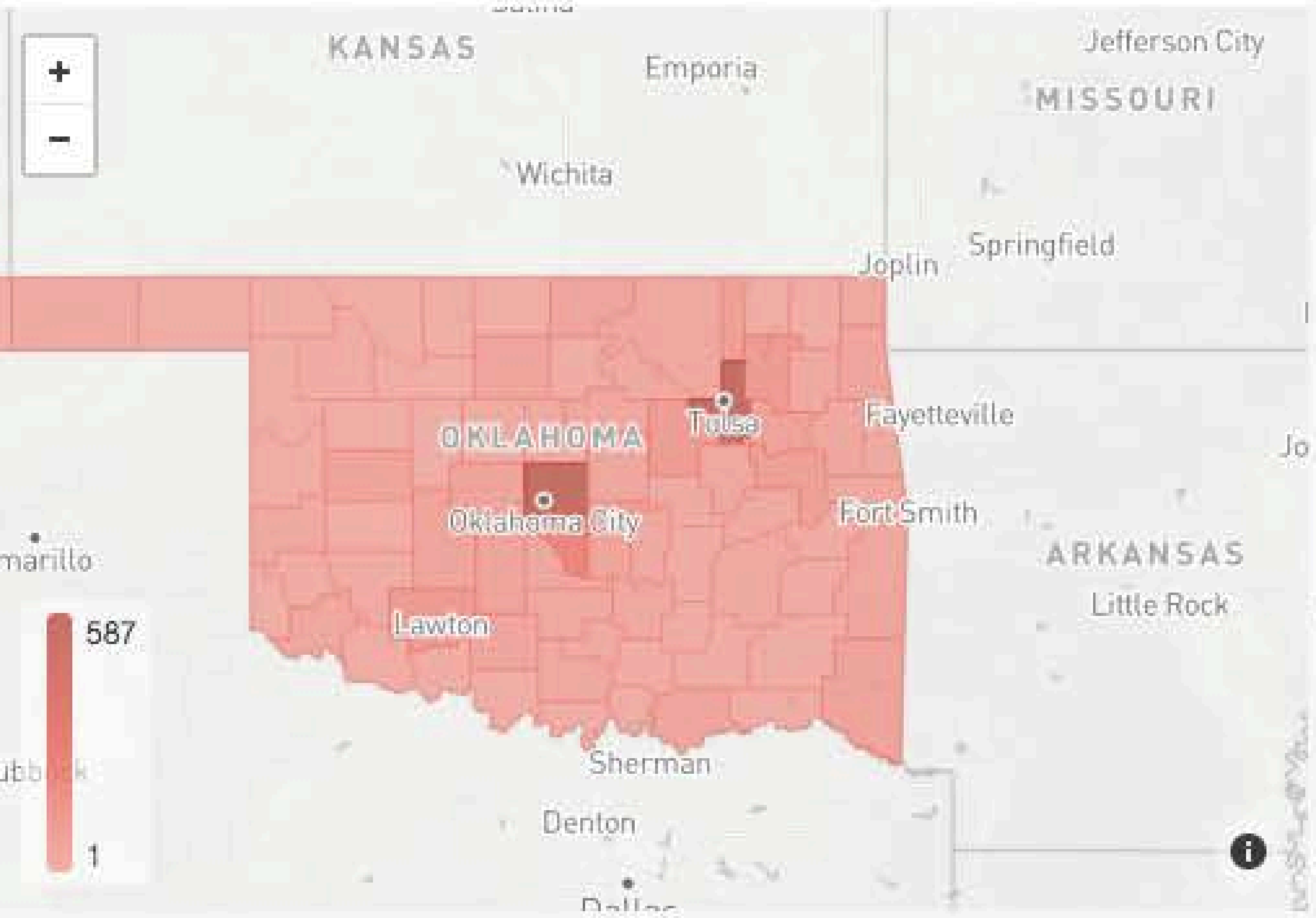

Deaths by City

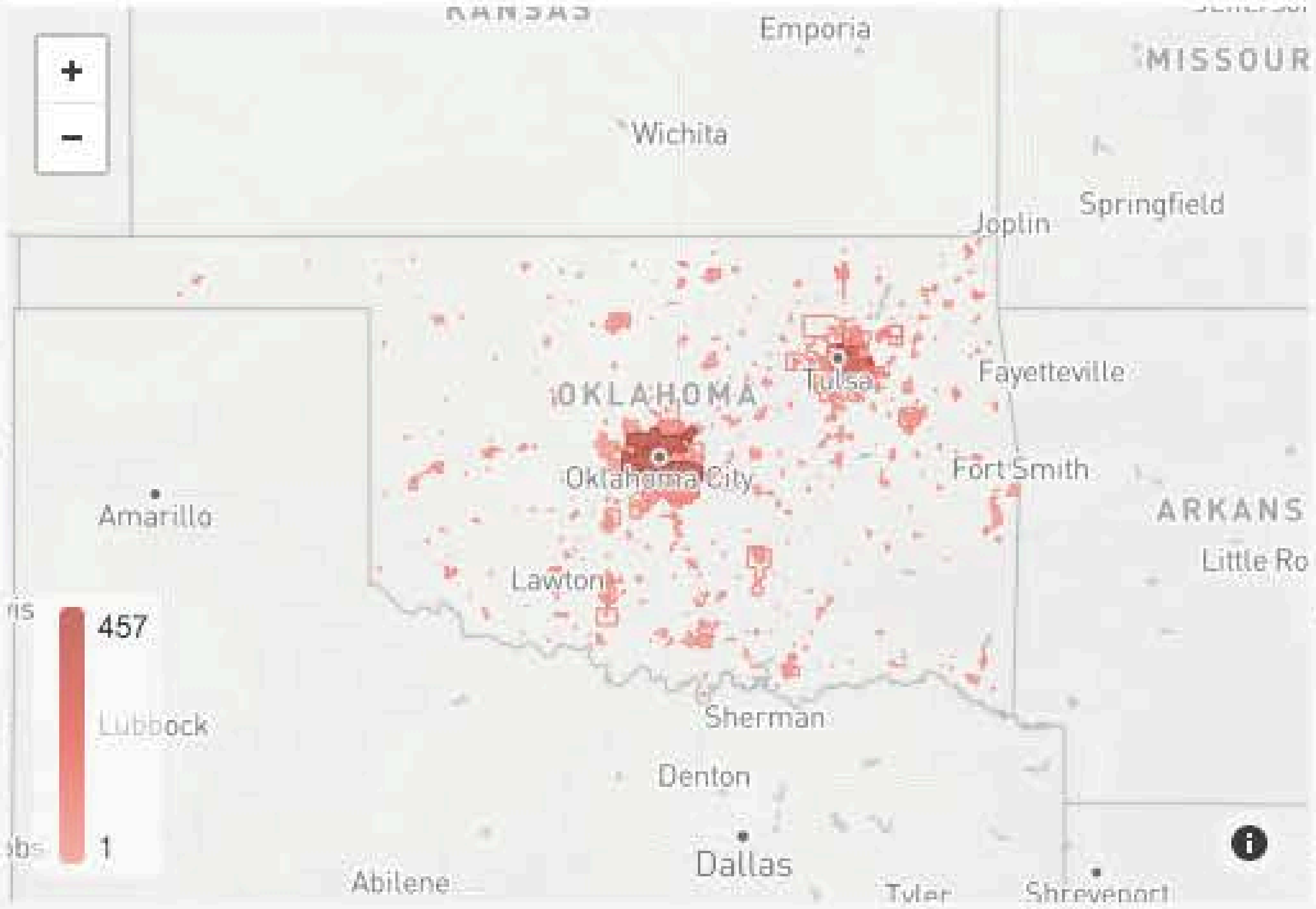

Deaths by Zip

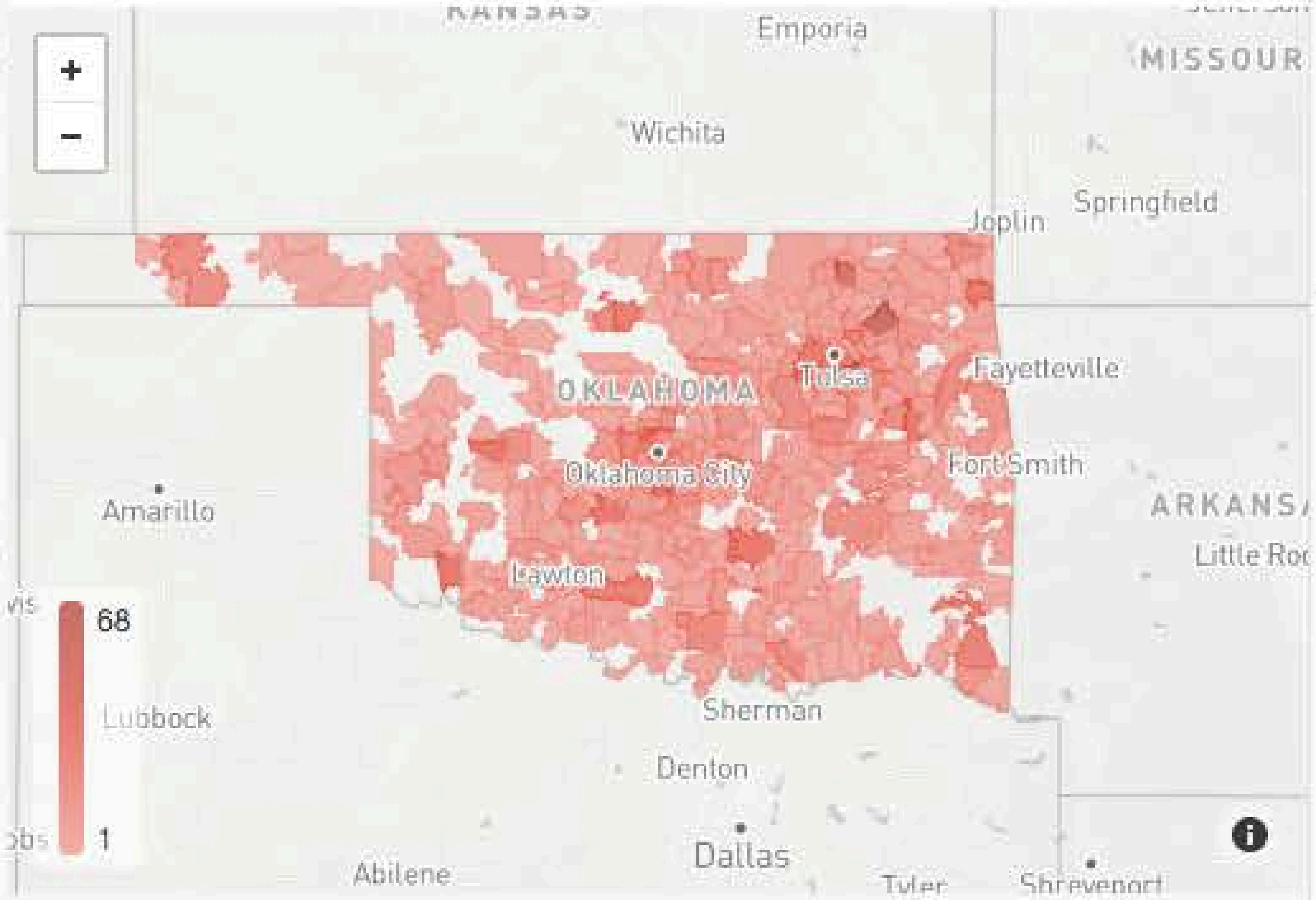

County Summary

Patient County	Total Deaths
Tulsa	587
Oklahoma	577
Cleveland	227
Comanche	106
Rogers	101
Creek	95
Muskogee	78
Washington	76
Wagoner	69
Canadian	65
Garfield	60
Custer	59
Grady	58

City Summary

Patient City	Total Deaths
Oklahoma City	457
Tulsa	383
Norman	119
Broken Arrow	116
Edmond	85
Lawton	82
Claremore	74
Bartlesville	63
Muskogee	59
Enid	55
Other***	50
Shawnee	41
Altus	38

Zip Summary

Patient Zip	Total Deaths
74012	68
74017	60
74006	50
Other***	49
74136	46
73071	45
74133	45
73505	43
73170	40
73072	40
73013	40
73521	38
73120	38

Powered by Looker

Oregon Health Authority

The Oregon Health Authority (OHA) is part of the statewide response working to address and reduce the effects of COVID-19 on the lives of Oregonians. Data presented here are from the OHA Public Health Division.

OHA COVID-19 Website | View All COVID-19 Data Dashboards

Summary Table

Contact Us

Total Cases	Deaths	Hospitalizations	Positive Tests	Negative Tests	Total Tests
142K	1,938	7,738	188K	3.0M	3.2M

Oregon's Epi Curve | COVID-19 Testing by County | COVID-19 Testing Over Time

Data current as of 12:01am 1/29/2021

Oregon's Epi Curve: COVID-19 cases

This chart shows the number of Oregonians who have been identified as COVID-19 cases and whether they were ever hospitalized for their illness.‡

Total Cases	Hospitalized	Not Hospitalized	Hospitalization Status Unknown
141,729	7,738	93,538	40,453

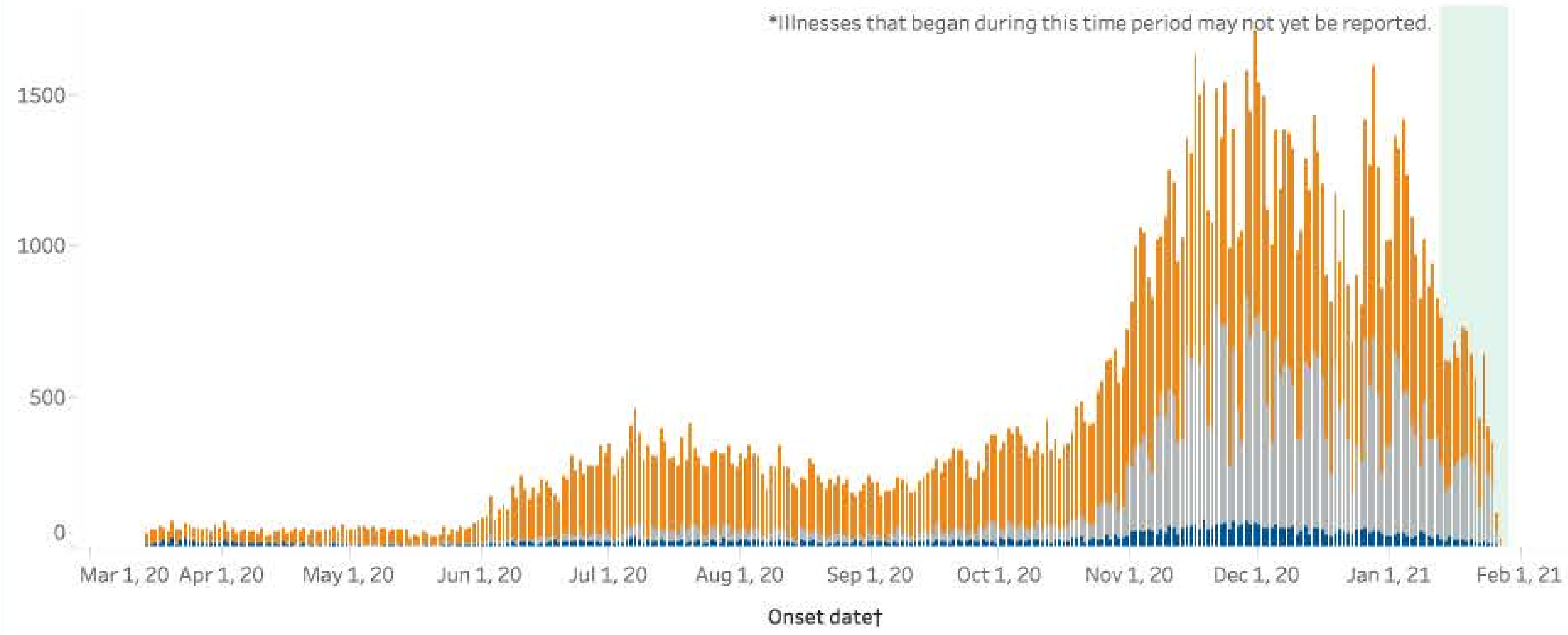

Enter or drag the cursors to change the onset date range.

March 14, 2020 — January 28, 2021

Not Hospitalized | Hospitalization Status Unknown | Hospitalized

When people say we want to "flatten the curve," this is the curve they are talking about.

We want to slow the number of new cases of COVID-19 so our healthcare system isn't overwhelmed and can provide care to everyone who needs it. Staying home except for essential needs and practicing social distancing are important ways we can flatten the curve.

Please note: The light blue shaded area shows the past 17 days, and not all people who got sick during this time period have been reported yet to OHA.

Notes

† Cases are counted by onset date (the date the person started having symptoms). When a person with symptoms did not report their onset date or when a person without symptoms is tested, their onset date is calculated as 2 days before the specimen was collected for testing.

‡ COVID-19 cases include both confirmed and presumptive cases.

COVID-19 data are provisional and subject to change.

+tableau

Oregon COVID-19 Case and Testing Counts Statewide

Viz Author: **Oregon Health Authority COVID-19**

Oregon Health Authority's dashboard on COVID-19 case and testing counts statewide. **#Oregon #OHA #COVID19 #coronavirus #PublicHealth #EpiCurve**

1,455,744 Views ☆98

More Detail

Originally Published: Apr 2, 2020

Last Updated: Jan 29, 2021

Workbook Details: 3 Sheets

Metadata: COVID-19 EPI Cases
Oregon's COVID-19 ELRs by County
Oregon's COVID-19 ELRs Over Time

© 2021 Tableau Software, LLC, a Salesforce Company. All Rights Reserved

Oregon Health Authority

The Oregon Health Authority (OHA) is part of the statewide response working to address and reduce the effects of COVID-19 on the lives of Oregonians. Data presented here are from the OHA Public Health Division.

OHA COVID-19 Website View All COVID-19 Data Dashboards

Summary Table

Contact Us

Total Cases	Deaths	Hospitalizations	Positive Tests	Negative Tests	Total Tests
142K	1,938	7,738	188K	3.0M	3.2M

Oregon's Epi Curve | COVID-19 Testing by County | COVID-19 Testing Over Time

Data current as of 12:01am 1/29/2021

Oregon COVID-19 Tests Over Time

Select a county from the drop down to filter the two charts below.

County
(All)

Positive and Negative COVID-19 test counts over time - All

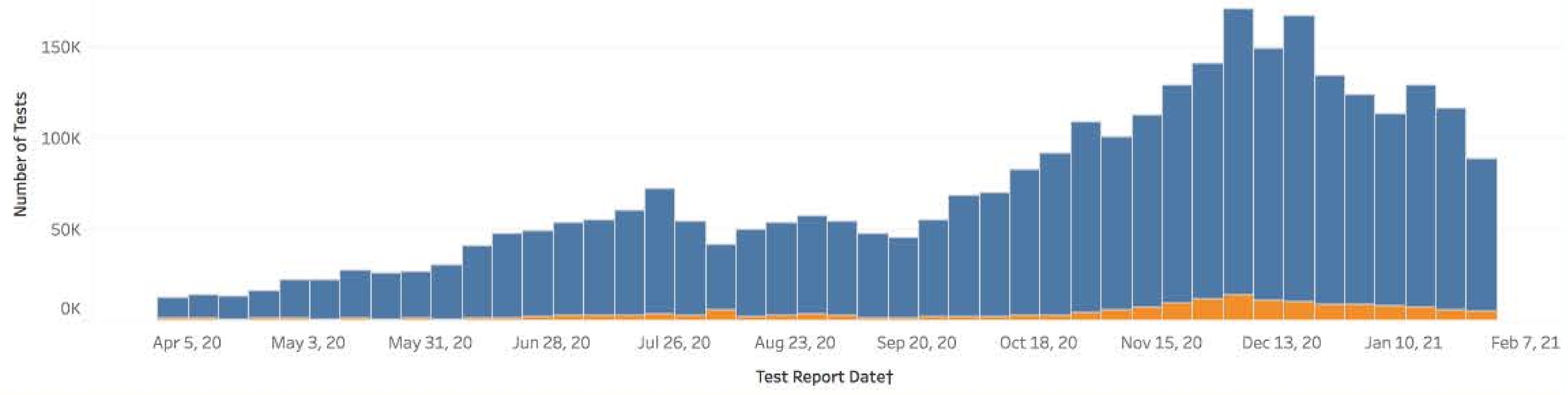

Test positivity over time - All

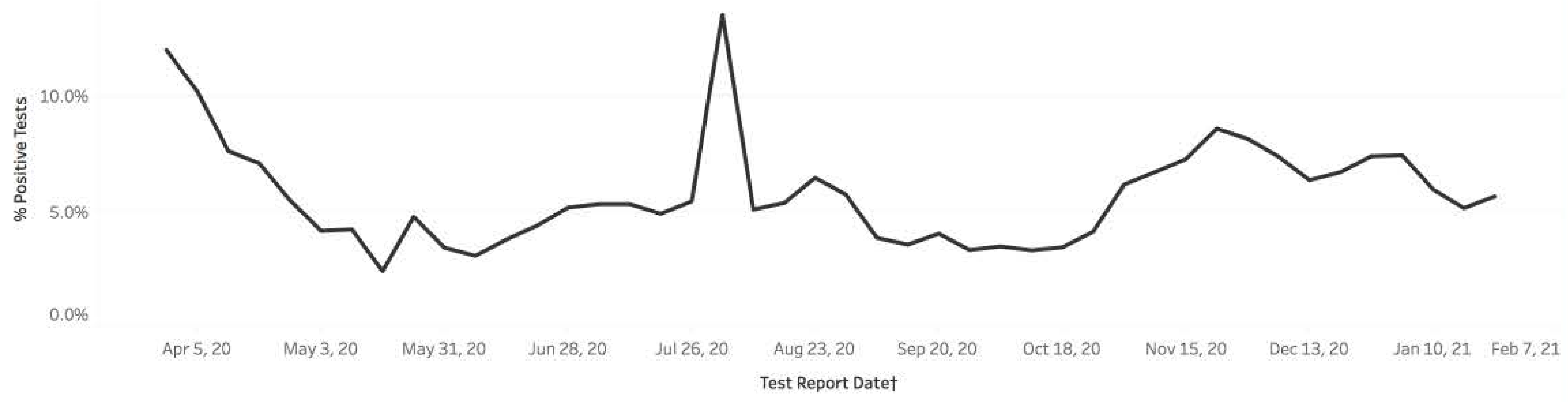

†Tests are counted by the date the electronic laboratory report (ELR) or other electronic test report was created. Counts reflect individual tests and not individual persons tested.

These are data from HL7 and CSV electronic laboratory reports and do not include results received via fax, eCR or the Oregon COVID-19 Reporting Portal. Non-antibody and non-'info only' COVID results are deduplicated based on medical record number (MRN), accession number, facility and result daily.

COVID-19 data are provisional and subject to change.

+ableau

Oregon COVID-19 Case and Testing Counts Statewide

Viz Author: **Oregon Health Authority COVID-19**

Oregon Health Authority's dashboard on COVID-19 case and testing counts statewide. **#Oregon #OHA #COVID19 #coronavirus #PublicHealth #EpiCurve**

1,455,744 Views ☆ 98

More Detail

Originally Published: Apr 2, 2020

Last Updated: Jan 29, 2021

Workbook Details: 3 Sheets

Metadata: COVID-19 EPI Cases
Oregon's COVID-19 ELRs by County
Oregon's COVID-19 ELRs Over Time

© 2021 Tableau Software, LLC, a Salesforce Company. All Rights Reserved

Oregon's COVID-19 Update

New cases and deaths are reported from the previous day. NOTE: Monday's report includes weekend data.

Daily Statewide Numbers On January 29, 2021

New Cases	7 Day Daily Average of Cases	COVID-19 Patients Hospitalized†	ELR Tests Reported	ELR Test Positivity	New Deaths
976 ▲*	707.9	288 ▼	29,072	6.1%	8

* Arrows indicate an increase or decrease from the previous day. †Hospitalization data from Oregon's Hospital Capacity Web System (HOSCAP).

Daily Cases and 7 Day Moving Average over the Previous Six Weeks

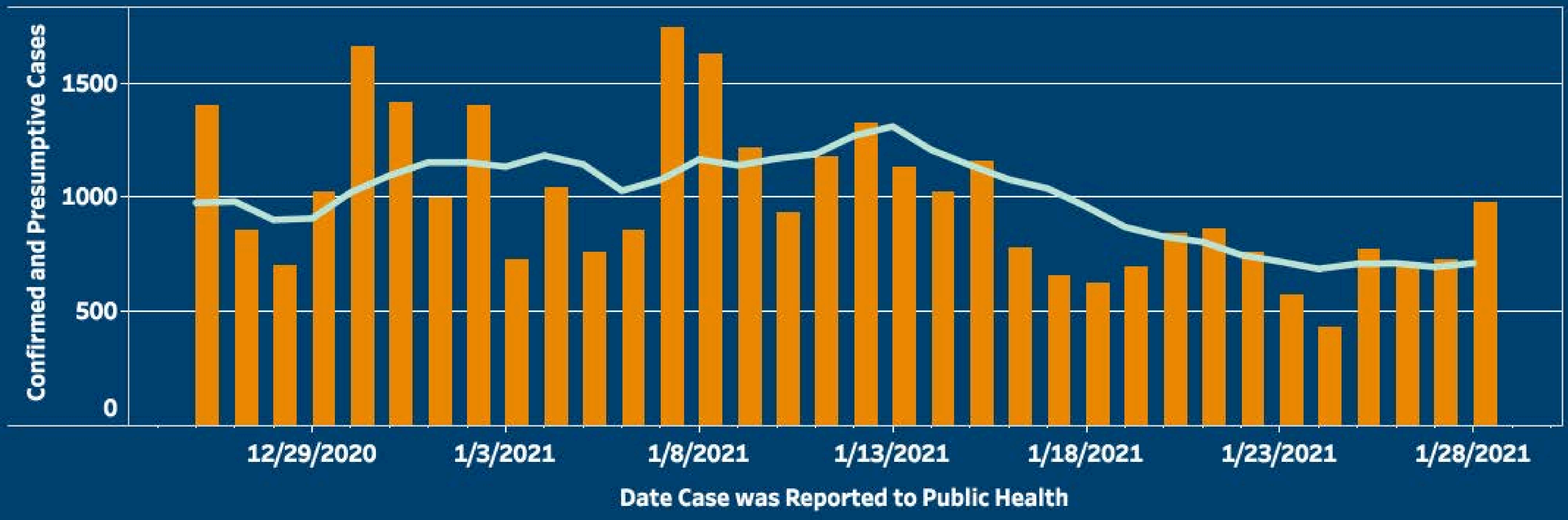

Daily and Cumulative County Numbers On January 29, 2021

County	New Daily Cases	7 Day Daily Average of New Cases	Daily ELR Percent Positivity	Deaths Reported Today	Cumulative Cases	Cumulative Deaths
Baker	2	1	29.7%	0	589	5
Benton	27	14	2.2%	0	1,891	14
Clackamas	97	67	6.0%	3	12,410	145
[illegible]	[illegible]	[illegible]	[illegible]	[illegible]	[illegible]	[illegible]

COVID-19 Vaccination Trends

Oregon Health Authority

OHA's COVID-19 Data Dashboards

OHA's COVID-19 Vaccine Website

Contact Us

View Statewide Data | County

Data current as of 12:01am 1/30/2021

People Vaccinated by County*

The map shows the number of people vaccinated per 10,000 by county of residence. For privacy purposes, counties with the number of vaccinated people between 1 and 5 will be shown as "Suppressed."

Click on a county below to filter the data on the right. Click again to clear the filter.

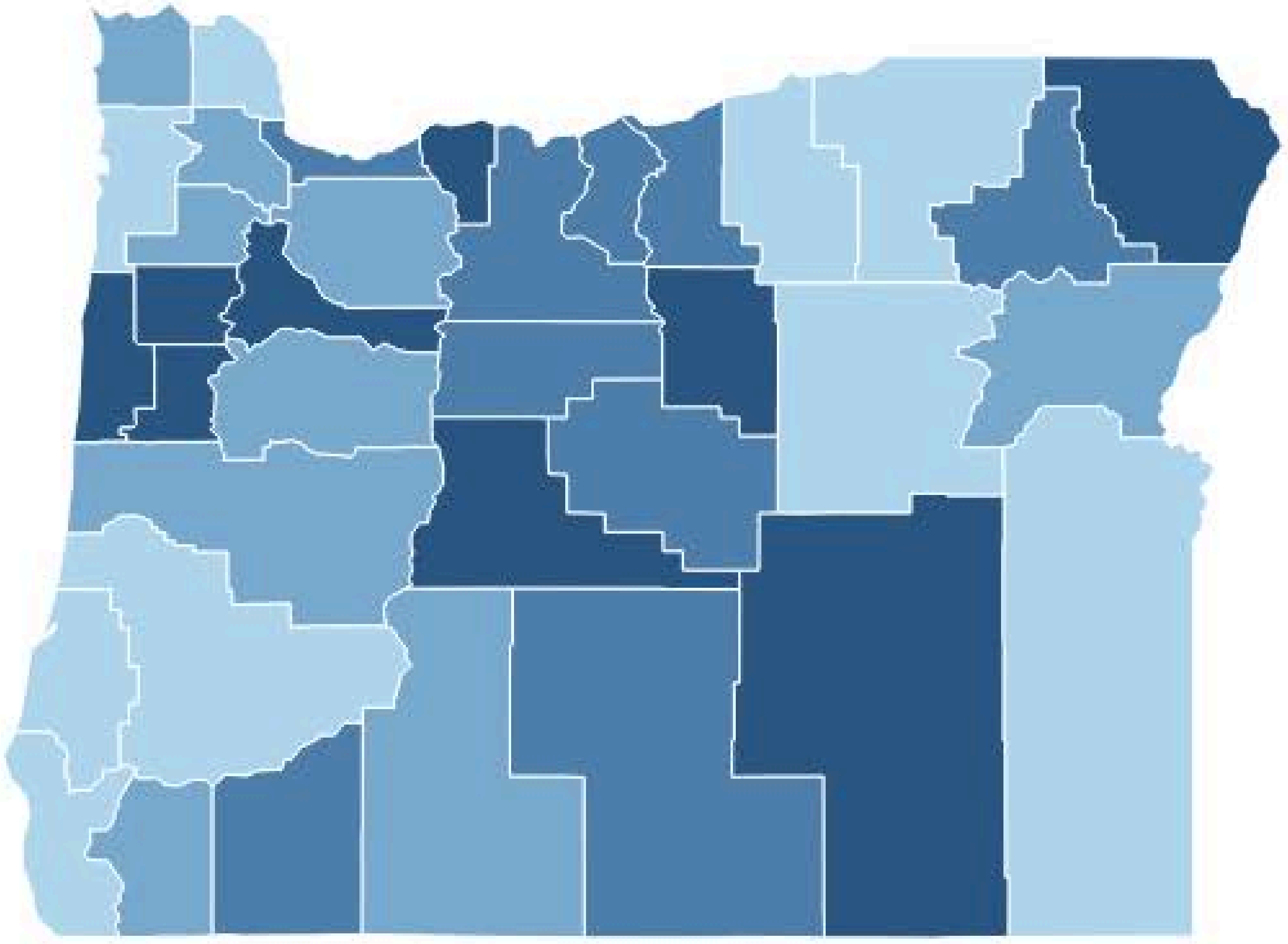

People vaccinated per 10,000: lighter colors represent counties with lower vaccination rates, while darker colors represent counties with higher vaccination rates.

People Vaccinated — ALL LOCATIONS

Series In Progress	Fully Vaccinated	Total People
259,938	71,747	331,685

Doses Administered by Day

*Doses administered during this time may not yet be reported.

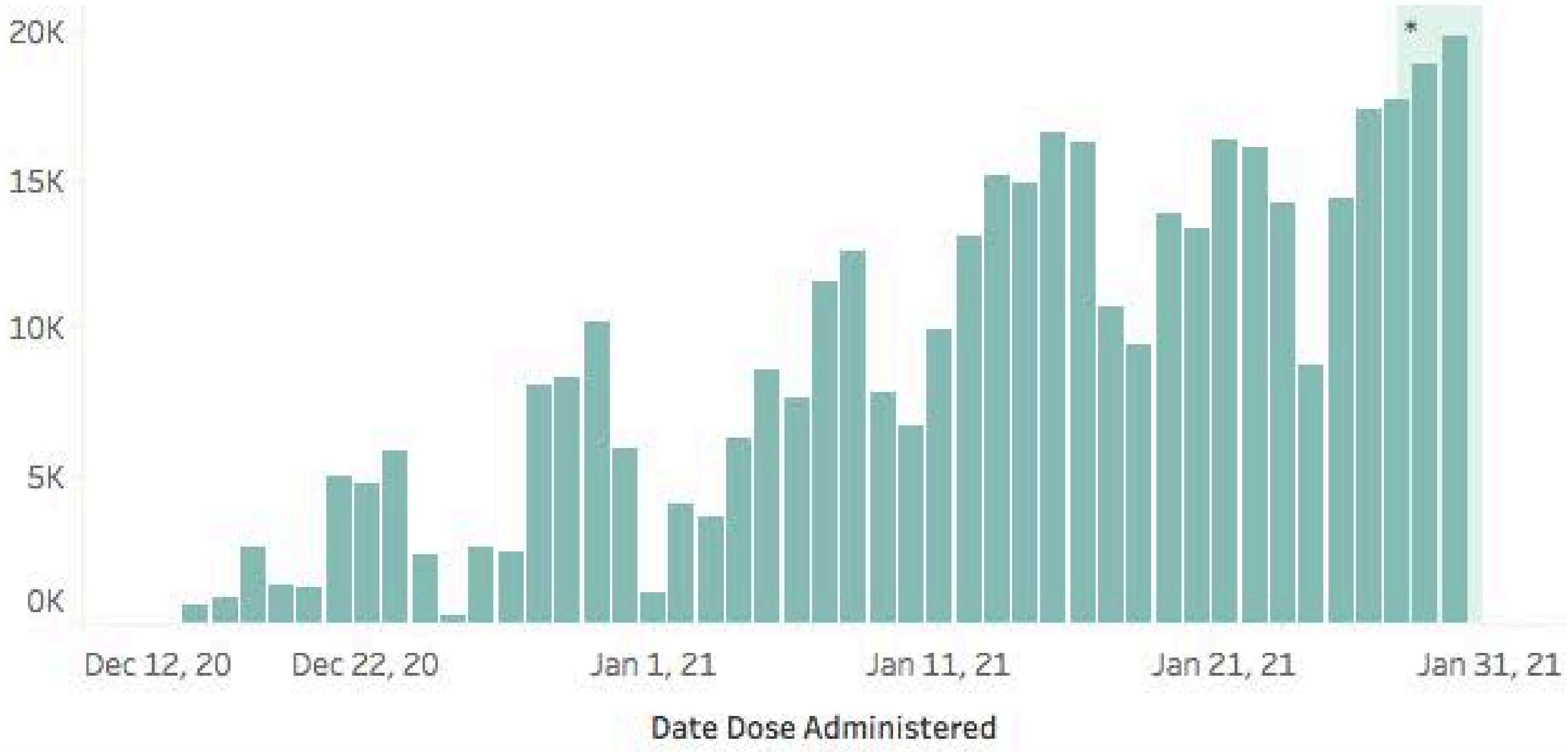

Data sources:

*Non-Oregon residents may receive a vaccination in Oregon. These people are not reflected in this map.

Oregon ALERT Immunization Information System (IIS) is a statewide, lifespan immunization registry.

Data is reported and updated Monday through Friday, excluding holidays.

COVID-19 vaccination data are provisional and subject to change.

Pennsylvania COVID-19 Dashboard

Confirmed Cases

733,743

Probable Cases

109,481

Negative Tests

3,637,151

Deaths

21,661

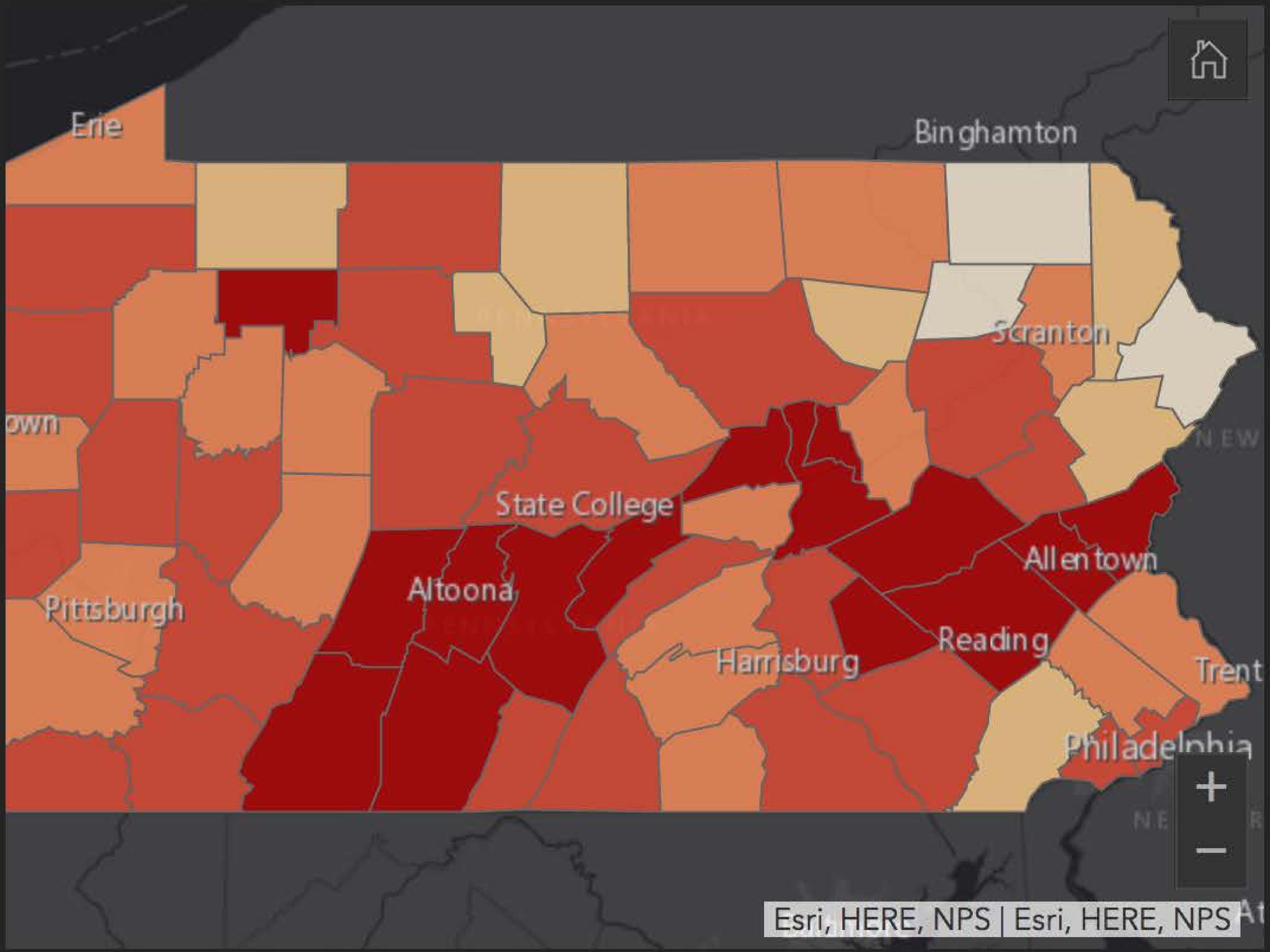

Cases per 100,000 Population

0 - 4,500 | > 4,500 - 5,500 | > 5,500 - 6,500 | > 6,500 - 7,500 | > 7,500

Information last updated at 12:00 noon on 01/31/2021. Case count data, map and new cases per day data from PA-NEDSS. Deaths by day graph data from EDRS.

New Cases Per Day

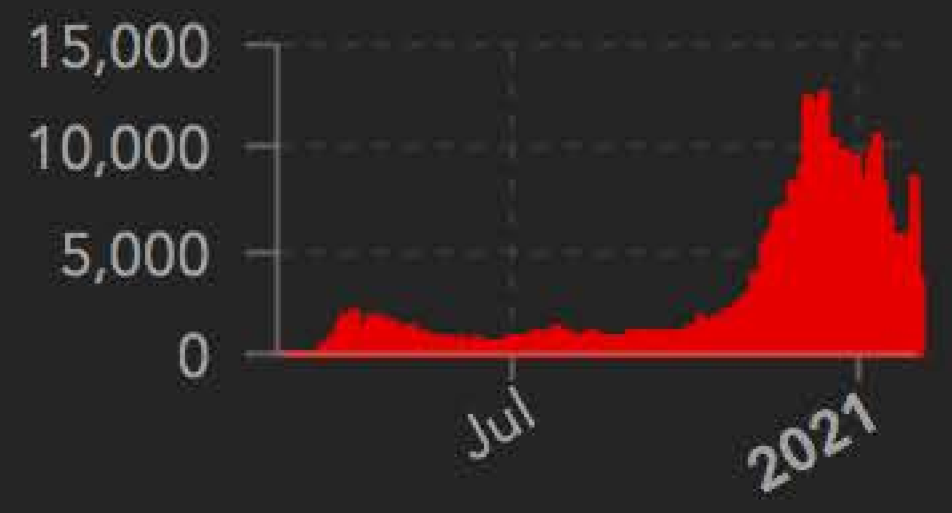

Deaths by Day

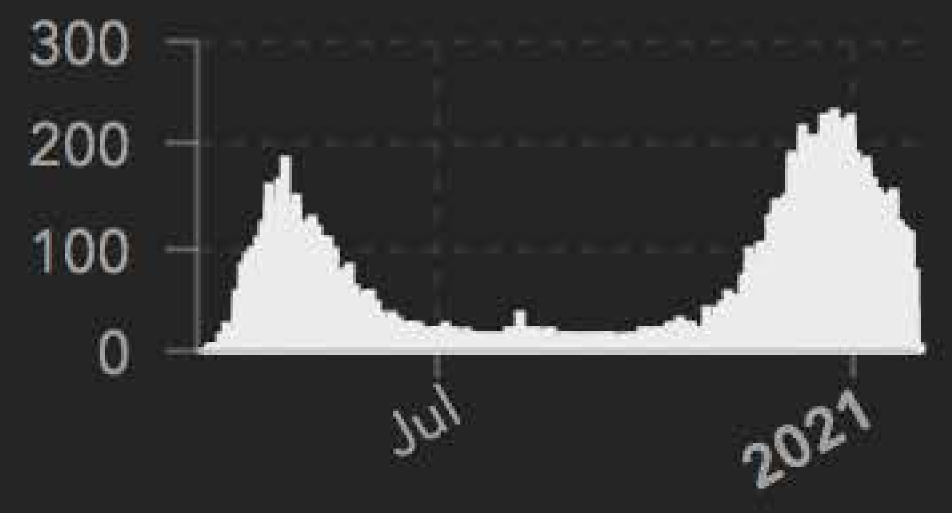

Daily PCR Tests

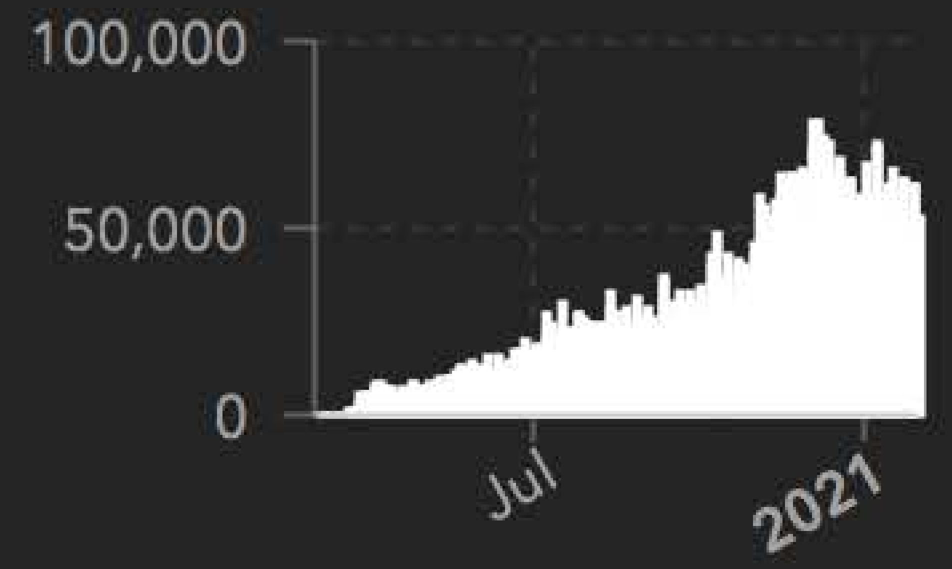

**Case Counts in the charts above include confirmed and*

Pennsylvania Cases

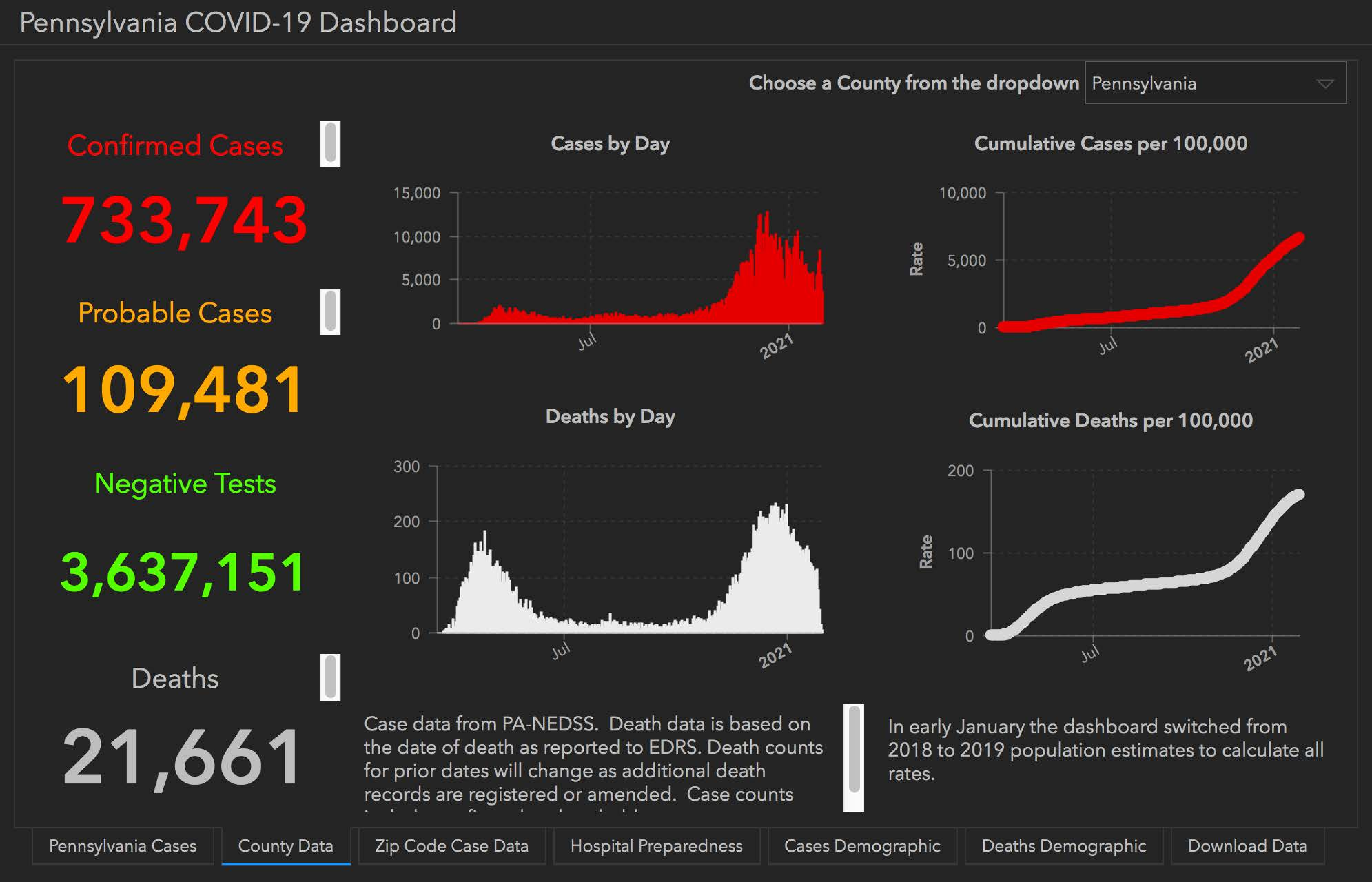
Pennsylvania COVID-19 Dashboard
Choose a County from the dropdown
Pennsylvania
Confirmed Cases
733,743
Probable Cases
109,481
Negative Tests
3,637,151
Deaths
21,661
Cases by Day
15,000
10,000
5,000
0
Jul
2021
Cumulative Cases per 100,000
10,000
5,000
0
Rate
Jul
2021
Deaths by Day
300
200
100
0
Jul
2021
Cumulative Deaths per 100,000
200
100
0
Rate
Jul
2021
Case data from PA-NEDSS. Death data is based on the date of death as reported to EDRS. Death counts for prior dates will change as additional death records are registered or amended. Case counts
In early January the dashboard switched from 2018 to 2019 population estimates to calculate all rates.
Pennsylvania Cases
County Data
Zip Code Case Data
Hospital Preparedness
Cases Demographic
Deaths Demographic
Download Data

Cases by Gender

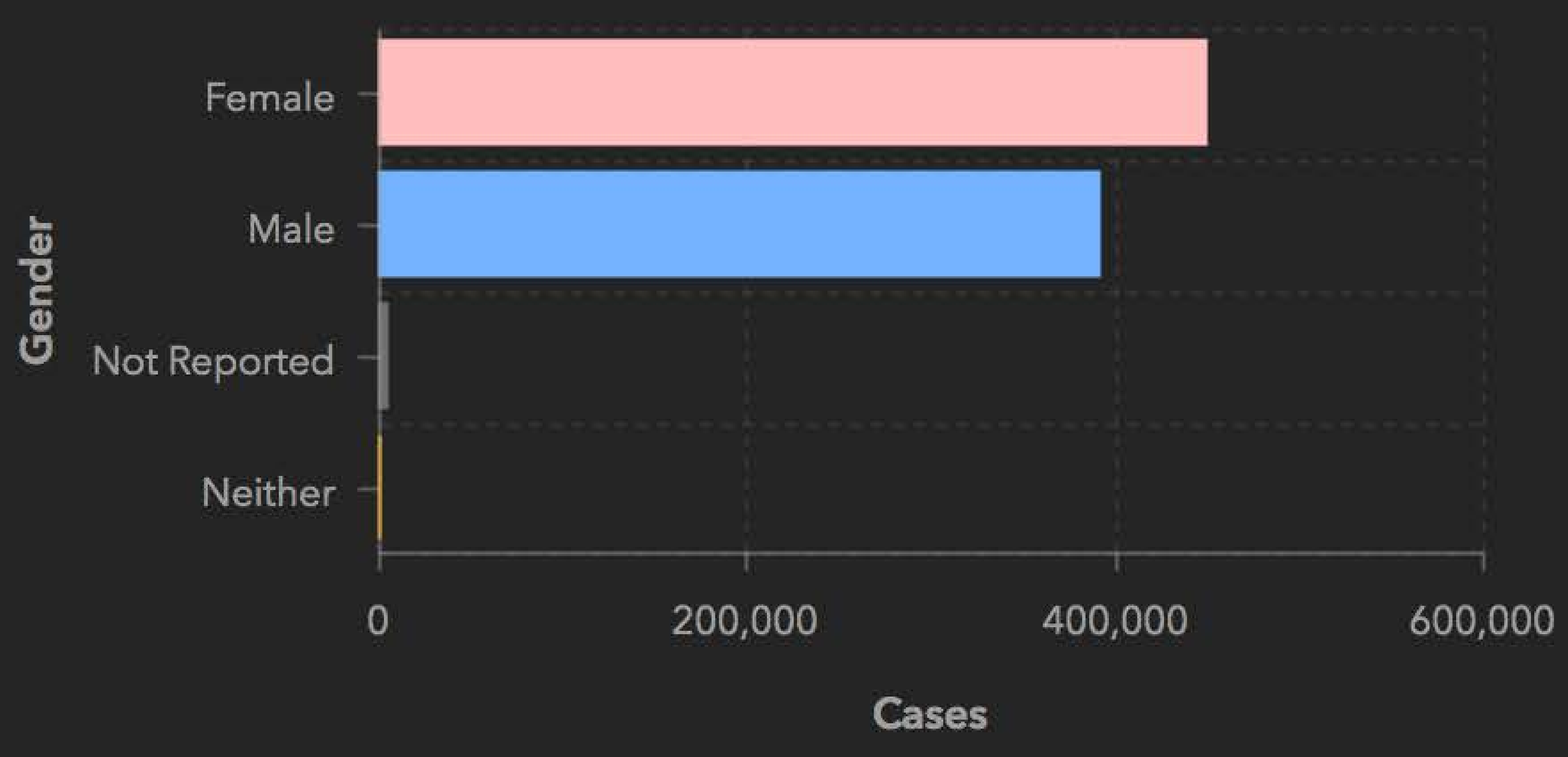

Age Distribution by Cases

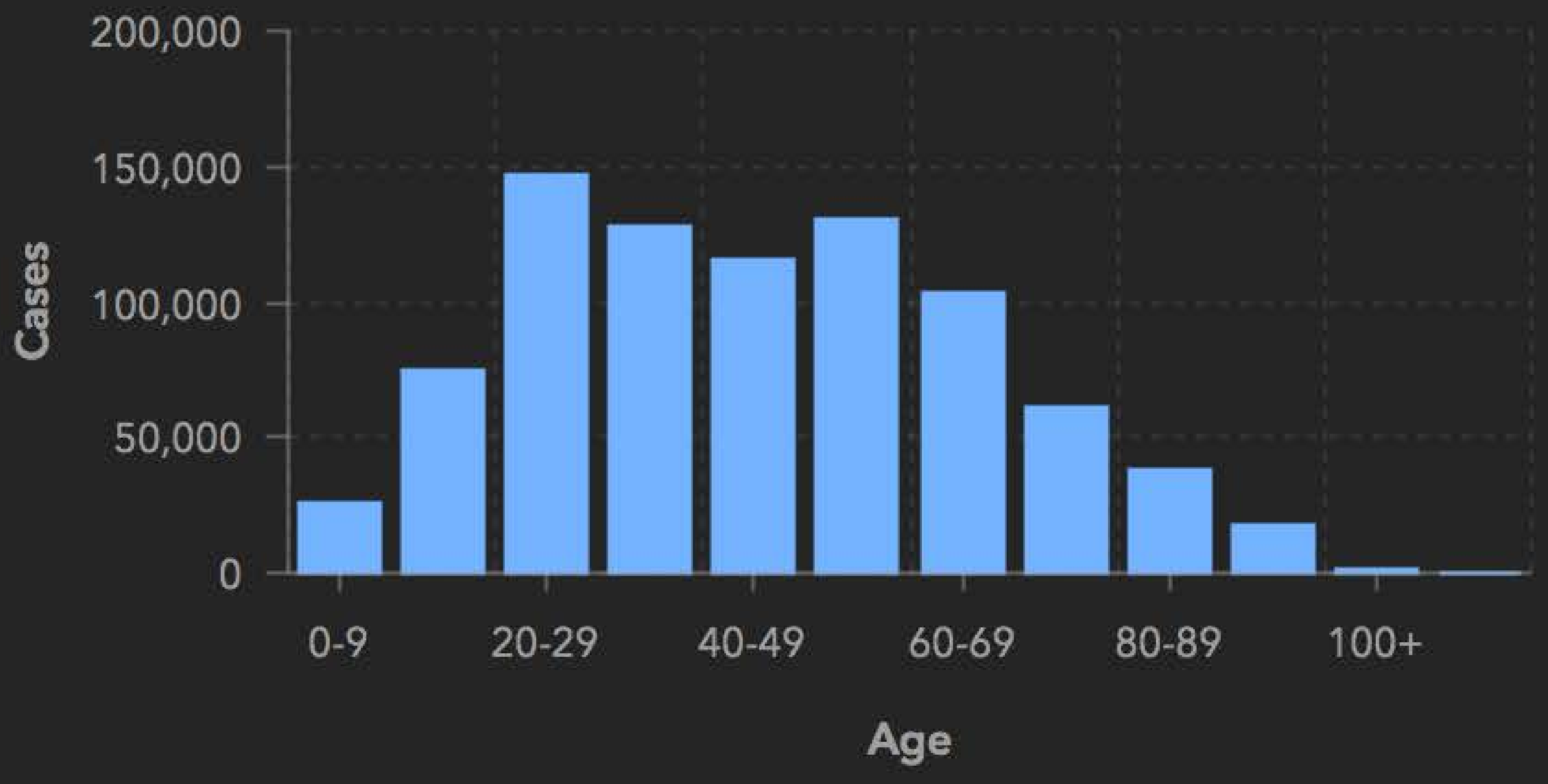

Cases by Ethnicity

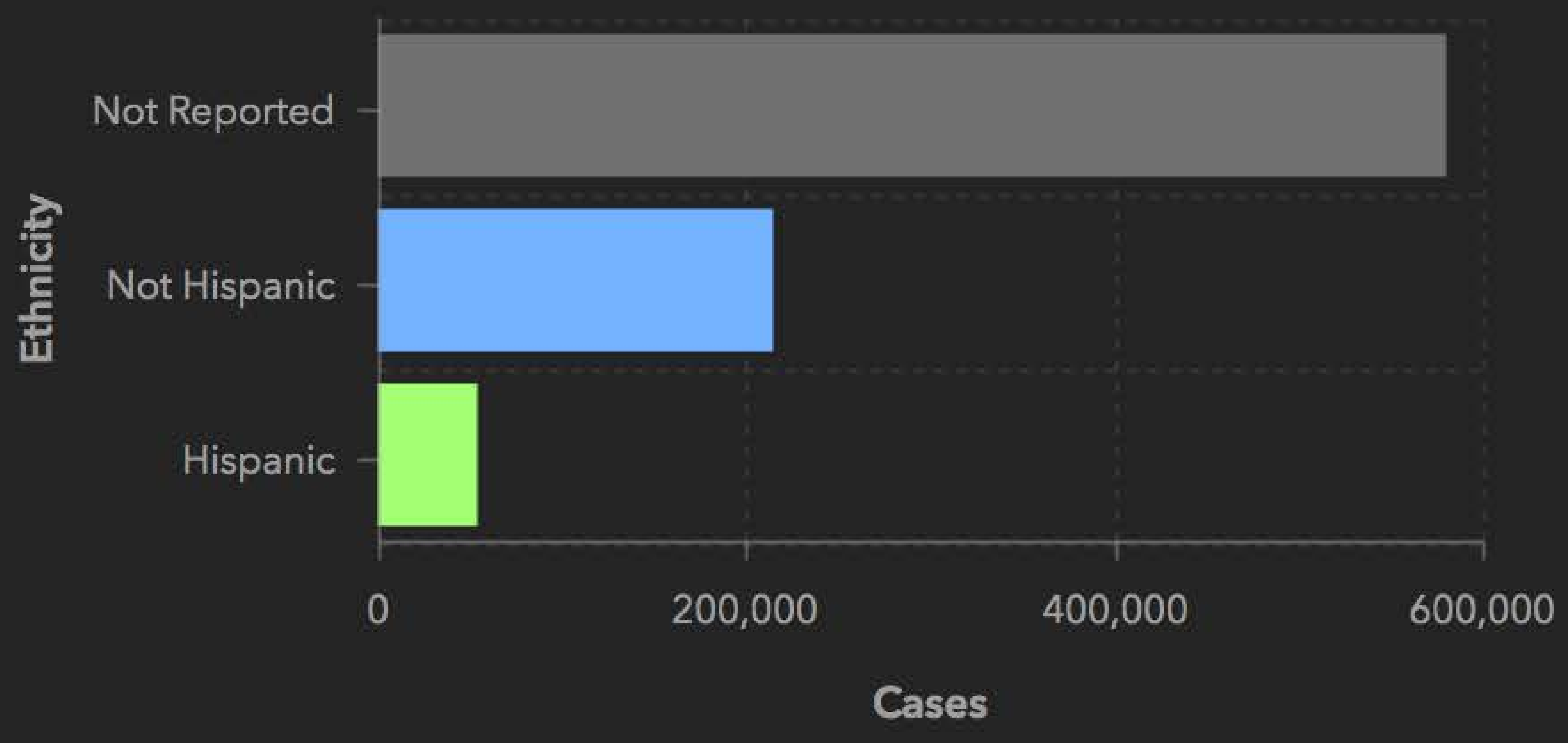

Cases by Race

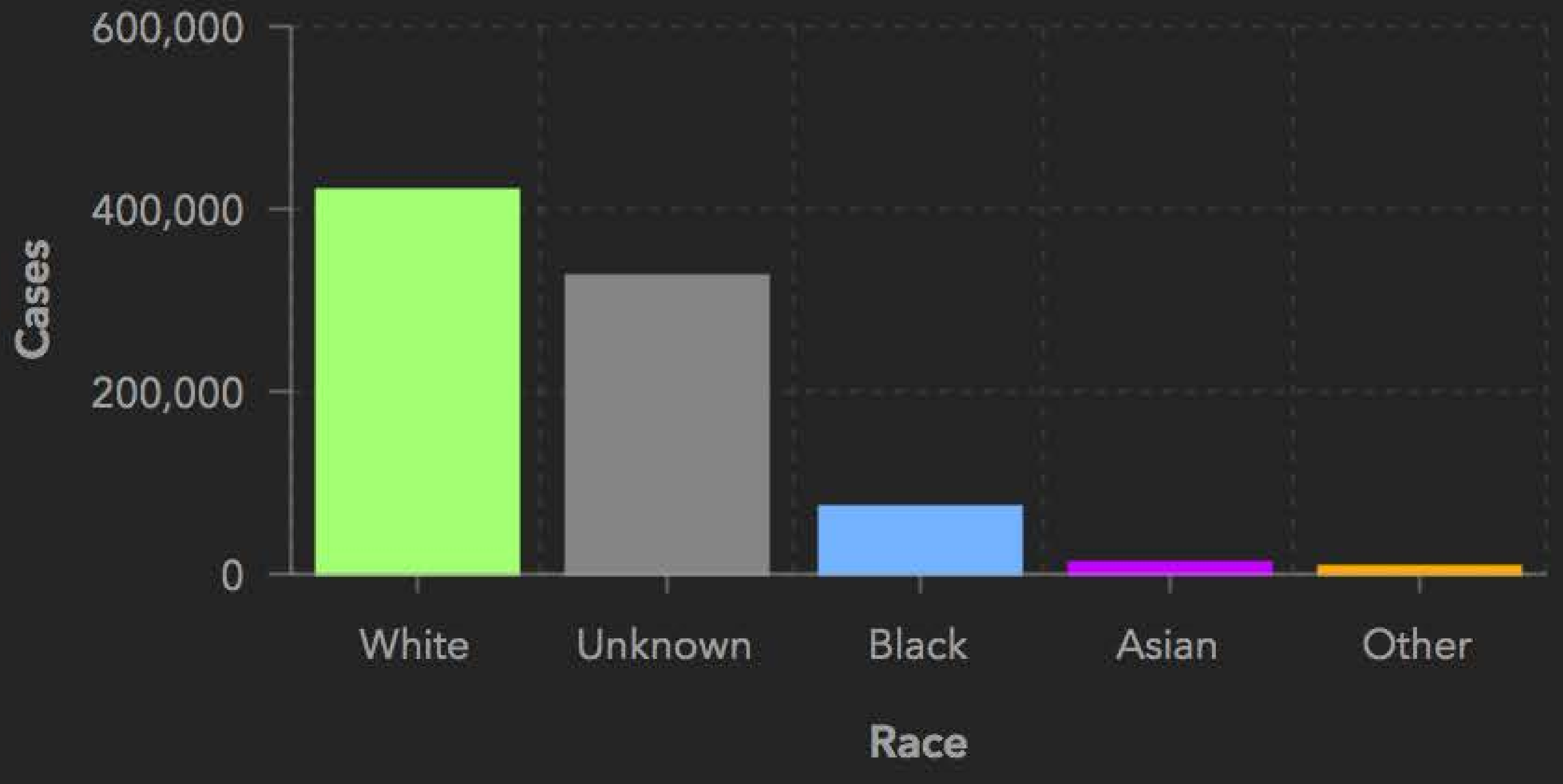

pennsylvania
DEPARTMENT OF HEALTH

Page Navigation

Demographics

COVID-19 Vaccine Dashboard

Updated on 1/31/2021

Number of People Vaccinated

County	Partially Covered	Fully Covered
Adams	2,934	966
Allegheny	58,076	21,176
Armstrong	2,705	806
Beaver	4,001	1,426
Bedford	2,003	311
Berks	18,580	5,845
Blair	4,701	2,378
Bradford	3,150	858
Bucks	26,236	8,243
Butler	8,159	3,724
Cambria	6,426	2,940
Cameron	442	47
Carbon	4,592	1,136
Centre	7,246	2,113
Chester	22,653	4,674
Clarion	1,654	733
Clearfield	3,553	2,020
Clinton	1,818	467
Columbia	5,750	1,633
Crawford	5,436	3,220
Cumberland	9,105	2,689
Dauphin	8,668	3,009
Delaware	27,136	6,268
**Total**	**604,381**	**191,325**

Number of People Receiving Partially Covered Vaccinations, by County

Partial Full

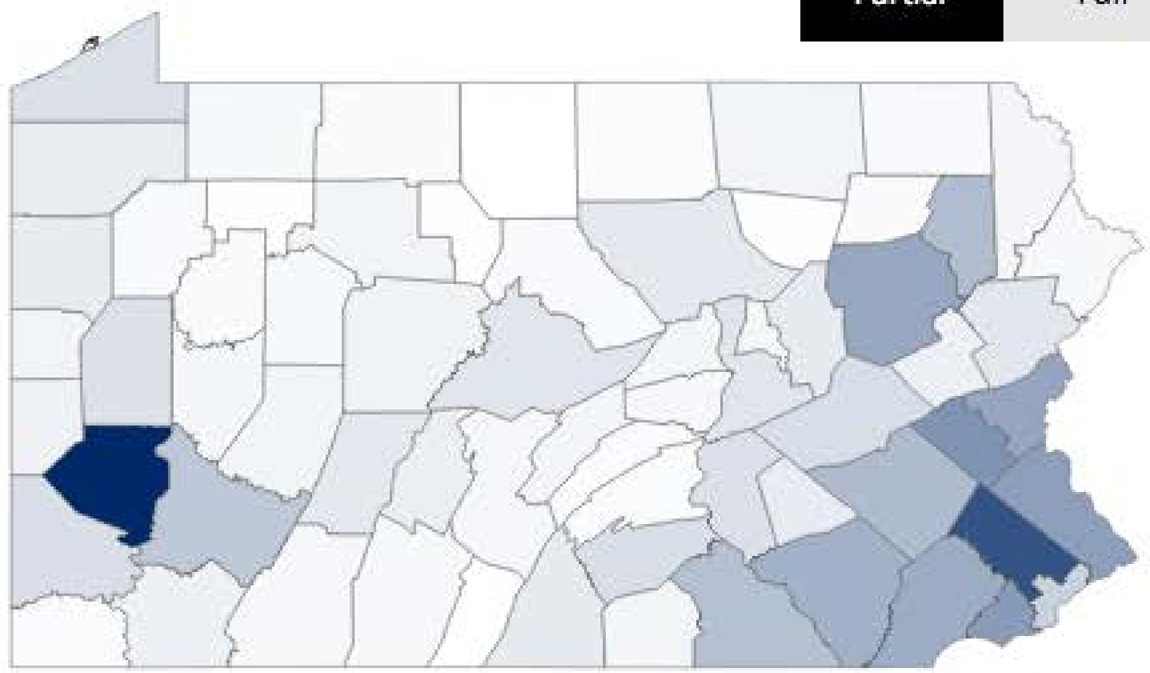

County/State

Pennsylvania

Number of Vaccinations Administered, by Date

● Number of full vaccinations ● Number of partial vaccinations

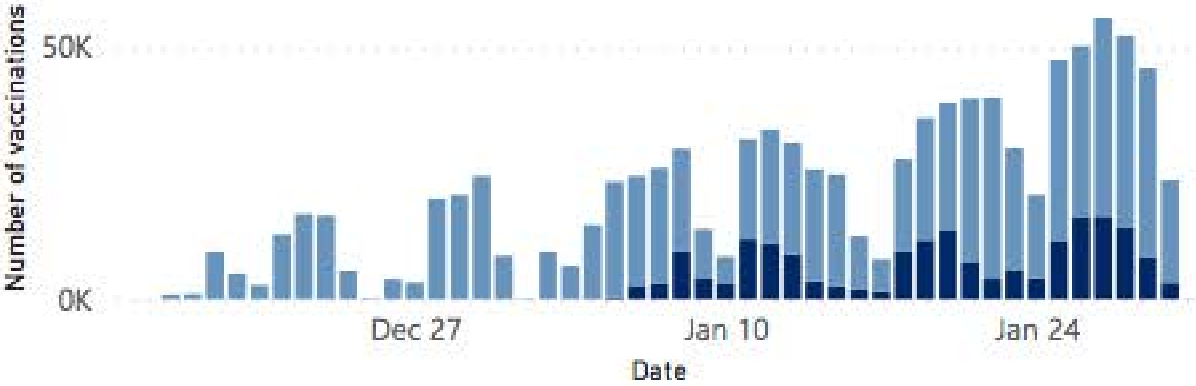

795,706
Partial vaccinations administered

191,325
Full vaccinations administered

pennsylvania
DEPARTMENT OF HEALTH

Updated on 1/29/2021

Page Navigation

Vaccines, by County

Number of People Vaccinated, by Gender

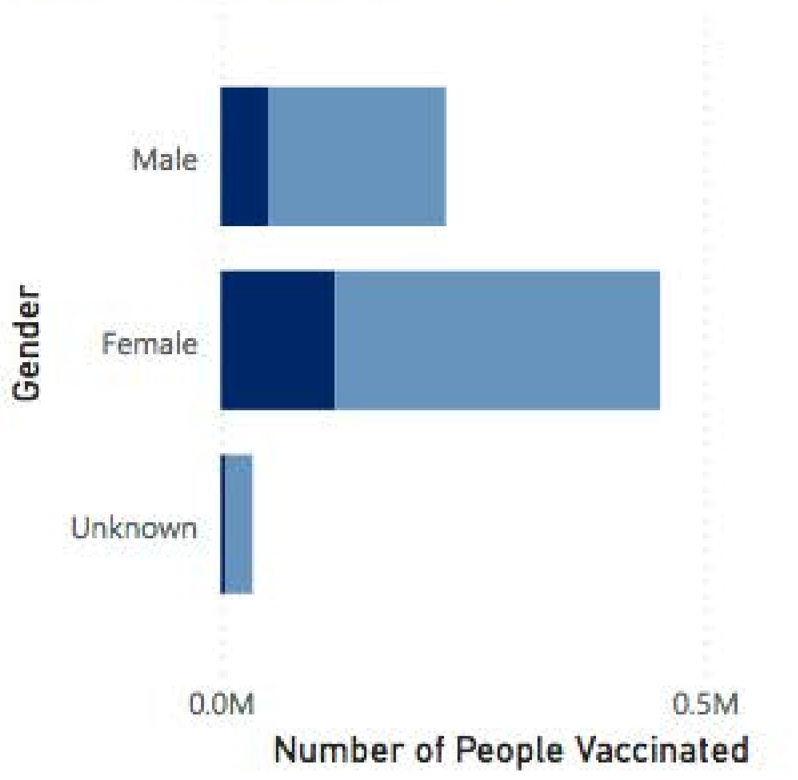

Number of People Vaccinated, by Age

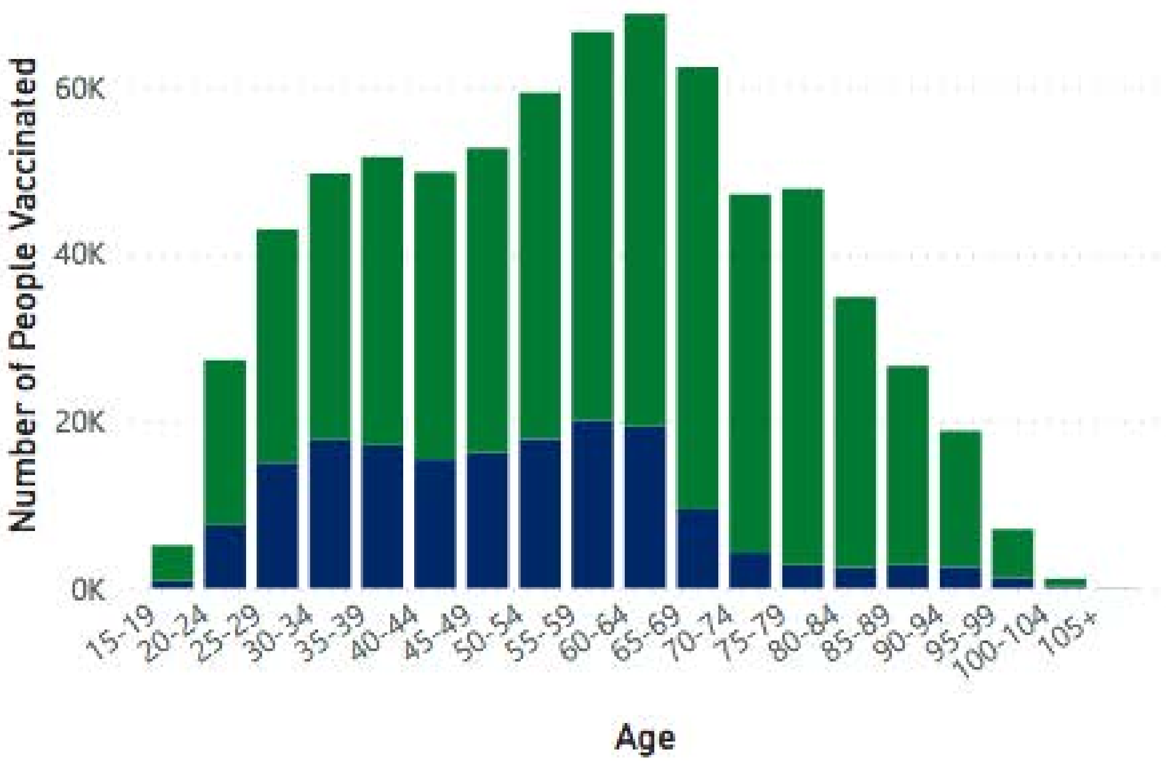

Number of People Vaccinated, by Ethnicity

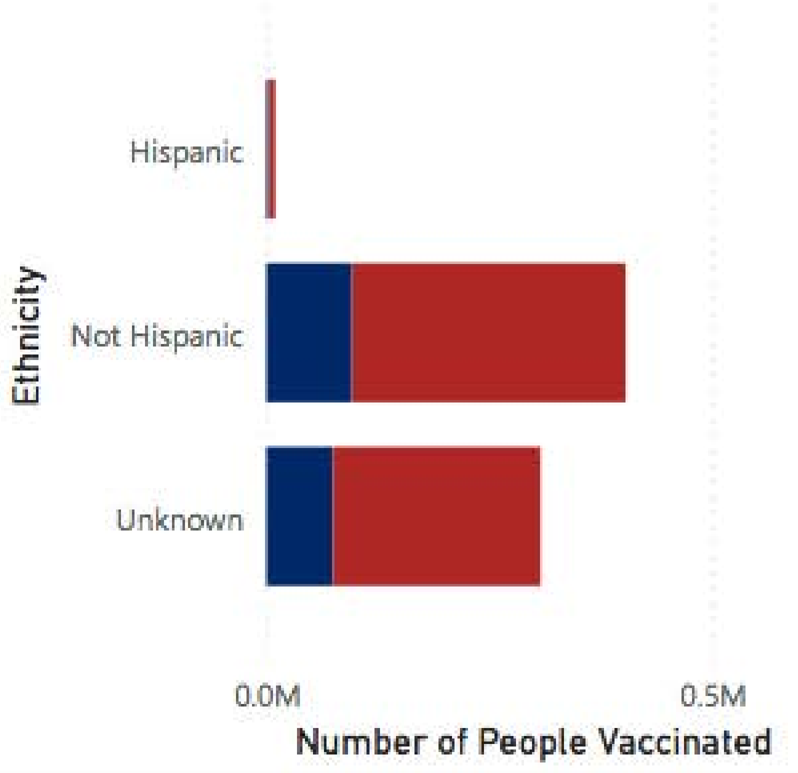

Number of People Vaccinated, by Race

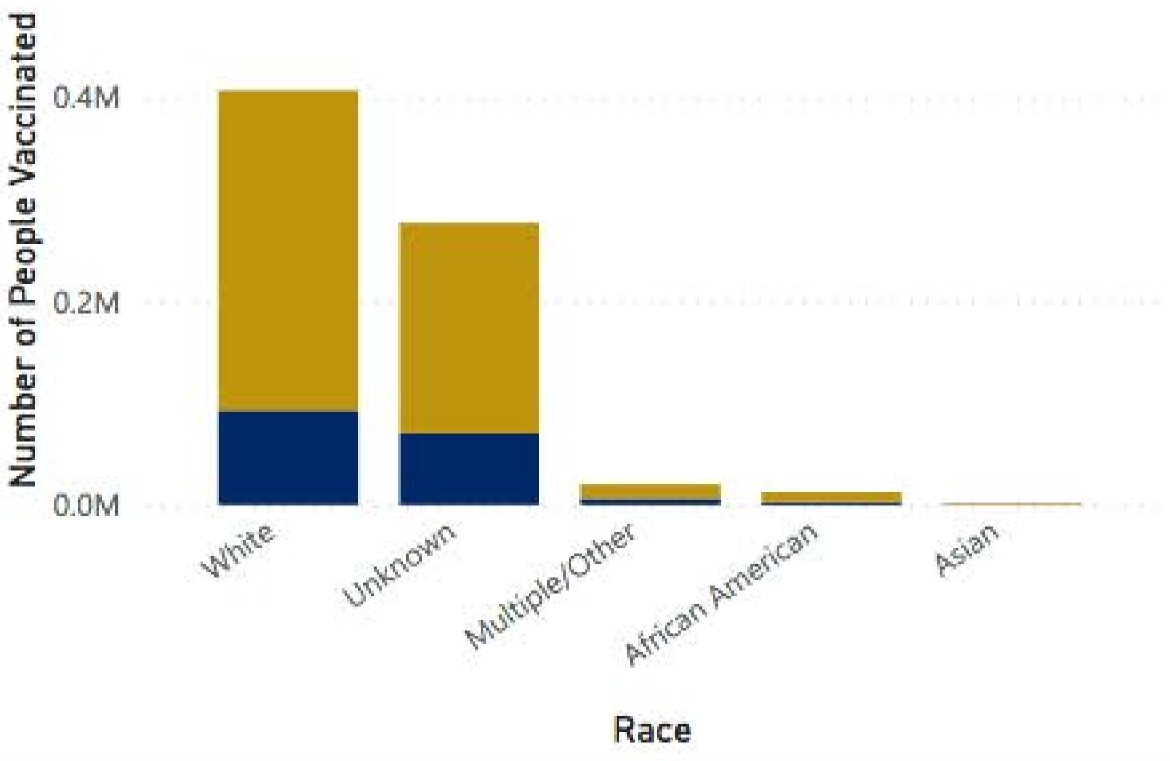

Data tracker daily data updated Monday through Friday by approximately 1 p.m.

Jan 29, 2021

Rhode Island Department of Health COVID-19 Data Tracker by Date

Total Tests	2,497,271
Total Positive Cases	114,438
Tests Prior Day	19,057
New Positive Cases Prior Day	550
Percent Positive Prior Day	2.9%
Total Fatalities	2,154
New Fatalities Prior Day	10
People Hospitalized	324

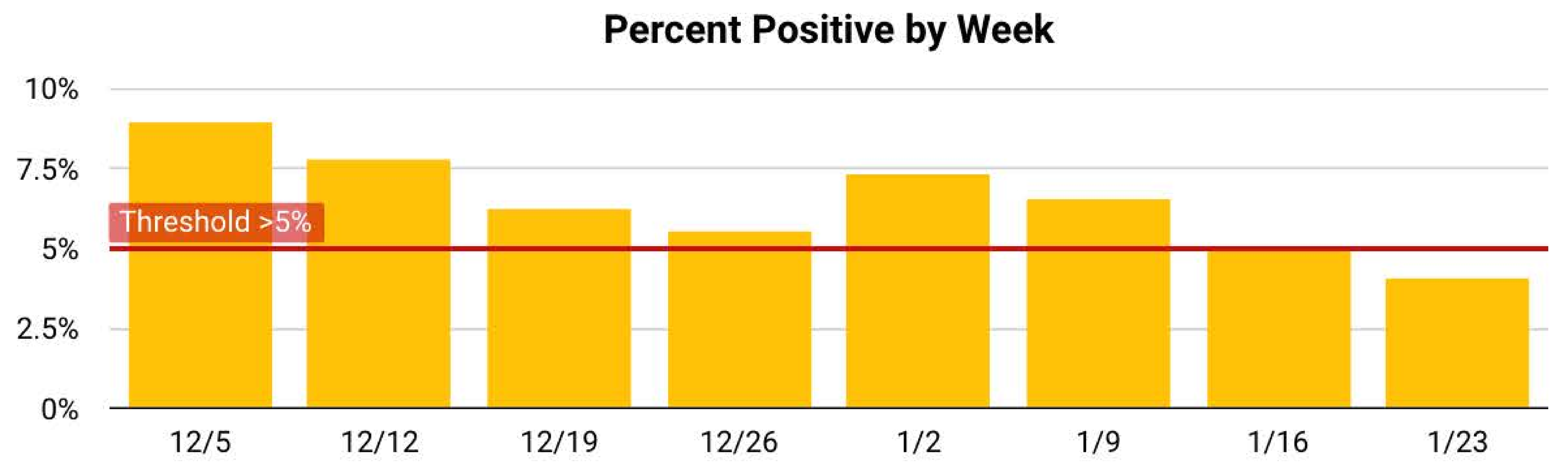

This week: 4.1%

Last Week: 5.0%

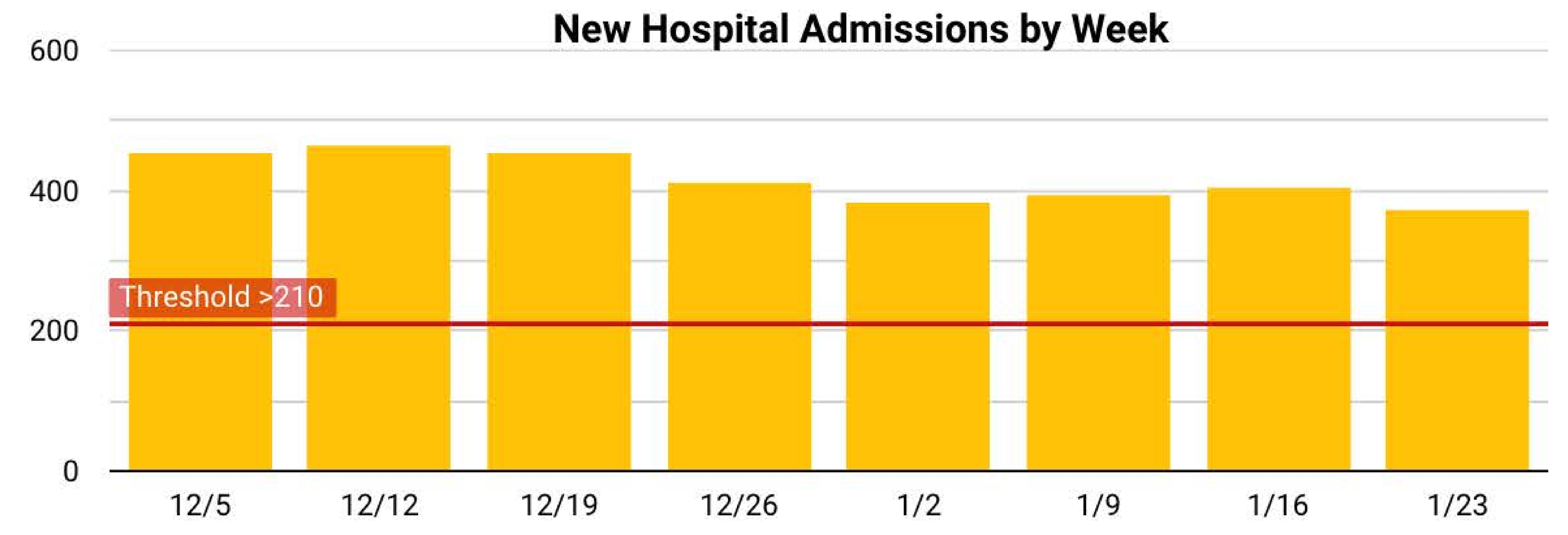

This week: 374

Last Week: 407

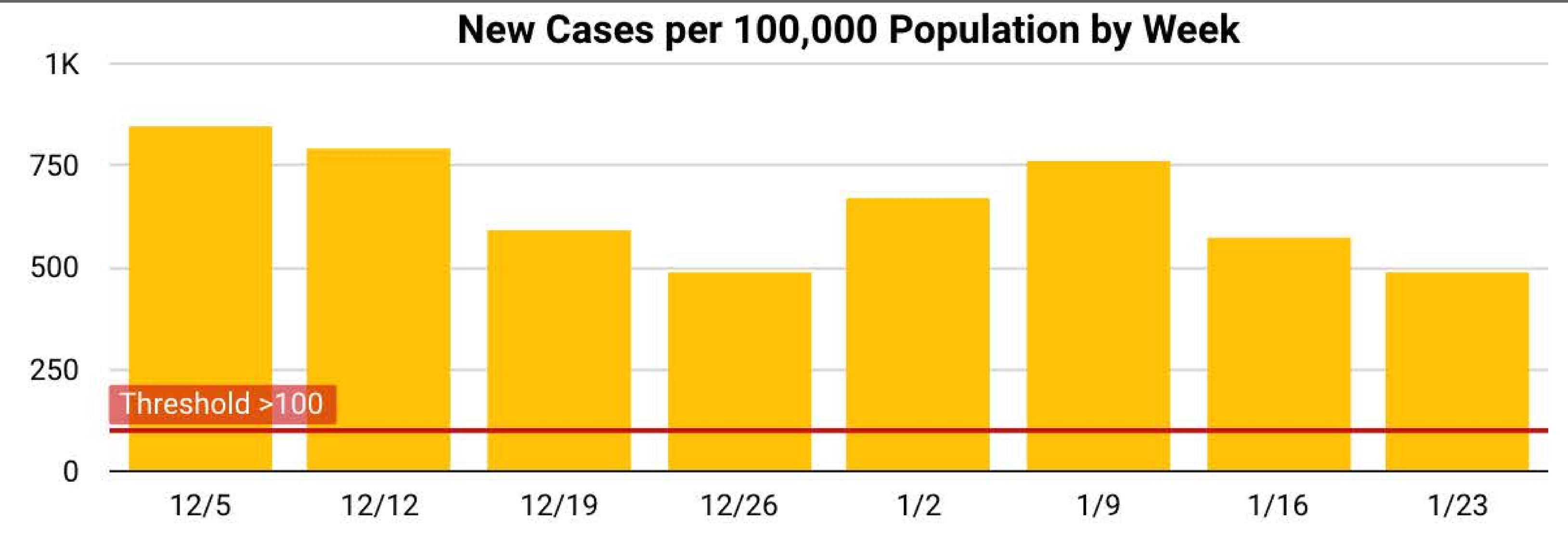

This week: 491

Last Week: 576

Exceeding these thresholds may result in greater restrictions for businesses, gatherings, etc

Data tracker weekly data updated Tuesdays by approximately 1 p.m.

Vaccine Administration (Cumulative since 12/14)

First Doses Administered	Second Doses Administered	People Fully Vaccinated	Total Doses Administered
66,778	22,983	22,983	89,761

Google Data Studio

Daily data are provisional and are subject to change.

New Positive Cases	People Hospitalized	New Fatalities
550	324	10

Total Tests	Total Positive Cases	Total Negative Tests
2,497,271	114,438	2,360,768

Total Fatalities	People in ICU	People on a Ventilator
2,154	50	31

Source: Rhode Island Department of Health

Google Data Studio

* Please see our Data FAQ for definitions and explanations for our different data fields

Rate of COVID-19 Cases per 100,000 Population, by Municipality of Residence and Week of First Positive Test Date - Excluding Congregate Setting Residents

Select town: RHODE ISLAND

Clear all selections

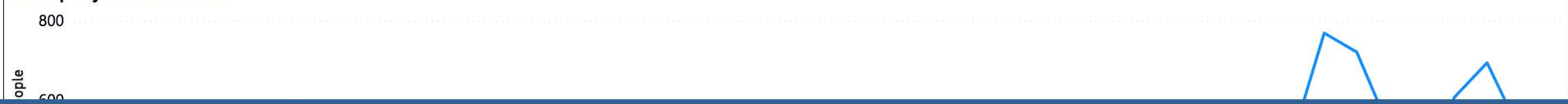

Sign In

Rhode Island COVID-19 Response Data [Click here] to return to the RIDOH COVID-19 Home Page

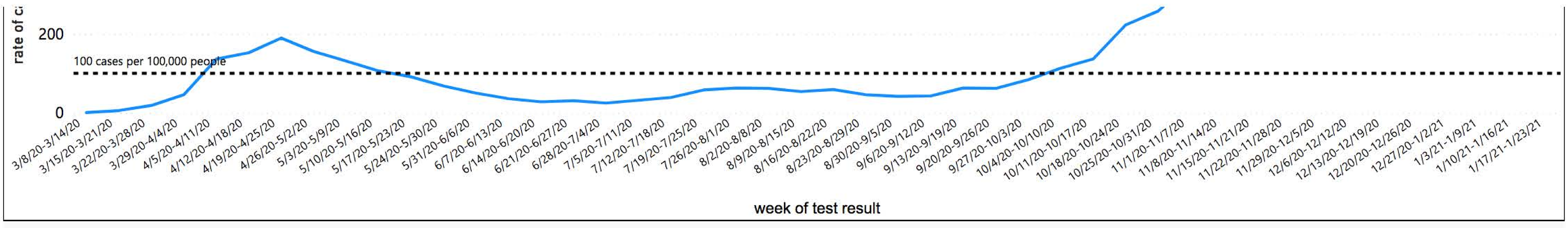

Note: rates are suppressed if count of cases is <5

Microsoft Power BI

Coronavirus COVID-19 Cases in South Carolina

Filter By County: None

Rate per 100k population

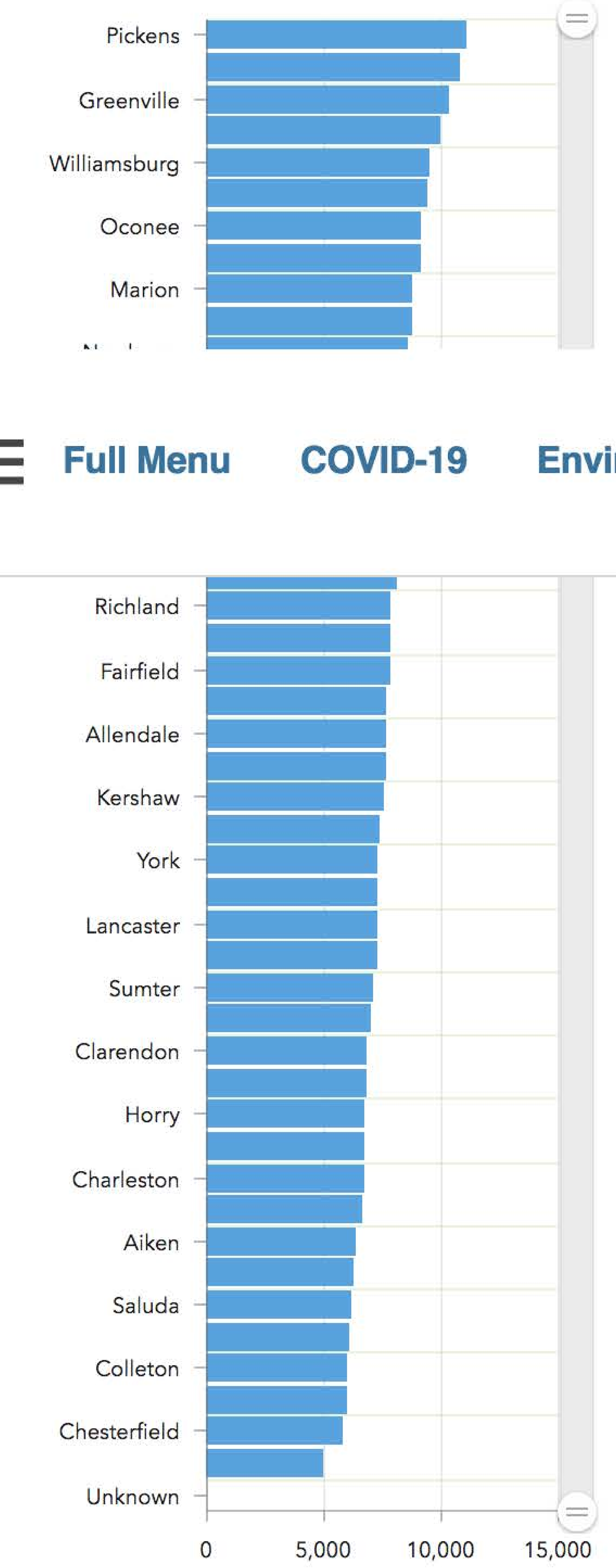

Rate (per 100k) | Deaths

Deaths in Individuals with COVID-19 infection

Confirmed: 6,355
Probable: 687

Read more

Total Positive Cases

Confirmed: 396,712
Probable: 46,674

Read more

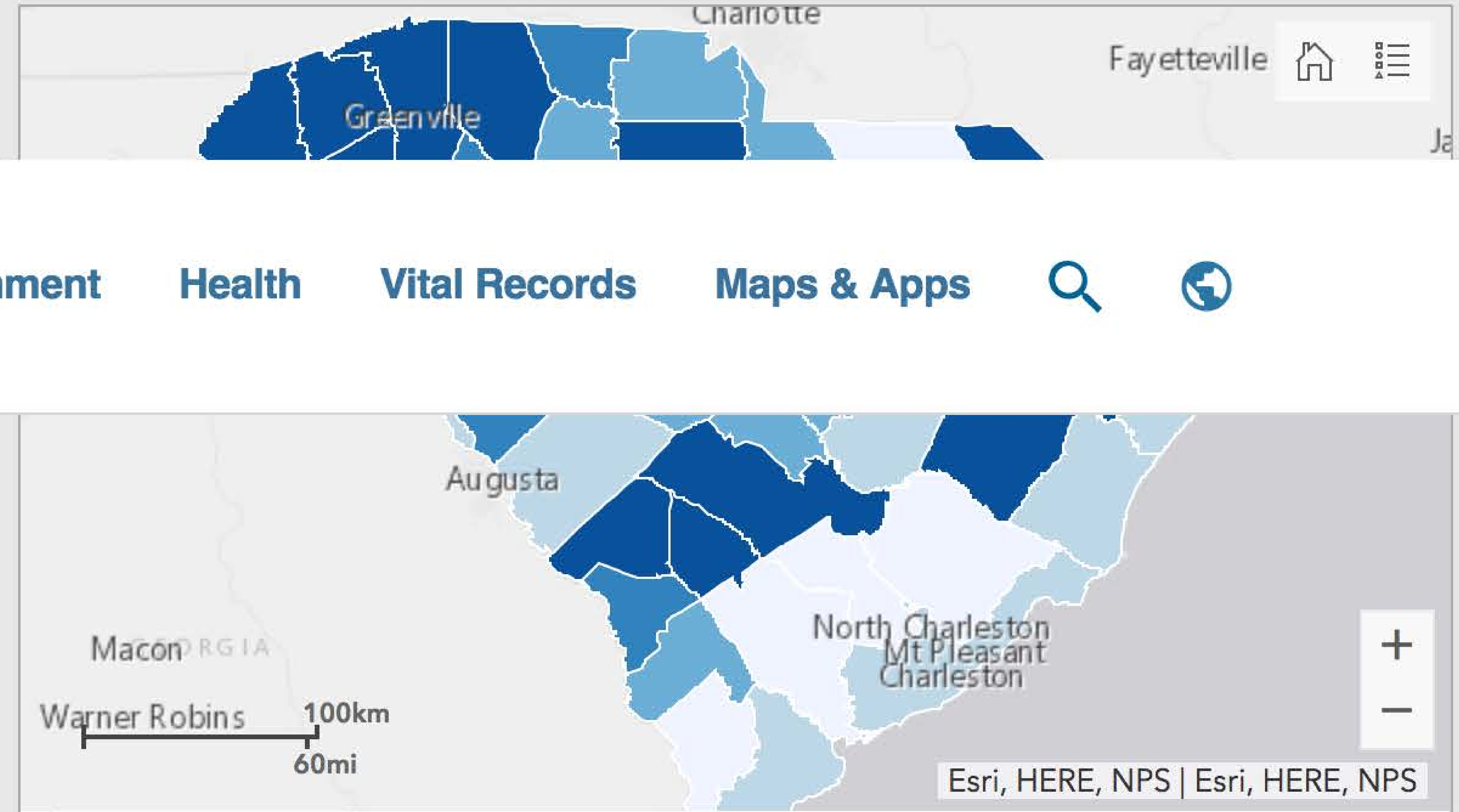

Coronavirus Map | Hospital | Specimen Collection Site

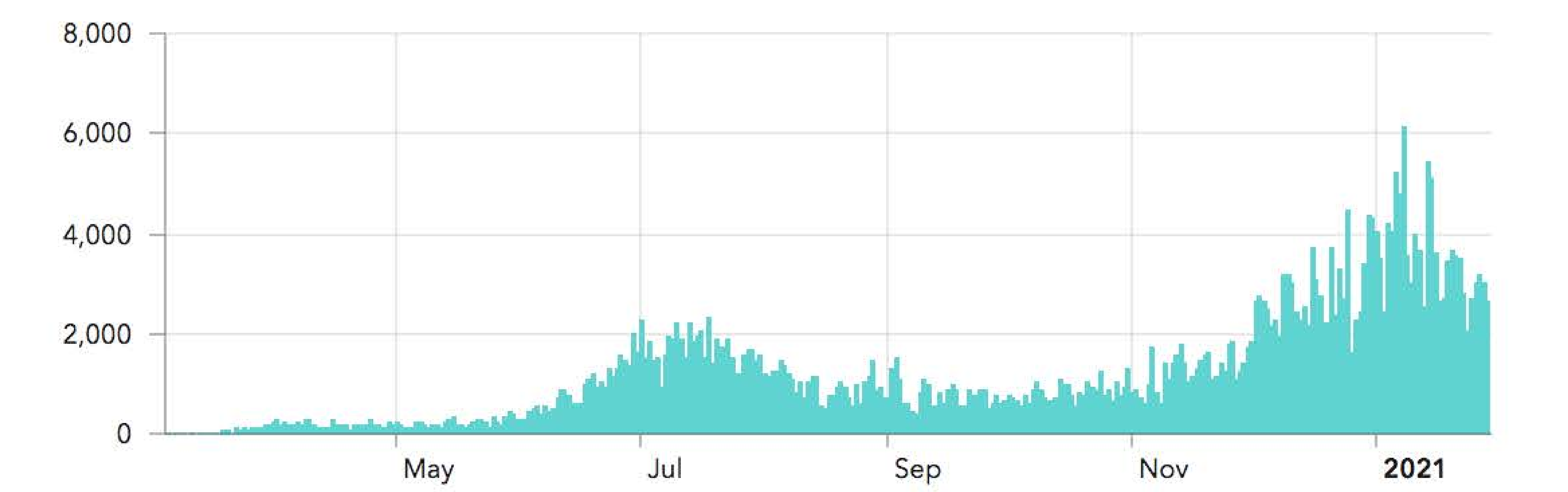

New Cases Per Day (Epi Curve) | Cumulative Cases

Data sources: DHEC Feature layer: Here.
Disclaimer: The **hospitals** displayed on the map are provided so that people can search for their nearest health facility. They are not indicative of coronavirus cases. The **alternate care sites (ACS)** and **specimen collection sites** [illegible]

County Cases Information

Read more

Bamberg
Rate (per 100k): 8,531.21
Cases--Confirmed positives: 1,200, Probable: 49
Deaths--Confirmed: 43, Probable: 0

Barnwell
Rate (per 100k): 9,096.14
Cases--Confirmed positives: 1,898,

[illegible]

Probable: 943
Deaths--Confirmed: 139, Probable: 7

Berkeley
Rate (per 100k): 4,926.13
Cases--Confirmed positives: 11,227, Probable: 1,472
Deaths--Confirmed: 127, Probable: 15

Calhoun
Rate (per 100k): 7,263.11
Cases--Confirmed positives: 1,057, Probable: 62
Deaths--Confirmed: 26, Probable: 3

Charleston
Rate (per 100k): 6,657.17
Cases--Confirmed positives: 27,388, Probable: 5,449
Deaths--Confirmed: 340, Probable: 44

Cherokee
Rate (per 100k): 7,616.06
Cases--Confirmed positives: 4,364, Probable: 166
Deaths--Confirmed: 112, Probable: 4

Chester

Cases

Interactive: COVID-19 curves for each South Carolina county

by Nicholas Papantonis | Monday, March 16th 2020

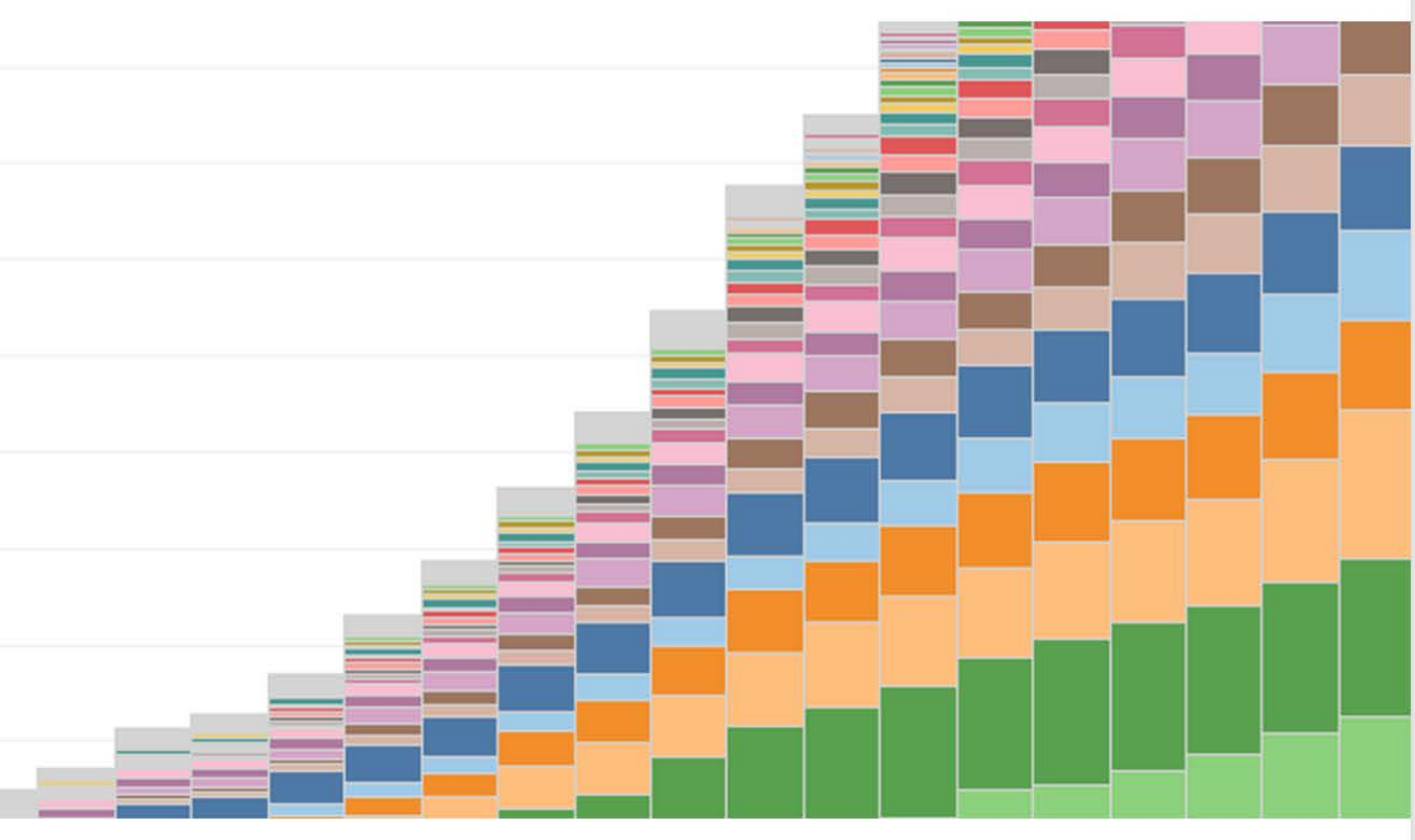

Screen shot from a Tableau chart put together by ABC 15 to track the coronavirus in South Carolina.

CONWAY, S.C. (WPDE) — Welcome to the COVID-19 data center, where you can track and compare efforts to flatten the curve in South Carolina and each of its counties.

A quickly spreading virus can overwhelm the state's health care system, forcing doctors and nurses to choose which patients to care for as resources like beds and ventilators run scarce.

By keeping people relatively isolated from each other, the virus has a more difficult time transmitting from one person to another. The practice and effects were simulated by The Washington Post.

You can check out the current number of cases and deaths in South Carolina (and along the North Carolina border belt) in an interactive map updated with each new case.

DHEC epidemiologists warn the number of confirmed cases is far fewer than the actual number of cases, since so many people are asymptomatic or only experience minor symptoms.

You can see a break down of the daily state tally in this chart below.

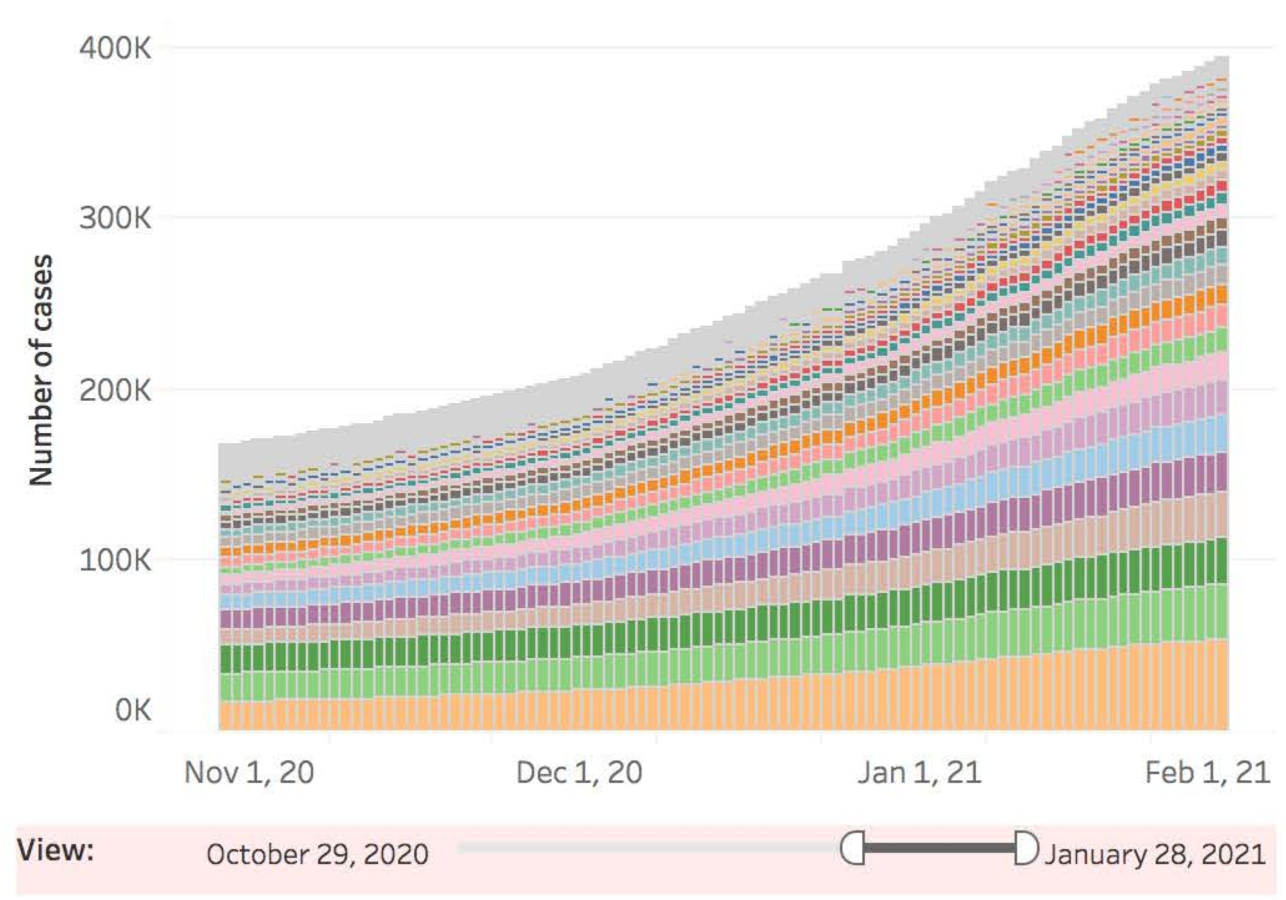

Can't see the chart on mobile? Tap here.

Next, we can see how each county is managing its curve.

Of course, smaller, more rural counties tend to have fewer cases, and gain new cases slower than larger, urban counties. That makes for a flatter curve.

You can view each county's curve over time in the third chart below. While the default view is set to all counties, you can look at each county one at a time, or compare multiple counties using the drop down to the right.

For reference, the following county pairings have similar populations:

- Horry County and Spartanburg County
- Charleston County and Richland County
- Florence County and Dorchester County
- Berkeley County and York County
- Lexington County and Spartanburg County
- Beaufort County and Anderson County
- Sumter County and Lancaster County
- Darlington, Georgetown, Laurens and Kershaw Counties

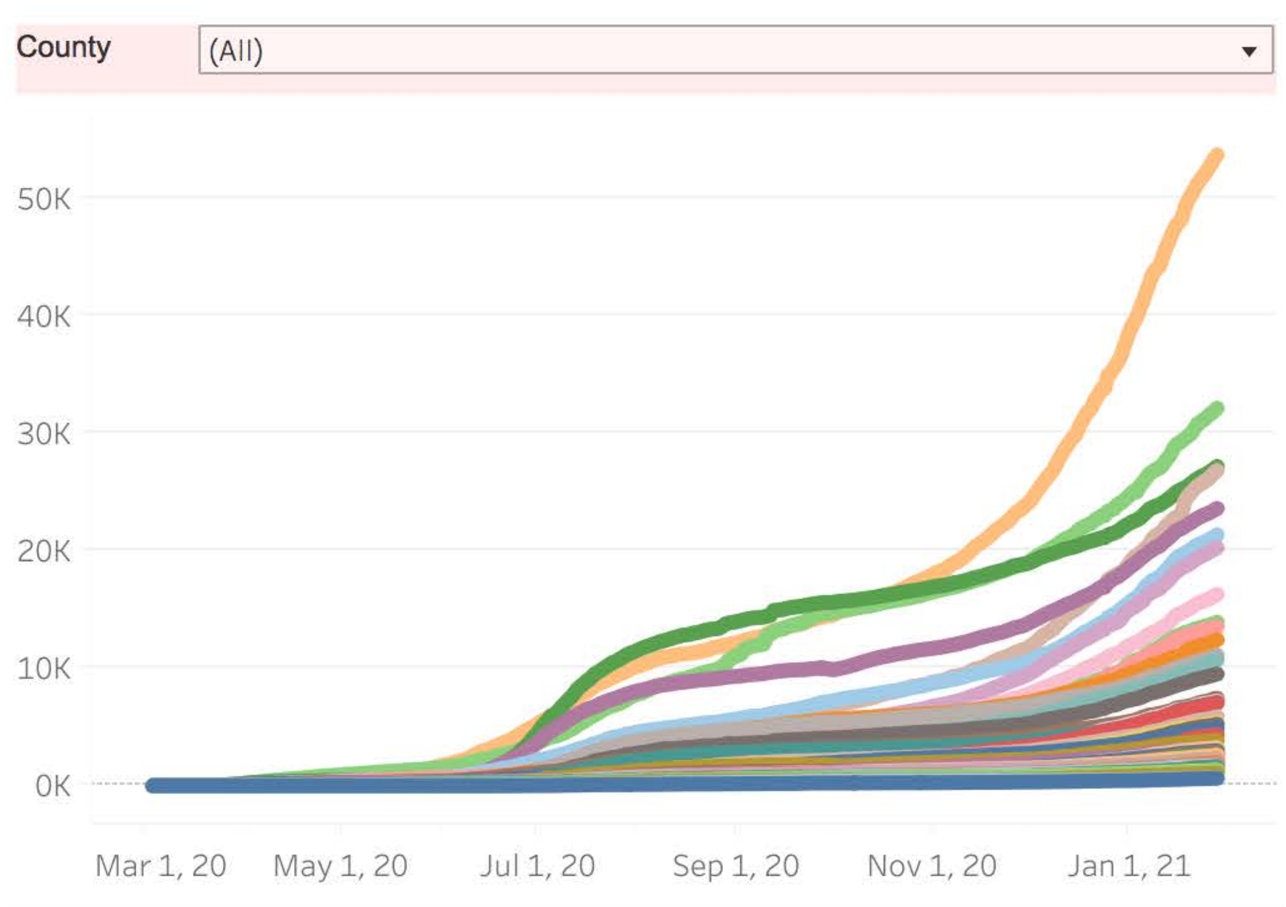

Can't see the chart on mobile? Tap here.

All of these charts show one set of data: the running totals for cases in South Carolina, which can be slightly more difficult to comprehend.

In making a decision to open or close businesses and schools, health experts and government leaders often rely on a "moving average" of the number of new cases, or an average of the past few days to smooth out the wild day-to-day swings in numbers.

The average lags behind the number of new cases, meaning that while cases are on the rise, the average appears lower than the daily case count. When cases are falling, the average appears higher. This helps by building a few days' worth of data into the trend line to prevent any sudden decisions from being made.

You can view the 7-day moving average of South Carolina's new cases, deaths and hospitalizations below:

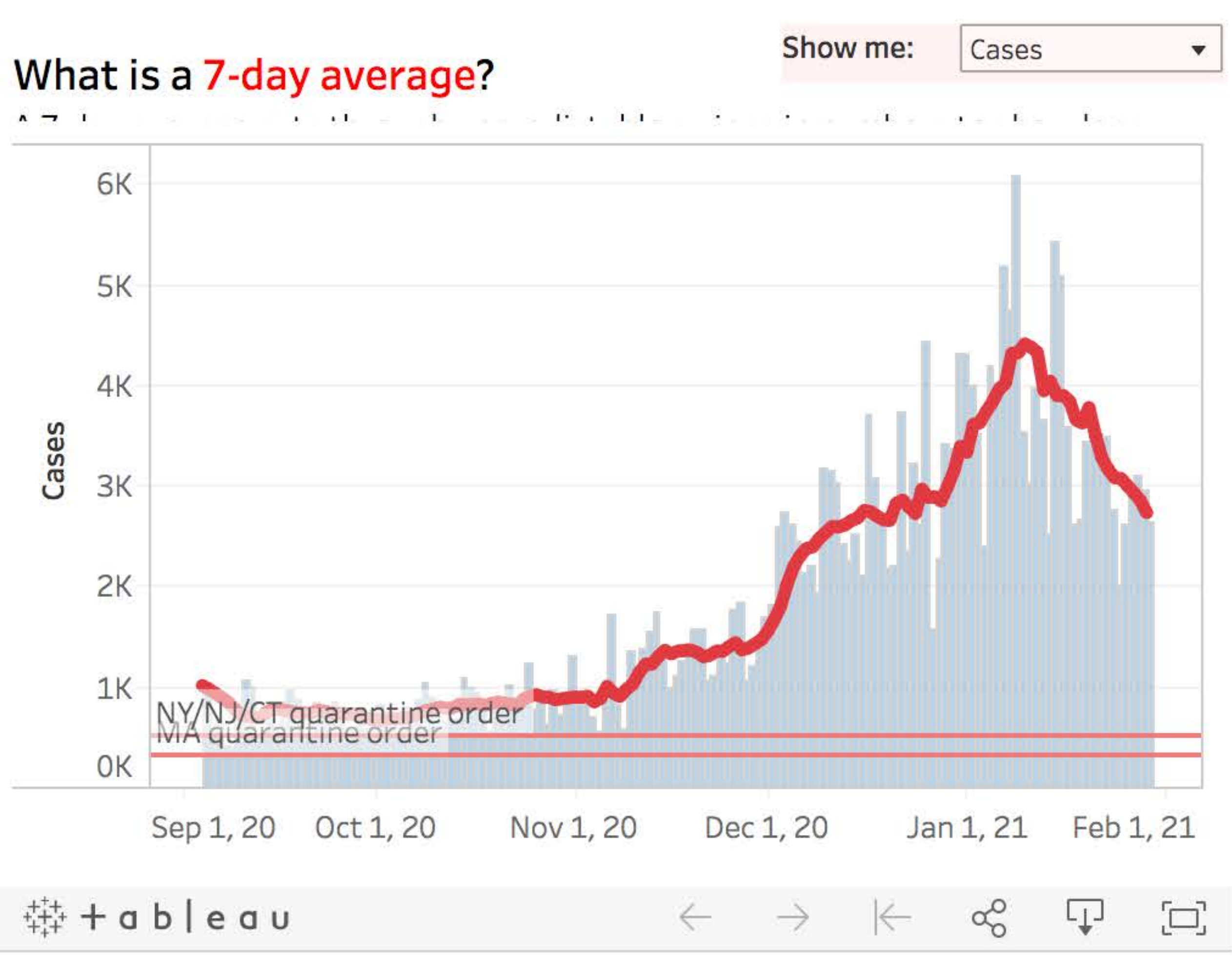

Can't see the chart on mobile? Tap here.

Related: Interactive map: tracking COVID-19 cases by county and zip code

We have also built a chart to track deaths in South Carolina over time. Because most of the counties have relatively few deaths, they appear in clusters and have fairly jagged lines.

You can use the drop down menu to filter counties individually or in small groups.

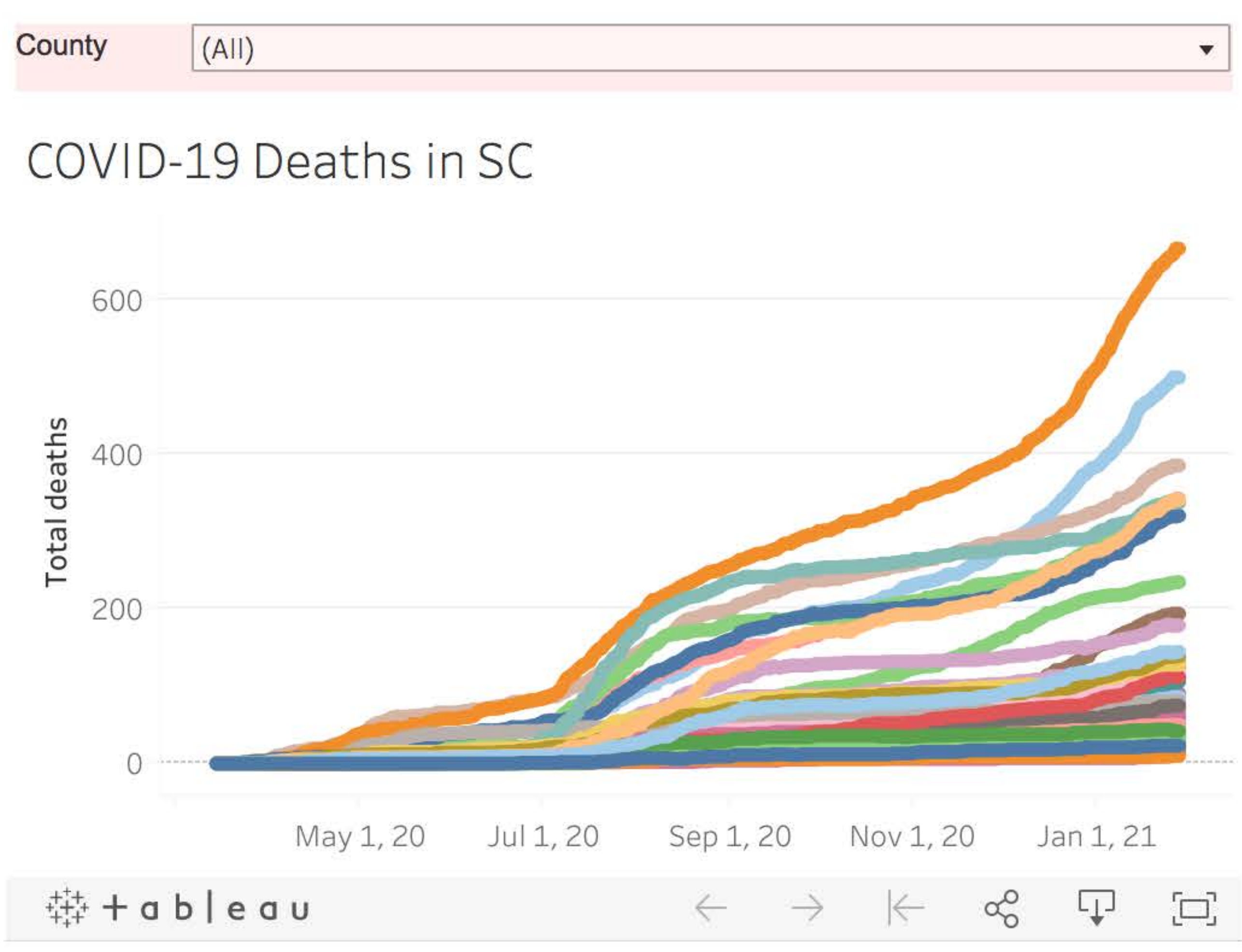

And finally, here's how the state is doing compared to the United States.

In this chart, we've scaled down the US population to 1.6% to match

EWS NEWS WEATHER CORONAVIRUS SPOTLIGHT CHIME IN WATCH

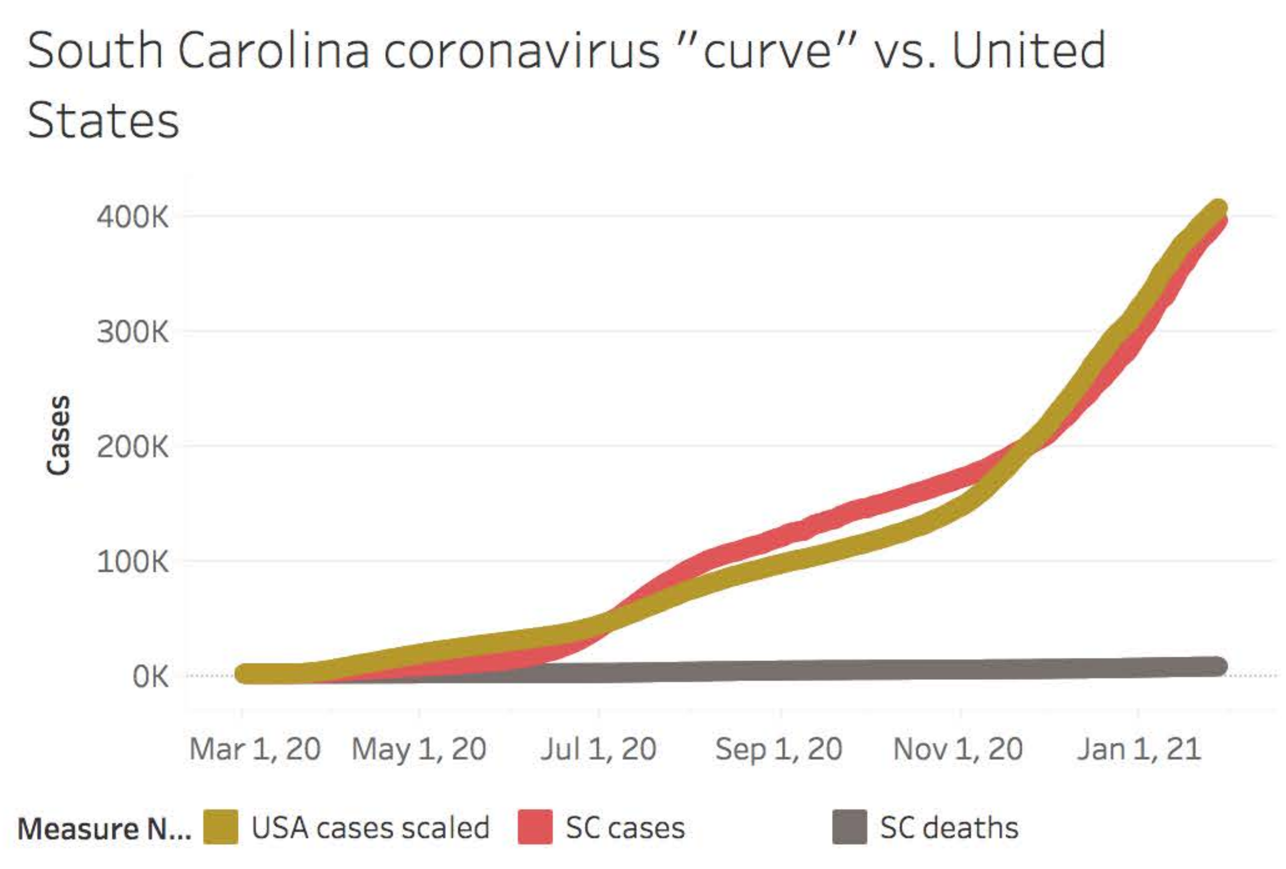

SOUTH DAKOTA DEPARTMENT OF HEALTH doh.sd.gov/covid/

SOUTH DAKOTA COVID-19 DASHBOARD

SOUTH DAKOTA COVID-19 UPDATES

Please wait. The data dashboard below takes a moment to load and is best viewed on desktop or in landscape mode on mobile (i.e. holding the phone sideways).

SD Overview | Demographics | Trends | Hospital Capacity | Hospitalized & ILI | Tables | Vaccines | Deaths

New Confirmed Cases	New Probable Cases	Active Cases	Recovered Cases	Currently Hospitalized
132	47	2,833	103,639	125

Community Spread Map by County of Residence

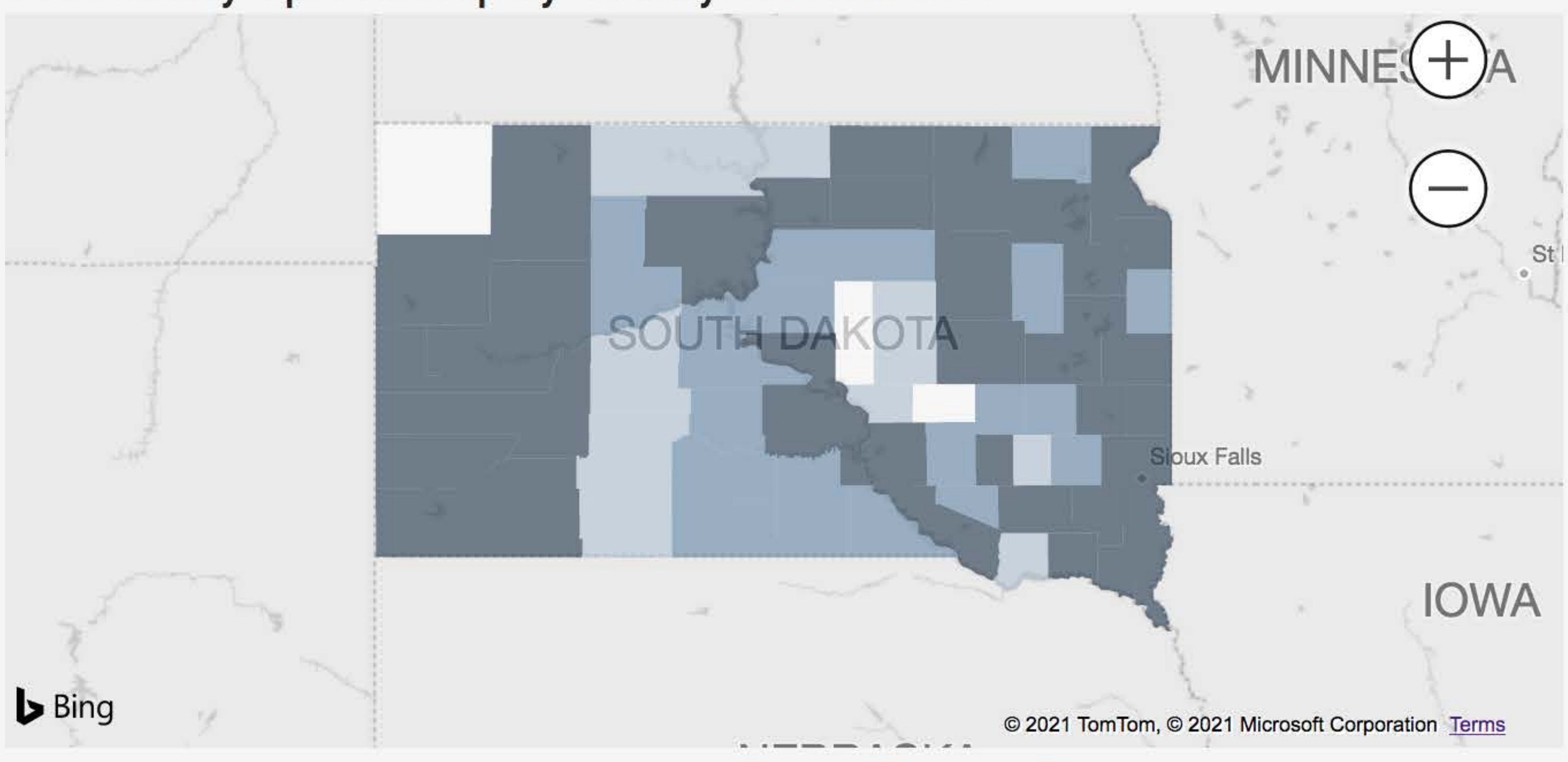

Community Spread None Minimal Moderate Substantial

Hover over a county to see its details, or click county to update the orange boxes.

Total Confirmed Cases	Total Probable Cases	RT-PCR Test Positivity Rate, Last 1 Day	Total Persons Tested	Total Tests
96,683	11,567	8.1%	402,613	876,143

Ever Hospitalized	Deaths Among Cases	% Progress (November Goal: 44233 Tests)	% Progress (December Goal: 44233 Tests)	% Progress (January Goal: 44233 Tests)
6,290	1,778	402%	345%	239%

Cases by County of Residence

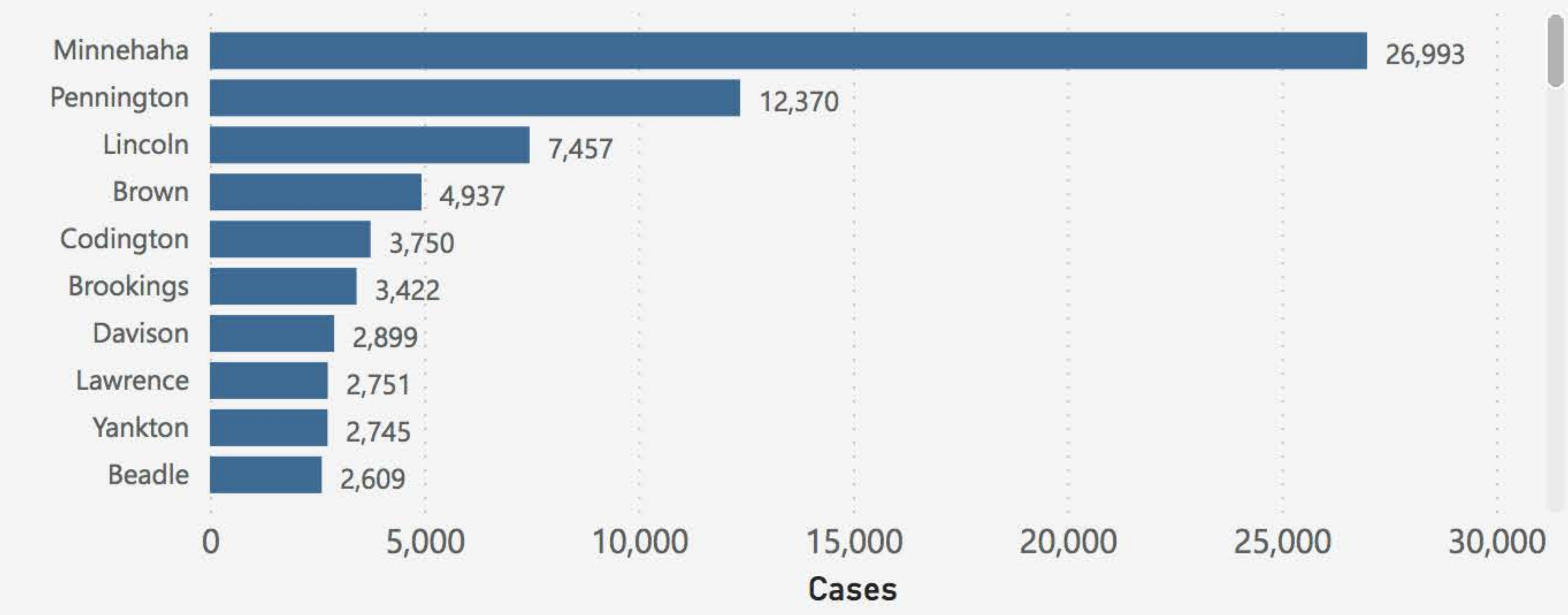

Cases: *Persons who meet the national surveillance case definition for COVID-19. According to the case definition, Confirmed Cases includes persons with a positive RT-PCR test and Probable Cases includes persons with a positive antigen test for the SARS-CoV-2 virus that causes COVID-19. A positive case moves from Active to Recovered status based on the CDC guidelines for removal from isolation.*

Hospitalized: *Currently Hospitalized numbers include COVID-19 cases and suspects that are in the hospital under transmission-based precautions. The number may include out-of-state cases and suspects. SD-DOH reports on data directly entered by hospitals into the US Department of Health and Human Services TeleTracking system. Federal entities, such as the Veterans Affairs Medical Centers, are not subject to reporting. Ever Hospitalized include COVID-19 cases that were, at some point during their infection, hospitalized under transmission-based precautions. The number of persons ever hospitalized are cumulative and includes only South Dakota resident cases. Data are reported from the Maven electronic disease surveillance system.*

Deaths among Cases: *This data includes deaths reported by death certificate or deaths within 20 days of a COVID-19 infection. SD-DOH does not report deaths until a certified death record has been filed with COVID-19 as a cause or contributing factor. By law, a death record must be filed within 5 days of the date of death. For more information see the Deaths Tab.*

Tests: *% Progress shows the percentage of RT-PCR and antigen tests completed toward the monthly goal of 44,233. The monthly goal for South Dakota is equal to 5% of the state's population. The federal government established a 2% goal for SARS-CoV-2 testing for each state. Total tests are the number of tests performed, not the number of individuals tested, and include tests conducted by the South Dakota Public Health Laboratory and hospital, commercial, Indian Health Service, Veteran Affairs, and other medical laboratories.*

Community spread is defined as:
None: *0 cases per 100,000 OR 0 cases**
Minimal: *Less than 10 cases per 100,000 OR a minimum of 1 case**
Moderate: *10-100 cases per 100,000 OR a minimum of 4 cases**
Substantial: *100 cases or more per 100,000 OR a minimum of 10 cases**
**Counties that have a population of less than 35,000 use a combination of the rate and minimum number of cases.*

Community Spread *is updated every Monday based on the number/rate of new cases per week (Sun-Sat), calculated from the data reported the previous two weeks.*

% RT-PCR Test Positivity Rate *is equal to the number of positive RT-PCR tests divided by total RT-PCR tests, within the specified period of time. Weekly % RT-PCR Test Positivity Rate is updated every Monday based on the number of new RT-PCR tests from the previous week (Sun-Sat).*

Note: *Data are provisional and subject to change. Data are updated daily by about 12 p.m. and include cases reported*

LAST UPDATED
1/31/2021 10:44:09 AM

Microsoft Power BI 1 of 8

Note:

- The Community Impact Map uses the **definitions** for none, minimal, moderate, and substantial community transmission. The level of spread is aligned with the **CDC's Community Mitigation Framework**.
- The number of persons positive for COVID-19 and number of tests positive for COVID-19 reflect only South Dakota residents, regardless of where testing was conducted (in-state or out-of-state).
- Investigation of a person positive for COVID-19 is completed by the public health agency in the state where the person resides. A South Dakota resident testing positive in another state is reported to SD-DOH for investigation.
- States notify the public health agency in which close contacts of a positive case reside so notification can be made. A South Dakota resident will be notified if they were a close contact to a confirmed case by the SD-DOH.
- A public notice is issued by SD-DOH when a person positive for COVID-19 is unable to identify all close contacts (spent 15 minutes or more within 6 feet) where there may be risk of exposure so that individuals can monitor themselves for symptoms and seek guidance from their medical provider.
- A person is considered recovered after they have been released from isolation by SD-DOH upon meetingthefollowing: (1) at least 10 days have elapsed after their symptoms began, and (2) at least 24 hours haveelapsed after their fever resolvedand their other symptoms have improved.
- COVID-19 cases within **South Dakota K-12 Schools and Colleges, Universities, and Technical Schools** will be updated weekly on Monday.
- **Monthly number of total tests reported and total persons tested for SARS-CoV-2**. Data will be updated monthly.
- **Number of COVID-19 Vaccine distributed to South Dakota**: https://covid.cdc.gov/covid-data-tracker/#vaccinations

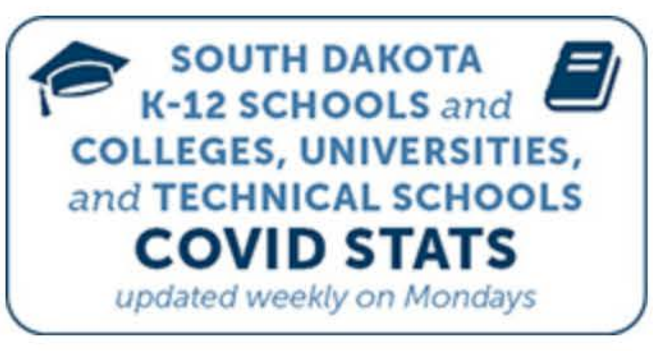

BACK TO TOP

COVID-19
Collapse All | Expand All
Dashboard
Your Health
Community, Work & School
+ Vaccination
Healthcare Providers

SOUTH DAKOTA DEPARTMENT OF HEALTH doh.sd.gov/covid/

SOUTH DAKOTA COVID-19 DASHBOARD

SOUTH DAKOTA COVID-19 UPDATES

Please wait. The data dashboard below takes a moment to load and is best viewed on desktop or in landscape mode on mobile (i.e. holding the phone sideways).

SD Overview | Demographics | Trends | Hospital Capacity | Hospitalized & ILI | Tables | Vaccines | Deaths

County
All

RT-PCR Test Positivity Rate, Last 1 Day	RT-PCR Test Positivity Rate, Last 7 Days	RT-PCR Test Positivity Rate, Last 14 Days	RT-PCR Test Positivity Rate, Cumulative
8.2%	7.7%	9.1%	14.9%
01/29/2021	1/23/2021 - 1/29/2021	1/16/2021 - 1/29/2021	3/10/2020 - 1/29/2021

RT-PCR Test Positivity Rate: *This rate is equal to the number of positive RT-PCR tests divided by total RT-PCR tests, within the specified period of time. Date range includes last full day (24 hours) captured in the data set.*

Positive Cases and 14-Day Trend Line by Date Reported to SD-DOH

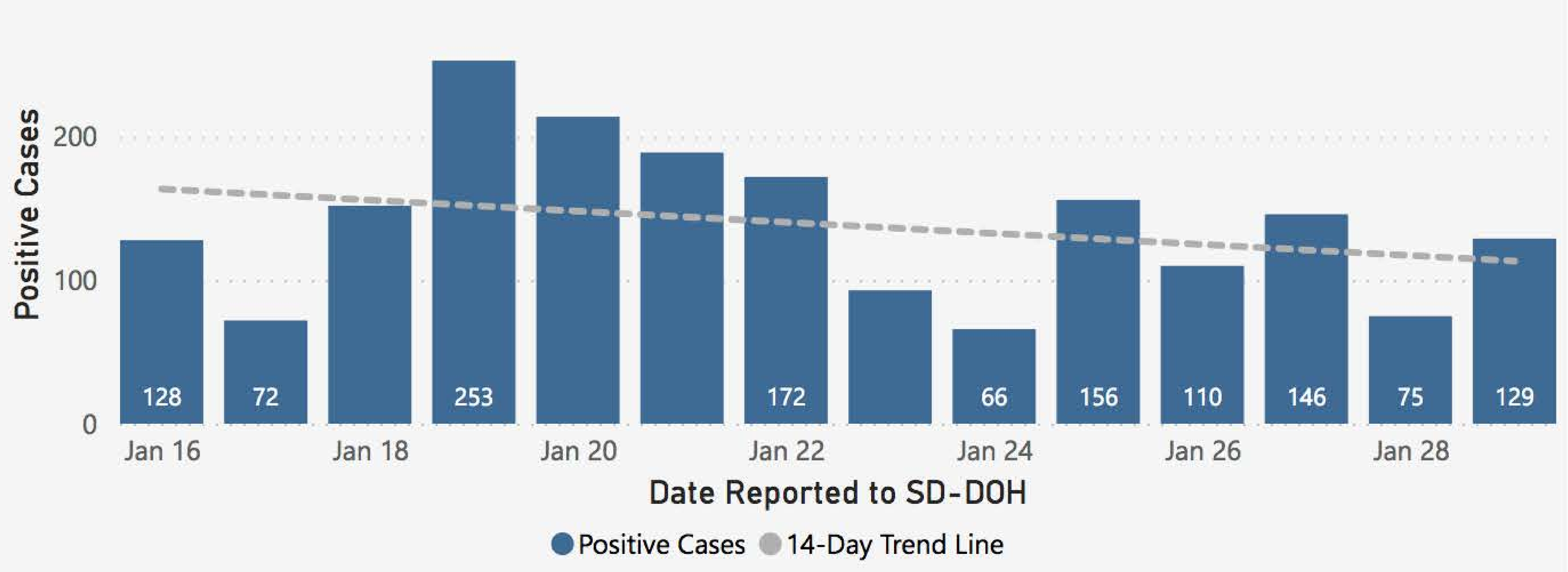

14-Day Trend of Positive Cases by Date Reported to SD-DOH: *This graph shows the trend (increasing or decreasing) of COVID-19 positive cases (persons who meet the national surveillance case definition of a confirmed case for COVID-19) at the state or county level.*

Rate of Cases, Last 7 Days	Rate of Cases, Cumulative
214	10,991
(per 100,000)	(per 100,000)

Rate of Cases: *Number of positive cases (persons who meet the national surveillance case definition for a confirmed case of COVID-19) reported to SD-DOH in the last 7 days (or cumulative) divided by the state or county population multiplied by 100,000. This is done to compare disease burden across geographic areas (eg. state or county).*

Date Reported to SD-DOH

3/10/2020 | 1/29/2021

Positive Cases by Date Reported to SD-DOH

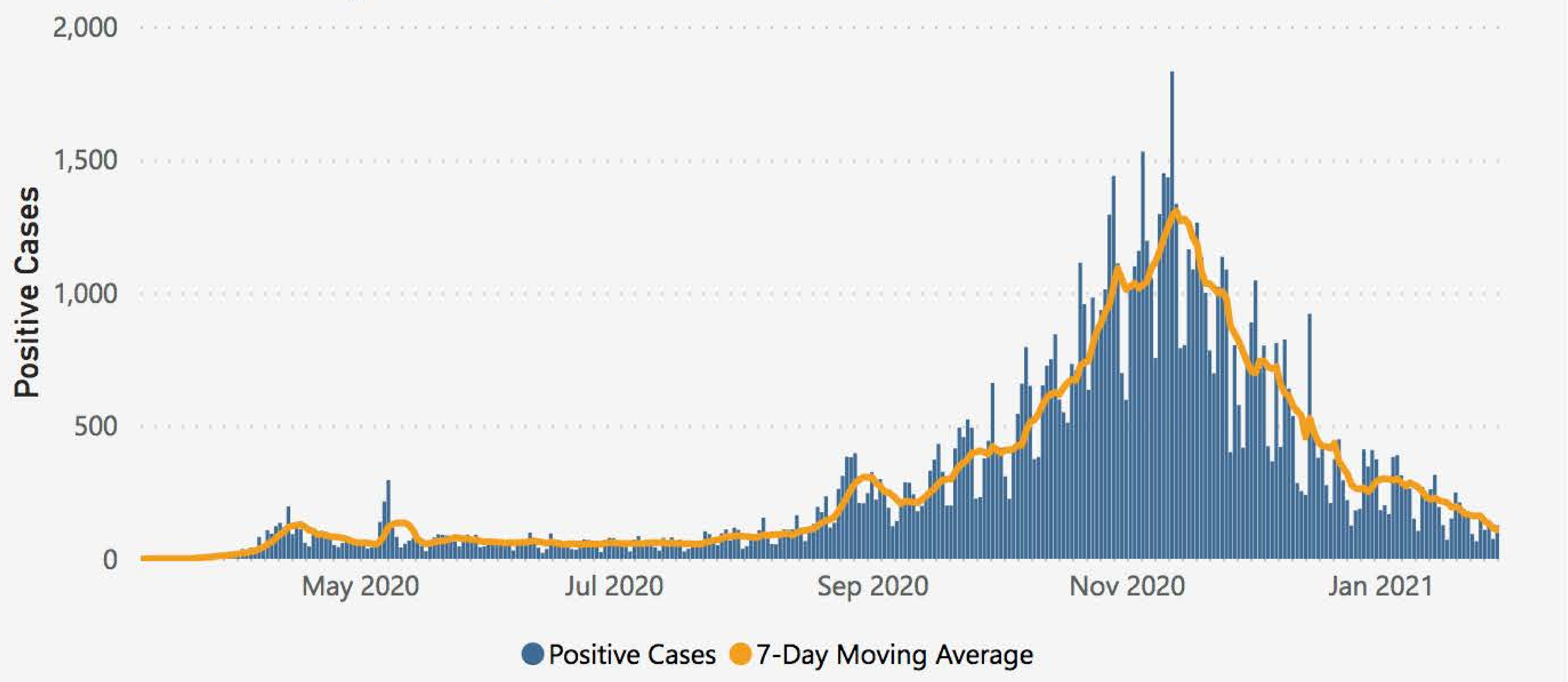

Positive Cases by Date Reported to SD-DOH: *This graph shows positive cases (persons who meet the national surveillance case definition for a confirmed case of COVID-19) over time includes results received by midnight and may not align with the daily data update.*

% RT-PCR Test Positivity Rate by Date Reported to SD-DOH

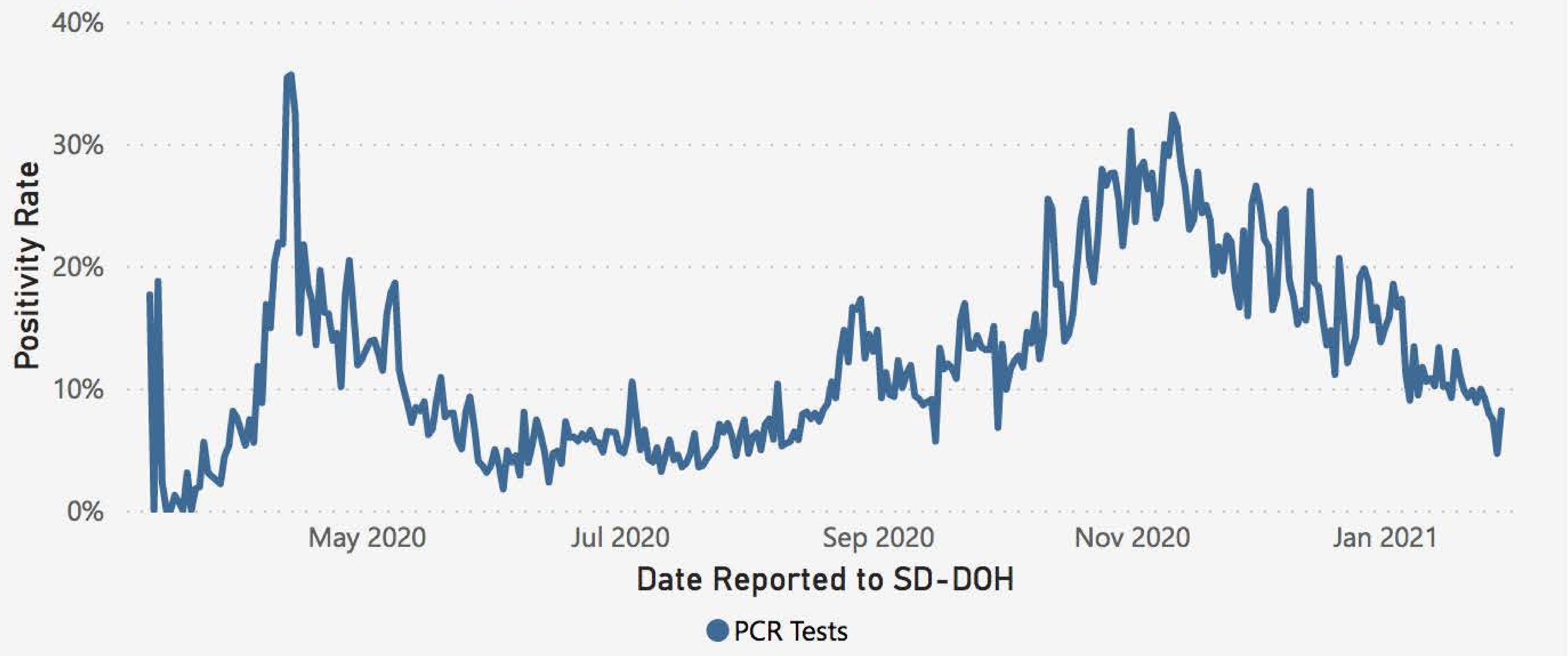

% RT-PCR Test Positive Rate by Date Reported to SD-DOH: *This graph shows % RT-PCR Test Positivity Rate which is equal to the number of positive RT-PCR tests divided by total RT-PCR tests, within the specified period of time.*

Note: *Data are provisional and subject to change. Data are updated daily by about 12 p.m. Results include tests conducted by the South Dakota Public Health Laboratory and hospital, commercial, Indian Health Service, Veteran Affairs, and other medical laboratories.*

LAST UPDATED
1/31/2021 10:44:09 AM

Note:

SOUTH DAKOTA DEPARTMENT OF HEALTH doh.sd.gov/covid/

SOUTH DAKOTA COVID-19 DASHBOARD

SOUTH DAKOTA COVID-19 UPDATES

Please wait. The data dashboard below takes a moment to load and is best viewed on desktop or in landscape mode on mobile (i.e. holding the phone sideways).

SD Overview | Demographics | Trends | Hospital Capacity | Hospitalized & ILI | Tables | Vaccines | Deaths

Total Doses Administered

99,560

Total Persons Administered a Vaccine

71,025

Manufacturer	Number of Doses
Moderna	53,302
Pfizer	46,258

Doses	Number of Recipients
Moderna - 1 dose	26,856
Moderna - Series Complete	13,223
Pfizer - 1 dose	15,634

Doses & Persons Administered Vaccine by County of Residence

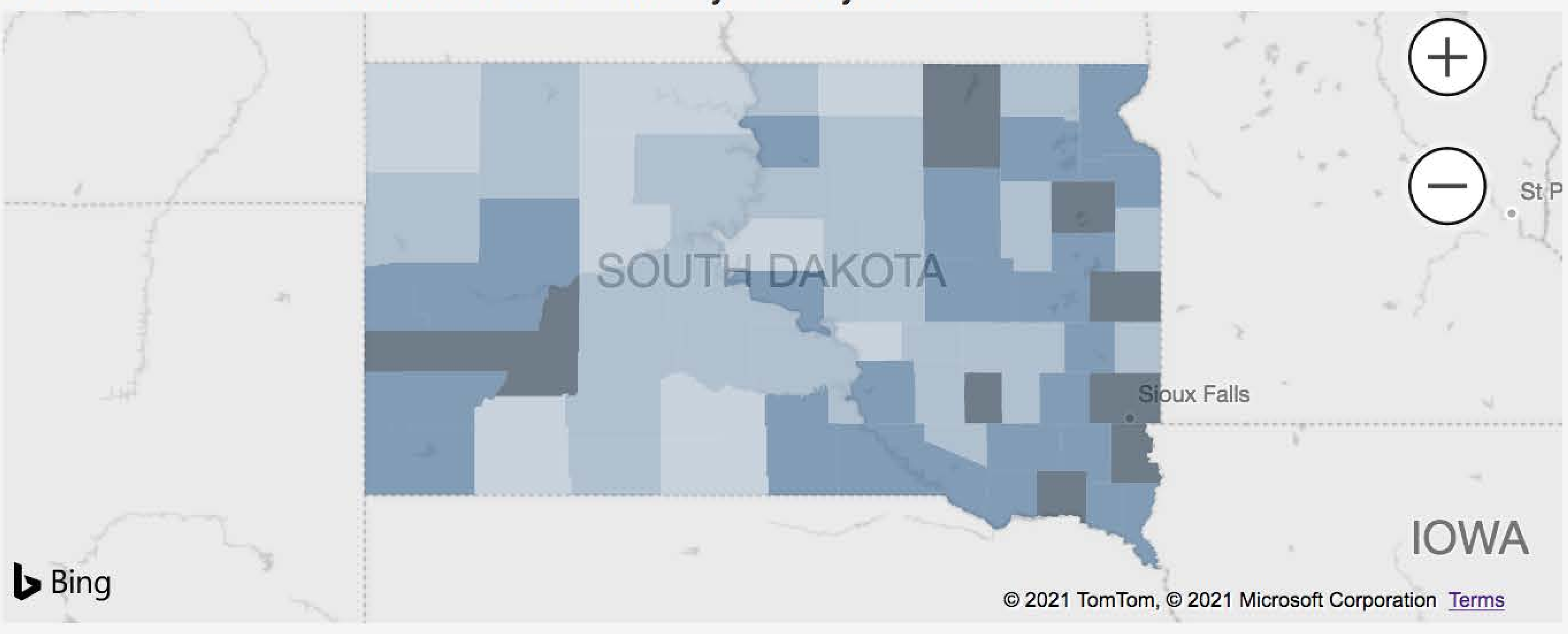

Doses Administered Range: 1-99, 100-499, 500-2,499, 2,500+

Counties with tribes receiving vaccine through Indian Health Services (IHS). Doses administered by IHS or federal entities, such as the Veterans Affairs Medical Centers, are not reported to the South Dakota Immunization Information System and and are not shown.

Doses Administered and Count of Recipients per South Dakota County

County	# Doses	# Persons (1 dose)	# Persons (2 doses)	Total # Persons
Minnehaha	26905	8,819	9,043	**17,862**
Pennington*	10389	6,433	1,978	**8,411**
Lincoln	9783	2,925	3,429	**6,354**
Brown	4834	1,956	1,439	**3,395**
Codington*	3488	1,588	950	**2,538**
Yankton	3561	1,387	1,087	**2,474**
Brookings	2769	1,001	884	**1,885**
Hughes*	2433	1,295	569	**1,864**
Lawrence	2061	1,561	250	**1,811**
[illegible]	[illegible]	[illegible]	[illegible]	[illegible]

Cumulative Number of Persons Vaccinated by Week

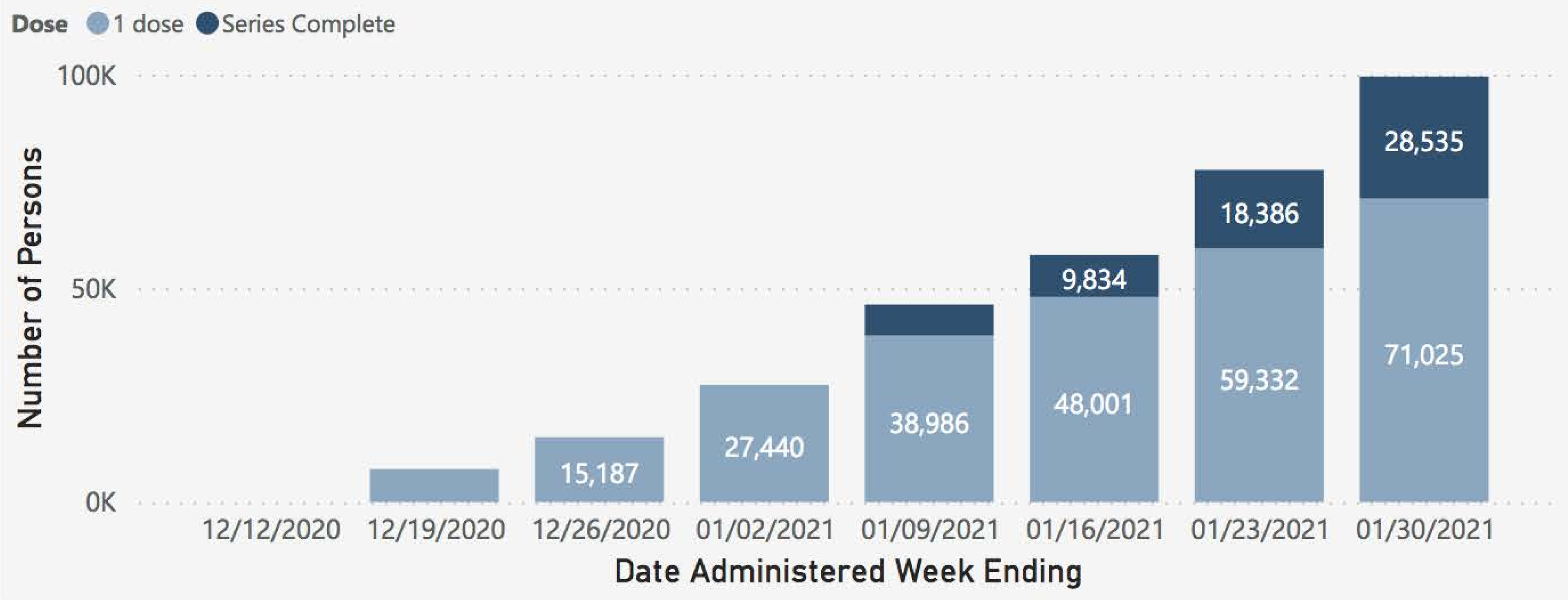

Number of Persons Vaccinated by Sex

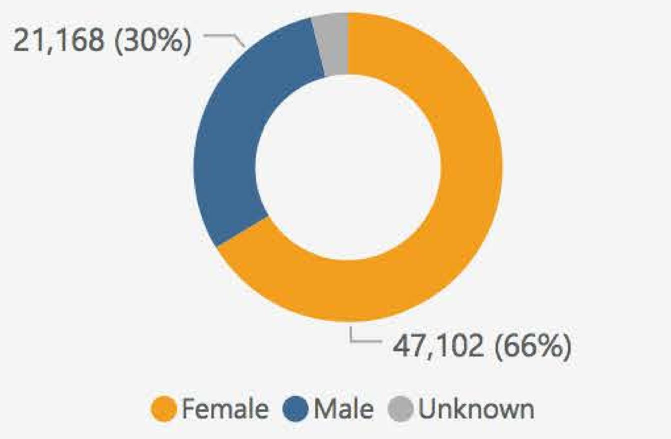

Serving	Vaccine Priority Group Description
1A	Frontline Healthcare Workers; Long-Term Care Facility Healthcare Workers
1B	Long-Term Care Residents
1C	Other healthcare workers, including laboratory and clinic staff; Law Enforcement
1D	Persons Aged 65 Years and Older (Starting with those 80 and over); Persons with 2 or More Underlying Medical Conditions; Residents in Congregate Settings; Teachers and Other School/College Staff; Funeral Service Workers

Number of Persons Vaccinated by Age Group

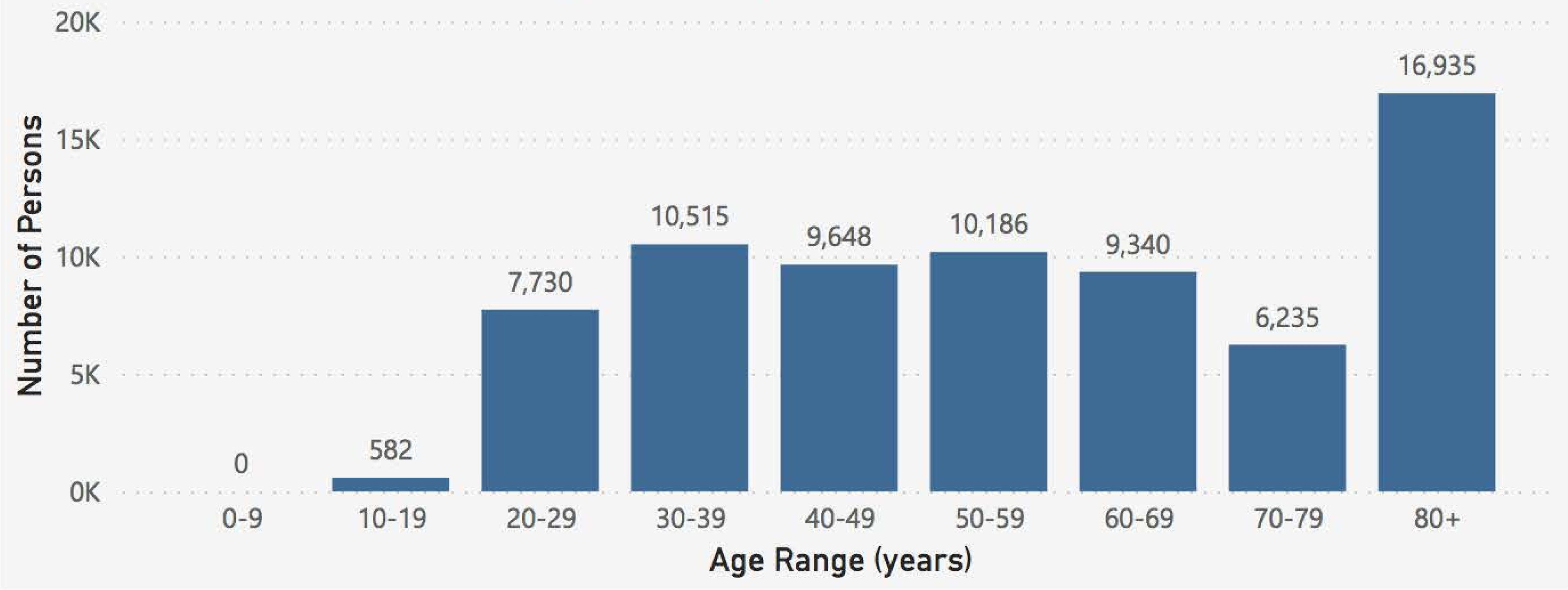

***Note:** Data are provisional and subject to change. Data are updated daily by about 12 p.m. and include doses reported through 10 a.m. the previous day from the South Dakota Immunization Information System (SDIIS), doses administered to IHS or federal entities, such as the Veterans Affairs Medical Centers, are not reported in the SDIIS. For more information on the State Vaccination Plan: visit https://doh.sd.gov/documents/COVID19/SD_COVID-19VaccinationPlan.pdf.*

LAST UPDATED

1/31/2021 10:44:09 AM

Novel Coronavirus (COVID-19) Unified Command Tennessee State Government

1/30/2021

County Snapshots

click county name to toggle map visibility

Shelby
Cases: 207 new, 80,588 total
Deaths: 11 new, 1,248 total
Hospitalizations: -1 new
View Dashboard

Davidson
Cases: 219 new, 74,643 total
Deaths: 2 new, 728 total
Hospitalizations: 2 new
View Dashboard

Knox
Cases: 105 new, 41,204 total
Deaths: 7 new, 487 total
Hospitalizations: 3 new
View Dashboard

Hamilton
Cases: 90 new, 36,908 total
Deaths: 3 new, 361 total
Hospitalizations: 4 new
View Dashboard

Rutherford
Cases: 93 new, 34,951 total
Deaths: 2 new, 314 total
Hospitalizations: 2 new
View Dashboard

Williamson
Cases: 56 new, 22,845 total
Deaths: 0 new, 171 total
Hospitalizations: 0 new
View Dashboard

Sumner
Cases: 54 new, 19,210 total
Deaths: 5 new, 256 total
Hospitalizations: 0 new
View Dashboard

Montgomery
Cases: 39 new, 15,652 total
Deaths: 2 new, 164 total
Hospitalizations: 5 new
View Dashboard

Wilson
Cases: 21 new, 15,153 total
Deaths: 1 new, 179 total

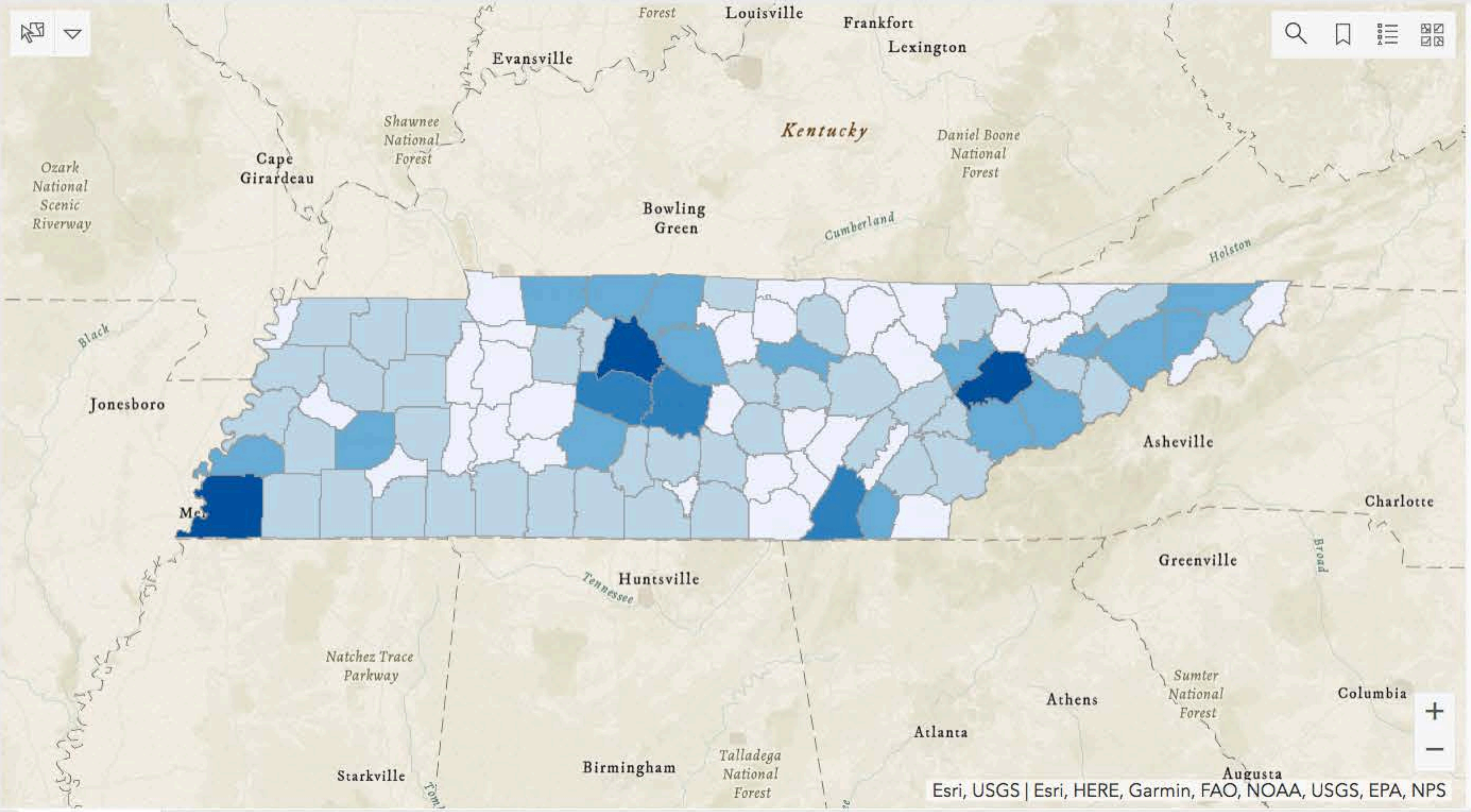

Affected Counties

Total Cases:

- > 40,000 - 85,000
- > 20,000 - 40,000
- > 6,000 - 20,000
- > 2,500 - 6,000
- 0 - 2,500

County Cases | Testing Locations

Statewide: 1/30/2021

724,742 CONFIRMED CASES

9,574 FATALITIES

17,092 HOSPITALIZATIONS

680,847 RECOVERED

Daily: 1/30/2021

2,251 NEW CASES

113 NEW FATALITIES

42 NEW HOSPITALIZATIONS

3,711 NEW RECOVERED

Statewide New Cases In Last 14 Days

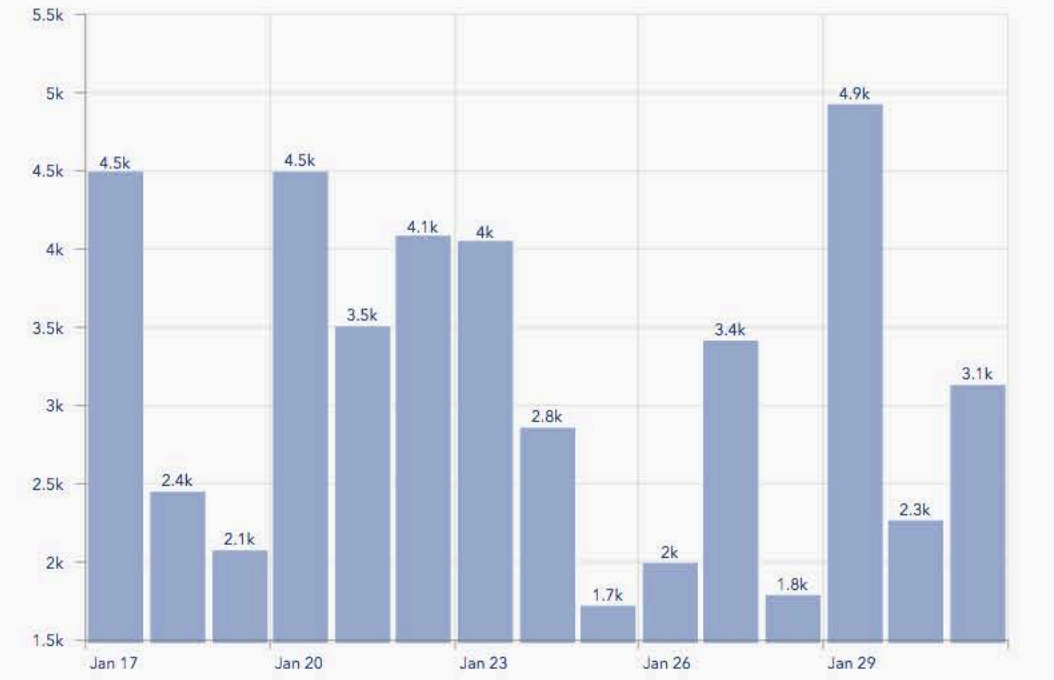

Confirmed Cases By Age

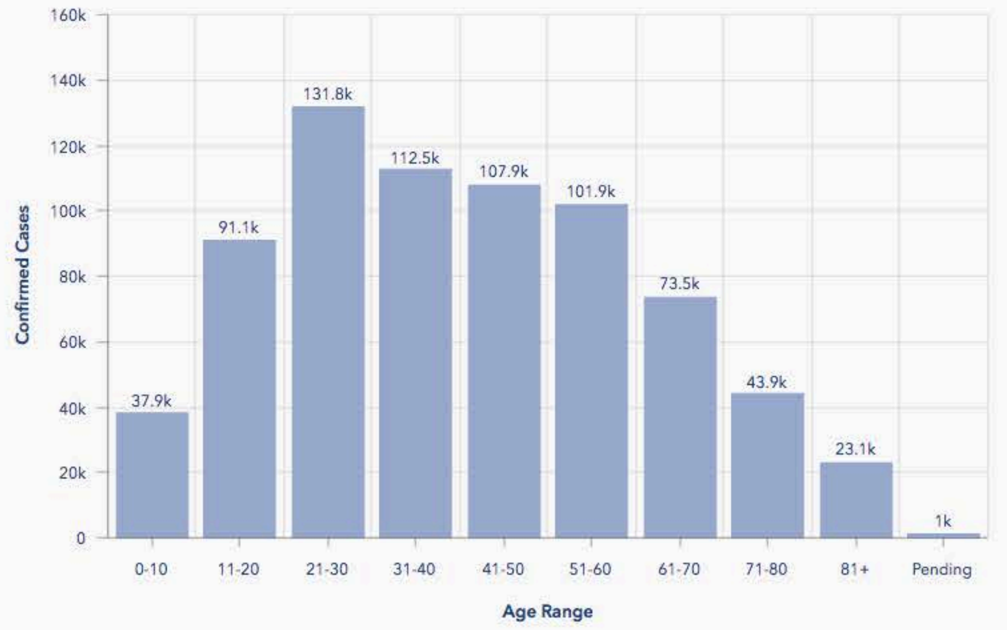

Cases by Age | New Daily Cases by Age

County Data Snapshot

TN Department of Health

COVID-19 County Data Snapshot
January 31, 2021

Please select a county: Anderson

7,239 Cases (1.0% of all cases statewide)	149 Hospitalized (0.9% of all hospitalizations)	128 Deaths (1.3% of deaths statewide)	6,758 Inactive/ Recovered (1.0% of all inactive/recovered)

Number of COVID-19 Cases Reported by Day (with 7d average)

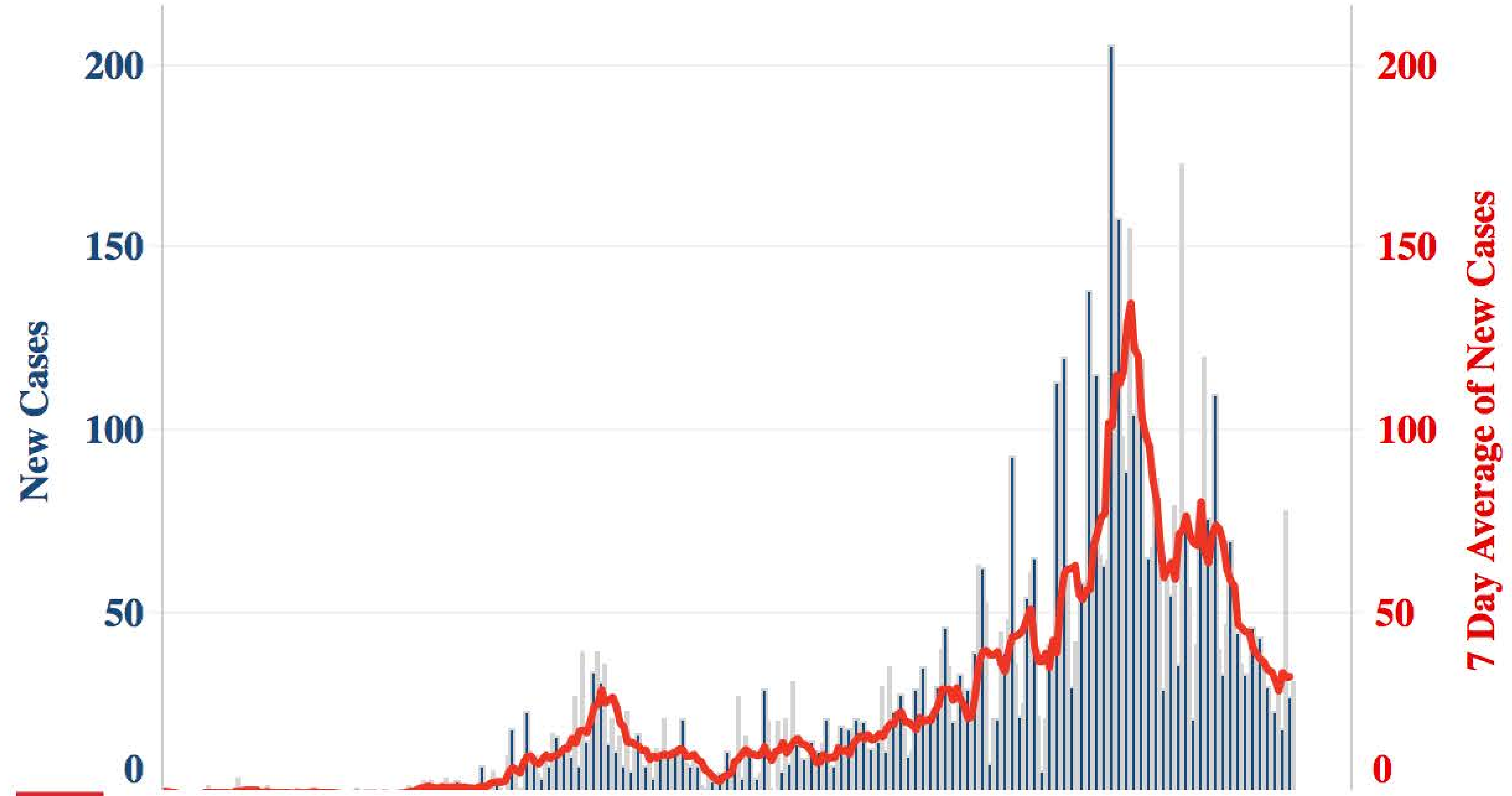

- To date, **Anderson County** has **9,404.0** cases per 100,000 residents.
- Over the last 7 days (1/25-1/31), **Anderson County** had a daily case rate of **41.9** cases per 100,000 residents per day.
- Over the last 14 days (1/18-1/31), **Anderson County** has averaged **34.3** new cases reported per day. For the 14 days prior (1/04-1/17), the average

Number and Percent Positive of Tests Reported Daily (Positive / Negative)

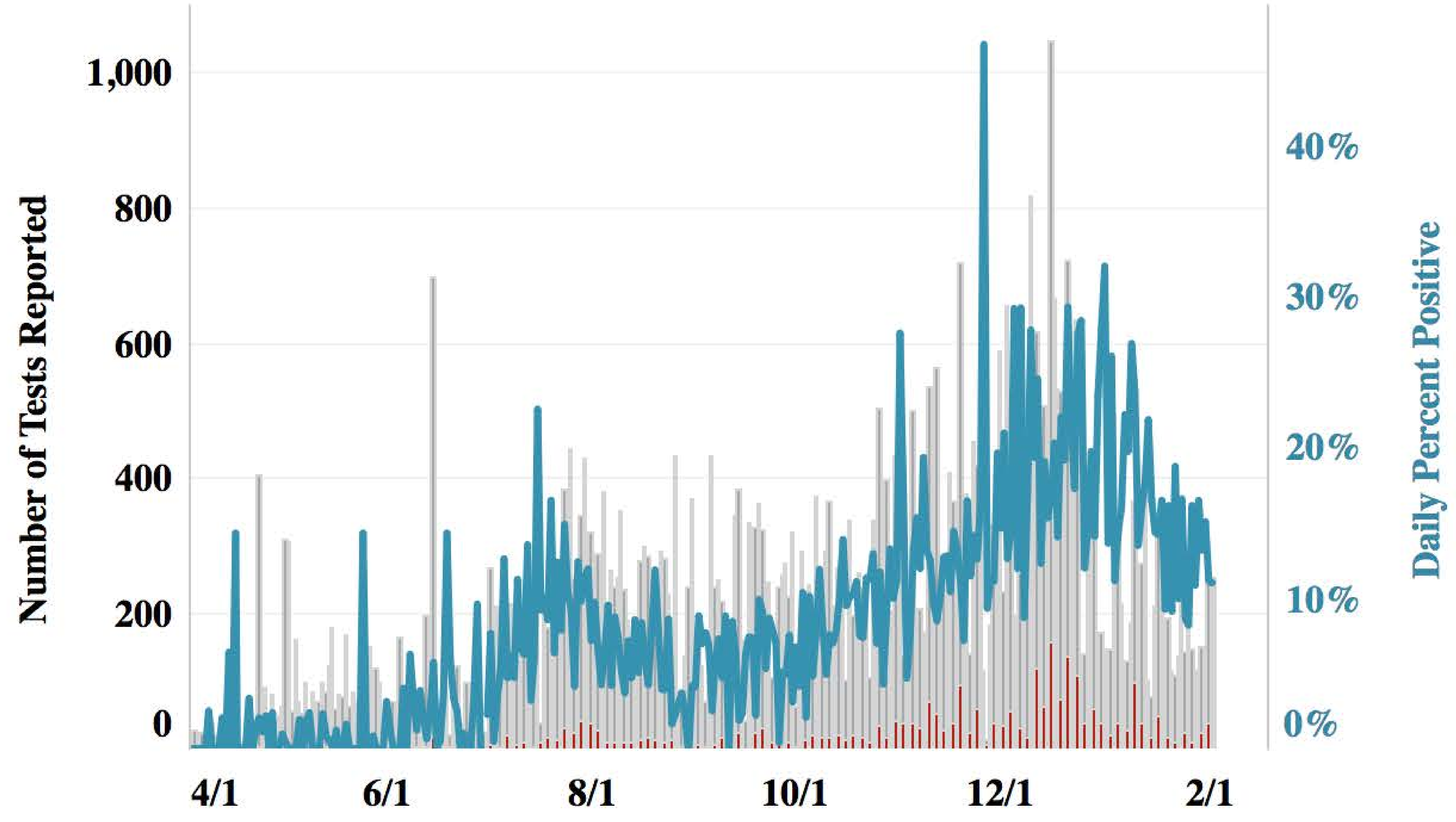

- Over the last 7 days, **Anderson County** has averaged **118.4** tests per day (**15.4** tests per 10,000 residents per day).
- Over the last 7 days, the average percent positive was **14.4%**.

Download a printer-friendly version!

PDF

As of January 24, 2021, Anderson County is in the Red Zone.
See the White House Task Force Map for more information.

New Cases by Region | New Cases by County | New Cases by MMWR Week

TN Department of Health

New Cases by Public Health Report Date, Region

Sunday, January 31, 2021

Select a Region

(All)

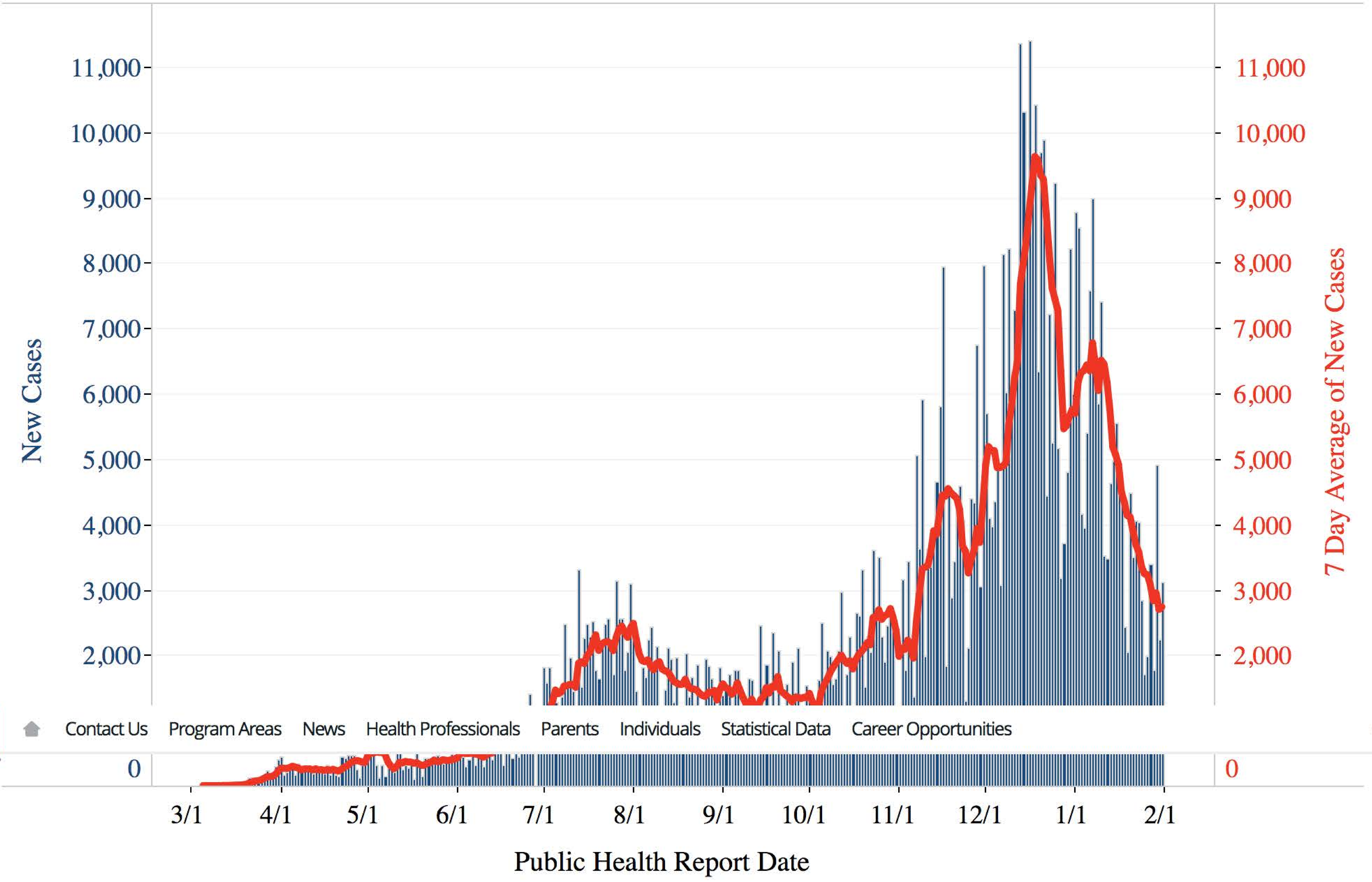

TN Contact Us Program Areas News Health Professionals Parents Individuals Statistical Data Career Opportunities

The Morbidity and Mortality Weekly Report (MMWR) week is the number of weeks since January 1, and is commonly used for public health reporting by states and CDC.

Category Filter

Maps Tab

- (selected) Confirmed Cases
- Probable Cases
- Fatalities
- Active Cases (Estimated)
- Recovered (Estimated)

Demographics Tab

- (selected) Confirmed Cases
- Fatalities

On Dec. 11, probable case counts were added, and the dashboard was reconfigured to improve performance. For more information about probable cases, watch this video from Chief State Epidemiologist Jennifer Shuford, MD.

TEXAS Health and Human Services

Texas Department of State Health Services

Cameron County confirmed cases were overreported by 120 on 1/30. The county and statewide case totals for 1/30 have been corrected.
In addition to 9,903 new confirmed cases, DSHS is also reporting 305 older confirmed cases reported by labs: Austin

New Confirmed Cases

9,903

New Probable Cases

1,252

Newly Reported Fatalities

171

as of 1/31/2021

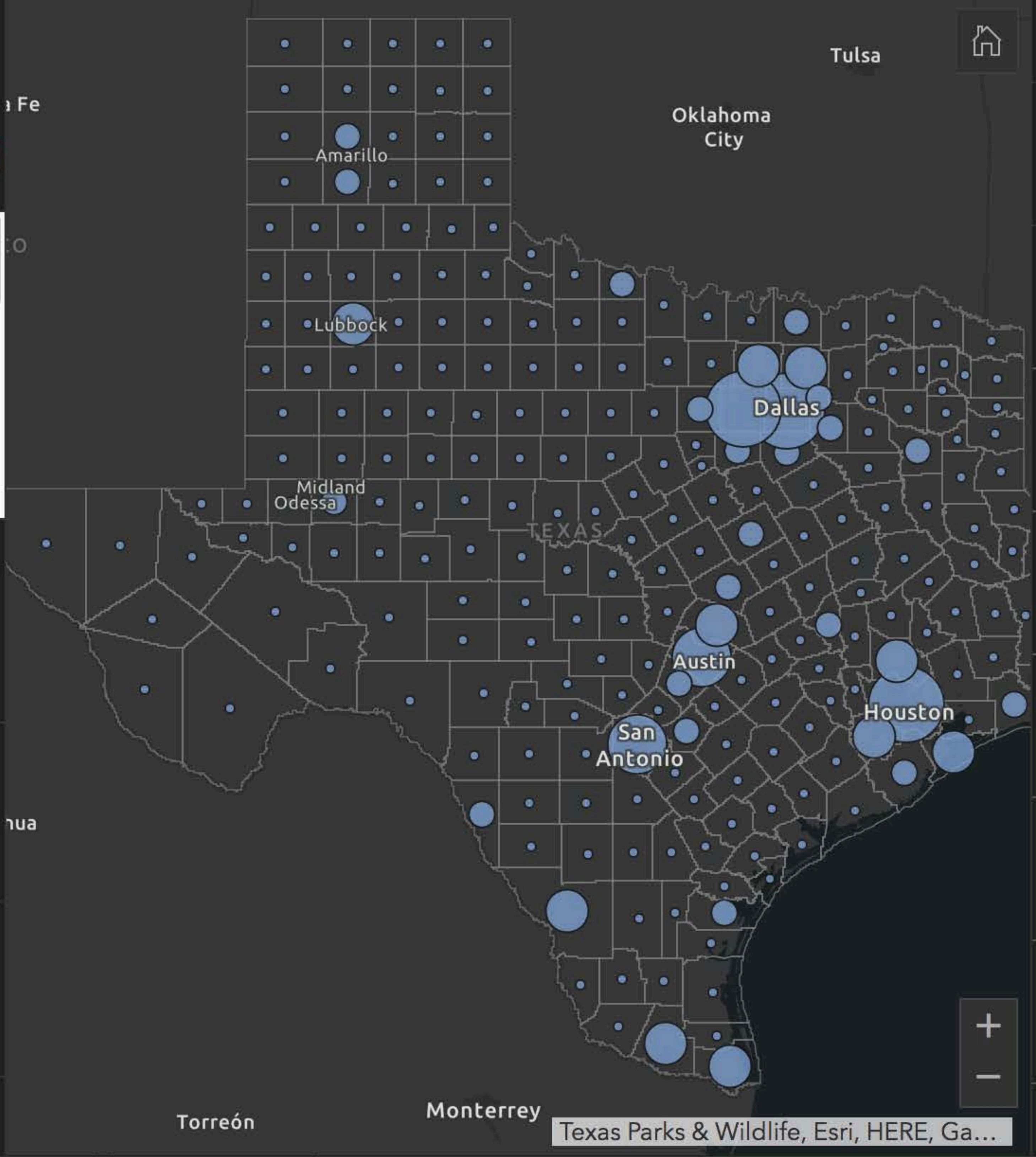

Confirmed

2,059,143

Harris County
Confirmed Cases
314,697

Dallas County
Confirmed Cases
226,452

Tarrant County
Confirmed Cases
187,038

Bexar County
Confirmed Cases
142,444

El Paso County
Confirmed Cases
113,637

Travis County
Confirmed Cases
68,290

Collin County

Maps | Trends | Demographics | About the Data

Disclaimer: All data are provisional and subject to change.
Fatality data come from death certificates and may include both confirmed and probable cases.
This dashboard will be updated daily by 4:00PM.

COVID-19 Test and Hospital Data

Last Updated
1/31/2021 1:05PM

Microsoft office 365
http://office365.uky.edu/

Cate[illegible]

Maps Tab

- Confirmed Cases
- Probable Cases
- Fatalities
- Active Cases (Estimated)
- Recovered (Estimated)

Demographics Tab

- Confirmed Cases
- Fatalities

New Confirmed Cases

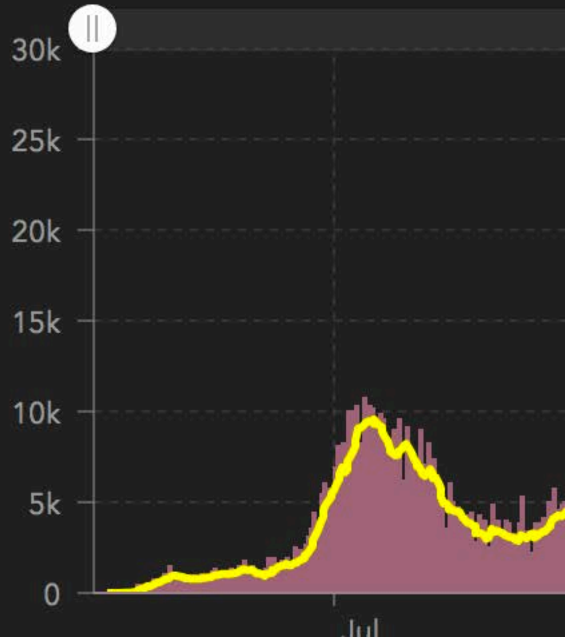

st Popular
Houston 'first daughter' Ashley Turner is engaged
2. Texas expecting more than half a million COVID-19 vaccine doses this week
3. Essay: Is Lina Hidalgo too principled for politics?
4. The athlete (Deshaun Watson) sends another message
5. New Caney wom to assaulting 13-y police say

CORONAVIRUS

Tracking coronavirus: Interactive maps, charts show spread of COVID-19 across Houston, rest of Texas

Jordan Ray-Hart, Jordan Rubio, Matt Dempsey, Stephanie Lamm

Jan. 25, 2021 | Updated: Jan. 29, 2021 12:22 p.m.

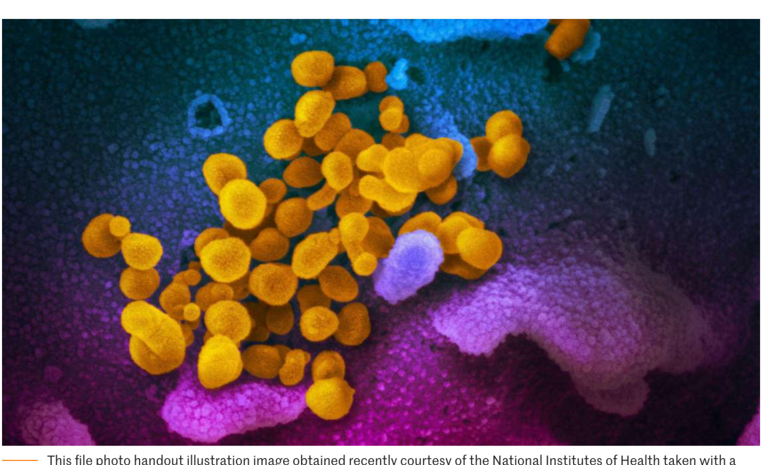

This file photo handout illustration image obtained recently courtesy of the National Institutes of Health taken with a scanning electron microscope shows the virus that causes the disease COVID-19 isolated from a patient in the U.S., emerging from the surface of cells (blue/pink) cultured in the lab.
HANDOUT / National Institutes of Health / AFP via Getty Images

To keep you up-to-date with the ever-growing number of COVID-19 cases in Houston, Texas and the rest of the world, the Houston Chronicle has come up with a few eye-catching, but easy-to-use interactives, based on our reporting and official data from the Texas Department of State Health Services.

CORONAVIRUS UPDATES: Stay informed with accurate reporting you can trust

Related COVID-19 Stories

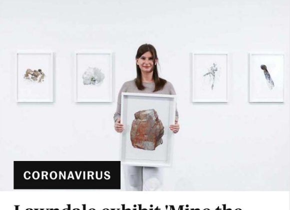

CORONAVIRUS
Lawndale exhibit 'Mine the Gap' emerges from pandemic
By Andrew Dansby

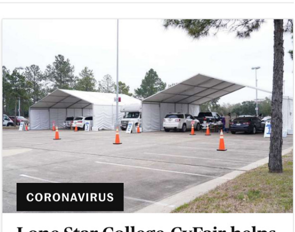

CORONAVIRUS
Lone Star College-CyFair helps host vaccine delivery site...
By David Taylor

With the U.S. Food and Drug Administration approving vaccines for COVID-19, you may find these charts on vaccines being distributed in Texas useful. First off, here's a chart showing how many vaccine doses have been distributed across Texas and how many have been administered.

COVID-19 vaccine distribution in Texas

This shows how many doses of the COVID-19 vaccine have been distributed and administered to people.

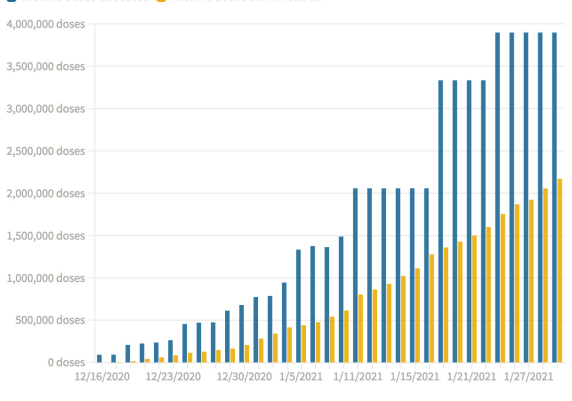

Source: Department of State Health Services • Created by Jordan Rubio

A Flourish chart

The next chart shows how many people have received at least one dose of a vaccine and how many have been fully vaccinated. It's important to remember that some vaccines, like the Pfizer one, require two doses to be effective.

Number of people who've received the COVID-19 vaccine

This shows how many people have gotten at least one dose of the COVID-19 vaccine as well as how many people are fully vaccinated against the virus. For some vaccines, like Pfizer, people need two doses for it to work.

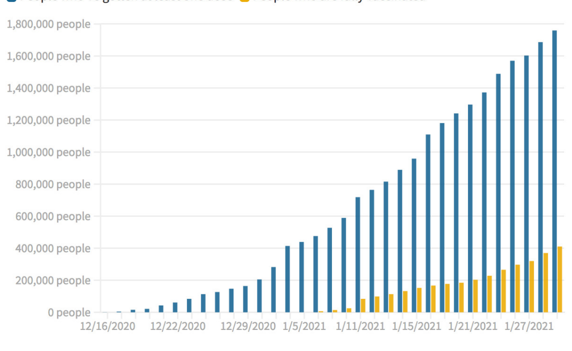

Source: Department of State Health Services • Created by Jordan Rubio

A Flourish chart

If you're mostly concerned with the spread of coronavirus specifically in Texas, you may find these next few charts helpful. First, let's take a look at the positive test rate for the state.

Positive test rate

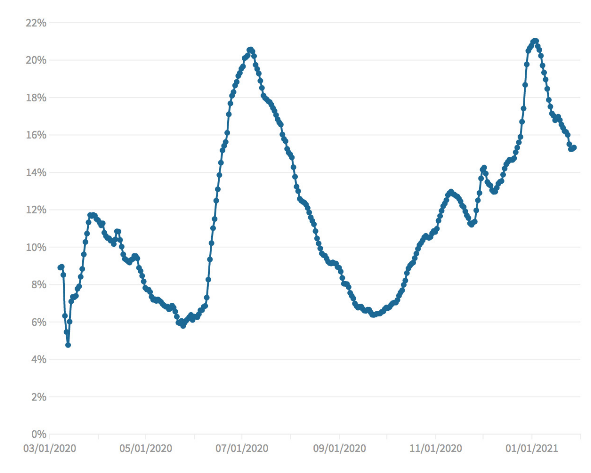

Starting May 19, antibody tests are excluded from the denominator

A Flourish chart

Next, the seven-day rolling average of new cases around the Lone Star State.

7-day average of daily new cases

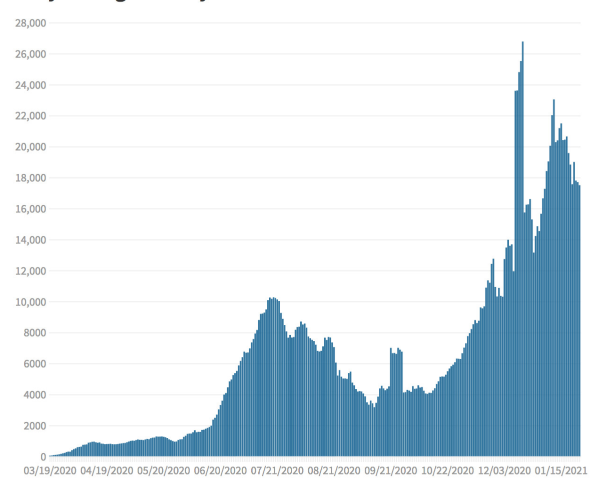

A Flourish chart

For an overview of the Lone Star State, we've got the Texas Coronavirus Map, one of the only comprehensive case trackers for the region. The map includes the number of confirmed cases, deaths and recoveries.

2,335,136 confirmed coronavirus cases, 36,002 deaths in Texas.

We are tracking cases of coronavirus disease using data collected from the Centers for Disease Control and Prevention and state and local public health departments and our own reporting.

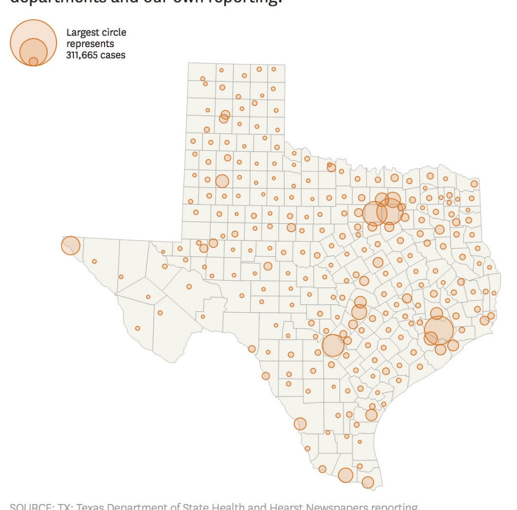

SOURCE: TX: Texas Department of State Health and Hearst Newspapers reporting

You can also get an in-depth look at the cumulative number of deaths in Texas by date below.

Deaths due to the coronavirus in Texas by date

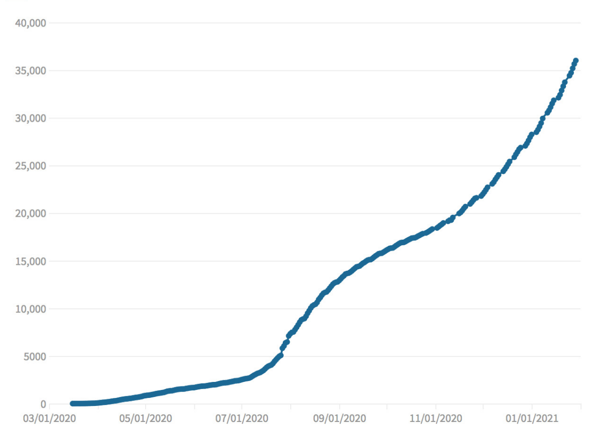

Source: Texas Department of State Health Services, Hearst Newspaper reporting

A Flourish chart

This GIF shows the spread of confirmed and probable COVID cases through Texas.

Confirmed and probable cases per 10,000 people

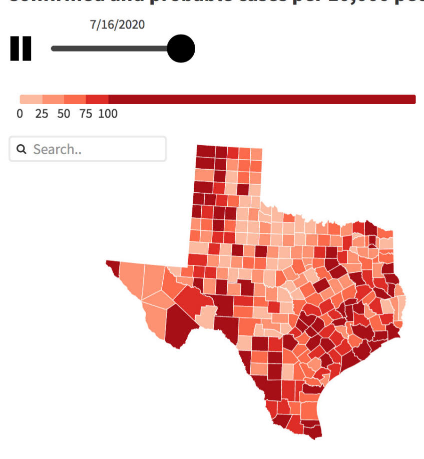

Source: Texas Department of State Health and Hearst Newspapers reporting • By Ryan Serpico

A Flourish data visualization

Pivoting to Houston-area hospitals, you can see the number of confirmed or suspected COVID-19 patients below.

Confirmed or suspected COVID patients in Houston region hospitals

Daily COVID-related patient admissions in Houston-area hospitals

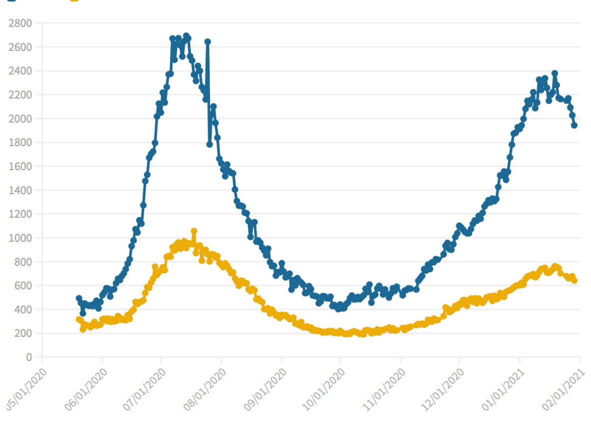

A Flourish chart

LATEST LOCAL NE
Driver arrested in injured 8
Suspect in Cypre early Sunday
Erratic driver cau with HPD officers
Push to reopen sc millions of studen
'Something really might help catch

The next two charts highlight deaths in the Houston region. The first shows the number of cumulative deaths by date.

Confirmed coronavirus deaths in the Houston region by date

These are all the known deaths in Harris, Fort Bend, Montgomery, Waller, Liberty, Brazoria, Galveston and Chambers counties since April 1.

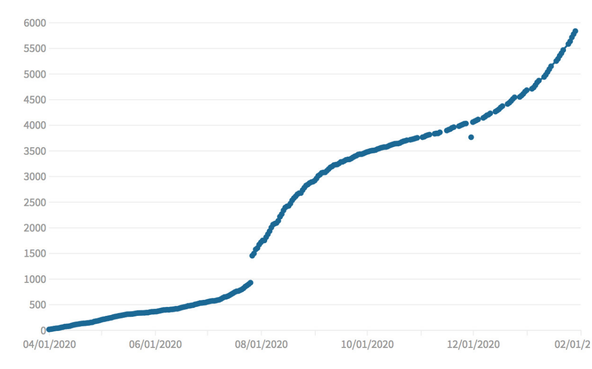

Source: Texas Department of State Health Services, Hearst Newspaper reporting • On June 9, Brazoria removed TCDJ cases and deaths from their reports, which removed 2 deaths and 220 cases from the region total.

A Flourish chart

The second shows the number of new deaths in the region by date.

New coronavirus deaths in the Houston region by date

These are the new deaths, by date, for the eight counties that comprise the Houston region.

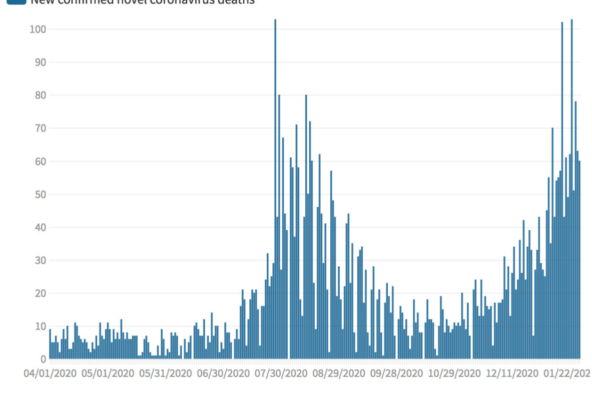

Source: Houston Chronicle reporting, county heath authorities • Created by Jordan Rubio

A Flourish chart

Finally, we've got a breakdown of national coronavirus cases in the style of a U.S. map, helping to show how the numbers in Texas compare to the rest of the country.

26,174,956 confirmed coronavirus cases that we know of, 441,200 deaths

U.S. reports of COVID-19 are tabulated by Johns Hopkins University, which is tracking cases using data collected from the Centers for Disease Control and Prevention and state and [illegible]

HOUSTON CHRONICLE

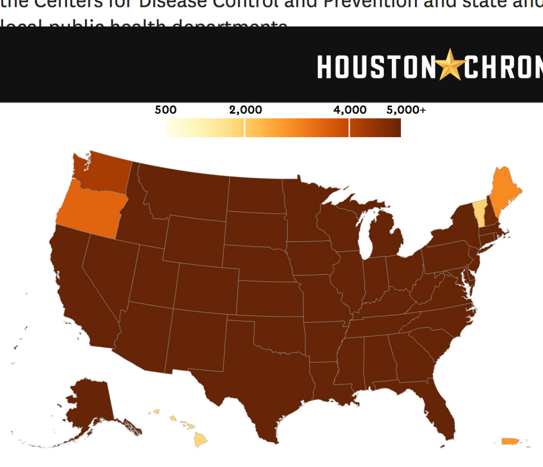

* 152 cases without available location data not mapped. Figures include presumed COVID-19 deaths.

Sources: The Center for Systems Science and Engineering at Johns Hopkins University, U.S. Census Bureau

Overview of COVID-19 Surveillance

This report is updated daily at approximately 1:00 p.m. Information on COVID-19 cases changes rapidly, and this report may not reflect updates made after 1:00 p.m. by local health departments or health care systems. Most of the tables, charts, and maps in this report are interactive. Tables can be sorted by clicking on column headings. Maps and charts can be sorted, zoomed, selected, etc. using the mouse cursor and data will appear when hovering or clicking the mouse cursor. Controls will appear at the top right corner of charts when the mouse cursor is placed on the chart. For response goals and operating metrics, please visit the Unified Response Scoreboard. The data used to create the charts on this dashboard are also available for download.

Report Date: January 31, 2021

346,624 COVID-19 Cases | **2,024,647** People Tested | **3,339,677** Tests Reported | **13,468** COVID-19 Hospitalizations | **1,665** COVID-19 Deaths | **310,692** Total Vaccines Administered

Total Number of Lab-Confirmed COVID-19 Cases Living in Utah

Jurisdiction	Cases	Hospitalizations	Deaths
Bear River	18580	697	60
Central Utah	7735	298	47
Davis County	32045	1058	120
San Juan	1756	157	36
Salt Lake County	130042	6011	679
Southeast Utah	2582	79	17
Southwest Utah	24720	1207	204
Summit County	4600	140	7
Tooele County	5920	174	23
TriCounty	3185	194	27
Utah County	84439	2236	286
Wasatch County	4074	92	16
Weber-Morgan	26137	1108	143
Under Investigation	809	17	0
State Total	346624	13468	1665

COVID-19 14-Day Case Rate per 100,000 Population by County

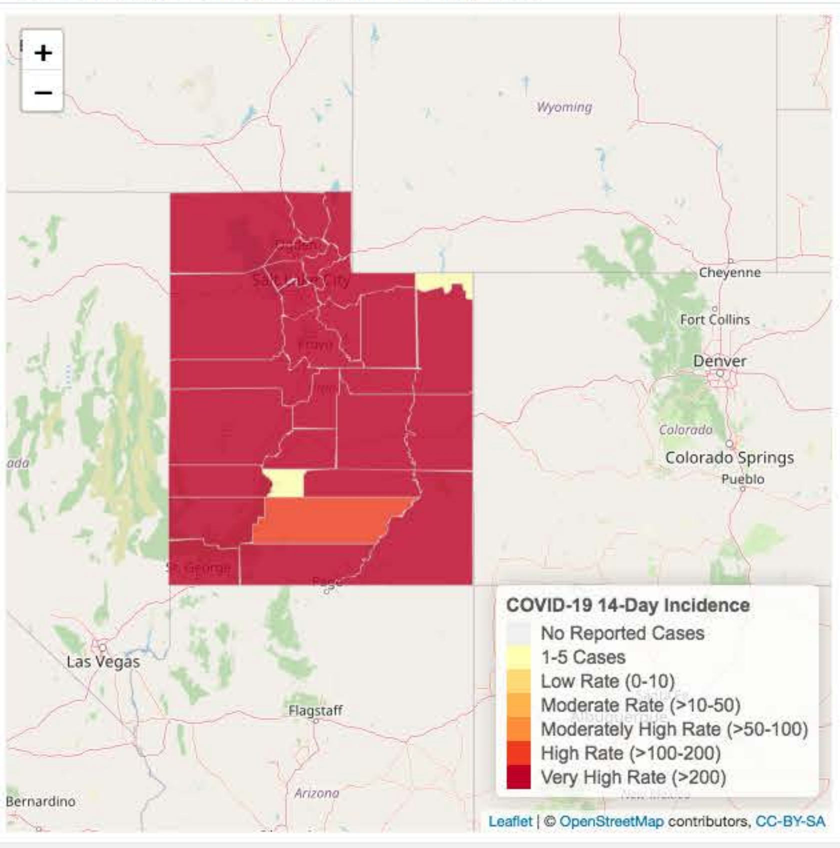

Cases Reported by Indian Health Service and Tribal Nations (these cases are also reflected in case counts by health district)

Reporting Jurisdiction	Cases
Navajo Nation Reservation (Utah) - Utah Navajo Health System	1039
Uintah and Ouray Reservation: Uintah & Ouray Indian Health Services (IHS)	319
State Total	346624

COVID-19 Cases by Test Report Date | COVID-19 Hospitalizations by Admission Date | COVID-19 Deaths by Date of Death | COVID-19 Cases by Symptom Onset or Diagnosis Date

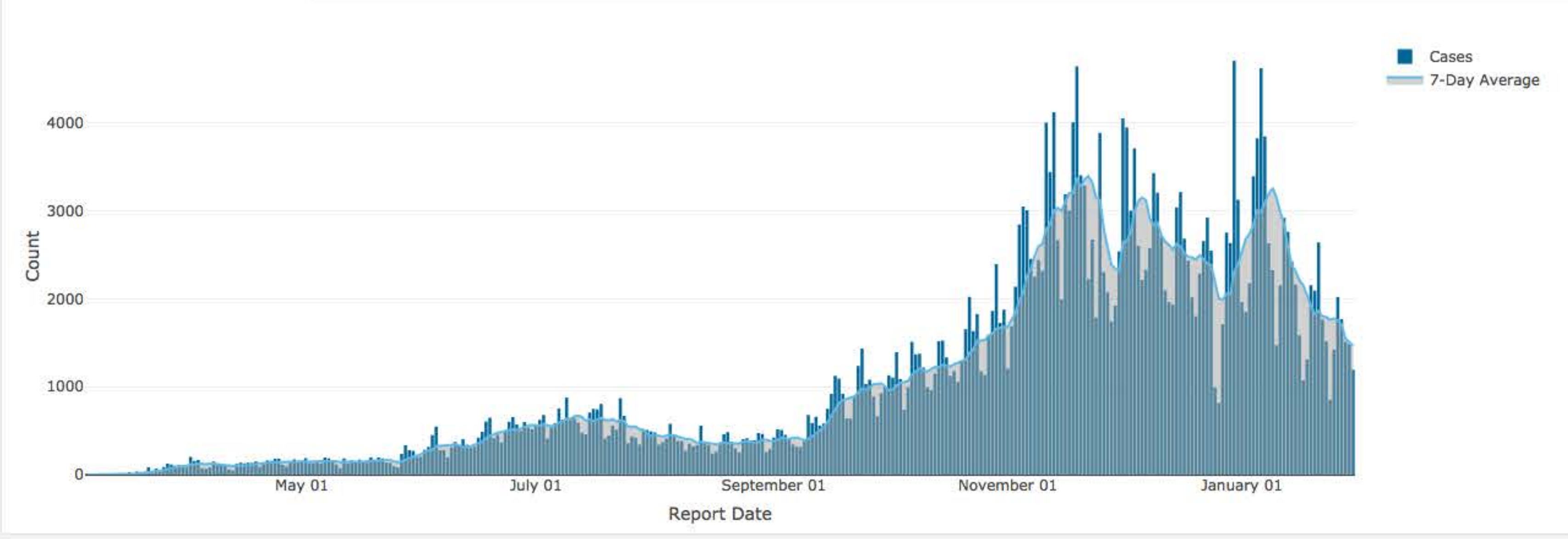

Total People Tested: Seven-Day Rolling Average Percent Positive Rates by Specimen Collection Date

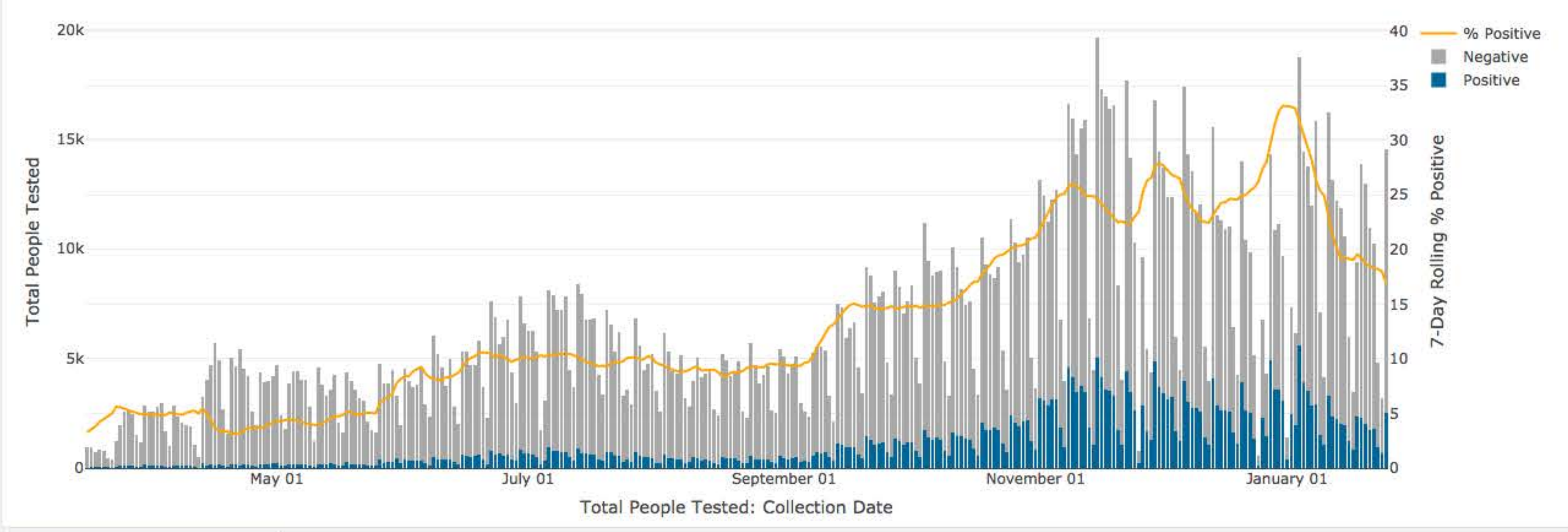

Number of People Tested by Date | Total Tests by Date

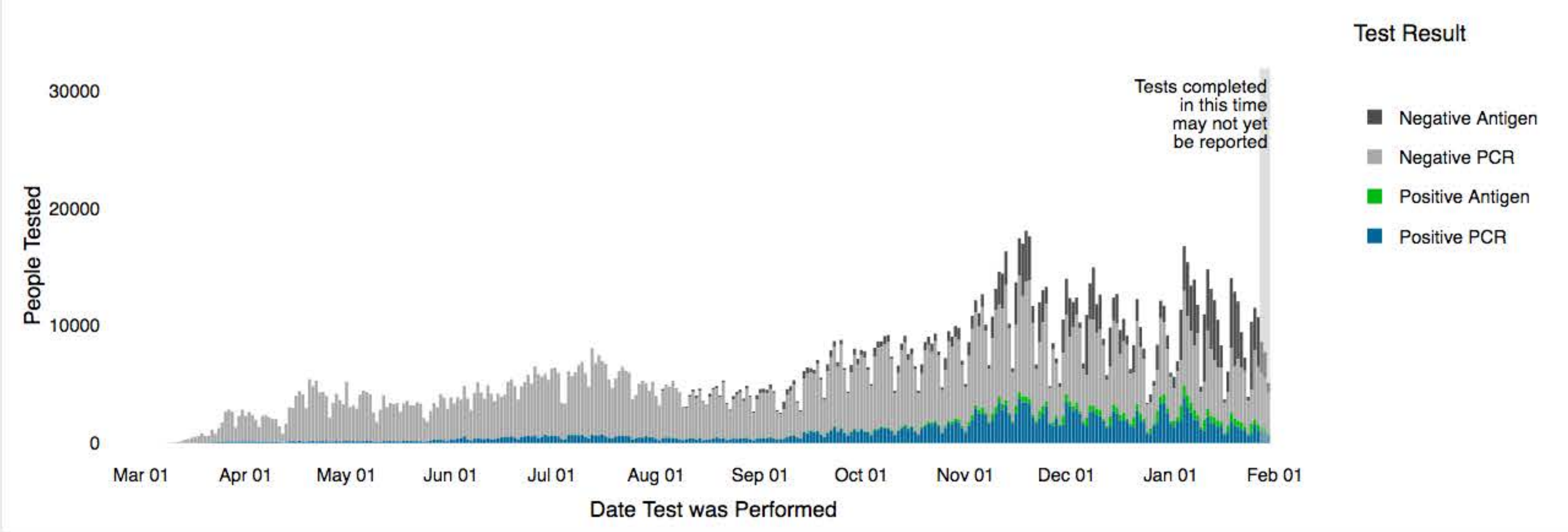

Cumulative COVID-19 Cases with Estimated Recoveries * (does not include the current day)

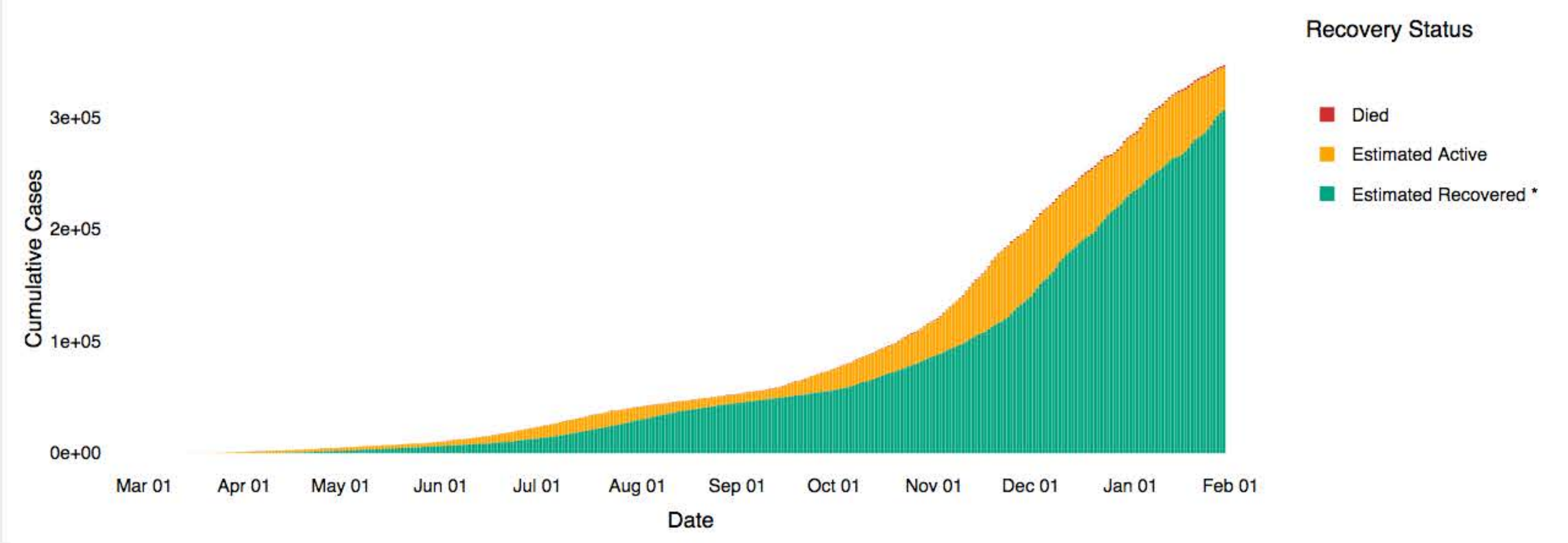

Small Area Case Rates

The below map shows the crude case rate per 100,000 people per Utah Small Area. Crude case rates show the number of COVID-19 cases in an area given its population. Case rates may be higher or lower depending on the age of people living in a small area and how much testing has occurred there. Small areas with low populations may have disproportionately high or low rates depending on few cases and the number of tests performed in an area. A high rate in an area does not necessarily indicate a current outbreak because rates are calculated from the beginning of the pandemic. "Utah Small Areas" are geographic areas with population sizes ranging from about 8,000 to 86,000. Small areas were created by UDOH and local health departments to assess health data and policies. More information on Utah Small Areas can be found at: ibis.health.utah.gov.

Note: Case counts by small area will not sum to the total statewide count due to missing address information for some cases.

Crude Case Rate Per 100,000 People in Utah by Small Area

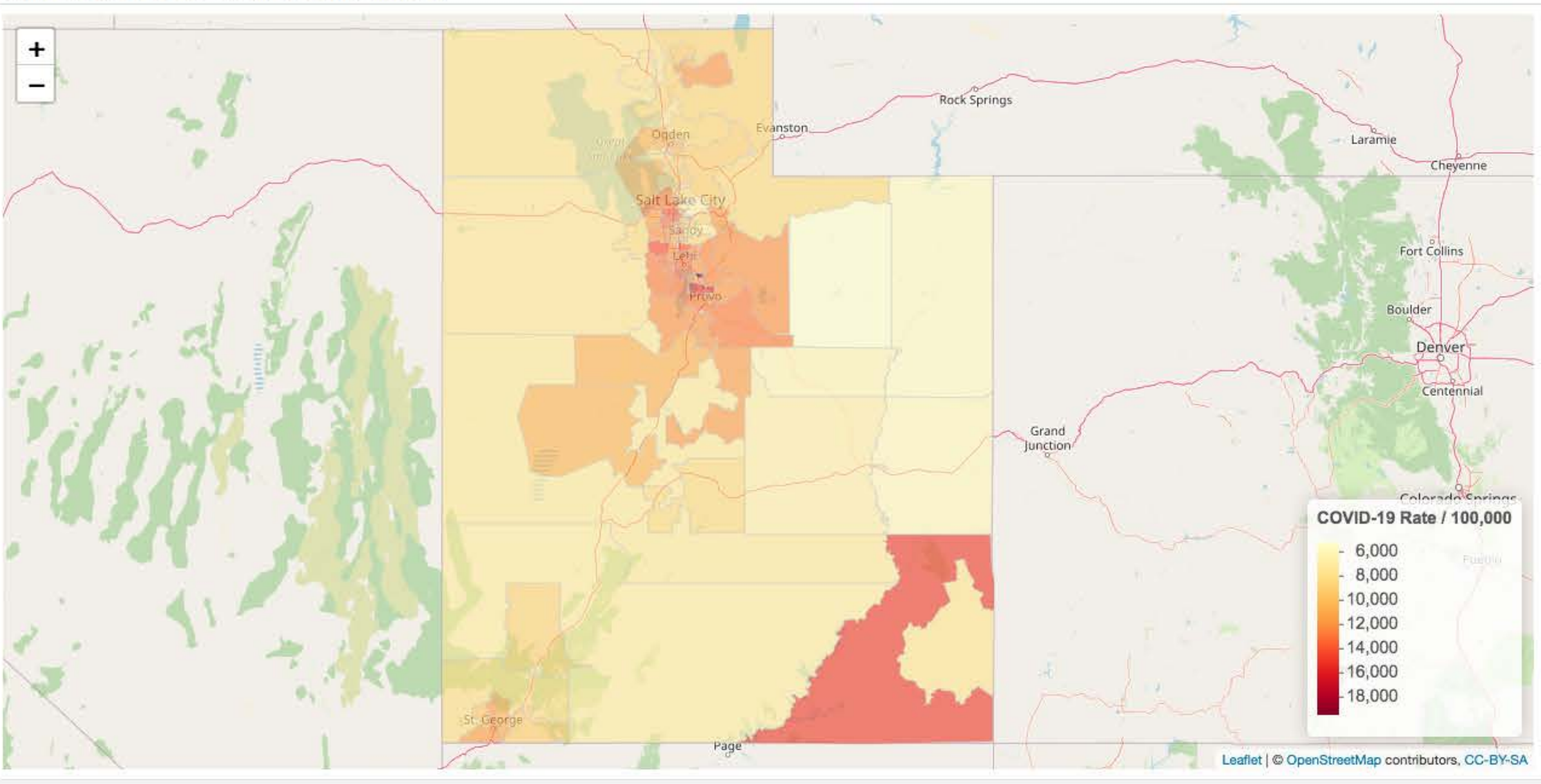

Cases by Local Health Department

Cases counts and total case rates by Local Health Department (LHD) are shown below. Utah's LHDs may also report their own case data. Local reports should be considered the most up to date and accurate information for their local area due to small differences in case classifications at any given time. The "Cumulative Case Rate per 100,000 population by LHD" shows the total number of laboratory-confirmed COVID-19 cases in an area given its population size since the start of the pandemic.

COVID-19 Cases by the Date a Positive Test was Reported to Public Health by LHD | Cumulative Case Rate per 100,000 Population by LHD

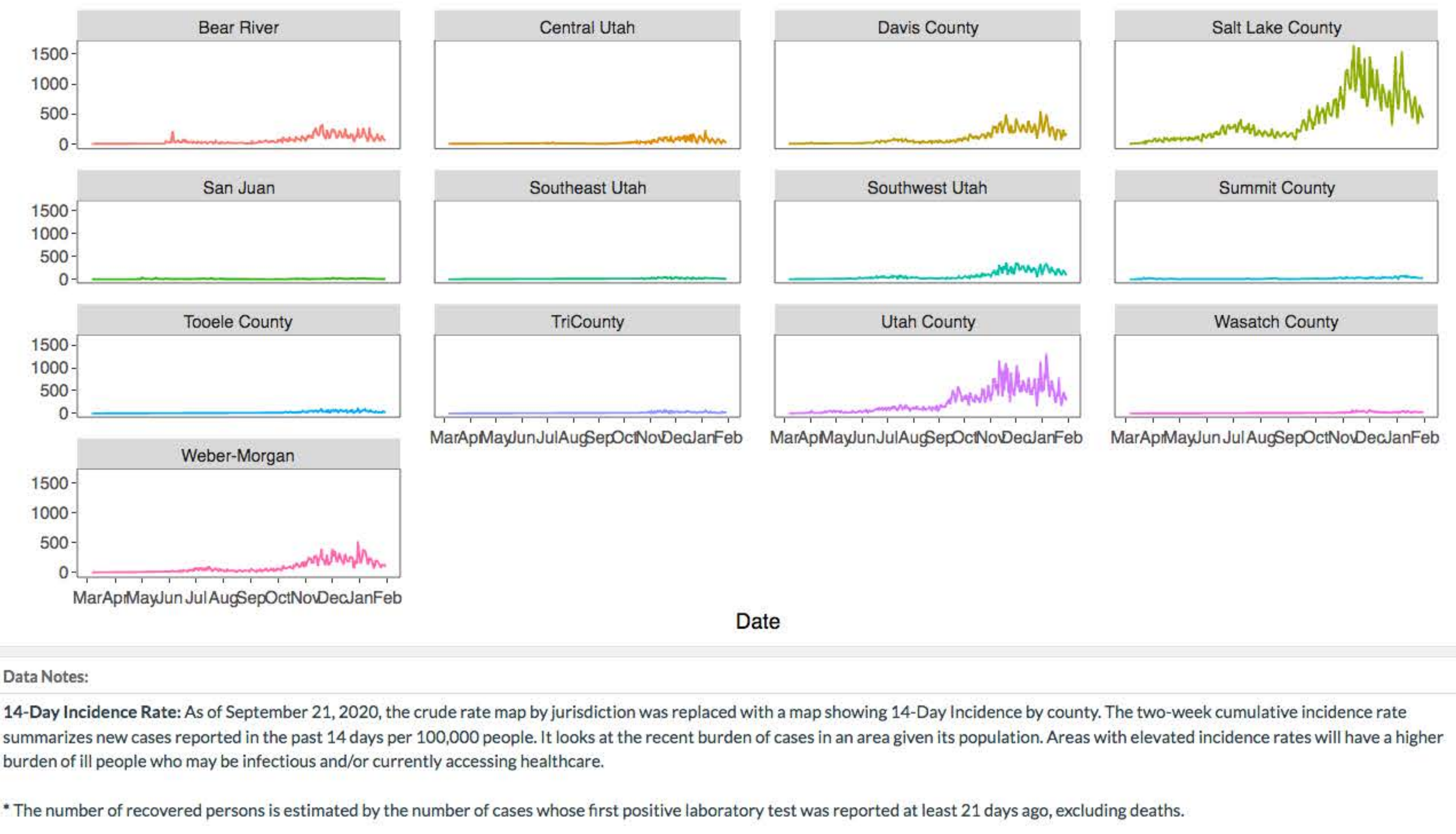

Data Notes:

14-Day Incidence Rate: As of September 21, 2020, the crude rate map by jurisdiction was replaced with a map showing 14-Day Incidence by county. The two-week cumulative incidence rate summarizes new cases reported in the past 14 days per 100,000 people. It looks at the recent burden of cases in an area given its population. Areas with elevated incidence rates will have a higher burden of ill people who may be infectious and/or currently accessing healthcare.

* The number of recovered persons is estimated by the number of cases whose first positive laboratory test was reported at least 21 days ago, excluding deaths.

Laboratory: The Utah Department of Health (UDOH) is currently reporting PCR and antigen test results and will begin reporting serology results in the coming weeks. Positive test results are reported immediately; negative test results may not be reported for 24-72 hours. Results prior to March 19, 2020 may be under-reported. Laboratory positives may not match confirmed cases due to ongoing investigations and confirmatory testing. When available, laboratory data is shown by the date a sample was tested. If unavailable, it is shown by the date reported to public health. Laboratory data are shown as both the total number of people tested, and as total number of tests performed. For the total number of people tested, one test per person is included by their earliest positive result. If there are no positive tests, their earliest negative result is used.

Case Dates: As of April 15, 2020, case data are displayed by both the date the first positive laboratory result (FPLR) is reported to public health and the self-reported symptom onset date. Previously, this was reported by the first day a person was reported (through laboratory results or contact tracing) to public health. The FPLR date will provide a more stable estimate of the new cases reported to public health; however, there will be small differences in cases by date as public health receives additional information. Onset date is taken preferentially from the following dates when available: 1) self-reported date of first symptoms, 2) date diagnosed by a clinician, 3) date first positive specimen was collected, 4) first report to public health. The actual number of cases in Utah is higher than what is reported due to mild cases not feeling ill enough to seek care, clinician judgment for testing, and expanding laboratory capacity. For more information on how COVID-19 deaths are registered in the state of Utah, visit coronavirus.utah.gov/COVID-19-deaths.

Death Counts: Deaths reported by UDOH include confirmed and probable cases as defined by the Council of State and Territorial Epidemiologists (CSTE) case definition. This includes: 1) confirmed cases with a positive COVID-19 PCR result and no alternative cause of death noted on the death certificate or reported by the Office of the Medical Examiner (OME), 2) probable cases where the death certificate lists COVID-19 disease or SARS-CoV-2 as a cause of death or a significant condition contributing to death and no alternative cause of death reported by the OME, and 3) probable cases with COVID-19 symptoms and close contact to a laboratory confirmed case and no alternative cause of death reported by the OME or the death certificate. Death counts are provisional and subject to change as investigations are completed.

Test Types

PCR Tests: COVID-19 PCR tests detect viral genetic material and are used to diagnose active infections.

Antigen Tests: COVID-19 antigen tests are rapid diagnostic tests that detect specific fragments of the virus. Like PCR tests, they are very specific to the SARS-CoV2 virus and detect active infections. Antigen tests can often be performed at point-of-care facilities. While all test results must be reported, as of July 10, 2020 some facilities had not yet fully implemented reporting for all negative antigen tests.

Antibody Tests: COVID-19 antibody tests, also called serological tests, detect the presence of antibodies to the virus in blood samples. They are typically used to identify people with prior infections. There is a lag between infection and antibody production by the immune system, so antibody tests usually cannot be used to detect active infections.

Case Definitions: UDOH assigns case status following the national case definition, with the exception of considering antigen positive tests as evidence of a confirmed case. A confirmed case is any person with a positive SARS-CoV2 PCR or antigen test.

Percent Positivity Calculation: CDC proposes three methods for calculating percent positivity. The UDOH uses the third method of number of people tested positive over the number of people tested. For a person who has taken more than one test, their first positive result is used and any future positive or negative results are not. If there is no positive result for that individual, then their first negative result is used. If a person is tested again and it has been at least 90 days after their last test, the UDOH will create a new record for the person and their most recent test result will also be included in the percent positivity calculations. If a person has two positive tests with at least 90 days between tests they will be investigated as a possible reinfection.

Data for this report were accessed on January 31, 2021 07:16 AM. Population data used for calculating rates are based on 2018 estimates from IBIS.

COVID-19 in Virginia: Cases

VIRGINIA'S HEALTH IS IN OUR HANDS. Do your part, stop the spread.

VDH VIRGINIA DEPARTMENT OF HEALTH

Dashboard Updated: 1/31/2021
Data entered by 5:00 PM the prior day.

Total Cases*		Total Hospitalizations**		Total Deaths	
504,779		21,409		6,464	
Confirmed†	Probable†	Confirmed†	Probable†	Confirmed†	Probable†
404,469	100,310	20,474	935	5,657	807

Select Measure
(Affects Map and Bar Chart)
- ◉ Cases
- ○ Hospitalizations
- ○ Deaths

Counts of Cases
- 48 - 142
- 143 - 427
- 428 - 1276
- 1277 - 3816
- 3817 - 11406
- 11407+

Select Counts or Rates
(Affects Map)
- ◉ Counts
- ○ Rates per 100,000

Rates that are based on counts less than 15 should be interpreted with caution

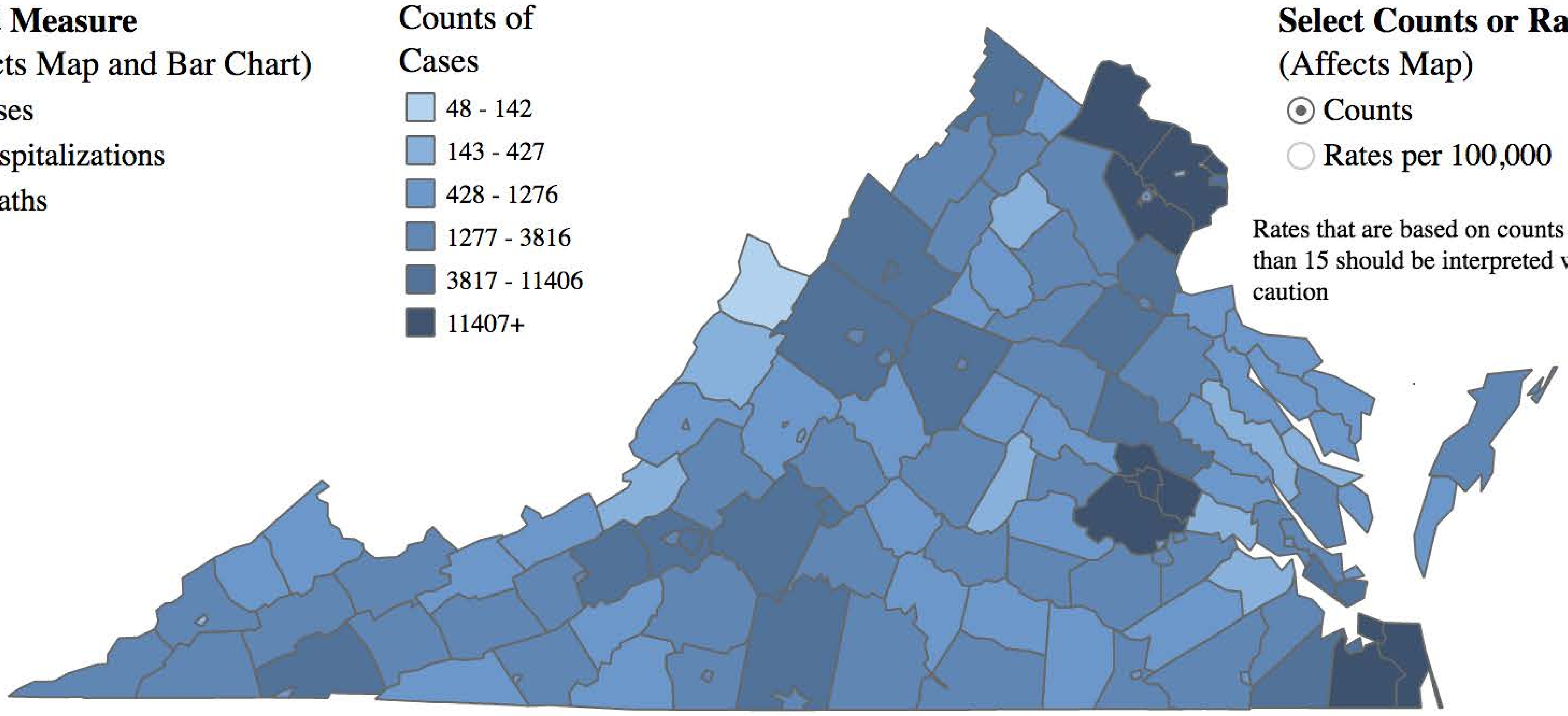

Number of Cases by Date of Symptom Onset

Number of cases by the day closest to when symptoms began.

Select Region
(Affects Bar Chart)
(All) ▾

Illness may not have been reported yet.

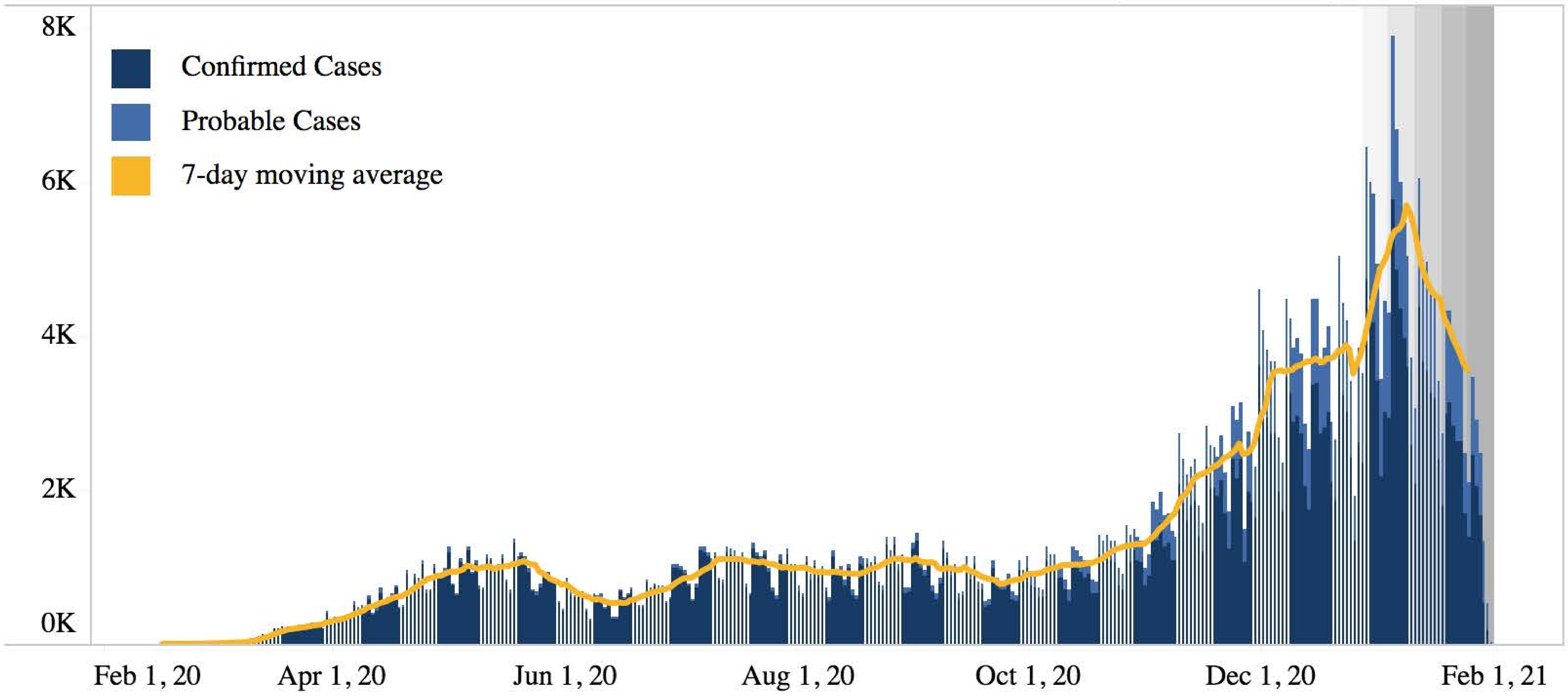

* Includes both people with a positive test (Confirmed), and symptomatic with a known exposure to COVID-19 (Probable).
** Hospitalization of a case is captured at the time VDH performs case investigation. This underrepresents the total number of hospitalizations in Virginia
† VDH adopted the updated CDC COVID-19 confirmed and probable surveillance case definitions on August 27, 2020. Found here: https://wwwn.cdc.gov/nndss/conditions/coronavirus-disease-2019-covid-19/case-definition/2020/08/05/
Source: Cases - Virginia Electronic Disease Surveillance System (VEDSS), data entered by 5:00 PM the prior day.

COVID-19 in Virginia:

Dashboard Updated: 1/31/2021

Data entered by 5:00 PM the prior day.

Cases, Hospitalizations and Deaths

Total Cases*		Total Hospitalizations**		Total Deaths	
504,779 (New Cases: 2,558)^		21,409		6,464	
Confirmed†	Probable†	Confirmed†	Probable†	Confirmed†	Probable†
404,469	100,310	20,474	935	5,657	807

* Includes both people with a positive test (Confirmed), and symptomatic with a known exposure to COVID-19 (Probable).
** Hospitalization of a case is captured at the time VDH performs case investigation. This underrepresents the total number of hospitalizations in Virginia.
^New cases represent the number of confirmed and probable cases reported to VDH in the past 24 hours.
† VDH adopted the updated CDC COVID-19 confirmed and probable surveillance case definitions on August 27, 2020. Found here: https://wwwn.cdc.gov/nndss/conditions/coronavirus-disease-2019-covid-19/case-definition/2020/08/05/

Outbreaks

Total Outbreaks*	Outbreak Associated Cases
2,319	57,348

* At least two (2) lab confirmed cases are required to classify an outbreak.

Testing (PCR Only)

Testing Encounters PCR Only*	Current 7-Day Positivity Rate PCR Only**
5,234,155	11.8%

* PCR" refers to "Reverse transcriptase polymerase chain reaction laboratory testing."
** Lab reports may not have been received yet. Percent positivity is not calculated for days with incomplete data.

Multisystem Inflammatory Syndrome in Children

Total Cases*	Total Deaths
16	0

*Cases defined by CDC HAN case definition: https://emergency.cdc.gov/han/2020/han00432.asp

COVID-19 in Virginia: Testing

VIRGINIA'S HEALTH IS IN OUR HANDS. Do your part, stop the spread.

VDH VIRGINIA DEPARTMENT OF HEALTH

Dashboard Updated: 1/31/2021
Data entered by 5:00 PM the prior day.

Select Health District from Drop Down Menu
(All)

Select PCR*, Antigen, Antibody, or Total Tests from Drop Down Me...
PCR Only

All Health Districts
Testing Encounters PCR Only

5,234,155

All Health Districts
Current 7-Day Positivity Rate PCR Only

11.8%

Testing Encounters by Health District, PCR Only

Fairfax	631,337
Prince William	282,230
Henrico	262,078
Virginia Beach	244,319
Chesterfield	230,981
Roanoke	178,242
Loudoun	201,843
Peninsula	174,155
Richmond	181,902
Central Shenandoah	171,847
Central Virginia	155,967
Norfolk	140,428
Arlington	160,823
Rappahannock	153,180
Lord Fairfax	152,766
Chesapeake	133,338
New River	125,409
Crater	118,576
Western Tidewater	96,571
Alexandria	108,157
Mount Rogers	85,339
Alleghany	94,851
Rappahannock Rapidan	89,133
Chickahominy	87,473
Three Rivers	81,055
Piedmont	71,513
Pittsylvania-Danville	63,282
Portsmouth	57,424
Southside	55,330
Hampton	61,000
West Piedmont	55,141
Lenowisco	37,781
Cumberland Plateau	37,473
Eastern Shore	26,963
Out of State	168,979
Unknown	17,847
Blue Ridge	239,422

Number of Testing Encounters, Number of Positive Testing Encounters, and Percent Positivity** by La Report Date - All Health Districts, PCR Only

Percent Positivity, 7-Day Moving Average
Positive Testing Encounters
Number of Testing Encounters

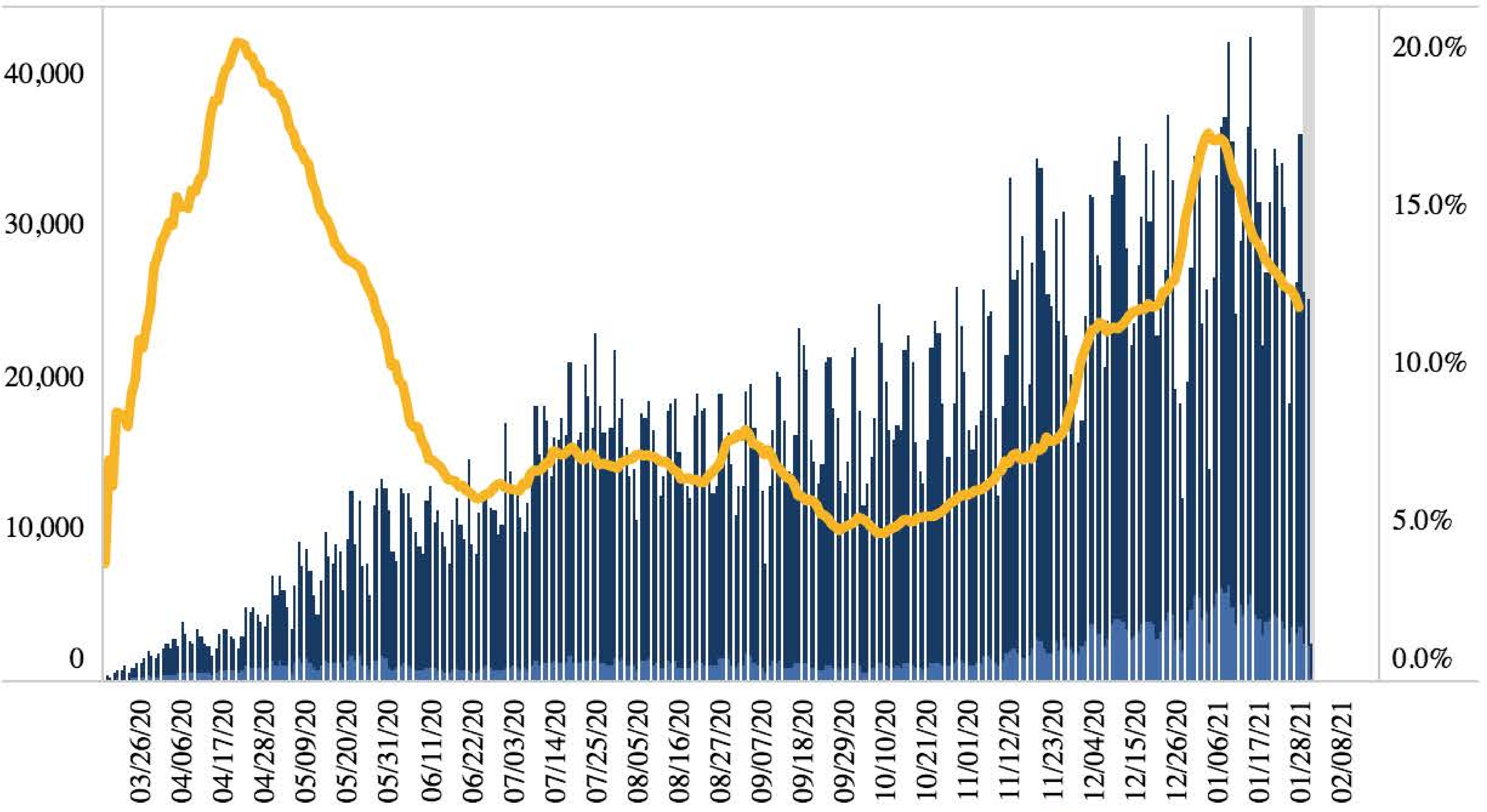

Number of Testing Encounters by Lab Report Date - All Health Districts, PCR Only

Number of Testing Encounters
7-Day Moving Average

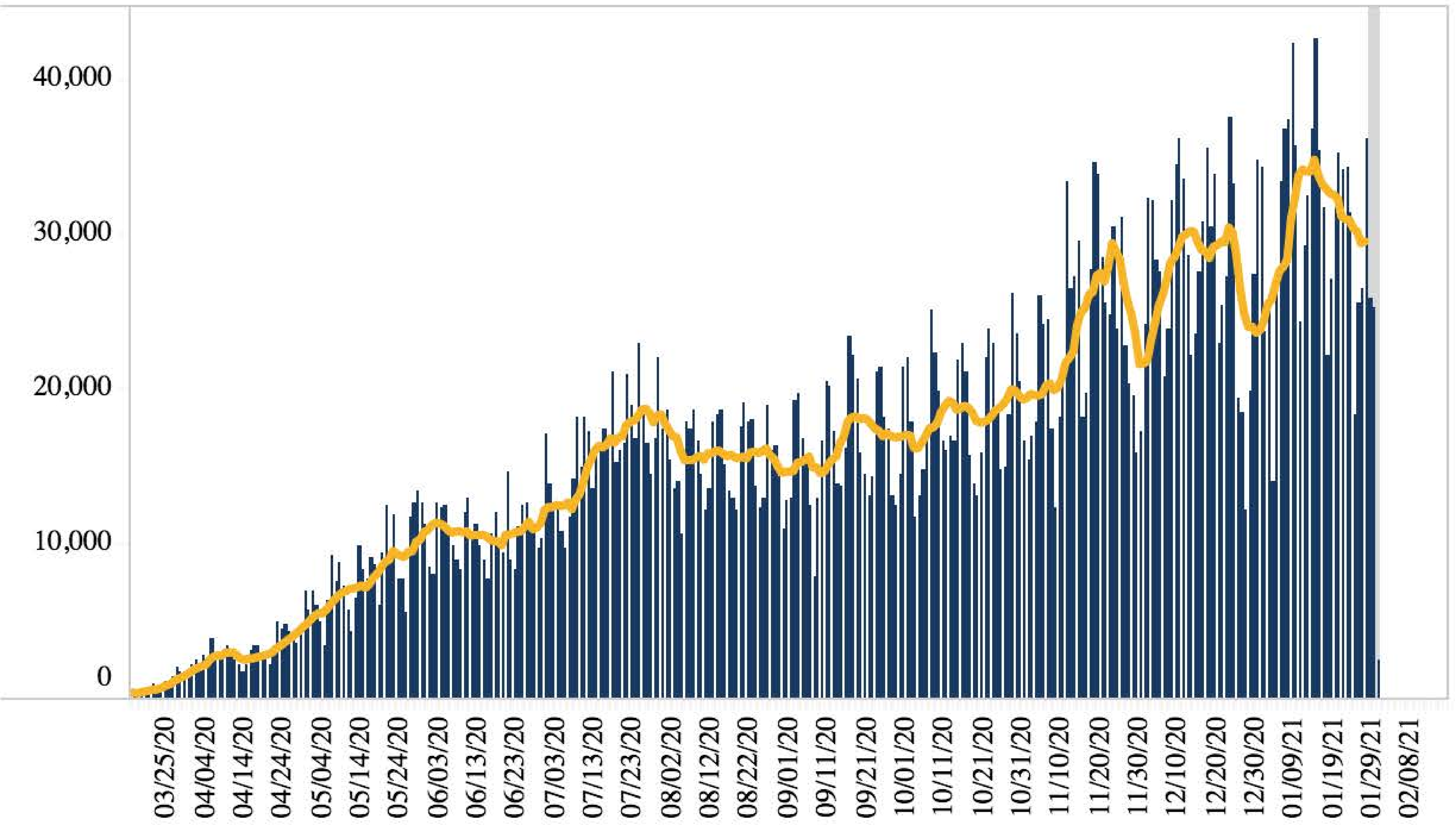

* PCR" refers to "Reverse transcriptase polymerase chain reaction laboratory testing."
** Lab reports may not have been received yet. Percent positivity is not calculated for days with incomplete data.

VIRGINIA'S HEALTH IS IN OUR HANDS. Do your part, stop the spread.

COVID-19 in Virginia: Vaccine Summary

VDH VIRGINIA DEPARTMENT OF HEALTH

Dashboard Updated: 1/31/2021

COVID-19 Vaccinations in Virginia

Total Doses Administered	People Vaccinated with at Least One Dose*	People Fully Vaccinated	% of the Population Vaccinated with at Least One Dose
805,695	688,561	117,134	8.1%

* Includes people vaccinated with 1 or 2 doses of a 2-dose vaccine, including doses administered through the Federal CDC Pharmacy Partnership for Long Term Care Facilities.

Vaccine Doses Administered by Locality of Residence - Rate per 100,000 Population

Doses Administered Rate per 100,000 Population

- 0
- 87 - 215
- 216 - 531
- 1314 - 3245
- 3246 - 8021
- 8022 - 19824
- 19825+

Select Counts or Rates for the M...

Rates per 100,000

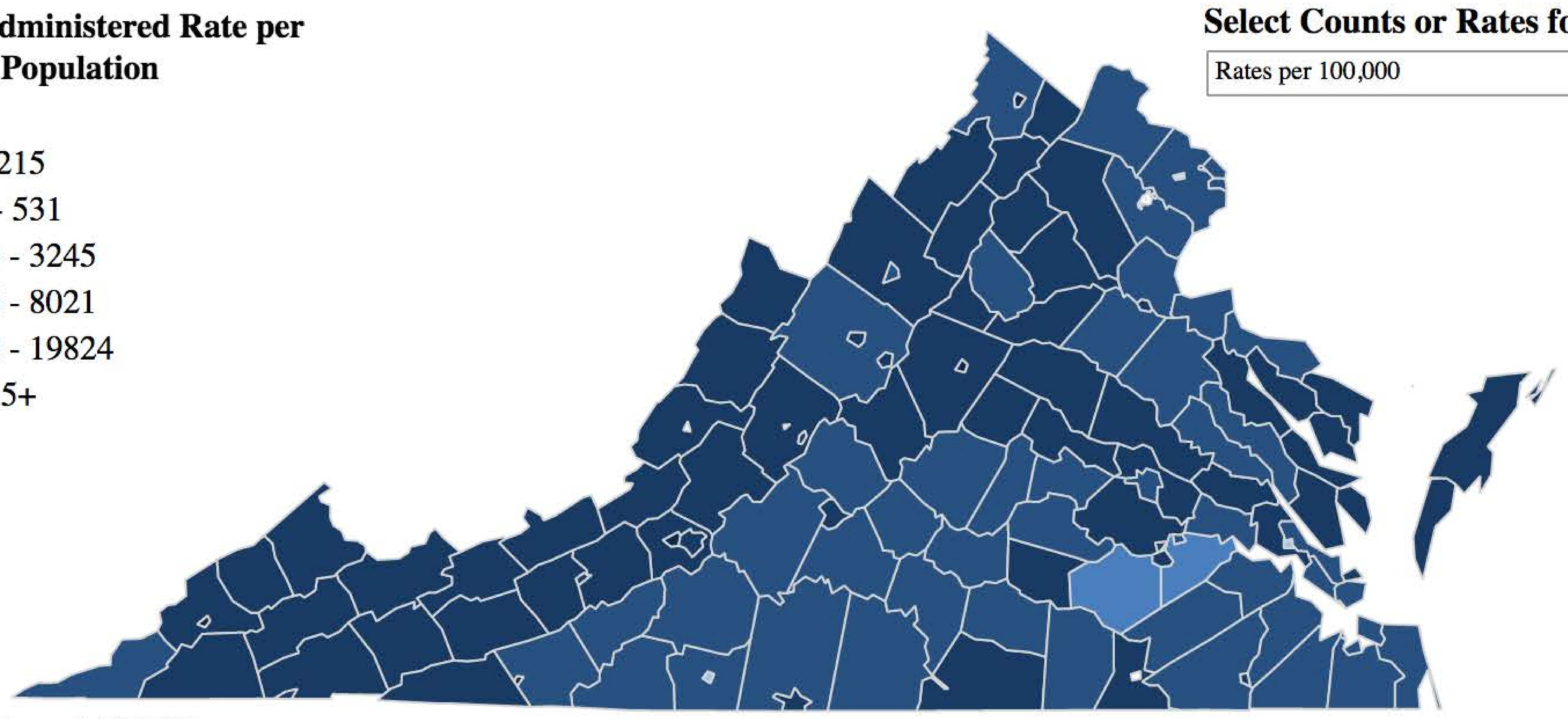

Doses Not Mapped: 101,789

COVID-19 Vaccine Doses Administered by Facility Type

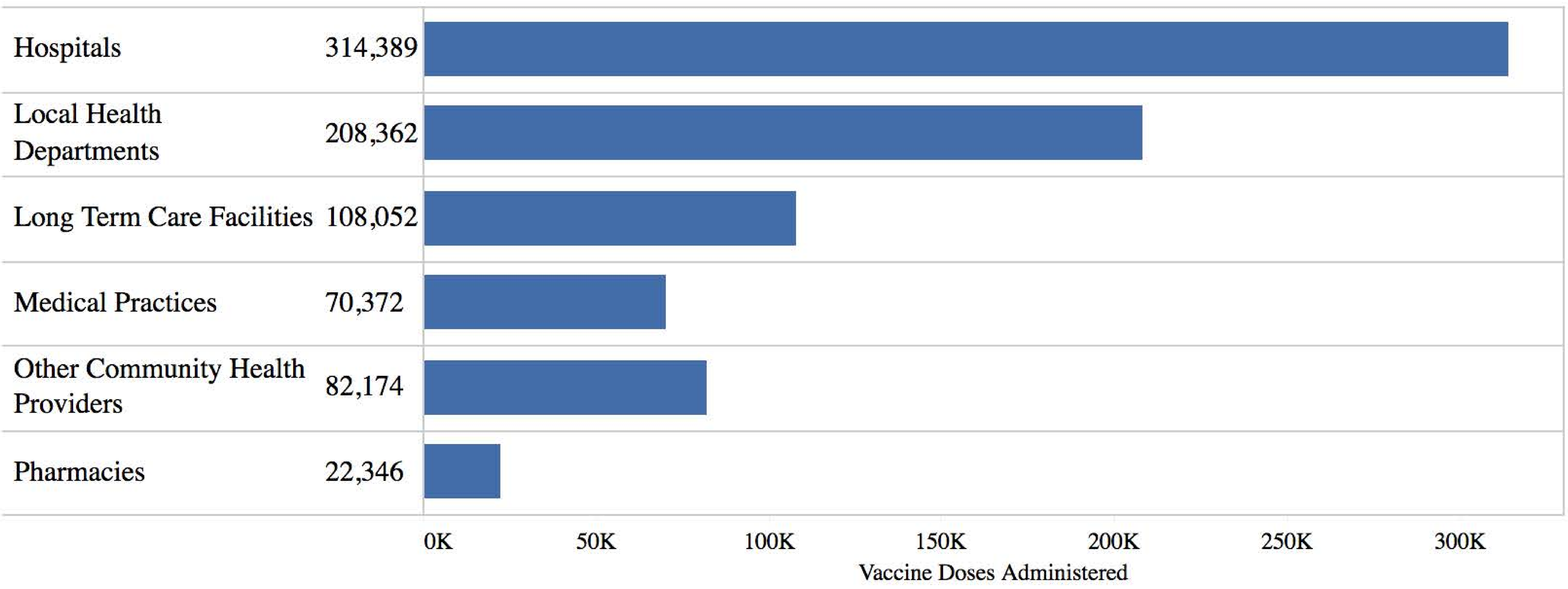

COVID-19 Vaccine Administration^ by Day

Average Doses Administered Each Day**

31,563

Moderna Pfizer Average Doses Administered Each Day**

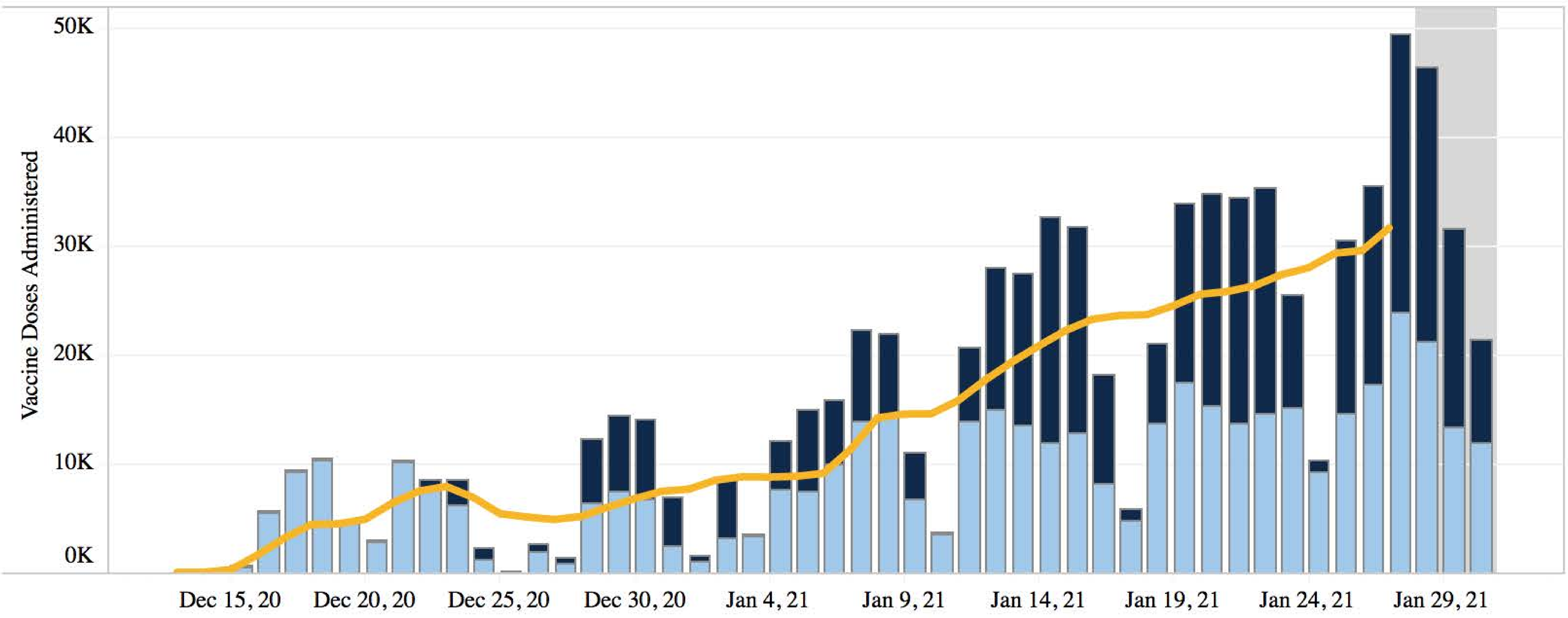

**The average doses administered each day takes the last 7 days of daily doses given, adds them up, and divides that number by 7. This is useful to get a clear picture of the data while taking into account reporting delays.

^Vaccine administrations may take up to 72 hours to be reported, shown by gray shaded area on graph.

COVID-19 in Virginia: Vaccine Demographics

VIRGINIA'S HEALTH IS IN OUR HANDS. Do your part, stop the spread.

VDH VIRGINIA DEPARTMENT OF HEALTH

Dashboard Updated: 1/31/2021

People Vaccinated with at least One Dose

688,561

People Fully Vaccinated

117,134

Vaccination Status

One Dose

Total Vaccinations by Age Group

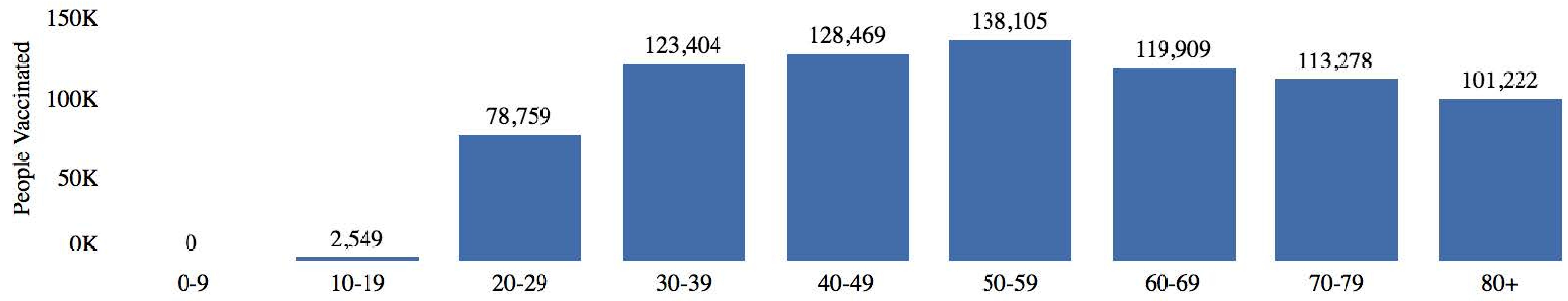

Not Reported: 0

Total Vaccinations by Race and Ethnicity

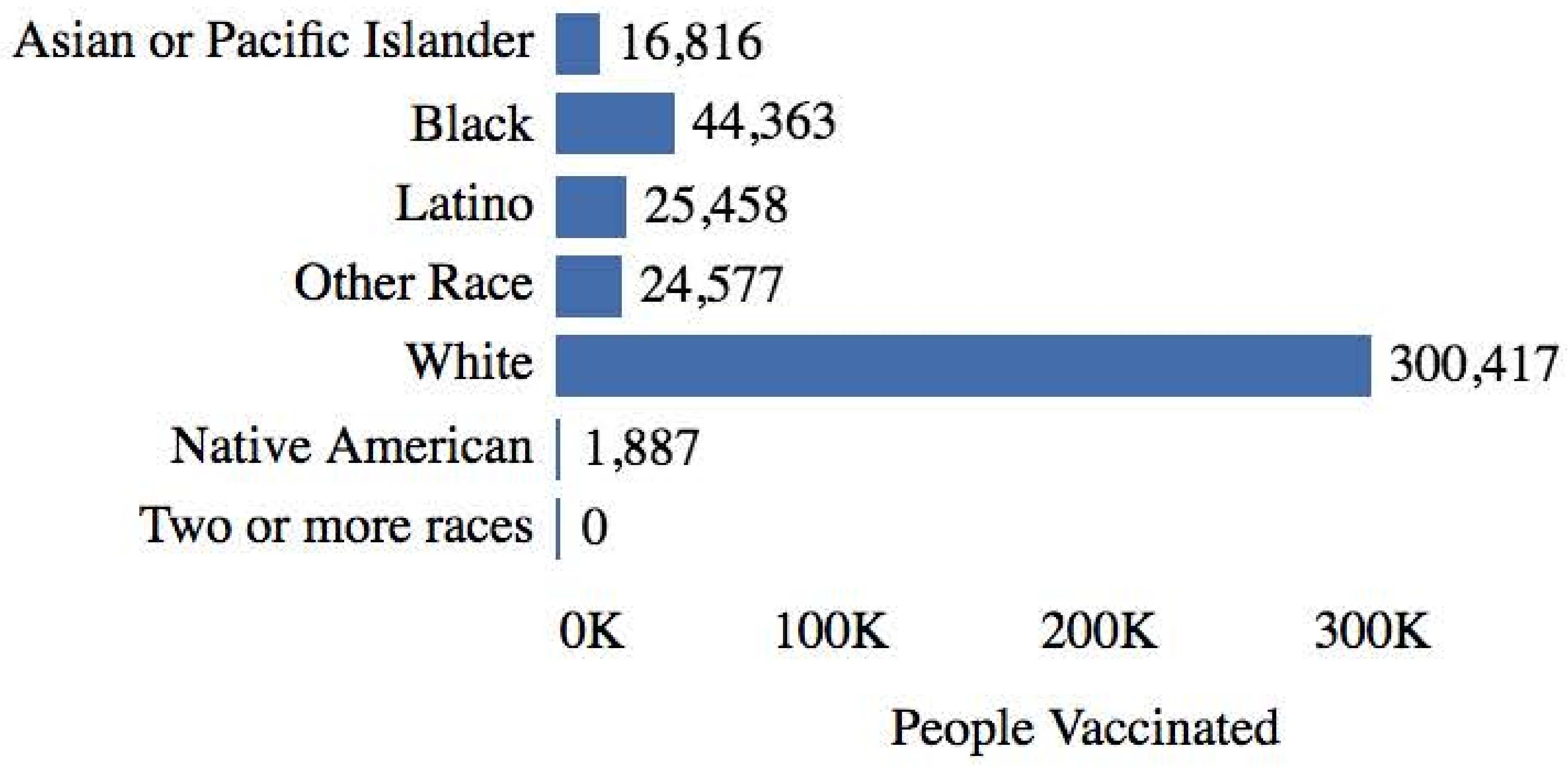

Not reported: 392,177

Total Vaccinations by Sex

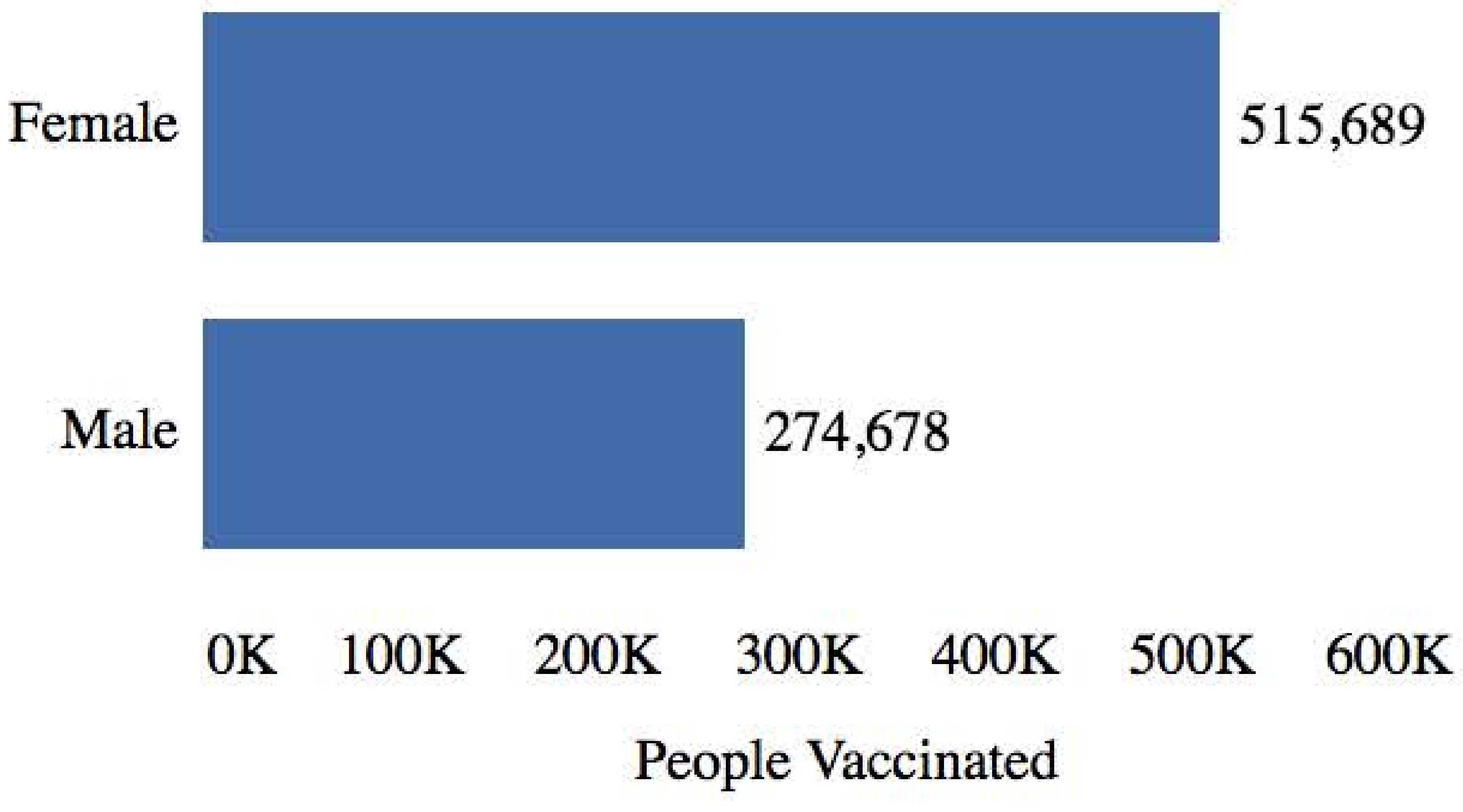

Not Reported: 15,328

COVID-19 in Vermont Vermont Department of Health (VDH)

New Cases
135
11,965 Total

Currently Hospitalized
63

Hospitalized In ICU
5

Hospitalized Under Investigation
5

Percent Positive 7-day Avg.
2%

People Tested
303,711

Total Tests
897,351

Recovered
8,254
69% of Cases

Deaths
174
1.5% of Cases

Travelers Monitored
159

Contacts Monitored
239

People Completed Monitoring
12,722

Last Updated: 1/31/2021, 10:49:14 AM

COVID-19 Positive Cases by County in Last 14 Days

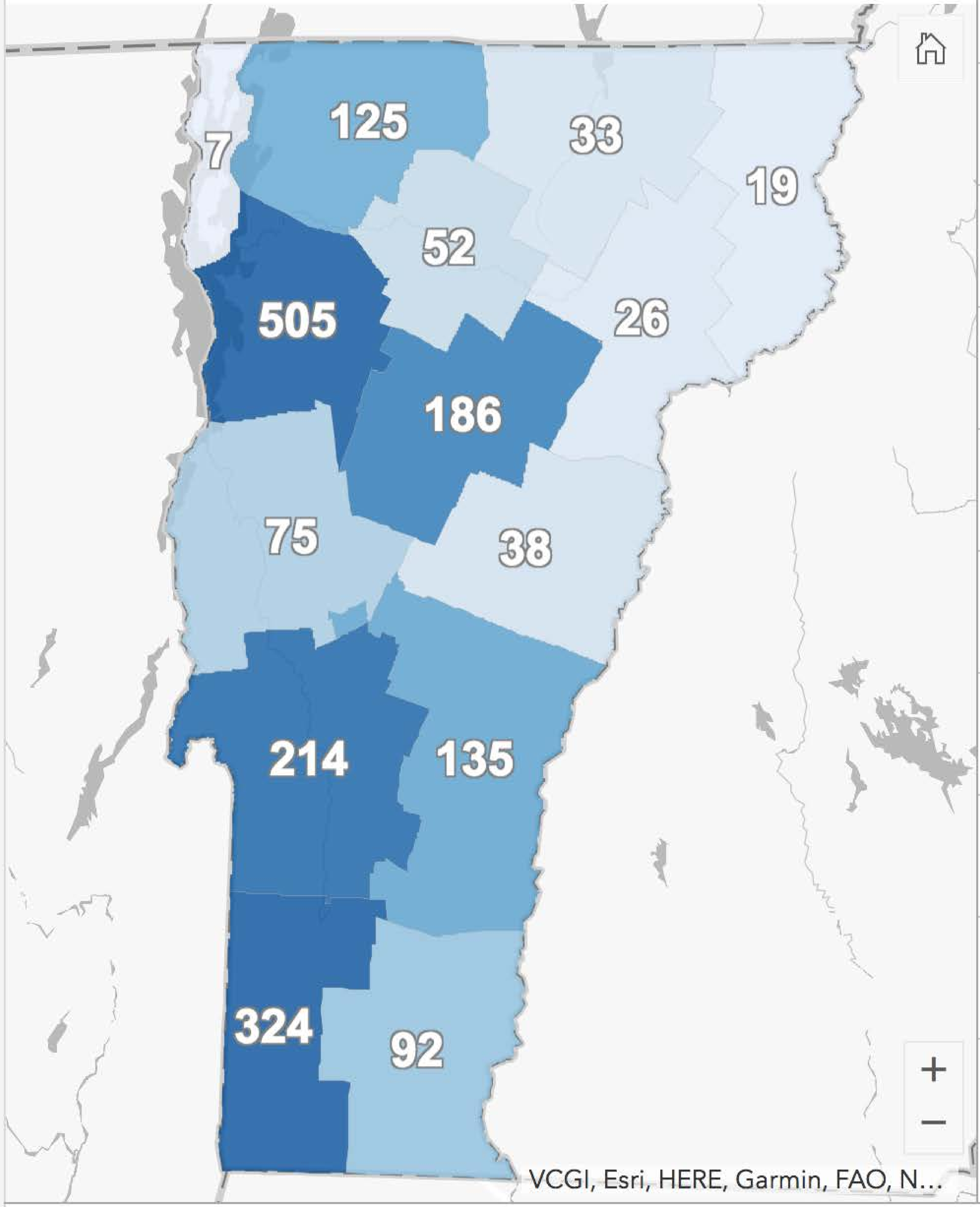

Addison County
New Cases: 6
Recent Cases 14 days: 75

Bennington County
New Cases: 38
Recent Cases 14 days: 324

Caledonia County
New Cases: 5
Recent Cases 14 days: 26

Chittenden County
New Cases: 19
Recent Cases 14 days: 505

Essex County
New Cases: 1
Recent Cases 14 days: 19

Franklin County
New Cases: 12
Recent Cases 14 days: 125

Grand Isle County
New Cases: 0
Recent Cases 14 days: 7

Lamoille County
New Cases: 0
Recent Cases 14 days: 52

Orange County
New Cases: 4
Recent Cases 14 days: 38

Orleans County
New Cases: 2
Recent Cases 14 days: 33

Click a County to highlight it on the map.

View more information by clicking on tab name or arrows below

Recent Cases

Support VTDigger
Rigorous nonprofit news for Vermont.
DONATE → Email SIGN UP
VTDIGGER
News in pursuit of truth

Covid new cases per day, rolling average:

Based on the latest Department of Health data, with a seven-day rolling average:

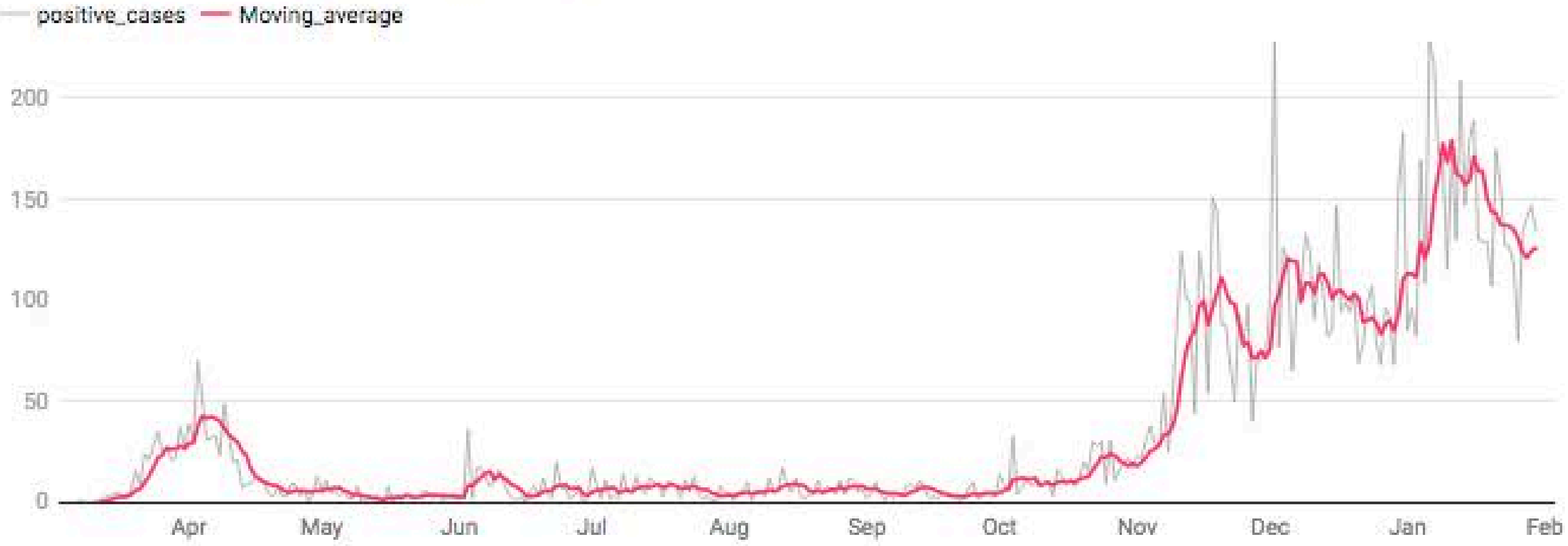

Chart: Erin Petenko • Source: VTDOH via Vermont Center for Geographic Information • Get the data • Created with Datawrapper

Covid new people tested per day, rolling average:

Based on the latest Department of Health data, with a seven-day rolling average:

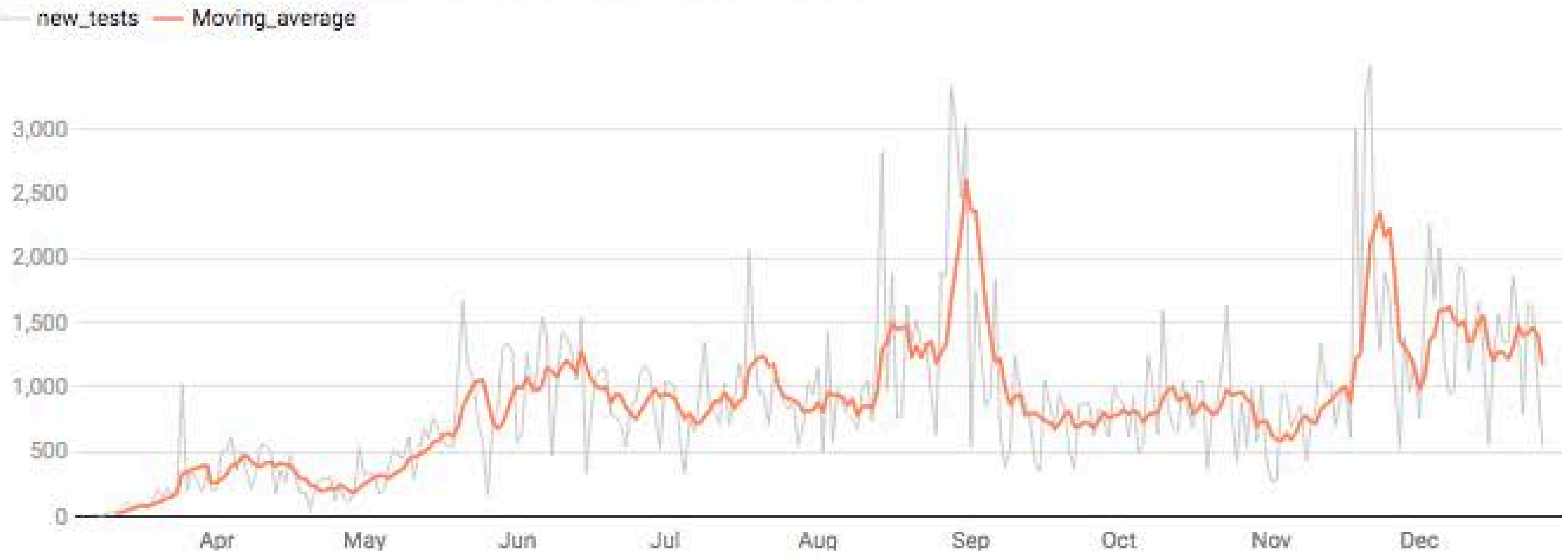

Chart: Erin Petenko • Source: VTDOH via Vermont Center for Geographic Information • Get the data • Created with Datawrapper

Recent cases, by county

Number of new cases in the past 14 days.
Click to see: Recent cases Total cases

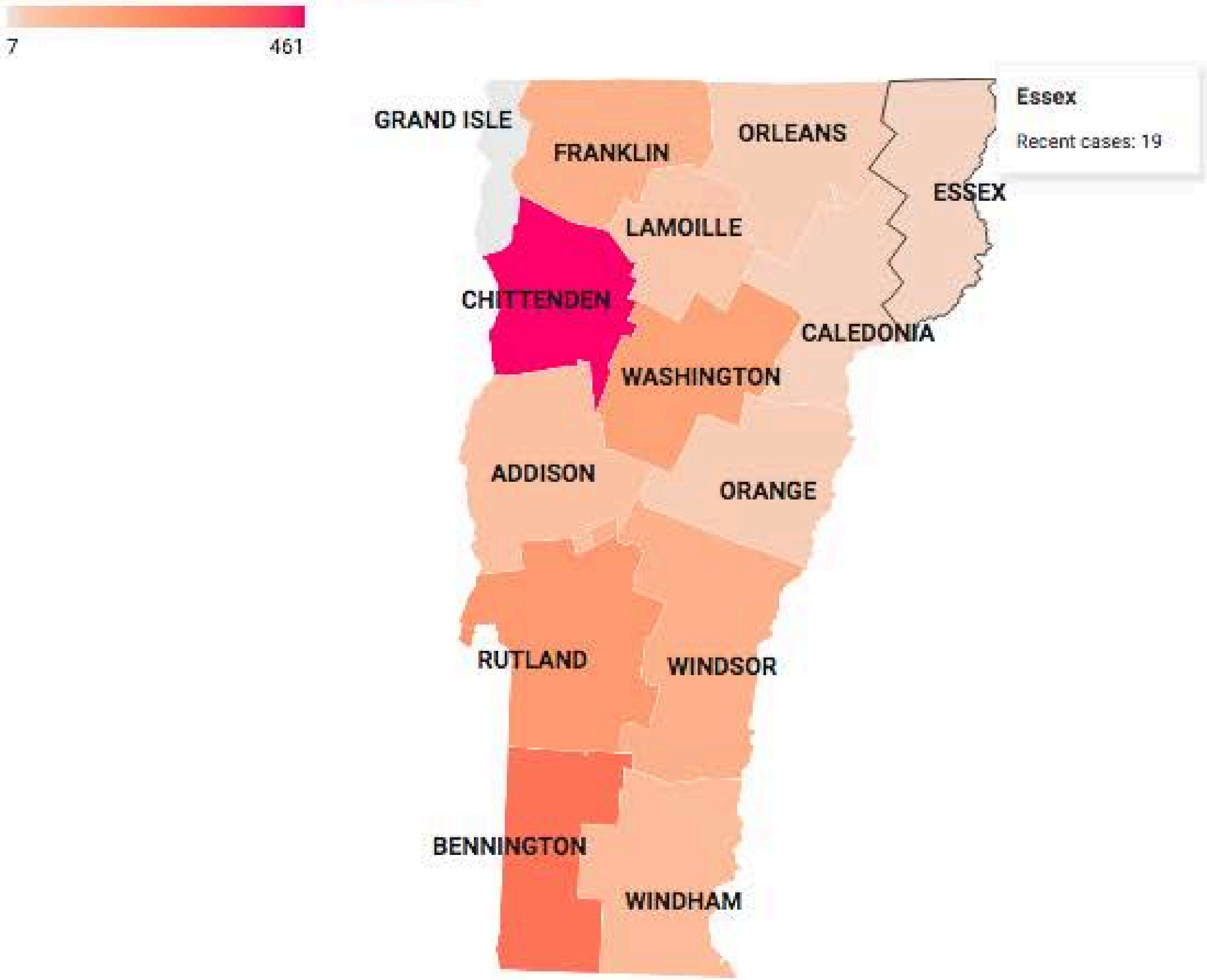

Current Status | Epidemiologic Curves | Cumulative Counts | Demographics | Testing | COVID-like Illness Hospitalizations | Vaccinations

Cases | Hospitalizations | Deaths | Vaccinations

COVID-19 IN WASHINGTON STATE

Cases, Hospitalizations, Deaths and Vaccinations by County

DATA AS OF 01/29/2021 11:59PM PT

Confirmed cases are individuals with a positive molecular test for COVID-19. Probable cases are individuals with a positive antigen test for COVID-19 and no positive molecular test. Hospitalizations and deaths are reported among confirmed and probable cases. Number of vaccine doses given includes all COVID-19 vaccine doses given in Washington facilities. **Learn More**

SELECT COUNTY

- Select all
- Adams County
- Asotin County
- Benton County
- Chelan County
- Clallam County
- Clark County
- Columbia County
- Cowlitz County
- Douglas County
- Ferry County
- Franklin County
- Garfield County
- Grant County
- Grays Harbor Co...
- Island County

Confirmed Cases	**297,513**
Probable Cases	**14,084**
Total Cases	**311,597**
Hospitalizations	**17,706**
Deaths	**4,285**
Percent of Deaths (deaths/total cases)	**1.4%**
Total Molecular Tests	**4,548,239**
Number of Vaccine Doses Given	**616,589**

*Please click **"Learn More"** for more information.*

CASES BY COUNTY

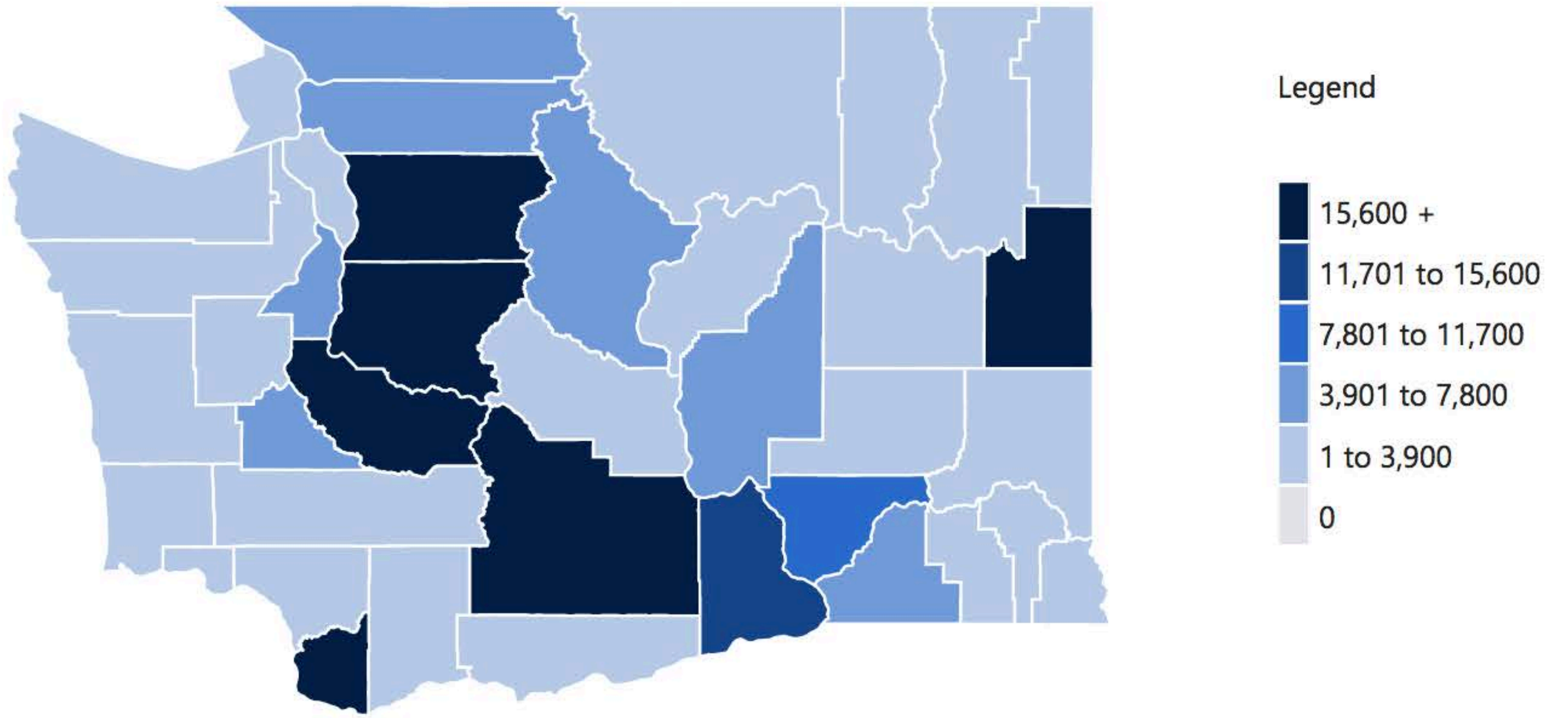

Tabular View

1,442 of 311,597 cases do not have an assigned county

County-level information can be found on Local Health Jurisdiction (LHJ) websites

Microsoft Power BI

Current Status | Epidemiologic Curves | Cumulative Counts | Demographics | Testing | COVID-like Illness Hospitalizations | Vaccinations

Cases | Hospitalizations | Deaths

COVID-19 IN WASHINGTON STATE

Cases and Deaths by Specimen Collection Date, and Hospitalizations by Admission Date

DATA AS OF 01/29/2021 11:59PM PT

This chart shows the progression of the COVID-19 outbreak in Washington by cases, hospitalizations and deaths over time and is known as an epidemiologic curve. The epidemiologic curve is the curve referred to in the phrase, "flatten the curve." **Learn More**

SELECT COUNTY

- Select all
- Adams County
- Asotin County
- Benton County
- Chelan County
- Clallam County
- Clark County
- Columbia County
- Cowlitz County
- Douglas County
- Ferry County
- Franklin County
- Garfield County
- Grant County

*Click on **"Tabular View"** to see and download the data.*

Tabular View

DEATHS COUNTS

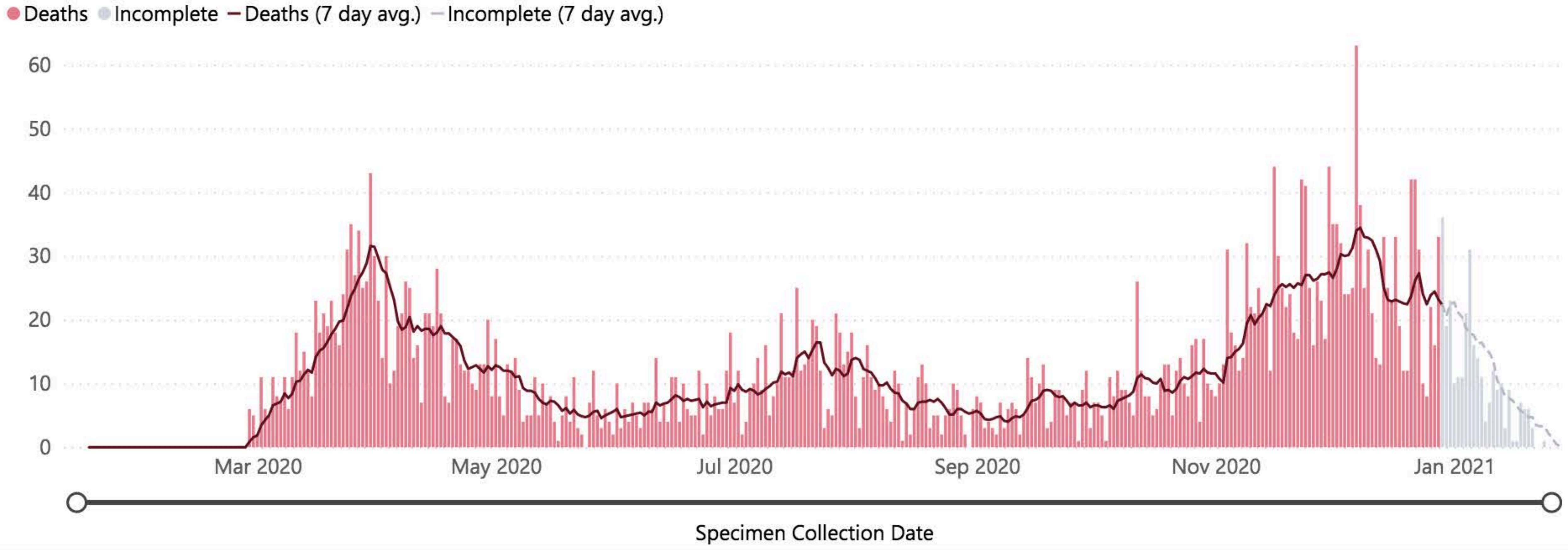

4 of 4,285 deaths do not have an assigned county. Deaths from the last 3-4 weeks may not yet be reported.

County-level information can be found on Local Health Jurisdiction (LHJ) websites

Current Status | Epidemiologic Curves | Cumulative Counts | Demographics | Testing | COVID-like Illness Hospitalizations | Vaccinations

COVID-19 IN WASHINGTON STATE **Number of Molecular Tests by Specimen Collection Date**

DATA AS OF 01/29/2021 11:59PM PT

Testing was initially limited, but since late June, we have averaged over 10,000 tests per day. We continue to work on increasing test availability and accessibility across Washington.

Learn More

SELECT COUNTY

- Select all
- Adams County
- Asotin County
- Benton County
- Chelan County
- Clallam County
- Clark County
- Columbia County
- Cowlitz County
- Douglas County
- Ferry County
- Franklin County
- Garfield County
- Grant County
- Grays Harbor Co...
- Island County
- Jefferson County

NUMBER OF MOLECULAR TESTS

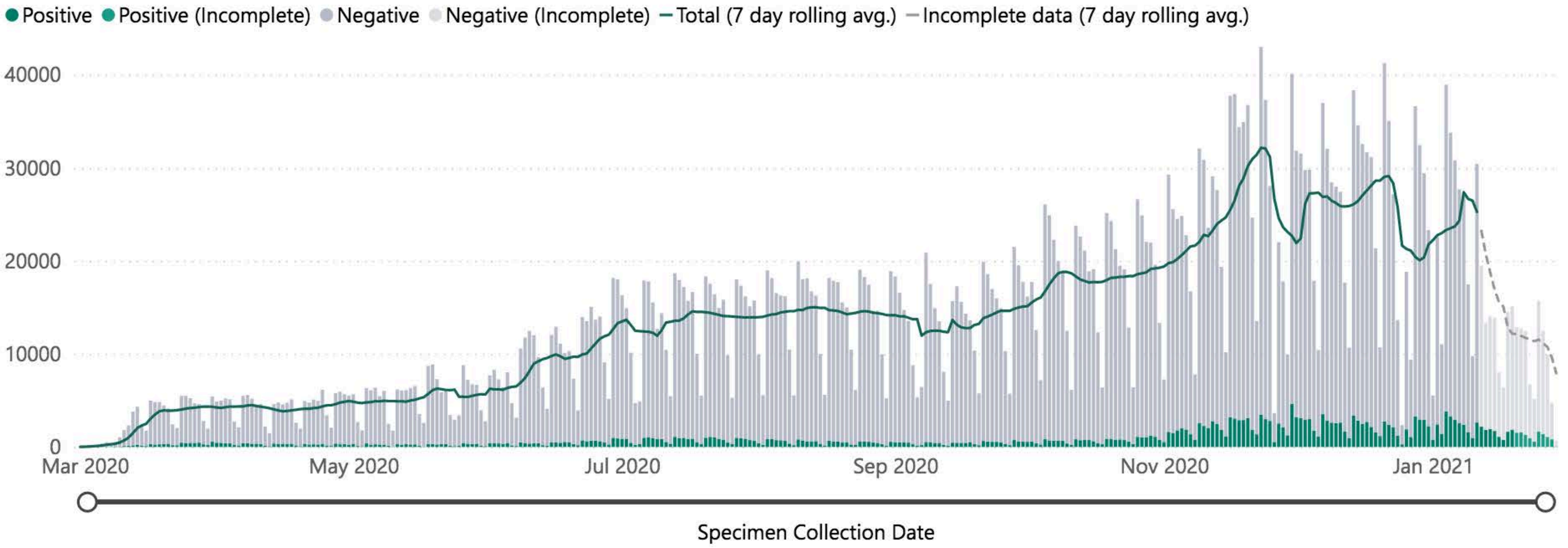

Download

647,626 out of 4,548,239 test results do not have an assigned county.

County-level information can be found on Local Health Jurisdiction (LHJ) websites

Microsoft Power BI

Pages

Current Status | Epidemiologic Curves | Cumulative Counts | Demographics | Testing | COVID-like Illness Hospitalizations | Vaccinations

Where and when are vaccinations being given? | How many people are getting vaccinated?

COVID-19 IN WASHINGTON STATE

Where and when are vaccinations being given?

DATA AS OF 1/27/2021 11:59 PM PT

This chart shows the number of COVID-19 vaccines given in Washington state facilities. Please note, the measures "vaccines given" and "people vaccinated" are not comparable. Vaccines given counts number of doses given in a location. People vaccinated counts number of Washington residents initiating vaccination and becoming fully vaccinated. Numbers may not always align since people may live in a different county than where they got vaccinated. **Learn More**

SELECT COUNTY

- Select all
- Adams County
- Asotin County
- Benton County
- Chelan County
- Clallam County
- Clark County
- Columbia County
- Cowlitz County
- Douglas County
- Ferry County
- Franklin County
- Garfield County
- Grant County
- Grays Harbor Co...

By Location

By Date

Vaccine Doses Given (statewide or selected counties)	**616,589**

Statewide Measures Only

Vaccine Doses Given in Washington	**616589**
Doses Delivered to Washington Providers	**853,325**
Doses Delivered for CDC long-term care vaccination program	**130,650**
Percentage of Delivered Doses Given	**62.66%**

VACCINE DOSES GIVEN PER DAY
Washington State

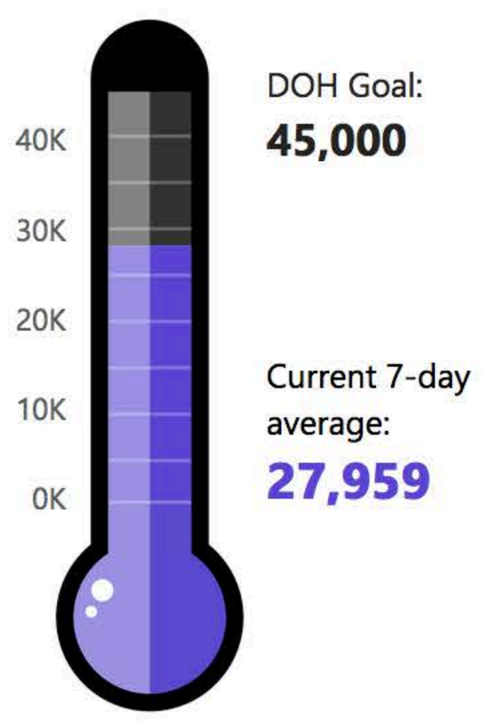

VACCINE DOSES GIVEN IN WASHINGTON COUNTIES

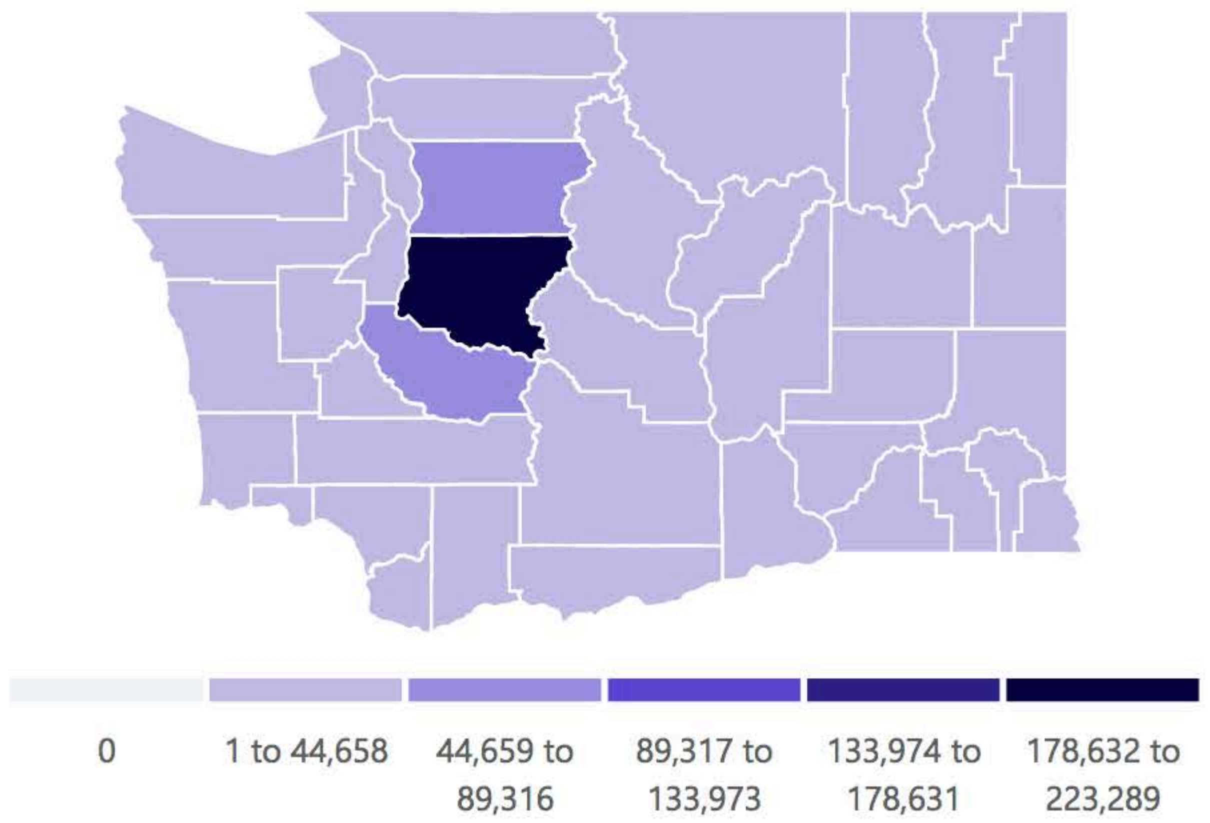

County data for doses given are based on the location of the facility giving the vaccine. 22,961 vaccine doses given do not have a county reported.

County-level information can be found on Local Health Jurisdiction (LHJ) websites

Power BI | Pages

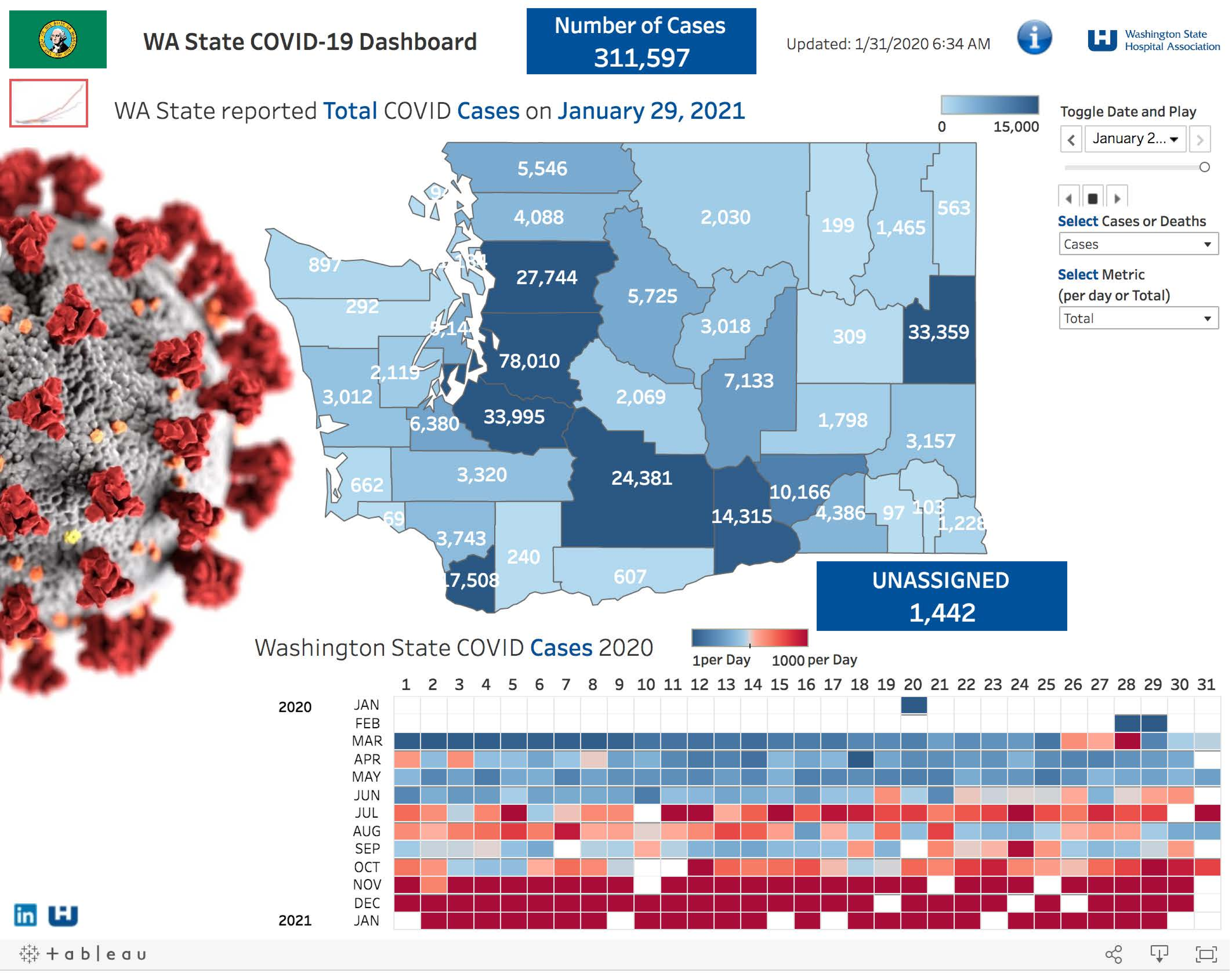
WA State COVID-19 Dashboard
Number of Cases
311,597
Updated: 1/31/2020 6:34 AM
Washington State Hospital Association
WA State reported Total COVID Cases on January 29, 2021
0
15,000
Toggle Date and Play
January 2...
Select Cases or Deaths
Cases
Select Metric
(per day or Total)
Total
5,546
4,088
2,030
199
1,465
563
897
27,744
5,725
292
3,018
309
33,359
78,010
2,119
7,133
3,012
2,069
6,380
33,995
1,798
3,157
3,320
24,381
662
10,166
14,315
4,386
97
103
1,228
3,743
240
607
UNASSIGNED
1,442
Washington State COVID Cases 2020
1per Day
1000 per Day
1 2 3 4 5 6 7 8 9 10 11 12 13 14 15 16 17 18 19 20 21 22 23 24 25 26 27 28 29 30 31
2020
JAN
FEB
MAR
APR
MAY
JUN
JUL
AUG
SEP
OCT
NOV
DEC
2021
JAN
tableau

West Virginia COVID-19

WEST VIRGINIA Department of Health & Human Resources BUREAU FOR PUBLIC HEALTH

Overview | County Alert System | 7-Day Trend | Cumulative Summary | Case and Lab Trends | Other Trends | Hospital | Long-Term Care | Vaccine Summary

121,001
Total Number of Cases
Includes Probable and Confirmed Cases (Click to View)

2,024
Deaths
Includes Probable and Confirmed Deaths (Click to View)

1,921,654
Confirmatory Lab Tests

5.61%
Cumulative Percent Positivity
Click here to view breakdown

661
New Cases Received in the Last 24 Hours
*Compared to yesterday's data

9
Deaths Reported in the Last 24 Hours
*Compared to yesterday's data

21,195
Current Active Cases

6.49%
Daily Percent Positivity
Click here to view breakdown

Vaccine

194,119
Total Number of First Doses Administered

68,230
People Fully Vaccinated

Note: Data posted on dashboard is from 12:00 midnight the previous day. All data are provisional and subject to change based on information obtained during public health investigations.

Click here to view CDC's definition on Confirmed and Probable cases

Updated 10:00 AM 1/31/2021

Microsoft Power BI

COVID-19: County Data

Jump to specific COVID-19 chart on this page:

- Cumulative total number of reported confirmed and probable cases by date of symptom onset or diagnosis
- Number of reported confirmed and probable cases by date of symptom onset or diagnosis
- Cumulative total number of reported confirmed and probable deaths by date of death
- Number of reported confirmed and probable deaths by date of death
- Cases and deaths by county table
- Learn how to download our data

To find COVID-19 maps by county and census tract, see the Wisconsin Summary webpage.

COVID-19: Data Pages

- Activity Level by Region and County
- Alternate Care Facility
- Cases
- Deaths
- Facility-Wide Investigations
- Frequently Asked Questions
- Hospitals
- Labs and Testing
- Racial and Ethnic Disparities
- Vaccines
- Wastewater Monitoring
- Wisconsin Summary
- COVID-19

Cumulative total number of reported confirmed and probable COVID-19 cases by date of symptom onset or diagnosis: Wisconsin

Updated: 1/31/2021 (Total: 592,140)

Select County: Wisconsin

Select Case Status:
- Confirmed
- Probable
- Confirmed and Probable

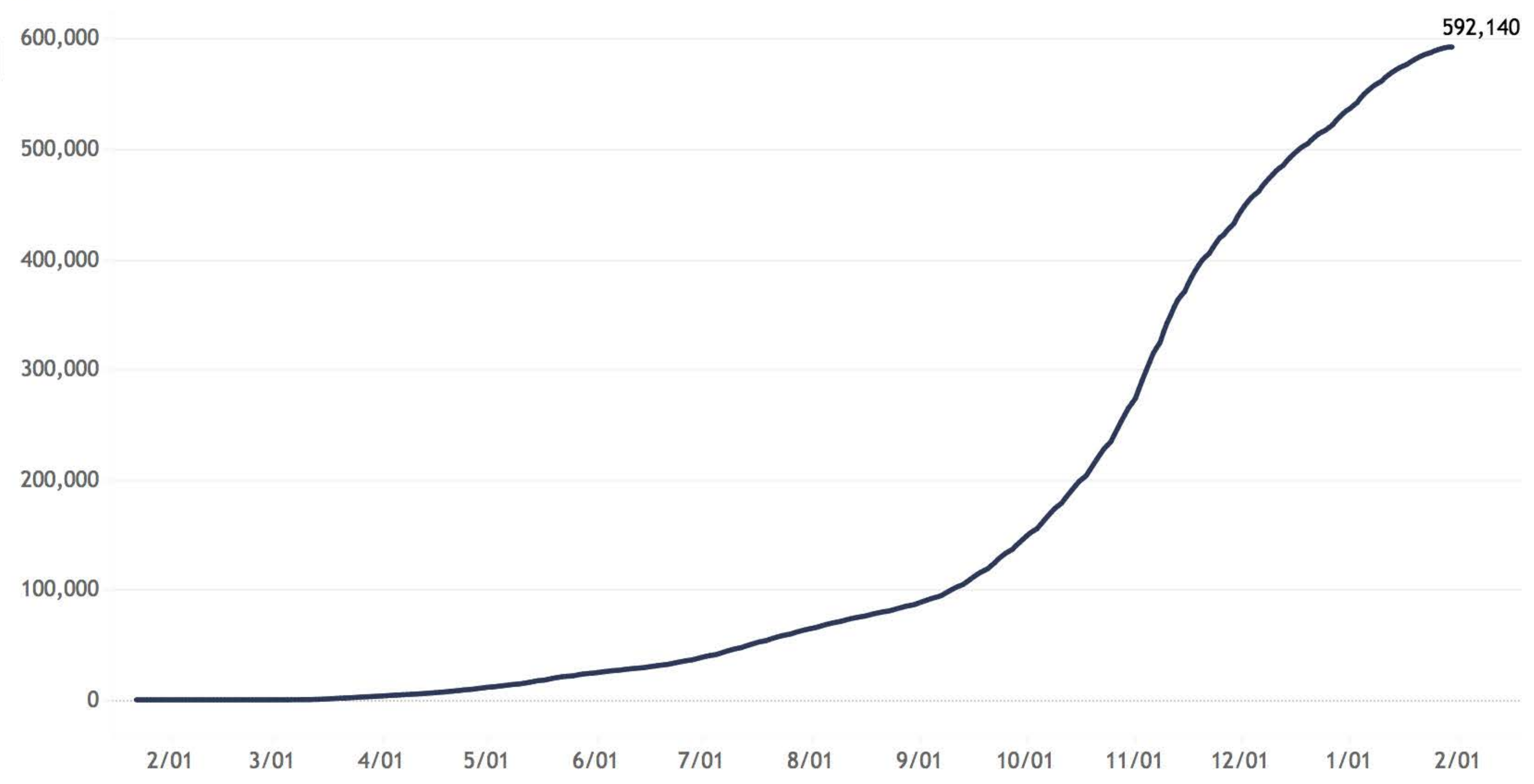

Undo Redo Revert Refresh Pause Share Download Full Screen

Understanding our data: What does this chart mean?

About our data: How do we measure this?

Back to a list of charts on this page.

Number of reported confirmed and probable COVID-19 cases by date of symptom onset or diagnosis: Wisconsin

Updated: 1/31/2021 (Total: 592,140)

Select County: Wisconsin

Select Case Status:
- Confirmed
- Probable
- Confirmed and Probable

Confirmed cases
Probable cases
7-day average

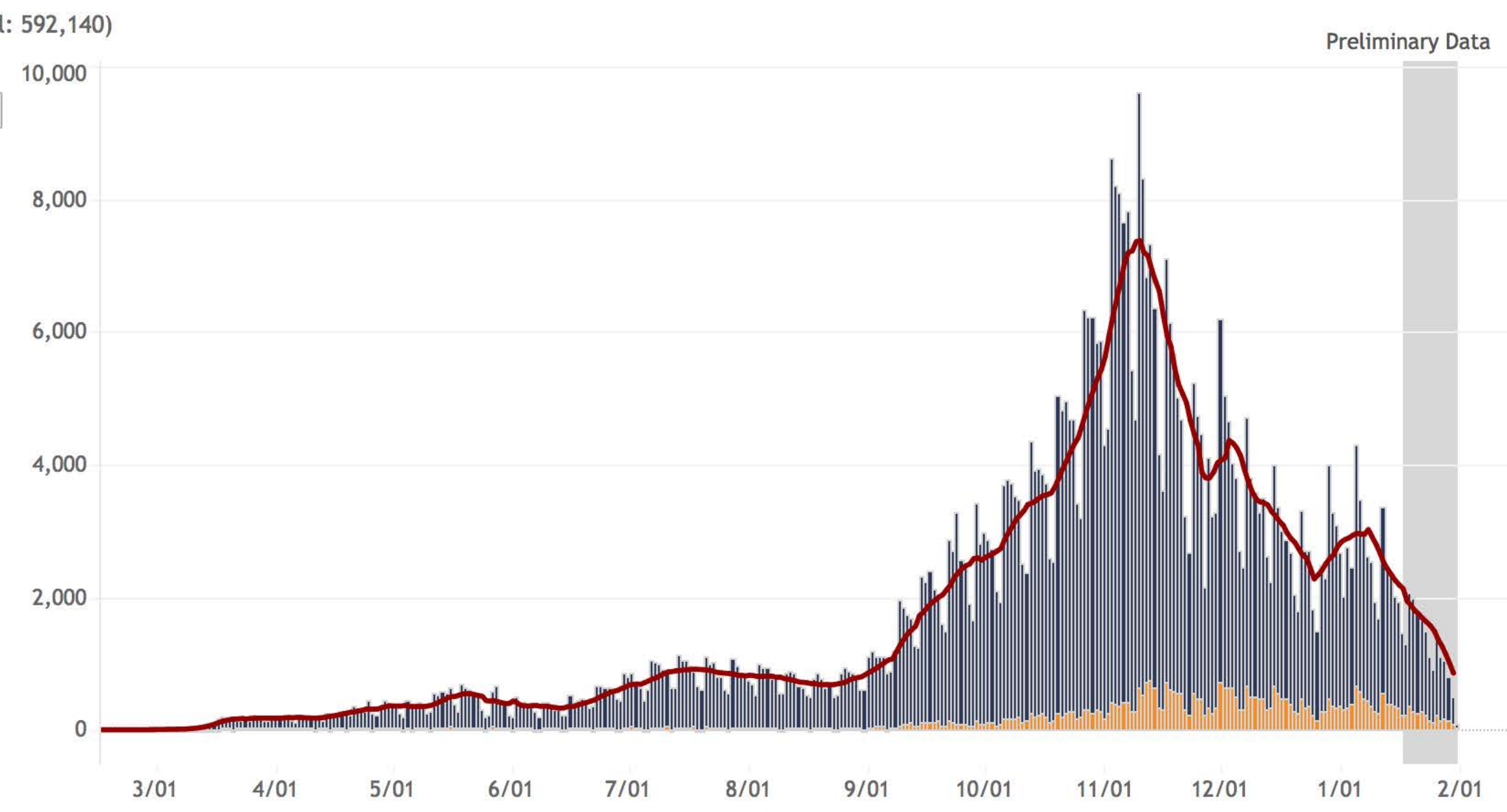

Undo Redo Revert Refresh Pause Share Download Full Screen

Understanding our data: What does this chart mean?

About our data: How do we measure this?

Back to a list of charts on this page.

Cumulative total number of reported COVID-19 deaths among confirmed and probable COVID-19 cases by date of death: Wisconsin

Updated: 1/31/2021 (Total: 6,430)

Select County: Wisconsin

Select Case Status:
- Confirmed
- Probable
- Confirmed and Probable

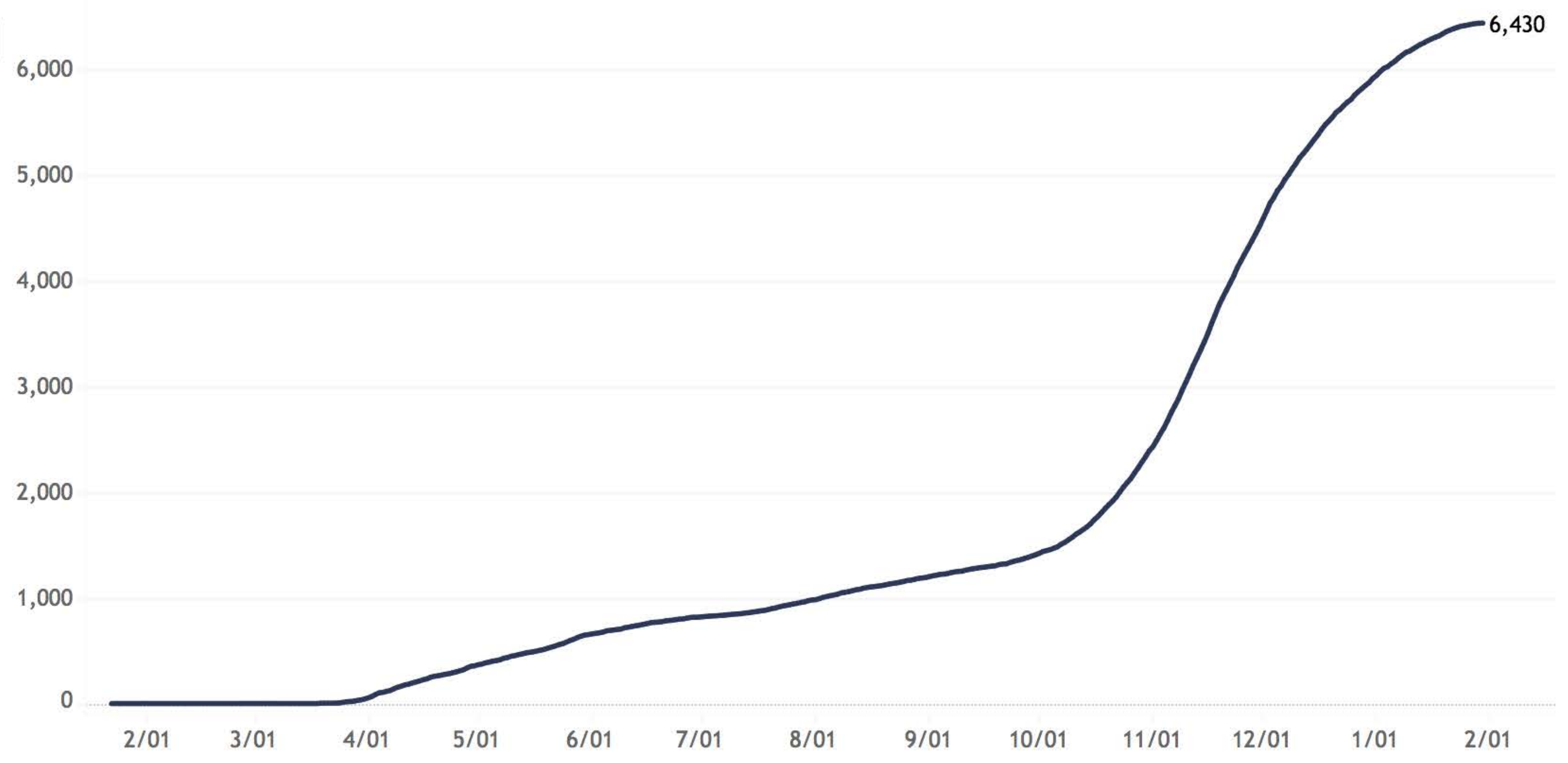

Undo Redo Revert Refresh Pause Share Download Full Screen

Understanding our data: What does this chart mean?

About our data: How do we measure this?

Back to a list of charts on this page.

Number of reported COVID-19 deaths among confirmed and probable cases of COVID-19 by date of death: Wisconsin

Updated: 1/31/2021 (Total: 6,430)

Select County: Wisconsin

Select Case Status:
- Confirmed
- Probable
- Confirmed and Probable

Confirmed case death
Probable case death

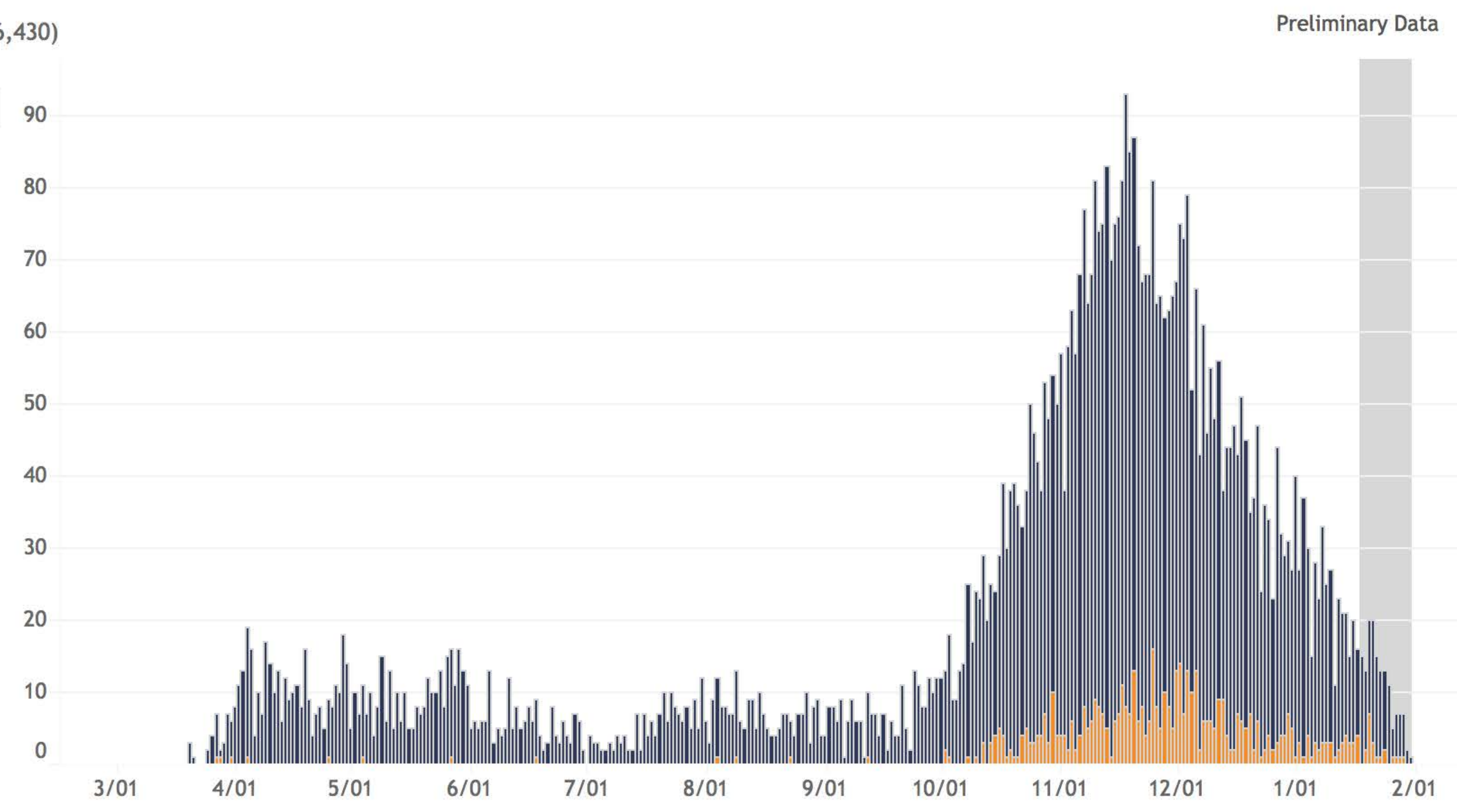

Undo Redo Revert Refresh Pause Share Download Full Screen

Understanding our data: What does this chart mean?

About our data: How do we measure this?

Back to a list of charts on this page.

Cases and deaths by county

Updated: 1/31/2021

Select a County: All Counties

County	Number of confirmed cases	Number of negatives*	Number of probable cases	Number of deaths †	Number of probable deaths ‡	Confirmed cases per 100,000 people	Deaths per 100,000 people**	Case fatality percentage ††
Wisconsin	542,415	2,507,600	49,725	5,896	538	9,382.6	102.0	1.1%
Adams	1,504	7,318	156	11	3	7,338.4	53.7	0.7%
Ashland	[illegible]	[illegible]	[illegible]	[illegible]	[illegible]	[illegible]	[illegible]	[illegible]

NOWCAST WISN 12 News at 5:30 pm Sunday Watch on Demand

Coronavirus: Tracking COVID-19 curve of cases, deaths in Wisconsin

We're tracking the curve of coronavirus cases and coronavirus-related deaths that have occurred in Wisconsin.

104
Shares

Infinite Scroll Enabled

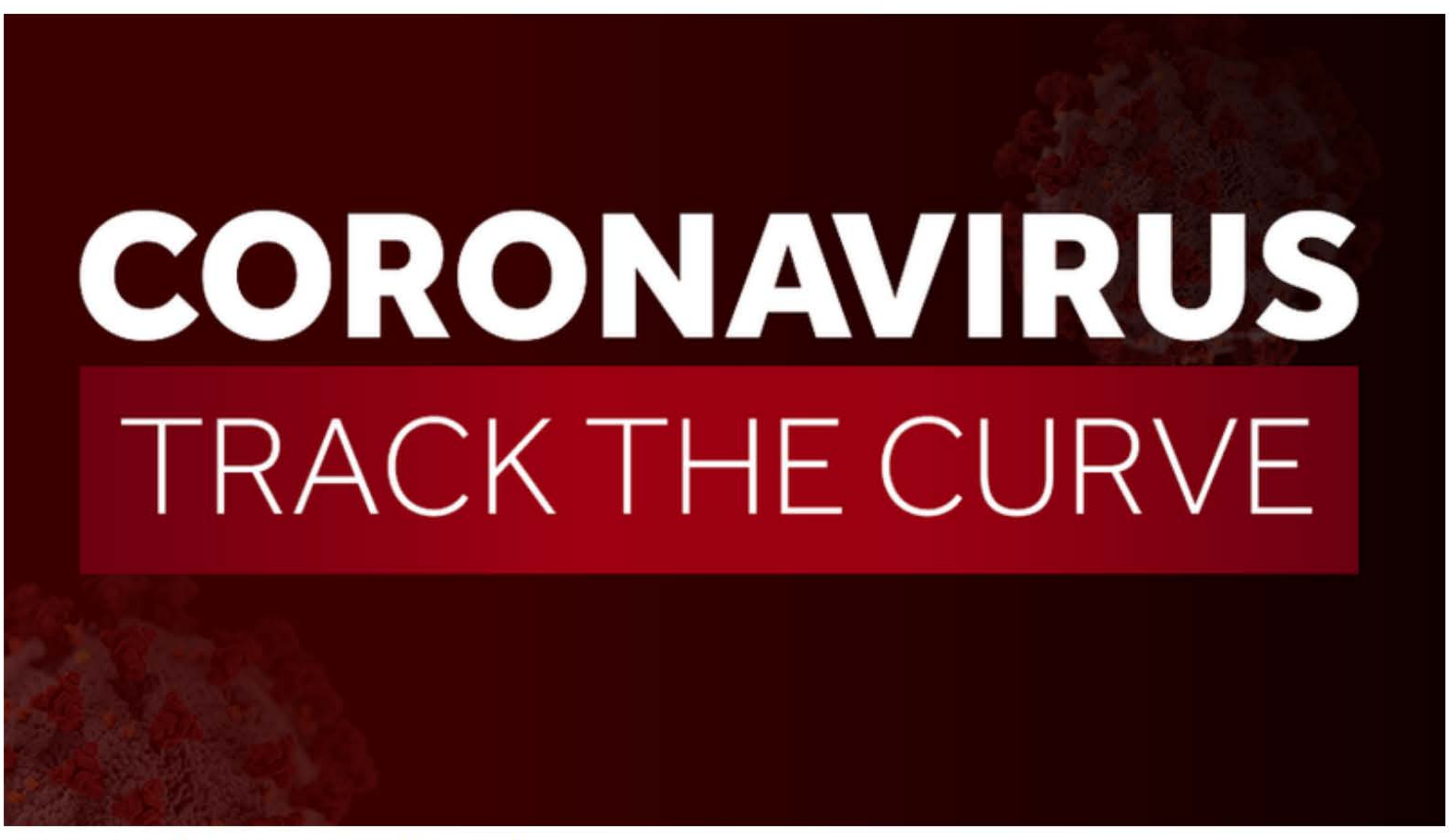

Coronavirus Flatten The Curve Wisconsin

DAILY CORONAVIRUS UPDATES BY EMAIL
Receive daily coronavirus & public health news straight to your inbox.
Your Email Address
SUBMIT
Privacy Notice

We're tracking the curve of coronavirus cases and coronavirus-related deaths that have occurred in Wisconsin.

Continuing Coverage: Coronavirus in Wisconsin

The data shows the total number of COVID-19 cases along with the number of COVID-19 cases per day.

In addition, it includes the total number of deaths related to COVID-19 complications along with the number of deaths from coronavirus occurring daily.

The coronavirus case numbers and death toll are updated daily.

App users click here

Tracking the COVID-19 curve in Wisconsin

COVID-19 Cases Total

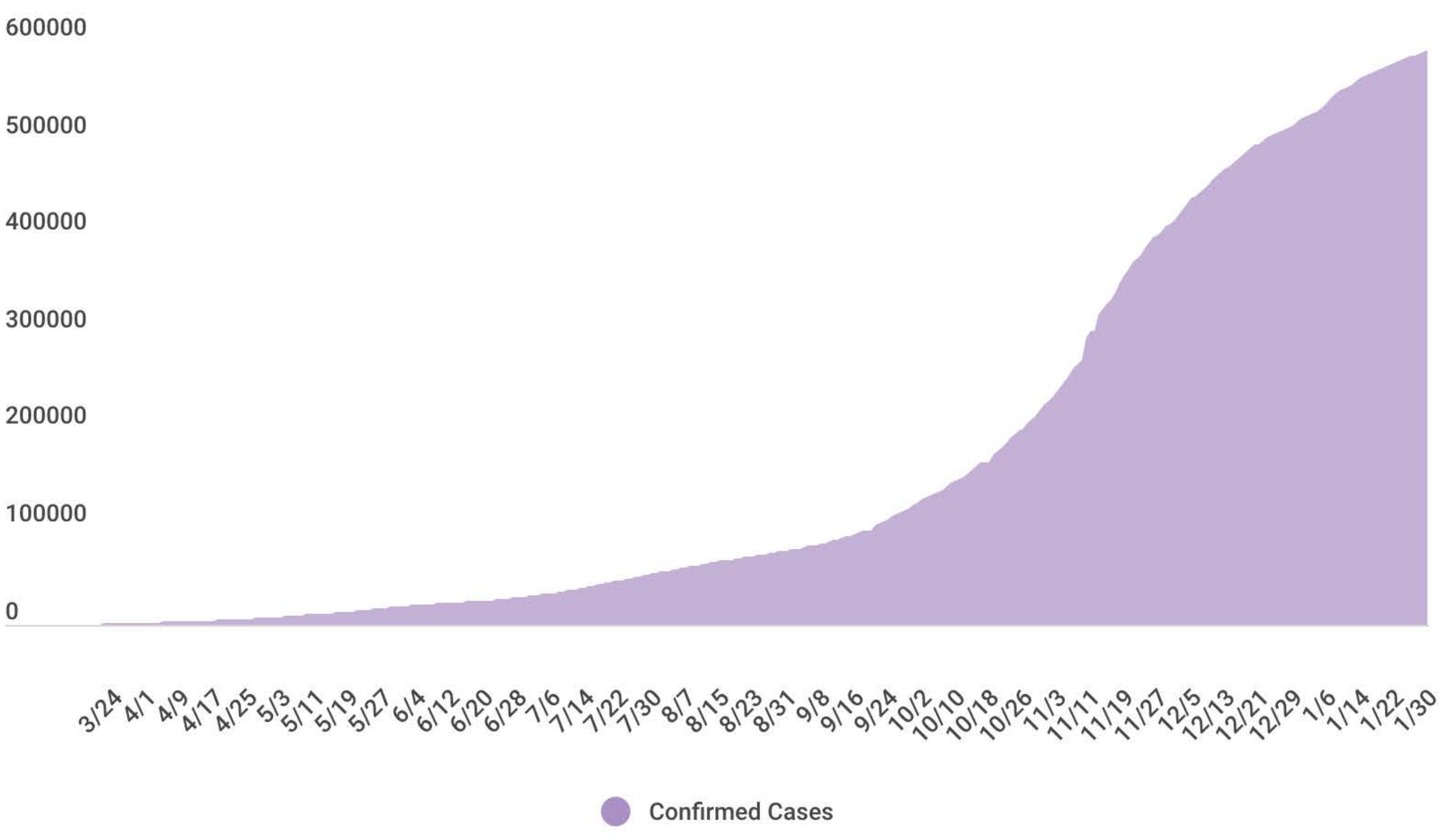

COVID-19 Cases Per Day

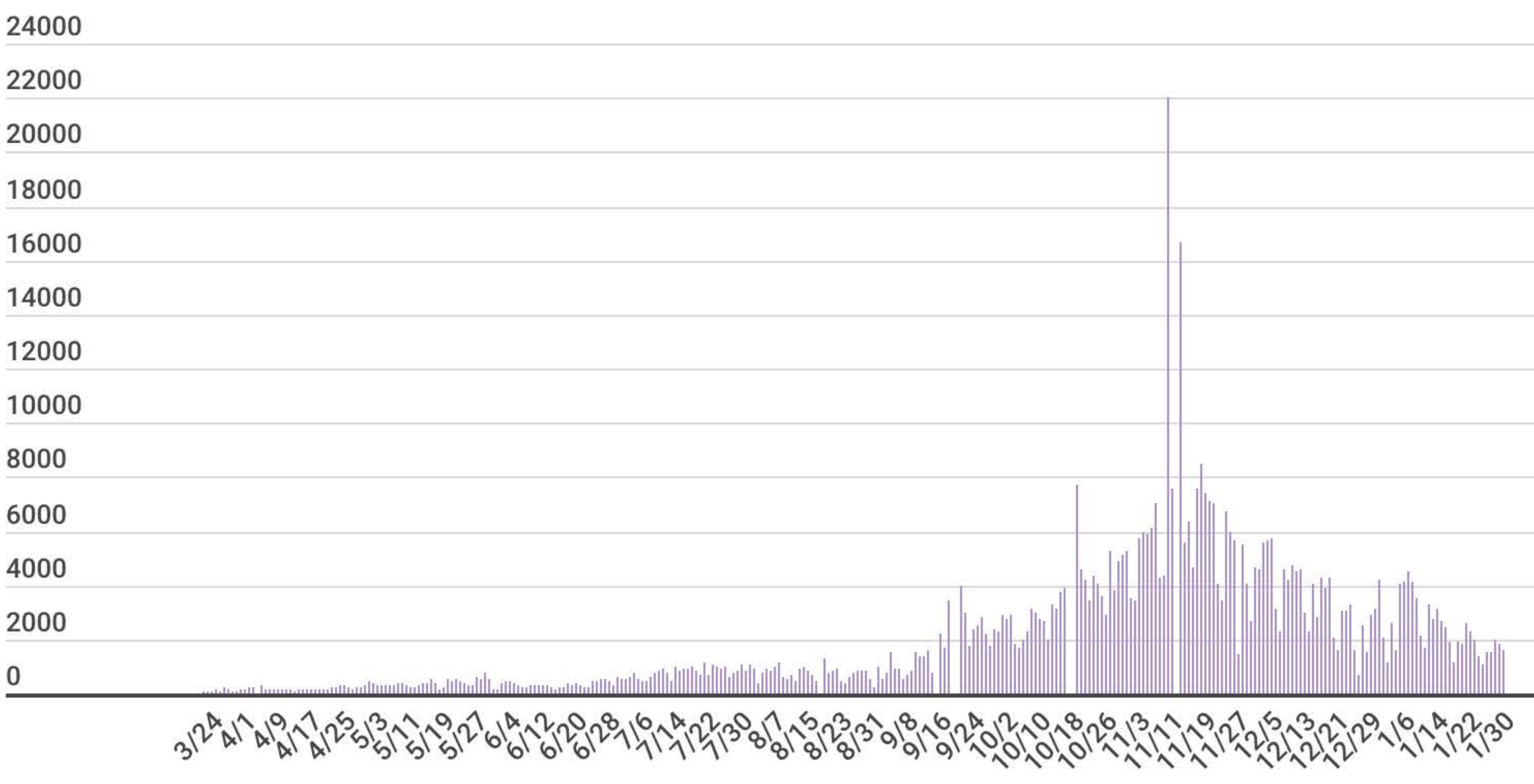

COVID-19 Deaths Total

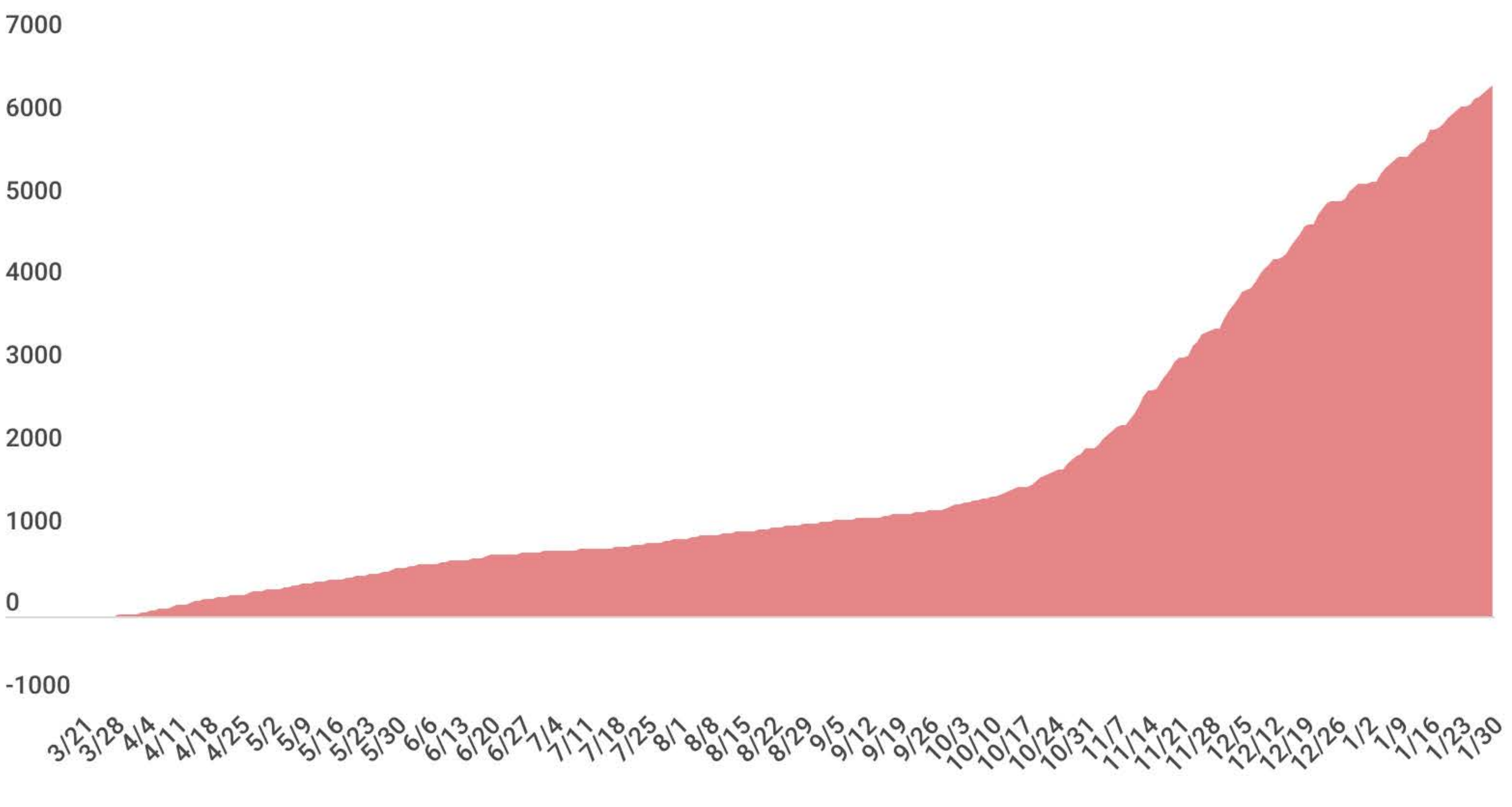

104
Shares

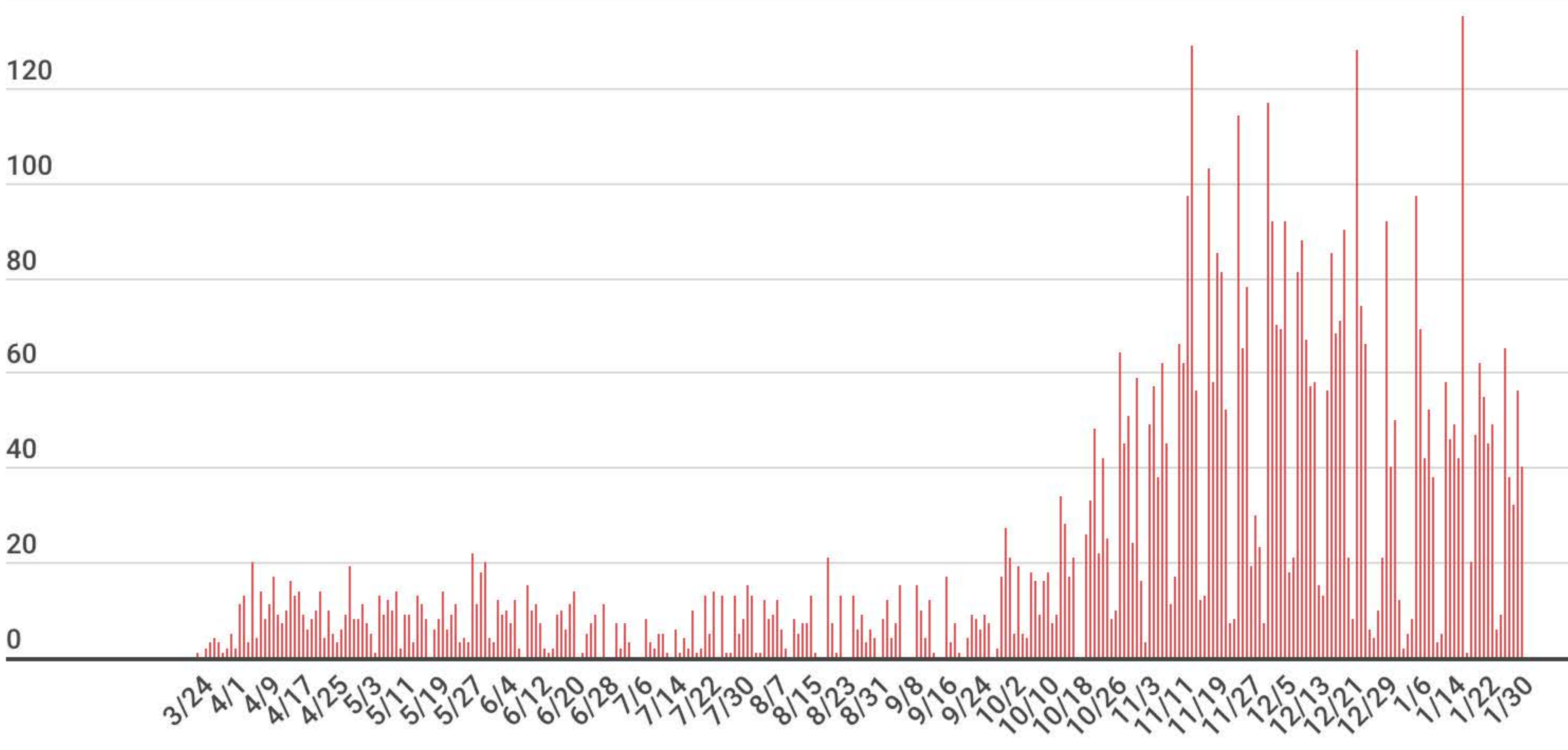

These charts are powered by data from Johns Hopkins University CSSE.
The coronavirus case numbers and death toll are updated daily.
This data may vary from state and local health department tallies.

COVID-19 County by County

HEARST Television

Infectious Disease Epidemiology
- Disease Information
  - Carbapenem-Resistant Enterobacteriaceae
  - Coronavirus Disease 2019 (COVID-19)
    - **COVID-19 Map and Statistics**
    - COVID-19 State and County Dashboards
    - COVID-19 Orders and Guidance
    - COVID-19 Testing Data
    - COVID-19 At-Home Testing
    - COVID-19 Workplace Testing
    - COVID-19 Information for Healthcare Providers
    - COVID-19 Media Campaign
    - COVID-19 Mental Health and Substance Abuse Provider Funding
  - Prion Diseases
  - Anthrax
  - Botulism
  - Brucellosis
  - Campylobacter
  - Colorado Tick Fever
  - Cryptosporidium
  - Ebola Virus Disease
  - E. Coli
  - Giardia
  - Hepatitis A
  - Influenza
  - Meningitis
  - MRSA
  - Norovirus
  - Ozone
  - Plague
  - Pertussis
  - Rabies
  - Rocky Mountain Spotted Fever
  - Salmonella
  - Tularemia
- Reportable Diseases and Conditions
- Reports and Statistics
- Staff
- Case Counts by County
- Foodborne Illness Complaint
- Food Safety
- Heathcare-Associated Infections

COVID-19 Map and Statistics

For an explanation of the graphs shown on this dashboard, please view the
Wyoming COVID-19 Map and Statistics Dashboard Interpretation

44,304 Lab Confirmed Cases	7,608 Probable Cases	596 deaths
42,691 Recovered	7,312 Recovered	Click here for COVID-19 Related Death Information

For testing data please see our COVID-19 Testing Data page:
https://health.wyo.gov/publichealth/infectious-disease-epidemiology-unit/disease/novel-coronavirus/covid-19-testing-data/

Wyoming COVID-19 Lab Confirmed Cases by County of Residence
Hover over a county name to see county level breakdowns | Date Updated 1/31/2021
Click here to view county numbers in a table

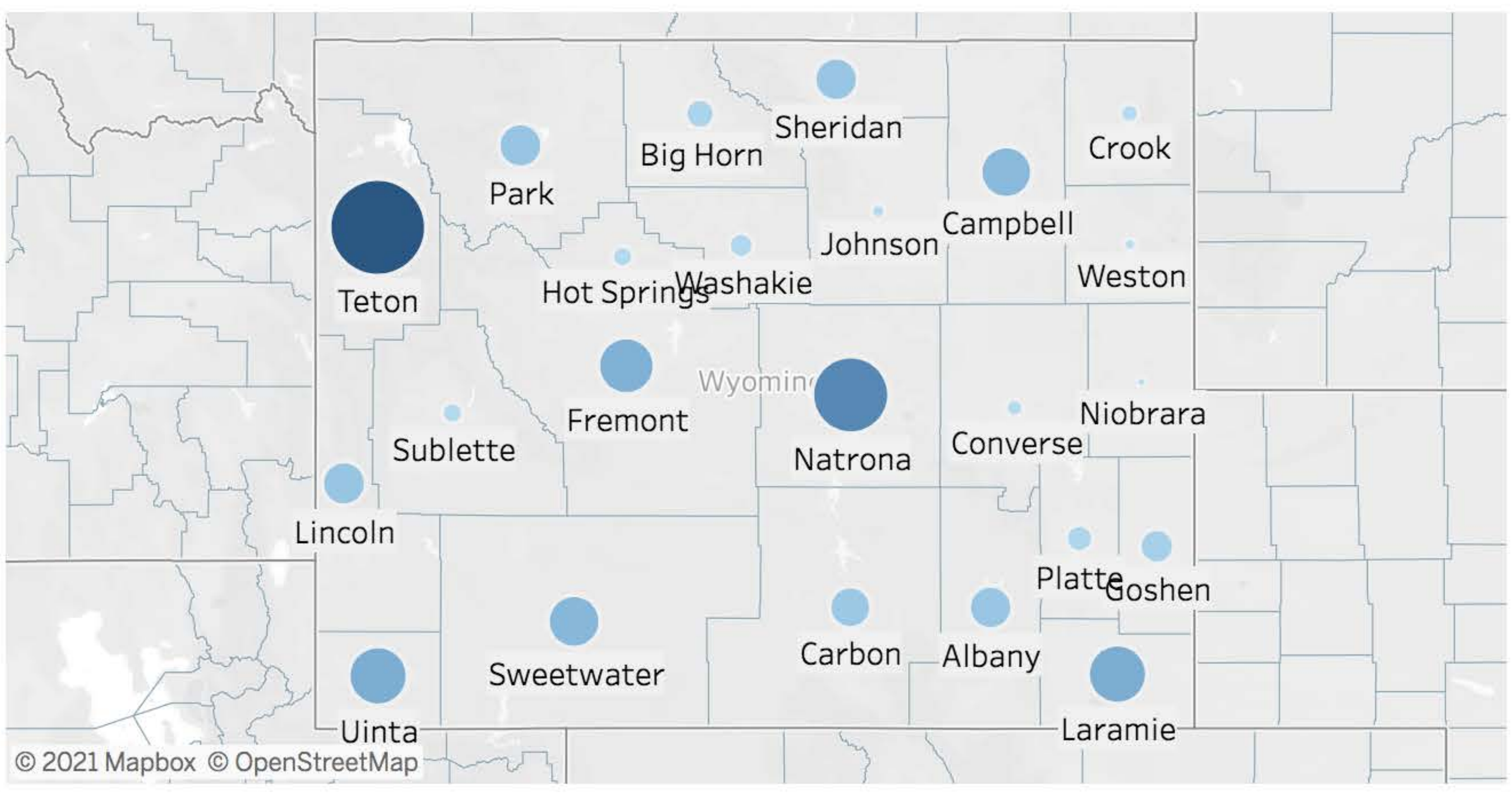

Click here to view cases by City/Town

select range of dates to view trend graphs:
11/1/2020 1/31/2021

Lab Confirmed Cases by Date of Symptom Onset

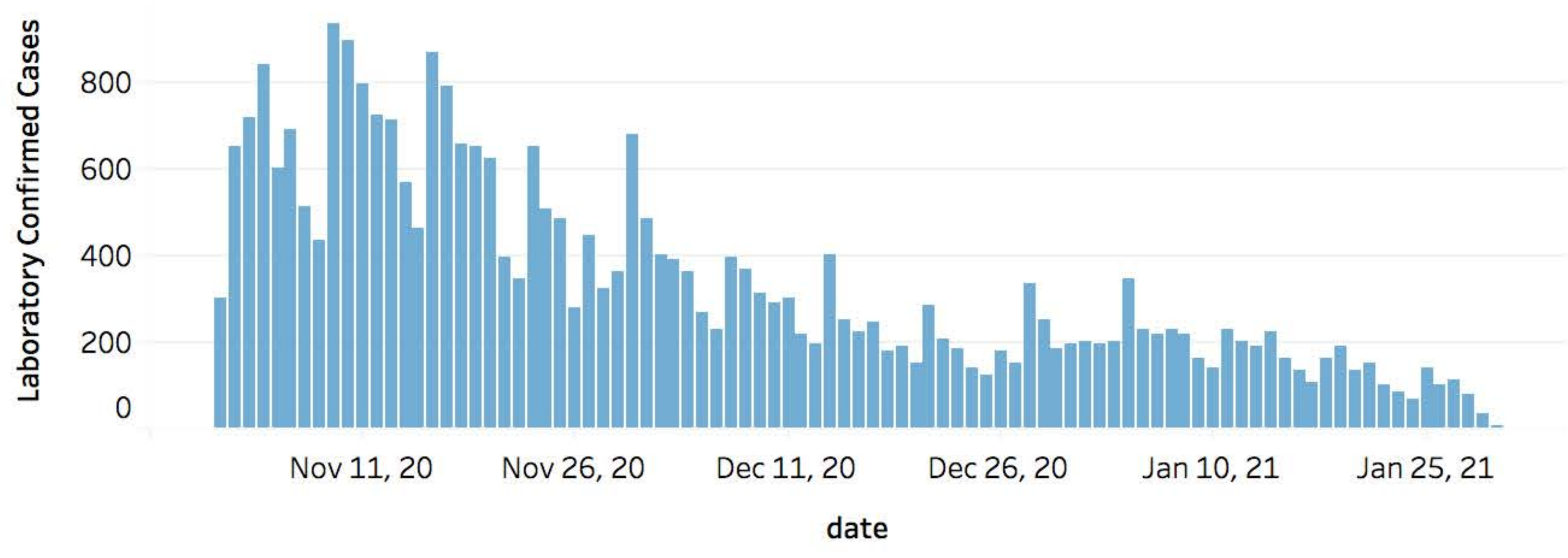

Lab Confirmed Cases by Date of Report, Total Number of **Recovered Lab Confirmed Cases**; and **Percent of Cases with Community Spread or Unknown Exposure**

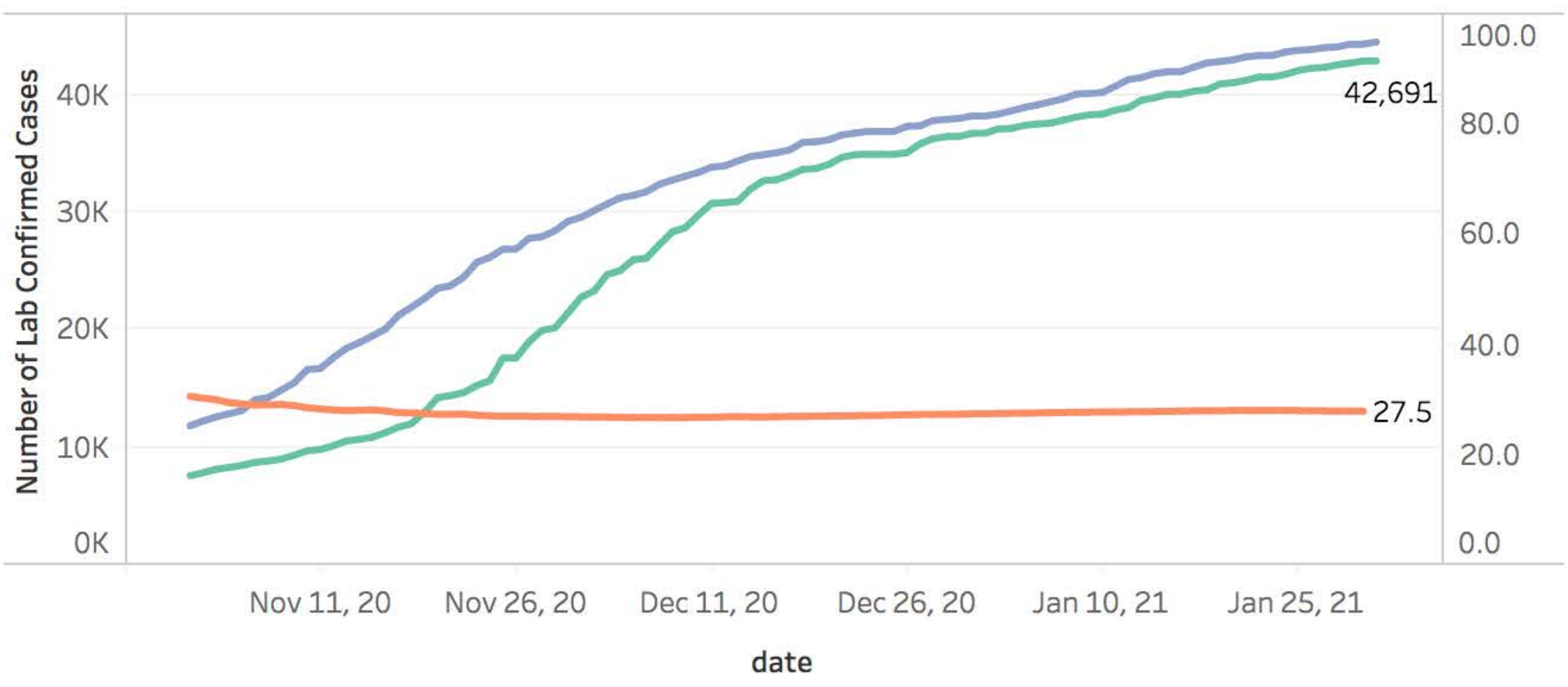

Average Number of Lab Confirmed Cases by Date of Report
Select length of time to compare:
14 day average

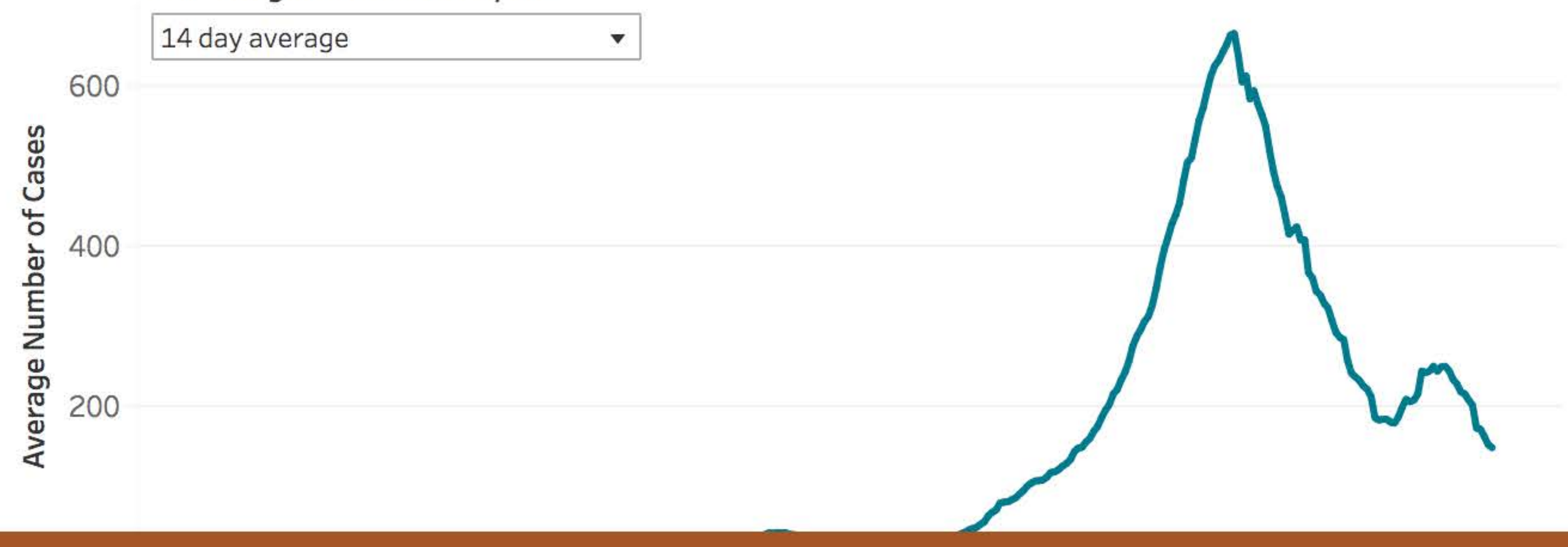

Select Language Powered by Google Translate

Lab Confirmed Cases by Age Group

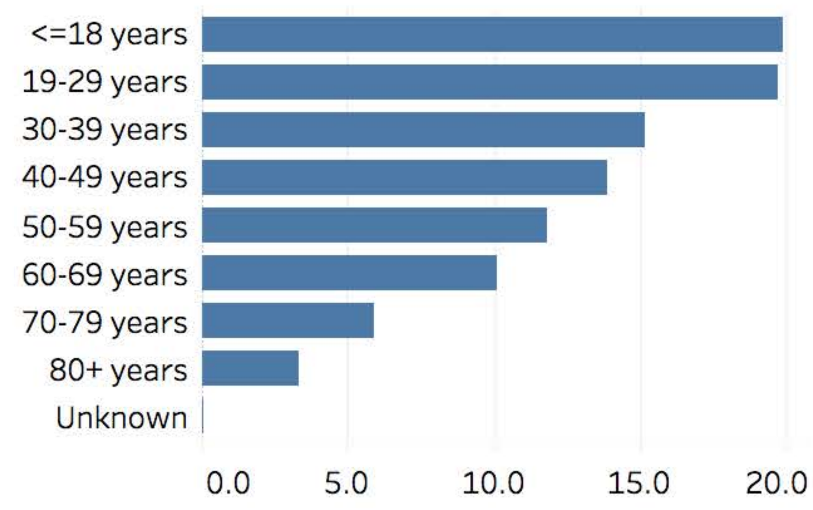

Lab Confirmed Cases by Exposure Risk

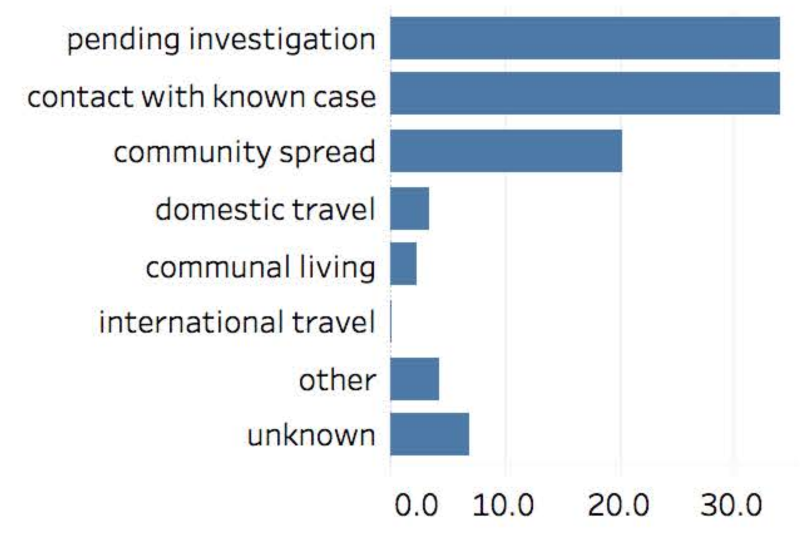

TRACK THE VACCINE: Get the latest map and county-by-county lists of COVID-19 vaccine providers.

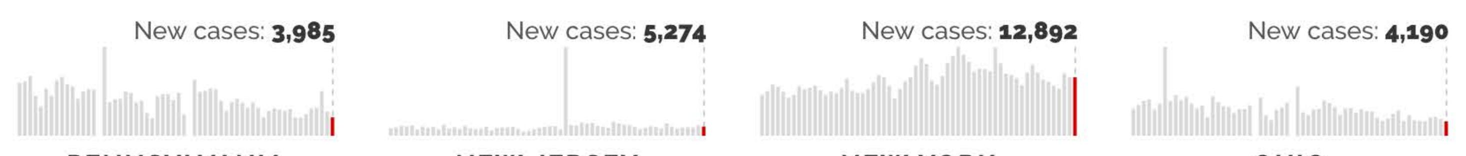

Coronavirus: Tracking The Spread

Since the first case of the coronavirus was identified in Pennsylvania on March 6, we've been tracking its spread. These numbers tell only part of the story. Tests are still not widely available, and you can have the virus — and transmit it — without feeling sick. The numbers shown here represent known cases. Because of limited testing, undiagnosed cases are probably widespread. Every day, we are compiling data from multiple sources, including state and county health departments, to provide a more comprehensive picture of the coronavirus in the region.

GET VACCINATED: Want to know where you can get vaccinated in Pennsylvania? Check out our provider map.

COVID-19 ALERTS

Sign up for a weekly update on the latest coronavirus data for your county.

Email address

Sign up

LAST UPDATED: 1/31/2021 • SOURCES: Pa. Department of Health; COVID Tracking Project

TOTAL TESTS
4,370,894 +16,584 from day before

TOTAL CASES
843,224 +3,985 from day before

TOTAL DEATHS
21,661 +59 from day before

REGIONAL TRENDS

This chart shows a 7-day moving average of new cases added each day per 100,000 people.

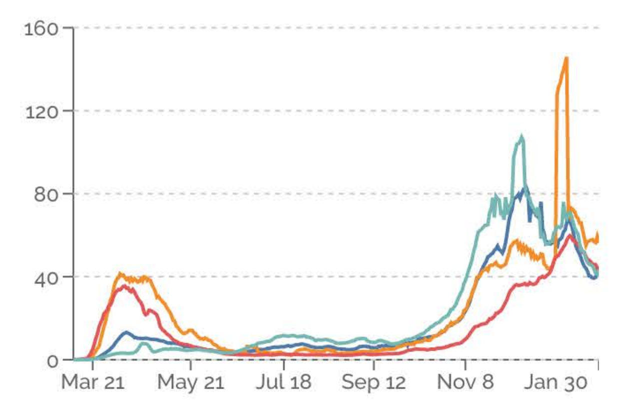

CASES | DEATHS

Per capita | Total

Total reported cases per 100,000 people over past 14 days

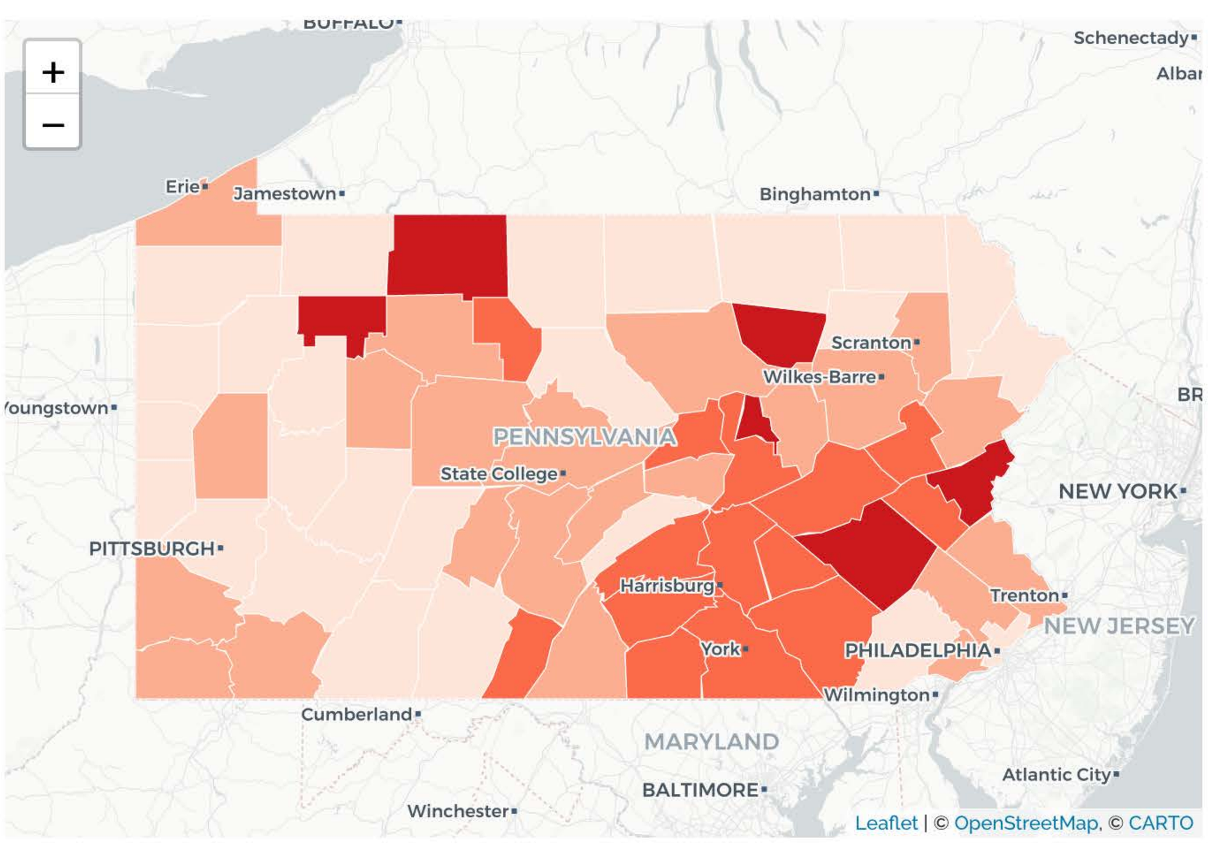

SINCE MARCH | PAST 14 DAYS

Cases by county

Over the past two weeks, 3 counties in Pennsylvania saw a rise in new cases, while 35 were falling. 29 were statistically unchanged or had an unclear trend. The table below shows data since **March 5**. To see data for only the past two weeks, use the toggle above.

Search by county

SORT BY: Name 14-day Trend Cases Deaths Tests

County	Past 14-day trend*	Cases Total	Cases Per 100k people	Deaths Total	Deaths Per 100k people	Tests Total	Tests Per 100k people
Philadelphia	Unclear	104,211	6,579	2,908	184	610,772	38,557
Allegheny	Falling	69,368	5,704	1,520	125	392,112	32,245
Montgomery	Unclear	48,612	5,850	1,389	167	335,192	40,340
Bucks	Unclear	40,193	6,397	1,037	165	235,317	37,455
Lancaster	Unclear	38,485	7,052	921	169	189,687	34,759
Delaware	Unclear	37,339	6,588	1,136	200	227,274	40,101
Berks	Unclear	32,577	7,735	802	190	129,803	30,820
York	Unclear	32,142	7,158	634	141	148,611	33,094
Lehigh	Unclear	28,135	7,618	688	186	141,379	38,281
Chester	Unclear	27,405	5,220	660	126	191,261	36,431

SHOW ALL

NOTE: Past 14-day trend is derived from a linear regression analysis of the 7-day moving average of new cases added each day over the past 14 days. Care should be taken in interpreting 'rising' or 'falling' trends for rural counties with low numbers of new daily cases. Read our FAQ for more information about our methodology.
SOURCES: Pa. Department of Health; Spotlight Pa/Philadelphia Inquirer analysis

DAILY TOTALS | CUMULATIVE TOTALS

Cases and deaths in Pennsylvania

There have been an average of 5,613 new cases and 150 deaths each day over the past week in Pennsylvania. Below are 7-day moving averages since March 5.

CASES

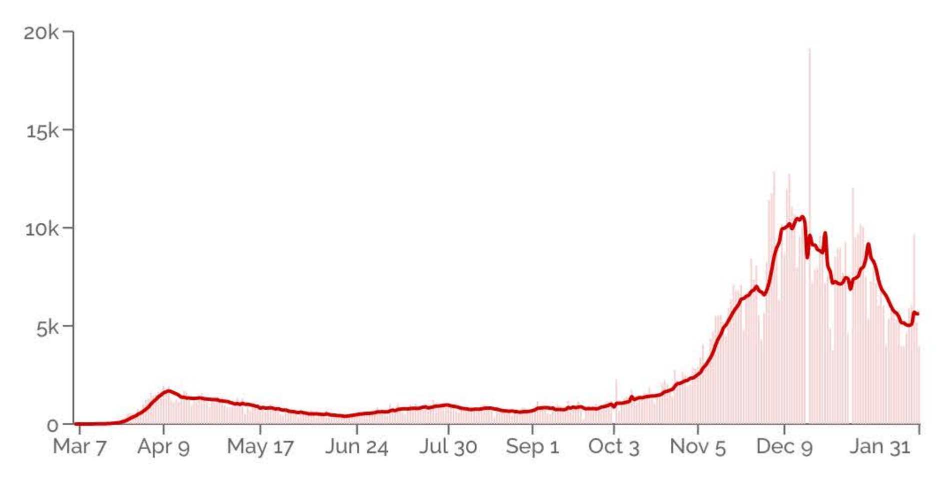

DEATHS

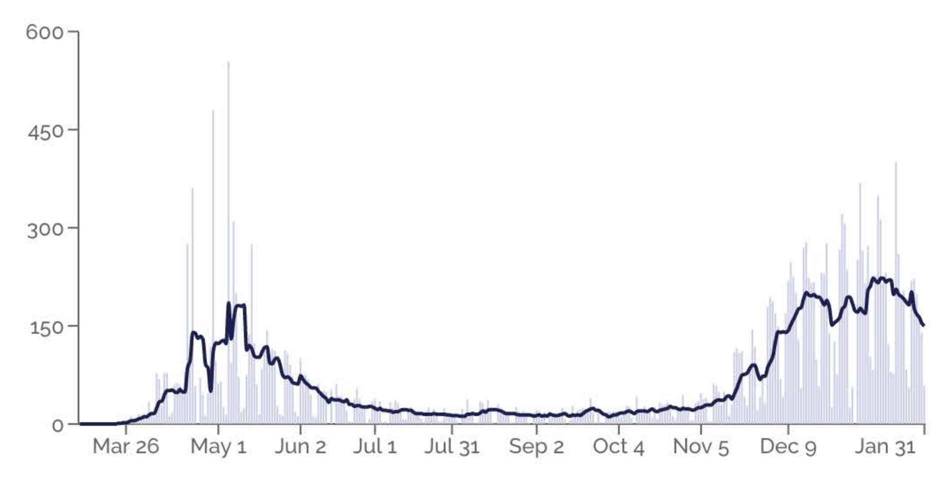

NOTE: Cases include both lab-confirmed positive results and cases deemed probable based on federal criteria. Several large spikes are present in death totals in April and May due to changes the Pa. Department of Health made to how it counts COVID-19 deaths .
SOURCE: Pa. Department of Health

DAILY TOTALS | MORE DATA

Testing in Pennsylvania

Pennsylvania reported an average of 14,982 new test results each day over the past week and had a positivity rate of 25.2%. The positivity rate is the average daily number of new positive tests over the past seven days divided by the average daily number of new tests over the same period.

TESTS PER DAY

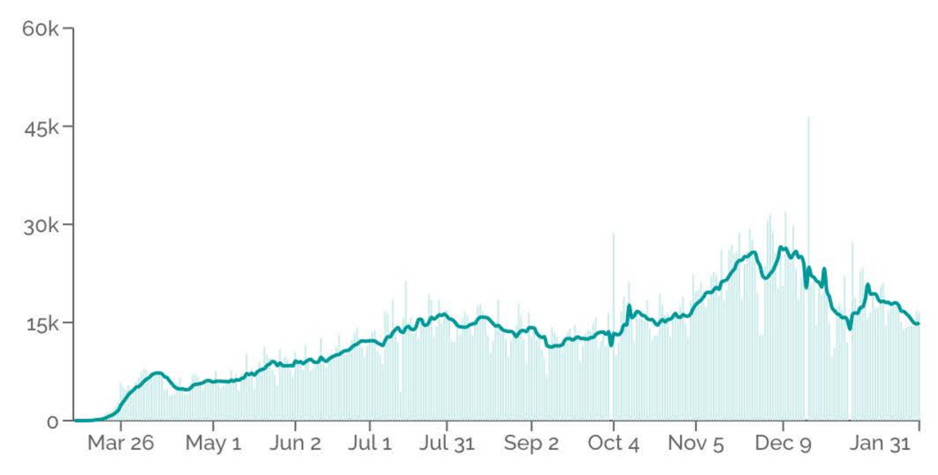

POSITIVITY RATE

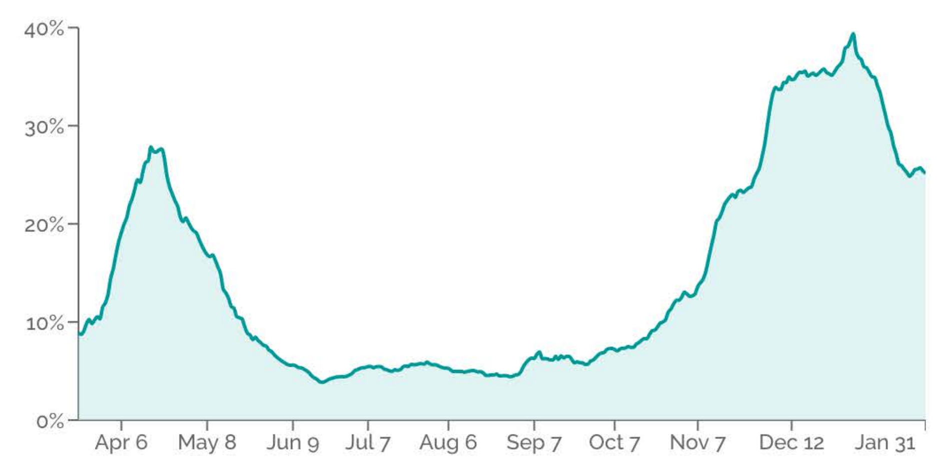

NOTE: Total test numbers are calculated by adding together positive and negative lab results. Note that 'total' and 'positive' numbers prior to July 13 include a small number of probable cases in addition to lab-confirmed results. Probable cases are cases that are determined likely to be COVID-19 based on federal criteria rather than lab results. Please note that the positivity rate above may differ from the rate calculated by the Pa. Department of Health due to differences in methodology. Please read our FAQ for more details.
SOURCE: Pa. Department of Health

Hospitalizations in Pennsylvania

As of January 31, 3370 people in Pennsylvania were in the hospital due to the coronavirus and 428 people were on ventilators. There have been an average of 3,663 people in the hospital and 440 people on ventilators each day over the past week.

PEOPLE IN HOSPITAL

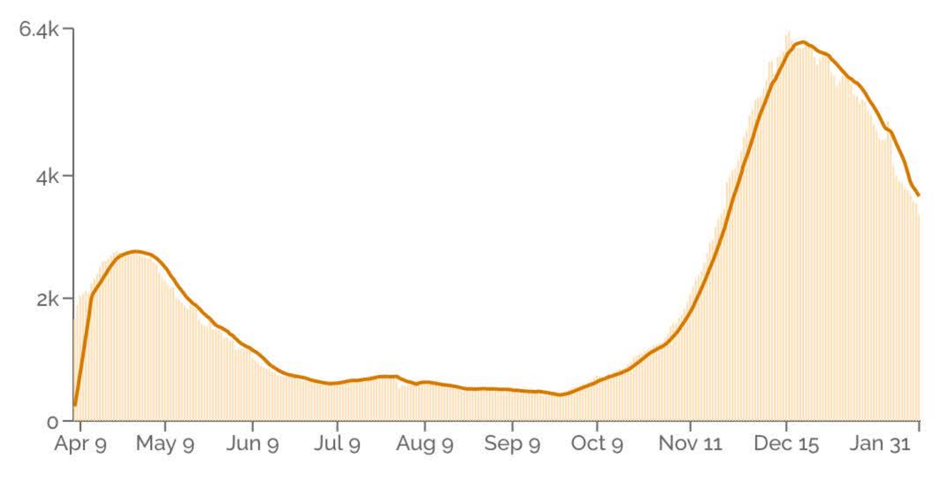

PEOPLE ON VENTILATORS

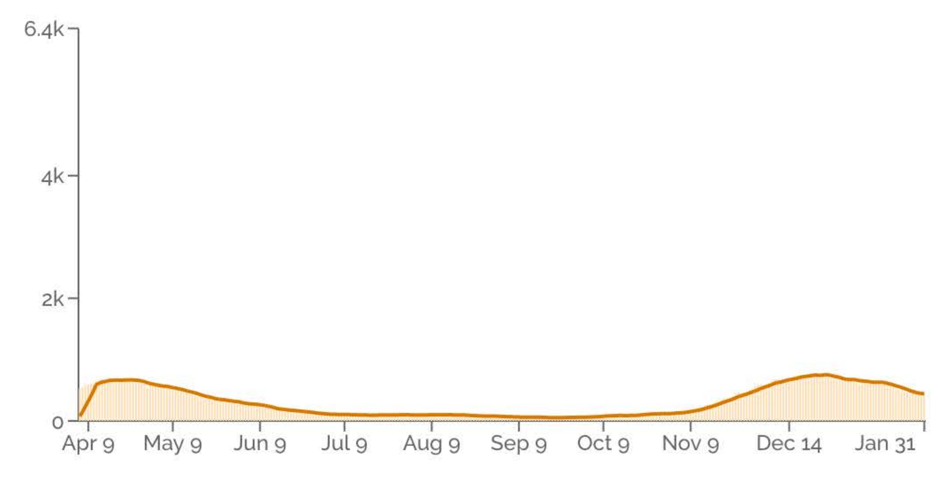

NOTE: Gaps in hospitalization and ventilator numbers may exist for certain periods of time. These gaps may be due to a lack of publicly-available data or because the data was not being compiled by the COVID Tracking Project at that time.

Tracking Coronavirus in Pennsylvania

Cases, deaths, and tests data from the Pa. Department of Health is compiled daily by Spotlight PA/The Philadelphia Inquirer from the department's interactive dashboard. The data in the department's dashboard may not always be consistent with the numbers reported in its press materials or other pages of its website. When major discrepancies exist, Spotlight PA/The Philadelphia Inquirer either defers to the numbers reported in its press materials or contacts the department for clarification. Read our FAQ for more information about our sourcing and methodology.

CREDITS

Graphics: Daniel Simmons-Ritchie, *Spotlight PA* and John Duchneskie, Dylan Purcell and Dominique DeMoe, *Philadelphia Inquirer*.

Developers: Daniel Simmons-Ritchie, *Spotlight PA* and Garland Potts and Dain Saint, *Philadelphia Inquirer*.

Editors: Chris Baxter, *Spotlight PA* and Jessica Parks, *Philadelphia Inquirer*.

COVID-19 TRACKING: Charts & Interactive Data

The very latest visual data on cases and statistics of coronavirus in Rhode Island and Massachusetts

NOTE: This tracking page includes the most current data available. The R.I. Department of Health is now only updating data Monday through Friday. Some categories — including geographic and demographic data — are updated intermittently.

COVID-19 in Rhode Island

Total Cases	Total Deaths	Currently Hospitalized	Currently in ICU	Currently on Ventilator	Total Hospital Discharges
114,438	2,154	324	50	31	6,941

Digital graphic created by Target 12 * Total cases cumulative since March 1, 2020

Interactive content by Flourish

How Many People Have Been Vaccinated in Rhode Island

(RI pop. ~ 1 million)

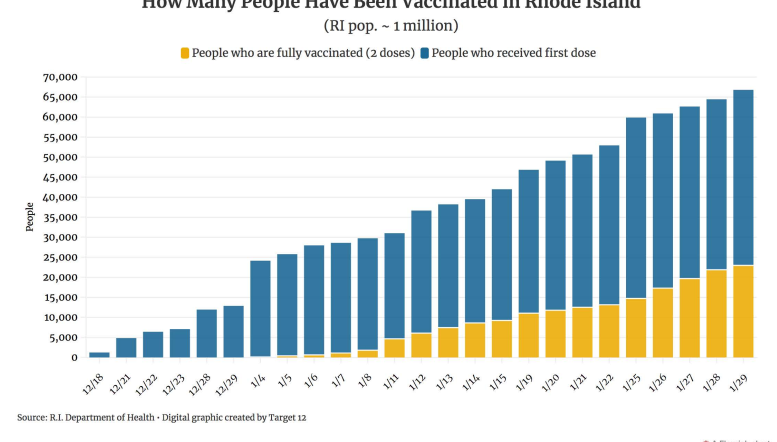

Source: R.I. Department of Health • Digital graphic created by Target 12

A Flourish chart

Vaccine Doses Administered by Week in Rhode Island

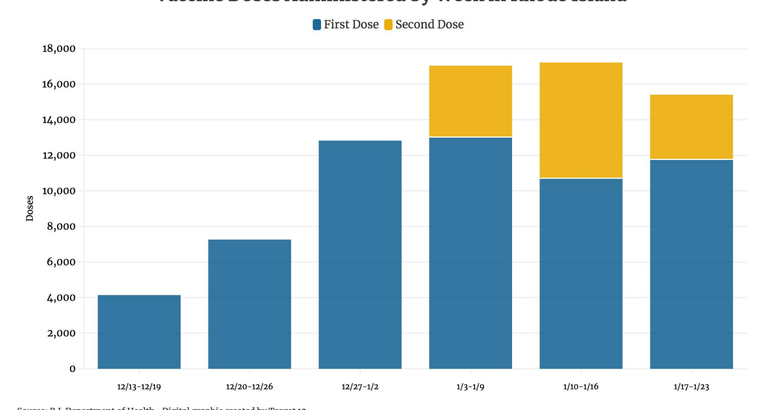

Source: R.I. Department of Health • Digital graphic created by Target 12

A Flourish chart

Vaccine Supply and Redistribution in Rhode Island

(RI data updated weekly)

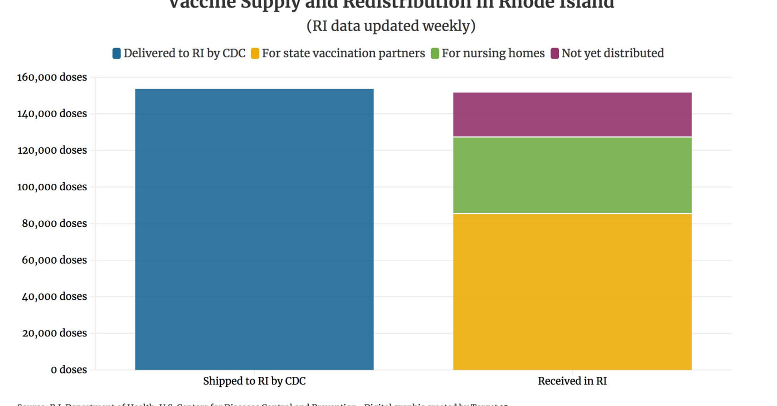

Source: R.I. Department of Health, U.S. Centers for Disease Control and Prevention • Digital graphic created by Target 12

A Flourish chart

7-Day Average of New Positive Cases in Rhode Island

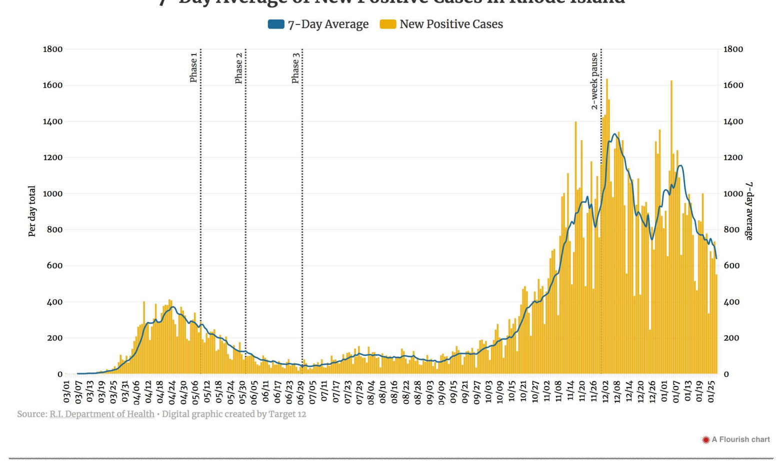

Source: R.I. Department of Health • Digital graphic created by Target 12

A Flourish chart

COVID-19 Deaths in Rhode Island

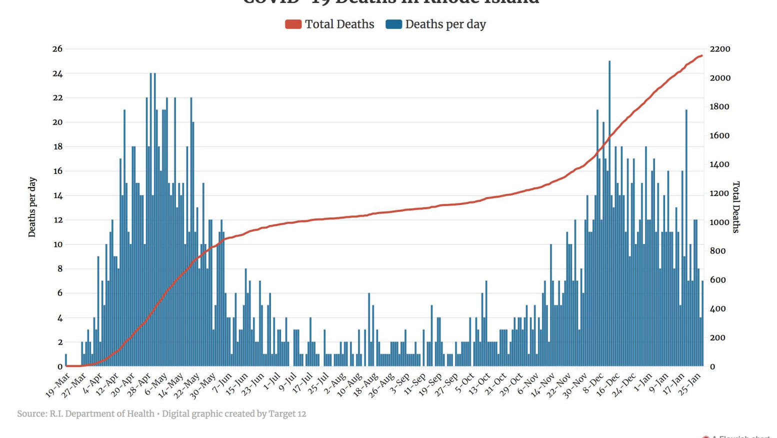

Source: R.I. Department of Health • Digital graphic created by Target 12

A Flourish chart

Total COVID-19 Hospitalizations in Rhode Island

(RI data revised daily with a two-day lag in reporting)

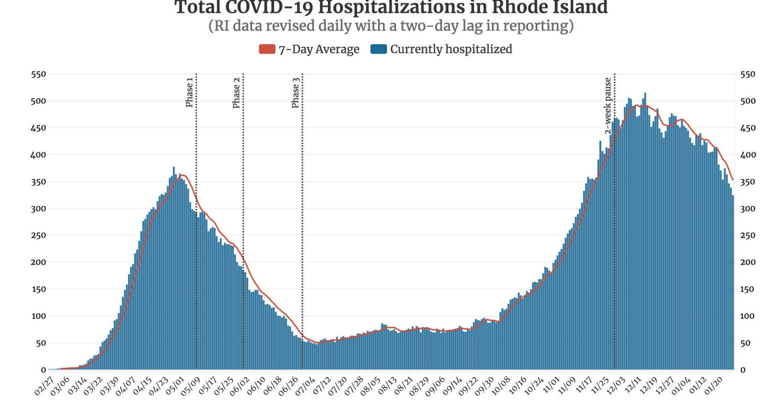

Source: R.I. Department of Health • Digital graphic created by Target 12

A Flourish chart

New Hospital Admissions in Rhode Island

(RI data revised daily with a two-day lag)

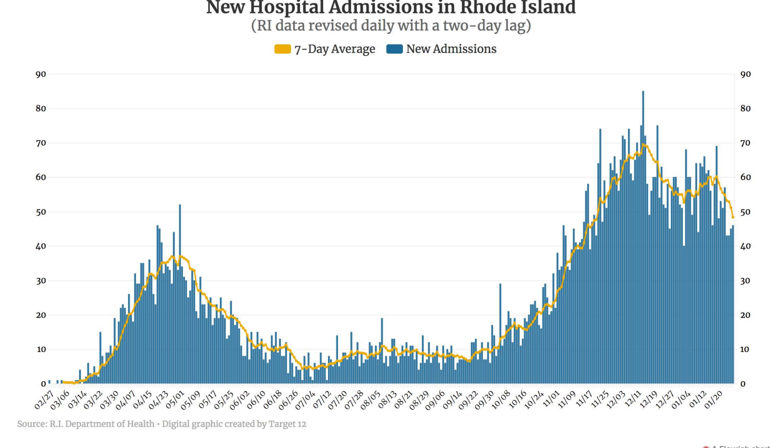

Source: R.I. Department of Health • Digital graphic created by Target 12

A Flourish chart

COVID-19 Intensive Care in Rhode Island

(RI data revised daily with a two-day lag)

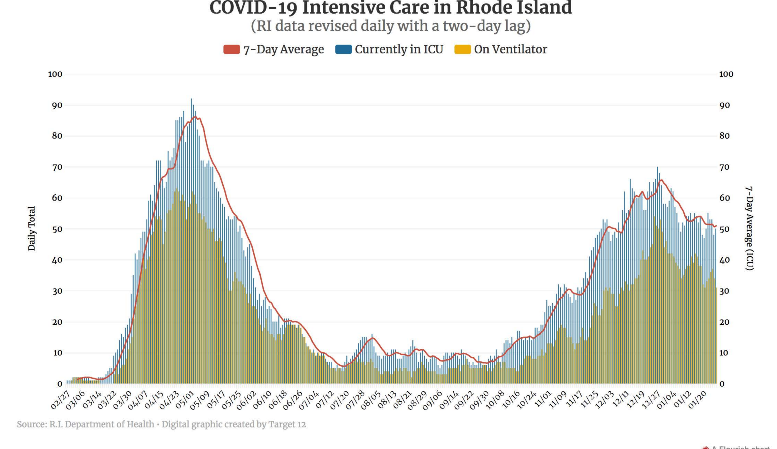

Source: R.I. Department of Health • Digital graphic created by Target 12

A Flourish chart

Rhode Island Positivity Rate

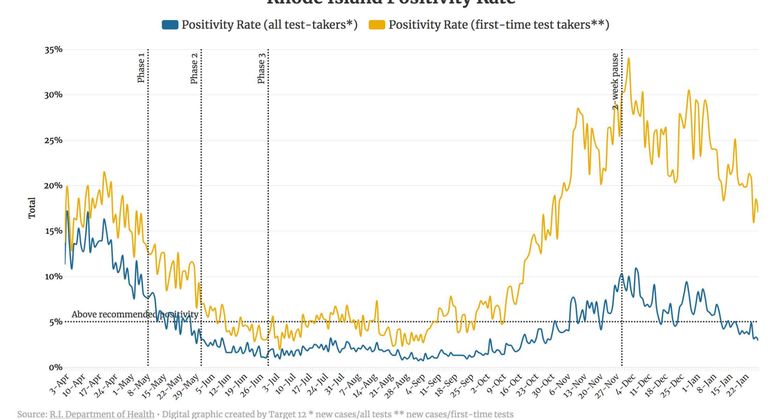

Source: R.I. Department of Health • Digital graphic created by Target 12 * new cases/all tests ** new cases/first-time tests

A Flourish chart

COVID-19 Cases by School

In-person and hybrid learning (last updated 1/20)

Search here...

School	LEA	New Student Cases in Past 7 Days†	Cumulative Student Cases since 9/14/2020	New Staff Cases in Past 7 Days†	Cumulative Staff Cases since 9/14/2020
AF Providence Mayoral Academy Middle	Achievement First Rhode Island	0	0	0	Fewer than five
Achievement First Iluminar Mayoral Academy	Achievement First Rhode Island	0	Fewer than five	0	Fewer than five
Achievement First Promesa Elementary	Achievement First Rhode Island	0	Fewer than five	0	Fewer than five
Achievement First Providence Mayoral Academy	Achievement First Rhode Island	0	10 to 14	0	Fewer than five
Barrington High School	Barrington	0	15 to 19	0	5 to 9
Barrington Middle School	Barrington	Fewer than five	25 to 29	0	5 to 9
Hampden Meadows School	Barrington	0	10 to 14	0	5 to 9
Nayatt School	Barrington	0	10 to 14	0	5 to 9
Primrose Hill School	Barrington	Fewer than five	10 to 14	0	5 to 9
Sowams Elementary School	Barrington	0	Fewer than five	0	0

1 / 44

"Data Sources: RIDOH, RIDE Notes: Data reflect any student or staff association with school and does not indicate in-school transmission of COVID-19 as cases may have been exposed outside of school. School association is determined based on school scheduling portal and case investigation. Counts less than five are suppressed in line with RIDOH's Small Numbers Policy. Data are current as of 11/12/2020 9am. †New cases in past 7 days include positive cases between 11/1/2020 and 11/7/2020 ‡""Virtual Cases"" include cases who have not been in school 14 days prior to symptom onset, or if asymptomatic, testing date. * ""Other"" includes cases home-schooled, associated with out-of-state schools and cases associated with multiple schools or districts."
Digital graphic created by Target 12

A Flourish data visualization

COVID-19 Cases by School

Virtual learning (last updated 1/13)

Search here...

School	LEA	New Student Cases in Past 7 Days†	Cumulative Student Cases since 9/14/2020	New Staff Cases in Past 7 Days†	Cumulative Staff Cases since 9/14/2020
AF Iluminar Mayoral Academy Middle School	Achievement First Rhode Island	Fewer than five	Fewer than five	0	0
Achievement First Iluminar Mayoral Academy	Achievement First Rhode Island	Fewer than five	10 to 14	0	Fewer than five
Achievement First Promesa Elementary	Achievement First Rhode Island	0	Fewer than five	0	0
Achievement First Providence Mayoral Academy	Achievement First Rhode Island	Fewer than five	35 to 39	Fewer than five	Fewer than five
Barrington High School	Barrington	5 to 9	40 to 44	0	Fewer than five
Barrington Middle School	Barrington	Fewer than five	5 to 9	Fewer than five	Fewer than five
Hampden Meadows School	Barrington	0	Fewer than five	0	0
Nayatt School	Barrington	0	5 to 9	0	0
Primrose Hill School	Barrington	0	Fewer than five	0	0
Sowams Elementary School	Barrington	0	Fewer than five	0	0

1 / 38

"Data Sources: RIDOH, RIDE Notes: Data reflect any student or staff association with school and does not indicate in-school transmission of COVID-19 as cases may have been exposed outside of school. School association is determined based on school scheduling portal and case investigation. Counts less than five are suppressed in line with RIDOH's Small Numbers Policy. Data are current as of 11/12/2020 9am. †New cases in past 7 days include positive cases between 11/1/2020 and 11/7/2020 ‡""Virtual Cases"" include cases who have not been in school 14 days prior to symptom onset, or if asymptomatic, testing date. * ""Other"" includes cases home-schooled, associated with out-of-state schools and cases associated with multiple schools or districts."
Digital graphic created by Target 12

A Flourish data visualization

7-Day Average of Daily Testing in Rhode Island

Includes all negative and positive tests from each day

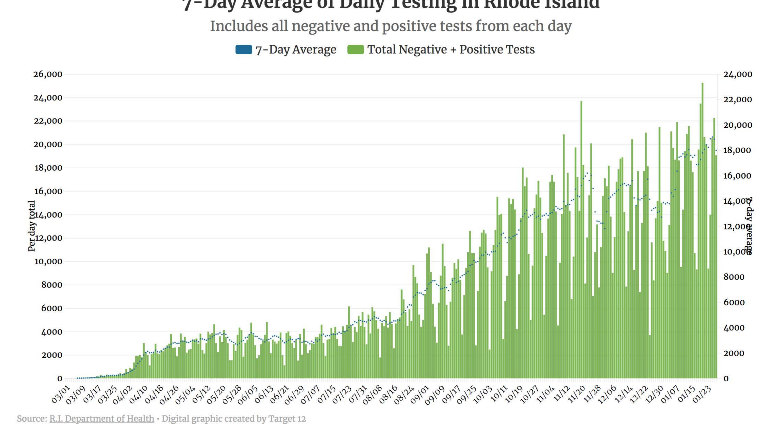

Source: R.I. Department of Health • Digital graphic created by Target 12

A Flourish chart

RI Cases by Age Each Week

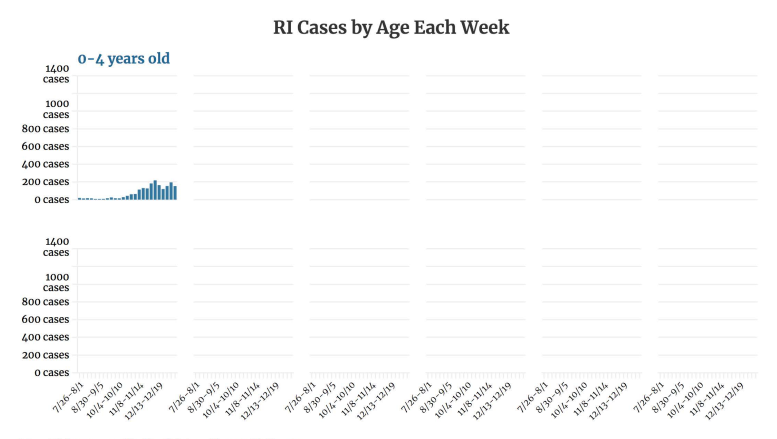

Source: R.I. Department of Health • Digital graphic created by Target 12

A Flourish chart

Total COVID-19 Cases by Age in Rhode Island

(Since March 1)

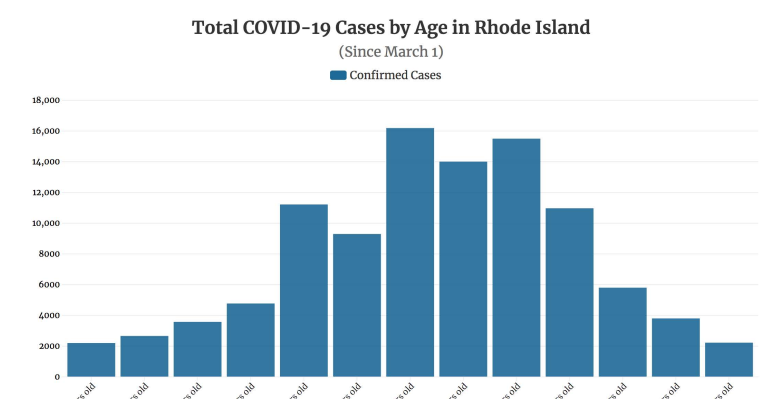

Source: R.I. Department of Health • Digital graphic created by Target 12

A Flourish chart

COVID-19 Hospitalizations & Deaths by Age in RI*

*1 = Fewer than 5

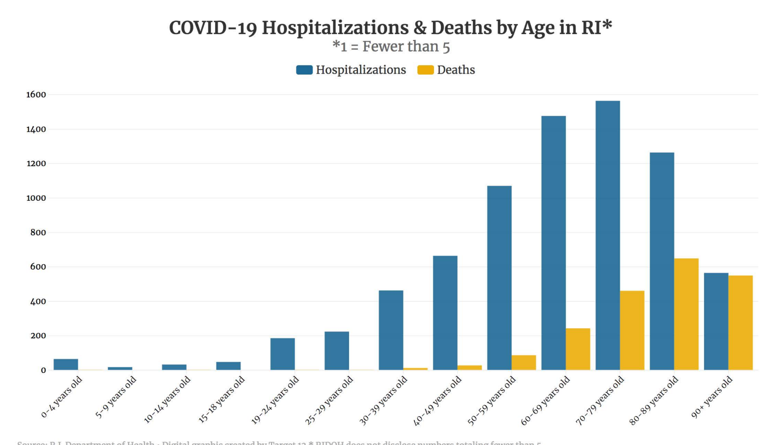

Source: R.I. Department of Health • Digital graphic created by Target 12 * RIDOH does not disclose numbers totaling fewer than 5

A Flourish chart

COVID-19 Cases, Hospitalizations and Deaths by Sex in Rhode Island

(Sex assigned at birth)

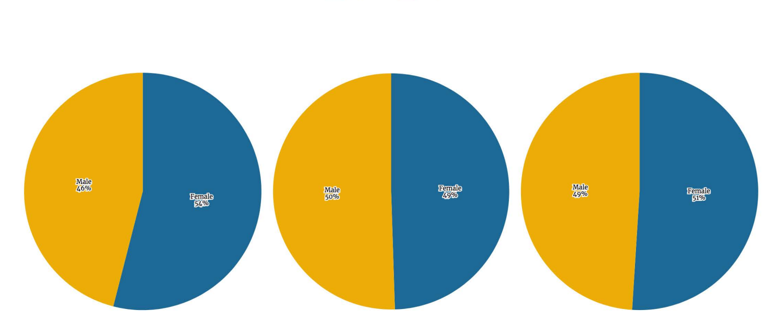

Source: R.I. Department of Health • Digital graphic created by Target 12

A Flourish chart

COVID-19 by Race and Ethnicity in Rhode Island

Percentages based on known cases*

1% = 1% or less than 1%

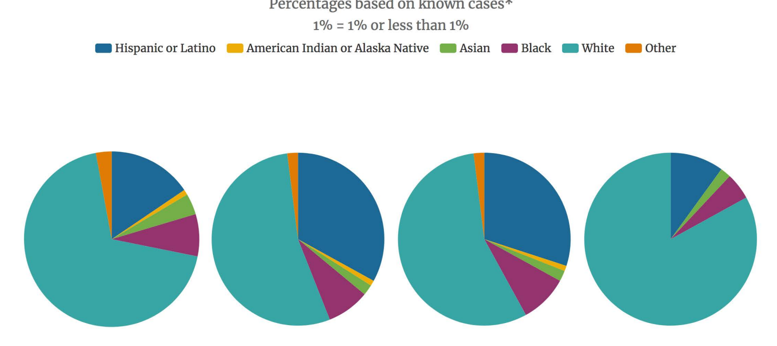

Source: R.I. Department of Health • Digital graphic created by Target 12 * Total may not equal 100% due to rounding

A Flourish chart

For maps of cases, hospitalizations and deaths by community in Rhode Island and Massachusetts,

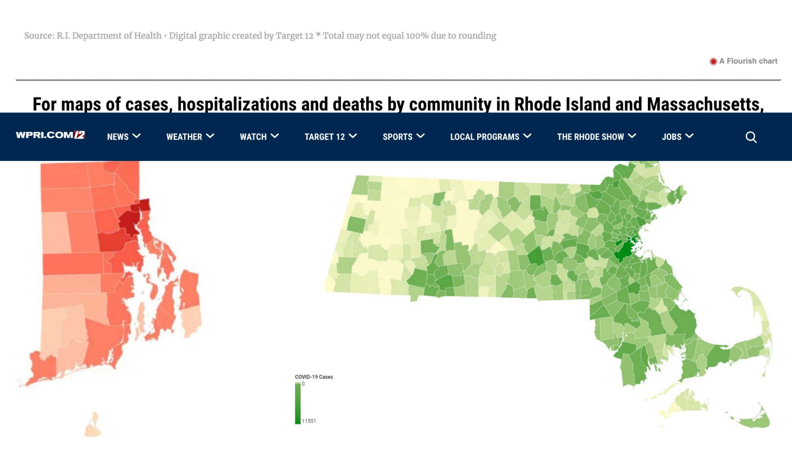

Bay Area		California		United States	
373,002 cases	4,216 deaths	3,297,726 cases	40,869 deaths	26,178,803 cases	441,282 deaths
397 on Jan. 31	7 on Jan. 31	7,760 on Jan. 31	169 on Jan. 31	142,091 on Jan. 30	2,731 on Jan. 30

Coronavirus Tracker

The Chronicle is mapping every reported coronavirus case in the Bay Area, California and the U.S. We are tallying the number of confirmed cases, deaths, hospitalizations and tests reported across the state by day.

By CHRONICLE DIGITAL TEAM | LAST UPDATED: Jan. 31, 2021 1:40 p.m.

Total cases | New cases

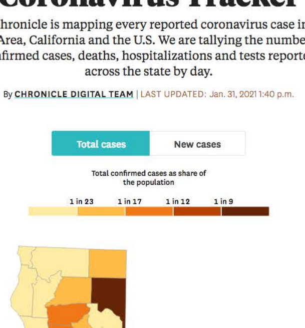

Hover over the map to see details for each county

California cases are organized by reporting county. Cases are based on reports from the U.S. Centers for Disease Control and Prevention, the California Department of Public Health and county public health departments, plus the Chronicle / Los Angeles Times data collaboration. For more information on the data, please read about our methodology.

What are the latest developments?

Charts show that California seems to be emerging from a "surge on top of a surge," but the battle against the coronavirus is far from over. With the state beginning to reopen and more variants being discovered all the time, experts say it remains vital for people to wear masks, avoid gatherings and follow safety protocols. Amid the positive signs like declining case rates and increased vaccinations have come some grim milestones: virus-related deaths have topped 40,000 in California with more than one-third of them reported in January, and Santa Clara County recently became the first in the Bay Area to reach 100,000 reported cases. Although the regional stay-at-home order was lifted nearly a week ago, many weary Bay Area businesses are reopening slowly, hoping to avoid another shutdown. The return of outdoor dining has many people excited, but experts say you should consider these risks before rushing out for that long-awaited meal.

Vaccine details: County-by-county look at when and where vaccines should be available; Crowdsourced website tracks where to get vaccinated

For the latest news and developments, read The Chronicle's coronavirus live updates or sign up for our coronavirus newsletter.

New reported cases and deaths in the Bay Area, by day

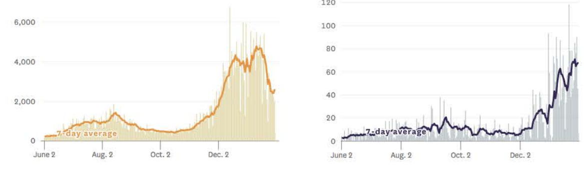

New reported cases and deaths in California, by day

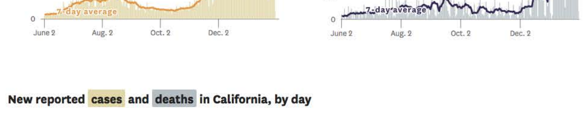

Sources: U.S. Centers for Disease Control and Prevention, California Department of Public Health and county public health departments, Chronicle reporting

The seven-day trailing average represents the average number of cases or deaths reported each day over the weeklong span ending on the given day. It is used to account for daily variances in reporting and large one-off changes in daily counts, such as April 20 when nearly 1,500 cases were reported in L.A. County as the result of a lab clearing a large backlog. Some counties do not provide daily updates which, combined with daily variances in the number of tests given, could result in randomly higher or lower counts for daily reported cases.

How many people are hospitalized?

The number of people hospitalized in the Bay Area due to COVID-19 dropped by 11.2% compared to the previous week and 13.6% statewide. There were fewer than 17,500 COVID patients as of Jan. 24, down from the high of nearly 22,000 on Jan. 6. The number of ICU patients in the Bay Area remained steady for the past week but that number was fewer than 500 for four consecutive days for the first time since Dec. 27. Statewide, the number of ICU patients fell by nearly 7%. Although the regional stay-at-home order was lifted Monday based on four-week ICU availability projections, the state was back to reporting 0% availability on Tuesday with both the Bay Area region and the Greater Sacramento region reporting less than 10% availability. The Southern California and San Joaquin regions were both reporting 0% availability.

ICU capacity by hospital service area

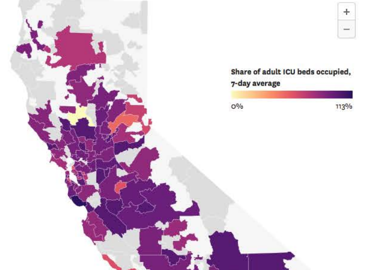

Source: U.S. Department of Health and Human Services

Data is updated every Monday and represents a seven-day period ending on the previous Thursday. It excludes psychiatric hospitals, rehabilitation facilities, Indian Health Service facilities, Veterans Affairs facilities, Defense Health Agency facilities and religious non-medical facilities.

Confirmed ICU and non-ICU COVID-19 patients in Bay Area hospitals

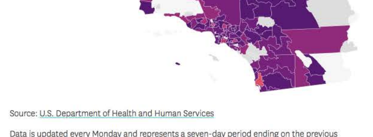

Source: California Department of Public Health

Confirmed ICU and non-ICU COVID-19 patients in California hospitals

Source: California Department of Public Health

Due to limited testing capacity, not all hospitalized patients with COVID-19 symptoms have been tested. While not all suspected cases will ultimately test positive, including these patients may give a more comprehensive picture of the demands on hospitals. Even with suspected cases included, hospitals have not seen a surge in virus-related patients as feared.

Total confirmed and suspected COVID-19 patients in California hospitals

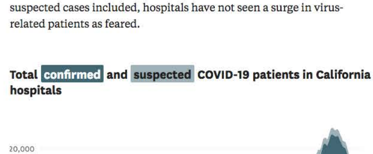

Source: California Department of Public Health

ADVERTISEMENT

Where do Bay Area counties stand in reopening?

Due to four-week projections that put all regions above the 15% ICU availability threshold, the regional stay-at-home order that has been in effect for most of the state since early December was lifted on Monday in all regions. The Bay Area region, which includes the nine Bay Area counties plus Monterey and Santa Cruz, was showing 23.4% ICU availability on Monday morning after being below 5% the past two weeks. The figure was back below 10% later in the day. Reopening across the state now returns to the color-coded tier system with all nine counties in the Bay Area in the most-restrictive purple tier. However, outdoor dining and personal services like hair and nail salons are allowed to reopen, though San Francisco officials have delayed their reopening until at least Thursday. Check the reopening tracker to see what's allowed in each tier and each county.

Find the Bay Area reopening checklist, a detailed list of what's open and what's not for each Bay Area county and the reopening status for each county in California in The Chronicle's reopening tracker

What are the current trends in the Bay Area?

The total number of coronavirus cases reported in the Bay Area last week dropped by 30% compared to the previous week. The Bay Area averaged 3,084 new cases per day last week compared to more than 4,400 two weeks ago. The seven-day average for cases on Jan. 25 was more than 45% lower than the average reported two weeks prior on Jan. 11. The number of virus-related deaths reported in the Bay Area hit a new high last week with 466 total, including 118 on Jan. 21, the first time the state has reported more than 100 deaths on a single day. In October and November, the Bay Area was reporting fewer than 100 deaths per week.

Cases and deaths by Bay Area county

The new case curve represents the 7-day moving average which accounts for variances in daily reporting. The change in new cases compares the number of cases in the past seven days to the previous seven days.

County	Total deaths	Total cases	New cases curve	Weekly change in new cases	
Santa Clara	1,395	101,453	730 (June 1 – Jan. 30)	▲239	+5%
Alameda	953	71,112	557	▼104	-3%
Contra Costa	529	57,021	372	▼238	-8%
San Mateo	382	35,466	248	▼792	-31%
San Francisco	324	31,111	192	▼38	-3%
Solano	122	27,706	173	▼628	-34%
Sonoma	260	25,992	176	▼269	-18%
Marin	172	12,284	51	▼114	-24%
Napa	52	8,198	62	▼167	-28%

Sources: U.S. Centers for Disease Control and Prevention, the California Department of Public Health and county public health departments, Chronicle / Los Angeles Times data collaboration

The Marin County figures include the San Quentin state prison outbreak that began June 20 and totaled more than 2,200 cases.

Bay Area cases by city or region

Some Bay Area county health departments are now providing details on where people who have tested positive reside, either by region, city or jurisdiction. Use the drop down to search counties (more will be added if data becomes available). Tables will be updated Tuesday and Friday.

Alameda County

Place	Total cases
Alameda	1,971
Albany	264
Berkeley	2,583
Castro Valley MAC	2,130
Dublin	1,359
Eden Area MAC	5,020

Show more

What is the status of testing in California?

State labs processed more than 2 million results during the week ending Jan. 24 with a positivity rate of 8.1%, which was down significantly from 11.3% the previous week. The positivity rate had been near 14% in early January. The rate was below 4.0% early in November. Nationally, the positivity rate has fallen to less than 9% from a high of 13.7%. The state used 8% as a goal for reopening in its original plan and now uses positivity rate along with case rate (adjusted based on testing levels) to determine tier status. As more people get tested, the positive test rate provides a more clear picture of how many people are carrying the virus, including those without symptoms. There are several different types of tests now available in California with at-home versions slowly becoming available. Here's a look at your options.

Daily test results reported in California

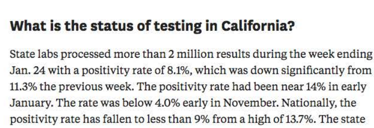

Positive test rate in California

The positive test rate, or positivity rate, is the percentage of total tests that come back positive each day. A lower positivity rate can indicate that enough tests are being conducted. Read more about the positive test rate here.

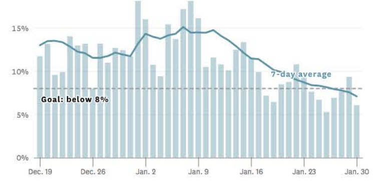

Source: The COVID Tracking Project

Tests are reported by the California Department of Public Health. On April 22, the CDPH began reporting total number of tests conducted, rather than individual people tested, causing test numbers to jump because some individuals are tested more than once.

On July 1, the state reported a backlog of nearly 4,000 positive test results which elevated the positivity rate for that day. This type of variation is accounted for by the trailing seven-day average.

How does California compare to the rest of the nation?

The seven-day average for new cases in the U.S. was down to 170,000 on Jan. 24, a 30% drop over the past 14 days and the lowest level since Dec. 2. Total U.S. cases, however, surpassed 25 million with more than 11 million reported just since the beginning of December. In California, the daily average decreased from about 40,000 to just over 27,000, which is a drop of more than 32%. Total cases have surpassed 3 million with more than 37,000 deaths, including more than 3,500 last week. Global estimates are that 55% of the 100 million patients who tested positive have recovered. Figures for recoveries are not available for the Bay Area or California.

26,178,803 confirmed cases in the U.S.
441,282 deaths

Cases per 100,000 people

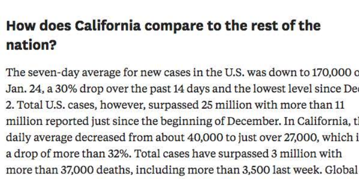

Figures include presumed COVID-19 deaths.

Sources: The Center for Systems Science and Engineering at Johns Hopkins University, U.S. Census Bureau

U.S. reports of COVID-19 are tabulated by Johns Hopkins, which is tracking cases using data collected from the Centers for Disease Control and Prevention and state and local public health departments. Numbers from Johns Hopkins for California may not match the numbers from the top map, which are being compiled by The Chronicle using county reports, county public health departments and Chronicle reporting.

Findings signal earlier start to U.S. outbreak

An autopsy report showing that the first death linked to COVID-19 happened Feb. 6 in Santa Clara County and not Feb. 26 in Washington supports research indicating that the outbreak began in the United States much earlier than we knew and before health officials began tracking data.

There were 300 cases reported in the Bay Area when shelter-in-place orders were put in place on March 16. But studies and disease models suggest that more than 10,000 people in the Bay Area likely were infected by then. That research, along with the earlier death identified in Santa Clara County, indicates how far behind the U.S. was in recognizing the outbreak of the virus.

The data used here examines the spread of the virus and potential containment going back to when officials knew the virus had arrived in the United States. While this data is limited and flawed in its ability to give a full history of the virus, it is all health officials have and underscores the importance of increased testing to provide a clearer picture of the current situation.

HAVE MORE QUESTIONS?

We're answering our readers' most common questions here. Below you can find even more answers for issues specific to the Bay Area.

- Where can I get tested?
- When do I need to wear a face mask?
- Reopening is confusing. What's happening in my county?
- How is school going to work?
- There's a state watchlist? What does that mean?
- I'm out of work. Is anyone hiring?
- Can I get COVID-19 from food?
- What are my grocery delivery options?
- Which restaurants are offering takeout and delivery?

FIFTH & MISSION PODCAST

Fifth & Mission is diving into all the ways COVID-19 is impacting life in the Bay Area. Listen by selecting your preferred podcast service below.

APPLE | SPOTIFY | OVERCAST | GOOGLE | STITCHER | POCKET

TELL US YOUR TOP CONCERNS

ASSIGNMENT EDITOR

We want to report the stories that matter to you. Tell us the questions you have about the pandemic's impact on life in the Bay Area, and the issues you want to see covered.

Tell us your questions; what should we investigate?

NEXT

ABOUT THE DATA

Update: Starting Sept. 28, the data powering the charts and maps on our Coronavirus Tracker come from a collaboration between journalists at The Chronicle and the Los Angeles Times.

The Chronicle is compiling an up-to-date case count from several sources. The Centers for Disease Control and Prevention updates its confirmed case count on its website. That is a broad list that breaks down all U.S. cases and deaths. The California Department of Public Health reports new cases in the state daily. Individual counties in California report new cases as they are confirmed by testing. The tracker may not match case counts from the California Department of Public Health due to 24-hour lags in reporting at the state level. Errors in data from state or county reporting agencies will be corrected as soon as possible and may result in changes to daily case totals.

The Chronicle tracker includes only cases of people who have tested positive for coronavirus – there are almost certainly many more cases among California residents who have not been tested. Maps show cumulative case counts, and do not differentiate between people who have recovered and people who are actively ill.

Up to 20 people who tested positive for the coronaviurs while under quarantine at Travis Air Force Base and were transferred to Northern California counties for care are not included on the California map. On March 12, The Chronicle changed how some of those individuals are counted, and removed 10 cases from county totals. The change in counting is reflected in the overall total for the Bay Area as well as some counties.

CREDITS

HEALTH REPORTER

Erin Allday · eallday@sfchronicle.com · @erinallday

STAFF WRITER

Mike Massa · mmassa@sfchronicle.com

NEWSROOM DEVELOPERS

Audrey DeBruine · audrey.debruine@sfchronicle.com · @acdebruine

Kazi Awal · kazi.awal@sfchronicle.com · @superKazi

Eric Blom · eric.blom@sfchronicle.com · @ejblom

Evan Wagstaff · evan.wagstaff@sfchronicle.com · @evanwagstaff

Hilary Fung · hilary.fung@sfchronicle.com · @hil_fung

ART DIRECTOR

Danielle Mollette-Parks · dmollette-parks@sfchronicle.com · @daniellemparks

EXECUTIVE PRODUCER, DIGITAL

Brittany Schell · bschell@sfchronicle.com · @brittlynns

MANAGING EDITOR, DIGITAL

Tim O'Rourke · torourke@sfchronicle.com · @TimothyORourke

©2021 Hearst

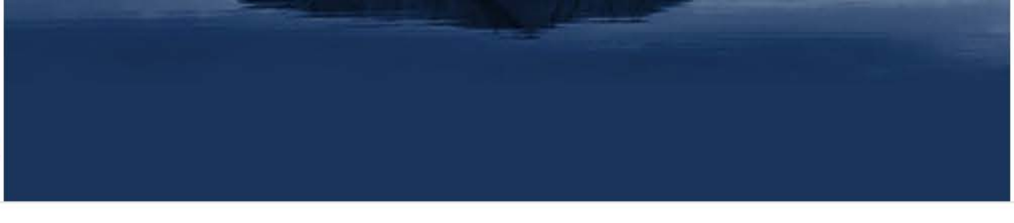

CORONAVIRUS, HEALTH

Coronavirus in Maine: Data Dashboard

Explore trends in Maine's COVID-19 cases by county, age and sex of the afflicted individuals and more in charts updated daily with data from Maine's CDC.

BY DARREN FISHELL | APRIL 2, 2020

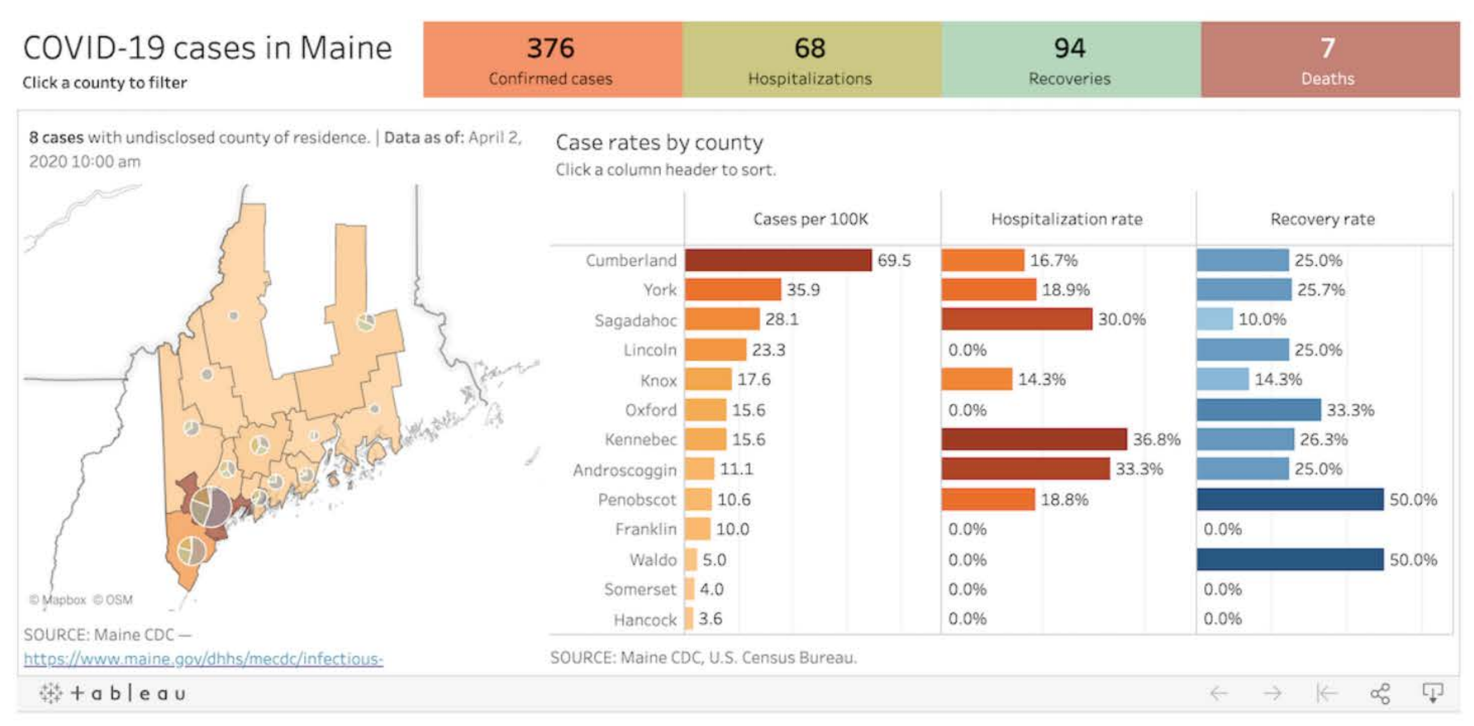

COVID-19, the illness caused by the novel coronavirus, continues to spread throughout Maine.

The Maine Center for Disease Control and Prevention is publishing details about each confirmed case in the state, which are visualized below.

As the CDC publishes these numbers, its director, Dr. Nirav Shah, has noted that it's unclear how much the data lags behind the real spread of the virus.

"What we know about outbreaks is that we are often just detecting the tip of the iceberg," Shah told reporters on March 22.

Total cases

The Maine CDC breaks out cases at the county level by deaths, hospitalizations, recoveries and total confirmed cases. Each of the categories subtracted from total confirmed cases results in the other confirmed cases, shown below.

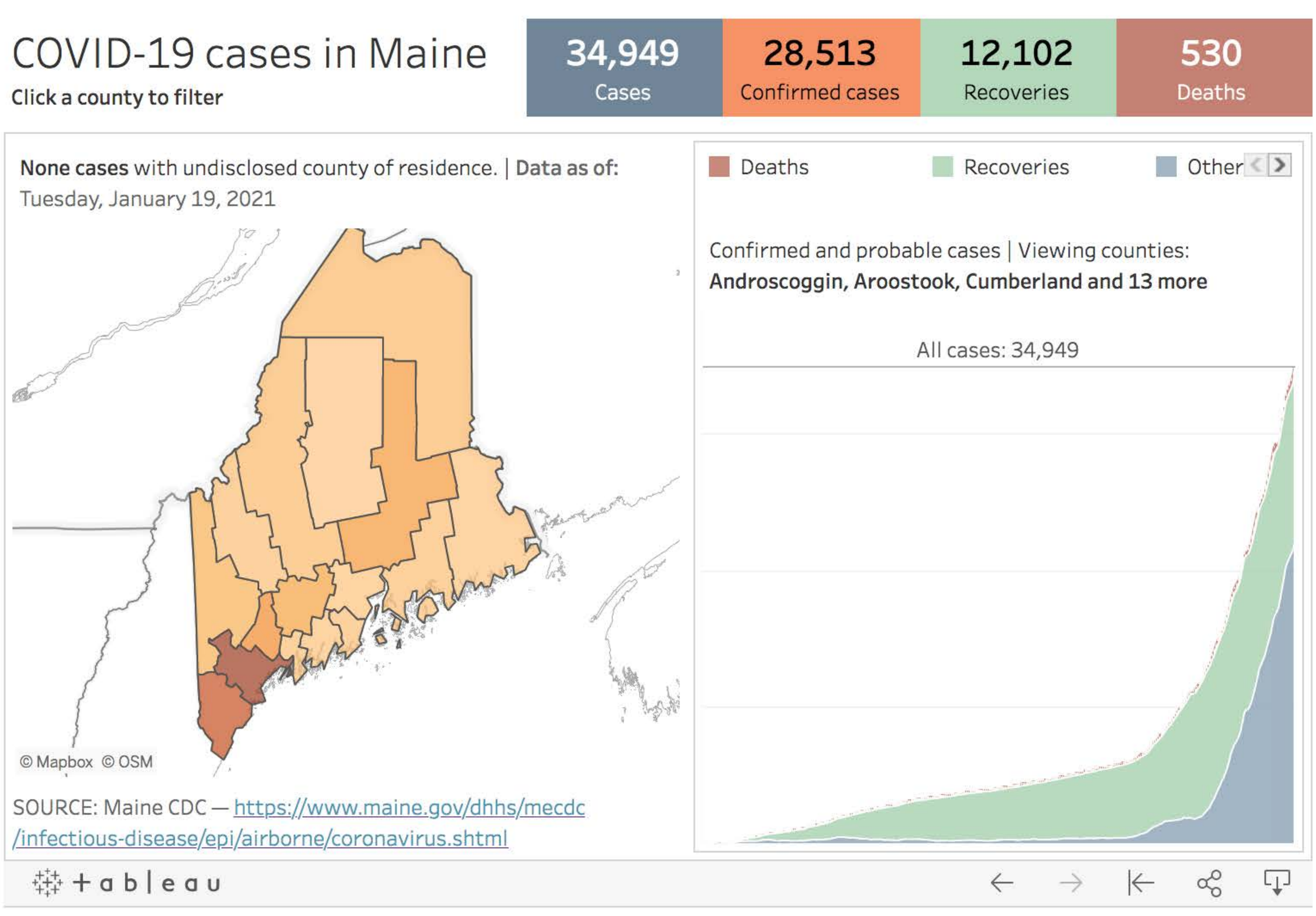

New cases

The rate of growth of cases is important to watch. Social distancing measures intend to slow the spread of the disease so that fewer people are sick all at once, which stands to overwhelm the healthcare system.

Cumberland County has seen the largest spikes in new cases, hitting a high for new cases each day. The early numbers could be impacted by delays in testing, as well.

The chart also illustrates some of the path the disease has taken through Maine counties, starting from March 18. Piscataquis, Aroostook and Washington counties had not reported a case as of April 1.

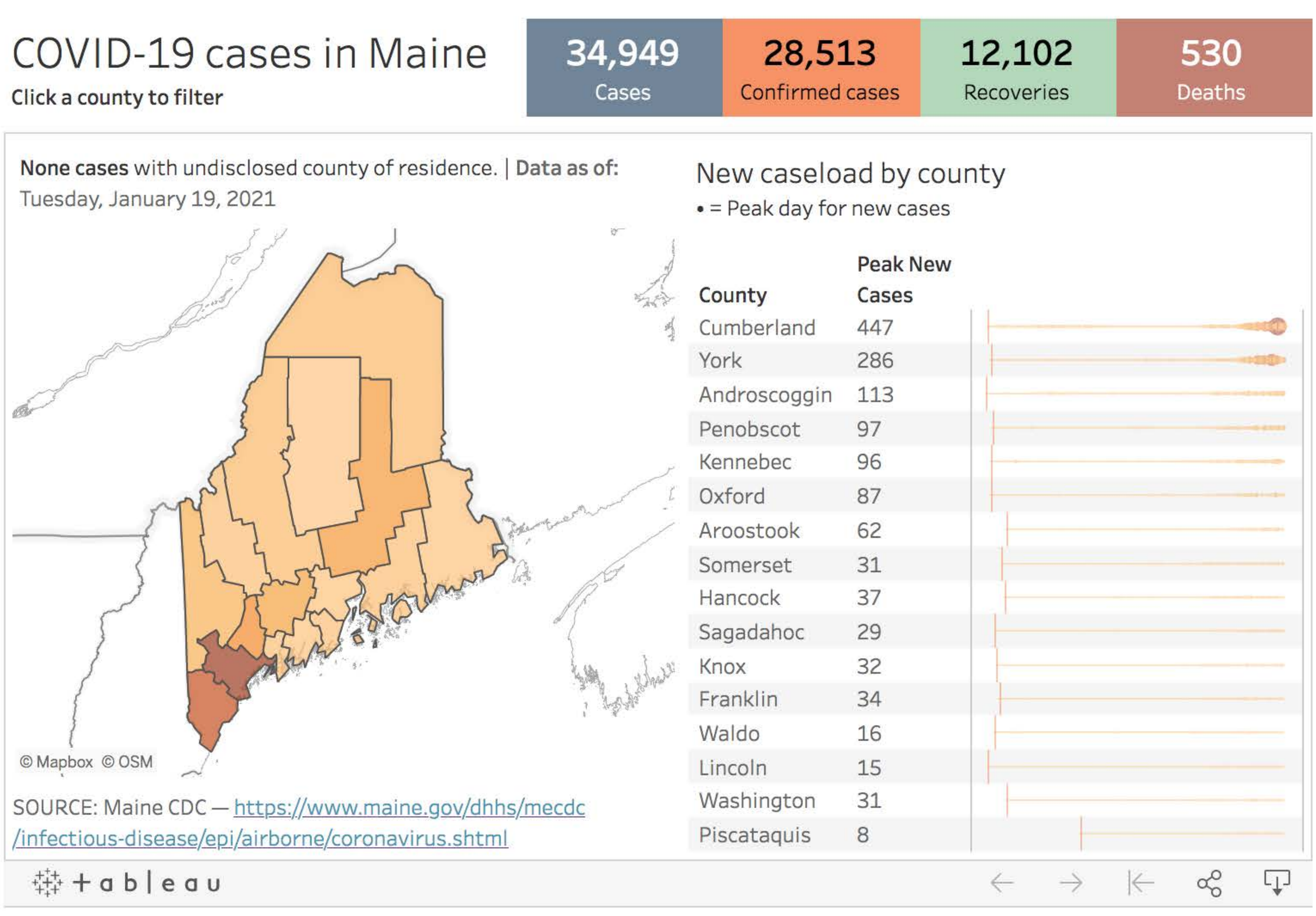

Case rates

For overall metrics, Cumberland and York will have the highest case counts because they have the most people.

The charts below adjust for population, showing the counties that have the highest current rates of confirmed infection.

These are shown alongside the latest calculations for the share of confirmed cases that have resulted in either recovery or hospitalization. These numbers only provide a small snapshot of the current status of testing and the disease in Maine.

In other words, those numbers reflect the situation on the ground in Maine and not necessarily a broader set of insights about the illness, COVID-19.

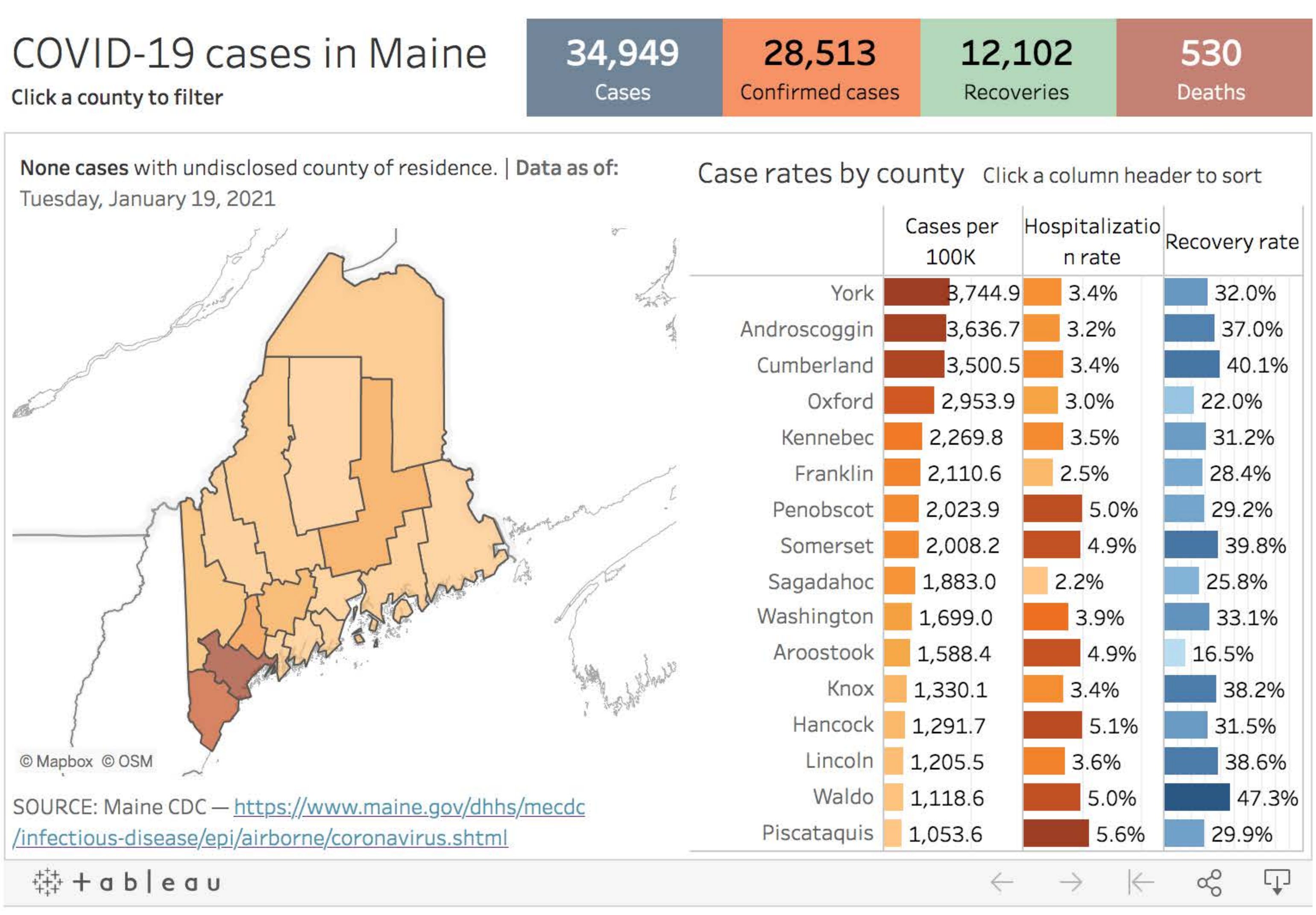

Age trends

The majority of Mainers infected so far have been in their 60s, followed by people in their 50s.

The latest studies of the disease have found that it is more deadly for older people, especially people over 80.

That has not meant that younger people are in the clear — the disease can still be fatal. The line chart shows how cases have trended among Mainers of different ages.

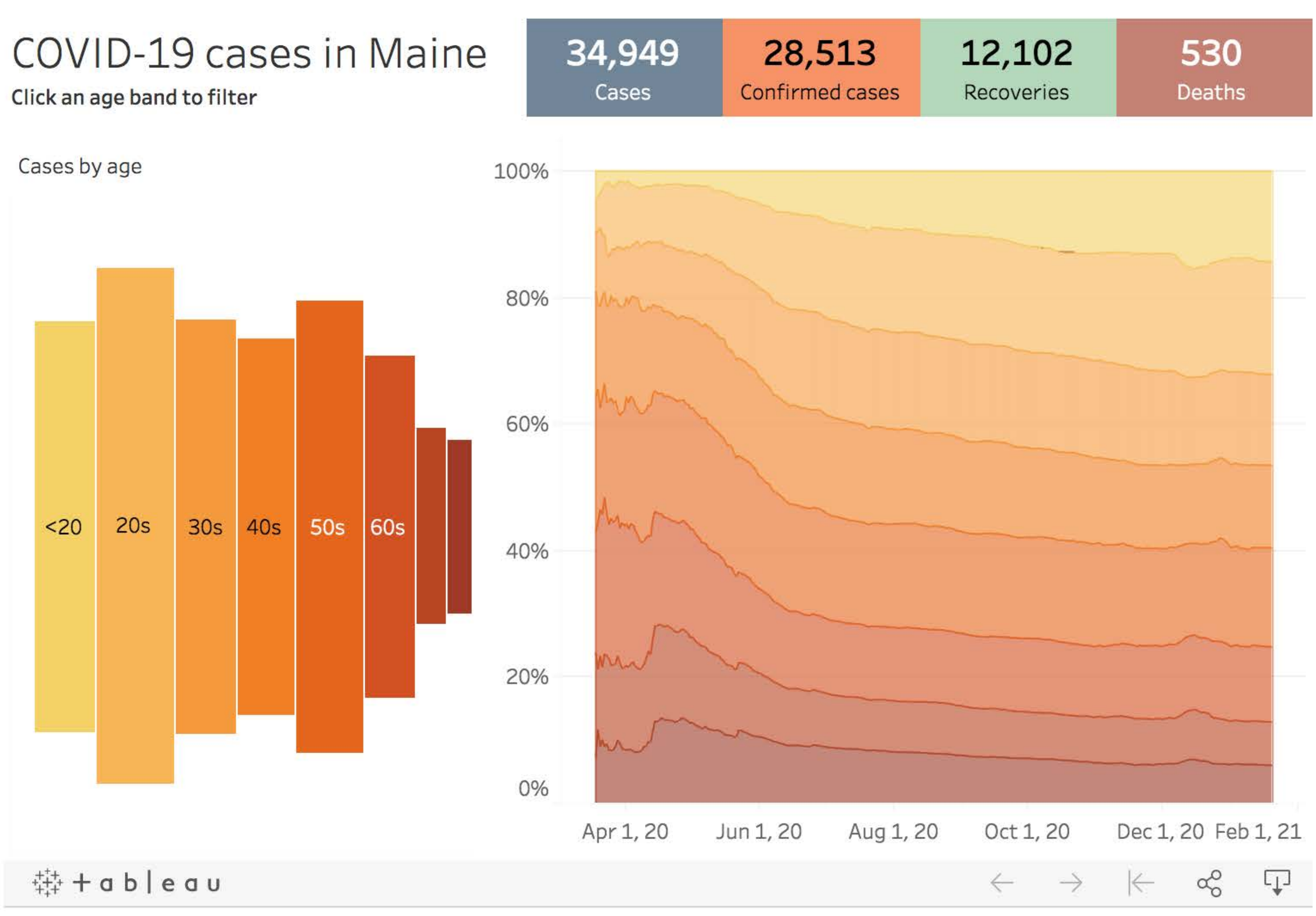

Comparing Maine with other states

The big question is: Can America stop the spread of this virus?

At the state level, this is reflected by looking at the rate of cases day by day. How quickly are they increasing? How many days is it taking for cases to double?

The goal is to slow that growth by: keeping six feet of distance from people you do not live with; washing your hands frequently and thoroughly; and disinfecting regularly touched surfaces.

The chart below uses a logarithmic y-axis, where the values double at every tick on the axis. As The New York Times explained, this makes it easier to tell when case growth begins to slow:

> Unconstrained, the coronavirus spreads exponentially, the caseload doubling at a steady rate. That curve, plotted linearly, is a skyrocketing curve. Plotted logarithmically, however, it transforms into a straight line — which means that deviations from the exponential spread of the virus become much easier to discern.

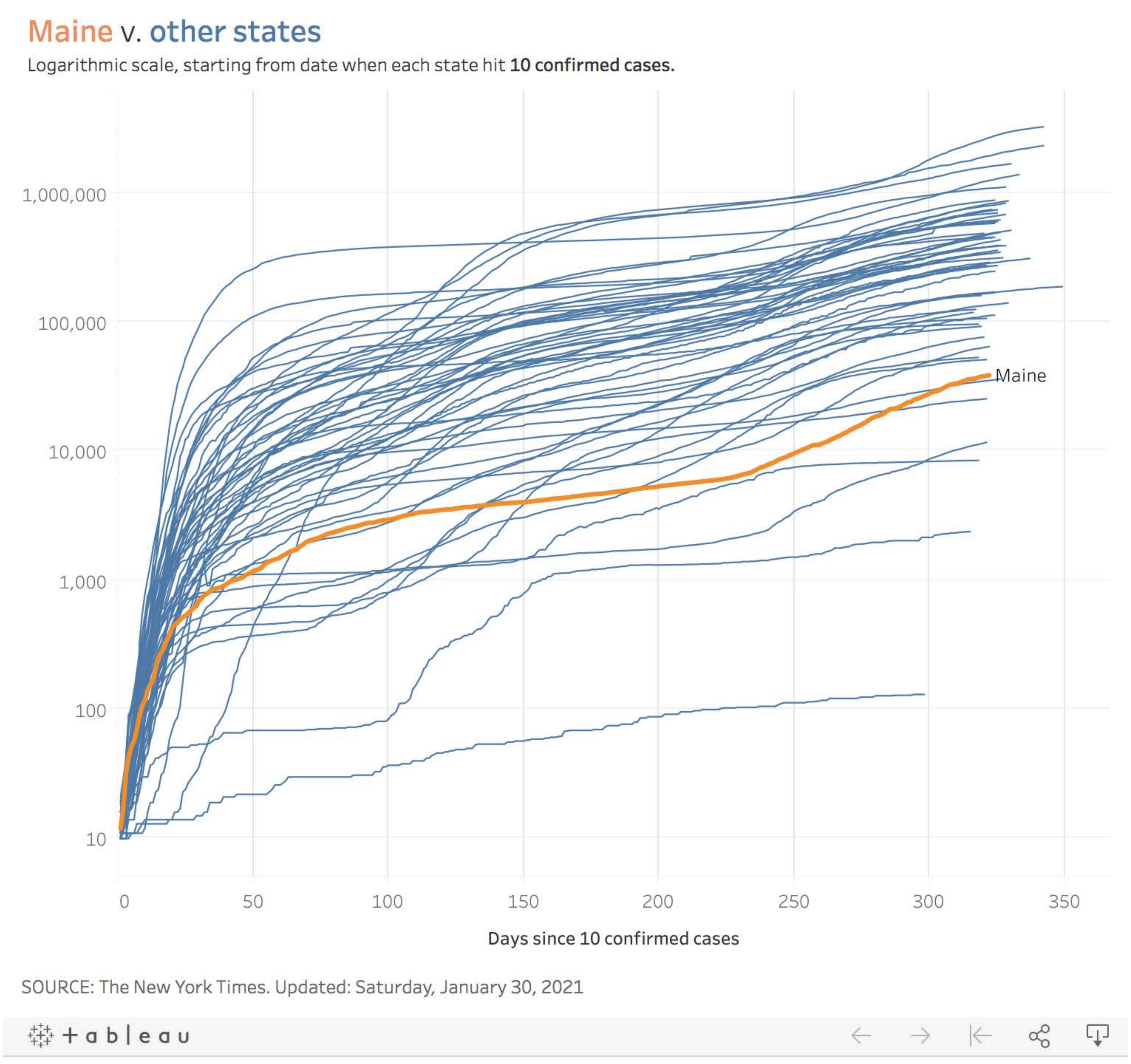

Like 2.2K Share

Coronavirus at a glance

The latest numbers, maps and charts to keep you up to date.

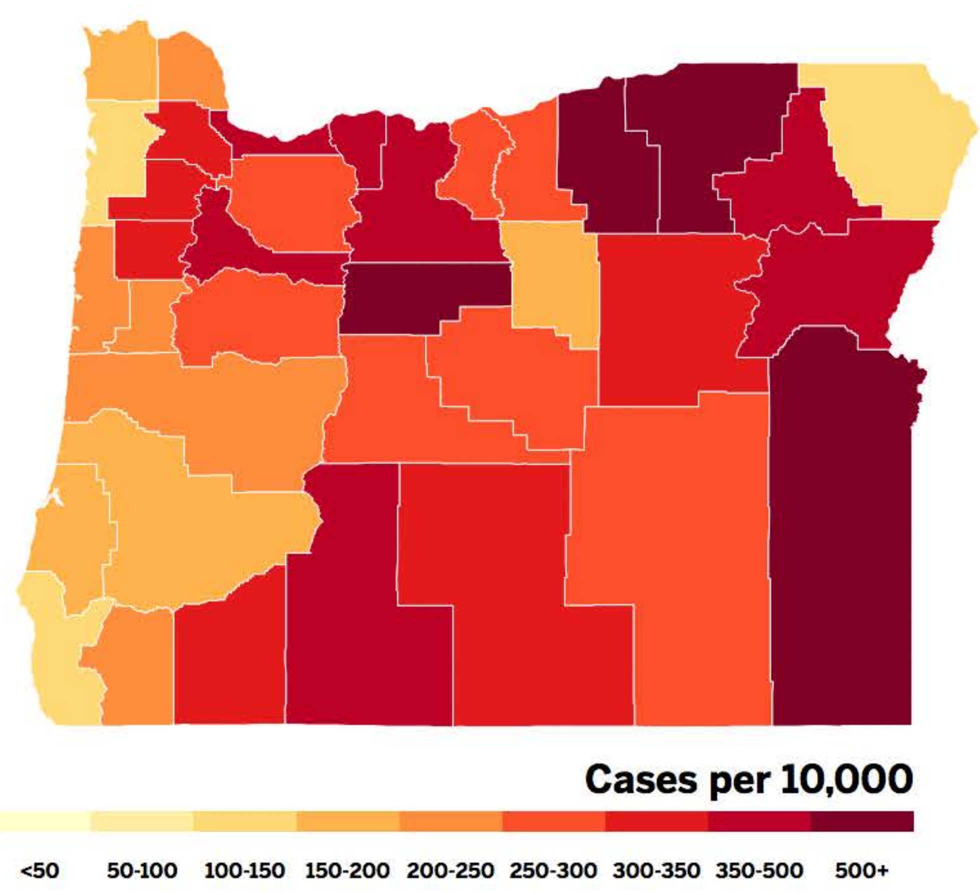

142,416
CASES REPORTED

688
AVG. NEW CASES PER DAY

1,957
DEATHS

208
HOSPITALIZATIONS

67
ICU PATIENTS

82%
REGIONAL ICU OCCUPANCY
Clackamas, Clatsop, Columbia, Multnomah, Tillamook, Washington

407,869
VACCINE DOSES ADMINISTERED

See county-level numbers »

Metro cases

MULTNOMAH: 29,920

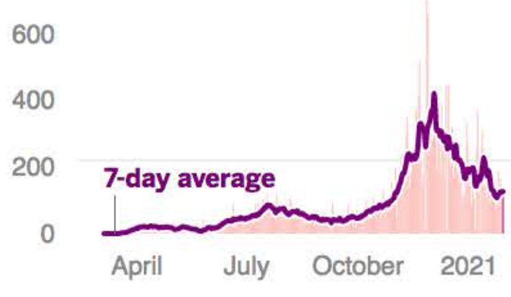

WASHINGTON: 19,769

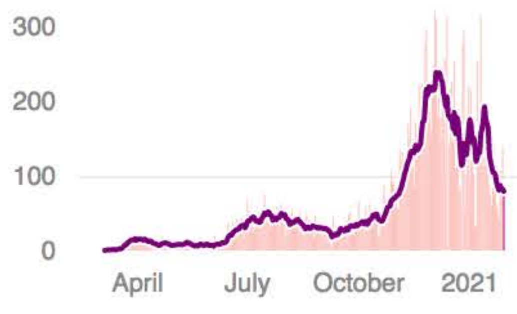

CLACKAMAS: 12,469

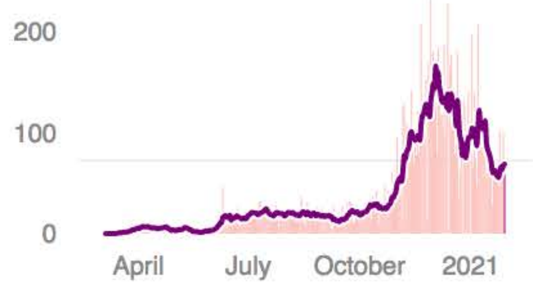

Daily new cases

Daily confirmed and presumptive coronavirus cases reported in Oregon and the 7-day rolling average.

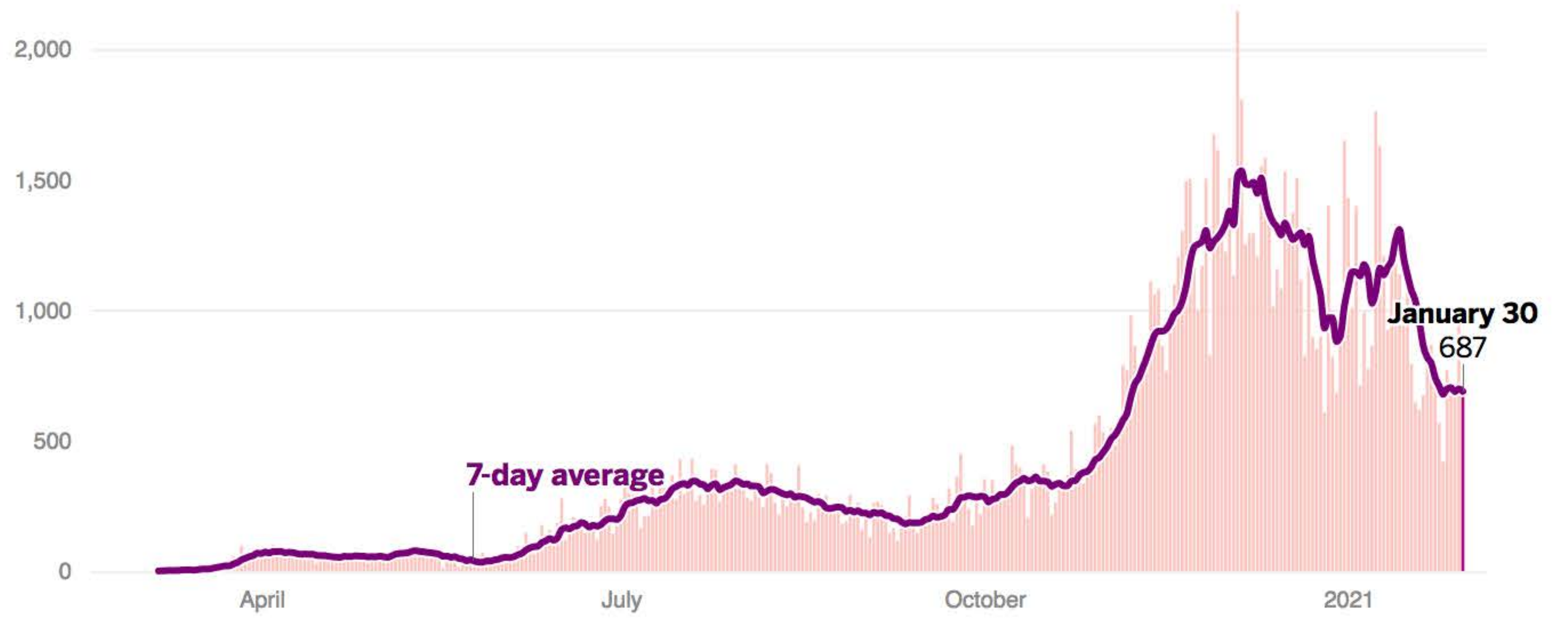

Daily positive test rates

The proportion of daily reported tests in Oregon that have resulted in a positive test result.

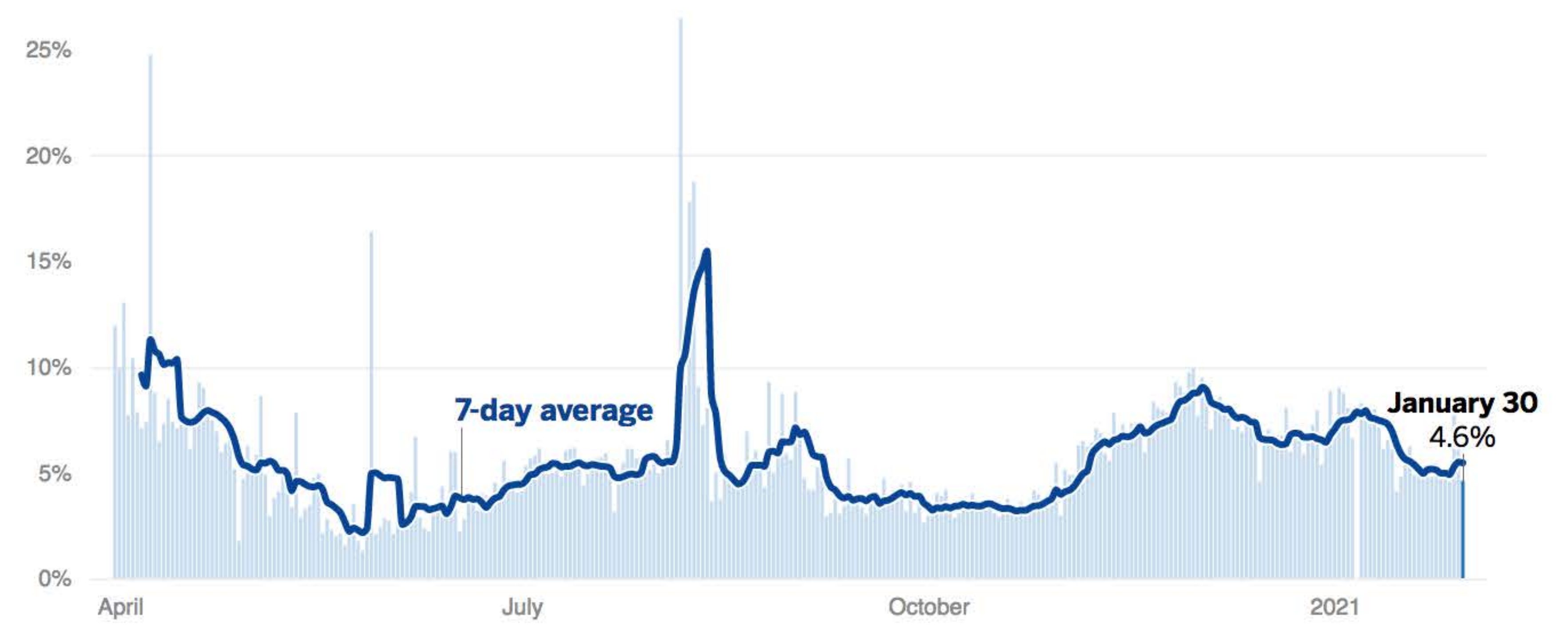

Daily testing

The number of daily new test results reported in Oregon and the 7-day rolling average.

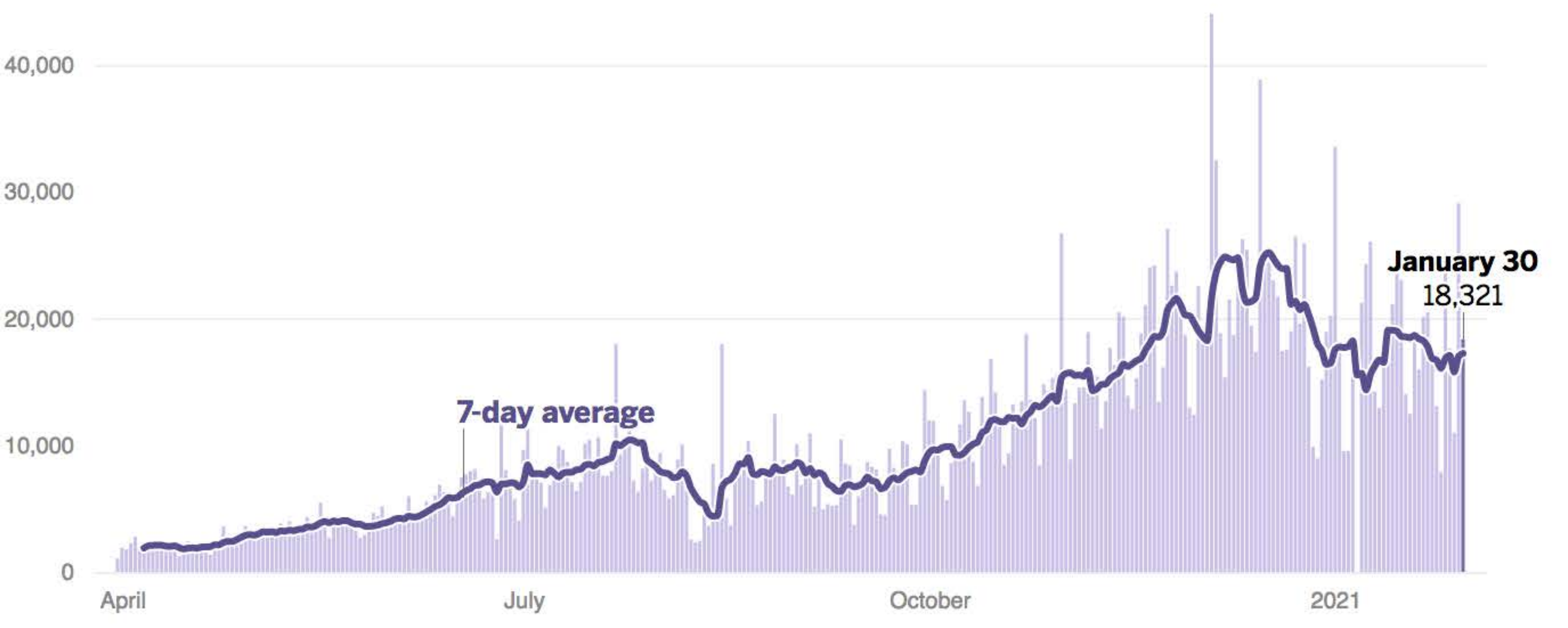

Daily hospitalization status

Number of confirmed coronavirus patients in Oregon who are **hospitalized**, in the **ICU** or on a **ventilator**

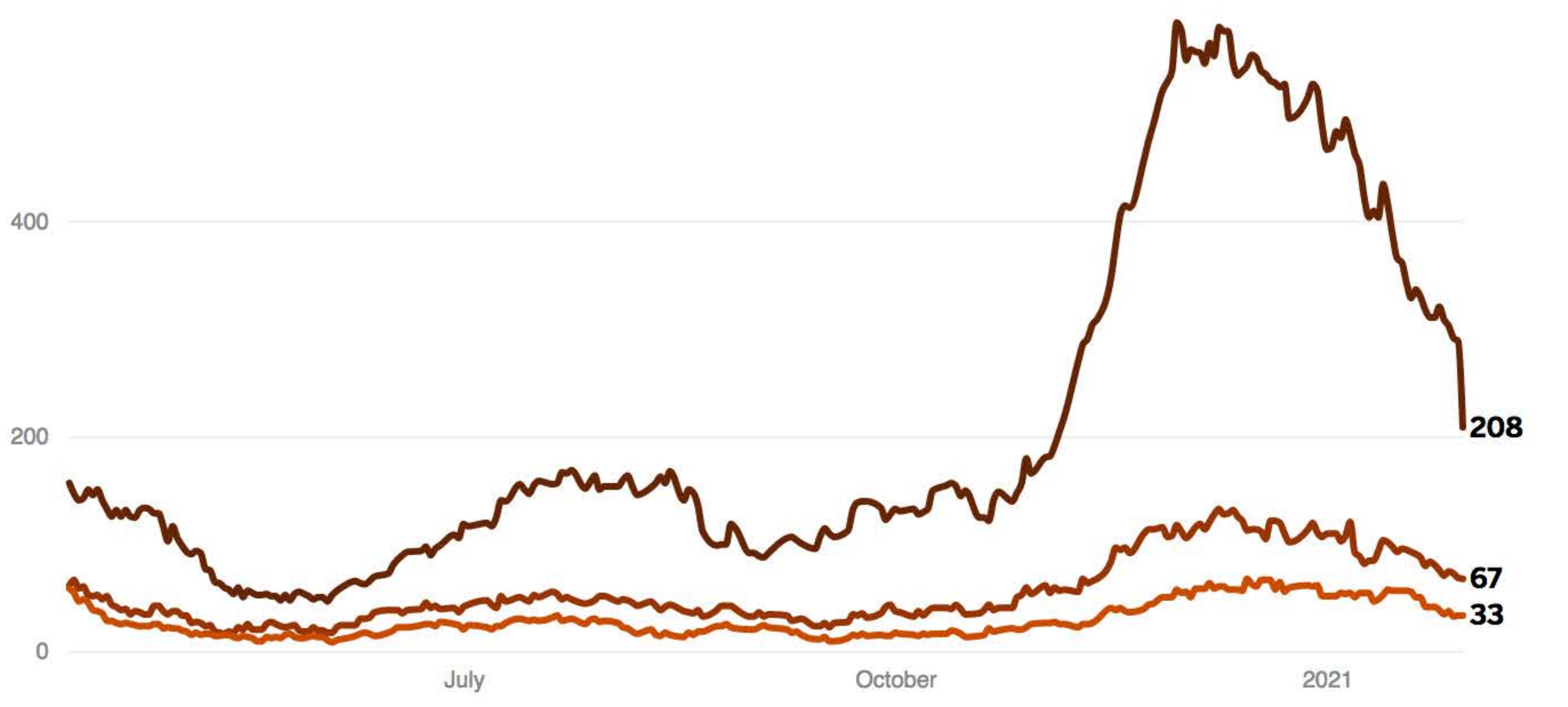

Daily new deaths

Daily coronavirus deaths reported in Oregon and the 7-day rolling average.

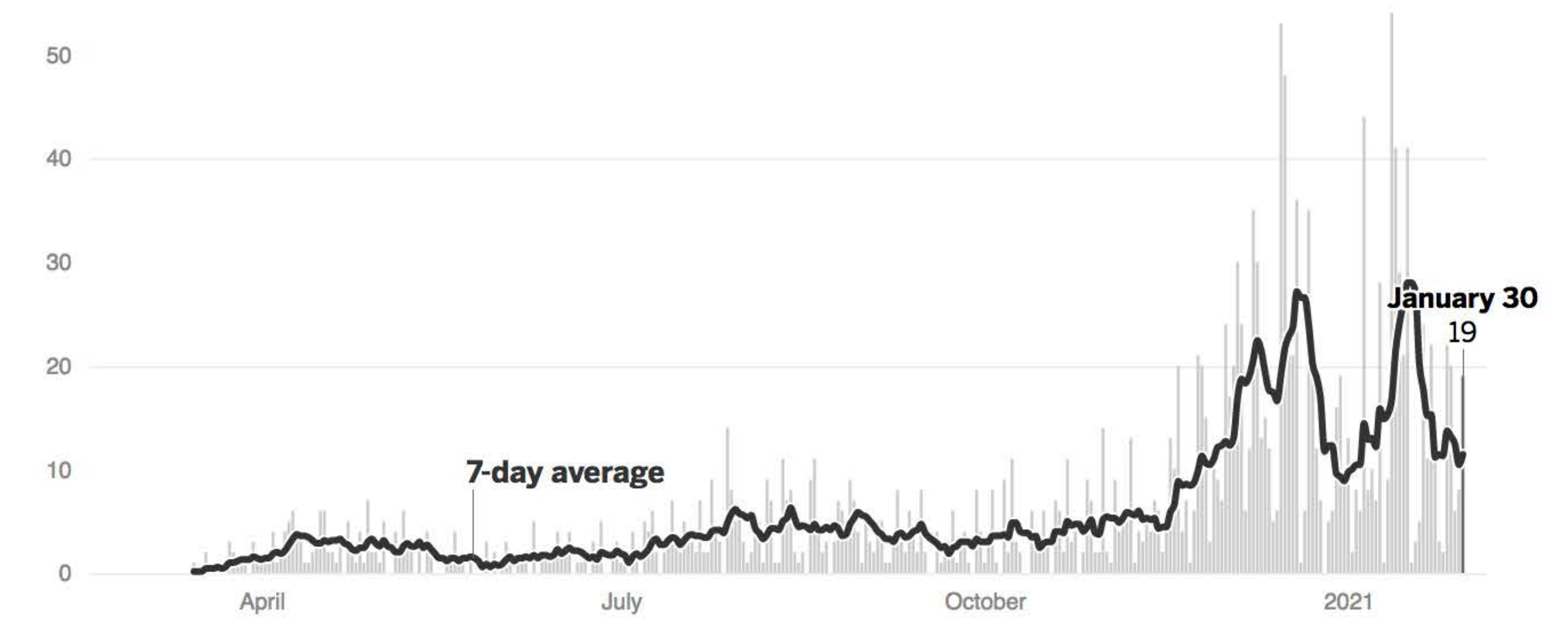

Interactives by Mark Friesen, David Cansler and Mark Graves/staff

Sources: Oregon Health Authority, Oregon Military Department Office of Emergency Management, county health departments, Center for Systems Science and Engineering at Johns Hopkins University, The COVID Tracking Project

Arkansas' first presumptive case of covid-19, the illness caused by the coronavirus, was detected in Pine Bluff on Wednesday, March 11, 2020. The patient had an out-of-state travel history.

There have been **294,387 cases** of covid-19 and **4,838 deaths** across the state of Arkansas since the pandemic began. Hover over the counties below for more details. On Jan. 1, 2021, Arkansas recorded its highest single-day jump in cases with an increase of 4,304.

On March 26, 2020, Hutchinson declared the state a disaster area, limiting indoor social gatherings. The declarations have been renewed periodically, and since July have included a mandate for masks in public places.

Total cases | Deaths | Per capita | Active cases

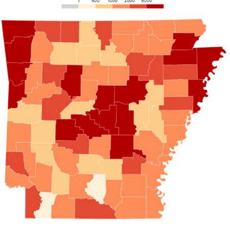

294,387 total cases

59,482 probable cases

17,608 active cases

4,838 deaths

271,911 recoveries

2,476,470 negative tests

* 2,885 cases with missing county information.

Symptoms of covid-19, including fever, cough and shortness of breath, have been mild for most people but severe or even deadly for some. The elderly and people with underlying health conditions considered most at risk.

The disease is thought to spread mainly from person to person through droplets produced when an infected person coughs or sneezes. Spread generally occurs between people who are in close contact (within about 6 feet).

People can also catch covid-19 by touching a contaminated surface and then touching their face.

There is also evidence suggesting people can spread covid-19 without developing symptoms, although the Centers for Disease Control and Prevention believes that people are most contagious when they have symptoms.

Arkansas covid-19 cases distribution

By age | By race | By gender | By health concerns

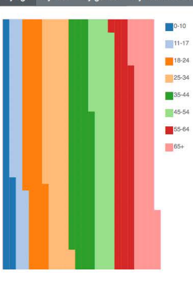

The first doses of the coronavirus vaccine were administered in Arkansas on Dec. 14, 2020. Walgreens and CVS pharmacies contracted with the federal Department of Health and Human Services in October, and their inoculations of long-term care facilities began in Arkansas, and nationwide, on Dec. 28. The companies then plan to offer the vaccine at pharmacies, possibly in February 2021.

For more information on the virus' symptoms and treatment, visit our frequently asked questions page. If you would like to share your experience with coronavirus and the changes it has brought to daily life or to submit your own questions about covid-19, please use this form.

Active cases | Deaths | Hospitalizations | New cases

New cases by day

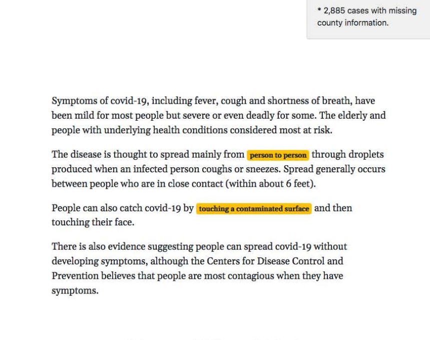

Created with Datawrapper

Covid-19 cases in Arkansas counties

Search in table

	County	Total cases	Active cases	Deaths	Recoverie
1	Pulaski	33,624	2,786	503	30,333
2	Washington	27,620	1,380	302	25,935
3	Benton	25,025	1,453	345	23,225
4	Sebastian	13,517	834	227	12,453
5	Craighead	12,123	477	166	11,479
6	Faulkner	10,596	786	134	9,675
7	Saline	10,370	710	147	9,511
8	Garland	8,354	630	203	7,519
9	Jefferson	8,041	483	148	7,408
10	Pope	7,167	378	85	6,704
11	White	6,467	352	89	6,025
12	Crawford	6,163	413	89	5,660
13	Lonoke	5,939	550	103	5,286
14	Greene	5,482	284	65	5,133
15	Crittenden	5,358	206	84	5,066
16	Mississippi	5,234	266	105	4,862
17	Hot Spring	4,382	218	62	4,102
18	Independence	3,451	152	112	3,187
19	Miller	3,363	99	29	3,235
20	Boone	3,329	255	74	2,999

+ Show 55 more

Created with Datawrapper

School districts statewide are working to slow the virus's spread, prevent large outbreaks, and protect students, teachers and staff members. The state health and education departments have provided recommendations to districts.

Gov. Asa Hutchinson and Education Secretary Johnny Key in July 2020 unveiled guidelines for school districts on how to respond to outbreaks. Before the beginning of the 2020-21 school year, Key said schools had a "state responsibility" to open five days a week for in-person classes. However, the guidelines allow for a pivot to remote learning in times of critical response, and a number of schools have made that shift in the 2020-21 school year.

Universities in Arkansas also began reporting covid-19 cases with individual dashboards in August. For the spring 2021 semester, the University of Arkansas, like many other colleges in the state, was offering a mix of in-person, remote and hybrid courses.

Covid-19 cases in Arkansas school districts and colleges

K-12 school districts | Colleges

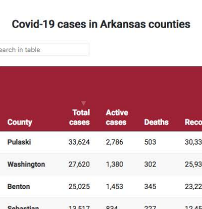

Show 10 entries   Search:

School district	Cumulative cases	Active cases	Cumulative student cases	Cumulative faculty/staff cases
Academics Plus Public Charter Schools	58	7	42	14
Alma	140	9	101	34
Arkadelphia	94	20	70	19
Augusta	20	5	15	5
Bald Knob	80	34	56	24
Barton-Lexa	30	5	19	10
Batesville	137	10	71	64
Bay	78	27	53	19
Bearden	29	18	--	--
Beebe	110	14	86	20

Showing 1 to 10 of 156   Previous 1 2 3 4 5 … 16 Next

* The Arkansas Department of Health reports K-12 cases each Monday and Thursday. The reports only list districts with five or more active cases.

The Arkansas Democrat-Gazette compiles its own database on coronavirus cases detected at Arkansas colleges using information posted on each school's website.

Nursing homes across the state have been in lockdown under federal orders to protect their residents. The state has adopted federal guidelines on visits, prohibiting visits at nursing homes where a resident or worker has tested positive in the past 28 days.

Covid-19 cases in Arkansas long-term care facilities

* The Arkansas Department of Health reports nursing home cases once a week. The reports only list facilities that have had a new or additional positive case in the past 14 days.

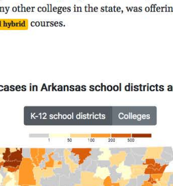

Show 10 entries   Search:

Facility	Positive residents	Positive staff members	Total cases	Total deaths
Alma Health & Rehabilitation Center	49	40	89	15
Arkansas Convalescent Center	75	39	114	13
Arkansas Nursing & Rehab	69	50	119	3
Arkansas State Veterans Home-Fayetteville	27	57	84	9
Arkansas State Veterans Home-North Little Rock	20	35	55	0
Ash Flat Healthcare and Rehabilitation Center	42	28	70	5
Barrow Creek Health & Rehab	76	50	126	20
Bear Creek Healthcare	3	16	19	0
Bradford House Nursing & Rehab	57	46	103	6
Brookdale Conway	4	3	7	0

Showing 1 to 10 of 102 entries   Previous 1 2 3 4 5 … 11 Next

Prisons, jails and juvenile lockups were identified early in the pandemic as sites where covid-19 could spread rapidly.

Officials have struggled to keep the virus contained even when they identified outbreaks, while prisoners and their families have accused the Arkansas Department of Corrections of bungling their response to the virus. The Marshall Project, a nonprofit covering criminal justice, has said Arkansas is among the worst states in per capita infections at prisons.

Covid-19 cases in Arkansas correctional facilities

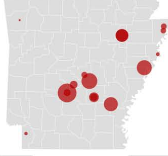

Show 10 entries   Search:

Facility	Positive inmate	Positive staff members	Total cases	Total deaths
ADC Barbara Ester Unit-Pine Bluff	186	6	192	0
ADC Benton Unit Work Release	207	2	209	0
ADC Central AR Community Corrections (CAC)	79	42	121	0
ADC East AR Regional Unit (EARU)	892	54	946	6
ADC East Central AR CCC-Women's Unit (ECCC)	28	3	31	0
ADC Grimes Unit-Newport AR	652	14	666	1
ADC McPherson Unit	645	9	654	0
ADC Mississippi Co Work Release Center (MCWR)	96	4	100	0
ADC Northeast Arkansas Community Corrections (NEACC)	96	0	96	4
ADC Northwest Arkansas Community Corrections (NWACC)	3	0	3	2

Showing 1 to 10 of 16 entries   Previous 1 2 Next

People who think they may have covid-19 should call their health care providers or schedule a test at a local health unit, according to the state Department of Health.

A health care provider will decide if a test is appropriate based on the symptoms and possible exposure to the coronavirus.

The University of Arkansas for Medical Sciences is also offering free screenings online and over the phone at (800) 632-4502.

For children, Arkansas Children's Hospital is offering drive-thru screenings and phone screenings at (800) 743-3616.

Coronavirus testing and screening locations in Arkansas

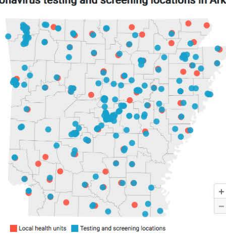

Created with Datawrapper

How Arkansas compares in testing

* States sized by population.

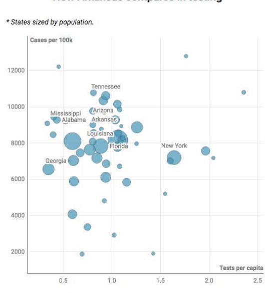

Created with Datawrapper

Cumulative covid-19 cases in the United States by state

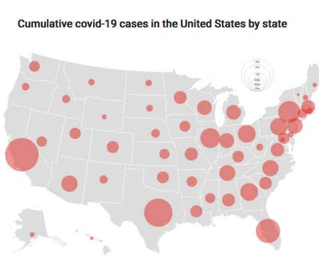

26,065,678 confirmed cases 439,405 deaths 9,004,738 recoveries

Covid-19 cases in the United States by state

Search in table   Page 1 of 4

	State	Total cases	New cases curve	Cases per 100k	Deaths	Total tests	% of positive
1	North Dakota	97,562		12,8…	1,442	1,344,399	7.3%
2	South Dakota	108,070		12,…	1,775	401,617	26.9%
3	Rhode Island	114,438		10,…	2,154	2,497,271	4.6%
4	Utah	345,430		10,…	1,663	2,598,403	13.3%
5	Tennessee	724,742		10,…	9,574	6,404,722	11.3%
6	Arizona	753,379		10,…	13,098	6,643,932	11.3%
7	Wisconsin	590,997		10,…	6,430	6,154,838	9.6%
8	Nebraska	190,570		9,…	1,920	2,090,962	9.1%
9	Oklahoma	386,590		9,…	3,504	3,181,931	12.1%
10	Arkansas	294,387		9,…	4,838	2,781,116	10.6%
11	Kansas	274,685		9,…	3,779	1,168,723	23.5%
12	Alabama	455,582		9,…	7,566	2,127,308	21.4%
13	Indiana	624,959		9,…	9,967	6,976,408	9.0%
14	Mississippi	274,190		9,…	6,018	1,568,678	17.5%
15	Idaho	162,355		9,…	1,725	601,646	27.0%

Created with Datawrapper

The outbreak in the United States first emerged in Washington state after a resident tested positive on January 20, 2020.

The first coronavirus-related death occurred in Northern California's Santa Clara County on February 6, 2020.

Cases have been discovered in all 50 states after West Virginia reported its first on March 17, 2020, although testing and reporting discrepancies exist among different states.

Tens of thousands of new cases are now being detected across the country each day.

Countries with the most covid-19 cases

Countries	Cases: Total (Daily Change)	Cases: Trend (vs. Prev. Week)	Deaths: Total (Daily Change)
U.S.	26,178,803 (+150,670)	↘ -14%	440,658 (+3,140)
India	10,746,183 (+13,090)	↘ -5%	154,274 (+130)
Brasil	9,204,731 (+51,530)	→ -0%	224,504 (+1,070)
United Kingdom	3,828,183 (+25,610)	↘ -31%	106,367 (+1,180)
Russia	3,808,348 (+18,830)	↘ -13%	72,029 (+520)
France	3,255,920 (+20,440)	↘ -12%	76,201 (+430)
Spain	2,743,119 (+34,790)	→ -2%	58,319 (+410)
Italy	2,553,032 (+12,370)	→ +0%	88,516 (+450)
Turkey	2,477,463 (+6,650)	↗ +7%	25,993 (+130)
Germany	2,225,659 (+12,460)	↘ -12%	57,163 (+750)

*Daily Change: 7-day average of new confirmed cases.

Created with Datawrapper

102,520,738 confirmed cases worldwide 2,217,668 deaths 56,699,004 recoveries

Cumulative covid-19 cases 350 days since 100th case

Europe | Asia | U.S. | South America

* This chart is plotted on a logarithmic scale.

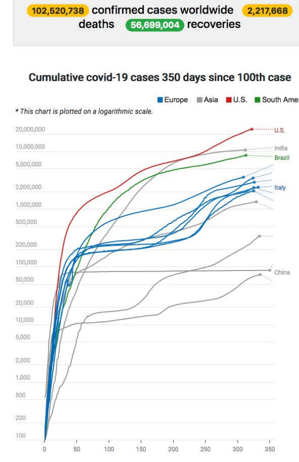

Supplement: Multimedia Appendix 7 [file humanfactors_v10i1e43819_app7.pdf]
