# Supplementary material for: Web-Based COVID-19 Dashboards and Trackers in the United States: Survey Study
Source: JMIR Hum Factors. 2023 Mar 20;10:e43819. doi: 10.2196/43819 (PMC10029858; doi:10.2196/43819)
Supplement: Multimedia Appendix 8 [file humanfactors_v10i1e43819_app8.pdf]

Appendix 8. Screenshots from dashboards and trackers — *Nation-wide coverage* *(captured February 26, 2021)*

Page	Ref	Host
2–4	N-1	US Centers for Disease Control and Prevention
5–6	N-2	US Centers for Disease Control and Prevention
7–8	N-3	The COVID Tracking Project / The Atlantic
9	N-4	New York Times
10	N-5	NPR
11	N-6	Johns Hopkins Centers for Civic Impact
12	N-7	Johns Hopkins Centers for Civic Impact
14	N-8	COVID-19 Health Equity Interactive Dashboard / Emory University
14	N-9	USAFacts
15	N-10	COVID Mapping Project / Standard Co
16	N-11	SAE Industry Technologies Consortia

Data Tracker Home
COVID Data Tracker Weekly Review
Your Community +
Vaccinations +
Cases & Deaths −
Cases and Deaths by State
Daily and Total Trends
State Trend Comparison
Global Counts and Rates
Global Percent Change
Global Trends
Demographic Trends +
Healthcare Systems +
Testing and Seroprevalence +
People at Increased Risk +
Communications Resources
COVID-19 Home

Get Email Updates

Sign up to receive the COVID Data Tracker Weekly Review.

Email Address:

Email Address

What's this? Submit

United States COVID-19 Cases and Deaths by State

Maps, charts, and data provided by the CDC, updated daily by 8 pm ET†

TOTAL CASES	AVERAGE DAILY CASES PER 100K IN LAST 7 DAYS	TOTAL DEATHS
28,212,548	20.2	506,834
+73,610 New Cases		+3,240 New Deaths

CDC | Updated: Feb 26 2021 12:24PM

View: Cases / Deaths
Time period: Last 7 Days / Since Jan 21, 2020
Metric: Count / Rate per 100,000

This shows the number of COVID-19 cases for every 100,000 people over the last 7 days, allowing you to compare areas with different population sizes.

US COVID-19 Average Daily Case Rate in Last 7 Days, by State/Territory (cases per 100K)

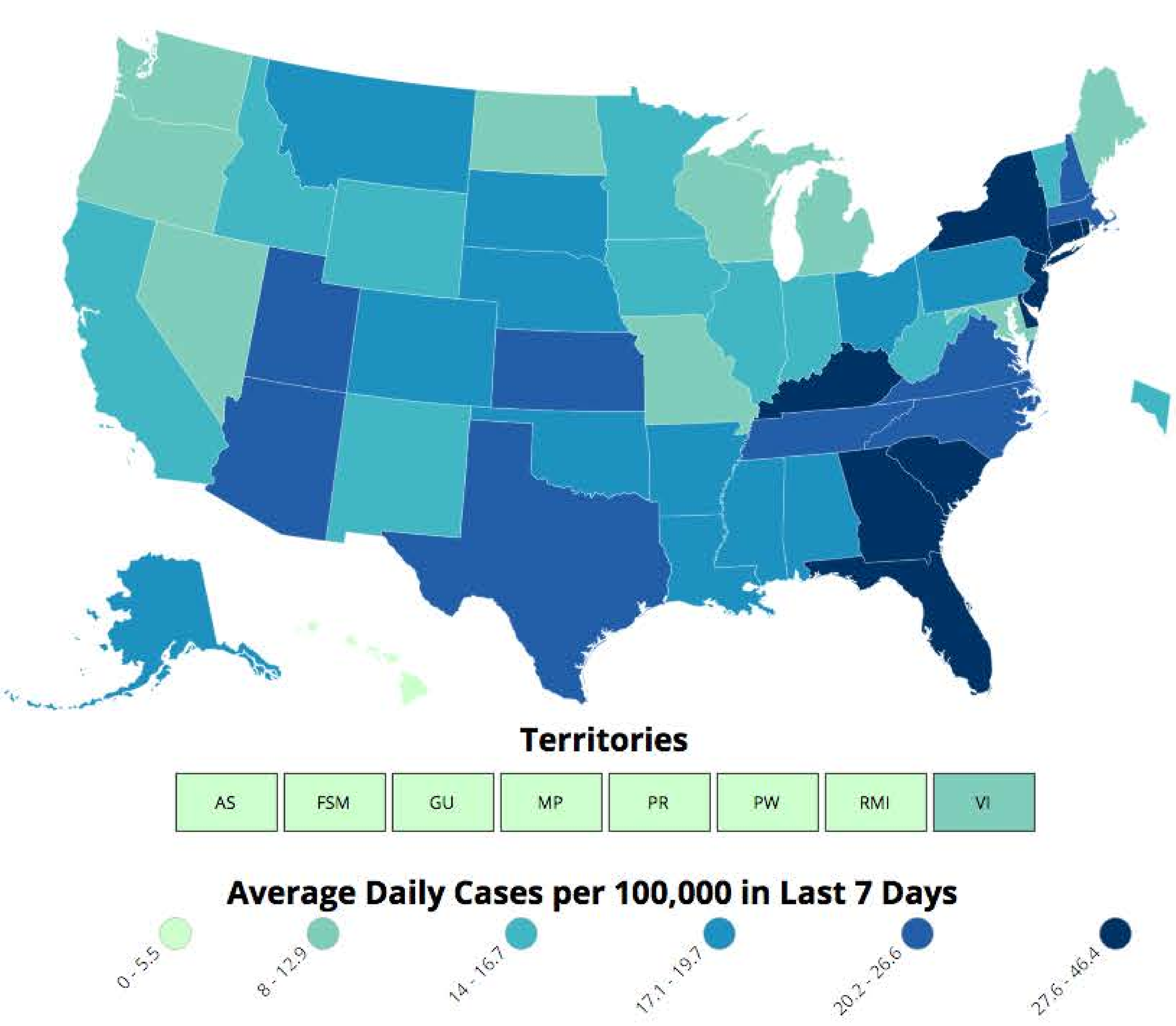

View Historic Case and Death Data | Download Map

Data Table for Average Daily Cases per 100k in Last 7 Days +

Wondering what all the data mean?

CDC's new COVID Data Tracker Weekly Review helps you stay up-to-date on the pandemic with weekly visualizations, analysis, and interpretations of key data and trends.

How does COVID-19 Spread?

Learn more

Information on US COVID-19 Cases Caused by Variants

Learn more here

View and Download COVID-19 Case Surveillance Public Use Data

†Data will update as soon as they are reviewed and verified, oftentimes before 8 pm ET. However, daily updates might be delayed due to delays in reported data.

On 4 February 2021, a state reported 1,507 new deaths. CDC is working with the state to assess the time period from which these data cover. This may temporarily impact death counts, rates and averages.

Data Sources, References & Notes: The case classifications for COVID-19, a nationally notifiable disease, are described in an an updated interim COVID-19 position statement and case definition issued by the Council of State and Territorial Epidemiologists on August 5, 2020 . However, there is some variation in how jurisdictions implement these case classifications. More information on how CDC collects COVID-19 case surveillance data can be found at CDC's COVID-19 FAQ webpage.

Total cases are based on aggregate counts of COVID-19 cases reported by state and territorial jurisdictions to the Centers for Disease Control and Prevention (CDC) since January 21, 2020, with the exception of persons repatriated to the United States from Wuhan, China, and Japan. All displayed counts include confirmed COVID-19 cases and deaths as reported by U.S. states, U.S. territories, New York City (NYC), and the District of Columbia from the previous day. Counts for certain jurisdictions also include probable COVID-19 cases and deaths. Counts for NYC and New York State are shown separately; data for New York State show total cases and deaths for the state excluding data for NYC. COVID-19 case and death data that are not available to CDC are denoted by N/A. For aggregate state level data, CDC calculates the number of new cases or deaths each day by calculating the difference in cumulative counts reported by the state from the day before. Historical data are not typically updated unless requested by the state. Therefore, the number of historical cases and deaths presented on CDC's website reflect the date the data was reported to CDC and not necessarily the date the case or death was recorded in the state.

The map can be modified to show cases and deaths per 100,000 people in the last 7 days, total new cases and deaths in the last 7 days, total cases and deaths since January 21, 2020, and rates for cases (cases/100,000 people) and deaths (deaths/100,000). The average daily rate per 100,000 people in the last 7 days is calculated as the 7-day moving average of new cases or deaths (current day + 6 preceding days divided by 7) per 100,000 people using the US Census Bureau Population Estimates Program (https://www.census.gov/data/datasets/time-series/demo/popest/2010s-counties-total.html). The 7-day cumulative rate is calculated as (current day + 6 preceding days) per 100,000 people using the US Census Bureau Population Estimates Program (https://www.census.gov/data/datasets/time-series/demo/popest/2010s-counties-total.html). Rates per 100,000 are calculated as the total cases or deaths per 100,000 people using the US Census Bureau Population Estimates Program (https://www.census.gov/data/datasets/time-series/demo/popest/2010s-counties-total.html).

*2018 population estimates are still used for American Samoa, Federated States of Micronesia, Guam, New York City, Northern Mariana Islands, Palau, Republic of Marshall Islands and United States Virgin Islands.

CDC's overall COVID-19 case and death numbers are validated through a confirmation process with each jurisdiction. COVID-19 case and death numbers reported on other websites may differ from what is posted on the CDC COVID Data Tracker due to the timing of reporting and COVID Data Tracker updates, which may differ by up to 24 hours. CDC COVID-19 counts from previous dates may be continually revised as more records are received and processed. Not all jurisdictions report counts daily; some counts are reported in batches and may increase COVID-19 case and death counts at different intervals and appear as spikes. The process used for finding and confirming COVID-19 cases and deaths displayed by other sites may differ.

On 18 December, Texas reported 171,505 historical counts of probable cases with dates between 1 November and 18 December. This raised the total number of new cases in both Texas and the U.S. during this time period and correspondingly affects the 7-day rolling average of new cases.

Data Tracker Home
COVID Data Tracker Weekly Review
Your Community +
Vaccinations +
Cases & Deaths +
Demographic Trends +
Healthcare Systems +
Testing and Seroprevalence −
Nationwide Testing (RT-PCR)
County Level Testing (RT-PCR)
National Seroprevalence
10-Site Seroprevalence
People at Increased Risk +
Communications Resources
COVID-19 Home

Get Email Updates
Sign up to receive the COVID Data Tracker Weekly Review.
Email Address:
Email Address
What's this? Submit

United States Laboratory Testing

Maps, charts, and data provided by the CDC, updated daily by 8 pm ET†

TOTAL TESTS REPORTED	TOTAL POSITIVE TESTS	TOTAL % POSITIVE
329,861,245	29,642,590	8.99%

National totals as of: Feb 24 2021

CDC | Updated: Feb 26 2021 12:24PM

View:
- Tests Performed
- Percent Positive (selected)

Time period:
- Last 7 Days (selected)
- Last 30 Days
- All Time

This shows the percentage of tests that were positive over the last 7 days.

COVID-19 Viral (RT-PCR) Laboratory Test 7-day Percent Positivity by State/Territory

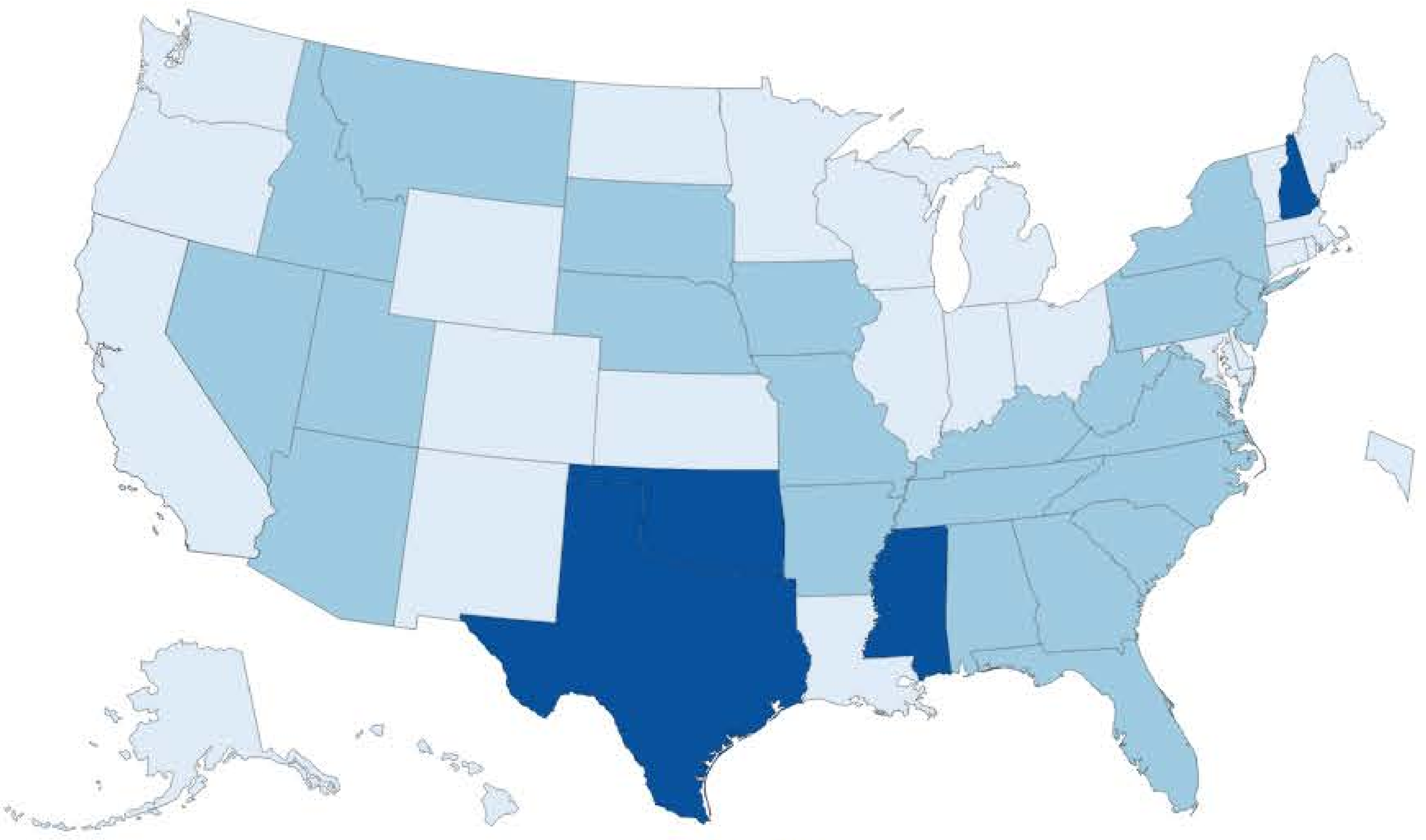

Download Map

Data table for COVID-19 Viral (RT-PCR) Laboratory Test 7-day Percent Positivity by State/Territory +

Do you need information on testing?

Find it here

View and Download COVID-19 Case Surveillance Public Use Data

†Data will update as soon as they are reviewed and verified, oftentimes before 8 pm ET. However, daily updates might be delayed due to delays in reported data.

Disclaimer: The data represent viral COVID-19 laboratory test (reverse transcription polymerase chain reaction) results from laboratories in the United States, including commercial and reference laboratories, public health laboratories, hospital laboratories, and other testing locations. The data represent laboratory test totals—not individual people—and exclude antibody and antigen tests. The data are provisional and subject to change. National total test counts reflect data from the previous day and may not match the sum of the data presented for all jurisdictions. Data reported for each state and territory may be delayed several days in order to mitigate discrepancies in daily test counts due to variation in jurisdiction reporting. The data may also not include results from all testing sites within a jurisdiction (e.g., point-of-care test sites) and therefore reflect the majority, but not all, COVID-19 tests in the United States. The data are reported to state and jurisdictional health departments in accordance with applicable state or local law and in accordance with the Coronavirus Aid, Relief, and Economic Security (CARES) Act (CARES Act Section 18115). The data for each state are sourced from either data submitted directly by the state health department via COVID-19 electronic laboratory reporting (CELR), or a combination of commercial, public health, and in-house hospital laboratories. CELR data are used for states that either submit line-level data or submit aggregate counts that do not include antigen or antibody tests. The data are available for public use on HealthData.Gov.

COVID Data Tracker

Cases in US 28,212,548 | Cases in US Last 30 Days | Total Vaccines Administered 70.5M | Deaths in US 506,834 | Deaths in US Last 30 Days

COVID-19 Vaccinations in the United States

Overall US COVID-19 Vaccine | Deliveries and Administration; Maps, charts, and data provided by the CDC, updated daily by 8 pm ET†

Represents all vaccine partners including jurisdictional partner clinics, retail pharmacies, long-term care facilities, Federal Emergency Management Agency and Health Resources and Services Administration partner sites, and federal entity facilities.

Total Vaccine Doses	
Delivered	94,300,910
Administered	70,454,064

Learn more about the distribution of vaccines.

People Vaccinated	Receiving 1 or More Doses	Receiving 2 Doses
Total	47,184,199	22,613,359
% of Total Population	14.2%	6.8%
Population ≥ 18 Years of Age	47,143,403	22,598,904
% of Population ≥ 18 Years of Age	18.5%	8.9%

Read more about how these data are reported.

CDC | Data as of: Feb 26 2021 6:00am ET | Posted: Feb 26 2021 12:24PM ET

View: Total Doses / People
Show: Administered / Delivered
Metric: Count / Rate per 100,000
Population: Total Population / Population ≥ 18 Years Of Age

This shows the number of doses administered within the state or territory for every 100,000 people of the total population. It does not reflect the residency of the person receiving the vaccine, but where they received it.

Total Doses Administered Reported to the CDC by State/Territory and for Select Federal Entities per 100,000 of the Total Population

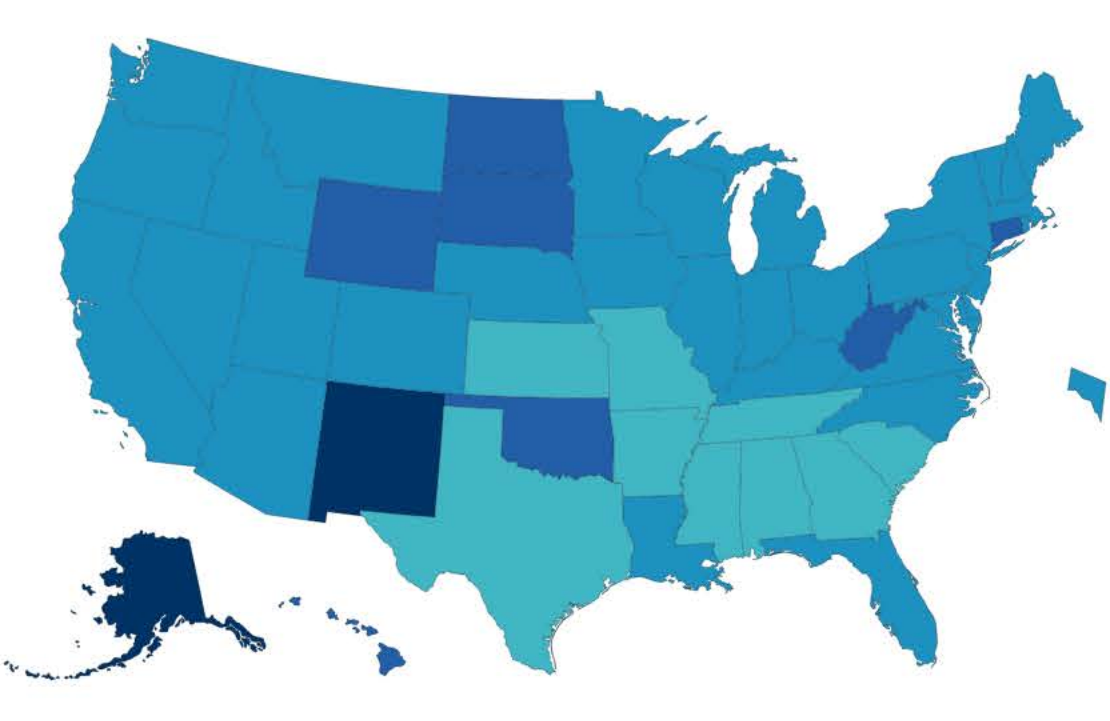

Territories

GU | AS | RP | FM | MP | PR | MH | VI

Federal Entities

BoP | DoD | IHS | VHA

* Data for Federal Entities are presented here and are also incorporated into the respective jurisdictional totals

Total Doses Administered per 100,000

No Data | 0 | 1 - 15,000 | 15,001 - 20,000 | 20,001 - 25,000 | 25,001 - 30,000 | 30,001+

U.S. COVID-19 Vaccine Administration by Vaccine Type

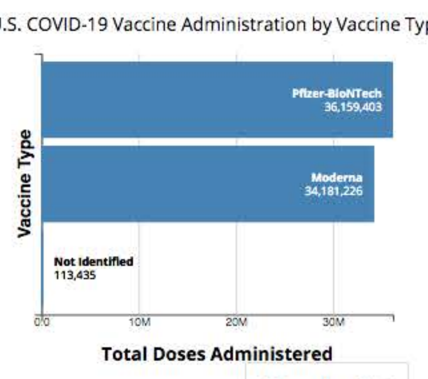

Download

Data Table for COVID-19 Vaccinations in the United States +

Want to know more about COVID-19 vaccinations?

Learn more about recommendations, product information, and more on CDC's COVID-19 Vaccination landing page.

Looking for Long-Term Care Facility vaccination data?

See the latest data for the Federal Pharmacy Partnership for Long-Term Care (LTC) Program.

Want to know more about trends in COVID-19 US vaccinations?

See the latest trends in the number of COVID-19 vaccinations given in the United States.

The content on this page is not accessible using Internet Explorer. Please use Chrome or Edge to access this page.

†Data will be updated after review and verification, usually before 8:00 pm ET.

Data on doses of vaccine distributed and administered include data received by CDC as of 6:00 am ET on the day of reporting. Vaccination data on CDC's COVID Data Tracker are updated daily between 1:30 pm and 8:00 pm ET. Updates will occur the following day when reporting coincides with a federal holiday. Note, daily updates might be delayed due to delays in reporting.

Vaccination data reported on the CDC COVID Data Tracker might differ from data reported by states and territories for several reasons:

- Data can be updated on different schedules and reflect data "as of" different dates or times of day. There can be a delay between the time a vaccination record appears in a state or jurisdictional system and when it is received by CDC.
- Occasionally, technical issues with data processing or transmission will occur. When technical issues arise, CDC works closely with tribes, states, territories, and federal entities to resolve them.
- Data on COVID-19 vaccine doses administered in the United States are collected by vaccination providers and reported to CDC through multiple sources, including jurisdictions, pharmacies, and federal entities, which use various reporting methods, including Immunization Information Systems, Vaccine Administration Management System, and direct data submission. When CDC applies validation and business rules to prevent data duplication, data presented on the COVID Data Tracker might differ from data listed in state immunization systems and dashboards. CDC makes every effort to reconcile doses administered that are reported through more than one system.
- Healthcare providers are expected to report doses administered to federal, state, territorial, tribal, and local agencies within 72 hours of administration. There could be additional lag for data to be transmitted from the federal, state, territorial, or local agency to CDC. During the 72 hours, a large difference between the number of doses distributed and the number of people who are vaccinated is expected due to several factors, including the time it takes for doses delivered to be administered, the time it takes for administered doses to be reported to CDC, and management of available vaccine stock by jurisdictions and federal pharmacy partners to meet local demands.

The "Rate per 100,000" metric displays as "n/a" for federal entities because population-based rates are not applicable. Data for federal entities will display when the "Total counts" metric is selected. Veterans Health Administration (VHA) totals include employees, veteran patients, and other federal partners vaccinated by VHA.

On February 15th, 2021 the COVID-19 Vaccine Tracker updated to reflect the revised Pfizer-BioTech EUA to allow the use of a sixth dose, if present in a vial, as authorized by the FDA (https://www.fda.gov/emergency-preparedness-and-response/mcm-legal-regulatory-and-policy-framework/pfizer-biontech-covid-19-vaccine-frequently-asked-questions). Pfizer-BioNTech deliveries to a jurisdiction on or after February 15, 2021 will reflect this increase to six doses per vial compared to five doses; historical data will not be updated. The change to the Pfizer-BioTech dosage increases the number of doses delivered relative to the number of doses administered.

On February 19, 2021 and February 20, 2021, the national aggregate doses delivered total reported were incorrect as deliveries to federal entities were inadvertently counted twice; the totals should have been 74,108,895 and 74,979,165, respectively.

Definitions:

Total doses delivered; total count is the total number of vaccine doses that have been delivered. Doses delivered include those delivered to vaccine providers by the jurisdiction, retail pharmacies, long-term care facilities, Federal Emergency Management Agency and Health Resources and Services Administration partner sites, and federal entity facilities in that state or territory. Data for each federal entity are also reflected at the national level in the agency call out boxes on the map; combining these data with jurisdiction-level doses delivered data will result in double counting.

As of February 23rd, 2021, the national doses delivered totals show larger-than-typical daily increases. This is an accurate reflection of the data and is the result of recent weather events causing a backlog of vaccine delivery to many parts of the United States.

- For U.S. states, Washington D.C., the US Virgin Islands, and Puerto Rico, total counts of COVID-19 vaccine doses include doses delivered since December 14, 2020.
- For Palau, Micronesia, Marshall Islands, Guam, American Samoa, and Northern Marianas Islands, total counts of COVID-19 vaccine doses include doses marked as shipped in VTrckS since December 13, 2020.

Doses delivered; rate per 100,000 is the total number of vaccine doses delivered for every 100,000 people (overall and per the population aged 18 years and older), allowing comparison between areas with different population sizes.

Total doses administered; total count is the total number of vaccine doses that have been given to people in the United States since December 14, 2020, the date when the first dose was administered to a person in the United States under the Emergency Use Authorization as a non-clinical trial. Doses administered in a state or territory include those administered in jurisdictional partner clinics, retail pharmacies, long-term care facilities, Federal Emergency Management Agency (FEMA) and Health Resources and Services Administration (HRSA) partner sites, and federal entity facilities in that state or territory. Data for each federal entity are also reflected at the national level in the agency call out boxes on the map; combining these data with jurisdiction-level doses administered data will result in double counting. For this measure, the CDC's COVID Data Tracker attributes each dose to the state or territory in which the person received the dose.

The total doses administered can be greater than the total doses distributed. This is because vials of the Pfizer-BioNTech COVID-19 vaccine officially contain at least 5 doses but can contain an additional sixth dose. Use of this sixth dose, if present, is authorized by the FDA. Prior to February 14, 2021 the sixth doses were not reflected in the doses delivered totals, however if administered, doses were reflected in the doses administered totals.

Total doses administered; rate per 100,000 is the total number of vaccine doses given for every 100,000 people (overall and per the population aged 18 years and older), allowing comparison between areas with different population sizes.

People receiving 1 or more doses; total count** represents the total number of people who received at least 1 dose of COVID-19 vaccine. This metric includes everyone who has received only 1 dose and those who received more than 1 dose. For this measure, CDC's COVID Data Tracker attributes each dose to the state or territory in which the person resides.

People receiving 1 or more doses; % of the population** represents the percent of people who received at least 1 dose of COVID-19 vaccine. This metric includes everyone who has received only 1 dose and those who received more than 1 dose. For this measure, CDC's COVID Data Tracker attributes each dose to the state or territory in which the person resides. This includes residents of, or individuals associated with Federal Emergency Management Agency (FEMA), Health Resources and Services Administration (HRSA) partner sites, and federal entity facilities based on their residency. Estimates for the total population and population of those aged 18 years and older are used as the denominators to calculate percentages.

People receiving 2 doses; total count** represents the number of people who have received the second dose in a 2-dose COVID-19 vaccine series. For this measure, CDC's COVID Data Tracker attributes each dose to the state or territory in which the person resides. Estimates for the total population and population of those aged 18 years and older are used as the denominators to calculate percentages.

People receiving 2 doses; % of the population** represents the number of people who have received the second dose in a 2-dose COVID-19 vaccine series. For this measure, CDC's COVID Data Tracker attributes each dose to the state or territory in which the person resides. This includes residents of, or individuals associated with Federal Emergency Management Agency (FEMA), Health Resources and Services Administration (HRSA) partner sites, and federal entity facilities based on their residency. Estimates for the total population and population of those aged 18 years and older are used as the denominators to calculate percentages.

**The number of people receiving 1 or more doses and the number of people receiving 2 doses were determined based on information reported to CDC by state, territorial, tribal, and local public health agencies and federal entities on dose number, administration date, recipient ID, and date of submission. Because the method used to determine dose numbers needs to be applied across multiple jurisdictions with different reporting practices, CDC's dose number estimates might differ from those reported by jurisdictions and federal entities. A dose number was determined for nearly all reported doses administered but, because there are some missing data for dose number, the sum of people receiving 1 or more doses and people receiving 2 doses will not exactly equal the total doses administered. People receiving doses are attributed to the jurisdiction in which the vaccine was administered.

Texas provides aggregate dose count data to CDC, therefore, we do not receive specific information at the individual level; this limits our ability to directly calculate certain people-based metrics.

As of 02/24/2020; to calculate people-based metrics CDC assumes: (1) all people receiving vaccinations in Texas are considered residents of Texas and (2) the percentage of people who received dose 1 and the percentage of people who received dose 2 that are aged =18 years are the same as the percentage of total doses that were administered to people aged =18 years and older.

Therefore, people-based metrics for the population aged =18 years of age were estimated by multiplying the counts for the total population receiving 1 or more doses and the total population receiving 2 doses by the percentage of total doses administered to persons aged =18 years in Texas.

Residents of Texas who receive a vaccination in a different state or territory are not attributed to Texas in their population based metrics, but are still included in national level metrics.

Rates per 100,000‡ represent the rate of total doses delivered, the rate of total doses administered, the rate of people receiving 1 or more doses, and the rate of people receiving 2 doses per 100,000. The total population and the population aged 18 years and older are used as estimates to calculate rates for total doses delivered and total doses administered. In some limited circumstances, people might receive vaccinations outside the state or territory where they live. These rates currently account for vaccinations occurring in the jurisdiction where the vaccination was administered.

Percent of the population‡ represents the percent of people receiving 1 or more doses and the percent of people receiving 2 doses. The total population and the population aged 18 years and older are used as denominators to calculate the percent of the total population and the percent of the population aged 18 years and older who have received 1 or more doses or 2 doses. The percent of the total population was derived using the location of residency.

‡For the rate per 100,000 and percent of the population metrics, measures of vaccination are calculated among the entire population (i.e., all ages) and the population aged 18 years and older. The metrics used for rate and percentage calculations use the US Census Bureau Annual Estimates of the Resident Population for the United States and Puerto Rico, 2019 population. US Census Bureau 2018 population estimates and CIA World Factbook estimates are used for American Samoa, Federated States of Micronesia, Guam, Northern Mariana Islands, Republic of Palau, Marshall Islands, and US Virgin Islands.

Emergency Use Authorization has been granted for use of the Pfizer-BioNTech vaccine among persons aged 16 and older and use of the Moderna vaccine among persons aged 18 and older. Therefore, vaccine use is limited among those under age 18 years, who represent approximately 22% of the US population. Inclusion of all age groups in these calculations helps to provide a better measure of community immunity. Jurisdictions may use more targeted population counts for the denominators in their rate calculations (i.e., persons over 18 years or over 16 years old), which would result in values different than those reported on the CDC COVID Data Tracker.

Data Tracker Home
COVID Data Tracker Weekly Review
Your Community –
County View
Forecasting
Vaccinations in the US
Community Profile Report
State Profile Report
Mobility
Pandemic Vulnerability Index
Social Impact
Vaccinations +
Cases & Deaths +
Demographic Trends +
Healthcare Systems +
Testing and Seroprevalence +
People at Increased Risk +
Communications Resources
COVID-19 Home

Get Email Updates

Sign up to receive the COVID Data Tracker Weekly Review.

Email Address:

Email Address

What's this? Submit

COVID-19 Integrated County View

Maps, charts, and data provided by the CDC, updated daily by 8 pm ET†

Select state and county to populate county-specific data:

State:
Select a State

County or metro area:

This site provides an integrated, county view of key data for monitoring the COVID-19 pandemic in the United States. It allows for the exploration of standardized data across the country. The footnotes describe each data source and the methods used for calculating the metrics. For the most complete and up-to-date data for any particular county or state, visit the relevant health department website. Additional data and features are forthcoming.

Map Data: Cases / Reported cases

Time Period: Thu Feb 18 2021 - Wed Feb 24 2021

+ -

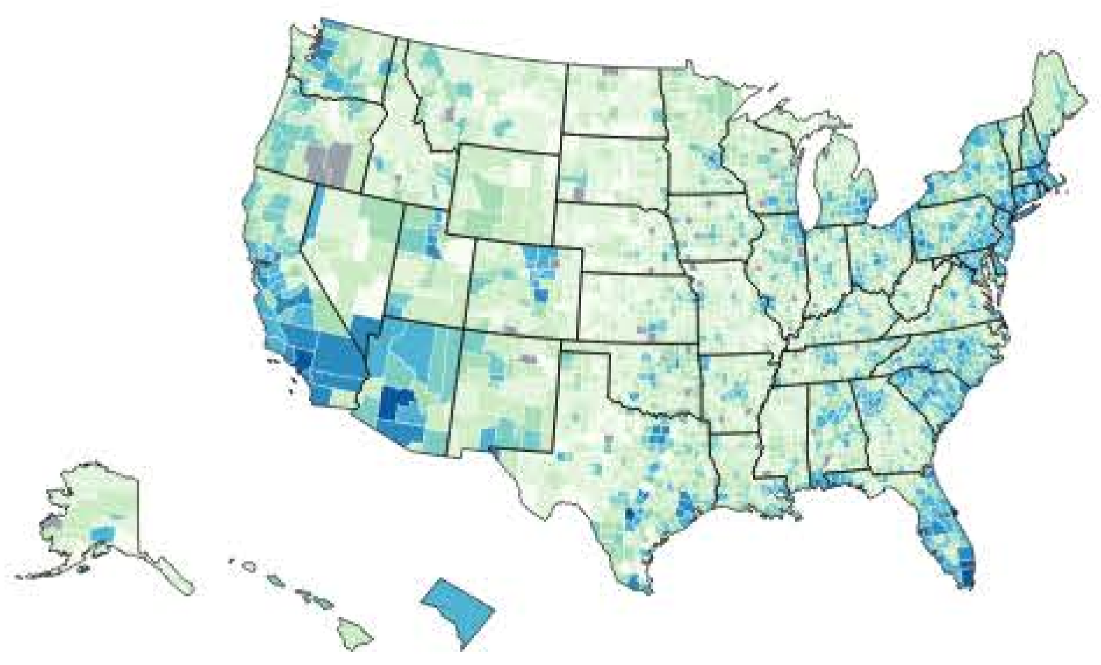

○0 ○1 to 10 ○11 to 50 ○51 to 100 ○101 to 250 ○251 to 1000 ○1001 to 5000 ○5000+
○No Data

How does this data compare to states?

Find case and death counts by state

View and Download COVID-19 Case Surveillance Public Use Data

†Data will update as soon as they are reviewed and verified, oftentimes before 8 pm ET. However, daily updates might be delayed due to delays in reported data.

*Data suppressed for confidentiality and stability

Data presented here may differ from data on state and local websites. This may be due to differences in how data were collected (e.g., date specimen obtained, or date reported for cases) or how the metrics are calculated. Data presented here use standard metrics across all counties in the United States. For the most accurate and up-to-date data for a specific county or state, visit the relevant state or local health department website.

The map can be modified to show various metrics for reported COVID-19 cases, deaths, and RT-PCR tests in U.S. states. Data for U.S. territories will be added as available. Reported metrics include total counts in last 7 days, total counts in last 7 days per 100,000 people, and percent change in counts from the previous 7 days. Totals per 100,000 people in last 7 days are calculated as the number of new COVID (cases or deaths) per 100k people in the last 7 days using the US Census Bureau Population Estimates Program. Additional RT-PCR metrics include overall percent positivity and absolute change in weekly percent positivity. Learn more about How CDC calculates percent positivity.

When a state and county is selected, the accompanying figures show 7-day totals and percent change for cases, deaths, percent positivity and testing volume. Total counts per 100,000 in last 7 days are also presented for cases, deaths and testing volume. At the bottom of the page, community characteristics are displayed for the selected county. 2019 Vintage Census Population Estimates and American Community Survey data were used to calculate county-level population density, household size, insurance status, poverty level, and percent of population ages 65 and over. Social Vulnerability Index (SVI) scores, which range from 0 to 1, are from CDC/ASTDR's Geospatial Research, Analysis & Service Program. COVID-19 Community Vulnerability Index (CCVI) scores, which also range from 0 to 1, are from Surgo Ventures.

Cases and Deaths: As of December 12, the COVID-19 case and death metrics are generated using a dataset managed by the CDC which is compiled from state and local health departments. Historical data were also updated with this dataset. To ensure data quality, daily data alerts are monitored for deviations in the data (e.g., decreases in cumulative values, no change in values, abnormal increases in values). These alerts are manually reviewed every day by checking the data against local government websites, state websites, and news sources, and the raw values are corrected as needed to reflect local government reports. Cases are based on date of report and not on date of symptom onset. Rates are calculated using the US Census Bureau Population Estimates Program.

Testing: The data represent results from United States laboratories on reverse transcription polymerase chain reaction (RT-PCR) tests for SARS-CoV-2, the virus that causes COVID-19. The data do not include results from all testing sites in a jurisdiction (e.g., point-of-care test sites are usually not included) and therefore reflect the majority of, but not all, COVID-19 tests in the United States. The majority of tests are from commercial and reference laboratories, but also include public health laboratories, hospital laboratories, and tests from other locations. The data represent laboratory test totals—not individual people—and exclude antigen and antibody tests. "Total Tests Reported" and "Total Positive Tests" counts reflect data through the "National totals as of" date. These totals might not match the sum of the numbers for each jurisdiction because data from some states and territories may be delayed by several days while officials reconcile discrepancies in counts due to variations in reporting by counties or cities. Each state includes the state "data as of" date when you hover over a given state. The data are provisional and subject to change.

The data are reported to state and jurisdictional health departments in accordance with applicable state or local law and in accordance with the Coronavirus Aid, Relief, and Economic Security (CARES) Act (CARES Act Section 18115). The data for each state and jurisdiction are sourced from either data submitted directly by the state or jurisdictional health department via COVID-19 electronic laboratory reporting (CELR), or by a combination of commercial, public health, and in-house hospital laboratory reports. CELR data are used for states and jurisdictions that submit data on each test result and for states and jurisdictions that submit aggregate counts that do not include antigen or antibody tests. The state-level data are available for public use on HealthData.Gov.

Learn more about How CDC calculates percent positivity.

Hospital Admissions and Capacity: Hospital data are reported to the US Department of Health and Human Services (HHS) either directly from facilities or via a state submission. Data for hospitals with the same Centers for Medicare & Medicaid Services (CMS) Certification Number (CCN) are aggregated. Three percent of CCNs contain multiple facilities that map to different counties. CCNs are assigned to counties based on the CMS Provider of Services files. Data presented here represent admissions and bed use among facilities within the selected county. Therefore, admissions and bed use are not limited to residents of the selected county.

Data include Veterans Administration, Defense Health Agency, and Indian Health Services hospitals and exclude psychiatric, rehabilitation, and religious non-medical hospitals.

Confirmed admissions are the sum of reported values for all hospitals reporting in the given timeframe. Data presented here include both adult and pediatric admissions. Due to inconsistent reporting and data errors, the number shown may not be a full representation of the true number of admissions in the area.

Data presented here include reported staffed adult beds and adult patients only. Total inpatient beds and ICU beds are calculated as an average among reports from each hospital in the given timeframe. Due to inconsistent reporting and impacts of staffing on the total number of beds at each hospital, variations may occur over time and the number shown may not be a full representation of the true number of resources in the area. Utilization metrics calculate the average utilization in the geography for the week.

These data are reported daily by more than 6,000 facilities across the country. While these data are reviewed for errors and corrected, some reporting errors may still exist within the data. To minimize errors in data reported here, outliers are removed before the metrics are calculated.

COVID Data Tracker

Cases in US 28,212,548 | Cases in US Last 30 Days | Total Vaccines Administered 70.5M | Deaths in US 506,834 | Deaths in US Last 30 Days

Data Tracker Home
COVID Data Tracker Weekly Review
Your Community
County View
Forecasting
Vaccinations in the US
Community Profile Report
State Profile Report
Mobility
Pandemic Vulnerability Index
Social Impact
Vaccinations
Cases & Deaths
Demographic Trends
Healthcare Systems
Testing and Seroprevalence
People at Increased Risk
Communications Resources
COVID-19 Home

Get Email Updates

Sign up to receive the COVID Data Tracker Weekly Review.

Email Address:
Email Address
What's this? Submit

COVID-19 Integrated County View

Maps, charts, and data provided by the CDC, updated daily by 8 pm ET†

Select state and county to populate county-specific data:

State: Kentucky

County or metro area: Fayette

This site provides an integrated, county view of key data for monitoring the COVID-19 pandemic in the United States. It allows for the exploration of standardized data across the country. The footnotes describe each data source and the methods used for calculating the metrics. For the most complete and up-to-date data for any particular county or state, visit the relevant health department website. Additional data and features are forthcoming.

Map Data: Deaths
Reported deaths

Back to U.S. Map

Time Period: Thu Feb 18 2021 - Wed Feb 24 2021

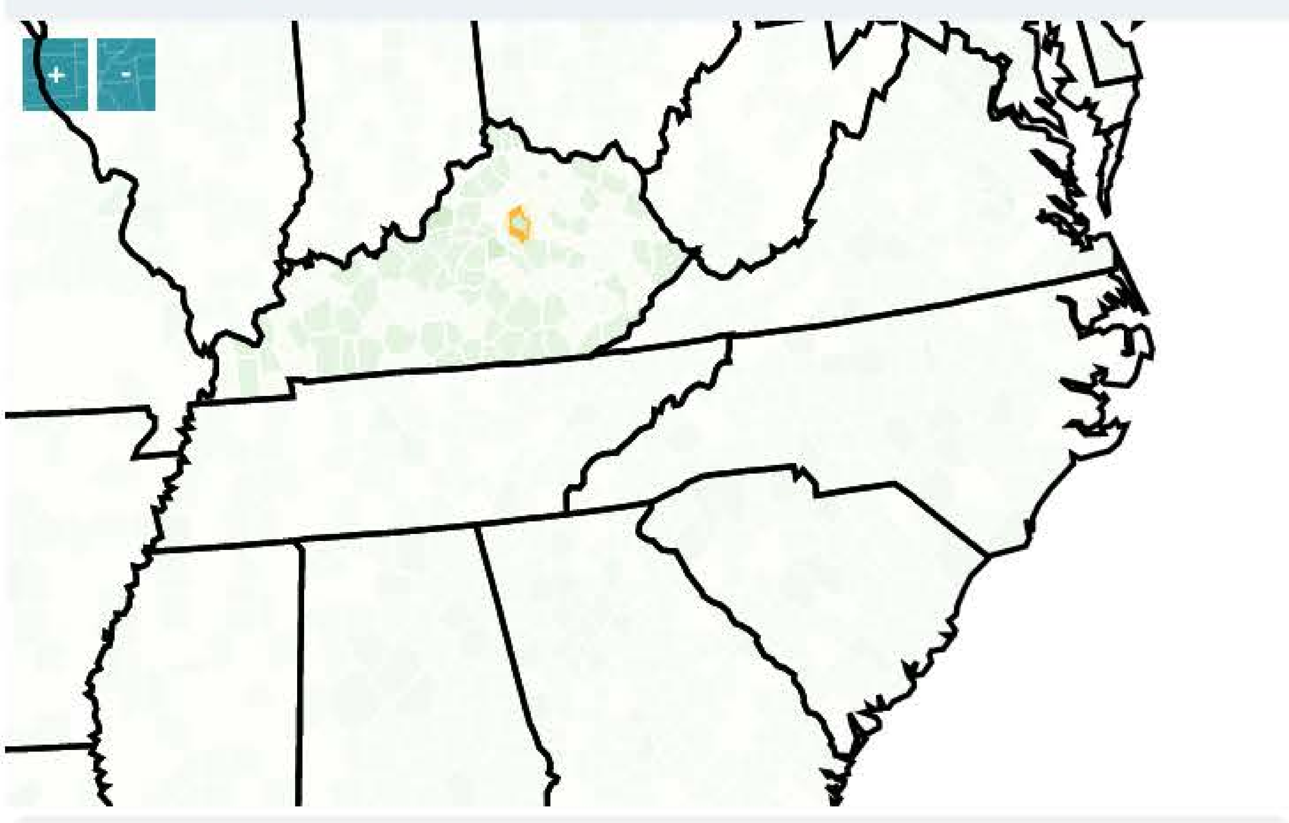

0 | <10 | 10 - 24 | 25 - 50 | 51 - 100 | 100 or more | No Data

7 Day Average
Wed Jan 22 2020 - Wed Feb 24 2021

7 Day total

Cases
598
(185.05 per 100k)
↓ -13.58 % change

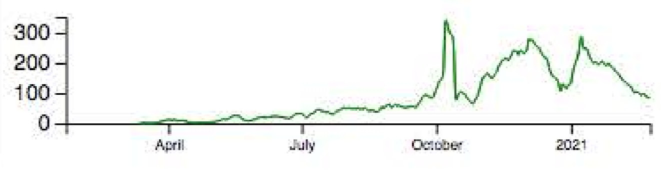

Deaths
12
(3.71 per 100k)
↑ 33.33 % change

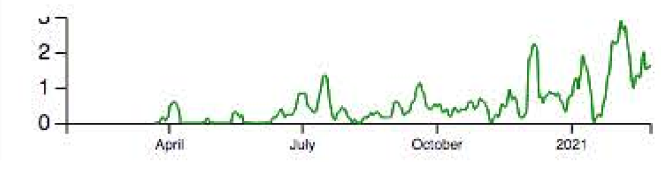

Percent Positivity
8.71
↑ 0.86 % change

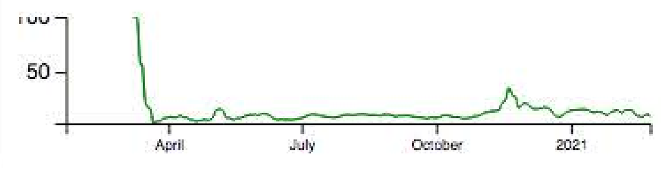

Testing Volume
6714
(2077.66 per 100k)
↓ -20.15 % change

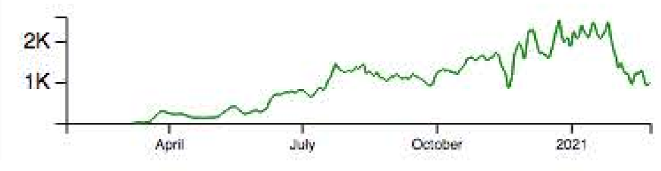

New Hospital Admissions (COVID)
72
(4.05 per 100 beds)
↑ 16.10 % change

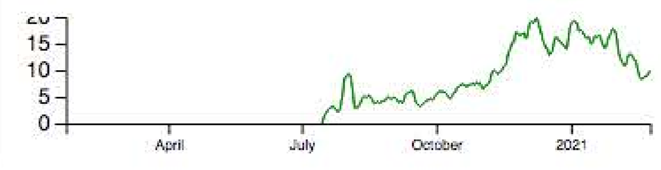

Percent Beds Used (COVID)
7.00 %
↓ -0.96 % change

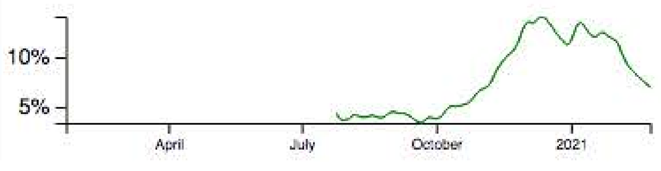

Percent ICU Beds Used (COVID)
11.13 %
↓ -3.76 % change

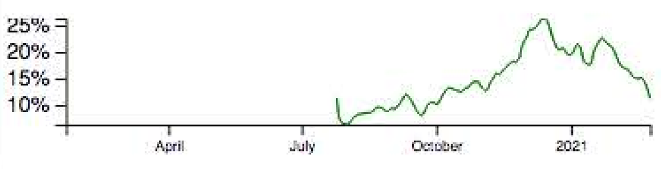

Community Characteristics

**Population density:**	1139.32 people per sq. mile
**Household size:**	2.38
**Percent uninsured:**	7.3%
**Percent living in poverty:**	17.8%
**Percent population 65yrs and over:**	13.87%
**Social Vulnerability Index (SVI):**	0.49
**COVID-19 Community Vulnerability Index (CCVI):**	0.13

How does this data compare to states?

Find case and death counts by state

View and Download COVID-19 Case Surveillance Public Use Data

†Data will update as soon as they are reviewed and verified, oftentimes before 8 pm ET. However, daily updates might be delayed due to delays in reported data.

*Data suppressed for confidentiality and stability

Data presented here may differ from data on state and local websites. This may be due to differences in how data were collected (e.g., date specimen obtained, or date reported for cases) or how the metrics are calculated. Data presented here use standard metrics across all counties in the United States. For the most accurate and up-to-date data for a specific county or state, visit the relevant state or local health department website.

The map can be modified to show various metrics for reported COVID-19 cases, deaths, and RT-PCR tests in U.S. states. Data for U.S. territories will be added as available. Reported metrics include total counts in last 7 days, total counts in last 7 days per 100,000 people, and percent change in counts from the previous 7 days. Totals per 100,000 people in last 7 days are calculated as the number of new COVID (cases or deaths) per 100k people in the last 7 days using the US Census Bureau Population Estimates Program. Additional RT-PCR metrics include overall percent positivity and absolute change in weekly percent positivity. Learn more about How CDC calculates percent positivity.

When a state and county is selected, the accompanying figures show 7-day totals and percent change for cases, deaths, percent positivity and testing volume. Total counts per 100,000 in last 7 days are also presented for cases, deaths and testing volume. At the bottom of the page, community characteristics are displayed for the selected county. 2019 Vintage Census Population Estimates and American Community Survey data were used to calculate county-level population density, household size, insurance status, poverty level, and percent of population ages 65 and over. Social Vulnerability Index (SVI) scores, which range from 0 to 1, are from CDC/ASTDR's Geospatial Research, Analysis & Service Program. COVID-19 Community Vulnerability Index (CCVI) scores, which also range from 0 to 1, are from Surgo Ventures.

Cases and Deaths: As of December 12, the COVID-19 case and death metrics are generated using a dataset managed by the CDC which is compiled from state and local health departments. Historical data were also updated with this dataset. To ensure data quality, daily data alerts are monitored for deviations in the data (e.g., decreases in cumulative values, no change in values, abnormal increases in values). These alerts are manually reviewed every day by checking the data against local government websites, state websites, and news sources, and the raw values are corrected as needed to reflect local government reports. Cases are based on date of report and not on date of symptom onset. Rates are calculated using the US Census Bureau Population Estimates Program.

Testing: The data represent results from United States laboratories on reverse transcription polymerase chain reaction (RT-PCR) tests for SARS-CoV-2, the virus that causes COVID-19. The data do not include results from all testing sites in a jurisdiction (e.g., point-of-care test sites are usually not included) and therefore reflect the majority of, but not all, COVID-19 tests in the United States. The majority of tests are from commercial and reference laboratories, but also include public health laboratories, hospital laboratories, and tests from other locations. The data represent laboratory test totals—not individual people—and exclude antigen and antibody tests. "Total Tests Reported" and "Total Positive Tests" counts reflect data through the "National totals as of" date. These totals might not match the sum of the numbers for each jurisdiction because data from some states and territories may be delayed by several days while officials reconcile discrepancies in counts due to variations in reporting by counties or cities. Each state includes the state "data as of" date when you hover over a given state. The data are provisional and subject to change.

The data are reported to state and jurisdictional health departments in accordance with applicable state or local law and in accordance with the Coronavirus Aid, Relief, and Economic Security (CARES) Act (CARES Act Section 18115). The data for each state and jurisdiction are sourced from either data submitted directly by the state or jurisdictional health department via COVID-19 electronic laboratory reporting (CELR), or by a combination of commercial, public health, and in-house hospital laboratory reports. CELR data are used for states and jurisdictions that submit data on each test result and for states and jurisdictions that submit aggregate counts that do not include antigen or antibody tests. The state-level data are available for public use on HealthData.Gov.

Learn more about How CDC calculates percent positivity.

Hospital Admissions and Capacity: Hospital data are reported to the US Department of Health and Human Services (HHS) either directly from facilities or via a state submission. Data for hospitals with the same Centers for Medicare & Medicaid Services (CMS) Certification Number (CCN) are aggregated. Three percent of CCNs contain multiple facilities that map to different counties. CCNs are assigned to counties based on the CMS Provider of Services files. Data presented here represent admissions and bed use among facilities within the selected county. Therefore, admissions and bed use are not limited to residents of the selected county.

Data include Veterans Administration, Defense Health Agency, and Indian Health Services hospitals and exclude psychiatric, rehabilitation, and religious non-medical hospitals.

Confirmed admissions are the sum of reported values for all hospitals reporting in the given timeframe. Data presented here include both adult and pediatric admissions. Due to inconsistent reporting and data errors, the number shown may not be a full representation of the true number of admissions in the area.

Data presented here include reported staffed adult beds and adult patients only. Total inpatient beds and ICU beds are calculated as an average among reports from each hospital in the given timeframe. Due to inconsistent reporting and impacts of staffing on the total number of beds at each hospital, variations may occur over time and the number shown may not be a full representation of the true number of resources in the area. Utilization metrics calculate the average utilization in the geography for the week.

These data are reported daily by more than 6,000 facilities across the country. While these data are reviewed for errors and corrected, some reporting errors may still exist within the data. To minimize errors in data reported here, outliers are removed before the metrics are calculated.

Currently hospitalized per 1 million people

Data updated February 26, 2021

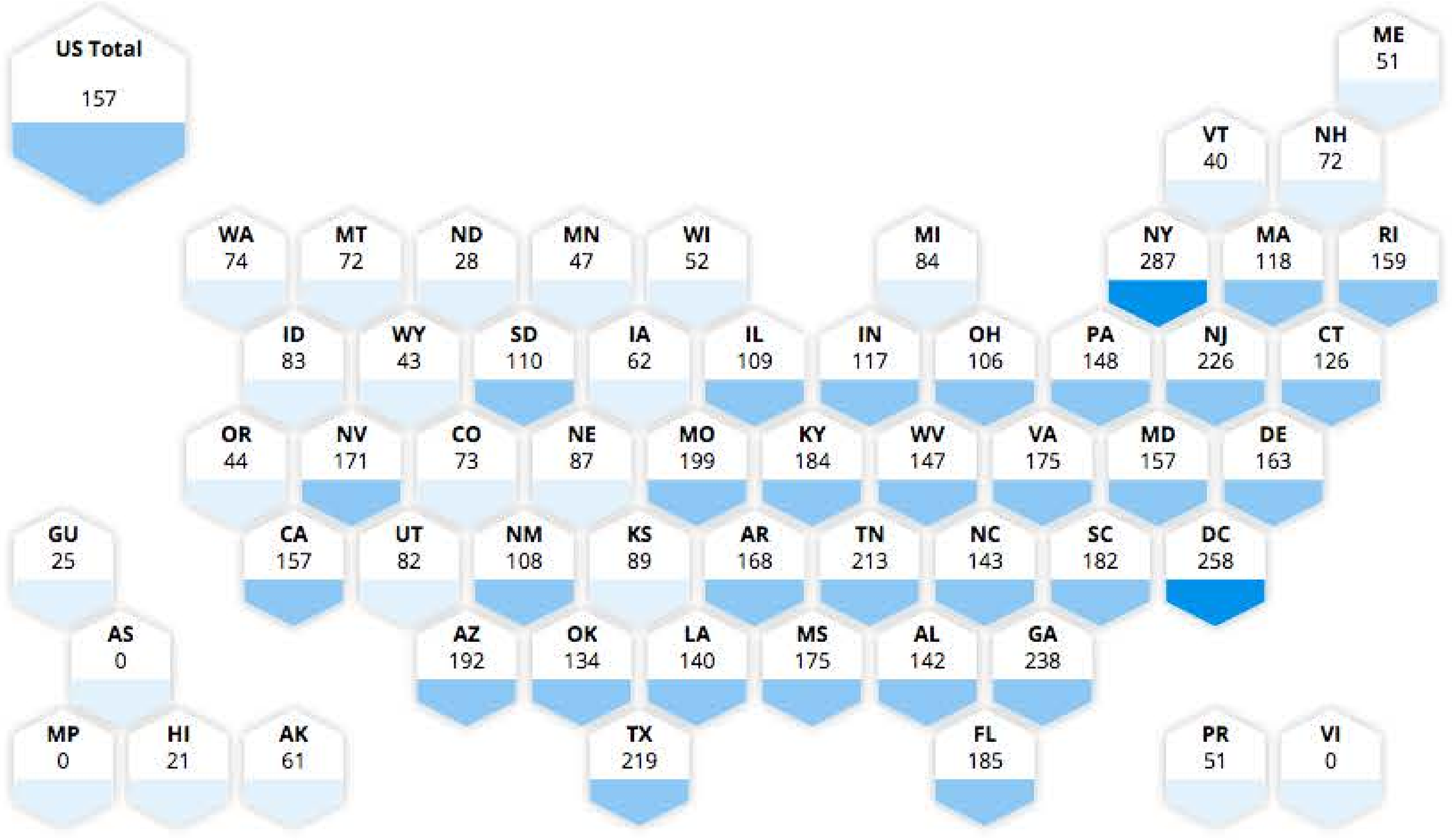

Under 100 | 100 - 250 | 250 - 500 | Over 500 hospitalizations per 1 million

About this data ↓

US Total

351,578,714
Total test results

28,225,167
Cases

51,116
Currently hospitalized

500,345
Deaths

February 1, 2021

It's Time: The COVID Tracking Project Will Soon Come to an End

Read the article →

The COVID Tracking Project collects and publishes the most complete data about COVID-19 in the US.

Every day, our volunteer teams compile the latest public health data from official US state, territorial, and federal sources.

See the data →
About the data →
Our analysis & updates →
About us →

Race & ethnicity data

The COVID Racial Data Tracker is a collaboration between the COVID Tracking Project and the Boston University Center for Antiracist Research. Together, we're gathering the most complete and up-to-date race and ethnicity data on COVID-19 in the United States.

All race & ethnicity data →

Jump to a state ↓

Long-term-care data

To date, the Long-Term Care COVID Tracker is the most comprehensive dataset about COVID-19 in US nursing homes and other long-term care facilities. It compiles crucial data about the effects of the pandemic on a population with extraordinary vulnerabilities to the virus due to age, underlying health conditions, or proximity to large outbreaks.

All long-term care data →

Jump to a state ↓

Our analysis & updates

February 18, 2021

More Good News: This Week in COVID-19 Data, Feb 18

Case numbers are dropping rapidly, and deaths are down 20 percent from last week. Vaccines are finally showing up in the data with dramatic declines in deaths from long-term-care facilities. We urge caution about interpreting wobbles in the data in the next week or two, given storm disruptions affecting Texas and elsewhere.

Read the article →

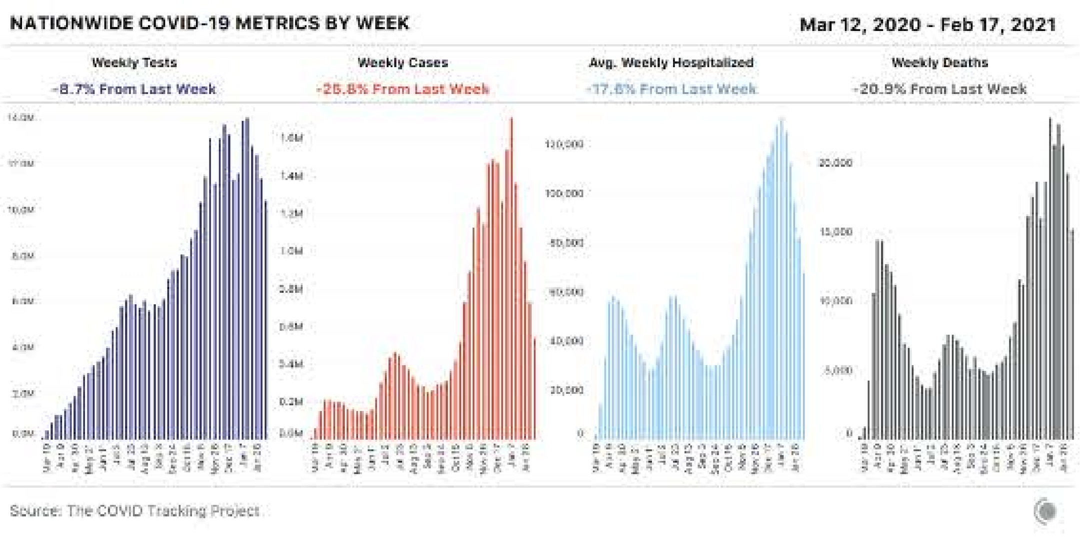

February 25, 2021

Good News Despite Data Wobbles: This Week in COVID-19 Data, Feb 25

Cases, hospitalizations, and deaths are still declining, though holiday reporting and winter storms have probably caused fluctuations in several metrics. We reiterate that deaths reported each day don't represent people who died that day—and they may even include deaths that occurred several months ago. And now is the time to switch over to federal data sources, because The COVID Tracking Project has only a little over a week of data compilation left.

Read the article →

February 25, 2021

Federal COVID Data 101: Working with Death Numbers

The CDC provides two different datasets regarding COVID-19 fatalities. Here's a walkthrough of how they compare to each other and to The COVID Tracking Project's data.

Read the article →

February 25, 2021

Variants 101: FAQ

Coronavirus variants have emerged worldwide. What does this really mean?

Read the article →

February 24, 2021

Federal COVID Data 101: Working with Case Data

Here's a walkthrough of the Centers for Disease Control and Prevention (CDC) daily case dataset and what you should know about it.

Read the article →

Our data API

Our public data API provides access to all of our data at a national and state level.

All API information →
Current API status →
API change log →

Who's using the data

The New York Times | The Washington Post | CNN | Vox | ProPublica | The Wall Street Journal | Politico | npr

CovidTracking.com Copyright © 2021 by The Atlantic Monthly Group. (CC BY 4.0)

The COVID Tracking Project will stop collecting data on March 7, 2021. ×

The Data

We update all our data each day between about 6pm and 7:30pm Eastern Time. We have recently begun publishing a bit later so as to capture Colorado and New Mexico data on the same day they update rather than on the following day.

Sign up for our newsletter to get our latest articles about this data by email.

Data for February 26, 2021 Get the data as: CSV API

Cases (Historical data)

Total cases (Definition)	28,225,167
New cases today (Calculated)	74,429
Change over 7 days (Calculated)	1.7%+

Tests (Historical data)

Total tests (Definition)	351,578,714
New tests today (Calculated)	1,793,570
Change over 7 days (Calculated)	2.9%+

Hospitalization (Historical data)

Currently hospitalized (Definition)	51,116
Currently in ICU (Definition)	10,466
Currently on ventilator (Definition)	3,466

See HHS hospitalization data on a map.

Outcomes (Historical data)

Deaths (Definition)	500,345

US states and territories report data in differing units and with varying definitions. Our national summary Cases, Tests, and Outcomes numbers are simple sums of the data states and territories provide, but because of the disparate metrics they include, they should be considered estimates.

Overview of National COVID-19 Data

Last 90 days | Historical

—— Solid line represents National 7-day average

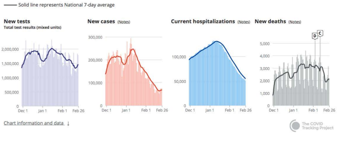

Chart information and data ↓

Data by State

Jump to a state
AL AK AS AZ AR CA CO CT DE DC FL GA GU HI ID IL IN IA KS KY LA ME MD MA MI MN MS MO MT NE NV NH NJ NM NY NC ND MP OH OK OR PA PR RI SC SD TN TX VI UT VT VA WA WV WI WY

Alabama

State's dataset was last updated February 26, 2021 11:00 am ET
Full state data including data sources and notes.

Cases (Historical data)

Total cases (Definition)	491,849
∟ Confirmed cases (Definition)	385,229
∟ Probable cases (Definition)	106,620
New cases today (Calculated)	739
Change over 7 days (Calculated)	1.4%+

Viral (PCR) tests (Historical data)

Total tests (test encounters) (Definition)	Not Reported
Total tests (specimens) (Definition)	Not Reported
Total tests (people) ⚠ Includes antigen (Definition)	2,281,648

Viral (PCR) tests (HHS data)

Total tests	3,853,300

Source: CELR data from states provided in line level format
This data is published by HHS. It may differ from state-provided data.
Data as of February 22, 2021

Antigen tests (Historical data)

Total tests (specimens) (Definition)	Not Reported
Total tests (people) (Definition)	Not Reported

Warning: Antigen reporting may significantly understate the true number of tests administered

Antibody tests (Historical data)

Total tests (specimens) (Definition)	Not Reported
Total tests (people) (Definition)	116,701

Hospitalization (Historical data)

Now hospitalized (Definition)	691
Now in ICU (Definition)	Not Reported
Now on ventilator (Definition)	Not Reported
Ever hospitalized (Definition)	45,428
Ever in ICU (Definition)	2,650
Ever on ventilator (Definition)	1,507

Hospitalization (HHS data)

See this data on a map.

Now hospitalized (confirmed + suspected) (Definition)	917
∟ Now hospitalized (confirmed only) (Definition)	777
Now in ICU (confirmed + suspected) (Definition)	250

This data is published by HHS.
Data as of February 25, 2021

Outcomes (Historical data)

Hospital discharges (Definition)	Not Reported
Total deaths (Definition)	9,869
∟ Probable deaths (Definition)	2,135
∟ Confirmed deaths (Definition)	7,734

Long-term-care facilities

Long-term care (LTC) (More data)

Total cases (Definition)	15,139
Total deaths (Definition)	1,625
Number of facilities affected (Definition)	0
Percent of state deaths from LTC facilities (Calculated)	16.5%
New cases since Feb 11 (Calculated)	0.6%
New deaths since Feb 11 (Calculated)	0%

View historical totals
Data as of February 18, 2021

Vaccinations

Total resident & staff doses administered	75,916
Staff & resident 1st dose administered	48,271
Staff &resident 2nd dose administered	27,379

Vaccinations administered through the Pharmacy Partnership for Long-Term Care Program
Data as of February 26, 2021

Race & ethnicity data

We compute the number of **cases and deaths per 100k people** for each race and ethnicity.

These numbers show the scale of outcomes compared to the size of each group's population. **These are not the number of cases or deaths**, rather the proportion of each demographic group who have been affected.

View more racial data →
Get shareable graphic of AL's racial data →
View AL's historical racial data →

Race & ethnicity: cases per capita

Cases per 100,000 people (Definition)	
∟ Black or African American	6,246
∟ Hispanic or Latino	5,814
∟ Asian	3,356
∟ White	4,730

(All data is calculated)
Data as of February 24, 2021

Race & ethnicity: deaths per capita

Deaths per 100,000 people (Definition)	
∟ Black or African American	164
∟ Hispanic or Latino	62
∟ Asian	35
∟ White	136

(All data is calculated)
Data as of February 24, 2021

Back to top.

Alaska

State's dataset was last updated February 25, 2021 3:59 am ET
Full state data including data sources and notes.

Cases (Historical data)

Total cases (Definition)	55,886
New cases today (Calculated)	0
Change over 7 days (Calculated)	1.2%+

Viral (PCR) tests (Historical data)

Total tests (test encounters) (Definition)	Not Reported
Total tests (specimens) ⚠ Includes antigen (Definition)	1,671,043
Total tests (people) (Definition)	Not Reported

Viral (PCR) tests (HHS data)

Total tests	1,768,758

Source: CELR data from states provided in line level format
This data is published by HHS. It may differ from state-provided data.
Data as of February 22, 2021

Antigen tests (Historical data)

Total tests (specimens) (Definition)	Not Reported
Total tests (people) (Definition)	Not Reported

Warning: Antigen reporting may significantly understate the true number of tests administered

Antibody tests (Historical data)

Total tests (specimens) (Definition)	Not Reported
Total tests (people) (Definition)	Not Reported

Hospitalization (Historical data)

Now hospitalized (Definition)	45
Now in ICU (Definition)	Not Reported
Now on ventilator (Definition)	5
Ever hospitalized (Definition)	1,271
Ever in ICU (Definition)	Not Reported
Ever on ventilator (Definition)	Not Reported

Hospitalization (HHS data)

See this data on a map.

Now hospitalized (confirmed + suspected) (Definition)	43
∟ Now hospitalized (confirmed only) (Definition)	38
Now in ICU (confirmed + suspected) (Definition)	12

This data is published by HHS.
Data as of February 25, 2021

Outcomes (Historical data)

Hospital discharges (Definition)	Not Reported
Deaths (Definition)	290

Long-term-care facilities

Long-term care (LTC) (More data)

Total cases (Definition)	144
Total deaths (Definition)	7
Number of facilities affected (Definition)	7
Percent of state deaths from LTC facilities (Calculated)	2.4%
New cases since Feb 11 (Calculated)	0.7%
New deaths since Feb 11 (Calculated)	0%

View historical totals
Data as of February 18, 2021

Vaccinations

Total resident & staff doses administered	6,202
Staff & resident 1st dose administered	4,120
Staff &resident 2nd dose administered	2,059

Vaccinations administered through the Pharmacy Partnership for Long-Term Care Program
Data as of February 26, 2021

Race & ethnicity data

We compute the number of **cases and deaths per 100k people** for each race and ethnicity.

These numbers show the scale of outcomes compared to the size of each group's population. **These are not the number of cases or deaths**, rather the proportion of each demographic group who have been affected.

View more racial data →
Get shareable graphic of AK's racial data →
View AK's historical racial data →

Race & ethnicity: cases per capita

Cases per 100,000 people (Definition)	
∟ Black or African American	6,098
∟ Hispanic or Latino	6,077
∟ Asian	5,157
∟ Native Hawaiian or Other Pacific Islander	16,189
∟ American Indian or Alaska Native	11,006
∟ White	3,758

(All data is calculated)
Data as of February 24, 2021

Race & ethnicity: deaths per capita

Deaths per 100,000 people (Definition)	
∟ Black or African American	37*
∟ Hispanic or Latino	23
∟ Asian	63
∟ Native Hawaiian or Other Pacific Islander	174
∟ American Indian or Alaska Native	95
∟ White	26

(All data is calculated)
* Based on <10 deaths among members of this race/ethnicity. Interpret with caution.
Data as of February 24, 2021

Back to top.

American Samoa

State's dataset was last updated December 1, 2020 12:00 am ET
Full state data including data sources and notes.

Cases (Historical data)

Total cases (Definition)	0
New cases today (Calculated)	0
Change over 7 days (Calculated)	N/A

Viral (PCR) tests (Historical data)

Total tests (test encounters) (Definition)	Not Reported
Total tests (specimens) ⚠ Unclear test type (Definition)	2,140
Total tests (people) (Definition)	Not Reported

Antigen tests (Historical data)

Total tests (specimens) (Definition)	Not Reported
Total tests (people) (Definition)	Not Reported

Warning: Antigen reporting may significantly understate the true number of tests administered

Antibody tests (Historical data)

Total tests (specimens) (Definition)	Not Reported
Total tests (people) (Definition)	Not Reported

Hospitalization (Historical data)

Now hospitalized (Definition)	Not Reported
Now in ICU (Definition)	Not Reported
Now on ventilator (Definition)	Not Reported
Ever hospitalized (Definition)	Not Reported
Ever in ICU (Definition)	Not Reported
Ever on ventilator (Definition)	Not Reported

Outcomes (Historical data)

Hospital discharges (Definition)	Not Reported
Deaths (Definition)	0

Long-term-care facilities

Long-term care (LTC) (More data)

Total cases (Definition)	0
Total deaths (Definition)	0
Number of facilities affected (Definition)	0
Percent of state deaths from LTC facilities (Calculated)	N/A
New cases since Feb 11 (Calculated)	N/A
New deaths since Feb 11 (Calculated)	N/A

View historical totals
Data as of February 18, 2021

Vaccinations

Total resident & staff doses administered	0
Staff & resident 1st dose administered	0
Staff &resident 2nd dose administered	0

Vaccinations administered through the Pharmacy Partnership for Long-Term Care Program
Data as of February 26, 2021

Race & ethnicity data

We compute the number of **cases and deaths per 100k people** for each race and ethnicity.

These numbers show the scale of outcomes compared to the size of each group's population. **These are not the number of cases or deaths**, rather the proportion of each demographic group who have been affected.

Race & ethnicity: cases per capita

Cases per 100,000 people

AS does not report this information. Help us get better data.

Race & ethnicity: deaths per capita

Deaths per 100,000 people

AS does not report this information. Help us get better data.

Back to top.

Arizona

State's dataset was last updated February 26, 2021 12:00 am ET
Full state data including data sources and notes.

Cases (Historical data)

Total cases (Definition)	814,528
∟ Confirmed cases (Definition)	759,626
∟ Probable cases (Definition)	54,902
New cases today (Calculated)	1,621
Change over 7 days (Calculated)	1.3%+

Viral (PCR) tests (Historical data)

Total tests (test encounters) (Definition)	Not Reported
Total tests (specimens) ⚠ Includes antigen (Definition)	7,596,552
Total tests (people) ⚠ Includes antigen (Definition)	3,740,067

Viral (PCR) tests (HHS data)

Total tests	5,970,129

Source: CELR data from states provided in line level format
This data is published by HHS. It may differ from state-provided data.
Data as of February 23, 2021

Antigen tests (Historical data)

Total tests (specimens) (Definition)	Not Reported
Total tests (people) (Definition)	Not Reported

Warning: Antigen reporting may significantly understate the true number of tests administered

Antibody tests (Historical data)

Total tests (specimens) (Definition)	437,195
Total tests (people) (Definition)	Not Reported

Hospitalization (Historical data)

Now hospitalized (Definition)	1,354
Now in ICU (Definition)	419
Now on ventilator (Definition)	225
Ever hospitalized (Definition)	57,460
Ever in ICU (Definition)	Not Reported
Ever on ventilator (Definition)	Not Reported

Hospitalization (HHS data)

See this data on a map.

Now hospitalized (confirmed + suspected) (Definition)	1,402
∟ Now hospitalized (confirmed only) (Definition)	1,089
Now in ICU (confirmed + suspected) (Definition)	354

This data is published by HHS.
Data as of February 25, 2021

Outcomes (Historical data)

Hospital discharges (Definition)	116,223
Total deaths (Definition)	15,897
∟ Probable deaths (Definition)	1,869
∟ Confirmed deaths (Definition)	14,028

Long-term-care facilities

Long-term care (LTC) (More data)

Total cases (Definition)	15,978
Total deaths (Definition)	2,402
Number of facilities affected (Definition)	975
Percent of state deaths from LTC facilities (Calculated)	15.1%
New cases since Feb 11 (Calculated)	1.7%
New deaths since Feb 11 (Calculated)	1.4%

View historical totals
Data as of February 18, 2021

Vaccinations

Total resident & staff doses administered	94,527
Staff & resident 1st dose administered	72,510
Staff &resident 2nd dose administered	21,747

Vaccinations administered through the Pharmacy Partnership for Long-Term Care Program
Data as of February 26, 2021

Race & ethnicity data

We compute the number of **cases and deaths per 100k people** for each race and ethnicity.

These numbers show the scale of outcomes compared to the size of each group's population. **These are not the number of cases or deaths**, rather the proportion of each demographic group who have been affected.

View more racial data →
Get shareable graphic of AZ's racial data →
View AZ's historical racial data →

Race & ethnicity: cases per capita

Cases per 100,000 people (Definition)	
∟ Black or African American	8,481
∟ Hispanic or Latino	10,888
∟ Asian or Pacific Islander	4,858
∟ American Indian or Alaska Native	14,427
∟ White	7,834

(All data is calculated)
Data as of February 24, 2021

Race & ethnicity: deaths per capita

Deaths per 100,000 people (Definition)	
∟ Black or African American	138
∟ Hispanic or Latino	203
∟ Asian or Pacific Islander	85
∟ American Indian or Alaska Native	470
∟ White	200

(All data is calculated)
Data as of February 24, 2021

Back to top.

Arkansas

State's dataset was last updated February 26, 2021 12:00 am ET
Full state data including data sources and notes.

Cases (Historical data)

Total cases (Definition)	318,638
∟ Confirmed cases (Definition)	251,711
∟ Probable cases (Definition)	66,927
New cases today (Calculated)	516
Change over 7 days (Calculated)	1.2%+

Viral (PCR) tests (Historical data)

Total tests (test encounters) (Definition)	Not Reported
Total tests (specimens) (Definition)	2,634,339
Total tests (people) (Definition)	Not Reported

Viral (PCR) tests (HHS data)

Total tests	2,420,583

⚠ Warning
Source: CELR data from states provided in line level format
This data is published by HHS. It may differ from state-provided data.
Data as of February 23, 2021

Antigen tests (Historical data)

Total tests (specimens) (Definition)	Not Reported
Total tests (people) (Definition)	444,073

Warning: Antigen reporting may significantly understate the true number of tests administered

Antibody tests (Historical data)

Total tests (specimens) (Definition)	Not Reported
Total tests (people) (Definition)	Not Reported

Hospitalization (Historical data)

Now hospitalized (Definition)	504
Now in ICU (Definition)	184
Now on ventilator (Definition)	98
Ever hospitalized (Definition)	14,747
Ever in ICU (Definition)	Not Reported
Ever on ventilator (Definition)	1,518

Hospitalization (HHS data)

See this data on a map.

Now hospitalized (confirmed + suspected) (Definition)	606
∟ Now hospitalized (confirmed only) (Definition)	502
Now in ICU (confirmed + suspected) (Definition)	182

This data is published by HHS.
Data as of February 25, 2021

Outcomes (Historical data)

Hospital discharges (Definition)	Not Reported
Total deaths (Definition)	5,407
∟ Probable deaths (Definition)	1,059
∟ Confirmed deaths (Definition)	4,348

Long-term-care facilities

Long-term care (LTC) (More data)

Total cases (Definition)	22,330
Total deaths (Definition)	2,040
Number of facilities affected (Definition)	311
Percent of state deaths from LTC facilities (Calculated)	37.7%
New cases since Feb 11 (Calculated)	1.9%
New deaths since Feb 11 (Calculated)	3.9%

View historical totals
Data as of February 18, 2021

Vaccinations

Total resident & staff doses administered	27,077
Staff & resident 1st dose administered	18,619
Staff &resident 2nd dose administered	8,364

Vaccinations administered through the Pharmacy Partnership for Long-Term Care Program
Data as of February 26, 2021

Race & ethnicity data

We compute the number of **cases and deaths per 100k people** for each race and ethnicity.

These numbers show the scale of outcomes compared to the size of each group's population. **These are not the number of cases or deaths**, rather the proportion of each demographic group who have been affected.

View more racial data →
Get shareable graphic of AR's racial data →
View AR's historical racial data →

Race & ethnicity: cases per capita

Cases per 100,000 people (Definition)	
∟ Black or African American	10,884
∟ Hispanic or Latino	15,132
∟ Asian	6,265
∟ Native Hawaiian or Other Pacific Islander	38,257
∟ American Indian or Alaska Native	5,031
∟ White	8,849

(All data is calculated)
Data as of February 24, 2021

Race & ethnicity: deaths per capita

Deaths per 100,000 people (Definition)	
∟ Black or African American	175
∟ Hispanic or Latino	82
∟ Asian	79
∟ Native Hawaiian or Other Pacific Islander	630
∟ American Indian or Alaska Native	93
∟ White	183

(All data is calculated)
Data as of February 24, 2021

Back to top.

California

State's dataset was last updated February 26, 2021 2:59 am ET
Full state data including data sources and notes.

Cases (Historical data)

Total cases (Definition)	3,465,726
∟ Confirmed cases (Definition)	3,465,726
New cases today (Calculated)	5,400
Change over 7 days (Calculated)	1.1%+

Viral (PCR) tests (Historical data)

Total tests (test encounters) (Definition)	Not Reported
Total tests (specimens) ⚠ Unclear test type (Definition)	47,995,576
Total tests (people) (Definition)	Not Reported

Viral (PCR) tests (HHS data)

Total tests	40,619,115

Source: CELR data from states provided in line level format
This data is published by HHS. It may differ from state-provided data.
Data as of February 20, 2021

Antigen tests (Historical data)

Total tests (specimens) (Definition)	Not Reported
Total tests (people) (Definition)	Not Reported

Warning: Antigen reporting may significantly understate the true number of tests administered

Antibody tests (Historical data)

Total tests (specimens) (Definition)	Not Reported
Total tests (people) (Definition)	Not Reported

Hospitalization (Historical data)

Now hospitalized (Definition)	6,152
Now in ICU (Definition)	1,725
Now on ventilator (Definition)	Not Reported
Ever hospitalized (Definition)	Not Reported
Ever in ICU (Definition)	Not Reported
Ever on ventilator (Definition)	Not Reported

Hospitalization (HHS data)

See this data on a map.

Now hospitalized (confirmed + suspected) (Definition)	6,179
∟ Now hospitalized (confirmed only) (Definition)	5,674
Now in ICU (confirmed + suspected) (Definition)	1,701

This data is published by HHS.
Data as of February 25, 2021

Outcomes (Historical data)

Hospital discharges (Definition)	Not Reported
Deaths (Definition)	51,382

Long-term-care facilities

Long-term care (LTC) (More data)

Total cases (Definition)	152,204
Total deaths (Definition)	12,653
Number of facilities affected (Definition)	2,181
Percent of state deaths from LTC facilities (Calculated)	24.6%
New cases since Feb 11 (Calculated)	0.4%
New deaths since Feb 11 (Calculated)	1.2%

View historical totals
Data as of February 18, 2021

Vaccinations

Total resident & staff doses administered	700,922
Staff & resident 1st dose administered	453,450
Staff &resident 2nd dose administered	244,321

Vaccinations administered through the Pharmacy Partnership for Long-Term Care Program
Data as of February 26, 2021

Race & ethnicity data

We compute the number of **cases and deaths per 100k people** for each race and ethnicity.

These numbers show the scale of outcomes compared to the size of each group's population. **These are not the number of cases or deaths**, rather the proportion of each demographic group who have been affected.

View more racial data →
Get shareable graphic of CA's racial data →
View CA's historical racial data →

Race & ethnicity: cases per capita

Cases per 100,000 people (Definition)	
∟ Black or African American	5,001
∟ Hispanic or Latino	9,604
∟ Asian	3,238
∟ Native Hawaiian or Other Pacific Islander	10,701
∟ American Indian or Alaska Native	6,206
∟ White	3,658

(All data is calculated)
Data as of February 24, 2021

Race & ethnicity: deaths per capita

Deaths per 100,000 people (Definition)	
∟ Black or African American	138
∟ Hispanic or Latino	146
∟ Asian	100
∟ Native Hawaiian or Other Pacific Islander	207
∟ American Indian or Alaska Native	117
∟ White	104

(All data is calculated)
Data as of February 24, 2021

Back to top.

Colorado

State's dataset was last updated February 26, 2021 1:59 am ET
Full state data including data sources and notes.

Cases (Historical data)

Total cases (Definition)	426,198
∟ Confirmed cases (Definition)	402,859
∟ Probable cases (Definition)	23,339
New cases today (Calculated)	1,521
Change over 7 days (Calculated)	1.8%+

Viral (PCR) tests (Historical data)

Total tests (test encounters) (Definition)	6,159,303
Total tests (specimens) (Definition)	Not Reported
Total tests (people) ⚠ Unclear test type (Definition)	2,565,792

Viral (PCR) tests (HHS data)

Total tests	5,707,064

Source: CELR data from states provided in line level format
This data is published by HHS. It may differ from state-provided data.
Data as of February 23, 2021

Antigen tests (Historical data)

Total tests (specimens) (Definition)	Not Reported
Total tests (people) (Definition)	Not Reported

Warning: Antigen reporting may significantly understate the true number of tests administered

Antibody tests (Historical data)

Total tests (specimens) (Definition)	413,552
Total tests (people) (Definition)	Not Reported

Hospitalization (Historical data)

Now hospitalized (Definition)	409
Now in ICU (Definition)	Not Reported
Now on ventilator (Definition)	Not Reported

Definition
733

122,773
80,538
41,666
(Calculated)
(Calculated)

These numbers show the scale of outcomes compared to the size of each group's population. **These are not the**

Get shareable graphic
CO
capita
Definition
16,120
*
122
100

State's dataset was last updated

Viral (PCR) tests
Definition
6,399,277
(Calculated)
Definition
Outcomes
Definition

156,484
76,413
49,564
(Calculated)

These numbers show the scale of outcomes compared to the size of each group's population. **These are not the**

Get shareable graphic
's historical

Delaware

(Calculated)
Definition
832,285
Antigen tests
Outcomes
Definition
Definition
(Calculated)
(Calculated)

We compute the number of for each race and ethnicity.

DE
capita
Definition

State's dataset was last updated

Viral (PCR) tests
1,215,331
Definition
Definition
(Calculated)
Antigen tests
Definition
Definition
(Calculated)
(Calculated)

These numbers show the scale of outcomes compared to the size of each group's population. **These are not the**

Get shareable graphic
DC
capita
Definition
10,884
20,749
233
154
N/A
N/A

Florida

Viral (PCR) tests
18,922,497
Definition
(Calculated)
1,024,780
Outcomes
Definition
(Calculated)

These numbers show the scale of outcomes compared to the size of each group's population. **These are not the**

Get shareable graphic
FL

(Calculated)
Definition
Antigen tests
Definition
474,319
Outcomes
Definition
17,219
(Calculated)
(Calculated)

We compute the number of for each race and ethnicity.

racial data
capita
Definition

State's dataset was last updated

Viral (PCR) tests
Definition
117,392
(Calculated)
Definition
(Calculated)
(Calculated)

These numbers show the scale of outcomes compared to the size of each group's population. **These are not the**

's historical
capita

Coronavirus in the U.S.: Latest Map and Case Count

Updated February 27, 2021, 12:36 A.M. E.T.
Leer en español

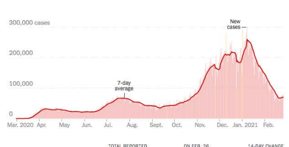

	TOTAL REPORTED	ON FEB. 26	14-DAY CHANGE
Cases	28.5 million+	78,262	–29%
Deaths	510,373	2,266	–20%
Hospitalized		51,116	–30%

Day with reporting anomaly. Hospitalization data from the Covid Tracking Project; 14-day change trends use 7-day averages.

Jump to: Map | Cases by state | Hot spots | Clusters

At least 2,266 new coronavirus deaths and 78,262 new cases were reported in the United States on Feb. 26. Over the past week, there has been an average of 69,483 cases per day, a decrease of 29 percent from the average two weeks earlier. As of Saturday morning, more than 28,509,300 people in the United States have been infected with the coronavirus according to a New York Times database.

See vaccinations by state on our new U.S. tracker page.

On Feb. 20, Iowa stopped reporting cases by county. Iowa still reports a statewide number of cases, which represent unique individuals known to have tested positive.

Hot spots | Total cases | Deaths | Per capita

Average daily cases per 100,000 people in past week

10 | 30 | 50 | 70 | 100 | 250 | Few or no cases

Double-click to zoom into the map.

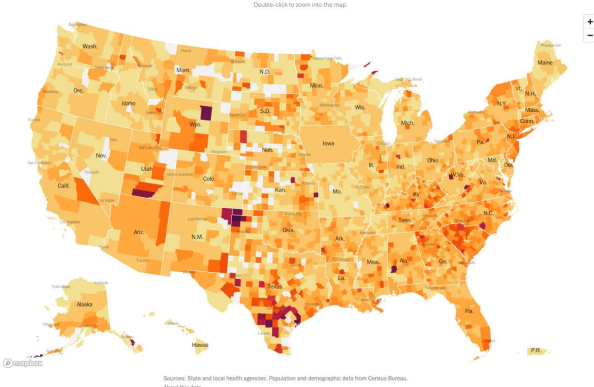

Sources: State and local health agencies. Population and demographic data from Census Bureau.
About this data

Covid-19 risk in your area ›
Find specific information about the county where you live.

Search for a county

The State of the Virus

Update for Feb. 26

- Case numbers remain about as low as they have been since late October. But after six weeks of rapid improvement, there are signs that reports of new infections are leveling off.
- The country surpassed 500,000 total deaths in recent days and California surpassed 50,000 deaths, the most of any state.
- New York and New Jersey continue to add cases at the second- and third-highest rates in the country. Scientists are examining a concerning new variant in New York City, where progress has largely stalled.
- National data was artificially low during the week of Feb. 14 because of storm-related testing and reporting problems in Texas. The state reported about 7,800 cases this Thursday, compared to around 2,400 cases a week prior.
- Cases have continued to emerge by the thousands at American colleges. About 120,000 cases have been linked to colleges in 2021, and more than 530,000 since the start of the pandemic.
- More than 70 million doses of coronavirus vaccine have been administered across the country. As supply has increased, states have expanded eligibility. Some teachers are now able to get shots in 32 states.

Where new cases are higher and staying high

States where new cases are higher had a daily average of at least 15 new cases per 100,000 people over the past week. Charts show daily cases per capita and are on the same scale. Tap a state to see detailed map page.

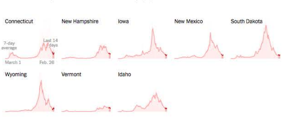

Where new cases are higher but going down

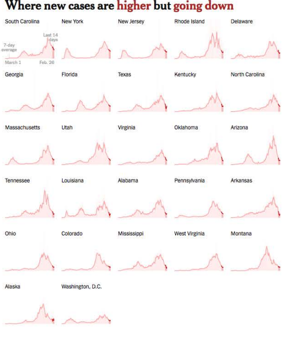

Where new cases are lower but going up

States where new cases are lower had a daily average of less than 15 new cases per 100,000 people over the past week. Charts show daily cases per capita and are on the same scale. Tap a state to see detailed map page.

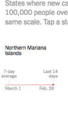

Where new cases are lower and staying low

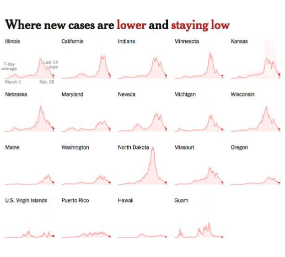

Where new deaths are increasing

Charts show daily deaths per capita and are on the same scale. States are sorted by deaths per capita for the most recent day. Tap a state to see detailed map page.

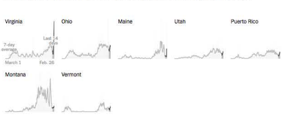

These states have had the highest growth in newly reported deaths over the last 14 days. Deaths tend to rise a few weeks after a rise in infections, as there is typically a delay between when people are infected, when they die and when deaths are reported. Some deaths reported in the last two weeks may have occurred much earlier because of these delays.

Cases and deaths by state and county

This table is sorted by places with the most cases per 100,000 residents in the last seven days. Charts are colored to reveal when outbreaks emerged.

Cases | Deaths | Search counties

	TOTAL CASES	PER 100,000	DAILY AVG. IN LAST 7 DAYS	▼ PER 100,000	WEEKLY CASES PER CAPITA (FEWER–MORE; March 1–Feb. 26)
+ South Carolina ›	513,295	9,969	2,194	43	
+ New York ›	1,628,255	8,370	7,257	37	
+ New Jersey ›	782,829	8,813	3,048	34	
+ Rhode Island ›	125,622	11,858	354	33	
+ Delaware ›	86,098	8,842	274	28	
+ Georgia ›	976,400	9,196	2,954	28	
+ Florida ›	1,898,215	8,838	5,971	28	
+ Texas ›	2,644,221	9,119	7,990	28	
+ Connecticut ›	279,946	7,852	978	27	
+ Kentucky ›	406,795	9,105	1,161	26	

Show all

About this data

New reported cases by day

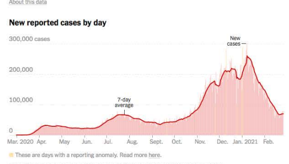

These are days with a reporting anomaly. Read more here.
Note: The seven-day average is the average of a day and the previous six days of data.

New reported deaths by day

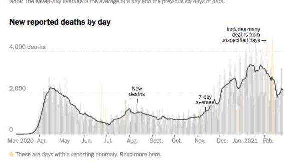

These are days with a reporting anomaly. Read more here.

Hospitalized Covid-19 patients by day

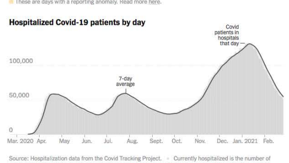

Source: Hospitalization data from the Covid Tracking Project. · Currently hospitalized is the number of patients with Covid-19 reported by states to be in a hospital on that day. Dips and spikes could be due to inconsistent reporting by hospitals.

The New York Times has found that official tallies in the United States and in more than a dozen other countries have undercounted deaths during the coronavirus outbreak because of limited testing availability.

The New York Times is engaged in a comprehensive effort to track information on every coronavirus case in the United States, collecting information from federal, state and local officials around the clock. The numbers in this article are being updated several times a day based on the latest information our journalists are gathering from around the country. The Times has made that data public in hopes of helping researchers and policymakers as they seek to slow the pandemic and prevent future ones.

The places hit hardest

The coronavirus has moved across the country in distinct phases, devastating one region, then another.

Variants are spreading rapidly in the United States, raising concerns about the path ahead.

The Northeast experienced the worst last spring, as temporary morgues were deployed in New York City. Over the summer, cases spiked across the Sun Belt, prompting many states to tighten restrictions just weeks after reopening. In the fall, the virus filled rural hospitals in the Midwest and West as it devastated communities that had for months avoided the pandemic's worst.

By the time cases finally started to fall in early 2021, every part of the country had been devastated.

Hot spots: Counties with the highest number of recent cases per resident

Search counties

[illegible]	[illegible]	PER [illegible]	DAILY AVG. IN LAST [illegible]	PER [illegible]	WEEKLY CASES PER CAPITA (March 1–Feb. 26)
[illegible]	[illegible]	[illegible]	[illegible]	[illegible]	
La Salle, Texas ›	932	12,394	18	237	
Cimarron, Okla. ›	175	8,189	5	214	
Hartley, Texas ›	473	8,483	10	185	
Calhoun, Texas ›	1,991	9,352	36	168	
Aleutians West Census Area, Alaska	555	9,851	9	167	
Johnson, Wyo. ›	712	8,431	14	164	
Marengo, Ala. ›	2,337	12,389	30	158	
Kane, Utah ›	565	7,165	11	143	
Petersburg Borough, Alaska ›	79	2,419	4	136	

Show all

Note: Recent cases are from the last seven days.

Because outbreaks in group settings where large numbers of people are in close quarters have been a major driver of the pandemic, The Times has paid special attention to cases in nursing homes, food processing plants, correctional facilities and colleges.

Information on these cases comes directly from official releases by governments, companies and institutions. The tables below show cases that have been identified since the beginning of the pandemic, and with the exception of the table for colleges and universities, only show groups of cases where 50 or more are related to a specific site.

Cases at colleges and universities

Some universities have decided to hold most or all classes online, but many others have reopened their campuses, often with extensive procedures and rules governing behavior and testing. In August and September, as the fall term began, college towns saw some of the highest per capita case growth in the country. And by November, as cases surged across the country, tens of thousands more cases emerged at universities.

More than 6,600 cases have emerged in college athletic departments.

More than 530,000 cases among college students and employees over the course of the pandemic, according to a Times database, including more than 130,000 in the first two months of 2021. At least 100 deaths have been reported, largely in 2020, and most of them among employees, not students.

	CASES LOCATION
+ Texas	31,157 cases at 84 schools
+ Ohio	19,842 cases at 62 schools
+ Florida	18,810 cases at 129 schools
+ Pennsylvania	17,369 cases at 113 schools
+ Indiana	16,505 cases at 35 schools
+ Wisconsin	15,666 cases at 31 schools
+ Illinois	15,148 cases at 50 schools
+ Michigan	14,631 cases at 52 schools
+ New York	14,364 cases at 192 schools
+ Georgia	14,131 cases at 37 schools

See the complete list and details about Covid-19 cases at colleges and universities »

Cases in jails and prisons

In American jails and prisons, more than 626,000 people have been infected and at least 2,790 inmates and correctional officers have died. During interviews with dozens of inmates across the country, many said they were frightened and frustrated by what prison officials have acknowledged has been an uneven response to the virus.

Cases are low in Mississippi prisons, but so is testing.

Michael Scronic, 49, an inmate at Fort Dix federal prison in New Jersey, who says he has high blood pressure and has previously had skin cancer, said successive outbreaks of the virus had led to despair among inmates.

"We're on our third wave. What's going to happen in wave four?" Mr. Scronic said. "The biggest frustration is that there's no end in sight. It appears to be just a continuation of these waves, and then we just have to wait out the next wave."

Search prisons

	CASES	LOCATION
Fresno County jail	3,985	Fresno, Calif.
Harris County jail	3,805	Houston, Texas
Avenal State Prison	3,630	Avenal, Calif.
Substance Abuse Treatment Facility and State Prison	3,625	Corcoran, Calif.
Soledad prison	3,067	Soledad, Calif.
California Men's Colony prison	2,989	San Luis Obispo, Calif.
San Quentin State Prison	2,675	San Quentin, Calif.
High Desert State Prison	2,593	Susanville, Calif.
Marion Correctional Institution	2,490	Marion, Ohio
California Rehabilitation Center prison	2,476	Norco, Calif.

Show all

Cases at nursing homes and long-term care facilities

Coronavirus cases have been reported in more than 31,000 nursing homes and other long-term care facilities, according to data collected by The New York Times from states, counties, the federal government and facilities themselves. More than 1.3 million residents and employees of those homes have been infected through late February, and more than 172,000 have died. That means, as of Feb. 26, more than 30 percent of deaths from the virus in the United States had been tied to nursing homes and other long-term care facilities.

At a West Virginia nursing home, vaccines brought some normalcy back to isolated residents.

"This disease creates the potential for a perfect storm in a long-term care facility — large groups of vulnerable people living together and a highly transmissible virus that may not cause symptoms in those who care for them," said Dr. Daniel Rusyniak, the chief medical officer for Indiana's state social services agency.

	CASES	LOCATION
Carrara	627	Plano, Texas
West Side Campus of Care	586	White Settlement, Texas
The Carlyle at Stonebridge Park	568	Southlake, Texas
North Ridge Health and Rehab	541	New Hope, Minn.
Hebrew Home of Greater Washington	539	Rockville, Md.
Brighton Rehabilitation & Wellness Center	496	Beaver, Pa.
Traymore Nursing Center	480	Dallas, Texas
Fair Acres Geriatric Center	473	Lima, Pa.
Corner View Nursing and Rehabilitation Center	460	Pitttsburgh, Pa.
Hearthstone Nursing and Rehabilitation	451	Round Rock, Texas

The counts in this table of coronavirus cases at individual nursing homes were last updated as recently as Jan. 12, 2021. Since then, we have continued to update state-level totals for cases and deaths in long-term care facilities.

Cases at food production facilities

Early in the pandemic, cases emerged by the hundreds in food processing facilities. The outbreaks disrupted the country's meat supply and led some of the hardest-hit plants to temporarily close.

In July, the Centers for Disease Control and Prevention reported more than 16,000 infections and 86 deaths tied to meat and poultry processing. But those numbers are almost certainly an undercount. Only 28 states provided data to the C.D.C., and many states and food processing companies have refused to provide case totals. Other large outbreaks have emerged on farms, in fruit or vegetable processing facilities and at plants where pet food is made.

Search facilities

	CASES	LOCATION
Smithfield Foods pork processing facility	1,098	Sioux Falls, S.D.
Tyson Foods pork processing facility	1,031	Waterloo, Iowa
Tyson Foods pork processing facility	900	Logansport, Ind.
Tyson Foods beef processing facility	786	Dakota City, Neb.
Smithfield Foods Farmer John meat processing facility	767	Vernon, Calif.

Show all

Other significant clusters

The coronavirus has followed Americans wherever they gathered, spreading early this year, on cruise ships and at business conferences. As the country has reopened, new clusters have emerged at churches, restaurants and workplaces. Read more here about some of the country's less-noticed coronavirus clusters. Because many states do not provide information about where the virus spread, no listing of clusters and local outbreaks will be complete.

Search clusters

	CASES	LOCATION
U.S.S. Theodore Roosevelt	1,271	Guam
Los Alamos National Laboratory	750	Los Alamos, N.M.
Savannah River Site nuclear reservation	686	Savannah River Site, S.C.
University of New Mexico Hospital	665	Albuquerque, N.M.
Newport News Shipbuilding	632	Newport News, Va.

Show all

About the data

In data for the United States, The Times uses reports from state, county and regional health departments. Most governments update their data on a daily basis, and report cases and deaths based on an individual's residence.

Not all governments report these the same way. The Times uses the total of confirmed and probable counts when they are available individually or combined. To see whether a state includes probable cases and deaths, visit the individual state pages listed at the bottom of this page.

For more, see answers to our Frequently Asked Questions about the methodology behind how we are collecting this data.

The Times has identified the following reporting anomalies or methodology changes in the data:

- **June 25:** New Jersey began reporting probable deaths, including those from earlier in the pandemic, causing a jump in the number of total deaths.
- **June 30:** New York City released deaths from earlier periods but did not specify when they were from.
- **July 27:** Texas began reporting deaths based on death certificates, causing a one-day increase.
- **Sept. 21:** Officials in Texas reported thousands of undated, backlogged cases, causing a spike in the state and national data.
- **Nov. 4:** Georgia began reporting probable deaths, causing a one-day increase.
- **Nov. 26:** Cases and deaths were lower because fourteen states reported no new data, and six states had only incomplete data from select counties.
- **Dec. 11:** Texas began reporting probable cases, resulting in a one-day increase of about 44,000 cases.
- **Dec. 25:** The daily count is artificially low because many states and local jurisdictions did not announce new data on Christmas.
- **Jan. 1:** The daily count is artificially low because many states and local jurisdictions did not announce new data on New Year's Day.
- **Jan. 2:** The daily count is artificially high because many states and local jurisdictions announced backlogged data after not announcing new data on New Year's Day.
- **Feb. 4:** Indiana announced about 1,500 deaths from previous months after reconciling records.
- **Feb. 11:** Ohio added many backlogged deaths from recent months.
- **Feb. 12:** Ohio added many backlogged deaths from recent months.
- **Feb. 13:** Ohio added many backlogged deaths from recent months.
- To see a detailed list of all reporting anomalies, visit the individual state pages listed at the bottom of this page.

The U.S. data includes cases and deaths that have been identified by public health officials as confirmed coronavirus patients, and also includes probable coronavirus cases and deaths when governments report them. **Confirmed cases and deaths,** which are widely considered to be an undercount of the true toll, are counts of individuals whose coronavirus infections were confirmed by a molecular laboratory test. **Probable cases and deaths** count individuals who meet criteria for other types of testing, symptoms and exposure, as developed by national and local governments.

Governments often revise data or report a single-day large increase in cases or deaths from unspecified days without historical revisions, which can cause an irregular pattern in the daily reported figures. The Times is excluding these anomalies from seven-day averages when possible.

on
GitHub

United States

See guidance for your local area

The true toll of the pandemic in the U.S.

World

Latest Maps and Data

Global Vaccinations

The true toll of coronavirus around

Health

Track their development

Treatments

safety

Brazil | Mexico | United States
U.K.

States, Territories and Cities

Iowa
Tennessee
Texas
Colorado
Connecticut
Massachusetts
Delaware
Oklahoma
West Virginia
Georgia
Oregon
Wisconsin
Missouri
Wyoming
Idaho
Puerto Rico
Indiana

seems that there are : how

person; whether that person expels viral droplets on or near you; Here is a guide to

following some

Wear a mask outside your home. A mask protects others from your

who wear masks, the more we all stay safer.

Wash your hands often. Anytime you come in contact with a

rinse and then dry your hands with a clean towel.

Avoid touching your face. The virus can spread when our hands come into contact with the virus, and we touch our nose, mouth or

just recently washed them.

answers to your current questions

, Sarah
, Matthew Conlen, Lindsey Cook, Gabriel
Gianordoli,
Richard A. Oppel Jr.
Anjali Singhvi Mitch
Albert Sun Jin Wu
was contributed by Jeff Arnold, , Brillian Bao,

Fortis, Matt Furber, Robert Gebeloff, Thomas Gibbons-Neff,

Steven Moity, Paul Moon, Derek M. Norman, Anahad O'Connor, Ashlyn O'Hara, Azi Paybarah, Elian Peltier, Sean Plambeck, Laney Pope, Elisabetta Povoledo, Cierra S. Queen, Savannah
Kai Schultz
Sarena Snider, Brandon Thorp, Alex Traub, Maura Turcotte, Tracey Tully, Lisa Waananen Jones,

Josh Williams
Rumsey Taylor, Nina Pavlich, Carmen Cincotti, Ben Smithgall, Andrew Fischer,
Blacki Migliozzi
Speicher, Hugh Mandeville, Robin Berjon, Thu Trinh, Carolyn Price, James G. Robinson, Phil Wells, Yanxing Yang, Michael Beswetherick, Michael Robles, Nikhil Baradwaj, Ariana Giorgi, Bella Virgilio, Dylan Momplaisir, Avery Dews, Bea Malsky and Ilana Marcus.

:
The map key in an earlier version of this article was mislabeled. The key showed the average number of new cases in each county per capita per day, not the total number of cases per

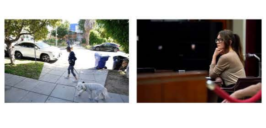

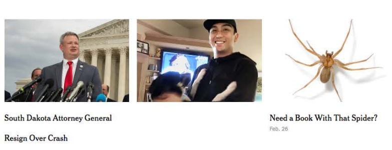

South Dakota Attorney General

Resign Over Crash

Need a Book With That Spider?
Feb. 26

Coronavirus in the U.S.: Latest Map and Case Count

Updated February 27, 2021, 12:36 A.M. E.T.
Leer en español

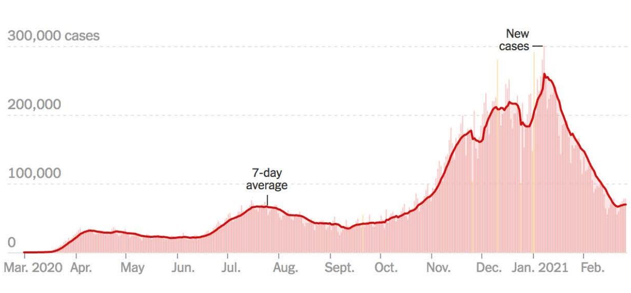

	TOTAL REPORTED	ON FEB. 26	14-DAY CHANGE
Cases	28.5 million+	78,262	–29%
Deaths	510,373	2,266	–20%
Hospitalized		51,116	–30%

Day with reporting anomaly. Hospitalization data from the Covid Tracking Project; 14-day change trends use 7-day averages.

Jump to: Map | Cases by state | Hot spots | Clusters

At least 2,266 new coronavirus deaths and 78,262 new cases were reported in the United States on Feb. 26. Over the past week, there has been an average of 69,483 cases per day, a decrease of 29 percent from the average two weeks earlier. As of Saturday morning, more than 28,509,300 people in the United States have been infected with the coronavirus according to a New York Times database.

See vaccinations by state on our new U.S. tracker page.

On Feb. 20, Iowa stopped reporting cases by county. Iowa still reports a statewide number of cases, which represent unique individuals known to have tested positive.

Hot spots | Total cases | Deaths | Per capita

Average daily cases per 100,000 people in past week

10 | 30 | 50 | 70 | 100 | 250 | Few or no cases

Double-click to zoom into the map.

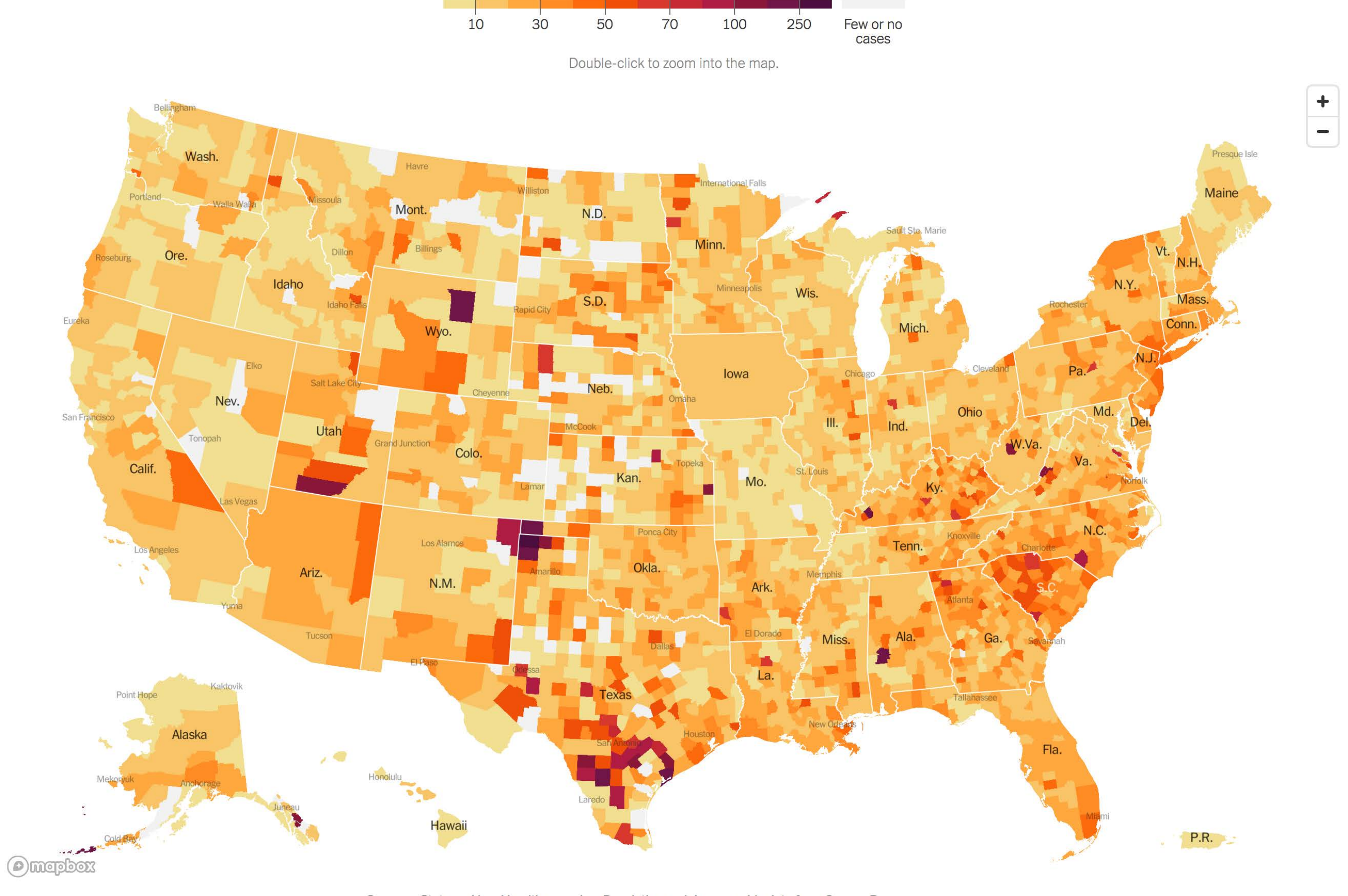

Sources: State and local health agencies. Population and demographic data from Census Bureau.
About this data

Covid-19 risk in your area ›
Find specific information about the county where you live.

Search for a county

The State of the Virus

Update for Feb. 26

- Case numbers remain about as low as they have been since late October. But after six weeks of rapid improvement, there are signs that reports of new infections are leveling off.
- The country surpassed 500,000 total deaths in recent days and California surpassed 50,000 deaths, the most of any state.
- New York and New Jersey continue to add cases at the second- and third-highest rates in the country. Scientists are examining a concerning new variant in New York City, where progress has largely stalled.
- National data was artificially low during the week of Feb. 14 because of storm-related testing and reporting problems in Texas. The state reported about 7,800 cases this Thursday, compared to around 2,400 cases a week prior.
- Cases have continued to emerge by the thousands at American colleges. About 120,000 cases have been linked to colleges in 2021, and more than 530,000 since the start of the pandemic.
- More than 70 million doses of coronavirus vaccine have been administered across the country. As supply has increased, states have expanded eligibility. Some teachers are now able to get shots in 32 states.

Where new cases are higher and staying high

States where new cases are higher had a daily average of at least 15 new cases per 100,000 people over the past week. Charts show daily cases per capita and are on the same scale. Tap a state to see detailed map page.

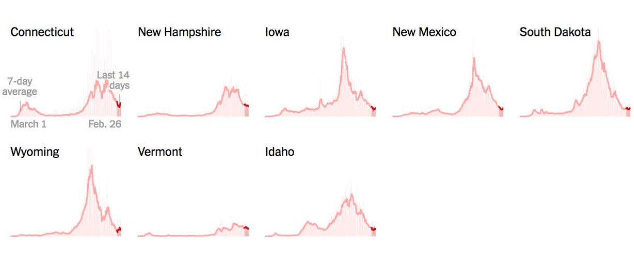

Where new cases are higher but going down

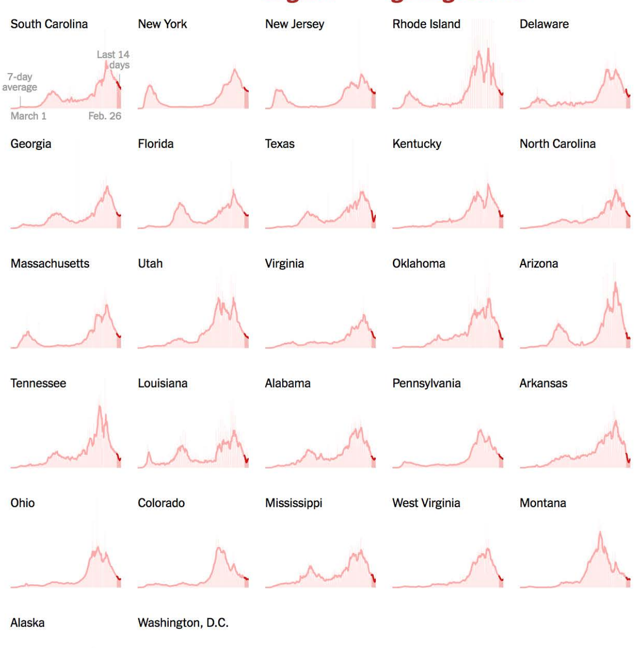

Where new cases are lower but going up

States where new cases are lower had a daily average of less than 15 new cases per 100,000 people over the past week. Charts show daily cases per capita and are on the same scale. Tap a state to see detailed map page.

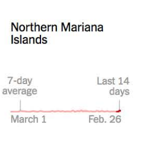

Where new cases are lower and staying low

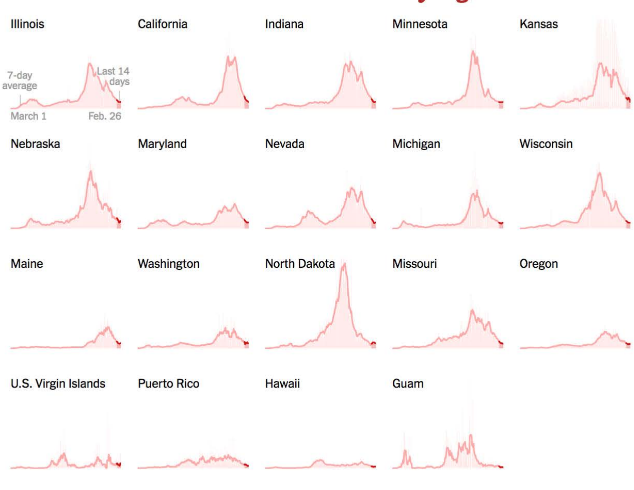

Where new deaths are increasing

Charts show daily deaths per capita and are on the same scale. States are sorted by deaths per capita for the most recent day. Tap a state to see detailed map page.

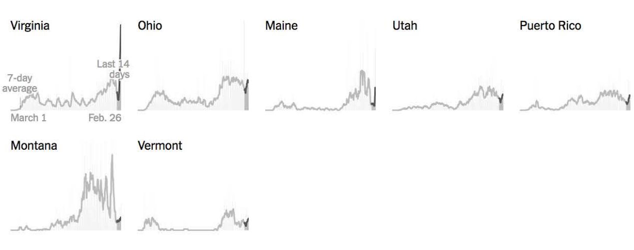

These states have had the highest growth in newly reported deaths over the last 14 days. Deaths tend to rise a few weeks after a rise in infections, as there is typically a delay between when people are infected, when they die and when deaths are reported. Some deaths reported in the last two weeks may have occurred much earlier because of these delays.

Cases and deaths by state and county

This table is sorted by places with the most cases per 100,000 residents in the last seven days. Charts are colored to reveal when outbreaks emerged.

Cases | Deaths | Search counties

	TOTAL CASES	PER 100,000	DAILY AVG. IN LAST 7 DAYS	▼ PER 100,000	WEEKLY CASES PER CAPITA (FEWER – MORE)
+ South Carolina ›	513,295	9,969	2,194	43	March 1 – Feb. 26
+ New York ›	1,628,255	8,370	7,257	37	
+ New Jersey ›	782,829	8,813	3,048	34	
+ Rhode Island ›	125,622	11,858	354	33	
+ Delaware ›	86,098	8,842	274	28	
+ Georgia ›	976,400	9,196	2,954	28	
+ Florida ›	1,898,215	8,838	5,971	28	
+ Texas ›	2,644,221	9,119	7,990	28	
+ Connecticut ›	279,946	7,852	978	27	
+ Kentucky ›	406,795	9,105	1,161	26	

Show all

About this data

New reported cases by day

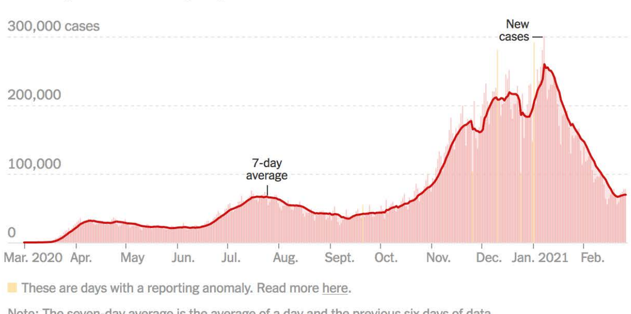

These are days with a reporting anomaly. Read more here.

Note: The seven-day average is the average of a day and the previous six days of data.

New reported deaths by day

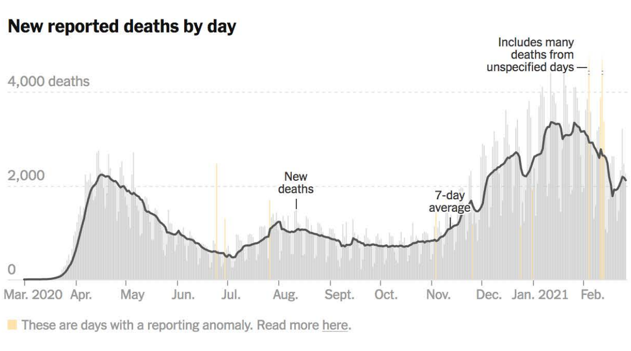

These are days with a reporting anomaly. Read more here.

Hospitalized Covid-19 patients by day

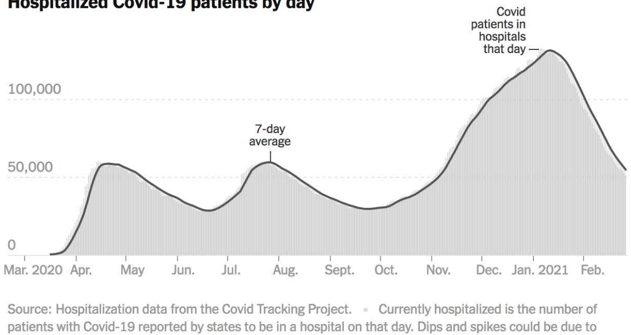

Source: Hospitalization data from the Covid Tracking Project. • Currently hospitalized is the number of patients with Covid-19 reported by states to be in a hospital on that day. Dips and spikes could be due to inconsistent reporting by hospitals.

The New York Times has found that official tallies in the United States and in more than a dozen other countries have undercounted deaths during the coronavirus outbreak because of limited testing availability.

The New York Times is engaged in a comprehensive effort to track information on every coronavirus case in the United States, collecting information from federal, state and local officials around the clock. The numbers in this article are being updated several times a day based on the latest information our journalists are gathering from around the country. The Times has made that data public in hopes of helping researchers and policymakers as they seek to slow the pandemic and prevent future ones.

The places hit hardest

The coronavirus has moved across the country in distinct phases, devastating one region, then another.

Variants are spreading rapidly in the United States, raising concerns about the path ahead.

The Northeast experienced the worst last spring, as temporary morgues were deployed in New York City. Over the summer, cases spiked across the Sun Belt, prompting many states to tighten restrictions just weeks after reopening. In the fall, the virus filled rural hospitals in the Midwest and West as it devastated communities that had for months avoided the pandemic's worst.

By the time cases finally started to fall in early 2021, every part of the country had been devastated.

Hot spots: Counties with the highest number of recent cases per resident

Search counties

COUNTY	TOTAL CASES	PER 100,000	DAILY AVG. IN LAST 7 DAYS	PER 100,000	WEEKLY CASES PER CAPITA (FEWER – MORE)
[illegible]	[illegible]	[illegible]	[illegible]	[illegible]	March 1 – Feb. 26
La Salle, Texas ›	932	12,394	18	237	
Cimarron, Okla. ›	175	8,189	5	214	
Hartley, Texas ›	473	8,483	10	185	
Calhoun, Texas ›	1,991	9,352	36	168	
Aleutians West Census Area, Alaska	555	9,851	9	167	
Johnson, Wyo. ›	712	8,431	14	164	
Marengo, Ala. ›	2,337	12,389	30	158	
Kane, Utah ›	565	7,165	11	143	
Petersburg Borough, Alaska ›	79	2,419	4	136	

Show all

Note: Recent cases are from the last seven days.

JOHNS HOPKINS UNIVERSITY & MEDICINE | CORONAVIRUS RESOURCE CENTER

Home | Tracking | Testing | Tracing | Vaccines | By Region | Events & News | About

Tracking Home | Critical Trends | Global Map | U.S. Map | Data in Motion

COVID-19 United States Cases by C... Johns Hopkins Unive...

States/Territories: Please select from list | County (or Equivalent): Please select from ...

Top 50 Confirmed Cases by County

1,189,232 confirmed
Los Angeles

508,997 confirmed
Maricopa

473,194 confirmed
Cook

408,096 confirmed
Miami-Dade

348,125 confirmed
Harris

289,450 confirmed
Riverside

286,077 confirmed
San Bernardino

279,834 confirmed
Dallas

260,721 confirmed
Orange

258,982 confirmed
San Diego

241,005 confirmed
Tarrant

226,120 confirmed
Clark

208,445 confirmed
Queens

207,366 confirmed
Kings

195,237 confirmed

Last Updated on:
2021-02-26

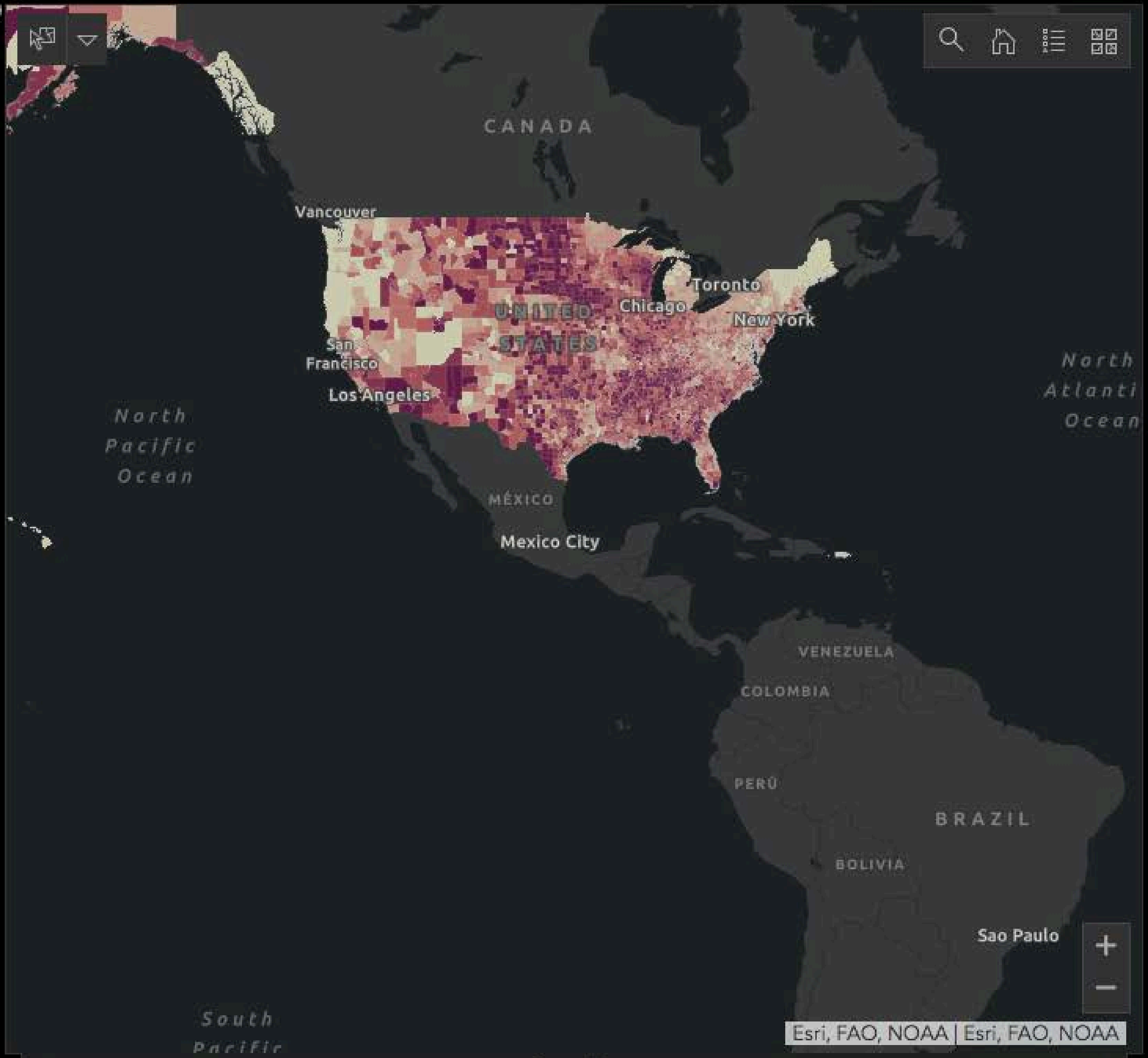

Confirmed by Pop

Data is updated once per day to allow the system to pull county-level data. For the most up-to-date confirmed cases and deaths, please see the COVID-19 Global Map. New York City borough deaths data does not include Probable COVID-19 deaths, as this data is not reported.

Map Visualization: Centers for Civic Impact. Automation Support: Esri Living Atlas team, JHU APL, and JHU

Top 20 Counties by Number of Deaths

21,241 deaths
Los Angeles

9,339 deaths
Cook

9,061 deaths
Maricopa

9,051 deaths
Kings

8,724 deaths
Queens

5,835 deaths
Bronx

5,396 deaths
Miami-Dade

5,019 deaths
Harris

4,131 deaths
Wayne

3,982 deaths
Orange

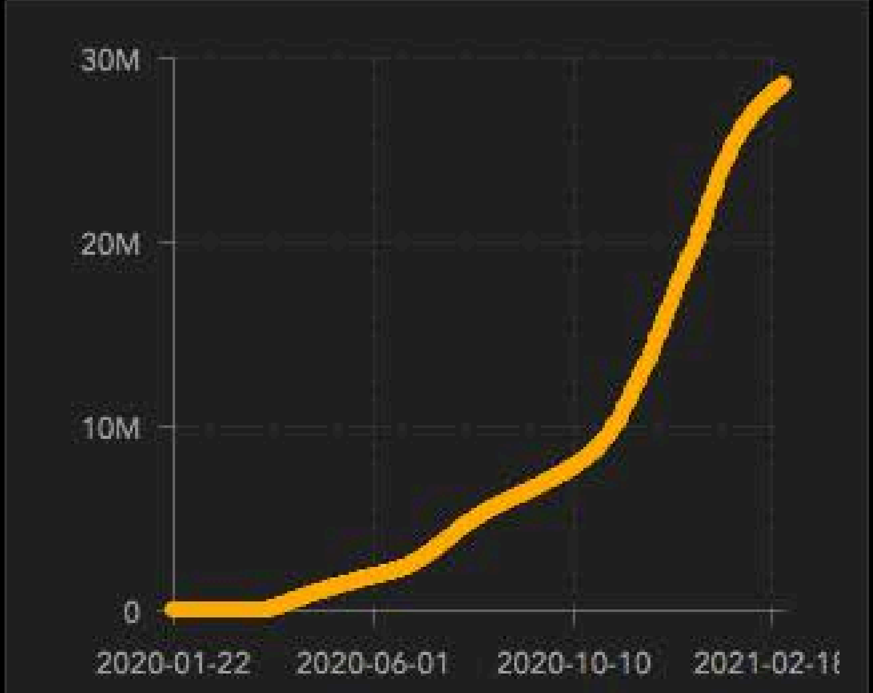

Confirmed | Deaths

MAPS & TRENDS

IMPACT OF OPENING AND CLOSING DECISIONS BY STATE

A look at how social distancing measures may have influenced trends in COVID-19 cases and deaths

SHARE:

ALABAMA

Select another state

Alabama

How to read this graphic

New Confirmed Cases | New Deaths

RECENT OPENING AND CLOSING POLICY DECISIONS

Restriction/closing · Opening · Deferring decisions to county · Other

Previous | Next

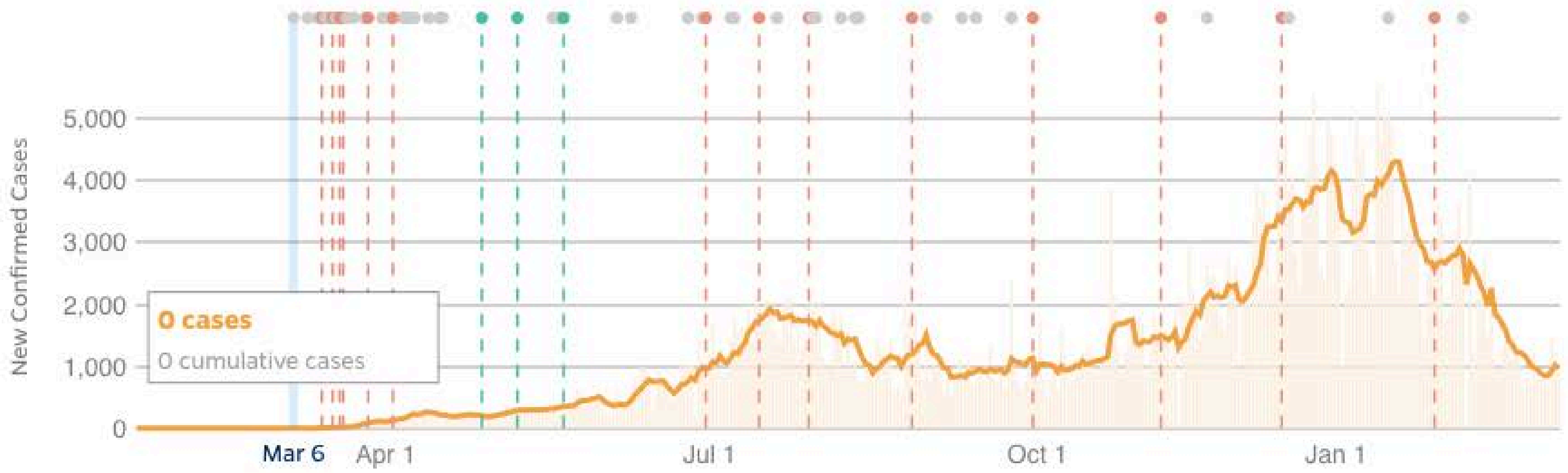

Mar 06, 2020 — 0 CUMULATIVE CASES | 0 CUMULATIVE DEATHS

- Governor Ivey announced the formation of a Coronavirus (COVID-19) Task Force of cabinet and state agency officials, as well as disease specialists in an abundance of caution.

Data Sources: Cases and deaths data from JHU CSSE. As of August 1, 2020, policy events are drawn from from various state-specific sources. Prior to that, the data source for policy events was the National Governor's Association.

ABOUT THIS PAGE:

This page was last updated on Friday, February 26, 2021 at 3:00 AM EST.

This timeline shows the major infection control measures and re-openings undertaken by states after the first COVID-19 cases appeared in the United States, alongside the number of new cases and deaths in each state over the same time period.

Dots on the timeline indicate key events in each state – closings (in red), openings (in green) and other policy changes and events (in grey). Clicking on a dot will display event details below the chart. Additionally, the PREVIOUS and NEXT buttons move users through key events by day, allowing them to be viewed in chronological order. The timeline can be viewed against either new cases or new deaths. To see current cumulative case and death counts and daily counts for days with no policy events, click here.

The Latest on this Dashboard

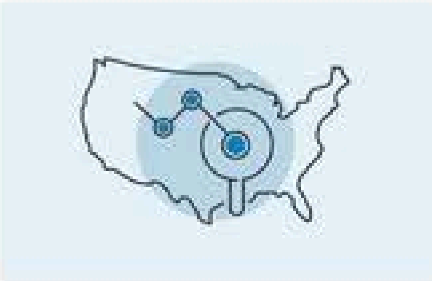

COVID-19 Vaccination Tracker
The COVID-19 Vaccination Tracker tab takes you to an overview of current vaccination status in the U.S. and in each state. Check out some FAQs about COVID-19 Vaccines...for more.

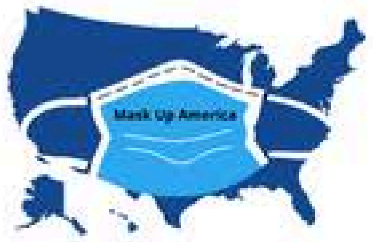

Statewide Mask Mandates in the United States
Implementing state-wide mask mandate in the early stages of the pandemic may have been a clever move for US states resulting in lower case rates during the third wave of the pandemic compared to ...for more.

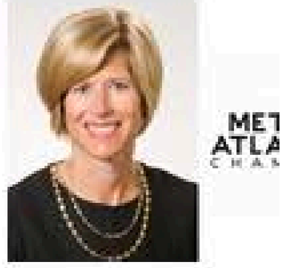

United States /

See Dashboard Guide (PDF / YouTube)

COVID-19 is affecting every community differently. Some areas are much harder-hit than others. What is happening where you live?

Average Daily COVID-19 Cases per 100,000 ▾

0 8 12 16 21 28 347
Low High
None Reported

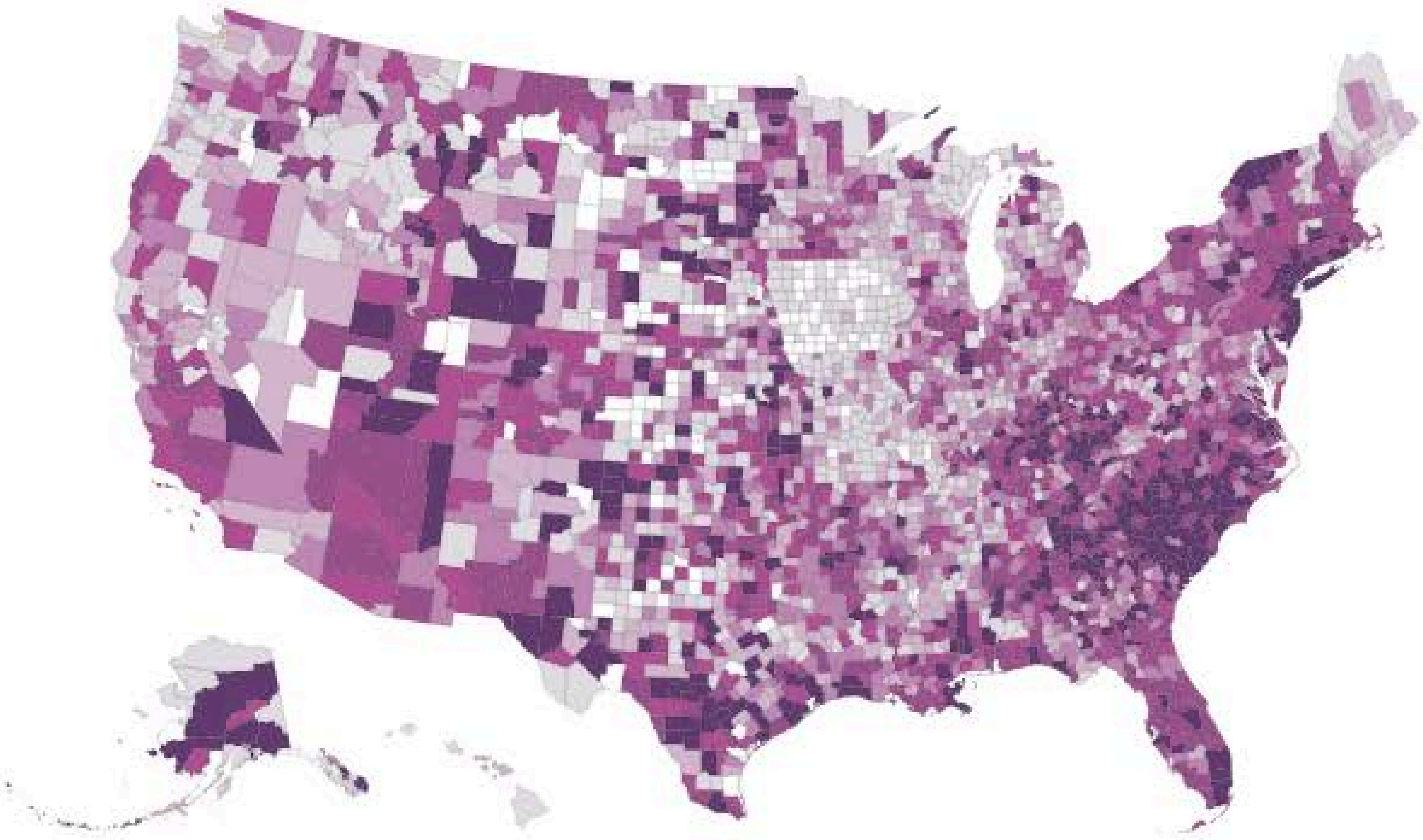

▾ About the data

Average Daily COVID-19 Cases per 100,000 helps us understand the average number of positive cases for COVID-19 infection over the last seven days by population of the county per 100,000 people.
For a complete table of definitions, click here.

Current Cases and Deaths in The United States

Daily Cases	Daily Deaths
77,804 ↓ 32% 14-day change	2,465 ↓ 22% 14-day change

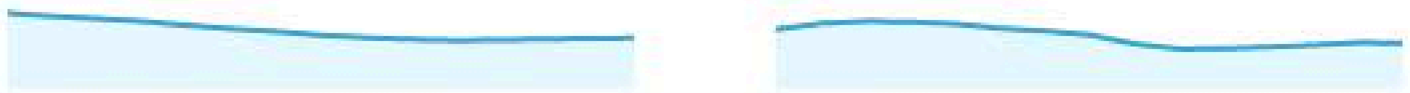

*14-day change trends use 7-day averages.

Disparities in COVID-19 Mortality **Nation**

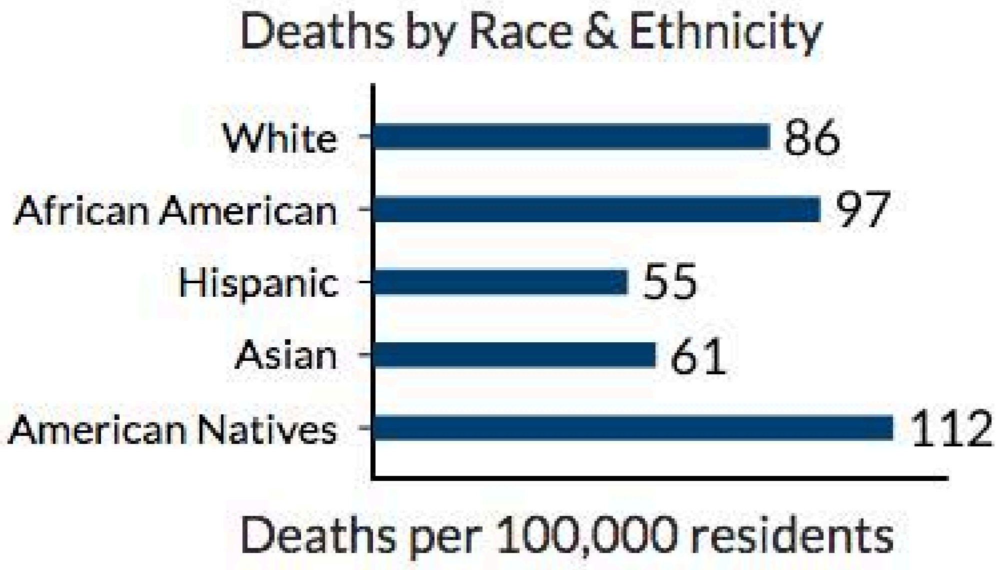

The United States reports deaths by combined race and ethnicity groups. The chart shows race and ethnicity groups that constitute at least 1% of the state population and have 30 or more deaths. Race and ethnicity data are known for 74% of deaths in the nation.

Data source: The CDC
Data last updated: 02/25/2021, updated every weekday.

	Total Reported	On Feb 25	7-Day Average
**Known cases**	27,911,671	67,572	67,123
**Deaths**	499,209	3,215	2,146

NEW KNOWN CASES PER DAY

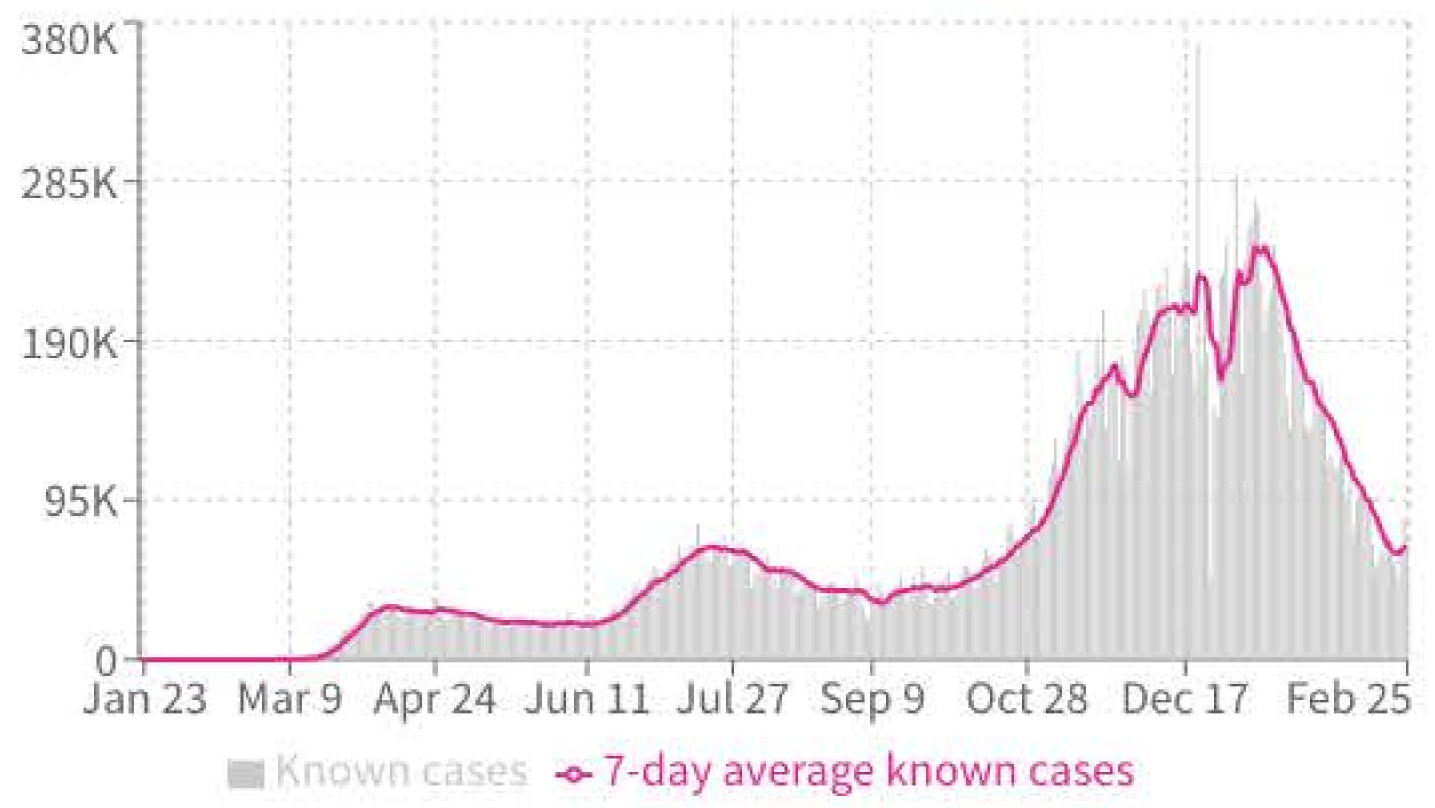

NEW KNOWN DEATHS PER DAY

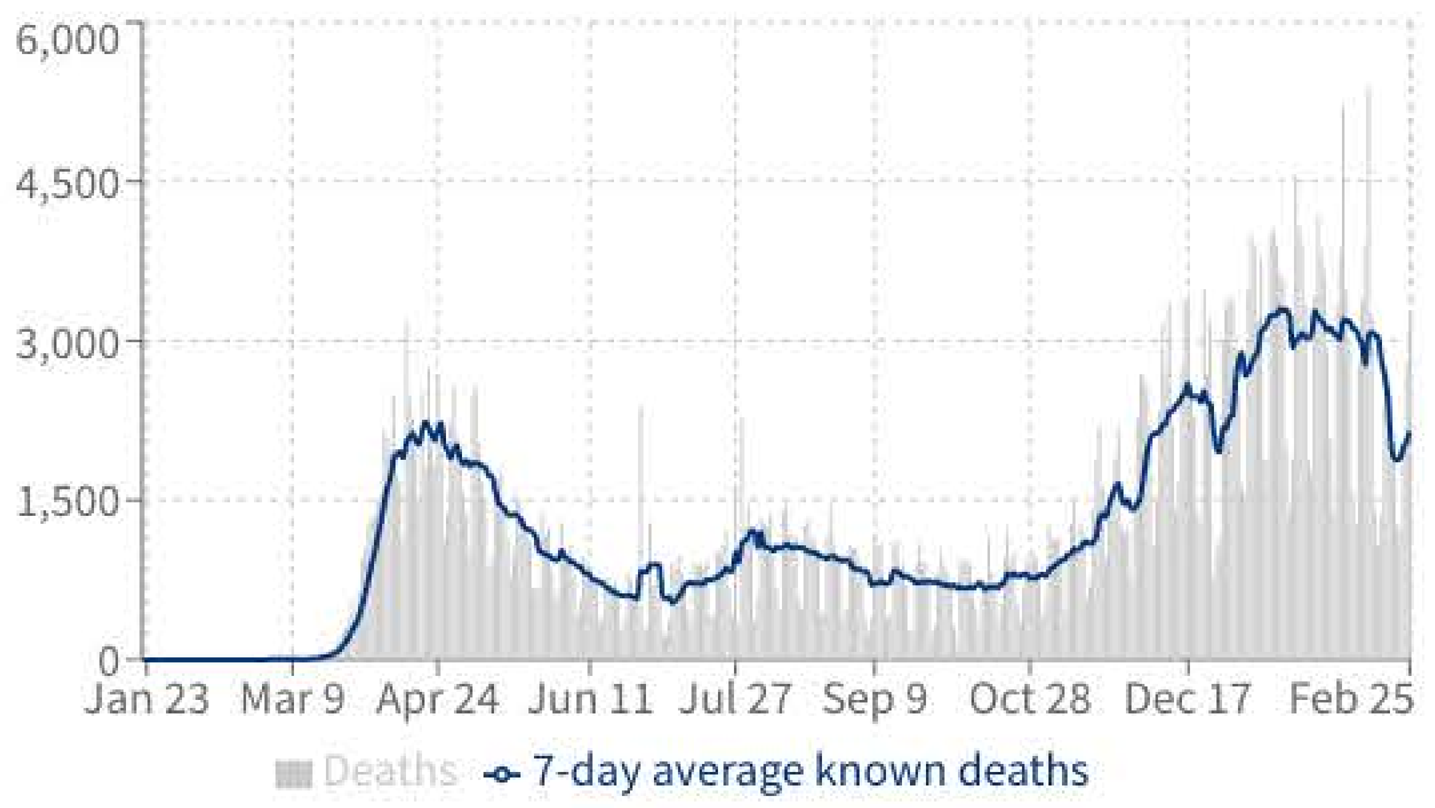

In the United States, there were 67,572 newly reported COVID-19 cases and 3,215 newly reported COVID-19 deaths on Feb 25, 2021

HOT SPOTS | PER 100K | TOTAL

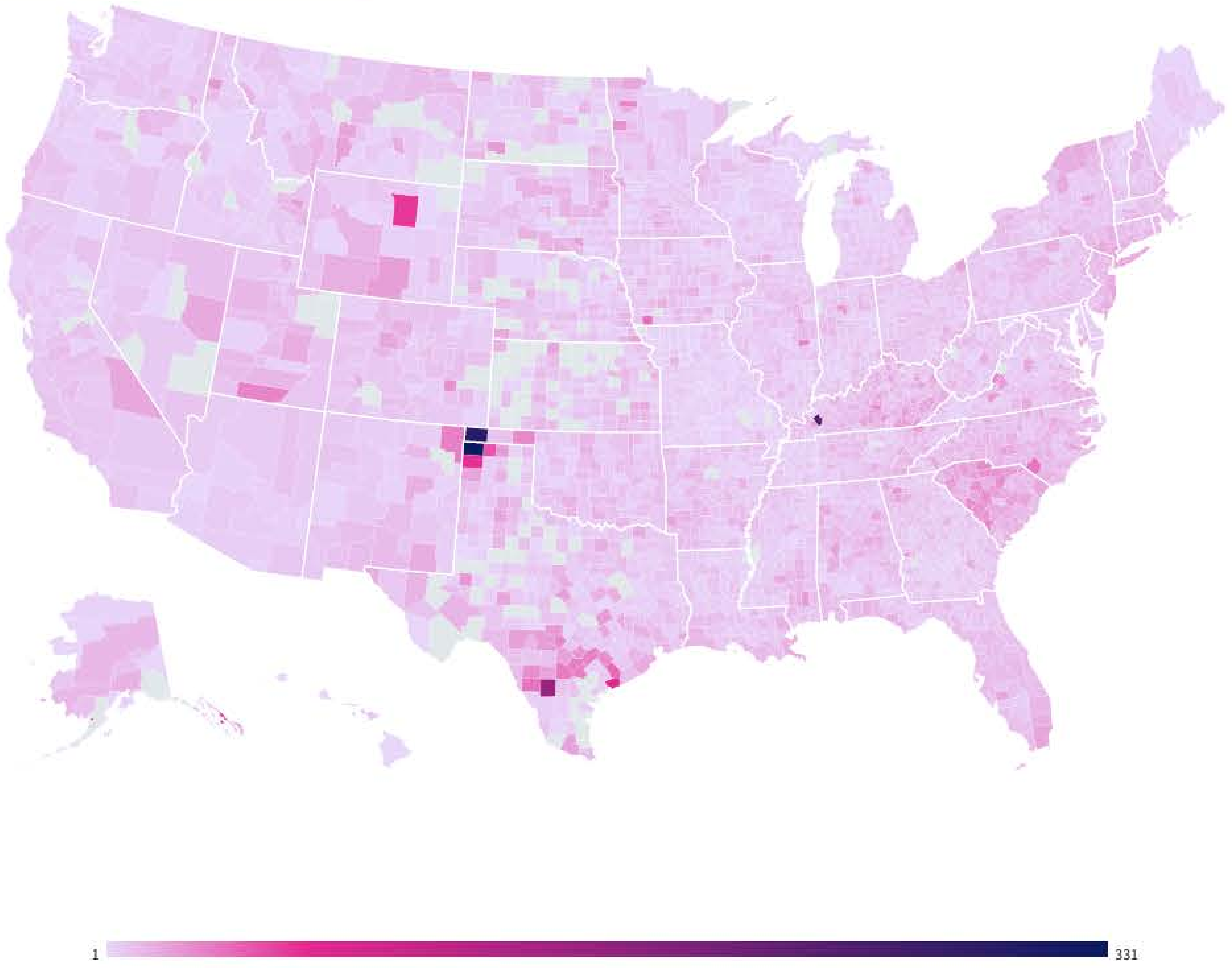

7-DAY AVERAGE KNOWN CASES PER 100K

From cities to rural towns, stay informed on where COVID-19 is spreading to understand how it could affect families, commerce, and travel. Follow new cases found each day and the total number of cases and deaths in the US. The county-level tracker makes it easy to

NATIONAL

US Coronavirus Cases & Deaths by State

Track COVID-19 data daily by state and county

Data Updated Feb 26, 2021

State ↑	7-day average known cases	7-day average known deaths	Known cases	Deaths
Alabama	964	58	491,116	9,835
Alaska	121	0	55,847	278
Arizona	1,761	89	814,528	15,897
Arkansas	524	11	318,135	5,400
California	5,517	438	3,460,338	50,991
Colorado	1,038	9	424,683	5,932
Connecticut	1,037	17	279,159	7,614
Delaware	126	11	84,732	1,406
District of Columbia	117	2	40,122	1,005

Kentucky

Overview | Case Studies | Counties

State Public Health Information

Statistics

402.9k
Number of Cases

402.9k
Number of Tests

4.6k
Number of Deaths

19k
Total Hospitalized

Cases Per Day

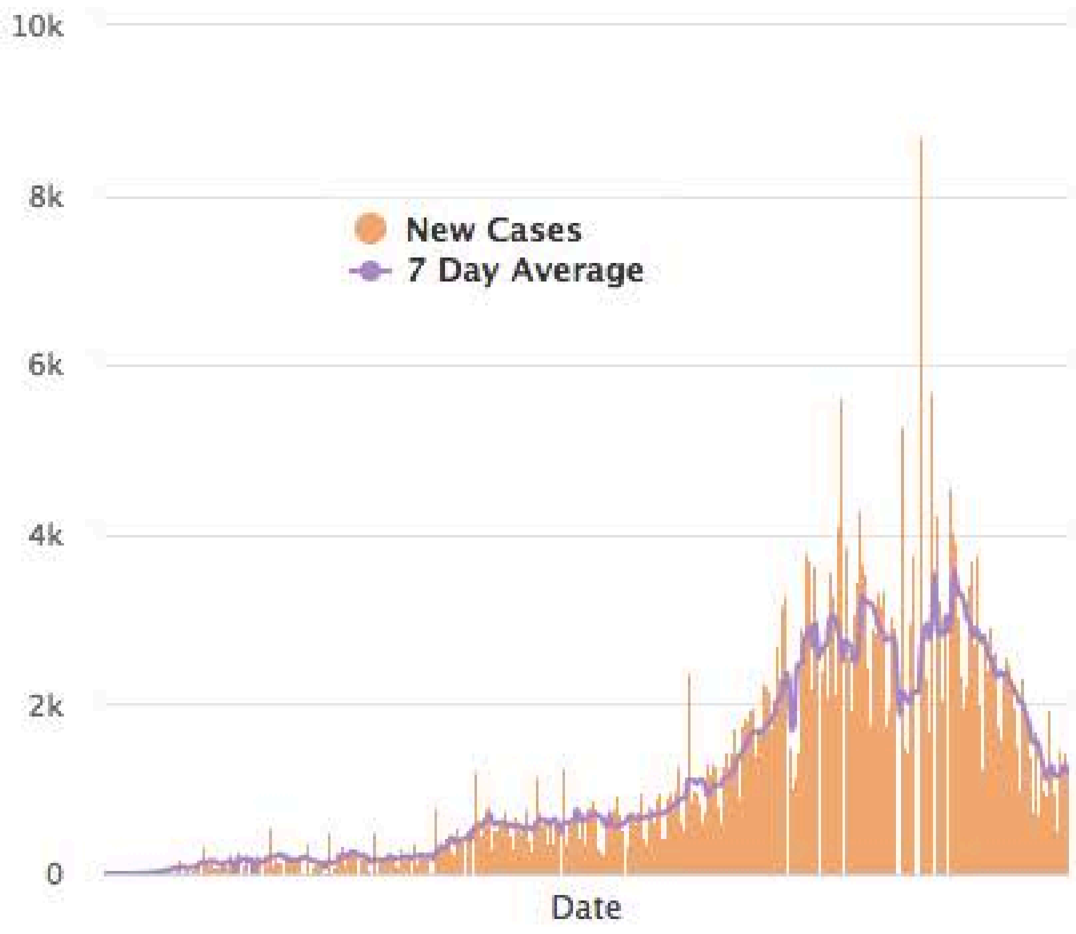

Deaths Per Day

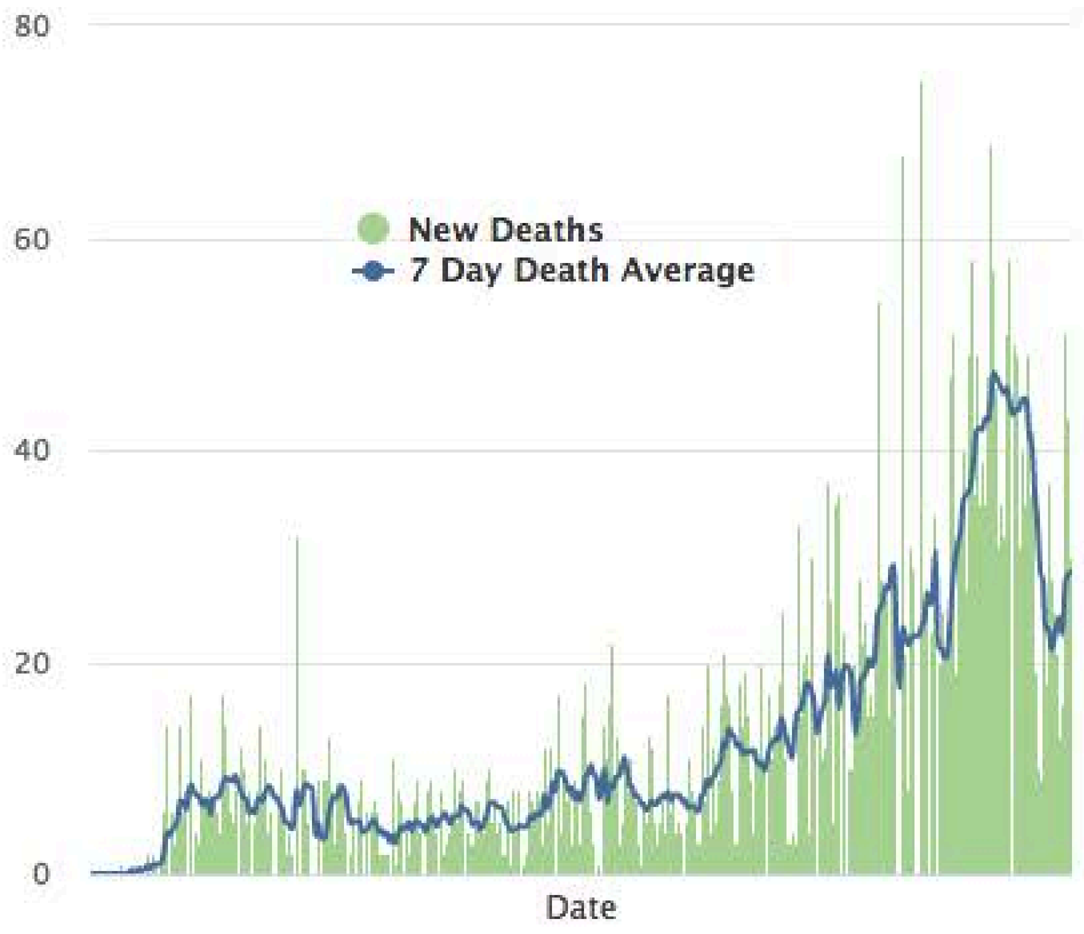

New Tests Per Day

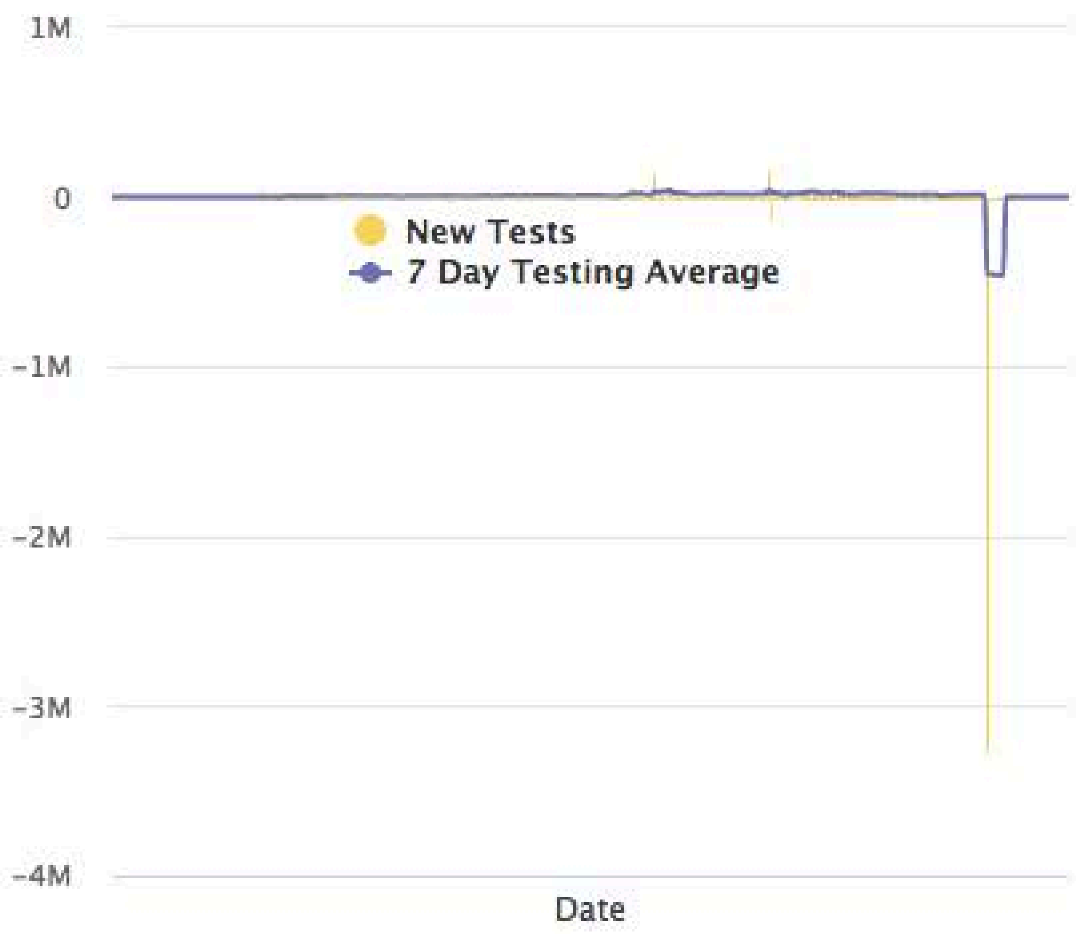

Hospitalized Per Day

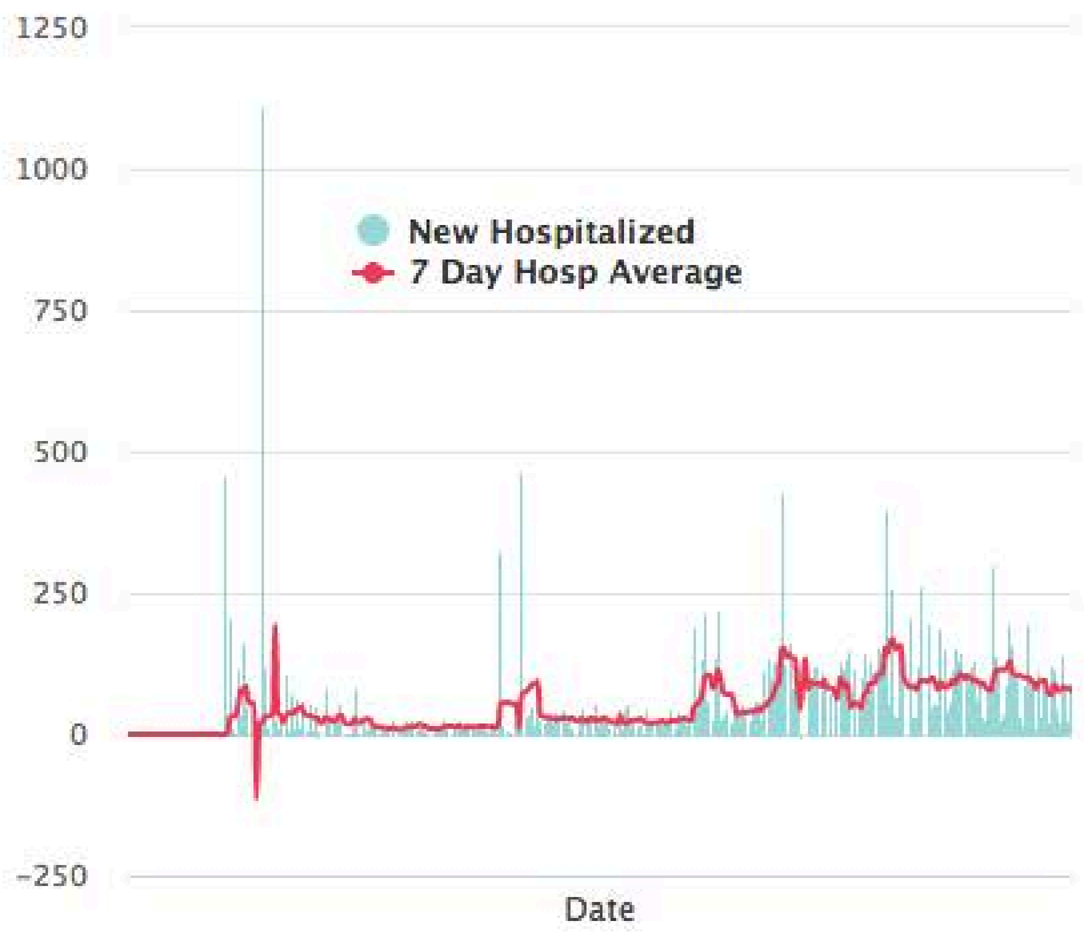

DASHBOARD

United States Coronavirus (COVID-19) Tracker

Infection Map (hide)

Click to autodetect your location

Inform your friends & family:

Share | Share | Email | Tweet | Share

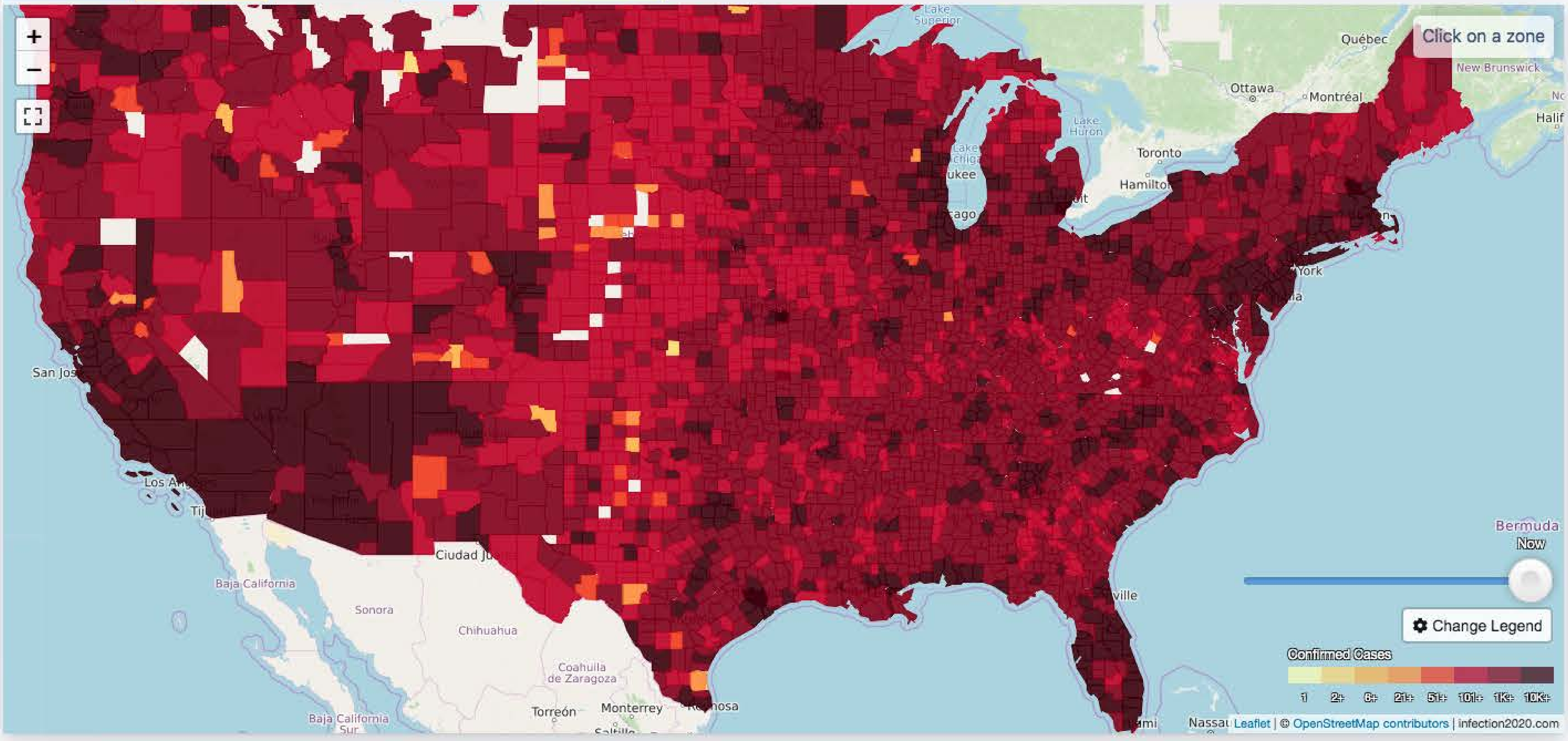

Nationwide Live Data

Note: As of 4/17, the number of cases and deaths now include probable cases to match the new CDC guidelines. This contributed to the spike in new deaths displayed for 4/17, which includes 4,226 probable deaths.

CASES	DEATHS	CASE FATALITY RATE	RECOVERED
28,106,206	505,519	1.80%	19,593,781
↑ 0% (+75,969 past 24hr)	↑ 0% (+2,247 past 24hr)	-	↑ 0% (+95,724 past 24hr)

Cases by County (hide)

Tap a state row to show its counties.
Tap the blue chart icon to see historical data.
Scroll down to see more.

State	Cases	Deaths
CA	3,560,765 Cases	51,917 Deaths
TX	2,621,758 Cases	43,055 Deaths
FL	1,894,761 Cases	30,625 Deaths
NY	1,628,255 Cases	46,917 Deaths
IL	1,147,766 Cases	21,888 Deaths
OH	964,391 Cases	17,190 Deaths
GA	954,937 Cases	16,391 Deaths
PA	931,099 Cases	23,983 Deaths

Nationwide Cases & Deaths

Cases | Deaths | All | linear | log

Touch the chart to view datapoints.
Tap on legend to hide specific dataset.

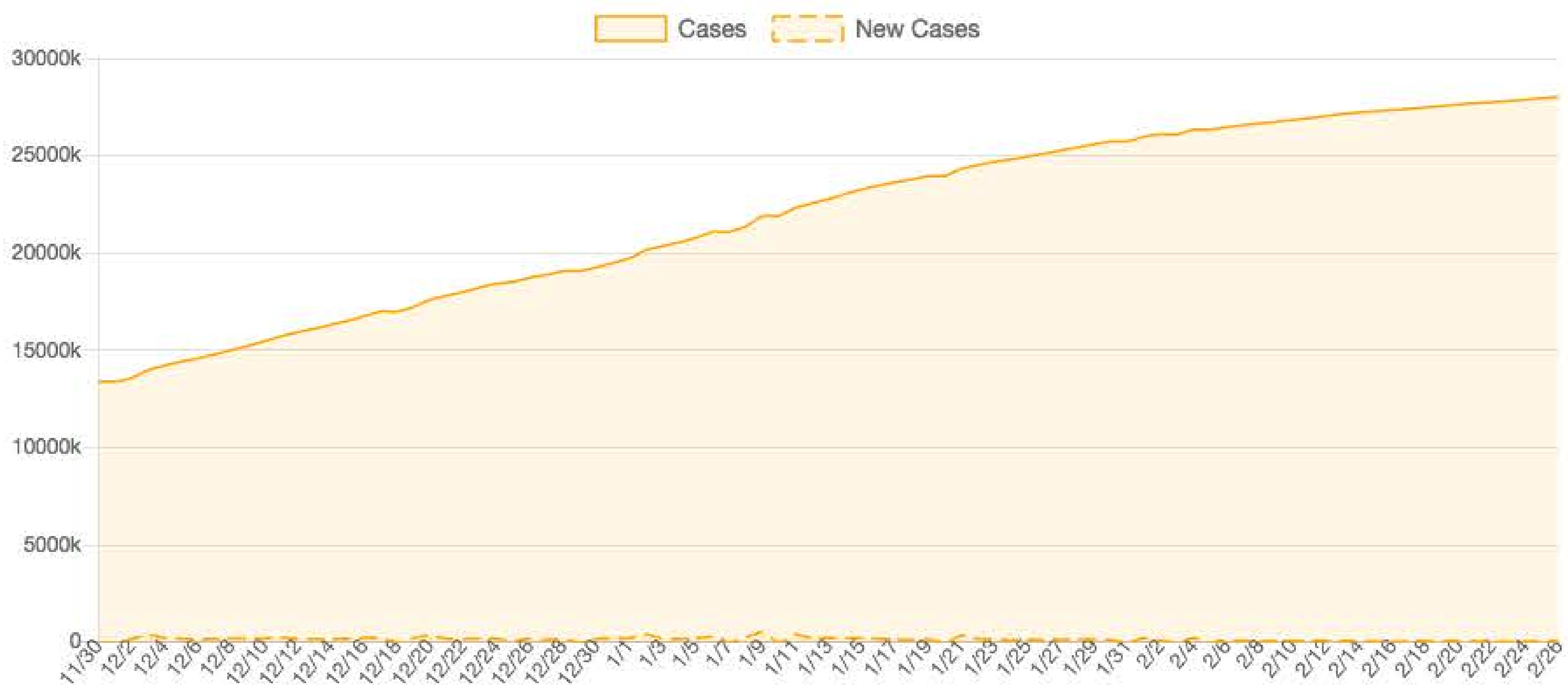

14 days | All days

Data sources: CDC, WHO, The New York Times, JHU, Corona Data Scraper, and official state and county health agencies.
Please contact us if you have any suggestions or feedback.
If you would like to volunteer, please fill out this form.
Big thanks to Netlify and Mapbox for supporting us during this outbreak.
Disclaimer

We need your help to cover ongoing operational and maintenance costs. Please consider making a contribution today to keep us running. Thank you.

Donate
