# Supplementary material for: Web-Based COVID-19 Dashboards and Trackers in the United States: Survey Study
Source: JMIR Hum Factors. 2023 Mar 20;10:e43819. doi: 10.2196/43819 (PMC10029858; doi:10.2196/43819)
Supplement: Multimedia Appendix 9 [file humanfactors_v10i1e43819_app9.pdf]

Appendix 9. Screenshots from dashboards and trackers — *Global coverage* *(captured morning of March 1, 2021)*

Page	Ref	Host
2	G-1	New York Times
3	G-2	CNN
4	G-3	Stat / AppliedXL
5	G-4	Johns Hopkins Centers for Civic Impact
6	G-5	Biocomplexity Institute, University of Virginia
7	G-6	HGIS Lab, University of Washington
8	G-7	HealthMap / various universities
9–11	G-8	World Health Organization
12	G-9	Kaiser Family Foundation
13–16	G-10	Our World in Data
17	G-11	DXY
18	G-12	Worldometer
19	G-14	Microsoft
20–21	G-15	1Point3Acres
22–23	G-16	Tableau

Coronavirus World Map: Tracking the Global Outbreak

Updated March 1, 2021, 8:19 A.M. E.T.

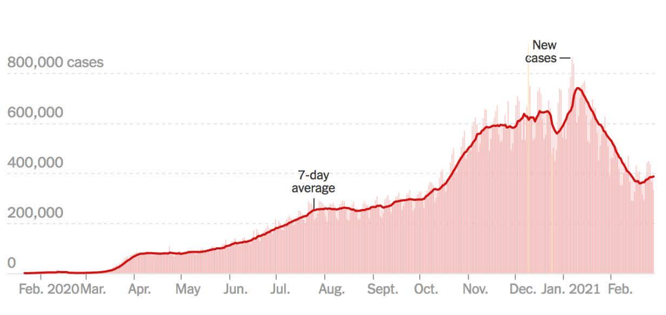

	TOTAL REPORTED	ON FEB. 28	14-DAY CHANGE
Cases	114.1 million+	333,883	Flat
Deaths	2.5 million+	5,832	–23%

Day with reporting anomaly. 14-day change trends use 7-day averages.

Jump to: Map | Country table | New cases | Tips

The coronavirus pandemic has sickened more than 114,183,100 people, according to official counts. As of Monday morning, at least 2,532,000 people have died from coronavirus.

See vaccinations by country on our new world tracker page.

Hot spots | Total cases | Deaths | Per capita

Average daily cases per 100,000 people in past week

4 | 12 | 20 | 28 | 40 | 68 | Few or no cases

Double-click to zoom into the map.

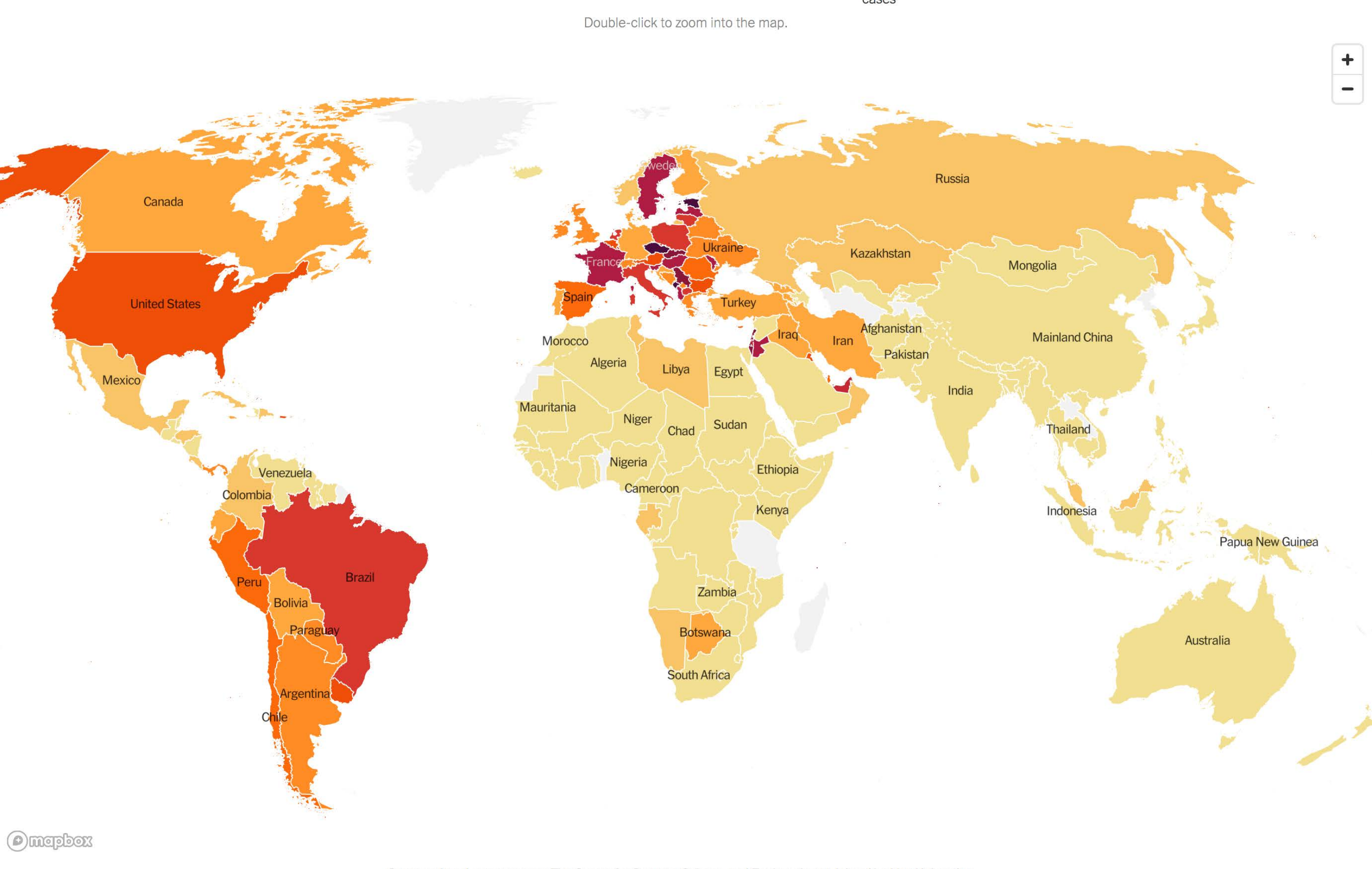

Sources: Local governments; The Center for Systems Science and Engineering at Johns Hopkins University; National Health Commission of the People's Republic of China; World Health Organization.
About this data

The coronavirus pandemic is ebbing in some of the countries that were hit hard early on, and the number of new cases has steadily declined since early January.

Cases | Deaths | Search countries

	TOTAL CASES	PER 100,000	DAILY AVG. IN LAST 7 DAYS	▼ PER 100,000	WEEKLY CASES PER CAPITA (FEWER – MORE; Jan. 22 – Feb. 28)
Czech Republic	1,240,051	11,670	11,760	111	
San Marino	3,716	10,999	35	103	
Montenegro	75,834	12,185	548	88	
Estonia	66,628	5,044	1,141	86	
Malta	22,657	4,686	227	47	
Serbia	459,259	6,578	3,231	46	
Slovakia	308,083	5,656	2,277	42	
Bahrain	122,394	7,799	655	42	
Lebanon	375,050	5,476	2,854	42	
Israel	778,172	8,759	3,681	41	

Show all

Weekly cases per capita shows the share of population with a new reported case for each week. Weeks without a reported case are shaded gray.

The virus continues to affect every region of the world, but some countries are experiencing high rates of infection, while others appear to have mostly controlled the virus.

Where new cases are higher and staying high

Countries where new cases are higher had a daily average of at least four new cases per 100,000 people over the past week. The charts, which are all on the same scale, show daily cases per capita and are of countries with at least five million people.

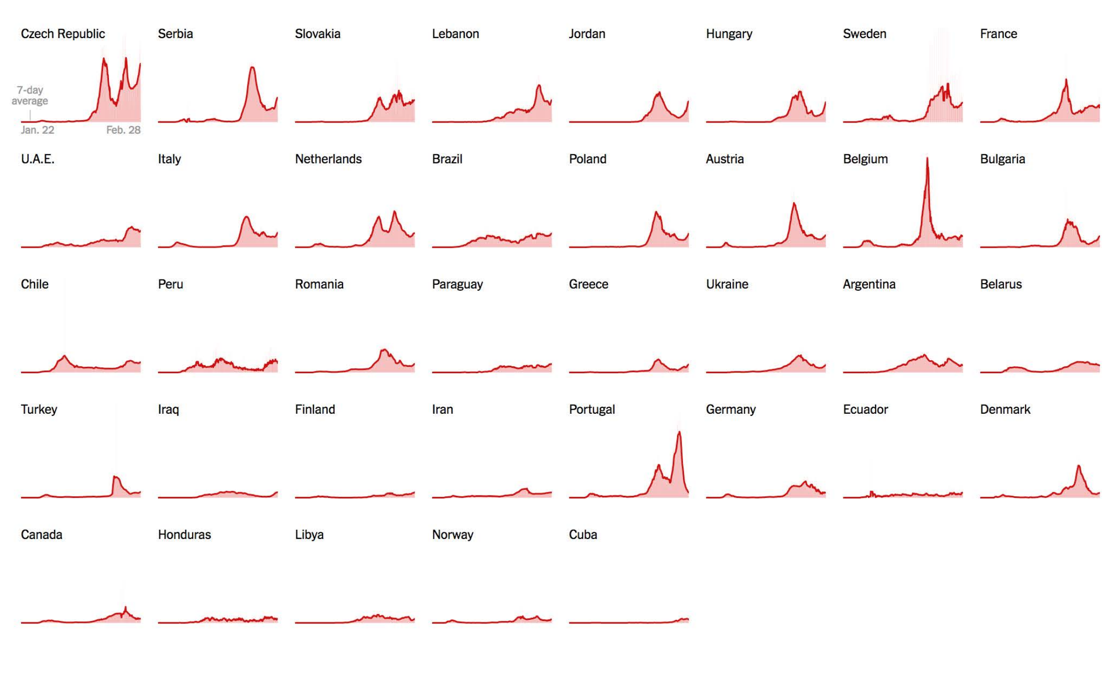

Where new cases are higher but going down

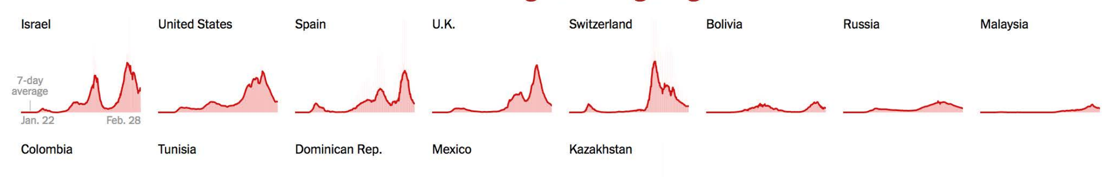

Where new cases are lower but going up

Countries where new cases are lower had a daily average of less than four new cases per 100,000 people over the past week. The charts, which are all on the same scale, show daily cases per capita and are of countries with at least five million people.

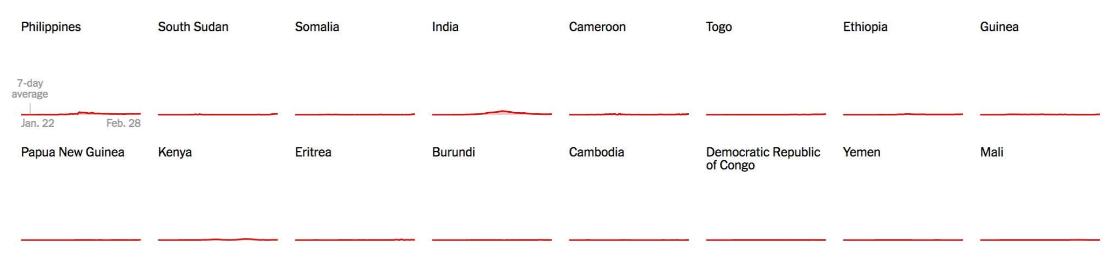

Where new cases are lower and staying low

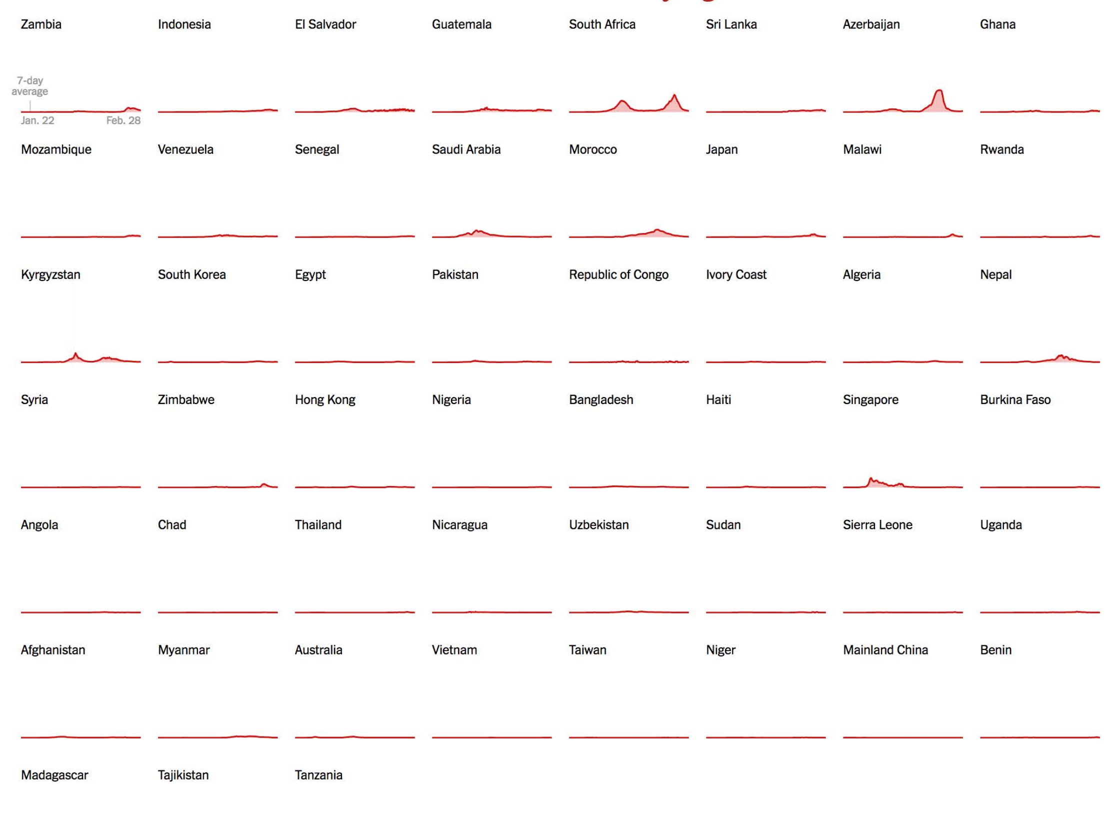

Where new deaths are increasing

The charts, which are all on the same scale, show daily deaths per capita and are of countries with at least five million people.

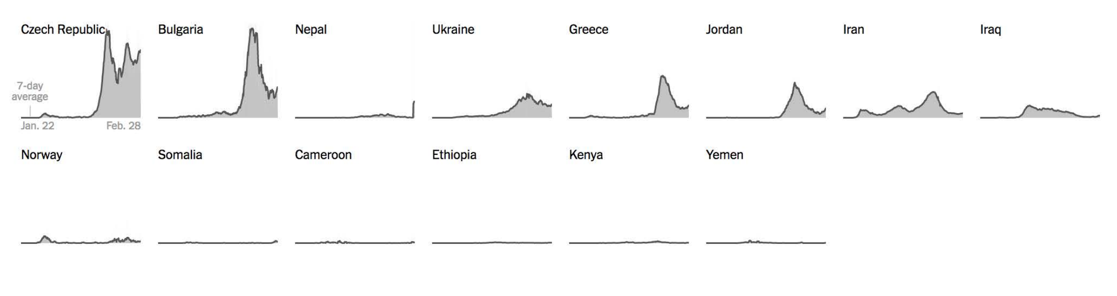

These countries have had the highest growth in newly reported deaths over the last 14 days. Deaths tend to rise a few weeks after a rise in infections, as there is typically a delay between when people are infected, when they die and when deaths are reported. Some deaths reported in the last two weeks may have occurred much earlier because of these delays.

The outbreak was initially defined by a series of shifting epicenters — including Wuhan, China; Iran; northern Italy; Spain; and New York. But the pandemic has now reached nearly every country in the world.

New reported cases by day across the world

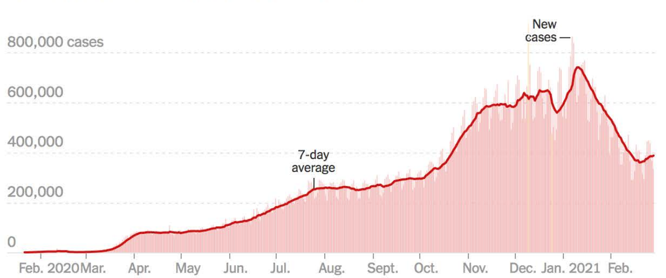

These are days with a reporting anomaly. Read more here.

Note: The seven-day average is the average of a day and the previous six days of data.

Reported deaths by day across the world

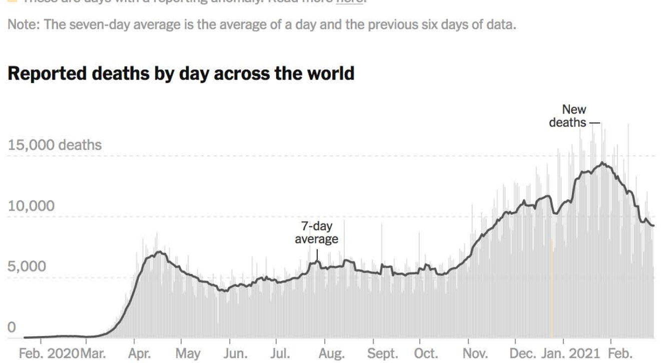

These are days with a reporting anomaly. Read more here.

Note: Scale for deaths chart is adjusted from cases chart to display trend.

The New York Times has found that official tallies in the United States and in more than a dozen other countries have undercounted deaths during the coronavirus outbreak because of limited testing availability.

United States

The number of known coronavirus cases in the United States continues to grow. As of Monday morning, at least 28,622,900 people across every state, plus Washington, D.C., and four U.S. territories, have tested positive for the virus, according to a New York Times database, and at least 512,900 patients with the virus have died.

Reported cases in the United States

Average daily cases per 100,000 people in the past week

← Fewer | More →

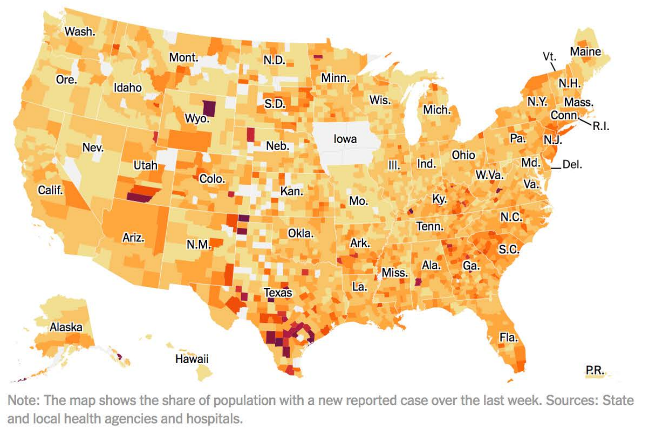

Note: The map shows the share of population with a new reported case over the last week. Sources: State and local health agencies and hospitals.

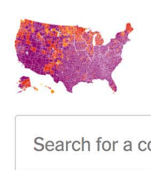

Covid-19 risk in your area ›
Find specific information about the county where you live.

Search for a county

The New York Times is engaged in an effort to track the details of every reported case in the United States, collecting information from federal, state and local officials around the clock. The numbers in this article are being updated several times a day based on the latest information our journalists are gathering from around the country. The Times has made that data public in hopes of helping researchers and policymakers as they seek to slow the pandemic and prevent future ones.

Read more about the methodology and download county-level data for coronavirus cases in the United States from The New York Times on [illegible]

About the data

The Times has identified the following reporting anomalies or methodology changes in the data:

- **Dec. 10:** Turkey announced the total number of reported cases, including asymptomatic cases since the end of July, reaching more than 1.7 million cases.
- **Dec. 25:** Several countries did not publish data on Dec. 25.

Governments often revise data or report a single-day large increase in cases or deaths from unspecified days without historical revisions, which can cause an irregular pattern in the daily reported figures. The Times is excluding these anomalies from seven-day averages when possible.

Tracking Covid-19's global spread

The disease has spread to every continent and case numbers continue to rise

By Henrik Pettersson, Byron Manley and Sergio Hernandez, CNN

Last updated: March 1, 2021 at 11:45 a.m. ET

Authorities in 219 countries and territories have reported about 114.2 million Covid-19 cases and 2.5 million deaths since China reported its first cases to the World Health Organization (WHO) in December 2019.

Cases per 100K | Deaths per 100K | Total cases | Total deaths

0 | 0 to 1,232 | 1,232 to 3,541 | 3,541 to 5,976 | 5,976 to 11,063 | 11,063 to 14,086

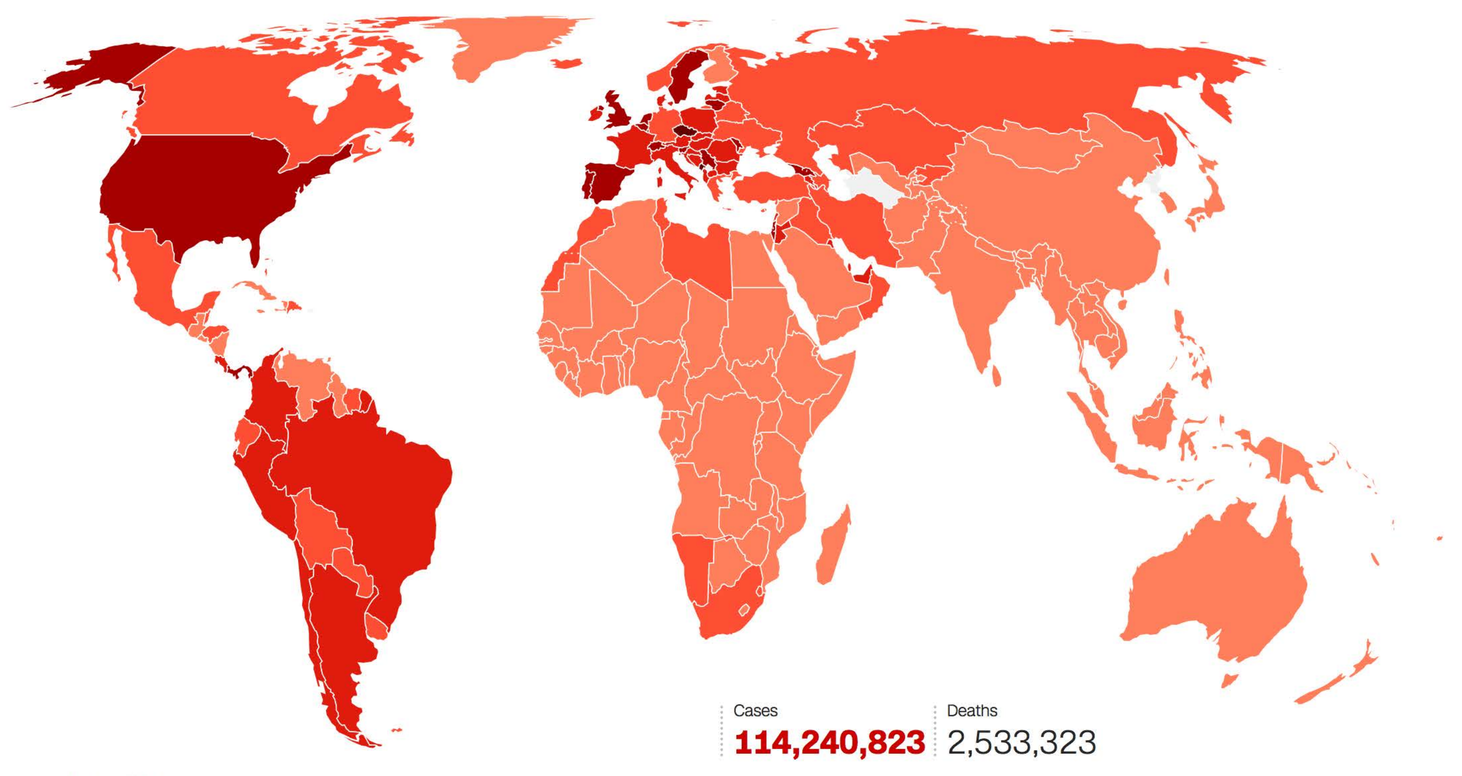

Cases **114,240,823** | Deaths 2,533,323

Last updated: March 1, 2021 at 11:45 a.m. ET
Source: Johns Hopkins University Center for Systems Science and Engineering

Reported cases and deaths

The figures below are based on data from the Johns Hopkins University Center for Systems Science and Engineering. These numbers are updated every 15 minutes but may differ from other sources due to differences in reporting times. For up-to-the-minute updates, follow our live coverage.

Location	Cases	...per 100K people	Deaths	...per 100K people
United States	28,609,645	8,716	513,112	156
India	11,112,241	813	157,157	12
Brazil	10,551,259	4,999	254,942	121
Russia	4,209,850	2,916	85,025	59
United Kingdom	4,176,558	6,249	122,849	184
France	3,732,426	5,566	85,980	128
Spain	3,188,553	6,773	69,142	147
Italy	2,925,265	4,851	97,699	162
Turkey	2,701,588	3,238	28,569	34
Germany	2,454,298	2,952	70,218	84
Colombia	2,251,690	4,473	59,766	119
Argentina	2,107,365	4,689	51,965	116
Mexico	2,086,938	1,636	185,715	146
Poland	1,711,772	4,508	43,793	115
Iran	1,639,679	1,978	60,181	73
South Africa	1,513,393	2,584	49,993	85
Ukraine	1,399,934	3,154	27,480	62
Indonesia	1,341,314	496	36,325	13
Peru	1,329,805	4,090	46,494	143
Czech Republic	1,240,051	11,622	20,469	192

Show more

* Cases identified on cruise ships.

Last updated: March 1, 2021 at 11:45 a.m. ET
Sources: Johns Hopkins University Center for Systems Science and Engineering. Population data from World Bank and United Nations.

Since then, cases have been reported on every continent. The vast majority of cases and deaths are now outside mainland China, where the outbreak began.

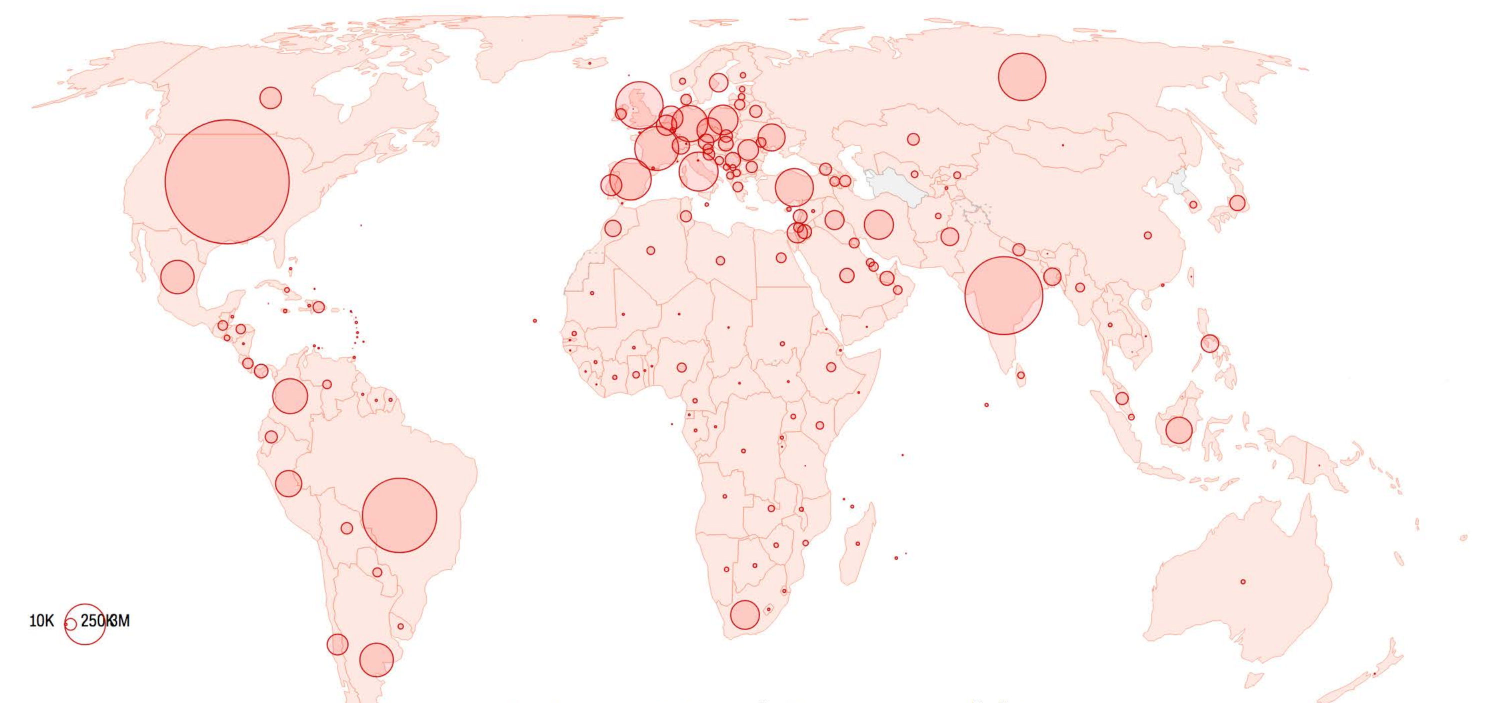

February 17 | Cases **109,815,894** | Deaths 2,427,982

Last updated: March 1, 2021 at 11:45 a.m. ET
Source: Johns Hopkins University Center for Systems Science and Engineering

As the pandemic has spread across the globe, the virus has left a trail of deaths in its wake. Deaths in Europe and North America now outnumber Asia's. In Latin America, South America and the Caribbean, the share of global deaths is still rising.

Daily deaths and cases by region

This chart uses rolling, seven-day averages. This approach makes trends clearer and smooths out anomalies, such as the lack of reporting during the weekend.

Deaths | Cases | Proportional | Actual

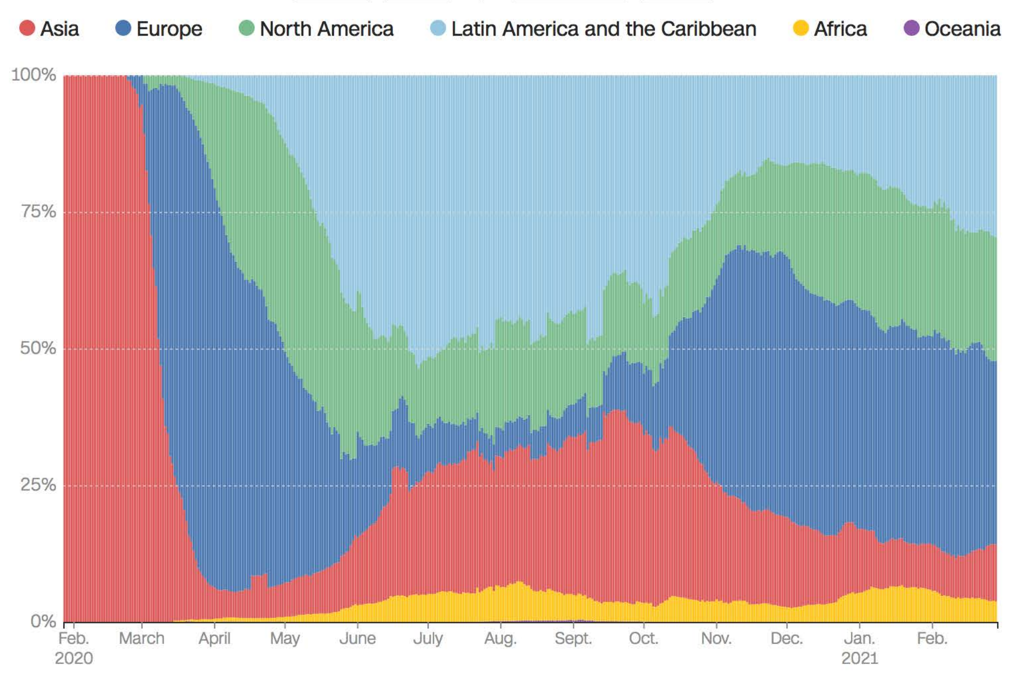

Regions are based on United Nations definitions. Americas have been broken down into subregions (Latin America and the Caribbean and North America).
Last updated: March 1, 2021 at 11:45 a.m. ET
Source: Johns Hopkins University Center for Systems Science and Engineering

Daily reported deaths by country

This timeline of new reported deaths shows how countries and regions have been affected so far.

Asia	Total	Latest
India	157,157	110 ↑
Iran	60,073	84 ↑
Indonesia	36,166	240 ↑
Turkey	28,569	73 ↓
Iraq	13,406	19 ↓

Expand

Europe	Total	Latest
United Kingdom	122,849	324 ↓
Italy	97,699	283 ↓
France	85,141	184 ↓
Russia	84,700	396 ↓
Germany	70,152	315 ↓

Expand

North America	Total	Latest
United States	513,091	2,012 ↓
Canada	21,990	45 ↓
Bermuda	12	0 →

Latin America and the Caribbean	Total	Latest
Brazil	254,942	1,205 ↑
Mexico	185,715	801 ↑
Colombia	59,766	133 ↓
Argentina	51,965	110 ↓
Peru	46,299	172 ↓

Expand

Africa	Total	Latest
South Africa	49,993	134 ↓
Egypt	10,688	48 ↓
Morocco	8,623	10 →
Tunisia	8,001	30 ↓
Algeria	2,983	3 →

Expand

Oceania	Total	Latest
Australia	909	0 →
French Polynesia	139	0 →
New Zealand	26	0 →
Papua New Guinea	12	0 →

Timeline: Jan. 28 – Feb. 28

Based on 7-day rolling averages | ↑↓ Trend in rolling average | 1 | 10 | 100 | 500 | 1,000

Different countries' epidemics have followed different trajectories. The disease has hit the United States especially hard. About 28.6 million cases have been reported in the country and 513,112 patients have died.

Covid-19 case growth rates

The chart below uses a logarithmic scale to show how quickly the number of known Covid-19 cases is growing in each region and territory. Select a location or hover over each line to see how quickly the number of known cases is doubling in that country or territory.

In **Yemen** the number of known cases is doubling about **every three months**.

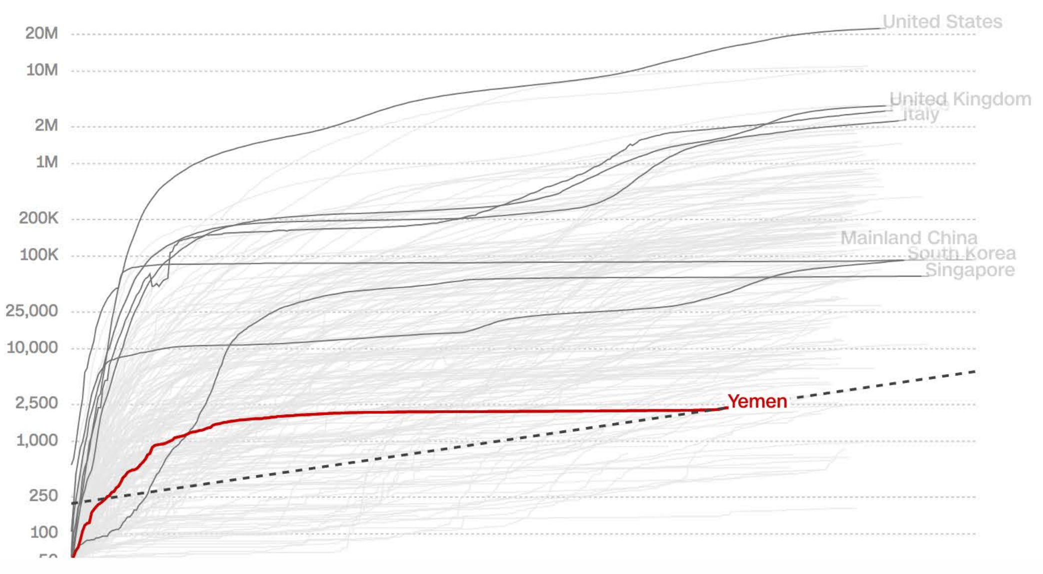

Note: Doubling times are based on the previous week.
Last updated: March 1, 2021 at 11:45 a.m. ET
Source: Johns Hopkins University Center for Systems Science and Engineering

APPLIED XL

In partnership with STAT

Covid-19 Worldwide

Last updated: March 01, 2021 at 11:29 AM EST

Search by country or region

Total Number of Cases | Cases Per 100k Population

Worldwide	114,229,586
United States	28,606,892
India	11,112,241
Brazil	10,551,259
Russia	4,209,850
United Kingdom	4,188,827
France	3,815,639
Spain	3,188,553
Italy	2,925,265
Turkey	2,701,588
Germany	2,453,909

TOTAL CASES	FIRST CASE	RECOVERED	DEATHS
114,229,586	426 DAYS AGO	75,118,436	2,533,256

2 WEEK AVERAGE CHANGE IN NEW CASES -9.4%

Average New Cases Yesterday	376,296
Average New Cases 2 Weeks Ago	415,491

2 WEEK AVERAGE CHANGE IN DEATHS -24.0%

Average Deaths Yesterday	9,338
Average Deaths 2 Weeks Ago	12,281

2 Week Trend | Cases Over Time

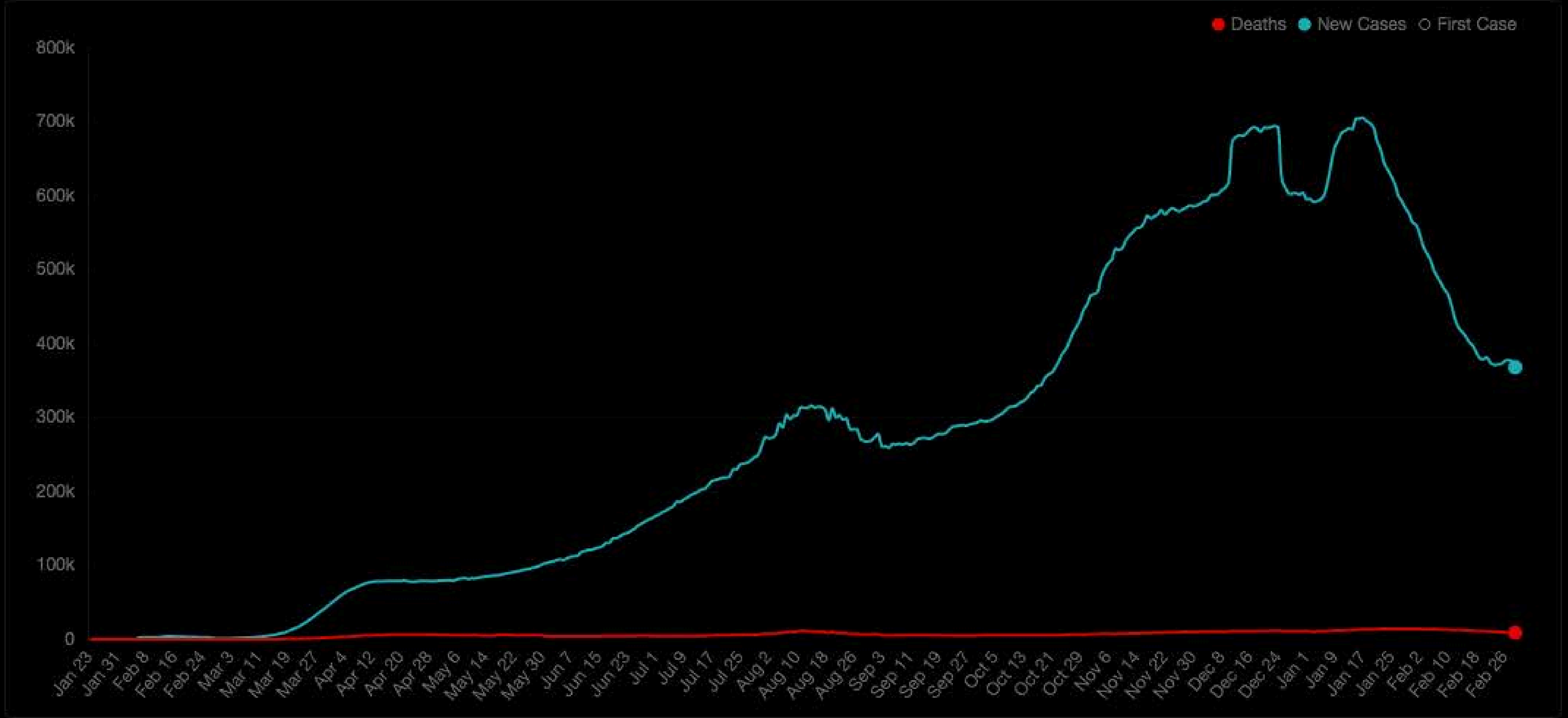

The datasets are drawn from over 15 sources and can be reviewed **here**. Data is updated regularly but may fluctuate based on variations in self-reported source data from each region.

JOHNS HOPKINS UNIVERSITY & MEDICINE | CORONAVIRUS RESOURCE CENTER

Home | **Tracking** | Testing | Tracing | Vaccines | By Region | Events & News | About

Tracking Home | Critical Trends | **Global Map** | U.S. Map | Data in Motion

COVID-19 Dashboard by the Center for Systems Science and Engineering (CSSE) at Johns Hopkins University (JHU)

Global Cases
114,240,823

Cases by Country/Region/Sovereignty

28,609,645 US
11,112,241 India
10,551,259 Brazil
4,209,850 Russia
4,188,827 United Kingdom
3,815,639 France
3,188,553 Spain
2,925,265 Italy
2,701,588 Turkey
2,454,298 Germany
2,251,690 Colombia
2,107,365 Argentina
2,086,938 Mexico
1,711,772 Poland
1,639,679 Iran
1,513,393 South Africa
1,399,934 Ukraine
1,341,314 Indonesia
1,329,805 Peru
1,240,051 Czechia
1,107,326 Netherlands
872,706 Canada
[illegible]

Admin0 | Admin1 | Admin2

Last Updated at (M/D/YYYY)
3/1/2021, 11:24 AM

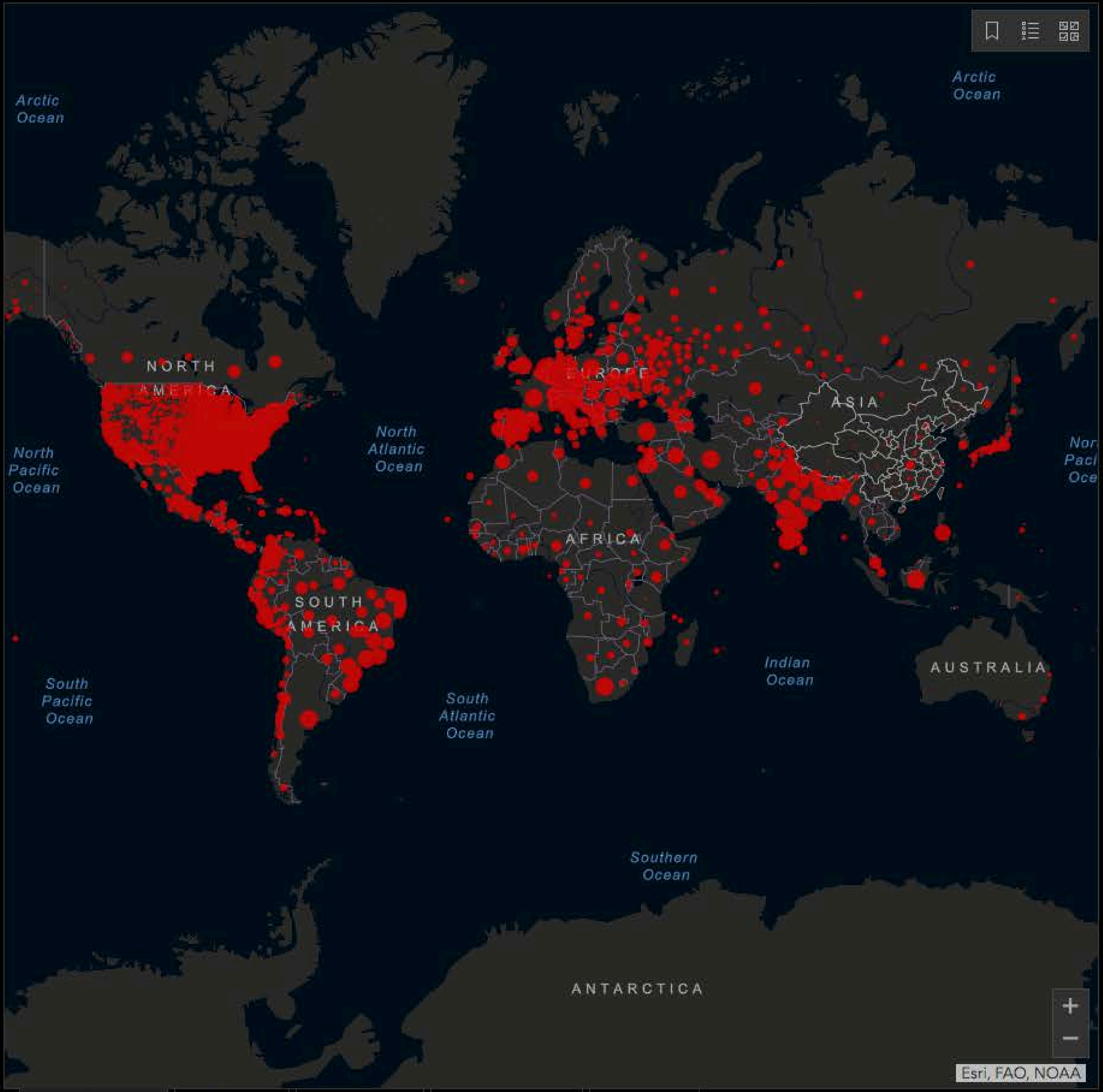

Cumulative Cases | Active Cases | Incidence Rate | Case-Fatality Ratio | Testing Rate

192
countries/regions

Lancet Inf Dis Article: Here. Mobile Version: Here. Data sources: Full list. Downloadable database: GitHub, Feature Layer.
Lead by JHU CSSE. Technical Support: Esri Living Atlas team and JHU APL. Financial Support: JHU, NSF, Bloomberg Philanthropies and Stavros Niarchos Foundation. Resource support: Slack, Github and AWS.
Click *here* to **donate** to the CSSE dashboard team, and other JHU COVID-19 Research Efforts. FAQ. Read more in this blog. Contact US.

Global Deaths
2,533,323

513,112 deaths
US
254,942 deaths
Brazil
185,715 deaths
Mexico
157,157 deaths
India
123,083 deaths
United Kingdom
97,699 deaths
Italy
86,580 deaths
France
85,025 deaths
Russia
70,218 deaths
Germany
69,142 deaths
Spain

Global Deaths

US State Level Deaths, Recovered

52,158 deaths, recovered
California US
47,615 deaths, recovered
New York US
43,697 deaths, 2,422,369 recovered
Texas US
30,852 deaths, recovered
Florida US
23,971 deaths, 836,727 recovered
Pennsylvania US
23,252 deaths, recovered
New Jersey US
22,735 deaths, recovered
Illinois US
17,297 deaths, 909,524 recovered
Ohio US
17,295 deaths, recovered
Georgia US

US Deaths, Recovered

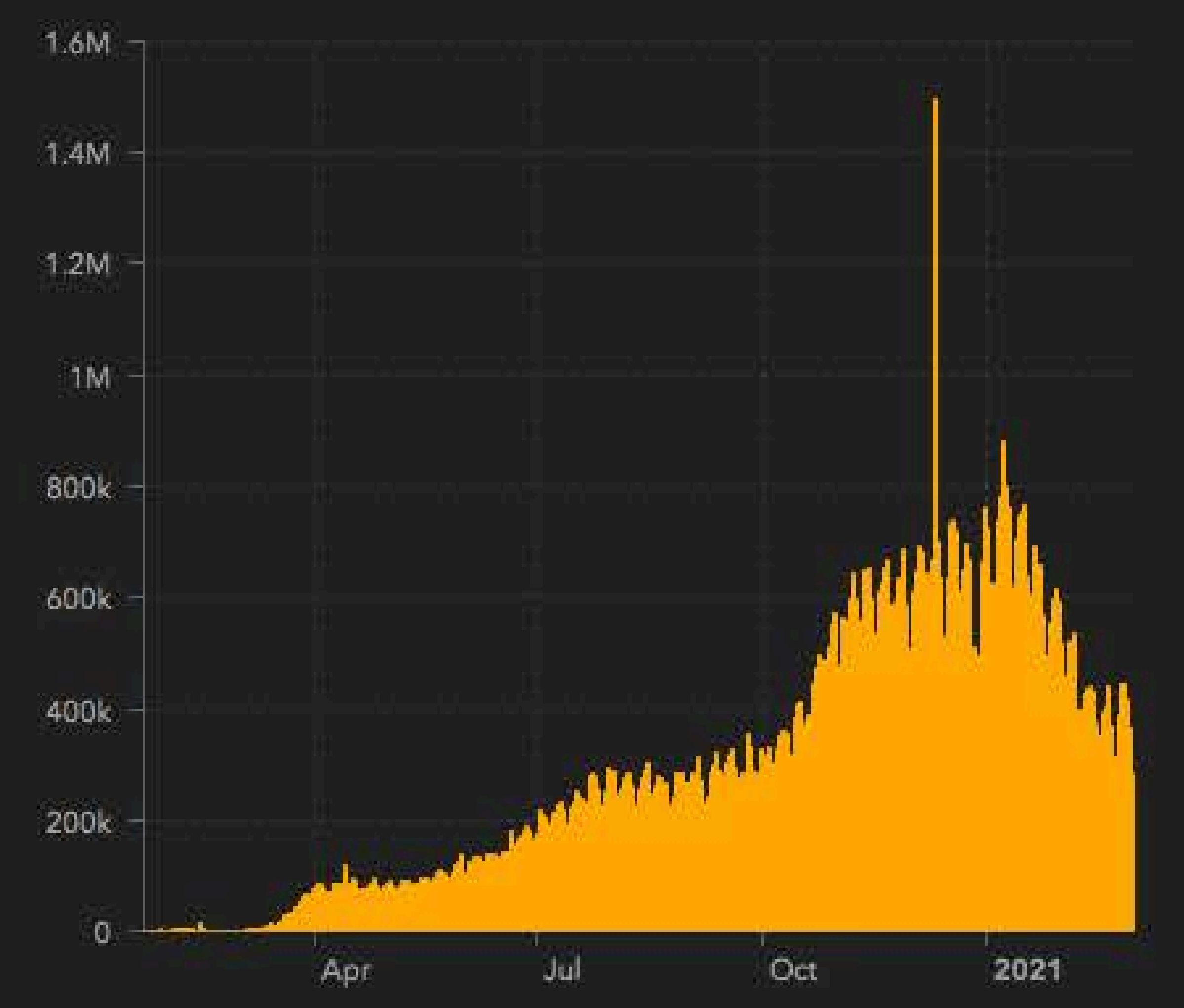

Daily Cases

UNIVERSITY of VIRGINIA BIOCOMPLEXITY INSTITUTE

COVID-19 Surveillance Dashboard 1.6.0

Vaccine Data Available Now

More Options

03-01-2021 | All regions | Download All | FAQ

Cases | Tests | Vaccine | Est.Active | Confirmed | Deaths | Est.Recovered

United States: Est.Active: 1,255,216 Conf: 28,605,333 Deaths: 512,783 Est.Rec: 26,837,334

Time Slider from *Jan 22 2020 to Mar 01 2021* Selected Date *Mar 01 2021*

1/22/2020 3/2/2020 4/11/2020 5/21/2020 6/30/2020 8/9/2020 9/18/2020 10/28/2020 12/7/2020 1/16/2021 2/25/2021

All regions

-180,723	81,064	1,855	259,932
6,029,217	114,227,593	2,533,365	105,665,011
Est.Active	Confirmed	Deaths	Est.Recovered

Notices: Vaccine Data Available | Change on USA Data Sources

Tutorials: USA county | Query and filter | Advanced click | USA testing data & toggles | more

Analytics | Chart | Data

Last 30 days | Type : Daily

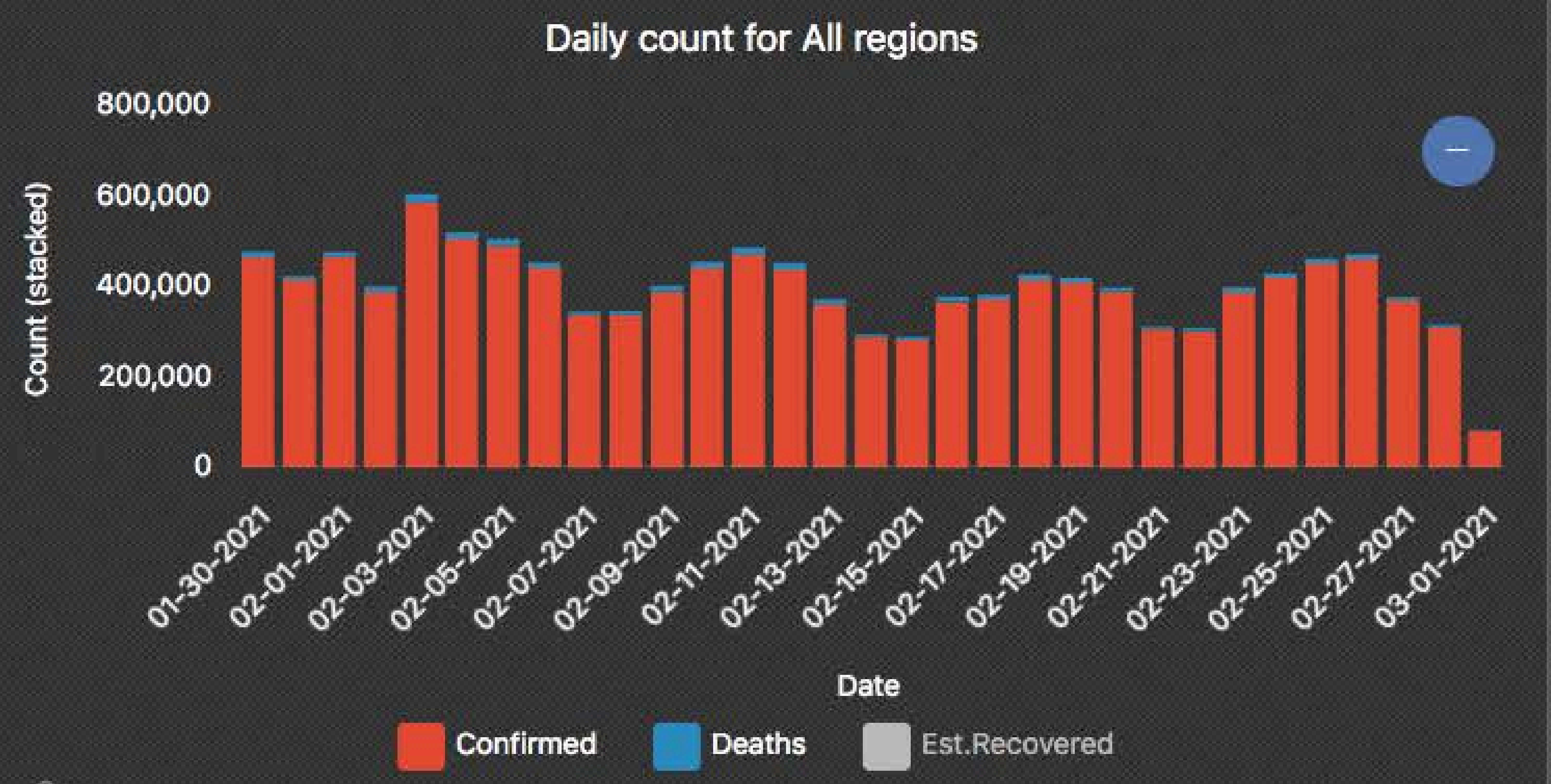

Copyright © 2021, UVA; ALL RIGHTS RESERVED. THE CONTENTS OF THIS DASHBOARD--INCLUDING DATA, MAPS, AND PLOTS--IS PROVIDED TO THE PUBLIC SOLELY FOR RESEARCH AND ACADEMIC PURPOSES. UVA PROVIDES NO WARRANTIES, CLAIMS OR REPRESENTATIONS–WHETHER EXPRESS, IMPLIED, OR STATUTORY–WITH RESPECT TO THIS DASHBOARD, INCLUDING, WITHOUT LIMITATION, WARRANTIES OF QUALITY, PERFORMANCE, NON-INFRINGEMENT, MERCHANTABILITY, ACCURACY, ADEQUACY, VALIDITY, RELIABILITY, COMPLETENESS, OR FITNESS FOR A PARTICULAR PURPOSE, AND DISAVOWS ANY REPRESENTATIONS OR WARRANTIES CREATED BY COURSE OF DEALING, COURSE OF PERFORMANCE, TRADE USAGE OR OTHERWISE. UNDER NO CIRCUMSTANCES WILL UVA HAVE ANY LIABILITY FOR ANY LOSS OR DAMAGE INCURRED AS A RESULT OF THE USE OF THIS DASHBOARD OR RELIANCE ON ANY INFORMATION PROVIDED HEREIN. THIS DASHBOARD MAY INCORPORATE DATA OR OTHER CONTENT BELONGING TO OR ORIGINATING FROM THIRD PARTIES. WE DO NOT WARRANT, ENDORSE, GUARANTEE, OR ASSUME RESPONSIBILITY FOR THE ACCURACY OR RELIABILITY OF ANY THIRD-PARTY INFORMATION.

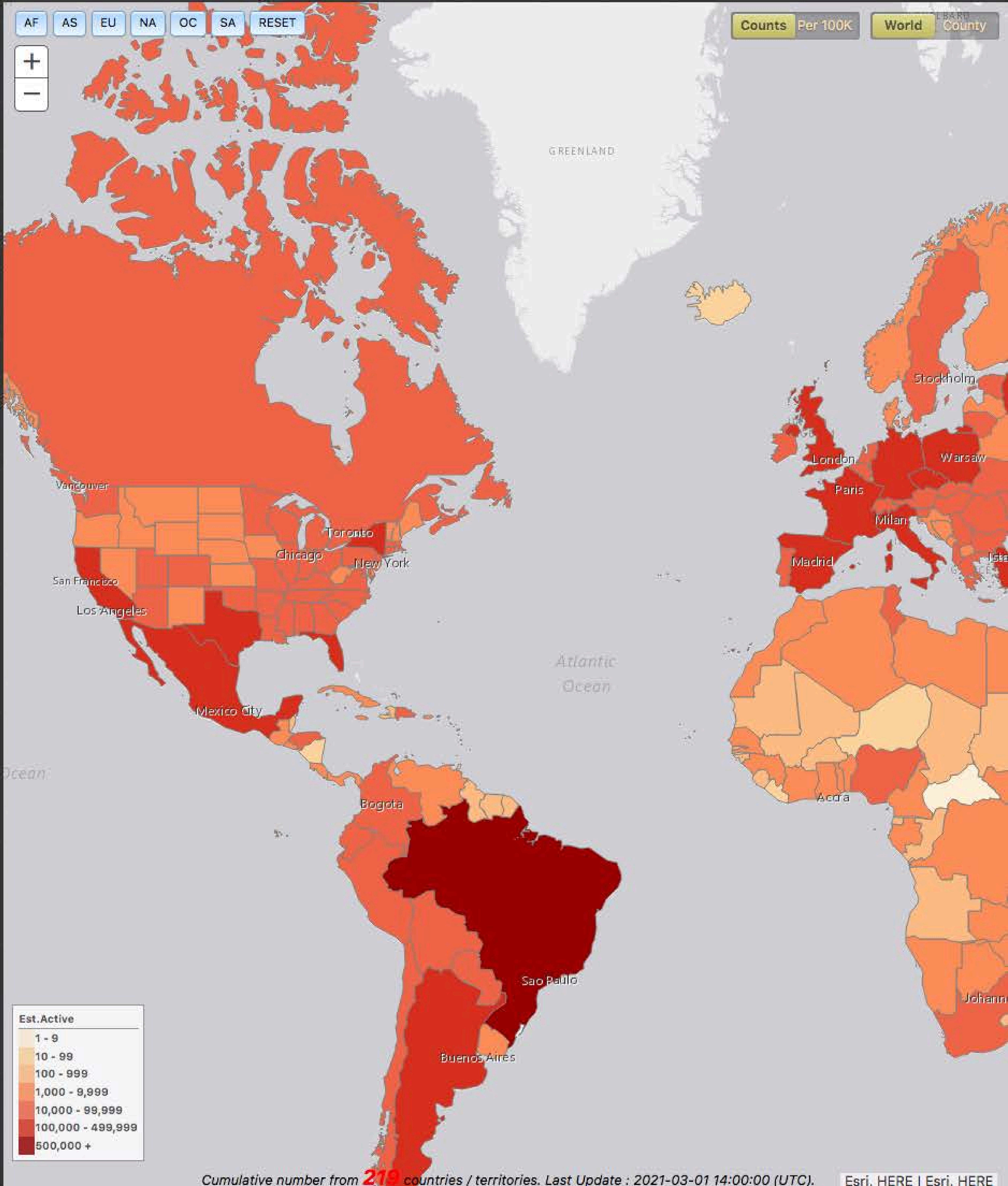

Novel Coronavirus (COVID-19) Infection Map

GLOBAL TREND

Last update: 2021-02-18 15:45:05 PST *Click a place to review local trend.*

Source: WHO, New York Times, NBC News, CDC, Baidu, Wikipedia, and etc.

Aggregated Confirmed	Death
111,222,783 +336,378	2,258,873 +8,951
**Active Confirmed**	**Recovered**
36,602,189 +108,867	72,361,721 +218,560

linear option — toggle on/off the curve or scroll on the curves to change the date range

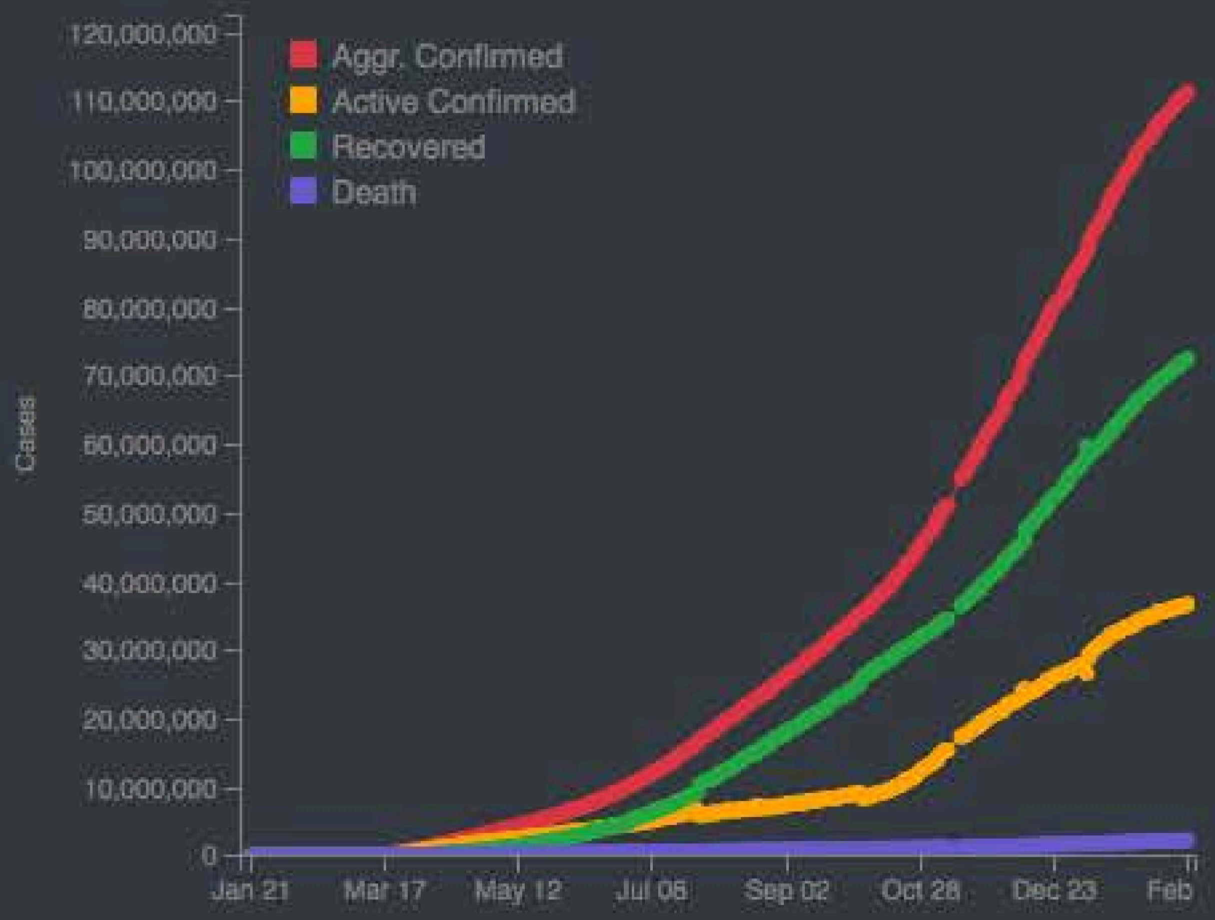

World or U.S. Rankings » Situational Heatmap

In the main map, an country or region's inflection level is measured by the # of today's active/remaining confirmed cases. The gray slash texture indicates no previous cases are discovered; the green texture means the region in which all the infected cases have recovered.

Infection Case(s) indicates one or several confirmed infected individual(s) in the U.S., excluding those were identified from the Diamond Princess. The dataset of the infected cases are continuously enriched from different data portals such as 1point3acres and state officials. Notably, each case was geocoded to an approximate location within the county which reported the case. The location is by no means identical to the exact address where the case was discovered.

This map is made by the Humanistic GIS Lab at University of Washington - Seattle. The data are mainly collected from WHO, CDC, PHA , China CDC, NBC News, Wikipedia, New York Times, and Baidu. Regarding the state level data in the U.S., they are collected from CDC, state officials, New York Times, and NBC News. The computational resources are provided

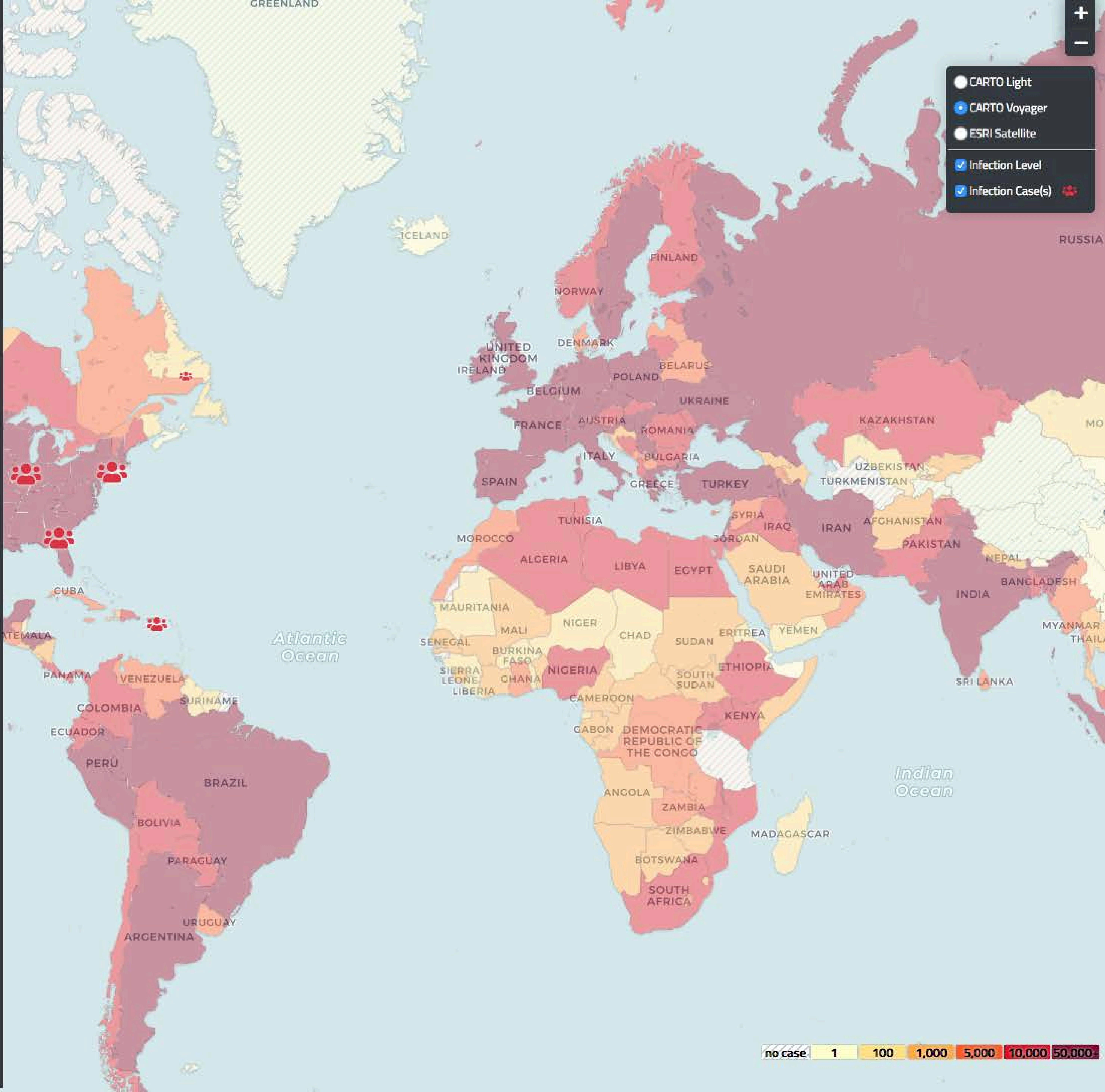

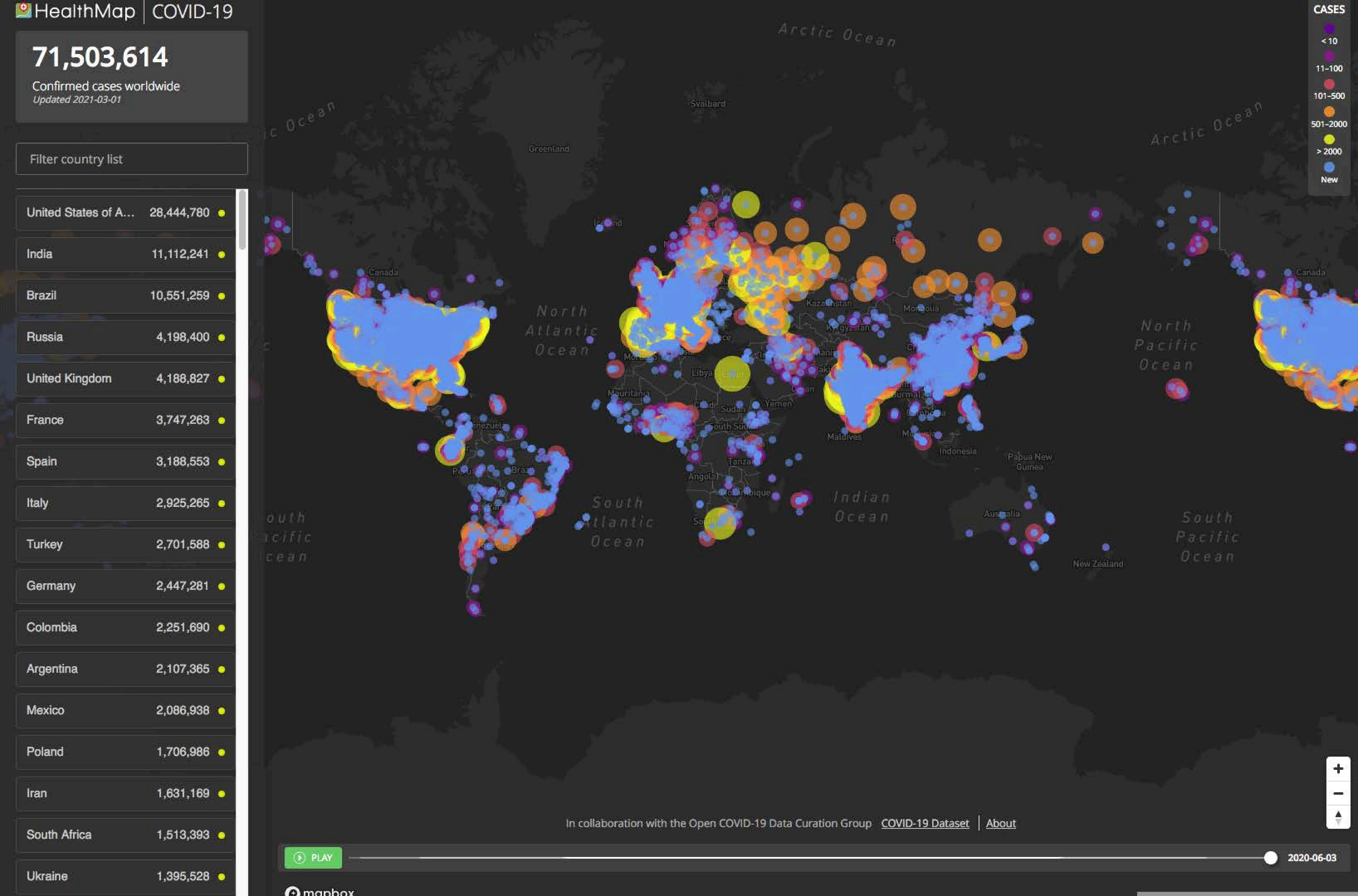
HealthMap | COVID-19
71,503,614
Confirmed cases worldwide
Updated 2021-03-01
Filter country list
United States of A... 28,444,780
India 11,112,241
Brazil 10,551,259
Russia 4,198,400
United Kingdom 4,188,827
France 3,747,263
Spain 3,188,553
Italy 2,925,265
Turkey 2,701,588
Germany 2,447,281
Colombia 2,251,690
Argentina 2,107,365
Mexico 2,086,938
Poland 1,706,986
Iran 1,631,169
South Africa 1,513,393
Ukraine 1,395,528
CASES
< 10
11–100
101–500
501–2000
> 2000
New
Arctic Ocean
North Atlantic Ocean
South Atlantic Ocean
Indian Ocean
North Pacific Ocean
South Pacific Ocean
Greenland
Svalbard
Canada
Kazakhstan
Mongolia
Libya
Mauritania
Sudan
Yemen
Maldives
Indonesia
Papua New Guinea
Australia
New Zealand
In collaboration with the Open COVID-19 Data Curation Group COVID-19 Dataset | About
PLAY
2020-06-03
mapbox

World Health Organization

Search by Country, Territory, or Area

Covid-19 Response Fund

Donate

WHO Coronavirus Disease (COVID-19) Dashboard

Data last updated: 2021/3/1, 4:03pm CET

Overview | Data Table | Explore

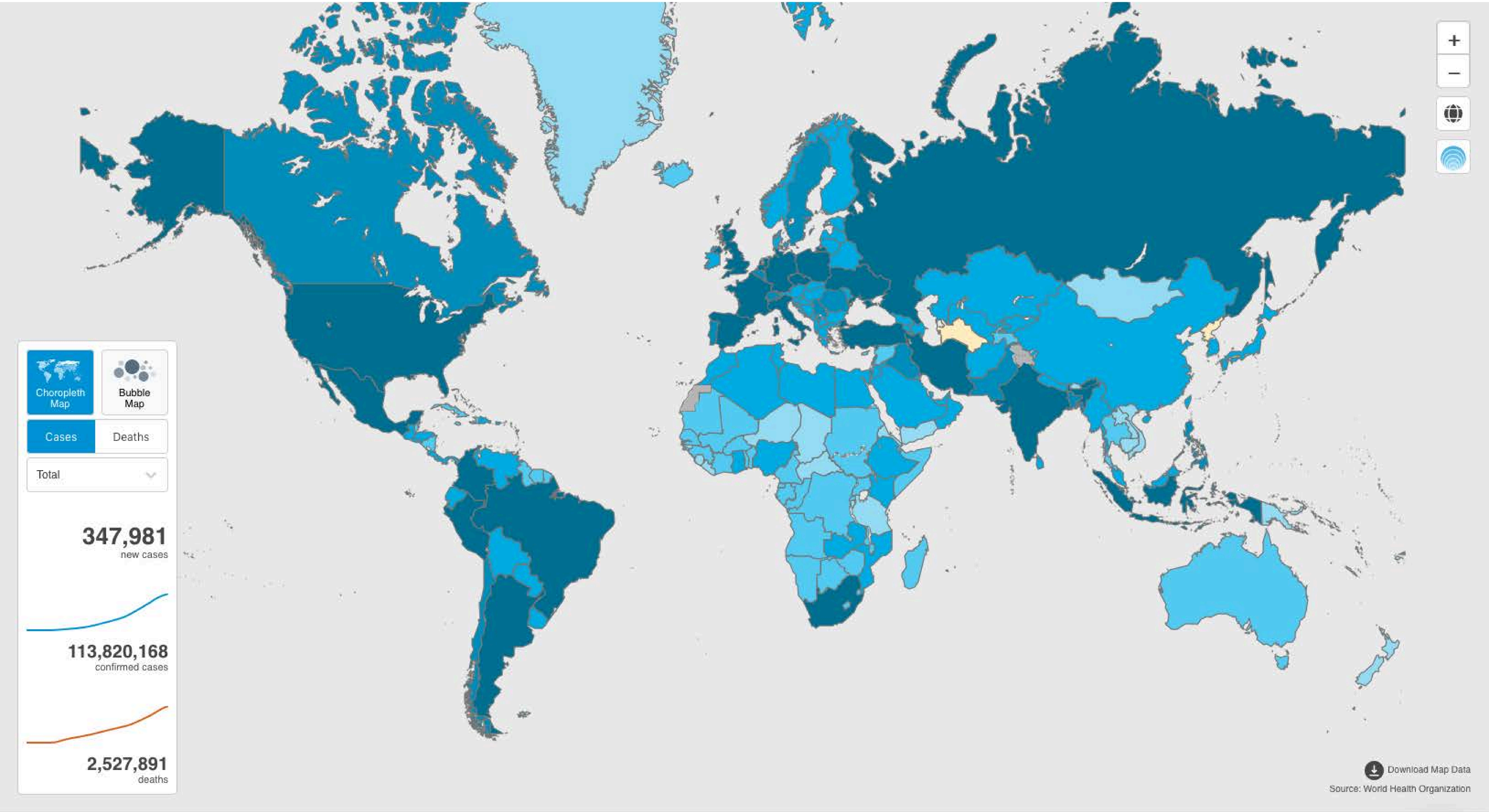

Globally, as of **4:03pm CET, 1 March 2021**, there have been **113,820,168 confirmed cases** of COVID-19, including **2,527,891 deaths**, reported to WHO.

World Health Organization

Search by Country, Territory, or Area

Covid-19 Response Fund

Donate

WHO Coronavirus Disease (COVID-19) Dashboard

Data last updated: 2021/3/1, 4:03pm CET

Overview | Data Table | Explore

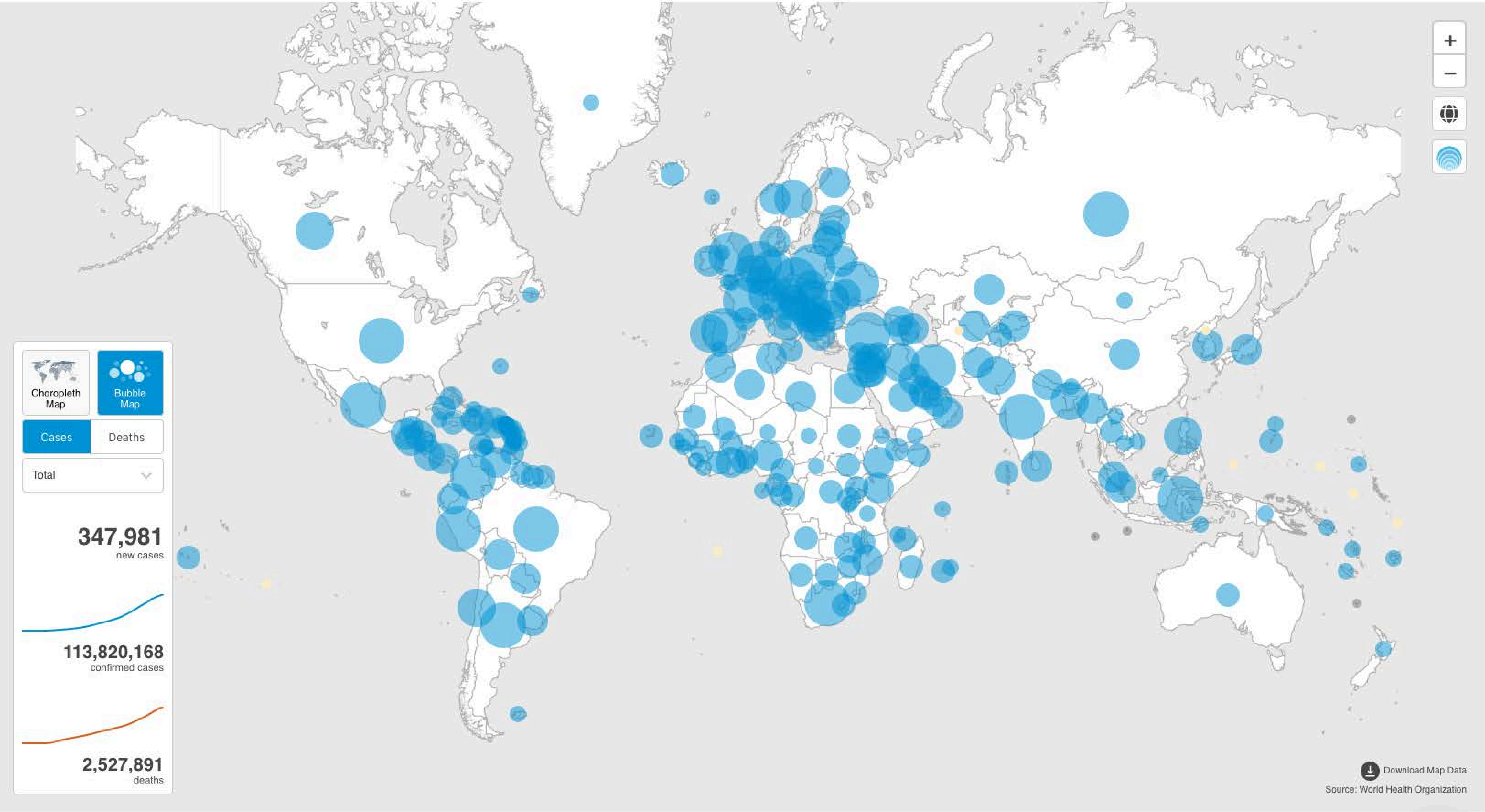

Globally, as of **4:03pm CET, 1 March 2021**, there have been **113,820,168 confirmed cases** of COVID-19, including **2,527,891 deaths**, reported to WHO.

World Health Organization

Search by Country, Territory, or Area

Covid-19 Response Fund

Donate

WHO Coronavirus Disease (COVID-19) Dashboard

Data last updated: 2021/3/1, 4:03pm CET

Overview | Data Table | Explore

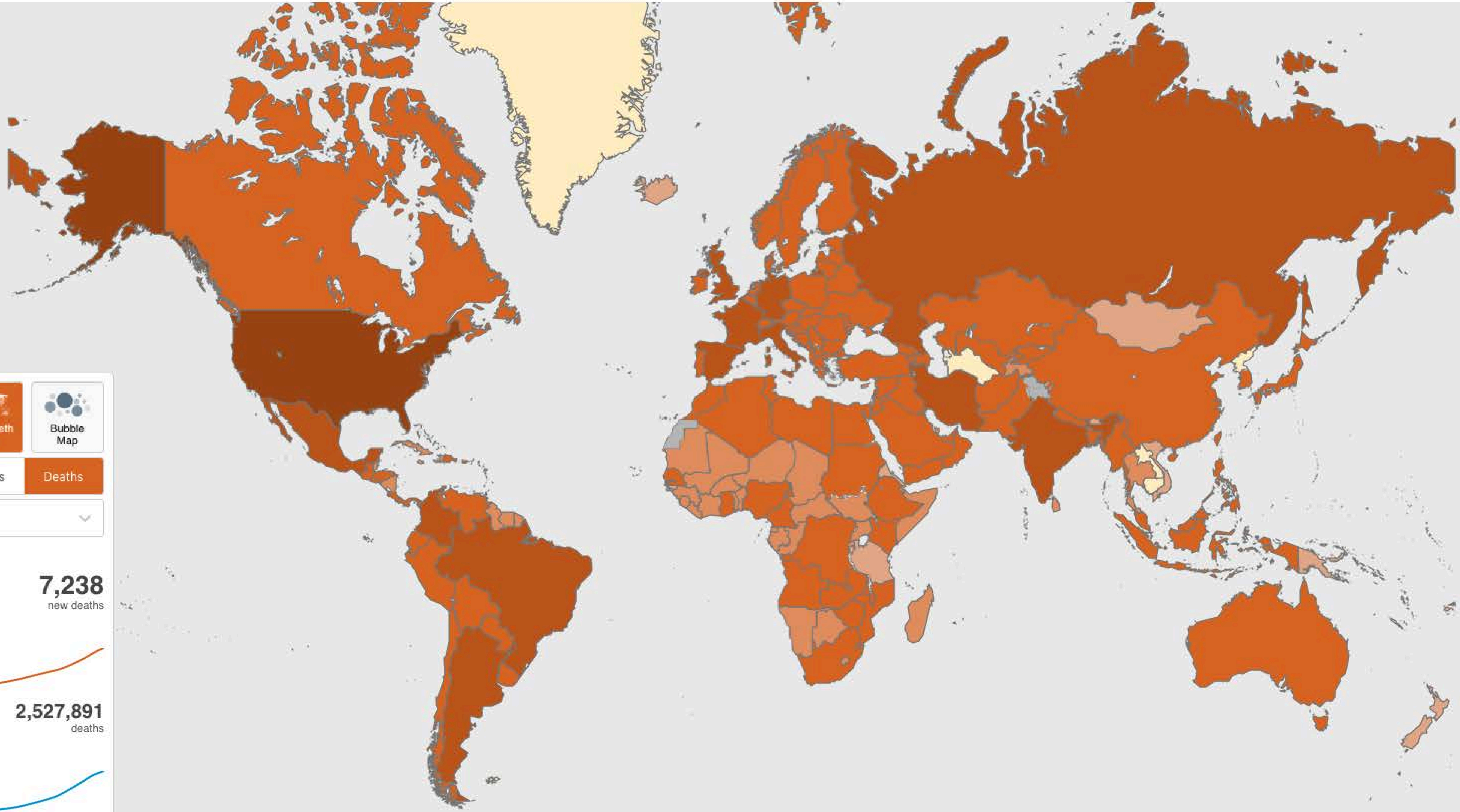

Choropleth Map | Bubble Map

Cases | Deaths

Total

7,238
new deaths

2,527,891
deaths

113,820,168
confirmed cases

Download Map Data

Source: World Health Organization

Globally, as of **4:03pm CET, 1 March 2021**, there have been **113,820,168 confirmed cases** of COVID-19, including **2,527,891 deaths**, reported to WHO.

COVID-19: Confirmed Cases & Deaths by Country

as of 28 February, 2021

KFF

Overview | Analyze Trends | View Data Table | Government Actions

Total Cases

114,067,970

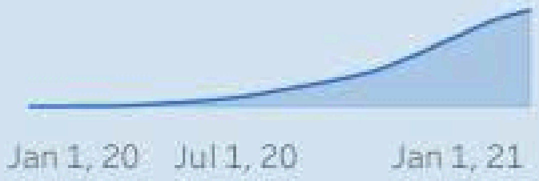

New Cases on 2/28/2021

283,244

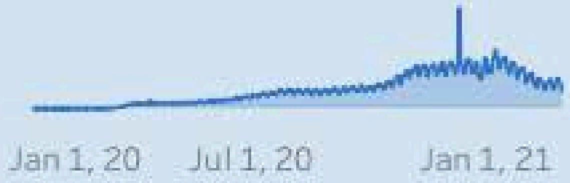

Total Deaths

2,530,714

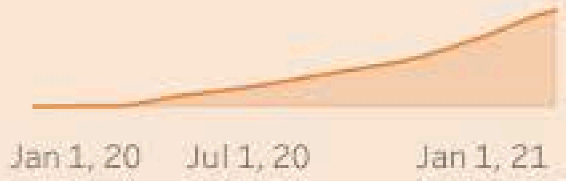

New Deaths on 2/28/2021

5,315

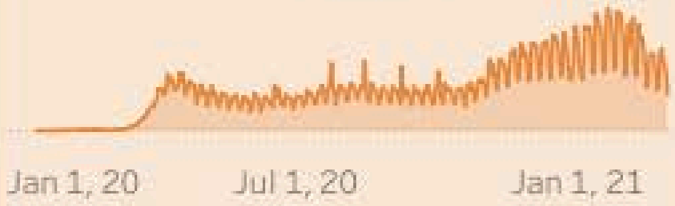

Select Metric: Cases

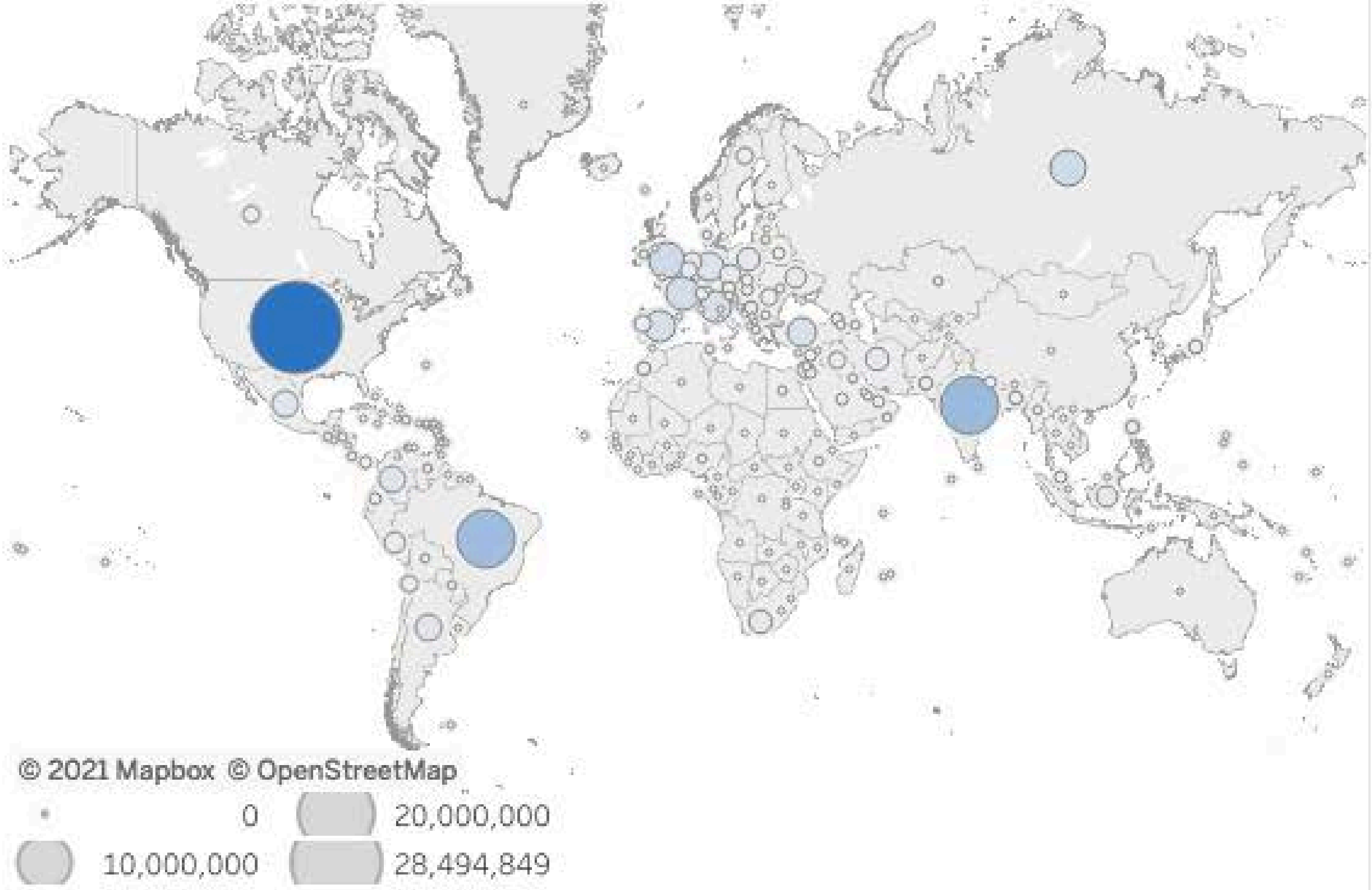

Select a Country to the left to highlight it on the chart below

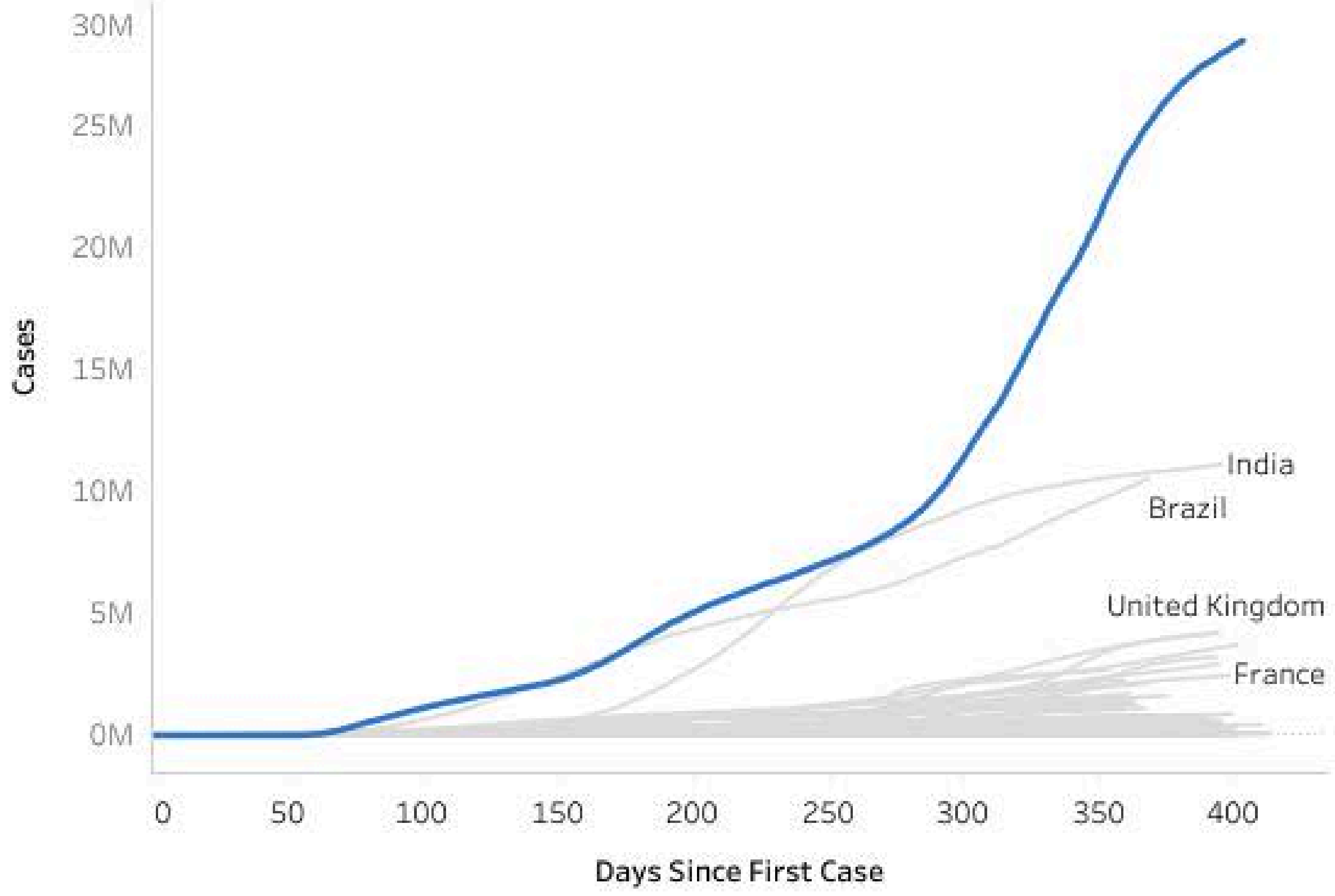

Source: Johns Hopkins University (JHU) Coronavirus Resource Center; last updated with data from 2/28/2021. Data prior to January 22, 2020 are from the World Health Organization's (WHO) Coronavirus disease (COVID-2019) situation reports. Notes: Cumulative case totals include both laboratory confirmed and clinically diagnosed cases; prior to February 14, 2020, totals include only laboratory confirmed cases. Japan's totals include cases that have been identified on the Diamond Princess cruise ship (except in cases that have been re-categorized by a reporting country).

+ a b | e a u

Statistics and Research

Coronavirus Pandemic (COVID-19)

Research and data: Hannah Ritchie, Esteban Ortiz-Ospina, Diana Beltekian, Edouard Mathieu, Joe Hasell, Bobbie Macdonald, Charlie Giattino, and Max Roser
Web development: Breck Yunits, Ernst van Woerden, Daniel Gavrilov, Matthieu Bergel, Shahid Ahmad, Jason Crawford, and Marcel Gerber

The data on the coronavirus pandemic is updated daily. Last update: **an hour ago.**

Reuse our work freely   Cite this research

Coronavirus › **By country** **Data explorer** Deaths Cases Tests Hospitalizations Vaccinations Mortality risk Excess mortality Policy responses Exemplars All charts

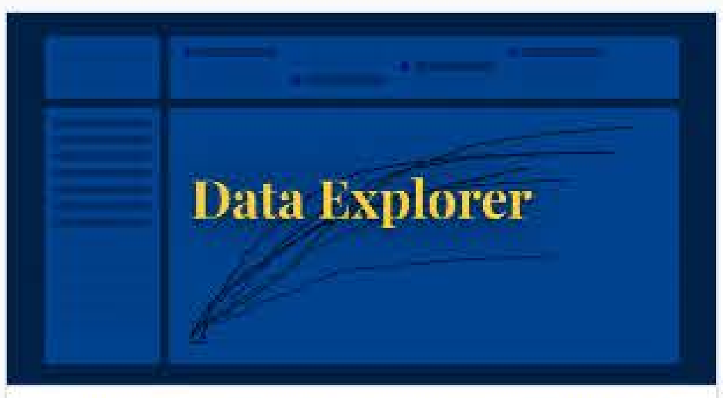

Explore all metrics – including cases, deaths, testing, and vaccinations – in one place.

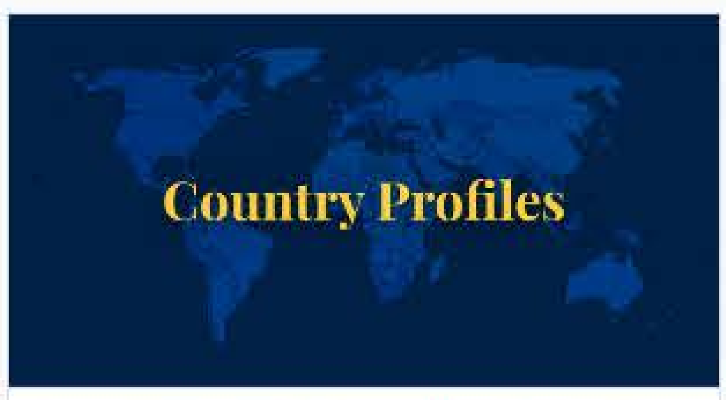

Get an overview of the pandemic for any country on a single page.

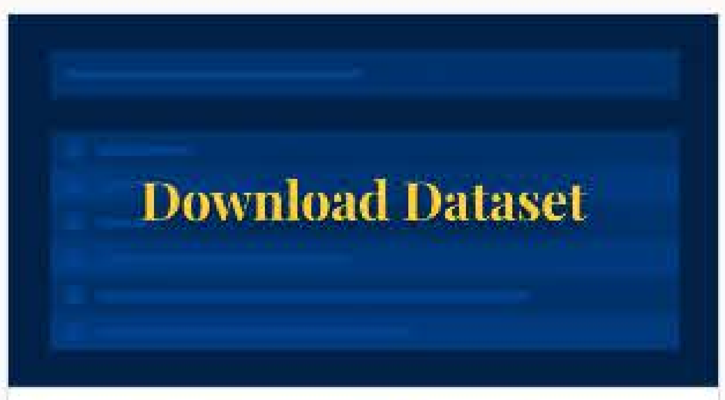

Download our complete dataset of COVID-19 metrics on GitHub. It's open access and free for anyone to use.

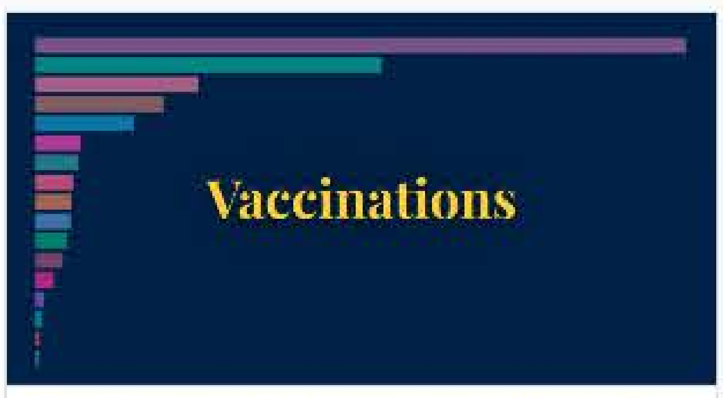

Explore our global dataset on COVID-19 vaccinations.

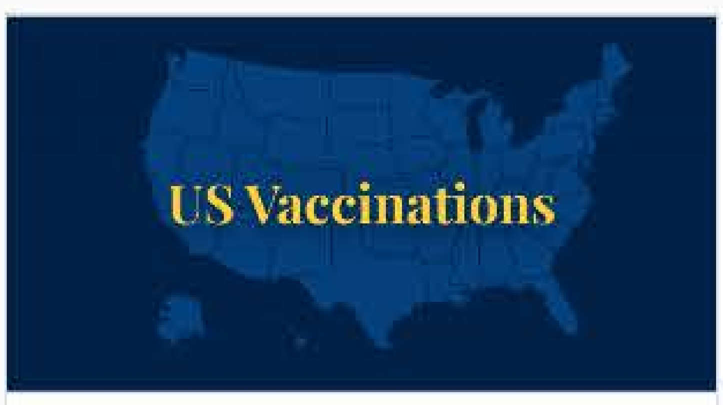

See state-by-state data on vaccinations in the United States.

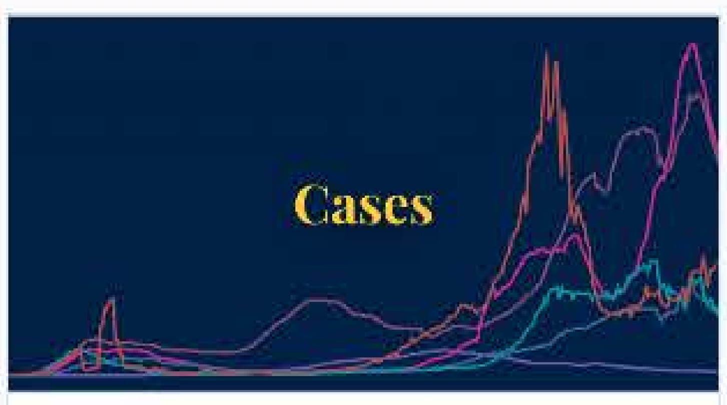

Explore the data on confirmed COVID-19 cases for all countries.

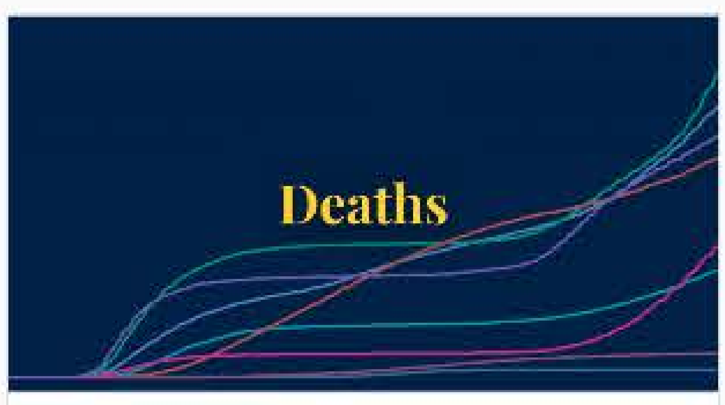

Explore the data on confirmed COVID-19 deaths for all countries.

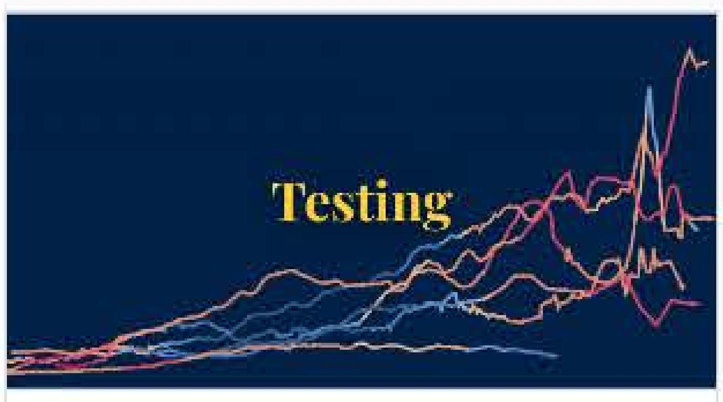

Explore our data on COVID-19 testing to see how confirmed cases compare to actual infections.

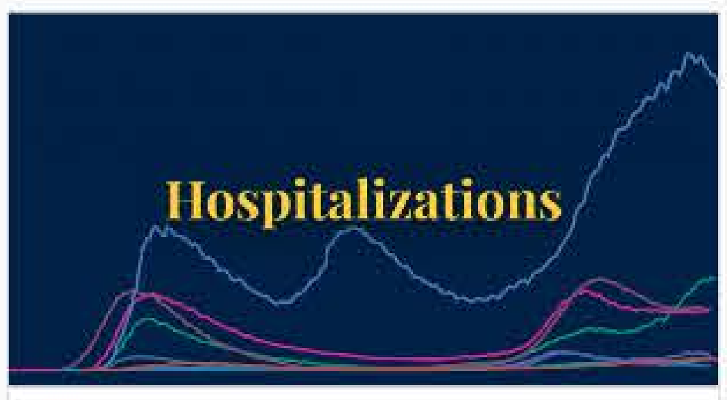

See data on how many people are being hospitalized for COVID-19.

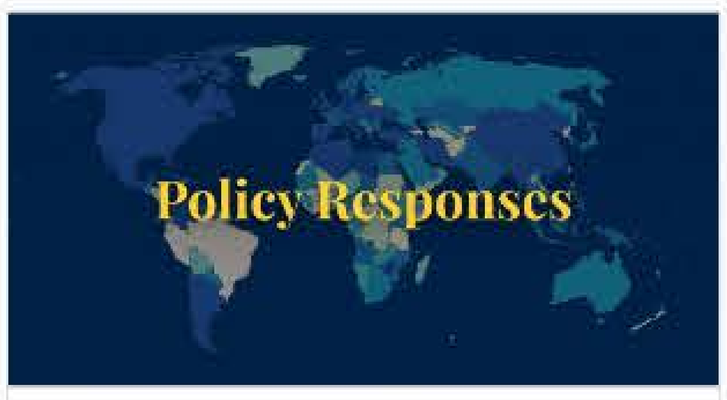

See how government policy responses – on travel, testing, vaccinations, face coverings, and more – vary across the world.

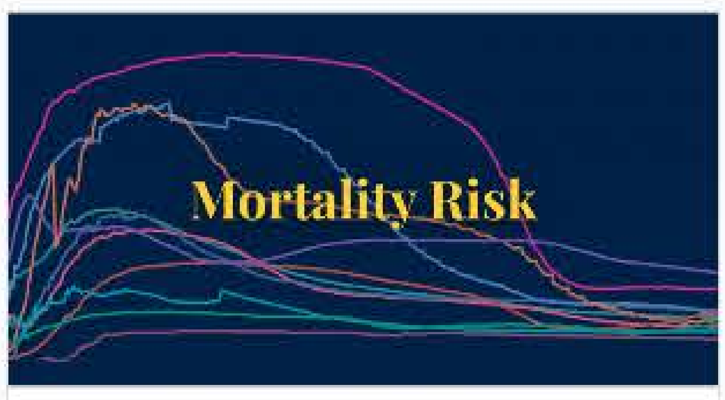

Learn what we know about the mortality risk of COVID-19 and explore the data used to calculate it.

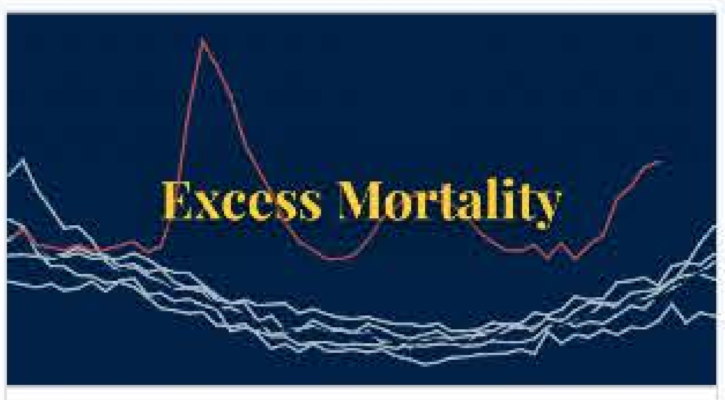

Compare the number of deaths from all causes during COVID-19 to the years before to gauge the total impact of the pandemic on deaths.

How can we make progress against the pandemic?
An overview of the COVID-19 pandemic ›

Explore the global situation

Coronavirus Pandemic
Data Explorer
Download the complete *Our World in Data* COVID-19 dataset.

METRIC: Confirmed cases (selected) · Tests · Confirmed deaths · Share of positive tests · Case fatality rate · Vaccinations
INTERVAL: 7-day rolling average
COUNT: Per million people (checked)
TIMELINE: Align outbreaks — Days since the total confirmed cases per million people reached 1

Type to add a country...

Sort by New cases (per 1M)

☑ United States	154.7
☑ United Kingdom	89.2
☑ Germany	73.0
☑ Canada	62.2
☑ India	11.2
☑ Japan	7.9
☐ Estonia	905.4
☐ Montenegro	799.3
☐ Czechia	736.3
☐ Lithuania	661.9
☐ Malta	595.6
☐ Hungary	462.6
☐ Jordan	450.3
☐ Serbia	412.8
☐ Maldives	362.6
☐ Bahrain	362.0
☐ Palestine	333.8
☐ [illegible]	[illegible]

✕ Clear selection

Daily new confirmed COVID-19 cases per million people

Shown is the rolling 7-day average. The number of confirmed cases is lower than the number of actual cases; the main reason for that is limited testing.

LINEAR LOG

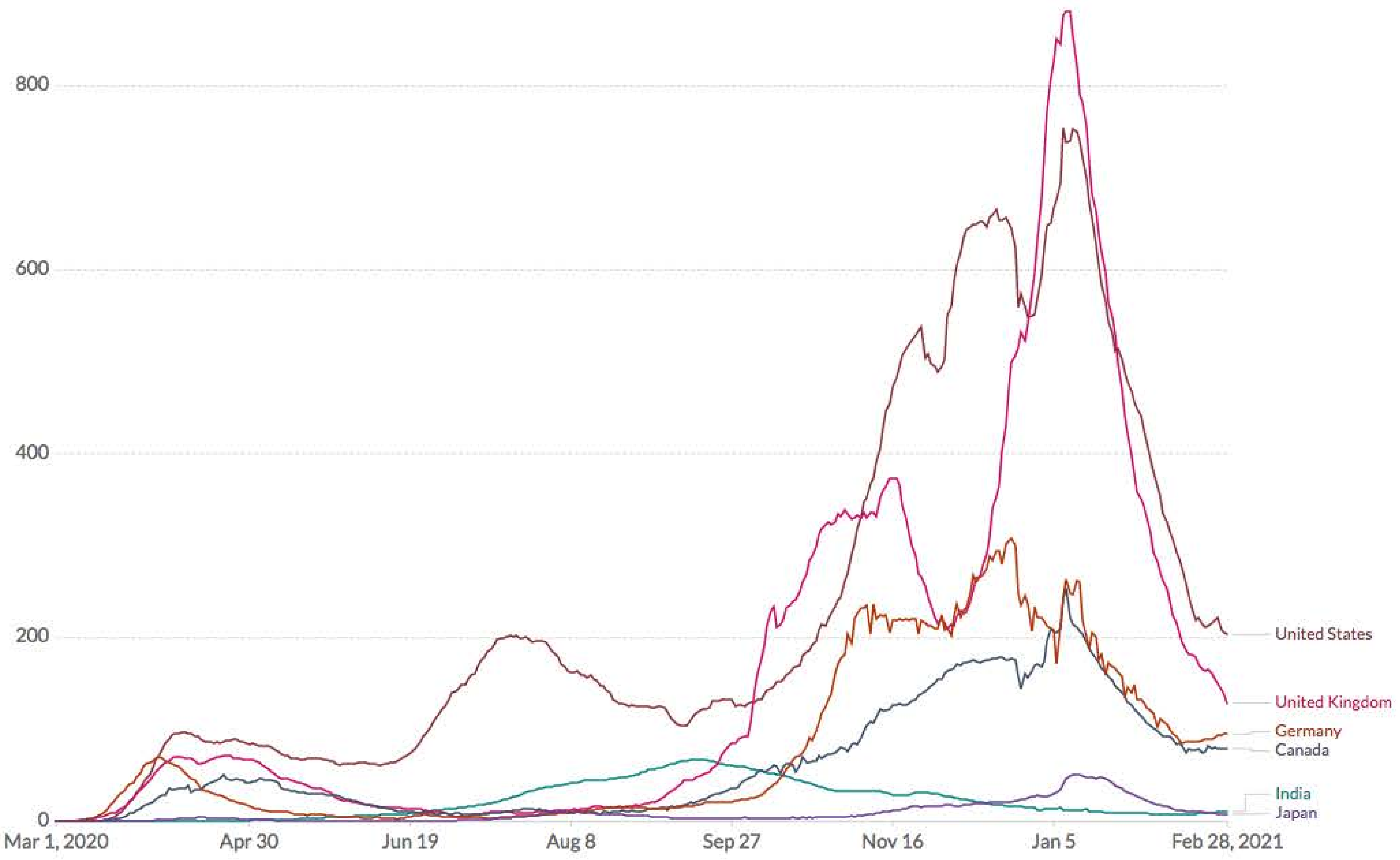

Source: Johns Hopkins University CSSE COVID-19 Data – Last updated 1 March, 06:02 (London time)

CC BY

Jan 23, 2020 — Feb 28, 2021

CHART MAP TABLE SOURCES DOWNLOAD

→ Open the Data Explorer in a new tab.

Explore the global situation

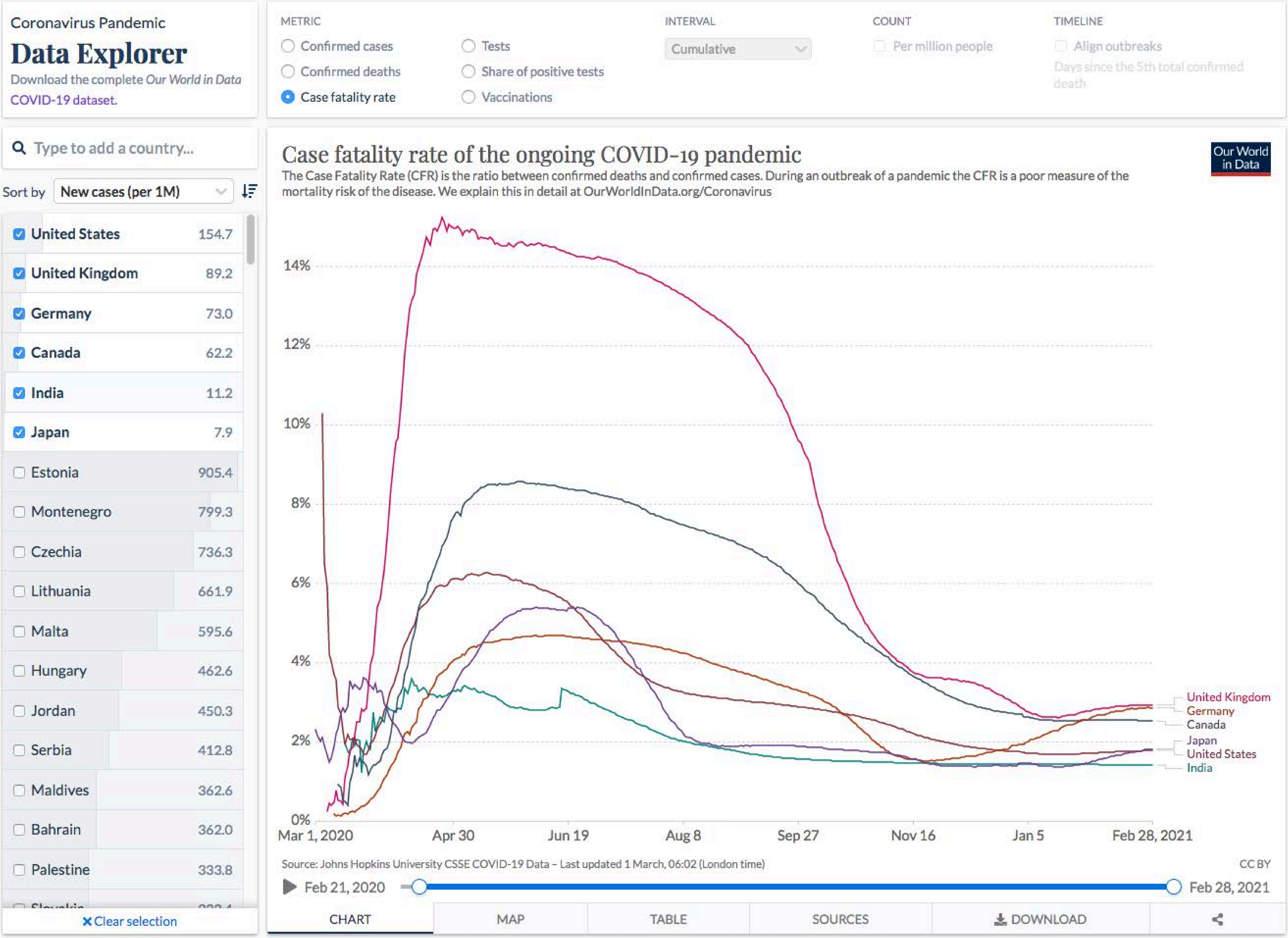

→ Open the Data Explorer in a new tab.

Explore the global situation

Coronavirus Pandemic

Data Explorer

Download the complete *Our World in Data* COVID-19 dataset.

METRIC
- Confirmed cases
- Confirmed deaths
- Case fatality rate
- Tests
- Share of positive tests
- Vaccinations

INTERVAL
7-day rolling average

COUNT
Per 1,000 people

TIMELINE
Align outbreaks
Days since total confirmed deaths reached 0.1 per million

Type to add a country...

Sort by New cases (per 1M)

United States	154.7
United Kingdom	89.2
Germany	73.0
Canada	62.2
India	11.2
Japan	7.9
Estonia	905.4
Montenegro	799.3
Czechia	736.3
Lithuania	661.9
Malta	595.6
Hungary	462.6
Jordan	450.3
Serbia	412.8
Maldives	362.6
Bahrain	362.0
Palestine	333.8
[illegible]	[illegible]

✕ Clear selection

Daily new COVID-19 tests per 1,000 people

Shown is the rolling 7-day average.

LINEAR LOG

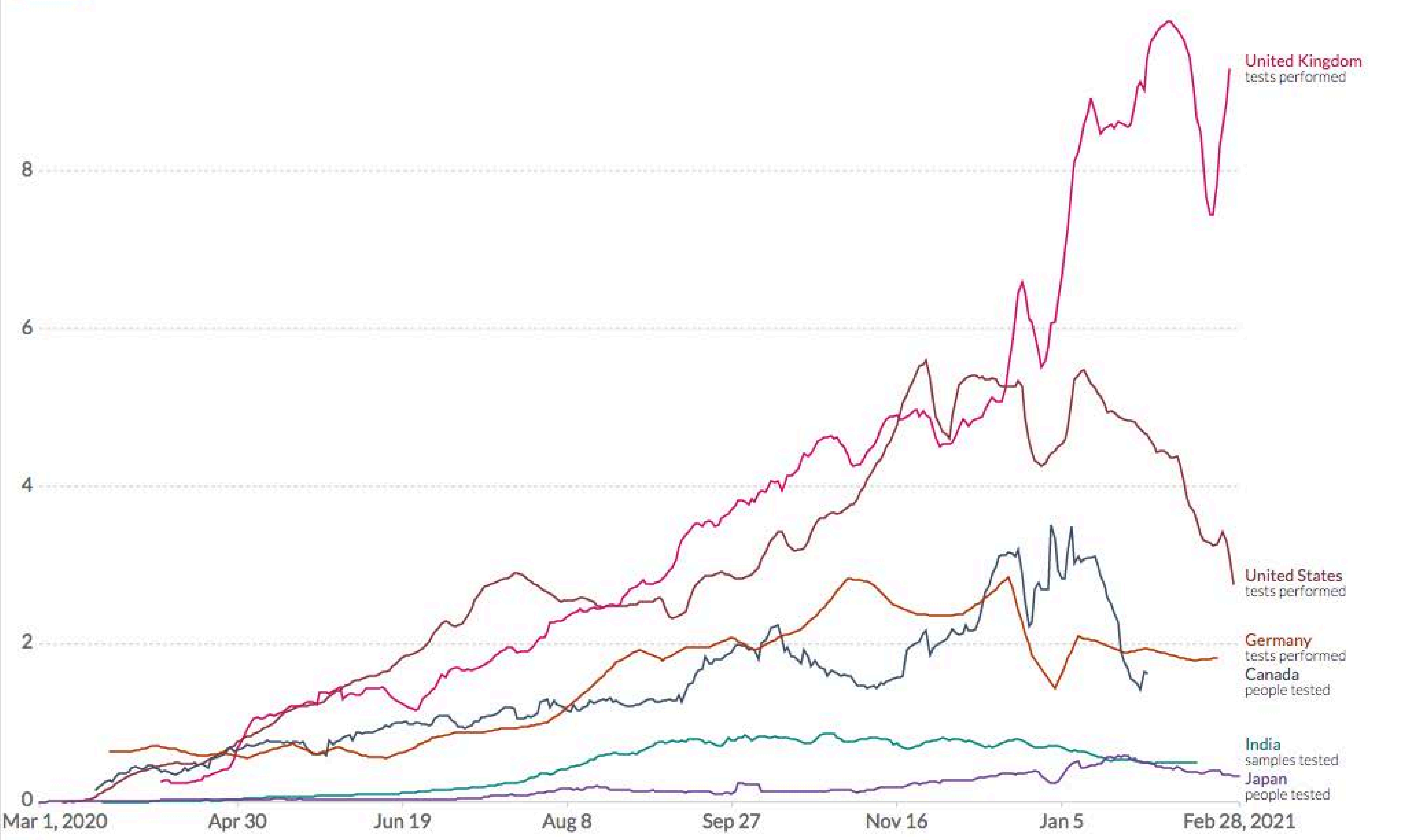

Source: Official data collated by Our World in Data – Last updated 1 March, 16:10 (London time)
Note: For testing figures, there are substantial differences across countries in terms of the units, whether or not all labs are included, the extent to which negative and pending tests are included and other aspects. Details for each country can be found on ourworldindata.org/covid-testing.

CC BY

Feb 12, 2020 — Feb 28, 2021

CHART | MAP | TABLE | SOURCES | DOWNLOAD

→ Open the Data Explorer in a new tab.

Explore the global situation

Coronavirus Pandemic

Data Explorer

Download the complete *Our World in Data* COVID-19 dataset.

METRIC
- Confirmed cases
- Confirmed deaths
- Case fatality rate
- Tests
- Share of positive tests
- Vaccinations

INTERVAL
7-day rolling average

COUNT
Per 100 people

TIMELINE
Align outbreaks
Days since total confirmed deaths reached 0.1 per million

Type to add a country...

Sort by New cases (per 1M)

Country	Value
United States	154.7
United Kingdom	89.2
Germany	73.0
Canada	62.2
India	11.2
Japan	7.9
Estonia	905.4
Montenegro	799.3
Czechia	736.3
Lithuania	661.9
Malta	595.6
Hungary	462.6
Jordan	450.3
Serbia	412.8
Maldives	362.6
Bahrain	362.0
Palestine	333.8

Clear selection

Daily new COVID-19 vaccination doses administered per 100 people

Shown is the rolling 7-day average. This is counted as a single dose, and may not equal the total number of people vaccinated, depending on the specific dose regime (e.g. people receive multiple doses).

Our World in Data

LINEAR LOG

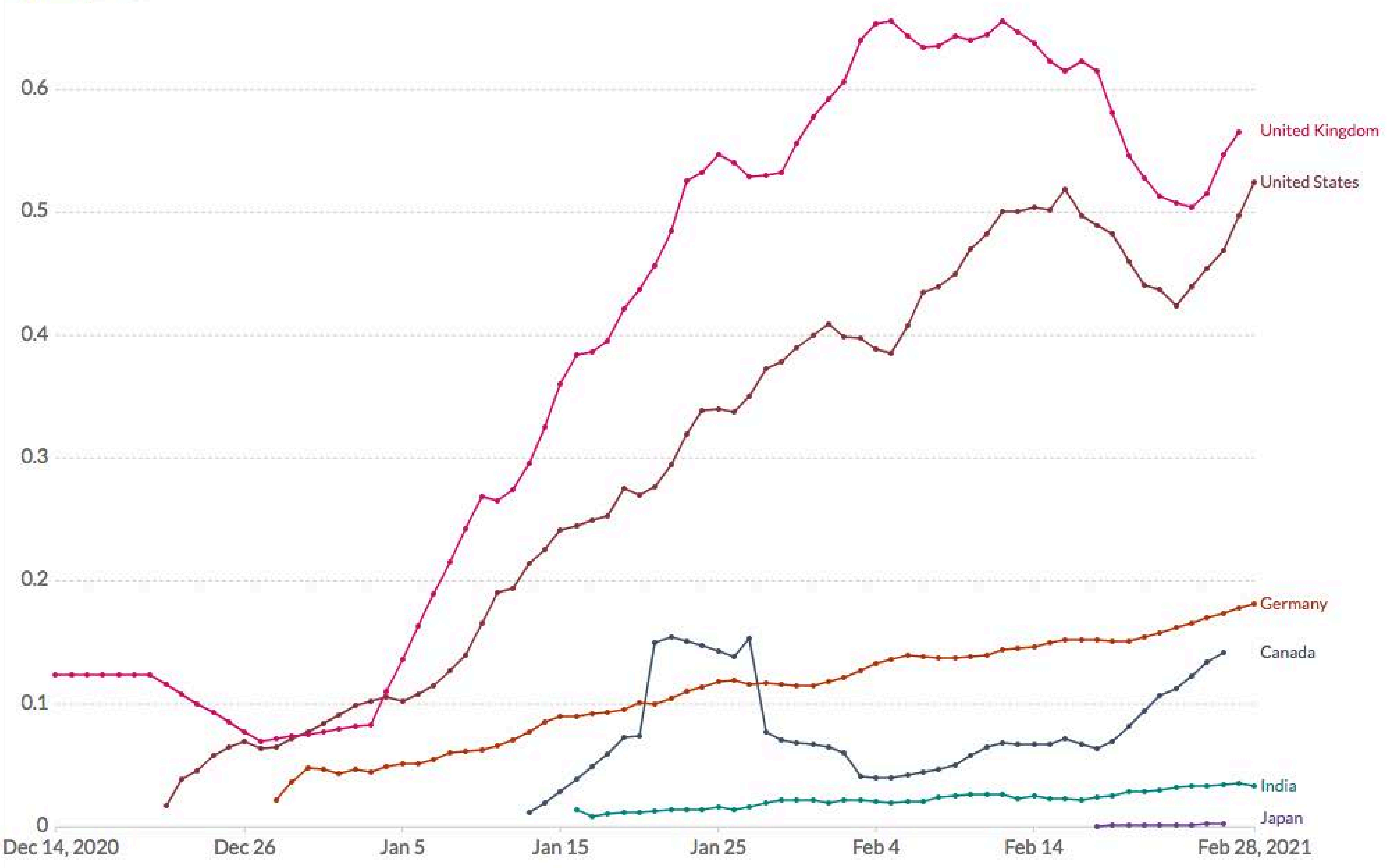

Source: Official data collated by Our World in Data – Last updated 1 March, 11:10 (London time)

CC BY

Dec 14, 2020 — Feb 28, 2021

CHART | MAP | TABLE | SOURCES | DOWNLOAD

→ Open the Data Explorer in a new tab.

⇌ 中文版

DXY · DX Doctor

COVID-19
Global Pandemic Real-time Report

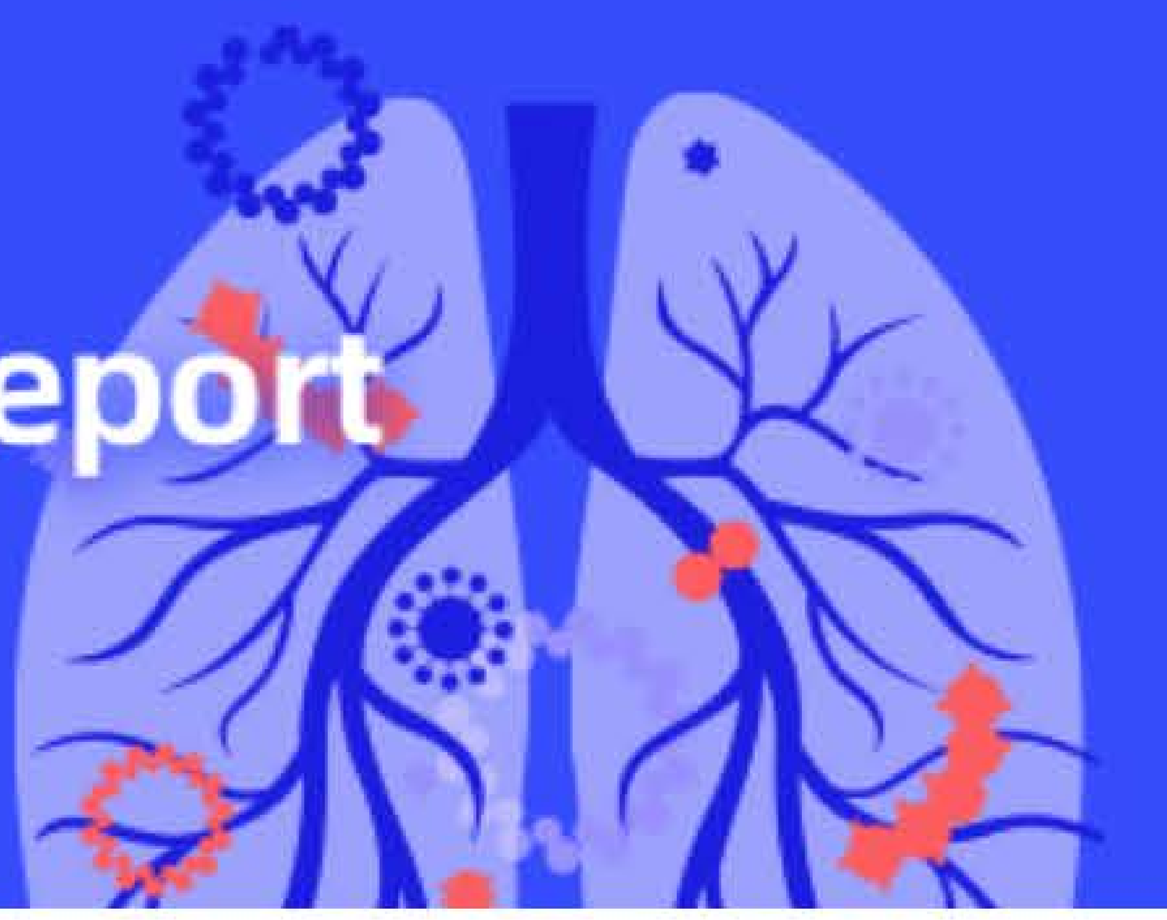

4,509,496,104 Views

Global statistics by UTC+8 2021-03-01 19:09

Data explanations

Active Cases	Total Confirmed	Deaths	Recovered
New +2329	New +237965	New +3980	New +231656
27,960,407	114,094,994	2,532,030	83,602,557

COVID-19 Global Pandemic Trend

Before Mar. 19, data source: WHO. Time zone: CET 10 AM.
After Mar. 19, data source: WHO, JHU CSSE, Local Media and DXY. Time zone: UTC 10 AM.
Since April 24th, the Spanish Ministry of Health has adjusted the statistical standard of confirmed Covid-19 data, only counting those confirmed by PCR tests.

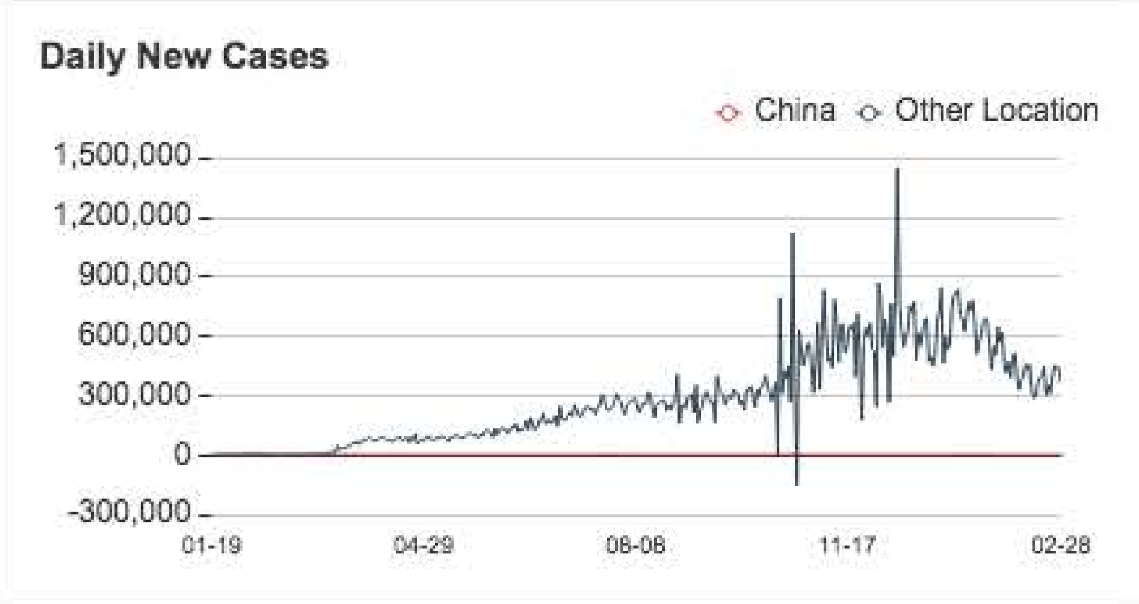

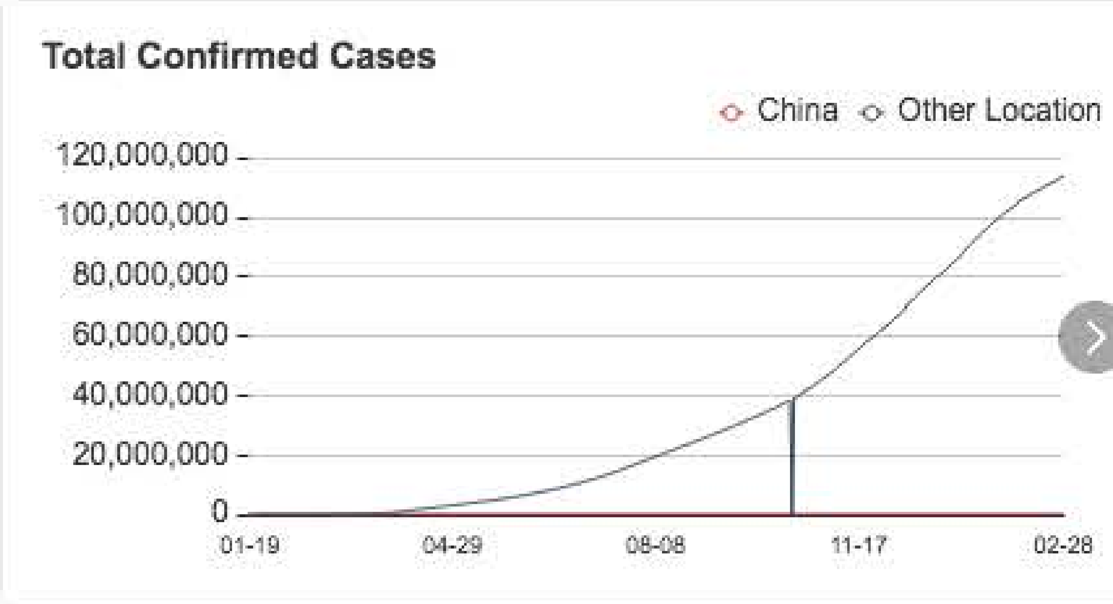

Daily New Cases | Total Confirmed Cases | Total Deaths Cases

Global Mapping | COVID-19 Knowledge

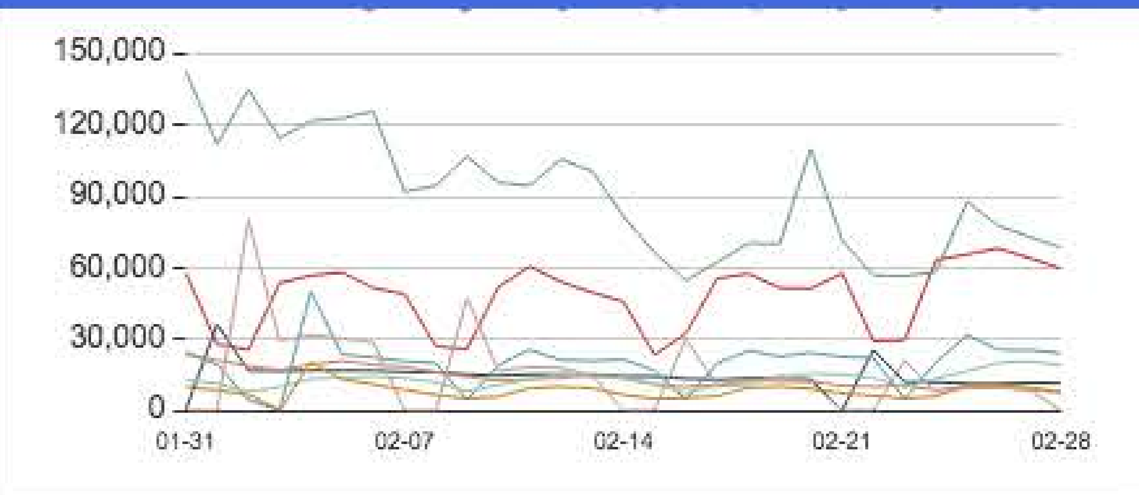

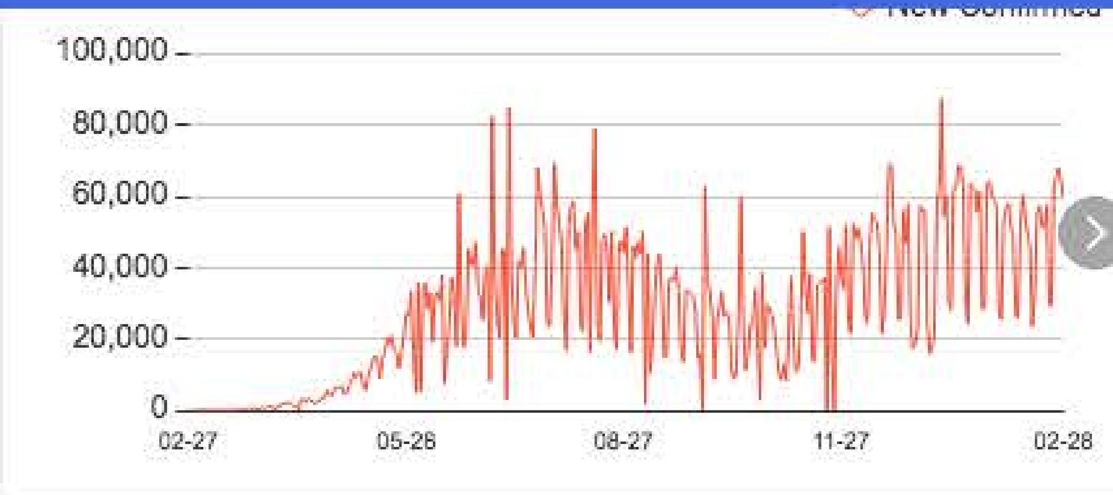

Daily New Cases | Brazil | Russian Federation | France
The United Kingdom | Italy | United States of America | Germany
Spain

Country/Region/ Territory	Active Cases	Total Confirmed	Total Deaths	Total Recovered	More
▼ **Europe(49)**	**16,022,701**	**34,209,105**	**814,211**	**17,372,193**	
The United Kingdom	4,047,279	4,170,523	122,705	-	More ›
France	3,408,365	3,755,968	86,454	-	More ›
Spain	2,969,035	3,188,553	69,142	150,376	More ›
Netherlands	1,061,241	1,088,690	15,563	-	More ›
Belgium	728,123	769,414	22,052	19,239	More ›
Show more ⌄					
▼ **North America(40)**	**9,025,769**	**33,013,252**	**748,792**	**23,238,691**	
United States of America	8,398,233	28,605,661	513,091	19,694,337	More ›
Mexico	268,869	2,084,128	185,257	1,630,002	More ›
Honduras	99,166	169,754	4,141	66,447	More ›
Puerto Rico	98,012	100,044	2,032	0	More ›
Dominican Republic	45,179	239,617	3,100	191,338	More ›
Show more ⌄					
▼ **South America(15)**	**1,276,975**	**17,984,514**	**466,641**	**16,240,898**	
Brazil	885,208	10,551,259	255,018	9,411,033	More ›
Argentina	150,364	2,107,312	51,965	1,904,983	More ›

COVID-19 CORONAVIRUS PANDEMIC

Last updated: March 01, 2021, 17:05 GMT

Graphs - Countries - News

Coronavirus Cases:

114,822,646

view by country

Deaths:

2,545,435

Recovered:

90,407,075

ACTIVE CASES

21,870,136

Currently Infected Patients

21,779,719 (99.6%) in Mild Condition

90,417 (0.4%) Serious or Critical

Show Graph

CLOSED CASES

92,952,510

Cases which had an outcome:

90,407,075 (97%) Recovered / Discharged

2,545,435 (3%) Deaths

Show Graph

daily linear logarithmic

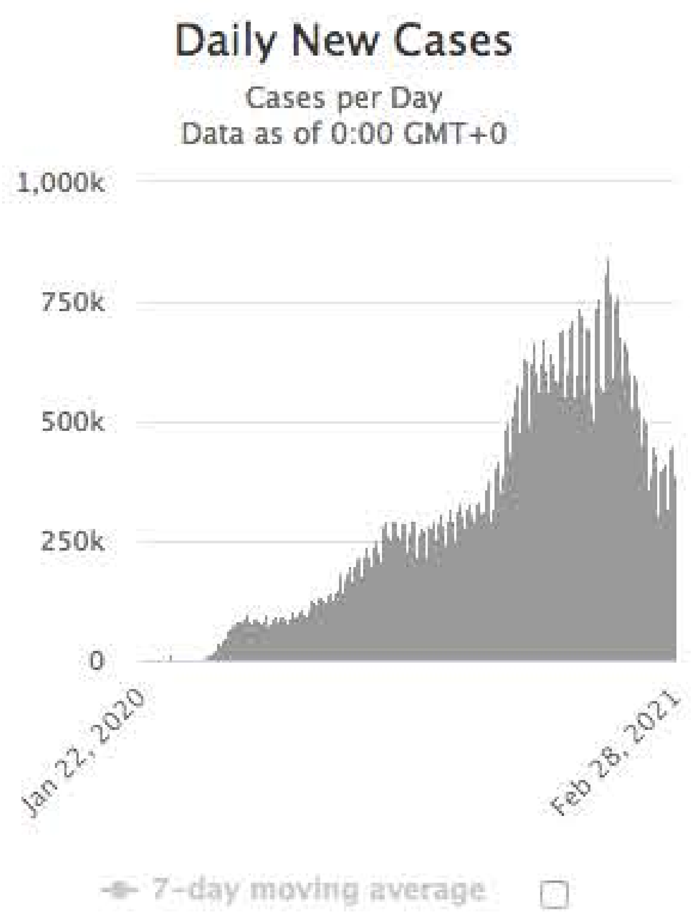

daily linear logarithmic

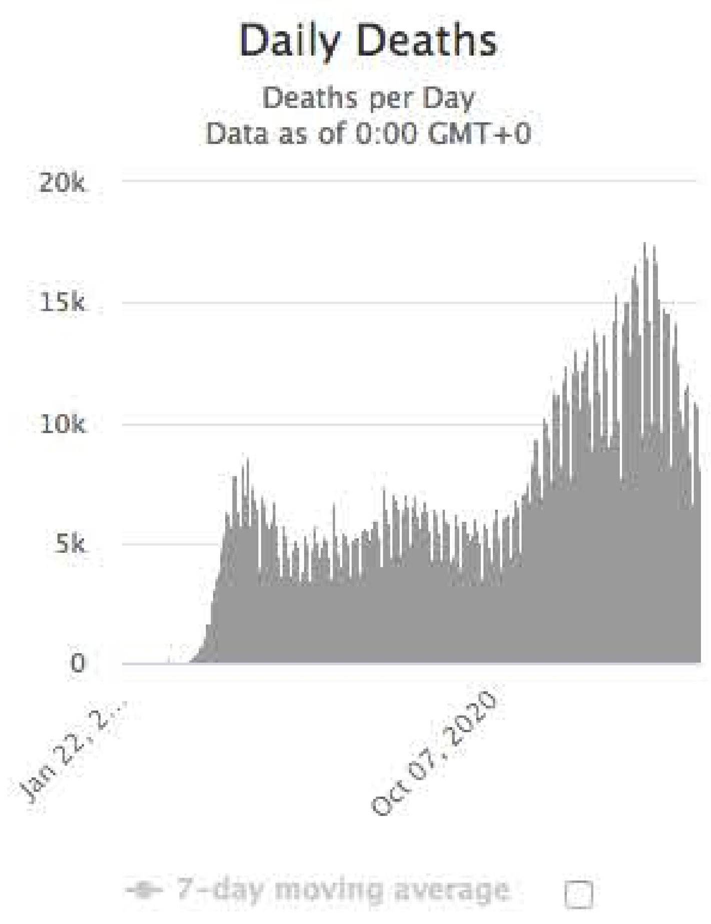

The charts above are updated after the close of the day in GMT+0. See more graphs

Reported Cases and Deaths by Country or Territory

The **coronavirus** COVID-19 is affecting **219 countries and territories. The day is reset after midnight GMT+0**. The list of countries and their regional classification is based on the United Nations Geoscheme. Sources are provided under "Latest News." **Learn more about Worldometer's COVID-19 data**

Report coronavirus cases

Now Yesterday 2 Days Ago Columns ▾ Search:

All Europe North America Asia South America Africa Oceania

#	Country, Other	Total Cases	New Cases	Total Deaths	New Deaths	Total Recovered	Active Cases	Serious, Critical	Tot Cases/ 1M pop	Deaths/ 1M pop	Total Tests	Tests/ 1M pop	Pop
	World	114,822,646	+141,300	2,545,435	+2,608	90,407,075	21,870,136	90,417	14,731	326.6			
1	USA	29,259,111	+3,746	525,795	+17	19,694,344	9,038,972	14,781	88,053	1,582	359,045,212	1,080,522	33
2	India	11,122,986	+10,930	157,257	+62	10,796,164	169,565	8,944	8,008	113	216,858,774	156,129	1,38
3	Brazil	10,551,259		255,018		9,411,033	885,208	8,318	49,407	1,194	28,600,000	133,920	21
4	Russia	4,257,650	+11,571	86,455	+333	3,823,074	348,121	2,300	29,167	592	111,300,000	762,454	14
5	UK	4,182,009	+5,455	122,953	+104	2,959,884	1,099,172	1,971	61,390	1,805	90,739,196	1,332,004	6
6	France	3,755,968		86,454		254,868	3,414,646	3,430	57,458	1,323	51,853,607	793,240	6
7	Spain	3,188,553		69,142		2,647,446	471,965	3,021	68,180	1,478	39,313,189	840,622	4
8	Italy	2,938,371	+13,114	97,945	+246	2,416,093	424,333	2,289	48,646	1,622	40,303,520	667,247	6
9	Turkey	2,701,588		28,569		2,572,234	100,785	1,191	31,806	336	33,175,016	390,575	8
10	Germany	2,452,378	+2,016	70,769	+82	2,255,500	126,109	2,848	29,208	843	43,950,029	523,454	8

COVID-19 Tracker

Search the web with Bing

Related Searches: coronavirus testing prevention treatment symptoms work from home jobs

Give with Bing

Track vaccination progress

Total confirmed cases

Updated 18 min ago

114,067,962 +203,947

- Active cases 47,118,777
- Recovered cases 64,418,462 +109,997
- Fatal cases 2,530,723 +3,930

Browse Sort

Filter to a location

Global	114,067,962
United States	28,897,871
California	3,568,991
Texas	2,653,285
Florida	1,909,221
New York	1,644,124
Illinois	1,189,922
Georgia	1,006,521
Ohio	967,422
Pennsylvania	935,911
North Carolina	862,635
Arizona	817,232
New Jersey	789,356

United States

Cases | Vaccines NEW | News & Videos | Graphs

Overview

28,897,871 +64,832

- Active cases 28,379,151
- Recovered cases -
- Fatal cases 518,720 +1,516

Trends

Daily spread trends Expand

New cases

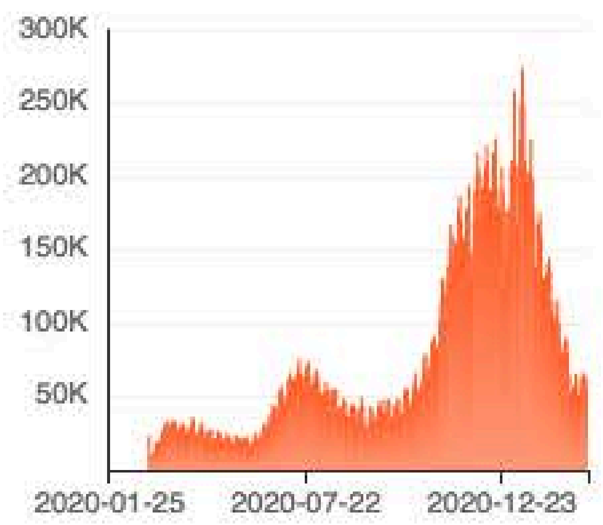

See more charts

News

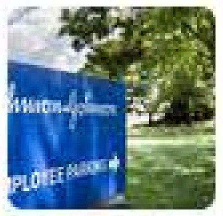

J&J looks for partners to ramp up supply of COVID-19 vaccine

Bloomberg · 25m

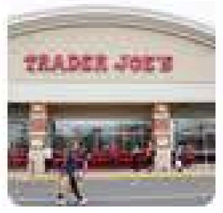

Trader Joe's employee says he was fired for requesting better Covid

The Guardian · 47m

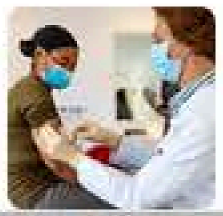

Here's Everything We Know About the Side Effects of the COVID-19

PopSugar · 2h

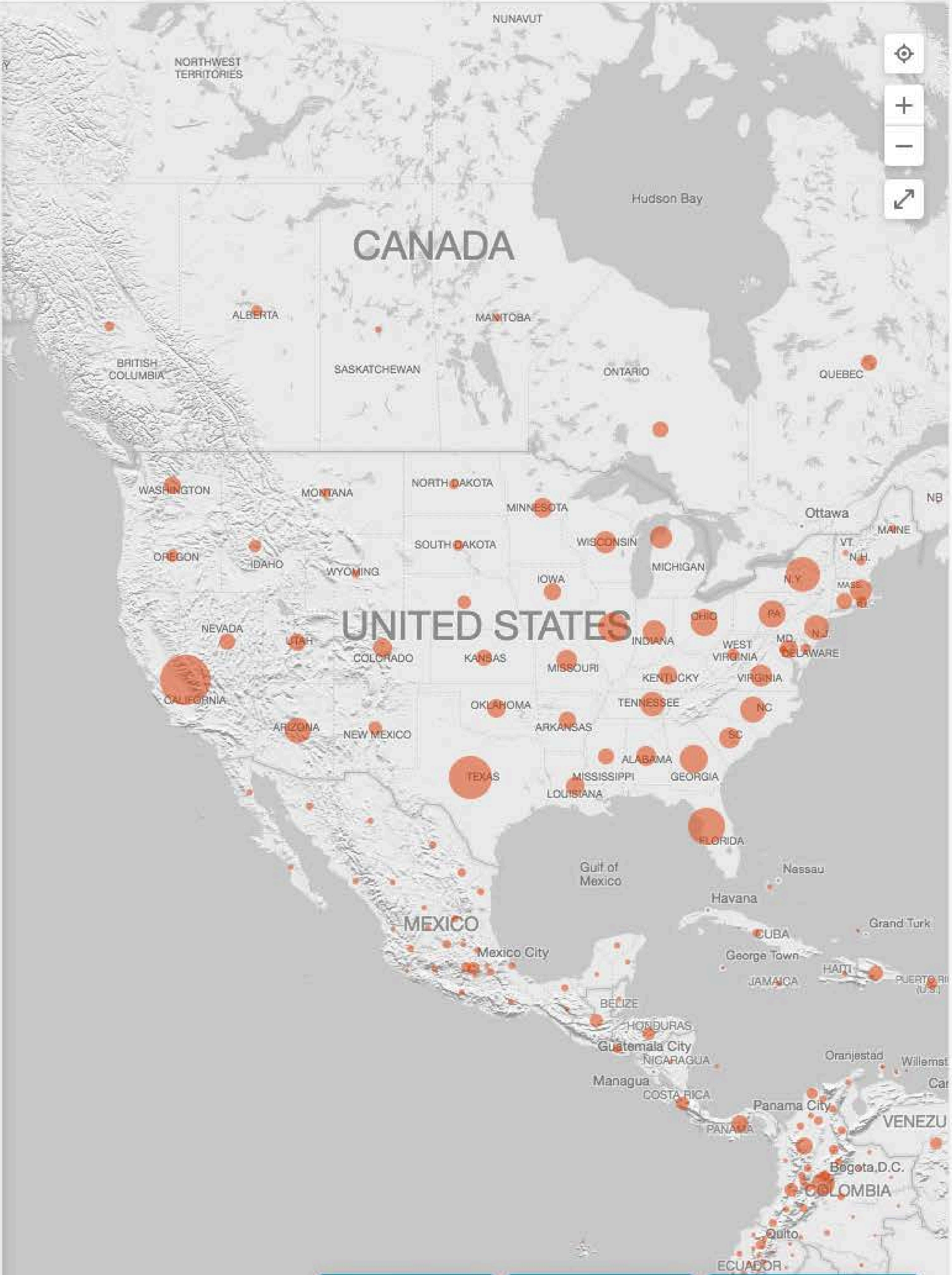

COVID-19 Timeline | Track with extension | Ask about COVID-19

© 2021 TomTom, © 2021 Microsoft Corporation Terms

I have donated ✕

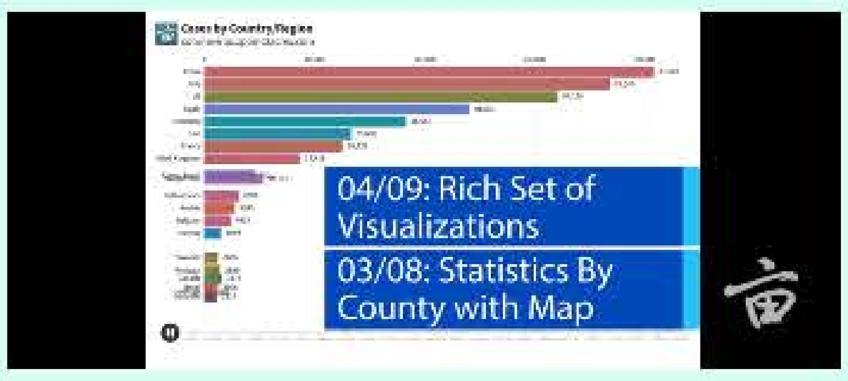

DEAR USERS: We created this website to increase awareness and bring transparency to the public, and we are glad many of you frequented our tracker and found it useful. CDC and JHU COVID-19 World Map etc. use our data, free of charge. However, without any funds, we pay all expenses out of pocket to keep this high traffic website alive and have reached a point that it's not sustainable anymore. If 10% of all users reading this is willing to donate us a boba tea, we would be able to retain our employees and provide FREE information for as long as needed. **Please help**

Our paper has been accepted for presentation at BIOKDD 2020 on Aug 24, 2020. Check it here.

Case Summary

Last updated at : 2021-03-01 12:00 EDT

Data source

Location	Cases	Deaths	Hospitalized	Test Pos% ⓘ
United States	+6,663<br>29,089,396	+295<br>521,208	-1,519<br>47,352	-0.2%<br>4.6%
World	+283,244<br>114,067,979	+5,315<br>2,530,716		

Check out other countries and regions Click to view more

Vaccine Summary

Data source

Location	Vaccine doses administered	People vaccinated (one dose)	People vaccinated (two doses)
United States	+1,914,219<br>69,440,058	+5,388<br>20,413,688	+863,247<br>19,903,423

(Some states are not reporting data related to people vaccinated.)

Filter the graph by selecting a state from the map below

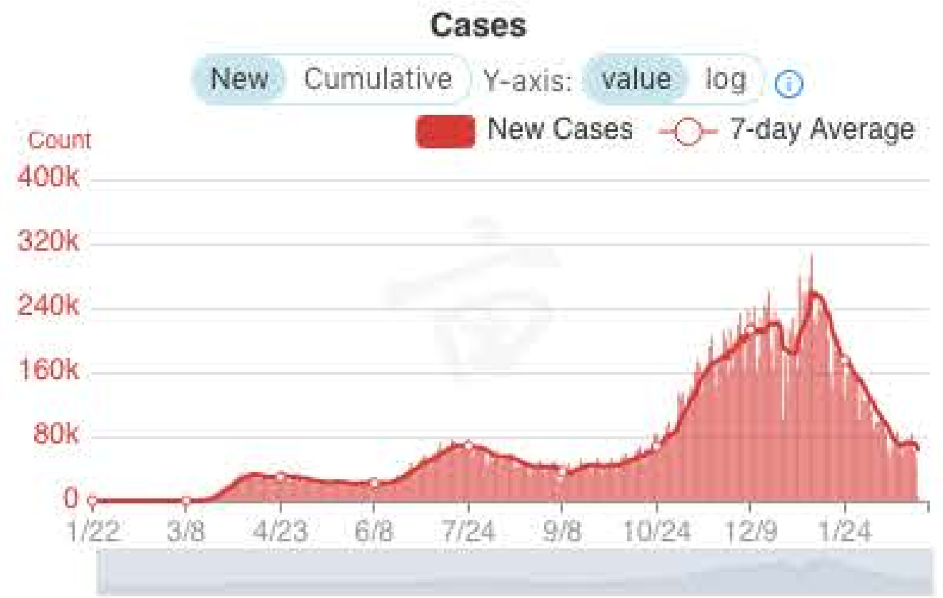

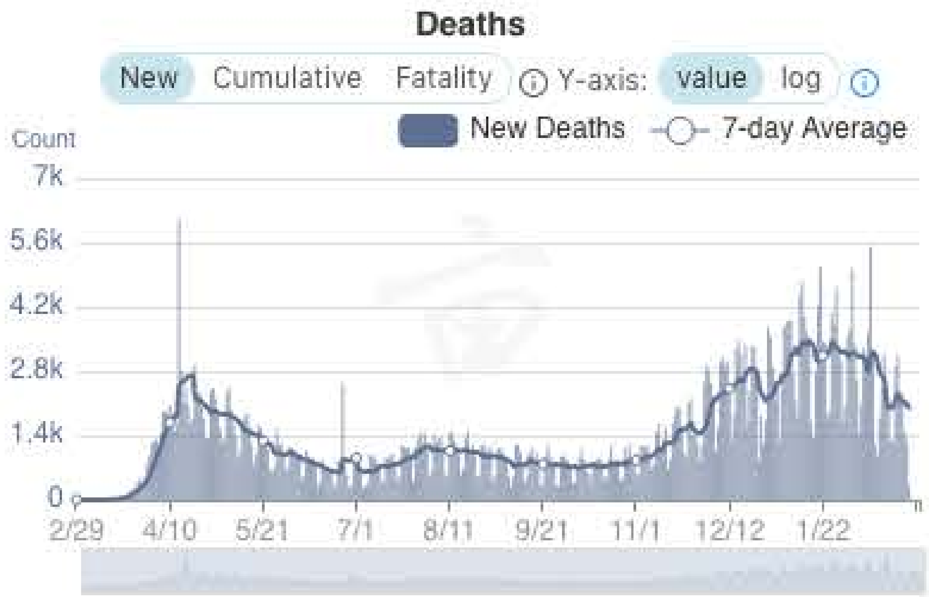

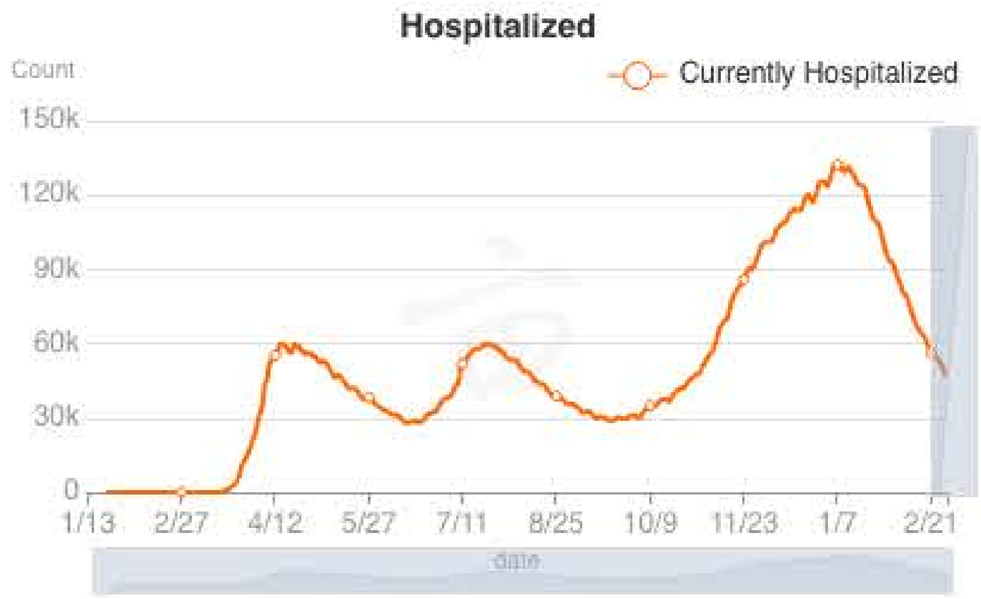

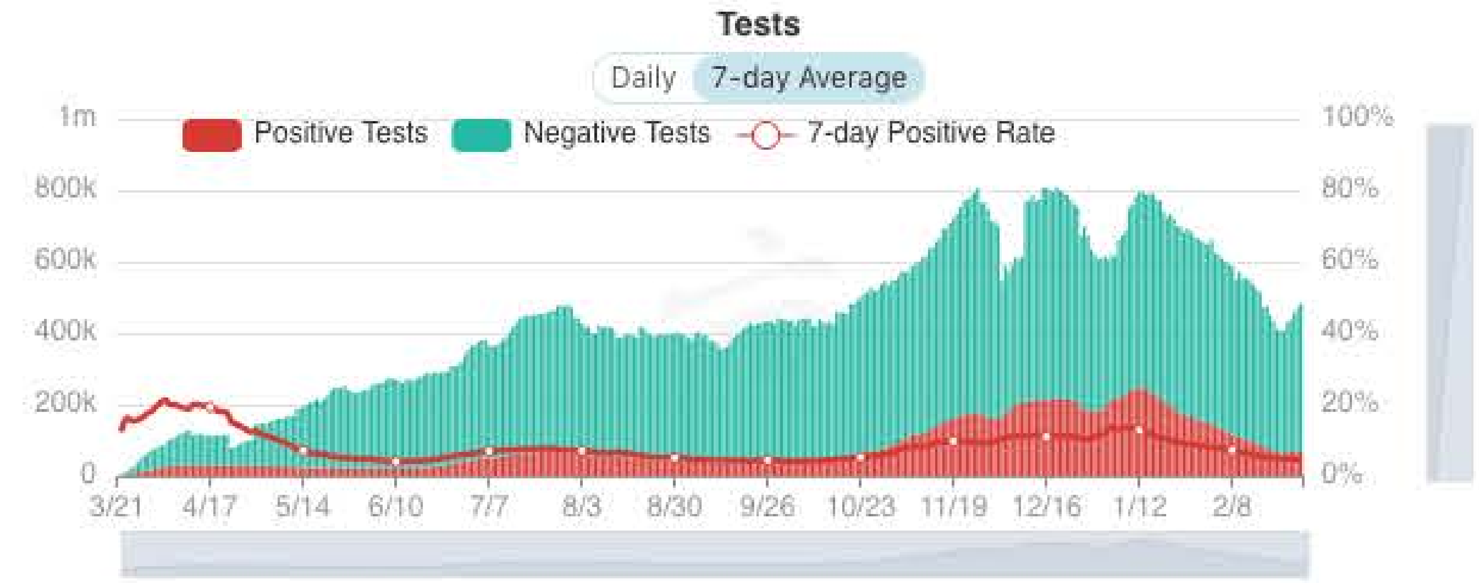

Case Map

Report Error

New Cases | Total Cases | Vaccine Distribution | Vaccination | Tests | Pop | Cases/M | Tests/M | Deaths/10M

Home Testing World News Job Life PPE About Get Data Support Us

Summary Map Cases News Supplies Trends

Viewing the data of: United States

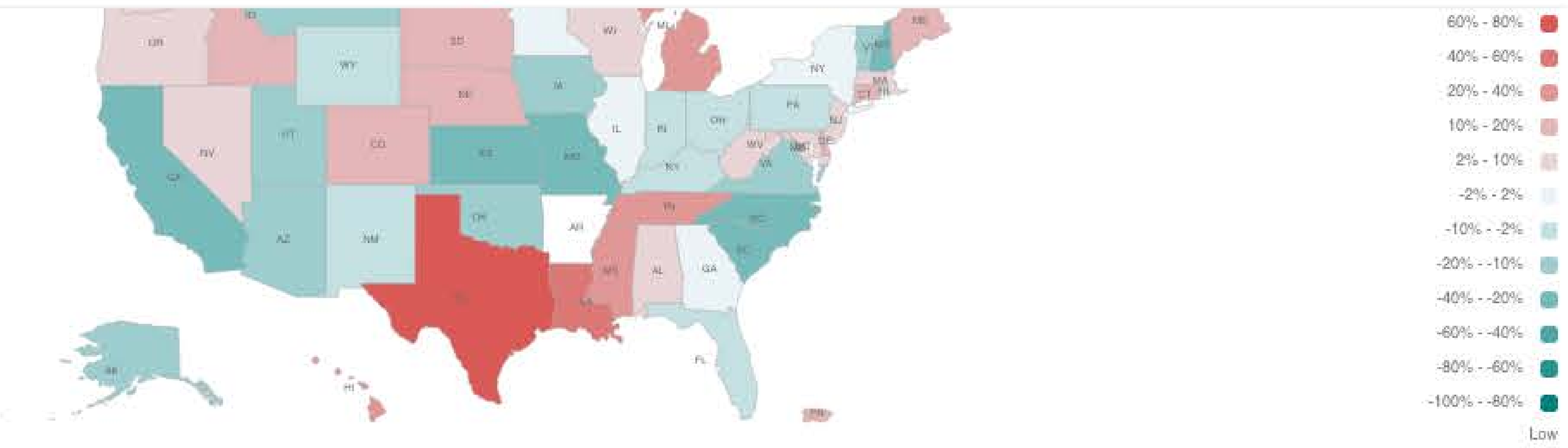

Filter cases by clicking or zooming the map.
Repatriation/Cruise ship cases are separated from TX/CA/NE cases count, consistent with CDC

US Cases

Sort by: Cases

Click here to check state level testing data/location

Location	Cases	Deaths	Fatality	7-Day New Cases ⓘ	Vaccine doses administered
United States<br>CDC	+6,663<br>29,089,396	+295<br>521,208	1.8%	487,694<br>2%	
▸ California	+585<br>3,558,844	+19<br>52,215	1.5%	39,719<br>-29.5%	+239,542<br>8,003,210
▸ Texas	+912<br>2,653,427	+9<br>42,972	1.6%	52,336<br>+63.8%	+177,190<br>4,864,976
▸ Florida	1,909,222	30,853	1.6%	40,450<br>-2.3%	+93,469<br>4,378,096
▸ New York	1,644,522	46,960	2.9%	52,035<br>+0.9%	3,985,558
▸ Illinois	1,186,703	20,519	1.7%	12,287<br>-0.1%	+130,021<br>2,440,950

Trends

Cases | Mobility | Employment

Cases | Death | Total | Daily new per million people | US | Canada | World

Coronavirus Case Trajectories in US

You can Customize/ScreenShot, then share with your friends!

AK AL AR AZ CA CO CT DC DE FL GA HI IA ID IL IN KS KY LA MA MD ME MI MN MO MS MT NC ND NE NH NJ NM NV NY OH OK OR PA PR RI SC SD TN TX UT VA VI VT WA WI WV WY

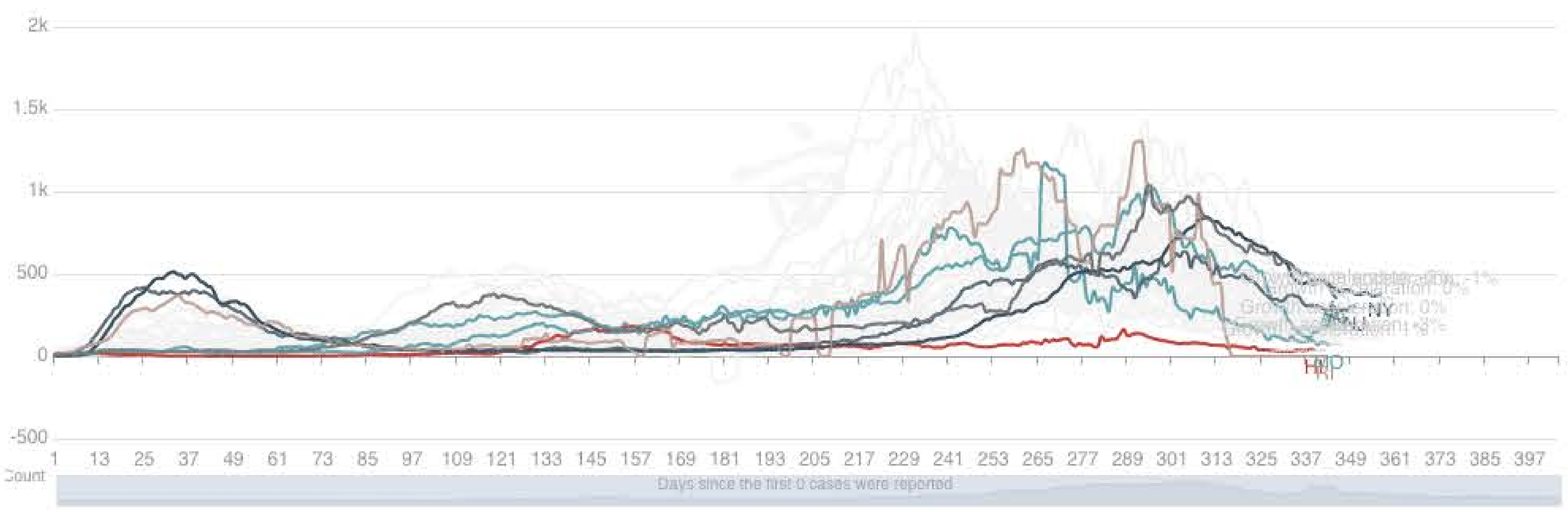

Counting each region from the first day when 100+ cases were reported. China/Hubei started on the 4 days after the first 400-500 cases were reported to align the graph.
Data based on Johns Hopkins University COVID-19 dataset. Inspired by Coronavirus tracked: the latest figures as the pandemic spreads

value | log | New cases based on average of recent | 1 day | 3 days | 5 days | 7 days

Coronavirus Case Trajectories in US

You can Customize/ScreenShot, then share with your friends!

AK AL AR AZ BOP CA CO CT DC DE Diamond Princess FL GA GU Grand Princess HI IA ID IL IN KS KY LA MA MD ME MI MN MO MP MS MT Military NC ND NE NH NJ NM NV NY OH OK OR PA PR RI SC SD TN TX US UT Unassigned VA VI VT Veteran Affair WA WI WV WY Wuhan Evacuee

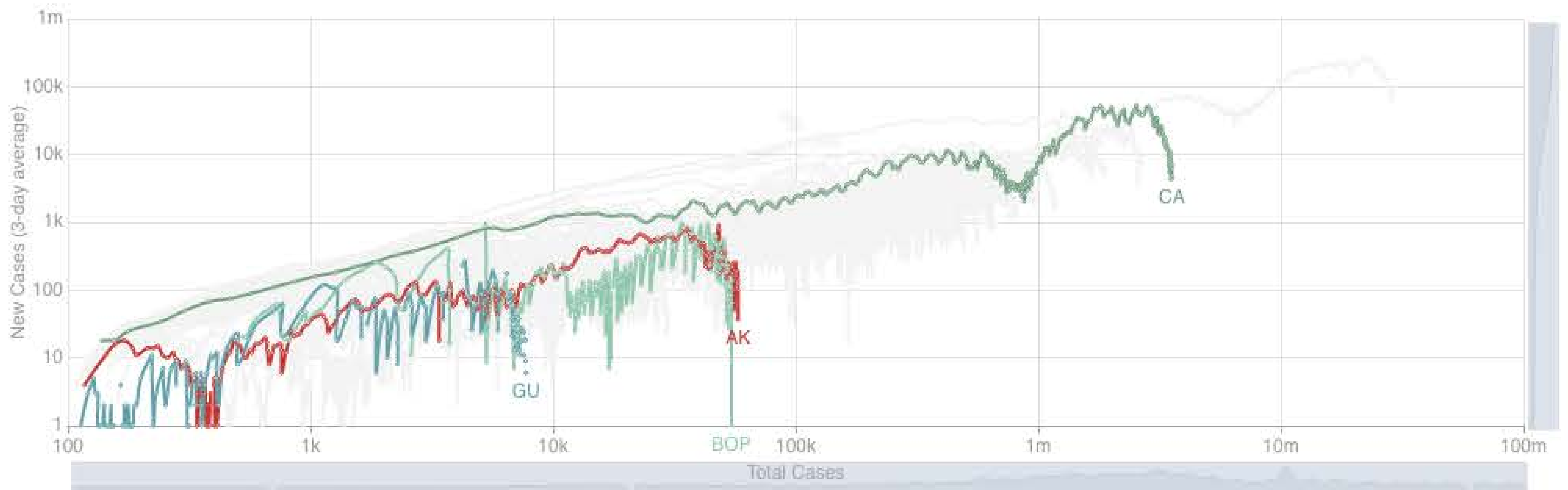

How to read this graph Inspired by https://aatishb.com/covidtrends/

Cases by Country/Region

coronavirus.1point3acres.com/en

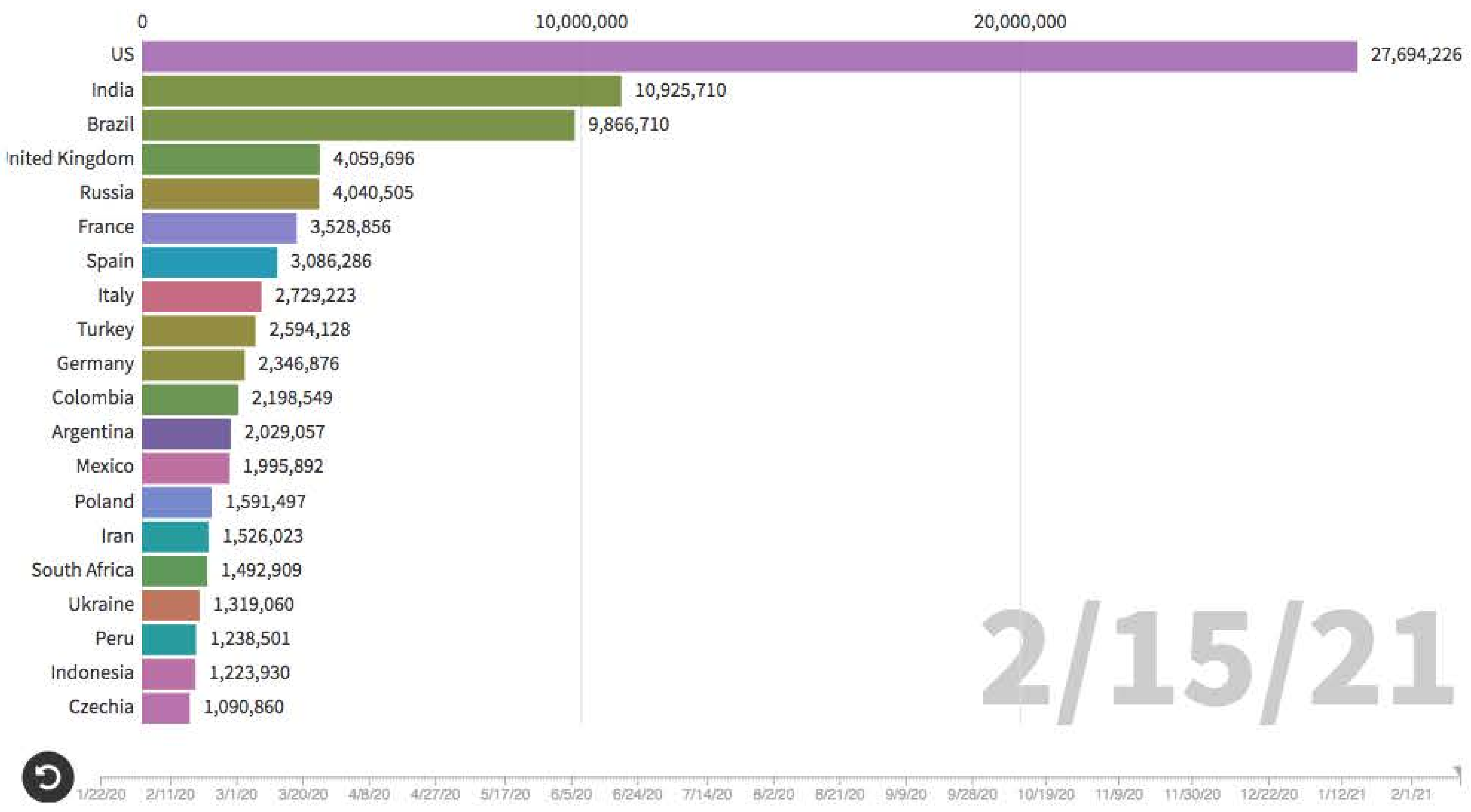

Source: Johns Hopkins University (JHU)

A Flourish bar chart race

Active Cases by Country/Region

coronavirus.1point3acres.com/en

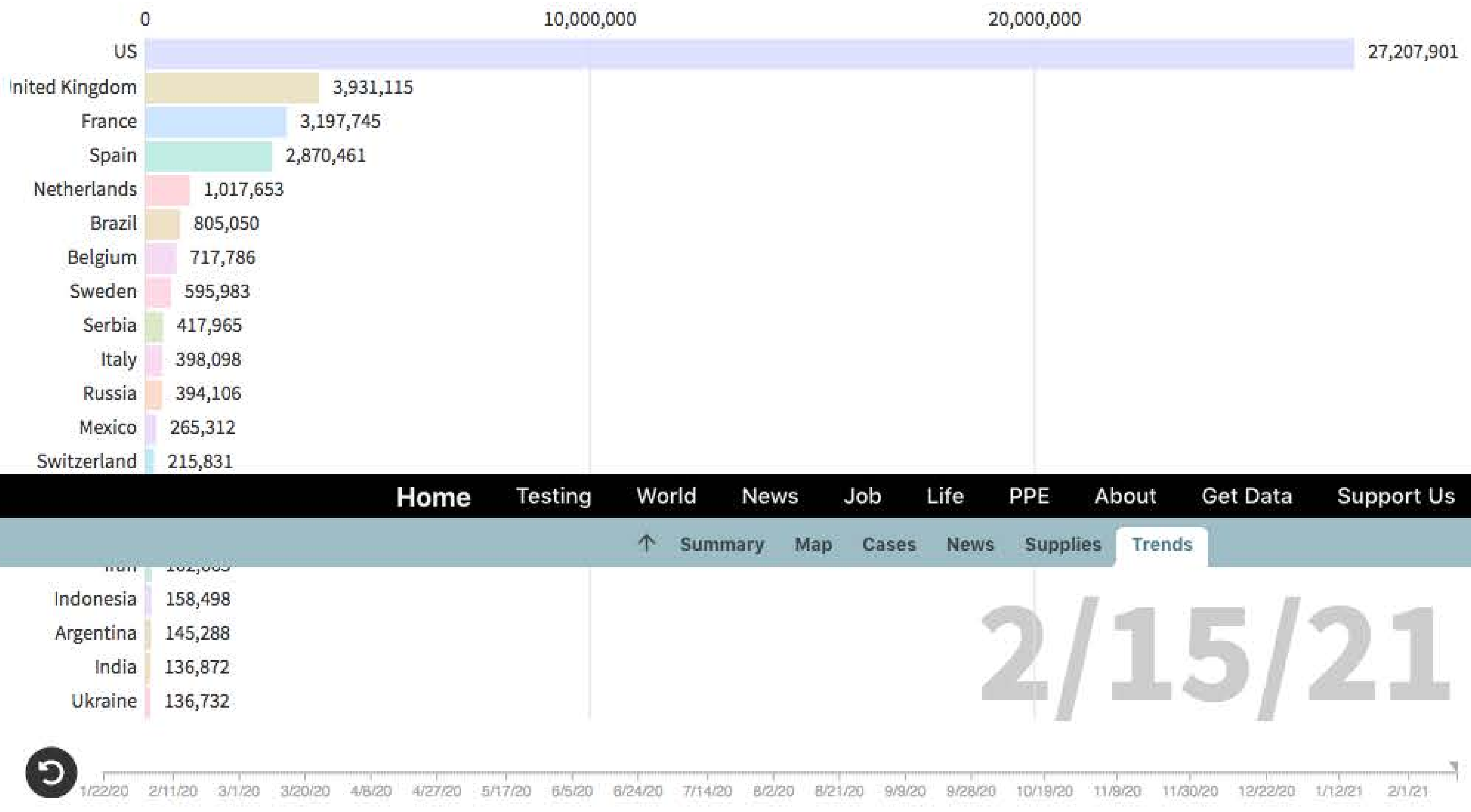

Home | Testing | World | News | Job | Life | PPE | About | Get Data | Support Us

Summary | Map | Cases | News | Supplies | Trends

Source: Johns Hopkins University (JHU) • Active Cases = Total Cases - Deaths - Recovery

A Flourish bar chart race

Cases by State in US

coronavirus.1point3acres.com/en

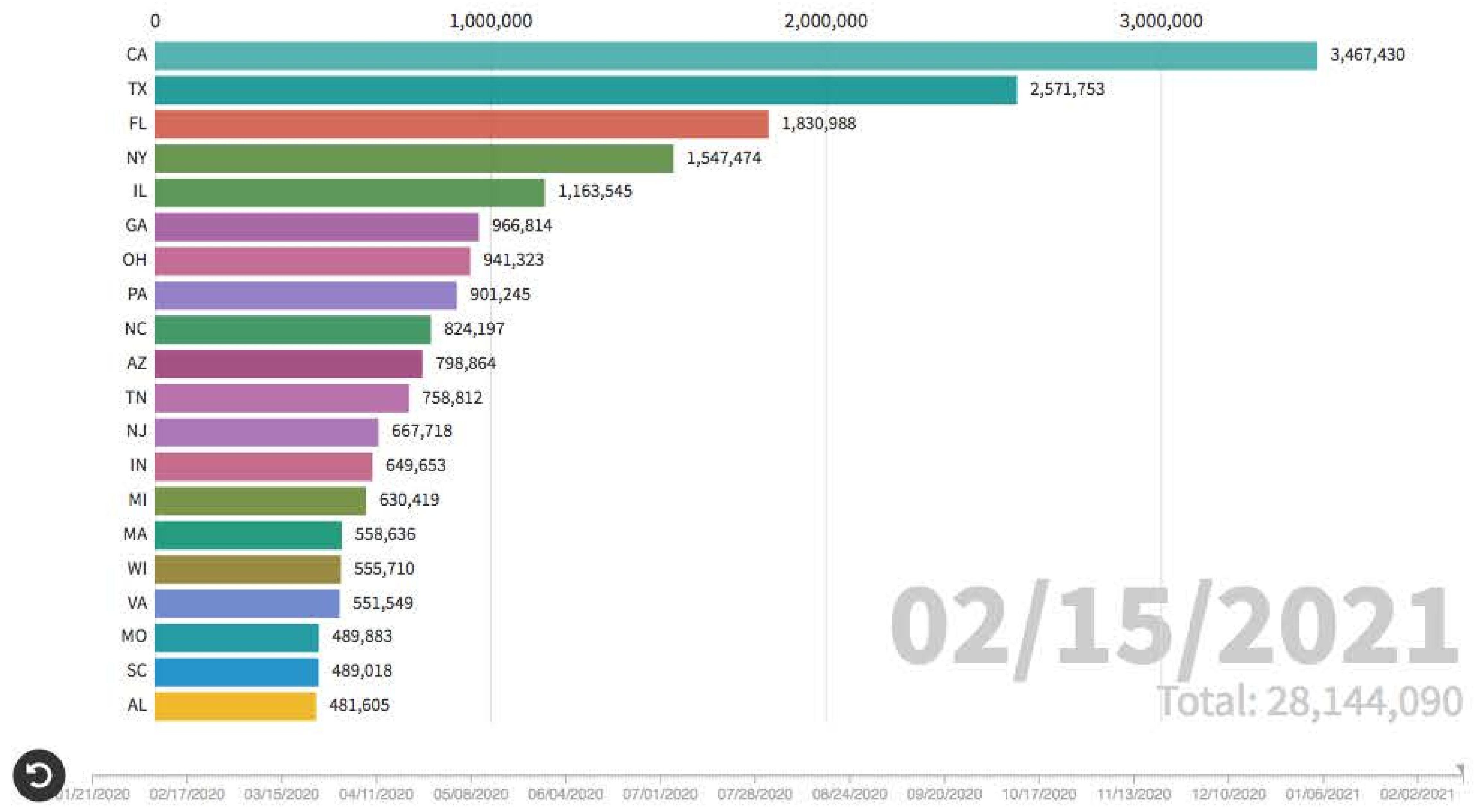

Source: 1point3acres • Data thanks to the excellent work from the 1point3acres team. Please visit them and donate.

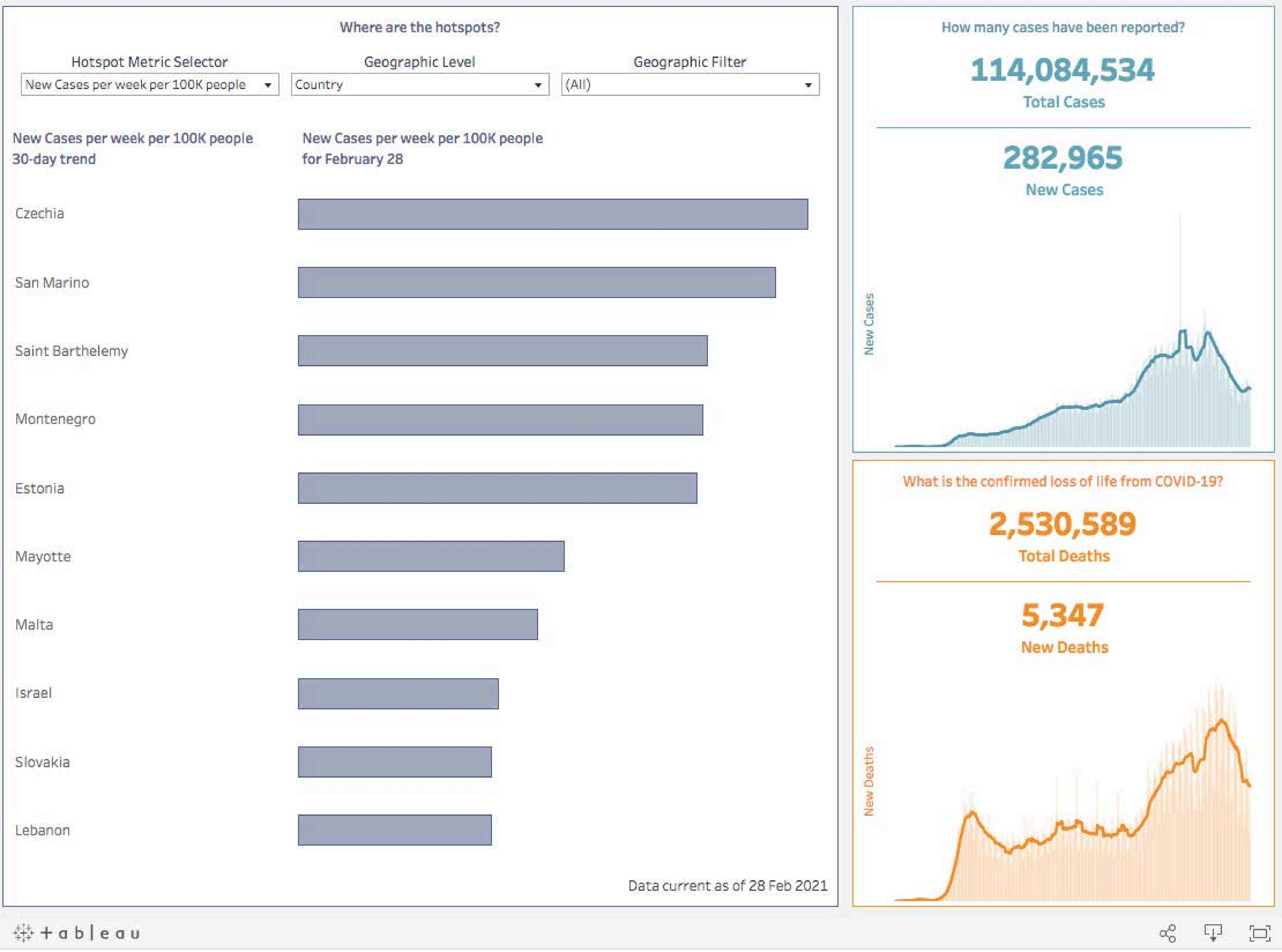

Where are the hotspots?
Hotspot Metric Selector
New Cases per week per 100K people
Geographic Level
Country
Geographic Filter
(All)
New Cases per week per 100K people 30-day trend
New Cases per week per 100K people for February 28
Czechia
San Marino
Saint Barthelemy
Montenegro
Estonia
Mayotte
Malta
Israel
Slovakia
Lebanon
Data current as of 28 Feb 2021
How many cases have been reported?
114,084,534
Total Cases
282,965
New Cases
New Cases
What is the confirmed loss of life from COVID-19?
2,530,589
Total Deaths
5,347
New Deaths
New Deaths
+ a b | e a u

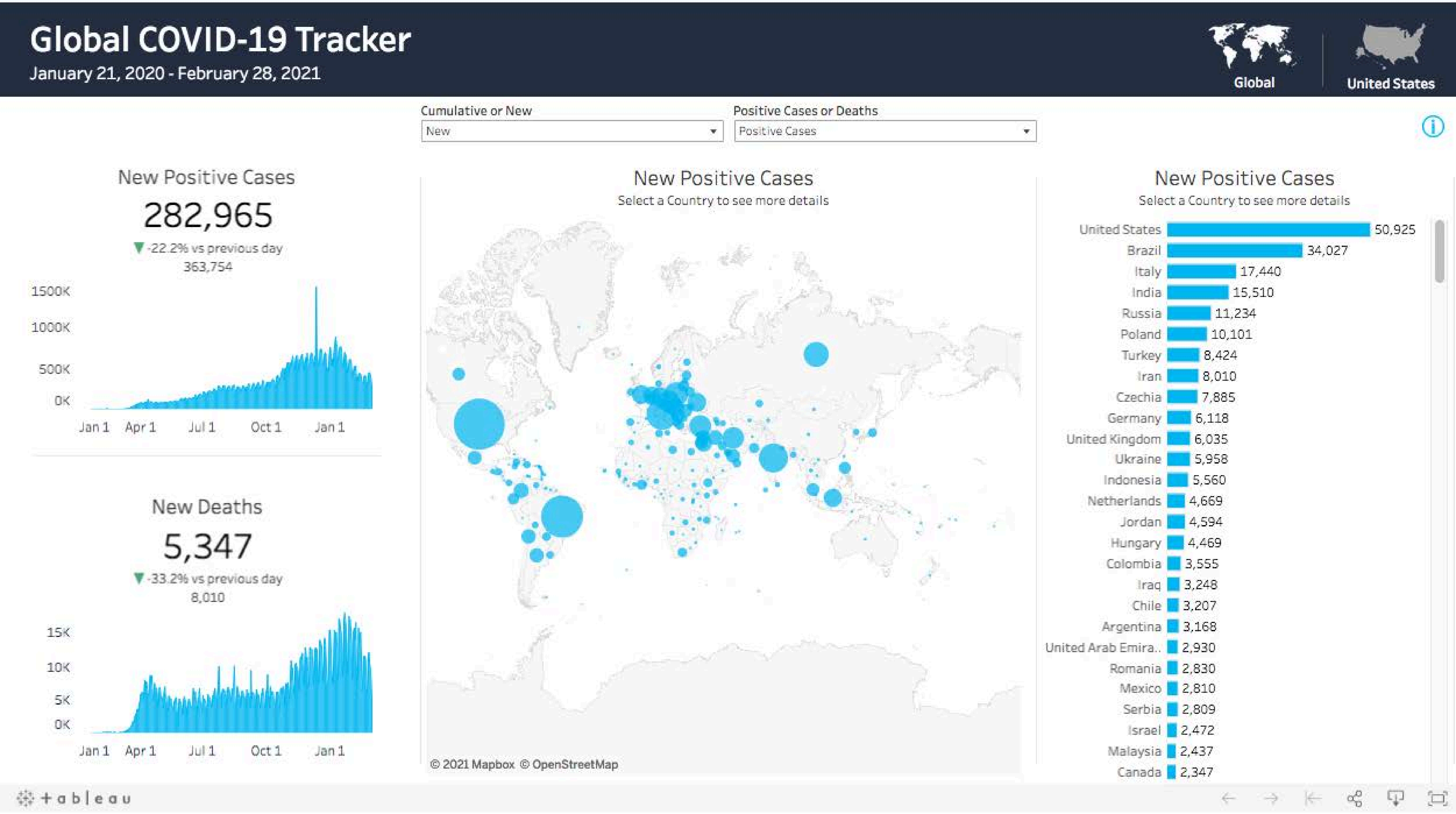
Global COVID-19 Tracker
January 21, 2020 - February 28, 2021
Global
United States
Cumulative or New
New
Positive Cases or Deaths
Positive Cases
New Positive Cases
282,965
-22.2% vs previous day
363,754
1500K
1000K
500K
0K
Jan 1
Apr 1
Jul 1
Oct 1
Jan 1
New Deaths
5,347
-33.2% vs previous day
8,010
15K
10K
5K
0K
Jan 1
Apr 1
Jul 1
Oct 1
Jan 1
New Positive Cases
Select a Country to see more details
© 2021 Mapbox © OpenStreetMap
New Positive Cases
Select a Country to see more details
United States 50,925
Brazil 34,027
Italy 17,440
India 15,510
Russia 11,234
Poland 10,101
Turkey 8,424
Iran 8,010
Czechia 7,885
Germany 6,118
United Kingdom 6,035
Ukraine 5,958
Indonesia 5,560
Netherlands 4,669
Jordan 4,594
Hungary 4,469
Colombia 3,555
Iraq 3,248
Chile 3,207
Argentina 3,168
United Arab Emira.. 2,930
Romania 2,830
Mexico 2,810
Serbia 2,809
Israel 2,472
Malaysia 2,437
Canada 2,347
+ a b | e a u
