# Supplementary material for: Web-Based COVID-19 Dashboards and Trackers in the United States: Survey Study
Source: JMIR Hum Factors. 2023 Mar 20;10:e43819. doi: 10.2196/43819 (PMC10029858; doi:10.2196/43819)
Supplement: Multimedia Appendix 10 [file humanfactors_v10i1e43819_app10.pdf]

Appendix 10. Screenshots from selected dashboards and trackers for vaccine distribution — *State, national, and global coverage* *(captured January 31 and February 27, 2021)*

Page	Ref	Host	Date captured
2	Svac-1	Alabama Dept of Public Health	Jan
3	Svac-2	David Marconnet, CTO of OneTeam.net	Jan
4	Svac-3	California Dept of Public Health	Jan
5–6	Svac-4	Government of the District of Columbia	Jan + Feb
7	Svac-5	CT Post	Jan
8–9	Svac-6	Chicago Tribune	Jan + Feb
10	Svac-7	Indiana State Dept of Health	Feb
11	Svac-8	Kansas Dept of Health and Environment	Jan
12–15	Svac-9	Michigan Dept of Health and Human Services	Jan + Feb
16–17	Svac-10	Minnesota Dept of Health	Jan
18	Svac-11	North Dakota Dept of Health	Jan
19	Svac-12	New Mexico Dept of Health	Jan
20	Svac-13	Ohio Dept of Health	Jan + Feb
21–22	Svac-14	Tennessee Dept of Health	Jan
23–25	Svac-15	Texas Health and Human Services	Jan
26	Svac-16	Vermont Dept of Health	Jan
27	Svac-17	Wisconsin Dept of Health Services	Jan
28	Nvac-1	New York Times	Feb
29	Nvac-2	Springfield News-Leader	Feb
30	Nvac-3	Washington Post	Feb
31	Nvac-4	COVID-19 Health Equity Interactive Dashboard / Emory University	Feb
32	Gvac-1	Our World in Data	Feb
33	Gvac-2	CNN	Feb
34–35	Gvac-3	Brown School of Public Health	Feb

Alabama's COVID-19 Vaccine Distribution Dashboard

ALABAMA PUBLIC HEALTH

Alabama Department of Public Health | Immunization Division | Updates Monday - Friday by 2:00 p.m.

Vaccine Doses Administered

(Most recent week may be incomplete)

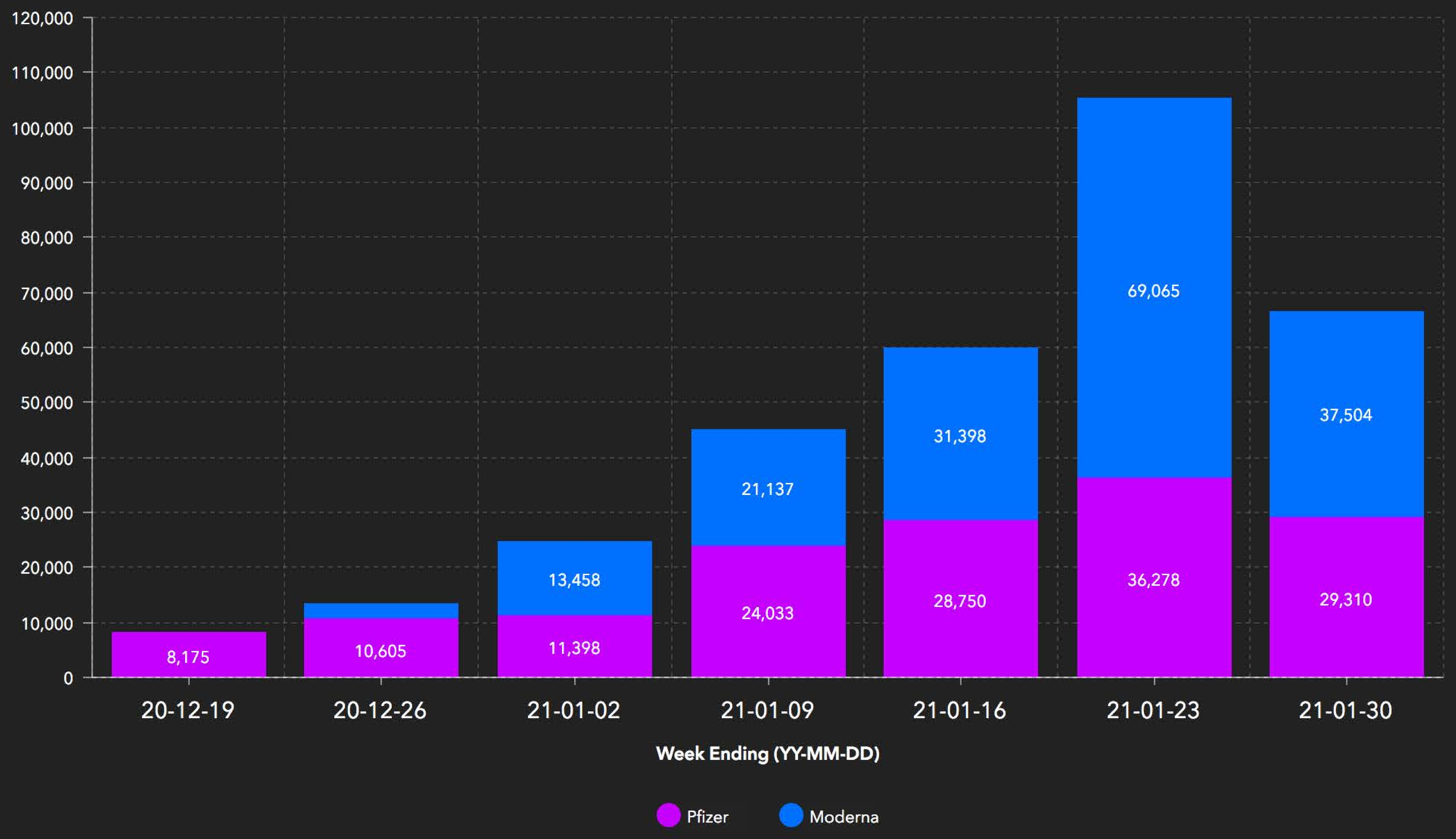

Doses Administered

323,875

Doses Administered By Federal LTC Programs

37,501

*This number will be updated as soon as it is available from the Federal LTC Program

Doses Delivered to Alabama

772,275

Doses Delivered to **690,375** Alabama Providers

Doses Delivered to **81,900** Federal LTC Program

Doses Administered | Vaccine Providers | About the Data

Alabama COVID-19 Vaccine Data

Doses Delivered	Doses Admin.	Percent Admin.	Fed LTC Admin.	Moderna Admin.	Pfizer Admin.
772,275	323,875	41.94%	37,501	175,326	148,549
690,375 AL 81,900 Fed LTC	46,268 per week	Of delivered	45.79% of Delivered	25,047 per week	21,221 per week

Administered per Week

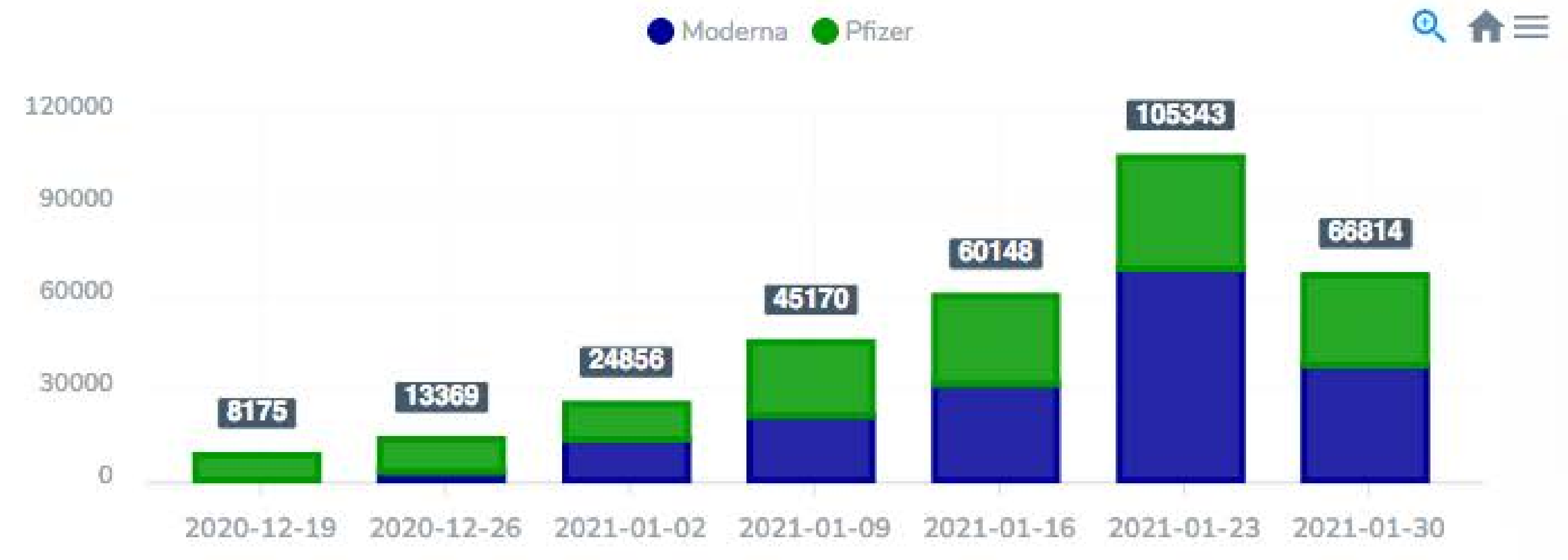

Moderna vs Pfizer Doses

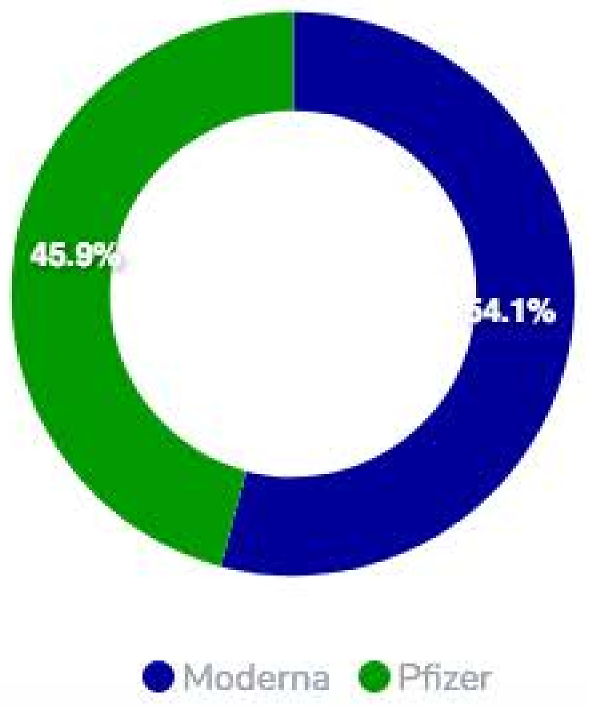

Percent Administered over time

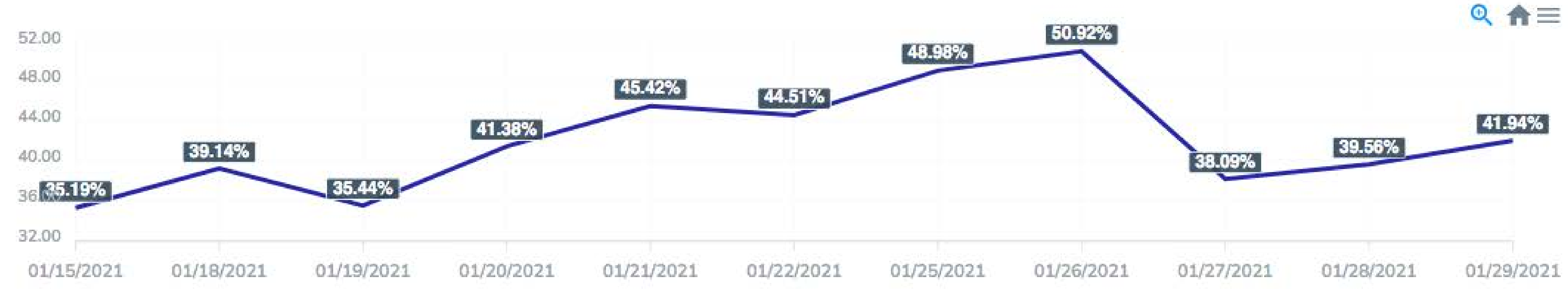

The following data is tracked from the ADPH Vaccine Distribution Dashboard and will update weekly on Mondays. For more information on the COVID-19 Vaccine, check out the ADPH COVID-19 Vaccine Page.

2020 - 2021 © Magic & Wires LLC. This website is for educational and academic research purposes only. Do not rely upon the content of this website as a basis for making any business, legal or health decisions. COVID-19 Data collected from ADPH, CDC & HHS.

Terms & Conditions

COVID-19: Vaccine Dashboard

Map View | **Summary View**

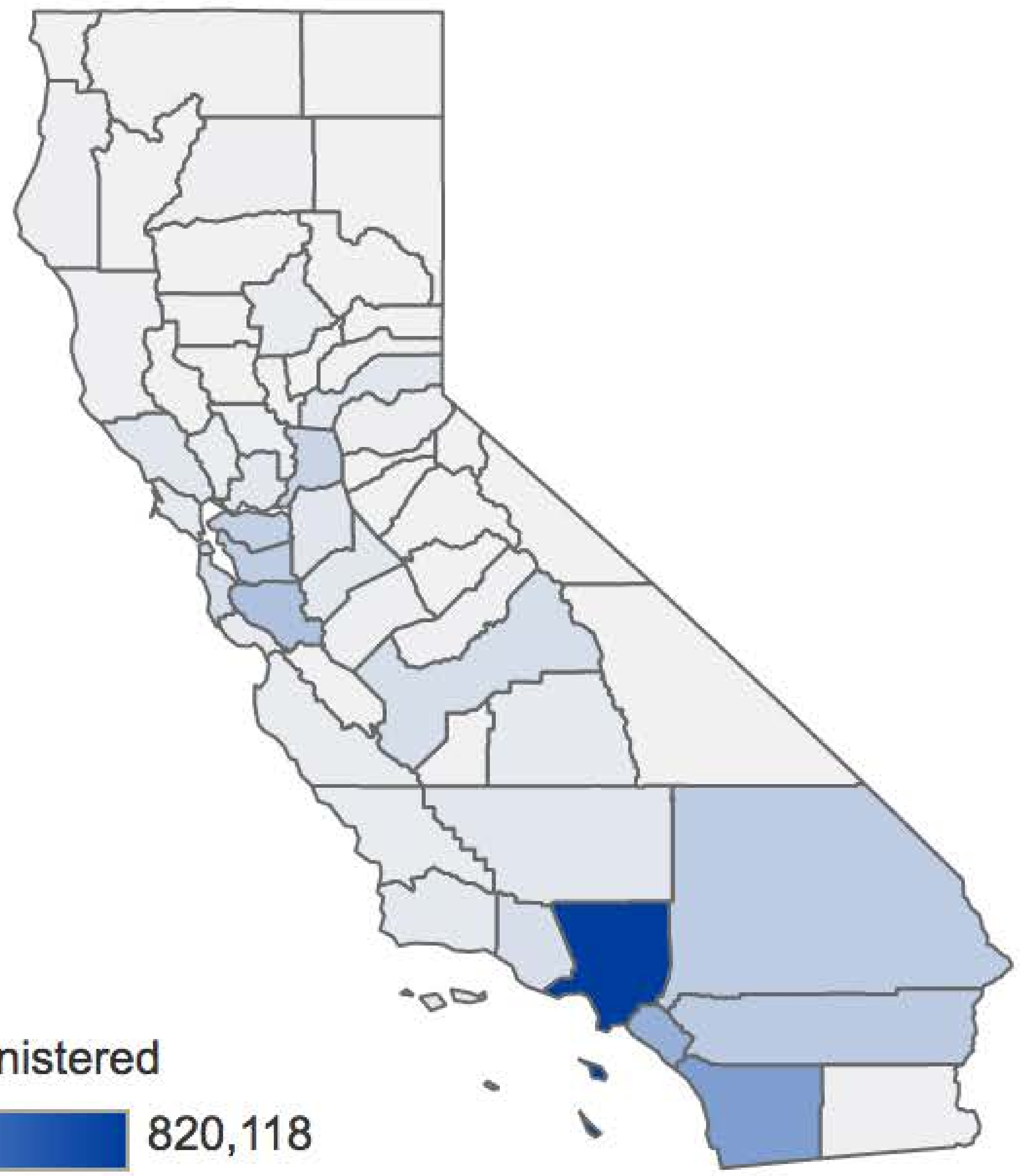

Total Doses Administered

0 – 820,118

Total Doses Administered

3,266,914

Find a County (County of Residence)

Highlight County

Click on the map for updated metrics

Data Last Updated on 1/30/2021 11:59pm

+ a b l e a u

Vaccination Data (As of 1/23/21)

VACCINE DOSE METRICS		
	TOTAL	% OF TOTAL
Doses Delivered	68,750	
Doses Administered	51,421	75%
Additional Doses Expected for the Week	9,475	

Data Definitions
Doses Delivered: The number of First Dose COVID-19 Vaccine physically received by healthcare providers in the District of Columbia as of 6:00a on the Wednesday before.
Doses Administered: The number of First Dose COVID-19 Vaccine administrations reported to the District of Columbia Immunization Information System (DOCIIS) as of the Saturday before.

Doses Administered

The data below provide information on how many COVID-19 vaccine doses were given to residents and non-residents in DC. COVID-19 vaccine information is included by Ward for all DC residents as well as for those who 65 years and older.

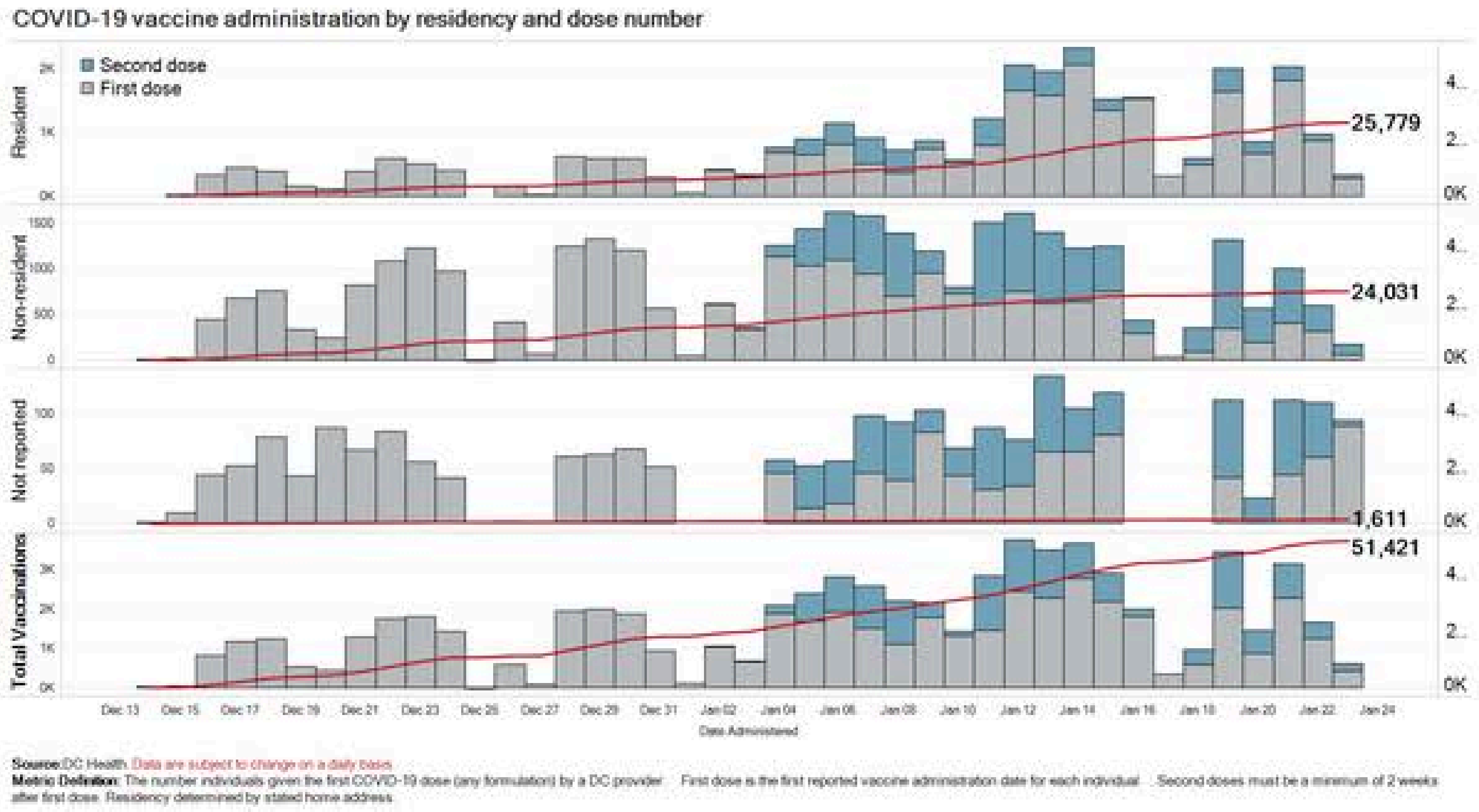

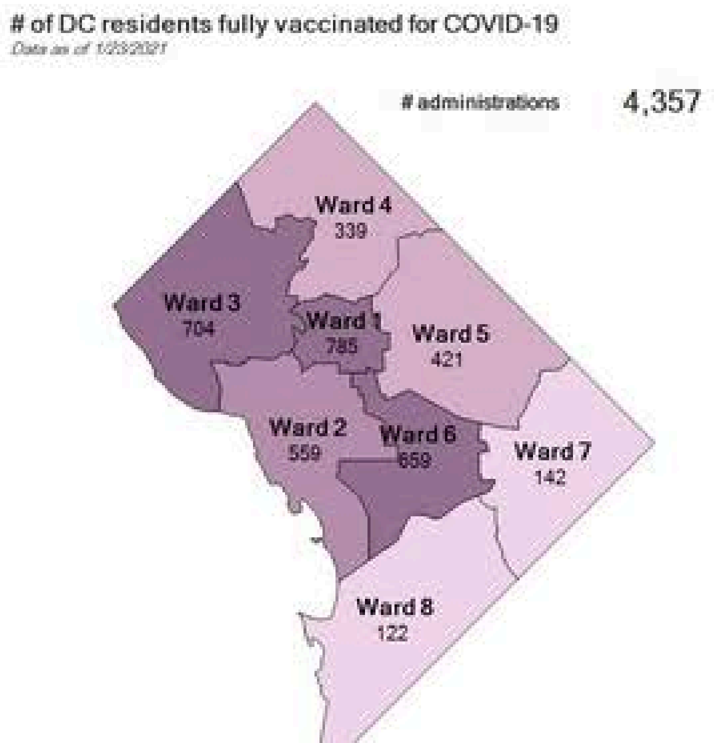

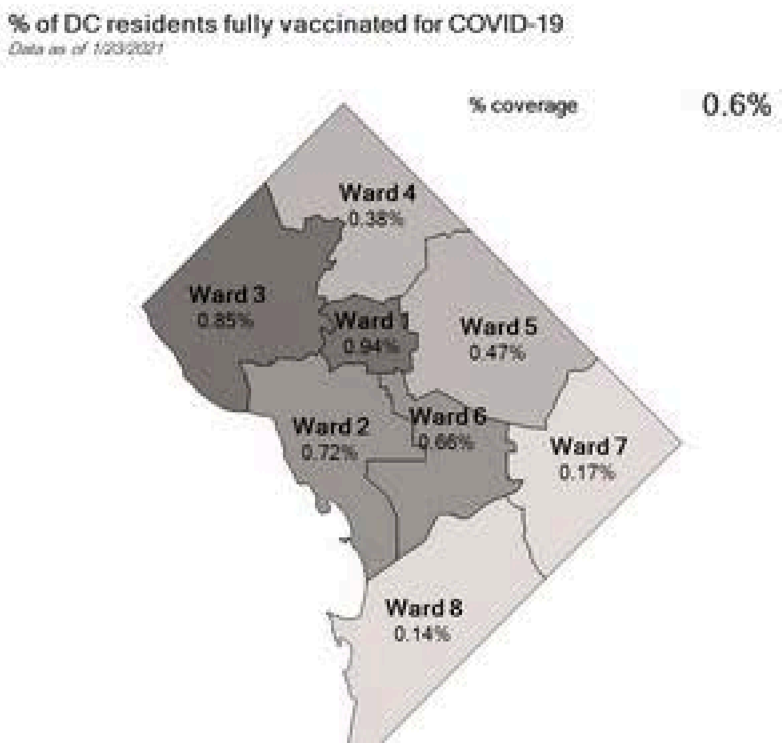

Listen

Vaccination Data

Summary statistics:	% RESIDENTS PARTIALLY OR FULLY VACCINATED	% RESIDENTS FULLY VACCINATED	TOTAL DOSES ADMINISTERED
	7.7%	3.1%	139,516

Individuals Partially or Fully Vaccinated by Administration Date

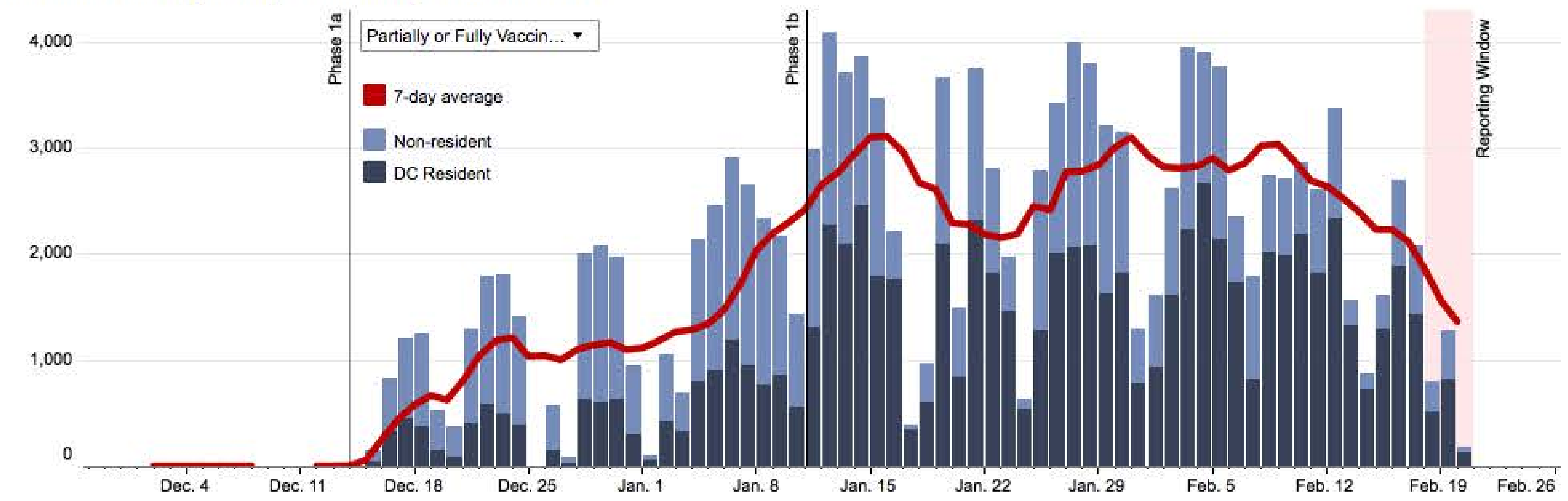

	PARTIALLY/FULLY VACCINATED	FULLY VACCINATED	TOTAL DOSES ADMINISTERED	DOSE 7-DAY AVERAGE
**DC Resident**	54,559	22,073	76,632	974
**Non-resident**	38,046	24,838	62,884	390
**Total**	92,605	46,911	139,516	1,363

Source: DC Health. Data are subject to change.
Metric Definition: Number of administrations reported by DC-area providers by date of vaccine administration. The 7-day rolling average represents the average number of administrations including the past 6 days.
Data Considerations: There may be a lag time between vaccine administration and provider report. This may impact the reporting of vaccine administered, especially in the three most recent days of report, highlighted in red above. DC residents may be vaccinated outside of the DC-area and are not included in this report. Non-DC residents may be vaccinated within DC, especially those who fall into prioritized non-resident categories. Administration may be impacted by holidays and weekends, and is impacted by the size of prioritized groups and vaccine supply.

Last Updated: 2/20/2021

# of DC residents aged 65+ received first dose of COVID-19 vaccine by Ward
Data as of 2/14/2021

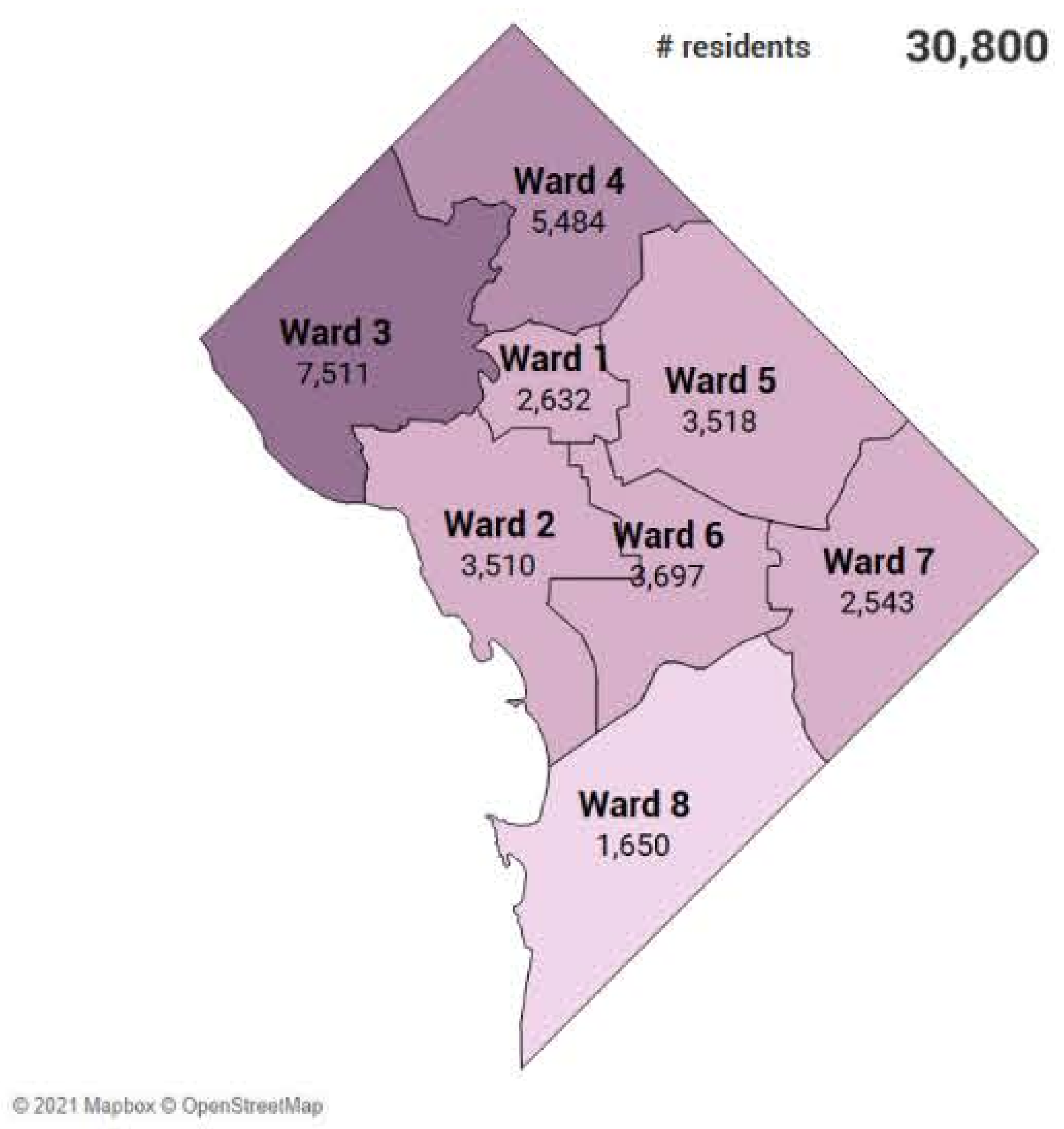

# of DC residents aged 65+ received first dose of COVID-19 vaccine by Ward
Data as of 2/14/2021

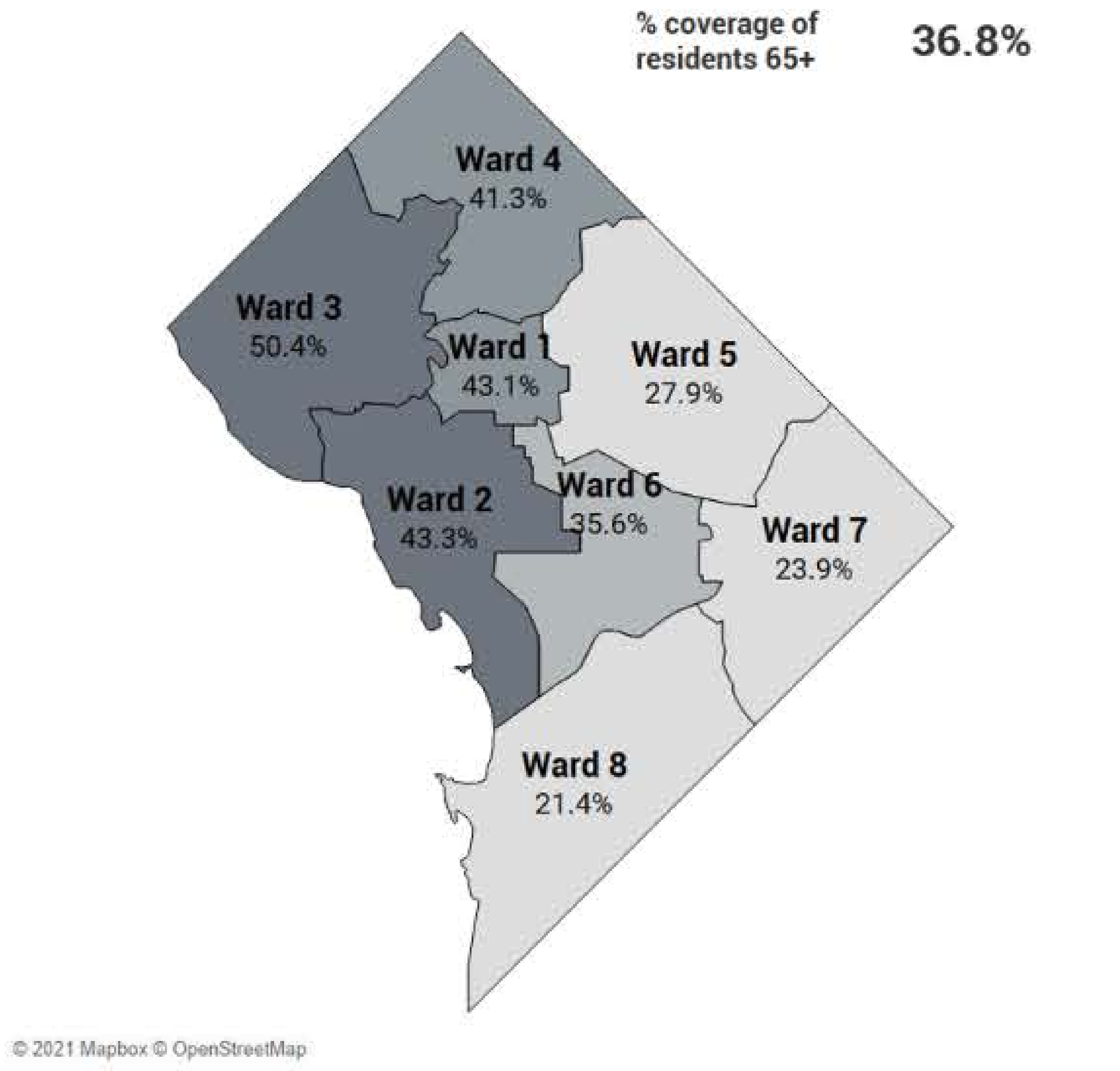

Source: DC Health.
Metric Definition: The number individuals 65 years of age and above given the first dose by Ward. Age is by self-report. Ward is determined by stated address. Coverage is a percentage calculated by dividing the number of persons 65+ given the first dose by the ACS 2019 ward-level population estimate for the 65+ age group.

Mayor Bowser Proclaims 'A Day of Remembrance for Lives Lost to COVID-19'
DC Entered Phase Two of Reopening on June 22. View the Guidance.

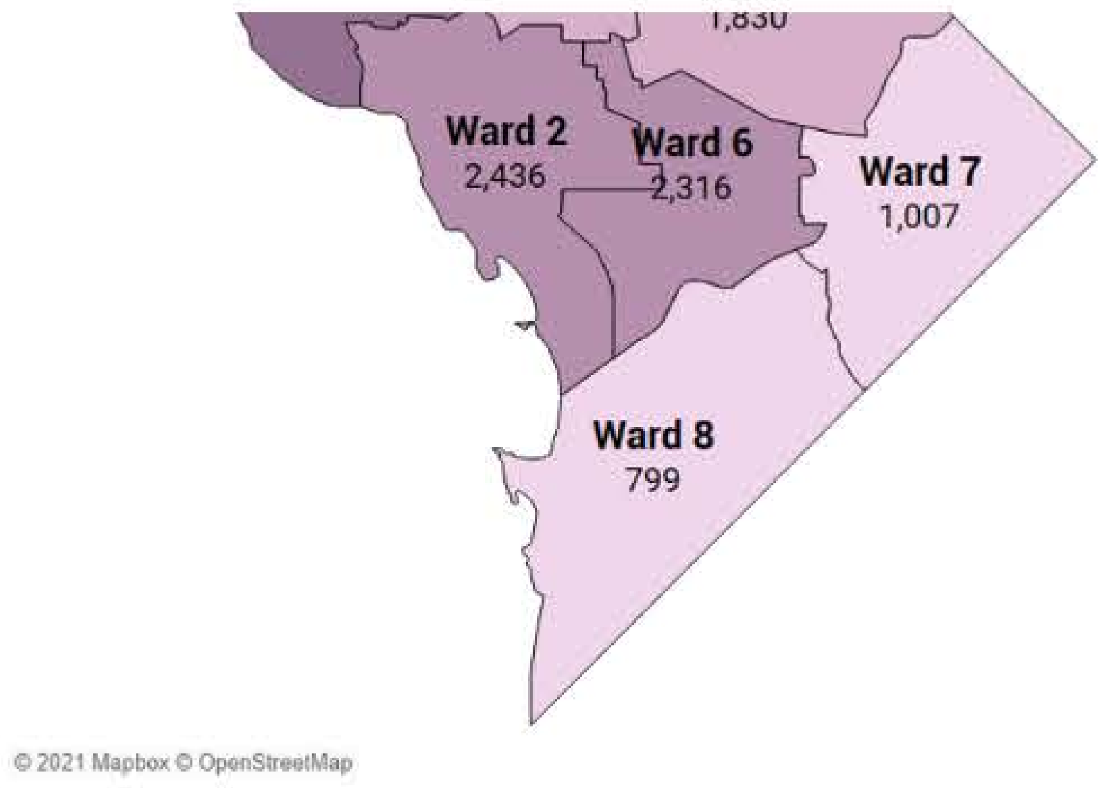

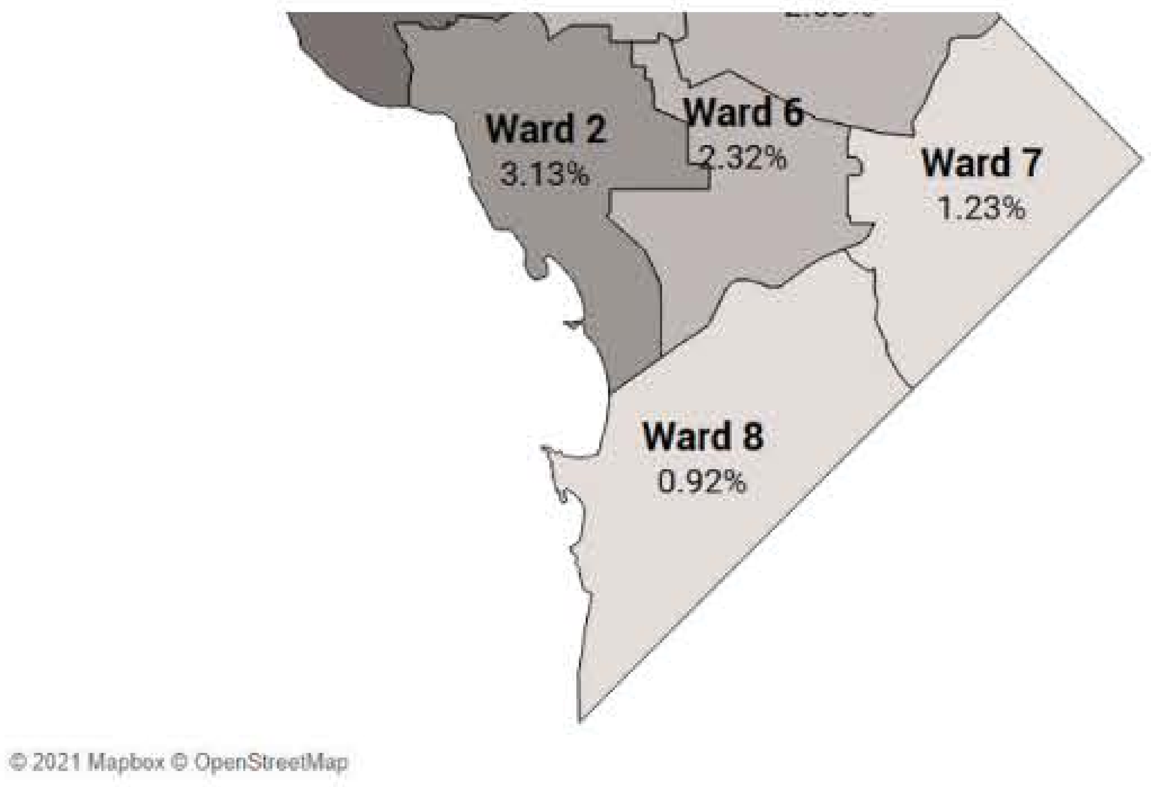

Source: DC Health.
Metric Definition: The number individuals given the both the first and second dose by Ward. Ward is determined by stated address. Coverage is a percentage calculated by dividing the number of persons vaccinated by the ACS 2019 ward-level population estimate.

Connecticut		United States	
578,600	340,886	49,932,850	24,064,165
doses distributed	first shots administered	doses distributed	first shots administered

Source: Centers for Disease Control and Prevention, updated Jan. 30, 2021, 5:15 p.m.

Connecticut COVID-19 Vaccine Tracker

By Eric Blom, Derek Turner, Paula Friedrich and Lidia Ryan

Dec. 2, 2020 4:44 p.m. | Updated: Jan. 29, 2021 8:32 a.m.

Stay informed on the latest local and statewide updates as well as major regional, national and global information about the coronavirus vaccine.

SUPPORTED BY
Hartford HealthCare
More COVID vaccine answers - click here

340,886 people in Connecticut have gotten their first shot

All vaccines currently authorized by the FDA require two doses and are authorized for people 16 years and older. Connecticut has administered 421,191 vaccine doses in total. Here's Connecticut's progress toward vaccinating its population.

340,886
first shots administered
9.6%
0 — 3,565,287 people

73,706
second shots administered
2.1%
0 — 3,565,287 people

Source: CDC
Note: 254,044 doses in the United States, including 6,599 doses in Connecticut, have been reported without specification of first or second dose.

Name ▲	First shots administered ▼	Second shots administered ▼	Population ▼	Percent vaccinated with at least one dose ▼	Percent fully vaccinated ▼
United States	24,064,165	5,259,693	331,996,199	7.2%	1.6%
Connecticut	340,886	73,706	3,565,287	9.6%	2.1%
Alabama	288,368	53,742	4,903,185	5.9%	1.1%
Alaska	91,794	23,320	731,545	12.5%	3.2%
American Samoa	8,926	997	55,689	16.0%	1.8%

Show more

Last updated: Jan. 30, 2021, 5:15 p.m.
Source: CDC

About 73% of vaccine doses shipped to Connecticut have been administered

National distributors have shipped 578,600 vaccine doses to Connecticut. The state has administered 421,191 of those doses to people.

72.8%
0 — 578,600 shipped doses

Name ▲	Doses distributed ▼	Shots administered ▼	Percent of shipped doses administered ▼
United States	49,932,850	29,577,902	59.2%
Connecticut	578,600	421,191	72.8%
Alabama	659,400	342,436	51.9%
Alaska	193,150	115,268	59.7%
American Samoa	18,450	9,925	53.8%

Show more

Last updated: Jan. 30, 2021, 5:15 p.m.
Source: CDC

In what order will Connecticut distribute vaccines?

Connecticut is rolling out vaccinations to COVID-19 in phases. The state has a goal for all residents to have access to the vaccination by late spring or early summer 2021. has started Phase 1b.

UNDERWAY
Phase 1a
December - February

- Custodial, dietary, administrative and support staff working in patient care settings
- Death care workers entering healthcare settings, homes or with exposure to decedents
- Dentists, dental hygienists and other oral health staff

Show more

UNDERWAY
Phase 1b
Begins Jan. 18

	Status
Individuals 75 years of age and older	Currently scheduling
Individuals between the ages of 65 and 74	Likely February
Front line essential workers	Likely March

Show more

PROPOSED
Phase 1c
Likely by spring

- Details yet to be provided by state

PROPOSED
Future phases
Summer and fall

- General public

Connecticut Department of Public Health

Authorized vaccines and others we're watching

The vaccines that the FDA has authorized both use messenger RNA technology, but they have different storage requirements.

PFIZER
Effectiveness **95%** (initial results)
Status: Authorized for emergency use by the FDA
Age: For people 16 years and older
Storage: Must be kept frozen at temperatures nearing 100 degrees Fahrenheit below zero
Shots: Two shots 21 days apart

MODERNA
Effectiveness **94.5%** (initial results)
Status: Authorized for emergency use by the FDA
Age: For people 18 years and older
Storage: Can be stored above freezing
Shots: Two shots 28 days apart

ASTRAZENECA
Effectiveness **60%** (initial results)
Status: In Phase 3 trials
Age: For people 18 years and older
Storage: Can be stored above freezing
Shots: Two doses four to 12 weeks apart

JOHNSON & JOHNSON
Effectiveness **66%** (initial results)
Status: In Phase 3 trials
Age: For people 18 years and older
Storage: Can be stored in a refrigerator
Shots: 1 dose

ADVERTISEMENT

Progress toward FDA authorization

PHASE 1	PHASE 2	PHASE 3	APPLIED	AUTHORIZED
[illegible]	[illegible]	[illegible]	[illegible] FDA	[illegible]
COVAXX	SANOFI	ASTRAZENECA	NONE	PFIZER
VAXART	IMMUNITYBIO	JOHNSON & JOHNSON		MODERNA
		NOVAVAX		

Each drug company has an independent data and safety monitoring board that reviews the Phase 3 clinical trial data. Once companies complete trials, they will submit the data to the Food and Drug Administration. The agency's Vaccines and Related Biological Products Advisory Committee will meet publicly to discuss the data. The FDA's career scientists will review the data and decide whether to grant the vaccine emergency use authorization.

Phase 1 trials are done in a small number of people to test for safety and dosing. Phase 2 trials are done in hundreds of people to further test for safety. Phase 3 trials typically include tens of thousands of people and are the final stage of testing before a vaccine can be submitted for review by the FDA.

Illinois COVID-19 vaccine tracker: 1,829,575 doses delivered, 981,988 administered, average 42,931 given a day

By CHICAGO TRIBUNE STAFF
CHICAGO TRIBUNE | JAN 31, 2021 AT 12:35 PM

With vaccinations underway across the U.S. and the world, here's where Illinois stands:

Average shots a day: 42,931

Total administered: 981,988

Total doses delivered: 1,829,575

See Coronavirus data on **deaths, cases and tests** here.

See Coronavirus data by **ZIP code** here.

Illinois vaccinations by day

Here are the number of vaccinations recorded across the state on each date, and the average. Data as of Jan. 30.

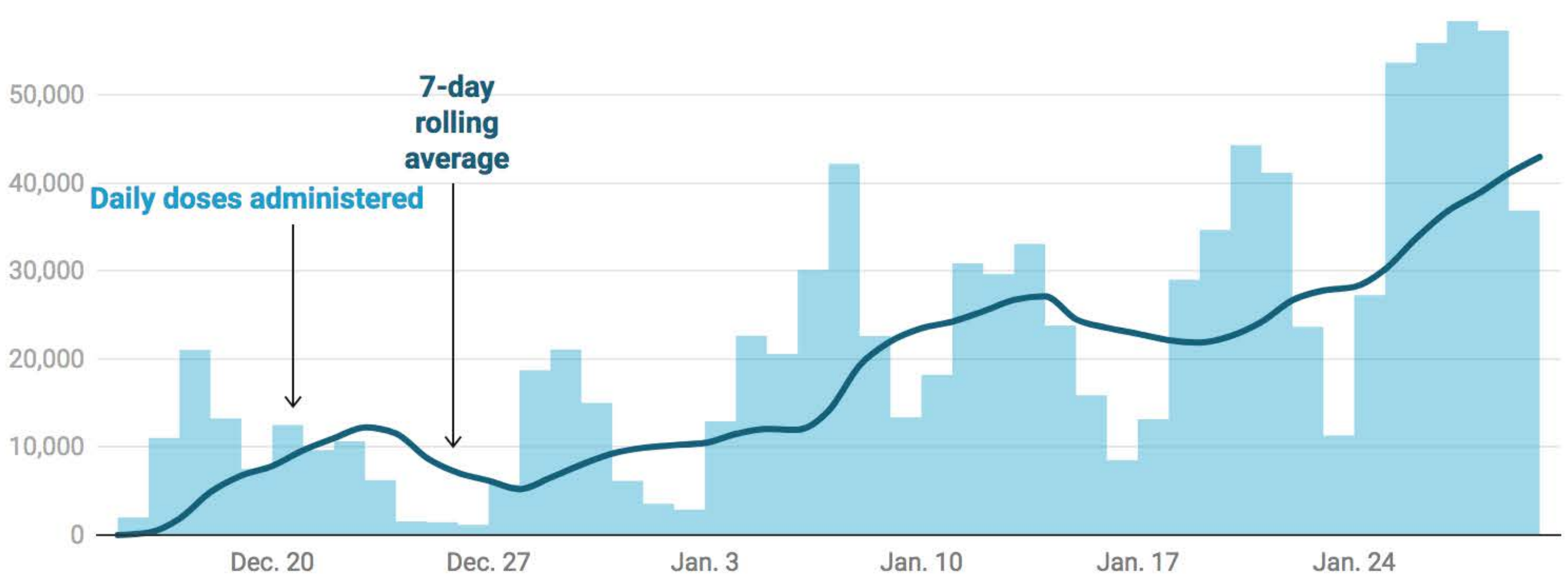

Chart: Chicago Tribune dataviz team •
Source: Tribune analysis of IDPH data. Doses administered include second doses as well as first doses. Figures for date shot reported to state, not necessarily date given. Rolling average calculated by state.
• Get the data • Created with Datawrapper

Illinois vaccinations by county

Here is the percentage of residents in each county who have received at least one shot, based on the most recently released state data.

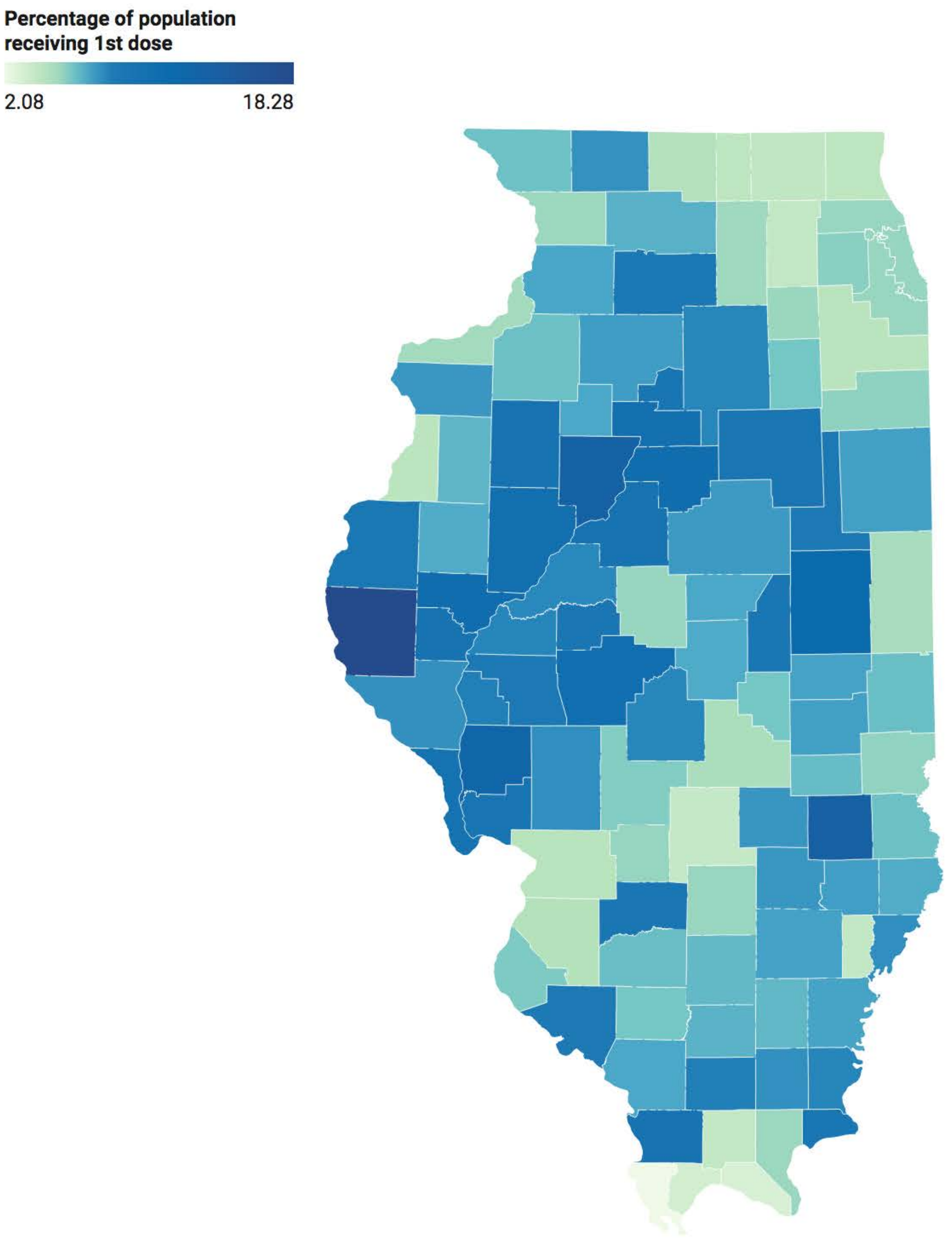

Map: Chicago Tribune dataviz department •
Source: Tribune analysis of IDPH data. Map does not include doses given to people whose addresses were not registered or lived out-of-state. •
Get the data • Created with Datawrapper

Vaccinations by state

The Centers for Disease Control and Prevention tracks COVID-19 vaccinations by state, including the rate of doses administered. The data may not account for the most recent figures uploaded by states.

[illegible]

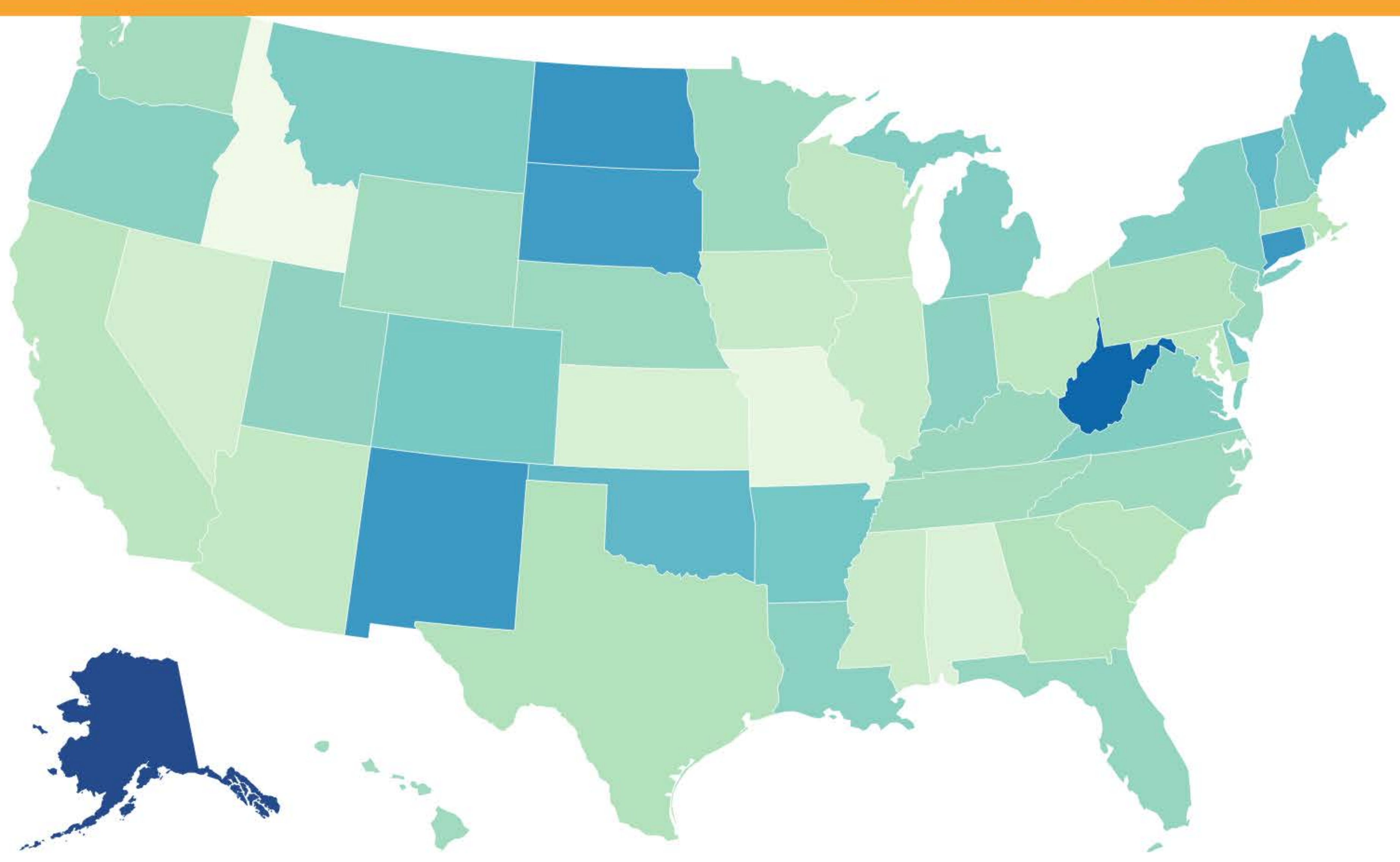

Map: Chicago Tribune dataviz team • Source: Centers for Disease Control and Prevention • Get the data • Created with Datawrapper

LATEST STORIES

Rep. Adam Kinzinger launches 'country first' movement as Trump criticism puts focus on Illinois GOP divide amid search for new leadership
20m

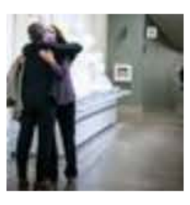

Third stimulus check updates: Senate Republicans urge Biden to consider less costly COVID-19 relief package
26m

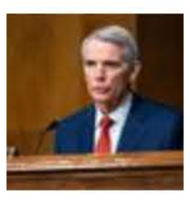

What is allowed and not allowed in Illinois' COVID tiers plan? And where does your region stand?
37m

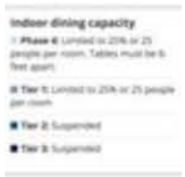

COVID-19 in Illinois by the numbers: Here's a daily update on cases, positivity rate and hospital data in your area
37m

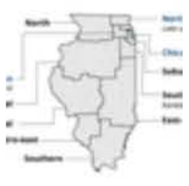

COVID-19 cases in Illinois by ZIP code: Search for your neighborhood
42m

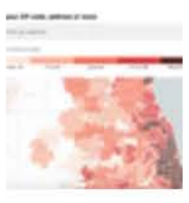

ECTIONS SEARCH Chicago Tribune 99¢ FOR 8 WEEKS Offer ends 2/8 LOG

CORONAVIRUS IN ILLINOIS UPDATES

MOST READ • COVID-19 VACCINE

1. Walgreens, Jewel-Osco, Walmart and Cook County scheduling COVID-19 vaccine appointments, though demand is great and doses scarce

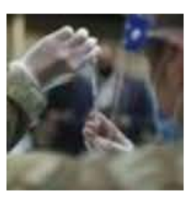

2. How do I schedule a second COVID-19 vaccine shot? Why hasn't my health system contacted me yet? Common Illinois vaccine questions answered.

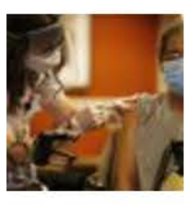

Illinois COVID-19 vaccine tracker: 46,946 additional people fully vaccinated for a total of 772,410 — 6.06% of the state's population. 83,048 new doses administered Friday.

By CHICAGO TRIBUNE STAFF
CHICAGO TRIBUNE | FEB 27, 2021 AT 2:01 PM

With vaccinations underway across the U.S. and the world, here's where Illinois stands:

Average shots a day: 69,736

New doses administered yesterday: 83,048

Total administered: 2,626,668

Total doses supplied: 3,183,805

Reported inventory: 404,757

Doses supplied yesterday: 57,650

New people fully vaccinated: 46,946

Total people fully vaccinated: 772,410

Percentage of Illinois population vaccinated: 6.06%

See Coronavirus data on **deaths, cases and tests** here.

See Coronavirus data by **ZIP code** here.

Illinois vaccinations by day

Here are the number of vaccinations recorded across the state on each date, and the average.

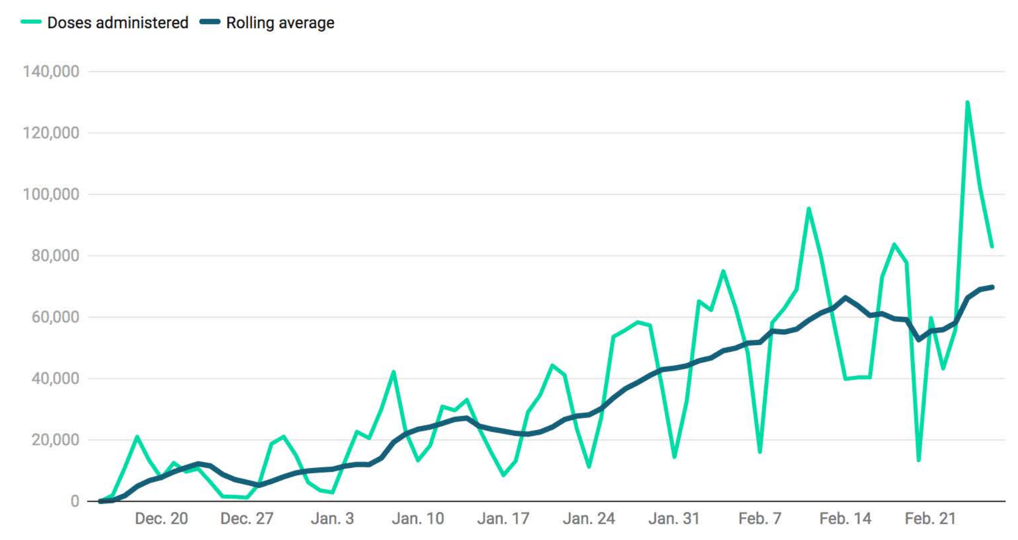

Chart: Chicago Tribune dataviz team •
Source: Tribune analysis of IDPH data. Doses administered include second doses as well as first doses. Figures for date shot reported to state, not necessarily date given. Rolling average calculated by state.
• Get the data • Created with Datawrapper

Illinois vaccinations by county

Here is the percentage of residents in each county who have received at least one shot, based on the most recently released state data.

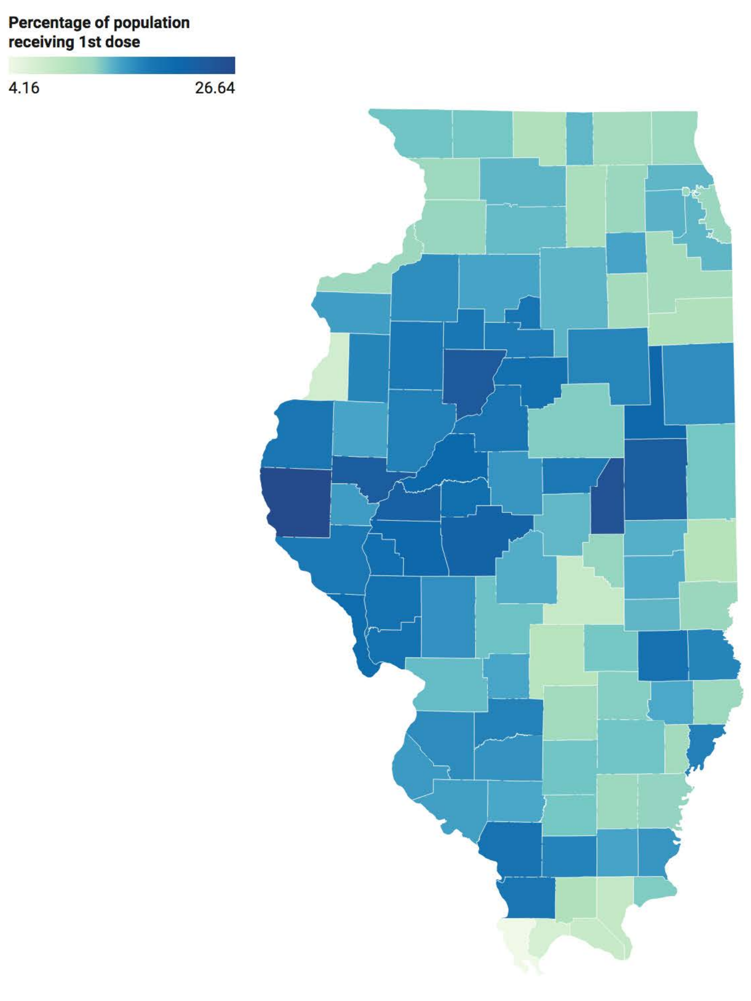

Map: Chicago Tribune dataviz department •
Source: Tribune analysis of IDPH data. Map does not include doses given to people whose addresses were not registered or lived out-of-state. •
Get the data • Created with Datawrapper

Doses of COVID-19 vaccine supplied to Illinois by day

The number of COVID-19 vaccine supplied to Illinois has varied widely.

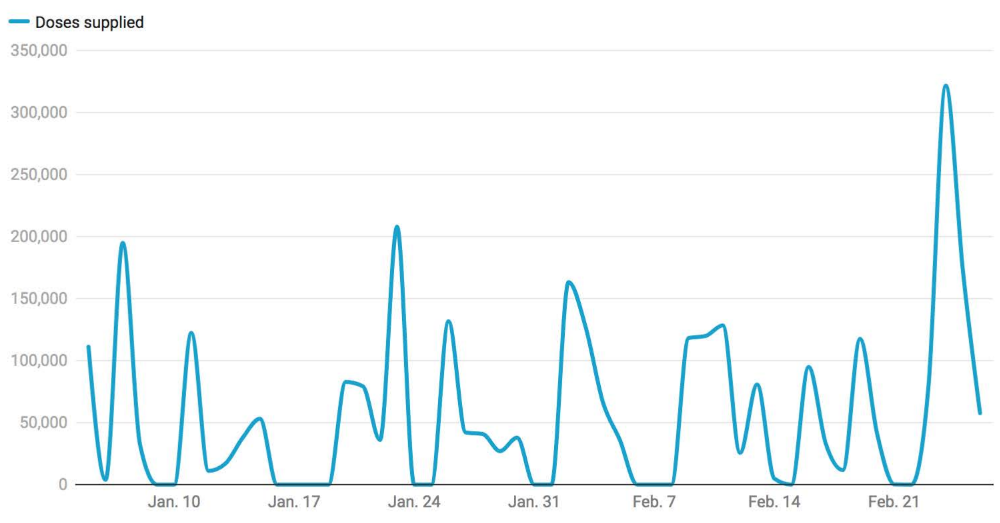

Chart: Chicago Tribune dataviz team • Source: Centers for Disease Control via the Associated Press • Get the data • Created with Datawrapper

Vaccinations by race by geography

Percentage of residents receiving 1st shot and fully vaccinated, by race/ ethnicity. Updated weekly.

Search in table   Page 1 of 12 >

County	Status	Hispanic	White	Black
Illinois	1st dose	6%	14%	7%
Illinois	Fully vaccinated	2%	5%	2%
Chicago	1st dose	7%	13%	7%
Chicago	Fully vaccinated	2%	6%	3%
Cook	1st dose	6%	14%	7%
Cook	Fully vaccinated	2%	4%	2%
DuPage	1st dose	4%	12%	5%
DuPage	Fully vaccinated	2%	4%	2%
Kane	1st dose	3%	13%	5%
Kane	Fully vaccinated	1%	4%	2%
Kendall	1st dose	6%	15%	5%
Kendall	Fully vaccinated	2%	4%	2%
Lake	1st dose	4%	12%	4%
Lake	Fully vaccinated	1%	3%	1%
McHenry	1st dose	4%	10%	4%
McHenry	Fully vaccinated	1%	3%	2%
Will	1st dose	6%	10%	5%
Will	Fully vaccinated	2%	3%	2%

Race/ethnicity figures are based on populations. The figures for unknown race/ethnicity are based on tests performed.
Table: Chicago Tribune dataviz team • Source: Illinois Department of Public Health • Get the data • Created with Datawrapper

Percentage 1st dose for those ages 65 and over

Percentage of those 65 and older getting vaccinated as of Feb. 12.

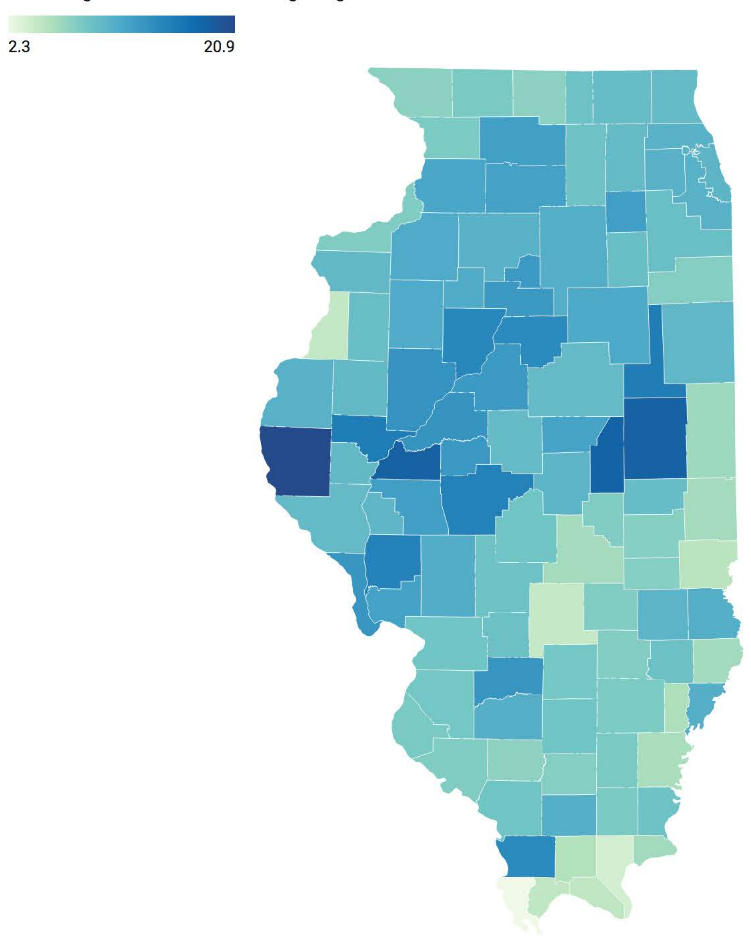

Map: Chicago Tribune dataviz team • Source: Illinois Department of Public Health • Get the data • Created with Datawrapper

Vaccinations by state

The Centers for Disease Control and Prevention tracks COVID-19 vaccinations by state, including the rate of doses administered. The data may not account for the most recent figures uploaded by states.

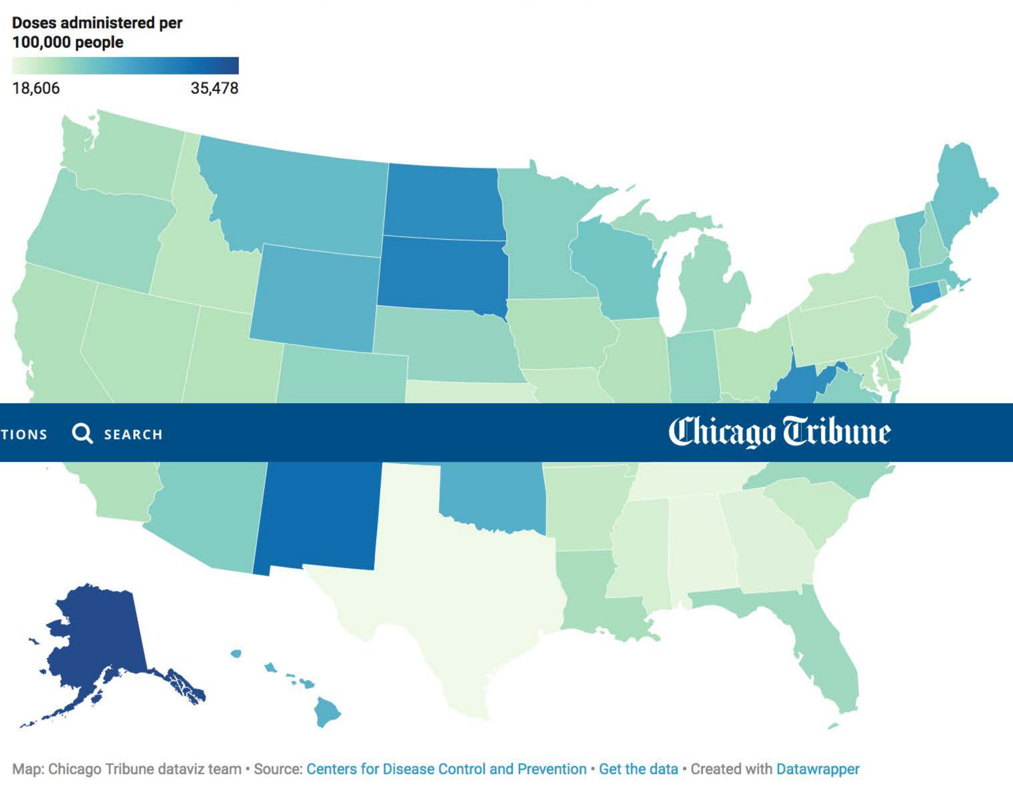

SECTIONS SEARCH Chicago Tribune $1 FOR 10 WEEKS OFFER ENDS SOON LOG IN

Map: Chicago Tribune dataviz team • Source: Centers for Disease Control and Prevention • Get the data • Created with Datawrapper

LATEST STORIES

Johnson & Johnson COVID-19 vaccine: What you need to know about the single-dose inoculation
31m

Coronavirus in Illinois updates: 83,048 vaccine doses administered, 1,780 new COVID-19 cases and 34 additional deaths reported Saturday
39m

States rush to open mass COVID vaccination sites as US expects vaccine supply to swell
1h

COVID-19 in Illinois by the numbers: Here's a daily update on cases, positivity rate and hospital data in your area
1h

COVID-19 cases in Illinois by ZIP code: Search for your neighborhood
1h

MOST READ • COVID-19 VACCINE

1. Where are you in the vaccine line? We charted out who is eligible and who is not in as much detail as we could find.

2. Illinois COVID-19 vaccine tracker: 46,946 additional people fully vaccinated for a total of 772,410 — 6.06% of the state's population. 83,048 new

Vaccine Dashboard

Indiana COVID-19 Vaccination Dashboard

Updated daily at 12:00 PM EST. Data current as of 02/27/2021 at 5:00 AM EST.

Newly Reported Vaccinated Individuals

Reported within the past 24 hours

First Dose Administered

18,601

During the previous 24 hours, number of individuals who were reported to have received their first dose of the COVID-19 vaccine.

Fully Vaccinated

20,287

During the previous 24 hours, number of individuals who were reported to be fully vaccinated for COVID-19.

Total Individuals Vaccinated

Reported total (all-time): 12/14/2020 - 02/27/2021

First Dose Administered

981,769

Total number of individuals who received their first dose of COVID-19 vaccine.

Fully Vaccinated

552,241

Total number of individuals who are considered to be fully vaccinated for COVID-19.

County Distribution

Select county or group of counties to filter dashboard.

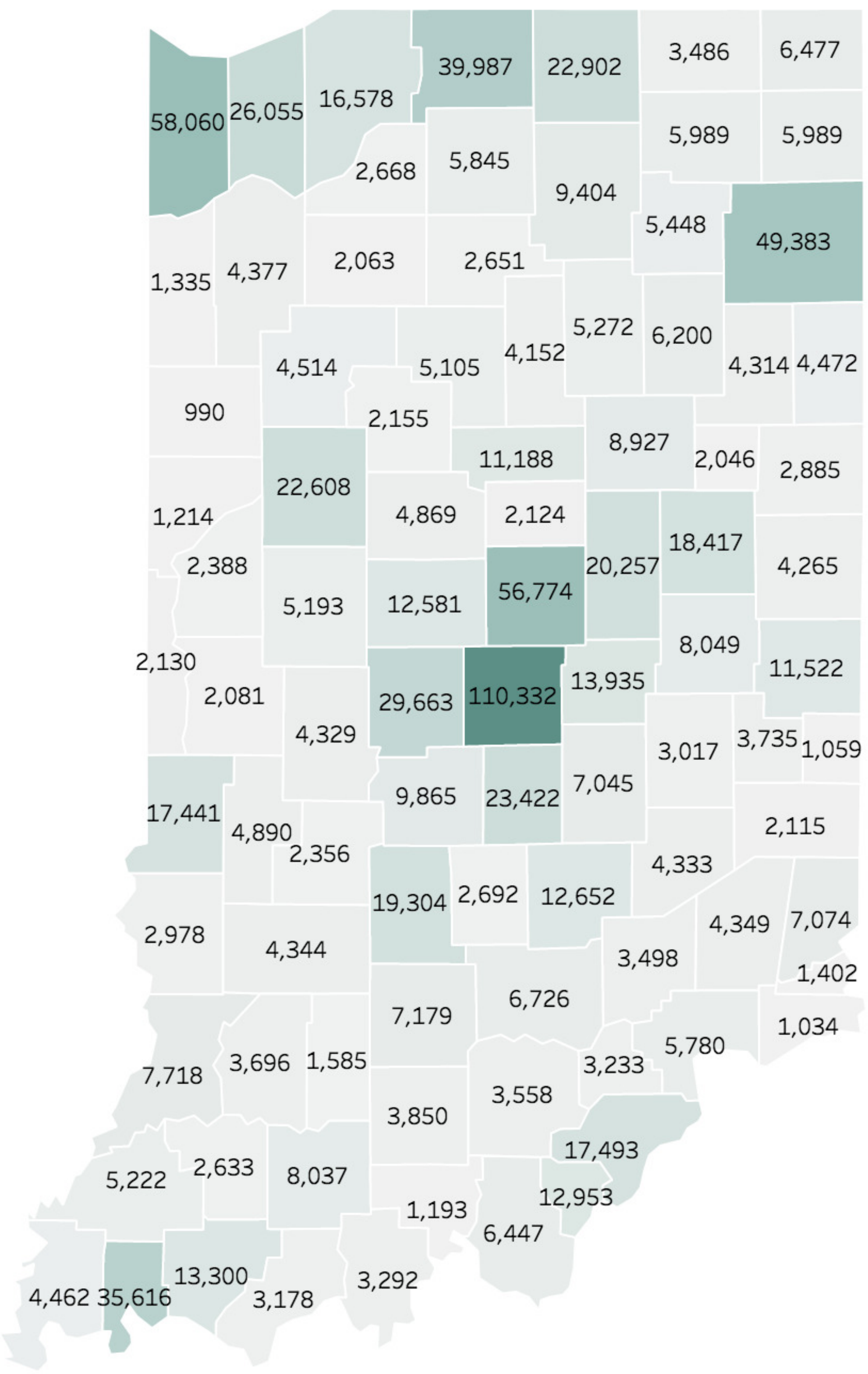

Vaccination Sequence
- (•) First Dose Administered
- ( ) Fully Vaccinated

Timeframe
- (•) All Time
- ( ) Previous Day

2,331 individuals did not report a county of residence and are excluded from this map.

20,034 individuals reported a county of residence outside of Indiana and are excluded from this map.

Statewide Demographics for Vaccinated Individuals

Demographic counts of less than 5 are suppressed within these visuals.

Vaccination Sequence
- (•) First Dose Administered
- ( ) Fully Vaccinated

Age Group

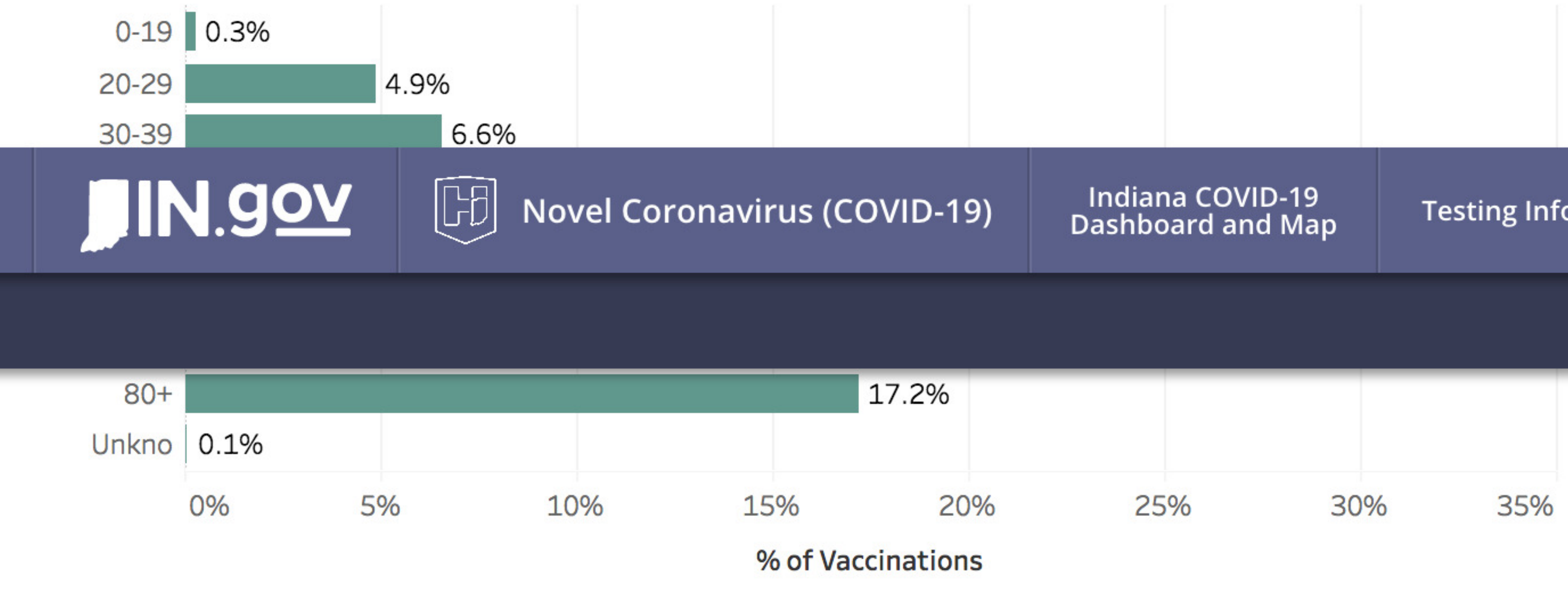

Race

	% of Vaccinations	% of Indiana Population
White	87.0%	85.1%
Unknown	3.9%	0.0%

Gender

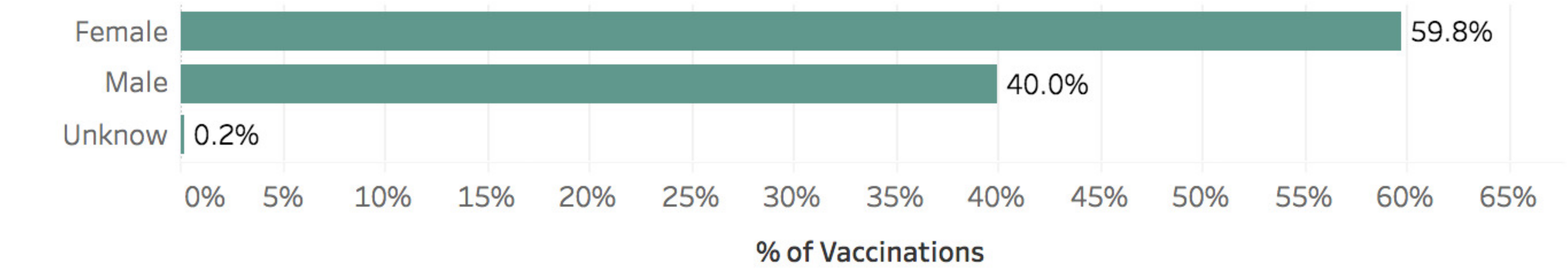

Ethnicity

	% of Vaccinations	% of Indiana Population
Not Hispanic or Latino	89.9%	92.9%
Hispanic or Latino	2.1%	7.1%
Unknown	7.9%	0.0%

All numbers are provisional and reflect only those reported to IDOH. Numbers should not be characterized as a comprehensive total and may change as more data is reported.

Data

Dashboard Information

The COVID-19 vaccine dashboard is updated Monday, Wednesday and Friday by 12:30 p.m. To maximize your experience, use a device with a larger screen, such as a tablet, laptop or desktop.

Kansas COVID-19
Vaccination Overview

168,341
People Vaccinated

205,114
Total Doses Reported as Administered
First Dose: 168,341
Second Dose: 36,773

412,375
Total Doses Distributed
State Distributed: 311,950
Federal LTC Program: 100,425

5.8%
Percent of Kansans Vaccinated

Vaccines Administered by Dose and Manufacturer as Reported in KS WebIZ

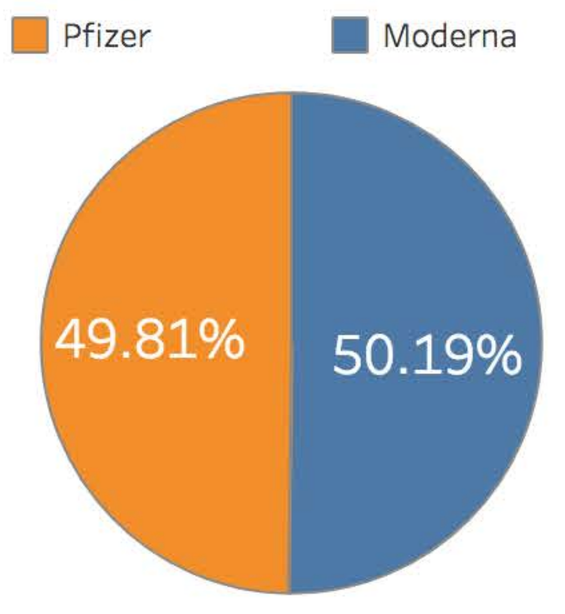

Sort by Dose Number
(All)

Vaccines Administered by Age Group

Younger than 18	365 (0.2%)
18 to 24	10,063 (6.0%)
25 to 34	24,658 (14.6%)
35 to 44	27,195 (16.2%)
45 to 54	23,996 (14.3%)
55 to 64	27,084 (16.1%)
65 to 74	23,559 (14.0%)
75 to 84	17,063 (10.1%)
85+	14,358 (8.5%)
Total	168,341 (100%)

Vaccines Administered by Sex

Female	114,490 (68.0%)
Male	53,469 (31.8%)
Unknown/Not Reported	382 (0.2%)
Total	168,341 (100%)

COVID-19 Vaccines Administered by First and Second Dose
(as reported into KS WebIZ)

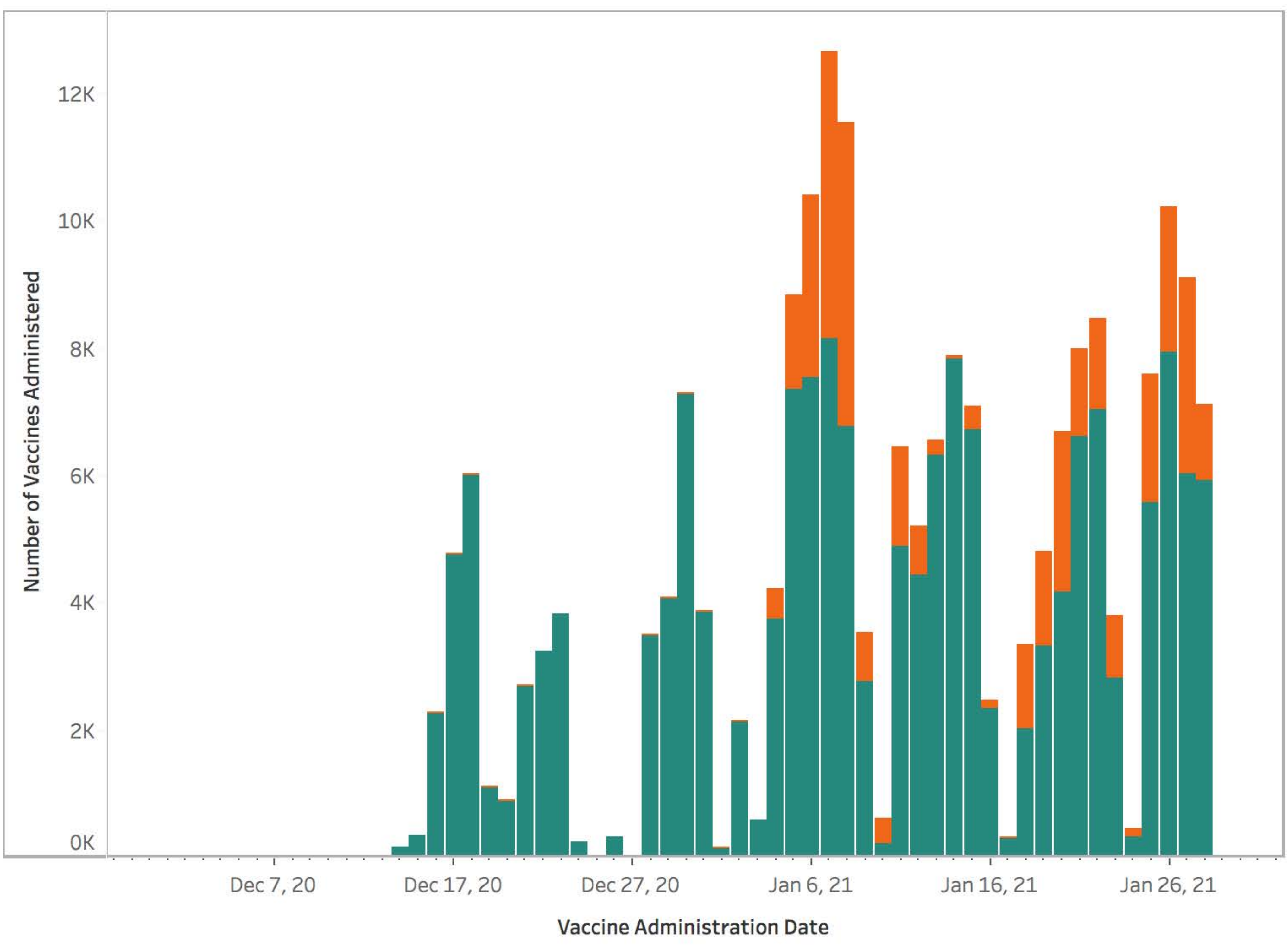

Number of Doses Distributed by County
(Week of January 25, 2021)

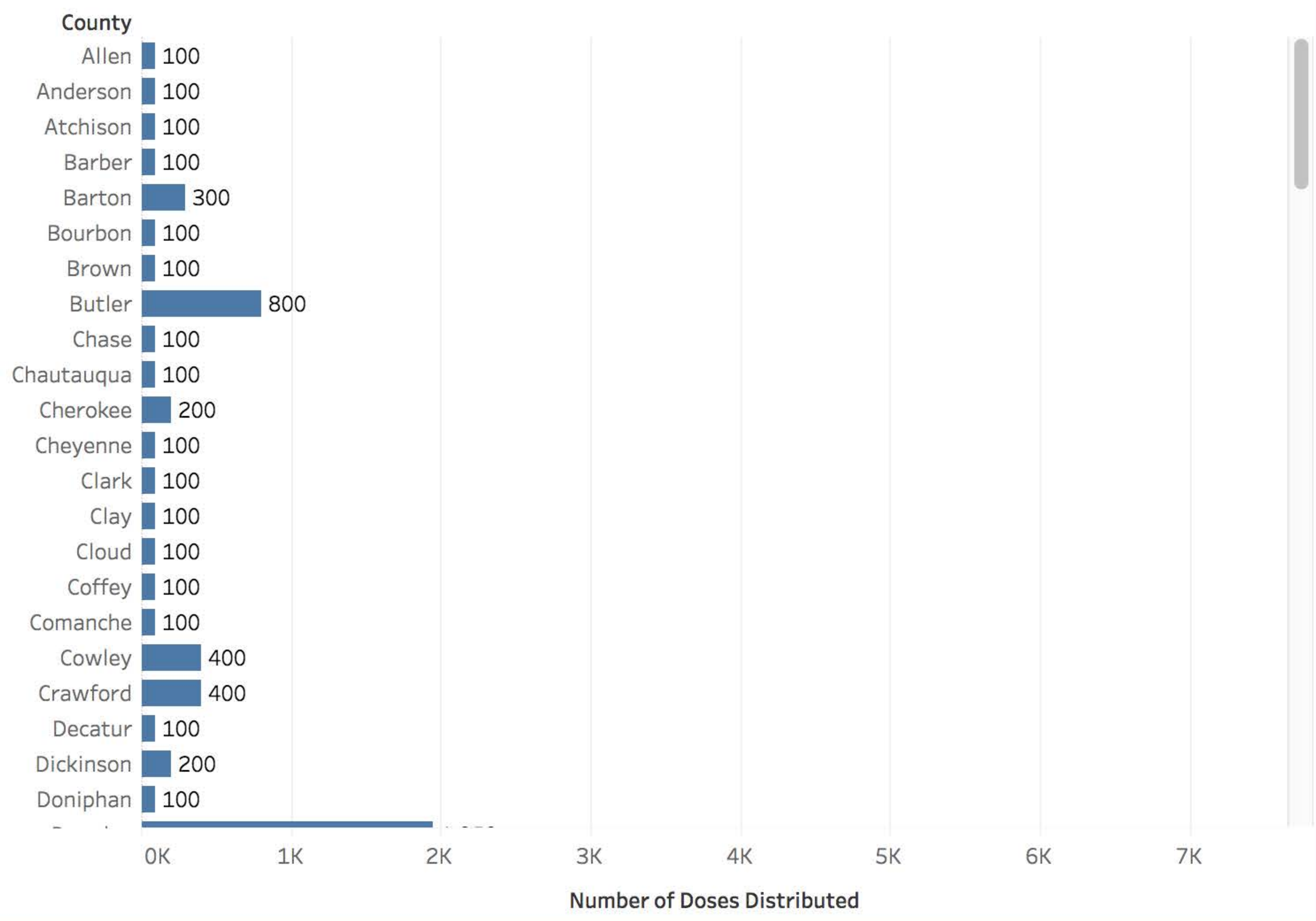

Data are preliminary and subject to quality improvement and quality assurance validation.
Data source: Kansas Immunization Information System, KS WebIZ.
Last updated: 1/29/2021 at 12:00pm

+ t a b l e a u

Total Doses Administered represents the total number of all COVID-19 vaccine doses (e.g. first dose, second dose) that have be[illegible] given to people in Kansas as reported in KS WebIZ.

Total Doses Distributed represents the total number of all COVID-19 vaccine doses that have been delivered to vaccine administration sites throughout Kansas. This includes all first and second doses. Second doses are delivered prior to completion of the appropriate interval.

Percent of Kansans Vaccinated represents the number of people with at least one dose of COVID-19 vaccine reported in KS WebIZ as a fraction of the total population of Kansas (2019 Bridged-Race Population Estimates, as reorganized in VSDA Population Master File). It is important to note that not all Kansans may be eligible for vaccination (e.g., Phase 3 recipients, individuals younger than 16).

Current Week Doses Distributed by County: Totals displayed in this dashboard represent first doses that are being allocated to each county for the current week. This represents the total number of doses expected this week. Local health departments and other distribution sites may have already scheduled the use of these doses. Please check with your local health department for more information about your county. Dose allocations are made taking multiple factors into consideration. More information can be found in the provider manual.

Vaccines Shipped | Distribution Trend | Doses Administered | Doses by Vaccine | Doses Metrics | Enrolled Providers | Learn More

COVID Vaccine Distribution

Dashboard updated: January 29, 2021. Date when the vaccine is shipped by the distributor, NOT the date of receipt by the provider. Includes first and second dose allotments. Doses are distributed to vaccine providers within the LHD jurisdiction.

Michigan

Preparedness Region: All

Local Health Dept. Jurisdiction: All

Provider County: All

Data as of :
1/28/21

Total Vaccines Distributed*

1,479,975

*430,200 doses were allocated to the Federal LTC Program

Distribution Date Range: 12/14/2020 – 1/28/2021

Facility Type: All

Year: All

Vaccine Type: All

Pfizer	734,175
Moderna	745,800

COVID Vaccine Distribution by County

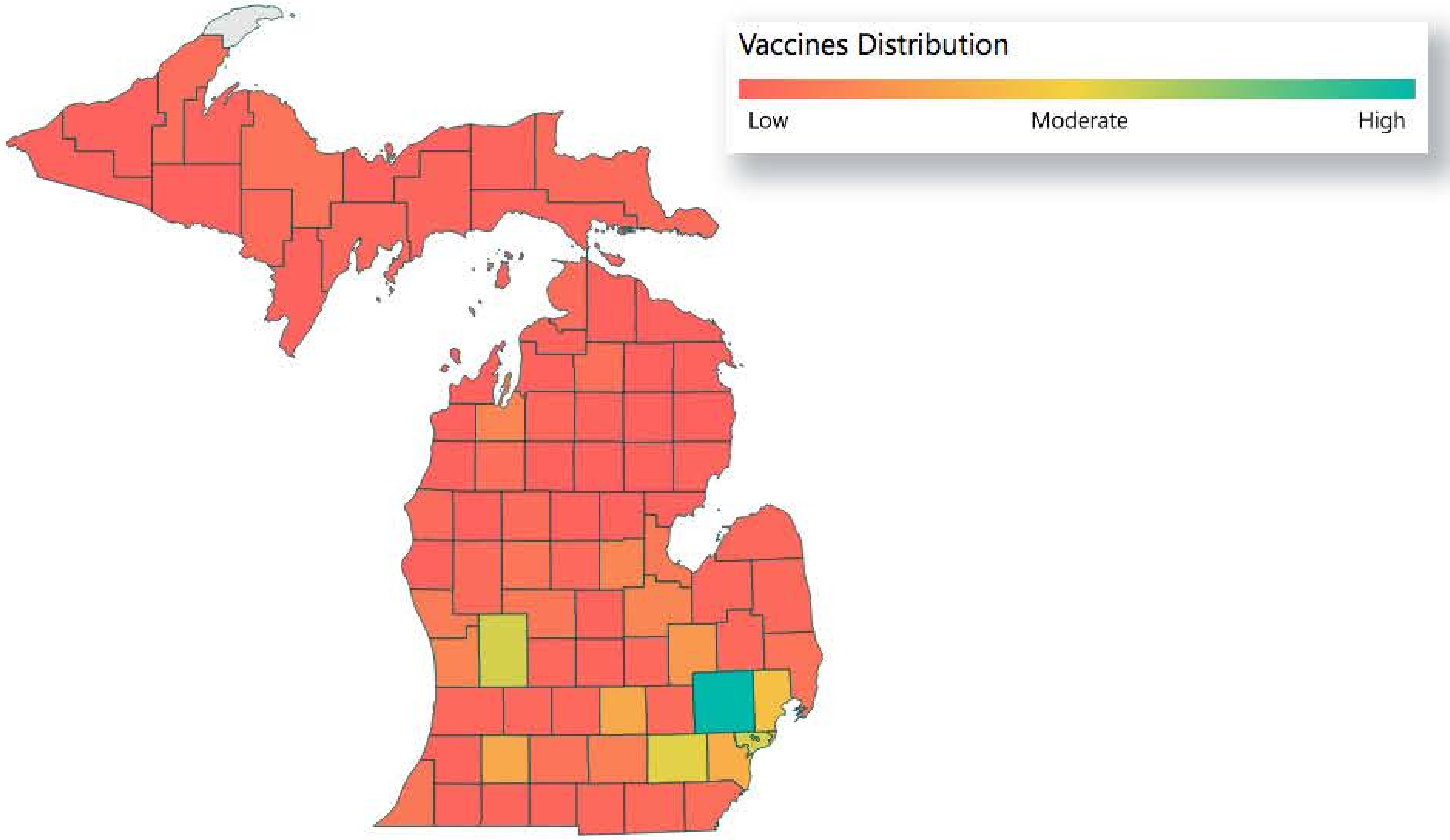

Vaccines Distributed in Preparedness Region (units in K = Thousand)

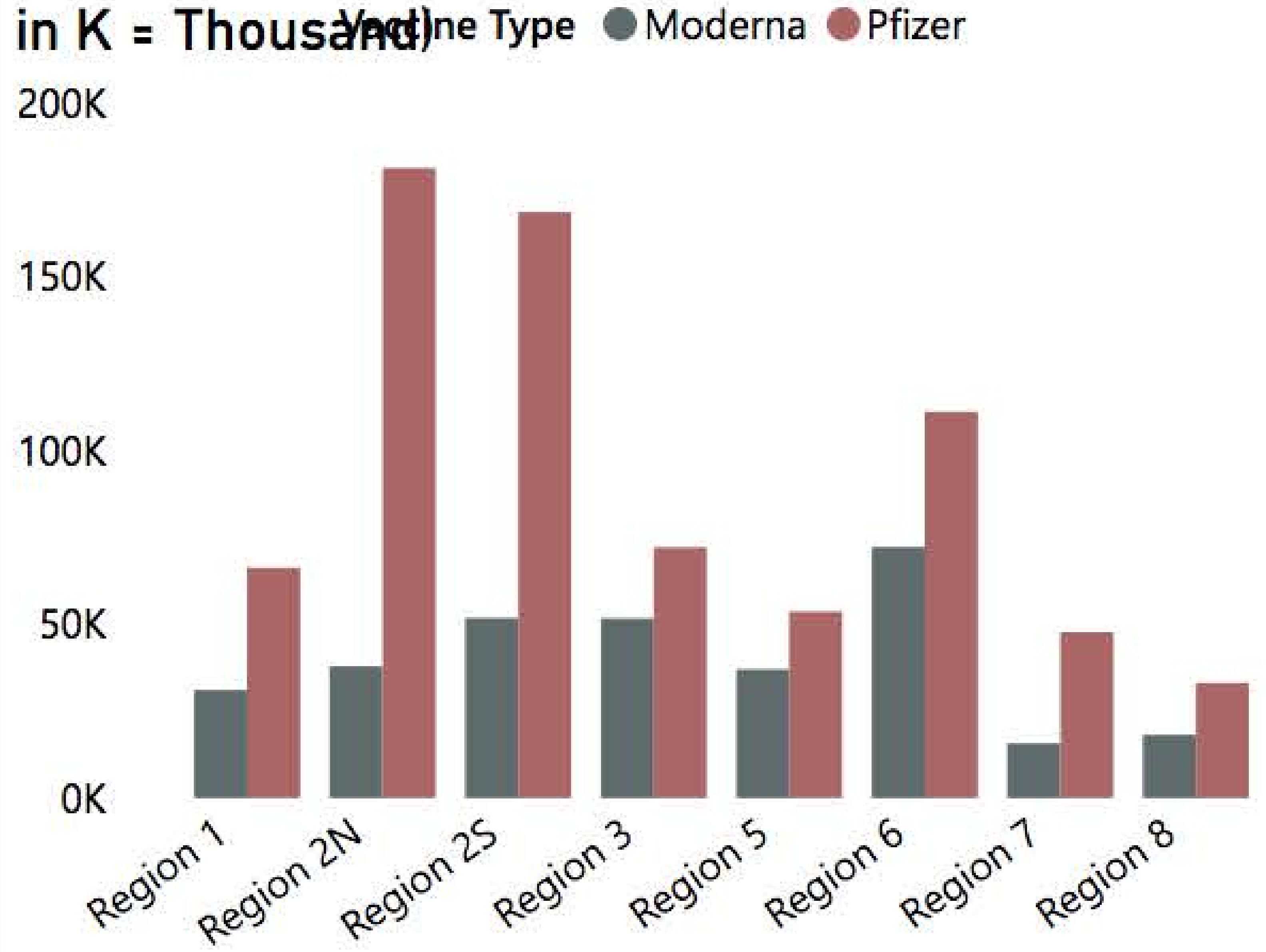

Microsoft Power BI

Vaccines Shipped | Distribution Trend | Doses Administered | Doses by Vaccine | Doses Metrics | Enrolled Providers | Learn More

COVID Vaccine Doses Administered - Vaccine Type and Provider Facility Type

Dashboard Updated: January 29, 2021. Doses administered are reported according to where the patient lives and the date they were vaccinated. Providers have 24 hours to report vaccinations to MDHHS. MDHHS pulls the data each morning for cleaning and then posting to website by 3 pm. Monday through Friday.

Provider Facility Type

All

Age Group

All

Doses Administration Date Range

12/14/2020 1/28/2021

Number of COVID Doses Administered

909,038

Vaccine Type

All

Pfizer
623,848
Doses Administered

Moderna
285,190
Doses Administered

Data as of:
01/28/21

COVID Vaccine Doses Administered by Provider Facility Type (units in K=1000)

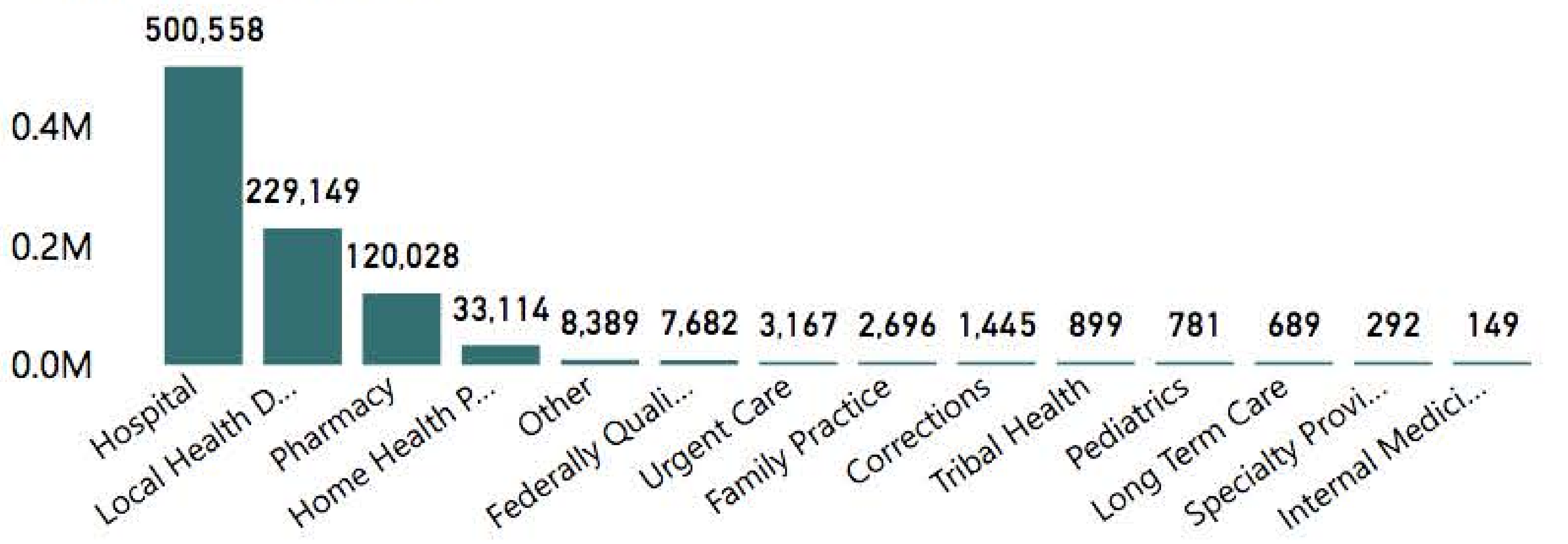

Some of the Pharmacy doses may be administered through the LTC program

COVID Vaccine Doses Administered by Vaccine Type (units in K = 1000)

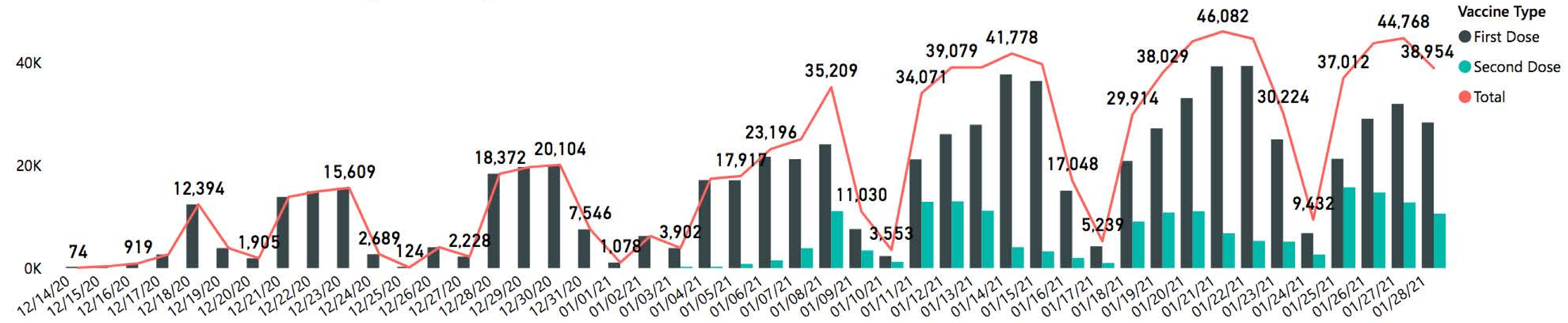

COVID-19 Vaccine Dashboard

Vaccines Shipped	Distribution Trend	Doses Administered	Doses by Vaccine	Doses Metrics	Coverage	Coverage Metrics	State Level Metrics	Enrolled Providers	Learn More

COVID Vaccine Distribution by Date and Facility Type

Dashboard updated: February 26, 2021. Date when the vaccine is shipped by the distributor, NOT the date of receipt by the provider. Includes first and second dose allotments. Doses are distributed to vaccine providers within the LHD jurisdiction.

Region 1

Preparedness Region: All

Local Health Department Jurisdiction: All

Provider County: All

Facility Type: All

Distribution Date Range: 12/14/202C – 2/25/2021

Data as of : 2/14/21

Facility Type	Moderna	Total
Federally Qualified Health Center	4,000	4,000
Hospital	52,200	52,200
Local Health Department	94,500	94,500
**Total Doses**	**150,700**	**150,700**

Vaccines Distributed by Date / Week Ending Date (K = Thousand, M = Million)

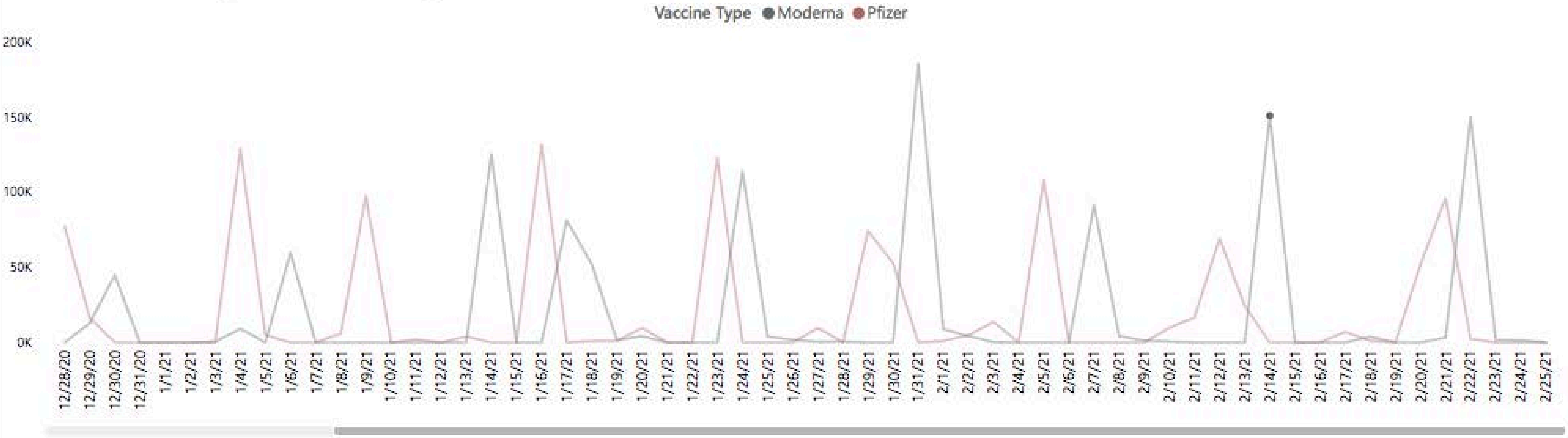

Microsoft Power BI

COVID-19 Vaccine Dashboard

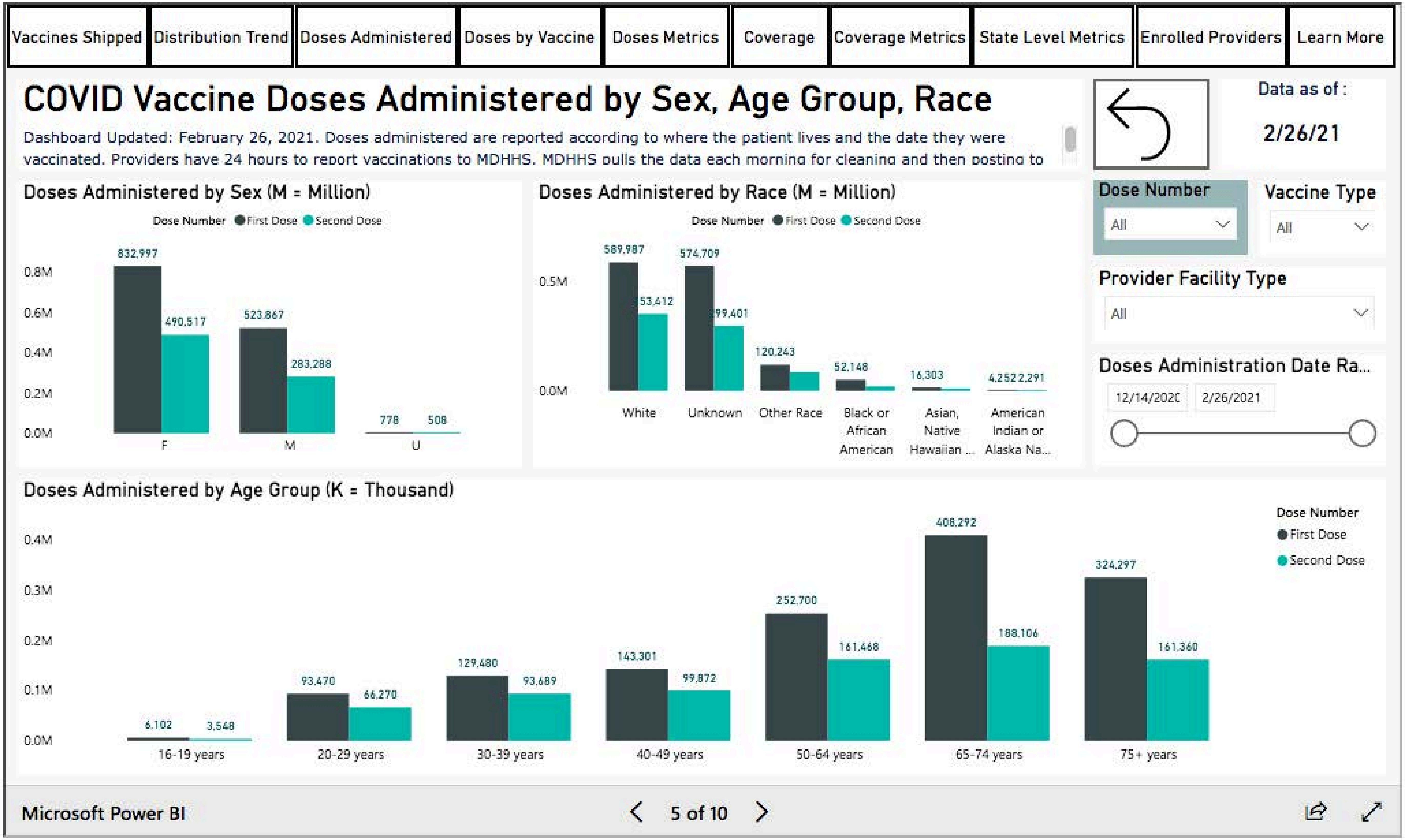

Vaccine Data

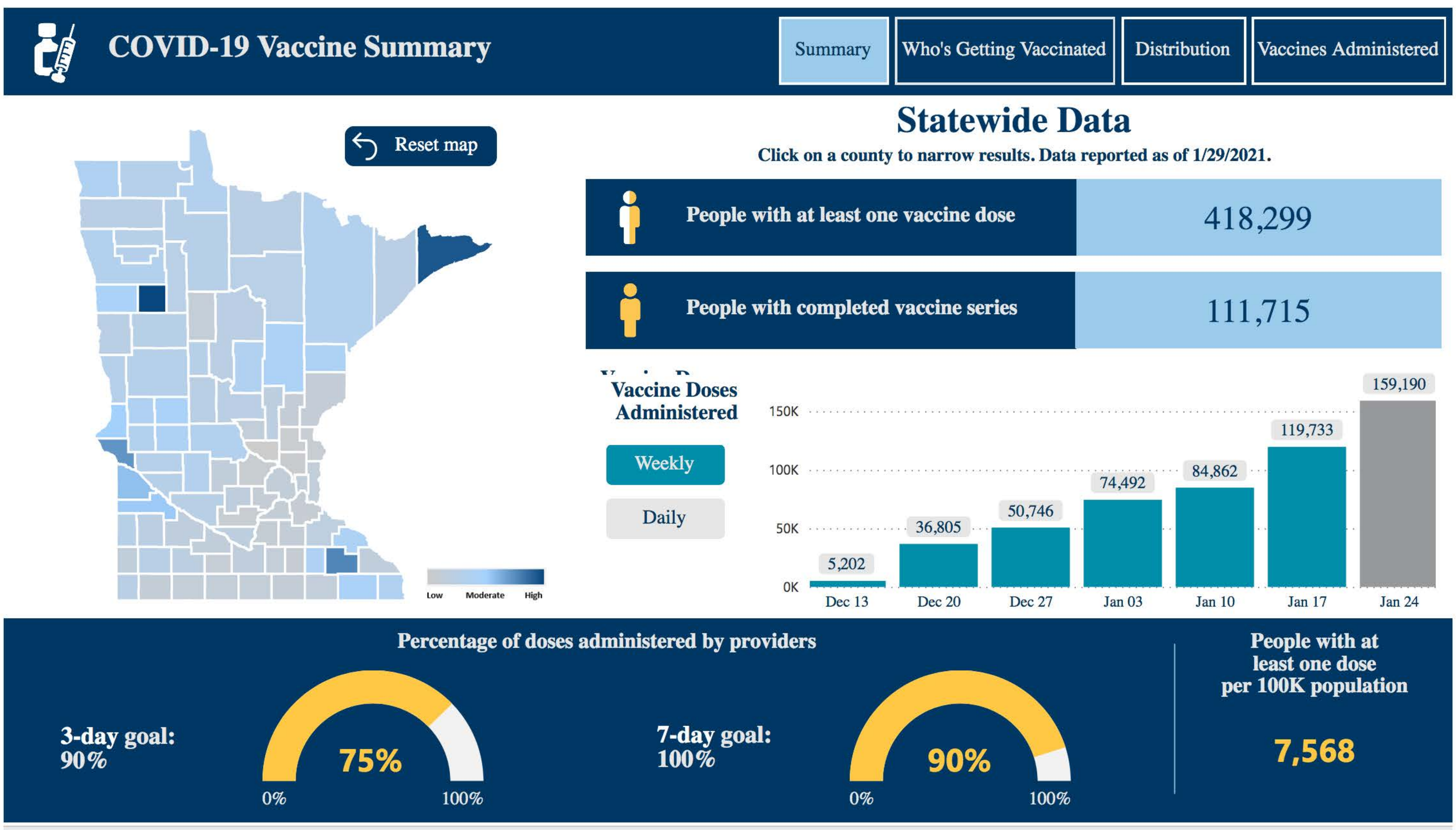

Microsoft Power BI

Data is updated daily at 11 a.m. unless otherwise specified, but there is a delay between a vaccine being given and when it is reported to the Minnesota Department of Health (MDH). This happens because the state must validate and process the data before reporting. Visit the Vaccine Distribution and Administration page to learn more about how a vaccine dose gets from the federal government, into a Minnesotan's arm, and reported back to the state.

Data Dashboard Definitions

Summary

- **Percentage of doses administered within 3-day goal:** Seven day rolling average of the percentage of doses shipped to providers that were administered within three days of receipt, not including second doses that cannot be administered yet due to interval timing. One day for data entry is allowed. Includes doses for the CDC's Pharmacy Partnership Program for vaccination in long-term care settings.
- **Percentage of doses administered within 7-day goal:** Seven day rolling average of the percentage of doses shipped to providers that were administered within seven days of receipt, not including second doses that cannot be administered yet due to interval timing. One day for data entry is allowed. Includes doses for the CDC's Pharmacy Partnership Program for vaccination in long-term care settings.
- **People with at least one dose per 100K population**: Number of Minnesotans with at least one dose of vaccine per 100,000 residents.
- **People with at least one vaccine dose**: Number of people in Minnesota who have received either a first or second dose of any COVID-19 vaccine.
- **People with completed vaccine series**: Number of people in Minnesota who have completed a COVID-19 vaccine series. For the vaccines that are currently available, Pfizer and Moderna, a complete series is two doses.
- **Vaccine doses administered**:
  - By week: Number of doses administered by week.
  - 7-day: Number of doses administered, by day, during the past seven days.

Who's Getting Vaccinated

- **Percent of people with at least one vaccine dose**: Percent of Minnesotans who have received at least one dose of any COVID-19 vaccine. Vaccine administration data are based on vaccination records in the Minnesota Immunization Information Connection (MIIC), a confidential immunization information system. Immunization records in MIIC are submitted by participating health care providers. Providers vaccinating with doses through the State of Minnesota are supposed to submit COVID-19 vaccination information to MIIC within 24 hours; pharmacies have 72 hours. Providers getting vaccine directly from the federal government are not required to submit information to MIIC, but some may.
- **Percent of people with completed vaccine series**: Percent of people in Minnesota who have completed a COVID-19 vaccine series. For the vaccines that are currently available, Pfizer and Moderna, a complete series is two doses.

Distribution

- **Doses shipped to Minnesota providers:** Number of doses that have been shipped to providers in Minnesota from the federal government's allocation for the state. This does not include doses that have been set aside for the CDC's Pharmacy Partnership Program for vaccination in long-term care settings. This is a cumulative count of COVID-19 vaccine doses recorded as shipped to Minnesota providers in the Centers for Disease Control and Prevention's (CDC) Vaccine Tracking System (VTrckS) since Dec. 13, 2020.
- **Doses shipped for CDC long-term care vaccination program**: Doses from the federal allocation for Minnesota that are shipped to pharmacies participating in the CDC's Pharmacy Partnership Program for vaccination in long-term care settings. Doses for CDC's long-term care vaccination program (also known as the Pharmacy Partnership Program or PPP) is a cumulative count of COVID-19 vaccine doses that have been transferred out of the state allocation to the federal Pharmacy Partnership Program. These doses are shipped to three large pharmacy chains, CVS, Walgreens, and Thrifty White, who vaccinate staff and residents within long-term care facilities.
- **Total provider sites receiving vaccine**: Number of Minnesota provider sites that are receiving vaccines from the federal allocation for Minnesota, including doses for the Pharmacy Partnership Program (PPP), by provider type. Provider type is self-reported.

Vaccines Administered

- **Total vaccine doses administered**: Number of vaccine doses that have been administered to Minnesotans and reported to the Minnesota Immunization Information Connection (MIIC). Doses include those from the federal allocation for Minnesota (including PPP) and from the allotment delivered to federal agencies in Minnesota directly (including Veteran's Affairs, Indian Health Services, the Bureau of Prisons, and the Department of Defense). Age and gender are self-reported at the time of vaccination.
- **Vaccines by product**: Total doses administered by vaccine brand, as reported to MIIC.
- **Percentage of doses administered within 3-day goal:** Seven day rolling average of the percentage of doses shipped to providers that were administered within three days of receipt, not including second doses that cannot be administered yet due to interval timing. One day for data entry is allowed. Includes doses for the CDC's Pharmacy Partnership Program for vaccination in long-term care settings.
- **Percentage of doses administered within 7-day goal:** Seven day rolling average of the percentage of doses shipped to providers that were administered within seven days of receipt, not including second doses that cannot be administered yet due to interval timing. One day for data entry is allowed. Includes doses for the CDC's Pharmacy Partnership Program for vaccination in long-term care settings.

Downloadable Datasets (.CSV)

Updated January 31, 2021

- Doses Administered, Total
- Doses Administered, by Week
- Doses Administered, by Age
- Doses Administered, by Gender
- Doses Administered, by Provider
- People With at Least One Vaccine Dose, by County
- People With at Least One Vaccine Dose, by Age
- People With at Least One Vaccine Dose, by Gender
- Doses Shipped to Minnesota Providers, by Vaccine Product
- Doses Shipped for the CDC Long-Term Care Vaccination Program, by Vaccine Product
- Provider Sites Receiving Vaccines, by Type
- Percentage of Doses Administered by Providers, 3-Day Goal
- Percentage of Doses Administered by Providers, 7-Day Goal
- People 50+ with at Least One Vaccine Dose, Percent of Age Group Population
- People by Gender with a Least One Vaccine Dose, Percent of Gender Group Population

About the data

All denominators based on American Community Survey (ACS) data: 2018 ACS 1-year Estimates. All data by county is based on residence.

The COVID-19 vaccination data can be used to monitor state- and county-level immunization coverage and progress towards national, state, and local coverage goals. The data can also help to identify at-risk populations and encourage public health actions and policies aimed at increasing immunization. The data cannot tell us why people are not vaccinated. Some people may not have ready access to the vaccine, and some people may choose not to vaccinate. Also, some people may have received vaccinations that are not recorded in MIIC, such as those who were vaccinated in another state.

Due to data collection and reporting timing, there may be temporary discrepancies between data from the Minnesota Department of Health (MDH) and other sources.

State-level metrics include all doses administered to Minnesotans that are reported to MIIC. This includes doses administered in other states and with doses from the other states' allocations, as well as doses administered to federal workers and agencies in Minnesota. Doses administered by Tribal Health/IHS are included in the state-level metrics if the person vaccinated lives in Minnesota. Systems-level metrics include all doses administered by Minnesota providers, regardless of the address of the person receiving the vaccine. Systems-level metrics also includes doses administered to non-Minnesotans, but not doses administered to Minnesotans out of state.

Although MIIC is a useful resource for real-time assessments of state and county immunization percentages in Minnesota, MIIC data may underestimate the actual number of people receiving COVID-19 vaccine due to several limitations:

- Some data may not be submitted to MIIC properly or there could be errors in the data that result in missing immunization information. MDH staff are working closely to monitor and address any lapses in data exchange to ensure that timely and accurate COVID-19 vaccination information is submitted.
- MIIC is not set up to capture a person's occupation or comorbidities.
- Under Minnesota's Immunization Data Sharing Law (MN Statute 144.3351), race or ethnicity information can only be entered into the state's immunization information system with the patient's permission; we do not always receive that information and may have incomplete data.

Total vaccine doses administered 531,048

Product	%	Doses Administered
MODERNA	34.1%	180,966
PFIZER	65.8%	349,626
Unknown/missing	0.1%	456
**Total**	**100.0%**	**531,048**

Percentage of doses administered by providers

3-day goal: 90% | 7-day goal: 100%

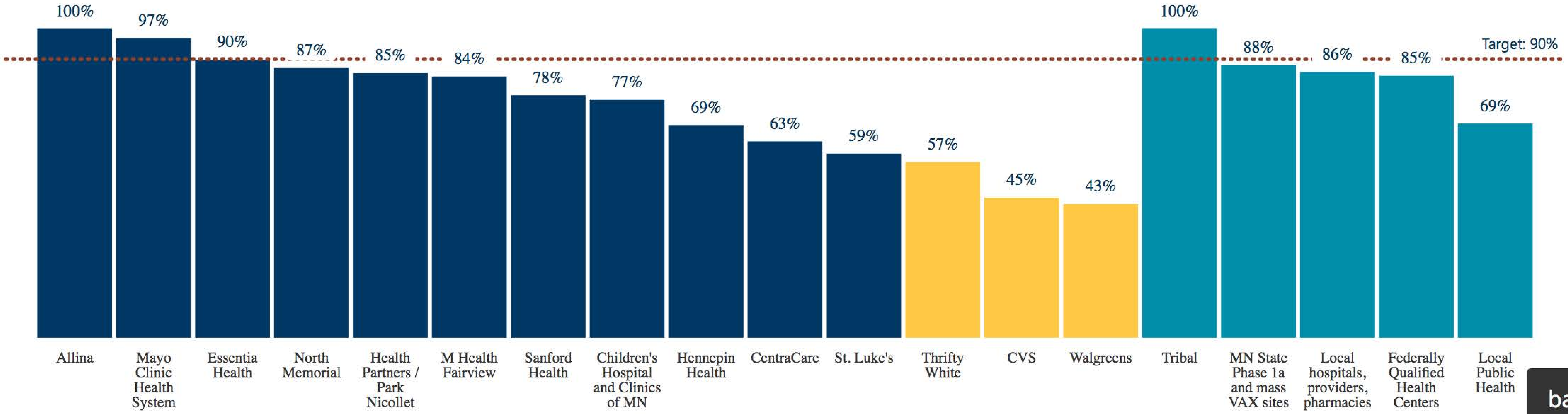

Data reported as of: **1/29/2021**

Microsoft Power BI
Pages
COVID-19 questions?
Chat with **our COVID-19 bot**

COVID19 Vaccine Dashboard

The COVID-19 vaccine data dashboard provides daily vaccine doses administered and weekly vaccine coverage rates for North Dakota.

Dashboard data will be updated by 10am Monday through Friday.

Data as of Thursday, 1/28/2021

COVID-19 Vaccine Doses Administered

96,291

Doses Administered

112,525

COVID-19 Doses Received

Cumulative Total COVID-19 Vaccine Doses Administered

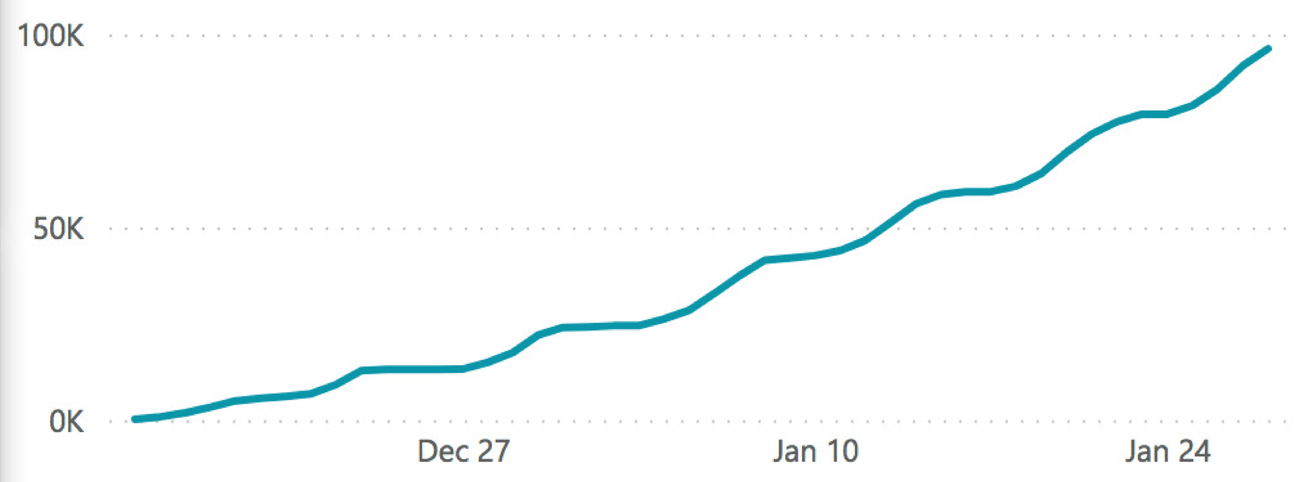

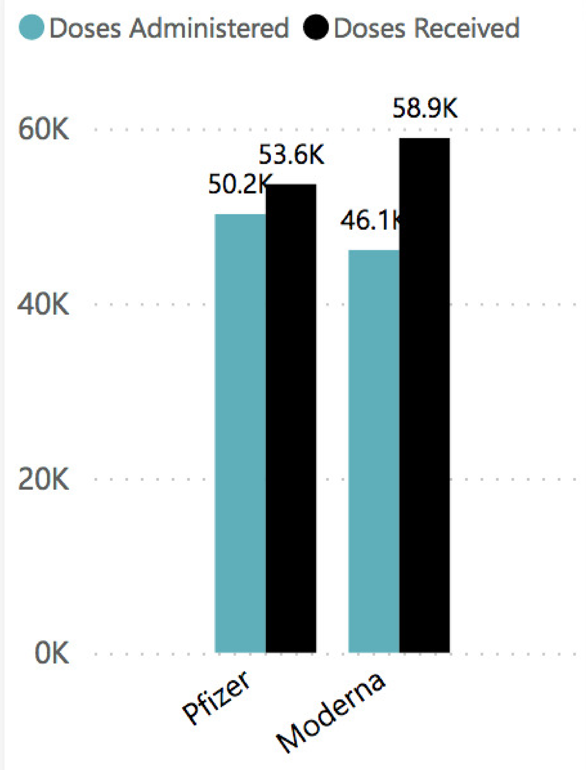

Hover over county to view Total COVID-19 Vaccine Doses Administered

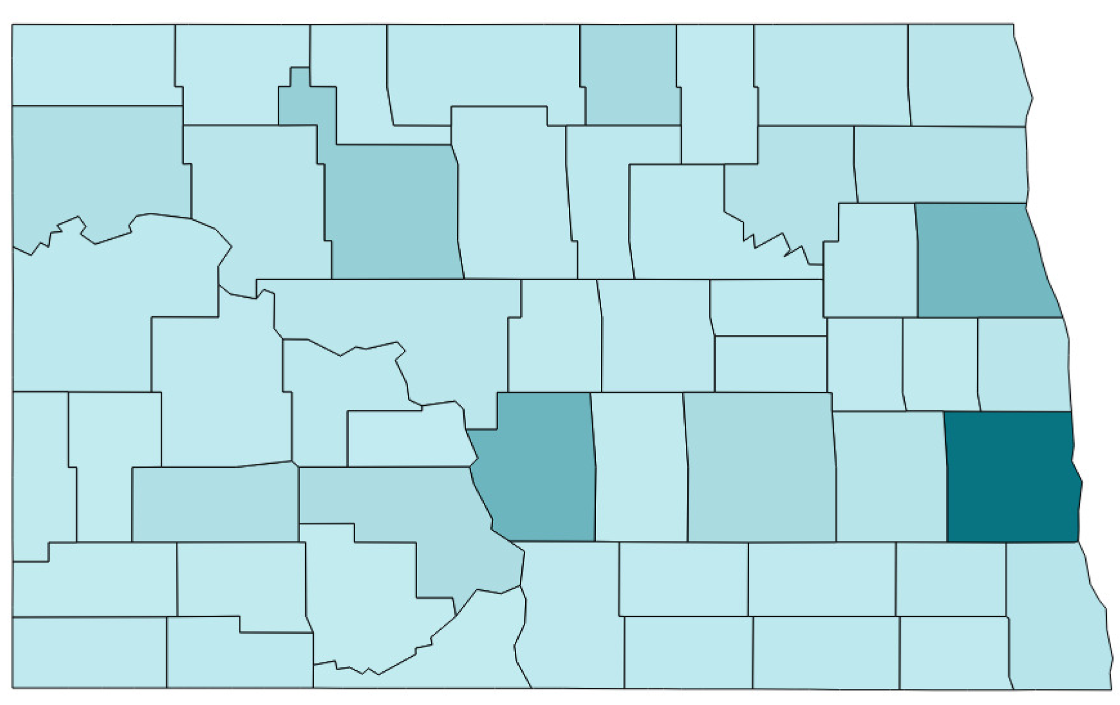

COVID-19 Vaccine Coverage Rates by Week

9.1%

1 Dose Coverage Rate

66,408 ND residents received 1st dose

3.0%

2 Dose Coverage Rate

21,806 ND residents received 2nd dose

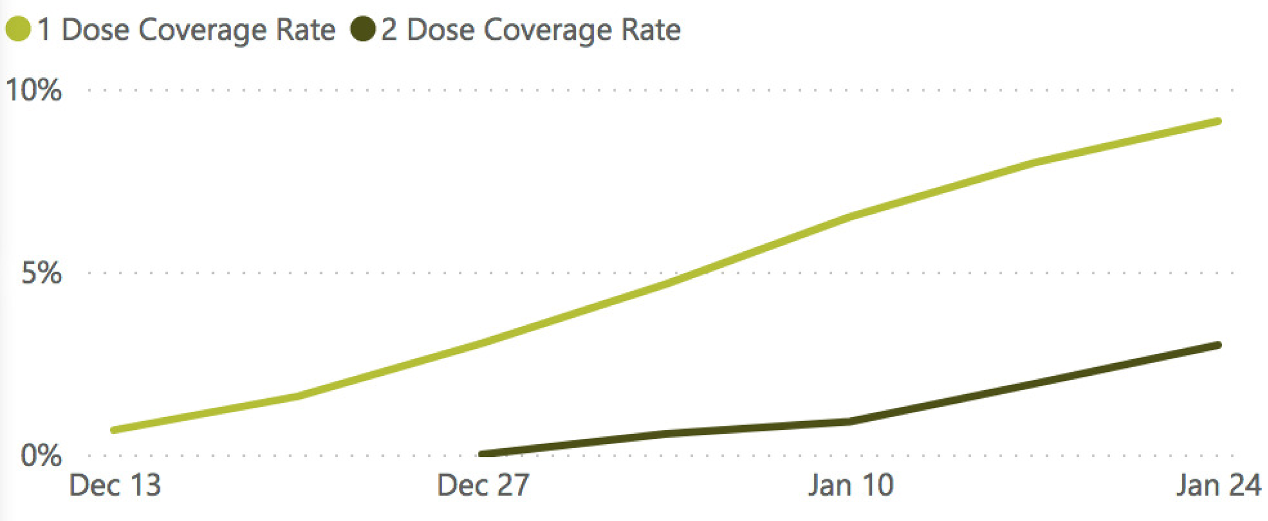

Select 1 Dose or 2 Dose Coverage Rate

1 Dose Coverage Rate | 2 Dose Coverage Rate

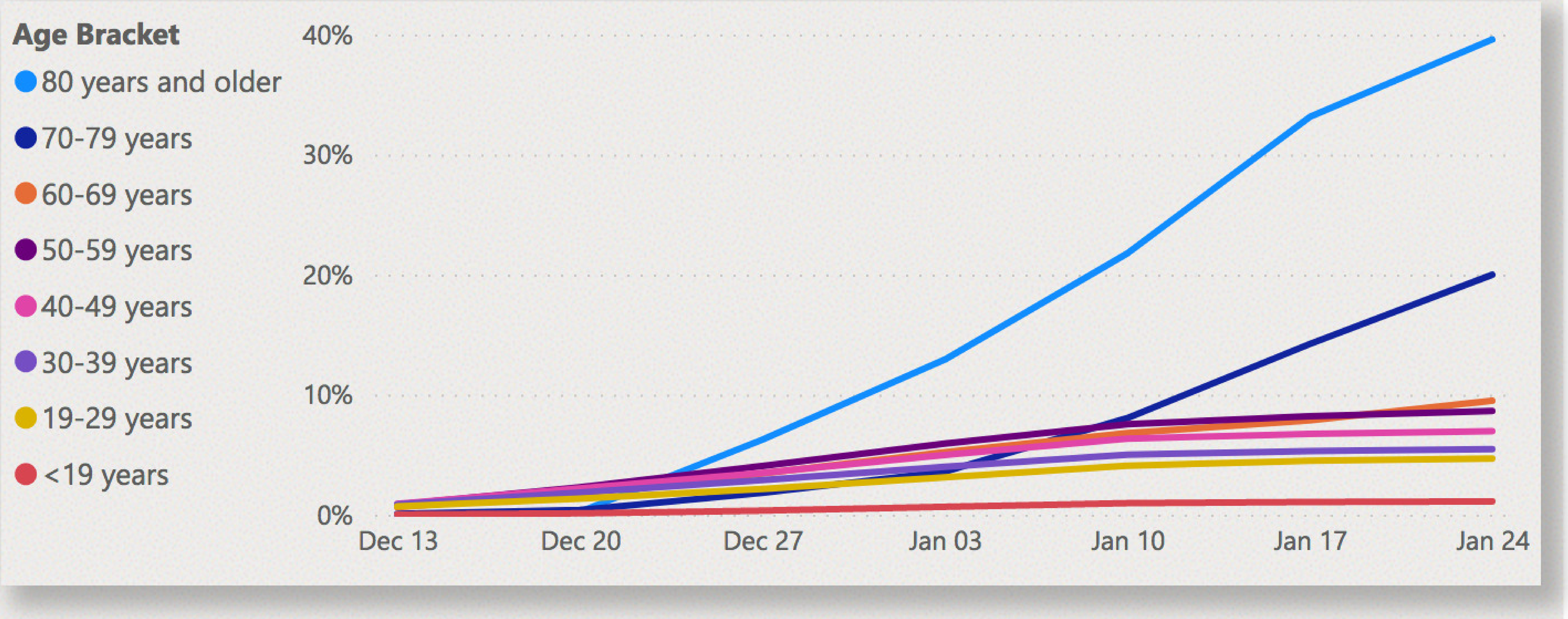

Hover over county to view COVID-19 Vaccine 1 Dose Coverage Rate

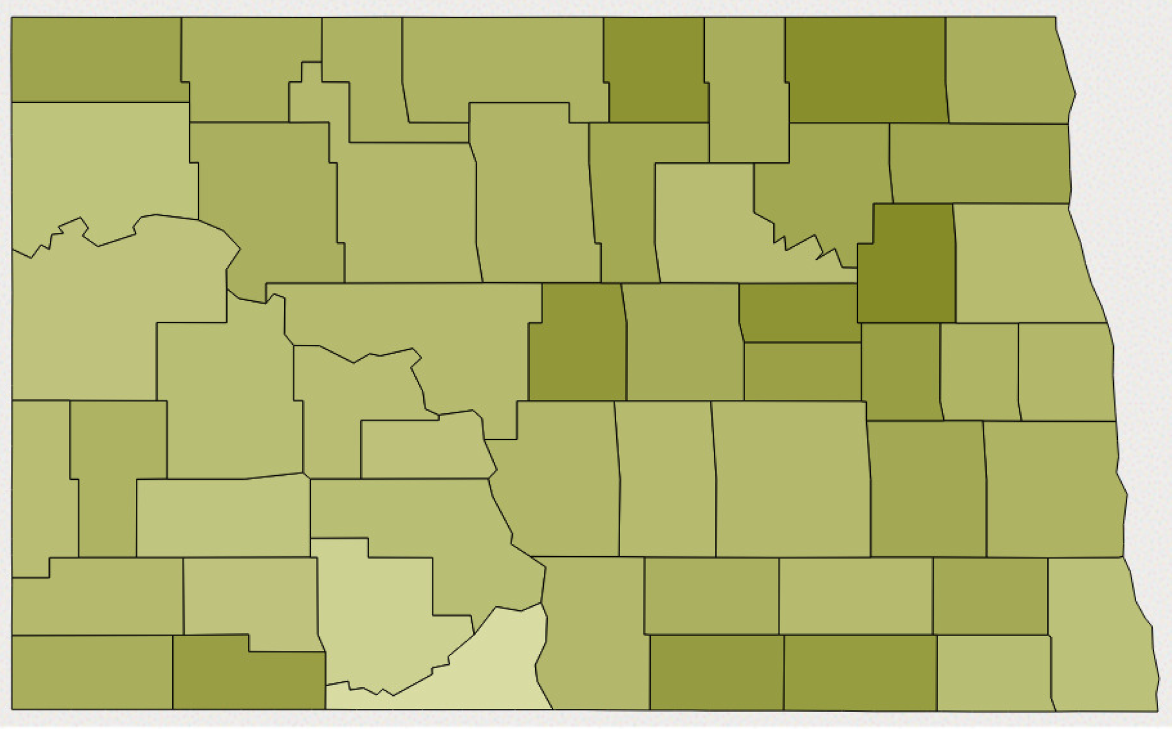

Coverage Rates by Ethnicity

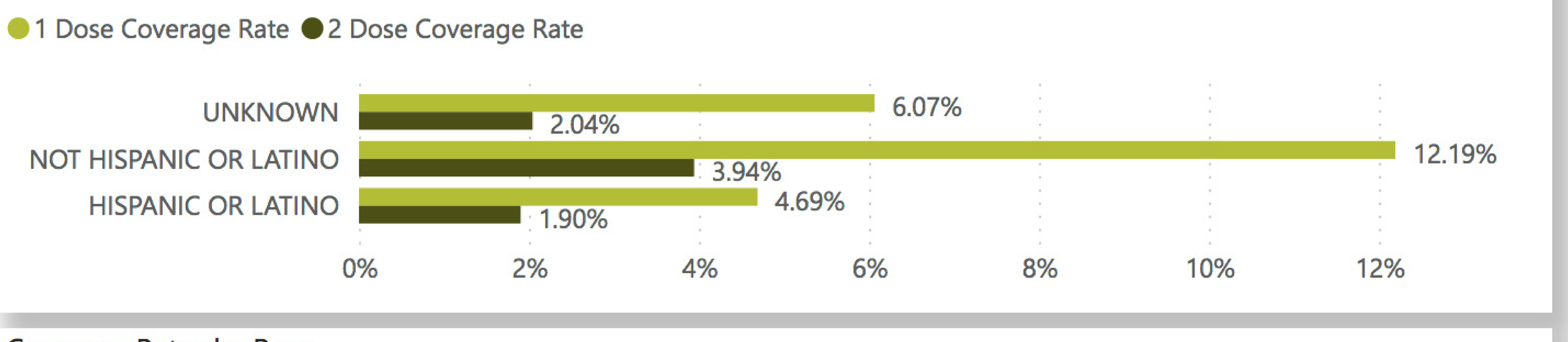

Coverage Rates by Race

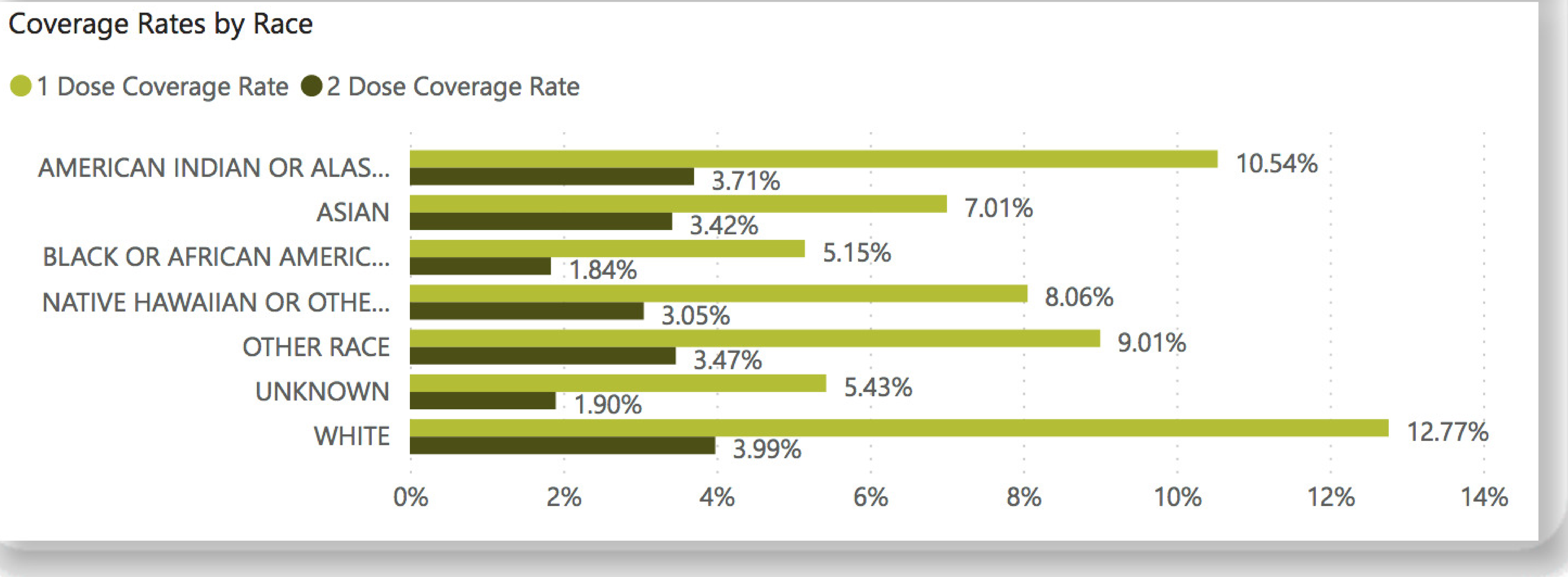

COVID-19 Vaccine Enrolled Provider Sites

397

Enrolled Provider Sites

Hover over the map to view the total number of provider sites located in the county that are enrolled to receive COVID-19 vaccine through the North Dakota Department of Health Division of Immunizations

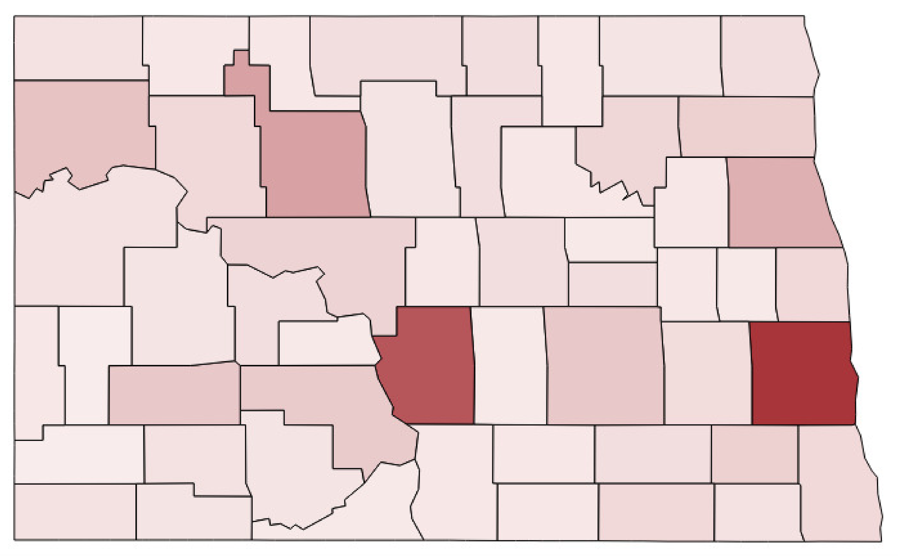

***COVID-19 vaccine doses administered, coverage rate, and exemption data is from the North Dakota Immunization Information System (NDIIS). North Dakota immunization providers who are not receiving COVID-19 vaccine allocations through the North Dakota Department of Health Division of Immunizations may not be entering COVID-19 vaccine information into the NDIIS.**

NMDOH

New Mexico Department of Health

COVID-19 Vaccine Dashboard

The New Mexico Department of Health (NMDOH) is leading the State of New Mexico's COVID-19 Vaccination Preparedness Planning in close collaboration with other state agencies, public, private and tribal partners throughout the state.

Learn More

COVID-19 Vaccine Dashboard

Last Updated: 1/30/2020

Cumulative Doses Administered
263,568

Total Primary Doses Administered
207,273

Total Booster Doses Administered
56,295

Doses Administered in the Last 7 Days
56,256

Total Doses Received
278,800

Total Registrants
551,845

BASE LAYER

Doses Adminstered Per 100 Residents

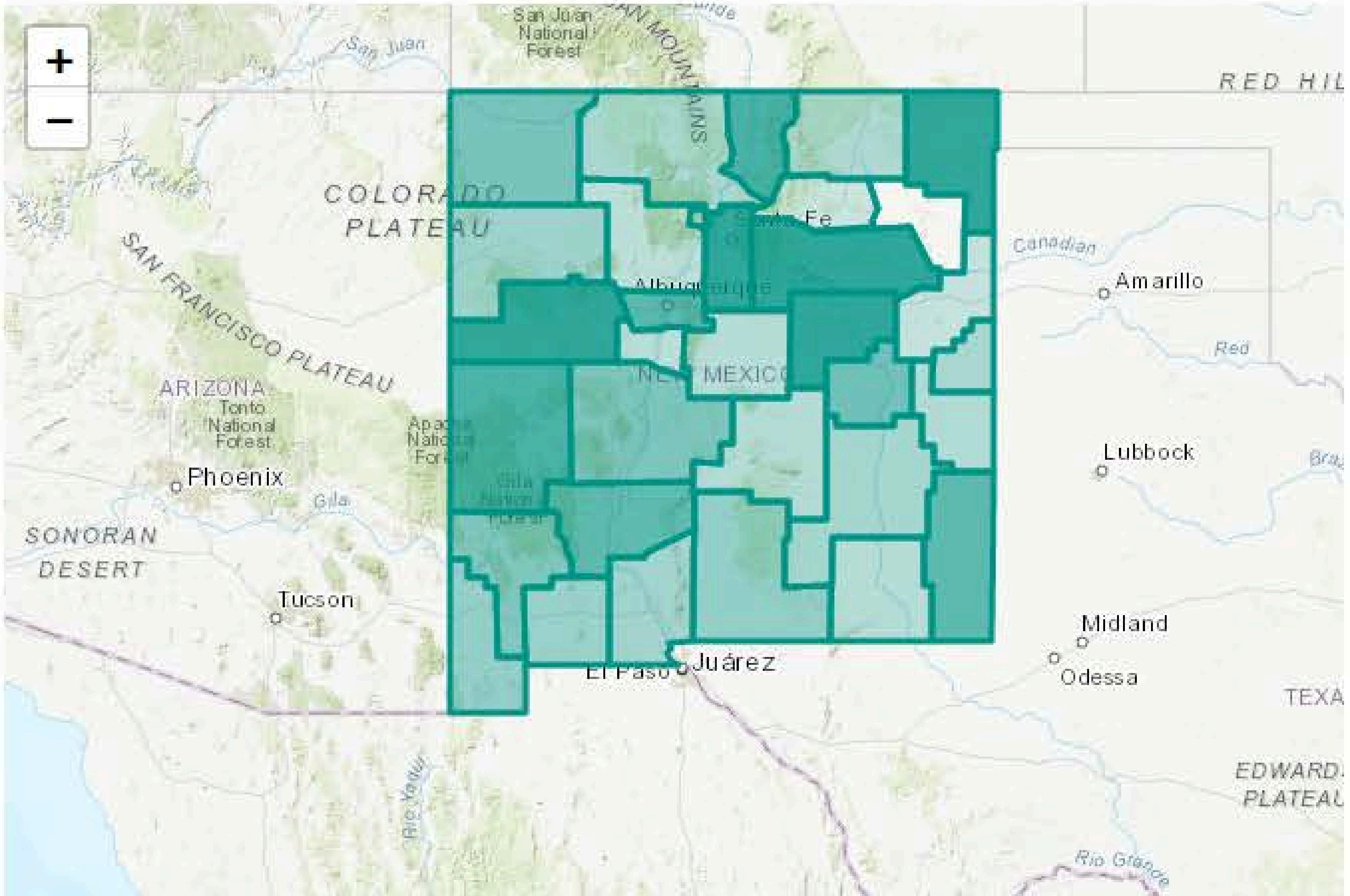

Rank	County	Doses Adminstered Per 100 Residents
1.	Union	38.8
2.	Guadalupe	37.7
3.	Cibola	30.6
4.	San Miguel	28.9
5.	Santa Fe	23.7
6.	Taos	17.3
7.	Catron	17.2
8.	Bernalillo	15.6
9.	Lea	15.4
10.	Sierra	15.2
11.	De Baca	13.4
12.	San Juan	12.5

Ohio | Department of Health

Coronavirus (COVID-19)

Search

Responsible RestartOhio | COVID-19 Vaccination Program | Equity and High-Risk Groups | Families and Individuals | Healthcare Providers and Local Health Districts

COVID-19 / Dashboards / COVID-19 Vaccine / COVID-19 Vaccination Dashboard

COVID-19 Dashboard

Overview | Current Trends | Key Metrics | Schools and Children | Long-Term Care Facilities | Demographics | COVID-19 Vaccine | Other Resources

COVID-19 Vaccination Dashboard

The COVID-19 Vaccination Dashboard displays the most recent data reported to the Ohio Department of Health (ODH) regarding the number of individuals that have started and completed the COVID-19 vaccination series by various demographics and county of residence.

The COVID-19 Vaccination Dashboard displays the most recent data reported to the Ohio Department of Health (ODH) regarding the number of individuals that have started and completed the COVID-19 vaccination series by various demographics and county of residence. "Vaccination started" indicates that the individual has received at least one valid dose of COVID-19 vaccine. The number listed as "vaccination completed" is a subset of the number included in "vaccination started," indicating that those individuals within that group have received all recommended COVID-19 vaccine doses and are considered fully immunized. ODH is making COVID-19 data available for public review while also protecting privacy. This dashboard will be updated daily. Please see footnotes below for more details.

- Download the summary data (CSV)

Note: A full screen option for this dashboard can be found on the lower right corner. To exit full screen mode press the 'Esc' key.

State of Ohio | COVID-19 Vaccine Dashboard

Last Updated: 2021-02-27

CSV

View By
Select to view by Vaccine Started or Completed
Vaccine Started*

Statewide: Vaccine Status
By Total and % of Population

Vaccine Started*, Change from Last 24 Hours****	Vaccine Started* By Population	Vaccine Started*, Total
48,350	13.86%	1,620,452

Click County to Filter Metrics
Counties are colored by % of Population with Vaccine Started*

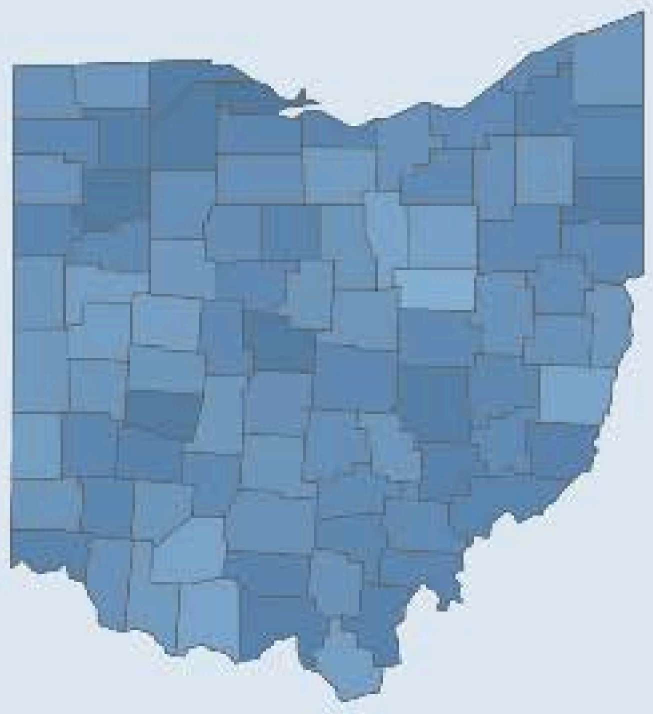

Residency Not Reported
31,492

Residents of Other States - Vaccinated in Ohio
33,030

Key Metrics
Select to view key demographic groups
Age Group

0-19	0.25%	7,356
20-29	8.70%	104,021
30-39	9.87%	145,822
40-49	11.07%	153,507
50-59	11.28%	173,984
60-64	11.77%	93,743
65-69	31.05%	207,083
70-74	44.45%	232,219
75-79	54.67%	195,497
80+	61.51%	307,218

View Count By
Select to view counts by daily or cumulative
Daily Total

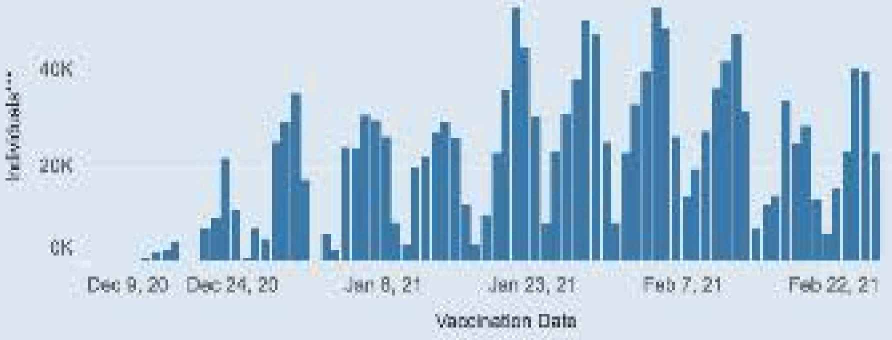

* Indicates that an individual has received at least one valid dose of COVID-19 vaccine.
** Indicates that an individual has received all recommended COVID-19 vaccine doses and is considered fully immunized; specific requirements (number of doses, spacing, age restrictions) may vary depending on COVID-19 vaccine brand.
***A person is counted in the 'Vaccine Started' category on the day that they receive their first valid dose of COVID-19 vaccine. A person is counted in the 'Vaccine Completed' category on the day that they get their final recommended valid dose of COVID-19 vaccine. All dates on this dashboard indicate the date the vaccine was administered.
****The number of confirmed valid vaccines identified by ODH over the last 24 hours ending at 6 am daily. Although most vaccines are identified within 24 hours of the vaccine administration date, some may take longer to be reported.

Population data as reported by the US Census Bureau. There are no population estimates for "Unknown" or "Other" demographic groups, so there is no percentage of population for those groups. Data reported to the Ohio Department of Health. All data displayed are preliminary and subject to change as more information is reported to ODH.

+ a b l e a u

TN Department of Health

Tennessee COVID-19 Vaccination Reporting

Last Updated 1/31/2021

Click Here For:
Summary
1st Dose Map
2nd Dose Map

Total Vaccinations Reported	Vaccinations Reported on **1/30/2021**	Vaccinations Reported Since **1/24/2021**	% of People Statewide With at Least One Dose
654,537	18,902	163,627	6.98%

Providers administering COVID-19 vaccines are expected to report vaccine doses to the state immunization information system (TennIIS) within 24 hours of administration and are required to report doses no later than 72 hours after administration.

Number of People with **1 Dose Only** vs **2 Doses**	1 Dose Only	2 Doses
	**281,864**	**185,977**

Age Group

	People Vaccinated	% of People Vaccinated
16-20 years	2,869	0.61
21-30 years	41,325	8.83
31-40 years	53,743	11.49
41-50 years	57,160	12.22
51-60 years	61,594	13.17
61-70 years	51,246	10.95
71-80 years	115,112	24.60
81+ years	84,738	18.11
Pending	54	0.01
Total	467,841	99.99

Patient Race

	People Vaccinated	% of People Vaccinated
Asian	4,536	1.09
Black or African American	27,704	5.81
White	294,063	65.11
Other/Multiracial	45,592	8.77
Unknown	95,946	19.22
Total	467,841	100.00

Note: An update was made to how data were being categorized, causing a decrease for the Other/Multiracial category. Individuals with multiple races where one was "Unknown" were removed from this category.

Patient Ethnicity

	People Vaccinated	% of People Vaccinated
Hispanic or Latino	8,520	1.87
Not Hispanic or Latino	357,650	76.44
Unknown	101,671	21.69
Total	467,841	100.00

Patient Sex

	People Vaccinated	% of People Vaccinated
Female	293,435	62.72
Male	173,635	37.11
Other	49	0.01
Unknown	722	0.15
Total	467,841	99.99

Vaccination Date by Site Type

Site Type: Public Health, Other Providers

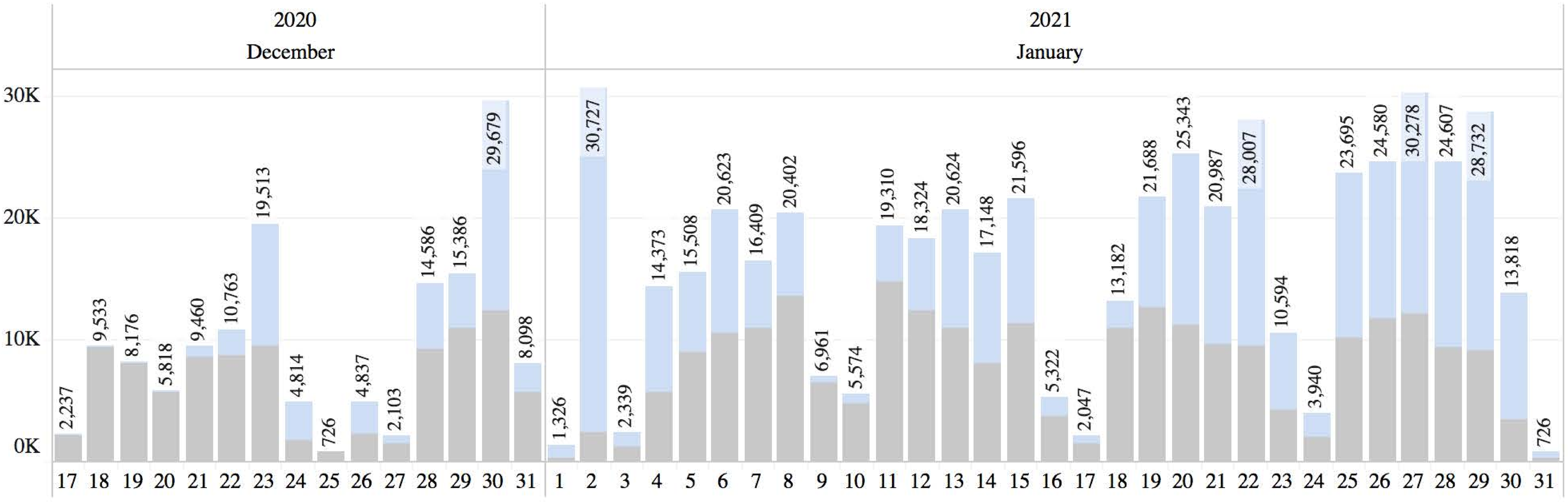

Public Health: local, regional, and metro health departments; **Other Providers:** hospitals, pharmacies, private practice, etc

Total Doses Delivered

**Public Health**	Moderna	253,700		
	Pfizer	201,825		
**Other Providers**	Moderna	74,800		
	Pfizer	333,450		
**LTCF Pharmacy Partnership**	Moderna		96,200	192,400
Total		863,775	96,200	192,400

LTCF Federal Pharmacy Program*

	[illegible]	[illegible]	[illegible]
CVS**	189	16,343	842
Walgreens***	359	30,252	4,213
Total	548	46,595	5,055

* Data from Tiberius (delayed 48-72h+)
** CVS publishes the most up-to-date LTCF data at https://cvshealth.com/covid-19/vaccine-information/vaccination-data
*** Walgreens publishes the most up-to-date LTCF data at https://www.walgreens.com/topic/findcare/long-term-care-facility-covid-vaccine.jsp#stayinformed

TN Contact Us Program Areas News Health Professionals Parents Individuals Statistical Data Career Opportunities

Downloadable COVID-19 Vaccine Datasets

SUMMARY | 1ST DOSE | 2ND DOSE

TN Department of Health

Tennessee COVID-19 Vaccination Reporting

Last Updated 1/31/2021

Click Here For: Summary | 1st Dose Map | 2nd Dose Map

Reported % of County Population With First Dose (Only)

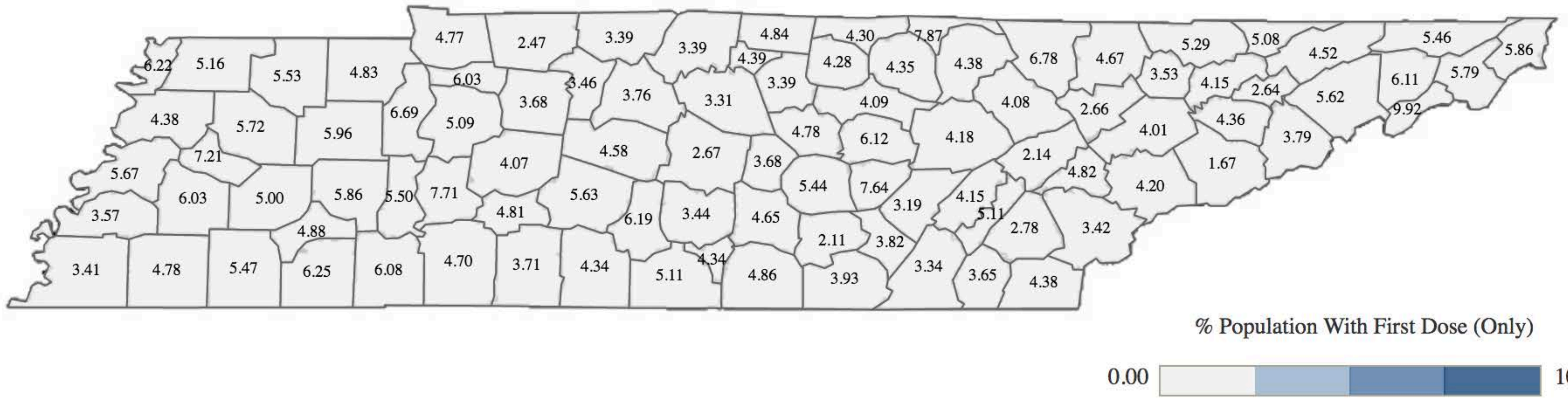

Based on reported doses to the state immunization information system (TennIIS) and 2019 county population data.

Summary | Vaccine Doses Allocated | Vaccine Doses Administered | People Vaccinated | About the Data

COVID-19 Vaccination in Texas

DSHS is partnering with vaccine providers across the state to ensure vaccine will be accessible to all Texans at no cost.

This dashboard is updated around 4 p.m. each day with data as of 11:59 the previous night. See the About the Data tab for more information on data sources.

TEXAS Health and Human Services | Texas Department of State Health Services

Vaccination of frontline and vulnerable populations

COVID-19 vaccination is currently available only to frontline and vulnerable populations due to limited supply. Texas will move through several phases to ensure those who need the vaccine most receive it first. We need every Texan's help to make this a success.

Visit the DSHS COVID-19 Vaccine Information page for more details on phases and tiers, and other vaccine info: https://www.dshs.texas.gov/coronavirus/immunize/vaccine.aspx

Choose population groups from the list below to see them by county in the red boxes.

Population Group - Phases 1A and 1B

1A - Healthcare (Tier 1)

Population Groups by County

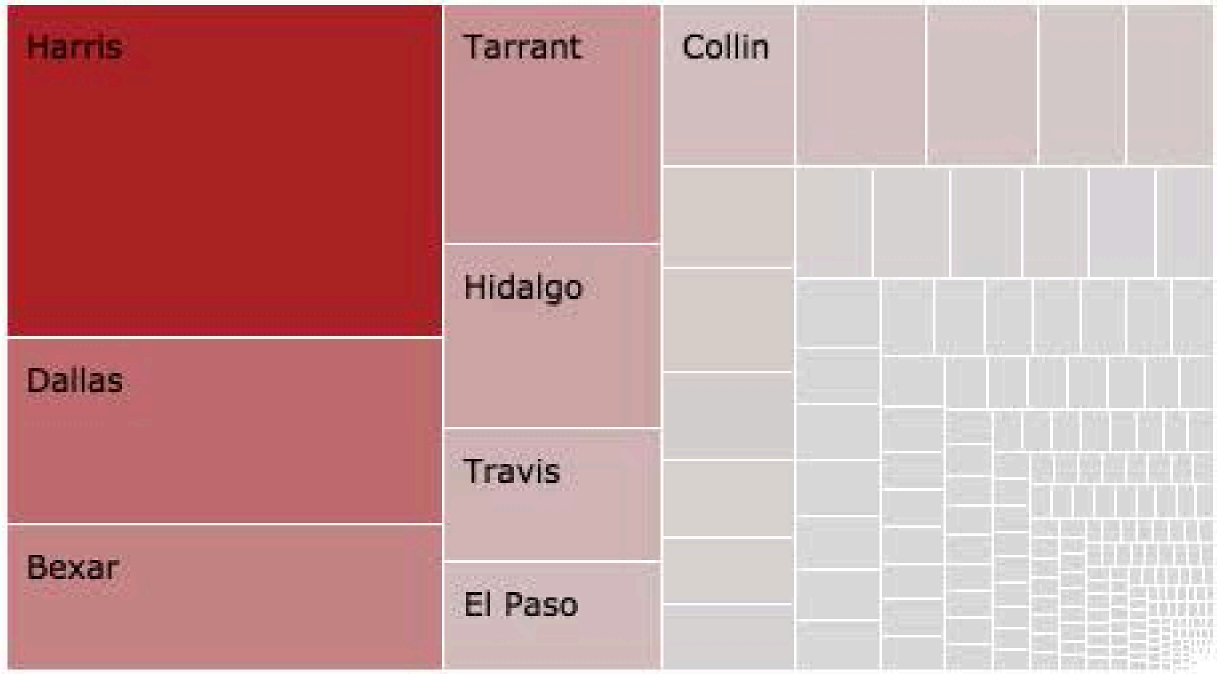

Texans Vaccinated for COVID-19

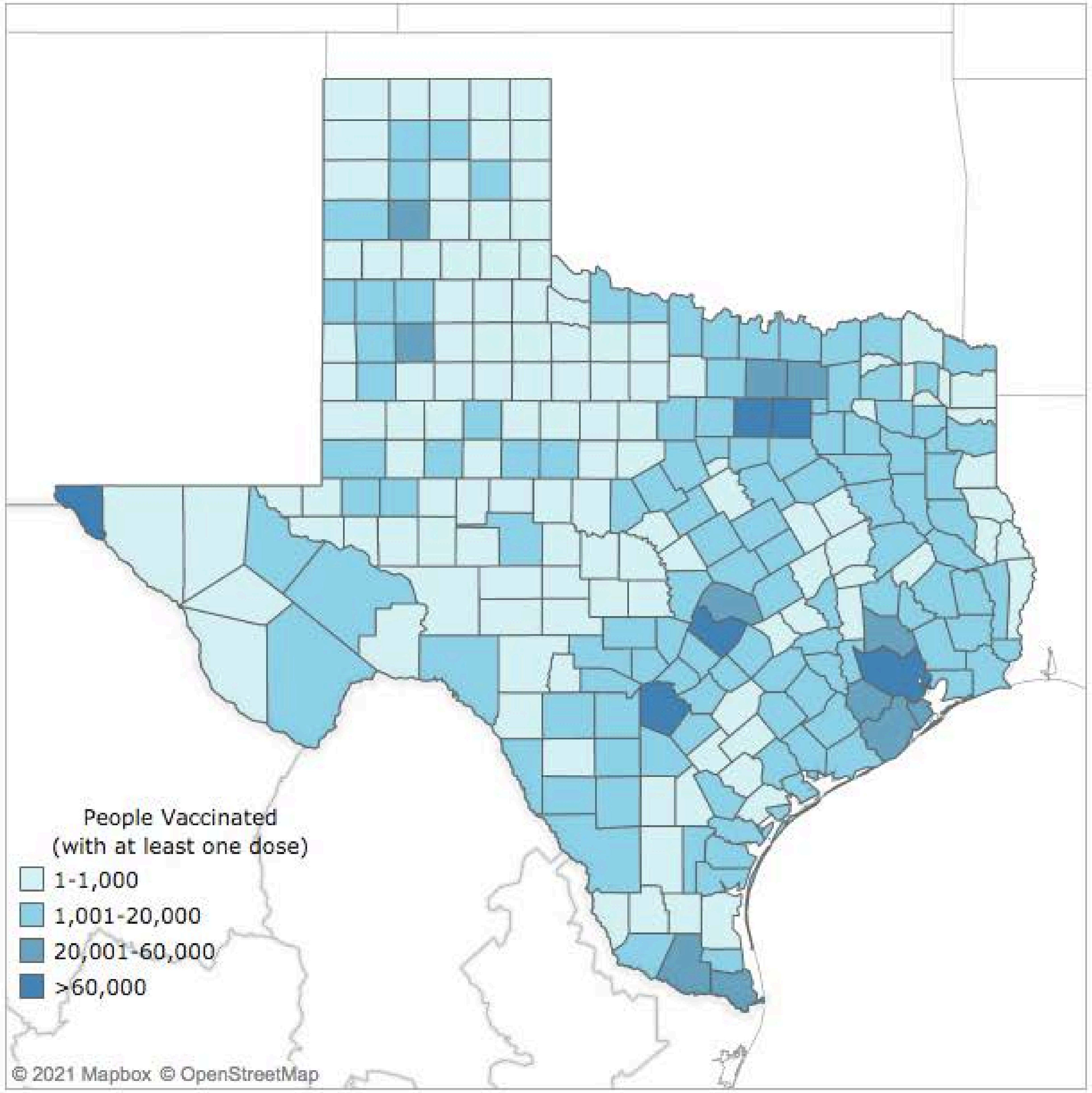

Doses Allocated

3,898,700

Doses Shipped

3,590,125

People Vaccinated with at least One Dose

1,883,862

People Fully Vaccinated

475,461

Doses Administered

2,359,323

Last Updated:
1/31/2021 12:26:52 PM

Summary | Vaccine Doses Allocated | Vaccine Doses Administered | People Vaccinated | About the Data

TEXAS Health and Human Services | Texas Department of State Health Services

COVID-19 Vaccination in Texas

Doses administered are doses providers have entered into ImmTrac2, the Texas Immunization Registry, by the recipient's county of residence and the date administered.

Providers have up to 24 hours to report doses to ImmTrac2, and dashboard is updated with data reported as of 11:59 the previous night.

Use the filter below to select a Texas county and view the number of vaccinations administered to persons living there, by week. Alternatively, click on a county on the map. Click the Revert button at the bottom of the screen to remove filters.

Search for a County

(All)

Doses Administered

2,359,323

Doses Administered by Organization Type

Emergency Services	86,465
Health Center (Community, Federally Qualified, Migrant, Rural)	102,951
Hospital	1,042,745
Long-Term Care	963
Medical Clinic/Doctor's Office	213,646
Public Health/Government	371,369
Pharmacy	403,468
Other	137,716

Vaccinations by Week in Texas

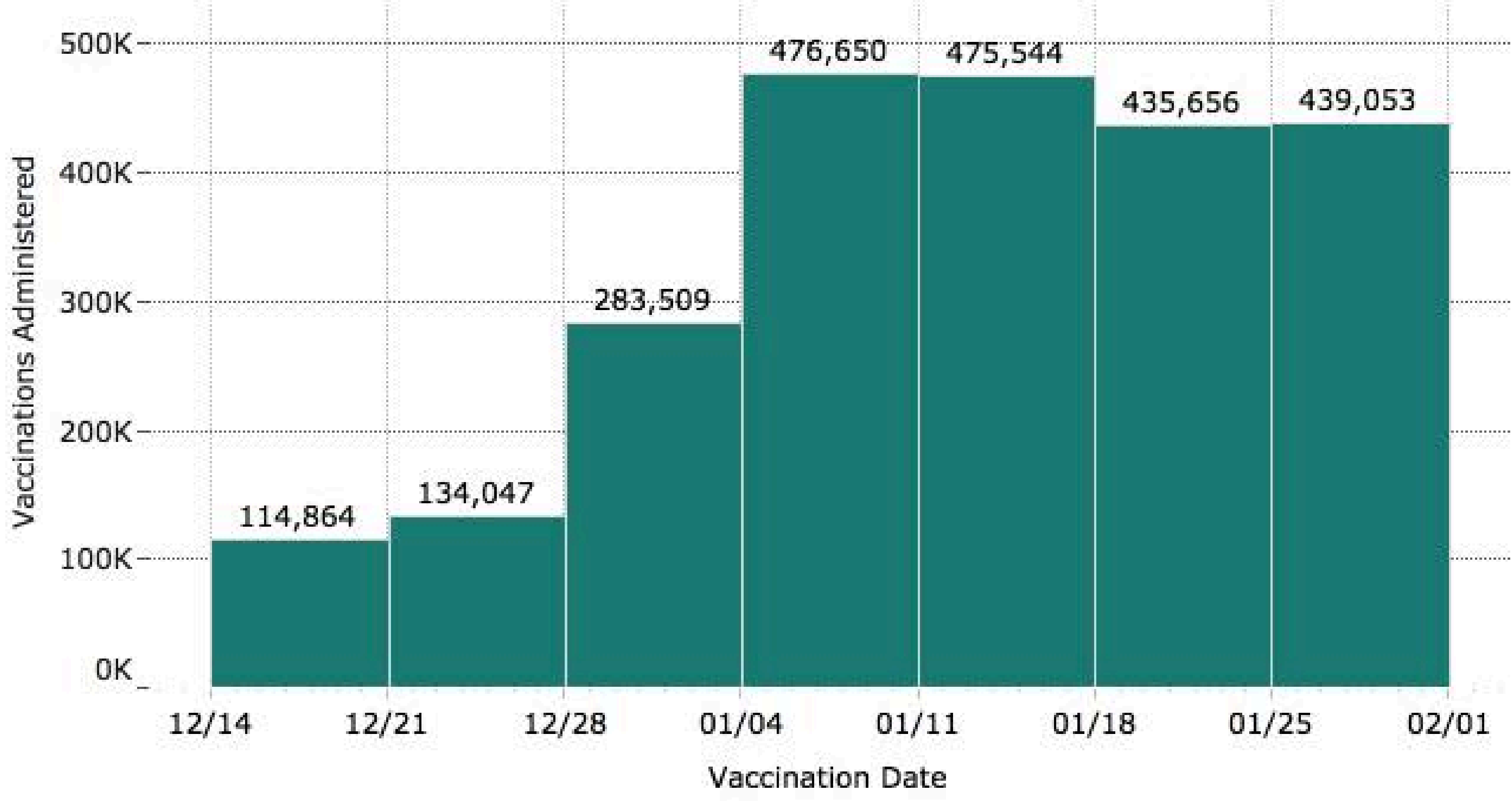

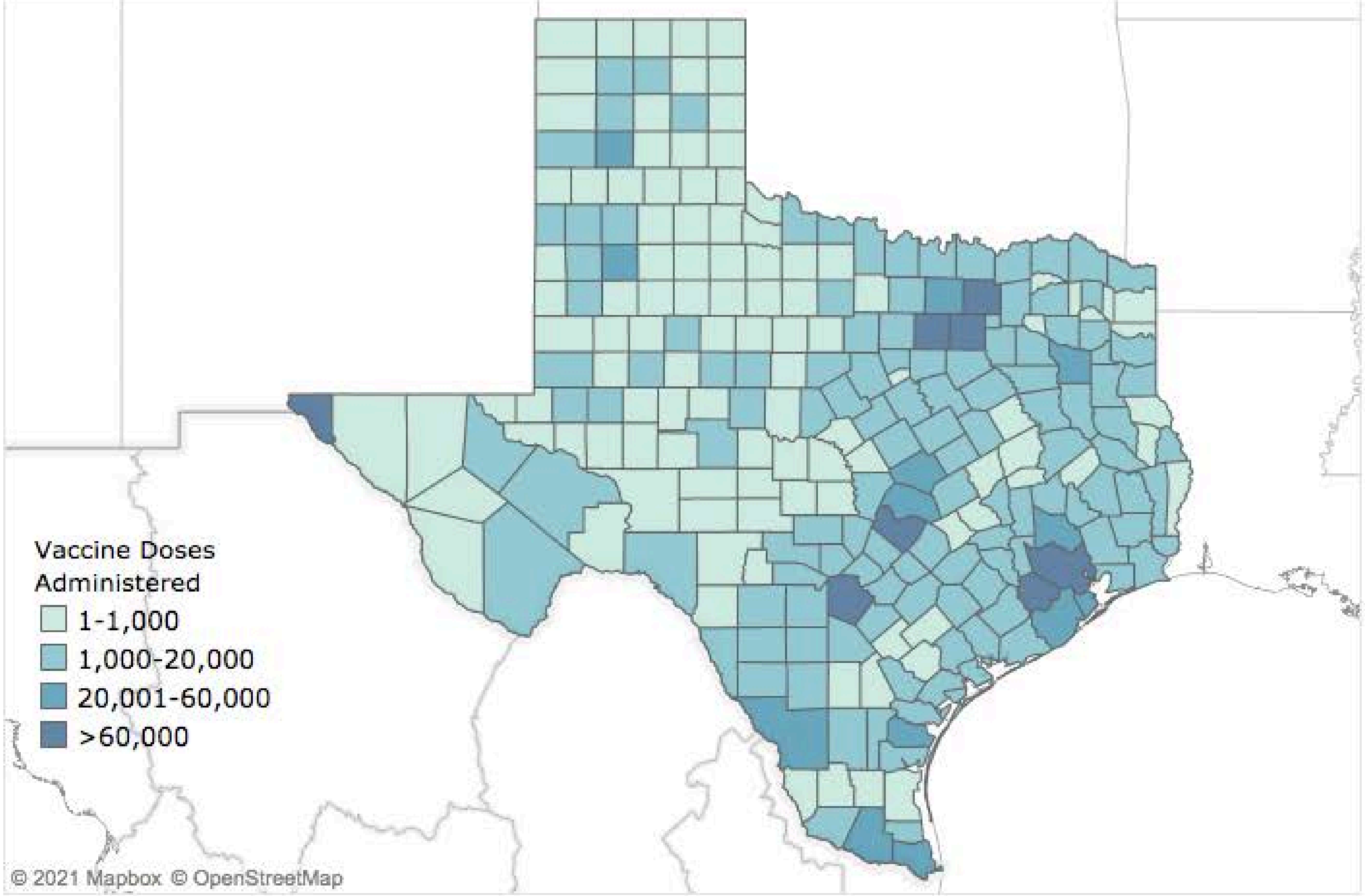

Dashboard is updated daily by 4 p.m. CST with data as of 11:59 the previous night.

For inquiries about the data, email COVIDVaccineQs@dshs.texas.gov.

Summary | Vaccine Doses Allocated | Vaccine Doses Administered | People Vaccinated | About the Data

TEXAS Health and Human Services | Texas Department of State Health Services

COVID-19 Vaccination in Texas

Select a Texas county below to view the number people who have received at least one dose of COVID-19 vaccine and those who have received the number of doses as outlined by the CDC (People Fully Vaccinated).

Alternatively, click on a county on the map. Click the Revert button at the bottom of the screen to remove filters.

People Vaccinated with at least One Dose	People Fully Vaccinated
**1,883,862**	**475,461**

Measures to Show

- (●) Vaccinated with at least One Dose
- ( ) Fully Vaccinated

Search for a County

(All) ▾

Number of People Vaccinated in Texas

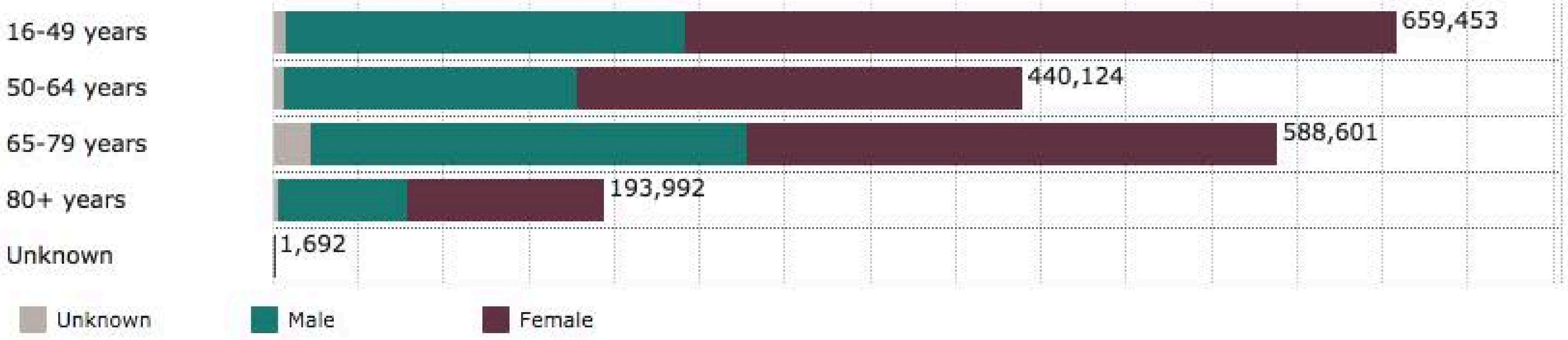

Race/Ethnicity	People Vaccinated	Percent of People Vaccinated
Asian	92,792	4.93%
Black	74,093	3.93%
Hispanic	162,674	8.64%
White	523,608	27.79%
Other	167,081	8.87%
Unknown	863,614	45.84%

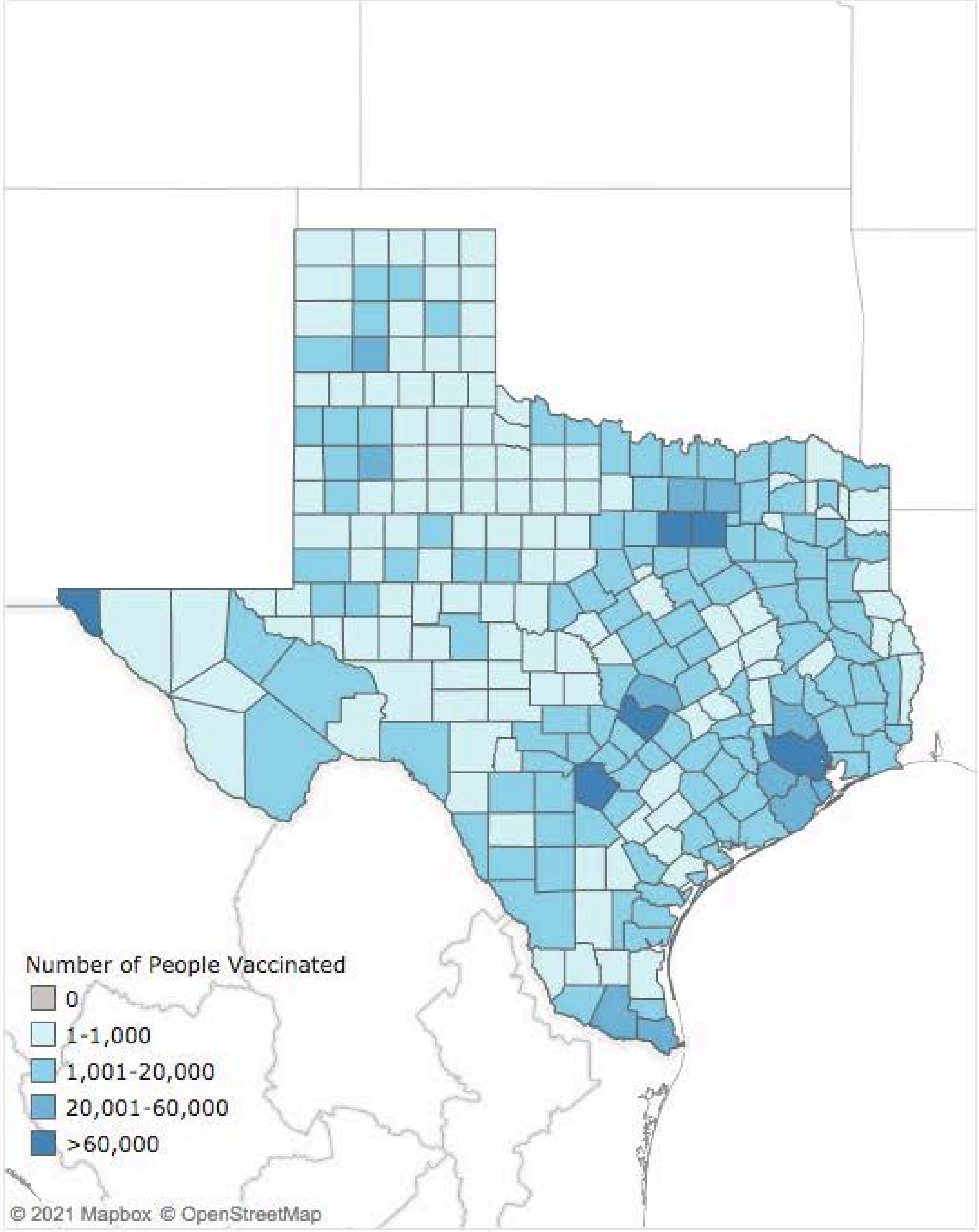

Dashboard is updated daily by 4 p.m. CST with data as of 11:59 the previous night. For inquiries about the data, email COVIDVaccineQs@dshs.texas.gov.

COVID-19 VACCINE DASHBOARD

The COVID-19 Vaccine Dashboard is updated Tuesdays through Saturdays.

The data in the dashboard includes the vaccines available in Vermont (currently, Pfizer-BioNTech and Moderna). Data is based on date reported to the Vermont Immunization Registry and does not reflect date of vaccination. Information reflects counts reported by the end of the previous day. Data is subject to change.

Read the notes below to learn more about what is reflected in dashboard.

Reset filters

Overall progress (age 16+)
People who have received at least one dose of the vaccine
8.8%

Vaccine doses received
99.8K

Vaccine doses administered
67.0K

Total people started
33.6K

Total people completed
16.7K

Select County to Filter

Overall progr... 3.7% 10.6%

- Addison
- Bennington
- Caledonia
- Chittenden
- Essex
- Franklin
- Grand Isle
- Lamoille
- Orange
- Orleans
- Rutland
- Washington
- Windham
- Windsor

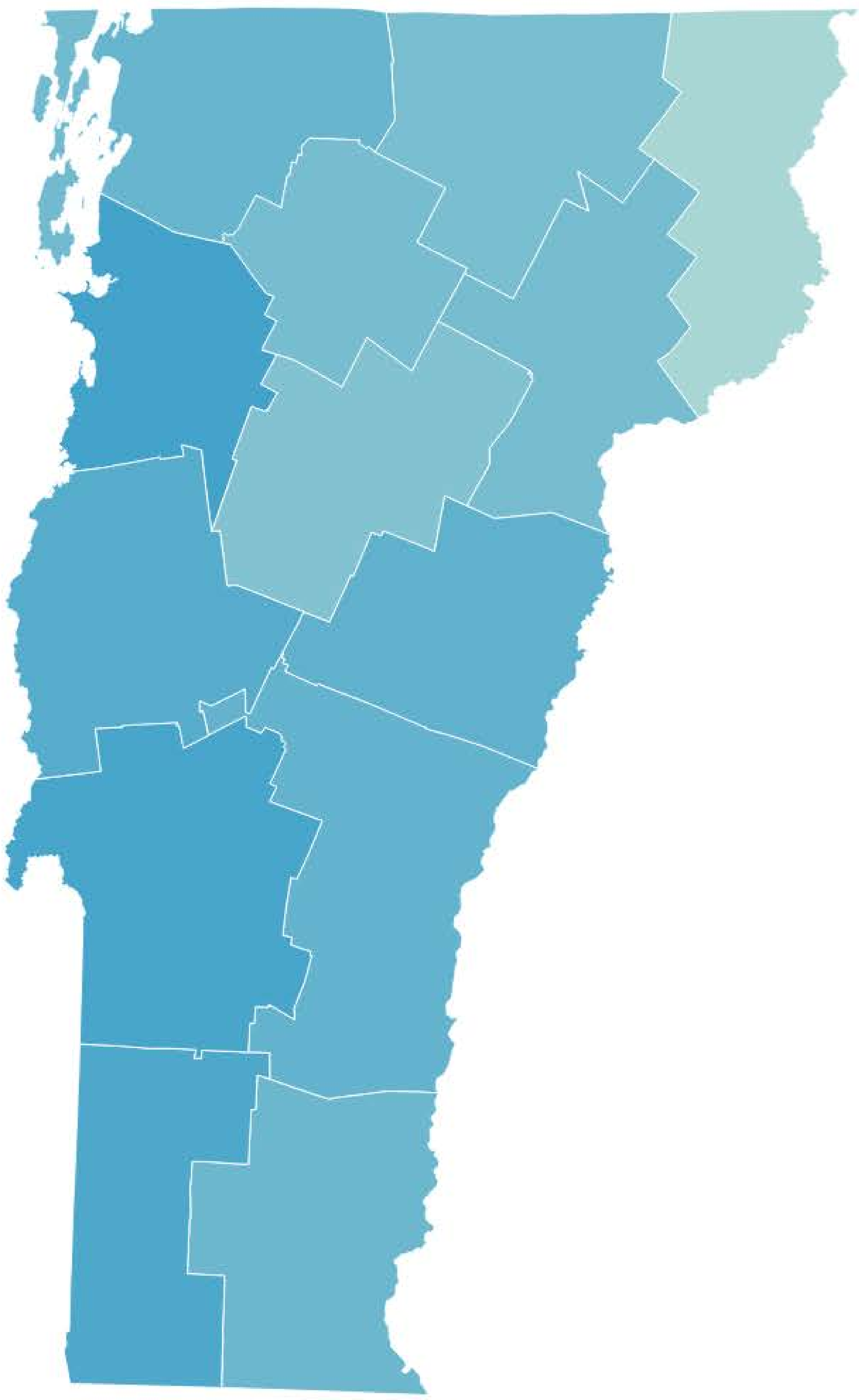

Updated 1/30/2021 8:42:47 AM

Total People Vaccinated

Completed Started

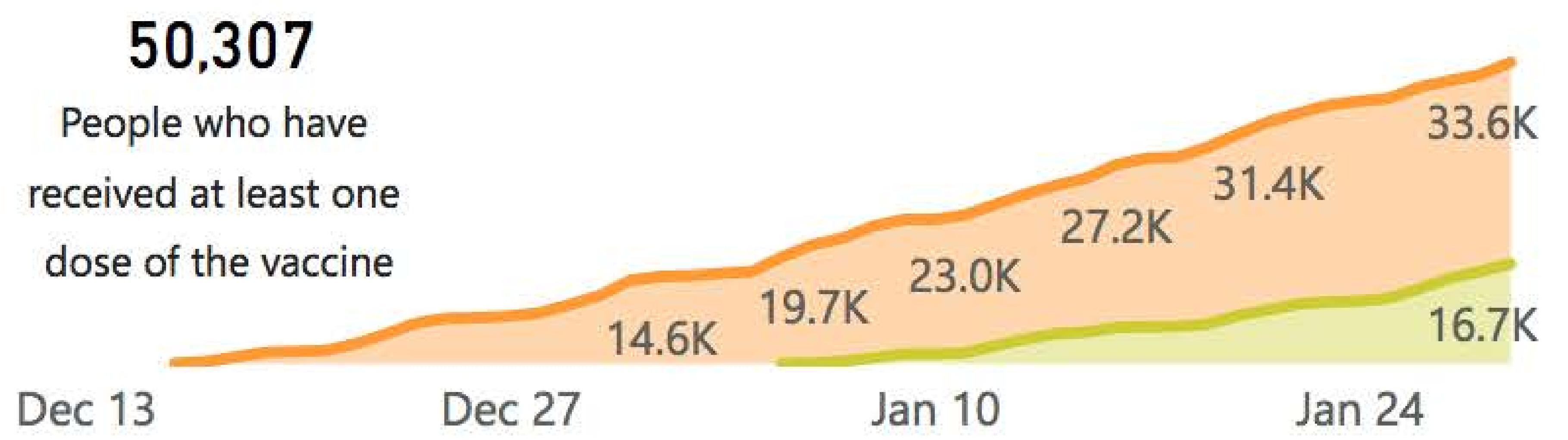

Show rates by Race/Ethnicity | **Show rates by Age/Sex**

By Race - Statewide

The percent of the Statewide population by race that has has received at least one dose of the vaccine..

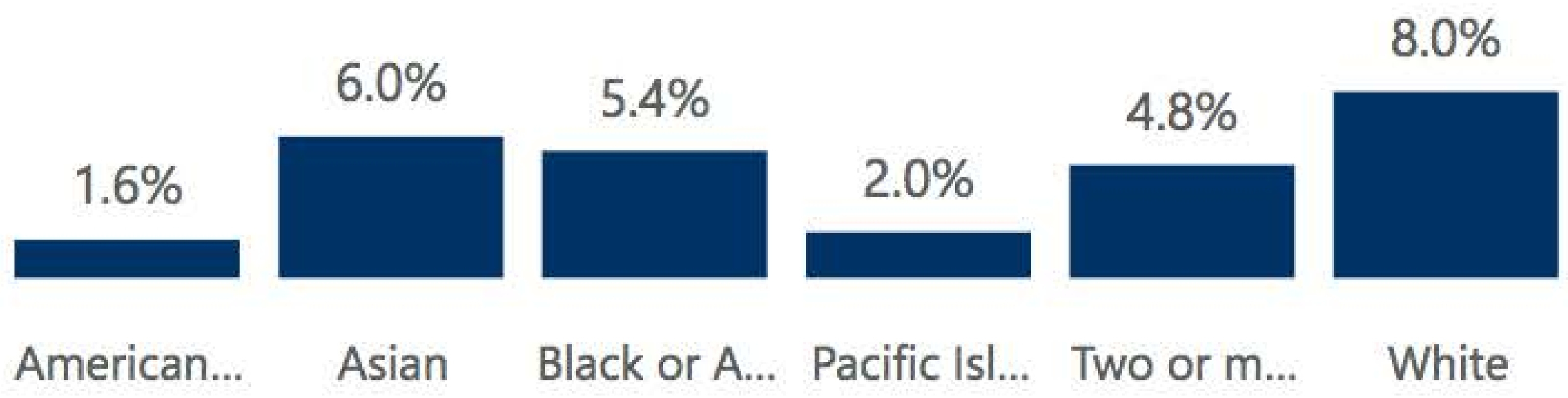

Race information is missing for 14% of people vaccinated.

By Ethnicity - Statewide

The percent of the Statewide population by ethnicity that has has received at least one dose of the vaccine.

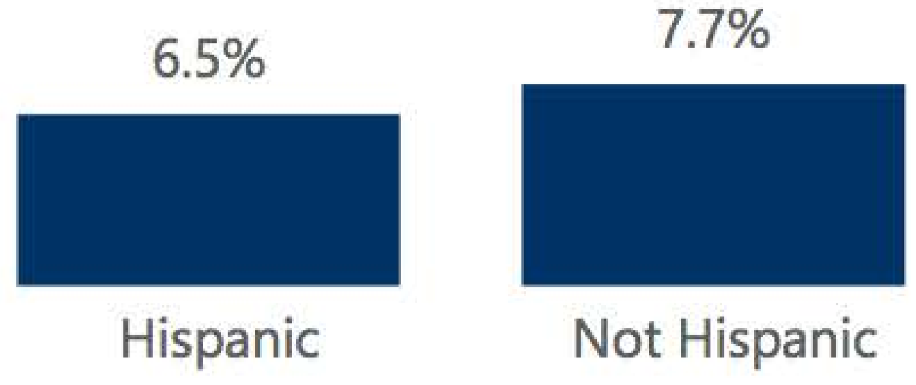

Ethnicity information is missing for 22% of people vaccinated.

Microsoft Power BI

COVID-19: Vaccine Data

Jump to specific COVID-19 chart on this page:

- Vaccine summary
- Vaccine doses administered by day
- Cumulative total residents completing vaccination series
- Percent of residents who received a dose, by age
- Number of residents who received a dose, by age
- Percent of residents who received a dose, by sex
- Number of residents who received a dose, by sex
- Learn how to download our data

The federal government allocates COVID-19 vaccine to Wisconsin based on population size. Once the vaccine is allocated, Wisconsin places an order with the federal government so they know exactly where to send the vaccine. Orders may not include the entire federal allocation due to limited storage space or to ensure hospitals and clinics are not overwhelmed with more doses than can safely be administered in a week. For the Pfizer COVID-19 vaccine, doses are shipped first to regional hospitals (called hubs) before going on to locations where they will ultimately be administered. Moderna COVID-19 vaccine is shipped directly to locations that will give vaccine.* Similar to ordering a package online, once vaccine is shipped it can still take several days to reach its final destination. Once the vaccine arrives at a location, they are able to begin vaccinating the populations recommended by the Advisory Committee on Immunization Practices (ACIP) and State Disaster and Medical Advisory Committee (SDMAC) as soon as they are able.

*As part of the federal Pharmacy Partnership Program, DHS is using a portion of Wisconsin's Moderna vaccine allocation to initiate part A of the program, which provides vaccinations for skilled nursing facility residents and staff. For part B of the program, DHS has reserved additional doses from the state's Moderna vaccine allocation for long-term care facilities. These doses are directed to vaccinating locations by the federal government and are therefore not reflected in data for shipped doses. These numbers help, in part, to explain the difference between the allocated and shipped data displayed below.

COVID-19: Data Pages
- Activity Level by Region and County
- Alternate Care Facility
- Cases
- County-Level
- Deaths
- Facility-Wide Investigations
- Frequently Asked Questions
- Hospitals
- Labs and Testing
- Racial and Ethnic Disparities
- Wastewater Monitoring
- Wisconsin Summary
- COVID-19

The remaining allocated doses are made available for populations that qualify for vaccination under Phase 1A. To learn more about vaccination data, visit our Frequently Asked Questions (FAQ) webpage.

We plan to update allocation and shipment data every Tuesday at 2 p.m and administration data daily at 2 p.m.

Vaccine Distribution Summary
Updated weekly, last updated: 1/26/2021

Allocated	Ordered	In Transit	Administered
846,300	641,150	167,850	544,234

Pharmacy Partnership Program

Skilled Nursing Facilities	Assisted Living Facilities
57,000	105,000

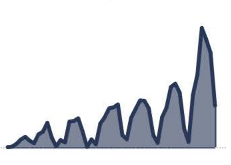

Undo Redo Revert Refresh Pause Share Download Full Screen

Understanding our data: What does this chart mean?

About our data: How do we measure this?

Back to a list of charts on this page.

COVID-19 vaccines administered per day to Wisconsin residents
Updated: 1/31/2021 (Total: **536,579**)

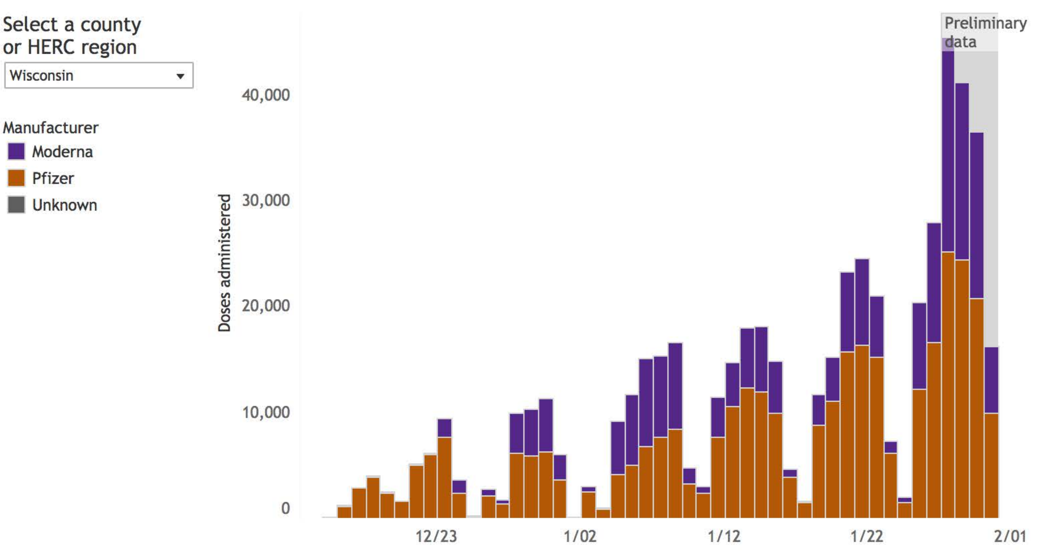

Undo Redo Revert Refresh Pause Share Download Full Screen

Understanding our data: What does this chart mean?

About our data: How do we measure this?

Back to a list of charts on this page.

Cumulative total Wisconsin residents who have completed the COVID-19 vaccination series
Updated: **1/31/2021** (Total: **101,219**)

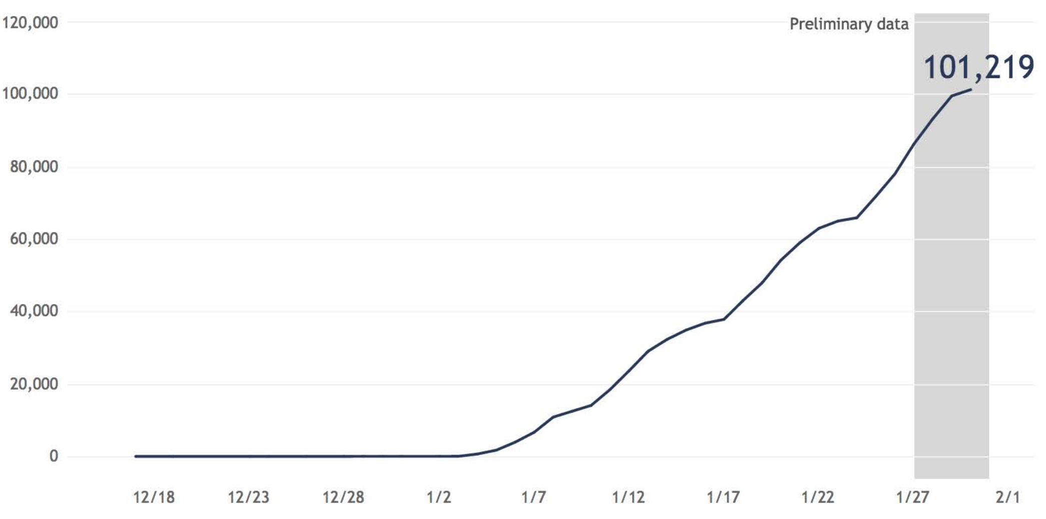

Undo Redo Revert Refresh Pause Share Download Full Screen

Understanding our data: What does this chart mean?

About our data: How do we measure this?

Back to a list of charts on this page.

Percent of Wisconsin population who have received at least one COVID-19 vaccine dose, by age group
Updated: 1/31/2021

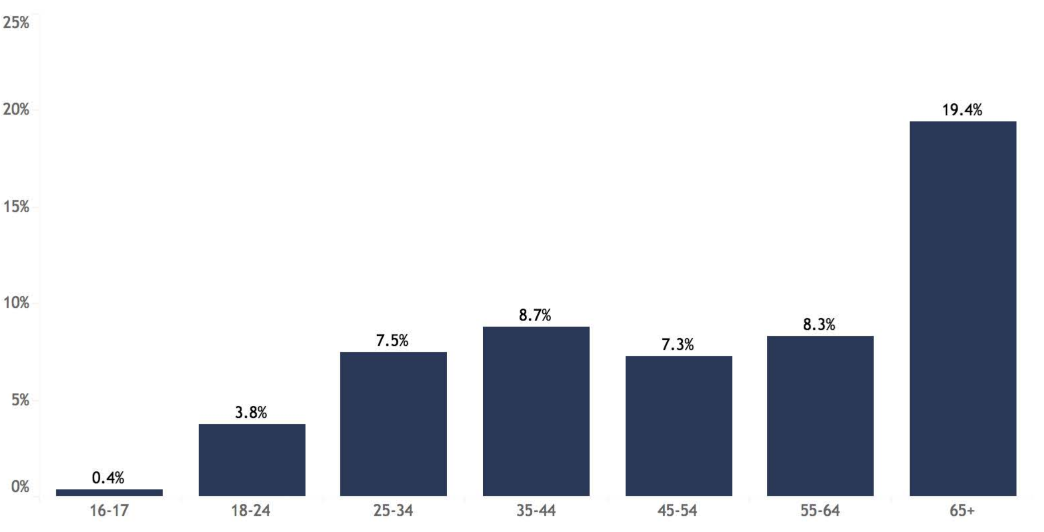

Undo Redo Revert Refresh Pause Share Download Full Screen

Understanding our data: What does this chart mean?

About our data: How do we measure this?

Back to a list of charts on this page.

Number of Wisconsin residents who have received at least one COVID-19 vaccine dose, by age group
Updated: 1/31/2021

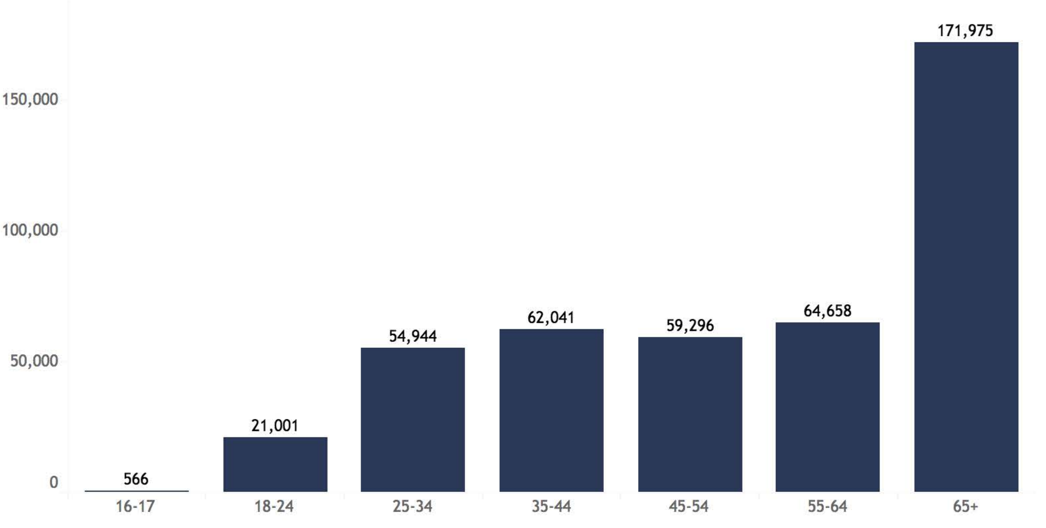

Undo Redo Revert Refresh Pause Share Download Full Screen

Understanding our data: What does this chart mean?

About our data: How do we measure this?

Back to a list of charts on this page.

Percent of Wisconsin population who have received at least one COVID-19 vaccine dose, by sex
Updated: 1/31/2021

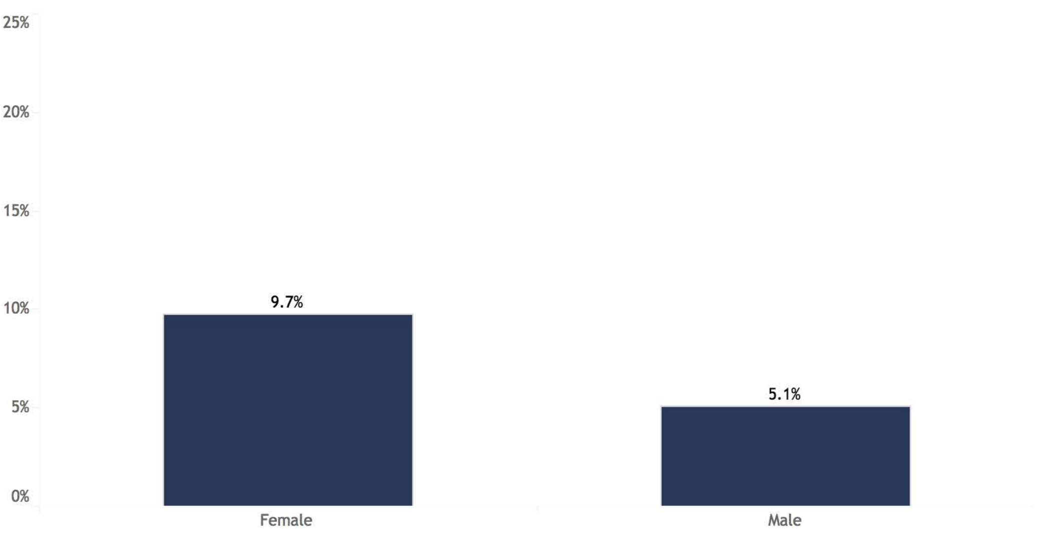

Undo Redo Revert Refresh Pause Share Download Full Screen

Understanding our data: What does this chart mean?

About our data: How do we measure this?

Back to a list of charts on this page.

Number of Wisconsin residents who have received at least one COVID-19 vaccine dose, by sex
Updated: 1/31/2021

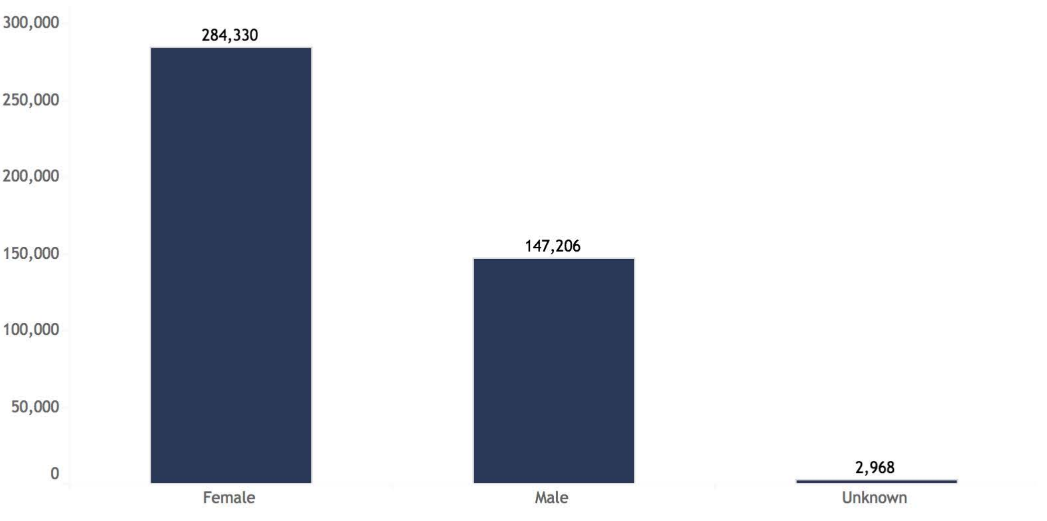

Undo Redo Revert Refresh Pause Share Download Full Screen

Understanding our data: What does this chart mean?

About our data: How do we measure this?

Back to a list of charts on this page.

See How the Vaccine Rollout Is Going in Your State

Updated Feb. 27, 2021

At least one dose | Two doses

Share of population that has gotten at least one shot

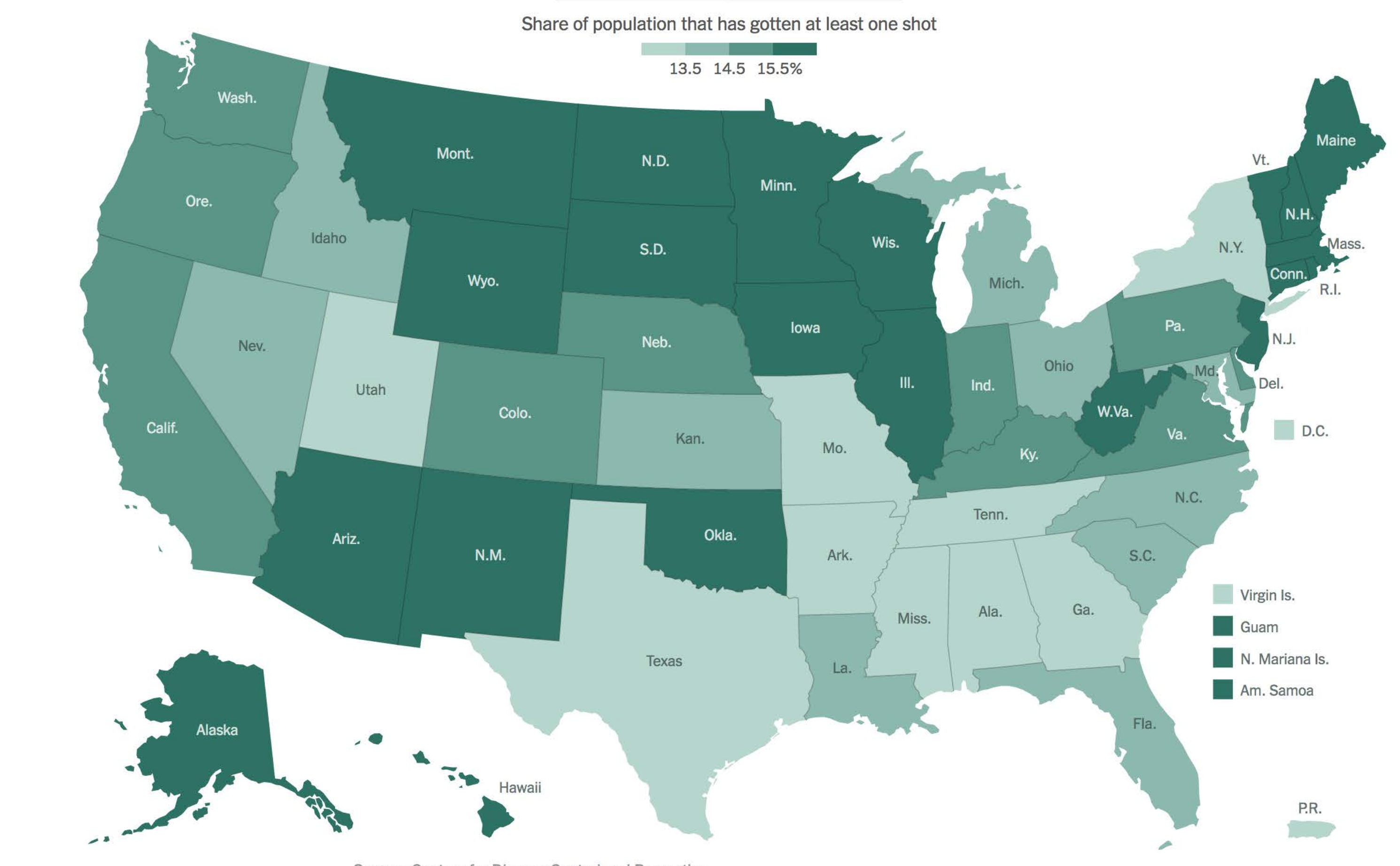

Source: Centers for Disease Control and Prevention

As of Feb. 23, the C.D.C. reports these figures based on where individuals reside, rather than the vaccine provider's location. This led to an especially noticeable change in Washington, D.C.

The Centers for Disease Control and Prevention said on Saturday about 48.4 million people have received at least one dose of a Covid-19 vaccine, including about 23.7 million people who have been fully vaccinated.

The work to distribute the vaccine comes as more than 510,000 people in the United States have died after contracting the virus.

How Quickly Are Shots Going in Arms?

Providers are administering about 1.65 million doses per day on average. Figures show the date shots were reported, rather than the date shots were given.

New reported doses administered by day

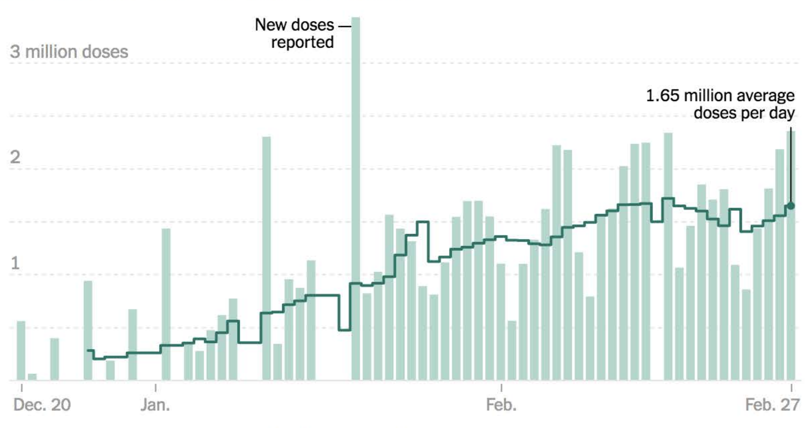

Source: Centers for Disease Control and Prevention | Note: Line shows a seven-day average. Data not updated on some weekends and holidays.

President Biden has promised to administer 100 million vaccines by his 100th day in office. He set a goal shortly after taking office last month for the United States to administer more than 1.5 million doses a day, despite criticism that it was not ambitious enough.

Federal regulators have given emergency approval to vaccines developed by Pfizer-BioNTech and Moderna. Both vaccines require patients to receive two doses spaced weeks apart.

The federal government has delivered about 96.4 million doses to states, territories and federal agencies.

When Might a Majority of People Have Been Vaccinated?

Some experts have estimated that 70 to 90 percent of the population needs to acquire resistance to the coronavirus to reach herd immunity, when transmission of the virus substantially slows because enough people have been protected through infection or vaccination.

A number of factors will determine how quickly this threshold is met, especially the pace at which newly vaccinated people join those who are immune after past infections. But the presence of more transmissible virus variants could complicate that progress.

The projection below only shows the share of the total population with at least one shot based on the current rate of vaccination, but it provides a rough indication of when the virus's spread could begin to stall.

When a given share of the U.S. population might be at least partially vaccinated

The current vaccination rate is based on average daily increase in first doses administered over the past week.

Average daily first doses in last 7 days: 803,706

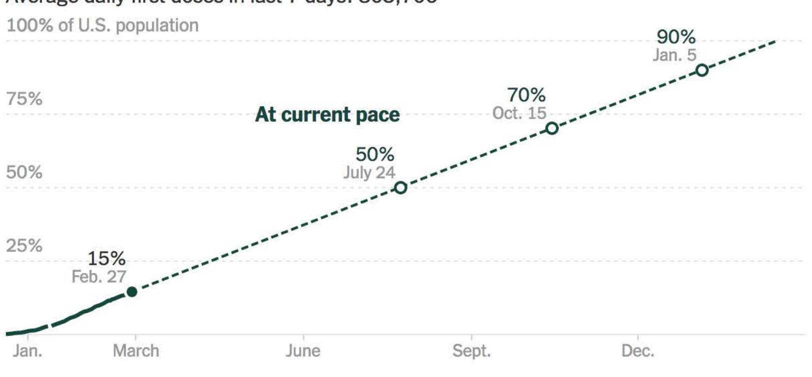

Source: Centers for Disease Control and Prevention | Note: Data from Dec. 20 to Jan. 12 are for all doses administered. Data for Jan. 13 is unavailable. Projections could change if additional vaccines are authorized.

If the country maintains its current pace of administering first doses, about half of the total population would be at least partially vaccinated around late July, and nearly all around early January, assuming supply pledges are met and vaccines are eventually available to children.

When Could the United States Reach Herd Immunity? It's Complicated.

How Is Each State Performing?

Some jurisdictions have been more efficient than others at administering their doses from the federal government. The table below includes states, territories, federal agencies and three countries with special agreements with the United States: Palau, Micronesia and the Marshall Islands.

Name	Pct. of people given: At least one shot ▾	Pct. of people given: Two shots	Doses delivered	Shots given	Doses used
**U.S. total***	15%	7.1%	**96,402,290**	**72,806,180**	76%
Palau	29%	17%	9,200	8,143	89%
American Samoa	25%	16%	29,450	20,338	69%
Alaska	22%	13%	342,535	259,538	76%
New Mexico	21%	11%	738,705	678,087	92%
Guam	20%	12%	51,300	54,244	106%
South Dakota	20%	10%	329,300	269,289	82%
Northern Mariana Islands	20%	14%	29,650	18,121	61%
Connecticut	19%	8.3%	1,198,495	980,211	82%
North Dakota	19%	9.8%	246,570	225,330	91%
West Virginia	18%	11%	605,255	526,059	87%

Show all

*Includes doses provided to Palau, Micronesia and the Marshall Islands.

Source: Centers for Disease Control and Prevention; U.S. Census Bureau | Note: Extra doses in Pfizer vials and data reporting inconsistencies may result in the percentage of doses used adding up to more than 100. Geographically isolated areas such as Alaska, American Samoa, Guam and the Northern Mariana Islands can place orders for multiple weeks at once.

There are many reasons for variation among the states and territories, including demand for the vaccine, lags in data reporting and other logistical challenges. Mr. Biden recently announced plans to expand supply and to give states more advance notice about the number of shots available to them each week.

Who Is Eligible for a Vaccine?

Health care workers and residents of long-term care facilities have been eligible for vaccination in every state for weeks.

The federal government recently recommended that states allow people 65 and older to get vaccinated. But states create and implement their own plans, and sometimes counties deviate from those plans, leading to a national patchwork of policies.

The New York Times periodically surveys all 50 states and has found that every state has begun widely giving shots to older people, though age ranges differ from place to place. The Times tracks statewide rules, and adds county-level discrepancies as that information arises.

Where older adults are eligible for vaccines

Every state has started widely vaccinating older adults, though the minimum eligibility age varies across the country.

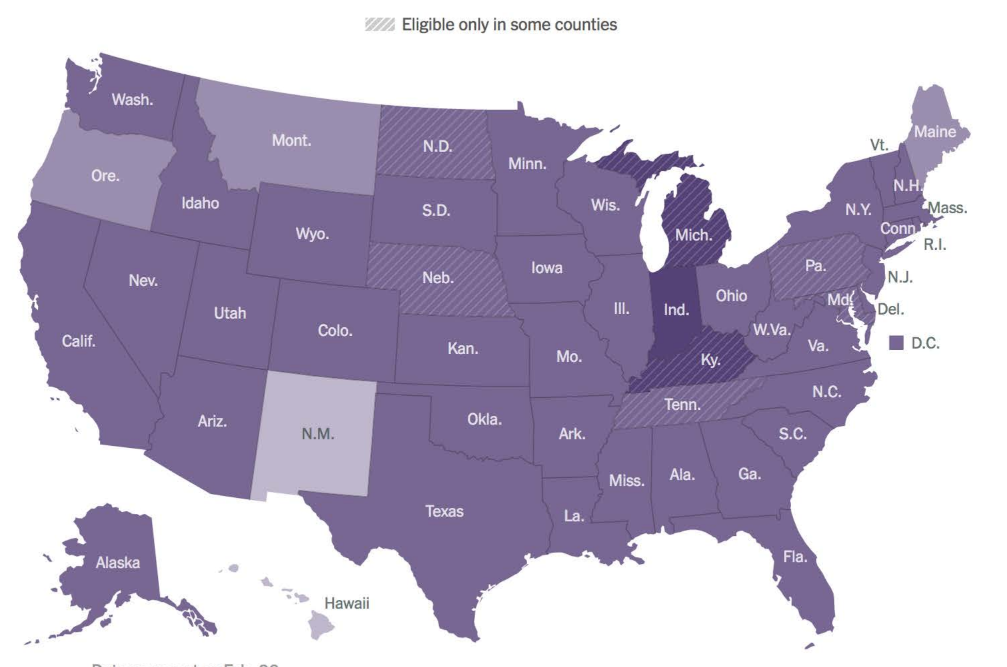

Data as recent as Feb. 26.

Sources: State and county health departments.

The table below, which will be updated regularly but may lag slightly, shows who is eligible for vaccination in each state, in

The New York Times U.S. | See How the Vaccine Rollout Is Going in Your State 40

many instances, people may be eligible for a vaccine but unable to make an appointment. And some counties may delay or pause eligibility for certain groups even after a state expands access.

The Times regularly tracks statewide eligibility based on age, occupation and underlying health conditions, and notes states where that information is known to vary by county.

Who is currently eligible for the vaccine in each state

All states are vaccinating health care workers and residents of long-term care facilities, and have started expanding eligibility to other groups. Click on a state for more information.

Eligible only in some counties

Location	Older adults	Teachers	Grocery workers	Some other essential workers	High-risk adults
Alabama ›	65+	Yes	Yes	Yes	
Alaska ›	65+	Yes	50+	Yes	50+
Arizona ›	65+	Yes	Yes	Yes	Yes
Arkansas ›	65+	Yes		Yes	
California ›	65+	Yes	Yes	Yes	Yes
Colorado ›	65+	Yes		Yes	
Connecticut ›	65+			Yes	

Data Central / COVID-19 Vaccine Tracker

Coronavirus (COVID-19)

COVID-19 Vaccine Tracker

This is your state and county equivalent level look at how many have gotten a dose or doses of the COVID-19 vaccine. Click on a state to see how many vaccines have been administered. In Florida, Illinois, Michigan, Minnesota, New Jersey, North Carolina, Ohio, Tennessee, Texas and Vermont, you can drill down to the county level and see a bar chart that tracks the running total. Data for this interactive map is provided by Johns Hopkins University and state health departments and will be updated automatically when states update data.

Map

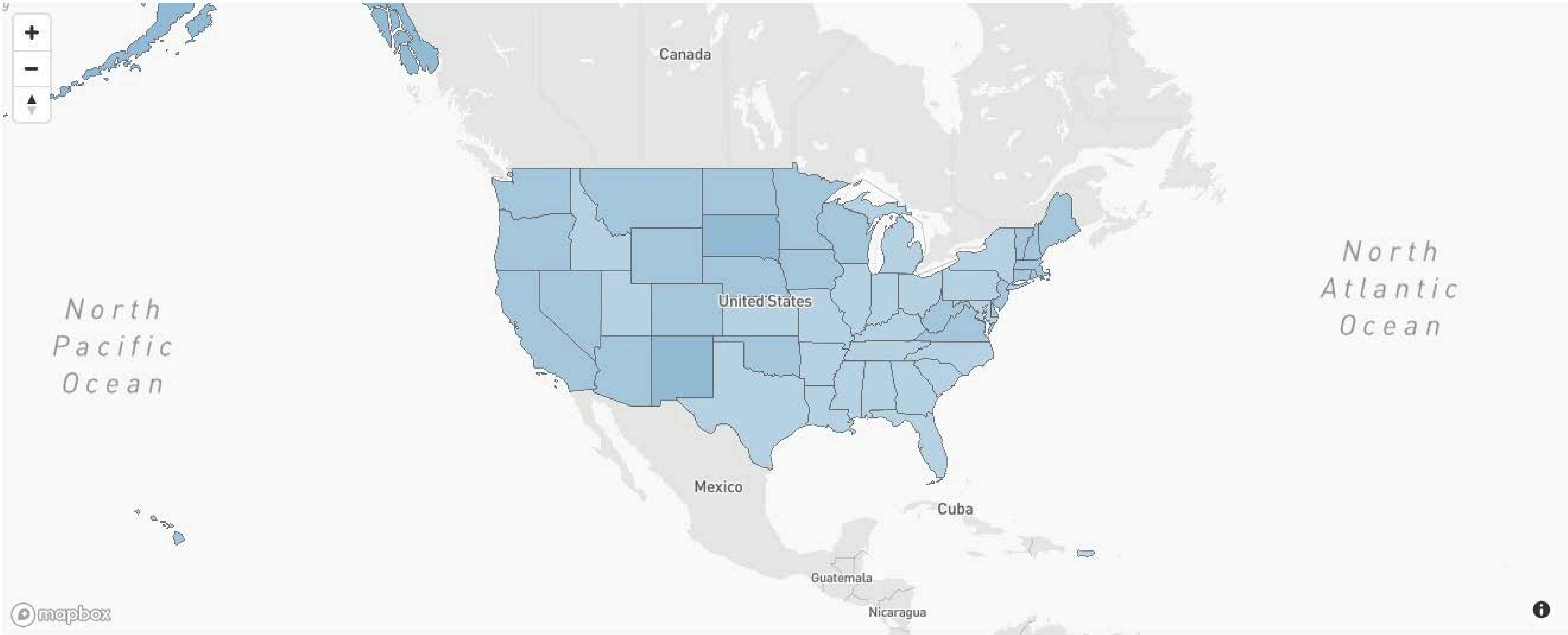

Source: State and county health departments, JHU Coronavirus Resource Center

To provide our community with important public safety information, the Springfield News-Leader is making this page free to read. To support important local journalism like this, please consider becoming a digital subscriber.

COVID-19 Vaccination

Area	Population	Doses Administered	People Vaccinated with at least One Dose	People Fully Vaccinated
United States	322,903,030	72,806,180	48,435,536 15.00%	23,698,627 7.34%
Alabama	4,864,680	931,090	628,583 12.92%	300,548 6.18%
Alaska	738,516	259,538	163,443 22.13%	95,485 12.93%

More

Tracking the Coronavirus Contagion

This map is your interactive guide to confirmed cases, recoveries and deaths from the coronavirus around the state, nation and world.

Comparing the Curve

Has the curve flatten in my town? Compare the spread of the infection with other areas in the U.S.

COVID-19 Hospital Capacity

Check out bed occupancy rate of hospitals.

Coronavirus Unemployment Tracker

Health

At least 48.4 million people have received one or both doses of the vaccine in the U.S.

This includes more than **23.7 million** people who have been fully vaccinated. **96.4 million** doses have been distributed.

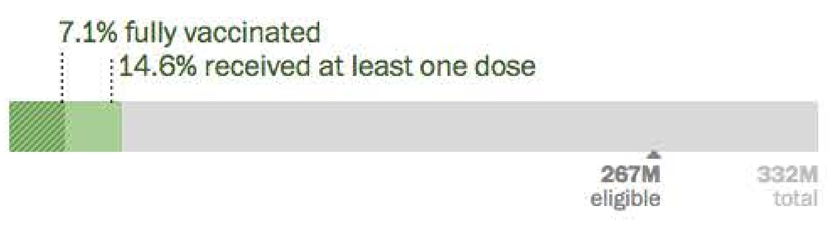

Data as of Feb. 27 at 6:14 p.m.

PLEASE NOTE

The Washington Post is providing this story for free so that all readers have access to this important information about the coronavirus. For more free stories, sign up for our Coronavirus Updates newsletter.

Vaccination in the United States is on the verge of big changes with manufacturers pledging larger supplies of the existing two-dose coronavirus treatments and a new one-dose vaccine close to emergency approval.

The larger supply that should arrive by April is raising hopes of more normal life returning by summer.

In the meantime, vaccine seekers continue to overrun signup websites and phone lines as states have expanded eligibility to more age groups and people with medical conditions that put them at greater risk from covid-19.

Reported doses administered by day

In the last week, an average of **1.57 million** doses per day were administered, a **13%** increase ↑ over the week before.

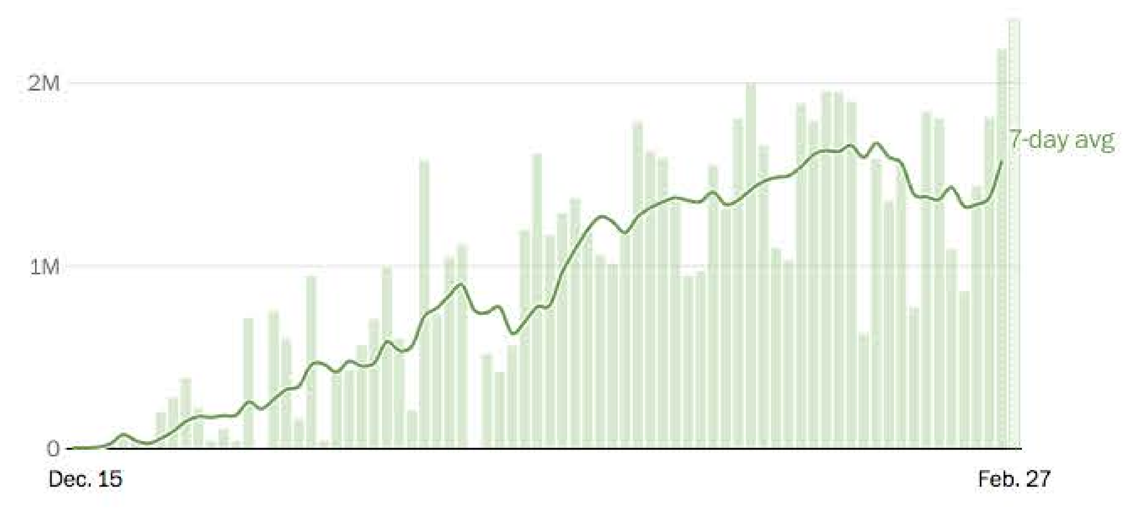

Sources: State dashboards and the CDC

Jump to a state

Select a state

Both the Pfizer and Moderna vaccines require a follow-up shot three or four weeks after the first dose. Allocation data displayed here is for the first dose. Vaccines that are expected to be delivered for the second dose are not included. The Johnson & Johnson vaccine seeking approval requires only one dose but no allocation data for that vaccine has been released.

Share of population that received one or both doses

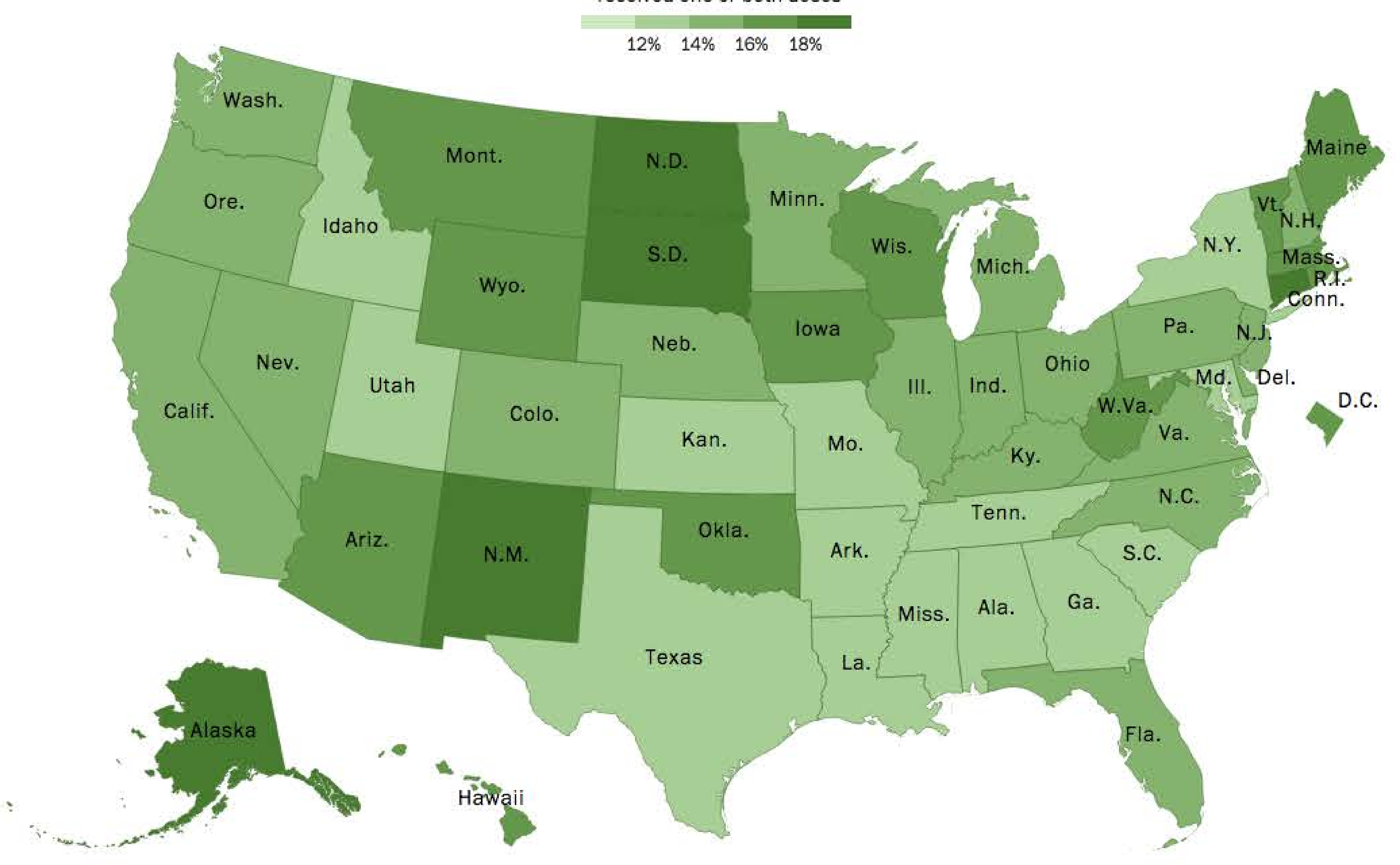

Jurisdiction	People who have received the first dose	Percent of pop. that has received the first dose	Percent of pop. that has completed vaccination
**U.S.**	**48,435,536**	**14.6%**	**7.1%**
Republic of Palau	8,143	45.5%	17.1%
American Samoa	20,338	36.5%	14.4%
Alaska	163,443	22.3%	13.1%
New Mexico	439,875	21%	11%
Guam	34,215	20.6%	11.8%
South Dakota	178,535	20.2%	10.2%
North Dakota	146,046	19.2%	10.1%
Connecticut	674,181	18.9%	8.3%
Northern Mariana [illegible]			

See all

Note: The U.S. total includes doses provided to Republic of Palau, Federated States of Micronesia and the Marshall Islands, as US-affiliated Pacific Islands.

[Tracking coronavirus cases, deaths and vaccinations worldwide]

Alabama

Reported doses administered by day in Alabama

In the last week, an average of **22.0k** doses per day were administered, a **12% decrease** ↓ over the week before.

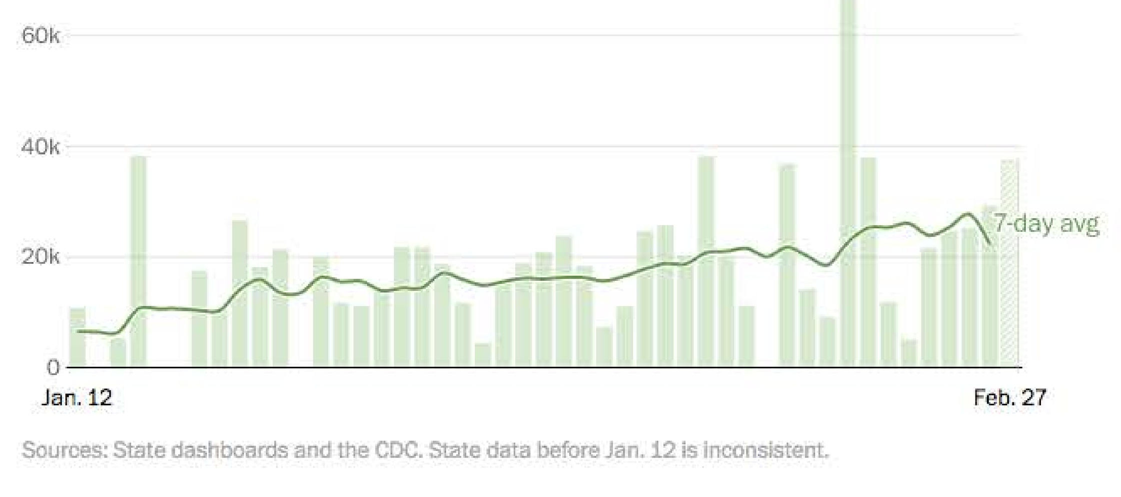

Sources: State dashboards and the CDC. State data before Jan. 12 is inconsistent.

Alabama has administered at least **628,583** first doses, covering **24.2%** of the prioritized population ...

Select a state

The state has been allocated **842,000** first doses,

Home Share 423

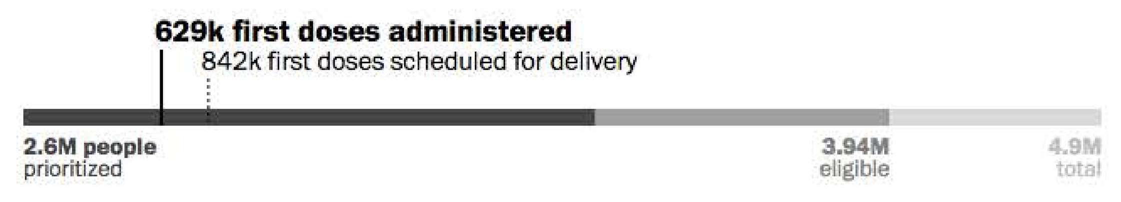

Note: The prioritized population includes everyone the state has identified so far as a priority. State's population includes people ages 15 and under who are not yet eligible.

Groups prioritized for the vaccine in Alabama

- High-risk front-line health-care workers
- Residents of long-term care facilities
- Adults over the age of 75
- First responders
- Critical workforce at highest risk for work-related exposure
- Persons working or living in congregate settings
- Adults over the age of 65
- Persons 16-64 years with high risk medical conditions
- Critical workforce not covered in previous phases

Not all priority populations listed are currently eligible for the vaccine. For the most recent information and to check your eligibility, go to the state page.

Over the last week, Alabama has seen an average of **957** confirmed cases and **49** deaths per day. Alabama has the **19th** most confirmed cases per capita among states and D.C. during the same period.

COVID-19 Vaccination Tracker

USA Vaccination Tracker
State Vaccination Tracker
State COVID-19 Burden
COVID-19 Vaccines FAQ
General Information
Vaccine Development
Vaccine Safety
Getting Vaccinated
After You Are Vaccinated

THE UNITED STATES

Total doses distributed	Number received first dose	Number received second dose	Newly distributed per 100,000 on 02/26
94,300,910	47,184,199	22,613,359	955

Percent of population partially vaccinated (one dose received)

14%

Percent of population fully vaccinated (two doses received)

7%

• About the data

COVID-19 VACCINATION BY STATE

Click on a state.

% of population partially vaccinated (one dose received)

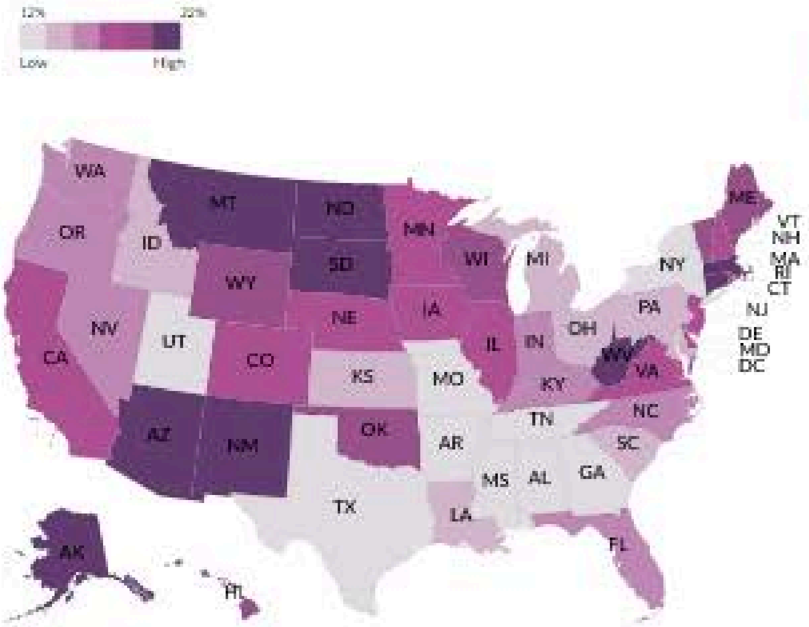

Vaccination Status in The United States

	Select State	U.S.
Number received first dose		47,184,199
Percent received first dose		14.42%
Number received second dose		22,613,359
Percent received second dose		6.91%
Newly distributed per 100,000		955

• About the data

COVID-19 BURDEN IN THE UNITED STATES

Daily Cases

70,768 ↓ 35% 14-day change

Daily Deaths

3,210 ↓ 26% 14-day change

• About the data

*14-day change trends use 7-day averages.
Daily COVID-19 Cases is the new number of cases as of the date the data is updated.
Daily COVID-19 Deaths is the new number of deaths as of the date the data is updated.

Average Daily COVID-19 Cases /100,000

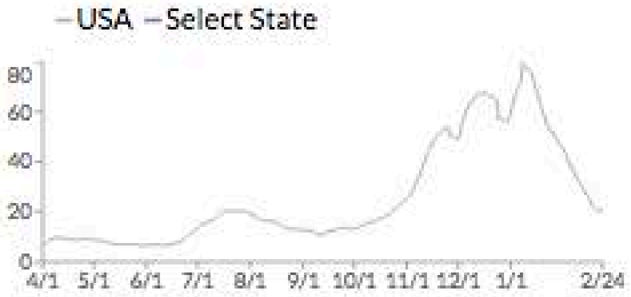

Disparities in COVID-19 Mortality
Nation

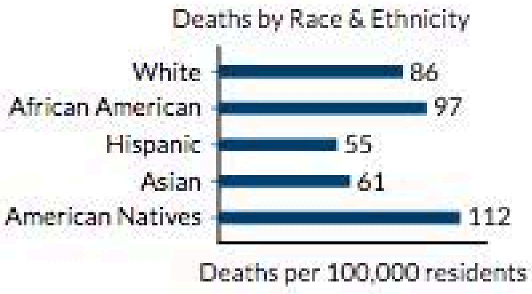

Average Daily COVID-19 Deaths /100,000

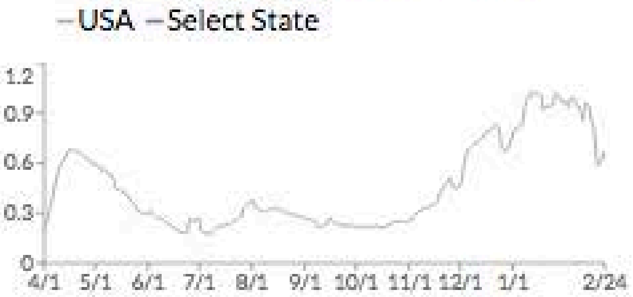

• About the data

For a complete table of definitions, click here.

The United States reports deaths by combined race and ethnicity groups. The chart shows race and ethnicity groups that constitute at least 1% of the state population

• About the data

Average Daily COVID-19 Cases per 100,000 helps us understand the average number of positive cases for COVID-19 infection over the last seven days by population of the county per 100,000 people.
Average Daily COVID-19 Deaths per 100,000 helps us

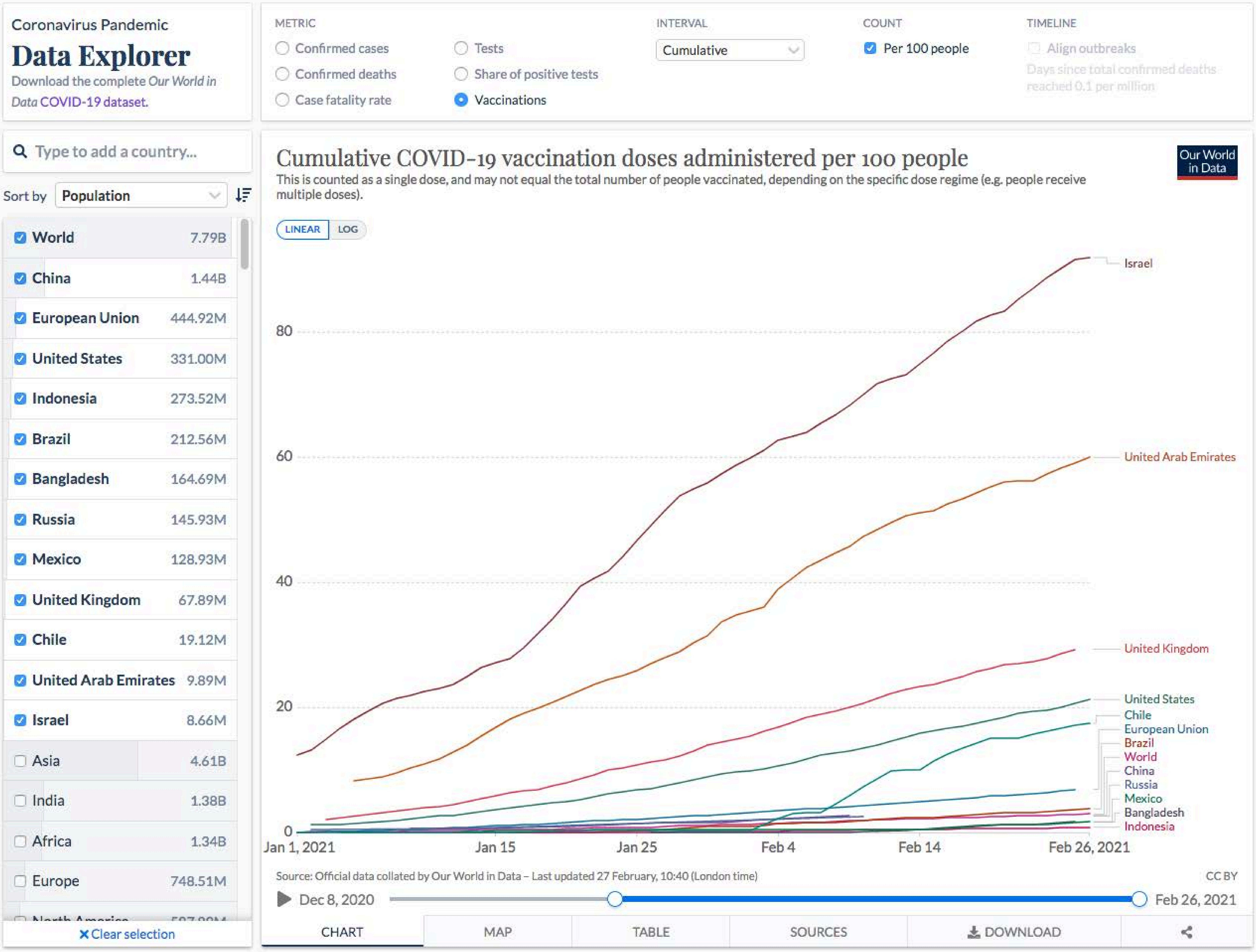

Coronavirus Pandemic
Data Explorer
Download the complete Our World in Data COVID-19 dataset.
METRIC
Confirmed cases
Confirmed deaths
Case fatality rate
Tests
Share of positive tests
Vaccinations
INTERVAL
Cumulative
COUNT
Per 100 people
TIMELINE
Align outbreaks
Days since total confirmed deaths reached 0.1 per million
Type to add a country...
Sort by
Population
World 7.79B
China 1.44B
European Union 444.92M
United States 331.00M
Indonesia 273.52M
Brazil 212.56M
Bangladesh 164.69M
Russia 145.93M
Mexico 128.93M
United Kingdom 67.89M
Chile 19.12M
United Arab Emirates 9.89M
Israel 8.66M
Asia 4.61B
India 1.38B
Africa 1.34B
Europe 748.51M
Clear selection
Cumulative COVID-19 vaccination doses administered per 100 people
This is counted as a single dose, and may not equal the total number of people vaccinated, depending on the specific dose regime (e.g. people receive multiple doses).
Our World in Data
LINEAR
LOG
80
60
40
20
0
Israel
United Arab Emirates
United Kingdom
United States
Chile
European Union
Brazil
World
China
Russia
Mexico
Bangladesh
Indonesia
Jan 1, 2021
Jan 15
Jan 25
Feb 4
Feb 14
Feb 26, 2021
Source: Official data collated by Our World in Data – Last updated 27 February, 10:40 (London time)
CC BY
Dec 8, 2020
Feb 26, 2021
CHART
MAP
TABLE
SOURCES
DOWNLOAD

Tracking Covid-19 vaccinations worldwide

By Henrik Pettersson, Byron Manley, Sergio Hernandez and Deidre McPhillips, CNN

Last updated: February 27, 2021 at 5:47 p.m. ET

At least 108 countries and territories have administered more than 234 million doses of a Covid-19 vaccine, barely a year after the first Covid-19 case was reported in China. Several different vaccines have been developed at record speed, in large part due to years of research on related viruses and billions of dollars in investment.

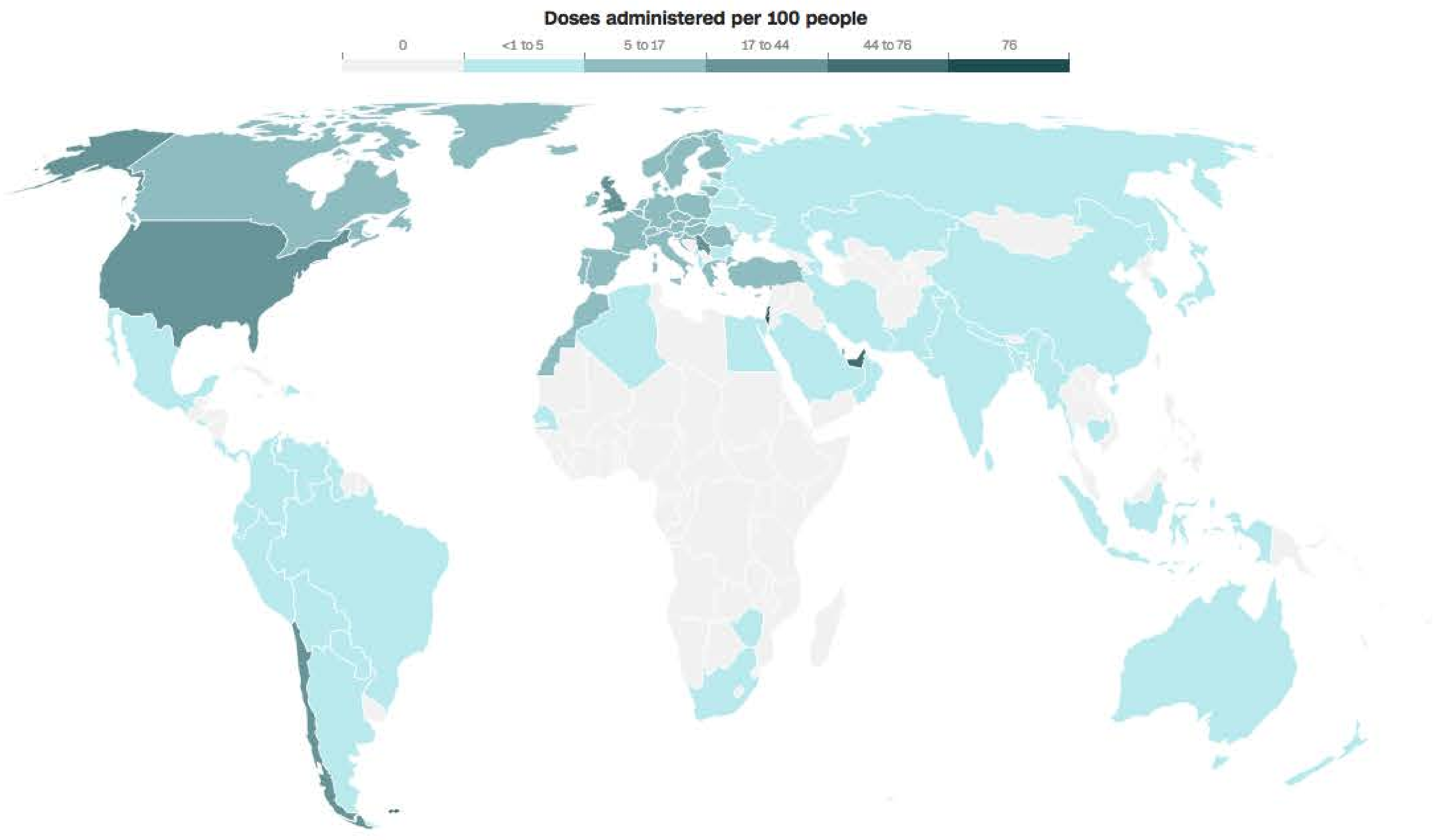

Last updated: February 27, 2021 at 5:47 p.m. ET
Source: Our World in Data

In December 2020, the first dose of a fully tested vaccine — manufactured by Pfizer/BioNTech — was administered in the United Kingdom. Now, multiple vaccines have been authorized for use around the world and dozens of countries and territories have joined the race to vaccinate their residents.

Rate of global vaccination campaign

Currently, the global 7-day moving average of administered doses is about 4.7 million per day.

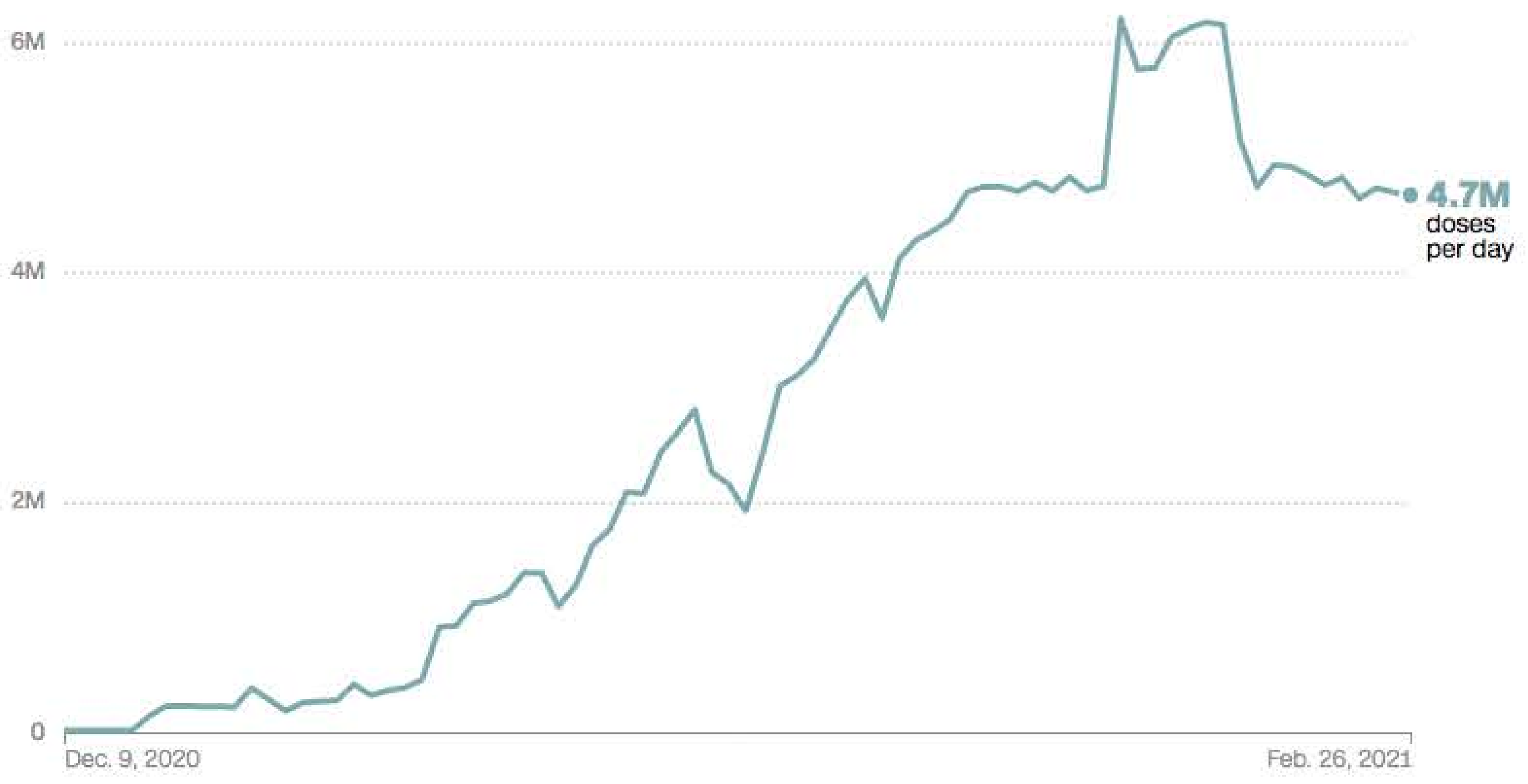

Last updated: February 27, 2021 at 5:47 p.m. ET
Source: Our World in Data

The vaccine rollout strategy varies from country to country. Some have favored vaccinating as many people as possible as quickly as possible, while others have tried to prioritize vaccinating specific vulnerable groups.

Leaders at the World Health Organization have emphasized the need for international cooperation in vaccination campaigns, noting that a global pandemic requires global efforts to end it.

Comparing vaccination rates

At least 108 countries and territories have started their vaccination rollouts. Different countries have different processes for approving vaccines, and not all countries have deals to obtain doses from manufacturers yet. As a result, countries and territories are at different stages of their vaccination campaigns.

Filter by number of days since first reported administered dose:

All | <14 days | 14-29 days | 30+ days

Location	Total doses	Doses administered per 100 people	Days since first dose
Gibraltar	34,600	103	46
Israel	7,957,787	92	68
Seychelles	75,096	76	46
United Arab Emirates	5,933,299	60	56
Falkland Islands	1,515	44	17
Cayman Islands	21,106	32	48
Guernsey	20,047	30	70
United Kingdom	19,913,592	29	79
Jersey	29,312	29	74

CNN health

Location	Total doses	Doses administered per 100 people	Days since first dose
United States	70,454,064	21	73
Serbia	1,393,086	20	63
Anguilla	2,762	18	20
Maldives	99,419	18	24
Chile	3,345,027	18	63
Bahrain	292,917	17	70
Malta	73,644	17	60
Turks and Caicos Is.	6,433	17	45
Faroe Islands	6,713	14	57

Show more

Last updated: February 27, 2021 at 5:47 p.m. ET
Source: Our World in Data, CNN research

Measuring countries' progress is challenging because many are using vaccines that require two doses. Inconsistent data makes it hard to know how many people have been fully or only partially immunized.

The race to get fully vaccinated

Most countries and territories are using vaccines that require two doses to provide the greatest protection. Measuring countries' progress is difficult, because reported data varies from country to country. Many report only the number of doses administered, but at least 54 break down how many people have received one or two doses.

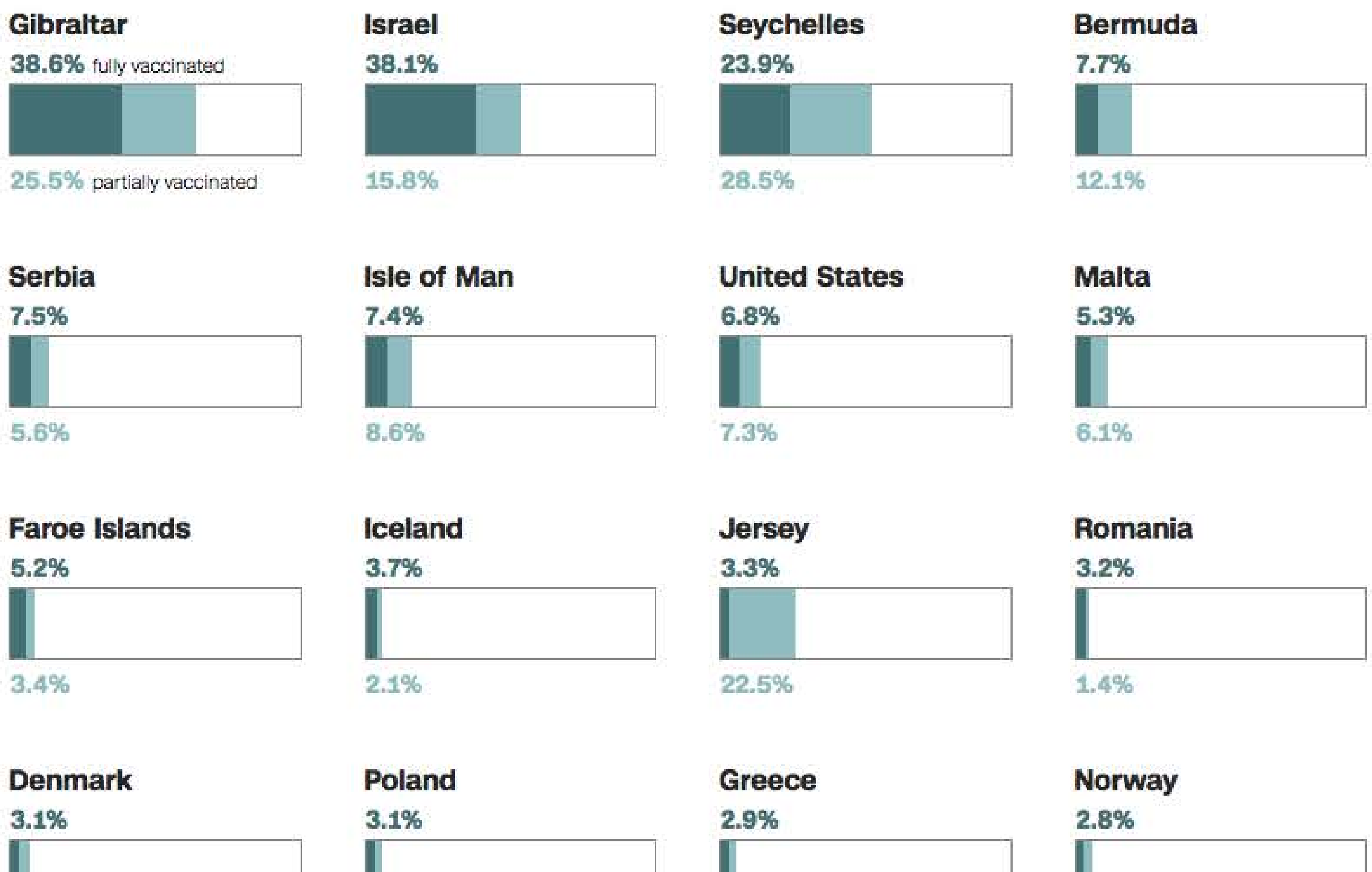

U.S. and Worldwide Vaccination Tracker

COVID-19 Vaccinations
Download Data

68,006,227
Doses Administered

Data last updated 02/26/21

6.8% of people fully vaccinated
At the current rate, 22.5% of people fully vaccinated by 4/30/21

Geolocation:

Worldwide | United States

- [ ] Alabama
- [ ] Alaska
- [ ] Arizona
- [ ] Arkansas
- [ ] California
- [ ] Colorado
- [ ] Connecticut
- [ ] Delaware
- [ ] District of Columbia
- [ ] Florida
- [ ] Georgia
- [ ] Hawaii
- [ ] Idaho
- [ ] Illinois
- [ ] Indiana
- [ ] Iowa
- [ ] Kansas
- [ ] Kentucky
- [ ] Louisiana
- [ ] Maine
- [ ] Maryland

Where and when are vaccinations being given? | **How many people are getting vaccinated?** | Tabular

Measures: Administered Doses | Delivered Doses | % Delivered Doses that were Administered

View: By Location | By Date

This chart shows the number of individual COVID-19 vaccination doses administered throughout the United States. In addition to tracking the overall doses administered, this chart tracks the progress to the **Federal Goal of 100M** doses administered in 100 days. **Learn more**

Vaccine Doses Given Per Day
United States

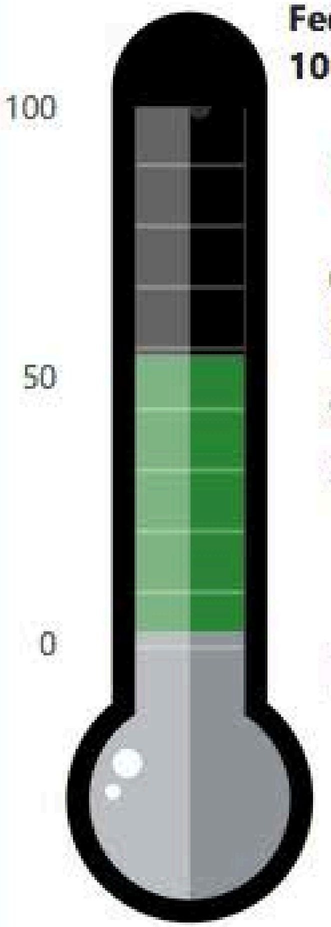

Federal Goal:
100M in 100 days

On track

Current:
52,298,457

% of Goal:
52.3%

Doses administered prior to 1/21/21: 15,707,770

Vaccine Doses Administered Per 100K:
United States

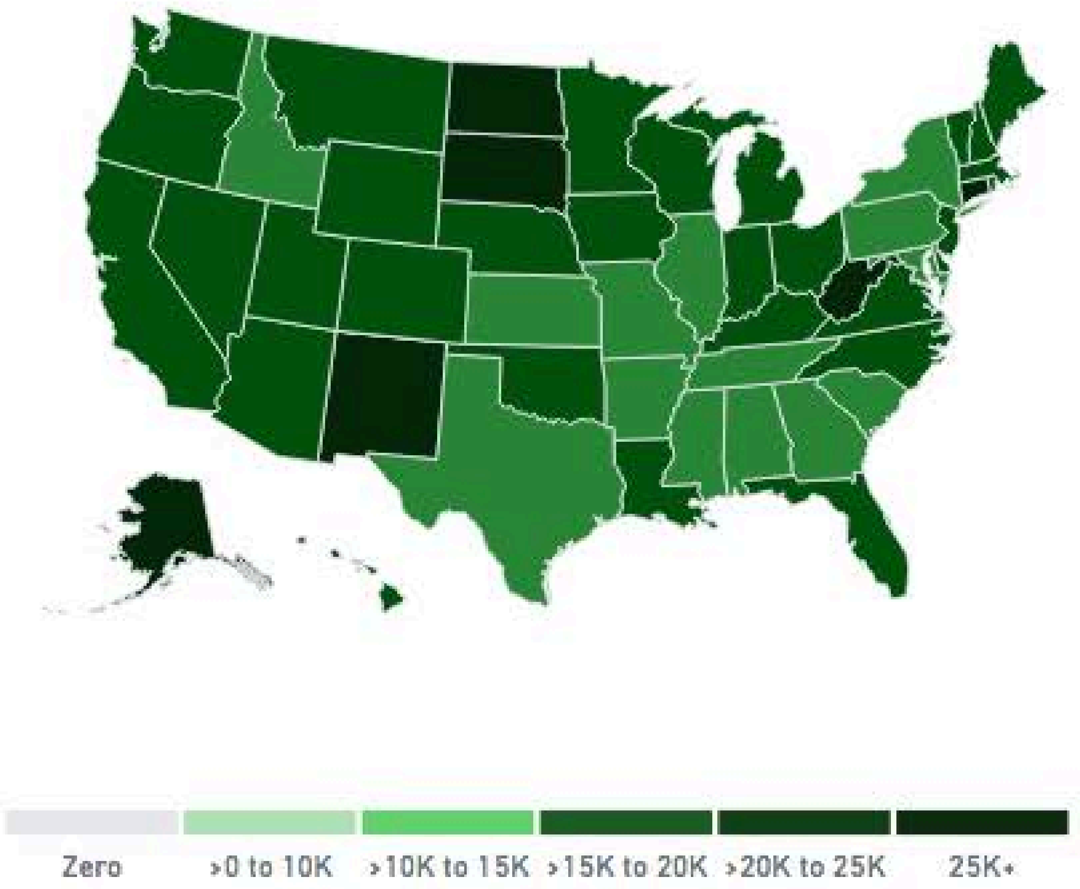

State	Doses Administered / 100K	Doses Administered
Alaska	34,114	249,559
New Mexico	30,276	634,845
South Dakota	28,284	250,216
West Virginia	27,092	485,535
North Dakota	26,758	203,911
Connecticut	25,971	925,946
Hawaii	24,642	348,897
District of Columbia	24,517	173,030
Wyoming	24,457	141,545
Oklahoma	24,451	967,522
Vermont	23,691	147,828
Montana	23,606	252,295
Massachusetts	22,875	1,576,657
Maine	22,719	305,397
Wisconsin	22,571	1,314,188
Colorado	22,474	1,294,236
Arizona	22,086	1,607,544
Nebraska	21,837	422,419
Indiana	21,676	1,459,296
Virginia	21,660	1,848,774
Minnesota	21,611	1,218,799

Microsoft
AI for Health

Information about Sources
Click Here

Contact us at:
ai4hc19@microsoft.com

licrosoft Power BI

U.S. and Worldwide Vaccination Tracker

COVID-19 Vaccinations
Download Data

227,513,434
Doses Administered

Data last updated 02/26/21

4,227,704 latest doses administered/day

Geolocation:

Worldwide | United States

- Albania
- Algeria
- Andorra
- Anguilla
- Argentina
- Australia
- Austria
- Azerbaijan
- Bahrain
- Bangladesh
- Barbados
- Belarus
- Belgium
- Bermuda
- Bolivia
- Brazil
- Bulgaria
- Cambodia
- Canada
- Cayman Islands
- Chile

Where and when are vaccinations being given? | **How many people are getting vaccinated?**

Measures: Administered Doses

View: By Location | By Date

This chart shows the cumulative number of individual COVID-19 vaccination doses administered worldwide. **Learn more**

Doses per 100K by Country/Region

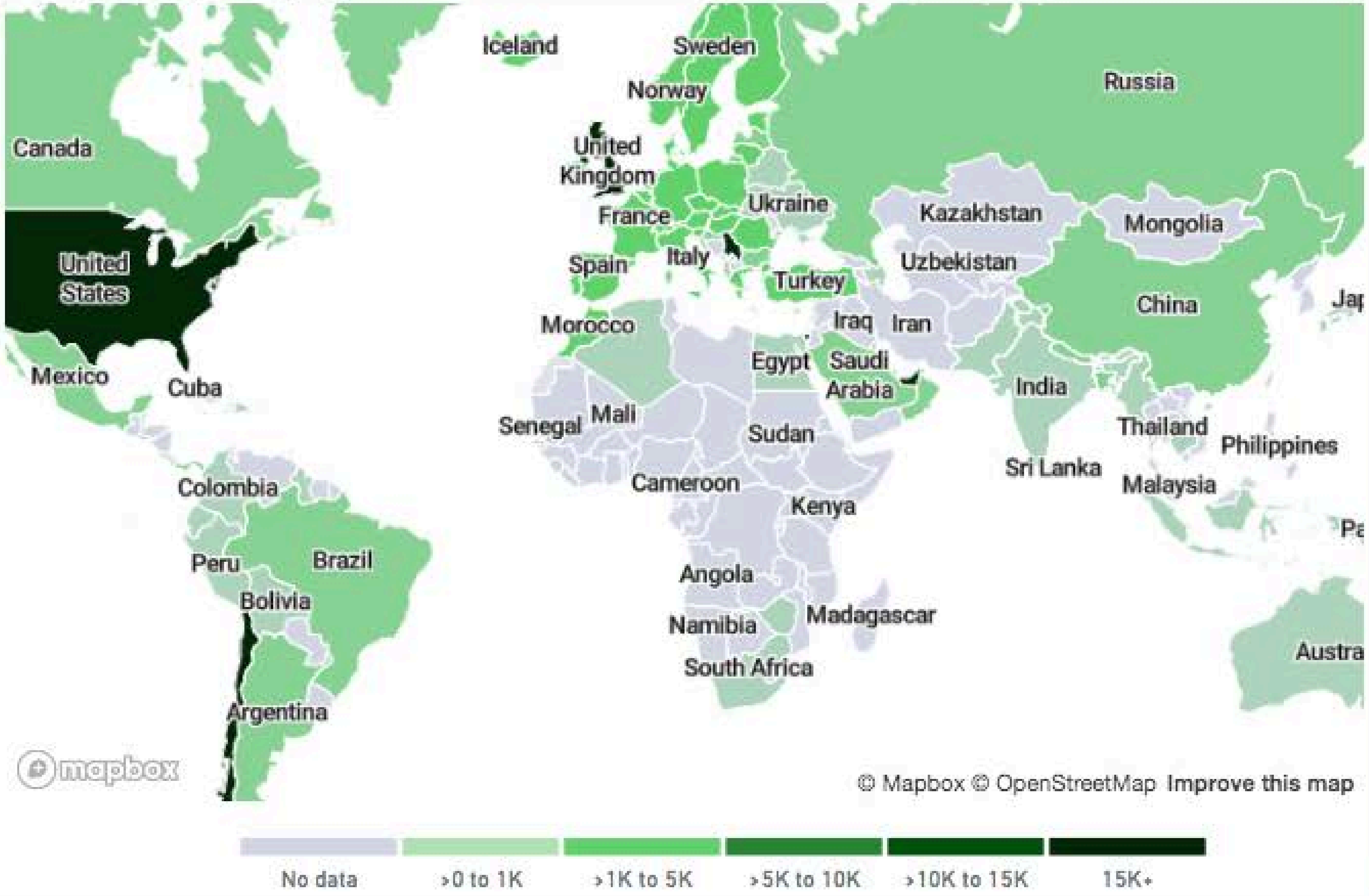

Country / Region	Doses Administered Per 100K	Doses Administered
Gibraltar	99,050	33,371
Israel	91,552	7,924,356
Seychelles	71,759	70,568
United Arab Emirates	59,108	5,846,036
Cayman Islands	32,115	21,106
Guernsey	30,572	20,047
United Kingdom	28,566	19,392,553
Jersey	27,446	29,312
Bermuda	27,416	17,073
Isle of Man	22,103	18,795
United States	20,723	68,006,227
Serbia	20,220	1,375,872
Anguilla	18,411	2,762
Maldives	18,187	98,311
Bahrain	17,049	290,103
Chile	16,781	3,207,932
Turks and Caicos Islands	16,615	6,433
Malta	16,207	71,562
Turkey	9,461	7,979,726

Microsoft
AI for Health

Information about Sources
Click Here

Contact us at:
ai4hc19@microsoft.com
