# Supplementary material for: Web-Based COVID-19 Dashboards and Trackers in the United States: Survey Study
Source: JMIR Hum Factors. 2023 Mar 20;10:e43819. doi: 10.2196/43819 (PMC10029858; doi:10.2196/43819)
Supplement: Multimedia Appendix 11 [file humanfactors_v10i1e43819_app11.pdf]

Appendix 11. Screenshots from dashboards and trackers — *State-focused coverage* *(captured December 11, 2021)*

Page	Ref	Host
2–4	S-1	Alabama Dept of Public Health
5	S-2	David Marconnet, CTO of OneTeam.net
6–8	S-4	Alaska Dept of Health and Social Services
9–11	S-6	Arizona Dept of Health Services
12–14	S-7	Arkansas Dept of Health
15	S-8	California Dept of Public Health
16–18	S-9	CA Open Data
19	S-11	CalMatters
20–24	S-12	Colorado Dept of Health & Environment
25	S-13	Coloradoan
26	S-14	The Denver Post
27	S-15	Connecticut's Official State Website
28	S-17	University of Connecticut Dept of Geography
29	S-18	Government of the District of Columbia
30	S-19	Delaware Environmental Public Health Tracking Network
31	S-23	Atlanta Journal-Constitution
32	S-24	State of Hawaii Dept of Health
33	S-25	Hawaii Data Collaborative
34–38	S-26	Idaho Division of Public Health
39	S-28	Illinois Dept of Public Health
40	S-29	Indiana State Dept of Health
41–42	S-31	Iowa Dept of Public Health
43–44	S-32	Kansas Dept of Health and Environment
45	S-33	Kentucky Dept of Public Health
46	S-34	WFPL 89.3
47	S-35	Louisiana Dept of Health
48	S-36	Maine Center for Disease Control & Prevention
49–50	S-37	Maine Geolibrary
51–52	S-38	Maryland Dept of Health
53–55	S-39	Massachusetts Dept of Public Health
56–58	S-40	Michigan Dept of Health and Human Services
59	S-41	Bridge Michigan
60	S-42	Minnesota Dept of Health
61	S-43	Mississippi State Dept of Health

Page	Ref	Host
62–64	S-44	Missouri Dept of Health and Senior Services
65–66	S-46	Montana State Library
67–69	S-47	North Carolina Dept of Health and Human Services
70–72	S-48	North Dakota Dept of Health
73–75	S-50	Nevada Dept of Health and Human Services
76–78	S-51	New Hampshire Dept of Health and Human Services
79	S-52	New Hampshire Public Radio
80–81	S-53	New Jersey Dept of Health
82	S-54	NJ Advance Media
83	S-55	New Mexico Dept of Health
84	S-56	New York State Dept of Health
85	S-57	Ohio Dept of Health
86–87	S-58	Oklahoma State Dept of Health
88	S-59	Oregon Health Authority
89–93	S-60	Pennsylvania Dept of Health
94	S-61	Rhode Island Dept of Health
95	S-62	South Carolina Dept of Health and Environment Control
96–98	S-64	South Dakota Dept of Health
99	S-65	Tennessee Dept of Health
100–101	S-66	Tennessee Dept of Health
102	S-68	Houston Chronicle
103	S-69	Utah Dept of Health
104–108	S-70	Virginia Dept of Health
109–110	S-71	Vermont Dept of Health
111	S-72	VTDigger
112–115	S-73	Washington State Dept of Health
116	S-74	Washington State Hospital Association
117	S-75	West Virginia Dept of Health & Human Resources
118	S-76	Wisconsin Dept of Health Services
119	S-77	WISN-12
120	S-79	Spotlight PA
121	S-80	WPRI-12
122	S-81	San Francisco Chronicle
123	S-82	The Maine Monitor
124	S-83	The Oregonian
125	S-84	Arkansas Democrat Gazette

Alabama's COVID-19 Data and Surveillance Dashboard

Alabama Department of Public Health | Division of Infectious Diseases & Outbreaks | Updates Daily by 10 a.m.

Alabama

Overall Level of Community Transmission

Click the county to change the data on the right and on tabs 1, 4, 6 and 10.

Alabama	SUBSTANTIAL
Autauga	MODERATE
Baldwin	MODERATE
Barbour	MODERATE
Bibb	MODERATE
Blount	SUBSTANTIAL
Bullock	SUBSTANTIAL
Butler	HIGH
Calhoun	MODERATE

Overall Level of Community Transmission	Indicator (Past 7 Days)	
	New cases per 100,000 persons	Percent positive
Low (Blue)	0-9.99	0-4.99%
Moderate (Yellow)	10-49.99	5-7.99%

-

Overall Commu
Transmission Le

LOW

MODERATE

SUBSTANTI

HIGH

Overall Level of Community Transmission - Past 7 Days

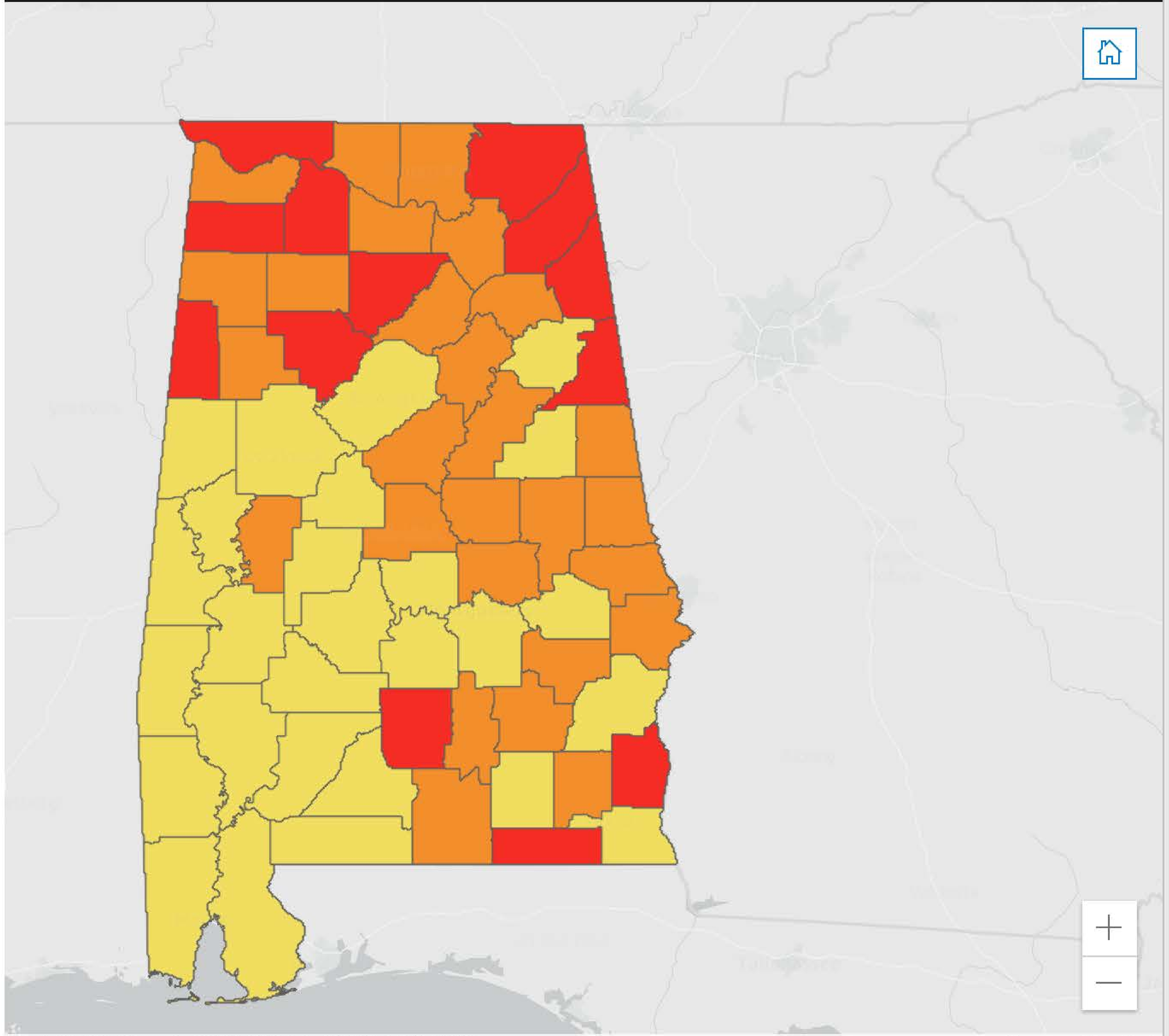

Esri, HERE, Garmin, FAO, NOAA, USGS, EPA, NPS

Powered by Esri

1 | 2 - Index | 3 | 4 | 5 | 6 | 7 | 8 | 9 | 10 | 11 | About | Notices

MOVING 7-DAY

5.5

% Positivity in Tests

CASES

851,950

TOTAL

387,575	464,375
2020	2021

DEATHS

16,265

TOTAL

7,185	9,080
2020	2021

LAST 7 DAYS - TOTAL TESTS

42,562	2,327
NAAT TESTS	POSITIVE NAAT TESTS

Alabama's COVID-19 Data and Surveillance Dashboard

ALABAMA PUBLIC HEALTH

Alabama Department of Public Health | Division of Infectious Diseases & Outbreaks | Updates Daily by 10 a.m.

Alabama

Overall Level of Community Transmission

Click the county to change the data on the right and on tabs 1, 4, 6 and 10.

Alabama	SUBSTANTIAL
Autauga	MODERATE
Baldwin	MODERATE
Barbour	MODERATE
Bibb	MODERATE
Blount	SUBSTANTIAL
Bullock	SUBSTANTIAL
Butler	HIGH
Calhoun	MODERATE

Overall Level of Community Transmission	Indicator (Past 7 Days)	
	New cases per 100,000 persons	Percent positive
Low (Blue)	0-9.99	0-4.99%
Moderate (Yellow)	10-49.99	5-7.99%

Daily and 7-Day Moving Average Number of Cases

(Please select Alabama or a specific county from the list on the left.)

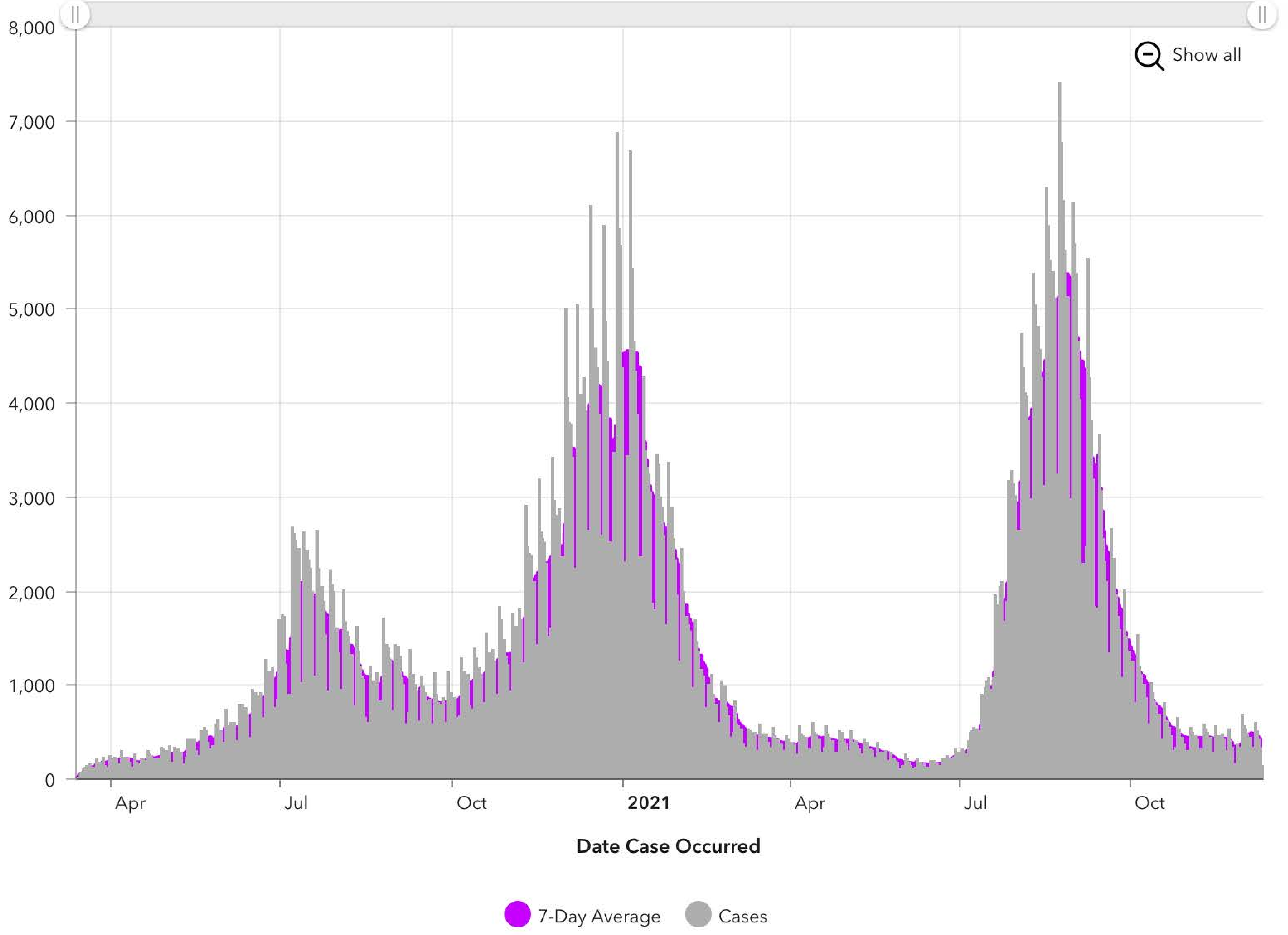

1 | 2 - Index | 3 | 4 | 5 | 6 | 7 | 8 | 9 | 10 | 11 | About | Notices

MOVING 7-DAY

5.5

% Positivity in Tests

CASES

851,950

TOTAL

387,575	464,375
2020	2021

DEATHS

16,265

TOTAL

7,185	9,080
2020	2021

LAST 7 DAYS - TOTAL TESTS

42,562	2,327
NAAT TESTS	POSITIVE NAAT TESTS

Alabama's COVID-19 Data and Surveillance Dashboard

Alabama Department of Public Health | Division of Infectious Diseases & Outbreaks | Updates Daily by 10 a.m.

Alabama

Overall Level of Community Transmission

Click the county to change the data on the right and on tabs 1, 4, 6 and 10.

Alabama	SUBSTANTIAL
Autauga	MODERATE
Baldwin	MODERATE
Barbour	MODERATE
Bibb	MODERATE
Blount	SUBSTANTIAL
Bullock	SUBSTANTIAL
Butler	HIGH
Calhoun	MODERATE

Overall Level of Community Transmission	Indicator (Past 7 Days)	
	New cases per 100,000 persons	Percent positive
Low (Blue)	0-9.99	0-4.99%
Moderate (Yellow)	10-49.99	5-7.99%

Statewide Percentage of Nucleic Acid Amplification Tests (NAATs) that were Positive During the Past 7 Days

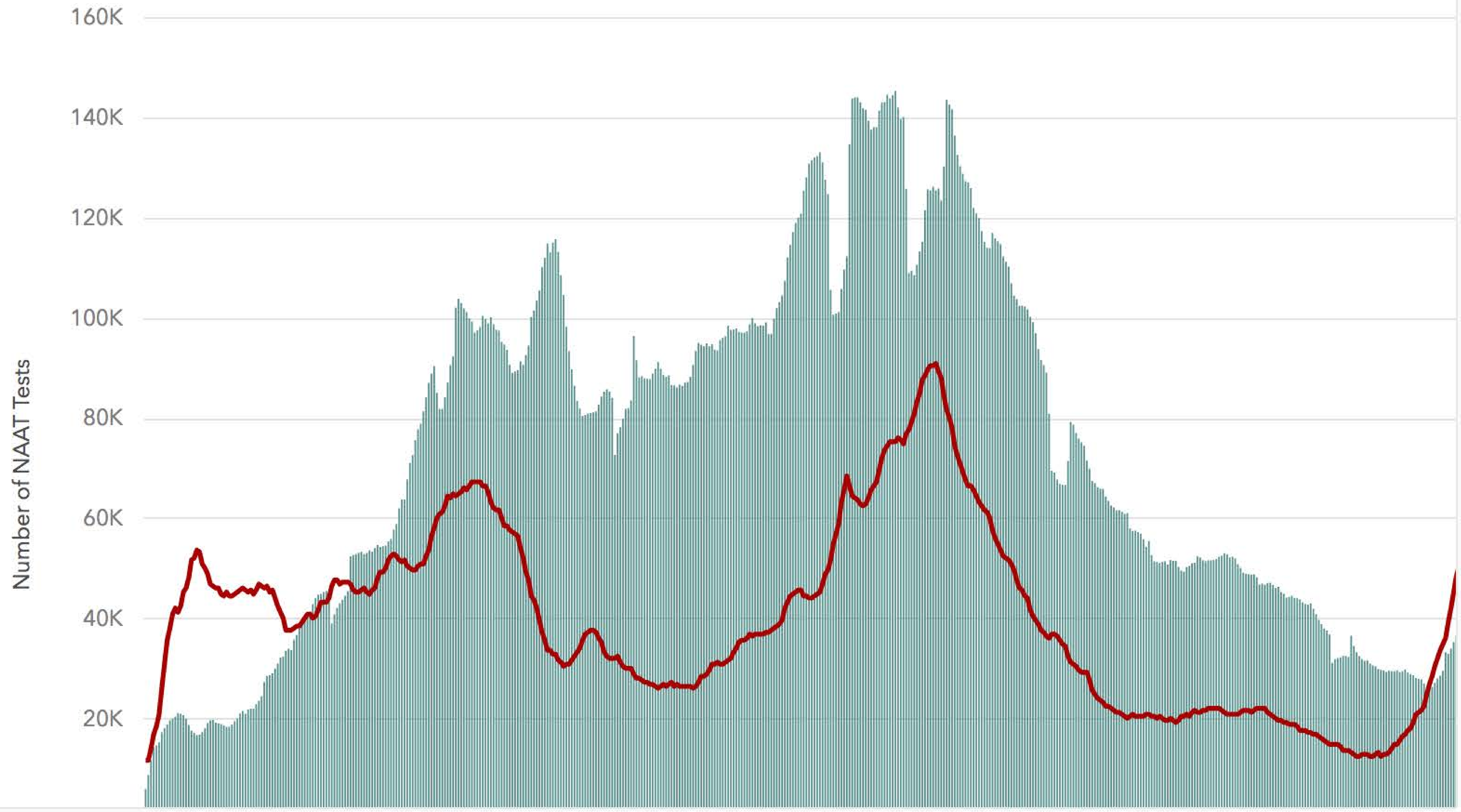

Laboratories are required by law to report both positive and negative COVID-19 test results to ADPH. To accurately calculate the percentage of positive test results, both numerator and denominator data are needed. Only nucleic acid amplification test (NAAT) methods are included in this calculation. A NAAT is a type of viral diagnostic test for SARS-CoV-2, the virus that causes COVID-19. NAATs detect genetic material (nucleic acids) of the virus. The date is assigned by the collection date and represents the percent that were positive during the past 7 days as of that date. Data are preliminary and subject to change.

1 | 2 - Index | 3 | 4 | 5 | 6 | 7 | 8 | 9 | 10 | 11 | About | Notices

MOVING 7-DAY

5.5

% Positivity in Tests

CASES

851,950

TOTAL

387,575	464,375
2020	2021

DEATHS

16,265

TOTAL

7,185	9,080
2020	2021

LAST 7 DAYS - TOTAL TESTS

42,562	2,327
NAAT TESTS	POSITIVE NAAT TESTS

BAMATRACKER

ALABAMA DAILY NEWS

COVID-19

- Main Tracker
- Charts
- Counties
- School Data
- Vaccine Data
- Maps
- Mortality
- Demographics
- National Totals

OTHER

- Updates

Alabama COVID-19 Tracking

As of 5/29/2021, Bama Tracker is no longer updating on a daily basis. The site will remain online as a resource for historical data collected from ADPH. For the latest Alabama COVID-19 counts, please refer to the ADPH COVID-19 Dashboard Hub.

If you are looking for a great, mobile friendly analysis of Alabama data, check out the AL Political Reporter COVID Tracker.

Total Cases
543,405
417,501 Conf — 125,904 Prob

Total Deaths
11,146
8,785 Conf — 2,361 Prob

New Cases
-
+0 Conf — +0 Prob

Recoveries
509,800
As of 2021-05-08 — 93.82%

Total Tested
2,611,777
+0 Yesterday — 139,480 Anti

People Vaccinated
1,318,446
2,851,388 Doses — 93.6% of AL Deliv

Last 14 Days
2,839
100,184 Tested — 2.83% Pos

Hospitalized
333
49,899 Total

Confirmed Cases Per Day

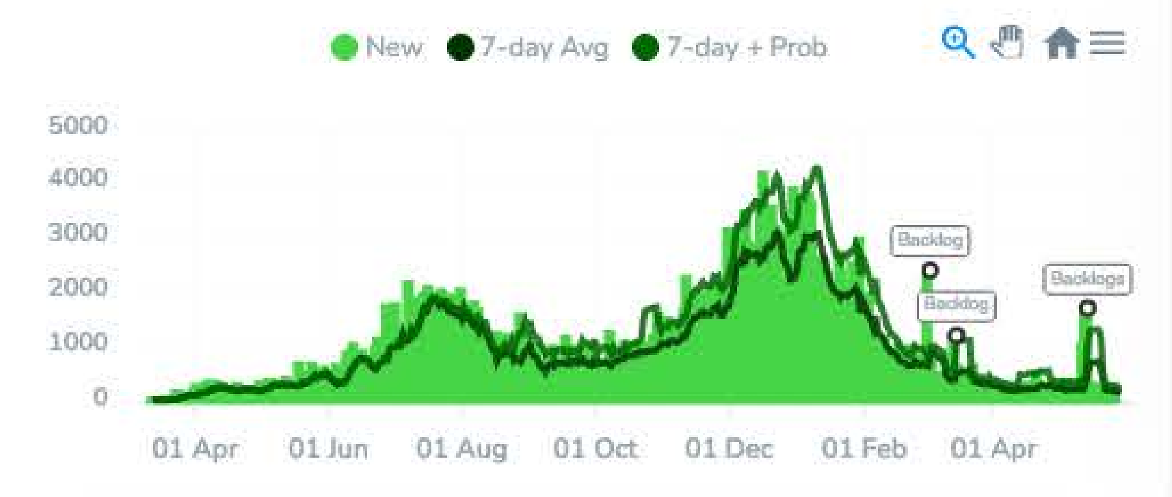

Deaths by Actual Date of Death

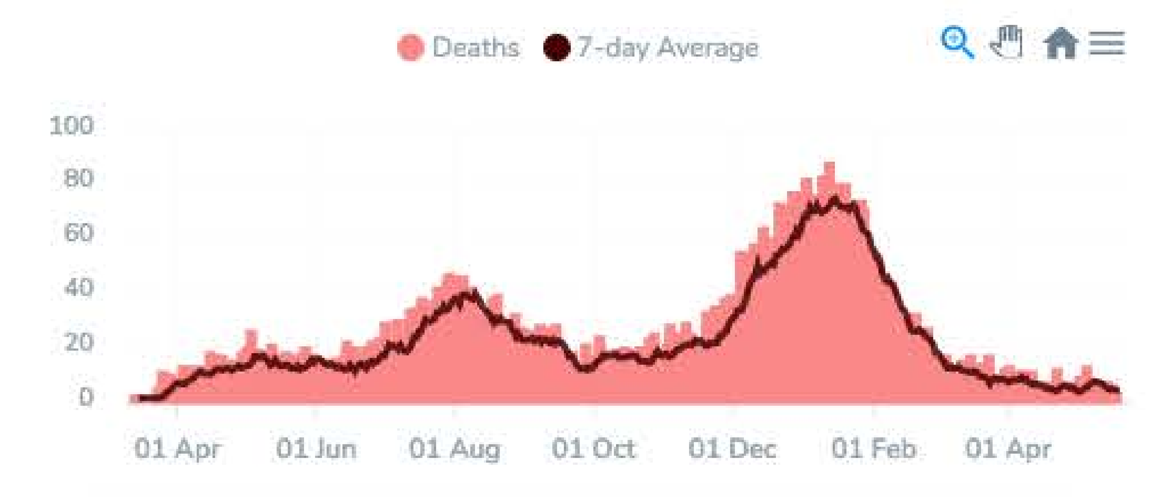

Confirmed Hospitalizations

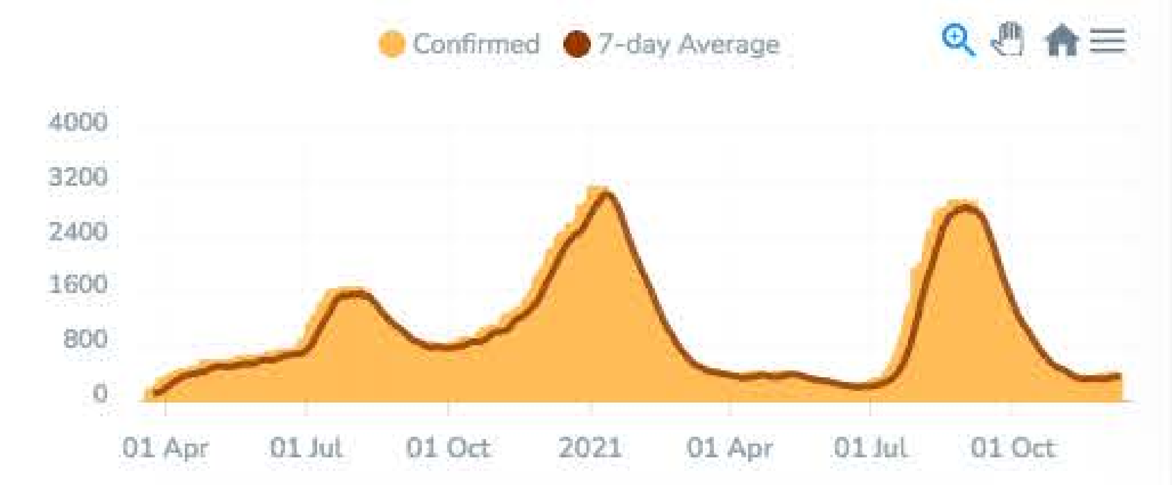

R_t Per Day

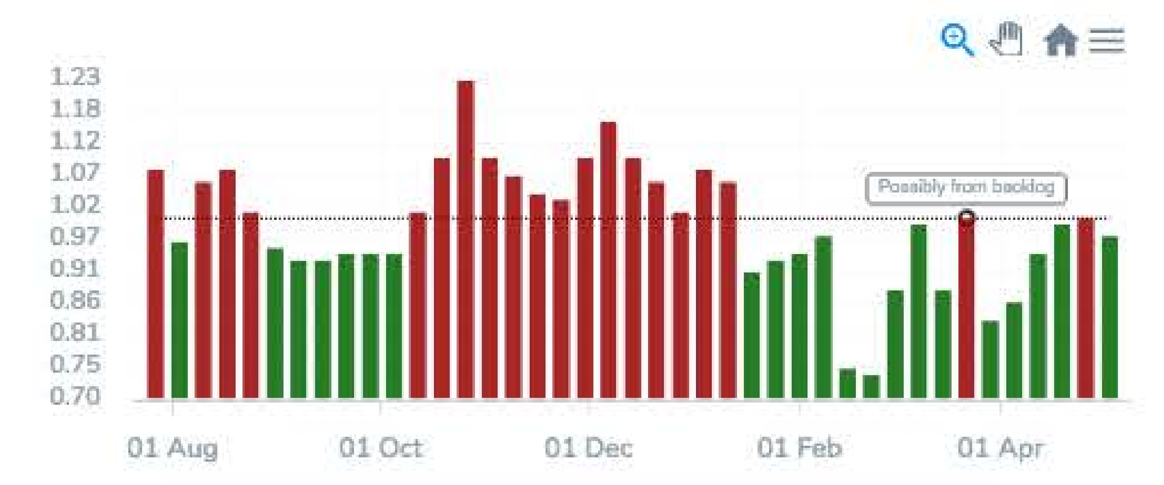

7-Day Avg. %Positive Per Day

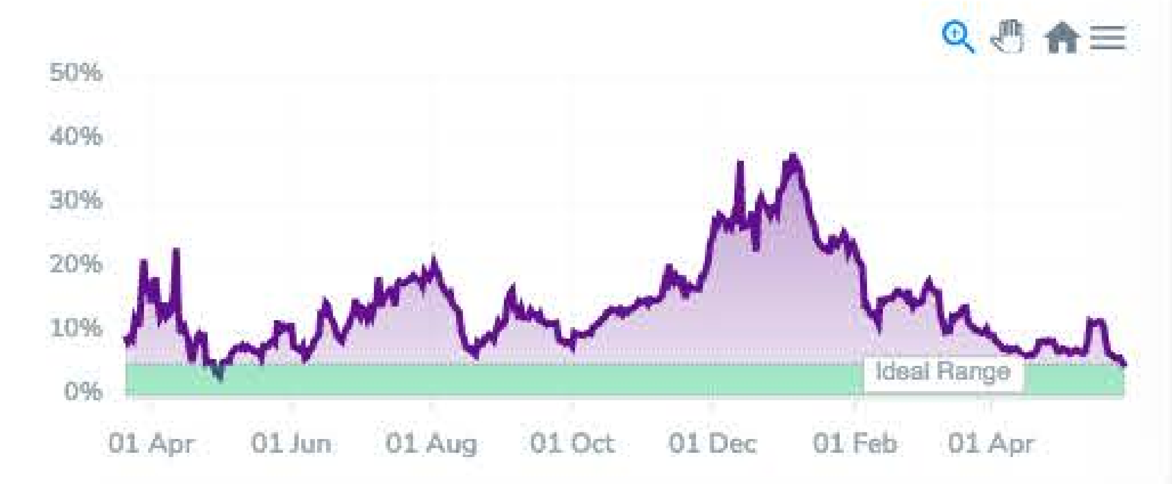

14-Day Counts %Positive Per Day

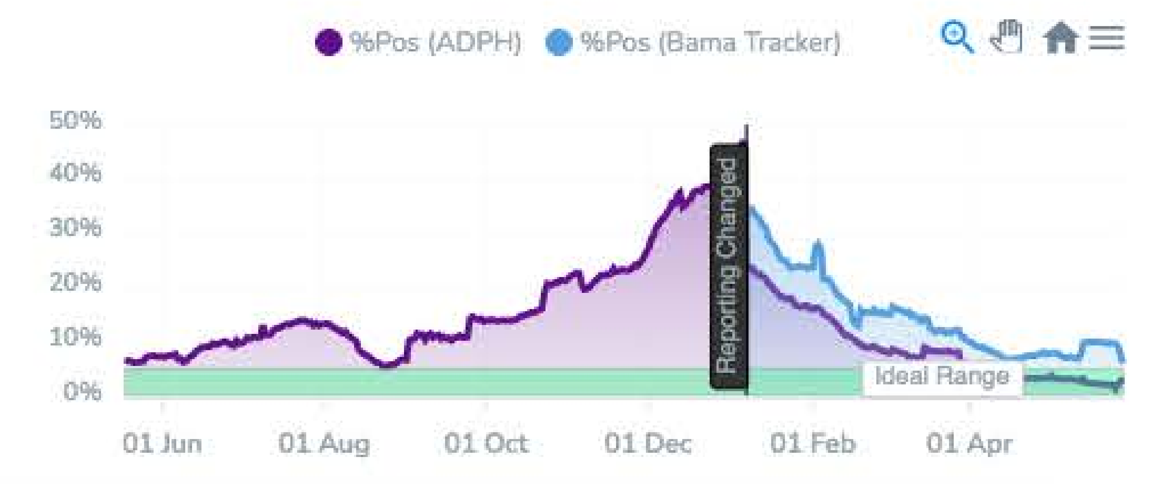

County Details

Show 25 entries

Search:

County	Cases	Change	Probable	Change	Deaths	Change	Tests	%Pos (14d)	7day Avg
Jefferson	69,627	0	10,568	0	1539	0	433,720	2.08%	17.00
Madison	30,462	0	4,882	0	513	0	204,101	2.70%	8.43
Mobile	29,085	0	12,466	0	815	0	203,239	3.12%	8.57
Montgomery	22,380	0	2,491	0	600	0	110,606	2.74%	6.71
Shelby	21,022	0	4,379	0	253	0	124,243	1.88%	5.71
Tuscaloosa	17,379	0	8,652	0	457	0	156,735	2.49%	3.71
Baldwin	15,190	0	6,430	0	311	0	110,257	4.69%	7.86
Morgan	12,108	0	2,412	0	283	0	54,394	3.94%	3.43
Etowah	11,104	0	2,877	0	359	0	61,039	2.09%	3.29
Calhoun	10,913	0	3,709	0	324	0	70,547	1.50%	2.86
Marshall	10,483	0	1,855	0	227	0	46,320	2.38%	2.00
Lee	9,017	0	7,098	0	173	0	87,155	3.85%	5.43
Limestone	8,528	0	1,590	0	156	0	38,104	2.86%	1.86
St. Clair	8,298	0	1,736	0	247	0	44,792	4.63%	6.71
DeKalb	7,612	0	1,284	0	188	0	31,070	2.15%	1.29
Elmore	7,526	0	2,707	0	208	0	50,346	2.20%	2.00
Cullman	6,653	0	3,190	0	198	0	42,338	3.20%	3.00
Lauderdale	6,017	0	3,494	0	245	0	42,582	3.65%	1.86
Autauga	5,963	0	1,179	0	110	0	28,012	2.29%	2.43
Walker	5,879	0	1,405	0	278	0	29,290	1.97%	1.43
Houston	5,848	0	4,866	0	285	0	51,616	3.39%	2.71
Talladega	5,833	0	2,540	0	180	0	37,397	1.34%	1.29
Jackson	5,705	0	1,161	0	112	0	30,465	3.54%	1.57
Blount	5,241	0	1,623	0	139	0	26,387	5.47%	3.14
Colbert	4,530	0	1,817	0	137	0	26,199	2.97%	1.57
**Total**	**417,501**	**0**	**125,904**	**0**	**11,146**	**0**	**2,611,777**		

Showing 1 to 25 of 67 entries

Previous 1 2 3 Next

2020 - 2021 © Magic & Wires LLC. This website is for educational and academic research purposes only. Do not rely upon the content of this website as a basis for making any business, legal or health decisions. COVID-19 Data collected from ADPH, CDC & HHS.

Terms & Conditions

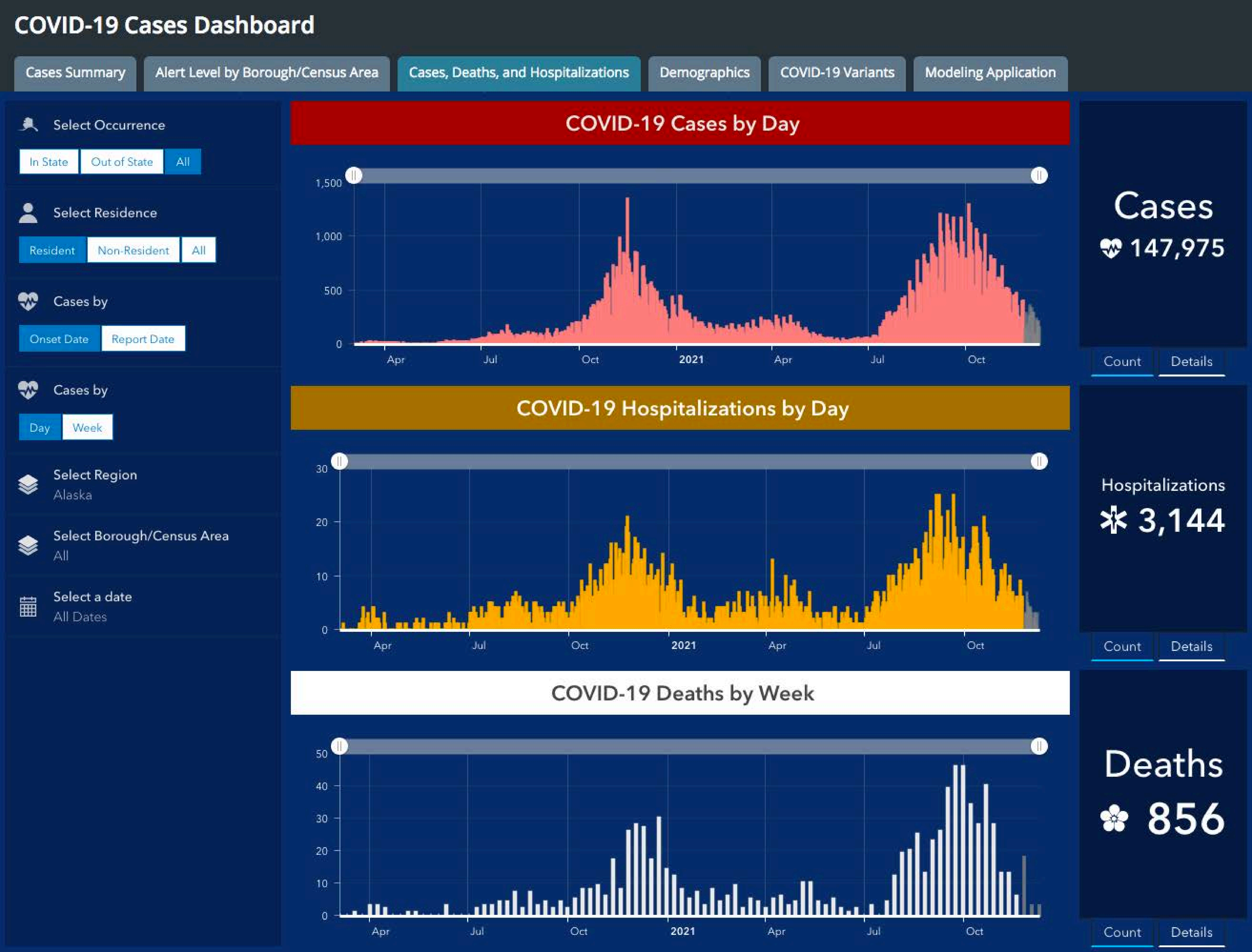
COVID-19 Cases Dashboard
Cases Summary
Alert Level by Borough/Census Area
Cases, Deaths, and Hospitalizations
Demographics
COVID-19 Variants
Modeling Application
Select Occurrence
In State
Out of State
All
Select Residence
Resident
Non-Resident
All
Cases by
Onset Date
Report Date
Cases by
Day
Week
Select Region
Alaska
Select Borough/Census Area
All
Select a date
All Dates
COVID-19 Cases by Day
1,500
1,000
500
0
Apr
Jul
Oct
2021
Apr
Jul
Oct
Cases
147,975
Count
Details
COVID-19 Hospitalizations by Day
30
20
10
0
Apr
Jul
Oct
2021
Apr
Jul
Oct
Hospitalizations
3,144
Count
Details
COVID-19 Deaths by Week
50
40
30
20
10
0
Apr
Jul
Oct
2021
Apr
Jul
Oct
Deaths
856
Count
Details

COVID-19 Cases Dashboard

Cases Summary | Alert Level by Borough/Census Area | Cases, Deaths, and Hospitalizations | Demographics | COVID-19 Variants | Modeling Application

Cumulative Cases

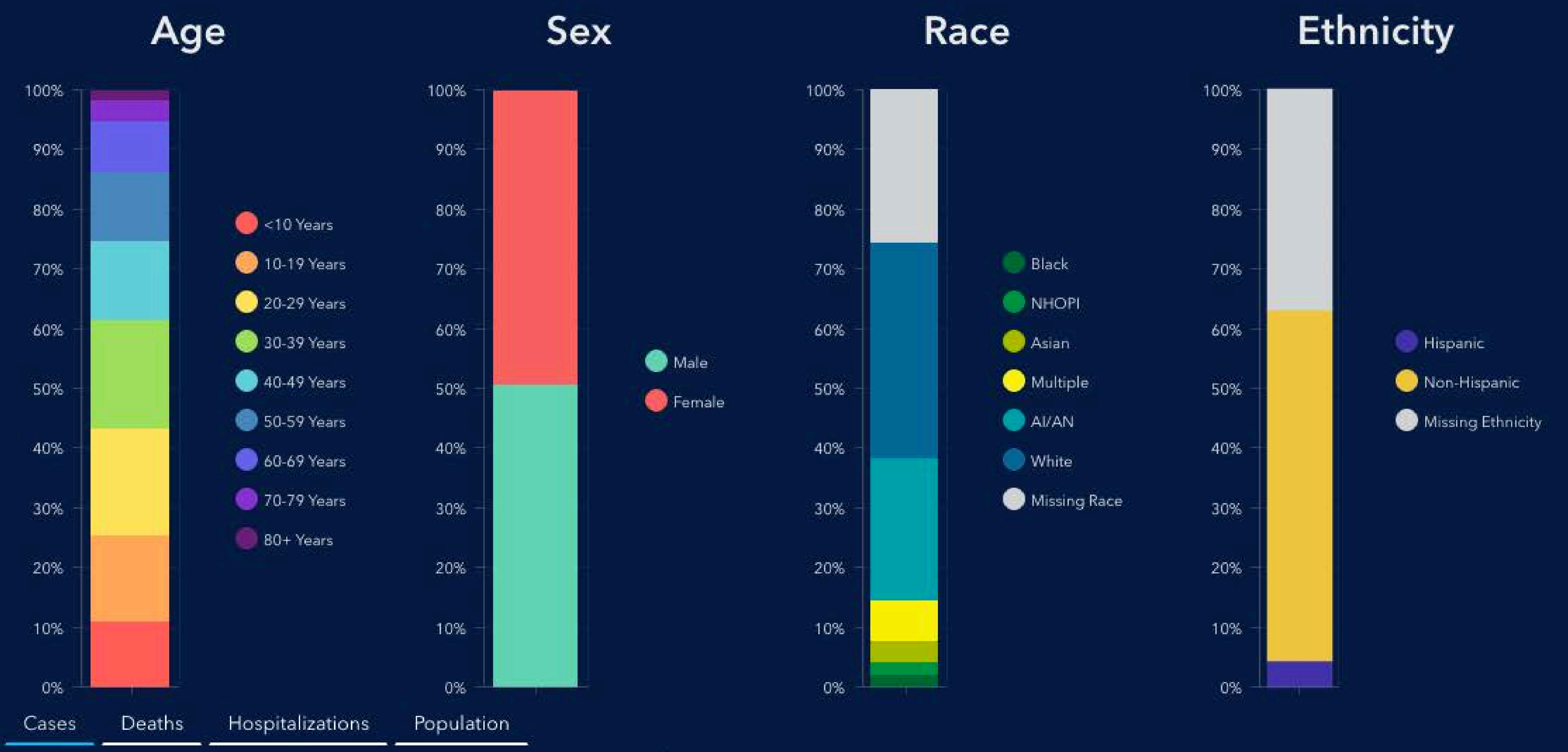

Demographic information may be self-reported. Missing Race and Missing Ethnicity contain cases under investigation, unknown race/ethnicity, and other race/ethnicity. For more information on a break out of missing race/ethnicity categories, click here

COVID-19 Cases Dashboard

Cases Summary | Alert Level by Borough/Census Area | Cases, Deaths, and Hospitalizations | Demographics | COVID-19 Variants | Modeling Application

Weekly Case Counts by Demographic Group

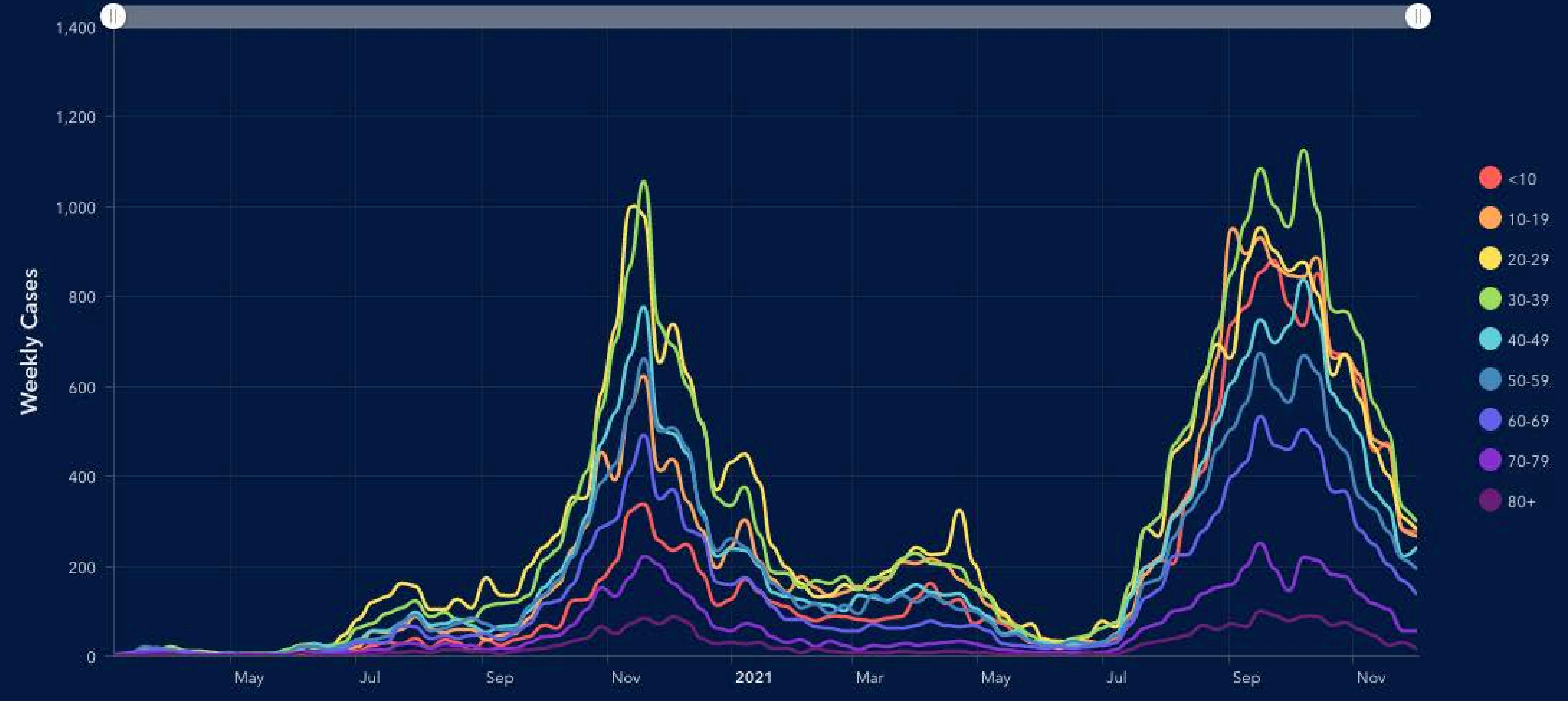

Age | Sex | Race | Ethnicity

Cases by race and ethnicity may be over or under represented due to missing information. Please interpret with caution. The past 7 days are excluded from this figure because cases with onset date during this period are unlikely to have been counted yet.

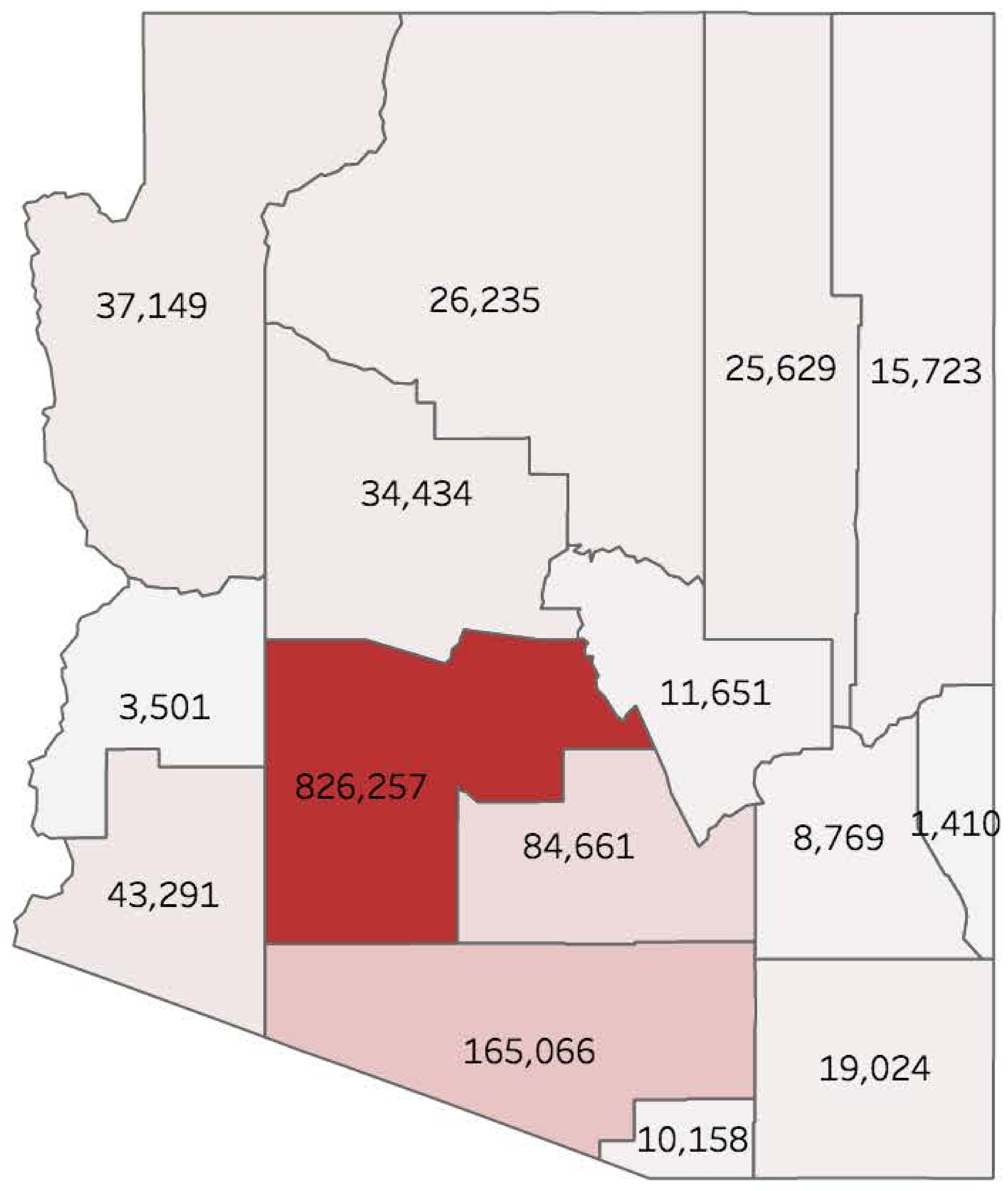

1,312,958	23,021	9,336,816
Number of New Cases reported *today**	Number of New Deaths reported *today**	Number of New Vaccine Doses reported *today** †
3,774	74	40,979
Rate of cases, per 100,000 population	Rate of fatalities, per 100,000 population	Percent of people vaccinated †
18,263.4	320.2	63.5%

Tests Conducted
using diagnostic tests**

Total Tests Conducted	New Tests reported *today**	Total Percent Positive***
14,999,371	38,613	9.9%

*Counts of new cases, deaths, tests conducted, and number of vaccine doses administered reflect increases in the total numbers compared to the previous day.

**Diagnostic tests include PCR and antigen testing.

***Percent positive is defined as the number of positive diagnostic results reported electronically, out of all COVID-19 diagnostic tests reported electronically.

† Vaccination numbers are displayed by the county of residence.

Recent COVID-19 Cases By Zip Code

Recent Case Count

RecentCaseCategory

- 0-50
- 51-200
- 201-500
- 501-700
- Greater Than 700
- Data Suppressed

Download Data

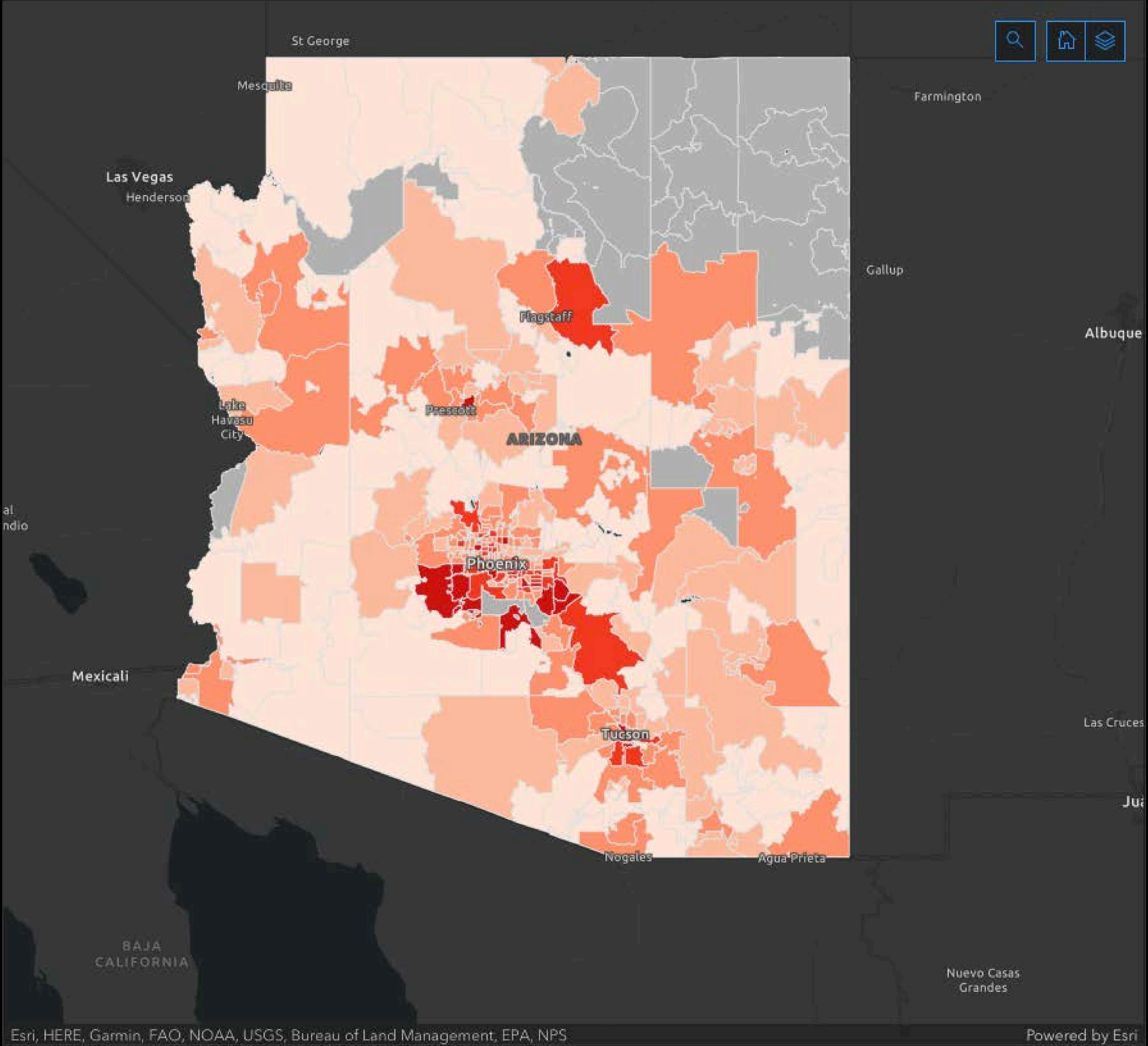

New! The *COVID-19 Cases by Zip Code* site will now feature recent cases as the default data layer. Recent cases are defined as new positive COVID-19 test results within the last month. Total case counts will still be available by clicking on individual zip codes, and within the data download.

Toggle between cumulative and recent visualizations by using the Layers button found in the upper right corner of the application

4,865,464	38,613	10.0%

Diagnostic tests by county
Select a county to filter the data.

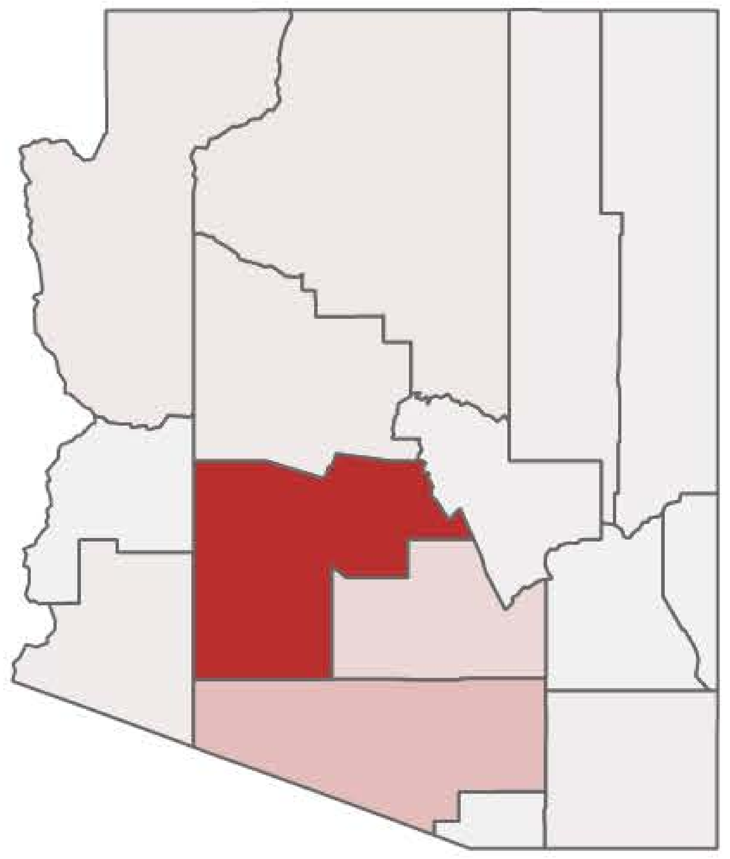

Diagnostic tests by date of collection

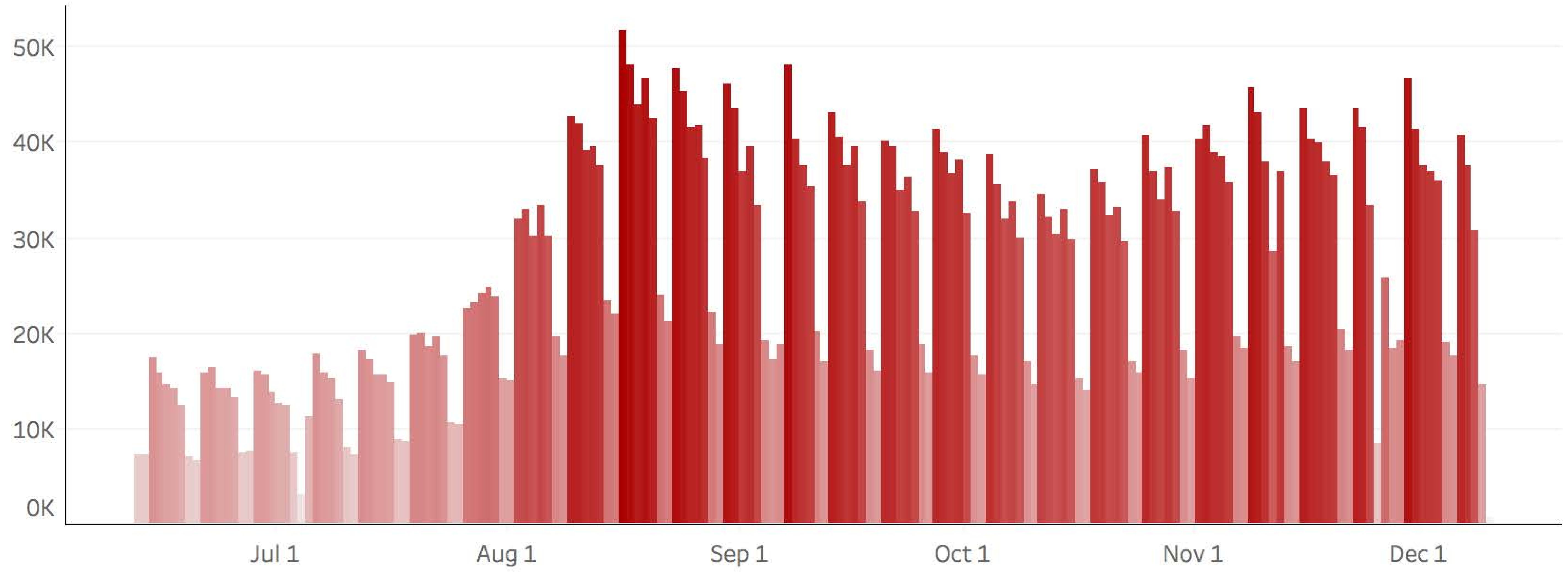

Total % Positive COVID-19 Diagnostic Tests: 10.0%

■ COVID-19 tests completed and ■ Percent positive by week
Percent positive is the number of positive tests in Electronic Laboratory Reporting (ELR), out of all COVID-19 tests reported via ELR.
Diagnostic tests include PCR and antigen testing.

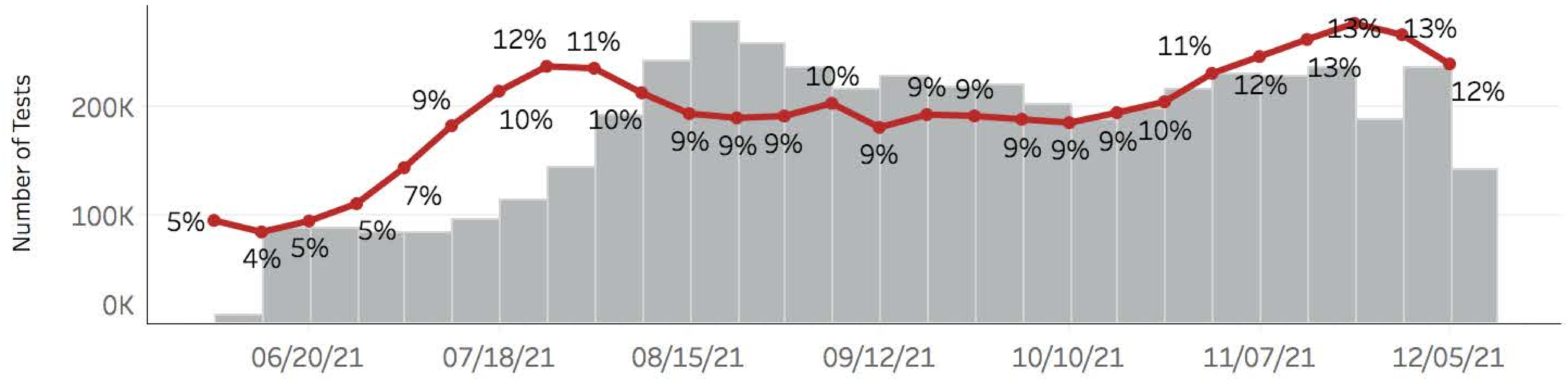

Diagnostic Testing by Age Group

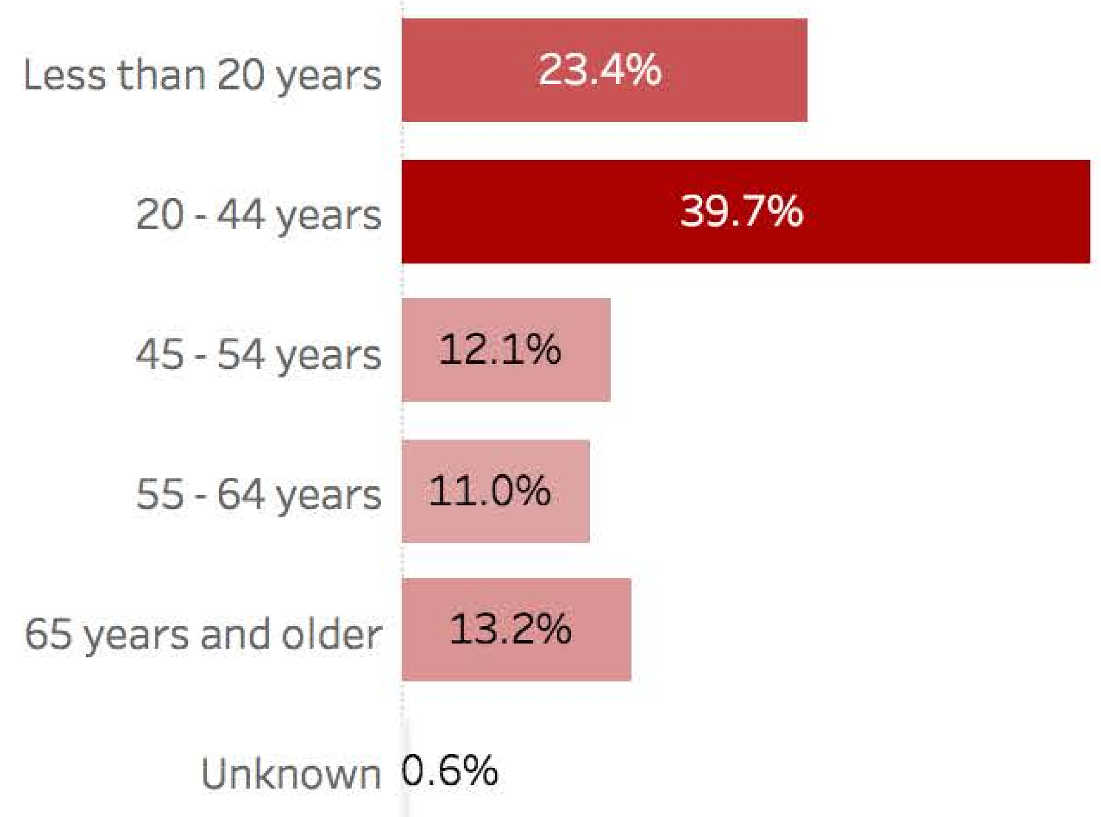

*NOTE: Results from the last 4-7 days may not be reported yet.

The Arkansas Department of Health COVID-19 Update

Total COVID-19 Cases
(*includes all confirmed and probable cases)

537,461

Recovered Cases

520,073

Cumulative Number of Cases
(last 14 days)

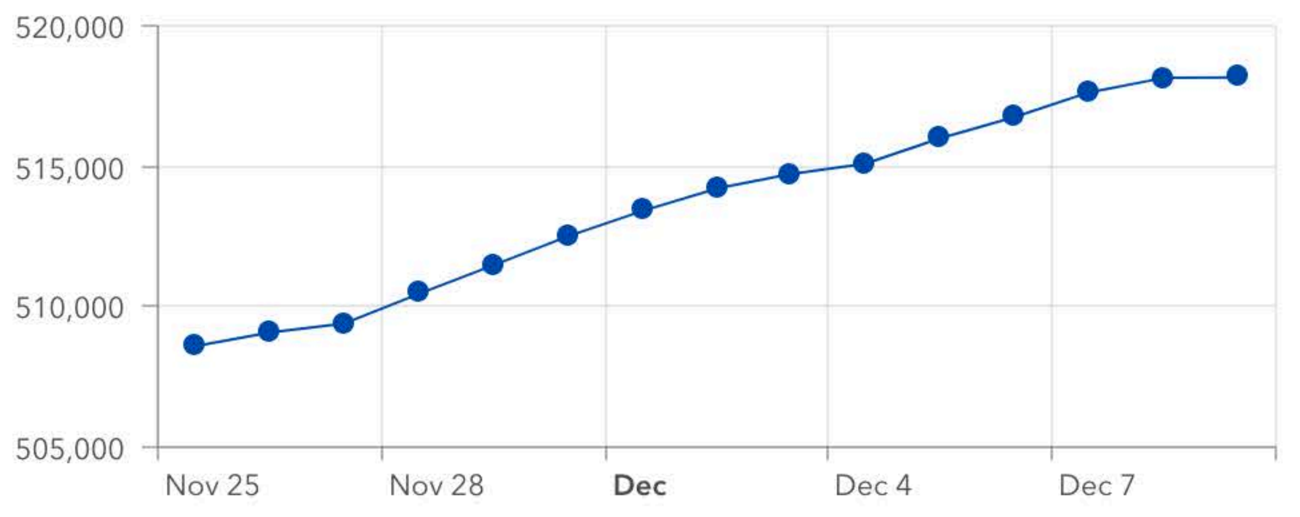

Number of COVID-19 Cases by County of Residence

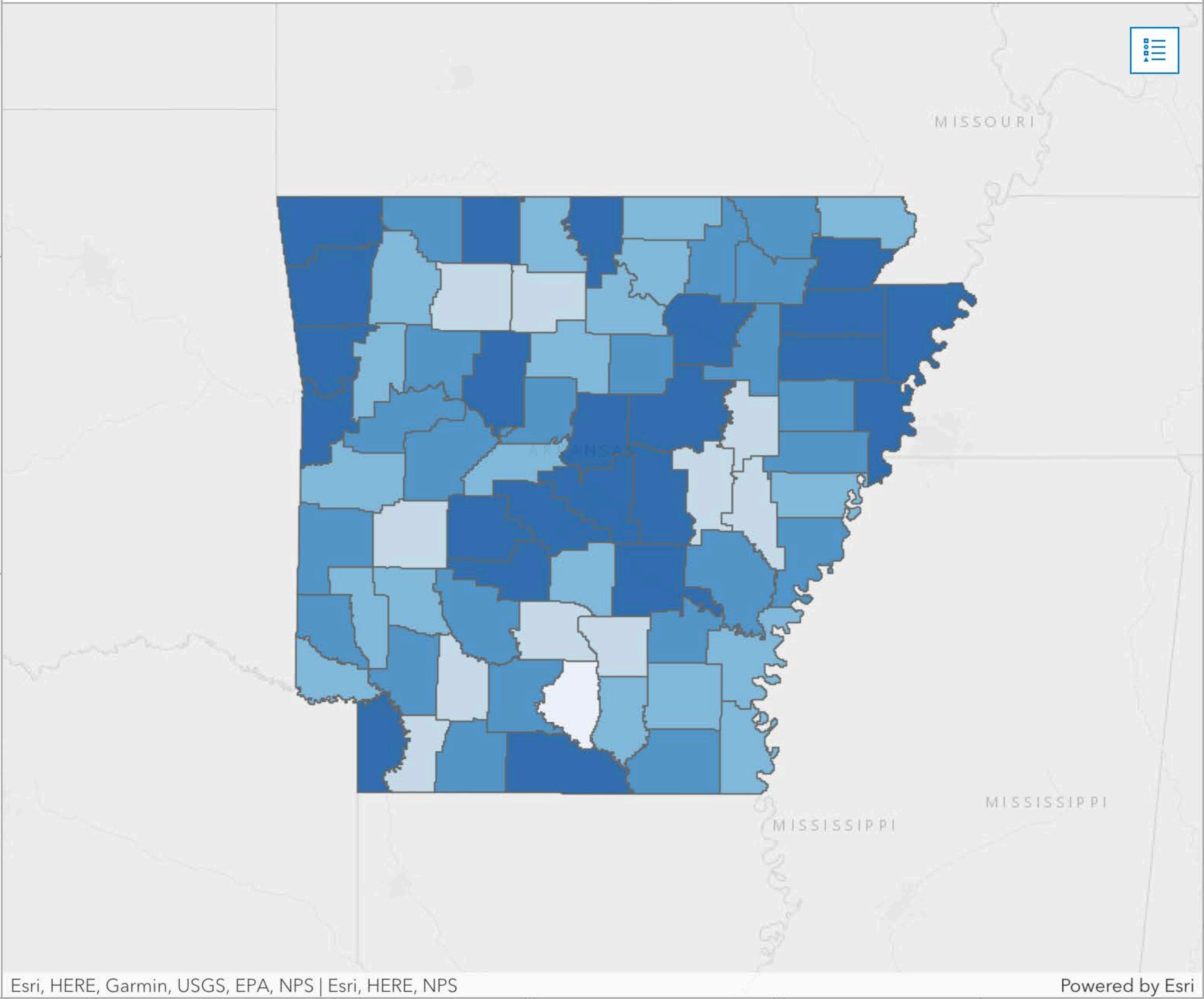

Vaccines | Total Cases | Active Cases | Pediatric Metrics | Deaths | Tests | Case Demographics | Health Metrics | Hospital Information | USA and Global

The Arkansas Department of Health COVID-19 Update

Totals Cases by Gender

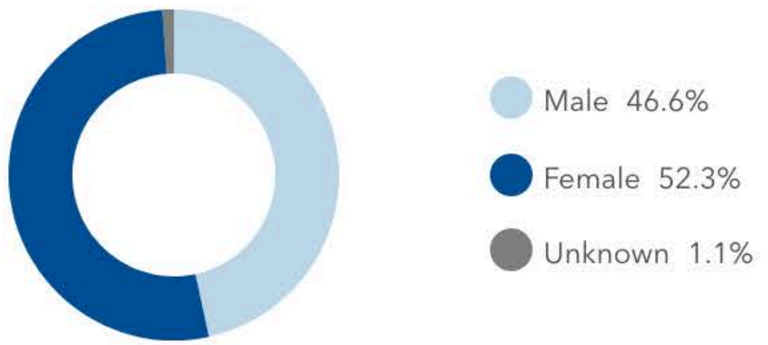

Cases by Race

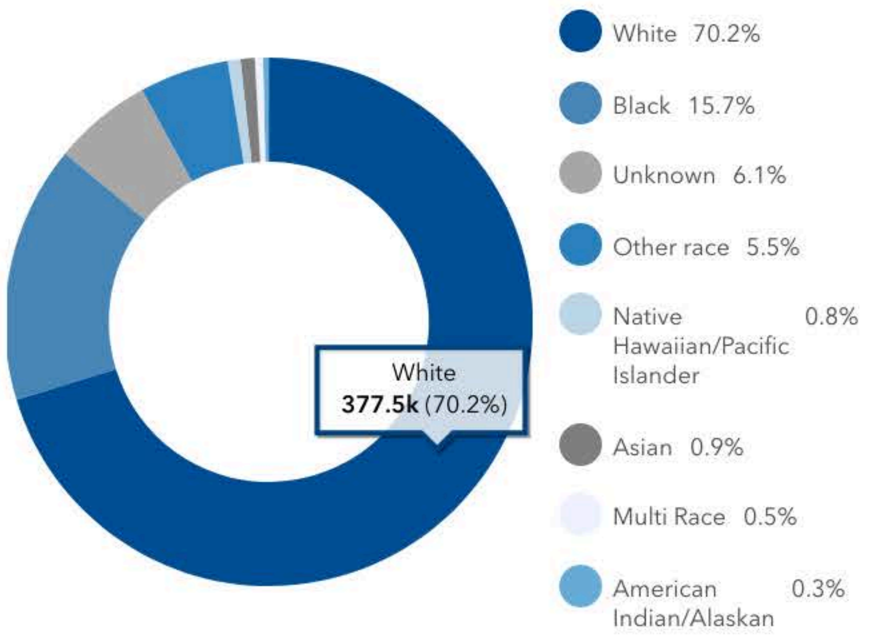

Cases by Ethnicity

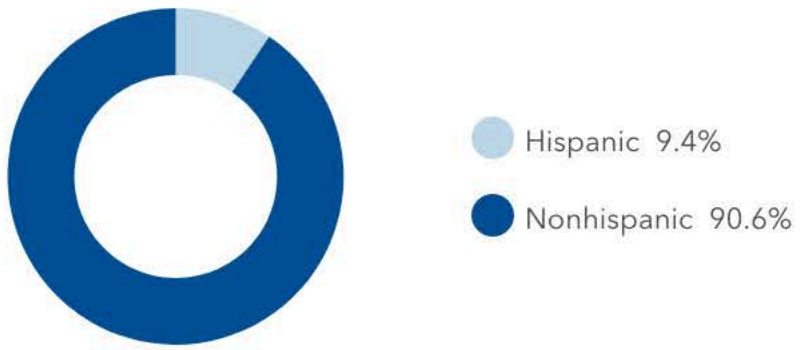

Cases by Age Category

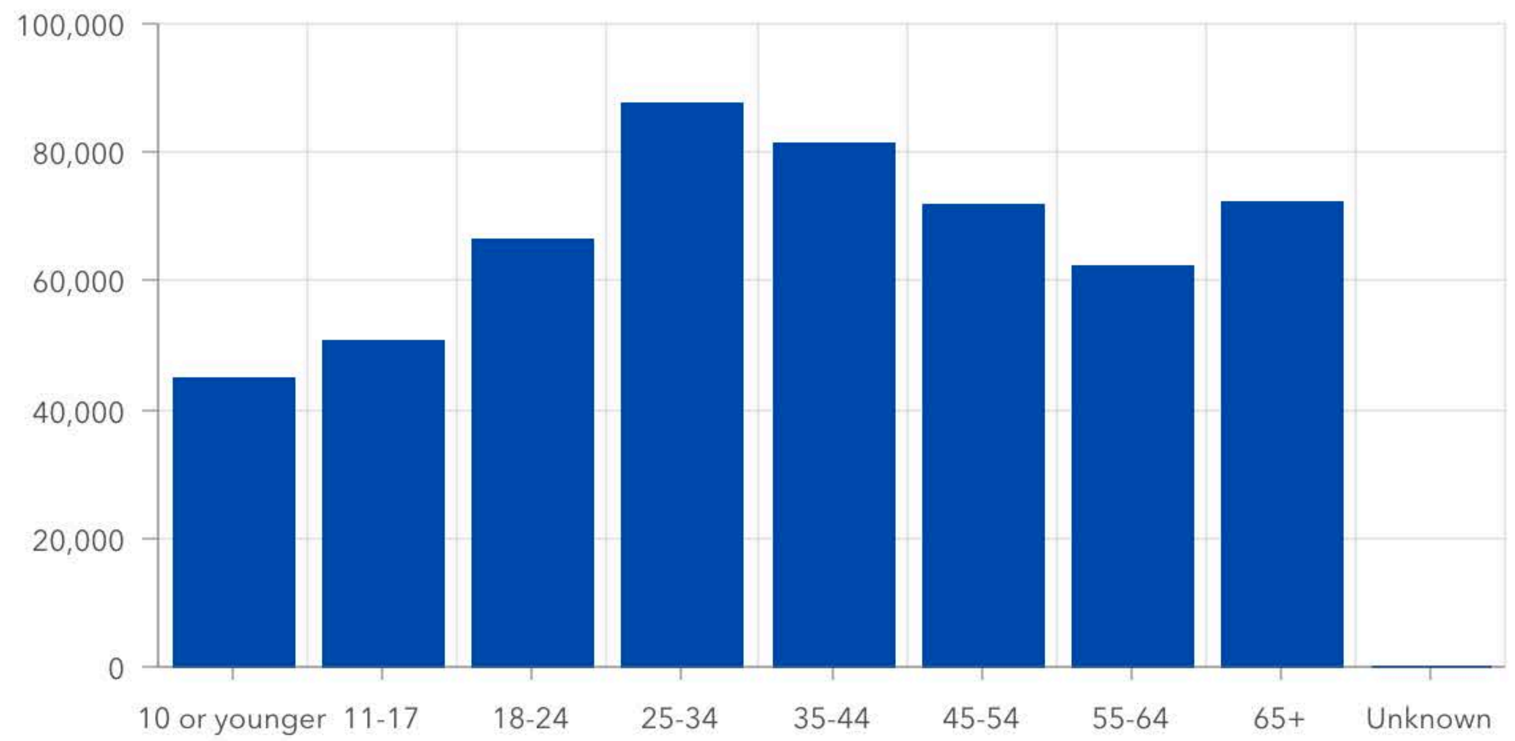

Cases by Underlying Health Concerns

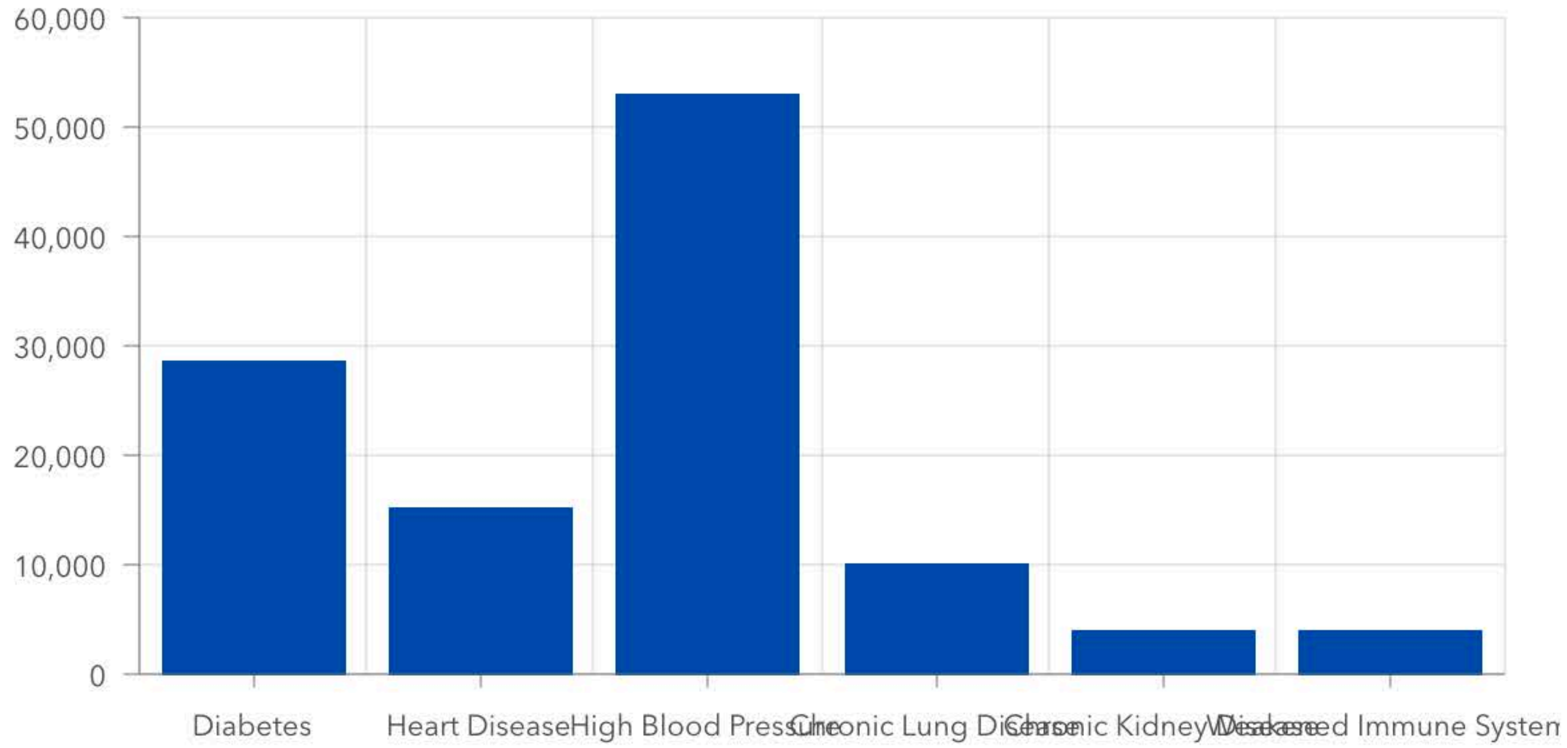

Percent of Cases That Are Healthcare Workers

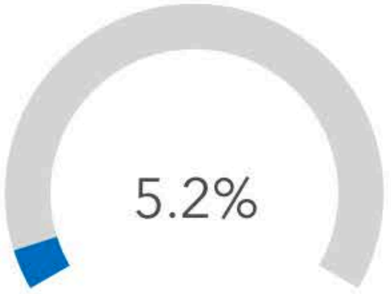

Percent of Cases In Nursing Homes

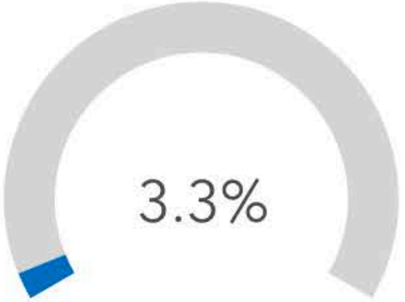

Percent of Cases In Correctional Facilities

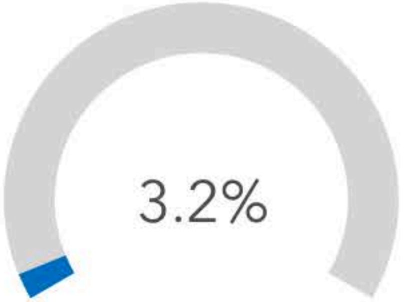

The Arkansas Department of Health COVID-19 Update

Total Tests Reported

5,585,376

Percent Positive PCR Tests

9.1%

Percent Positive Antigen Tests

13.1%

14 Day Testing Trend - PCR

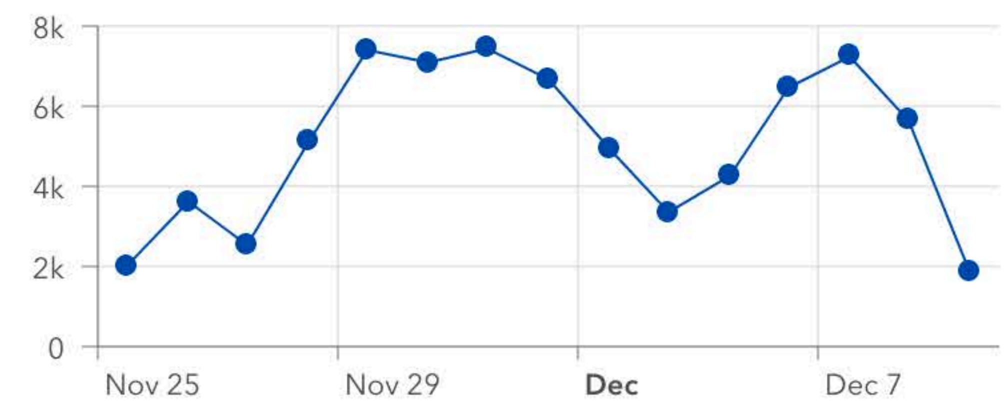

14 Day Testing Trend - Antigen

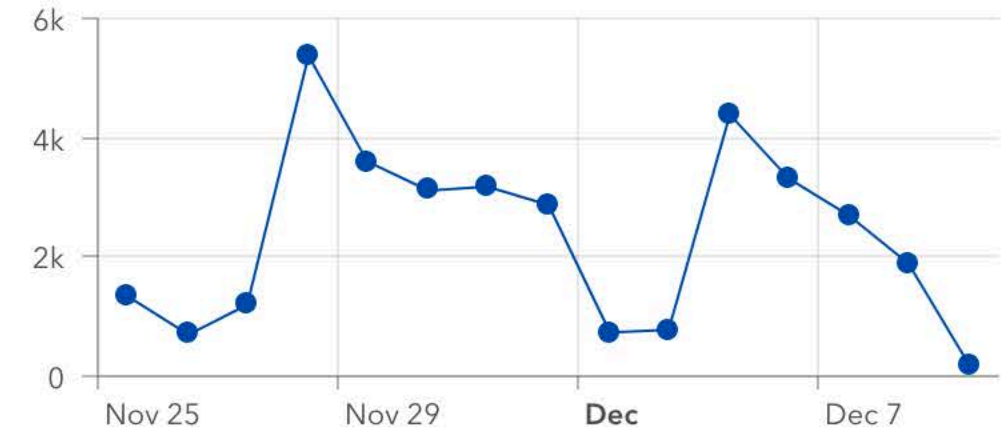

COVID-19 Tests Resulted per 1,000 Individuals

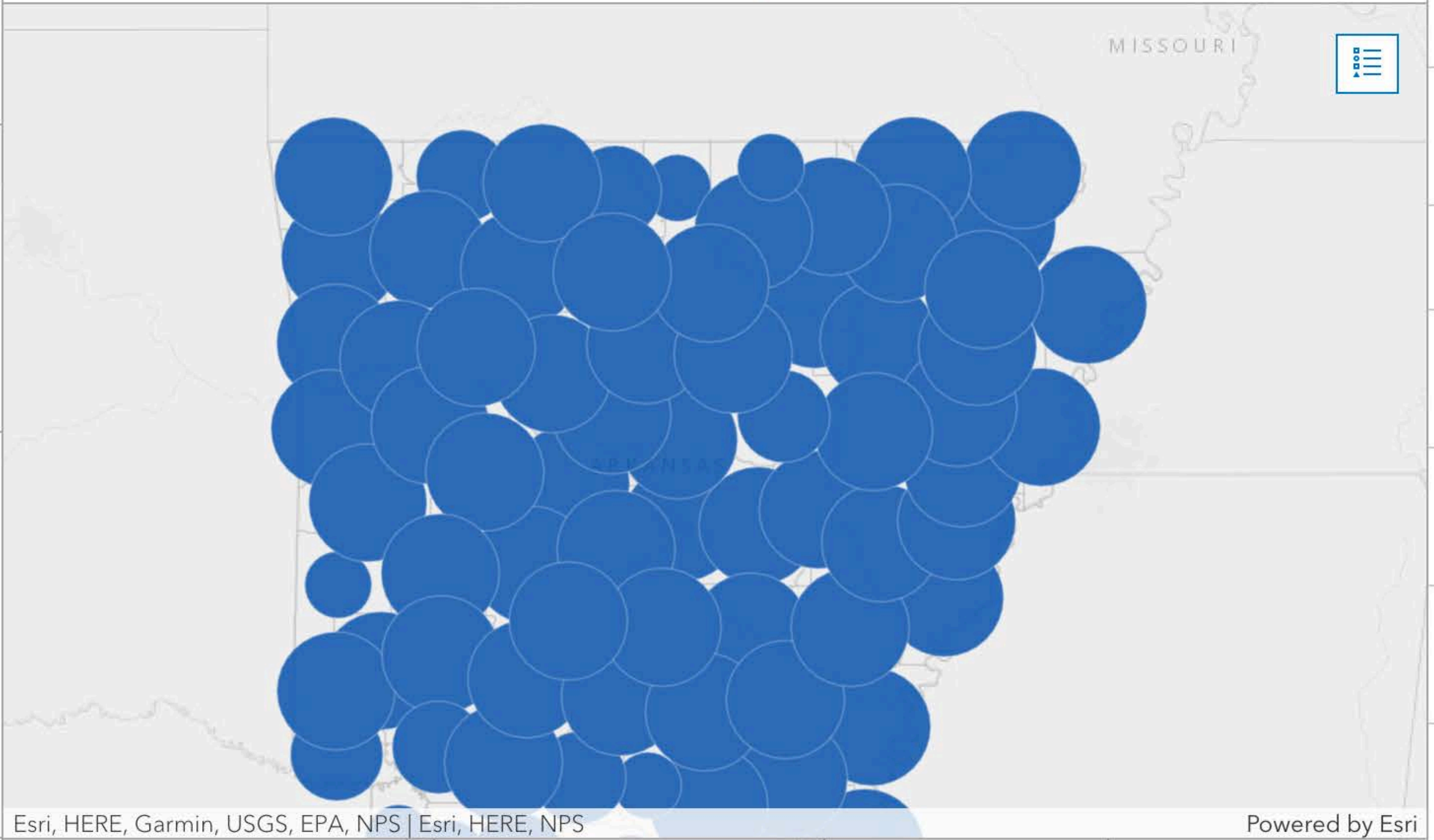

- **Arkansas** (40606 total tests)
- **Ashley** (29935 total tests)
- **Baxter** (47079 total tests)
- **Benton** (416700 total tests)
- **Boone** (69126 total tests)
- **Bradley** (19470 total tests)
- **Calhoun** (6051 total tests)
- **Carroll** (41309 total tests)
- **Chicot** (29065 total tests)
- **Clark** (50431 total tests)

COVID-19 Negative Tests	PCR Negatives	COVID-19 Positive Tests	PCR
5,023,900	3,902,609	561,476	392,196
	Antigen 1,121,291		Antigen 169,280

Tracking COVID-19 in California

Last updated December 11, 2021 at 10:00 AM

California is tracking data to understand the spread of COVID-19.

On this page you'll find:

- Today's update
- Unvaccinated and vaccinated data
- County and statewide data
- Cases and deaths by ethnicity, gender, and age
- Explore more data

Update for December 11, 2021

To date, California has confirmed a total of 4,867,604 COVID-19 cases and 74,509 deaths.

VACCINES ADMINISTERED
61,034,965 total
221,677 daily avg.
77.6% of population vaccinated (5+ with at least one dose)

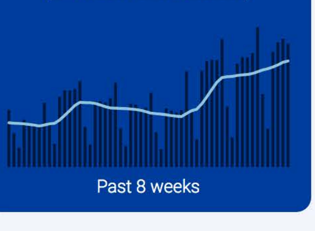

Past 8 weeks

CASES
4,867,604 total
4,909 daily avg.
13.4 new cases (per 100K)

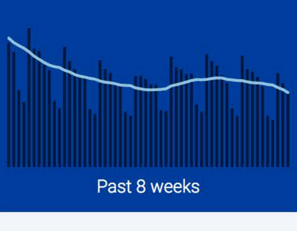

Past 8 weeks

DEATHS
74,509 total
50 daily avg.
0.1 new deaths (per 100K)

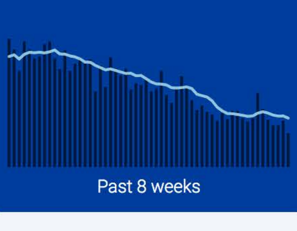

Past 8 weeks

TESTS
2.7% test positivity

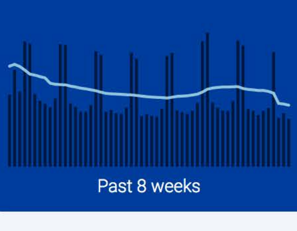

Past 8 weeks

Updated December 11, 2021, with data from December 10, 2021.

Vaccines administered source data and cases, deaths, and tests source data

Data notes

Unvaccinated and vaccinated data

Vaccines prevent serious illness, save lives, and reduce further spread of COVID-19. As more people are vaccinated, the virus is less likely to spread, mutate, and potentially become even more dangerous. Vaccines will help put an end to the pandemic.

Cases | **Hospitalizations** | **Deaths**

From November 21, 2021 to November 27, 2021, unvaccinated people were **7.1 times more likely** to get COVID-19 than fully vaccinated people.

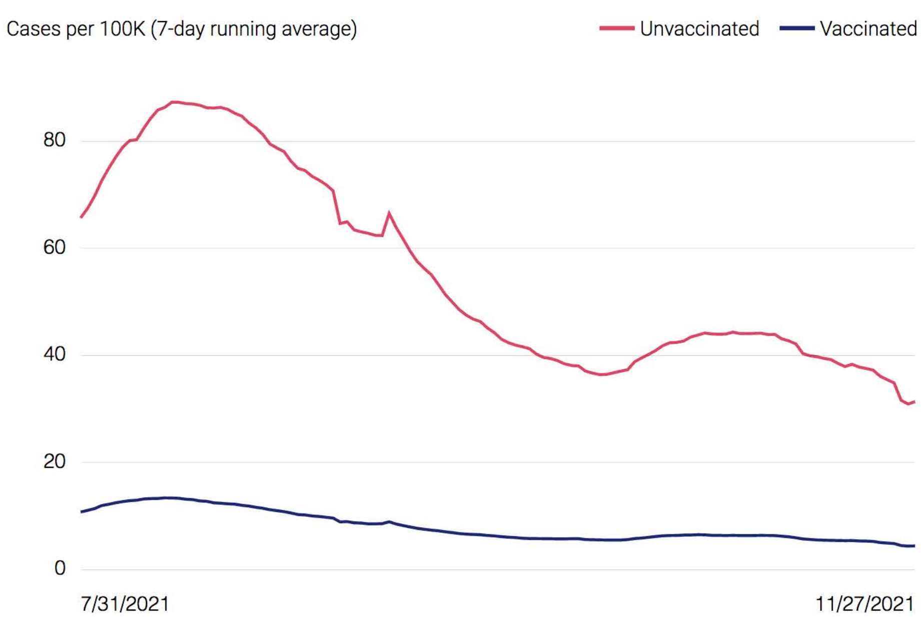

Unvaccinated and vaccinated cases, hospitalizations, and death source data

Chart information

Vaccines administered data

We're tracking the progress of vaccinating Californians across the state.

VIEW VACCINATION DATA

County and statewide data

Enter a county
Get county data

Cases and deaths

California has 4,867,604 confirmed cases of COVID-19, resulting in 74,509 deaths.

Confirmed cases in California

Episode date | Reported date

4,867,604 total confirmed cases
6,252 new cases (**0.1%** increase)
13.4 cases per 100K (7-day average)

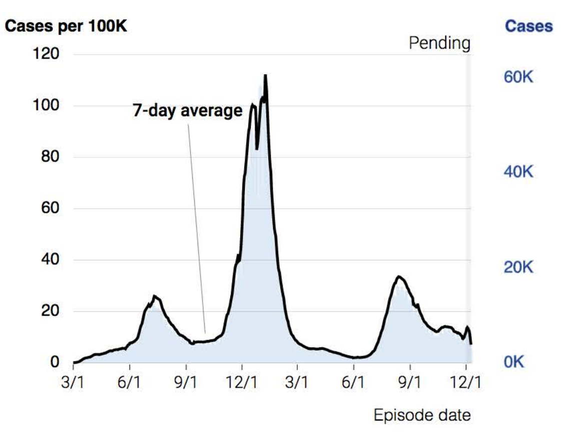

Confirmed deaths in California

Death date | Reported date

74,509 total confirmed deaths
77 new deaths (**0.1%** increase)
0.1 deaths per 100K (7-day average)

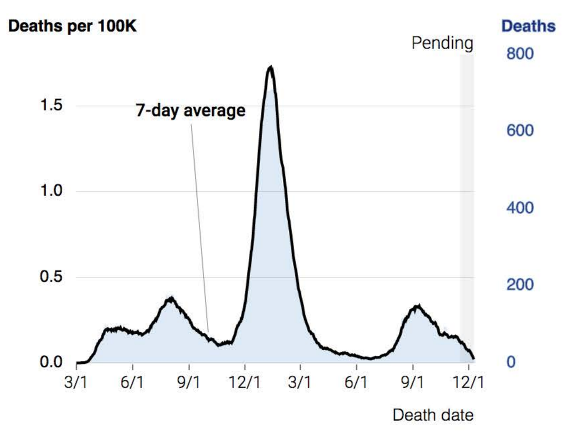

Confirmed cases and deaths source data

Chart information

Testing for COVID-19

The number of COVID-19 diagnostic test results in California reached a total of 114,042,927, an increase of — tests from the prior day total. The rate of positive tests over the last 7 days is 2.7%.

Total tests in California

Testing date | Reported date

114,042,927 total tests performed
356,488 new tests reported (**0.3%** increase)

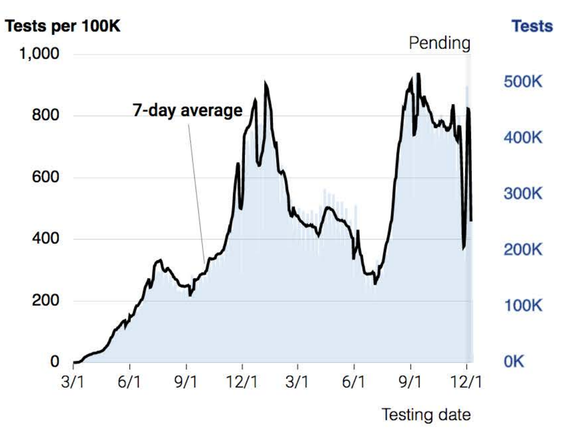

Positivity rate in California

2.7% test positivity (7-day rate)
0.4% increase from 7-days prior

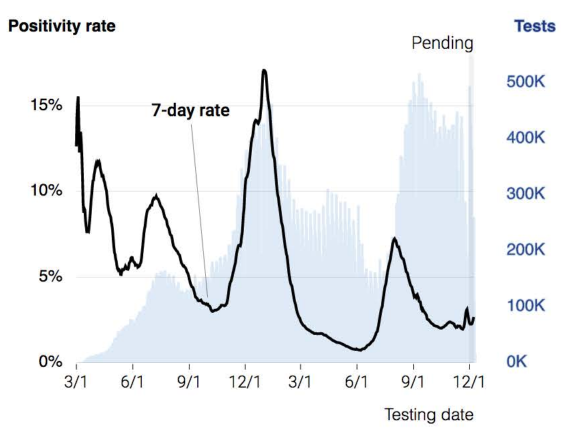

Total tests and positivity rate source data

Chart information

Confirmed and suspected hospitalizations

The number of hospitalizations due to confirmed and suspected COVID-19 cases in California reached a total of 3,819, an increase of 168 from the prior day total. The number of ICU patients due to confirmed and suspected COVID-19 cases in California reached a total of 927, an increase of 47 from the prior day total.

COVID-19 hospitalized patients in California

Hospitalized | ICU

3,819 COVID-19 hospitalized patients
168 more patients hospitalized from prior day total (4.6% increase)

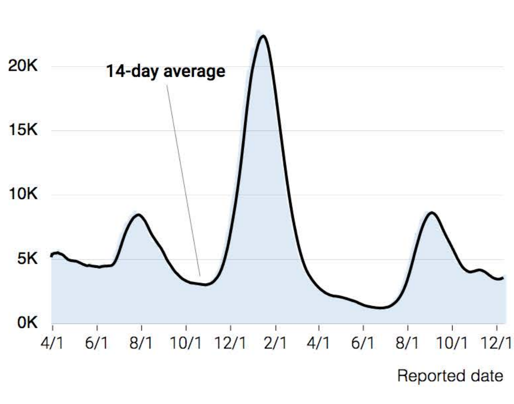

ICU beds in California

2,423 ICU beds available
800 more ICU beds available from prior day total (49.3% increase)

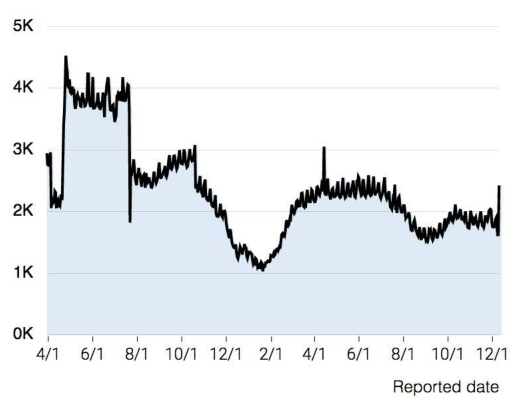

COVID-19 hospitalized patients and ICU source data

Cases and deaths by ethnicity, gender, and age

Race and Ethnicity | **Gender** | **Age**

The distribution of confirmed COVID-19 cases reveals significant disparities within California's overall racial and ethnic demographics, with Latino and Native Hawaiian / Pacific Islander groups having a disproportionate number of cases relative to their population in the state. Additional COVID-19 race and ethnicity data is available.

Confirmed cases by race and ethnicity in California

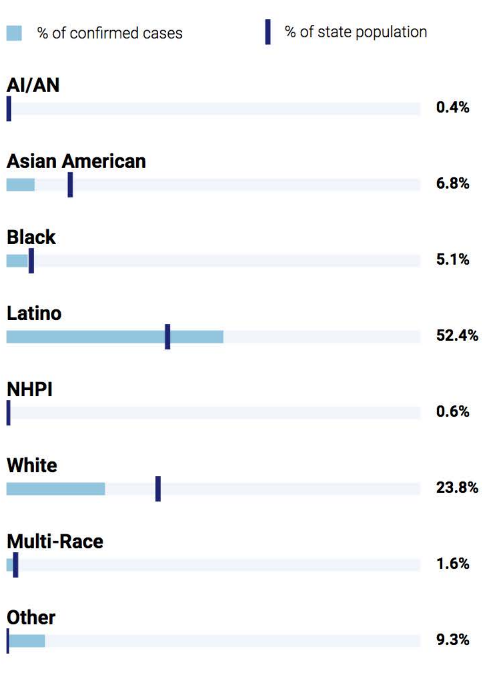

Confirmed deaths by race and ethnicity in California

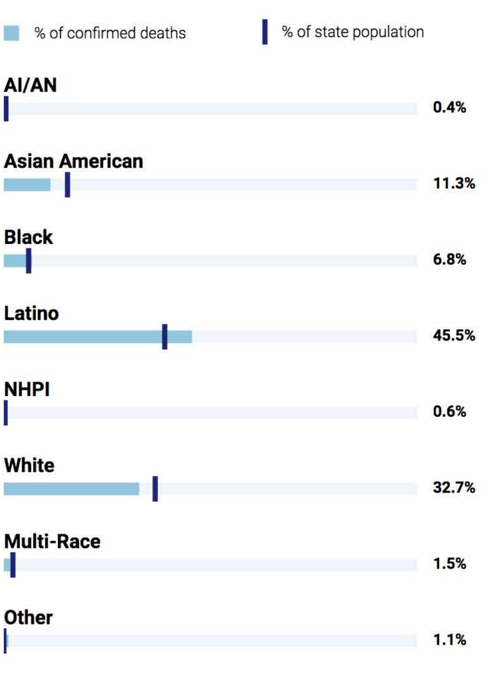

Updated December 11, 2021 with data from December 10, 2021. Note: Percentages may not add up to 100% due to rounding. "Other" race and ethnicity means those who do not fall under any listed race or ethnicity.

Confirmed cases and deaths by race and ethnicity, gender, and age source data

SEE MORE DETAILED DATA BY GROUP

COVID-19 Cases Dashboard v2.0 by CA Open Data

COVID-19: California Case Statistics

Cases | Testing | Demographics

Select Metric

Cases per 100K (7-day avg)

Cases per 100k (7-day avg) by County

Mono	57
Imperial	33
Kings	25
Mariposa	25
San Benito	23
Calaveras	23
Mendocino	22
Tuolumne	22
San Bernardino	21
San Diego	20
Riverside	19
Sierra	18
Madera	18
Inyo	18
Plumas	17
Nevada	17
Merced	17
Fresno	16
Humboldt	16
Del Norte	16
Glenn	15
Shasta	15
Placer	15
Stanislaus	14
Amador	14
Yuba	14
Kern	14
Santa Cruz	14
Santa Barbara	14
Marin	13

Cases (Statewide)

4,867,604 Total Confirmed Cases

13.4 Cases per 100K (7-day average)

+6,252 (+0.1%) Reported 12/9

US Total Cases: 38,150,911

Deaths (Statewide)

74,509 Total Confirmed Deaths

0.12 Deaths per 100K (7-day average)

+77 (+0.1%) Reported 12/9

US Total Deaths: 629,139

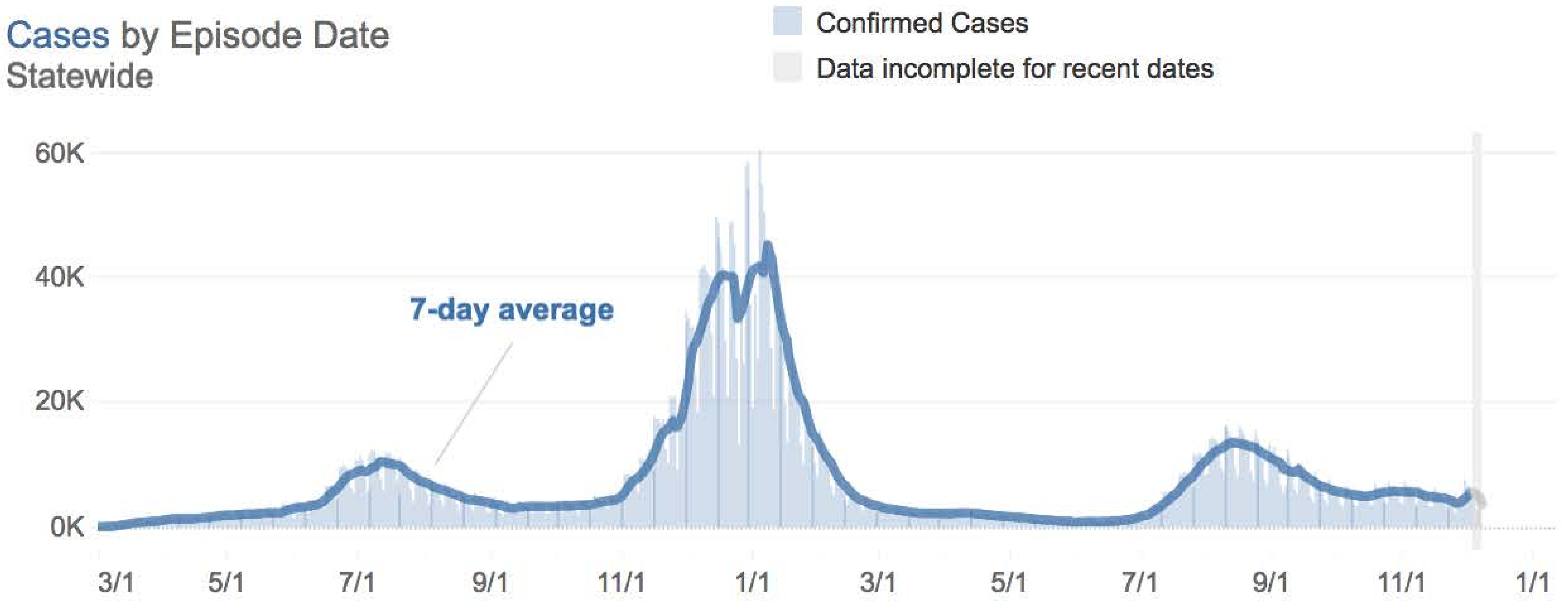

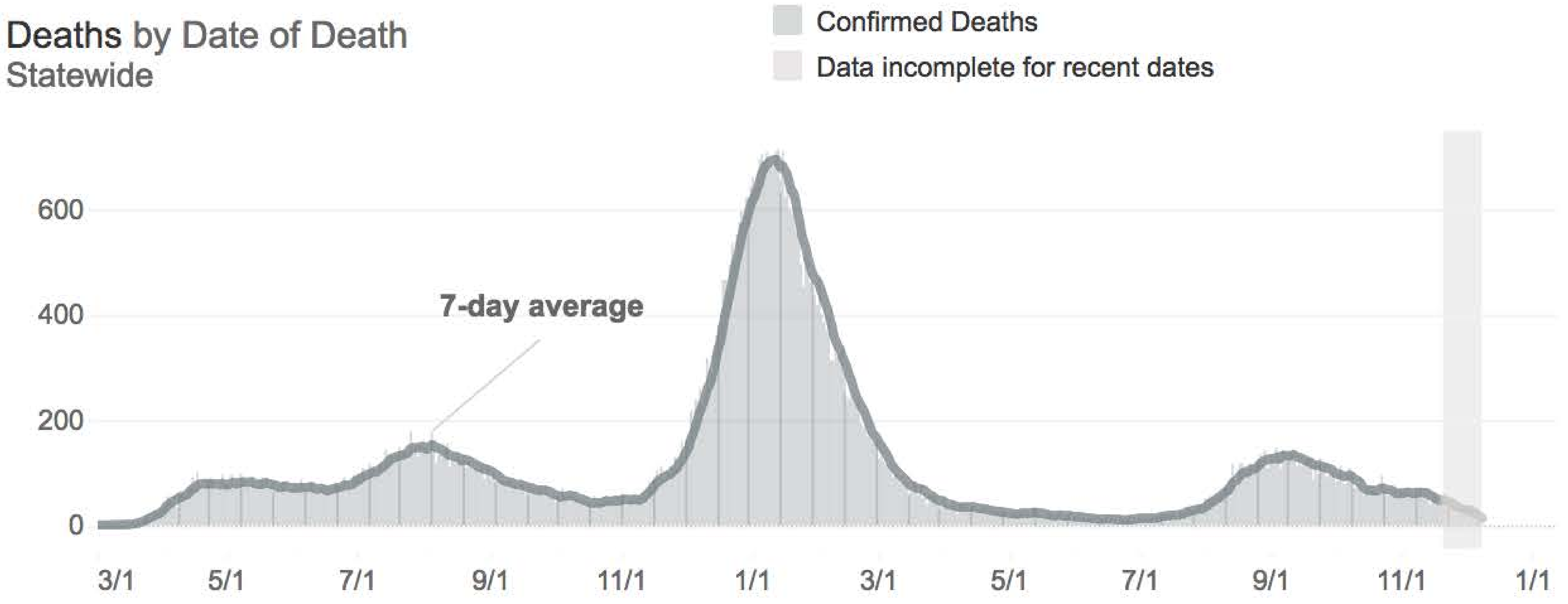

Last Updated 12/10/2021

+ableau

Details

24 2,069,728

COVID-19 Cases Dashboard v2.0

Published: Feb 19, 2021 **Updated:** Dec 10, 2021

COVID-19 Cases Dashboard v2.0 by CA Open Data

COVID-19: California Testing Statistics

Cases | Testing | Demographics

Sort by

Total Tests

Total Tests by County

Los Angeles	42,186,634
San Diego	8,345,485
Santa Clara	6,744,085
Orange	5,890,977
San Bernardino	4,777,506
Riverside	4,679,875
Alameda	4,621,428
Sacramento	3,333,849
San Francisco	3,132,912
San Mateo	2,717,496
Contra Costa	2,715,491
Ventura	2,227,162
Fresno	2,014,659
Kern	1,804,496
San Joaquin	1,672,706
Sonoma	1,183,863
Out of state	1,136,392
Solano	1,093,582
Stanislaus	1,063,598
Yolo	1,053,619
Santa Barbara	1,001,111
Tulare	946,972
Monterey	872,337
Santa Cruz	786,001
San Luis Obispo	769,873
Marin	767,800
Placer	714,780
Merced	581,648
Kings	575,035
Imperial	456,142
Madera	418,573
Napa	396,738
Shasta	367,635

Total Tests (Statewide)

114,042,927 Total Reported Tests

356,488 (+0.3%) 1-day change

2,512,159 (+2.3%) 7-day change

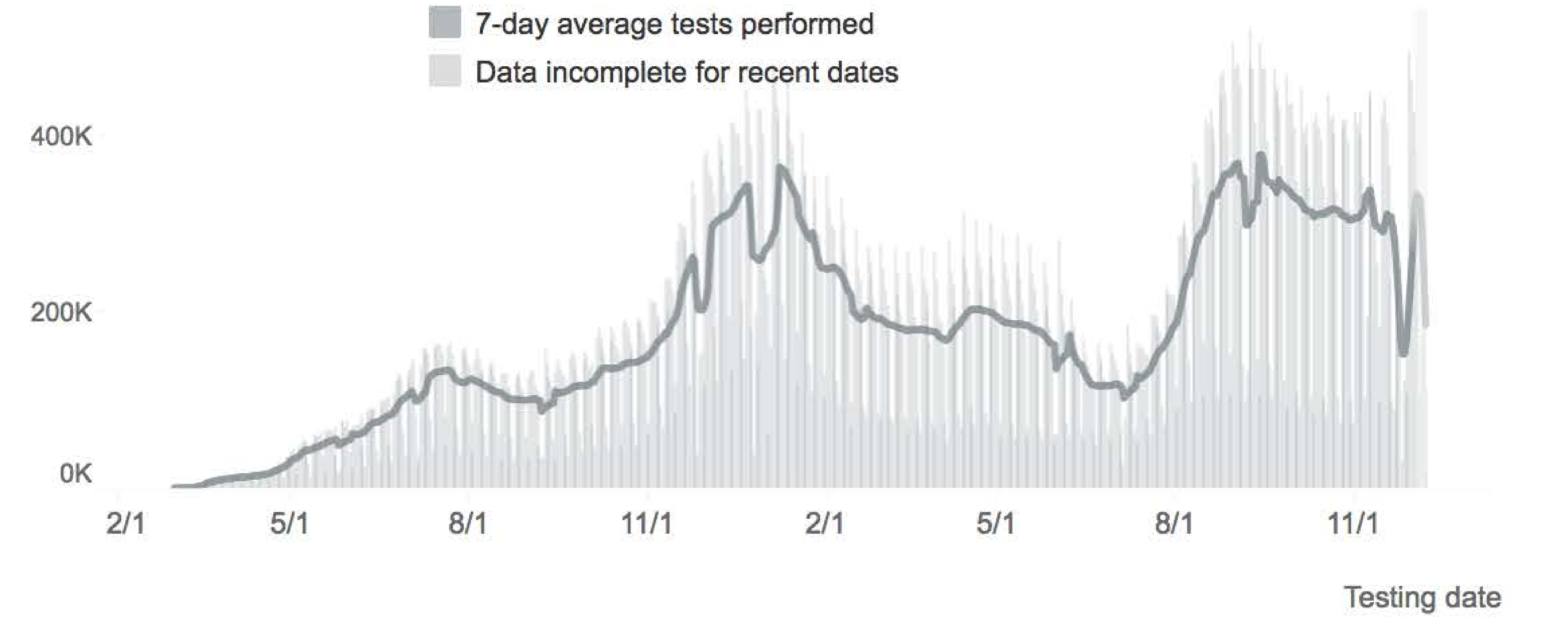

Test Positivity (Statewide)

2.7% 7-day rate

+0.4% 7-day change

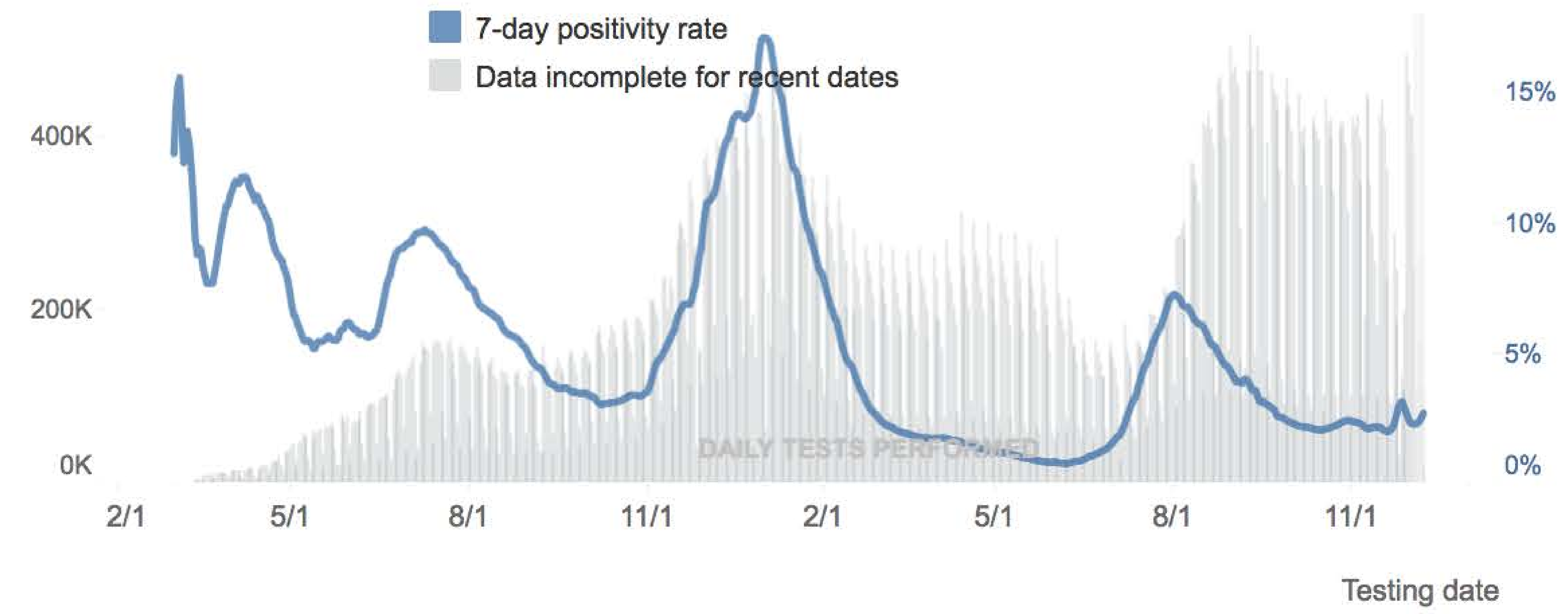

Last Updated 12/9/2021

+ a b | e a u

Details

24 2,069,728

COVID-19 Cases Dashboard v2.0

Published: Feb 19, 2021 **Updated:** Dec 10, 2021

COVID-19 Cases Dashboard v2.0 by CA Open Data

CA.GOV

COVID-19: California Demographic Statistics

Cases Testing **Demographics**

Confirmed Cases

| = California Population %

Race and Ethnicity	Case %	CA Pop %
AI/AN	0%	1%
Asian	7%	15%
Black	5%	6%
Latino	52%	39%
NH/PI	1%	0%
White	24%	37%
Multi-Race	2%	2%
Other	9%	0%

Gender		
Female	51%	50%
Male	48%	50%
Unknown	1%	0%

Age		
0-17	15%	23%
18-49	57%	44%
50-64	18%	18%
65+	10%	16%
Missing	0%	0%

Confirmed Deaths

| = California Population %

Race and Ethnicity	Death %	CA Pop %
AI/AN	0%	1%
Asian	11%	15%
Black	7%	6%
Latino	46%	39%
NH/PI	1%	0%
White	33%	37%
Multi-Race	2%	2%
Other	1%	0%

Gender		
Female	41%	50%
Male	58%	50%
Unknown	0%	0%

Age		
0-17	0%	23%
18-49	8%	44%
50-64	21%	18%
65+	71%	16%
Missing	0%	0%

Last Updated 12/9/2021

+ a b | e a u

Details

24 2,069,728

COVID-19 Cases Dashboard v2.0

Published: Feb 19, 2021 **Updated:** Dec 10, 2021

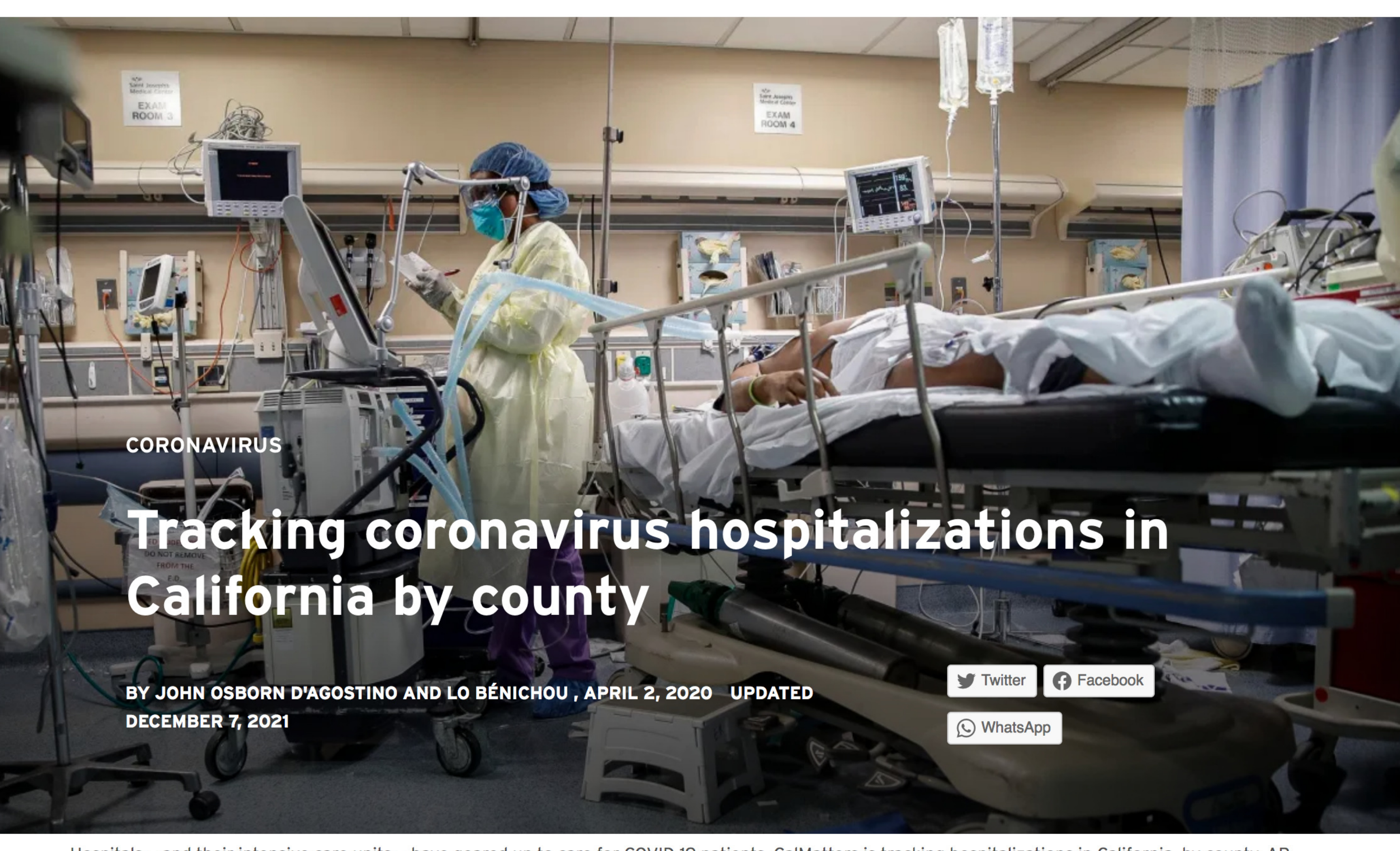

CORONAVIRUS

Tracking coronavirus hospitalizations in California by county

BY JOHN OSBORN D'AGOSTINO AND LO BÉNICHOU , APRIL 2, 2020 UPDATED DECEMBER 7, 2021

Hospitals – and their intensive care units – have geared up to care for COVID-19 patients. CalMatters is tracking hospitalizations in California, by county. AP Photo by John Minchillo

IN SUMMARY

Which California counties have the highest rates of coronavirus hospitalization? CalMatters is following confirmed and suspected cases. Some are a surprise.

Lea este artículo en español.

Keep tabs on the latest California policy and politics news

Enter your email

SUBSCRIBE

By clicking subscribe, you agree to share your email address with CalMatters to receive marketing, updates, and other emails from the site owner. Use the unsubscribe link in those emails to opt out at any time.

I'M NOT INTERESTED

CalMatters is tracking – and updating daily – California hospitalizations, by county, of people who have tested positive for the coronavirus that causes COVID-19, and people suspected of having the disease.

3,345
COVID-19 patients currently hospitalized
-56 (-1.6%) on December 09

847
COVID-19 patients in ICU
-14 (-1.6%) on December 09

Data retrieved on December 11, 2021 at 05:18 AM. The most recent data available is from December 09, 2021.

How many people are currently hospitalized with COVID-19?

Hospitalizations of confirmed COVID-19 patients in California

The number of confirmed patients in **ICU** and **non-ICU** settings began to climb in mid-June after California began to ease more of its shutdown restrictions. Here's the trend since the California Department of Public Health began releasing the data on April 1, 2020. As of December 09, there are **847** patients in ICUs, a change of **-14** from the day before, and **2,498** non-ICU patients, a change of **-42** from the day before.

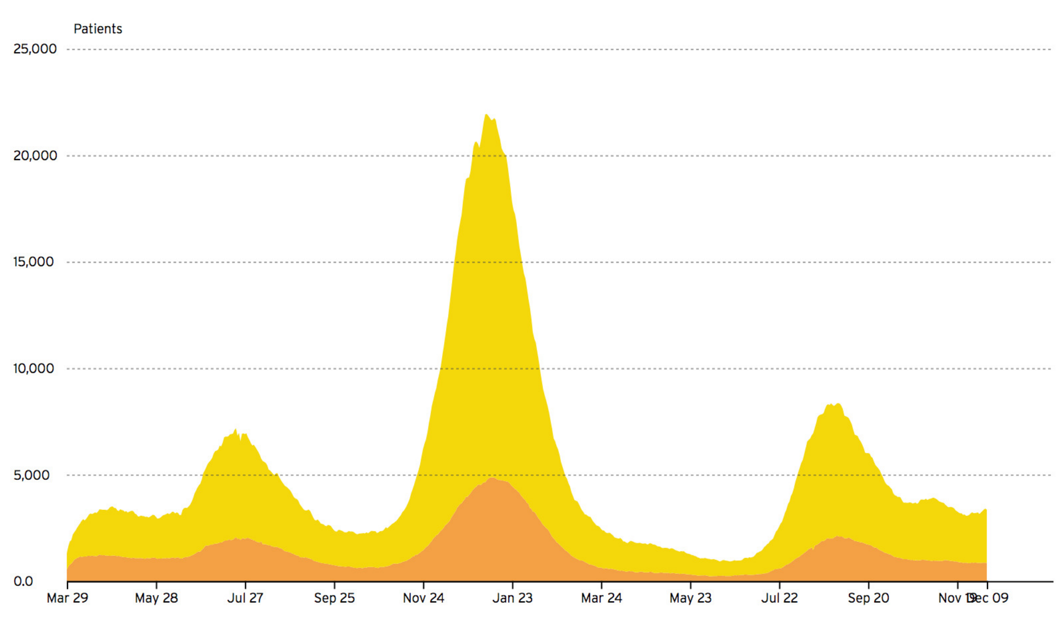

Hospitalized confirmed COVID-19 patients per county

Total number of confirmed COVID-19 patients per 100,000 people.

Note: Several rural counties do not have hospitals or ICU wards, so patients from those counties would go to neighboring counties for COVID-19 medical care. This includes Alpine, Sierra, Sutter and Trinity counties.

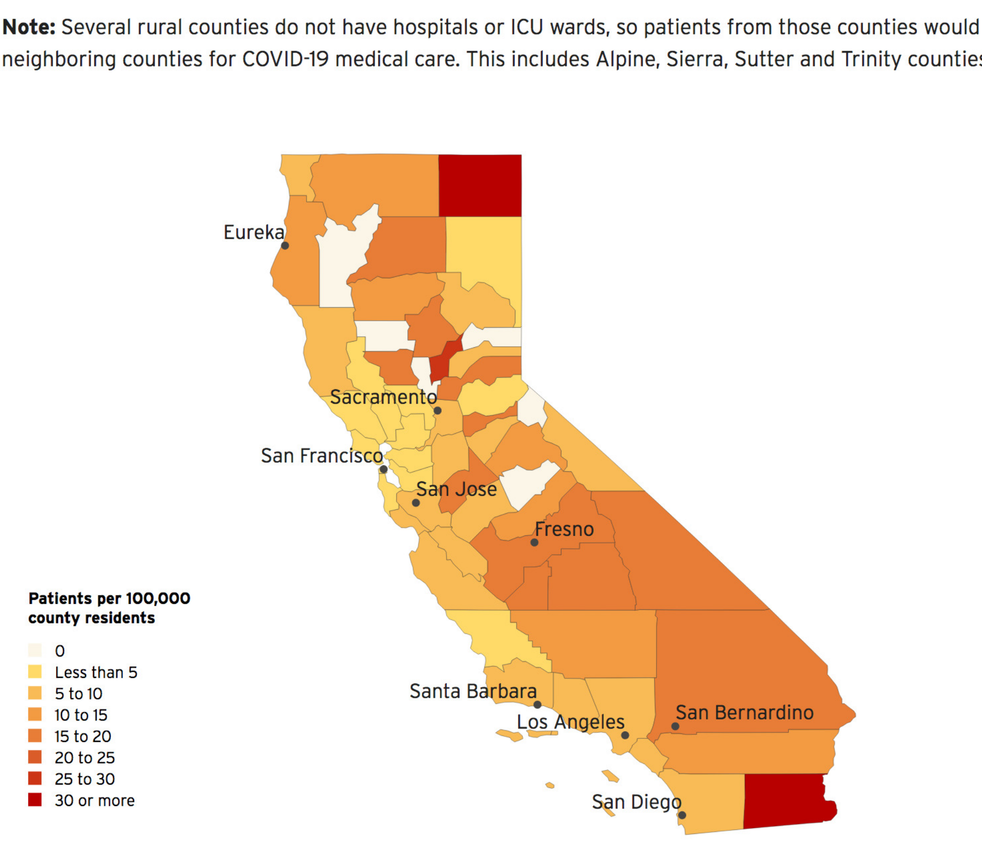

COUNTY	TOTAL PATIENTS	IN ICU	TREND*	DAILY CHANGE	PER 100,000 PEOPLE
Imperial	64	17		-2%	35.2
Modoc	3	0		-25%	34.2
Yuba	21	4		11%	26.9
Kings	30	4		3%	19.8

More Counties

* Trend lines show 7-day moving average of patients over time from March 31, 2020 to December 09, 2021.

Counties in depth

Alameda

Hospitalizations of confirmed COVID-19 patients in Alameda

Total number of confirmed patients in **ICU** and **non-ICU** settings per 100,000 people compared to **California's** – displayed per 100,000 people to enable visual comparison between very different sized total populations. Note the Y axis changes based on the county's highest reported patient count since March 31, 2020.

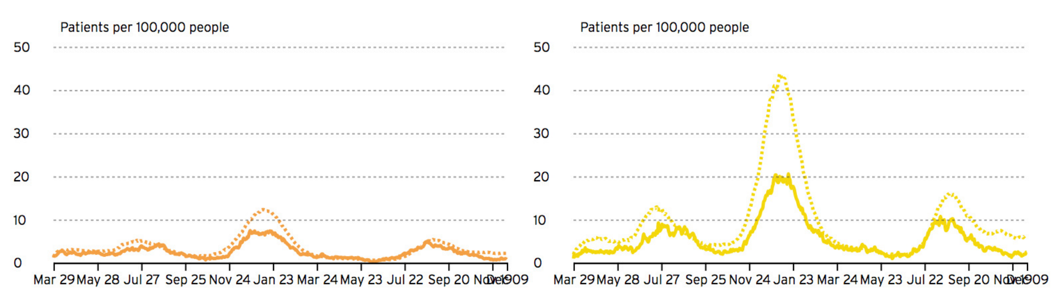

Confirmed COVID-19 patients hospitalizations over time

Total number of confirmed patients in ICU and non-ICU settings per 100,000 people since March 31, 2020.

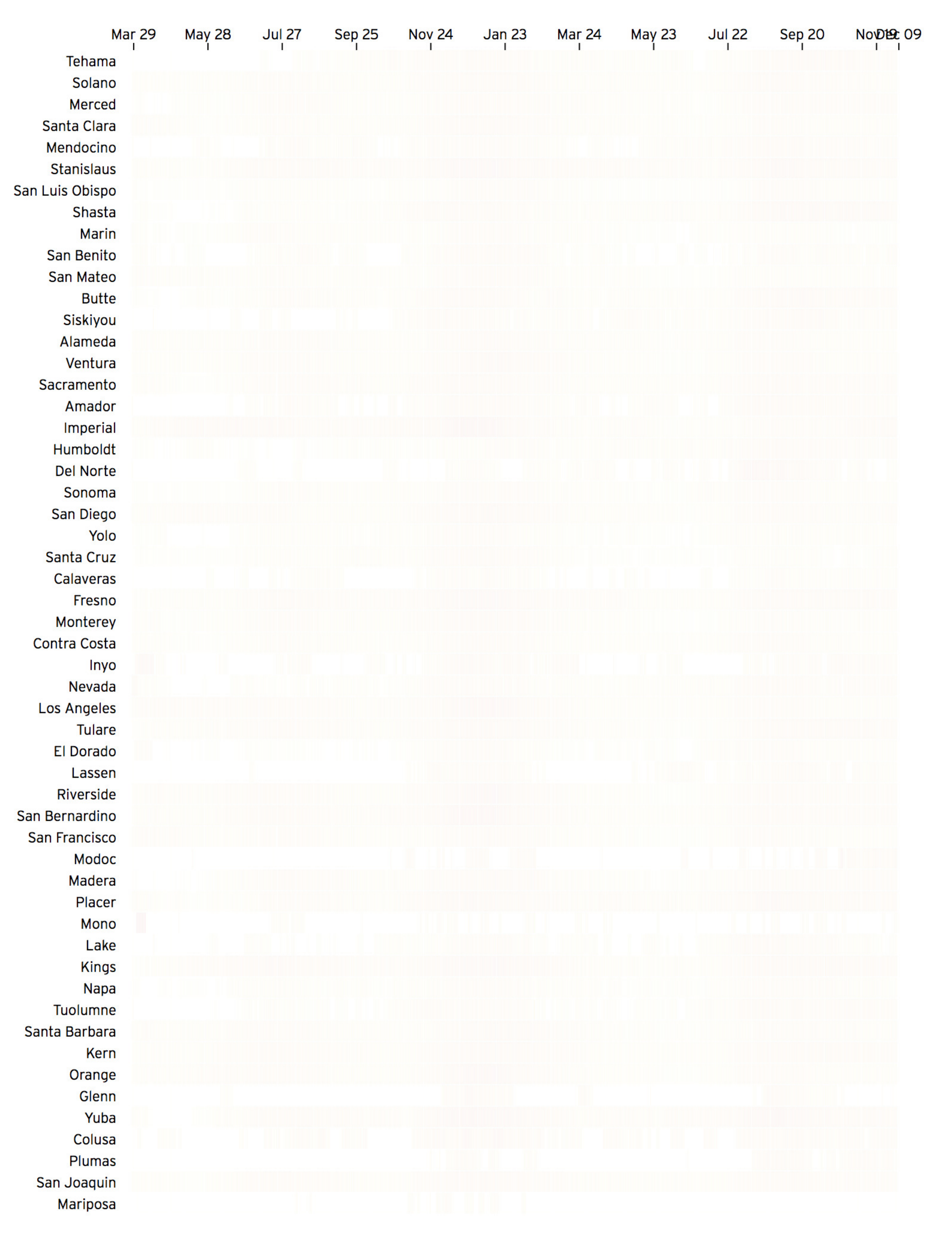

CAL MATTERS All gifts matched $10 $15 $25 OTHER DONATE

On April 1, 2020, the California Department of Public Health released a dashboard breaking down statewide hospitalizations of positive and suspected COVID-19 patients.

We adjusted the numbers for a few counties that reported small anomalies, such as a negative patient count instead of zero.

The data used in the following graphics are pulled from the California Health and Human Services database on a daily basis. Any questions about the data can be sent to CalMatters' data journalist and web developer John D'Agostino.

Population data for each county is based on the 2018 American Community Survey.

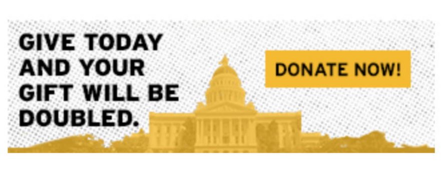

CalMatters investigative and data-driven reporting is supported by the Inasmuch Foundation, previously known as the Ethics and Excellence in Journalism Foundation.

Colorado COVID-19 Data

Data is updated daily by about 4 p.m. Monday through Friday and includes cases reported through the previous day.
All data, for days past and present, is recalculated daily.

COLORADO Department of Public Health & Environment

854,084 Cases	48,764 Hospitalized	64 Counties	4,181,264 People Tested	13,468,933 Test Encounters	9,627 Deaths Among Cases	9,874 Deaths Due to COVID-19	7,026 Outbreaks

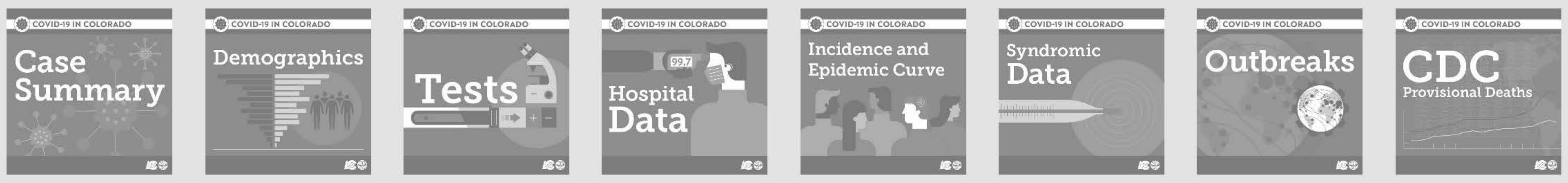

1,247 (▼ 999) Cases Reported Today	1,072 Cases Added with Previous Reported Date	1,914 (▼ 224) 7-Day Moving Average of Cases Reported	527.7 (▼ 1.0) 2-Week Cumulative Incidence Per 100K People
159 (▲ 24) New Hospital Admissions	140 (▼ 2) 7-Day Moving Average of New Hospitalizations	44% Adult Critical Care Ventilators in Use	19% Available Hospital Beds Occupied by Confirmed and Suspected COVID Patients
6.25% (▼ 1.05%) State Positivity Rate	7.21% (▼ 0.39%) State 7-Day Positivity Rate	7.20% (▼ 0.53%) Previous Week Emergency Department Syndromic COVID Visits	6.23% (▲ 1.36%) Previous Week Outpatient Syndromic COVID Visits
5.1 (▼ 1.4) Tests Per 1,000 People	5.5 (▼ 0.10) 7-Day Average of Tests Per 1,000 People	5,331 (▼ 1,547) New Individual People Tested	29,383 (▼ 8,271) New Test Encounters

Trouble viewing? Access the data from Tableau or view the data tables below.

Case summary snapshot
Hospital data snapshot
Cases and deaths by county

COVID-19 Variant Sentinel Surveillance

COLORADO Department of Public Health & Environment

0.00% B.1.1.529 (Omicron)	5.80% B.1.1.7 (Alpha)	0.06% B.1.351 (Beta)	0.32% B.1.427 (Epsilon)	1.03% B.1.429 (Epsilon)	88.31% B.1.617.2 (Delta)	0.03% AY.1 (Delta)	0.10% AY.2 (Delta)	0.37% P.1 (Gamma)	3.97% Other

Overall Proportion of Variants of Concern

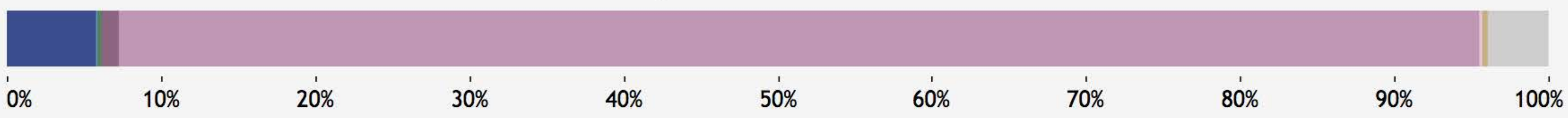

Current Variants of Concern
Hover to Highlight

B.1.617.2 Delta | AY.1 Delta | AY.2 Delta

Other Variants
Hover to Highlight

B.1.1.7 Alpha | B.1.351 Beta | B.1.427 Epsilon | B.1.429 Epsilon | P.1 Gamma | Other

Weekly Proportion of Variants of Concern by Specimen Collection Week

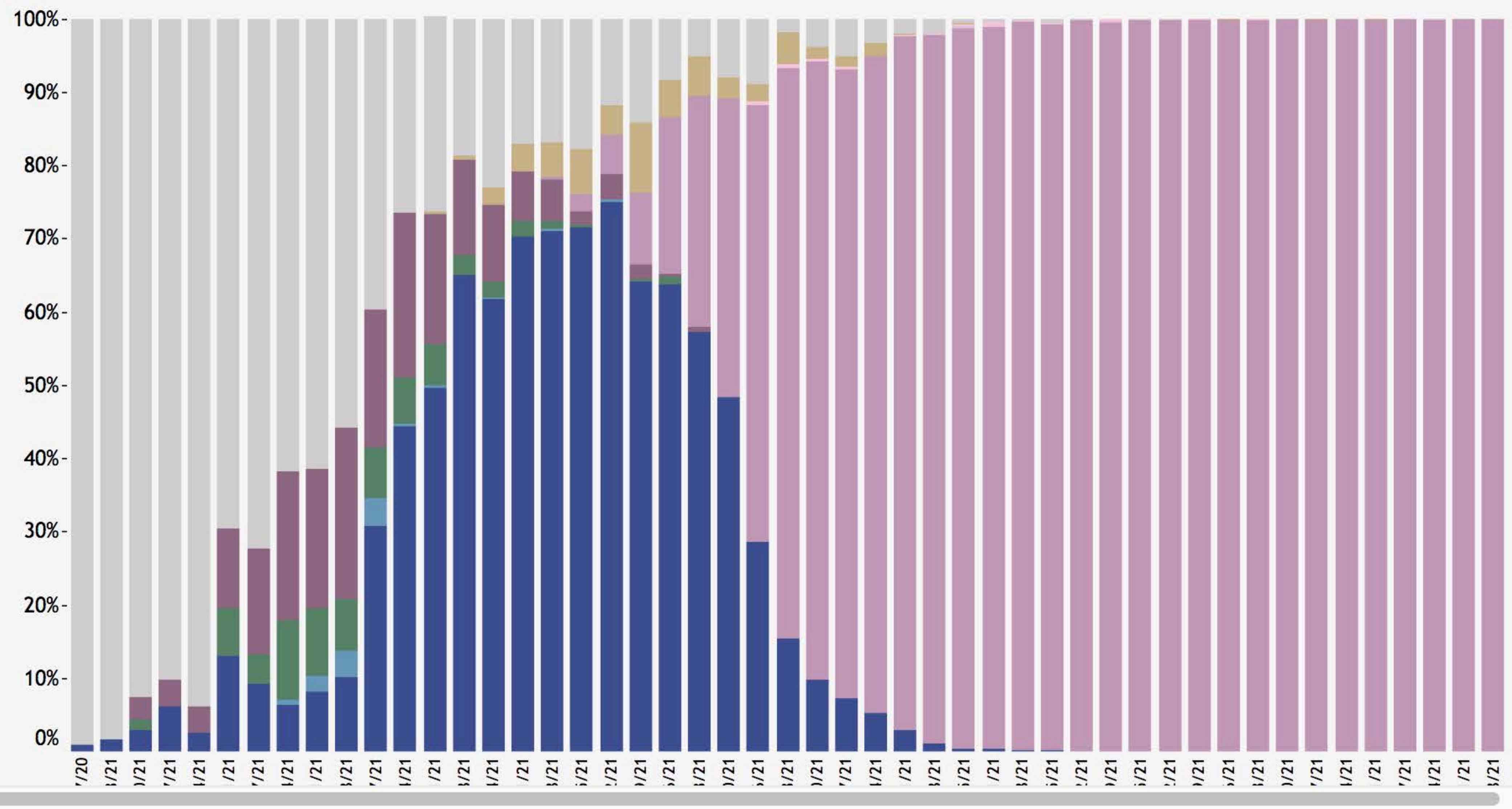

Colorado COVID-19 Data

Data is updated daily by about 4 p.m. Monday through Friday and includes cases reported through the previous day.
All data, for days past and present, is recalculated daily.

COLORADO
Department of Public Health & Environment

854,084 Cases	48,764 Hospitalized	64 Counties	4,181,264 People Tested	13,468,933 Test Encounters	9,627 Deaths Among Cases	9,874 Deaths Due to COVID-19	7,026 Outbreaks

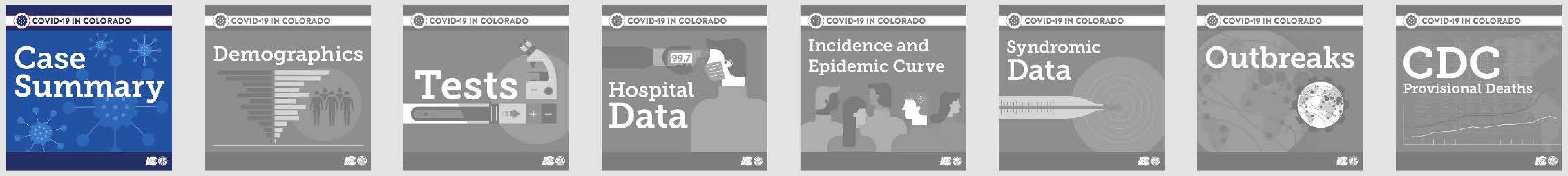

Cases | Deaths Among Cases | Maps

Reported Day | Reported Week | Onset Day | Onset Week

Counts | Cumulative | 3-Day Average | 7-Day Average

Cases of COVID-19 in Colorado by Date Reported to the State

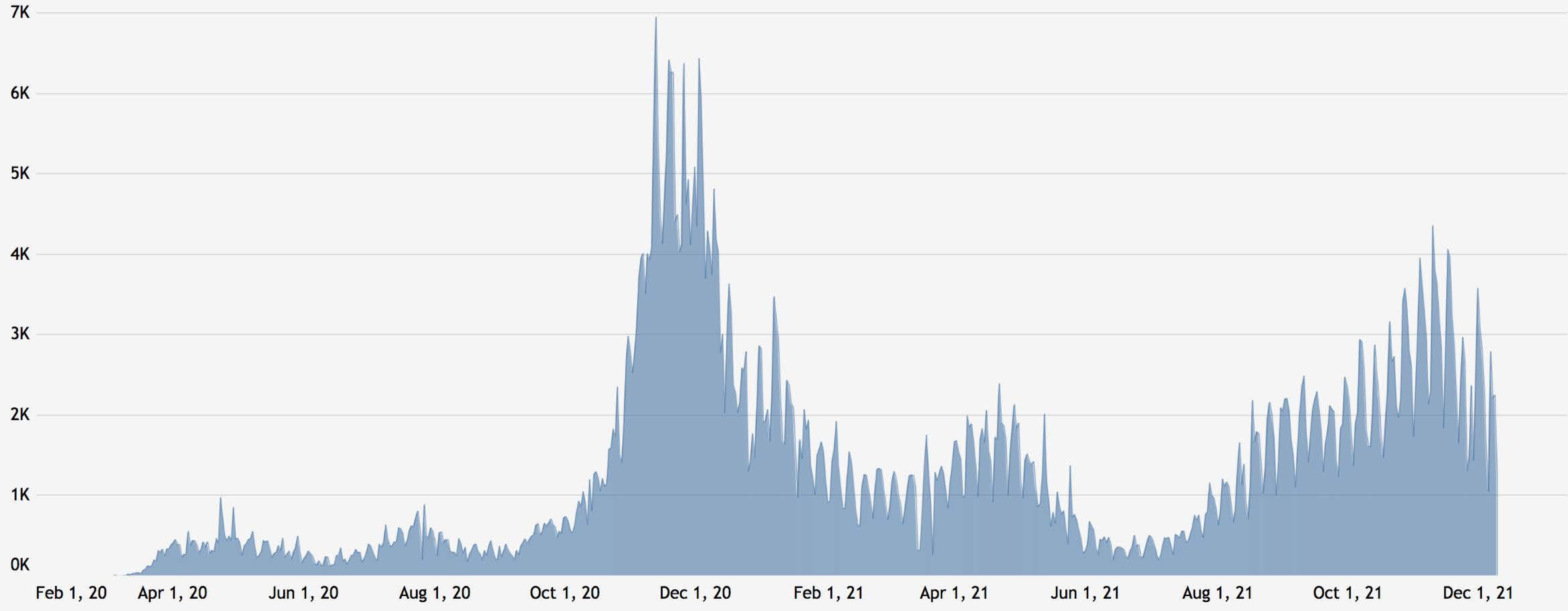

CDPHE

+ableau

Colorado COVID-19 Data

Data is updated daily by about 4 p.m. Monday through Friday and includes cases reported through the previous day.
All data, for days past and present, is recalculated daily.

COLORADO Department of Public Health & Environment

854,084 Cases	48,764 Hospitalized	64 Counties	4,181,264 People Tested	13,468,933 Test Encounters	9,627 Deaths Among Cases	9,874 Deaths Due to COVID-19	7,026 Outbreaks

Case Summary | Demographics | Tests | Hospital Data | Incidence and Epidemic Curve | Syndromic Data | Outbreaks | CDC Provisional Deaths

Cases | Deaths Among Cases | Maps

Daily | Weekly

Counts | Cumulative | 3-Day Average | 7-Day Average

7-Day Average of Deaths Among COVID-19 Cases in Colorado by Date of Death*

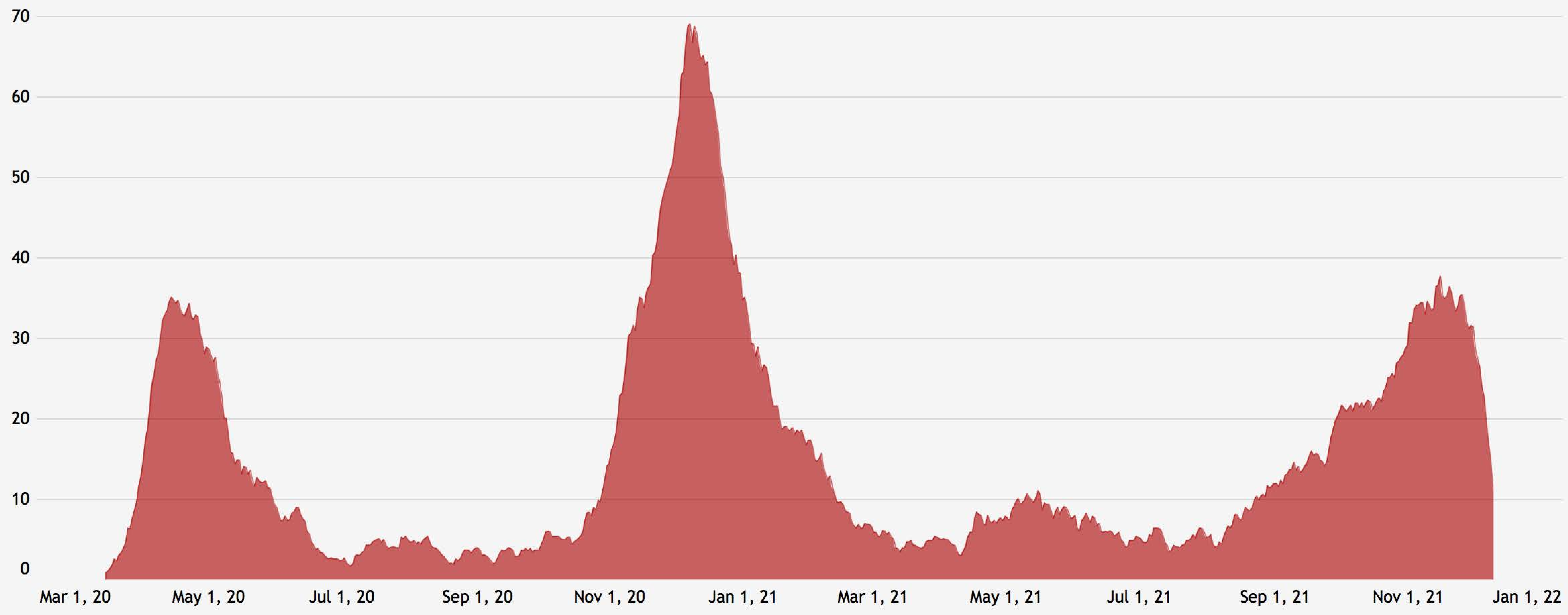

* This graph includes deaths among people with COVID-19. COVID-19 may or may not be listed as the cause of death on the death certificate

+ableau

Colorado COVID-19 Data

COLORADO
Department of Public Health & Environment

Data is updated daily by about 4 p.m. Monday through Friday and includes cases reported through the previous day.
All data, for days past and present, is recalculated daily.

854,084 Cases	48,764 Hospitalized	64 Counties	4,181,264 People Tested	13,468,933 Test Encounters	9,627 Deaths Among Cases	9,874 Deaths Due to COVID-19	7,026 Outbreaks

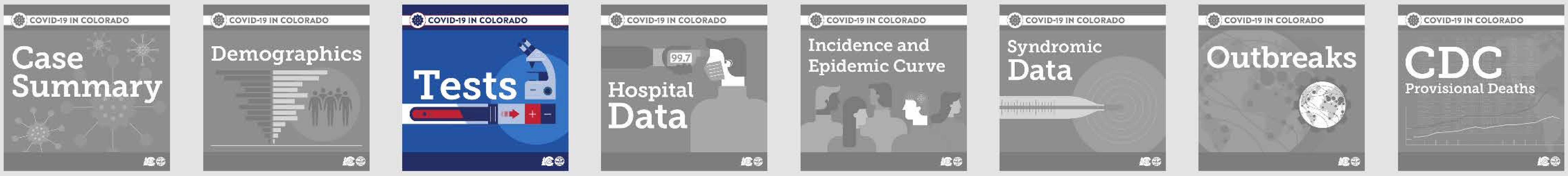

Daily | Weekly | Maps

PCR Tests | Antibody Tests | Daily Positivity | 3-Day Avg Positivity | 7-Day Avg Positivity

Daily Tests | Cumulative Tests

Daily COVID-19 PCR Test Data From Clinical Laboratories With 7-Day Average Positivity

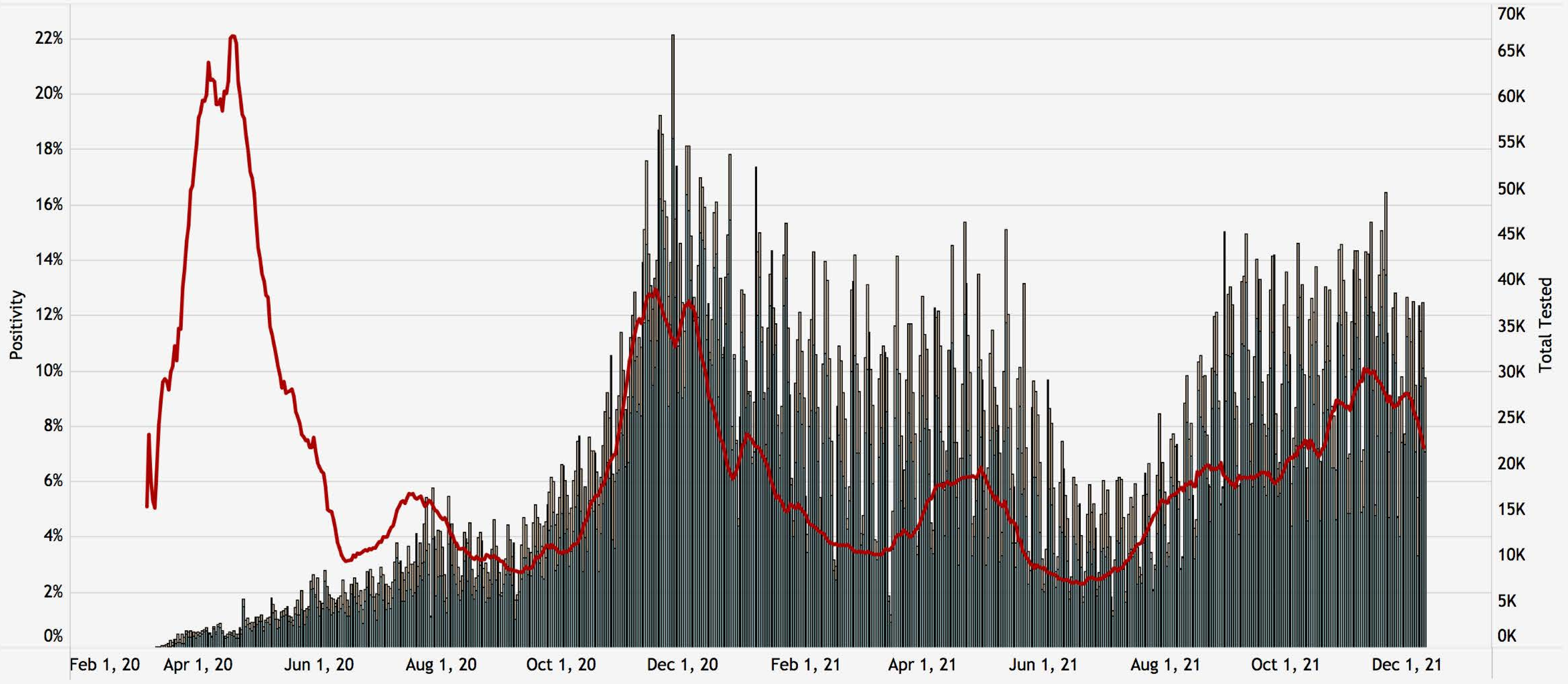

Colorado COVID-19 Data

COLORADO Department of Public Health & Environment

Data is updated daily by about 4 p.m. Monday through Friday and includes cases reported through the previous day.
All data, for days past and present, is recalculated daily.

854,084 Cases	48,764 Hospitalized	64 Counties	4,181,264 People Tested	13,468,933 Test Encounters	9,627 Deaths Among Cases	9,874 Deaths Due to COVID-19	7,026 Outbreaks

Daily COVID-19 Antibody Test Data From Clinical Laboratories With 7-Day Average Positivity

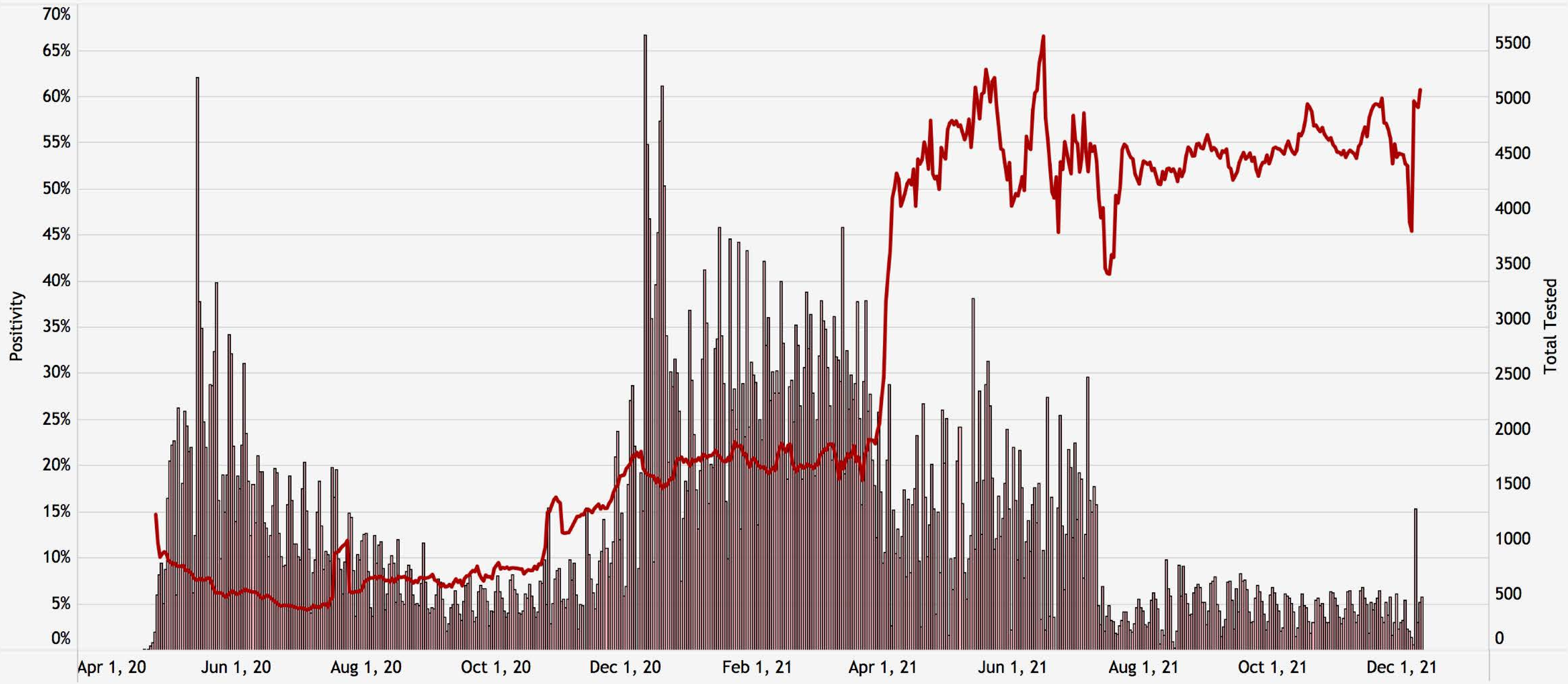

COVID-19 in Colorado tracker: Larimer and state case, death and hospital data for August

We're always working to improve your experience. Let us know what you think.

Coloradoan staff

Published 7:56 p.m. MT Aug. 1, 2020 | **Updated 4:35 p.m. MT Sept. 1, 2020**

View Comments

As Beavers Market says goodbye, here's how it came to be in Fort Collins
The little grocery store opened on Mountain Avenue in 1976. After 45 years, it's saying goodbye to the city that grew around it. *Erin Udell, Fort Collins Coloradoan*

As the number of confirmed coronavirus cases in Colorado grows, here are the latest updates and information.

Editor's note: *As the coronavirus outbreak continues to evolve, we don't want you to panic. In fact, quite the opposite. That's why the Coloradoan is committed to providing you with accurate, up-to-date information so you can make informed decisions on issues affecting you and the people you love. As such, this story, and many others, are being provided free for all to read. Help us continue this important work by subscribing to the Coloradoan.*

Here are the latest developments on coronavirus in Colorado:

Note: This tracker includes updates from August.

Looking for September updates?

► Find updates on the spread of COVID-19 in Colorado in September

Looking for past updates?

► A timeline of major updates on coronavirus in Colorado

► Find updates on the spread of coronavirus in Colorado from July

► Find updates on the spread of coronavirus in Colorado from May and June

► Early updates at the beginning of the pandemic in Colorado: March and April

Monday, Aug. 31, 2020

Larimer County cases, deaths:

- New cases reported by county: 27 (Note: This is the number of new confirmed and probable cases reported since The Coloradoan last shared information from the county. **In this case, that was Saturday, Aug. 29, 2020.** This may vary from the "new positive cases" listed on the county's COVID dashboard.)
- New deaths reported by county: 0
- Confirmed cases: 1,861 (positive swab test)
- Probable cases: 51 (people with two of the following: COVID-19 symptoms; epidemiological link to someone who tested positive; positive antibody test)
- Suspect cases: 433 (asymptomatic with positive antibody test)
- Total deaths: 36
- Risk Index in county (based on things such as hospital utilization, new cases, etc.): Medium
- Number of COVID-19 patients in hospitals: 5 (includes Banner and UCHealth)*
- Hospital utilization: 62% (low risk is <70%)*
- ICU utilization: 71% (low risk is <70%)*

Source: Larimer County Department of Health and Environment

Colorado cases, deaths:

- New cases reported by state **(since Saturday, Aug. 29)**: 383
- New deaths reported by state (caused by COVID-19; **since Saturday, Aug. 29**): 6
- Current hospitalizations: 148 (75% reporting)
- Hospital transfers or discharges in past 24 hours: 7
- Number of ventilators in use: 27%
- Hospitals anticipating a staff shortage in the next week: 5%
- Facilities anticipating personal protective equipment shortages in the next week: 1%
- Hospitals anticipating ICU bed shortages in the next week: 1%
- Total cases: 57,424
- Total hospitalizations: 7,028
- Total deaths due to coronavirus: 1,849
- Total outbreaks: 604 (reported at residential and nonhospital health care facilities, correctional settings and other facilities in Colorado, such as factories and group homes)

Source: Colorado Department of Public Health & Environment

Coronavirus cases in Larimer County

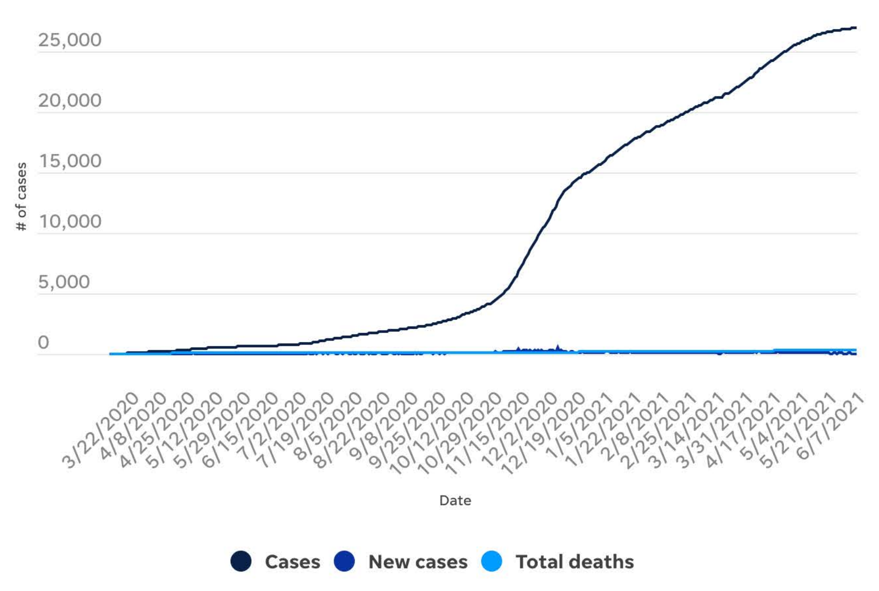

Last updated June 7, 2021
The county has three definitions for case types: confirmed, probable and suspect.

- **Confirmed case**: Someone who has tested positive using a molecular amplification detection test (such as a nasal swab tested using PCR testing). This test determines if someone is currently ill and able to spread COVID-19 to others.
- **Probable case**:
  - Has COVID-19 symptoms or clinical characteristics AND epidemiologic evidence with no test confirming the diagnosis
  - Has COVID-19 symptoms or clinical characteristics AND has tested positive using an antigen or serologic (antibody) test
  - Has an epidemiological link based on risk from a known exposure AND has tested positive using an antigen or serologic (antibody) test
  - Has a death certificate that lists COVID-19 disease or SARS-CoV-2 as a cause of death or significant condition contributing to death without positive test results to confirm the diagnosis.
- **Suspect case**: Someone who has tested positive using a serology (antibody) test but who did not have COVID-19 symptoms or clinical characteristics. Antibodies can take several weeks to develop after being exposed so someone who has tested positive for antibodies has likely recovered and is no longer able to spread the virus at the time they are tested for antibodies.

Coronavirus cases by demographic

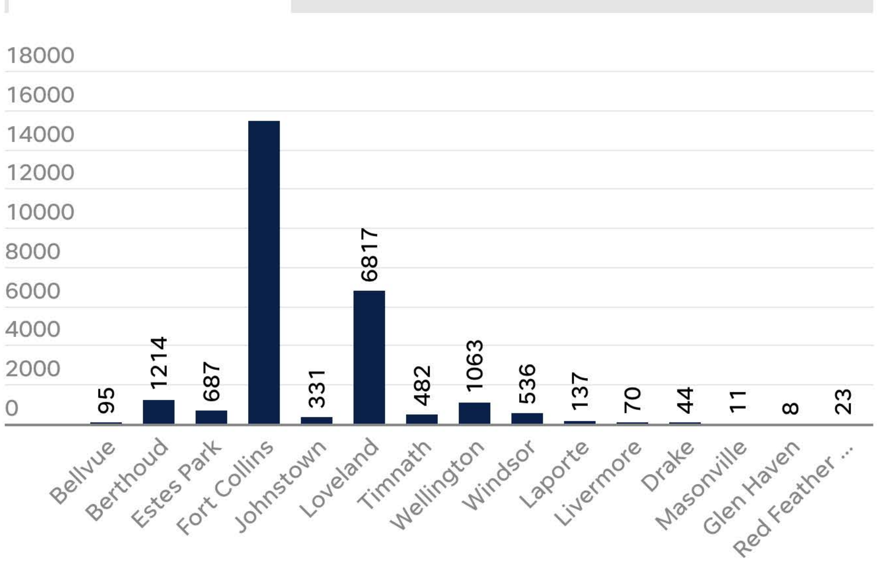

*Data for Windsor does not include cases from Weld County. Locations listed are those included in data from the Larimer County health department. Some locations may be outside of Larimer County.

Demographic data is for Confirmed and Probable cases only, and does not include antibody test results.

Share

Coronavirus cases in Colorado by day

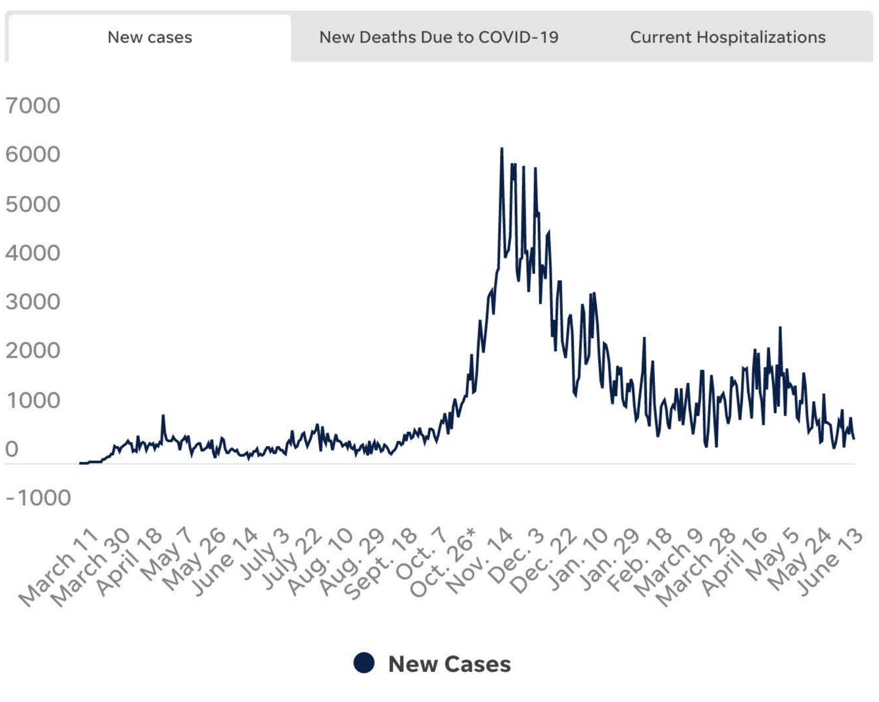

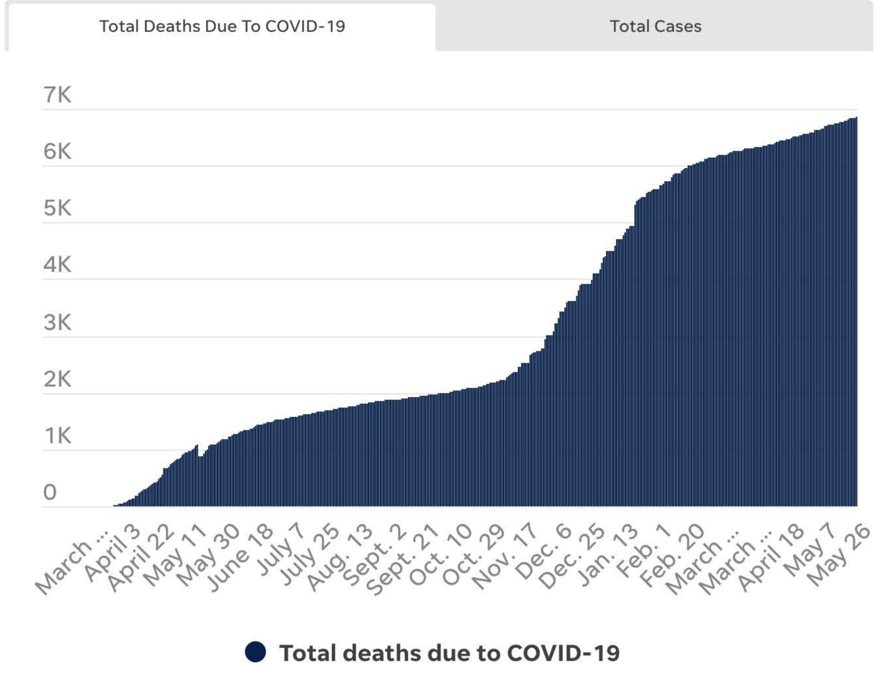

The Colorado health department releases data daily at 4 p.m. These charts reflect the dates cases were first reported by the state, not necessarily the date when cases were determined or first reported to the state.

*The new cases on March 13 and 14, 2021, were added on March 14. The Coloradoan tracked 3,748 cases over that weekend and split them evenly on this tracker to avoid the appearance of a spike on a particular day.

**The state health department added 462 backdated positive test results on Jan. 29, 2021, according to a news release.*

**For Oct. 24 and Oct. 26, the number of new cases reflects the addition of older cases from as early as Oct. 1 that had not previously been included in charts due to formatting issues at the state health department.*

**Starting with May 15, deaths are defined as people who died as a result of COVID-19. Prior to that date, deaths included anyone who had died and tested positive for coronavirus, regardless of their cause of death. This why you see a change of zero and a dip in total cases on May 15. Unfortunately, this data is not available retroactively.*

**The April 24-25 reporting figures for deaths may be skewed by retroactive tallying of deaths from coronavirus on previous dates that had not yet been accounted for.*

The state defines cases as "anyone who has tested positive for COVID-19 and anyone who is symptomatic and has had close contact with someone who tested positive."

Hospitalization data is provided to the state of Colorado by hospitals through the Colorado Hospital Association. Not all hospitals provide data, and the percentage of hospitals reporting data each day varies.

Share

Coronavirus cases in Colorado

Hover over the county to see how many confirmed cases there are in that location.

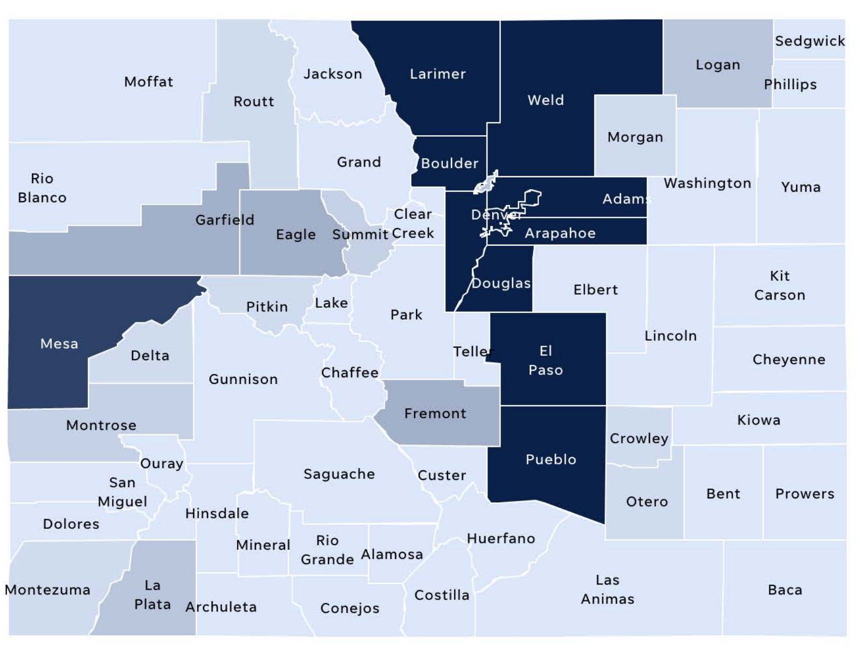

Download data

Last updated 6/7/2021

SUBSCRIBE NOW
$29 for one year. Save 76%.

Coloradoan.

SUBSCRIBE NOW
$29 for one year. Save 76%.

[News] Sports Business NoCo Life Opinion Obituaries E-Edition Legals 38°F Subscribe Sign In

Larimer case counts are based on state data, to give an accurate comparison by county.

Death data provided on the county level by the state includes all deaths among COVID-19 cases, regardless of if COVID-19 is listed as a cause of death. The state only provides the number of coronavirus-caused deaths as a total for the state as a whole.

From the health department: Please note initial information may change as more information is collected on each patient.

Share

NEWS > HEALTH • News

Colorado coronavirus tracker: How many new cases are being reported, and where are they?

The charts and maps will be updated daily

By KEVIN HAMM | khamm@denverpost.com | The Denver Post

PUBLISHED: March 6, 2020 at 5:41 p.m. | UPDATED: June 14, 2021 at 2:40 p.m.

102

COVID-19 in Colorado

Charts updated Tuesday, Dec. 7

On Dec. 3 we began using a different CDPHE data set for "reported" cases, deaths and hospital admissions, which resulted in a higher, but more accurate, picture of current deaths. The growing reporting lag time in the previous data set was beginning to present an inaccurate picture of current trends.

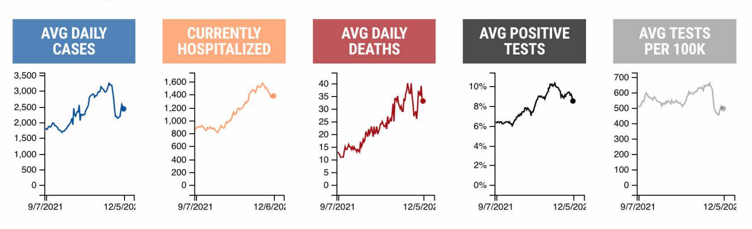

Vaccinations

Colorado population vaccinated through Dec 5

Current vaccine distribution phase: 2

ONE DOSE	FULLY VACCINATED
71.3%	64.2%
4,105,085 people vaccinated	*3,696,627 people vaccinated*

Vaccines administered

By week; current week will update daily

Hover/click chart for details; click legend item to filter chart

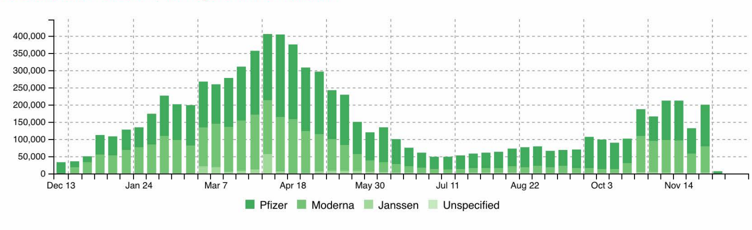

Vaccinations by county

Click county for details

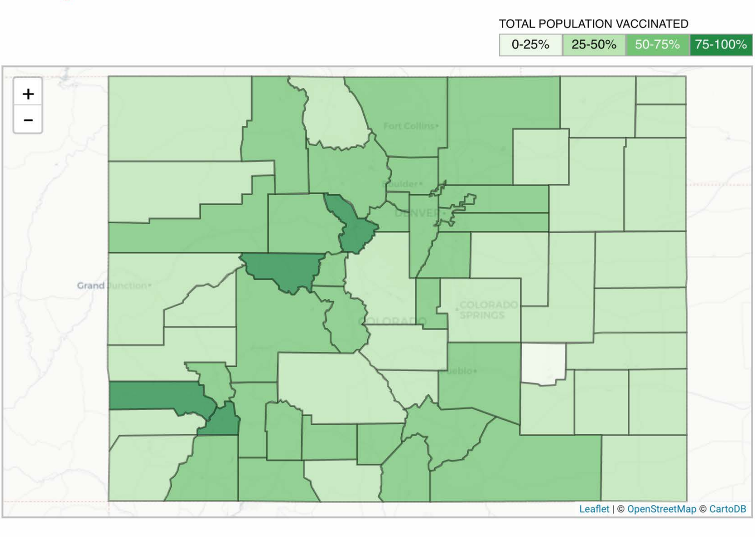

Cases, deaths and hospitalizations

HOW TO READ THE CHARTS

Please note that each chart uses two different data sets: The lines show data by the date it was reported to the Colorado Department of Public Health and Environment, and the bars show data by symptom onset date or date of death.

Lines: *Best for showing trend.* The line charts show when cases, deaths and hospitalizations were reported to the Colorado Department of Public Health and Environment. The lag time between when those occurred and when they were reported to the state can be several days. The chart uses a 7-day average to smooth out peaks and valleys in the data due to reporting lag times.

Bars: *Best for showing historical data.* The bar charts show when cases and hospitalizations occurred based on CDPHE's investigation into the onset date of symptoms, and when a death occurred based on the date on the death certificate. **Data for most recent two weeks is not shown because it will be signifcantly revised** with subsequent data releases as CDPHE investigations progress, and is not an accurate representation of recent cases, deaths and hospitalizations.

Totals: The total number of cases, deaths and hospitalizations that have been reported to the CDPHE.

Hover/click chart for details; click legend item to filter chart

Gray area indicates the statewide stay-at-home order

CASES 843,851

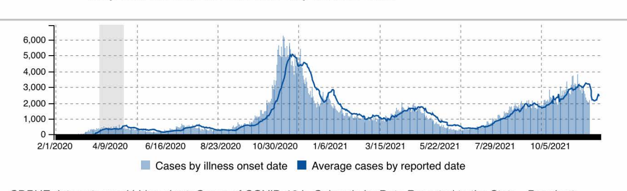

CDPHE data sets used | Line chart: Cases of COVID-19 in Colorado by Date Reported to the State • Bar chart: Cases of COVID-19 in Colorado by Date of Illness Onset

DEATHS 9,652

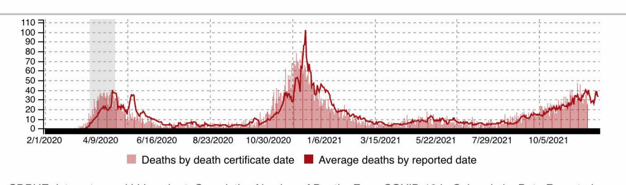

CDPHE data sets used | Line chart: Cumulative Number of Deaths From COVID-19 in Colorado by Date Reported to the State • Bar chart: Number of Deaths From COVID-19 in Colorado by Date of Death - By Day

HOSPITAL ADMISSIONS 47,949

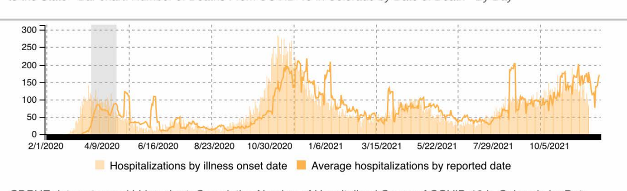

CDPHE data sets used | Line chart: Cumulative Number of Hospitalized Cases of COVID-19 in Colorado by Date Reported to the State • Bar chart: Cumulative Number of Hospitalized Cases of COVID-19 in Colorado by Date of Illness Onset

People currently hospitalized

This chart shows the number of people currently hospitalized with either a confirmed or suspected case of COVID-19.

Hover/click chart for details; click legend item to filter chart

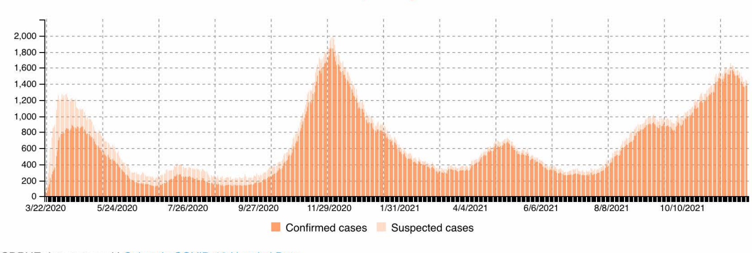

CDPHE data set used | Colorado COVID-19 Hospital Data

Testing rate and results

HOW TO READ THE CHARTS

Bar: The bar chart shows the 7-day moving average of the number of daily tests per 100,000 people, as reported to the CDPHE. Health officials say a testing rate of 152 per 100,000 is necessary to identify the majority of people who are infected.

Line: The line chart shows a 7-day moving average of the percentage of daily tests for COVID-19 that were positive.

Hover/click chart for details; click legend item to filter chart

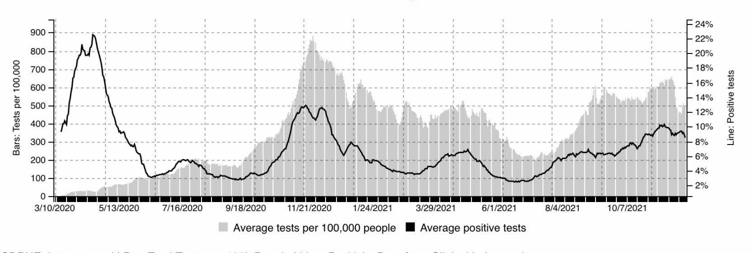

CDPHE data set used | Bar: Total Tests per 100k People | Line: Positivity Data from Clinical Laboratories

Daily cases in Colorado's largest counties

In July the CDPHE stopped breaking out daily cases for weekend days. We are now using a 10-day average to smooth out those spikes in reported cases.

These charts show the daily case count and 10-day average of reported COVID-19 cases for the state's 10 most-populous counties. **Note: Each chart's vertical axis range is unique to its data; change from a week ago is based on averages.**

Hover/click chart for details; click legend item to filter chart

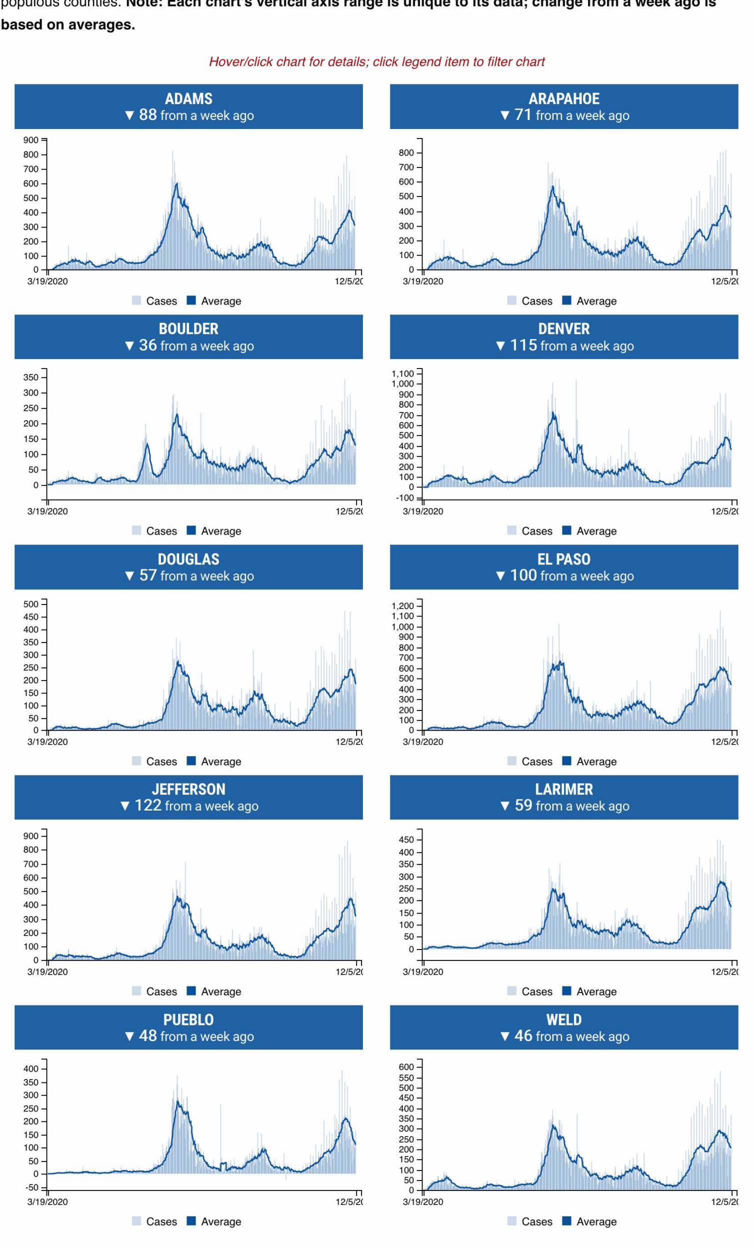

Case and death rates for counties

These charts compare the total case and death rates per 100,000 people for Colorado's 10 most-populous counties using a 7-day moving average of cases and deaths as they are reported to CDPHE. Rates are useful for comparing areas with different populations.

Hover/click chart for details; click legend item to filter chart

Cases per 100,000 people

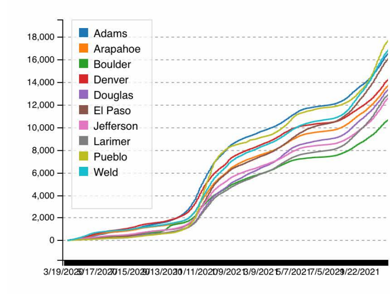

Deaths per 100,000 people

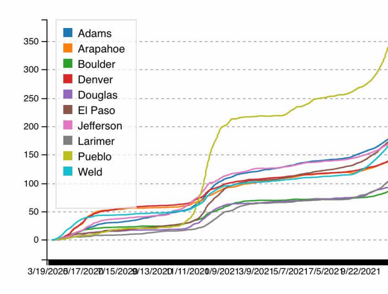

These charts compare total cases and deaths for Colorado's 10 most-populous counties.

Total cases

County (population rank)

Total deaths

County (population rank)

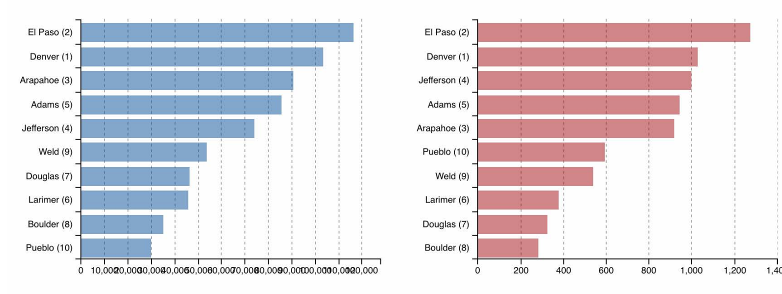

CDPHE data sets used | Colorado Case Counts by County, Number of Deaths by County

Maps: County cases and deaths

The first map shows the total number of cases and deaths by county and the second shows the case rate per 100,000 by county (rate will be slightly different than chart above because it is not a 7-day moving average).

Circles are centered in counties and are not meant to indicate an actual location; click circles for details

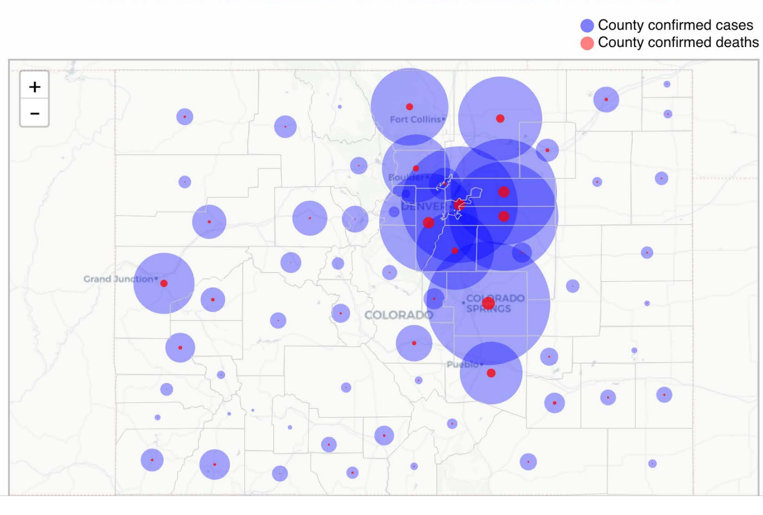

Connecticut COVID-19 Response

Get answers.
Get help.
Get involved.

DAILY DATA REPORT
FOR CONNECTICUT

LATEST UPDATE: DECEMBER 10, 2021 AT 3:00 P.M.

Weekly Extended Data reports are released every Thursday. Click here for an archive those weekly reports.

Reporting schedule: The State of Connecticut's COVID-19 metric report is issued once per day, every Monday through Friday, usually around 3:00 p.m. The report that is issued each Monday contains combined data that was collected on Friday, Saturday, and Sunday.

Open data portal: Supplemental data on COVID-19 in Connecticut can be found on the state's open data portal, data.ct.gov, in the COVID-19 data portal. Data presented on the open data portal is available in its rawest form.

Town Map

Average Daily Rate of COVID-19 Cases Among Persons Living in Community Settings per 100,000 Population By Town

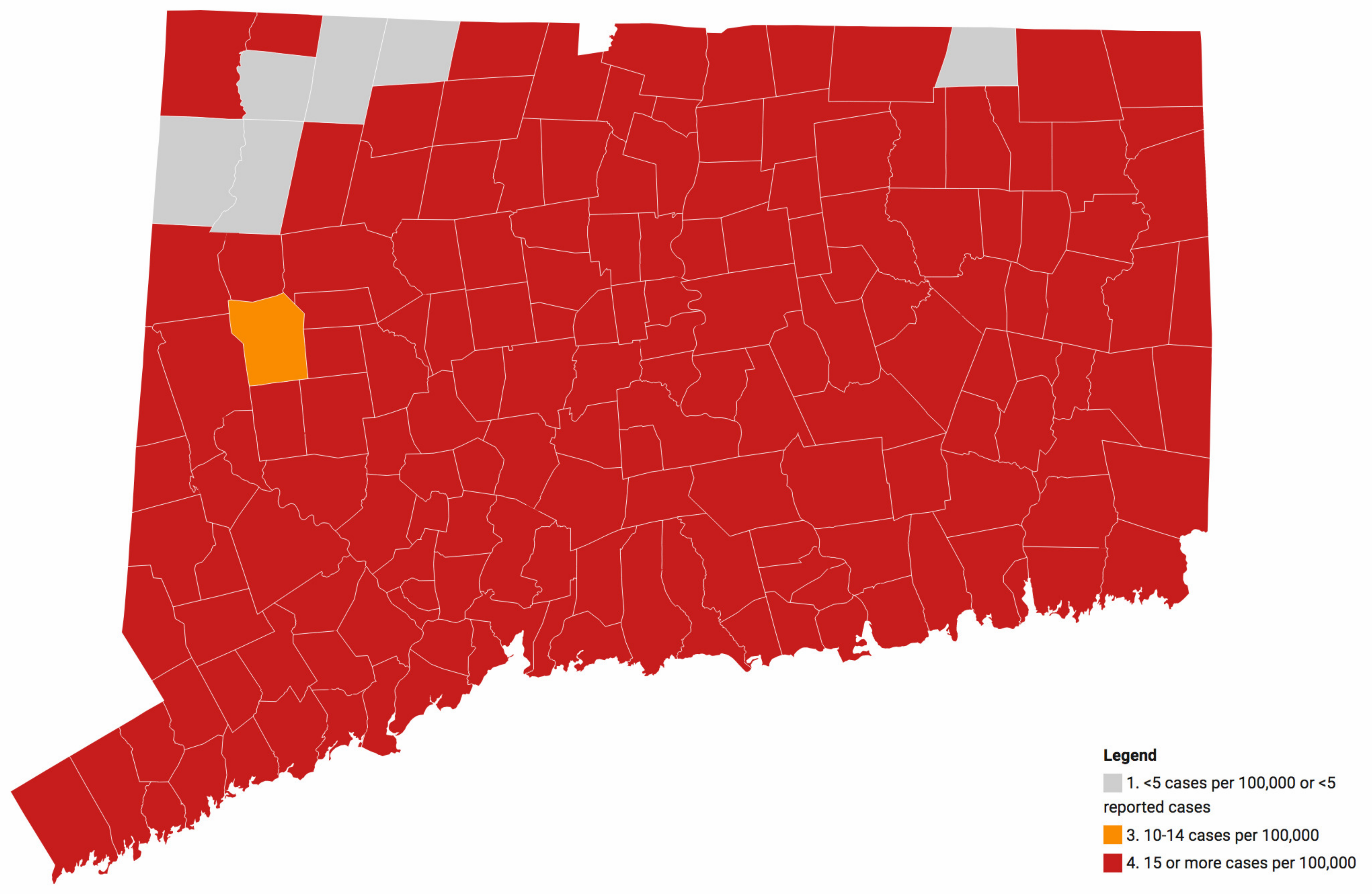

This map shows the average daily rate of new cases of COVID-19 by town during the past two weeks. Only cases among persons living in community settings are included in this map; the map does not include cases among people who reside in nursing home, assisted living, or correctional facilities.

Map: Ver 5.18.2021 • Source: CT Department of Public Health • Embed • Download image • Created with Datawrapper

Connecticut Department of Public Health Town-level COVID Response Framework

State Summary

Connecticut COVID-19 Summary

Summary for the most recent day of reporting. Includes confirmed plus probable cases; probable cases include persons with positive antigen results

Measure	Total	ChangeDirection	Change
COVID-19 Cases (confirmed and probable)	439,423	+	3,280
COVID-19 Tests Reported (molecular and antigen)	12,683,845	+	53,948
Daily Test Positivity*			6.08%
Patients Currently Hospitalized with COVID-19	585	+	9

Daily test positivity is the number of new positive molecular and antigen cases divided by the number of new molecular and antigen tests reported in the past 24 hours.

Table: Ver 5.11.2021 • Source: CT Department of Public Health • Embed • Download image • Created with Datawrapper

Age Group Chart

Number of COVID-19 Cases, Associated Deaths, and Rates per 100,000 by Age Groups

Total Number of Cases

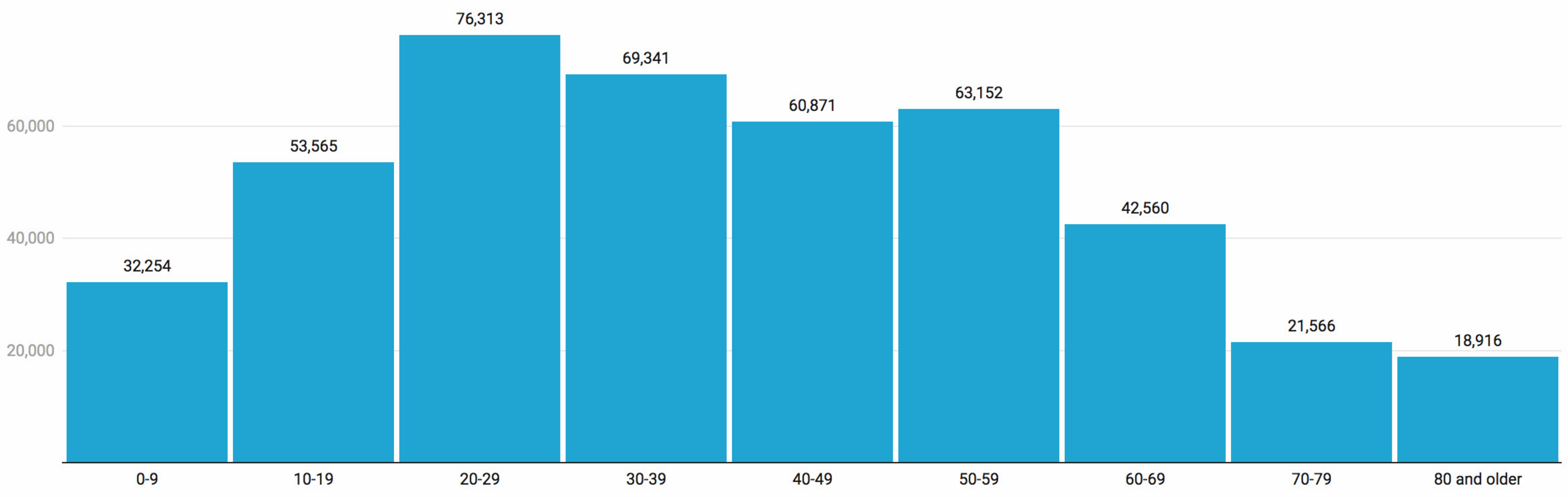

Total COVID-19 cases and associated deaths include confirmed plus probable.

Chart: Ver 5.11.2021 • Source: CT Department of Public Health • Embed • Download image • Created with Datawrapper

County Chart

COVID-19 Cases, Rate per 100,000, Associated Deaths, and Number Hospitalized by County

Cases and deaths are cumulative over time

Number of Total Cases

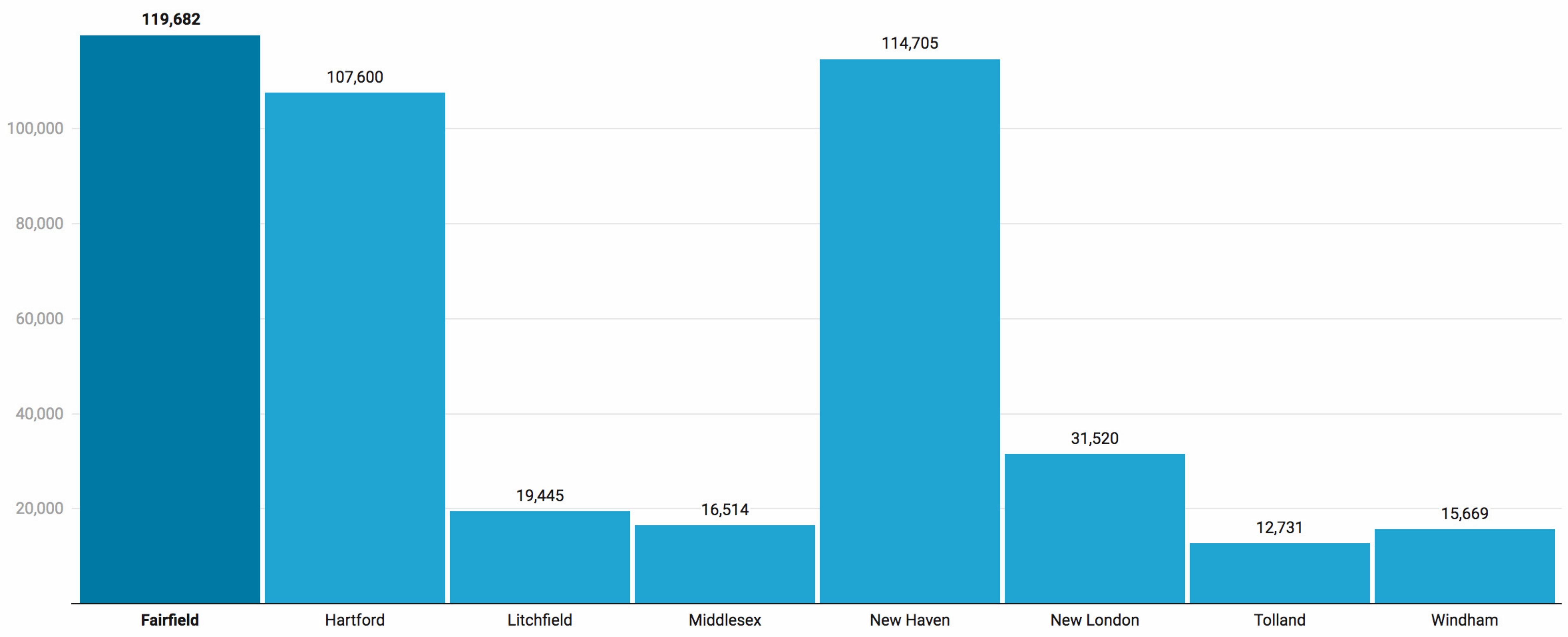

COVID-19 cases, rate per 100,000, and associated deaths are by county of residence. Hospitalization data are collected by CT Hospital Association and represent the number of patients currently hospitalized by location of the hospital and not the patient residence.

Chart: Ver 5.11.2021 • Source: CT Department of Public Health • Embed • Download image • Created with Datawrapper

Cases of COVID-19 Reported in Connecticut by Town by the Department of Geography at UConn

Last Updated at
12/10/2021, 6:30 PM

Total Confirmed
439,423

Increase Cases
3,280

as of previous day

Total Deaths
8,972

Change Deaths
26

as of previous day

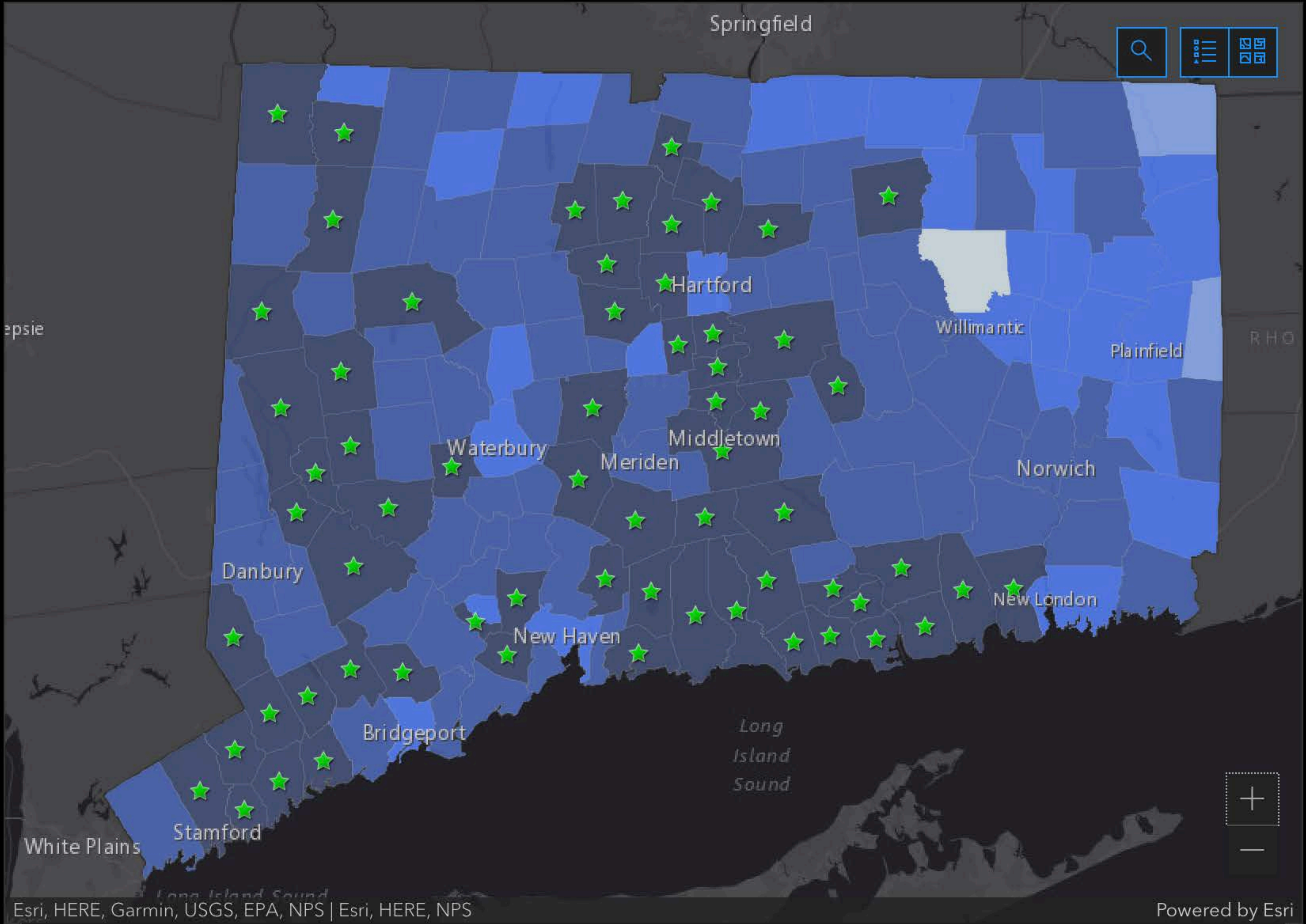

Covid-19 Vaccine Percent by Town

For sources and contact information please click upper right button in this box

Contact

Sources | Technical Note

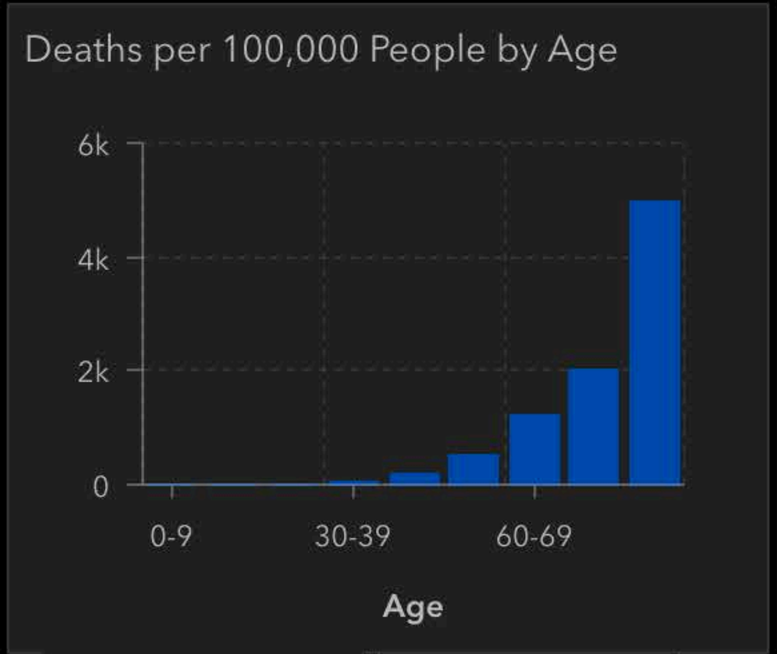

Deaths by Age | Cases by Age

Connecticut Towns

- 254, 118, 5, Andover
- 2,523, 177, 26, Ansonia
- 367, 122, 1, Ashford
- 1,325, 89, 13, Avon
- 256, 107, 3, Barkhamsted
- 756, 170, 2, Beacon Falls
- 2,160, 148, 19, Berlin
- 520, 126, 0, Bethany

Cases, Tests, Change from Previous day

Towns | Counties

Listen

COVID-19 Surveillance

COVID-19 Data Overview | Key Metrics | Outbreak Data | Vaccinations | K-12 Schools Data

December 9, 2021

- Total Overall Number of Tests: 2,473,461
- Total Number of DC Residents Tested: 676,626
- Total Positives: 68,702
- Total Lives Lost: 1,199
- Cleared from Isolation: 34,985

*All data are preliminary and are subject to change based on additional reporting

Download a copy of DC COVID-19 data

Other Data

- Public Safety Agency Data
- Human Services Agency Data
- DMV Agency Data
- Hospital Status Data
- DC Public Schools Data
- COVID-19 School Based Testing Program

Trends | Ward | Neighborhood | Demographics | Tables

Testing and Case Trends | Hospital Census and Bed Availability | Ventilator Use and Availability

Total Overall Tested	Total Residents Tested	Total Positives	Total Recovered	Total Lives Lost
2,410,575	668,611	67,173	34,985	1,197

Overall Tested | Positives | Recovered | Lives Lost | Residents Tested

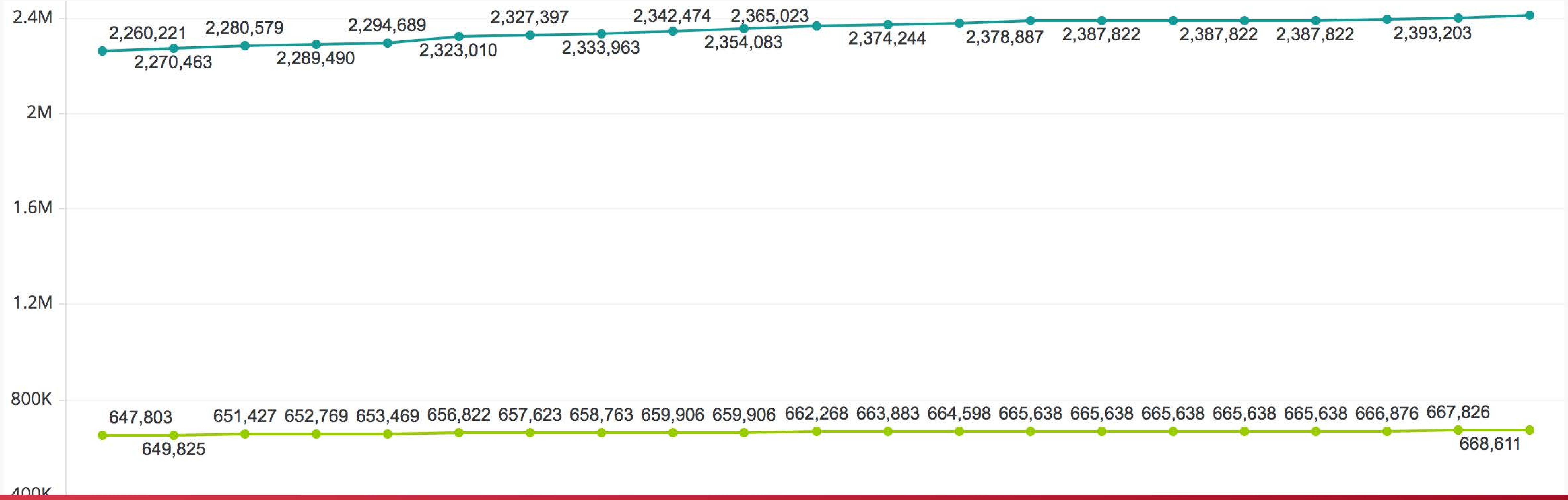

View Mayor Bowser's Presentation on DC's COVID-19 Situational Update for December 2, 2021

User Guide for Data Visualizations

View 2020 Coronavirus Data

Coronavirus (COVID-19) Data Dashboard

Download Coronavirus (COVID-19) Data Dashboard Data

SCHOOL & STUDENT CASES

COVID-19 In-Person Contagious School Cases Dashboard

This dashboard contains information about school and student COVID-19 cases.

View In-Person Contagious School Cases Dashboard >

IMPORTANT INFORMATION ABOUT THIS PAGE

Data are current as of 6pm the previous day. Last update: 12/10/2021 4:28 PM.

About the data

DOWNLOAD DATA AS .CSV

Overview

Select dataset (Use dropdown to select)

Percentage of Total Population Fully Vaccinated

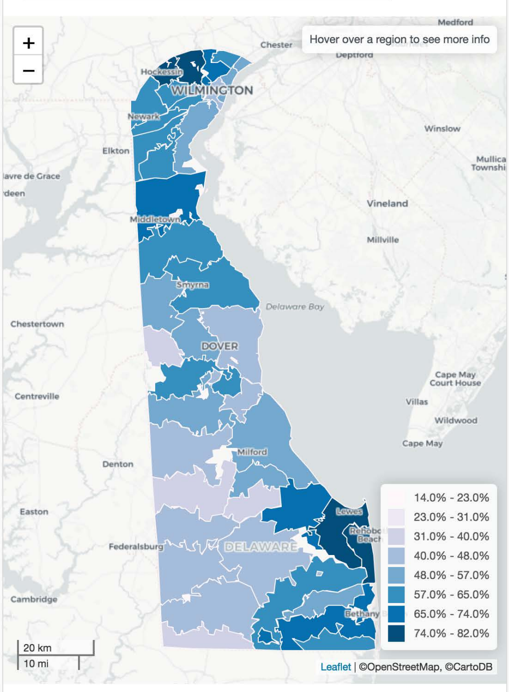

Percentage of 18+ Population Vaccinated With At Least One Dose

86.6% Data Source - CDC

Learn about how it differs from other DE data here

Percentage of Total Population Vaccinated With At Least One Dose

66.9%

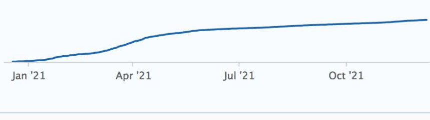

Percentage of Tests Positive

9.2% 7-Day Average

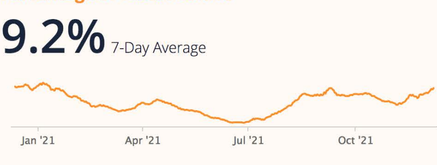

OTHER KEY COVID METRICS

New Positive Cases

603.9 7-Day Average

View more case data

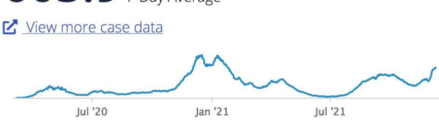

Total Vaccine Doses Administered

1,464,094

View more vaccine data

Current Hospitalizations

317 with 33 critical

View more hospital data

STATE OF DELAWARE / NOT AVAILABLE FOR SMALLER GEOGRAPHIES

Breakthrough Cases

Last Updated: 12/10/2021

Vaccine breakthrough cases are expected. COVID-19 vaccines are effective and are a critical tool to bring the pandemic under control. However, no vaccines are 100% effective at preventing illness in vaccinated people.

Of the **556,078 fully vaccinated Delawareans**, there have been **8,341 breakthrough cases** of COVID-19, roughly **1.50%**.

The number of breakthrough cases and the number of fully vaccinated Delawareans are updated once per week on Fridays when the data becomes available, which may be after the rest of the data is published.

Total Fully Vaccinated 556,078
62% of eligible population

Total Breakthrough Cases
1.50% of Delawareans who have been fully vaccinated
8,341

STATE OF DELAWARE

Trend Comparison: Fully Vaccinated and Deaths among All Delawareans

Last Updated: 12/10/2021 4:28 PM

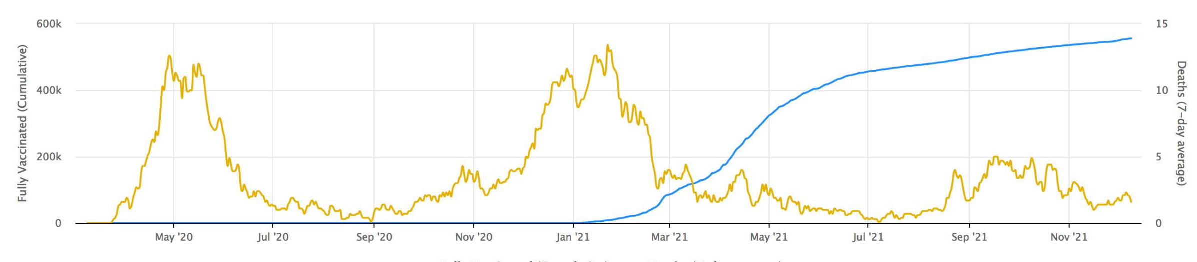

STATE OF DELAWARE

Positive Cases Data

Last Updated: 12/10/2021 4:28 PM

Total Positive Cases

159,399 1,727.3 per 10,000 people

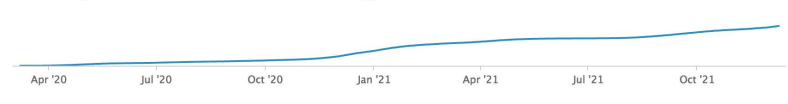

Cumulative Number of Confirmed Positive Cases
145,698

Cumulative Number of Probable Positive Cases
13,701

Cumulative Number of Long-Term Care Cases
3,017

Positive Cases by County

County	Positive Cases	
New Castle County	90,416	View New Castle County data
Kent County	29,583	View Kent County data
Sussex County	38,922	View Sussex County data
Unknown	478	

View more case data

Data are current as of 6pm the previous day. Last update: 12/09/2021

STATE OF DELAWARE

Deaths Data

Last Updated: 12/10/2021 4:28 PM

Total Deaths

2,211 17.8 per 10,000 people

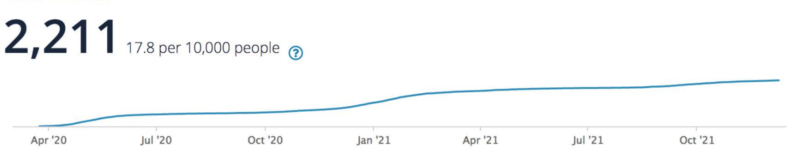

Confirmed Deaths
2,025

Probable Deaths
186

Long-Term Care Deaths
869

View more death data

Data are current as of 6pm the previous day. Last update: 12/09/2021

Trends

STATE OF DELAWARE OVER THE LAST 14 DAYS

14-day charts | 30-day charts | 90-day charts | All available data

Case counts and test data by date of report can vary significantly from day to day for a variety of reasons. In addition to changes due to actual changes in disease incidence, trends are strongly influenced by testing patterns (who gets tested and why), testing availability, lab analysis backlogs, lab reporting delays, new labs joining our electronic laboratory reporting system, mass screenings, etc.

STATE OF DELAWARE

Current Hospitalizations

Last Updated: 12/10/2021

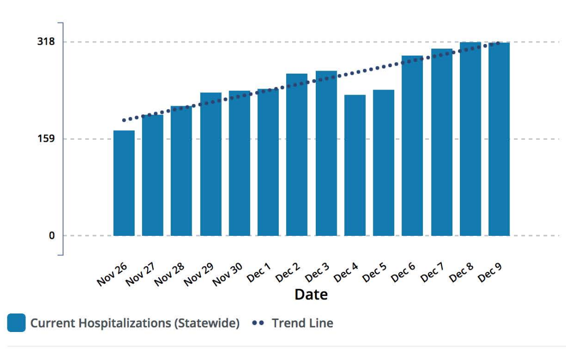

*When available, hospitalization estimates are provided by the University of Delaware if a hospital should fail to report.

STATE OF DELAWARE

New Hospital Admissions (Confirmed and Suspected with COVID-19)

Last Updated: 12/10/2021

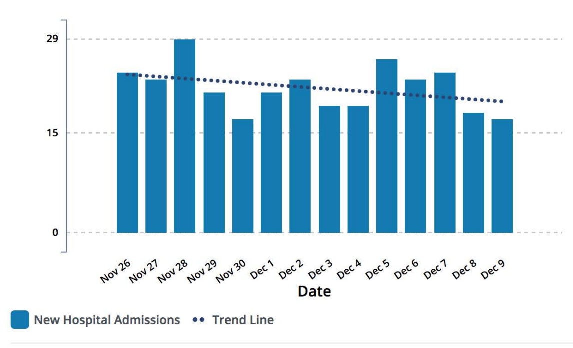

Data source: Delaware Health Information Network

More details

STATE OF DELAWARE

Percent Positive

Last Updated: 12/10/2021

Percent of persons testing positive, and percent of tests that are positive are helpful indicators in understanding the spread of infection in a community. Percent of persons testing positive indicate one result reported for each individual tested (positive or negative), even if that person is tested more than once with the same result. As a result, it often reflects higher positivity rates.

Percent of tests that are positive indicates the total number of specimens tested, including when an individual is tested more than once, and reflects higher reported test numbers and lower positivity rates. It is also more widely used by other states and the Federal government, and is therefore useful to compare Delaware to other jurisdictions.

Because of the increasing frequency of repeat testing, using person-based percent positives could be misleading on a daily basis. Therefore, using the percent of tests that are positive is a more useful measure to make operational decisions. Data for percent of tests that are positive are based on the testing date, while data for persons testing positive is based on the date results are reported to DPH. There is a two-day lag for presenting data related to percent of tests that are positive to account for the time delay between the date of the test and the date that DPH receives the test result.

Percent of persons testing positive | Percent of tests that are positive

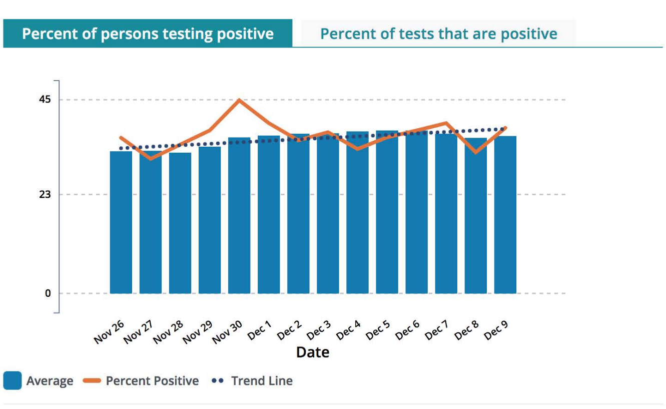

Averaging method: averages are a moving calculation that smooth unstable data. For each data point, seven values are averaged: the data point and the six preceding data points. Toward the beginning of the time series, when there are fewer than six values before a data point, instead of averaging seven values, only the available values are used in the calculation.

More details

STATE OF DELAWARE

New Positive Cases

Last Updated: 12/10/2021

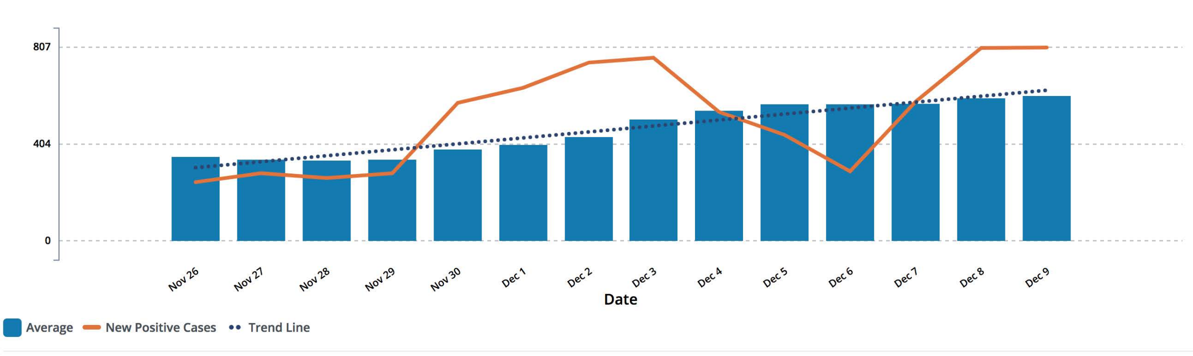

Averaging method: averages are a moving calculation that smooth unstable data. For each data point, seven values are averaged: the data point and the six preceding data points. Toward the beginning of the time series, when there are fewer than six values before a data point, instead of averaging seven values, only the available values are used in the calculation.

More details

STATE OF DELAWARE

Percentage of Emergency Department Visits for Influenza-Like Illness (ILI) and COVID-19-Like Illness (CLI) by Week

Last Updated: 12/10/2021

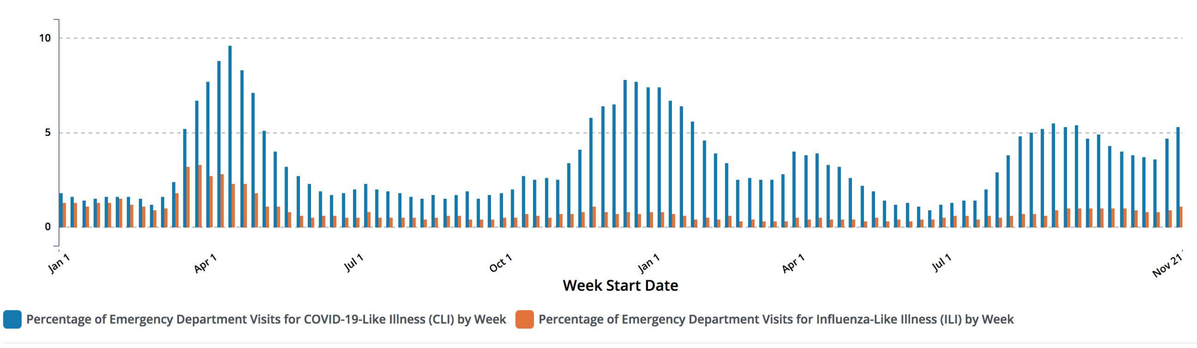

More details

STATE OF DELAWARE

Tests Performed

Last Updated: 12/10/2021

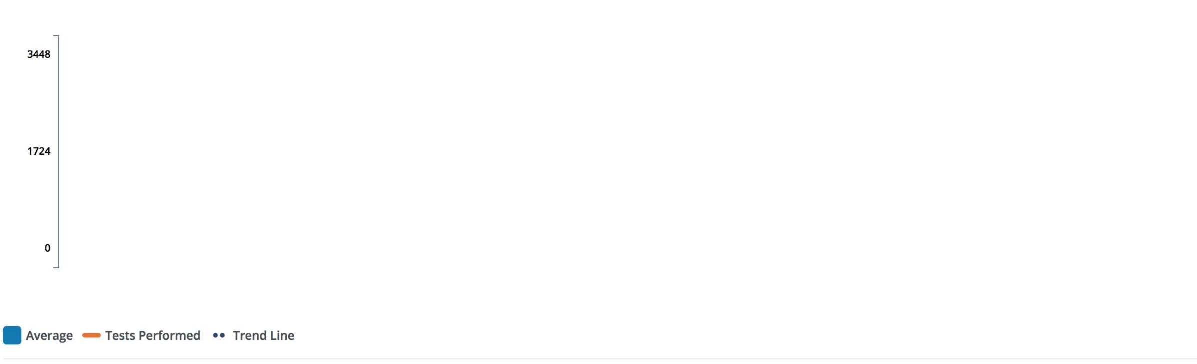

Averaging method: averages are a moving calculation that smooth unstable data. For each data point, seven values are averaged: the data point and the six preceding data points. Toward the beginning of the time series, when there are fewer than six values before a data point, instead of averaging seven values, only the available values are used in the calculation.

STATE OF DELAWARE

Cases by County (Cumulative)

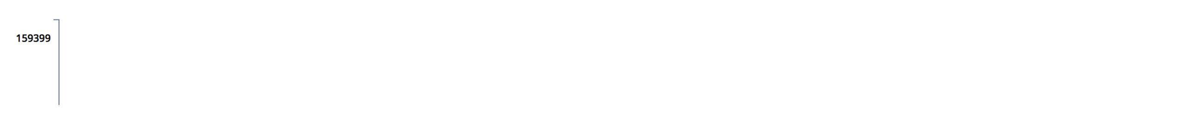

Change location and/or topic

Select a new location and/or topic then click "Go" at the bottom.

Share feedback | Close

State of Delaware

Select Topic

Coronavirus (COVID-19)

Locations

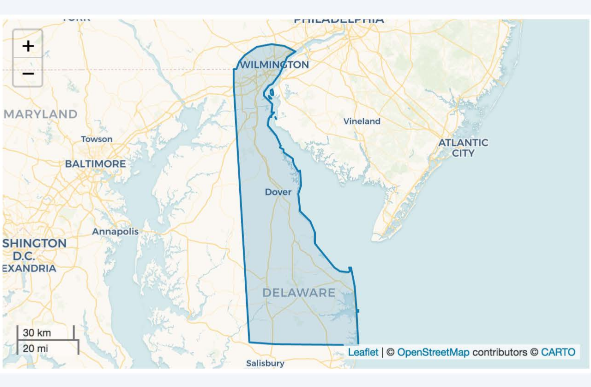

Go

365.4 | 8.1% | 24.4

Unlike other COVID data on My Healthy Community, historical data presented on the weekly community spread summary are not updated daily. These data are updated on Mondays. This is to allow for complete reporting of the week's test data.

SHOWING DATA FOR
State of Delaware
Population: 957,248

COVID-19 Overview | Vaccines | Schools | Cases | Deaths | More

	(per 100,000 people)	Percent of tests that are positive	(per 100,000 people)
11/27/2021 - 12/03/2021	365.4	8.1	24.4
11/20/2021 - 11/26/2021	248.4	7.7	17.1
11/13/2021 - 11/19/2021	256.8	7.6	15.9
11/06/2021 - 11/12/2021	196.1	5.6	15.2
10/30/2021 - 11/05/2021	169.9	5.1	16.5

Show/hide additional weeks

View more detailed data

Coronavirus in Georgia: COVID-19 Dashboard

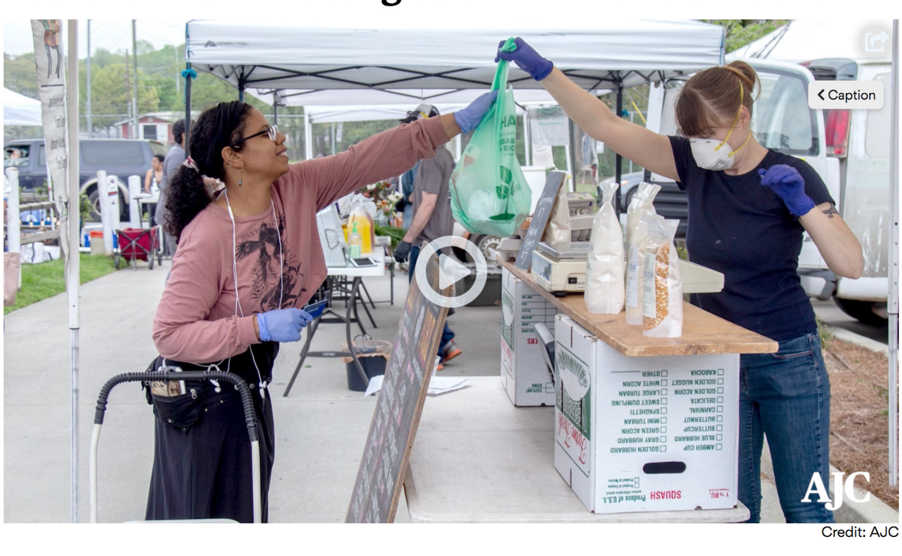

Credit: AJC

COVID-19

| UPDATED DAILY

Latest stats and the news on the coronavirus outbreak

This dashboard gives up-to-date counts for confirmed and probable cases, deaths and other key statewide measures for the novel coronavirus. We update it at 3:15 p.m. (Sources: Georgia Department of Public Health, Georgia Emergency Management & Homeland Security Agency, Atlanta Journal-Constitution analysis)

The dashboard reflects the day the state reported confirmed and probable cases and confirmed deaths. This means that the new confirmed cases could be reported a day or more after the test results came back, several days after the test was taken, and a week or more after the patient was infected. So new confirmed cases reflect the spread of actual infections from a week or more ago.

Georgia DPH tracks confirmed cases (via molecular PCR tests) and probable cases (typically rapid antigen test positive cases), and the AJC has updated its dashboard to reflect both varieties of test.

Explore — Current COVID-19 vaccination rates for Georgia counties

We have added additional charts that present the data based on first reported symptom for confirmed cases or date of death, as reported by the state. The AJC also tracks hospitalization data, rates of spread by county and computes transmission estimates, which are explained in detail below.

Jump to a specific chart: Cases | Deaths | Hospitalizations | Tests | County Data | Transmission Rates | Vaccines

Q: What is the latest on confirmed and probable coronavirus cases in Georgia?

Newly reported cases by day in Georgia

Here, new confirmed [1] and probable [2] cases of coronavirus are shown by the date they first appeared on the DPH website. Because of the lag between first symptoms and DPH reporting a positive test result, other charts representing symptom onset can be misleading without proper context, as they tend to show a recent steep decline due to incomplete data. Looking at newly reported cases in this chart adds context by providing a static snapshot of each day's reports.

1,295,855 TOTAL CONFIRMED CASES
1,677,947 TOTAL INCLUDING PROBABLE CASES

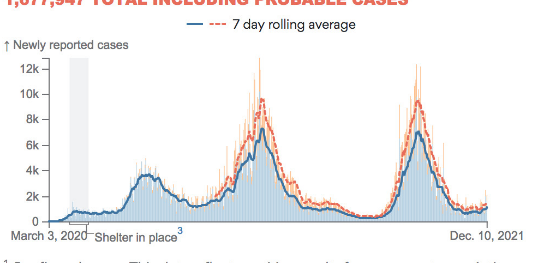

[1] Confirmed cases: This data reflects positive results from reverse-transcription polymerase chain reaction (PCR) tests.

[2] Probable cases: This data reflects positive results from rapid antigen tests, which are considered probable cases. DPH tracks cases by person, not individual tests. That means DPH moves antigen positive cases to the confirmed category if a person also receives a PCR positive. That change in data is updated daily. DPH first started reporting antigen positive cases on Nov. 4.

[3] Shelter in place: For most Georgians over age 65, shelter in place continued through June 12, 2020. Georgians who are residents of long term care facilities or are medically fragile must continue to shelter in place through at least March 31.

Source: Georgia Department of Public Health

Updated December 11 at 3:30 p.m.

[If you are on mobile and are not seeing our charts, please click here to reload full version of page]

Explore — See how cases are trending in your county

Coronavirus cases by date of symptom onset[1] in Georgia

Here, new confirmed [2] and probable [3] cases of coronavirus are shown by the date of symptom onset as reported by DPH. But because there is a lag between first symptoms and the date DPH reports a positive test result, more recent data is likely to be adjusted upward in the future.

While DPH may adjust the number of cases from any date, the dashed line and lighter colored bars represent the window when the most adjustments are likely to occur, according to our analysis.

1,294,590 TOTAL CONFIRMED CASES
1,677,034 TOTAL INCLUDING PROBABLE CASES

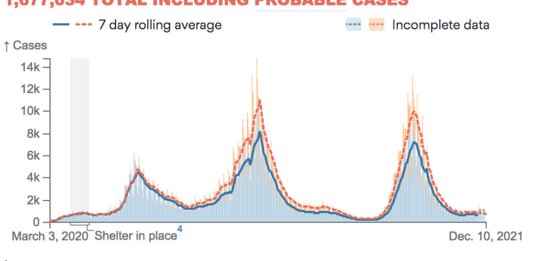

[1] Date of symptom onset: Date of test is used if date of symptom onset is unknown; date reported to DPH is used if none of the above are known. Cases where DPH is unable to determine a date are not included, which accounts for the discrepancy between the number of confirmed cases on this chart and the newly reported cases chart.

[2] Confirmed cases: This data reflects positive results from reverse-transcription polymerase chain reaction (PCR) tests.

[3] Probable cases: This data reflects positive results from rapid antigen tests, which are considered probable cases. DPH tracks cases by person, not individual tests. That means DPH moves antigen positive cases to the confirmed category if a person also receives a PCR positive. That change in data is updated daily.

[4] Shelter in place: For most Georgians over age 65, shelter in place continued through June 12, 2020. Georgians who are residents of long term care facilities or are medically fragile must continue to shelter in place through at least March 31.

Source: Georgia Department of Public Health

Updated December 11 at 3:30 p.m.

Q: What is the latest on coronavirus deaths in Georgia?

Newly reported deaths by day in Georgia

Here, new confirmed [1] and probable [2] deaths from coronavirus are shown by the date they first appeared on the DPH website. Because of the lag between the date of a death and DPH reporting it, other charts representing day of death can be misleading without proper context, as they tend to show a recent steep decline due to incomplete data. Looking at newly reported deaths in this chart adds context by providing a static snapshot of each day's reports.

25,940 TOTAL CONFIRMED DEATHS
30,823 TOTAL INCLUDING PROBABLE DEATHS

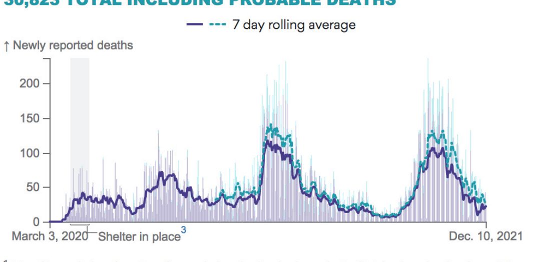

[1] Confirmed deaths: Confirmed deaths include only individuals who had positive reverse-transcription polymerase chain reaction (PCR) test results.

[2] Probable cases: This data reflects positive results from rapid antigen tests, which are considered probable. DPH first started reporting probable deaths on Nov. 4.

[3] Shelter in place: For most Georgians over age 65, shelter in place continued through June 12, 2020. Georgians who are residents of long term care facilities or are medically fragile must continue to shelter in place through at least March 31.

Source: Georgia Department of Public Health

Updated December 11 at 3:30 p.m.

Coronavirus deaths by day of death in Georgia

Here, new confirmed [1] and probable [2] deaths from coronavirus are shown by the date they occurred, as reported by DPH. But because there is a lag between the date of death and the date DPH reports it, more recent data is likely to be adjusted upward in the future.

While DPH may adjust the number of deaths from any date, the dashed line and lighter colored bars represent the window when the most adjustments are likely to occur, according to our analysis.

25,939 TOTAL CONFIRMED DEATHS
4,883 TOTAL PROBABLE DEATHS

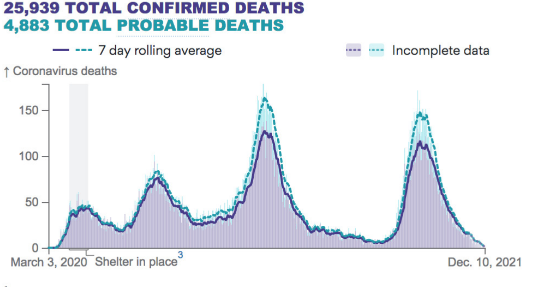

[1] Confirmed deaths: This data includes only individuals who had positive reverse-transcription polymerase chain reaction (PCR) test results.

[2] Probable deaths: This data includes individuals who tested antigen positive or who had compatible illness and known close contact to a case and were either reported to DPH as deceased; identified by death certificates with COVID-19 indicated as the cause of death; there is evidence that COVID contributed to the individual's death; or individuals with a death certificate that has COVID-19 indicated as the cause of death and there is no laboratory evidence for SARS-CoV-2.

[3] Shelter in place: For most Georgians over age 65, shelter in place continued through June 12, 2020. Georgians who are residents of long term care facilities or are medically fragile must continue to shelter in place through at least March 31.

Source: Georgia Department of Public Health

Updated December 11 at 3:30 p.m.

Explore — » Follow the full AJC coverage of the coronavirus outbreak

Q: What is the latest on people hospitalized for COVID-19 in Georgia?

Current hospitalizations by day in Georgia

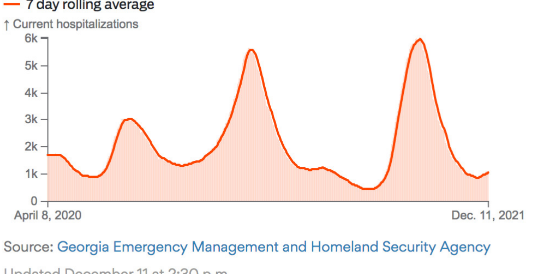

Source: Georgia Emergency Management and Homeland Security Agency

Updated December 11 at 2:30 p.m.

A: This is the number of people confirmed to have COVID-19 who are currently hospitalized, as published by the Georgia Department of Emergency Management & Homeland Security Agency. It began reporting this measure daily on May 2. It does not include those hospitalized who remain under investigation for suspected COVID. This number is updated daily.

Q: How many Georgians are testing positive for Coronavirus?

Percent testing positive for Coronavirus in Georgia

Each bar in this chart shows the percent testing positive for coronavirus each day as reported to the Georgia Department of Public Health (DPH). The line running through the bars shows the seven-day rolling average of the daily rates. The data does not include serologic tests, which identify if someone was exposed to the virus in the past and developed antibodies. The 5% line highlighted on the graph is the upper threshold identified by the White House Coronavirus Task Force for states to be in the green, or safe, zone for reopening.

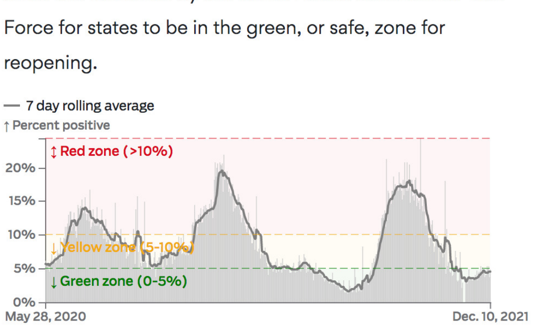

Note: Data on current infections is from electronic laboratory reporting to the state, which does not include all tests performed in Georgia. DPH excludes other testing sources because they do not consistently provide negative results, which must be factored in to arrive at positivity percentage. DPH also notes that, "People with a positive test often get retested and may test positive multiple times. These repeat positive tests will be counted as new positive tests."

Data: Georgia Department of Public Health

Zones: White House Coronavirus Task Force

Updated December 11 at 3:30 p.m.

Q: Does today's case data show how the virus is spreading right now in Georgia?

A: No. Epidemiologists agree that today's counts are a snapshot of what the virus did roughly two weeks ago. It can take a week or more for a person to become infected, show symptoms, get tested and have their results reported to public health officials. Deaths are generally reported to the state quickly, but it can take weeks for an infected person to succumb to the virus.

Q: What does the 7-day average line indicate?

A: This measure makes it easier to see trends in the spread of the virus that can be obscured by unrelated bumps and dips. For instance, Georgia's daily case counts tend to be lower on the weekends because reporting and testing slows down, not because fewer people are getting sick.

Q: Where is coronavirus spreading now?

Recently reported confirmed* coronavirus cases in Georgia

Rate of recently reported confirmed* coronavirus cases in each county by population (confirmed* cases reported in the last 14 days by the Georgia Department of Public Health based on each patient's reported county of [illegible], divided by the county's population and then multiplied by 100,000).

Log In
5

Tap each county for details on recently reported confirmed* and probable** cases there.

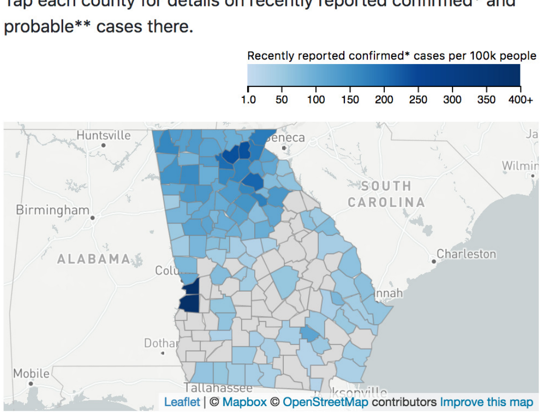

*This data reflects positive results from reverse-transcription polymerase chain reaction, or PCR, tests.
**This data reflects positive results from rapid antigen tests, which are considered probable cases. DPH tracks cases by person, not individual tests. That means DPH moves antigen positive cases to the confirmed category if a person also receives a PCR positive. That change in data is updated daily.
Source: Georgia Department of Public Health and the U.S. Census Bureau: *2019 American Community Survey 5-year estimates*

Updated November 30

State of Hawai'i – Department of Health
Disease Outbreak Control Division | COVID-19

Search

Home | News | What You Should Know | What You Can Do | For Clinicians | Resources | Testing Locations | Schools | DOCD Home

Home » Hawaii COVID-19 Data

Hawaii COVID-19 Data

Hawaii COVID-19 Summary Metrics

LAST UPDATED ON Saturday, December 11, 2021
DATA AS OF Thursday, December 9, 2021

Click info icon for notes on this data

NAVIGATE TO OTHER VIEWS: SUMMARY | MAP | EPI CURVE | COUNTY RATES | TESTING

STATEWIDE

Change from Previous Day	Daily Average Cases*	Average Test Positivity*
189	108▲	1.6%

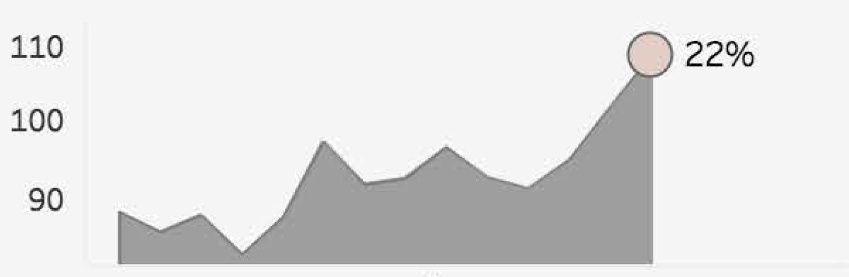

New cases w/ 14-day % change

HAWAII COUNTY

Change from Previous Day	7-day Avg Cases	Test Positivity
10	9▼	1.5%

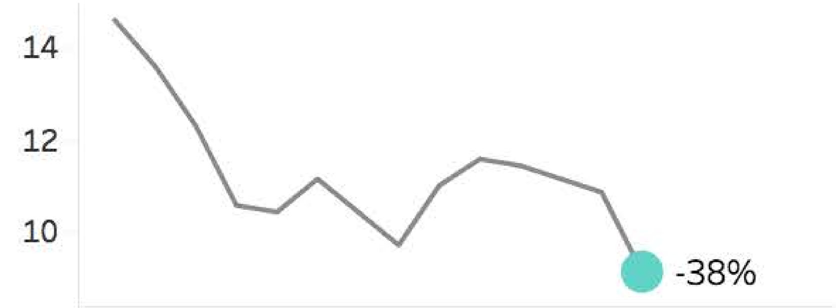

New cases w/ 14-day % change

HONOLULU COUNTY

Change from Previous Day	7-day Avg Cases	Test Positivity
134	75▲	1.8%

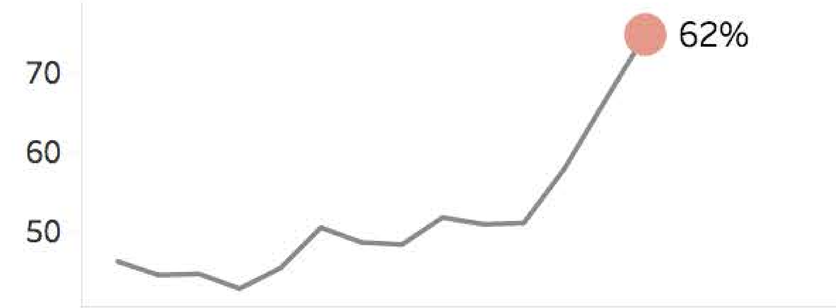

New cases w/ 14-day % change

COMPLETED VACCINATION

Statewide	Hawaii	Honolulu	Kauai	Maui
72.6%	65%	76%	70%	65%

KAUAI COUNTY

Change from Previous Day	7-day Avg Cases	Test Positivity
15	10▼	2.0%

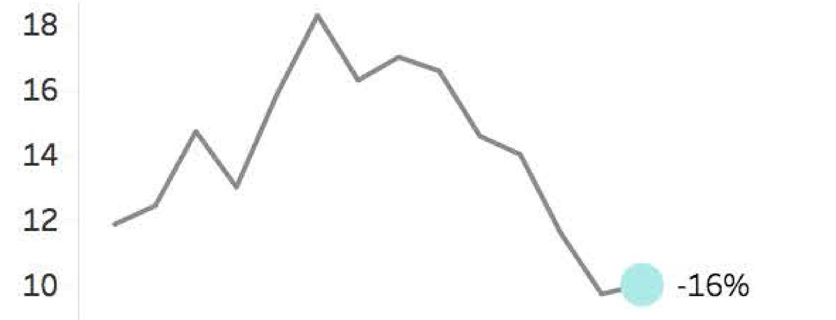

New cases w/ 14-day % change

MAUI COUNTY

Change from Previous Day	7-day Avg Cases	Test Positivity
24	14▲	1.3%

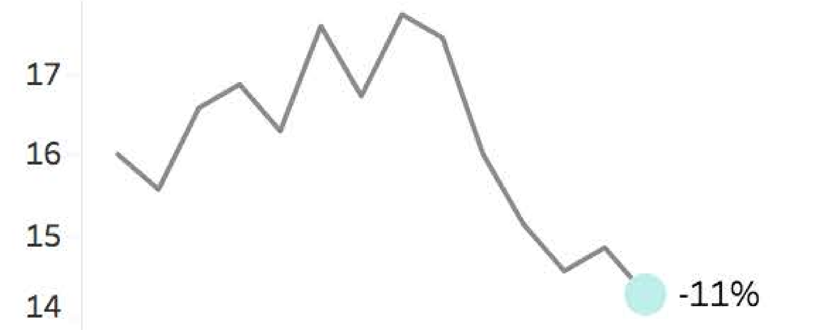

New cases w/ 14-day % change

*Metrics represent 7-day moving averages; Includes both confirmed and probable cases

% Change, Nov 26 to Dec 9

-100% 100%

OTHER DATA DASHBOARDS

New Case Counts

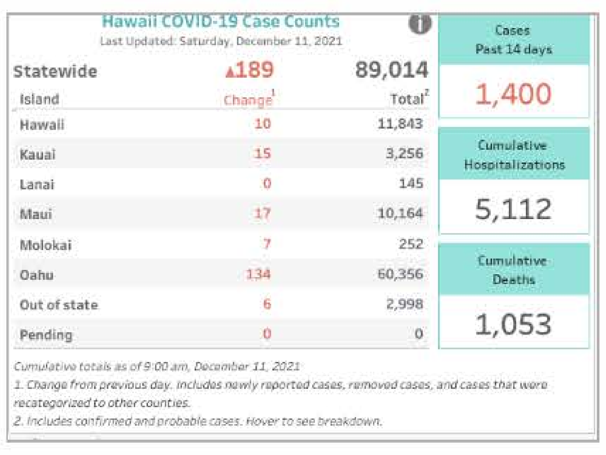

How many new cases were reported today on each island?
See a breakdown of total and newly reported cases by island and case status.

Vaccine Summary

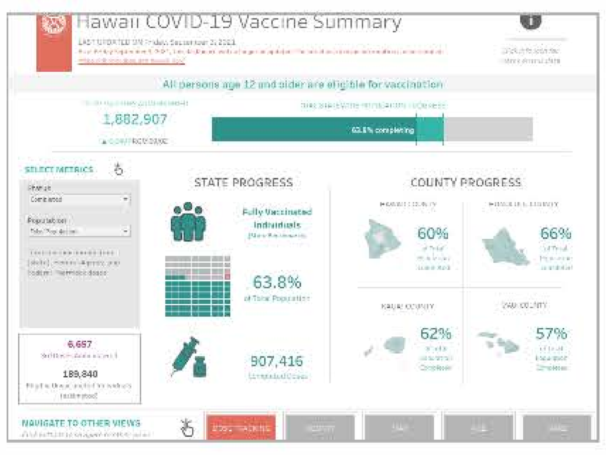

What are the current vaccination rates across the state?
Track vaccine administration by county, zip code, age, and race.

Travel Data

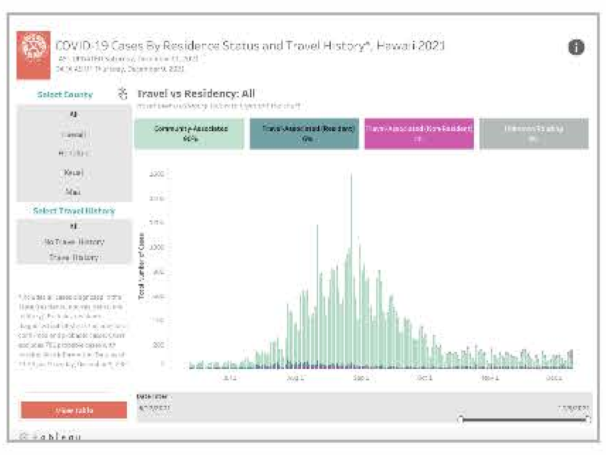

How does travel relate to newly reported cases?
View cases by travel history and residence status.

Mortality Data

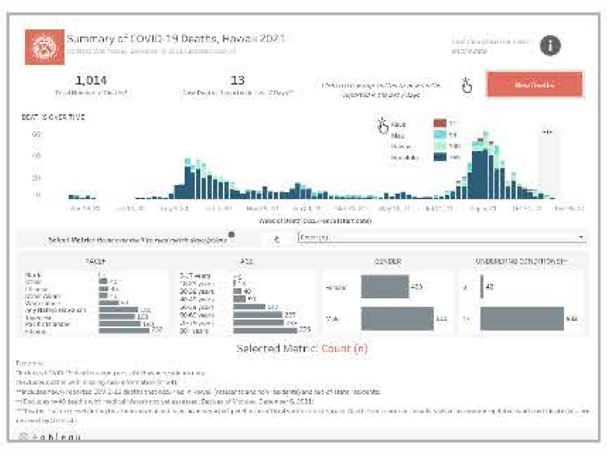

What are the characteristics of people who have died from COVID-19?
Explore trends and demographics like age, race, and gender of COVID-19 deaths.

Investigation Summary

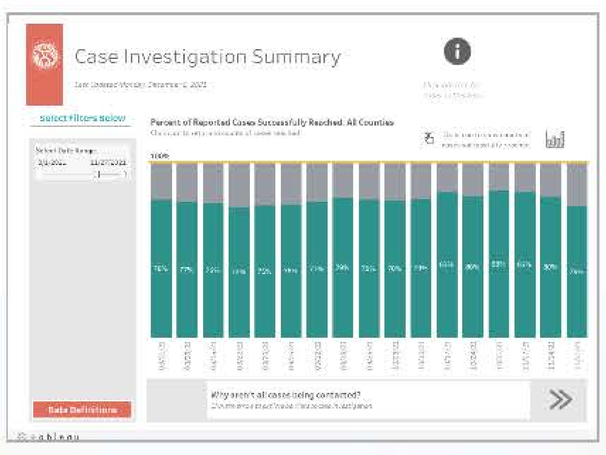

How successful is DOH in reaching newly reported cases?
Visualize key contact tracing and investigation indicators and barriers.

Outcome Data

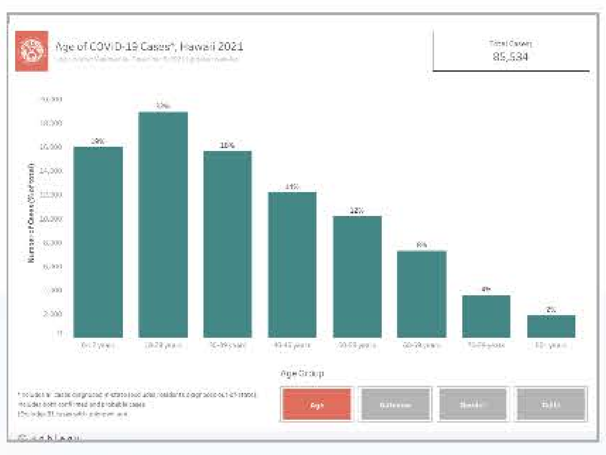

Who has been impacted by severe outcomes?
Understand COVID-19 hospitalizations and deaths by age and gender.

Age Trends

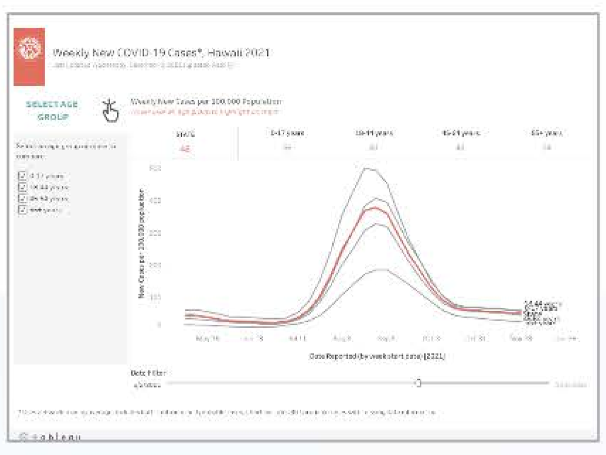

How are different age groups being affected by COVID?
Examine case rates by age group over time.

Race/Ethnicity Data

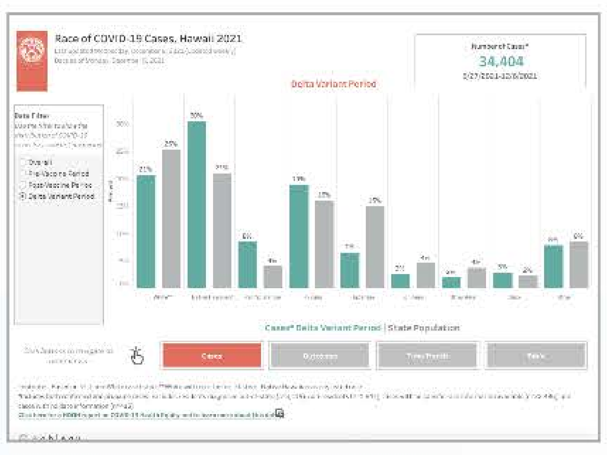

Which racial and ethnic groups have been most affected?
Compare cases, hospitalizations, and deaths by race/ethnicity and trends over time.

COVID Data Resources

With the onset of COVID in March 2020, we launched this page as a platform for providing consistent and accessible data relevant for responding to the pandemic. Since then, multiple public and private data resources have evolved to meet this need. As such, we have repurposed this page to serve as a directory of these resources while pivoting to support key COVID data partners going forward.

Overviews & Forecasts ↓ | Detailed COVID Data ↓ | Economic Recovery Data ↓

COVID-19 in Hawaii – Current Situation

State
98.4
(170 | 88,825 | 7.0)
14-Day Trend:

Additional State-Wide 7-Day Averages:

Hospitalizations	Deaths
7.0	1.7
(2 | 5,102 | 0.5)	(5 | 1,045 | 0.1)

14-Day Trend:

Honolulu 64.4 (118 | 60,222 | 6.7)

Hawaii 10.6 (10 | 11,833 | 5.2)

Maui 13.7 (24 | 10,537 | 8.2)

Kauai 9.7 (5 | 3,241 | 13.5)

Data Format:
7-Day Avg of New Cases
(New Cases | Total Cases | 7-Day Avg of Daily Cases/100k)

Updated with data as of Dec 10

Hawaii Data Collaborative

+ a b l e a u

Base data for this dashboard is pulled daily from DOH's Current Situation in Hawaii page.
Click here to access the data for this dashboard.

COVID Overviews & Forecasts

Summary Metrics

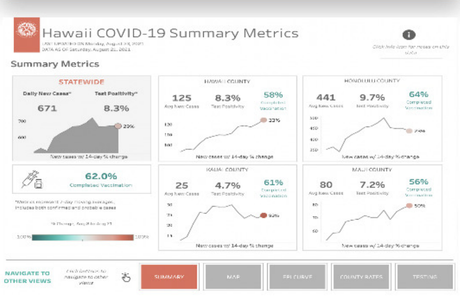

State and County current 7-day average of new cases and test positivity rate.
Source: DOH Disease Outbreak Control Division
Updated daily

COVID Tracking Project

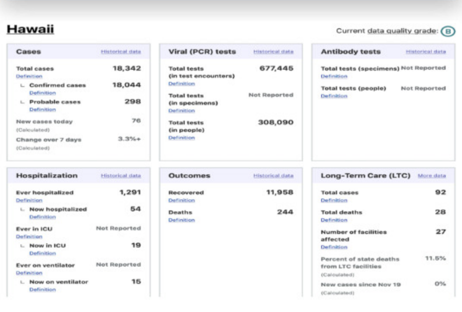

This project compiles and publishes various COVID indicators. Data API available.
Source: The COVID Tracking Project
Updated daily

HIEMA Dashboard

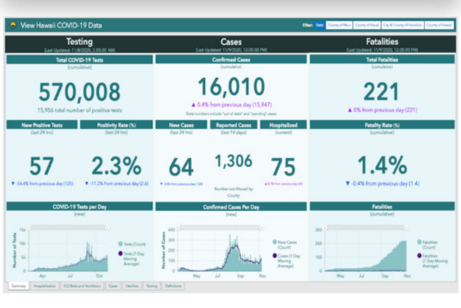

Dashboard of key indicators the State relies on to inform policy decisions.
Source: Hawaii Emergency Management Agency
Updated daily

COVID Pau Key Indicators

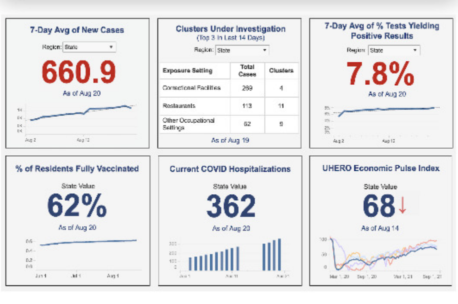

Overview of key indicators and easy-to-digest information and resources.
Source: COVID Pau
Updated daily

HiPAM Forecasts

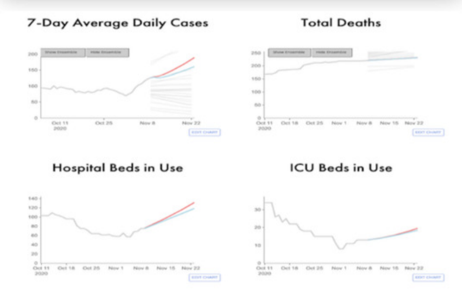

Forecasts of key indicators, based on pessimistic and expected inputs.
Source: Hawaii Pandemic Applied Modeling
Updated daily

CDC COVID Data Tracker

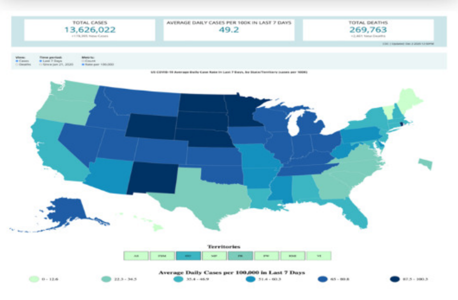

Maps, charts and downloadable data for various COVID indicators, by state.
Source: Centers for Disease Control and Prevention
Updated daily

Detailed COVID Data

Variant Report

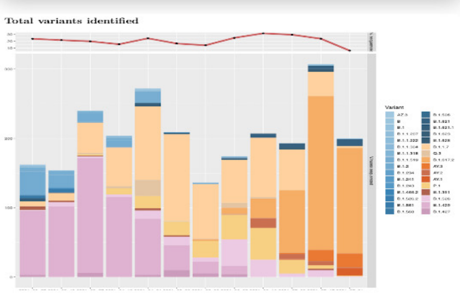

Highlights the prevalent variants in the state and in each of the counties.
Source: DOH Disease Outbreak Control Division
Updated weekly

Vaccinations

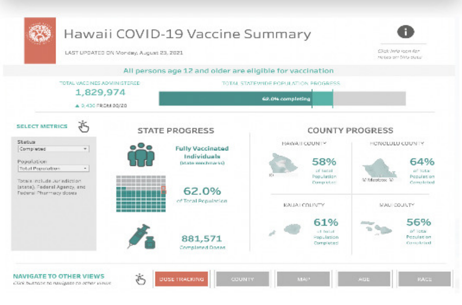

Vaccination rate by county, zip code, age and race.
Source: DOH Disease Outbreak Control Division
Updated daily

Outcomes

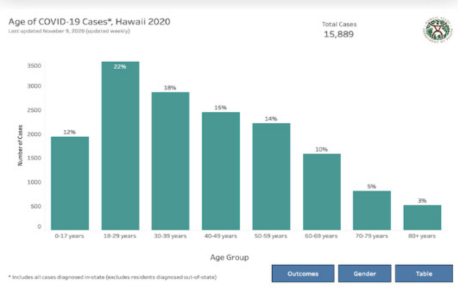

Cases by age group and gender, as well as deaths and hospitalizations by gender.
Source: DOH Disease Outbreak Control Division
Updated weekly

Age Trends

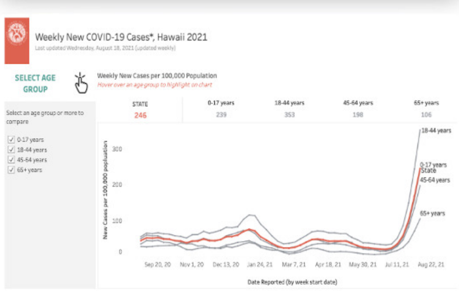

Weekly trends of new cases per 100,000 population by age group.
Source: DOH Disease Outbreak Control Division
Updated weekly

Race Trends

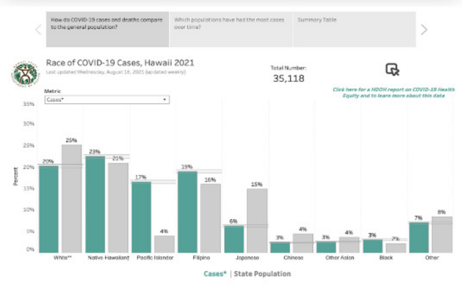

Breakdown of cases by race as it compares to the State's general population.
Source: DOH Disease Outbreak Control Division
Updated weekly

Mask Use

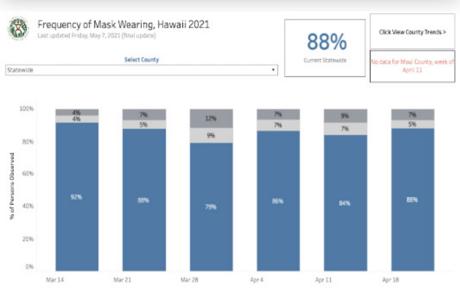

Percentage of people by county observed following mask wearing recommendations.
Source: DOH Disease Outbreak Control Division
Updated weekly

Risk Factor

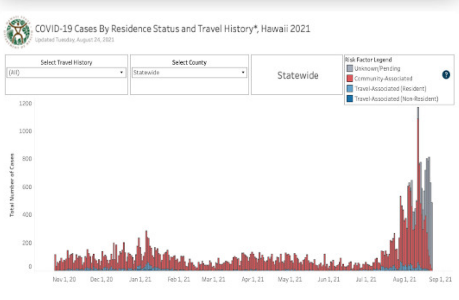

Number of COVID-19 cases over time by risk factor, statewide and by county.
Source: DOH Disease Outbreak Control Division
Updated daily

Case Investigation

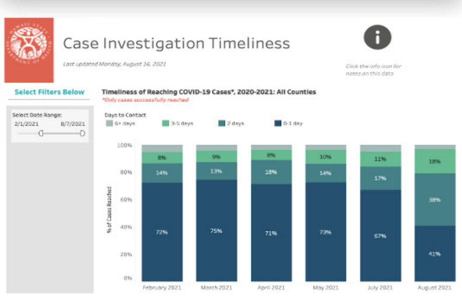

Percent of reported cases ever reached and contact tracing barriers and timeliness.
Source: DOH Disease Outbreak Control Division
Updated weekly

Long-Term Care Facilities

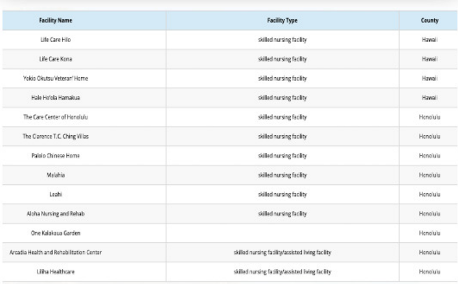

Long-term care facilities reporting cases in the past 14 days.
Source: DOH Disease Outbreak Control Division
Updated weekly

Cluster Reports

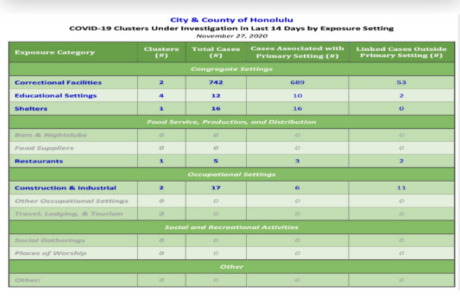

Summary of identified COVID-19 clusters that are actively under investigation.
Source: DOH Disease Outbreak Control Division
Updated weekly

BHHSURG PPE

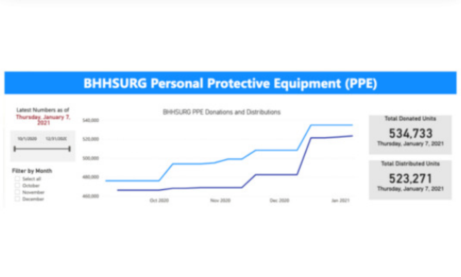

Summary of personal protective equipment donated to BHHSURG and distributed.
Source: DOH Behavioral Health & Homelessness Statewide Unified Response Group
Updated monthly

Isolation & Quarantine

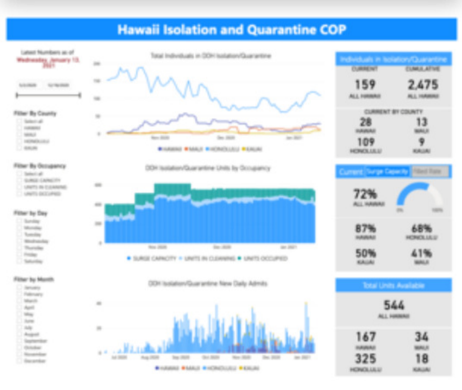

Individuals in DOH Isolation/Quarantine, statewide and by county.
Source: DOH Behavioral Health & Homelessness Statewide Unified Response Group
Updated weekly

Hawaii CARES

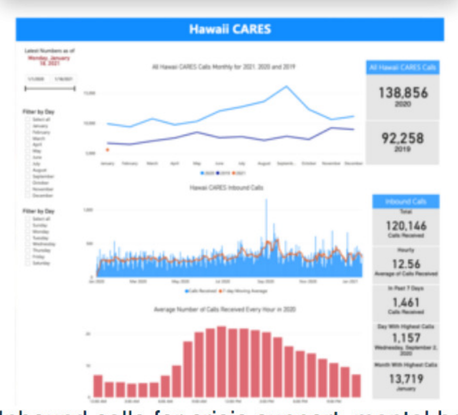

Inbound calls for crisis support, mental health resources, and substance use treatment services.
Source: DOH Behavioral Health & Homelessness Statewide Unified Response Group
Updated weekly

Economic Recovery Data

UHERO Economic Pulse

Passenger Arrivals

Unemployment Claims

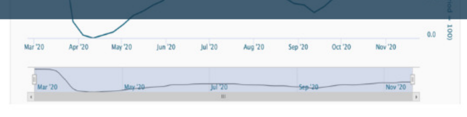

Indicates the extent of recovery from the trough of the COVID-recession.
Source: University of Hawaii Economic Research Organization
Updated weekly

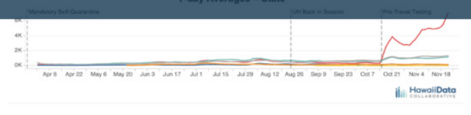

Daily counts of passenger arrivals by air, broken down by reason for travel.
Source: Hawaii Tourism Authority
Updated daily

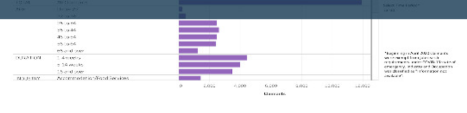

Unemployment insurance initial claims and weeks claimed, and claimant characteristics.
Source: Department of Labor & Industrial Relations
Updated weekly

Community Mobility

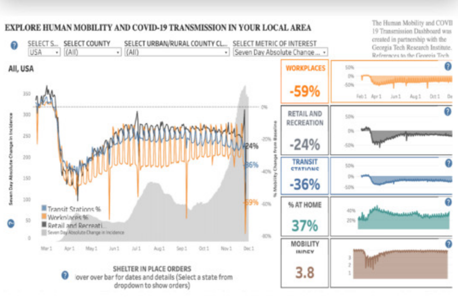

State and county trends of change in visits to different categories of places. Select "HI" from the state drop-down menu.
Source: Dashboard created by CDC, using data from Google's Community Mobility Reports
Updated weekly

Tourism

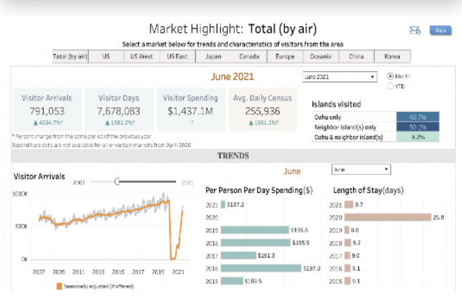

Visitor indicators, including length of stay, spending, and other trip characteristics.
Source: Department of Business, Economic Development & Tourism
Updated monthly

Safe Travels Program

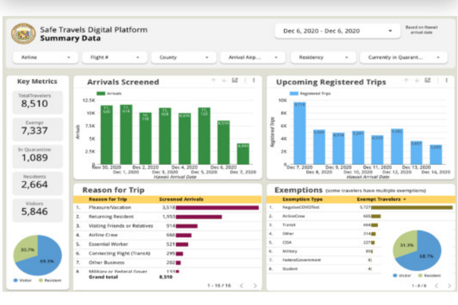

Summary data on incoming travelers collected through the Safe Travels Program.
Source: State of Hawaii
Updated daily

+tableau+public
DISCOVER BLOG RESOURCES ABOUT SIGN UP SIGN IN

DPH Idaho COVID-19 Dashboard by Idaho Division of Public Health

Statewide Cases*

311,521 Total Statewide Cases

(Daily Update: +371)

Trend *(Number of Cumulative Confirmed and Probable Cases)*

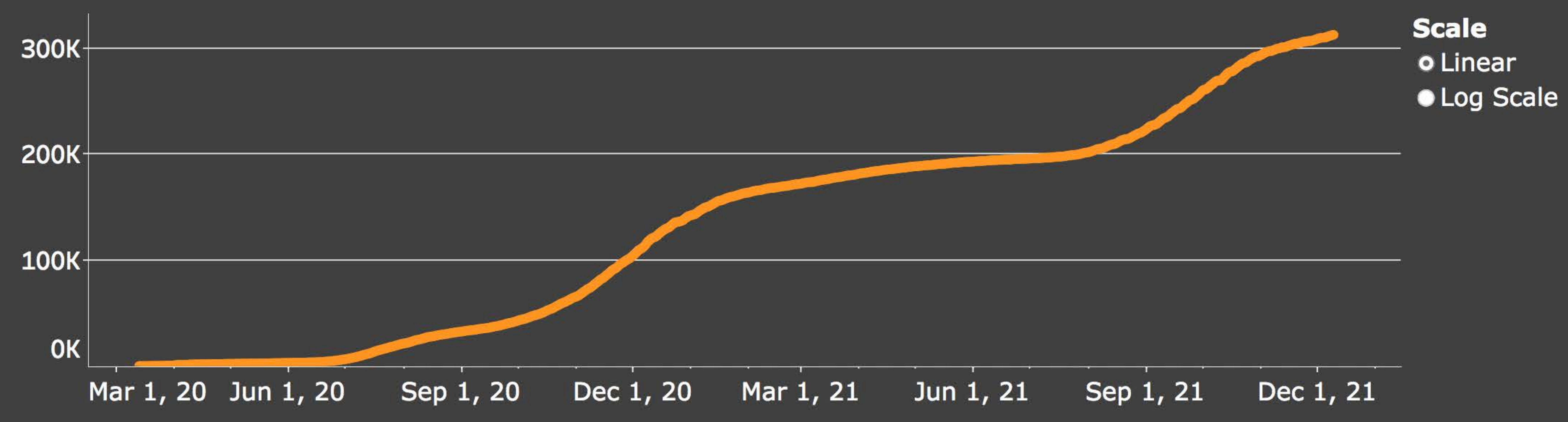

Daily Cases *(by Date of Official State Notification)*

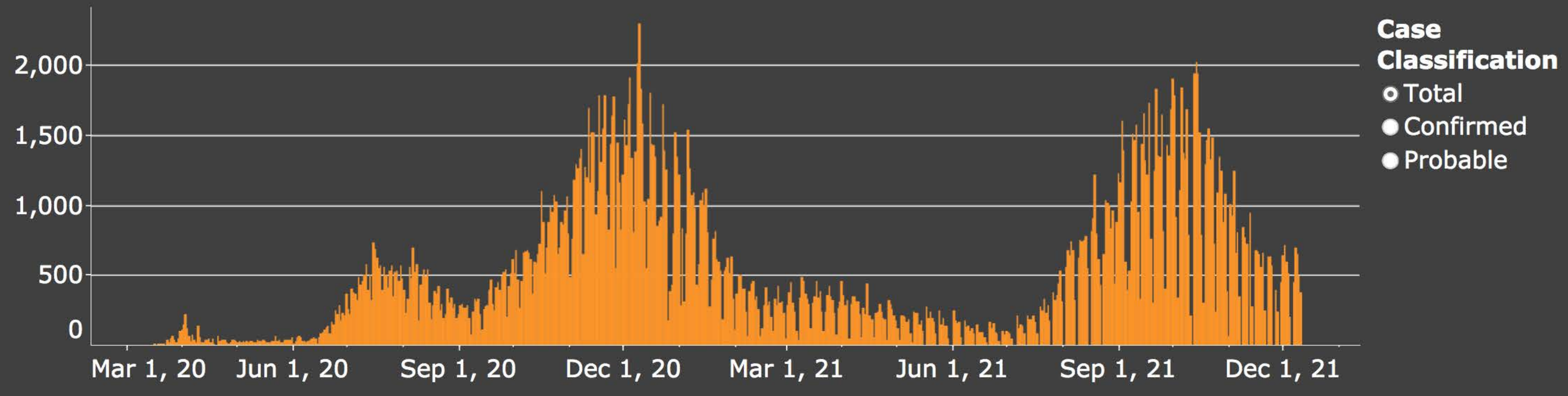

Onset Date of Cases

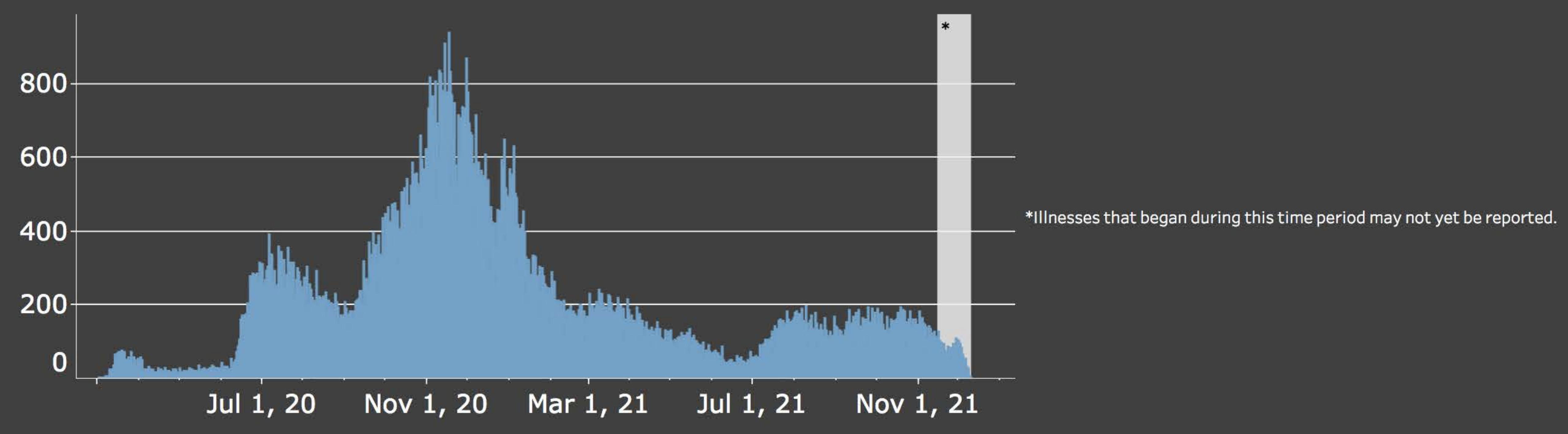

*For definitions of confirmed and probable cases, as well as details on the data release schedule, please visit the Notes page at the top right

IDAHO DEPARTMENT OF HEALTH & WELFARE

+tableau

Details

34 8,073,675

DPH Idaho COVID-19 Dashboard

Published: Sep 3, 2020 **Updated:** Dec 10, 2021

+tableau
© 2021 Tableau Software, LLC, a Salesforce Company. All Rights Reserved.

Products: Tableau Desktop, Tableau Server, Tableau Online, Tableau Prep, Tableau Public Free
Legal: Privacy Policy, Data Policy, Terms of Service, Patents & IP, Do Not Sell My Personal Information
About: What is Tableau Public?, Current Status, FAQ, Careers, Contact Us

English

+tableau public DISCOVER BLOG RESOURCES ABOUT SIGN UP SIGN IN

DPH Idaho COVID-19 Dashboard by Idaho Division of Public Health

Cases by County*

Select a county from the dropdown to view a trend of the daily 7-day moving average incidence rate per 100,000 population

Statewide

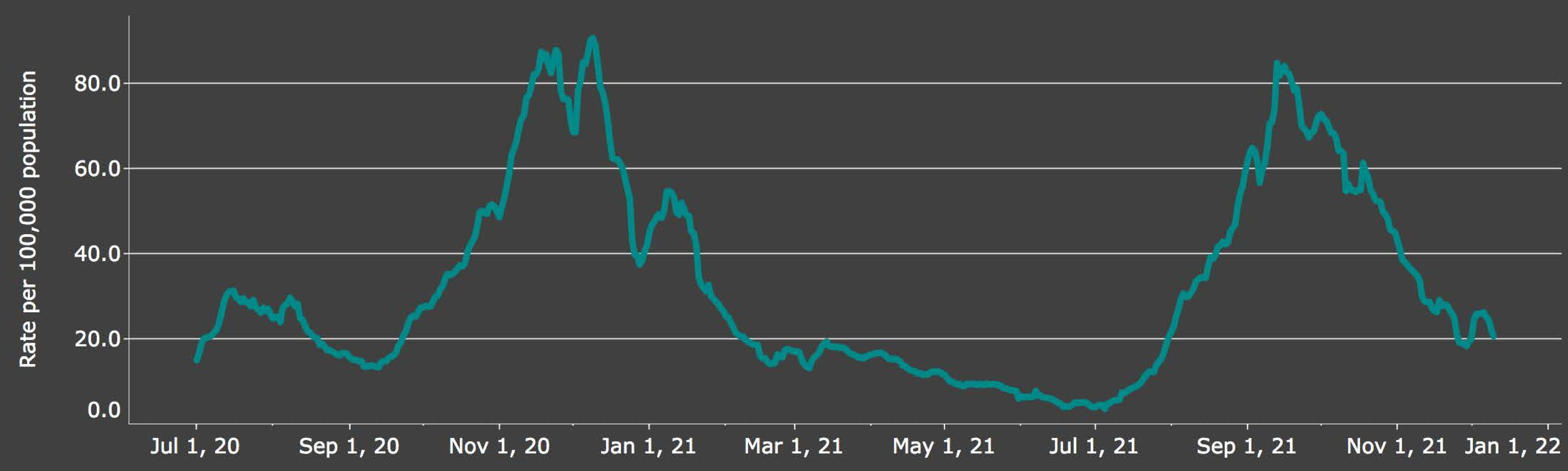

County	Confirmed	Probable	Total Cases	Cumulative Incidence Rate	Daily 7-Day Moving Average Incidence Rate	Deaths
Ada	74,590	12,073	86,663	17,529.0	18.6	847
Adams	443	113	556	12,502.8	10.0	14
Bannock	8,770	5,663	14,433	16,254.3	19.5	207
Bear Lake	534	207	741	12,062.5	4.7	15
Benewah	961	647	1,608	17,052.0	83.0	29
Bingham	3,947	3,334	7,281	15,425.2	16.8	130
Blaine	2,873	365	3,238	13,822.2	14.3	27
Boise	581	246	827	10,254.2	10.9	11
Bonner	5,456	1,163	6,619	14,138.0	30.6	138
Bonneville	15,082	6,823	21,905	17,935.2	22.0	242
Boundary	1,252	472	1,724	13,622.0	12.8	48
Butte	268	119	387	14,625.9	5.5	5
Camas	83	25	108	9,557.5	0.0	1
Canyon	38,182	5,822	44,004	18,562.9	16.7	593
Caribou	392	680	1,072	15,049.8	20.0	23
Cassia	3,128	896	4,024	16,575.4	16.6	43
Clark	63	27	90	10,563.4	16.9	0
Clearwater	1,010	722	1,732	19,579.5	65.3	27
Custer	265	141	406	9,555.2	13.2	6
Elmore	3,640	776	4,416	16,088.6	11.9	52
Franklin	1,152	633	1,785	12,557.2	18.5	26
Fremont	1,355	325	1,680	12,709.9	48.0	18
Gem	2,146	776	2,922	15,623.2	8.7	69
Gooding	1,504	876	2,380	15,238.8	12.2	44
Idaho	1,651	692	2,343	13,927.4	24.0	39
Jefferson	2,992	1,539	4,531	14,816.4	33.5	51
Jerome	2,929	1,497	4,426	18,008.0	20.5	55
Kootenai	27,313	4,065	31,378	18,389.7	25.9	461
Latah	4,459	342	4,801	11,758.5	17.5	41
Lemhi	620	363	983	12,205.1	28.5	21
Lewis	555	290	845	22,016.7	29.8	23
Lincoln	515	246	761	14,203.1	21.3	14
Madison	7,865	1,345	9,210	22,843.4	29.4	42
Minidoka	2,366	897	3,263	15,379.9	4.8	52
Nez Perce	5,615	860	6,475	15,887.6	22.3	105
Oneida	380	215	595	13,163.7	9.5	10
Owyhee	1,517	200	1,717	14,151.5	10.9	41
Payette	3,261	1,021	4,282	17,286.3	14.3	72
Power	635	479	1,114	14,575.4	9.3	17
Shoshone	1,919	270	2,189	16,954.5	47.7	66
Teton	912	858	1,770	14,158.9	37.6	7
Twin Falls	11,323	5,382	16,705	18,894.7	16.1	236
Valley	1,244	356	1,600	13,568.5	18.8	13
Washington	1,281	649	1,930	18,629.3	11.2	47

Cases by Date of Onset and Public Health District

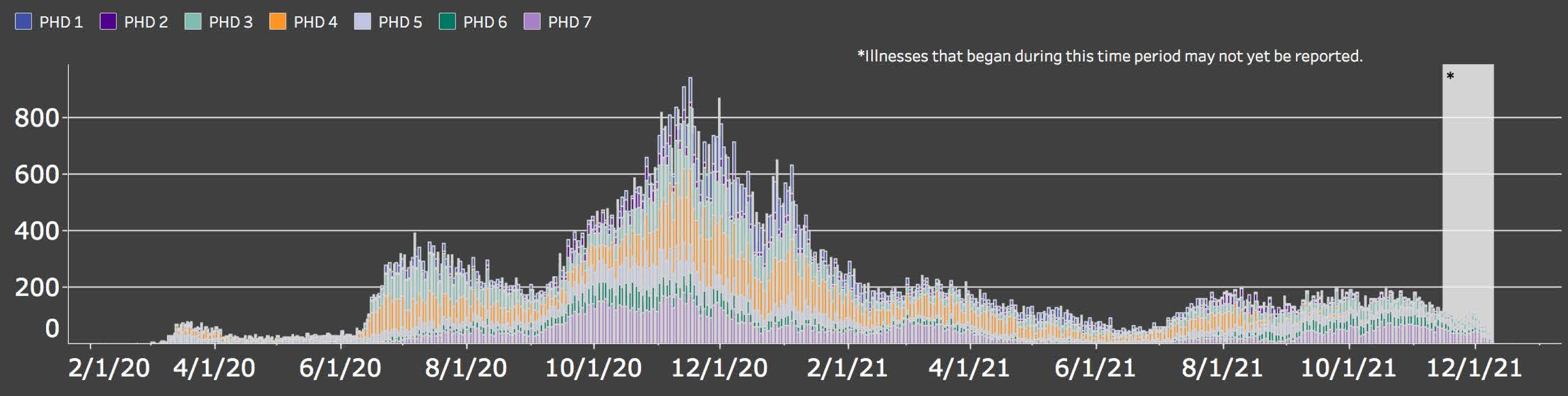

*For notes on county cases from the above visualizations, please visit the Notes page at the top right.

IDAHO DEPARTMENT OF HEALTH & WELFARE

+tableau

Details

34 8,073,675

DPH Idaho COVID-19 Dashboard

Published: Sep 3, 2020 **Updated:** Dec 10, 2021

+tableau+public DISCOVER BLOG RESOURCES ABOUT SIGN UP SIGN IN

DPH Idaho COVID-19 Dashboard by Idaho Division of Public Health

Laboratory Testing for SARS-CoV-2*

PCR/NAAT Tests — **2,339,107 Tests Performed** — **1,065,891 Idahoans Tested**

PCR/NAAT Tests Performed and Percent Positivity by Week of Specimen Collection

Select Jurisdiction: State

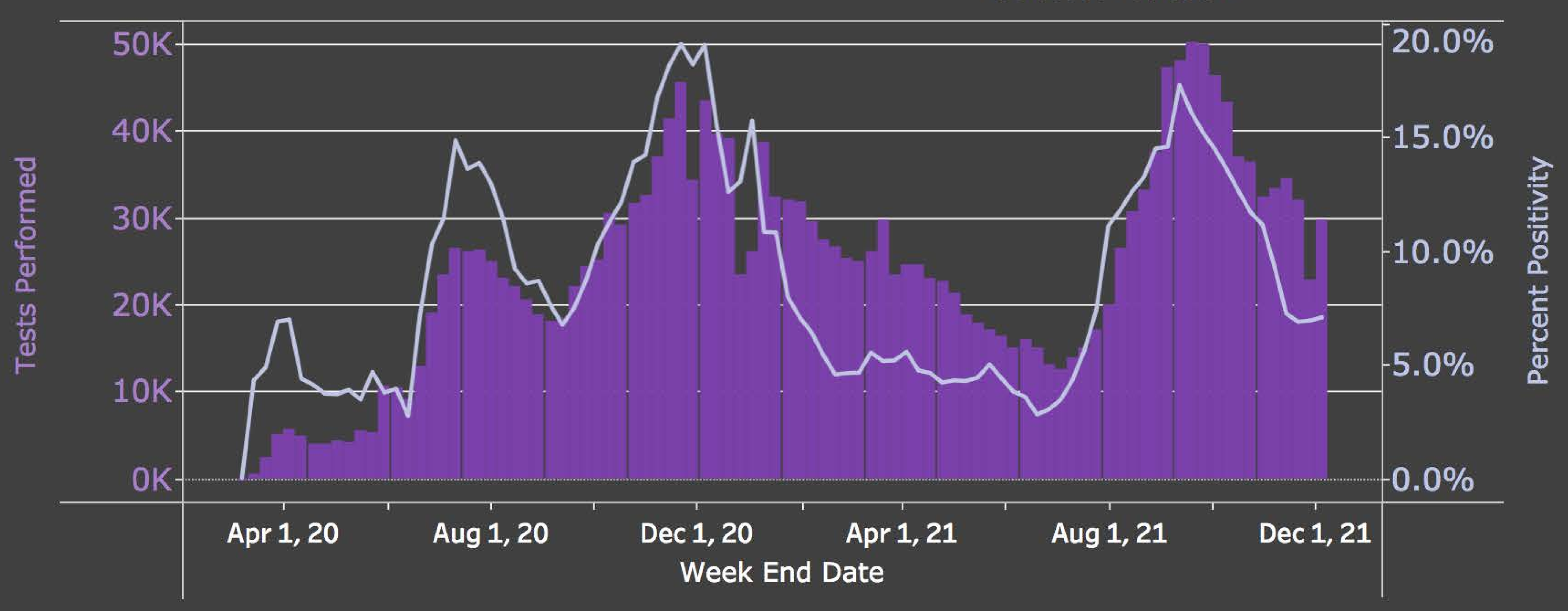

Variants of Concern and Interest

Variant of Concern or Interest Identified by County

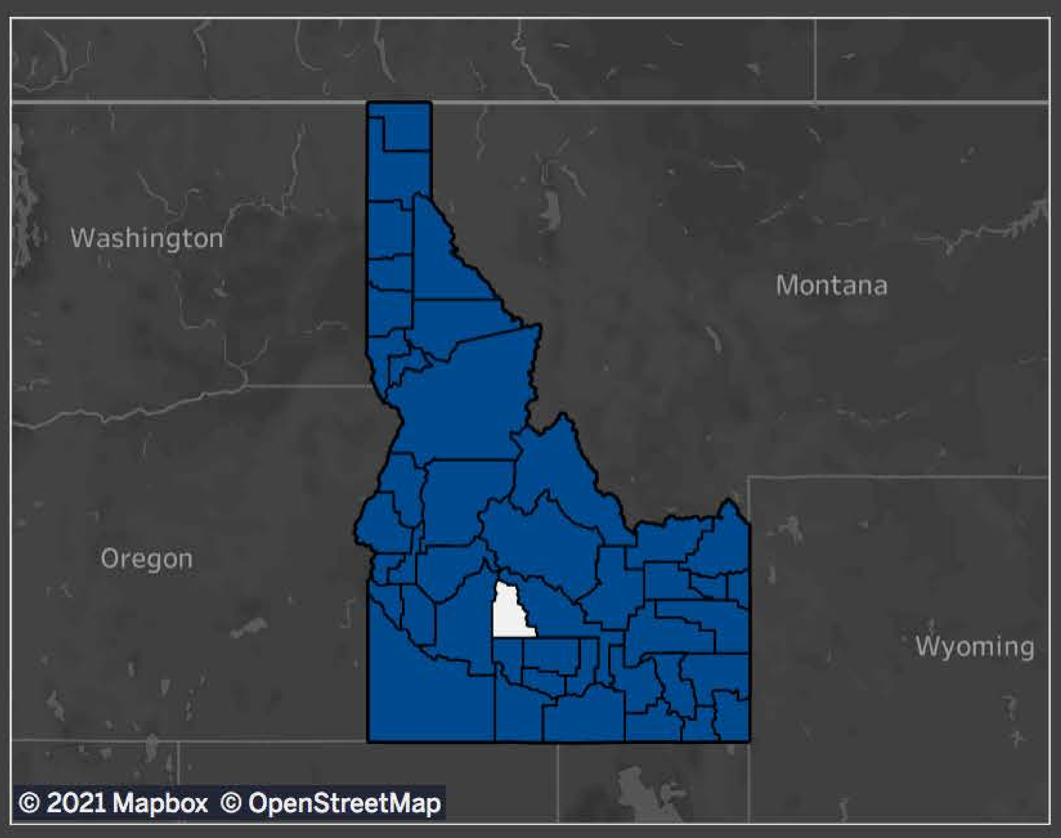

To view number of variants by county, click on the county in the map and refer to the chart below. To go back to statewide data click anywhere on the map outside Idaho.

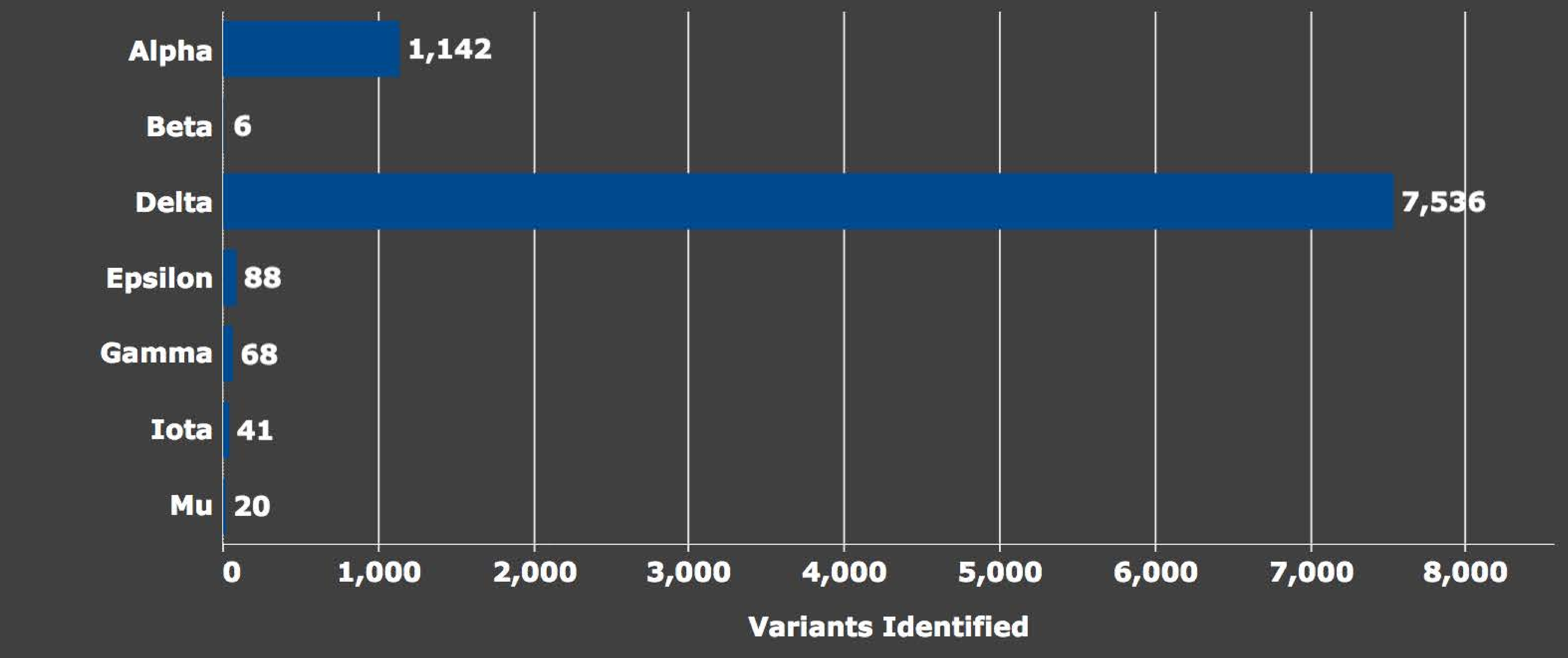

Proportion of Variants Identified by Specimen Collection Date

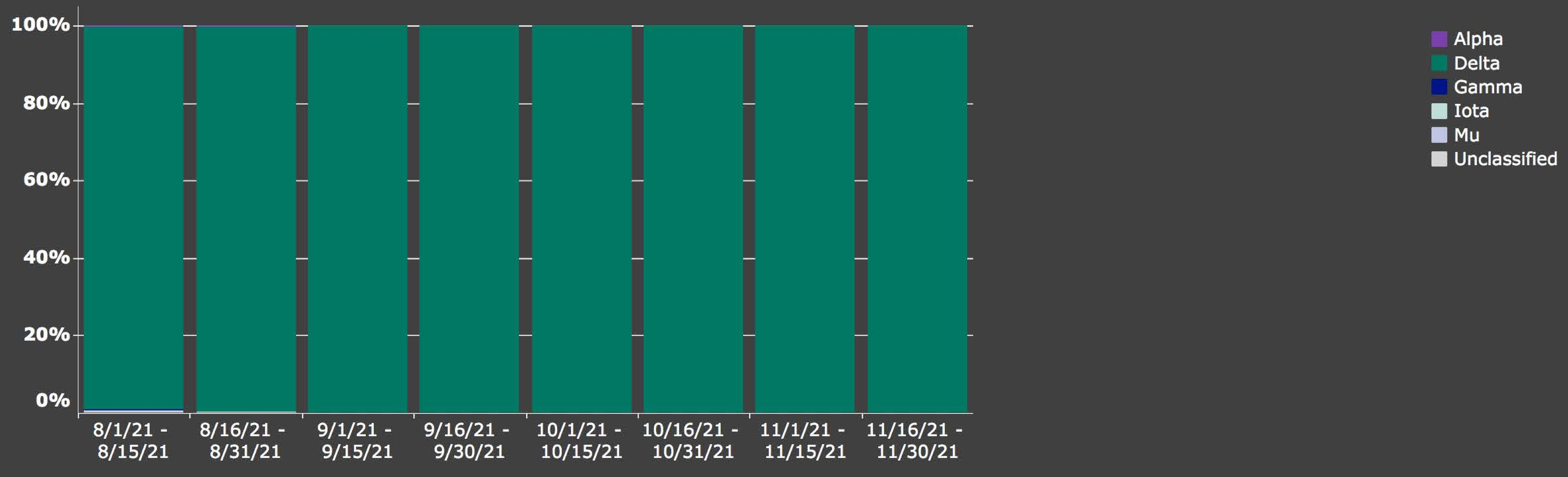

Antigen Tests — **417,280 Tests Performed**

Number of Positive Antigen Tests Reported by Week of Specimen Collection

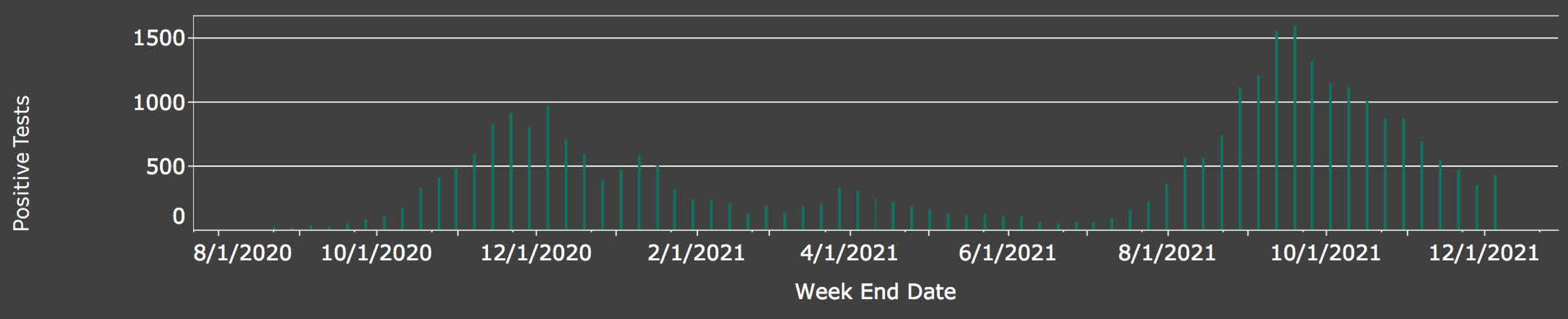

*For notes and details on variant data and testing calculations, please visit the Notes page at top right.

IDAHO DEPARTMENT OF HEALTH & WELFARE

+tableau

Details

34 8,073,675

DPH Idaho COVID-19 Dashboard

Published: Sep 3, 2020 **Updated:** Dec 10, 2021

DPH Idaho COVID-19 Dashboard by Idaho Division of Public Health

Demographics of COVID-19 Related Deaths*

Idaho Resident Deaths: 4,028 *(3,324 Confirmed Cases, 704 Probable Cases)*
220.5 Deaths per 100,000 Population

Age Group

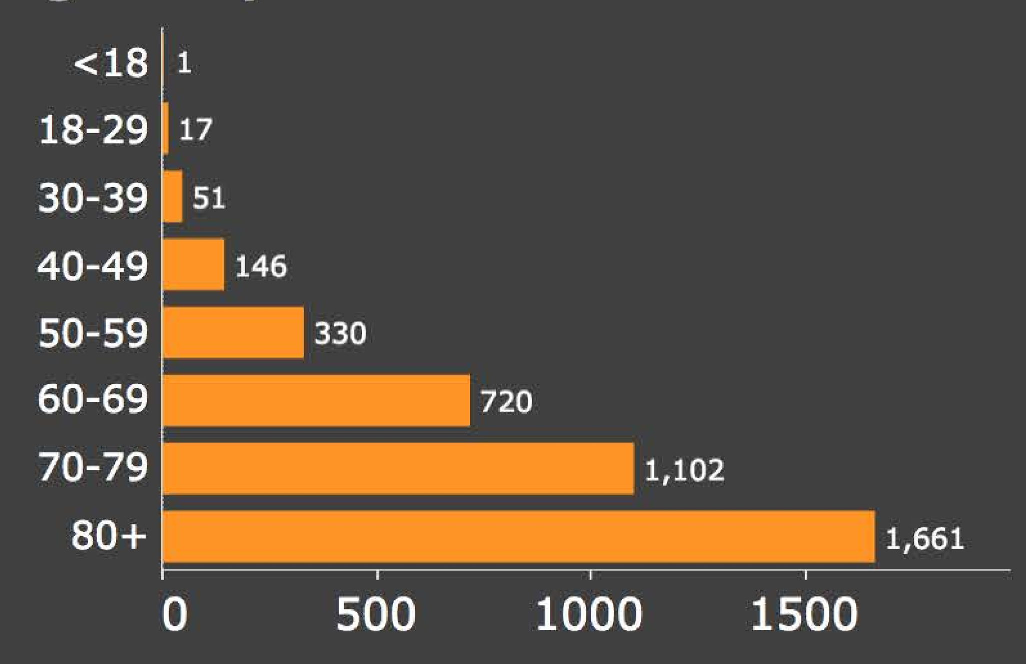

Mean Age

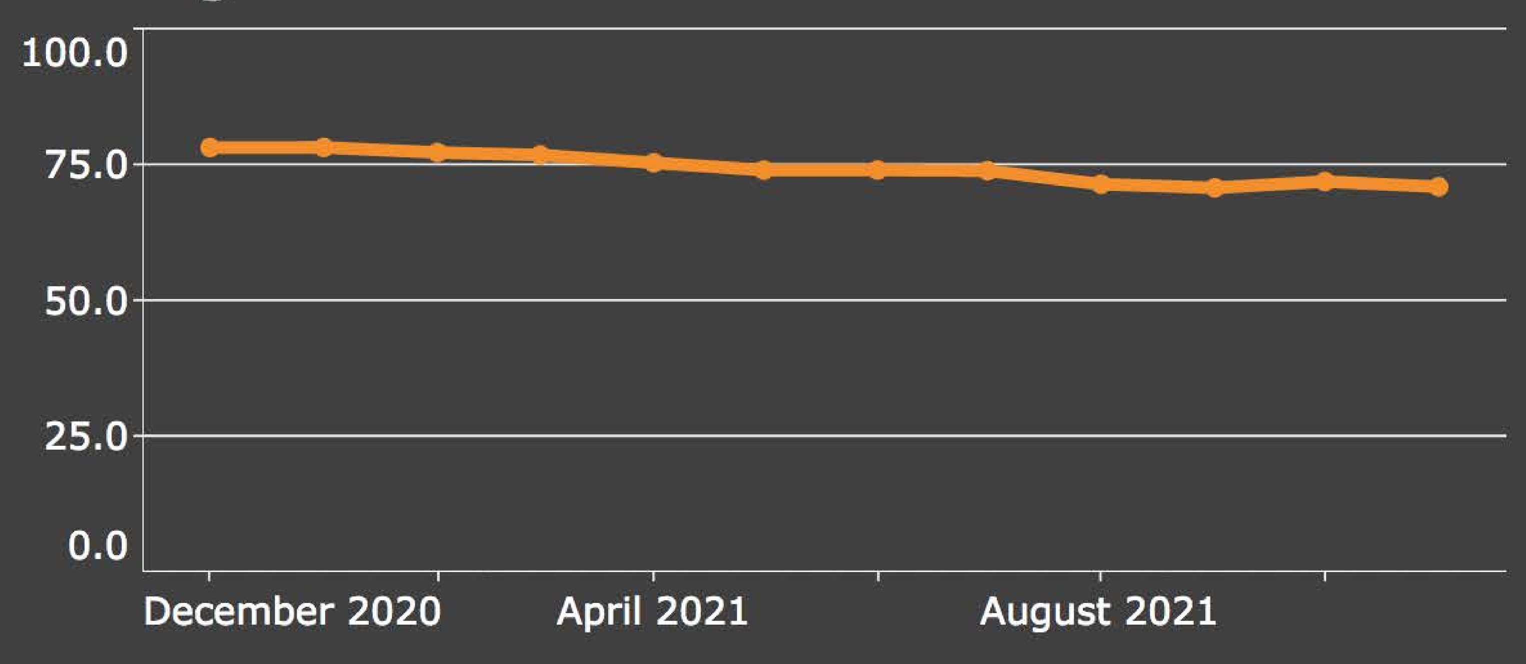

Sex

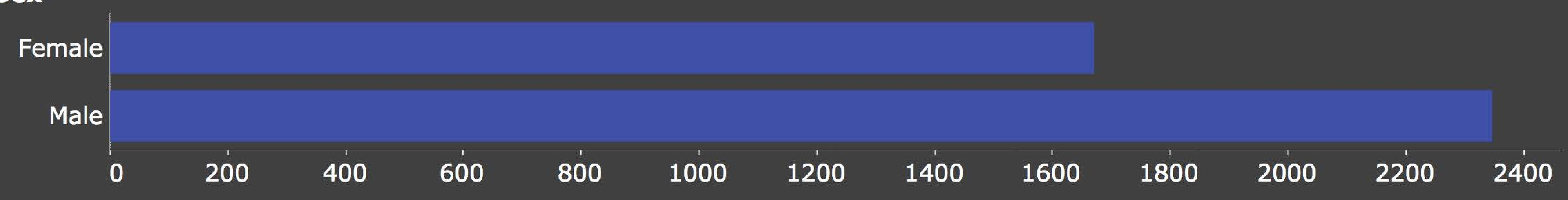

Race

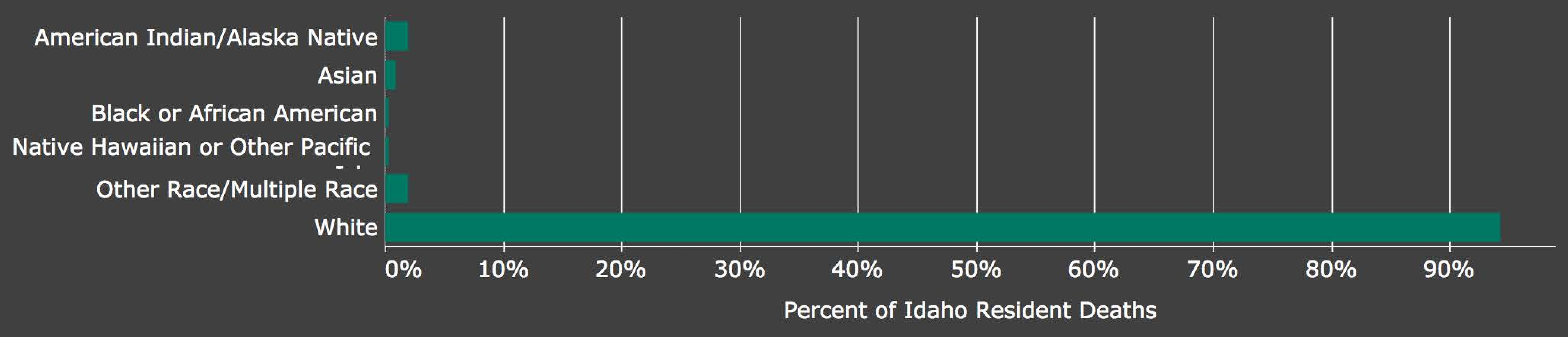

The race category for 10 deaths is pending

Ethnicity

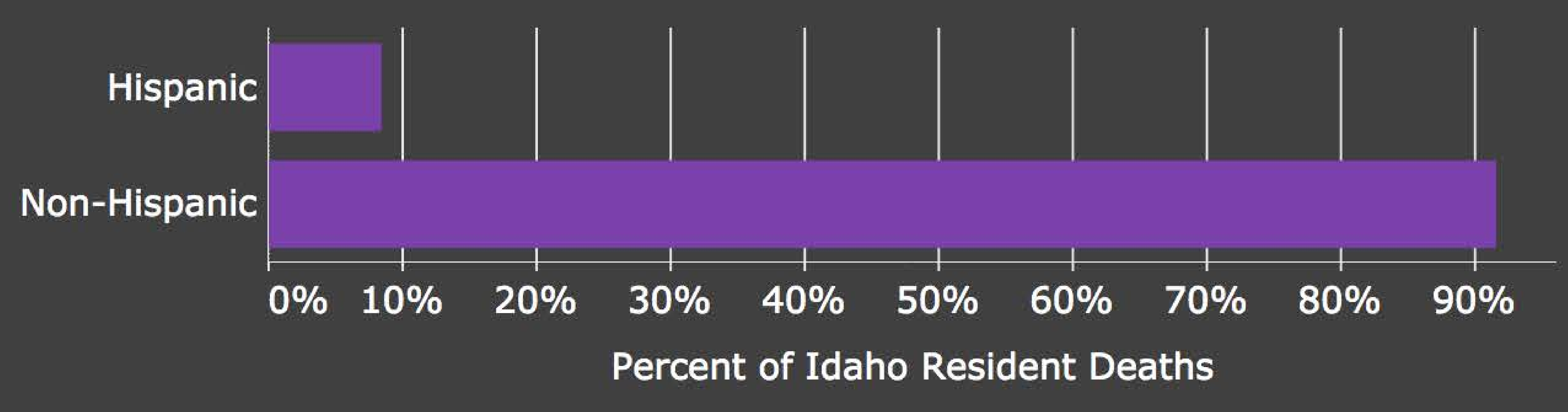

The ethnicity category for 6 deaths is pending

*For definitions, please visit the Notes page at the top right.

IDAHO DEPARTMENT OF HEALTH & WELFARE

+tableau

Details

34 8,073,675

DPH Idaho COVID-19 Dashboard

Published: Sep 3, 2020 **Updated:** Dec 10, 2021

COVID-19 Related Deaths Among Idaho Residents*

Idaho Resident Deaths: 4,028 *(3,324 Confirmed Cases, 704 Probable Cases)*
220.5 Deaths per 100,000 Population

COVID-19 Related Deaths by Public Health District

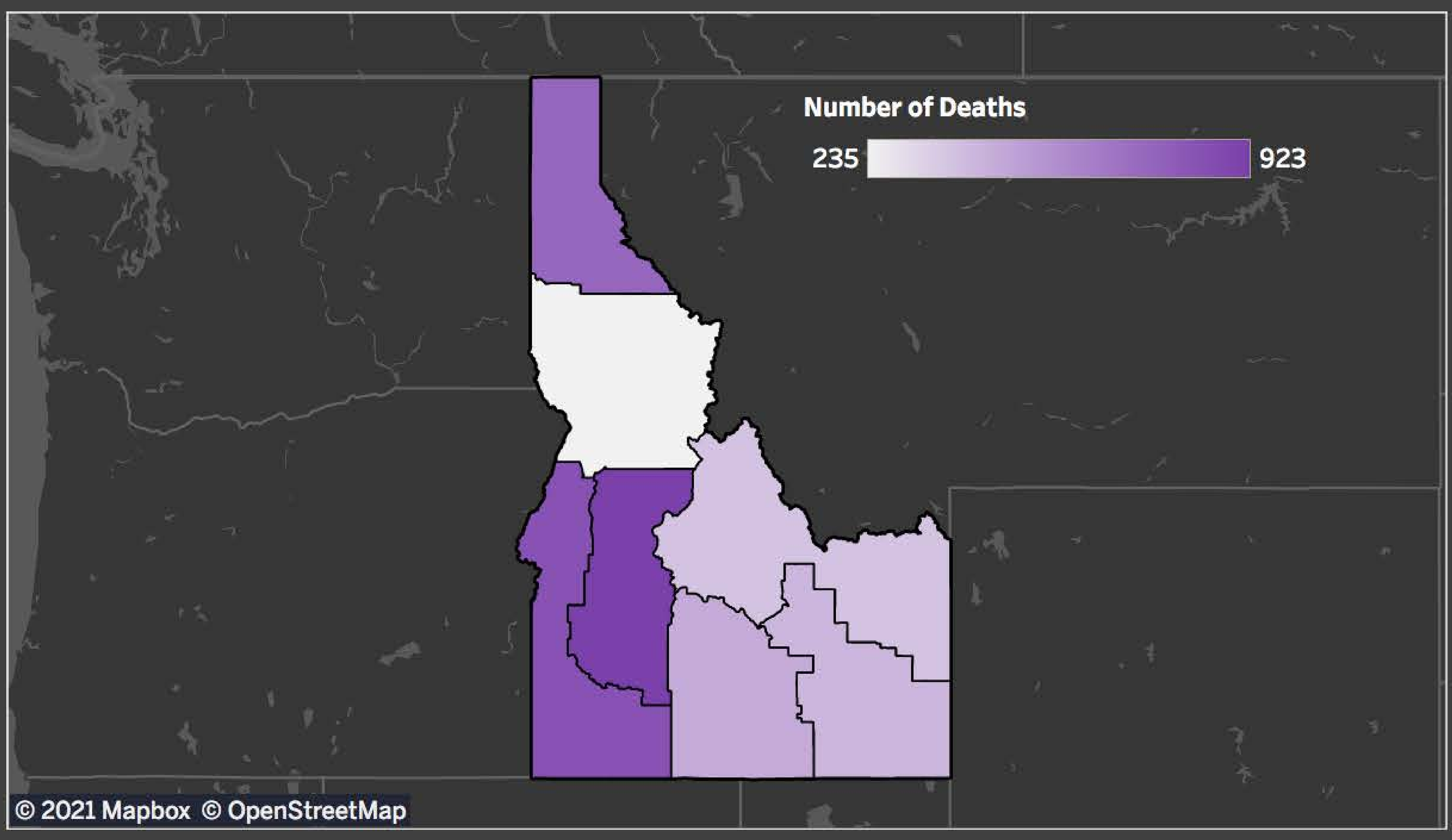

COVID-19 Related Deaths by County

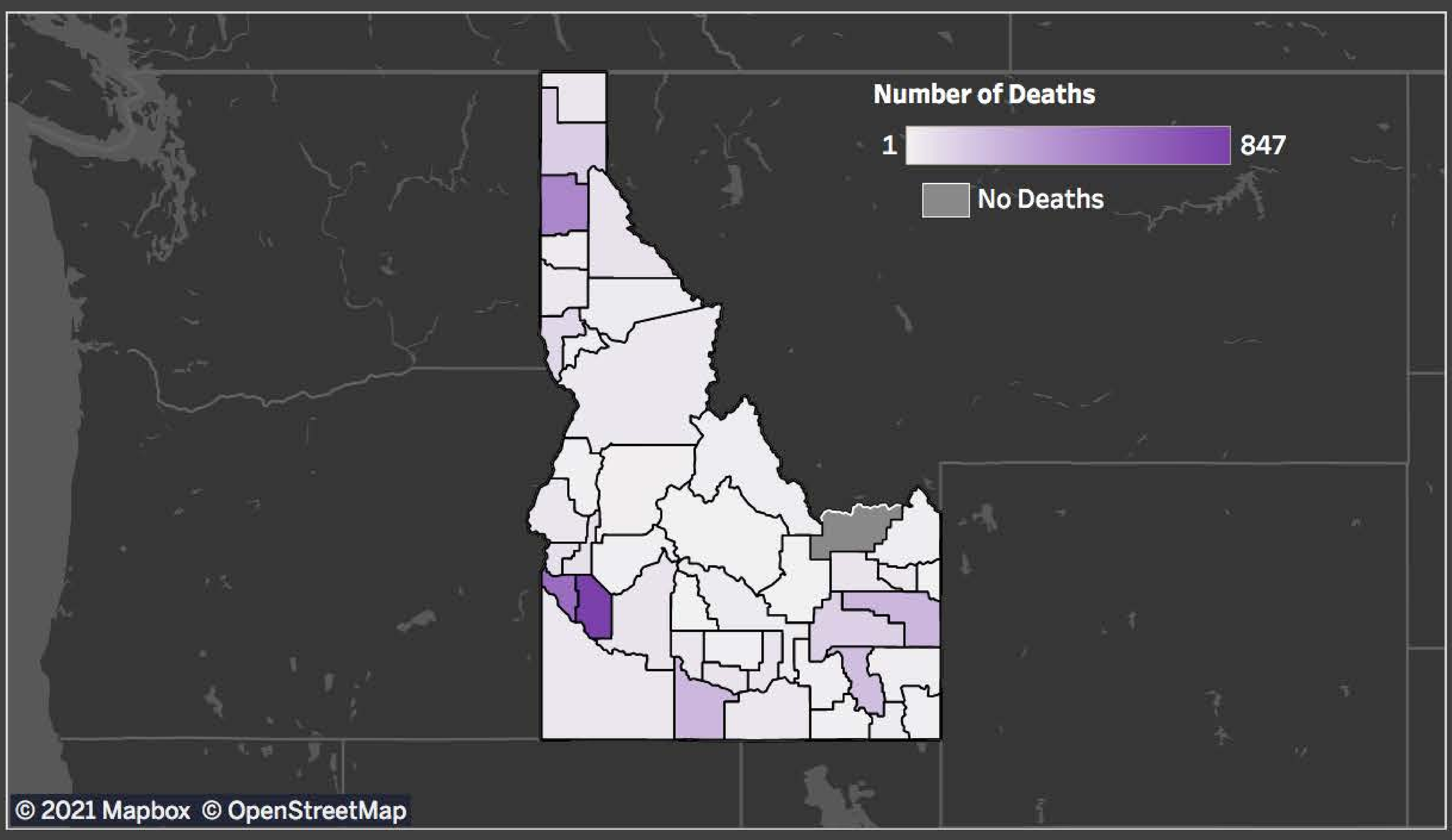

COVID-19-Related Deaths Among Idaho Residents by Date of Death

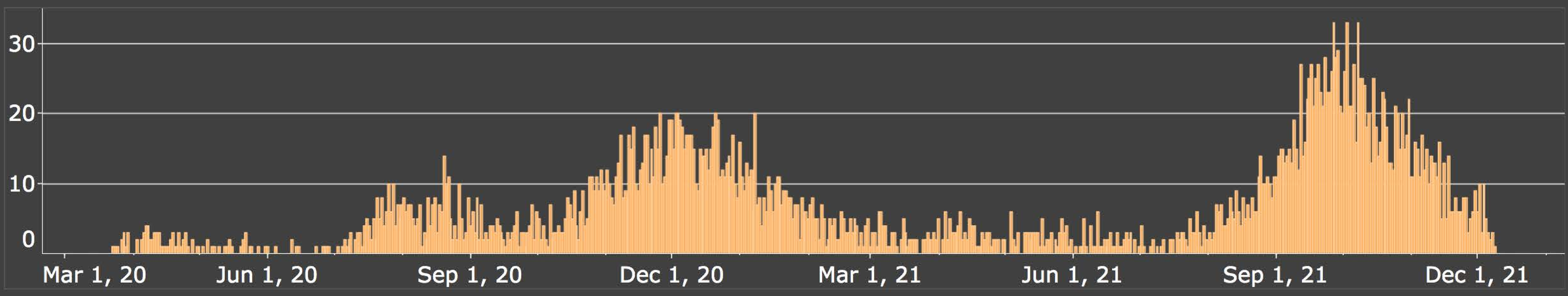

*For definitions and notes, please visit the Notes page at the top right.

IDAHO DEPARTMENT OF HEALTH & WELFARE

Details

34 8,073,675

DPH Idaho COVID-19 Dashboard

Published: Sep 3, 2020 **Updated:** Dec 10, 2021

Illinois Data
Statewide Metrics
Region Metrics
Contact Tracing
County Level Risk Metrics
Hospitalization Utilization
Syndromic Surveillance
Long-Term Care Data
Long-Term Care Facility Data
Community Transmission
Data Portal
Travel
Variant Proportions
Variants
School and Youth Data

Statewide Metrics

IDPH continues to monitor key indicators to identify early increases of virus transmission in Illinois, potentially signifying resurgence. These indicators are calculated for the entire state. IDPH will monitor if these indicators show an increase in the COVID-19 disease burden with a simultaneous decrease in hospital capacity.

Indicators are calculated daily for Illinois based on the most current, complete data. Each indicator is monitored for the trend over 28 days to evaluate the COVID-19 disease burden and hospital resource capacity. By applying the same metrics across Illinois, IDPH uses a standardized approach to monitor the state as a whole.

On June 11, 2021, Illinois moved into Phase 5 of the Restore Illinois Plan. IDPH will continue to track the vaccinations rates and hospital capacity metrics, and cases over 28-day monitoring periods. New evidence concerning variants, vaccine effectiveness, or waning immunity could substantially and quickly increase COVID-19 hospitalizations and will be taken into consideration, in addition to the metrics being monitored.

Data are provisional and are updated daily. Data Updated: 12/10/2021

New Hospital Admissions for COVID-19-like-Illness

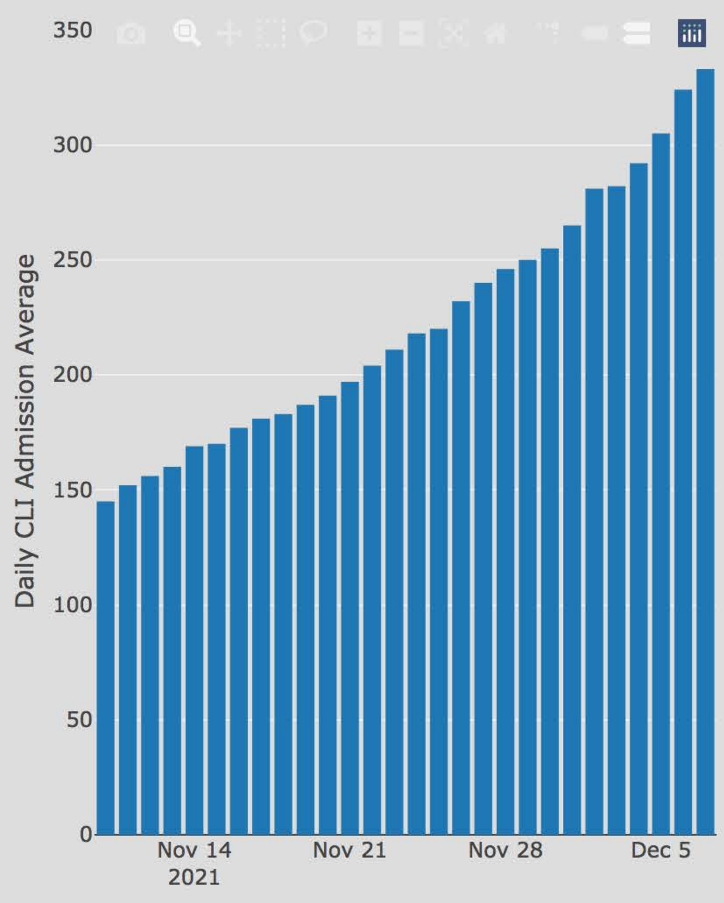

Hospitalized Patients with COVID-19

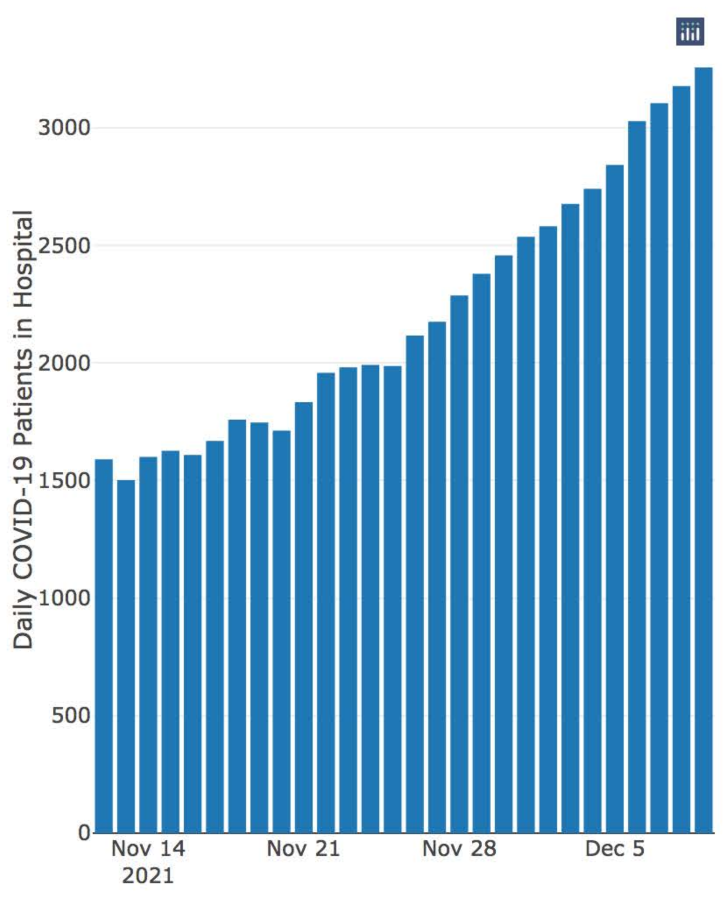

Average Available and Staffed ICU Beds

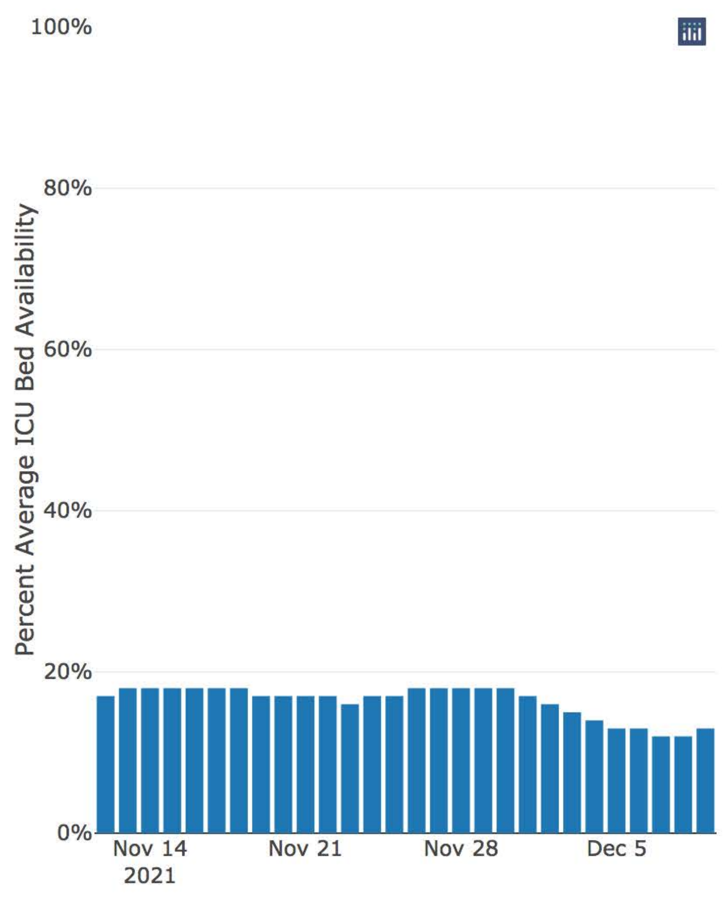

COVID-19 Daily Mortality

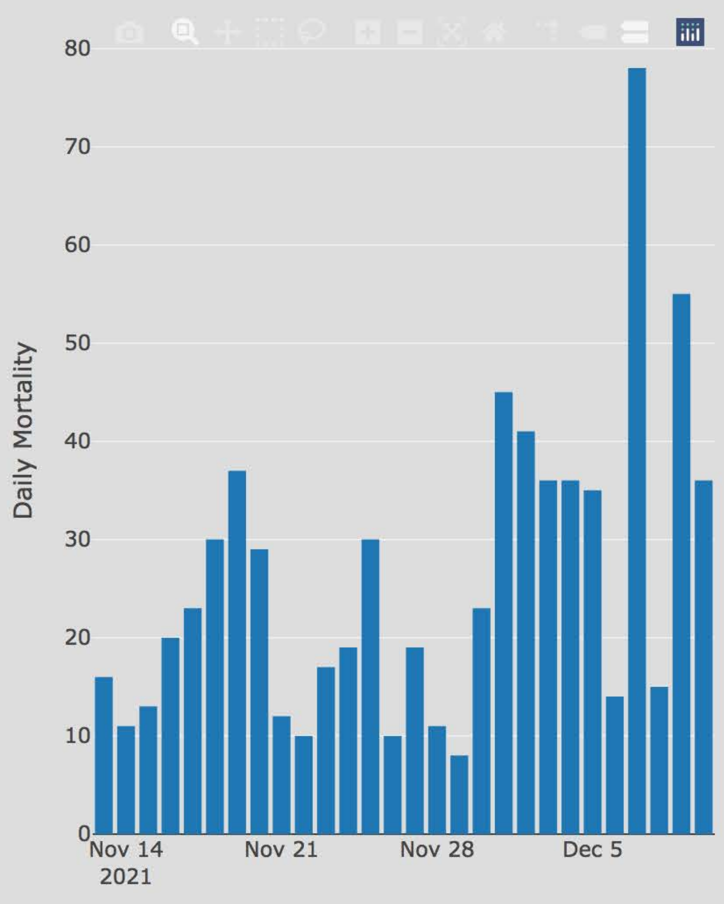

COVID-19 Daily Cases

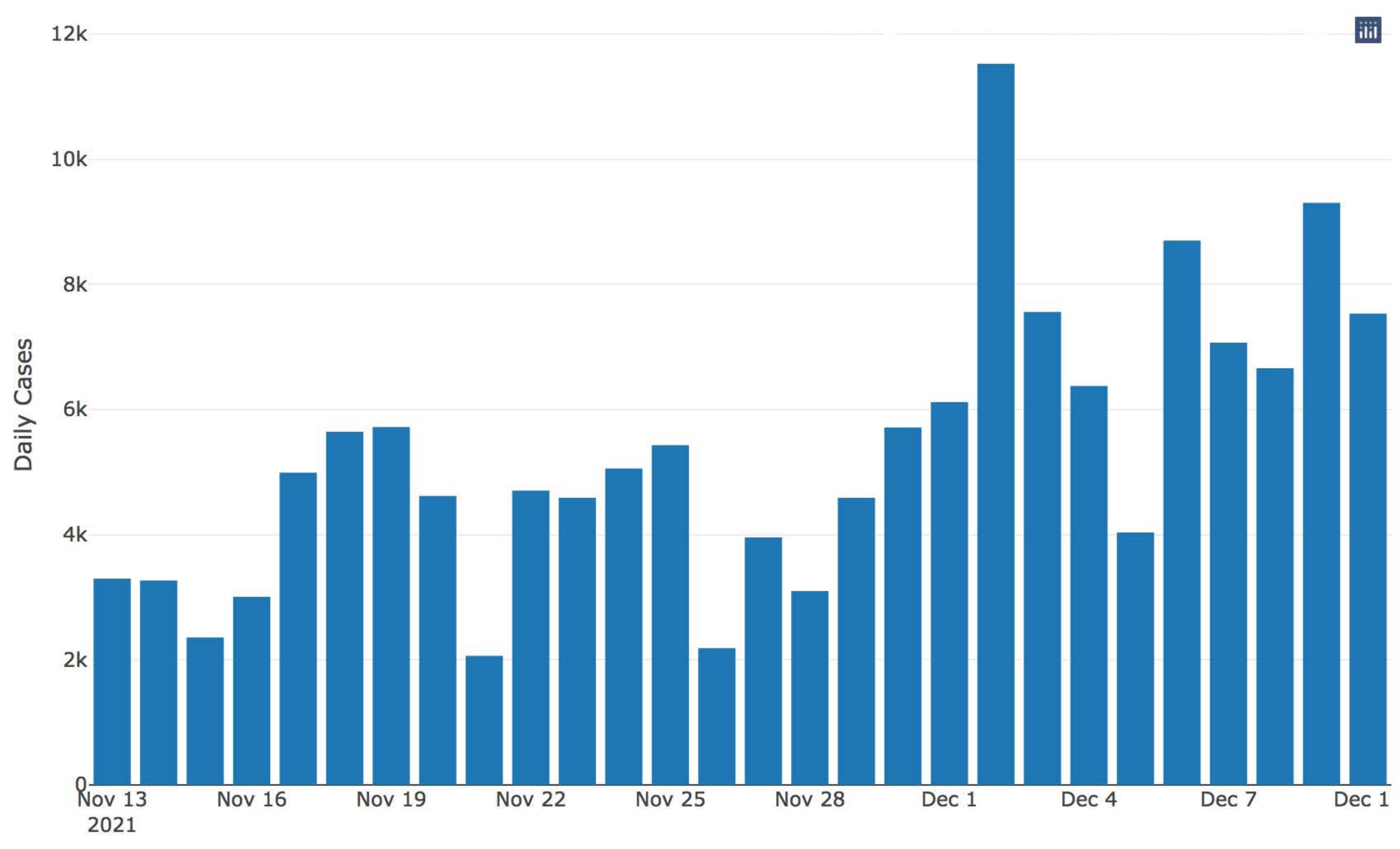

The following vaccination data are pulled from I-CARE and do not reflect federal efforts to vaccinate Illinoisans. CDC reports the total number of people who reside in Illinois who have been vaccinated, including Illinois residents who have been vaccinated in other states.

Illinois Population Vaccinated With At Least 1 Dose

At Least 1 Dose | Fully Vaccinated

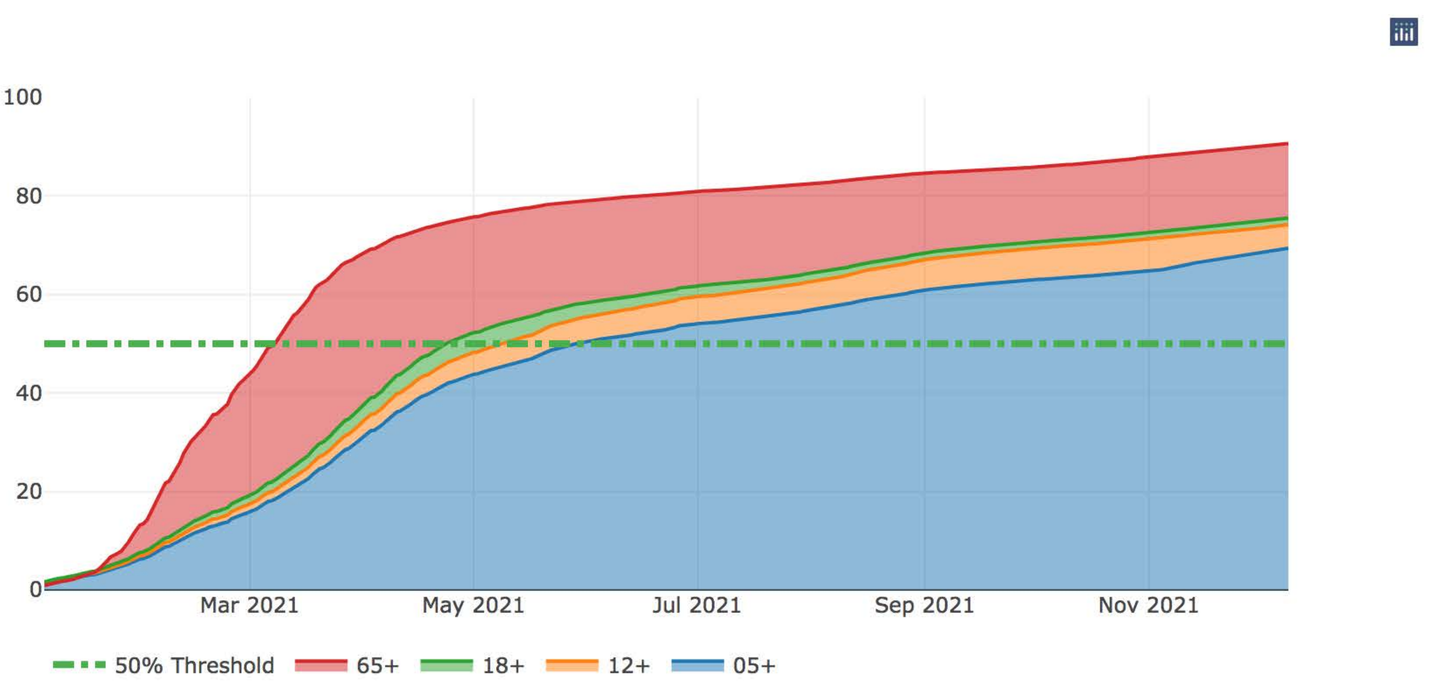

Methodology

Vaccination Rate (First Dose)

Vaccine administrations are reported to the Illinois-Comprehensive Automated Immunization Registry Exchange (I-CARE). Administered vaccines are reported based on the date IDPH receives the vaccination record.

Calculation: [Total First Dose of Vaccine Administered in persons 65+] / [population 65+]
Population 65+ = 1,992,961

COVID-19-like-Illness (CLI) Admissions

COVID-19-like-Illness (CLI) new hospital admissions are reported to the Illinois Syndromic Surveillance System. Visits are defined as CLI if the patient presents with a fever AND either cough, difficulty breathing or is given a diagnosis of COVID-19. Data is provisional, and a three-day lag after admission is used to allow time for IDPH to receive more complete data before analysis is performed.

Hospital Beds Occupied by a COVID-19 Patient

The number of COVID-19 patients in hospital is reported daily by each hospital to IDPH EMResouces and represents the daily total number of hospital beds occupied by a suspected or confirmed COVID-19 patient.

Number of Deaths

Deaths of lab-confirmed cases of COVID-19 are reported in the Illinois-National Electronic Disease Surveillance System (I-NEDSS) as the underlying cause of death or the contributing

Intensive Care Unit (ICU) Beds Percent Availability

Intensive Care Unit (ICU) beds availability is reported daily by each hospital to IDPH EMResources and represents the number of ICU hospital beds that are empty.

Calculation: ICU availability = [Sum of ICU beds available for 7 days] / [Total ICU beds for 7 days] x 100

Number of New Cases

COVID-19 cases are newly identified lab-confirmed cases of COVID-19 that are reported in the Illinois-National Electronic Disease Surveillance System (I-NEDSS).

Indiana COVID-19 Dashboard and Map

Indiana COVID-19 Data Report

Additional Resources

Dashboard | LTC | School

Below results are as of **12/09/2021, 11:59 PM**. Dashboard updated **by 5 p.m.** Monday through Friday.
New positive cases, deaths and tests have occurred over a range of dates but were reported to the state Department of Health in the last 24 hours.

Filters

Date Range: Last 30 days | Last 45 days | Last 60 days | Last 90 days

Newly Reported Confirmed COVID-19 Counts

New Tests Administered
47,606
04/24/2020 ... 12/09/2021
9,501 New Individuals Tested

New Positive Cases
5,315
12/02/2021 ... 12/09/2021

Positivity - All Tests
14.4 % 7-Day Rate
11/27/2021 ... 12/03/2021
8.8 % cumulative rate

New Deaths
49 Newly Reported Deaths
Date of death between 11/13/2021 ... 12/09/2021
7-Day Average - 29 Deaths

Total Confirmed COVID-19 Counts

Total Tests Administered
15,861,643
02/26/2020 ... 12/09/2021
4,605,271 Individuals Tested

Total Positive Cases
1,150,899
03/06/2020 ... 12/09/2021
6,363 Statewide Reinfection Cases since 9/1/2021

Positivity - Unique Individuals
26.4 % 7-Day Rate
11/27/2021 ... 12/03/2021
24.9 % cumulative rate

Total Deaths
17,400
03/16/2020 ... 12/09/2021

County Distributions

Select a county below by tap or click

Cases | Deaths | Tested | Positivity | County Metrics

Below results are as of **12/06/2021, 11:59 PM**. The county metrics map is updated Wednesdays at **12:00 PM** and reflects data through the previous Sunday.
Click here to learn more about the county metrics map

Map Filter: Weekly 2-Metric Score   IDOH District: Statewide

Advisory Level

Advisory level refers to the current guidelines the county must follow. A county must remain at a lower Weekly Two-Metric Score for two consecutive weeks to move down to a lower advisory level.

Weekly 2-Metric Score

- Blue (0 and .5)
- Yellow (1 and 1.5)
- Orange (2 and 2.5)
- Red (3)

Weekly Cases Per 100,000 Residents

- Less Than 10 new cases(0)
- 10 to 99 new cases(1)
- 100 to 199 new cases(2)
- 200 or more(3)

7-Day All Tests Positivity Rate

- Less than 5% (0)
- 5% to 9.9% (1)
- 10% to 14.9% (2)
- 15% or greater (3)

The ⚠ indicates a disclaimer alert for the county. Hover over the symbol for additional details.

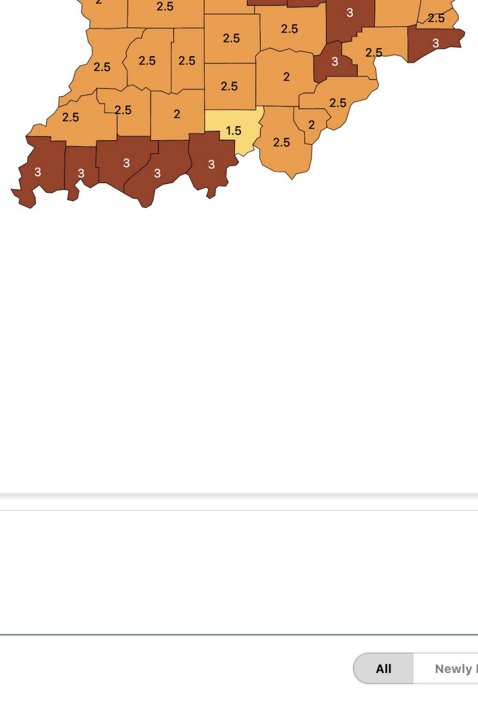

Positive Cases

Statewide Positive Cases by Day

All | Newly Reported

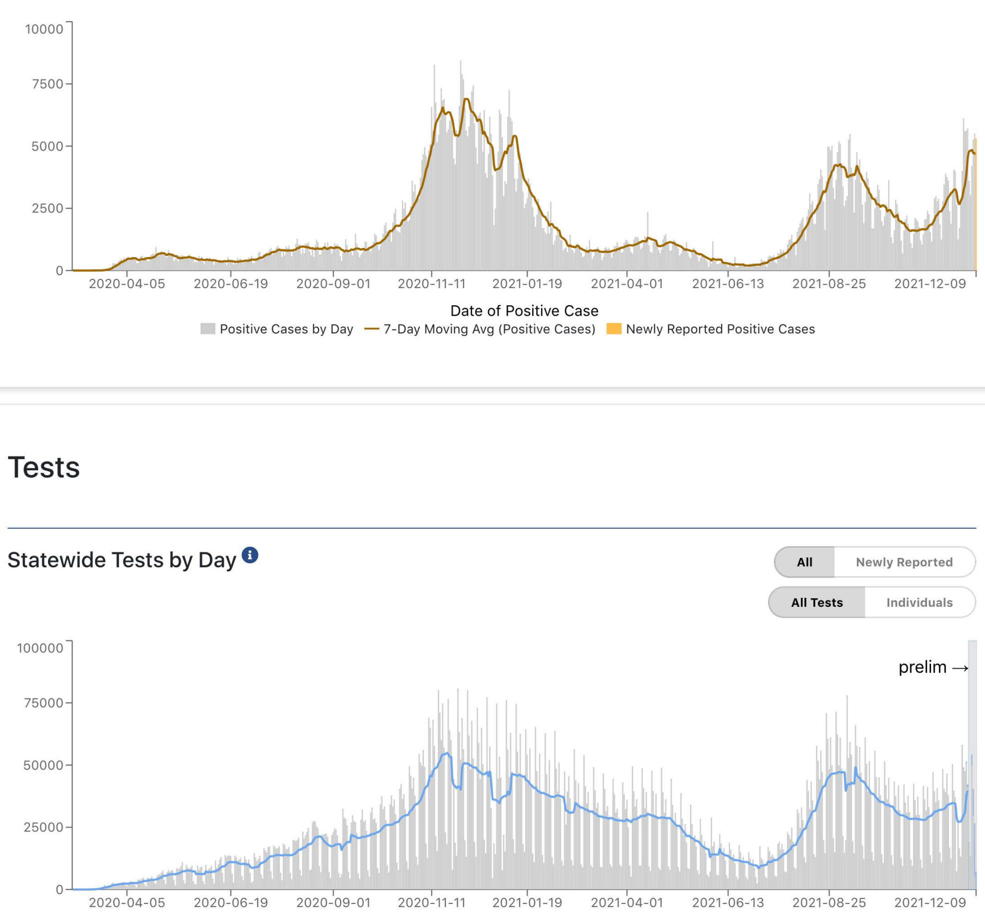

Tests

Statewide Tests by Day

All | Newly Reported
All Tests | Individuals

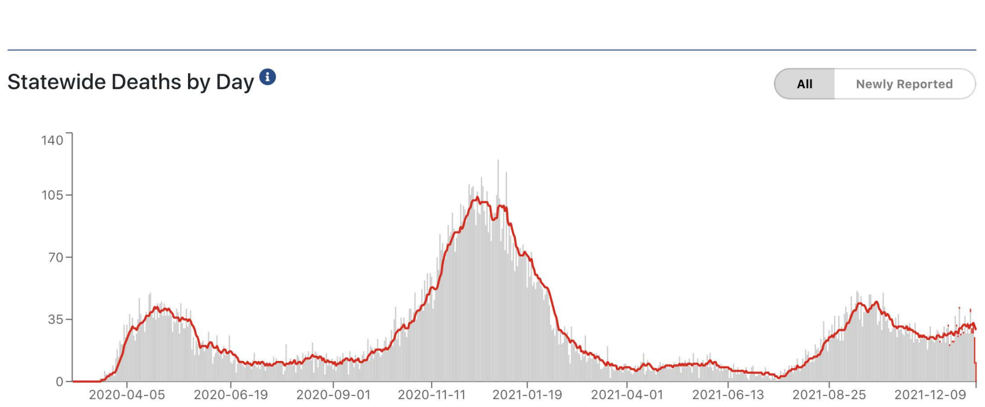

Deaths

Statewide Deaths by Day

All | Newly Reported

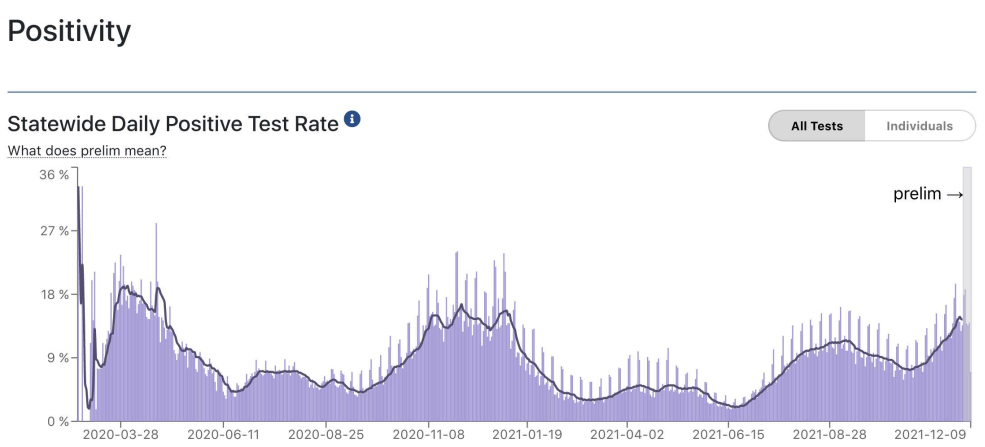

Positivity

Statewide Daily Positive Test Rate

What does prelim mean?

All Tests | Individuals

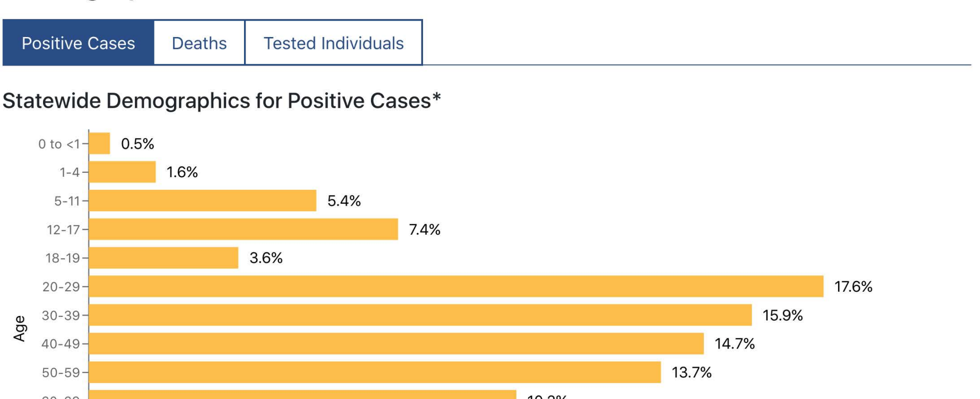

Demographic Distributions

Why do I see "Unknowns"?

Positive Cases | Deaths | Tested Individuals

Statewide Demographics for Positive Cases*

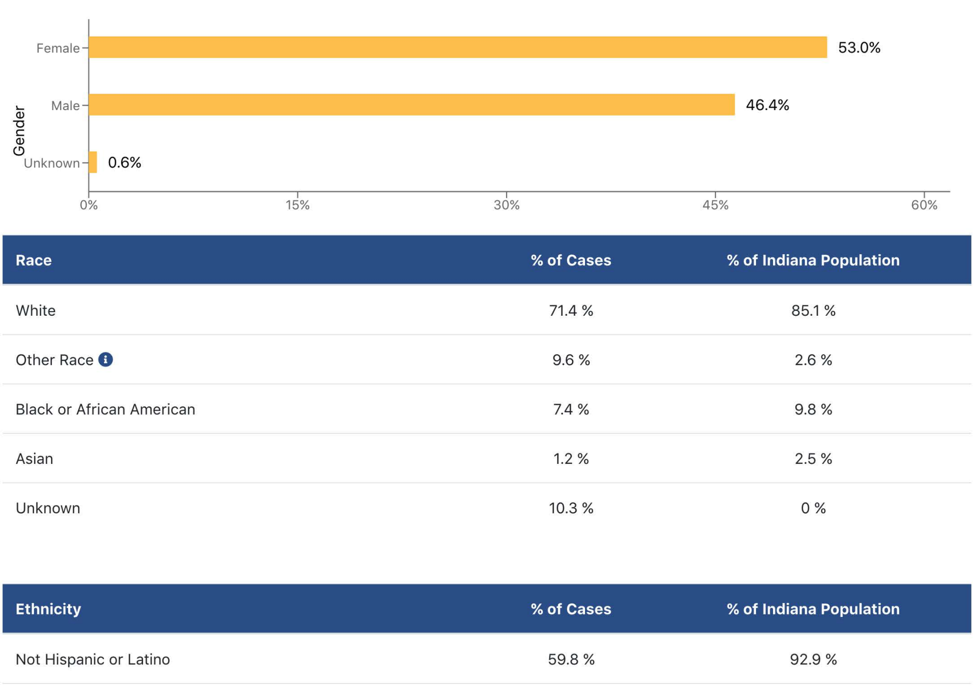

Race	% of Cases	% of Indiana Population
White	71.4 %	85.1 %
Other Race	9.6 %	2.6 %
Black or African American	7.4 %	9.8 %
Asian	1.2 %	2.5 %
Unknown	10.3 %	0 %

Ethnicity	% of Cases	% of Indiana Population
Not Hispanic or Latino	59.8 %	92.9 %
Hispanic or Latino	5.7 %	7.1 %
Unknown	34.5 %	0 %

** Indiana population percentages provided by 2019 U.S. Census Bureau; Population Estimate Program.*

Resource Usage

Today | By Day

Today's Statewide ICU Bed Usage

19.5% ICU Beds Available

29.8% ICU Beds in Use - COVID

50.7% ICU Beds in Use - Non-COVID

Today's Statewide Ventilator Usage

68.5% Ventilators Available

11.9% Ventilators in Use - COVID

19.6% Ventilators in Use - Non-COVID

Summary Dashboard

The dashboard on this page is updated every Monday, Wednesday and Friday.
A full site update will occur every Wednesday.

Data as of 12/9/2021

Iowa COVID-19 Summary

Testing Data

Positive Tests
by Day

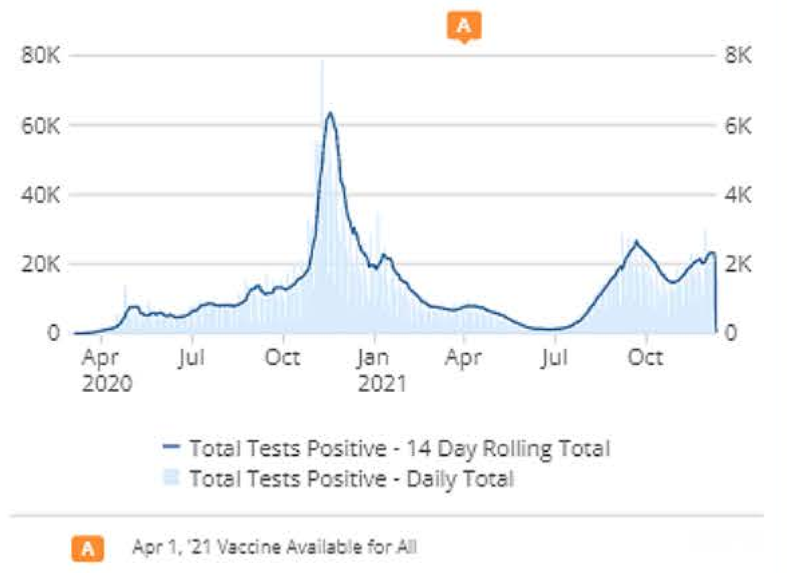

Hospitalization Data

COVID-19 Hospitalized Patients
by Day

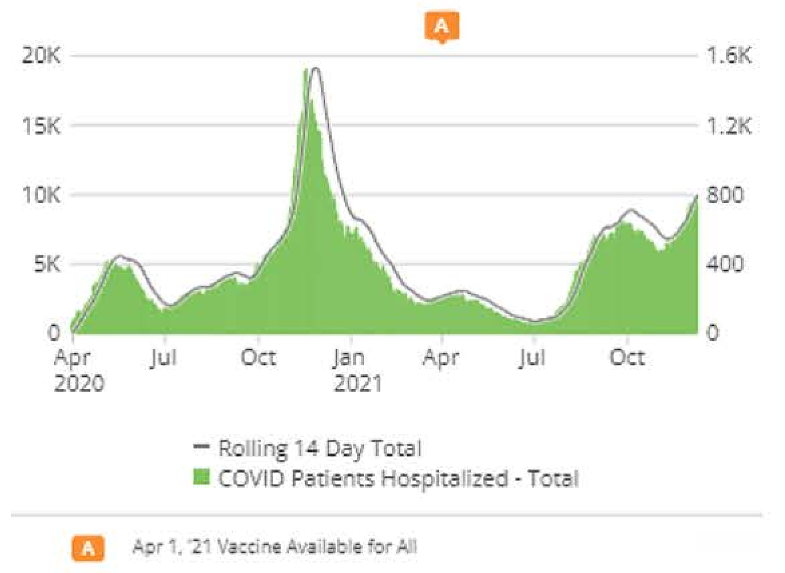

Death Data

Total Deaths
by Day

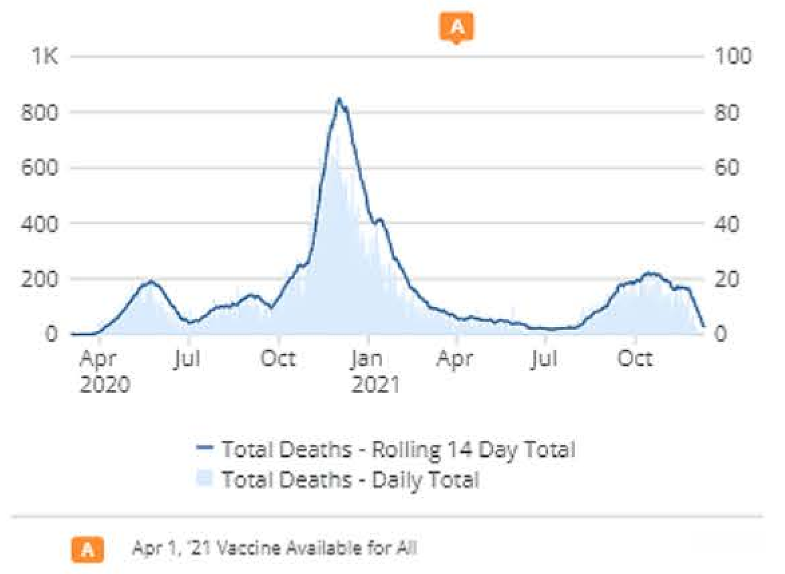

9,535
Positive Tests last 7 days[2]

589,445
Total Positive Tests

12.1%
Positive Tests (Past 14 Days)

806
Covid-19 Patients in Hospitals

179
Covid-19 Patients in ICU

132
Covid-19 Patients admitted last 24H

105
Deaths confirmed in the last 7 days and reported on 12/07/2021. Deaths occurred between 10/12/2021 - 12/01/2021[5]

7,550
Total Deaths

4,356,654
Total Doses Administered[1]

69.5%
18+ Fully Vaccinated[1]

73.0%
12+ with at least one dose[1]

Those not fully vaccinated account for...

81.6%
Covid-19 Patients in ICU[4]

74.7%
Patients Hospitalized because of Covid-19[3 4]

Long Term Care Data

16
Long Term Care Outbreaks

IDPH IOWA Department of PUBLIC HEALTH

COVID-19 in IOWA

[illegible] IRIS, Iowa's Immunization Registry Information System, is a secure, confidential, population-based computerized system that contains immunization information for individuals of all ages residing in the State of Iowa.

2. "Last 7 days" represent data as of the end of the day prior to the last update date on this website.

3. "Excludes patients hospitalized for another reason but are also positive for Covid-19"

4. Numbers reported on Monday are as of the previous Friday.

5. Total number of deaths confirmed and reported to IDPH is updated weekly on Wednesdays. Please refer to the specific dates listed in the graphic above for the time period in which the deaths occurred.
For information on deaths occurring by date, view the charts on Outcome Analysis Deaths.

Positive Case Analysis

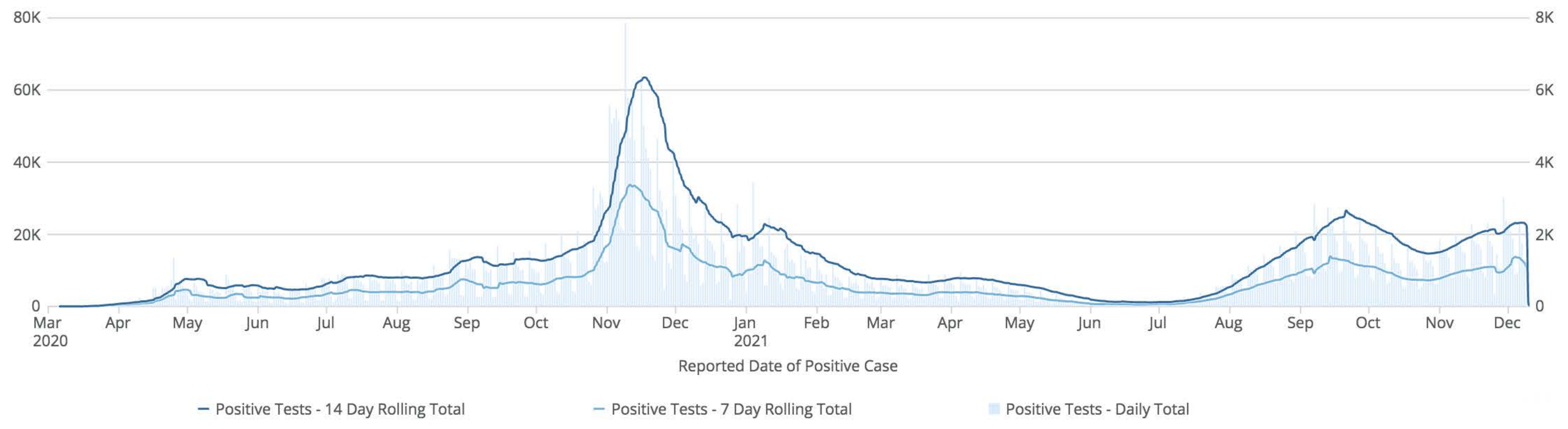

Total

Total Tests	Negative Tests
7,274,709	6,666,792

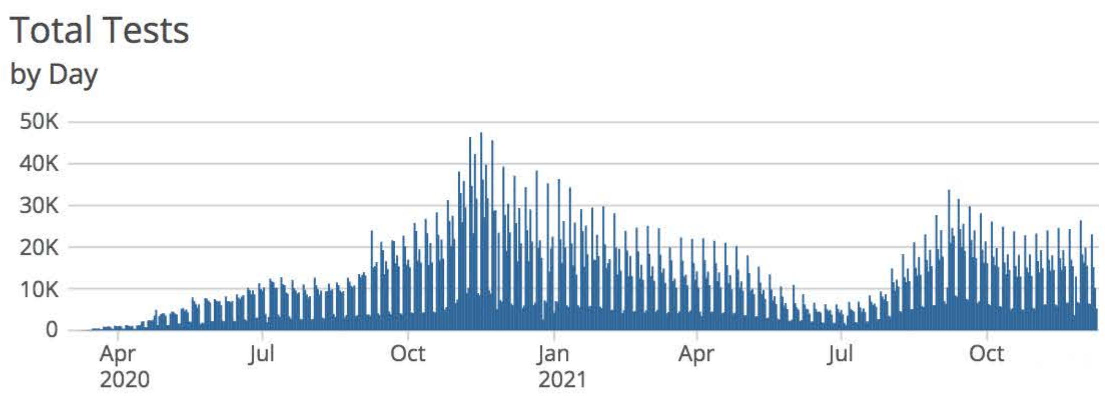

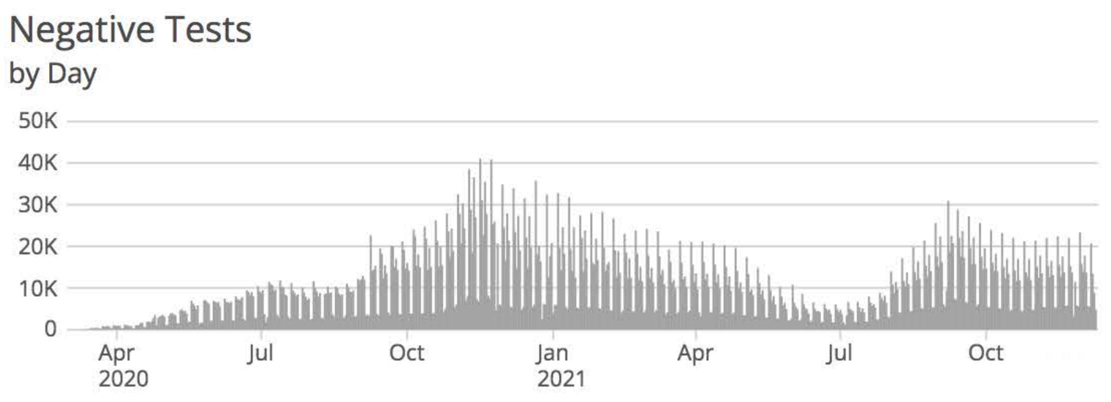

Positive Tests	% Positive Tests (Past 14 Days)
589,445	12.1%

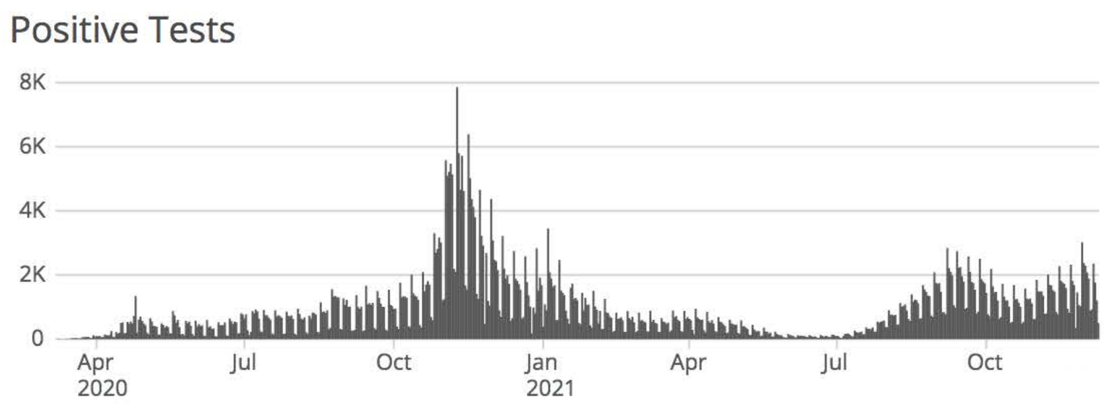

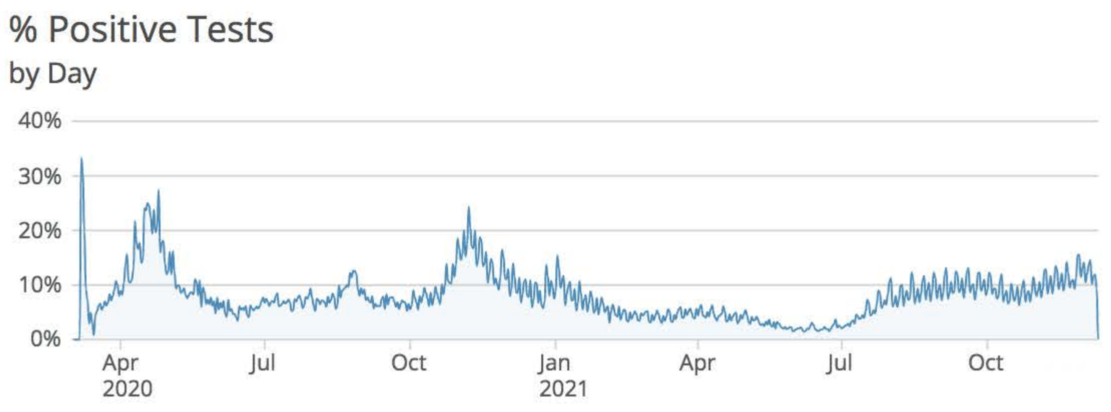

PCR

Inconclusive, indeterminate and invalid tests are included in Total Tests and Individuals Tested.

Total Tests	Negative Tests	Positive Tests
4,462,770	3,976,252	468,320
**Individuals Tested**	**Individuals Negative**	**Individuals Positive**
1,796,176	1,362,147	431,432

Antigen

Inconclusive, indeterminate and invalid tests are included in Total Tests and Individuals Tested.

Total Tests	Negative Tests	Positive Tests
2,811,939	2,690,540	121,125
**Individuals Tested**	**Individuals Negative**	**Individuals Positive**
432,010	317,599	114,372

% Positive Tests 14 Day and 7 Day Average

% Positive Test average is calculated by the sum total of Positive Tests divided by the sum total of Total Tests for the past period of days (14 or 7)

Past 14 Day Average	Past 7 Day Average
12.1%	11.6%

Positive Tests

This interactive dashboard allows you to explore tests by county. Select a county name within the map or table to filter to specific counties.

Positive Tests Last 7 Days

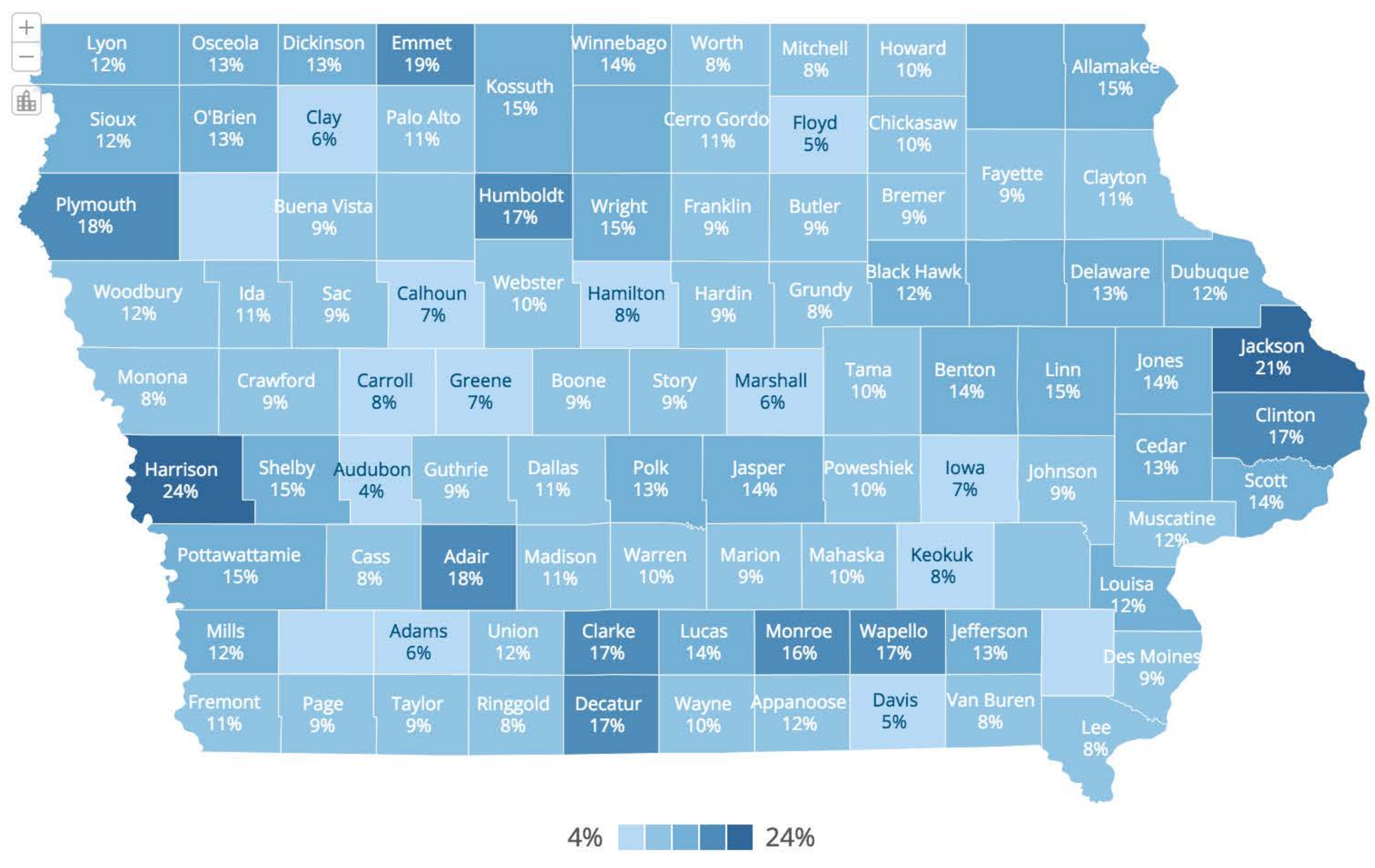

Positive Tests Last 7 days by Age Group

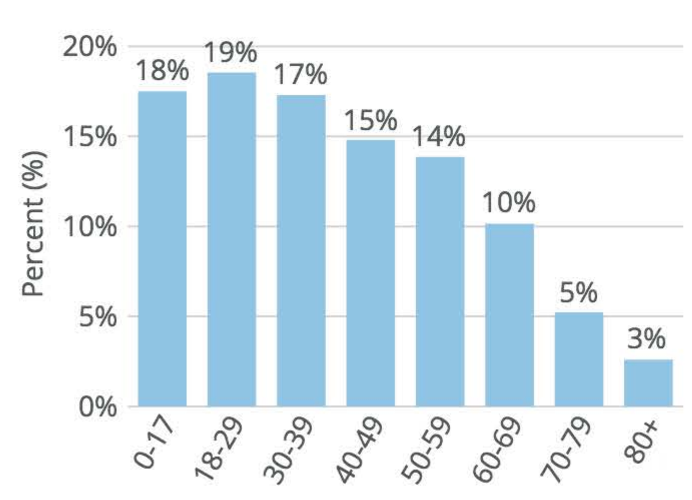

Positive Tests by Sex

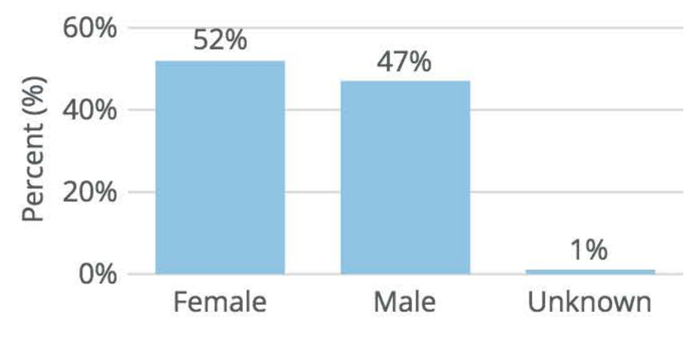

Positive Tests

Country	Total Positive Tests	Positive Tests Per 100,000 Population
Polk	94,164	19,211
Linn	36,690	16,184
Scott	30,173	17,447
Black Hawk	25,229	19,225
Woodbury	22,901	22,211
Johnson	22,218	14,700
Dubuque	21,594	22,191
Pottawattamie	18,431	19,774
Dallas	17,760	19,004
Story	15,407	15,864
Warren	9,629	18,709
Cerro Gordo	9,269	21,835
Clinton	9,175	19,761
Webster	8,065	22,463
Des Moines	7,882	20,227
Marshall	7,420	18,847
Muscatine	7,393	17,328
Wapello	7,352	21,024
Jasper	7,013	18,860
[illegible]	[illegible]	[illegible]
Buena Vista	5,912	30,133
Plymouth	5,401	21,452

Positive Tests by Race

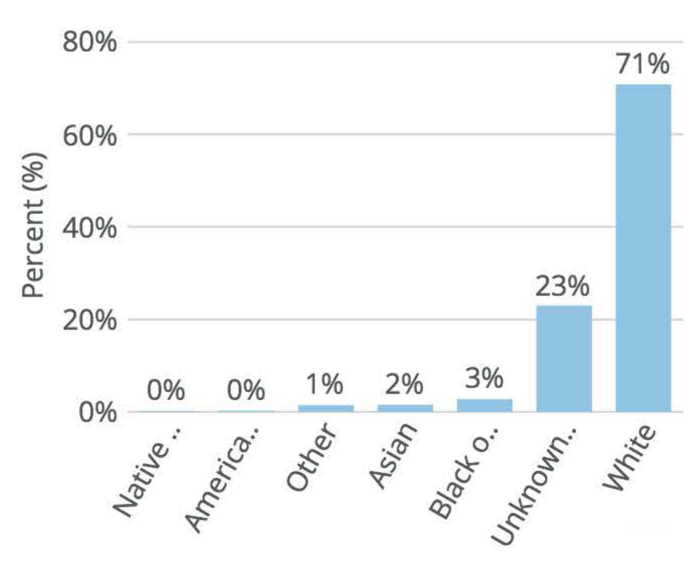

Positive Tests by Ethnicity

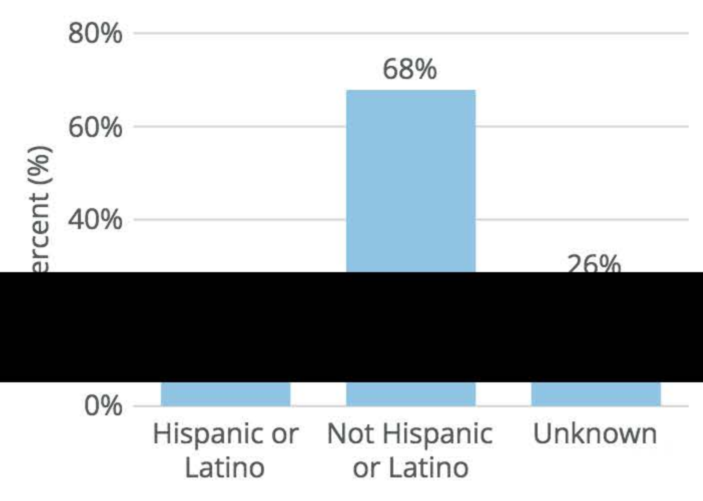

COVID-19 in IOWA

Last updated on: 2021-12-09

LATEST UPDATE **KDHE Recommends COVID-19 Booster Dose for Kansans ages 16 and Older —** KDHE supports CDC and FDA recommendation to expand booster eligibility to adolescents ages 16 and 17 for the Pfizer vaccine. Read on...

Home › COVID-19 in Kansas

COVID-19 Cases in Kansas

Map Information
The map is updated Monday, Wednesday and Friday by 12:30 p.m. The map contains a variety of data at the state and county level. Click on the gray buttons to access the data.

To maximize your experience, use a device with a larger screen, such as a tablet, laptop or desktop. The Map FAQ Document (PDF) includes detailed instructions on how to use and share the data.

COVID-19 Vaccine Data
Data about the COVID-19 vaccine can be found at KansasVaccine.gov.

Case Summary & Historical Data
The COVID-19 Summary (PDF) is published Monday, Wednesday and Friday at 12:30 p.m. and includes historical data. The summary may not be published during holiday observances. The 2020 Summary (PDF) information is available in a separate file.

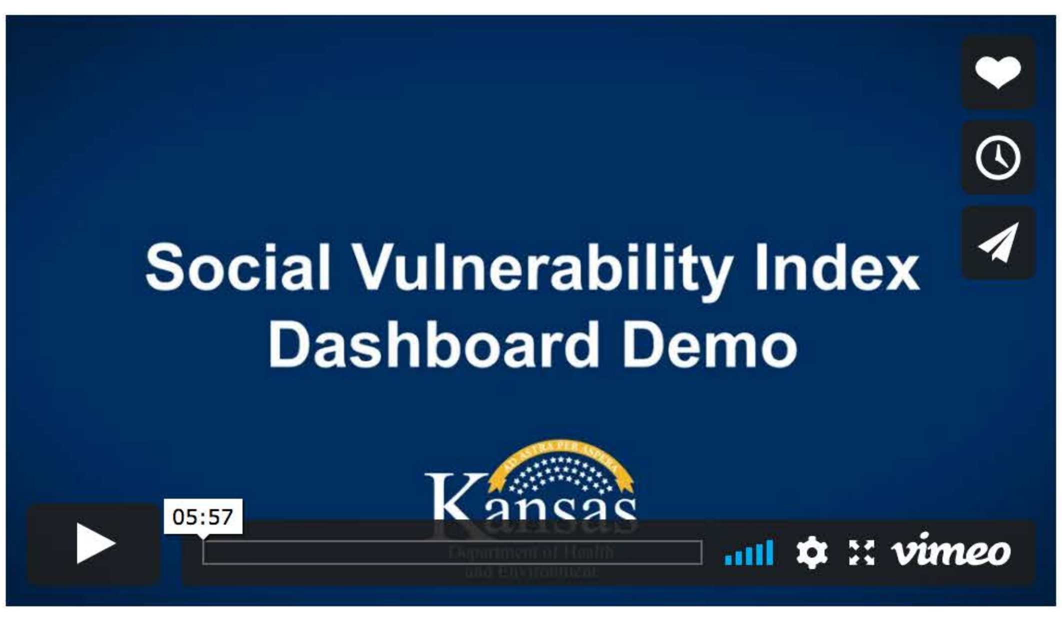

Former Secretary Lee Norman, M.D. demonstrates the Social Vulnerability Index dashboard. Explore this tool by clicking the gray SVI Explorer button.

Kansas County COVID-19 Rankings | Location Identification | COVID-19 Case Definition | Safer Classrooms Workgroup

Multiple factors impact COVID-19 vaccination and testing rates in Kansas communities. State, county and local leaders must work together to stop the spread of the dangerous Delta variant and keep businesses and schools open. The Kansas County COVID-19 Rankings data will be updated on Monday, Wednesday and Friday to empower local leaders to take timely actions to keep their communities safe. The summary may not be published during holiday observances.

Kansas Department of Health and Environment

KS COVID-19 Overview | Testing Rates | Hospital Capacity | Cluster Summary
Case Characteristics | SARS-CoV-2 Variants | Hospital Summary | Death Summary
Case Rates | COVID-19 Metrics | ED Summary | SVI Explorer

Kansas COVID-19: Overview

COVID-19 Cases	Hospitalizations	Statewide Deaths	MIS-C*
486,109	16,217	6,830	20

Data are preliminary and subject to quality improvement and quality assurance validation.
*MIS-C: Multisystem Inflammatory Syndrome in Children (MIS-C) associated with COVID-19.

Last updated: 12/10/2021 at 9:00am. There were 3,877 new cases, 62 new deaths, and 130 new hospitalizations reported since Wednesday, 12/08/2021.

State Map of COVID-19 Cases by County of Residence

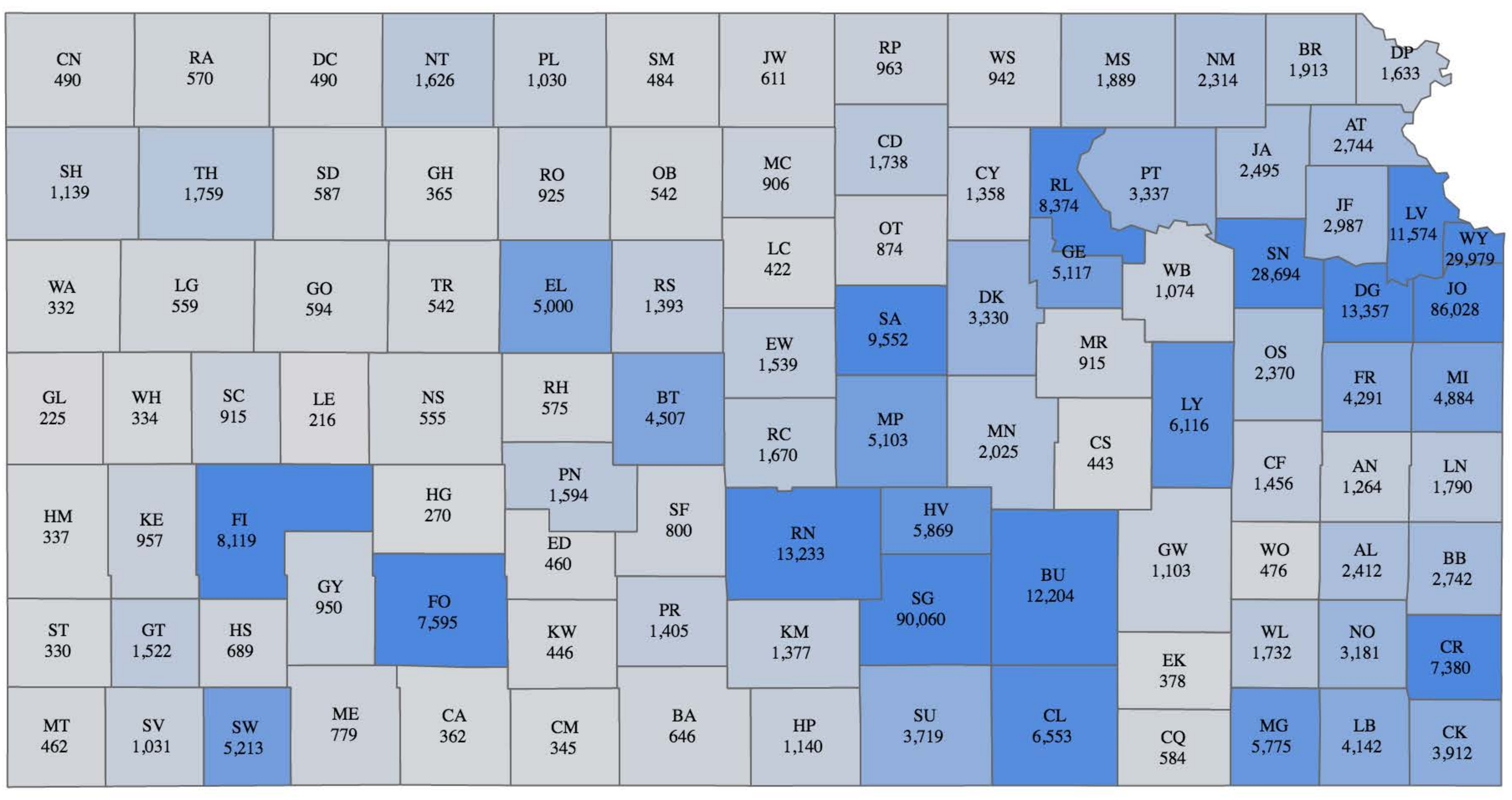

Statewide COVID-19 Cases by Symptom Onset Date

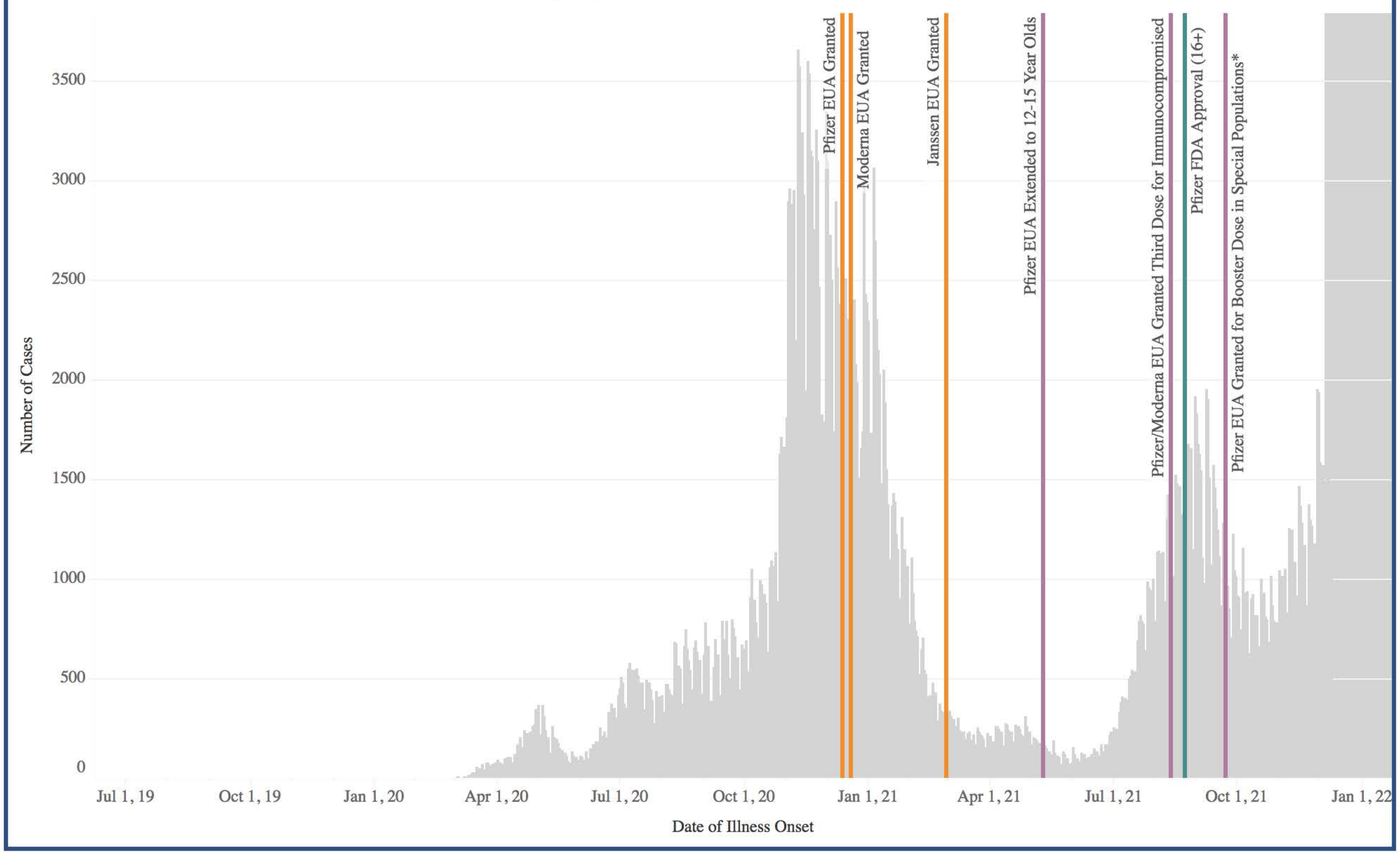

IMPORTANT NOTE: If symptom onset date is missing, the date of specimen collection is used as a proxy for the onset date.

Daily Counts of New Cases and Total Cases Reported by Date Diagnosed

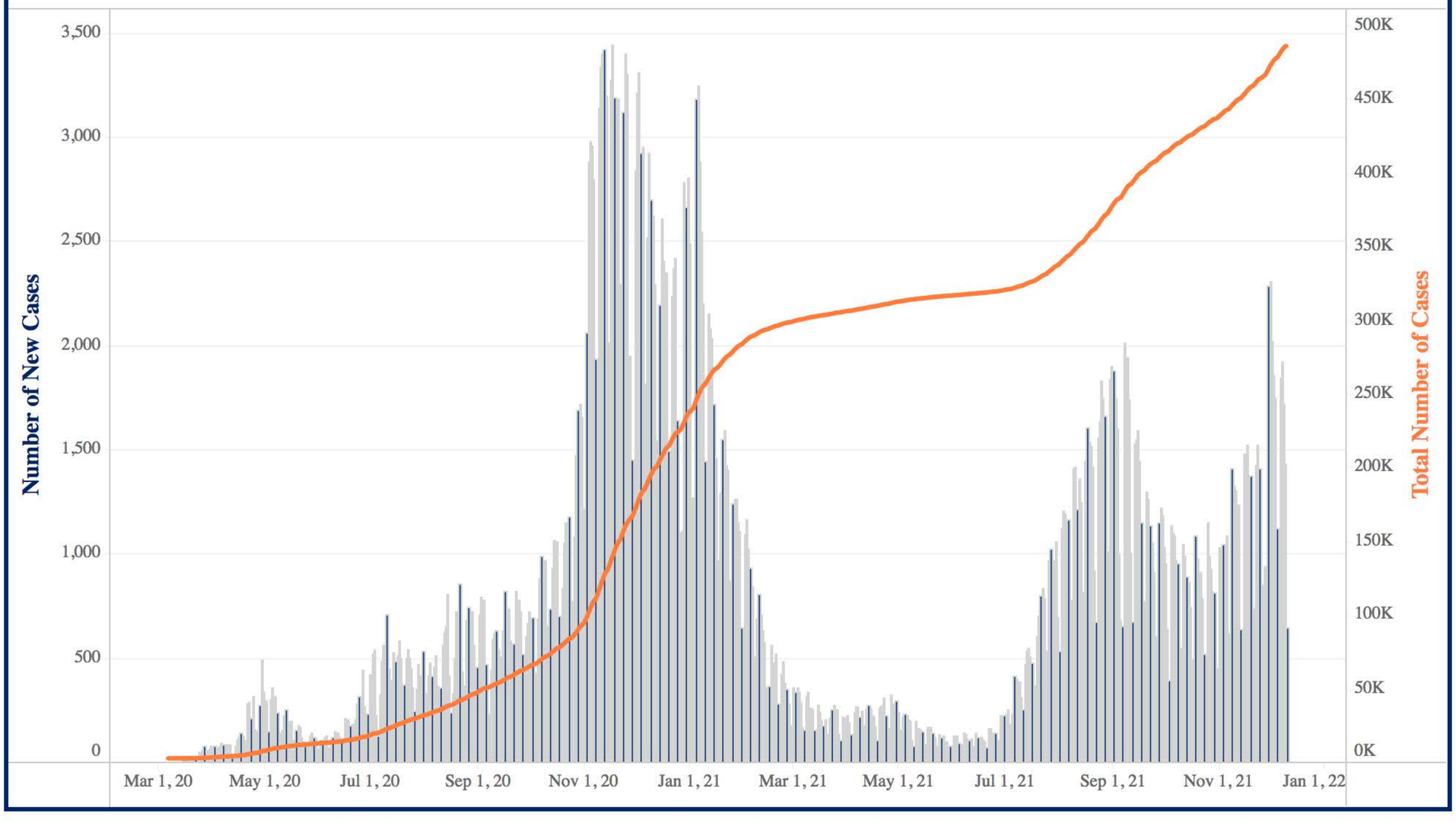

Cases by County

Undo | Redo | Revert | Refresh | Pause | Metrics | Share | Download | Full Screen

Select Language

LATEST UPDATE

KDHE Recommends COVID-19 Booster Dose for Kansans ages 16 and Older — KDHE supports CDC and FDA recommendation to expand booster eligibility to adolescents ages 16 and 17 for the Pfizer vaccine. Read on...

COVID-19 Cases in Kansas

Map Information

The map is updated Monday, Wednesday and Friday by 12:30 p.m. The map contains a variety of data at the state and county level. Click on the gray buttons to access the data.

To maximize your experience, use a device with a larger screen, such as a tablet, laptop or desktop. The Map FAQ Document (PDF) includes detailed instructions on how to use and share the data.

COVID-19 Vaccine Data

Data about the COVID-19 vaccine can be found at KansasVaccine.gov.

Case Summary & Historical Data

The COVID-19 Summary (PDF) is published Monday, Wednesday and Friday at 12:30 p.m. and includes historical data. The summary may not be published during holiday observances. The 2020 Summary (PDF) information is available in a separate file.

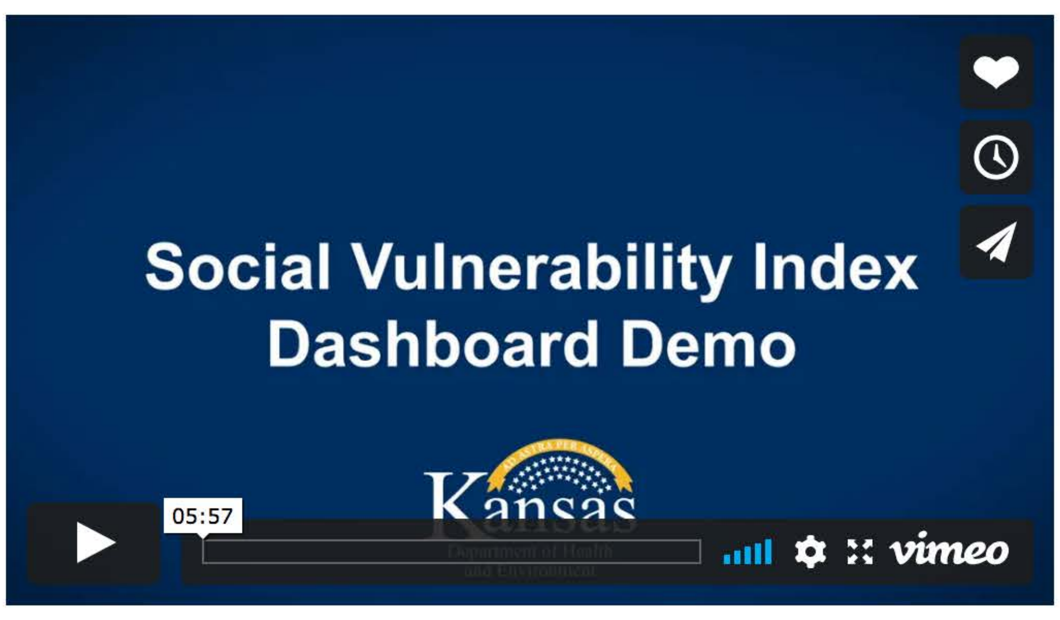

Former Secretary Lee Norman, M.D. demonstrates the Social Vulnerability Index dashboard. Explore this tool by clicking the gray SVI Explorer button.

Kansas County COVID-19 Rankings | Location Identification | COVID-19 Case Definition | Safer Classrooms Workgroup

Multiple factors impact COVID-19 vaccination and testing rates in Kansas communities. State, county and local leaders must work together to stop the spread of the dangerous Delta variant and keep businesses and schools open. The Kansas County COVID-19 Rankings data will be updated on Monday, Wednesday and Friday to empower local leaders to take timely actions to keep their communities safe. The summary may not be published during holiday observances.

Kansas Department of Health and Environment

KS COVID-19 Overview | Testing Rates | Hospital Capacity | Cluster Summary
Case Characteristics | SARS-CoV-2 Variants | Hospital Summary | Death Summary
Case Rates | COVID-19 Metrics | ED Summary | SVI Explorer

Kansas COVID-19: Testing Rates

Overall Testing	Negative Individuals	Monthly Percent Positive	Monthly Testing Rate
1,950,402 People<br>4,415,106 PCR Tests<br>1,689,240 Antigen Tests	1,464,293	12.6%<br>(December 2021 to date)	1,053 per 100,000<br>(December 2021 to date)

IMPORTANT NOTE: The number of people tested represents any Kansas resident that was tested for the SARS-CoV-2 virus by a diagnostic test (e.g., PCR, antigen). Data as reported by laboratories into the KDHE electronic disease surveillance system (EpiTrax). Data are preliminary and subject to quality improvement and quality assurance validation. As of July 27, 2020 the definition of number of people tested was updated to include only diagnostic testing. Monthly testing rate is per 100,000 population.

Positive PCR Tests by Performing Lab

Laboratory	Number of Cases	% of Cases
State Lab (KHEL)	47,773	12.7%
Private Labs	329,749	87.3%
Total	377,522	100.0%

Cases by Lab Type

Test Type	Percent of Cases
PCR	79.1%
Antigen	20.9%
Serology*	0.1%

*Cases with serology results are required to have either an epidemiologic link to a case or signs and symptoms compatible with COVID-19.

Race Testing Rates per 100,000

Race	Persons Tested	Rate per 100,000
White	1,286,447	50,553.56
Black or African American	106,073	48,657.12
Asian	41,518	38,687.25
American Indian or Alaska Native	13,821	21,328.37
Other Race	86,613	94,080.14
Not Reported/Missing	415,930	

Ethnicity Testing Rates per 100,000

Ethnicity	People Tested	Rate per 100,0..
Hispanic or Latino	158,874	45,595.41
Not Hispanic or Latino	1,166,762	45,522.10
Unknown or Missing	624,766	

Weekly Testing Rate by County and Age Group (All PCR Tests) (Per 1,000 Population)

Filter by Age Groups: (All)

Filter by County: All (Kansas)

Week of Lab Test	0-4 years	5-10 years	11-13 years	14-17 years	18-24 years	25-34 years	35-44 years	45-54 years	55-64 years	65-74 years	75-84 years	85+ years
12/05/2021	13.9	20.5	17.7	17.6	15.3	16.4	16.4	14.7	12.8	12.2	13.8	18.6
11/28/2021	17.6	22.0	18.5	19.5	22.0	24.6	24.5	20.9	18.9	18.1	19.4	30.6
11/21/2021	12.3	12.8	11.0	11.5	15.9	18.1	18.6	16.3	14.3	13.7	15.7	28.5
11/14/2021	16.6	23.2	19.0	18.4	20.1	21.6	22.2	19.5	16.8	15.4	18.6	34.2
11/07/2021	16.5	21.0	16.4	16.8	20.2	20.6	21.3	18.8	16.8	14.9	17.4	34.3
10/31/2021	15.0	17.2	14.4	17.0	20.4	20.8	20.9	18.5	16.7	14.9	17.6	34.7
10/24/2021	14.4	14.1	12.9	15.9	18.4	19.7	19.9	17.8	15.7	13.9	16.8	31.6
10/17/2021	14.6	15.5	14.2	17.1	20.1	21.3	21.4	18.4	16.7	15.1	17.3	31.2
10/10/2021	13.8	15.5	13.9	16.7	19.6	20.2	21.1	18.1	16.4	14.8	16.9	32.8
10/03/2021	15.6	16.9	16.8	19.3	22.7	23.1	23.1	19.5	17.4	15.6	17.3	33.4
09/26/2021	17.2	19.5	20.5	21.9	23.9	24.7	24.7	21.0	18.6	16.9	20.1	38.8
09/19/2021	19.8	24.2	24.1	25.6	28.2	27.6	27.9	23.0	19.7	17.9	19.9	38.8
09/12/2021	22.1	29.3	30.4	34.6	31.9	31.7	32.2	26.2	22.9	20.3	22.5	44.4
09/05/2021	22.2	30.1	29.6	32.7	28.9	28.7	29.4	23.3	20.1	17.8	22.5	52.8
08/29/2021	25.7	36.7	36.3	36.7	30.0	30.9	32.3	25.3	22.1	19.4	23.4	53.9
08/22/2021	23.5	29.5	28.7	30.7	28.7	30.1	29.6	24.9	22.1	19.5	24.4	49.3
08/15/2021	17.1	16.4	13.2	19.4	27.9	26.6	26.4	22.9	20.4	18.8	24.1	50.3
08/08/2021	15.3	10.4	9.6	15.9	26.3	25.3	24.8	22.0	19.4	18.0	22.4	47.5
08/01/2021	14.6	9.7	9.8	15.2	24.0	24.4	23.8	20.4	18.5	17.4	22.2	47.7
07/25/2021	13.4	9.3	9.2	14.0	20.3	21.0	20.7	17.5	15.5	14.4	19.0	40.1
07/18/2021	10.8	7.1	6.7	11.5	15.5	16.1	15.9	14.1	11.6	11.2	13.6	28.0
07/11/2021	8.0	5.9	5.8	8.8	12.7	12.7	12.5	11.1	9.6	8.7	11.3	25.0

Kansas Monthly Testing Rates

	New People Tested	Test Rate per 100,000
December 2021	30,689	1,053
November 2021	82,617	2,836
October 2021	78,380	2,690
September 2021	127,539	4,378
August 2021	119,324	4,096
July 2021	60,790	2,087
June 2021	32,154	1,104
May 2021	39,087	1,342
April 2021	50,368	1,729
March 2021	56,820	1,950
February 2021	70,839	2,432
January 2021	138,993	4,771
December 2020	185,303	6,361
November 2020	191,914	6,587
October 2020	132,958	4,564
September 2020	117,768	4,042
August 2020	125,732	4,316

Kansas Monthly Percent Positivity

Month, Year of La..	Percent Positivity
December 2021	12.6%
November 2021	9.8%
October 2021	7.5%
September 2021	8.2%
August 2021	10.0%
July 2021	8.8%
June 2021	3.1%
May 2021	2.6%
April 2021	3.2%
March 2021	2.8%
February 2021	4.6%
January 2021	8.4%
December 2020	11.4%
November 2020	16.6%
October 2020	8.9%
September 2020	6.7%
August 2020	7.7%

Total laboratory tests (the denominator) are the number of PCR tests performed, not the number of individuals tested (i.e. unit of measurement for percent positivity is the test, not a person). For each individual, only the first positive test result is included in the numerator and the denominator (multiple positive tests for a single person will not appear as multiple COVID-19 cases). All negative PCR tests are included in the denominator.

Percent Positive and Number of Negative and Positive COVID-19 Molecular Assay Testing (e.g. PCR) Performed by Laboratory Test Date

Specify County: All (Kansas)

Data Table

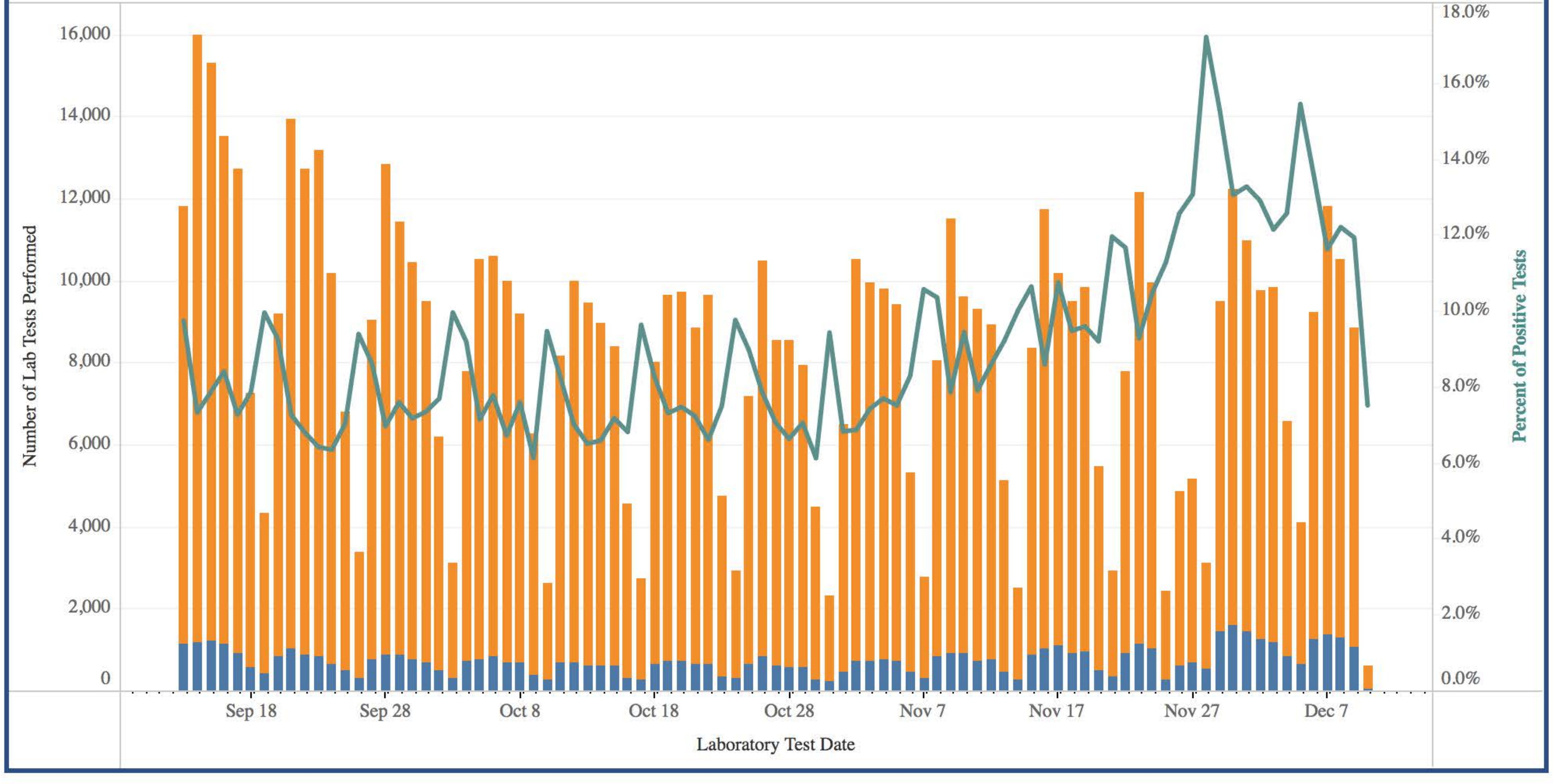

Undo | Redo | Revert | Refresh | Pause | Metrics | Share | Download | Full Screen

Select Language

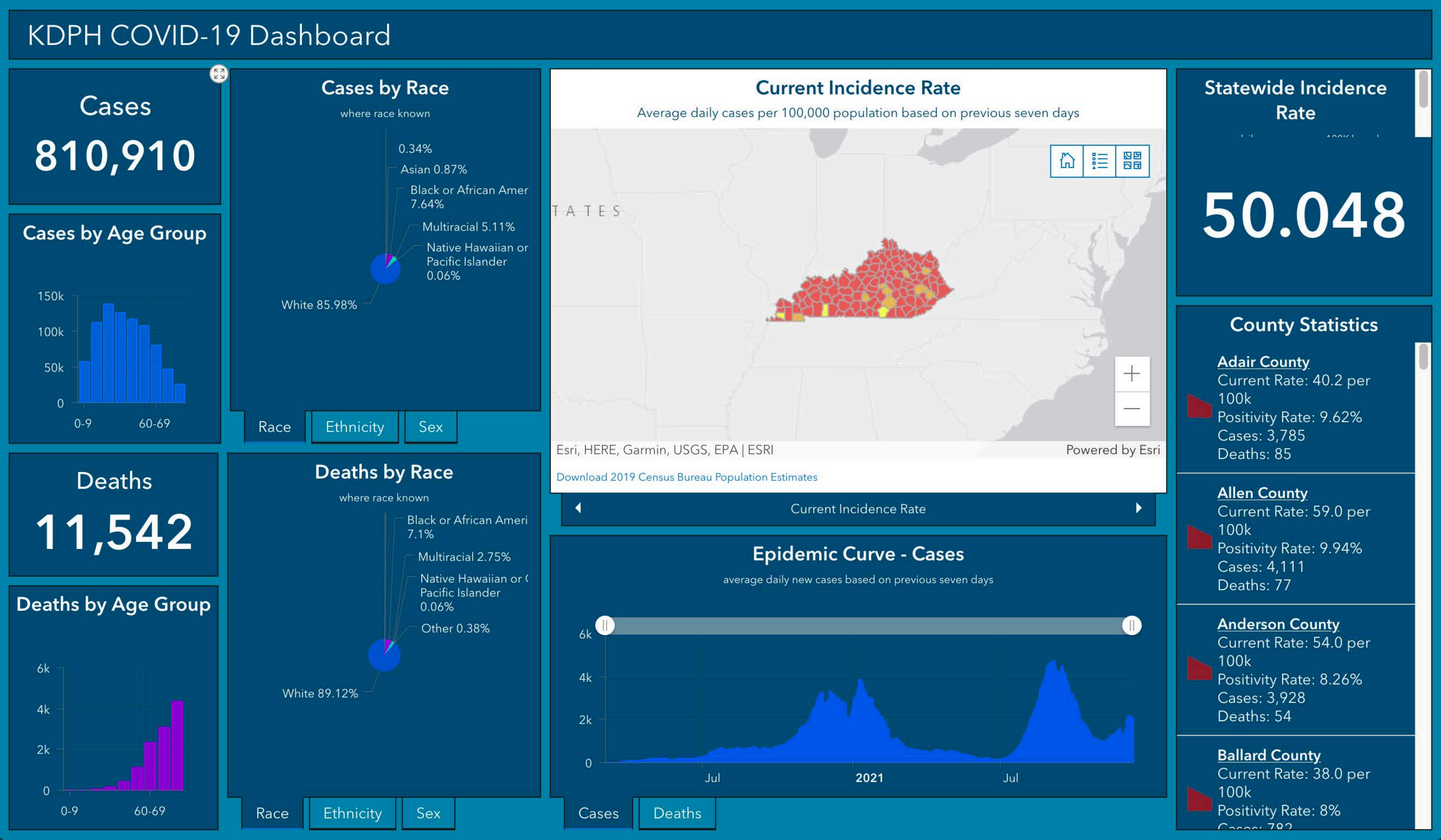
KDPH COVID-19 Dashboard
Cases
810,910
Cases by Age Group
150k
100k
50k
0
0-9
60-69
Cases by Race
where race known
0.34%
Asian 0.87%
Black or African Amer
7.64%
Multiracial 5.11%
Native Hawaiian or
Pacific Islander
0.06%
White 85.98%
Race
Ethnicity
Sex
Current Incidence Rate
Average daily cases per 100,000 population based on previous seven days
TATES
Esri, HERE, Garmin, USGS, EPA | ESRI
Powered by Esri
Download 2019 Census Bureau Population Estimates
Current Incidence Rate
Statewide Incidence Rate
50.048
County Statistics
Adair County
Current Rate: 40.2 per 100k
Positivity Rate: 9.62%
Cases: 3,785
Deaths: 85
Allen County
Current Rate: 59.0 per 100k
Positivity Rate: 9.94%
Cases: 4,111
Deaths: 77
Anderson County
Current Rate: 54.0 per 100k
Positivity Rate: 8.26%
Cases: 3,928
Deaths: 54
Ballard County
Current Rate: 38.0 per 100k
Positivity Rate: 8%
Deaths
11,542
Deaths by Age Group
6k
4k
2k
0
0-9
60-69
Deaths by Race
where race known
Black or African Ameri
7.1%
Multiracial 2.75%
Native Hawaiian or
Pacific Islander
0.06%
Other 0.38%
White 89.12%
Race
Ethnicity
Sex
Epidemic Curve - Cases
average daily new cases based on previous seven days
6k
4k
2k
0
Jul
2021
Jul
Cases
Deaths

Looking For County-Level Data On The Coronavirus? Here's Our Kentucky COVID-19 Tracker

By Alexandra Kanik

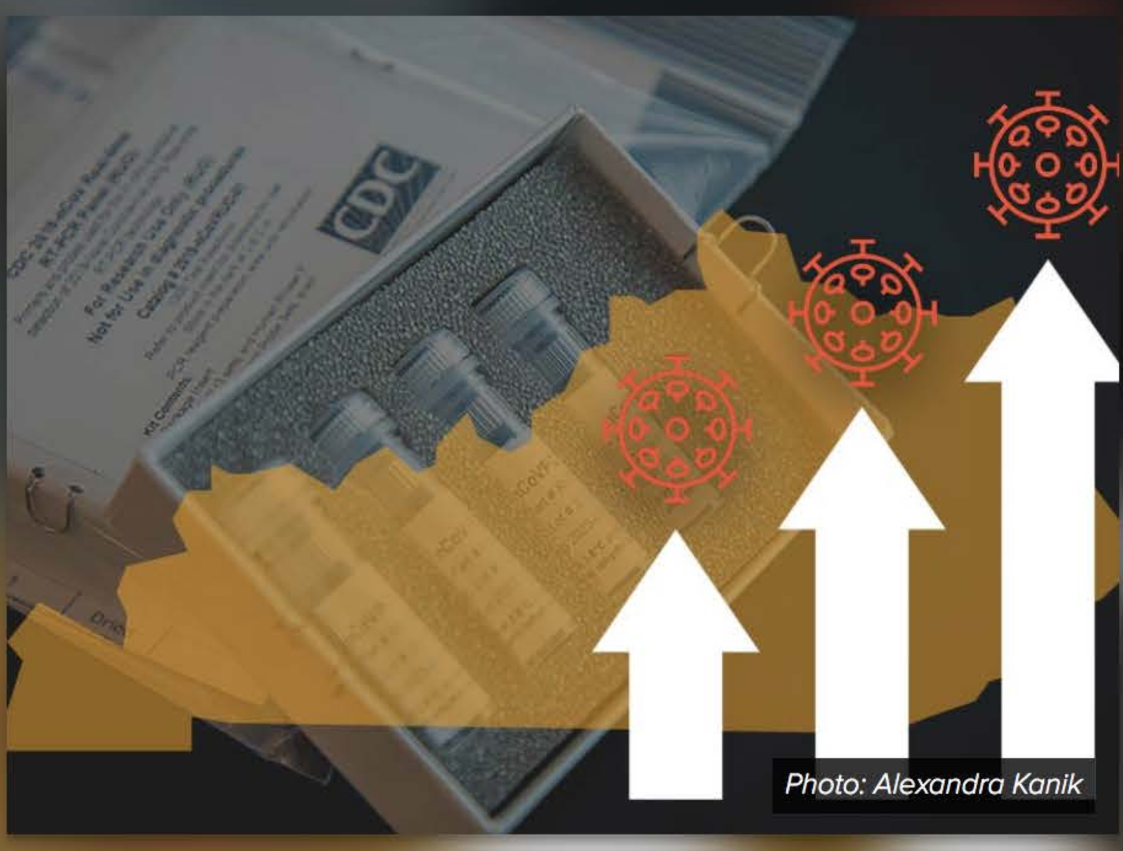

INTERACTIVE June 29, 2020

Kentucky COVID-19 Tracker

This dashboard provides a localized look at COVID-19-related cases and deaths in your county, as well as a risk assessment for your county's population.

Data last updated December 10, 2021.

Use the dropdown below to display more information.

Select a county...

Total cases

There have been **815,487 cases** of COVID-19 in Kentucky. The population of this region is 4,440,204. That's 18366 cases per 100,000 people.

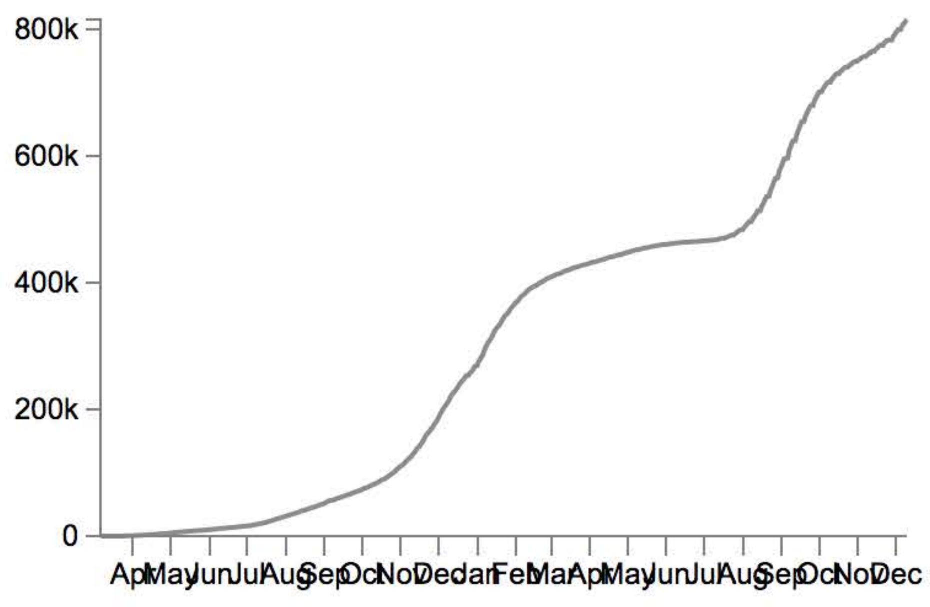

Newly reported cases

There were **2,701 newly reported cases** of COVID-19 on December 10, 2021 in Kentucky. Over time, new cases indicate how fast the virus is spreading in a community.

Seven-day Average: 2,284 Newly Reported Cases.

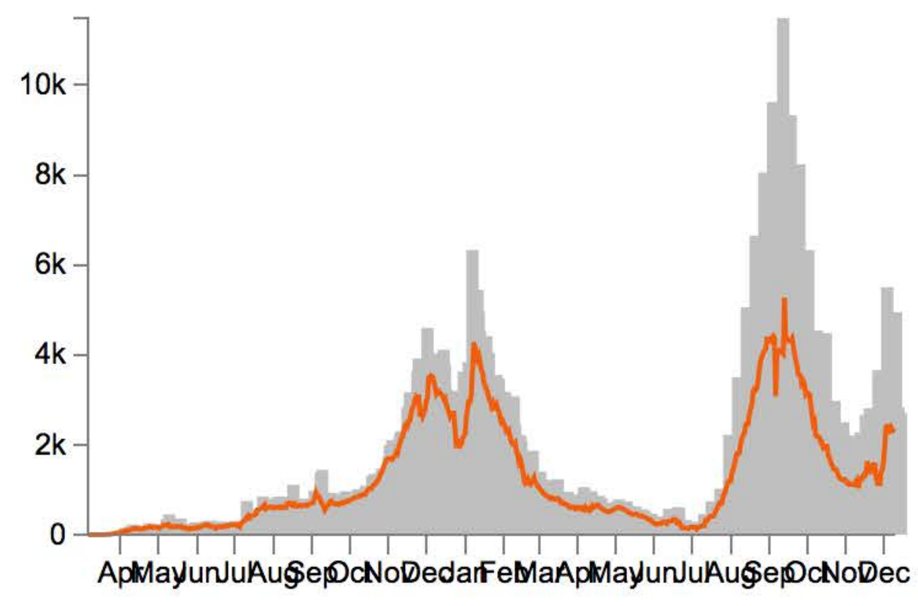

Total deaths

There have been **11,606 reported deaths** related to COVID-19 in Kentucky.

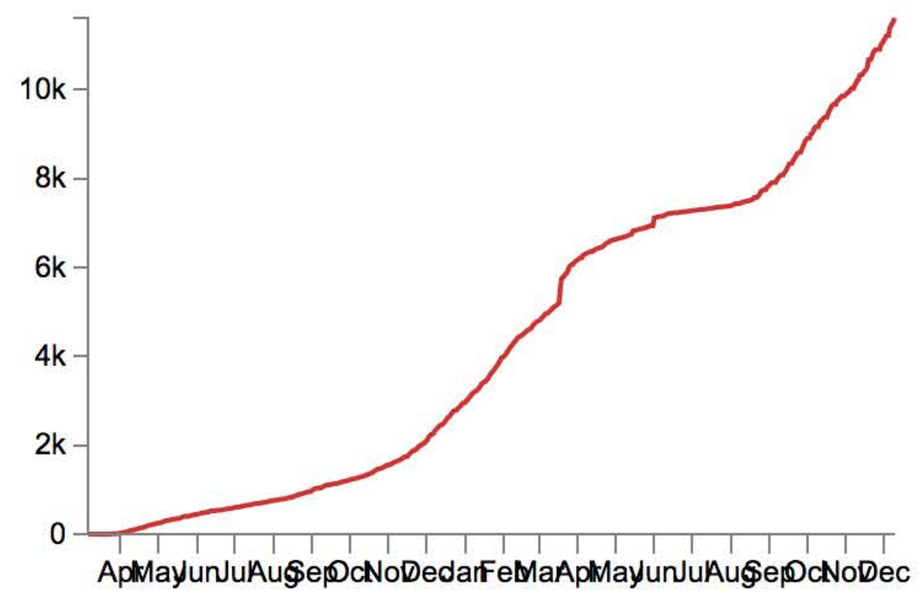

Newly reported deaths

There were **67 new deaths** related to COVID-19 reported on December 10, 2021 in Kentucky.

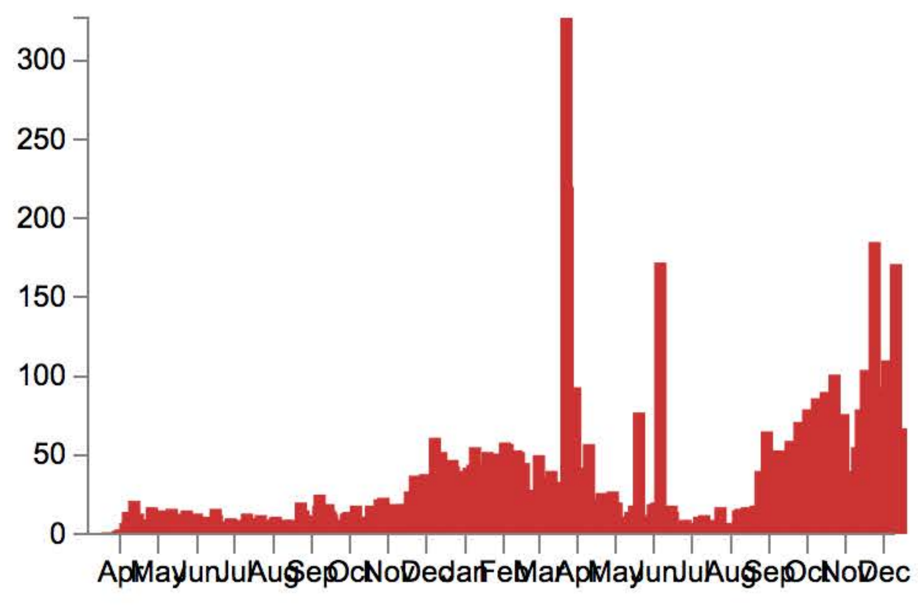

Disclaimer & Data Sources

Age data provided by the 2018 5 year American Community Survey. Disease data are from 2014 and are provided by the Institute for Health Metrics and Evaluation's Global Health Data Exchange

As of April 27, 2020, case and death data for Kentucky counties are provided by the New York Times county dataset. Read more about the data and potential caveats here.

The data presented here only includes cases in which a person has [illegible]

89.3 WFPL
Interactive
LISTEN LIVE
Weekend All Things Considered
Donate

Looking For County-Level Data On The Coronavirus? Here's Our Kentucky COVID-19 Tracker

in one day.

Credits

This dashboard was created by Allie Kanik at Louisville Public Media, Lisa Pickoff-White at KQED, Emily Zentner at CapRadio, and Dana Amihere at KPCC.

LOUISIANA DEPARTMENT OF HEALTH

BACK TO LDH

New Cases	New Deaths	COVID Patients in Hospitals
567	10	201
**Total Cases***	**Total Deaths***	**COVID Patients on Ventilators**
775,935	14,877	26

Those not fully vaccinated account for...

80%	87%	85%
of cases from 11/25 - 12/1	of deaths from 11/25 - 12/1	of current COVID hospitalizations

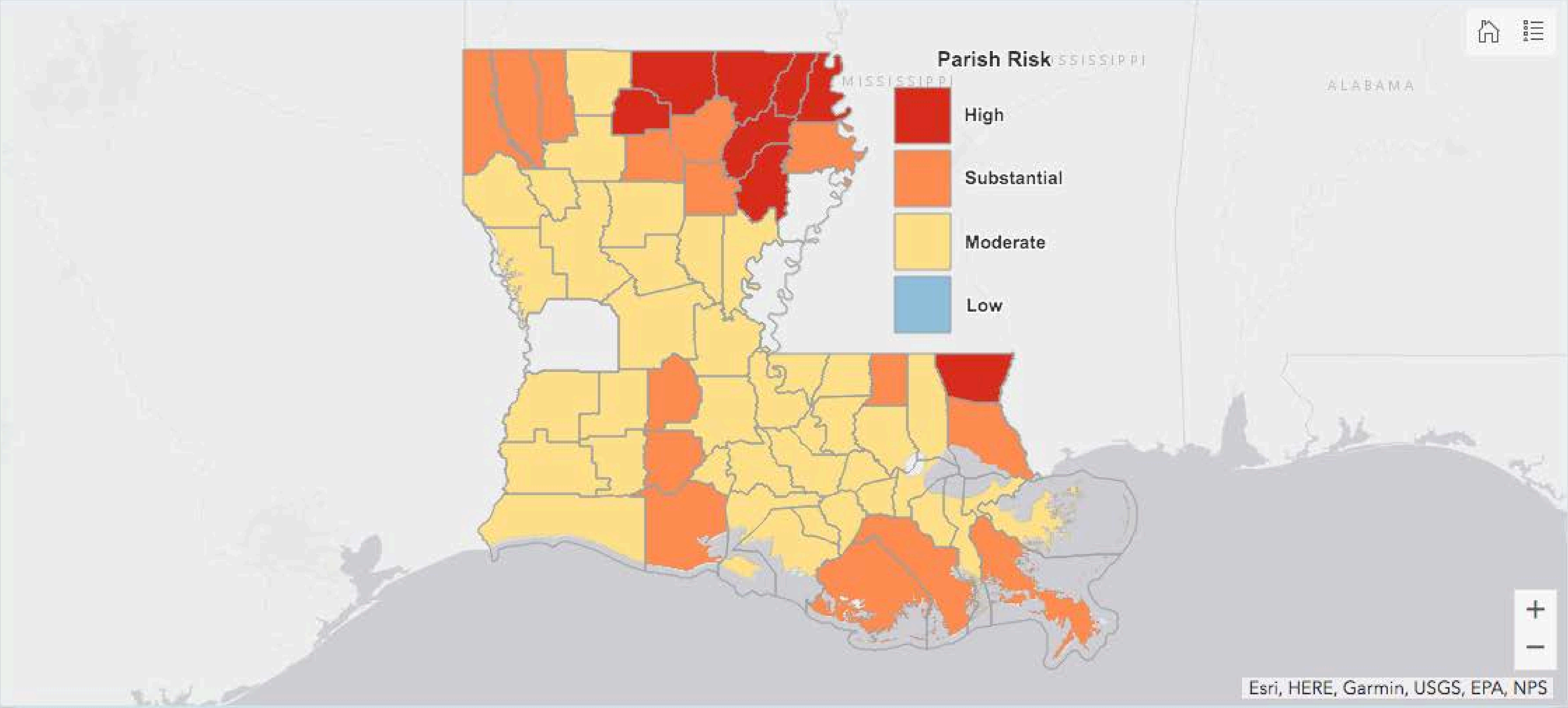

Community Risk by Parish

Click the < > arrows to navigate tabs containing other information and downloadable data

This dashboard is updated on weekdays by noon.

FEEDBACK

ACCESSIBILITY

Coronavirus Disease 2019 (COVID-19)
COVID-19 Homepage
Maine Data
General Information
Contact Tracing
Travelers
Healthcare Providers
Long Term Care Facilities and Congregate Living
Communities, Schools, and Workplaces
EPI Information
A-Z Index of Epidemiology Diseases
Contact Us
Disease Reporting
Request for Data

211 Social services help and information about COVID-19 in Maine, call 211, email info@211maine.org, text your ZIP code to 898-211, or if out-of-state call 1-877-463-6207.

COVID-19: Maine Data

Maine's state of civil emergency ended on June 30, 2021. Since July 3, 2021, Maine CDC has been conduting case investigations and contact tracing Monday through Friday. As a result, COVID-19 case data will be updated by 9:30 AM Tuesday through Saturday. Case data will not be updated Sunday and Monday or on holidays. All data are preliminary and may change as Maine CDC investigates cases. For more information about the data, please see the "Read Details About the Data" section below.

Maine CDC has focused its efforts to follow up with cases of COVID-19 on cases who are under the age of 19, over the age of 74, or who meet certain other criteria (including being hospitalized, identified as a minority, identified as having a disability, being a health care worker or first responder, living or working in congregate settings, or associated with a school or child care facility). Depending on available resources and case levels, Maine CDC may follow up with other cases who do not meet those criteria.

Download csv files with the most recent cumulative case data by zip code (CSV), county (CSV), age (CSV), sex (CSV), race (CSV), and ethnicity (CSV).

Download a csv file with historical case data by county and date.

Download a csv file with syndromic surveillance gate metrics by date.

View Maine's complete COVID-19 vaccination dashboard.

COVID-19 Case Trends

Daily lab results | New cases by date | Cases by age trend | Case rates by county | COVID-19 data by race | Maine v. other states

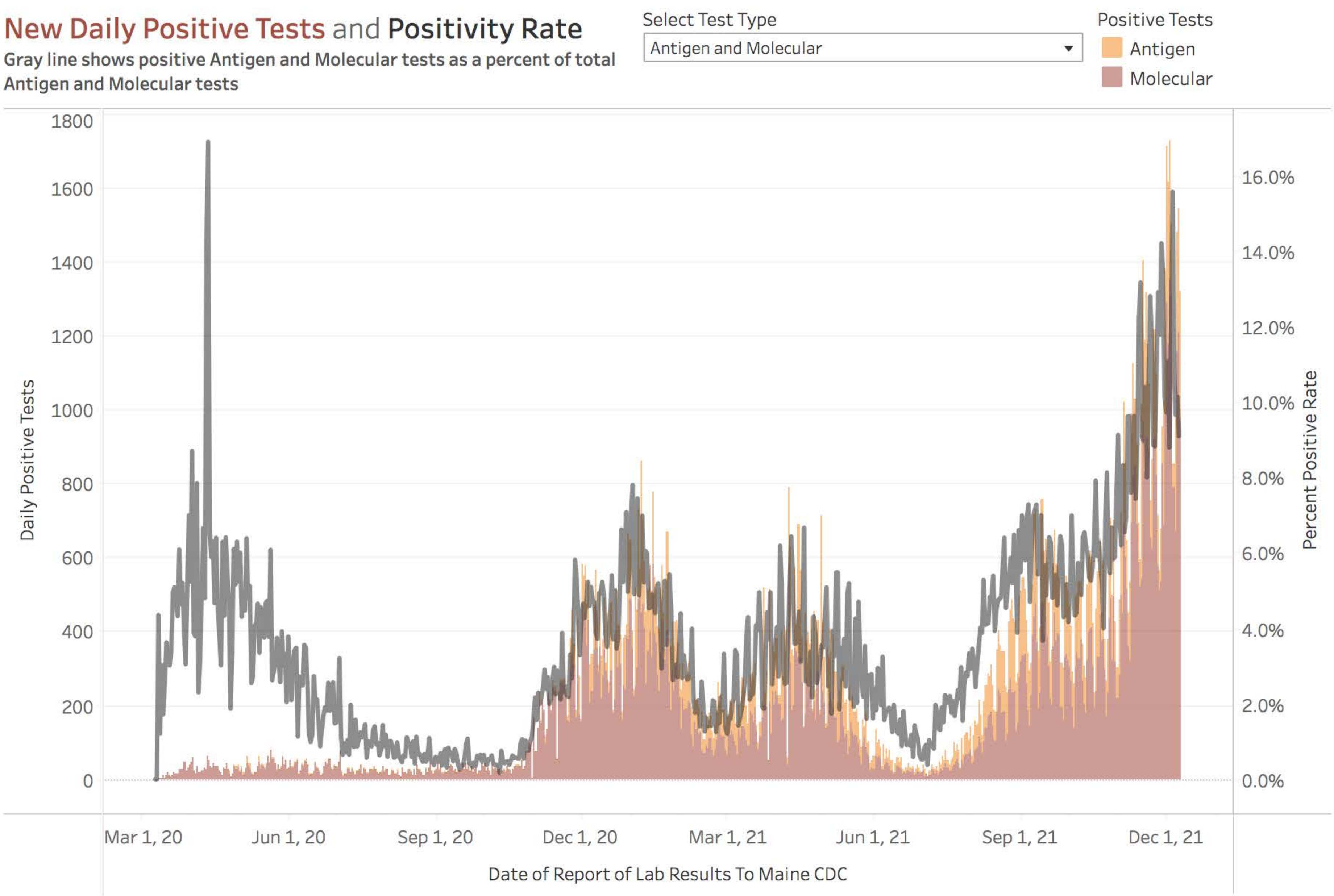

Undo Redo Revert Refresh Pause Metrics Share Download Full Screen

Download csv files with the most recent cumulative case data by zip code (CSV), county (CSV), age (CSV), sex (CSV), race (CSV), and ethnicity (CSV).

Download a csv file with historical case data by county and date.

Cumulative COVID-19 Cases by County

Export table to PDF

Cumulative COVID-19 Cases by County

Table updated: December 11, 2021
Data through: Friday, December 10, 2021 11:59 pm

Patient County	Cases	Confirmed cases	Probable cases	Deaths	Hospitalizations	Case rate (per 10K people)
**Statewide**	**129,997**	**93,040**	**36,957**	**1,367**	**3,226**	**971.3**
Cumberland	25,616	19,228	6,388	257	597	872.6
York	21,178	16,905	4,273	173	508	1,026.9
Penobscot	15,194	10,245	4,949	206	474	1,005.6
Androscoggin	14,051	9,266	4,785	131	345	1,304.9
Kennebec	12,783	7,592	5,191	133	279	1,047.1
Oxford	6,864	5,030	1,834	90	162	1,191.3
Aroostook	6,282	4,264	2,018	90	152	936.1
Somerset	5,732	3,977	1,755	66	177	1,133.0
Hancock	3,873	2,859	1,014	56	101	706.6
Franklin	3,291	2,696	595	35	69	1,100.8
Waldo	3,105	2,387	718	36	75	782.2
Sagadahoc	2,658	1,991	667	16	43	745.9
Knox	2,641	2,392	249	17	59	664.1
Washington	2,536	1,301	1,235	37	81	805.3
Lincoln	2,390	1,995	395	10	41	695.9
Piscataquis	1,773	894	879	14	63	1,055.4
Unknown	30	18	12	0	0	

Download a csv file with the most recent cumulative case data by county.

Download a csv file with historical case data by county and date.

Maine COVID-19 Reopening Gating Metrics

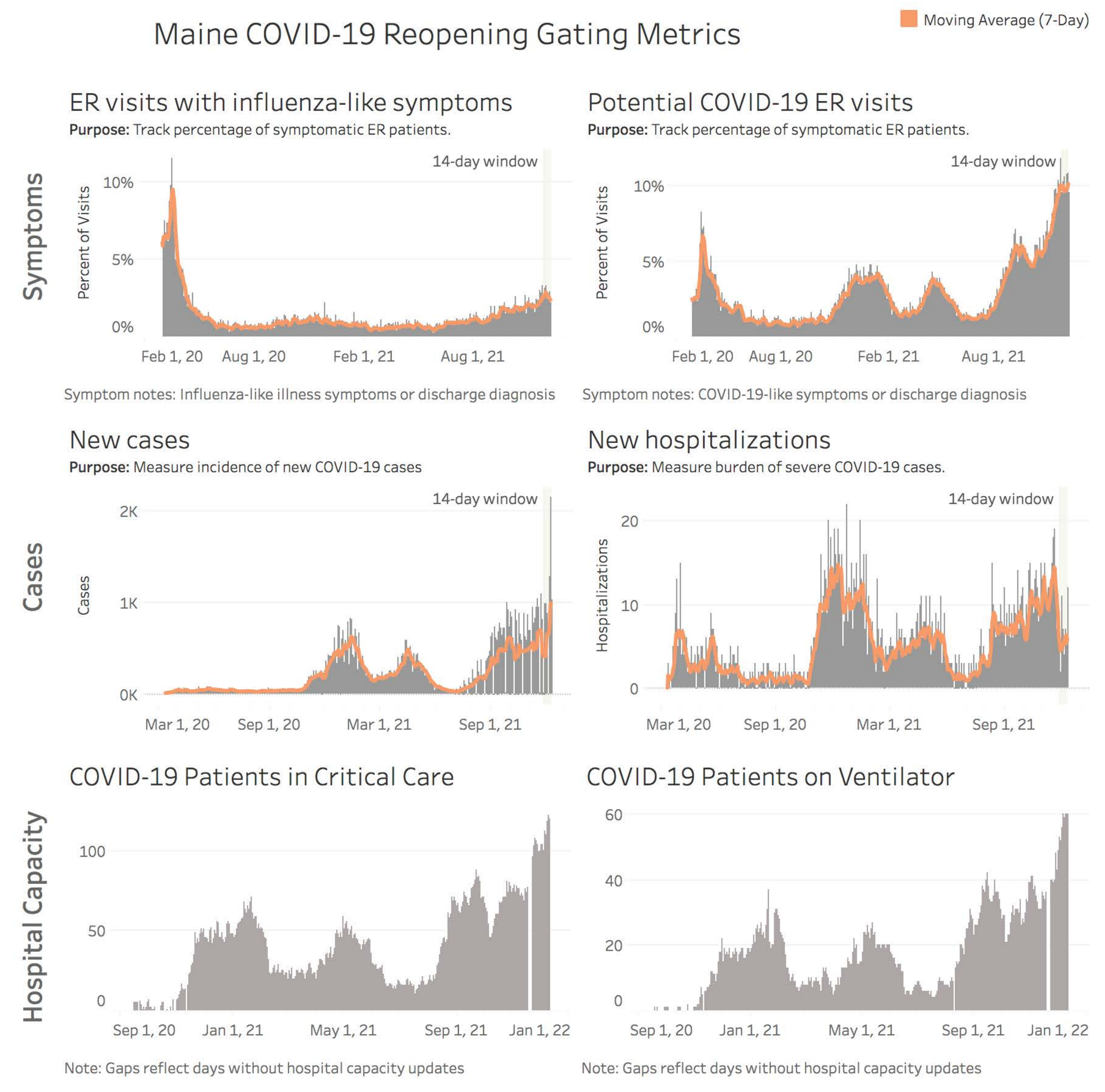

Undo Redo Revert Refresh Pause Metrics Share Download Full Screen

Cumulative COVID-19 Cases by ZIP Code

Cumulative COVID-19 cases by ZIP code

Hover for detail | **555** cases with unknown ZIP codes, **1,439** cases with unmappable ZIP codes
Data as of: December 5, 2021
Dashboard updated: December 7, 2021

Patient County
(All)
Highlight Patient Zip
Highlight Municipality

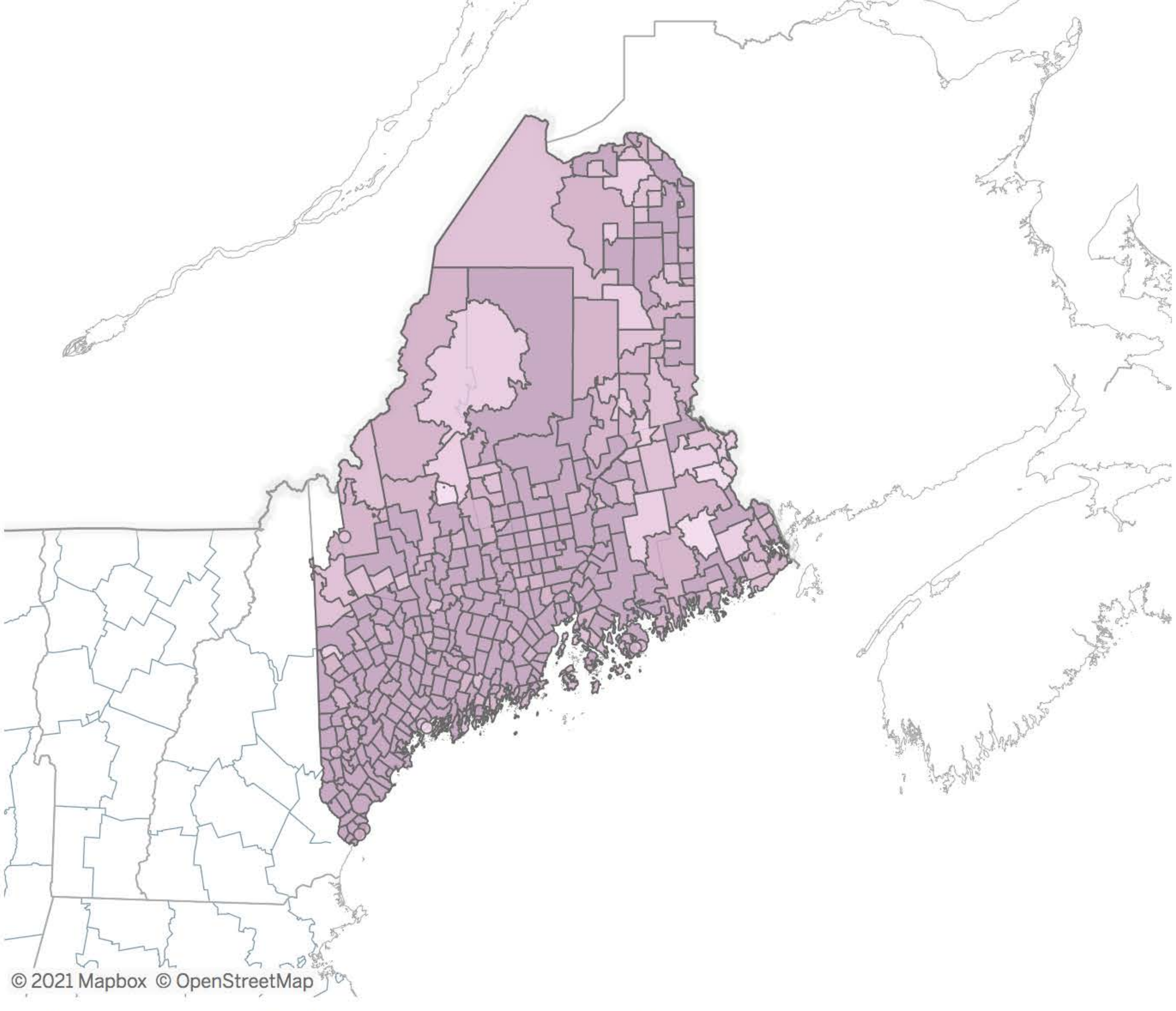

Color legend
Click to Highlight
1-5
6-19
20-49
50-99
>100
No detected cases

Per Maine CDC's privacy policy, 1) Case counts for ZIP codes in which the population is less than 50 or the population is unknown must be suppressed and are not shown on the map. 2) Exact case counts are suppressed in all zip codes with 5 or fewer cases or a population less than 2,000.

Download a csv file with the most recent cumulative case data by zip code.

Maine and COVID-19

Maine COVID-19 Centralized Dashboard

Maine and COVID-19 | Cumulative Case Data | Hospital Capacity Data | Population and Case Rate Data | 24-Hour Case Changes

Maine COVID-19 Cumulative County Case Data Last Update: Friday, December 10, 2021 at 3:00 PM EST

Notice: The Maine Office of GIS (MEGIS) updates COVID-19 content Monday through Friday. Maine CDC updates case data Tuesday through Saturday. The Monday MEGIS update will display data posted by MECDC on Saturday. MEGIS and MECDC will not update on holidays.

Total Cases*	Total Confirmed Cases	Hospitalizations	Deaths
128,981	92,313	3,223	1,365

*Total cases includes both confirmed and probable cases.

COVID-19 Positive Case Rate Map

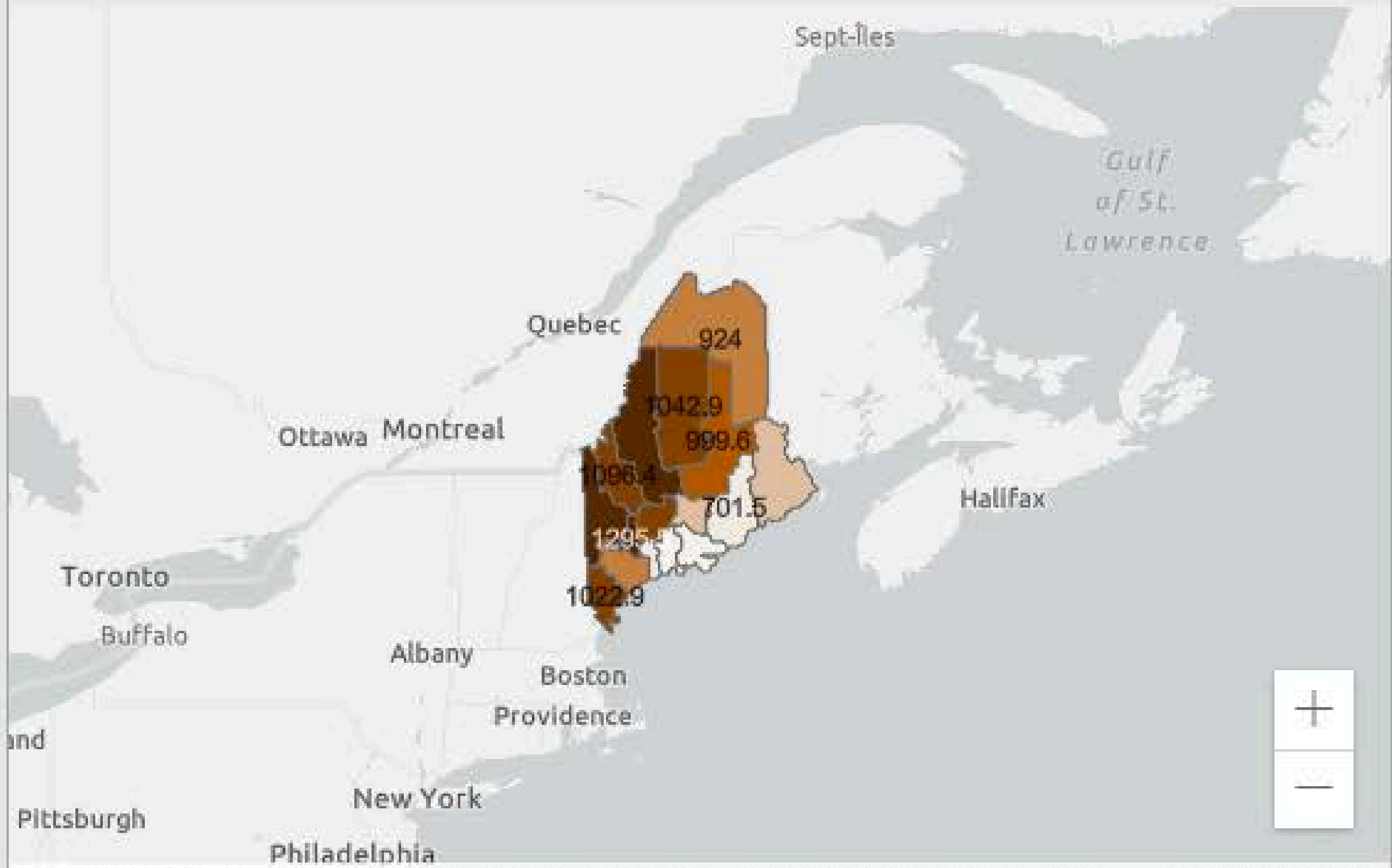

Province of New Brunswick, Esri, HERE, Garmin, FAO, NOAA, USGS, EPA, NRCan,... Powered by Esri

Note: The case distribution displayed on this map is likely an underrepresentation of the true number of cases in Maine since not all individuals are being tested. For individuals not considered to be at high risk, medical providers are currently advised to diagnose COVID-19 based on symptoms.

COVID-19 Progression in Maine

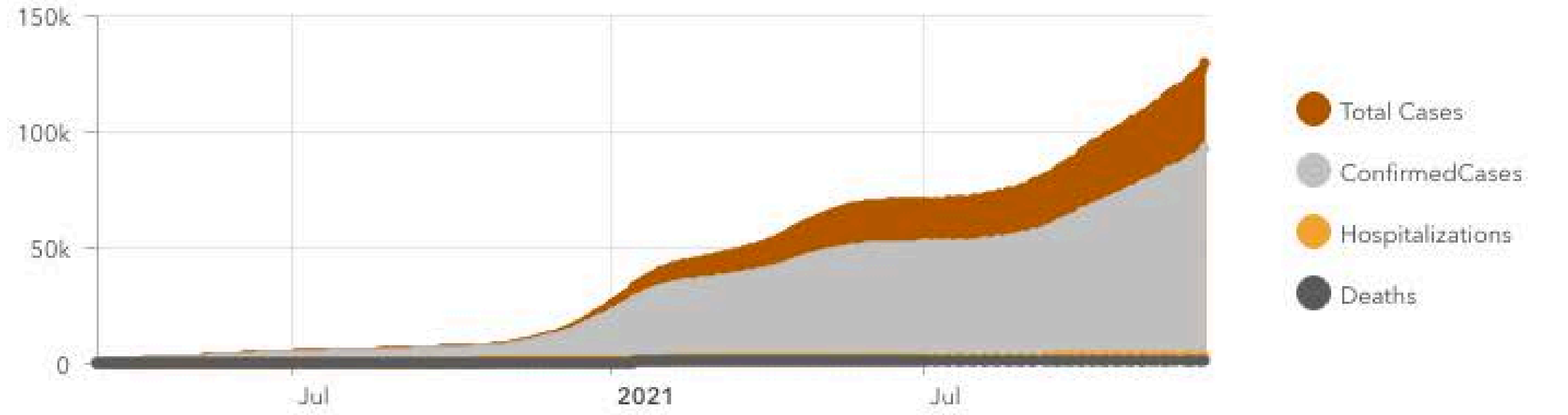

Case data are preliminary due to day-to-day changes in available information reported by the counties.

Note: As of July 1, 201, MECDC no longer reports data seven days a week.

Maine Counties Caseload Over Time

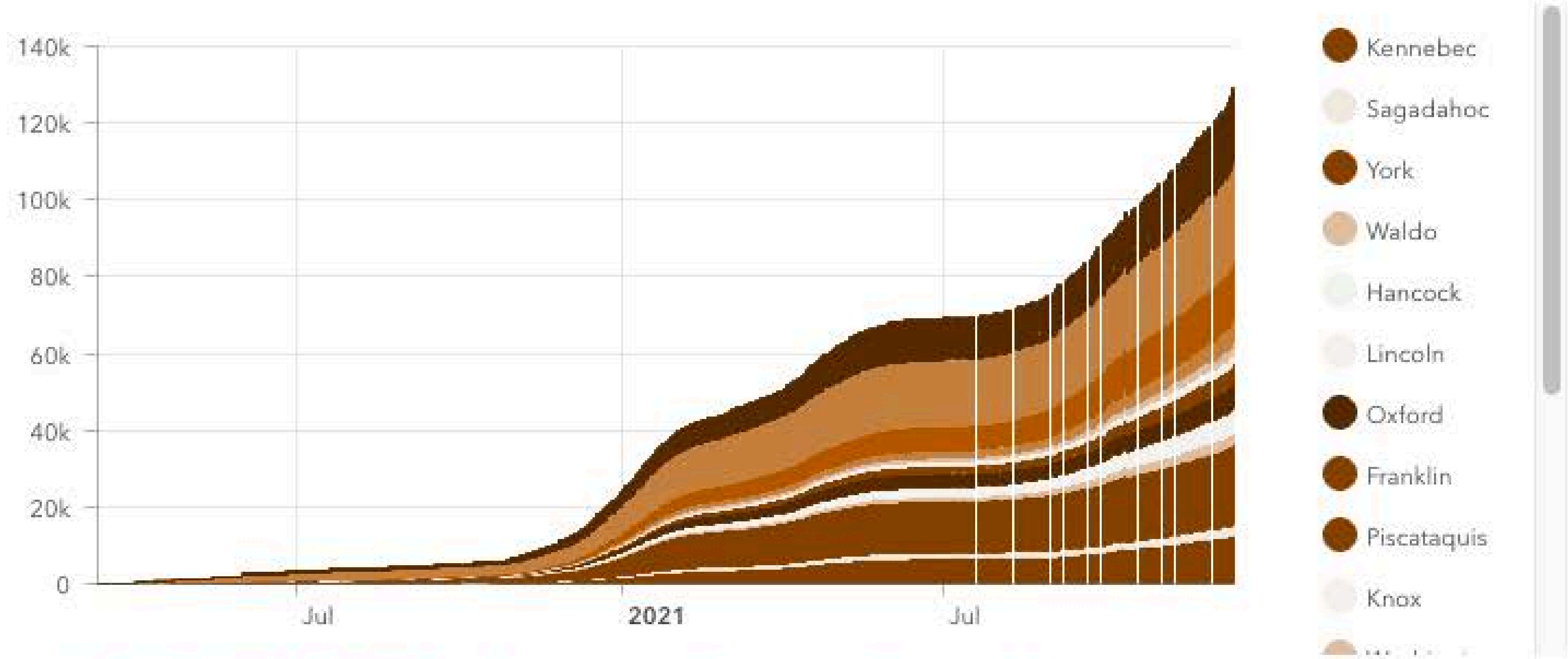

Note: As of July 1, 2021, MECDC no longer reports data seven days a week.

Maine and COVID-19

Maine COVID-19 Centralized Dashboard

Maine and COVID-19 | Cumulative Case Data | Hospital Capacity Data | Population and Case Rate Data | 24-Hour Case Changes

Maine COVID-19 Population and Case Rates Last Update: Friday, December 10, 2021 at 3:00 PM EST

Notice: The Maine Office of GIS (MEGIS) updates COVID-19 content Monday through Friday. Maine CDC updates case data Tuesday through Saturday. The Monday MEGIS update will display data posted by MECDC on Saturday. MEGIS and MECDC will not update on holidays.

Total Positive Cases

128,981

Total Tests

3,964,870

-- Only updated on Thursdays --

Statewide Cumulative Cases by Age

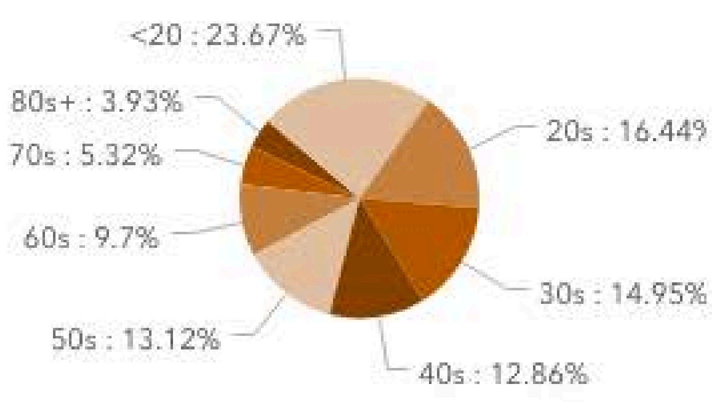

Statewide Cumulative Cases by Sex

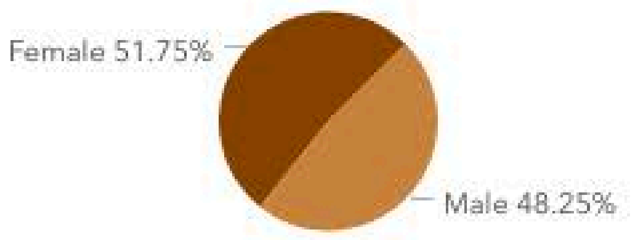

Maine COVID-19 Cases by Race

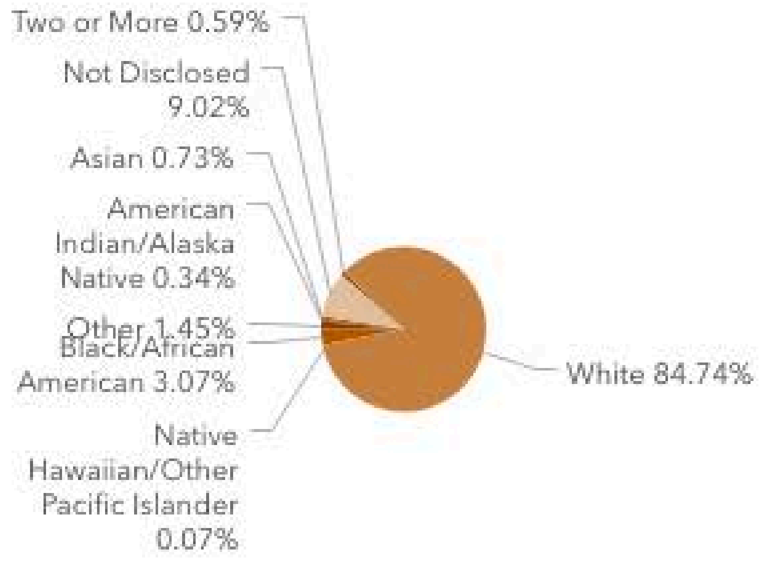

COVID-19 Case Rates by County

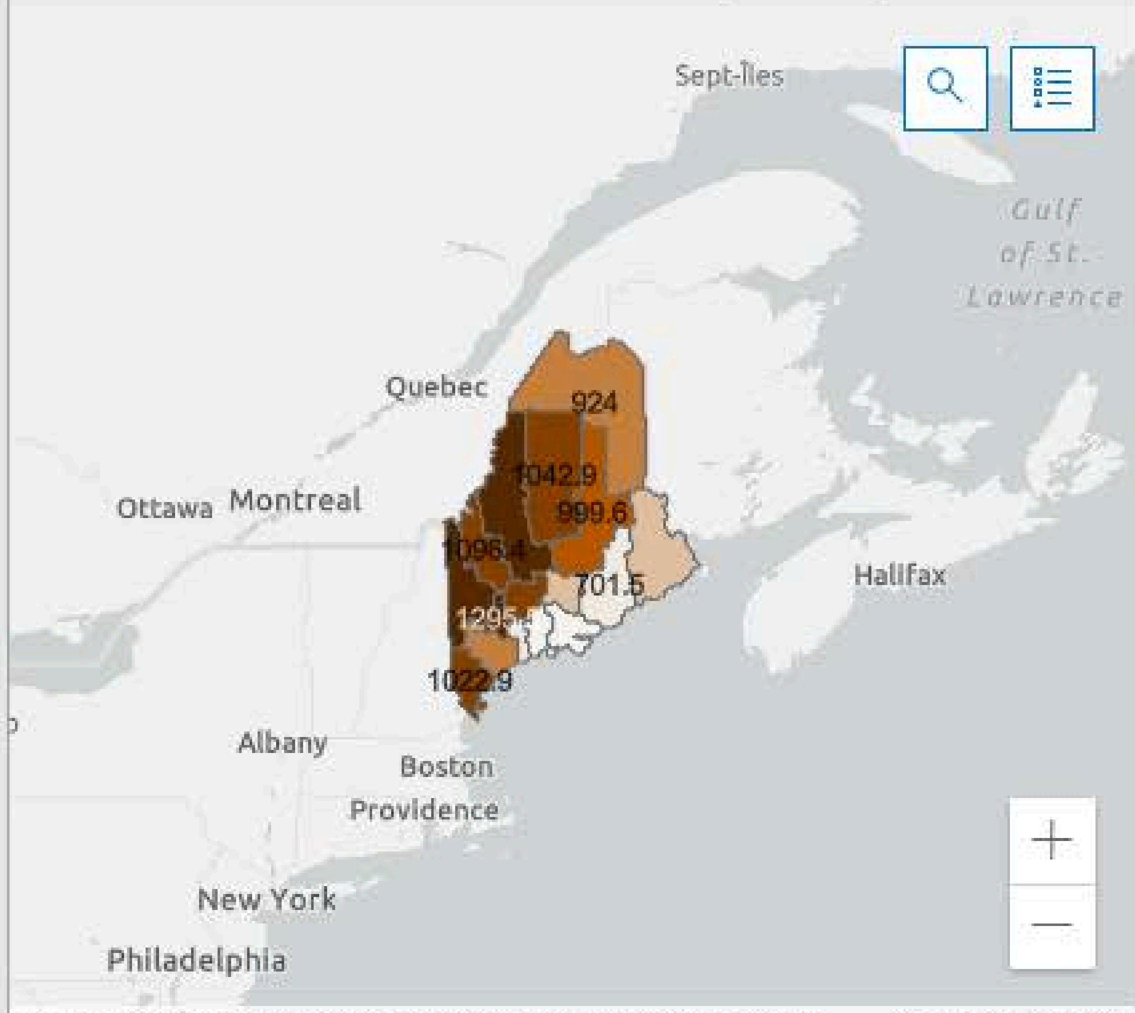

Province of New Brunswick, Esri, HERE, Garmin, FAO, N... Powered by Esri

Note: Case rates are calculated by dividing the total number of positive cases in a county by the total population of that county multiplied by 10,000. Confirmed data represent only those individuals with positive test results, which likely under-represents the true number of cases in Maine.

Rates (%) of Hospitalization and Deceased by County

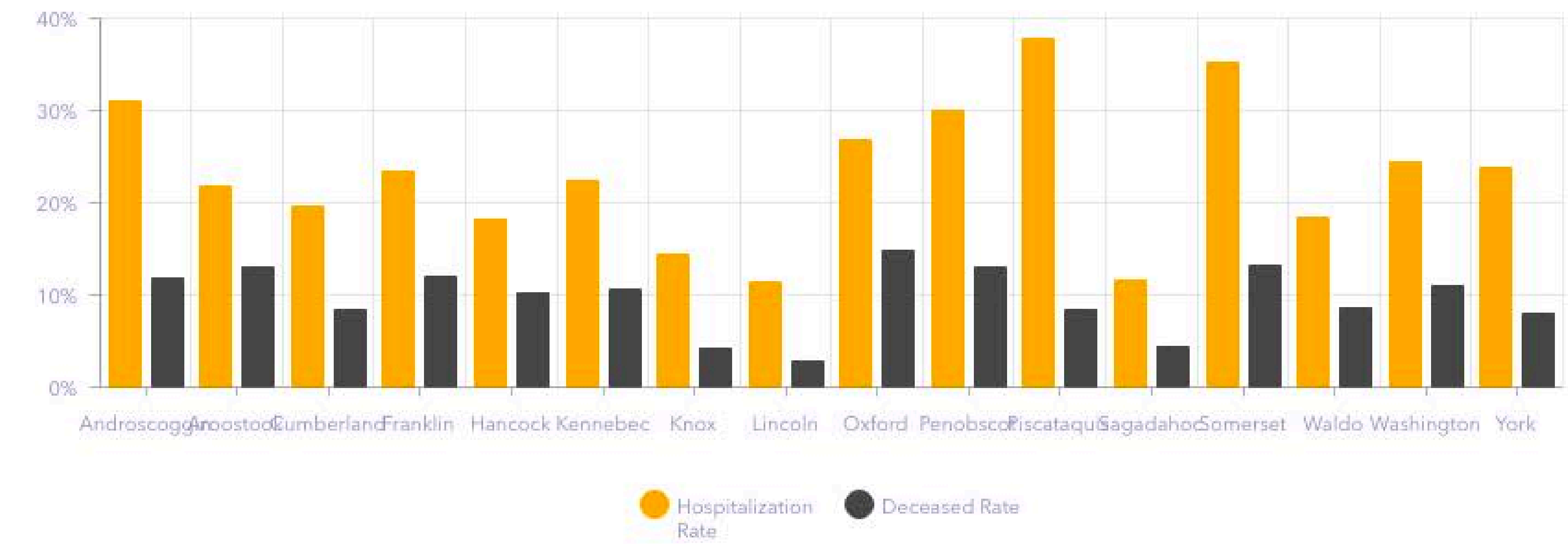

Calculation: (Case category divided by total county population) multiplied by 100

Maryland DEPARTMENT OF HEALTH
MARYLAND

Coronavirus Disease 2019 (COVID-19) Outbreak

This is a rapidly evolving situation and the Maryland Department of Health (MDH) will provide updates as they become available.

Notice of Non-Discrimination and Accessibility Statement

Additional COVID-19 Resources at CovidLINK

Search

Maryland COVID-19 Data Dashboard

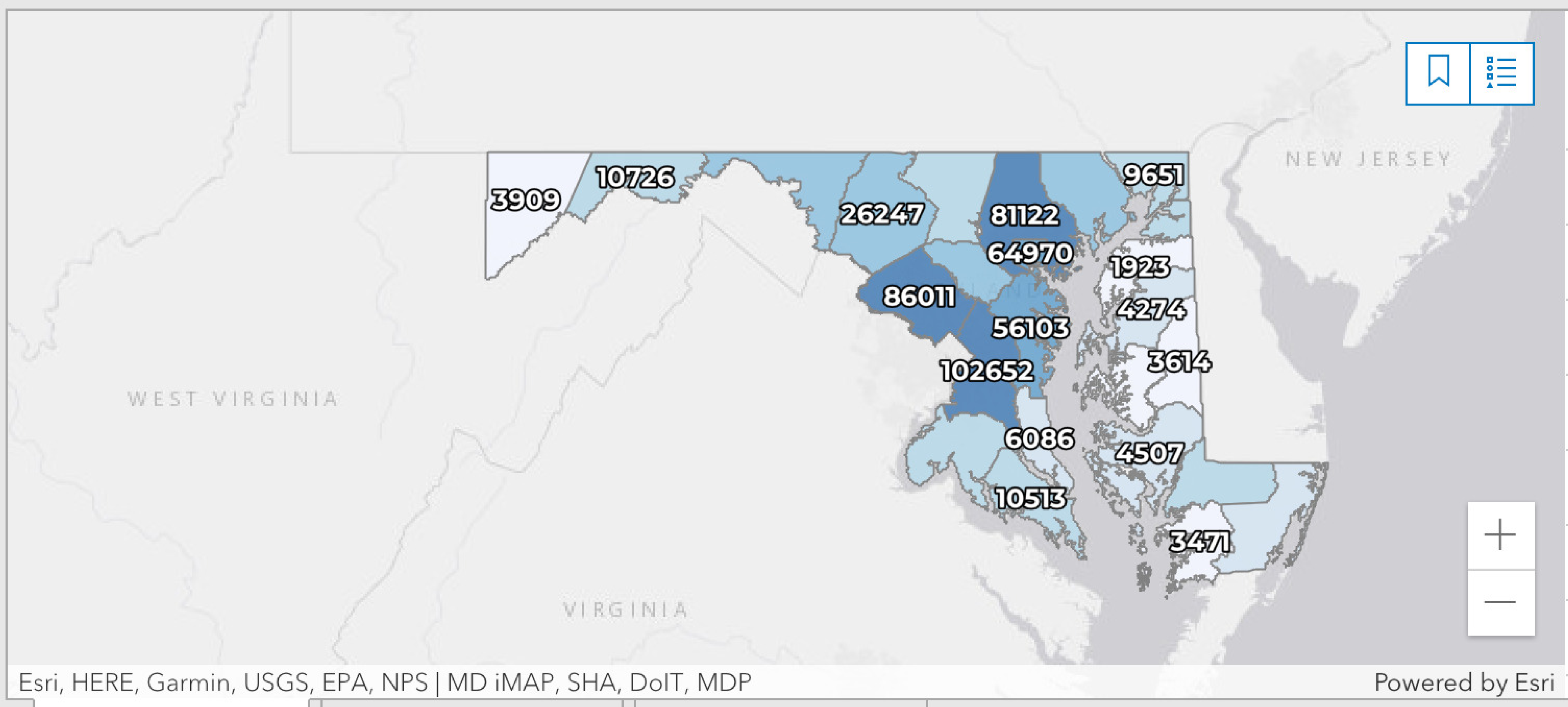

Affected Counties:

- Prince George's - 102,652
- Montgomery - 86,011
- Baltimore - 81,122
- Baltimore City - 64,970
- Anne Arundel - 56,103
- Frederick - 26,247
- Howard - 23,804
- Harford - 22,674

Cases by County | Cases by ZIP Code | Testing by County

ICU and Acute Hospital Beds for COVID-19, Currently in Use

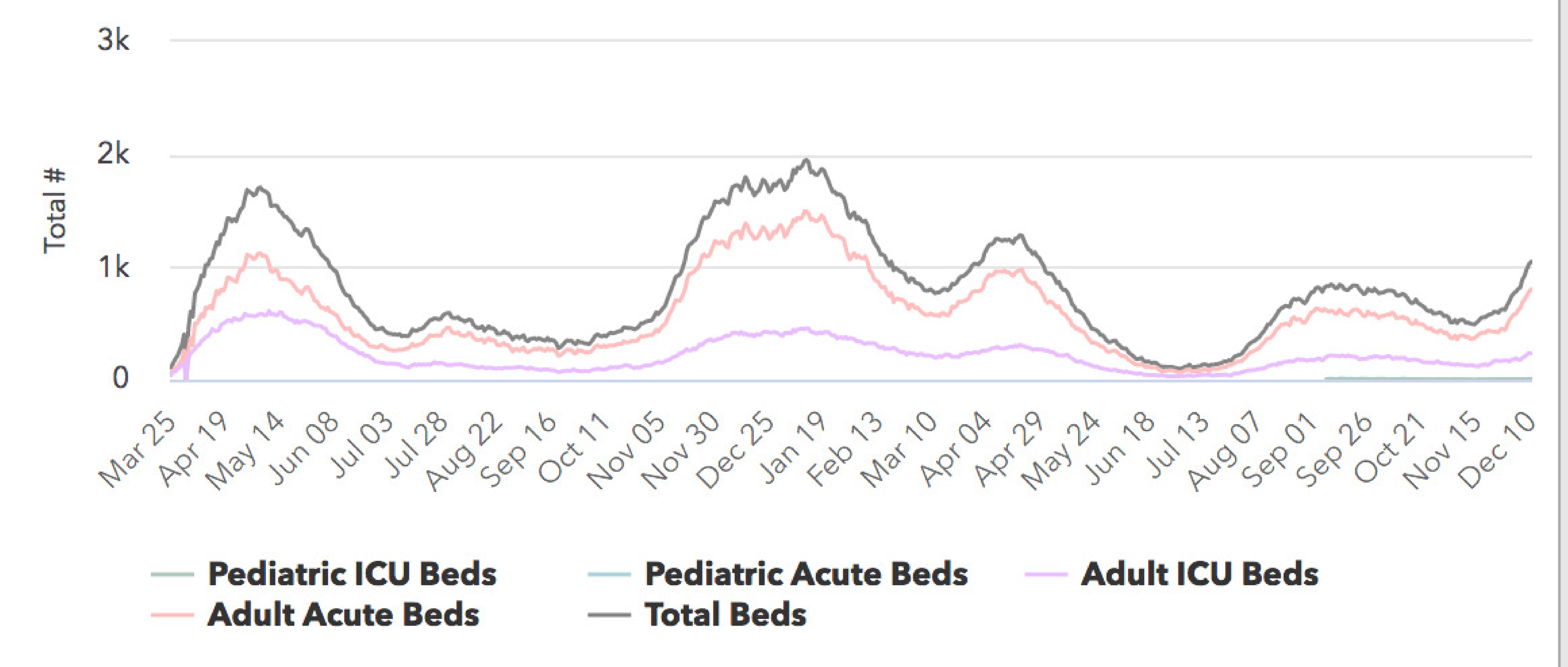

Testing Volume, Tests per Day and Percent Positive Rate (7-Day Avg)
- Methodology

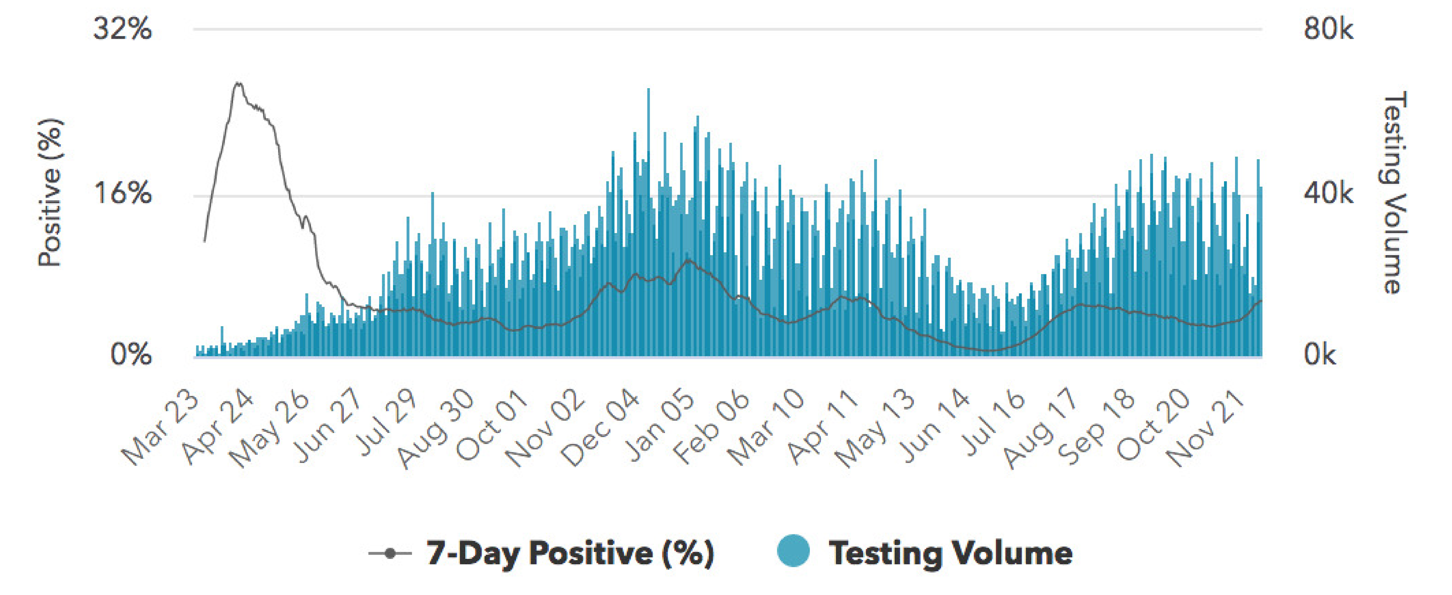

Maryland.gov

LANGUAGE ASSISTANCE SERVICES

Testing | Find a Vaccine | Vaccine Info | Vaccine Data | Contact Tracing Data | Nursing Home Data | Schools Data

Confirmed Cases	Persons Tested Negative	Testing Volume	Testing % Positive	Confirmed Deaths	Currently Hospitalized
592,679	4,636,177	15,1[illegible]	5.43%	11,022	1,048
24hr Change: +1,86[illegible]	24hr Change: +7,66[illegible]	24hr Change: +45,44[illegible]	24hr Change: -0.01	24hr Change: +1[illegible]	24hr Change: 21

12/11/2021: MDH is experiencing a server outage. Hospitalizations data are current. Other surveillance data will be updated as soon as possible. Vaccine data is updated as of 12/11/2021.

MDH will pause COVID-19 reporting on Nov. 25 and 26; Dec. 24 and 25; and Dec. 31 and Jan. 1 in observance of the holidays. When reporting resumes, updates will include both the days encompassed in the pause and the standard 24-hour changes in metrics.

Note: The Maryland Department of Health updates these data daily during the 10 a.m. hour. All data are preliminary and subject to change based on additional reporting. Case and death data reflect Maryland residents only. Death data are presented by both date of report and by date of death and are updated as amendments to the death record are received. Data for ZIP codes with 7 or fewer cases are suppressed. Hospitalization data reflect COVID-19 bed occupancy in Maryland hospitals. Testing volume data represent the static daily total of PCR COVID-19 tests electronically reported; this count does not include test results submitted by labs and other clinical facilities through non-electronic means. The percent positive rate is a seven day rolling average of positive results as a percentage of all tests. MDH is continuously evaluating its data and reporting systems and will make updates as more data becomes available. View the Maryland COVID-19 Data Dashboard FAQ.

COVID-19 Vaccination Dashboard

CDC: Population with at Least One Dose

Population Age 18+	Population Age 5+
90%	83.2%

Source: CDC Covid Data Tracker

Doses Administered

9,773,999

All Doses Administered | Vaccinations by Dose

Fully Vaccinated

4,180,901

Doses Distributed

12,713,380

Vaccinations Administered by Date

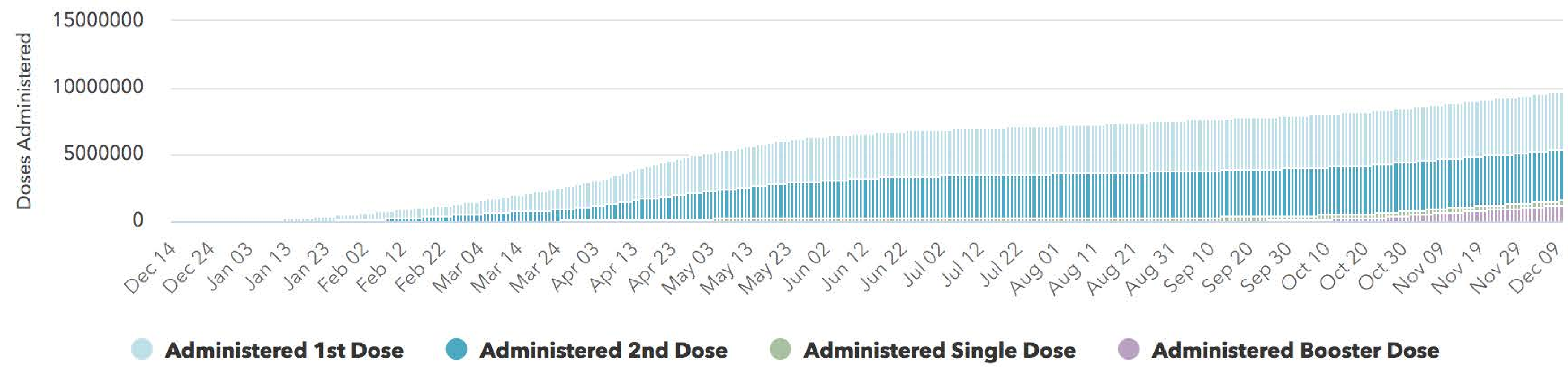

Vaccination Administered by Date

COVID-19 Vaccine Announcements

- Vaccine Milestone: Maryland Surpasses 1 Million COVID-19 Booster Shots *12/1/2021*
- Governor Hogan Statement on COVID-19 Omicron Variant *11/29/2021*
- Vaccine Milestones: State of Maryland Surpasses 88% of Adults, 99.9% of Seniors Vaccinated *11/22/2021*
- Governor Hogan Announces Immediate Expansion of COVID-19 Booster Eligibility to All Adults *11/19/2021*
- Vaccine Milestone: Governor Hogan Announces Maryland Surpasses 9 Million Vaccinations *11/18/2021*
- Vaccine Milestone: Governor Hogan Announces 99% of Maryland Seniors Vaccinated *11/13/2021*
- Vaccine Milestone: Maryland Surpasses 500,000 COVID-19 Booster Shots *11/8/2021*

Vaccinations Statewide by Age

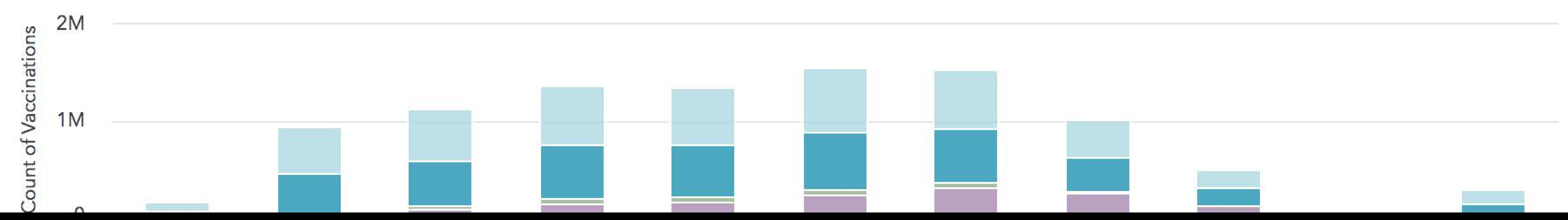

Tweets by @GoVAXMaryland

GoVAX Maryland @GoVAXMaryland

.@AACountySchools & @AAHealth are again teaming up to offer another round of COVID-19 [illegible] week. Click the link to see the schedule => [illegible].com/2021/12/10/aac... via @SoMDChronicle

Completed Vaccinations [illegible]

MDH will pause COVID-19 reporting on Nov. 25 and 26, Dec. 24 and 25, and Dec. 31 and Jan. 1 in observance of the holidays. When reporting resumes, updates will include both the days encompassed in the pause and the standard 24-hour changes in metrics.

Note: The Maryland Department of Health updates these data daily during the 10 a.m. hour. Data are based on facility reports to MDH, which may be revised if additional information becomes available. Some vaccination data may be unavailable due to the time lag between vaccine administration and data entry. All data are preliminary and subject to change based on additional reporting. Doses distributed and administered both account for the 16,000 doses of vaccine provided to the District of Columbia to vaccinate Maryland residents who work in DC. MDH is continuously evaluating its data and reporting systems and will make updates as more data becomes available. Vaccines administered in Maryland include Pfizer and Moderna, which are two dose vaccine regimens, and Johnson & Johnson, which is a one dose vaccine regimen. Percent of population vaccinated is calculated based on total population, not total population eligible to be vaccinated. ZIP code data is capped when values exceed 100% and are suppressed if vaccination total is less than 7; ZIP code data is updated once weekly on Wednesdays. "Received at least one dose" represents the total number of people who received at least one dose of COVID-19 vaccine, including those who received one dose of the single-shot Johnson & Johnson COVID-19 vaccine. "Fully vaccinated" represents the number of people who have received the second dose in a two-dose vaccine series or one dose of the single-shot COVID-19 vaccine. Race/ethnicity data previously displayed was taken from data gathered at the time of immunization, which is not always collected. As such, as of 3/13/2021, Maryland identifies missing race/ethnicity from the last two years of clinical history, to improve the percentage of fully identified race/ethnicity data. A reconciliation in vaccination data has resulted in a one-time adjustment made to the Maryland Covid-19 vaccine administration dashboard. A reduction of about 42,000 Veteran Affairs vaccine doses (combined first, second and single) has been made to bring reporting of the Maryland Department of Health and the U.S. Center for Disease Control and Prevention (CDC) into alignment. View additional notes: 11/12/21

COVID-19 Interactive Data Dashboard

Updated Monday-Friday by 5 p.m.

Data from Friday, Saturday and Sunday are included in the Monday dashboard data.

When Monday is a scheduled holiday, the Friday through Monday data will be posted on Tuesdays.

Massachusetts Department of Public Health | COVID-19 Dashboard
Today at a Glance

Released on: December 10, 2021
Data as of: December 9, 2021
Caution: recent data may be incomplete

Navigation

Today's Overview

Overview Trends

COVID-19 Cases

COVID-19 Testing

COVID-19 Interactive Data Dashboard

TOP

Table of Contents

COVID-19 Deaths

Higher Ed & LTCF

Patient Breakdown

City & Town Data

Resources

Data Archive

For data on COVID-19 variants, click here (CDC website)

Cases

Confirmed Cases

Today there were 5,007 new, confirmed cases reported bringing the total to 901,430 total confirmed cases.

probable cases reported bringing the total to 68,159 total probable cases.

Testing

Tests Reported

There were 104,625 new COVID-19 molecular tests reported, bringing the cumulative total to 34,474,478 tests.

positivity is [illegible]

Hospitalizations

Hospitalizations

There are 1,238 patients hospitalized for COVID-19.

Intensive Care Units (ICU) and 148 patients are intubated.

Deaths

Confirmed Deaths

There were 27 new, confirmed deaths reported. There have been 19,217 confirmed deaths in total.

deaths reported. There have been 417 probable deaths in total.

Average Age of Deaths

The average age of patients who died of COVID-19 was 75 years old.

Confirmed cases by age during the last two weeks *Data updated weekly

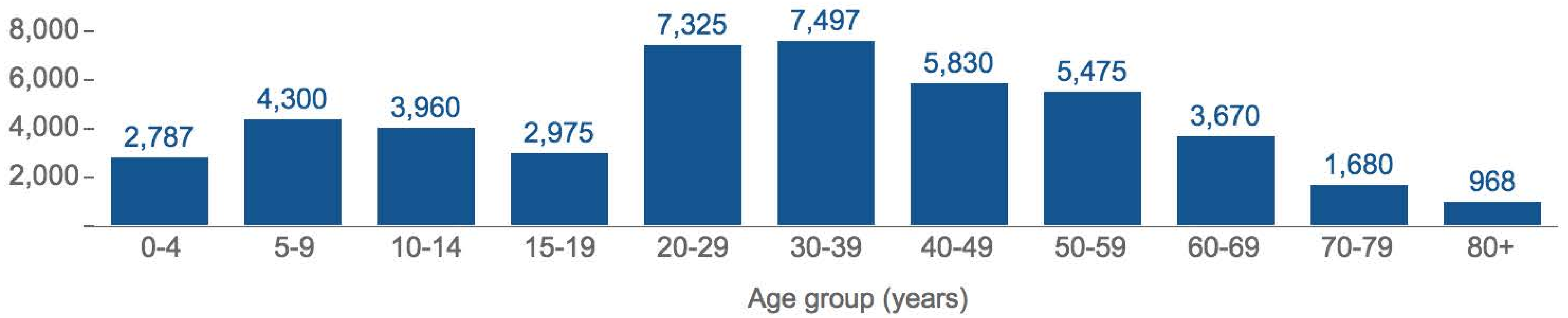

For details on the definitions of each indicator hover over the box or graph. All data included in this dashboard are preliminary and subject to change. Data Sources: COVID-19 Data provided by the Bureau of Infectious Disease and Laboratory Sciences and the Registry of Vital Records and Statistics; COVID-19 Hospitalization Data provided by the MDPH survey of hospitals (hospital survey data are self-reported); Created by the Massachusetts Department of Public Health, Bureau of Infectious Disease and Laboratory Sciences, Division of Surveillance, Analytics and Informatics.

Undo Redo Revert Refresh Pause Share Download Full Screen

Massachusetts Department of Public Health | COVID-19 Dashboard
Trends: 7-day Averages Over Time

Released on: December 10, 2021
Data as of: December 9, 2021
Caution: recent data may be incomplete

Navigation

- Today's Overview
- **Overview Trends**
- COVID-19 Cases
- COVID-19 Testing
- Hospitalizations
- COVID-19 Deaths
- Higher Ed & LTCF
- Patient Breakdown
- City & Town Data
- Resources
- Data Archive

Select dates:
3/1/2020 12/9/2021

Cases

7-day average of COVID-19 confirmed cases

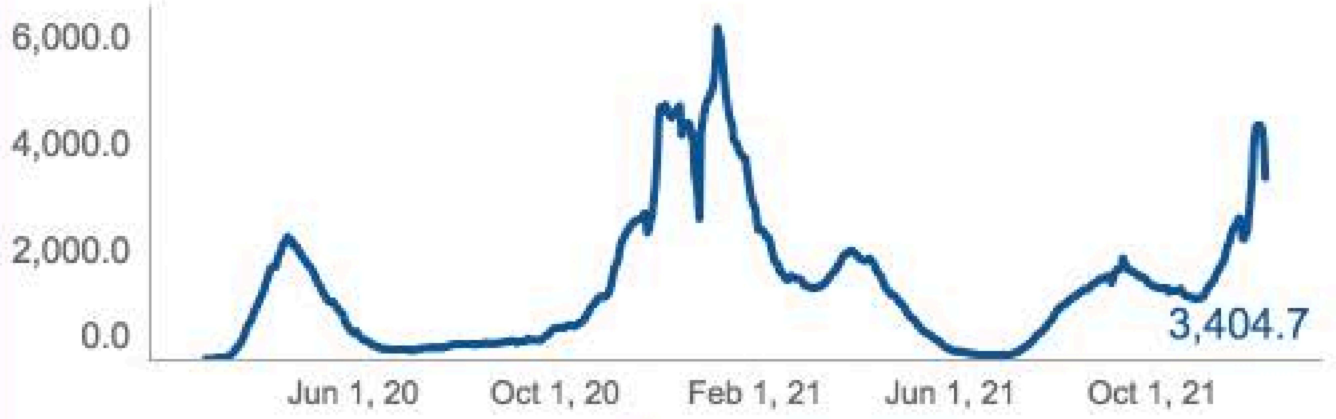

The lowest observed value was 64.1 on 6/25/2021.

Testing

7-day weighted average percent positivity

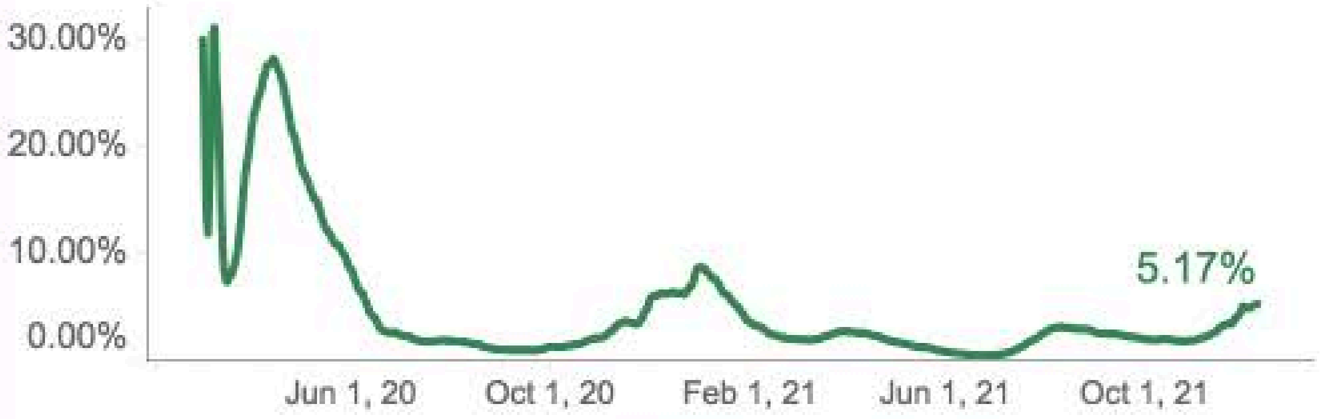

The lowest observed value was 0.31% on 6/25/2021.

Hospitalizations

7-day average of hospitalizations

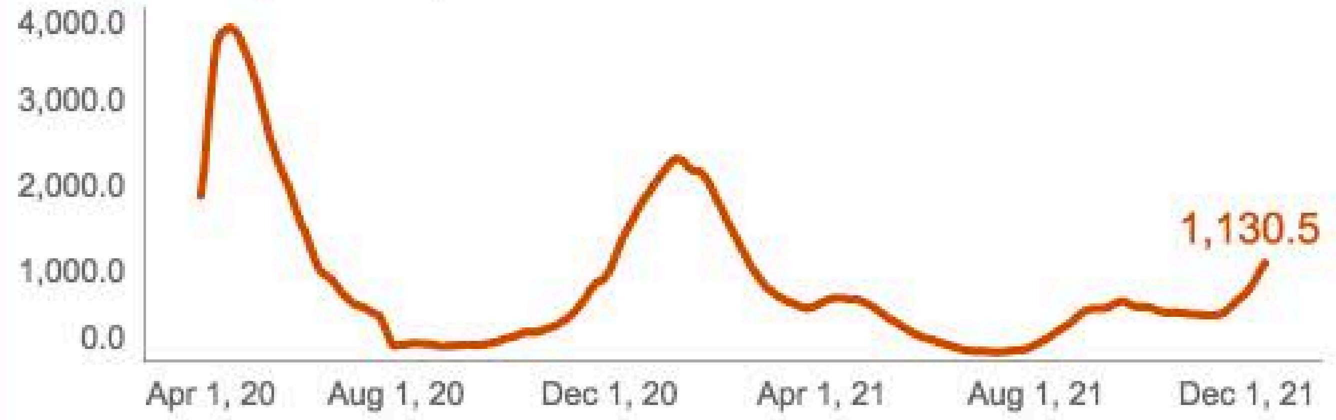

The lowest observed value was 84.8 on 7/9/2021.

Deaths

7-day average of confirmed deaths

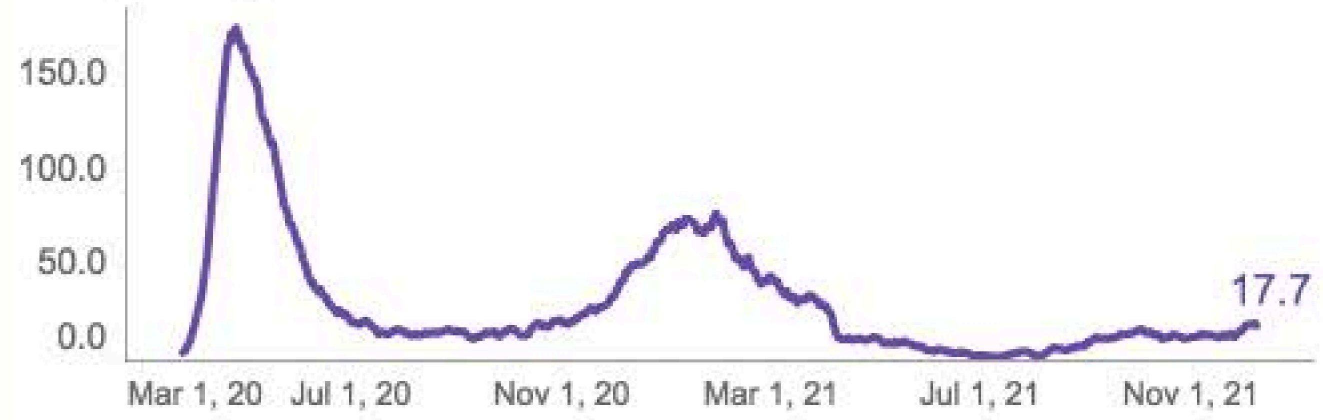

The lowest observed value was 1.3 on 7/11/2021.

The lowest observed value is since tracking of the lowest value began on April 15, 2020. For details on the definitions of each indicator please see the corresponding tab for that indicator. All data included in this dashboard are preliminary and subject to change. Data Sources: COVID-19 Data provided by the Bureau of Infectious Disease and Laboratory Sciences and the Registry of Vital Records and Statistics; Created by the Massachusetts Department of Public Health, Bureau of Infectious Disease and Laboratory Sciences, Division of Surveillance, Analytics and Informatics.

Massachusetts Department of Public Health | COVID-19 Dashboard
Cases of COVID-19

Released on: December 10, 2021
Data as of: December 9, 2021
Caution: recent data may be incomplete

COVID-19 Confirmed and Probable Cases

Select dates:
1/29/2020 – 12/9/2021

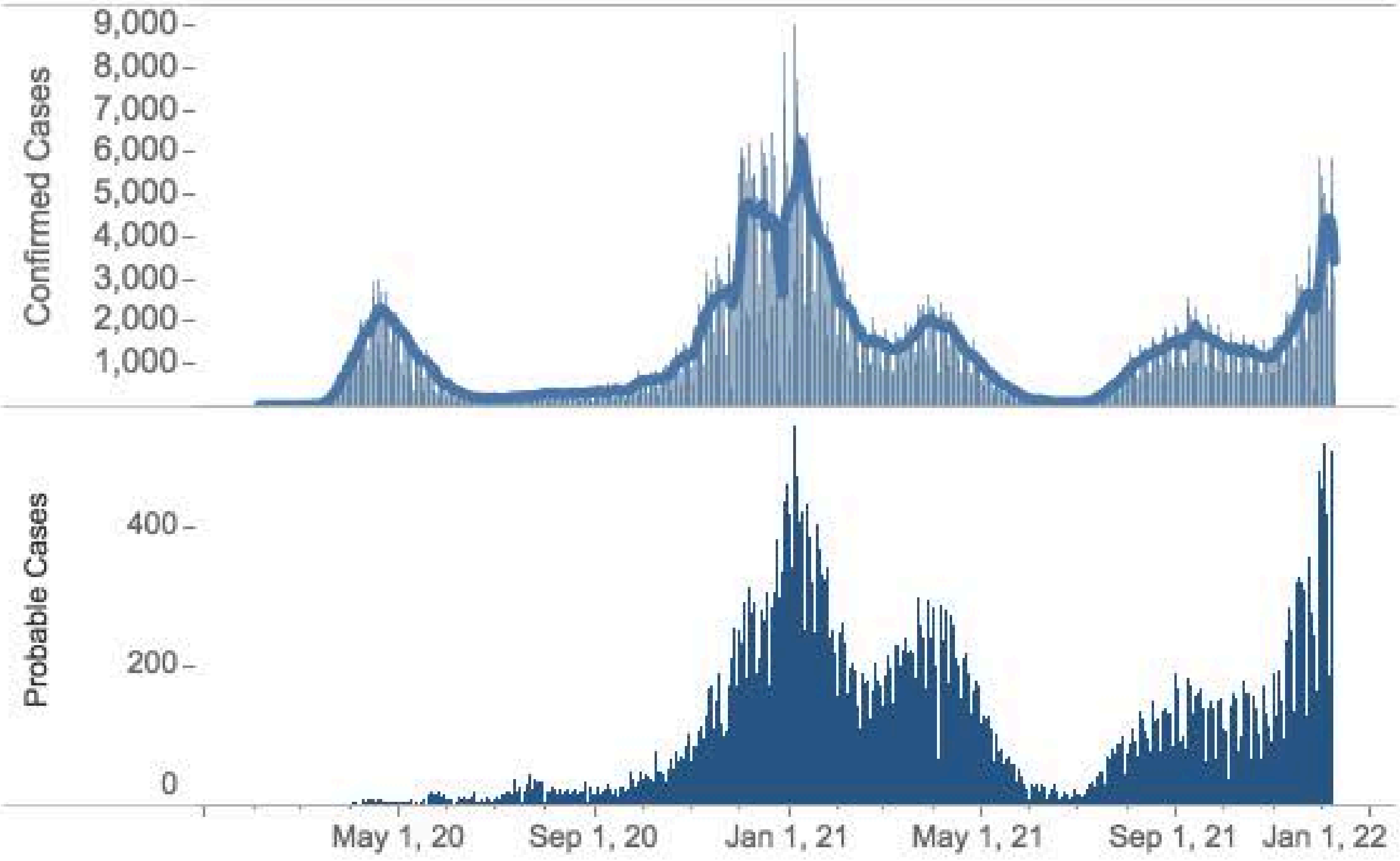

New confirmed cases reported by county and date

Select new or total cases: New confirmed cases

Select a date*: 12/10/2021

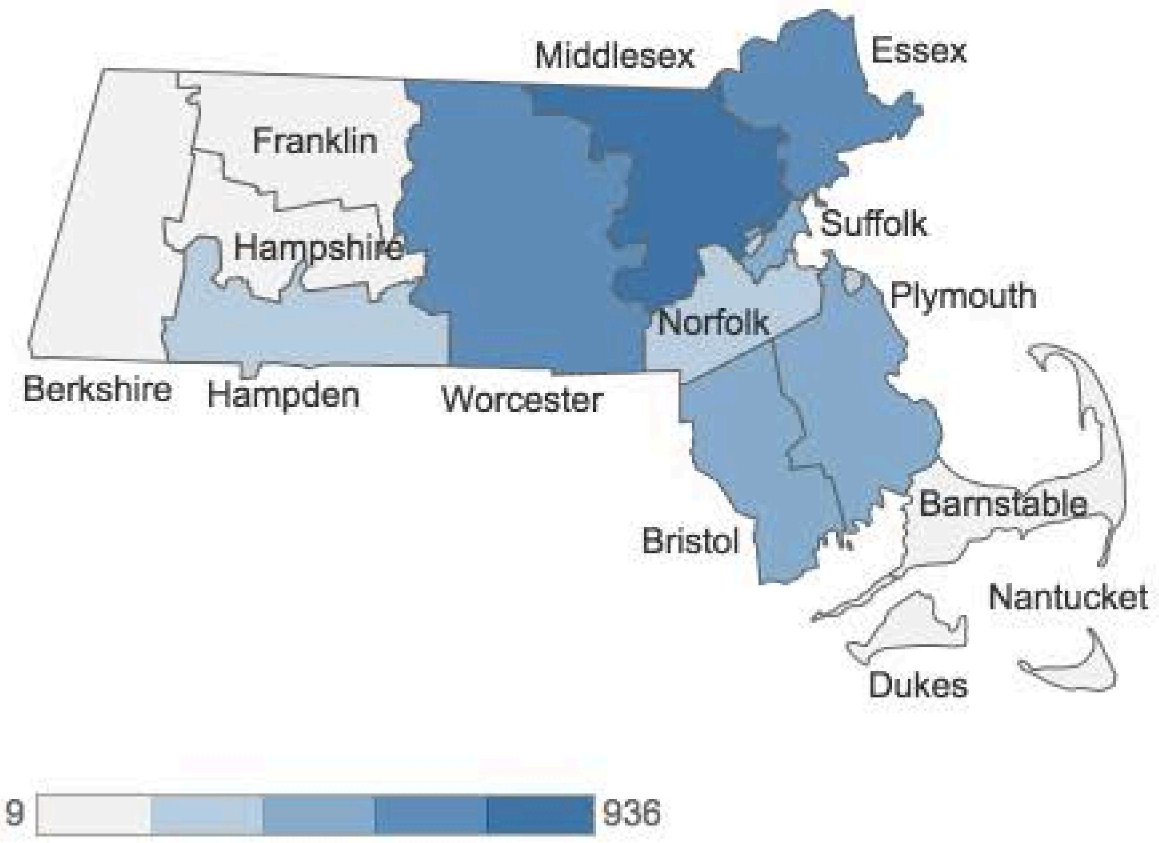

All data included in this dashboard are preliminary and subject to change. Data Sources: COVID-19 Data provided by the Bureau of Infectious Disease and Laboratory Sciences and the Registry of Vital Records and Statistics; Created by the Massachusetts Department of Public Health, Bureau of Infectious Disease and Laboratory Sciences, Division of Surveillance, Analytics and Informatics. . Case counts for specific cities, towns, and counties change as data cleaning occurs (removal of duplicate reports within the system) and new demographic information (assigning cases to their city or town of residence) is obtained.

*The most recent 30 days of data are viewable on this map. To view data outside of this range, please visit our data archive and download the raw data.

CURRENT STATUS | DAILY CASES | CUMULATIVE TRENDS | DEMOGRAPHICS | TOTAL TESTING | CONFIRMATORY TESTING | LEARN MORE

View Cases

View Deaths

View Table

Select Preparedness Region:

All

Select County:

All

This report is provisional and subject to change. City of Detroit and Wayne County are reported separately. MDHHS began tracking probable cases April 5, 2020. FCI indicates federal correctional institute. MDOC indicates Michigan Department of Corrections. Diagnostic tests, including nucleic acid amplification/real-time polymerase chain reaction (NAAT/RT-PCR) and rapid virus antigen detection tests, look for current infection. Serology (antibody) tests are used to look for the possibility of previous infection.

COVID-19 Cases by County

Confirmed cases include individuals with a positive NAAT/RT-PCR test for COVID-19. Probable cases include individuals with either a positive rapid antigen detection test OR COVID-19 symptoms and an epidemiologic link to a confirmed COVID-19 case, but no diagnostic test.

Cases by County

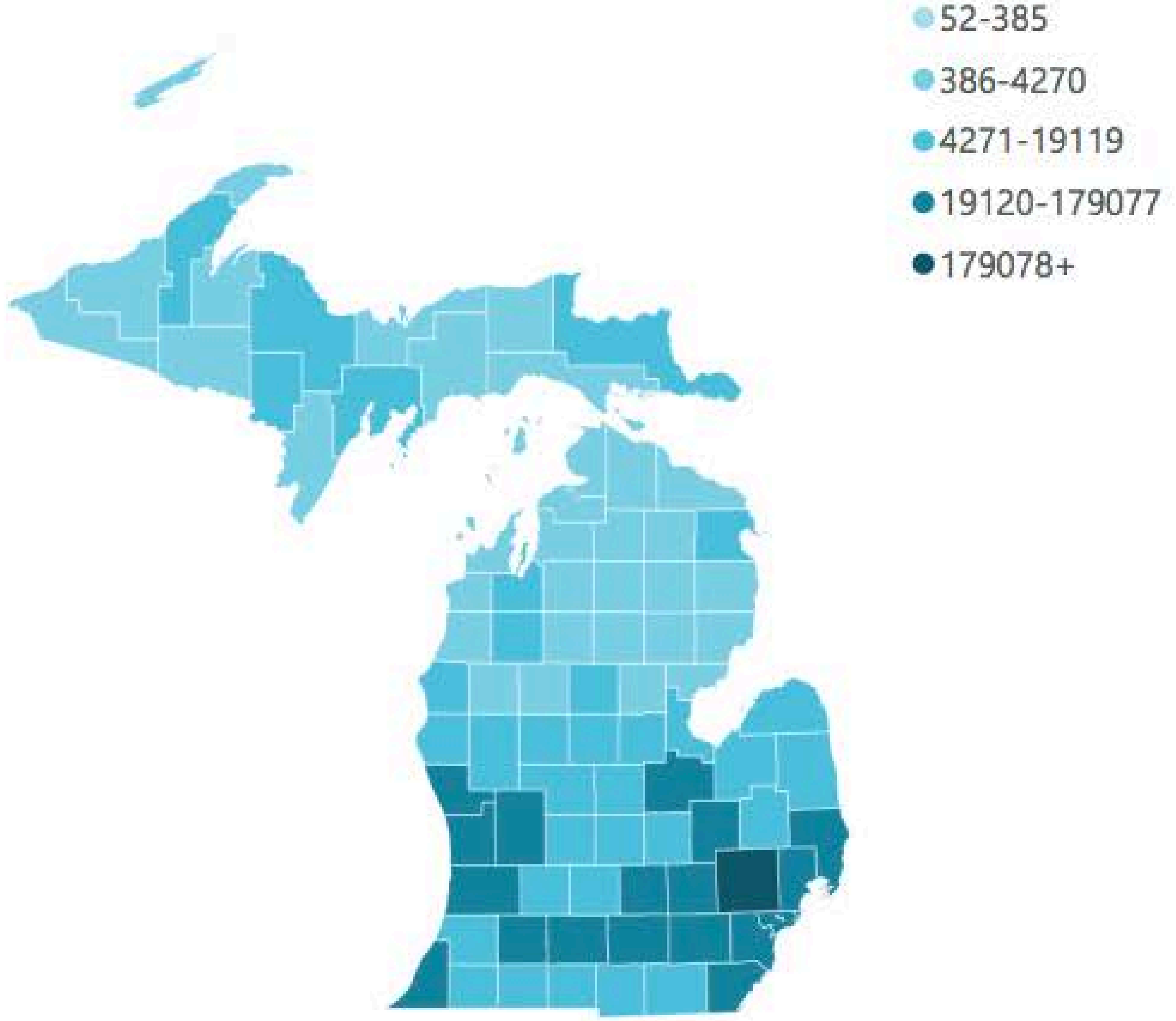

Michigan

Friday, December 10, 2021

Michigan (Statewide) Case Fatality Rate

1.8%

Current Totals

Case Status	Cases	Deaths
Confirmed	1,380,324	25,080
Probable	187,254	1,834
**Total**	**1,567,578**	**26,914**

Laboratory Testing

TestType	Tests Performed
Diagnostic	19,281,866
Serology	750,002
**Total**	**20,031,868**

Microsoft Power BI

1 of 7

CURRENT STATUS | DAILY CASES | CUMULATIVE TRENDS | DEMOGRAPHICS | TOTAL TESTING | CONFIRMATORY TESTING | LEARN MORE

Cases

Deaths

View Table

Select Preparedness Region:

All

Select County:

All

Cases are shown by onset date or by lab specimen collection date if onset date was unavailable. Deaths are shown by date of death or date or last status update if date of death was unavailable. Tracking of probable cases and deaths did not begin until April 5, 2020, therefore only probable cases referred for investigation on or after April 5, 2020 are displayed (onset may have been prior).

Notes: FCI indicates federal correctional institute. MDOC indicates Michigan Department of Corrections.

Daily Cases by Status

The number of daily confirmed cases represent the total number of people who had a positive NAAT/RT-PCR test for COVID-19. Cases are shown by the date of onset of symptoms.

Filter by Date:

3/1/2020 12/10/2021

Cases by Date of Onset

CASE_STATUS ● Confirmed ● Probable

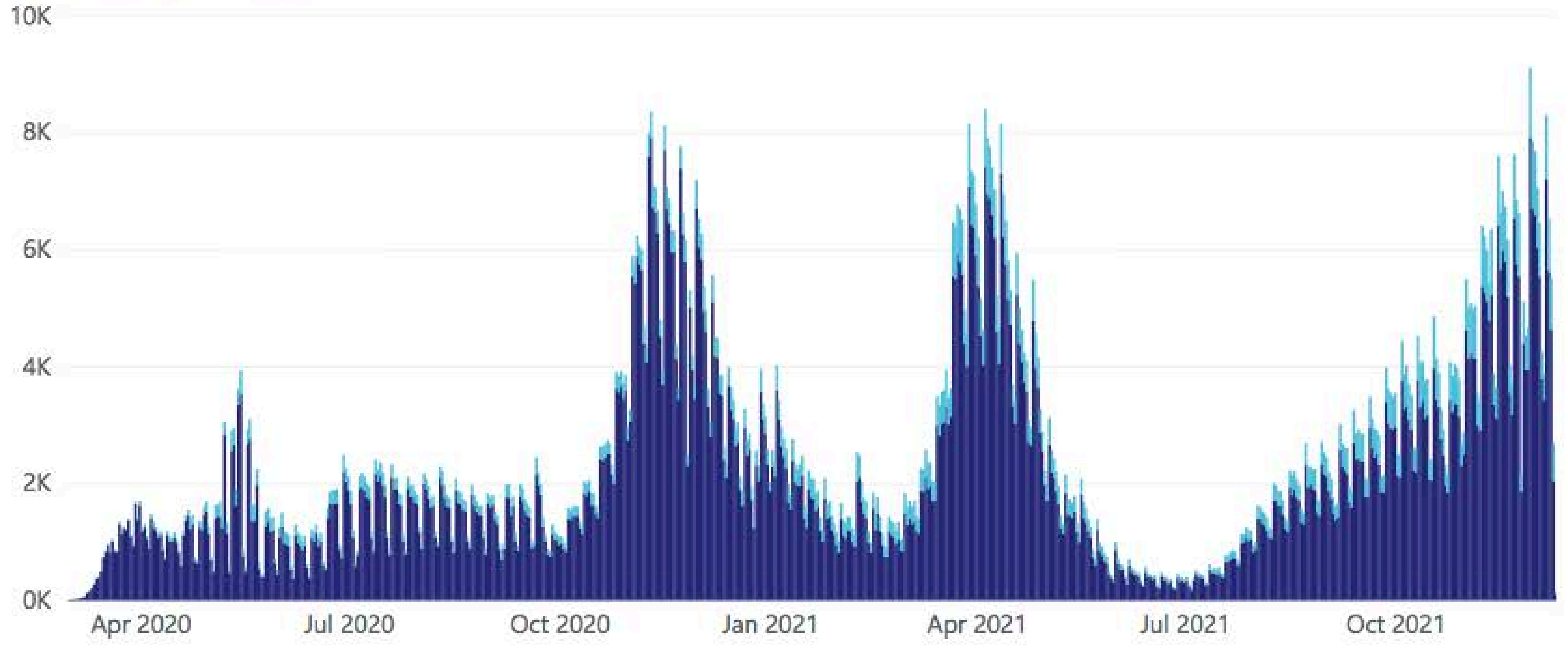

Microsoft Power BI

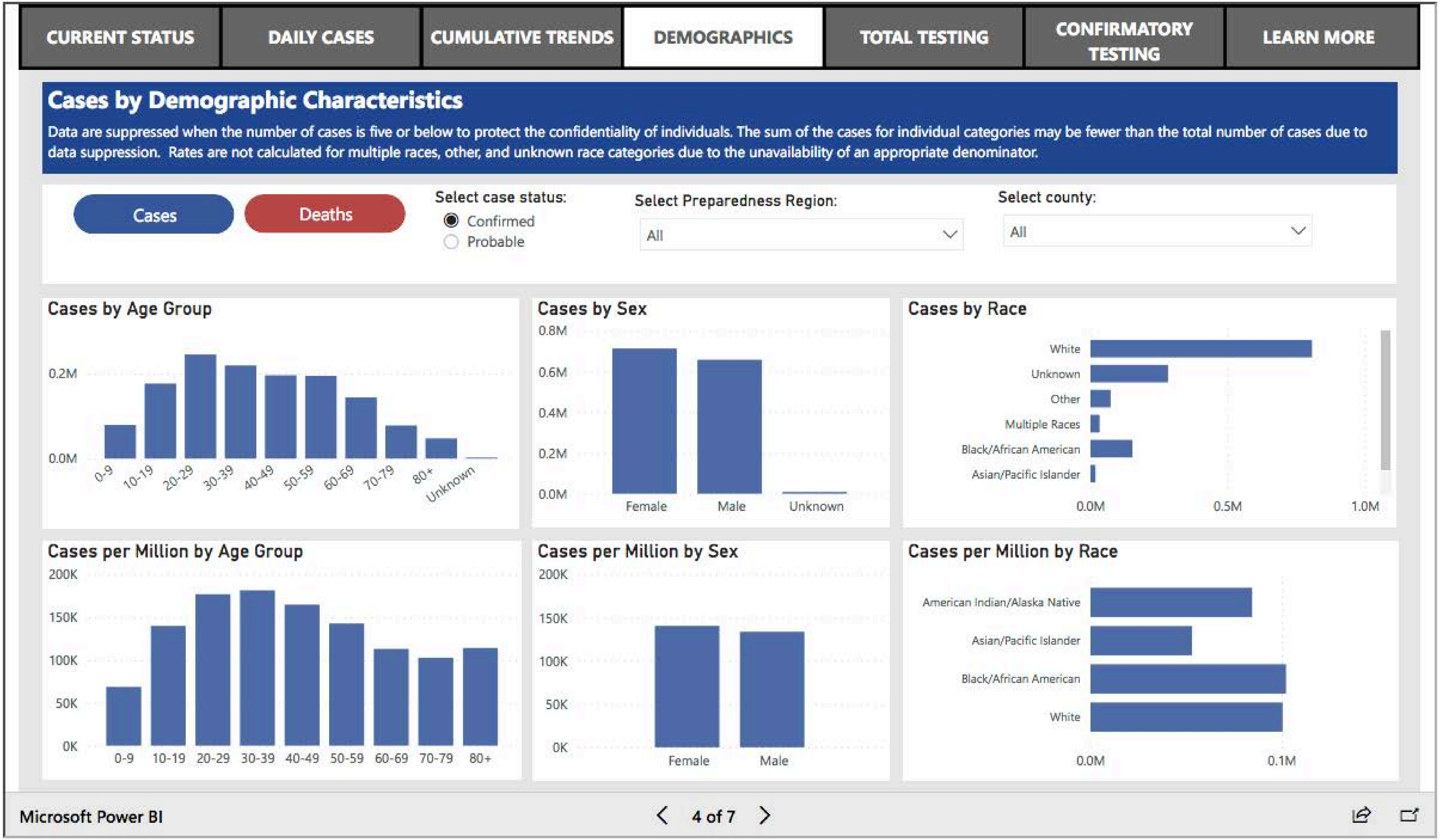
CURRENT STATUS
DAILY CASES
CUMULATIVE TRENDS
DEMOGRAPHICS
TOTAL TESTING
CONFIRMATORY TESTING
LEARN MORE
Cases by Demographic Characteristics
Data are suppressed when the number of cases is five or below to protect the confidentiality of individuals. The sum of the cases for individual categories may be fewer than the total number of cases due to data suppression. Rates are not calculated for multiple races, other, and unknown race categories due to the unavailability of an appropriate denominator.
Cases
Deaths
Select case status:
Confirmed
Probable
Select Preparedness Region:
All
Select county:
All
Cases by Age Group
0.2M
0.0M
0-9
10-19
20-29
30-39
40-49
50-59
60-69
70-79
80+
Unknown
Cases by Sex
0.8M
0.6M
0.4M
0.2M
0.0M
Female
Male
Unknown
Cases by Race
White
Unknown
Other
Multiple Races
Black/African American
Asian/Pacific Islander
0.0M
0.5M
1.0M
Cases per Million by Age Group
200K
150K
100K
50K
0K
0-9
10-19
20-29
30-39
40-49
50-59
60-69
70-79
80+
Cases per Million by Sex
200K
150K
100K
50K
0K
Female
Male
Cases per Million by Race
American Indian/Alaska Native
Asian/Pacific Islander
Black/African American
White
0.0M
0.1M
Microsoft Power BI
4 of 7

Bridge MICHIGAN CELEBRATING 10 YEARS | Michigan's nonpartisan, nonprofit news source

The gift that gives all year long. Please support our work.
DONATE TODAY

DISMISS

Our year-end campaign is happening now!

Happy holidays from the Bridge Michigan team! Feeling festive? Your tax-deductible support of our nonprofit newsroom will help Bridge continue to thrive and prepare for all of the events of 2022. Will you support the Michigan news you rely upon this giving season?

VISA MasterCard AMERICAN EXPRESS PayPal Donate Today

Michigan coronavirus dashboard: vaccines, cases, deaths and maps

Michigan releases new coronavirus data on Mondays, Wednesdays and Fridays.

Coronavirus in Michigan

Find coronavirus infections rates across much of the state. Click on a county to see the current day's* cases and the rate per 100,000 over the past week.

Daily cases per 100,000 people (average over last week)

- No cases
- Less than 10 cases
- 10 to 19 cases
- 20 to 29 cases
- 30 to 39 cases
- 40 to 49 cases
- More than 50

*As of 10 a.m. Dec. 10

	Cases reported today	Daily cases per 100,000
Michigan	5,891	63
Detroit**	737	55

** Detroit not included in Wayne County's numbers in map

Source: Michigan Department of Health & Human Services

Bridge — *Mike Wilkinson*

Cases and testing

You can use this graphic to check daily changes in cases and tests, as well as compare to the previous week. **NOTE: The state now releases data on Mondays, Wednesdays and Fridays with each day's totals covering two or three days.**

Cases | Testing

As of: Dec. 10	Cases reported today*	7-day average	Prior week avg.	Cases per 100,000 rate	Prior week rate
Oakland	1,343	730	883	57	69
Wayne	1,313	778	827	67	72
Macomb	1,216	691	737	78	84
Kent	794	395	504	60	77
Detroit City	737	350	353	55	55
Genesee	535	278	293	68	72
Kalamazoo	384	160	179	61	68
Ottawa	353	200	262	68	88
Washtenaw	328	173	195	46	52

* Cases reported cover either two or three days. The weekly averages encompass cases over seven-day increments. Also, in counties with state prisons, new cases are often first reported within the county. Later, they are removed and identified as having occurred within the Michigan Department of Corrections. This can create days where a county will have a negative number of new cases.

Bridge — *Mike Wilkinson*

Hospitalizations

This is hospitalizations as reported several times a week by Michigan public health officials.

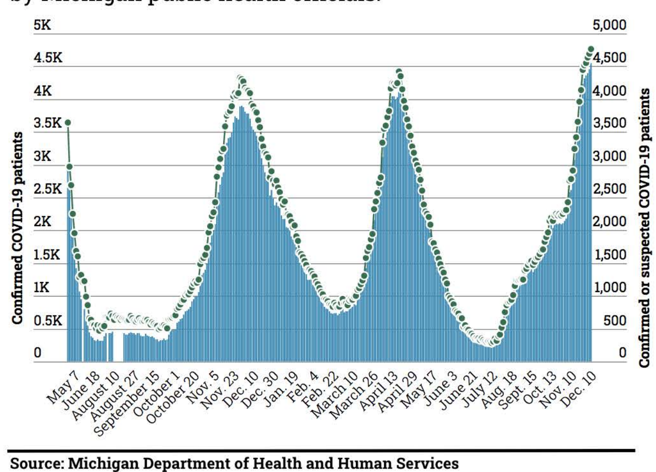

Source: Michigan Department of Health and Human Services and Western Michigan University Libraries.

Bridge — *Mike Wilkinson*

Daily cases

New daily cases since March, 2020

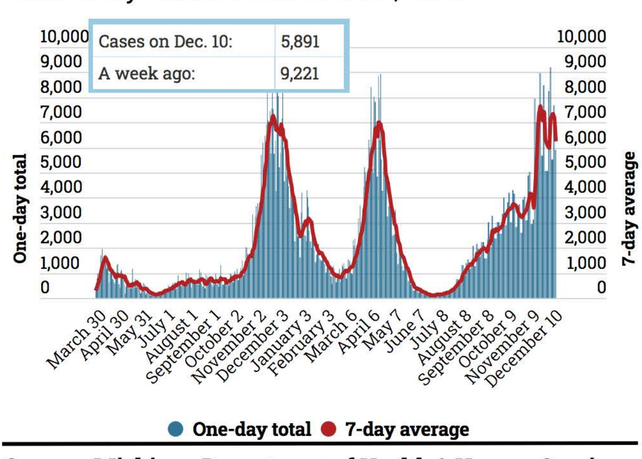

Source: Michigan Department of Health & Human Services

COVID-19 vaccines given, by date

Michigan health officials are trying to vaccinate as many people as possible with the limited quantities of vaccine that they have. Here's how it is going, by date.*

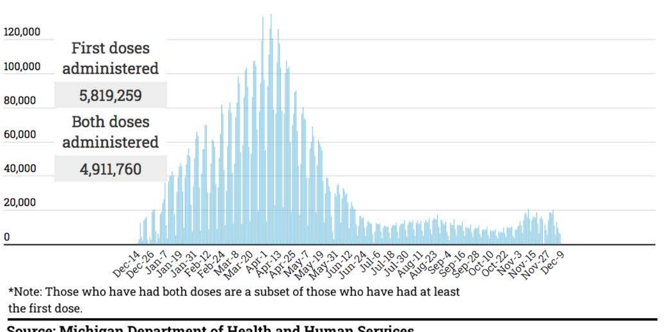

*Note: Those who have had both doses are a subset of those who have had at least the first dose.

Source: Michigan Department of Health and Human Services

Bridge — *Mike Wilkinson*

Coronavirus in Michigan

Click the tabs to see total numbers and per-capita rates by county.

	Confirmed	Probable
Total cases:	1,380,324	187,254
Total deaths:	25,080	1834

Cases per 100,000:	13,831
Deaths per 100,000:	251

Total cases (cases per 100,000 people)

- 1 case or none
- 2 to 49 (fewer than 50 cases per 100,000)
- 50 to 99 (50 to 99 cases per 100,000)
- 100 to 199 (100 to 199 cases per 100,000)
- 200 to 999 (200 to 299 cases per 100,000)
- Over 1,000 (300 or more cases per 100,000)

Who's getting the vaccine?

The vaccine rollout in Michigan has been bumpy, with elderly residents in some parts of the state unsuccessfully scrambling to get vaccines while some areas are vaccinating younger people as well. Search here to see what percent of those 12 years old and older have been vaccinated so far. Gov. Gretchen Whitmer has set a goal of vaccinating 70 percent of those 16 years old and older.

Search

Oakland	Initiated	Completed	Percent Initiated	Percent Completed
Emmet	22,760	20,878	77.2	70.8
Wayne	702,360	634,626	76.4	69.1
Kent	416,398	381,365	75.3	69
Kalamazoo	169,004	155,500	74.4	68.4
Mackinac	7,215	6,669	73.9	68.3
Benzie	11,693	10,719	74.1	68
Charlevoix	16,913	15,577	72.9	67.2
Marquette	41,893	38,835	71.2	66
Mason	18,155	16,683	71.7	65.9
Clinton	48,748	44,928	71.2	65.6
Alger	5,865	5,409	71	65.5

Source: Michigan Department of Health and Human Services

Bridge — *Mike Wilkinson*

Age breakdown

The coronavirus has infected and killed people of all ages in Michigan but has hit the elderly particularly hard. Here's the current breakdown.

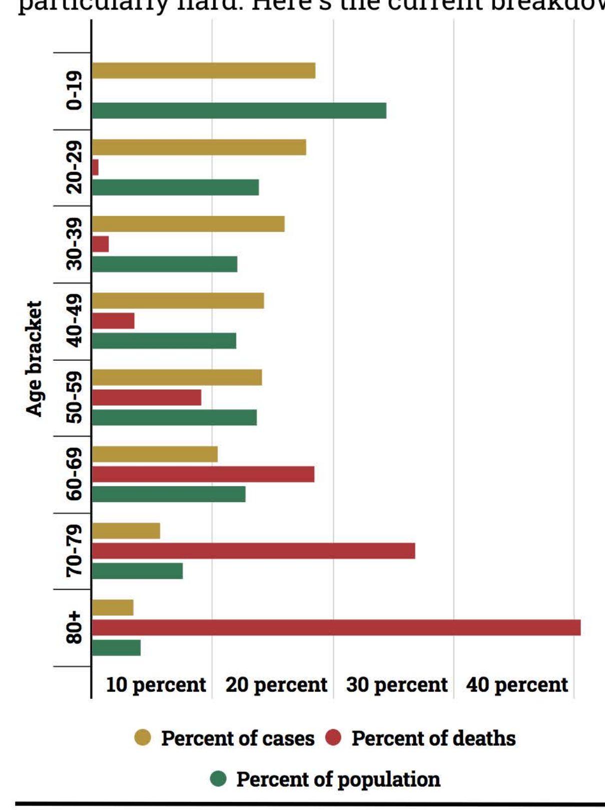

Source: Michigan Department of Health & Human Services

Daily testing results

Michigan health officials say the goal is to keep the positive test rate at 3 percent or lower.

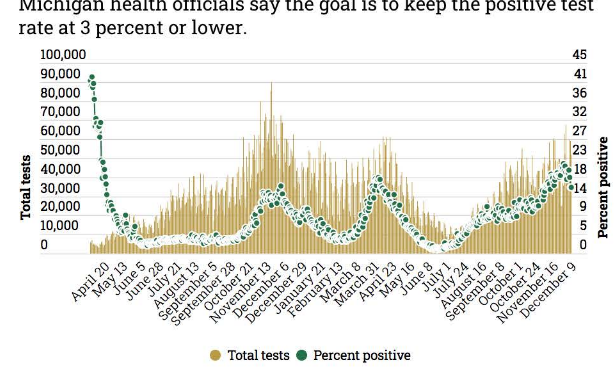

School outbreaks

Michigan public health officials have begun identifying coronavirus outbreaks at schools, including preschool, K-12 and higher ed institutions. **Report date: Dec. 6, 2021. In ongoing outbreaks, counts with asterisk (*) refer only to last 28 days.**

New outbreaks | Ongoing

Search

County (community)	School	Cases
Allegan (Holland)	Forest Elementary School	5 staff and students
Barry (Hasings)	Star Elementary	3 students
Bay (Bay City)	St Paul Lutheran School	5 students
Bay (Bay City)	State St Academy	6 students
Eaton (Eaton Rapids)	Eaton Rapids High School	6 students
Eaton (Charlotte)	Parkview Elementary	3 students
Eaton (Lansing)	Waverly Middle School	4 students
Genesee (Davison)	Davison Middle School	20 students
Ionia (Belding)	Faith Community Christian	4 staff and students
Kalamazoo (Portage)	Lake Center Elementary	3 students
Kalamazoo (Portage)	Portage 12th St Elementary	3 staff and students
Kent (Grand Rapids)	Childrens Workshop Preschool	4 students
Kent (Grandville)	Grandville Central Elementary School	4 students
Kent (Caledonia)	Kraft Meadows Middle School	6 students

Source: Michigan Department of Health and Human Services

Bridge — *Mike Wilkinson*

COVID-19 deaths in Michigan

This map shows home residence of those who have died from the coronavirus.

Total deaths: 25,080

- No deaths
- 1 to 25 deaths
- 26 to 49 deaths
- 50 to 99 deaths
- More than 100 deaths

*As of 10 a.m. Dec. 10

Source: Michigan Department of Health & Human Services

Share Bridge

Daily deaths

Deaths by day reported, since March 25

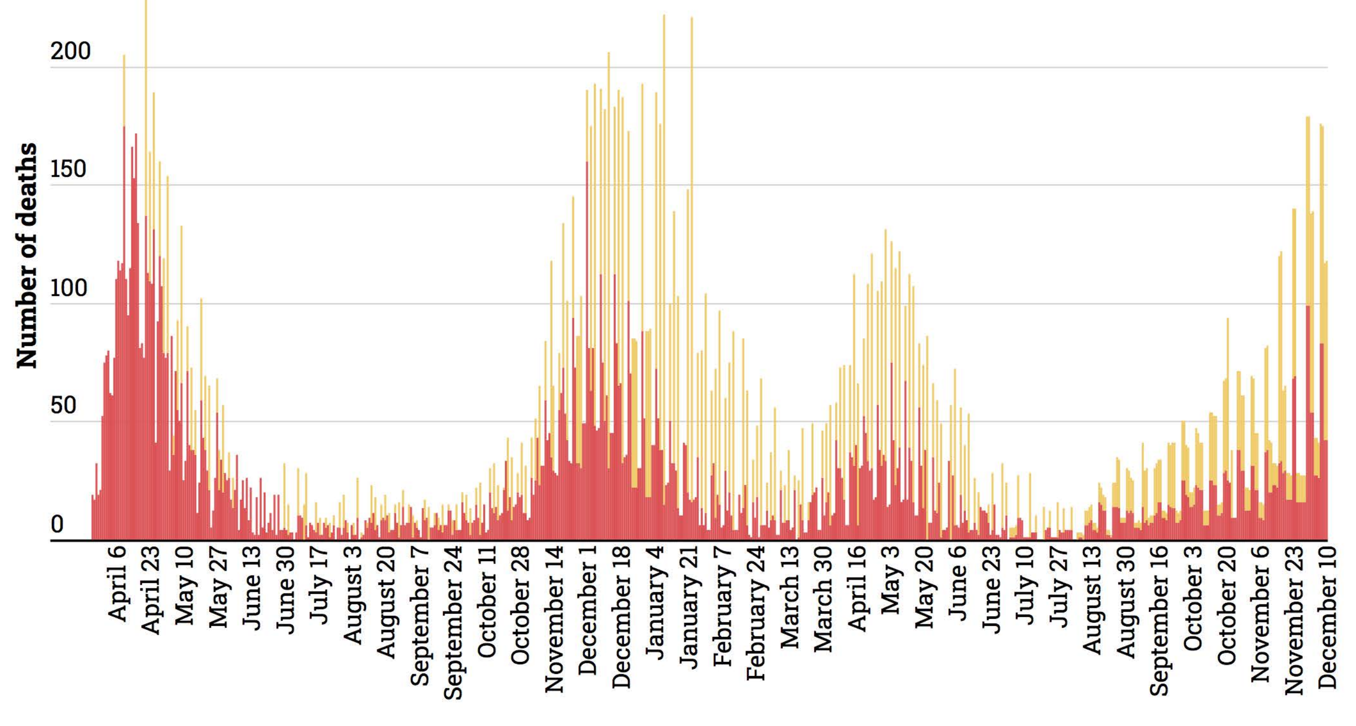

Added deaths are those added after a review of earlier death records.

Deaths by date

These are deaths by the **date** of death. Often, following a review of medical records, deaths are reported days and weeks after the actual death.

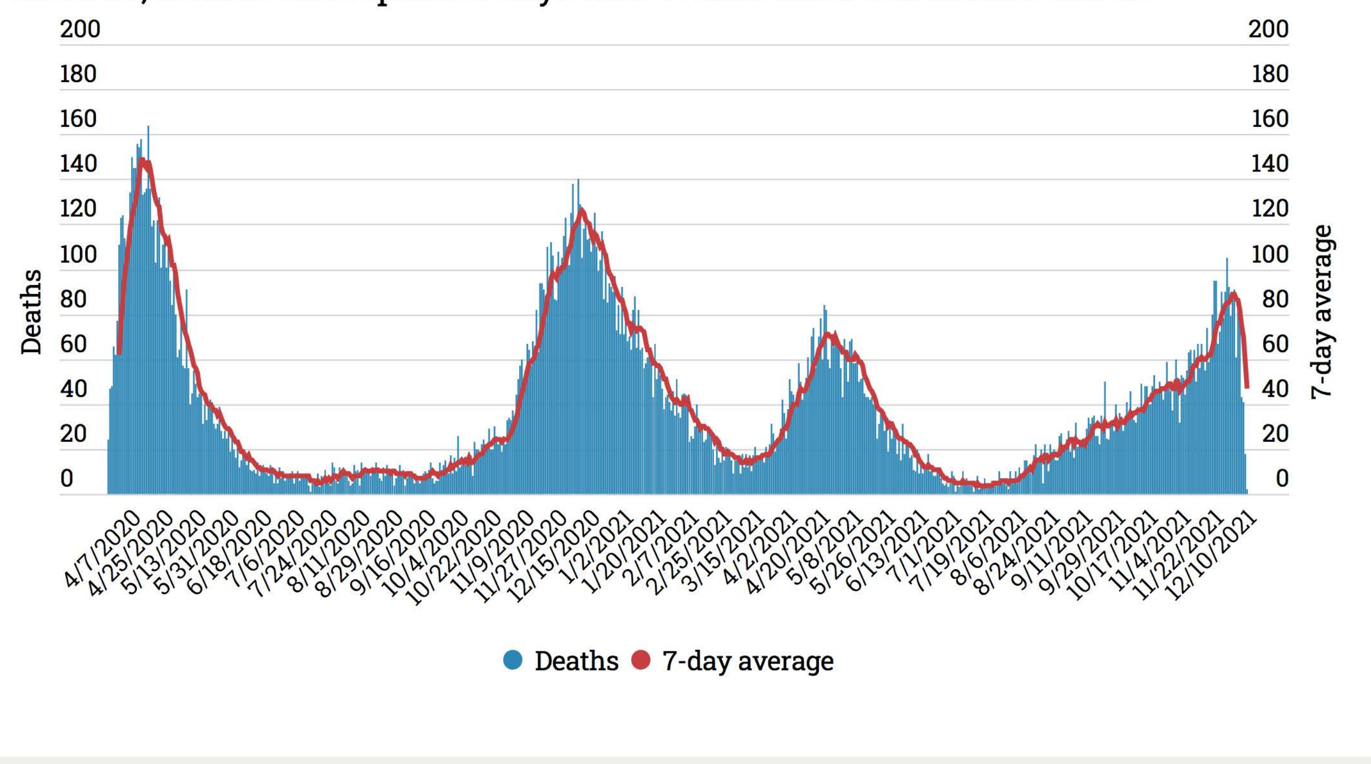

©2021 Bridge Michigan. All rights reserved.

Situation Update for COVID-19

Subscribe: COVID-19 updates
Sign up to receive email or mobile alerts when this data is updated, as well as other periodic COVID-19 updates.

Updated December 10, 2021
Updated weekdays at 11 a.m., with data current as of 4 a.m. the previous business day.

Data is for cases that were tested and returned positive. At-home test results are not counted by MDH.

All data is preliminary and may change as cases are investigated. Many data points are collected through an online survey or during case interviews; however, not all cases are interviewed. Case interviews are focused on people under 18 years old, hospitalizations, deaths, and people with vaccine breakthrough or variants. Data presented below is for all cases, regardless of interview status.

Positive PCR test results are considered confirmed cases. Positive antigen test results are considered probable cases. All probable cases get the same public health follow up and recommendations as cases confirmed by PCR tests.

On this page:
Daily Update
Testing
Minnesota Case Overview
Case Demographics
Residence
Weekly Report
Vaccine Breakthrough Report
Other COVID-19 Data and Statistics

Expand All Contract All

Daily Update:

Because all data is preliminary and some reports require verification before counting as a case, the change in number of cumulative positive cases and deaths from one day to the next may not exactly equal the newly reported cases or deaths.

We continuously receive case reports and work to confirm, process, and report them as quickly as possible. Newly reported cases may not include every case reported to us since the last data update.

Total positive cases, including reinfections (cumulative)	956,779
Total positive people (cumulative)	945,486
Newly reported cases	4,554

Newly reported cases detail

Total deaths (cumulative)	9,872
Newly reported deaths	58

Newly reported deaths detail

Testing

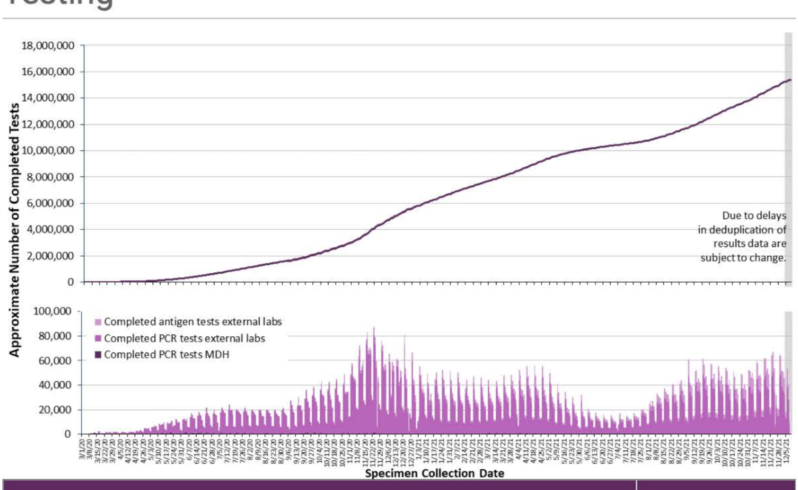

Total approximate completed tests (cumulative)	15,369,673
Total approximate number of completed PCR tests (cumulative)	13,348,200
Total approximate number of completed antigen tests (cumulative)	2,021,473

Testing data table

More about testing data

Minnesota Case Overview

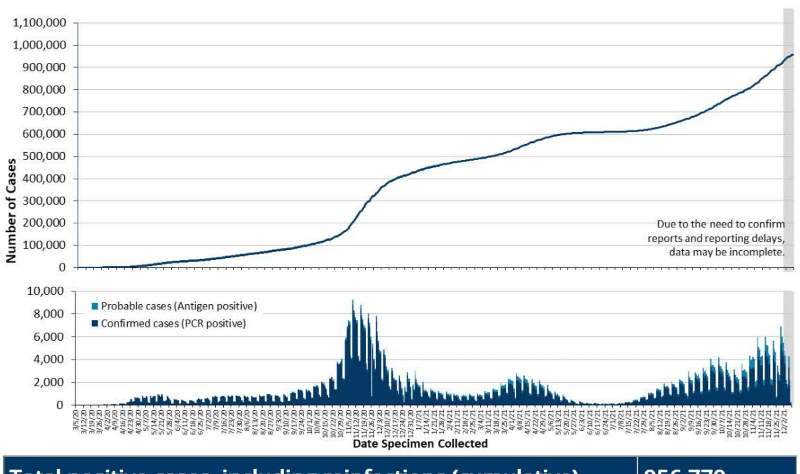

Total positive cases, including reinfections (cumulative)	956,779
Total confirmed cases (PCR positive) (cumulative)	851,378
Total probable cases (Antigen positive) (cumulative)	105,401

Positive cases by date specimen collected data table

Total positive people (cumulative)	945,486
Total number of reinfections (cumulative)	11,293

Reinfections by date specimen collected data table

More about positive cases, positive people, and reinfections

Cases no longer needing isolation

Cases no longer needing isolation (cumulative)	908,526

More about cases no longer needing isolation

Deaths

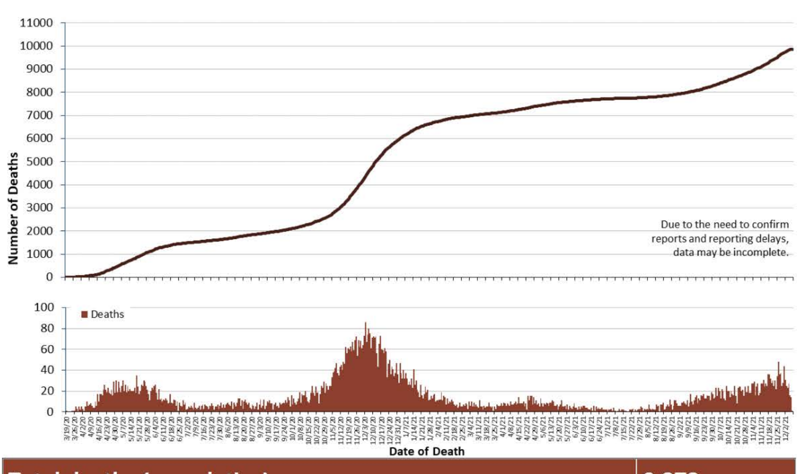

Total deaths (cumulative)	9,872
Deaths from confirmed cases (cumulative)	9,277
Deaths from probable cases (cumulative)	595

Deaths data table

Total non-laboratory-confirmed COVID-19 deaths (death certificate) (cumulative)	124
Deaths among cases that resided in long-term care or assisted living facilities (cumulative)	5,036

More about deaths

Hospitalizations

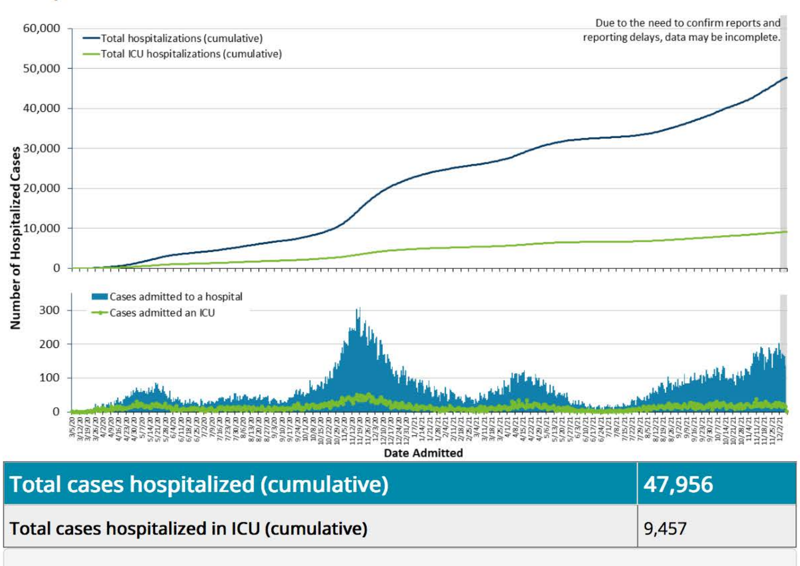

Total cases hospitalized (cumulative)	47,956
Total cases hospitalized in ICU (cumulative)	9,457

Hospitalization data table

More about hospitalizations

Case Demographics

Age

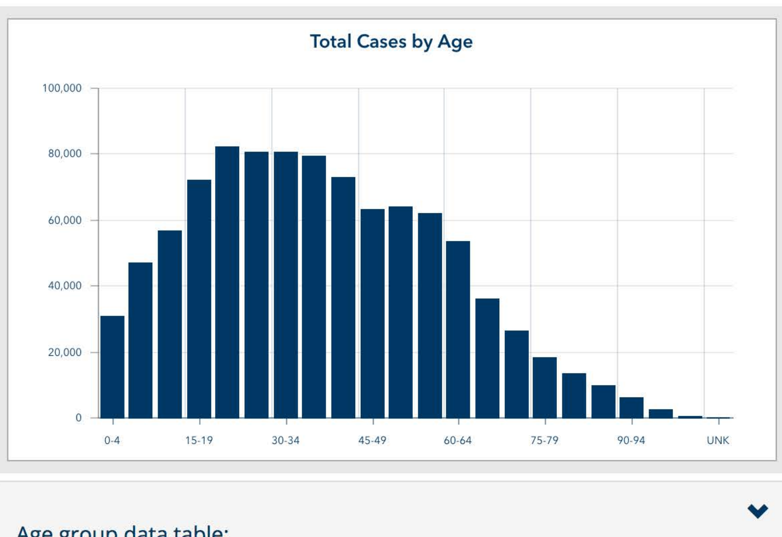

Age group data table:
Including age group of deaths

More about age

Gender

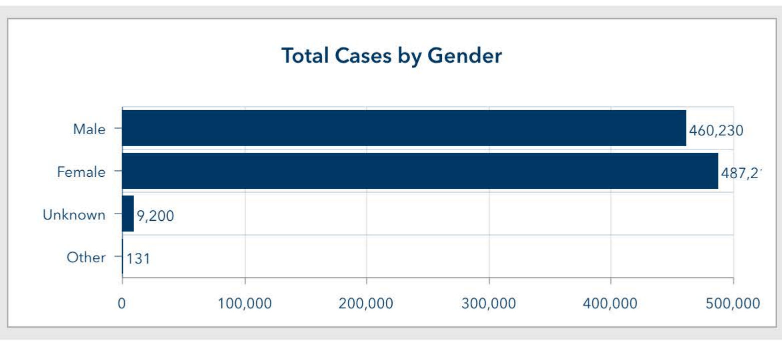

Gender data table

More about gender

Race & Ethnicity

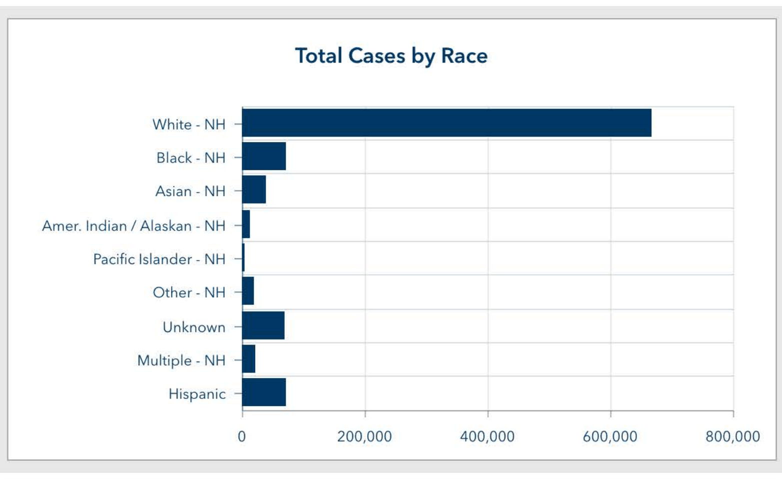

Race and ethnicity data table:
Including race and ethnicity of deaths

More about race and ethnicity

Residence

Cases by County of Residence

County of residence is confirmed during the case interview. At the time of this posting not all interviews have been completed.

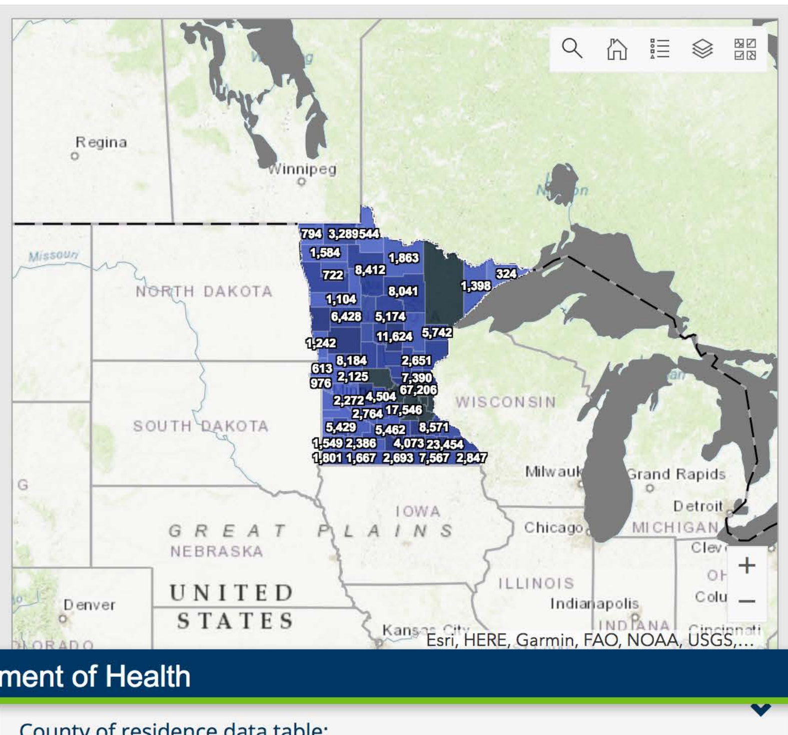

Minnesota Department of Health

County of residence data table:
Including county of residence of deaths

More about location data

As of 10/28/21, likely exposure and residence type are no longer collected for all cases and therefore no longer reported. Archived data can be found in the COVID-19 Weekly Report.

Share This

Subscribe: COVID-19 updates

STAY SAFE MN
Minnesota COVID-19 Response

COVID-19 VACCINE

COVID-19 MEDICATIONS

TESTING FOR COVID-19

PARENT & CAREGIVER INFO

COVID-19 Public Hotline:

For questions related to the COVID-19 pandemic, call 1-833-431-2053
Mon.-Fri.: 9 a.m. to 7 p.m.
Sat.: 10 a.m. to 6 p.m.

Mental Health Crisis Line - Call: **CRISIS (**274747)

COVID-19 information in other languages:

- Kab Mob Khaus Las Nas Vais Lav 2019 (COVID-19) (Hmong)
- Cudurka Koronafayras 2019 (COVID-19) (Somali)
- Enfermedad del Coronavirus 2019 (COVID-19) (Spanish)
- American Sign Language (ASL) Videos

Contact us:

If you have questions or comments about this page, use our IDEPC Comment Form or call 651-201-5414 for the MDH Infectious Disease Epidemiology, Prevention and Control Division.

E-Newsletter
Get COVID-19 updates and public health alerts when you subscribe to our e-mail newsletter.
email address
SUBSCRIBE

Interactive Maps: Mississippi COVID-19 Cases and Incidence

Select Language

More COVID-19 charts and data

Current COVID-19 Activity: Two-Week Case Incidence

Full screen

Mississippi COVID-19 Two-Week Cases* Incidence Rate**
over 11/16/21 - 11/29/21

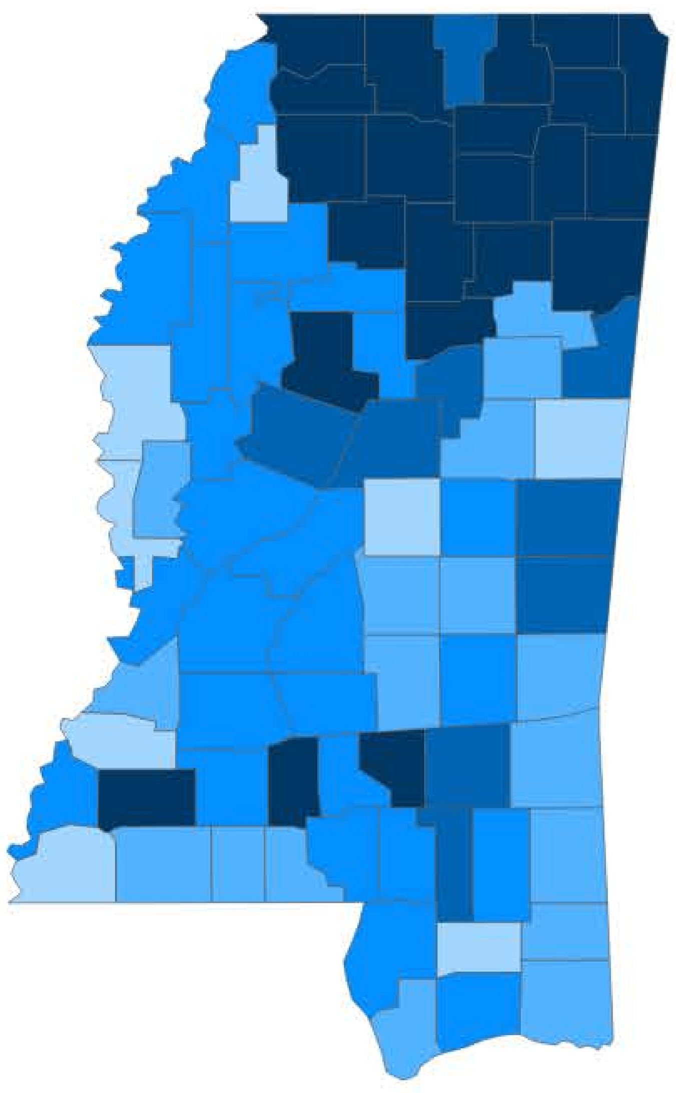

<45 45 to 90 90 to 135 135 to 180 >180

Mississippi

Two-Week New Cases*
4525

Two-Week Cases* Incidence Rate Per 100,000
152.0

Total Cases* through 11/29/21
515,313

Percent Change
0.9%

County	Two-Week New Cases	Two-Week Cases Rate Per 100,000	Cases to Date	Percent Change
UNION	168	583.0	6,432	3.0%
CHICKASAW	86	502.8	3,178	3.0%
ITAWAMBA	106	453.2	4,881	2.0%
PRENTISS	104	413.9	5,070	2.0%
TISHOMINGO	73	376.6	3,859	2.0%
LEE	320	374.5	16,435	2.0%
TIPPAH	82	372.5	4,954	2.0%
PONTOTOC	115	357.4	7,027	2.0%

* Cases based on **Event Date**. Event date is date of onset, date of collection, or date of report to MSDH; updated as cases are investigated.
** Rate calculation is based on cumulative cases of past two weeks per 100,000 population.

Microsoft Power BI

Cumulative COVID-19 Cases and Deaths

Full screen

Mississippi COVID-19
Cases and Deaths by Race with Ethnicity
as of 3 pm CT, December 9, 2021

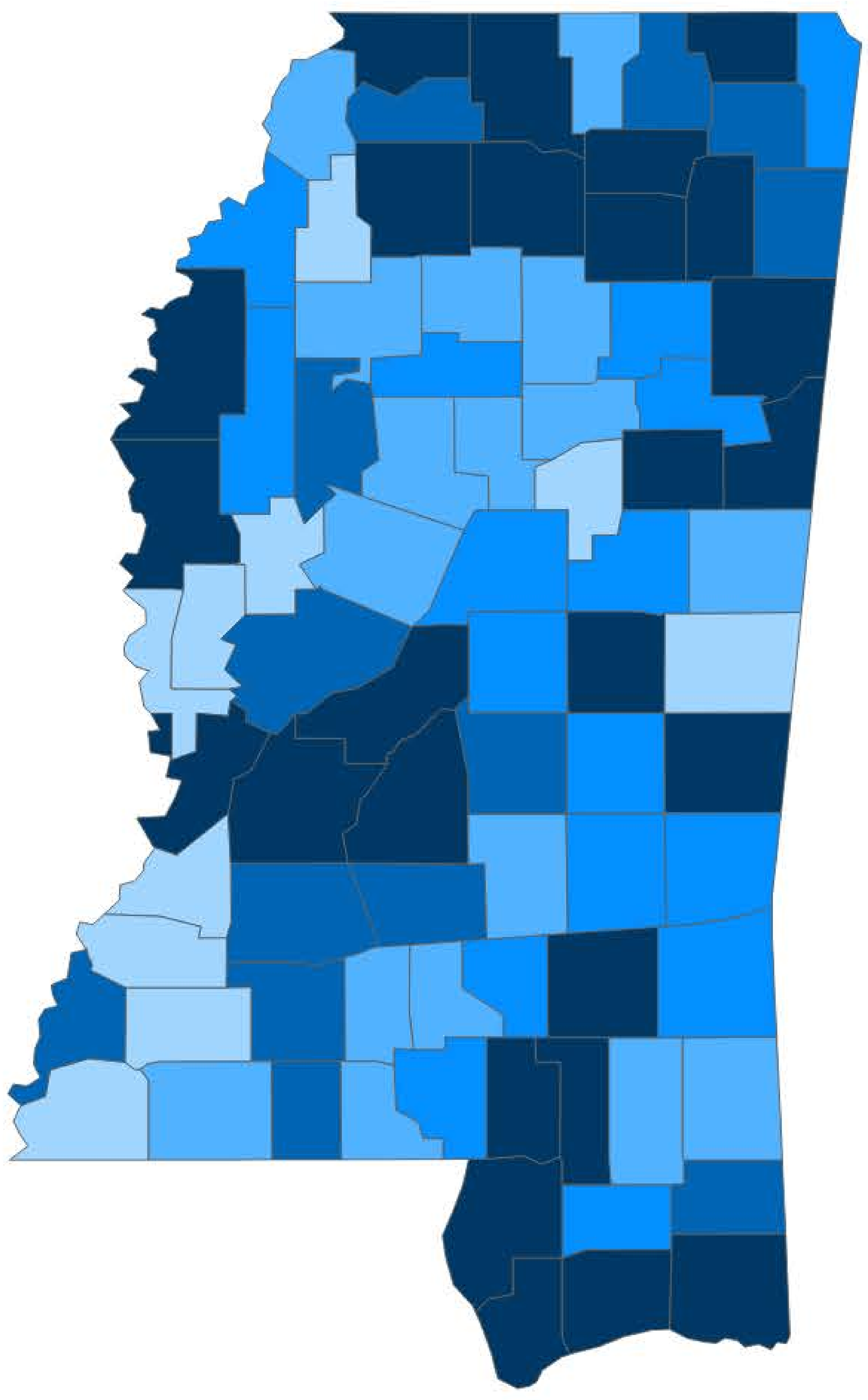

1 to 1500 1501 to 3000 3001 to 4500 4501 to 6000 >6000

Mississippi

Total Cases
519,115

	American Indian or Alaska Native	Asian	Black	White	Other	Unknown
Non Hispanic	1,813	1,848	137,817	226,236	5,424	5,572
Hispanic	120	84	832	3,432	5,938	1,285
Unknown Ethnicity	1,209	490	22,017	38,412	11,274	55,312

Total Deaths
10,326

	American Indian or Alaska Native	Asian	Black	White	Other	Unknown
Non Hispanic	100	20	3,466	5,034	70	24
Hispanic	1	2	10	51	53	5
Unknown Ethnicity	25	11	436	709	102	207

Microsoft Power BI

These Dashboards are a cross-government effort to bring you data about COVID-19 in Missouri. Have questions? **Send us an email.**

Details About Metrics | Download Data

COVID-19 in Missouri at a Glance

Cases, testing, and death data last updated in the afternoon of **12/11/2021**. The 7-day range reflects 12/2/2021 through 12/8/2021. A delay allows daily data to be accurate and complete. See FAQ for details.
Note: Rates based on low numbers are statistically unstable, please use discretion when comparing rates.

Metric	Past 7 Days	Average / Rate	Rate / Detail	Description
**Vaccinations**	Vaccine Doses Administered in the Past 7 Days **177,282**	Average of **25,326** per day		The number of vaccine doses administered in the past week. The Pfizer and Moderna vaccines both require two doses per person. See our new vaccine dashboard and the new vaccine website for more information **(links below)**.
**Confirmed Cases**	New Confirmed Cases in the Past 7 Days **13,701**	Average of **1,957** per day	**223** Confirmed cases per 100K residents	The number of total people with a positive PCR COVID-19 test or a positive genomic sequencing test in the past week. If a person has both a positive PCR/genomic sequence and Antigen test, they are only counted as a confirmed case.
**Probable Cases**	New Antigen Cases in the Past 7 Days **4,366**	Average of **618** per day	**71** Antigen cases per 100K residents	The number of total people with either a positive Antigen COVID-19 test, an epidemiological linkage or confirmation via vital records in the past week. If a person has **both** a positive PCR/genomic sequence **and** one of the three probable causes listed above, then they are only counted as a confirmed case.
**Reinfections** (New)	New Reinfection Cases in the Past 7 Days **297**	Average of **42** per day	**5** reinfection cases per 100K residents	The total number of individuals who tested positive with a PCR or Antigen test and tested positive again after 90 days with a PCR or Antigen test.
**Confirmed Deaths**	New Confirmed Deaths in the Past 7 Days **23** (see note)	Average of **3** per day (see note)	**0.4** deaths per 100K residents	The number of Missourians who died from COVID-19 in the past week with positive PCR test. Note: Deaths are often reported with a delay. We encourage you to look at historical 7-day averages on our Statewide and County dashboards **(links below)**.
**Probable Deaths** (New)	New Probable Deaths in the Past 7 Days **1** (see note)	Average of per **0** day (see note)	**0.0** deaths per 100K residents	The number of total people who died with either a positive Antigen COVID-19 test or had confirmation via vital records in the past week. If a person has **both** a positive PCR/genomic sequence **and** one of the two criteria for probable deaths listed above, then they are only counted as a confirmed death.
**PCR Tests**	New PCR Tests in the Past 7 Days **117,973**	Average of **16,853** per day	**1,922** PCR tests per 100K residents	The number of total PCR tests in the past week. Testing helps identify new cases so we can take actions to slow the spread of the virus.
**Antigen Tests**	New Antigen Tests in the Past 7 Days **65,457**	Average of **9,351** per day	**1,067** Antigen tests per 100K residents	The number of total Antigen tests in the past week. Testing helps identify new cases so we can take actions to slow the spread of the virus.
**PCR Positivity Rate**	Positivity Rate over the Past 7 Days (CDC method) **12.5%**			Positivity rate can be calculated multiple ways. The CDC method uses the number of positive PCR tests divided by the total number of PCR tests. **See FAQ for more details (link below).**
**Antigen Positivity Rate**	Positivity Rate over the Past 7 Days (Antigen Only) **7.5%**			Positivity rate can be calculated multiple ways. Our method uses the number of positive Antigen tests divided by the total number of Antigen tests. **See FAQ for more details (link below).**
**Hospitali-zations**	COVID-19 Patients in the Hospital **1,845** (as of 12/8/2021)	**412** in the ICU	**226** on ventilators	The number of Missourians in the hospital who are confirmed or suspected to have COVID-19. This number is important for assessing the overall strain on the Missouri health care system. **See note below for more context.**

COVID-19 Testing in Missouri

This dashboard was last updated in the afternoon of **12/11/2021** and includes data reported through **12/10/2021**.

Statewide PCR Positivity Rates Over Time

Calculated two different ways - Hover over the chart and read the tooltip to understand the methodology, and see the FAQ for more detail.

Moving 7-day PCR Positivity Rate over Time - Not Deduplicated to the Individual (CDC method)

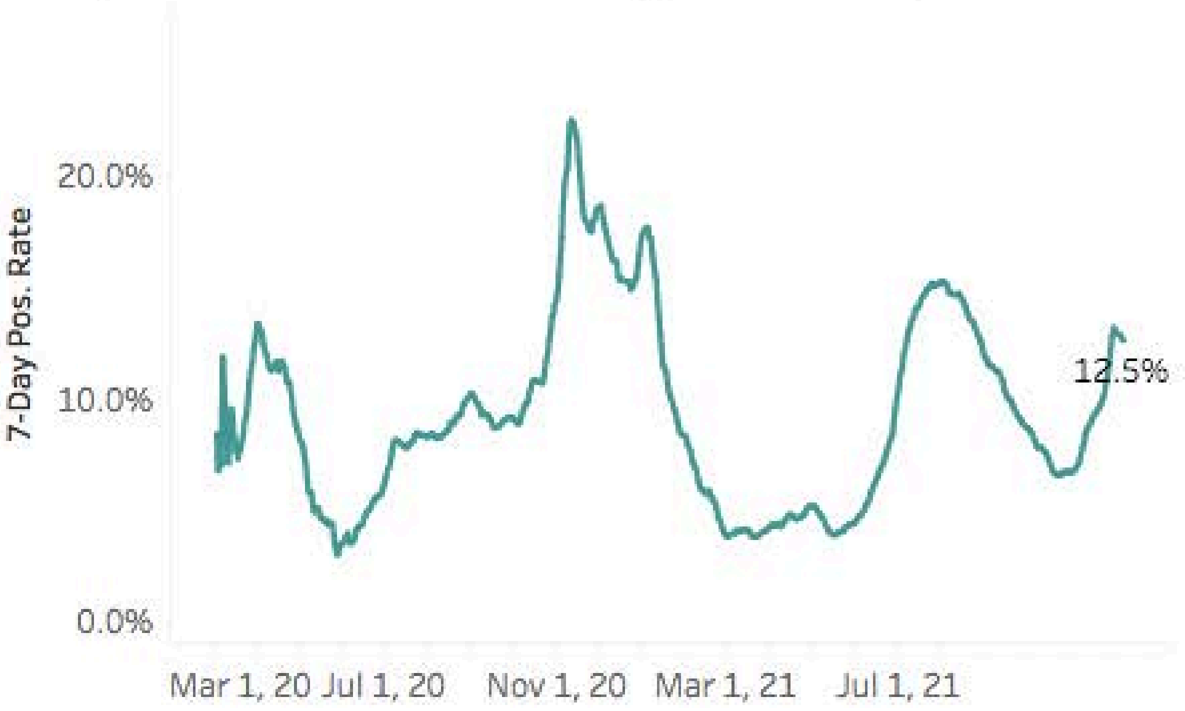

Cumulative PCR Positivity Rate Over Time - Deduplicated to the Individual

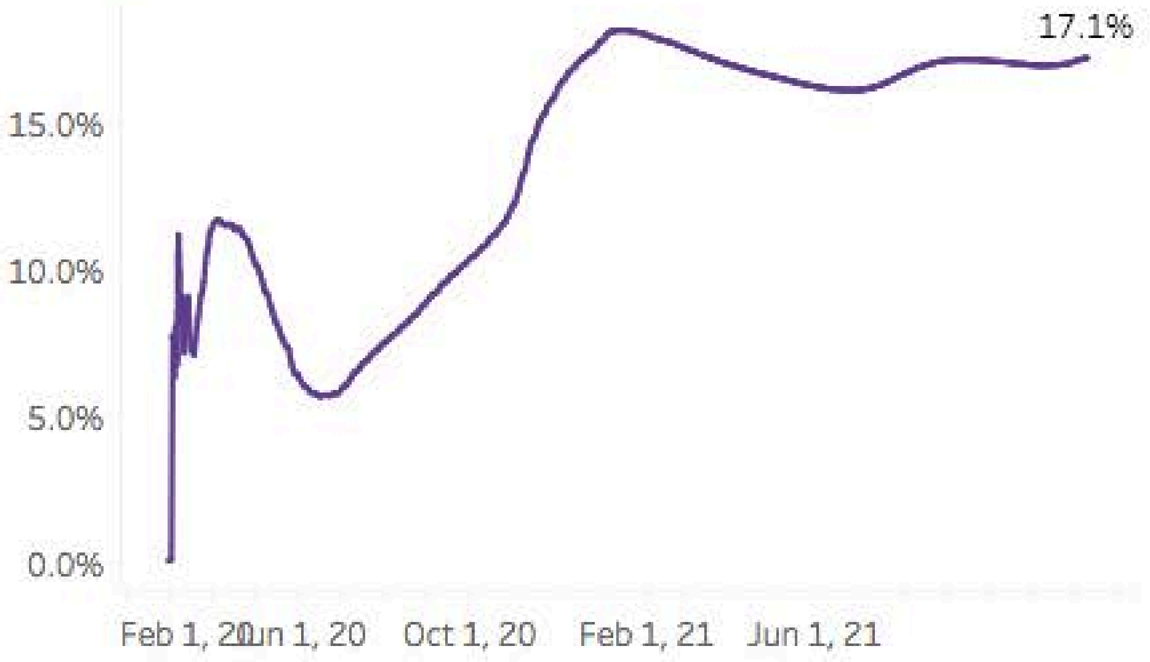

Test Results by Test Type

See the FAQ for more details about types of tests.

Test Type	Total Tests	Negative
PCR	8,066,488	7,211,964 89%
SEROLOGY	230,584	172,266 75%
ANTIGEN	3,394,875	3,189,931 94%

Individuals Tested by Test Type

This metric counts people, instead of tests.

Test Type	Total Individuals Tested
PCR	3,418,066
SEROLOGY	185,353
ANTIGEN	1,049,472

Result: Positive | Negative | Indeterminate

Weekly Total Testing by Test Type

See the FAQ for more details about types of tests.

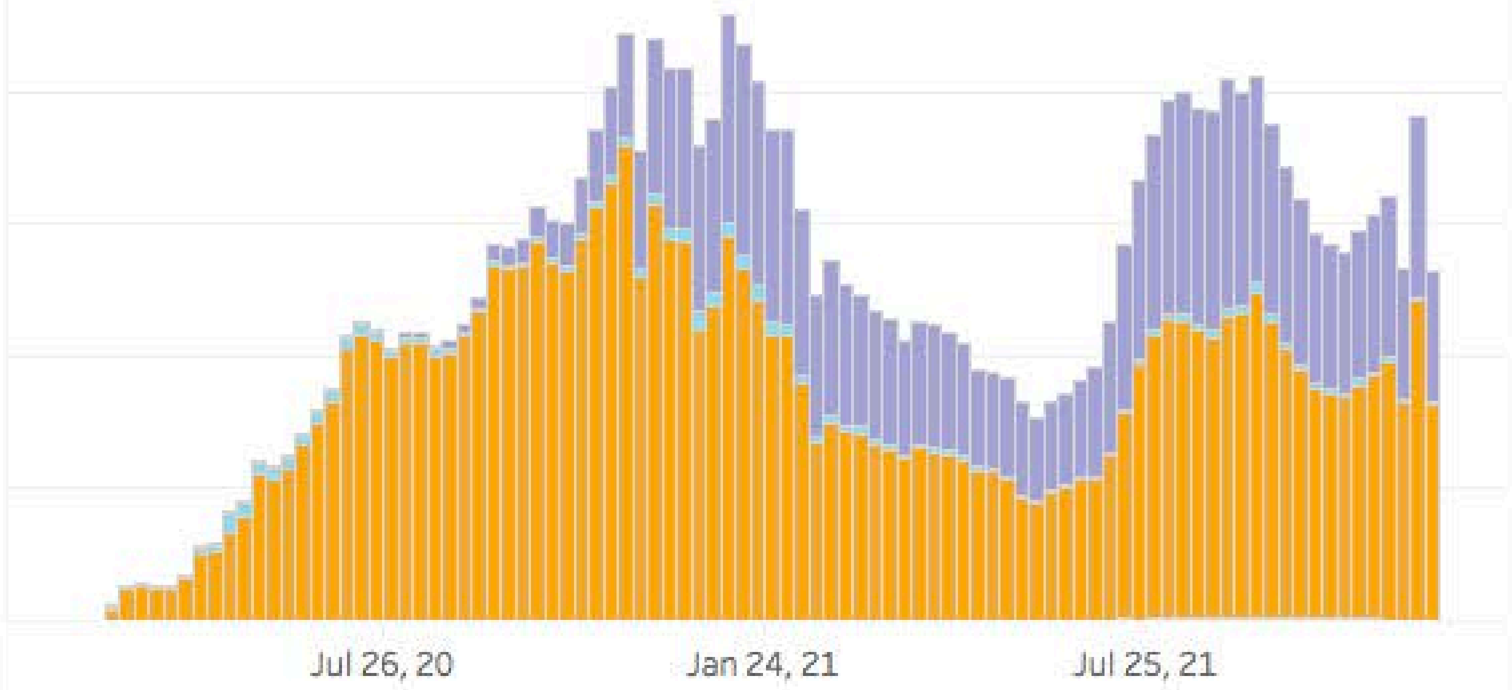

Test Type: ANTIGEN | SEROLOGY | PCR | Null

COVID-19 Vaccinations in Missouri

Overall Vaccinations in Missouri

This dashboard was last updated on **12/11/2021** and contains data on vaccinations adminstered through **12/10/2021**. Historical numbers, especially over the most recent few days, will update as providers report data to the state. Differences between this dashboard's numbers and those on CDC's dashboard are primarily due to timing.

COVID-19 Vaccine Dose Details

Total COVID-19 Doses Administered	**7,503,586**
First and Second Doses	**6,642,697**
Third Doses (starting 8/16/2021)	**860,889**

Doses Administered in past 7 days (12/2/2021 through 12/8/2021)	Daily Average Doses Administered (12/2/2021 through 12/8/2021)
**177,282**	**25,326**

Choose Age Group from Dropdown:

Total Population

Total Population Vaccination Details

Population Initiated Vaccination	**3,640,130**
Percent Initiating Vaccination	**59.3%**
Population Completed Vaccination	**3,216,984**
Percent Completing Vaccination	**52.4%**

Use Slider to Filter Date:

12/13/20 12/10/21

Total Doses Administered Over Time

Line represents 7-day average - hover over it to get detailed dose information (i.e. **first/second** doses, **third** doses, etc.) for each day. Note that data from the most recent days may be incomplete because of delays in reporting.

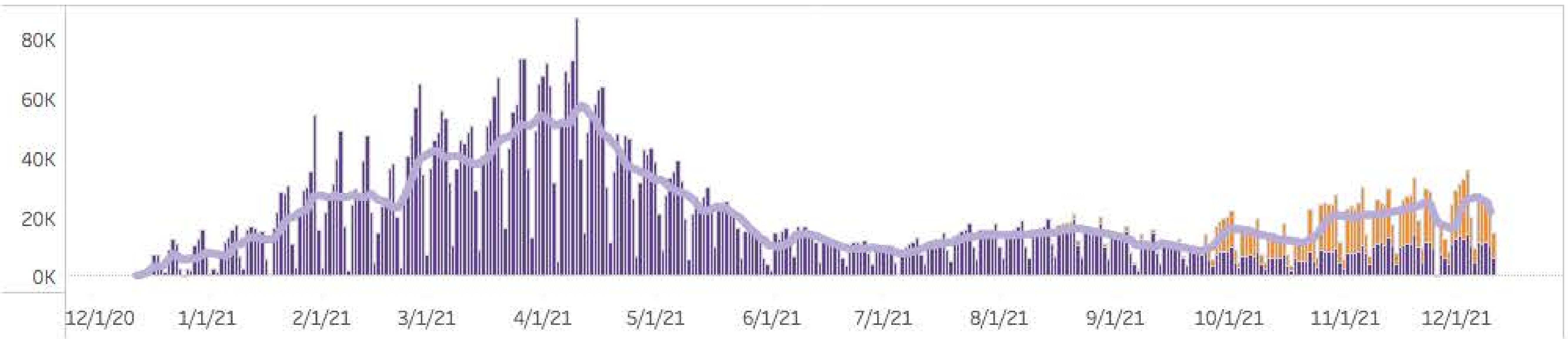

Who Has Initiated & Completed Vaccination?

These charts show vaccination progress by demographic group. The % rates reflect the share of the population group who has initiated and completed vaccination. These rates will increase over time as more people are vaccinated. Hover over the bar chart to see the number of persons who have initiated and completed vaccination for that population group.

Notes: These rates exclude records for which we have no sex, race, and/or ethnicity on file, so the actual rates may be higher. These rates include persons who list their address as outside of Missouri but received their vaccination in Missouri.

Choose a Demographic:

- ◉ Age Group
- ○ Sex
- ○ Race
- ○ Ethnicity

Share of Population by Age Group

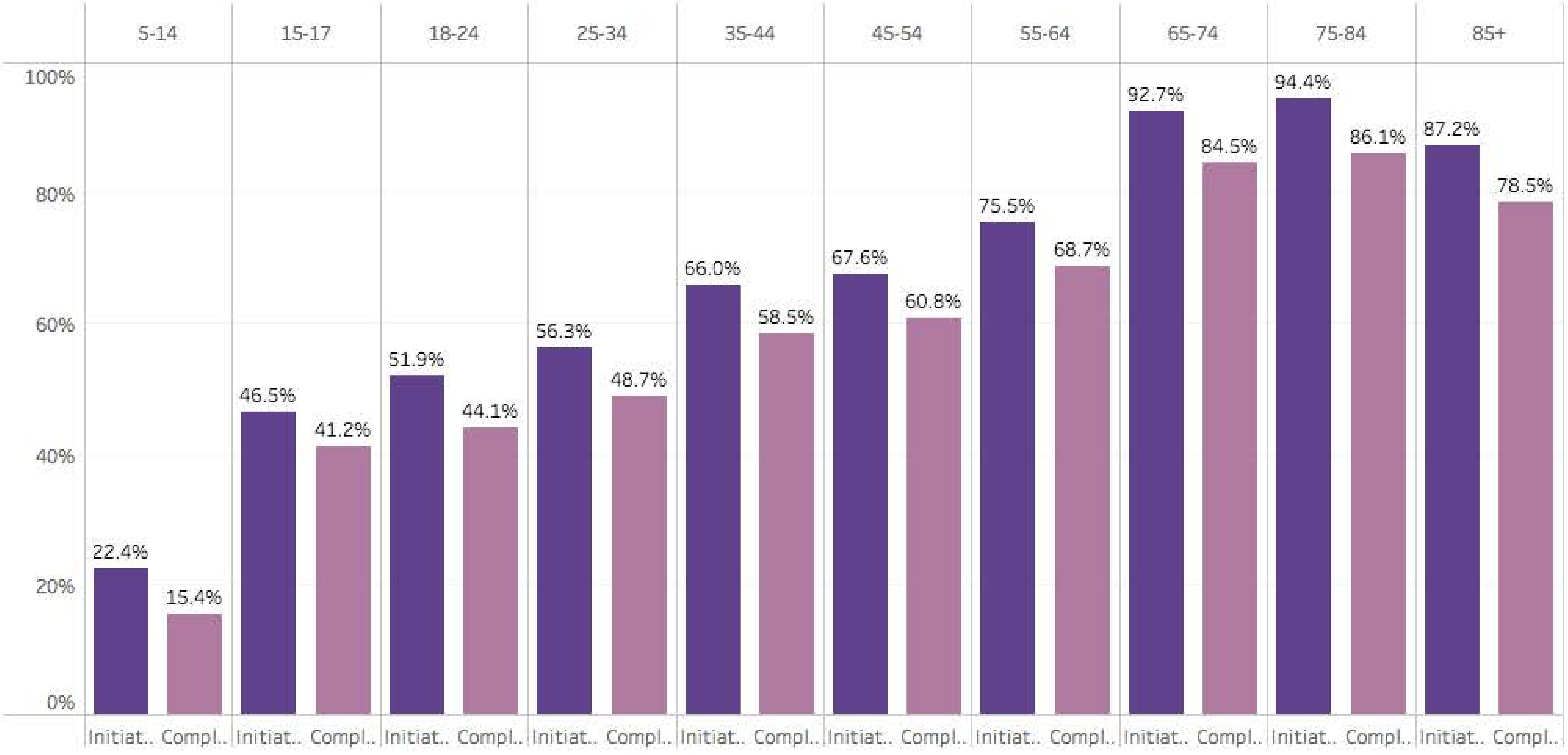

MONTANA RESPONSE: COVID-19 - Coronavirus - Global, National, and State Inforn

Montana Maps

State Library

COVID-19 Vaccine | COVID-19 Cases | Local Vaccine Contacts | COVID-19 Demographics | Montana COVID-19 Vaccine FAQs | Global Outbreak Dashboard

Cumulative Cases
193,718

202 New Cases

Cumulative, New Daily, Active
Cases by County

Yellowstone County Cases
31,626 Total | 37 New | 576 Active

Flathead County Cases
21,077 Total | 24 New | 372 Active

Gallatin County Cases
22,563 Total | 23 New | 195 Active

Lewis and Clark County Cases
12,434 Total | 17 New | 313 Active

Lake County Cases
4,740 Total | 13 New | 73 Active

Missoula County Cases

Montana Coronavirus Active Cases by County

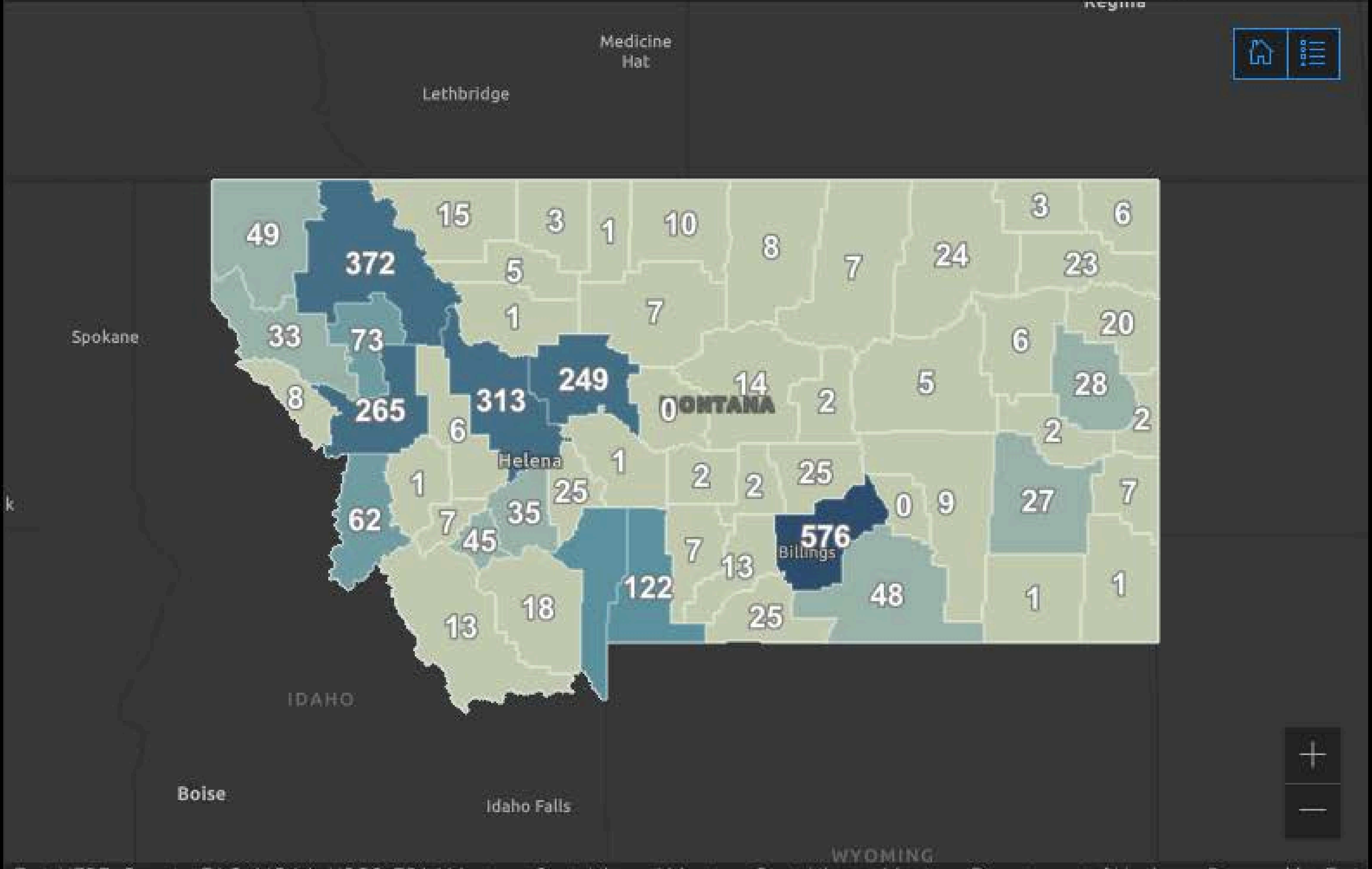

Esri, HERE, Garmin, FAO, NOAA, USGS, EPA | Montana State Library | Montana State Library, Montana Department of Healt... Powered by Esri

Active Cases | Cumulative Cases

Updates are made between 10:00 and 11:30 a.m. on weekdays. Information is reported based on the previous day. It is highly likely that the information on this dashboard and map will change based on further public health investigation. For information on demographics and nonresidents, click here. For assistance, please e-mail or call the Montana Joint Information Center 1-888-333-0461. Source Data: Montana Department of Health & Human Services, Montana State Library. Date and time of last update: 12/10/2021, 12:36 PM.

Total Deaths
2,821

Recovered
188,070

Active
2,827

Total Tests
2,212,449
New Tests: 4204

Active Hospitalizations
174

Total Hospitalizations
10,184

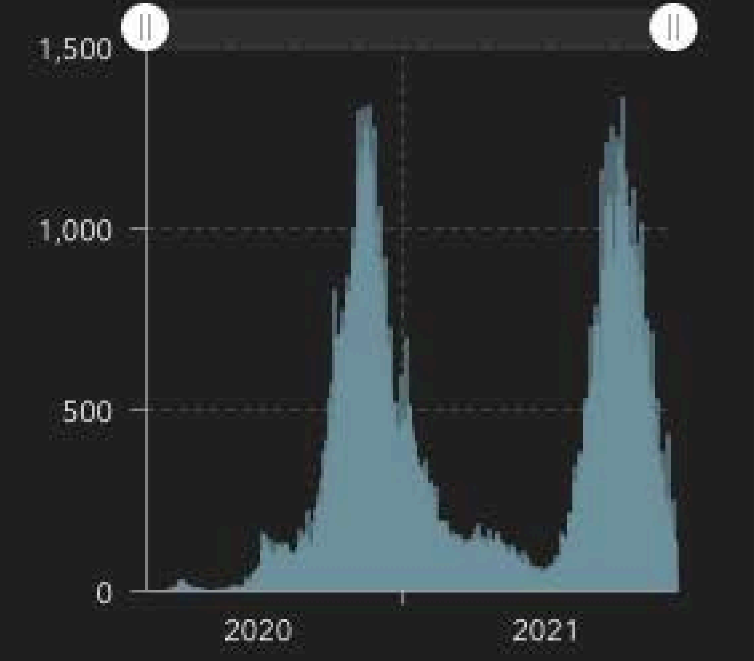

Cases by Diagnosis Date

MONTANA RESPONSE: COVID-19 - Coronavirus - Global, National, and State Inforn Montana Maps

COVID-19 Vaccine | COVID-19 Cases | Local Vaccine Contacts | COVID-19 Demographics | Montana COVID-19 Vaccine FAQs | Global Outbreak Dashboard

Total Doses Administered

1,266,756

Total Montanans Fully Immunized

528,862

Eligible Population Fully Vaccinated

51%

COVID-19 Vaccinations By County

Beaverhead County Vaccines:
10,684 Doses Given | **4,869 (54%)** Fully Immunized

Big Horn County Vaccines:
15,686 Doses Given | **6,715 (56%)** Fully Immunized

Blaine County Vaccines:
7,184 Doses Given | **3,021 (50%)** Fully Immunized

Broadwater County Vaccines:
4,399 Doses Given | **1,811 (30%)** Fully Immunized

COVID-19 Vaccinations by County of Residence: Percent of Eligible Population Fully Vaccinated

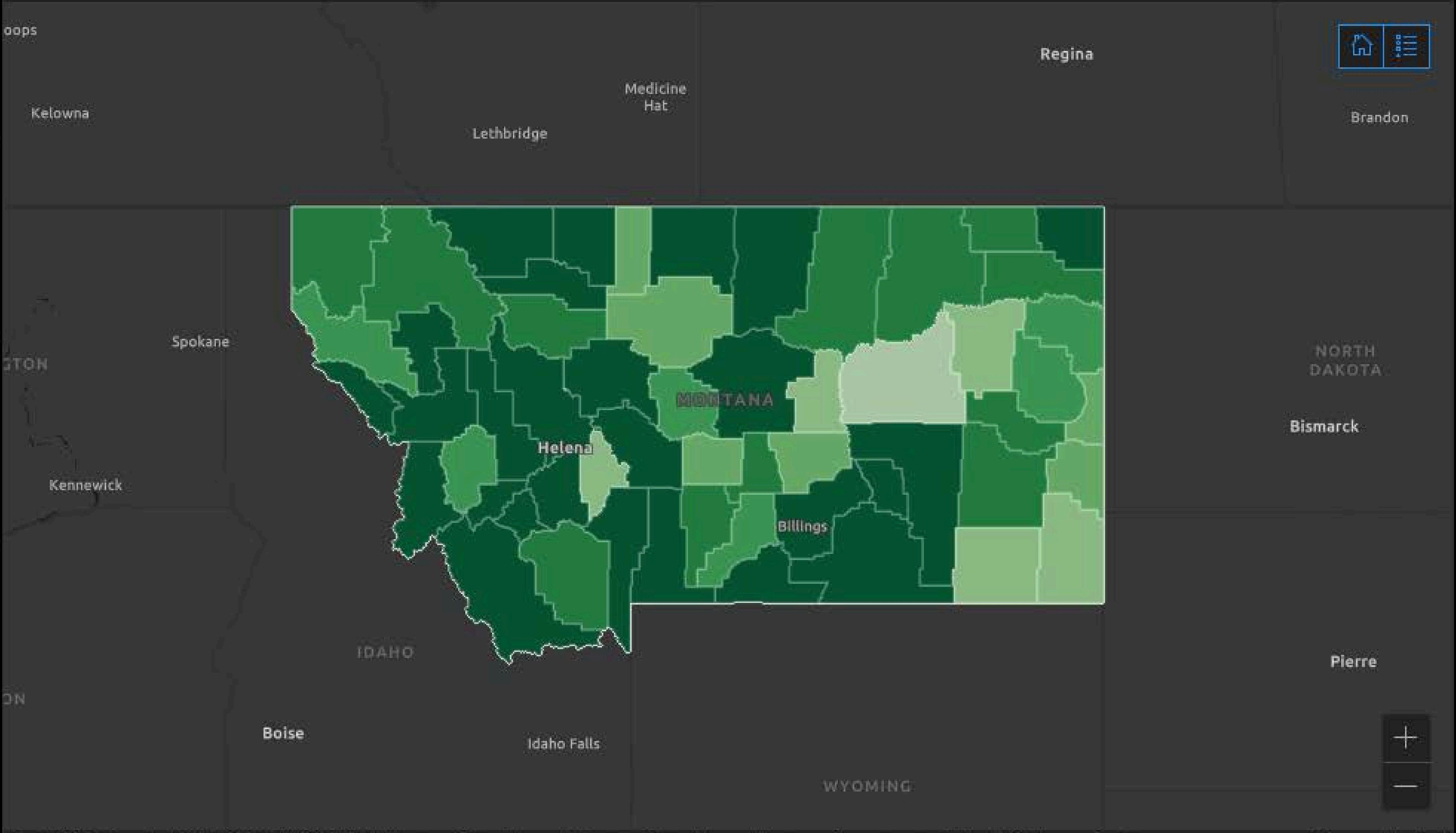

Esri, HERE, Garmin, FAO, NOAA, USGS, EPA | Montana State Library | Montana State Library, Montana Department of Health & Human Services Powered by Esri

Information is reported based on the previous day/week. Total Doses Administered and Total Montanans Fully Immunized are updated weekdays between 10:00 and 11:30 a.m. Last Updated: **12/10/2021, 12:37 PM**. County Vaccine Information are updated every Monday, between 10:00 and 11:30 a.m. For information on vaccines in Montana click here. For assistance, please e-mail or call the Montana Joint Information Center 1-888-333-0461. Source Data: Montana Department of Health & Human Services, Montana State Library.

COVID-19 North Carolina Dashboard

We want to hear from you! Take the NCDHHS Dashboard Survey today!

Knowing when to dial-up or down measures that slow the spread of the virus depends on North Carolina's testing, tracing and trends. This dashboard provides an overview on the metrics and capacities that the state is following.

View Larger

Updated Monday - Friday by approximately 12:00 p.m.
Last updated December 10, 2021 at 12:05 p.m.

Daily Cases

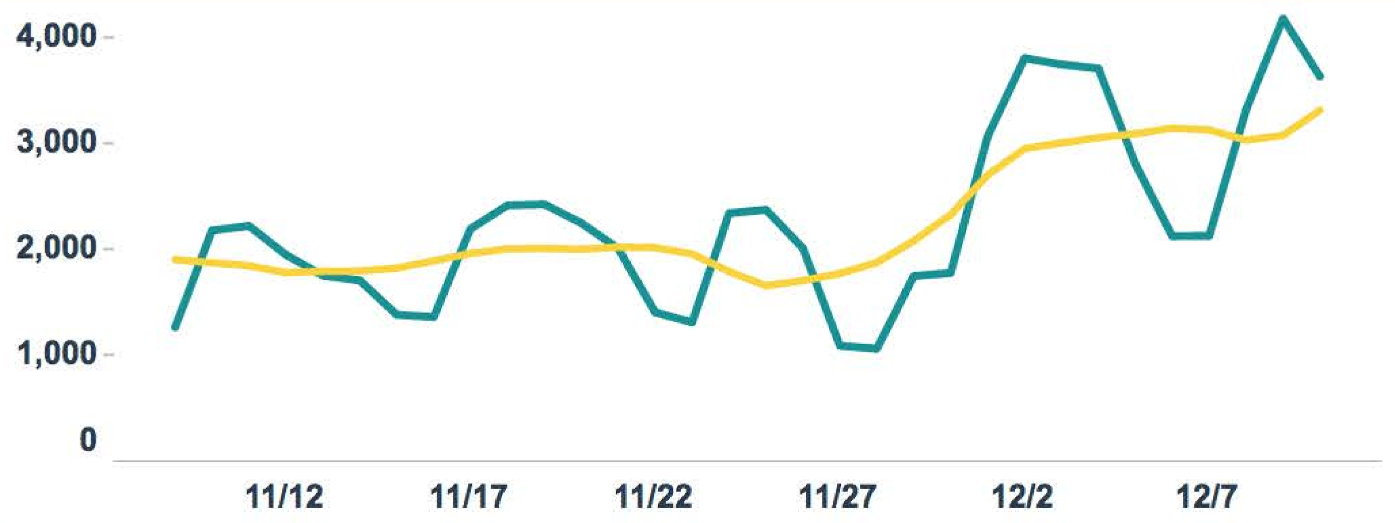

TOTAL CASES

1,566,269

Molecular (PCR) Positive Cases

1,313,902

Antigen Positive Cases

252,367

Daily Tests

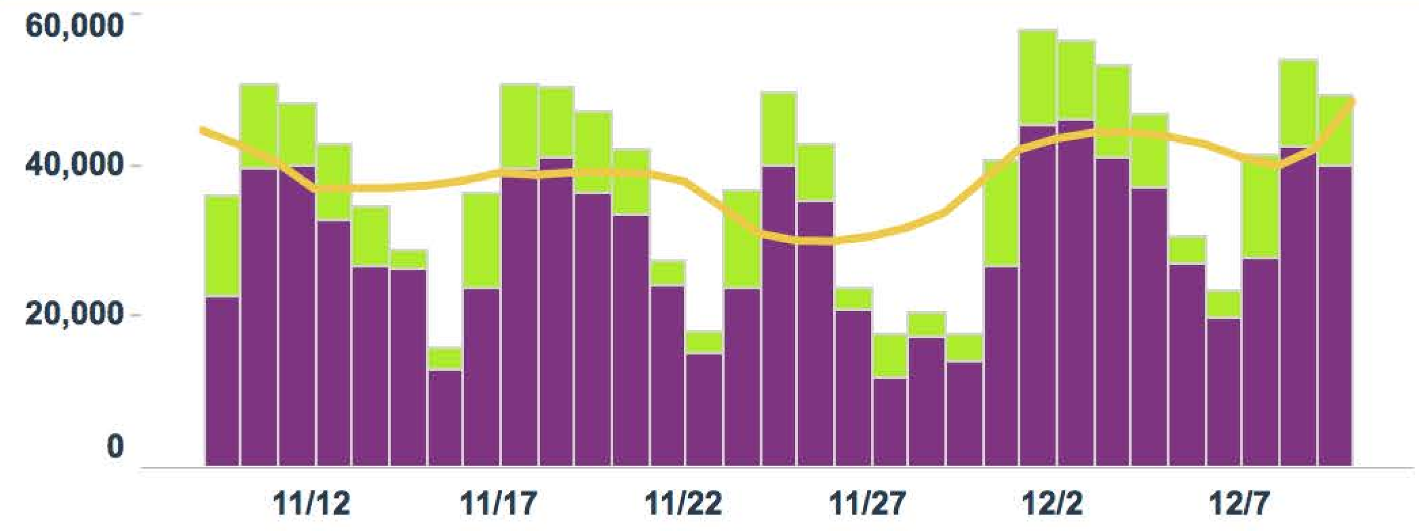

COMPLETED TESTS

20,736,153

Daily Number of People Currently Hospitalized

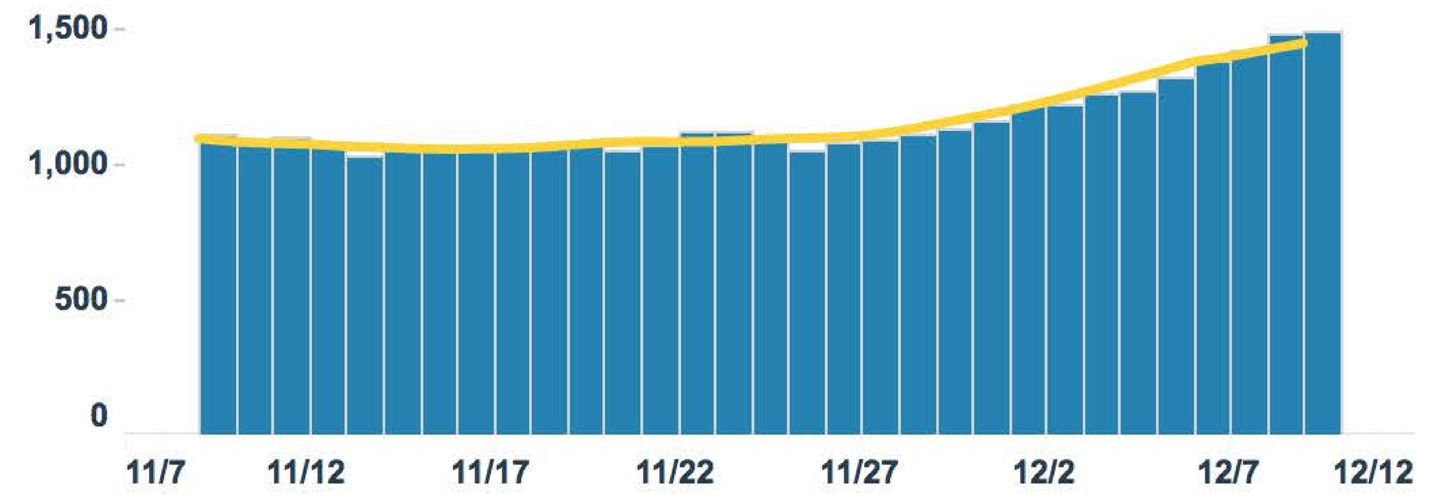

CURRENTLY HOSPITALIZED

1,493

County Map by Cases per 100,000 Residents (Molecular (PCR) and Antigen)
(Click to highlight map, unclick to revert map)

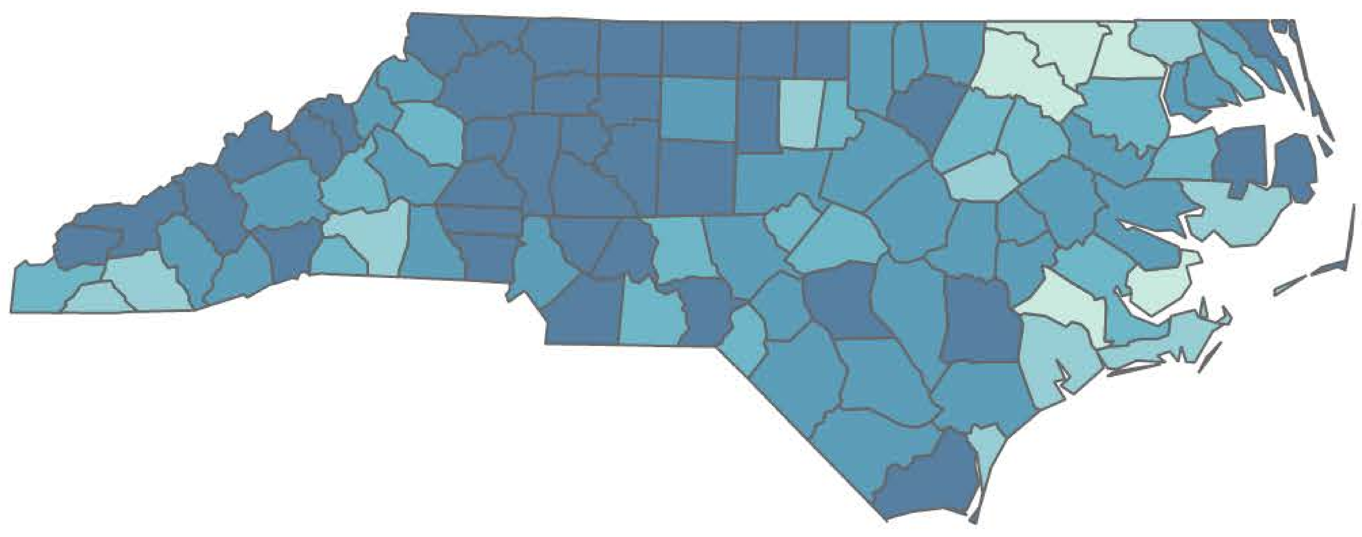

1-164
165-229
230-271
272-369
370+

Select Geography
- County
- ZIP Code

Search for County

Select Metric
- Cases
- Cases per 10,000 Residents
- Cases per 100,000 Residents

Date Range
Total Cases - Last 14 Days

Total County Cases and Deaths (Molecular (PCR) and Antigen)

Alexander	6,924	119
Alleghany	1,911	6
Anson	4,083	83
Ashe	3,776	56
Avery	2,919	39
Beaufort	7,619	136
Bertie	2,583	53

Hover over icons for information.

December 10, 2021

+ a b l e a u

Dashboard
- Summary
- County Transmission Data
- Vaccinations
- CLI Surveillance
- Cases
- Testing
- Hospitalizations
- Contact Tracing
- Outbreaks and Clusters
- Wastewater Monitoring
- Reports
- Data Behind the Dashboards

Testing

View Larger

Total Tests Reported

Is North Carolina increasing its testing capacity?

Testing is an important tool to help diagnose and isolate people who are infected. Testing is also important to help characterize the prevalence, spread and contagiousness of the disease.

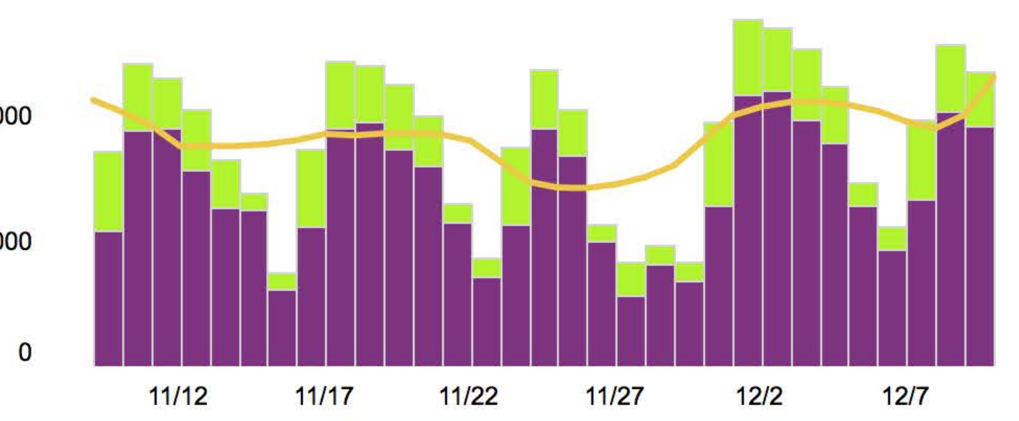

TOTAL TESTS

20,736,153

Labs Reported Electronically

Labs submit testing data to the State in two ways: at the patient-level, and in aggregate.

Patient-level testing data (positives and negatives) is submitted either electronically through NC COVID or manually via secure fax or electronic communications. Approximately 98% of total patient-level tests are now submitted electronically through NC COVID. The other 2% that are manually submitted must then be hand-entered into NC COVID.

Aggregate testing data is provided by labs that are not using electronic laboratory reporting. These labs submit the daily number of tests performed to NCDHHS using the electronic COVID-19 Aggregate Test Reporting (eCATR) survey tool.

Test totals are combined from these two sources of testing data to obtain the daily and cumulative report of total tests performed. NCDHHS has been working to improve data reporting processes to minimize redundancy with all labs to move to reporting exclusively through NC COVID to improve data integrity.

NCDHHS is working to transition away from manual reporting processes by working to onboard all labs into NC COVID

Positive Tests as a Percent of Total Tests

Is North Carolina seeing a 14-day downward trajectory or sustained leveling of positive tests as a percentage of total tests?

As we ramp up testing, there will be more positive tests. Looking at what percent of total tests are positive helps us understand whether *positive* Molecular (PCR) tests are increasing in comparison to the total number of PCR tests conducted.

Laboratory results received electronically for previous dates may cause slight variation in day-to-day reporting.

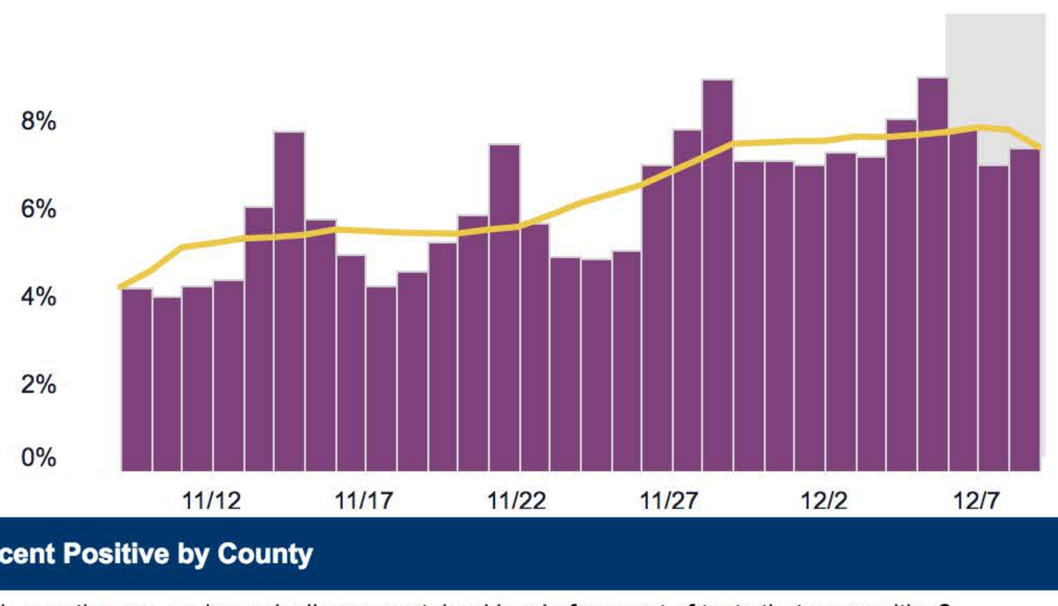

POSITIVE TESTS

7.4%

Percent Positive by County

Which counties are seeing a decline or sustained level of percent of tests that are positive?

It is important to recognize that the amount of testing being conducted varies across counties. Because the percent positive is calculated using only molecular (PCR) testing data reported electronically in to NC COVID, which is a subset of total tests reported, county-level percent positive rates may be impacted by how many of the total tests for residents in the county were reported electronically through NC COVID.

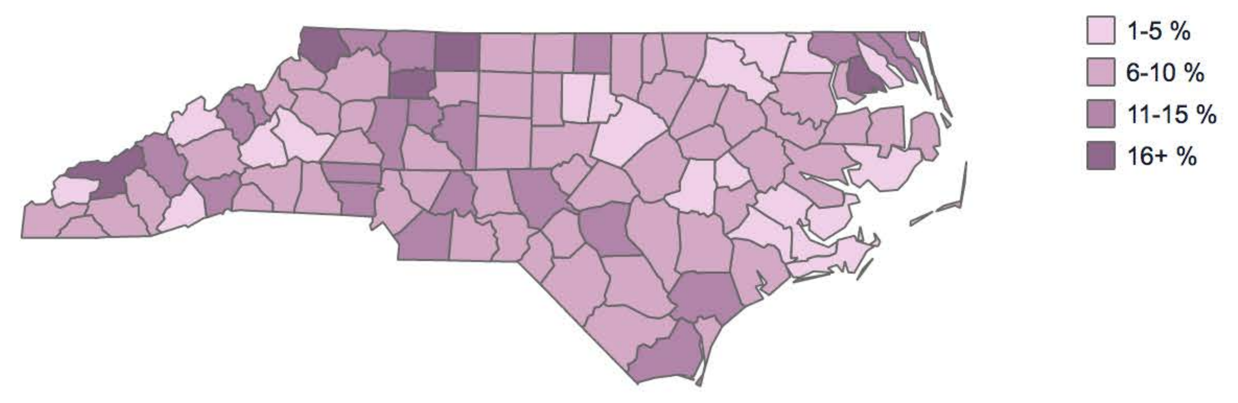

Testing Turnaround Time

There are multiple stages that make up the time it takes from when a person is tested to the time the person receives their results — the testing turnaround time The first stage is the time between when a specimen is collected to when it is received by a laboratory. Several factors can impact this timing, including how and when the specimen is transported to a laboratory. The second stage is the time between when the specimen is received and when the laboratory has a result. This first and second stage is shown in the dark purple in the graph below. The third stage is the time between when the laboratory determines a result and the laboratory electronically reported to NCDHHS. This third stage is shown in the light purple. The fourth stage, which is not represented in the graph below; is the time between when a laboratory reports a result and the patient is notified of their results. This fourth stage happens between the health care provider and patient and is not reported to NCDHHS. The yellow line shows the 7-day rolling average of the dark purple, when a specimen is collected to when the laboratory has a result.

Please note that the graph shows the average turnaround time across all laboratories reporting electronically to NCDHHS. Individual laboratories may have shorter or longer turnaround times and, therefore people's individual experience will vary. Antigen tests are excluded from testing turnaround calculations because antigen tests are designed to be rapid and are typically resulted within an hour or less. Including these tests in testing turnaround time calculations would potentially artificially decrease testing turnaround times for molecular (PCR) test results, which are the most widely used tests today. Laboratory results received electronically for previous dates may cause slight variation in day-to-day reporting.

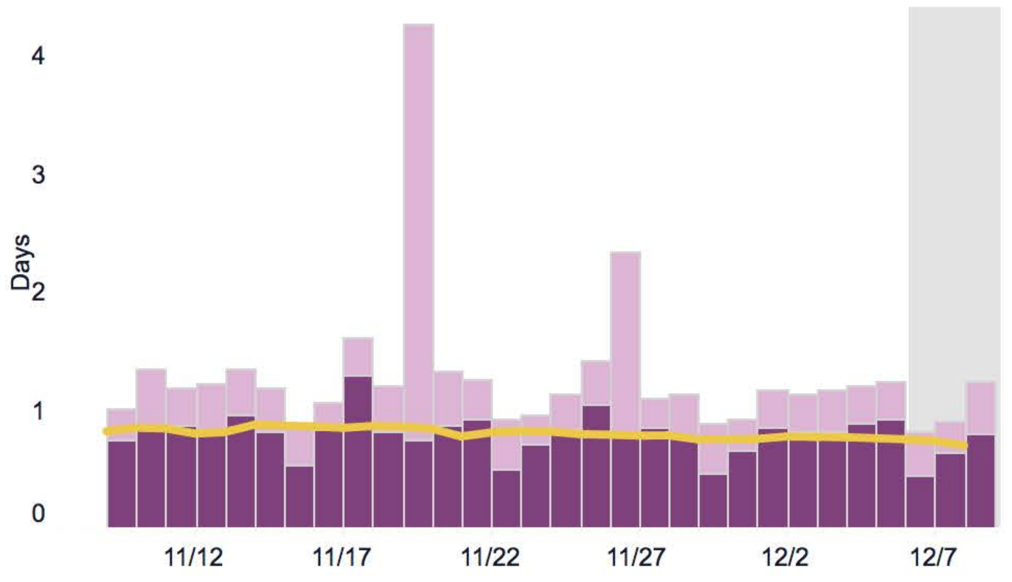

Last 32 Days

0.8

Average Days from Specimen Collection to Laboratory Result

+

0.5

Average Days from Laboratory Result to Electronically Reported to NCDHHS

=

1.3

Average Days of Turnaround Time for COVID-19 Testing

December 10, 2021

COVID-19 Testing

Labs submit testing data to the State in two ways: at the patient-level, and in aggregate.

Patient-level testing data (positives and negatives) is submitted either electronically through NC COVID or manually via secure fax or electronic communications. Approximately 80% of total patient-level tests are now submitted electronically through NC COVID. The other 20% that are manually submitted must then be hand-entered into NC COVID.

Aggregate testing data is provided by labs that don't have electronic laboratory reporting. These labs submit the daily number of tests performed to NCDHHS using the electronic COVID-19 Aggregate Test Reporting (eCATR) survey tool.

Test totals are combined from these two sources of testing data to obtain the daily and cumulative report of total tests performed. NCDHHS has been working to improve data reporting processes to minimize redundancy with all labs to move to reporting exclusively through NC COVID to improve data integrity with test totals from NC COVID to obtain the total number of tests conducted.

Tests can be reported in batches (e.g., a lab reports three days of testing data at once) and tests are not always reported on the day they occurred. When a new lab begins reporting, it may report a backlog of tests from the past days or weeks. When tests are reported in batches or a new laboratory begins reporting, those tests are assigned back to the correct date. Therefore, previous days of testing are updated as more tests are reported. The number of tests completed by day is a dynamic number and is dependent on reporting labs.

Total tests represent the number of tests reported to NCDHHS for that day. This tells us how much daily testing is increasing throughout the state. This includes both total tests that report daily through electronic laboratory reporting, and those that are submitted manually. Because tests can be reported in batches, dates may be backfilled, and data might fluctuate. Tests have been broken out to show molecular (PCR) vs. antigen tests completed starting with data on Sept. 22, 2020 on the "Total Tests Reported" chart. For dates prior to Sept. 22, 2020, test totals cannot be broken out according to test type due to data limitations in the process previously used for laboratory reporting of test totals. However, it is likely that antigen tests were included in aggregate totals submitted by laboratories in daily reports in the past.

positive tests we are seeing in comparison to how much testing is being done. All data are preliminary and may change.

To calculate **positive tests as a percent of total tests** NCDHHS only uses molecular (PCR) test results from laboratories that report both positives and negatives through electronic laboratory reporting in NC COVID. This ensures that the positive and negative tests were from the same day to calculate an accurate daily percent positive. This is because test totals that are not reported electronically can occur in batches, and it cannot be confirmed that the positives and negatives occurred on the same day. Antigen tests are excluded from percent positivity calculations at this time, but NCDHHS will be evaluating including them in calculations in the future.

Dashboard
Summary
County Transmission Data
Vaccinations
CLI Surveillance
Cases
Testing
Hospitalizations
Contact Tracing
Outbreaks and Clusters
Wastewater Monitoring
Reports
Data Behind the Dashboards

Vaccinations

We want to hear from you! Take the [NCDHHS Dashboard Survey](#) today!

This dashboard shows the total number of doses that have been administered by NC providers and through the federal pharmacy program. The information will be available on the tabs "Summary Data" and "Demographics Data" on the dashboard through a drop-down option that allows users to view data for NC Providers, Federal Pharmacy Programs, or All Programs. It is updated Monday through Friday.

Current vaccines may require either a single shot or two doses of the vaccine. Data are included for the total number of doses that have been administered through a single dose or two-dose vaccine series. Data on people who have been vaccinated with at least one dose and people who have been fully vaccinated are available by race, ethnicity, gender, and age group and can be filtered by county on the tab labeled "Demographics Data". Data includes information on the percent of total people that have been vaccinated broken down by demographic categories and information on the percent of the population that has been vaccinated with at least one dose, or fully vaccinated. Data shown are preliminary and may change. There can be a 72-hour lag in data reported to NCDHHS.

View Larger

Summary Data | Demographics Data | Additional NC D

Data: December 14, 2020 – December 10, 2021 at 4:00 a.m.
Vaccinations Data will be updated Monday - Friday

North Carolina's Vaccination Progress Summary

North Carolina's Vaccinations Progress Summary includes data from North Carolina's COVID-19 Vaccine Management System (CVMS), as well as data from the federal reporting platform Tiberius for providers that are not currently reporting to the state including Veterans Affairs, U.S. Department of Defense, Federal Bureau of Prisons, and Indian Health Service. **This federal data is included in the statewide and county-level estimates of percent of population by age group that has received at least one dose and those fully vaccinated. It is not available for data included in the "Doses Administered by Week" section below or on the "Demographics Data" tab. Data for these sections come only from North Carolina's COVID-19 Vaccine Management System (CVMS), which does not include data from providers not reporting data to the state. Therefore, these sections of the dashboard undercount the true number of doses administered to North Carolina residents.** Dashboards are updated every weekday afternoon with the most recently available numbers as of that morning.

First of 2 Doses Administered	Second of 2 Doses Administered	Single Shot Doses Administered	Additional/Booster Doses Administered
6,027,972	5,605,294	460,386	1,871,460

	Percent of Population Vaccinated with at Least One Dose	Percent of Population Fully Vaccinated
Total Population	62%	58%
5+ Years of Age	66%	61%
12+ Years of Age	71%	67%
18+ Years of Age	73%	69%
65+ Years of Age	94%	91%

People Vaccinated with at Least One Dose by County of Residence

Select Metric: People Vaccinated with at Least One Dose
Select Population: Total Population
Select County: Alamance County

100,601
People Vaccinated with at Least One Dose for Total Population
Alamance County

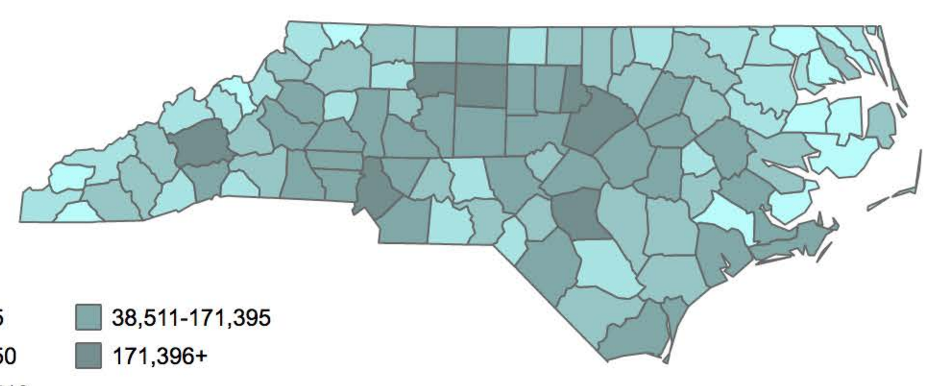

People Vaccinated with at Least One Dose for Total Population: 196,423 NC residents missing county information; 107,183 out of state residents

Doses Administered by Week

Data below come from North Carolina's COVID-19 Vaccine Management System (CVMS). This data does not include data from providers not reporting data to the state including Veterans Affairs, U.S. Department of Defense, Federal Bureau of Prisons, and Indian Health Service. Therefore, these sections of the dashboard undercount the true number of doses administered to North Carolina residents.

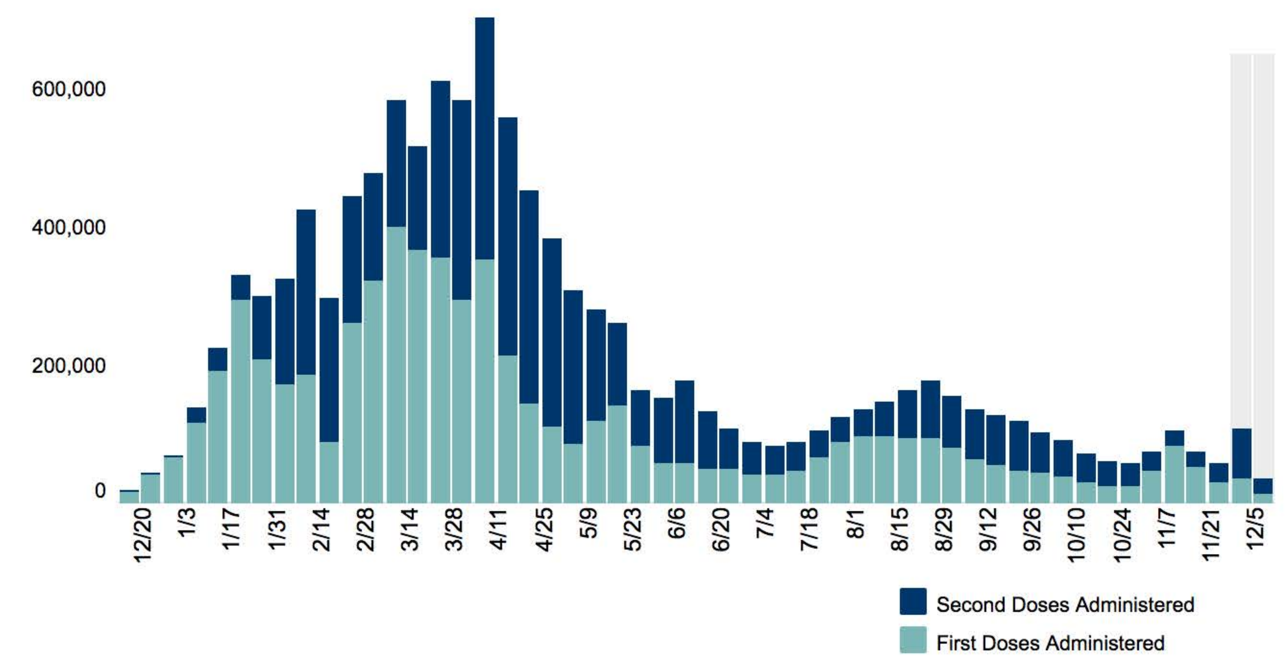

Date of Vaccination Administered is missing for 139 doses.

December 10, 2021

+ t a b l e a u

County vaccination data may change once residence is verified. All data are preliminary and may change.

NCDHHS applies the following rules to the COVID-19 data dashboards where data is at a county level and includes demographics data:

- Data will be stratified by county by one demographic group at a time (ethnicity, race, age group, gender).
- Population data sources, as listed in the [Data Behind the Dashboards](#) are used to find 'cell denominators' for county populations. Data for any county demographic sub-group that has a population of less than 500, will be suppressed for privacy.
- For county cell denominators that require suppression, all corresponding demographic data elements are attributed to the 'Suppressed' category. This method allows the display of actual values on the dashboards, while still suppressing which demographic sub-groups the data are attributed to.
- Additional data suppression is done for demographic sub-groups that can be discovered by subtracting row or column totals (e.g., if a County has only age group 18-24 that requires suppression, the next smallest age group by population is also suppressed).

'Missing or Undisclosed' may include data for people that did not provide demographic information, or where vaccine providers did not submit the demographic information. Numbers may not add up to 100% due to rounding.

Percent of population metrics are calculated using the entire North Carolina population (i.e., all ages). The state's dashboard is the source for the most accurate and timely information for vaccine data for the state.

Consistent with CDC practice, NCDHHS has capped percent of population metrics at 100%. Dashboard metrics for percent of population vaccinated could exceed 100% for a few reasons,

North Carolina's information on people vaccinated comes from the COVID-19 Vaccine Management System (CVMS), a secure, web-based system provided for free to all who administer COVID-19 vaccinations. It helps vaccine providers know who has had a first dose of which vaccine to make sure people get the second dose of the same vaccine at the right time. It also helps people register for vaccination at the appropriate time and allows the state to manage vaccine supply.

Information on vaccinations is confidential health information that is carefully managed to protect patient privacy. Information will not be shared except in accordance with state and federal law.

Visit [Here](#) for more information on the vaccines and vaccine distribution in North Carolina.

Dashboard
Summary
County Transmission Data
Vaccinations
CLI Surveillance
Cases
Testing
Hospitalizations
Contact Tracing
Outbreaks and Clusters
Wastewater Monitoring
Reports
Data Behind the Dashboards

NORTH Dakota | Health
Be Legendary.

Overview | Trends | Demographics | Hospitalizations | Breakthrough | County Analysis

Last Updated: 12/11/2021

POPULATION
762,062

County
All

% of POPULATION TESTED
72.26%

Hover over a value and click the icon for more information

TODAY

POSITIVITY RATE
5.80%

NEW TESTS

TOTAL PROCESSED
7,641

SUSCEPTIBLE ENCOUNTERS
6,934

NEW POSITIVES

TOTAL (PCR AND ANTIGEN TESTS)
402

CONFIRMED (PCR TESTS)
172

PROBABLE (ANTIGEN TESTS)
230

NEW RECOVERED

RECOVERED
496

ACTIVE POSITIVES
2,674

ACTIVE POSITIVES

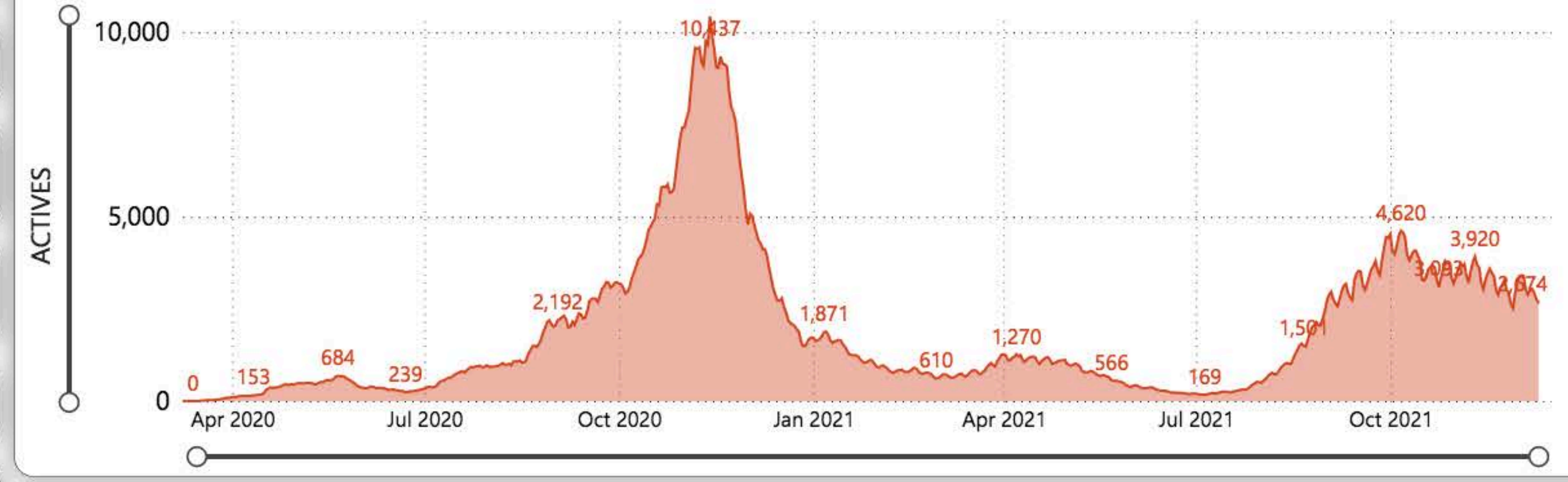

14 DAY ROLLING AVERAGE POSITIVITY RATE

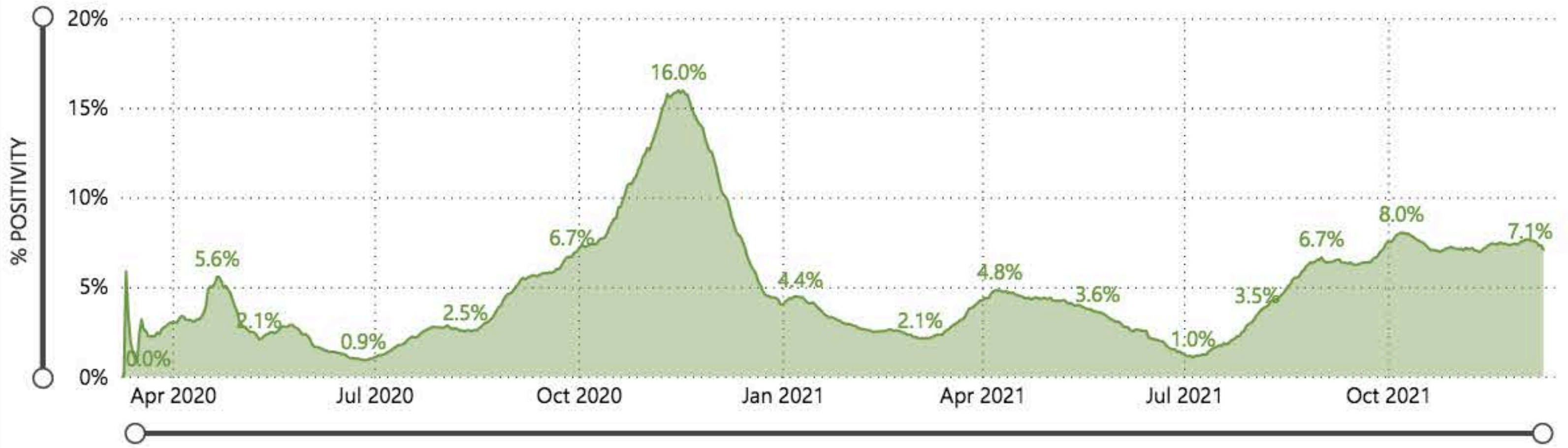

Calculation = (Sum of Past 14 Days of Positives / 14 Days of Tests)

CUMULATIVE

REINFECTIONS (SINCE JUNE 27, 2021)
1,497

TESTS

TOTAL PROCESSED
2,956,202

SUSCEPTIBLE ENCOUNTERS
2,676,289

UNIQUE INDIVIDUALS
550,643

POSITIVES

TOTAL (PCR AND ANTIGEN TESTS)
166,998

CONFIRMED (PCR TESTS)
137,863

PROBABLE (ANTIGEN TESTS)
29,135

RECOVERED and DEATHS

RECOVERED	DEATHS
162,376	1,948

DAILY

TOTAL POSITIVES

CONFIRMED POSITIVES | PROBABLE POSITIVES

14 DAY AVERAGE TOTAL PROCESSED TESTS

CUMULATIVE

TOTAL POSITIVES

CONFIRMED POSITIVES | PROBABLE POSITIVES

RECOVERED | DEATHS

Hover over a value and click the icon for more information

AGE COMPARISON

ACTIVE POSITIVES

DAILY POSITIVES

CUMULATIVE POSITIVES

Confirmed Positives

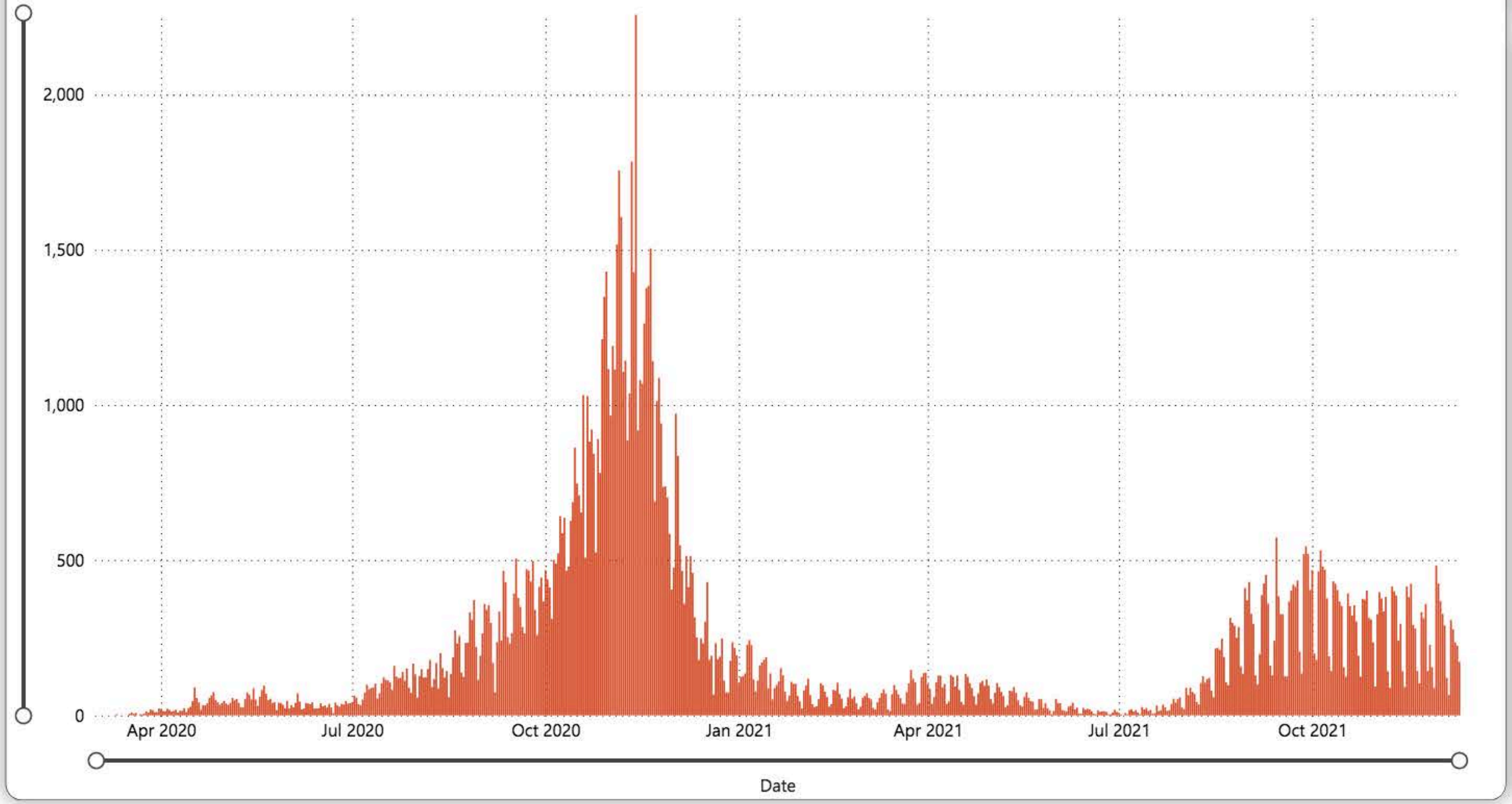

Cumulative Positives

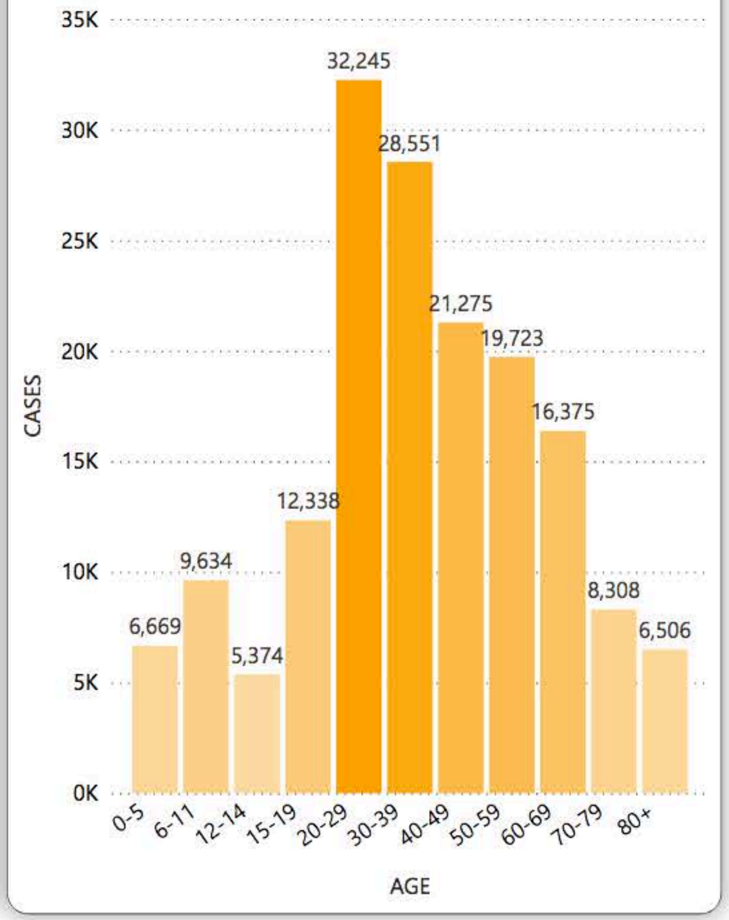

Hover over a value and click the icon for more information

PRESS FOR ACTIVE POSITIVES

PRESS FOR CUMULATIVE POSITIVES

GENDER

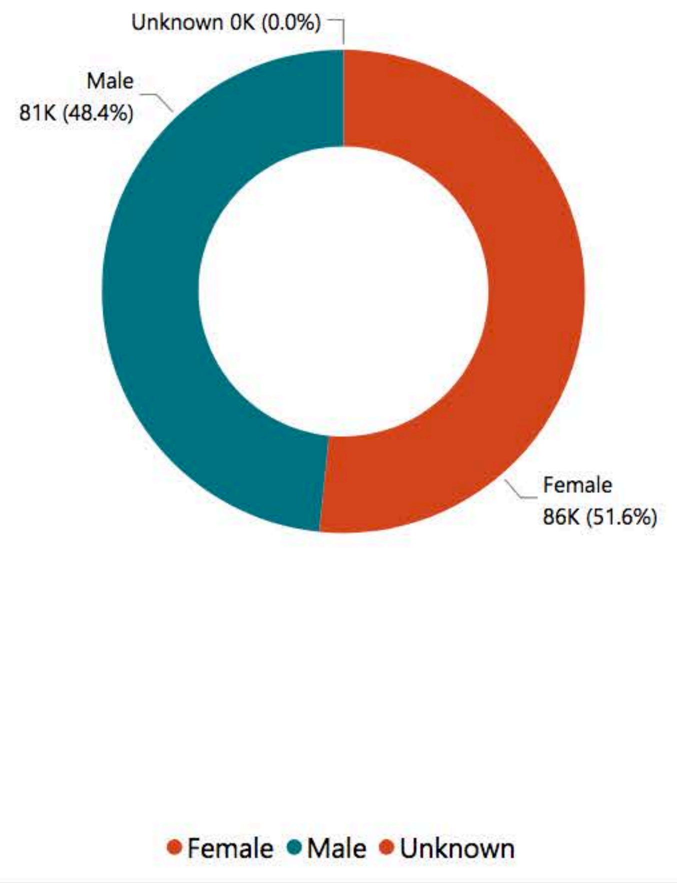

SPREADTYPE

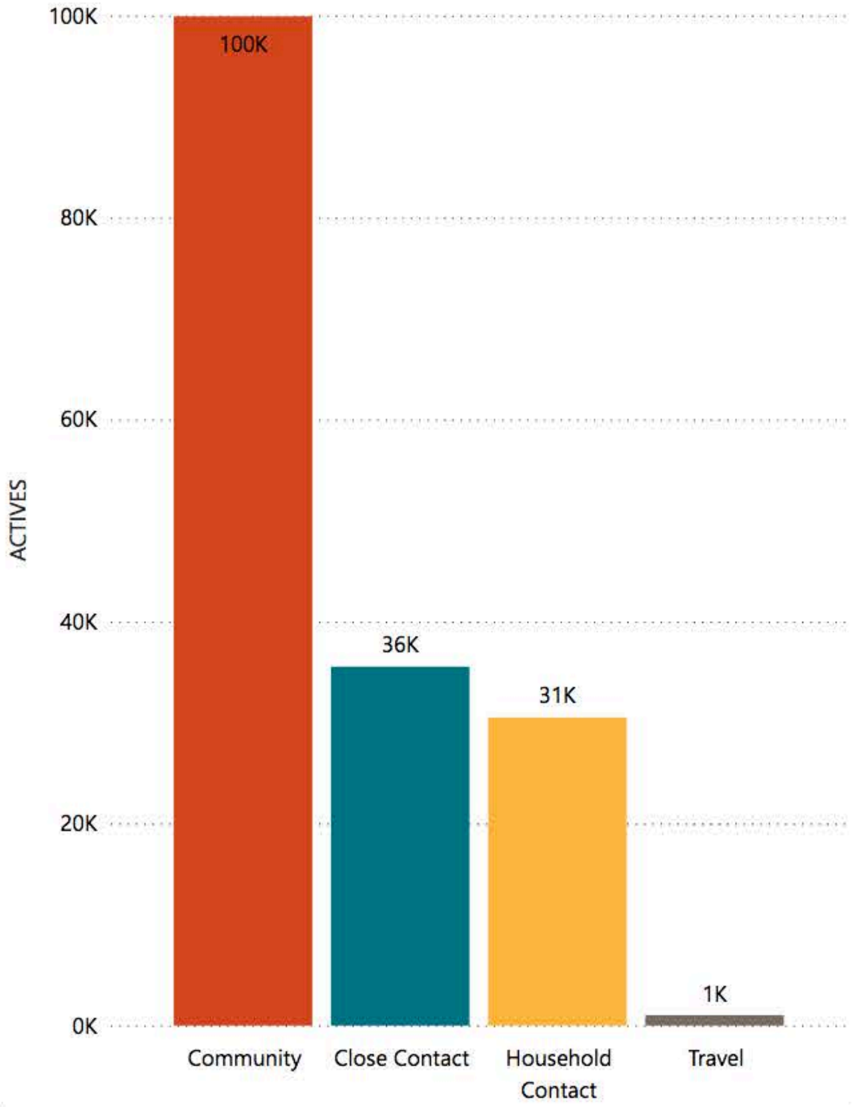

RACE

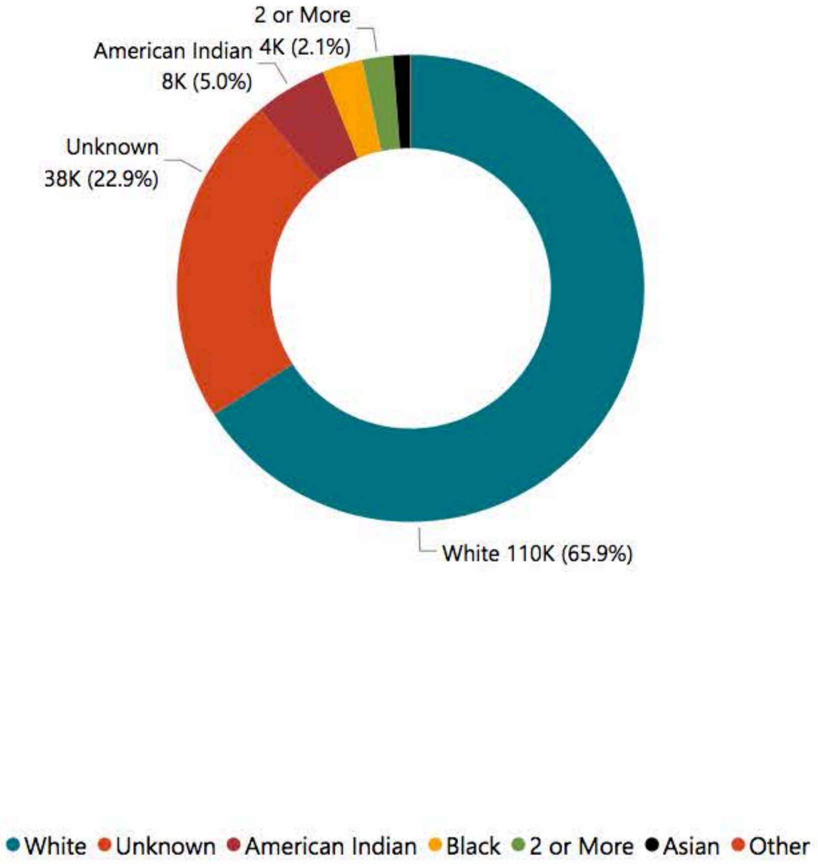

Current Status | Trends | Demographics | County Tracker | NV Health Response Homepage | Office of Analytics Homepage | **Download Data**

Total Cases | Testing | Hospitalizations | Deaths | Vaccinations

DASHBOARD UPDATED ON 12/10/2021

CURRENT STATUS - TOTAL CASES (MARCH 2020 - CURRENT)

DATA AS OF 12/9/2021

COVID-19 case data are summarized below and the key metrics are separated by confirmed and probable. Daily new cases are displayed as 14-day moving averages of all cases confirmed and probable. Cases per 100,000 are displayed for the most recent 30-day period and cumulatively. In the maps below, counties with higher rates are shaded darker. Probable cases may be artificially low due to inconsistent reporting and some counties did not start monitoring probable cases until May 10th, 2021. Results of at-home COVID-19 tests are not included in these data. **Learn more**

SELECT A COUNTY

Search

- North
  - Carson City
  - Washoe
- South
  - Clark
- Rural
  - Churchill
  - Douglas
  - Elko
  - Esmeralda
  - Eureka
  - Humboldt
  - Lander
  - Lincoln
  - Lyon
  - Mineral
  - Nye
  - Pershing
  - Storey
  - White Pine

KEY METRICS

494

NEW DAILY CONFIRMED CASES
14-day moving average

467 Confirmed Cases per 100K
30-day period

464,385 Cumulative Confirmed Cases

14,448 Confirmed Cases per 100K

31

NEW DAILY PROBABLE CASES
14-day moving average

23 Probable Cases per 100K
30-day period

TOTAL CASES PER 100K (30-day period)

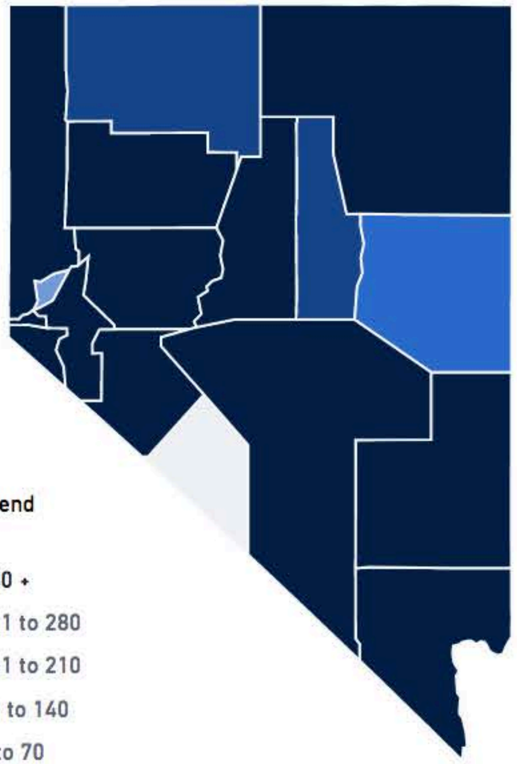

Legend

280 +
211 to 280
141 to 210
71 to 140
1 to 70
0

TOTAL DAILY NEW CASES (14-day moving avg.) STATEWIDE ONLY

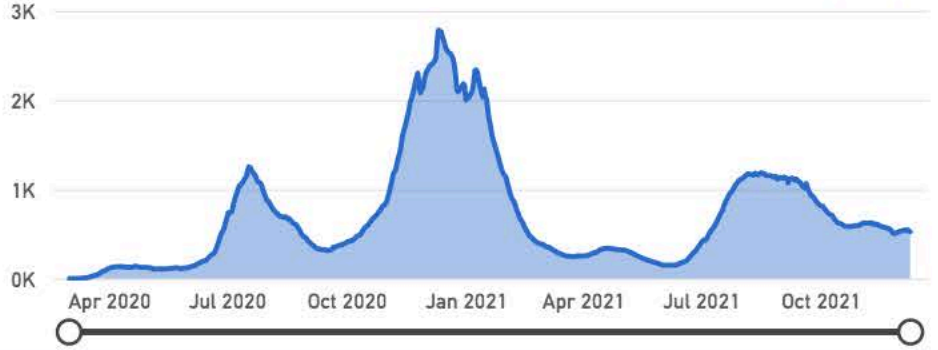

COUNTY	POPULATION	TOTAL CUMULATIVE CASES	TOTAL CUMULATIVE CASES PER 100K
Carson City	57,222	9,523	16,642
Churchill	26,242	4,331	16,504
Clark	2,378,903	358,791	15,082
Douglas	49,535	5,145	10,387
Elko	54,035	9,622	17,807
Esmeralda	971	62	6,385
Eureka	1,885	250	13,263
Humboldt	16,897	3,198	18,926
Lander	5,992	864	14,419
**Total**	**3,214,260**	**478,082**	**14,874**

This dashboard is updated Monday-Friday at approximately 10:00 am. Please note that updates apply incrementally and may be in process for up to an hour. The Nevada DHHS also maintains a facility-specific COVID-19 dashboard, available **Here**

Microsoft Power BI

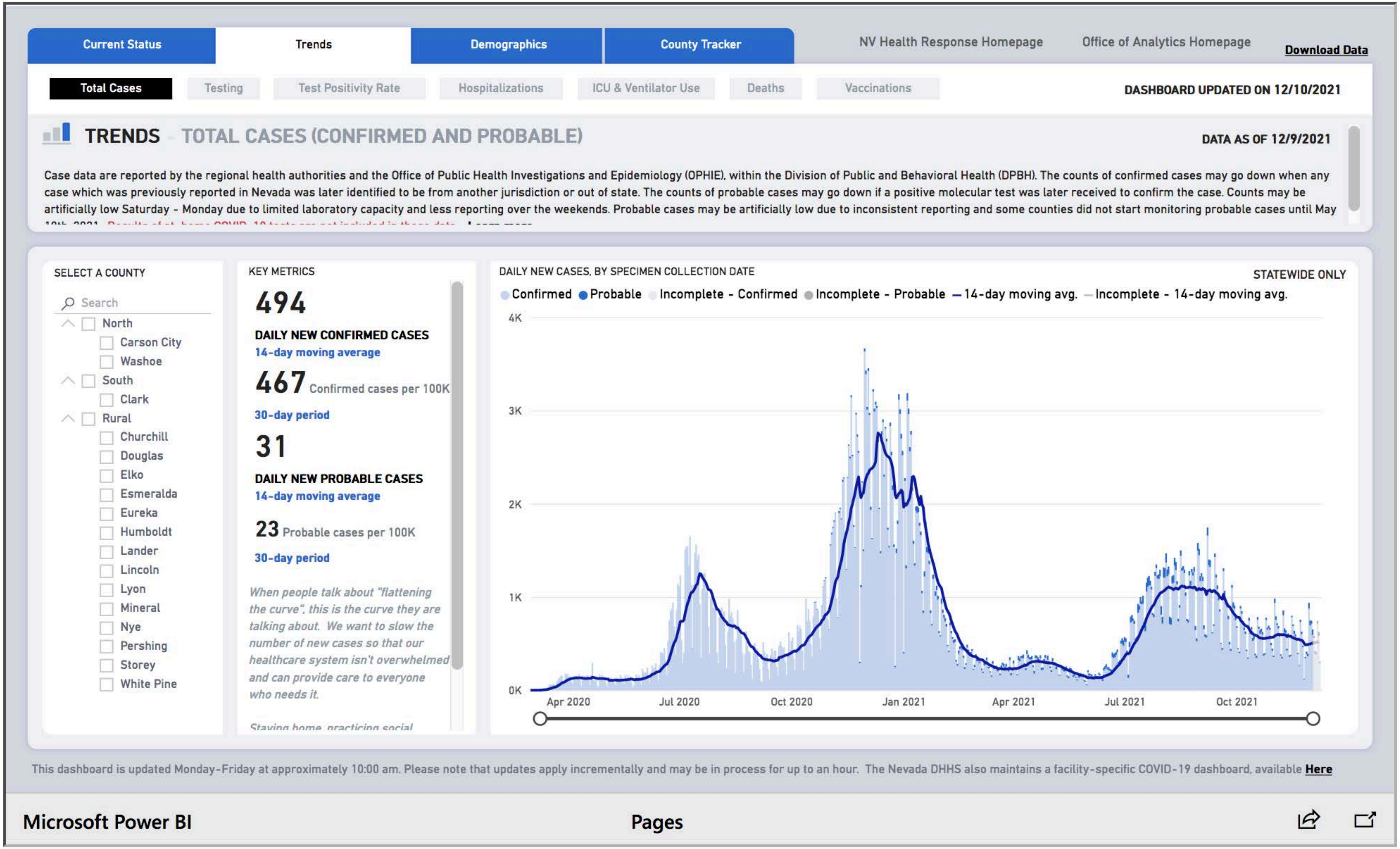
Current Status
Trends
Demographics
County Tracker
NV Health Response Homepage
Office of Analytics Homepage
Download Data
Total Cases
Testing
Test Positivity Rate
Hospitalizations
ICU & Ventilator Use
Deaths
Vaccinations
DASHBOARD UPDATED ON 12/10/2021
TRENDS - TOTAL CASES (CONFIRMED AND PROBABLE)
DATA AS OF 12/9/2021
Case data are reported by the regional health authorities and the Office of Public Health Investigations and Epidemiology (OPHIE), within the Division of Public and Behavioral Health (DPBH). The counts of confirmed cases may go down when any case which was previously reported in Nevada was later identified to be from another jurisdiction or out of state. The counts of probable cases may go down if a positive molecular test was later received to confirm the case. Counts may be artificially low Saturday - Monday due to limited laboratory capacity and less reporting over the weekends. Probable cases may be artificially low due to inconsistent reporting and some counties did not start monitoring probable cases until May
SELECT A COUNTY
Search
North
Carson City
Washoe
South
Clark
Rural
Churchill
Douglas
Elko
Esmeralda
Eureka
Humboldt
Lander
Lincoln
Lyon
Mineral
Nye
Pershing
Storey
White Pine
KEY METRICS
494
DAILY NEW CONFIRMED CASES
14-day moving average
467 Confirmed cases per 100K
30-day period
31
DAILY NEW PROBABLE CASES
14-day moving average
23 Probable cases per 100K
30-day period
When people talk about "flattening the curve", this is the curve they are talking about. We want to slow the number of new cases so that our healthcare system isn't overwhelmed and can provide care to everyone who needs it.
DAILY NEW CASES, BY SPECIMEN COLLECTION DATE
STATEWIDE ONLY
Confirmed
Probable
Incomplete - Confirmed
Incomplete - Probable
14-day moving avg.
Incomplete - 14-day moving avg.
4K
3K
2K
1K
0K
Apr 2020
Jul 2020
Oct 2020
Jan 2021
Apr 2021
Jul 2021
Oct 2021
This dashboard is updated Monday-Friday at approximately 10:00 am. Please note that updates apply incrementally and may be in process for up to an hour. The Nevada DHHS also maintains a facility-specific COVID-19 dashboard, available Here
Microsoft Power BI
Pages

Current Status | Trends | Demographics | County Tracker | NV Health Response Homepage | Office of Analytics Homepage | **Download Data**

Total Cases | Tests | Deaths | Vaccinations

DASHBOARD UPDATED ON 12/10/2021

DEMOGRAPHICS - TOTAL CASES (CONFIRMED AND PROBABLE)

DATA AS OF 12/9/2021

COVID-19 can affect people of all age groups, including children. However, adults in Nevada are more frequently tested and diagnosed with COVID-19. Children can spread COVID-19 despite the absence of signs or symptoms. Adults aged 60 years or above are more vulnerable to COVID-19-associated mortality as opposed to other age groups. The total number of cases for each demographic group may not be equal due to missing data. Results of at-home COVID-19 tests are not included in these data. **Learn more**

Group	CUMULATIVE CASES	CASES %	POPULATION %	POPULATION
**AGE**				
<10	22,879	5.0%	12.4%	398,565
10-19	50,785	11.1%	13.5%	432,709
20-29	87,790	19.1%	13.9%	447,369
30-39	85,814	18.7%	13.6%	436,407
40-49	73,213	16.0%	13.0%	419,139
50-59	63,995	13.9%	12.7%	407,113
60-69	41,072	9.0%	11.0%	353,238
70+	33,226	7.2%	9.9%	319,719
**GENDER**				
Female	236,659	51.5%	50.0%	1,607,496
Male	222,534	48.5%	50.0%	1,606,764
**RACE/ETHNICITY**				
Hispanic	123,202	34.0%	30.7%	986,526
Non-Hispanic American Indian or Alaska Native	2,469	0.7%	1.1%	36,119
Non-Hispanic Asian/Pacific Islander	29,777	8.2%	10.1%	325,604
Non-Hispanic Black	34,073	9.4%	9.0%	290,120
Non-Hispanic Other Race	28,588	7.9%	0.0%	0

This dashboard is updated Monday-Friday at approximately 10:00 am. Please note that updates apply incrementally and may be in process for up to an hour. The Nevada DHHS also maintains a facility-specific COVID-19 dashboard, available **Here**

New Hampshire announced **1,146 cases** for December 10. There were an average of **1,208 cases per day** over the most recent 7-day period (December 4 to December 10). This is a **6% increase** compared to the previous 7-day period.

174,456 Total Cases	55.3% Fully Vaccinated	11.8% 7-Day Test Positivity	466 Current Hosp.
10,138 Active Cases	61.4% At Least 1 Dose	10,564 Avg. Daily Tests	1,781 Total Deaths

Total Deaths by Age

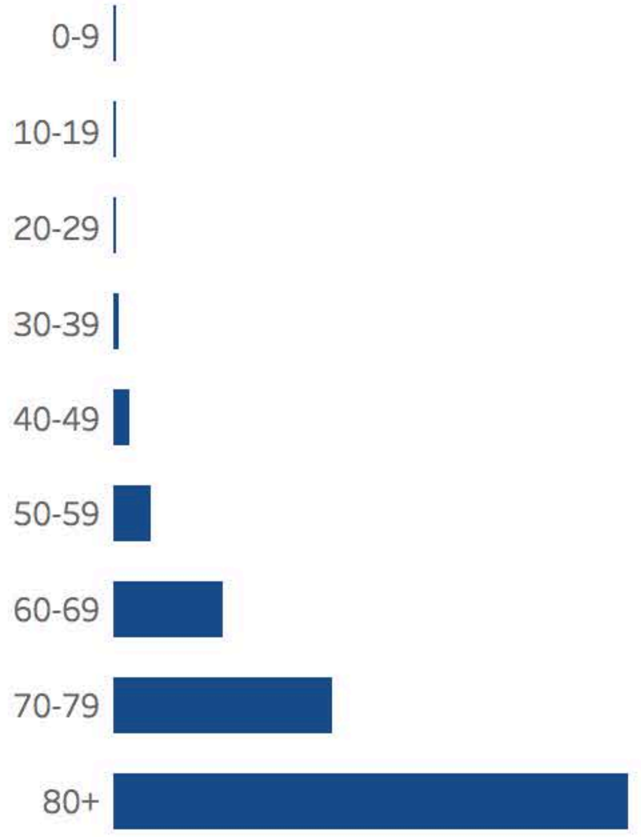

Data as of: 12/10/2021

New Cases and Vaccine Trend

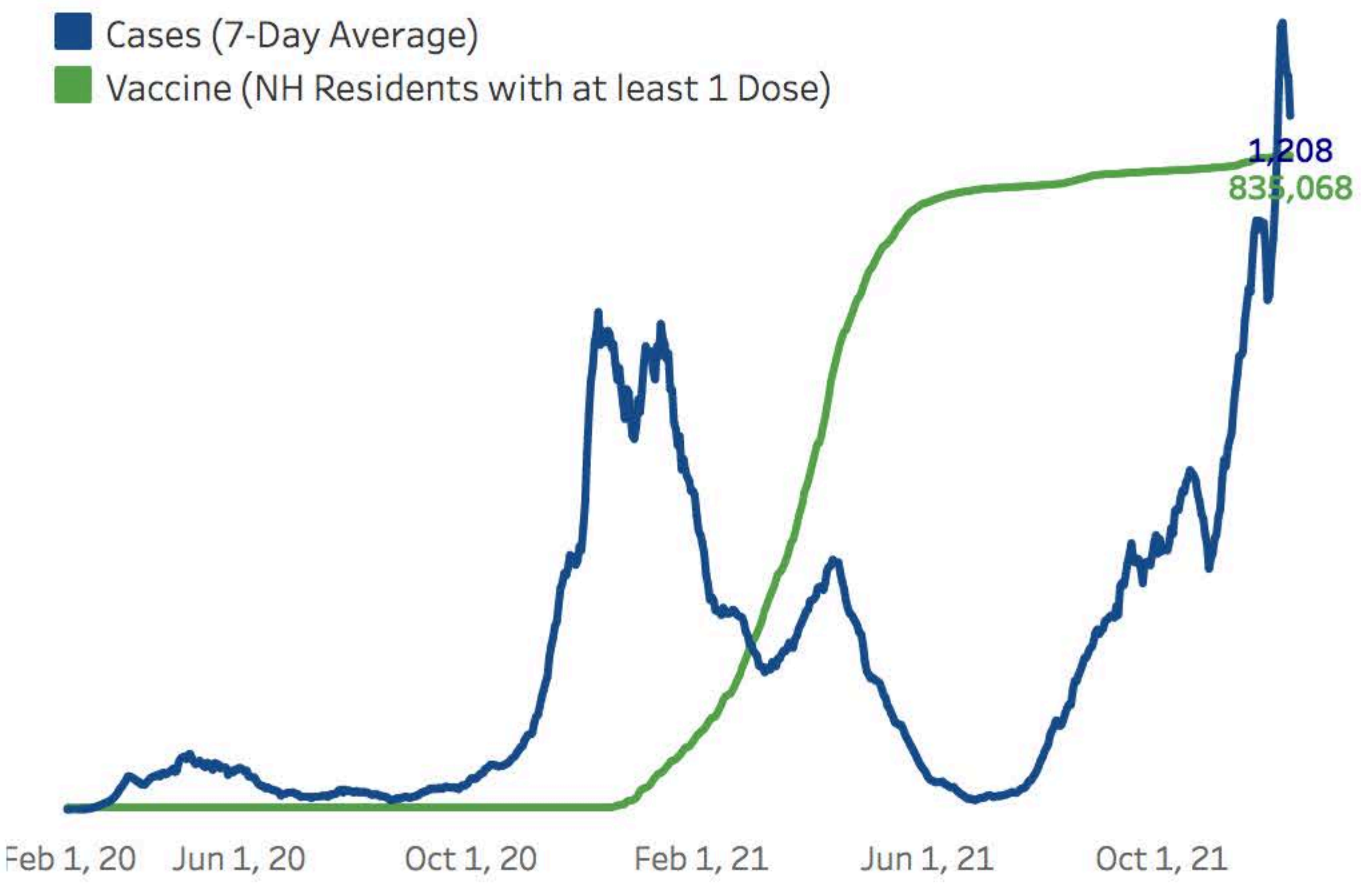

New Cases per 100k over 14 days

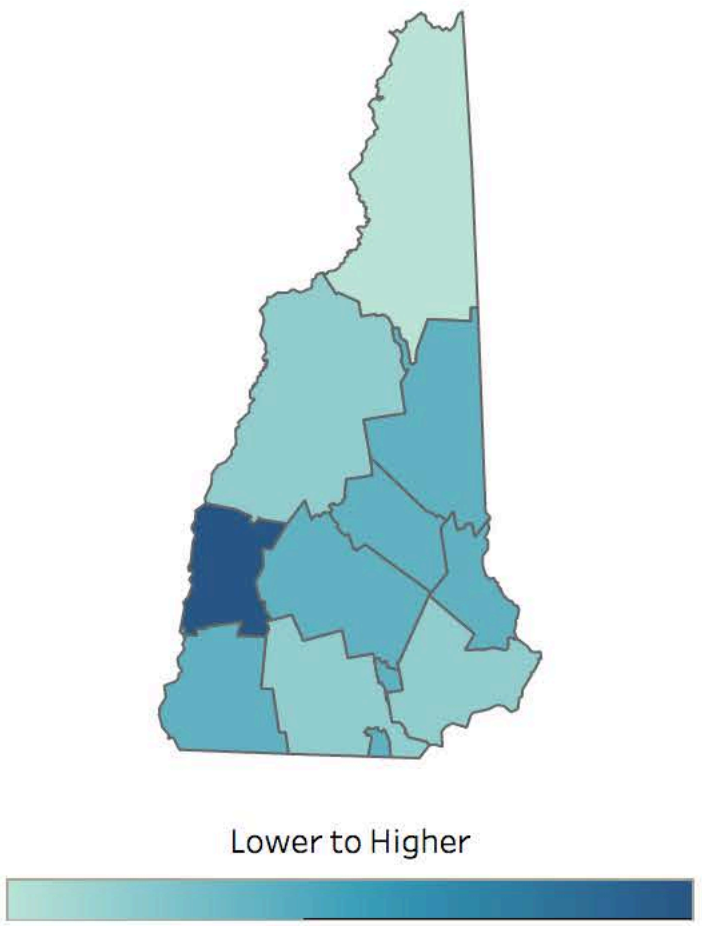

Undo Redo Revert Refresh Pause Metrics Share Download

COVID-19 Trends

Metrics

Select a Metric

- (●) New Cases by Report Date
- ( ) New Cases by Illness Onset Date
- ( ) Long-Term Care Facility Cases
- ( ) Non Long-Term Care Facility Cases
- ( ) Deaths
- ( ) Long-Term Care Facility Deaths
- ( ) Non Long-Term Care Facility Deaths
- ( ) Antigen and PCR Tests - Test Based
- ( ) PCR Tests - Test Based
- ( ) Antigen Tests - Test Based
- ( ) Antibody Tests - Test Based
- ( ) Individuals Fully Vaccinated
- ( ) Individuals with At Least 1 Dose

Filters

Sex

(All)

Age

(All)

- Daily Total
- 7-Day Average
- Cumulative Total

There are 0 COVID-19 related cases not currently plotted due to unknown date of report to NH DHHS.

Data as of: 12/10/2021

Daily Trends for Cases by Report Date

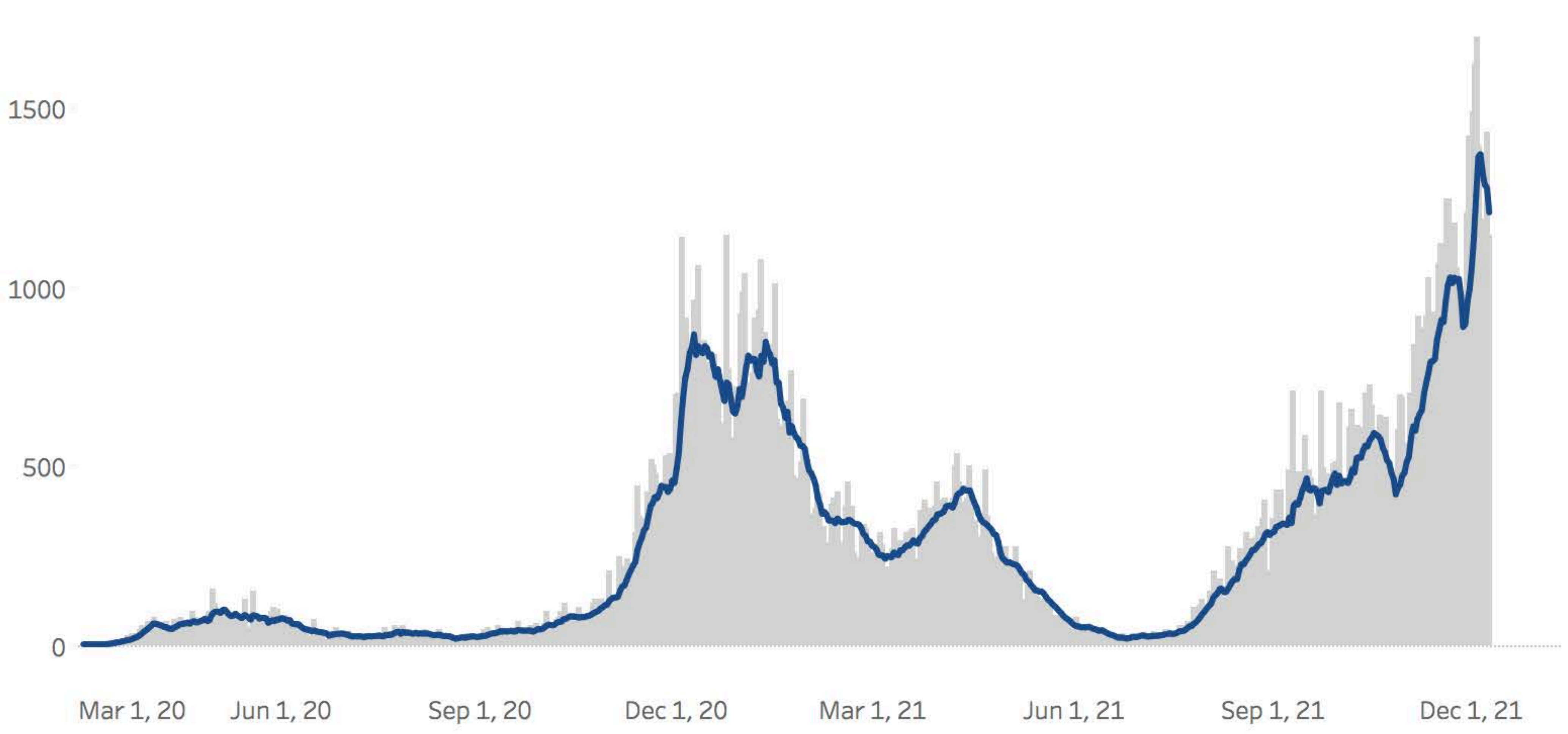

Cumulative Total for Cases by Report Date

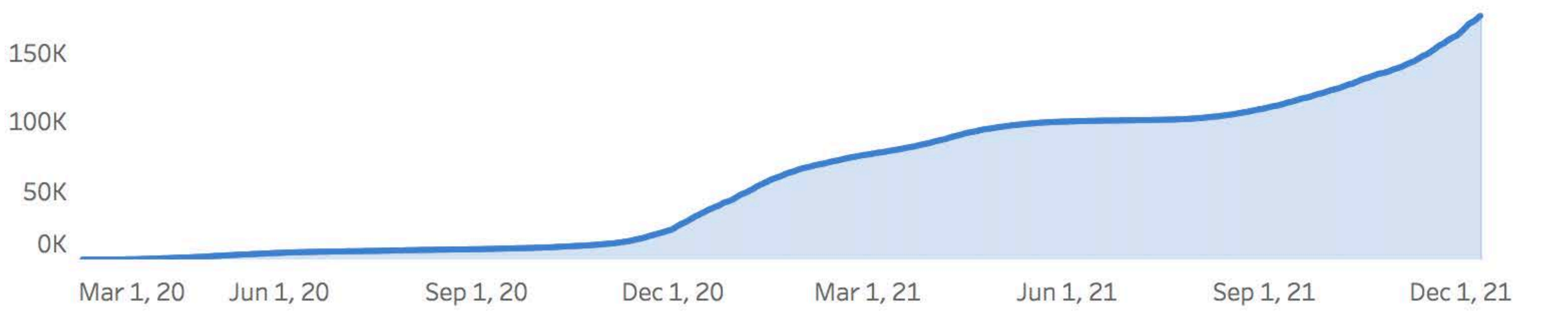

Undo Redo Revert Refresh Pause Metrics Share Download

COVID-19 Trends

Metrics

Select a Metric

- ○ New Cases by Report Date
- ○ New Cases by Illness Onset Date
- ○ Long-Term Care Facility Cases
- ○ Non Long-Term Care Facility Cases
- ○ Deaths
- ○ Long-Term Care Facility Deaths
- ○ Non Long-Term Care Facility Deaths
- ○ Antigen and PCR Tests - Test Based
- ○ PCR Tests - Test Based
- ○ Antigen Tests - Test Based
- ○ Antibody Tests - Test Based
- ◉ Individuals Fully Vaccinated
- ○ Individuals with At Least 1 Dose

Filters

Sex

(All)

Age

(All)

- Daily Total
- 7-Day Average
- Cumulative Total

There are 0 COVID-19 related individuals fully vaccinated not currently plotted due to unknown administered date.

Data as of: 12/10/2021

Daily Trends for Individuals Fully Vaccinated

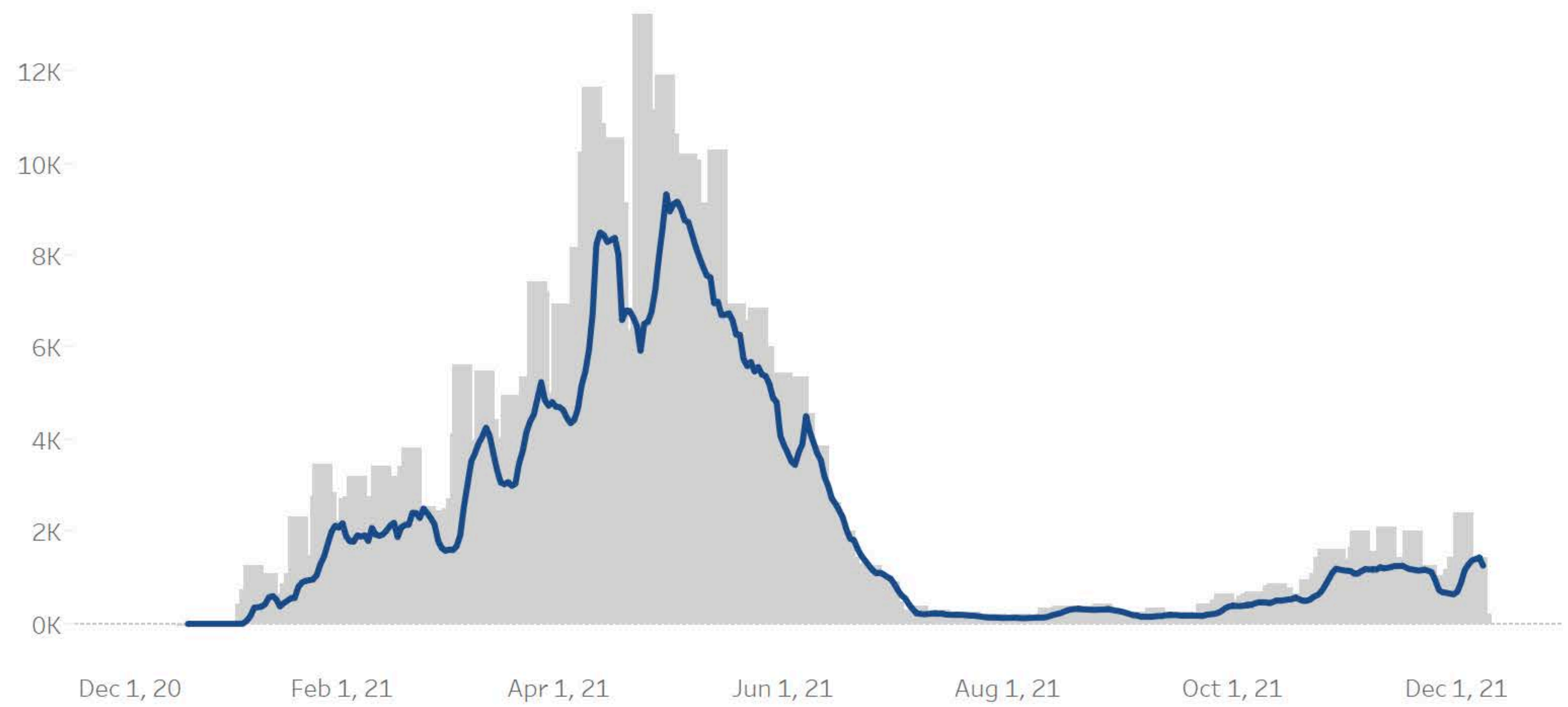

Cumulative Total for Individuals Fully Vaccinated

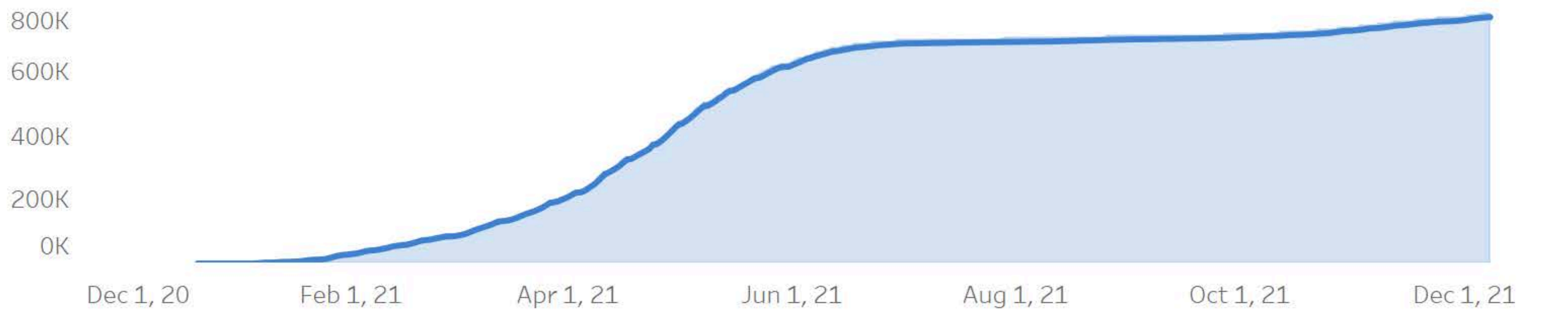

← Undo → Redo |← Revert Refresh Pause Metrics Share Download

Coronavirus Coverage

Explore the Data: Tracking COVID-19 in New Hampshire

New Hampshire Public Radio | By NHPR Staff
Published September 23, 2021 at 6:28 PM EDT

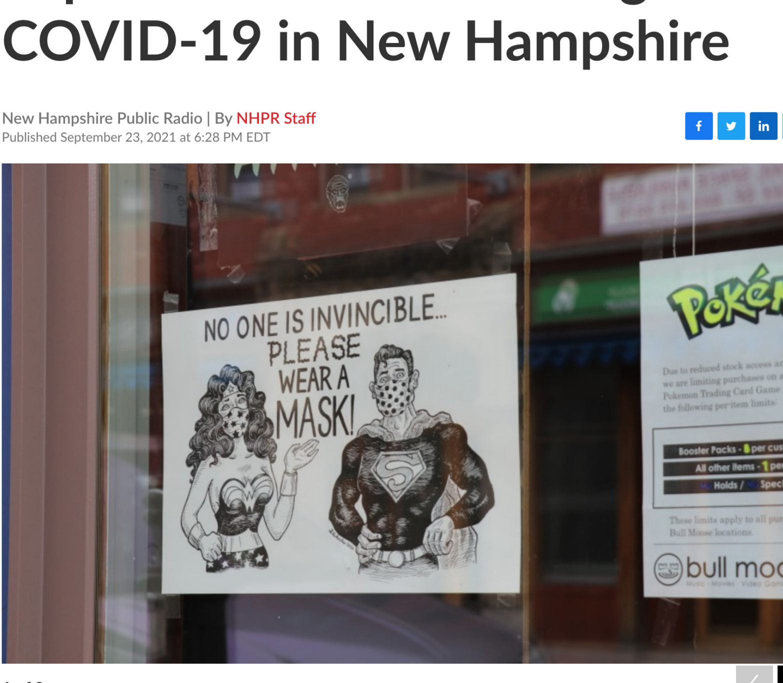

1 of 2

Dan Tuohy / NHPR

NHPR has been tracking the pandemic's impact on New Hampshire since March 2020, when COVID-19 was first detected in the state.

Along the way, we've adjusted our approach to this tracker as new data sources became available and as we've moved through different phases of the pandemic. As the pandemic continues to stretch into its second year, we're focused on continuing to provide this service, though at a smaller scale.

Starting in May 2021, we retooled this tracker to focus on the basics: a big-picture look at trends in vaccinations, case counts, hospitalizations and more. That means we're no longer including charts on some more detailed pieces of data, like bed capacity at individual hospitals. This has allowed us to focus our reporting efforts on covering new phases of the pandemic and other important issues affecting the state.

A word of caution: Recently, state officials disclosed that their vaccine data is likely inaccurate due to problems with New Hampshire's immunization tracking systems. The Centers for Disease Control and Prevention also recently shared that they are mistracking many of New Hampshire's booster shots and third shots as first doses of the COVID-19 vaccines. Please keep this in mind as you're reading the charts below.

Read more: N.H.'s COVID-19 vaccination data hasn't been accurate since June. Why?

If you have questions about this tracker or COVID-19 data, or if you think we made a mistake, please email us at coronavirus@nhpr.org.

To skip ahead to a specific section, click on the links below.

- Just the basics: Where do cases, hospitalizations and other key metrics stand?
- How prevalent is COVID-19 where you live, and what does that mean for mask recommendations?
- What do we know about breakthrough COVID-19 infections?
- How many people have been vaccinated?
- How many new cases are emerging each day?
- How many people have COVID-19 right now?
- How many people are hospitalized?
- How many people are dying?
- How is New Hampshire's test positivity?
- What do we know about COVID-19's impact across different racial/ethnic populations in New Hampshire?
- What do we know about COVID-19 cases in schools?
- Where can I find out about outbreaks in nursing homes, jails, treatment centers and other congregate living settings?
- How is New Hampshire doing compared to other states?
- Where can I find the raw data from New Hampshire's daily COVID-19 updates?
- Where can I find more information about testing, travel restrictions or other resources?

Just the basics: Total cases, hospitalizations, deaths and other key metrics for New Hampshire

The numbers below are pulled directly from New Hampshire's COVID-19 website. If you think there's an error, please email us at **coronavirus@nhpr.org** so that we can review and correct any mistakes as soon as possible.

171,538	People Have Been Diagnosed With COVID-19 Since March 2020
159,902	People Have Recovered From COVID-19 Since March 2020
1,768	People Have Died From COVID-19 Since March 2020
9,868	People Currently Have COVID-19
462	People Are Currently Hospitalized With COVID-19

Table: NHPR • Source: N.H. Department of Health and Human Services • Created with Datawrapper

How Prevalent is COVID-19 where you live —and what Does that mean for mask recommendations?

The information below comes from the Centers for Disease Control and Prevention's county-level COVID-19 dashboard. The CDC has halted some evictions in counties with substantial or high community transmission, and the public health agency also says that people in those areas should wear masks while in public indoor settings regardless of their vaccination status.

Hover over the map below for more details on transmission rates across New Hampshire, and what that means for mask recommendations at the county level.

This map previously included information on the CDC's eviction moratorium, but that policy is no longer in effect as of Aug. 26. More information on evictions and rental assistance is available on the New Hampshire Judicial Branch website.

Level of Community Transmission

High Substantial Moderate Low

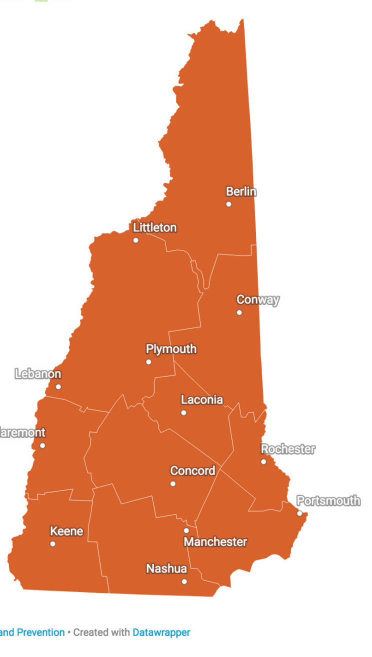

Source: Centers for Disease Control and Prevention • Created with Datawrapper

What do we know about breakthrough COVID-19 infections?

New Hampshire identified its first breakthrough case on Jan. 20, 2021. Since then, the vast majority of new cases, hospitalizations and deaths reported to state health officials have been among people who are unvaccinated.

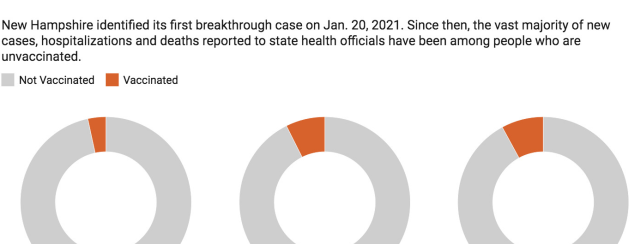

The numbers above reflect only COVID-19 cases reported to state health officials between Jan. 20 and Oct. 20, 2021.
Chart: NHPR • Source: N.H. Department of Health and Human Services • Created with Datawrapper

Read more: What We Know About The Vaccination Status Of People Testing Positive For COVID-19 In N.H.

We've been reporting updates on breakthrough cases as we receive them from the state. Recently, state officials disclosed that their data on this and other vaccine-related metrics are likely inaccurate due to problems with its immunization tracking systems — so keep that in mind as you're reviewing the numbers in this section. The state has been unable to provide more updated data on breakthrough cases due to issues with its immunization tracking systems.

Read more: N.H.'s COVID-19 vaccination data hasn't been accurate since June. Why?

- New Hampshire identified its first breakthrough case on Jan. 20, 2021. Between then and Oct. 20, the state recorded 2,441 infections among people who were vaccinated against COVID-19, according to the state Department of Health and Human Services. That represents about 3.4% of the more than 71,000 cases reported during the same time period.
- Out of the 635 people hospitalized for COVID-19 in New Hampshire between Jan. 20 and Oct. 20, 47 were confirmed to be vaccinated. That represents about 7.4% of hospitalizations during the same time period.
- Out of the 504 people who've died from COVID-19 in New Hampshire between Jan. 20 and Oct. 20, 40 have been vaccinated. That represents about 7.8% of deaths during the same time period.

Most New Hampshire residents didn't start receiving their first doses of the COVID-19 vaccine until this spring. With that in mind, NHPR has asked the state for data from a more recent time frame to better measure breakthrough infections at a time of higher vaccination rates. The state has not yet provided that data. Again, state officials say the above numbers might not represent all cases, citing limitations in its data systems.

We know many people are interested in this question, so we'll continue to ask the state health department for this information on a regular basis.

How many people have been vaccinated?

The Centers for Disease Control and Prevention has been tracking how many people are receiving new doses of the vaccine each day.

While we used to present the state's vaccine data in this chart, we switched to federal numbers in light of the state's recent disclosure that its vaccine numbers have likely been inaccurate since June 2021. However, it's likely that the CDC's data is inflating the number of first doses reported in recent months due to issues tracking New Hampshire's booster vaccine doses.

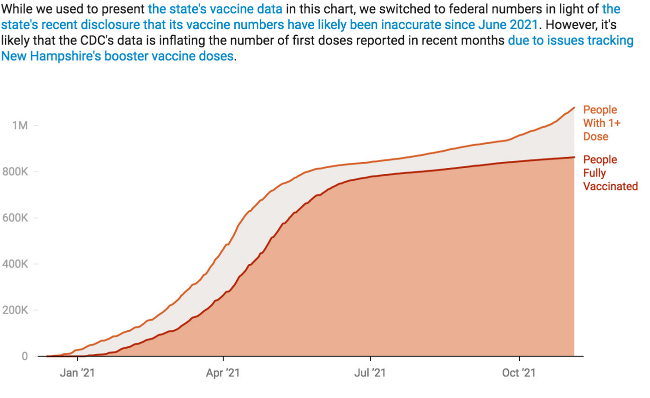

Chart: NHPR • Source: Centers for Disease Control and Prevention • Created with Datawrapper

Vaccine Coverage By Age Group

Until late March, the COVID-19 vaccine was only open to the general public above age 65 in New Hampshire. State officials say this data might not reflect the full picture of vaccination rates, especially for older adults, because it doesn't include shots given through a federal long-term care vaccination program.

It's also likely that the numbers below are not fully accurate due to recently disclosed problems with New Hampshire's immunization tracking systems.

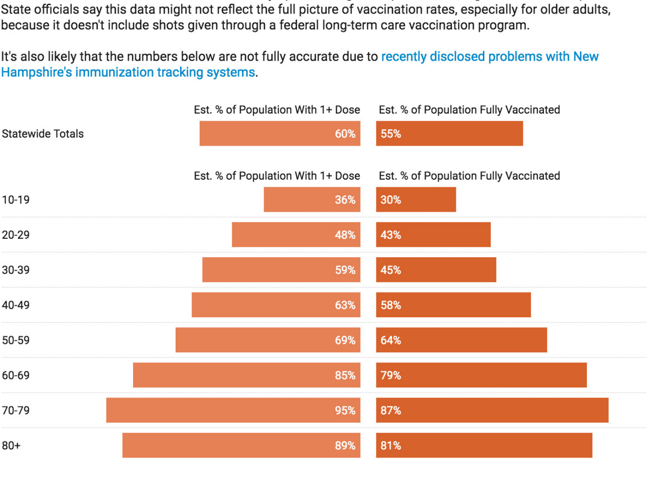

These numbers reflect data reported on New Hampshire's COVID-19 Vaccination Dashboard as of Oct. 25, 2021.
Chart: NHPR • Source: N.H. Department of Health and Human Services • Created with Datawrapper

Vaccine Coverage By Race/Ethnicity

State data shows that Black and Latino residents have been falling behind in New Hampshire's COVID-19 vaccine rollout for months. For the most up-to-date data on vaccine equity, check out the state's dashboard.

It's also likely that the numbers below are not fully accurate due to recently disclosed problems with New Hampshire's immunization tracking systems.

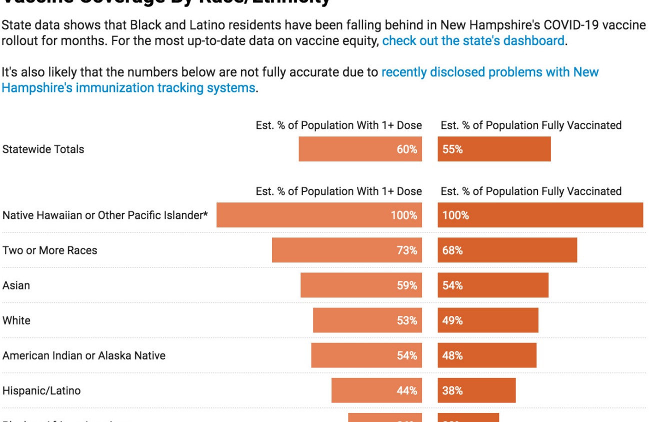

These numbers reflect data reported on New Hampshire's COVID-19 Vaccination Dashboard as of Oct. 25, 2021. According to the state, the number of people vaccinated who identify as Native Hawaiian or Other Pacific Islander exceeds Census population estimates and that data point is capped at 100%. The state says this is likely to happen in groups with smaller populations.
Chart: NHPR • Source: N.H. Department of Health and Human Services • Created with Datawrapper

How many new cases are emerging each day?

This shows how many new COVID-19 cases were announced each day and the seven-day average for the same metric. It doesn't reflect the number of new positive tests reported on each date or new infections by illness onset date, but more information on both of those data points can be found on the state's coronavirus dashboard.

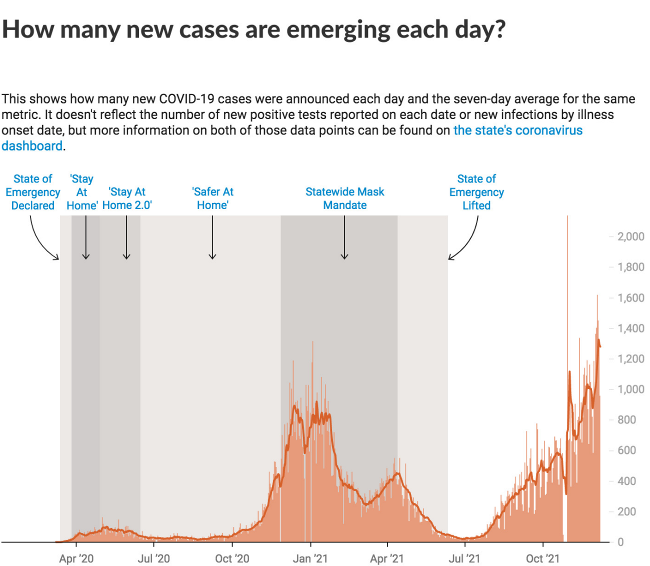

***Note:** New Hampshire began including positive antigen tests in addition to positive PCR tests in its case totals in early October 2020. The state also skipped reporting on several holidays, which could make it appear that cases decreased and then spiked more than they actually did after those dates.*
Chart: NHPR • Source: New Hampshire Department of Health and Human Services • Created with Datawrapper

How many people have COVID-19 right now?

This reflects the number of people in New Hampshire confirmed to have COVID-19 on each date, as reported by state health officials, plus the seven-day rolling average for the same metric.

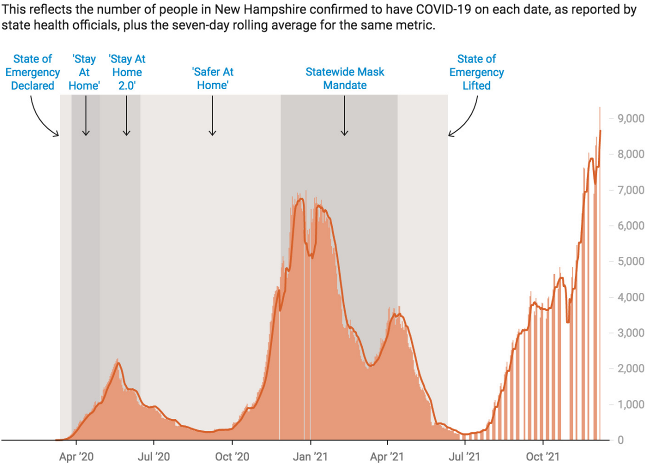

***Note:** The state skipped reporting on several holidays and, starting in June 2021, on weekends. This could make it appear that cases decreased and then spiked more than they actually did after those dates. We recently adjusted our formula for calculating weekly averages in response to the state's changes in its reporting schedule, which resulted in a slight shift in some of the numbers on this chart.*
Chart: NHPR • Source: New Hampshire Department of Health and Human Services • Created with Datawrapper

How many people are hospitalized?

This shows hospitalizations in New Hampshire for confirmed COVID-19 cases dating back to April 2020, when the state first started reporting that information. It also shows the rolling seven-day average for the same metric.

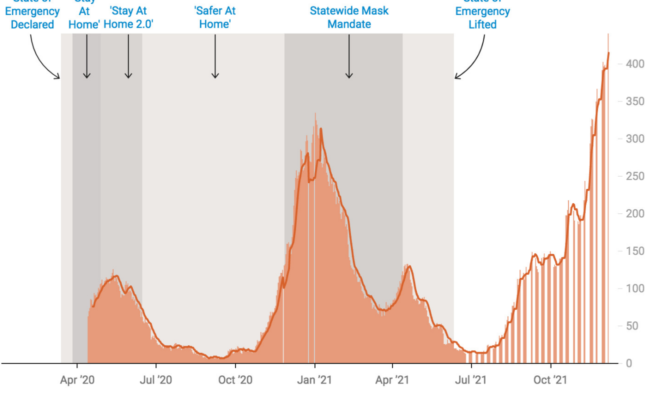

***Note:** The state skipped reporting on several holidays and, starting in June 2021, on weekends. This could make it appear that cases decreased and then spiked more than they actually did after those dates. We recently adjusted our formula for calculating weekly averages in response to the state's changes in its reporting schedule, which resulted in a slight shift in some of the numbers on this chart.*
Chart: NHPR • Source: New Hampshire Department of Health and Human Services • Created with Datawrapper

How many people are dying from COVID-19?

nhpr News from New Hampshire and npr
NHPR
World Cafe
NEXT U

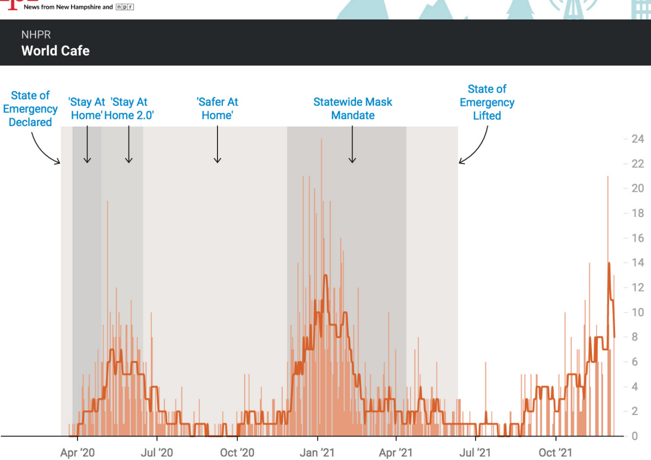

***Note:** The state skipped reporting on several holidays and, starting in June 2021, on weekends. This could make it appear that cases decreased and then spiked more than they actually did after those dates. We recently adjusted our formula for calculating weekly averages in response to the state's changes in its reporting schedule, which resulted in a slight shift in some of the numbers on this chart.*
Chart: NHPR • Source: New Hampshire Department of Health and Human Services • Created with Datawrapper

New Jersey COVID-19

NJ COVID-19 Hub

Vaccination Overview | Cases and Trends | Hospital Census | Long Term Care | Case and Mortality Summaries

COVID-19 Dashboard

Filter by County:
STATEWIDE

On April 26, 2021, New Jersey implemented an automated method to remove duplicate case reports, leading to a one-time drop of 10,442 confirmed cases from the total confirmed cumulative COVID-19 cases. Learn more.

Notes

1. "Confirmed cases" are individuals with a positive PCR test for COVID-19.
2. "Probable cases" are individuals with a positive antigen test for COVID-19 and no positive, confirmatory PCR test.
3. Data as-reported from NJHA facility survey, deaths are not yet confirmed to be COVID-19 related

More information about this data layer, including

Click a county to view its metrics

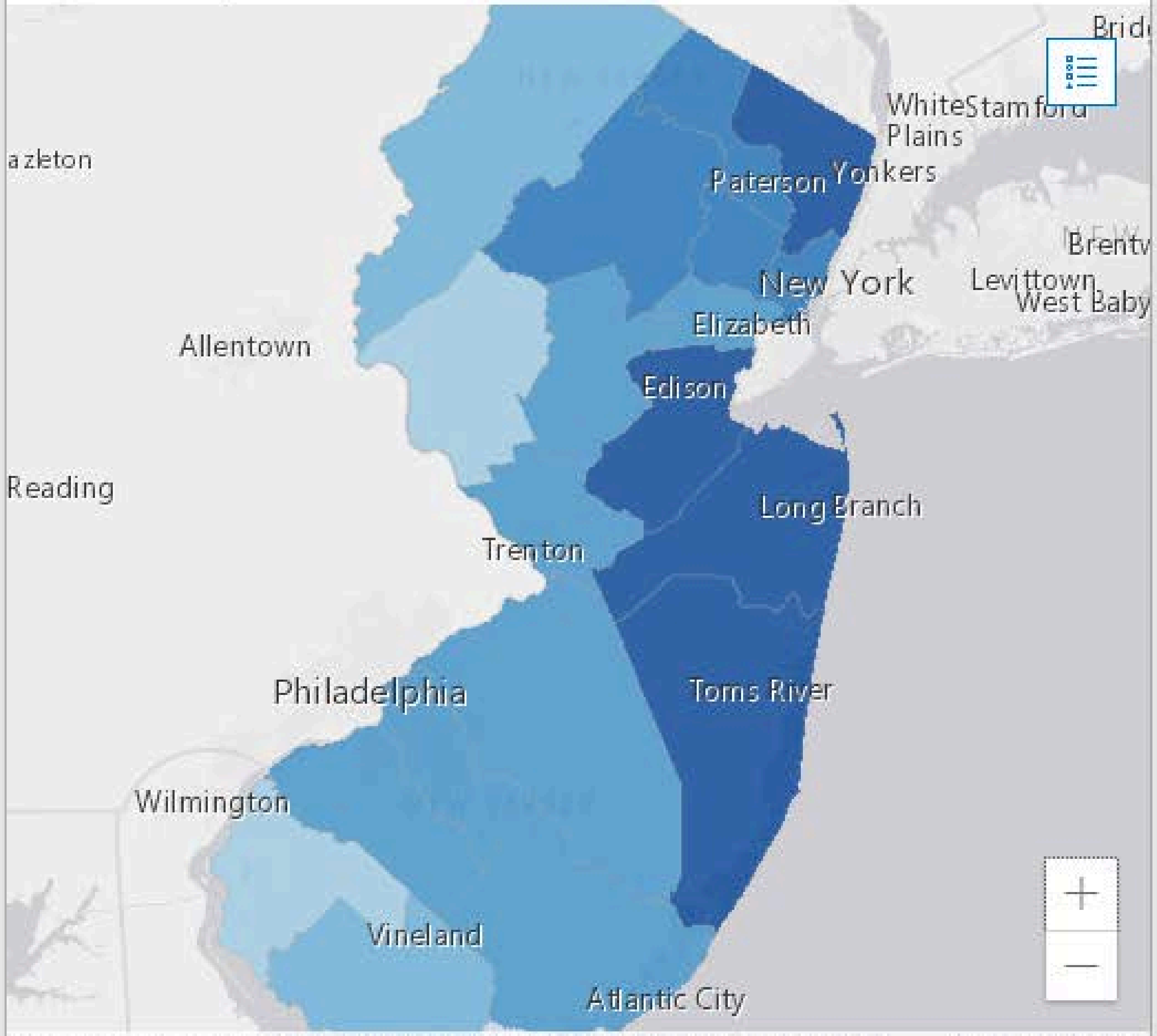

Esri, HERE, Garmin, USGS, EPA, NPS | New Jersey Department of Health -... Powered by Esri

499
Confirmed Cases - County Not Yet Assigned

Map | Case Counts | Variants

New Confirmed Cases[1]

4,198

Established via PCR testing

New Probable Cases[2]

928

Established via antigen testing

New Lab-Confirmed Deaths

25

Source: CDRSS

Hospital-reported Deaths (last 24h)[3]

14

Rate of Transmission (Rt)

1.41

Statewide

Daily Metrics

esri A Story Map

New Jersey COVID-19 | NJ COVID-19 Hub

Vaccination Overview | Cases and Trends | Hospital Census | Long Term Care | Case and Mortality Summaries

Vaccination Overvi...

Map Fil... All Ages | 12-17 | 12+ | 18+ | 30+ | 65+

Filter Demographics by Coun...
STATEWIDE

Due to a filtering error, the additional/3rd/booster doses were included in the counts for the People Vaccinated - Primary Series tab. These doses are now filtered out as of December 7th, 2021.

Notes

This dashboard is updated once daily, usually between 10:30-11:30 a.m.
Map of vaccination coverage refreshed weekly, current data as of 12/08/2021 and 11/10/2021.

[1] Individuals who have received a single dose from a one-dose vaccine course, e.g., the Johnson & Johnson vaccine, or their first dose from a two-dose vaccine course

[2] Individuals who have received a single dose from a one-dose vaccine course, e.g., the Johnson & Johnson vaccine, or their second dose from a two-dose vaccine course

*These numbers do not reflect New Jersey residents vaccinated out of state or by federal programs

**Total vaccine doses administered

Persons with at Least One Dose, % of population

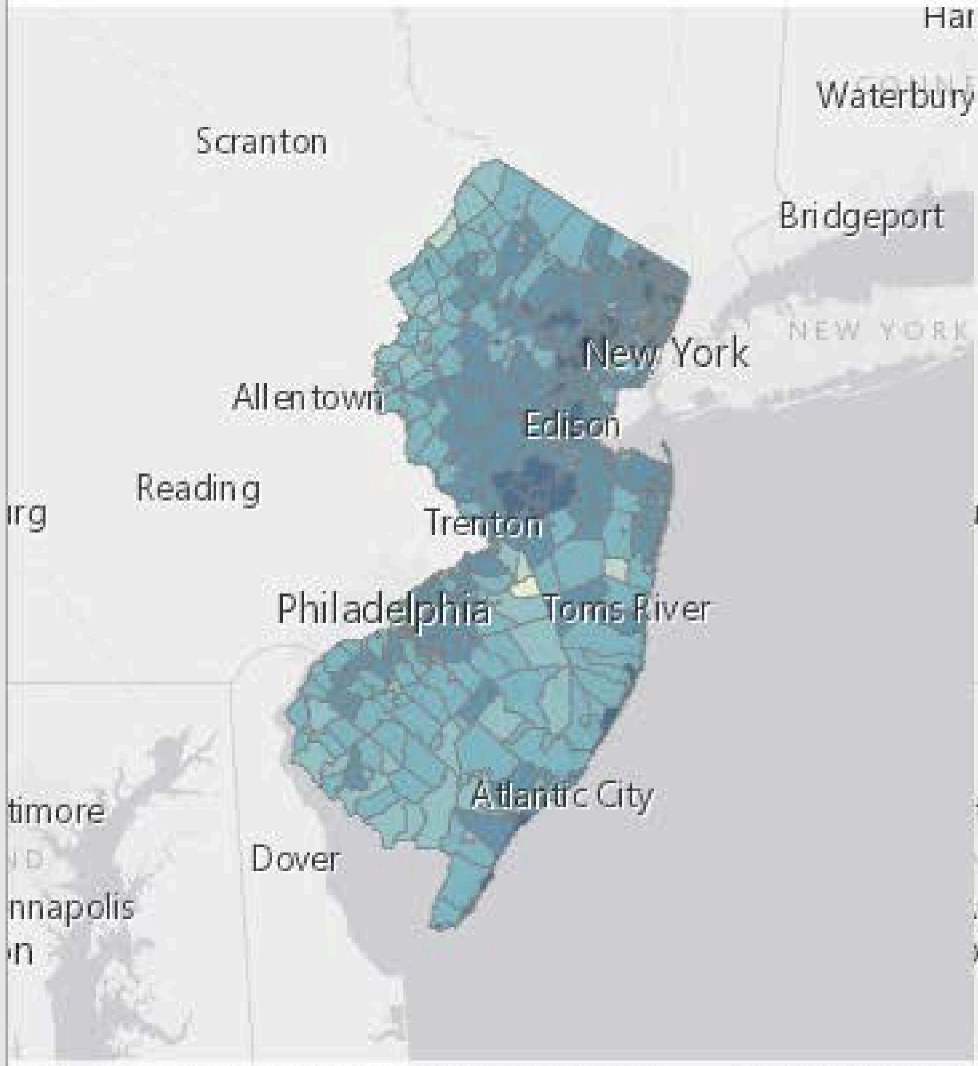

Esri, HERE, Garmin, USGS, EPA, NPS | Esri, H... Powered by Esri

Individuals who have received at least one dose of any vaccine (combines the population of those who have initiated courses or completed courses)

Persons with Vaccine Courses Complete, % of population

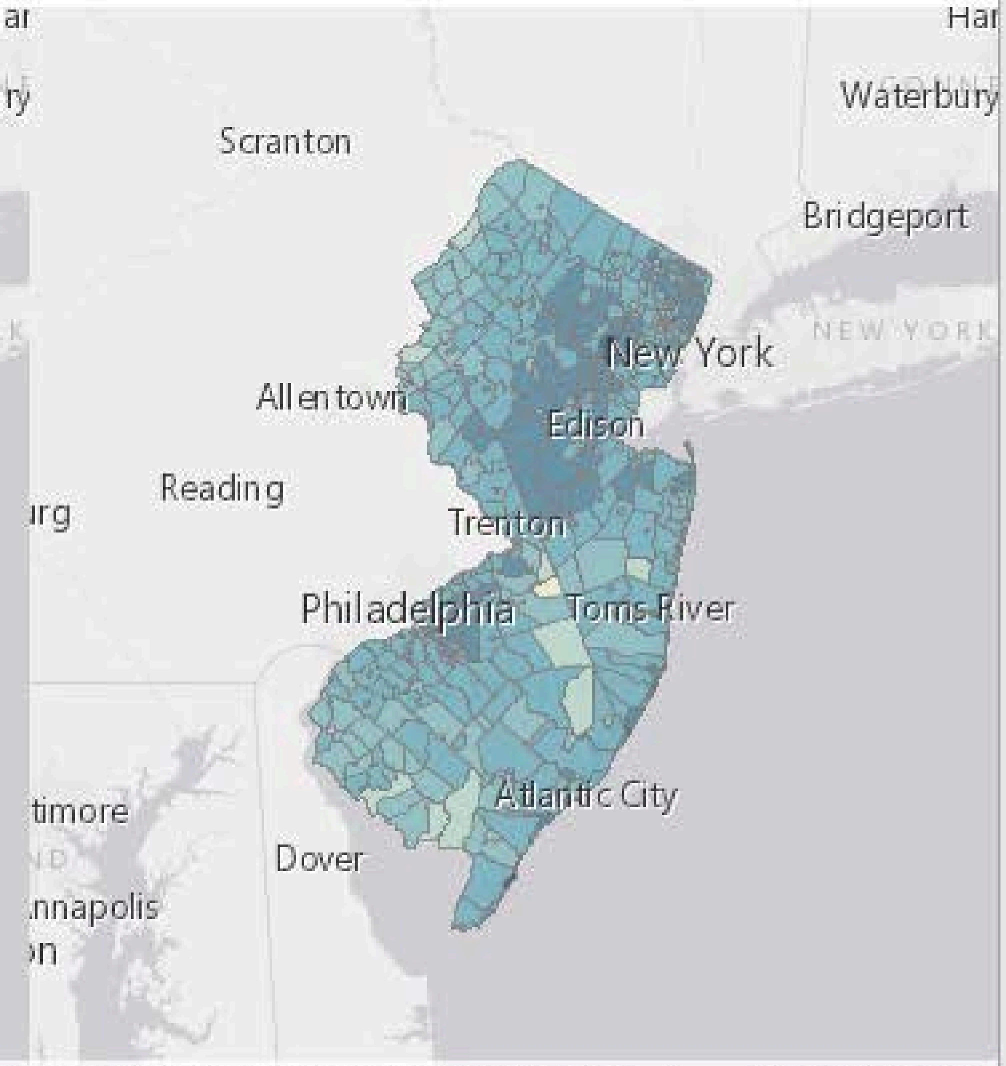

Esri, HERE, Garmin, USGS, EPA, NPS | Esri, H... Powered by Esri

Individuals who have received a single dose from a one-dose vaccine course, e.g., the J&J vaccine, or their second dose from a two-dose course

Map of Vaccination Coverage

esri A Story Map

Select a location: All N.J.

Daily Summary: All N.J.

New Cases	Total Cases
**218**	**1,124,980**
New Deaths	Total Deaths
**0**	**25,738**
New Tests	Total Tests
**-**	**21,324,528**
[illegible]	[illegible]

Cases: All N.J.

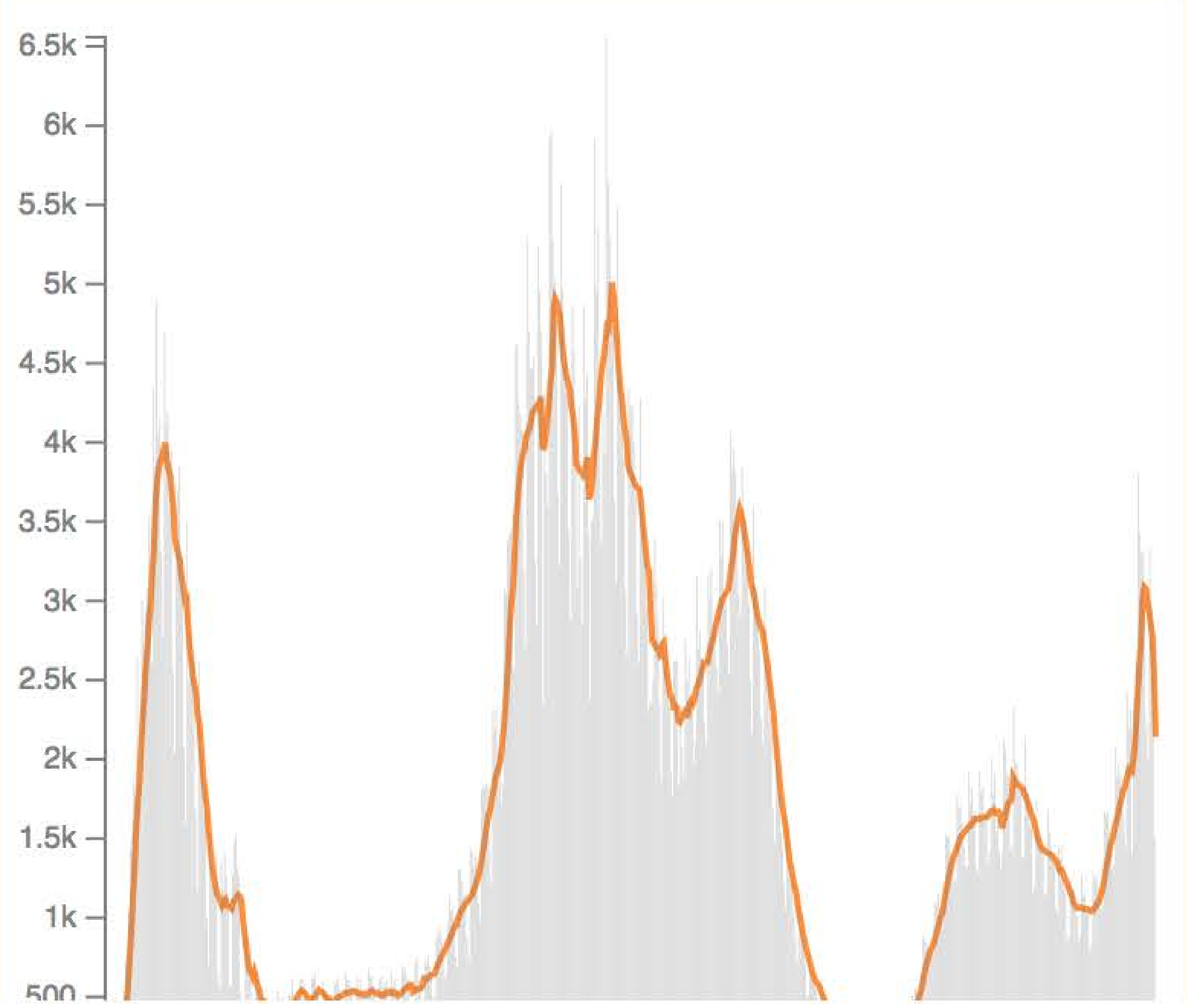

Deaths: All N.J.

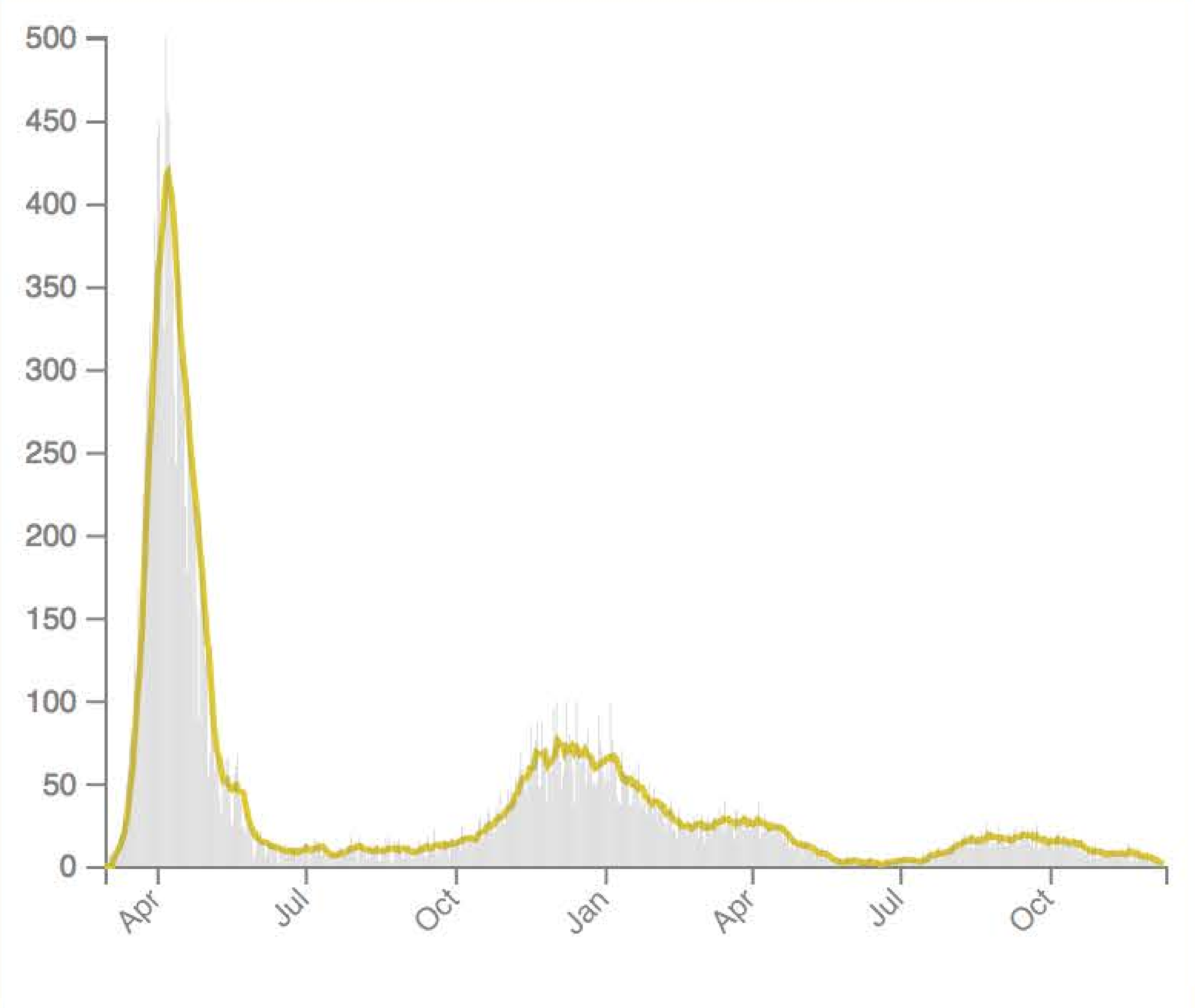

N.J. Coronavirus Tracker

subscribe

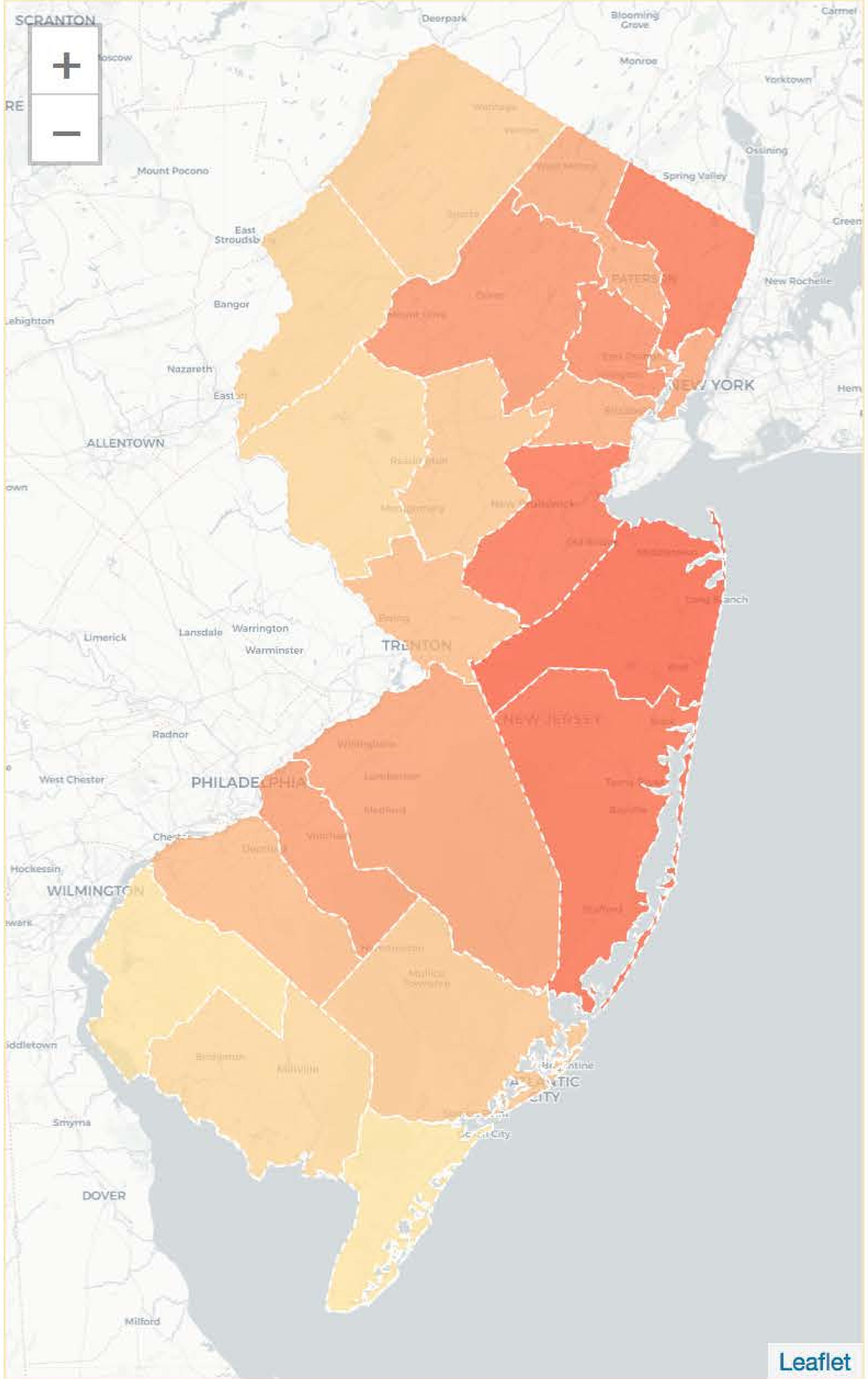

Total Cases & Deaths: All N.J.

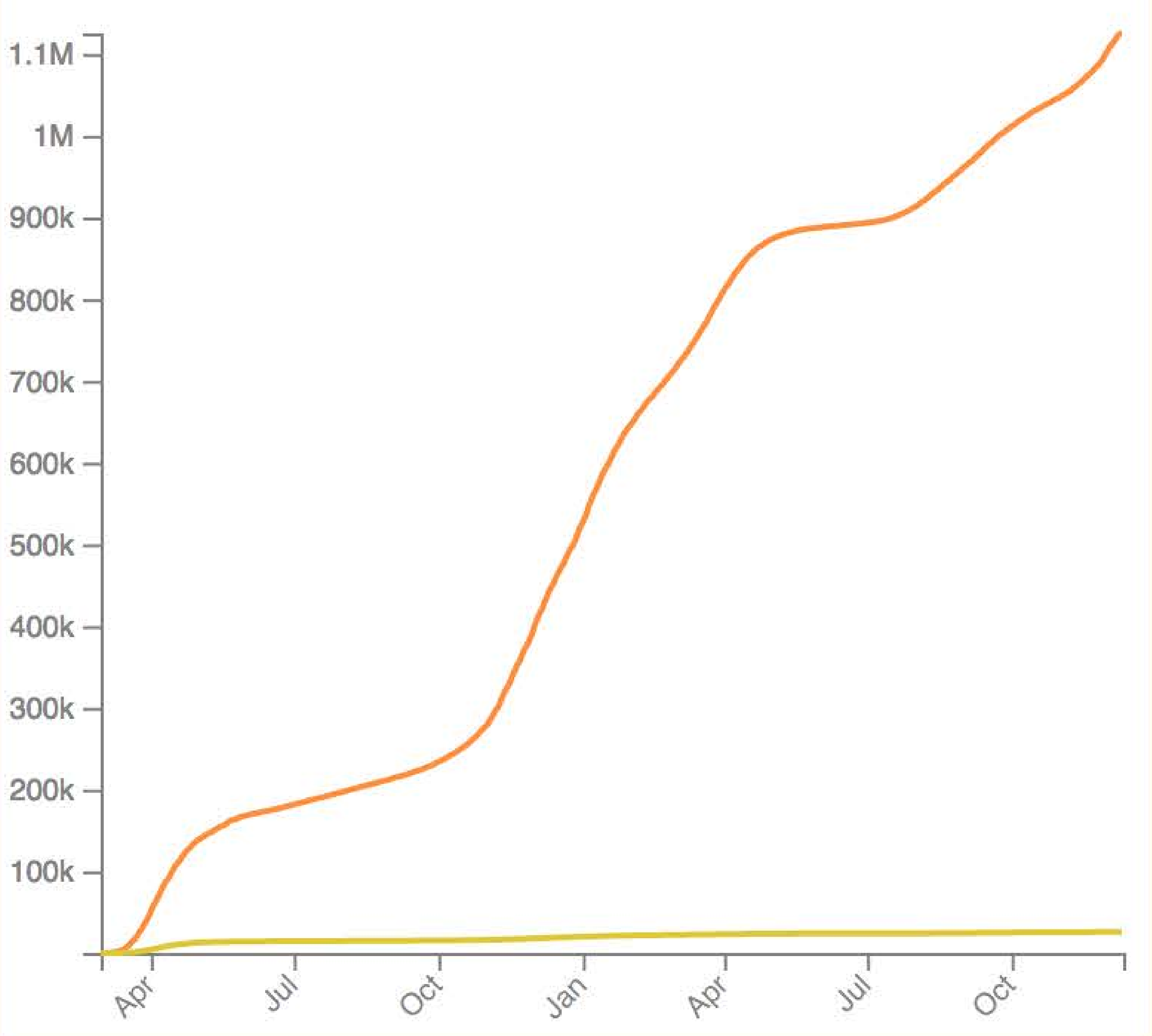

Daily Testing

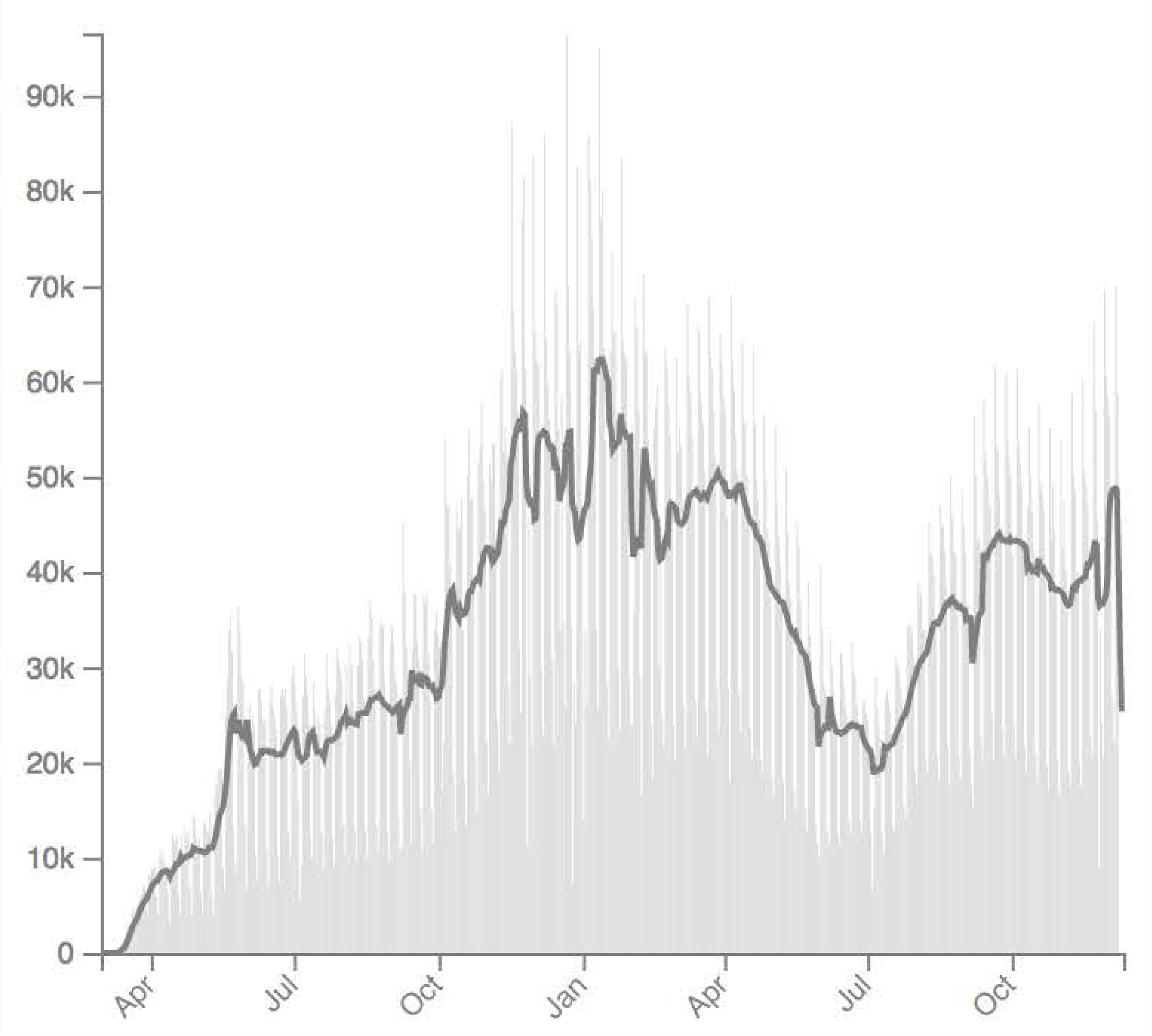

U.S. Summary

Cumulative Cases	Cumulative Deaths
**49,748,387**	**793,937**

Resources

- [Sign up for coronavirus text alerts, emails and other resources.]
- [See all N.J. coronavirus news.]

New Mexico Department of Health

COVID-19 in New Mexico

Back to CV.NMHEALTH.ORG

NUMBER OF CASES

329,778

TOTAL # OF TESTS PERFORMED

5,452,675

TOTAL HOSPITALIZATIONS

21,260

CURRENT HOSPITALIZATIONS

691

TOTAL DEATHS

5,472

TOTAL RECOVERED

278,030

Show Historical Statewide Data

Last Updated: 12/10/2021, 6:17:43 PM

Information about the Data

Current COVID case outlook 11/16/2021 - 11/29/2021

DATA IS UPDATED EVERY OTHER WEEK. NEXT UPDATE: 12/07/2021. DAILY CASE COUNT AND TEST NUMBERS ARE RAW DATA BASED ON INFORMATION THE STATE

Map Key: Fewer Cases More Cases View Map Key

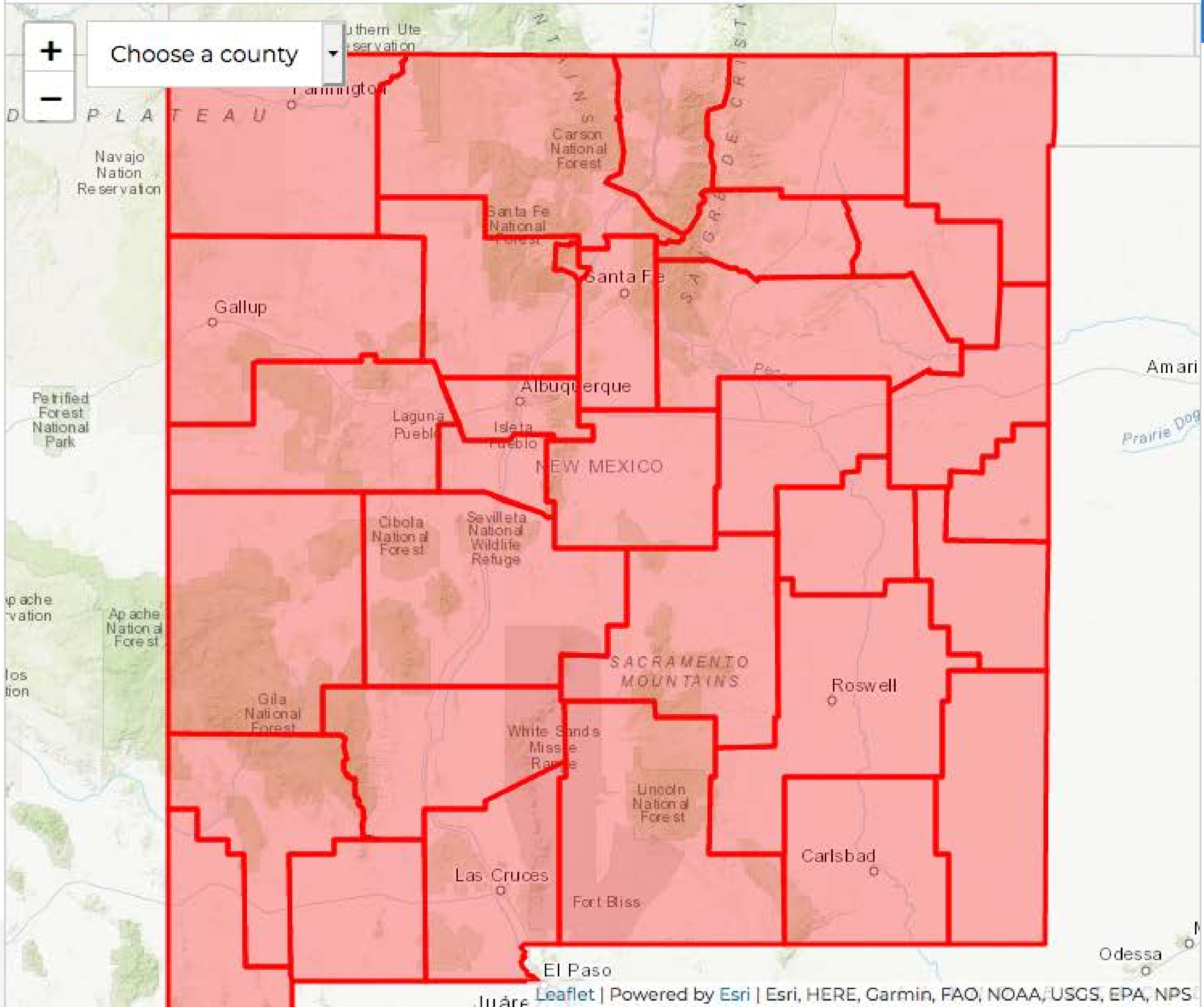

EXPAND DEMOGRAPHICS VIEW

Statewide Gender Breakdown

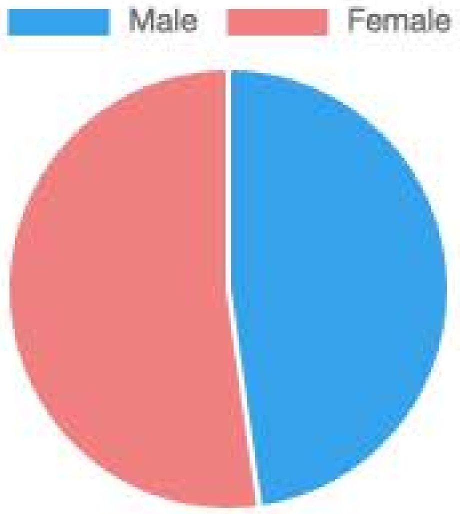

Gender	Cases
Male	47.75%
Female	52.25%

Statewide Age Breakdown

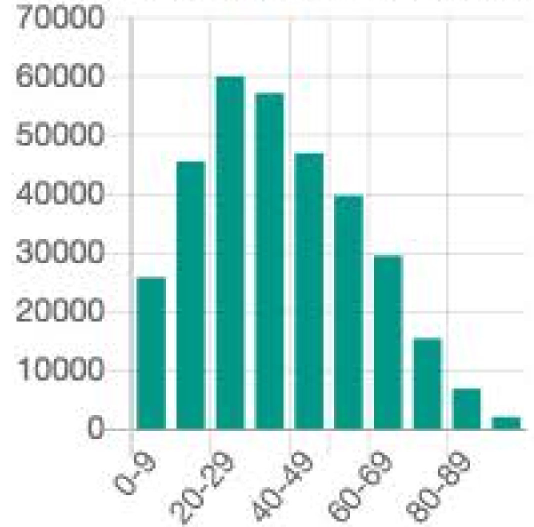

New York State Department of Health COVID-19 Tracker

NEW YORK STATE Department of Health

Testing data as of: 12/9/2021 2 PM
Testing data last updated on: 12/10/2021

Statewide

Tested Positive (12/09)
11,203

Total Tested (12/09)
268,372

Tested Positive (Cumulative)
2,803,007

Total Tested (Cumulative)
82,806,380

Tested Positive by Sex (Cumulative)

Female	Male	Unknown
51.0%	47.9%	1.2%

Positive Tests by County

- (selected) Cases per 100k, 7-day avg
- Test % Positive, 7-day avg

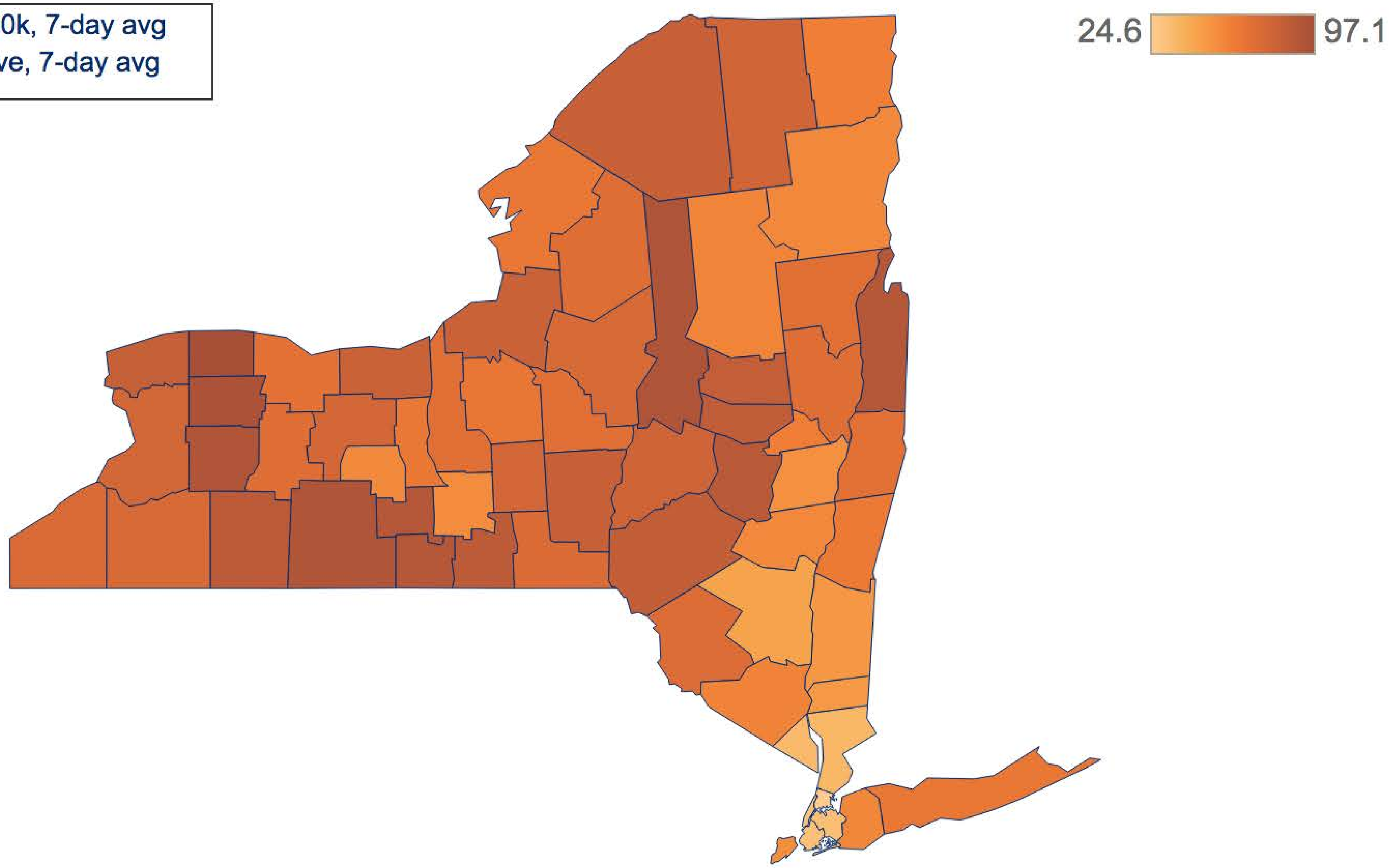

County Testing Details: **None**

Click County to See Detail
Click Again for Statewide

- Albany
- Allegany
- Bronx
- Broome
- Cattaraugus
- Cayuga
- Chautauqua
- Chemung
- Chenango
- Clinton
- Columbia
- Cortland
- Delaware
- Dutchess
- Erie
- Essex
- Franklin
- Fulton
- Genesee
- Greene
- Hamilton
- Herkimer
- Jefferson
- Kings
- Lewis
- Livingston
- Madison
- Monroe
- Montgomery

State of Ohio | COVID - 19 Dashboard

Last Updated: 12-11-21
Updated daily at 2 p.m.

Other states do not send death certificates to ODH's Bureau of Vital Statistics on a regular schedule and therefore fluctuations will be reflected in reported mortality data. Deaths are assigned to their appropriate date of death.

County Map | Cases
Select a County To Filter Dashboard

Case Classification Status — Filter all views by case classificati... — (All) Total Cases
View By — Pick an option to view in map — Cases
County Search — Search a county to highlight — Highlight County

County View: — View deaths by county — County of Residence
Ethnicity: — Select an Ethnicity to filter the vie... — (All)
Race — Select a race to filter the views — (All)

Start Date 1/1/2020
End Date 12/11/2021

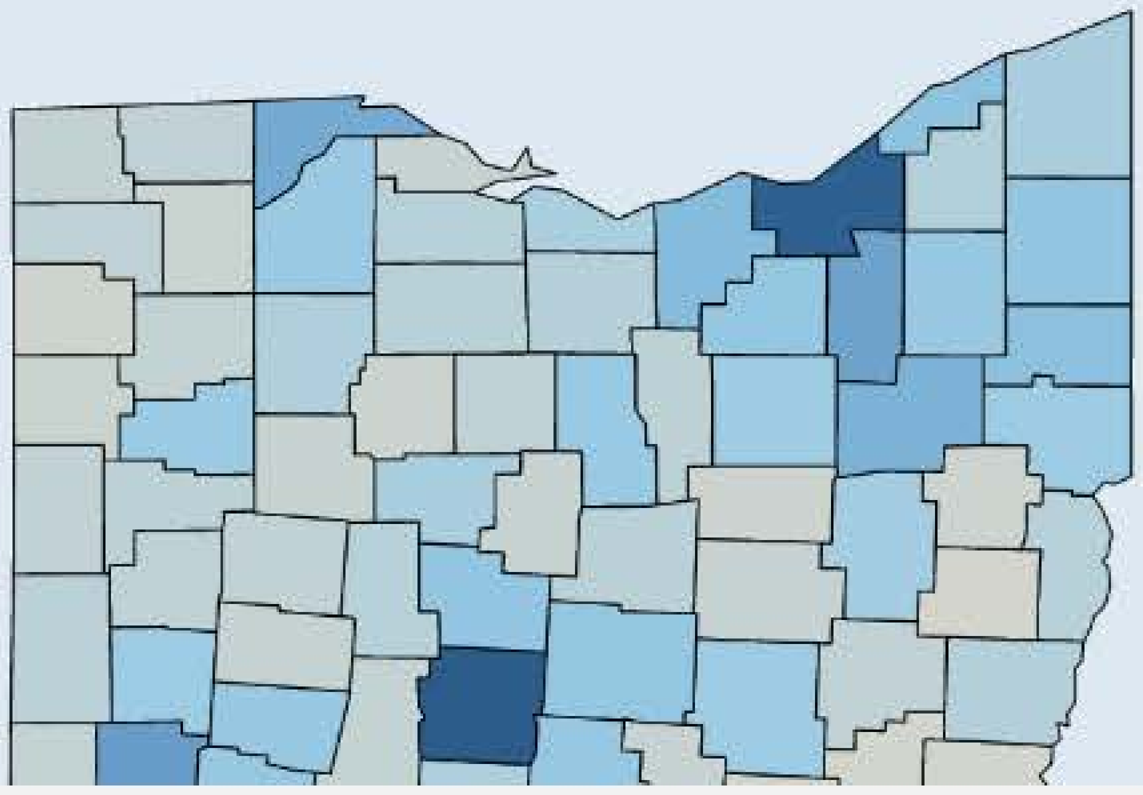

© OpenStreetMap

Metrics | Cumulative and Daily Count

View Count By:
Select to view counts by daily or cumulative
Daily Count

Cases
* Preliminary

1,776,439

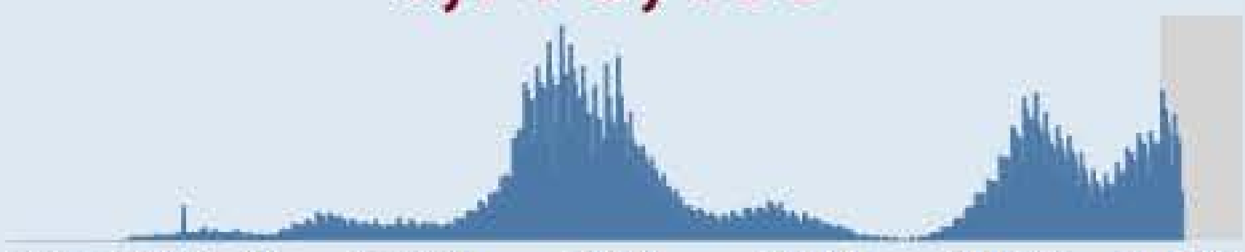

Hospitalizations
* Preliminary

90,045

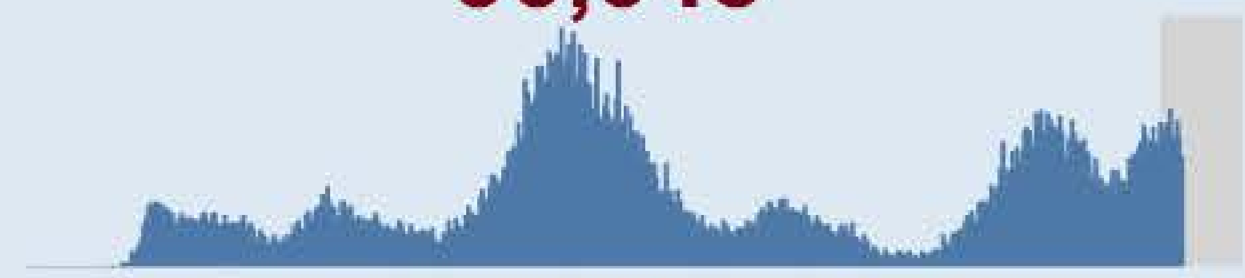

Deaths

27,371

* Preliminary

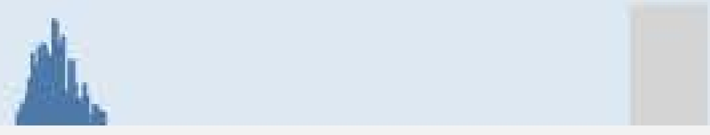

Language Translation

Ohio | Department of Health — Coronavirus (COVID-19)

Responsible RestartOhio | COVID-19 Vaccination Program | Equity and High-Risk Groups | Families and Individuals | He

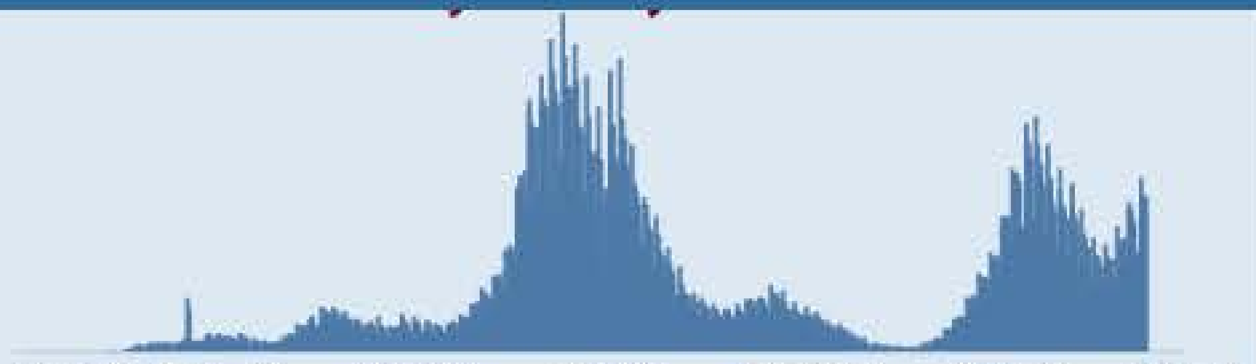

Key Metrics | By County

View By
Pick an option to change the view
County

View By	Case Count	Hospitalization Count	Death Count
Franklin	183,940	4,843	1,858
Cuyahoga	175,914	9,793	2,692
Hamilton	116,608	4,597	1,559
Montgomery	82,724	7,336	1,433
Summit	74,965	6,475	1,233
Lucas	65,001	3,426	1,044
Butler	58,487	2,470	829
Stark	55,935	3,192	1,204
Lorain	44,259	2,634	669
Mahoning	37,002	1,672	799
Warren	36,976	1,055	423
Lake	33,777	1,129	516
Clermont	32,244	1,172	374
Trumbull	28,579	1,762	633
Delaware	28,037	403	189
Licking	[illegible]	[illegible]	[illegible]

Note: Totals include Confirmed and CDC Expanded Case Definition (Probable)

All data displayed is preliminary and subject to change as more information is reported to ODH. Trends are based on the date of illness onset for cases, date of hospital admission for hospitalizations, and date of death for deaths. The date slider at the top of the dashboard will limit the graphical display of data using these date fields. The CSV download file will always show all dates, regardless of date slider settings. (For cases, if onset date is unknown, the earliest known date associated with the case is used as a substitute for the date of illness onset. For hospitalizations, if date of admission is not reported, these are omitted from the graphical display but are still included as counts in the CSV download file.)

* Preliminary case data in the shaded area is likely under-reported to ODH because of the delay between initial contact with a health care provider, testing, and diagnosis.

** Mortality data is verified by coded death certificate information received from the National Center for Health Statistics, and can take some time to receive. This information is not available daily, and is updated approximately twice per week as data is received.

*** Presumed Recovered is defined as cases with a symptom onset date >21 days prior who are not deceased.

+tableau

Goal: Vaccinate 70% of Oklahoma's eligible population.

Population (12+ yrs)
2,485,176 At Least 1 Dose

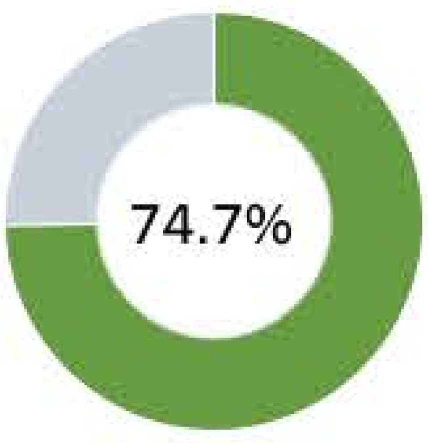

Total Population
2,520,319 At Least 1 Dose

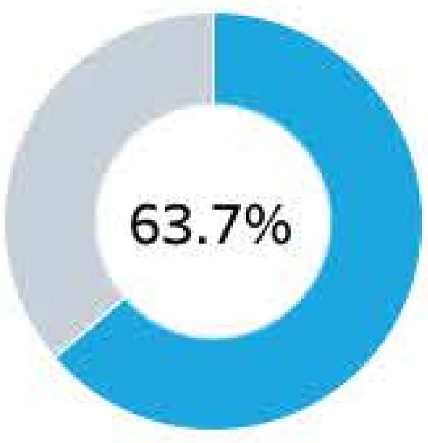

Population (12+ yrs)
2,050,555 Fully Vaccinated

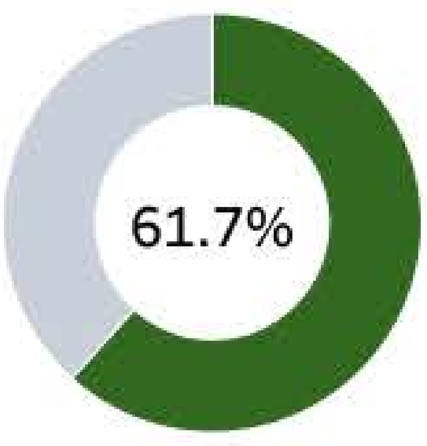

Total Population
2,062,867 Fully Vaccinated

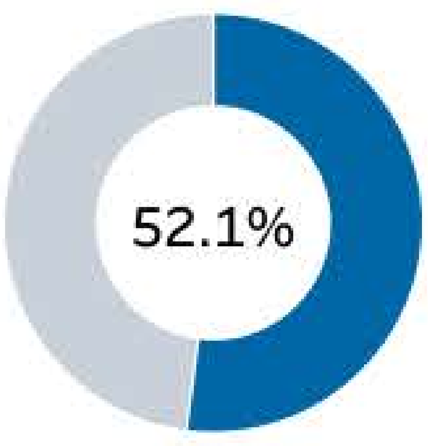

Vaccinations (Daily Doses Administered & Full Vaccinated People)

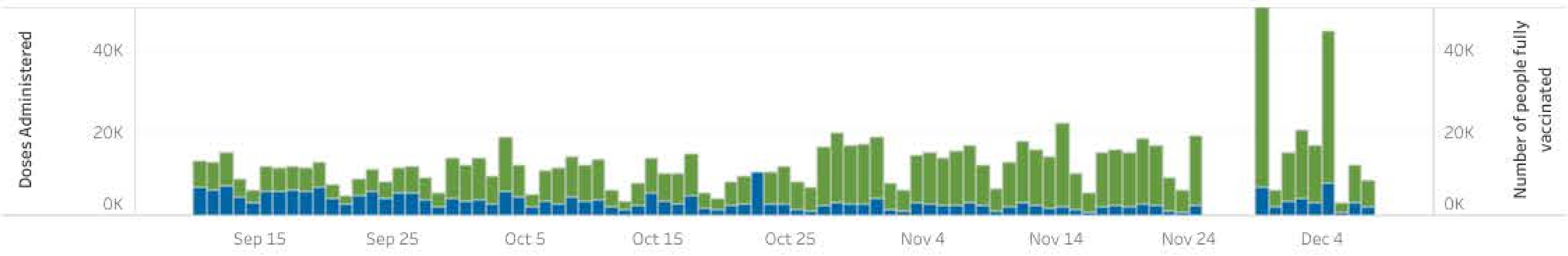

Vaccine Time Frame
Last 90 Days

Administered
Series Complete

Oklahoma Licensed Acute Care Hospitals

Source (unless otherwise noted): HHS Survey date **12/6/2021**
90 (84.9%) Facilities Reported
Response rate impact accuracy of data. Facilities may update previously reported information as necessary.

Hospitalizations by Vaccination Status,
Previous 30 days (as of November 28, 2021)
Source: OSDH Case Investigation Registry

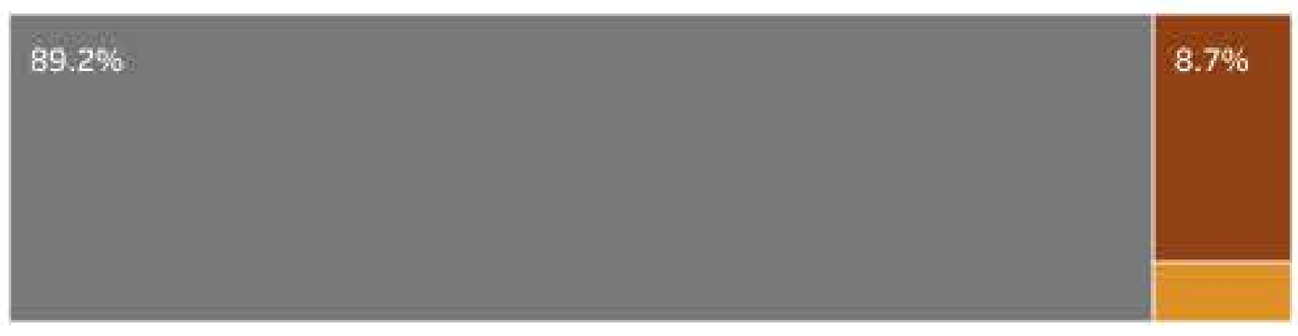

Non-Vaccinated
Fully Vaccinated
Partially Vaccinated

Most Recent Information Reported by Hosptials
(Response rates impact accuracy of regional totals)

Unoccupied Staffed Adult Inpatient Beds - 1,617
Unoccupied Staffed Adult ICU Beds - 98
Hospital Capacity Reports -
https://oklahoma.gov/covid19/newsroom/hhs-hospital-capacity-reports.html

COVID-19 Positive Adult Hospitalizations - 486
COVID-19 Positive Adults in the ICU - 163
COVID-19 Situation Updates and other News -
https://oklahoma.gov/covid19/newsroom.html

3 Day Average COIVD-19 Positive Adult **Hospitalizations** & **ICU**

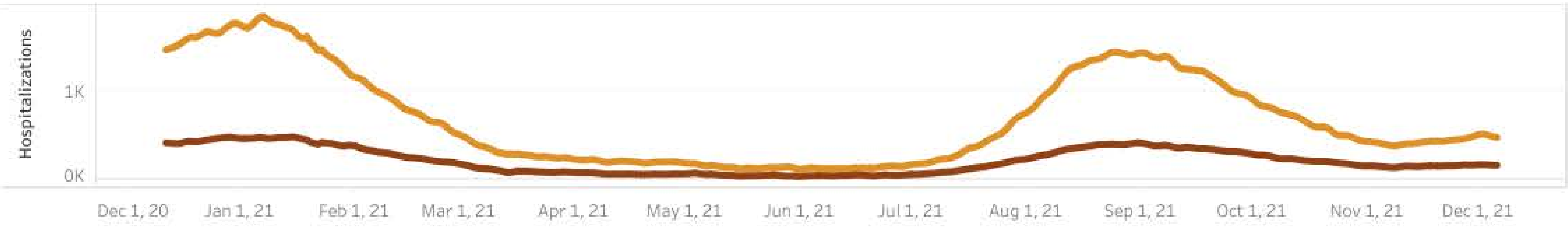

Case Information

Source (unless otherwise noted): OSDH Case Investigation Registry

7 Day Average Number of New Daily Cases 1,245

Total Cases 674,758 as of December 6, 2021
Newly Reported Cases 1,280

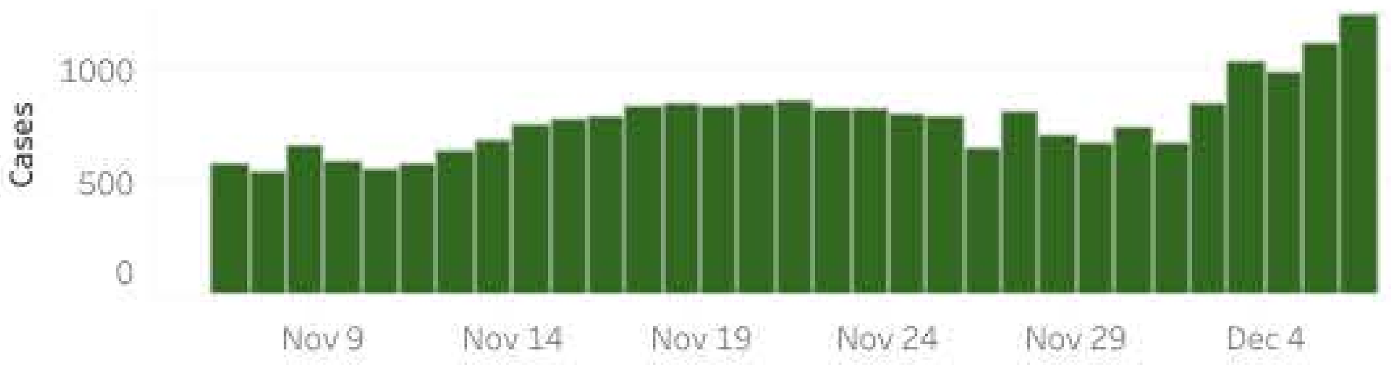

CDC Provisional Deaths Mentioning COVID-19 as of **12/7/2021**
Oklahoma Occurances - **12,028**
https://www.cdc.gov/nchs/nvss/vsrr/COVID19/index.htm

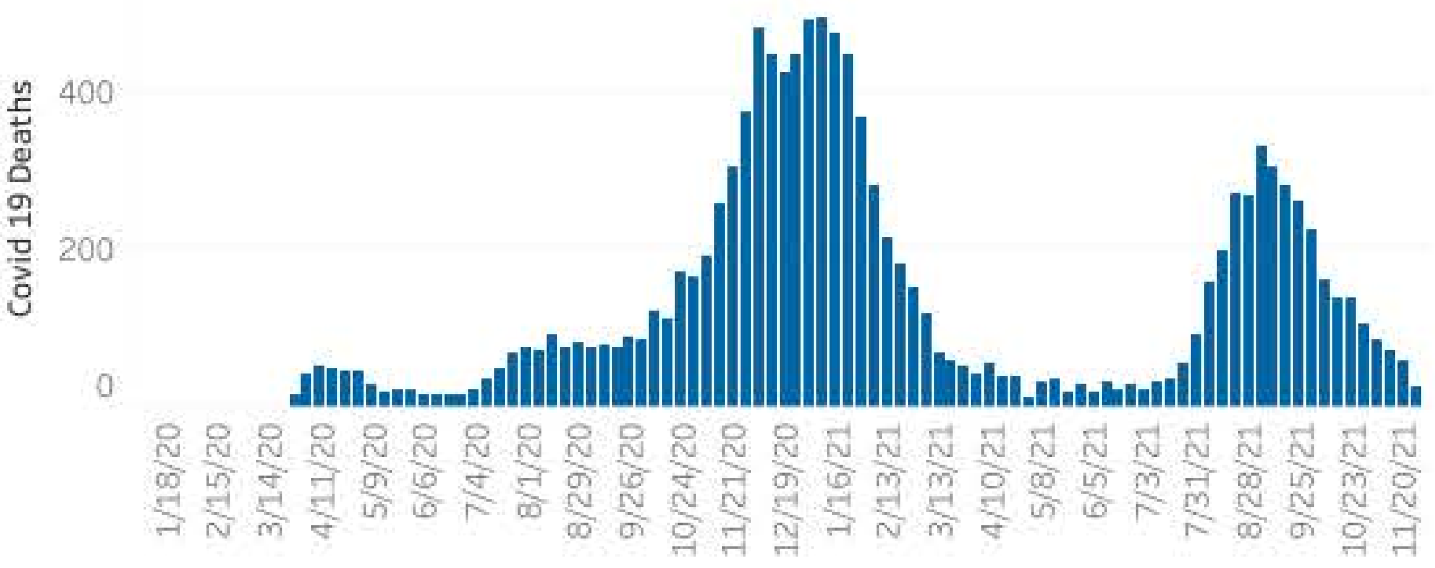

COVID-19 Cases per 100,000 Population (previous 30 days)
Click a County or select a Public Health District to see counts for that specific area.

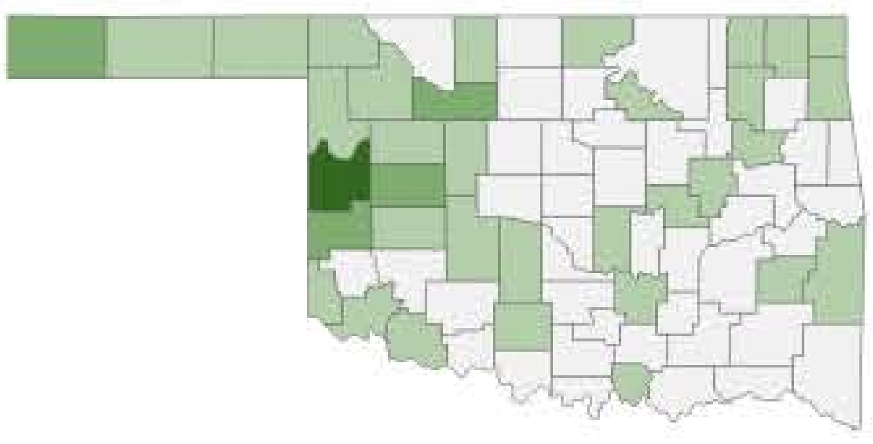

Age Distribution by Case Month

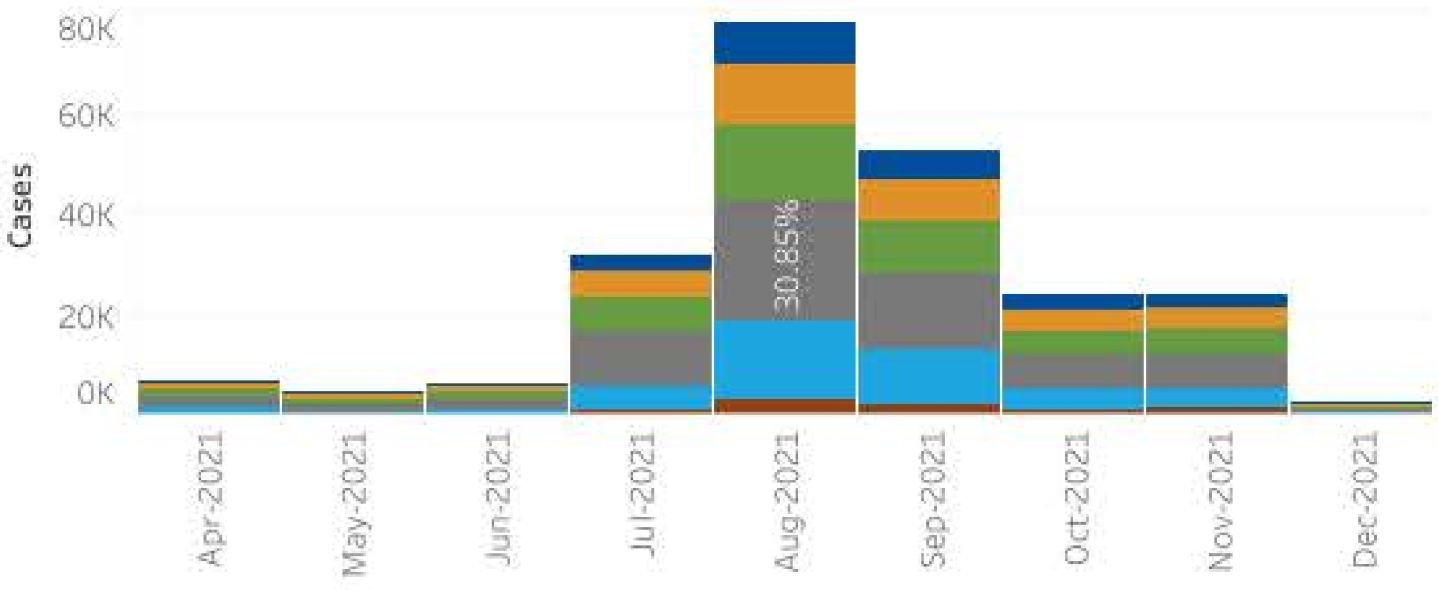

Public Health District
(All)

Age Group
65+ 50-64 36-49 18-35 05-17 00-04 UNK

Daily Cases Last 90 Days
Public Health District: All
County: All

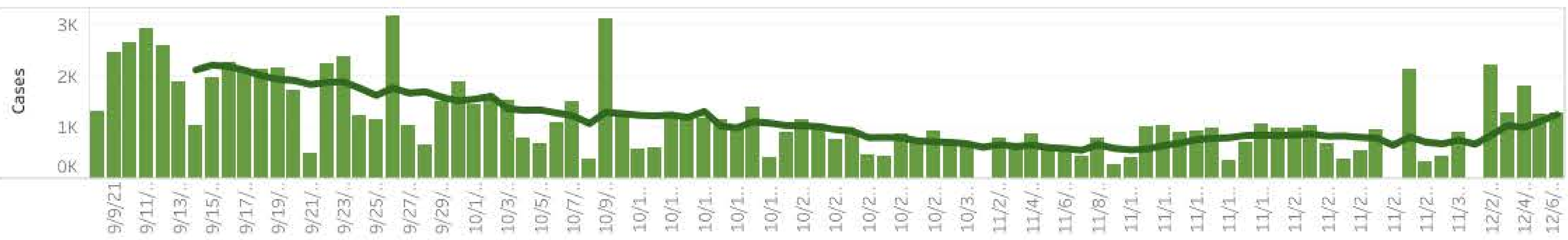

Case Time Frame
Last 90 Days

*Please note, data reported on 12.2.21 includes two days. This is a result of a data processing issue causing gaps in daily totals.
For additional COVID19 data please see the: **Weekly Epidemiology and Surveillance Report**-https://oklahoma.gov/covid19/newsroom/weekly-epidemiology-and-surveillance-report.html
Additional public health data, including a new COVID19 module, can be accessed through **OK2SHARE**-https://ok2share.health.ok.gov/

+ a b l e a u

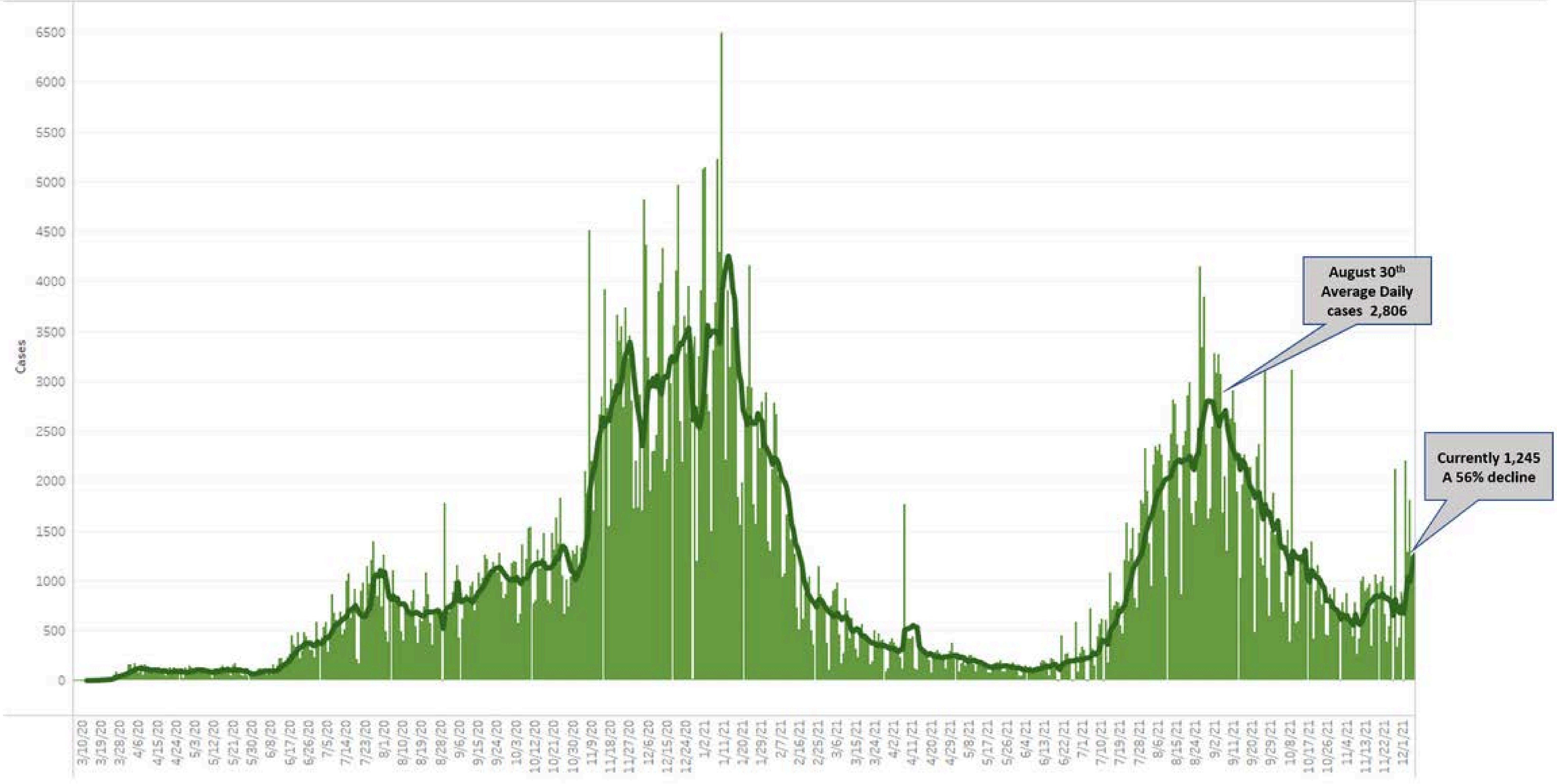

Cases
August 30th Average Daily cases 2,806
Currently 1,245 A 56% decline

Oregon COVID-19 Update by Oregon Health Authority COVID-19

Oregon Health Authority

The Oregon Health Authority (OHA) is part of the statewide response working to address and reduce the effects of COVID-19 on the lives of Oregonians. Data presented here are from the OHA Public Health Division.

OHA COVID-19 Website   View All COVID-19 Data Dashboards

Contact Us

Oregon's COVID-19 Update

New cases and deaths are reported from the previous day. The first business day following a weekend or holiday includes all newly reported data.

Daily Data Update | Case and Testing | Hospital Capacity | Emergency Department

Data Current as of 12:01am 12/10/2021

Statewide Numbers from 12/9/2021 to 12/9/2021

New Cases	7 Day Daily Average of Cases	Cases Per 100k in Previous 7 Days	COVID-19 Patients Hospitalized†	Tests Reported	Test Positivity	New Deaths
834 ▼*	789.9	129.5	392 ▼	23,050	4.3%	25

* Arrows indicate an increase or decrease from the previous day. †Hospitalization data from Oregon's Hospital Capacity Web System (HOSCAP).

Daily Cases and 7 Day Moving Average over the Previous Six Weeks

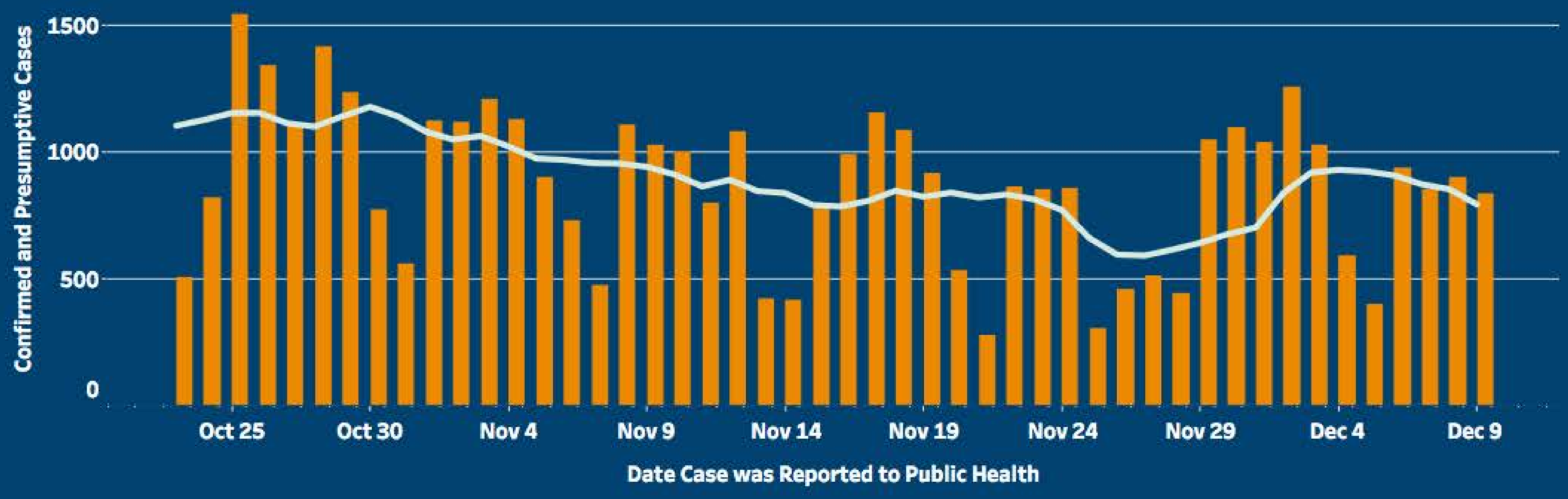

December 4, 2021 COVID-19 Variant Cumulative Case Counts**

Variants of Concern

Delta (B.1.617.2, AY Sublineages)	12,757
Omicron (B.1.1.529)	0

Variants of Interest

There are no Variants of Interest at this time.

Variants Being Monitored

Alpha (B.1.1.7, Q Sublineages)	3,109	Iota (B.1.526)	426
Beta (B.1.351 and sublineages)	233	Kappa (B.1.617.1)	0
Gamma (P.1 and sublineages)	1,187	B.1.617.3	0
Epsilon (B.1.427/B.1.429)	1,289	Mu (B.1.621)	53
Eta (B.1.525)	9	Zeta (P.2)	8

Variant Prevalence Past Six Weekss

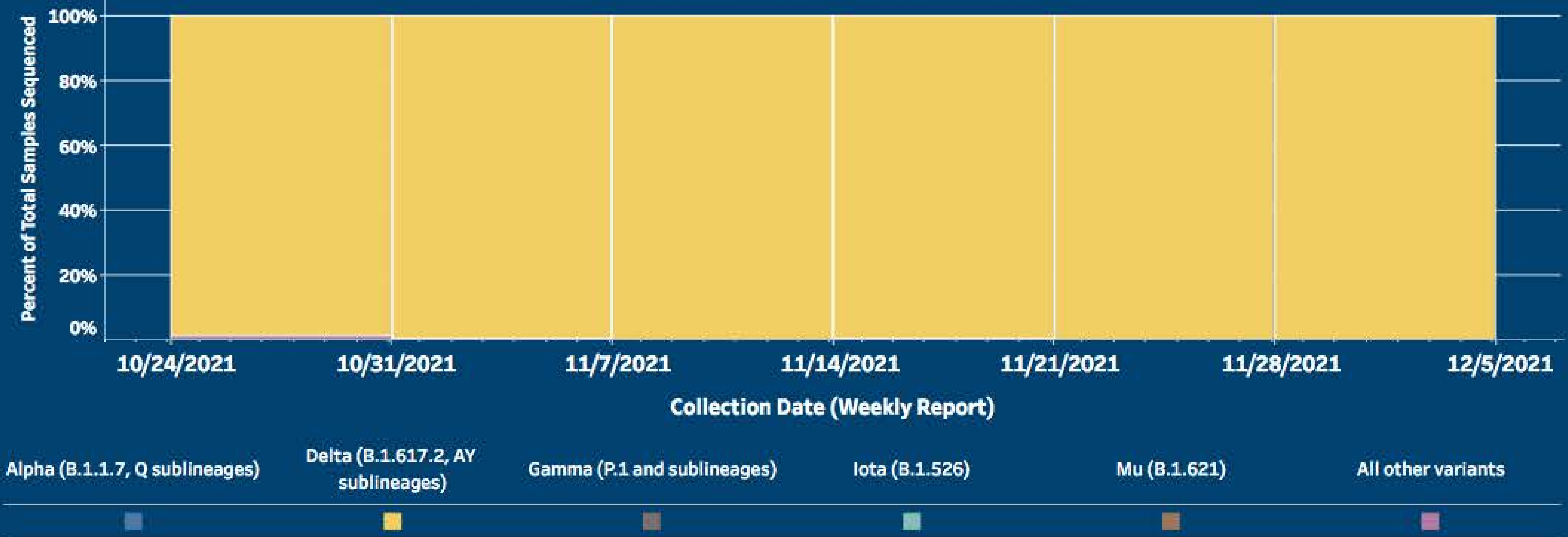

For more information on all variants go to:
https://public.tableau.com/app/profile/oregon.health.authority.covid.19/viz/GISAIDVariantDashboardUpdated/OregonVariantDashboard

Variant Data is from the open-source sequencing data platform GISAID and will be updated weekly on Wednesdays. For more info on GISAID click here.

Daily and Cumulative County Numbers from 12/9/2021 to 12/9/2021

Pennsylvania COVID-19 Dashboard

Confirmed Cases

1,504,746

Probable Cases

324,605

Negative Tests

5,972,235

Deaths

34,571

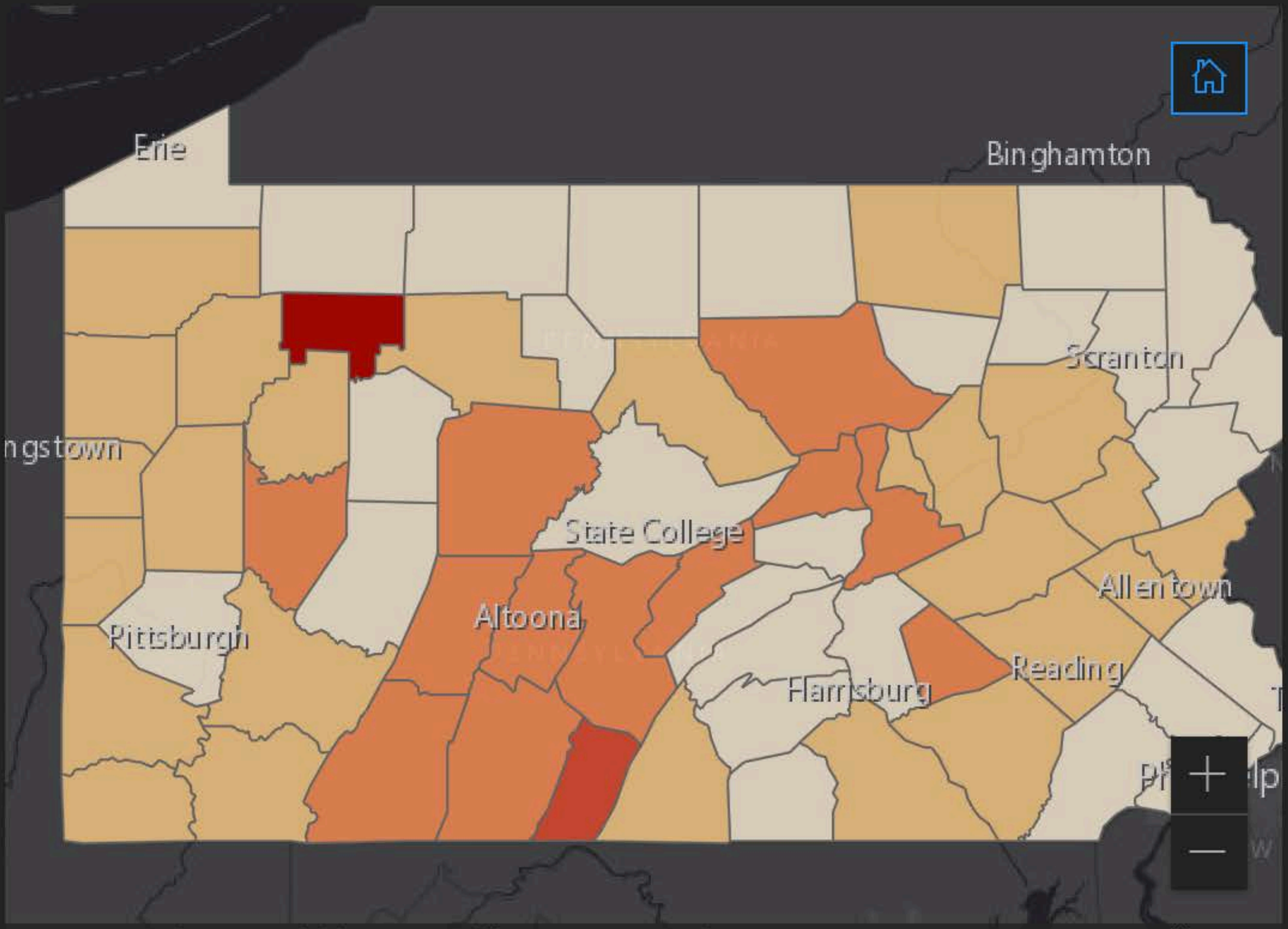

Cases per 100,000 Population

0 - 15,000 | > 15,000 - 17,000 | > 17,000 - 19,000 | > 19,000 - 21,000 | > 21,000 - 30,000

Case count data, map and new cases per day data from PA-NEDSS. Deaths by day graph data from EDRS.

Last Updated	December 11, 2021

New Cases Per Day

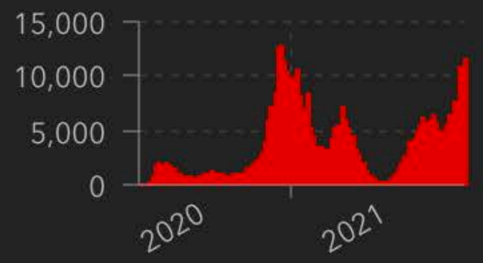

Deaths by Day

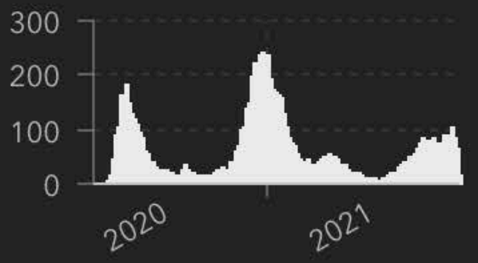

Daily PCR Tests

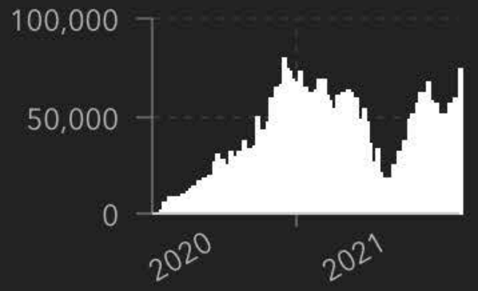

**Case Counts in the charts above include confirmed and*

Pennsylvania Cases

Pennsylvania COVID-19 Dashboard

Choose a County from the dropdown: Pennsylvania

Confirmed Cases

1,504,746

Probable Cases

324,605

Negative Tests

5,972,235

Deaths

34,571

Cases by Day

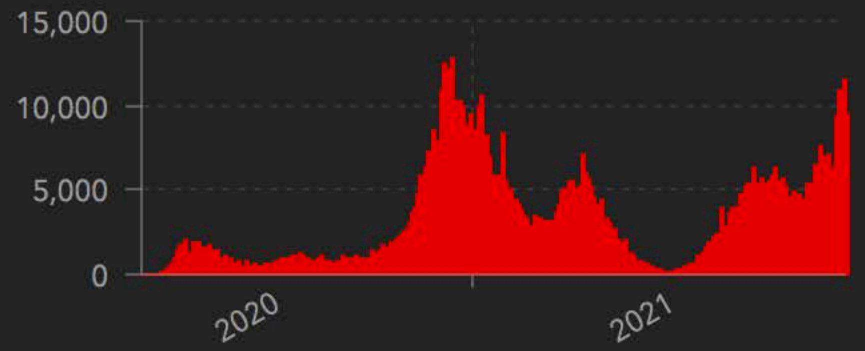

Cumulative Cases per 100,000

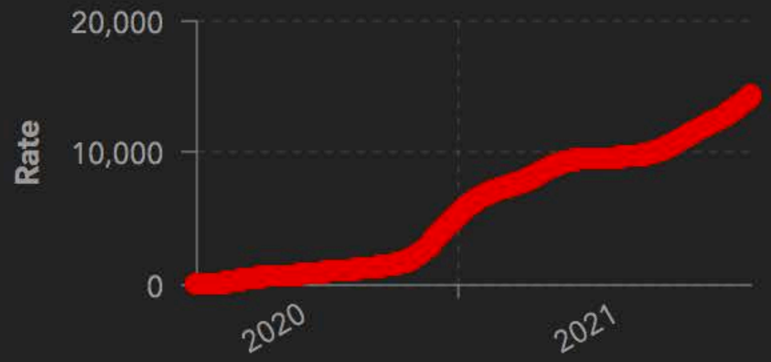

Deaths by Day

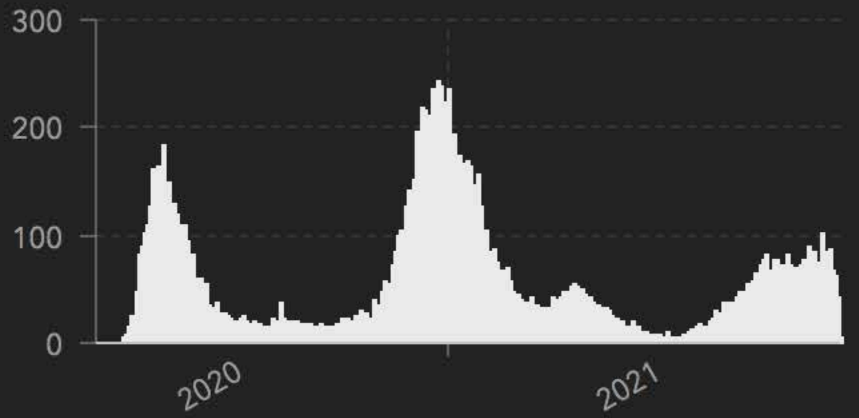

Cumulative Deaths per 100,000

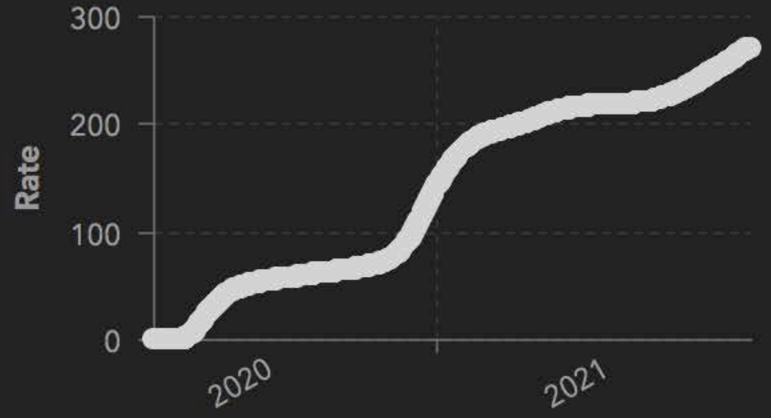

Case data from PA-NEDSS. Death data is based on the date of death as reported to EDRS. Death counts for prior dates will change as additional death records are registered or amended. Case counts [illegible]

In early January the dashboard switched from 2018 to 2019 population estimates to calculate all rates.

Pennsylvania Cases | County Data | Zip Code Case Data | Hospital Preparedness | Cases Demographic | Deaths Demographic | Download Data

Pennsylvania COVID-19 Dashboard

Select a category
Pennsylvania

Cases by Gender

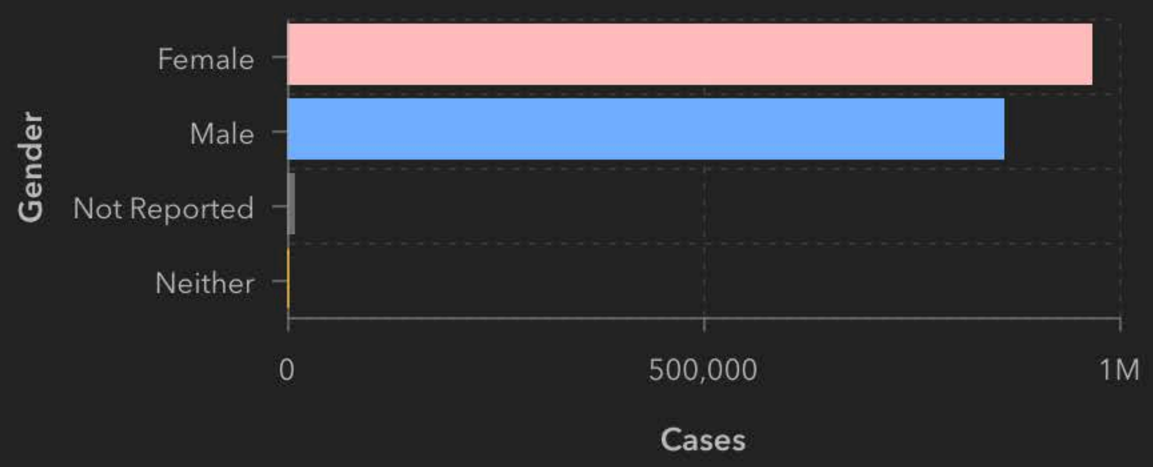

Age Distribution by Cases

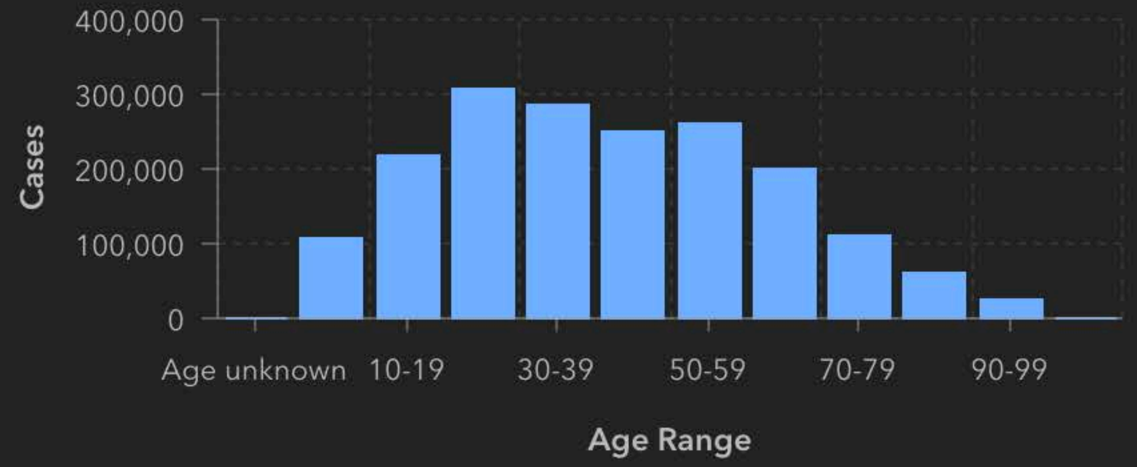

Cases by Ethnicity

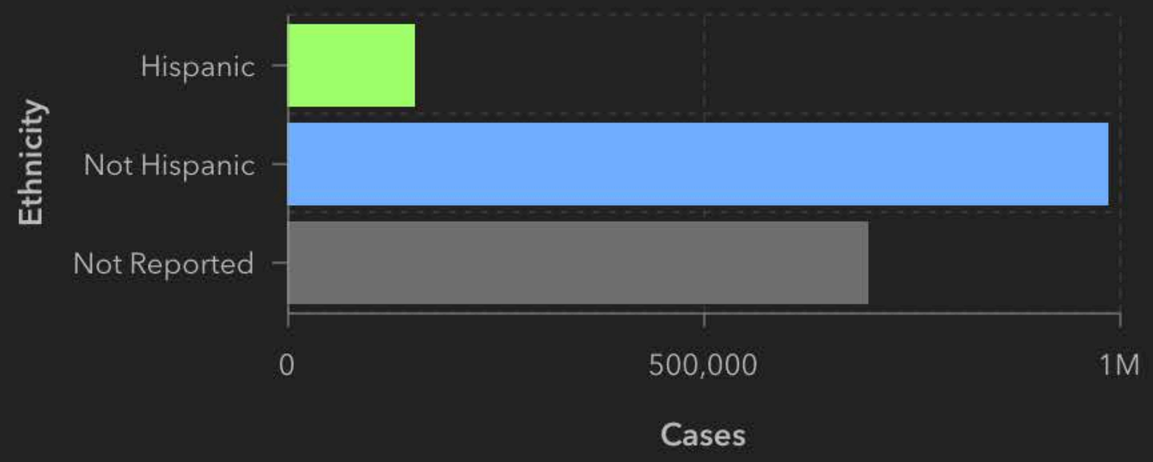

Cases by Race

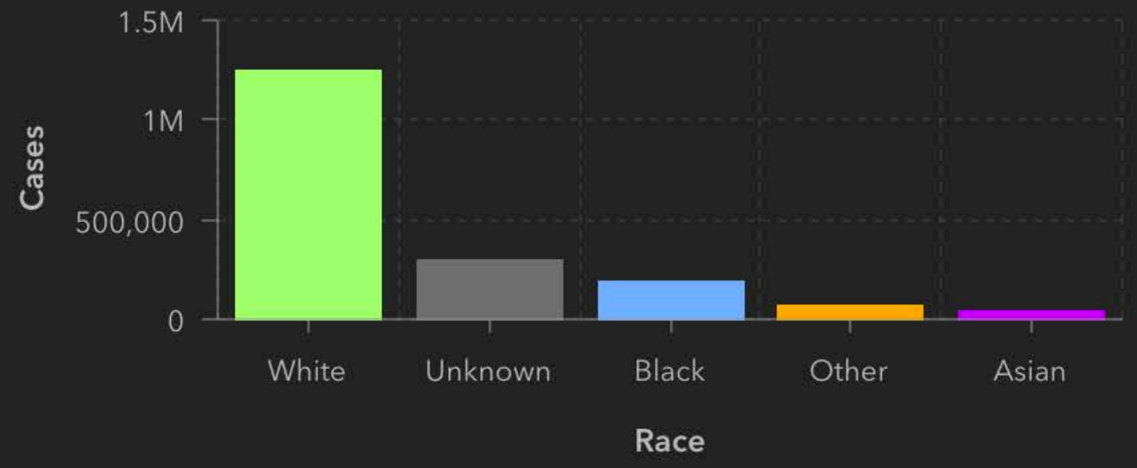

Pennsylvania Cases | County Data | Zip Code Case Data | Hospital Preparedness | Cases Demographic | Deaths Demographic | Download Data

Demographics

COVID-19 Vaccine Dashboard

Vaccinations Administered

Daily | Cumulative

County/State

Pennsylvania

Number of Vaccinations Administered to Pennsylvania Residents

Partial vaccinations administered	7,410,709
Full vaccinations administered	6,766,917
Additional doses administered since August 13, 2021	1,885,506
**Total vaccinations administered**	**16,063,132**

Number of Vaccinations Administered to Pennsylvania Residents, by Date

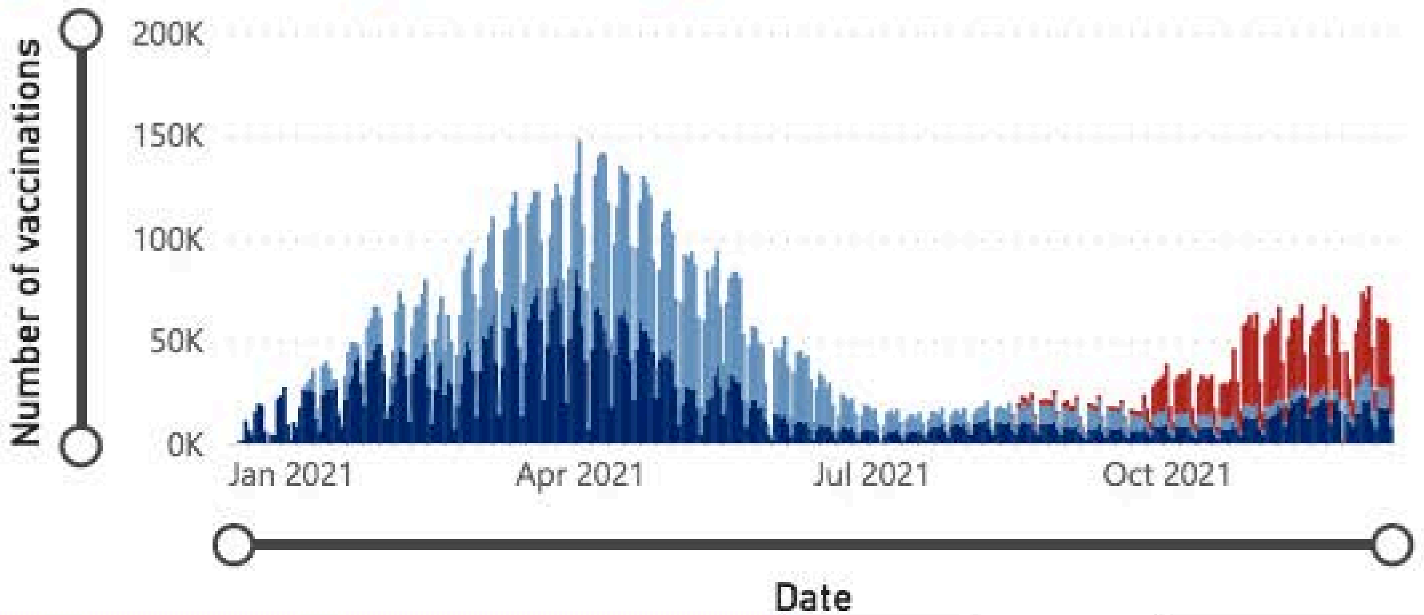

People Vaccinated

Partial | Full | Additional dose

Number of People Vaccinated, by County

County	Partially Covered	Fully Covered	Received an Additional Dose Since August 13, 2021
Adams	5,287	48,149	15,455
Allegheny	122,812	770,062	250,406
Armstrong	3,890	35,781	12,236
Beaver	12,159	82,586	27,585
Bedford	1,840	16,488	5,470
Berks	28,583	223,433	53,525
**Total**	**1,183,570**	**6,766,917**	**1,885,506**

Rate per 100,000 Residents Who Have Received Fully Covered Vaccinations, by County

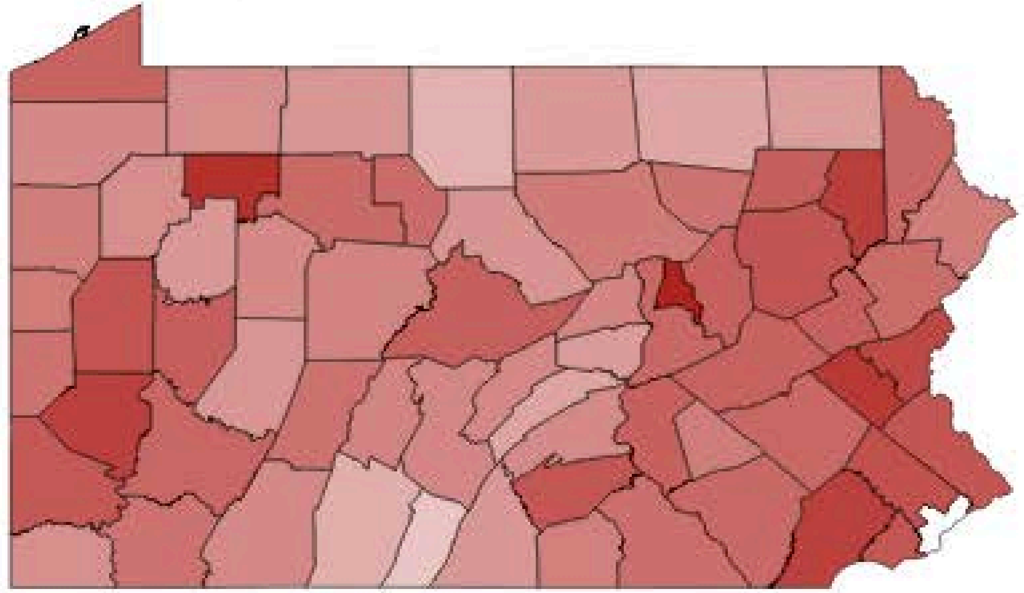

pennsylvania
DEPARTMENT OF HEALTH

Updated on 12/10/2021

View

Percent of the Population | Count

Select a demographic category

Race | Ethnicity | Gender | Age

Page Navigation

Download

Gender

All genders

Coverage

People with at least one vaccine dose

Partially Covered

Fully Covered

Percent of Pennsylvania Residents With at Least One Vaccine Dose, by County

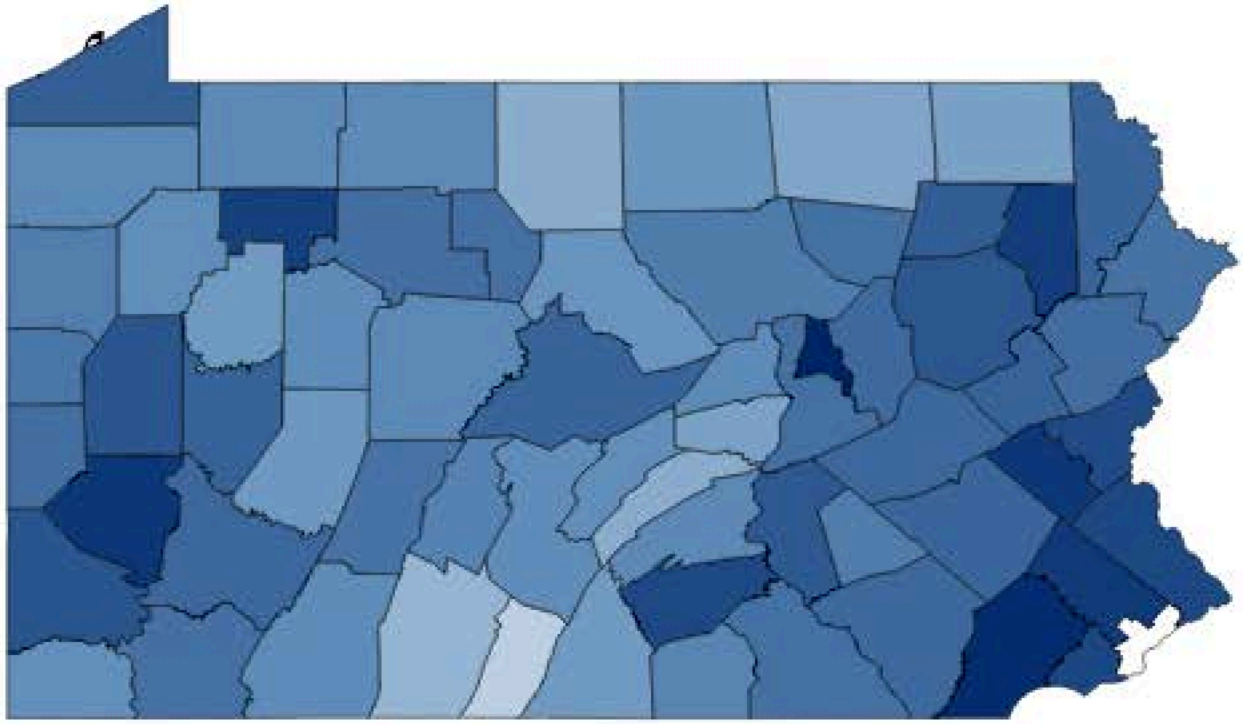

County Ranking of the Percent of Pennsylvania Residents With at Least One Vaccine Dose

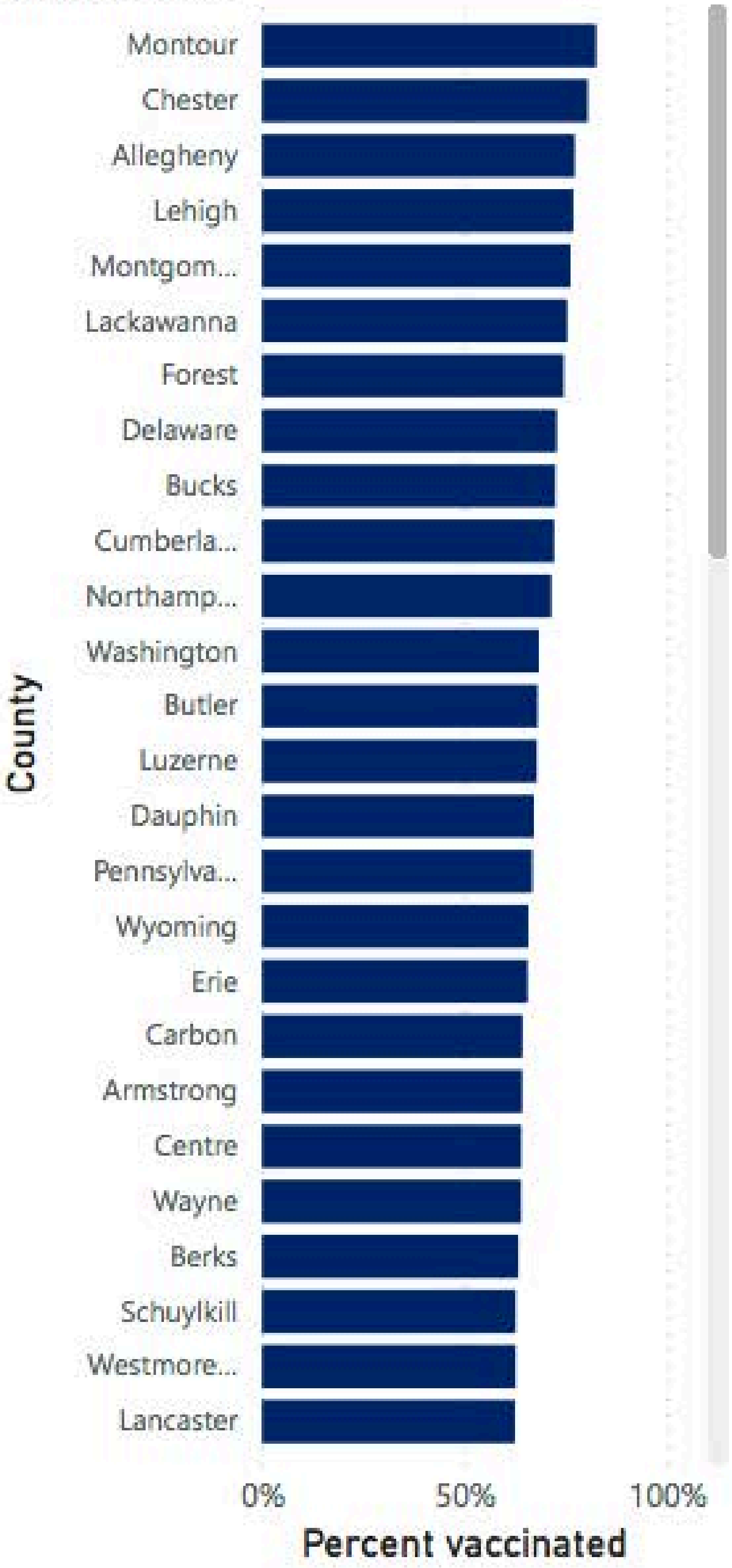

Gender	All genders			Male			Female	
County	People with at least one vaccine dose	Partially Covered	Fully Covered	People with at least one vaccine dose	Partially Covered	Fully Covered	People with at least one vaccine dose	Partia Cover
Pennsylvania**	66.8%	8.7%	58.1%	62.0%	8.2%	53.7%	69.8%	9
Adams	54.4%	5.4%	49.0%	50.9%	4.8%	46.1%	57.4%	5
Allegheny	77.3%	10.6%	66.7%	73.1%	10.3%	62.9%	80.0%	10
Armstrong	64.4%	6.3%	58.1%	57.7%	5.9%	51.7%	68.5%	6
Beaver	60.7%	7.8%	52.9%	56.1%	7.5%	48.7%	64.1%	8

Note: The number of people whose gender is unknown accounted for 1.4% of the total number of people vaccinated in Pennsylvania.

** Percentages for Pennsylvania were calculated excluding Philadelphia County and out-of-state residents (see Technical Notes).

Daily data updated Monday through Friday by approximately 3 p.m.
Weekly charts updated on Tuesdays.

Daily data last updated: Dec 10, 2021

Rhode Island Department of Health COVID-19 Data Tracker

CDC Indicator of Community Transmission of COVID-19

CDC COVID-19 Community Transmission Thresholds (Cases per 100,000 Population)	Low Transmission 0-9.99	Moderate Transmission 10-49.99	Substantial Transmission 50-99.99	High Transmission ≥100

High Transmission

595.9
Total new cases per 100,000 persons in the past 7 days

Total Tests
6,234,464

Total Positive Cases
201,275

Tests Prior Day
19,929

New Positive Cases Prior Day
1,066

Percent Positive Prior Day
5.3%

Total Fatalities
2,957

Fatalities Confirmed Prior Day
6

People Hospitalized
206

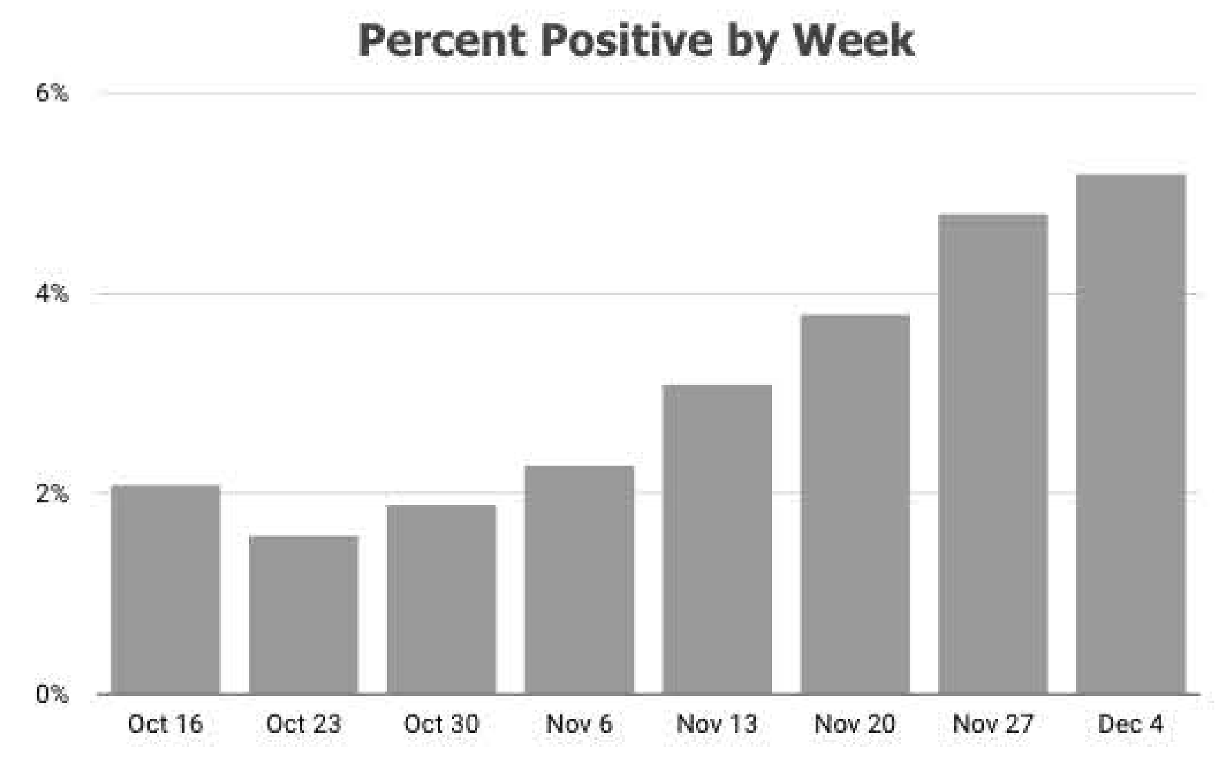

This week: 5.2%
Last Week: 4.8%

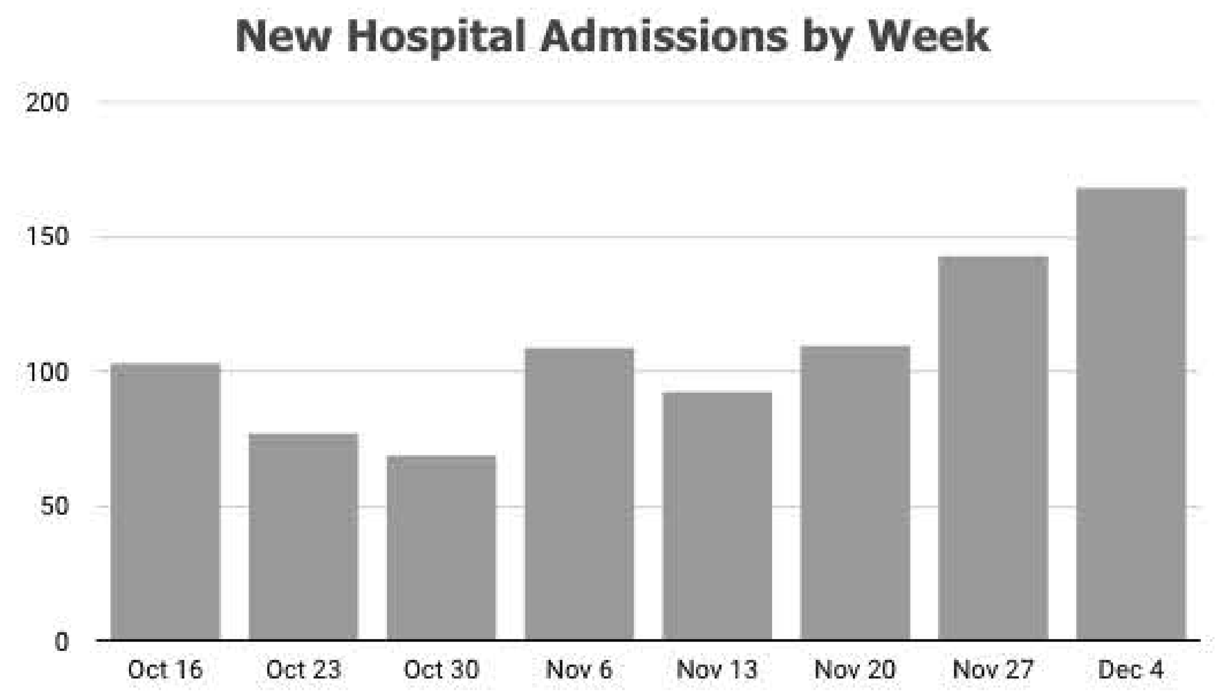

This week: 168
Last Week: 143

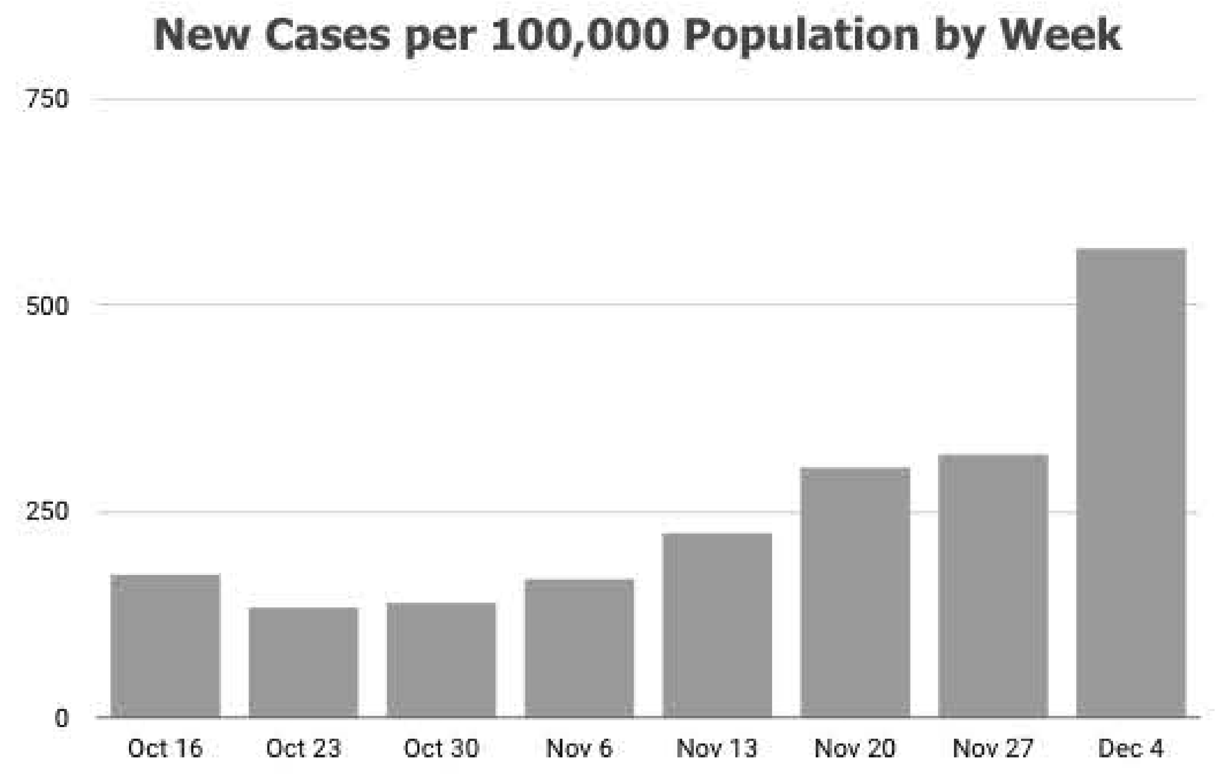

This week: 570
Last Week: 320

Vaccine Administration Data

Data cumulative since 12/14/20
Updated daily.

People At Least Partially Vaccinated in Rhode Island	People Who Have Completed Primary Vaccine Series in Rhode Island	Total Doses Administered in Rhode Island
866,126	782,613	1,822,438

Monoclonal Antibody (MAB) Treatments Administered by Providers in Rhode Island

Updated weekly on Wednesday
Last updated: Dec 9, 2021

Number of MAB Treatments Administered Week Prior	Number of MAB Treatments Administered Cumulatively
298	6,770

Notes: Data reflect MAB treatments administered by providers in Rhode Island, and may include those administered to out-of-state residents. Data include MABs administered as treatment for lab-confirmed COVID-19 and as post-exposure prophylaxis. Prior to 1/17/21, number of treatments administered were determined using inventory data. From 1/17/21 onward, data reported are based on the number of people who received monoclonal antibody treatments.

Google Data Studio

Data are provisional and subject to change.

Testing data reported on the RIDOH COVID-19 data hub include all detailed testing data reported to RIDOH. These detailed data enable RIDOH to report on data elements such as demographics and residence. The COVID-19 data hub testing data does not include asymptomatic K-12 and employer test results that are reported to RIDOH in aggregate form.

New Positive Cases	People Hospitalized	Newly Confirmed Fatalities
1,066	206	6

Total Tests	Total Positive Cases	Total Negative Tests
[illegible]	[illegible]	[illegible]

Sign In

Rhode Island COVID-19 Response Data [Click here] to return to the RIDOH COVID-19 Home Page

Total Fatalities	People in ICU	People on a Ventilator
2,957	36	22

Source: Rhode Island Department of Health

Google Data Studio

Rate of COVID-19 Cases per 100,000 Population, by Municipality of Residence and Week of Positive Test Date - Excluding Congregate Setting Residents

Select town: RHODE ISLAND
Select time period, by week ending date: 3/7/2020 – 12/4/2021
Clear all selections

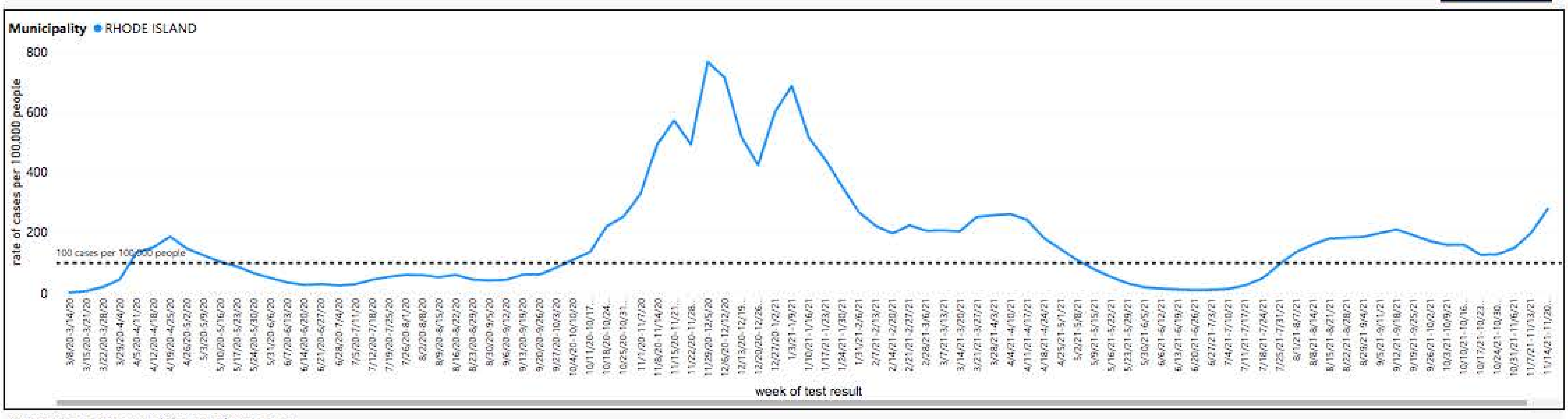

Note: rates are suppressed if count of cases is <5

Microsoft Power BI

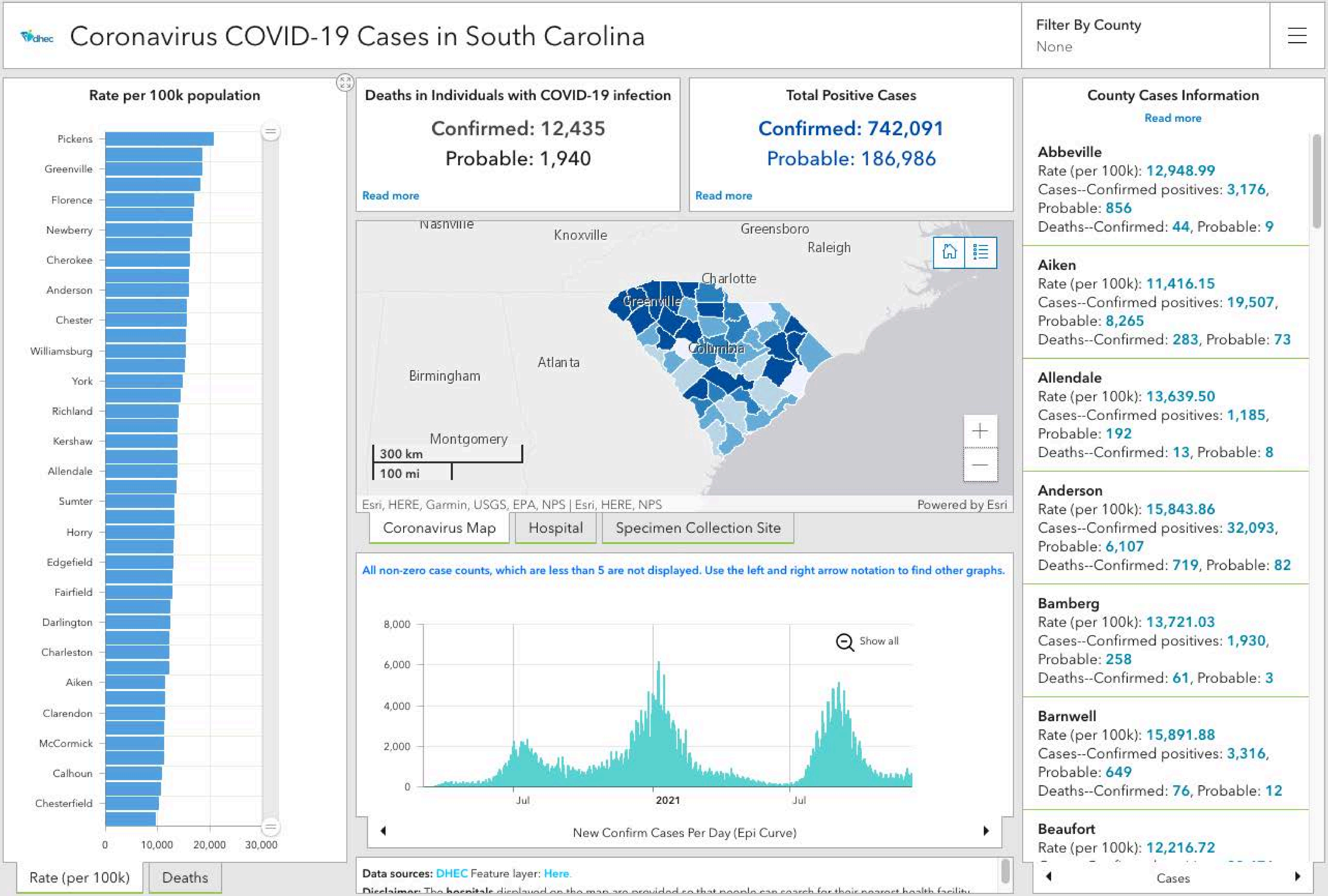

dhec
Coronavirus COVID-19 Cases in South Carolina
Filter By County
None
Rate per 100k population
Pickens
Greenville
Florence
Newberry
Cherokee
Anderson
Chester
Williamsburg
York
Richland
Kershaw
Allendale
Sumter
Horry
Edgefield
Fairfield
Darlington
Charleston
Aiken
Clarendon
McCormick
Calhoun
Chesterfield
0
10,000
20,000
30,000
Rate (per 100k)
Deaths
Deaths in Individuals with COVID-19 infection
Confirmed: 12,435
Probable: 1,940
Read more
Total Positive Cases
Confirmed: 742,091
Probable: 186,986
Read more
Nashville
Knoxville
Greensboro
Raleigh
Charlotte
Greenville
Columbia
Atlanta
Birmingham
Montgomery
300 km
100 mi
Esri, HERE, Garmin, USGS, EPA, NPS | Esri, HERE, NPS
Powered by Esri
Coronavirus Map
Hospital
Specimen Collection Site
All non-zero case counts, which are less than 5 are not displayed. Use the left and right arrow notation to find other graphs.
8,000
6,000
4,000
2,000
0
Show all
Jul
2021
Jul
New Confirm Cases Per Day (Epi Curve)
Data sources: DHEC Feature layer: Here.
County Cases Information
Read more
Abbeville
Rate (per 100k): 12,948.99
Cases--Confirmed positives: 3,176,
Probable: 856
Deaths--Confirmed: 44, Probable: 9
Aiken
Rate (per 100k): 11,416.15
Cases--Confirmed positives: 19,507,
Probable: 8,265
Deaths--Confirmed: 283, Probable: 73
Allendale
Rate (per 100k): 13,639.50
Cases--Confirmed positives: 1,185,
Probable: 192
Deaths--Confirmed: 13, Probable: 8
Anderson
Rate (per 100k): 15,843.86
Cases--Confirmed positives: 32,093,
Probable: 6,107
Deaths--Confirmed: 719, Probable: 82
Bamberg
Rate (per 100k): 13,721.03
Cases--Confirmed positives: 1,930,
Probable: 258
Deaths--Confirmed: 61, Probable: 3
Barnwell
Rate (per 100k): 15,891.88
Cases--Confirmed positives: 3,316,
Probable: 649
Deaths--Confirmed: 76, Probable: 12
Beaufort
Rate (per 100k): 12,216.72
Cases

SOUTH DAKOTA DEPARTMENT OF HEALTH doh.sd.gov/covid/

SOUTH DAKOTA COVID-19 DASHBOARD

SOUTH DAKOTA COVID-19 UPDATES

Please wait. The data dashboard below takes a moment to load and is best viewed on desktop or in landscape mode on mobile (i.e. holding the phone sideways).

- **NOTE:** Percent of the population with 1-dose, series complete, and booster dose has been updated to reflect the 5+ year old population. There was a decrease of 7% from the 12+ year old population. The change was made to align with the vaccine-eligible population, which now includes children ages 5-11 years.

SD Overview | Demographics | Trends | Hospital Capacity | Hospitalized & ILI | Tables | Vaccines | Deaths

New Confirmed Cases	New Probable Cases	Active Cases	Recovered Cases	Currently Hospitalized
343	105	7,692	161,158	261

Community Spread Map by County of Residence

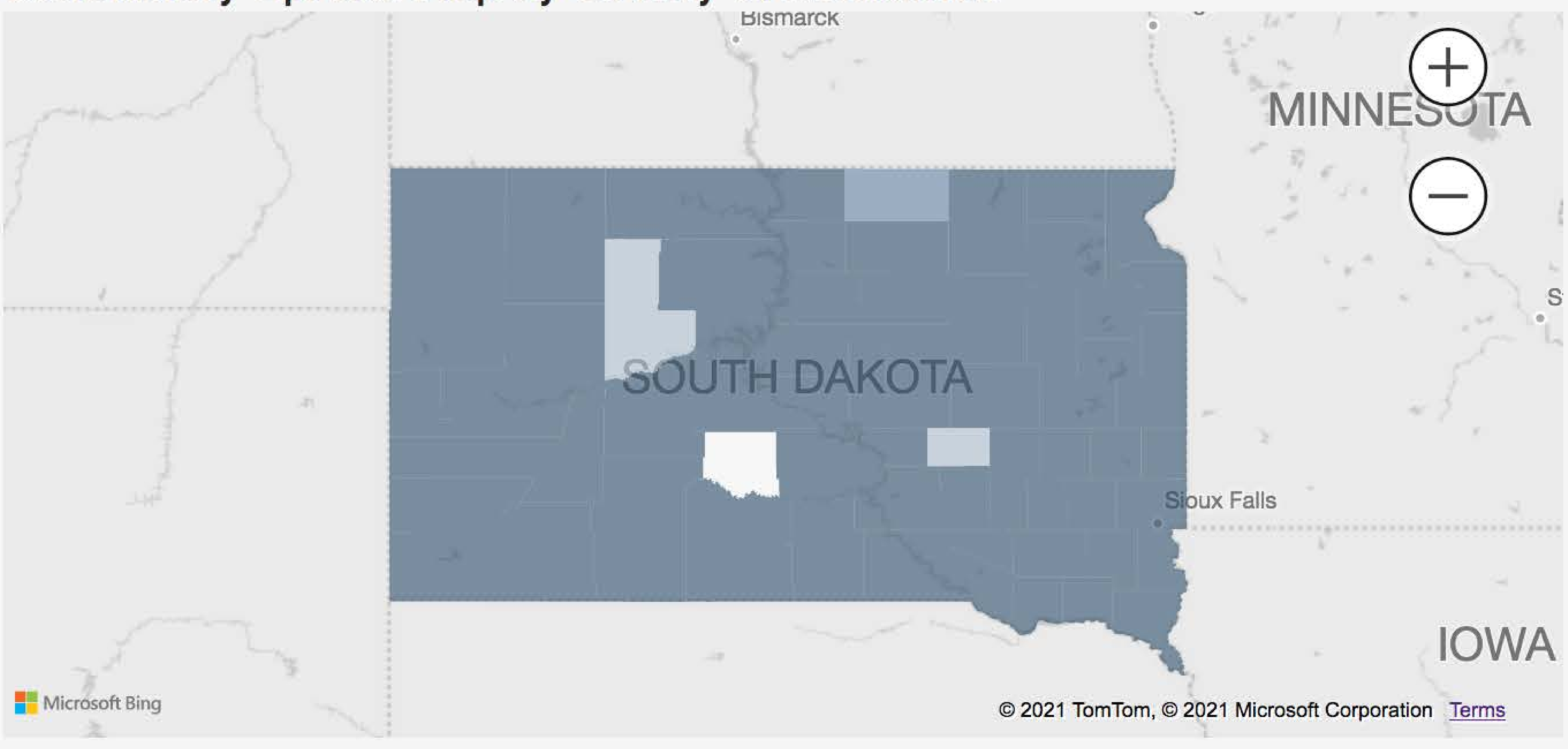

Community Spread Low Moderate Substantial High

Hover over a county to see its details, or click county to update the orange boxes.

Total Confirmed Cases	Total Probable Cases	PCR Test Positivity Rate, Last 7 Days	Total Tests
146,218	25,030	14.7% (12/2/2021 - 12/8/2021)	1,752,846

Ever Hospitalized	Deaths Among Cases	% Progress (October Goal: 44233 Tests)	% Progress (November Goal: 44233 Tests)	% Progress (December Goal: 44233 Tests)
8,689	2,398	233%	242%	76%

Cases by County of Residence

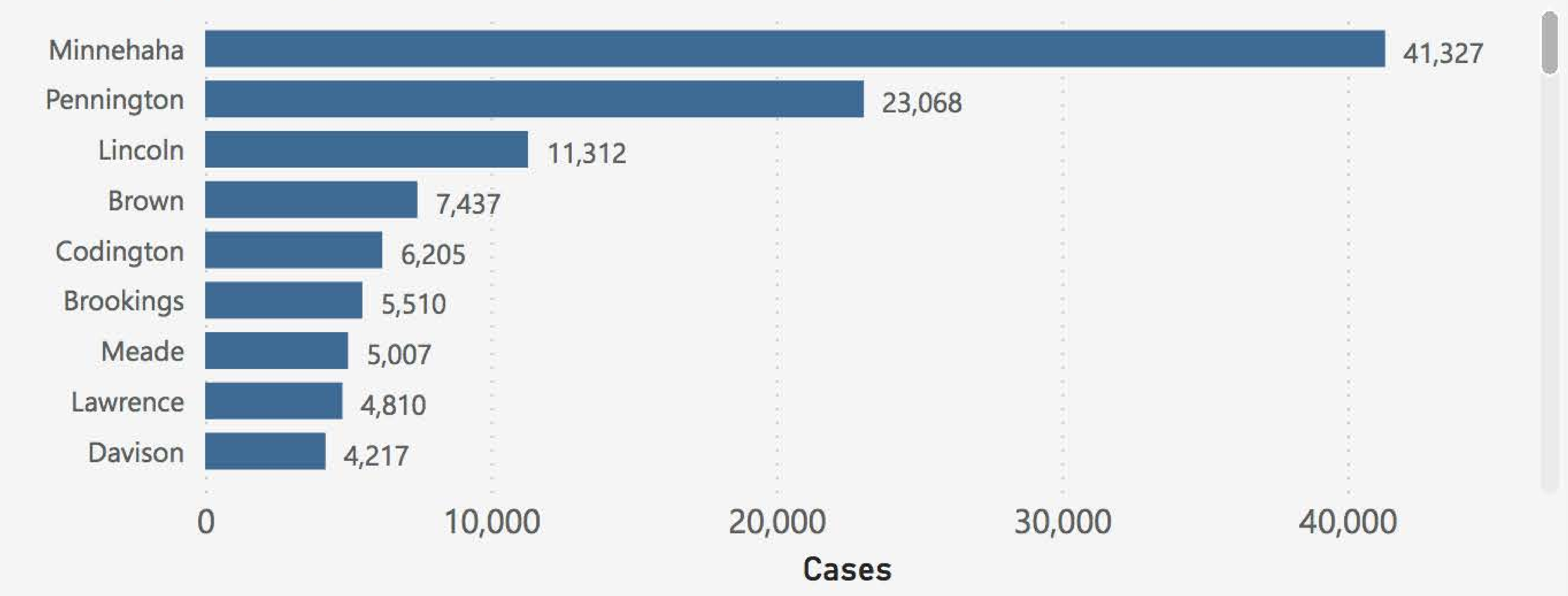

Cases: *Persons who meet the national surveillance case definition for COVID-19. According to the case definition, Confirmed Cases includes persons with a positive PCR test and Probable Cases includes persons with a positive antigen test for the SARS-CoV-2 virus that causes COVID-19. A positive case moves from Active to Recovered status based on the CDC guidelines for removal from isolation.*

Hospitalized: *Currently Hospitalized numbers include COVID-19 cases and suspects that are in the hospital under transmission-based precautions. The number may include out-of-state cases and suspects. SD-DOH reports on data directly entered by hospitals into the US Department of Health and Human Services TeleTracking system. Federal entities, such as the Veterans Affairs Medical Centers, are not subject to reporting. Ever Hospitalized include COVID-19 cases that were, at some point during their infection, hospitalized under transmission-based precautions. The number of persons ever hospitalized are cumulative and includes only South Dakota resident cases. Data are reported from the South Dakota Electronic Disease Surveillance System.*

Deaths among Cases: *This data includes deaths reported by death certificate or deaths within 20 days of a COVID-19 infection. SD-DOH does not report deaths until a certified death record has been filed with COVID-19 as a cause or contributing factor. By law, a death record must be filed within 5 days of the date of death. For more information see the Deaths Tab.*

Tests: *% Progress shows the percentage of PCR and antigen tests completed toward the monthly goal of 44,233. The monthly goal for South Dakota is equal to 5% of the state's population. The federal government established a 2% goal for SARS-CoV-2 testing for each state. Total tests are the number of tests performed, not the number of individuals tested, and include tests conducted by the South Dakota Public Health Laboratory and hospital, commercial, Indian Health Service, Veteran Affairs, and other medical laboratories.*

Community Spread *is updated every Monday based on two indicators: 1) the rate of new cases per week, and 2) the weekly % PCR test positivity rate - both calculated from the data reported during the previous calendar week (Sun-Sat). If the two indicators suggest different transmission levels for a county, the higher level is selected.*

Community spread is defined as:
Low: *Less than 10 cases per 100,000 AND weekly % PCR test positivity rate less than 5%*
Moderate: *10-49 cases per 100,000 OR weekly % PCR test positivity rate of 5-7.9%*
Substantial: *50-99 cases per 100,000 OR weekly % PCR test positivity rate of 8-9.9%*
High: *100 cases or greater per 100,000 OR weekly % PCR test positivity rate of 10% or greater*
**Small number of new cases (less than 10) reported during the previous calendar week*

% PCR Test Positivity Rate *is equal to the number of positive PCR tests divided by total PCR tests, within the specified period of time. Weekly % PCR Test Positivity Rate is updated every Monday based on the number of new PCR tests from the previous week (Sun-Sat).*

LAST UPDATED
12/10/2021 9:30:48 AM

COVID-19

Collapse All | Expand All

Dashboard
Your Health
Quarantine & Isolation Calculator
Community, Work, & School
Testing
+ COVID-19 Vaccine
+ Healthcare Providers
Resources

SOUTH DAKOTA DEPARTMENT OF HEALTH doh.sd.gov/covid/

SOUTH DAKOTA COVID-19 DASHBOARD

SOUTH DAKOTA COVID-19 UPDATES

Please wait. The data dashboard below takes a moment to load and is best viewed on desktop or in landscape mode on mobile (i.e. holding the phone sideways).

- **NOTE:** Percent of the population with 1-dose, series complete, and booster dose has been updated to reflect the 5+ year old population. There was a decrease of 7% from the 12+ year old population. The change was made to align with the vaccine-eligible population, which now includes children ages 5-11 years.

SD Overview | Demographics | Trends | Hospital Capacity | Hospitalized & ILI | Tables | Vaccines | Deaths

County

All

PCR Test Positivity Rate, Last 7 Days	PCR Test Positivity Rate, Last 14 Days	PCR Test Positivity Rate, Cumulative
14.7%	15.5%	13.3%
12/2/2021 - 12/8/2021	11/25/2021 - 12/8/2021	3/10/2020 - 12/8/2021

***PCR Test Positivity Rate:** This rate is equal to the number of positive PCR tests divided by total PCR tests, within the specified period of time. Date range includes last full day (24 hours) captured in the data set.*

Confirmed Cases and 14-Day Trend Line by Date Reported to SD-DOH

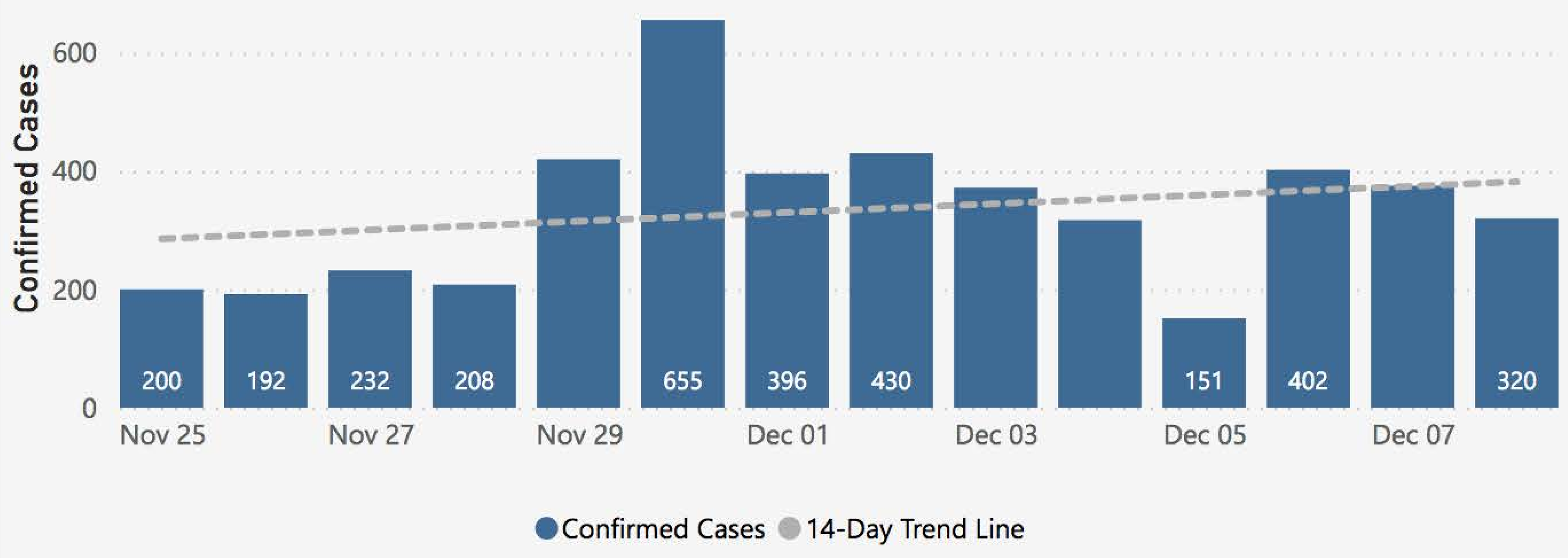

***14-Day Trend of Confirmed Cases by Date Reported to SD-DOH:** This graph shows the trend (increasing or decreasing) of COVID-19 confirmed cases (persons who meet the national surveillance case definition case for COVID-19) at the state or county level.*

Daily Rate of Total Cases, Last 7 Days	Rate of Total Cases, Cumulative
12/2/2021 - 12/8/2021	3/10/2020 - 12/8/2021
49.86	19,392
Daily Case Rate per Capita	Case Rate per Capita

***Rate of Cases:** Number of cases (persons who meet the national surveillance case definition for a confirmed or probable case of COVID-19) reported to SD-DOH in the last 7 days as a daily average (or cumulative) divided by the state or county population multiplied by 100,000. This is done to compare disease burden across geographic areas (eg. state or county).*

Date Reported to SD-DOH

3/10/2020 | 12/8/2021

Cases by Date Reported to SD-DOH

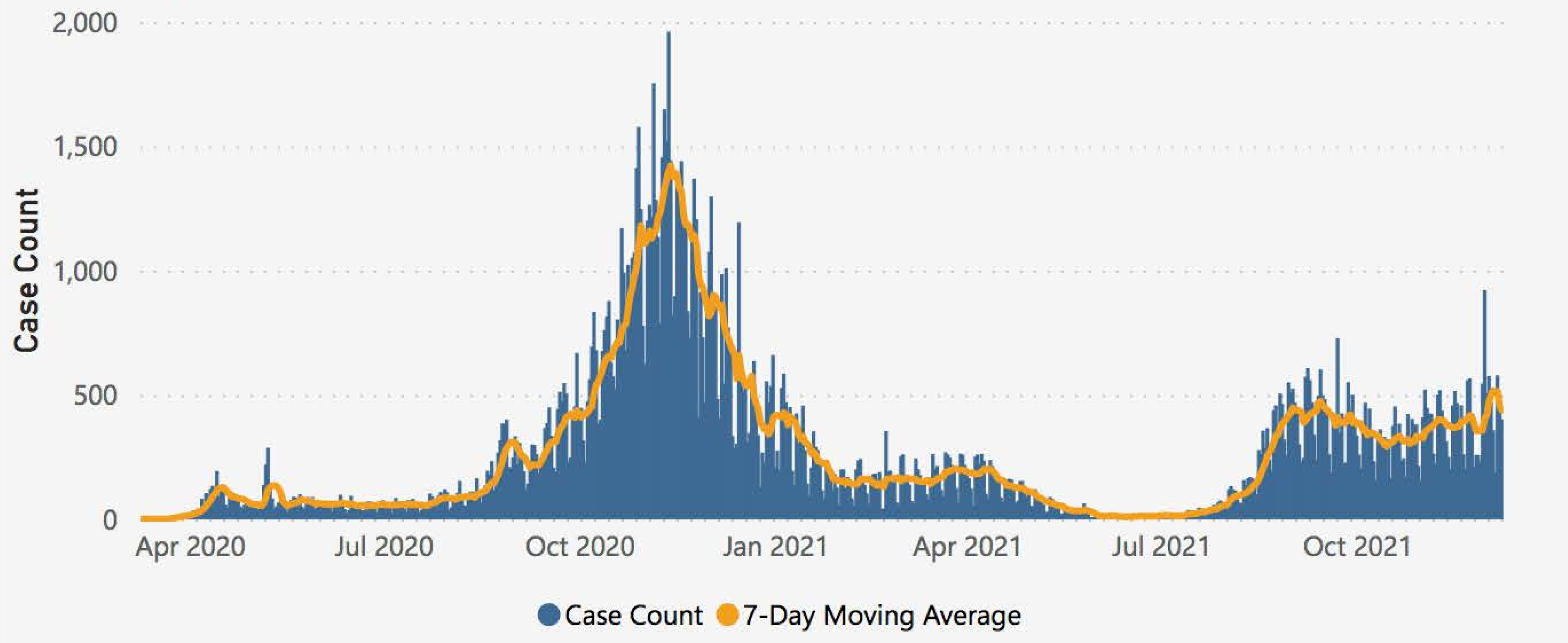

Confirmed | Probable

***Cases by Date Reported to SD-DOH:** This graph shows cases (persons who meet the national surveillance case definition for a confirmed or probable case of COVID-19) over time includes results received by midnight and may not align with the daily data update.*

PCR Test Positivity Rate by Date Reported to SD-DOH

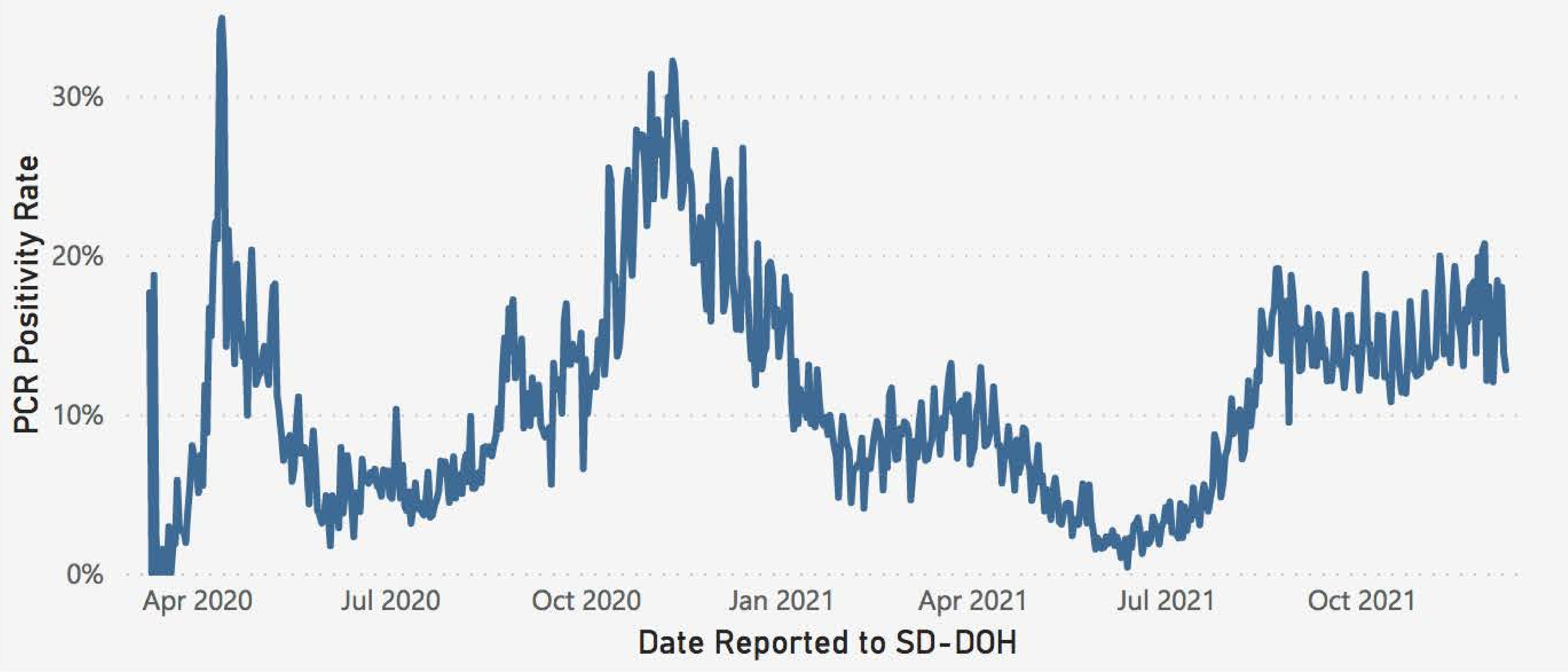

***PCR Test Positive Rate by Date Reported to SD-DOH:** This graph shows % PCR Test Positivity Rate which is equal to the number of positive PCR tests divided by total PCR tests, within the specified period of time. Table view data also shows total positive tests and total tests which includes combined antigen and PCR test data..*

***Note:** Data are provisional and subject to change. Data are updated daily (Mon-Fri) by about 12 p.m. Data from Saturday and Sunday will be included in Tuesday's update. Results include tests conducted by the South Dakota Public Health Laboratory and hospital, commercial, Indian Health Service, Veteran Affairs, and other medical laboratories.*

LAST UPDATED
12/10/2021 9:30:48 AM

SOUTH DAKOTA DEPARTMENT OF HEALTH doh.sd.gov/covid/

SOUTH DAKOTA COVID-19 DASHBOARD

SOUTH DAKOTA COVID-19 UPDATES

Please wait. The data dashboard below takes a moment to load and is best viewed on desktop or in landscape mode on mobile (i.e. holding the phone sideways).

- **NOTE:** Percent of the population with 1-dose, series complete, and booster dose has been updated to reflect the 5+ year old population. There was a decrease of 7% from the 12+ year old population. The change was made to align with the vaccine-eligible population, which now includes children ages 5-11 years.

SD Overview | Demographics | Trends | Hospital Capacity | Hospitalized & ILI | Tables | Vaccines | Deaths

Total Doses Administered*
1,041,313

Total Persons Administered a Vaccine*
485,461

Percent of State Population with at least 1 Dose**
66%

Manufacturer	# of Doses
Janssen	34,614
Moderna	420,925
Pfizer	585,774

Doses	# of Recipients
Janssen - Series complete	32,873
Janssen - Booster dose	1,738
Moderna - 1 dose	22,752
Moderna - Series complete	167,896
Moderna - 3rd/Booster dose	62,334
Pfizer - 1 dose	34,365
Pfizer - Series complete	236,155

Doses	% of Pop.
1 dose	65.86%
Series Complete	54.49%
Booster dose	18.38%

Based on 2019 Census Estimate for those aged 5+ years.

**Includes reports to SD Immunization Information System (SDIIS)*
***Includes aggregate federal data from IHS, VA, DoD, and BoP plus reports to SDIIS*

For COVID-19 Vaccine Coverage at 12+, 18+, or 65+ years click link below:
US Vaccine Coverage

Doses & Persons Administered Vaccine by County of Residence

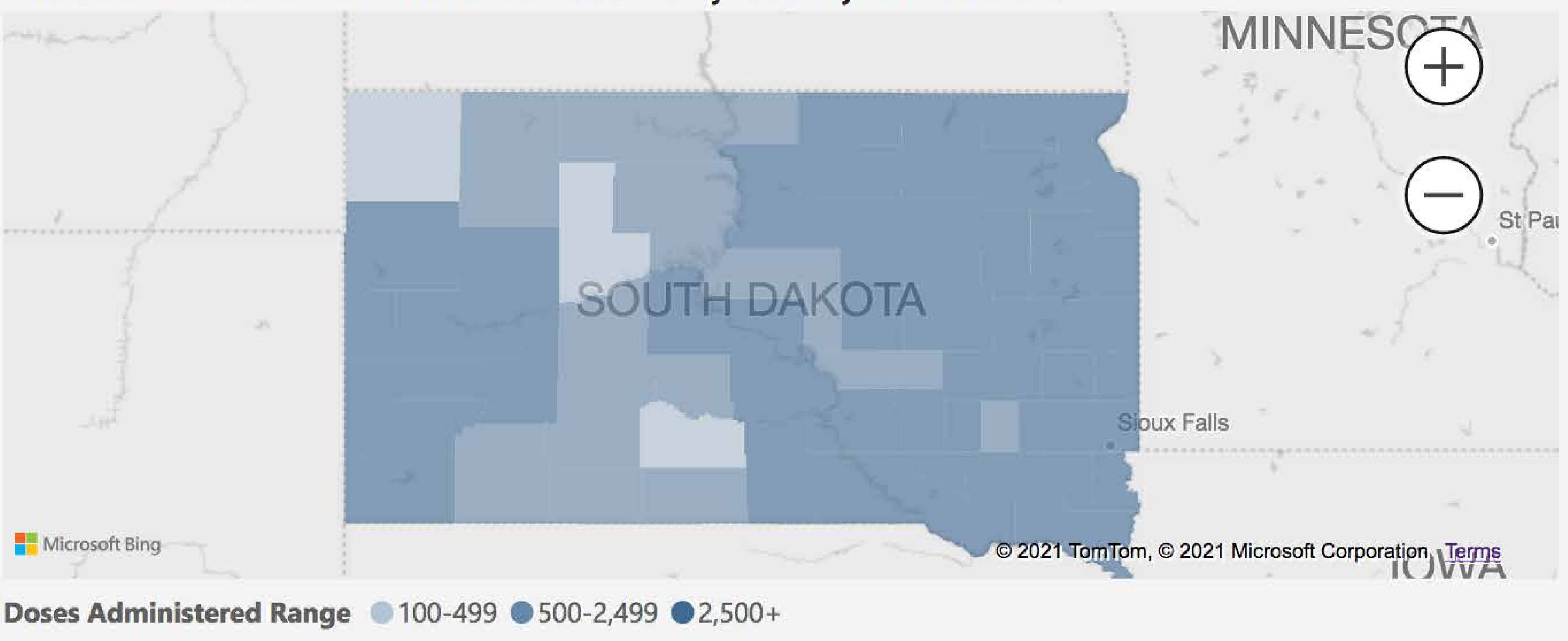

Hover over a county to see its details, or click county to update the orange boxes.

**Counties with tribes receiving vaccine through Indian Health Services (IHS). Doses administered by IHS, VA (Veteran's Affairs), DoD (Dept of Defense), and BoP (Bureau of Prisons) are not reported to SDIIS and are not shown, except for Percent of State Population with at least 1 Dose.*

County	# Doses	# Persons (1 dose)	# Persons (2 doses)	# Persons (3 doses)	Total # Persons
Minnehaha*	273,397	18,084	68,861	38,644	**125,220**
Pennington*	113,653	10,313	28,573	15,128	**53,706**
Lincoln	83,143	4,829	20,645	12,164	**37,546**
Other	39,068	10,361	11,228	1,989	**23,536**
Brown	49,573	2,799	12,392	7,296	**22,338**
Brookings	41,106	3,442	10,130	5,689	**19,133**
Codington*	31,759	1,793	8,006	4,592	**14,362**
Yankton	30,455	1,652	7,536	4,441	**13,598**

Cumulative Number of Persons Vaccinated by Week

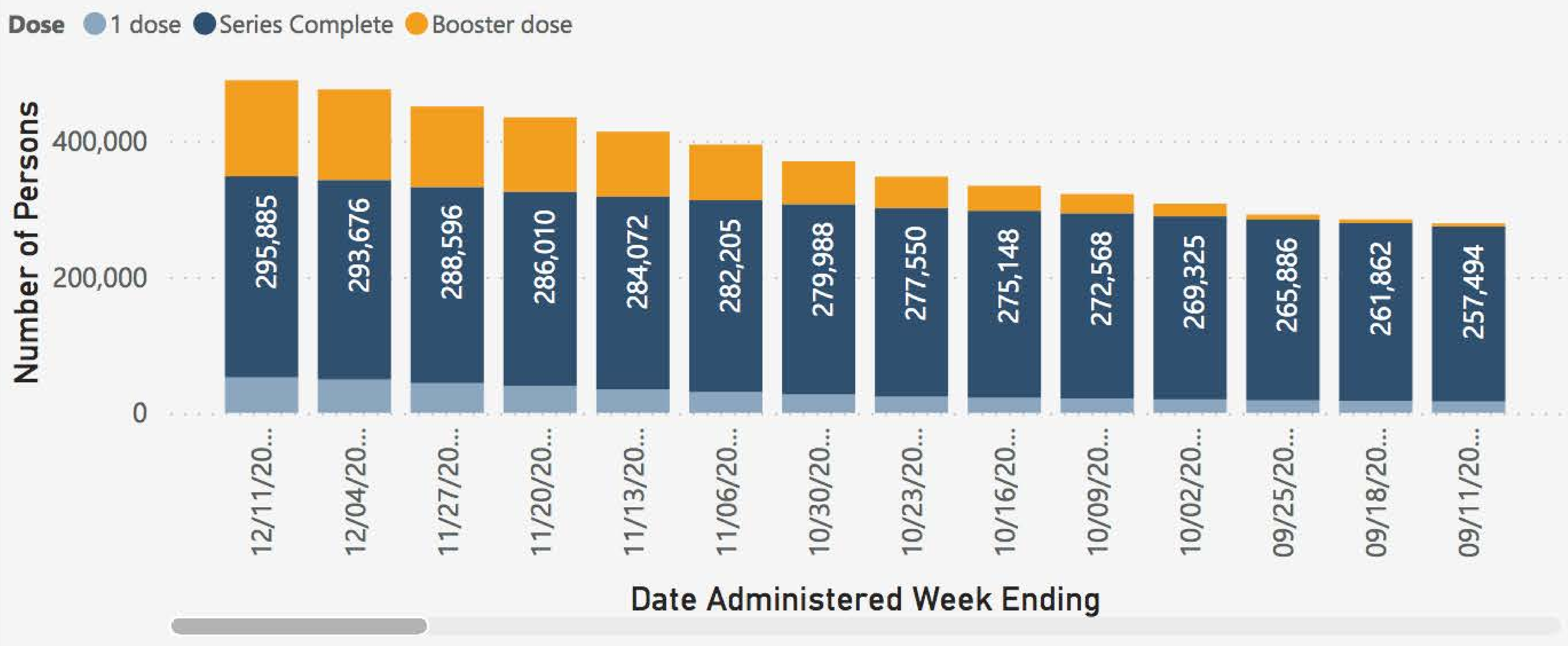

Age | Race/Ethnicity | Sex

Number of Persons Vaccinated by Age Group

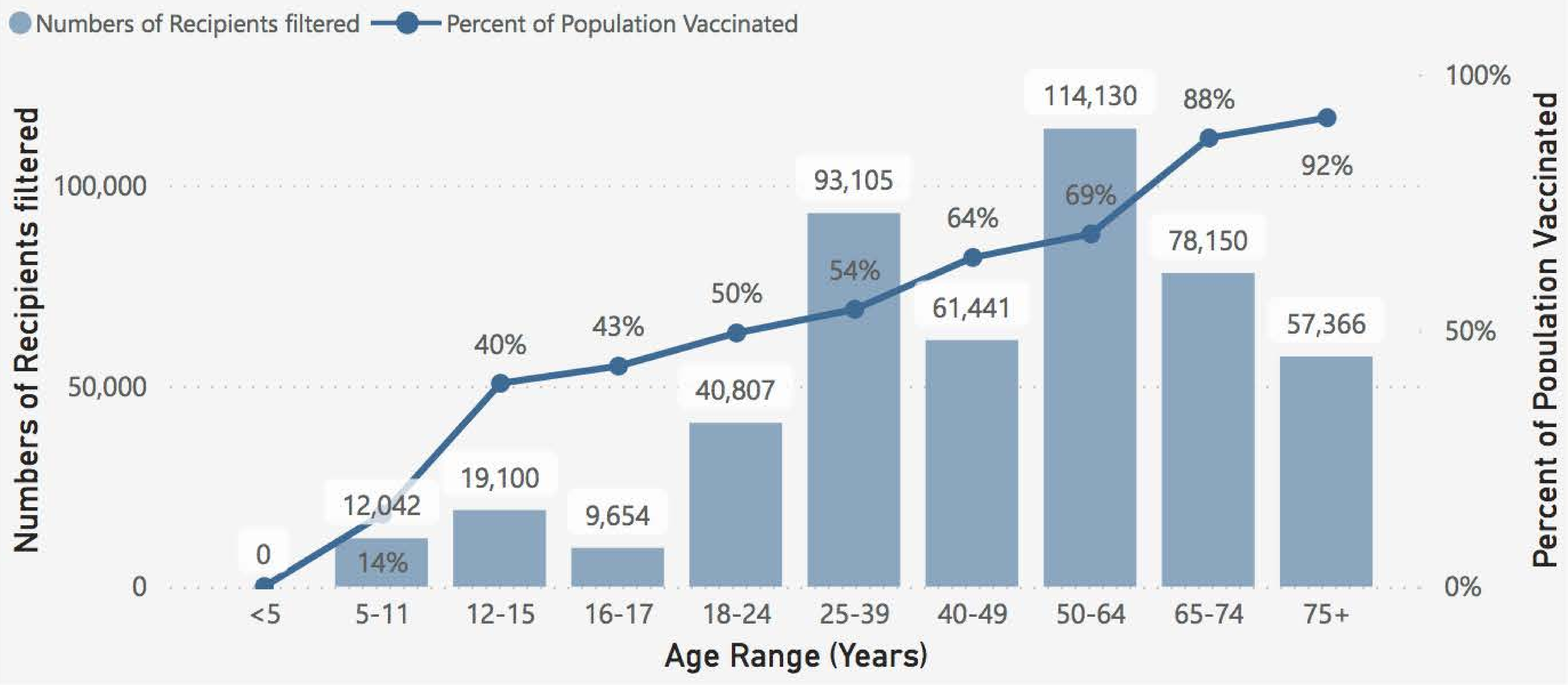

COVID-19 vaccine provided to veterans by the VA Medical Centers and American Indians by the Indian Health Service are not reported to SD-DOH and will lower the percentage of minority race/ethnicity represented when compared to the SD population. COVID-19 vaccine recipient race/ethnicity is a data element of the vaccination record reported to SD-DOH from hospitals, clinics, and other vaccination partners (e.g., federal pharmacy partners).

***Note:** Data are provisional and subject to change. Data are updated daily (Mon-Fri) by about 12 p.m. and include doses reported through 10 a.m. the previous day from SDIIS, data from Saturday and Sunday will be included in Tuesday's update. Doses administered by IHS, VA, DoD, and BoP are not reported to SDIIS and are not shown, except for Percent of State Population with at least 1 Dose.*

LAST UPDATED
12/10/2021 9:30:48 AM

Novel Coronavirus (COVID-19) Unified Command Tennessee State Government

Select a date
12/7/2021

County Snapshots

click county name to toggle map visibility

Shelby
Cases: 210 new, 149,587 total
Deaths: 0 new, 2,409 total
Hospitalizations: 0 new
View Dashboard

Davidson
Cases: 149 new, 125,840 total
Deaths: 0 new, 1,226 total
Hospitalizations: 1 new
View Dashboard

Knox
Cases: 167 new, 82,191 total
Deaths: 3 new, 977 total
Hospitalizations: 0 new
View Dashboard

Hamilton
Cases: 101 new, 66,111 total
Deaths: 5 new, 726 total
Hospitalizations: 0 new
View Dashboard

Rutherford
Cases: 91 new, 64,768 total

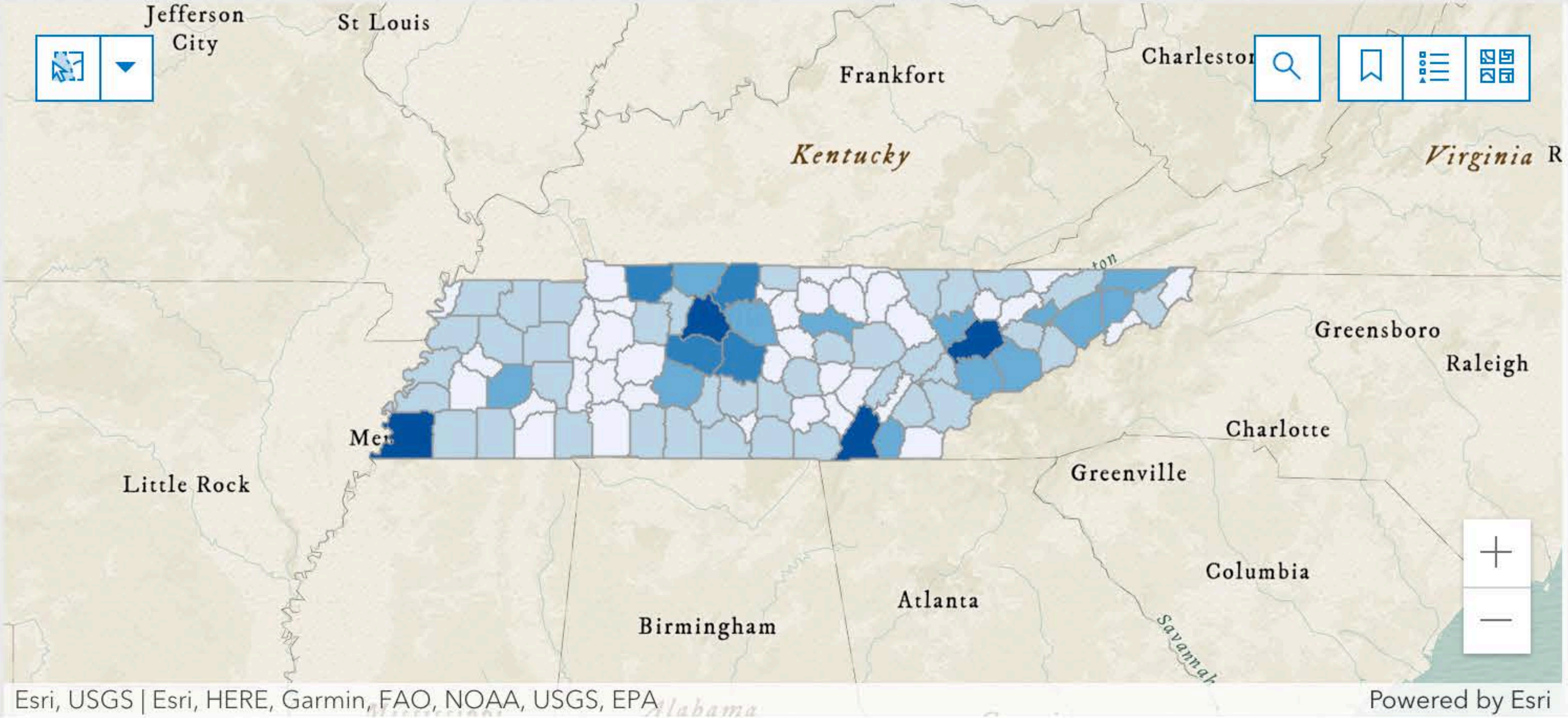

Affected Counties

Total Cases:
- > 65,000 - 150,000
- > 30,000 - 65,000
- > 12,000 - 30,000
- > 5,000 - 12,000
- 1 - 5,000

County Cases | Testing Locations

Statewide New Cases In Last 14 Days

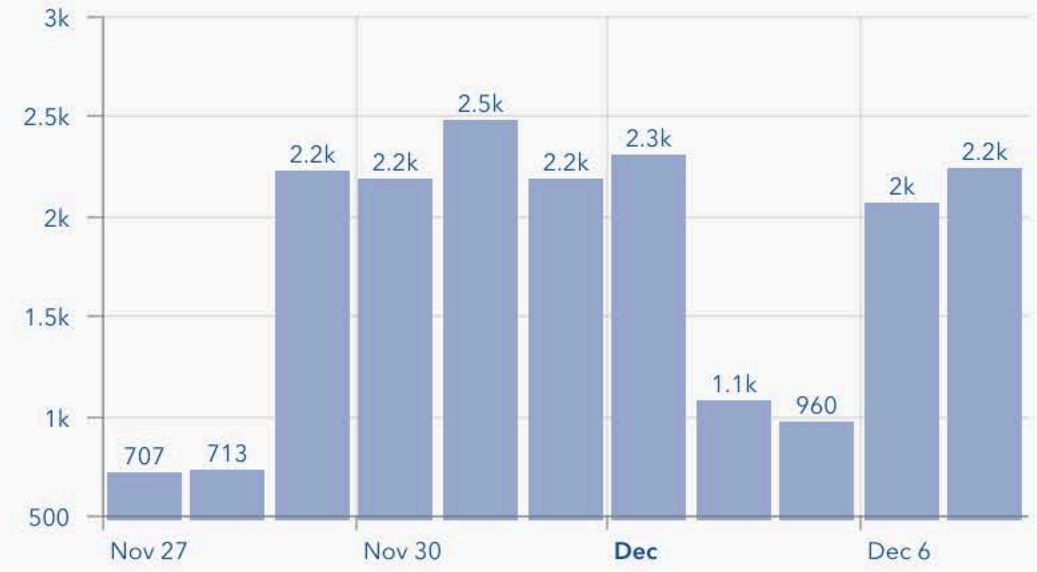

New Daily Cases By Age Range

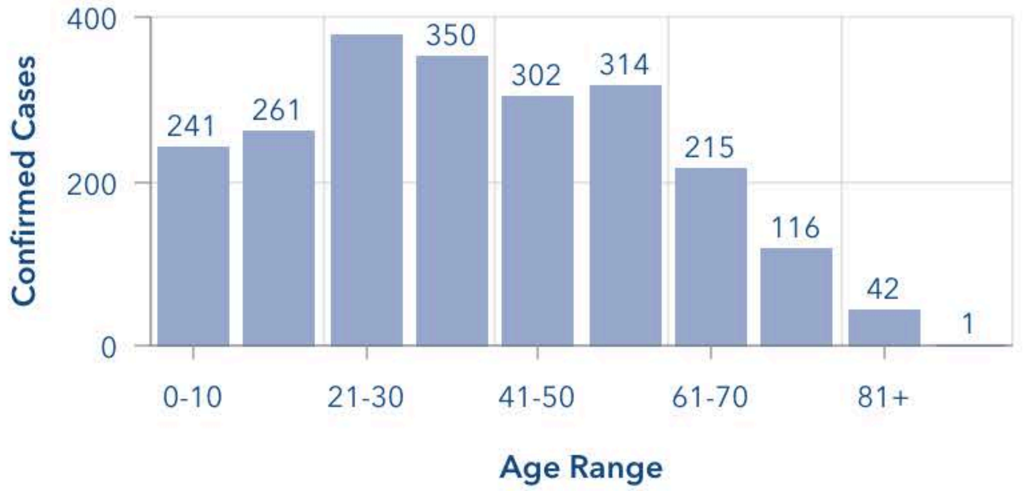

New Daily Cases by Age | Cumulative Cases By Age

Statewide: 12/7/2021

1,331,703 CASES

17,676 FATALITIES

38,925 HOSPITALIZATIONS

1,295,258 RECOVERED

Daily: 12/7/2021

2,219 NEW CASES

26 NEW FATALITIES

9 NEW HOSPITALIZATIONS

1,416 NEW RECOVERED

Tennessee COVID-19 - December 10, 2021
Epidemiology and Surveillance Data

TN Department of Health

PDF

Total Cases: **1,336,078** Confirmed Cases: **1,042,229** Probable Cases: **293,849** Total Inactive/Recovered: **1,297,313**

Total Deaths: **17,729** Confirmed Deaths: **13,984** Probable Deaths: **3,745**

Cases by County

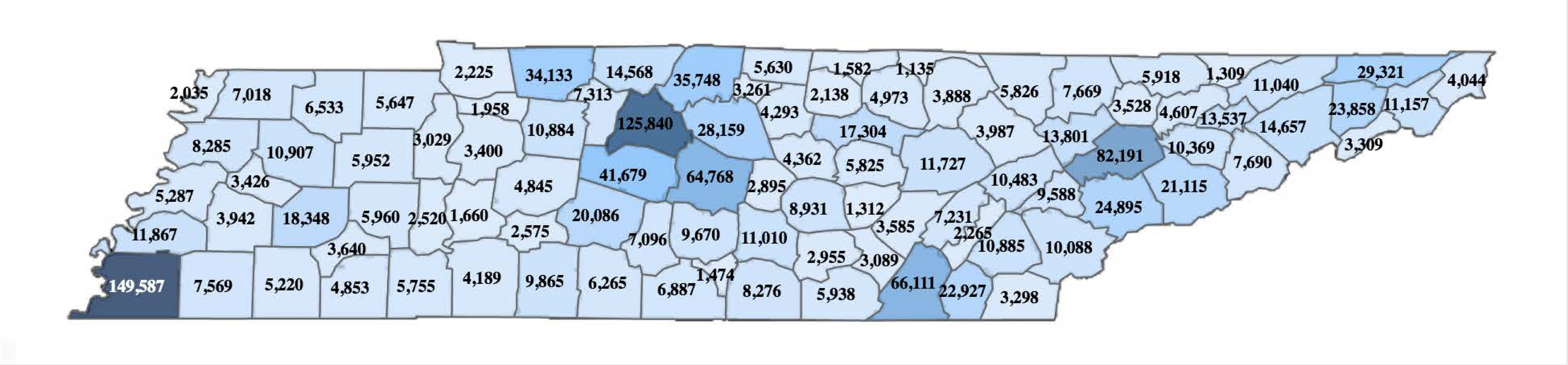

Daily Counts of Cases for Last 120 Days

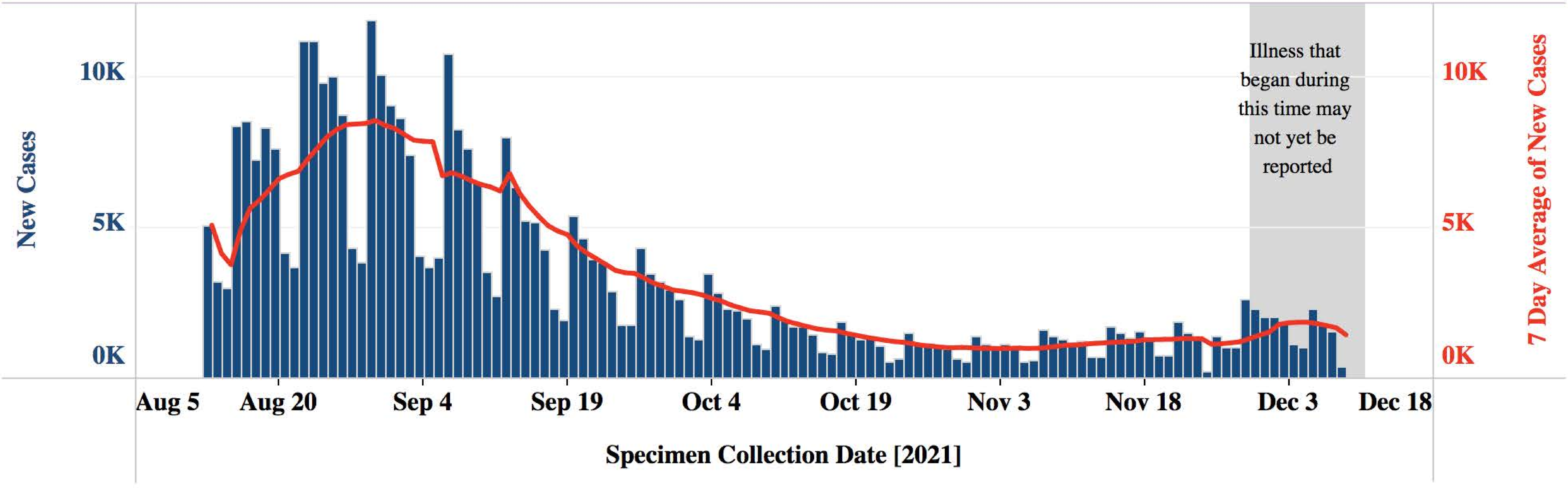

To see full epi curve visit: https://www.tn.gov/health/cedep/ncov/data/epi-curves.html

Demographics

Minimum Age	Average Age	Maximum Age
0	39	112

Age Range	Cases	% of Cases	Deaths	% of Deaths
0-10 years	106,629	8%	8	0%
11-20 years	196,019	15%	18	0%
21-30 years	231,831	17%	132	1%
31-40 years	208,615	16%	369	2%
41-50 years	190,589	14%	938	5%
51-60 years	174,250	13%	2,097	12%
61-70 years	122,875	9%	3,621	20%
71-80 years	70,242	5%	5,047	29%
[illegible]	[illegible]	[illegible]	[illegible]	[illegible]
Pending	1,422	0%	0	0%

TN Contact Us Program Areas News Health Professionals Parents Individuals Statistical Data Career Opportunities

Patient Sex	Cases	% of Cases	Deaths	% of Deaths
Female	706,798	53%	8,134	46%
Male	619,796	46%	9,586	54%
Pending	9,484	1%	9	0%

Patient Race	Cases	% of Cases	Deaths	% of Deaths
American Indian or Alaska Native	2,045	0%	16	0%
Asian	11,356	1%	95	1%
Black or African American	181,818	14%	3,158	18%
Native Hawaiian or Other Pacific Islander	1,180	0%	10	0%
White	853,765	64%	13,288	75%
Other/Multiracial	97,865	7%	595	3%
Pending	188,049	14%	567	3%

Patient Ethnicity	Cases	% of Cases	Deaths	% of Deaths
Not Hispanic or Latino	915,621	69%	15,833	89%
Hispanic or Latino	69,670	5%	445	3%
Pending	350,787	26%	1,451	8%

Outcomes

Ever Hospitalized	Cases	% of Cases
Yes	39,014	3%
No	483,780	36%
Pending	813,284	61%

Please note for hospitalizations: Information about hospitalization status is gathered at the time of diagnosis, therefore this information may be incomplete. This number indicates the number of patients that were ever hospitalized during their illness, it does not indicate the number of patients currently hospitalized.

Inactive/Recovered Cases	Cases	% of Cases
[illegible]	[illegible]	[illegible]

Please note for inactive/recovered: TDH defines "inactive/recovered" as people who are (1) at least 14 days beyond their symptom onset date, or are at least 14 days beyond the first test confirming their illness if asymptomatic, and (2) are not deceased.

Deceased	Cases	% of Cases
Yes	17,729	1%
No	1,318,349	99%

Please note for deaths: Some deaths may be reported by health care providers, hospitals, medical examiners, local health departments, or others before they are included in the statewide count.

Laboratory Tests

Test Type	Positive	Negative	Total
PCR	1,187,797	9,681,058	10,868,855
Antigen	328,108	3,645,067	3,973,175

*Please note, these data are meant to provide a **rough estimate** of testing volume. Due to different source data, numbers may differ slightly from day to day. Laboratory reports of positive cases are reported to metro and local health departments as soon as results are available. State numbers are updated weekdays before 5pm and there may be a lag in the reporting of cumulative numbers at the state level. Please note numbers within tables may not add up to total confirmed number of cases due to reporting delays and/or missing data. Percentages may not add up to 100.0% due to rounding.*

New Cases by Region | New Cases by County | New Cases by MMWR Week

TN Department of Health

New Cases by Public Health Report Date, Region

Friday, December 10, 2021

Select a Region

(All)

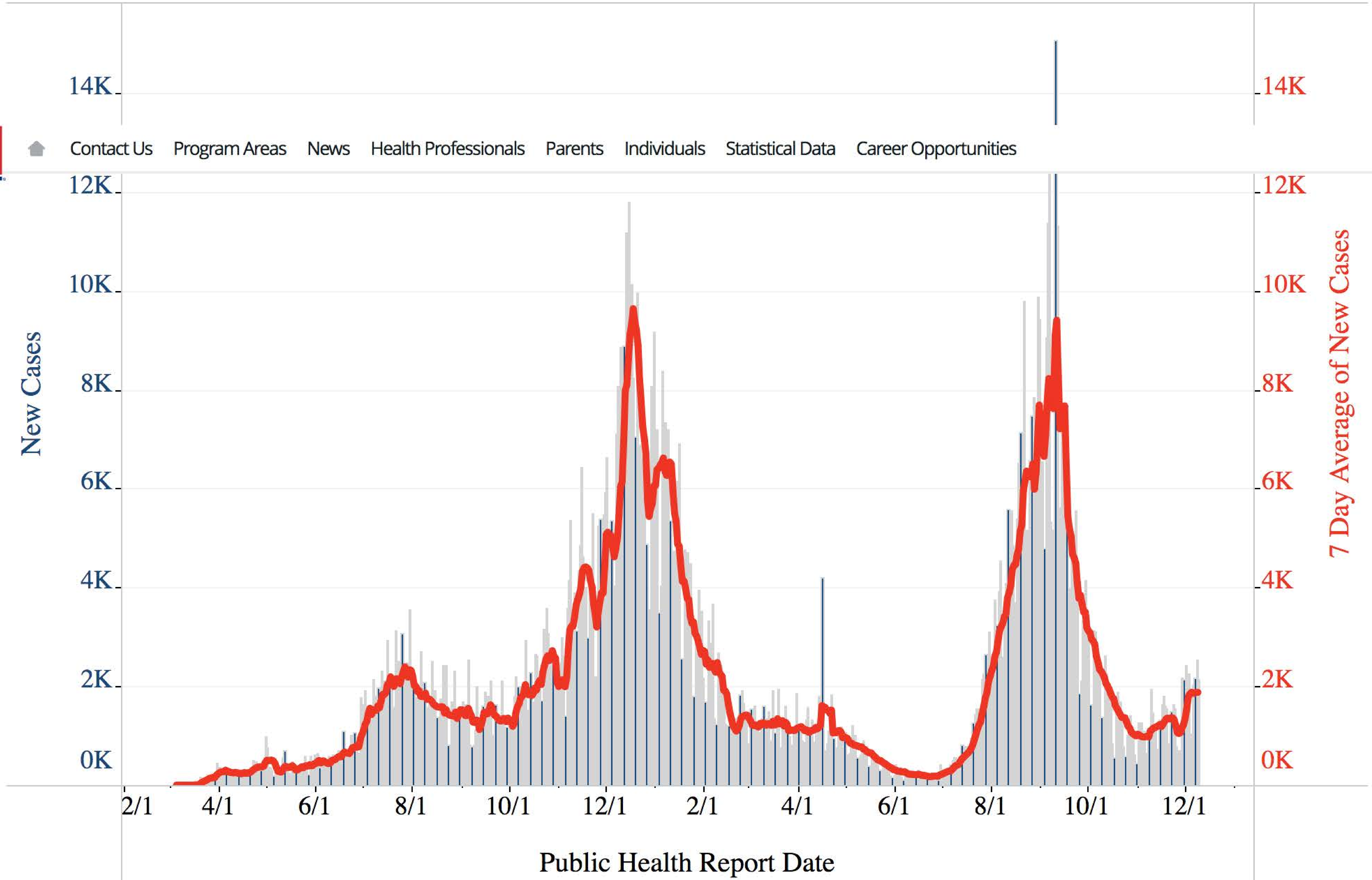

TN Contact Us Program Areas News Health Professionals Parents Individuals Statistical Data Career Opportunities

COVID

Tracking COVID: Interactive maps, charts show spread of virus across Houston, rest of Texas

Jordan Ray-Hart, Stephanie Lamm, Daniel Simmons-Ritchie, Kirkland An
Nov. 25, 2021

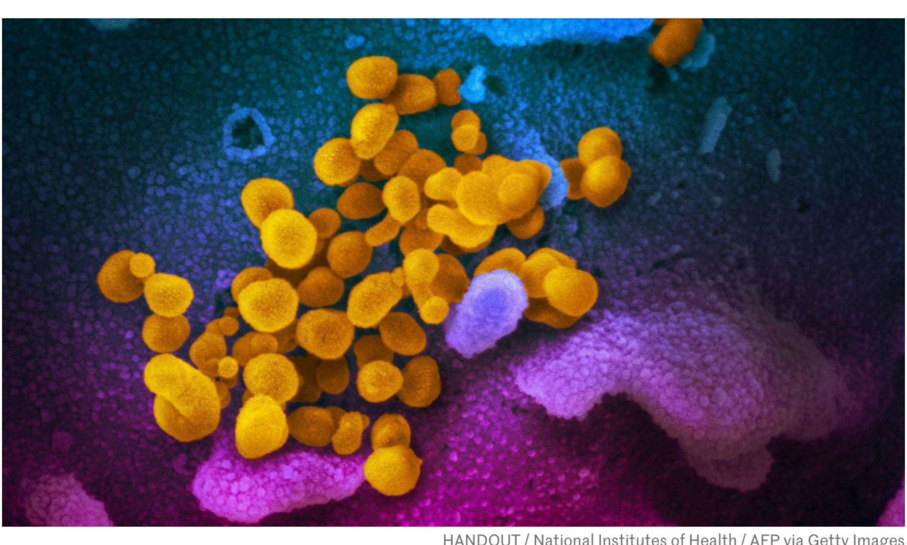

HANDOUT / National Institutes of Health / AFP via Getty Images

To keep you up-to-date with the ever-growing number of COVID-19 cases in Houston, Texas and the rest of the world, the Houston Chronicle has come up with a few eye-catching, but easy-to-use interactives, based on our reporting and official data from the Texas Department of State Health Services.

To start, let's take a look at the test positivity rates in Texas and, more specifically, Harris County.

Texas positive test rate

Percent of COVID-19 tests* that are positive for the virus

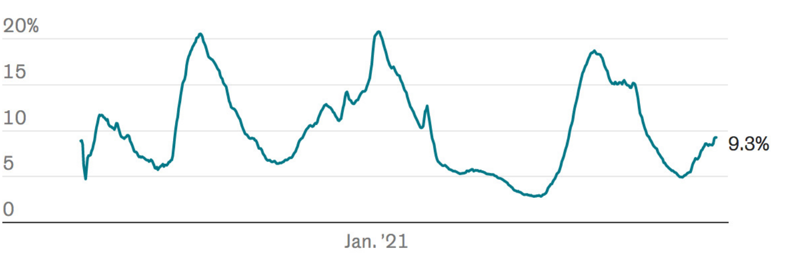

Chart: Texas Hearst Data Visualization Team • Source: Texas Department of State Health Services

*This chart uses molecular tests, which the state health department uses to count confirmed COVID-19 cases.

Harris County positive test rate

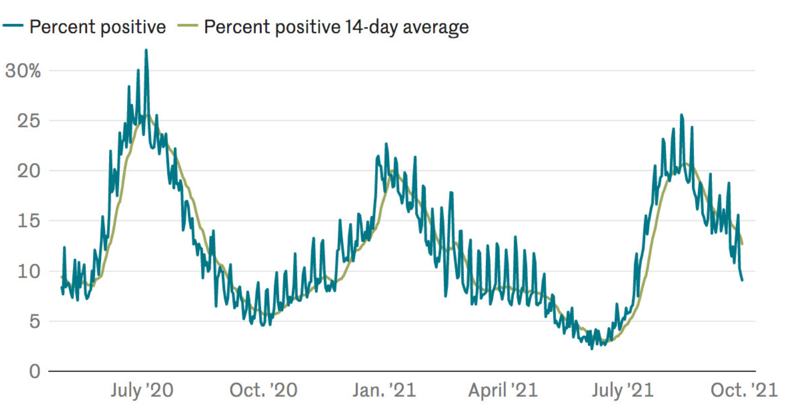

Chart: Texas Hearst Data Visualization Team • Source: Harris County

Next, you can check out our Texas Coronavirus Map to see the state's number of active cases per 1,000 residents.

Active COVID-19 cases per 1,000 residents

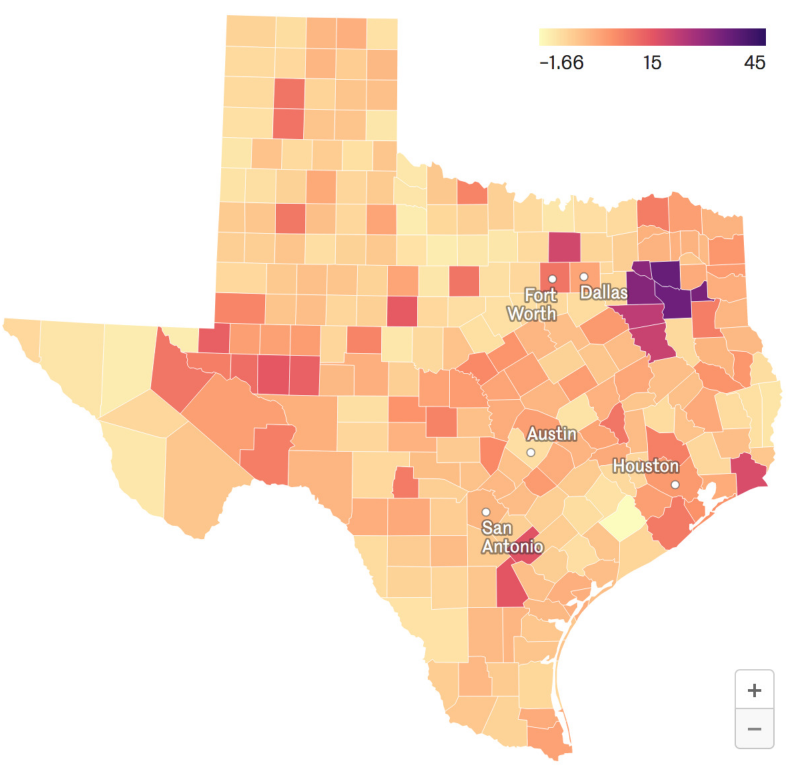

Map: Texas Hearst Data Visualization Team • Source: Texas Department of State Health Services

Active case totals only tell one part of the story though, so here's the seven-day rolling average of new cases around the Lone Star State.

Daily new cases in Texas

7-day average

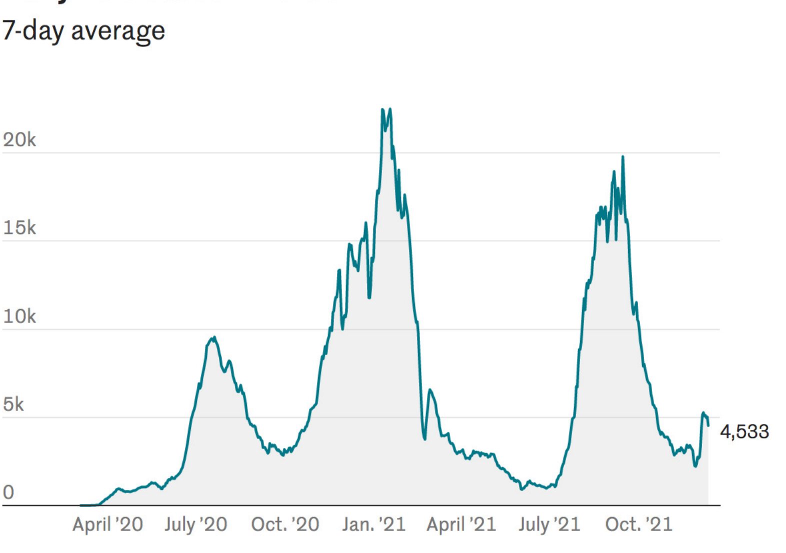

Chart: Texas Hearst Data Visualization Team • Source: Texas Department of State Health Services

Tens of thousands of Texans have died from COVID since the pandemic began. You can view the state death totals by date below.

Deaths due to the COVID-19 in Texas

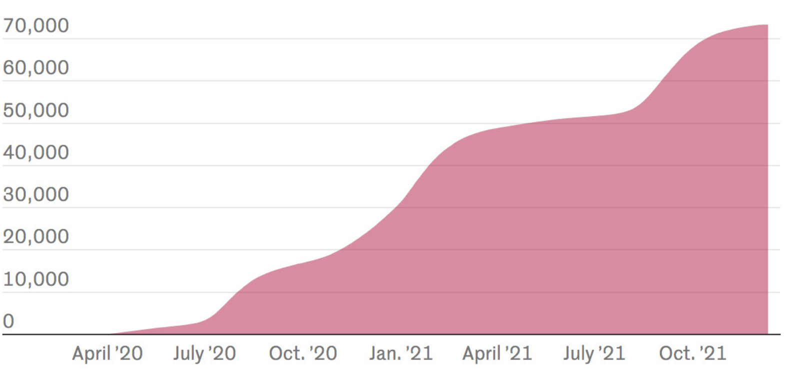

Chart: Texas Hearst Data Visualization Team • Source: Texas Department of State Health Services

The number of available hospital beds has loomed large during the recent delta variant surge, and the chart below shows the percentage of hospital beds occupied by COVID patients.

Percent of hospital beds occupied by COVID-19 patients

Hospital capacity fluctuates depending on staffing, beds available and other factors.

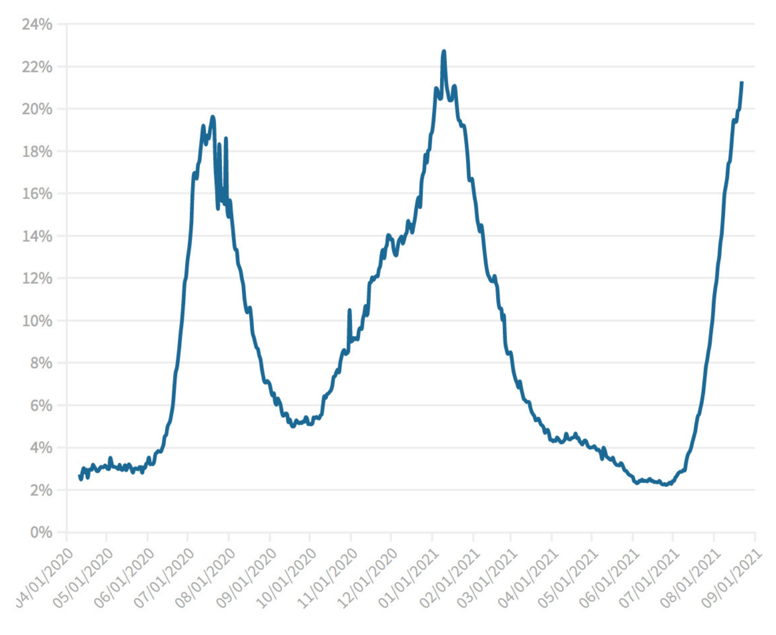

Source: Department of State Health Services • Created by Stephanie Lamm

Made with Flourish

Next up, we'll dive into vaccines and the ongoing fight to reach herd immunity. Here's a chart showing what percentage of eligible Texans have received both vaccine doses in each county.

Percent of eligible people fully vaccinated by county

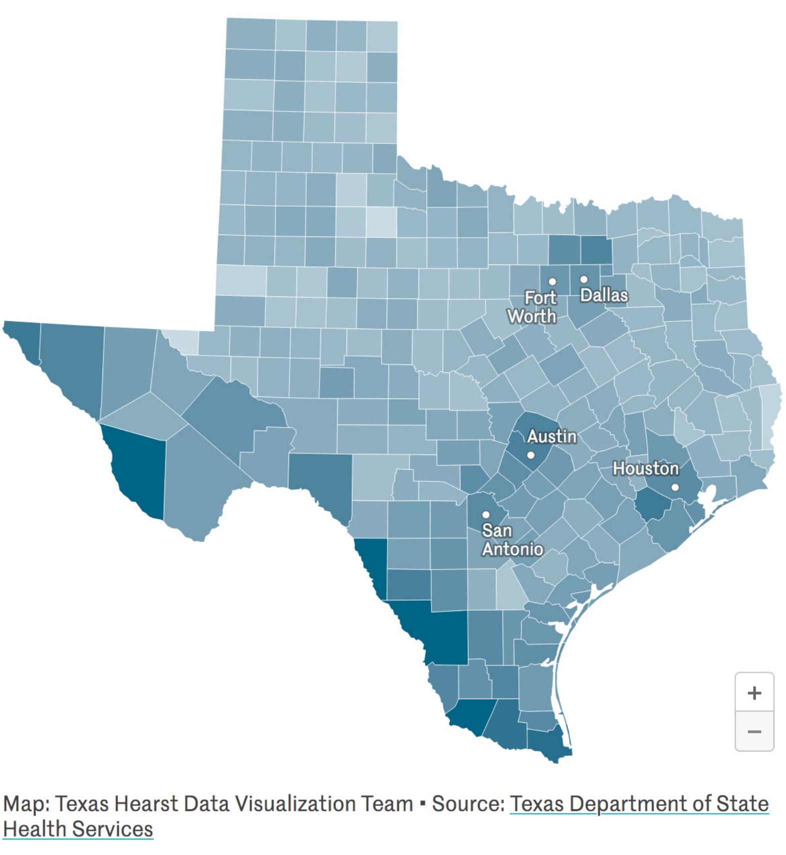

Map: Texas Hearst Data Visualization Team • Source: Texas Department of State Health Services

And now that school is back, here is a breakdown of reported COVID cases in Houston-area public schools.

COVID-19 cases in Houston-area school districts

September 6 - September 12, 2021

Search in table — Page 1 of 4

District Name	New Student Cases	New Staff Cases	Cumulative Student Cases	Cumulative Staff Cases	On Camp[illegible] Sourc[illegible]
Cypress-Fairbanks	699	129	1,676	453	58
Conroe	665	89	*	647	0
Katy	500	89	2,132	438	0
Klein	433	66	1,532	308	0
Humble	422	35	2,997	425	0
Fort Bend	377	53	2,562	309	0
Lamar	293	37	676	174	0
Alvin	272	29	786	119	23

HOUSTON CHRONICLE SUBSCRIBE Sign In

Table: Kirkland An • Source: Texas Department of State Health Services

*Data suppressed. See the Local Education Agency report for more on data suppression. Blank cells indicate that no data were reported.

As we create and tweak our interactives to best suit our audience, we'll be updating this list, so be sure to subscribe to HoustonChronicle.com for all the latest news and analysis. To stay on top of everything pandemic-related, you can also check out our COVID landing page.

Vaccination Status

COVID-19 vaccines have been proven to be safe and effective. However, no vaccines are 100% effective at preventing illness in vaccinated people. There will be a small percentage of fully vaccinated people who still get sick, are hospitalized, or die from COVID-19. These are called "breakthrough cases." Fortunately, there is evidence that vaccination may make breakthrough cases less severe and people who are fully vaccinated may be less likely to spread the disease to others. In the data below, breakthrough cases are defined as someone who has a positive test ≥14 days after they have completed the full series of an approved or authorized COVID-19 vaccine. The UDOH determines vaccine status for cases using two methods: by investigators linking known cases to vaccination records reported into the Utah Statewide Immunization Information System (USIIS), and through self-report by asking all cases if they have been fully vaccinated. Breakthrough cases may be under-represented if a case investigator cannot link a case to USIIS or reach a person for interview. The UDOH is working to update how cases are linked to USIIS records in the upcoming weeks, which may lead to additional identification of breakthrough cases.

Breakthrough cases: These counts and rates are the number of breakthrough infections that occurred in fully vaccinated people (at least 14 days after their final COVID-19 vaccine dose).

Unvaccinated cases: These counts and rates are the number of cases that occur in Utah residents who are not fully vaccinated. This includes cases in people who have only received one dose of the Moderna or Pfizer vaccine, or for whom 14 days have not passed between the last dose of their vaccine series and their positive test.

Since February 1, 2021, Unvaccinated Utahns had:

2.5x Greater Risk of Testing Positive for COVID-19

5.6x Greater Risk of Hospitalization

6.7x Greater Risk of Dying from COVID-19

Age Adjusted Unvaccinated and Vaccinated Incidence Rates in People Eligible for the Vaccine since February 1, 2021

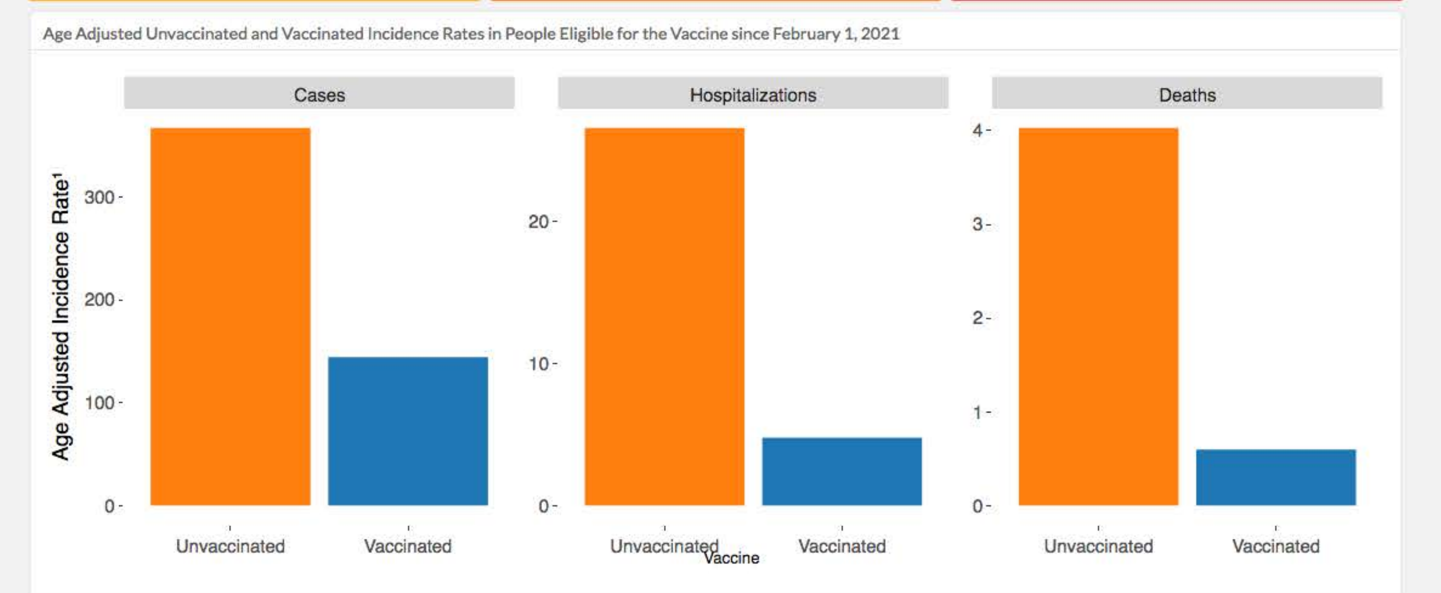

COVID-19 Case, Hospitalization, and Mortality Rates in Unvaccinated and Vaccinated Utahns by Age Group Since February 1, 2021

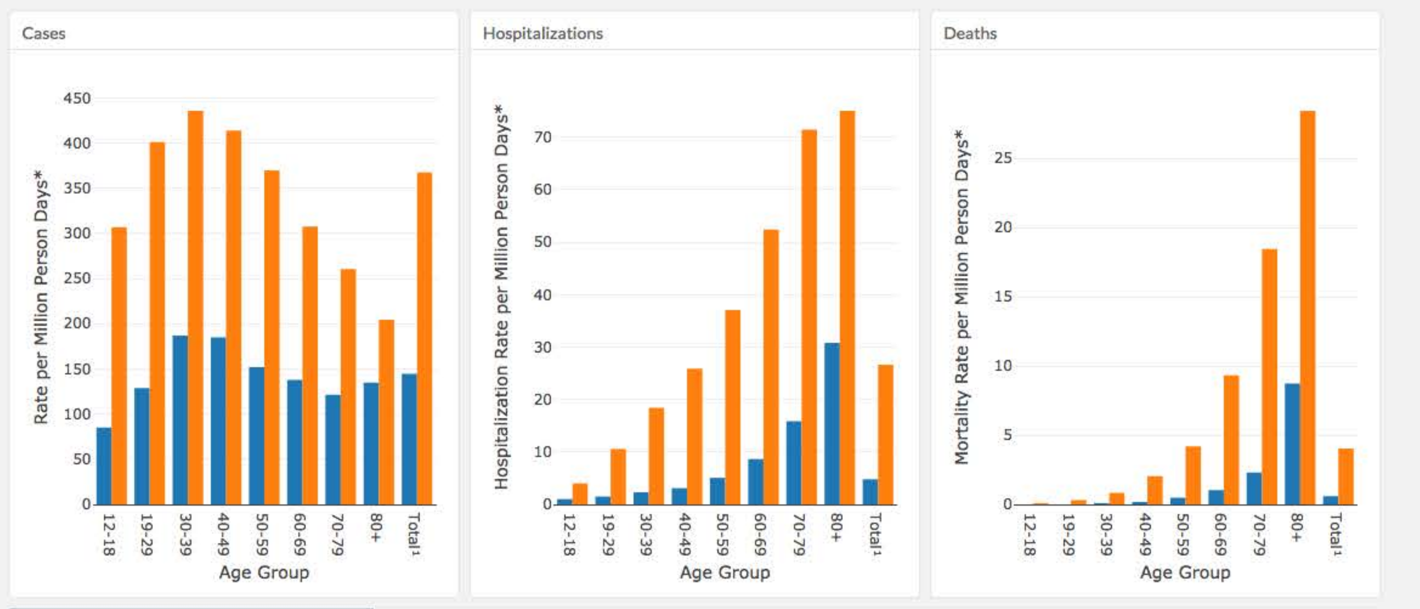

Vaccinated vs Unvaccinated COVID-19 Cases | Vaccinated vs Unvaccinated COVID-19 Hospitalizations | Vaccinated vs Unvaccinated COVID-19 Deaths

Age Group/ Total	Unvaccinated Cases	Breakthrough Cases	Vaccinated Rate*	Unvaccinated Rate*	Rate Ratio**	95% CI
12-18 Years	26945	2348	85.0	306.9	3.61	3.5-3.8
19-29 Years	46485	7927	128.7	401.1	3.12	3-3.2
30-39 Years	36978	10423	186.9	435.6	2.33	2.3-2.4
40-49 Years	28759	10247	184.9	413.9	2.24	2.2-2.3
50-59 Years	17070	7586	151.9	369.8	2.43	2.4-2.5
60-69 Years	10979	7008	137.7	307.6	2.23	2.2-2.3
70-79 Years	4348	4356	121.5	260.4	2.14	2.1-2.2
80+ Years	1740	2320	134.9	204.3	1.51	1.4-1.6
**Total (Age Adjusted)**	**173304**	**52215**	**144.5**	**367.3**	**2.54**	**2.5-2.6**

Seven-day Age Adjusted Case, Hospitalization, and Mortality Rates² in Unvaccinated and Vaccinated Utahns Aged 12+

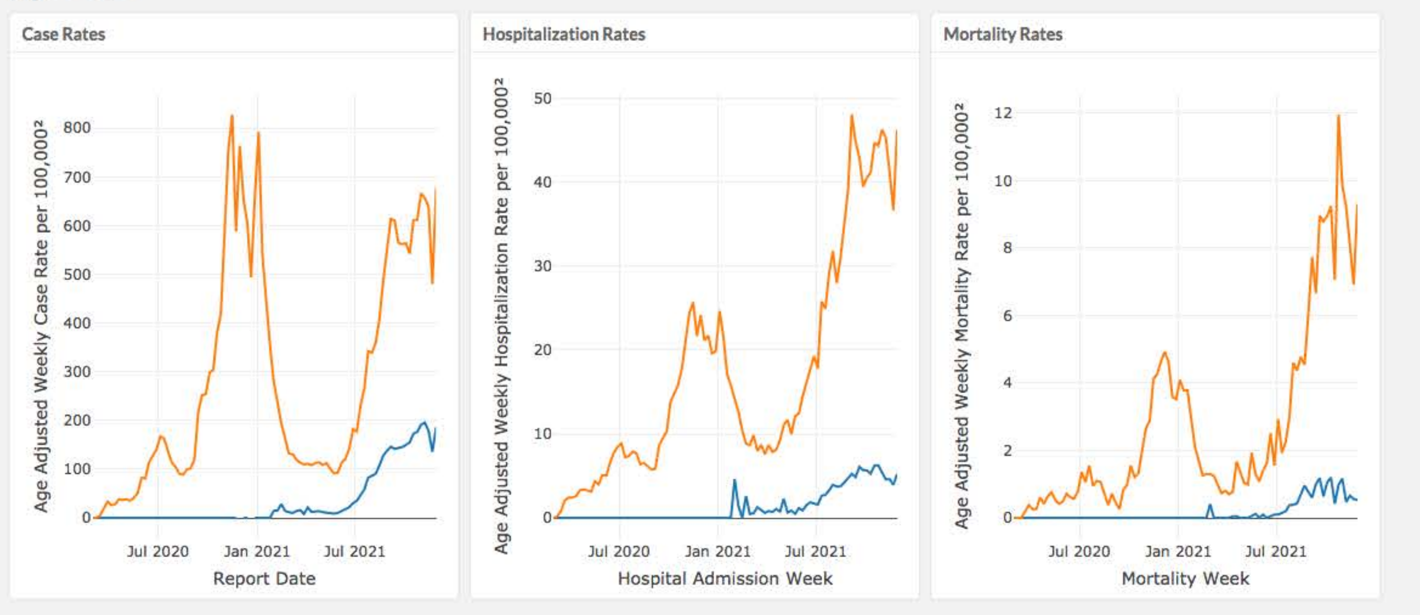

Vaccinated and Unvaccinated Rates in the Most Recent 28 Days

These rates show more up-to-date risk in vaccinated vs unvaccinated people given the recent trends in testing, transmission, and circulating lineages of COVID-19 (like Delta). **Please Note:** these data may change quickly as new cases are identified and should be considered preliminary.

28-Day Age Adjusted Unvaccinated and Vaccinated Incidence Rates in People Eligible for the Vaccine from 11/09 to 12/07/21

Total	Unvaccinated Count	Vaccinated Count	Unvaccinated Rate*¹	Vaccinated Rate*¹	Rate Ratio**	95% CI
Cases	21831	12577	874.4	243.1	3.6	3.5-3.7
Hospitalizations	1115	394	60.7	6.6	9.22	8.2-10.3
Deaths	183	47	11.7	0.8	15.34	11.1-21.1

Data Notes for Vaccinated and Unvaccinated Risk Analysis

These analyses exclude the last three days because people with COVID-19 in this time may not be interviewed yet and their vaccination status may not yet be known.

Person-day population estimates: To calculate the eligible Utah population 12 years and older who are fully vaccinated, the UDOH takes the cumulative total of people 14 days post-full vaccination reported into USIIS each day and sums all of the days. To calculate the eligible but unvaccinated population, the UDOH takes the cumulative total of people 14 days post-full vaccination minus the 2019 census population estimate for people 12 years and older each day and sums all of the days.

[1]Age adjustment for total rates: As of 10/5/2021, the UDOH age-adjusts total rates to better show the risk of people who are vaccinated and unvaccinated getting COVID-19, being hospitalized, or dying. Age adjusting is a way to control for the effects of age in two populations, in this case in vaccinated and unvaccinated Utahns. We age-adjust using the direct age standardization method with the Utah 2019 census population from IBIS as the standard population. To calculate age-adjusted rates, the UDOH calculates each age groups' individual case rate for vaccinated and unvaccinated people and then multiplies that rate by the proportion of that age group in the 2019 Utah population. The UDOH then sums each weighted rate to get the total age-adjusted rate for the state.

*Rates since February 1, 2021 are a cumulative incidence rate per million person-days and are calculated as the "sum of people observed with COVID-19 outcome each day" divided by the "sum of people vaccinated or unvaccinated each day" times 1,000,000.

**The rate ratio is the "incidence rate of unvaccinated people" divided by "incidence rate of vaccinated people." The rate ratio is interpreted as how many times more the risk is for unvaccinated people than vaccinated people. If the lower bound of the 95% confidence interval (CI) is greater than 1, then the vaccines have a statistically significant effect on risk. Groups that don't have overlapping 95% confidence intervals have statistically significant differences in risk, while groups whose confidence intervals do overlap are not statistically different.

[2] Seven-day age-adjusted rates are calculated using the number of vaccinated and unvaccinated cases per age group and dividing by the median vaccinated and unvaccinated population during that week. The total weekly rates are age-adjusted to the Utah 2019 census population estimates available on IBIS. Seven-day rates exclude the current calendar week as cases reported in this time may not yet be investigated. Hospitalizations and deaths will backfill as these events are reported to the UDOH.

There may be large daily changes in recent case rates and age-specific hospitalizations and deaths because of small case counts. These changes do not indicate differences in vaccine efficacy but instead reflect the UDOH identifying additional hospitalizations and deaths in this time window, which often lag behind case reports.

This analysis relies on surveillance data and does not control for individual differences in risk, location, or testing practices so these data should be considered in conjunction with clinical trials and other vaccine efficacy studies that show the vaccine is very effective at preventing symptomatic illness and severe disease. Breakthrough cases may be under-represented if a case investigator cannot link a case to a USIIS record or reach a person for interview.

Total Vaccine Breakthroughs

Total Vaccine Breakthroughs | Daily Case Counts by Vaccination Status

Type	Breakthrough Cases	People 14 Days Post Full Vaccination	Rate per 100k Vaccinated	% Fully Vaccinated to Get COVID-19
Cases	52,990	1,798,334	2946.6	2.94662%
Hospitalizations	2,232	1,798,334	124.1	0.12411%
Deaths	319	1,798,334	17.7	0.01774%

Pre-Existing Conditions

Data on pre-existing conditions are gathered from a variety of sources, including case interviews and medical records (when available). Case interviews are conducted by public health investigators across state and local health departments to determine what potential medical risk factors cases may have had prior to developing COVID-19. Data gathered through interviews is self-reported by cases and is dependent on their willingness to share this information with health departments. This is not a complete list of conditions and rates may change as we collect more data and performs additional analysis.

Data collection forms used by the Utah Department of Health and Utah's Local Health Departments have been updated to collect information on additional pre-existing conditions: autoimmune conditions, disabilities, hypertension, severe/morbid obesity, psychological/psychiatric conditions, and substance abuse. The previous neurologic pre-existing condition has been moved into the broader disability condition, which includes neurological, neurodevelopmental, intellectual, and physical disabilities. Additionally, investigators are now able to distinguish between type 1 and type 2 diabetes. All analyses of these new conditions are based on the subset of cases that use the new forms. These forms no longer collect information on specific conditions from cases without that condition or where the status is unknown; this information remains available for the overall 'Any Pre-Existing Condition' question.

Examples of persons with compromised immune systems include those with cancer and transplant patients who are taking certain immunosuppressive drugs, persons living with HIV/AIDS, and those with inherited diseases that affect the immune system. Chronic pulmonary conditions include uncontrolled asthma, emphysema, and COPD. Examples of persons with a disability include those with dementia, seizure disorders, cognitive impairment, and Alzheimer's disease. Psychological/psychiatric conditions include schizophrenia, major depressive disorder, and bipolar disorder.

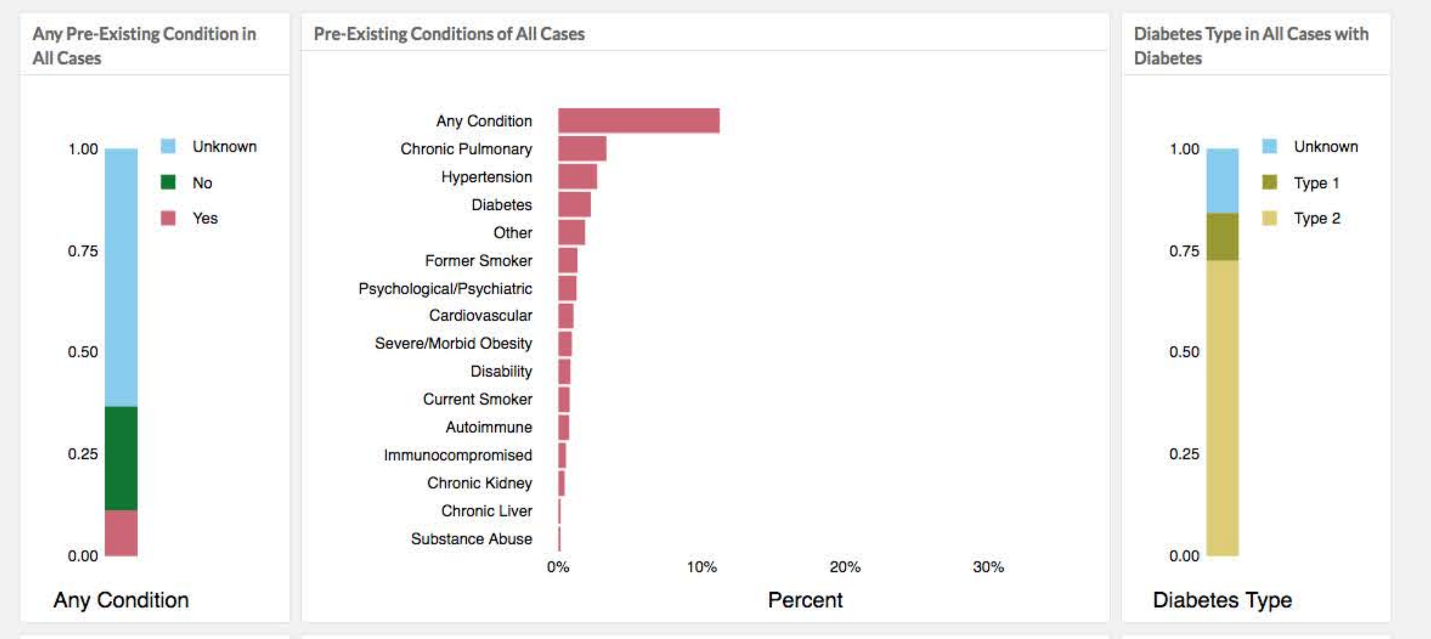

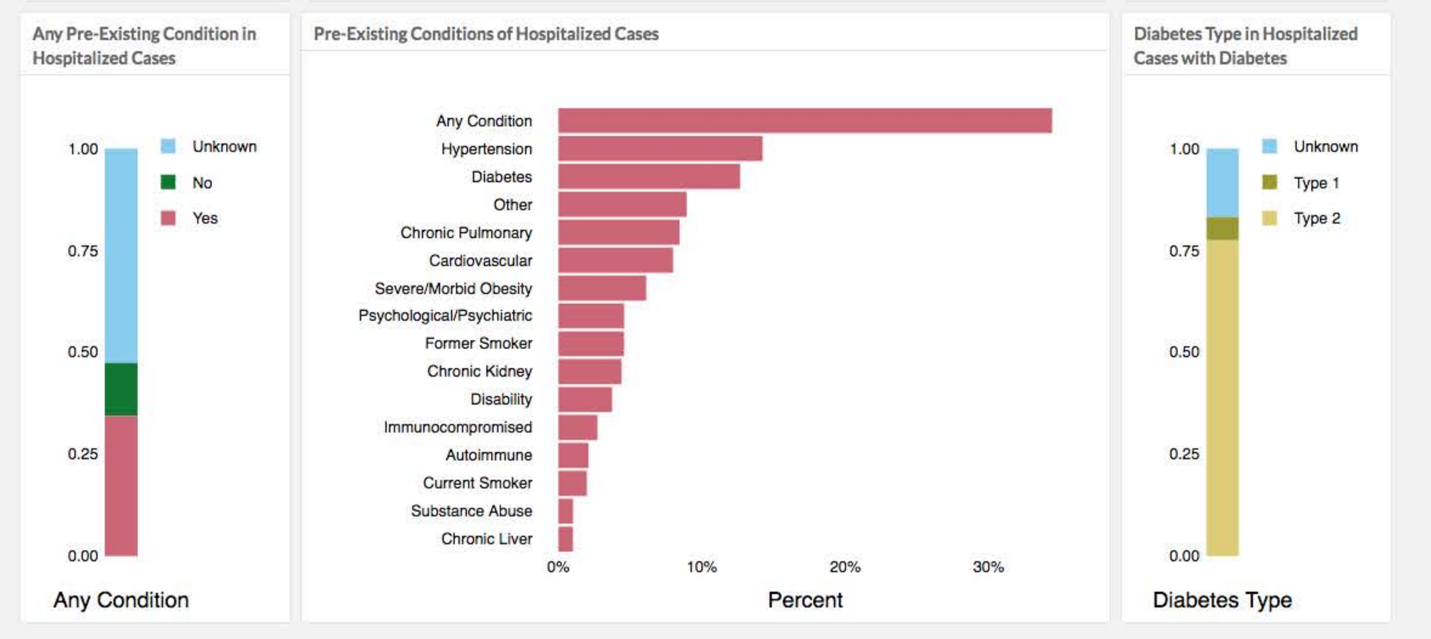

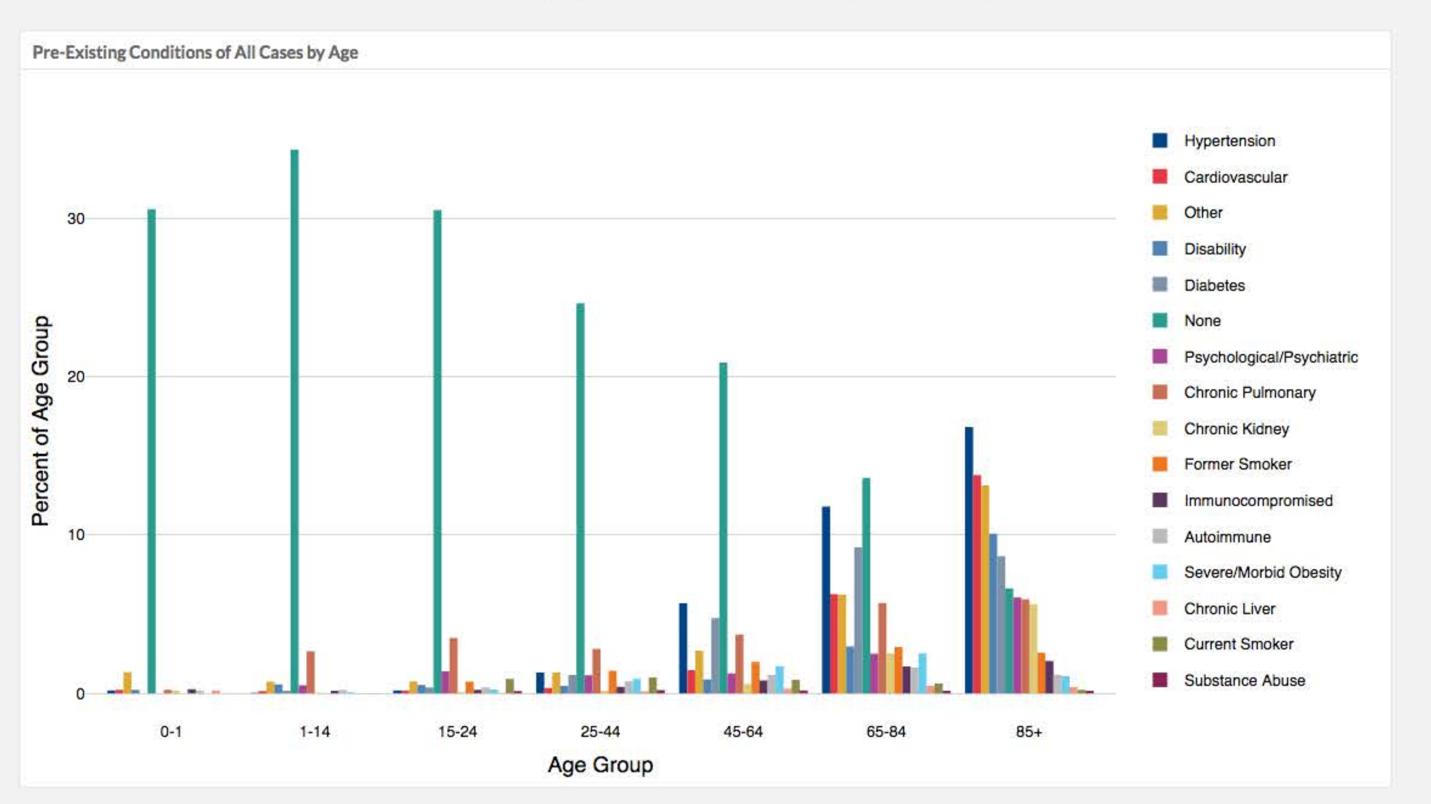

Potential Exposures

The charts below show if the public health investigator has determined a case to have potentially acquired COVID-19 through known contact to another confirmed case, or through "Community Spread", where a single source of infection cannot be identified. In this analysis, "Known Contact" represents any case with a known contact to a confirmed case in the 14 days before experiencing symptoms through contact tracing, self-report, or outbreak identification. "Community" is any case with a completed investigation, but no indication of a known contact. "Unknown/ Pending" are cases who either could not be contacted for an interview, would not provide contact information, are recently reported and not yet interviewed, or where incomplete information has been entered by the investigator. Data will be updated weekly on Wednesday for the past calendar week and may backfill significantly as investigations are completed and outbreaks are identified.

Potential exposures are identified through case investigations and are not mutually exclusive. "Household" exposures are defined as any case identified through contact tracing of household contacts, or who self-reports a confirmed case of COVID-19 in the household. "Social" exposures are defined as any case identified through contract tracing of social contacts (like church, group gathering, dating, friends, and coworkers outside of the worksite) or who self-report known social contact with a confirmed case. "Workplace" exposures are defined as any case who reports known contact to a confirmed case in the workplace, or is part of an identified worksite outbreak (two or more cases associated with a worksite within 14 days). Finally, Travel exposures are any case who reports known contact to a confirmed case while traveling outside of the state of Utah, or with known contact to a confirmed case through CDC contact tracing of flights. Often, cases have multiple exposures in the categories or, in some instances, no exposures in these categories.

Known Contact and Community Spread

Infection Source	Count	Percent
Community	106538	17.6
Known Contact	307475	50.9
Unknown/Pending	189795	31.4

Reported Exposures (categories are not mutually exclusive)

Potential Exposure	Count	Percent
Household	175390	29
Social	48443	8
Travel	124	0
Workplace	36405	6

Known Contact and Community Spread By Week - Counts | Known Contact and Community Spread By Week - Percent

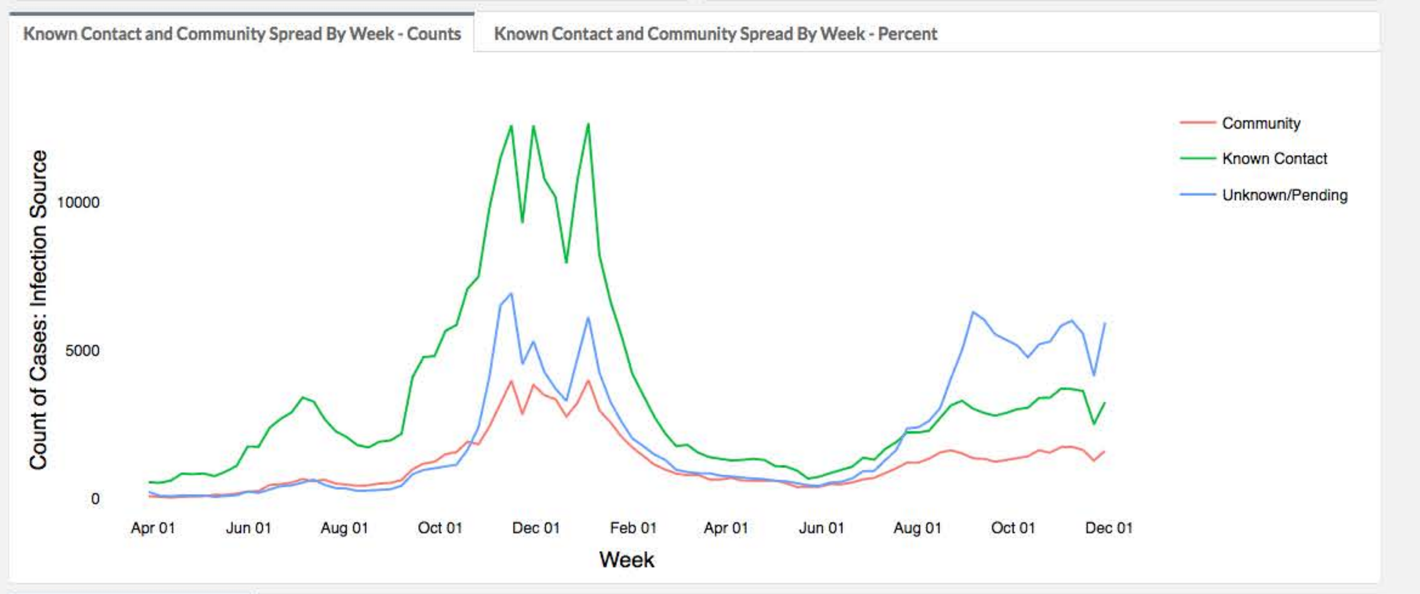

Potential Exposures - Counts | Potential Exposures - Percent

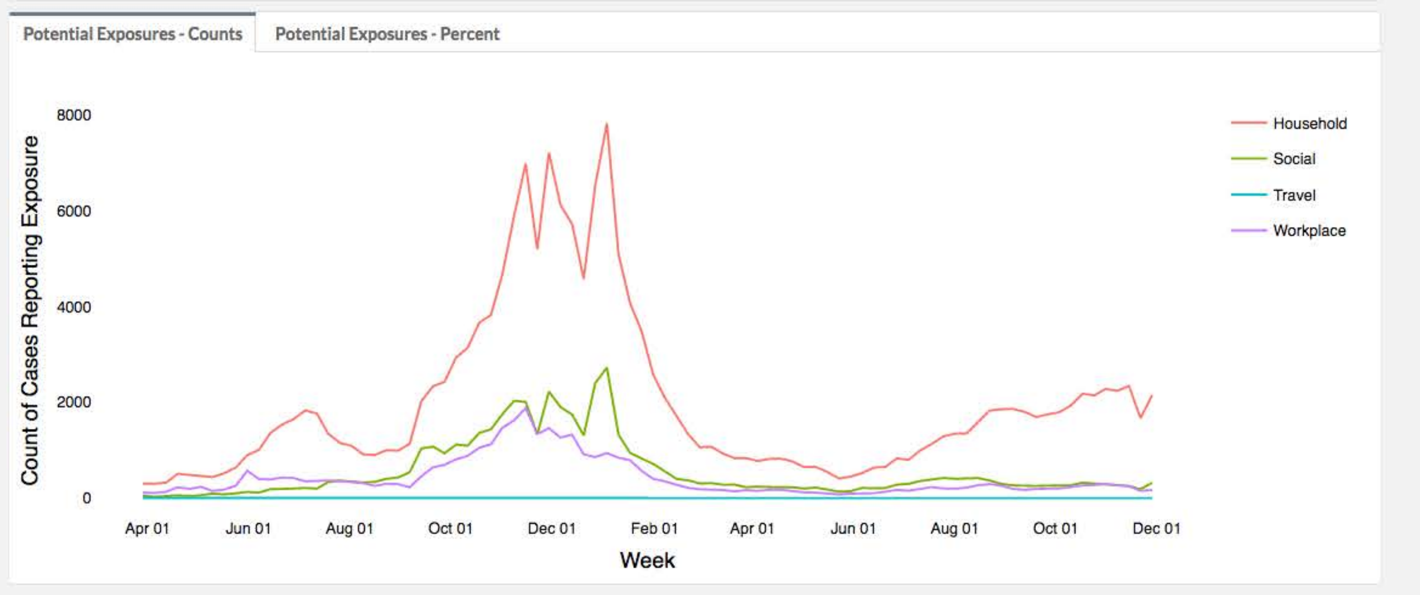

Mask Wearing Compliance

The Utah Department of Health collects information on mask compliance through the Behavioral Risk Factor Surveillance System (BRFSS) where Utah residents are asked to rank how often they wear a mask in public or when unable to socially distance. The response options are always, usually, sometimes, rarely, or never.

The BRFSS is a random-digit dial telephone survey of adults aged 18 and older. In 2020, about 25% of completed interviews came from landline phones and 75% from cellular phones in Utah. In order to have timely estimates, the data are downloaded weekly from the system and are not adjusted to reflect unequal sampling or population characteristics. However, sampling is done proportionally to population and the broad coverage of telephones makes this data sufficient for state and local estimates.

Note: The UDOH has transitioned all mask wearing surveillance data to the BRFSS methodology. The BRFSS is more robust and representative of Utah's population than the Physical Mask Wearing Survey data. Additionally, the data now show the three most recently completed months, plus the current month, rather than all available data. This is important for understanding current trends in mask wearing.

BRFSS: Mask Wearing Compliance – Past 3 Months

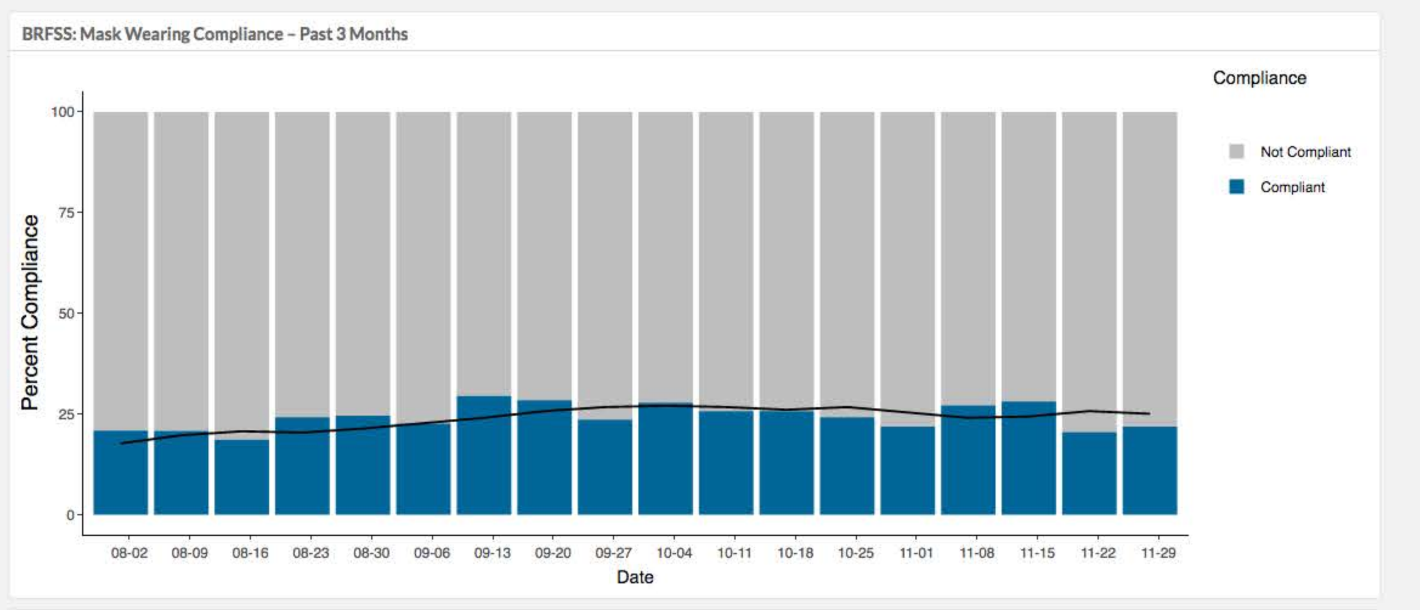

BRFSS Survey: Mask Wearing Compliance by Age and Sex – Past 3 Months

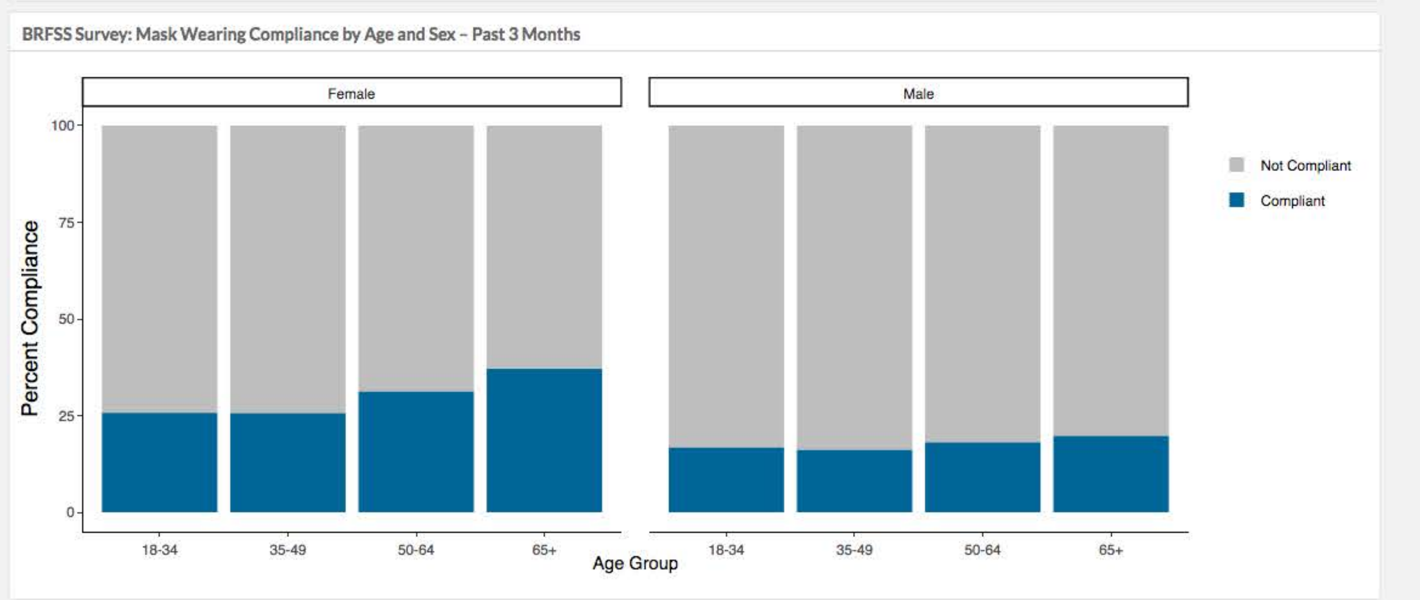

BRFSS Survey: Mask Wearing Compliance by County – Past 3 Months

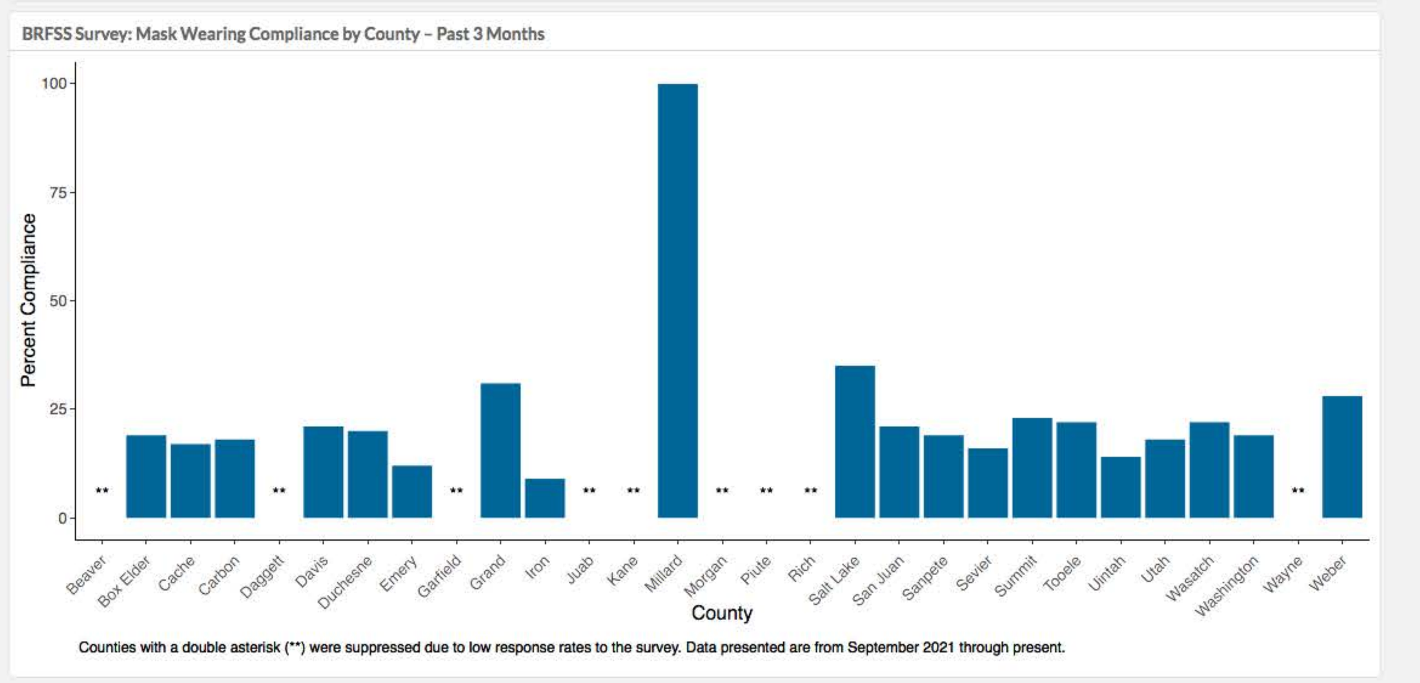

Healthcare Workers

These data reflect any individual who reports being an employee in the healthcare setting in any capacity such as cooks, environmental services, administrative roles, support staff, physicians, nurses, respiratory therapists, pharmacists, home health and personal care aids, etc. Healthcare workers also include those who commonly have patient interactions such as dentists, physical therapists, EMTS, firefighters, phlebotomists, and other technical occupations. These data are not intended to represent the amount of transmission occurring within the healthcare setting. Rather, the data can help us have a better understand the burdenour healthcare systems due to employees testing positive for COVID-19.

Total number of cases, hospitilizations, and deaths among healthcare workers

Jurisdiction	Cases	Hospitalizations	Deaths
State Total	23697	592	26

Number of Cases Among Healthcare Workers by Day

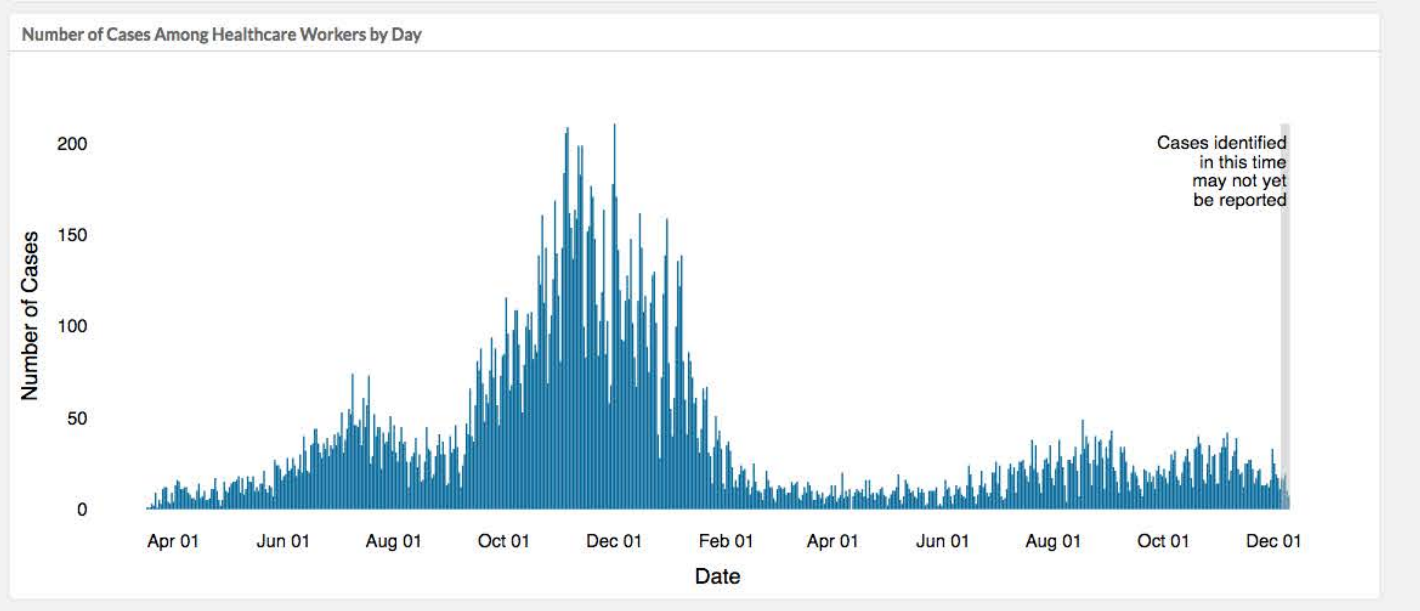

Data for this report were accessed on December 10, 2021 06:46 AM.

VIRGINIA'S HEALTH IS IN OUR HANDS. Do your part, stop the spread.

COVID-19 in Virginia: Cases

VDH VIRGIN[illegible] DEPART[illegible] OF HEA[illegible]

Dashboard Updated: 12/10/2021
Data entered by 5:00 PM the prior day.

Total Cases*		Total Hospitalizations**		Total Deaths	
994,069		40,346		14,895	
Confirmed†	Probable†	Confirmed†	Probable†	Confirmed†	Probable†
730,718	263,351	37,959	2,387	12,513	2,382

Note on Filters
Select measures to see the data change on one or more of the graphics on the dashboard. Refresh the page to reset the view.

Select Measure
(Affects Map and Bar Chart)
- (•) Cases
- ( ) Hospitalizations
- ( ) Deaths

Select Counts or Rates
(Affects Map)
- (•) Counts
- ( ) Rates per 100,000

Counts of Cases
- 108 - 284
- 285 - 747
- 748 - 1965
- 1966 - 5171
- 5172 - 13604
- 13605 - 35787
- 35788+

Rates that are based on counts l[illegible] than 15 should be interpreted w[illegible] caution

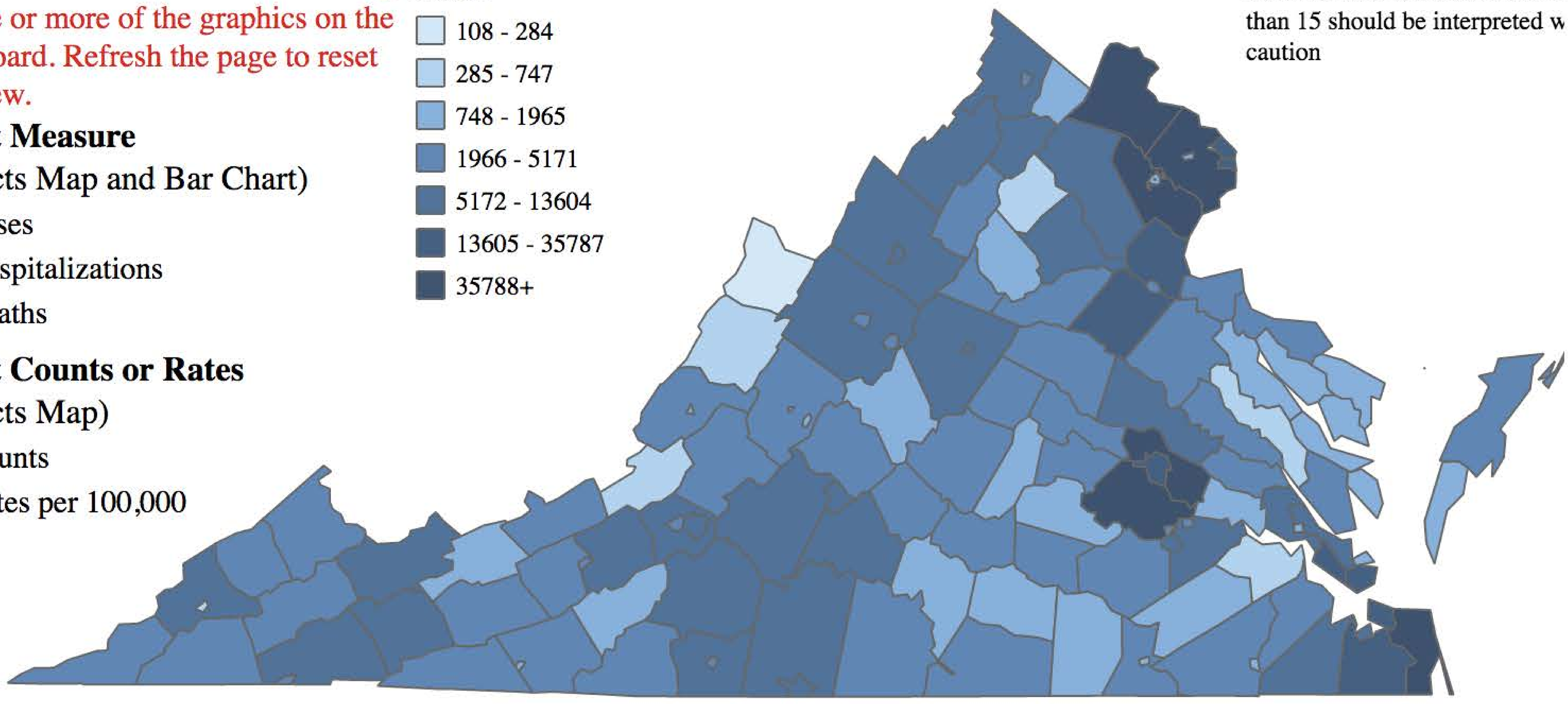

Number of Cases by Date of Symptom Onset

Number of cases by the day closest to when symptoms began.

VDH Locality N...
(Affects Bar Chart)
(All)

Daily counts by Report Date can be found on the Locality dashboard

Select Date Range
(Affects Bar Chart)
Past 13 Weeks

Confirmed Cas[illegible]
Probable Cases

Gray shaded area indicates illness may not have been reporte[illegible]

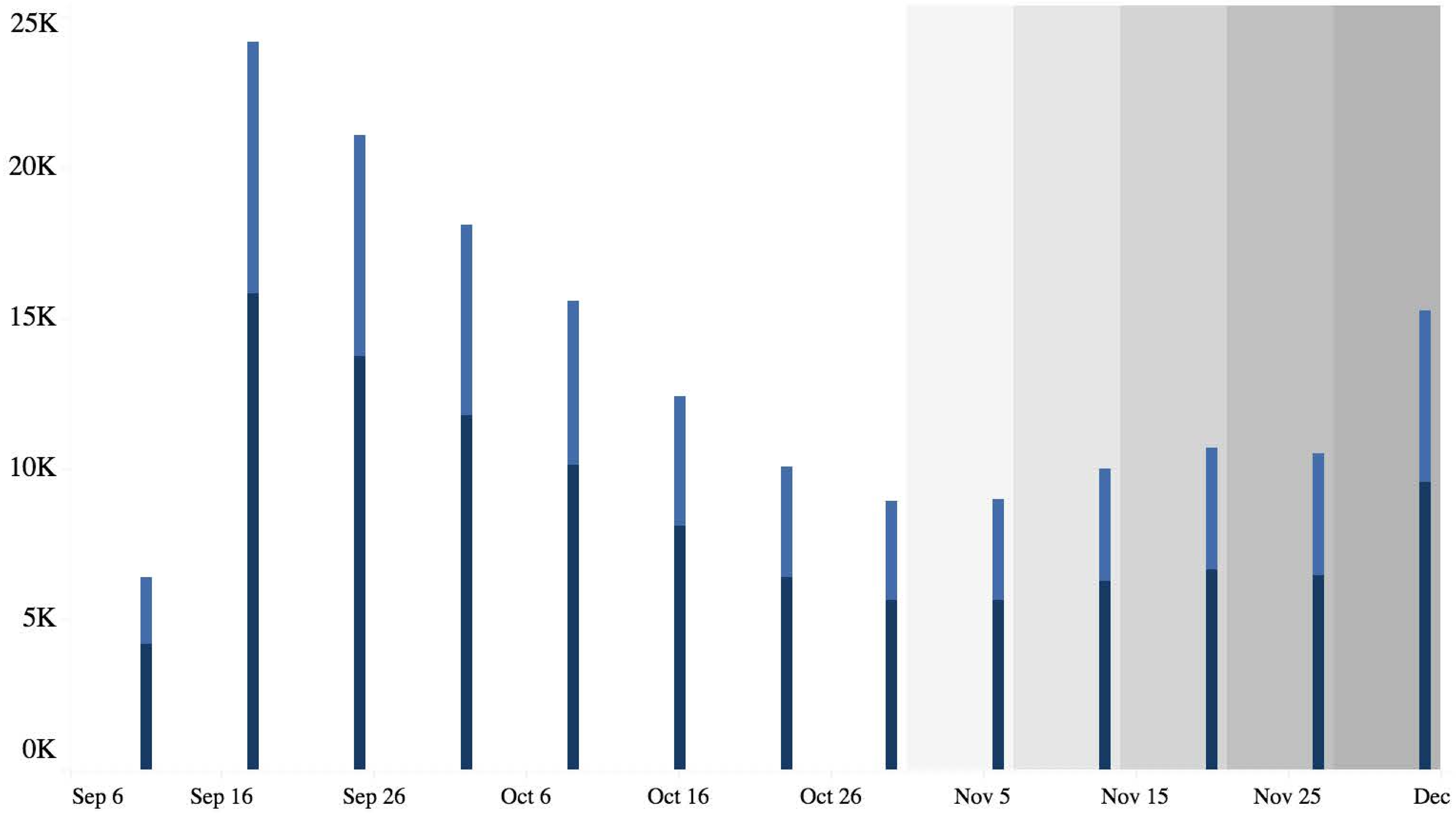

* Includes people with either a positive molecular/PCR test (Confirmed), positive antigen test (Probable) or symptomatic with known exposure to COVID-19 (Probable).
** Hospitalization of a case is captured at the time VDH performs case investigation. This underrepresents the total number of hospitalizations in Vi[illegible]
† VDH adopted the updated CDC COVID-19 2021 Surveillance Case Definition on September 1, 2021 which is found here: --https://ndc.services.c[illegible] /case-definitions/coronavirus-disease-2019-2021/
Source: Cases - Virginia Electronic Disease Surveillance System (VEDSS), data entered by 5:00 PM the prior day.

VIRGINIA'S HEALTH IS IN OUR HANDS. Do your part, stop the spread.

VDH

COVID-19 in Virginia: Summary

Dashboard Updated: 12/10/2021
Data entered by 5:00 PM the prior day.

Cases, Hospitalizations and Deaths

Total Cases*		Total Hospitalizations**		Total Deaths	
994,069		40,346		14,895	
(New Cases: 2,848)^					
Confirmed†	Probable†	Confirmed†	Probable†	Confirmed†	Probable†
730,718	263,351	37,959	2,387	12,513	2,382

* Includes people with either a positive molecular/PCR test (Confirmed), positive antigen test (Probable) or symptomatic with known exposure to COVID-19 (Probable).
** Hospitalization of a case is captured at the time VDH performs case investigation. This underrepresents the total number of hospitalizations in Virginia.
^New cases represent the number of confirmed and probable cases reported to VDH in the past 24 hours.
† VDH adopted the updated CDC COVID-19 2021 Surveillance Case Definition on September 1, 2021 which is found here: --https://ndc.services.cdc.gov/case-definitions/coronavirus-disease-2019-2021/
Source: Cases - Virginia Electronic Disease Surveillance System (VEDSS), data entered by 5:00 PM the prior day.

Outbreaks

Total Outbreaks*	Outbreak Associated Cases
5,734	94,458

* At least two (2) lab confirmed cases are required to classify an outbreak.

Testing (PCR Only)

Testing Encounters PCR Only*	Current 7-Day Positivity Rate PCR Only**
10,698,256	8.1%

* PCR" refers to "Reverse transcriptase polymerase chain reaction laboratory testing."
** Lab reports may not have been received yet. Percent positivity is not calculated for days with incomplete data.

Multisystem Inflammatory Syndrome in Children

Total Cases*	Total Deaths
123	1

*Cases defined by CDC HAN case definition: https://emergency.cdc.gov/han/2020/han00432.asp

Undo Redo Revert Refresh Pause Download

COVID-19 in Virginia: Vaccine Demographics

Dashboard Updated: 12/11/2021

People Vaccinated with at Least One Dose	People Fully Vaccinated	People Vaccinated with Booster/ Third Dose
**6,432,287**	**5,652,422**	**1,552,182**

Federal doses administered are included in the number of People Vaccinated with at Least One Dose (342,061), People Fully Vaccinated (307,121) , 5+, 12+, 18+ KPI but are not included in the other demographic groups below.

Locality Name (Includes Out-of-State and Not Reported Optio… (All)

Vaccination Status At Least One Dose

Age Group Type Vaccine

Vaccination Count By Age Group

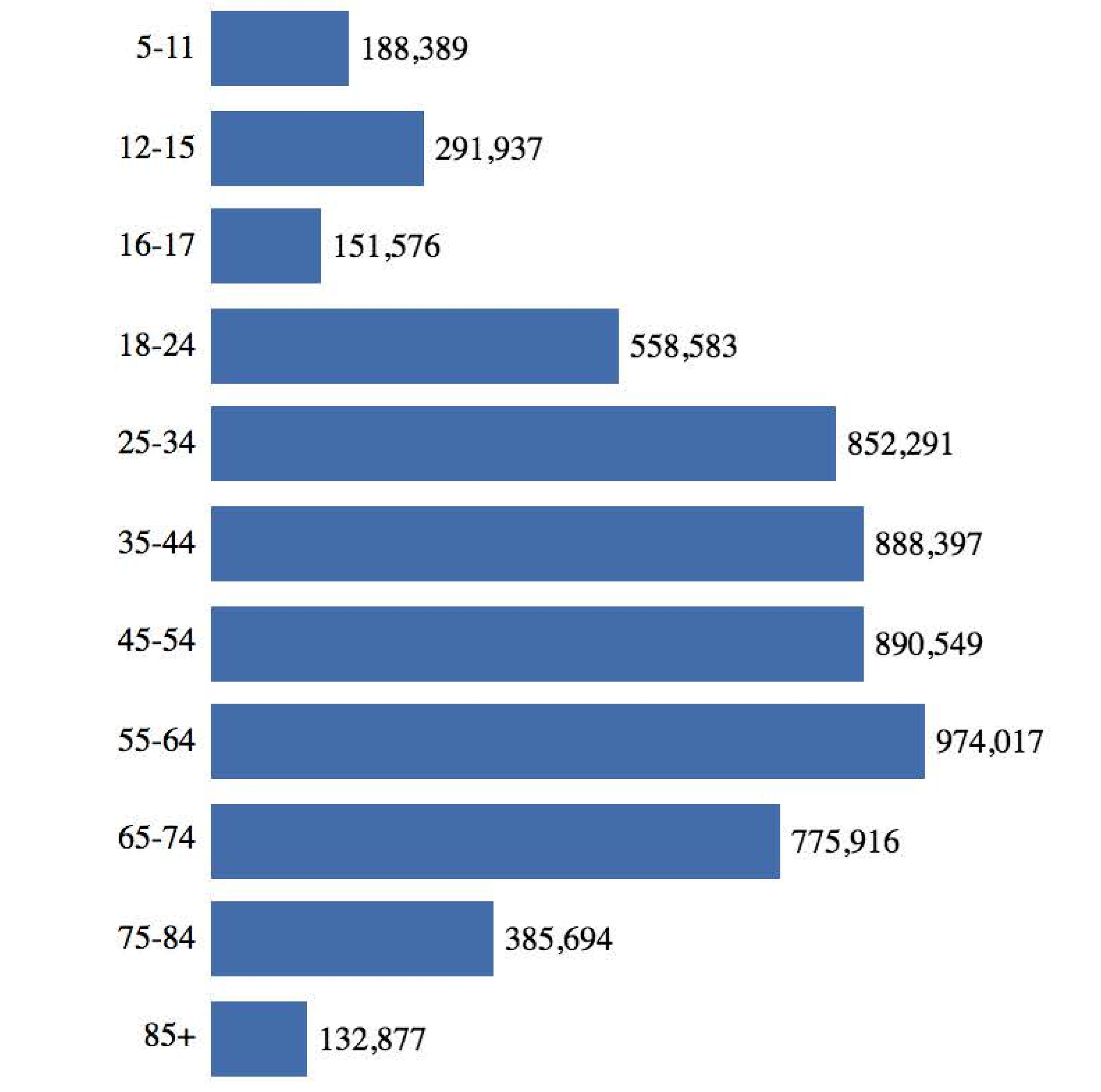

Percent of the Population Vaccinated with At Least One Dose - By Age Group

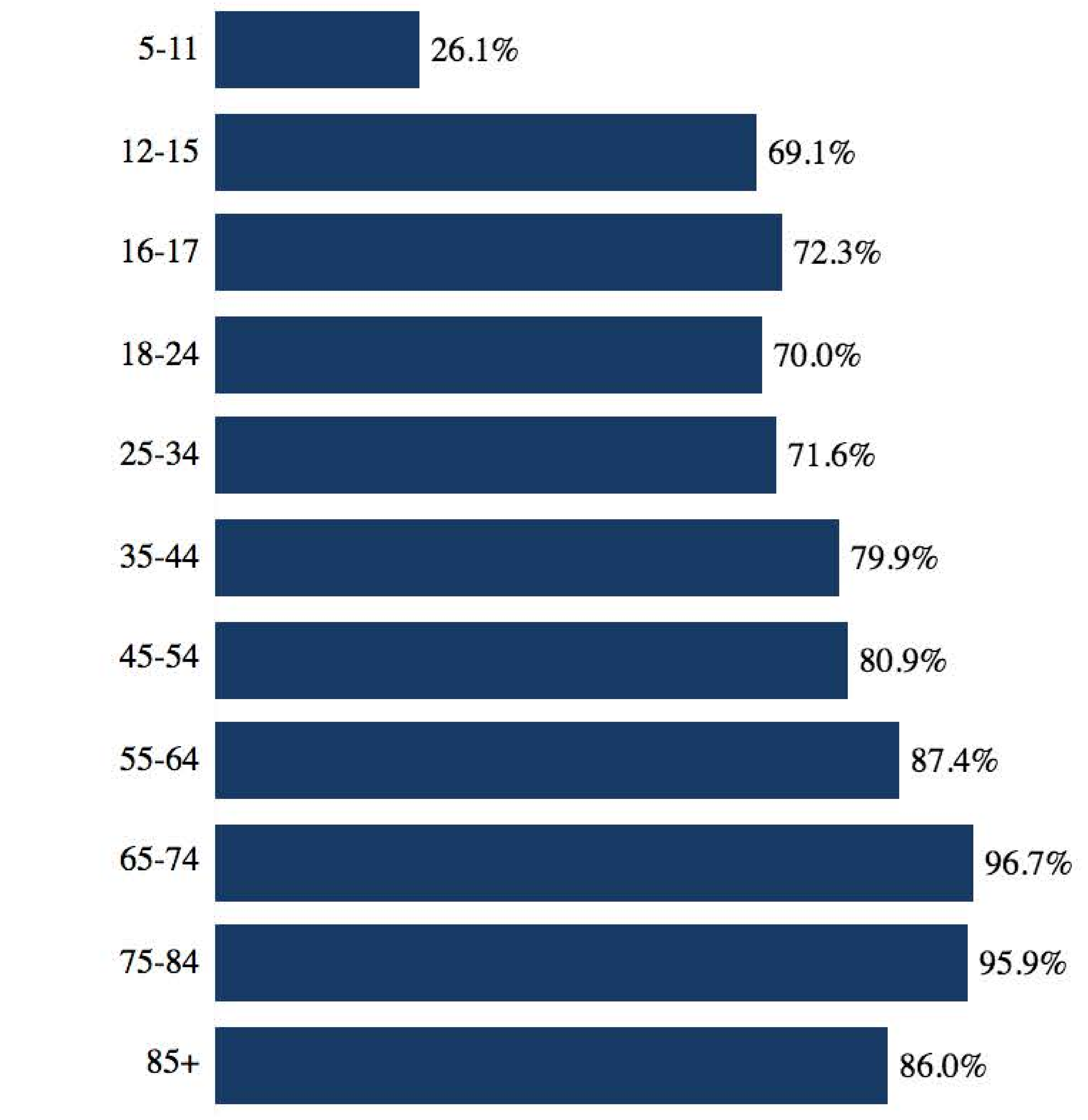

Not Reported: 342,061

Ages : 5-17	Ages : 5+	Ages : 12+	Ages : 18+	Ages : 65+
People Vaccinated: 631,902	People Vaccinated: 6,432,286	People Vaccinated: 6,243,897	People Vaccinated: 5,800,384	People Vaccinated: 1,294,486
% Vaccinated: 46.6%	% Vaccinated: 80.1%	% Vaccinated: 85.5%	% Vaccinated: 86.9%	% Vaccinated: 95.3%

Population Type Eligible Population

Vaccination Count By Race and Ethnicity

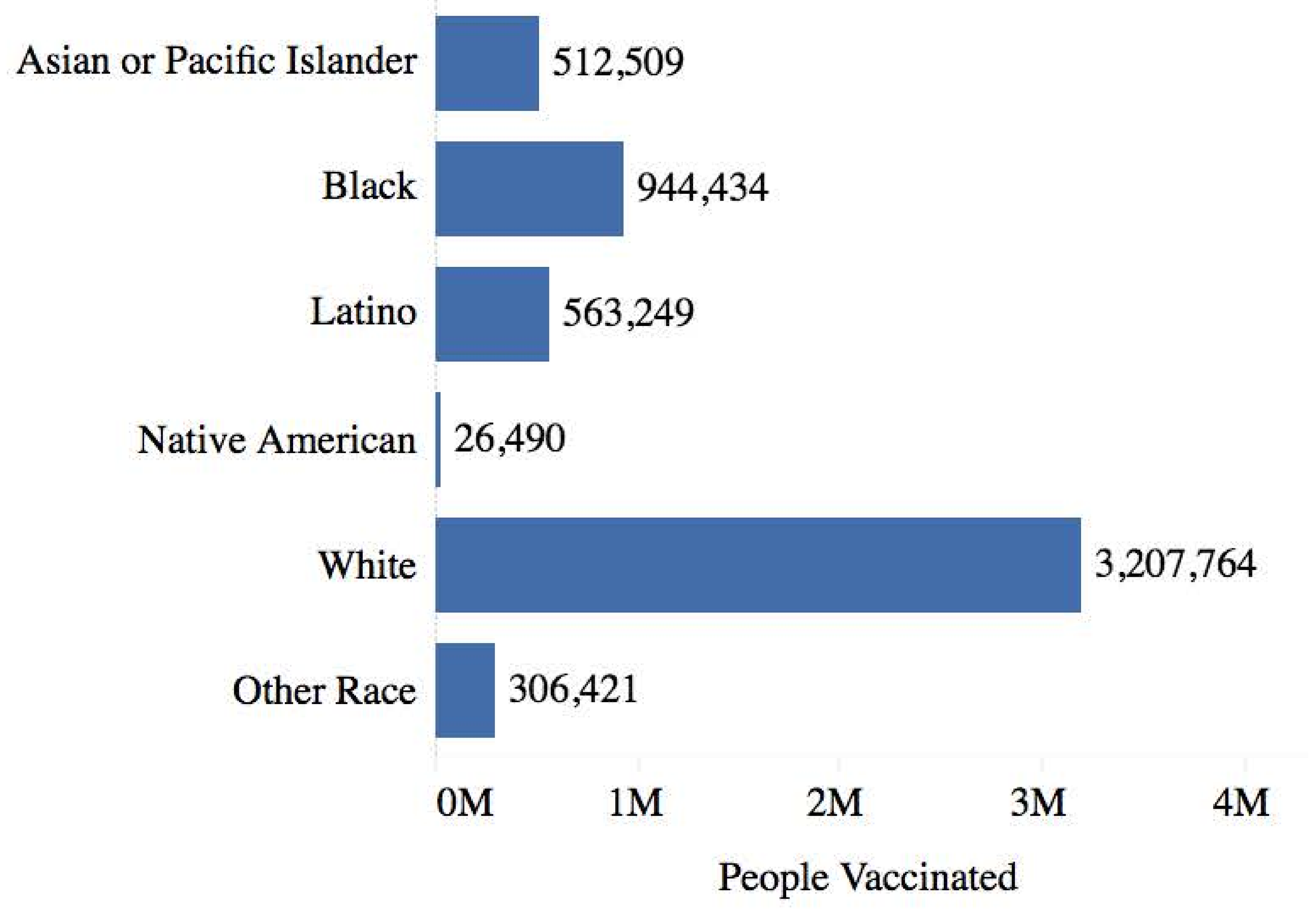

Percent of the Eligible Population Vaccinated with At Least One Dose - By Race and Ethnicity*

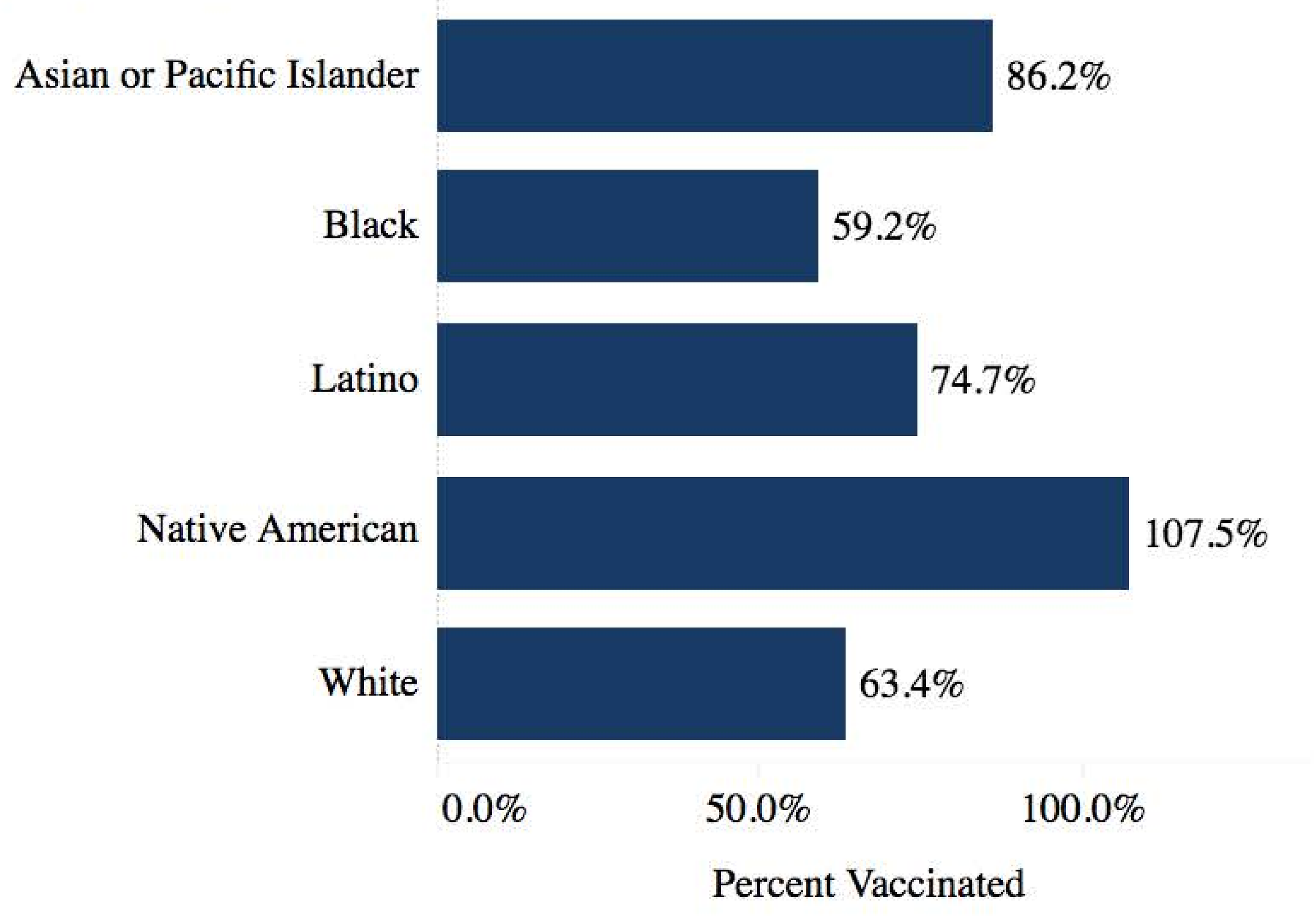

Not Reported: 871,420

Vaccination Count By Sex

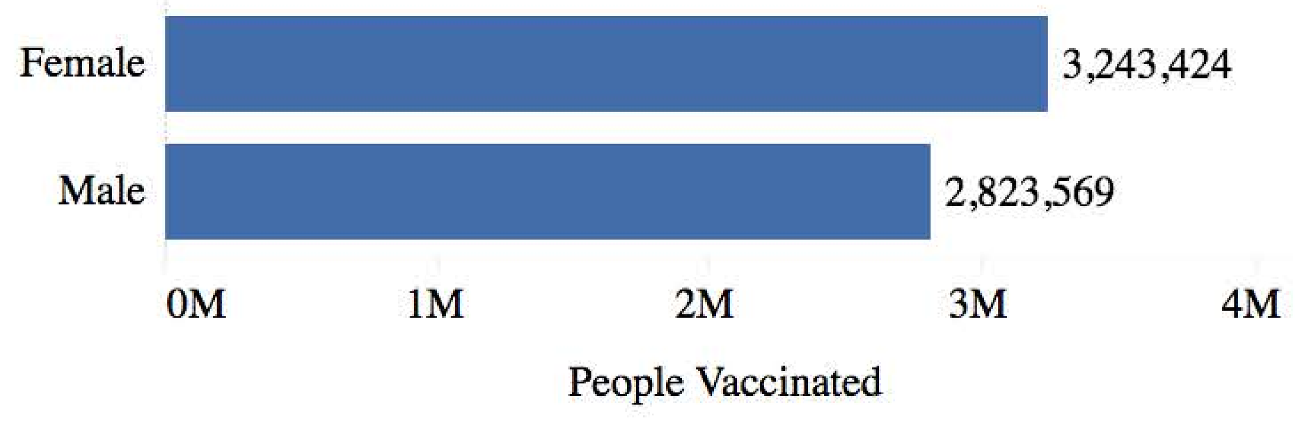

Percent of the Eligible Population Vaccinated with At Least One Dose - By Sex

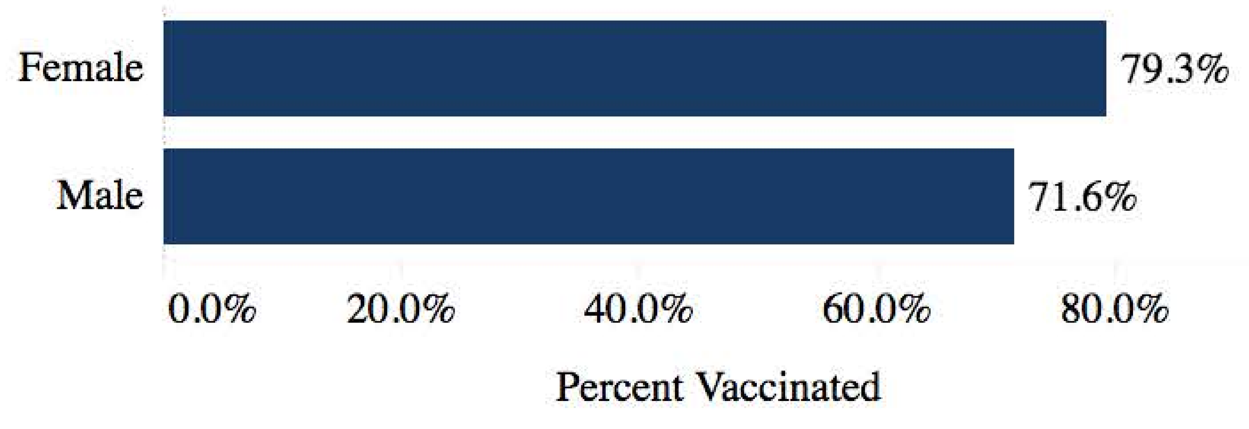

Not Reported: 365,294

Population estimates data source: 2019 National Center for Health Statistics (NCHS) Bridged-Race population estimates.
*This data source does not include population estimates for the Other Race category. No population estimates are available for out-of-state individuals or those without a reported locality. More information about the population estimates can be found at this link - https://www.cdc.gov/nchs/nvss/bridged_race.htm

904,754 People with at least one dose and 722,847 people fully vaccinated are out-of-state residents or reported without a health district of residence. These are included in Virginia state vaccine totals.

This Virginia Immunization Information System (VIIS) is a live data set that continually receives new information. Data review processes are ongoing to improve the quality of the information. On July 7, 2021, the vaccine dashboard was updated to include additional information on race. This update affected approximately 2 million records. Previously the race field for many records was "unknown" or "not reported".

VIRGINIA'S HEALTH IS IN OUR HANDS. Do your part, stop the spread.

COVID-19 in Virginia: Vaccine Summary

VDH VIRGINIA DEPARTMENT OF HEALTH

Dashboard Updated: 12/11/2021

COVID-19 Vaccinations in Virginia

Total Doses Administered - 13,247,127

People Vaccinated with at Least One Dose*	% of the Population Vaccinated with at Least One Dose	People Fully Vaccinated^	% of the Population Fully Vaccinated	People Vaccinated with Booster/ Third Dose**
6,432,287	75.4%	5,652,422	66.2%	1,552,182

% of the Adult (18+) Population Vaccinated with at Least One Dose **86.9%**

% of the Adult (18+) Population Fully Vaccinated **77.3%**

* People vaccinated with one dose of a two-dose vaccine and one dose of a single dose vaccine, including doses administered through the Federal CDC Pharmacy Partnership. Doses on the Federal Doses Administered dashboard are included.

^ People vaccinated with two doses of a two-dose vaccine and one dose of a single dose vaccine, including doses administered through the Federal CDC Pharmacy Partnership. Doses on the Federal Doses Administered dashboard are included.

**People vaccinated with a booster or third dose after the primary vaccine series, including doses administered through the Federal CDC Pharmacy Partnership. Doses on the Federal Doses Administered dashboard are not included

People Vaccinated by Locality of Residence and Vaccination Status - Percent of the Population

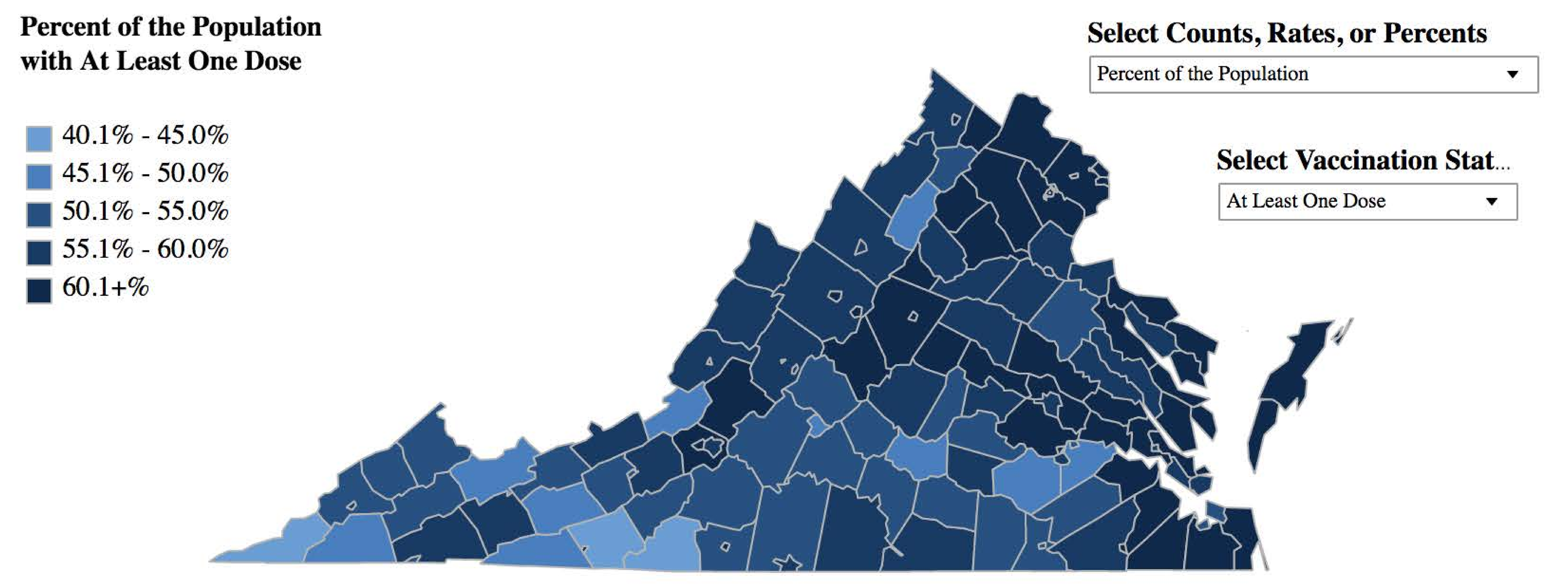

People Not Mapped : 904,754

COVID-19 Vaccine Doses Administered by Facility Type

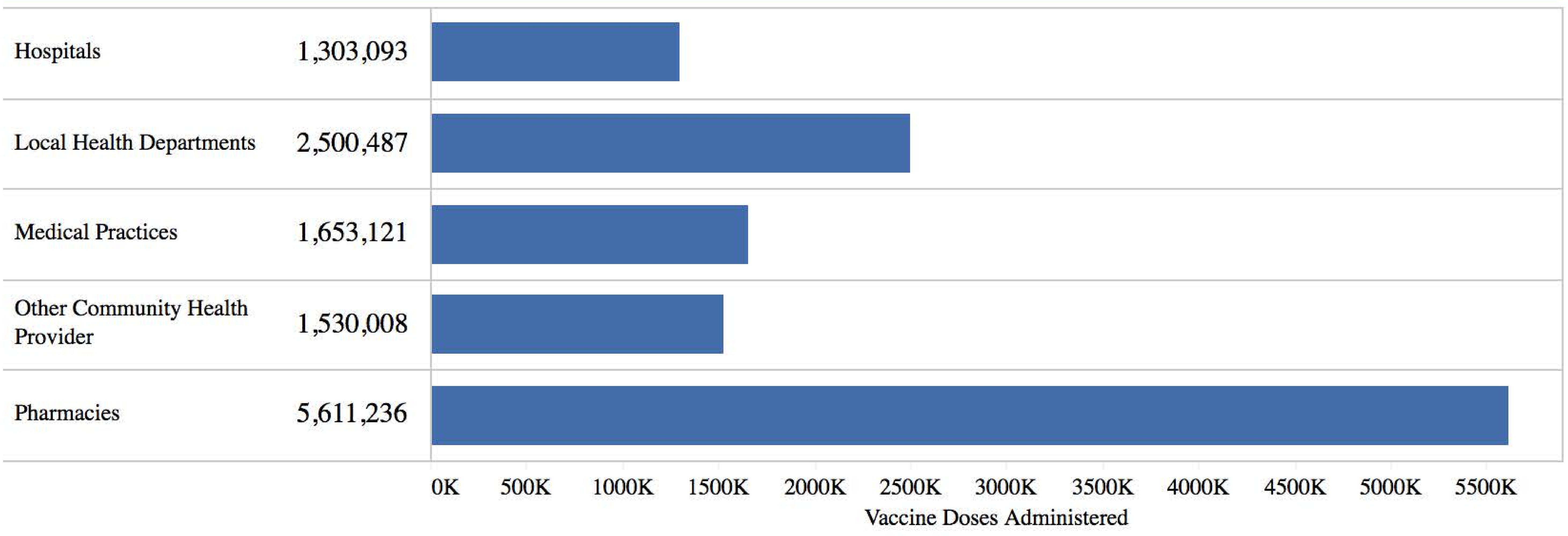

COVID-19 Vaccine Administration^ by Day

Average Doses Administered Each Day**

48,807

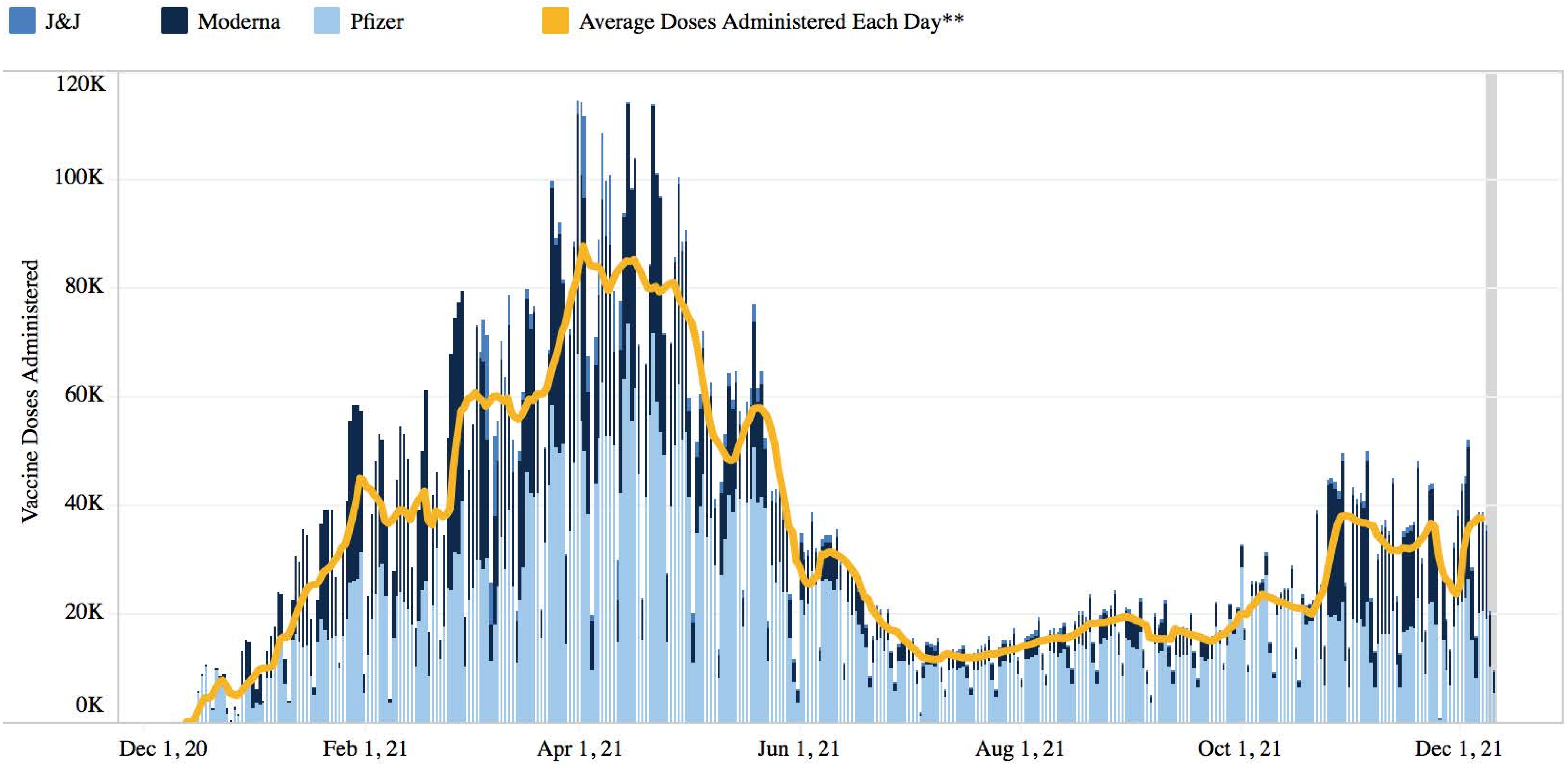

**The average doses administered each day takes the last 7 days of daily doses given, adds them up, and divides that number by 7. This is useful to get a clear picture of the data while taking into account reporting delays.

^Vaccine administrations may take up to 72 hours to be reported, shown by gray shaded area on graph.

VIRGINIA'S HEALTH IS IN OUR HANDS. Do your part, stop the spread.

COVID-19 in Virginia: Testing

VDH VIRGINIA DEPARTMENT OF HEALTH

Dashboard Updated: 12/10/2021
Data entered by 5:00 PM the prior day.

Select Health District from Drop Down Menu
(All)

Select PCR*, Antigen, Antibody, or Total Tests from Drop Down Me...
PCR Only

All Health Districts Testing Encounters PCR Only

10,698,256

All Health Districts Current 7-Day Positivity Rate PCR Only

8.1%

Testing Encounters by Health District, PCR Only

Fairfax	1,416,074
Blue Ridge	638,721
Prince William	564,813
Henrico	540,904
Virginia Beach	476,038
Chesterfield	476,679
Loudoun	459,758
Richmond	348,130
Central Shenandoah	348,737
Arlington	431,127
Peninsula	343,234
Roanoke	332,936
Rappahannock	335,658
Lord Fairfax	301,743
Central Virginia	250,502
New River	259,014
Norfolk	257,228
Chesapeake	263,747
Crater	256,640
Alexandria	274,451
Western Tidewater	184,952
Rappahannock Rapidan	181,024
Alleghany	165,963
Chickahominy	172,493
Mount Rogers	169,995
Three Rivers	148,519
Piedmont	130,292
Hampton	132,714
Pittsylvania-Danville	106,362
Portsmouth	112,614
Southside	100,365
West Piedmont	93,168
Lenowisco	83,227
Cumberland Plateau	73,315
Eastern Shore	49,527
Out of State	190,793
Unknown	26,799

Percent Positivity, 7-Day Moving Average
Positive Testing Encounters
Number of Testing Encounters

Select Date Range for Charts
90 Days

Number of Testing Encounters, Number of Positive Testing Encounters, and Percent Positivity** by Lab Report Date - All Health Districts, PCR Only

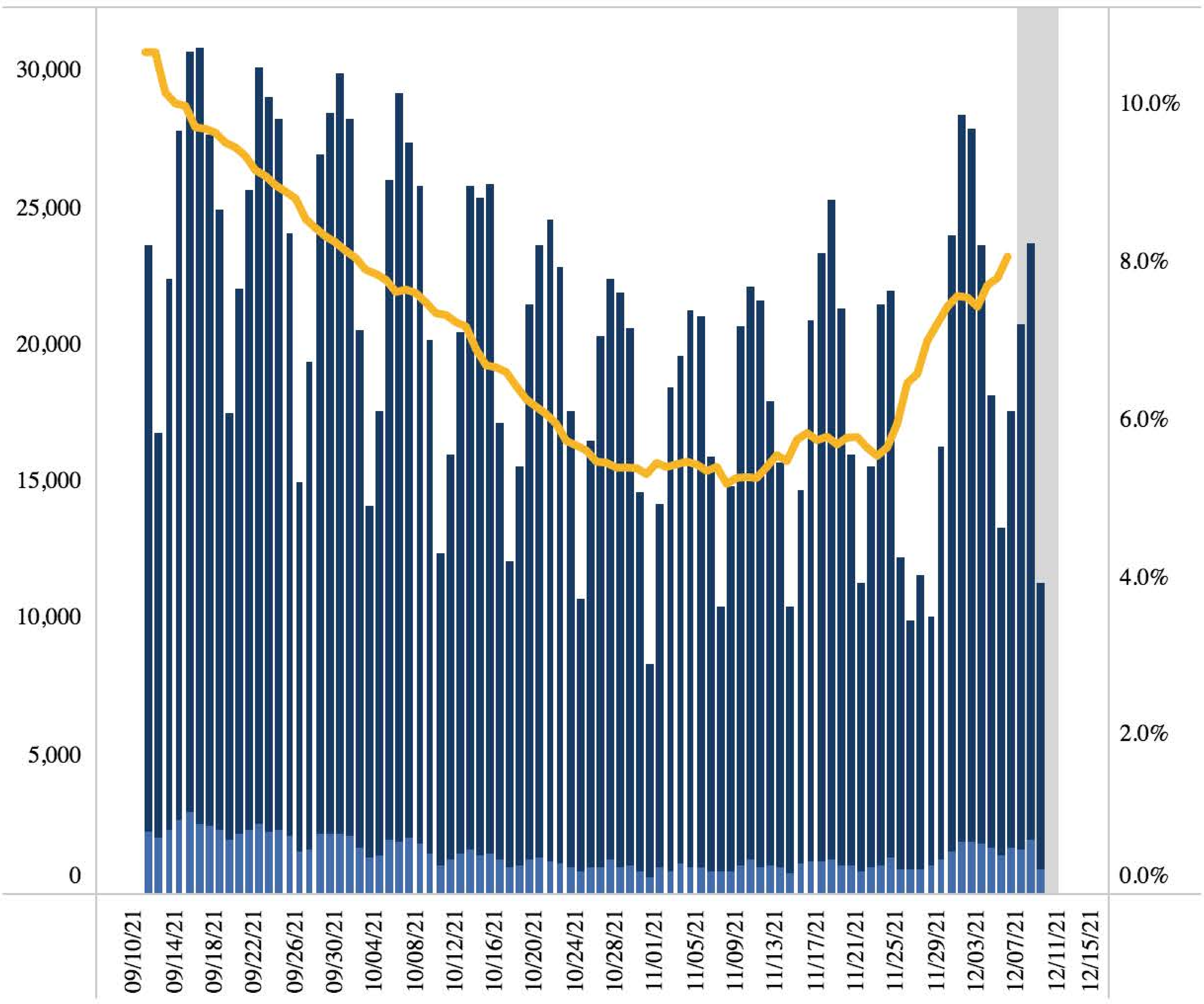

Number of Testing Encounters
7-Day Moving Average

Number of Testing Encounters by Lab Report Date - All Health Districts, PCR Only

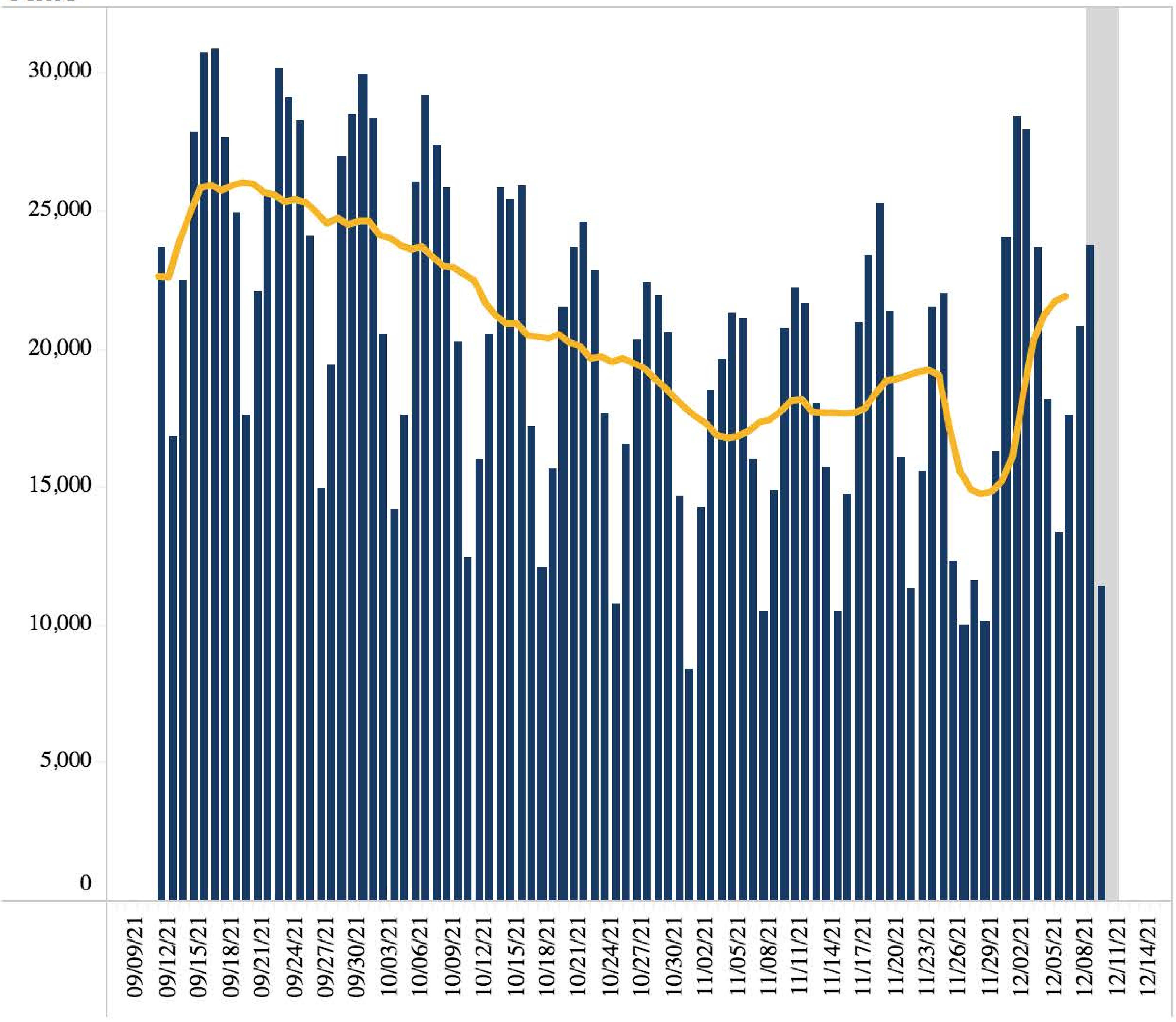

* PCR" refers to "Reverse transcriptase polymerase chain reaction laboratory testing."
** Lab reports may not have been received yet. Percent positivity is not calculated for days with incomplete data.
As of February 1, 2021, only diagnostic tests (molecular, including PCR and antigen) and antibody test results received electronically will be included in the data and calculations presented on this Testing dashboard.

Undo Redo Revert Refresh Pause Download

COVID-19 in Vermont Vermont Department of Health (VDH)

New Cases
260
55,317 Total

Currently Hospitalized
76

Hospitalized In ICU
21

Hospitalized Under Investigation
0

Percent Positive 7-day Avg.
4.8%

People Tested
556,563

Total Tests
2,679,538

Recovered
44,457
80.4% of Cases

Deaths
429
0.8% of Cases

Last Updated: 12/11/2021, 10:58:27 AM

COVID-19 Positive Cases by County in Last 14 Days

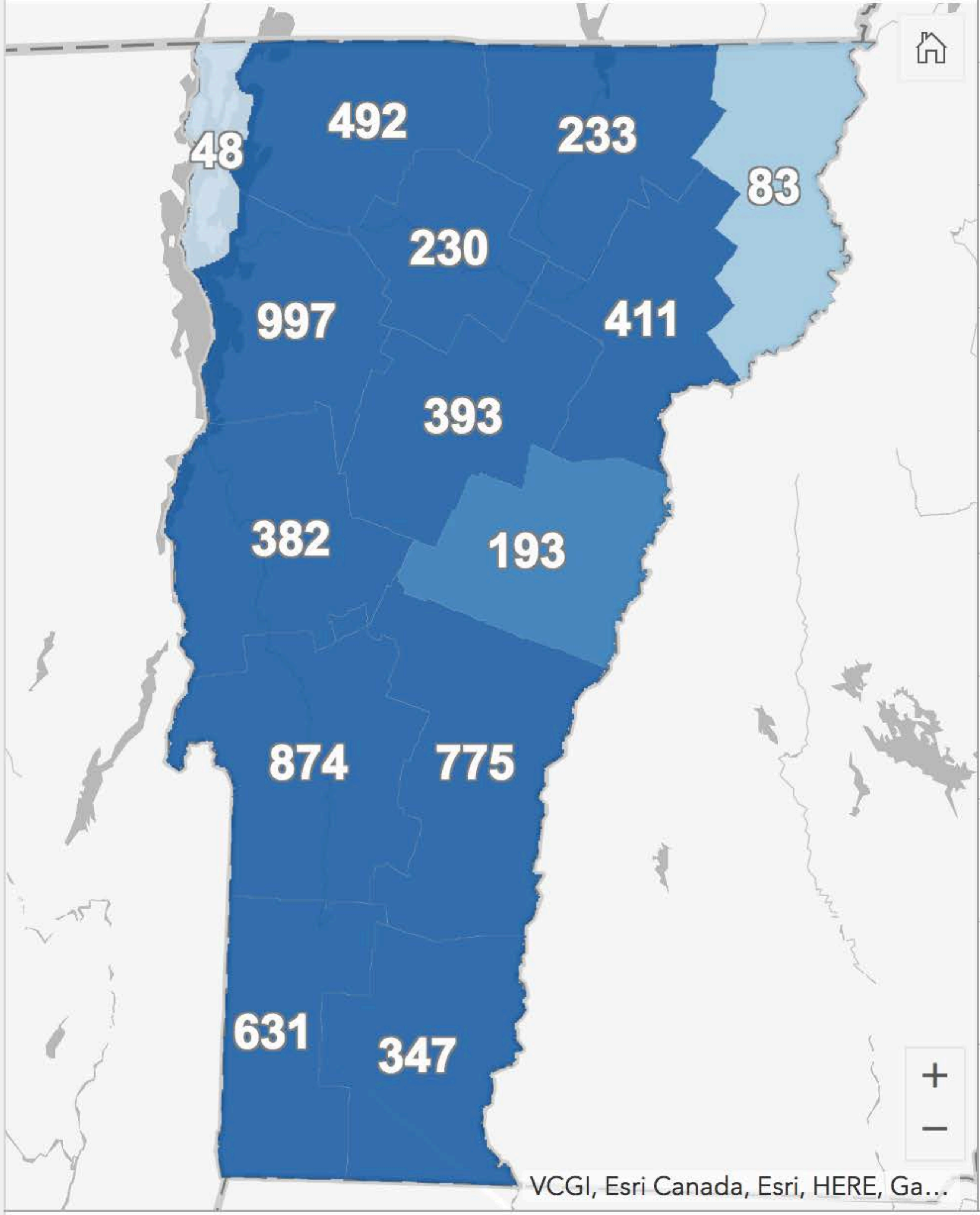

Addison County
New Cases: **17**
Recent Cases 14 days: **382**

Bennington County
New Cases: **30**
Recent Cases 14 days: **631**

Caledonia County
New Cases: **8**
Recent Cases 14 days: **411**

Chittenden County
New Cases: **45**
Recent Cases 14 days: **997**

Essex County
New Cases: **1**
Recent Cases 14 days: **83**

Franklin County
New Cases: **32**
Recent Cases 14 days: **492**

Grand Isle County
New Cases: **5**
Recent Cases 14 days: **48**

Lamoille County
New Cases: **6**
Recent Cases 14 days: **230**

Orange County
New Cases: **4**
Recent Cases 14 days: **193**

Orleans County
New Cases: **1**
Recent Cases 14 days: **233**

Click a County to highlight it on the map.

View more information by clicking on tab name or arrows below

Recent Cases

Vermont Vaccination Data

Overall | Statewide | By County | By County - Additional

Updated 12/11/2021 9:08 AM

Data notes

Show rates by: Overall | Race/Ethnicity | Ethnicity | Age | Sex

Select vaccine status: At least one dose | Completed

Vaccination rate by county

The percent of the county population age 5+ that has received at least one dose of vaccine by county.

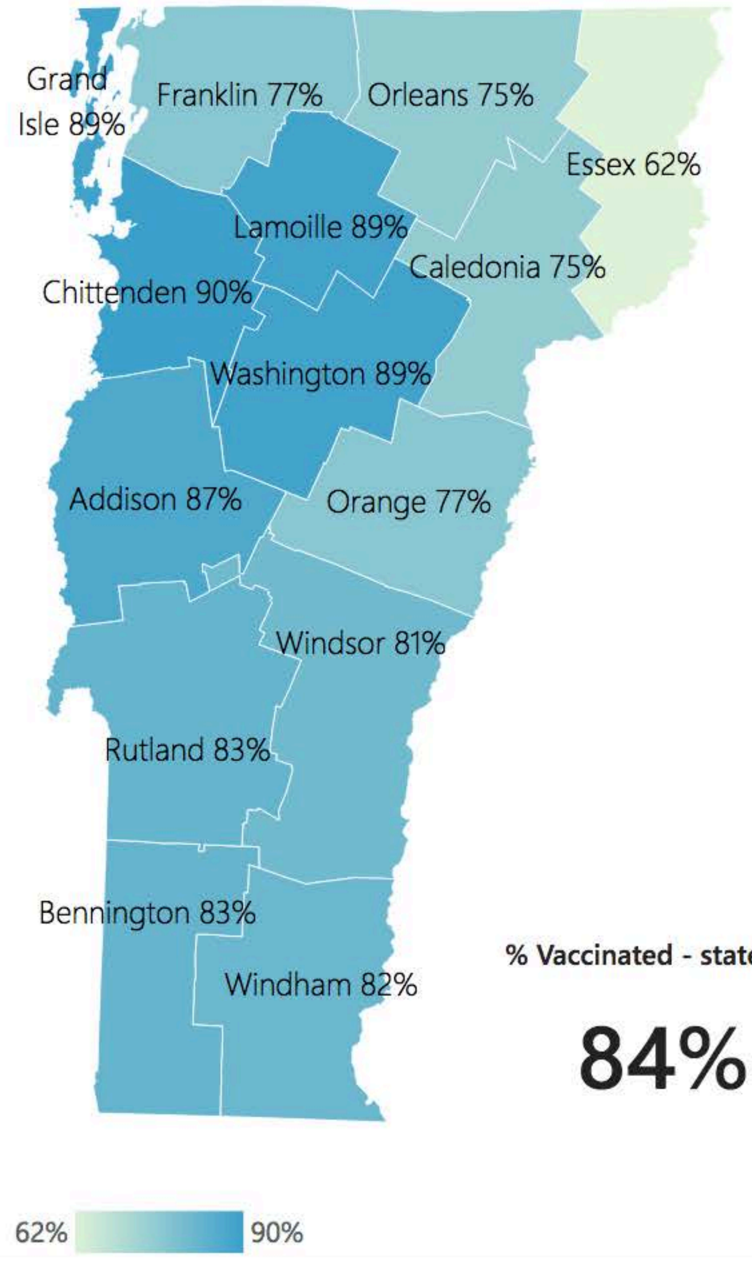

% Vaccinated - statewide

84%

County	Overall progress	5+ pop estimate
Addison	87%	35,214
Bennington	83%	33,828
Caledonia	75%	28,570
Chittenden	90%	156,317
Essex	62%	5,887
Franklin	77%	46,515
Grand Isle	89%	6,900
Lamoille	89%	24,075
Orange	77%	27,542
Orleans	75%	25,742
Rutland	83%	55,626
Washington	89%	55,680
Windham	82%	40,377
Windsor	81%	52,673
**VT**	**84%**	**594,946**

Source: Vermont Immunization Registry, VDH Population Estimates (2019)

Coronavirus in Vermont

VTDigger is closely tracking the effects of Covid-19 in Vermont, from the spread of the Delta variant to the ongoing disruption to the state's economy and schools. This page includes live-updating public health data and VTDigger's complete coverage of the pandemic.

Vermont Coronavirus report

Saturday, December 11, 2021
260 new cases reported today, with 76 hospitalized (21 in ICU)
4.8% seven-day test positivity rate
Most recent death reported December 9, totaling 429 deaths statewide
94.5% of Vermonters aged 12+ are partially or fully vaccinated
48% of Vermont children aged 5-11 have received at least one dose

Data retrieved from Vermont Department of Health, which is updated daily representing the previous day's report. Vaccination percentage is based on the number of Vermonters aged 12 and above, per Vermont Department of Health Population numbers (2019).

See more: Breakthrough infections • Cases by county • School cases • All Covid-19 stories

---

Covid new cases per day, rolling average:

Based on the latest Department of Health data, with a seven-day rolling average:

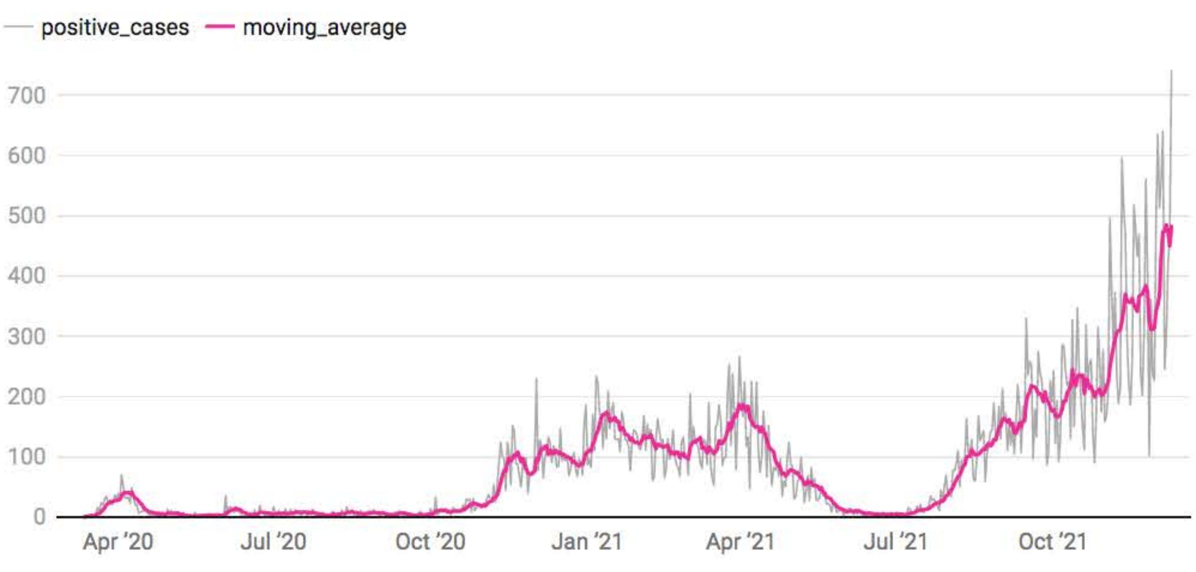

Chart: Erin Petenko • Source: VTDOH via Vermont Center for Geographic Information • Get the data • Download image • Created with Datawrapper

People hospitalized for Covid over time

Number of people currently hospitalized for Covid:

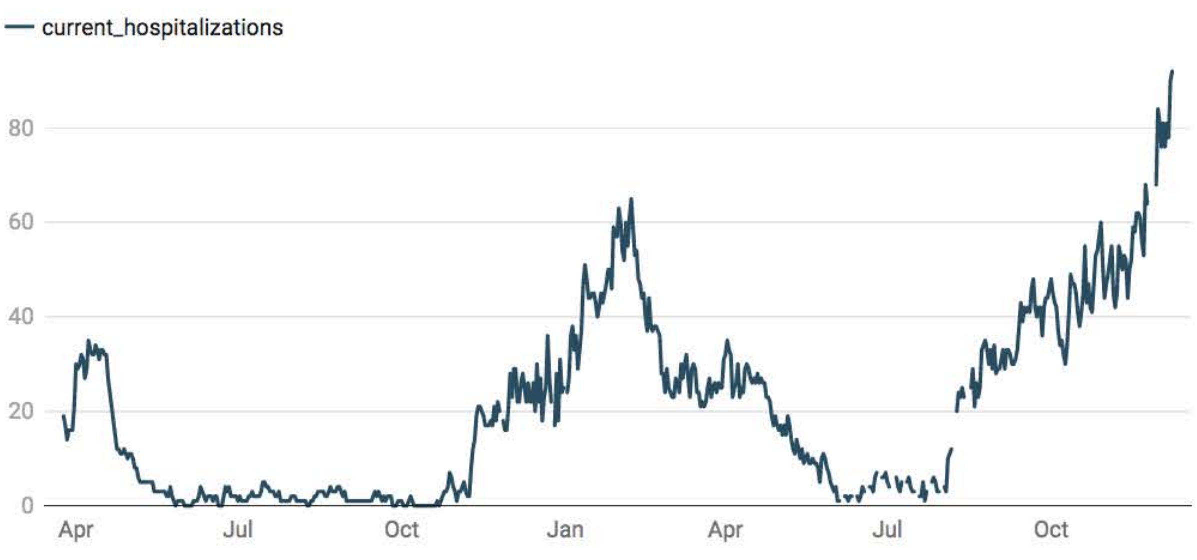

Does not include people hospitalized under investigation for Covid.
Chart: Erin Petenko • Source: VTDOH via VCGI • Get the data • Created with Datawrapper

Fund local journalism. Feed the community.
If you rely on our timely and rigorous news coverage to keep you informed, please support it during our annual membership drive. When you give, VTDigger will send 10 meals on your behalf to the Vermont Foodbank to help neighbors facing hunger. Will you join us?

Covid Cases by vaccination status

How Vermont rates of infection, hospitalization and death compare by vaccination status

	Infection rate (per 100k)		
	Vaccinated	Unvaccinated	
Two weeks ending Nov. 17	373	1491	4x higher for unvaccinated people
Two weeks ending Dec. 1	370	1625	4.4x
**Delta wave to date (July 1 - Dec. 1)**	**1982**	**8600**	**4.3x**
	Hospitalization rate (per 100k)		
	Vaccinated	Unvaccinated	
Two weeks ending Nov. 17	8.9	36.0	4x higher for unvaccinated people
Two weeks ending Dec. 1	8.1	38.9	4.8x
**Delta wave to date (July 1 - Dec. 1)**	**49.8**	**229.1**	**4.6x**
	Death rate (per 100k)		
	Vaccinated	Unvaccinated	
Two weeks ending Nov. 17	3.0	3.5	1.2x higher for unvaccinated people
Two weeks ending Dec. 1	2.2	2.1	1x
**Delta wave to date (July 1 - Dec. 1)**	**19.5**	**31.4**	**1.6x**

"Vaccinated" refers to fully vaccinated people. "Unvaccinated" includes people who are partially vaccinated as well as out-of-state residents with unknown vaccination status.
Table: Mike Dougherty/VTDigger • Source: Vermont Department of Health • Get the data • Created with Datawrapper

Covid totals by county: map and two-week trends

COVID-19 cases, by county

Mouse over or click on a county to view deaths and the rate by population. Updated by the VT Department of Health by 1 p.m. daily.

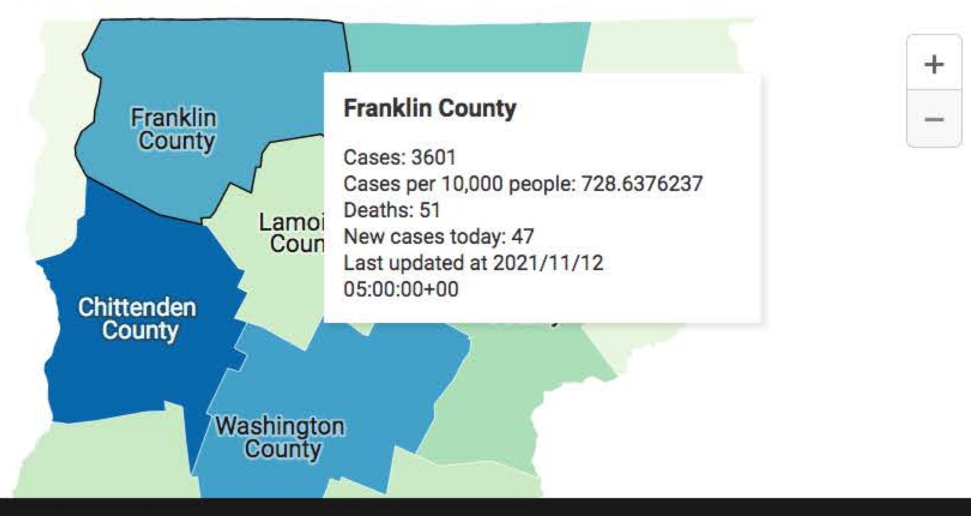

enter search term

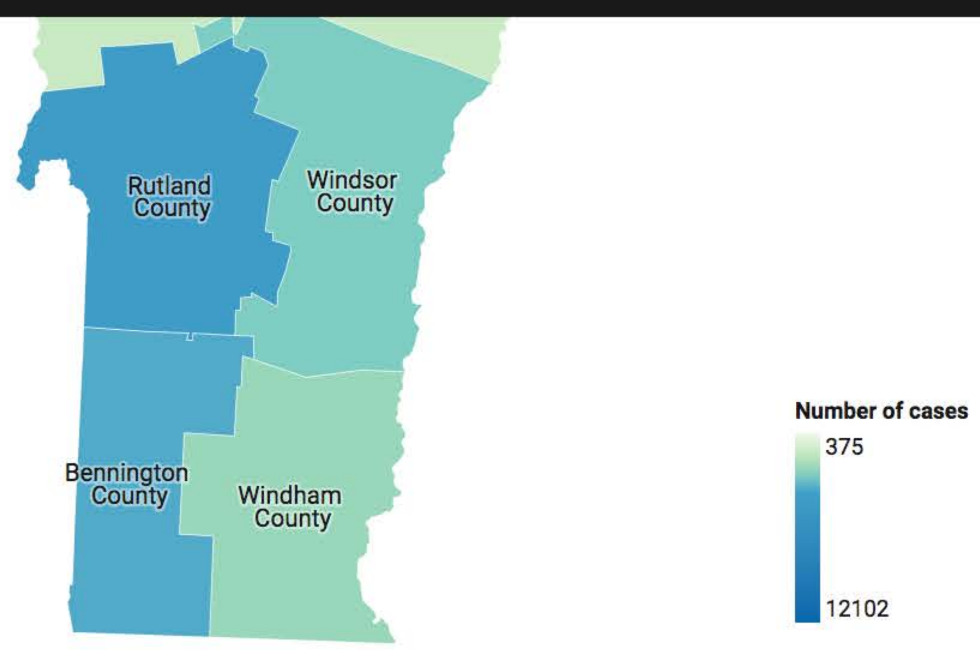

Map: Erin Petenko • Source: VTDOH via VT Center for Geographic Information • Get the data • Created with Datawrapper

New Covid infections per day, by county

Vermont's daily new Covid-19 infections by county, tracking the past 14 days. Last updated December 10, 2021.

	New cases by date, November 26-December 9	Total new cases past 14 days	Percent change from two weeks ago	Recent cases per 10,000 people
Addison County	1 … 51	348	75.76%	93.14
Bennington County	18 … 28	615	25.51%	164.67
Caledonia County	2 … 26	387	11.21%	128.01
Chittenden County	10 … 93	926	6.31%	55.01
Essex County	1 … 8	83	−18.63%	140.20
Franklin County	7 … 40	449	−6.07%	89.90
Grand Isle County	1 … 6	42	−12.5%	57.59
Lamoille County	3 … 18	223	37.65%	85.95
Orange County	2 … 19	189	21.15%	64.56
[illegible] Validation	0 … [illegible]	152	2071.43%	
Rutland County	21 … 90	858	0.94%	141.65
Washington County	9 … 49	368	6.98%	61.53
Windham County	6 … 52	332	37.76%	72.32
Windsor County	16 … 99	753	53.36%	130.38
**Statewide total**	**102 … 740**	**5,953**	**17.9%**	**92.57**

Support VTDigger
DONATE NEWSLETTER SHOP
VTDIGGER

Table: Erin Petenko & Stacey Peters, VTDigger • Source: Vermont Department of Health • Get the data • Created with Datawrapper

Current Status | Epidemiologic Curves | Cumulative Counts | Demographics | Testing | COVID-like Illness Hospitalizations | Healthcare System Readiness | Vaccinations

Cases | Hospitalizations | Deaths | Vaccinations

COVID-19 IN WASHINGTON STATE

Cases, Hospitalizations, Deaths and Vaccinations by County

DATA AS OF 12/09/2021 11:59PM PT

Confirmed cases are individuals with a positive molecular test for COVID-19. Probable cases are individuals with a positive antigen test for COVID-19 and no positive molecular test. Hospitalizations and deaths are reported among confirmed and probable cases. Number of vaccine doses given includes all COVID-19 vaccine doses given and reported in Washington facilities. **Learn More**

SELECT COUNTIES

- Select all
- Adams County
- Asotin County
- Benton County
- Chelan County
- Clallam County
- Clark County
- Columbia County
- Cowlitz County
- Douglas County
- Ferry County
- Franklin County
- Garfield County
- Grant County
- Grays Harbor Co...
- Island County
- Jefferson County
- King County
- Kitsap County

Confirmed Cases	**694,617**
Probable Cases	**95,795**
Total Cases	**790,412**
Hospitalizations	**43,670**
Deaths	**9,554**
Percent of Deaths (deaths/total cases)	**1.2%**
Total Molecular Tests	**9,302,096**
Total Molecular+ Antigen Tests	**9,958,651**
Statewide ICU Occupancy by COVID-19 Patients	**18.7%**
Number of Vaccine Doses Given	**11,195,694**

CASES BY COUNTY

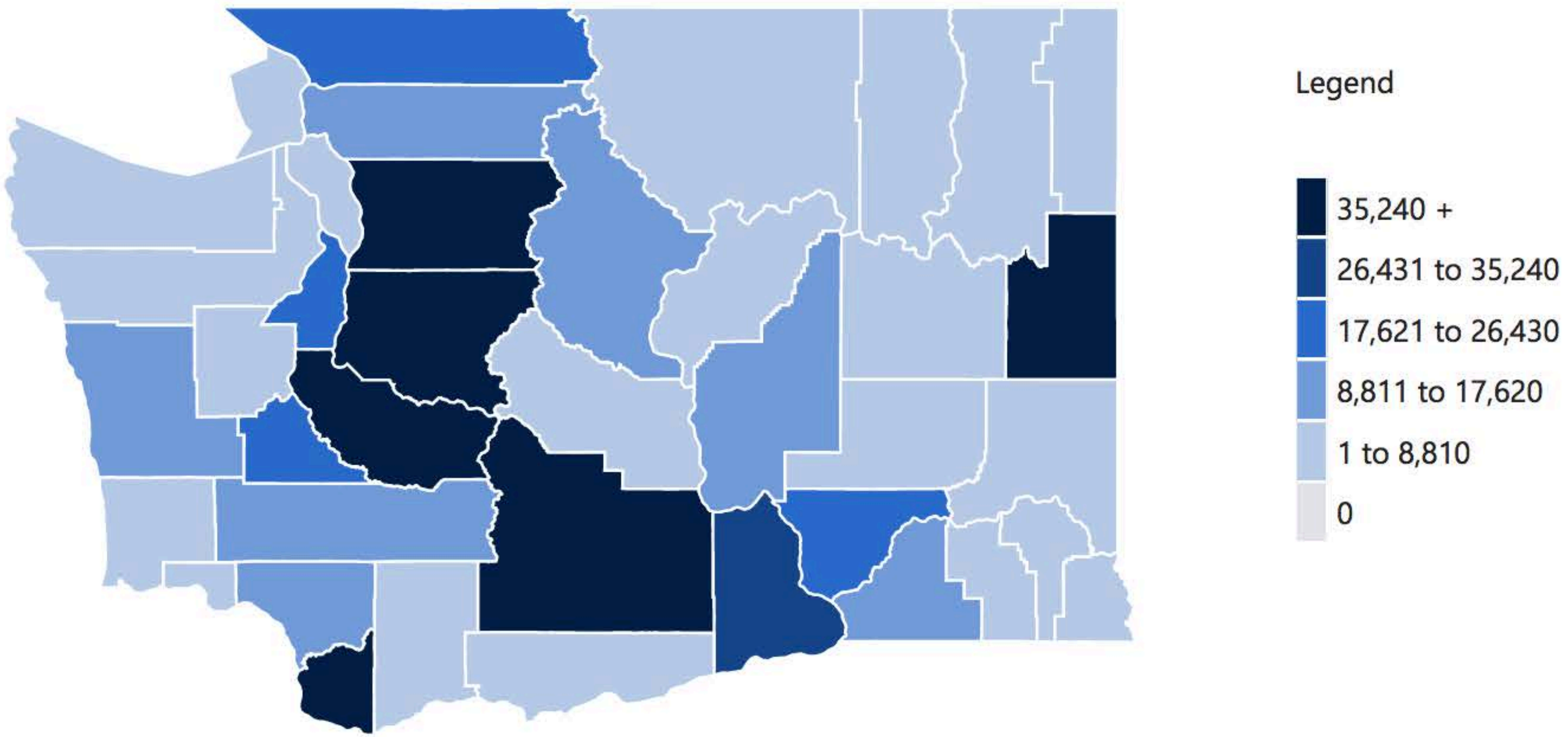

Tabular View

*Please click **"Learn More"** for more information.*

1,542 of 790,412 cases do not have an assigned county

County-level information can be found on Local Health Jurisdiction (LHJ) websites

Current Status | Epidemiologic Curves | Cumulative Counts | Demographics | Testing | COVID-like Illness Hospitalizations | Healthcare System Readiness | Vaccinations

Cases | Hospitalizations | **Deaths** | Vaccinations

COVID-19 IN WASHINGTON STATE

Cases, Hospitalizations, Deaths and Vaccinations by County

DATA AS OF 12/09/2021 11:59PM PT

Confirmed cases are individuals with a positive molecular test for COVID-19. Probable cases are individuals with a positive antigen test for COVID-19 and and no positive molecular test. Hospitalizations and deaths are reported among confirmed and probable cases. Number of vaccine doses given includes all COVID-19 vaccine doses given and reported in Washington facilities. **Learn More**

SELECT COUNTIES

- Select all
- Adams County
- Asotin County
- Benton County
- Chelan County
- Clallam County
- Clark County
- Columbia County
- Cowlitz County
- Douglas County
- Ferry County
- Franklin County
- Garfield County
- Grant County
- Grays Harbor Co...
- Island County
- Jefferson County
- King County
- Kitsap County

Confirmed Cases	**694,617**
Probable Cases	**95,795**
Total Cases	**790,412**
Hospitalizations	**43,670**
Deaths	**9,554**
Percent of Deaths (deaths/total cases)	**1.2%**
Total Molecular Tests	**9,302,096**
Total Molecular+ Antigen Tests	**9,958,651**
Statewide ICU Occupancy by COVID-19 Patients	**18.7%**
Number of Vaccine Doses Given	**11,195,694**

DEATHS BY COUNTY

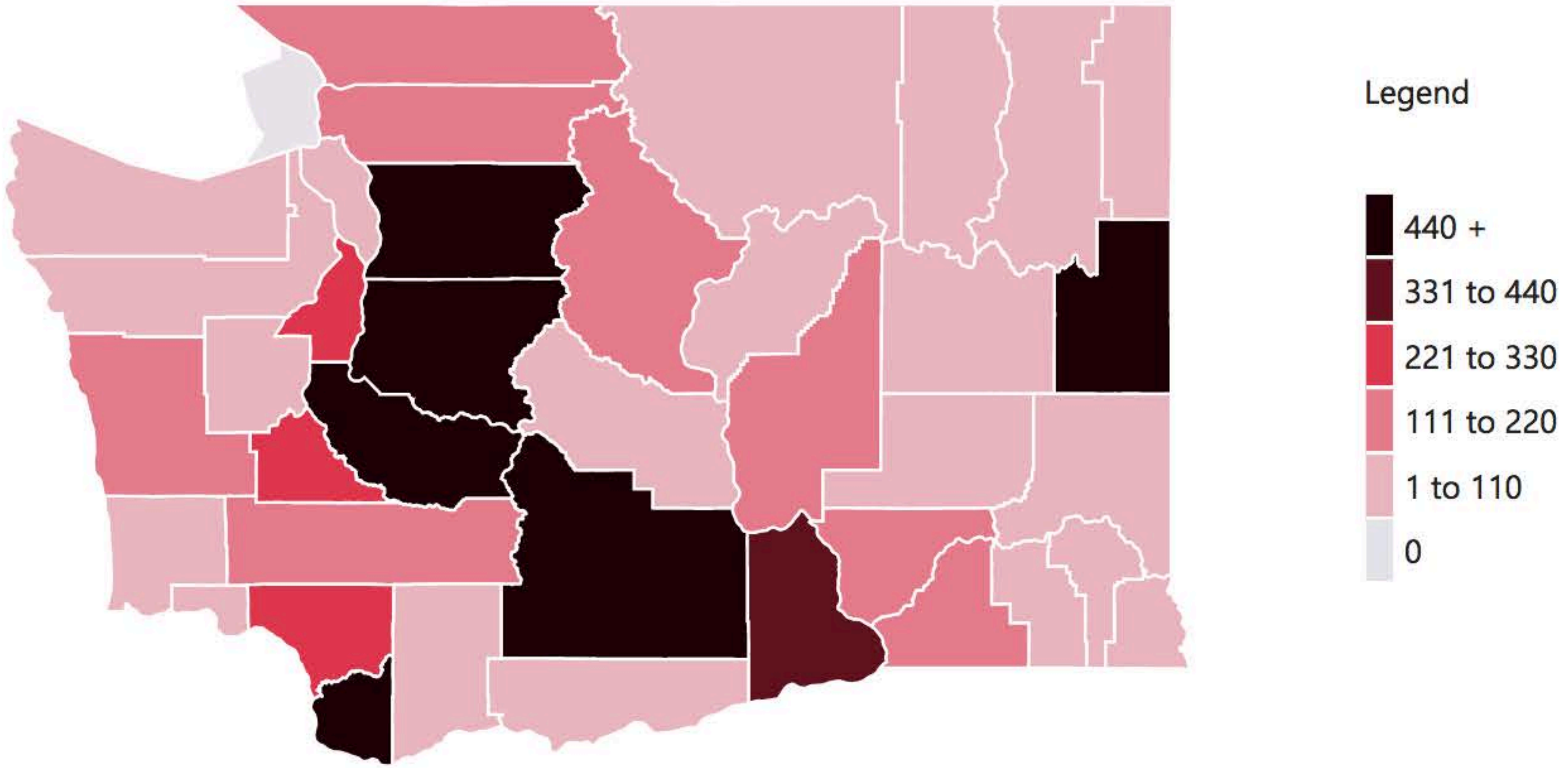

Tabular View

*Please click **"Learn More"** for more information.*

4 of 9,554 deaths do not have an assigned county

County-level information can be found on Local Health Jurisdiction (LHJ) websites

Current Status | Epidemiologic Curves | Cumulative Counts | Demographics | Testing | COVID-like Illness Hospitalizations | Healthcare System Readiness | Vaccinations

Current Status

Percent Positive

Testing Rate

Select Test Type:

Molecular

COVID-19 IN WASHINGTON STATE

Number of Tests by Specimen Collection Date

DATA AS OF 9/14/2021 11:39:49 AM

Molecular testing started in Washington in February 2020, and antigen testing started in June 2020. **Learn More**

SELECT COUNTIES

- Select all
- Adams County
- Asotin County
- Benton County
- Chelan County
- Clallam County
- Clark County
- Columbia County
- Cowlitz County
- Douglas County
- Ferry County
- Franklin County
- Garfield County
- Grant County
- Grays Harbor Co...
- Island County
- Jefferson County
- King County
- Kitsap County

Download

NUMBER OF TESTS

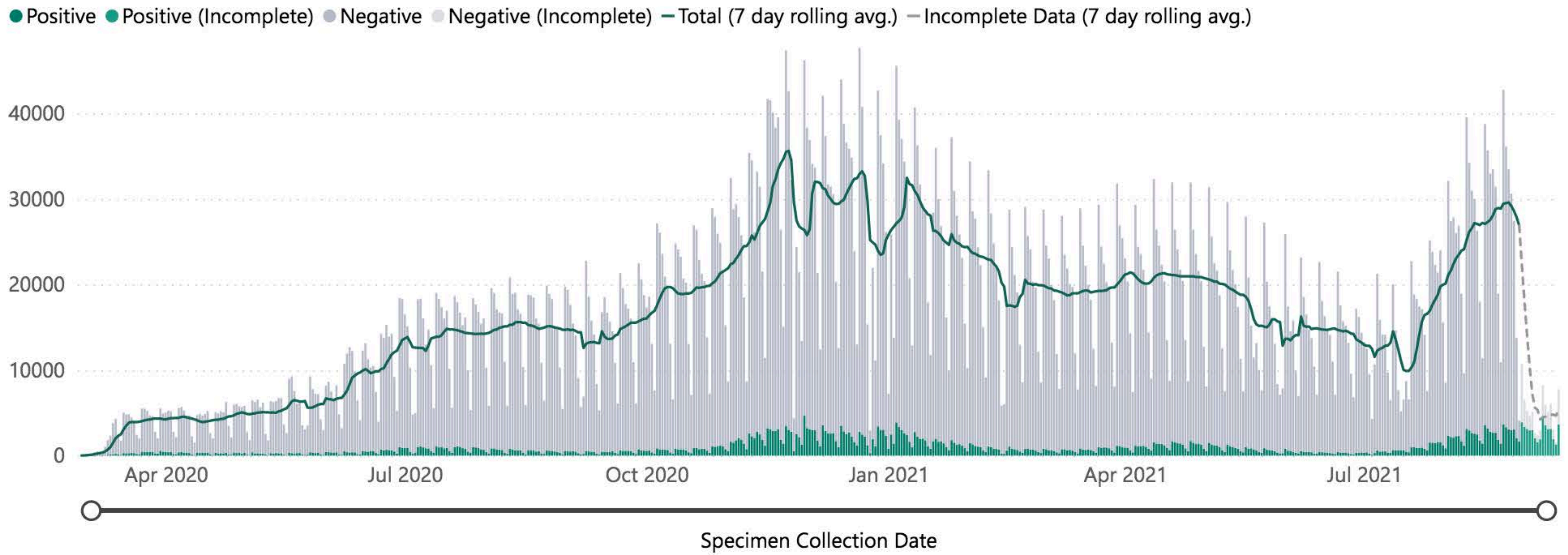

2,488,246 out of 9,353,004 test results do not have an assigned county.

County-level information can be found on Local Health Jurisdiction (LHJ) websites

Current Status | Epidemiologic Curves | Cumulative Counts | Demographics | Testing | COVID-like Illness Hospitalizations | Healthcare System Readiness | Vaccinations

Where and when are vaccinations being given? | How many people are getting vaccinated? | Who is getting vaccinated?

COVID-19 IN WASHINGTON STATE

Where and when are vaccinations being given?

DATA AS OF 12/8/2021 11:59:00 PM PT

This chart shows the number of COVID-19 vaccines given in Washington state facilities and reported to the state. Not all COVID-19 vaccines given in Washington are reported to the state. Vaccine doses given reflects the data reported to the state as of the latest update date. Please note, the measures "vaccines given" and "people vaccinated" are not comparable. Vaccines given counts number of doses given in a location. People vaccinated counts number of Washington residents initiating vaccination and becoming fully vaccinated. Numbers may not always align since people may live in a different county than where they got vaccinated. Vaccination data are updated on Mondays, Wednesdays, and Fridays only. **Learn More**

SELECT COUNTIES

- Select all
- Adams County
- Asotin County
- Benton County
- Chelan County
- Clallam County
- Clark County
- Columbia County
- Cowlitz County
- Douglas County
- Ferry County
- Franklin County
- Garfield County
- Grant County
- Grays Harbor Co...
- Island County
- Jefferson County

By Location

By Date

Vaccine Doses Given and Reported (statewide or selected counties)	**11,195,694**

Statewide Measures Only

Vaccine Doses Given and Reported in Washington	**11,195,694**
Doses Delivered to Washington Providers	**7,570,840**
Doses Delivered for Federal Partnership Programs	**5,209,280**
Percentage of Delivered Doses Given	**87.6%**
Current Complete 7-Day Average	**37,721**

VACCINE DOSES GIVEN AND REPORTED IN WASHINGTON COUNTIES

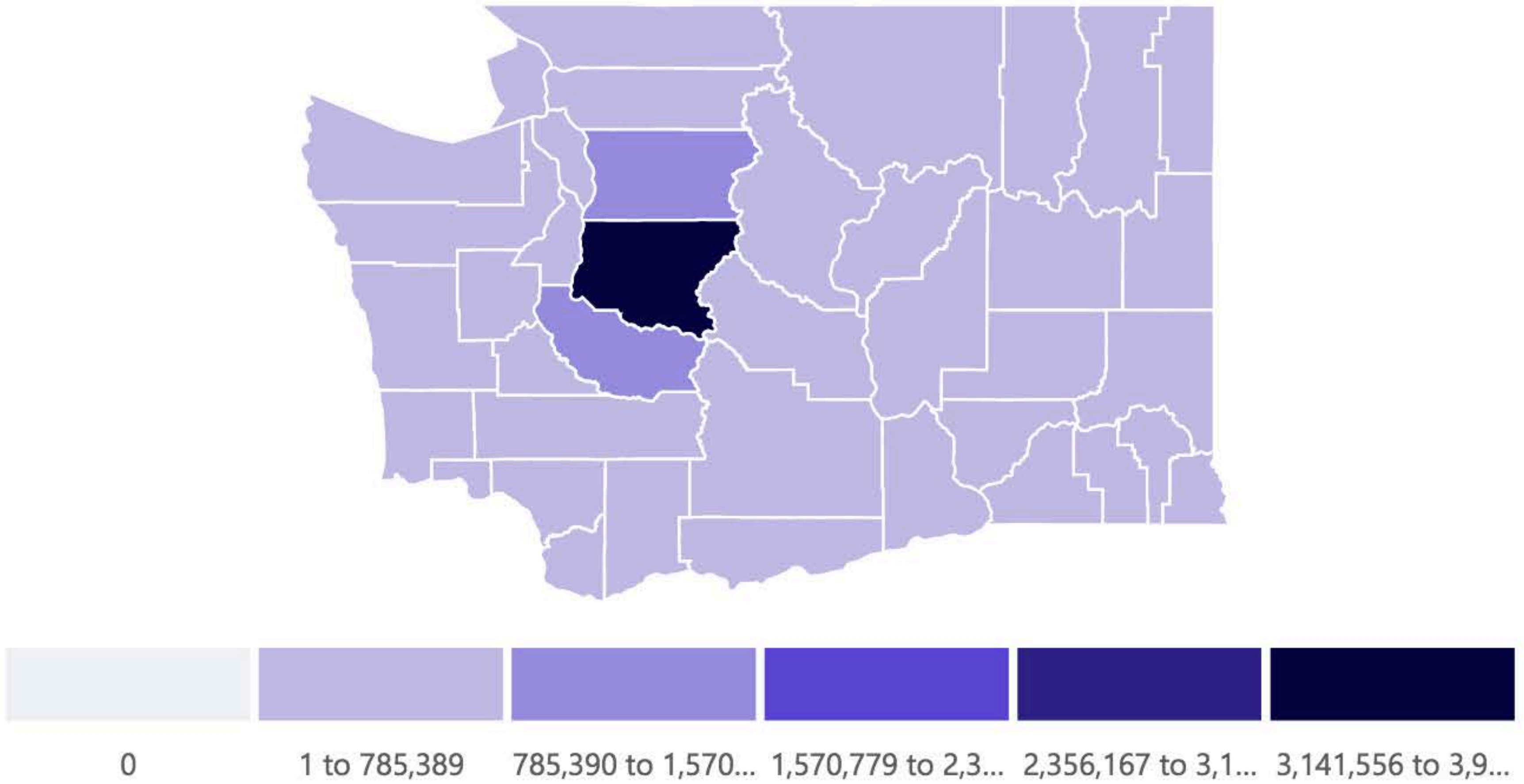

County data for doses given are based on the location of the facility giving the vaccine. 83,809 vaccine doses given do not have a county reported.

County-level information can be found on Local Health Jurisdiction (LHJ) websites

This tracker is updated at least daily using data from the Washington State Department of Health. For a more detailed update on COVID-19 in Washington State, please review the DOH dashboard.

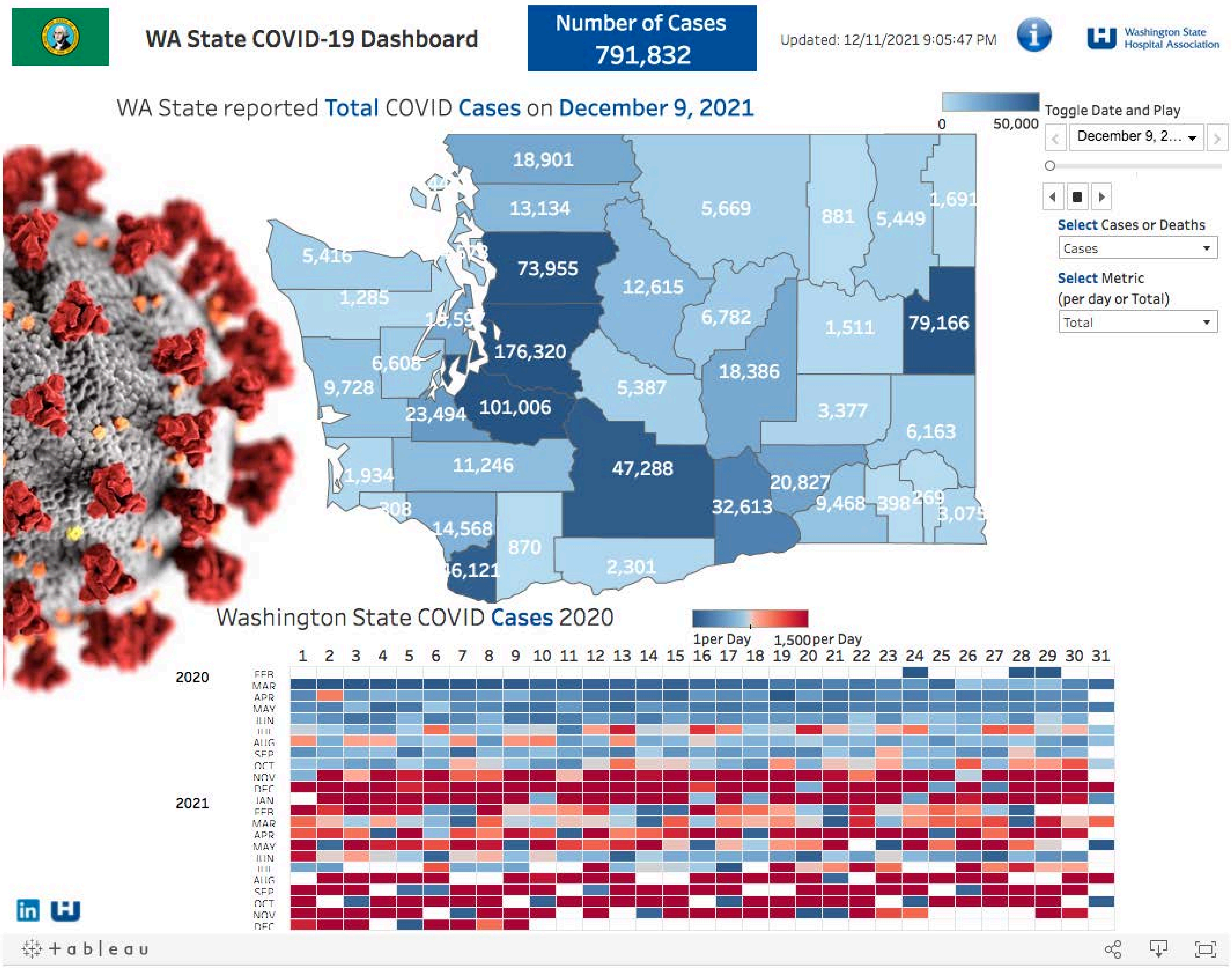

West Virginia COVID-19

WEST VIRGINIA Department of Health & Human Resources BUREAU FOR PUBLIC HEALTH

Overview | County Alert | 7-Day Trend | Cumulative Summary | Breakthrough Data | Long-Term Care | Hospital | Other Trends ▾ | Vaccine Summary

Click here to view variant data

306,552
Total Number of Cases
Click here to view Breakthrough Cases and Total Cases

5,085
Deaths
Click here to view Breakthrough Deaths and Total Deaths

1,134
New Cases Received Since Last Update
0
Deaths Reported Since Last Update

9,138
Current Active Cases

7.88%
Daily Percent Positivity
Click here to view breakdown

6.30%
Cumulative Percent Positivity
Click here to view breakdown

Note: Boost Doses % is calculated with respect to the number of people fully vaccinated.

Vaccine Doses Administered Into Arms of West Virginia Residents

Key Metric	Doses	Population	%
At Least One Dose	1,079,141	1,792,147	60.2%
Fully Vaccinated	904,547	1,792,147	50.5%
Boost Doses	262,756	904,547	29.0%
5+ At Least One Dose	1,079,141	1,699,122	63.5%
5+ Fully Vaccinated	904,547	1,699,122	53.2%
5+ Boost Doses	262,756	904,547	29.0%
18+ At Least One Dose	1,000,204	1,432,580	69.8%
18+ Fully Vaccinated	844,147	1,432,580	58.9%
18+ Boost Doses	261,372	844,147	31.0%
50+ At Least One Dose	605,265	736,823	82.1%
50+ Fully Vaccinated	525,889	736,823	71.4%
50+ Boost Doses	205,627	525,889	39.1%
65+ At Least One Dose	330,190	367,011	90.0%
65+ Fully Vaccinated	290,084	367,011	79.0%
65+ Boost Doses	132,270	290,084	45.6%
[illegible]nts	2[illegible]		

Data Source: HHS, CDC and WV DHHR Updated 10:00 AM 12/10/2021

Individuals counted are West Virginia residents vaccinated in-state or out-of-state through State Allocations, Federal Pharmacy Program, Federal FQHC Program and Federal Entities

Note: Data posted on dashboard is from 12:00 midnight the previous day. All data are provisional and subject to change based on information obtained during public health investigations.

Click here to view CDC's definition on Confirmed and Probable cases

Updated 10:00 AM 12/11/2021

Microsoft Power BI

COVID-19: County Data

Jump to specific COVID-19 chart on this page:

- Cumulative total number of reported confirmed and probable cases by date of symptom onset or diagnosis
- Number of reported confirmed and probable cases by date of symptom onset or diagnosis
- Cumulative total number of reported confirmed and probable deaths by date of death
- Number of reported confirmed and probable deaths by date of death
- Cases and deaths by county table
- Learn how to download our data

To find COVID-19 maps by county and census tract, see the Wisconsin Summary webpage.

COVID-19: Data Pages

- Activity Level by Region and County
- Cases
- Deaths
- Facility-Wide Investigations
- Frequently Asked Questions
- Hospitals
- Illness After Vaccination
- Racial and Ethnic Disparities
- Vaccines
- Variants
- Wastewater Monitoring
- Wisconsin Summary
- COVID-19

Cumulative total number of reported confirmed and probable COVID-19 cases by date of symptom onset or diagnosis: Wisconsin

Updated: 12/10/2021 (Total: 1,024,094)

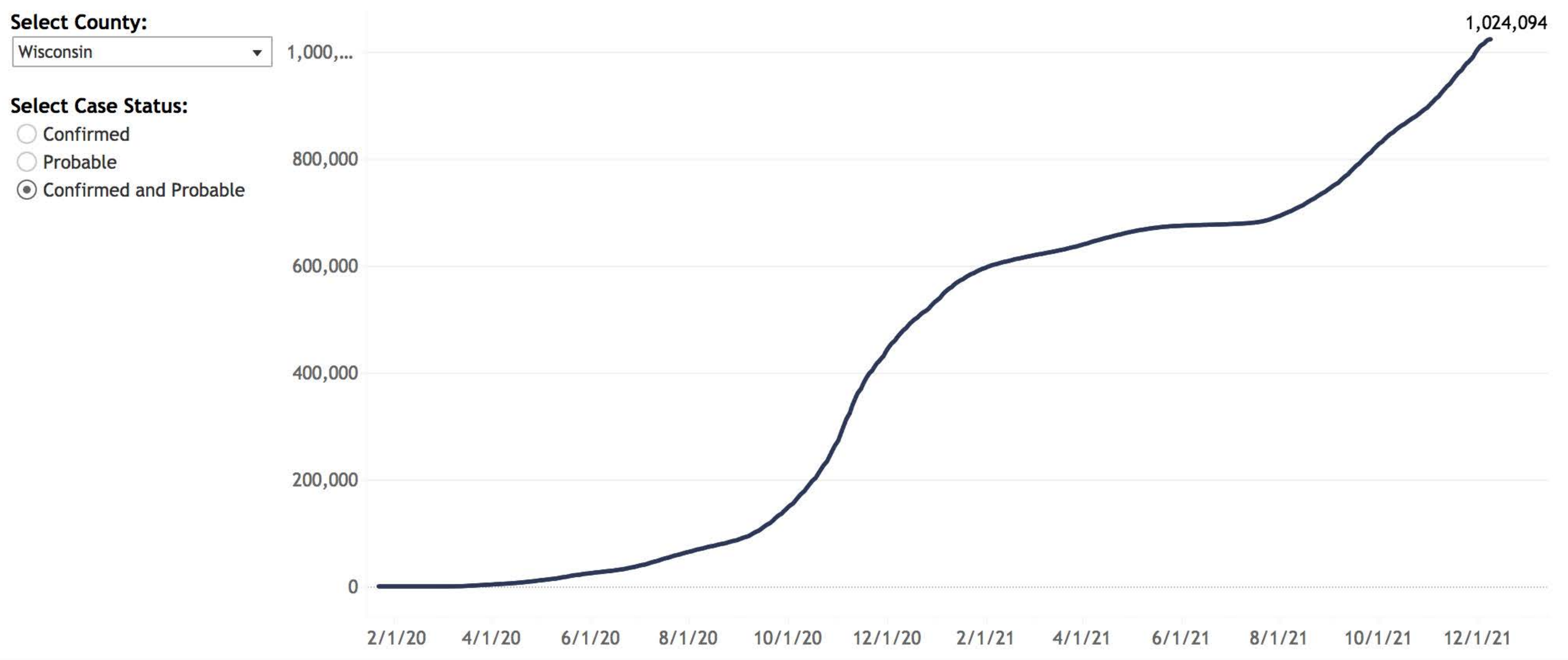

Undo Redo Revert Refresh Pause Metrics Share Download Full Screen

Understanding our data: What does this chart mean?

About our data: How do we measure this?

Back to a list of charts on this page.

Number of reported confirmed and probable COVID-19 cases by date of symptom onset or diagnosis: Wisconsin

Updated: 12/10/2021 (Total: 1,024,094)

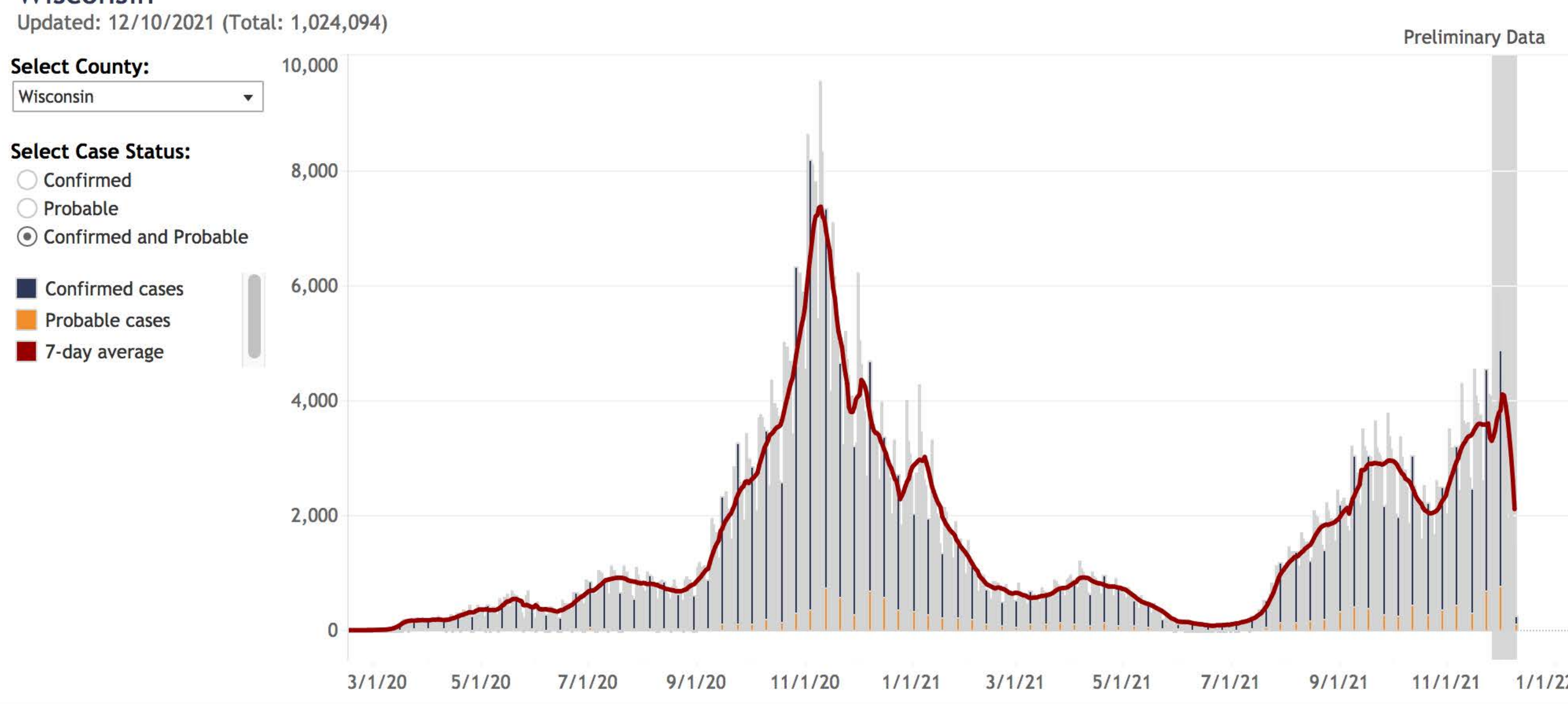

Undo Redo Revert Refresh Pause Metrics Share Download Full Screen

Understanding our data: What does this chart mean?

About our data: How do we measure this?

Back to a list of charts on this page.

Cumulative total number of reported COVID-19 deaths among confirmed and probable COVID-19 cases by date of death: Wisconsin

Updated: 12/10/2021 (Total: 10,386)

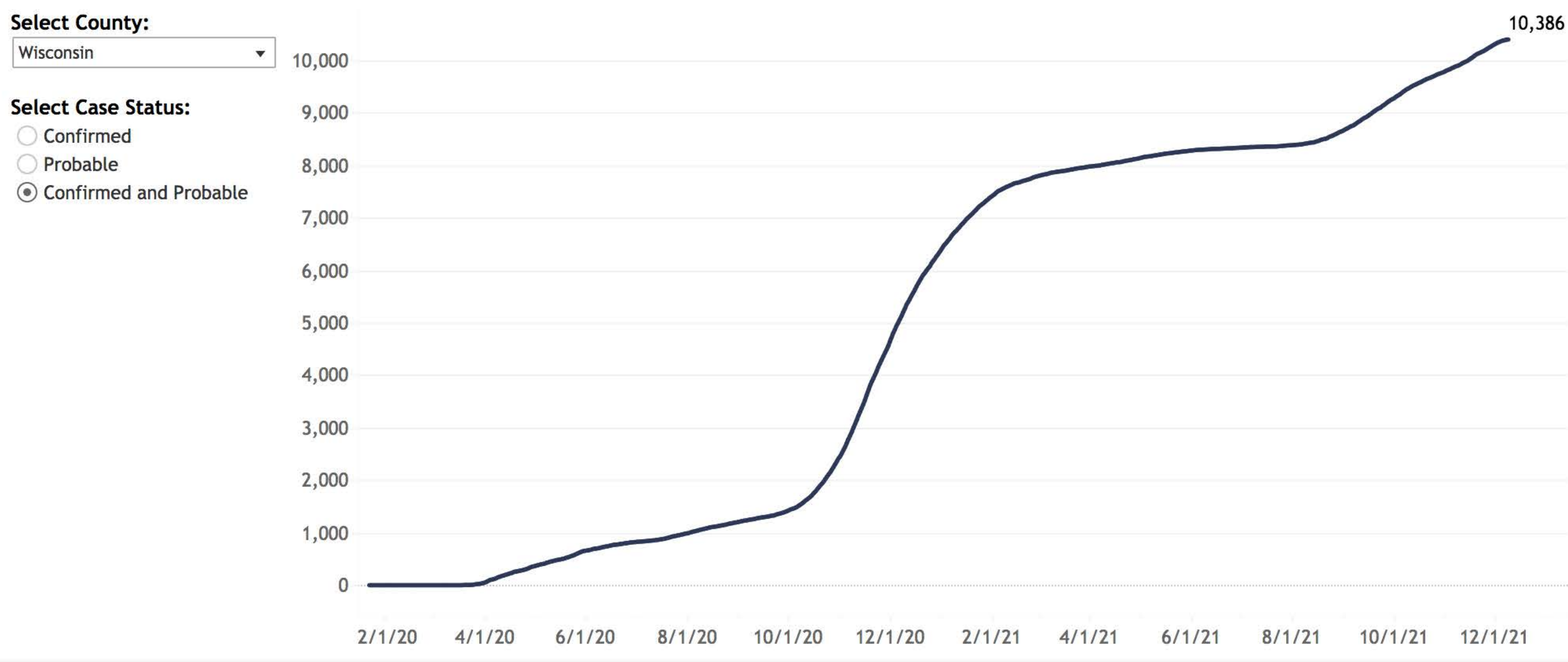

Undo Redo Revert Refresh Pause Metrics Share Download Full Screen

Understanding our data: What does this chart mean?

About our data: How do we measure this?

Back to a list of charts on this page.

Number of reported COVID-19 deaths among confirmed and probable cases of COVID-19 by date of death: Wisconsin

Updated: 12/10/2021 (Total: 10,386)

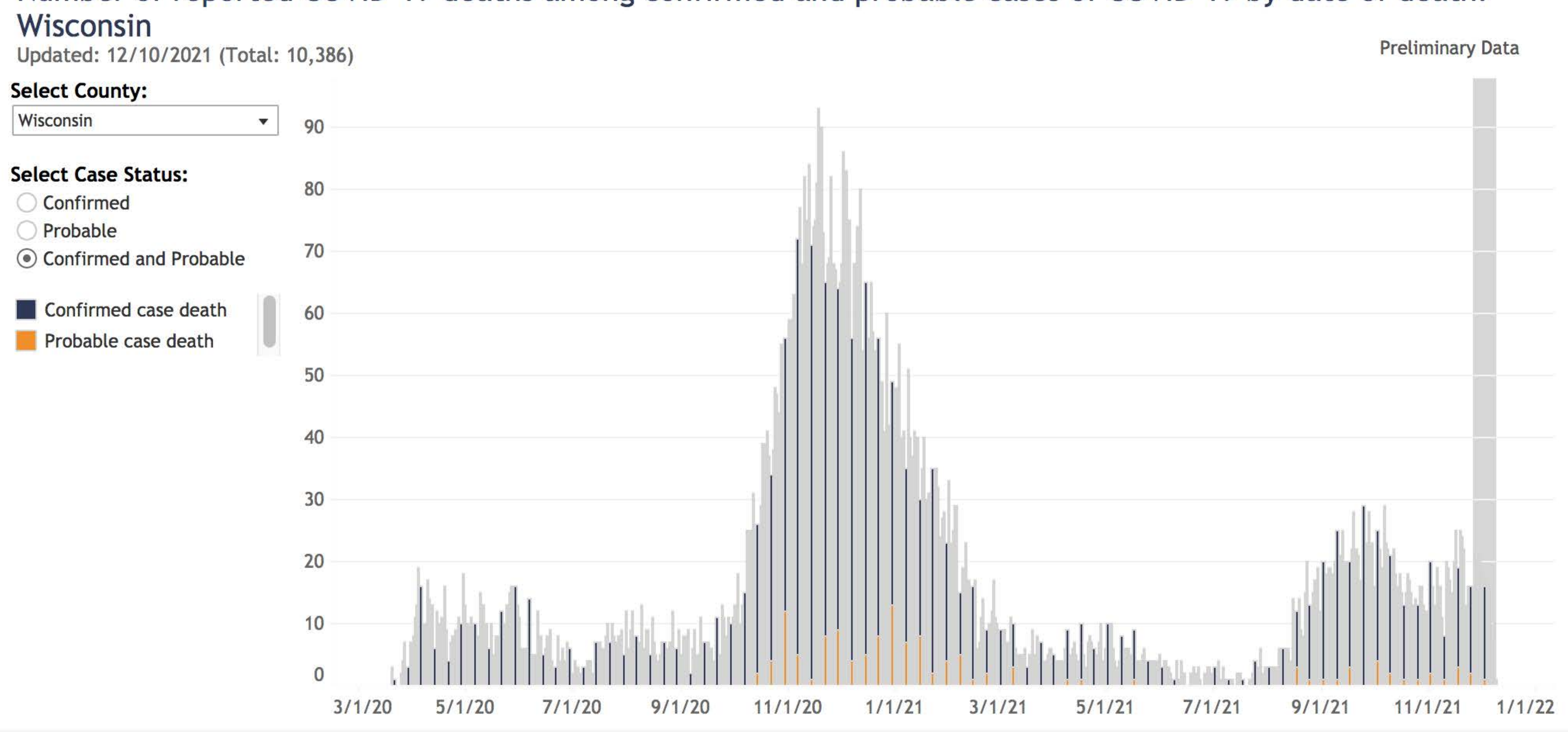

Undo Redo Revert Refresh Pause Metrics Share Download Full Screen

Understanding our data: What does this chart mean?

About our data: How do we measure this?

Back to a list of charts on this page.

Cases and deaths by county

Updated: December 10, 2021

Select a County: All Counties

County	Number of confirmed cases	Number of negatives*	Number of probable cases	Number of deaths among confirmed cases	Number of probable deaths	Cases per 100,000 people (state)	Deaths per 100,000 people (state)	Case fatality percentage
Wisconsin	911,568	3,736,317	112,526	9,344	1,042	15,656.1	160.5	1.0%
[illegible]	[illegible]	[illegible]	[illegible]	[illegible]	[illegible]	[illegible]	[illegible]	[illegible]
Oneida	5,723	22,740	302	95	8	16,078.1	266.9	1.7%
Outagamie	29,464	118,207	6,910	266	29	15,681.9	141.6	0.9%
Ozaukee	12,509	59,553	1,671	118	10	14,020.2	132.3	0.9%
Pepin	1,314	4,548	56	12	0	18,032.1	164.7	0.9%
Pierce	6,585	23,303	1,379	49	6	15,402.1	114.6	0.7%
Polk	8,512	26,565	265	90	12	19,441.3	205.6	1.1%
Portage	10,281	36,933	1,328	117	4	14,526.9	165.3	1.1%
Price	2,150	7,484	180	16	0	16,103.7	119.8	0.7%
Racine	31,391	123,620	5,097	455	36	15,990.4	231.8	1.4%
Richland	2,437	10,046	176	29	4	14,125.9	168.1	1.2%
Rock	22,972	100,675	3,235	236	31	14,062.7	144.5	1.0%

* Total number of people with negative confirmatory diagnostic test results (does not include negative antibody or negative antigen results).
† Total number of COVID-associated deaths among confirmed cases of COVID-19.
‡ Number of COVID-associated deaths among probable cases of COVID-19.
** Total number of deaths among confirmed cases per 100,000 people.
†† Percent of total confirmed cases who died of COVID-19.

Undo Redo Revert Refresh Pause Metrics Share Download Full Screen

Understanding our data: What does this chart mean?

About our data: How do we measure this?

Tracking the COVID-19 curve in Wisconsin

COVID-19 Cases Total

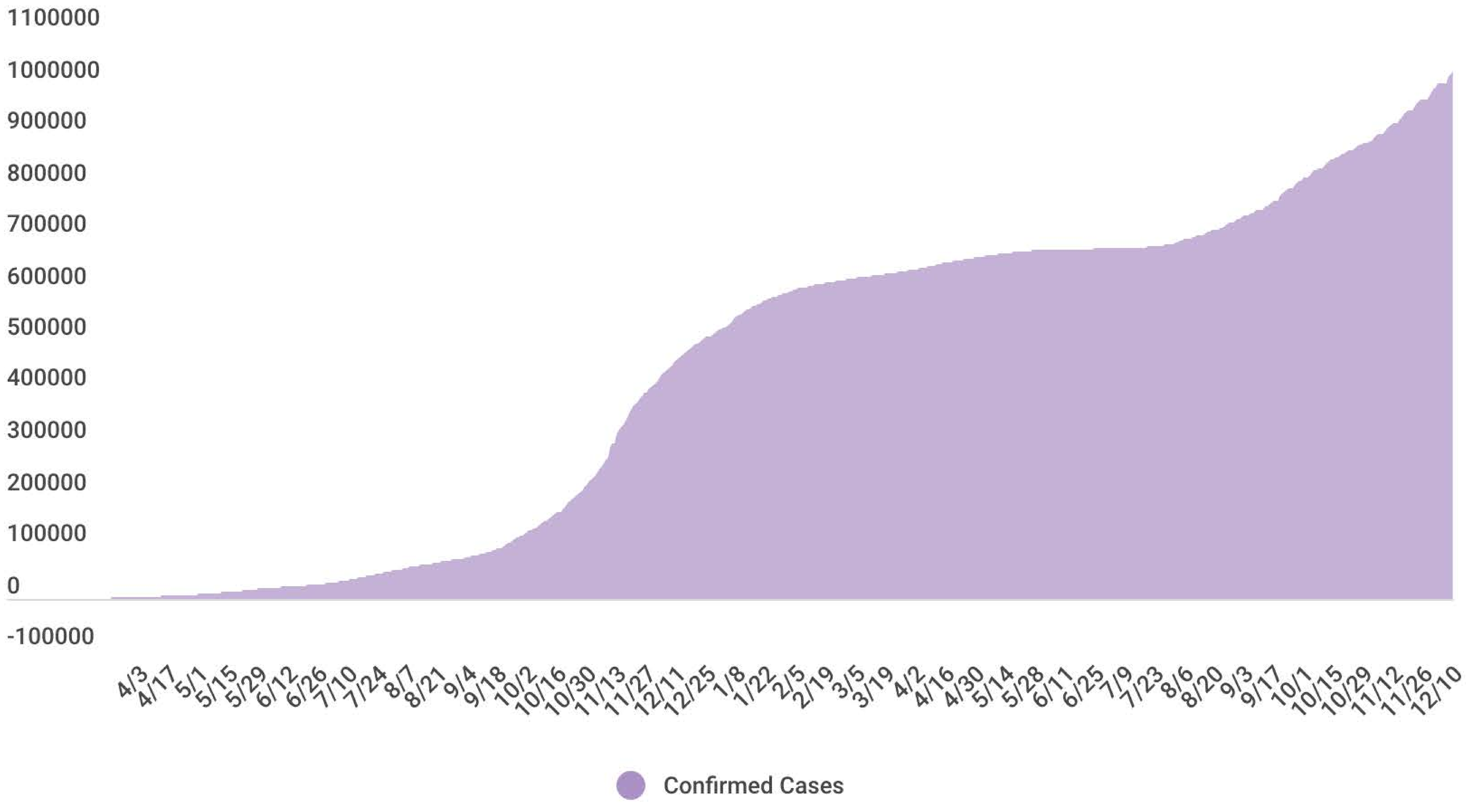

COVID-19 Cases Per Day

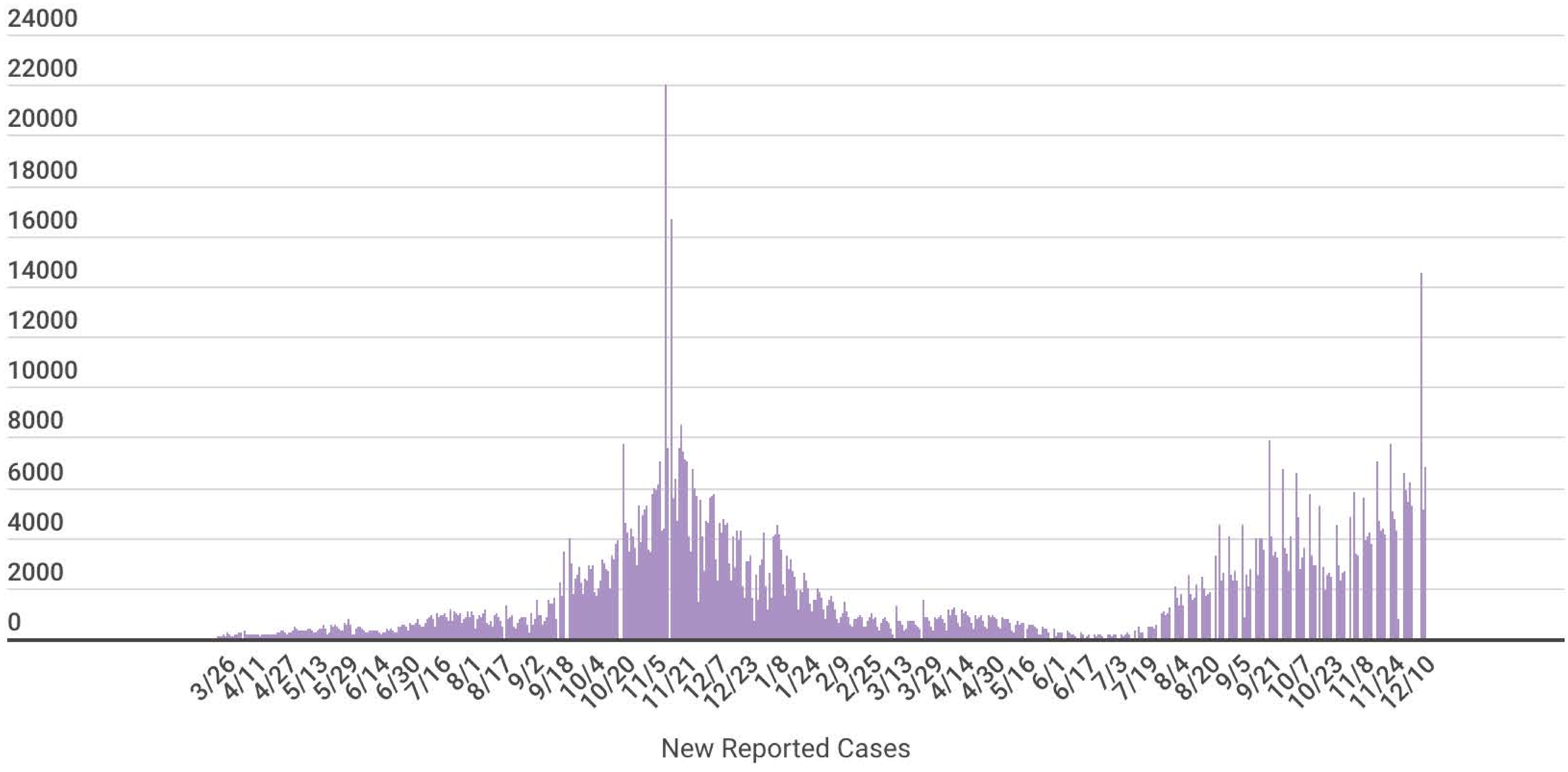

COVID-19 Deaths Total

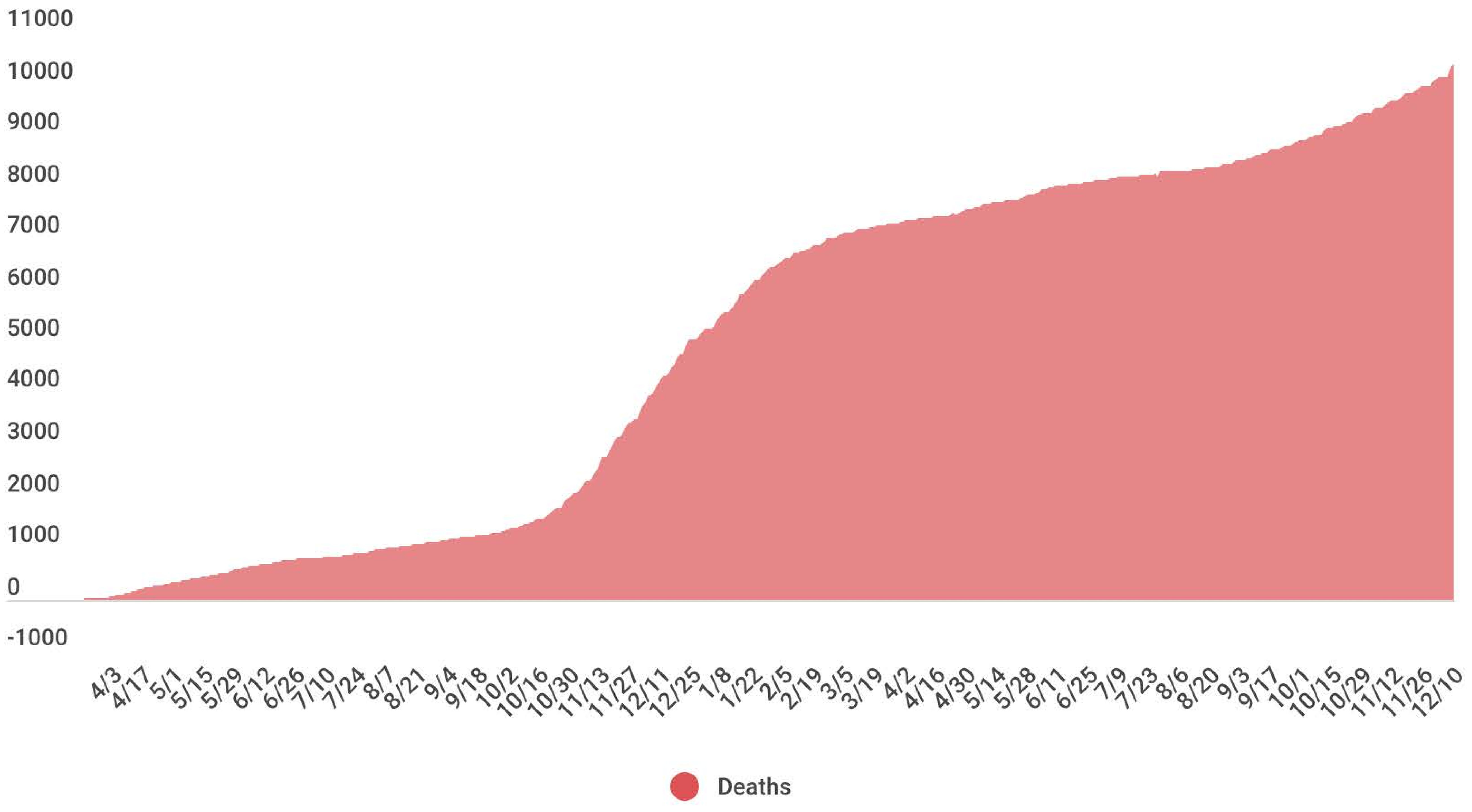

COVID-19 Deaths Per Day

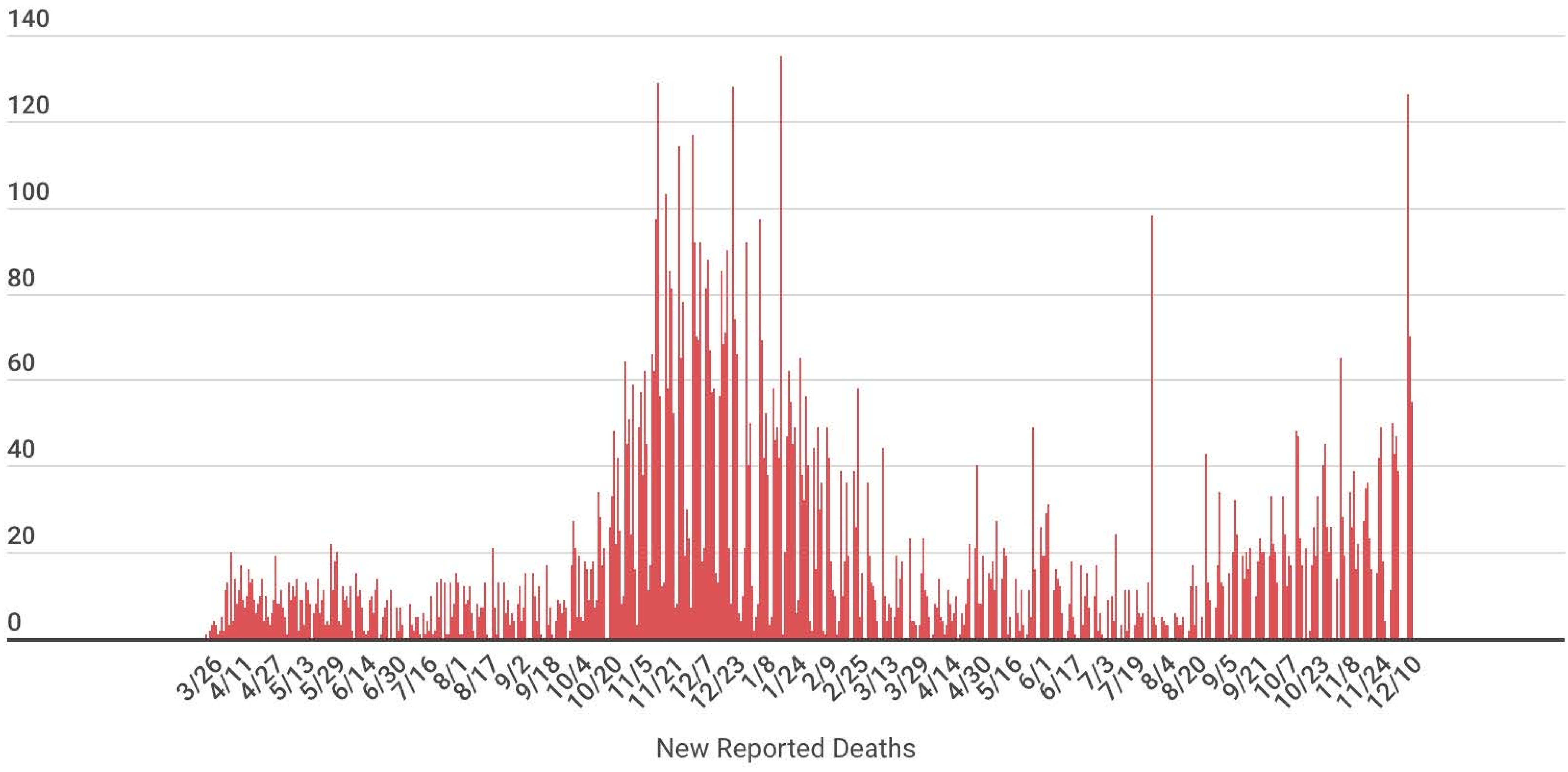

These charts are powered by data from Johns Hopkins University CSSE.
The coronavirus case numbers and death toll are updated daily.
This data may vary from state and local health department tallies.

COVID-19 County by County

HEARST television

ALL GIFTS TRIPLED
GIVE NOW
"We need your essential public service more than ever"
— Gail P., Spotlight PA member
$53,599
of $70,000 goal by 12/31
77%
of goal

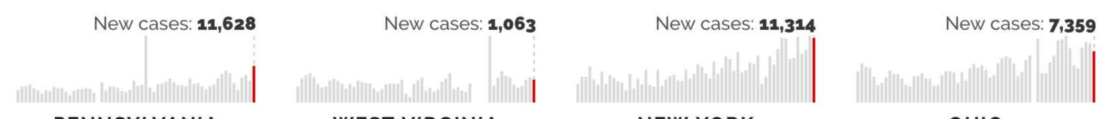

Coronavirus: Tracking The Spread

Since the first case of the coronavirus was identified in Pennsylvania on March 6, 2020, we've been tracking its spread. Every day, we compile data from multiple sources, including state and federal health departments, to provide the most comprehensive picture possible on the virus. Keep in mind, however, that the numbers below tell only part of the story. Due to limited testing during much of the pandemic, it has been difficult to gauge the true scale of the the virus's spread. In addition, data collection and reporting by health departments has often been inconsistent, sometimes resulting in unusual spikes or dips in daily totals. Please read our FAQ for more details.

GET VACCINATED: Want to know where you can get vaccinated in Pennsylvania? Check out Vaccines.gov.

LAST UPDATED: 12/10/2021 • SOURCES: New York Times; U.S. Department of Health & Human Services

TOTAL TESTS
21,284,347 +11,431 from day before

TOTAL CASES
1,818,299 +11,628 from day before

TOTAL DEATHS
34,415 +144 from day before

NOTE: Total tests as of Dec 8, 2021; cases, deaths as of Dec 10, 2021.

REGIONAL TRENDS

This chart shows a 7-day moving average of new cases added each day per 100,000 people.

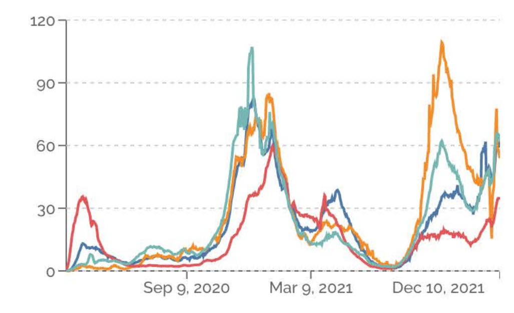

CASES | DEATHS

Per capita | Total

Total reported cases per 100,000 people over past 14 days

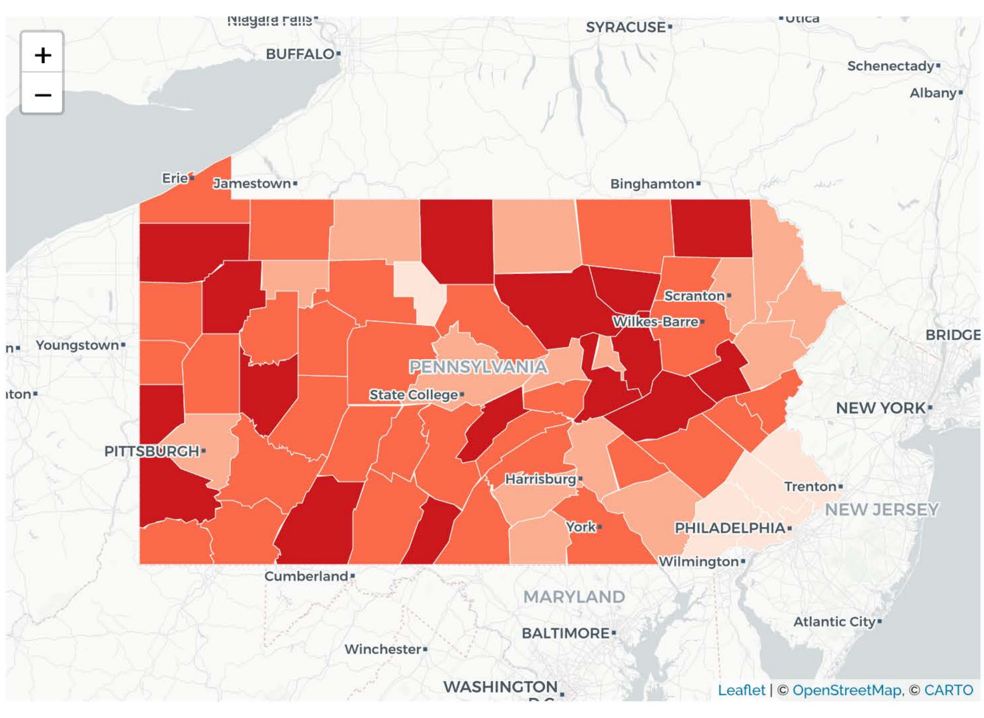

SINCE MARCH | PAST 14 DAYS

Cases by county

As of Dec 10, 2021, 29 counties in Pennsylvania saw a rise in new cases over the past two weeks, while 2 were falling. 36 were statistically unchanged or had an unclear trend. The table below shows data since **Mar 6, 2020**. To see data for only the past two weeks, use the toggle above.

Search by county

SORT BY: Name 14-day Trend Cases Deaths

County	Past 14-day trend*	Cases: Total	Cases: Per 100k people	Deaths: Total	Deaths: Per 100k people
Philadelphia	Rising	197,431	12,464	4,145	262
Allegheny	Unclear	157,410	12,944	2,560	211
Montgomery	Rising	96,653	11,632	1,934	233
Lancaster	Rising	81,990	15,024	1,449	266
Bucks	Rising	81,974	13,048	1,499	239
York	Unclear	74,081	16,497	1,100	245
Delaware	Rising	68,413	12,071	1,536	271
Berks	Rising	68,000	16,146	1,231	292
Chester	Rising	57,893	11,027	916	174
Lehigh	Rising	55,725	15,089	985	267

DAILY CASES TREND (Mar 6 – Dec 10)

SHOW ALL

NOTE: Cases and deaths as of Dec 10, 2021. Past 14-day trend is derived from a linear regression analysis of the 7-day moving average of new cases added each day over the past 14 days. Care should be taken in interpreting 'rising' or 'falling' trends for rural counties with low numbers of new daily cases. Read our FAQ for more information about our methodology.
SOURCE: New York Times

Vaccinations in Pennsylvania

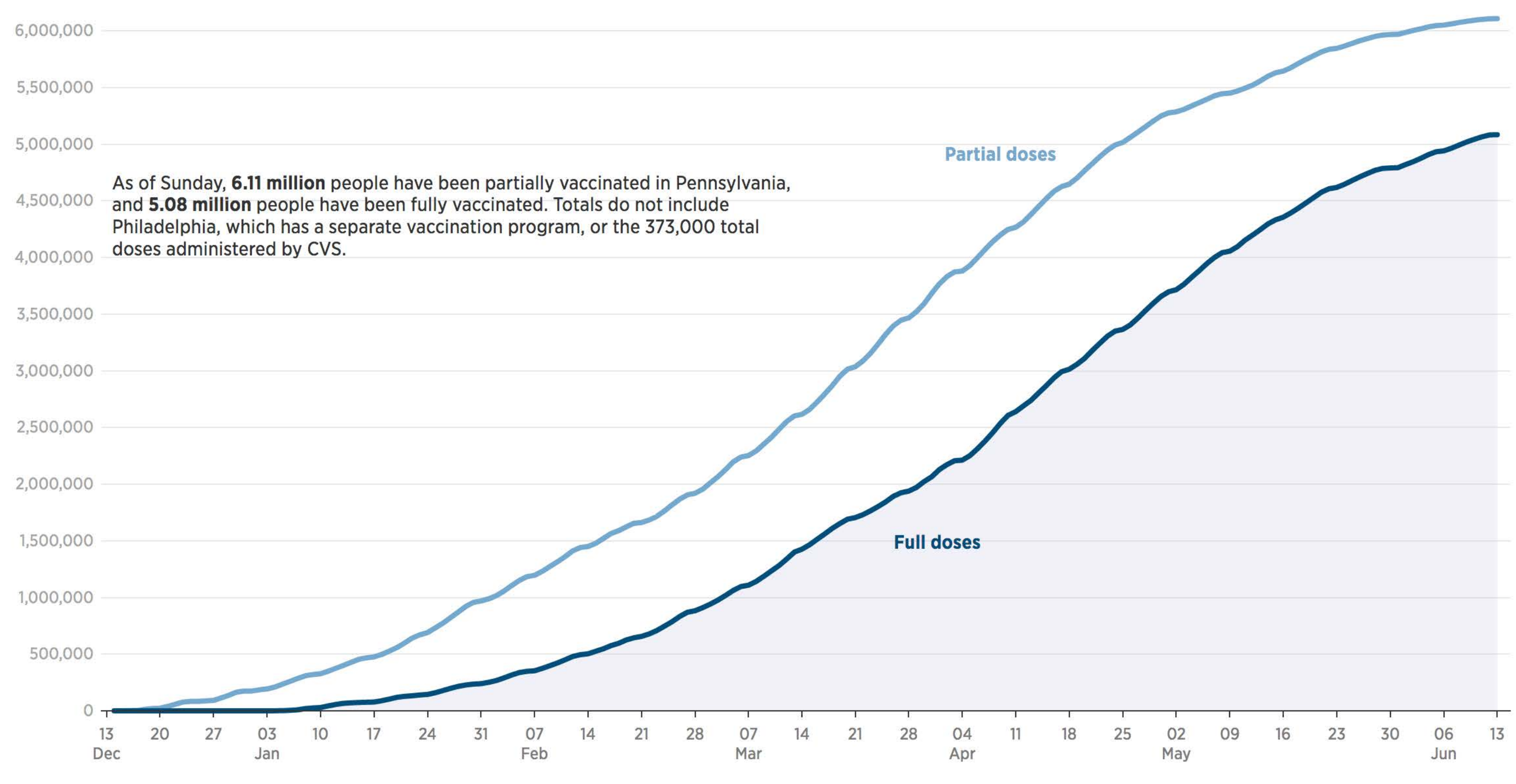

Chart: JOHN DUCHNESKIE / Staff Artist • Source: Pa. Dept. of Health

DAILY TOTALS | CUMULATIVE TOTALS

Cases and deaths in Pennsylvania

As of Dec 10, 2021, there have been an average of 7,786 new cases and 96 new deaths each day over the past week in Pennsylvania. Below are 7-day moving averages since Mar 6, 2020.

CASES

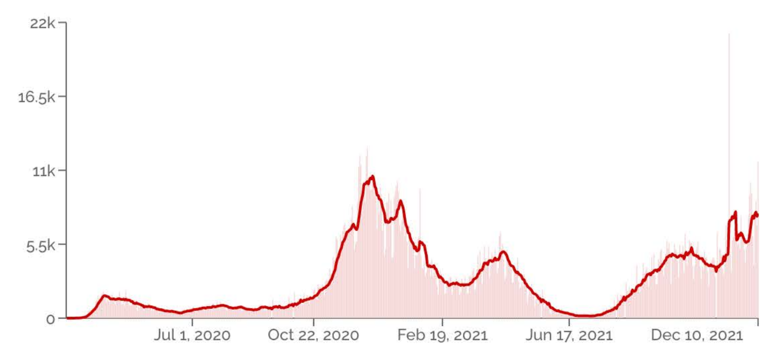

DEATHS

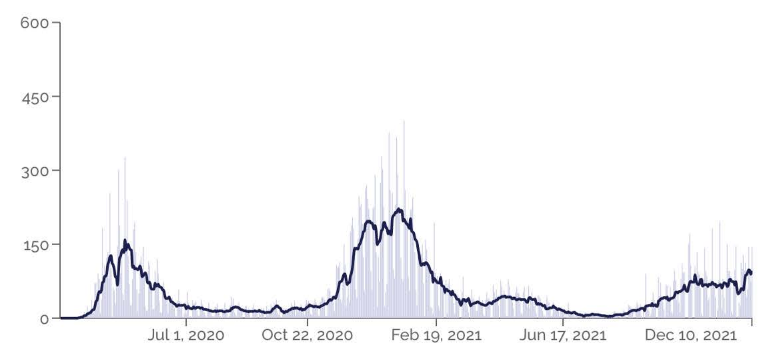

NOTE: Cases include both lab-confirmed positive results and cases deemed probable based on federal criteria.
SOURCE: New York Times

DAILY TOTALS | MORE DATA

Testing in Pennsylvania

As of Dec 8, 2021, Pennsylvania reported an average of 39,650 new viral specimens tested each day over the past week and had a positivity rate of 14.2%. The positivity rate is the average daily number of specimens that tested positive over the past seven days divided by the average daily number of specimens tested over the same period.

TESTS PER DAY

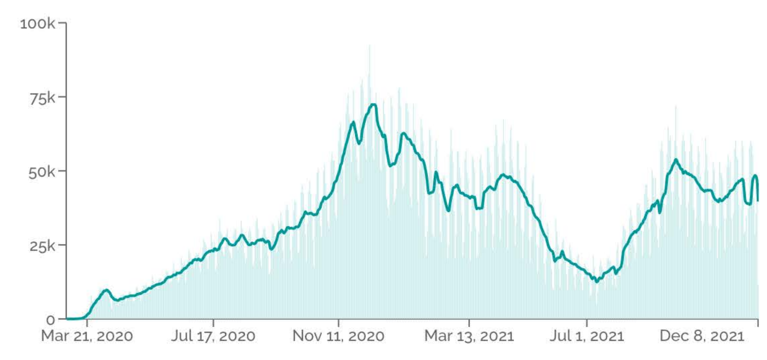

POSITIVITY RATE

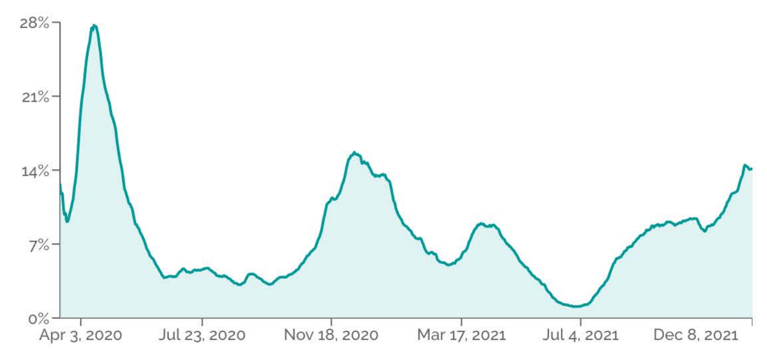

NOTE: Test numbers represent viral COVID-19 laboratory test (PCR) results from laboratories and testing locations. Data is representative of diagnostic specimens being tested, not individual people, and serology tests are excluded where possible. Data might not represent the most current counts for the most recent three days due to the time it takes to report testing information. Data are provisional and subject to change. Also note that the positivity rate above is calculated by the Philadelphia Inquirer/Spotlight PA and may differ from the rate calculated by state officials due to methodological differences. Please read our FAQ for more details.
SOURCE: U.S. Department of Health & Human Services

Hospitalizations in Pennsylvania

As of Dec 10, 2021, 4,962 people in Pennsylvania were in hospital inpatient beds due to a confirmed or suspected case of the coronavirus and 976 adults were in ICU beds. There have been an average of 4,800 people in inpatient hospital beds and 953 adults in ICU beds each day over the week prior.

PEOPLE IN HOSPITAL

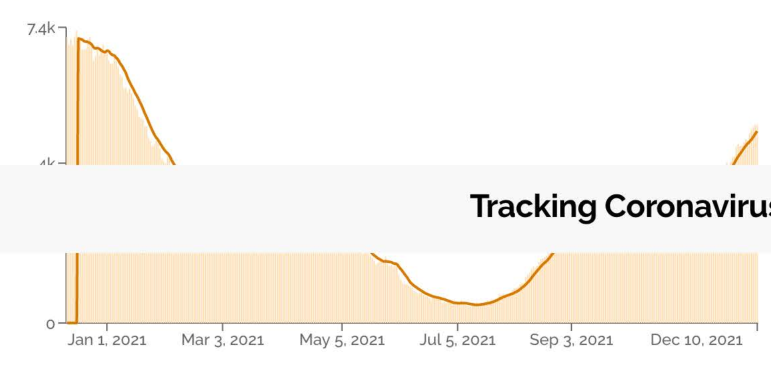

ADULTS IN ICU

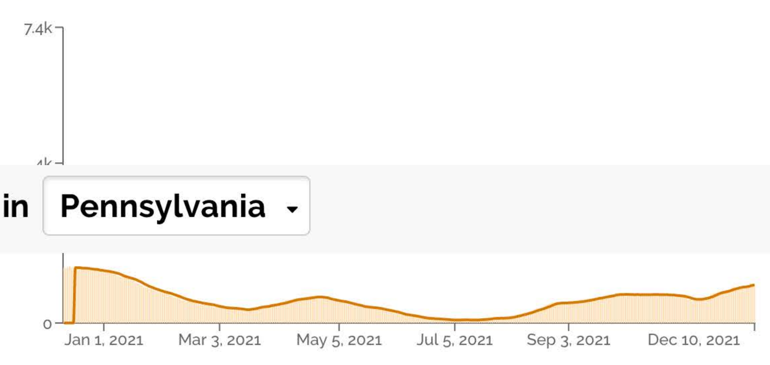

Tracking Coronavirus in Pennsylvania

NOTE: Hospitalization and adults in ICU beds data is updated weekly. Please read our FAQ for more information about this data.
SOURCE: U.S. Department of Health & Human Services (compiled by Carnegie Mellon University)

For more information about the sourcing and methodology on this page, please read our FAQ.

COVID-19 TRACKING: Charts & Interactive Data

The very latest visual data on cases and statistics of coronavirus in Rhode Island and Massachusetts

NOTE: This tracking page includes the most current data available. The R.I. Department of Health updates data Monday through Friday. Some categories — including demographic, school and nursing home data — are updated intermittently.

Percent of Population Fully Vaccinated in Rhode Island

(RI pop. ~ 1.1 million)

% At Least Partially Vaccinated % Fully Vaccinated

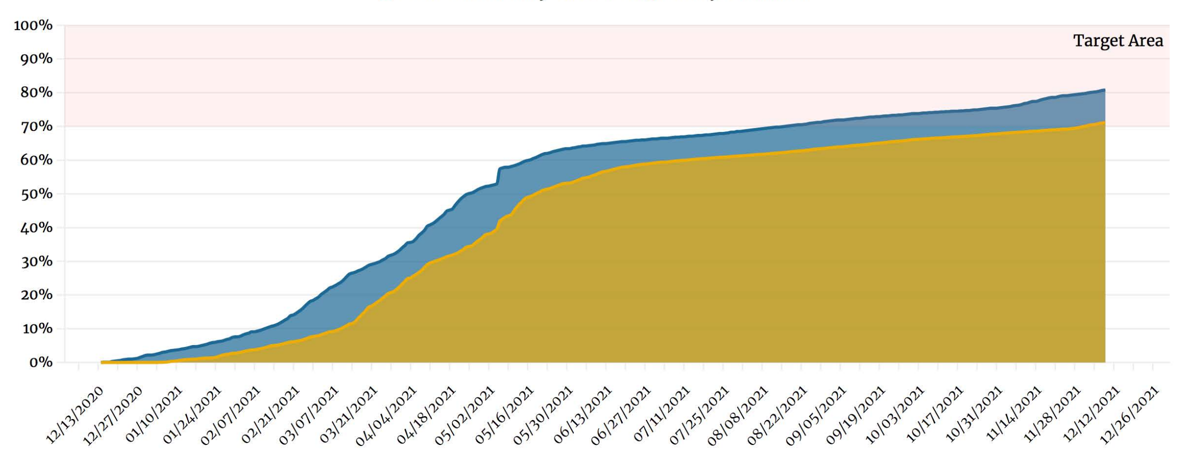

Source: R.I. Department of Health • Digital graphic created by Target 12
*Target area based on public comments made by public health officials and Gov. Dan McKee, who estimate the threshold for herd immunity could range anywhere from 70-90% * Population numbers updated with new Census data 4/27/2021

A Flourish chart

Rhode Islanders Getting COVID-19 Vaccine For First Time

7-Day Average

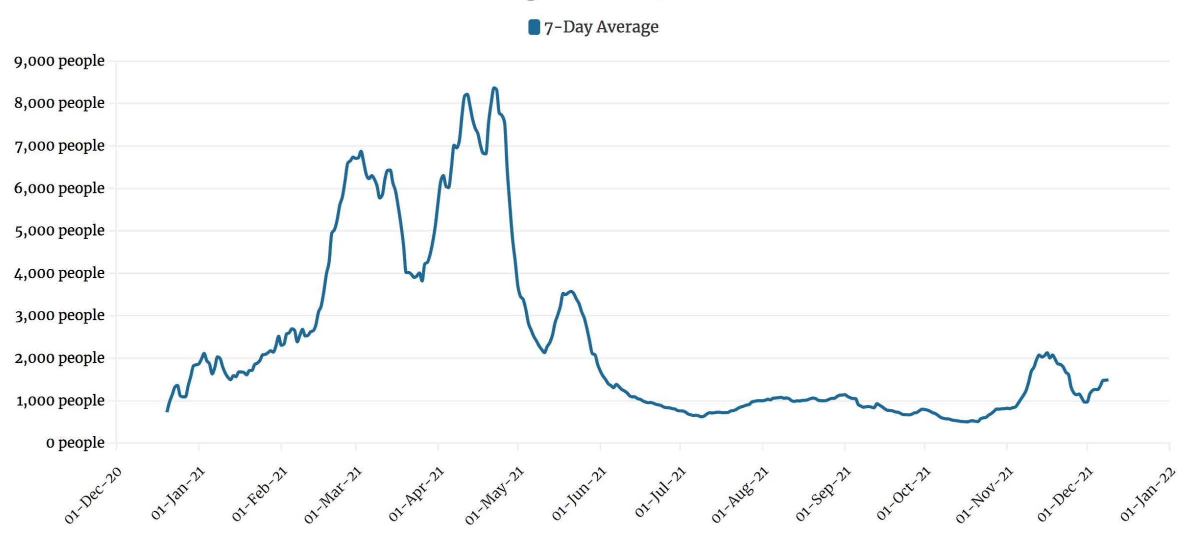

Source: R.I. Department of Health • Digital graphic created by Target 12
Data excludes doses administered at federal facilities in Rhode Island

A Flourish chart

7-Day Average of New Positive Cases in Rhode Island

7-Day Average New Positive Cases

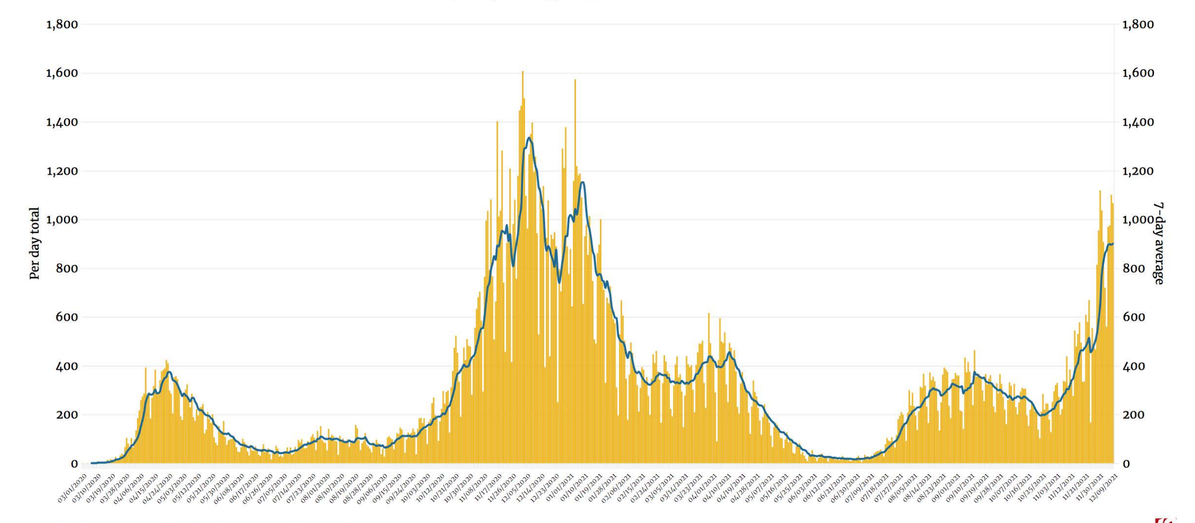

Source: R.I. Department of Health • Digital graphic created by Target 12

A Flourish chart

COVID-19 Cases (2020 v. 2021)

7-day average

2020 2021

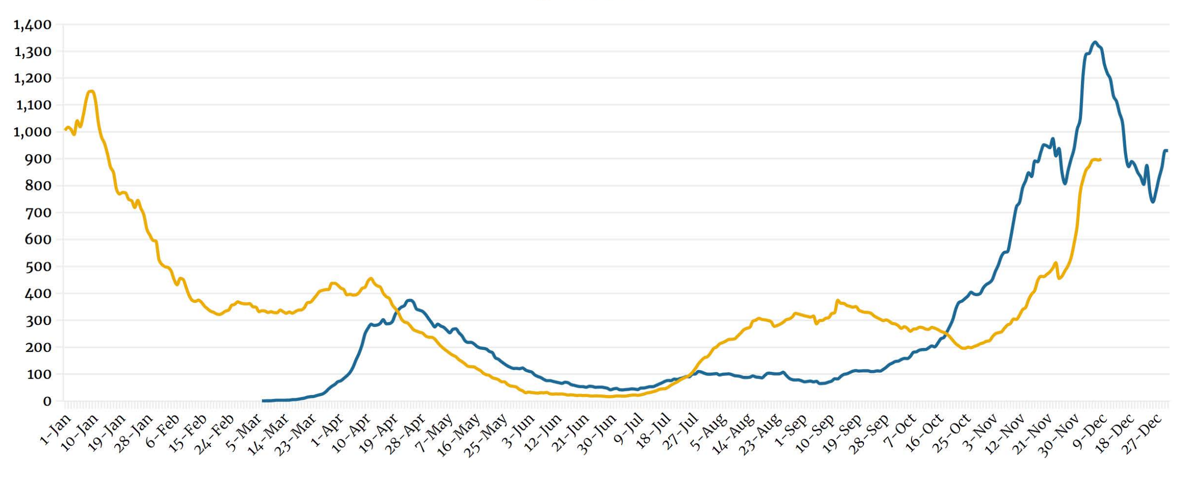

Source: R.I. Department of Health • Digital graphic created by Target 12

A Flourish chart

Weekly Case Rate by Vaccination Status in Rhode Island

Per 100,000 people

(1 = <5)

Vaccinated Unvaccinated

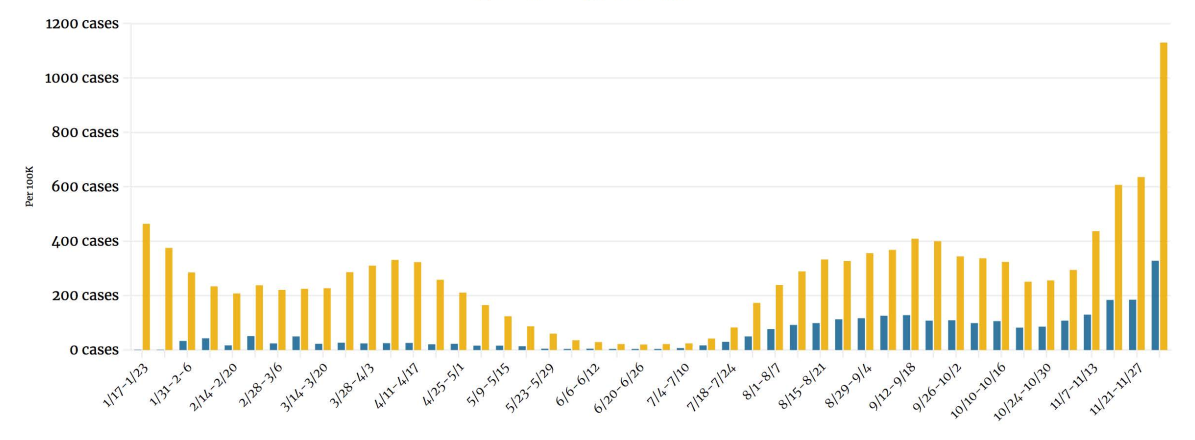

Source: R.I. Department of Health • Digital graphic created by Target 12 * Based on 2018 Census data . Out of state residents are excluded.
Notes: Vaccinated cases include cases that have been diagnosed at least 14 days after completing the primary series of a COVID-19 vaccine

A Flourish chart

COVID-19 Deaths in Rhode Island

Total Deaths Deaths per day

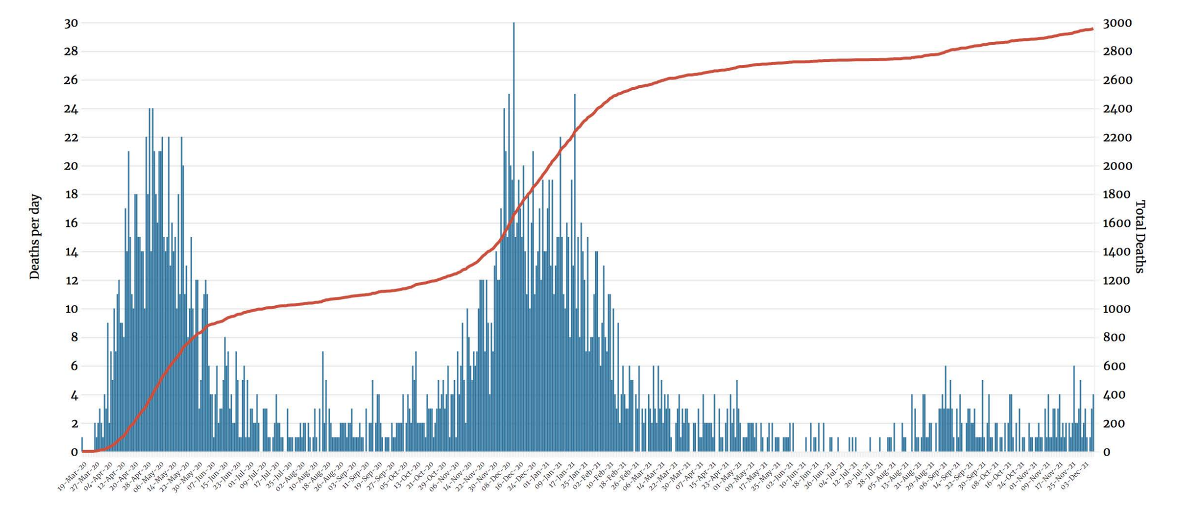

Source: R.I. Department of Health • Digital graphic created by Target 12

A Flourish chart

COVID-19 Deaths (2020 v. 2021)

7-day average

2020 2021

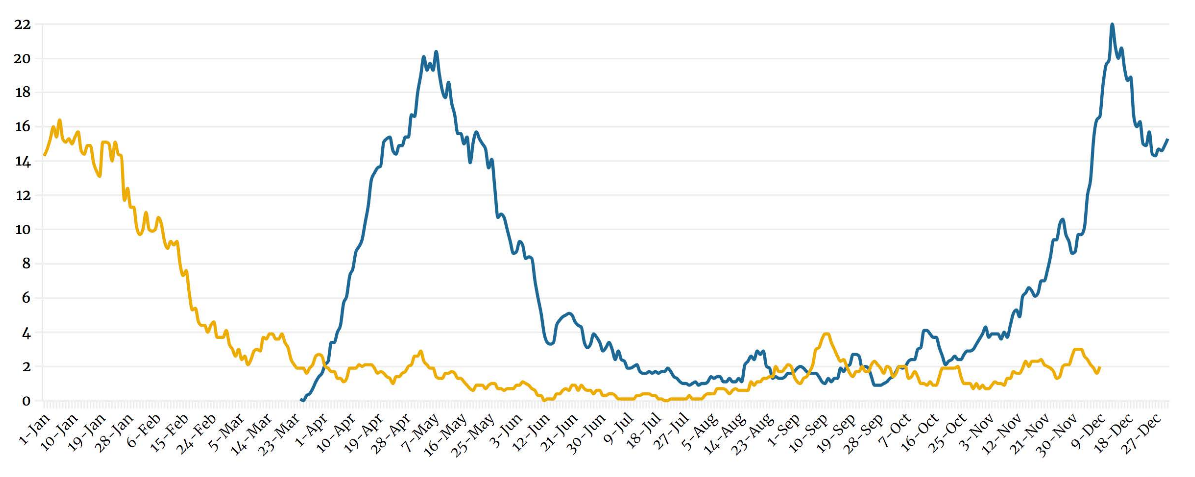

Source: R.I. Department of Health • Digital graphic created by Target 12

A Flourish chart

Total COVID-19 Hospitalizations in Rhode Island

(RI data revised daily with a two-day lag in reporting)

7-Day Average Currently hospitalized

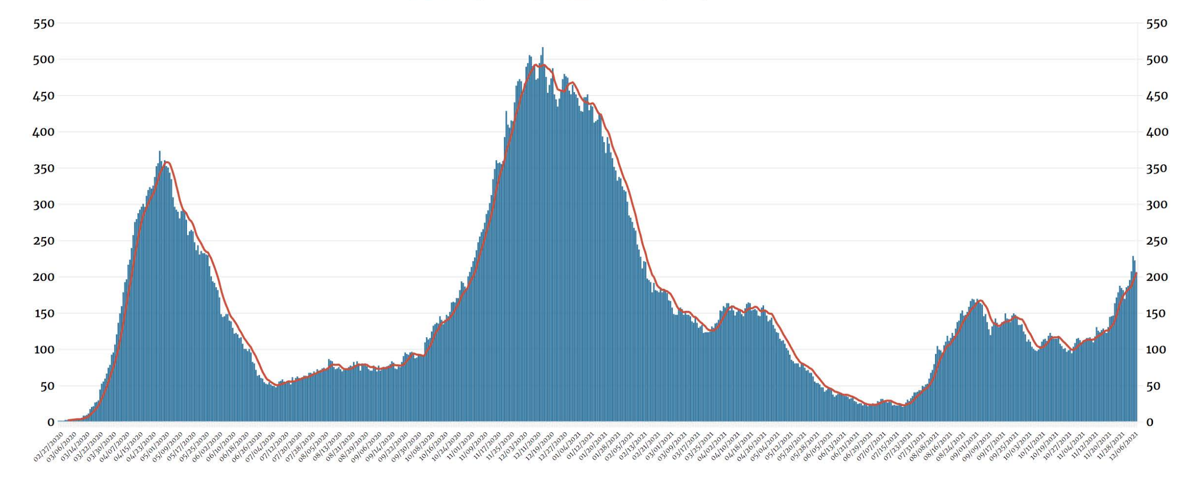

Source: R.I. Department of Health • Digital graphic created by Target 12

A Flourish chart

COVID-19 Hospitalizations (2020 v. 2021)

7-day average

2020 2021

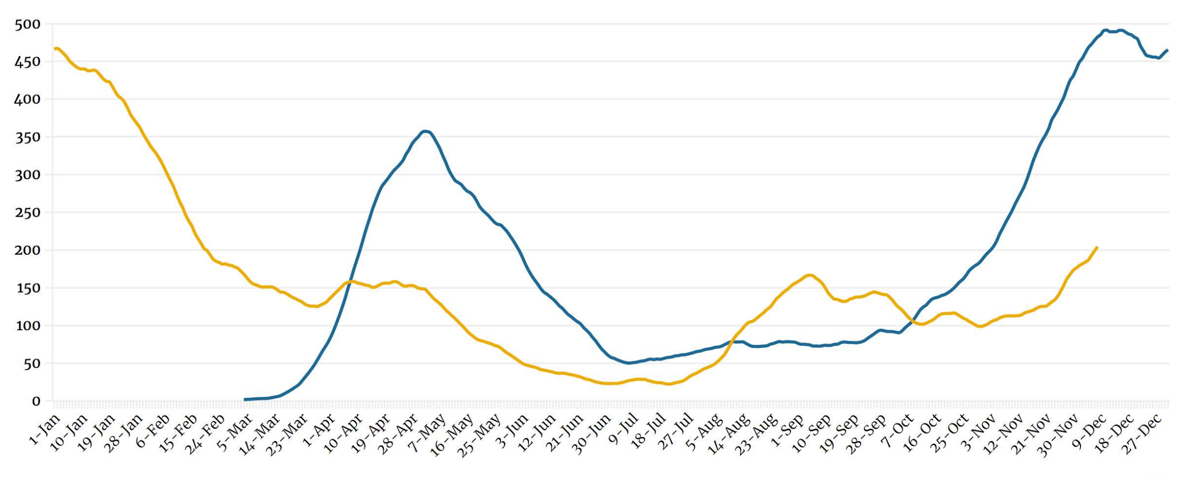

Source: R.I. Department of Health • Digital graphic created by Target 12

A Flourish chart

New Hospital Admissions in Rhode Island

(RI data revised daily with a two-day lag)

7-Day Average New Admissions

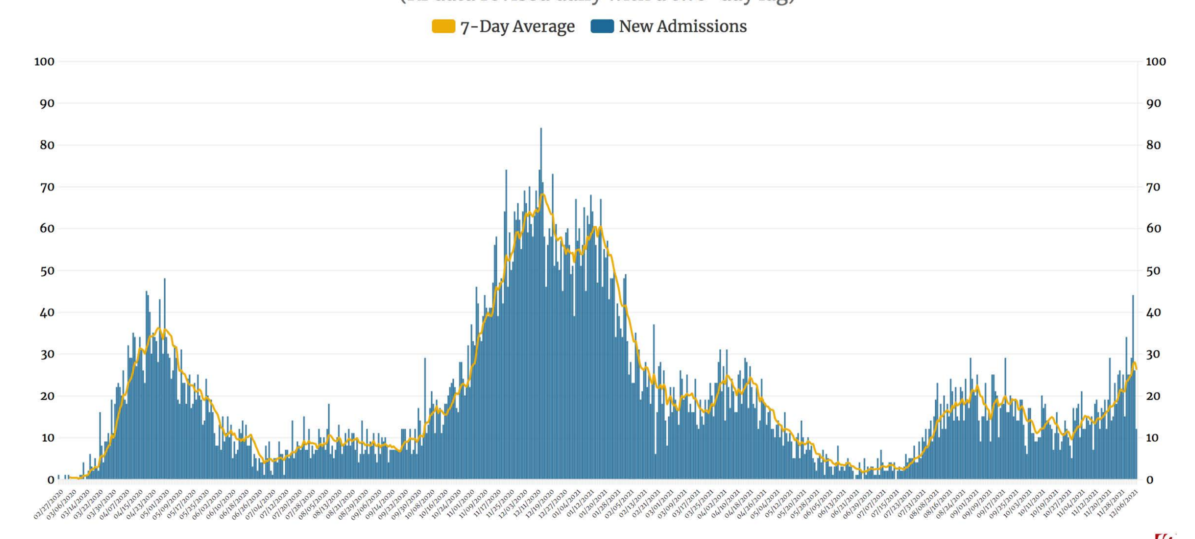

Source: R.I. Department of Health • Digital graphic created by Target 12

A Flourish chart

Rhode Island Positivity Rate

Positivity Rate Positivity Rate (first-time test takers**)

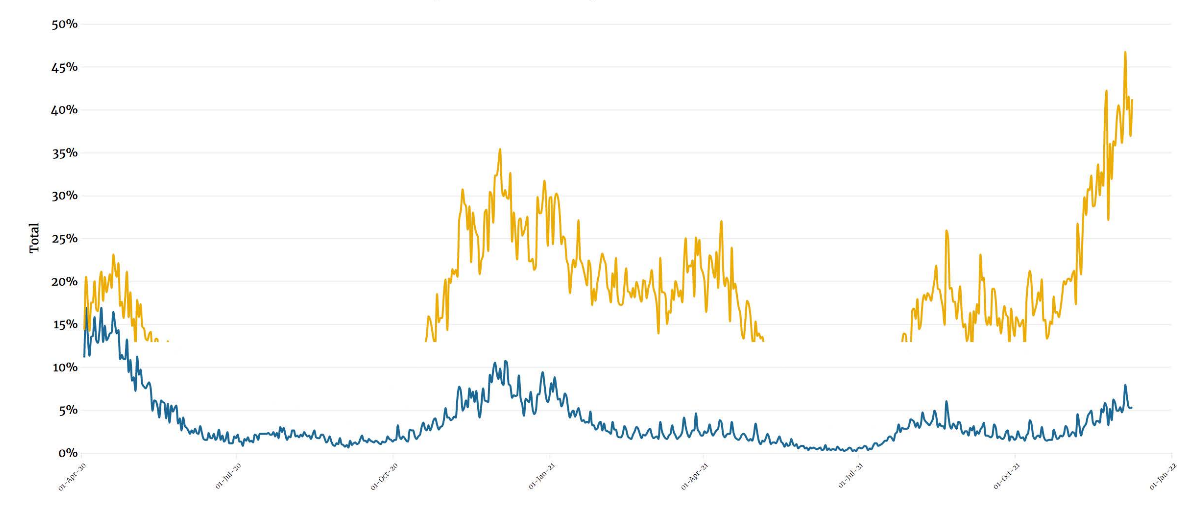

Source: R.I. Department of Health • Digital graphic created by Target 12. Positivity rate = new cases/all tests

A Flourish chart

7-Day Average of Daily Testing in Rhode Island

Includes all negative and positive tests from each day

7-Day Average Total Negative + Positive Tests

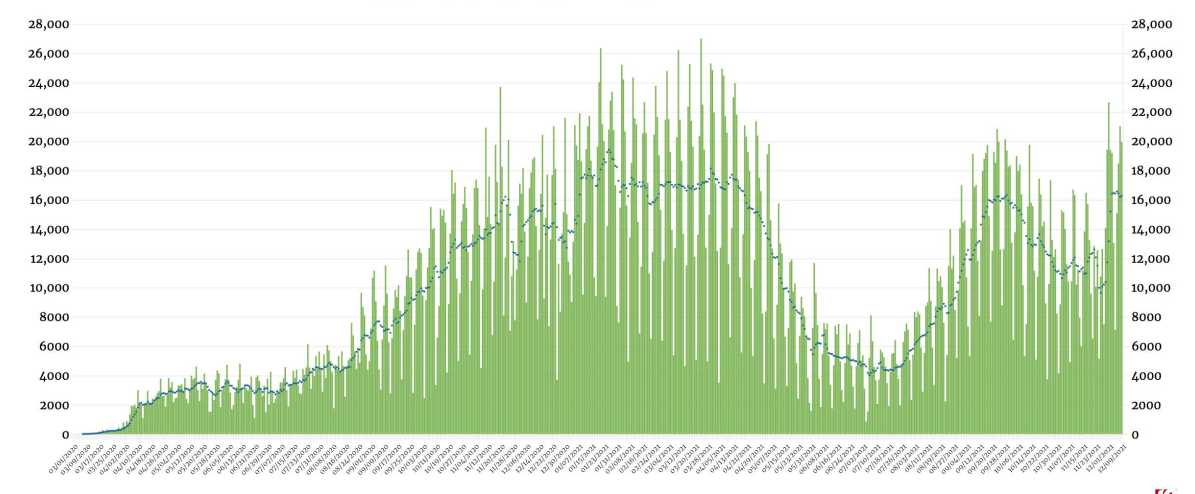

Source: R.I. Department of Health • Digital graphic created by Target 12

A Flourish chart

For the demographics of COVID-19 in Rhode Island, click on the image below

COVID-19 Cases, Hospitalizations and Deaths by Sex Assigned at Birth in Rhode Island

(Sex assigned at birth)

Female Male

Cases Hospitalizations Deaths

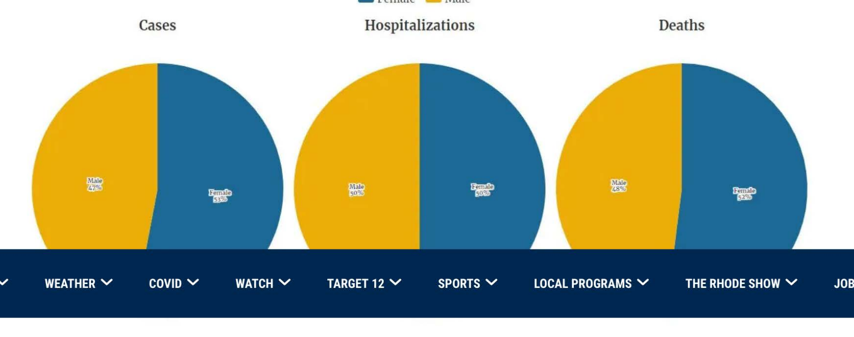

Source: R.I. Department of Health • Digital graphic created by Target 12

A Flourish chart

Bay Area		California		United States	
625,811 cases	6,884 deaths	5,133,036 cases	74,509 deaths	49,875,592 cases	797,128 deaths

Coronavirus Tracker

The Chronicle is mapping every reported coronavirus case in the Bay Area, California and the U.S. We are tallying the number of confirmed cases, deaths, hospitalizations and tests reported across the state by day.

By CHRONICLE DIGITAL TEAM | LAST UPDATED: Dec. 11, 2021 4:38 p.m.

New cases | Total cases

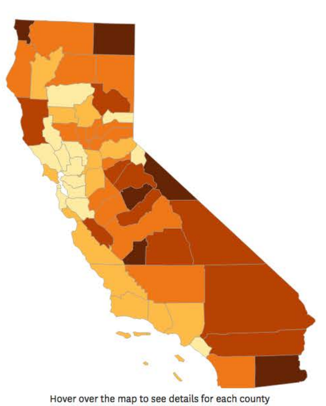

Hover over the map to see details for each county

California cases are organized by reporting county. Cases are based on reports from the U.S. Centers for Disease Control and Prevention, the California Department of Public Health and county public health departments, plus the Chronicle / Los Angeles Times data collaboration. For more information on the data, please read about our methodology.

Update
Starting Sept. 3, the California Data Coalition switched its source for county virus data to counts released the California Department of Public Health. A one-time change in charts and totals occurred on the date of that change. Read more about this change below.

Latest coronavirus news

Due to parish worries, S.F. church delays Archbishop Cordileone's visit after he reveals he's unvaccinated Dec. 10, 2021

Bay Area winter surge arrives, even as omicron numbers stay low Dec. 9, 2021

COVID cases are rising quickly in highly vaccinated Marin. Here's why the mask mandate isn't coming back Dec. 7, 2021

New reported cases and deaths in the Bay Area, by day

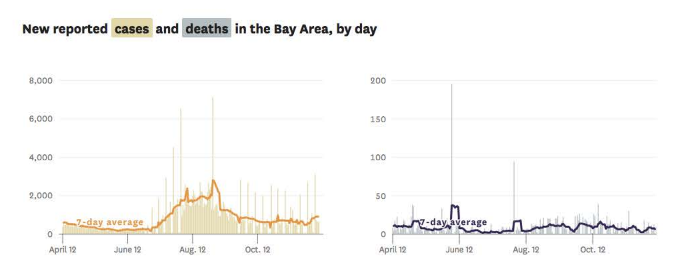

New reported cases and deaths in California, by day

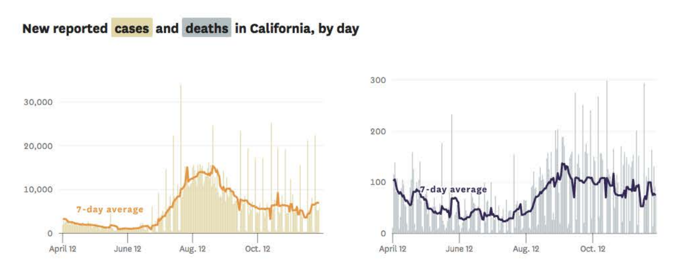

Sources: U.S. Centers for Disease Control and Prevention, California Department of Public Health and county public health departments, Chronicle reporting

The seven-day trailing average represents the average number of cases or deaths reported each day over the weeklong span ending on the given day. It is used to account for daily variances in reporting and large one-off changes in daily counts, such as April 20 when nearly 1,500 cases were reported in L.A. County as the result of a lab clearing a large backlog. Some counties do not provide daily updates which, combined with daily variances in the number of tests given, could result in randomly higher or lower counts for daily reported cases.

How many people are hospitalized?

The number of people hospitalized with COVID-19 in California and in the Bay Area still is far below the totals reached during the winter surge when more than 20,000 Californians were admitted with virus-related symptoms. Hospitalizations and ICU availability were key numbers used to determine the level of restrictions while California was shut down and continue to be monitored as the region experiences a fourth surge driven by the delta variant.

Confirmed ICU and non-ICU COVID-19 patients in Bay Area hospitals

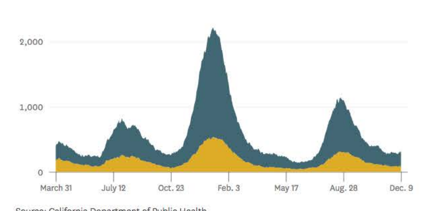

Source: California Department of Public Health

Confirmed ICU and non-ICU COVID-19 patients in California hospitals

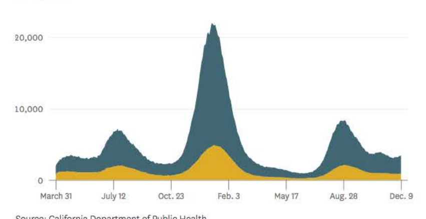

Source: California Department of Public Health

Due to limited testing capacity, not all hospitalized patients with COVID-19 symptoms have been tested. While not all suspected cases will ultimately test positive, including these patients may give a more comprehensive picture of the demands on hospitals. Even with suspected cases included, hospitals have not seen a surge in virus-related patients as feared.

Total confirmed and suspected COVID-19 patients in California hospitals

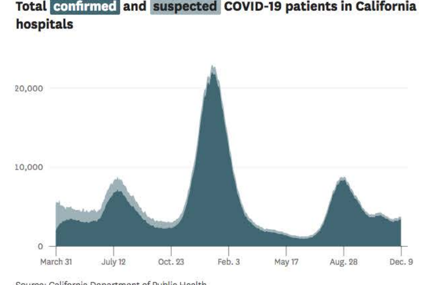

Source: California Department of Public Health

ADVERTISEMENT

What are the current trends in the Bay Area?

The delta variant has caused a sharp increase in case rates across California. Cases have increased more than fivefold in the Bay Area and statewide since California dropped almost all public health restrictions on June 15. COVID hospitalizations in the state have more than tripled. The Bay Area has relatively high vaccination rates, but the delta variant is spreading rapidly among the unvaccinated, according to county and state data, driving surges across counties. California public health officials have recommended universal masking for state residents in all public indoor settings, regardless of vaccination status, following similar guidance issued by the U.S. Centers for Disease Control a day earlier.

Cases and deaths by Bay Area county

The new case curve represents the 7-day moving average which accounts for variances in daily reporting. The change in new cases compares the number of cases in the past seven days to the previous seven days.

County	Total deaths	Total cases	New cases curve	Weekly change in new cases	
Santa Clara	1,941	154,536	255 (April 11 – Dec. 10)	▲466	+35%
Alameda	1,496	126,740	168	▲454	+63%
Contra Costa	1,061	105,074	126	▲151	+21%
San Francisco	678	57,770	88	▲209	+51%
San Mateo	580	57,192	79	▲132	+31%
Solano	365	48,211	51	▲21	+6%
Sonoma	407	43,811	67	▲113	+31%
Marin	249	18,895	42	▲97	+49%
Napa	107	13,582	15	▲44	+70%

Sources: U.S. Centers for Disease Control and Prevention, the California Department of Public Health and county public health departments, Chronicle / Los Angeles Times data collaboration

The Marin County figures include the San Quentin state prison outbreak that began June 20 and totaled more than 2,200 cases.

Bay Area cases by city or region

Some Bay Area county health departments are now providing details on where people who have tested positive reside, either by region, city or jurisdiction. Use the drop down to search counties (more will be added if data becomes available). Last updated Dec. 1.

Alameda County

Place	Total cases	Cases last month
Alameda	3,674	128
Albany	526	30
Berkeley	5,266	221
Castro Valley MAC	3,862	148
Dublin	2,737	90
Eden Area MAC	7,620	191

Show more

What is the status of testing in California?

The volume of testing has slowly declined with the increase in vaccinations. Before vaccines were widely available, the World Health Organization had established a recommended positivity rate of 5% for reopening. California was well below that since the end of the winter surge. The state has risen above that marker during the current surge of cases driven by the delta variant.

Daily test results reported in California

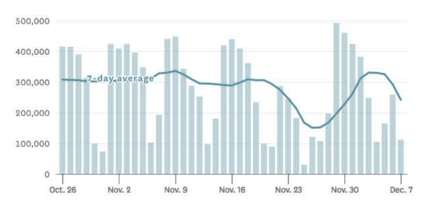

Positive test rate in California

The positive test rate, or positivity rate, is the percentage of total tests that come back positive each day. A lower positivity rate can indicate that enough tests are being conducted. Read more about the positive test rate here.

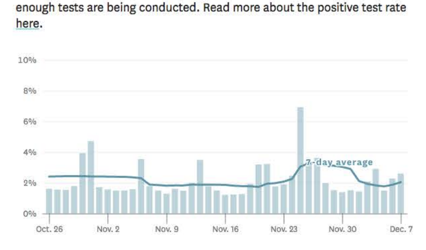

Source: California Department of Public Health, Los Angeles Times

Tests are reported by the California Department of Public Health. On April 22, the CDPH began reporting total number of tests conducted, rather than individual people tested, causing test numbers to jump because some individuals are tested more than once.

On July 1, the state reported a backlog of nearly 4,000 positive test results which elevated the positivity rate for that day. This type of variation is accounted for by the trailing seven-day average.

Total cases and deaths in the U.S.

The U.S. has the highest reported total COVID-19 cases of any country. And while California has the highest totals in the nation in terms of cases and deaths, but on a per-capita basis, the total number of cases and deaths is below the national average.

49,875,592 confirmed cases in the U.S.
797,128 deaths

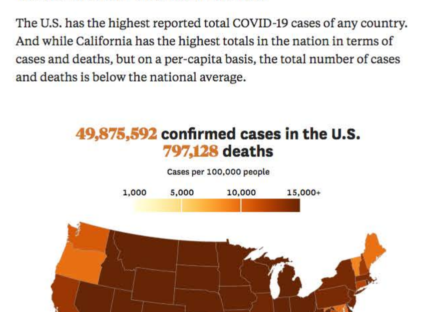

Figures include presumed COVID-19 deaths.

Sources: The Center for Systems Science and Engineering at Johns Hopkins University, U.S. Census Bureau

U.S. reports of COVID-19 are tabulated by Johns Hopkins, which is tracking cases using data collected from the Centers for Disease Control and Prevention and state and local public health departments. Numbers from Johns Hopkins for California may not match the numbers from the top map, which are being compiled by The Chronicle using county reports, county public health departments and Chronicle reporting.

HAVE MORE QUESTIONS?

We're answering our readers' most common questions here. Below you can find even more answers for issues specific to the Bay Area.

Where can I get tested?

When and where do I need a mask?

How does the digital vaccine record work?

Should I keep wearing a mask?

What if I don't get the second dose?

How do the vaccines work?

How dangerous is the delta variant?

Will UC system require vaccinations?

When will the pandemic be over?

How will the pandemic be remembered?

FIFTH & MISSION PODCAST

Fifth & Mission is diving into all the ways COVID-19 is impacting life in the Bay Area. Listen by selecting your preferred podcast service below.

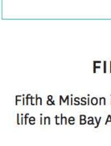

APPLE | SPOTIFY | OVERCAST | GOOGLE | STITCHER | POCKET

TELL US YOUR TOP CONCERNS

ASSIGNMENT EDITOR

We want to report the stories that matter to you. Tell us the questions you have about the pandemic's impact on life in the Bay Area, and the issues you want to see covered.

Tell us your questions; what should we investigate?

NEXT

ABOUT THE DATA

Update: Cases and deaths data come from files released by the California Department of Public Health, collected from 61 county and city health departments statewide.

Before September 2021, The Chronicle and the Los Angeles Times led a data collection partnership of California media organizations, including the San Diego Union-Tribune, KQED, KPCC, CapRadio, Calmatters and Big Local News at Stanford University.

Data collection at the outset of the pandemic was completed manually, four times each day. This count was able to collect figures far ahead of any state-led tally. Once the state's data improved, the coalition moved to end its own collection. The source update on Sept. 3 resulted in a one-time change to charts and figures on this page. Questions can be sent to our team here.

Findings signal earlier start to U.S. outbreak: An autopsy report showing that the first death linked to COVID-19 happened Feb. 6 in Santa Clara County and not Feb. 26 in Washington supports research indicating that the outbreak began in the United States much earlier than we knew and before health officials began tracking data.

There were 300 cases reported in the Bay Area when shelter-in-place orders were put in place on March 16. But studies and disease models suggest that more than 10,000 people in the Bay Area likely were infected by then. That research, along with the earlier death identified in Santa Clara County, indicates how far behind the U.S. was in recognizing the outbreak of the virus.

CREDITS

HEALTH REPORTER

Erin Allday · eallday@sfchronicle.com · @erinallday

STAFF WRITER

Mike Massa · mmassa@sfchronicle.com

NEWSROOM DEVELOPERS

Audrey DeBruine · @acdebruine

Kazi Awal · @superKazi

Eric Blom · eric.blom@sfchronicle.com · @ejblom

Katlyn Alo · katlyn.alo@sfchronicle.com · @kat_alo

Evan Wagstaff · evan.wagstaff@sfchronicle.com · @evanwagstaff

Hilary Fung · hilary.fung@sfchronicle.com · @hil_fung

ART DIRECTOR

Danielle Mollette-Parks · dmollette-parks@sfchronicle.com · @daniellemparks

EXECUTIVE PRODUCER, DIGITAL

Brittany Schell · bschell@sfchronicle.com · @brittlynns

MANAGING EDITOR, DIGITAL

Tim O'Rourke · tororke@sfchronicle.com · @TimothyORourke

HEARST newspapers
©2021 Hearst

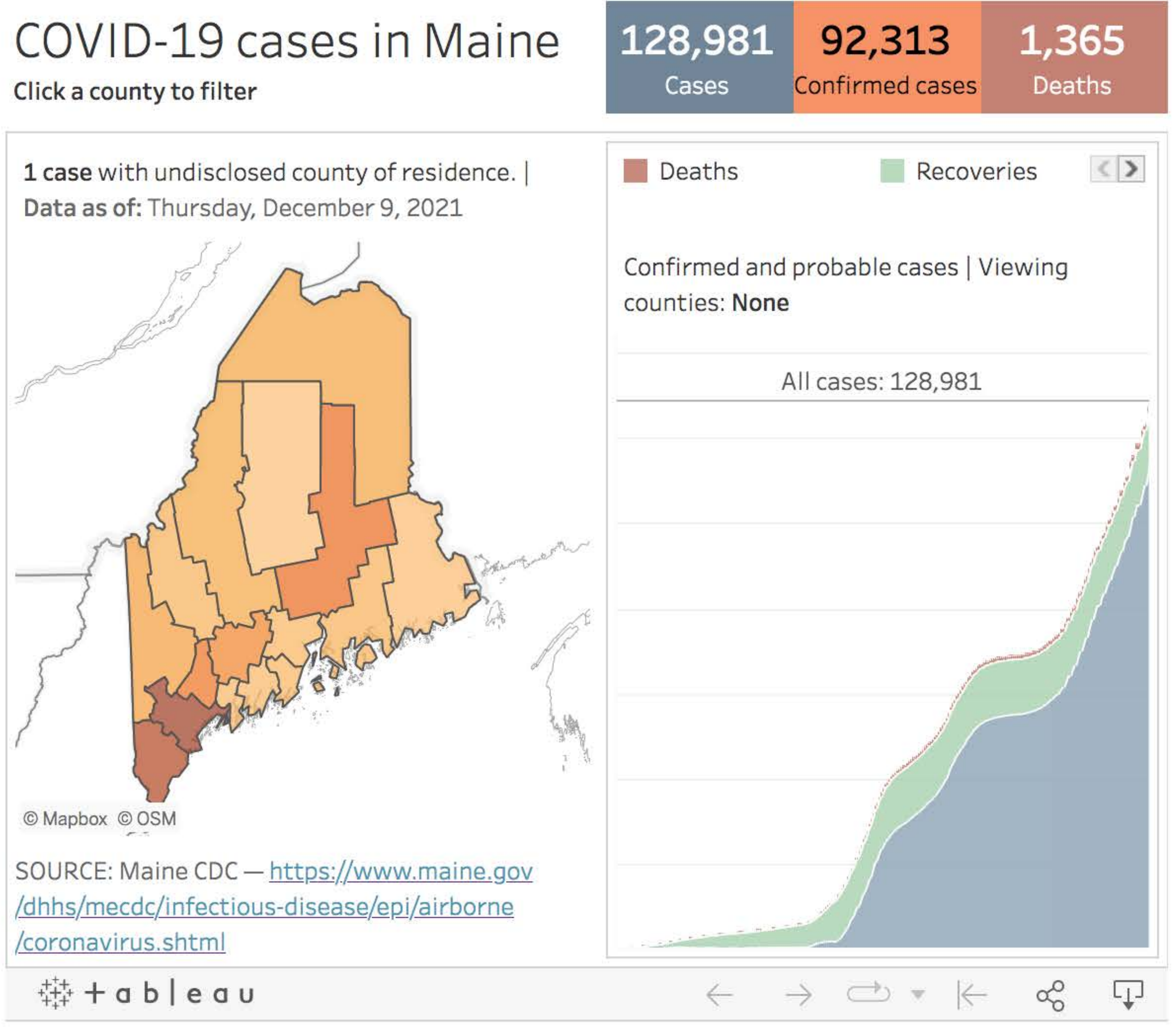

New cases

The rate of growth of cases is important to watch. Social distancing measures intend to slow the spread of the disease so that fewer people are sick all at once, which stands to overwhelm the healthcare system.

Cumberland County has seen the largest spikes in new cases, hitting a high for new cases each day. The early numbers could be impacted by delays in testing, as well.

The chart also illustrates some of the path the disease has taken through Maine counties, starting from March 18. Piscataquis, Aroostook and Washington counties had not reported a case as of April 1.

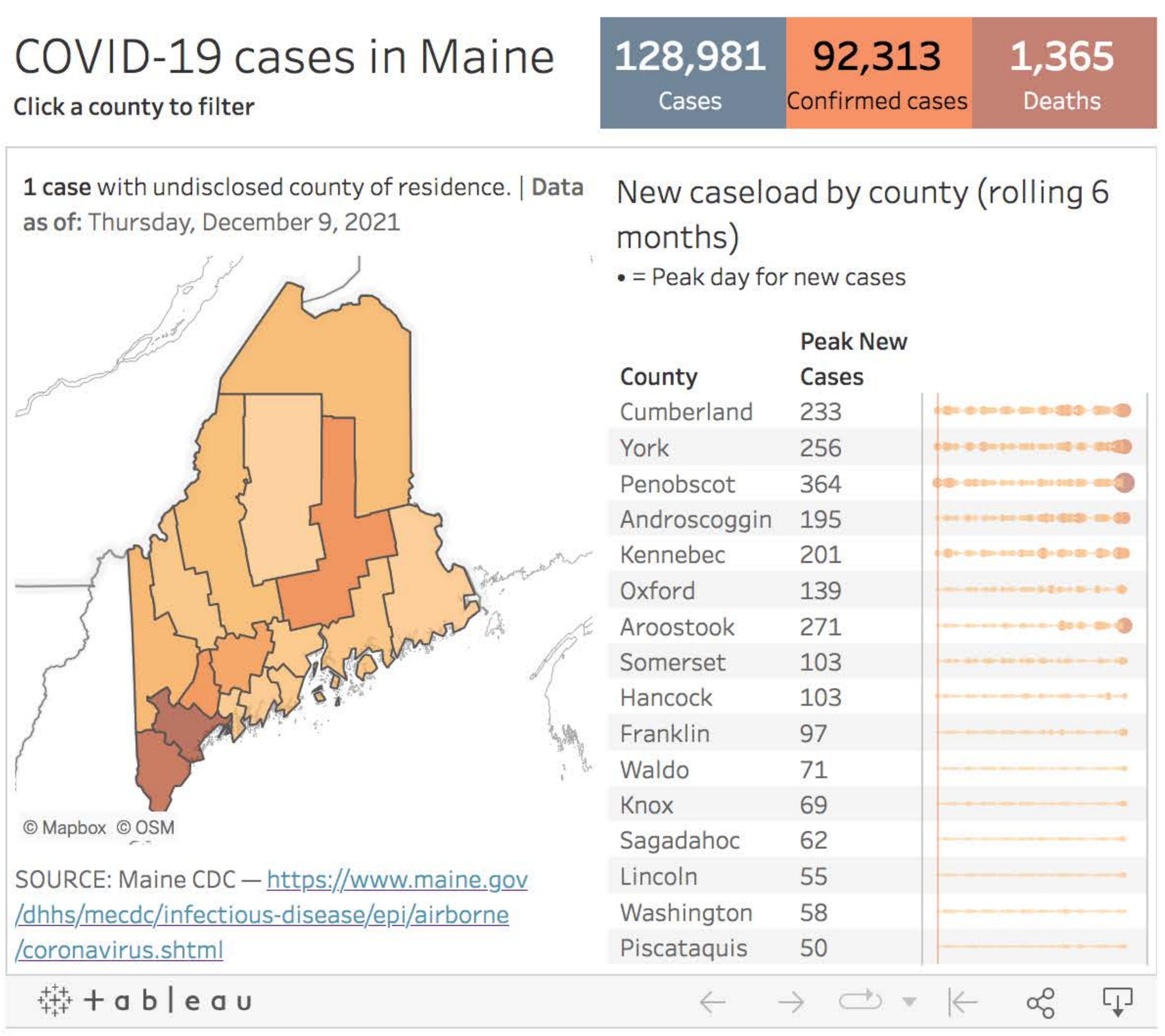

Case rates

For overall metrics, Cumberland and York will have the highest case counts because they have the most people.

The charts below adjust for population, showing the counties that have the highest current rates of confirmed infection.

These are shown alongside the latest calculations for the share of confirmed cases that have resulted in either recovery or hospitalization. These numbers only provide a small snapshot of the current status of testing and the disease in Maine.

In other words, those numbers reflect the situation on the ground in Maine and not necessarily a broader set of insights about the illness, COVID-19.

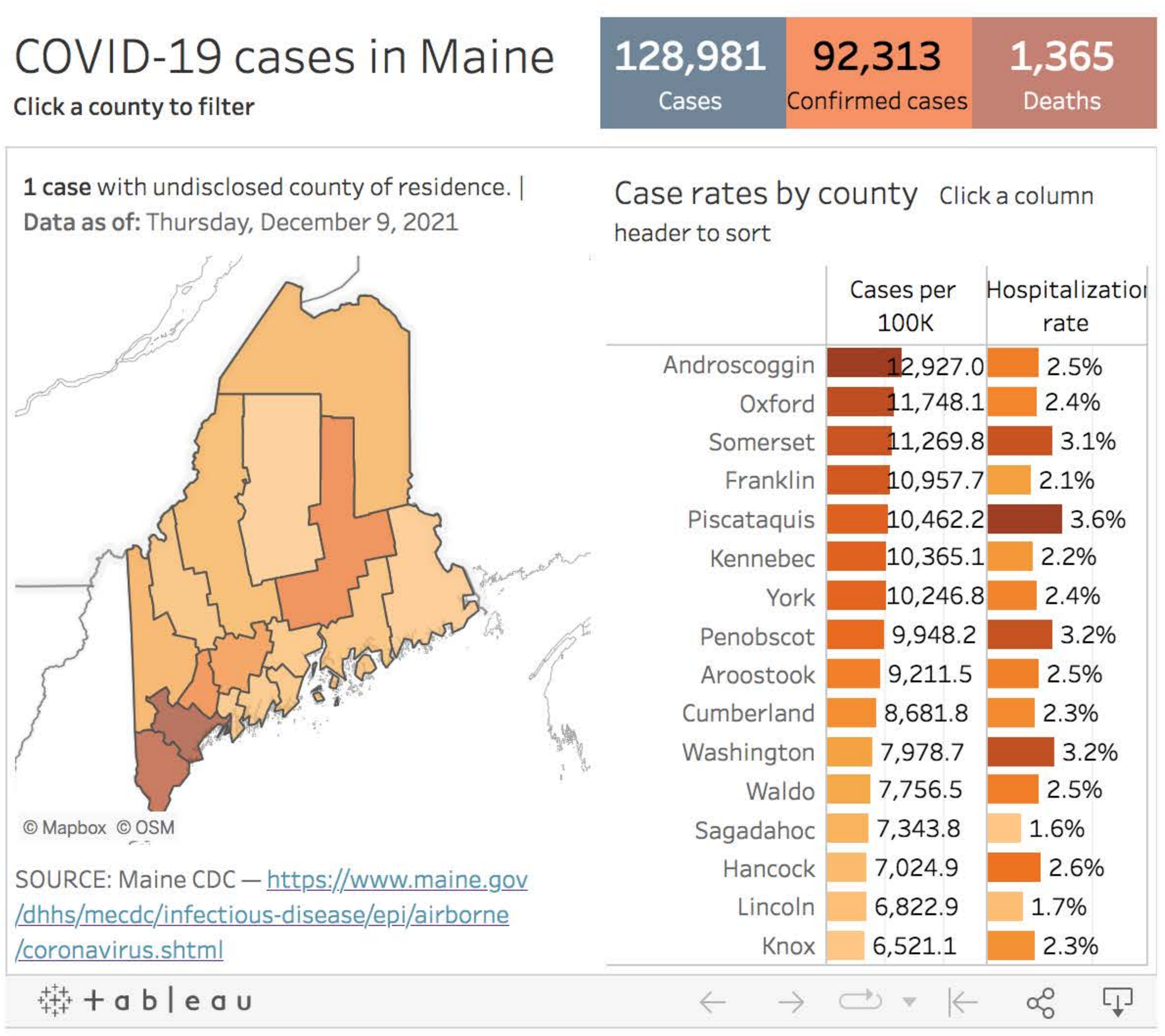

Age trends

The majority of Mainers infected so far have been in their 60s, followed by people in their 50s.

The latest studies of the disease have found that it is more deadly for older people, especially people over 80.

That has not meant that younger people are in the clear — the disease can still be fatal. The line chart shows how cases have trended among Mainers of different ages.

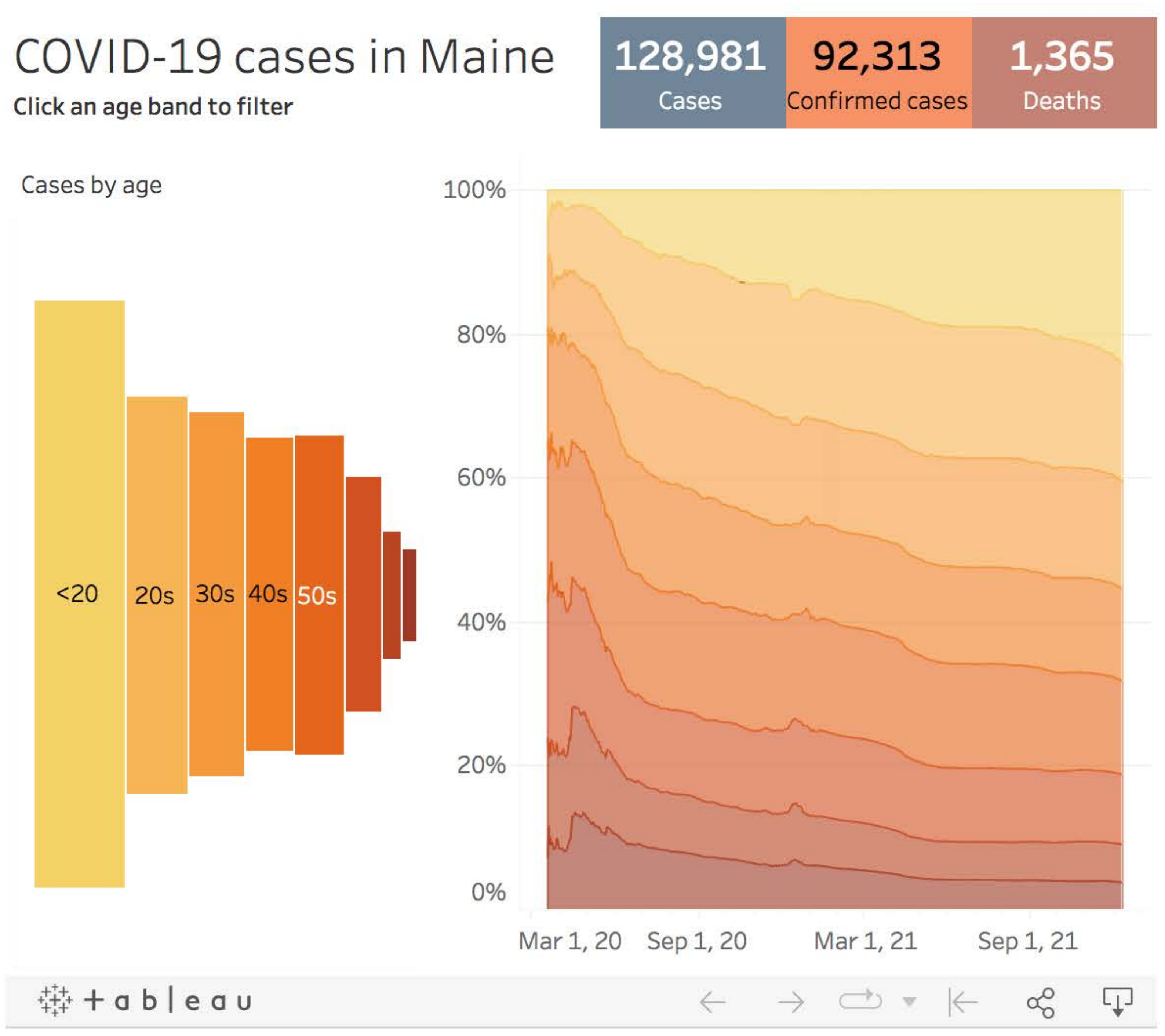

Comparing Maine with other states

The big question is: Can America stop the spread of this virus?

At the state level, this is reflected by looking at the rate of cases day by day. How quickly are they increasing? How many days is it taking for cases to double?

The goal is to slow that growth by: keeping six feet of distance from people you do not live with; washing your hands frequently and thoroughly; and disinfecting regularly touched surfaces.

The chart below uses a logarithmic y-axis, where the values double at every tick on the axis. As The New York Times explained, this makes it easier to tell when case growth begins to slow:

> Unconstrained, the coronavirus spreads exponentially, the caseload doubling at a steady rate. That curve, plotted linearly, is a skyrocketing curve. Plotted logarithmically, however, it transforms into a straight line — which means that deviations from the exponential spread of the virus become much easier to discern.

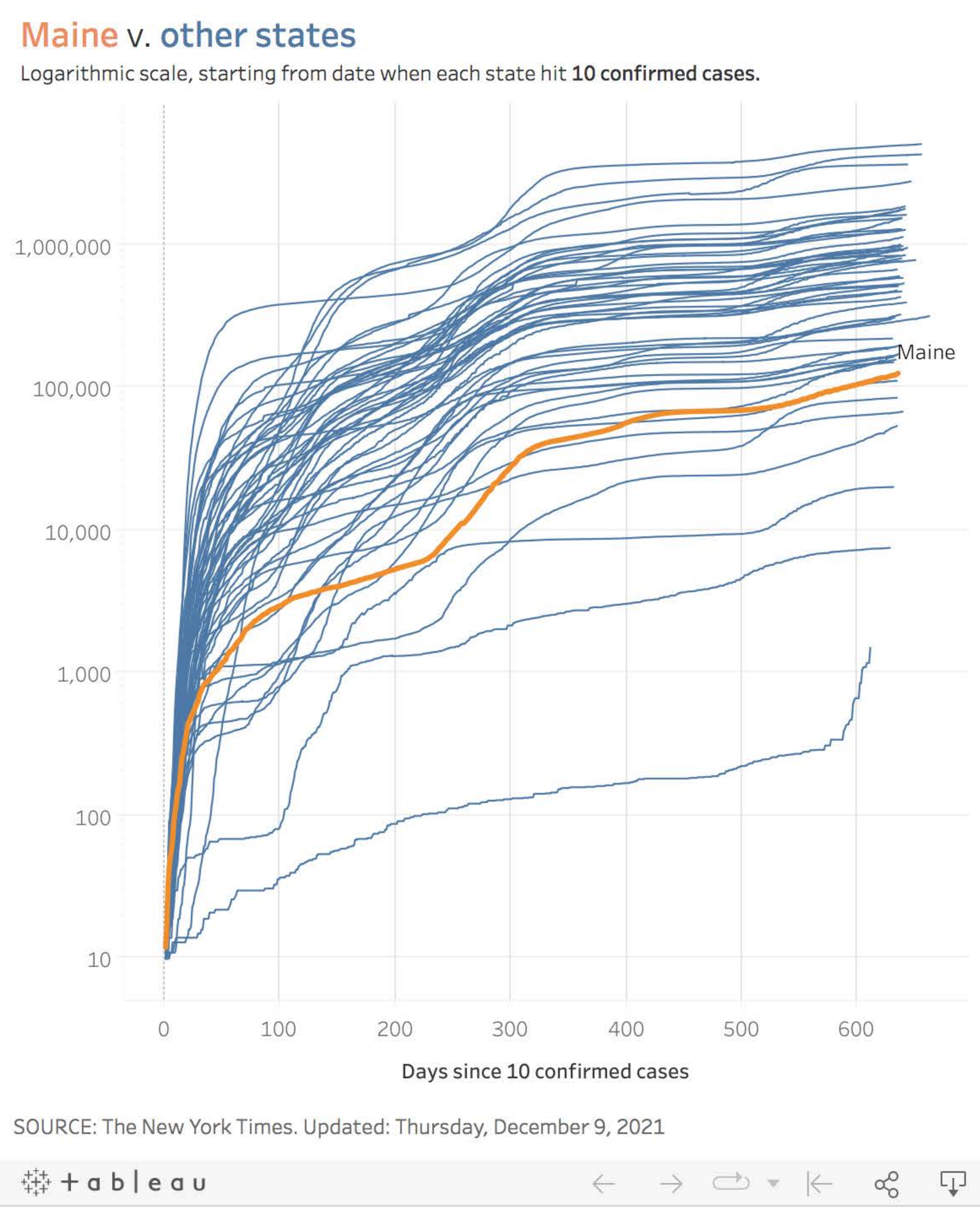

Coronavirus at a glance

The latest Oregon COVID-19 numbers, maps and charts to keep you up to date.

New cases by county

Seven-day case total per 10,000 residents

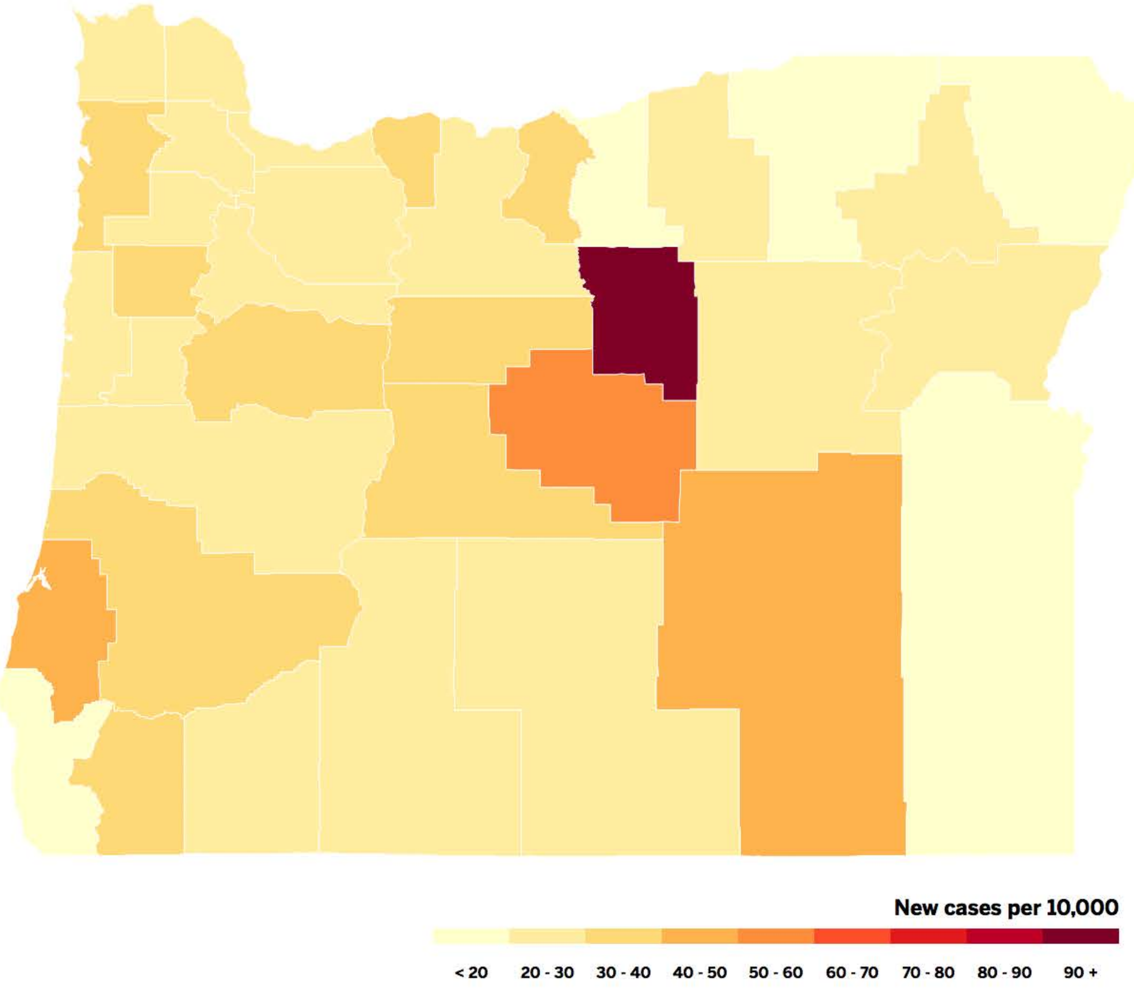

399,361
CASES REPORTED

876
AVG. NEW CASES PER DAY

5,356
DEATHS

404
HOSPITALIZATIONS

94
ICU PATIENTS

93%
REGIONAL ICU OCCUPANCY
Clackamas, Clatsop, Columbia, Multnomah, Tillamook, Washington

See county case trends »

See vaccination numbers »

Metro cases

MULTNOMAH: 61,622

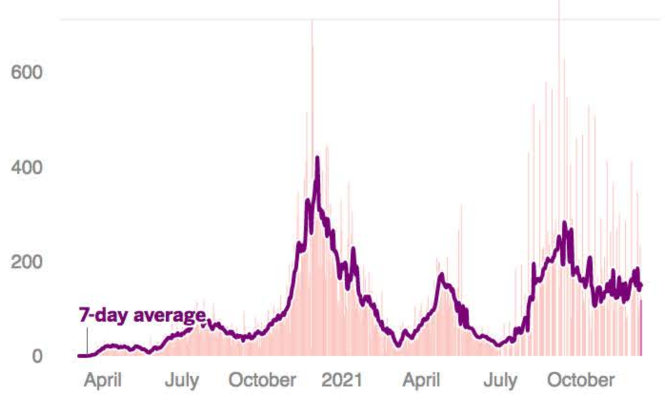

WASHINGTON: 42,997

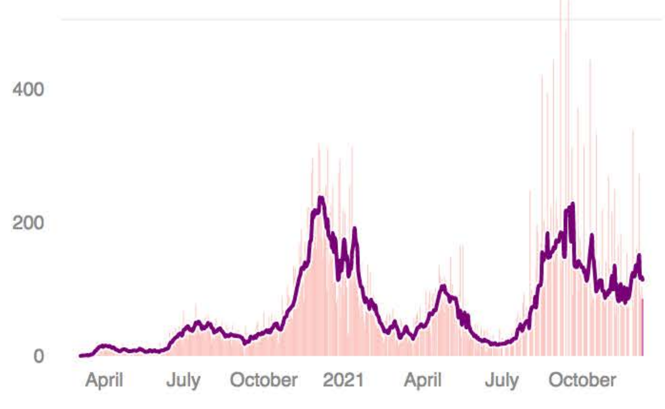

CLACKAMAS: 33,553

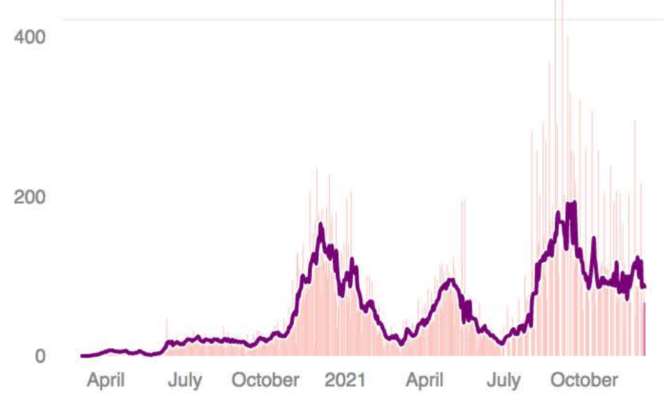

Daily new cases

Daily confirmed and presumptive coronavirus cases reported in Oregon and the 7-day rolling average.

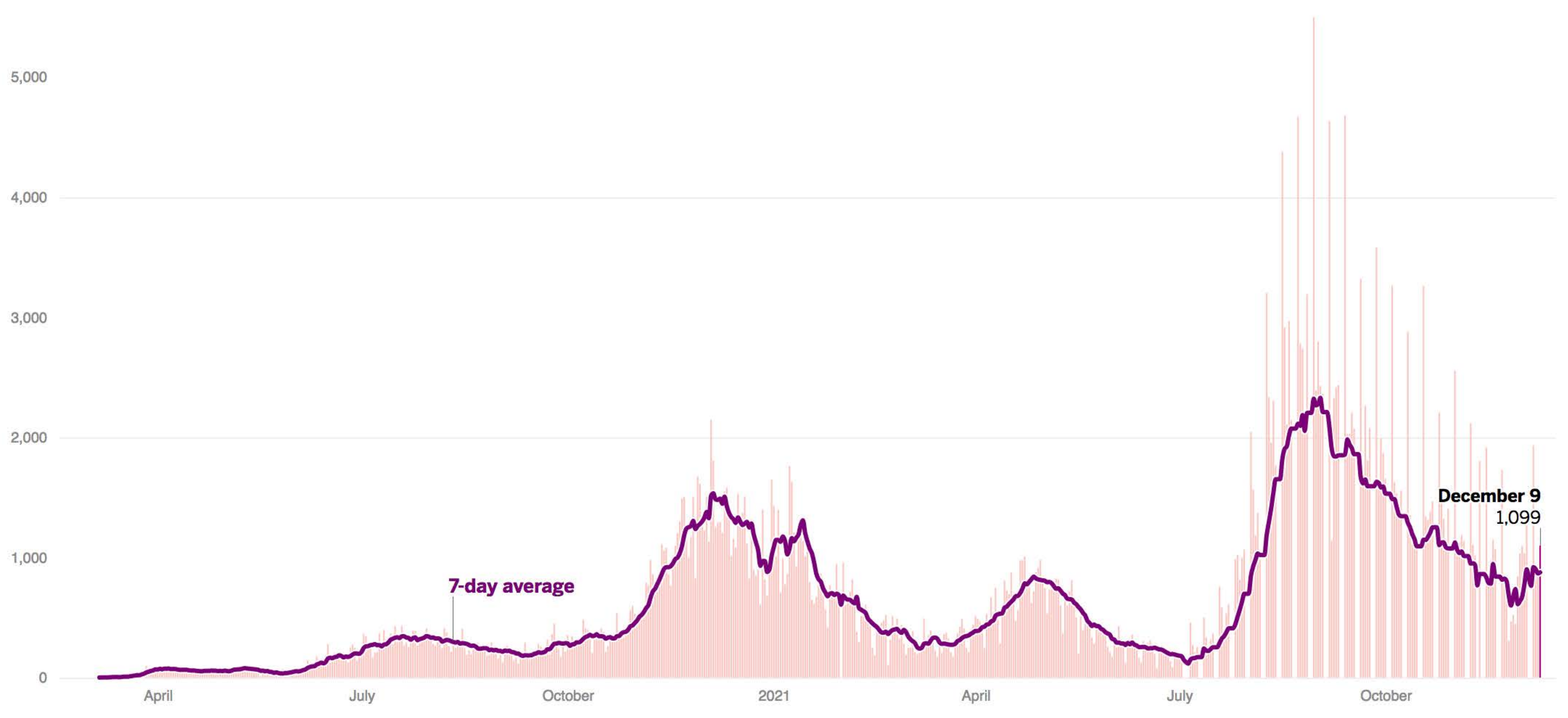

Daily positive test rates

The proportion of daily reported tests in Oregon that have resulted in a positive test result.

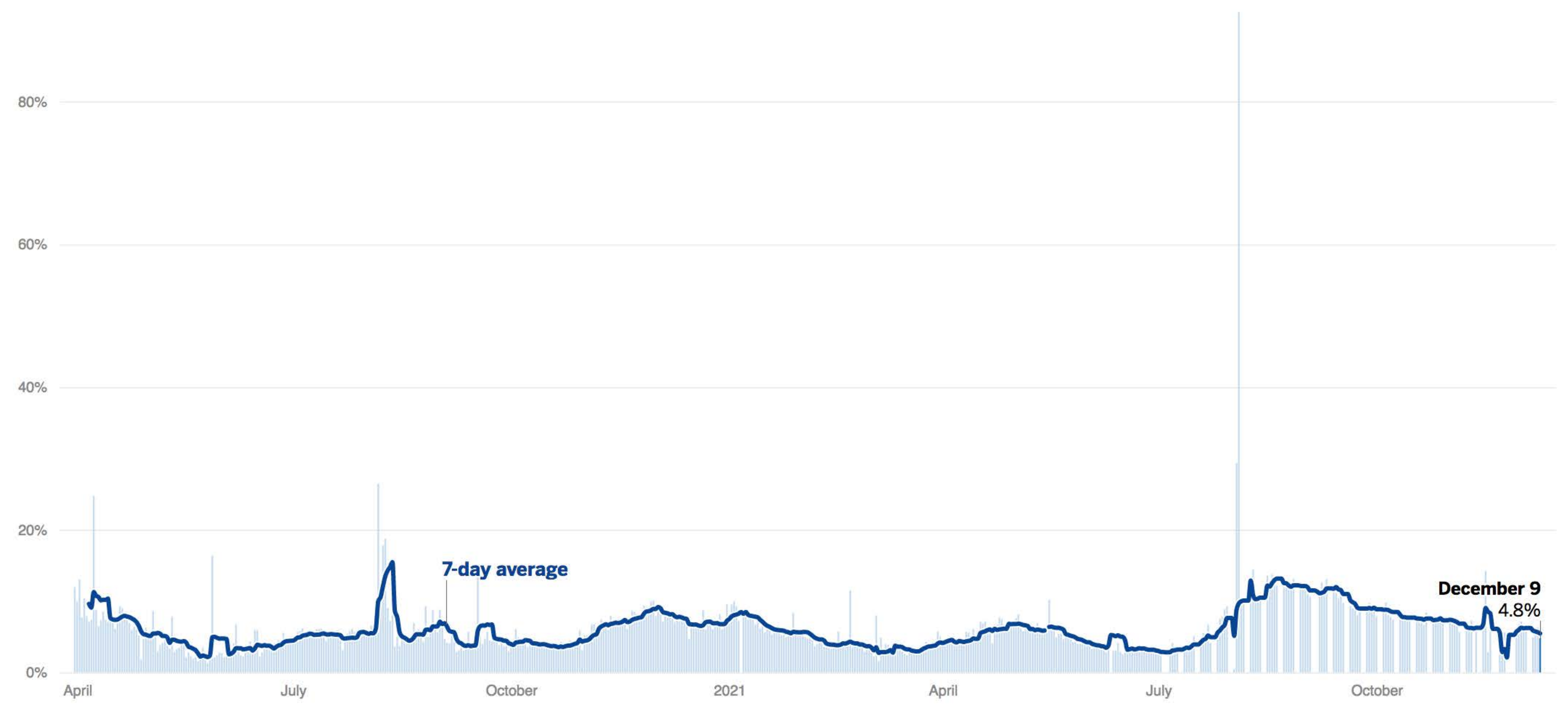

Daily vaccine doses administered

The number of daily new vaccine doses reported administered in Oregon and the 7-day rolling average.

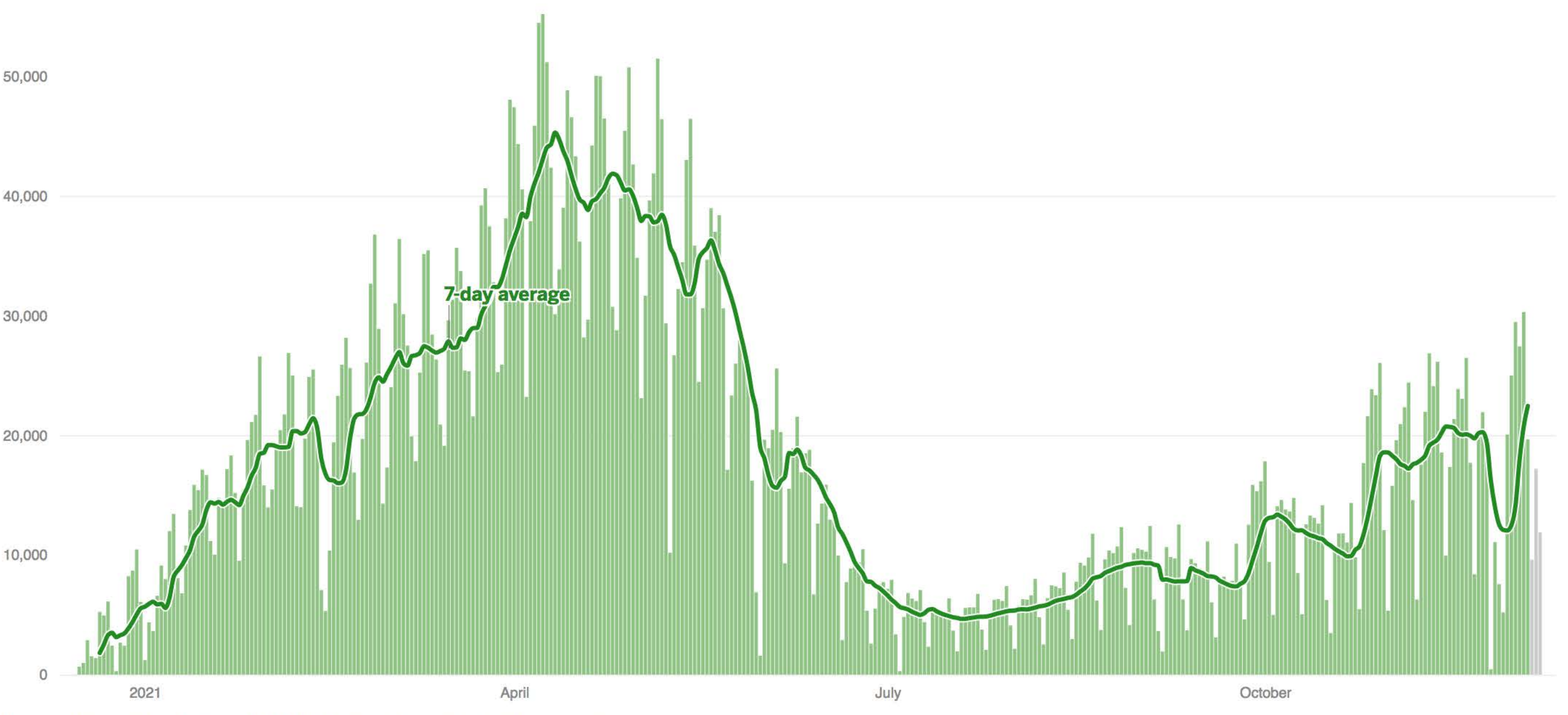

Due to delays in data gathering, dose totals from most recent days are incomplete.

Daily testing

The number of daily new test results reported in Oregon and the 7-day rolling average.

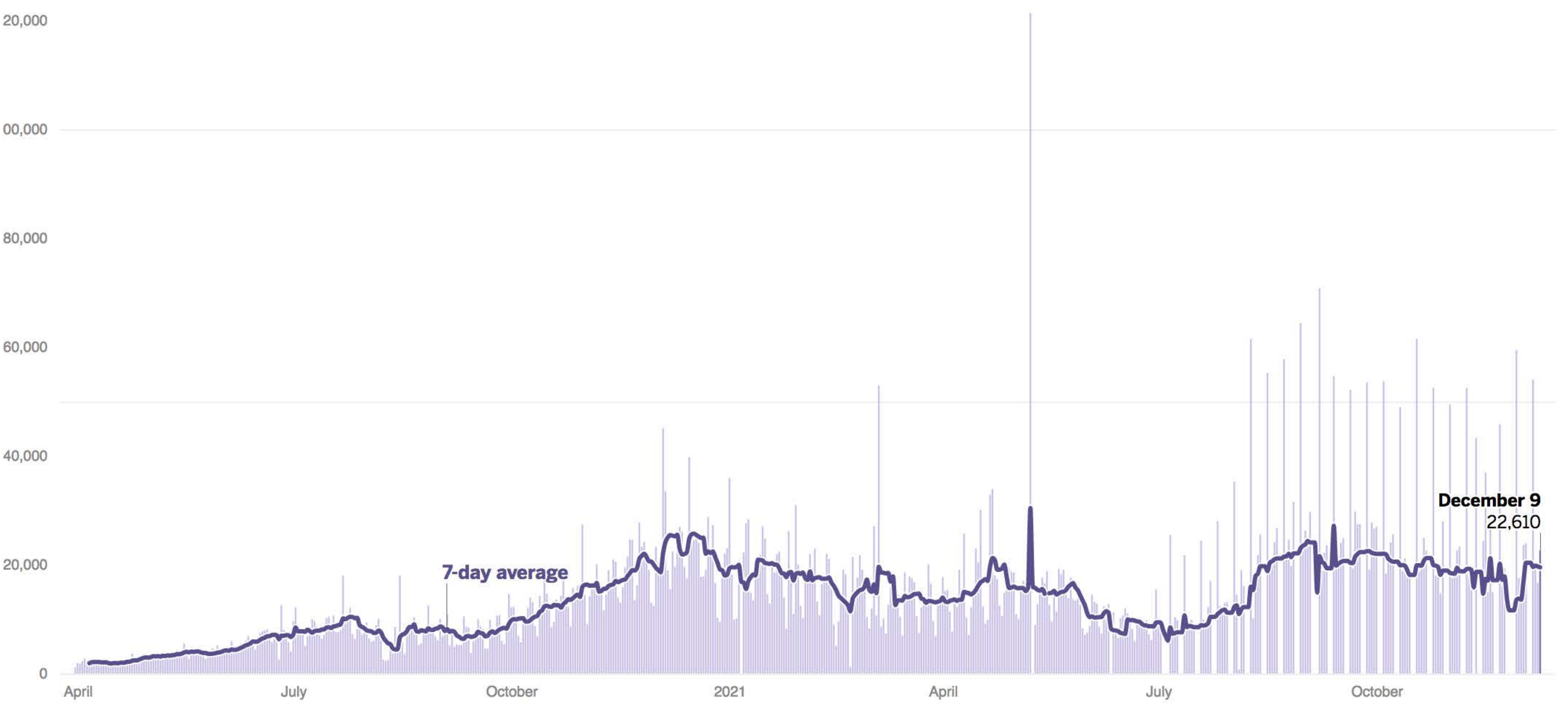

Daily hospitalization status

Number of confirmed coronavirus patients in Oregon who are **hospitalized,** in the **ICU** or on a **ventilator**

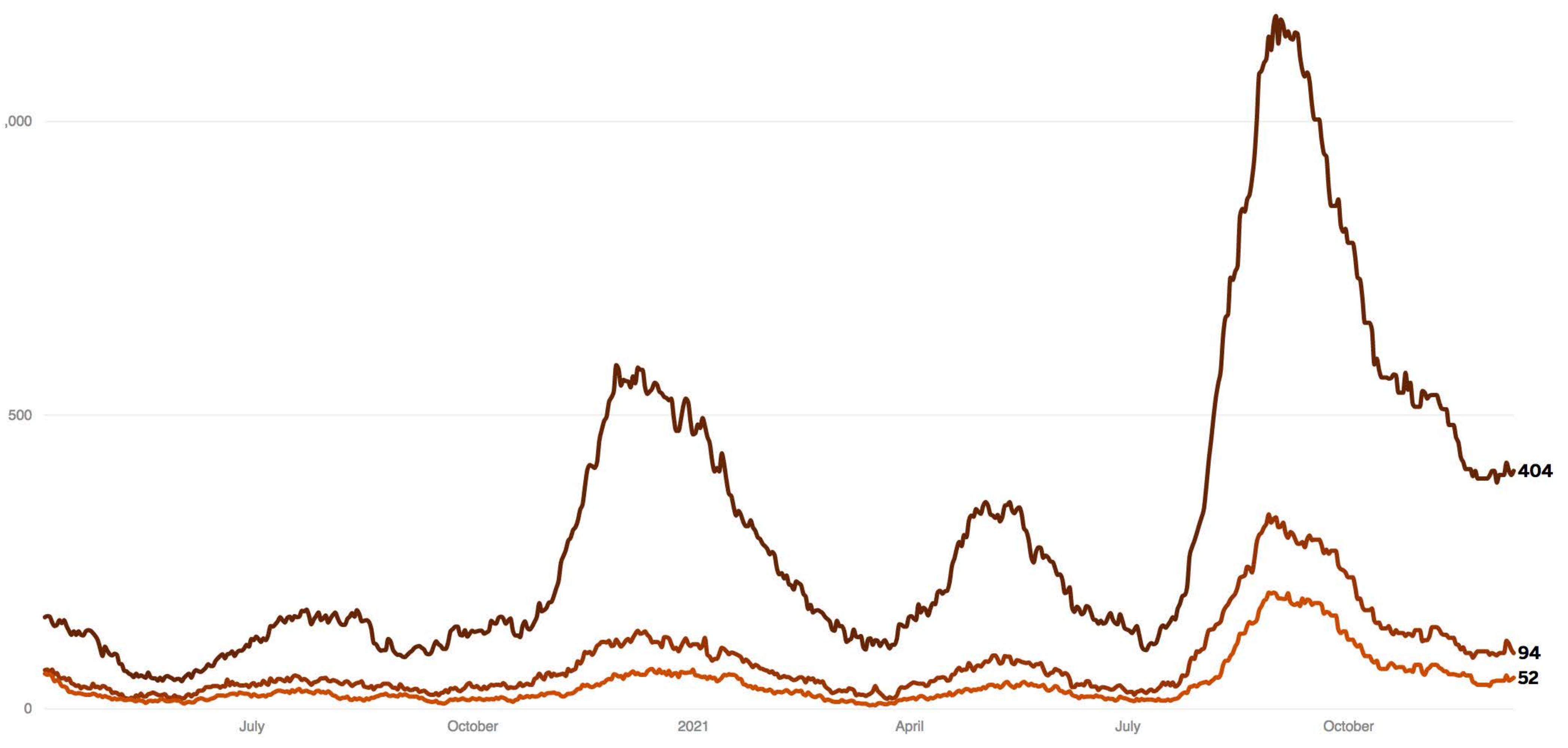

Daily new deaths

Daily coronavirus deaths reported in Oregon and the 7-day rolling average.

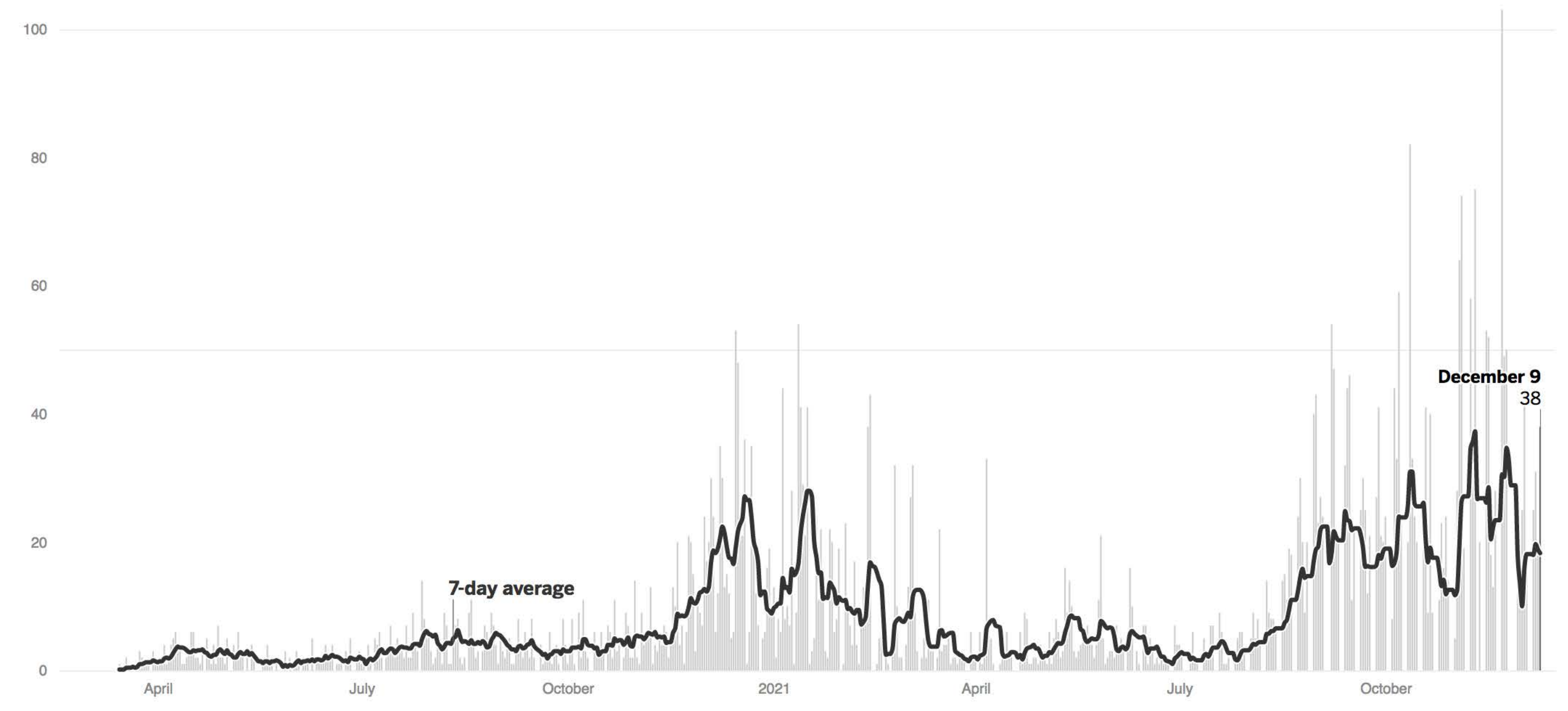

Interactives by Mark Friesen and David Cansler/staff

Sources: Oregon Health Authority, Centers for Disease Control and Prevention

Total cases | Deaths | Per capita | Active cases

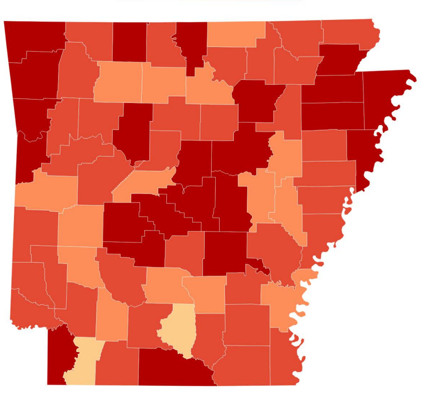

537,461 total cases

8,396 active cases

8,831 deaths

520,073 recoveries

5,023,900 negative tests

* Arkansas recorded 79,569 probable cases and 2,997 cases with missing county information through July 8, 202

Symptoms of covid-19, including fever, cough and shortness of breath, have been mild for most people but severe or even deadly for some. The elderly and people with underlying health conditions considered most at risk.

The disease is thought to spread mainly from **person to person** through droplets produced when an infected person coughs or sneezes. Spread generally occurs between people who are in close contact (within about 6 feet).

People can also catch covid-19 by **touching a contaminated surface** and then touching their face.

There is also evidence suggesting people can spread covid-19 without developing symptoms, although the Centers for Disease Control and Prevention believes that people are most contagious when they have symptoms.

Arkansas covid-19 cases distribution

By age | By race | By gender | By health concerns

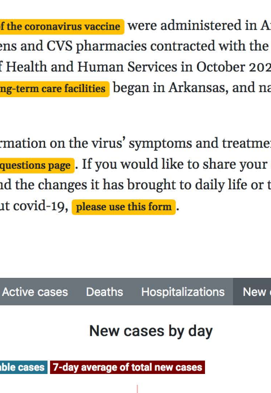

The **first doses of the coronavirus vaccine** were administered in Arkansas on Dec. 14, 2020. Walgreens and CVS pharmacies contracted with the federal Department of Health and Human Services in October 2020, and their **inoculations of long-term care facilities** began in Arkansas, and nationwide, on Dec. 28.

For more information on the virus' symptoms and treatment, visit our **frequently asked questions page**. If you would like to share your experience with coronavirus and the changes it has brought to daily life or to submit your own questions about covid-19, **please use this form**.

Active cases | Deaths | Hospitalizations | New cases

New cases by day

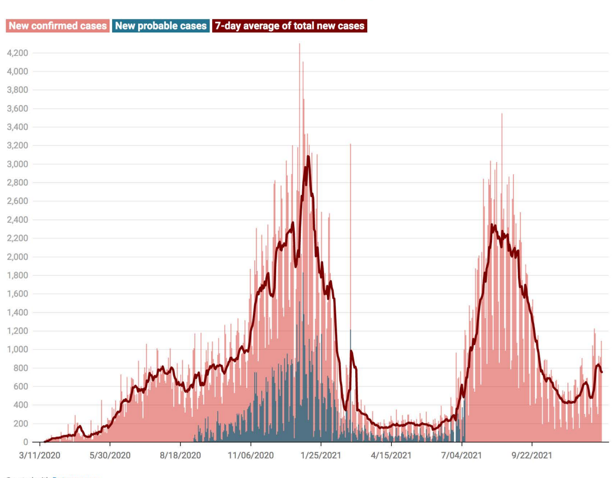

Created with Datawrapper

Covid-19 cases in Arkansas counties

Search in table

	County	Total cases	Active cases	Deaths	Recoveries	Total tests
1	Pulaski	60,912	695	943	59,226	727,794
2	Washington	46,280	514	502	45,260	437,545
3	Benton	44,667	686	649	43,330	416,700
4	Sebastian	23,574	371	401	22,791	241,062
5	Craighead	22,653	455	251	21,945	225,418
6	Faulkner	20,718	407	228	20,080	223,985
7	Saline	19,197	249	252	18,688	212,392
8	Garland	16,301	115	407	15,777	168,193
9	White	14,175	278	207	13,684	106,427
10	Lonoke	12,631	234	177	12,212	119,973
11	Jefferson	12,545	77	233	12,226	159,886
12	Pope	12,460	172	159	12,127	126,584
13	Crawford	11,190	136	183	10,866	113,993
14	Greene	10,615	194	129	10,290	98,810
15	Crittenden	9,089	87	185	8,814	82,880
16	Mississippi	8,852	173	153	8,525	73,506
17	Hot Spring	7,842	73	112	7,654	105,255
18	Independence	7,081	202	165	6,714	90,244
19	Miller	6,734	57	151	6,524	49,473
20	Boone	6,661	191	132	6,338	69,126

+ Show 55 more

Created with Datawrapper

School districts statewide are working to slow the virus's spread, prevent large outbreaks, and protect students, teachers and staff members. The state health and education departments have provided **recommendations** to districts.

Hutchinson and Education Secretary Johnny Key in July 2020 **unveiled guidelines** for school districts on how to respond to outbreaks. Before the beginning of the 2020-21 school year, Key said schools had a "state responsibility" to **open five days a week** for in-person classes. However, the guidelines allow for a **pivot to remote learning** in times of critical response, and a number of schools **made that shift** in the 2020-21 school year.

Universities in Arkansas also **began reporting** covid-19 cases with individual dashboards in August 2020. For the spring 2021 semester, the University of Arkansas, like many other colleges in the state, was offering a mix of **in-person, remote and hybrid** courses.

Covid-19 cases in Arkansas school districts and colleges

K-12 school districts | Colleges

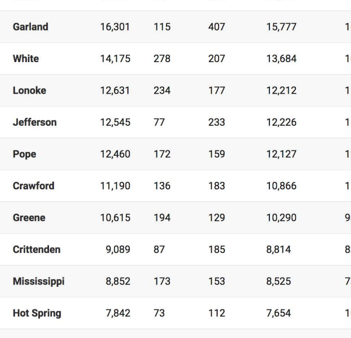

School district	Cumulative cases	Active cases	Cumulative student cases	Cumulative faculty/staff cases

* The Arkansas Department of Health reports K-12 cases each Monday and Thursday. The reports only list districts with five or more active cases.

The Arkansas Democrat-Gazette compiles its own database on coronavirus cases detected at Arkansas colleges using information posted on each school's website.

Nursing homes across the state have been in lockdown under federal orders to protect their residents. The state has adopted **federal guidelines on visits**, prohibiting visits at nursing homes where a resident or worker has tested positive in the past 28 days.

Covid-19 cases in Arkansas long-term care facilities

* The Arkansas Department of Health reports nursing home cases once a week. The reports only list facilities that have had a new or additional positive case in the past 14 days.

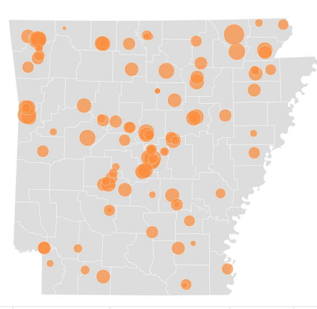

Facility	Positive residents	Positive staff members	Total cases	Total deaths

Prisons, jails and juvenile lockups were identified early in the pandemic as sites where covid-19 could spread rapidly.

Officials have struggled to keep the virus contained even when they identified outbreaks, while prisoners and their families have accused the Arkansas Department of Corrections of **bungling their response** to the virus. The Marshall Project, a nonprofit covering criminal justice, has said Arkansas is among the worst states in **per capita infections** at prisons.

Covid-19 cases in Arkansas correctional facilities

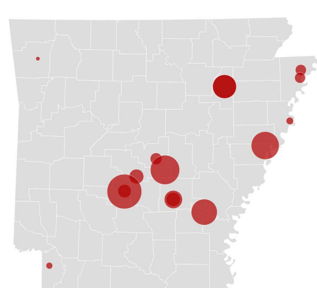

Facility	Positive inmate	Positive staff members	Total cases	Total deaths

People who think they may have covid-19 should call their health care providers or schedule a test at a **local health unit**, according to the state Department of Health.

A health care provider will decide if a test is appropriate based on the symptoms and possible exposure to the coronavirus.

The University of Arkansas for Medical Sciences is also offering **free screenings** online and over the phone at (800) 632-4502.

For children, Arkansas Children's Hospital is offering drive-thru screenings and phone screenings at (800) 743-3616.

Coronavirus testing and screening locations in Arkansas

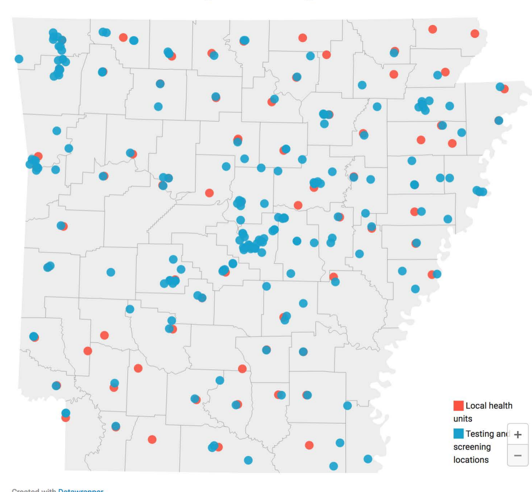

Created with Datawrapper

How Arkansas compares in testing

* States sized by population.

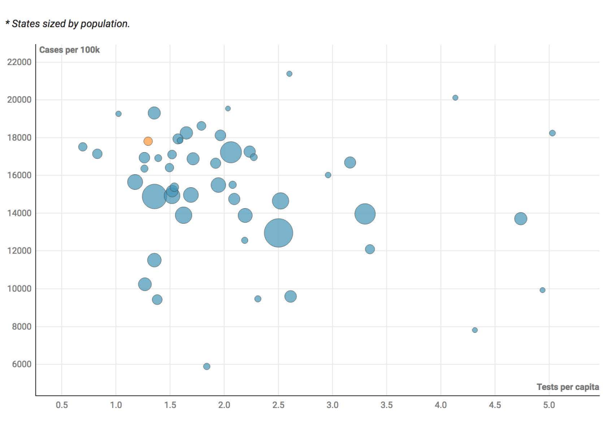

Created with Datawrapper

Cumulative covid-19 cases in the United States by state

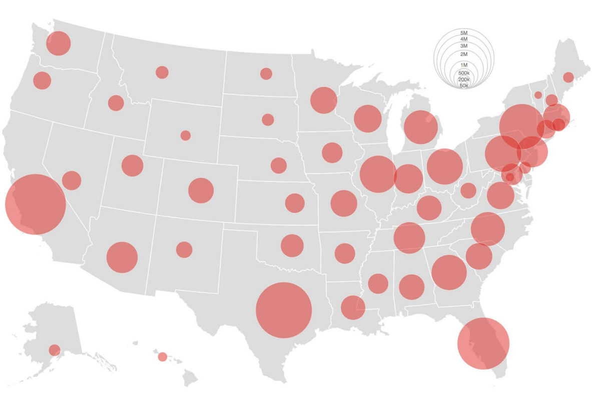

49,831,534 confirmed cases in the United States 796,733 U.S. deaths

Covid-19 cases in the United States by state

Search in table

	State	Total cases	New cases curve	Cases per 100k	Deaths	Total tests	% of positive	Tests per capita
1	North Dakota	166,600	510 … 359	21384	1,944	2,026,941	8.2%	2.60
2	Alaska	147,535	554 … 184	20117	856	3,032,161	4.9%	4.13
3	Wyoming	112,731	328 … 167	19542	1,472	1,173,321	9.6%	2.03
4	Tennessee	1,334,278	976 … 2K	19307	17,696	9,351,491	14.3%	1.35
5	South Dakota	170,800	381 … 434	19263	2,392	907,441	18.8%	1.02
6	Utah	609,351	2K … 1K	18625	3,633	5,853,063	10.4%	1.79
7	Arizona	1,305,260	3K … 4K	18252	22,854	11,800,380	11.1%	1.65
8	Rhode Island	200,197	278 … 926	18243	2,951	5,520,696	3.6%	5.03
9	South Carolina	927,658	639 … 1K	18124	14,348	10,056,322	9.2%	1.96
10	Kentucky	808,148	856 … 2K	17936	11,479	7,085,149	11.4%	1.57
11	Montana	193,543	622 … 231	17851	2,788	1,725,592	11.2%	1.59
12	Arkansas	536,368	430 … 768	17811	8,810	3,905,392	13.7%	1.30
13	Mississippi	518,670	316 … 495	17515	10,317	2,053,637	25.3%	0.69
14	Wisconsin	1,017,301	3K … 4K	17261	10,330	13,170,080	7.7%	2.23
15	Florida	3,710,624	1K … 2K	17228	62,007	44,408,723	8.4%	2.06
16	Oklahoma	678,815	651 … 1K	17145	11,272	3,277,330	20.7%	0.83
17	Iowa	545,804	1K … 2K	17108	7,550	4,843,307	11.3%	1.52
18	West Virginia	304,355	740 … 991	16968	5,055	4,076,927	7.5%	2.27
19	Alabama	851,239	604 … 572	16943	16,243	6,343,214	13.4%	1.26
20	Idaho	311,150	667 … 428	16919	4,013	2,556,482	12.2%	1.39
21	Indiana	1,145,633	2K … 5K	16883	17,954	11,617,439	9.9%	1.71
22	Minnesota	952,232	3K … 4K	16687	9,938	18,046,331	5.3%	3.16
23	Louisiana	775,368	412 … 437	16647	14,867	8,944,356	8.7%	1.92
24	Kansas	482,232	900 … 2K	16414	6,768	4,388,953	11.0%	1.49
25	Nebraska	320,943	806 … 1K	16362	3,260	2,476,583	13.0%	1.26
26	Delaware	158,600	248 … 598	16021	2,210	2,929,411	5.4%	2.96
27	Georgia	1,676,787	935 … 1K	15653	30,798	12,601,633	13.3%	1.18
28	New Mexico	328,332	1K … 1K	15505	5,459	4,398,567	7.5%	2.08
29	Michigan	1,561,184	5K … 8K	15492	26,699	19,606,072	8.0%	1.95
30	Nevada	477,407	769 … 713	15377	8,153	4,777,646	10.0%	1.54
31	Missouri	933,717	1K … 3K	15170	15,562	9,349,554	10.0%	1.52
32	North Carolina	1,562,663	2K … 3K	14969	18,955	17,659,950	8.8%	1.69
33	Ohio	1,761,008	4K … 8K	14924	27,011	17,905,479	9.8%	1.52
34	Texas	4,338,790	3K … 3K	14887	73,227	39,520,764	11.0%	1.36
35	Colorado	851,785	3K … 2K	14753	9,554	12,080,279	7.1%	2.09
36	Illinois	1,877,213	3K … 7K	14651	29,793	32,286,400	5.8%	2.52
37	New York	2,820,999	4K … 10K	13964	57,523	66,675,847	4.2%	3.30
38	Pennsylvania	1,806,671	4K … 8K	13895	34,271	21,121,206	8.6%	1.62
39	New Jersey	1,289,542	1K … 4K	13882	28,526	20,371,146	6.3%	2.19
40	Massachusetts	963,923	2K … 5K	13712	19,606	33,310,971	2.9%	4.74
41	California	5,123,390	6K … 7K	12958	74,432	98,907,364	5.2%	2.50
42	New Hampshire	173,089	650 … 1K	12565	1,773	3,015,441	5.7%	2.19
43	Connecticut	436,143	342 … 2K	120…	8,946	12,064,000	3.6%	3.35
44	Virginia	994,069	1K … 2K	115…	14,895	11,689,842	8.5%	1.35
45	Washington	788,877	2K … 1K	10238	9,535	9,763,035	8.1%	1.27
46	District of Columbia	68,460	79 … 184	9928	1,197	3,406,088	2.0%	4.94
47	Maryland	592,679	818 … 267	9595	11,255	16,138,736	3.7%	2.61
48	Maine	128,974	639 … 962	9467	1,365	3,147,569	4.1%	2.31
49	Oregon	399,361	952 … 876	9425	5,356	5,850,985	6.8%	1.38
50	Vermont	50,238	281 … 419	7812	405	2,774,582	1.8%	4.31
51	Hawaii	85,676	109 … 101	5887	1,029	2,675,519	3.2%	1.84

Created with Datawrapper
