# Supplementary material for: Web-Based COVID-19 Dashboards and Trackers in the United States: Survey Study
Source: JMIR Hum Factors. 2023 Mar 20;10:e43819. doi: 10.2196/43819 (PMC10029858; doi:10.2196/43819)

Appendix 12. Screenshots from dashboards and trackers — *Nation-wide coverage* *(captured December 11, 2021)*

Page	Ref	Host
2–4	N-1	US Centers for Disease Control and Prevention
5	N-4	New York Times
6	N-5	NPR
7	N-6	Johns Hopkins Centers for Civic Impact
8	N-7	Johns Hopkins Centers for Civic Impact
9	N-8	COVID-19 Health Equity Interactive Dashboard / Emory University
10	N-9	USAFacts
11	N-10	COVID Mapping Project / Standard Co

United States At a Glance

Cases Total 49,748,387 Last 30 Days

Deaths Total 793,937 Last 30 Days

76.4% of People 5+ with At Least One Vaccination

Community Transmission High

Data Tracker Home
COVID Data Tracker Weekly Review
Your Community +
Health Equity Data
Pediatric Data
Pregnancy Data
Vaccination Delivery and Coverage +
Vaccine Effectiveness and Breakthrough Surveillance +
Cases, Deaths, and Testing —
Cases, Deaths, and Testing
Daily and Total Trends
Compare Trends Across States and Regions
Global Counts and Rates
Global Percent Change
Global Trends
Demographic Trends +
Health Care Settings +
Variants and Genomic Surveillance +
Antibody Seroprevalence +
People at Increased Risk +
Multisystem Inflammatory Syndrome in Children (MIS-C)
Prevention Measures and Social Impact +
Additional COVID-related Data +
Communications Resources
COVID-19 Home

United States COVID-19 Cases, Deaths, and Laboratory Testing (NAATs) by State, Territory, and Jurisdiction

Maps, charts, and data provided by CDC, updated Mon-Sat by 8 pm ET†

View Footnotes and Download Data

TOTAL CASES	7 DAY CASE RATE PER 100,000	TOTAL DEATHS
49,748,387	250	793,937
+152,970 New Cases		+1,823 New Deaths

CDC | Data as of: December 11, 2021 2:21 PM ET. Posted: December 11, 2021 3:36 PM ET

View:
- Level of Community Transmission
- (selected) Cases
- Deaths
- Tests Performed
- Percent Positive

Time period:
- (selected) Last 7 Days
- Since Jan 21, 2020

Metric:
- Count
- (selected) Rate per 100,000

This shows the number of COVID-19 cases for every 100,000 people over the last 7 days, allowing you to compare areas with different population sizes.

US COVID-19 7-Day Case Rate per 100,000, by State/Territory

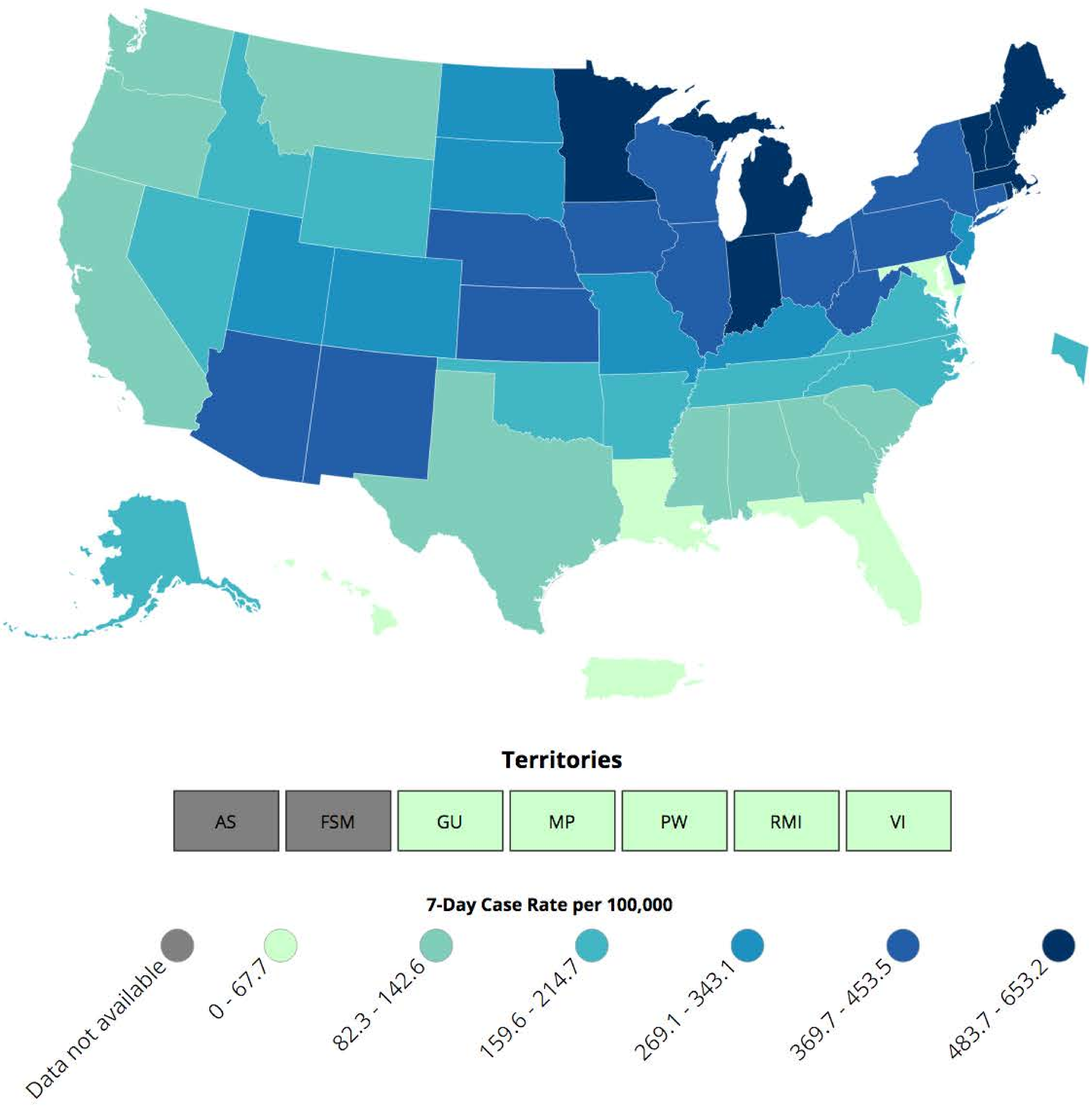

View Historic Case and Death Data | Download Image

Data Downloads and Footnotes

Expand each accordion to view data table and download data

Data Table for Cumulative Cases per 100k in Last 7 Days +

Footnotes +

Data Tracker Home

COVID Data Tracker Weekly Review

Your Community +

Health Equity Data

Pediatric Data

Pregnancy Data

Vaccination Delivery and Coverage +

Vaccine Effectiveness and Breakthrough Surveillance +

Cases, Deaths, and Testing —

Cases, Deaths, and Testing

Daily and Total Trends

Compare Trends Across States and Regions

Global Counts and Rates

Global Percent Change

Global Trends

Demographic Trends +

Health Care Settings +

Variants and Genomic Surveillance +

Antibody Seroprevalence +

People at Increased Risk +

Multisystem Inflammatory Syndrome in Children (MIS-C)

Prevention Measures and Social Impact +

Additional COVID-related Data +

Communications Resources

COVID-19 Home

Get Email Updates

Sign up to receive the COVID Data Tracker Weekly Review.

United States COVID-19 Cases, Deaths, and Laboratory Testing (NAATs) by State, Territory, and Jurisdiction

Maps, charts, and data provided by CDC, updated Mon-Sat by 8 pm ET†

View Footnotes and Download Data

TOTAL TESTS REPORTED	TOTAL POSITIVE TESTS	7-DAY % POSITIVITY
680,420,172	51,223,222	7.64%

National totals as of: Dec 08 2021. National postivity date as of: Dec 08 2021 CDC | Updated: Dec 11 2021 2:18PM

View:
- Level of Community Transmission
- Cases
- Deaths
- (selected) Tests Performed
- Percent Positive

Metric:
- Count
- (selected) Rate per 100,000

Time period:
- (selected) Last 7 Days
- Last 30 Days
- All Time

This shows the number of tests performed for every 100,000 people over the last 7 days, allowing you to compare areas with different population sizes.

COVID-19 Nucleic Acid Amplification Tests (NAATs) Performed in Last 7 Days per 100k by State/Territory

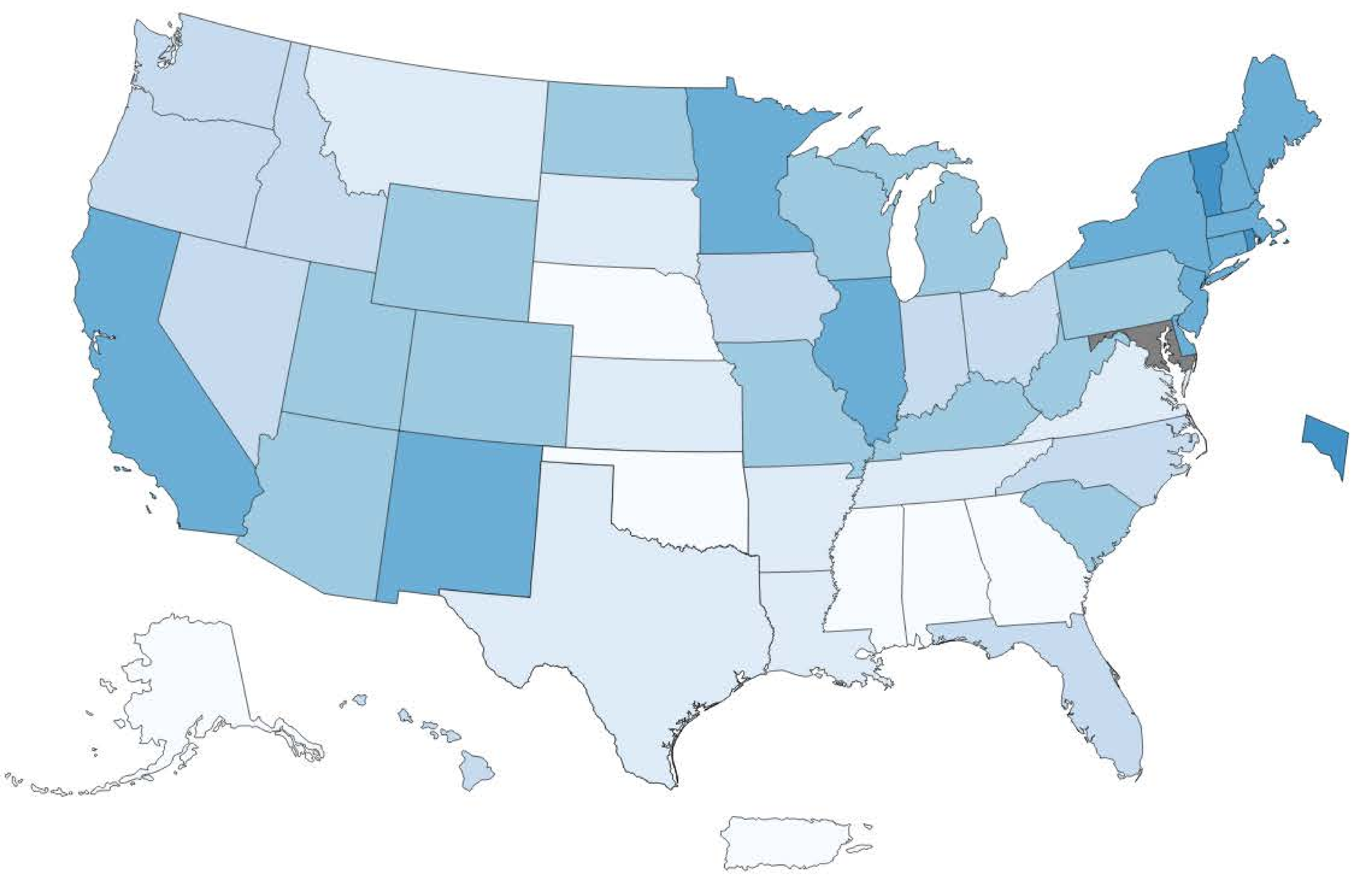

Territories

AS | FSM | GU | MP | PW | RMI | VI

Tests Performed Last 7 Days per 100,000

Data not available | 284.9 - 1.1k | 1.2k - 1.8k | 1.9k - 2.4k | 2.4k - 3.6k | 3.8k - 8.0k | 10.7k - 13.9k

Download Image

Data Downloads and Footnotes

Expand each accordion to view data table and download data

Data Table for COVID-19 Nucleic Acid Amplification Tests (NAATs) Performed in Last 7 Days per 100k by State/Territory +

Footnotes +

COVID Data Tracker

United States
At a Glance

Cases Total 49,748,387
Last 30 Days

Deaths Total 793,937
Last 30 Days

76.4% of People 5+ with At Least One Vaccination

Community Transmission High

Data Tracker Home
COVID Data Tracker Weekly Review
Your Community +
Health Equity Data
Pediatric Data
Pregnancy Data
Vaccination Delivery and Coverage −
US, States, and Jurisdictions
By County
Trends
By Race/Ethnicity, Age, and Sex
Trends by Race/Ethnicity, Age, and Sex
Equity
Pregnant People
Confidence and Coverage Estimates
People with Disabilities
Nursing Homes
Dialysis Facilities
Global
Vaccine Effectiveness and Breakthrough Surveillance +
Cases, Deaths, and Testing +

Trends in Number of COVID-19 Vaccinations in the US

Maps, charts, and data provided by CDC, updates daily by 8 pm ET[†]

Learn how CDC estimates vaccination coverage

View Footnotes and Download Data

About these data

Select a Location: United States

Metric:
- (●) Total Doses Administered
- ( ) People Receiving Dose 1
- ( ) People Fully Vaccinated
- ( ) People Receiving a Booster Dose

View:
- (●) Daily Count
- ( ) Cumulative
- ( ) Daily Change Reported

Show:
- [x] 7-Day moving average

Bars shown in the darker blue shade represent the most recent five days of reporting where the number of vaccine administrations might be impacted the most due to delays in reporting. All reported numbers might change over time as historical data are reported to CDC.

How Do I Find a COVID-19 Vaccine?

Daily Count of Total Doses Administered and Reported to CDC by Date Administered, United States

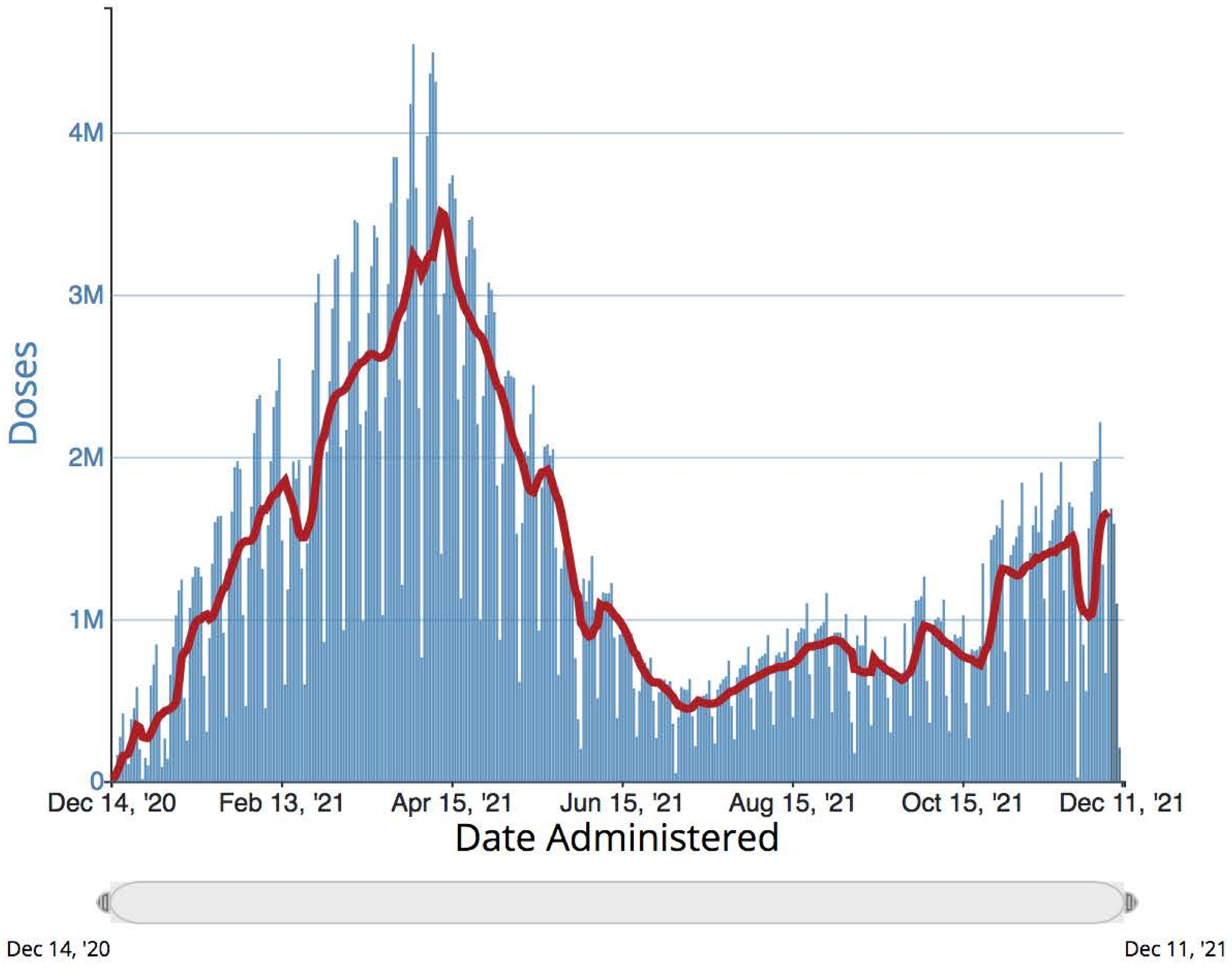

Download Chart View Historic Vaccination Data

Coronavirus in the U.S.: Latest Map and Case Count

Updated Dec. 11, 2021

Get the latest updates on the Omicron variant.

New reported cases

All time | Last 90 days

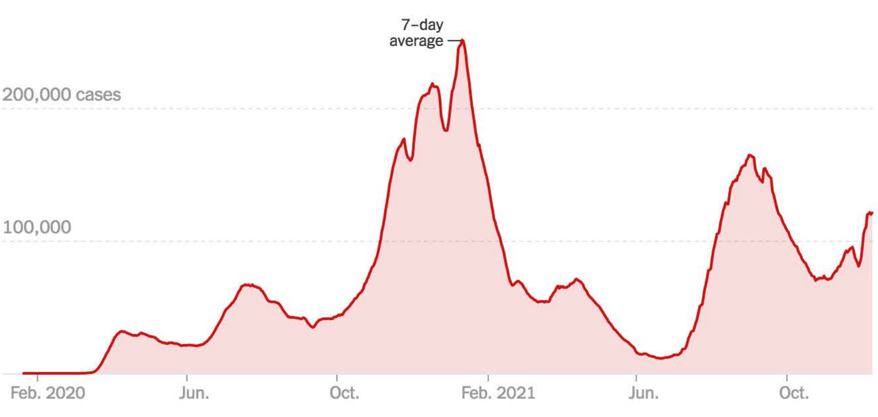

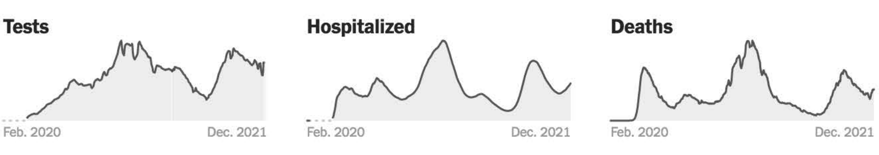

	DAILY AVG. ON DEC. 10	14-DAY CHANGE	TOTAL REPORTED
Cases	120,967	+38%	49,792,430
Tests	1,388,061	+12%	—
Hospitalized	63,995	+22%	—
Deaths	1,310	+26%	795,301

About this data

Vaccinations

	AT LEAST ONE DOSE	FULLY VACCINATED
All ages	72%	61%
12 and up	82%	70%
65 and up	95%	87%

See more details ›

About this data

State of the virus

Update for Dec. 9

- More than 120,000 coronavirus cases are emerging each day as conditions continue to worsen, especially in the Great Lakes region and in the Northeast.
- Cases, deaths and hospitalizations are rising nationally, but remain below the levels seen during the summer and during last winter's peak.
- New Hampshire leads the country in recent cases per capita. More coronavirus patients are hospitalized in that state than at any other point in the pandemic.
- Michigan has the country's highest hospitalization rate. Federal medical teams have been sent there to help handle the surge in patients.
- The Southwest has also struggled in recent weeks. Arizona and New Mexico have hospitalization rates that are among the worst in the country.
- The country is reporting more than 1,200 deaths a day, on average, and is on pace to surpass 800,000 total deaths in the coming days.
- The current uptick is being driven by the Delta variant. It is not yet known how the Omicron variant, which continues to emerge in more states, might affect those trends in the coming weeks and months.
- About 60 percent of Americans are fully vaccinated. Around 1.9 million doses are being administered each day, a figure that includes booster doses for vaccinated people.

The Coronavirus in a Tiny Drop

A new simulation shows how the virus survives inside tiny airborne particles of water — and gives clues about how the Delta variant became dominant.

Cases by region

This chart shows how average daily cases per capita have changed in different parts of the country. The state with the highest recent average cases per capita is shown.

All time | Last 90 days

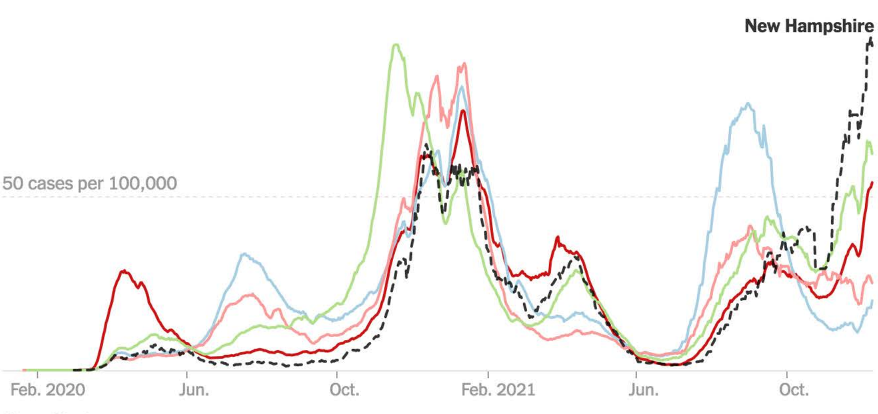

About this data

Rates for vaccinated and unvaccinated

Data from the Centers for Disease Control and Prevention shows that people who are unvaccinated are at a much greater risk than those who are fully vaccinated to test positive or die from Covid-19. These charts compare age-adjusted average daily case and death rates for vaccinated and unvaccinated people in the 22 states and two cities that provide this data.

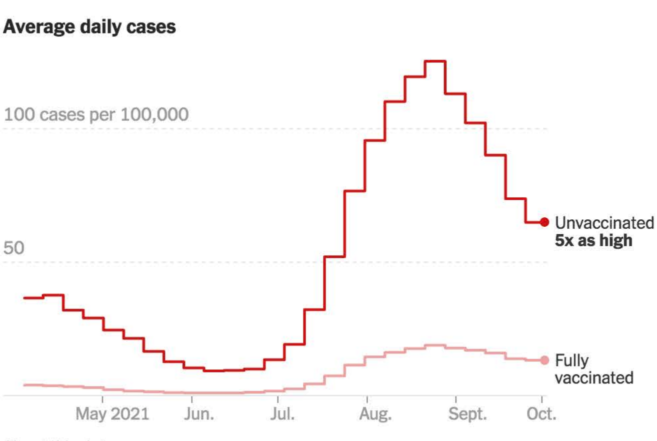

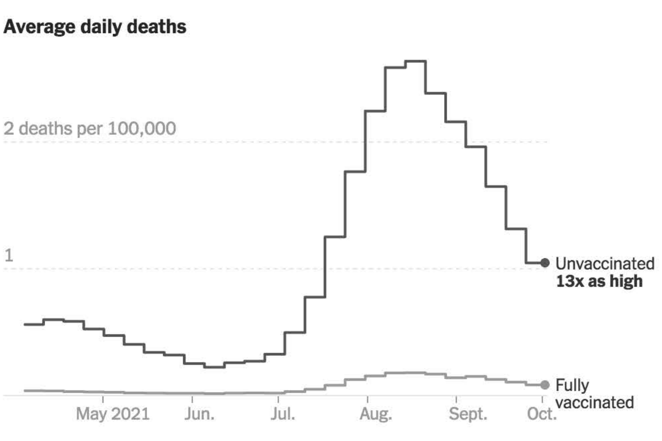

About this data

Hot spots

AVERAGE DAILY CASES PER 100,000 PEOPLE IN PAST WEEK

10 30 50 70 100 250 | FEW OR NO CASES | MISSING DATA

Hot spots | Hospitalized | Vaccinations | Risk levels | Total cases | Deaths | Per capita

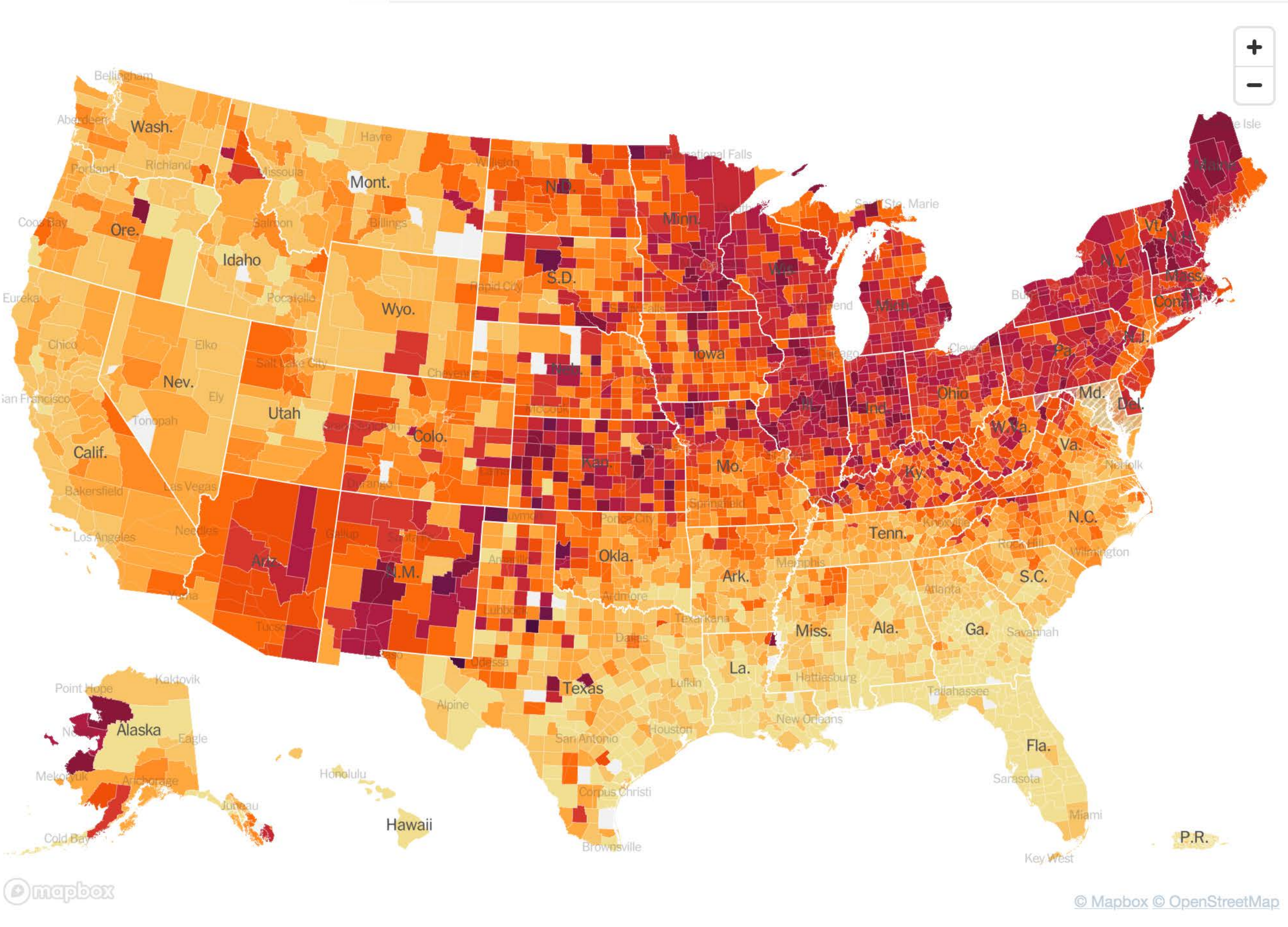

About this data

State trends

This table is sorted by places with the most cases per 100,000 residents in the last seven days. Charts show change in daily averages and are each on their own scale. Select a table header to sort by another metric.

Recent trends | All time | Search states

	CASES DAILY AVG.	PER 100,000	14-DAY CHANGE	HOSPITALIZED DAILY AVG.	PER 100,000	14-DAY CHANGE	DEATHS DAILY AVG.	PER 100,000	FULLY VACCINATED
United States	120,967	36	+38%	63,995	19	+22%	1,310.0	0.39	61%
Northern Mariana Islands ›	65	120	+96%	—	—	—	0.3	0.53	70%
New Hampshire ›	1,269	93	+27%	450	33	+26%	6.0	0.44	65%
Rhode Island ›	917	87	+94%	206	19	+69%	2.3	0.22	75%
Vermont ›	490	78	+29%	86	14	+36%	1.9	0.30	75%
Minnesota ›	4,264	76	+24%	1,777	32	+13%	36.6	0.65	64%
Michigan ›	7,166	72	−15%	4,656	47	+16%	114.7	1.15	56%
Maine ›	963	72	+52%	378	28	+17%	4.7	0.35	74%
Massachusetts ›	4,821	70	+78%	1,027	15	+54%	20.7	0.30	73%
New Mexico ›	1,457	69	+4%	756	36	+12%	9.4	0.45	65%
Indiana ›	4,655	69	+44%	2,648	39	+47%	43.1	0.64	51%

Show all | About this data

Daily new hospital admissions by age

This chart shows for each age group the number of people per 100,000 that were newly admitted to a hospital with Covid-19 each day, according to data reported by hospitals to the U.S. Department of Health and Human Services.

All time | Last 90 days

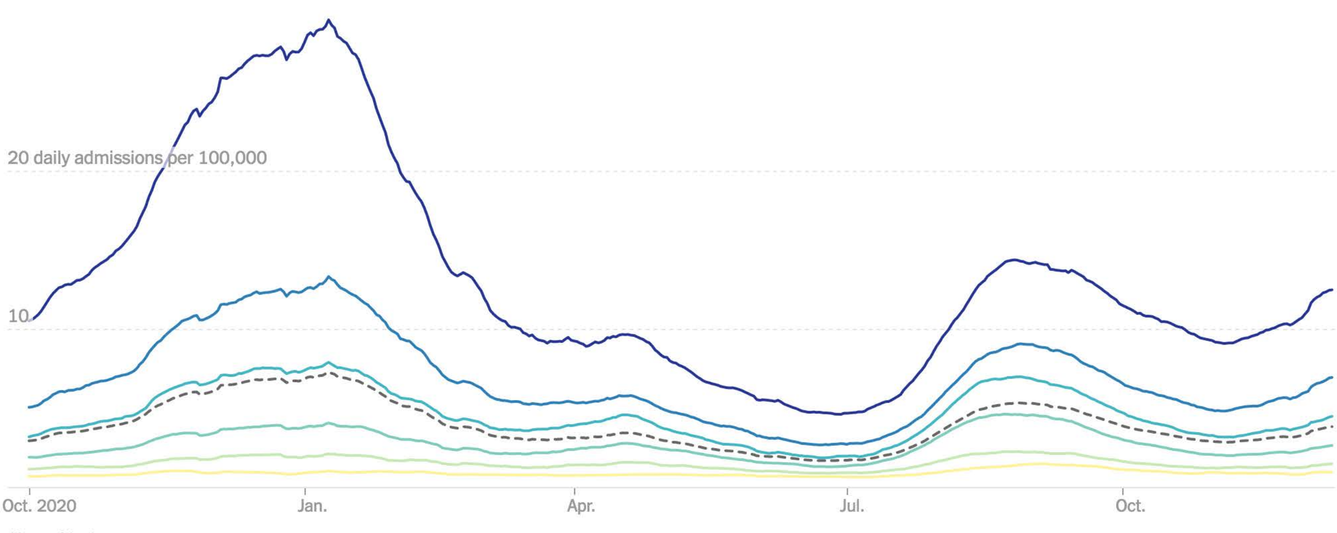

About this data

U.S. trends

All time | Last 90 days

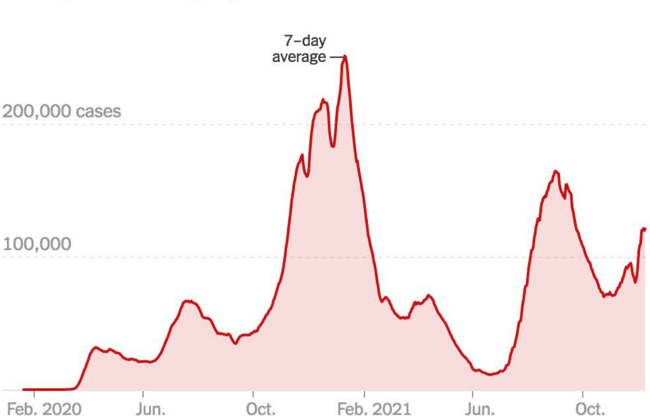

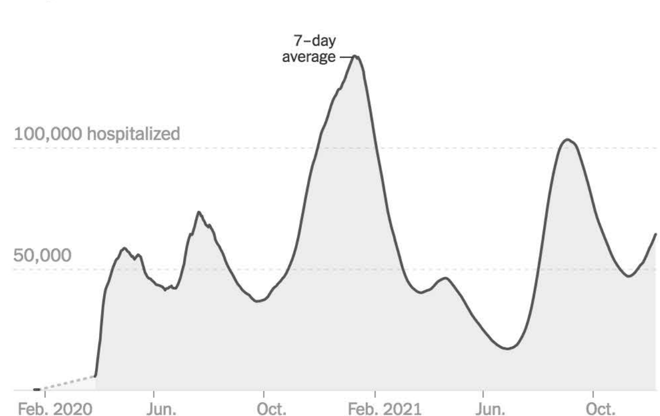

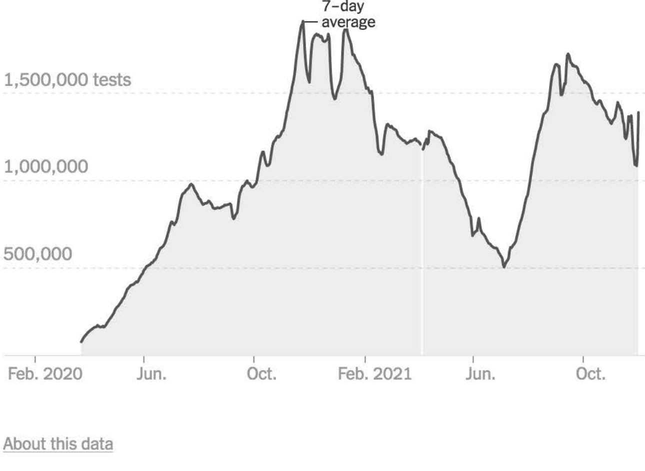

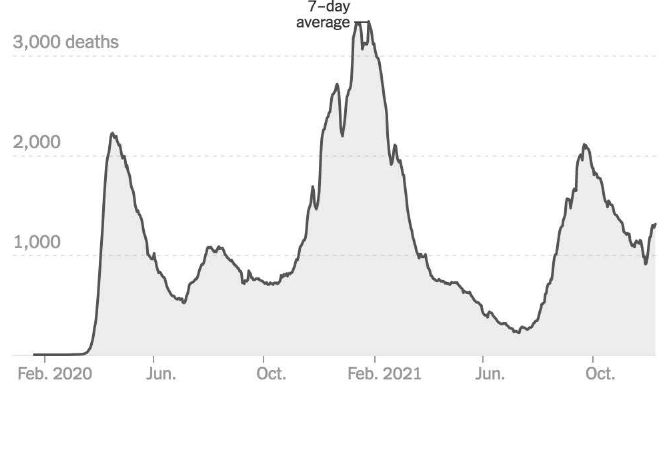

About this data

County trends

This table is sorted by places with the most cases per 100,000 residents in the last seven days. Charts show change in daily averages and are each on their own scale. Select a table header to sort by another metric.

	CASES DAILY AVG.	PER 100,000	14-DAY CHANGE	HOSPITALIZED AVG. PER 100,000	14-DAY CHANGE	DEATHS DAILY AVG.	PER 100,000	FULLY VACCINATED
Waseca, Minn. ›	37	199	+241%	30	+43%	0.2	1.07	53%
Seward, Kan. ›	38	177	+171%	49	+70%	0	—	42%
Allen, Kan. ›	20	165	+123%	20	+343%	0	—	42%
Edgar, Ill. ›	25	146	+169%	27	+54%	0.1	0.83	45%
Vermilion, Ill. ›	107	141	+74%	24	+64%	0.6	0.75	43%
Aroostook, Maine ›	92	138	+51%	30	+99%	0.2	0.30	64%
Piatt, Ill. ›	22	135	+38%	23	+56%	0.1	0.87	56%
Fayette, Ind. ›	31	134	+196%	9	—	0.6	2.71	37%
Sullivan, N.H. ›	57	132	+6%	33	+43%	0	—	59%
Clay, Ill. ›	17	131	+53%	17	+143%	0	—	37%

Show more | About this data

CORONAVIRUS BY THE NUMBERS

Tracking the coronavirus around the U.S.: See how your state is doing

Updated December 9, 2021 · 10:45 AM ET

STEPHANIE ADELINE | CONNIE HANZHANG JIN | ALYSON HURT | THOMAS WILBURN | DANIEL WOOD | RUTH TALBOT

TRACKING THE CORONAVIRUS
U.S. cases | County map | Vaccines | Hospitals | World cases

Data as of 6:22 a.m. ET on Dec. 9

U.S. CASES

TOTAL	DAILY AVERAGE	2-WEEK TREND
49,538,798	+121,084	+26.7%

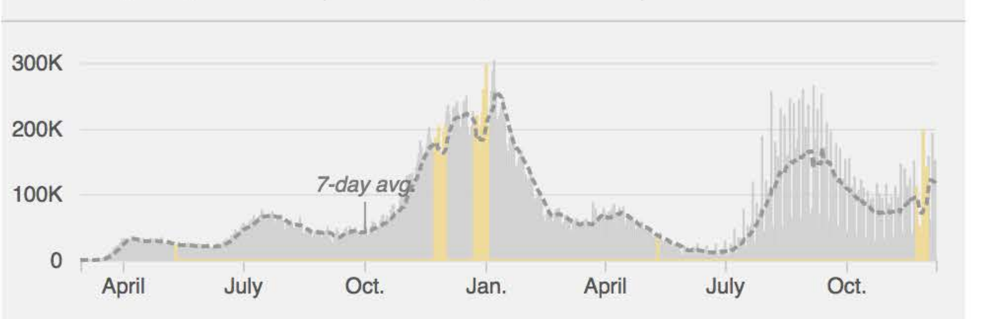

U.S. DEATHS

TOTAL	DAILY AVERAGE	2-WEEK TREND
793,225	+1,529	+37.3%

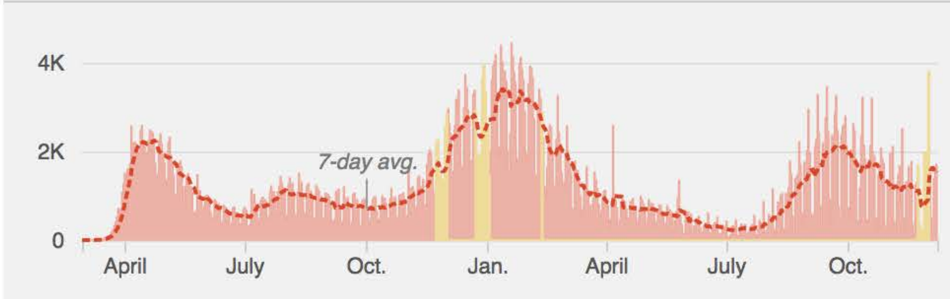

Data irregularities: Data reporting is often delayed on weekends and around major U.S. holidays. Additionally, some states have scaled back how often they report new data, creating more spikes and dips in the daily data.
The daily averages are 7-day averages of new cases and deaths for the week ending December 8. The 2-week trend refers to the percent change in the 7-day average vs. 2 weeks prior.

Source: Center for Systems Science and Engineering at Johns Hopkins University

This page is updated on Mondays and Thursdays.

More than 49 million people in the U.S. have had confirmed coronavirus infections and more than 785,000 have died of COVID-19. Thousands of new cases are reported daily nationwide. In the graphics below, explore the trends in your state.

View the data via a **heat map** (immediately below), **curve charts, a table** of state-by-state trends over four weeks, or a **map** of total cases and deaths.

37 Places Are At The Highest COVID-19 Risk Level

Data as of December 8

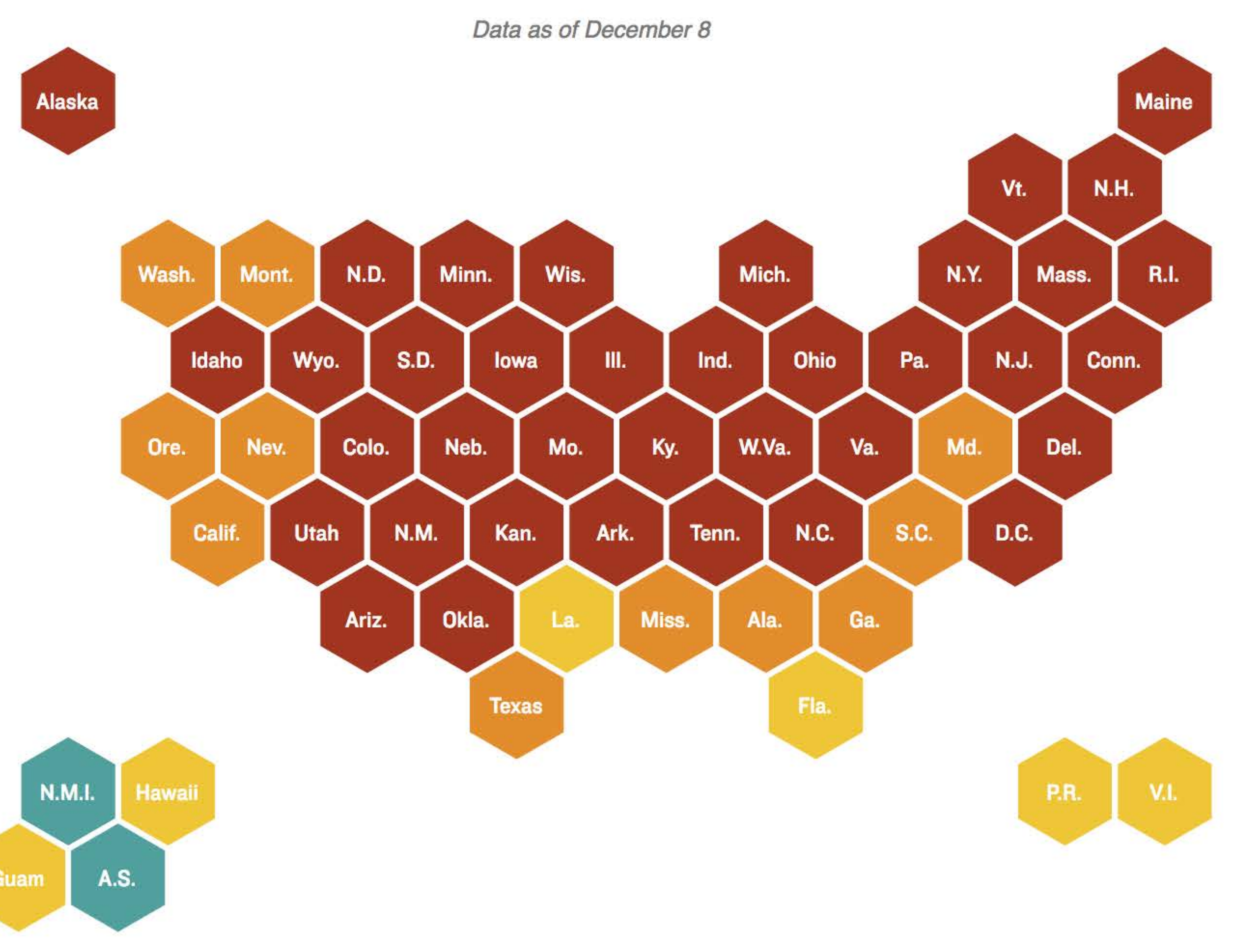

RED
Threshold: 25+ daily new cases per 100,000 people
Indicates: unchecked community spread

ORANGE
Threshold: 10-24 daily new cases per 100,000 people
Indicates: escalating community spread

YELLOW
Threshold: 1-9 daily new cases per 100,000 people
Indicates: potential community spread

GREEN
Threshold: <1 daily new case per 100,000 people
Indicates: close to containment

THE HIGHEST-RISK PLACES INCLUDE:

STATE	RISK LEVEL	AVG. THIS WEEK	PER 100K	2 WEEK TREND
**New Hampshire**	RED	1,279 new cases/day	94 per 100K	+28%
**Michigan**	RED	8,405 new cases/day	84 per 100K	-1%
**Minnesota**	RED	4,650 new cases/day	82 per 100K	+9%
**Rhode Island**	RED	855 new cases/day	81 per 100K	+81%
**Vermont**	RED	475 new cases/day	76 per 100K	+25%

Note: Daily cases are a 7-day average to smooth out day-to-day variations in the data. Risk levels are based on a scale developed by the Harvard Global Health Institute and a collaboration of top scientists at institutions around the country.

Source: Center for Systems Science and Engineering at Johns Hopkins University, Census Bureau 2019 population estimates, 2010 Census (U.S. territories)

The map above shows the risk of infection in each state based on new daily cases per capita. The consortium of researchers and public health experts who developed these risk levels advises states in the red category to issue stay-home orders. Orange states should consider stay-home orders, along with increased testing and contact tracing. Yellow states need to keep up social distancing and mask usage, and all states should continue testing and contact tracing.

Are New Cases And Deaths Still Growing In Your State?

Data as of December 8

NEW DAILY CASES | NEW DAILY DEATHS

All States

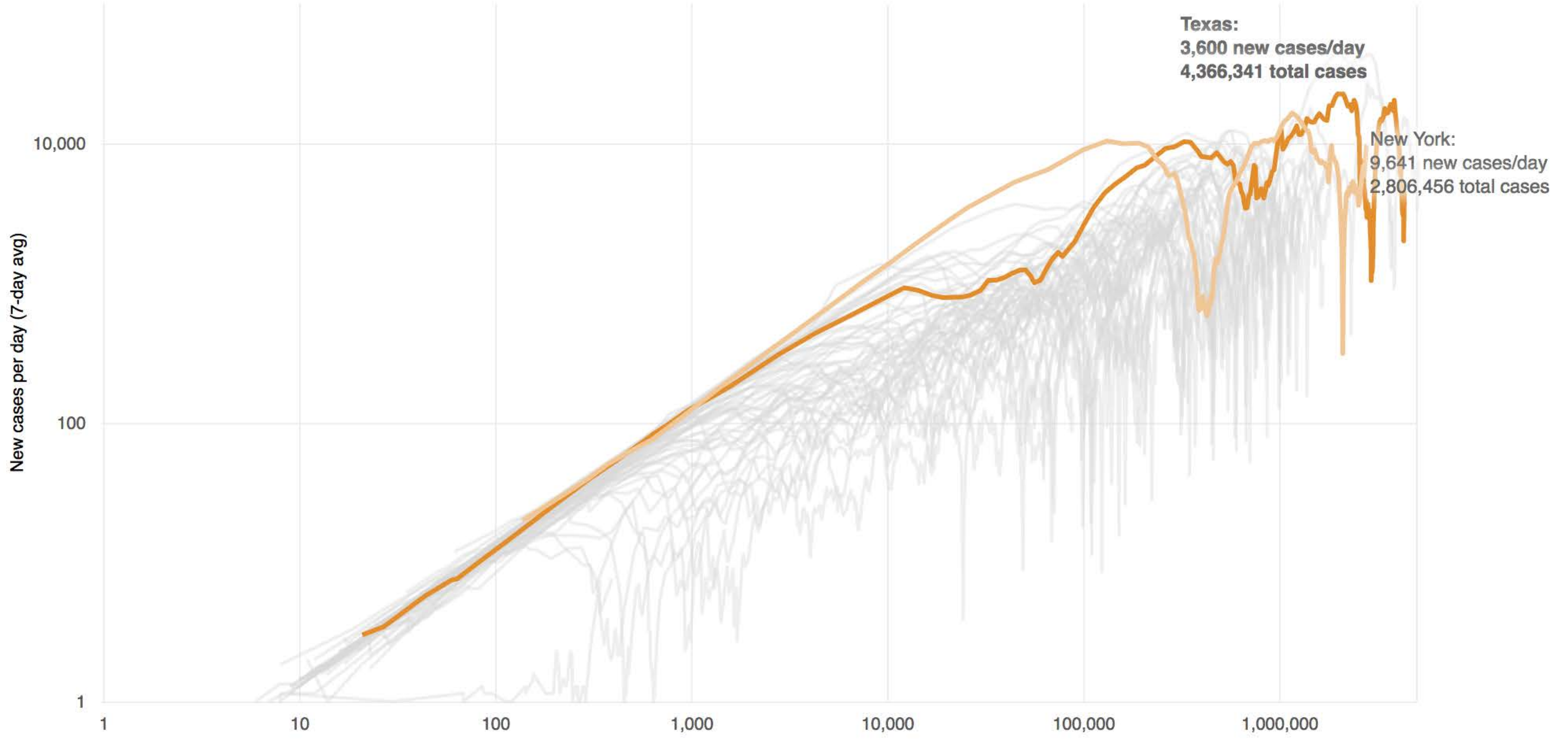

Notes

This chart uses a logarithmic scale, which is meant to emphasize rate of change and allows for the display of a wide range of numbers. Every axis line is 10 times greater than the previous one. The x-axis uses total confirmed cases or deaths on a logarithmic scale rather than date to underscore the overall size of a state's outbreak relative to its daily growth.

Source: Center for Systems Science and Engineering at Johns Hopkins University

To compare state outbreaks, the chart above graphs trend lines for average new daily cases and deaths against each state's totals to date. This type of visualization highlights a state's daily growth or decline relative to the overall size of its outbreak.

When both new and total case and death counts grow quickly, the curves bend upward. As new cases and deaths slow, the curves level or bend down.

Total Cases And Deaths, State By State

Data as of 6:22 a.m. ET on Dec. 11

CASES | CASES PER 100,000 | DEATHS | DEATHS PER 100,000

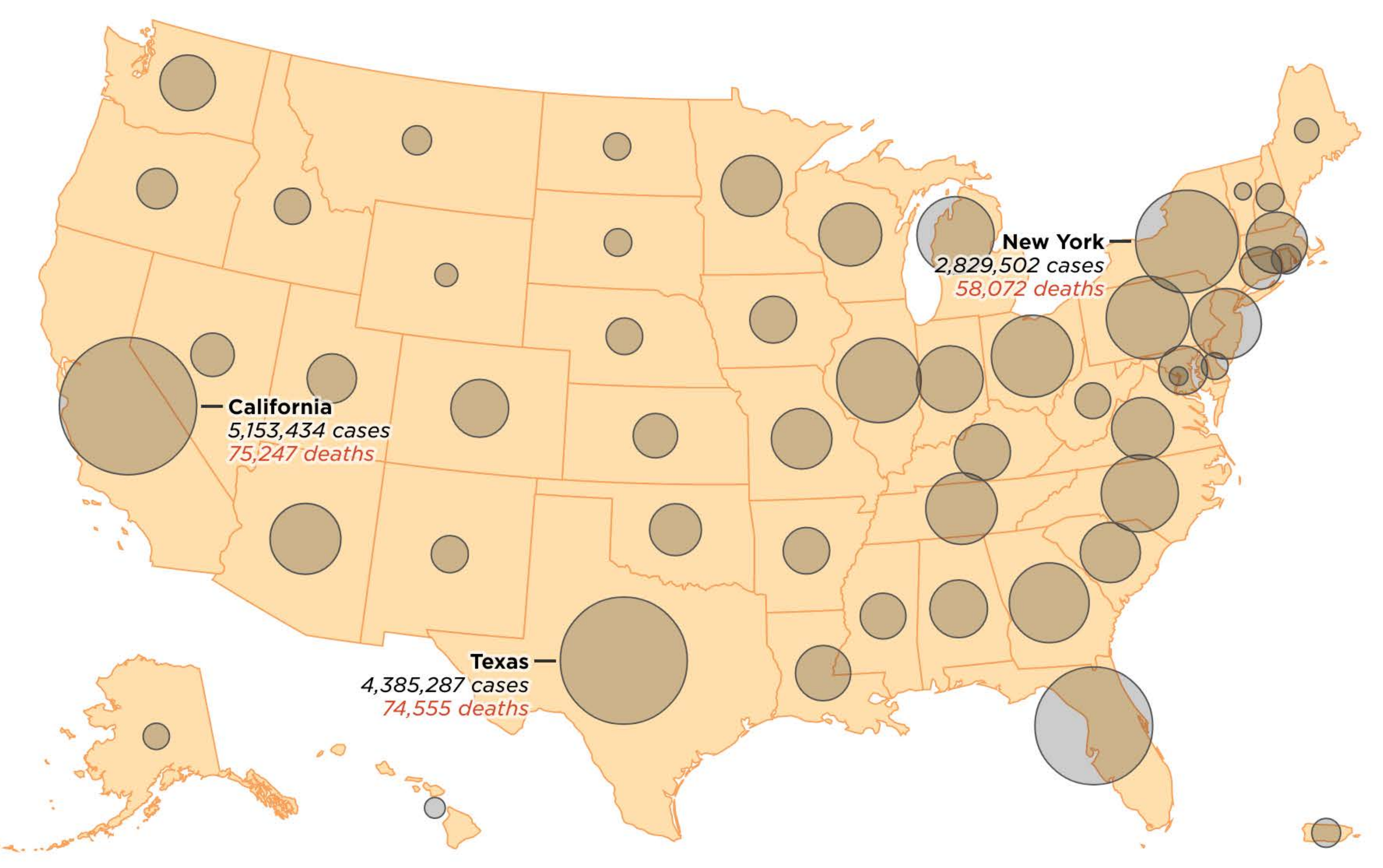

Source: Center for Systems Science and Engineering at Johns Hopkins University

Explore the map above to see totals and per capita figures around the country for both new confirmed cases and reported deaths from COVID-19. New York was the original epicenter of the pandemic in the U.S. As of June 2021, California, Florida and Texas surpass New York for total cases to date.

Click here to see a global map of confirmed cases and deaths.

To show trends, the table below shows the change in average new cases per day in each state, week over week for the last 28 days. States marked in shades of red have growing outbreaks; those in shades of green, are declining.

Total Cases And Weekly Trends, By State And Territory

Totals as of 6:22 a.m. ET on Dec. 11. Daily averages as of December 8.

PERCENT CHANGE VS. PREVIOUS WEEK
-100% -50% -5% +5% +50% +100%

	AVG. NEW CASES/DAY				TOTALS SINCE JAN. 2020	
STATE	3 weeks ago	2 weeks ago	Last week	This week	CASES	DEATHS
**United States**	**87,325**	**95,602**	**85,505**	**121,084**	**49,833,632**	**796,762**
California	5,097	4,405	4,308	7,461	5,153,434	75,247
Texas	3,342	3,479	2,912	3,600	4,385,287	74,555
Florida	1,472	1,413	1,270	1,523	3,754,042	62,026
New York	5,763	6,852	6,689	9,641	2,829,502	58,072
[illegible]	[illegible]	[illegible]	[illegible]	[illegible]	[illegible]	[illegible]

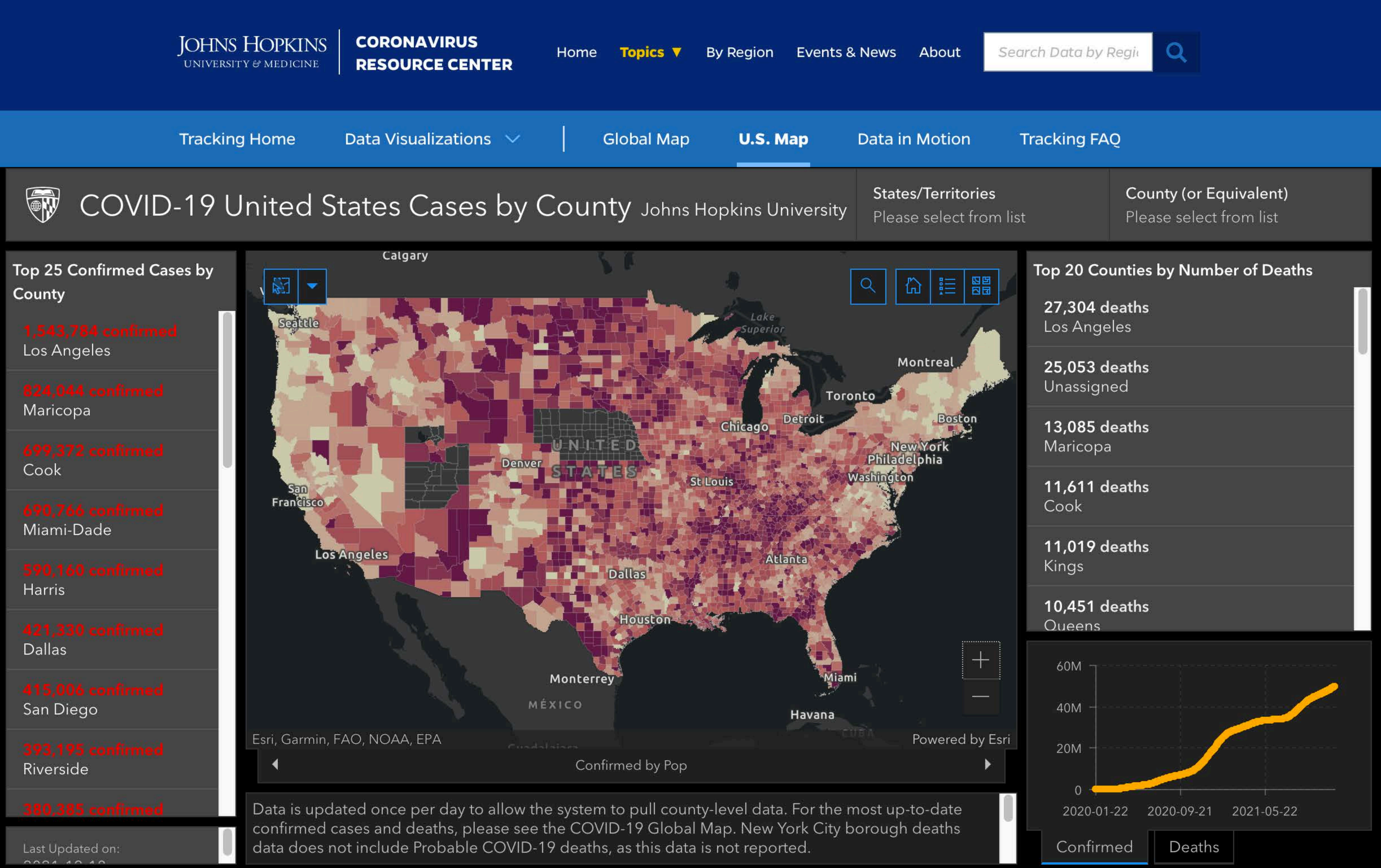
JOHNS HOPKINS
UNIVERSITY & MEDICINE
CORONAVIRUS RESOURCE CENTER
Home
Topics
By Region
Events & News
About
Search Data by Regi
Tracking Home
Data Visualizations
Global Map
U.S. Map
Data in Motion
Tracking FAQ
COVID-19 United States Cases by County Johns Hopkins University
States/Territories
Please select from list
County (or Equivalent)
Please select from list
Top 25 Confirmed Cases by County
1,543,784 confirmed
Los Angeles
824,044 confirmed
Maricopa
699,372 confirmed
Cook
690,766 confirmed
Miami-Dade
590,160 confirmed
Harris
421,330 confirmed
Dallas
415,006 confirmed
San Diego
393,195 confirmed
Riverside
380,385 confirmed
Last Updated on:
Calgary
Seattle
Lake Superior
Montreal
Toronto
Boston
Detroit
Chicago
New York
Philadelphia
Washington
UNITED STATES
Denver
St Louis
San Francisco
Los Angeles
Atlanta
Dallas
Houston
Monterrey
MÉXICO
Miami
Havana
Esri, Garmin, FAO, NOAA, EPA
Powered by Esri
Confirmed by Pop
Data is updated once per day to allow the system to pull county-level data. For the most up-to-date confirmed cases and deaths, please see the COVID-19 Global Map. New York City borough deaths data does not include Probable COVID-19 deaths, as this data is not reported.
Top 20 Counties by Number of Deaths
27,304 deaths
Los Angeles
25,053 deaths
Unassigned
13,085 deaths
Maricopa
11,611 deaths
Cook
11,019 deaths
Kings
10,451 deaths
Queens
60M
40M
20M
0
2020-01-22
2020-09-21
2021-05-22
Confirmed
Deaths

Alabama

How to read this graphic

New Confirmed Cases New Deaths

RECENT OPENING AND CLOSING POLICY DECISIONS

● Restriction/closing ● Opening ● Deferring decisions to county ● Other

← Previous | Next →

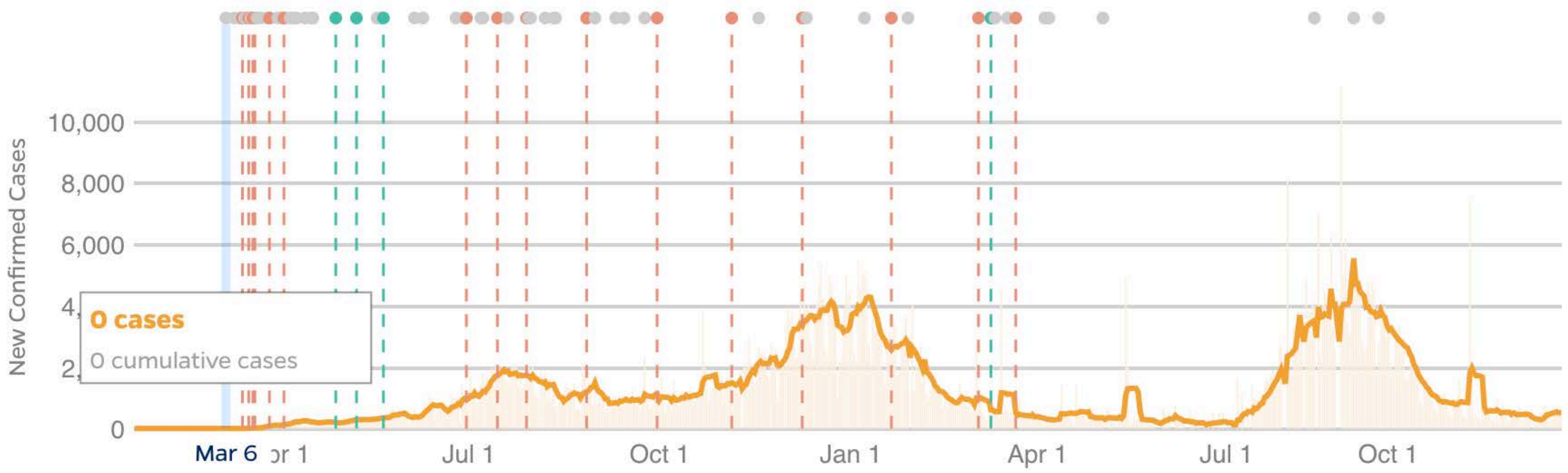

Mar 06, 2020 **0** CUMULATIVE CASES | **0** CUMULATIVE DEATHS

- Governor Ivey announced the formation of a Coronavirus (COVID-19) Task Force of cabinet and state agency officials, as well as disease specialists in an abundance of caution.

Data Sources: Cases and deaths data from JHU CSSE. As of August 1, 2020, policy events are drawn from from various state-specific sources. Prior to that, the data source for policy events was the National Governor's Association.

The Latest on this Dashboard

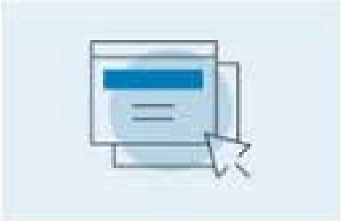

National Report
The National Report offers a detailed overview of the impact of COVID-19 in the U.S.. How has the pandemic been trending? Who are the most vulnerable communities...click to access.

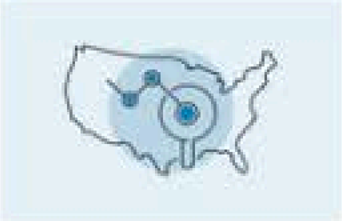

COVID-19 Vaccination Tracker
The COVID-19 Vaccination Tracker tab takes you to an overview of current vaccination status in the U.S. and in each state. For FAQs on COVID-19 Vaccines...click to access.

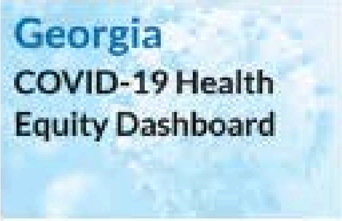

Georgia COVI
The Georgia C
dashboard is a
compare the b
counties in Ge

United States /

See Dashboard Guide (PDF / YouTube)

COVID-19 is affecting every community differently.
Some areas are much harder-hit than others.
What is happening where you live?

Average Daily COVID-19 Cases per 100K

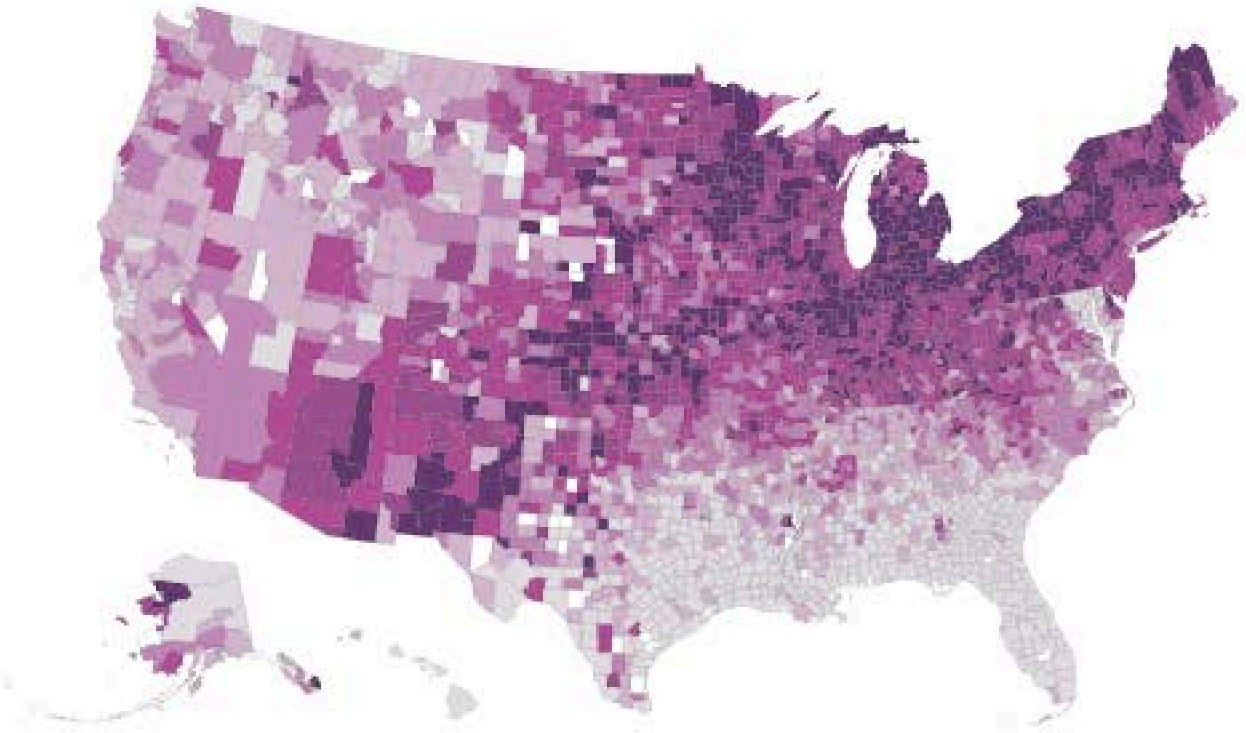

Current Cases and Deaths in The United States

Daily Cases	Daily Deaths
149,041 ↑ 25% 14-day change	1,851 ↓ 9% 14-day change

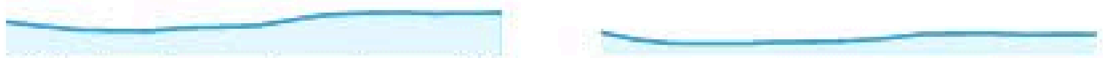

*14-day change trends use 7-day averages.

Disparities in COVID-19 Mortality **Nation**

COVID-19 Death Rates | COVID-19 Death and Population

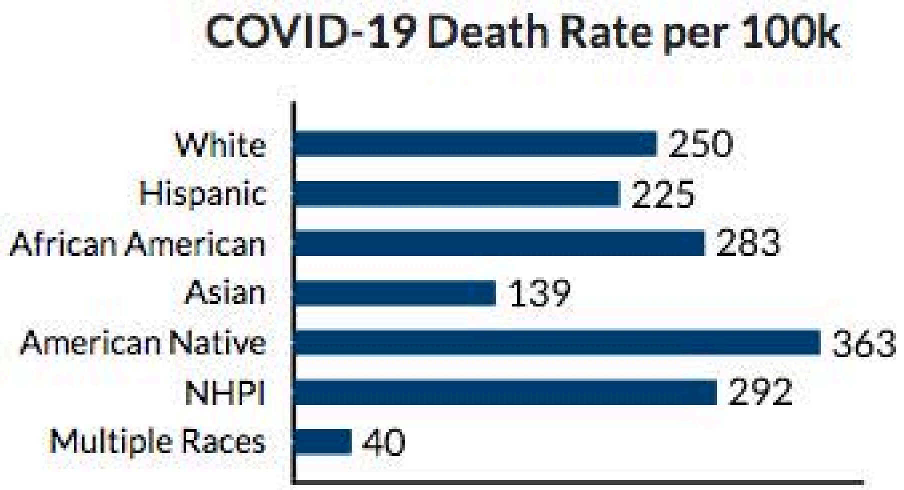

Data as of: 12/09/2021, updated every weekday.

▸ About the data

The chart shows race and ethnicity groups that constitute at least 1% of the state population and have 30 or more deaths. Race and ethnicity data are known for 95% of deaths in the nation. Rates are not reported for race & ethnic groups with < 30 deaths recorded or groups that constitute at least 1% of the state population. NHPI: Native Hawaiians and Pacific Islanders.

Data source: The CDC
Deaths by Race & Ethnicity data as of: 12/08/2021.

USAFACTS

US COVID-19 cases and deaths by state

How is the nation faring against COVID-19, both overall and at a state level? Get the answers here, with data on hotspots and infection rates. This map tracks the history of coronavirus cases in America, both recent and all-time.

Data Updated Dec 9

	Total Reported	On Dec 09	7-Day Average
Cases	48,610,670	102,999	113,657
Deaths	786,531	797	1,176

CASES DEATHS

1 Month | 3 Month | 6 Month | All time

NEW CASES PER DAY

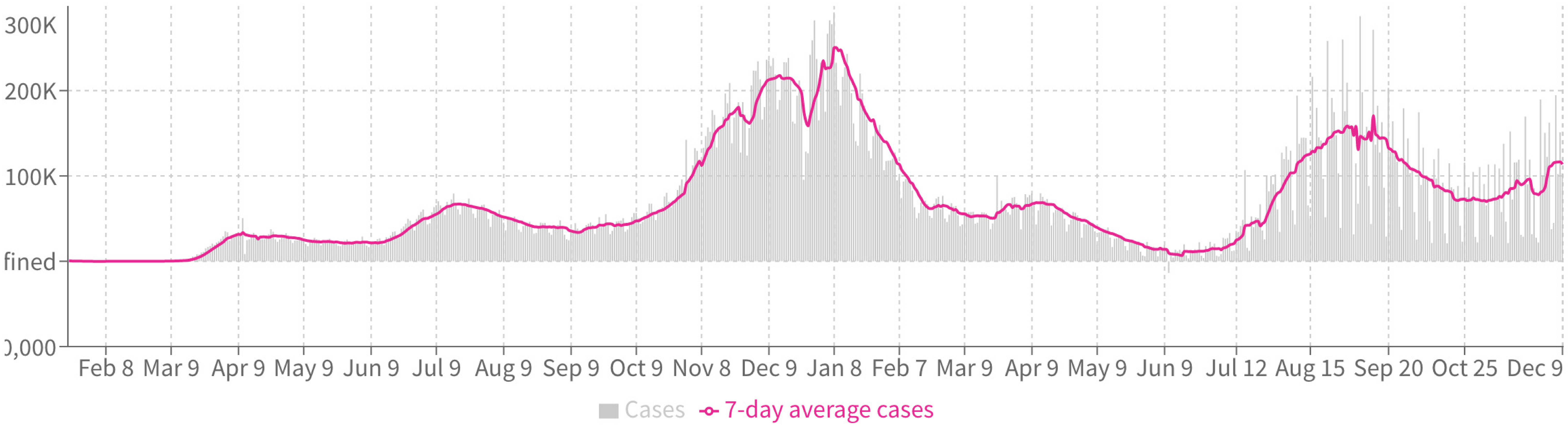

In the United States, there were 102,999 newly reported COVID-19 cases and 797 newly reported COVID-19 deaths on Dec 09, 2021

HOT SPOTS PER 100K TOTAL

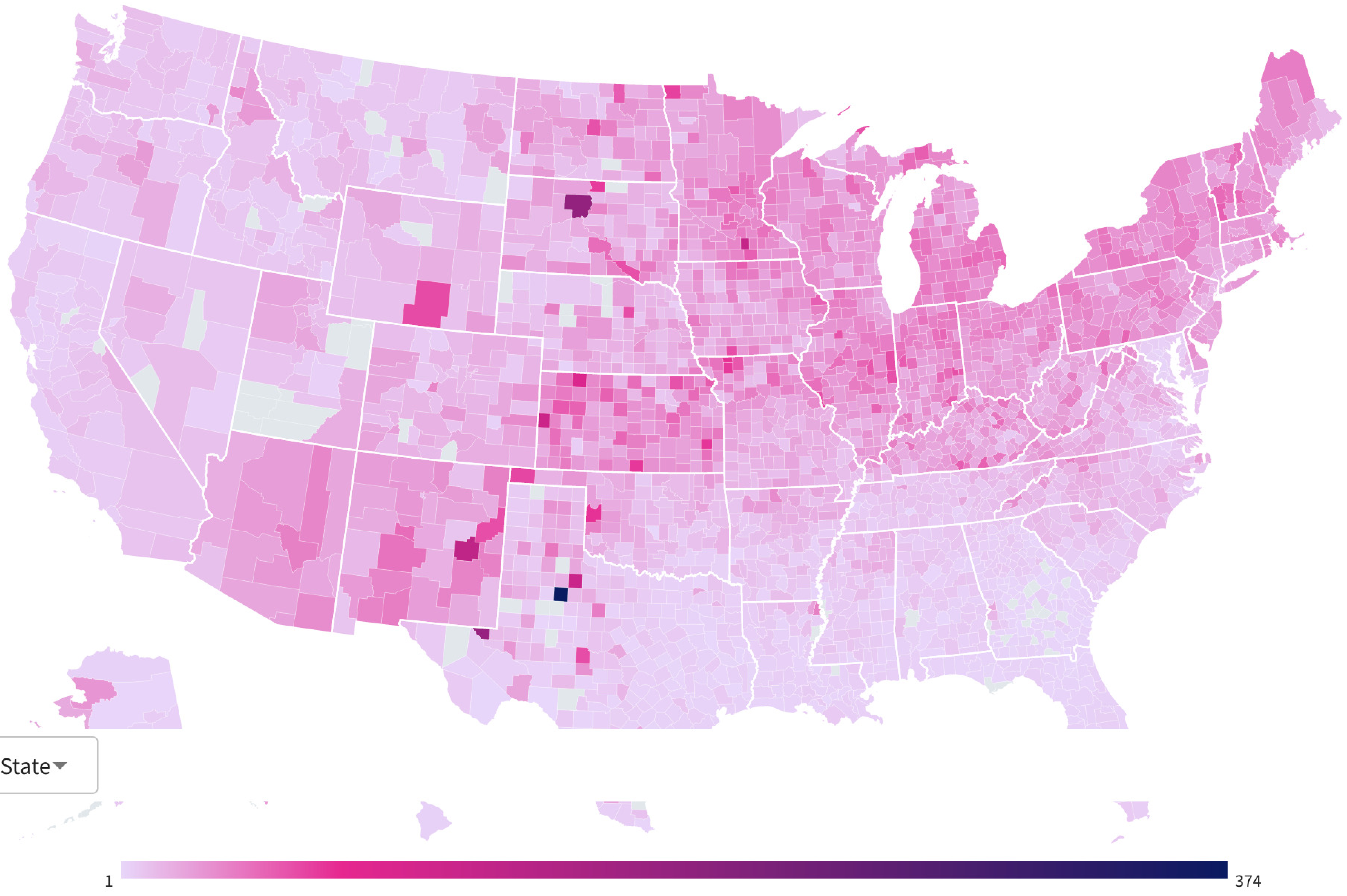

NATIONAL / State

7-DAY AVERAGE CASES PER 100K

From cities to rural towns, stay informed on where COVID-19 is spreading to understand how it could affect families, commerce, and travel. Follow new cases found each day and the number of cases and deaths in the US. The county-level tracker makes it easy to follow COVID-19 cases on a granular level, as does the ability to break down infections per 100,000 people. This county visualization is unique to USAFacts and will be updated with the most recent data as frequently as possible. The underlying data is available for download below the US county map and has helped government agencies like Centers for Disease Control and Prevention in its nationwide efforts.

Kentucky

State Public Health Information

Statistics

810.9k	7.9M	11.5k	N/A
Number of Cases	Number of Tests	Number of Deaths	Total Hospitalized

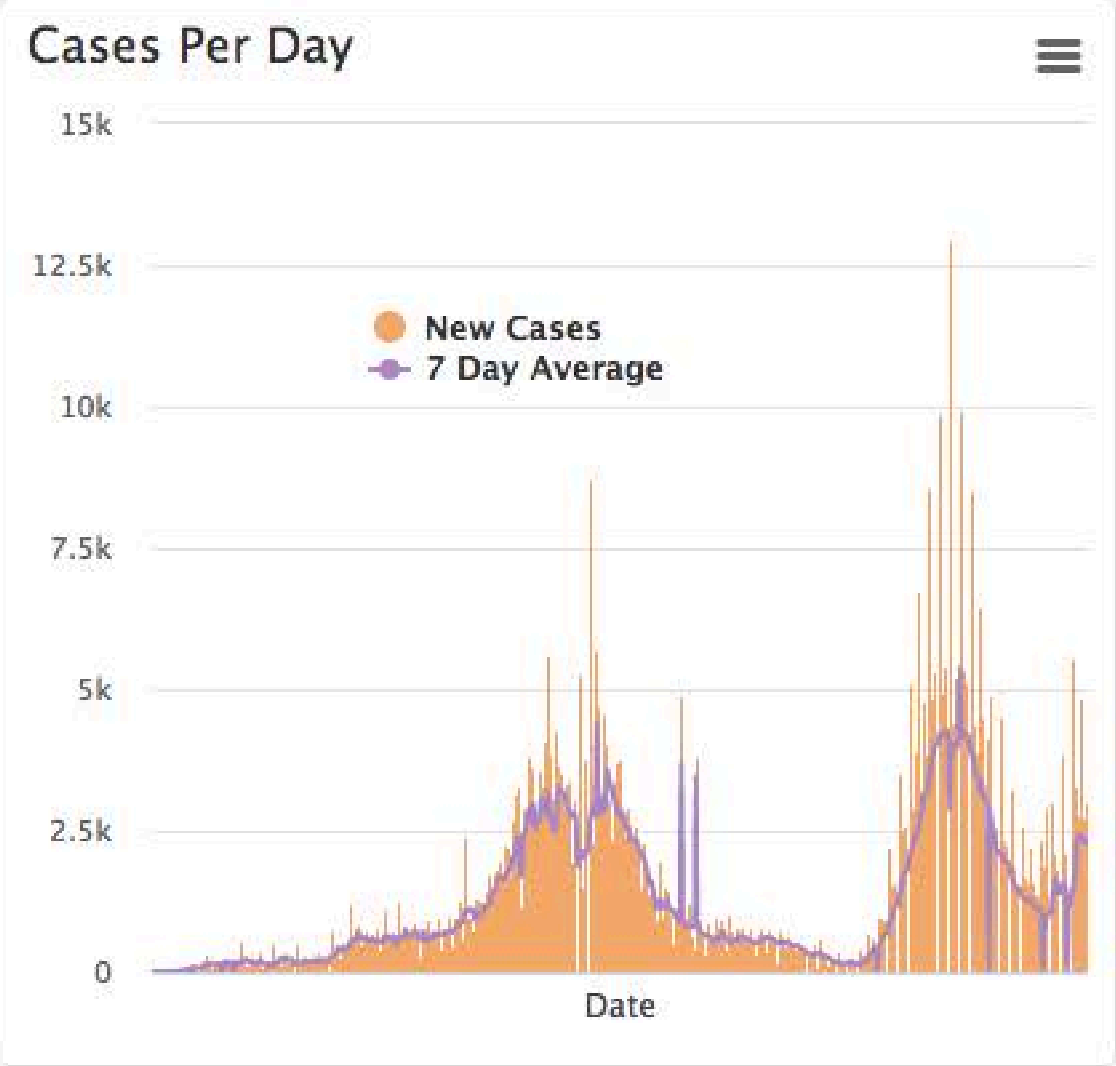

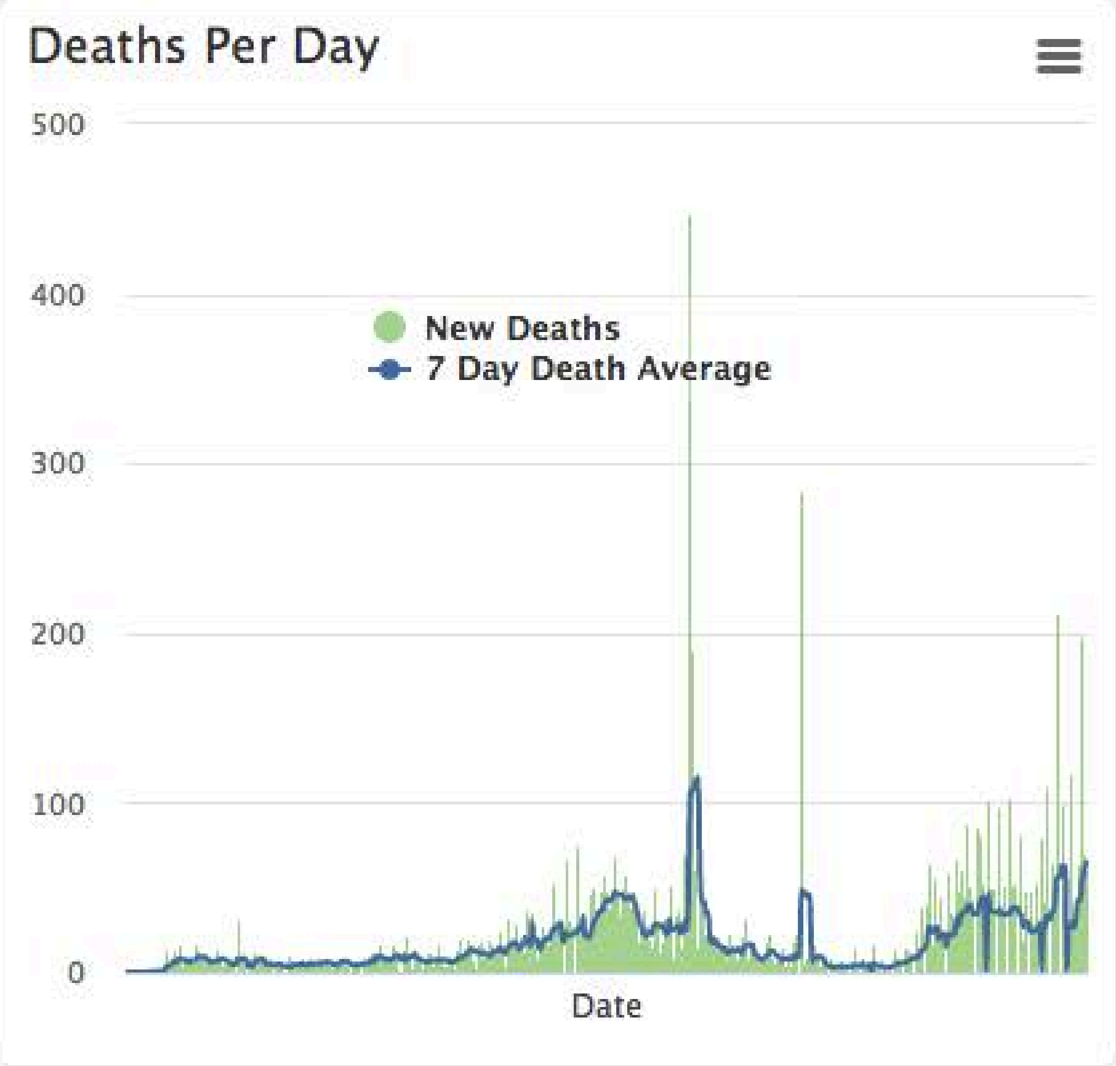

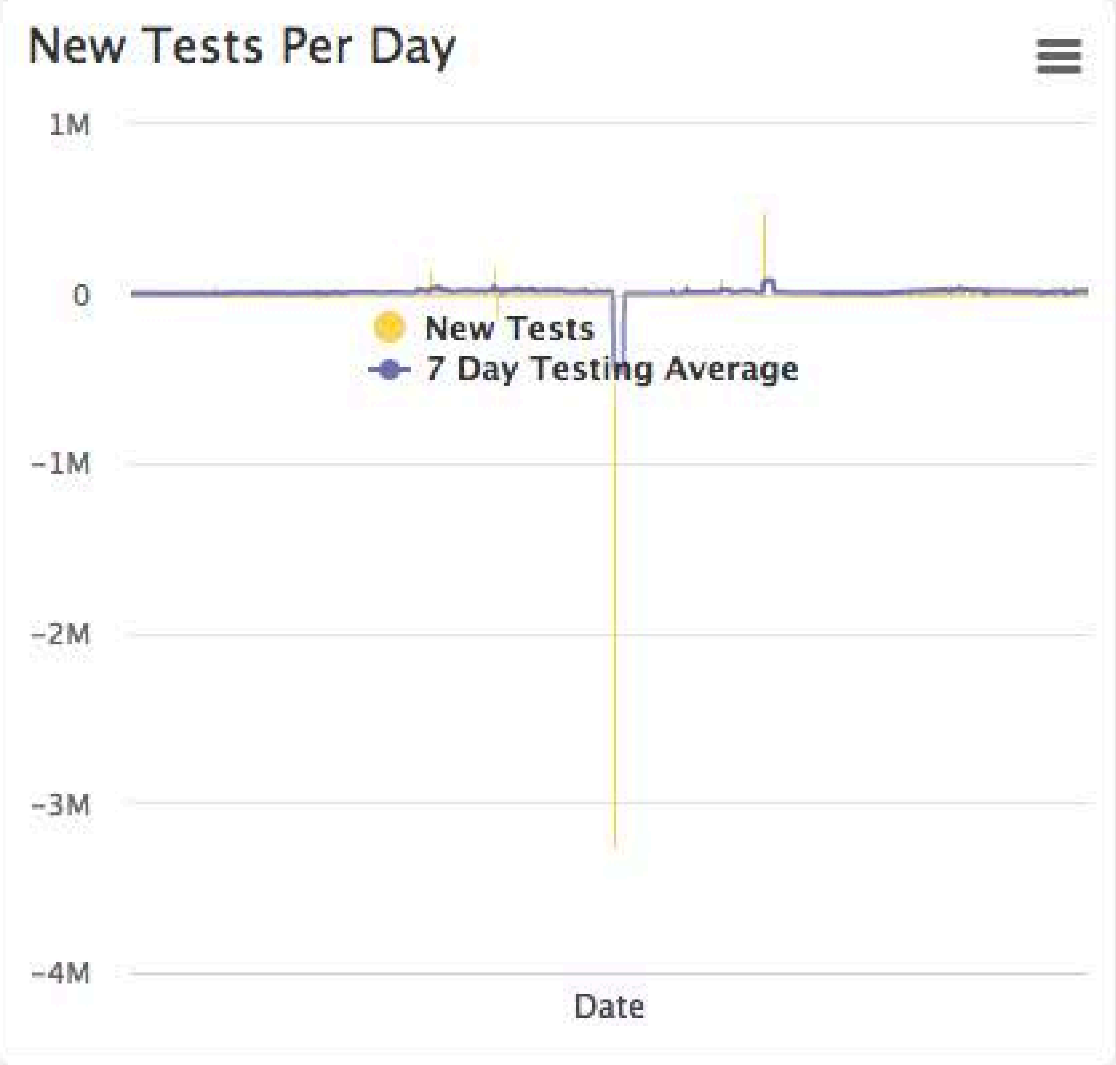

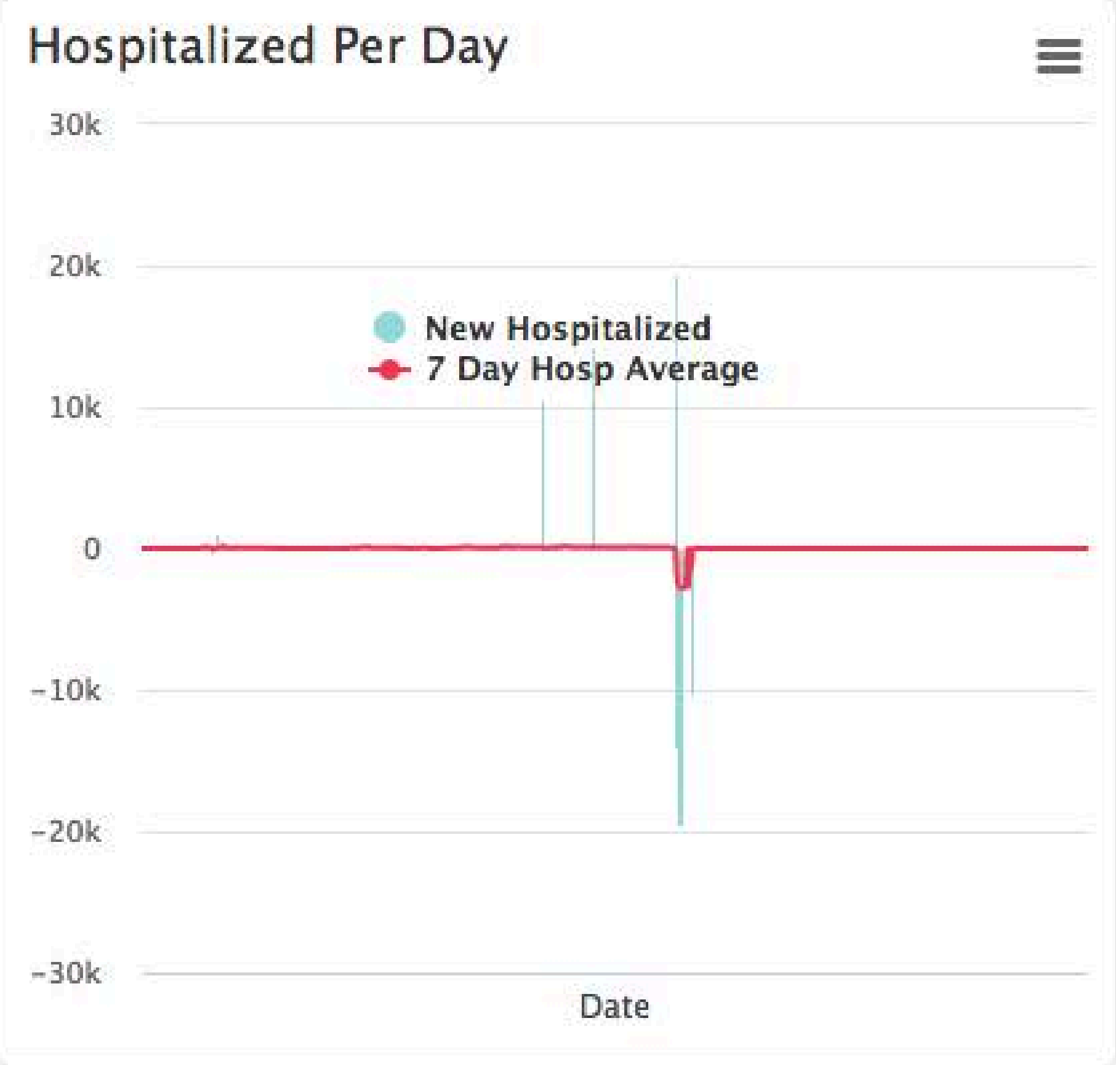

Supplement: Multimedia Appendix 12 [file humanfactors_v10i1e43819_app12.pdf]
