# Supplementary material for: Web-Based COVID-19 Dashboards and Trackers in the United States: Survey Study
Source: JMIR Hum Factors. 2023 Mar 20;10:e43819. doi: 10.2196/43819 (PMC10029858; doi:10.2196/43819)
Supplement: Multimedia Appendix 13 [file humanfactors_v10i1e43819_app13.pdf]

Appendix 13. Screenshots from dashboards and trackers — *Global coverage* *(captured December 11, 2021)*

Page	Ref	Host
2	G-1	New York Times
3	G-2	CNN
4	G-3	Stat / AppliedXL
5	G-4	Johns Hopkins Centers for Civic Impact
6	G-5	Biocomplexity Institute, University of Virginia
7	G-7	HealthMap / various universities
8	G-8	World Health Organization
9	G-9	Kaiser Family Foundation
10–13	G-10	Our World in Data
14	G-11	DXY
15	G-12	Worldometer
16	G-14	Microsoft
17	G-16	Tableau

Coronavirus World Map: Tracking the Global Outbreak

Updated Dec. 11, 2021

Get the latest updates on the Omicron variant.

New reported cases

All time | Last 90 days

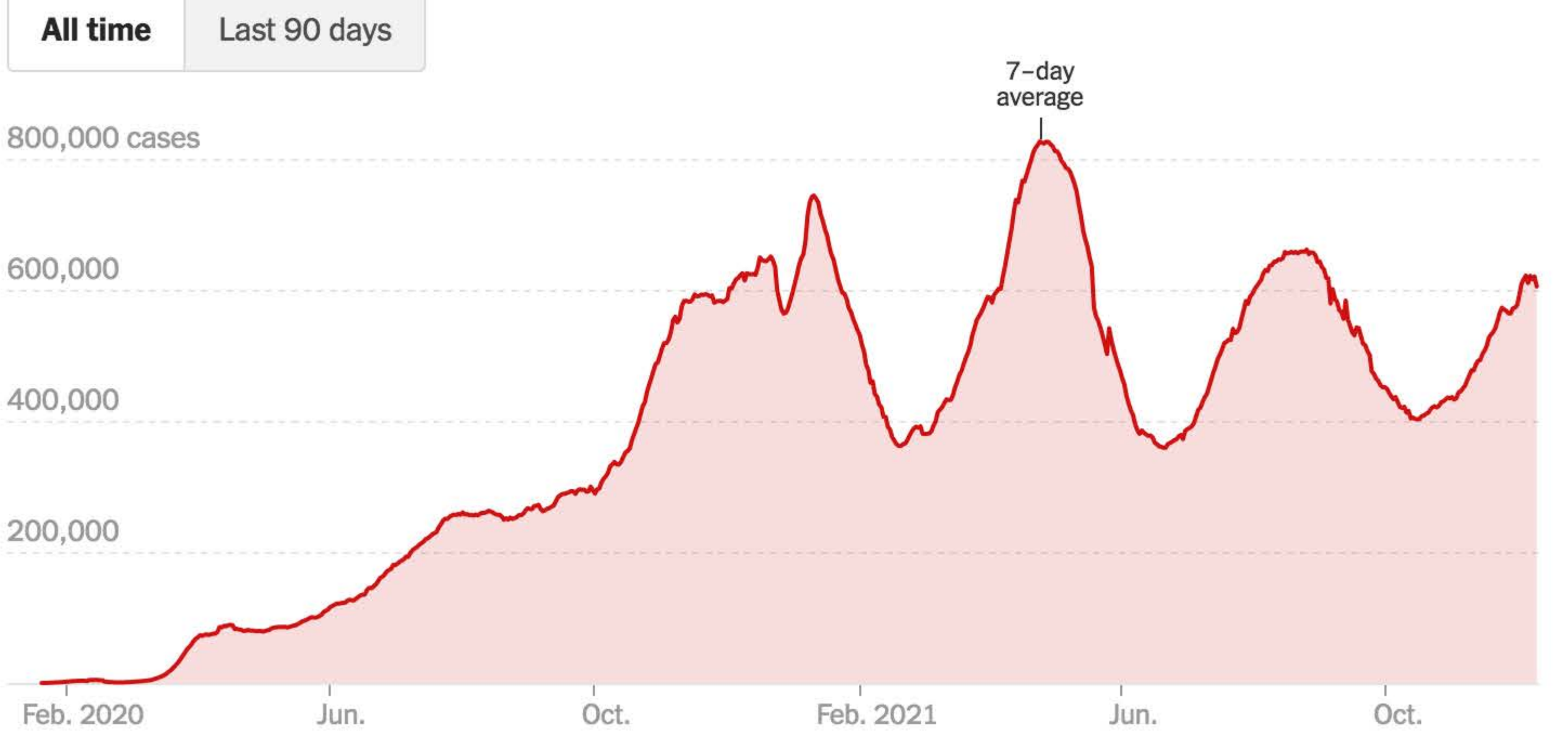

	DAILY AVG. ON DEC. 10	14-DAY CHANGE	TOTAL REPORTED
Cases	605,676	+7%	269,172,181
Deaths	7,494	+7%	5,294,627

About this data

Cases by region

This chart shows how cases per capita have changed in different parts of the world.

All time | Last 90 days

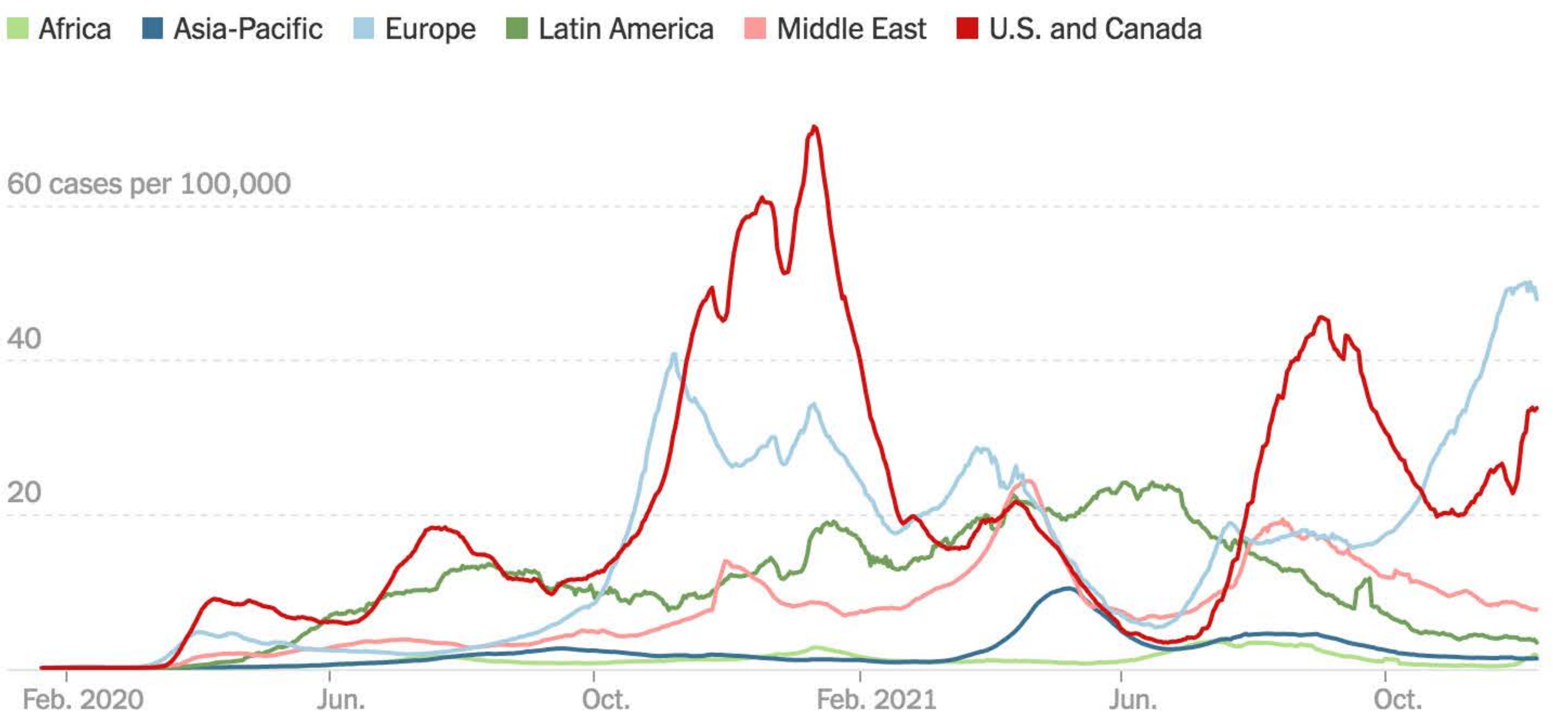

About this data

State of the virus

Update for Dec. 8

- Countries around the world tightened restrictions on international travel and public life. The policies are intended to curb the fast-spreading new Omicron variant of the coronavirus and to increase pressure on people to get vaccinated.
- Just two weeks after it was announced to the world, Omicron has reached dozens of countries and almost half the states in the U.S. Experts caution that it is too soon to reach conclusions about the new variant, but early reports from South Africa, where Omicron is prevalent, indicate that it spreads faster than previous variants but may cause less serious illness.
- Germany became the second Western democracy, after Austria, to shut unvaccinated people out of much of public life, requiring proof of vaccination or of recent recovery from Covid to visit most stores, restaurants and other indoor spaces. Europe is suffering through its biggest wave of cases yet, and there are fears that Omicron will prolong it.
- About 57 percent of the world's population has received at least one vaccine dose, but the rates remain far lower in many poor countries, including many in Africa. Track each country's vaccination progress here.

What Data Shows About Vaccine Supply and Demand in the Most Vulnerable Places

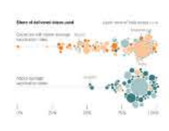

Among the least vaccinated countries, some have used nearly all of the Covid vaccine doses available to them, while others have been able to distribute just a fraction of their available doses.

Hot spots

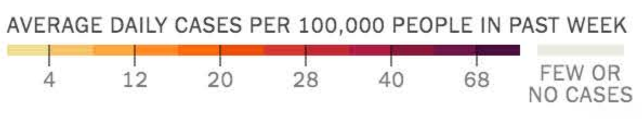

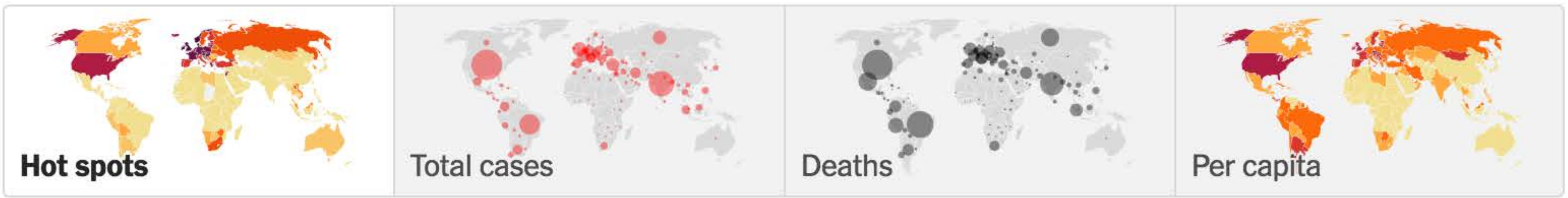

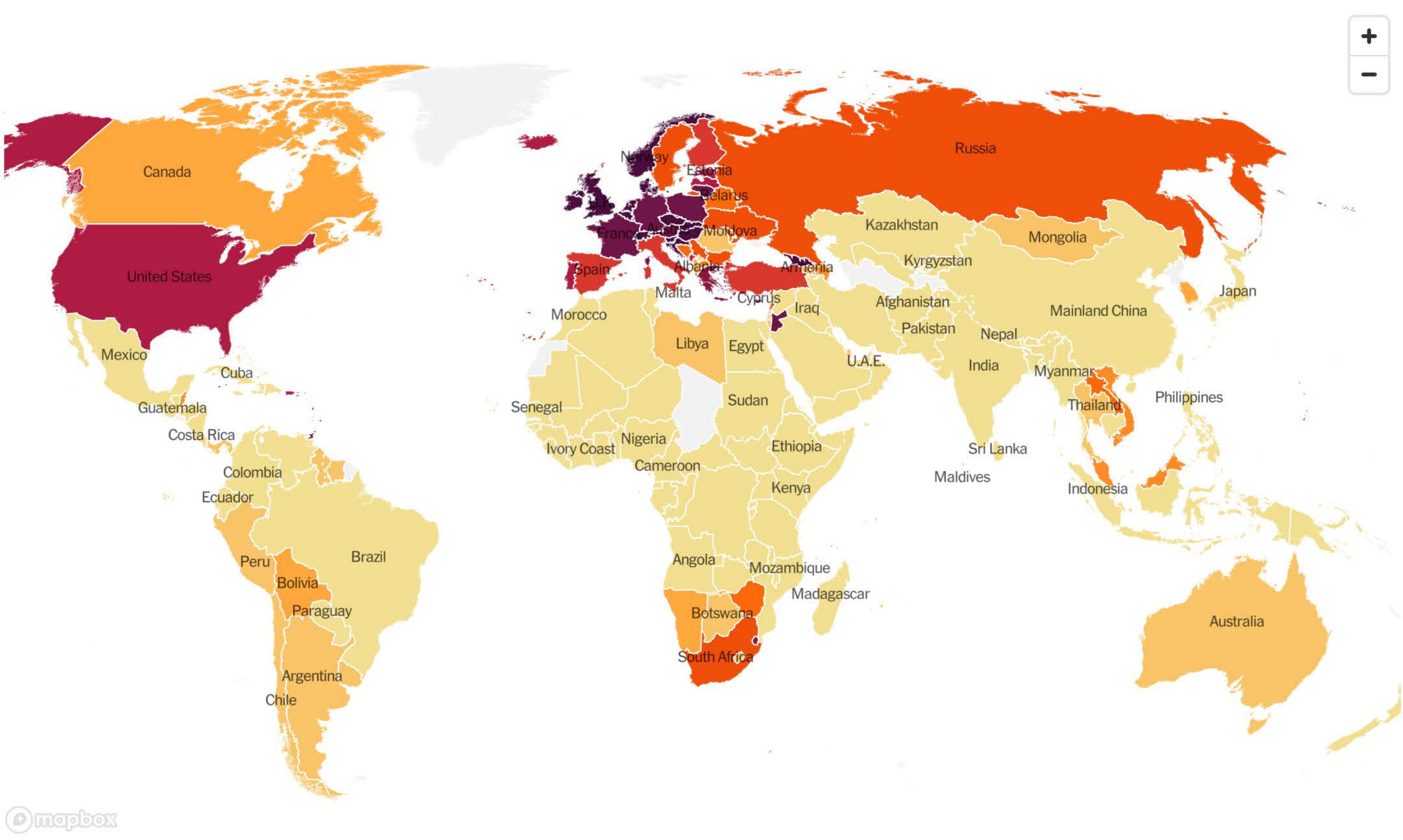

About this data

Reported cases, deaths and vaccinations by country

This table is sorted by places with the most cases per 100,000 residents in the last seven days. Charts show change in daily averages and are each on their own scale. Select a table header to sort by another metric.

Recent trends | All time | Search countries

	CASES DAILY AVG.	PER 100,000	14-DAY CHANGE	DEATHS DAILY AVG.	PER 100,000	FULLY VACCINATED
Andorra	204	265	+128%	0.1	0.19	65%
Slovakia	9,002	165	–21%	77.1	1.41	43%
Liechtenstein	62	163	+26%	0.7	1.88	66%
Czech Republic	14,395	135	–24%	113.1	1.06	61%
San Marino	45	133	+84%	0.1	0.42	70%
Belgium	14,615	127	–15%	48.1	0.42	76%
Channel Islands	205	119	–12%	0.4	0.25	—
Isle of Man	97	114	+38%	0	—	77%
Netherlands	20,033	114	–10%	60.6	0.34	74%
Switzerland	9,613	112	+48%	26.2	0.31	67%

Show all   About this data

How global trends have changed

All time | Last 90 days

New reported cases by day

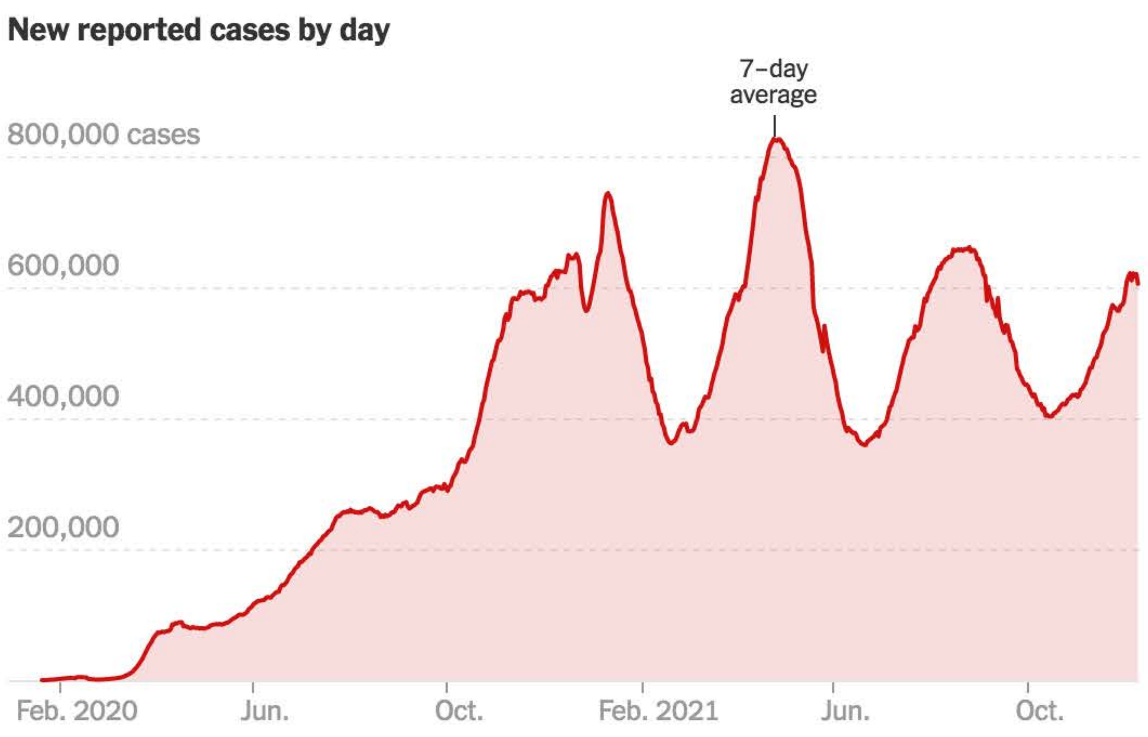

New reported deaths by day

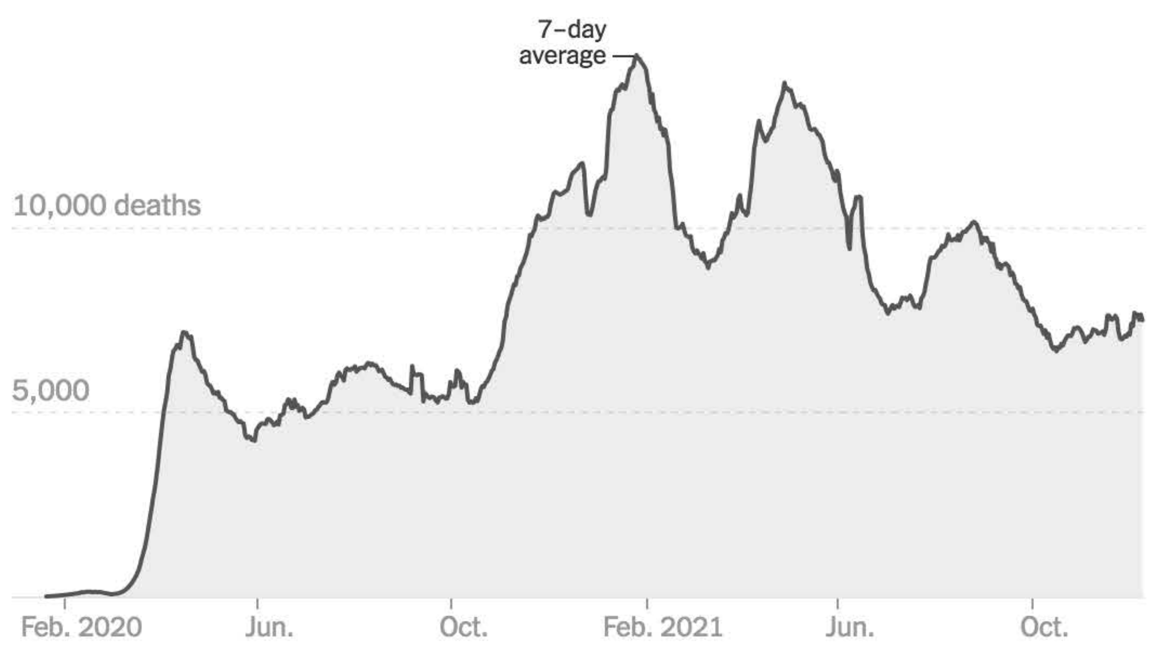

About this data

Tracking Covid-19's global spread

The disease has spread to every continent and case numbers continue to rise

By Henrik Pettersson, Byron Manley and Sergio Hernandez, CNN

Last updated: December 11, 2021 at 7:45 p.m. ET

Authorities in 223 countries and territories have reported about 269.7 million Covid-19 cases and 5.3 million deaths since China reported its first cases to the World Health Organization (WHO) in December 2019.

Cases per 100K | Deaths per 100K | Total cases | Total deaths

0 | 0 to 3,056 | 3,056 to 7,730 | 7,730 to 13,669 | 13,669 to 21,034 | 21,034 to 25,659

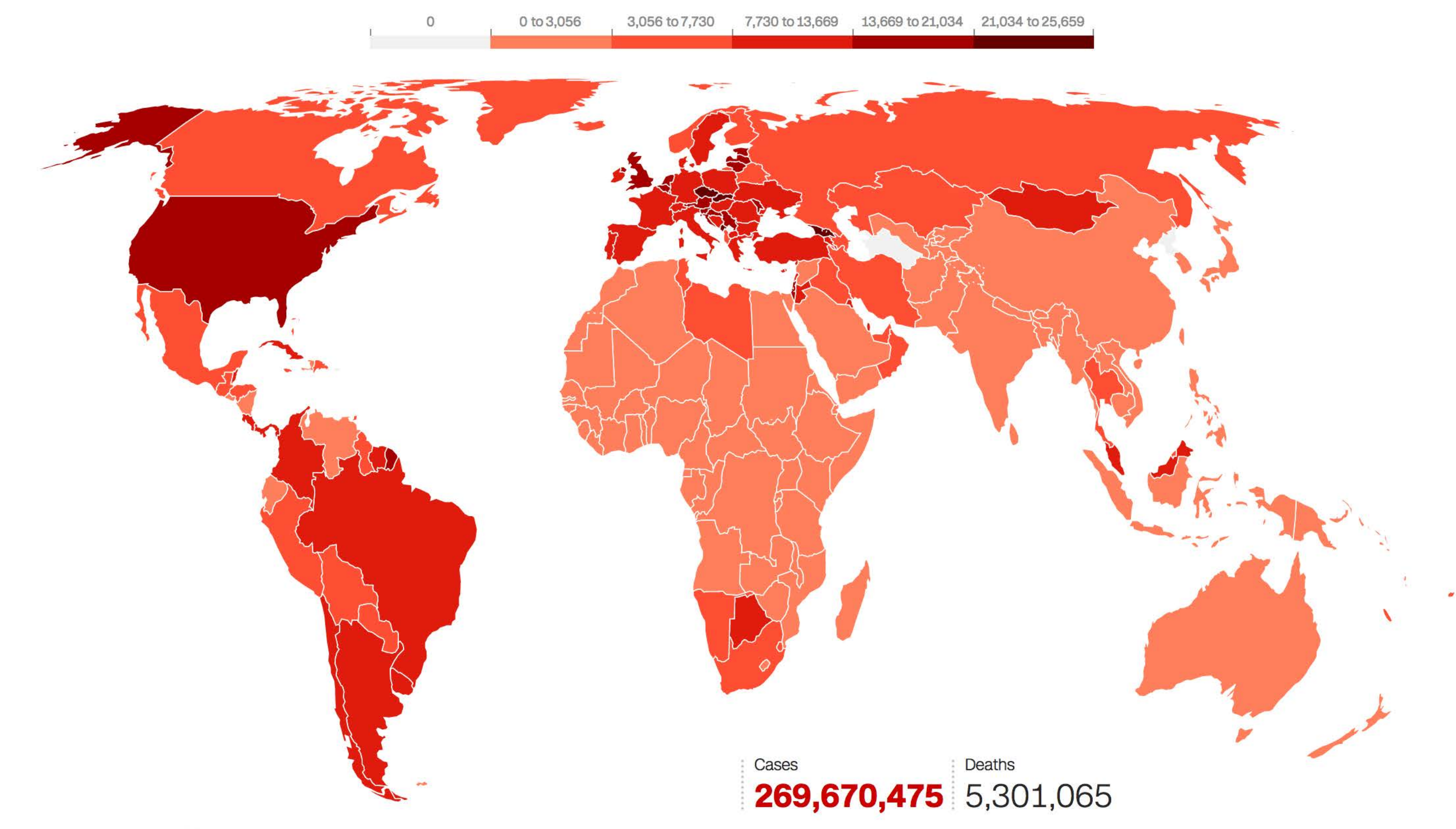

Cases
269,670,475

Deaths
5,301,065

Last updated: December 11, 2021 at 7:45 p.m. ET
Source: Johns Hopkins University Center for Systems Science and Engineering

Reported cases and deaths

The figures below are based on data from the Johns Hopkins University Center for Systems Science and Engineering. These numbers are updated every 15 minutes but may differ from other sources due to differences in reporting times. For up-to-the-minute updates, follow our live coverage.

Location	Cases	...per 100K people	Deaths	...per 100K people
United States	49,875,763	15,195	797,129	243
India	34,682,736	2,538	475,128	35
Brazil	22,177,059	10,508	616,457	292
United Kingdom	10,771,448	16,117	146,387	219
Russia	9,812,538	6,797	282,713	196
Turkey	9,024,193	10,817	78,969	95
France	8,021,334	11,961	117,918	176
Germany	6,501,289	7,820	105,536	127
Iran	6,152,524	7,420	130,661	158
Argentina	5,356,885	11,920	116,760	260
Spain	5,290,190	11,237	88,381	188
Italy	5,206,305	8,634	134,765	224
Colombia	5,091,508	10,114	129,056	256
Indonesia	4,258,980	1,574	143,929	53
Mexico	3,914,706	3,069	296,385	232
Poland	3,808,798	10,031	88,414	233
Ukraine	3,728,246	8,400	96,427	217
South Africa	3,129,622	5,344	90,116	154
Netherlands	2,863,838	16,523	20,074	116
Philippines	2,836,360	2,623	50,096	46

Show more

* Cases identified on cruise ships.

Last updated: December 11, 2021 at 7:45 p.m. ET
Sources: Johns Hopkins University Center for Systems Science and Engineering. Population data from World Bank and United Nations.

Since then, cases have been reported on every continent. The vast majority of cases and deaths are now outside mainland China, where the outbreak began.

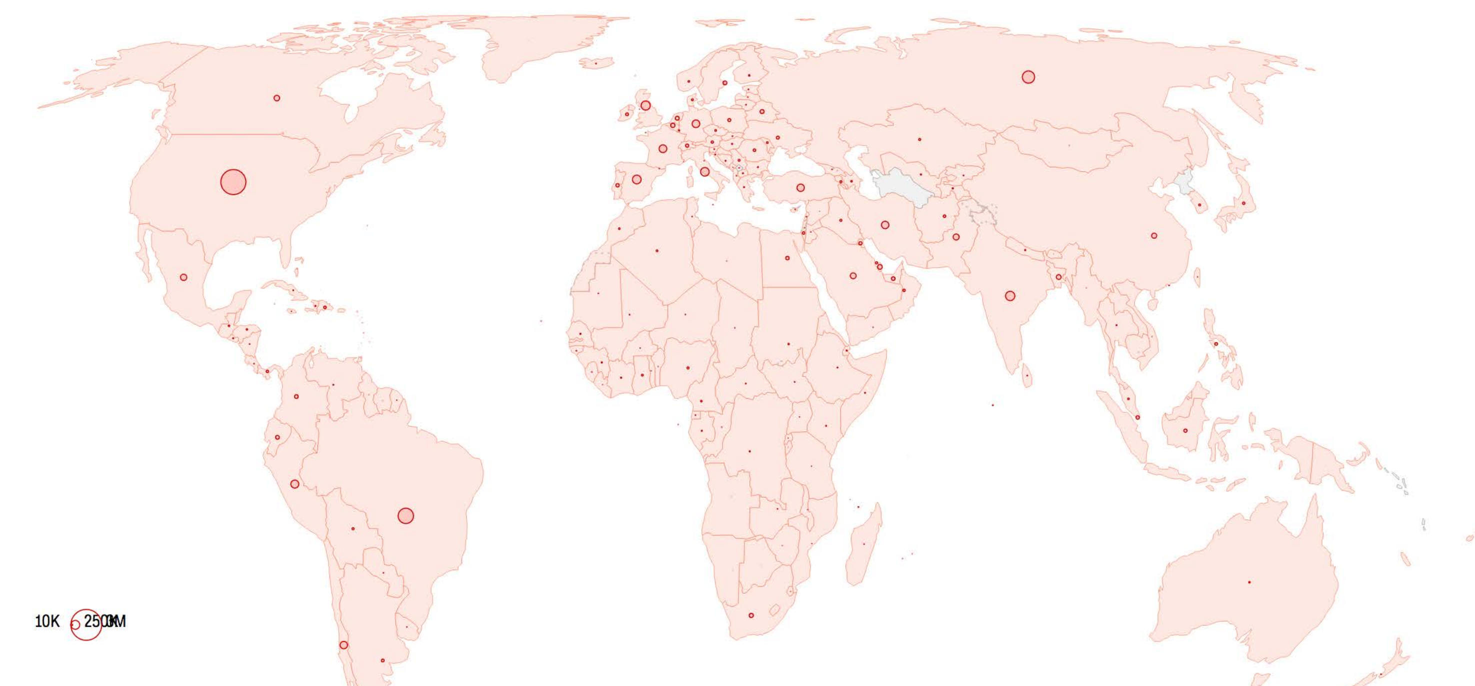

June 10

Cases
7,231,869

Deaths
431,831

Last updated: December 11, 2021 at 7:45 p.m. ET
Source: Johns Hopkins University Center for Systems Science and Engineering

As the pandemic has spread across the globe, the virus has left a trail of deaths in its wake. Deaths in Europe and North America now outnumber Asia's. In Latin America, South America and the Caribbean, the share of global deaths is still rising.

Daily deaths and cases by region

This chart uses rolling, seven-day averages. This approach makes trends clearer and smooths out anomalies, such as the lack of reporting during the weekend.

Deaths | Cases | Proportional | Actual

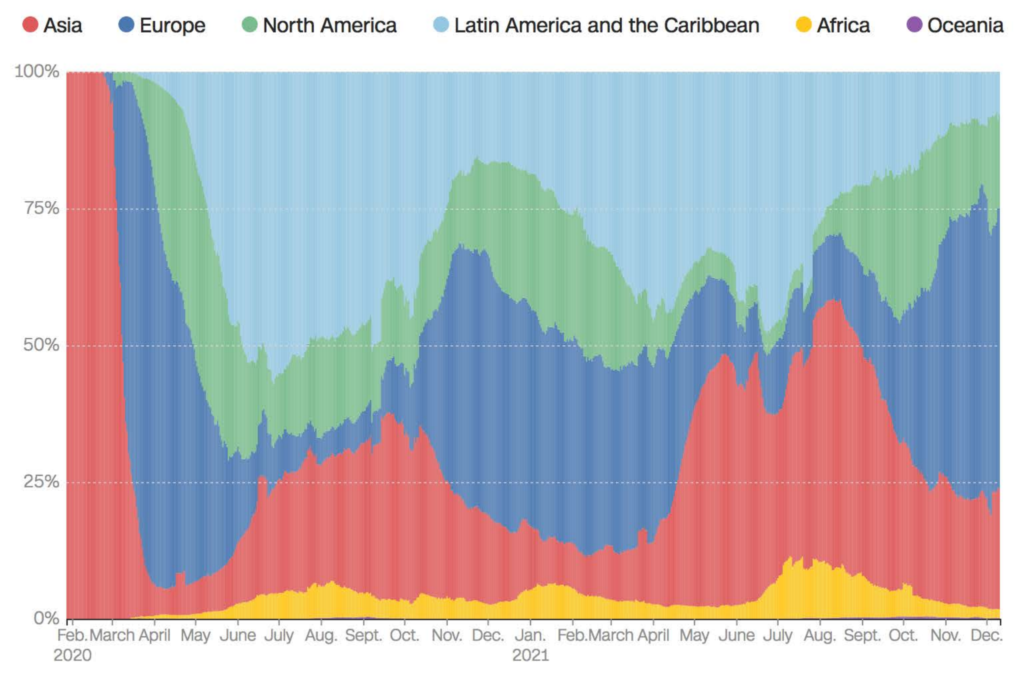

Regions are based on United Nations definitions. Americas have been broken down into subregions (Latin America and the Caribbean and North America).
Last updated: December 11, 2021 at 7:45 p.m. ET
Source: Johns Hopkins University Center for Systems Science and Engineering

Daily reported deaths by country

This timeline of new reported deaths shows how countries and regions have been affected so far.

Asia	Total	Jan. 28, 2020 – Dec. 10, 2021	Latest
India	474,872		620 ↓
Indonesia	143,923		9 ↓
Iran	130,603		77 →
Turkey	78,778		194 ↓
Philippines	49,961		139 ↓

Expand

Europe	Total	Jan. 28, 2020 – Dec. 10, 2021	Latest
Russia	281,571		1,158 ↓
United Kingdom	146,255		119 ↓
Italy	134,669		85 ↑
France	117,706		99 ↓
Germany	105,510		366 ↑

Expand

North America	Total	Jan. 28, 2020 – Dec. 10, 2021	Latest
United States	796,764		1,273 ↑
Canada	29,953		20 →
Bermuda	106		0 →

Latin America and the Caribbean	Total	Jan. 28, 2020 – Dec. 10, 2021	Latest
Brazil	616,457		151 ↓
Mexico	296,188		183 ↓
Peru	201,650		46 ↑
Colombia	129,011		47 →
Argentina	116,748		16 ↑

Expand

Africa	Total	Jan. 28, 2020 – Dec. 10, 2021	Latest
South Africa	90,080		19 ↓
Tunisia	25,419		4 ↓
Egypt	20,966		46 →
Morocco	14,795		2 →
Ethiopia	6,822		5 →

Expand

Oceania	Total	Jan. 28, 2020 – Dec. 10, 2021	Latest
Australia	2,100		8 ↑
Fiji	697		0 →
French Polynesia	636		0 →
Papua New Guinea	573		3 →
New Caledonia	280		0 ↓

Expand

Based on 7-day rolling averages | ↑↓ Trend in rolling average | 1 10 100 500 1,000

Different countries' epidemics have followed different trajectories. The disease has hit the United States especially hard. About 49.9 million cases have been reported in the country and 797,129 patients have died.

Covid-19 case growth rates

The chart below uses a logarithmic scale to show how quickly the number of known Covid-19 cases is growing in each region and territory. Select a location or hover over each line to see how quickly the number of known cases is doubling in that country or territory.

In **the United States** the number of known cases is doubling about **every nine months**.

CNN health

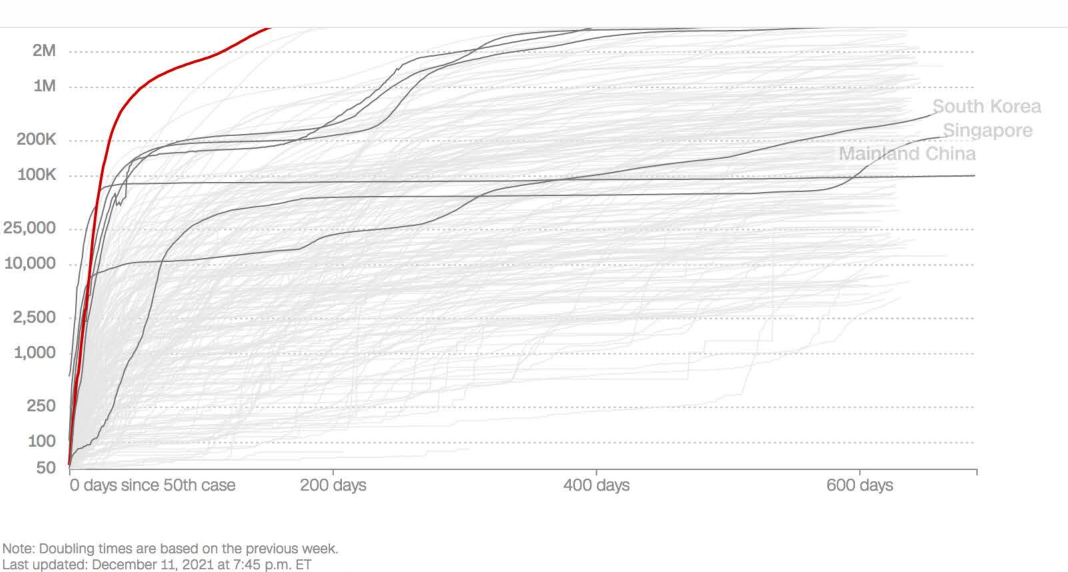

Note: Doubling times are based on the previous week.
Last updated: December 11, 2021 at 7:45 p.m. ET
Source: Johns Hopkins University Center for Systems Science and Engineering

APPLIED XL

In partnership with STAT

Covid-19 Worldwide

Last updated: December 11, 2021 at 7:08 PM EST

Search by country or region

Total Number of Cases | Cases Per 100k Population

▸ United Kingdom	10,833,033
Russia	9,812,538
Turkey	9,024,193
▸ France	8,318,995
▸ Germany	6,501,289
Iran	6,152,524
▸ Argentina	5,356,885
▸ Spain	5,290,273
▸ Italy	5,206,305
▸ Colombia	5,091,508
Indonesia	4,258,980

TOTAL CASES
269,669,416

FIRST CASE
711 DAYS AGO

RECOVERED
144,502,154

DEATHS
5,301,065

2 WEEK AVERAGE CHANGE IN NEW CASES 4.2%

Average New Cases Yesterday 604,995

Average New Cases 2 Weeks Ago 580,430

2 WEEK AVERAGE CHANGE IN DEATHS 5.8%

Average Deaths Yesterday 7,583

Average Deaths 2 Weeks Ago 7,170

2 Week Trend | Cases Over Time

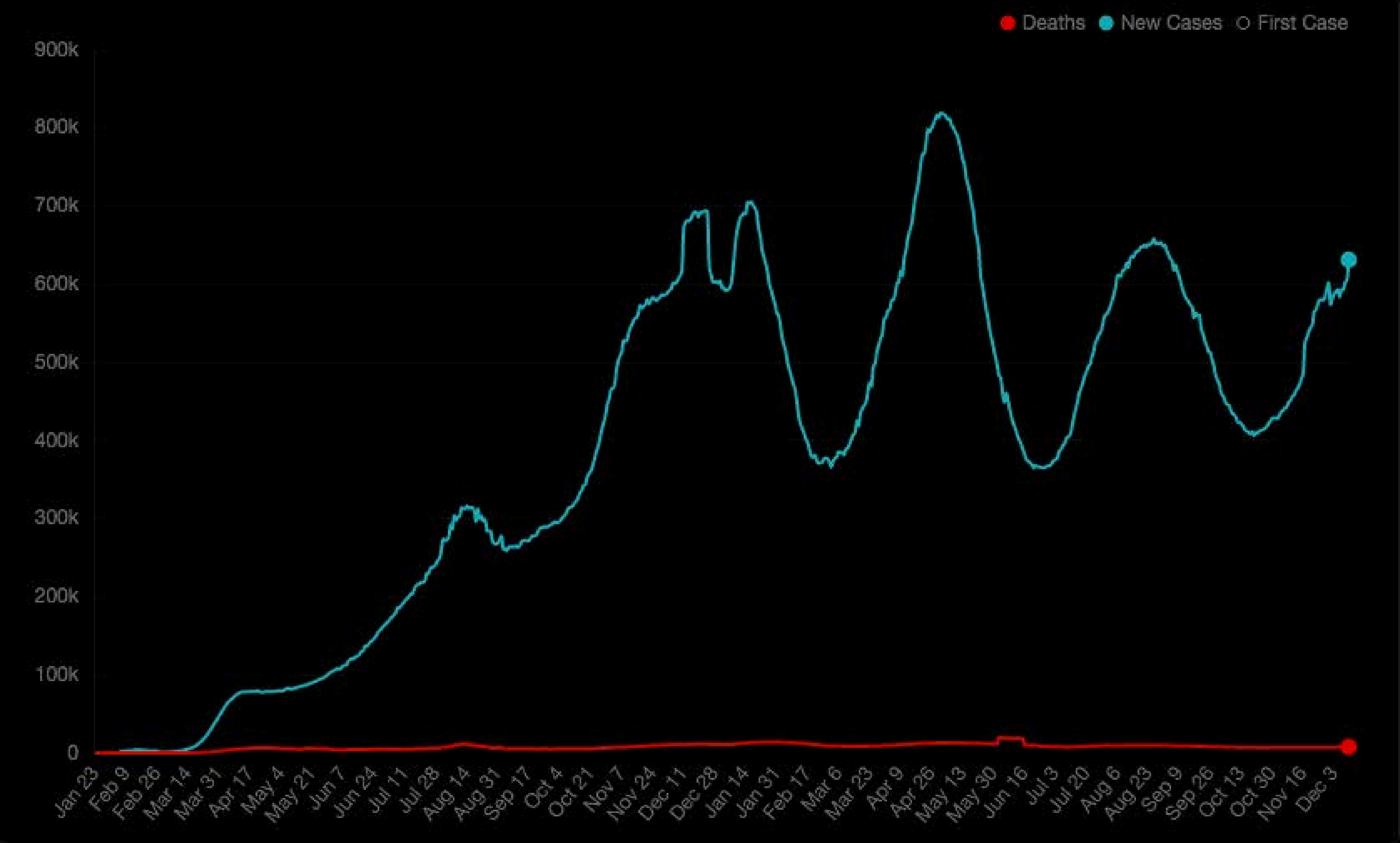

The datasets are drawn from over 15 sources and can be reviewed **here**. Data is updated regularly but may fluctuate based on variations in self-reported source data from each region.

About | **Privacy policy** | **Built at** _NEWLAB

Johns Hopkins University & Medicine | CORONAVIRUS RESOURCE CENTER

Home | Topics ▼ | By Region | Events & News | About | Search Data by Regi

Tracking Home | Data Visualizations | Global Map | U.S. Map | Data in Motion | Tracking FAQ

COVID-19 Dashboard by the Center for Systems Science and Engineering (CSSE) at Johns Hopkins University (JHU)

Last Updated at (M/D/YYYY)	Total Cases	Total Deaths	Total Vaccine Doses Administered
12/11/2021, 7:22 PM	269,670,475	5,301,065	8,413,955,346
	**28-Day Cases**	**28-Day Deaths**	**28-Day Vaccine Doses Administered**
	16,206,114	206,111	916,225,507

Cases | Deaths by Country/Region /Sovereignty

US
28-Day: 2,818,651 | 33,359
Totals: 49,875,763 | 797,129

Germany
28-Day: 1,493,412 | 7,887
Totals: 6,501,289 | 105,536

United Kingdom
28-Day: 1,246,066 | 3,596
Totals: 10,833,033 | 146,844

Russia
28-Day: 939,485 |

Admin0

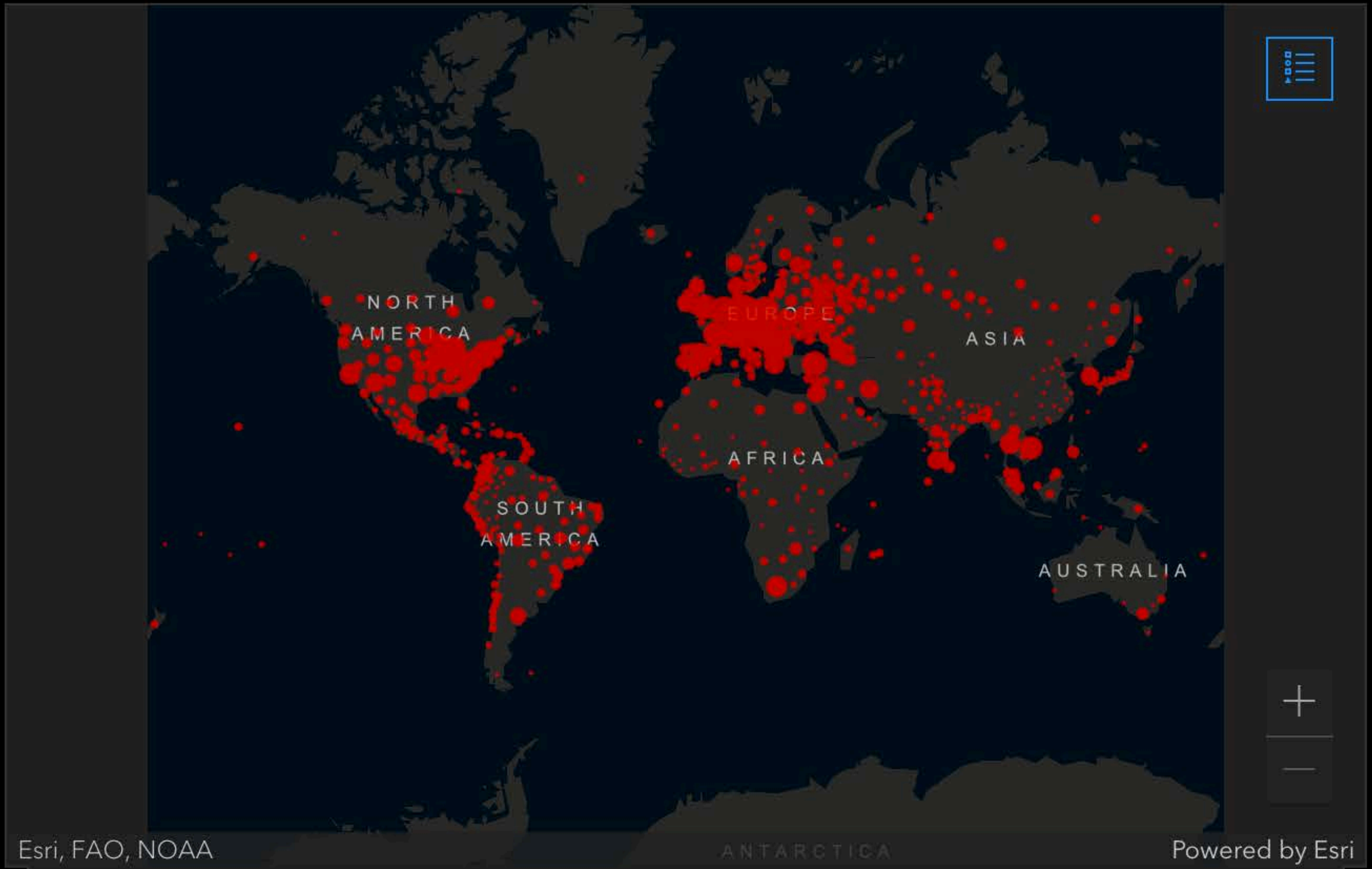

28-Day

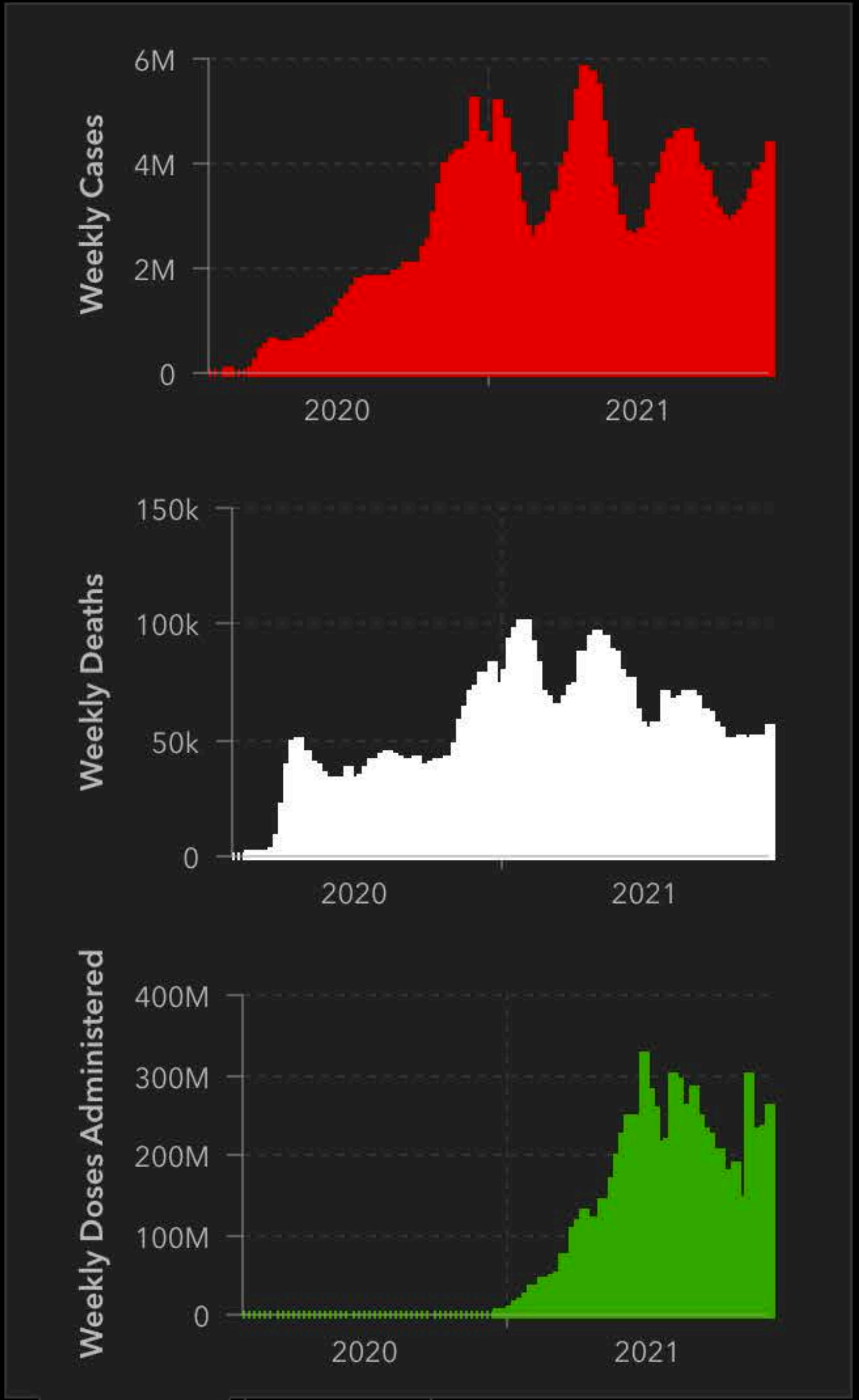

Weekly | 28-Day

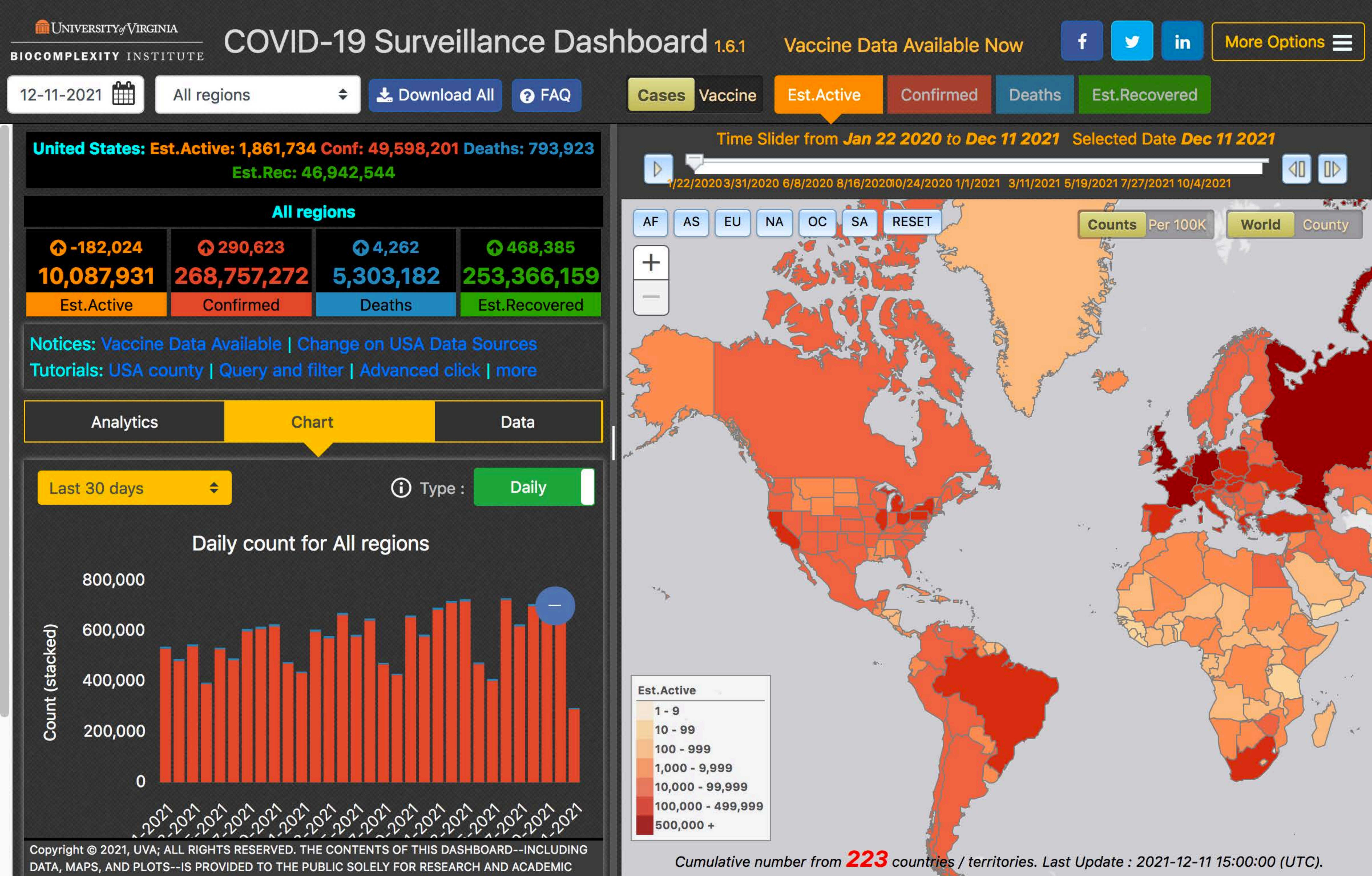
UNIVERSITY of VIRGINIA
BIOCOMPLEXITY INSTITUTE
COVID-19 Surveillance Dashboard 1.6.1
Vaccine Data Available Now
More Options
12-11-2021
All regions
Download All
FAQ
Cases
Vaccine
Est.Active
Confirmed
Deaths
Est.Recovered
United States: Est.Active: 1,861,734 Conf: 49,598,201 Deaths: 793,923
Est.Rec: 46,942,544
All regions
-182,024
10,087,931
Est.Active
290,623
268,757,272
Confirmed
4,262
5,303,182
Deaths
468,385
253,366,159
Est.Recovered
Notices: Vaccine Data Available | Change on USA Data Sources
Tutorials: USA county | Query and filter | Advanced click | more
Analytics
Chart
Data
Last 30 days
Type :
Daily
Daily count for All regions
Count (stacked)
800,000
600,000
400,000
200,000
0
Time Slider from Jan 22 2020 to Dec 11 2021 Selected Date Dec 11 2021
1/22/2020 3/31/2020 6/8/2020 8/16/2020 10/24/2020 1/1/2021 3/11/2021 5/19/2021 7/27/2021 10/4/2021
AF
AS
EU
NA
OC
SA
RESET
Counts
Per 100K
World
County
Est.Active
1 - 9
10 - 99
100 - 999
1,000 - 9,999
10,000 - 99,999
100,000 - 499,999
500,000 +
Cumulative number from 223 countries / territories. Last Update : 2021-12-11 15:00:00 (UTC).
Copyright © 2021, UVA; ALL RIGHTS RESERVED. THE CONTENTS OF THIS DASHBOARD--INCLUDING DATA, MAPS, AND PLOTS--IS PROVIDED TO THE PUBLIC SOLELY FOR RESEARCH AND ACADEMIC

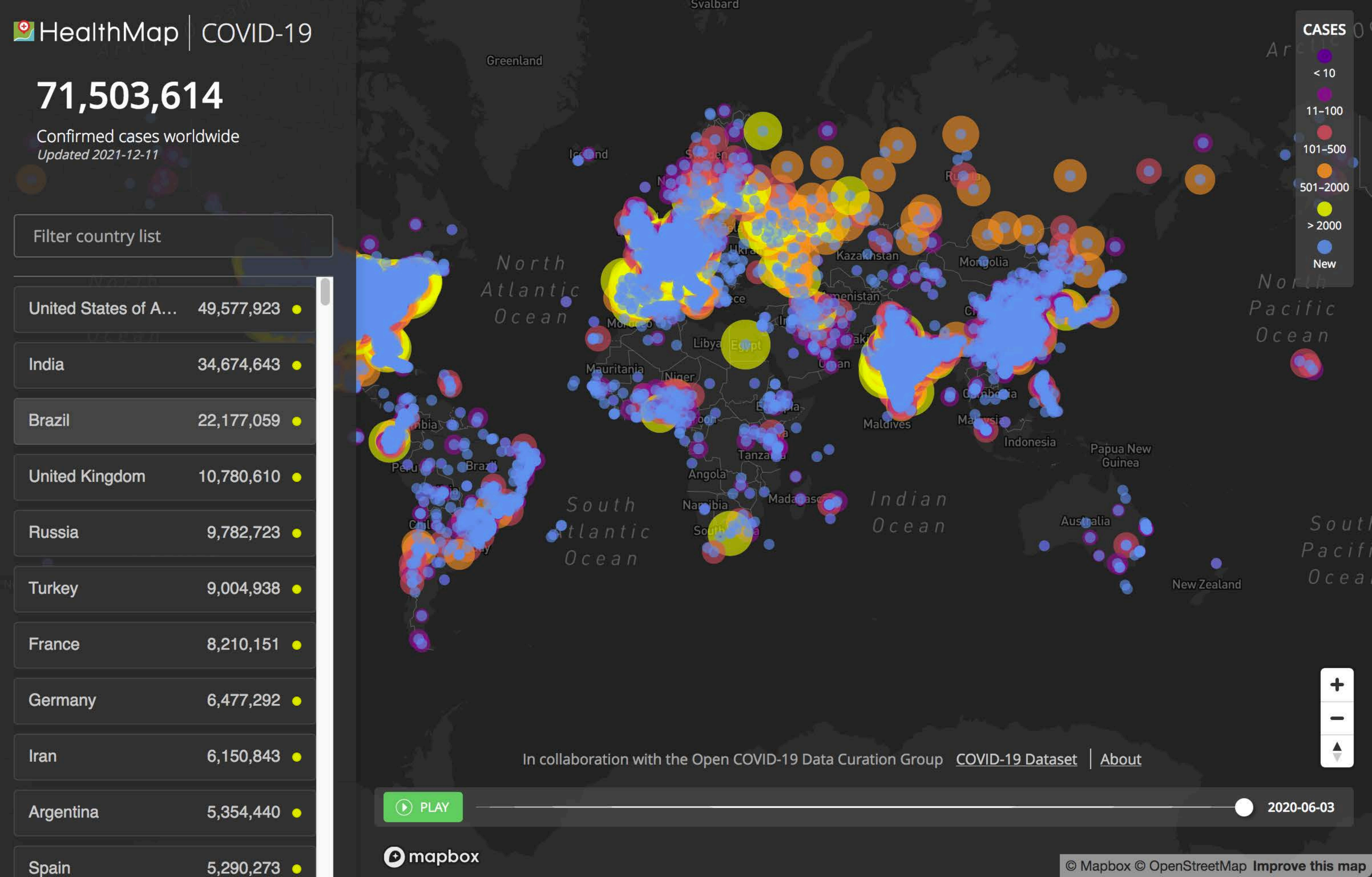
HealthMap | COVID-19
71,503,614
Confirmed cases worldwide
Updated 2021-12-11
Filter country list
United States of A... 49,577,923
India 34,674,643
Brazil 22,177,059
United Kingdom 10,780,610
Russia 9,782,723
Turkey 9,004,938
France 8,210,151
Germany 6,477,292
Iran 6,150,843
Argentina 5,354,440
Spain 5,290,273
CASES
< 10
11–100
101–500
501–2000
> 2000
New
In collaboration with the Open COVID-19 Data Curation Group
COVID-19 Dataset
About
PLAY
2020-06-03
mapbox
© Mapbox © OpenStreetMap Improve this map

World Health Organization

Search by Country, Territory, or Area

Covid-19 Response Fund

Donate

WHO Coronavirus (COVID-19) Dashboard

Overview | Measures | Data Table | Explore

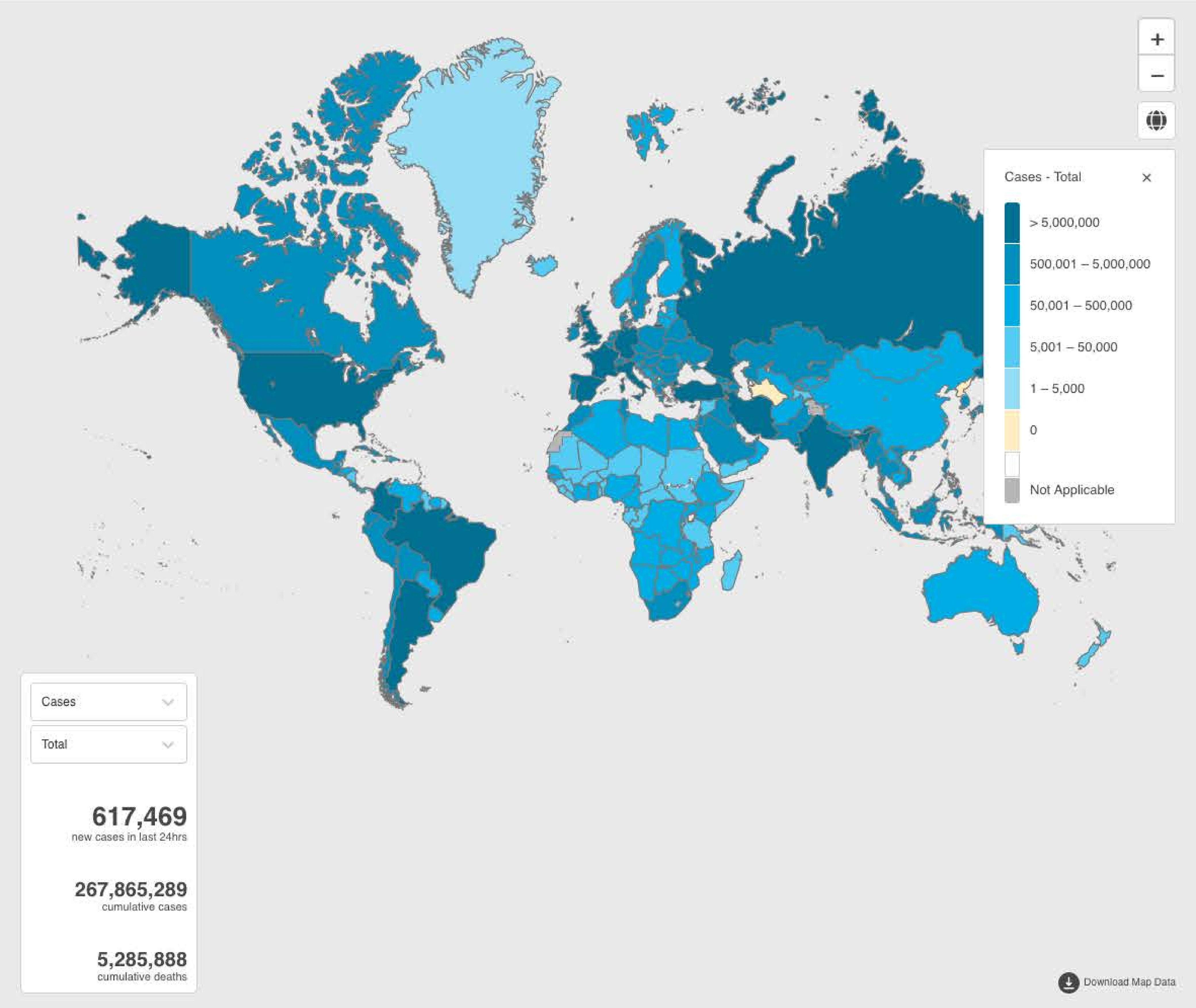

Globally, as of **4:48pm CET, 10 December 2021**, there have been **267,865,289 confirmed cases** of COVID-19, including **5,285,888 deaths**, reported to WHO. As of **9 December 2021**, a total of **8,158,815,265 vaccine doses** have been administered.

Global Situation

Daily | Weekly

267,865,289
confirmed cases

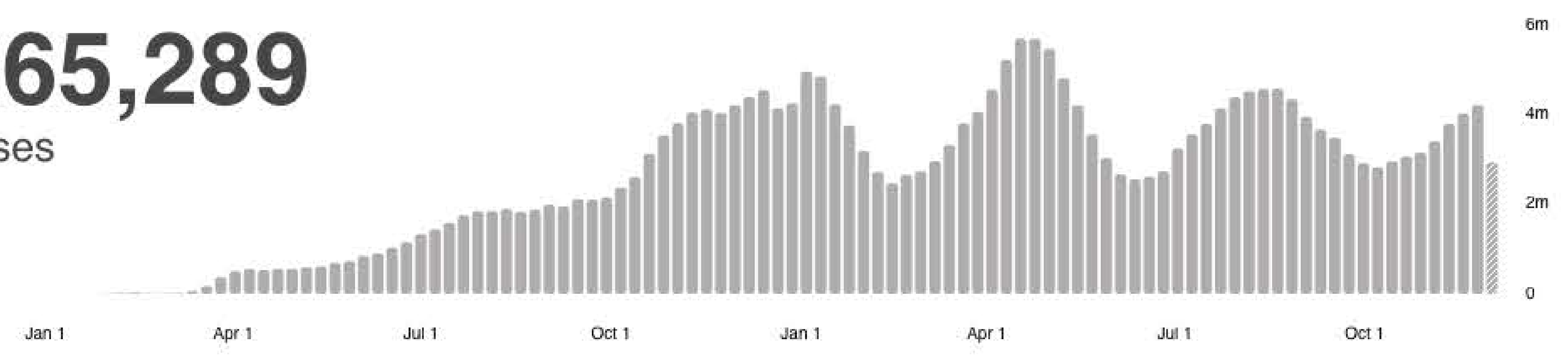

5,285,888
deaths

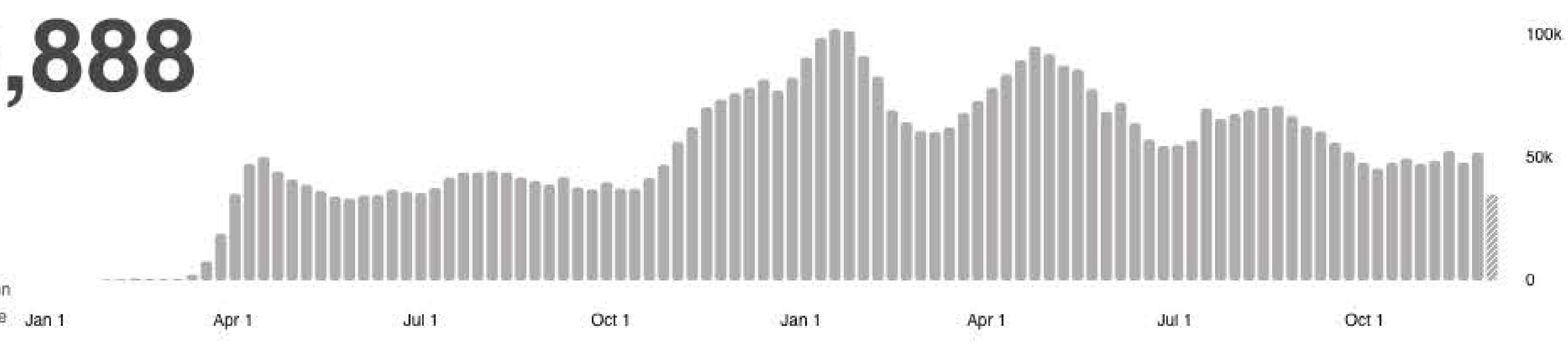

Source: World Health Organization
Data may be incomplete for the current day or week.

Situation by WHO Region

Daily | Weekly | Cases | Deaths | Count

Americas 98,346,191 confirmed

Europe 90,914,526 confirmed

South-East Asia 44,708,535 confirmed

Eastern Mediterranean 16,907,000 confirmed

Western Pacific 10,519,944 confirmed

Africa 6,468,329 confirmed

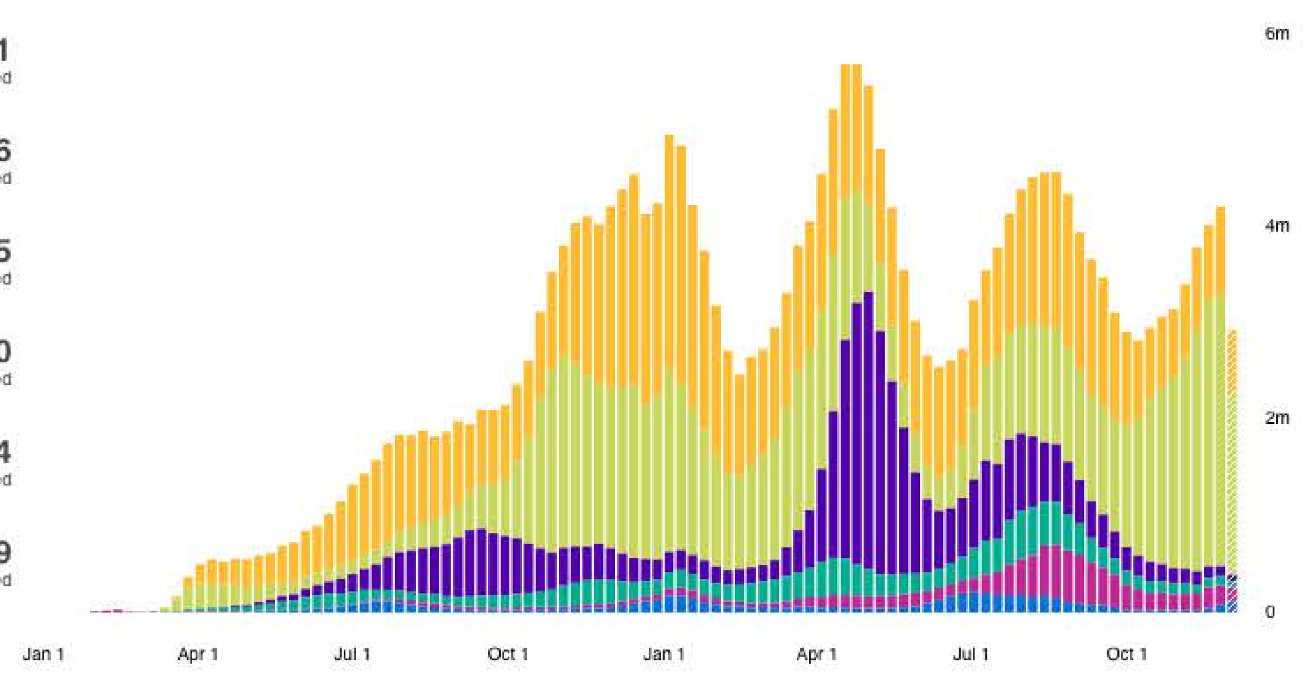

Source: World Health Organization
Data may be incomplete for the current day or week.

Americas
98,346,191
confirmed cases

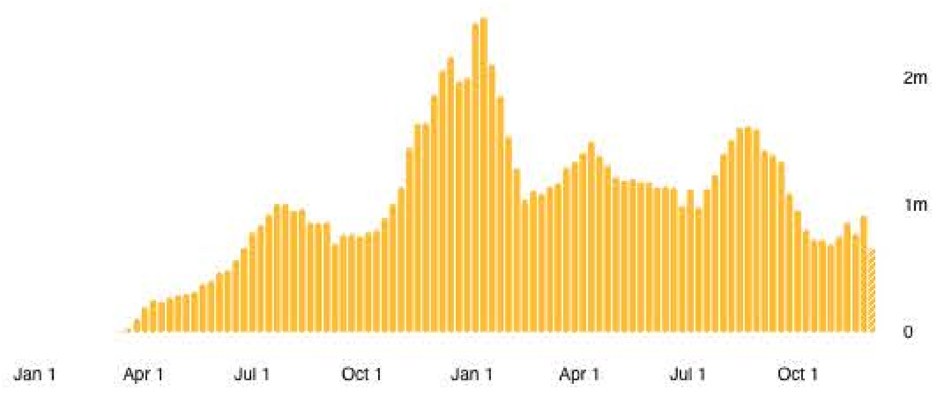

Europe
90,914,526
confirmed cases

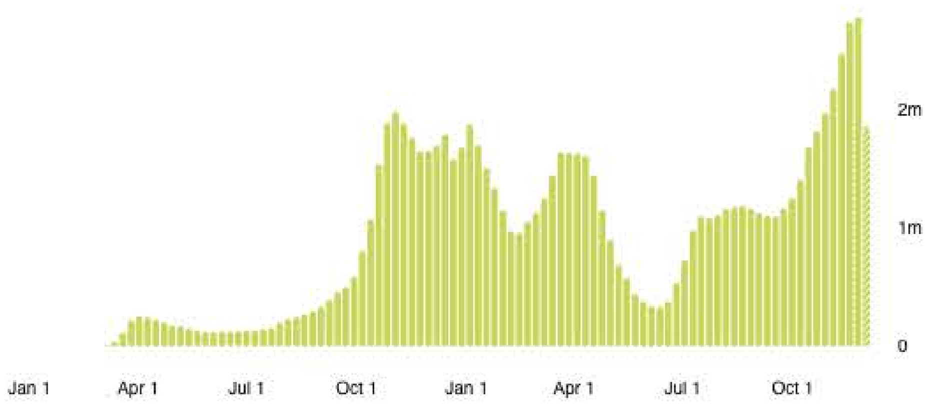

South-East Asia
44,708,535
confirmed cases

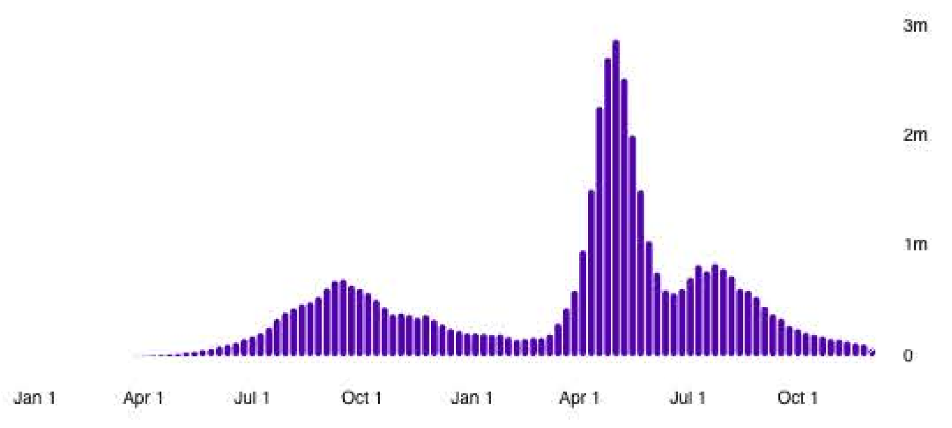

Eastern Mediterranean
16,907,000
confirmed cases

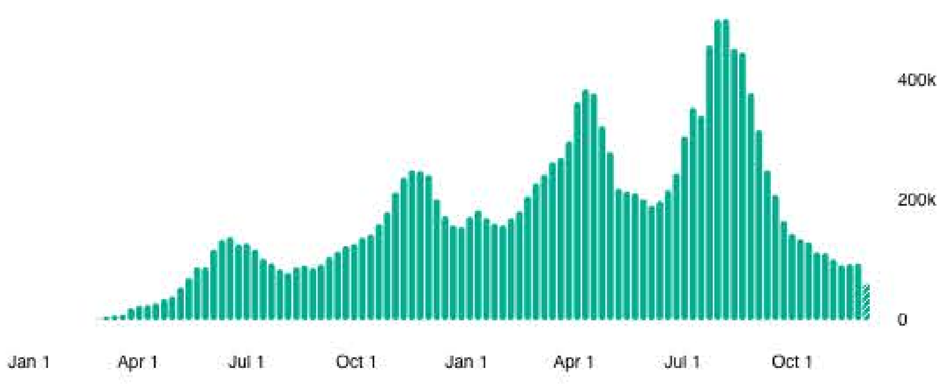

Western Pacific
10,519,944
confirmed cases

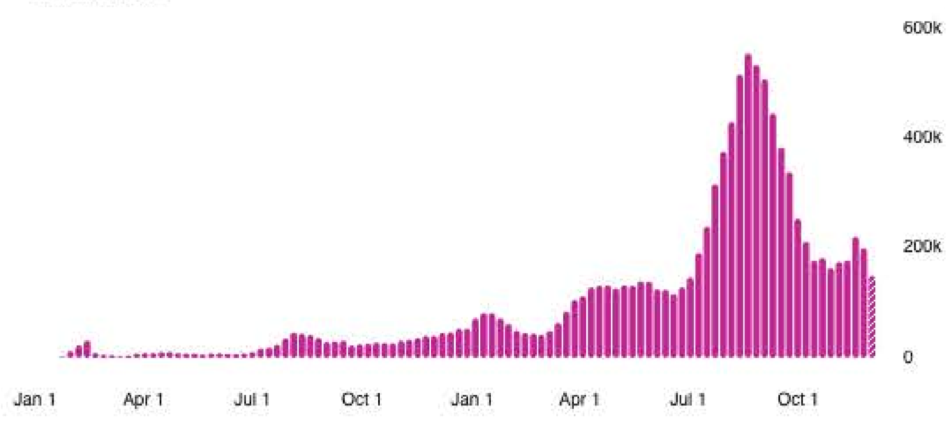

Africa
6,468,329
confirmed cases

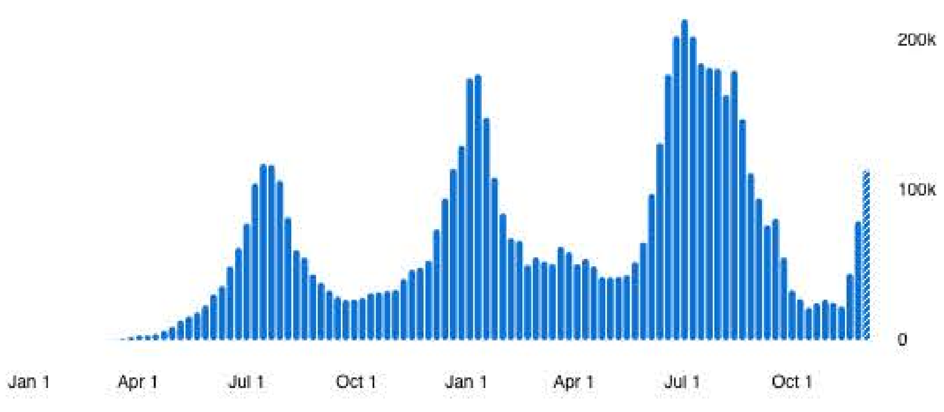

Source: World Health Organization
Data may be incomplete for the current day or week.

Situation by Country, Territory or Area

Cases | Deaths | Total

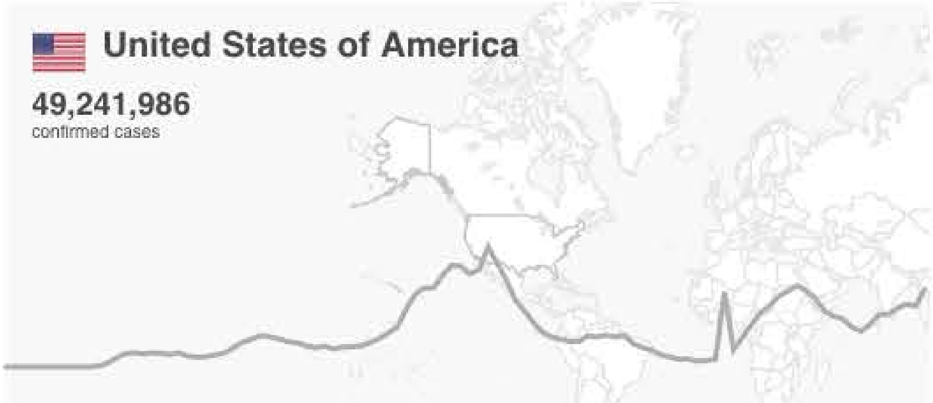

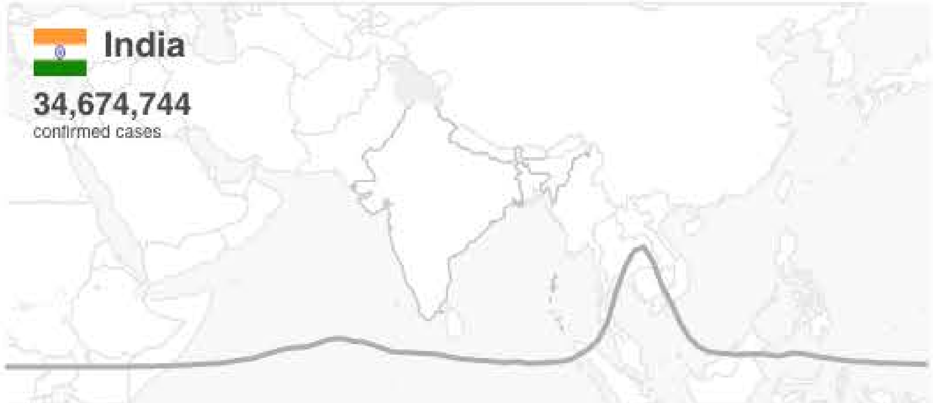

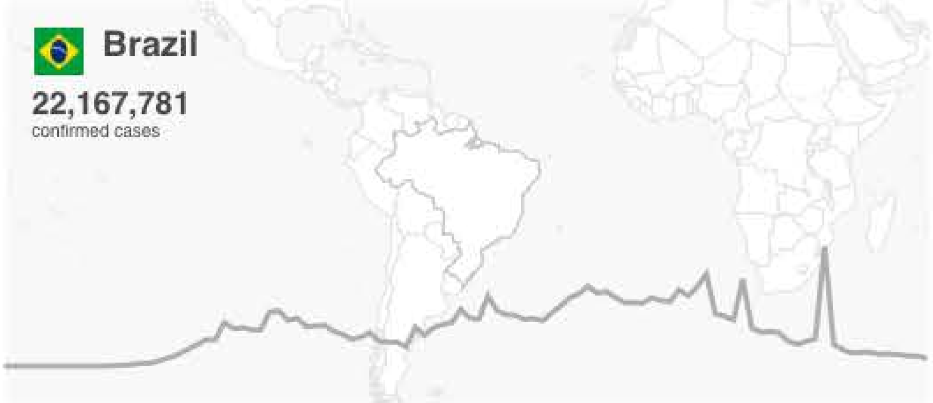

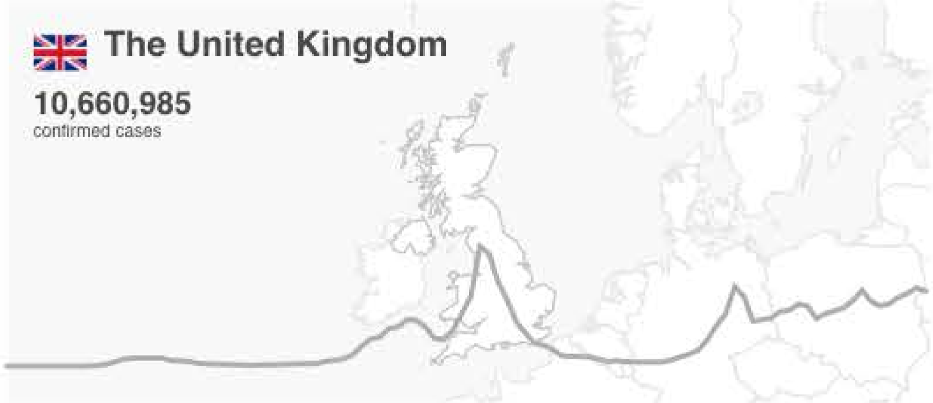

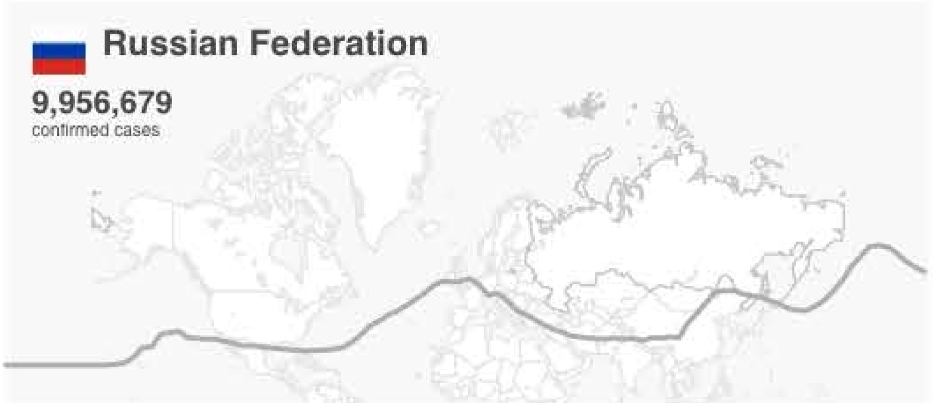

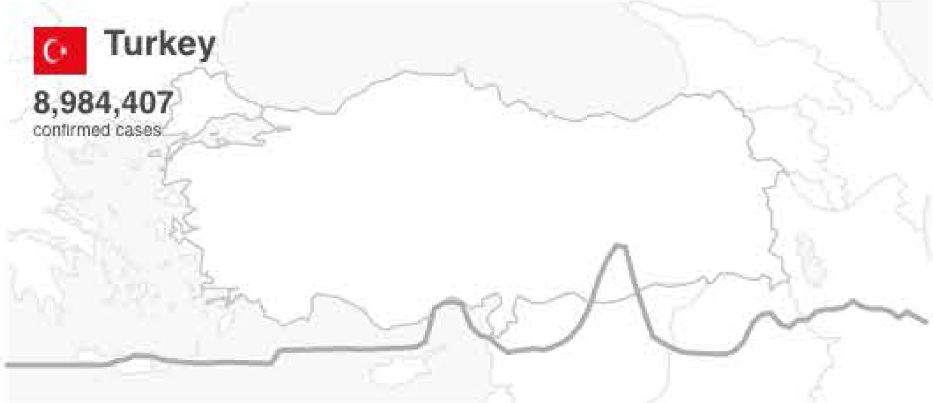

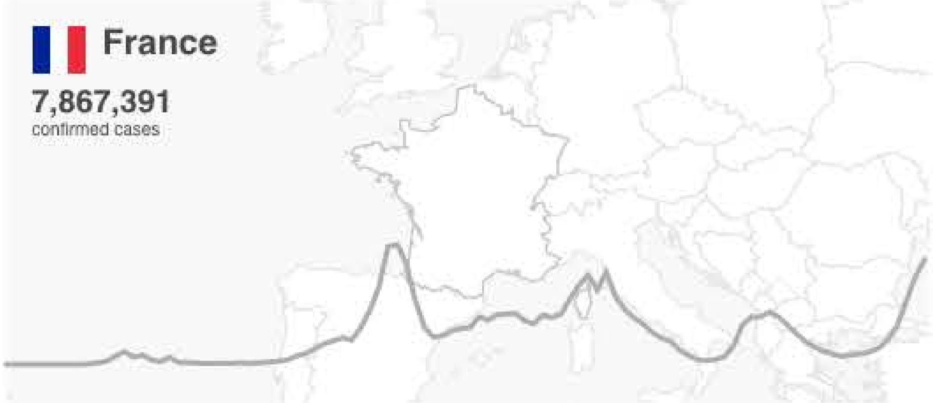

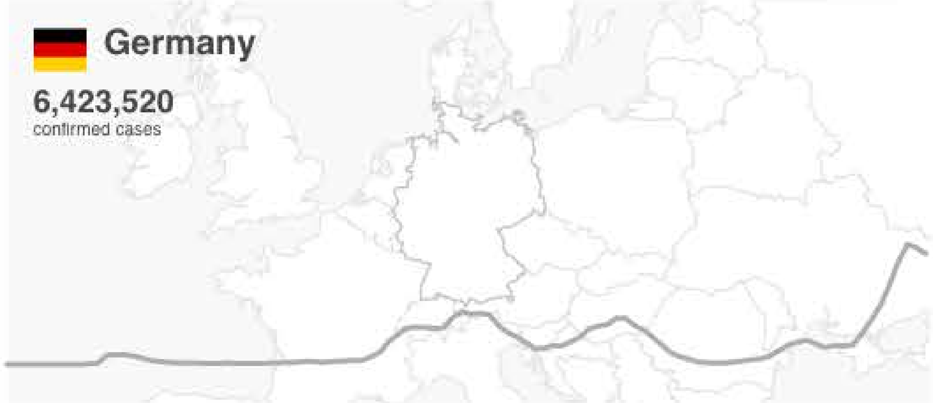

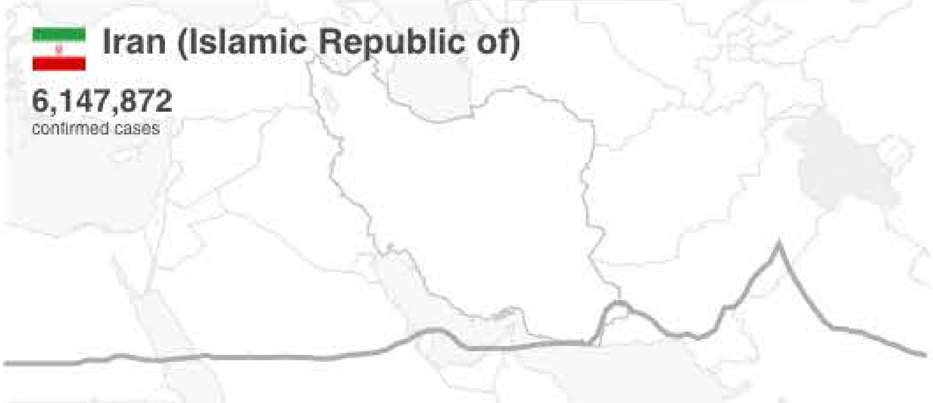

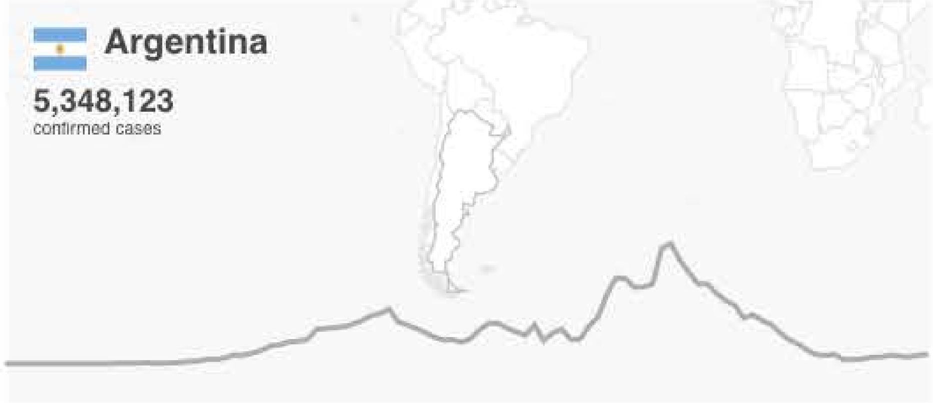

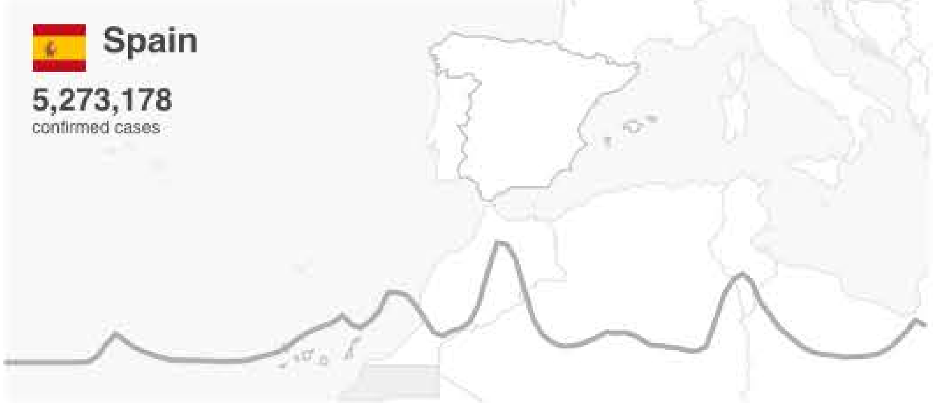

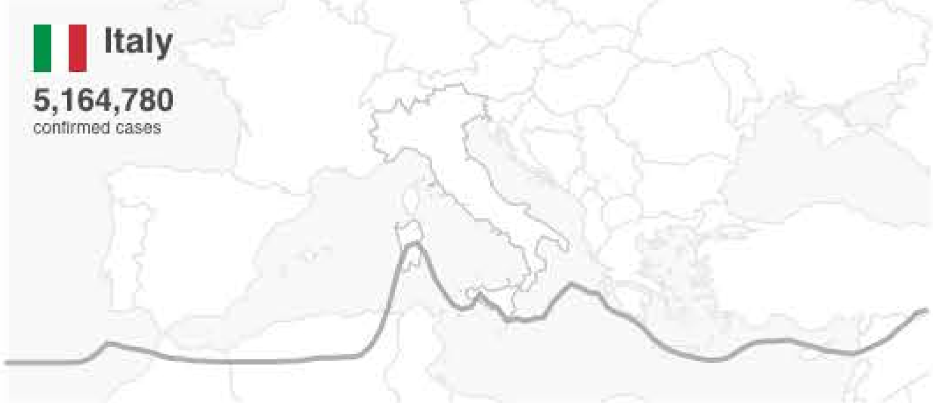

Source: World Health Organization

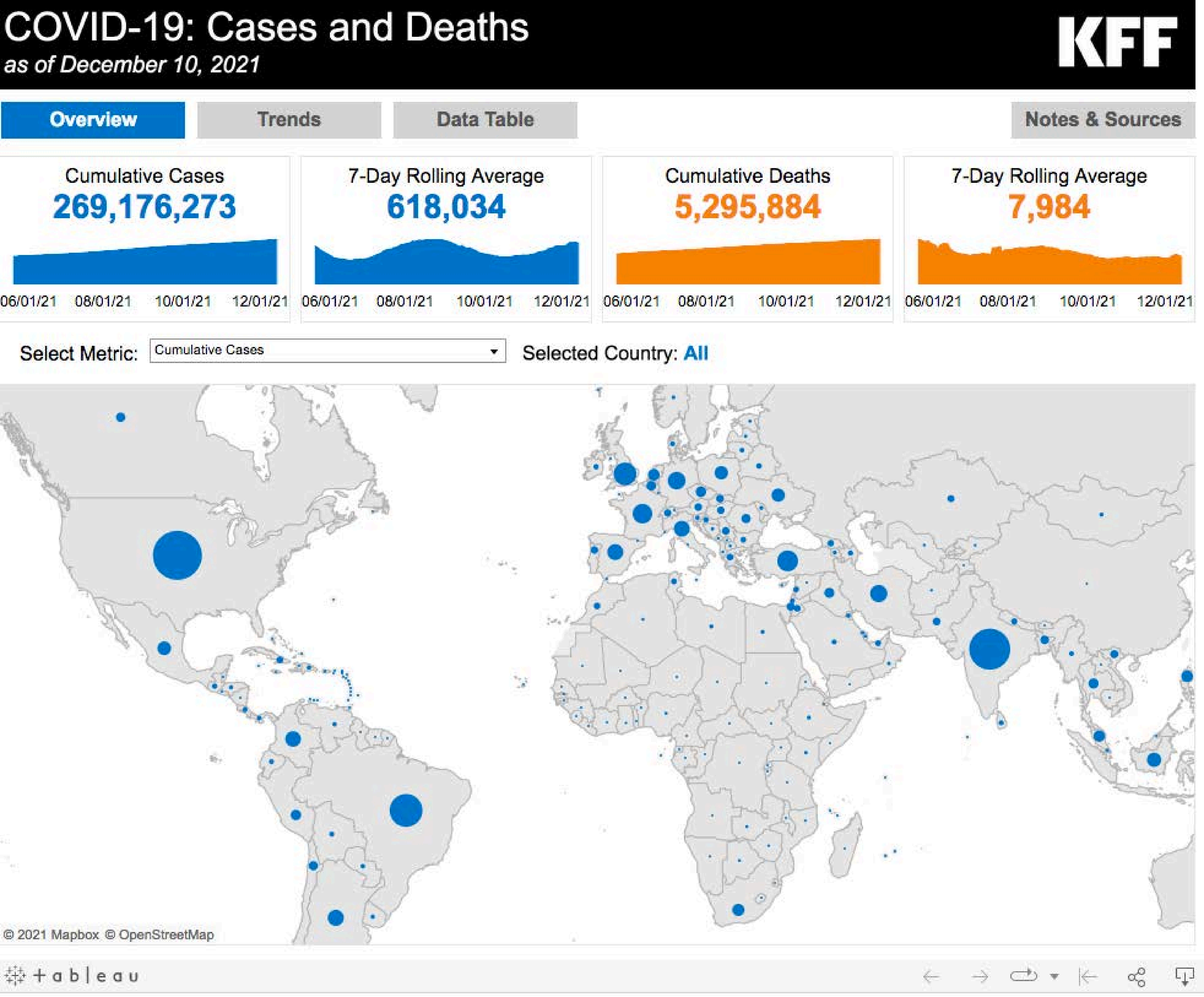

COVID-19: Cases and Deaths
as of December 10, 2021
KFF
Overview
Trends
Data Table
Notes & Sources
Cumulative Cases
269,176,273
06/01/21 08/01/21 10/01/21 12/01/21
7-Day Rolling Average
618,034
06/01/21 08/01/21 10/01/21 12/01/21
Cumulative Deaths
5,295,884
06/01/21 08/01/21 10/01/21 12/01/21
7-Day Rolling Average
7,984
06/01/21 08/01/21 10/01/21 12/01/21
Select Metric:
Cumulative Cases
Selected Country: All
© 2021 Mapbox © OpenStreetMap
tableau

Statistics and Research

Coronavirus Pandemic (COVID-19)

Research and data: Hannah Ritchie, Edouard Mathieu, Lucas Rodés-Guirao, Cameron Appel, Charlie Giattino, Esteban Ortiz-Ospina, Joe Hasell, Bobbie MacDonald, Diana Beltekian, Saloni Dattani and Max Roser
Web development: Lars Yencken, Daniel Bachler, Ernst van Woerden, Daniel Gavrilov, Marcel Gerber, Matthieu Bergel, and Jason Crawford

The data on the coronavirus pandemic is updated daily. Last update: **9 hours ago**.
Reuse our work freely   Cite this research

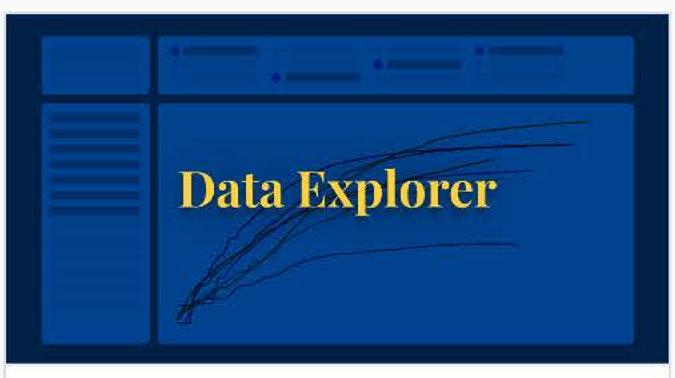

Explore all metrics – including cases, deaths, testing, and vaccinations – in one place.

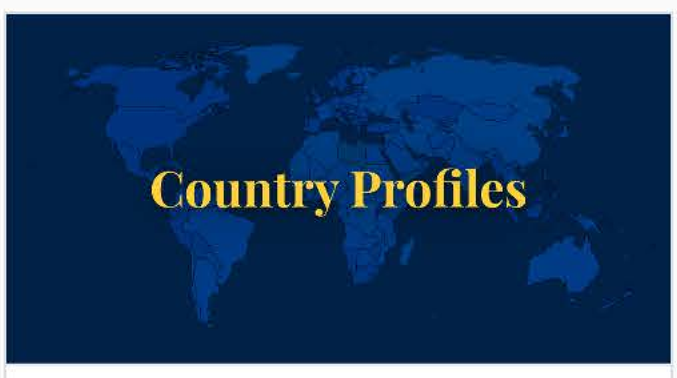

Get an overview of the pandemic for any country on a single page.

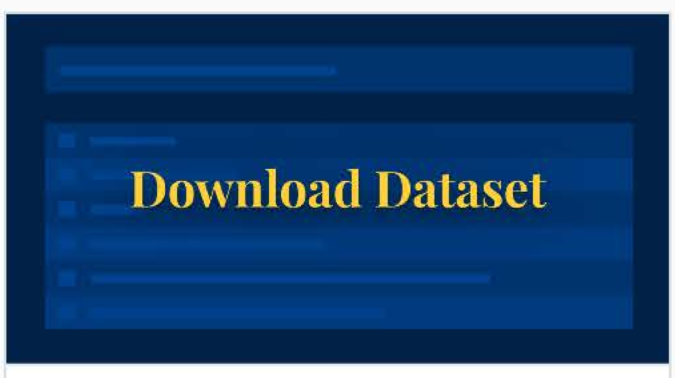

Download our complete dataset of COVID-19 metrics on GitHub. It's open access and free for anyone to use.

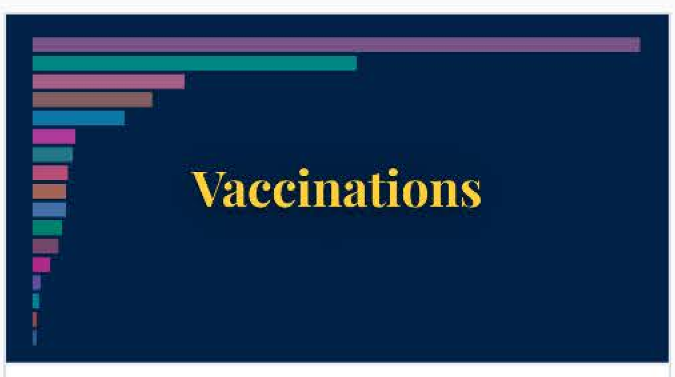

Explore our global dataset on COVID-19 vaccinations.

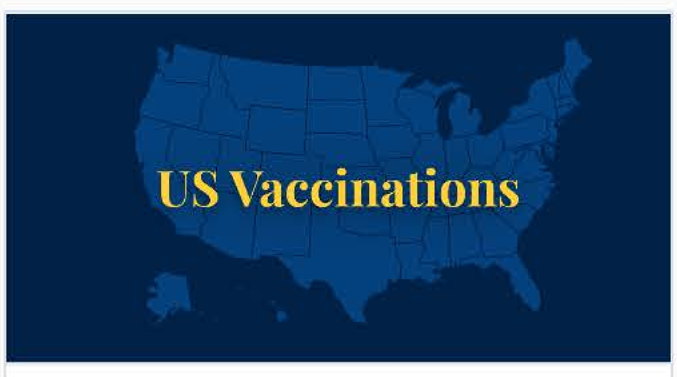

See state-by-state data on vaccinations in the United States.

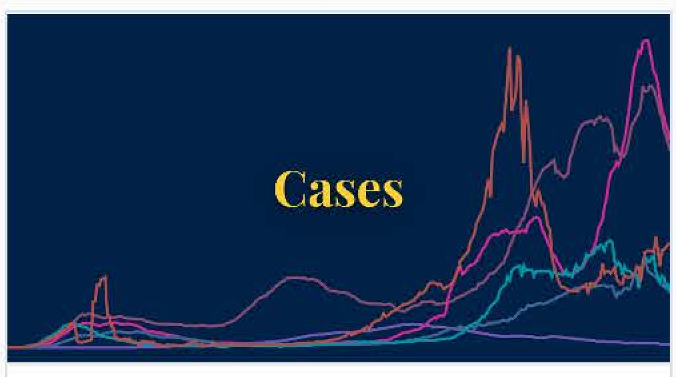

Explore the data on confirmed COVID-19 cases for all countries.

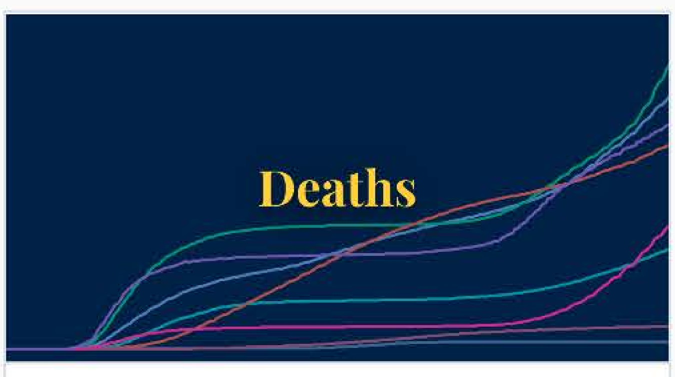

Explore the data on confirmed COVID-19 deaths for all countries.

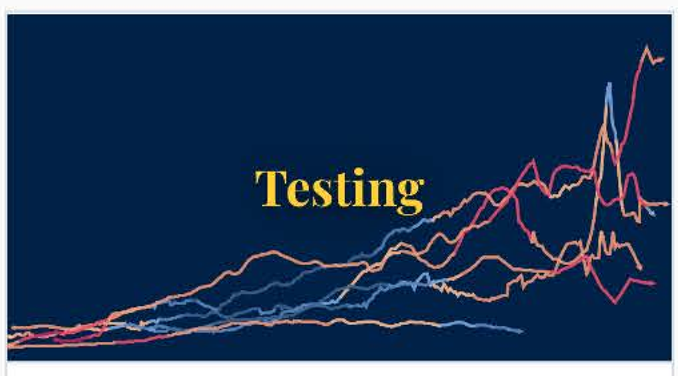

Explore our data on COVID-19 testing to see how confirmed cases compare to actual infections.

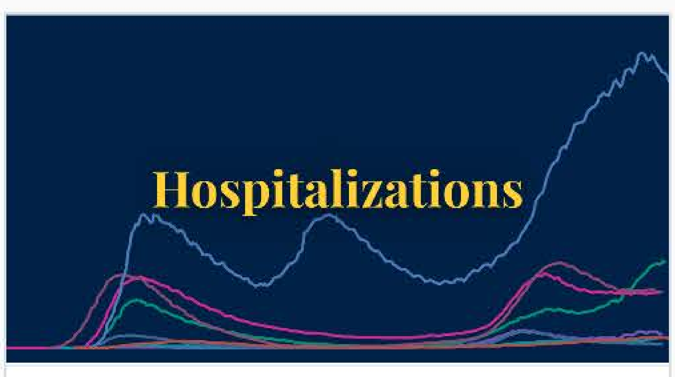

See data on how many people are being hospitalized for COVID-19.

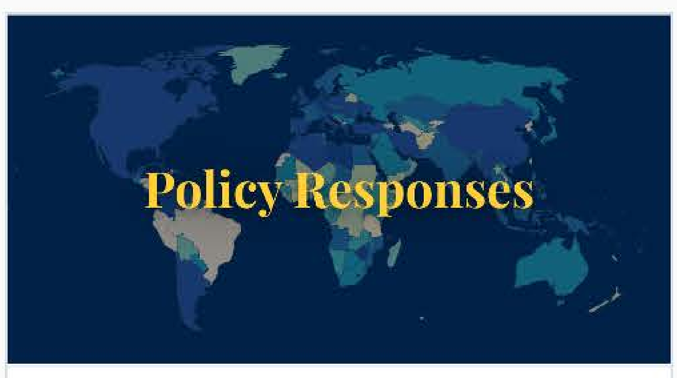

See how government policy responses – on travel, testing, vaccinations, face coverings, and more – vary across the world.

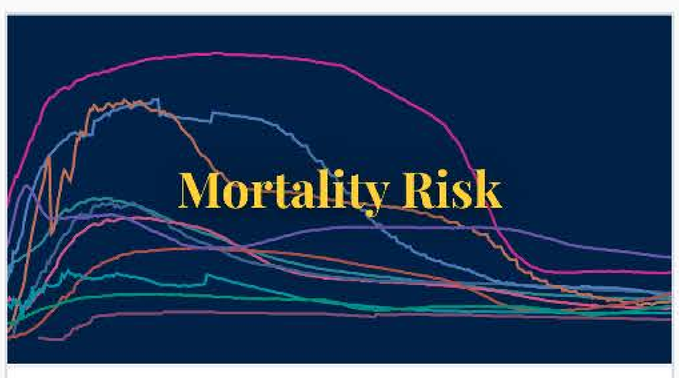

Learn what we know about the mortality risk of COVID-19 and explore the data used to calculate it.

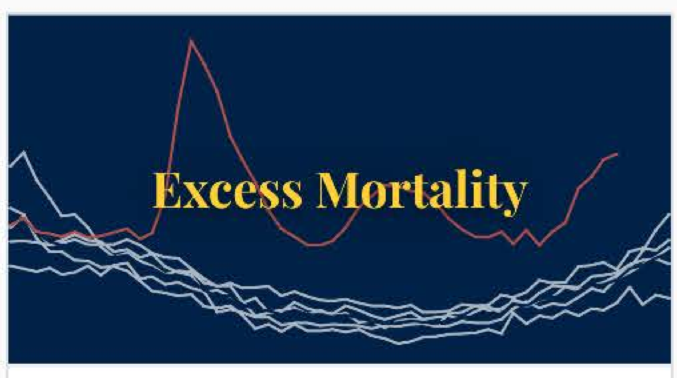

Compare the number of deaths from all causes during COVID-19 to the years before to gauge the total impact of the pandemic on deaths.

Explore the global situation

COVID-19 Data Explorer
Download the complete *Our World in Data* COVID-19 dataset.

METRIC: Confirmed c... INTERVAL: 7-day rolling... ☑ Relative to Population ☐ Align outbreaks

Type to add a country..
Sort by Select...

☑ United Kingdom	858.08
☑ Germany	635.23
☑ United States	517.53
☑ Canada	84.8
☑ India	5.73
☑ Japan	1.08
☐ San Marino	3.15k
☐ Andorra	2.17k
☐ Liechtenstein	1.78k
☐ Slovakia	1.45k
☐ Czechia	1.33k
☐ Belgium	1.31k
☐ Monaco	1.21k

✕ Clear selection

Daily new confirmed COVID-19 cases per million people

7-day rolling average. Due to limited testing, the number of confirmed cases is lower than the true number of infections.

LINEAR LOG

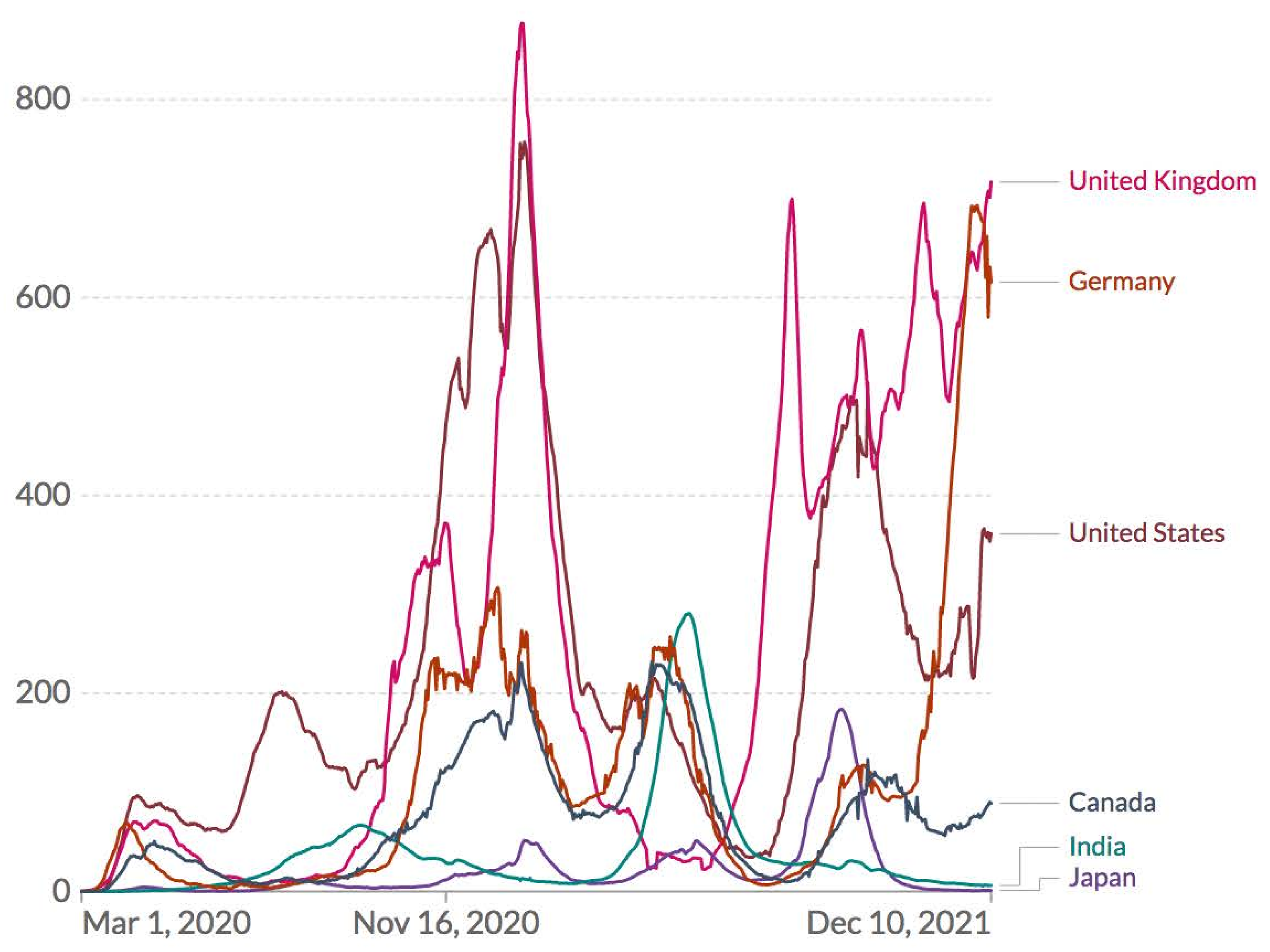

Source: Johns Hopkins University CSSE COVID-19 Data   CC BY

Jan 28, 2020 — Dec 10, 2021

CHART MAP TABLE SOURCES DOWNLOAD

→ Open the Data Explorer in a new tab.

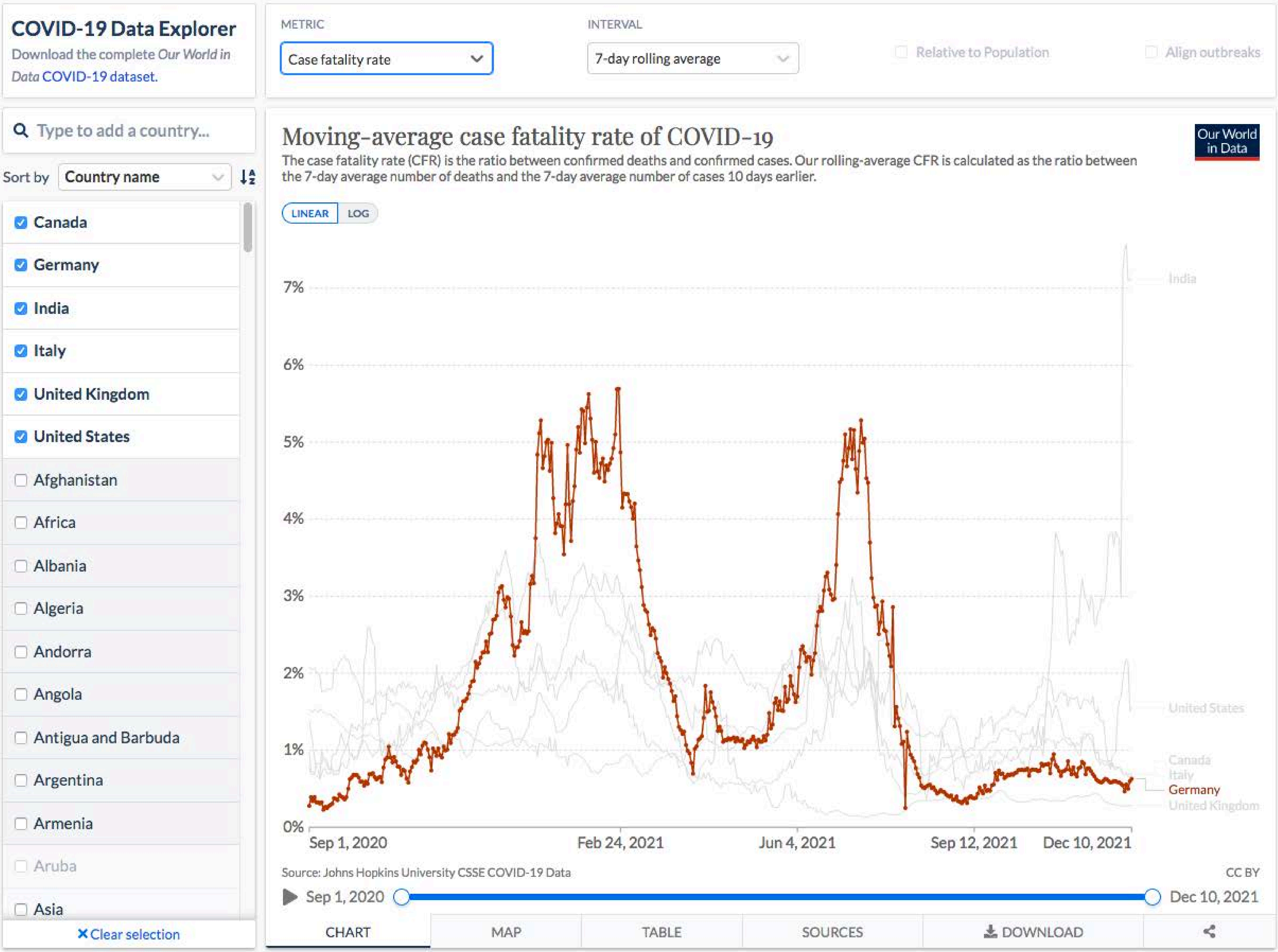

COVID-19 Data Explorer
Download the complete Our World in Data COVID-19 dataset.
METRIC
Case fatality rate
INTERVAL
7-day rolling average
Relative to Population
Align outbreaks
Type to add a country...
Sort by
Country name
Canada
Germany
India
Italy
United Kingdom
United States
Afghanistan
Africa
Albania
Algeria
Andorra
Angola
Antigua and Barbuda
Argentina
Armenia
Aruba
Asia
Clear selection
Moving-average case fatality rate of COVID-19
The case fatality rate (CFR) is the ratio between confirmed deaths and confirmed cases. Our rolling-average CFR is calculated as the ratio between the 7-day average number of deaths and the 7-day average number of cases 10 days earlier.
Our World in Data
LINEAR
LOG
7%
6%
5%
4%
3%
2%
1%
0%
India
United States
Canada
Italy
Germany
United Kingdom
Sep 1, 2020
Feb 24, 2021
Jun 4, 2021
Sep 12, 2021
Dec 10, 2021
Source: Johns Hopkins University CSSE COVID-19 Data
CC BY
Sep 1, 2020
Dec 10, 2021
CHART
MAP
TABLE
SOURCES
DOWNLOAD

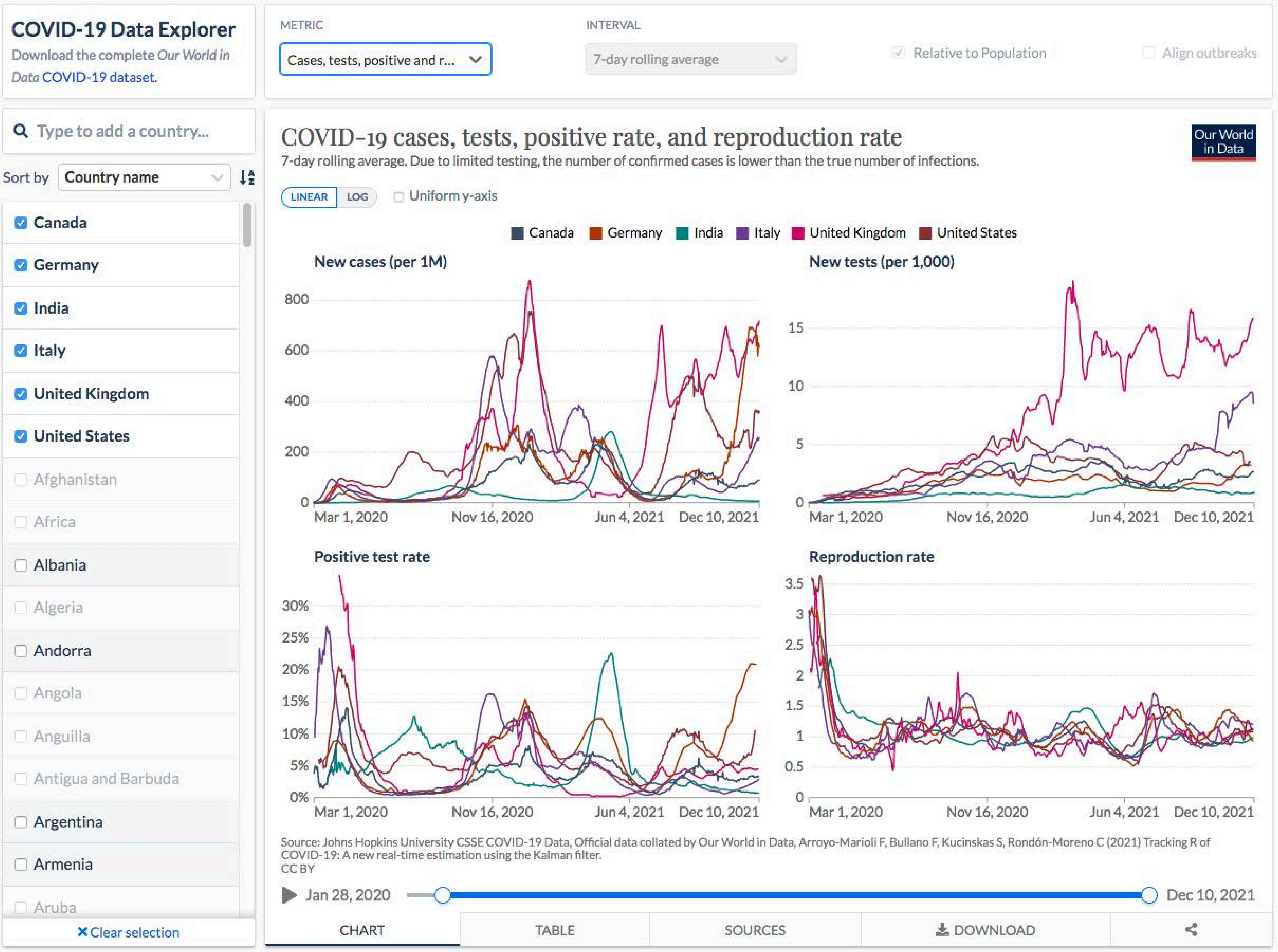
COVID-19 Data Explorer
Download the complete Our World in Data COVID-19 dataset.
METRIC
Cases, tests, positive and r...
INTERVAL
7-day rolling average
Relative to Population
Align outbreaks
Type to add a country...
Sort by
Country name
Canada
Germany
India
Italy
United Kingdom
United States
Afghanistan
Africa
Albania
Algeria
Andorra
Angola
Anguilla
Antigua and Barbuda
Argentina
Armenia
Aruba
Clear selection
COVID-19 cases, tests, positive rate, and reproduction rate
7-day rolling average. Due to limited testing, the number of confirmed cases is lower than the true number of infections.
Our World in Data
LINEAR
LOG
Uniform y-axis
Canada
Germany
India
Italy
United Kingdom
United States
New cases (per 1M)
800
600
400
200
0
Mar 1, 2020
Nov 16, 2020
Jun 4, 2021
Dec 10, 2021
New tests (per 1,000)
15
10
5
0
Mar 1, 2020
Nov 16, 2020
Jun 4, 2021
Dec 10, 2021
Positive test rate
30%
25%
20%
15%
10%
5%
0%
Mar 1, 2020
Nov 16, 2020
Jun 4, 2021
Dec 10, 2021
Reproduction rate
3.5
3
2.5
2
1.5
1
0.5
0
Mar 1, 2020
Nov 16, 2020
Jun 4, 2021
Dec 10, 2021
Source: Johns Hopkins University CSSE COVID-19 Data, Official data collated by Our World in Data, Arroyo-Marioli F, Bullano F, Kucinskas S, Rondón-Moreno C (2021) Tracking R of COVID-19: A new real-time estimation using the Kalman filter.
CC BY
Jan 28, 2020
Dec 10, 2021
CHART
TABLE
SOURCES
DOWNLOAD

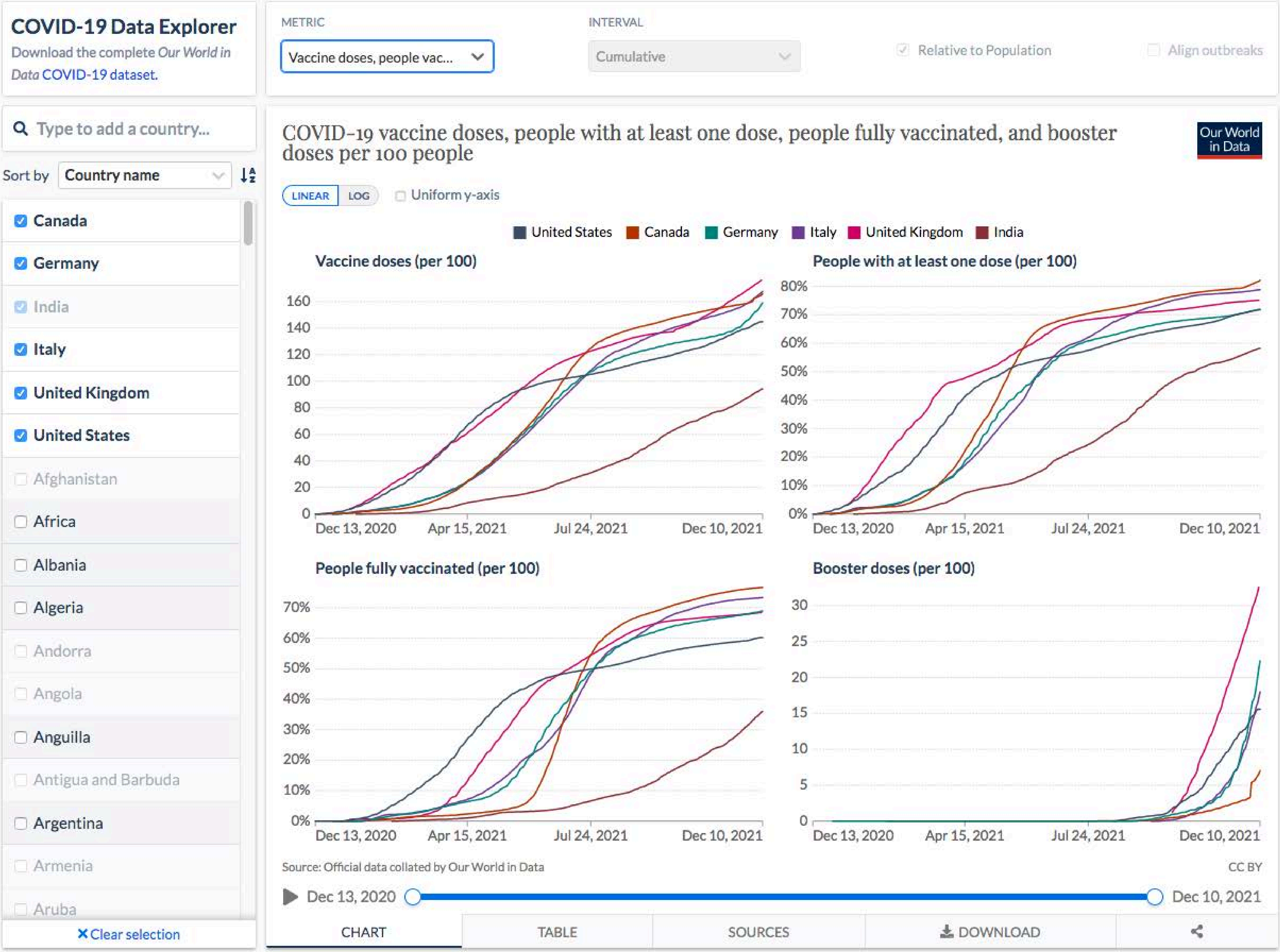
COVID-19 Data Explorer
Download the complete Our World in Data COVID-19 dataset.
METRIC
Vaccine doses, people vac...
INTERVAL
Cumulative
Relative to Population
Align outbreaks
Type to add a country...
Sort by
Country name
Canada
Germany
India
Italy
United Kingdom
United States
Afghanistan
Africa
Albania
Algeria
Andorra
Angola
Anguilla
Antigua and Barbuda
Argentina
Armenia
Aruba
Clear selection
COVID-19 vaccine doses, people with at least one dose, people fully vaccinated, and booster doses per 100 people
Our World in Data
LINEAR
LOG
Uniform y-axis
United States
Canada
Germany
Italy
United Kingdom
India
Vaccine doses (per 100)
160
140
120
100
80
60
40
20
0
Dec 13, 2020
Apr 15, 2021
Jul 24, 2021
Dec 10, 2021
People with at least one dose (per 100)
80%
70%
60%
50%
40%
30%
20%
10%
0%
Dec 13, 2020
Apr 15, 2021
Jul 24, 2021
Dec 10, 2021
People fully vaccinated (per 100)
70%
60%
50%
40%
30%
20%
10%
0%
Dec 13, 2020
Apr 15, 2021
Jul 24, 2021
Dec 10, 2021
Booster doses (per 100)
30
25
20
15
10
5
0
Dec 13, 2020
Apr 15, 2021
Jul 24, 2021
Dec 10, 2021
Source: Official data collated by Our World in Data
CC BY
Dec 13, 2020
Dec 10, 2021
CHART
TABLE
SOURCES
DOWNLOAD

⇌ 中文版

DXY · DX Doctor

COVID-19
Global Pandemic Real-time Report

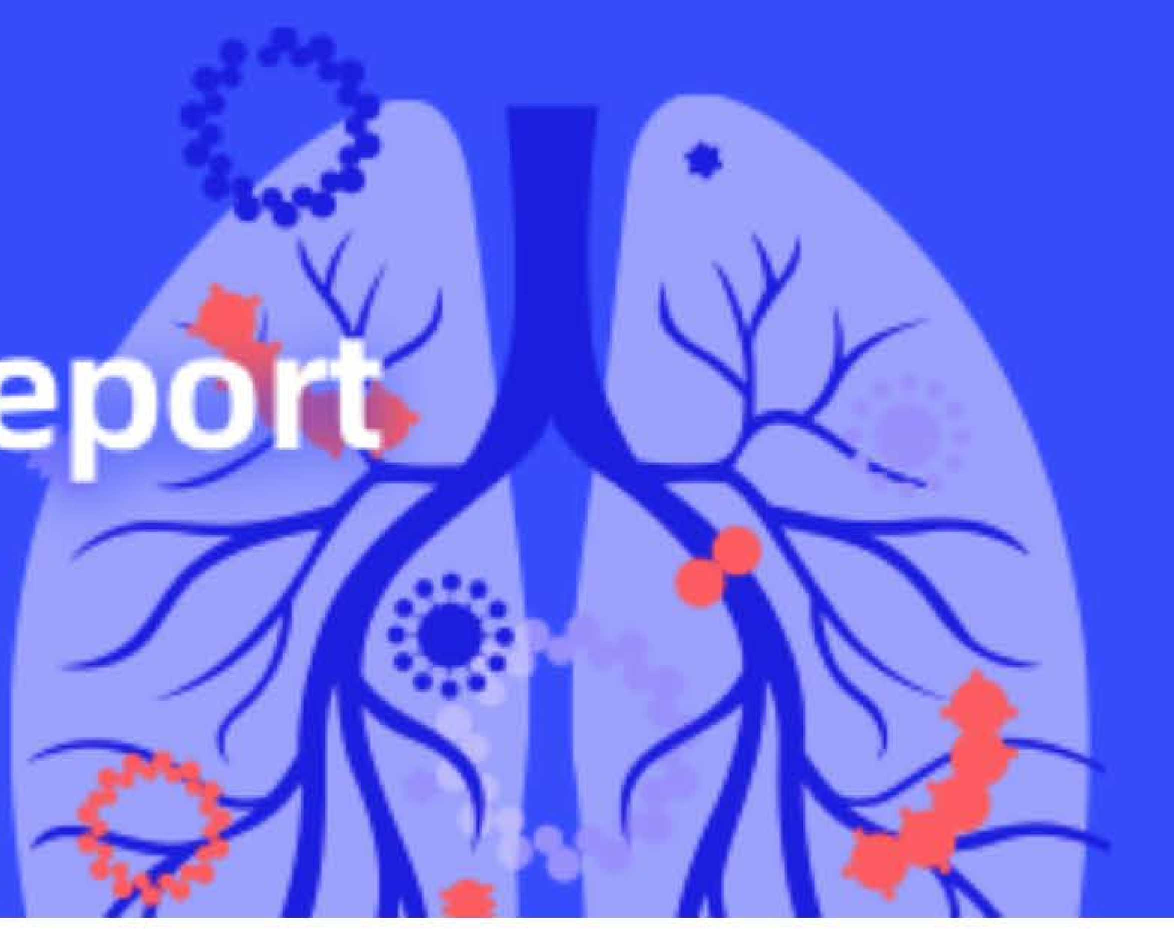

4,627,349,083 Views

Global statistics by UTC+8 2021-12-12 09:28

Data explanations

47,917,355	270,034,970	5,307,170	216,810,445
Active Cases	Total Confirmed	Deaths	Recovered

COVID-19 Global Pandemic Trend

Before Mar. 19, data source: WHO. Time zone: CET 10 AM.
After Mar. 19, data source: WHO, JHU CSSE, Local Media and DXY. Time zone: UTC 10 AM.
Since April 24th, the Spanish Ministry of Health has adjusted the statistical standard of confirmed Covid-19 data, only counting those confirmed by PCR tests.

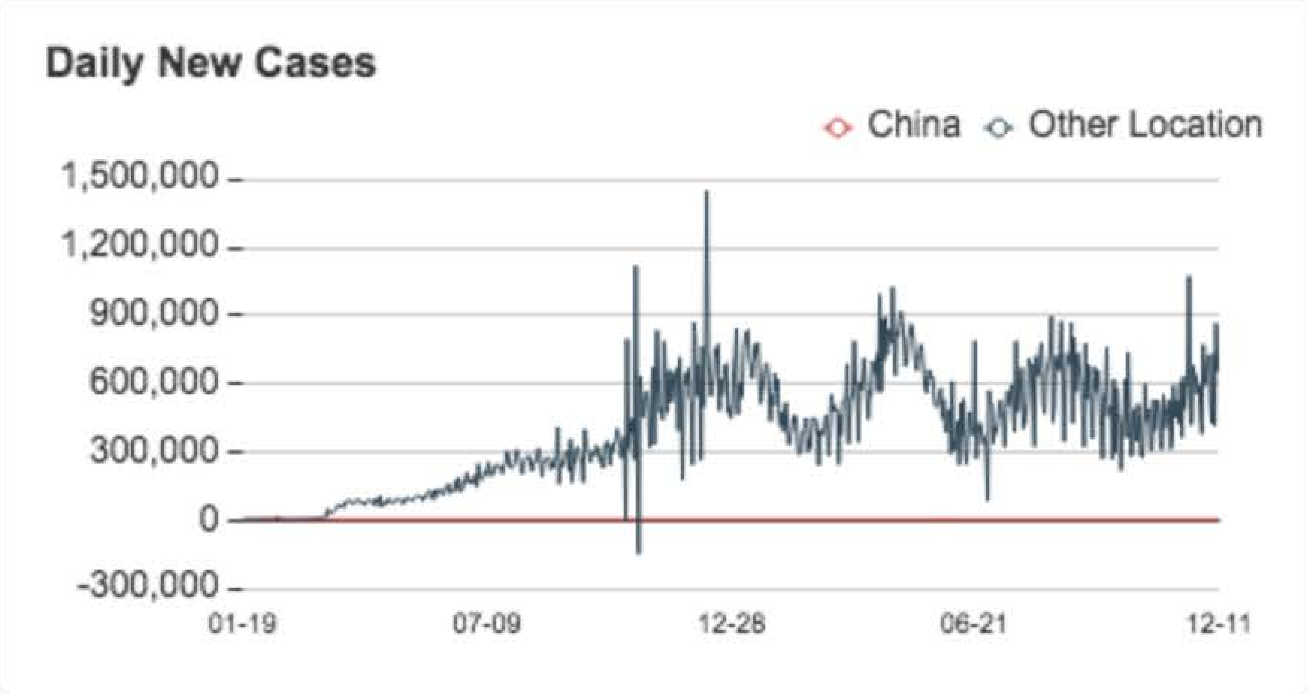

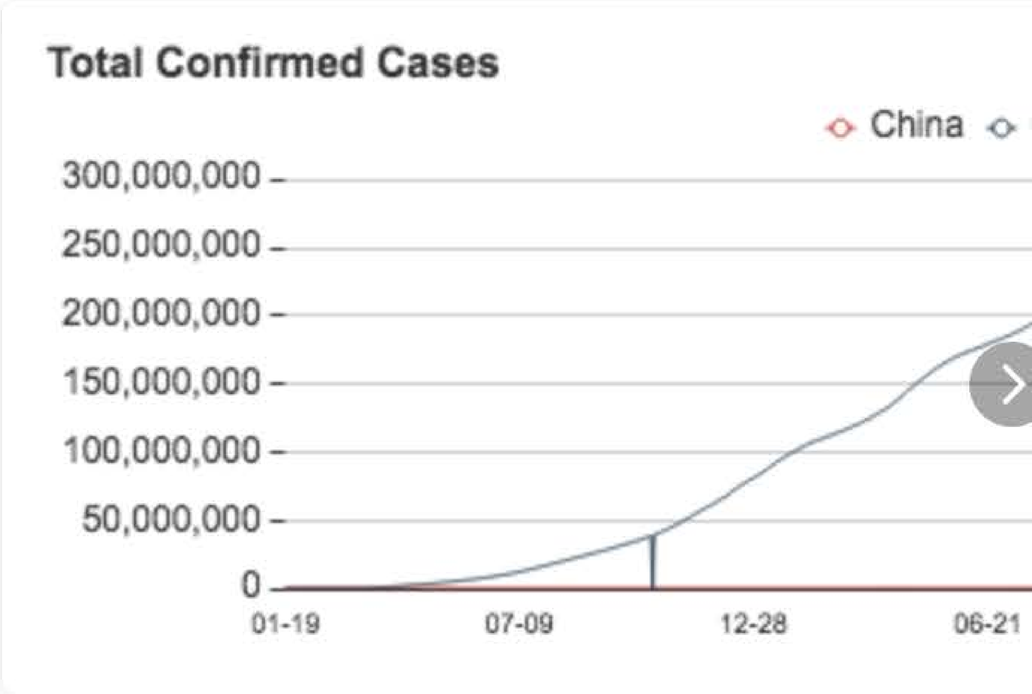

Daily New Cases | Total Confirmed Cases | Total Deaths Cases

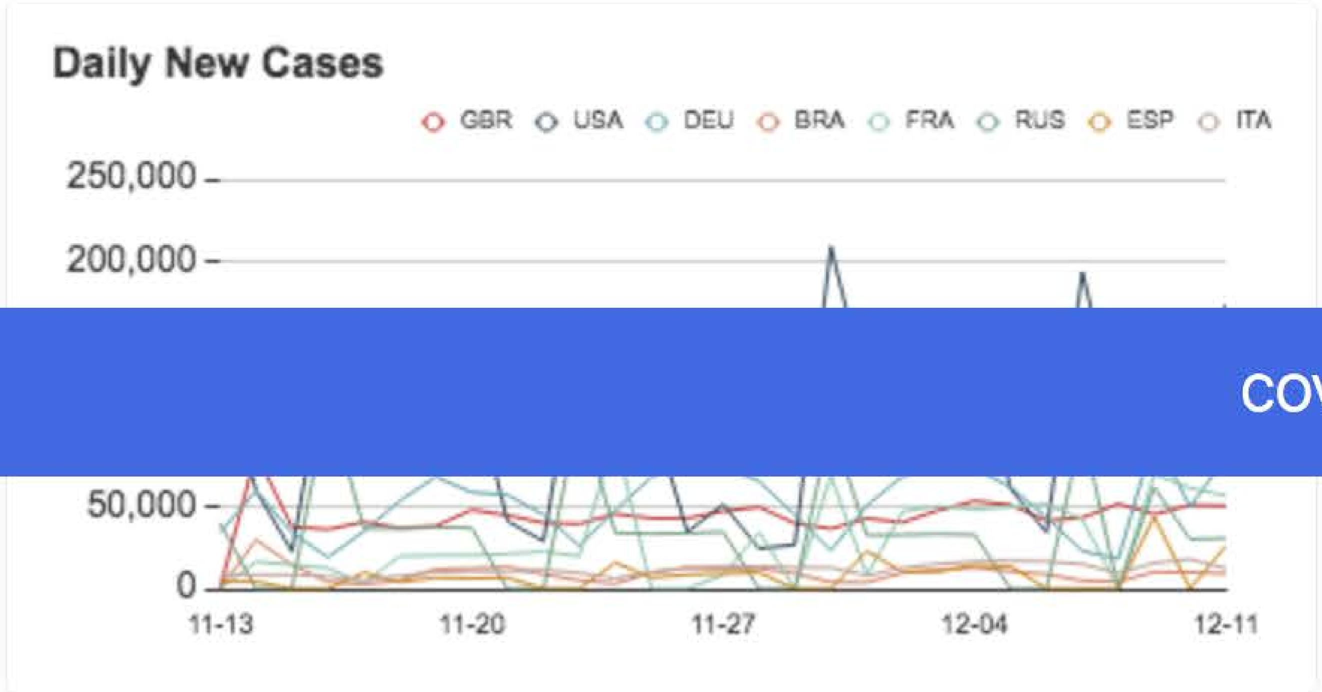

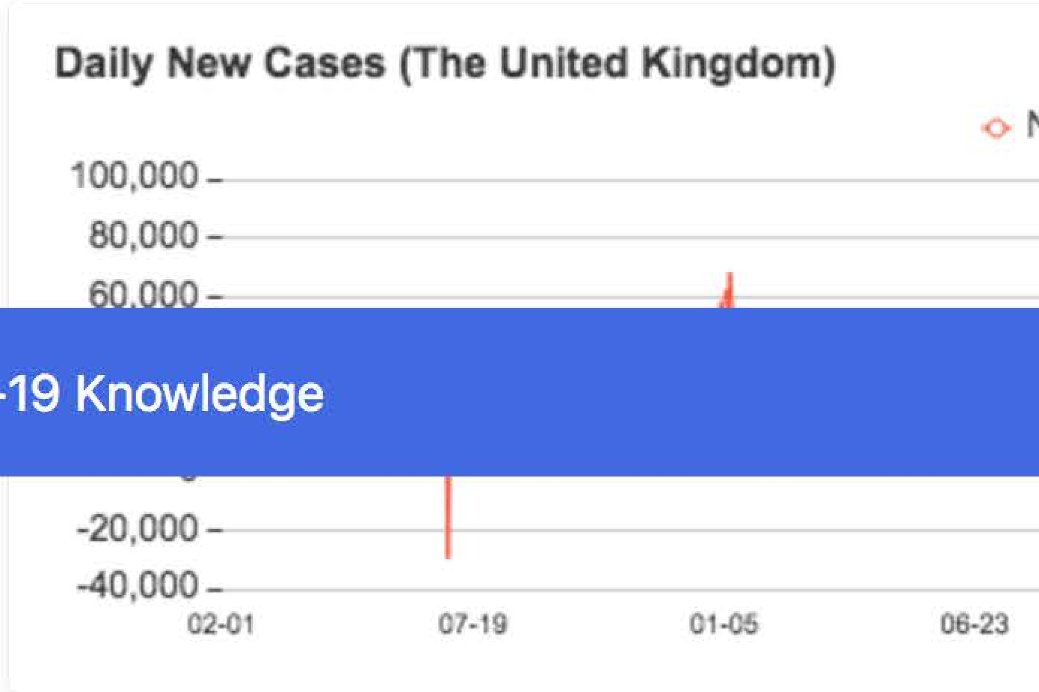

Global Mapping | COVID-19 Knowledge

Daily New Cases | The United Kingdom | United States of America | Germany
Brazil | France | Russian Federation | Spain
Italy

Country/Region/ Territory	Active Cases	Total Confirmed	Total Deaths	Total Recovered	More
**▼ Europe(49)**	**28,609,292**	**78,302,173**	**1,464,042**	**48,228,839**	
France	7,720,735	8,209,911	121,153	-	More ›
The United Kingdom	4,084,422	10,722,083	146,592	-	More ›
Netherlands	2,838,201	2,870,459	20,372	-	More ›
Germany	2,009,446	6,442,846	105,000	4,328,400	More ›
Belgium	1,867,825	1,914,527	27,463	19,239	More ›
Show more ⌄					
**▼ North America(40)**	**10,693,521**	**59,813,872**	**1,194,006**	**47,926,345**	
United States of America	9,085,326	49,833,784	796,765	39,951,693	More ›
Mexico	482,259	3,911,714	296,188	3,133,267	More ›
Honduras	245,416	378,423	10,416	122,591	More ›

COVID-19 CORONAVIRUS PANDEMIC

Last updated: December 12, 2021, 01:14 GMT

Weekly Trends - Graphs - Countries - News

Coronavirus Cases:

269,982,622

view by country

Deaths:

5,317,616

Recovered:

242,711,542

ACTIVE CASES | CLOSED CASES

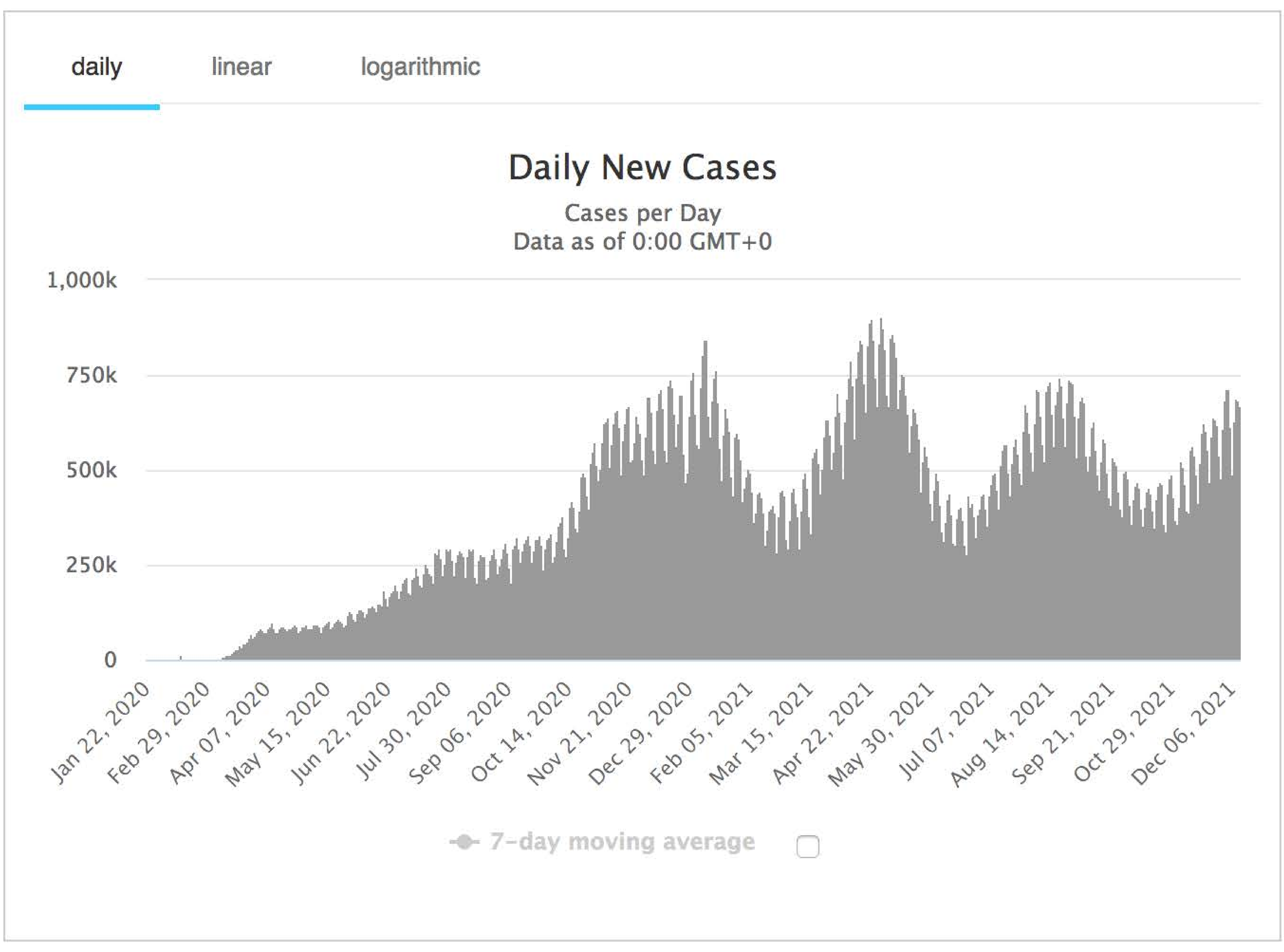

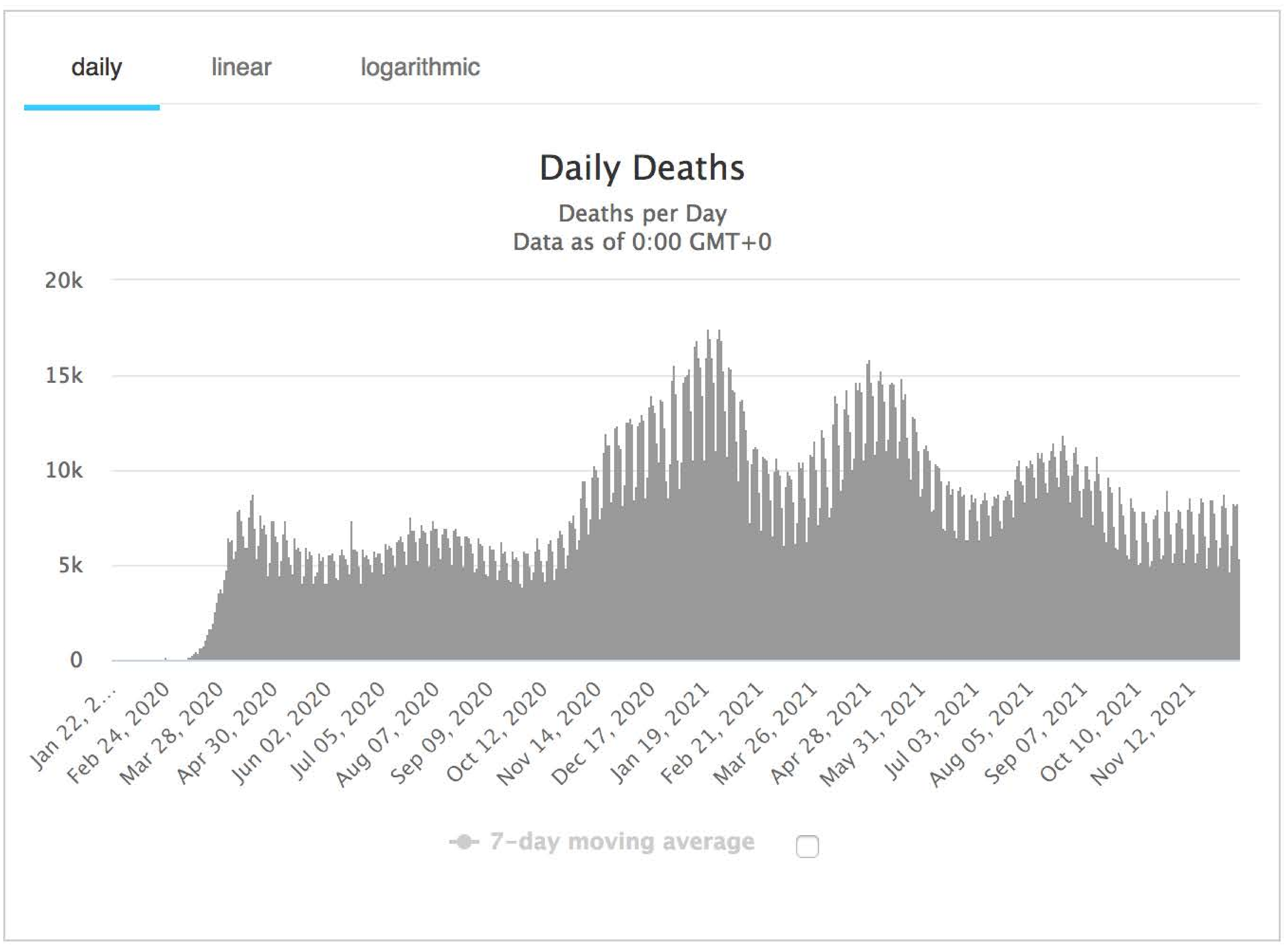

The charts above are updated after the close of the day in GMT+0. See more graphs

Reported Cases and Deaths by Country or Territory

The **coronavirus** COVID-19 is affecting **222 countries and territories. The day is reset after midnight GMT+0.** The list of countries and their regional classification is based on the United Nations Geoscheme. Sources are provided under "Latest News." **Learn more about Worldometer's COVID-19 data**

Report coronavirus cases

MAIN | WEEKLY TRENDS

Now | Yesterday | 2 Days Ago | Columns | Search:

All | Europe | North America | Asia | South America | Africa | Oceania

#	Country, Other	Total Cases	New Cases	Total Deaths	New Deaths	Total Recovered	New Recovered	Active Cases	Serious, Critical
	World	269,982,622	+14,781	5,317,616	+302	242,711,542	+10,709	21,953,464	88,8[illegible]
1	USA	50,762,671		817,789		39,986,483		9,958,399	14,9[illegible]
2	India	34,684,396		475,128		34,114,331		94,937	8,9[illegible]
3	Brazil	22,188,179		616,859		21,407,699		163,621	8,3[illegible]
4	UK	10,771,444		146,387		9,453,429		1,171,628	9[illegible]
5	Russia	9,986,967		288,351		8,709,964		988,652	2,3[illegible]
6	Turkey	9,022,223		78,969		8,592,094		351,160	1,1[illegible]

COVID-19 Tracker

Search the web with Bing

Give with Bing

Related Searches: coronavirus vaccine registration prevention

Vaccine doses administered worldwide

8,406,197,502

Confirmed cases worldwide

268,558,266 +728,504

- Active cases *No data*
- Fatal cases 5,287,902 +8,702

Updated 11 min ago

Browse Sort

Filter to a location

Global	268,558,266
United States	49,815,374

Click '+' to drill-down to a state or a city

+ India	34,682,736
+ Brazil	22,188,179
+ United Kingdom	10,722,083

United States ☆

Cases Vaccines News & Videos Graphs

Overview

Vaccine doses administered

482,695,970

- Fully vaccinated 56.75%
- Received at least one dose 71.89%

Total confirmed cases

49,815,374 +133,958

- Active cases *No data*
- Fatal cases 795,584 +1,713

Trends

Daily spread trends Expand

Cases Deaths

400K

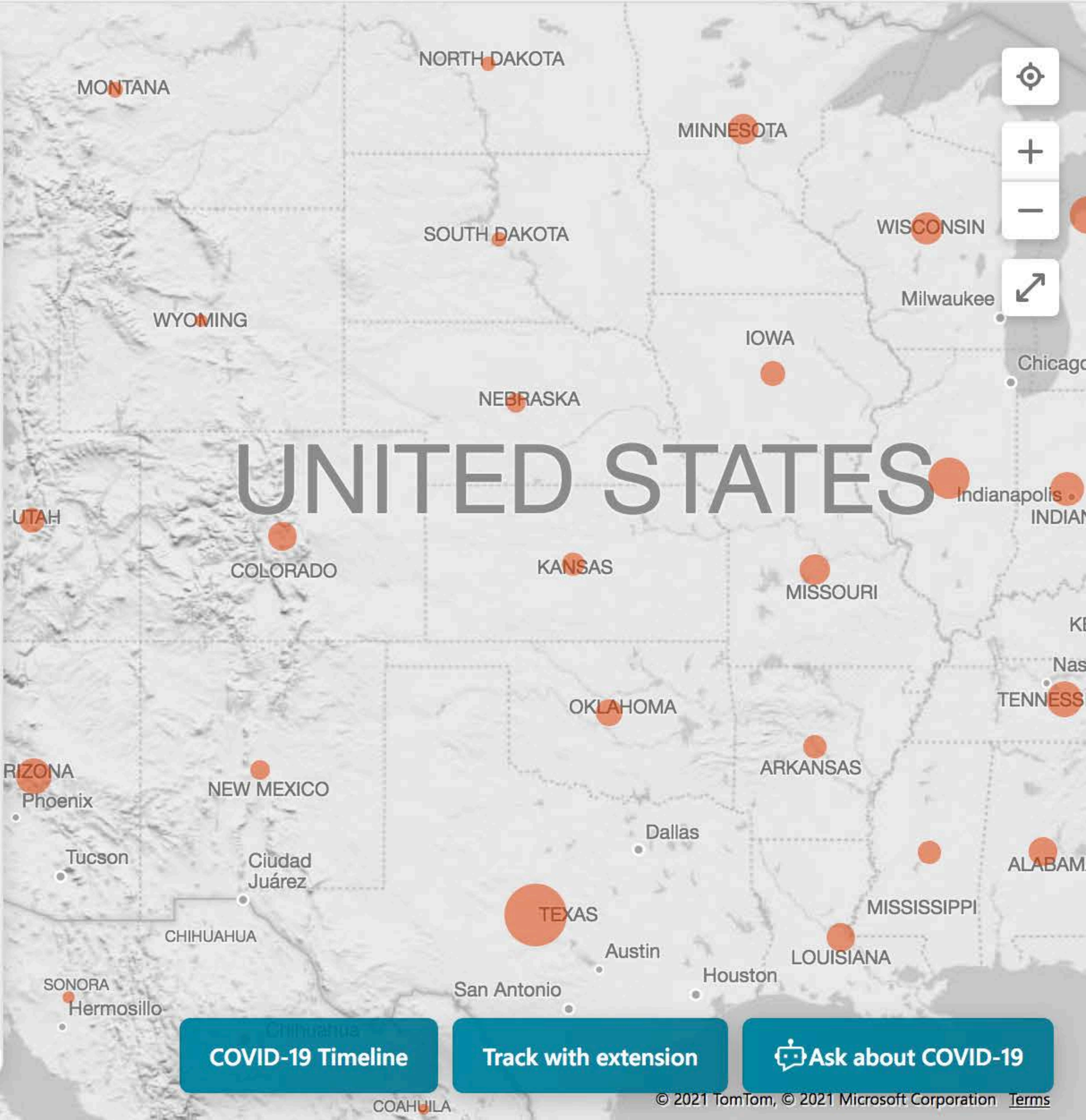

COVID-19 Timeline Track with extension Ask about COVID-19

Coronavirus (COVID-19) Cases by Coronavirus (COVID-19) Data Hub

Global COVID-19 Tracker

January 21, 2020 - December 10, 2021

Global United States

Cumulative or New: New

Positive Cases or Deaths: Positive Cases

New Positive Cases

609,344

▼ -16.5% vs previous day 729,810

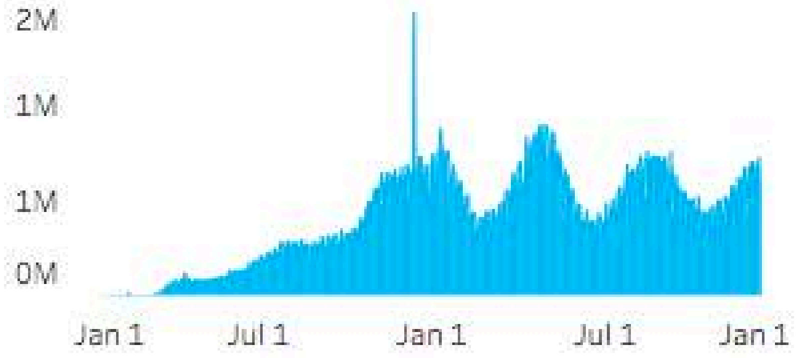

New Deaths

7,943

▼ -7.4% vs previous day 8,575

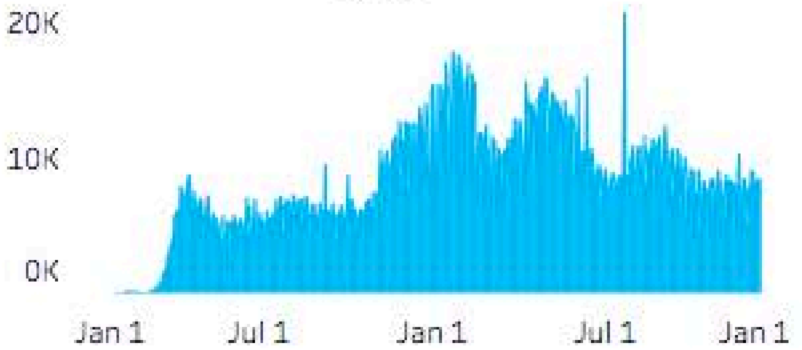

New Positive Cases

Select a Country to see more details

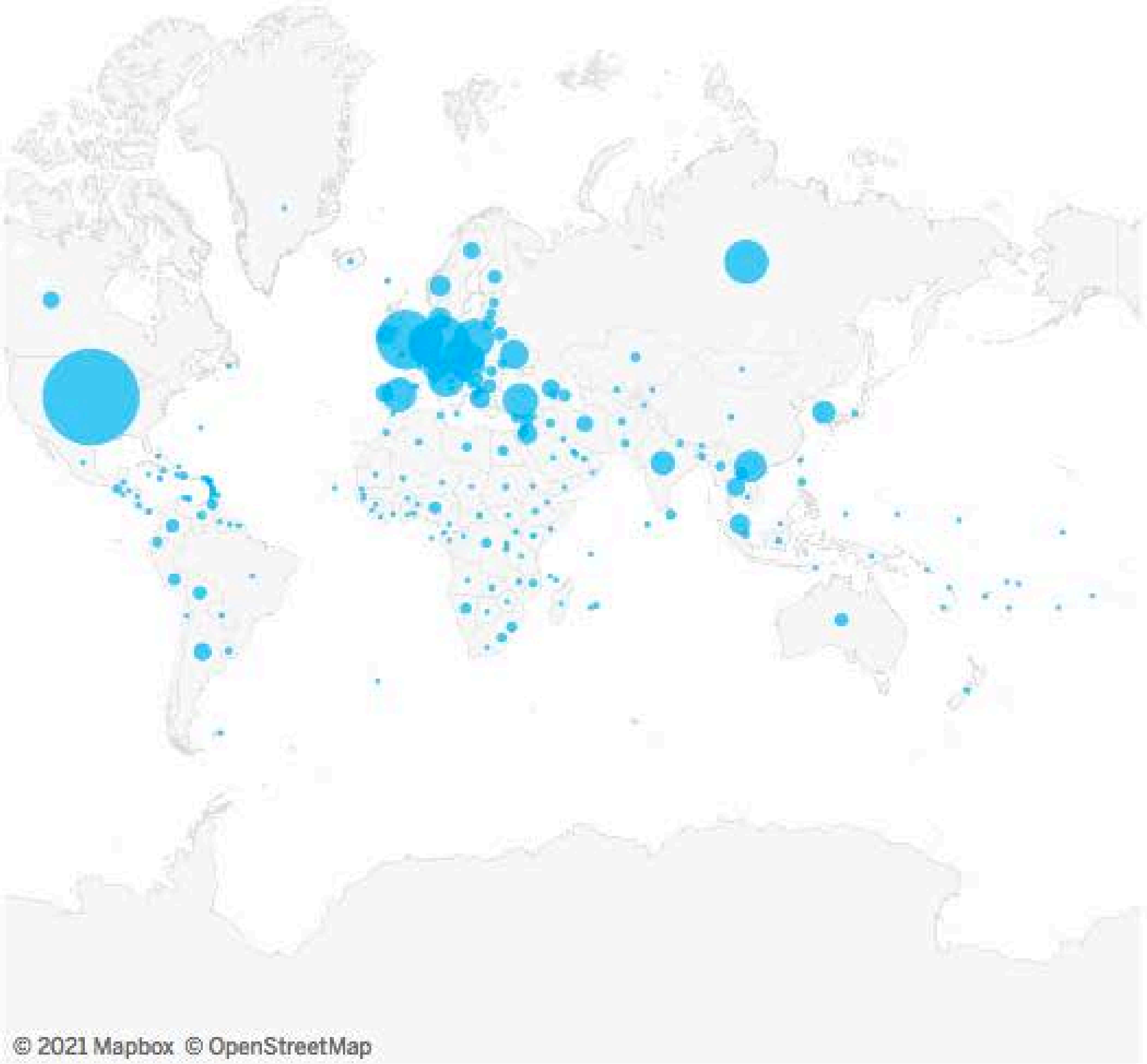

New Positive Cases

Select a Country to see more details

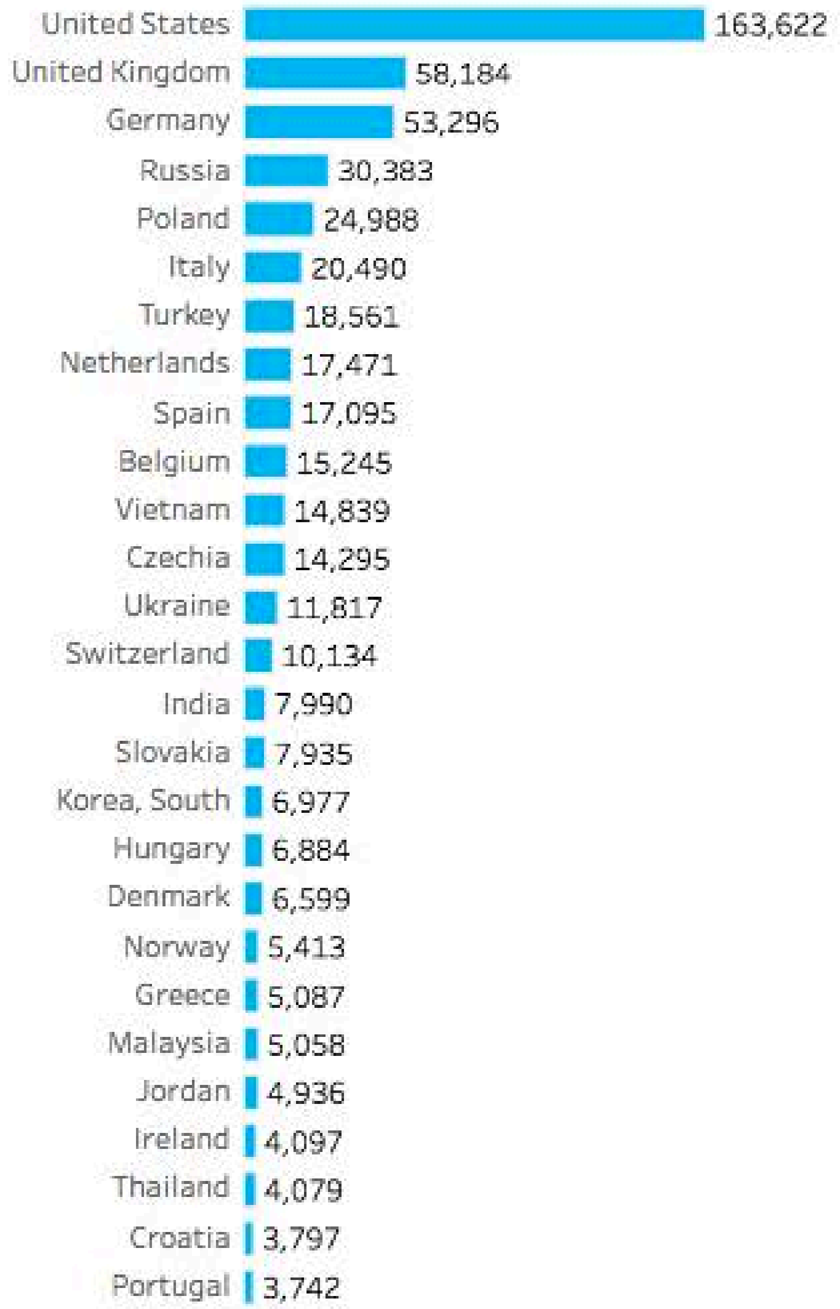

+tableau

Details

810 156,846

#COVID19 #Coronavirus

Published on: www.tableau.com

Published: Mar 12, 2020 **Updated:** Dec 11, 2021
