# Supplementary material for: Web-Based COVID-19 Dashboards and Trackers in the United States: Survey Study
Source: JMIR Hum Factors. 2023 Mar 20;10:e43819. doi: 10.2196/43819 (PMC10029858; doi:10.2196/43819)
Supplement: Multimedia Appendix 14 [file humanfactors_v10i1e43819_app14.pdf]

Appendix 14. Screenshots from selected dashboards and trackers for vaccine distribution — *State, national, and global coverage* *(captured January 3, 2022)*

Page	Ref	Host
2	Svac-1	Alabama Dept of Public Health
3	Svac-2	David Marconnet, CTO of OneTeam.net
4–5	Svac-4	Government of the District of Columbia
6	Svac-6	Chicago Tribune
7	Svac-7	Indiana State Dept of Health
8–11	Svac-8	Kansas Dept of Health and Environment
12	Svac-9	Michigan Dept of Health and Human Services
13–16	Svac-10	Minnesota Dept of Health
17–20	Svac-11	North Dakota Dept of Health
21	Svac-12	New Mexico Dept of Health
22	Svac-13	Ohio Dept of Health
23	Svac-14	Tennessee Dept of Health
24–26	Svac-15	Texas Health and Human Services
27–28	Svac-16	Vermont Dept of Health
29–30	Svac-17	Wisconsin Dept of Health Services
31	Nvac-1	New York Times
32	Nvac-2	Springfield News-Leader
33	Nvac-4	COVID-19 Health Equity Interactive Dashboard / Emory University
34	Gvac-1	Our World in Data
35	Gvac-2	CNN

Alabama's COVID-19 Vaccine Distribution Dashboard

Alabama Department of Public Health | Immunization Division | Updates Tuesday & Thursday by 2:00 p.m.

Select a county
Alabama

Vaccine Doses Administered

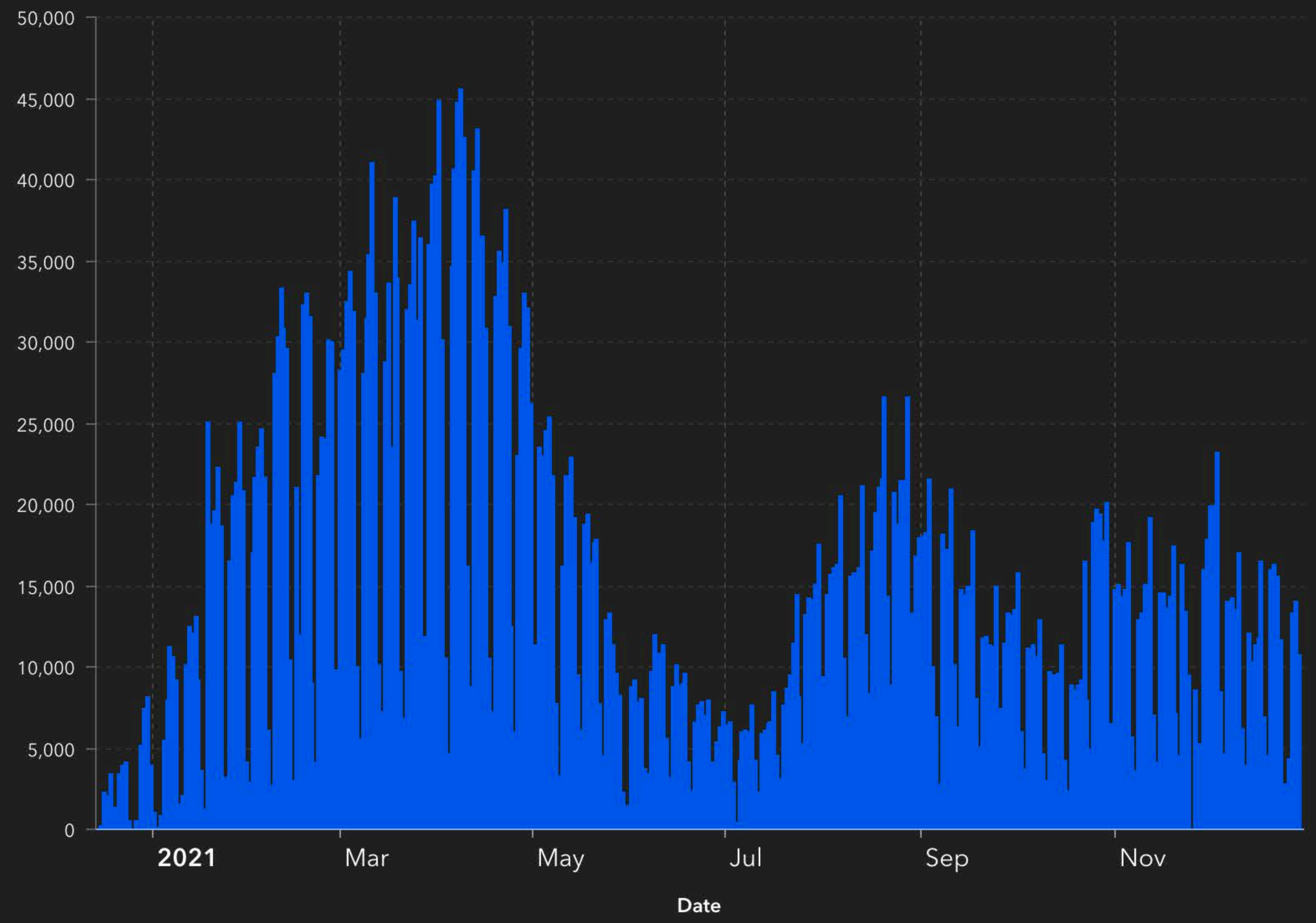

Doses Administered

Doses Administered

5,426,566

Number of people
2,722,869
receiving one or more doses

Number of people
2,218,757
who have completed vaccine series

Additional Doses

574,203
Administered

Doses Delivered to Alabama

7,827,125

Doses Delivered to
4,289,825
Alabama Providers

Doses Delivered to
3,537,300
Federal Programs

Alabama COVID-19 Vaccine Data

As of 5/24/2021, this section is no longer updating. The data shown below is for historical tracking purposes. For the most current data, please check the ADPH Vaccine Distribution Dashboard.

Doses Delivered
4,206,825
3,046,235 AL 1,181,890 Fed LTC

Doses Admin.
2,851,368
93.6% of AL delivered

People Vaccinated
1,625,262
1 or More Doses

People Vaccinated
1,318,446
Completed Vaccine Series

Administered per Day

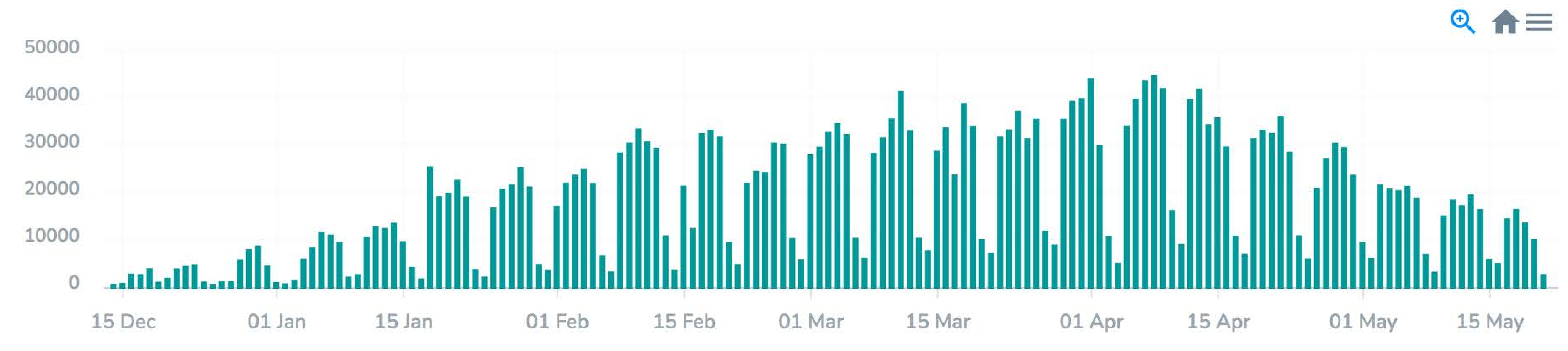

People Vaccinated over time

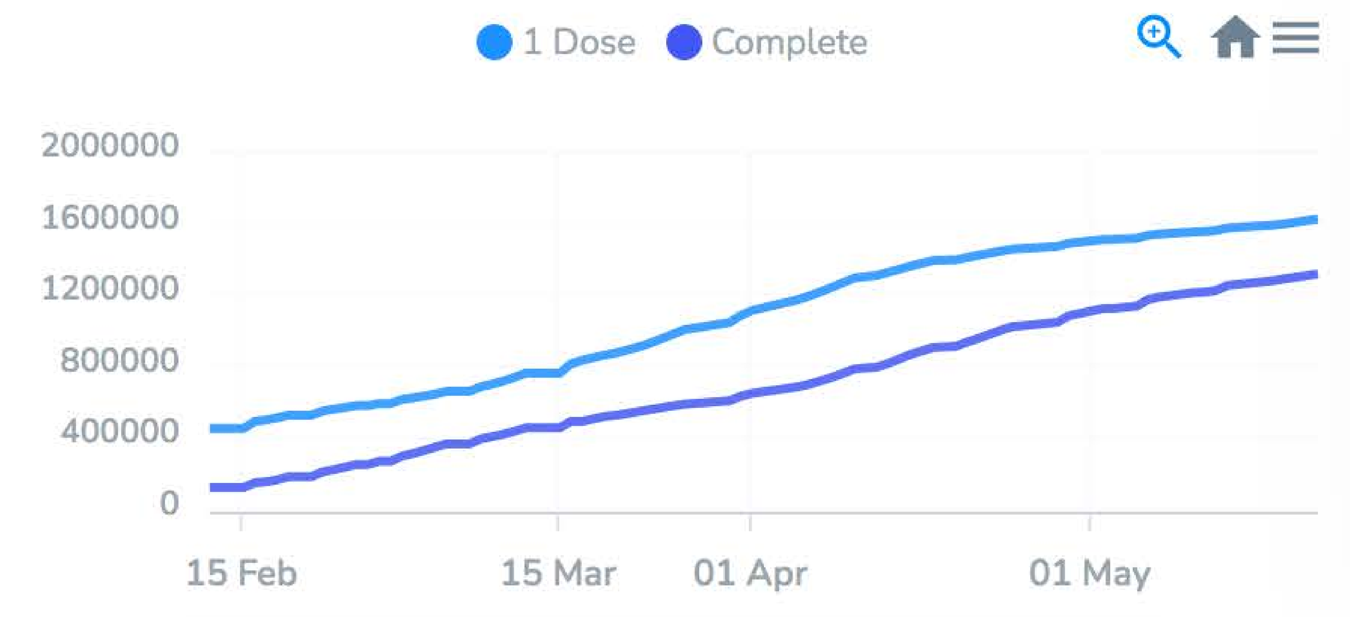

Delivered over time

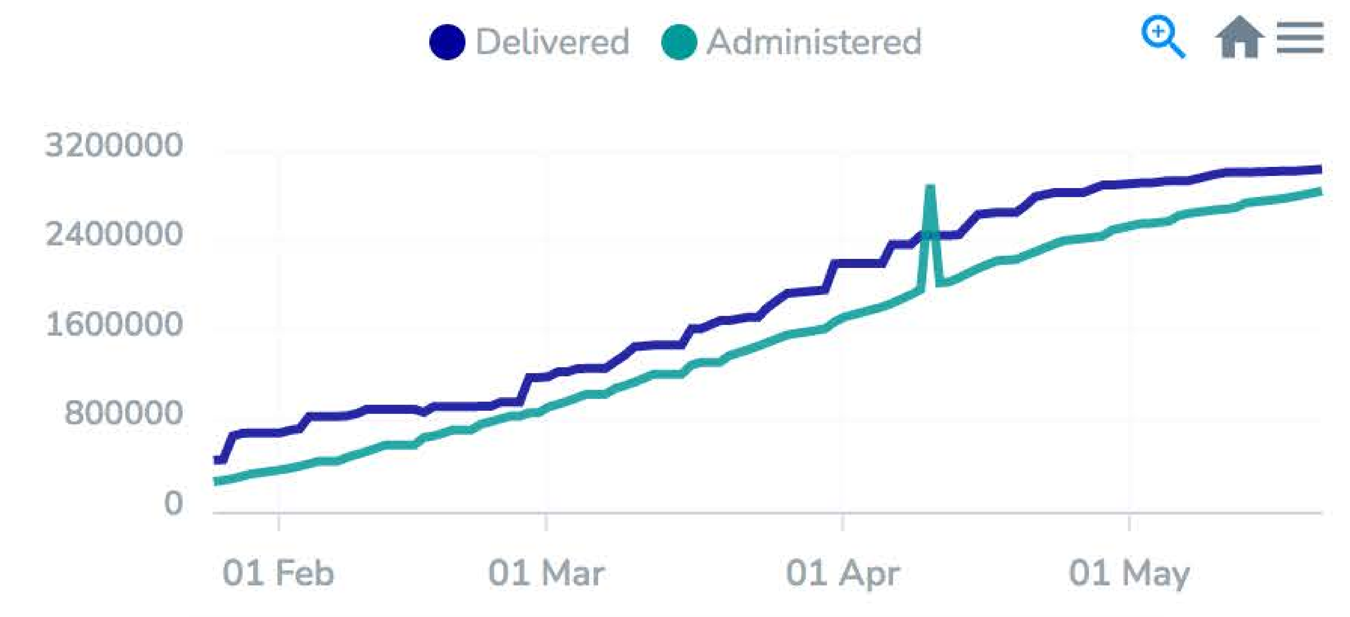

People Complete Change Per Day

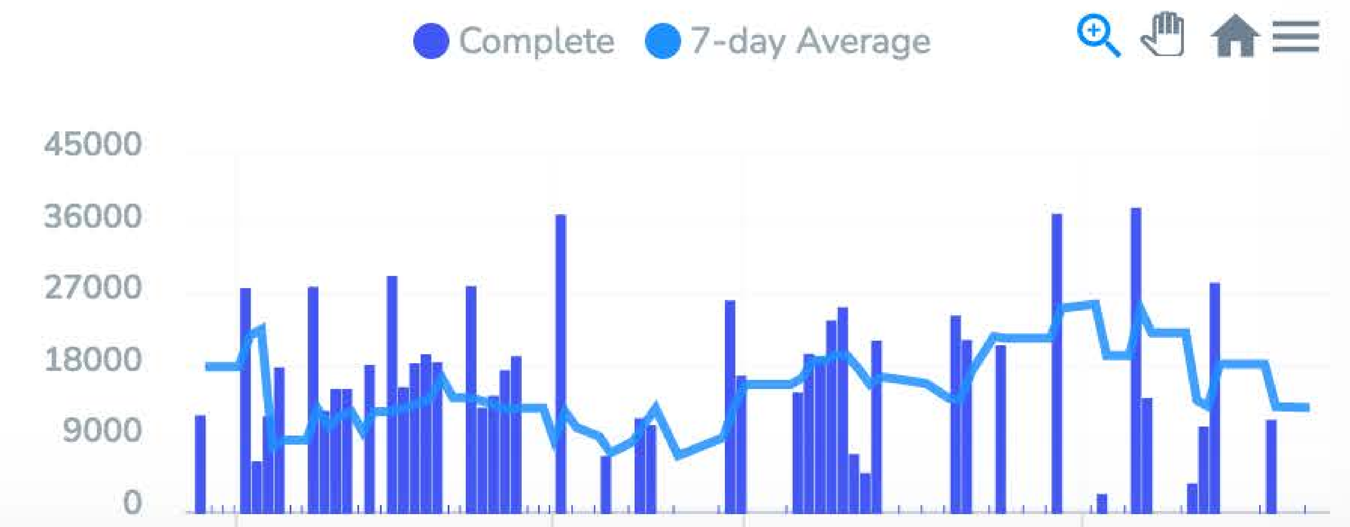

Delivered Change Per Day

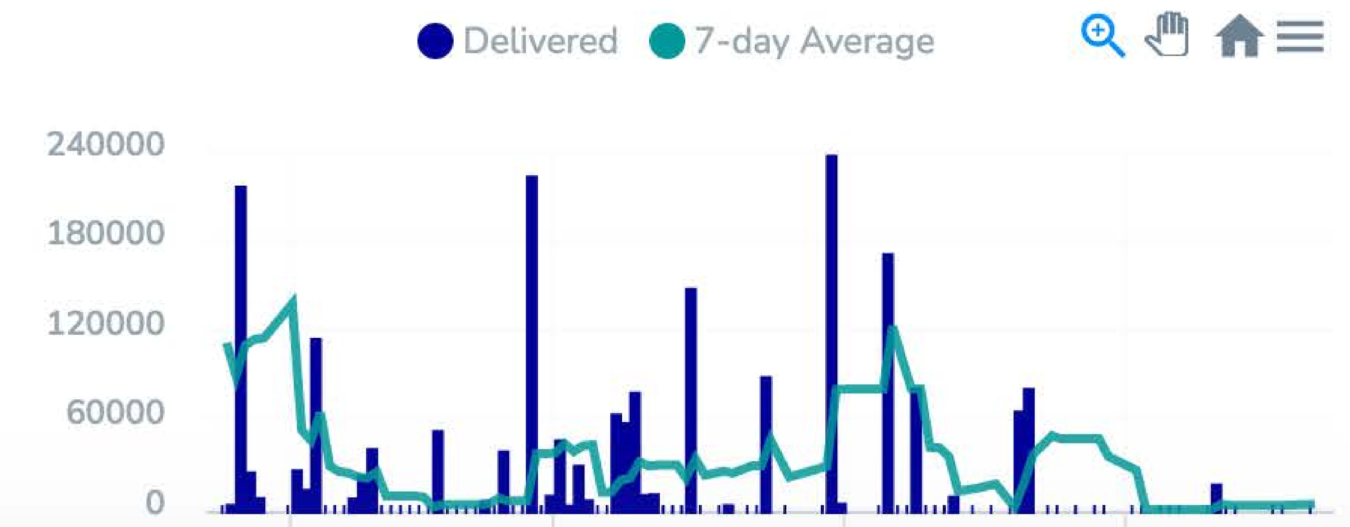

Percent Admin over time (Admin / Total Delivered)

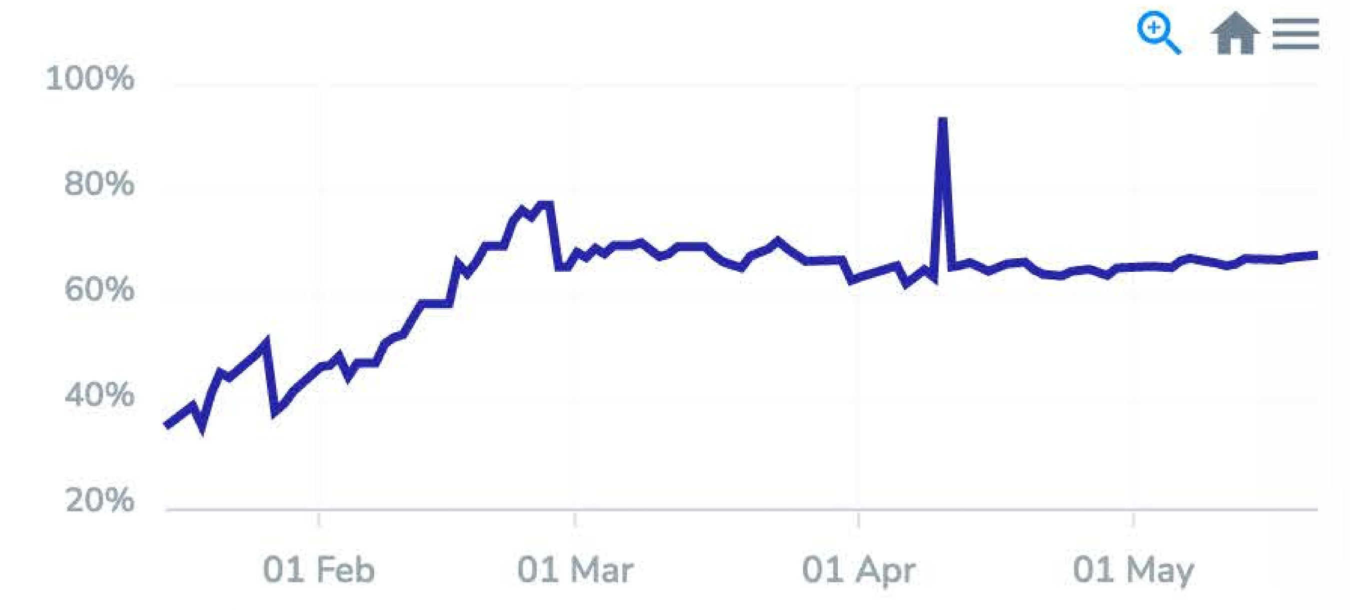

Percent Population over time

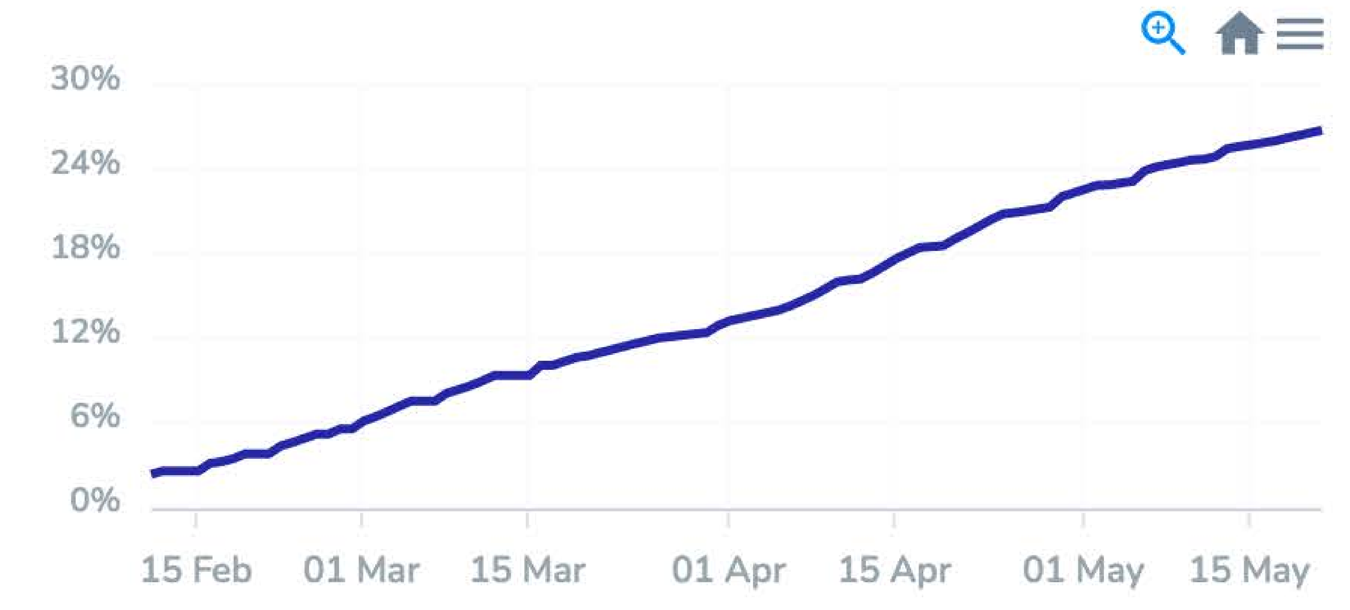

County Details

Show 25 entries

Search:

County	People Complete	Percent Complete	1 or More Doses	Admin	7-day Avg Admin	Pop > 16
Jefferson	201,608	30.61%	253,458	440,804	1408.71	658,573
Madison	116,392	31.21%	139,093	245,639	1056.57	372,909
Mobile	104,516	25.29%	122,251	222,947	491.29	413,210

Neighborhood | Ward

Count or coverage:

Coverage (%)

Mapped: 521,016
Not mapped: 11,251

Ward - Age Group

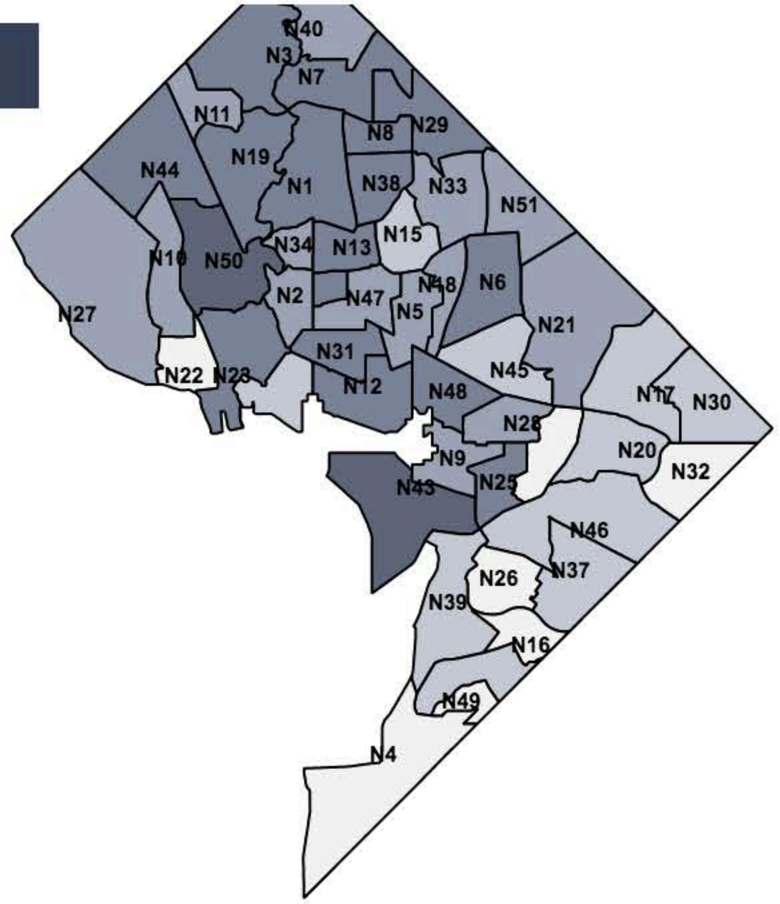

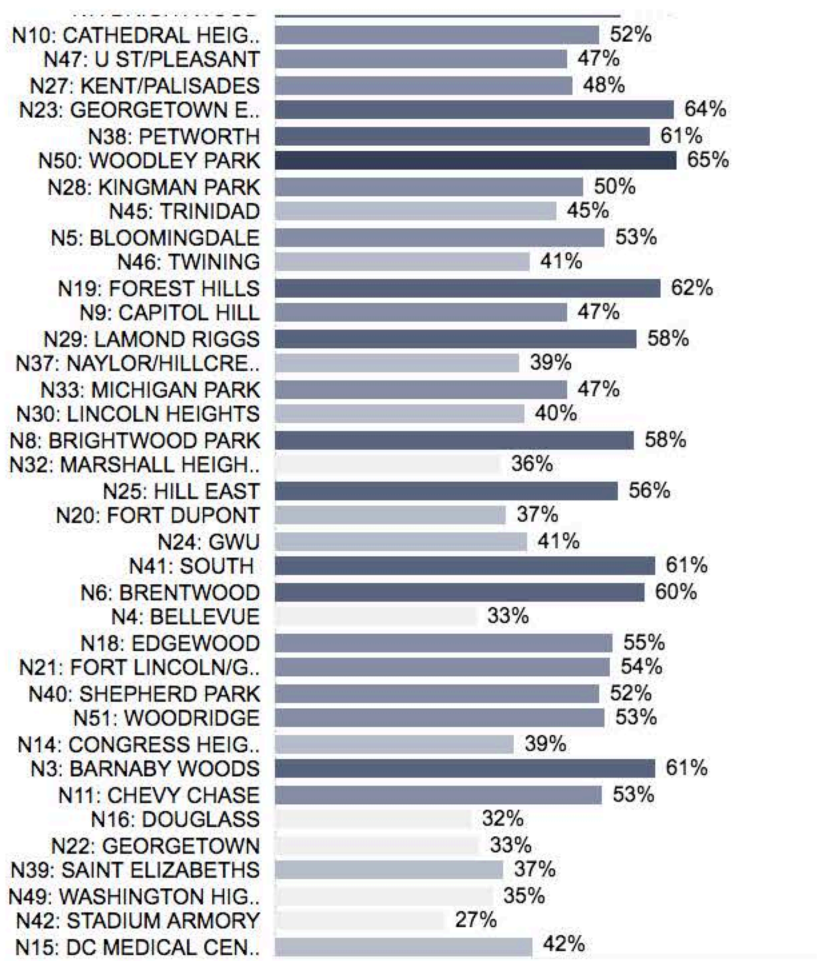

Source: DC Health. Data are subject to change.

Metric Definition: Number of residents who completed the vaccine regimen for COVID-19. Coverage is defined as the number as a proportion of the number of residents in each neighborhood, as reported by the total population reported in the ACS 2019 census tract estimates, including residents who may be ineligible to receive the vaccine.

Data Considerations: The proportion of fully vaccinated residents does not translate to population immunity. Residents who are partially vaccinated may have a some level of immunity, immunity may change over time, and non-residents may not be included in the population. Vaccine administration data is reported by facilities and may not be complete. Individuals who receive single dose regimens such as J&J are considered fully vaccinated. These numbers do not include DC residents who have received vaccination outside of DC. Charts and table consists of data from DC Health and summary bar was calculated from combination of data sources and can not be compared. **Total doses administered within DC and estimated rates of fully/partially vaccinated DC residents are calculated using both administered

Last Updated: 12/27/2021

TOTAL DOSES ADMINISTERED WITHIN DC	ESTIMATED % RESIDENTS PARTIALLY OR FULLY VACCINATED**	ESTIMATED % RESIDENTS FULLY VACCINATED**	ESTIMATED % OF BREAKTHROUGH CASES***
1,248,562	86.9%	67.2%	2.47%

View Mayor Bowser's Presentation on DC's COVID-19 Situational Update for December 29, 2021
DC launches citywide vaccination entry requirement (VaxDC), calls for many establishments to verify patrons are vaccinated against COVID-19

GOVERNMENT OF THE DISTRICT OF COLUMBIA
DC MURIEL BOWSER, MAYOR

Select Language
Powered by Google Translate
Search

Home | Testing | Vaccine | DC CAN | Recovery | Food | Utilities | Data | Health Guidance | Operating Status

	At Least One Dose	Fully Vaccinated	Booster Dose	Total Administrations	Dose 7-day Average
DC Resident (within DC)	437,971	361,404	91,222	864,787	1,312
DC Resident (outside DC)	141,716	80,477	17,815	229,879	
DC Resident (Federal Entity)	33,448	32,147	7,717	72,791	
Total DC Resident	613,135	474,028	116,754	1,167,457	1,312
Non DC Resident (within DC)	161,489	103,280	13,742	272,242	523
Non DC Resident (Federal [illegible]	15,906	15,867	18	38,742	
Total Non DC Resident	177,395	119,147	13,760	310,984	523

Source: DC Health and DC Residents (outside DC and Federal Entity) is an estimate value from Tiberius (Product of Health and Human Services) **Data are subject to change.**

Metric Definition: Number of administrations reported by DC-area providers by date of vaccine administration for DC Residents and Non DC Residents. The 7-day rolling average represents the average number of administrations including the past 6 days. Listed in Summary include the Total Doses administered within DC include residents and non DC residents.

Data Considerations: There may be a lag time between vaccine administration and provider report. This may impact the reporting of vaccine administered, especially in the three most recent days of report, highlighted in red above. Non DC residents may be vaccinated within DC, especially those who fall into prioritized non-resident categories. Administration may be impacted by holidays and weekends, and is impacted by the size of prioritized groups and vaccine supply. Individuals who receive single dose regimens such as J&J are considered fully vaccinated.The Chart consists of data from DC Health and table data consists combination of DC Health and Tiberius Data. Tiberius data are provided in cumulative numbers and have granular representation and only listed in the table and included in Summary bar

Last Updated: 12/27/2021

COVID-19 VACCINE | CORONAVIRUS

Illinois COVID-19 vaccine tracker: Daily data updates on the state's vaccination effort

By CHICAGO TRIBUNE STAFF
CHICAGO TRIBUNE | AUG 31, 2021

Illinoisans ages 12 and over are now eligible for the coronavirus vaccine. Below are key statistics about the vaccination effort in Illinois.

Here, the Tribune gets your COVID-19 **questions answered** from experts.

See Coronavirus data on **deaths, cases and tests** here.

Illinois COVID-19 vaccination rates by county

How Cook County stacks up

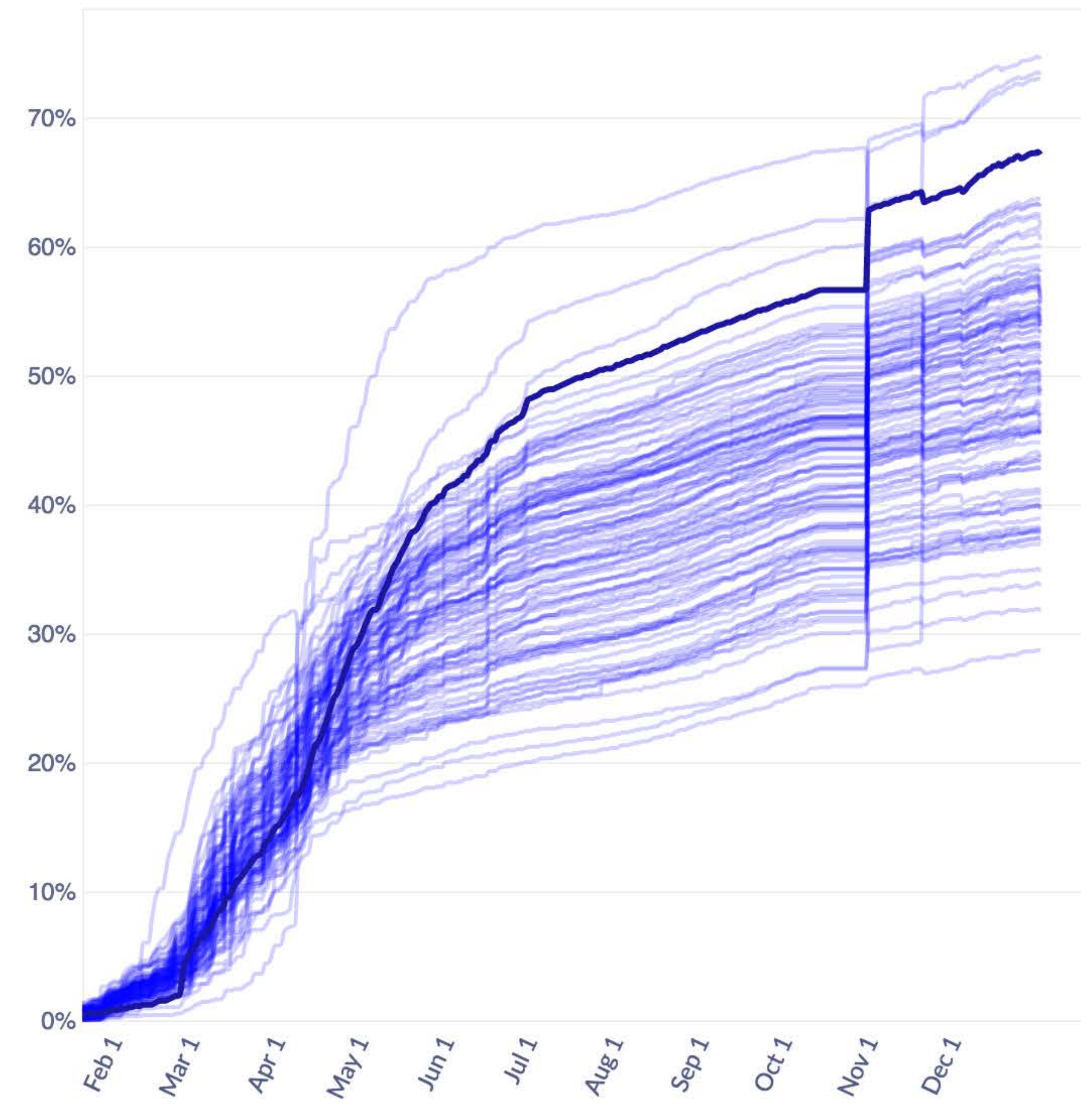

Source: T H

Illinois COVID-19 vaccination rates

Updated January 3, 2022

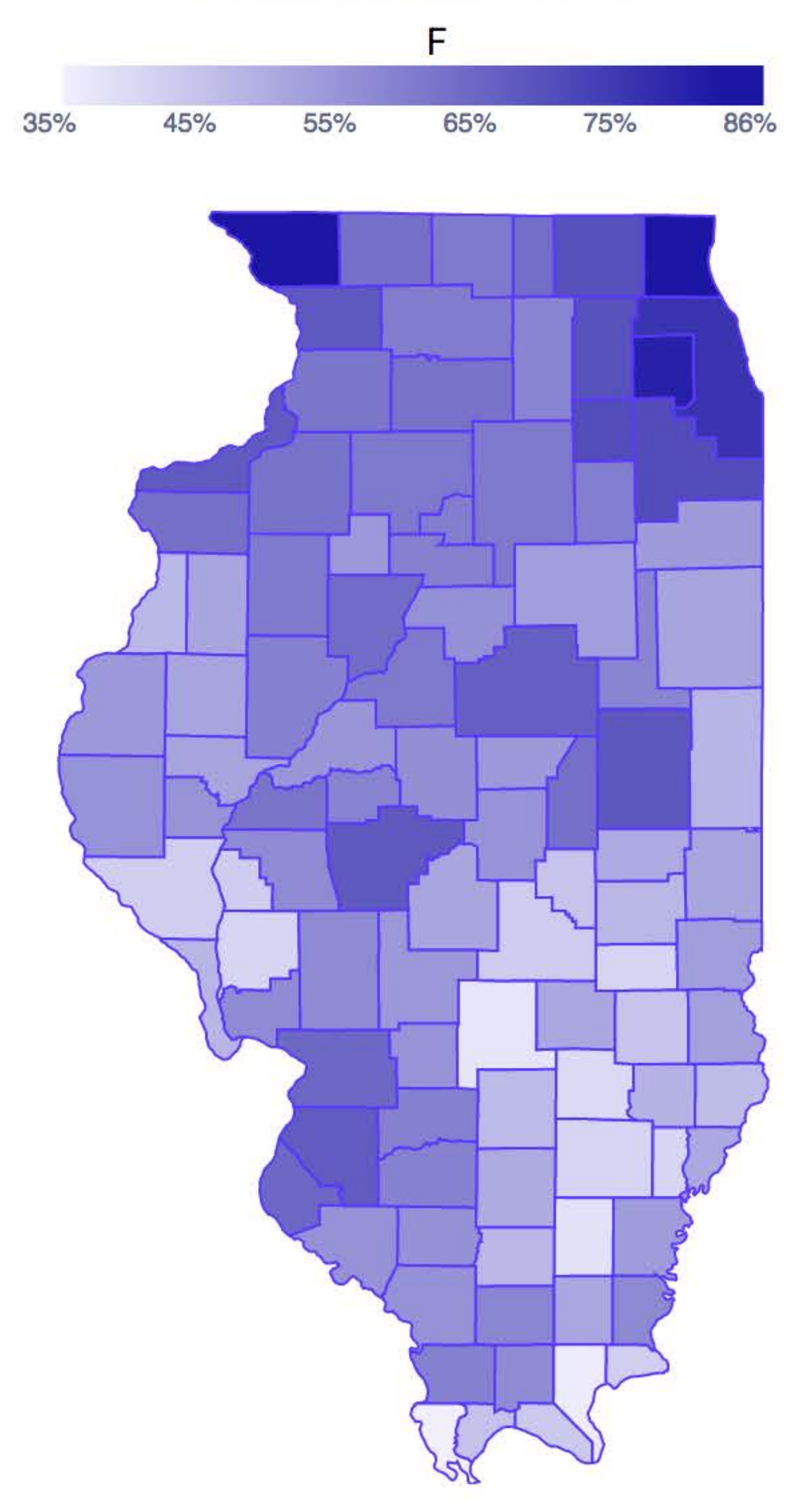

Source: H

COVID-19 vaccination rates for the U.S.

Last Updated on Thursday 12/30/21

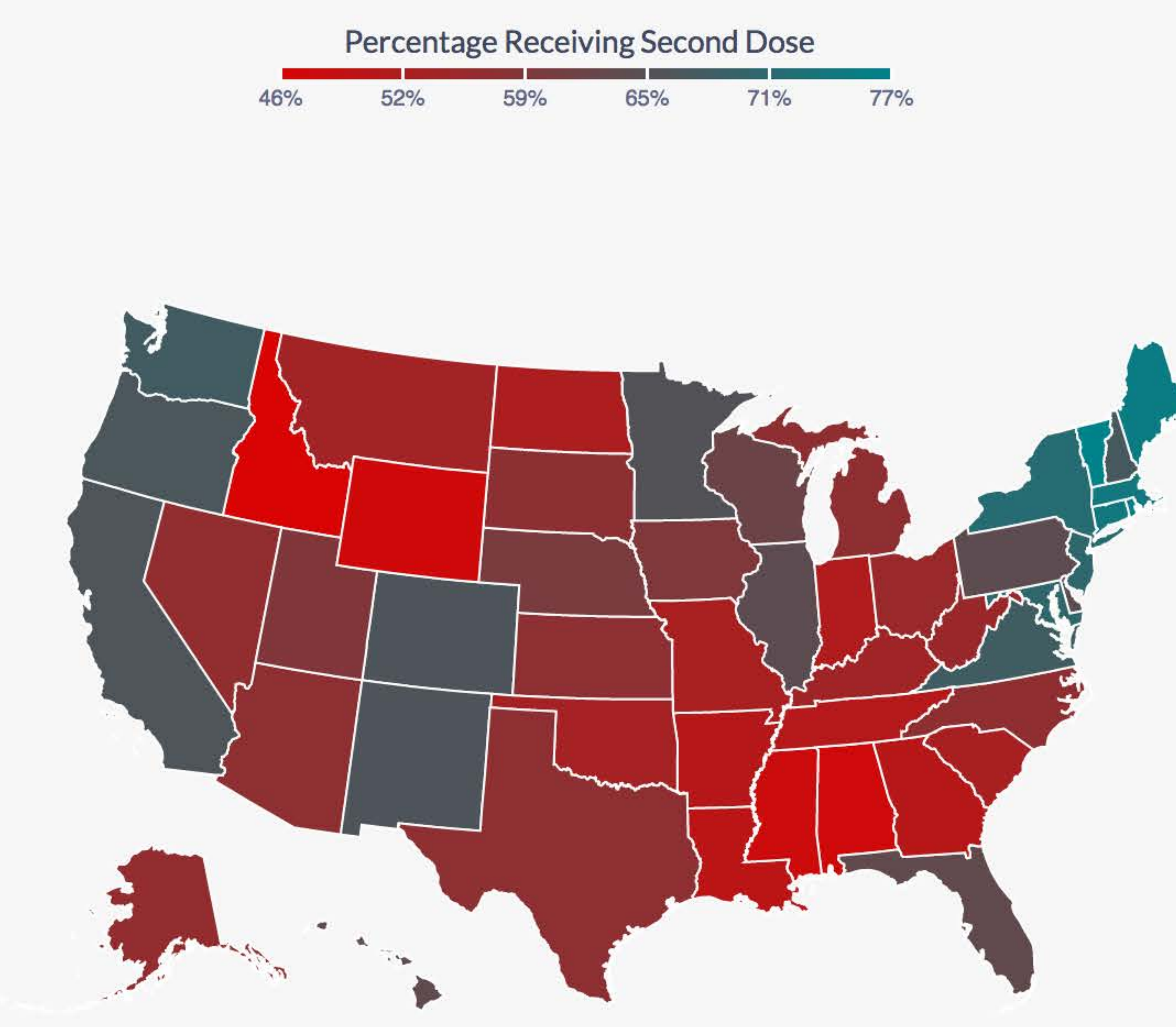

Source: CDC COVID Data Tracker Vaccinations

By DATAHERALD

COVID-19 vaccination rates by state

How Illinois stacks up

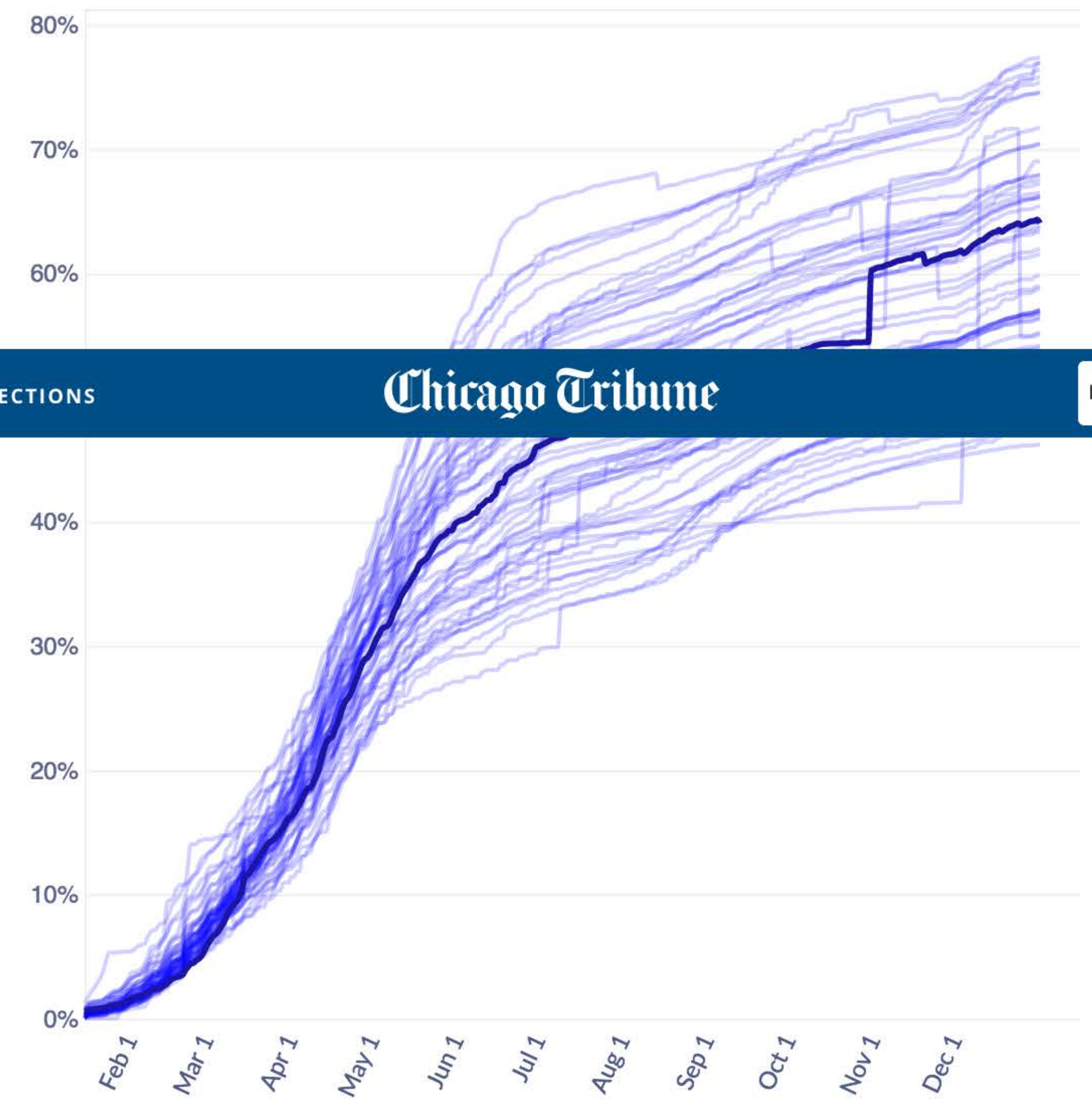

SECTIONS Chicago Tribune LOG IN

Source: T H

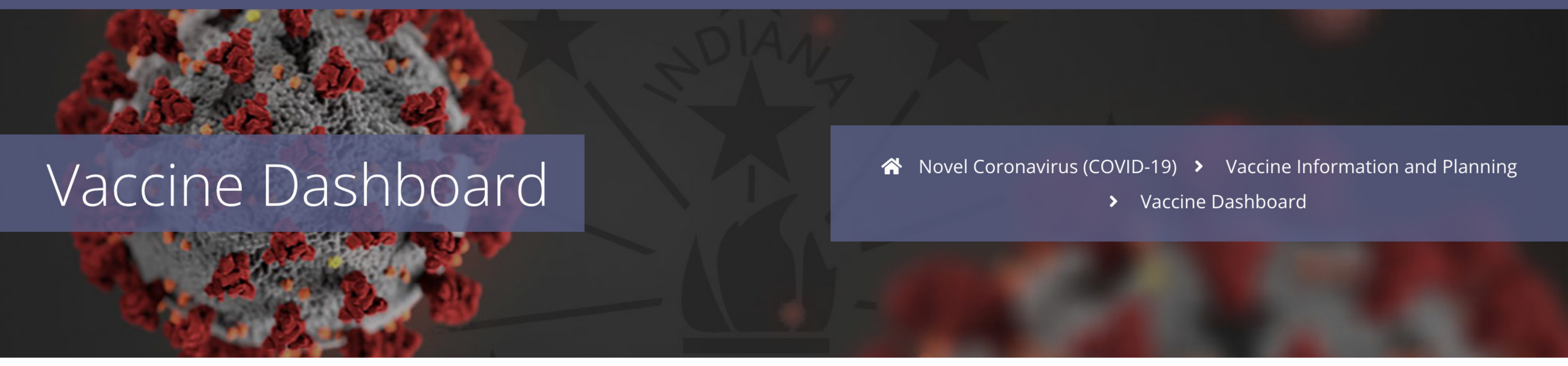

Indiana COVID-19 Vaccination Dashboard

Updated Monday through Friday by 5:00 PM EST. Data current as of 01/03/2022 at 5:00 AM EST.

Newly Reported Vaccinations

Reported within the past 24 hours

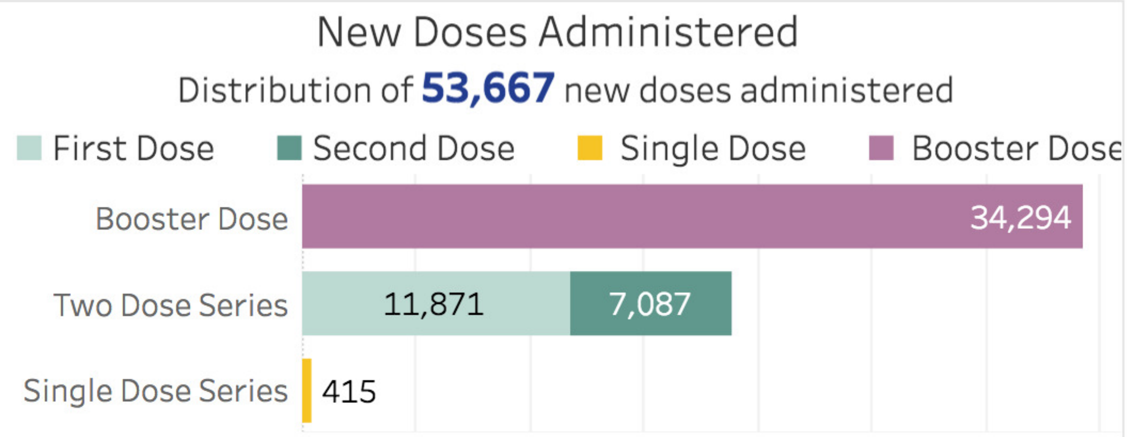

New Fully Vaccinated Individuals

7,501

During the previous 24 hours, number of individuals reported to be fully vaccinated for COVID-19 (receiving a single-dose vaccine or the second dose of the two-dose vaccine).

Total Vaccinations

Reported total (all-time): 12/14/2020 - 01/03/2022

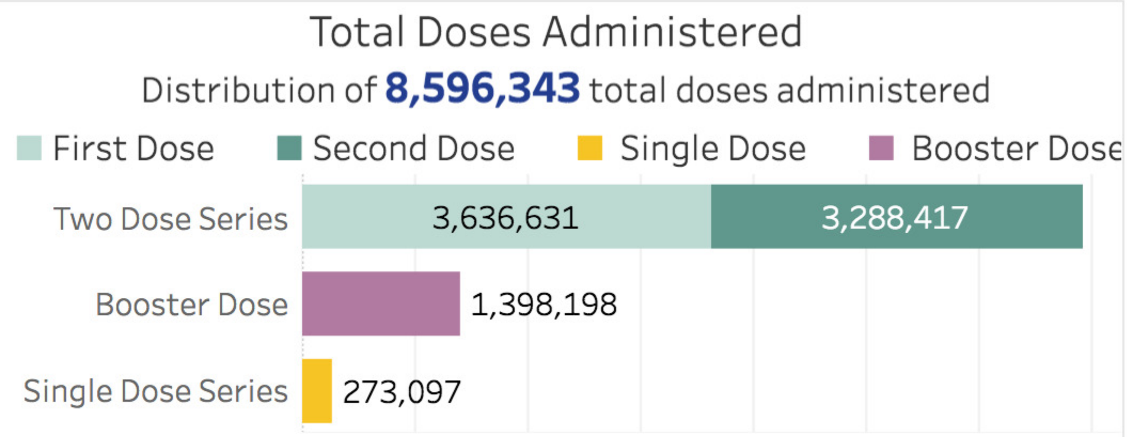

Fully Vaccinated Individuals

3,561,514

Total number of individuals who are considered to be fully vaccinated for COVID-19 (receiving a single-dose vaccine or the second dose of the two-dose vaccine).

Vaccinations by Date

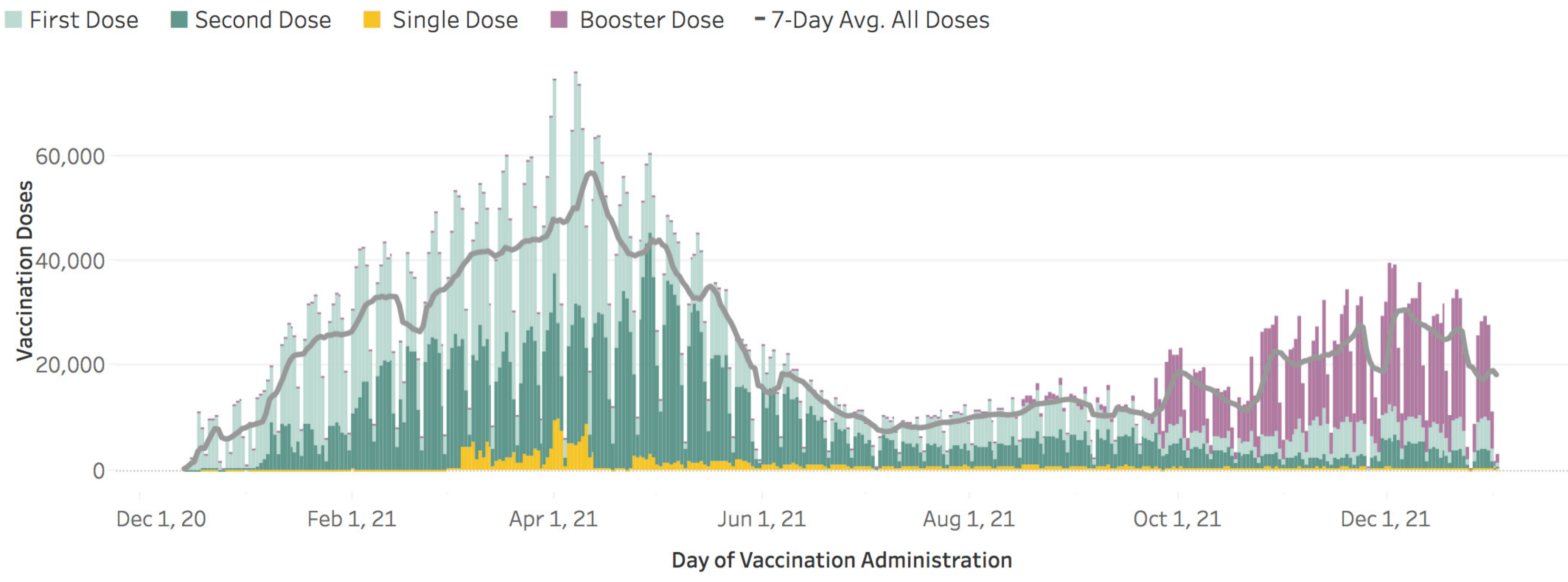

County Distribution

Select county or group of counties to filter dashboard. Hover over the map to learn more about the counts.

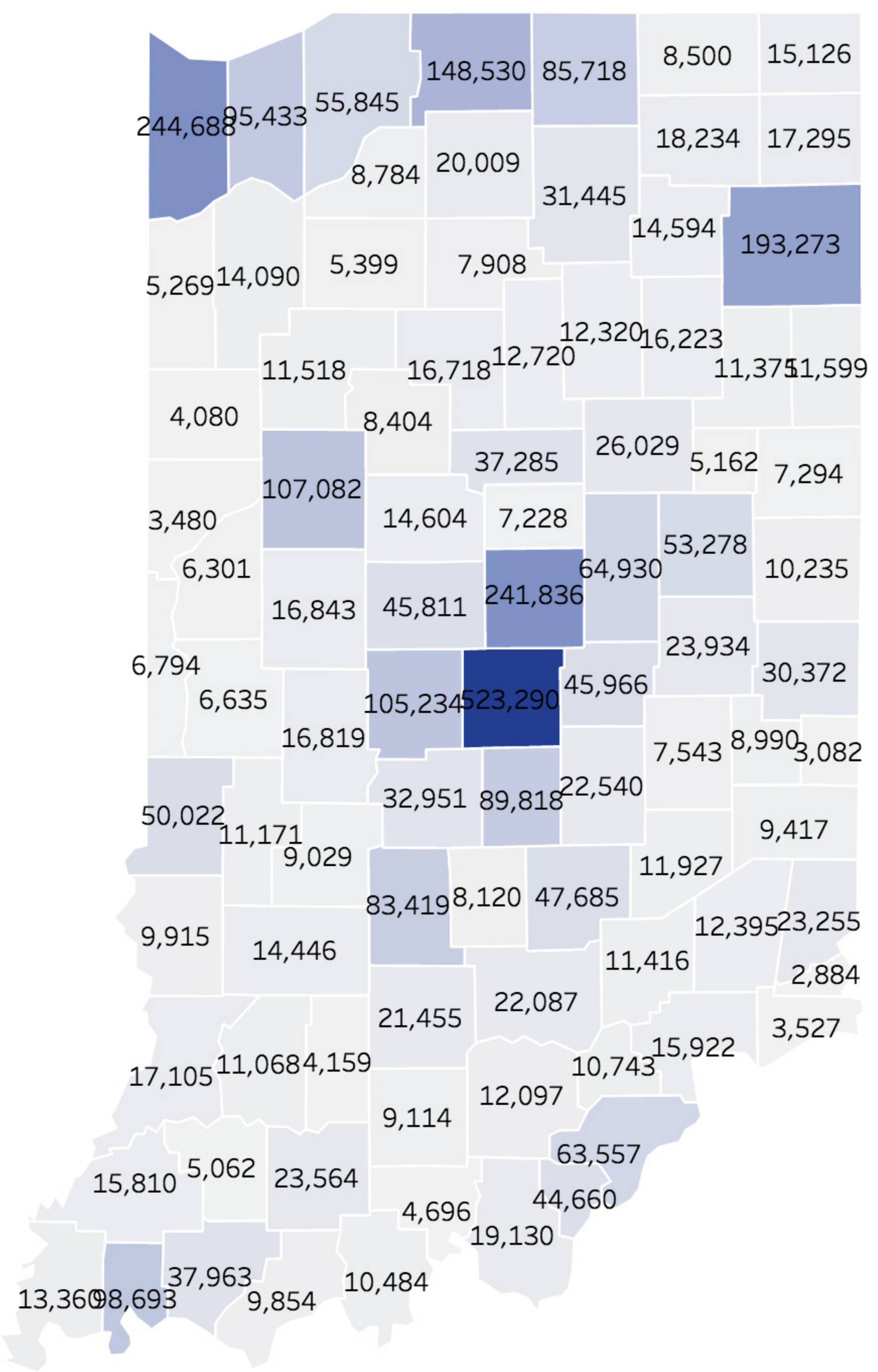

Vaccination Sequence
- (•) Fully Vaccinated
- ( ) First Dose Administered
- ( ) Second Dose Administered
- ( ) Single Dose Administered

Timeframe
- (•) All Time
- ( ) Previous Day

Metric
- (•) Total Count
- ( ) Per 100,000 Residents (Age 5+)

11,434 individuals did not report a county of residence and are excluded from this map.

104,406 individuals reported a county of residence outside of Indiana and are excluded from this map.

Statewide Demographics for Vaccinated Individuals

Individuals reporting a county of residence outside of Indiana are excluded from this section. Demographic counts of less than 5 are suppressed within these visuals. Hover over the charts to learn more about the counts.

Vaccination Sequence
- (•) Fully Vaccinated
- ( ) First Dose Administered
- ( ) Second Dose Administered

Gender

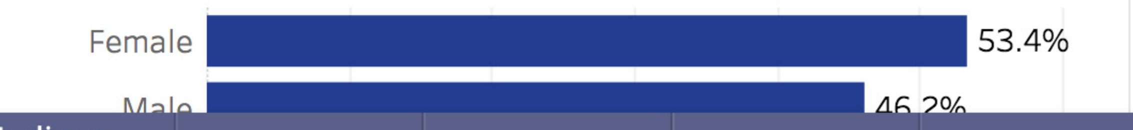

MENU | IN.gov | Novel Coronavirus (COVID-19) | Indiana COVID-19 Dashboard and Map | COVID-19 Testing Information | Find a Vaccination Site | Public Resources | Professional Resources | SEARCH
Gov. Eric J. Holcomb

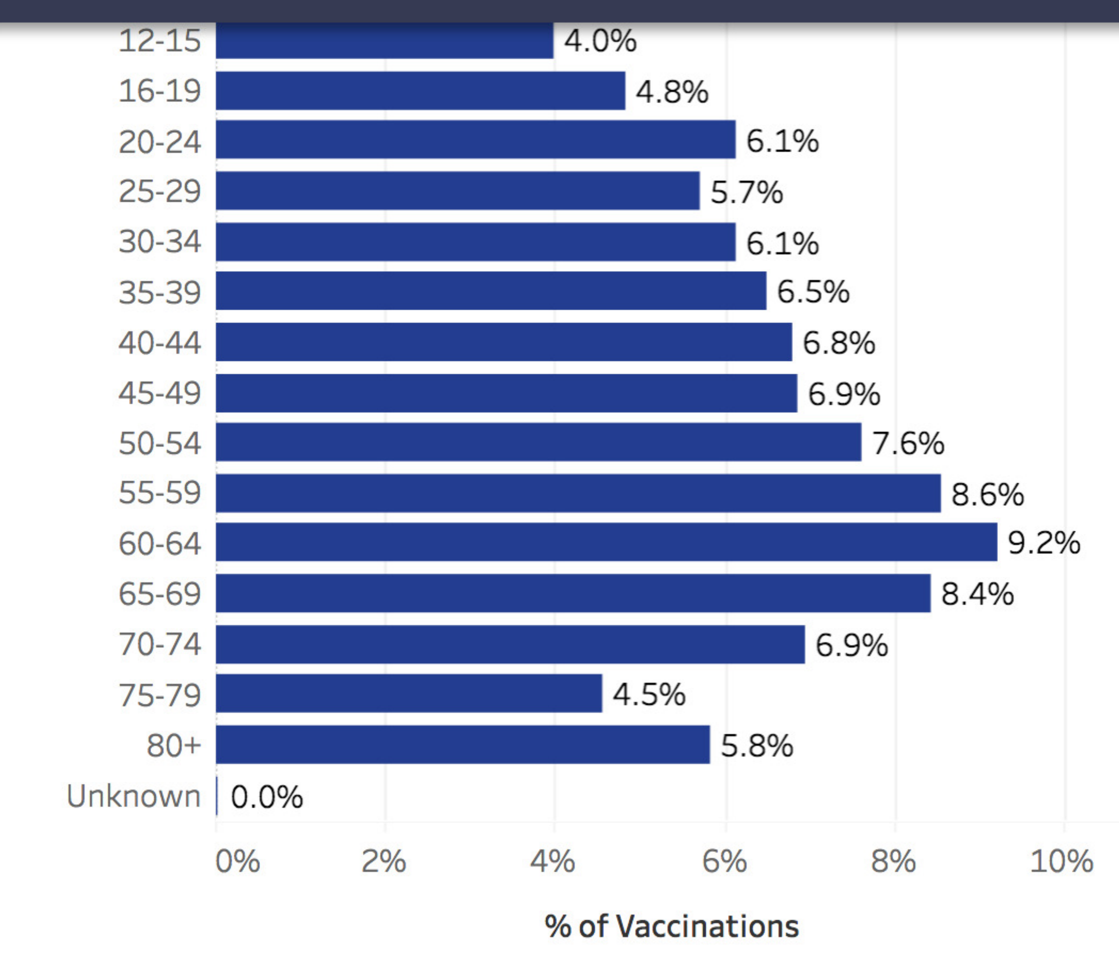

Race

	% of Vaccinations	% of Eligible IN Population
White	66.7%	85.1%
Black or African American	6.2%	9.8%
Other Race	21.1%	2.5%
Asian	2.7%	2.6%
Unknown	3.3%	0.0%

Ethnicity

	% of Vaccinations	% of Eligible IN Population
Not Hispanic or Latino	87.3%	93.0%
Hispanic or Latino	6.5%	7.0%
Unknown	6.2%	0.0%

All numbers are provisional and reflect only those reported to IDOH. Numbers should not be characterized as a comprehensive total and may change as more data is reported.

Data

Dashboard Information

The COVID-19 vaccine dashboard is updated Monday, Wednesday and Friday by 12:30 p.m. To maximize your experience, use a device with a larger screen, such as a tablet, laptop or desktop.

November 17 Update
The eligible population of the vaccine administration tab was updated to include the recently approved pediatric population of 5 to 11 years of age and should be interpreted as Kansans 5 years of age and older. Due to this change in population, the percent of eligible population vaccinated with at least one dose and eligible population fully vaccinated has decreased compared to 11/15/2021.

Kansas COVID-19
Vaccination Overview

Data are preliminary and subject to quality improvement and quality assurance validation.
Last updated: 1/3/2022 at 9:00am
* Third dose includes all instances of 3 doses reported in KSWebIZ. On 8/12/2021, a third dose was approved for those who are severely immunocompromised. On 9/24/2021, booster doses were approved for select populations 6 months following completion of a Pfizer initial series. On 10/21/2021, booster doses for Moderna were approved for select populations 6 months following initial series completion. Johnson and Johnson booster doses were approved for all recipients 2 months after initial series.

1,853,537
People Vaccinated with One Dose

3,889,946
Total Doses Reported as Administered
First Dose: 1,853,537
Second Dose: 1,484,778
Third Dose*: 551,632

4,879,630
Total Doses Distributed
State Distributed: 2,838,220
Federal Pharmacy Program: 2,041,410

59.4%
Percent of Kansans Vaccinated with One Dose

51.4%
Percent of Kansans Completed COVID-19 Vaccine Series

Vaccine Administration

County Vaccination Rates

Demographics

Understanding the Numbers

Data Source: The data displayed on this dashboard come from the Kansas Immunization Registry (KSWebIZ). As providers in Kansas administer immunizations, including COVID-19 immunizations, they are required by law to submit vaccination information to KSWebIZ. KSWebIZ data are constantly updated and reviewed to ensure data quality and completeness. Vaccine providers outside of Kansas and through federal partnerships do not report to KSWebIZ and are not captured in these data. All states and federal partners report all doses administered to CDC; however, Kansas data used in the CDC COVID-19 Data Tracker has not been updated to reflect removal of duplicates and other updates to data originally submitted. For COVID-19 immunization data that includes federally administered vaccines, please see the CDC COVID-19 Data Tracker https://covid.cdc.gov/covid-data-tracker/#county-view.

Total Doses Administered: Represents the total number of all COVID-19 vaccine doses (e.g. first dose, second dose) that have been given to people in Kansas as reported in KSWebIZ.

Total Doses Distributed: Represents the total number of all COVID-19 vaccine doses that have been delivered to vaccine administration sites throughout Kansas. This includes all first and second doses. Second doses are delivered prior to completion of the appropriate interval.

Population Estimate Totals: U.S. Census Bureau, June 2019 County Characteristics Resident Population Estimates

People Vaccinated: Represents the total number of individuals with a vaccination record reported in the Kansas

KSWebIZ as a fraction of the total population of Kansas (2019 Bridged-Race Population Estimates, as reorganized in VSDA Population Master File).

Select Language

Eligible Population: COVID-19 vaccinations are currently approved under Emergency Use Authorization for individuals 12 years of age and older.

County Vaccination Rate: County vaccination rates are calculated based on vaccine recipient county of residence. KDHE is currently only able to report on doses given in Kansas and reported into the Kansas Immunization Registry (KSWebIZ). Kansas residents that have been vaccinated in surrounding states or through federal agencies are not currently included in this data. Vaccination rates for counties bordering other states or with close ties to federal agencies may have higher vaccination rates than reported using KSWebIZ. Addresses missing zip codes are unable to be associated with a county. Addresses with incomplete or inaccurate zip codes may be associated with an incorrect county. Data cleaning processes are in place to correct these mistakes over time.

Race/Ethnicity Information: Race and ethnicity data is reported into the Kansas Immunization Registry (KSWebIZ) by vaccine providers using patient self-identified race and ethnicity. Multiple races are available for selection with the primary race listed first followed by other selected races. Race is reported into the demographics section of a patient record and can be updated as needed; updated race information will apply to all previous and future vaccine administered to that individual.

Data

Dashboard Information

The COVID-19 vaccine dashboard is updated Monday, Wednesday and Friday by 12:30 p.m. To maximize your experience, use a device with a larger screen, such as a tablet, laptop or desktop.

November 17 Update

The eligible population of the vaccine administration tab was updated to include the recently approved pediatric population of 5 to 11 years of age and should be interpreted as Kansans 5 years of age and older. Due to this change in population, the percent of eligible population vaccinated with at least one dose and eligible population fully vaccinated has decreased compared to 11/15/2021.

Kansas COVID-19
Vaccine Administration

Data are preliminary and subject to quality improvement and quality assurance validation.
Data Source: Kansas Immunization Information System, KSWebIZ

county
(All)

Return to Vaccine Overview

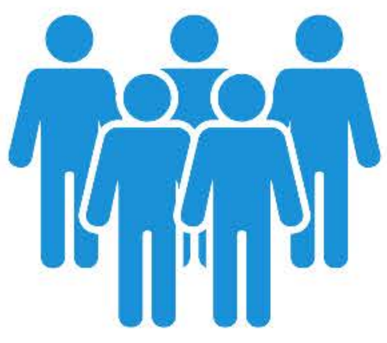

1,730,871 | 63.45%
Eligible Population with at least one dose

1,496,107| 54.84%
Eligible Population Completed COVID-19 Vaccine Series

Eligible Population Vaccinated with One Dose

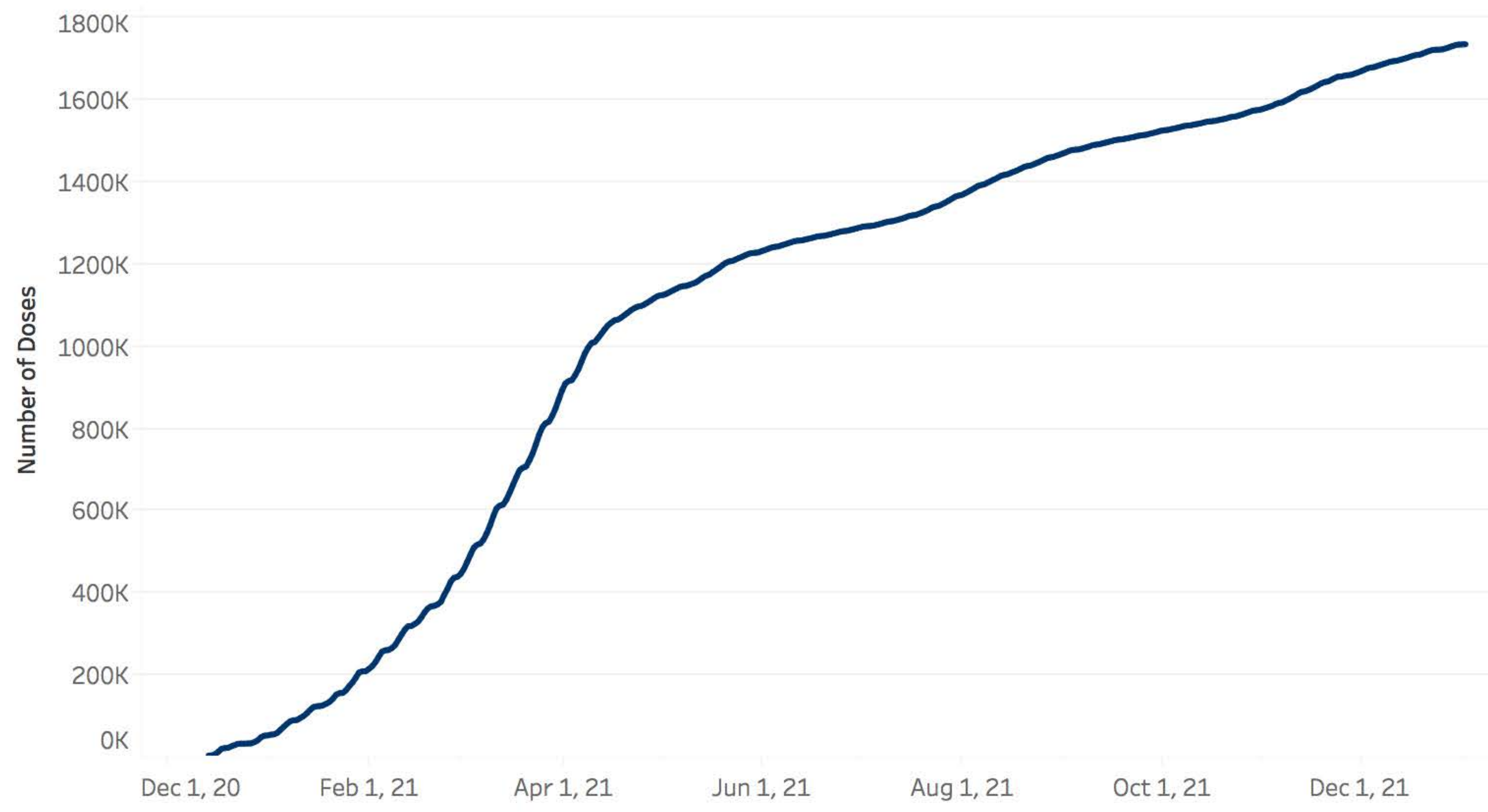

Eligible Population Completed COVID-19 Vaccine Series

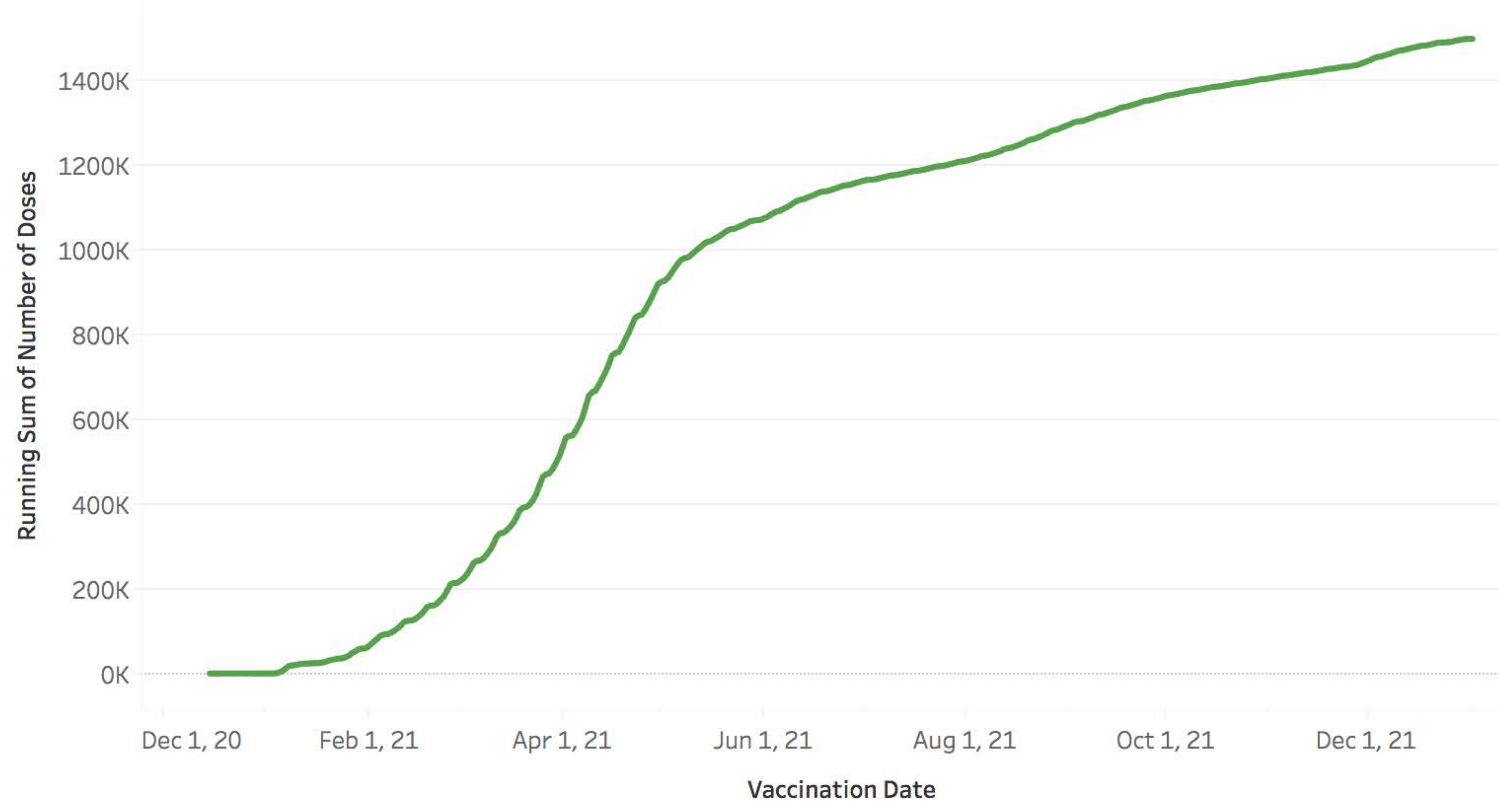

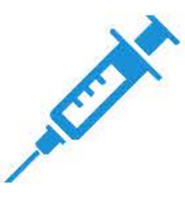

3,889,946
Total Doses Administered
First Dose: 1,853,537
Second Dose: 1,484,778
Third Dose*: 551,632

* Third dose includes all instances of 3 doses reported in KSWebIZ. On 8/12/2021, a third dose was approved for those who are severely immunocompromised. On 9/24/2021, a booster dose was approved for select populations 6 months following completion of an initial series of Pfizer. On 10/21/2021, booster doses for Moderna were approved for select populations 6 months following completion of initial series. Johnson and Johnson booster doses were approved for all recipients 2 months after initial series.

Vaccines Administered by Dose and Manufacturer

Sort by Dose Number
(All)

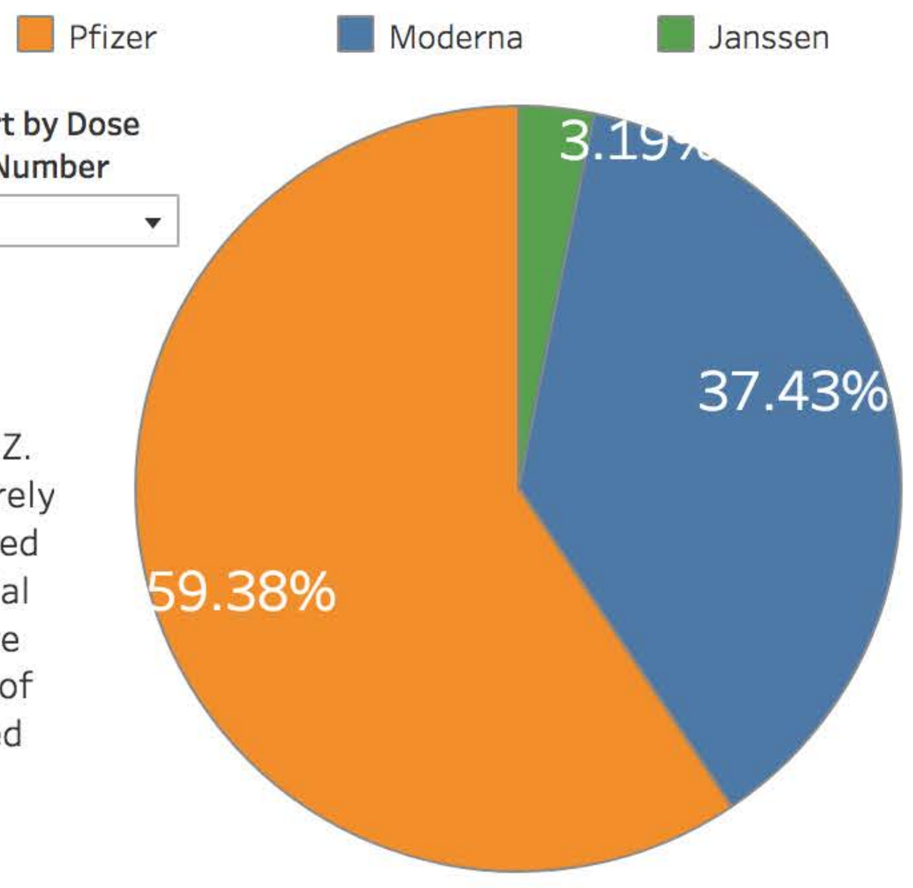

COVID-19 Vaccines Administered by Dose Number

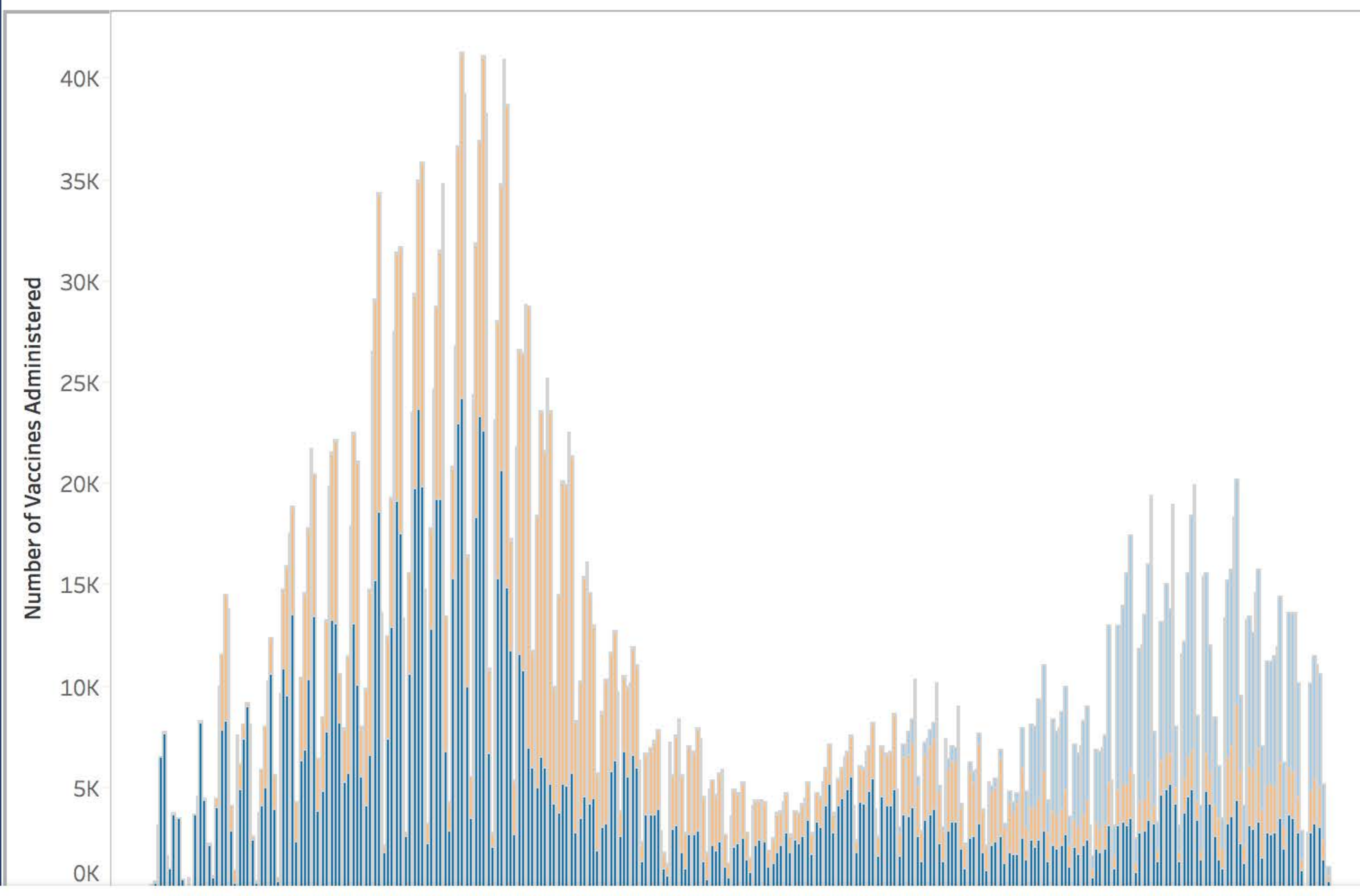

Data

Dashboard Information

The COVID-19 vaccine dashboard is updated Monday, Wednesday and Friday by 12:30 p.m. To maximize your experience, use a device with a larger screen, such as a tablet, laptop or desktop.

November 17 Update

The eligible population of the vaccine administration tab was updated to include the recently approved pediatric population of 5 to 11 years of age and should be interpreted as Kansans 5 years of age and older. Due to this change in population, the percent of eligible population vaccinated with at least one dose and eligible population fully vaccinated has decreased compared to 11/15/2021.

Kansas COVID-19
Demographics

Data are preliminary and subject to quality improvement and quality assurance validation. Race and ethnicity data is reported into the Kansas Immunization Registry (KSWebIZ) by vaccine providers using patient self-identified race and ethnicity. Multiple races are available for selection with the primary race listed first followed by other selected races. Race is reported into the demographics section of a patient record and can be updated as needed; updated race information will apply to all previous and future vaccine administered to that individual.

Data Source: Kansas Immunization Information System, KSWebIZ

^Younger than 18 includes all individuals younger than 18 years of age while the vaccine is only approved for persons 5 years of age and older. This rate includes people who are currently ineligible for the vaccine.

Return to Vaccine Overview

People Vaccinated by Sex
(rate per 1,000 population)

	People Vaccinated	Rates per 1,000
Female	920,567	629.83
Male	807,590	556.31
Unknown/Not Reported	2,859	
Total	1,731,016	

People Vaccinated by Age Group
(rate per 1,000 population)

	People Vaccinated	Rate per 1000
5 to 11	54,135	195.94
12 to 17	122,394	512.90
18 to 24	156,413	531.73
25 to 34	220,087	574.76
35 to 44	240,684	666.66
45 to 54	231,364	705.46
55 to 64	281,446	757.58
65 to 74	248,314	912.13
75 to 84	123,654	898.82
85+	52,380	797.53
Grand Total	1,730,871	

People Vaccinated by Race
(rate per 1,000 population)

race	People with at least 1 ..	Rate of at least 1	People fully vaccinated	Rate fully vaccinated ..
White	1,152,439	458.44	1,405,129	558.96
Black or African Amerian	67,237	376.20	71,980	402.74
American Indian or Alaskan Native	6,185	176.40	6,208	177.05
Asian	48,801	525.52	52,326	563.48
Native Hawaiian or Pacific Islander	1,340	363.44	1,227	332.79
Two or more Races	237,710	2,666.97	320,331	3,593.93
Other	72,832		67,426	
Not Reported/Missing	144,472		137,391	

People Vaccinated by Ethnicity
(rate per 1,000 population)

Ethnicity	People with at least 1	Ethnicity Rate	People fully vaccinated	Ethnicity Rate complete
Hispanic or Latino	174,113	488.98	171,214	480.84
Not Hispanic or Latino	1,342,094	524.82	1,684,926	658.88
Unknown or Missing	214,809		205,878	

Specify Race
(Multiple values)

Vaccination Rate by Age Group and Race
(rate per 1,000 population)

Data Table

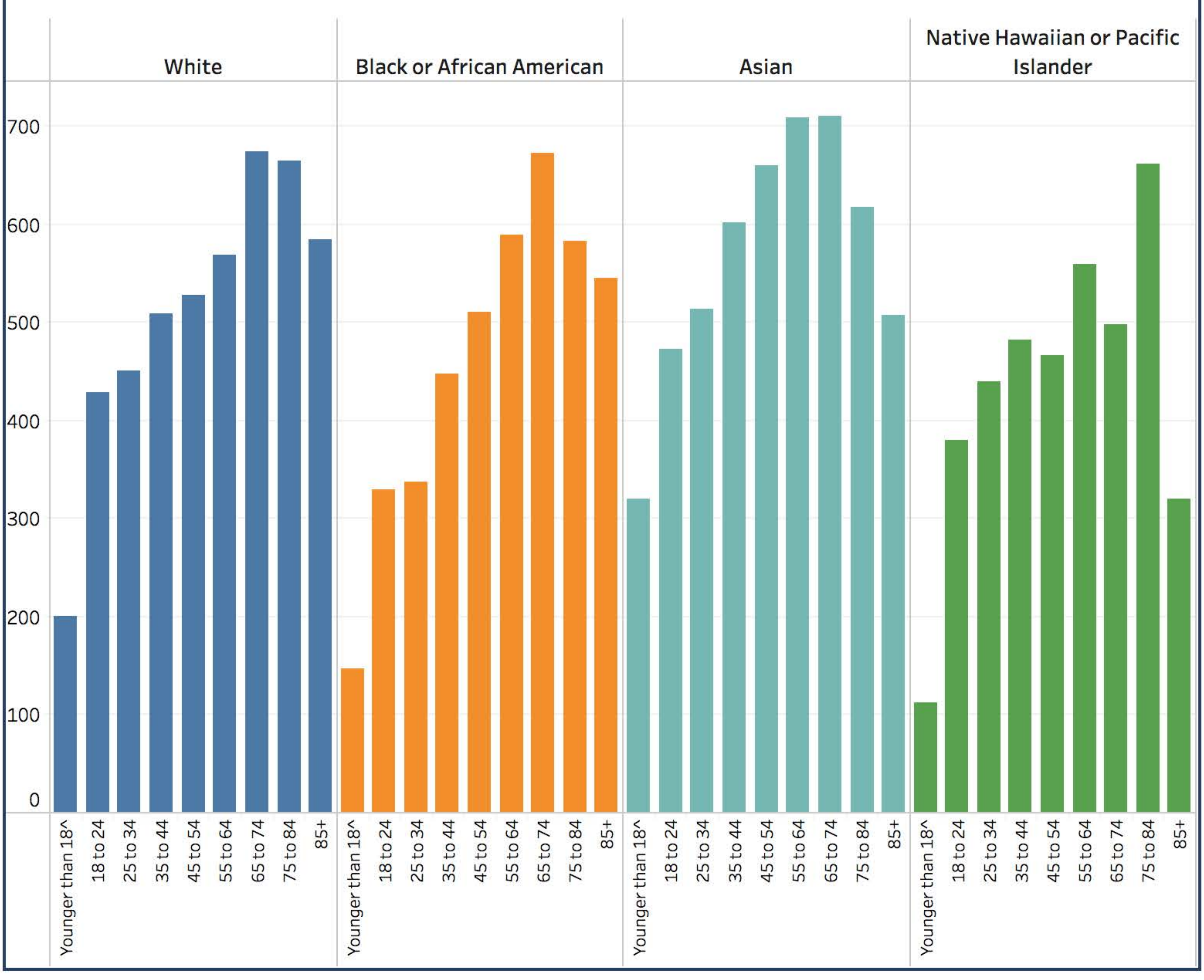

Vaccination Rate by Age Group and Ethnicity
(rate per 1,000 population)

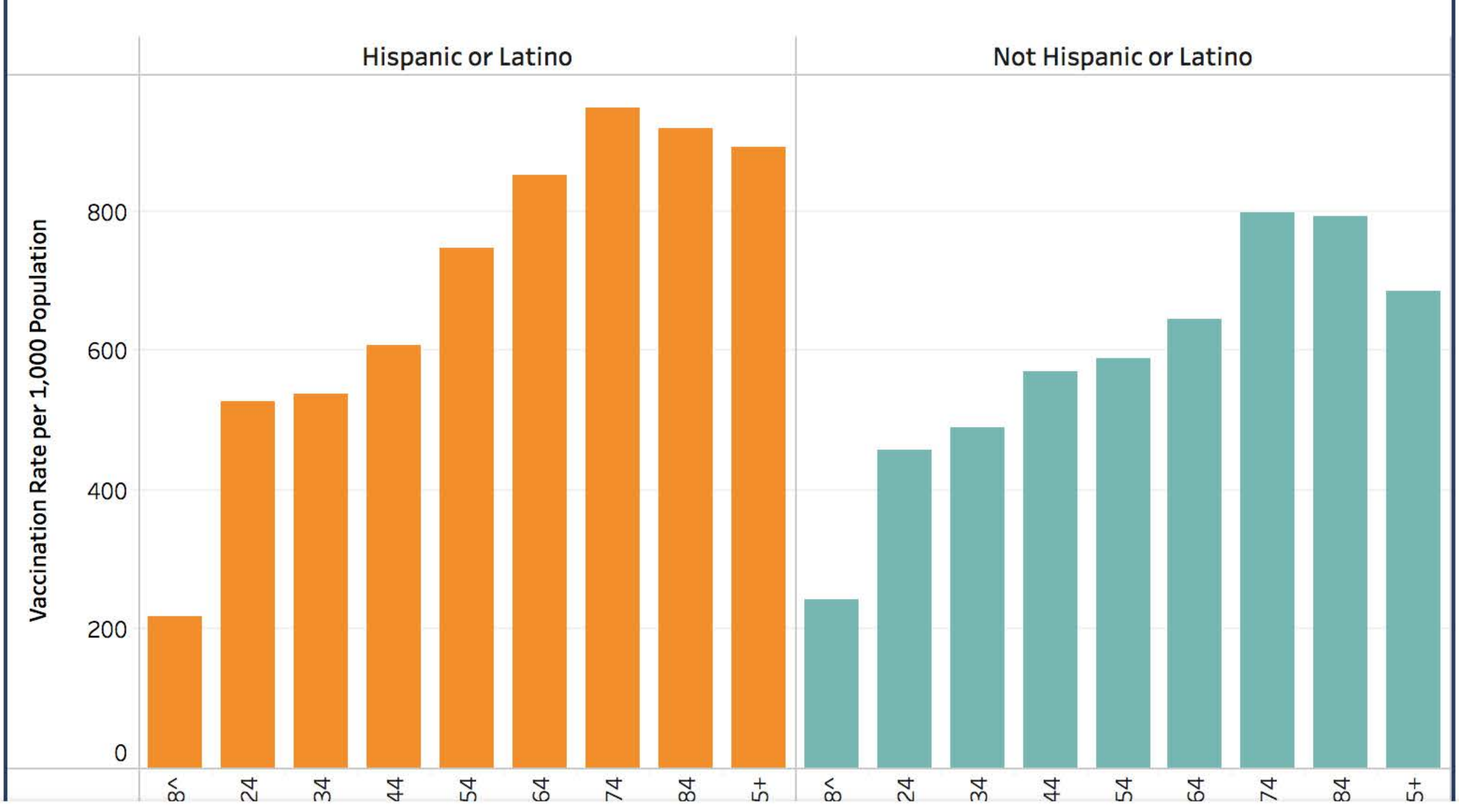

oviders Vaccine Info Data Find My Vaccine Español
Search
Undo Redo Revert Refresh Pause Metrics Share Do
Select Language

Home › Data

Data

Dashboard Information

The COVID-19 vaccine dashboard is updated Monday, Wednesday and Friday by 12:30 p.m. To maximize your experience, use a device with a larger screen, such as a tablet, laptop or desktop.

November 17 Update

The eligible population of the vaccine administration tab was updated to include the recently approved pediatric population of 5 to 11 years of age and should be interpreted as Kansans 5 years of age and older. Due to this change in population, the percent of eligible population vaccinated with at least one dose and eligible population fully vaccinated has decreased compared to 11/15/2021.

Kansas COVID-19 Vaccination Rates

Information is provided below on vaccine coverage for people 5 years and older. Information is also provided on vaccine coverage for the total population as a supplement for determining herd immunity.
Vaccine coverage for at least 1 dose includes all individuals who have received their first dose of COVID-19 vaccine. Vaccine coverage for series completion includes all individuals who have completed 2 doses of Pfizer-BioNTech or Moderna vaccine or 1 dose of Johnson and Johnson/Janssen.
Data Source: Kansas Immunization Information System, KSWebIZ

Additional Age Groups | Return to Vaccine Overview

Below Charts Use 5 and Older County Population For Rates

Rate of People ≥ 5 Years of Age Vaccinated with at least 1 Dose

(rate per 1,000 population ≥ 5 years of age; as reported in KSWebIZ)

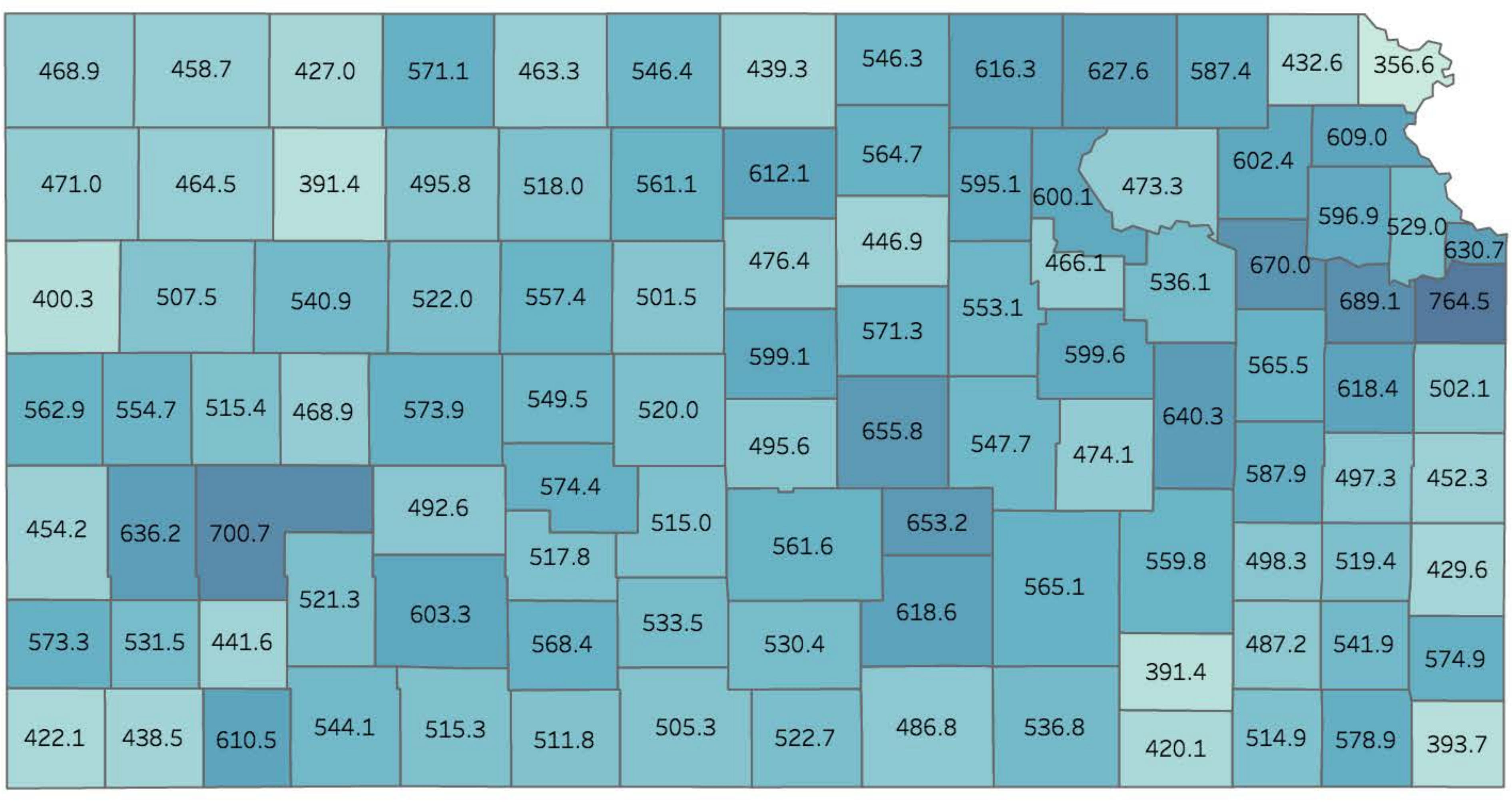

Rate of People ≥ 5 Years of Age with Completed Vaccine Series

(rate per 1,000 population ≥ 5 years; as reported in KSWebIZ)

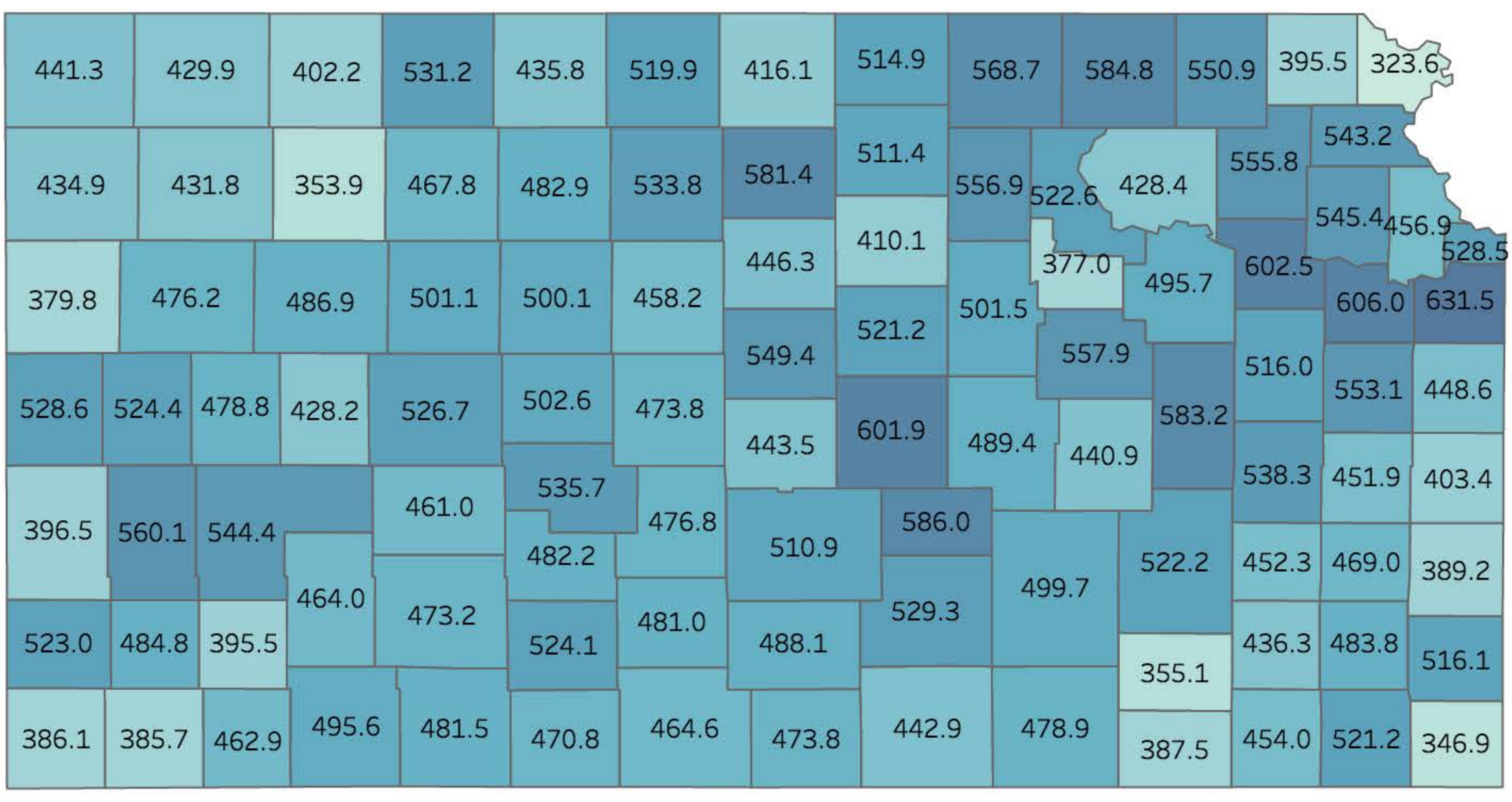

Below Charts Use Total County Population for Rates

Rate of People Vaccinated with at least 1 Dose

(rate per 1,000 total population; as reported in KSWebIZ)

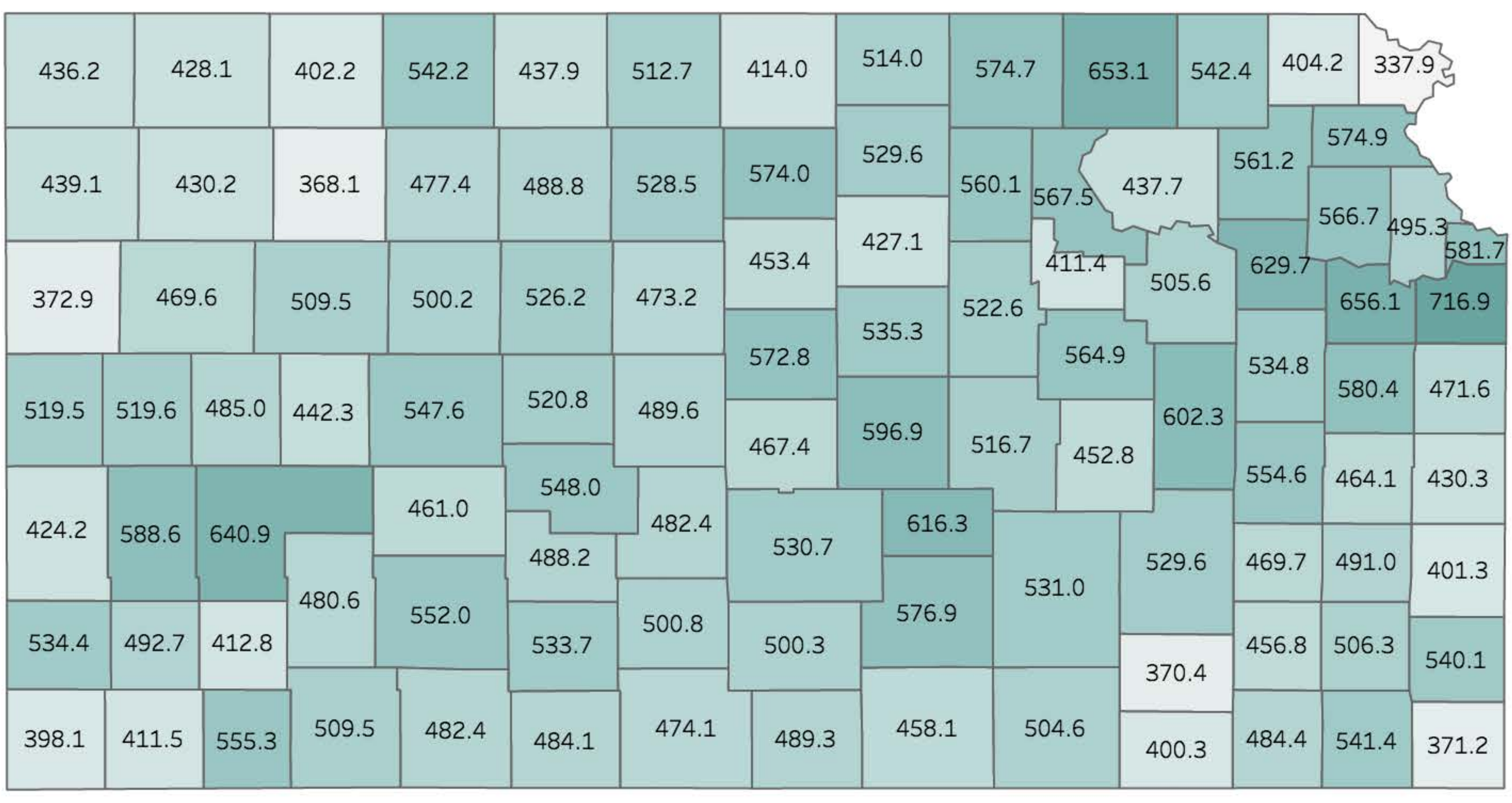

Rate of People with Completed Vaccine Series

(rate per 1,000 total population; as reported in KSWebIZ)

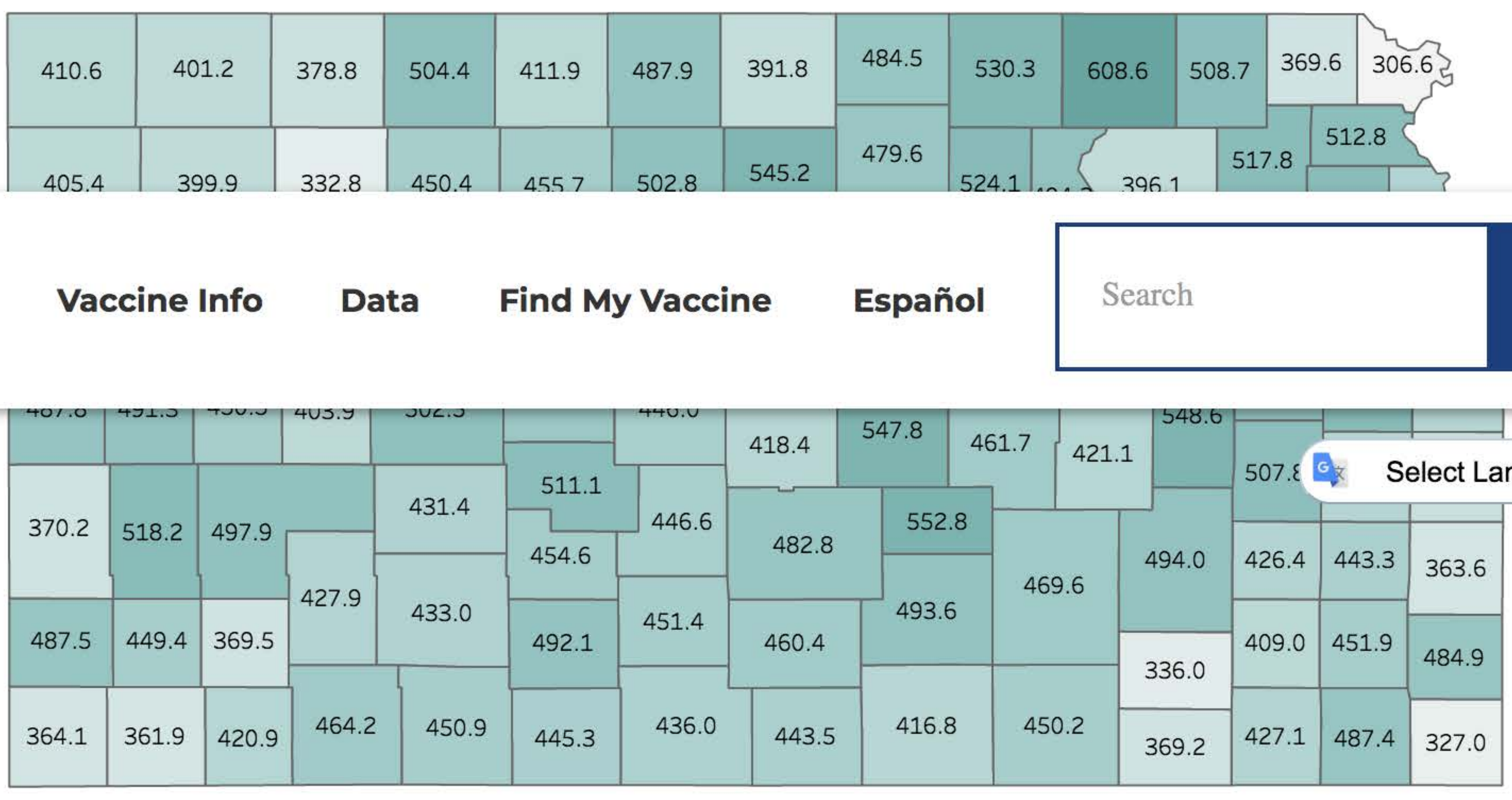

Undo Redo Revert Refresh Pause Metrics Share Download Full Screen

COVID-19 Vaccine Dashboard

Looking for COVID-19 Case Data? Click here.

Michigan First-Dose Tracker

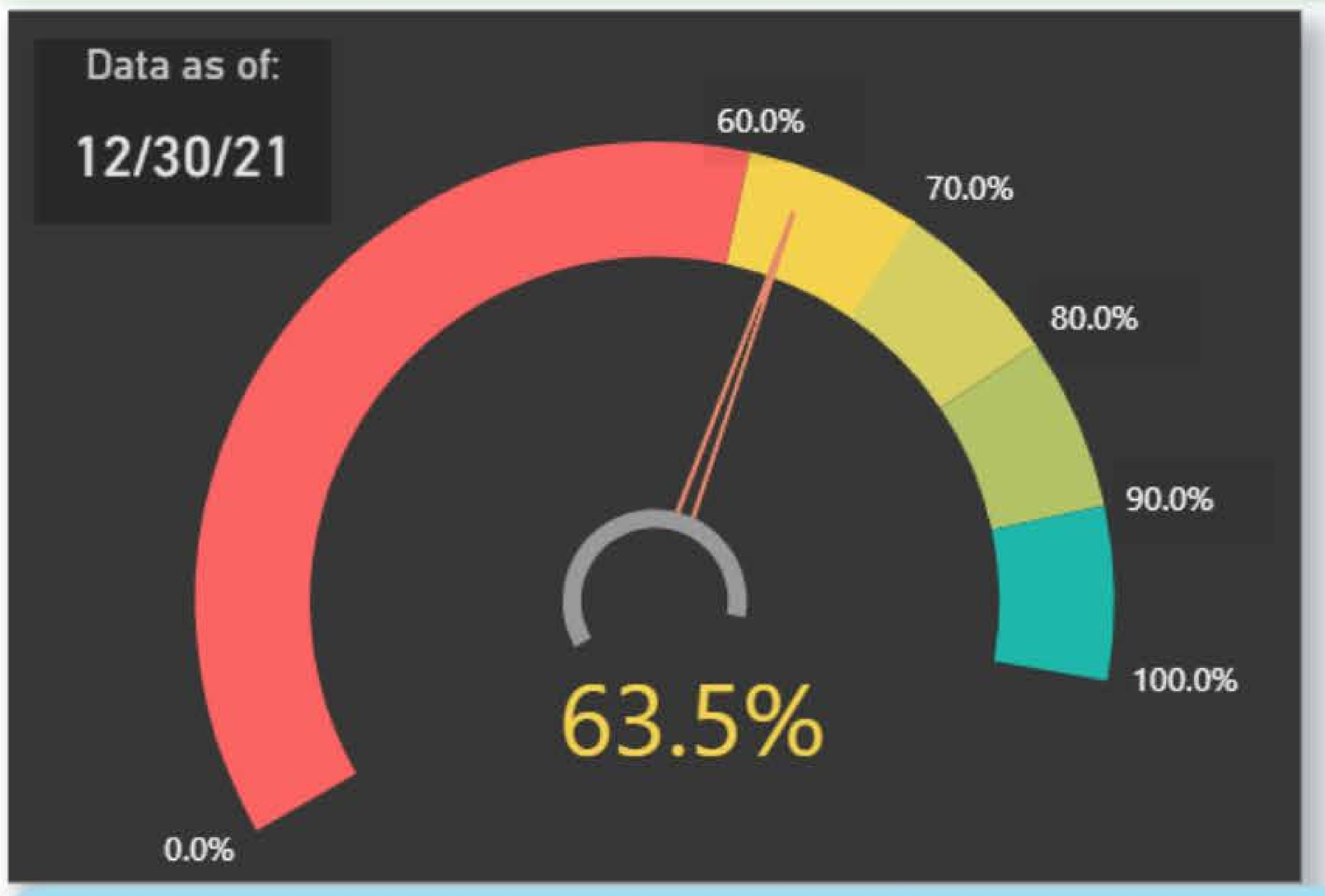

6,337,587 MI Residents with 1+ dose

÷

9,984,503 Total MI Population

=

63.5%

For Reference, 70% is equal to 6,989,152 Michigan Residents
80% is equal to 7,987,602 Michigan Residents
90% is equal to 8,986,053 Michigan Residents

The dial tracker was updated based on data from December 30th because the CDC data tracker on which it is based was not updated since December 30, 2021

Progress is based on the CDC data tracker, which includes MI residents vaccinated by providers not currently reporting to the state dashboard below: Veterans Affairs, Department of Defense, Bureau of Prisons, and most out-of-state providers. Data provided in the Michigan COVID-19 Dashboard below slightly undercounts the true number of doses administered to MI residents. See the "Learn More" tab below for details.

First-dose tracker above updated 3pm daily, except when CDC's data tracker updates later; then it is updated the following morning.

First-dose tracker above is for entire Michigan Population. Coverage maps below are for age 5+.

Vaccine Delivered | Delivery Trend | Primary Doses Adm | Ethnicity | Enrolled Providers

COVID Vaccination Coverage - Initiation

Dashboard Updated: December 28, 2021. "Completion" is the percentage of Michigan residents receiving 2 doses of Pfizer or Moderna or 1 dose of J&J. "Initiation" is the percentage who have received either 1 or more doses of ANY vaccine. See the "Learn More" page to learn how percentages are calculated.

Michigan

Preparedness Region: All | Local Health Dept. Jurisdiction: All | County: All

Initiation | Completion | Coverage Slicers

COVID Vaccine Coverage by County

Week Ending Date

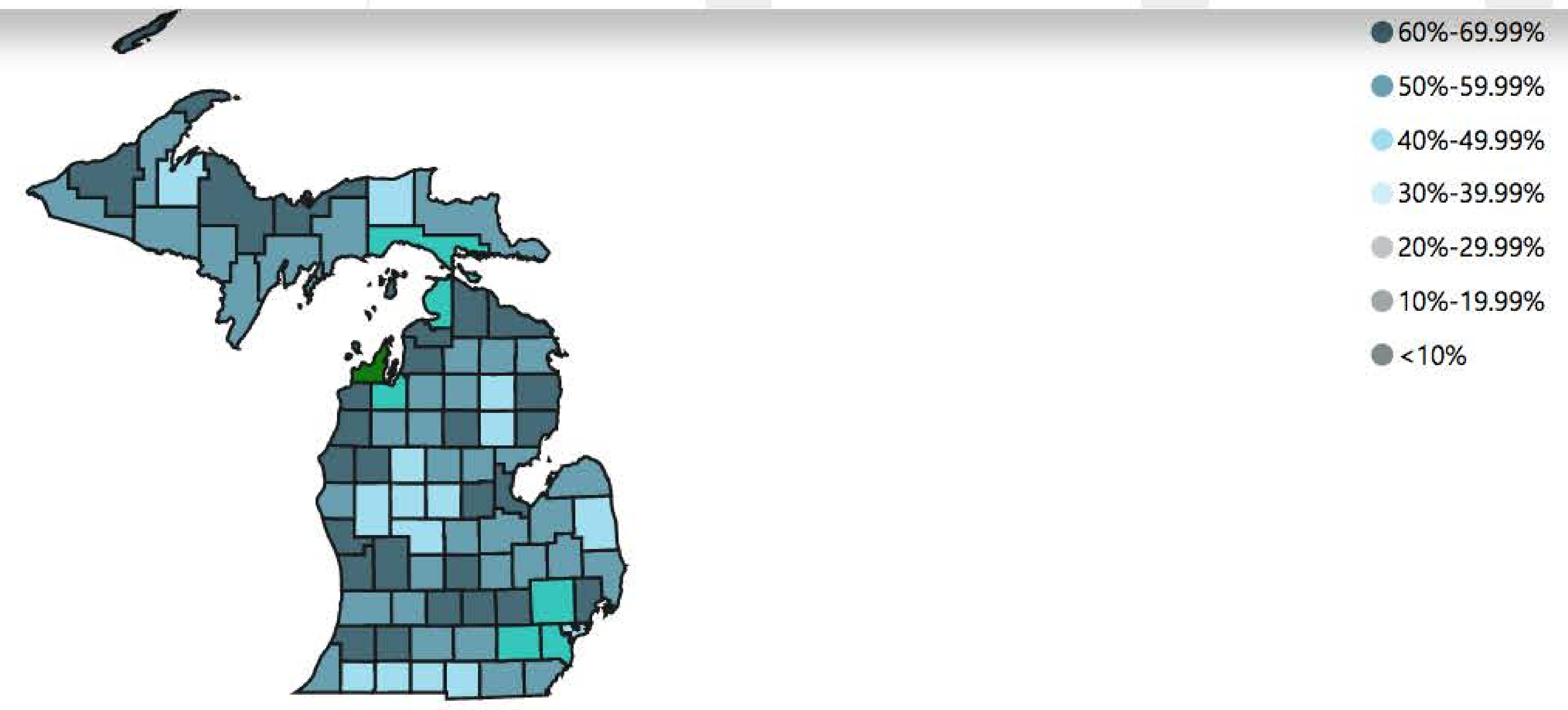

Residents Vaccinated
5,933,585

Coverage (% of Residents Vaccinated)
63.0%

MI Population*
9,420,414

*2019 US Census estimates for persons 5 years of age and older

Vaccine Data

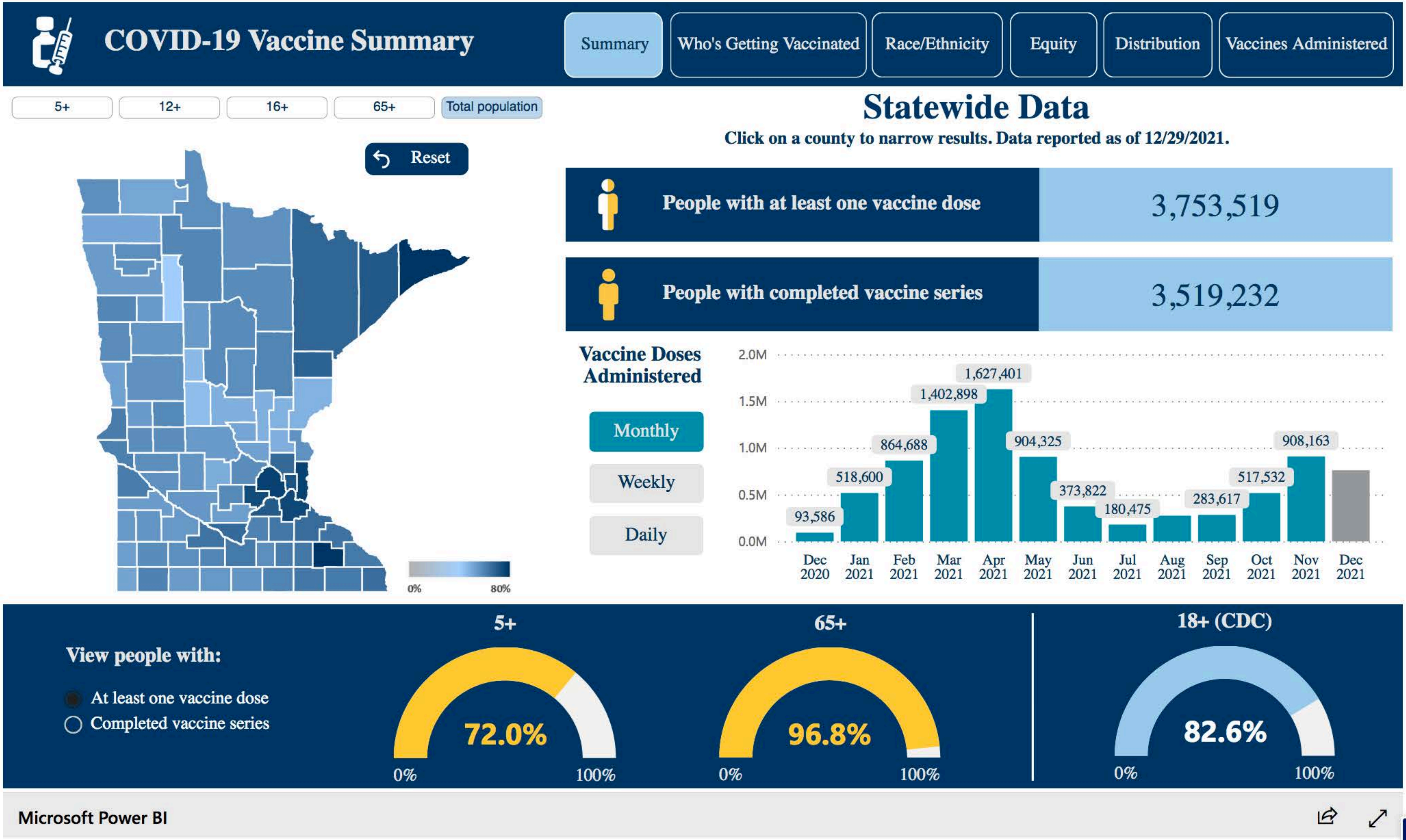

Vaccine Data

Who's getting vaccinated

Summary | Who's Getting Vaccinated | Race/Ethnicity | Equity | Distribution | Vaccines Administered

5+ | 12+ | 16+ | 65+ | Total population

At least one vaccine dose | Completed vaccine series

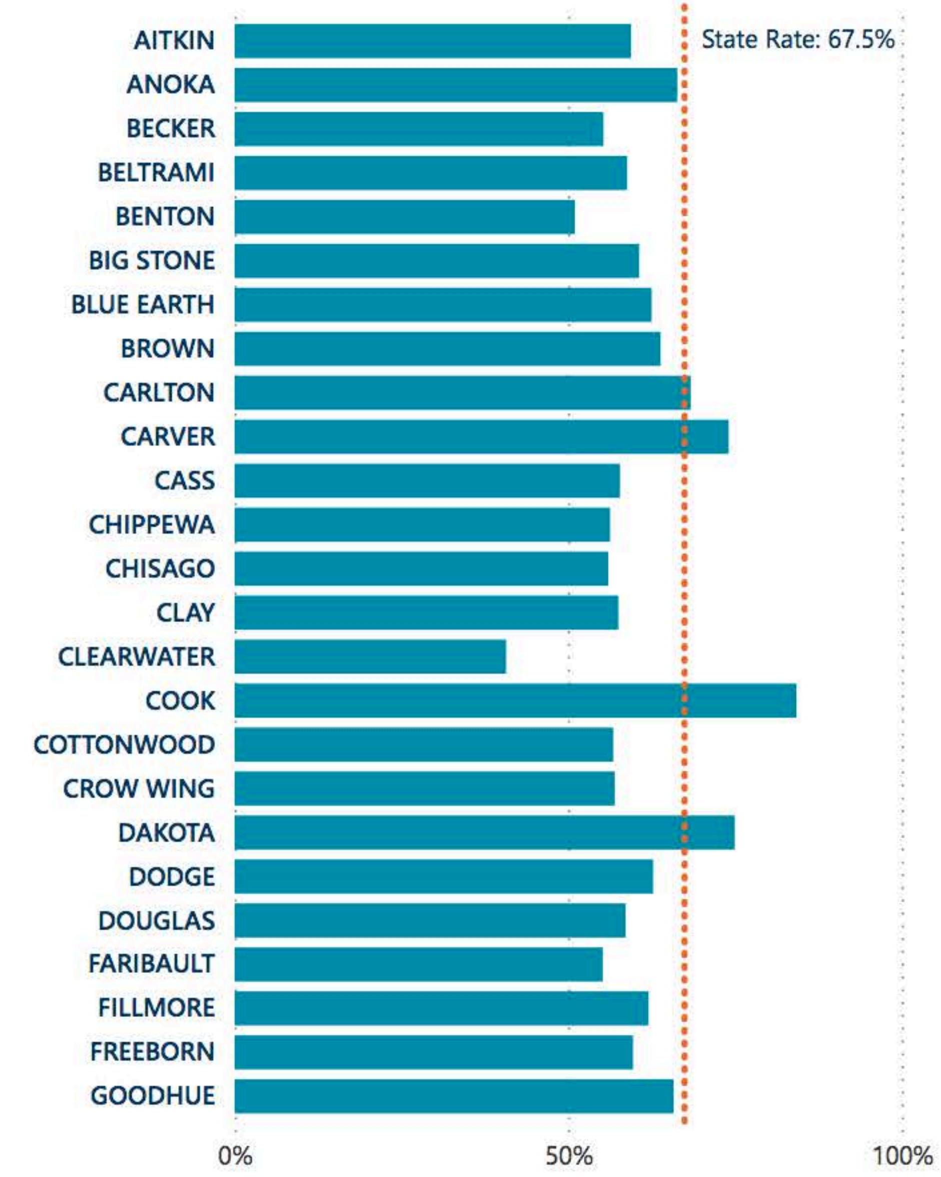

Statewide Data

Click on a county to narrow results. Data reported as of 12/29/2021.

**People with at least one vaccine dose**	3,753,519	**67.5%**
**People with completed vaccine series**	3,519,232	**63.3%**

Age group | Gender

Age group	People with at least one vaccine dose	People with completed vaccine series
5-11	159,530	117,742
12-15	181,605	167,049
16-17	94,356	88,694
18-49	1,612,974	1,504,888
50-64	873,929	841,917
65+	830,881	798,925
Unknown/missing	244	17
**Total**	**3,753,519**	**3,519,232**

Proportion of People Vaccinated by Age Group Population

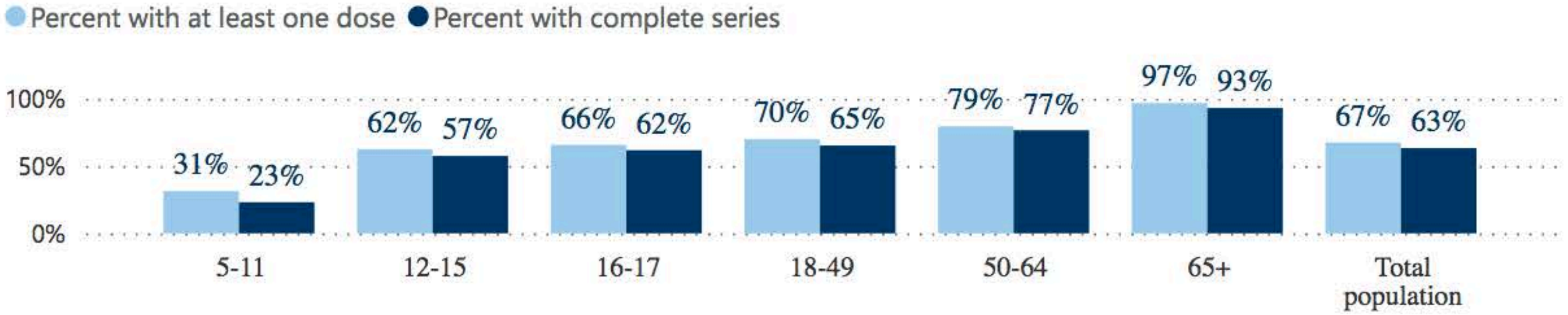

Vaccine Data

Race and Ethnicity

Summary | Who's Getting Vaccinated | Race/Ethnicity | Equity | Distribution | Vaccines Administered

Reset filter

View by people with:

- At least one vaccine dose
- Completed vaccine series

View by age group:

- 5+
- 5-11
- 12+
- 12-18
- 19-44
- 45-64
- 65+

The race/ethnicity data provided on this dashboard accounts for 93% of the people who have a COVID-19 immunization record with MDH. It also includes immunizations from VA records which are not included in immunization records with MDH. For the remaining records, race/ethnicity data is not available, unknown, or missing. This percentage may vary slightly for each age demographic.

Data reported as of: **12/11/2021**
Updated weekly on Friday.

Progress with Vaccination by Race and Ethnicity, Age 5+

Totals To Date | Weekly Progress

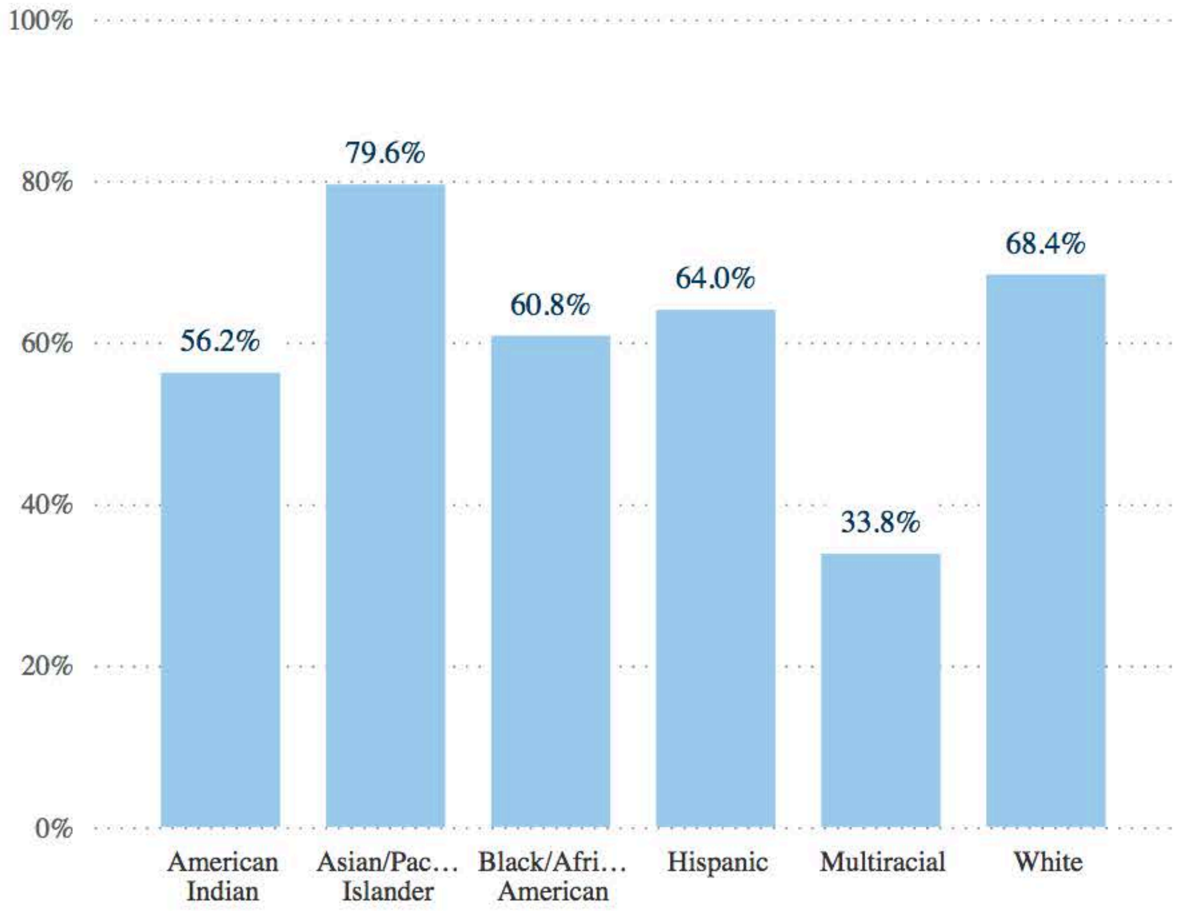

Vaccination Progress by Race and Ethnicity, Age 5+

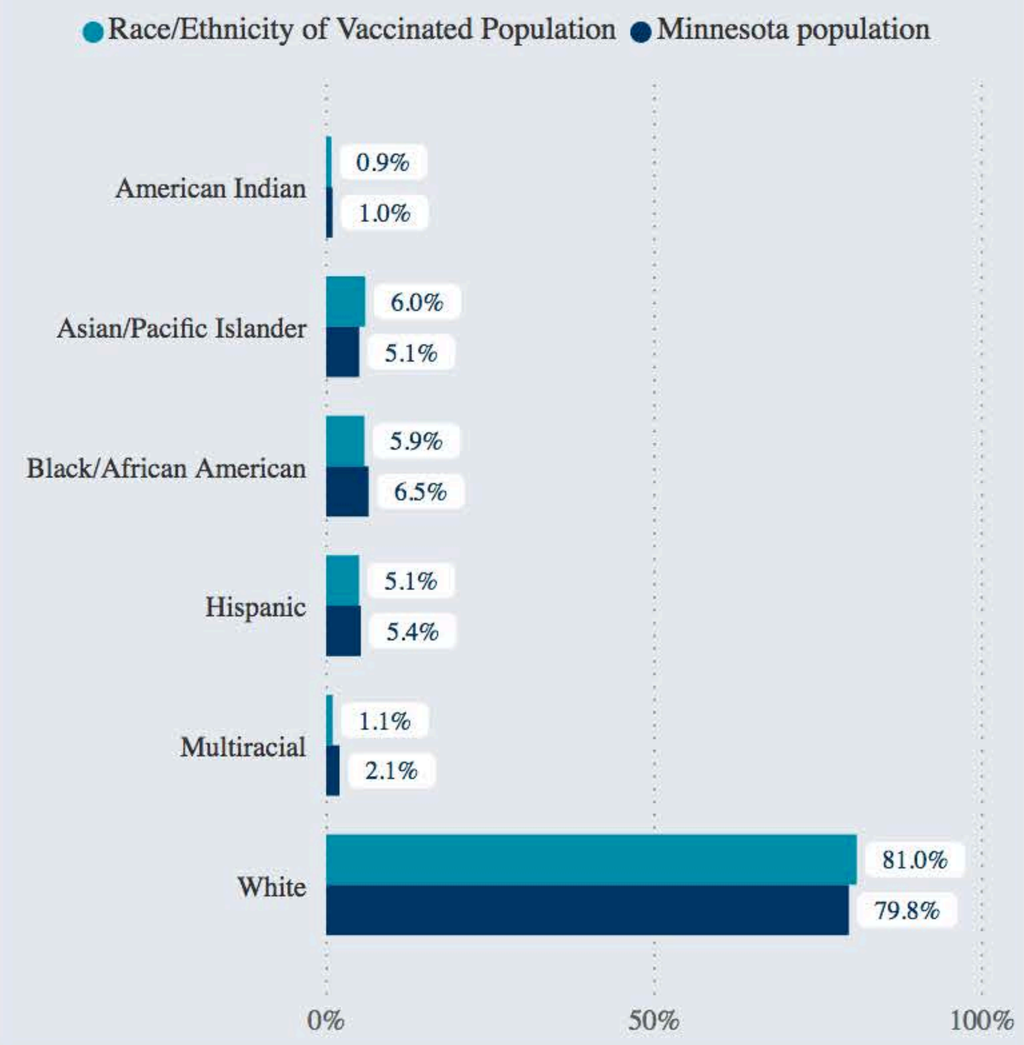

View additional information about COVID-19 cases by Race/Ethnicity

Microsoft Power BI   Pages

Vaccine Data

Vaccines Administered

Summary | Who's Getting Vaccinated | Race/Ethnicity | Equity | Distribution | Vaccines Administered

Total vaccine doses administered

8,711,845

Product	Percent	Doses Administered
Pfizer	59.5%	5,184,252
Moderna	36.7%	3,195,420
Johnson & Johnson	3.8%	330,090
Unknown/missing	0.0%	2,083
**Total**	**100.0%**	**8,711,845**

Third doses and booster doses administered

1,712,538

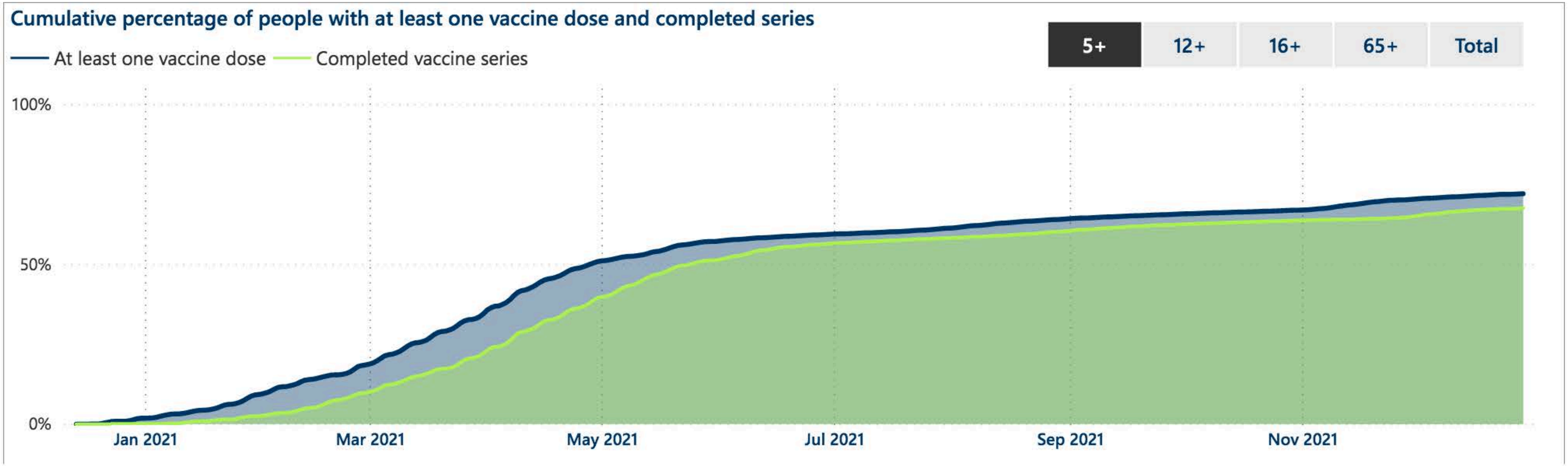

Data reported as of **12/29/2021**

Microsoft Power BI Pages

Dashboard Last Updated Monday, 1/3/2022

Data as of Sunday, 1/2/2022

Coverage Rates | Doses Administered | Demographics | County Level | Enrolled Providers

COVID-19 Vaccine Coverage Rates

Select year and month to view data within a specific time-frame.

	5 and older	12 and older	18 and older	65 and older
At least one dose	54.8% 412,077 ND residents	59.6% 399,499 ND residents	61.8% 375,301 ND residents	84.3% 102,831 ND residents
Primary series complete	51.7% 388,793 ND residents	56.8% 380,347 ND residents	59.0% 358,247 ND residents	80.3% 98,339 ND residents
Booster dose			42.7% 153,063 ND residents	69.9% 66,917 ND residents

Select Year: All | Select Month: All | Select County: All

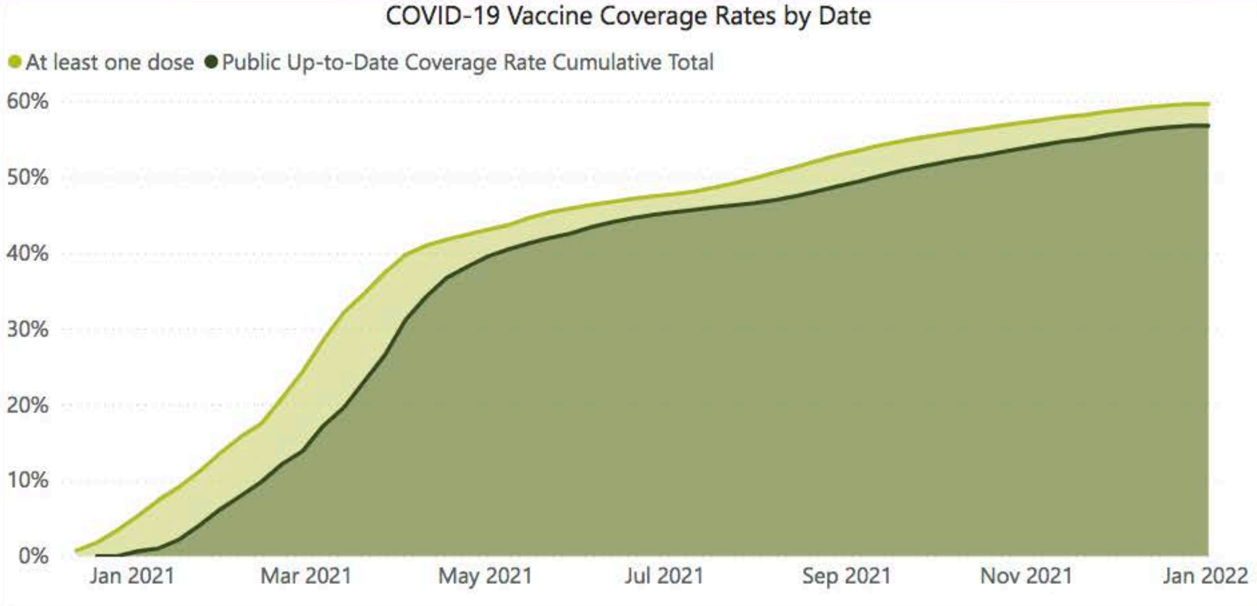

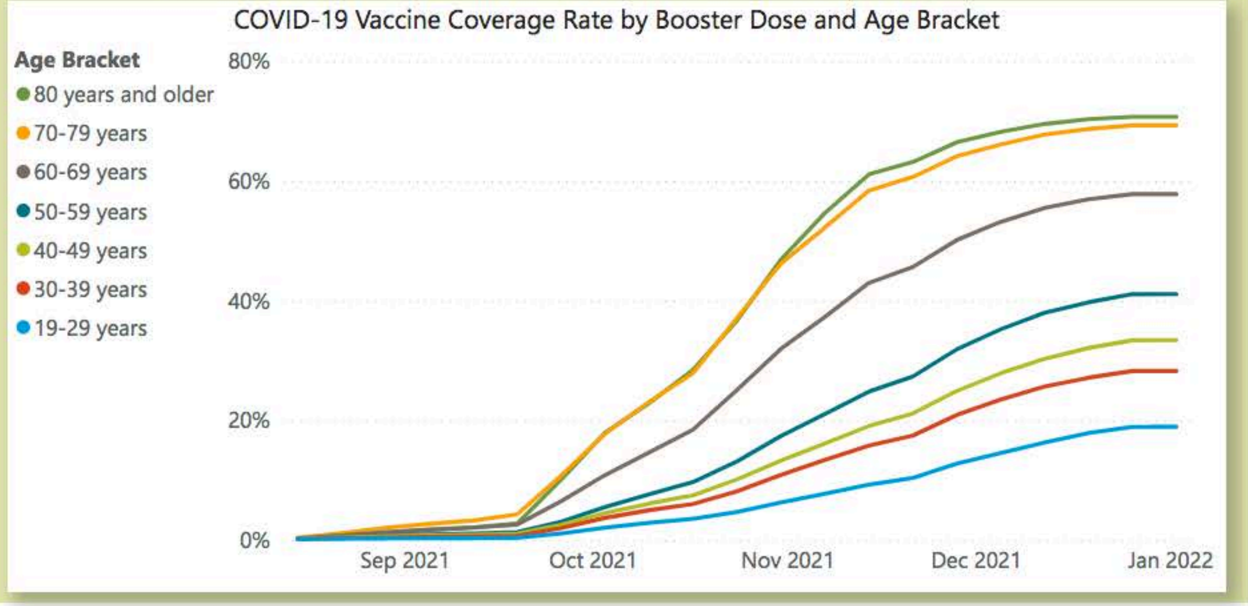

Select View: At least one dose | Primary series complete

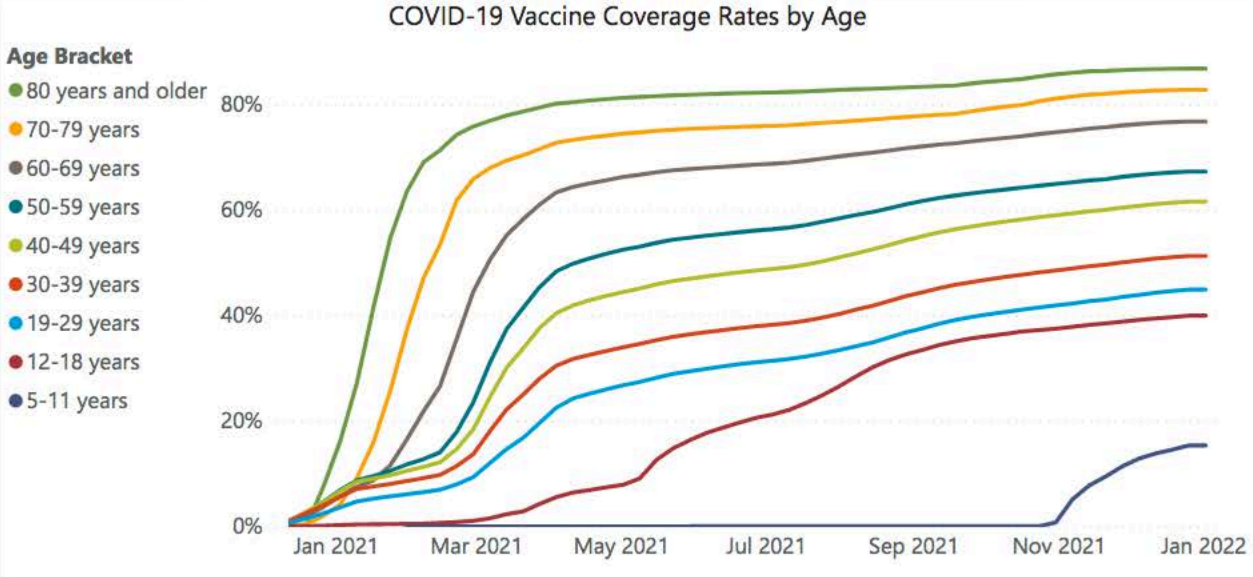

Dashboard Last Updated Monday, 1/3/2022

Data as of Sunday, 1/2/2022

Coverage Rates | Doses Administered | Demographics | County Level | Enrolled Providers

COVID-19 Vaccine Doses Administered

976,906
Total Doses Administered

562,555
Pfizer 12+ Doses

23,159
Pfizer 5-11 Doses

353,641
Moderna Doses

37,534
Janssen Doses

Select Year: All | **Select Month:** All

Cumulative Daily Total COVID-19 Vaccine Doses Administered

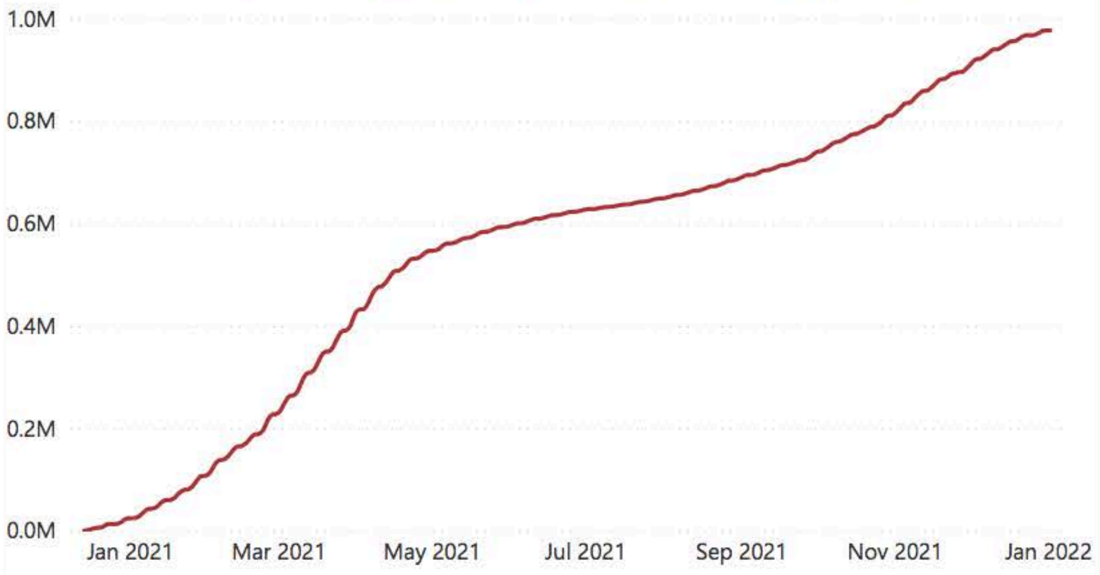

Cumulative Daily Doses Administered by Dose Number

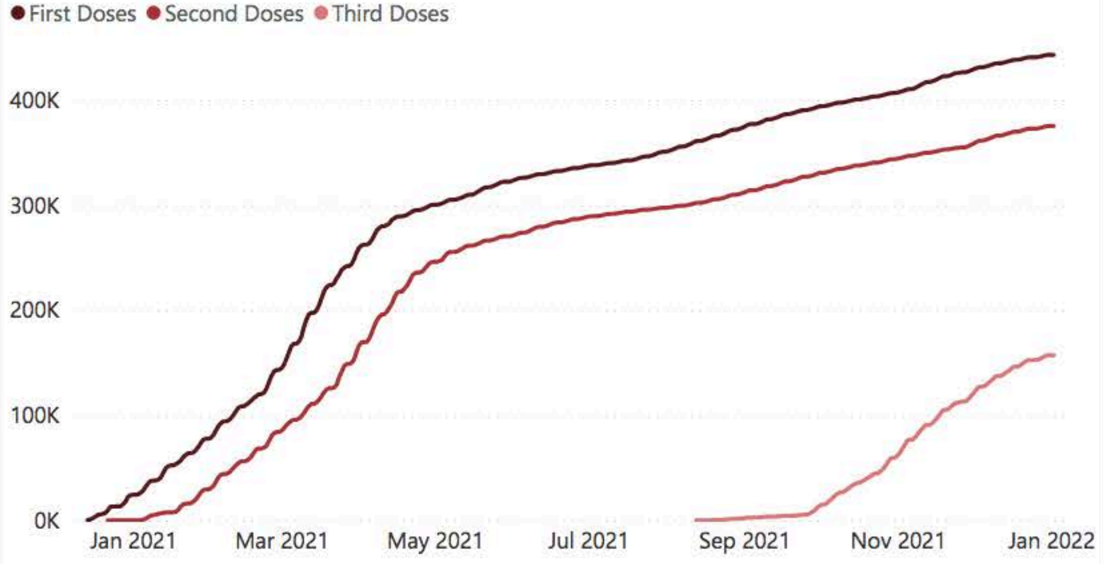

Comparison of the Previous and Current Month Doses Administered

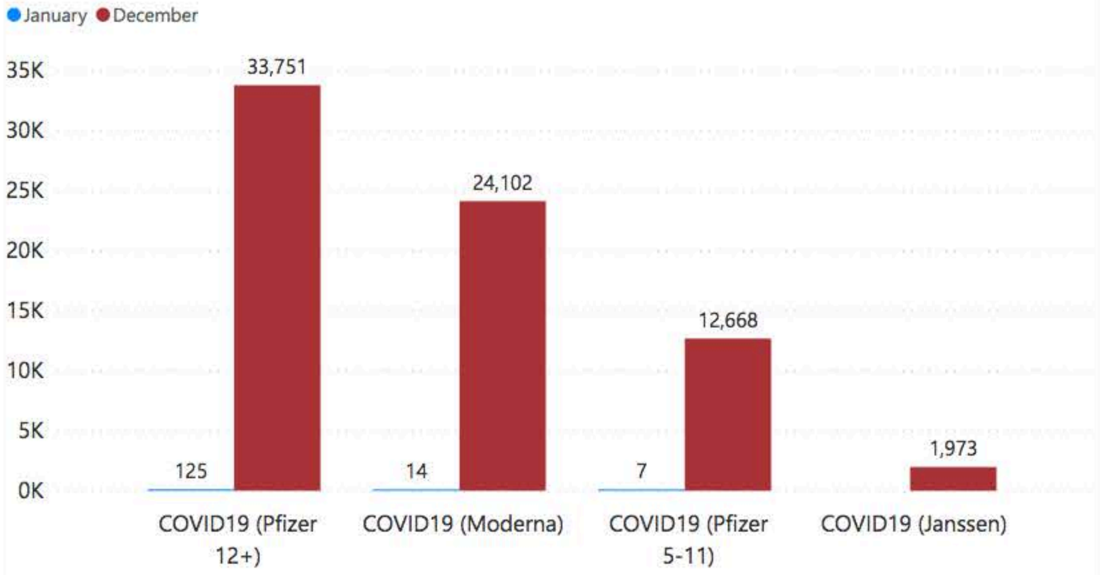

Doses Total Administered by Day for the Past 14 days

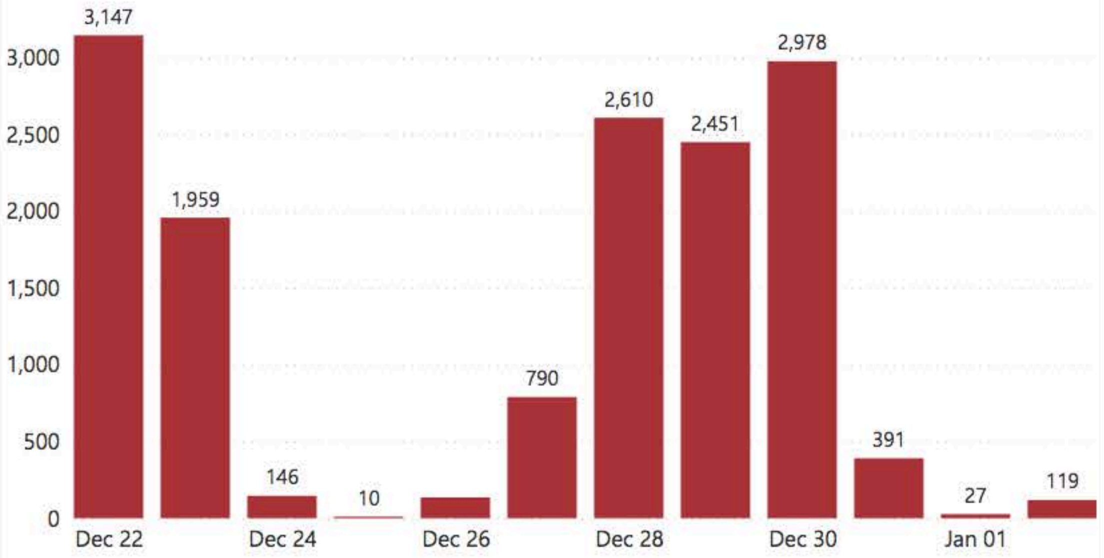

Dashboard Last Updated Monday, 1/3/2022

Data as of Sunday, 1/2/2022

Coverage Rates | Doses Administered | Demographics | County Level | Enrolled Providers

COVID-19 Vaccine Demographics

Select Year: All | **Select Month:** All

Coverage Rates by Age

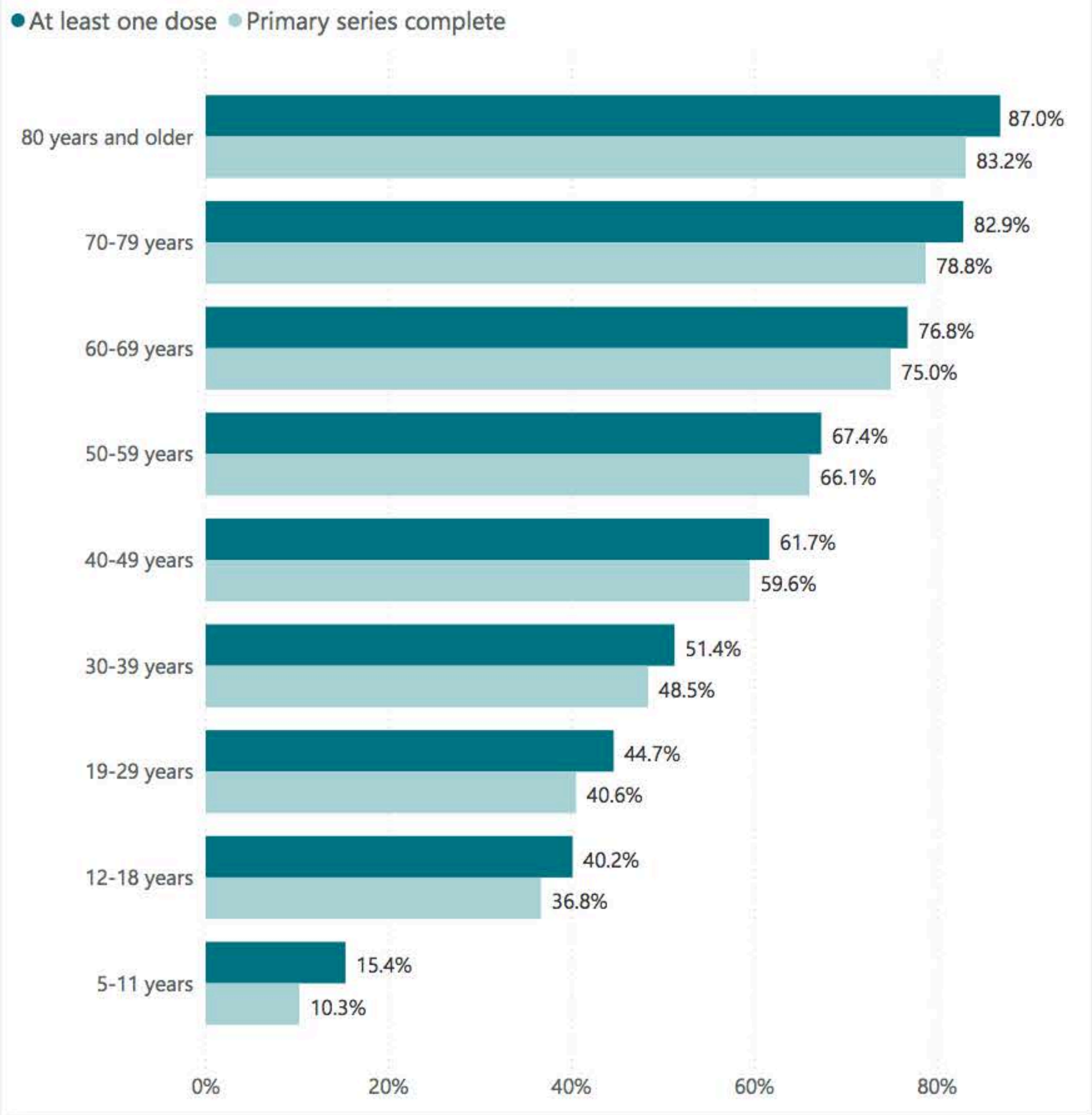

Coverage Rates by Sex

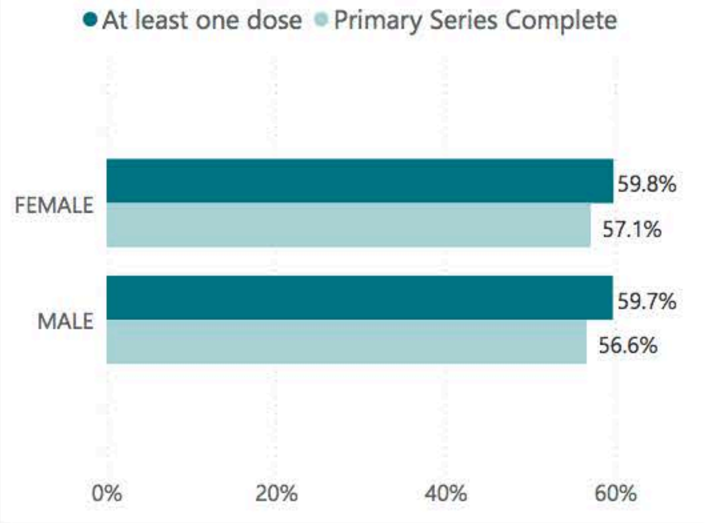

Coverage Rates by Ethnicity

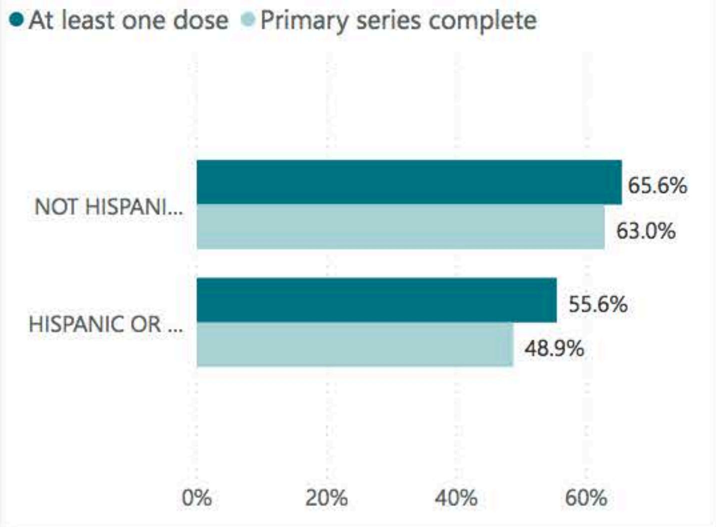

Coverage Rates by Race

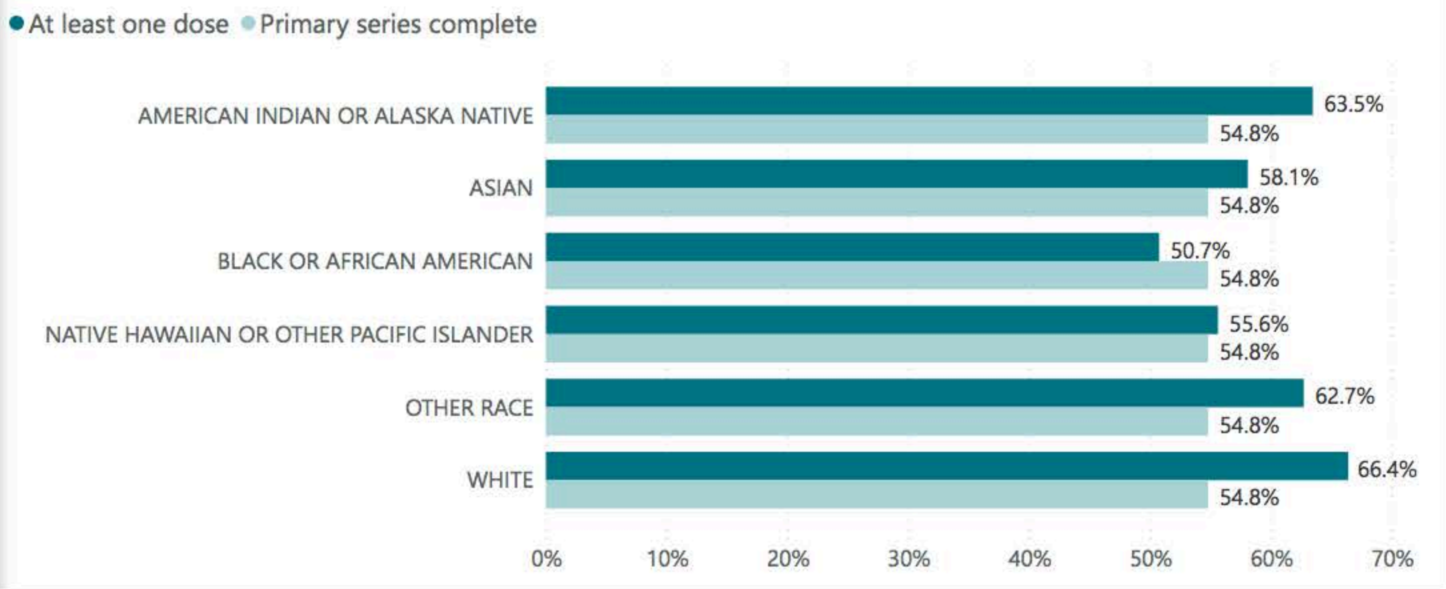

Dashboard Last Updated Monday, 1/3/2022

Data as of Sunday, 1/2/2022

Coverage Rates | Doses Administered | Demographics | County Level | Enrolled Providers

COVID-19 Vaccine County Level Coverage

976,906
Total Doses Administered

562,555
Pfizer 12+ Doses

23,159
Pfizer 5-11 Doses

353,641
Moderna Doses

37,534
Janssen Doses

Select Year: All

Select Month: All

Select At least one dose or Primary series complete

At least one dose | Primary series complete

Hover over county to view Total COVID-19 Vaccine Doses Administered

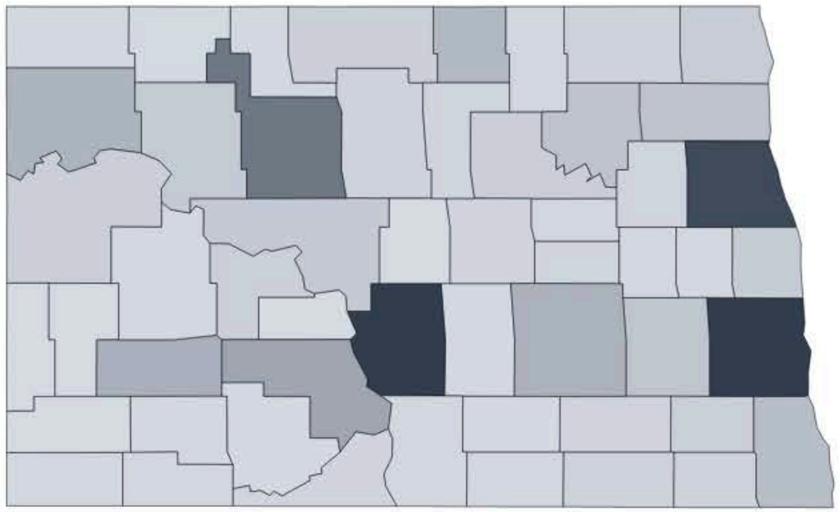

Scroll down to view Total COVID-19 Vaccine Doses Administered

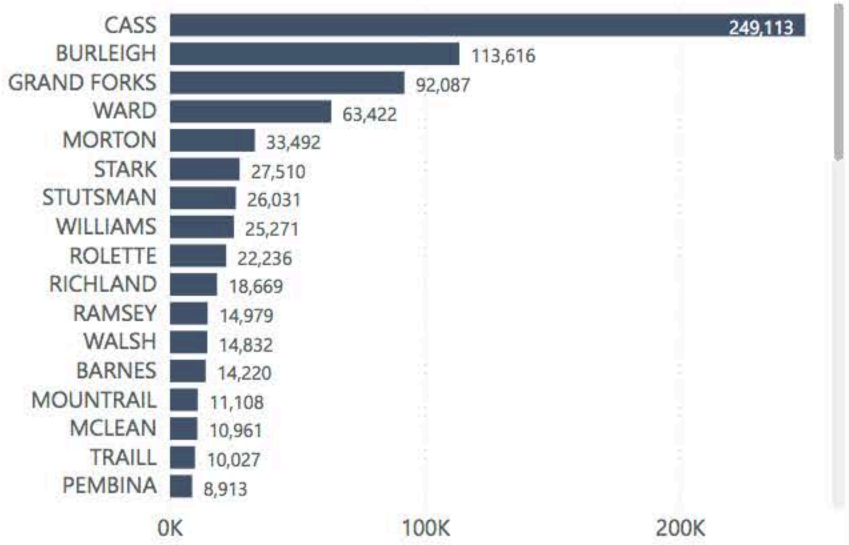

5 and older
54.8%
412,077 ND residents

12 and older
59.6%
399,499 ND residents

18 and older
61.8%
375,301 ND residents

65 and older
84.3%
102,831 ND residents

Hover over county to view COVID-19 Vaccine At least one dose

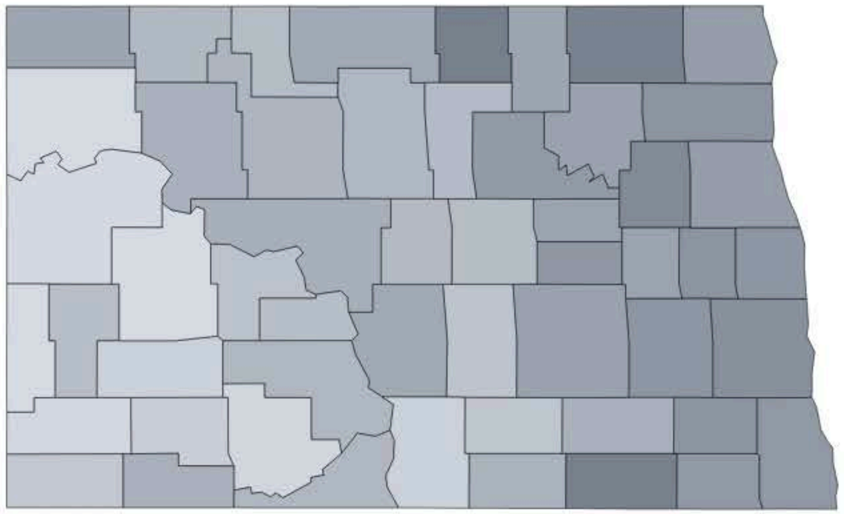

Scroll down to view COVID-19 Vaccine Coverage Rates (5 and older)

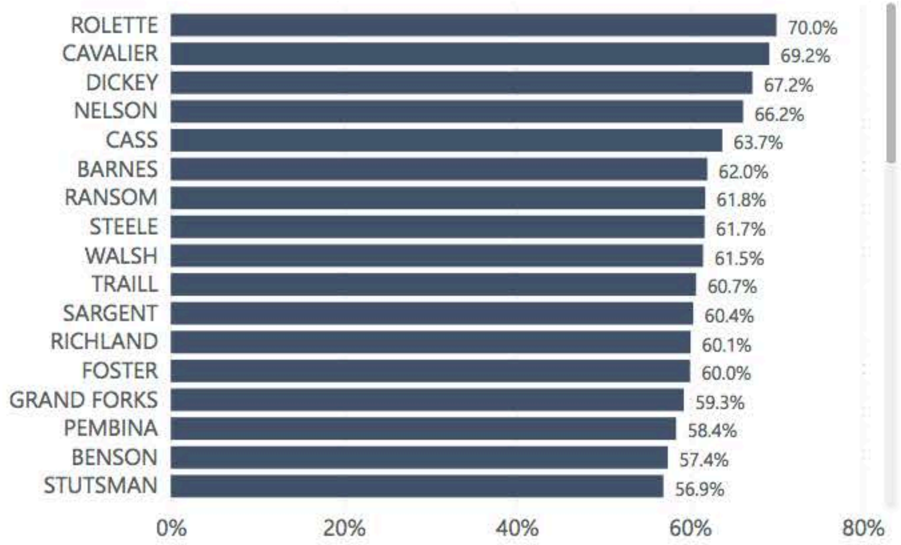

New Mexico Department of Health

COVID-19 Vaccine Dashboard

The New Mexico Department of Health (NMDOH) is leading the State of New Mexico's COVID-19 Vaccination Preparedness Planning in close collaboration with other state agencies, public, private and tribal partners throughout the state.

Learn More

Interested in Phase-Specific Vaccine Data?

If you are interested in information on New Mexico's progress through the phase-based distribution plan, please click here.

Doses Administered By County (State And Federal Data Combined)

Due to changes in the federal vaccine database and/or updated information shared with NMDOH, changes to the attribution of federal data by county may occur.

County-level data is based on the 18+ population.

County-level data will be updated weekly. Includes data through 1/03/2022

BASE LAYER

Percent Of Residents With Vaccination Series Completed

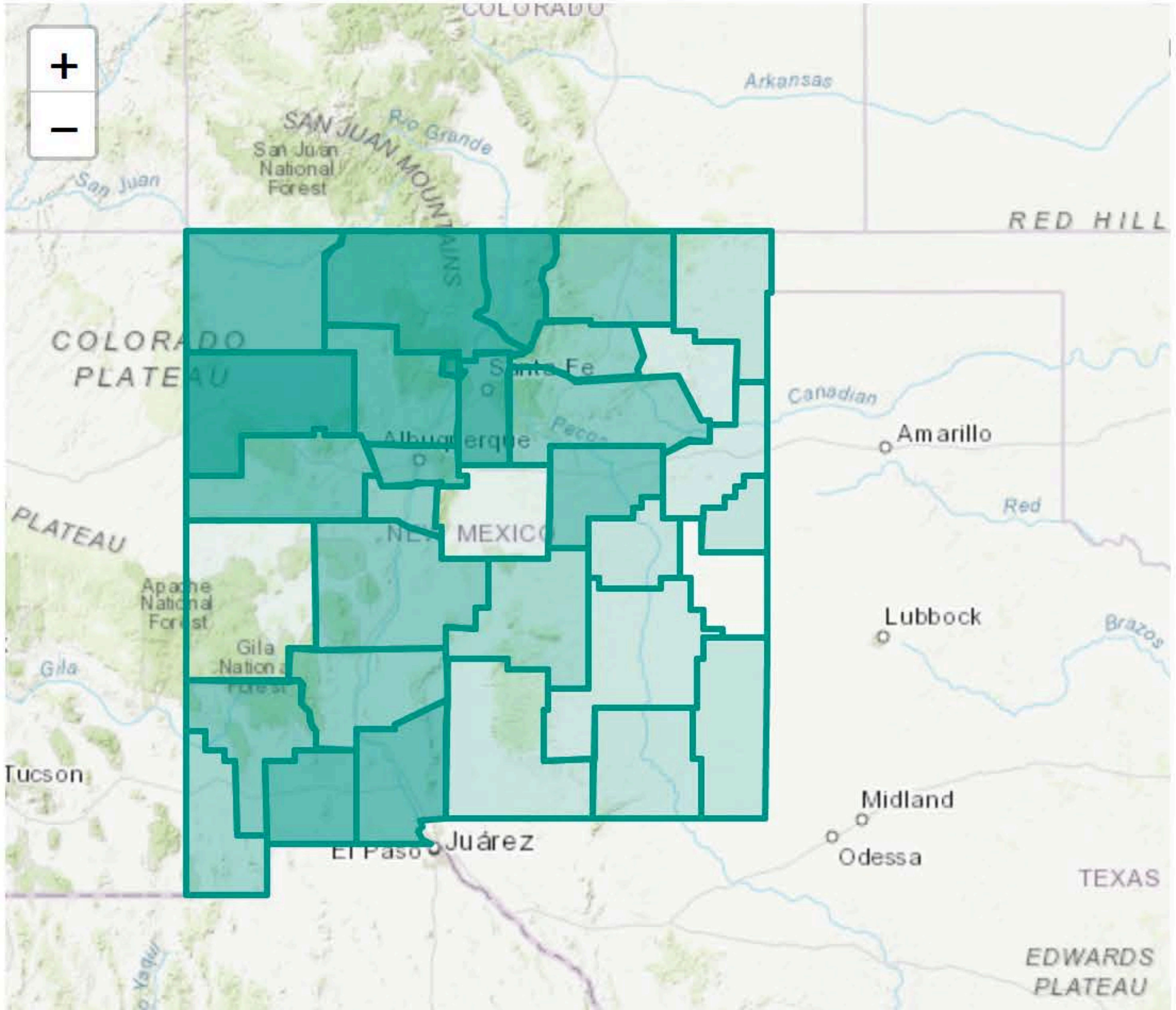

Find a vaccine near you.

*Please register for vaccine at vaccinenm.org. **Please do not show up at provider sites without an appointment.***

STEP 1:

REGISTER AT VACCINENM.ORG

Create a profile by filling out the registration form. Once the form is complete, you will receive a confirmation code.

STEP 2:

COMPLETE YOUR PROFILE

Use your confirmation code to access your profile and enter your chronic medical conditions, employer information, insurance information, and demographic information.

STEP 3:

SCHEDULE YOUR APPOINTMENT

Once you receive a notification, enter your special event code and select a location, day, and schedule your COVID-19 vaccine appointment.

State of Ohio | COVID-19 Vaccine Dashboard

Last Updated: 2022-01-03

CSV

View By
Select to view by Vaccine Started or Completed
Vaccine Started*

Statewide: Vaccine Status
By Total and % of Population

Vaccine Started*	All Ages	5+	12+	18+
% of Population	59.93%	63.69%	68.03%	70.15%
# Recipients	7,004,764	7,004,732	6,803,420	6,391,811

Click County to Filter Metrics
Counties are colored by % of Population with Vaccine

Vaccine Started*, Change from Last 24 Hours****	Vaccine Started* By Population	Vaccine Started*, Total
1,806	59.93%	7,004,764

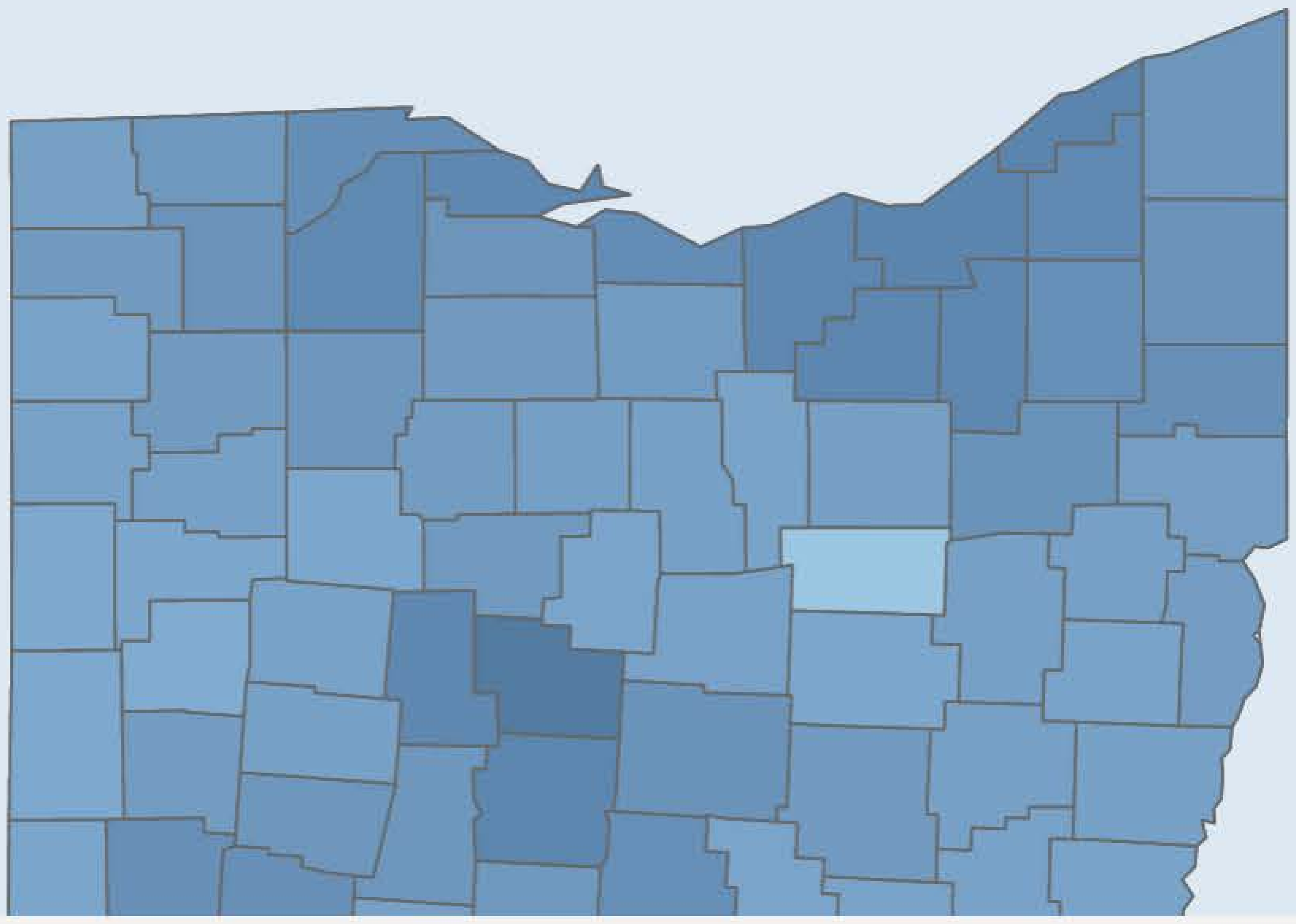

Key Metrics
Select to view key demographic groups
Age Group

0-19	27.16%	784,084
20-29	54.55%	847,414
30-39	61.06%	901,909
40-49	65.85%	913,563
50-59	72.09%	1,111,714
60-64	80.56%	641,727
65-69	87.77%	585,270
70-74	92.32%	482,281
75-79	88.09%	315,031
80+	84.43%	421,771

View Count By
Select to view counts by daily or cumulative
Daily Total

Language Translation
An Official Site of Ohio.g

Ohio Department of Health
Coronavirus (COVID-19)
Search

Responsible RestartOhio
COVID-19 Vaccination Program
Equity and High-Risk Groups
Families and Individuals
Healthcare Providers and Local Heal Districts

Residency Not Reported
8,825

Residents of Other States - Vaccinated in Ohio
256,379

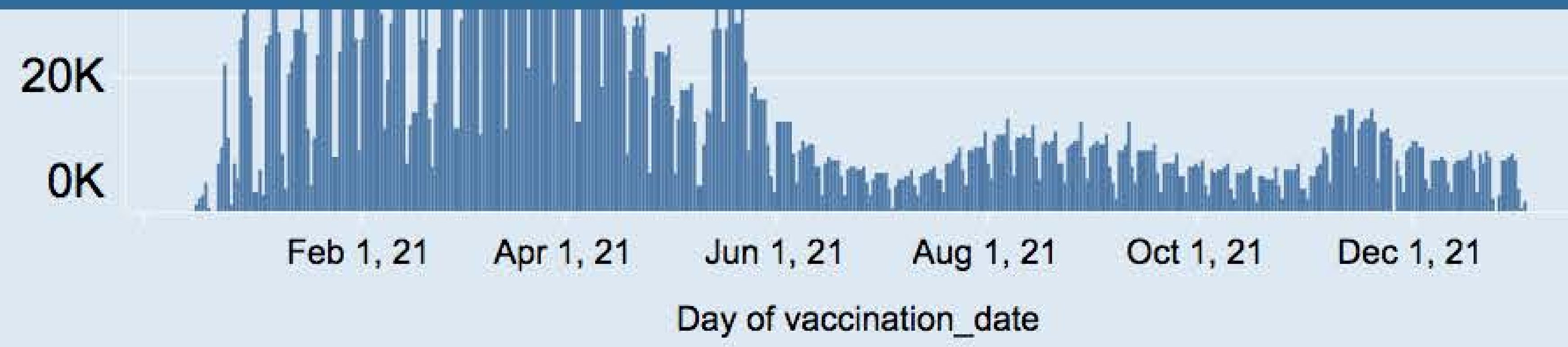

* Indicates that an individual has received at least one reported dose of COVID-19 vaccine.
** Indicates that all recommended COVID-19 vaccine doses have been reported for an individual, and the person is considered fully immunized. Status accounts for the total number of doses needed to be fully immunized only, which is completion of the original vaccine series; other specific requirements (dose spacing, age restrictions) vary depending on COVID-19 vaccine product and the latest CDC/ACIP administration guidance.
***A person is counted in the "Vaccine Started" category on the day the first COVID-19 vaccination dose was administered. A person is counted in the "Vaccine Completed" category on the day the primary COVID-19 vaccination series was completed.
****The number of vaccines reported to ODH over the previous 24 hours ending at 6 a.m. daily. Although most vaccines are identified within 24 hours of the vaccine administration date, some may take longer to be reported.

***** The number of fully vaccinated people (from Vaccine Completed) who received an additional dose of any COVID-19 vaccine on or after **August 13, 2021**.
This definition differs from CDC guidance on administration (**see link #1**) of additional doses, but follows CDC's approach for estimating additional doses (**see link #2**) for purposes of the CDC COVID Data Tracker.

Link #1: CDC guidance on administration

Link #2: CDC's approach for estimating additional doses

Population data as reported by the US Census Bureau. There are no population estimates for "Unknown" or "Other" demographic groups, so there is no percentage of population for those groups. Data reported to the Ohio Department of Health. All data displayed are preliminary and subject to change as more information is reported to ODH.

+ a b | e a u

TN Department of Health

Tennessee COVID-19 Vaccination Reporting

Last Updated 1/3/2022

Click Here For: Population Metrics

Total Vaccinations Reported	Vaccinations Reported Since 12/27/2021	% of People Statewide With at Least One Dose	% of People Statewide Fully Vaccinated
8,678,565	105,819	57.9%	51.3%

Number of People with:
Series Initiation: 1 dose of Pfizer / Moderna
Series Completion: 2 doses of Pfizer / Moderna OR 1 dose of Janssen
Additional Dose: 3 doses of Pfizer / Moderna

Series Initiation	Series Completion	Additional Dose
456,245	2,319,003	1,210,744

Notes:
* Providers administering COVID-19 vaccines are expected to report vaccine doses to the state immunization information system (TennIIS) within 24 hours of administration and are required to report doses no later than 72 hours after administration.
* Dashboard does not contain data for COVID-19 vaccinations administered by the Bureau of Prisons (BOP), Department of Defense (DOD), Indian Health Service (IHS) and Veterans Health Administration (VHA).
* The number of all vaccine recipients is the sum of Series Initiation, Series Completion, and Additional Dose. The number of fully vaccinated recipients is the sum of Series Completion and Additional Dose.
* The *Summary Page* includes all vaccinations reported to TennIIS from Tennessee providers, regardless of patient's state of residence. These numbers will differ from the *Population Page*, which contains data on Tennessee residents only.

Age Group

	People Vaccinated	% of People Vaccinated
5-11 years	78,634	2.0
12-15 years	142,020	3.6
16-20 years	209,373	5.3
21-30 years	504,976	12.7
31-40 years	542,093	13.6
41-50 years	564,041	14.1
51-60 years	653,774	16.4
61-70 years	663,335	16.6
71-80 years	441,927	11.1
81+ years	187,472	4.7
Pending	460	0.0
Total	3,988,105	100.0

Patient Race

	People Vaccinated	% of People Vaccinated
Asian	73,555	1.8
Black or African American	474,147	11.4
White	2,404,965	60.6
Other/Multiracial	898,824	23.6
Unknown	136,614	2.7
Total	3,988,105	100.0

Patient Ethnicity

	People Vaccinated	% of People Vaccinated
Hispanic or Latino	229,239	5.2
Not Hispanic or Latino	3,424,299	87.6
Unknown	334,567	7.2
Total	3,988,105	100.0

Patient Sex

	People Vaccinated	% of People Vaccinated
Female	2,155,757	54.1
Male	1,826,504	45.8
Other	1,400	0.0
Unknown	4,444	0.1
Total	3,988,105	100.0

COVID-19 Vaccinations Administered and Reported to TennIIS

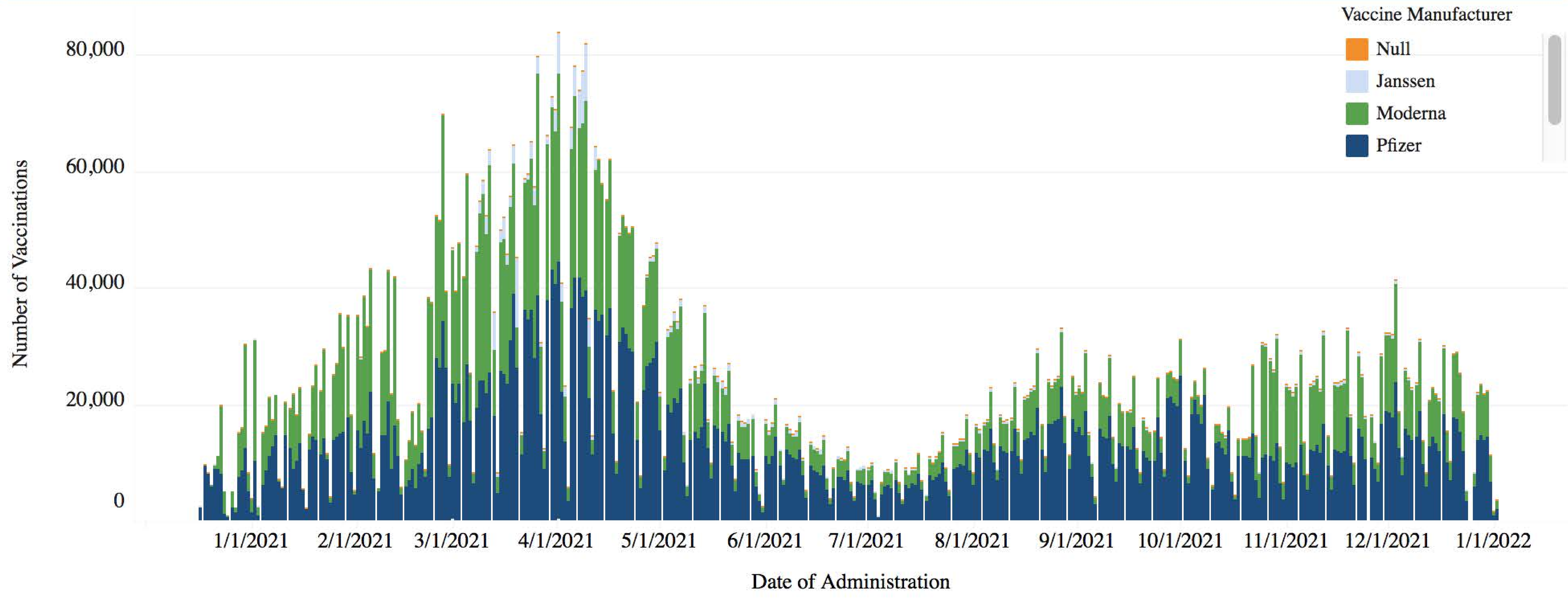

Reported Vaccinations by Patient County

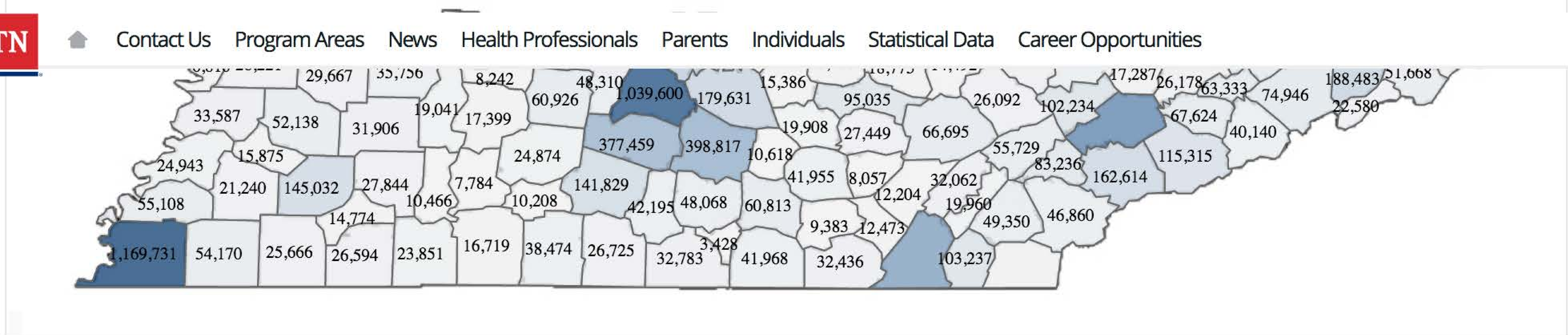

Hover over each county in the map for: county name, number of vaccinations reported, % of county population with at least one dose (any manufacturer), % of county population with series initiation (1 dose of Pfizer / Moderna), % of county population with series completion (2 doses of Pfizer / Moderna OR 1 dose of Janssen).

Based on reported doses to the state immunization information system (TennIIS) and 2019 county population data.

Summary | Vaccine Doses Administered | People Vaccinated | Vaccine Doses Allocated | About the Data

COVID-19 Vaccination in Texas

TEXAS Health and Human Services | Texas Department of State Health Services

DSHS is partnering with vaccine providers across the state to ensure vaccine will be accessible to all Texans at no cost. Visit the DSHS COVID-19 Vaccine Information page for more details on phases and tiers, and other vaccine info here:

Percentage Measure to Show

- (●) Percentage of Population 5+ Vaccinated with At Least One Dose
- ( ) Percentage of Population 5+ Fully Vaccinated
- ( ) Percentage of Population 65+ Vaccinated with At Least One Dose
- ( ) Percentage of Population 65+ Fully Vaccinated

71.97% of Texas State Population Vaccinated

Percentage of County Population Vaccinated

23.79% — 99.99%

This dashboard is updated by 4:00 pm each day with data as of 11:59 pm the previous night. See the About the Data tab for more information on data sources.

Percentage of Population Vaccinated for COVID-19

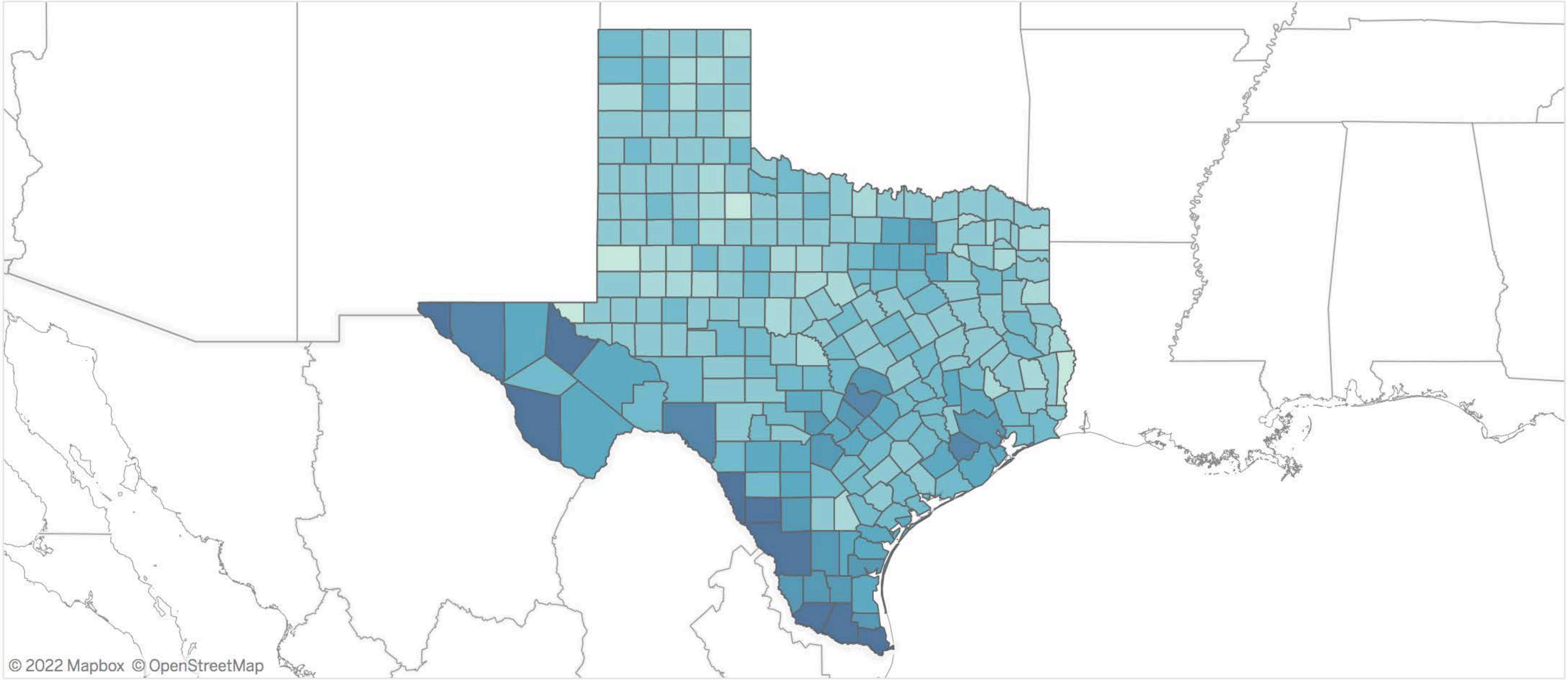

Doses Administered

39,431,870

People Vaccinated

19,407,637

People Fully Vaccinated

16,504,702

People Vaccinated With a Booster Dose

4,836,084

State Doses Shipped

28,927,970

Last Updated:
01/03/2022 1:00 pm

Undo | Redo | Revert | Refresh | Pause | Share | Download | Full Screen

Summary | Vaccine Doses Administered | People Vaccinated | Vaccine Doses Allocated | About the Data

TEXAS Health and Human Services | Texas Department of State Health Services

COVID-19 Vaccination in Texas

Doses administered are doses providers have entered into ImmTrac2, the Texas Immunization Registry, by recipient county of residence and date administered.

Use the filter below to select a Texas county and view the number of vaccinations administered to persons living there, by week. Alternatively, click on a county on the map. Click the Revert button at the bottom of the screen to remove filters.

Doses Administered

39,431,870

County Name

(All)

Vaccinations by Week in Texas

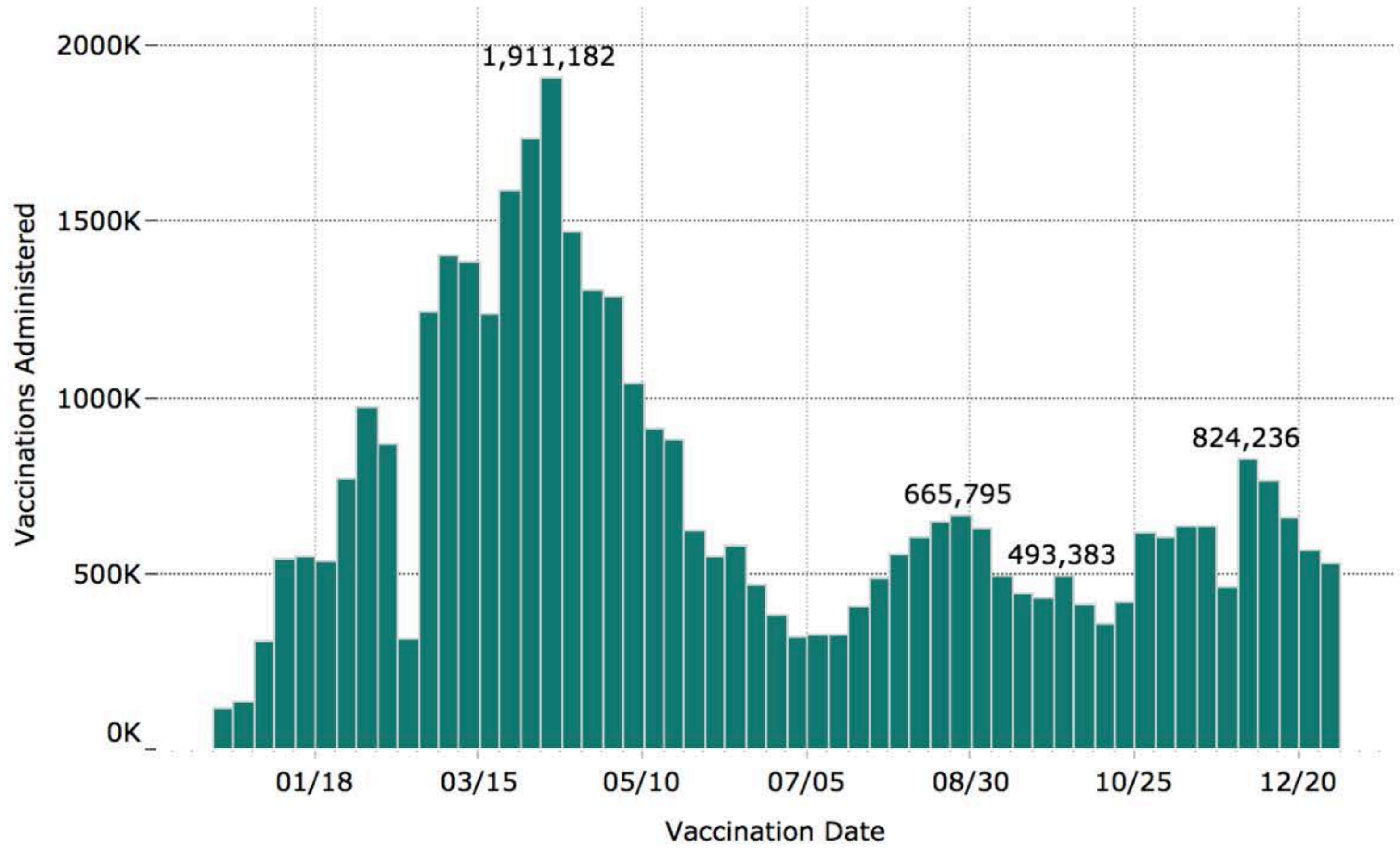

Doses Administered by Organization Type

Emergency Services	1,416,978
Health Center (Community, Federally Qualified, Migrant, Rural)	1,476,097
Hospital	7,229,033
Long-Term Care	17,957
Medical Clinic/Doctor's Office	2,930,087
Public Health/Government	5,775,345
Pharmacy	19,194,199
Other	1,392,174

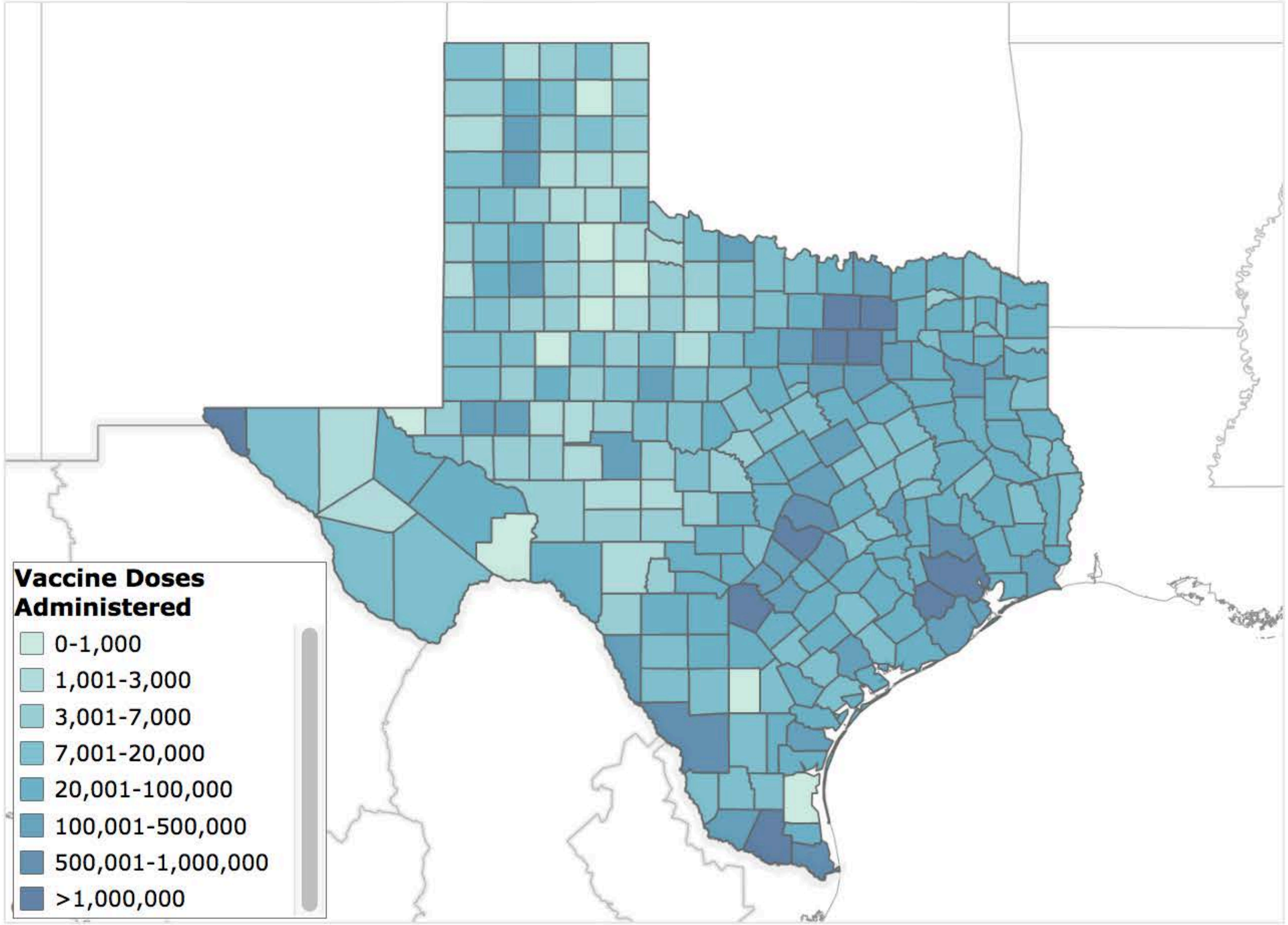

Dashboard is updated daily by 4 p.m. CST with data as of 11:59 the previous night.

For inquiries about the data, email COVIDVaccineQs@dshs.texas.gov.

Summary | Vaccine Doses Administered | People Vaccinated | Vaccine Doses Allocated | About the Data

TEXAS Health and Human Services | Texas Department of State Health Services

COVID-19 Vaccination in Texas

Select a Texas county below to view the number people who have received at least one dose of COVID-19 vaccine and those who are fully vaccinated as outlined by the CDC.

Alternatively, click on a county on the map. Click the Revert button at the bottom of the screen to remove filters.

People Vaccinated with at least One Dose	People Fully Vaccinated	People with Booster Dose
19,407,637	16,504,702	4,836,084

Measures to Show

- ○ Vaccinated with at least One Dose
- ○ Fully Vaccinated
- ◉ Booster Dose

County Name

(All)

Number of People Vaccinated in Texas

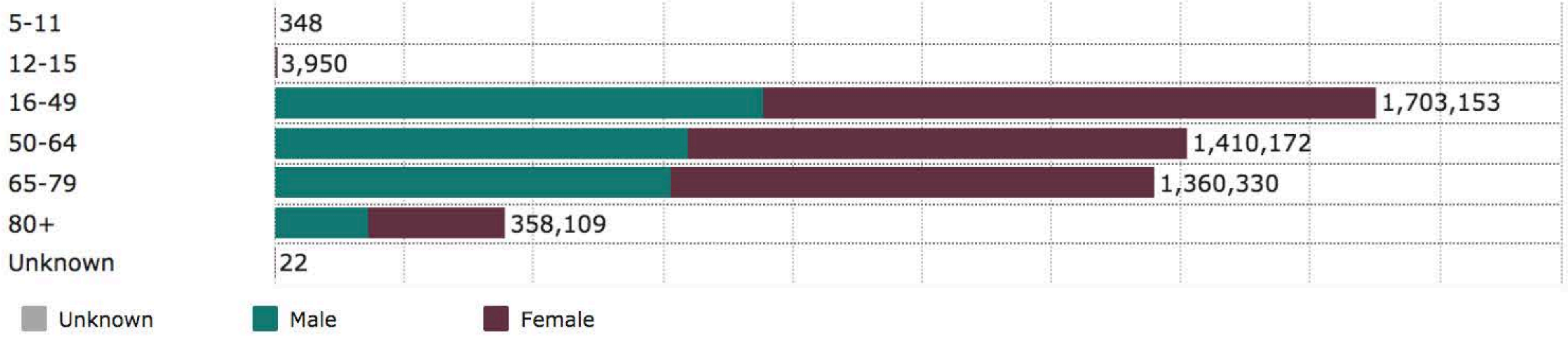

Race/Ethnicity	People Vaccinated	Percent of People Vaccinated
Asian	292,168	6.04%
Black	340,503	7.04%
Hispanic	1,174,831	24.29%
Other	1,140,327	23.58%
Unknown	107,769	2.23%
White	1,780,486	36.82%

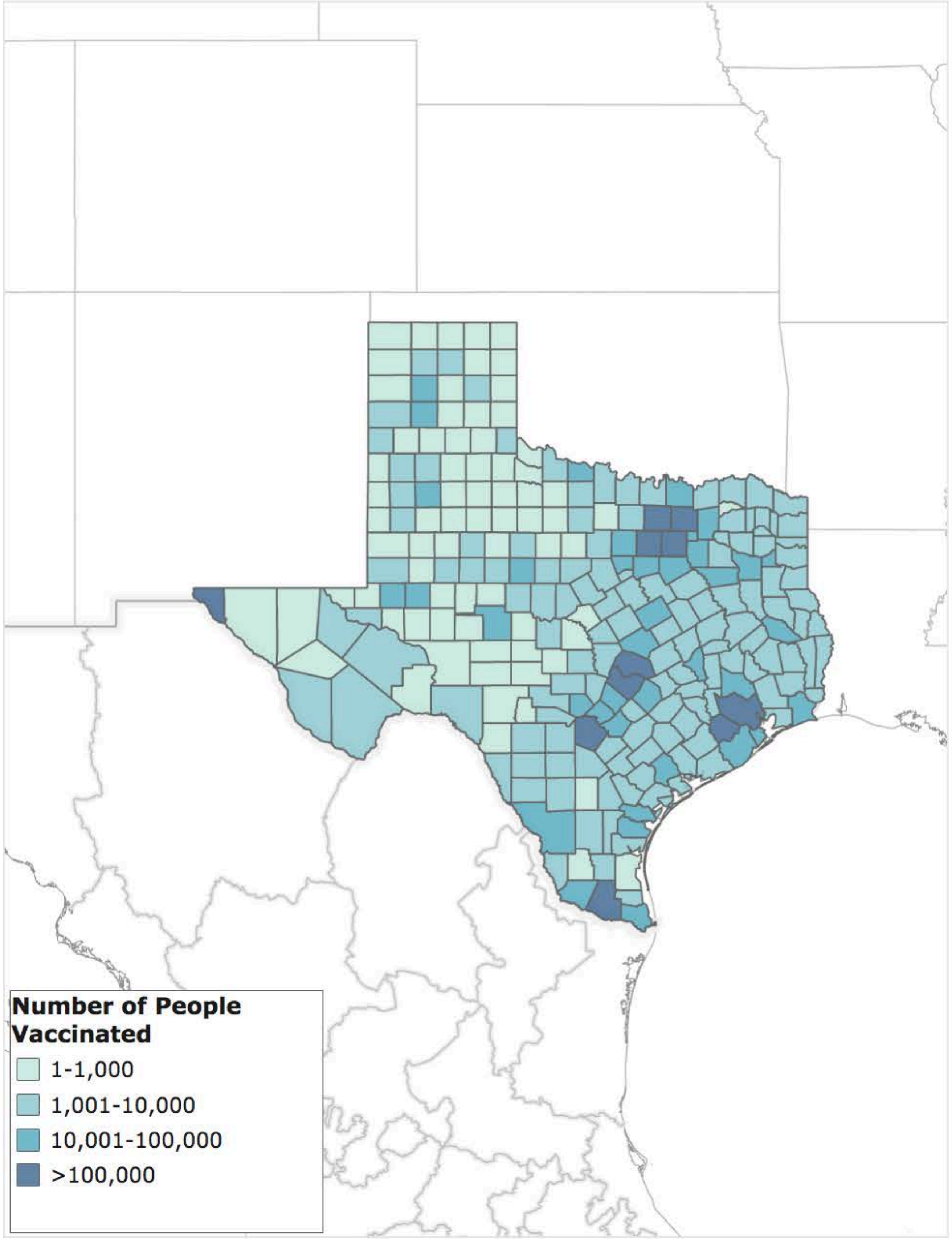

Dashboard is updated daily by 4 p.m. CST with data as of 11:59 the previous night. For inquiries about the data, email COVIDVaccineQs@dshs.texas.gov.

Vermont Vaccination Data

Overall | Statewide | By County | By County - Additional

Updated 01/01/2022 9:10 AM

People vaccinated

At least one dose | Completed | Additional dose

510.3K
At least one dose

86%
% of Vermonters age 5+ who have received at least one vaccine

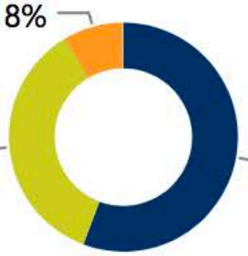

Show VT Residents Only
VT Only

Percent of the statewide population **age 5+ of each subgroup that has received at least one dose of vaccine**

Age	%
5 - 11	55%
12 - 17	82%
18 - 29	68%
30 - 39	91%
40 - 49	88%
50 - 59	89%
60 - 64	95%
65 - 69	100%
70 - 74	100%
75+	100%

Race	%
Asian	77%
Black or African American	80%
Native American, Indigenous, or First Nation	28%
Pacific Islander	28%
Two or more races	79%
White	84%

Ethnicity	%
Hispanic	100%
Not Hispanic	82%

Sex	%
Female	88%
Male	84%

Race*	5-11	12-30	31-64	65+	Age 5+
BIPOC	51%	77%	94%	100%	**83%**
Non-Hispanic White	52%	68%	86%	99%	**82%**

Source: Vermont Immunization Registry, VDH Population Estimates (2019)

Data notes

Statewide numbers and percentages are capped at 100%. To protect the identity of individuals, data is suppressed when there are fewer than six people vaccinated in a subgroup.

Race information is not reported for 3% of people vaccinated.

Race/ethnicity information is not reported for 4% of people vaccinated.

Ethnicity information is not reported for 4% of people vaccinated.

BIPOC refers to Black, Indigenous, and people of color.

Sex information is not reported for 556 people vaccinated. The categorization of **male/female** for some people is based on sex assignment at birth, while for others, it is based on gender. This is due to the varying sources and ways the information is reported.
Sex not reported may mean the provider did not collect that information, the patient did not provide it, or the provider or the patient selected a category other than male or female.

Data on this page is sourced from the Vermont Immunization Registry and

Microsoft Power BI

Vermont Vaccination Data

Overall | Statewide | By County | By County - Additional

Updated 01/01/2022 9:10 AM

Data notes

Show rates by: Overall | Race/Ethnicity | Ethnicity | Age | Sex

Select vaccine status: At least one dose | Completed

Vaccination rate by county

The percent of the county population age 5+ that has received at least one dose of vaccine by county.

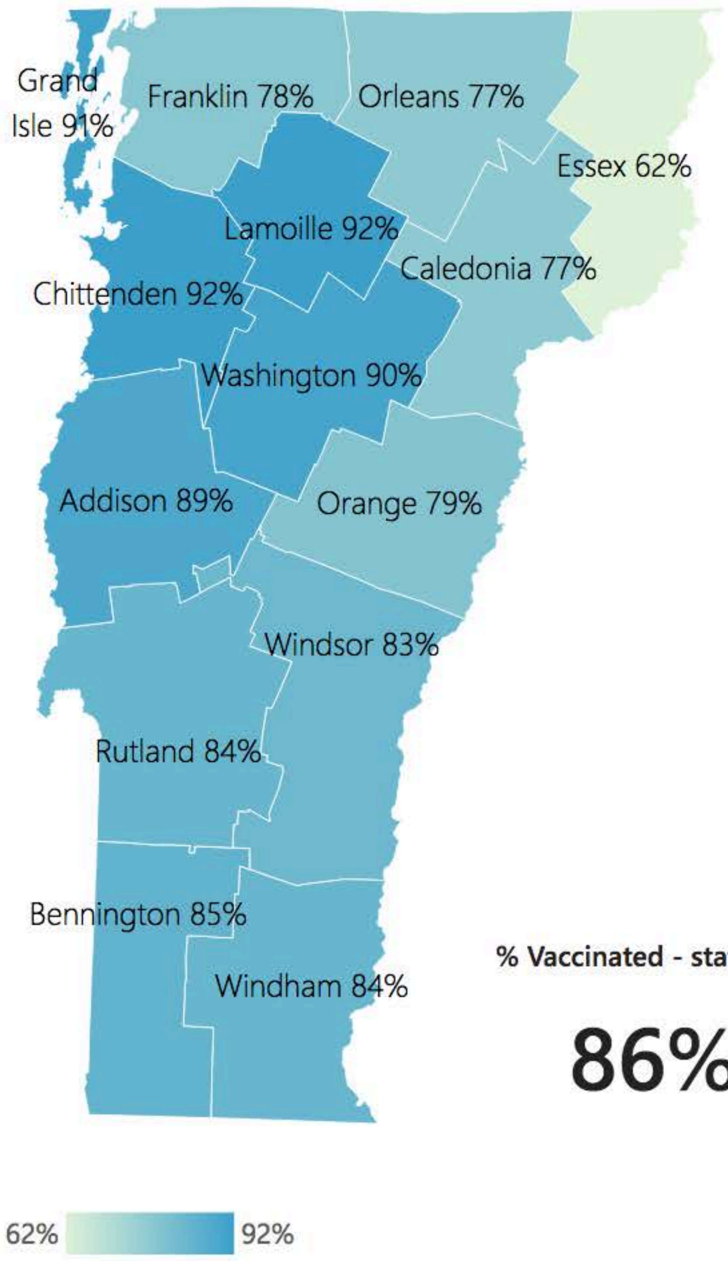

% Vaccinated - statewide

86%

County	Overall progress	5+ pop estimate
Addison	89%	35,214
Bennington	85%	33,828
Caledonia	77%	28,570
Chittenden	92%	156,317
Essex	62%	5,887
Franklin	78%	46,515
Grand Isle	91%	6,900
Lamoille	92%	24,075
Orange	79%	27,542
Orleans	77%	25,742
Rutland	84%	55,626
Washington	90%	55,680
Windham	84%	40,377
Windsor	83%	52,673
**VT**	**86%**	**594,946**

Source: Vermont Immunization Registry, VDH Population Estimates (2019)

Administration | Providers

Total vaccine doses administered
Last updated: 1/3/2022

8,625,777

Johnson & Johnson	Moderna	Pfizer
330,645 doses administered	3,312,943 doses administered	4,982,189 doses administered

Total vaccine doses administered in Wisconsin by day

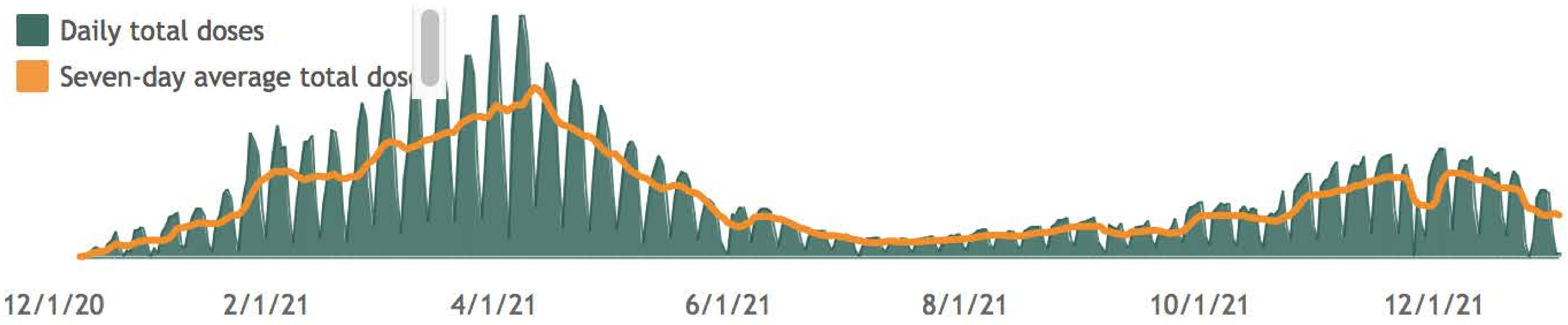

Total additional/booster doses administered

1,620,411

Johnson & Johnson	Moderna	Pfizer
21,242 doses administered	706,163 doses administered	893,006 doses administered

Additional/booster vaccine doses administered in Wisconsin by day

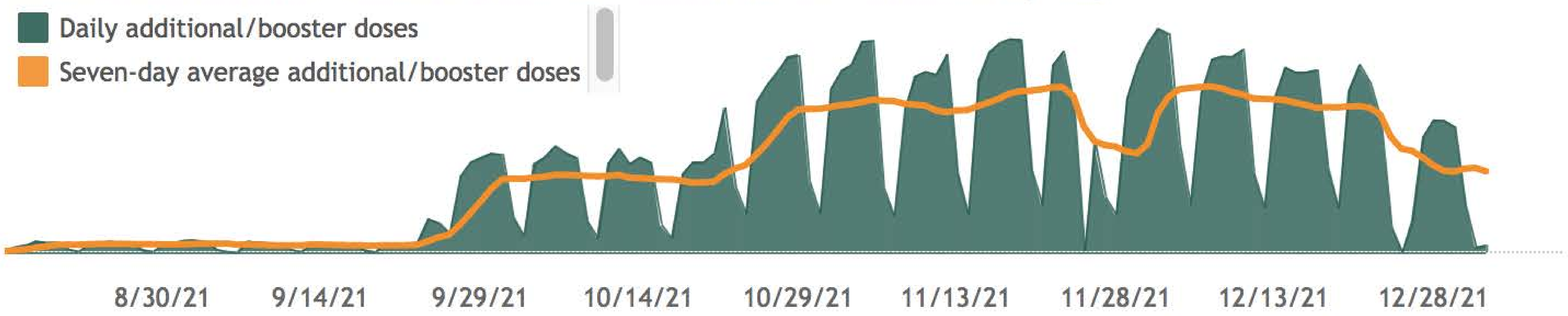

Undo Redo Revert Refresh Pause Metrics Share Download Full Screen

Understanding our data: What does this chart mean?

About our data: How do we measure this?

Back to a list of charts on this page.

COVID-19 Vaccines for Wisconsin residents
Updated: 1/3/2022

HERC region data

- (•) Total population who have received at least one dose
- ( ) Total population who have completed the series

Percent of Wisconsin residents who have received at least one dose by county

Click a county to filter data

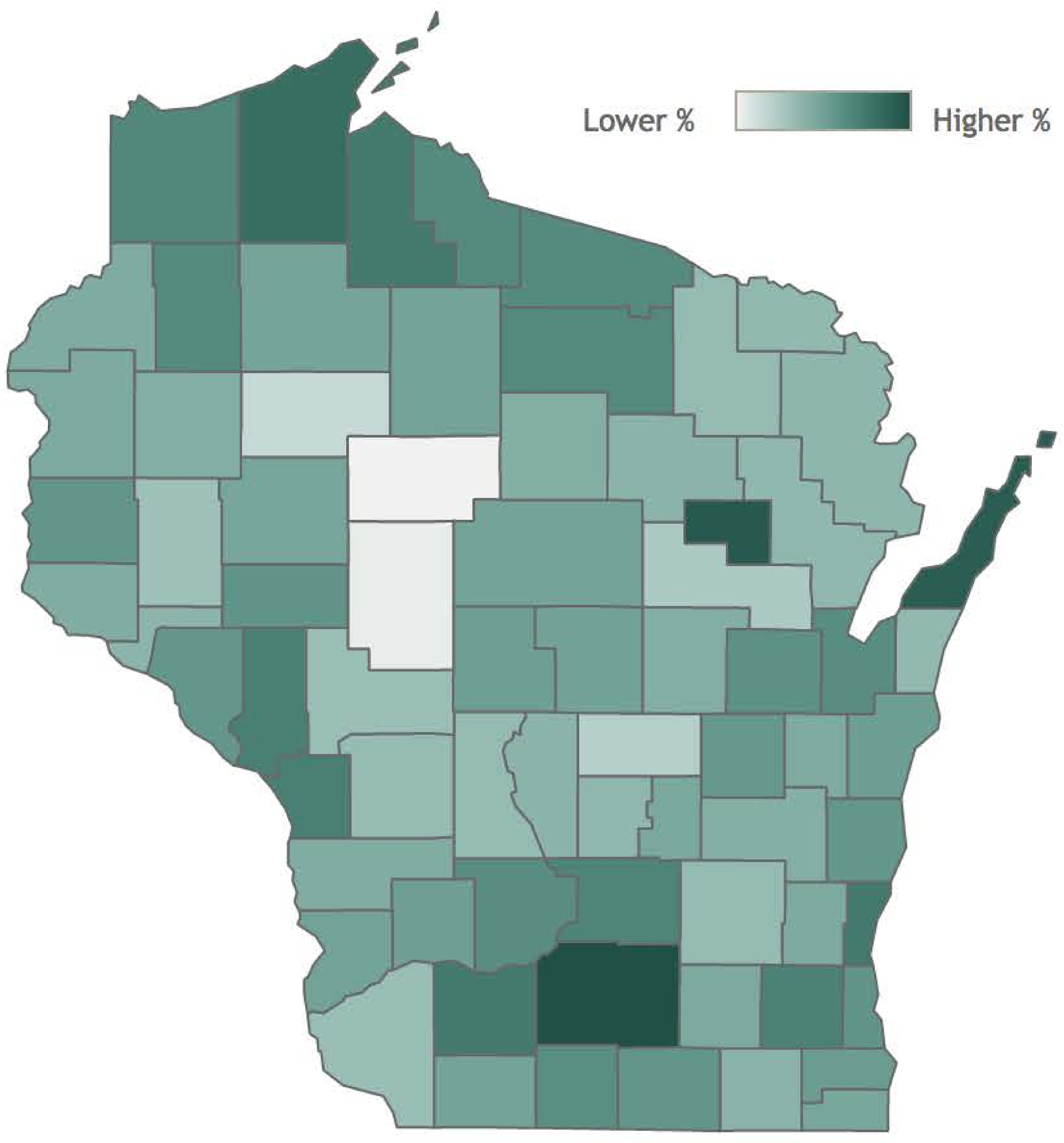

Percent of Wisconsin residents who have received at least one dose

The **orange** represents the population for whom the vaccine is authorized.
The **gray** indicates the population under 5 years of age for whom the vaccines are not authorized.

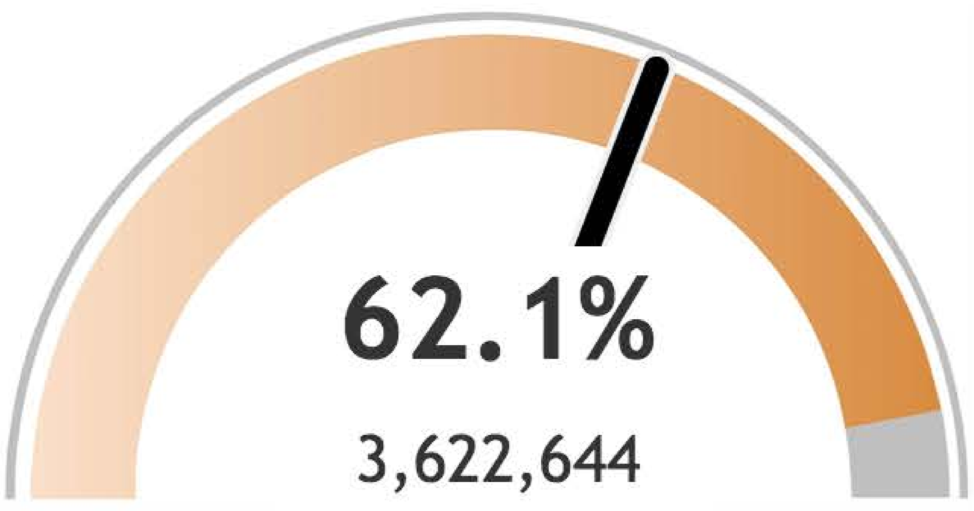

Age	
5-11	21.2%
12-17	57.3%
18-24	56.8%
25-34	61.3%
35-44	67.2%
45-54	70.0%
55-64	76.9%
65+	85.5%

Race	
American Indian	45.9%
Asian	61.6%
Black	39.6%
White	56.7%

*6.9% of records reported a race of "Other"

*3.9% of records reported an unknown race

Sex	
Female	65.1%
Male	58.5%

*0.4% of records were reported without sex.

Ethnicity	
Hispanic	52.9%
Non-hispanic	60.3%

*3.8% of records were reported without ethnicity.

View more data on racial and ethnic disparities in Wisconsin

Vaccine doses for Wisconsin residents by week
(Total: 6,817,782)

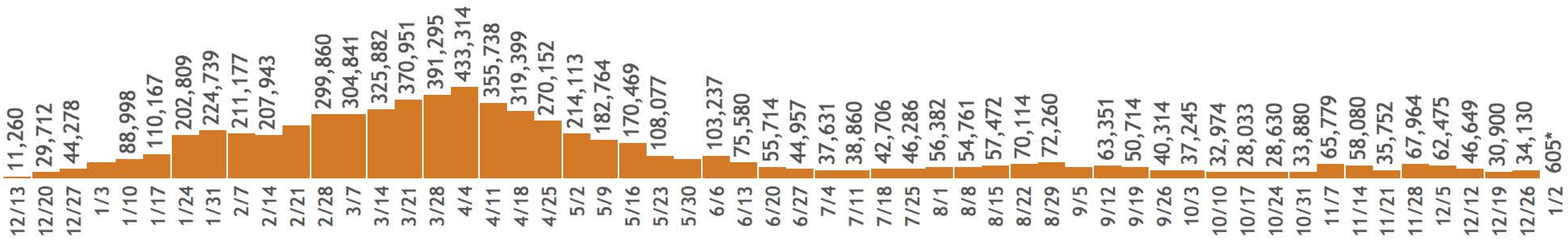

*Current week may be incomplete.

Undo Redo Revert Refresh Pause Metrics Share Download Full Screen

Census Tract | Municipality | School District | Zip Code Tabulation Area (ZCTA)

COVID-19 Vaccinations by Municipality | Completed Vaccine Series

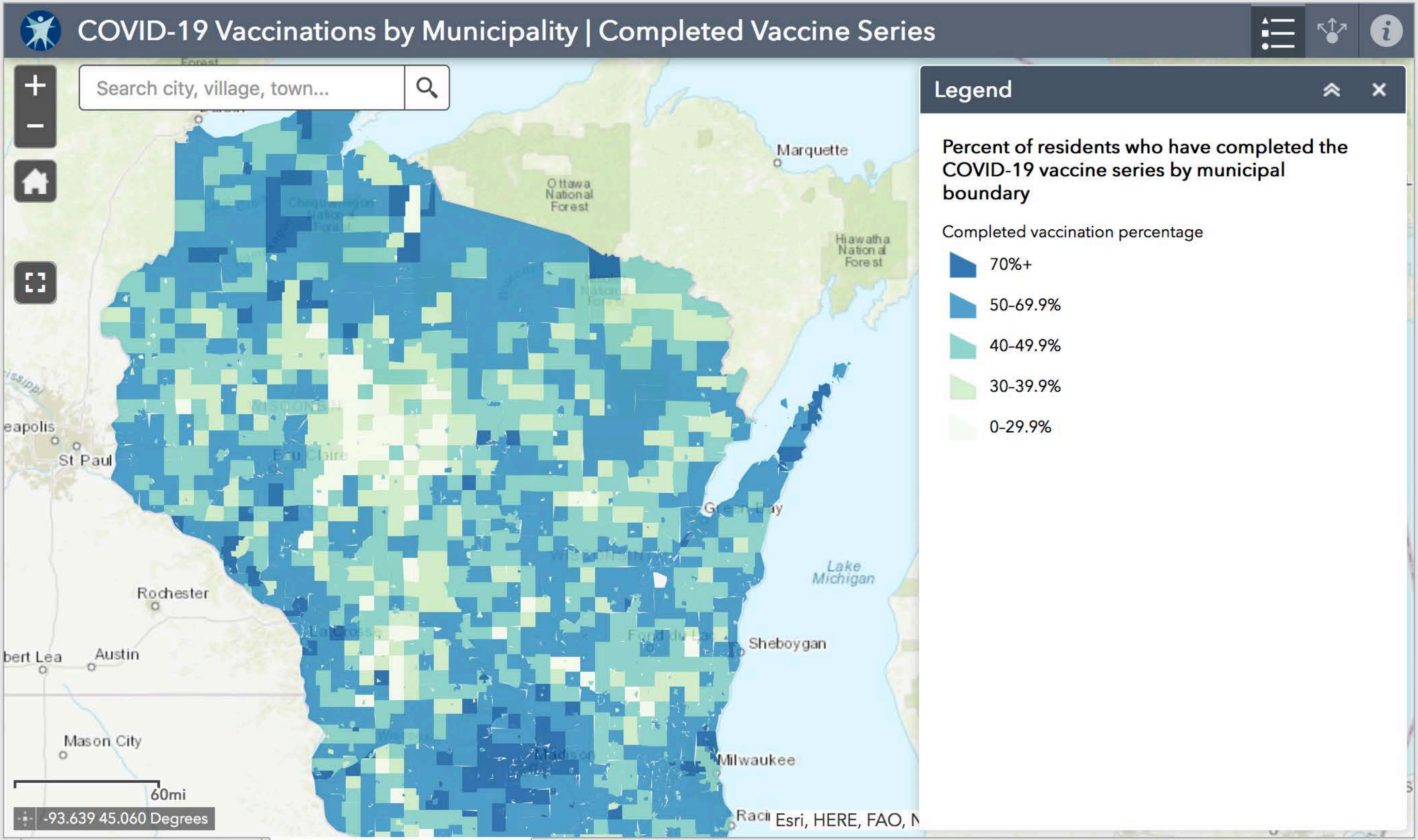

At Least 1 Vaccine Dose | Completed Vaccine Series

See How Vaccinations Are Going in Your County and State

Updated Dec. 30, 2021

12+ 18+ 65+

Pct. of residents age 12+ who are fully vaccinated

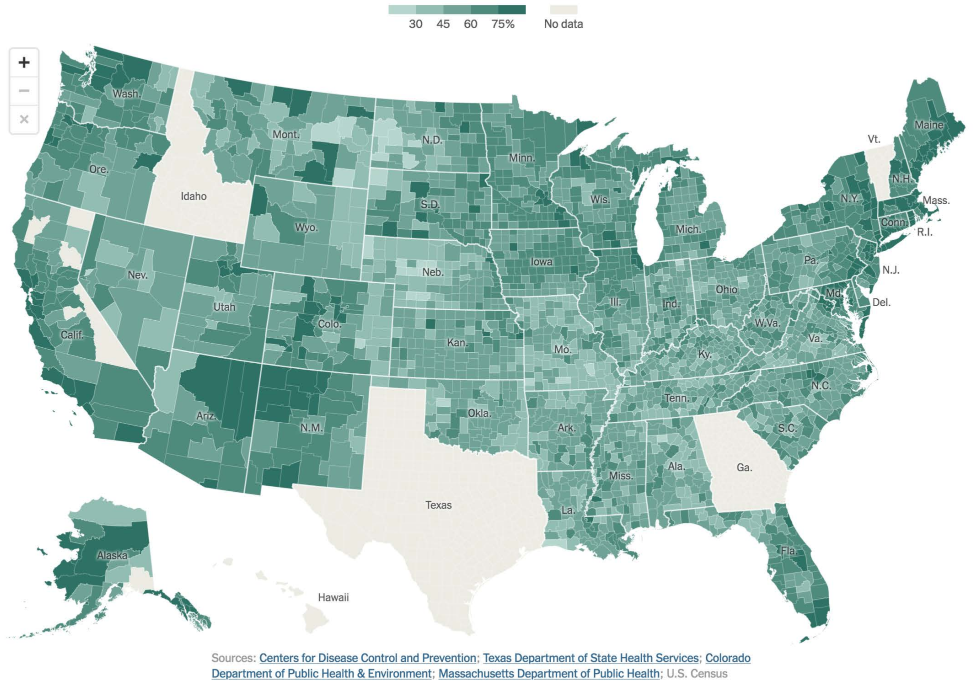

Sources: Centers for Disease Control and Prevention; Texas Department of State Health Services; Colorado Department of Public Health & Environment; Massachusetts Department of Public Health; U.S. Census Bureau | Note: No C.D.C. data available for some counties. Georgia and Vermont were excluded because more than a quarter of data is missing. Data from Texas and Colorado excludes shots given by most federal agencies. On Dec. 9, the C.D.C. capped its vaccination rate figures at 95 percent.

Jump to: New doses | Projection | County inequities | Doses by state | Eligibility

DATA ISSUES UNDER INVESTIGATION

- The C.D.C.'s vaccination rates for West Virginia are much higher than the state's figures because of reporting issues. The state says its data is more accurate.
- Boosters can be misclassified as first doses, which may overcount first dose coverage among U.S. adults, according to the C.D.C.

The Centers for Disease Control and Prevention said on Thursday about 243.5 million people have received at least one dose of a Covid-19 vaccine, including about 205.8 million people who have been fully vaccinated by Johnson & Johnson's single-dose vaccine or the two-dose series made by Pfizer-BioNTech and Moderna.

United States vaccinations

	At least one dose*	Fully vaccinated
All ages	73%	62%
5 and up	78%	66%
12 and up	84%	71%
18 and up	86%	73%
65 and up	95%	88%

*The C.D.C. reported on Nov. 30 that booster doses are sometimes misclassified as first doses, which may overestimate first dose coverage among adults.

Sources: Centers for Disease Control and Prevention, U.S. Census Bureau | Note: Figures include the U.S. territories and three countries with special agreements.

The C.D.C. also reported that about 68.8 million fully vaccinated people have received an additional vaccine dose since Aug. 13, the day after the F.D.A. opened up eligibility for third shots for some people with weakened immune systems. This figure also includes people who have gotten a booster dose, which the F.D.A. authorized on Sept. 22 for Pfizer recipients over 65 or at high risk of severe Covid-19.

On Oct. 20, the F.D.A. authorized booster shots for Johnson & Johnson recipients, as well as Moderna recipients over 65 or at high risk of severe Covid-19. It will also allow providers to boost people with a different Covid-19 vaccine than the one they initially received. On Nov. 19, the federal agency authorized booster shots of the Pfizer and Moderna vaccines for everyone 18 and older, and on Dec. 9 authorized Pfizer boosters for 16- and 17-year-olds.

The number of people who have gotten an additional dose does not include an estimated 1.2 million people in the U.S. who may have received unauthorized additional doses prior to mid-August, according to the C.D.C.

New reported people vaccinated

Each line shows the seven-day average.

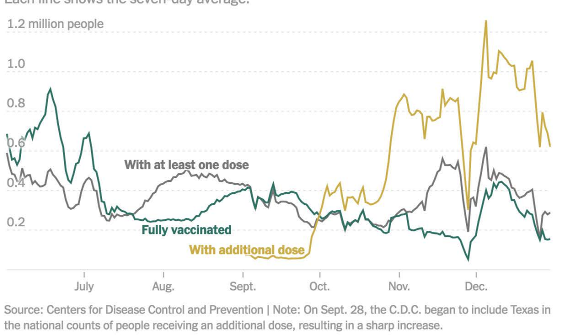

Source: Centers for Disease Control and Prevention | Note: On Sept. 28, the C.D.C. began to include Texas in the national counts of people receiving an additional dose, resulting in a sharp increase.

How Quickly Are Shots Going in Arms?

Providers are administering about 1.06 million doses per day on average, including first, second and additional doses, about a 69 percent decrease from the peak of 3.38 million reported on April 13.

New reported doses administered by day

U.S. Total*

See daily doses in Kentucky

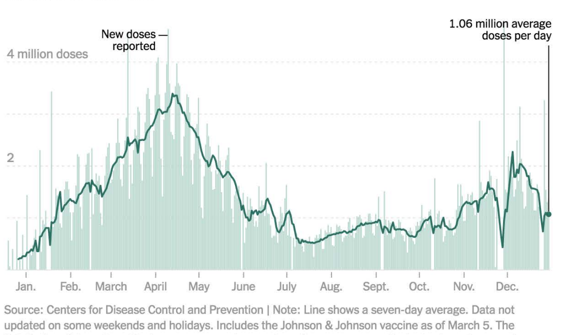

Source: Centers for Disease Control and Prevention | Note: Line shows a seven-day average. Data not updated on some weekends and holidays. Includes the Johnson & Johnson vaccine as of March 5. The C.D.C., in collaboration with the states, sometimes revises data or reports a single-day large increase in vaccinations from previous dates, which can cause an irregular pattern in the daily reported figures.

Figures show the date shots were reported, rather than the date shots were given and include first and second doses of Pfizer and Moderna, and single doses of Johnson & Johnson.

Daily reported doses given by manufacturer

Each line shows the seven-day average.

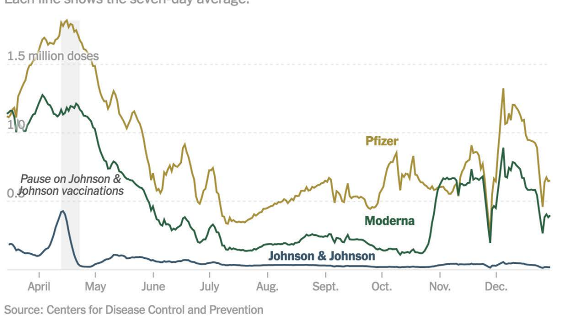

Source: Centers for Disease Control and Prevention

In December 2020, federal regulators gave emergency use authorization to two-dose vaccines developed by Pfizer and Moderna. Regulators authorized Johnson & Johnson's one-dose vaccine in February, but recommended a pause in its use on April 13 because of reports of blood clots in a small number of patients. All 50 states paused or recommended that providers pause those vaccinations. The government ended the Johnson & Johnson pause on April 23, clearing the way for states to resume vaccinations.

On Aug. 12, regulators gave emergency use authorization for people with weakened immune systems to get a third dose of the Pfizer and Moderna vaccines, and expanded that authorization in September and October to include booster shots for many Americans.

On Aug. 23, the federal government approved the Pfizer vaccine for those 16 and older, the first full approval of a Covid-19 vaccine in the country.

The F.D.A. cleared the Pfizer vaccine for children, ages 5 to 11, on Oct. 29. Emergency use authorization of the vaccine continues for those ages 5 to 15 and for those receiving additional doses.

Global vaccinations tracker ›
How many have been vaccinated, by country

YOUR CORONAVIRUS TRACKER: *We'll send you the latest data for places you care about each day.* Sign Up

When Might Nearly Everyone Be Vaccinated?

Some experts have estimated that 90 percent or more of the total population — adults and children — would need to be fully vaccinated for the country to reach a possibly elusive threshold of protection against the coronavirus known as herd immunity, now that the outbreak is driven by the highly contagious Delta variant.

A number of factors will determine if and when this threshold is met, including the pace at which newly vaccinated people join those who are immune after past infections. But the presence of more transmissible virus variants could complicate that progress. And children, who became eligible for the Pfizer vaccine in late October, may be key to reaching herd immunity, experts say.

The projection below shows the share of the total population that is fully vaccinated based on the current rate of vaccination, and provides a rough indication of when the virus's spread could begin to stall.

The pace of vaccinations has been rising slowly since the summer

Based on the seven-day average of people becoming fully vaccinated each day.

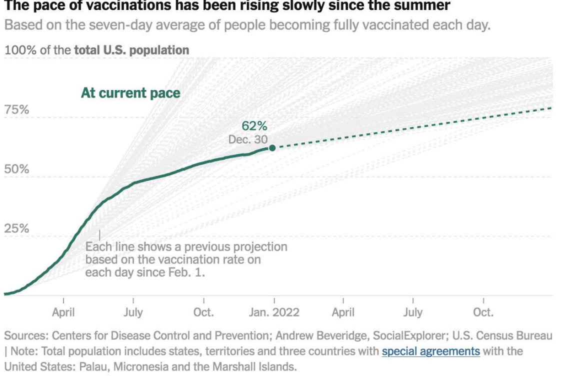

Sources: Centers for Disease Control and Prevention; Andrew Beveridge, SocialExplorer; U.S. Census Bureau | Note: Total population includes states, territories and three countries with special agreements with the United States: Palau, Micronesia and the Marshall Islands.

But the pace of vaccinations varies across the country. Many states in the South and West, for example, have fully vaccinated a smaller share of their population than in other regions.

On Dec. 9, the C.D.C capped its vaccination rate figures at 95 percent, after acknowledging that it may be overestimating first doses of the vaccine and underestimating booster shots. As a result, even locations that have exceeded that vaccination rate will remain at 95 percent.

The C.D.C. said on its website that the "cap helps address potential overestimates of vaccination coverage due to first, second, and booster doses that were not linked." Other possible reasons for overestimation, according to the agency, include census population data that excludes part-time residents or reporting errors.

How each state compares to the national share of fully vaccinated people

Share of the state population fully vaccinated.

Above U.S. share   Below U.S. share

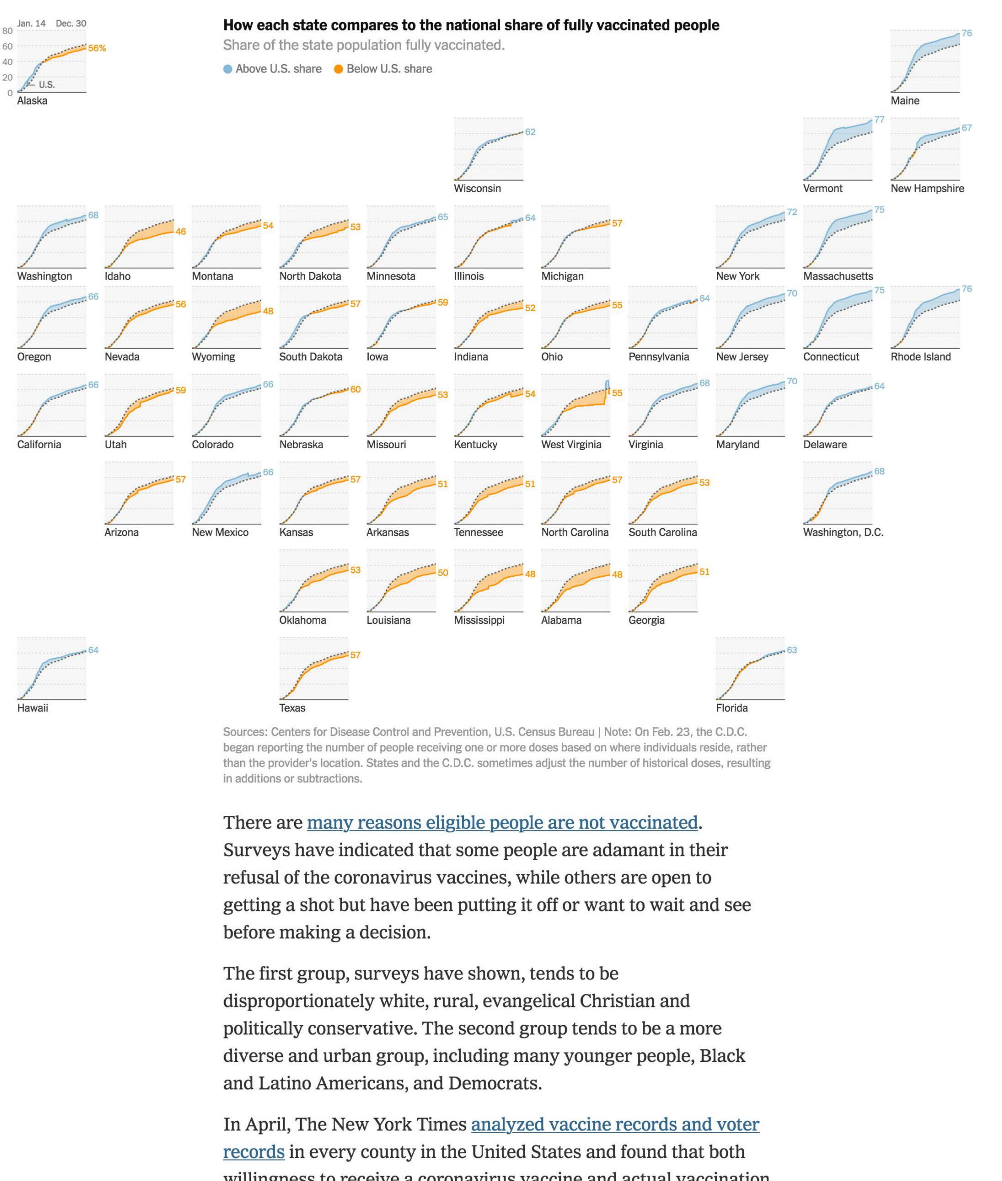

Sources: Centers for Disease Control and Prevention, U.S. Census Bureau | Note: On Feb. 23, the C.D.C. began reporting the number of people receiving one or more doses based on where individuals reside, rather than the provider's location. States and the C.D.C. sometimes adjust the number of historical doses, resulting in additions or subtractions.

There are many reasons eligible people are not vaccinated. Surveys have indicated that some people are adamant in their refusal of the coronavirus vaccines, while others are open to getting a shot but have been putting it off or want to wait and see before making a decision.

The first group, surveys have shown, tends to be disproportionately white, rural, evangelical Christian and politically conservative. The second group tends to be a more diverse and urban group, including many younger people, Black and Latino Americans, and Democrats.

In April, The New York Times analyzed vaccine records and voter records in every county in the United States and found that both willingness to receive a coronavirus vaccine and actual vaccination rates were lower, on average, in counties where a majority of residents voted to re-elect former President Donald J. Trump in 2020.

Are The Most Vulnerable Counties Being Vaccinated?

Speed isn't the only priority for the country's vaccination campaign. The Biden administration has also committed to distributing shots equitably to the communities most affected by the pandemic.

Yet many months into the rollout, the most socially vulnerable counties in the U.S. have a lower vaccination rate on average than the nation's least vulnerable. The majority of the most disadvantaged counties with the fewest fully vaccinated people are in the South, while the most vaccinated, least vulnerable counties are in the Midwest and Northeast.

Vaccination rates by county social vulnerability

Share of total population fully vaccinated. Circles sized by county population.

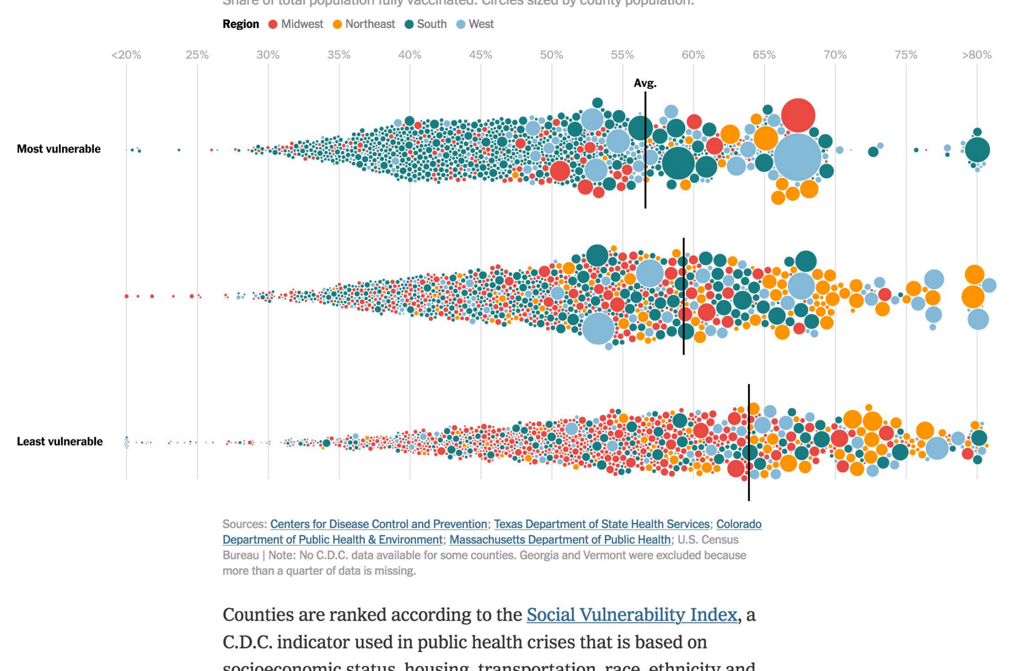

Sources: Centers for Disease Control and Prevention; Texas Department of State Health Services; Colorado Department of Public Health & Environment; Massachusetts Department of Public Health; U.S. Census Bureau | Note: No C.D.C. data available for some counties. Georgia and Vermont were excluded because more than a quarter of data is missing.

Counties are ranked according to the Social Vulnerability Index, a C.D.C. indicator used in public health crises that is based on socioeconomic status, housing, transportation, race, ethnicity and language. Each county's vaccination rate is its share of all residents that have been fully vaccinated, a figure that does not reflect those who have only received one dose of a two-shot vaccine.

How Is Each State Doing?

Some jurisdictions have been more efficient than others at administering their doses from the federal government.

At least one dose | Fully vaccinated

Pct. of all residents that are fully vaccinated

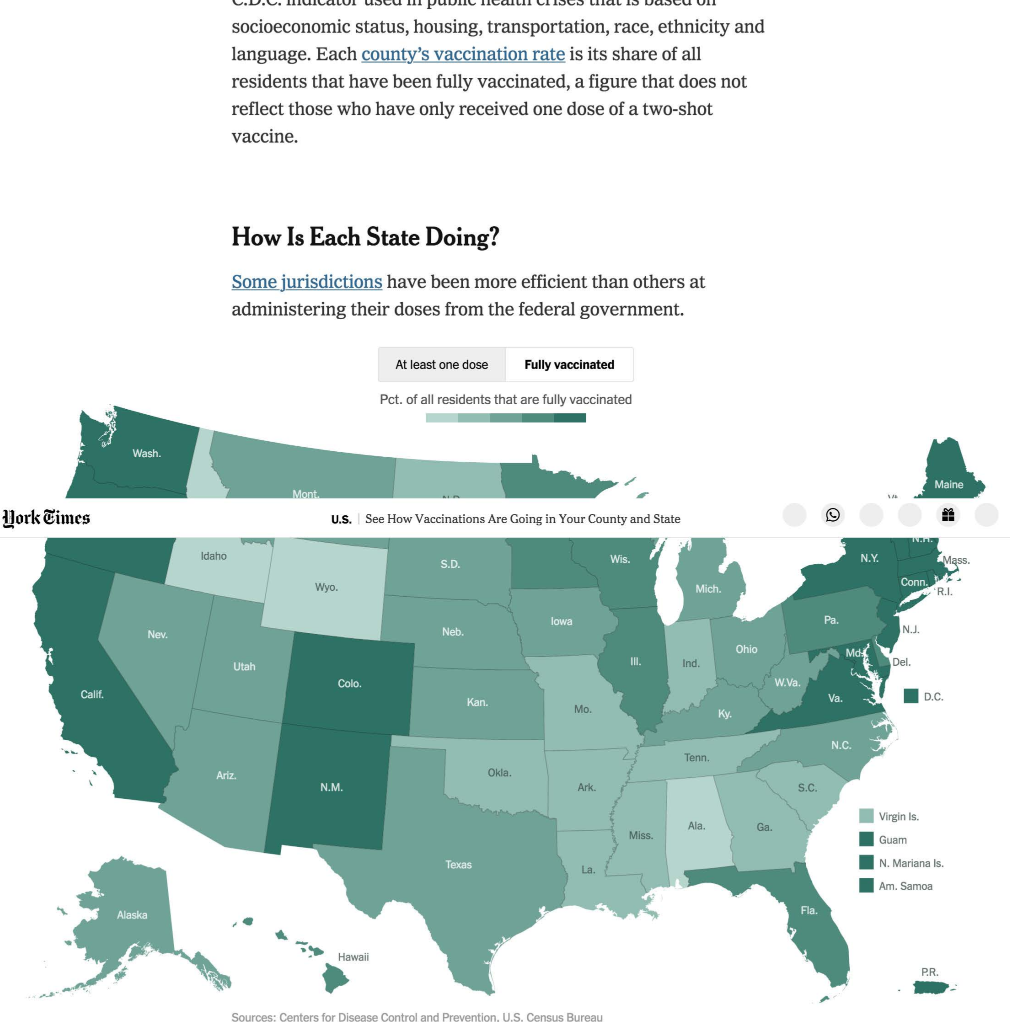

Sources: Centers for Disease Control and Prevention, U.S. Census Bureau

Coronavirus (COVID-19)

COVID-19 Vaccine Tracker

This is your state and county equivalent level look at how many have gotten a dose or doses of the COVID-19 vaccine. Click on a state to see how many vaccines have been administered and you can drill down to the county level and see a bar chart that tracks the running total. Data for this interactive map is provided by the Centers for Disease Control and Prevention (CDC) and state health departments and will be updated automatically when states update data.

Map

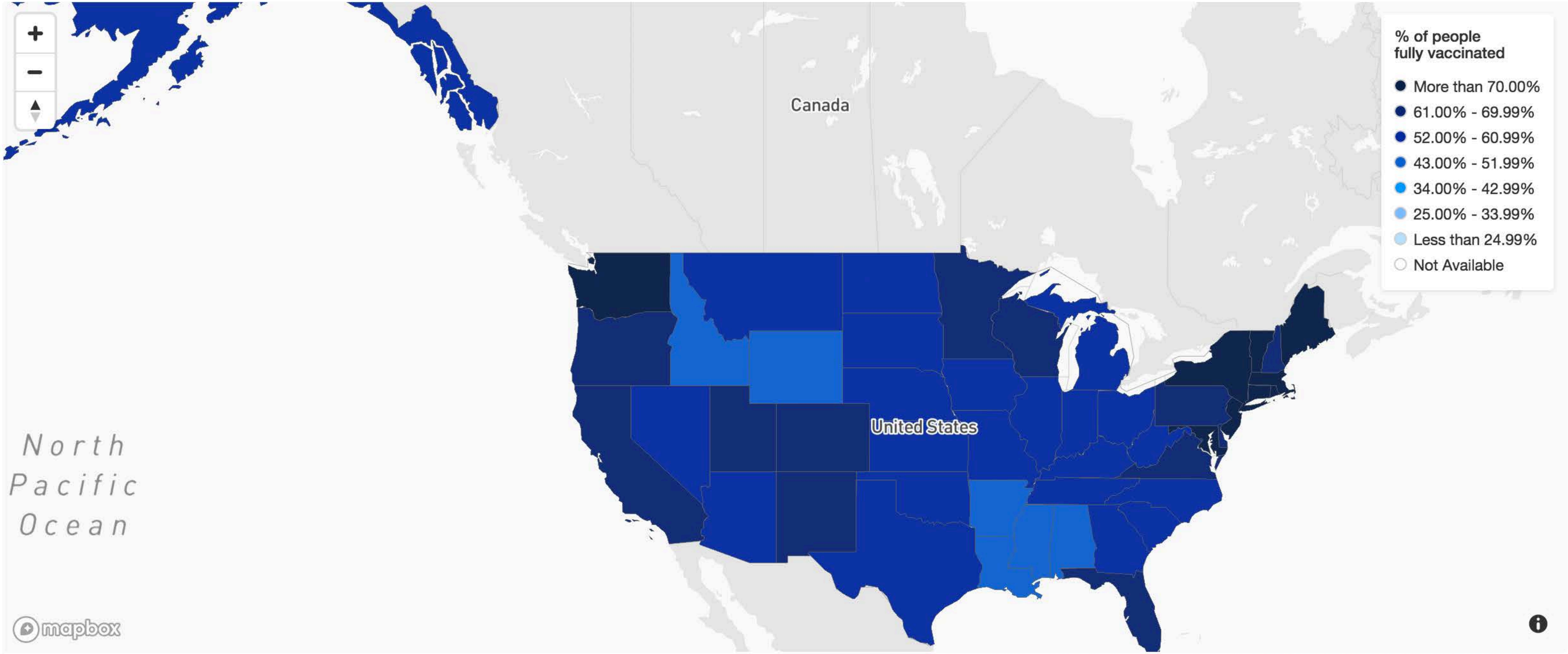

Source: State and county health departments, Centers for Disease Control and Prevention

COVID-19 Vaccination Tracker

THE UNITED STATES

Total doses delivered	Total doses administered	Number received at least one dose	Number fully vaccinated
615,358,465	507,657,980	243,527,564	205,811,394

Percent of the U.S. population partially vaccinated

One of two doses of Pfizer or Moderna vaccine received

11.4%

Percent of the U.S. population fully vaccinated

Both doses of Pfizer or Moderna vaccine or one and only dose of Johnson and Johnson received

62%

Percent of the U.S. population that received at least one dose

One or more doses of any of the authorized vaccines received

73.4%

About the data

COVID-19 VACCINATION BY RACE & ETHNICITY

Comparing the racial and ethnic breakdown of all vaccinated individuals, recently vaccinated individuals, and the total US population can help identify groups that require addition efforts to achieve full vaccination coverage.

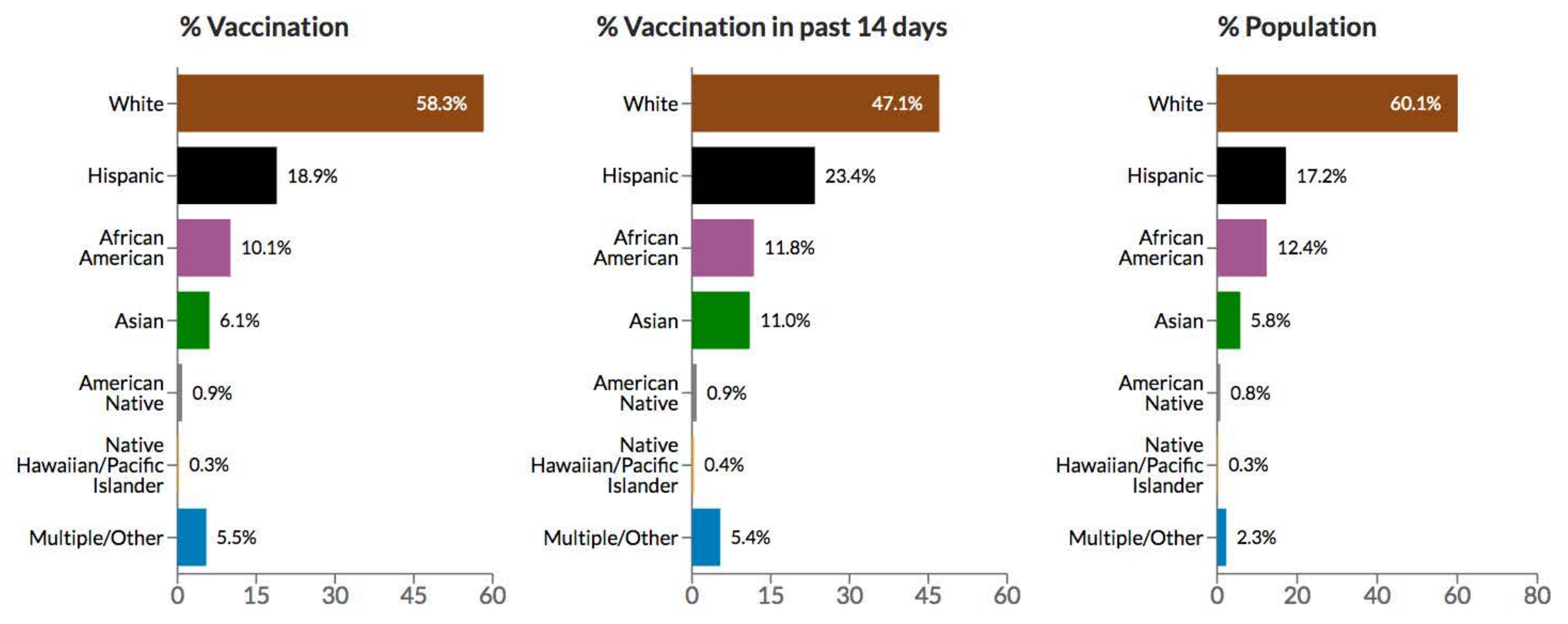

About the data About the data About the data

- White Americans make up 58.3 % of those fully vaccinated, 47.1 % vaccinated in past 14 days, 61.2 % population.
- Hispanic Americans make up 18.9 % of those fully vaccinated, 23.4 % vaccinated in past 14 days, 17.2 % population.
- African Americans make up 10.1 % of those fully vaccinated, 11.8 % vaccinated in past 14 days, 12.4 % population.
- Asian Americans make up 6.1 % of those fully vaccinated, 11.8 % vaccinated in past 14 days, 5.8 % population.
- Native Americans make up 0.9 % of those fully vaccinated, 0.9 % vaccinated in past 14 days, 0.8 % population.
- Pacific Islanders make up 0.3 % of those fully vaccinated, 0.4 % vaccinated in past 14 days, 0.3 % population.
- Others make up 5.5 % of those fully vaccinated, 5.4 % vaccinated in past 14 days, 2.3 % population.

About the data

COVID-19 VACCINATION BY STATE

State Vaccination Status | State Vaccination Trends

Click on a state.

% of population partially vaccinated (one dose received)

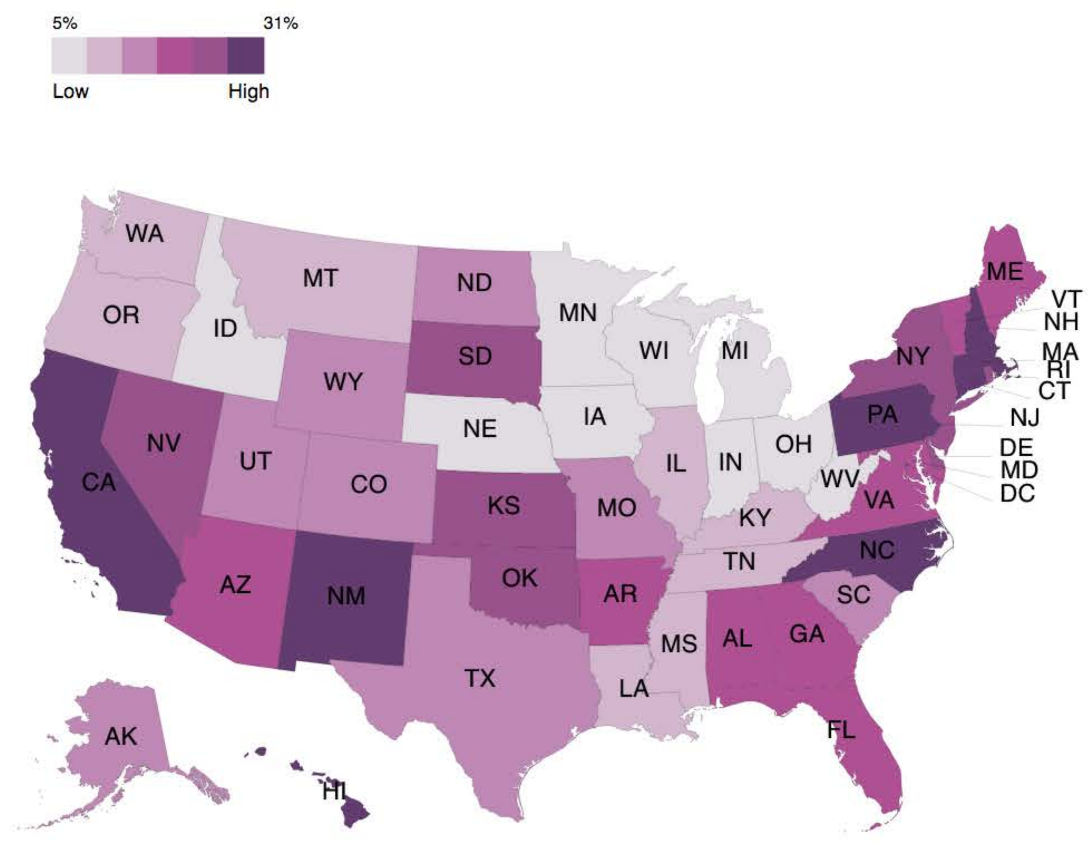

Vaccination Status in
The United States

	Select State	U.S.
Percent fully vaccinated		62%
Percent of the U.S population that received at least one dose		73.4%
Distributed on 12/30/2021		1,096,200

About the data

COVID-19 VACCINATION BY COUNTY

County Vaccination Trends | County Vaccination Map

African American | Hispanic | Age 65+ | Underlying condition | In poverty | Minority | Native American | Uninsured | Region | Urbanicity | College

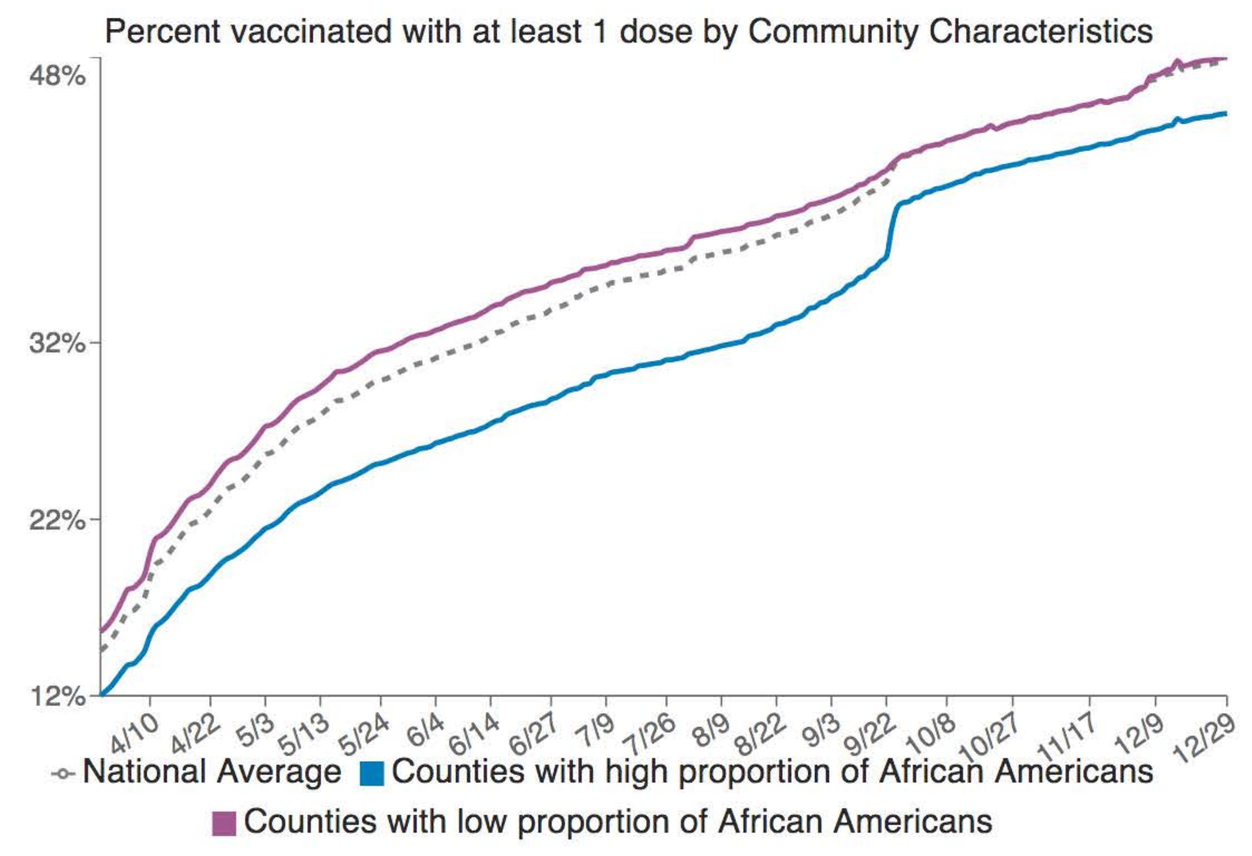

Date Updated: 12/30/2021

About the data

COVID-19 BURDEN IN THE UNITED STATES

Daily Cases	Daily Deaths
585,013 ↑181% 14-day change	1,420 0% 14-day change

Average Daily COVID-19 Cases / 100K

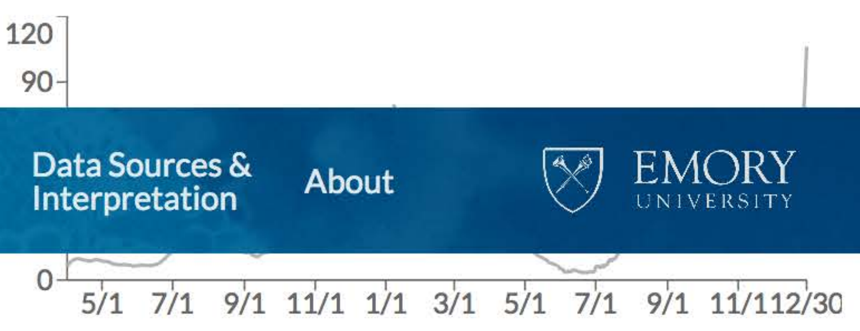

USA Vaccination Tracker
Vaccination by Race & Ethinicity
State Vaccination Tracker
State COVID-19 Burden
COVID-19 Vaccines FAQ
General Information
Vaccine Development
Vaccine Safety
Getting Vaccinated
After You Are Vaccinated

Disparities in COVID-19 Mortality
Nation

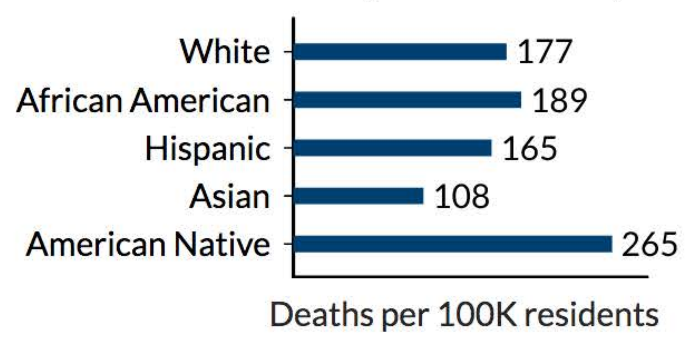

Average Daily COVID-19 Deaths / 100K

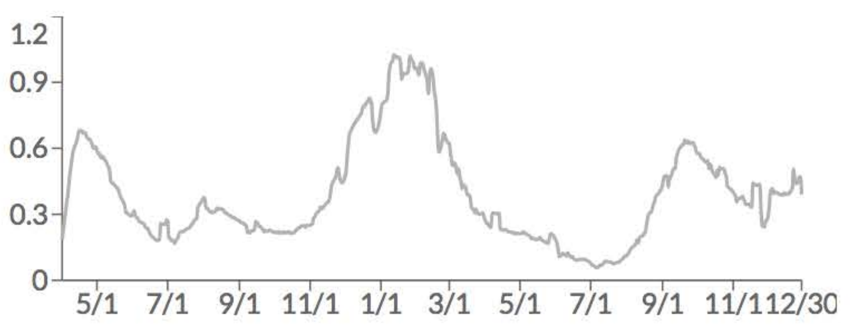

About the data About the data

Statistics and Research

Coronavirus (COVID-19) Vaccinations

58.3% of the world population has received at least one dose of a COVID-19 vaccine.
9.21 billion doses have been administered globally, and **29.17 million** are now administered each day.
Only **8.5%** of people in low-income countries have received at least one dose.

Our international COVID-19 vaccination dataset is updated each morning (London time), with data up to the previous day. Last update: **16 hours ago.** Reuse our work freely Cite this research

Our vaccination dataset uses the most recent official numbers from governments and health ministries worldwide. Population estimates for per-capita metrics are based on the United Nations World Population Prospects. Income groups are based on the World Bank classification. A full list of our country-specific sources is available at the bottom of this page, and we also answer frequently-asked questions there.

In our Data Explorer you can see all of our data on COVID-19 vaccinations (doses administered, people with at least 1 dose, and people fully vaccinated).

COVID-19 Data Explorer
Download the complete *Our World in Data* COVID-19 dataset.

METRIC: People vaccinated (by dose) | INTERVAL: Cumulative | Relative to Population | Align outbreaks

Type to add a country...
Sort by Country name

- Bangladesh
- Brazil
- Canada
- Chile
- China
- Cuba
- Egypt
- Ethiopia
- France
- Germany
- India
- Indonesia
- Italy

Contents
Clear selection

Share of people vaccinated against COVID-19, Jan 2, 2022

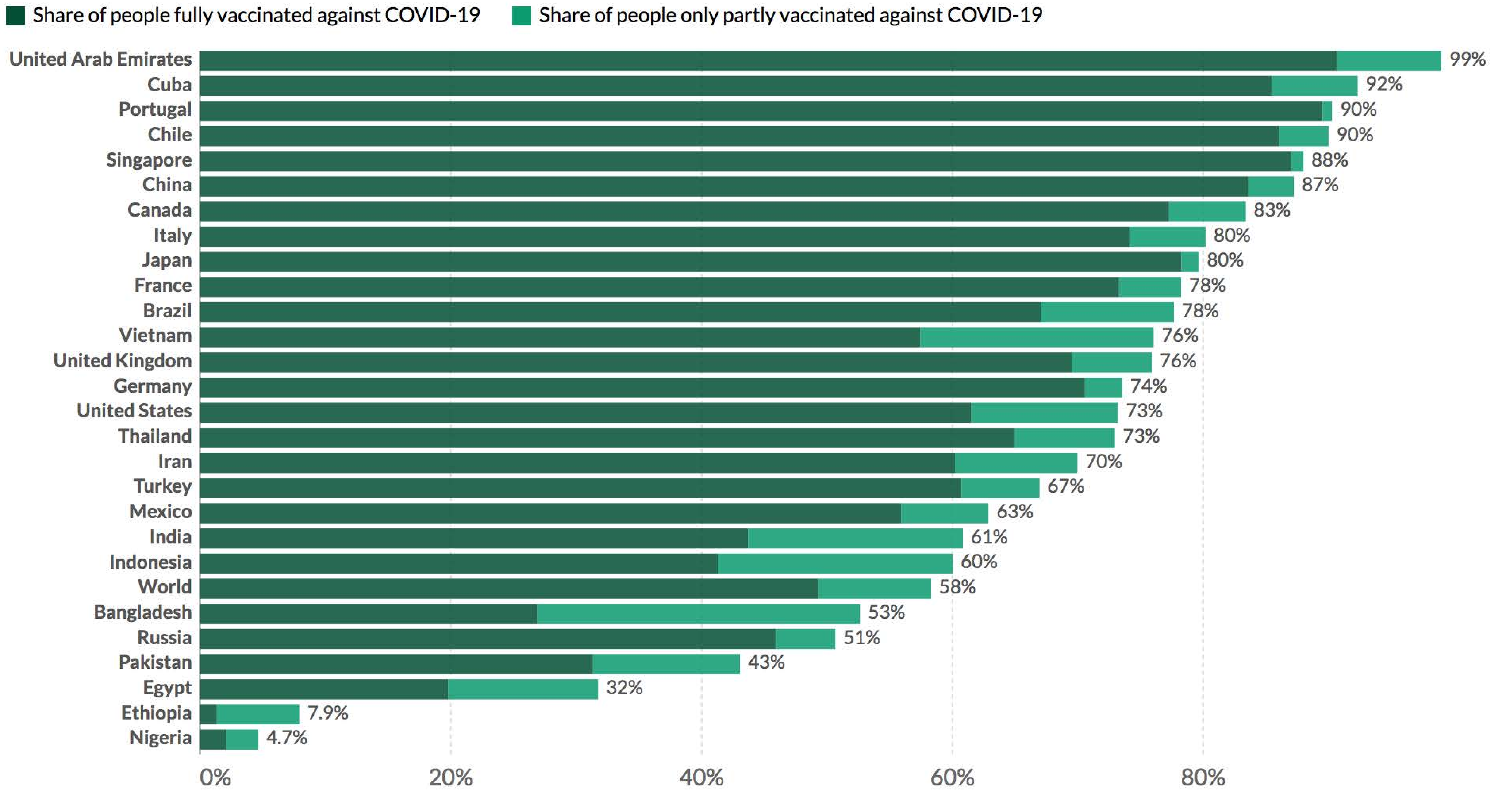

Source: Official data collated by Our World in Data
Note: Alternative definitions of a full vaccination, e.g. having been infected with SARS-CoV-2 and having 1 dose of a 2-dose protocol, are ignored to maximize comparability between countries.

CC BY

Dec 13, 2020 — Jan 2, 2022

CHART | TABLE | SOURCES | DOWNLOAD

Tracking Covid-19 vaccinations worldwide

By Henrik Pettersson, Byron Manley, Sergio Hernandez, Deidre McPhillips and Tatiana Arias, CNN

Last updated: January 3, 2022 at 7:50 p.m. ET

At least 218 countries and territories have administered more than 9 billion doses of a Covid-19 vaccine, since the first Covid-19 case was reported in China in late 2019. Several different vaccines have been developed at record speed, in large part due to years of research on related viruses and billions of dollars in investment.

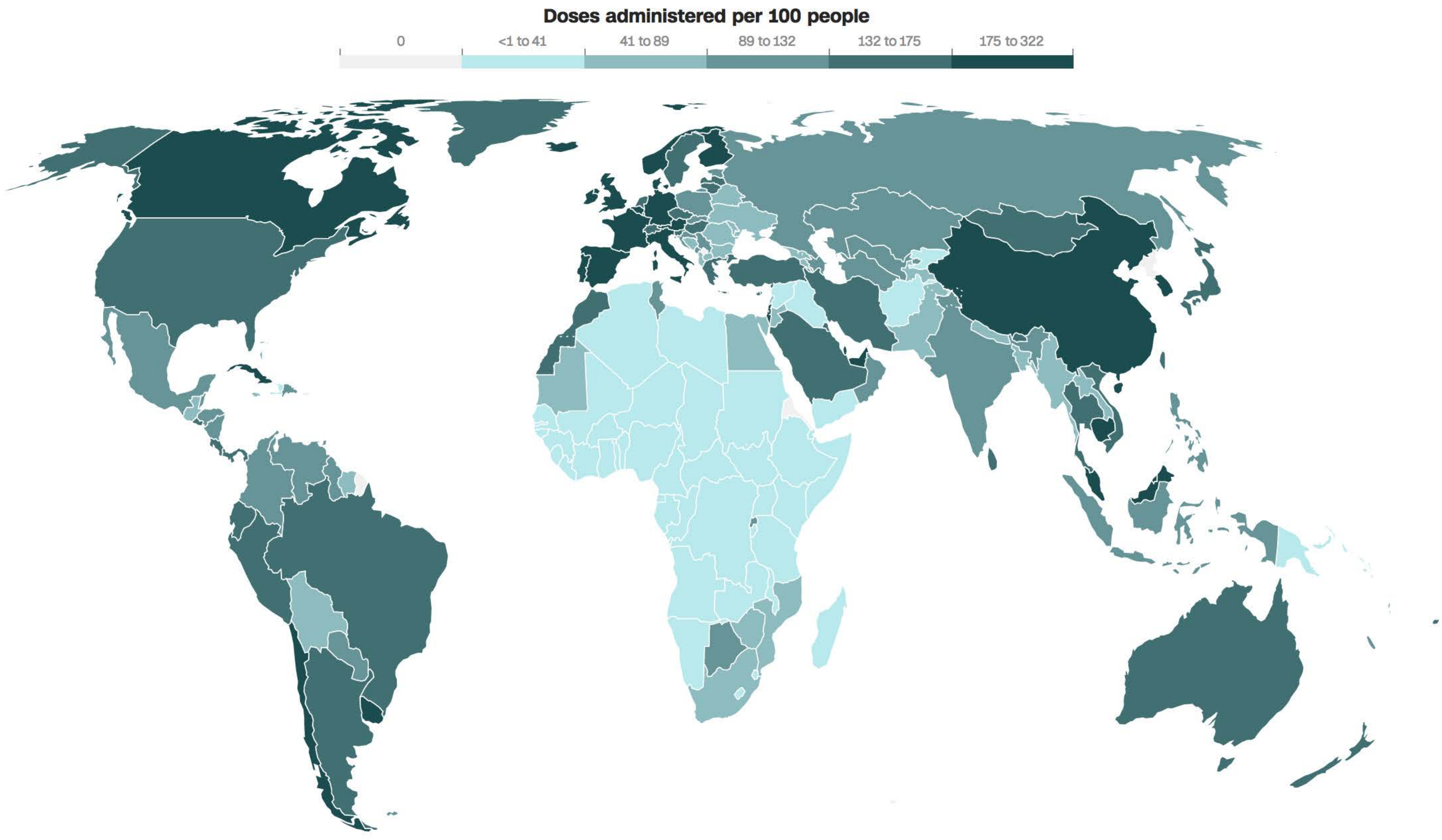

Last updated: January 3, 2022 at 7:50 p.m. ET
Source: Our World in Data

In December 2020, the first dose of a fully tested vaccine — manufactured by Pfizer/BioNTech — was administered in the United Kingdom. Now, multiple vaccines have been authorized for use around the world and dozens of countries and territories have joined the race to vaccinate their residents.

Rate of global vaccination campaign

Currently, the global 7-day moving average of administered doses is about 29.2 million per day.

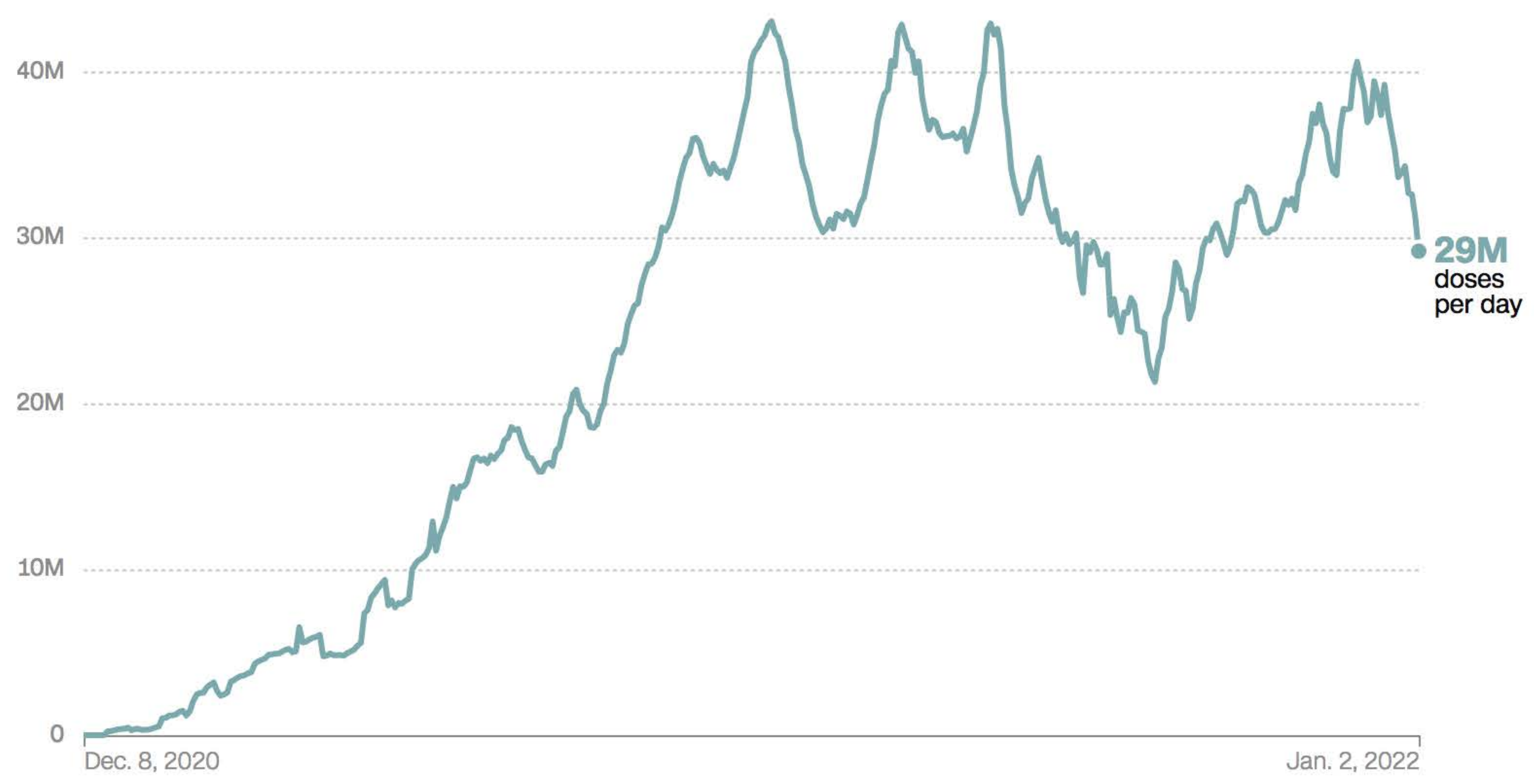

Last updated: January 3, 2022 at 7:50 p.m. ET
Source: Our World in Data

The vaccine rollout strategy varies from country to country. Some have favored vaccinating as many people as possible as quickly as possible, while others have tried to prioritize vaccinating specific vulnerable groups.

Leaders at the World Health Organization have emphasized the need for international cooperation in vaccination campaigns, noting that a global pandemic requires global efforts to end it.

Comparing vaccination rates

At least 218 countries and territories have started their vaccination rollouts. Different countries have different processes for [illegible] are at different stages of their vaccination campaigns.

CNN health

Filter by number of days since first reported administered dose:

All | <14 days | 14–29 days | 30+ days

Location	Total doses	Doses administered per 100 people	Days since first dose
Gibraltar	108,323	322	359
Cuba	30,874,045	273	239
Chile	44,166,888	230	376
United Arab Emirates	22,406,584	224	369
Iceland	716,926	209	371
Denmark	12,136,265	209	373
Isle of Man	177,816	208	365
Malta	1,073,046	208	373
Jersey	206,976	205	387
South Korea	103,872,155	202	312
Pitcairn	94	200	231
Uruguay	6,964,663	200	309
Mainland China	2,847,898,000	197	385
Cayman Islands	130,608	196	361
Faroe Islands	96,235	196	370
United Kingdom	133,112,711	195	392
Ireland	9,664,812	194	371
Portugal	19,379,608	191	373
Belgium	21,609,656	186	372
Seychelles	183,472	185	359

Show more

Last updated: January 3, 2022 at 7:50 p.m. ET
Source: Our World in Data, CNN research
