# Supplementary material for: Web-Based COVID-19 Dashboards and Trackers in the United States: Survey Study
Source: JMIR Hum Factors. 2023 Mar 20;10:e43819. doi: 10.2196/43819 (PMC10029858; doi:10.2196/43819)
Supplement: Multimedia Appendix 15 [file humanfactors_v10i1e43819_app15.pdf]

Appendix 15. Screenshots from dashboards and trackers — *State-focused coverage* *(captured June 12, 2022)*

Page	Ref	Host
2–3	S-1	Alabama Dept of Public Health
4–6	S-4	Alaska Dept of Health and Social Services
7–8	S-6	Arizona Dept of Health Services
9–11	S-7	Arkansas Dept of Health
12	S-8	California Dept of Public Health
13–15	S-9	CA Open Data
16	S-11	CalMatters
17–21	S-12	Colorado Dept of Health & Environment
22	S-13	Coloradoan
23	S-14	The Denver Post
24–27	S-16	CTData Collaborative
28	S-17	University of Connecticut Dept of Geography
29	S-18	Government of the District of Columbia
30	S-19	Delaware Environmental Public Health Tracking Network
31	S-23	Atlanta Journal-Constitution
32	S-24	State of Hawaii Dept of Health
33–36	S-26	Idaho Division of Public Health
37	S-28	Illinois Dept of Public Health
38–40	S-29	Indiana State Dept of Health
41–42	S-32	Kansas Dept of Health and Environment
43	S-33	Kentucky Dept of Public Health
44	S-35	Louisiana Dept of Health
45	S-36	Maine Center for Disease Control & Prevention
46–47	S-38	Maryland Dept of Health
48–50	S-39	Massachusetts Dept of Public Health
51–53	S-40	Michigan Dept of Health and Human Services
54	S-41	Bridge Michigan
55	S-42	Minnesota Dept of Health
56	S-43	Mississippi State Dept of Health

Page	Ref	Host
57	S-44	Missouri Dept of Health and Senior Services
58–59	S-46	Montana State Library
60–62	S-47	North Carolina Dept of Health and Human Services
63–65	S-48	North Dakota Dept of Health
66–68	S-50	Nevada Dept of Health and Human Services
69–71	S-51	New Hampshire Dept of Health and Human Services
72	S-52	New Hampshire Public Radio
73–74	S-53	New Jersey Dept of Health
75	S-55	New Mexico Dept of Health
76	S-56	New York State Dept of Health
77	S-57	Ohio Dept of Health
78	S-58	Oklahoma State Dept of Health
79	S-59	Oregon Health Authority
80–82	S-60	Pennsylvania Dept of Health
83	S-61	Rhode Island Dept of Health
84–85	S-62	South Carolina Dept of Health and Environment Control
86–88	S-64	South Dakota Dept of Health
89–90	S-66	Tennessee Dept of Health
91	S-68	Houston Chronicle
92	S-69	Utah Dept of Health
93–95	S-70	Virginia Dept of Health
96	S-72	VTDigger
97	S-73	Washington State Dept of Health
98	S-74	Washington State Hospital Association
99	S-75	West Virginia Dept of Health & Human Resources
100–102	S-76	Wisconsin Dept of Health Services
103	S-77	WISN-12
104	S-80	WPRI-12
105	S-81	San Francisco Chronicle
106	S-82	The Maine Monitor
107	S-83	The Oregonian

Alabama's COVID-19 Data and Surveillance Dashboard

ALABAMA PUBLIC HEALTH

Alabama Department of Public Health | Division of Infectious Diseases & Outbreaks | Updates Monday - Friday by 10 a.m.

Alabama

Click the county to change the data on the right and on tabs 6 and 10.

- Alabama
- Autauga
- Baldwin
- Barbour
- Bibb
- Blount
- Bullock
- Butler
- Calhoun
- Chambers
- Cherokee
- Chilton
- Choctaw
- Clarke
- Clay
- Cleburne
- Coffee
- Colbert
- Conecuh

Daily and 7-Day Moving Average Number of Cases in 2022

(Please select Alabama or a specific county from the list on the left.)

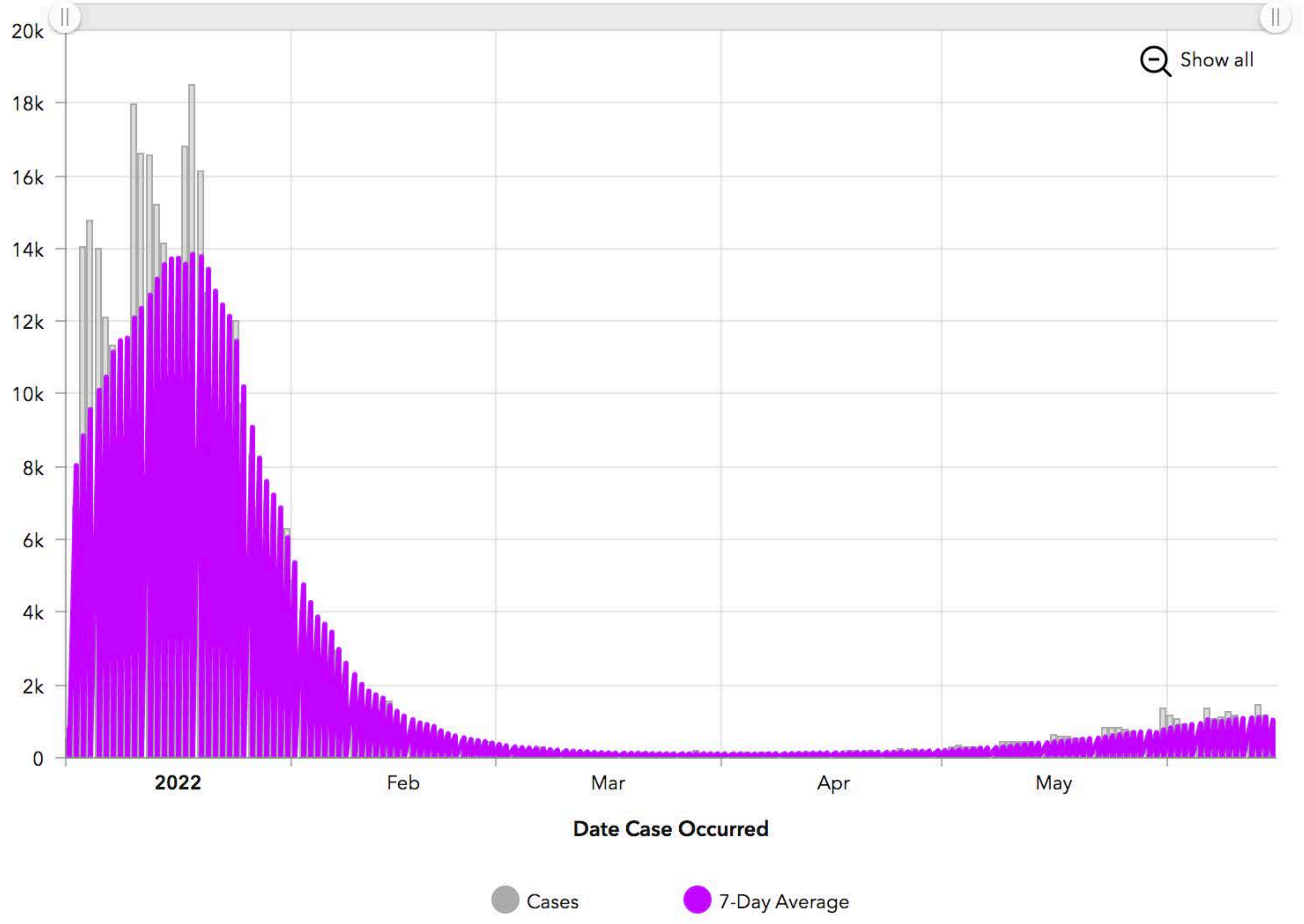

Notices | Index | 3 | 5 | 6-22 | 6-21 | 6-20 | 7 | 8 | 9 | 10 | 11 | About

MOVING 7-DAY

20.1

% Positivity in Tests

CASES

1,333,137

TOTAL

387,828	538,460	406,849
2020	2021	2022

DEATHS

19,696

TOTAL

7,189	9,555	2,952
2020	2021	2022

LAST 7 DAYS - TOTAL TESTS

30,192	6,070
NAAT TESTS	POSITIVE NAAT TESTS

Alabama's COVID-19 Data and Surveillance Dashboard

Alabama Department of Public Health | Division of Infectious Diseases & Outbreaks | Updates Monday - Friday by 10 a.m.

Alabama

Click the county to change the data on the right and on tabs 6 and 10.

- Alabama
- Autauga
- Baldwin
- Barbour
- Bibb
- Blount
- Bullock
- Butler
- Calhoun
- Chambers
- Cherokee
- Chilton
- Choctaw
- Clarke
- Clay
- Cleburne
- Coffee
- Colbert
- Conecuh

Impact of COVID-19 on Cases and Deaths in Alabama the Past Four Weeks

Age (in years)	Cases	Deaths	Population
0-4 years	3.9%	0.0%	5.9%
5-11 years	2.2%	0.0%	8.6%
12-17 years	2.4%	0.0%	7.6%
18-24 years	9.0%	0.0%	9.1%
25-49 years	33.6%	0.0%	31.5%
50-64 years	21.2%	20.0%	19.6%
65-74 years	10.5%	28.0%	10.5%
≥75 years	7.0%	52.0%	7.2%
Age unknown	10.1%	0.0%	0.0%

Race	Cases	Deaths	Population
American Indian or Alaska	0.4%	0.0%	0.5%
Asian	2.0%	4.0%	1.3%
Black or African-American	23.6%	28.0%	26.9%
Native Hawaiian or Other Pacific	0.1%	0.0%	0.0%
White	52.1%	52.0%	67.8%
Other race	5.6%	4.0%	1.5%
Two or more races	0.4%	0.0%	1.9%
Race unknown	15.8%	12.0%	0.0%

Ethnicity	Cases	Deaths	Population
Hispanic or Latino	0.7%	0.0%	4.5%
Not Hispanic or Latino	80.3%	88.0%	95.5%
Ethnicity unknown	19.0%	12.0%	0.0%

MOVING 7-DAY

20.1
% Positivity in Tests

CASES

1,333,137
TOTAL

2020	2021	2022
387,828	538,460	406,849

DEATHS

19,696
TOTAL

2020	2021	2022
7,189	9,555	2,952

LAST 7 DAYS - TOTAL TESTS

NAAT TESTS	POSITIVE NAAT TESTS
30,192	6,070

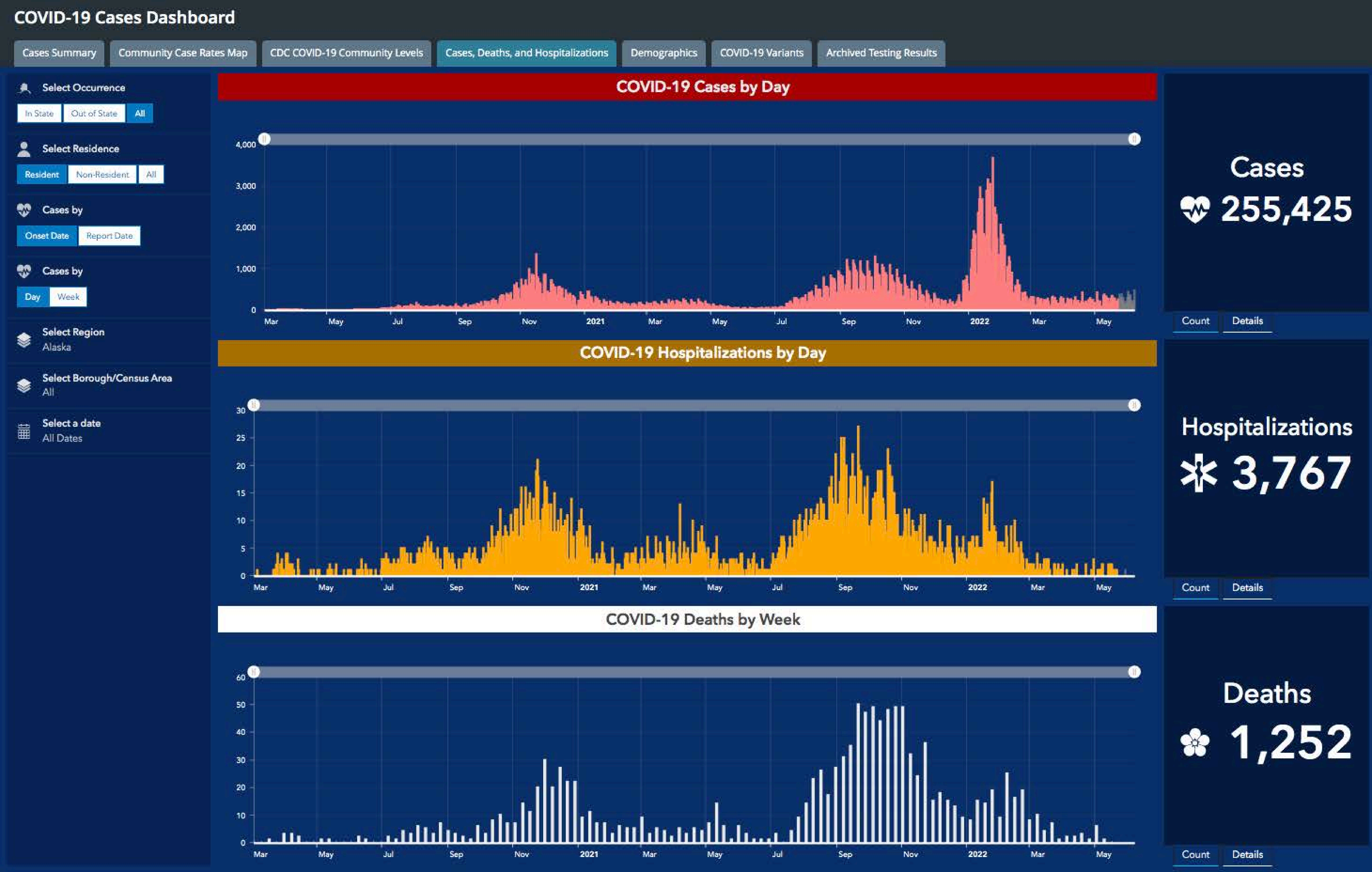

COVID-19 Cases Dashboard
Cases Summary
Community Case Rates Map
CDC COVID-19 Community Levels
Cases, Deaths, and Hospitalizations
Demographics
COVID-19 Variants
Archived Testing Results
Select Occurrence
In State
Out of State
All
Select Residence
Resident
Non-Resident
All
Cases by
Onset Date
Report Date
Cases by
Day
Week
Select Region
Alaska
Select Borough/Census Area
All
Select a date
All Dates
COVID-19 Cases by Day
4,000
3,000
2,000
1,000
0
Mar May Jul Sep Nov 2021 Mar May Jul Sep Nov 2022 Mar May
Cases
255,425
Count
Details
COVID-19 Hospitalizations by Day
30
25
20
15
10
5
0
Mar May Jul Sep Nov 2021 Mar May Jul Sep Nov 2022 Mar May
Hospitalizations
3,767
Count
Details
COVID-19 Deaths by Week
60
50
40
30
20
10
0
Mar May Jul Sep Nov 2021 Mar May Jul Sep Nov 2022 Mar May
Deaths
1,252
Count
Details

COVID-19 Cases Dashboard

Cases Summary | Community Case Rates Map | CDC COVID-19 Community Levels | Cases, Deaths, and Hospitalizations | Demographics | COVID-19 Variants | Archived Testing Results

Cases, Hospitalizations, and Deaths by Age

Cases

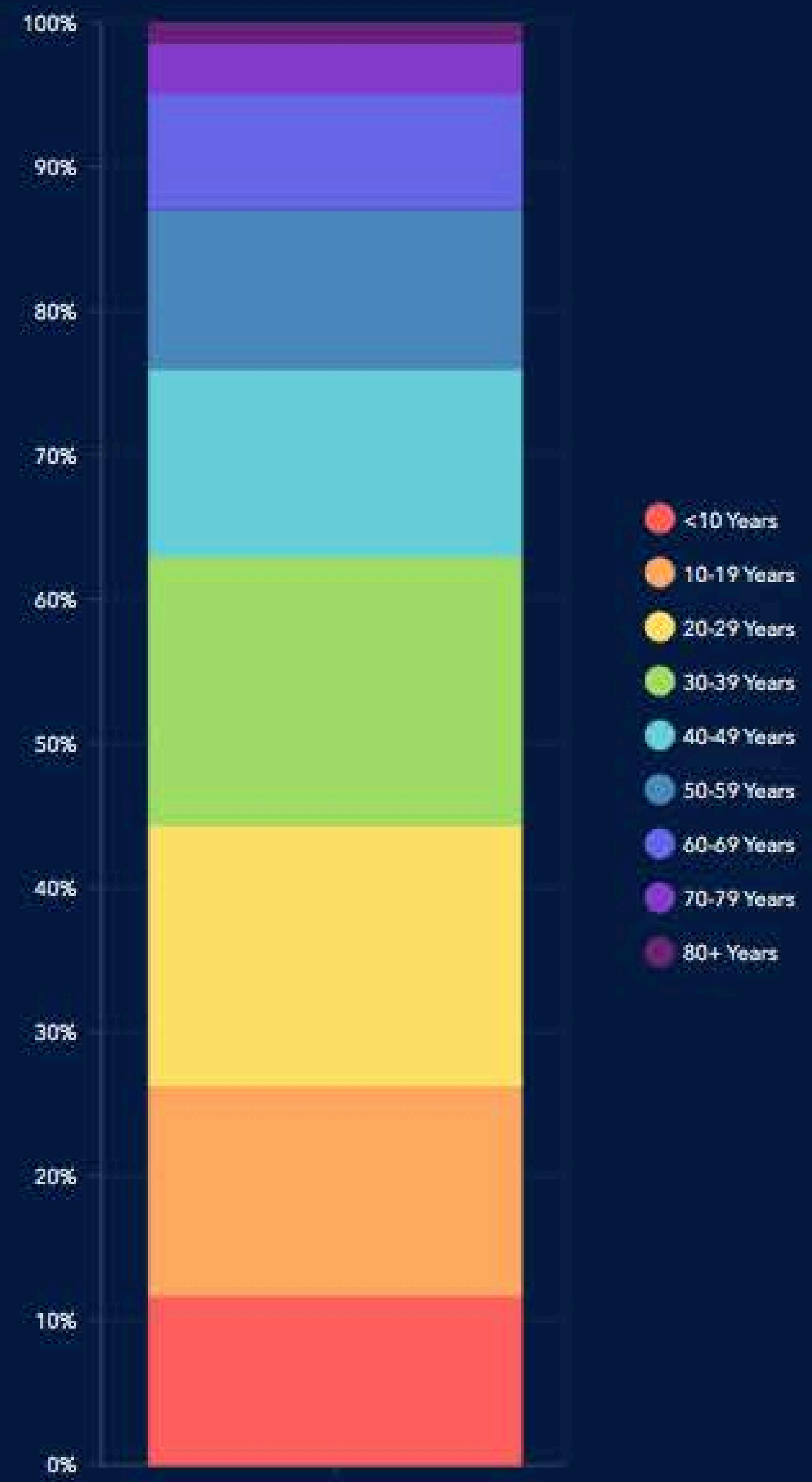

Hospitalizations

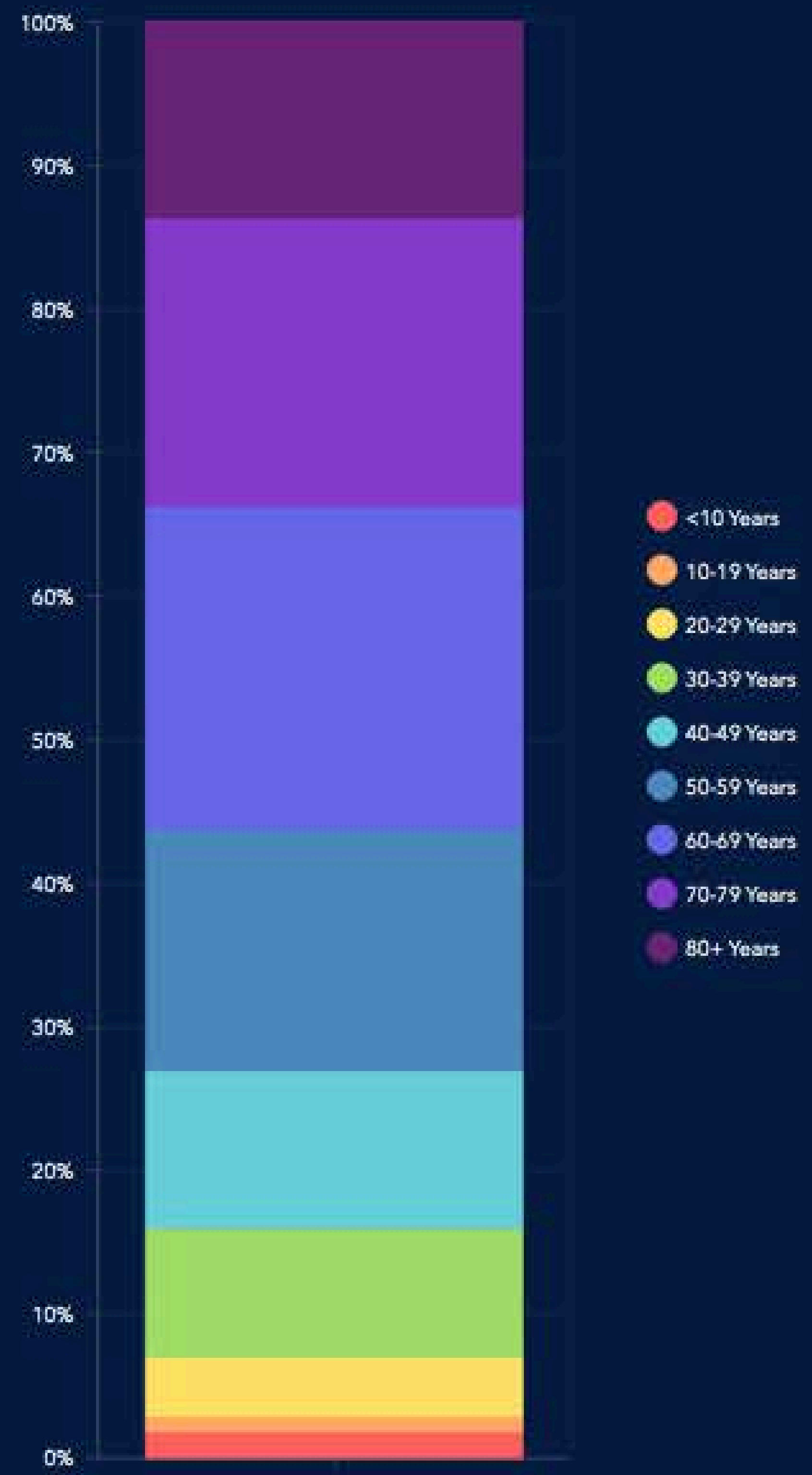

Deaths

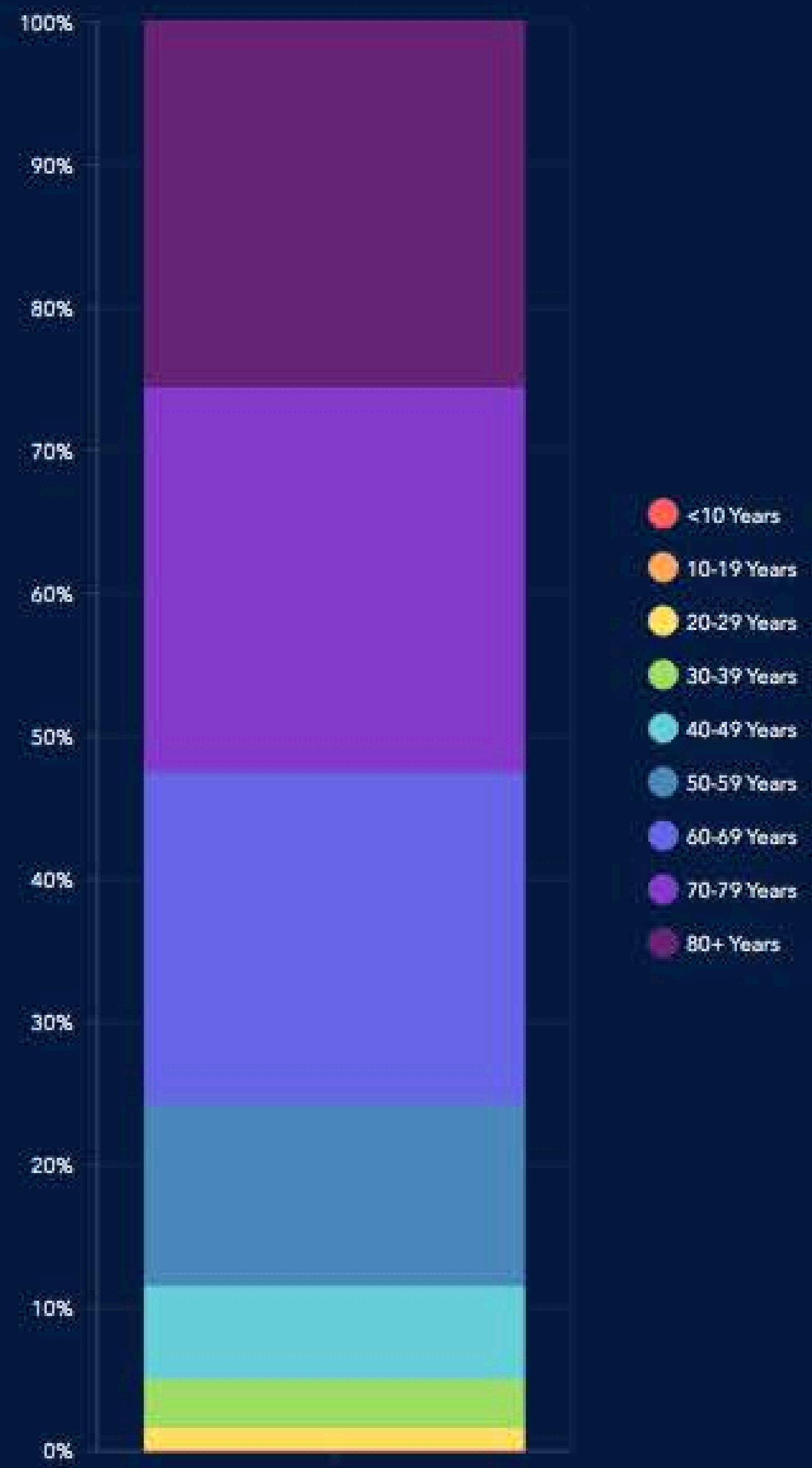

Population Comparison

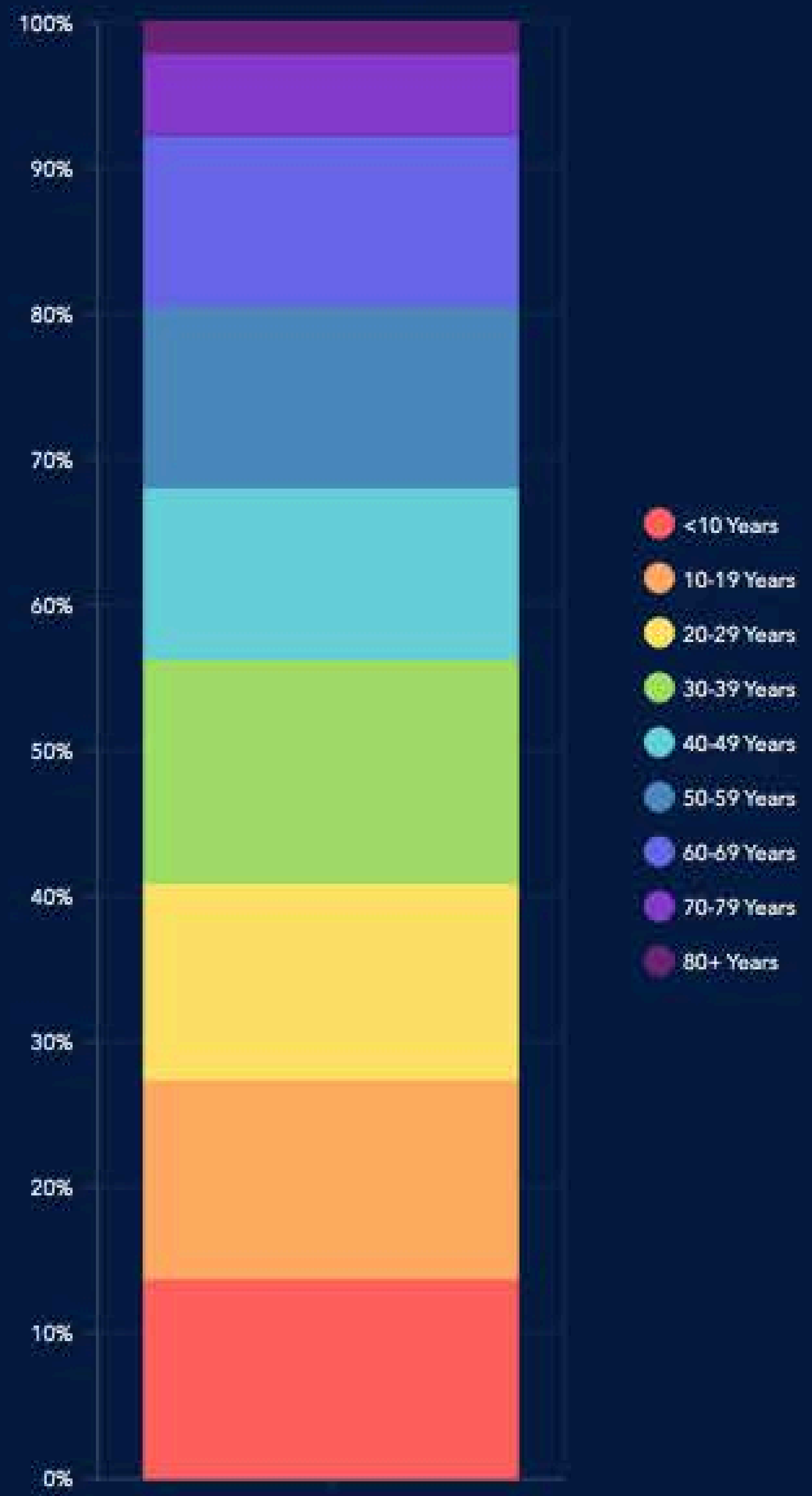

Age | Sex | Race | Ethnicity

Demographic information may be self-reported. Missing Race and Missing Ethnicity contain cases under investigation, unknown race/ethnicity, and other race/ethnicity. For more information on a break out of missing race/ethnicity categories, click here

COVID-19 Cases Dashboard

Cases Summary | Community Case Rates Map | CDC COVID-19 Community Levels | Cases, Deaths, and Hospitalizations | Demographics | COVID-19 Variants | Archived Testing Results

7-Day Case Rate by Demographic Group

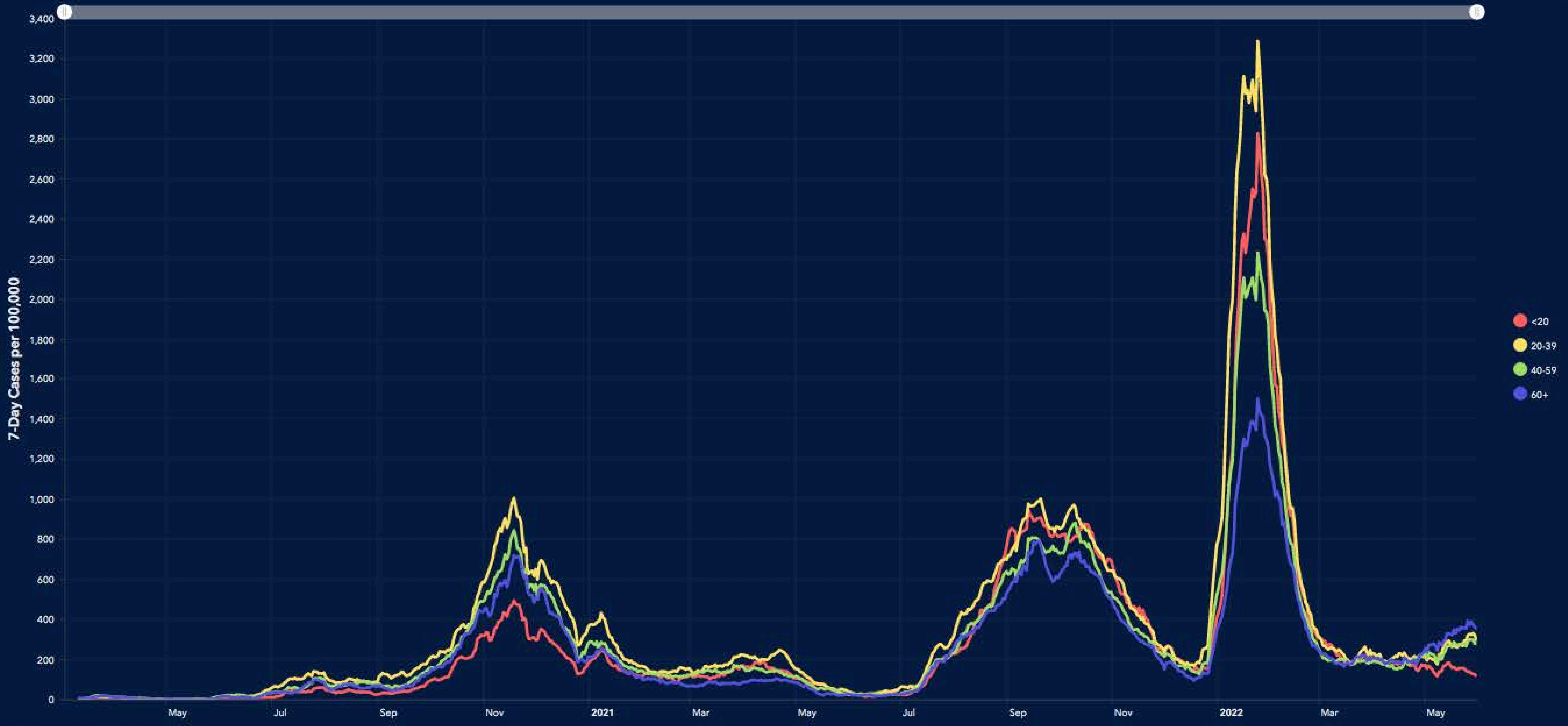

Age | Sex | Race | Ethnicity

Cases rates by race and ethnicity may be over or under represented due to missing information. Please interpret with caution. The past 7 days are excluded from this figure because cases with onset date during this period are unlikely to have been counted yet.

Data currently shown for **All Time**
Date Updated: 6/8/2022

Select time frame: **All Time** | Last 6 Months

Show case counts or population rates in the map below?

Cases ▾

Select a county to filter the other numbers.

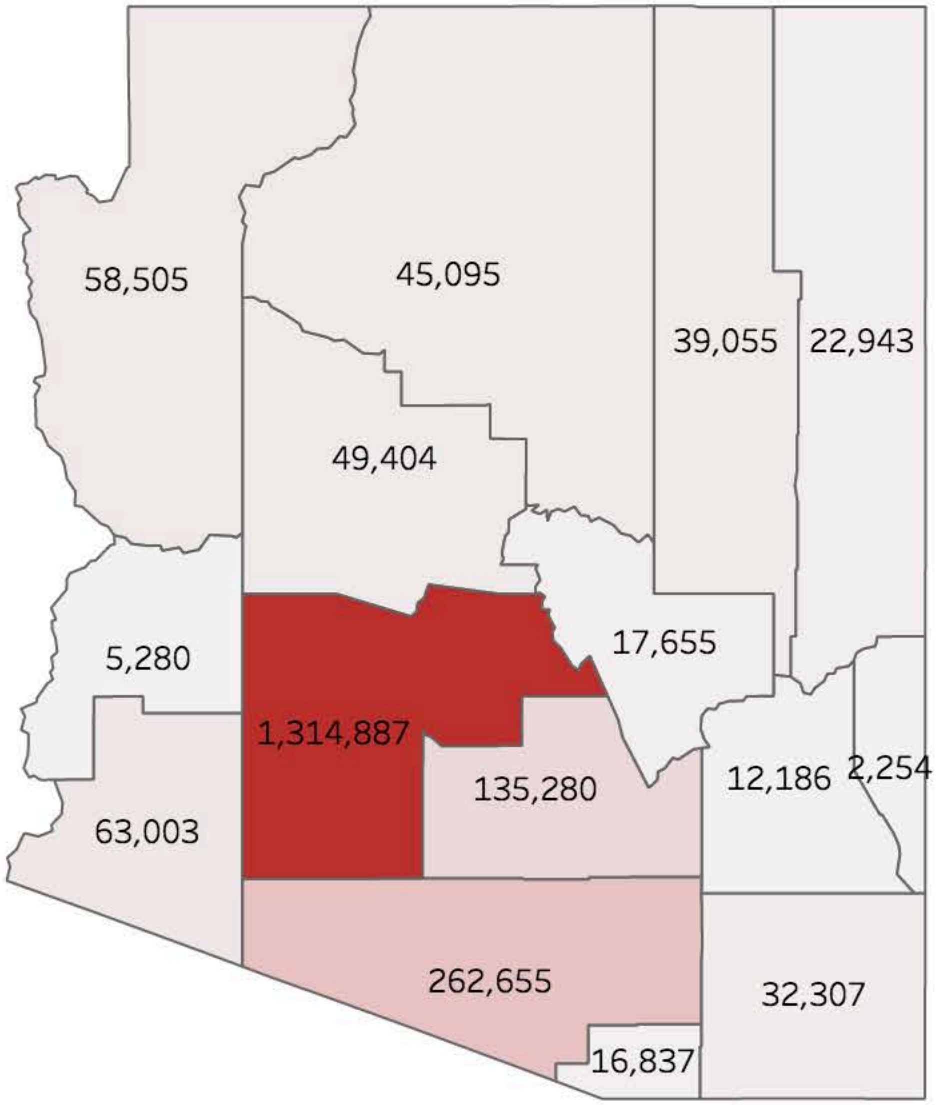

Number of Cases	Number of Deaths *	Number of Vaccine Doses Administered **
**2,077,346**	**30,372**	**11,837,009**

Number of New Cases reported since last week***	Number of New Deaths reported since last week***	Number of New Vaccine Doses reported since last week** ***
**14,677**	**40**	**43,237**

Rate of cases, per 100,000 population	Rate of fatalities, per 100,000 population	Percent of people vaccinated **
**28,896.1**	**422.5**	**71.4%**

Tests Conducted
using diagnostic tests †

Total Tests Conducted	New Tests reported since last week***	Total Percent Positive ††
**19,712,761**	**90,300**	**12.1%**

* Deaths among cases with specimen collection in the past four weeks are excluded to allow more complete information to be received.

Total Number of COVID-19 **Cases** (Last 6 Months): 747,998

Show cases or rates?

Cases

Select a county to filter the other graphs.

Graphs will not be displayed for counties with fewer than 10 cases.

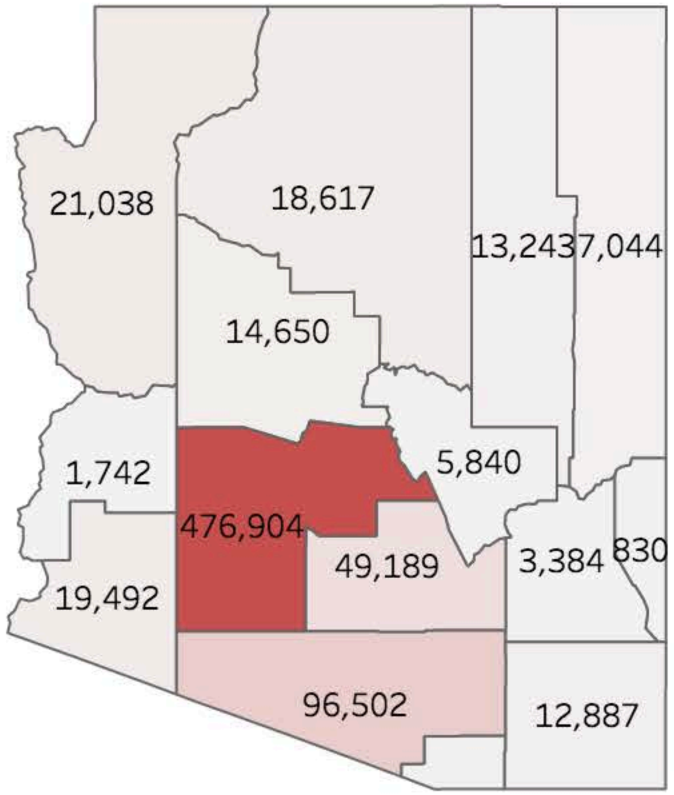

Age Groups and Sex at Birth

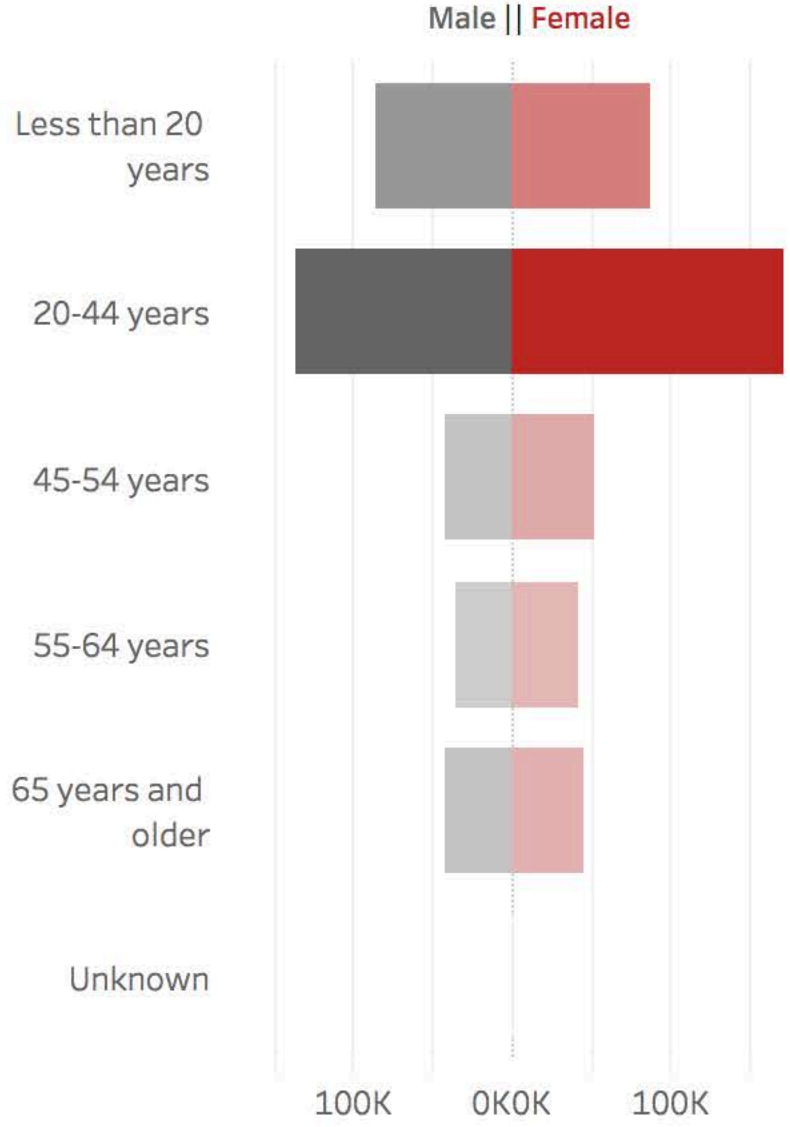

COVID-19 Cases by Race/Ethnicity

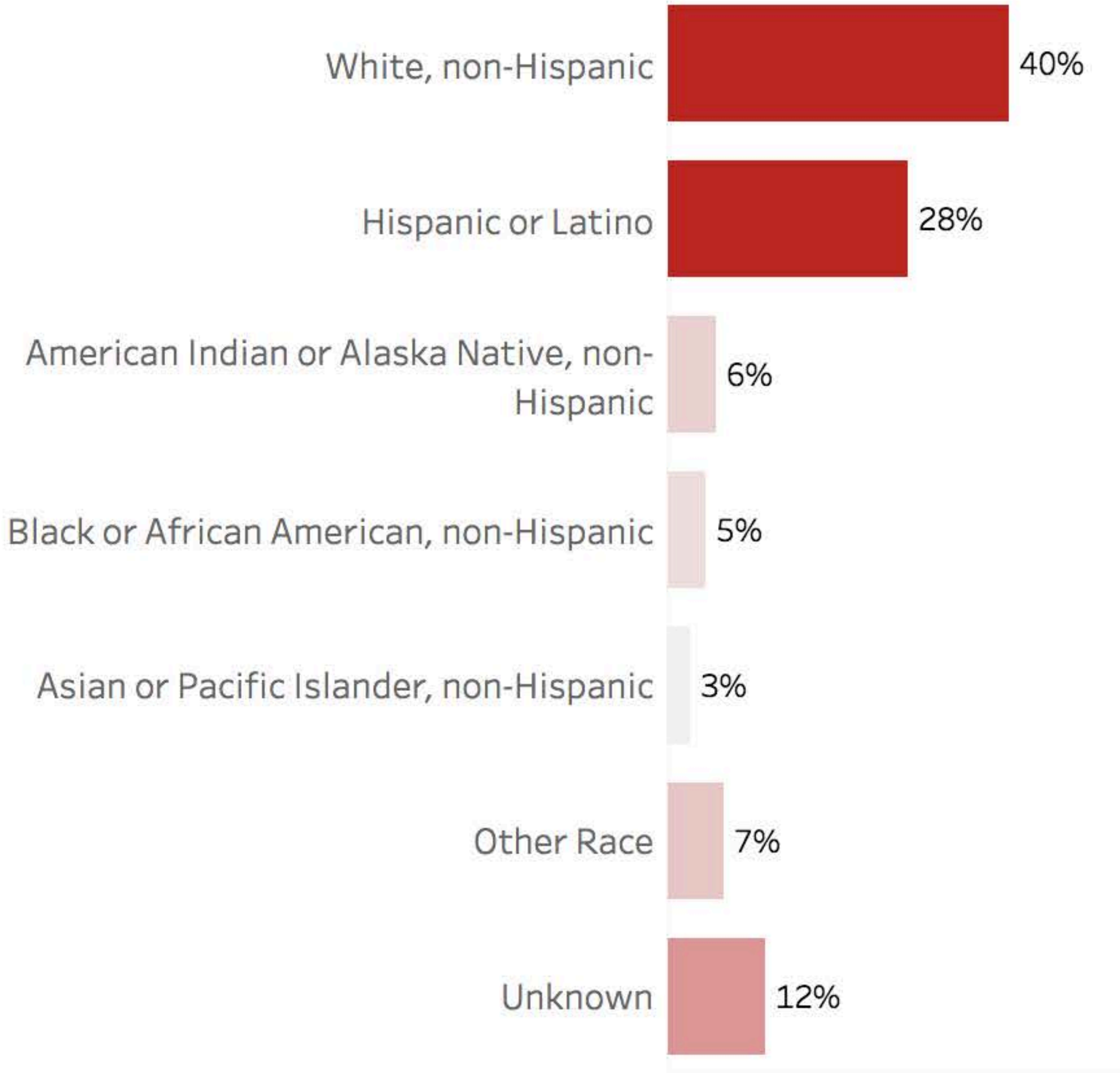

COVID-19 Cases by Sex at Birth

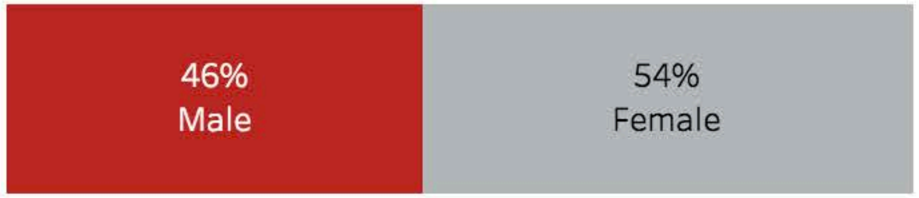

Chronic Medical Condition

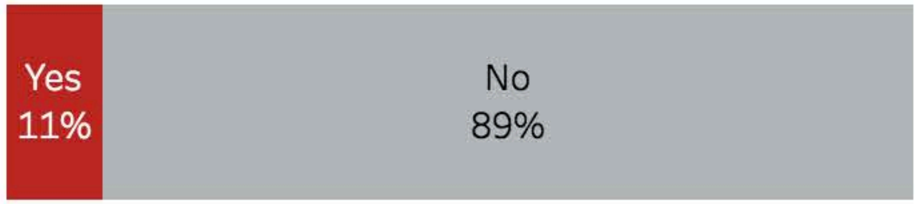

High-risk

(65+ years OR 1+ chronic medical condition)

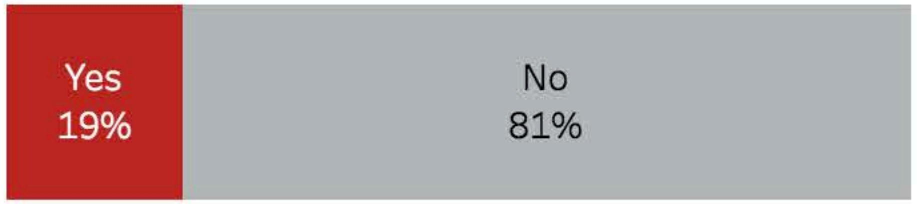

Chronic medical conditions include diabetes, cardiac disease, hypertension, chronic pulmonary disease, chronic kidney disease, chronic liver disease, or obesity.

Data are updated on Wednesdays and include data through **6/4/2022.**

The Arkansas Department of Health COVID-19 Update

Total COVID-19 Cases
(*includes all confirmed and probable cases)

850,203

Number of New Cases
(reported in the last 24 hours)

449

Recovered Cases

831,597

Cumulative Number of Cases
(last 14 days)

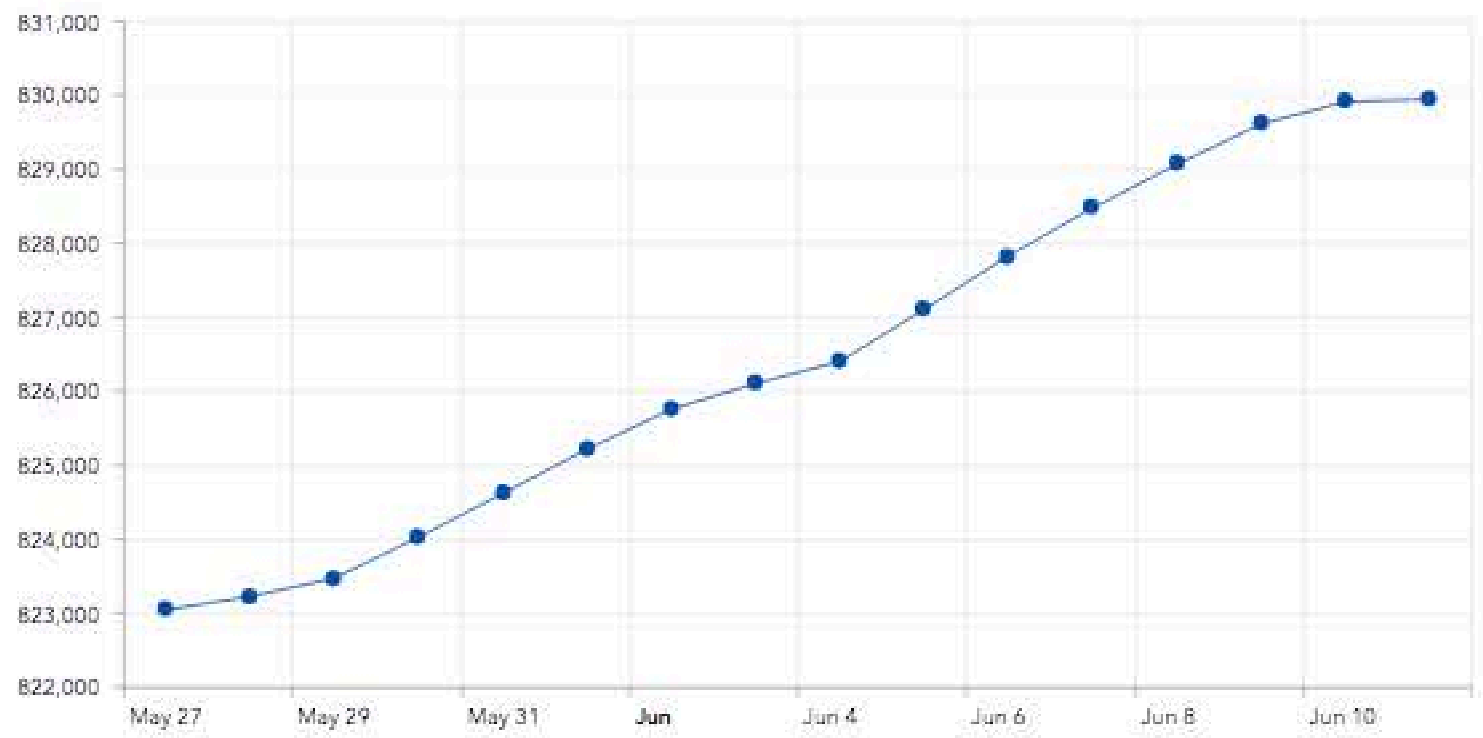

Number of COVID-19 Cases by County of Residence

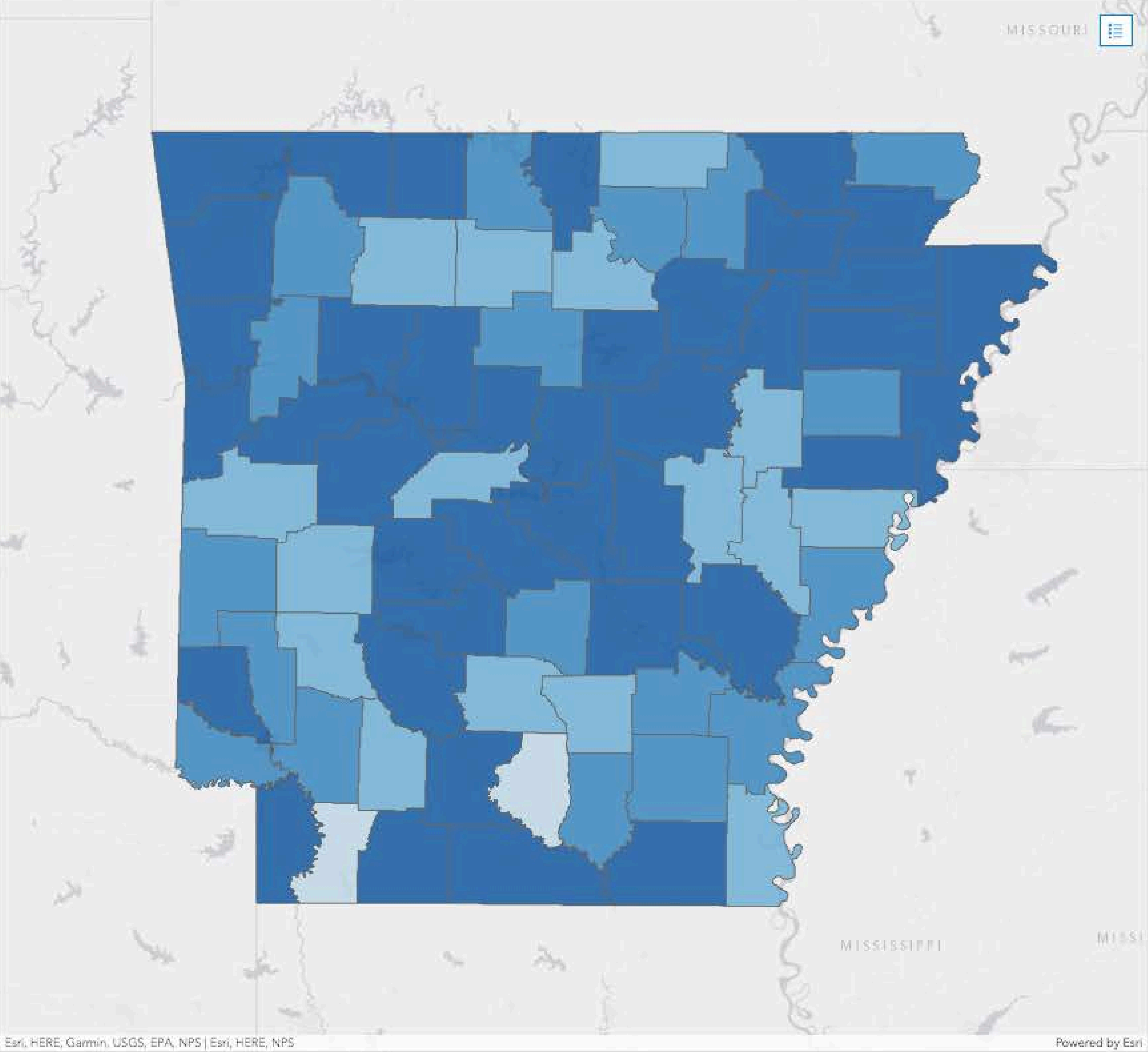

Vaccines | Total Cases | Active Cases | Pediatric Metrics | Deaths | Tests | Case Demographics | Health Metrics | Hospital Information | USA and Global

The Arkansas Department of Health COVID-19 Update

Totals Cases by Gender

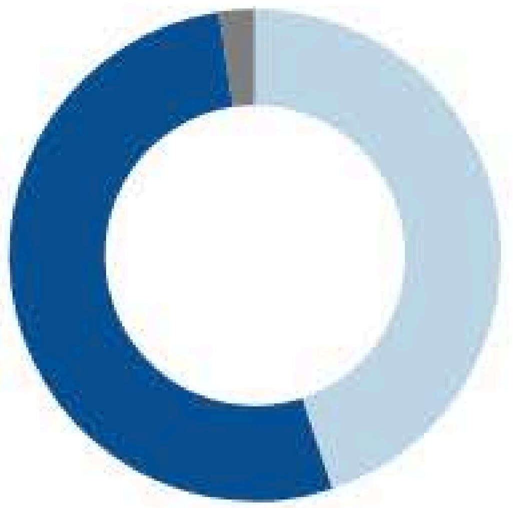

Cases by Race

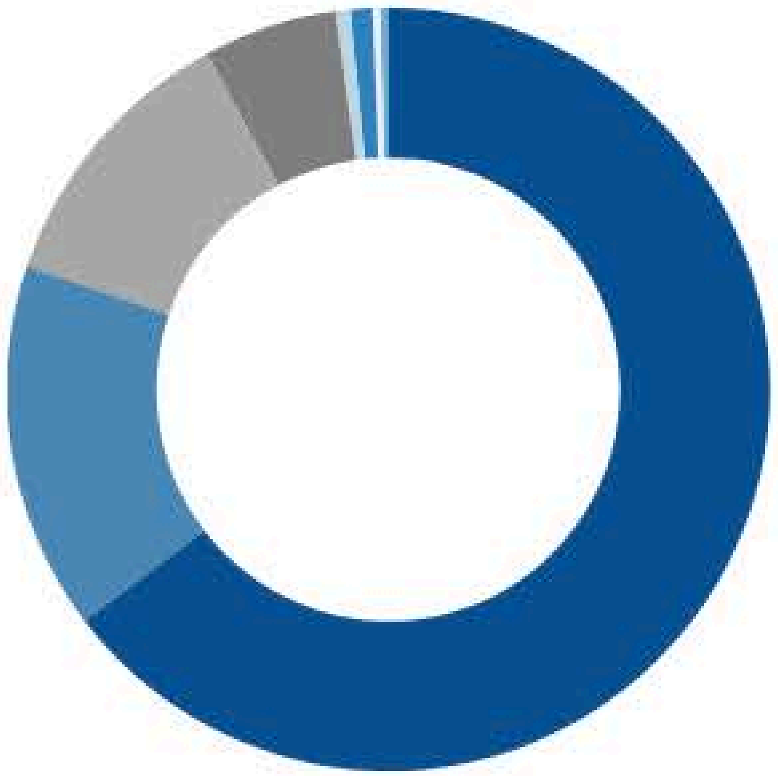

Cases by Ethnicity

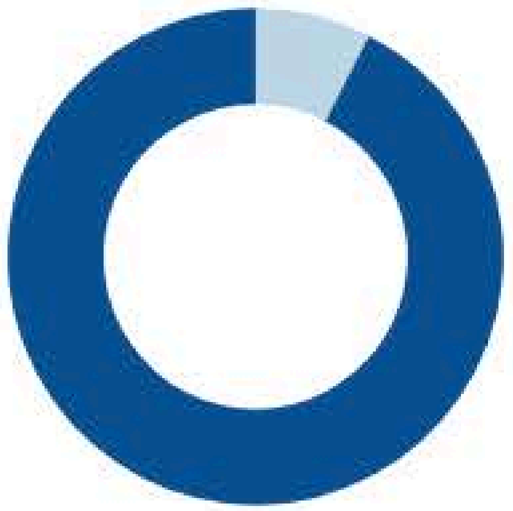

Cases by Age Category

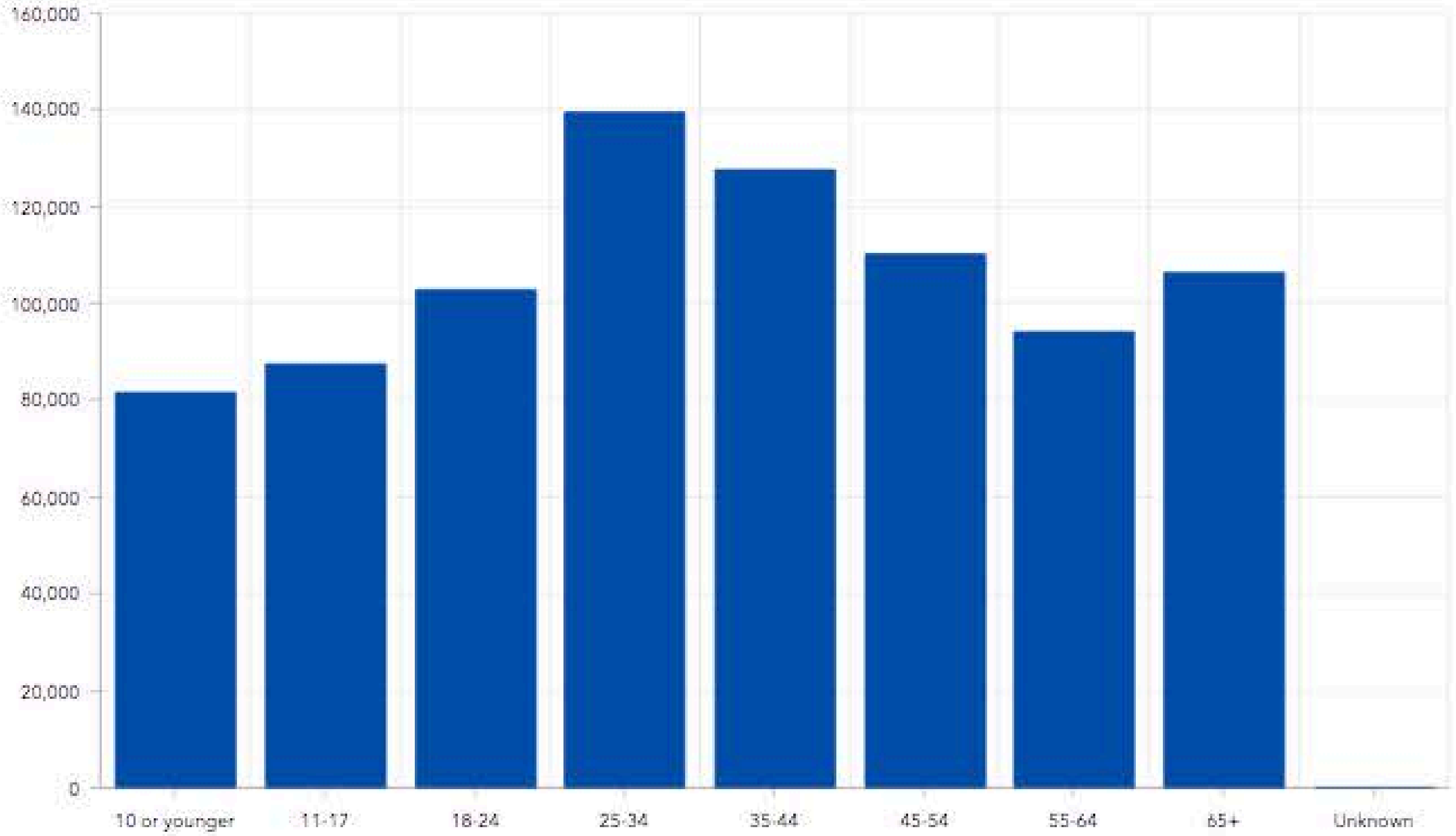

Cases by Underlying Health Concerns

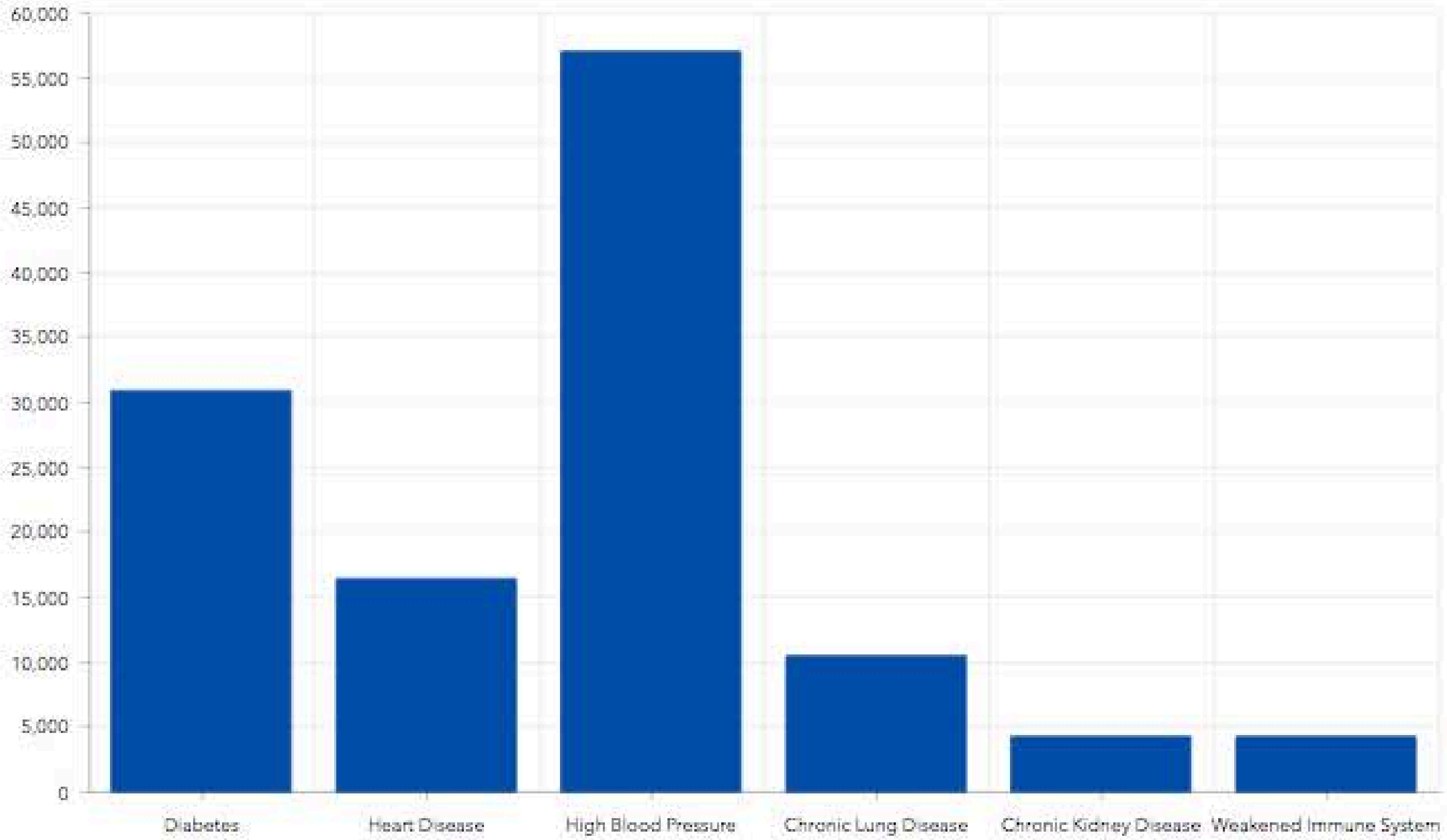

Percent of Cases That Are Healthcare Workers

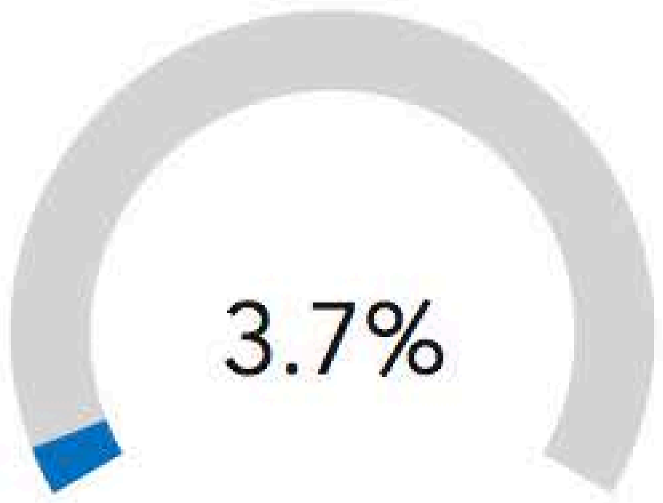

Percent of Cases In Nursing Homes

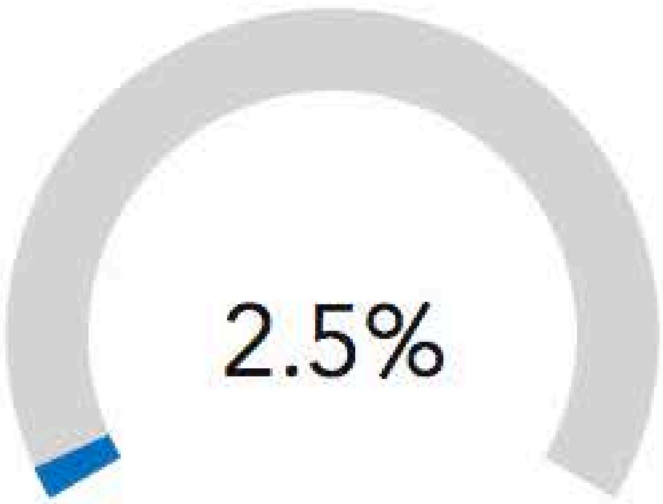

Percent of Cases In Correctional Facilities

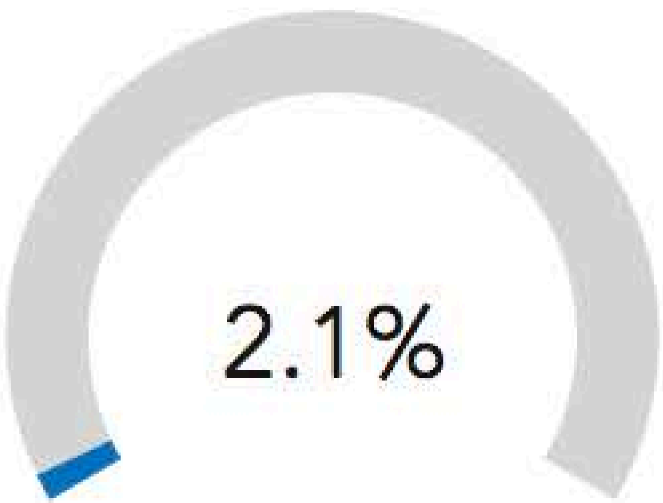

Total Tests Reported

6,992,431

Percent Positive PCR Tests	Percent Positive Antigen Tests
**11.5%**	**15.5%**

14 Day Testing Trend - PCR

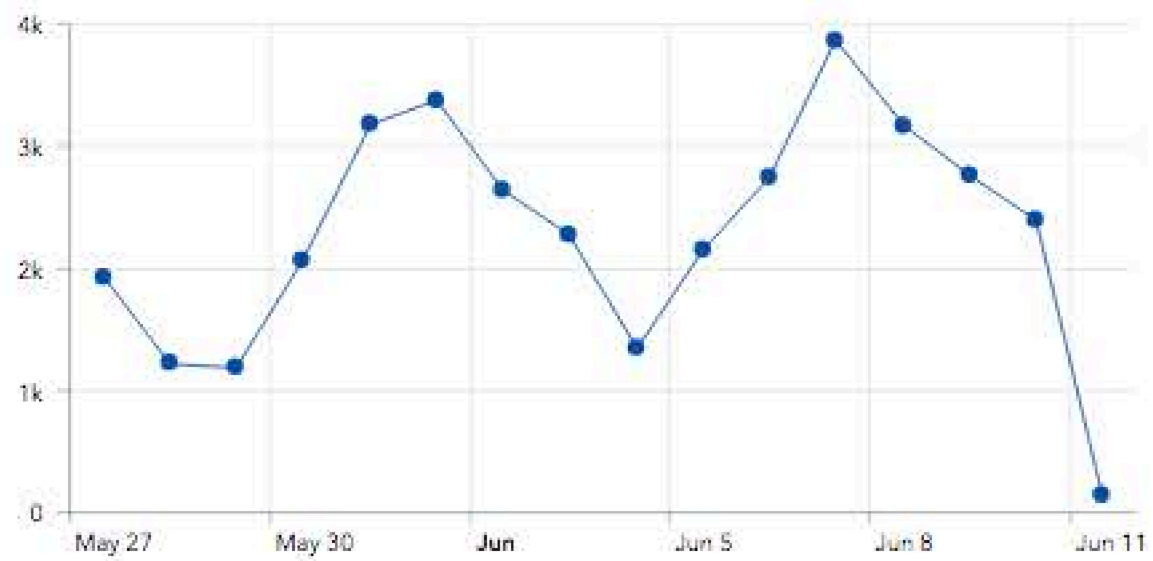

14 Day Testing Trend - Antigen

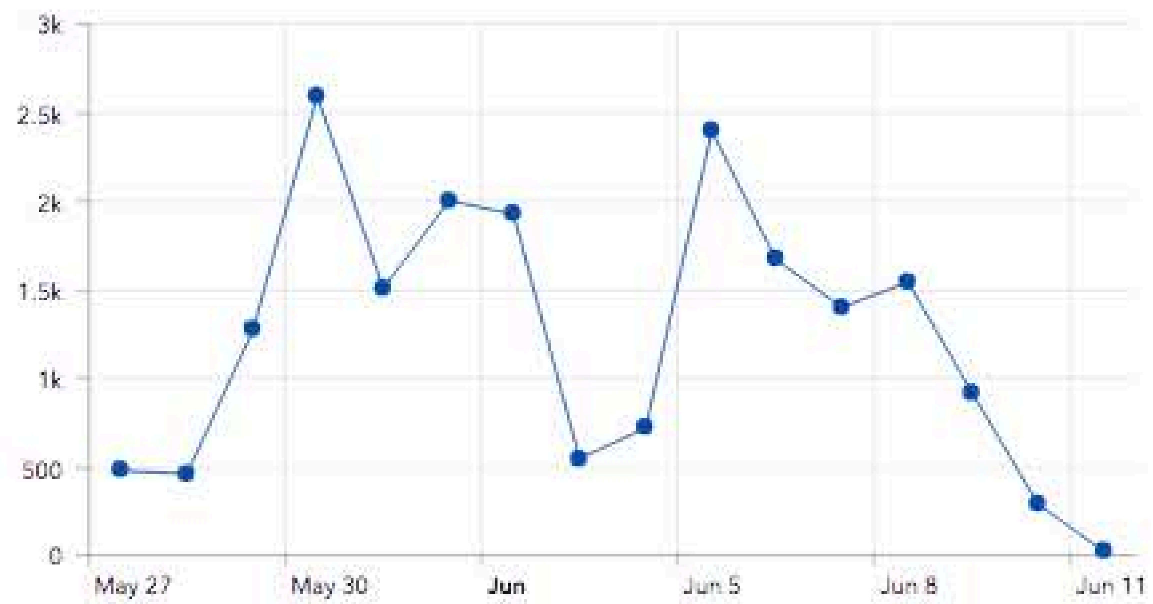

COVID-19 Tests Resulted per 1,000 Individuals

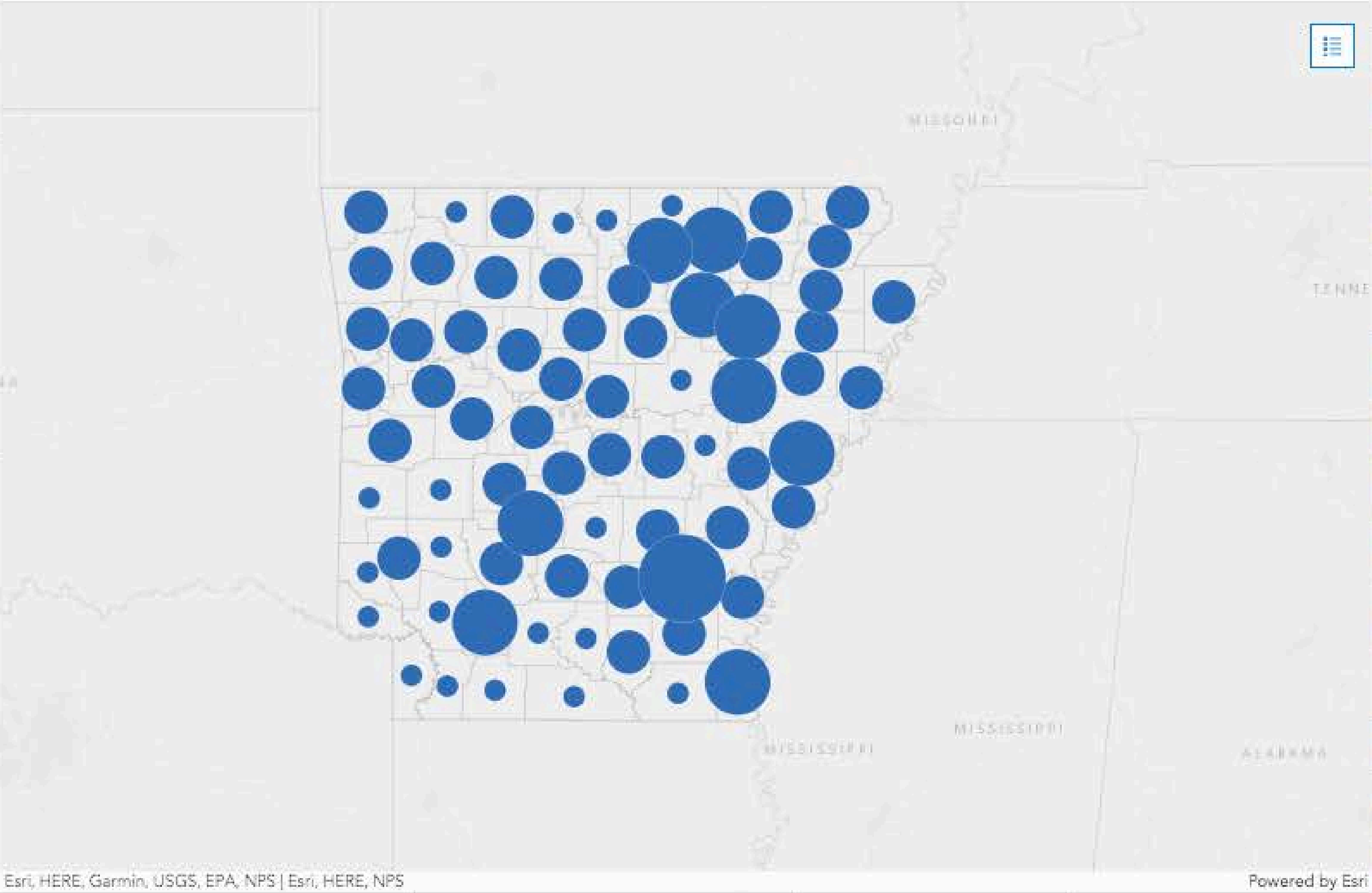

- **Arkansas** (52253 total tests)
- **Ashley** (36055 total tests)
- **Baxter** (60614 total tests)
- **Benton** (519141 total tests)
- **Boone** (87524 total tests)
- **Bradley** (24454 total tests)
- **Calhoun** (7624 total tests)
- **Carroll** (51433 total tests)
- **Chicot** (33186 total tests)
- **Clark** (64967 total tests)
- **Clay** (39256 total tests)
- **Cleburne** (52305 total tests)
- **Cleveland** (20286 total tests)
- **Columbia** (47562 total tests)
- **Conway** (47108 total tests)
- **Craighead** (293768 total tests)
- **Crawford** (140720 total tests)
- **Crittenden** (106878 total tests)
- **Cross** (35283 total tests)
- **Dallas** (17162 total tests)
- **Desha** (24093 total tests)
- **Drew** (44855 total tests)
- **Faulkner** (292211 total tests)
- **Franklin** (40479 total tests)
- [illegible]

COVID-19 Negative Tests		COVID-19 Positive Tests	
**6,119,050**	PCR Negatives **4,620,314**	**873,381**	PCR Positives **598,137**
	Antigen Negatives **1,498,736**		Antigen Positives **275,244**

COVID19.CA.GOV
Your actions save lives

SEARCH Search this site

Select language

Safety in daily life | Fighting COVID-19 | California's response | Services

Tracking COVID-19 in California

Last updated June 10, 2022 at 10:00 AM

California is tracking data to understand the spread of COVID-19.

On this page you'll find:

- Latest update
- Unvaccinated and vaccinated data
- County and statewide data
- Cases and deaths by ethnicity, gender, and age
- Explore more data

Update for June 10, 2022

VACCINES ADMINISTERED
76,821,177 total
46,090 daily avg.
83.7% of population vaccinated (5+ with at least one dose)

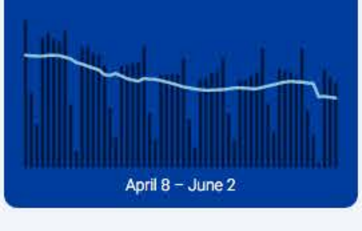

CASES
9,106,031 total
13,903 daily avg.
34.6 new cases (per 100K)

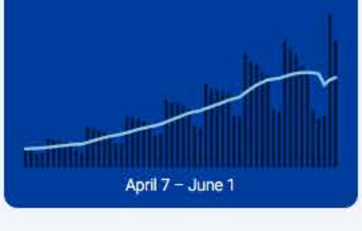

DEATHS
91,006 total
14 daily avg.
0.04 new deaths (per 100K)

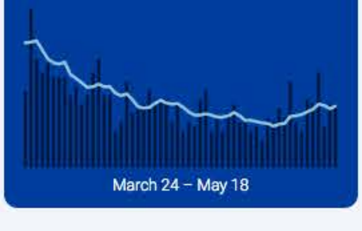

TESTS
8.6% test positivity

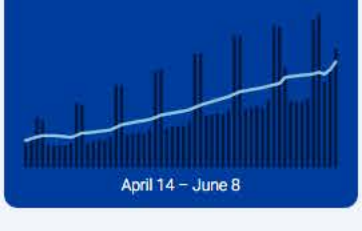

Updated June 10, 2022 at 9:36 AM, with data from June 9, 2022. Data is updated on Tuesdays and Fridays.

Vaccines administered source data and cases, deaths, and tests source data

Data notes

Unvaccinated and vaccinated data

Vaccines prevent serious illness, save lives, and reduce further spread of COVID-19. As more people are vaccinated, the virus is less likely to spread, mutate, and potentially become even more dangerous. Vaccines will help put an end to the pandemic.

Cases | Hospitalizations | Deaths

From May 16, 2022 to May 22, 2022, unvaccinated people were **5.0 times more likely** to get COVID-19 than people who received their booster dose.

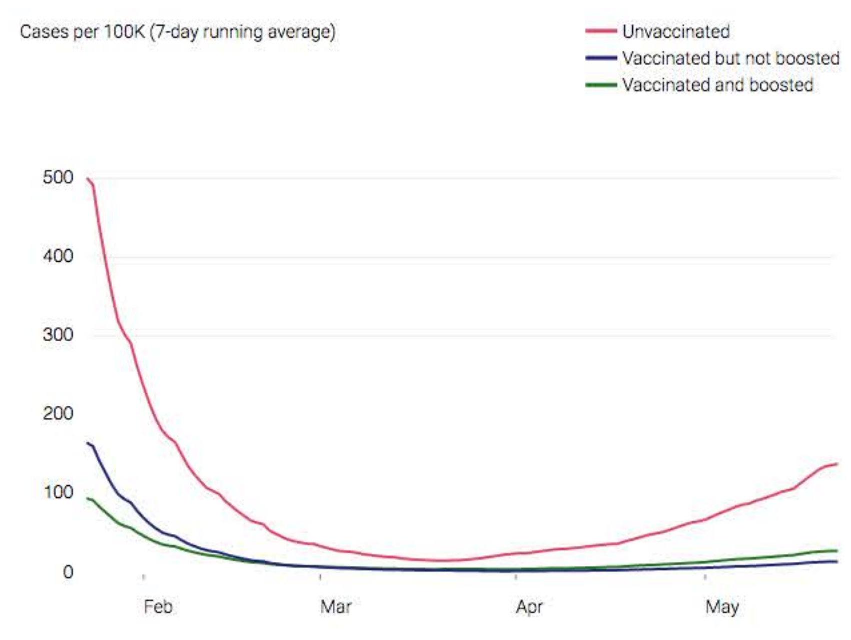

Unvaccinated and vaccinated cases, hospitalizations, and death source data. Data is updated on Fridays.

Chart information

Vaccines administered data

We're tracking the progress of vaccinating Californians across the state.

VIEW VACCINATION DATA

County and statewide data

Enter a county | Get county data

Cases and deaths

California has 9,106,031 confirmed cases of COVID-19, resulting in 91,006 deaths.

Confirmed cases in California

Episode date | All time

9,106,031 total confirmed cases
47,129 new cases (**0.5%** increase)
34.6 cases per 100K (7-day average)

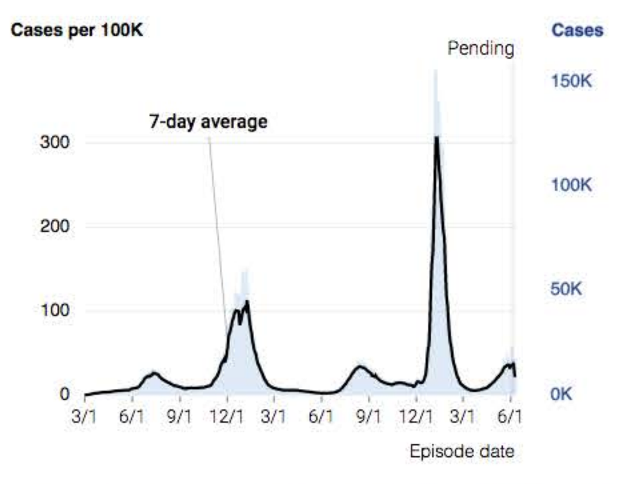

Confirmed deaths in California

Death date | All time

91,006 total confirmed deaths
114 new deaths (**0.1%** increase)
0.04 deaths per 100K (7-day average)

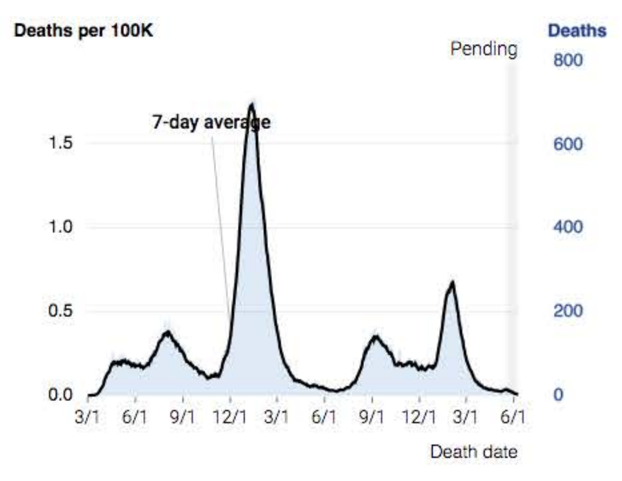

Confirmed cases and deaths source data. Data is updated on Tuesdays and Fridays.

Chart information

Testing for COVID-19

The number of COVID-19 diagnostic test results in California reached a total of 171,841,582, an increase of 969,129 tests from the prior day total. The rate of positive tests over the last 7 days is 8.6%.

Total tests in California

Testing date | All time

171,841,582 total tests performed
969,129 new tests reported (**0.6%** increase)

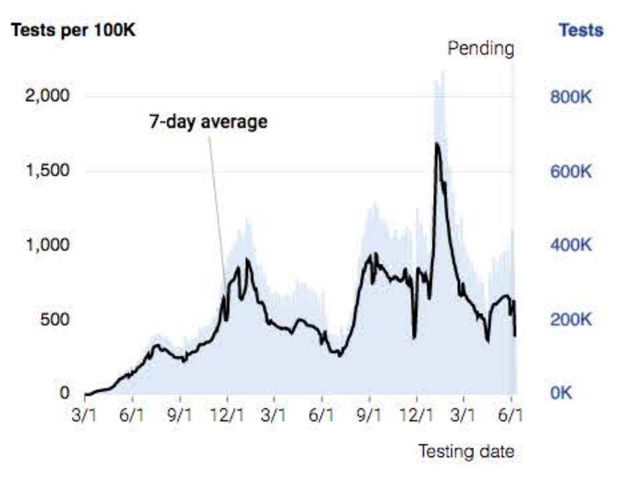

Positivity rate in California

All time

8.6% test positivity (7-day rate)
1.2% increase from 7-days prior

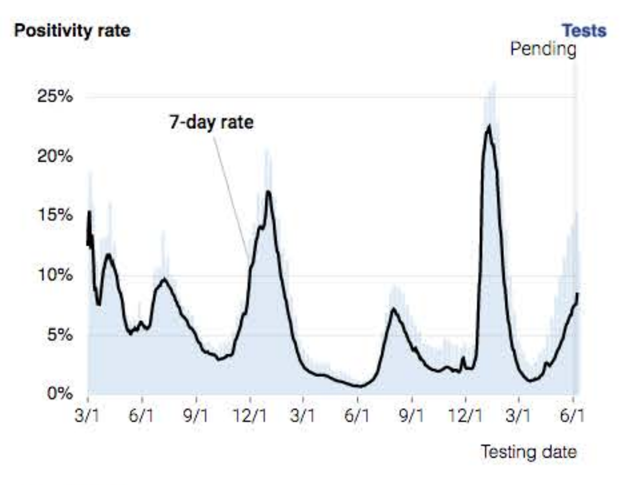

Total tests and positivity rate source data. Data is updated on Tuesdays and Fridays.

Chart information

Hospitalizations

The number of hospitalizations due to confirmed COVID-19 cases in California reached a total of 2,687, an increase of 28 from the prior day total. The number of ICU patients due to confirmed COVID-19 cases in California reached a total of 294, a decrease of 1 from the prior day total.

COVID-19 hospitalized patients in California

Hospitalized | All time

2,687 COVID-19 hospitalized patients
28 more patients hospitalized from prior day total (1.1% increase)

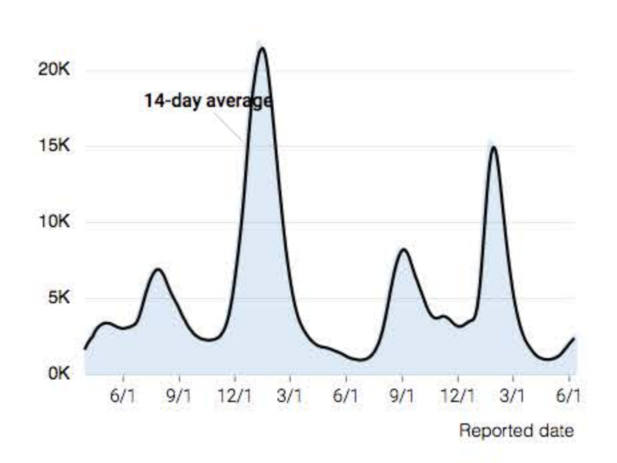

ICU beds in California

All time

2,125 ICU beds available
15 fewer ICU beds available from prior day total (0.7% decrease)

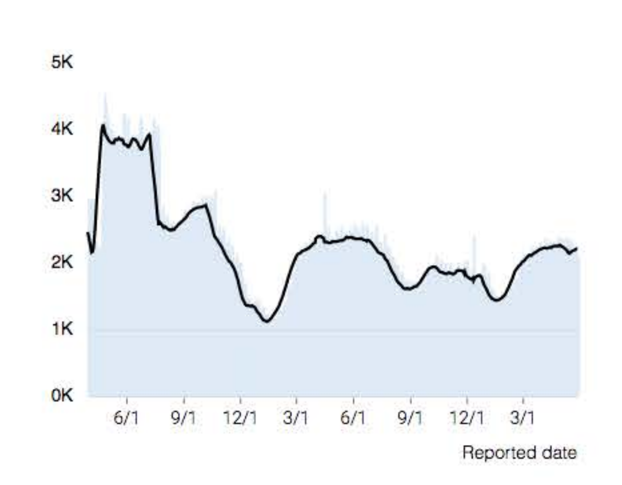

COVID-19 hospitalized patients and ICU source data. Data is updated on Tuesdays and Fridays.

Chart information

Cases and deaths by ethnicity, gender, and age

Race and Ethnicity | Gender | Age

The distribution of confirmed COVID-19 cases reveals significant disparities within California's overall racial and ethnic demographics, with Latino and Native Hawaiian / Pacific Islander groups having a disproportionate number of cases relative to their population in the state. Additional COVID-19 race and ethnicity data is available.

Confirmed cases by race and ethnicity in California

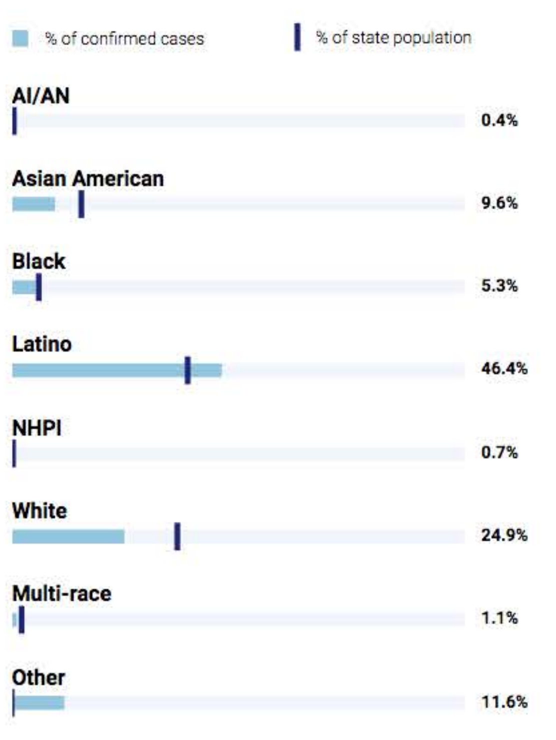

Confirmed deaths by race and ethnicity in California

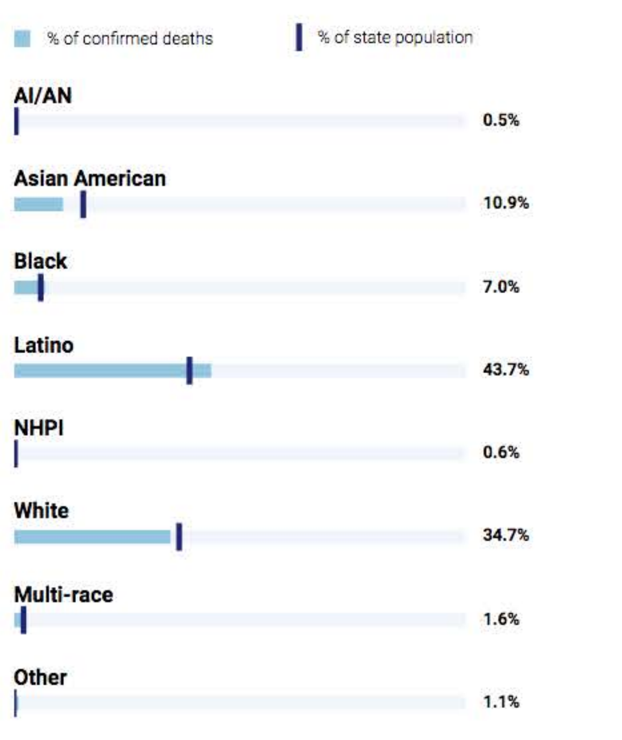

Updated June 10, 2022 with data from June 9, 2022. Note: Data is updated on Tuesdays and Fridays. Percentages may not add up to 100% due to rounding. "Other" race and ethnicity means those who do not fall under any listed race or ethnicity.

Confirmed cases and deaths by race and ethnicity, gender, and age source data

SEE MORE DETAILED DATA BY GROUP

Explore more data

Vaccination data
State and county data about vaccination, including by race and ethnicity and age

Variants
Data about which variants are in California, including Delta and Omicron

Health equity data
How COVID-19 has affected different communities across the state

Data and tools
In-depth models, dashboards, databases, and information about California's COVID-19 data reporting

Did you find what you were looking for?

Yes | No

COVID-19 Cases Dashboard v2.0 by CA Open Data

COVID-19: California Case Statistics

Cases · Testing · Demographics

Select Metric

Cases per 100K (7-day avg)

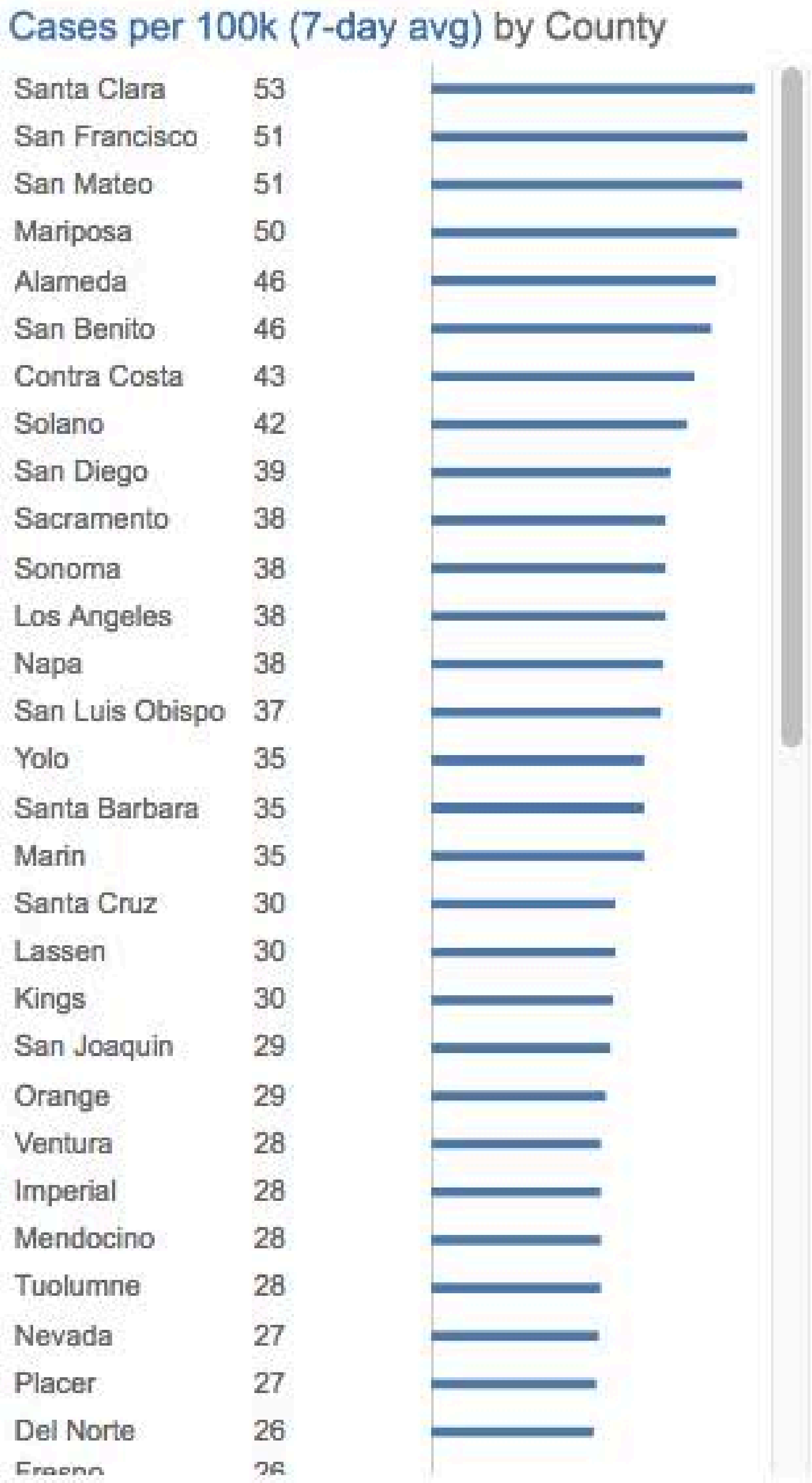

Cases (Statewide)

9,106,031 Total Confirmed Cases

34.6 Cases per 100K (7-day average)

+47,129 (+0.5%) Reported 6/9

US Total Cases: 85,084,715

Deaths (Statewide)

91,006 Total Confirmed Deaths

0.04 Deaths per 100K (7-day average)

+114 (+0.1%) Reported 6/9

US Total Deaths: 1,005,823

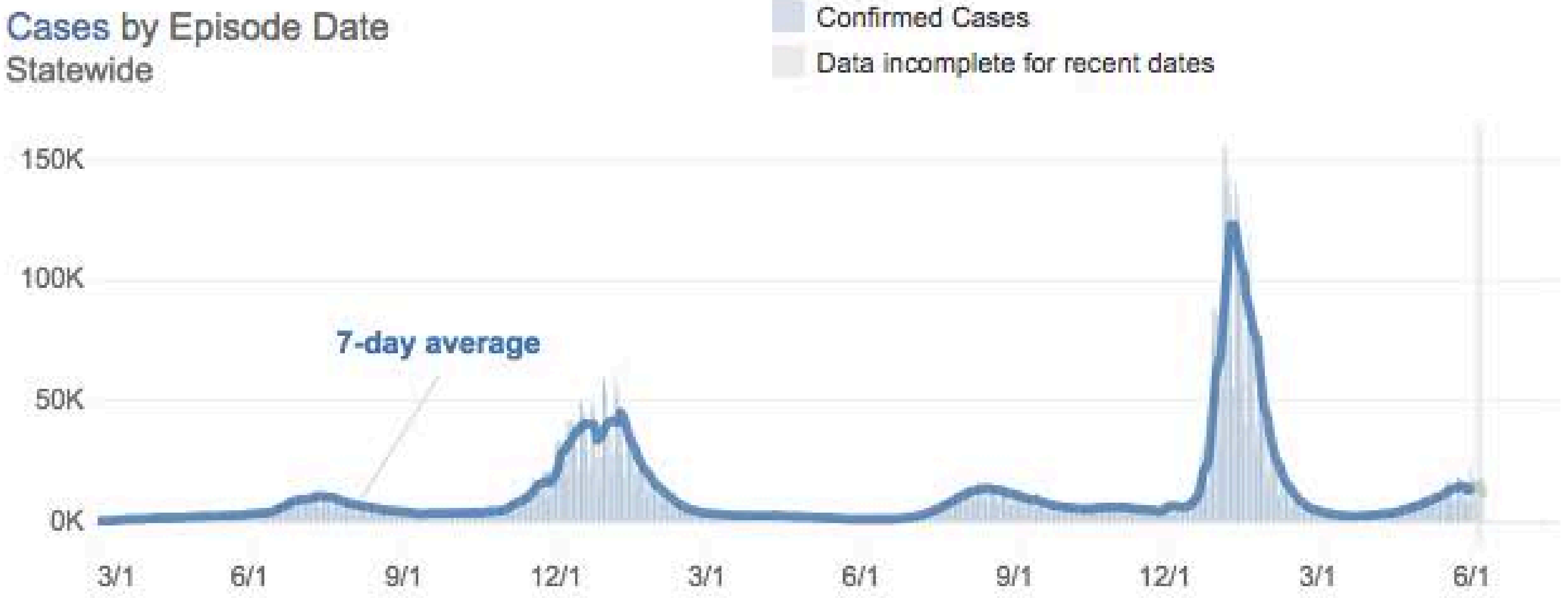

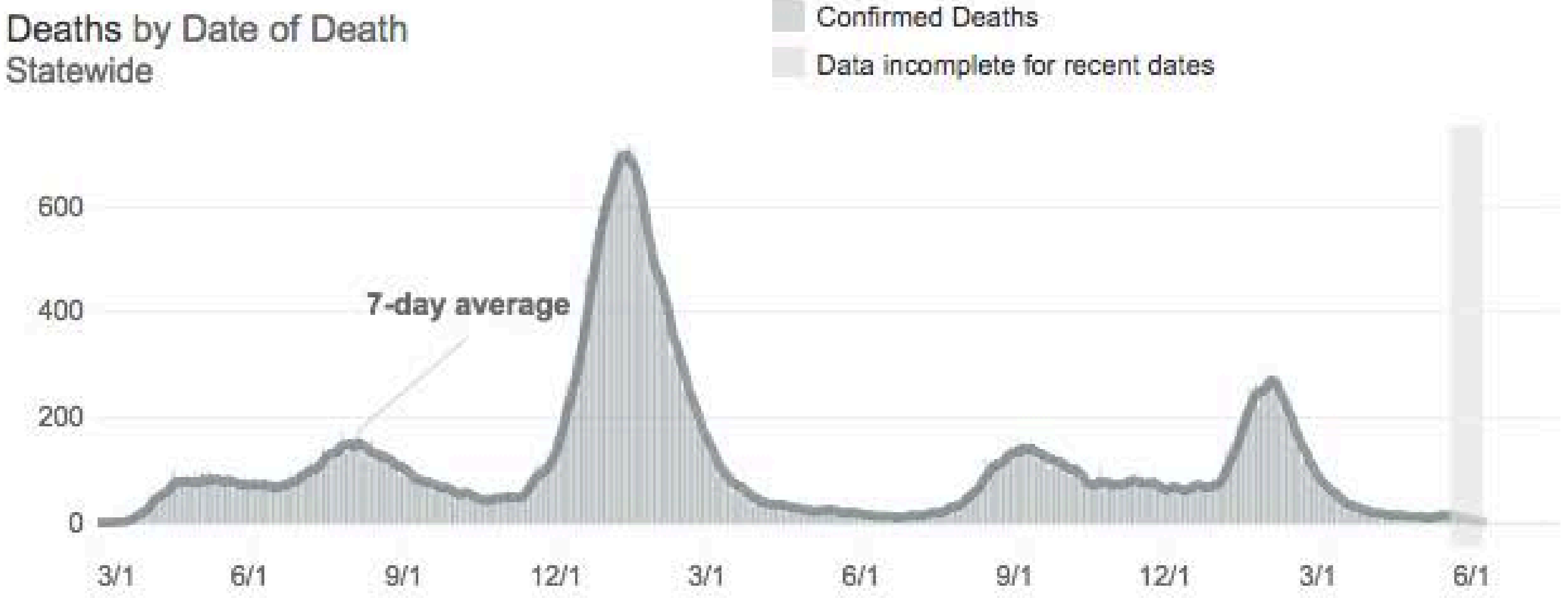

Last Updated 6/10/2022

+ableau

Details

38 · 3,184,962

COVID-19 Cases Dashboard v2.0

Published: Feb 19, 2021 **Updated:** Jun 10, 2022

COVID-19 Cases Dashboard v2.0 by CA Open Data

COVID-19: California Testing Statistics

Cases | Testing | Demographics

Sort by

Total Tests

Total Tests by County

Los Angeles	68,328,186
San Diego	11,762,233
Santa Clara	10,071,475
Orange	9,168,738
Alameda	7,020,703
San Bernardino	6,725,832
Riverside	6,647,609
Sacramento	4,678,655
San Francisco	4,453,880
San Mateo	4,097,004
Contra Costa	3,842,996
Ventura	3,117,733
Fresno	2,779,335
Kern	2,502,151
San Joaquin	2,373,058
Yolo	1,713,208
Sonoma	1,708,566
Out of state	1,607,136
Solano	1,553,392
Santa Barbara	1,553,008
Stanislaus	1,419,157
Santa Cruz	1,403,076
Tulare	1,285,902
Monterey	1,271,253
Marin	1,033,084
San Luis Obispo	1,024,135
Placer	973,181
Kings	761,964
Merced	735,847
Imperial	612,341
Madera	557,777
Napa	551,304
Shasta	501,969

Total Tests (Statewide)

171,841,582 Total Reported Tests

969,129 (+0.6%) 1-day change

1,830,908 (+1.1%) 7-day change

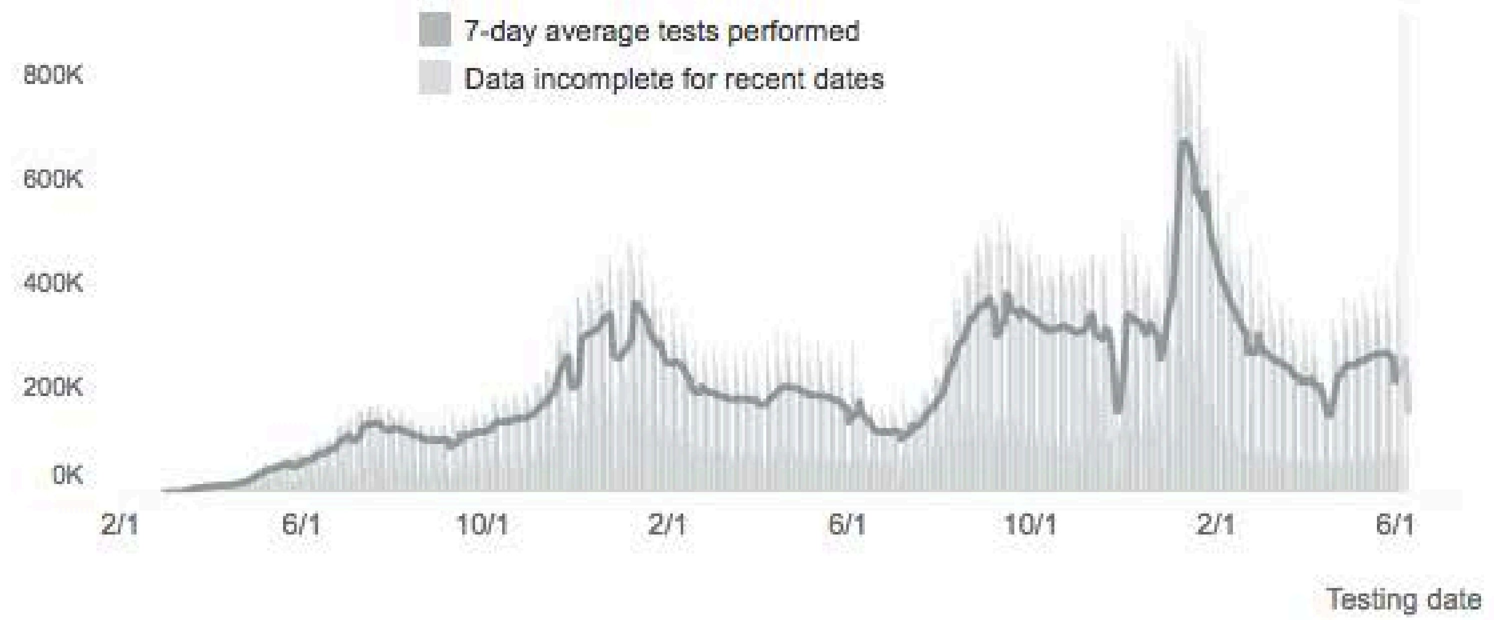

Test Positivity (Statewide)

8.6% 7-day rate

+1.2% 7-day change

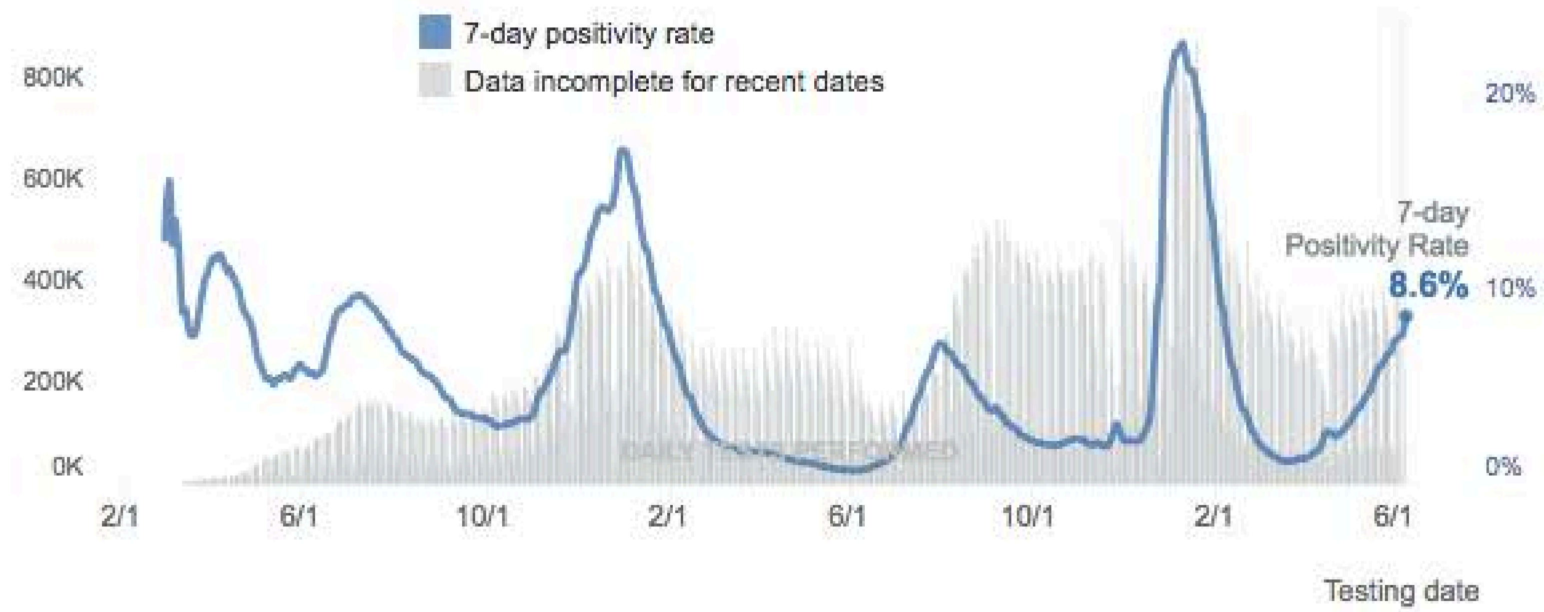

Last Updated 6/9/2022

+ a b l e a u

Details

38 3,184,962

COVID-19 Cases Dashboard v2.0

Published: Feb 19, 2021 **Updated:** Jun 10, 2022

COVID-19 Cases Dashboard v2.0 by CA Open Data

COVID-19: California Demographic Statistics

Cases | Testing | Demographics

Confirmed Cases

| = California Population %

Race and Ethnicity	Case %	CA Pop %
AI/AN	0%	1%
Asian	10%	15%
Black	5%	6%
Latino	46%	39%
NH/PI	1%	0%
White	25%	37%
Multi-Race	1%	2%
Other	12%	0%

Gender	Case %	CA Pop %
Female	52%	50%
Male	47%	50%
Unknown	2%	0%

Age	Case %	CA Pop %
0-17	19%	23%
18-49	55%	44%
50-64	17%	18%
65+	9%	16%
Missing	0%	0%

Confirmed Deaths

| = California Population %

Race and Ethnicity	Death %	CA Pop %
AI/AN	1%	1%
Asian	11%	15%
Black	7%	6%
Latino	44%	39%
NH/PI	1%	0%
White	35%	37%
Multi-Race	2%	2%
Other	1%	0%

Gender	Death %	CA Pop %
Female	41%	50%
Male	58%	50%
Unknown	0%	0%

Age	Death %	CA Pop %
0-17	0%	23%
18-49	8%	44%
50-64	21%	18%
65+	71%	16%
Missing	0%	0%

Last Updated 6/9/2022

+ a b | e a u

Details

38 3,184,962

COVID-19 Cases Dashboard v2.0

Published: Feb 19, 2021 **Updated:** Jun 10, 2022

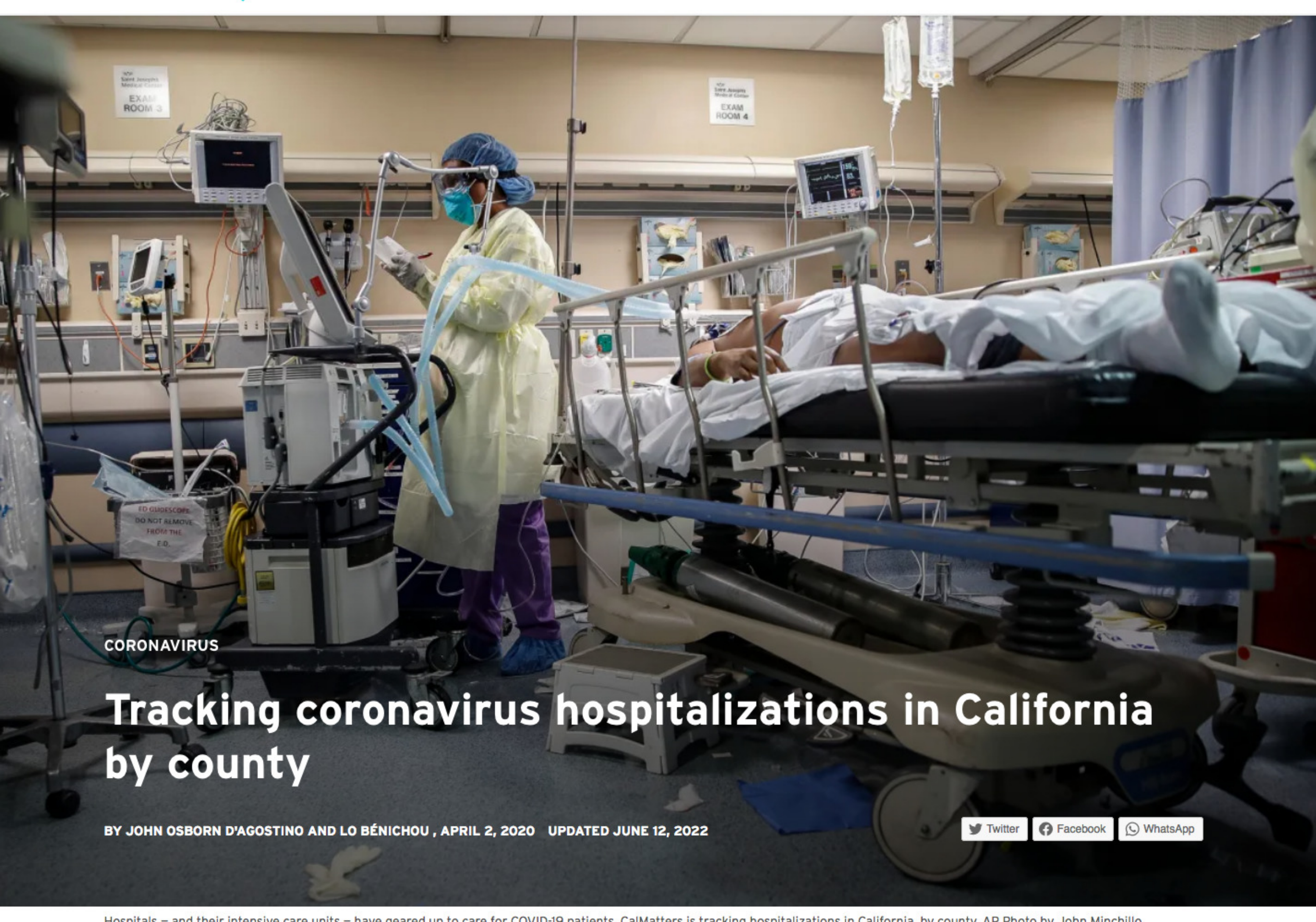

CORONAVIRUS

Tracking coronavirus hospitalizations in California by county

BY JOHN OSBORN D'AGOSTINO AND LO BÉNICHOU , APRIL 2, 2020 UPDATED JUNE 12, 2022

Twitter Facebook WhatsApp

Hospitals – and their intensive care units – have geared up to care for COVID-19 patients. CalMatters is tracking hospitalizations in California, by county. AP Photo by John Minchillo

IN SUMMARY

Which California counties have the highest rates of coronavirus hospitalization? CalMatters is following confirmed and suspected cases. Some are a surprise.

Lea este artículo en español.

CalMatters is tracking – and updating daily – California hospitalizations, by county, of people who have been infected or suspected of being infected with COVID-19.

KEEP TABS ON THE LATEST CALIFORNIA POLICY AND POLITICS NEWS

Enter your email address SUBSCRIBE

By clicking subscribe, you agree to share your email address with CalMatters to receive marketing, updates, and other emails.

Winters have proven to be brutal for California hospitals. In January 2022, hospitals saw another wave of admissions ignited by the omicron variant. The mid-January high of COVID-positive hospitalizations surpassed that of last summer's delta surge – although it didn't quite reach last winter's peak. Since then, admissions have once again started to decline.

As hospitals start to emerge from the worst of the omicron wave, the state is scheduled to relax some of its mask rules and soon announce an endemic strategy. **NOTE: The California Department of Public Health is no longer updating data daily. Updates will now happen on Tuesdays and Fridays.**

2,614
COVID-19 patients currently hospitalized
-73 *(-2.7%) on June 10*
+287.8% *compared to last year*

273
COVID-19 patients currently in ICU
-21 *(-7.1%) on June 10*
+13.3% *compared to last year*

Data retrieved on June 11, 2022 at 05:09 PM. The most recent data available is from June 10, 2022.

How many people are currently hospitalized with COVID-19?

Hospitalizations of confirmed COVID-19 patients in California

The number of confirmed patients in ICU and non-ICU settings began to climb in mid-June after California began to ease more of its shutdown restrictions. Here's the trend since the California Department of Public Health began releasing the data on April 1, 2020. As of June 10, there are **273** patients in ICUs, a change of **-21** from the day before, and **2,341** non-ICU patients, a change of **-52** from the day before.

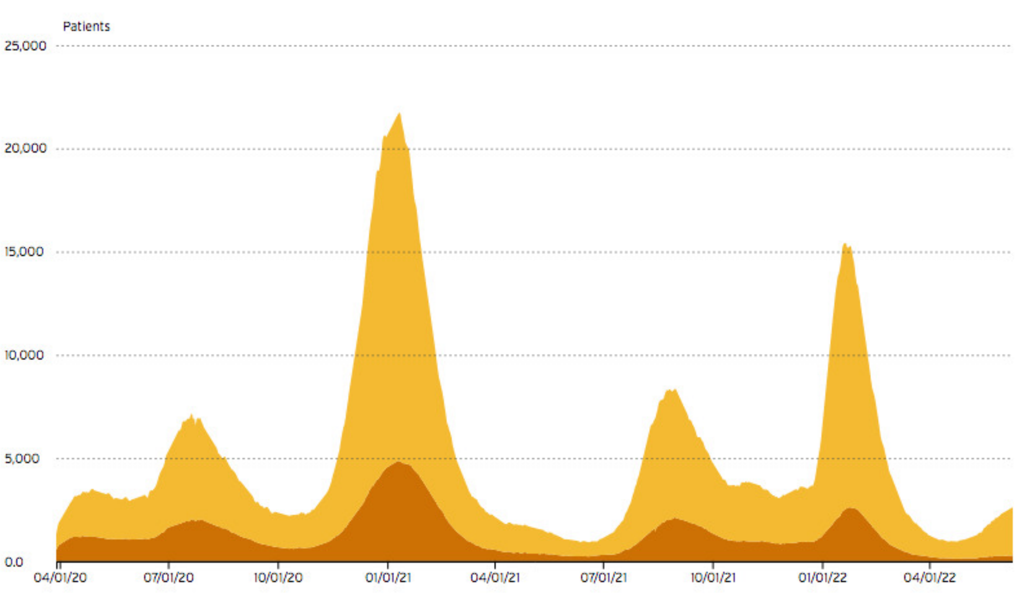

Hospitalized confirmed COVID-19 patients per county

Total number of confirmed COVID-19 patients per 100,000 people.

Note: Several rural counties do not have hospitals or ICU wards, so patients from those counties would go to neighboring counties for COVID-19 medical care. This includes Alpine, Sierra, Sutter and Trinity counties.

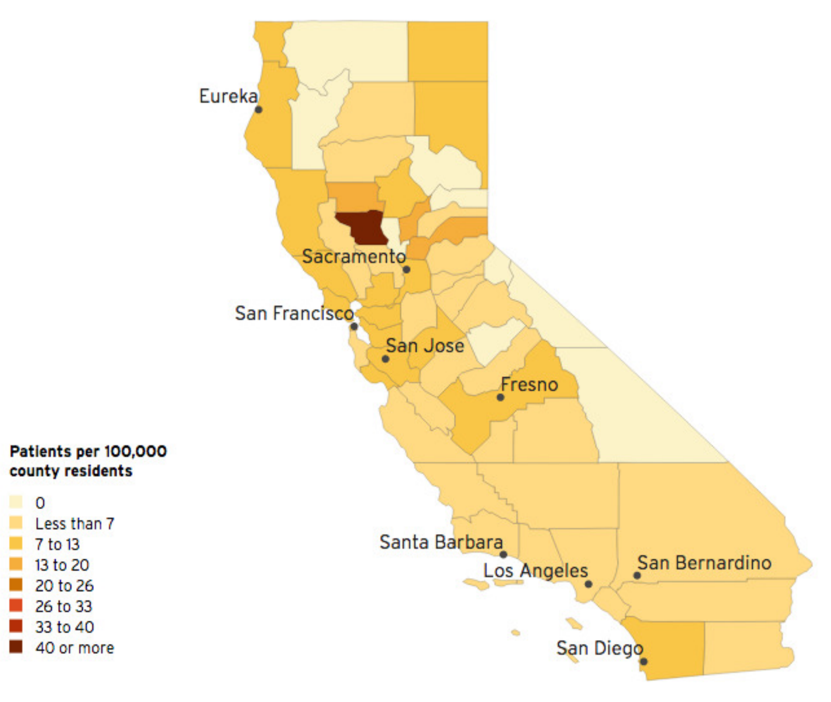

COUNTY	TOTAL PATIENTS	IN ICU	TREND*	DAILY CHANGE	PER 100,000 PEOPLE
Colusa	10	0		0%	46.2
Placer	75	6		10%	19.1
Glenn	5	0		0%	17.8
Yuba	13	0		-7%	16.7

More Counties

* Trend lines show 7-day moving average of patients over time from March 31, 2020 to June 10, 2021.

Counties in depth

Alameda

Hospitalizations of confirmed COVID-19 patients in Alameda

Total number of confirmed patients in **ICU** and **non-ICU** settings per 100,000 people compared to **California's** – displayed per 100,000 people to enable visual comparison between very different sized total populations. Note the Y axis changes based on the county's highest reported patient count since March 31, 2020.

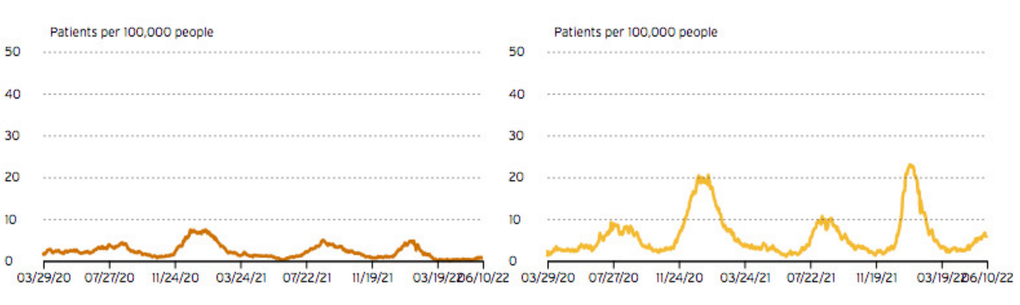

Post-vaccination infections

Breakdown of hospitalized patients by vaccination status

The California Department of Public Health publishes a weekly analysis of post-vaccination infections based on individual case reports from local health departments and hospitals. The analysis breaks out patients at least 12-years old who are **unvaccinated**, **vaccinated but not boosted**, and **vaccinated and boosted**; partially vaccinated patients are excluded from the analysis. Patients are only counted once for a single day, even if they stayed in the hospital multiple days. Data lags about 14 days from present day and updates every Wednesday.

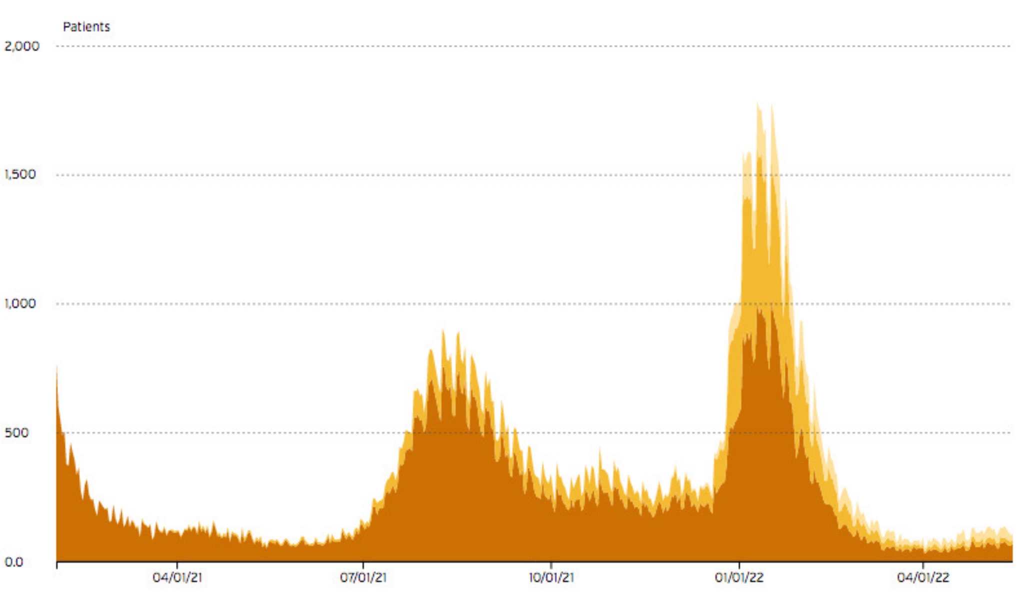

About the Data

On April 1, 2020, the California Department of Public Health released a dashboard breaking down statewide hospitalizations of positive COVID-19 patients.

We adjusted the numbers for a few counties that reported small anomalies, such as a negative patient count instead of zero.

The data used in the following graphics are pulled from the California Health and Human Services database on a daily basis. Any questions about the data can be sent to CalMatters' data journalist and web developer John D'Agostino.

Population data for each county is based on the 2018 American Community Survey.

Number of deaths due to COVID-19 comes from the California Department of Public Health. Post-vaccination infection data comes from the California Department of Public Health.

CalMatters investigative and data-driven reporting is supported by the Inasmuch Foundation, previously known as the Ethics and Excellence in Journalism Foundation.

MORE ON THE CORONAVIRUS IN CALIFORNIA:

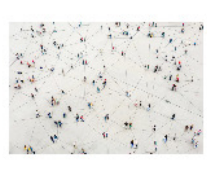

Coronavirus detectives: Here's how counties try to track everyone exposed

California needs thousands of contact tracers. But counties and cities are overwhelmed and understaffed. "Woefully inadequate," said one public health director.

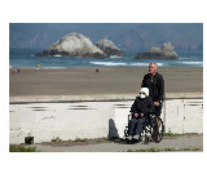

California's response to coronavirus, explained

Gov. Gavin Newsom says the state appears to be flattening the curve. We unravel the response to the coronavirus outbreak and look at what lies ahead.

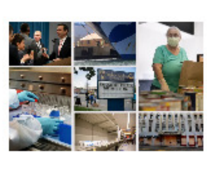

Timeline: California reacts to coronavirus

This timeline tracks how California state and local governments tackled the evolving COVID-19 crisis since the first case was detected.

WE WANT TO HEAR FROM YOU

Want to submit a guest commentary or reaction to an article we wrote? You can find our submission guidelines here. Please contact CalMatters with any commentary questions: commentary@calmatters.org

The story you just read was funded by people like you.

CalMatters is a nonprofit newsroom and your tax-deductible donations help us keep bringing you and every Californian essential, nonpartisan information.

DONATE NOW

"I was filling out my ballot and realized that **I use CalMatters every time I vote**. Everything else is behind paywalls. I also appreciate that CalMatters is one of the more objective news sources in California.

Rod, Sacramento
FEATURED CALMATTERS MEMBER

John Osborn D'Agostino
john@calmatters.org

John Osborn D'Agostino is an award-winning data journalist, web developer and game designer. Previously, he's worked with The Hechinger Report, EdSource, the East Bay Express, Berkeleyside, and the North... More by John Osborn D'Agostino

Lo Bénichou
lo@calmatters.org

Lo Bénichou is a visual journalist at CalMatters. Prior to joining the team, they worked at Mapbox and at a number of media organizations like WIRED, Youth Radio, KQED, NPR. At Mapbox, they supported... More by Lo Bénichou

Datos del COVID-19 en Colorado

Estatus del COVID-19 en los Condados

Colorado public school COVID-19 vaccination rates

Colorado COVID-19 Data

Data is updated daily by about 4 p.m. Monday through Friday and includes cases reported through the previous day.
All data, for days past and present, is recalculated daily.

COLORADO
Department of Public Health & Environment

1,484,356 Cases	63,488 Hospitalized	64 Counties	4,951,563 People Tested	18,555,496 Test Encounters	12,625 Deaths Among Cases	13,349 Deaths Due to COVID-19	9,069 Outbreaks

Case Summary | Demographics | Tests | Hospital Data | Incidence and Epidemic Curve | Syndromic Data | Outbreaks | CDC Provisional Deaths

3,314 (▲ 521) Cases Reported Today	439 Cases Added with Previous Reported Date	2,387 (▲ 30) 7-Day Moving Average of Cases Reported	526.6 (▲ 12.2) 2-Week Cumulative Incidence Per 100K People
91 (▲ 10) New Hospital Admissions	81 (▲ 4) 7-Day Moving Average of New Hospitalizations	Adult Critical Care Ventilators in Use	4% Available Hospital Beds Occupied by Confirmed and Suspected COVID Patients
14.00% (▲ 2.67%) State Positivity Rate	12.65% (▲ 0.25%) State 7-Day Positivity Rate	4.97% (▲ 0.95%) Previous Week Emergency Department Syndromic COVID Visits	16.00% (▲ 8.79%) Previous Week Outpatient Syndromic COVID Visits
4.1 (▼ 0.4) Tests Per 1,000 People	3.4 (▼ 0.10) 7-Day Average of Tests Per 1,000 People	2,847 (▲ 399) New Individual People Tested	23,922 (▼ 1,933) New Test Encounters

+ a b | e a u

Trouble viewing? Access the data from Tableau or view the data tables below.

Case summary snapshot

Hospital data snapshot

Cases and deaths by county

COVID-19 Variant Sentinel Surveillance

COLORADO
Department of Public Health & Environment

5.10% Alpha	0.02% Beta	52.54% Delta	0.42% Epsilon	0.14% Gamma	40.55% Omicron	1.23% Other

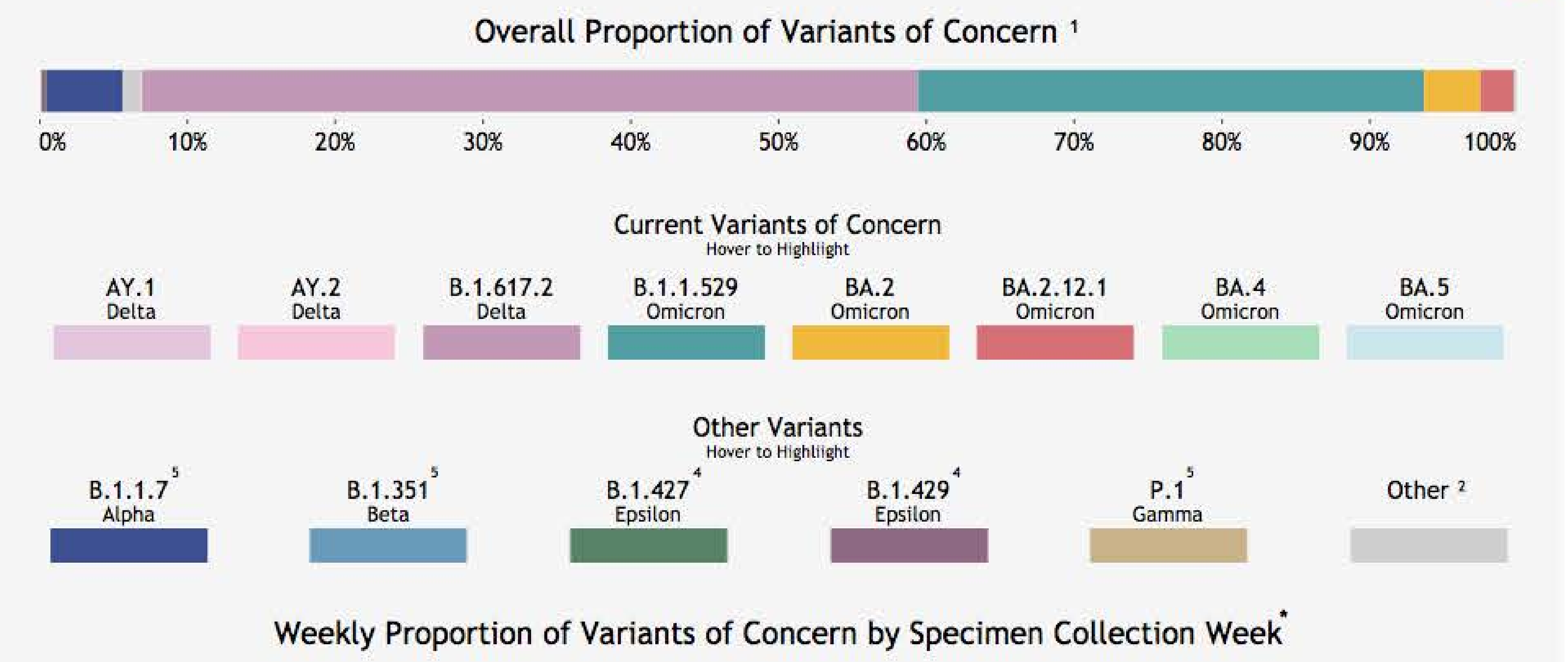

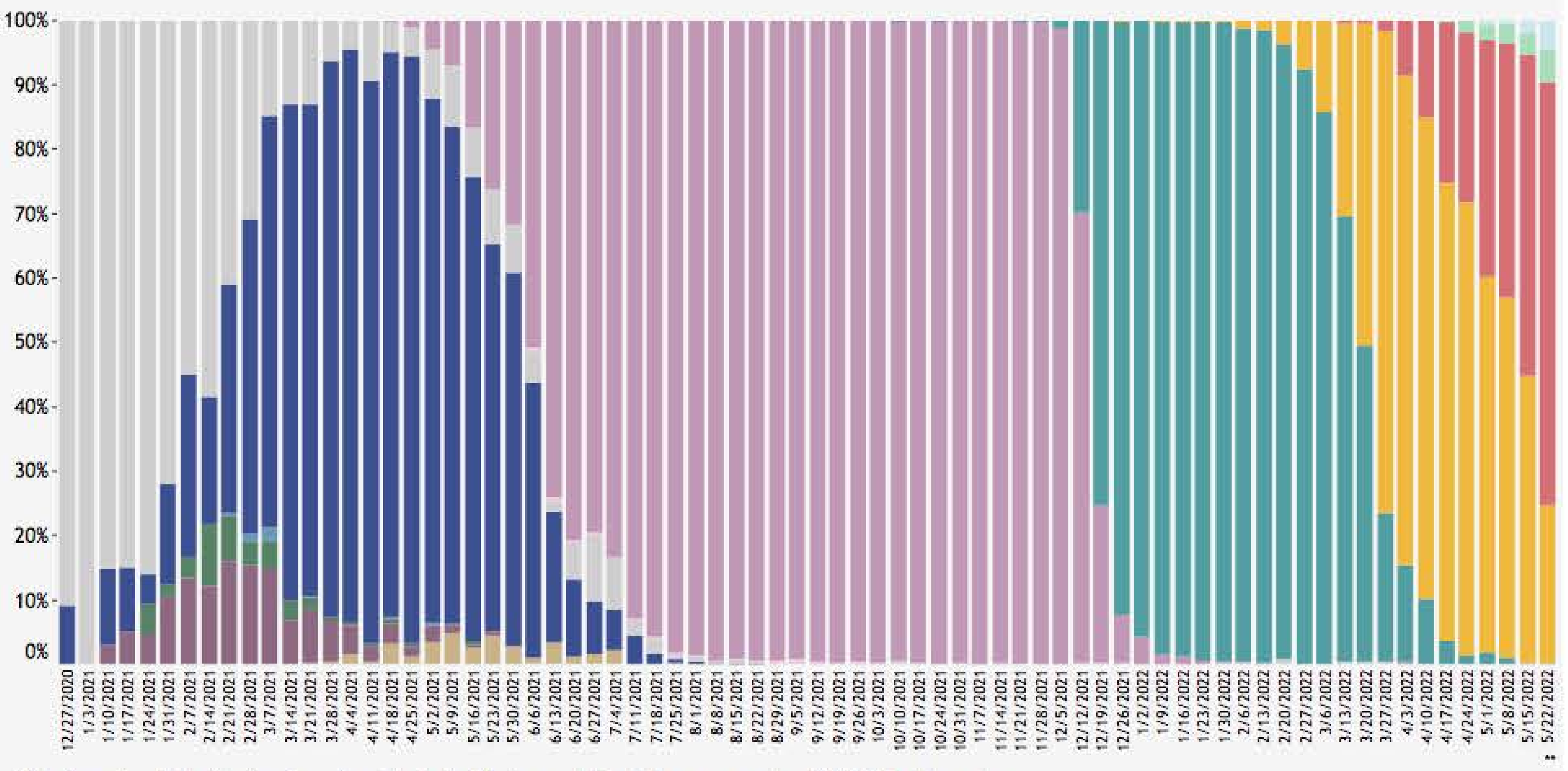

* Variant proportions data is subject to a two-week reporting lag due to the time required for whole genome sequencing of SARS-CoV-2 positive specimens.
** Data for this week represent results from a limited number of specimens and are subject to change. CDPHE is working to strike a balance between data timeliness and data completeness

1. Variants of Concern: B.1.617.2, AY.1, AY.2, B.1.1.519, BA2, BA.2.12.1, BA.4, and BA.5.
2. "Other" category includes all non-variants, variants of interest/variants under investigation and variants being monitored.
3. Not all samples in the state of Colorado are able to be whole genome sequenced. This is a random sample of specimens submitted each week through the Colorado SARS-CoV-2 Variant Sentinel Surveillance Program and provides estimates of total variant of concern prevalence.
4. As of June 28, 2021, B.1.427 and B.1.429 are no longer variants of concern. Counts after 7/6/2021 are included in "Other".

+ a b | e a u

The omicron variant has rapidly replaced the delta variant in Colorado and the U.S. At this time nearly all new cases can be attributed to omicron. With the current large volume of cases, statewide sentinel surveillance data provides the most reliable picture of variant transmission.

Datos del COVID-19 en Colorado

Estatus del COVID-19 en los Condados

[Colorado public school COVID-19 vaccination rates]

Colorado COVID-19 Data

Data is updated daily by about 4 p.m. Monday through Friday and includes cases reported through the previous day.
All data, for days past and present, is recalculated daily.

COLORADO
Department of Public Health & Environment

1,484,356 Cases	63,488 Hospitalized	64 Counties	4,951,563 People Tested	18,555,496 Test Encounters	12,625 Deaths Among Cases	13,349 Deaths Due to COVID-19	9,069 Outbreaks

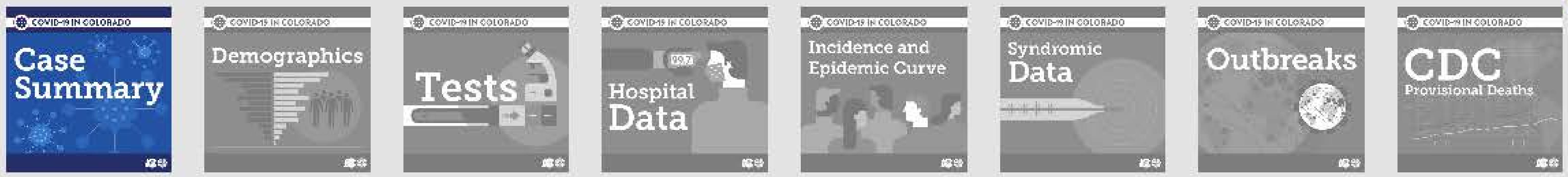

Cases | Deaths Among Cases | Maps

Reported Day | Reported Week | Onset Day | Onset Week

Counts | Cumulative | 3-Day Average | 7-Day Average

Cases of COVID-19 in Colorado by Date Reported to the State

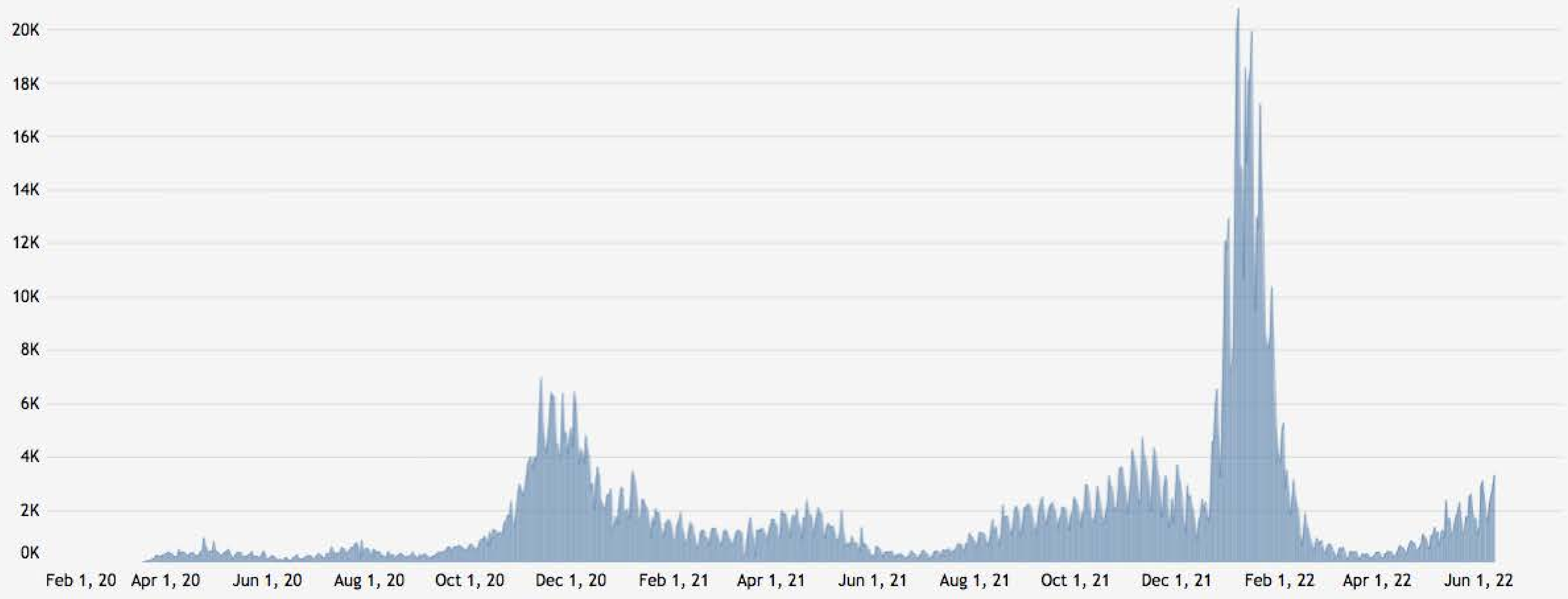

* Due to reporting lags created by high case volume, case counts between December 27, 2021 and February 1, 2022 are likely to change.

CDPHE

+ a b | e a u

COLORADO
Department of Public Health & Environment

COLORADO
State Emergency Operations Center

Search

For Coloradans › | For businesses & communities › | Public health orders › | Data › | News & media resources › | LPHAs & providers › | Español ›

Datos del COVID-19 en Colorado

Estatus del COVID-19 en los Condados

[Colorado public school COVID-19 vaccination rates]

Colorado COVID-19 Data

Data is updated daily by about 4 p.m. Monday through Friday and includes cases reported through the previous day.
All data, for days past and present, is recalculated daily.

COLORADO
Department of Public Health & Environment

1,484,356 Cases	63,488 Hospitalized	64 Counties	4,951,563 People Tested	18,555,496 Test Encounters	12,625 Deaths Among Cases	13,349 Deaths Due to COVID-19	9,069 Outbreaks

Case Summary | Demographics | Tests | Hospital Data | Incidence and Epidemic Curve | Syndromic Data | Outbreaks | CDC Provisional Deaths

Cases | Deaths Among Cases | Maps

Daily | Weekly

Counts | Cumulative | 3-Day Average | 7-Day Average

Deaths Among COVID-19 Cases in Colorado by Date of Death*

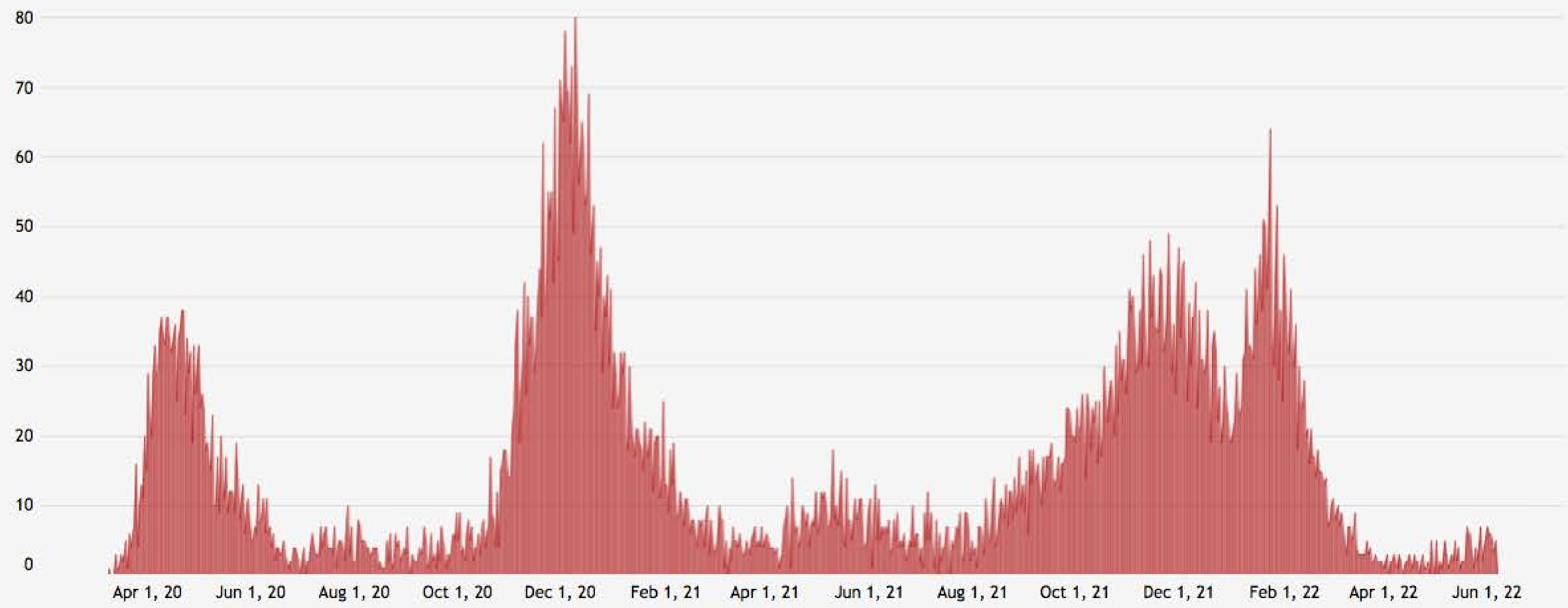

* This graph includes deaths among people with COVID-19. COVID-19 may or may not be listed as the cause of death on the death certificate

+ a b l e a u

Datos del COVID-19 en Colorado

Estatus del COVID-19 en los Condados

[Colorado public school COVID-19 vaccination rates]

Colorado COVID-19 Data

COLORADO
Department of Public Health & Environment

Data is updated daily by about 4 p.m. Monday through Friday and includes cases reported through the previous day.
All data, for days past and present, is recalculated daily.

1,484,356 Cases	63,488 Hospitalized	64 Counties	4,951,563 People Tested	18,555,496 Test Encounters	12,625 Deaths Among Cases	13,349 Deaths Due to COVID-19	9,069 Outbreaks

Case Summary · Demographics · Tests · Hospital Data · Incidence and Epidemic Curve · Syndromic Data · Outbreaks · CDC Provisional Deaths

Daily · Weekly · Maps

PCR Tests · Antibody Tests · Daily Positivity · 3-Day Avg Positivity · 7-Day Avg Positivity

Daily Tests · Cumulative Tests

Daily COVID-19 PCR Test Data From Clinical Laboratories With 7-Day Average Positivity

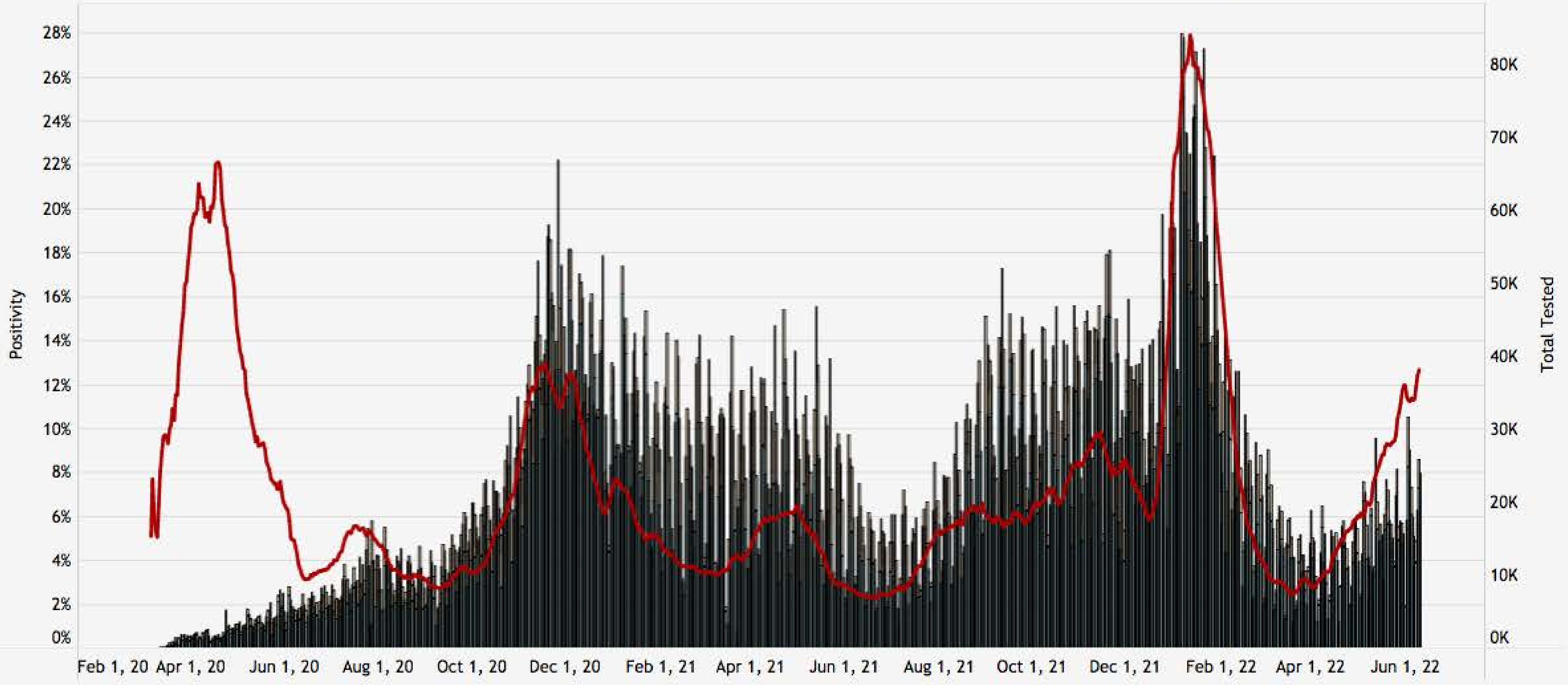

tableau

COLORADO
Department of Public Health & Environment

COLORADO
State Emergency Operations Center

Search

For Coloradans › For businesses & communities › Public health orders › Data › News & media resources › LPHAs & providers › Español ›

Datos del COVID-19 en Colorado

Estatus del COVID-19 en los Condados

Colorado public school COVID-19 vaccination rates

Colorado COVID-19 Data

Data is updated daily by about 4 p.m. Monday through Friday and includes cases reported through the previous day.
All data, for days past and present, is recalculated daily.

COLORADO
Department of Public Health & Environment

1,484,356 Cases	63,488 Hospitalized	64 Counties	4,951,563 People Tested	18,555,496 Test Encounters	12,625 Deaths Among Cases	13,349 Deaths Due to COVID-19	9,069 Outbreaks

Case Summary | Demographics | Tests | Hospital Data | Incidence and Epidemic Curve | Syndromic Data | Outbreaks | CDC Provisional Deaths

Daily | Weekly | Maps

PCR Tests | Antibody Tests | Daily Positivity | 3-Day Avg Positivity | 7-Day Avg Positivity

Daily Tests | Cumulative Tests

Daily COVID-19 Antibody Test Data From Clinical Laboratories With 7-Day Average Positivity

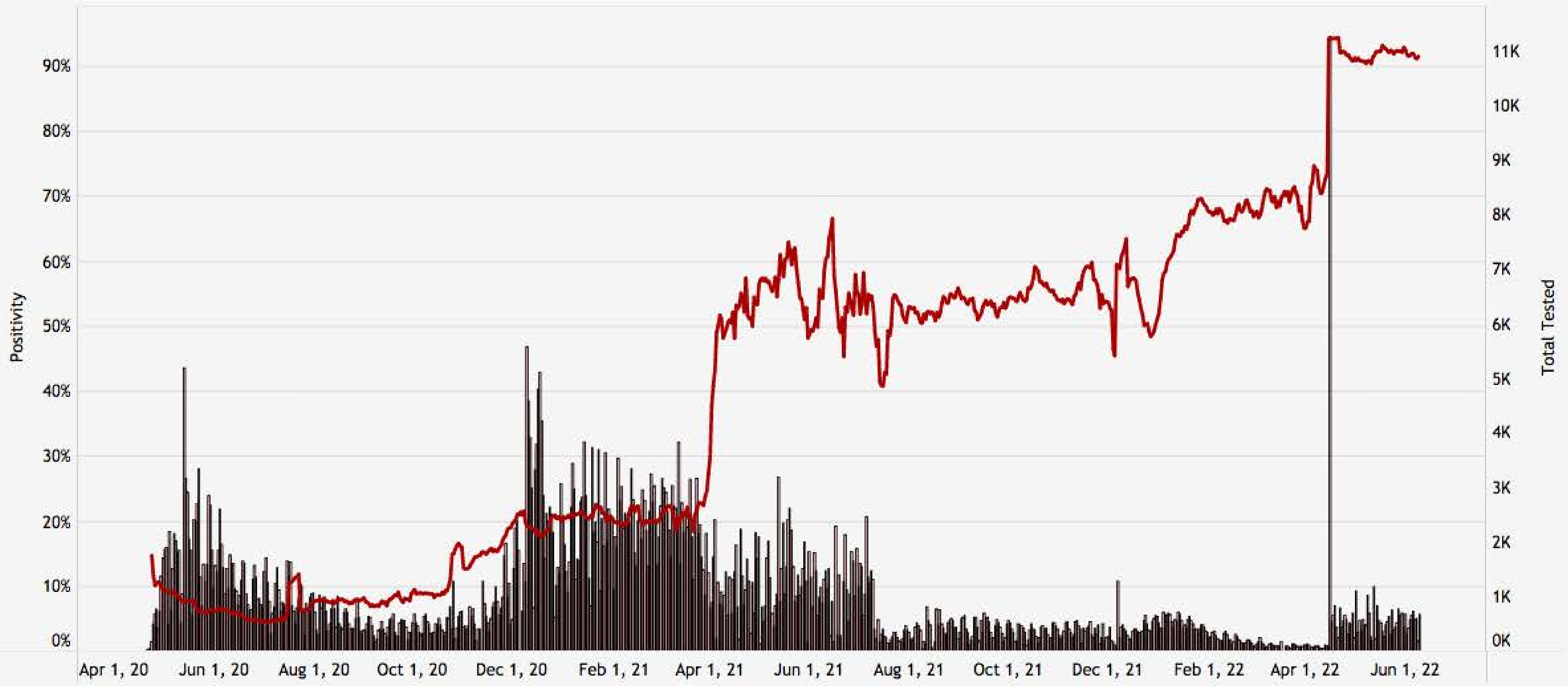

tableau

NEWS

COVID-19 in Colorado tracker: Larimer and state case, death and hospital data for August

Coloradoan staff

Published 7:56 p.m. MT Aug. 1, 2020 | **Updated 4:35 p.m. MT Sept. 1, 2020**

View Comments

Colorado State University Chancellor Tony Frank addresses Joyce McConnell's...
Colorado State University Chancellor Tony Frank addresses President Joyce McConnell's resignation on June 9, 2022, during a Board of Governors meeting *Colorado State University System*

We're always working to improve your experience. Let us know what you think.

As the number of confirmed coronavirus cases in Colorado grows, here are the latest updates and information.

Editor's note: *As the coronavirus outbreak continues to evolve, we don't want you to panic. In fact, quite the opposite. That's why the Coloradoan is committed to providing you with accurate, up-to-date information so you can make informed decisions on issues affecting you and the people you love. As such, this story, and many others, are being provided free for all to read. Help us continue this important work by subscribing to the Coloradoan.*

Here are the latest developments on coronavirus in Colorado:

Note: This tracker includes updates from August.

Looking for September updates?

► Find updates on the spread of COVID-19 in Colorado in September

Looking for past updates?

► A timeline of major updates on coronavirus in Colorado

► Find updates on the spread of coronavirus in Colorado from July

► Find updates on the spread of coronavirus in Colorado from May and June

► Early updates at the beginning of the pandemic in Colorado: March and April

Monday, Aug. 31, 2020

Larimer County cases, deaths:

- New cases reported by county: 27 (Note: This is the number of new confirmed and probable cases reported since The Coloradoan last shared information from the county. **In this case, that was Saturday, Aug. 29, 2020.** This may vary from the "new positive cases" listed on the county's COVID dashboard.)
- New deaths reported by county: 0
- Confirmed cases: 1,861 (positive swab test)
- Probable cases: 51 (people with two of the following: COVID-19 symptoms; epidemiological link to someone who tested positive; positive antibody test)
- Suspect cases: 433 (asymptomatic with positive antibody test)
- Total deaths: 36
- Risk Index in county (based on things such as hospital utilization, new cases, etc.): Medium
- Number of COVID-19 patients in hospitals: 5 (includes Banner and UCHealth)*
- Hospital utilization: 62% (low risk is <70%)*
- ICU utilization: 71% (low risk is <70%)*

Source: Larimer County Department of Health and Environment

Colorado cases, deaths:

- New cases reported by state **(since Saturday, Aug. 29)**: 383
- New deaths reported by state (caused by COVID-19; **since Saturday, Aug. 29**): 6
- Current hospitalizations: 148 (75% reporting)
- Hospital transfers or discharges in past 24 hours: 7
- Number of ventilators in use: 27%
- Hospitals anticipating a staff shortage in the next week: 5%
- Facilities anticipating personal protective equipment shortages in the next week: 1%
- Hospitals anticipating ICU bed shortages in the next week: 1%
- Total cases: 57,424
- Total hospitalizations: 7,028
- Total deaths due to coronavirus: 1,849
- Total outbreaks: 604 (reported at residential and nonhospital health care facilities, correctional settings and other facilities in Colorado, such as factories and group homes)

Source: Colorado Department of Public Health & Environment

Coronavirus cases in Larimer County

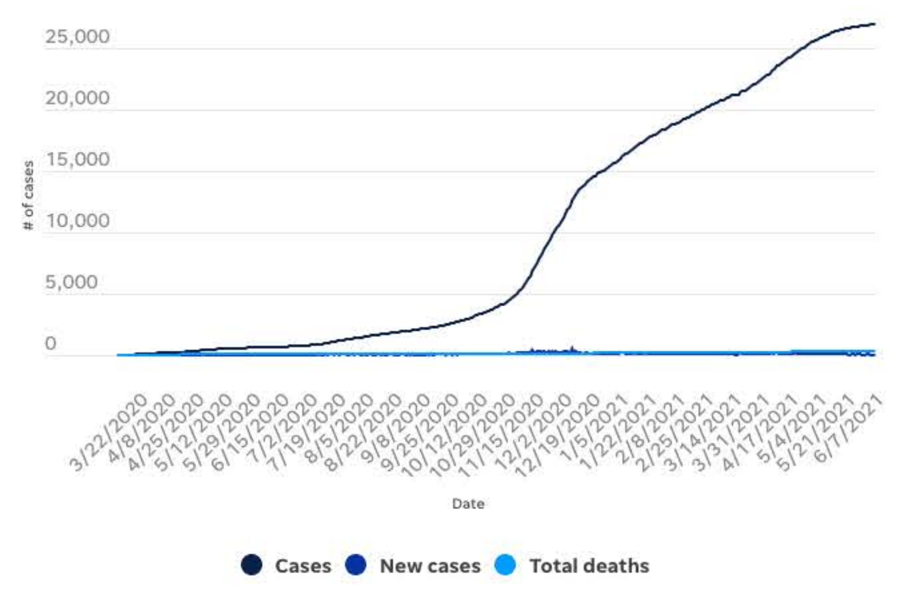

Last updated June 7, 2021
The county has three definitions for case types: confirmed, probable and suspect.

- **Confirmed case**: Someone who has tested positive using a molecular amplification detection test (such as a nasal swab tested using PCR testing). This test determines if someone is currently ill and able to spread COVID-19 to others.
- **Probable case**:
  - Has COVID-19 symptoms or clinical characteristics AND epidemiologic evidence with no test confirming the diagnosis
  - Has COVID-19 symptoms or clinical characteristics AND has tested positive using an antigen or serologic (antibody) test
  - Has an epidemiological link based on risk from a known exposure AND has tested positive using an antigen or serologic (antibody) test
  - Has a death certificate that lists COVID-19 disease or SARS-CoV-2 as a cause of death or significant condition contributing to death without positive test results to confirm the diagnosis.
- **Suspect case**: Someone who has tested positive using a serology (antibody) test but who did not have COVID-19 symptoms or clinical characteristics. Antibodies can take several weeks to develop after being exposed so someone who has tested positive for antibodies has likely recovered and is no longer able to spread the virus at the time they are tested for antibodies.

Coronavirus cases by demographic

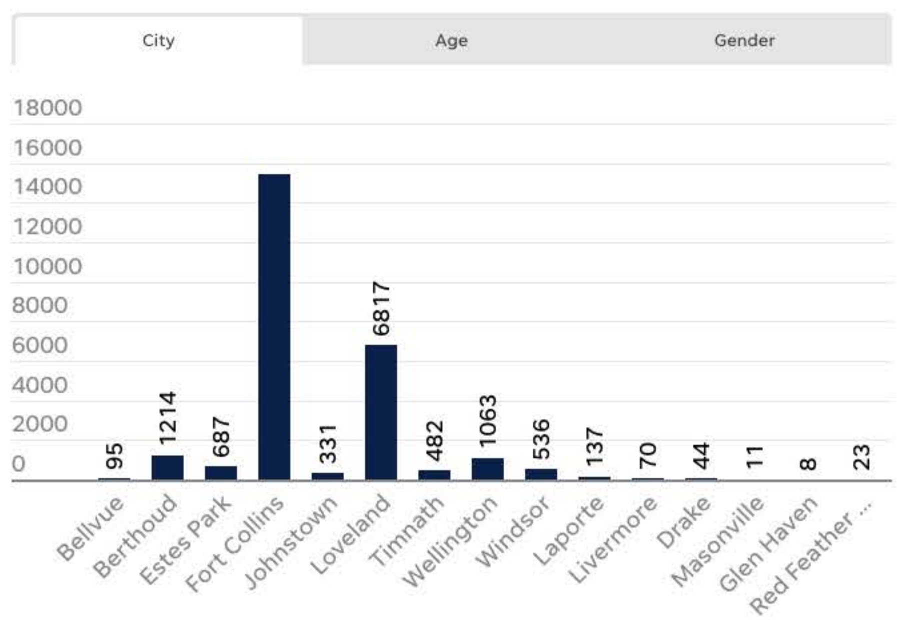

*Data for Windsor does not include cases from Weld County. Locations listed are those included in data from the Larimer County health department. Some locations may be outside of Larimer County.

Demographic data is for Confirmed and Probable cases only, and does not include antibody test results.

Share

Coronavirus cases in Colorado by day

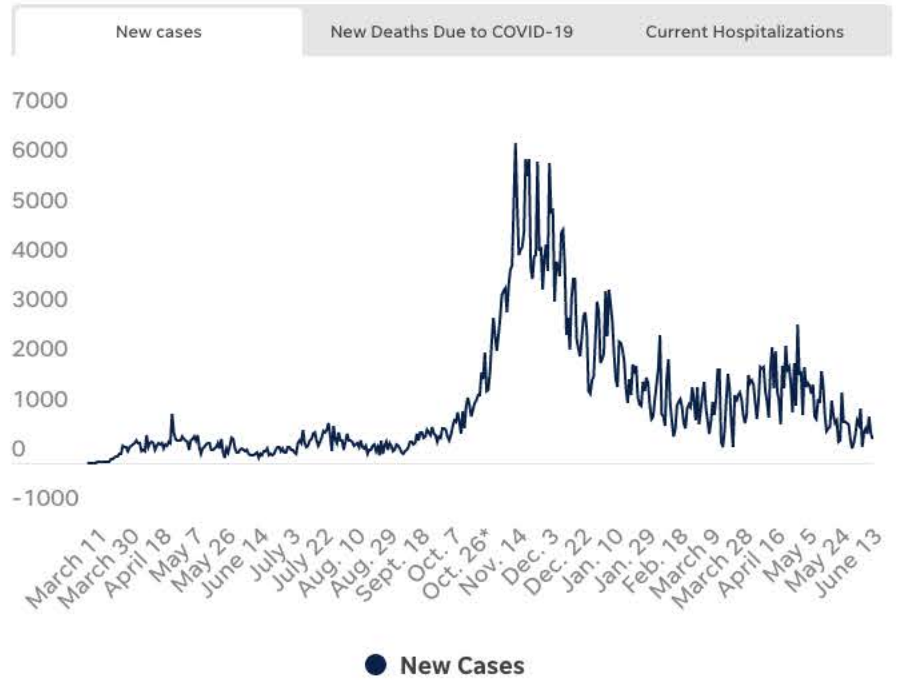

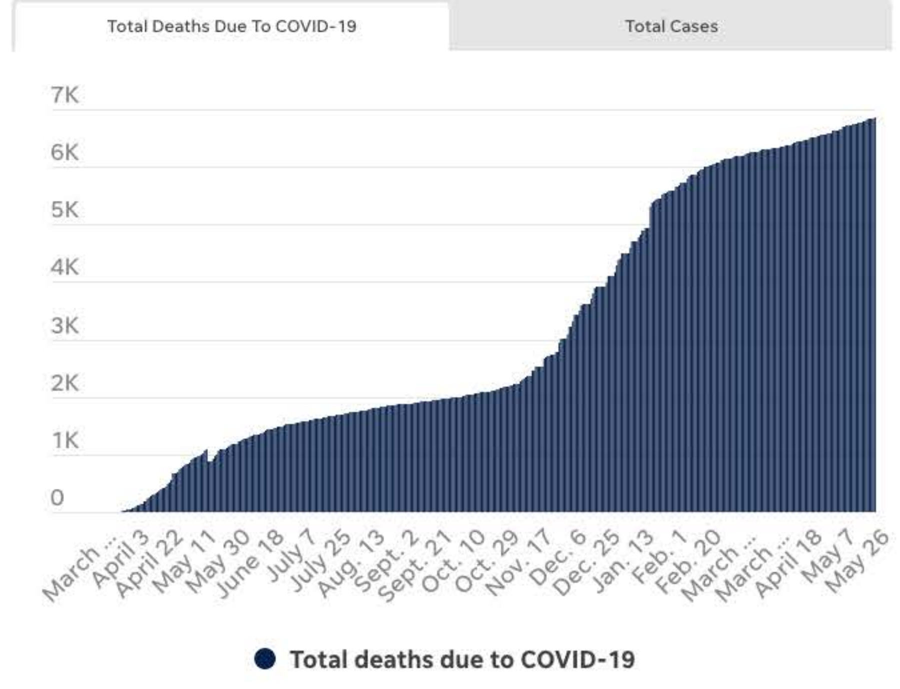

The Colorado health department releases data daily at 4 p.m. These charts reflect the dates cases were first reported by the state, not necessarily the date when cases were determined or first reported to the state.

*The new cases on March 13 and 14, 2021, were added on March 14. The Coloradoan tracked 3,748 cases over that weekend and split them evenly on this tracker to avoid the appearance of a spike on a particular day.

**The state health department added 462 backdated positive test results on Jan. 29, 2021, according to a news release.*

**For Oct. 24 and Oct. 26, the number of new cases reflects the addition of older cases from as early as Oct. 1 that had not previously been included in charts due to formatting issues at the state health department.*

**Starting with May 15, deaths are defined as people who died as a result of COVID-19. Prior to that date, deaths included anyone who had died and tested positive for coronavirus, regardless of their cause of death. This why you see a change of zero and a dip in total cases on May 15. Unfortunately, this data is not available retroactively.*

**The April 24-25 reporting figures for deaths may be skewed by retroactive tallying of deaths from coronavirus on previous dates that had not yet been accounted for.*

The state defines cases as "anyone who has tested positive for COVID-19 and anyone who is symptomatic and has had close contact with someone who tested positive."

Hospitalization data is provided to the state of Colorado by hospitals through the Colorado Hospital Association. Not all hospitals provide data, and the percentage of hospitals reporting data each day varies.

Share

Coronavirus cases in Colorado

Hover over the county to see how many confirmed cases there are in that location.

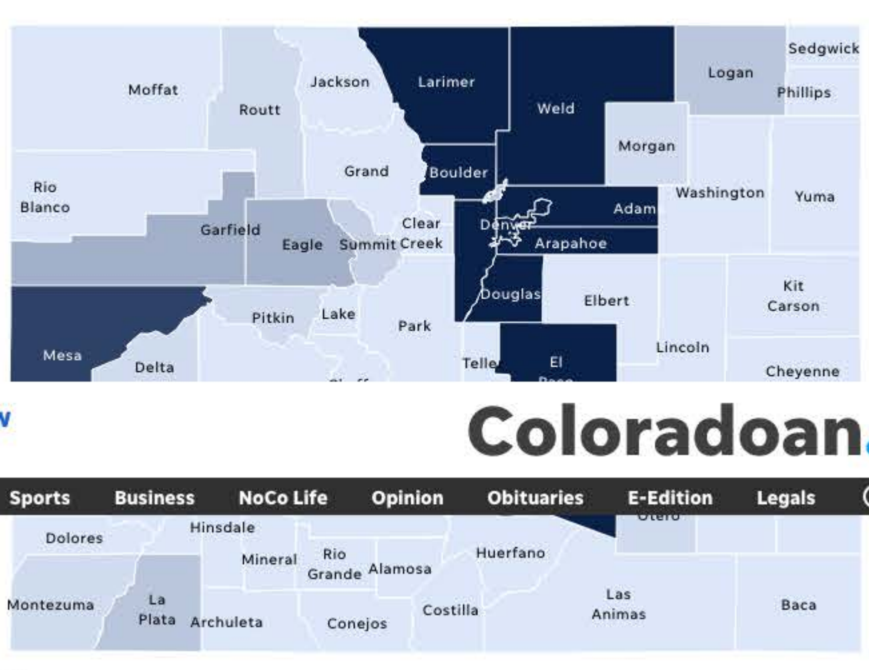

SUBSCRIBE NOW
$1 for 6 Months
Coloradoan.
News Sports Business NoCo Life Opinion Obituaries E-Edition Legals
Subscribe Sign In
SUBSCRIBE NOW
$1 for 6 Months

Download data

Last updated 6/7/2021

The health department notes 72 cases as international and therefore not reflected on this map.

Larimer case counts are based on state data, to give an accurate comparison by county.

Death data provided on the county level by the state includes all deaths among COVID-19 cases, regardless of if COVID-19 is listed as a cause of death. The state only provides the number of coronavirus-caused deaths as a total for the state as a whole.

From the health department: Please note initial information may change as more information is collected on each patient.

Share

Colorado coronavirus tracker: How many new cases are being reported, and where are they?

The charts will be updated daily

By KEVIN HAMM | khamm@denverpost.com | The Denver Post
PUBLISHED: March 6, 2020 at 5:41 p.m. | UPDATED: June 10, 2022 at 9:59 a.m.

Sign up for Newsletters and Alerts
SUBSCRIBE
Follow Us

02

Data updated 06/09/2022

Cases 1,480,663

All daily cases, 7-day average

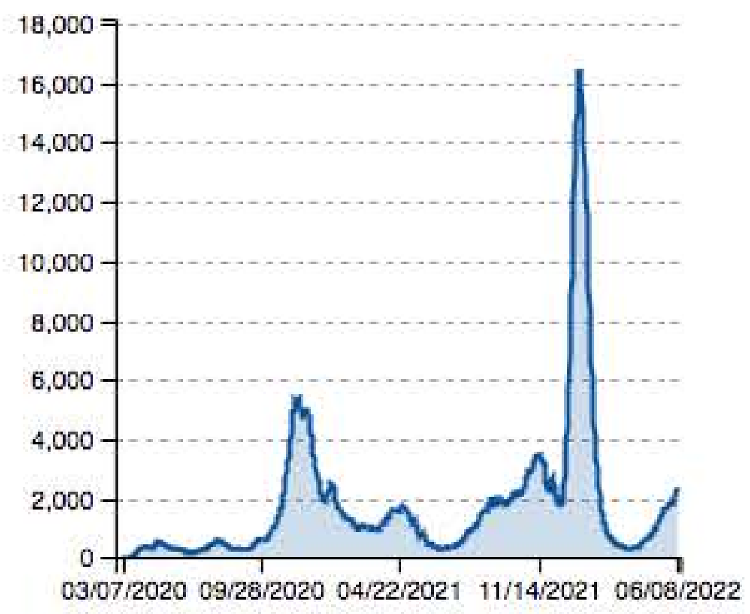

Weekly cases by vaccination status, per 100,000 people

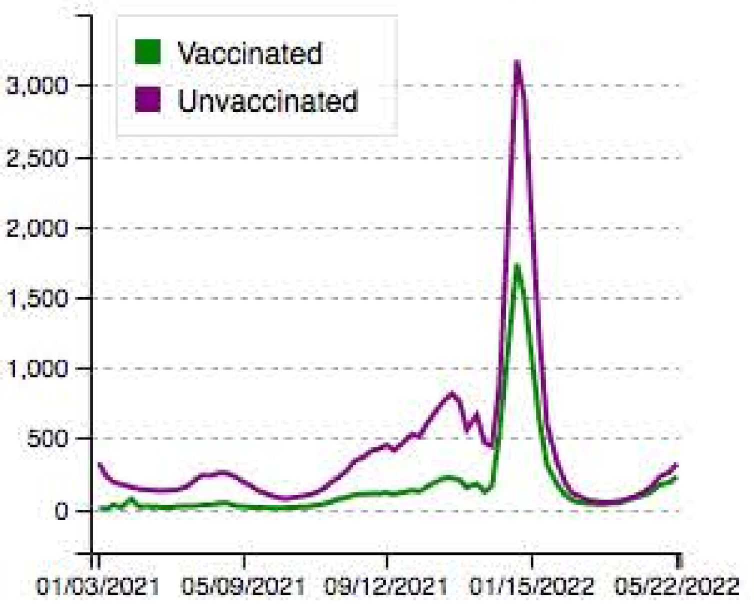

Hospitalizations 63,424

Currently hospitalized, by week

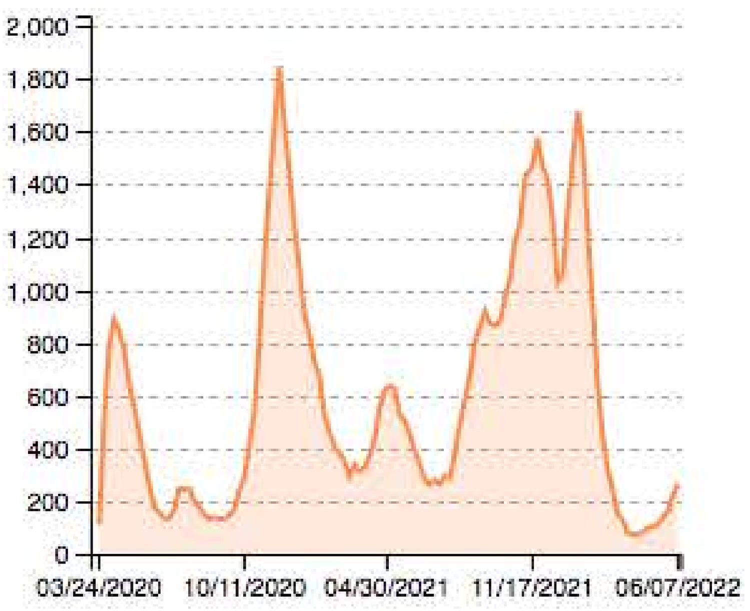

Weekly hospitalizations by vaccination status, per 100,000 people

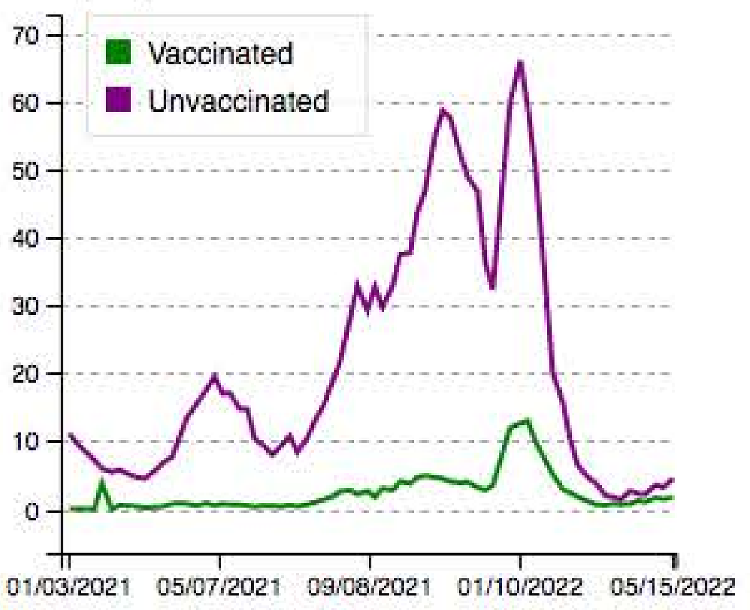

Deaths 13,349

All daily deaths, 7-day average

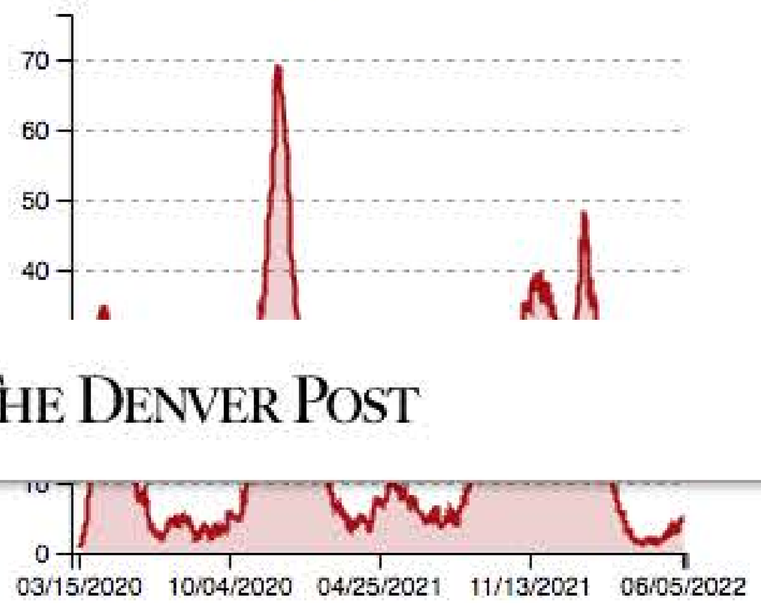

Monthly deaths by vaccination status, per 100,000 people

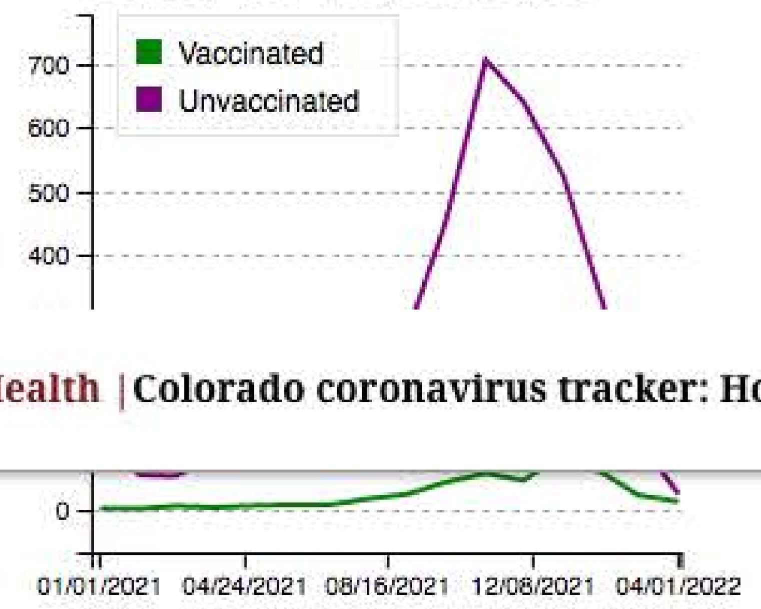

Positivity rate & testing

Daily positivity rate, 7-day average

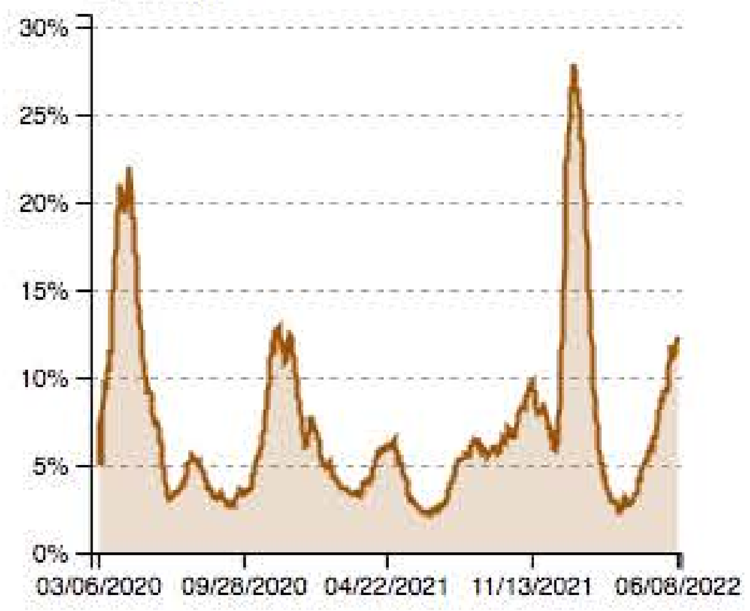

Weekly PCR tests

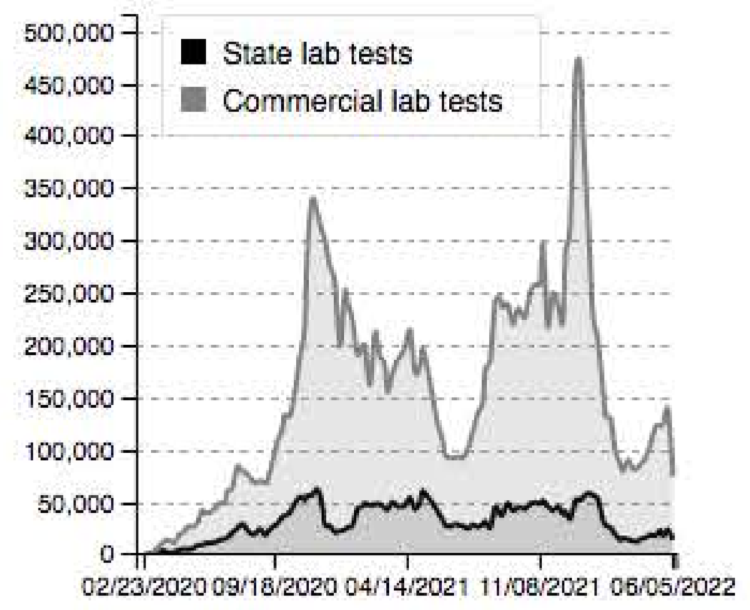

County cases and deaths

Show only large counties | Show all counties

County	Cases	Case rate per 100K	Deaths	Death rate per 100K	Population
Adams	142,476	27,395.2	1,321	254.0	520,076
Alamosa	4,724	28,930.1	62	379.7	16,329
Arapahoe	165,727	25,300.0	1,210	184.7	655,047
Archuleta	3,005	22,479.1	16	119.7	13,368
Baca	879	25,287.7	14	402.8	3,476
Bent	2,596	46,456.7	47	841.1	5,588
Boulder	73,574	22,237.3	371	112.1	330,859

MOST POPULAR

1 Kickin' It with Kiz: A frustrated Rockies fan's dream? Walmart heir makes Dick Monfort $4.65 billion offer he can't refuse.
2 Downtown Denver residents complain of being "terrorized" by scooter riders. Councilman weighs action
3 Denver will lose another favorite, decades-old diner at the end of June
4 Kiszla: Why do Broncos need a new stadium? Short answer: They don't.
5 Avalanche goalie Darcy Kuemper is 100% for Stanley Cup Finals; forwards Nazem Kadri and Andrew Cogliano aren't ruled out
6 Chambers: Wayne Gretzky is right about Cale Makar. He is the best defenseman since Bobby Orr
7 Keeler: Darcy Kuemper or Pavel Francouz? For Avalanche coach Jared Bednar, it's simple. Here's why.
8 Which team should Avalanche prefer t play in Cup Finals: Rangers or Lightning?
9 Plagiarism, policy and money woes: Republicans Heidi Ganahl and Greg Lopez race to challenge Colorado Gov. Jared Polis
10 Three injured in three-vehicle crash on I-25 in northern Douglas County

TRENDING NATIONALLY

1 Denver cocktail bar named one of Nort America's "50 Best"
2 Cops called as Britney Spears' ex, Jason Alexander, crashes wedding to Sam Asghari: report
4 Tesla Shanghai workers can leave the factory for first time in weeks
5 Hopkins doctor: This COVID surge not a bad as the last but prepare for another And monkeypox.

Connecticut COVID-19 Update

Connecticut Daily COVID-19 Daily Update for 06/10/22

As of **June 09, 2022** , the total of laboratory-confirmed and probable COVID-19 cases reported among Connecticut residents

is **816356** ; **3246** have been reported in the past 7 days. **Two hundred fifty-nine** patients are currently hospitalized with

laboratory-confirmed COVID-19; of these , **78** (30.12 %) are not fully vaccinated.

Table: Ver 5.18.2021 • Source: Connecticut Department of Public Health • Embed • Download image • Created with Datawrapper

As of June 2, 2022, the provisional 2020 census data* is being used to calculate age-adjusted COVID-19 case and mortality rates in the extended weekly COVID-19 report published on Thursdays. The 2020 estimates will replace 2018 estimates that have been used. Caution should be taken when making comparisons of percentages calculated using the 2018 and 2020 census estimates since observed difference may result from the shift in the denominator.

* DPH Provisional State and County Characteristics Estimates April 1, 2020. Hayes L, Abdellatif E, Jiang Y, Backus K (2022) Connecticut DPH Provisional April 1, 2020 State Population Estimates by 18 age groups, sex, and 6 combined race and ethnicity groups. Connecticut Department of Public Health, Health Statistics & Surveillance, SAR, Hartford, CT.

Click here for an archive of PDF reports on COVID-19 from the CT Department of Public Health, including detailed reports released every Thursday.

Connecticut COVID-19 Summary

Overall Summary	Cumulative (except for hospital census)	Past 7 days*	DateUpdated
Positive PCR/NAAT Tests	884,760	3203	June 10, 2022
All PCR/NAAT Tests	14,623,890	36424	June 10, 2022
Test Positivity (pos/all PCR/NAAT)		8.79%	June 10, 2022
Patients currently hospitalized with COVID-19	259	-70	June 10, 2022

Test positivity is calculated as a rolling 7-day test positivity by specimen collection date; all positive molecular (PCR/NAAT) test results are divided by all molecular (PCR/NAAT) test results (positive and negative) for the last 7 days and multiplied by 100 to reach a percentage.
Table: Ver 4.4.2022 • Source: CT Department of Public Health • Embed • Download image • Created with Datawrapper

The Past 7 Days column indicates all PCR/NAAT tests by specimen collection date from the past 7 days. Test positivity is calculated as a rolling 7-day test positivity by specimen collection date; all positive molecular (PCR/NAAT) test results are divided by all molecular (PCR/NAAT) test results (positive and negative) for the last 7 days and multiplied by 100 to reach a percentage. Hospitalizations over the past 7 days indicates the change in the number of patients hospitalized with COVID-19 over that period. Deaths over the past 7 days indicates the number of new COVID-19 associated deaths reported; deaths are reported once weekly.

As of April 4th 2022, negative rapid antigen and rapid PCR test results for SARS-CoV-2 are no longer required to be reported to the Connecticut Department of Public Health. Negative test results from laboratory based molecular (PCR/NAAT) results are still required to be reported as are all positive test results from both molecular (PCR/NAAT) and antigen tests. Both positive molecular (PCR/NAAT) and antigen tests will continue to be used for determining case status.

Risk Associated with Being Unvaccinated for COVID-19 in Connecticut

Metric	Result
**As of June 08, 2022**	
The number of cases of COVID-19 among fully vaccinated persons in Connecticut:	231,997
The Number of fully vaccinated persons in Connecticut:	2,731,409
Percent of fully vaccinated persons who have contracted COVID-19:	8

Case and test data obtained from CT EDSS. Vaccination data obtained from CT WiZ. All data are preliminary and subject to change.
Table: Ver 12.6.21 • Source: CT Department of Public Health • Get the data • Created with Datawrapper

National COVID-19 statistics and information about preventing spread of COVID-19 are available from the Centers for Disease Control and Prevention.

Day-to-day changes reflect newly reported cases, deaths, and tests that occurred over the last several days to week. All data in this report are preliminary; data for previous dates will be updated as new reports are received and data errors are corrected. Hospitalization data were collected by the Connecticut Hospital Association. Deaths reported to either the Office of the Chief Medical Examiner or DPH are included in the daily COVID-19 update.

COVID-19 Cases, Hospitalizations, Deaths and Tests Over Time

In Connecticut during the early months of this pandemic, it became increasingly clear that it would be necessary to track probable COVID-19 cases and deaths, in addition to laboratory-confirmed (e.g. RT-PCR) cases and deaths. This was needed to better measure the burden and impact of this disease in our communities and is now part of the national surveillance case definition for COVID-19. Prior to June 1, probable and confirmed cases were reported together.

Number of Confirmed and Probable COVID-19 Cases by Date

Test results may be reported several days after the result. Data are incomplete for the most recent days. Data from previous dates are routinely updated.

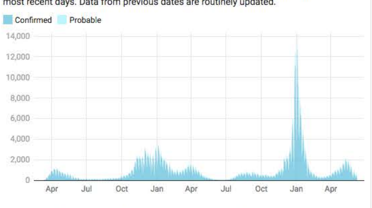

Date of Specimen Collection (confirmed) or Symptom Onset (probable)
Chart: Ver 5.11.2021 • Source: CT Department of Public Health • Embed • Download image • Created with Datawrapper

COVID-19 Cases over Time

This chart shows the number of COVID-19 cases reported to CT DPH by day of first positive specimen collection or onset of illness.

More information about hospitalized cases of COVID-19 in New Haven and Middlesex Counties is available from COVID-NET.

Number of COVID-19 Associated Deaths by Date of Death

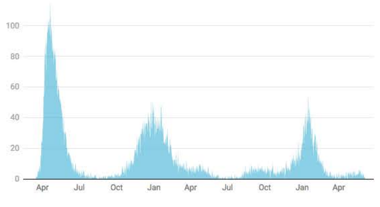

All data are preliminary and subject to change. Data from previous dates are routinely updated.
Chart: Ver 5.11.2021 • Source: Connecticut Department of Public Health • Embed • Download image • Created with Datawrapper

COVID-19 Associated Deaths

This chart shows the number of COVID-19-associated deaths among Connecticut residents by date of death. COVID-19-associated deaths involve persons who tested positive for COVID-19 around the time of death (confirmed), and those who were not tested for COVID-19 whose death certificates list COVID-19 disease or SARS-CoV-2 infection as a cause of death or a significant condition contributing to death (probable).

Number of COVID-19 Tests by Date of Specimen Collection

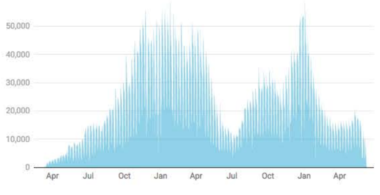

Data are preliminary and subject to change. Data from previous dates are routinely updated.
Chart: Ver 5.11.2021 • Source: Connecticut Department of Public Health • Embed • Download image • Created with Datawrapper

Number of COVID-19 Tests by Date of Specimen Collection

Testing for COVID-19 has increased over the course of the pandemic. Both the availability of supplies needed to perform the tests and the number of laboratories performing tests have increased. There are different kinds of tests for COVID-19. This graph shows the number of molecular and antigen tests reported to CT DPH by the date the specimen was collected. Fewer people are tested on Saturdays and Sundays compared to Monday through Friday.

Cumulative COVID-19 Cases, Deaths, Tests, and Rate per 100,000 by Town Among People Living in Community Settings

Includes confirmed plus probable cases and deaths. Tests includes persons with positive antigen results. All data are preliminary and are subject to change.

Search in table Page 1 of 17

Town	DateUpdated	Total Cases	Total Deaths	Rate per 100,000	Number of Tests	Number of Positive Tests
Andover	10 June 2022	504	6	15,575	9,283	579
Ansonia	10 June 2022	4,865	48	26,080	76,370	5,989
Ashford	10 June 2022	735	5	17,274	13,051	885
Avon	10 June 2022	2,975	81	16,278	73,962	3,488
Barkhamsted	10 June 2022	518	8	14,365	10,945	610
Beacon Falls	10 June 2022	1,291	11	20,749	23,017	1,565
Berlin	10 June 2022	4,057	51	19,852	74,725	4,909
Bethany	10 June 2022	984	4	17,736	23,149	1,203
Bethel	10 June 2022	4,057	71	20,490	74,085	4,891
Bethlehem	10 June 2022	612	2	17,989	11,766	742

Table: Ver 5.11.2021 • Source: CT Department of Public Health • Embed • Download image • Created with Datawrapper

Cumulative COVID-19 Cases and Deaths by County, Town, Age, Gender, and Race/Ethnicity

The following graphs show COVID-19 data by the Connecticut counties.

COVID-19 Cases, Rate per 100,000, Associated Deaths, and Number Hospitalized by County

Cases and deaths are cumulative over time

Number of Total Cases

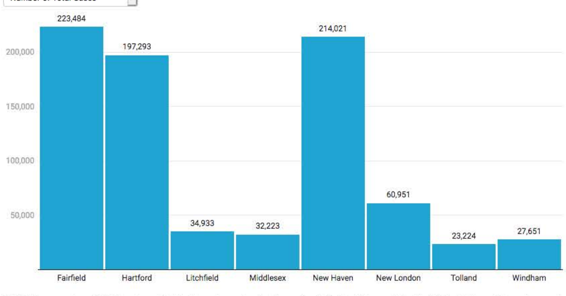

COVID-19 cases, rate per 100,000, and associated deaths are by county of residence. Hospitalization data are collected by CT Hospital Association and represent the number of patients currently hospitalized by location of the hospital and not the patient residence.
Chart: Ver 5.11.2021 • Source: CT Department of Public Health • Embed • Download image • Created with Datawrapper

COVID-19 by Age Groups

As with many other illnesses, age plays an important role in COVID-19. The graph below shows the number of COVID-19 cases and associated deaths by age groups. Because rates put disease frequency in the perspective of the size of the population, rates are particularly useful to compare disease frequency in different locations, at different times, or among different groups of people with different sized populations.

Both Case counts and rates among older age groups have declined since early in 2021 as individuals in those age groups become vaccinated, thus skewing the distribution of infections towards younger age groups over time. The number of COVID-19 cases is currently highest among adults age 20-39 compared to other age groups, as is the rate of cases per 100,000 population.

Number of COVID-19 Cases, Associated Deaths, and Rates per 100,000 by Age Groups

Total Number of Cases

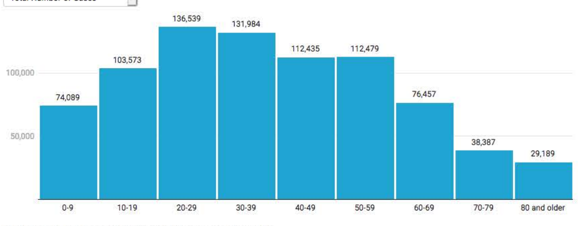

Total COVID-19 cases and associated deaths include confirmed plus probable.
Chart: Ver 5.11.2021 • Source: CT Department of Public Health • Embed • Download image • Created with Datawrapper

COVID-19 by Race and Ethnicity

The following graphs show the number and rate of cases and deaths by race and ethnicity. Categories are mutually exclusive. The category "multiracial" includes people who answered 'yes' to more than one race category. Approximately 30% of COVID-19 reports are missing information on the patient's race and ethnicity.

Additionally, the graphs show the rate of COVID-19 cases and COVID-19-associated deaths per 100,000 population by race and ethnicity. Crude rates represent the total cases or deaths per 100,000 people. Age-adjusted rates consider the age of the person at diagnosis or death when estimating the rate and use a standardized population to provide a fair comparison between population groups with different age distributions. Age-adjustment is important in Connecticut because the median age of among the non-Hispanic white population is 47 years, whereas it is 34 years among non-Hispanic blacks, and 29 years among Hispanics. Because most non-Hispanic white residents who died were over 75 years of age, the age-adjusted rates are lower than the unadjusted rates. In contrast, Hispanic residents who died tend to be younger than 75 years of age which results in higher age-adjusted rates.

The 2018 Connecticut DPH Population Statistics and 2000 US Standard Million populations were used for age adjustment. Categories are mutually exclusive. Cases missing data on race/ethnicity are excluded from calculation of rates. NH=Non-Hispanic

Cases and Deaths by Race and Ethnicity

Total Cases

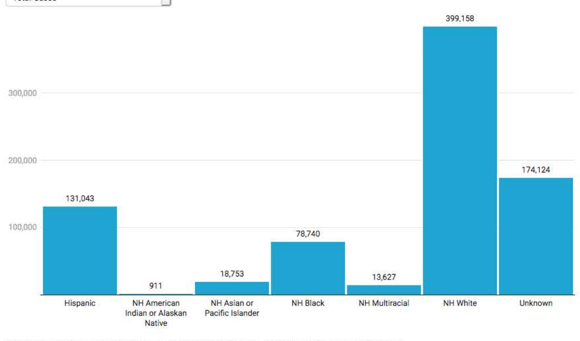

All data are preliminary and subject to change. Data from previous dates are routinely updated. NH = Non-Hispanic
Chart: Ver 5.11.2021 • Source: CT Department of Public Health • Embed • Download image • Created with Datawrapper

Community COVID-19 Cases and Tests in the Past 2 Weeks

This map shows the average daily rate of new COVID-19 cases per 100,000 population by town during the past two weeks. This map is updated weekly on Thursdays. Hover over a town to see the number of new cases, case rate, which is the average daily number of new cases per 100,000 population during the past two weeks, and the date range for the two week report period.

This map only includes cases among persons living in community-settings and excludes cases among persons residing in nursing homes, assisted living, and correctional facilities. Cases pending address validation are excluded from the map.

Average Daily Rate of COVID-19 Cases Among Persons Living in Community Settings per 100,000 Population By Town

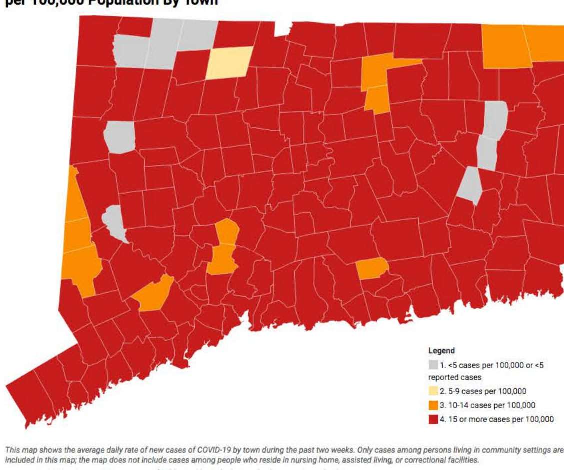

This map shows the average daily rate of new cases of COVID-19 by town during the past two weeks. Only cases among persons living in community settings are included in this map; the map does not include cases among people who reside in nursing home, assisted living, or correctional facilities.
Map: Ver 5.18.2021 • Source: CT Department of Public Health • Embed • Download image • Created with Datawrapper

COVID-19 Cases, Deaths, and Tests by Town

This map shows the distribution of COVID-19 cases, deaths, and tests since the beginning of the pandemic. Darker colors indicate towns with more cases.

Cumulative Number of COVID-19 Cases by Town

Click Town for Additional Information

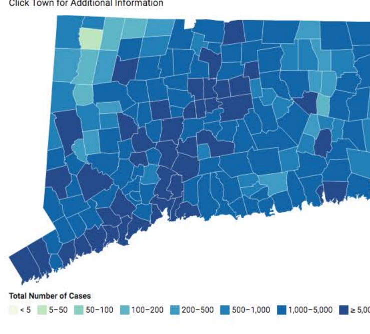

Map: Ver 5.11.2021 • Source: Connecticut Department of Public Health • Embed • Download image • Created with Datawrapper

Data Quality and Status

CT DPH and its partners go to great lengths to provide high quality data that is free of errors. Because of the nature of public health surveillance, there are times when data updates are necessary due to a variety of reasons. These include, but are not limited to, errors contained in the data CT DPH receives, updates to existing data as additional or new information becomes available, and the on-going de-duplication of cases and/or laboratory reports. CT DPH and your Local Health Department or District staff are all working to ensure only the best data and information are available for state and local decision makers.

Where Can I Get the Data?

CT DPH in partnership with the CT Office of Policy and Management (CT OPM) publishes data on the state's open data portal. You can find the data, charts, graphs, and maps here.

Connecticut Covid-19 Case Tracking by Connecticut State Data Center

814,328 (+652) CONFIRMED CASES	294 (+1) HOSPITALIZATIONS	10,998 (+27) DEATHS
14,597,127 (+5,260) TESTS COMPLETED	12.40% (+3.55%) POSITIVE TEST RATE	0.98 TRANSMISSION RATE

Select an indicator box above

Connecticut Covid-19 Tracking

This dashboard is updated daily with Covid-19 data from Connecticut Department of Public Health. Data only includes confirmed and reported numbers and are preliminary and subject to change.

As of August 26, 2020, this dashboard has replaced our previous Covid-19 dashboard.

Questions? Comments? Contact Jason Cheung: jc@ctdata.org

Navigate Our Dashboard

Main Dashboard

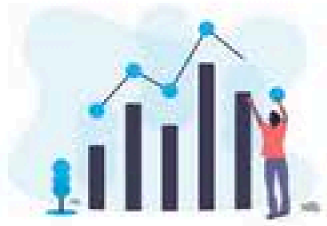

Town and County

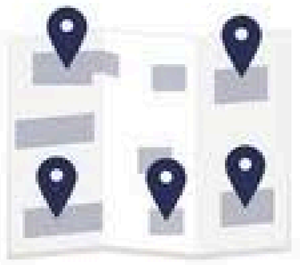

Demographics

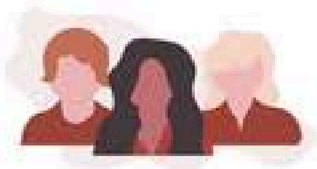

Daily Confirmed and Probable Cases

This chart shows new Covid-19 cases reported by CT DPH. The **blue 7-day moving average line** provides the best insight about state trends since daily counts may vary as a result of various reasons. Please focus on the **7-day moving average** rather than daily increases. Fluctuations in daily increases can be a result of many factors, such as errors in data collection, CT DPH not reporting on weekends as of July 2020 (resulting in larger increases on Mondays), and more.*

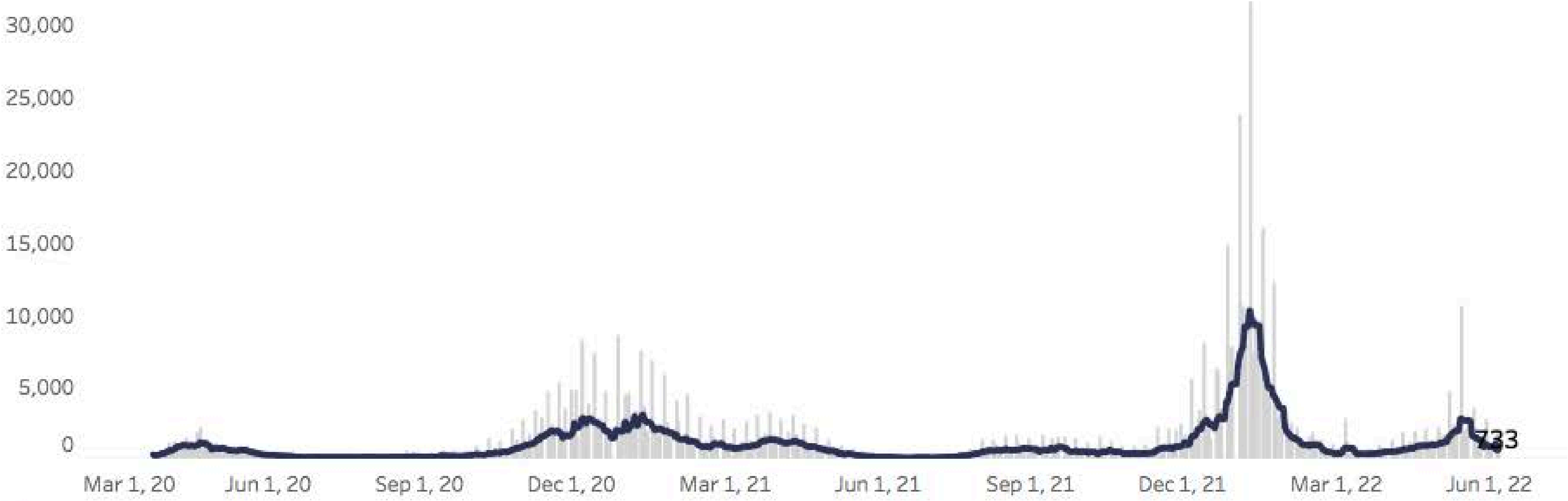

* The Date of Specimen Collection data is recommended by epidemiologists at CT DPH as it provides a more accurate daily increase in Covid-19 cases based on test date. **However, daily increases may be undercounted for days or weeks depending on the time it takes to receive test results.** To provide an accurate and recent snapshot of Covid-19, CTData has decided to use the Daily Report data with a focus on the **7-day moving average**.

+ableau

Details

37 166,905

Connecticut Covid-19 Case Tracking

Published: Mar 31, 2020 **Updated:** Jun 8, 2022

Connecticut Covid-19 Case Tracking by Connecticut State Data Center

814,328 (+652) CONFIRMED CASES	294 (+1) HOSPITALIZATIONS	10,998 (+27) DEATHS
14,597,127 (+5,260) TESTS COMPLETED	12.40% (+3.55%) POSITIVE TEST RATE	0.98 TRANSMISSION RATE

Select an indicator box above

Connecticut Covid-19 Tracking

This dashboard is updated daily with Covid-19 data from Connecticut Department of Public Health. Data only includes confirmed and reported numbers and are preliminary and subject to change.

As of August 26, 2020, this dashboard has replaced our previous Covid-19 dashboard.

Questions? Comments? Contact Jason Cheung: jc@ctdata.org

Navigate Our Dashboard

Main Dashboard

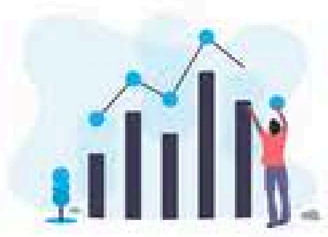

Town and County

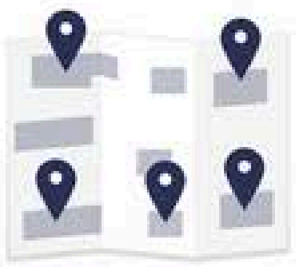

Demographics

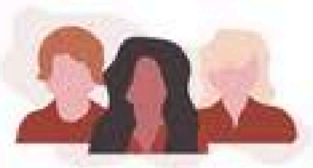

Current Hospitalizations

The number of patients currently hospitalized with Covid-19, as collected and reported by the CT Hospital Association

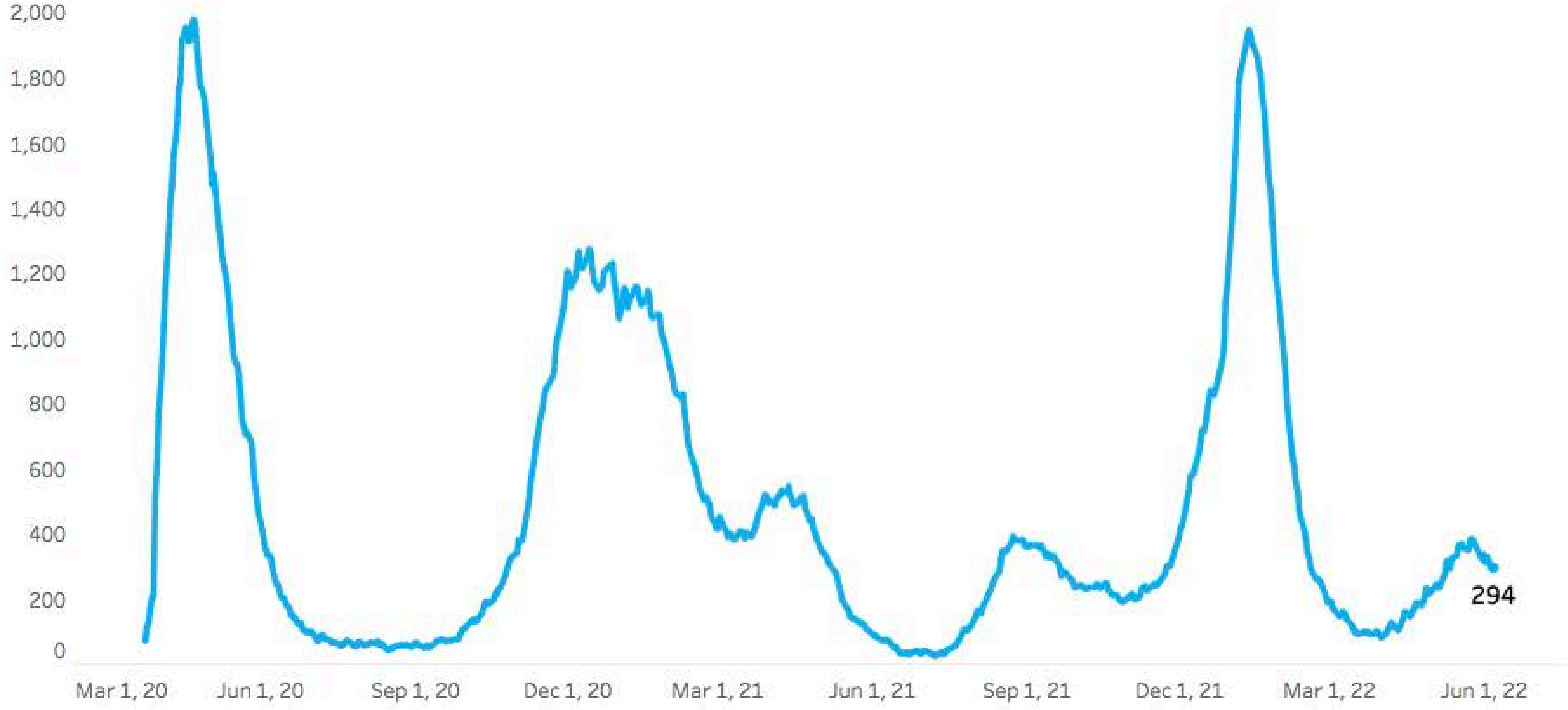

+ a b l e a u

Details

37 166,905

Connecticut Covid-19 Case Tracking

Published: Mar 31, 2020 **Updated:** Jun 8, 2022

Connecticut Covid-19 Case Tracking by Connecticut State Data Center

814,328 (+652) CONFIRMED CASES	294 (+1) HOSPITALIZATIONS	10,998 (+27) DEATHS
14,597,127 (+5,260) TESTS COMPLETED	12.40% (+3.55%) POSITIVE TEST RATE	0.98 TRANSMISSION RATE

Select an indicator box above

Connecticut Covid-19 Tracking

This dashboard is updated daily with Covid-19 data from Connecticut Department of Public Health. Data only includes confirmed and reported numbers and are preliminary and subject to change.

As of August 26, 2020, this dashboard has replaced our previous Covid-19 dashboard.

Questions? Comments? Contact Jason Cheung: jc@ctdata.org

Navigate Our Dashboard

Main Dashboard

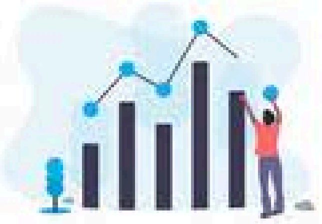

Town and County

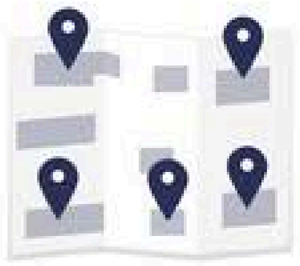

Demographics

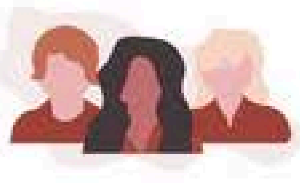

Daily Deaths

The number of confirmed and probable Covid-19 deaths, as reported to either the Office of the Chief Medical Examiner or CT DPH

Please focus on the **7-day moving average** rather than daily increases. Fluctuations in daily increases can be a result of many factors, such as errors in data collection, CT DPH not reporting on weekends as of July 2020 (resulting in larger increases on Mondays), and more.

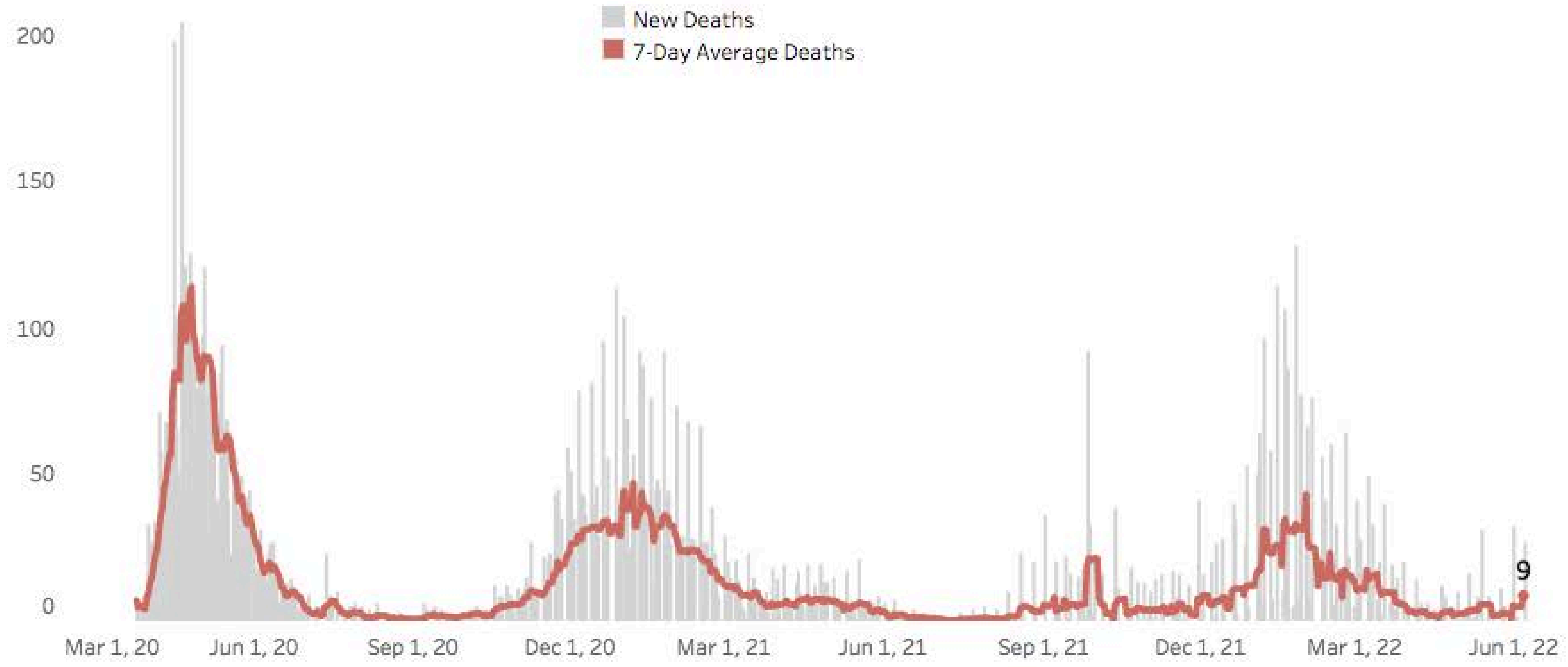

+ a b l e a u

Details

37 166,905

Connecticut Covid-19 Case Tracking

Published: Mar 31, 2020 **Updated:** Jun 8, 2022

Cases of COVID-19 Reported in Connecticut by Town by the Department of Geography at UConn

Last Updated at 6/10/2022, 6:30 PM

Total Confirmed Cases and Deaths

816,356

11,000

Increase Cases and Deaths

696

0

as of previous day

Daily Positivity Rate

8.79%

Previous day change: 0%

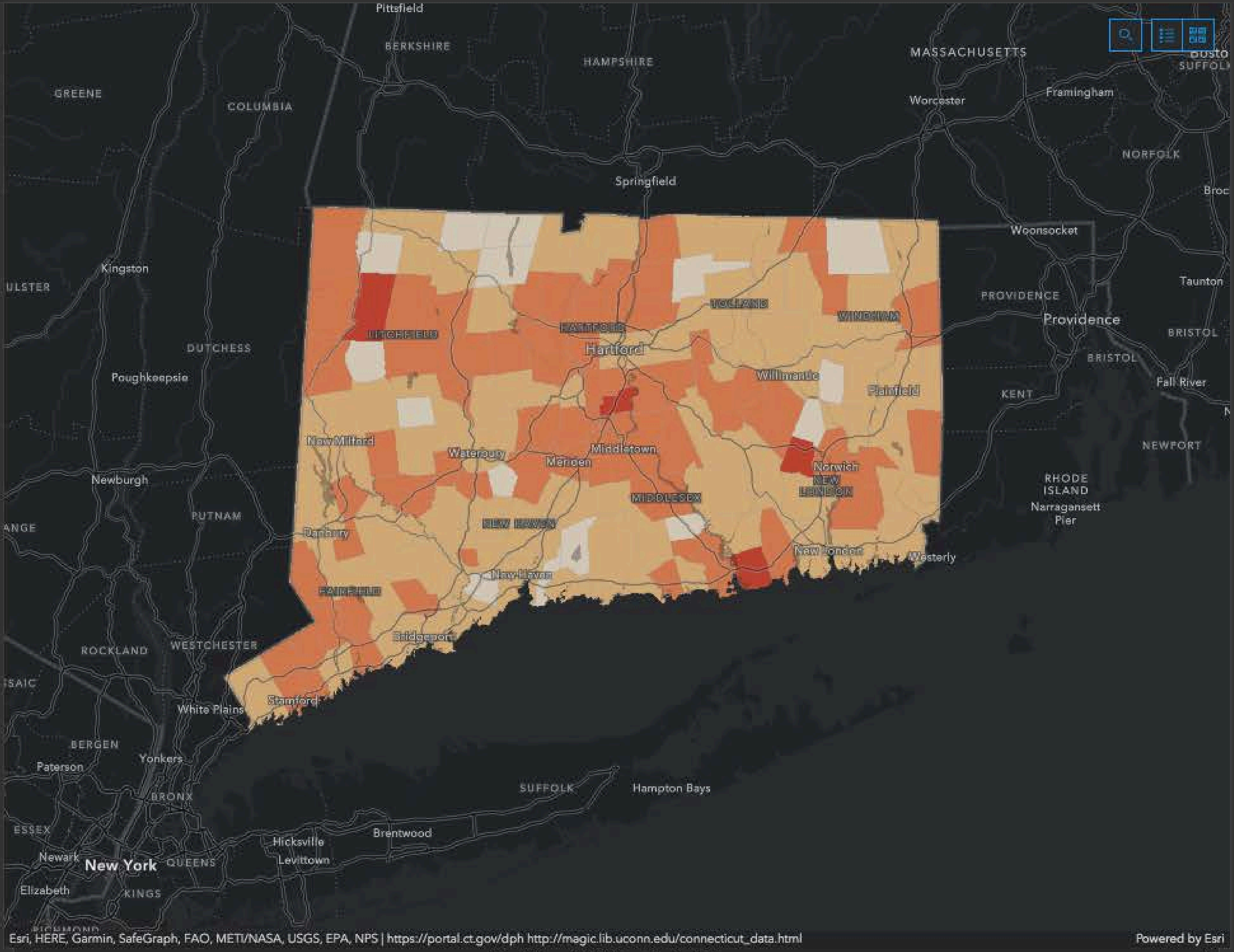

Esri, HERE, Garmin, SafeGraph, FAO, METI/NASA, USGS, EPA, NPS | https://portal.ct.gov/dph http://magic.lib.uconn.edu/connecticut_data.html Powered by Esri

Case Rate per 10,000 People in Past 14-Days

For sources and contact information please click upper right button in this box

Contact
Debarchana (Debs) Ghosh, PhD
Email: debarchana.ghosh@uconn.edu

Sources | Technical Note

Deaths per 100,000 People by Age

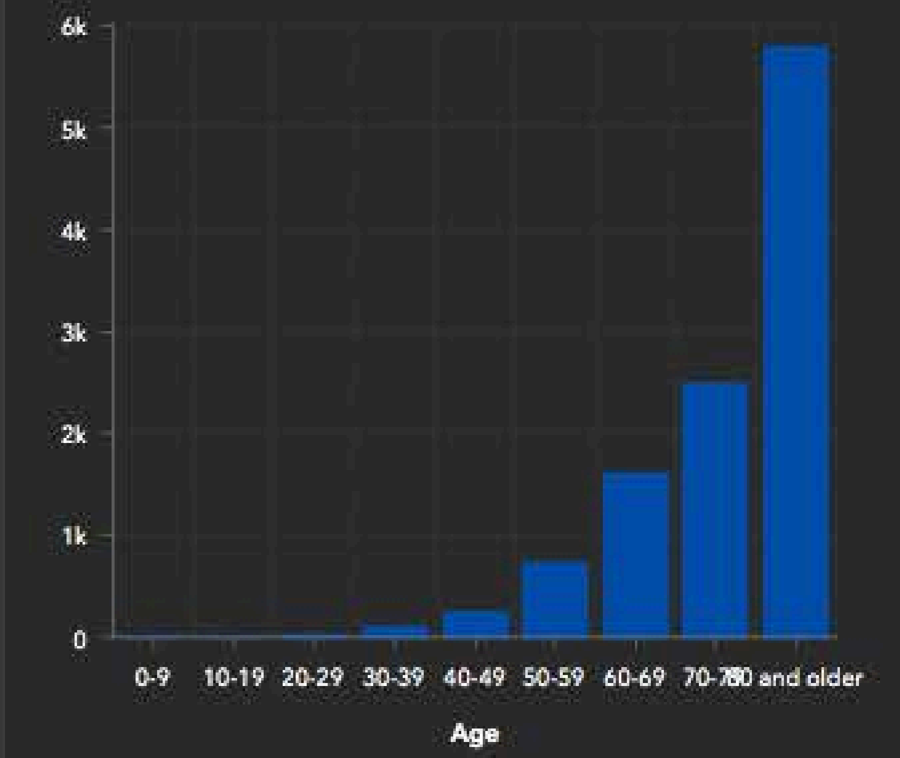

Deaths by Age | Cases by Age

Connecticut Towns

504, 208, 62, Andover
4,865, 299, 268, Ansonia
735, 217, 51, Ashford
2,975, 175, 304, Avon
518, 193, 35, Barkhamsted
1,291, 264, 80, Beacon Falls
4,057, 246, 355, Berlin
984, 212, 67, Bethany
4,057, 225, 219, Bethel
612, 220, 28, Bethlehem
4,907, 243, 353, Bloomfield
747, 189, 74, Bolton
603, 272, 36, Bozrah
5,852, 230, 353, Branford
42,433, 304, 1,100, Bridgeport
200, 140, 6, Bridgewater
14,339, 274, 666, Bristol
3,696, 234, 188, Brookfield
[illegible]

Cases, Tests, Change from Previous day

Towns | Counties

Key Metrics

Key Metrics | Vaccinations | K-12 Schools Data | School Surveillance Testing | Variant Surveillance

District of Columbia monitors key public health signs of multiple communicable diseases as a part of routine surveillance activities and disseminates health communications to decrease community spread. The following subset of key metrics, aligned with CDC's Community Levels, can be used in conjunction with wider public health messaging to support residents in personal risk decision-making.

Key Metrics data are scheduled to be released on a weekly basis every Wednesday by 6pm ET. Data are grouped by week and display the previous Sunday through Saturday.

Current COVID-19 Community Level

District of Columbia

Medium

Week of May 29, 2022 to June 04, 2022

Moderate impact on the healthcare system
Moderate levels of disease severity

Legend

Low | Medium | High

DC HEALTH
GOVERNMENT OF THE DISTRICT OF COLUMBIA

COVID-19 Community Levels | Use the Highest Level that Applies to Your Community

New COVID-19 Cases (per 100,000 people in the last 7 days)	Indicators	Level: LOW	Level: MEDIUM	Level: HIGH
FEWER THAN 200	New COVID-19 admissions per 100,000 population (7-day total)	<10.0	10.0-19.9	≥20.0
	Percent of staffed inpatient beds occupied by COVID-19 patients (7-day average)	<10.0%	10.0-14.9%	≥15.0%
200 OR MORE	New COVID-19 admissions per 100,000 population (7-day total)	NA	<10.0	≥10.0
	Percent of staffed inpatient beds occupied by COVID-19 patients (7-day average)	NA	<10.0%	≥10.0%

The COVID-19 Community Level is determined by the higher of the new admissions and inpatient beds metrics, based on the current level of new cases.

Dashboard Last Refreshed: 6/8/2022 4:01:03 PM

District of Columbia COVID-19 Weekly Case Rate

DC residents, per 100,000 population

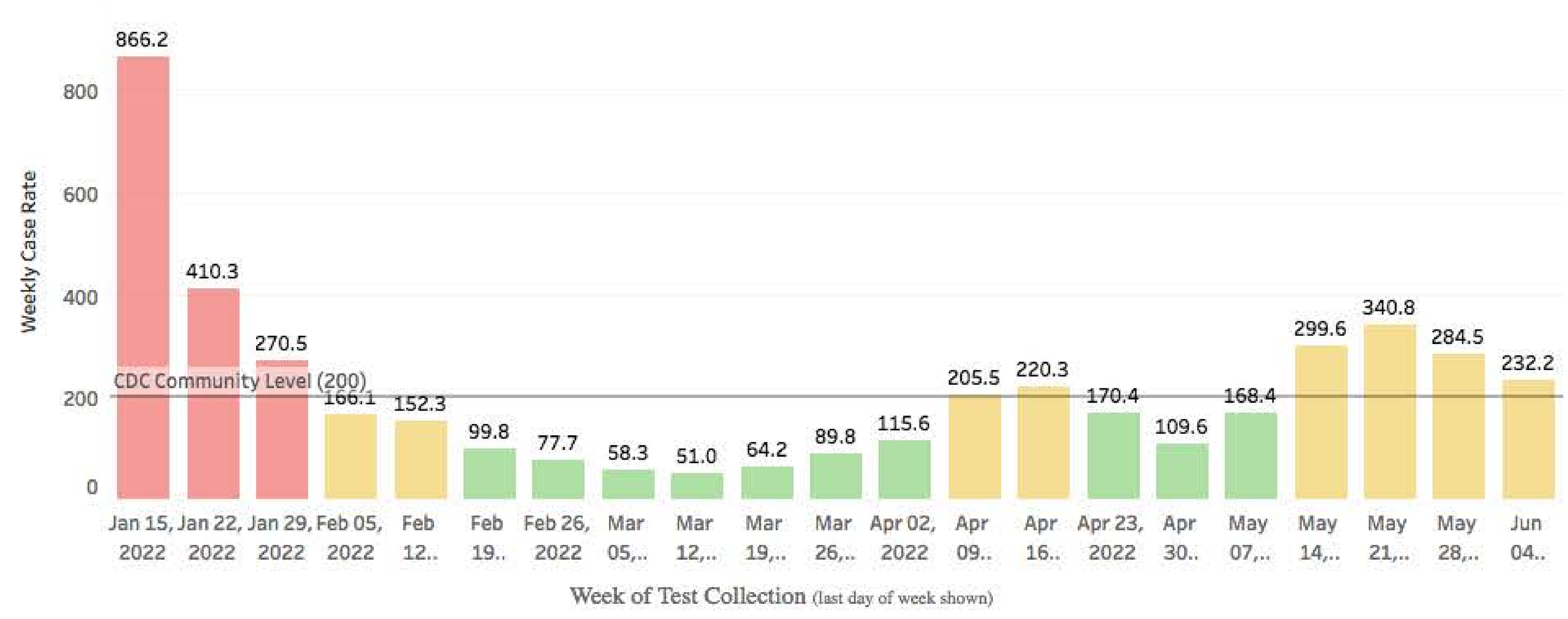

New Weekly Hospital Admissions due to COVID-19

DC Residents, per 100,000 population

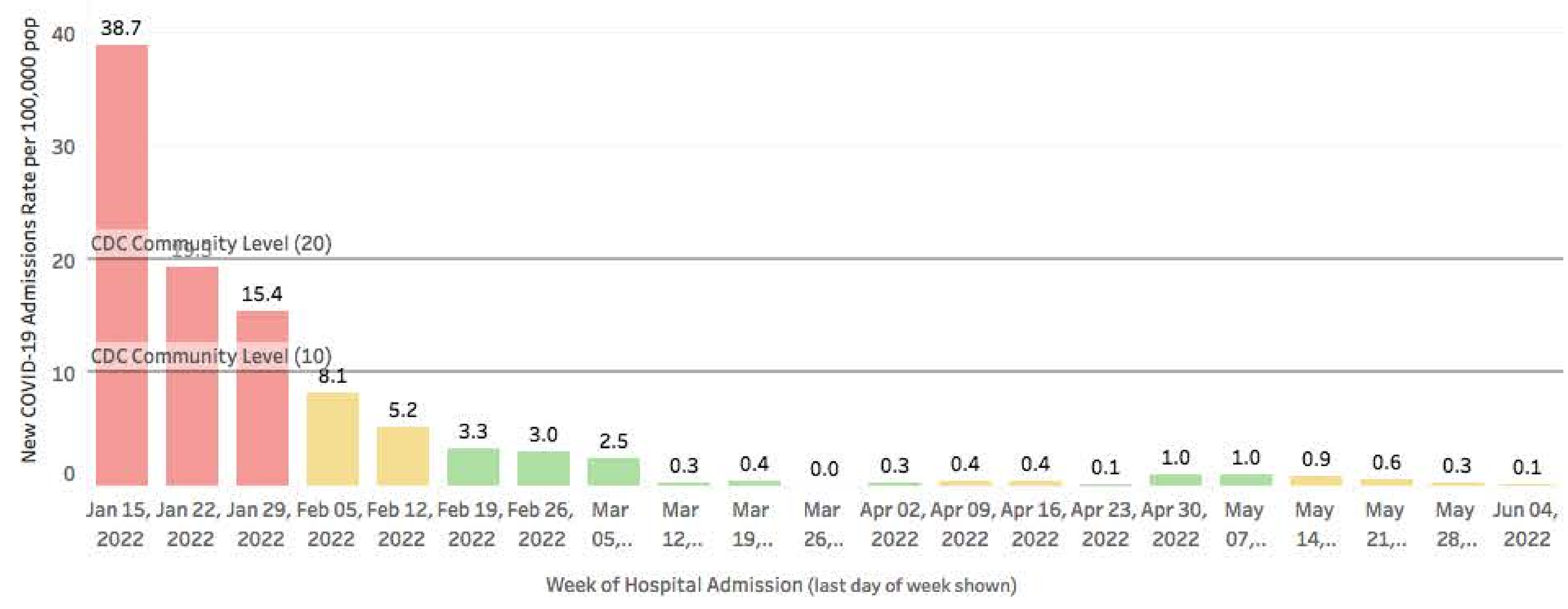

Percent Staffed Beds Occupied by Patients with COVID-19 in the District of Columbia

All jurisdictions, by week

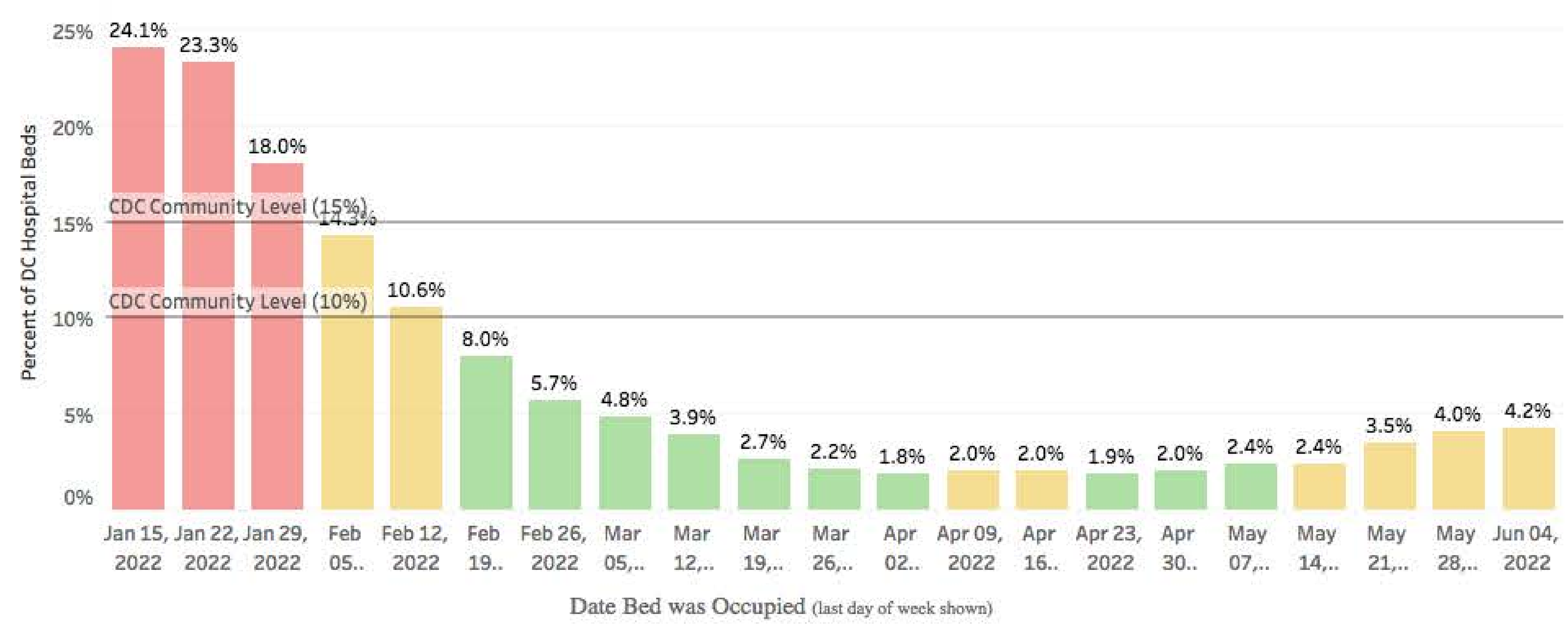

Percent of COVID-19 Confirmed Positive Cases who were Hospitalized due to COVID-19

DC Residents, by week

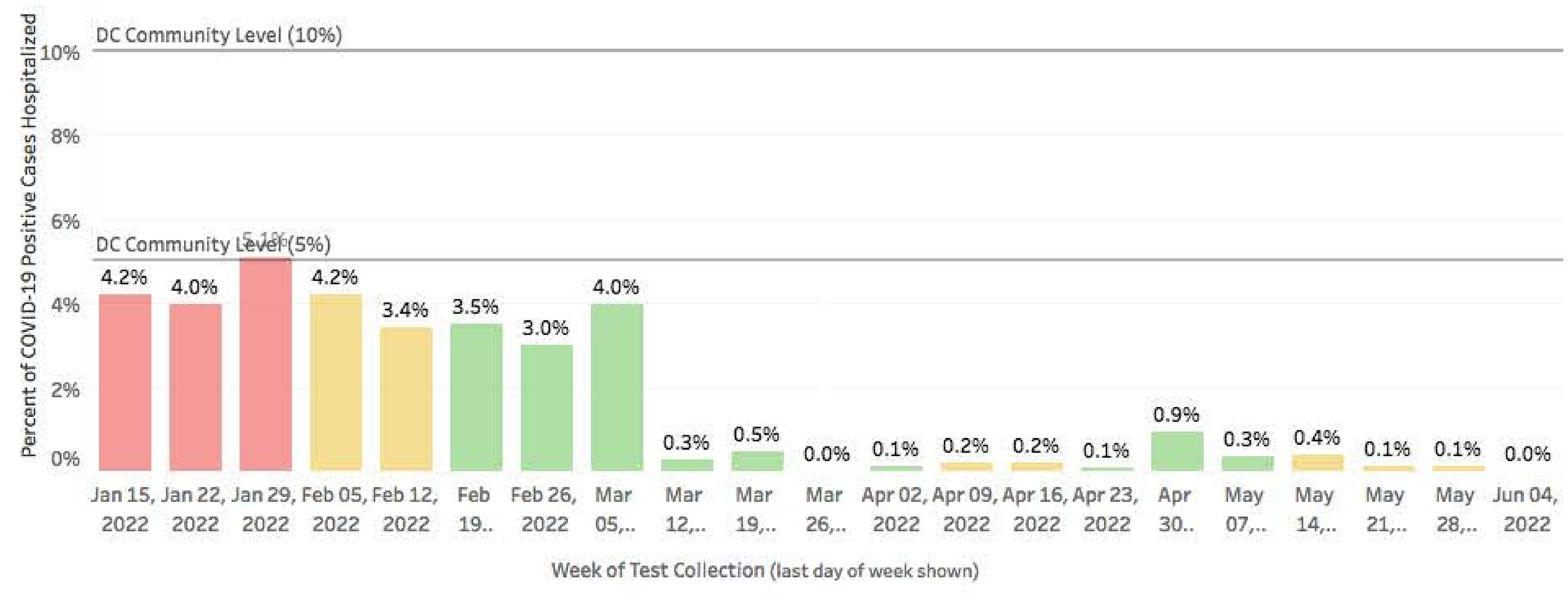

Data Sources and Notes

Weekly Case Rate: **Data Source:** DC Health via the Notifiable Disease Surveillance System; US Census.

Data Notes: Data may change on a daily basis. Total of new confirmed positive cases over 7 days, calculated per 100,000 DC resident population.

New COVID-19 Admission Rate of DC Residents: **Data Source:** DC Health via the Notifiable Disease Surveillance System; Chesapeake Regional Information System for Our Patients (CRISP); US Census.

Data Notes: Data may change on a daily basis. Total of new DC resident COVID-19-related hospital admissions over 7 days, calculated per 100,000 DC resident population. Dependent upon accurate reporting by hospitals into CRISP. Only confirmed positive COVID-19 cases are included. CDC values may differ as they do not distinguish differences in residency in admissions data. Historically, about half of DC hospital inpatients are non-DC residents.

Percent Hospital Beds Occupied by those with COVID-19: **Data Source:** DC Health via EMResource Hospital Reporting System and SHPDA.

Data Notes: Data may change on a daily basis. Current hospital census of all patients who have COVID-19 and are hospitalized in a DC acute-care facility on a given day, calculated as a percent of available staffed hospital beds. Dependent upon accurate reporting by hospitals into the EMResource system. Data include both DC and non-DC resident inpatients in DC hospitals.

Percent of Positive Cases who were Hospitalized: **Data Source:** DC Health via the Notifiable Disease Surveillance System; Chesapeake Regional Information System for Our Patients (CRISP).

Data Notes: Data may change on a daily basis. Percent of new DC resident COVID-19 confirmed positive cases who were hospitalized in DC hospitals. Dependent upon accurate reporting by hospitals into CRISP.

[illegible]

Coronavirus (COVID-19) Data Dashboard

Download Coronavirus (COVID-19) Data Dashboard Data

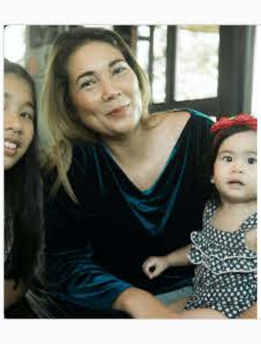

COVID-19 Vaccinations in the Hispanic Community

Hispanic Delawareans were hit hard during the pandemic's early months and slow to be vaccinated. Now they top the vaccination tables.

What happened?

View data story >

IMPORTANT INFORMATION ABOUT THIS PAGE

Data are current as of 9pm the previous weekday. Last update: 06/10/2022 3:59 PM.

About the data

DOWNLOAD DATA AS .CSV

Overview

Select dataset (Use dropdown to select)

Percentage of Total Population Fully Vaccinated

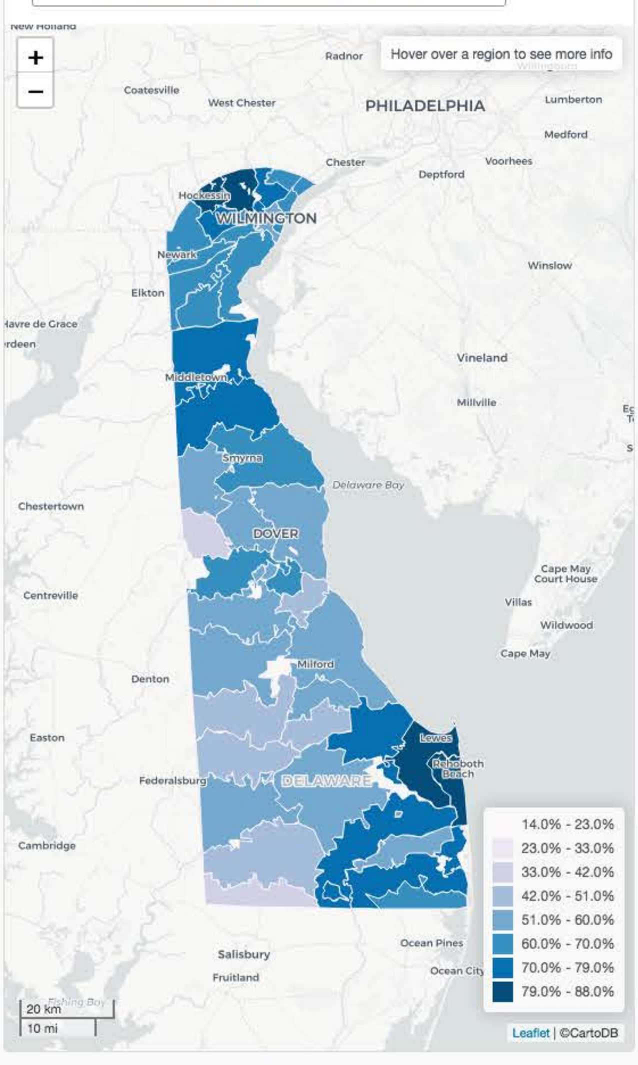

Percentage of 18+ Population Vaccinated With At Least One Dose

95.0% Data Source - CDC

Learn about how it differs from other DE data here

Percentage of Total Population Vaccinated With At Least One Dose

74.0%

Percentage of Tests Positive

16.1%
7-Day Average

OTHER KEY COVID METRICS

New Positive Cases
356.4
7-Day Average
View more case data

Total Vaccine Doses Administered
1,829,610
View more vaccine data

Current Hospitalizations
121 with 12 critical
View more hospital data

State of Delaware / *Not available for smaller geographies*

Variant Proportions

Last Updated: 06/08/2022

Virus mutation is common. The data presented here include thousands of specimens sequenced by the Delaware Public Health Laboratory each month, as well as sequencing results submitted by several other laboratories.

The variant data is not complete and will be updated later due to a technical issue. Please check back.

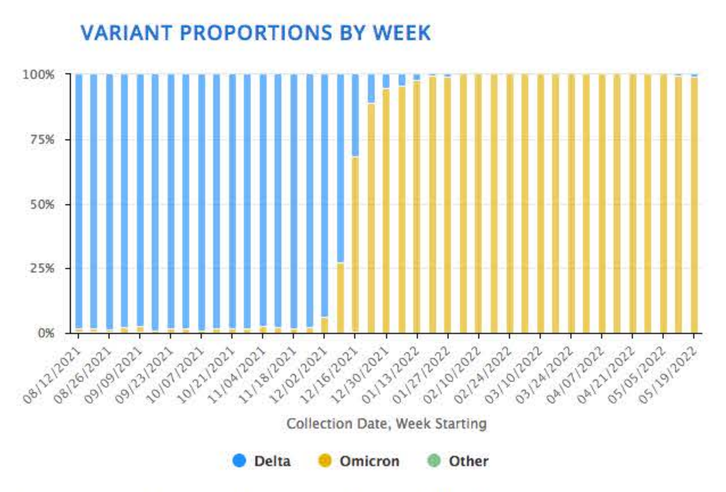

Dates are based on date of specimen collection. This chart is updated every Thursday.

Due to the length of time between sample collection (when a person is tested) and when samples are sequenced by laboratories, which can be lagged by two weeks or longer, variant data are provided based on sample collection date rather than sequencing date. This more accurately reflects the proportion of specific variants that are circulating in the community at various points in time.

State of Delaware / *Not available for smaller geographies*

Breakthrough Cases

Last Updated: 04/08/2022

Vaccine breakthrough cases are expected. COVID-19 vaccines are effective and are a critical tool to bring the pandemic under control. However, no vaccines are 100% effective at preventing illness in vaccinated people.

Of the **610,309 fully vaccinated Delawareans**, there have been **23,037 breakthrough cases** of COVID-19, roughly **3.77%**.

Total Fully Vaccinated **610,309**
68% of eligible population

Total Breakthrough Cases **23,037**
3.77% of Delawareans who have been fully vaccinated

The total number of breakthrough cases shown is based on verified information provided by interviewed cases. Efforts are underway to provide more complete information by data matching positive cases (regardless of ability to be reached for an interview) with the state's vaccine database, DelVAX. Therefore, the cumulative number of breakthrough cases may be updated in the future to reflect new and more complete information. A note announcing this change will be posted when implemented.

DPH will replace the below metric on breakthrough cases, with data showing the percent of individuals with COVID who are not up to date on their vaccines as it is a more useful indicator. Until that is implemented, we will no longer update the breakthrough case data.

State of Delaware

Trend Comparison: Fully Vaccinated and Deaths among All Delawareans

Last Updated: 06/10/2022 3:59 PM

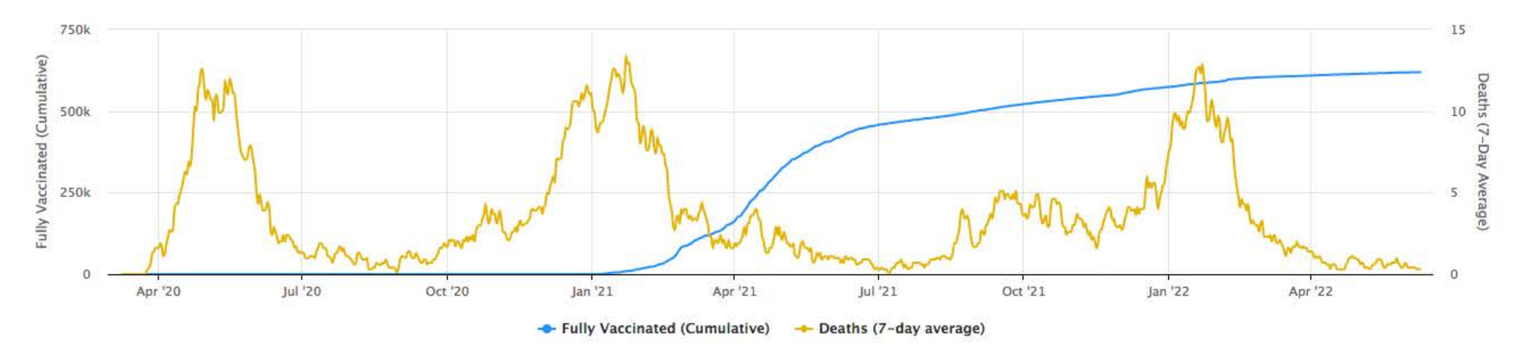

State of Delaware

Positive Cases Data

Last Updated: 06/10/2022 3:59 PM

Due to a technical issue causing a large number of lab results from previous months not to be added to My Healthy Community, the number of daily positive cases displayed on 06/07 has been updated to reflect additional positive cases. This along with additional technical issues causing MHC not to be updated during the last few days, will also cause the increase in overall cumulative total positive cases to be higher than the total reported cases for the prior days.

Total Positive Cases

280,252 3,049.1 per 10,000 people

Cumulative Number of Confirmed Positive Cases	Cumulative Number of Probable Positive Cases	Cumulative Number of Long-Term Care Cases
254,884	25,368	4,049

Positive Cases by County

County	Positive Cases	
New Castle County	159,057	View New Castle County data
Kent County	54,385	View Kent County data
Sussex County	65,854	View Sussex County data
Unknown	956	

View more case data

Data are current as of 9pm the previous weekday. Last update: 06/09/2022

State of Delaware

Deaths Data

Last Updated: 06/10/2022 3:59 PM

Total Deaths

2,970 24.0 per 10,000 people

Confirmed Deaths	Probable Deaths	Long-Term Care Deaths
2,668	302	962

View more death data

Data are current as of 9pm the previous weekday. Last update: 06/09/2022

Trends

STATE OF DELAWARE OVER THE LAST 14 DAYS

14-day charts | 30-day charts | 90-day charts | All available data

Case counts and test data by date of report can vary significantly from day to day for a variety of reasons. In addition to changes due to actual changes in disease incidence, trends are strongly influenced by testing patterns (who gets tested and why), testing availability, lab analysis backlogs, lab reporting delays, new labs joining our electronic laboratory reporting system, mass screenings, etc.

State of Delaware

Current Hospitalizations

Last Updated: 06/10/2022

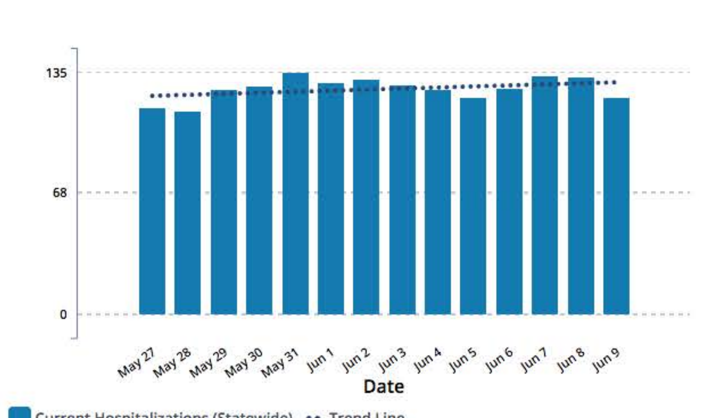

*When available, hospitalization estimates are provided by the University of Delaware if a hospital should fail to report.

State of Delaware

New Hospital Admissions (Confirmed and Suspected with COVID-19)

Last Updated: 06/10/2022

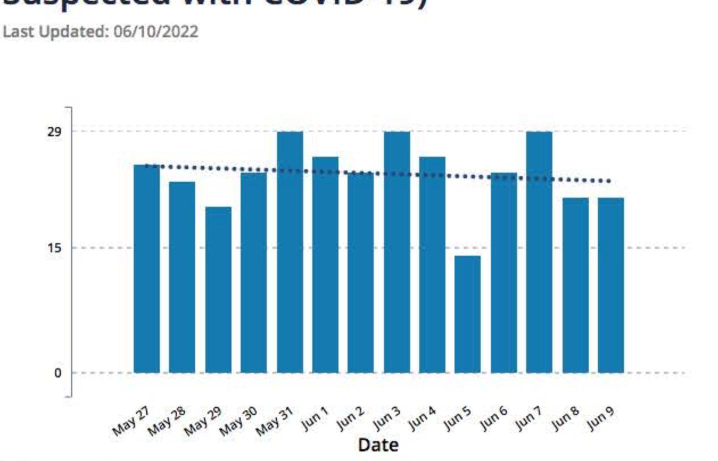

Data source: Delaware hospitals, Delaware Health Information Network

More details

State of Delaware

Percent Positive

Last Updated: 06/10/2022

Percent of tests that are positive is a helpful indicator in understanding the spread of infection in a community. It indicates the total number of specimens tested, including when an individual is tested more than once. This metric is widely used by other states and the Federal government, and is therefore useful to compare Delaware to other jurisdictions.

Data for percent of tests that are positive are based on the testing date. There is a two-day lag for presenting data related to percent of tests that are positive to account for the time delay between the date of the test and the date that DPH receives the test result.

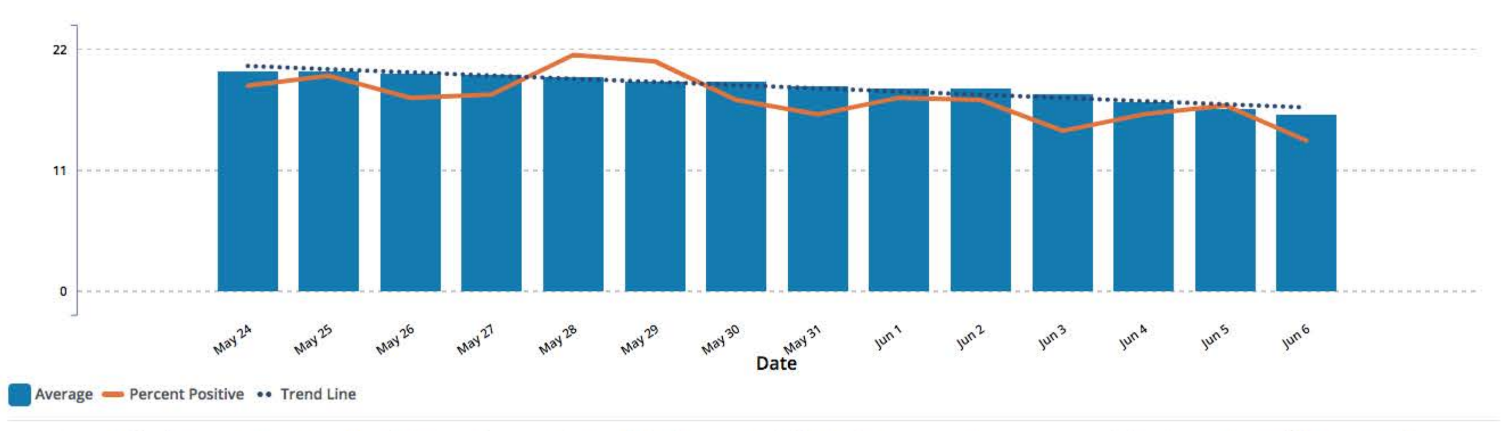

Averaging method: averages are a moving calculation that smooth unstable data. For each data point, seven values are averaged: the data point and the six preceding data points. Toward the beginning of the time series, when there are fewer than six values before a data point, instead of averaging seven values, only the available values are used in the calculation.

More details

State of Delaware

New Positive Cases

Last Updated: 06/10/2022

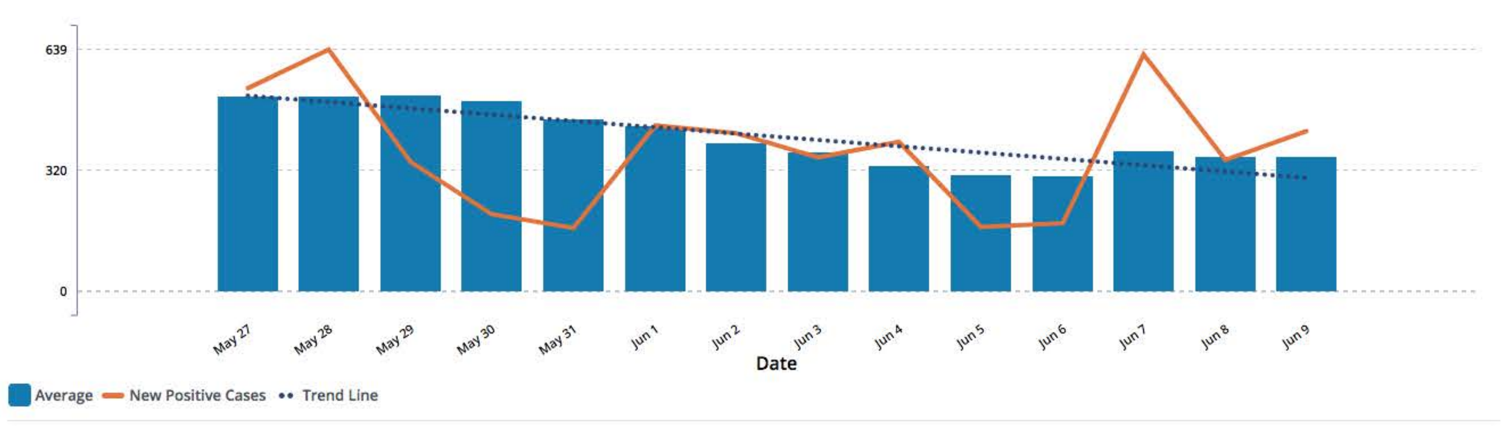

Averaging method: averages are a moving calculation that smooth unstable data. For each data point, seven values are averaged: the data point and the six preceding data points. Toward the beginning of the time series, when there are fewer than six values before a data point, instead of averaging seven values, only the available values are used in the calculation.

More details

State of Delaware

Percentage of Emergency Department Visits for Influenza-Like Illness (ILI) and COVID-19-Like Illness (CLI) by Week

Last Updated: 06/10/2022 3:59 PM

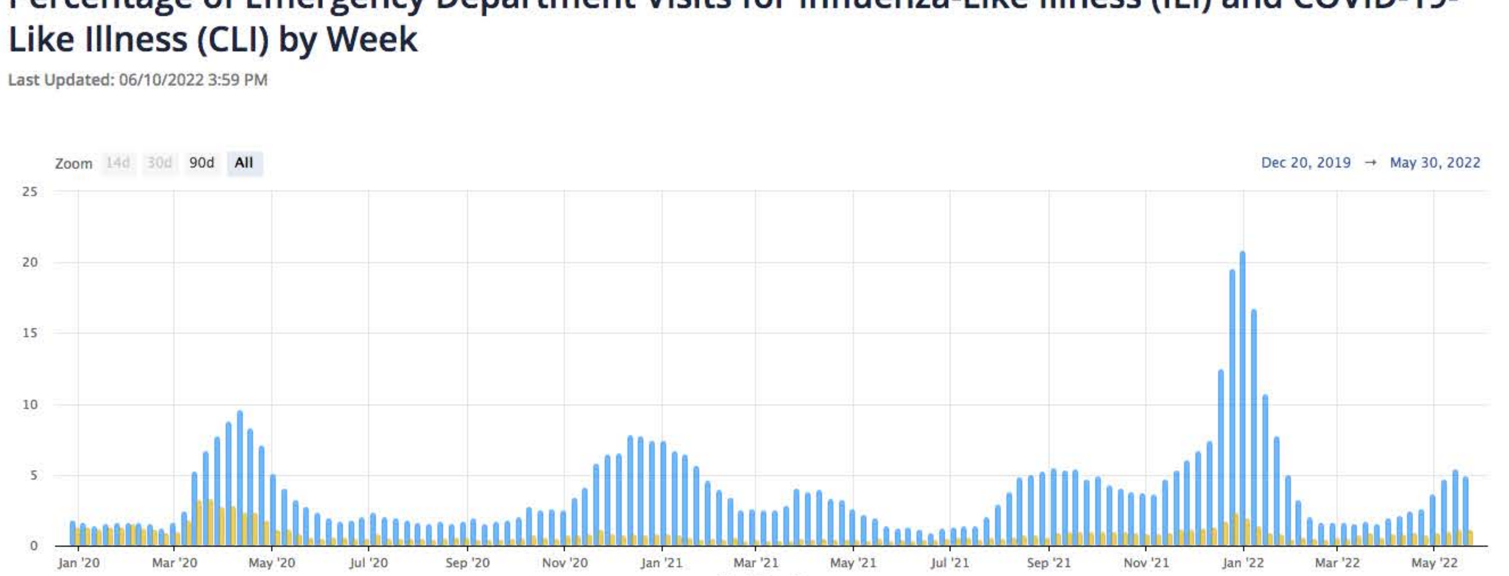

Data source: Delaware syndromic surveillance data from emergency department and freestanding emergency centers.

More details

State of Delaware

Tests Performed

Last Updated: 06/10/2022

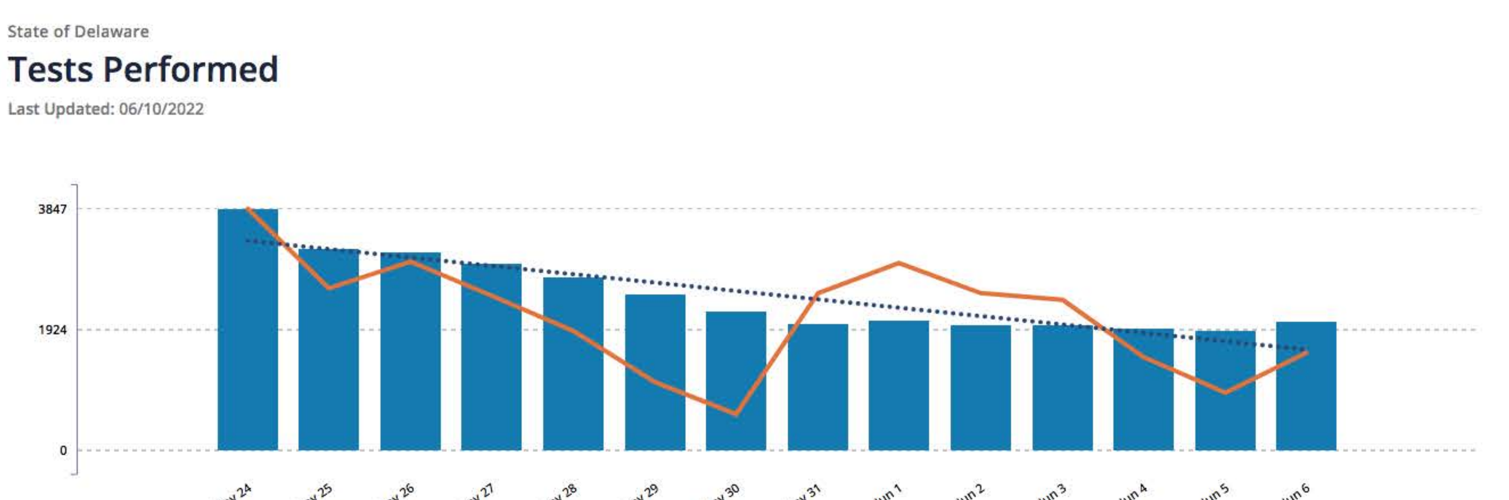

Averaging method: averages are a moving calculation that smooth unstable data. For each data point, seven values are averaged: the data point and the six preceding data points. Toward the beginning of the time series, when there are fewer than six values before a data point, instead of averaging seven values, only the available values are used in the calculation.

STATE OF DELAWARE

Cases by County (Cumulative)

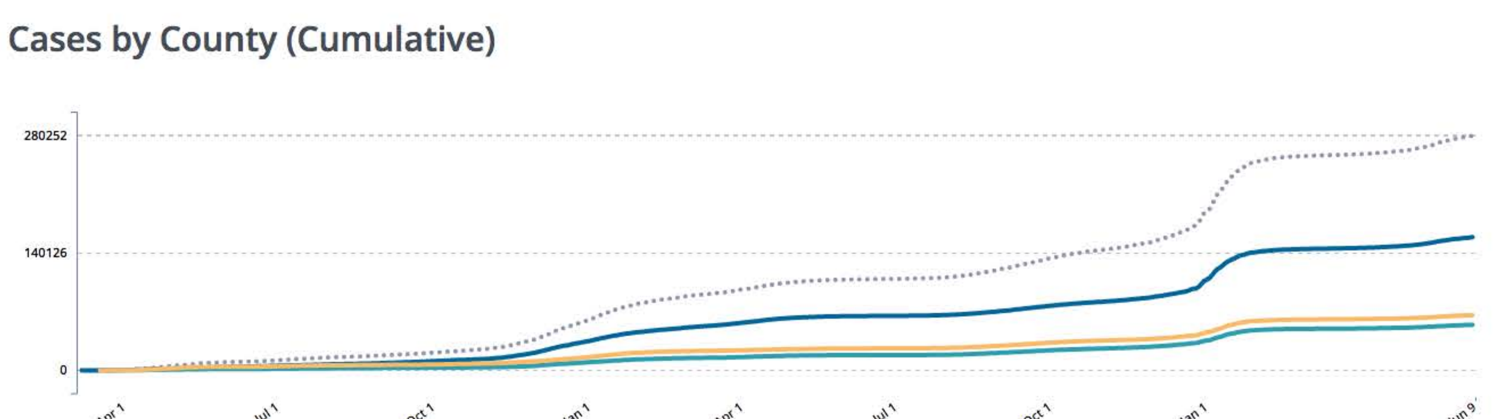

Weekly Community Spread Summary

About This Section

This section provides a weekly snapshot measuring the level of COVID-19 community spread in Delaware. It is based on three key indicators: Case rate, percent of tests that are positive, and hospitalizations. The change in these weekly indicators can be viewed over time. In addition, additional detail and a County-level summary can be viewed by clicking on "View more detailed data."

Breakdown of This Week's Status
Updated: Every Monday

05/28/2022 - 06/03/2022

New cases rate (per 100,000 people)	Percent of tests that are positive	Avg. daily hospitalizations (per 100,000 people)
258.5	18.0%	13.0

Unlike other COVID data on My Healthy Community, historical data presented on the weekly community spread summary are not updated daily. These data are updated on Mondays. This is to allow for complete reporting of the week's test data.

Status by Week

	New cases rate *(per 100,000 people)*	Percent of tests that are positive	Avg. daily hospitalizations *(per 100,000 people)*
05/28/2022 - 06/03/2022	258.5	18.0	13.0
05/21/2022 - 05/27/2022	365.7	19.3	12.8
05/14/2022 - 05/20/2022	417.2	19.7	13.3
05/07/2022 - 05/13/2022	312.6	16.9	10.7
04/30/2022 - 05/06/2022	213.7	12.6	8.7

Show/hide additional weeks

View more detailed data →

Coronavirus in Georgia: COVID-19 Dashboard

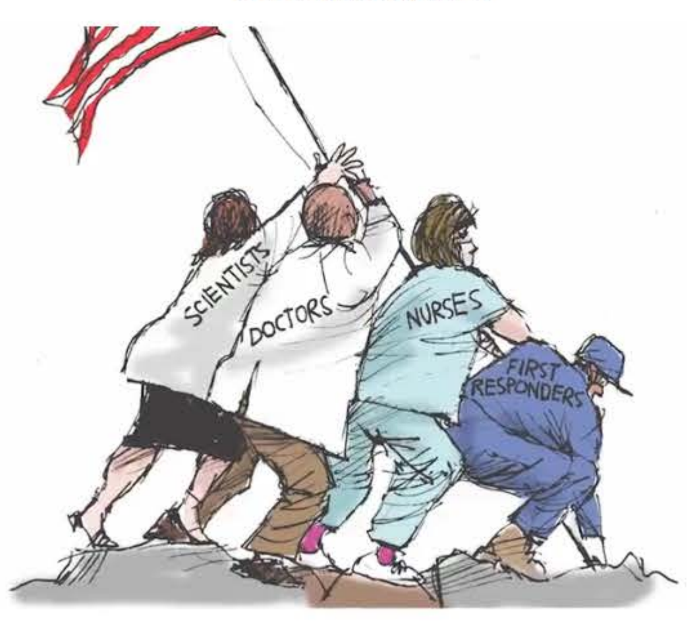

‹ Caption

Credit: AJC

COVID-19

Latest stats and the news on the coronavirus outbreak

NOTE: *As of April 18, the Georgia Department of Public Health releases its coronavirus reports once a week on Wednesdays at 3 p.m. The data on this page will follow those weekly updates.*

This dashboard gives up-to-date counts for confirmed and probable cases, deaths and other key statewide measures for the novel coronavirus. (Sources: Georgia Department of Public Health, Georgia Emergency Management & Homeland Security Agency, Atlanta Journal-Constitution analysis)

The dashboard may reflect a lag between confirmed and probable cases and confirmed deaths. So new confirmed cases reflect the spread of actual infections from a week or more ago.

Georgia DPH tracks confirmed cases (via molecular PCR tests) and probable cases (typically rapid antigen test positive cases), and the AJC has updated its dashboard to reflect both varieties of test.

Explore Current COVID-19 vaccination rates for Georgia counties

We have added additional charts that present the data based on first reported symptom for confirmed cases or date of death, as reported by the state. The AJC also tracks hospitalization data, rates of spread by county and computes transmission estimates, which are explained in detail below.

Jump to a specific chart: Cases | Deaths | Hospitalizations | Tests | County Data | Transmission Rates | Vaccines

Q: What is the latest on confirmed and probable coronavirus cases in Georgia?

Note: The DPH made a data correction beginning on March 30 and over the next several days to clear a backlog of cases from a laboratory that were not previously recorded. The spike in two charts — "Total Confirmed Cases" chart and the "Percent testing positive for coronavirus in Georgia" — on those dates reflect this correction of older data and not a spike in daily cases.

Newly reported cases by day in Georgia

Here, new **confirmed** [1] and **probable** [2] cases of coronavirus are shown by the date they first appeared on the DPH website. Because of the lag between first symptoms and DPH reporting a positive test result, other charts representing symptom onset can be misleading without proper context, as they tend to show a recent steep decline due to incomplete data. Looking at newly reported cases in this chart adds context by providing a static snapshot of each day's reports.

2,007,615 TOTAL CONFIRMED CASES
2,588,073 TOTAL INCLUDING PROBABLE CASES

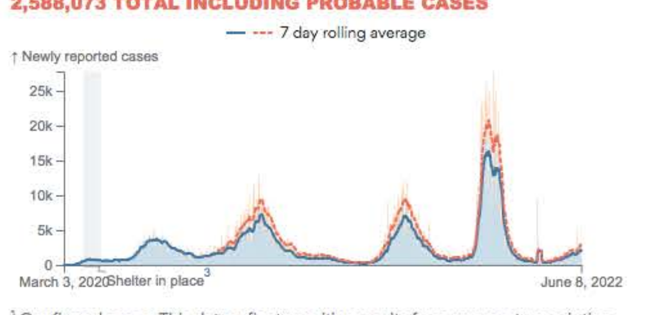

[1] Confirmed cases: This data reflects positive results from reverse-transcription polymerase chain reaction (PCR) tests.

[2] Probable cases: This data reflects positive results from rapid antigen tests, which are considered probable cases. DPH tracks cases by person, not individual tests. That means DPH moves antigen positive cases to the confirmed category if a person also receives a PCR positive. That change in data is updated daily. DPH first started reporting antigen positive cases on Nov. 4.

[3] Shelter in place: For most Georgians over age 65, shelter in place continued through June 12, 2020. Georgians who are residents of long term care facilities or are medically fragile must continue to shelter in place through at least March 31.

Source: Georgia Department of Public Health

Updated June 12 at 4:20 p.m.

[If you are on mobile and are not seeing our charts, please click here to reload full version of page]

Explore See how cases are trending in your county

Coronavirus cases by date of symptom onset [1] in Georgia

Here, new **confirmed** [2] and **probable** [3] cases of coronavirus are shown by the date of symptom onset as reported by DPH. But because there is a lag between first symptoms and the date DPH reports a positive test result, more recent data is likely to be adjusted upward in the future.

While DPH may adjust the number of cases from any date, the dashed line and lighter colored bars represent the window when the most adjustments are likely to occur, according to our analysis.

2,005,983 TOTAL CONFIRMED CASES
2,586,241 TOTAL INCLUDING PROBABLE CASES

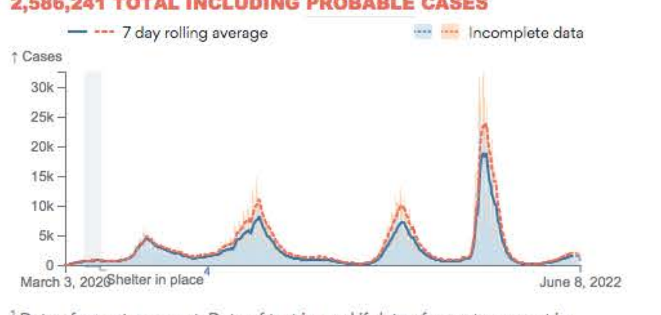

[1] Date of symptom onset: Date of test is used if date of symptom onset is unknown; date reported to DPH is used if none of the above are known. Cases where DPH is unable to determine a date are not included, which accounts for the discrepancy between the number of confirmed cases on this chart and the newly reported cases chart.

[2] Confirmed cases: This data reflects positive results from reverse-transcription polymerase chain reaction (PCR) tests.

[3] Probable cases: This data reflects positive results from rapid antigen tests, which are considered probable cases. DPH tracks cases by person, not individual tests. That means DPH moves antigen positive cases to the confirmed category if a person also receives a PCR positive. That change in data is updated daily.

[4] Shelter in place: For most Georgians over age 65, shelter in place continued through June 12, 2020. Georgians who are residents of long term care facilities or are medically fragile must continue to shelter in place through at least March 31.

Source: Georgia Department of Public Health

Updated June 12 at 4:20 p.m.

Q: What is the latest on coronavirus deaths in Georgia?

Newly reported deaths by day in Georgia

Here, new **confirmed** [1] and **probable** [2] deaths from coronavirus are shown by the date they first appeared on the DPH website. Because of the lag between the date of a death and DPH reporting it, other charts representing day of death can be misleading without proper context, as they tend to show a recent steep decline due to incomplete data. Looking at newly reported deaths in this chart adds context by providing a static snapshot of each day's reports.

31,885 TOTAL CONFIRMED DEATHS
38,290 TOTAL INCLUDING PROBABLE DEATHS

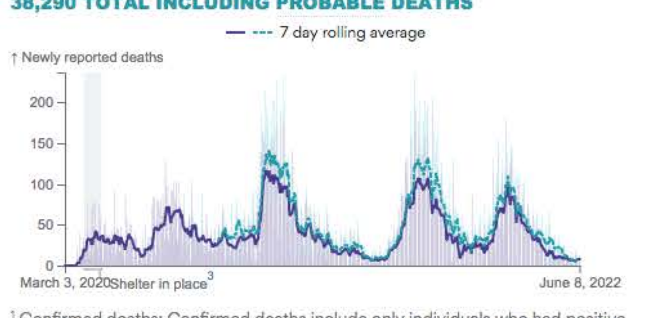

[1] Confirmed deaths: Confirmed deaths include only individuals who had positive reverse-transcription polymerase chain reaction (PCR) test results.

[2] Probable cases: This data reflects positive results from rapid antigen tests, which are considered probable. DPH first started reporting probable deaths on Nov. 4.

[3] Shelter in place: For most Georgians over age 65, shelter in place continued through June 12, 2020. Georgians who are residents of long term care facilities or are medically fragile must continue to shelter in place through at least March 31.

Source: Georgia Department of Public Health

Updated June 12 at 4:20 p.m.

Coronavirus deaths by day of death in Georgia

Here, new **confirmed** [1] and **probable** [2] deaths from coronavirus are shown by the date they occurred, as reported by DPH. But because there is a lag between the date of death and the date DPH reports it, more recent data is likely to be adjusted upward in the future.

While DPH may adjust the number of deaths from any date, the dashed line and lighter colored bars represent the window when the most adjustments are likely to occur, according to our analysis.

31,885 TOTAL CONFIRMED DEATHS
6,405 TOTAL PROBABLE DEATHS

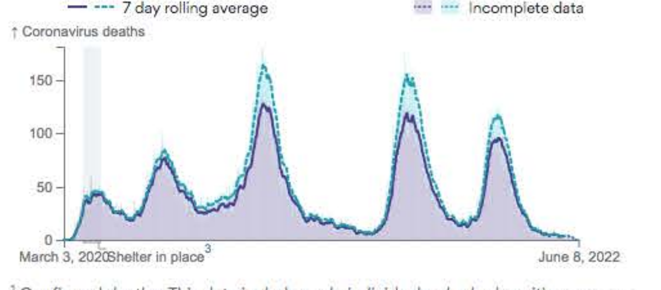

[1] Confirmed deaths: This data includes only individuals who had positive reverse-transcription polymerase chain reaction (PCR) test results.

[2] Probable deaths: This data includes individuals who tested antigen positive or who had compatible illness and known close contact to a case and were either reported to DPH as deceased; identified by death certificates with COVID-19 indicated as the cause of death; there is evidence that COVID contributed to the individual's death; or individuals with a death certificate that has COVID-19 indicated as the cause of death and there is no laboratory evidence for SARS-CoV-2.

[3] Shelter in place: For most Georgians over age 65, shelter in place continued through June 12, 2020. Georgians who are residents of long term care facilities or are medically fragile must continue to shelter in place through at least March 31.

Source: Georgia Department of Public Health

Updated June 12 at 4:20 p.m.

Explore » Follow the full AJC coverage of the coronavirus outbreak

Q: What is the latest on people hospitalized for COVID-19 in Georgia?

Current hospitalizations by day in Georgia

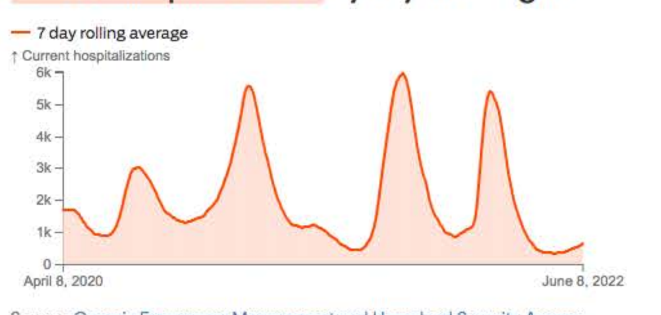

Source: Georgia Emergency Management and Homeland Security Agency

Updated June 12 at 3:30 p.m.

A: This is the number of people confirmed to have COVID-19 who are currently hospitalized, as published by the Georgia Department of Emergency Management & Homeland Security Agency. It began reporting this measure daily on May 2. It does not include those hospitalized who remain under investigation for suspected COVID. This number is updated daily.

Q: How many Georgians are testing positive for Coronavirus?

Percent testing positive for Coronavirus in Georgia

Each bar in this chart shows the **percent testing positive** for coronavirus each day as reported to the Georgia Department of Public Health (DPH). The line running through the bars shows the seven-day rolling average of the daily rates. The data does not include serologic tests, which identify if someone was exposed to the virus in the past and developed antibodies. The 5% line highlighted on the graph is the upper threshold identified by the White House Coronavirus Task Force for states to be in the green, or safe, zone for reopening.

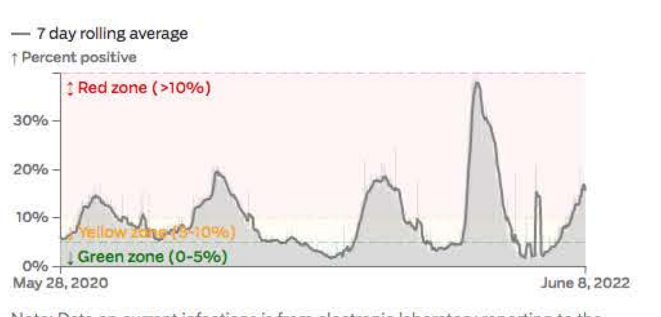

Note: Data on current infections is from electronic laboratory reporting to the state, which does not include all tests performed in Georgia. DPH excludes other testing sources because they do not consistently provide negative results, which must be factored in to arrive at positivity percentage. DPH also notes that, "People with a positive test often get retested and may test positive multiple times. These repeat positive tests will be counted as new positive tests."

Data: Georgia Department of Public Health

Zones: White House Coronavirus Task Force

Updated June 12 at 4:20 p.m.

Q: Does this week's case data show how the virus is spreading right now in Georgia?

A: No. Epidemiologists agree that this week's counts are a snapshot of what the virus did roughly two weeks ago. It can take a week or more for a person to become infected, show symptoms, get tested and have their results reported to public health officials. Deaths are generally reported to the state quickly, but it can take weeks for an infected person to succumb to the virus.

Q: What does the 7-day average line indicate?

A: This measure makes it easier to see trends in the spread of the virus that can be obscured by unrelated bumps and dips. For instance, Georgia's daily case counts — when they were reported — tended to be lower on the weekends because reporting and testing slowed down, not because fewer people are getting sick.

Q: Where is coronavirus spreading now?

Recently reported confirmed* coronavirus cases in Georgia

Rate of recently reported confirmed* coronavirus cases in each county by population (confirmed* cases reported in the last 14 days by the Georgia Department of Public Health based on each patient's reported county of residence, divided by the county's population and then multiplied by 100,000).

Tap each county for details on recently reported confirmed* and probable** cases there.

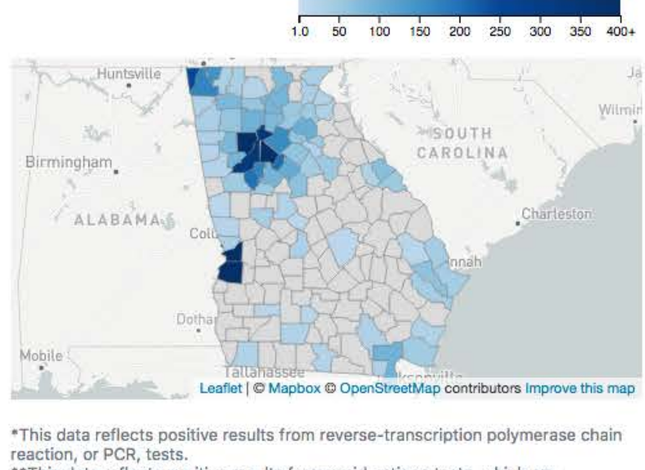

*This data reflects positive results from reverse-transcription polymerase chain reaction, or PCR, tests.
**This data reflects positive results from rapid antigen tests, which are considered probable cases. DPH tracks cases by person, not individual tests. That means DPH moves antigen positive cases to the confirmed category if a person also receives a PCR positive. That change in data is updated daily.
Source: Georgia Department of Public Health and the U.S. Census Bureau: *2019 American Community Survey 5-year estimates*

Updated April 12

Explore See how cases are trending in your county

Q: Are there are other measures that can indicate how the virus is spreading?

A: Yes: the effective reproduction number, or Rt.

Rt is an epidemiological statistic that represents transmissibility, or the number of people a sick person infects at a given stage in the epidemic. If each person gets one person sick, the epidemic neither grows nor shrinks. Less than one means each person infects, on average, fewer than one person, so the epidemic shrinks. If Rt is larger than one, the epidemic grows. As Rt moves farther from 1, the epidemic shrinks or grows more rapidly.

results from all were very similar, we're displaying a method designed by the Imperial College of London that uses epidemic curves to produce Rt estimates. The college's method is unrelated to its COVID-19 estimates.

As with other data presented on this dashboard, Rt values lag because of testing lags.

Transmission estimates in Georgia

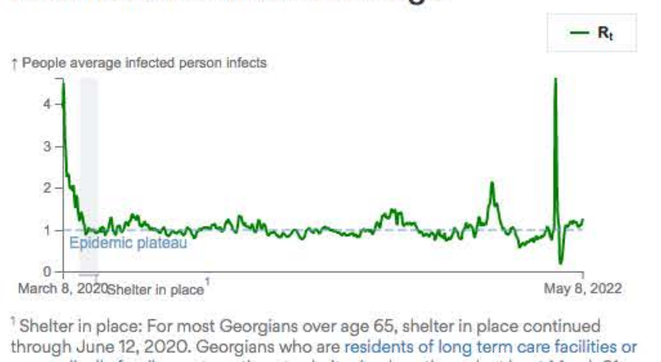

[1] Shelter in place: For most Georgians over age 65, shelter in place continued through June 12, 2020. Georgians who are residents of long term care facilities or are medically fragile must continue to shelter in place through at least March 31.

Source: Georgia Department of Public Health

Updated May 16 at 10:57 a.m.

Hawaii COVID-19 Data

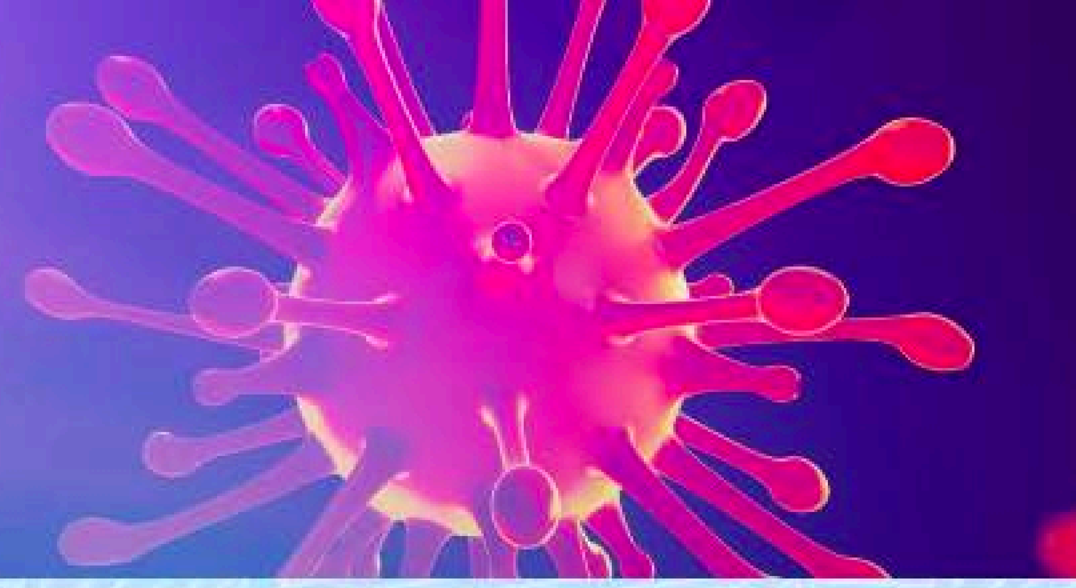

Hawai'i COVID-19 Summary Metrics by State and County

Hawaii COVID-19 Summary Metrics

LAST UPDATED ON Wednesday, June 8, 2022 (Updated Weekly)
DATA AS OF Monday, June 6, 2022

Click info icon for notes on this data

NAVIGATE TO OTHER VIEWS

SUMMARY | MAP | EPI CURVE | COUNTY RATES | TESTING

STATEWIDE

Change from Previous Week	Daily Average Cases*	Average Test Positivity*
8,033	1,085	19.2%

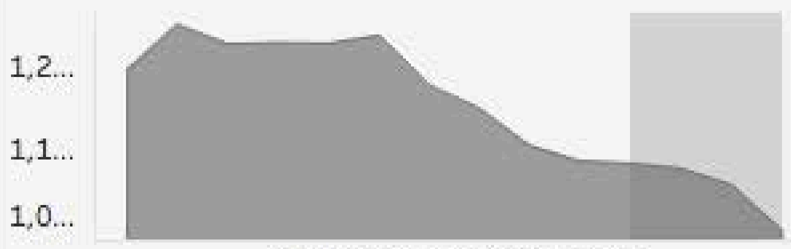

COMPLETED VACCINATION

77.7% Statewide

Hawaii	Honolulu
69%	81%
**Kauai**	**Maui**
72%	71%

*Metrics represent 7-day moving averages;
Includes both confirmed and probable cases

HAWAII COUNTY

841	114	13.1%
Change from Previous Week	7-day Avg Cases	Test Positivity

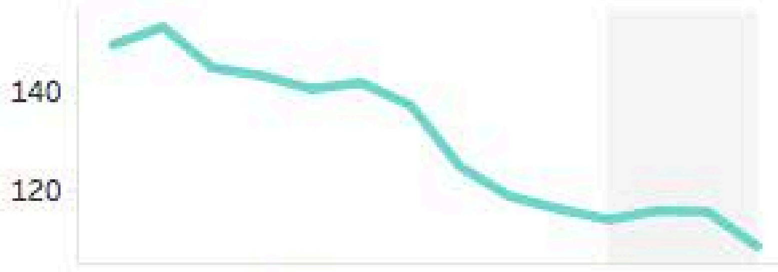

HONOLULU COUNTY

5,794	804	21.9%
Change from Previous Week	7-day Avg Cases	Test Positivity

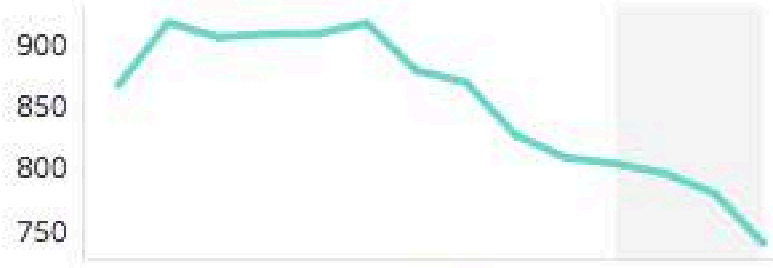

KAUAI COUNTY

435	65	26.5%
Change from Previous Week	7-day Avg Cases	Test Positivity

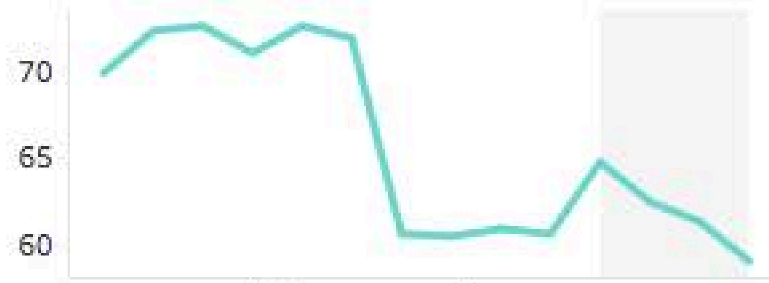

MAUI COUNTY

873	103	17.2%
Change from Previous Week	7-day Avg Cases	Test Positivity

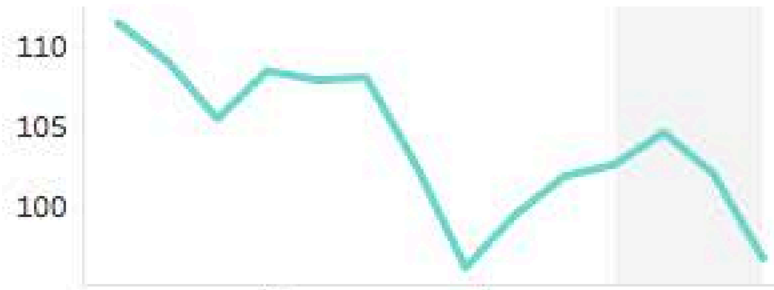

+tableau

DPH Idaho COVID-19 Dashboard by Idaho Division of Public Health

Statewide Cases*

455,427 Total Statewide Cases

Cumulative Trend

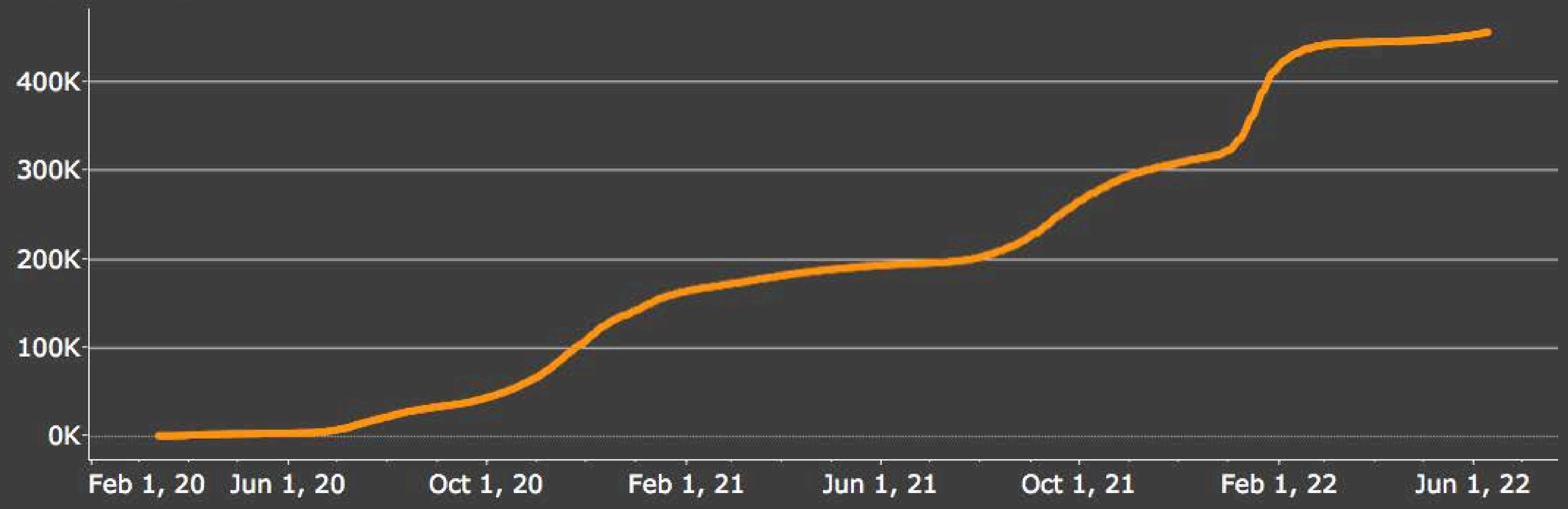

Daily Cases

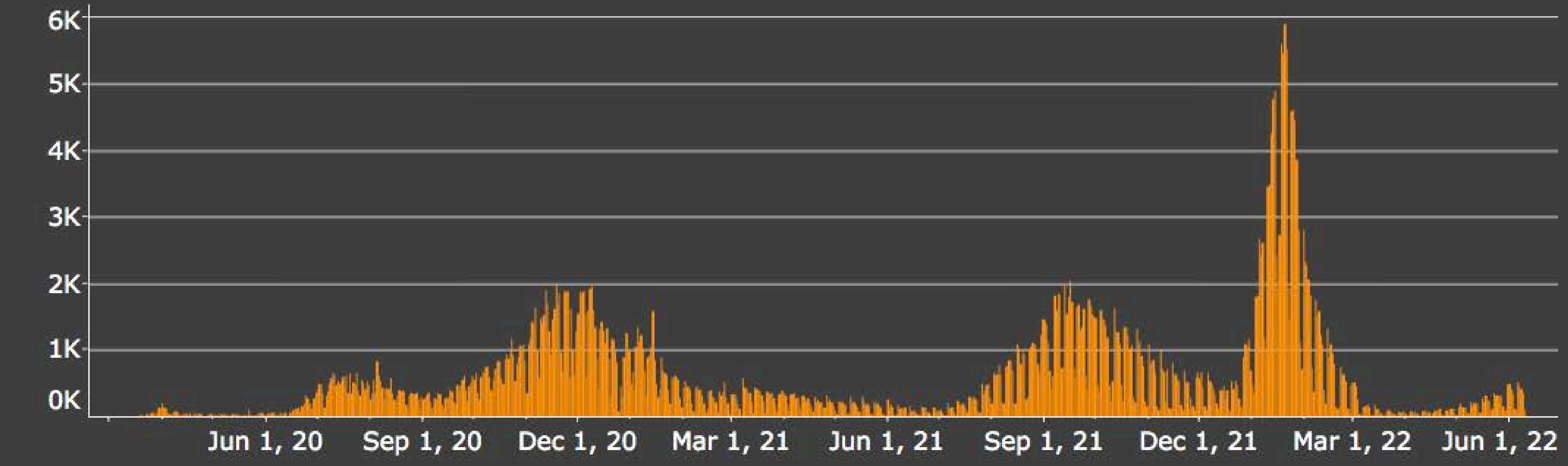

*For notes and details on the data release schedule, please visit the Notes page at the top right.

IDAHO DEPARTMENT OF HEALTH & WELFARE

Cases by County*

Select a county from the dropdown to view a trend of the daily 7-day moving average incidence rate per 100,000 population

Statewide

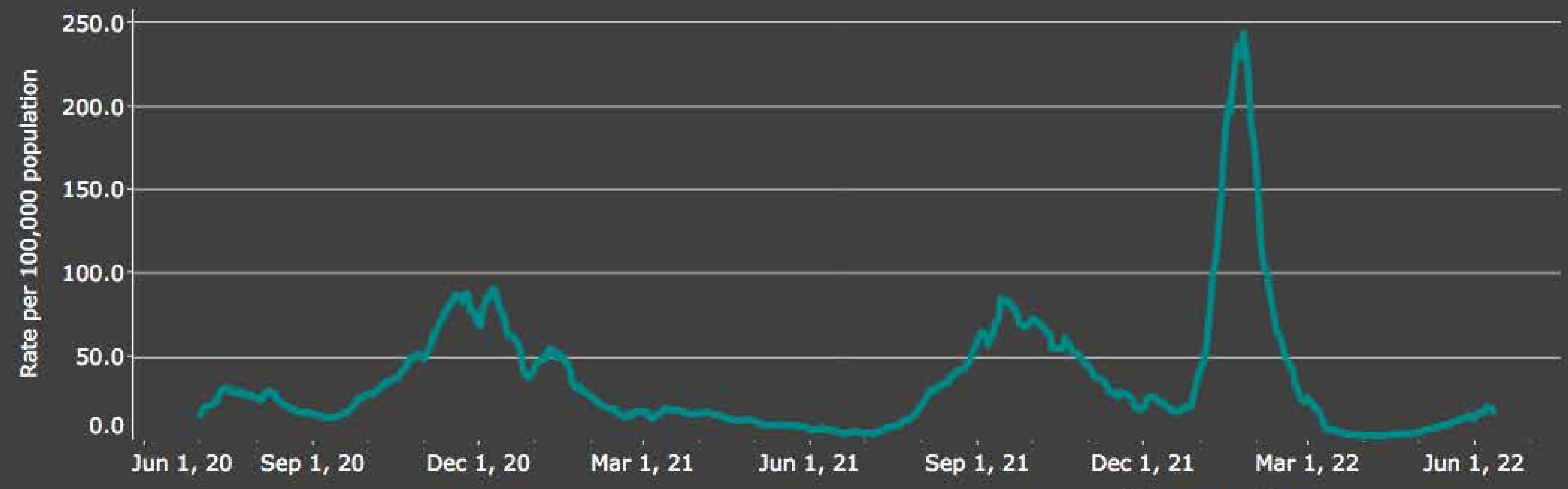

County	Confirmed	Probable	Total Cases	Cumulative Incidence Rate	Daily 7-Day Moving Average Incidence Rate	Deaths
Ada	116,441	22,184	138,625	28,039.1	25.4	1,025
Adams	578	138	716	16,100.7	10.0	15
Bannock	13,123	8,044	21,167	23,838.1	17.1	249
Bear Lake	674	267	941	15,318.2	7.0	16
Benewah	1,642	779	2,421	25,673.4	1.5	41
Bingham	5,783	4,183	9,966	21,113.5	18.3	156
Blaine	5,468	748	6,216	26,534.6	23.0	30
Boise	762	358	1,120	13,887.2	7.3	14
Bonner	7,518	1,567	9,085	19,405.3	14.1	189
Bonneville	20,719	10,570	31,289	25,618.6	13.6	286
Boundary	1,614	532	2,146	16,956.4	10.5	63
Butte	325	154	479	18,102.8	5.5	9
Camas	121	37	158	13,982.3	0.0	2
Canyon	52,842	9,562	62,404	26,324.9	13.2	687
Caribou	492	1,009	1,501	21,072.6	10.0	26
Cassia	3,769	1,285	5,054	20,818.1	1.2	53
Clark	76	34	110	12,910.8	16.9	1
Clearwater	1,340	1,012	2,352	26,588.3	22.8	33
Custer	362	184	546	12,850.1	16.6	9
Elmore	6,286	1,200	7,486	27,273.4	36.9	70
Franklin	1,343	1,024	2,367	16,651.4	10.3	29
Fremont	1,691	486	2,177	16,470.0	6.5	28
Gem	2,697	1,018	3,715	19,863.1	3.9	79
Gooding	2,085	1,250	3,335	21,353.6	10.4	56
Idaho	2,194	842	3,036	18,046.7	12.9	52
Jefferson	3,906	2,180	6,086	19,901.2	10.0	65
Jerome	3,905	2,259	6,164	25,079.3	9.9	65
Kootenai	36,702	5,978	42,680	25,013.5	13.4	597
Latah	6,376	605	6,981	17,097.7	13.2	49
Lemhi	780	519	1,299	16,128.6	24.9	25
Lewis	762	391	1,153	30,041.7	14.9	29
Lincoln	718	396	1,114	20,791.3	8.0	15
Madison	10,981	1,824	12,805	31,760.0	15.8	51
Minidoka	2,820	1,449	4,269	20,121.6	8.1	65
Nez Perce	7,698	1,379	9,077	22,272.1	7.1	148
Oneida	545	270	815	18,031.0	12.6	11
Owyhee	1,957	337	2,294	18,907.1	8.5	53
Payette	4,326	1,322	5,648	22,800.9	13.7	87
Power	783	696	1,479	19,351.0	1.9	20
Shoshone	2,387	379	2,766	21,423.6	4.4	76
Teton	1,352	1,408	2,760	22,078.2	12.9	9
Twin Falls	16,438	8,040	24,478	27,686.6	17.1	295
Valley	2,212	503	2,715	23,024.1	18.8	16
Washington	1,581	851	2,432	23,474.9	5.6	57

Cases by Public Health District

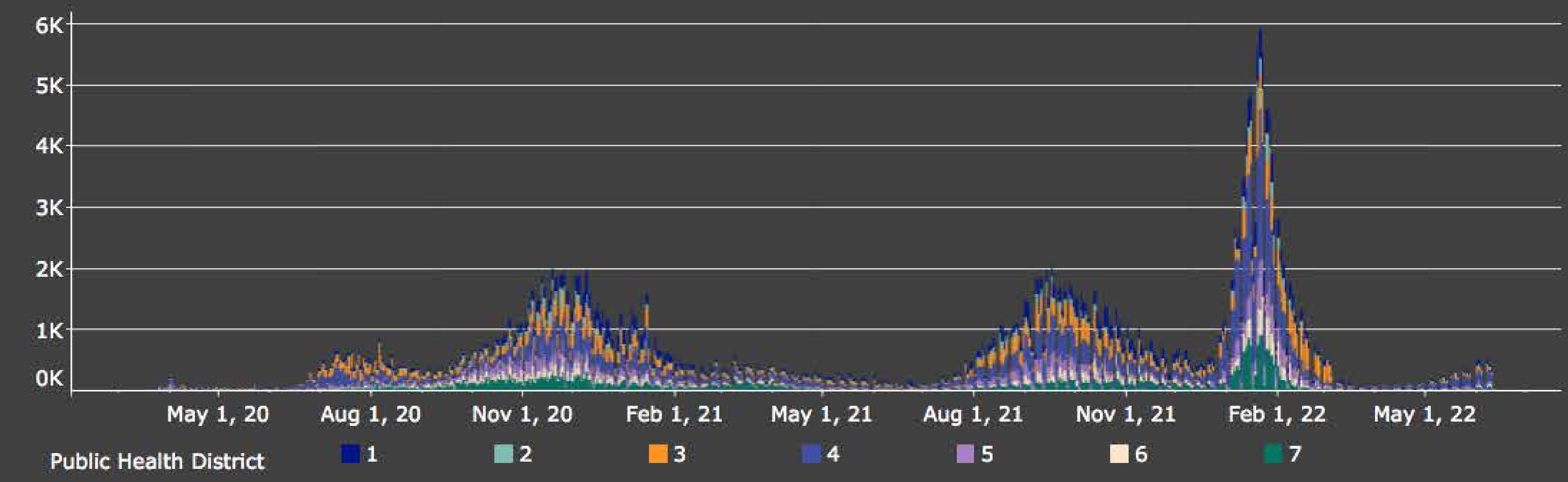

*For notes on county cases from the above visualizations, please visit the Notes page at the top right.

IDAHO DEPARTMENT OF HEALTH & WELFARE

+ a b l e a u

Details

37 9,226,068

DPH Idaho COVID-19 Dashboard

Published: Sep 3, 2020 **Updated:** Jun 10, 2022

DPH Idaho COVID-19 Dashboard by Idaho Division of Public Health

HOME | Statewide Cases | Case Characteristics | Cases by County | Weekly Hotspots & Local Trends | Emergency Department Visits | Laboratory Testing | COVID-19 Related Deaths | Deaths by Demographics | Hospitalizations | NOTES

Laboratory Testing for SARS-CoV-2*

PCR/NAAT Tests

2,962,654 Tests Performed

1,236,544 Idahoans Tested

PCR/NAAT Tests Performed and Percent Positivity by Week of Specimen Collection

Select Jurisdiction: State

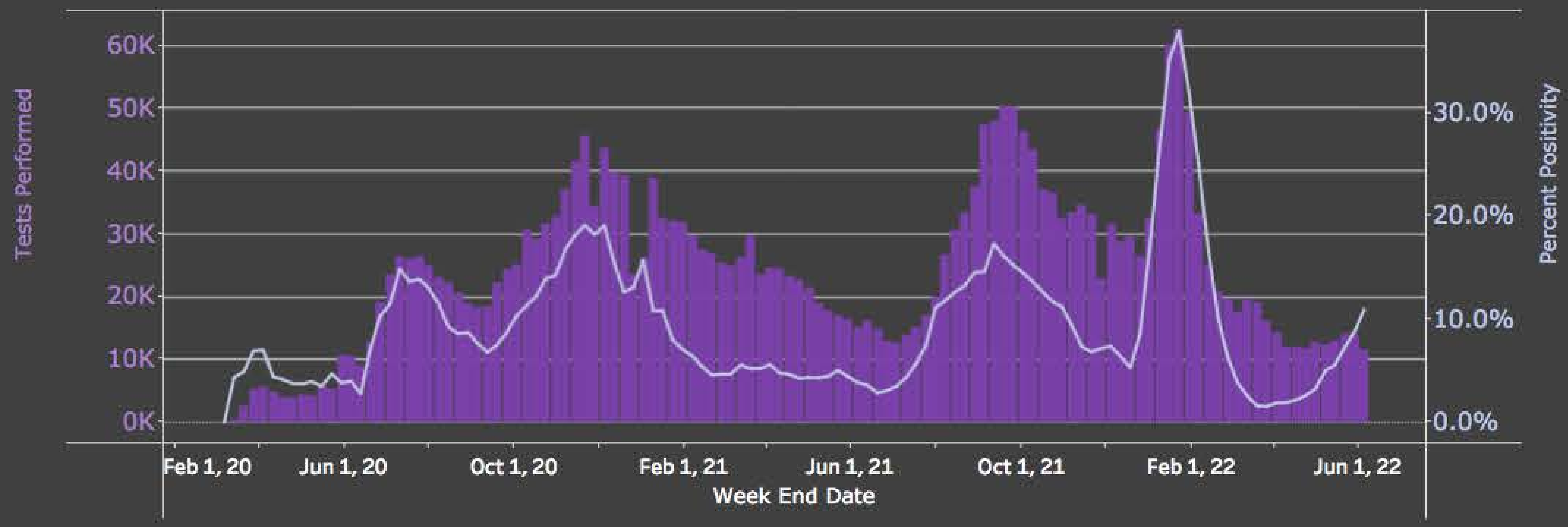

Variants of Concern and Interest

Total Number of Variants of Concern or Interest Identified by County

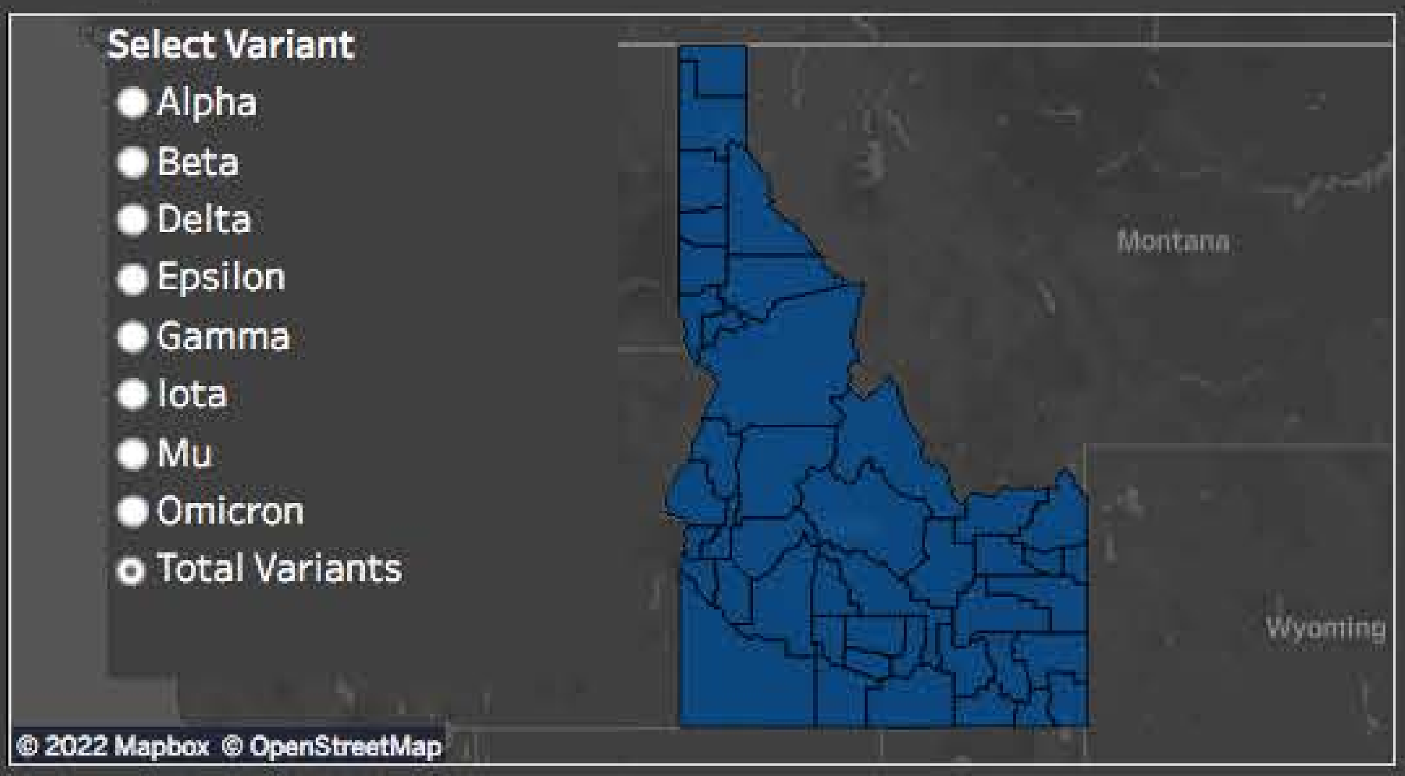

To view number of variants by county, click on the county in the map and refer to the chart below. To go back to statewide data click anywhere on the map outside Idaho.

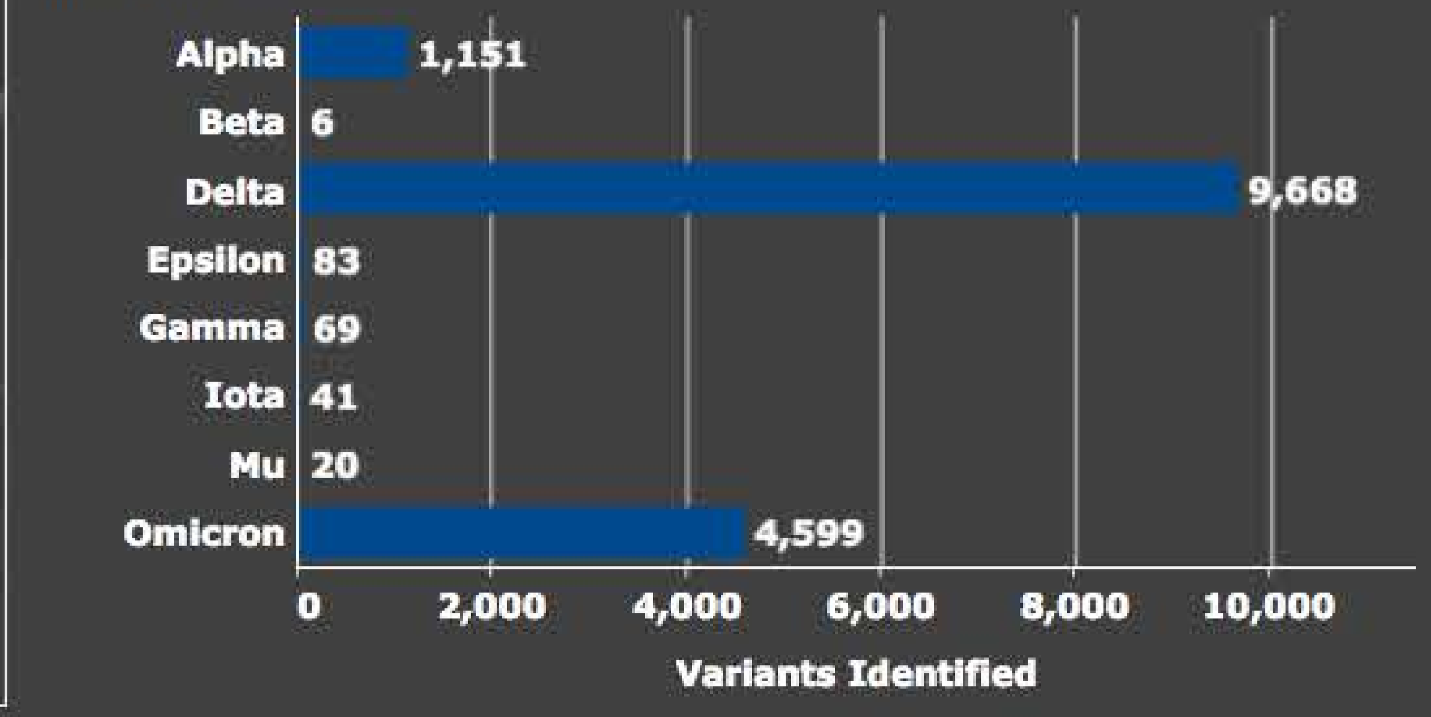

Proportion of Variants Identified by Specimen Collection Date

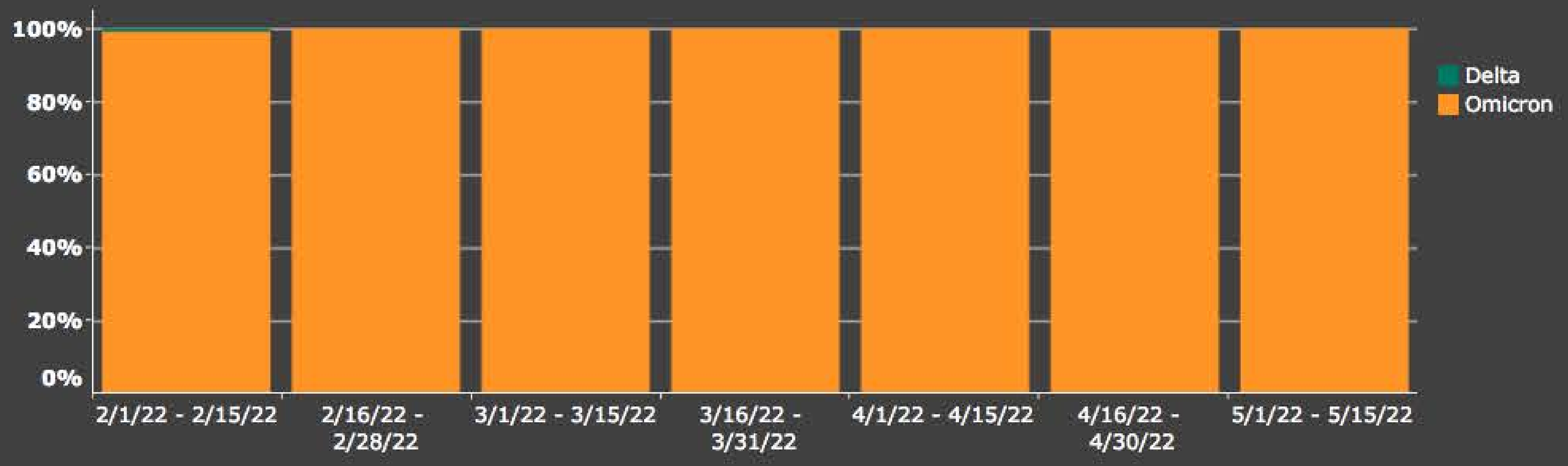

Antigen Tests

Number of Positive Antigen Tests Reported by Week of Specimen Collection

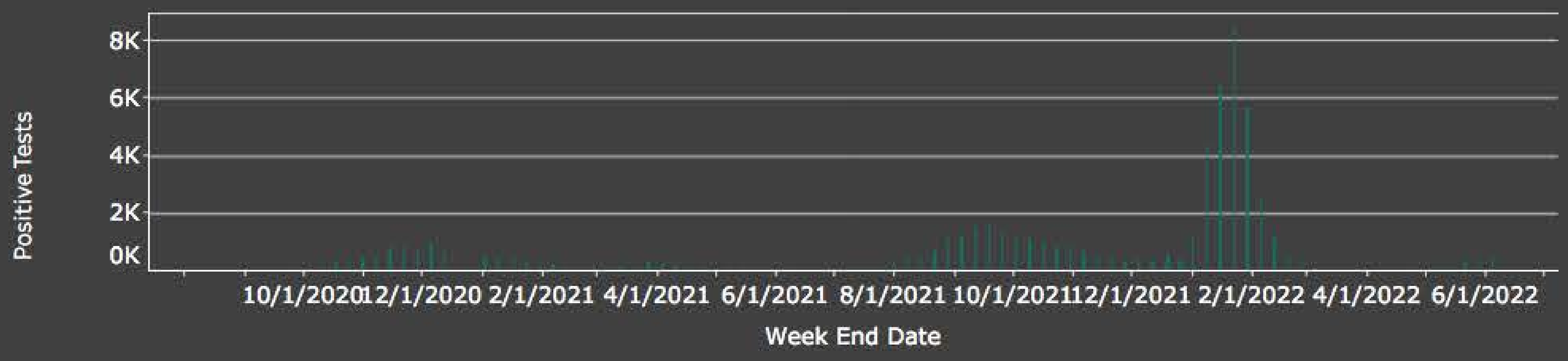

*For notes and details on variant data and testing calculations, please visit the Notes page at top right.

IDAHO DEPARTMENT OF HEALTH & WELFARE

+ a b l e a u

Details

37 9,226,068

DPH Idaho COVID-19 Dashboard

Published: Sep 3, 2020 **Updated:** Jun 10, 2022

COVID-19 Related Deaths Among Idaho Residents*

Idaho Resident Deaths: 4,951 *(4,032 Confirmed Cases, 919 Probable Cases)*
271.0 Deaths per 100,000 Population

COVID-19 Related Death Rates by Public Health District

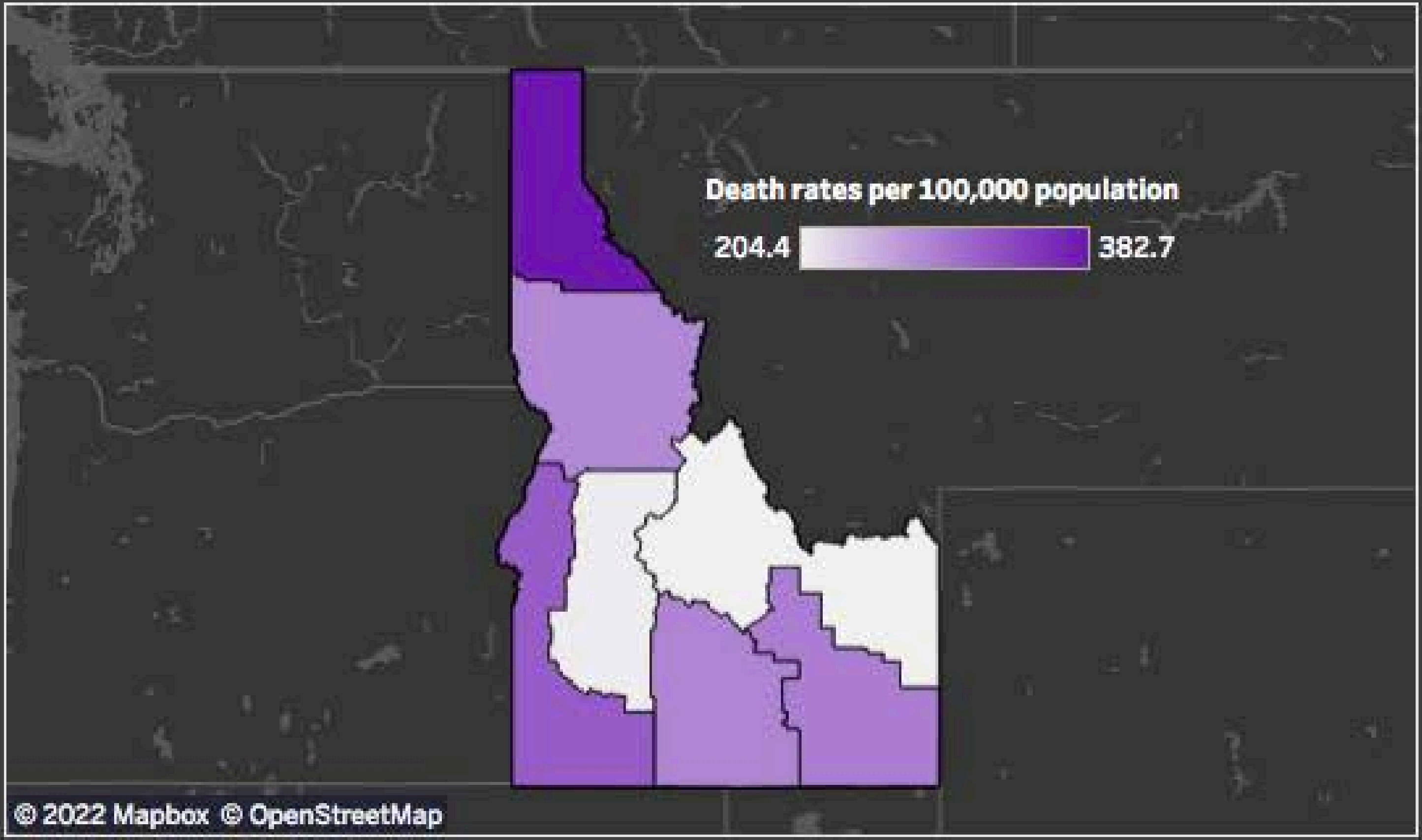

COVID-19 Related Death Rates by County

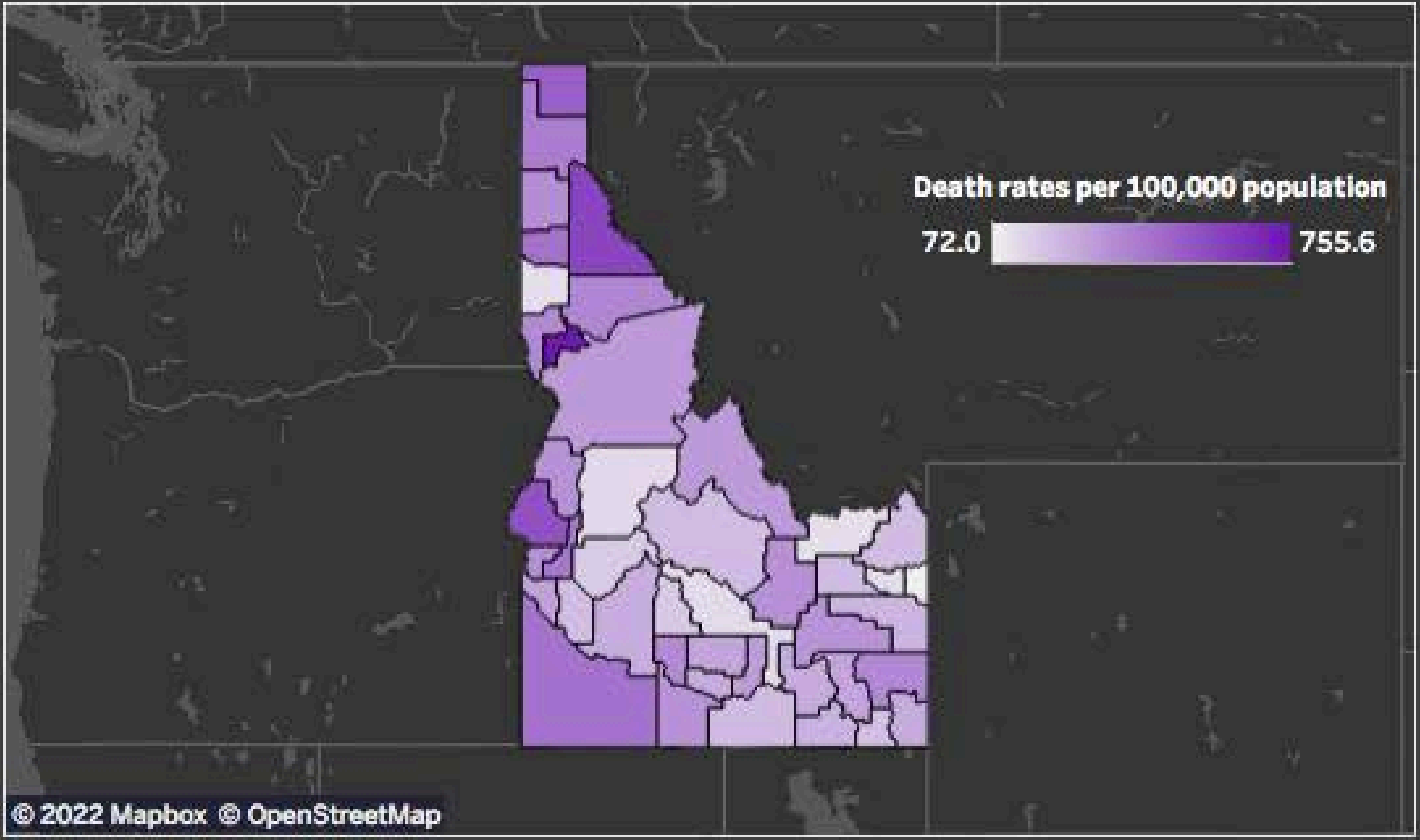

COVID-19-Related Deaths Among Idaho Residents by Date of Death

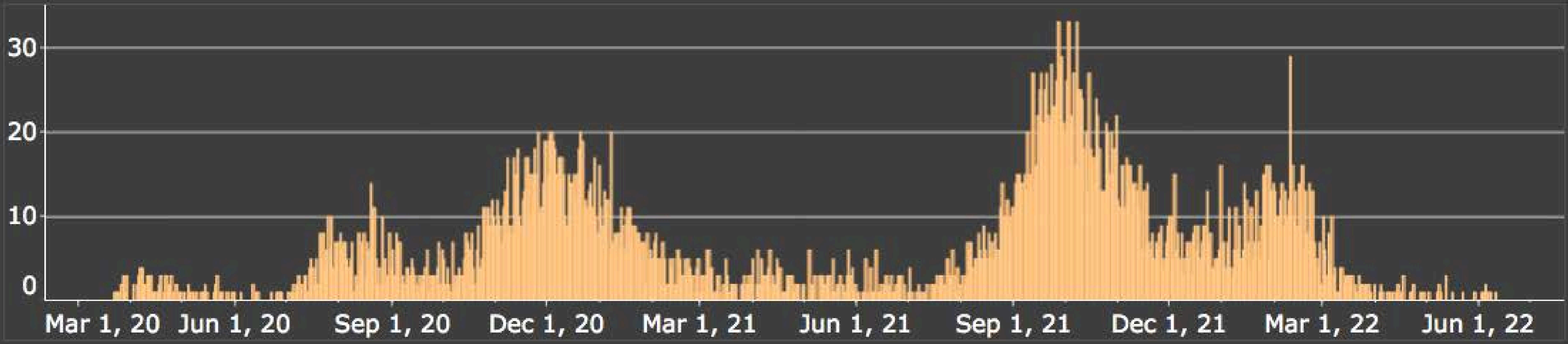

*For definitions and notes, please visit the Notes page at the top right.

IDAHO DEPARTMENT OF HEALTH & WELFARE

Illinois Data

- Surveillance
- Community Level
- Hospitalization Utilization
- Long-Term Care Data
- Long-Term Care Facility Data
- School and Youth Data
- Variants
- Data Portal
- Contact Tracing
- Travel

COVID-19 Statistics

Data Last Updated: 6/10/2022

Total Cases	Weekly Case Rate
3,352,983	267 per 100k
**Deaths**	**Probable Deaths**
33,926	4,385

On September 1, 2021, IDPH adopted the updated 2021 COVID-19 national case definition. As part of this case definition, IDPH began including in its total case counts individuals who previously were reported as a confirmed or probable case, but have become infected again. Therefore, total case counts no longer represent unique individuals as those individuals meeting the new criteria** may be counted more than once. These changes will apply only to cases with new positive test results on or after September 1, 2021.

**Criteria to Distinguish a New Case from an Existing Case – New confirmed or probable cases will be enumerated if SARS-CoV-2 sequencing results from a new positive specimen and a positive specimen from the most recent previous case demonstrate a different lineage OR if the person was most recently counted as a confirmed or probable case with onset date (if available) or first positive specimen collection date for that classification more than 90 days prior. The entire national case definition can be found at: https://ndc.services.cdc.gov/case-definitions/coronavirus-disease-2019-2021/

*Total molecular and antigen tests performed and reported electronically for testing of COVID-19 at IDPH, commercial or hospital laboratories. All numbers displayed are provisional and will change.

By Zip | By County

Select

Illinois

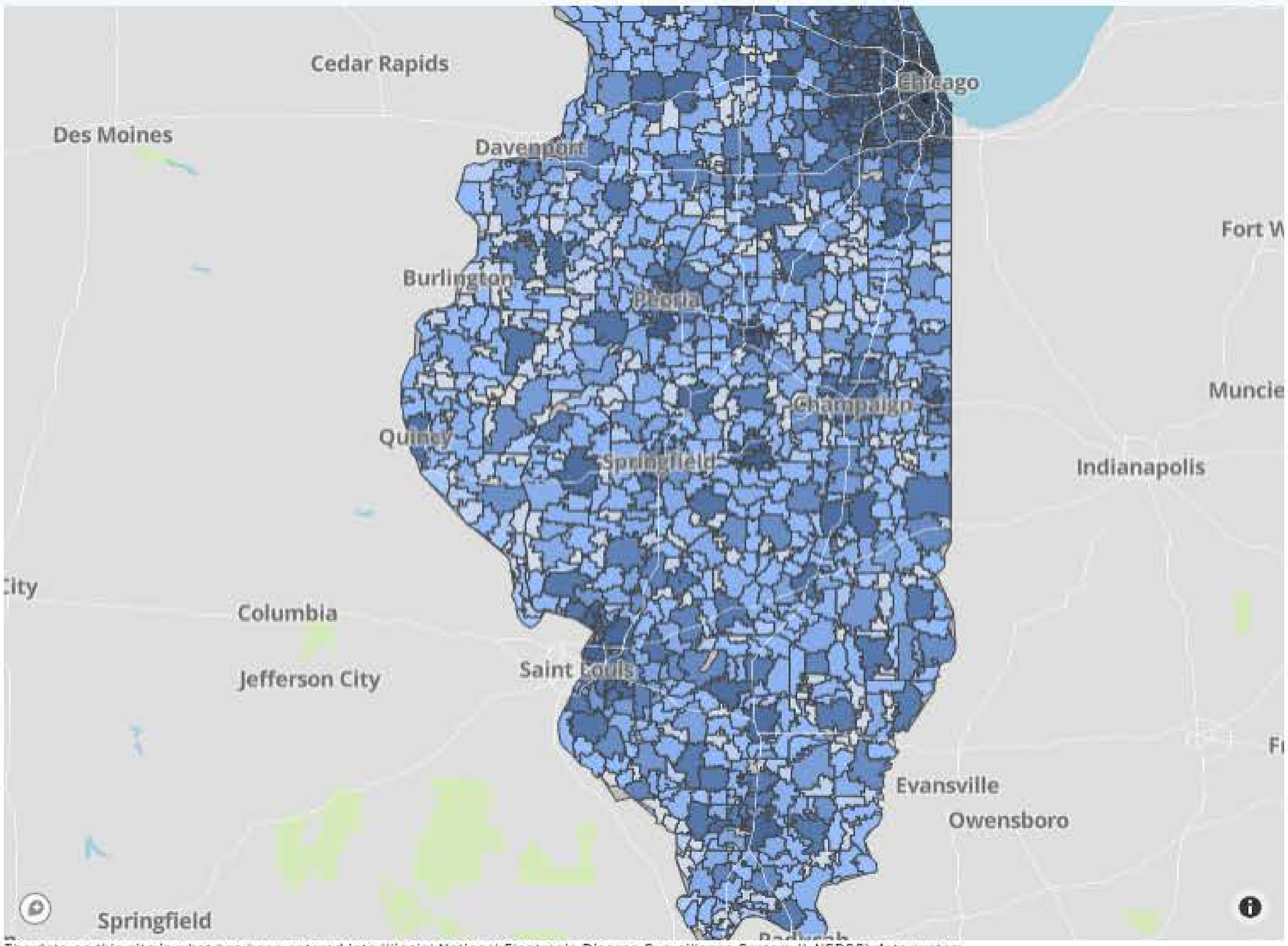

The data on this site is what has been entered into Illinois' National Electronic Disease Surveillance System (I-NEDSS) data system.
The data is constantly being entered and may change as cases are investigated. Therefore, numbers may vary from other sites where data is published.

*Data shown is >5 cases per zip code which is consistent with Collection, Disclosure, and Confidentiality of Health Statistics (77 Ill. Adm. Code 1005).
It should be assumed that COVID-19 exposure can occur in every county in IL.
Zip code is zip code of residence, which may not be location of exposure.

Indiana COVID-19 Home Dashboard

Below results are as of 06/09/22, 11:59 PM. Dashboard updates by 5 p.m. every Monday, Wednesday, and Friday.

Return to Landing Page

CASES | DEATHS | TESTS

Page Filter

Timeframe: Previous Month

7-Day Average COVID-19 Counts *(Previous Month Total Counts in Italics)*

Cases	Reinfections	Deaths	###########
1,343 (↑251)	16.6% of Cases	3 (↓1)	
34,514 Total Count	*5,423 Total Count*	*92 Total Count*	
		10 Probable Deaths	

Statewide Cases for Previous Month

By Specimen Collection Day

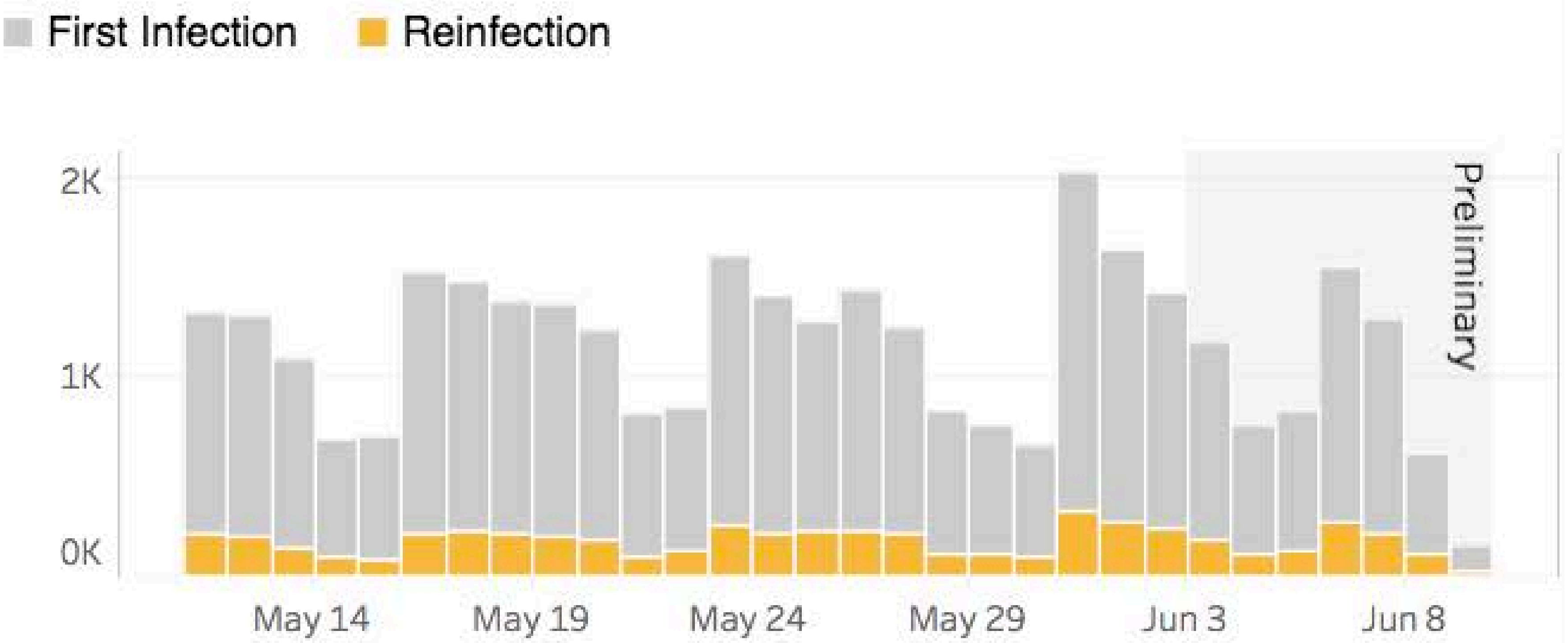

Statewide Case Demographics

Demographic: Age

Total

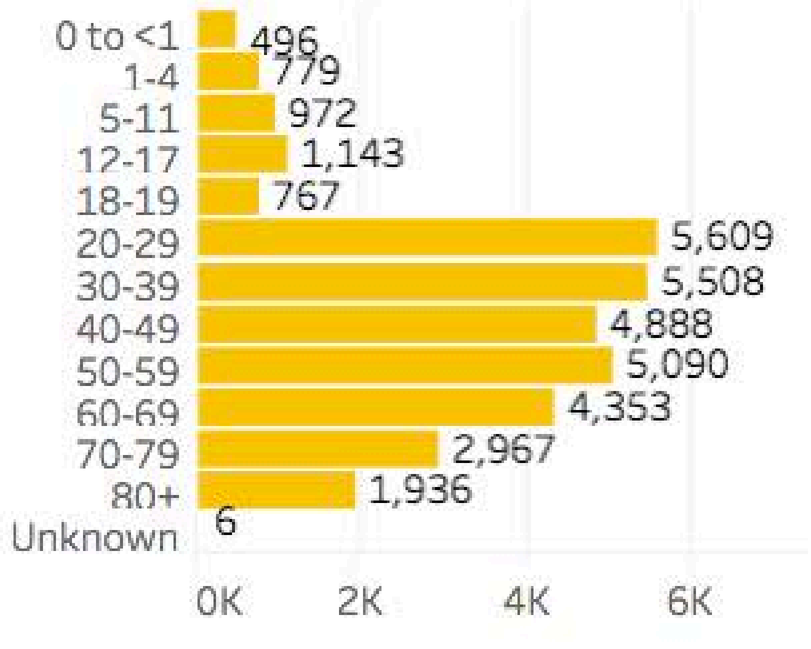

Per 10,000 Residents

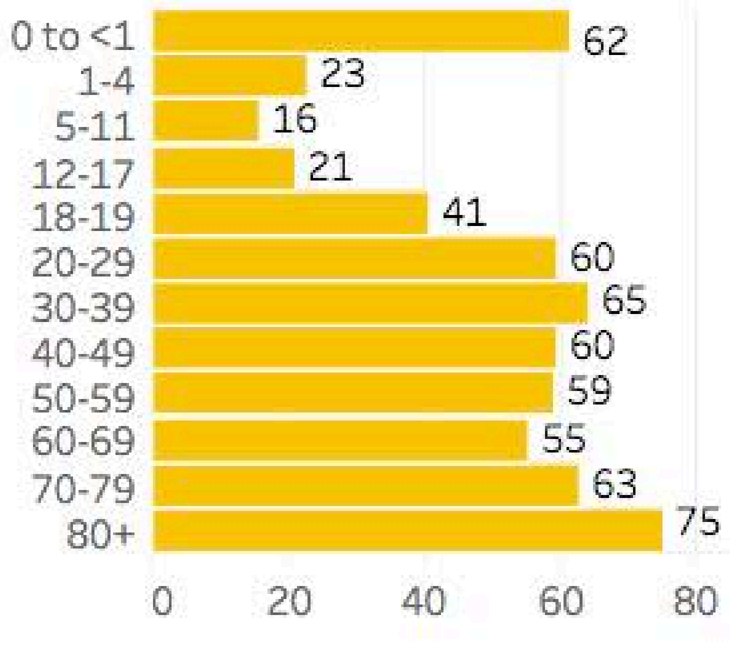

County Distribution

Select a county (or counties) to filter whole page.

Metric (Filters Map Only): Per 10k

Click here for CDC COVID reporting by county.

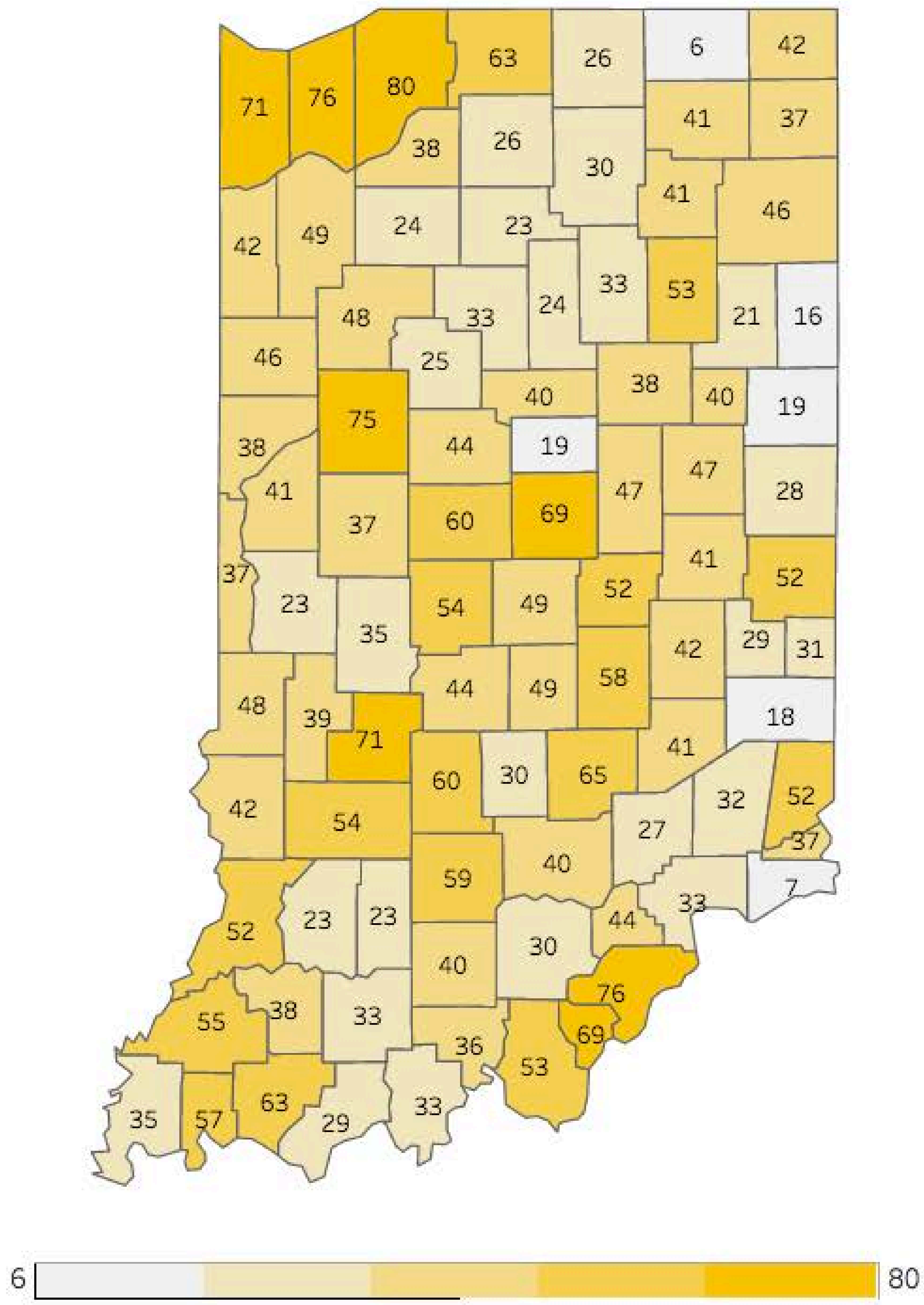

All numbers are provisional and reflect only those reported to IDOH. Numbers should not be characterized as a comprehensive total and may change as more data is reported.

Indiana COVID-19 Home Dashboard

Below results are as of 06/09/22, 11:59 PM. Dashboard updates by 5 p.m. every Monday, Wednesday, and Friday.

Page Filter

Timeframe: Previous Month

7-Day Average COVID-19 Counts *(Previous Month Total Counts in Italics)*

Cases	Reinfections	Deaths	###########
1,343 (↑251)	16.6% of Cases	3 (↓1)	
34,514 Total Count	*5,423 Total Count*	*92 Total Count*	
		10 Probable Deaths	

Statewide Deaths for Previous Month

By Day of Death

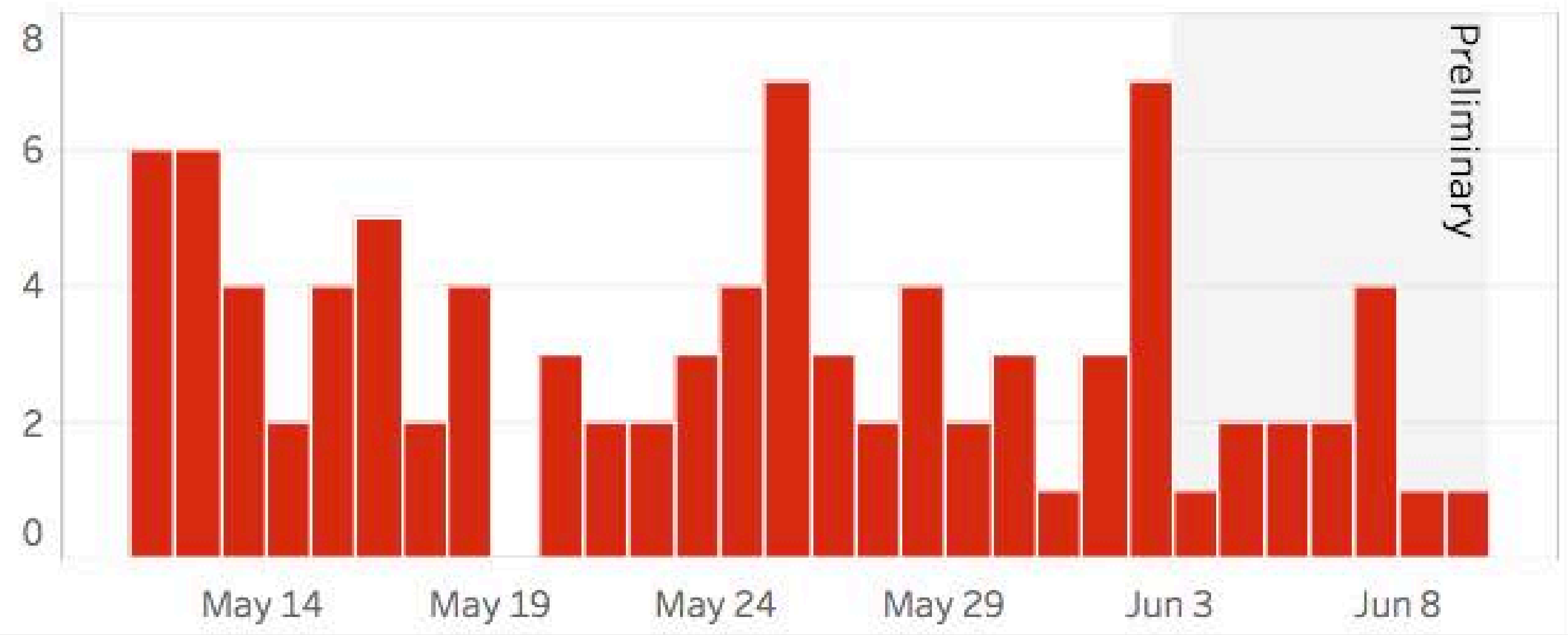

Statewide Death Demographics

Demographic: Age

Total

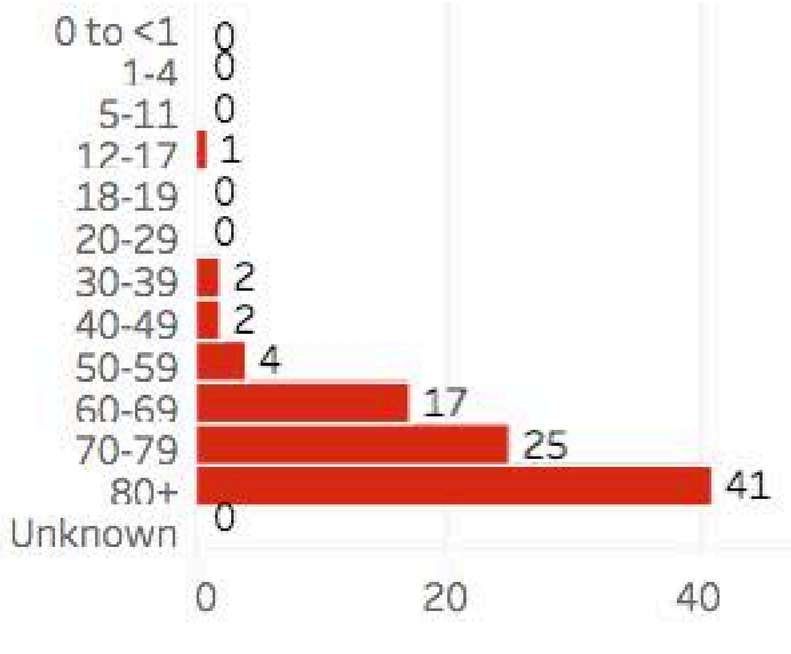

Per 10,000 Residents

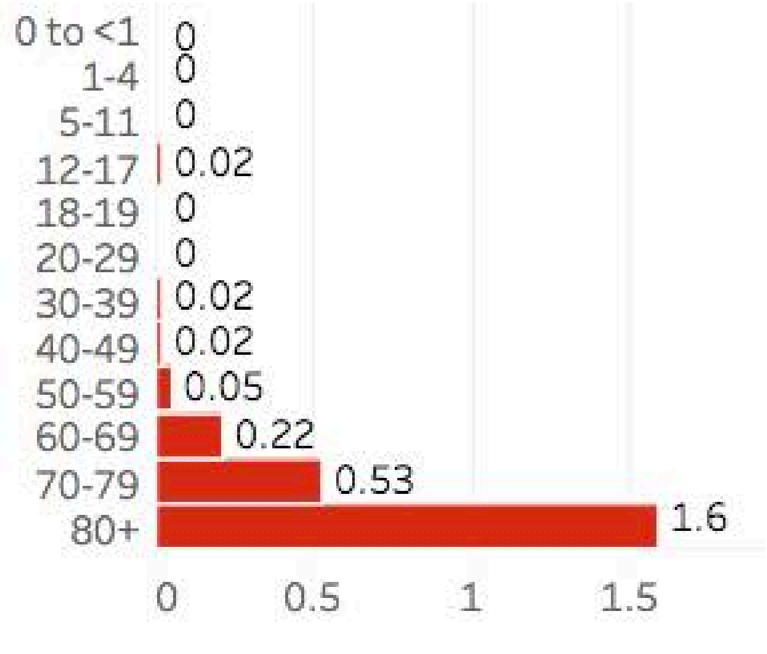

County Distribution

Select a county (or counties) to filter whole page.

Click here for CDC COVID reporting by county.

Metric (Filters Map Only): Per 10k

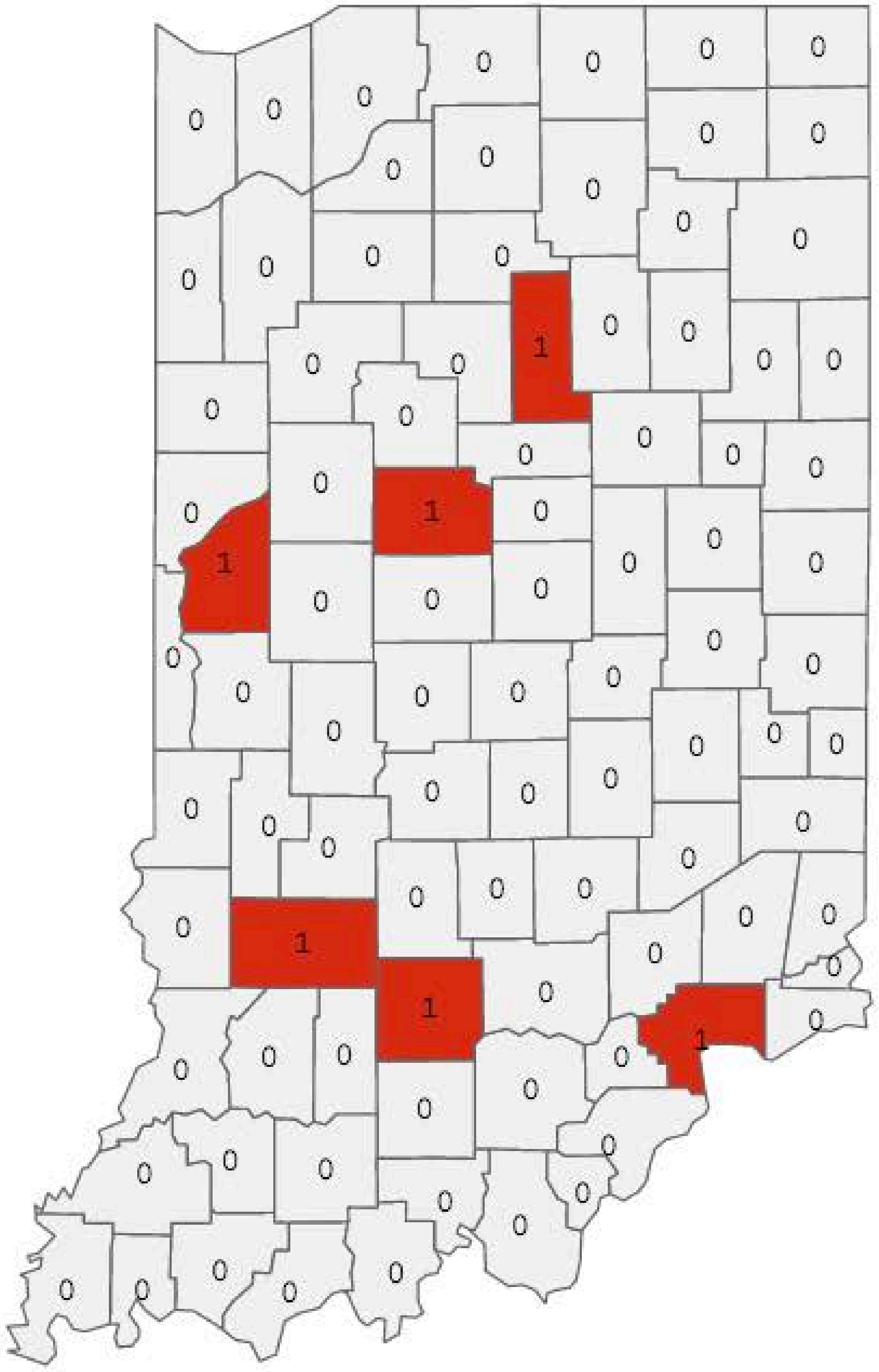

All numbers are provisional and reflect only those reported to IDOH. Numbers should not be characterized as a comprehensive total and may change as more data is reported.

Indiana COVID-19 Home Dashboard

Below results are as of 06/09/22, 11:59 PM. Dashboard updates by 5 p.m. every Monday, Wednesday, and Friday.

Return to Landing Page

CASES | DEATHS | TESTS

Page Filter

Timeframe: Previous Month

7-Day Average COVID-19 Counts *(Previous Month Total Counts in Italics)*

Cases	Reinfections	Deaths	###########
1,343 (↑251)	16.6% of Cases	3 (↓1)	
34,514 Total Count	*5,423 Total Count*	*92 Total Count*	
		10 Probable Deaths	

Statewide Tests Administered for Previous Month

By Specimen Collection Day

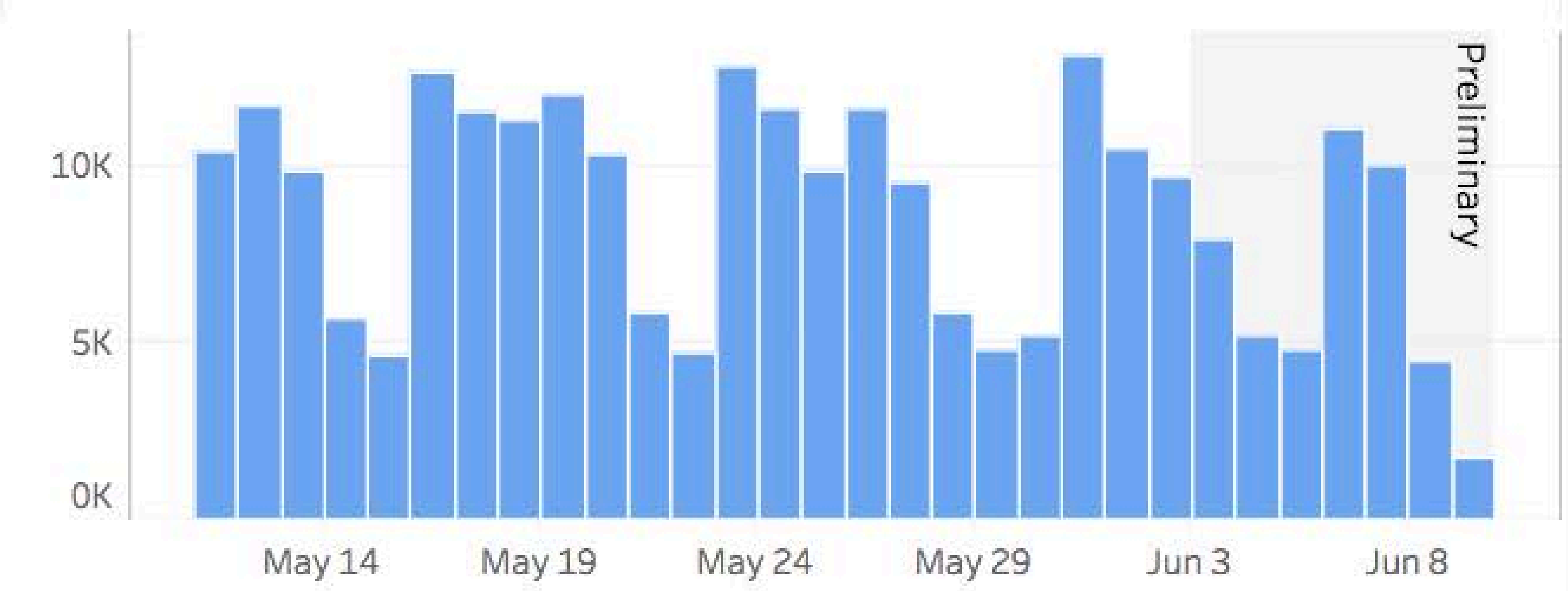

Statewide Tests Administered

Demographic: Age

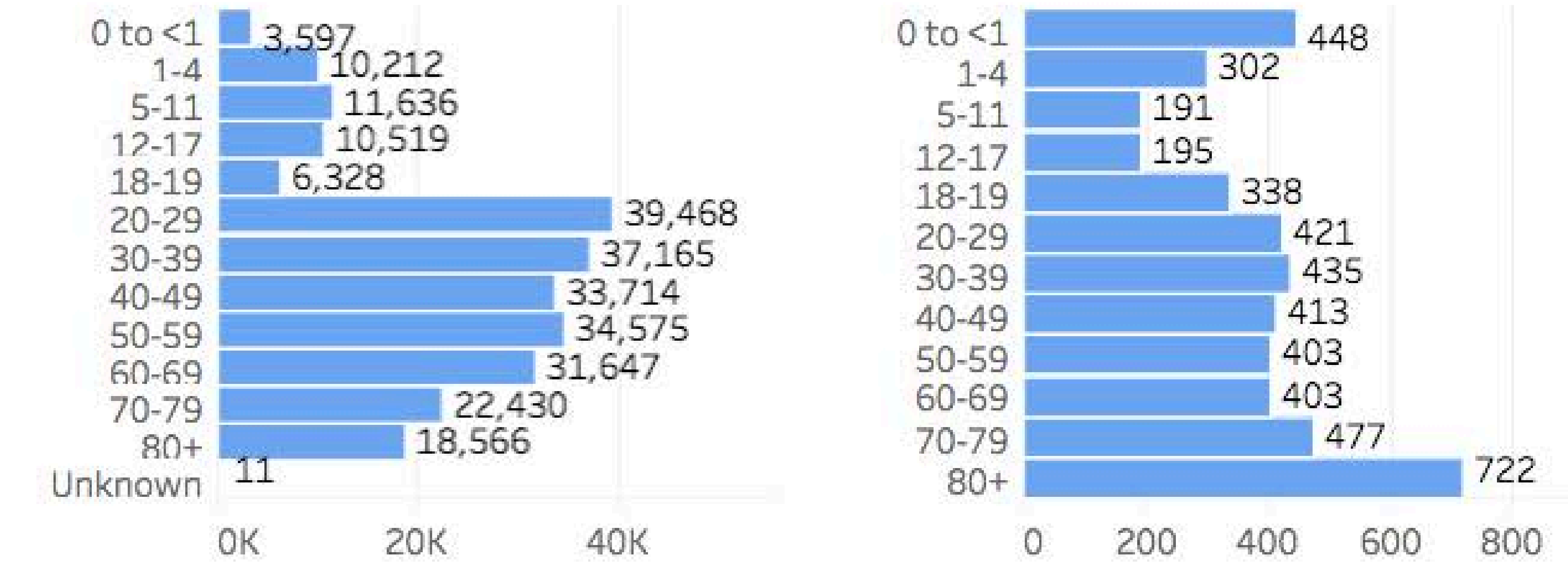

County Distribution

Select a county (or counties) to filter whole page.

Click here for CDC COVID reporting by county.

Metric (Filters Map Only): Per 10k

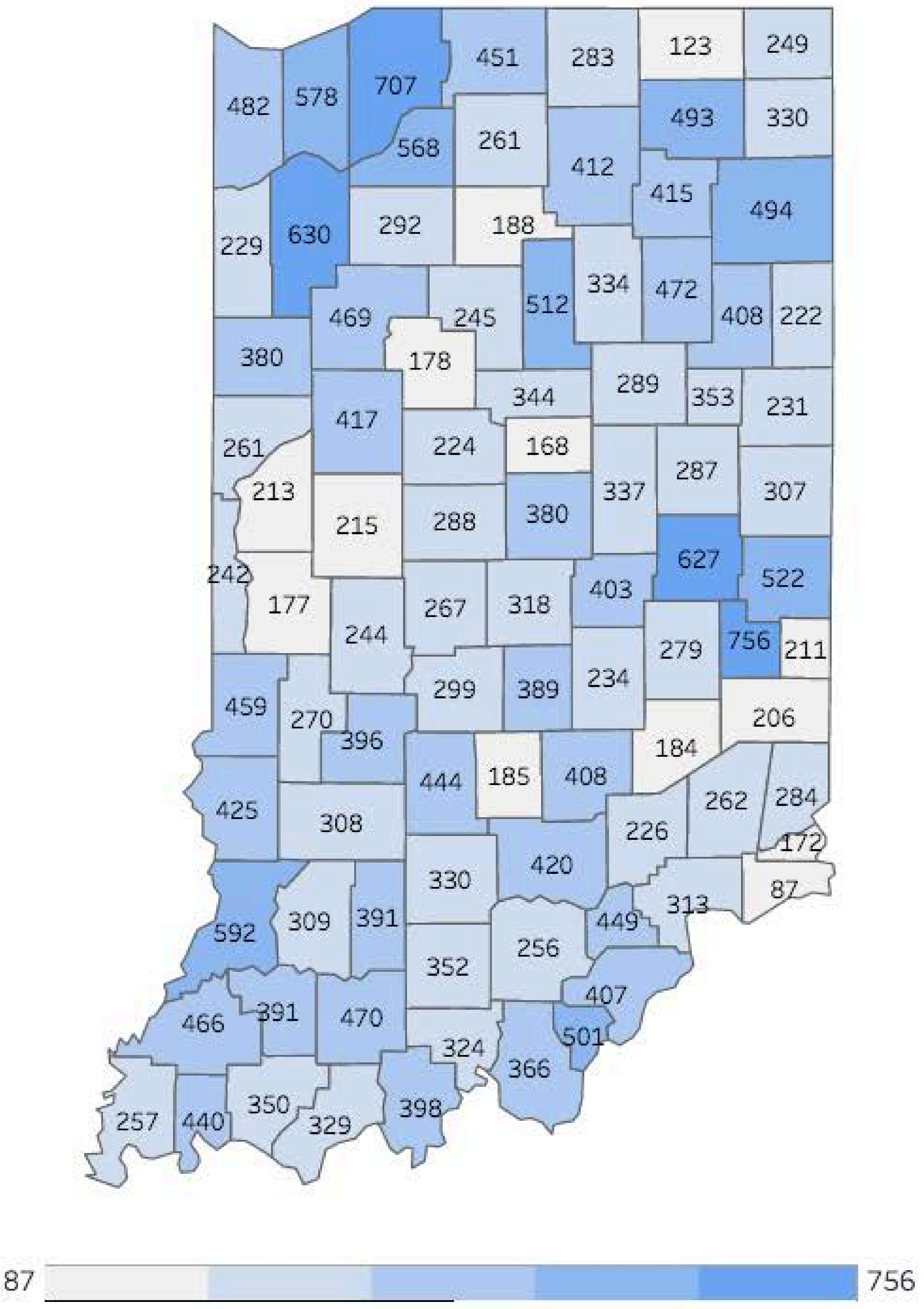

All numbers are provisional and reflect only those reported to IDOH. Numbers should not be characterized as a comprehensive total and may change as more data is reported.

KS COVID-19 Overview | Case Demographics | Case Rates | Sequencing Data | Other Resources | Hospital Capacity | ED Summary | Death Summary | SVI Explorer

Kansas COVID-19: Overview

COVID-19 Cases	7-Day Average Cases	COVID-19 Deaths	MIS-C* Cases
793,833	538 New Cases (as of June 6, 2022)	8,946	49

Data are preliminary and subject to quality improvement and quality assurance validation.
*MIS-C: Multisystem Inflammatory Syndrome in Children (MIS-C) associated with COVID-19. **UPDATE: Due to reconcilliation and review of 2021 data, a number of new MIS-C cases have been identified.**

Last updated: 6/8/2022 at 9:00am. There were 4,706 new cases and 3 new deaths reported since Wednesday, 6/1/2022.

UPDATE: Beginning May 18, the COVID-19 dashboard will be published once a week, Wednesday by 12:30pm.

Daily Number of New Cases and 7-Day Rolling Average of Daily New Cases by Symptom Onset Date All (Kansas)

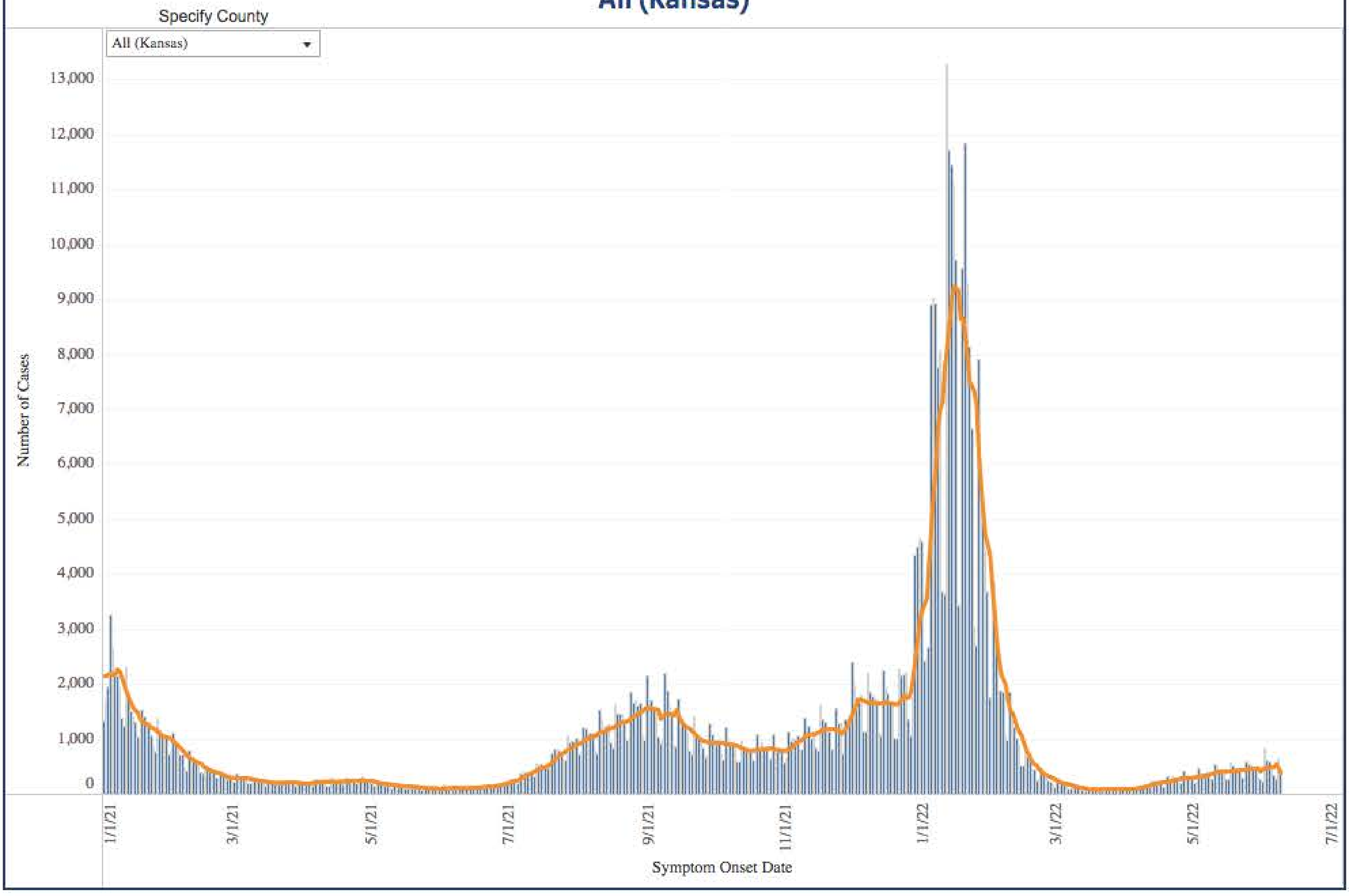

Weekly Cumulative Incidence Rate
Starting 5/28/2022 and ending 6/3/2022.

The most recent five days are not included as data is expected to be incomplete.

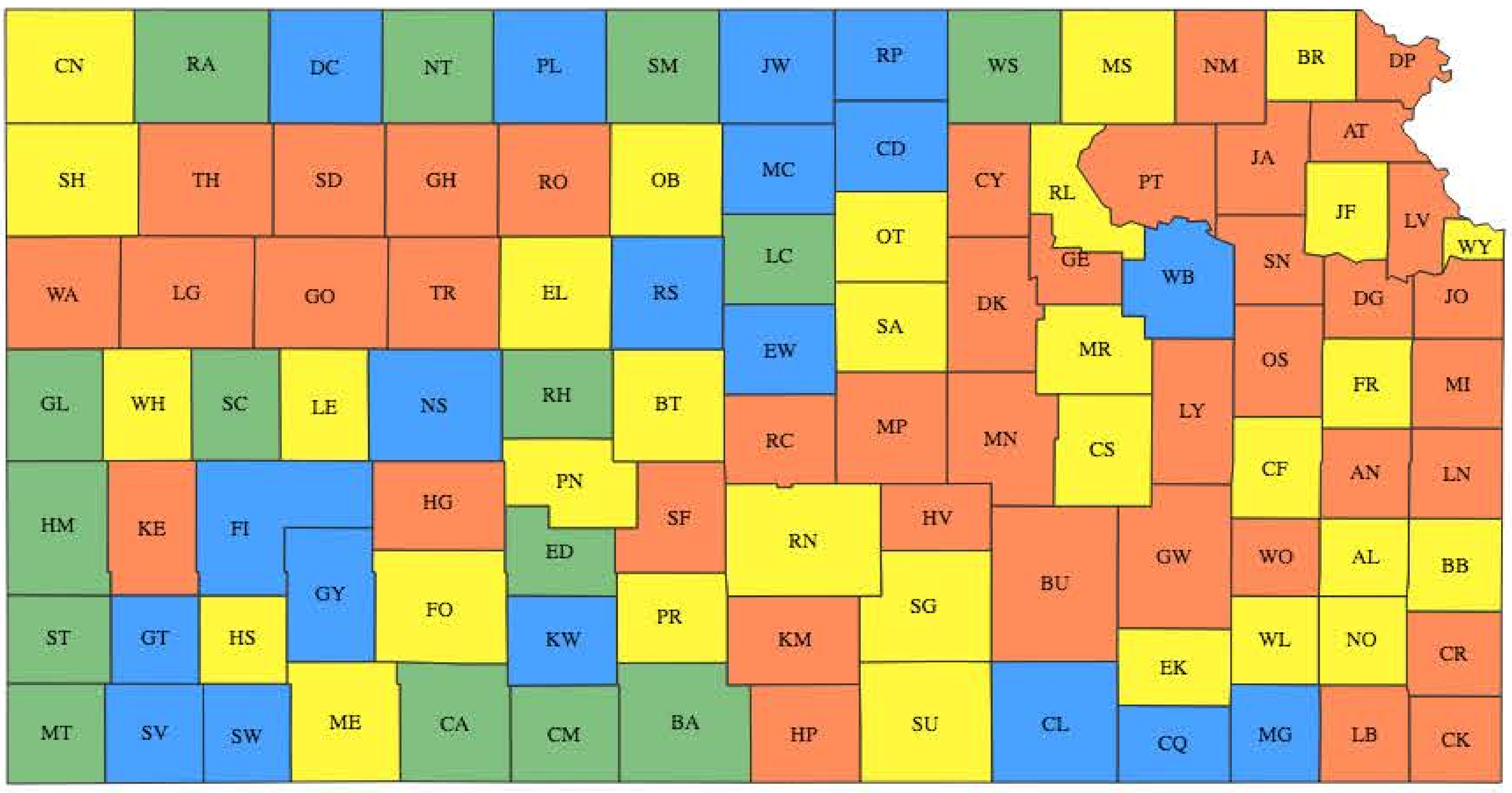

Low	Moderate	Substantial	High
<10 cases per 100,000	10-49 cases per 100,000	50-99 cases per 100,000	≥100 cases per 100,000

Data Note: The Weekly New Case Rate is also known as the Weekly Cumulative Incidence Rate. KDHE calculates the weekly cumulative incidence rate by summing the number of new COVID-19 cases with a symptom onset date in the week displayed divided by the population in the county and multiplied by 100,000.

The colors on this map will not necessarily correspond with the colors on the CDC Community Transmission or Community Level Maps. The CDC maps uses three different indicators for ranking counties as low, moderate, substantial, or high transmission counties. The Weekly New Case Rate is one of the three indicators the CDC Community Transmission Map uses.

Kansas Department of Health and Environment

KS COVID-19 Overview | Sequencing Data | Hospital Capacity | Death Summary
Case Demographics | Other Resources | ED Summary | SVI Explorer
Case Rates

Kansas COVID-19: Case Rates

Overall State Weekly Trends

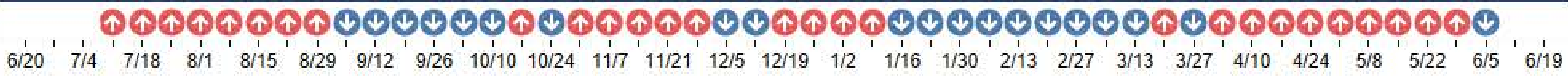

Weekly State Trends by Age Group

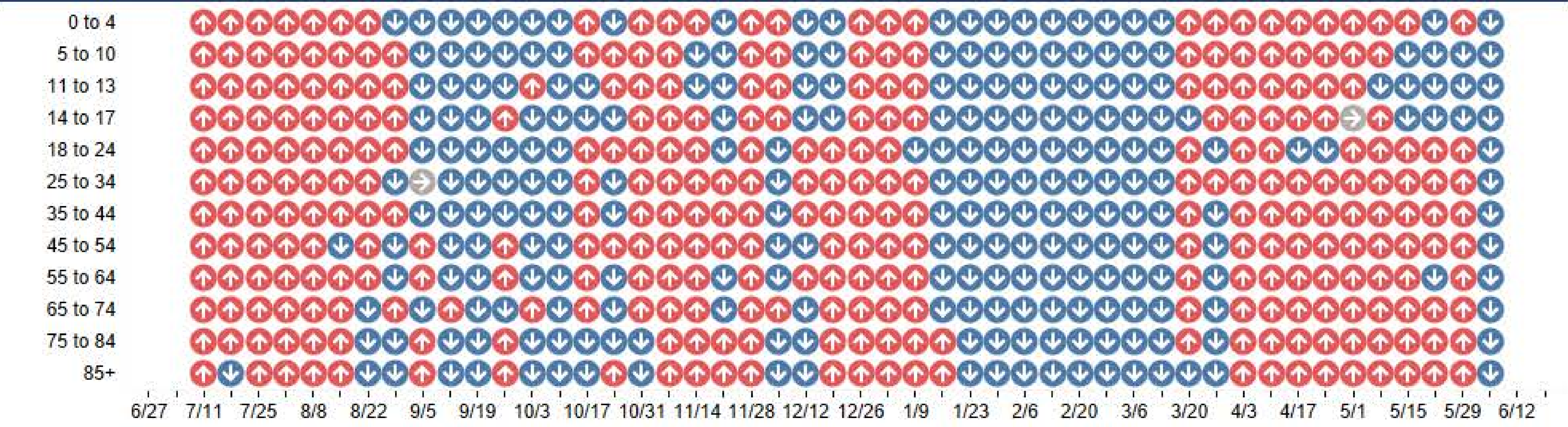

Select a County or Counties
(All)

Select Age Groups
(All)

Weekly Trends by County

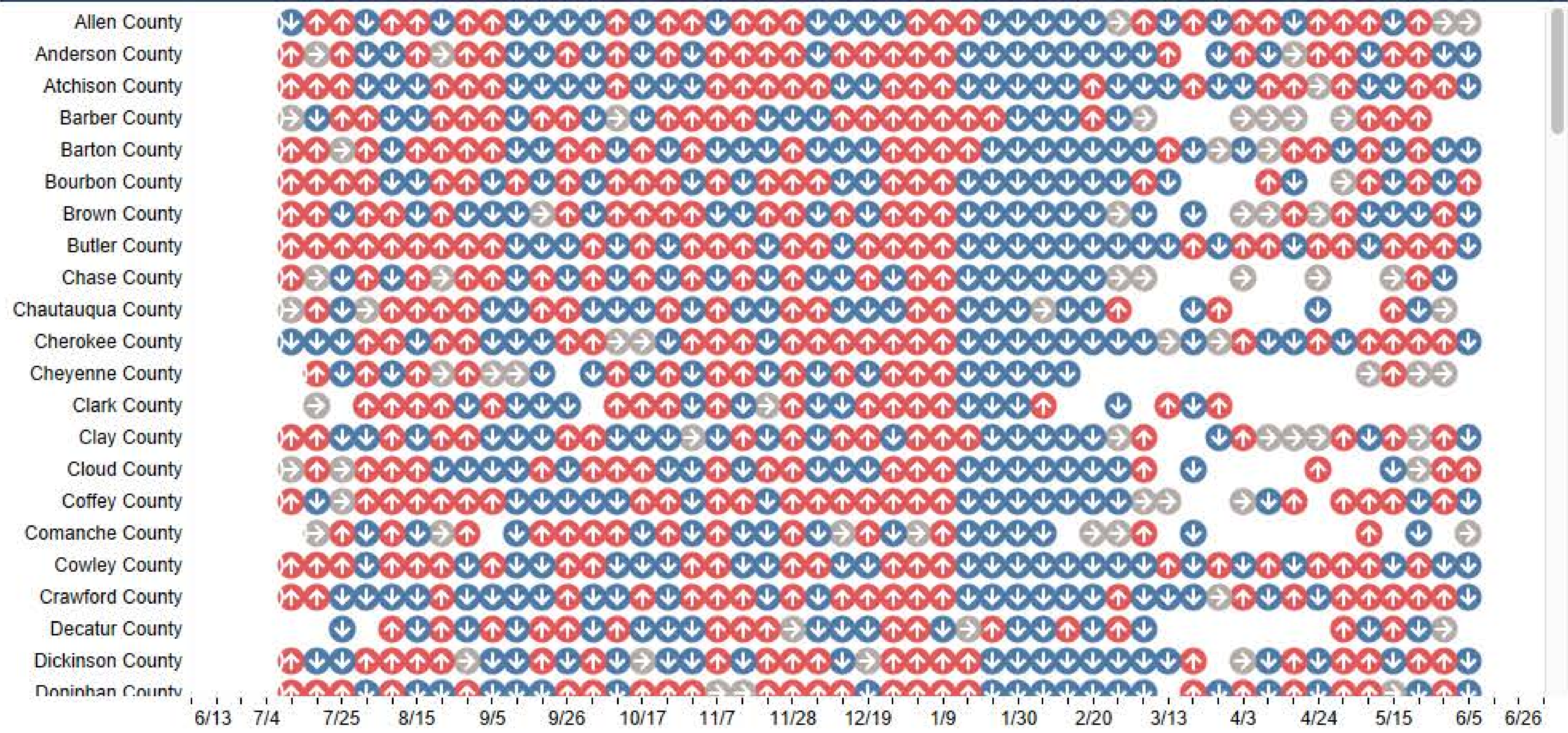

Weekly Trends by County and Age Group

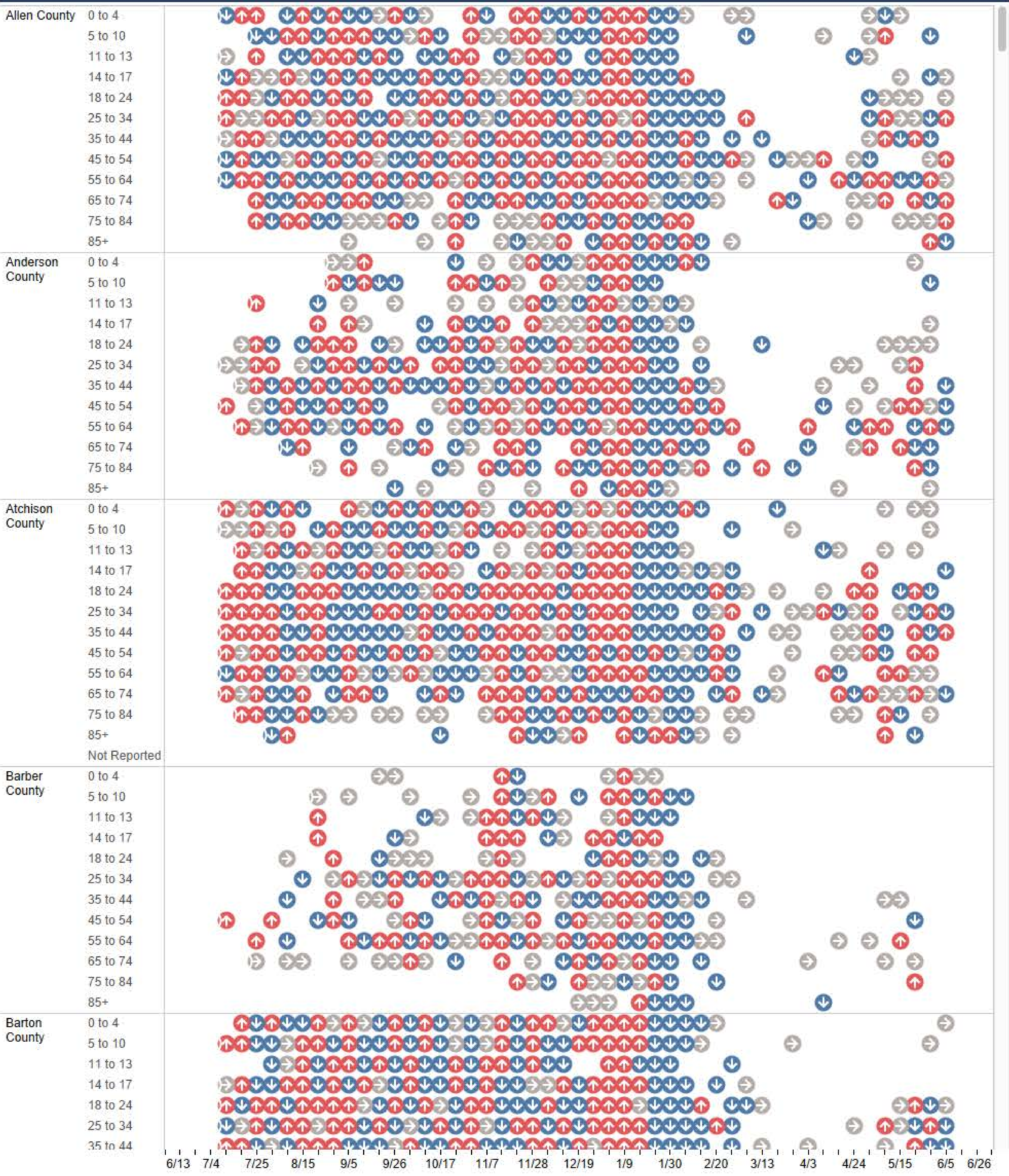

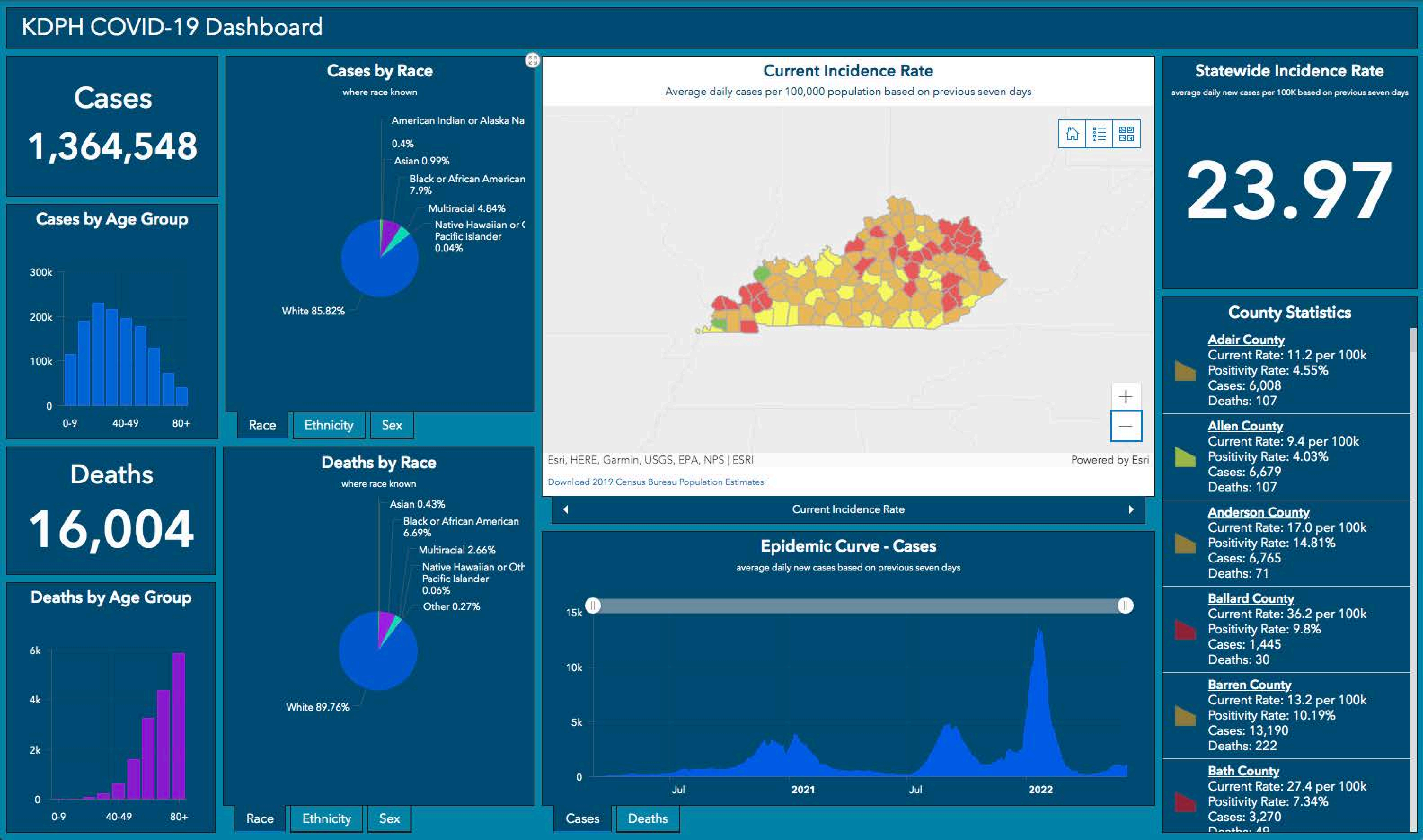
KDPH COVID-19 Dashboard
Cases
1,364,548
Cases by Age Group
300k
200k
100k
0
0-9
40-49
80+
Deaths
16,004
Deaths by Age Group
6k
4k
2k
0
0-9
40-49
80+
Cases by Race
where race known
American Indian or Alaska Na
0.4%
Asian 0.99%
Black or African American 7.9%
Multiracial 4.84%
Native Hawaiian or Pacific Islander 0.04%
White 85.82%
Race
Ethnicity
Sex
Deaths by Race
where race known
Asian 0.43%
Black or African American 6.69%
Multiracial 2.66%
Native Hawaiian or Oth Pacific Islander 0.06%
Other 0.27%
White 89.76%
Race
Ethnicity
Sex
Current Incidence Rate
Average daily cases per 100,000 population based on previous seven days
Esri, HERE, Garmin, USGS, EPA, NPS | ESRI
Powered by Esri
Download 2019 Census Bureau Population Estimates
Current Incidence Rate
Epidemic Curve - Cases
average daily new cases based on previous seven days
15k
10k
5k
0
Jul
2021
Jul
2022
Cases
Deaths
Statewide Incidence Rate
average daily new cases per 100K based on previous seven days
23.97
County Statistics
Adair County
Current Rate: 11.2 per 100k
Positivity Rate: 4.55%
Cases: 6,008
Deaths: 107
Allen County
Current Rate: 9.4 per 100k
Positivity Rate: 4.03%
Cases: 6,679
Deaths: 107
Anderson County
Current Rate: 17.0 per 100k
Positivity Rate: 14.81%
Cases: 6,765
Deaths: 71
Ballard County
Current Rate: 36.2 per 100k
Positivity Rate: 9.8%
Cases: 1,445
Deaths: 30
Barren County
Current Rate: 13.2 per 100k
Positivity Rate: 10.19%
Cases: 13,190
Deaths: 222
Bath County
Current Rate: 27.4 per 100k
Positivity Rate: 7.34%
Cases: 3,270

COVID-19 Information | Vaccination Information

LOUISIANA DEPARTMENT OF HEALTH

*Filtered to: Louisiana

New Cases	New Deaths	COVID Patients in Hospitals
1,981	1	275
**Total Cases***	**Total Deaths***	**COVID Patients on Ventilators**
1,274,798	17,356	10

Those not fully vaccinated account for...

53% of cases from 5/26 - 6/1	40% of deaths from 5/26 - 6/1	68% of current COVID hospitalizations

Covid-19 Community Levels by Parish

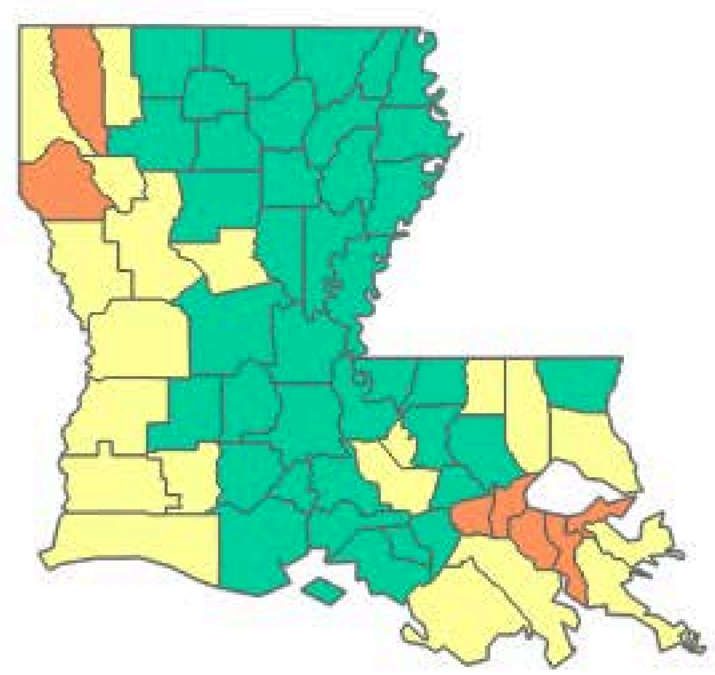

Last Updated: 6/10/2022 **Community Level Last Updated:** 6/10/2022
Data are updated on weekdays by noon.
Covid-19 Community Levels are updated noon on Friday. If Friday is a holiday, the update will occur on the following non-holiday business dav.

COVID-19 Community Level

Click the < > arrows to navigate tabs containing other information and downloadable data
This dashboard is updated on weekdays by noon.

2-1-1 Social services help and information about COVID-19 in Maine, call 211, email info@211maine.org, text your ZIP code to 898-211, or if out-of-state call 1-877-463-6207.

COVID-19: Maine Data

Maine's state of civil emergency ended on June 30, 2021. Since July 3, 2021, Maine CDC has been conducting case investigations and contact tracing Monday through Friday. As a result, COVID-19 case data will be updated by 9:30 AM Tuesday through Saturday. Case data will not be updated Sunday and Monday or on holidays. All data are preliminary and may change as Maine CDC investigates cases. For more information about the data, please see the "Read Details About the Data" section below.

Maine CDC has focused its efforts to follow up with cases of COVID-19 on cases who are under the age of 19, over the age of 74, or who meet certain other criteria (including being hospitalized, identified as a minority, identified as having a disability, being a health care worker or first responder, living or working in congregate settings, or associated with a school or child care facility). Depending on available resources and case levels, Maine CDC may follow up with other cases who do not meet those criteria.

Download csv files with the most recent cumulative case data by zip code (CSV), county (CSV), age (CSV), sex (CSV), race (CSV), and ethnicity (CSV).

Download a csv file with historical case data by county and date.

Download a csv file with historical COVID-associated death counts by county, age group, gender, and date.

Download a csv file with syndromic surveillance gate metrics by date.

View Maine's complete COVID-19 vaccination dashboard.

COVID-19 Daily Lab Results, New Daily Deaths, Hospitalized Patients, and Syndromic Data

Lab Results | Deaths | Hospitalizations | Emergency Room Visits

New Daily Positive Tests and Positivity Rate

Gray line shows positive Antigen and Molecular tests as a percent of total Antigen and Molecular tests

Select Test Type: Antigen and Molecular

All | Last 30 Days | Last 60 Days | Last 90 Days | Last 180 Days | Last 365 Days

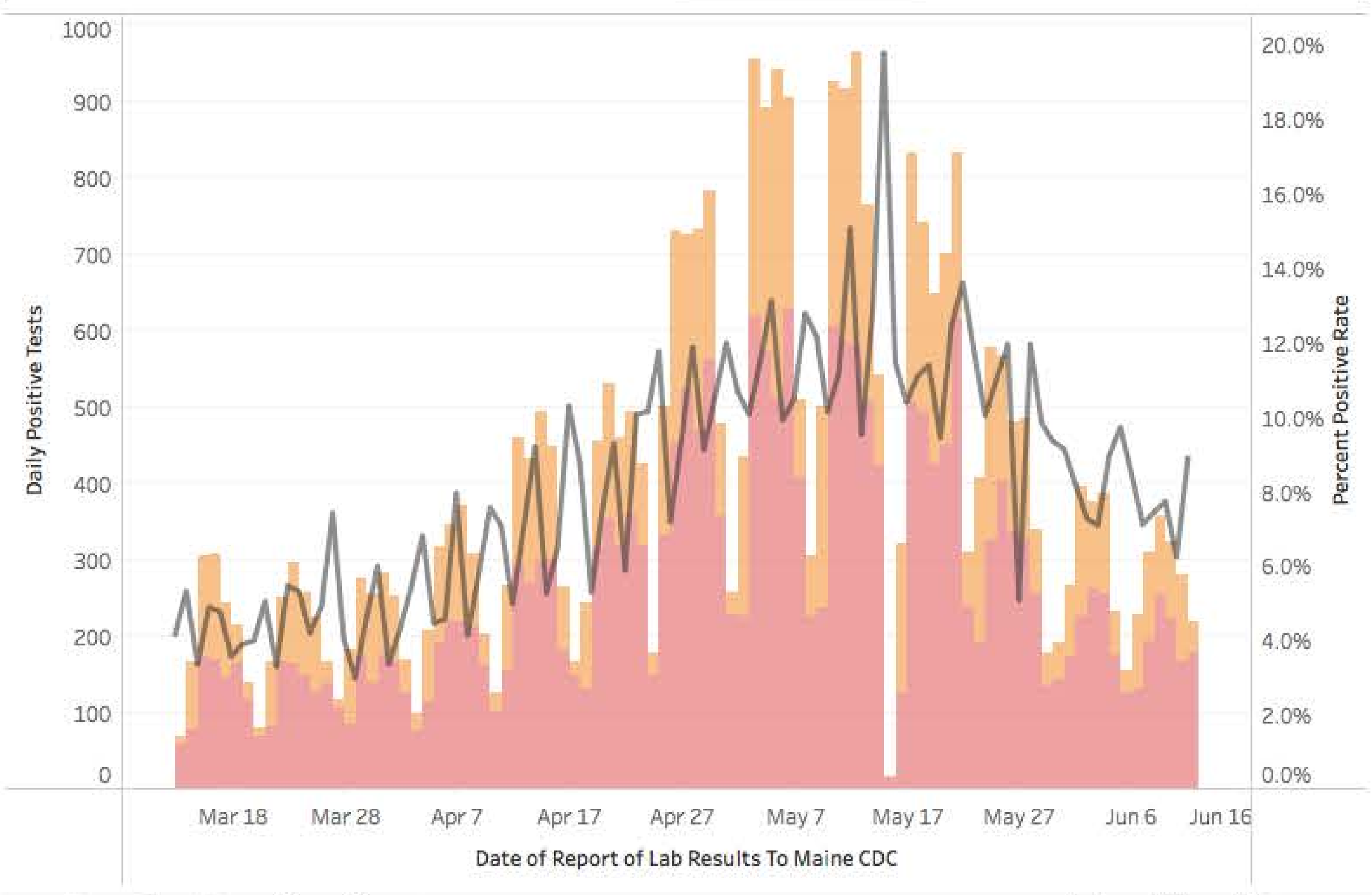

Share

COVID-19 Case Trends

New cases by date | Cases by age trend | Case rates by county | COVID-19 data by race

New daily COVID-19 cases

Positive lab results are reviewed by Maine CDC as soon as possible to determine if it is a new case of COVID-19 and whether or not it meets the criteria for follow-up by Maine CDC. The bars on this chart show case counts according to the date they were reviewed, and whether or not they were assigned for follow-up. The gray line on this chart shows the case count according to the date Maine CDC received the initial information. This metric is only available for dates before the oldest date in the existing backlog of lab results that Maine CDC has yet to review to determine

All | Last 30 Days | Last 60 Days | Last 90 Days | Last 180 Days | Last 365 Days

Showing: Case rate per 10,000 people
Date Range: March 13, 2022 to June 11, 2022
Case Status: All
Click a county to filter

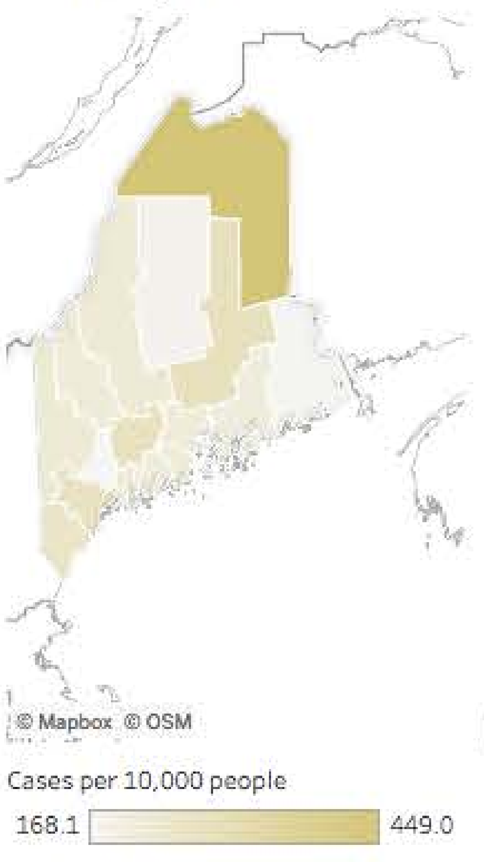

County: All | Case status: All | Date Range: March 13, 2022 to June 11, 2022

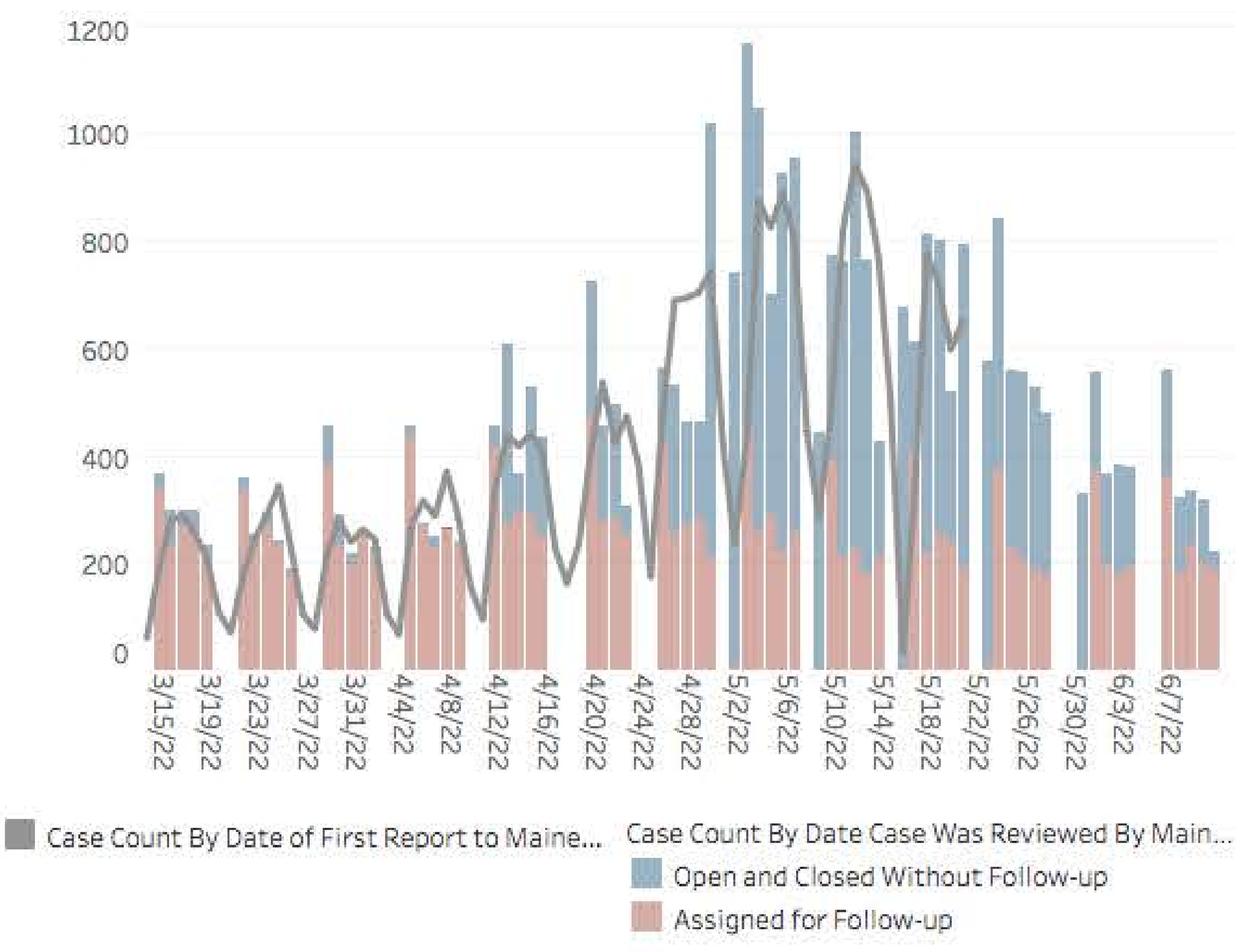

Share

Download csv files with the most recent cumulative case data by zip code (CSV), county (CSV), age (CSV), sex (CSV), race (CSV), and ethnicity (CSV).

Download a csv file with historical case data by county and date.

Cumulative COVID-19 Cases by County

Export table to PDF

Cumulative COVID-19 Cases by County

Table updated: June 12, 2022
Data through: Saturday, June 11, 2022 11:59 pm

Patient County	Cases	Confirmed cases	Probable cases	Deaths	Hospitalizations	Case rate (per 10K people)
**Statewide**	**266,105**	**191,792**	**74,313**	**2,403**	**5,057**	**1,988.2**
Cumberland	55,489	42,051	13,438	379	861	1,890.2
York	44,062	35,258	8,804	295	759	2,136.6
Penobscot	31,052	21,142	9,910	340	760	2,055.1
Kennebec	25,715	15,995	9,720	252	447	2,106.4
Androscoggin	24,011	15,298	8,713	289	532	2,229.9
Aroostook	15,253	9,987	5,266	174	293	2,272.8
Oxford	12,322	9,111	3,211	149	249	2,138.6
Somerset	10,516	7,318	3,198	112	300	2,078.6
Hancock	8,392	6,393	1,999	90	173	1,531.1
Waldo	6,792	5,273	1,519	61	115	1,711.1
Knox	6,686	5,876	810	40	90	1,681.1
Franklin	6,260	5,053	1,207	62	105	2,093.9
Lincoln	5,812	4,893	919	36	81	1,692.4
Sagadahoc	5,746	4,218	1,528	35	86	1,612.5
Washington	4,737	2,305	2,432	58	119	1,504.3
Piscataquis	3,233	1,608	1,625	31	87	1,924.4
Unknown	27	13	14	0	0	

Download a csv file with the most recent cumulative case data by county.

Download a csv file with historical case data by county and date.

Cumulative COVID-19 Cases by ZIP Code

Cumulative COVID-19 cases by ZIP code

Hover for detail | 256 cases with unknown ZIP codes, 2,689 cases with unmappable ZIP codes
Data as of: June 5, 2022

Patient County: (All)
Highlight Patient Zip
Highlight Municipality

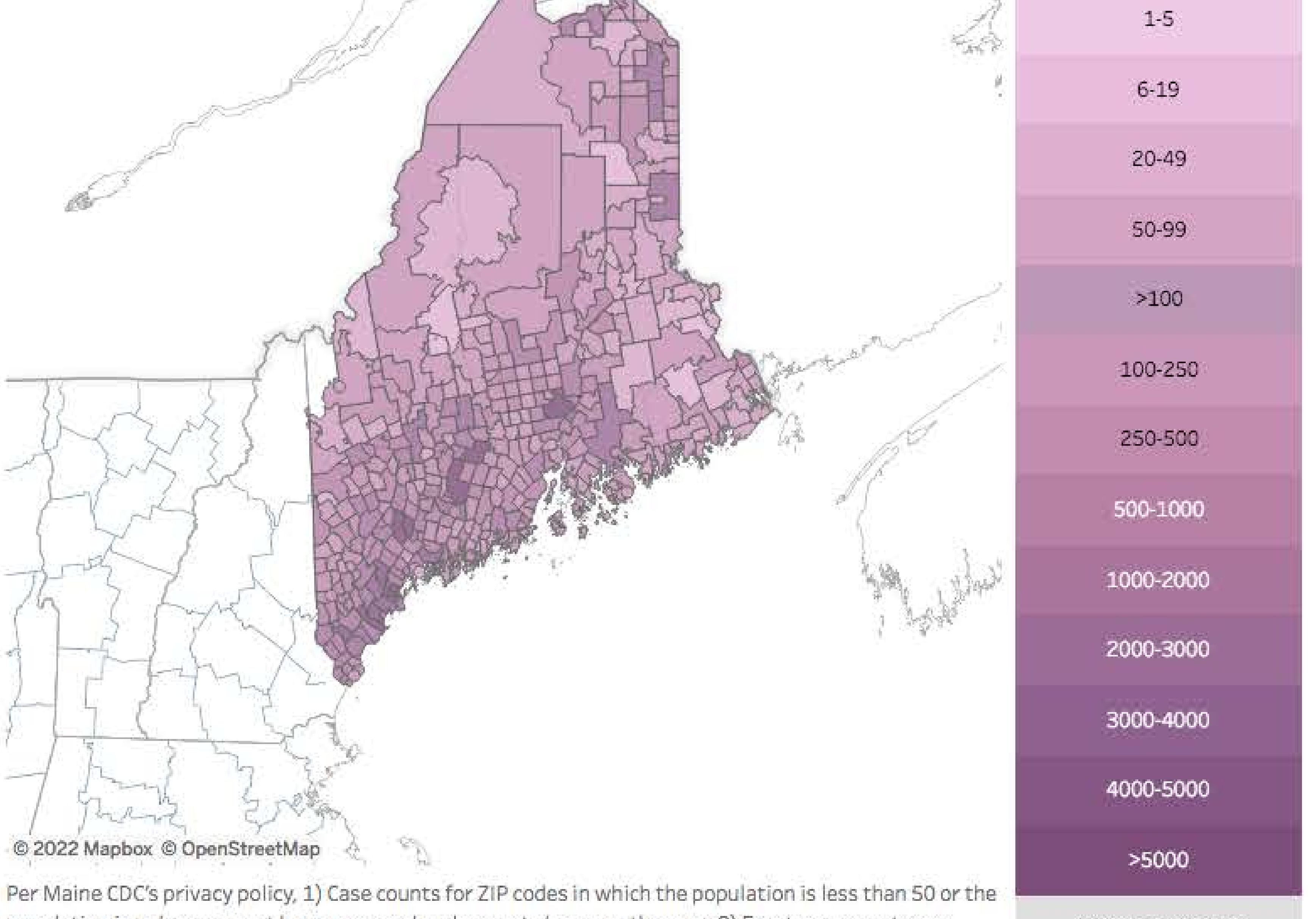

Per Maine CDC's privacy policy, 1) Case counts for ZIP codes in which the population is less than 50 or the population is unknown must be suppressed and are not shown on the map. 2) Exact case counts are suppressed in all zip codes with 5 or fewer cases or a population less than 2,000.

Maryland MARYLAND DEPARTMENT OF HEALTH

Coronavirus Disease 2019 (COVID-19) Outbreak

This is a rapidly evolving situation and the Maryland Department of Health (MDH) will provide updates as they become available.

Notice of Non-Discrimination and Accessibility Statement

Additional COVID-19 Resources at CovidLINK

Search

Maryland COVID-19 Data Dashboard

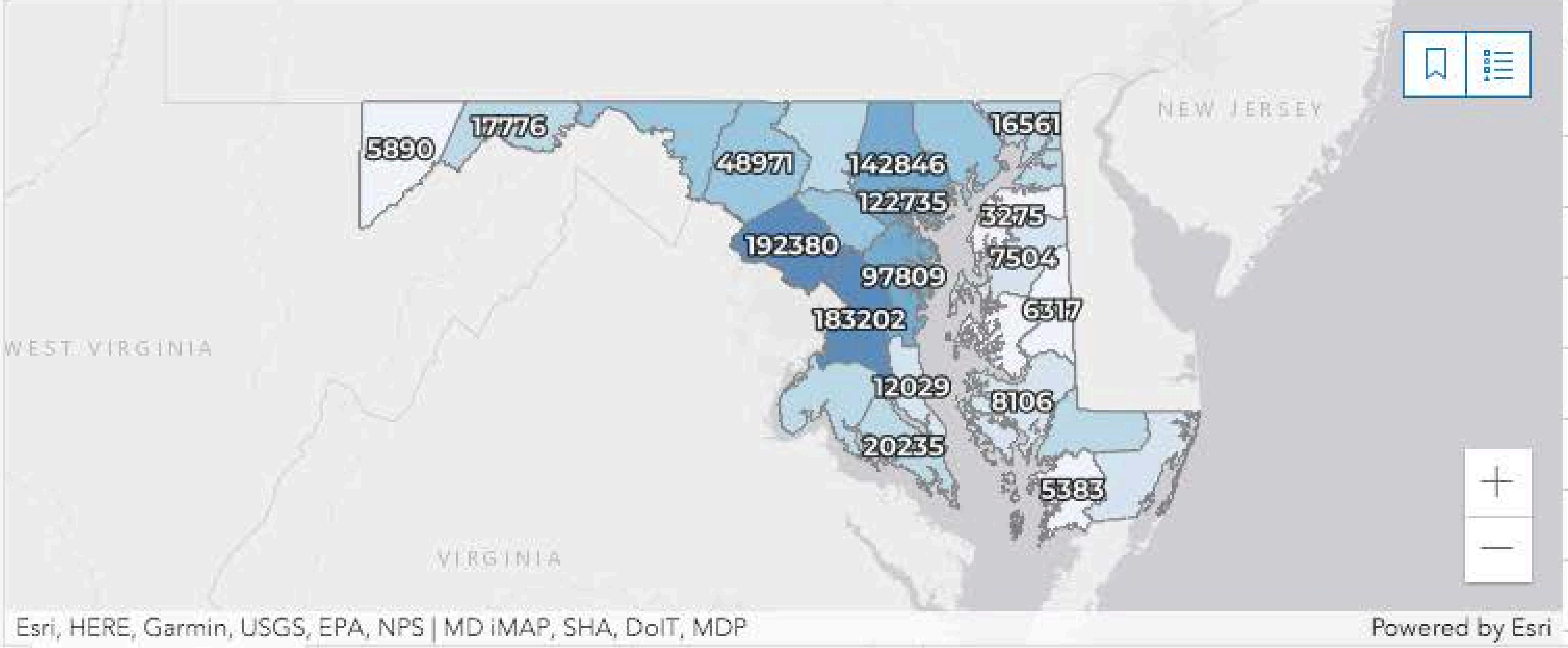

Affected Counties:

- Montgomery - 192,380
- Prince George's - 183,202
- Baltimore - 142,846
- Baltimore City - 122,735
- Anne Arundel - 97,809
- Howard - 49,573
- Frederick - 48,971
- Harford - 41,090

Cases by County | Cases by ZIP Code | Testing by County

ICU and Acute Hospital Beds for COVID-19, Currently in Use

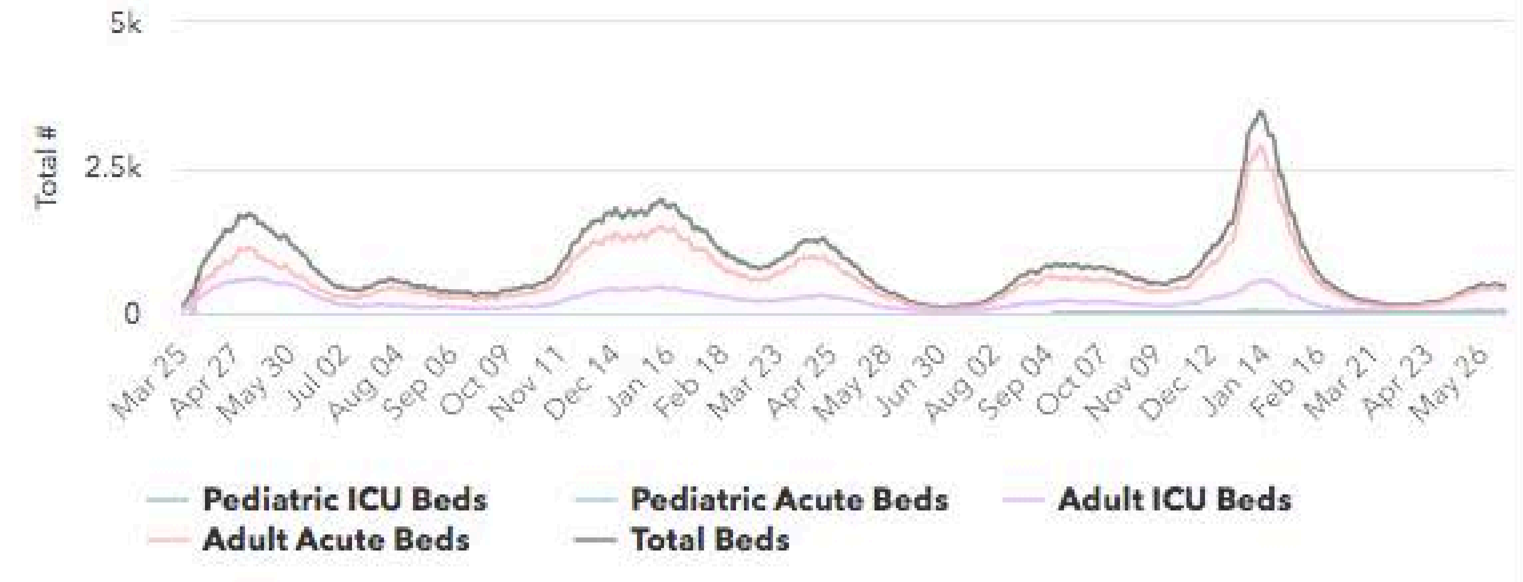

Hospitalizations, Current

Testing Volume, Tests per Day and Percent Positive Rate (7-Day Avg) - Methodology

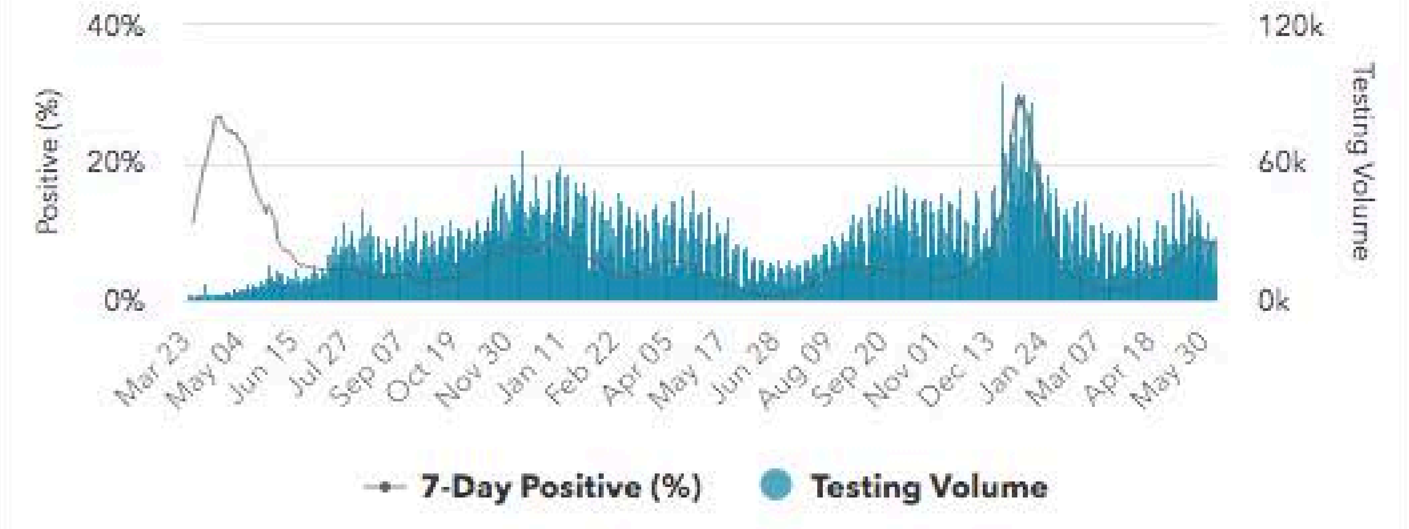

Positivity Rate

Maryland.gov LANGUAGE ASSISTANCE SERVICES

Testing | Find a Vaccine | Vaccine Info | Vaccine Data | Nursing Home Data | Schools Data

Vaccinations in Maryland

COVID-19 Vaccination Dashboard

CDC: Population with at Least One Dose

Population Age 18+	Population Age 5+
95%	92.7%

Source: CDC Covid Data Tracker

Doses Administered

11,923,585

All Doses Administered in Maryland | Vaccinations by Dose in Maryland

Fully Vaccinated

4,656,738

Doses Distributed

16,566,570

Vaccinations Administered in Maryland by Date

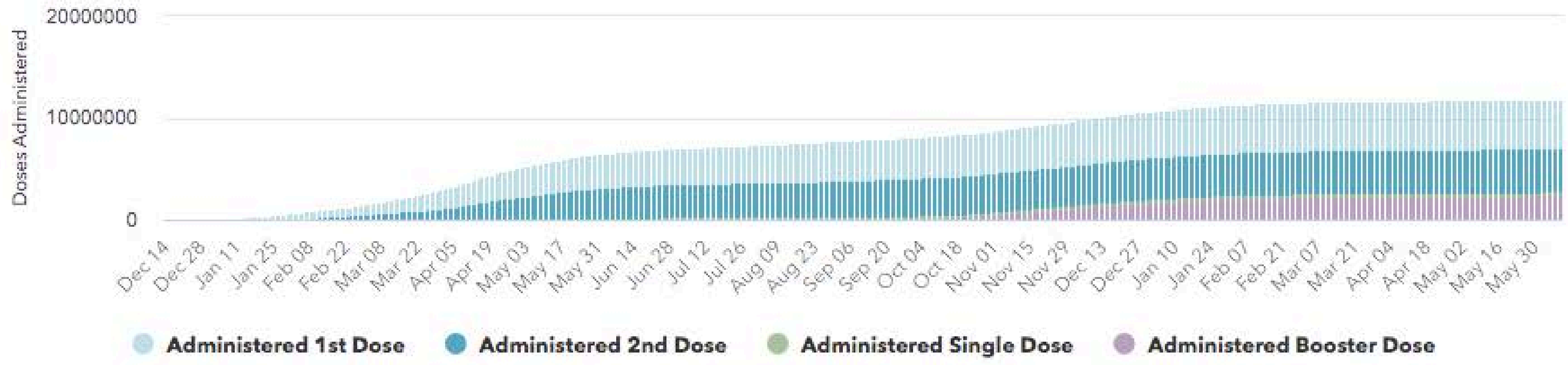

Vaccinations Administered in Maryland by Date

Vaccinations Statewide by Age

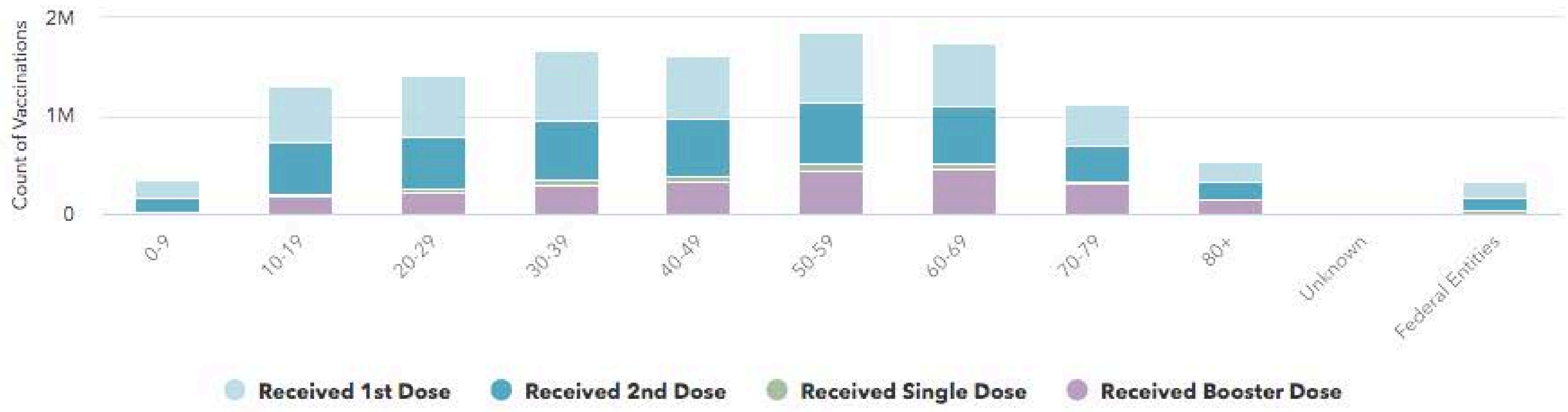

Completed Vaccinations by Age

COVID-19 Vaccine Announcements

PHOTO RELEASE: Governor Hogan Tours COVID-19 Booster Clinic, Visits Local Businesses on the Eastern Shore 2/19/2022

Get A Boost: Governor Hogan Announces New Casino-Based Clinics Offering Booster Shots For Marylanders 2/17/2022

COVID-19 Positivity Rate Drops Below 5%, State Officials Encourage Marylanders to Get Boosted This Weekend 2/11/2022

VACCINE MILESTONE: Maryland Surpasses 95% of Adults Vaccinated Against COVID-19 2/8/2022

Maryland Surpasses 2 Million COVID-19 Booster Shots Administered, Hospitalizations Decline Below Pre-Omicron Peak 1/29/2022

Maryland COVID-19 Hospitalizations Drop Below 2,000, State Surpasses 11 Million Vaccinations 1/28/2022

Tweets by @GoVAXMaryland

GoVAX Maryland
@GoVAXMaryland

Dr. Jinlene Chan said the state is poised to begin vaccinating children under 5 years of age, pending federal approval. Chan said the state recently completed a pre-order for 65,400 doses. #COVIDReadyMD wtop.com/maryland/2022/...

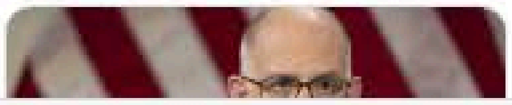

The Maryland Department of Health provides COVID-19 data reporting on business days at 10 a.m.

Massachusetts Department of Public Health | COVID-19 Dashboard
Today at a Glance

Released on: June 10, 2022
Data as of: June 9, 2022
Caution: recent data may be incomplete

Cases

Confirmed Cases

Today there were 2,050 new, confirmed cases reported bringing the total to 1,738,138 total confirmed cases.

Probable Cases

Today, there were 321 new, probable cases reported bringing the total to 156,031 total probable cases.

Testing

Tests Reported

There were 35,543 new COVID-19 molecular tests reported, bringing the cumulative total to 45,490,916 tests.

Percent Positivity

The 7-day average of percent positivity is 6.53%.

Hospitalizations

Hospitalizations

There are 533 patients hospitalized with COVID-19.

ICU & Intubated Patients

There are 54 patients in Intensive Care Units (ICU) and 18 patients are intubated.

Deaths

Confirmed Deaths

There were 7 new, confirmed deaths reported. There have been 19,538 confirmed deaths in total.

Probable Deaths

There were 2 new, probable deaths reported. There have been 1,197 probable deaths in total.

Average Age of Deaths

The average age of patients who died of COVID-19 was 80 years old.

Confirmed cases by age during the last two weeks *Data updated weekly

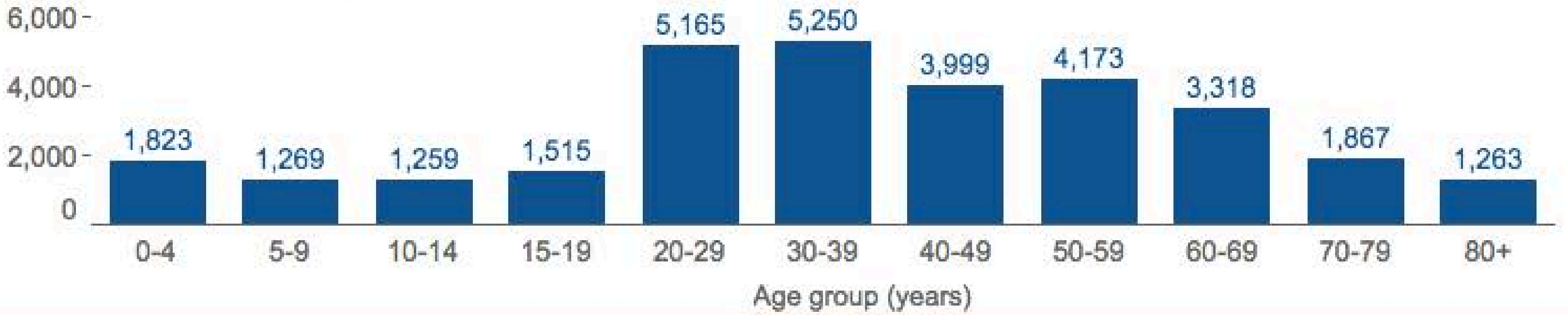

For details on the definitions of each indicator hover over the box or graph. All data included in this dashboard are preliminary and subject to change. Data Sources: COVID-19 Data provided by the Bureau of Infectious Disease and Laboratory Sciences and the Registry of Vital Records and Statistics; COVID-19 Hospitalization Data provided by the MDPH survey of hospitals (hospital survey data are self-reported); Created by the Massachusetts Department of Public Health, Bureau of Infectious Disease and Laboratory Sciences, Division of Surveillance, Analytics and Informatics.

Massachusetts Department of Public Health | COVID-19 Dashboard
Trends: 7-day Averages Over Time

Released on: June 10, 2022
Data as of: June 9, 2022
Caution: recent data may be incomplete

Cases

7-day average of COVID-19 confirmed cases

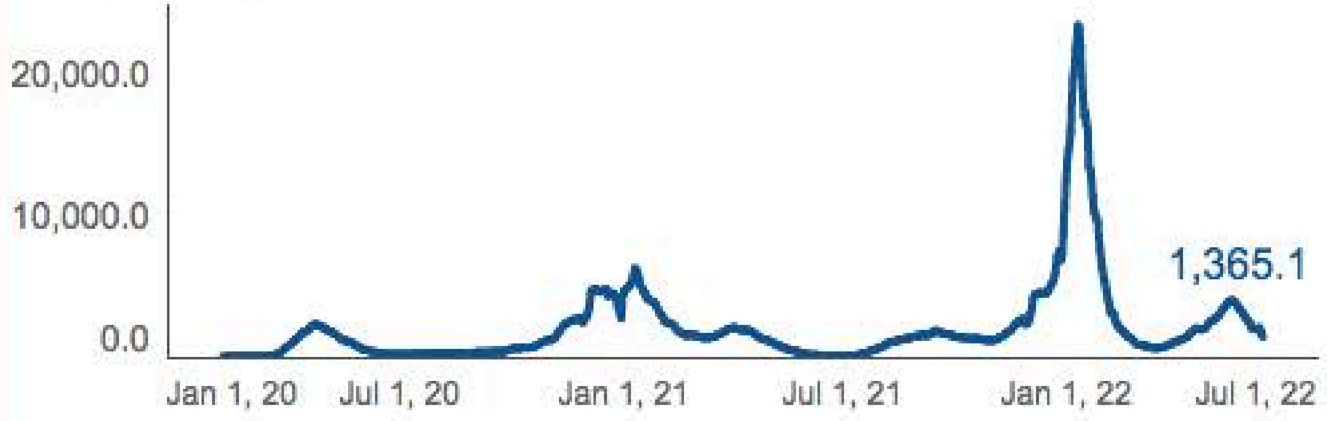

The lowest observed value was 64.1 on 6/25/2021.

Testing

7-day weighted average percent positivity

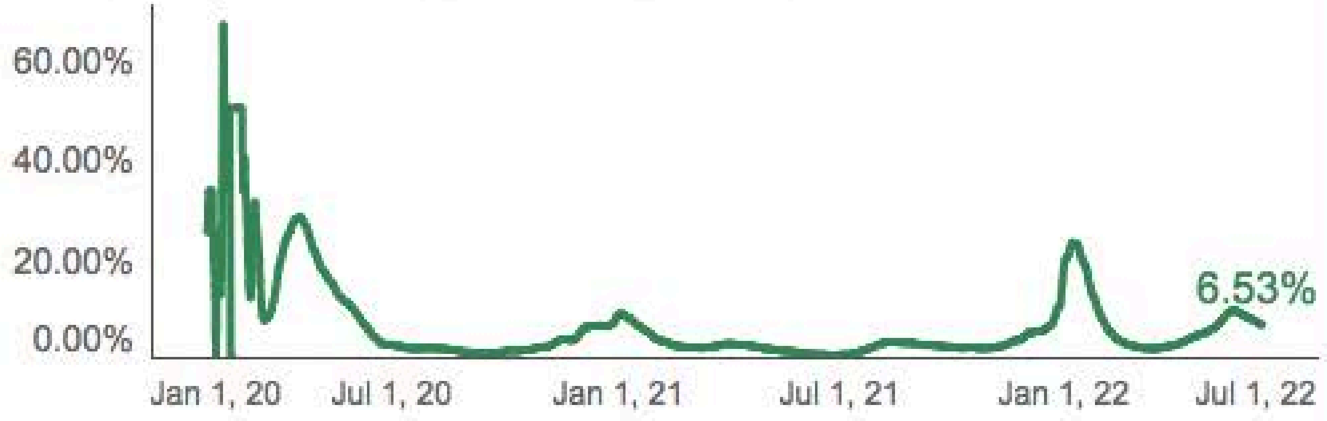

The lowest observed value was 0.31% on 6/25/2021.

Hospitalizations

7-day average of hospitalizations

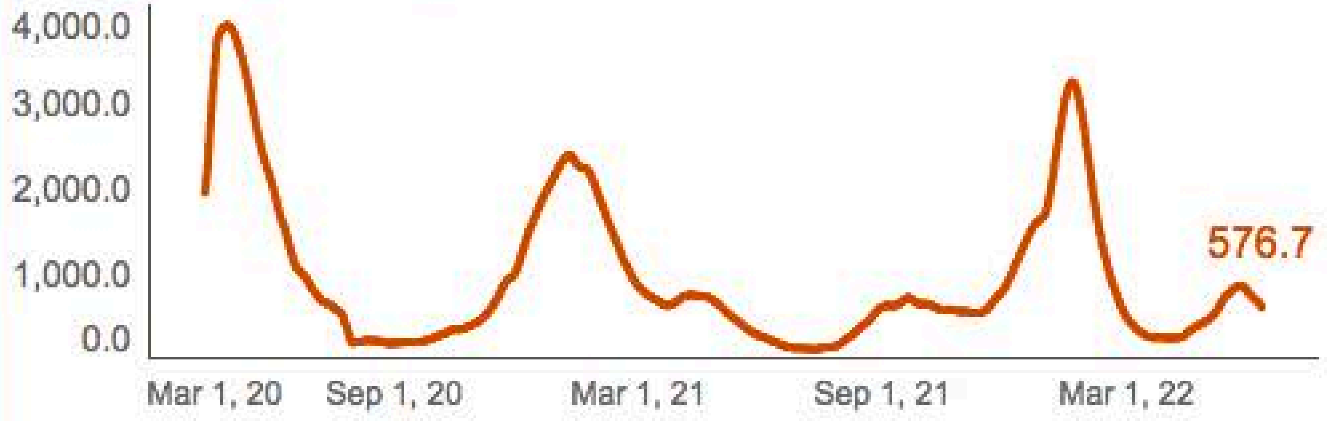

The lowest observed value was 84.3 on 7/8/2021.

Deaths

7-day average of confirmed deaths

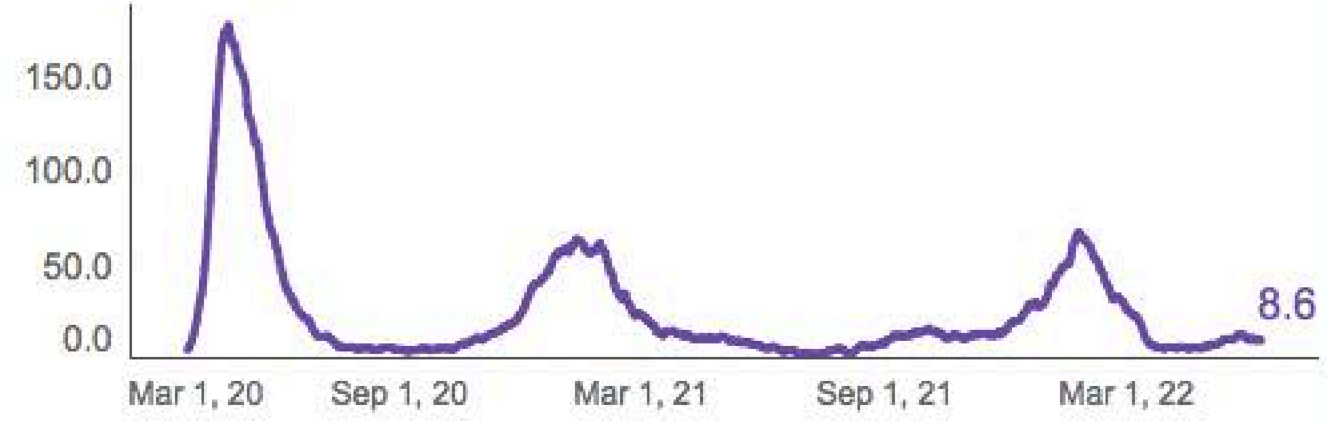

The lowest observed value was 1.0 on 7/11/2021.

Select a time period
All time

The lowest observed value is since tracking of the lowest value began on April 15, 2020. For details on the definitions of each indicator please see the corresponding tab for that indicator. All data included in this dashboard are preliminary and subject to change. Data Sources: COVID-19 Data provided by the Bureau of Infectious Disease and Laboratory Sciences and the Registry of Vital Records and Statistics; Created by the Massachusetts Department of Public Health, Bureau of Infectious Disease and Laboratory Sciences, Division of Surveillance, Analytics and Informatics.

Undo Redo Replay Revert Refresh Pause Share Download Full Screen

Massachusetts Department of Public Health | COVID-19 Dashboard
Cases of COVID-19

Released on: June 10, 2022
Data as of: June 9, 2022
Caution: recent data may be incomplete

Navigation

- Today's Overview
- Overview Trends
- **COVID-19 Cases**
- COVID-19 Testing
- Hospitalizations
- COVID-19 Deaths
- Higher Ed & LTCF
- Patient Breakdown
- City & Town Data
- Resources
- Data Archive

Cases Over Time | Contact Tracing | Clusters

COVID-19 Confirmed and Probable Cases: All time

Select a time period: All time

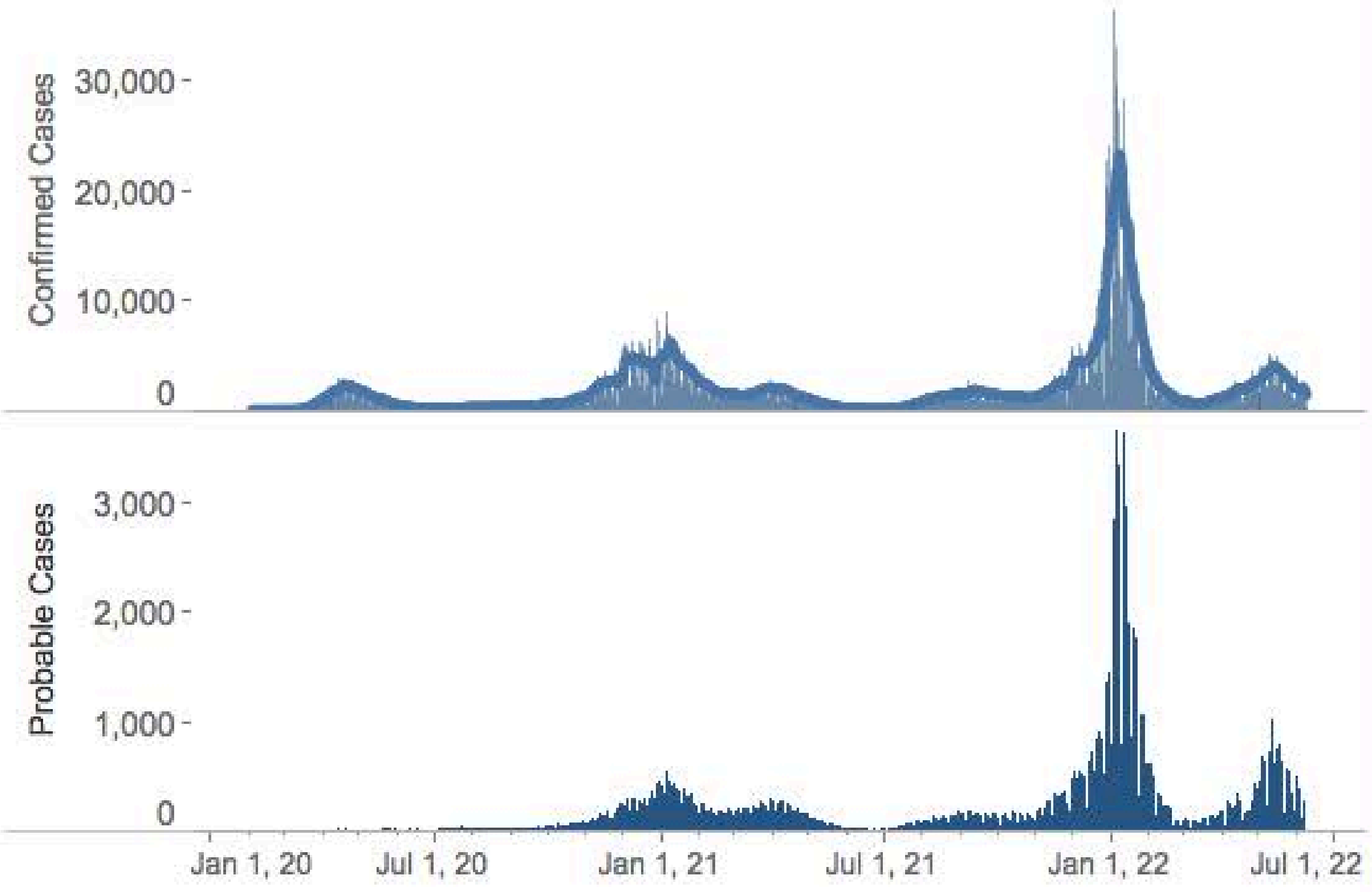

New confirmed cases reported by county, June 10, 2022

Select new or total cases: New confirmed cases

Select a date*: 6/10/2022

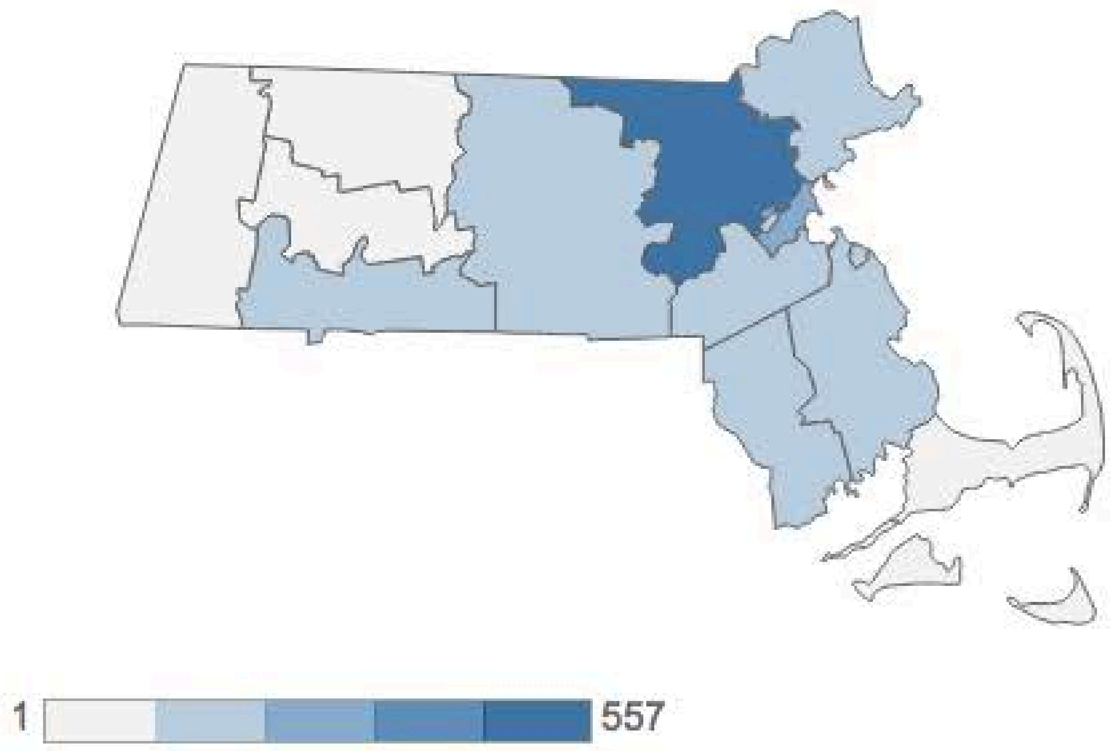

All data included in this dashboard are preliminary and subject to change. Data Sources: COVID-19 Data provided by the Bureau of Infectious Disease and Laboratory Sciences and the Registry of Vital Records and Statistics; Created by the Massachusetts Department of Public Health, Bureau of Infectious Disease and Laboratory Sciences, Division of Surveillance, Analytics and Informatics. . Case counts for specific cities, towns, and counties change as data cleaning occurs (removal of duplicate reports within the system) and new demographic information (assigning cases to their city or town of residence) is obtained.

*The most recent 30 days of data are viewable on this map. To view data outside of this range, please visit our data archive and download the raw data.

Undo | Redo | Replay | Revert | Refresh | Pause | Share | Download | Full Screen

CURRENT STATUS | DAILY CASES | CUMULATIVE TRENDS | DEMOGRAPHICS | TOTAL TESTING | CONFIRMATORY TESTING | LEARN MORE

View Cases

View Deaths

View Table

Select Preparedness Region: All

Select County: All

This report is provisional and subject to change. City of Detroit and Wayne County are reported separately. MDHHS began tracking probable cases April 5, 2020. FCI indicates federal correctional institute. MDOC indicates Michigan Department of Corrections. Diagnostic tests, including nucleic acid amplification/real-time polymerase chain reaction (NAAT/RT-PCR) and rapid virus antigen detection tests, look for current infection. Serology (antibody) tests are used to look for the possibility of previous infection.

COVID-19 Cases by County

Confirmed cases include individuals with a positive NAAT/RT-PCR test for COVID-19. Probable cases include individuals with either a positive rapid antigen detection test OR COVID-19 symptoms and an epidemiologic link to a confirmed COVID-19 case, but no diagnostic test.

Cases by County

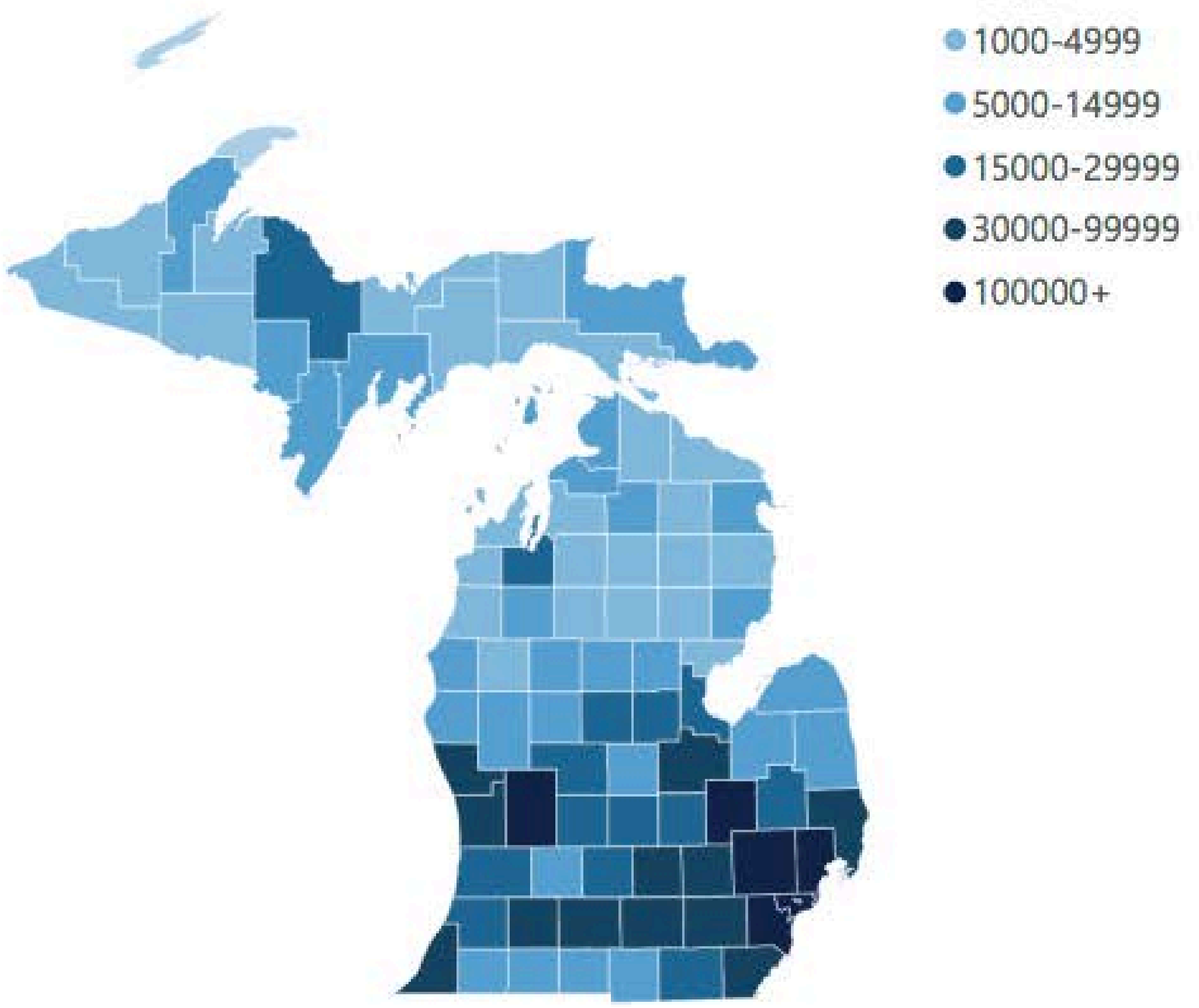

Michigan

Wednesday, June 08, 2022

Michigan (Statewide) Case Fatality Rate
1.5%

Current Totals

Case Status	Cases	Deaths
Confirmed	2,229,169	33,566
Probable	336,650	2,972
**Total**	**2,565,819**	**36,538**

Laboratory Testing

TestType	Tests Performed
Diagnostic	24,861,921
Serology	817,892
**Total**	**25,679,813**

- + 89%

Microsoft Power BI

CURRENT STATUS | DAILY CASES | CUMULATIVE TRENDS | DEMOGRAPHICS | TOTAL TESTING | CONFIRMATORY TESTING | LEARN MORE

Cases

Deaths

View Table

Select Preparedness Region:

All

Select County:

All

Cases are shown by onset date or by lab specimen collection date if onset date was unavailable. Deaths are shown by date of death or date or last status update if date of death was unavailable. Tracking of probable cases and deaths did not begin until April 5, 2020, therefore only probable cases referred for investigation on or after April 5, 2020 are displayed (onset may have been prior).

Notes: FCI indicates federal correctional institute. MDOC indicates Michigan Department of Corrections.

Daily Cases by Status

The number of daily confirmed cases represent the total number of people who had a positive NAAT/RT-PCR test for COVID-19. Cases are shown by the date of onset of symptoms.

Filter by Date:

3/1/2020 6/8/2022

Cases by Date of Onset

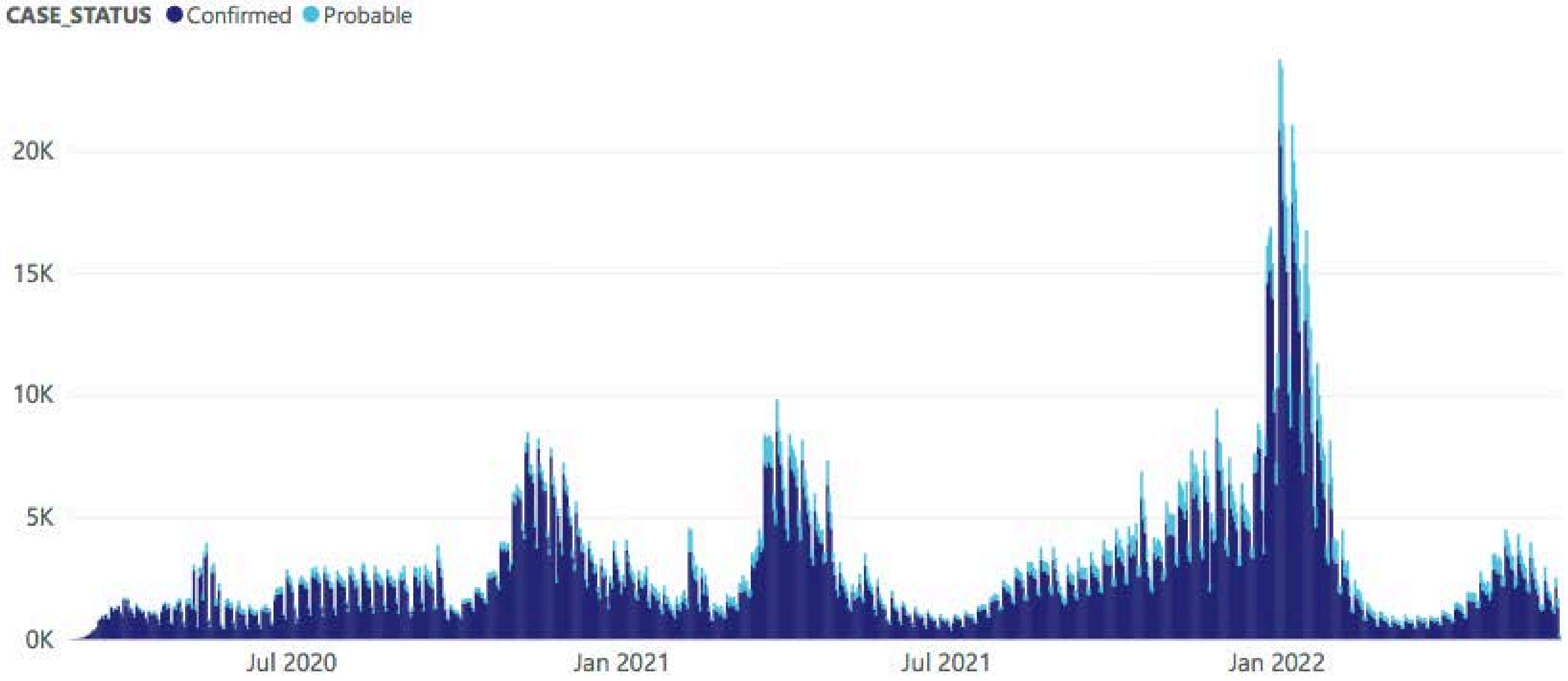

- 89%

Microsoft Power BI

CURRENT STATUS | DAILY CASES | CUMULATIVE TRENDS | DEMOGRAPHICS | TOTAL TESTING | CONFIRMATORY TESTING | LEARN MORE

Cases by Demographic Characteristics

Data are suppressed when the number of cases is five or below to protect the confidentiality of individuals. The sum of the cases for individual categories may be fewer than the total number of cases due to data suppression. Rates are not calculated for multiple races, other, and unknown race categories due to the unavailability of an appropriate denominator.

Cases | Deaths

Select case status: (●) Confirmed ( ) Probable

Select Preparedness Region: All

Select county: All

Cases by Age Group

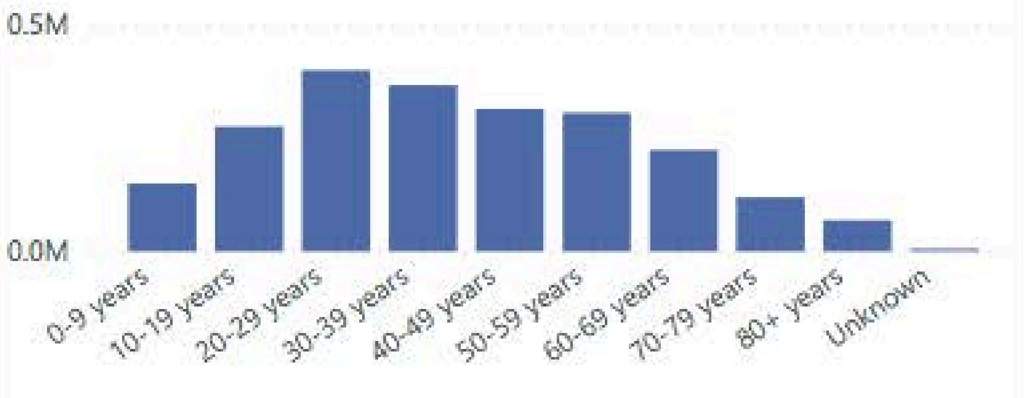

Cases by Sex

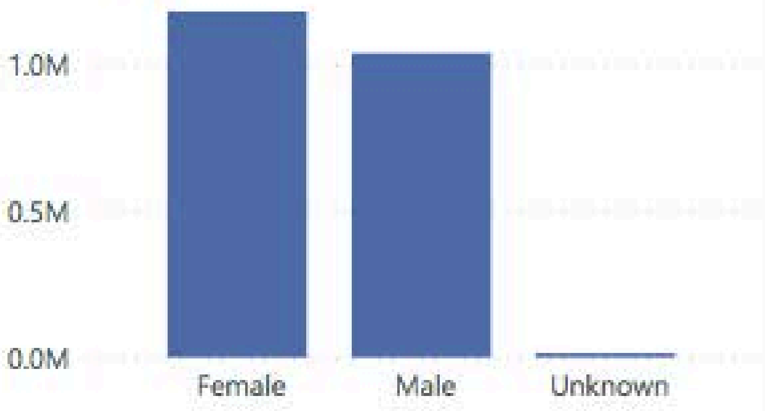

Cases by Race

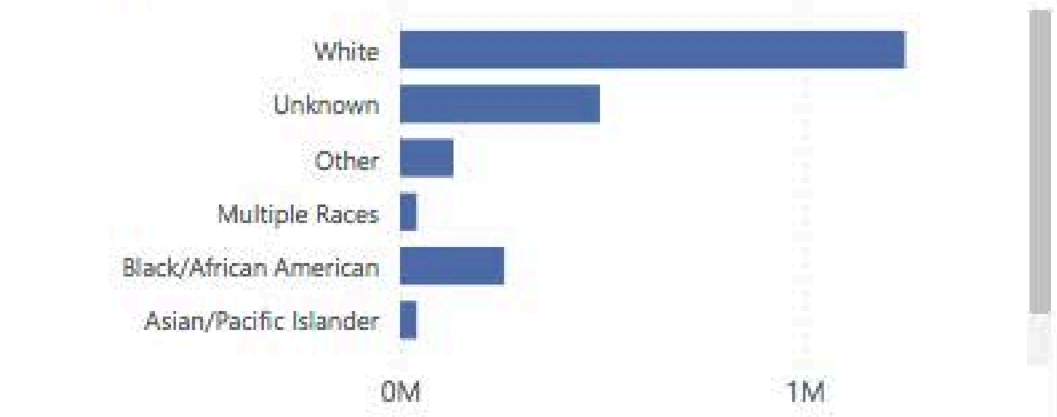

Cases per Million by Age Group

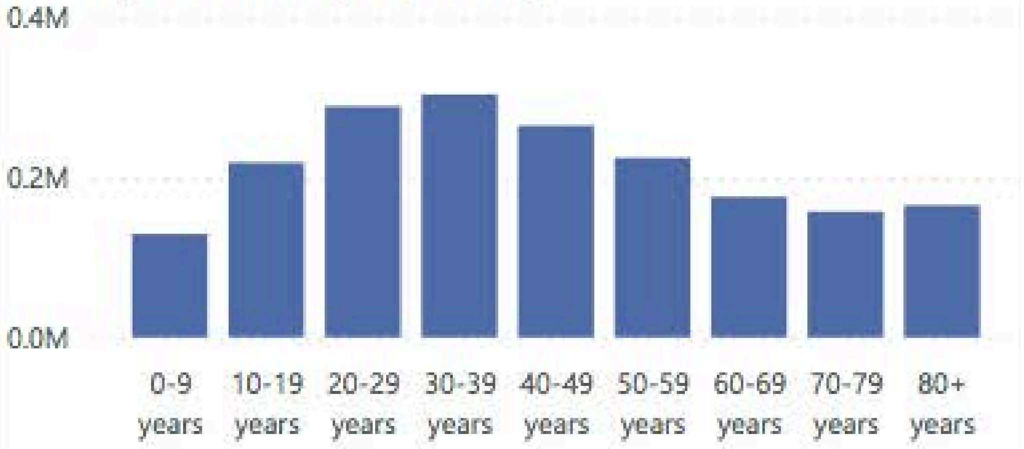

Cases per Million by Sex

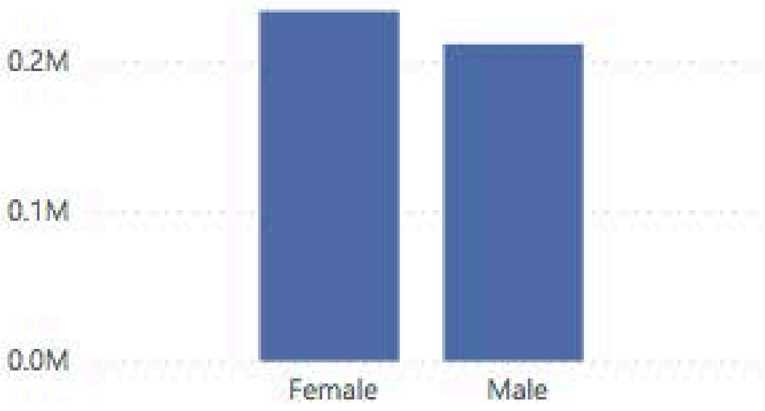

Cases per Million by Race

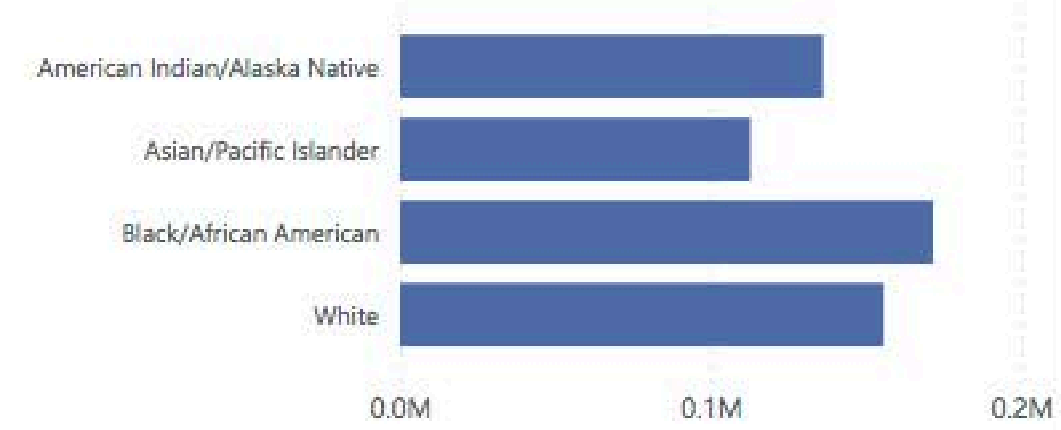

89%

Michigan coronavirus dashboard: vaccines, cases, deaths and maps

Michigan public health officials release new coronavirus data on cases, deaths and testing on Wednesdays. Hospital data is released three days a week.

COVID-19 cases

New cases since March, 2020

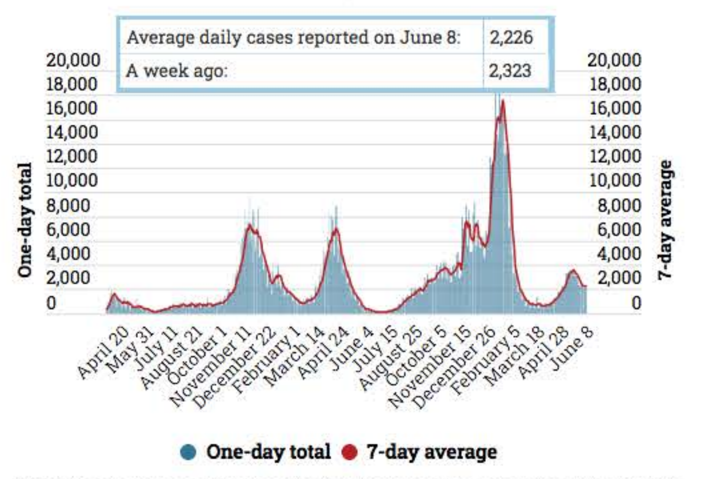

Note: The daily cases represent a daily average based on the state's totals over the past week. Michigan is now reporting case data only on Wednesdays.

Source: Michigan Department of Health & Human Services

Coronavirus in Michigan

Find coronavirus infections rates across much of the state. Click on a county to see the current day's* cases and the rate per 100,000 over the past week.

Daily cases per 100,000 people (average over last week)

- No cases
- Less than 10 cases
- 10 to 19 cases
- 20 to 29 cases
- 30 to 39 cases
- 40 to 49 cases
- More than 50

*As of 10 a.m. June 8

	Cases reported today	Daily cases per 100,000
Michigan	2,226	22
Detroit**	1,138	26

** Detroit not included in Wayne County's numbers in map

Note: Multiple days are included in each day's total.

Source: Michigan Department of Health & Human Services

Bridge — Mike Wilkinson

Hospitalizations

This is hospitalizations as reported several times a week by Michigan public health officials.

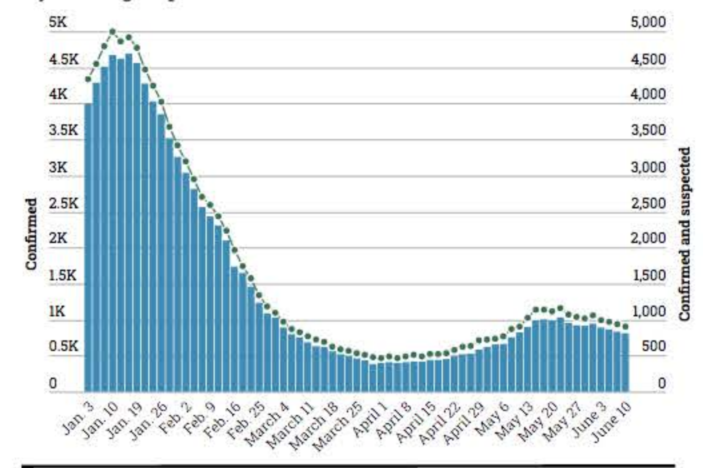

Source: Michigan Department of Health and Human Services and Western Michigan University Libraries.

Bridge — Mike Wilkinson

CDC's 'community risk' levels in Michigan

Indoor masking is now considered optional for healthy people in Michigan counties deemed "low" or "medium" risk, under new CDC guidance. The risk levels are based on hospital capacity, hospital COVID admissions and COVID-19 case rates. Updated info as of June 2, 2022.

CDC 'risk' levels
- Low (masks optional)
- Medium (masks optional)
- High

Source: Centers for Disease Control and Prevention

— Mike Wilkinson Bridge

COVID-19 vaccines given, by date

Michigan health officials are trying to vaccinate as many people as possible with the limited quantities of vaccine that they have. Here's how it is going, by date.*

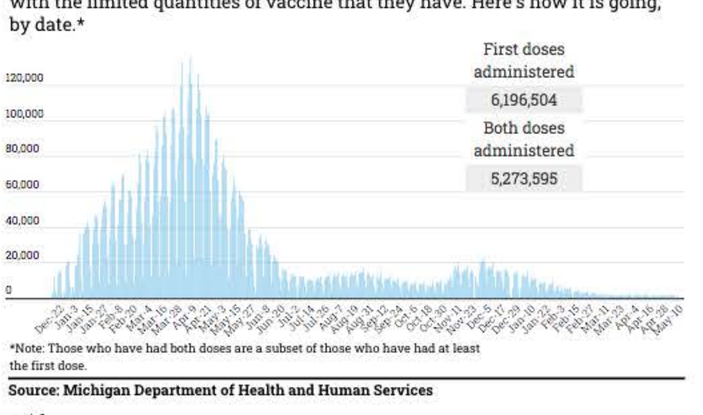

*Note: Those who have had both doses are a subset of those who have had at least the first dose.

Source: Michigan Department of Health and Human Services

Bridge — Mike Wilkinson

Who's getting the vaccine?

The vaccine rollout in Michigan has been bumpy, with elderly residents in some parts of the state unsuccessfully scrambling to get vaccines while some areas are vaccinating younger people as well. Search here to see what percent of those 12 years old and older have been vaccinated so far. Gov. Gretchen Whitmer has set a goal of vaccinating 70 percent of those 16 years old and older.

Search

Alcona	Initiated	Completed	Percent Initiated	Percent Completed
Keweenaw	1,311	1,213	67.9	62.8
Otsego	14,408	13,400	67.5	62.8
Delta	21,033	19,485	67.2	62.3
Jackson	91,188	84,608	66.8	62
Saginaw	110,390	101,759	67.3	62
Roscommon	14,440	13,417	66.3	61.6
Huron	17,743	16,665	65.5	61.5
Wexford	18,692	17,553	65.5	61.5
Berrien	89,750	80,900	68.1	61.4
Cheboygan	15,069	13,934	66.2	61.2

Source: Michigan Department of Health and Human Services

Bridge — Mike Wilkinson

Cases and testing

You can use this graphic to check daily changes in cases and tests, as well as compare to the previous week. **NOTE: The state now releases data on Mondays, Wednesdays and Fridays with each day's totals covering two or three days.**

Cases | **Testing**

As of: June 8	Cases reported today*	7-day average	Prior week avg.	Cases per 100,000 rate	Prior week rate
Oakland	2,571	367	382	29	30
Wayne	2,272	325	332	28	29
Macomb	1,534	219	233	25	26
Detroit City	1,138	163	162	26	25
Kent	943	135	138	21	21
Washtenaw	800	114	141	31	38
Genesee	462	66	69	16	17
Kalamazoo	454	65	72	25	28
Ingham	367	52	62	18	22

* Cases reported cover either two or three days. The weekly averages encompass cases over seven-day increments. Also, in counties with state prisons, new cases are often first reported within the county. Later, they are removed and identified as having occurred within the Michigan Department of Corrections. This can create days where a county will have a negative number of new cases.

Bridge — Mike Wilkinson

Daily testing results

Michigan health officials say the goal is to keep the positive test rate at 3 percent or lower. Results are for the last three months.

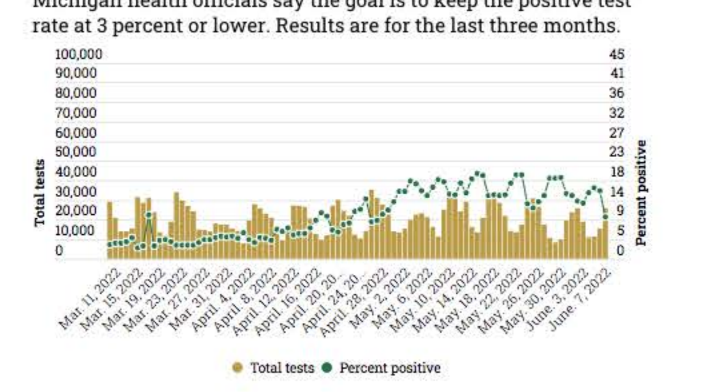

COVID-19 deaths in Michigan

This map shows home residence of those who have died from the coronavirus.

Total deaths: 33,566

- No deaths
- 1 to 25 deaths
- 26 to 49 deaths
- 50 to 99 deaths
- More than 100 deaths

*As of 10 a.m. June 8

Source: Michigan Department of Health & Human Services

Share Bridge

Age breakdown

The coronavirus has infected and killed people of all ages in Michigan but has hit the elderly particularly hard. Here's the current breakdown.

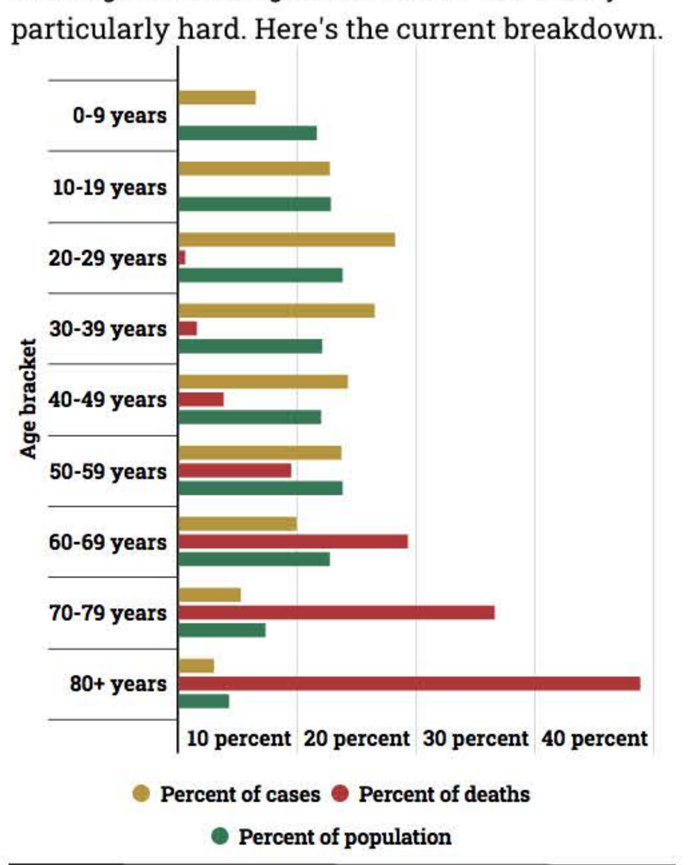

Source: Michigan Department of Health & Human Services

Daily deaths

Deaths by day reported, since March 25

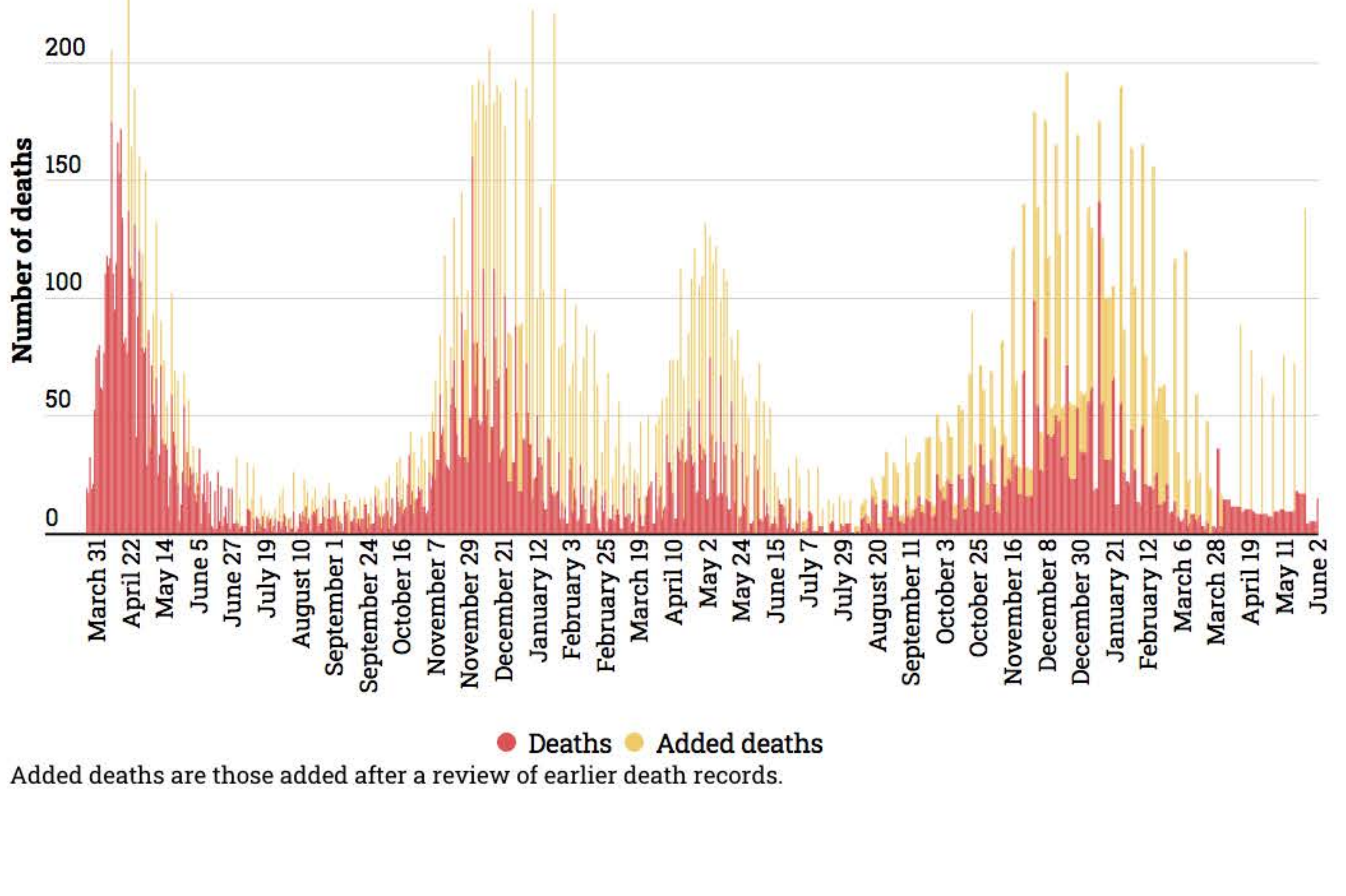

Added deaths are those added after a review of earlier death records.

Deaths by date

These are deaths by the **date** of death. Often, following a review of medical records, deaths are reported days and weeks after the actual death.

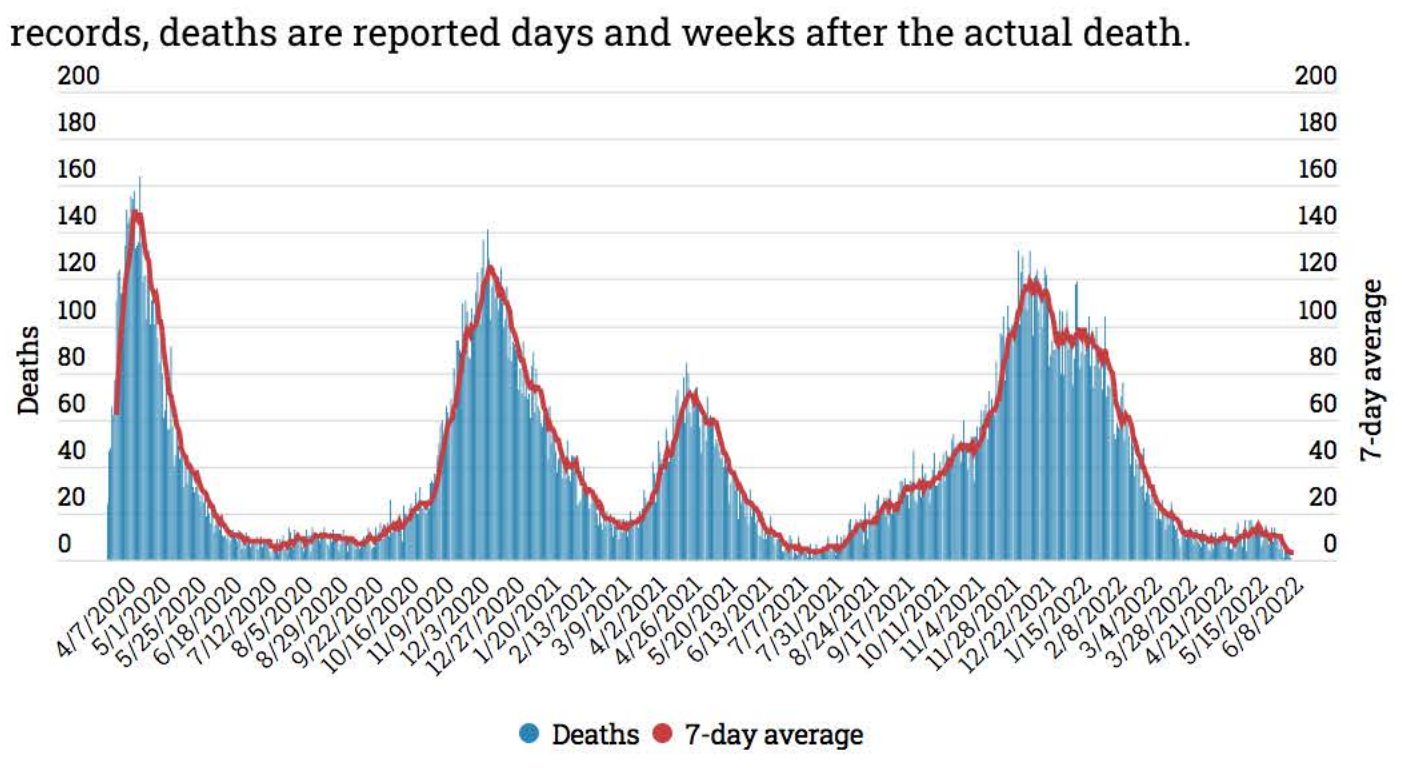

Situation Update for COVID-19

Subscribe: COVID-19 updates
Sign up to receive email or mobile alerts when this data is updated, as well as other periodic COVID-19 updates.

Updated June 10, 2022
Updated weekdays at 11 a.m., with data current as of 4 a.m. the previous business day.

Data is for cases that were tested and returned positive. At-home test results are not counted by MDH.

All data is preliminary and may change as cases are investigated. Many data points are collected through an online survey or during case interviews; however, not all cases are interviewed. Case interviews are focused on people under 18 years old, hospitalizations, deaths, and people with vaccine breakthrough or variants. Data presented below is for all cases, regardless of interview status.

Positive PCR test results are considered confirmed cases. Positive antigen test results are considered probable cases. All probable cases get the same public health follow up and recommendations as cases confirmed by PCR tests.

On this page:
Daily Update
Minnesota Case Overview
Case Demographics
Residence
Weekly Report
Vaccine Breakthrough Report
Other COVID-19 Data and Statistics

Expand All Contract All

Daily Update:

Because all data is preliminary and some reports require verification before counting as a case, the change in number of cumulative positive cases and deaths from one day to the next may not exactly equal the newly reported cases or deaths.

We continuously receive case reports and work to confirm, process, and report them as quickly as possible. Newly reported cases may not include every case reported to us since the last data update.

Total positive cases, including reinfections (cumulative)	1,527,145
Total positive people (cumulative)	1,451,355
Newly reported cases	2,028

Newly reported cases detail

Total deaths (cumulative)	12,713
Newly reported deaths	12

Newly reported deaths detail

Minnesota Case Overview

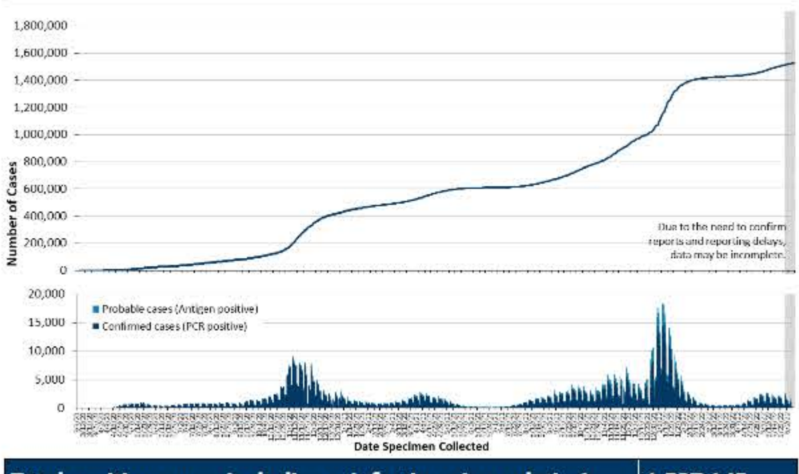

Total positive cases, including reinfections (cumulative)	1,527,145
Total confirmed cases (PCR positive) (cumulative)	1,312,773
Total probable cases (Antigen positive) (cumulative)	214,372

Positive cases by date specimen collected data table

Total positive people (cumulative)	1,451,355
Total number of reinfections (cumulative)	75,790

Reinfections by date specimen collected data table

More about positive cases, positive people, and reinfections

Cases no longer needing isolation

Cases no longer needing isolation (cumulative)	1,500,603

More about cases no longer needing isolation

Deaths

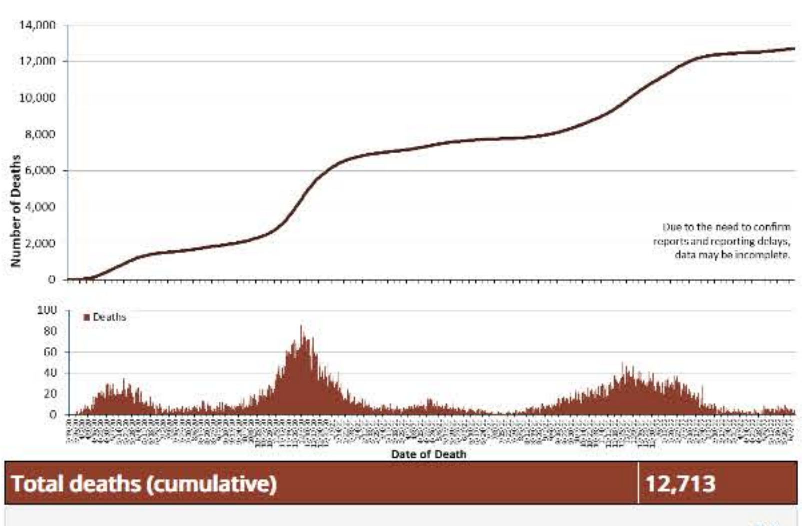

Total deaths (cumulative)	12,713

Deaths data table

Total non-laboratory-confirmed COVID-19 deaths (death certificate) (cumulative)	316
Deaths among cases that resided in long-term care or assisted living facilities (cumulative)	5,808

More about deaths

Hospitalizations

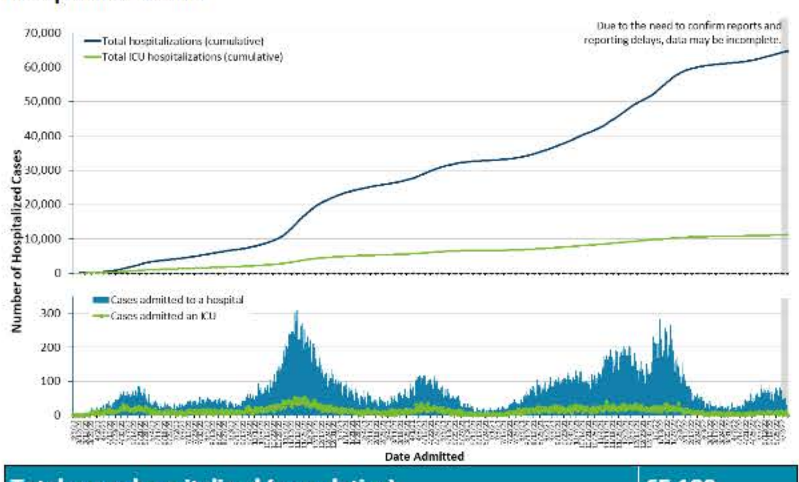

Total cases hospitalized (cumulative)	65,180
Total cases hospitalized in ICU (cumulative)	11,789

Hospitalization data table

More about hospitalizations

Case Demographics

Age

Age group data table:
Including age group of deaths

More about age

Gender

Gender data table

More about gender

Race & Ethnicity

Race and ethnicity data table:
Including race and ethnicity of deaths

More about race and ethnicity

Residence

Cases by County of Residence

County of residence is confirmed during the case interview. At the time of this posting not all interviews have been completed.

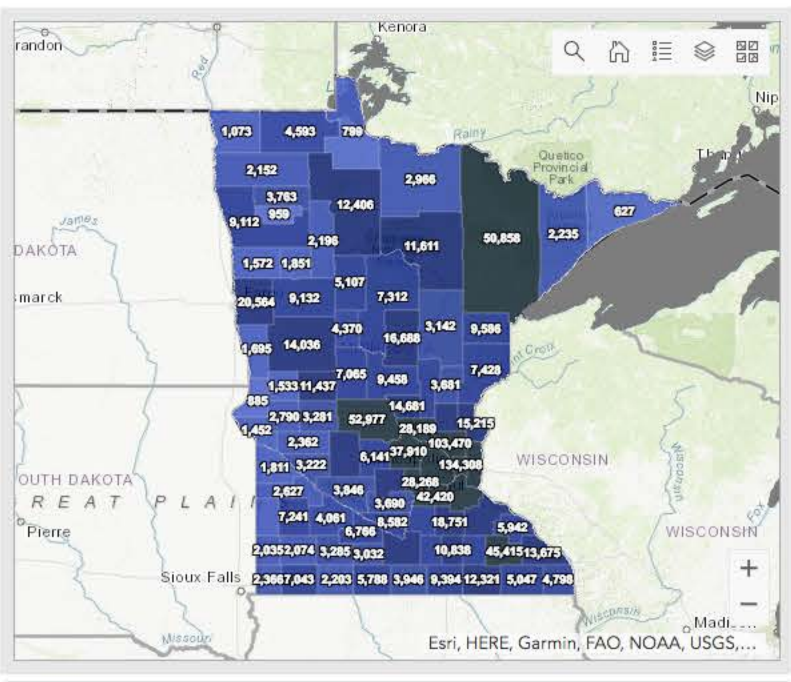

County of residence data table:
Including county of residence of deaths

More about location data

As of 10/28/21, likely exposure and residence type are no longer collected for all cases and therefore no longer reported. Archived data can be found in the COVID-19 Weekly Report.

Weekly Report

The COVID-19 Weekly Report includes more detailed information on testing, demographics, syndromic surveillance, variants, and more. Updated Thursdays.

- Weekly COVID-19 Report: 6/9/2022 (PDF)
  - COVID-19 Weekly Report
    Archive of past weekly reports and some downloadable data.

As of 10/28/21, Cases in Pre-K through Grade 12 School Buildings, by County and Congregate Care Facilities with Exposures, by County can be found with the COVID-19 Weekly Report.

Vaccine Breakthrough Report

This report includes information on vaccine breakthrough cases in Minnesota. Updated Mondays.

- COVID-19 Vaccine Breakthrough Weekly Update
  Cases in people fully vaccinated for COVID.

Other Statistics

- Minnesota COVID-19 Public Dashboard
  - Response and Preparation Capacity
  - Public Health Risk Measures (Dial Back Dashboard)
  - Minnesota COVID-19 Modeling
  - Vaccine Data
- CDC: COVID Data Tracker
- Metropolitan Council: Tracking COVID-19 Prevalence with Metro Plant Wastewater
- U of M: Wastewater SARS-CoV2 Surveillance Study
- Multisystem Inflammatory Syndrome In Children (MIS-C) Statistics
- COVID-19 Vaccine Data: Provider Registration

Dynamic images and maps will not work if your browser is in compatibility mode and may update after the data on the page.

COVID-19 Public Hotline:
1-833-431-2053
Mon.-Fri.: 9 a.m. to 7 p.m.
Sat.: 10 a.m. to 6 p.m.
ASL option at Contact Us

Mental Health Crisis Line - Call: **CRISIS (**274747)

COVID-19 information in other languages:
- Kab Mob Khaus Las Nas Vais Lav 2019 (COVID-19) (Hmong)
- Cudurka Koronafayras 2019 (COVID-19) (Somali)
- Enfermedad del Coronavirus 2019 (COVID-19) (Spanish)
- American Sign Language (ASL) Videos

Contact us:
If you have questions or comments about this page, use our IDEPC Comment Form or call 651-201-5414 for the MDH Infectious Disease Epidemiology, Prevention and Control Division.

E-Newsletter
Get COVID-19 updates and public health alerts when you subscribe to our e-mail newsletter.
email address
SUBSCRIBE

Interactive Maps: Mississippi COVID-19 Cases and Incidence

Select Language

▸ More COVID-19 charts and data

Current COVID-19 Activity: Two-Week Case Incidence

Full screen

Mississippi COVID-19 Two-Week Cases* Incidence Rate**
over 2/15/22 - 2/28/22

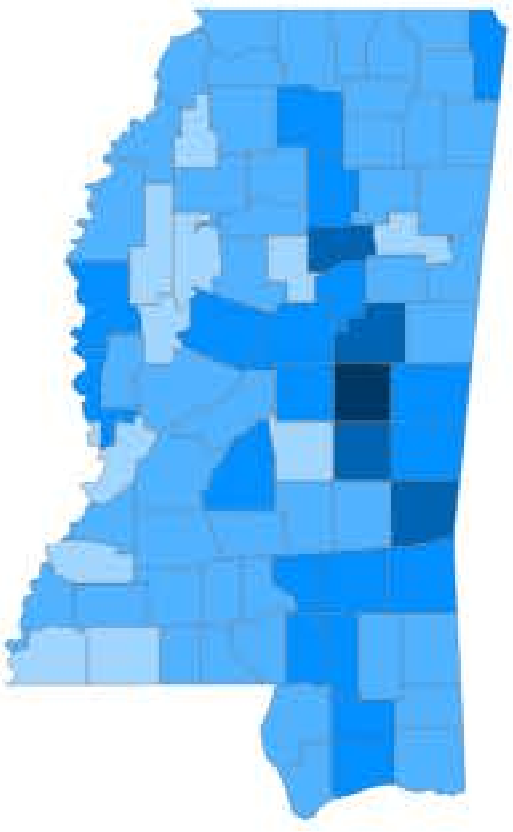

Mississippi

Two-Week New Cases* 5231

Two-Week Cases* Incidence Rate Per 100,000 175.8

Total Cases* through 2/28/22 790,964

Percent Change 0.7%

County	Two-Week New Cases	Two-Week Cases Rate Per 100,000	Cases to Date	Percent Change
NESHOBA	185	635.3	10,555	2.0%
NEWTON	83	394.9	5,978	1.0%
WINSTON	69	384.3	5,194	1.0%
WEBSTER	34	350.9	3,374	1.0%
CLARKE	47	302.4	4,205	1.0%
COVINGTON	55	295.1	7,052	1.0%
LAUDERDALE	201	271.2	18,706	1.0%
[illegible]	[illegible]	[illegible]	[illegible]	[illegible]

* Cases based on **Event Date**. Event date is date of onset, date of collection, or date of report to MSDH, updated as cases are investigated.

** Rate calculation is based on cumulative cases of past two weeks per 100,000 population.

- + 61%

Microsoft Power BI

Cumulative COVID-19 Cases and Deaths

Full screen

Mississippi COVID-19
Cases and Deaths by Race with Ethnicity
as of 11:59 PM CT, March 7, 2022

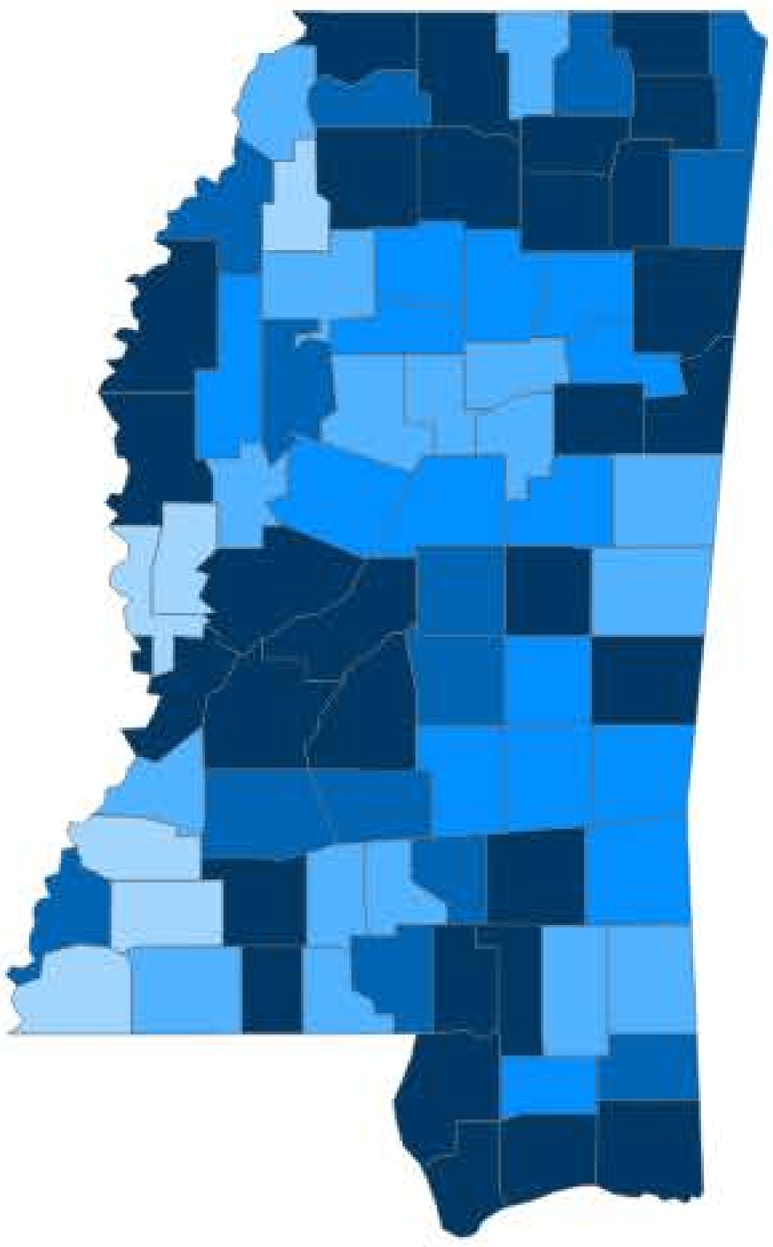

Mississippi

Total Cases

791,966

	American Indian or Alaska Native	Asian	Black	White	Other	Unknown
Non Hispanic	6,526	3,443	215,232	346,926	9,549	9,549
Hispanic	204	152	1,168	5,114	8,242	2,265
Unknown Ethnicity	1,428	754	31,548	52,763	17,710	79,393

Total Deaths

12,206

	American Indian or Alaska Native	Asian	Black	White	Other	Unknown
Non Hispanic	107	25	4,012	6,174	96	31
Hispanic	1	2	12	55	60	5
Unknown Ethnicity	26	11	463	789	116	221

- + 92%

Microsoft Power BI

Public Health

DHSS Home » Healthy Living » Health Conditions & Diseases » Communicable Diseases » COVID-19 » Data » public-health

Have questions? Here are answers to the most **frequently asked questions** about our data and methodology. Data for download is also available **here**.

Learn more about Missouri's COVID-19 response

Missouri Weekly COVID-19 Activity Report

Dashboard is updated weekly every Friday morning. Data last updated on ***6/10/2022*** *and the 7-day range reflects* ***6/2/2022*** *through* ***6/8/2022****. All data presented are provisional and subject to change as additional reports are received.*

COVID-19 Community Levels

Regional COVID-19 Community Levels:*

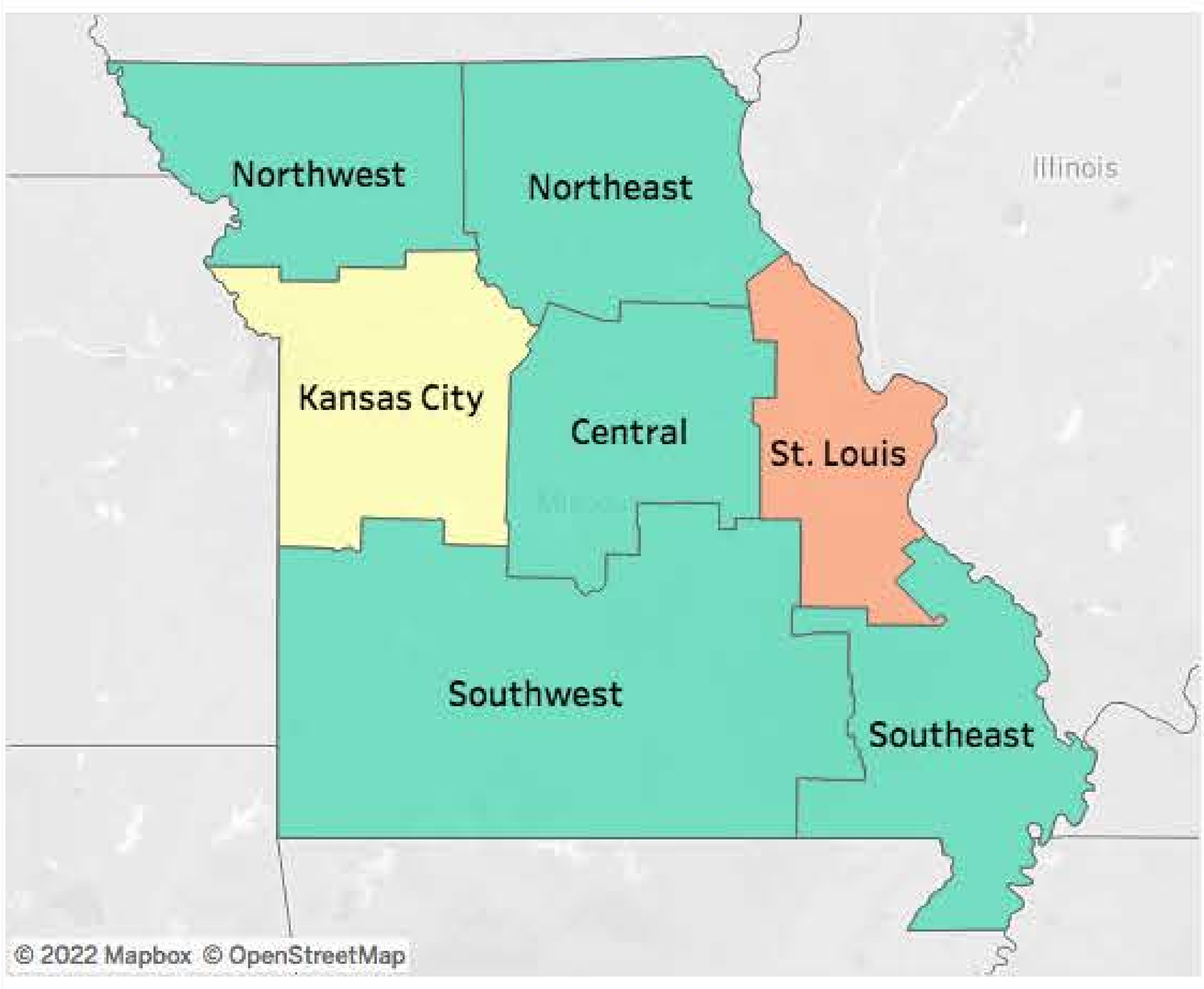

**Regional data provided by the Missouri Hospital Association.*

County COVID-19 Community Levels:**

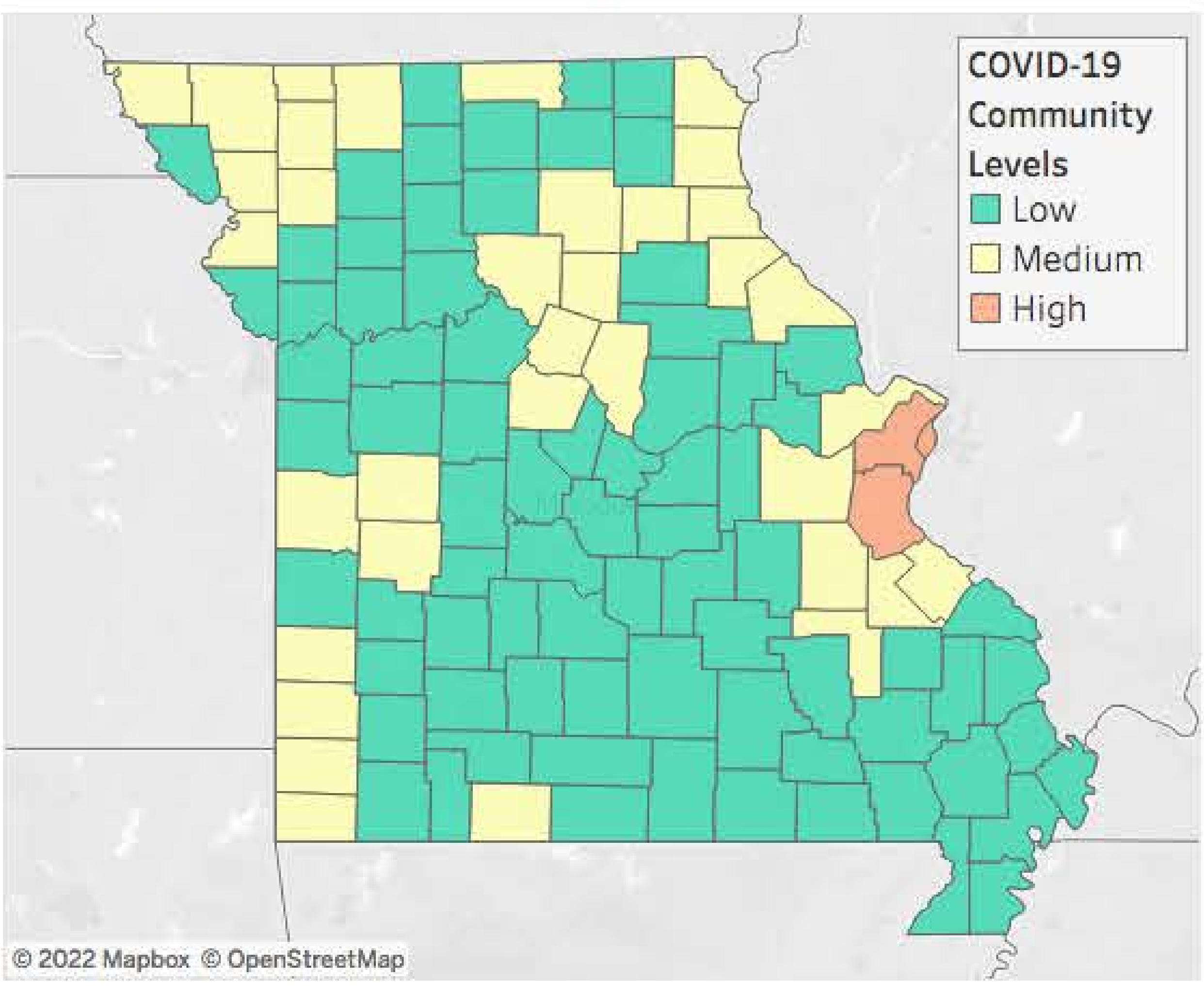

*** County data provided by the CDC. For more information about COVID-19 community levels, please see the* CDC website here

Hospitalizations

Current COVID-19 Hospitalized Patients (as of 6/8/2022):
680

Choose Hospitalization Status:
- COVID-19 Hospitalized Patients
- COVID-19 ICU Patients

Use Slider to Filter Dates Below:
7/11/2020 — 6/8/2022

COVID-19 Hospitalized Patients by Day

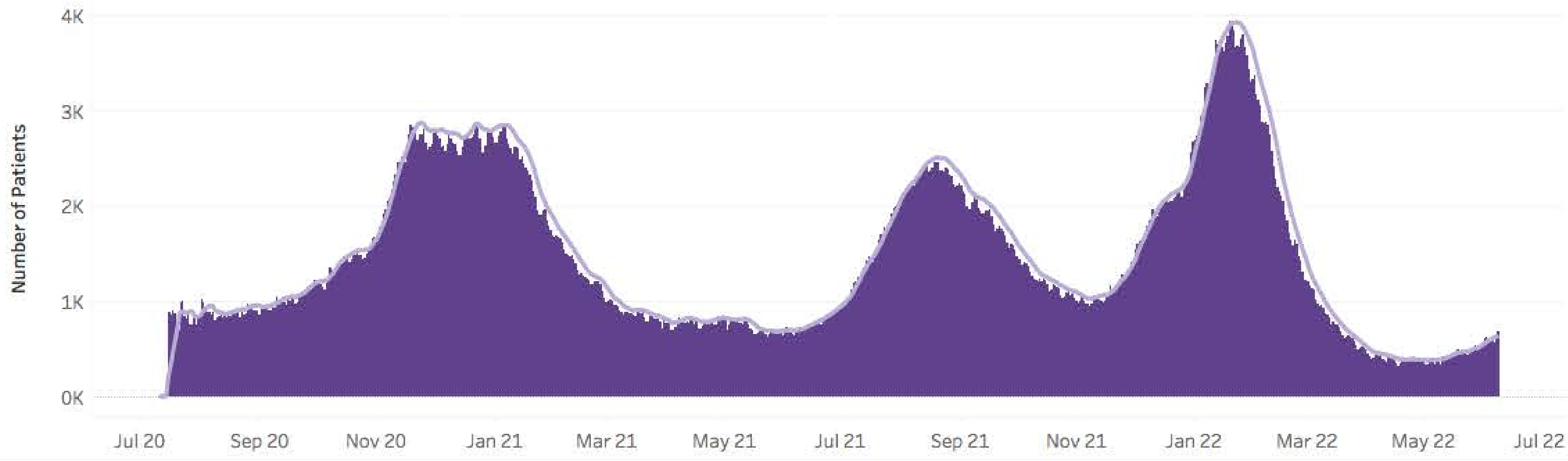

Cases and Deaths

Cases in the Last 7 Days: 9,731

Cases in the Last 7 Days, by Age Group:

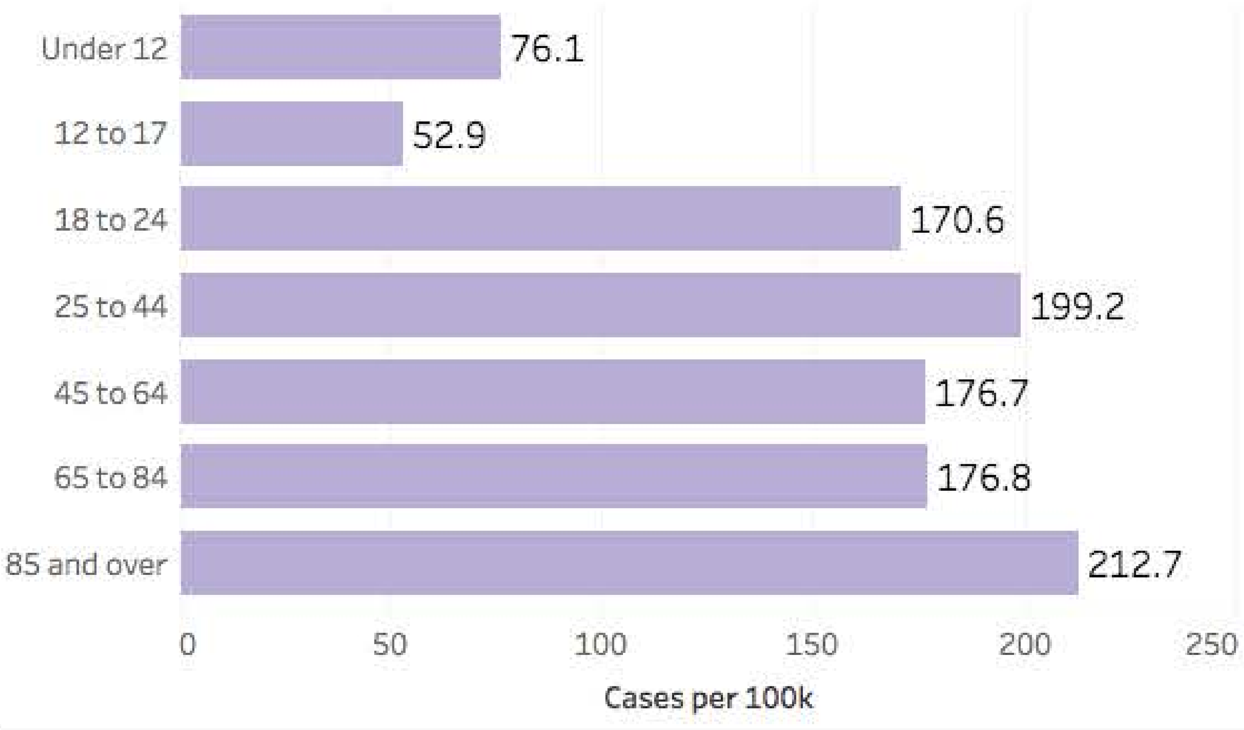

Case Rate per 100k in the Last 7 Days, by Jurisdiction of Residence:

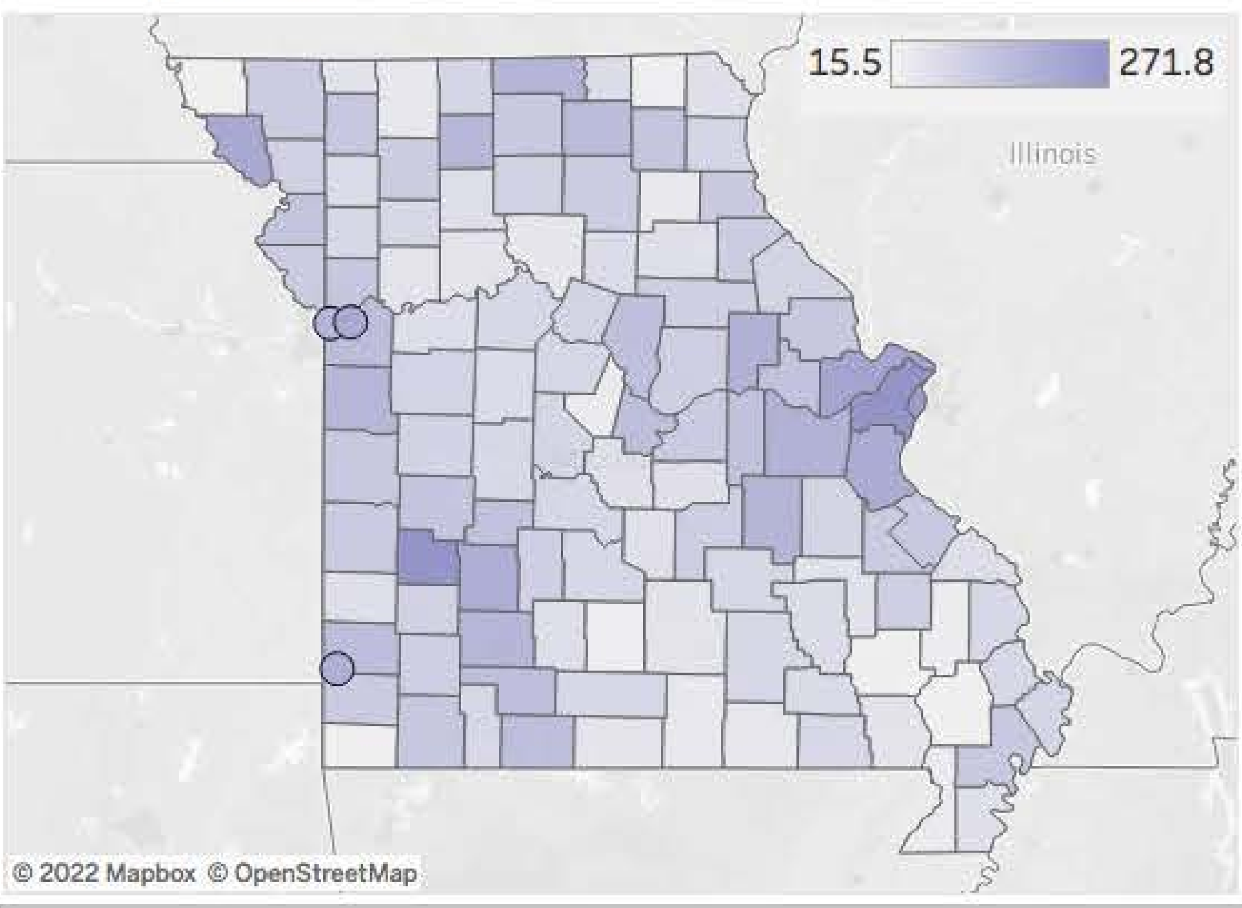

Statewide COVID-19 Cases, by Week:

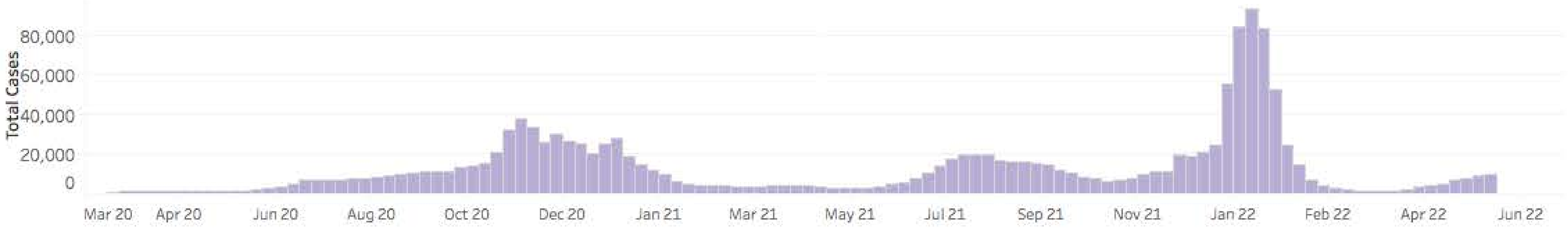

Statewide COVID-19 Deaths, by Week:

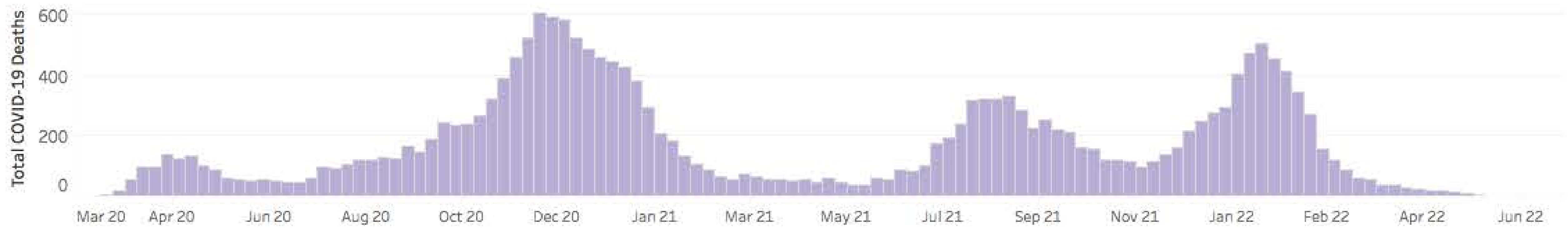

Variants

Select a Week Below to View Variant Details:
5/22/2022

Variant Details for Week 5/22/2022:

Variant	Lineage	
Omicron	BA.2	66.7%
	BA.2.12.1	33.3%

SARS-CoV2 Variants in Missouri, by Week:

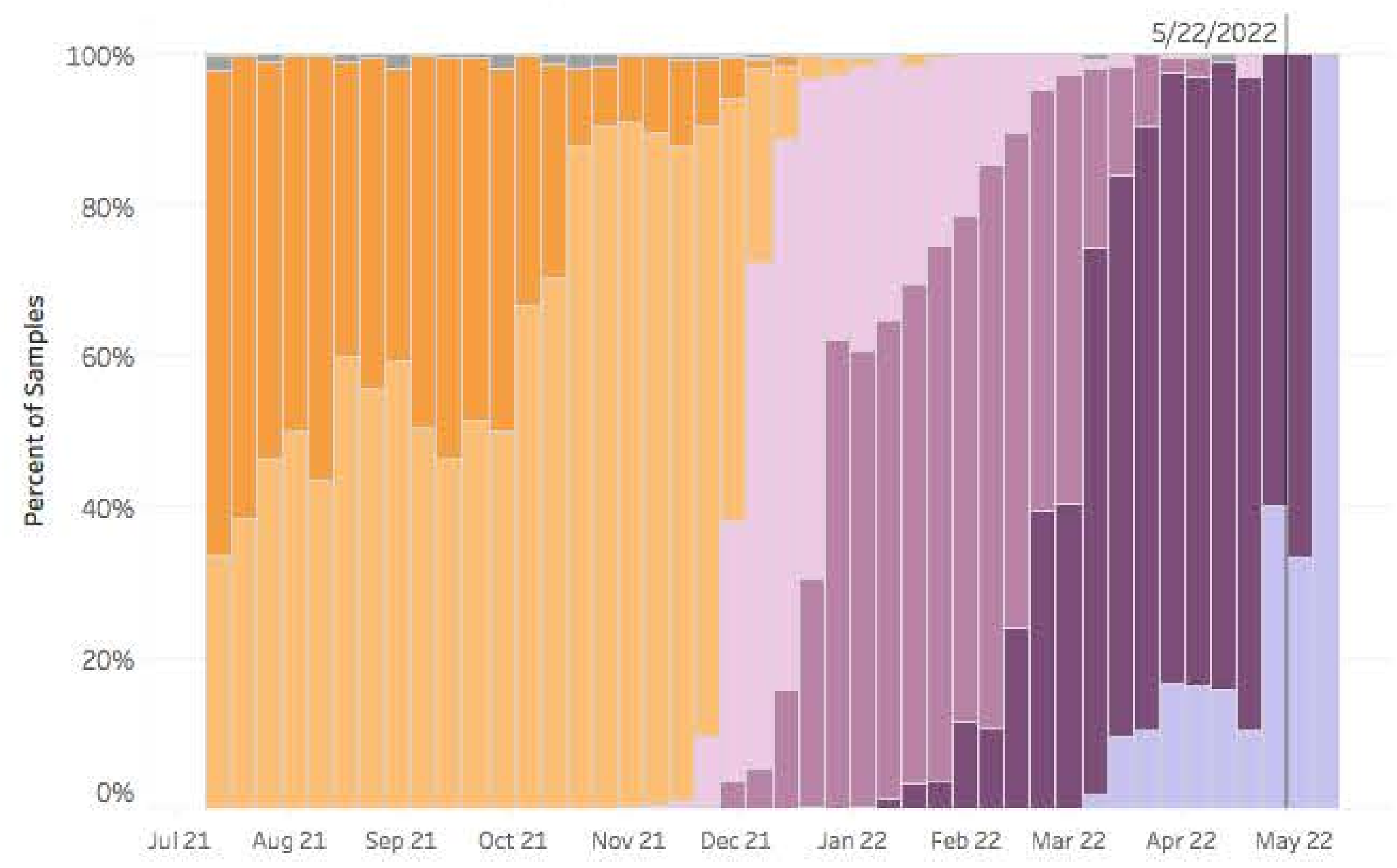

MONTANA RESPONSE: COVID-19 - Coronavirus - Global, National, and State Information Resources

Montana Maps

COVID-19 Community Levels and Cases | COVID-19 Vaccine | Local Vaccine Contacts | COVID-19 Demographics | Global Outbreak Dashboard | GIS Data Hub

Applications
Melissa
Downloads
Devices
Melissa's MacBoo...
Shared

Last updated: 6/10/2022, 12:07 PM

Cumulative Cases

280,965

Active Cases

2,123

Newly Reported Cases

1,964

in the last week

Cumulative, New Weekly Reported, Active Cases by County

Gallatin County Cases:
36,060 Total | 424 Newly Reported | 579 Active

Missoula County Cases:
28,440 Total | 219 Newly Reported | 272 Active

Lewis and Clark County Cases:
18,257 Total | 211 Newly Reported | 78 Active

Yellowstone County Cases:
43,713 Total | 210 Newly Reported | 308 Active

Flathead County Cases:
29,787 Total | 160 Newly Reported | 81 Active

Cascade County Cases:
25,594 Total | 128 Newly Reported | 178 Active

Lake County Cases:
6,787 Total | 63 Newly Reported | 54 Active

Park County Cases:
4,445 Total | 55 Newly Reported | 36 Active

Silver Bow County Cases:
8,629 Total | 42 Newly Reported | 52 Active

Ravalli County Cases:
7,198 Total | 37 Newly Reported | 56 Active

Glacier County Cases:
4,076 Total | 29 Newly Reported | 14 Active

Lincoln County Cases:
4,872 Total | 29 Newly Reported | 45 Active

Hill County Cases:
4,635 Total | 26 Newly Reported | 17 Active

Blaine County Cases:

Montana Coronavirus Community Levels by County

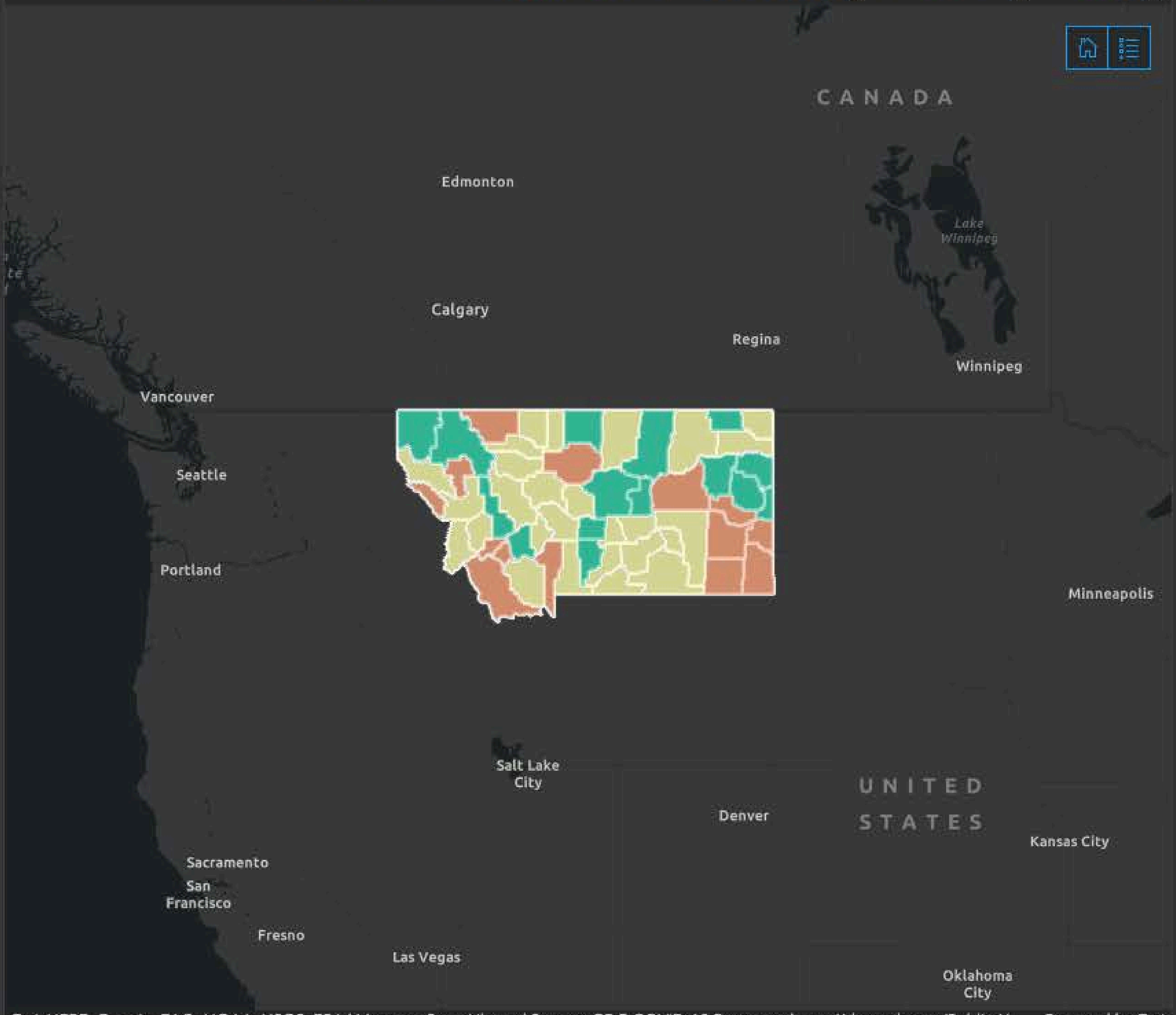

Esri, HERE, Garmin, FAO, NOAA, USGS, EPA | Montana State Library | Source: CDC COVID-19 Response https://data.cdc.gov/Public-H... Powered by Esri

Community Levels | Active Cases | Cumulative Cases | More Information

Total Deaths

3,434

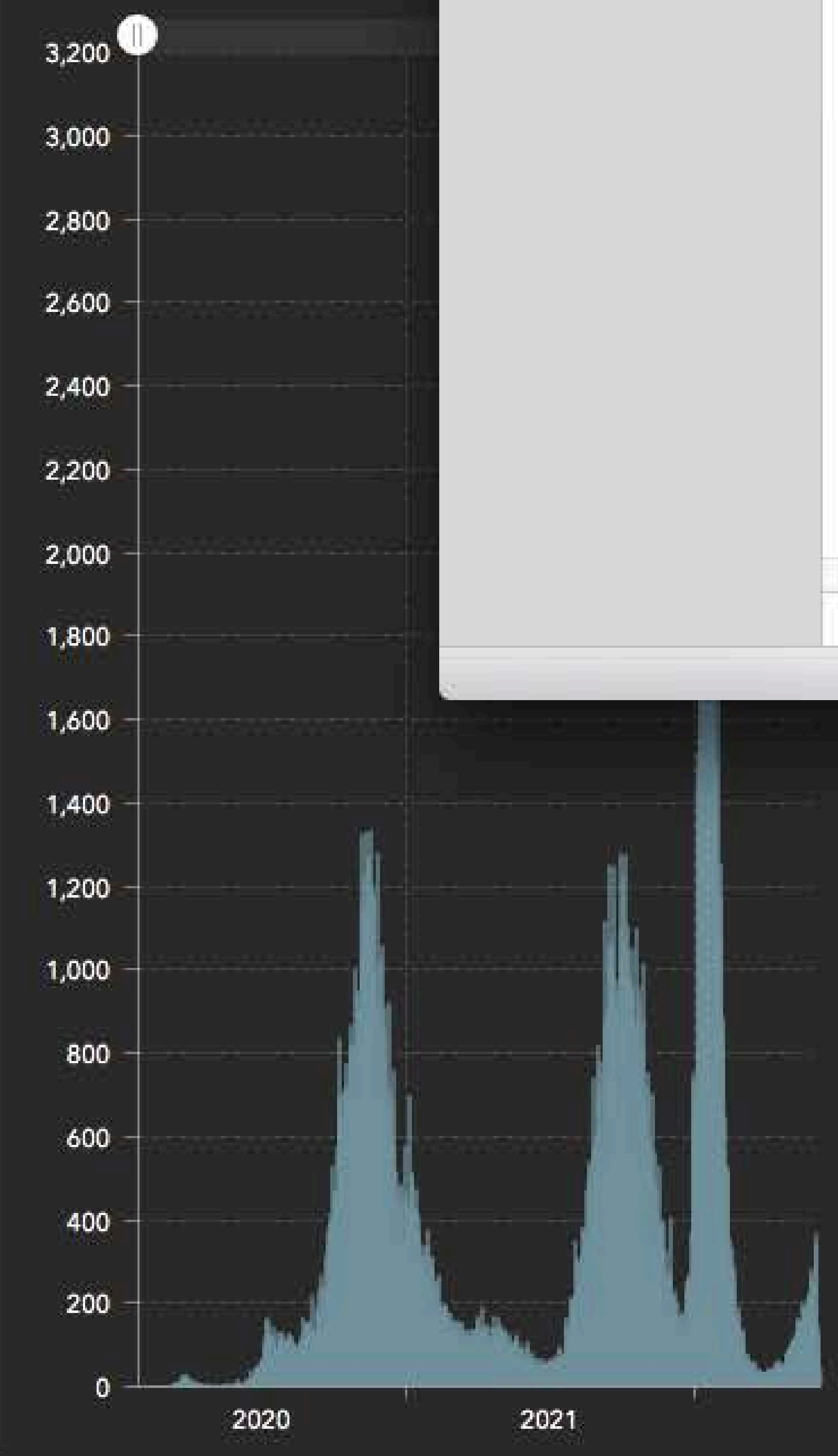

Cases by Diagnosis Date

Hospitalizations and ICU Bed Occupancy

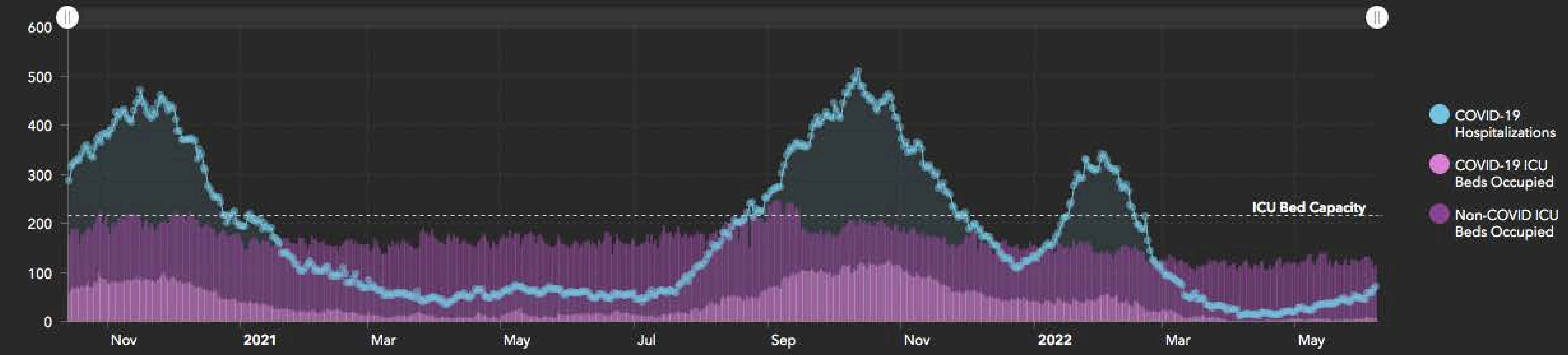

MONTANA RESPONSE: COVID-19 - Coronavirus - Global, National, and State Information Resources

Last updated: 6/10/2022, 12:08 PM

Total Doses Administered

1,486,799

Total Montanans Fully Immunized

566,205

Eligible Population Fully Vaccinated

55%

COVID-19 Vaccinations By County

Beaverhead County Vaccines:
12,966 Doses Given | **5,153 (57%)** Fully Immunized

Big Horn County Vaccines:
18,625 Doses Given | **7,549 (63%)** Fully Immunized

Blaine County Vaccines:
8,704 Doses Given | **3,383 (56%)** Fully Immunized

Broadwater County Vaccines:
5,326 Doses Given | **2,013 (33%)** Fully Immunized

Carbon County Vaccines:
13,537 Doses Given | **5,174 (49%)** Fully Immunized

Carter County Vaccines:
908 Doses Given | **360 (32%)** Fully Immunized

Cascade County Vaccines:
99,381 Doses Given | **38,710 (51%)** Fully Immunized

Chouteau County Vaccines:
5,645 Doses Given | **2,084 (38%)** Fully Immunized

Custer County Vaccines:
12,470 Doses Given | **4,902 (46%)** Fully Immunized

Daniels County Vaccines:
1,795 Doses Given | **710 (46%)** Fully Immunized

Dawson County Vaccines:

COVID-19 Vaccinations by County of Residence: Percent of Eligible Population Fully Vaccinated

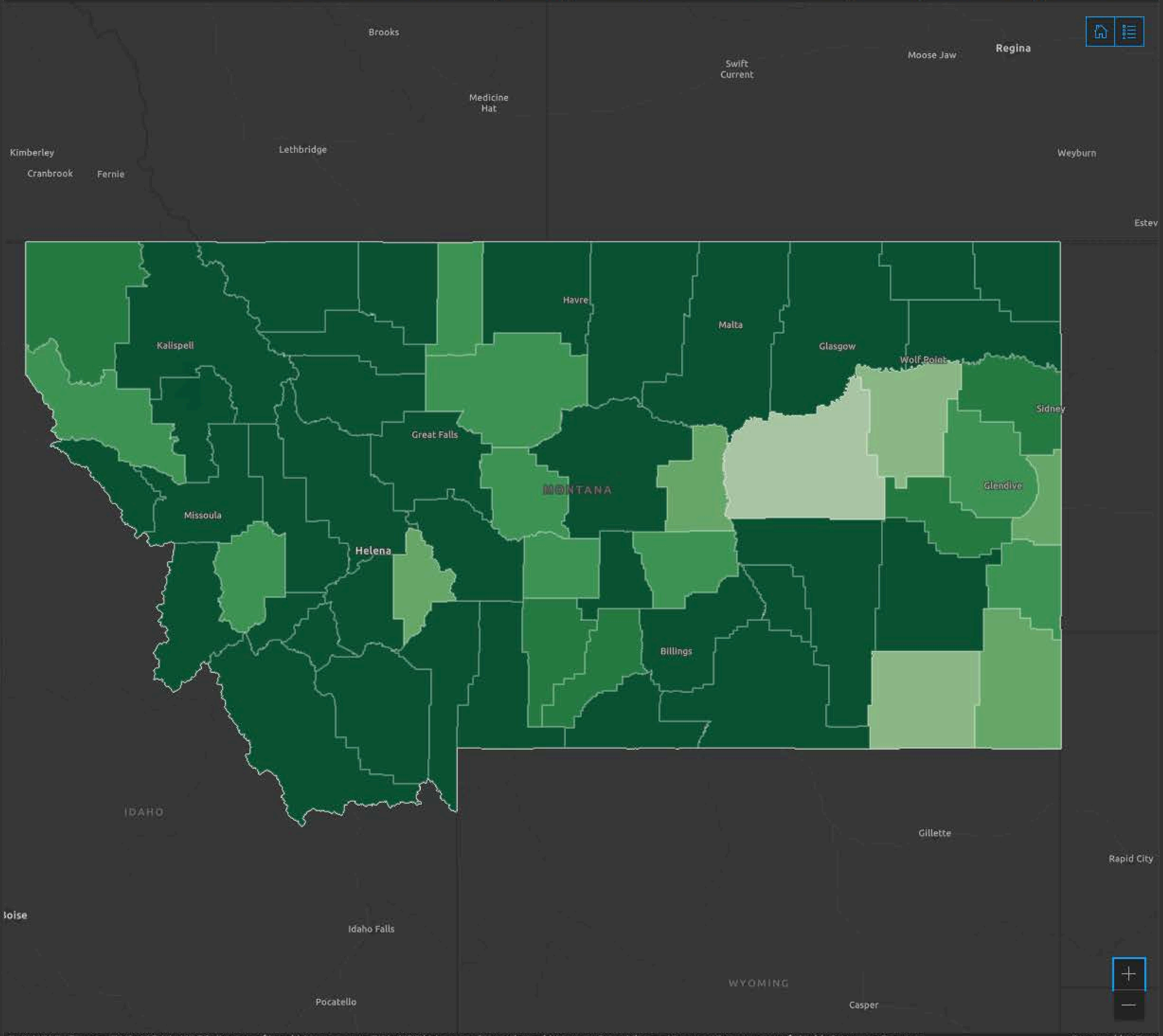

Esri, HERE, Garmin, FAO, NOAA, USGS, Bureau of Land Management, EPA, NPS | Montana State Library | Montana State Library, Montana Department of Health & Human Services

Powered by Esri

Vaccines | More Information

Cases and Deaths Dashboard

Delays in laboratories reporting tests to NCDHHS may occur as laboratories are processing unprecedented numbers of COVID-19 tests.

We want to hear from you! Take the NCDHHS Dashboard Survey today!

Dashboards are best viewed on a desktop or laptop. However, if viewing dashboards on a mobile device, please rotate your device horizontally, to landscape view, for a better mobile experience.

View Larger

All COVID-19 Dashboards

- Summary
- Vaccinations
- Respiratory Virus Surveillance
- Cases and Deaths
- Hospitalizations
- Outbreaks
- Wastewater Monitoring
- Reports
- Data Behind the Dashboards

Weekly Cases and Deaths Summary

Weekly Cases and Deaths Summary Data: March 1, 2020 – June 4, 2022

Updated every Wednesday

Cases and Deaths Summary | Cases and Deaths Demographics

Cases and Deaths by Week

Select County: North Carolina

Select Week Ending Date: 3/7/2020

Cases by Week of Specimen Collection for North Carolina - 3/7/2020 through Week Ending 6/4/2022

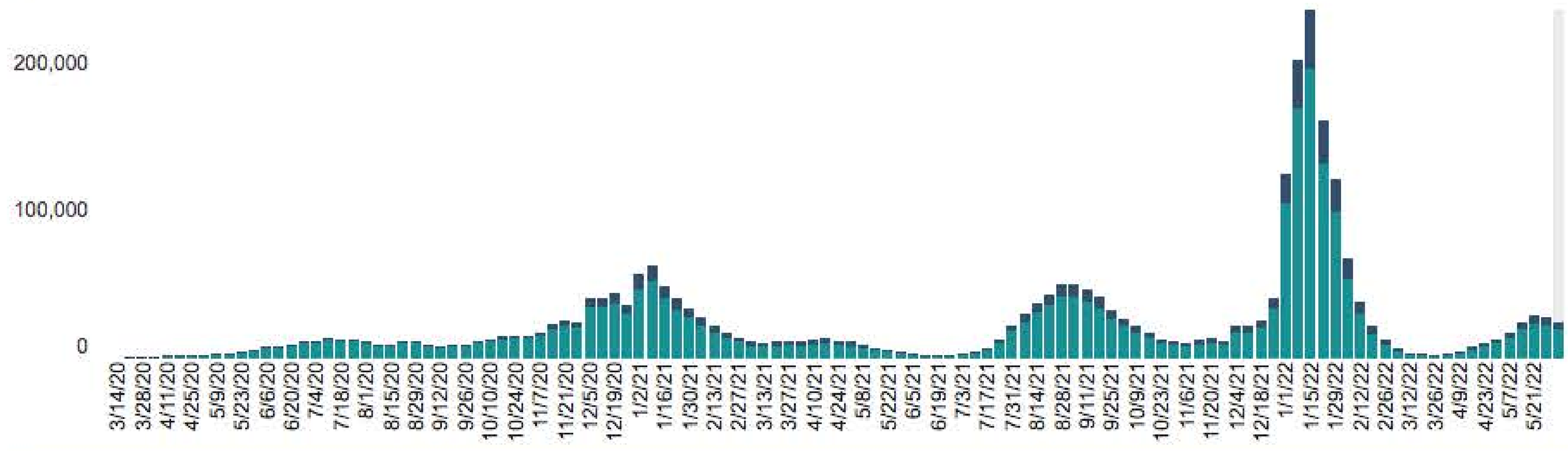

North Carolina
Total Cases
2,799,515

Molecular (PCR) Positive Cases
2,330,604

Antigen Positive Cases
468,911

Specimen collection date missing for 109 of the total cases.

Deaths by Week of Death for North Carolina - 3/7/2020 through Week Ending 6/4/2022

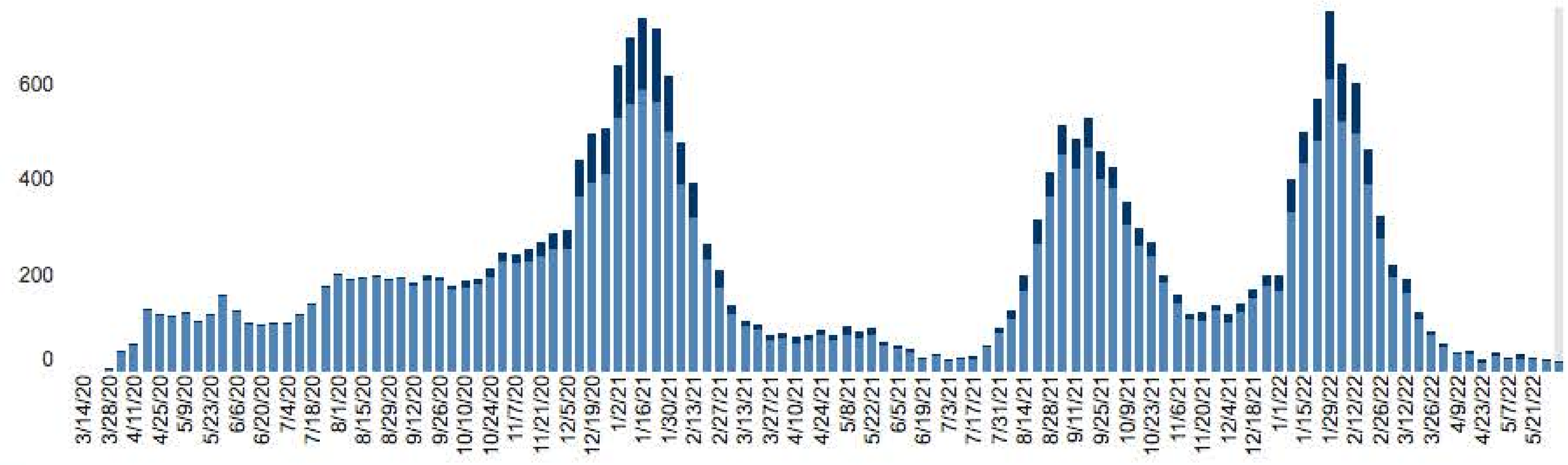

North Carolina
Total Deaths
25,094

Molecular (PCR) Positive Deaths
21,864

Antigen Positive Deaths
3,230

Date of death missing for 2 of the total deaths.

County Map by Cases per 100,000 Residents (Molecular (PCR) and Antigen)
(Click to highlight map, unclick to revert map)

Select Geography
- County
- ZIP Code

Select Date Range: Total Cases - Last Two Weeks

Search for County

Select Metric
- Cases
- Cases per 10,000 Residents
- Cases per 100,000 Residents

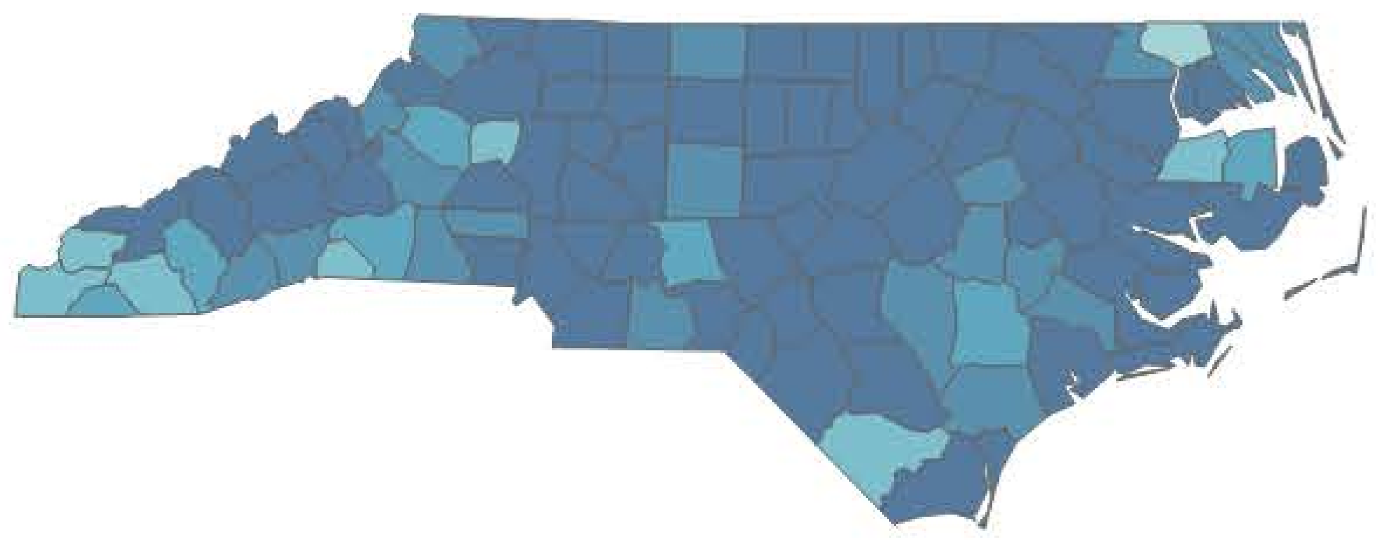

0 | 1 - 169 | 170 - 214 | 215 - 276 | 277 - 352 | 353+

Total County Cases and Deaths (Molecular (PCR) and Antigen)
(Click to highlight map, unclick to revert map)

County	Cases	Deaths
Alamance	49,661	496
Alexander	10,635	145
Alleghany	3,065	17
Anson	6,707	102
Ashe	6,613	84
Avery	4,722	46
Beaufort	13,029	177
Bertie	4,397	70
[illegible]	[illegible]	[illegible]

Molecular (PCR) positive cases represent confirmed cases, and antigen positive cases represent probable cases of COVID-19, in accordance with CDC case classification guidelines. The terms "confirmed" and "probable" are used nationally to standardize case classifications for public health surveillance but should not be used to interpret the utility or validity of any laboratory test type.

Total Cases do not represent all people with COVID-19 in North Carolina as some people do not get tested and many people use at-home tests that are not reported to the State.

Hover over icons for information. June 8, 2022

+ableau

To download tabular data displayed in the NCDHHS dashboards please go to Data Behind the Dashboards.

North Carolina COVID-19 Dashboard

North Carolina has relied on science, timely data, and key metrics to guide its pandemic response. Because no one metric captures everything, we use a combination of metrics to guide our approach. The most important of these are below.

As trends rise and fall, you can choose to add layers of protection to mitigate your risk. Get tips on layering protection.

Update: This dashboard's new format launched on March 23, 2022 and will be updated every Wednesday. More about the new design.

For more information on the data shown here and why they're important, see "These Measures and Why They Were Chosen," below.

We want to hear from you! Take the NCDHHS Dashboard Survey today!

All COVID-19 Dashboards

- **Summary**
- Vaccinations
- Respiratory Virus Surveillance
- Cases and Deaths
- Hospitalizations
- Outbreaks
- Wastewater Monitoring
- Reports
- Data Behind the Dashboards

Updated Every Wednesday by approximately 12:00 p.m.
Last updated June 8, 2022 at 12:15 p.m.

Early Warning Indicators

Rising levels of these can be an early sign of community spread and illness.

27.3 Million → Previous Week 26.5 Million

COVID-19 Virus Particles Found in Wastewater

COVID-19 virus particles appearing in wastewater can signal how quickly the virus is spreading, even if people don't get tested or have symptoms.

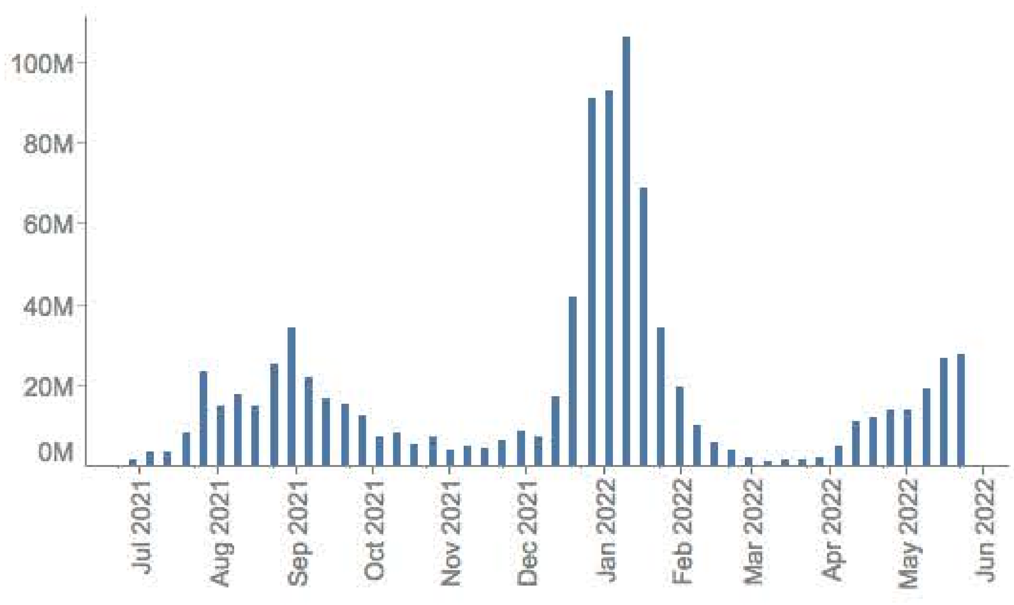

Average COVID-19 virus copies found per person per week from participating North Carolina wastewater treatment plants. More Info

5% → Previous Week 5%

Emergency Room Visits for COVID Symptoms

The percentage of all emergency department visits that are for COVID-like symptoms can signal how much illness there is in a community.

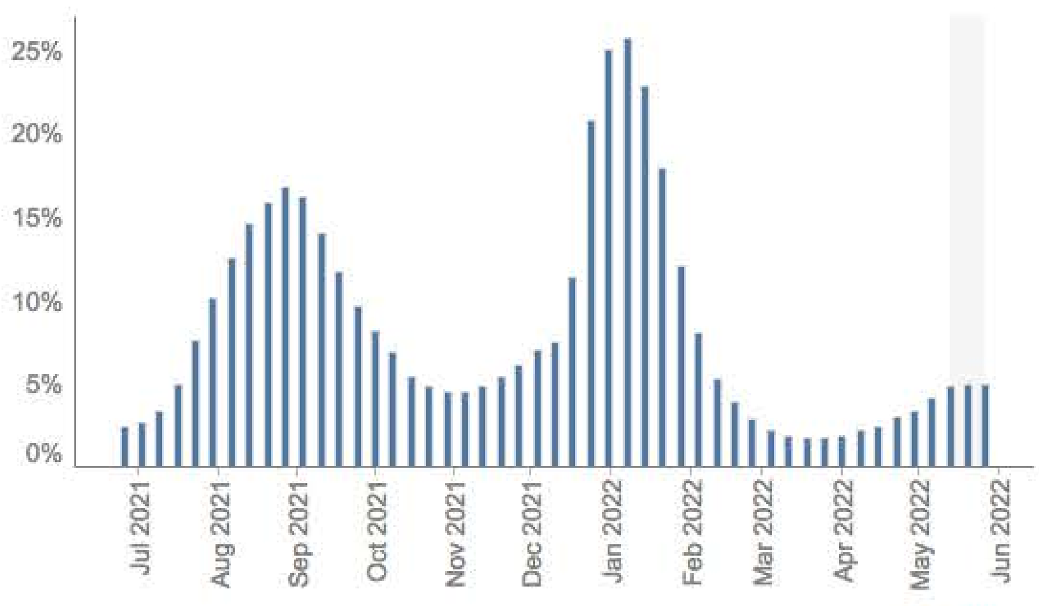

Emergency department visits that are for COVID-like illnesses (CLI). More Inf..

Health System Capacity

Rising levels of these can indicate strain on the health care system.

24,941 → Previous Week 27,552

COVID-19 Reported Cases by Week of Specimen Collection

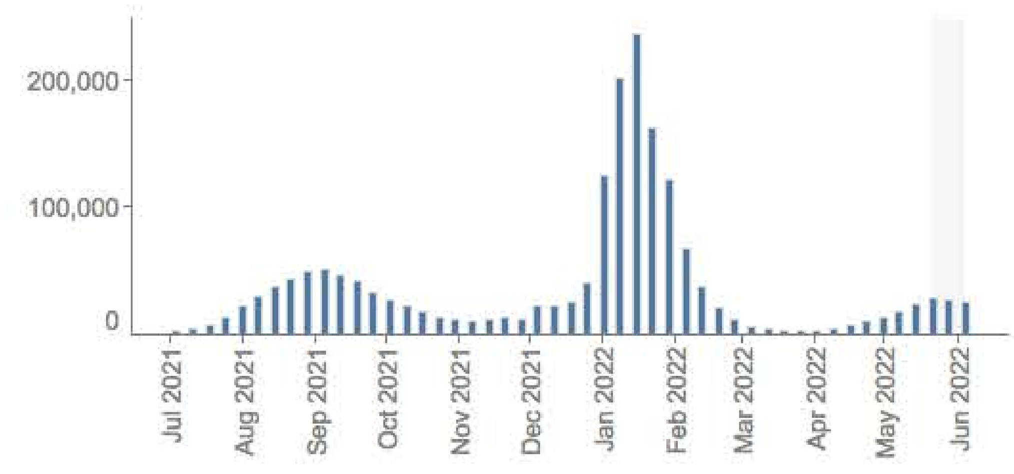

Number of new cases reported to the state each week, shown by the date specimen was collected. More Info

833 → Previous Week 735

Hospital Admissions - COVID-19 Patients by Week

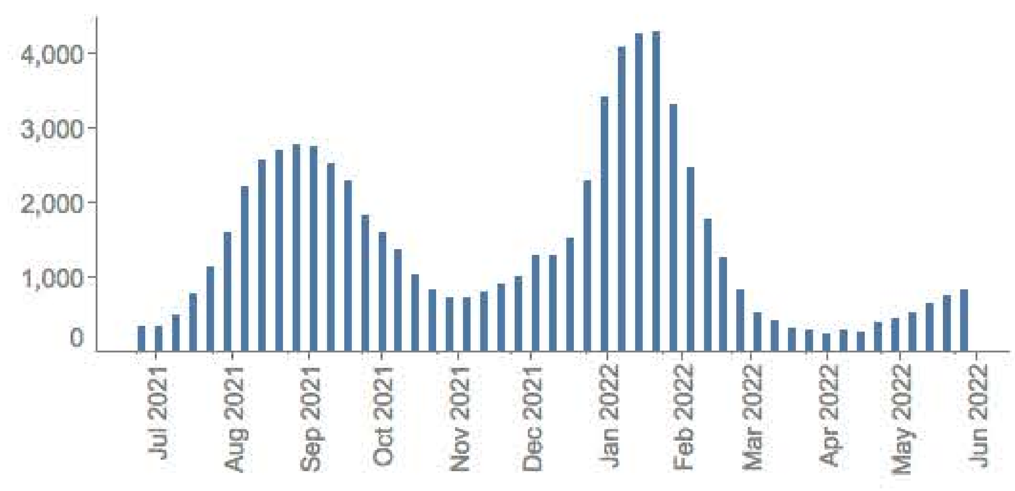

Number of confirmed COVID-19 patients admitted to hospitals each week. More info

Vaccines and Boosters

These numbers help us measure protection in the state. More info

56%
Boosted
Percent of Total Vaccinated Population with at Least One Booster or Additional Dose

77%
Adults Vaccinated
Percent of Total Adult Population Vaccinated with at Least One Dose

38%
Children and Teens Vaccinated
Percent of Population Ages 5-17 Vaccinated with at Least One Dose

Percent of Total Vaccinated Population with at Least One Booster or Additional Dose

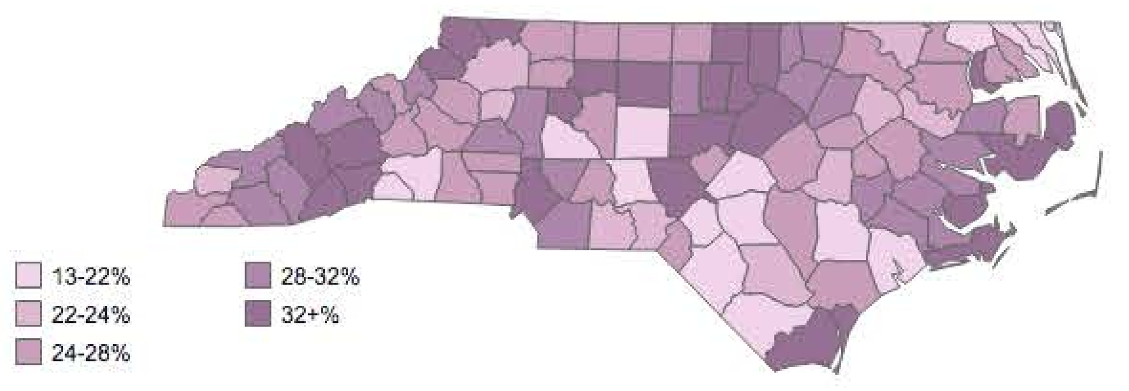

Surveillance of Variants

What variants are being detected in North Carolina?

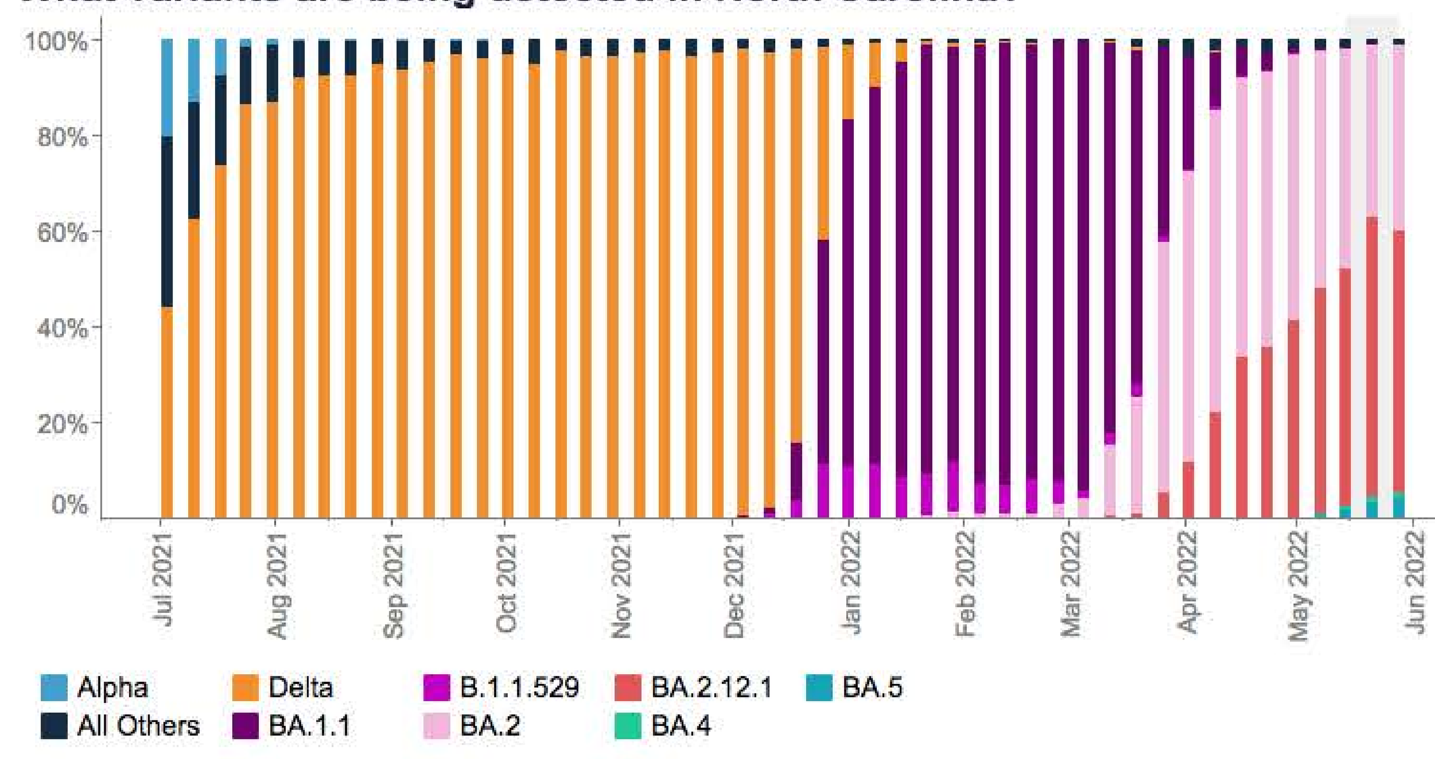

Last Two Weeks

May 15, 2022 - May 28, 2022

Category	Type	Sequenced Cases	% of Total
Omicron	BA.1.1	1	0.06%
	BA.2	575	36.79%
	BA.2.12.1	905	57.90%
	BA.4	16	1.02%
	BA.5	53	3.39%
Other	All Others	13	0.83%

Percentage of variants reported each week by laboratories that sequence to identify COVID-19 variants and report sequence data to GISAID. Sequencing is only performed on a small proportion of positive tests statewide and not all laboratories that sequence report results to GISAID.

Note: NCDHHS is currently working to improve processes for receiving sequencing data from labs. In the meantime, this dashboard will report variant information reported to GISAID instead of data reported electronically to NCDHHS. National and regional variant proportions are available at covid.cdc.gov/covid-data-tracker/#variant-proportions. More info

+ a b l e a u

CDC COVID-19 Community Levels by County

24/100 Previous Week 13/100

Counties with a high risk of illness and strain on the health care system

The Centers for Disease Control assign a community risk level for every county. More info *or* find your county

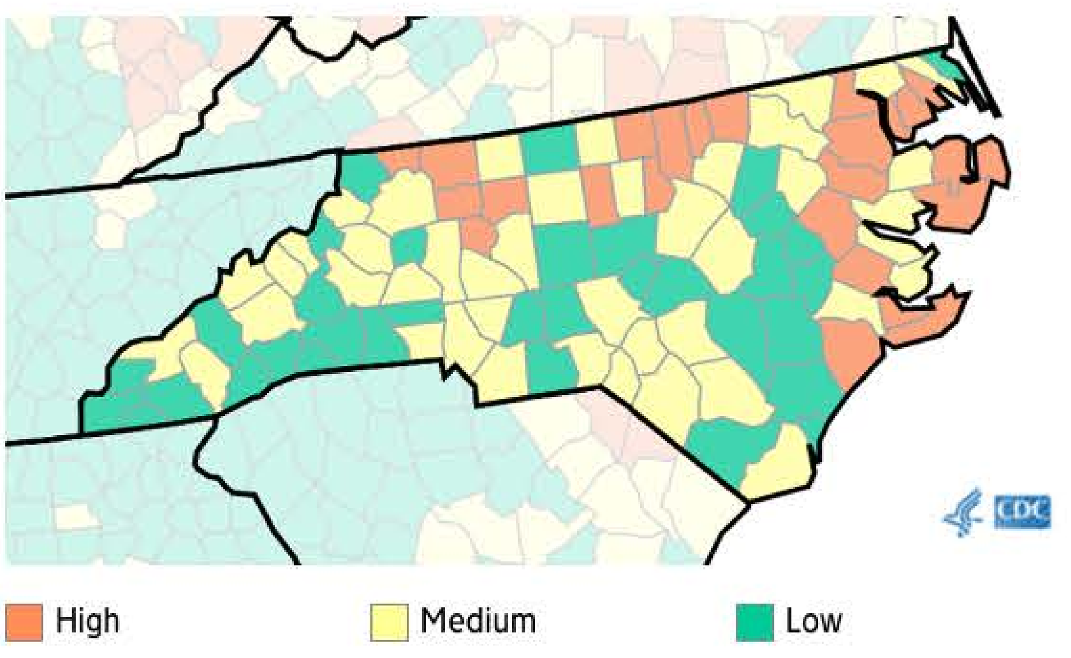

Vaccinations Dashboard

We want to hear from you! Take the [NCDHHS Dashboard Survey] today!

This dashboard shows the total number of doses that have been administered by NC providers and through the federal pharmacy program. The information will be available on the tabs "Summary Data" and "Demographics Data" on the dashboard through a drop-down option that allows users to view data for NC Providers, Federal Pharmacy Programs, or All Programs. It is updated every Wednesday.

Current vaccines may require either a single shot or two doses of the vaccine. Data are included for the total number of doses that have been administered through a single dose or two-dose vaccine series. Data on people who have been vaccinated with at least one dose and people who have been vaccinated with two doses or one dose J&J are available by race, ethnicity, gender, and age group and can be filtered by county on the tab labeled "Demographics Data". Data includes information on the percent of total people that have been vaccinated broken down by demographic categories and information on the percent of the population that has been vaccinated with at least one dose, or vaccinated with two doses or one dose J&J. Data shown are preliminary and may change. There can be a 72-hour lag in data reported to NCDHHS.

Dashboards are best viewed on a desktop or laptop. However, if viewing dashboards on a mobile device, please rotate your device horizontally, to landscape view, for a better mobile experience.

View Larger

All COVID-19 Dashboards

- Summary
- Vaccinations
- Respiratory Virus Surveillance
- Cases and Deaths
- Hospitalizations
- Outbreaks
- Wastewater Monitoring
- Reports
- Data Behind the Dashboards

Summary Data | Demographics Data | Additional NC Demographic Data

Data: December 14, 2020 – June 8, 2022 at 4:00 a.m.
Vaccinations Data will be updated every Wednesday

North Carolina's Vaccination Progress Summary

North Carolina's Vaccinations Progress Summary includes data from North Carolina's COVID-19 Vaccine Management System (CVMS), as well as data from the federal reporting platform Tiberius for providers that are not currently reporting to the state including Veterans Affairs, U.S. Department of Defense, Federal Bureau of Prisons, and Indian Health Service. **This federal data is included in the statewide and county-level estimates of percent of population by age group that has received at least one dose and those vaccinated with two doses or one dose J&J. It is not available for data included in the "Doses Administered by Week" section below or on the "Demographics Data" tab. Data for these sections come only from North Carolina's COVID-19 Vaccine Management System (CVMS), which does not include data from providers not reporting data to the state. Therefore, these sections of the dashboard undercount the true number of doses administered to North Carolina residents.** Dashboards are updated every weekday afternoon with the most recently available numbers as of that morning.

First of 2 Doses Administered	Second of 2 Doses Administered	Single Shot Doses Administered	Additional/Booster Doses Administered
6,447,910	6,026,440	494,021	3,653,338

Percent of Population Vaccinated with at Least One Dose

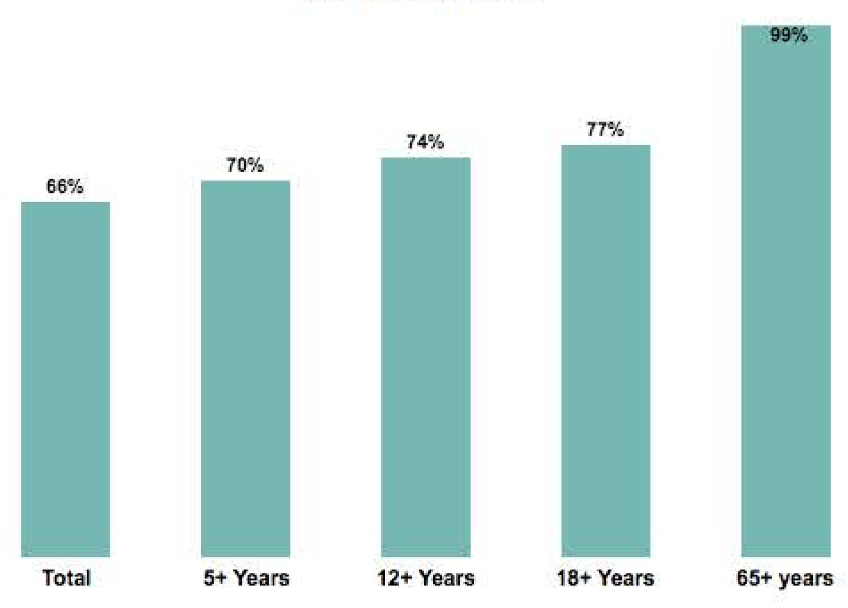

Percent of Population Vaccinated with Two Doses or One Dose J&J

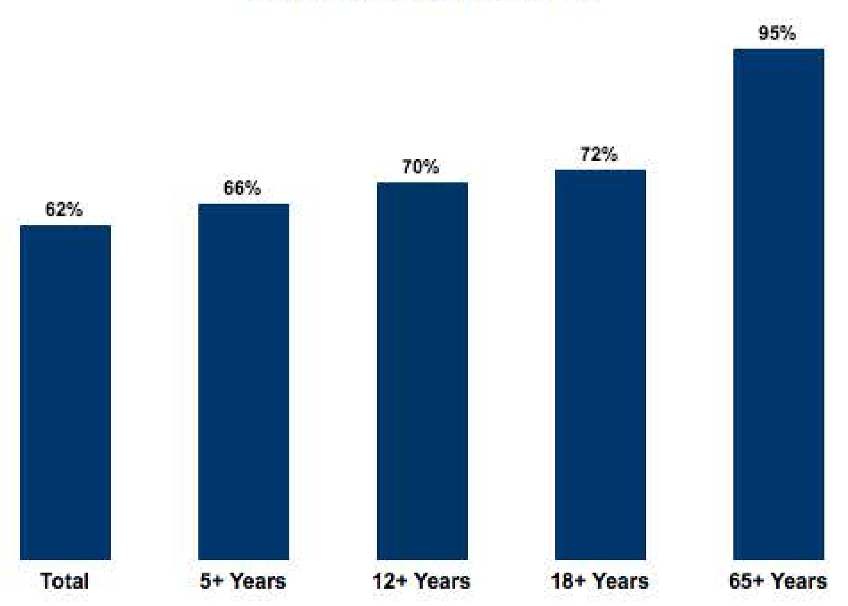

Percent of Vaccinated Population with at Least One Booster/Additional Dose

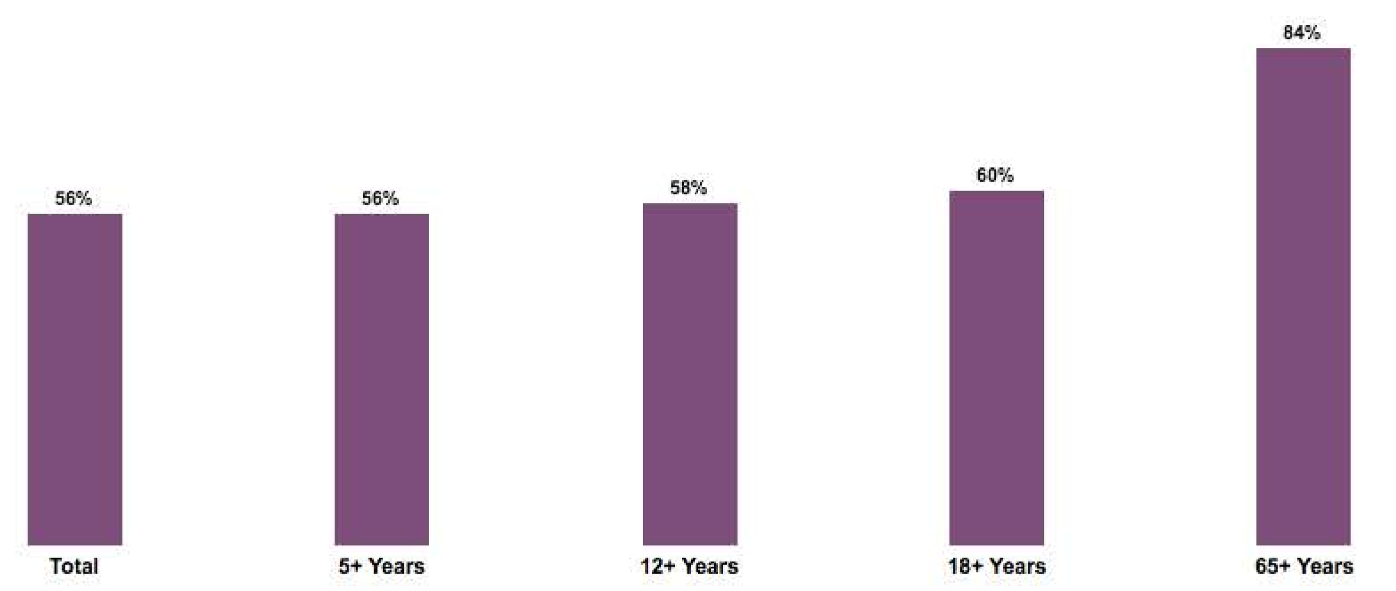

People Vaccinated with at Least One Dose by County of Residence

Select Population: Total Population
Select Metric: People Vaccinated with at Least One Dose
Select County: Alamance County

106,069
People Vaccinated with at Least One Dose
Total Population
Alamance County

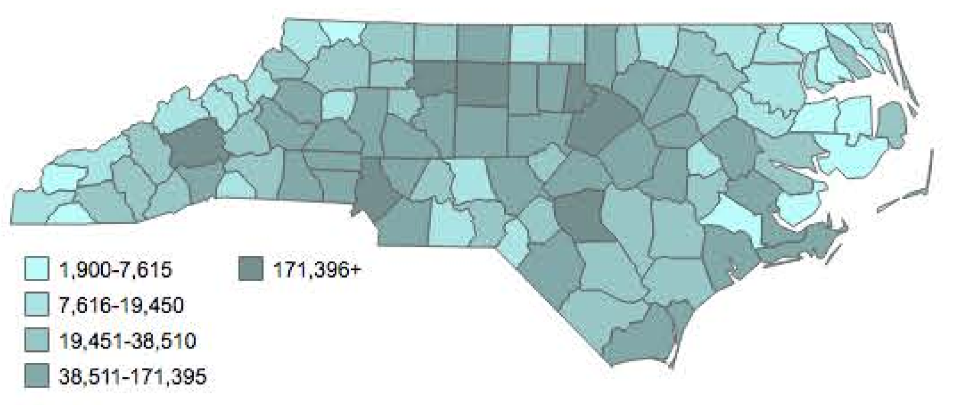

People Vaccinated with at Least One Dose for Total Population: 280,588 NC residents missing county information; 117,329 out of state residents

Doses Administered by Week

Data below come from North Carolina's COVID-19 Vaccine Management System (CVMS). This data does not include data from providers not reporting data to the state including Veterans Affairs, U.S. Department of Defense, Federal Bureau of Prisons, and Indian Health Service. Therefore, these sections of the dashboard undercount the true number of doses administered to North Carolina residents.

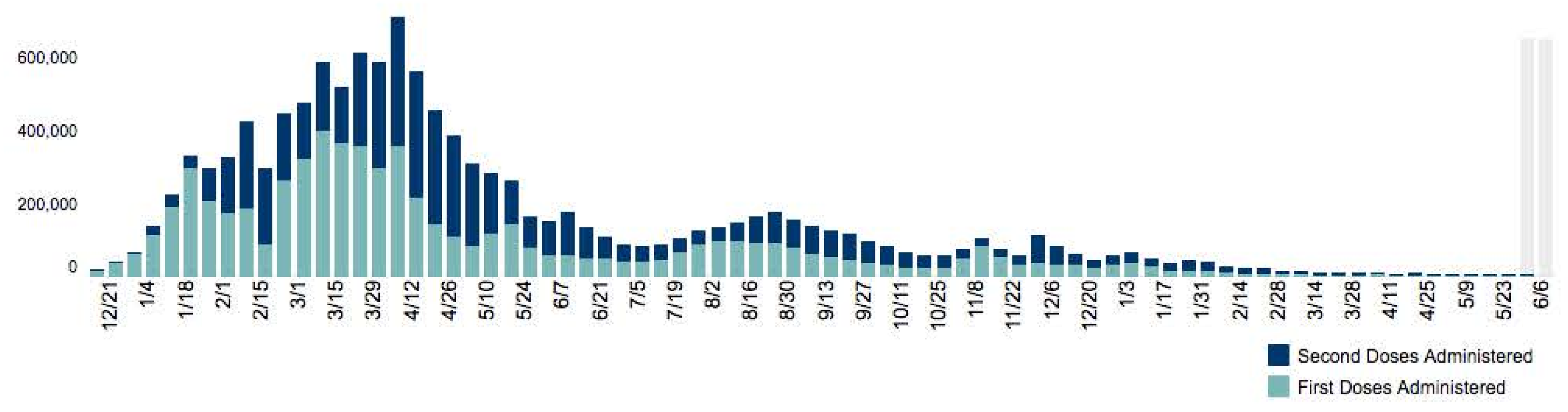

Date of Vaccination Administered is missing for 39 doses.

June 8, 2022

+ableau

To download tabular data displayed in the NCDHHS dashboards please go to [Data Behind the Dashboards].

County vaccination data may change once residence is verified. All data are preliminary and may change.

NCDHHS applies the following rules to the COVID-19 data dashboards where data is at a county level and includes demographics data:

- Data will be stratified by county by one demographic group at a time (ethnicity, race, age group, gender).
- Population data sources, as listed in the Data Behind the Dashboards are used to find 'cell denominators' for county populations. Data for any county demographic sub-group that has a population of less than 500, will be suppressed for privacy.
- For county cell denominators that require suppression, all corresponding demographic data elements are attributed to the 'Suppressed' category. This method allows the display of actual values on the dashboards, while still suppressing which demographic sub-groups the data are attributed to.
- Additional data suppression is done for demographic sub-groups that can be discovered by subtracting row or column totals (e.g., if a County has only age group 18-24 that requires suppression, the next smallest age group by population is also suppressed).

'Missing or Undisclosed' may include data for people that did not provide demographic information, or where vaccine providers did not submit the demographic information. Numbers may not add up to 100% due to rounding.

Percent of population metrics are calculated using the entire North Carolina population (i.e., all ages). The state's dashboard is the source for the most accurate and timely information for vaccine data for the state.

Consistent with CDC practice, NCDHHS has capped percent of population metrics at 100%. Dashboard metrics for percent of population vaccinated could exceed 100% for a few reasons, including census denominator data not including all individuals that currently reside in the county (e.g., part time residents or residents that have moved to the county since the population data source was compiled).

North Carolina's information on people vaccinated comes from the COVID-19 Vaccine Management System (CVMS), a secure, web-based system provided for free to all who administer COVID-19 vaccinations. It helps vaccine providers know who has had a first dose of which vaccine to make sure people get the second dose of the same vaccine at the right time. It also helps people register for vaccination at the appropriate time and allows the state to manage vaccine supply.

Information on vaccinations is confidential health information that is carefully managed to protect patient privacy. Information will not be shared except in accordance with state and federal law.

Visit Here for more information on the vaccines and vaccine distribution in North Carolina.

Promoting COVID-19 Vaccine Equity in North Carolina reports the share of vaccinations in the past week going to Black/African American, Hispanic/Latinx, and American Indian or Alaskan Native populations as well as key metrics for earning trust, embedding equity in vaccine operations, and promoting accountability through data transparency. The report, updated every two weeks, also highlights best practices to promote equitable access to vaccinations. Another tool to help North Carolina reach its goal of vaccinating as many North Carolinians as quickly and fairly as possible is a map that shows census tracts in North Carolina with the highest rates of social vulnerability and the lowest rates of COVID-19 vaccination. Social vulnerability is measured by Social Vulnerability Index (SVI); the higher the score, the higher the social vulnerability. The map also includes the ability to see where COVID-19 vaccine providers and community-based organizations that have offered to support vaccine events are located.

Tabular data for the COVID-19 Vaccinations Dashboard will be updated on the Data Behind the Dashboards page.

Overview | Hospital Capacity | Demographics | County Map

COVID-19 Cases Overview

Last Updated: 6/10/2022

POPULATION
762,062

COUNTY
All

Hover over a value and click the icon for more information

THIS WEEK
6/3/2022 - 6/9/2022

WEEKLY NEW POSITIVE CASES
1084

7 DAY CASE RATE PER 100,000 INDIVIDUALS
142

CUMULATIVE

TOTAL POSITIVE CASES
246,544

DEATHS
CLICK TO VIEW AT VITAL RECORDS

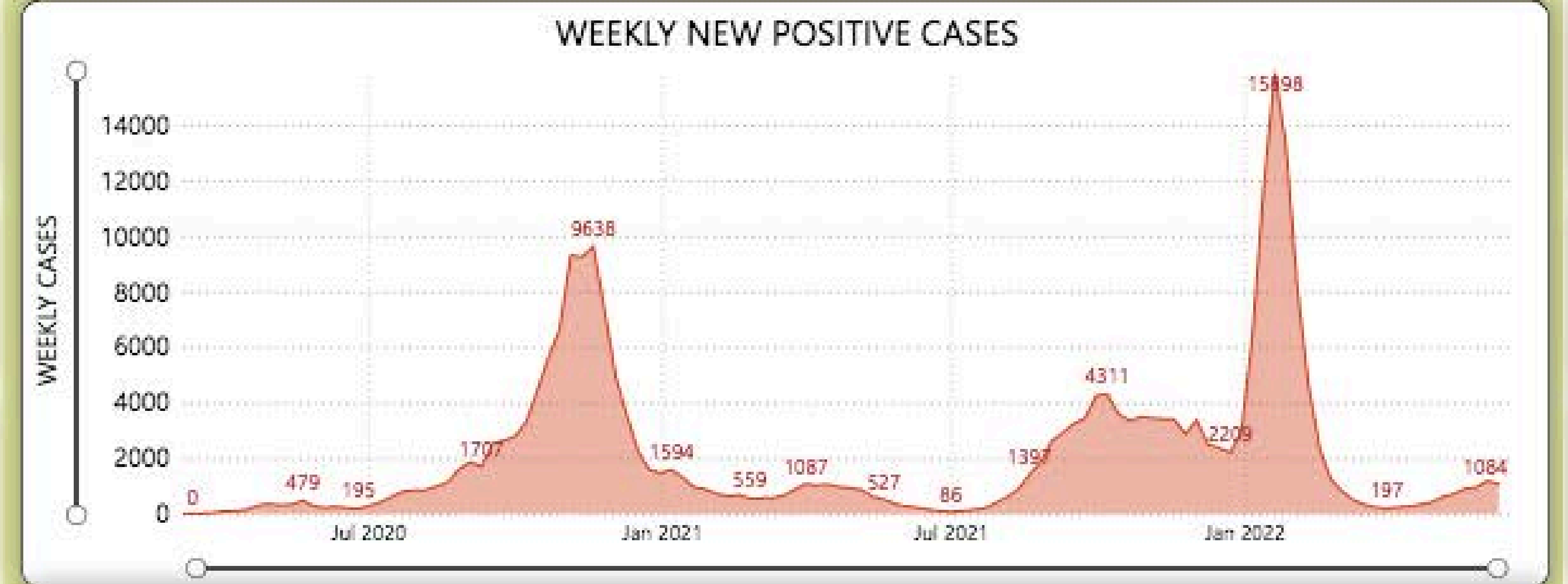

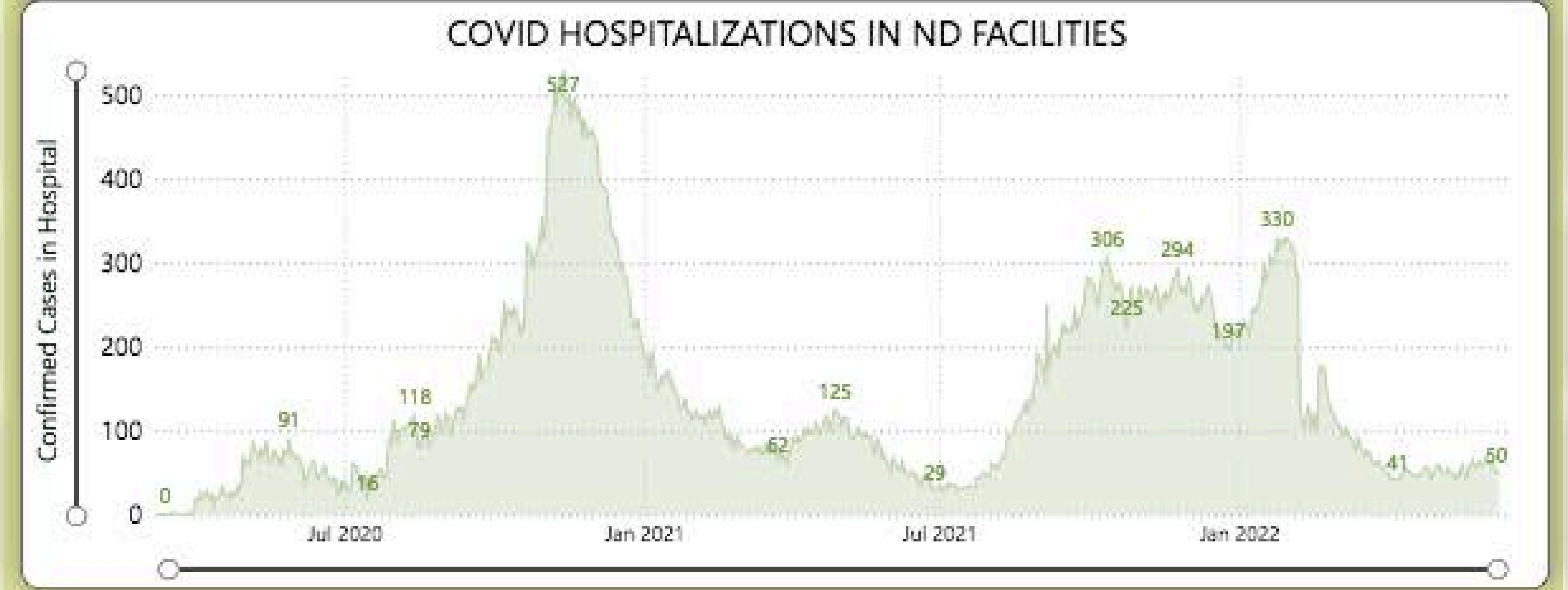

STATEWIDE HOSPITAL CAPACITY

PERCENT INPATIENT BEDS OCCUPIED
77%

PERCENT ICU BEDS OCCUPIED
53%

INPATIENT BEDS OCCUPIED WITH COVID
46

ICU BEDS OCCUPIED WITH COVID
4

WEEKLY COVID ADMISSIONS
50

Overview | Hospital Capacity | Demographics | County Map

COVID-19 Case Demographics

Hover over a value and click the icon for more information

WEEKLY NEW CASES | CUMULATIVE CASES

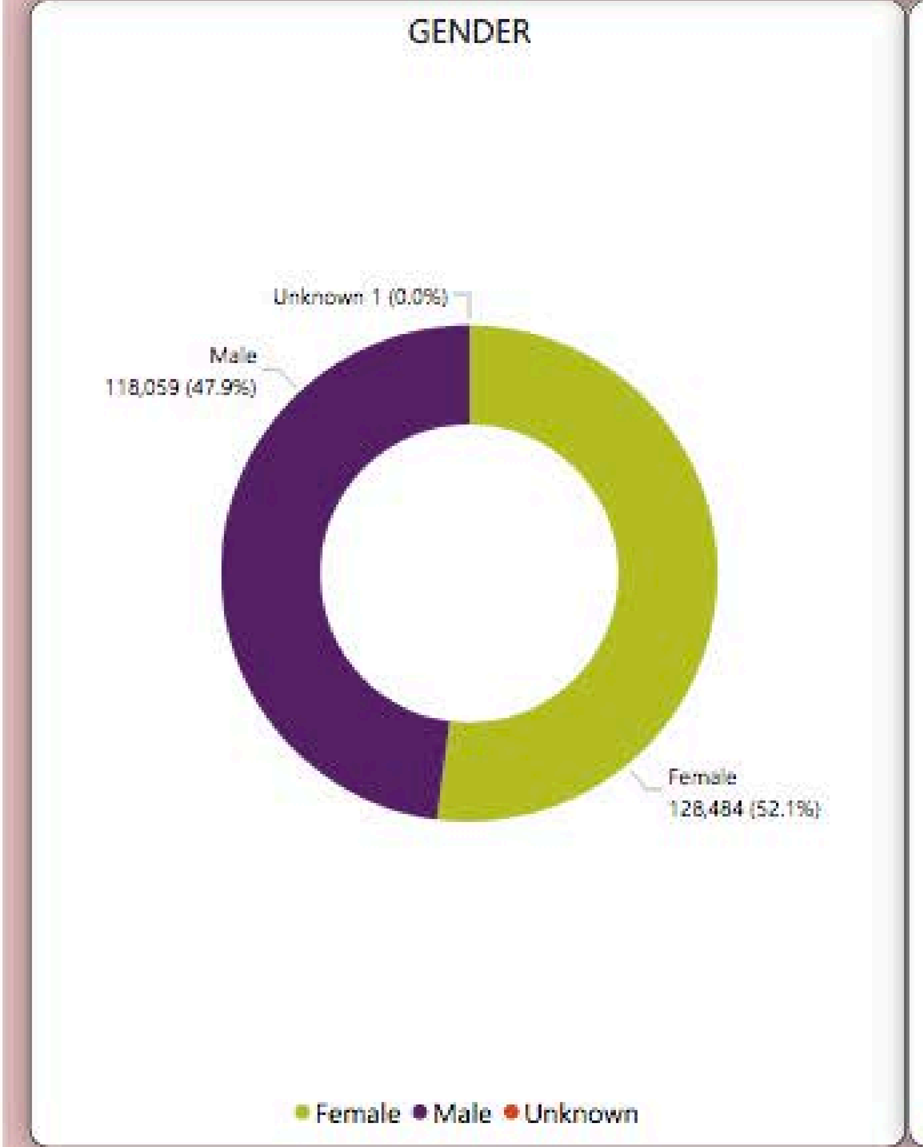

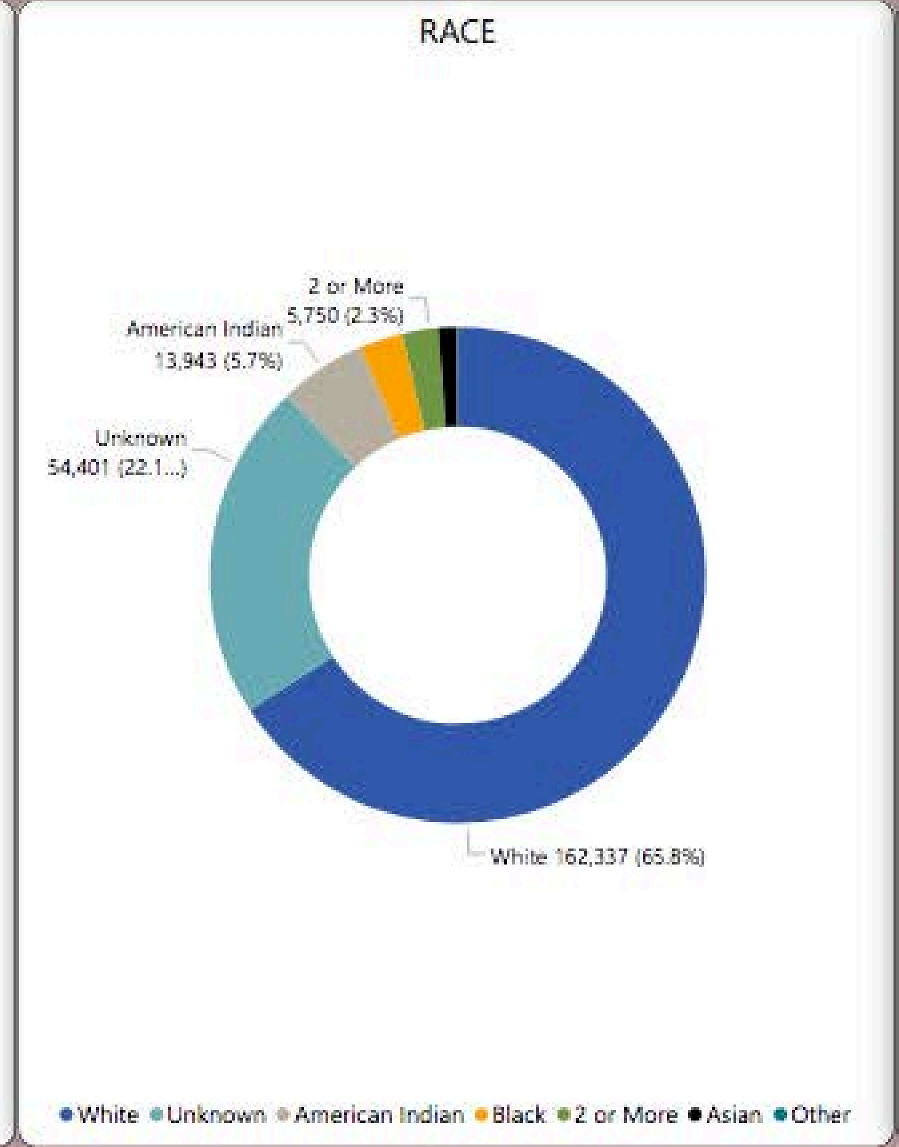

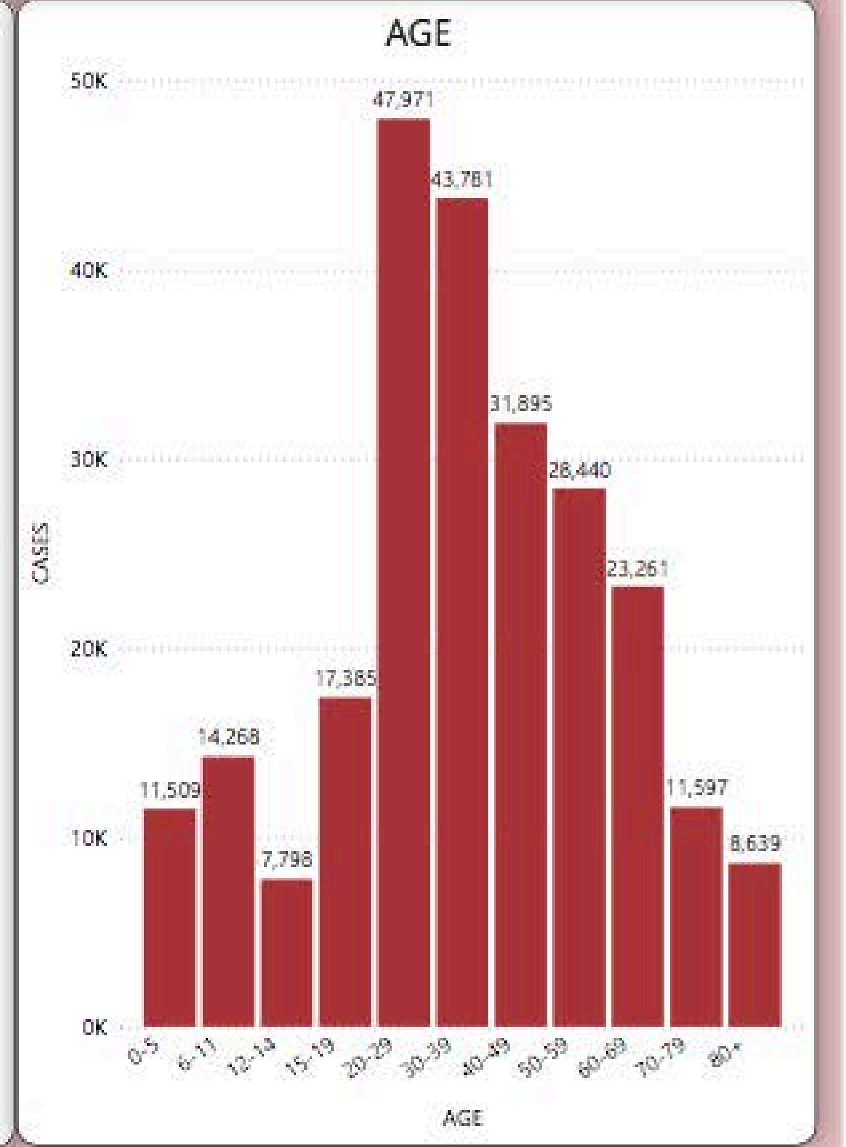

Overview | Hospital Capacity | Demographics | County Map

COVID-19 Case County Map

Hover over a value and click the icon for more information

NEW CASES THIS WEEK

6/3/2022 - 6/9/2022

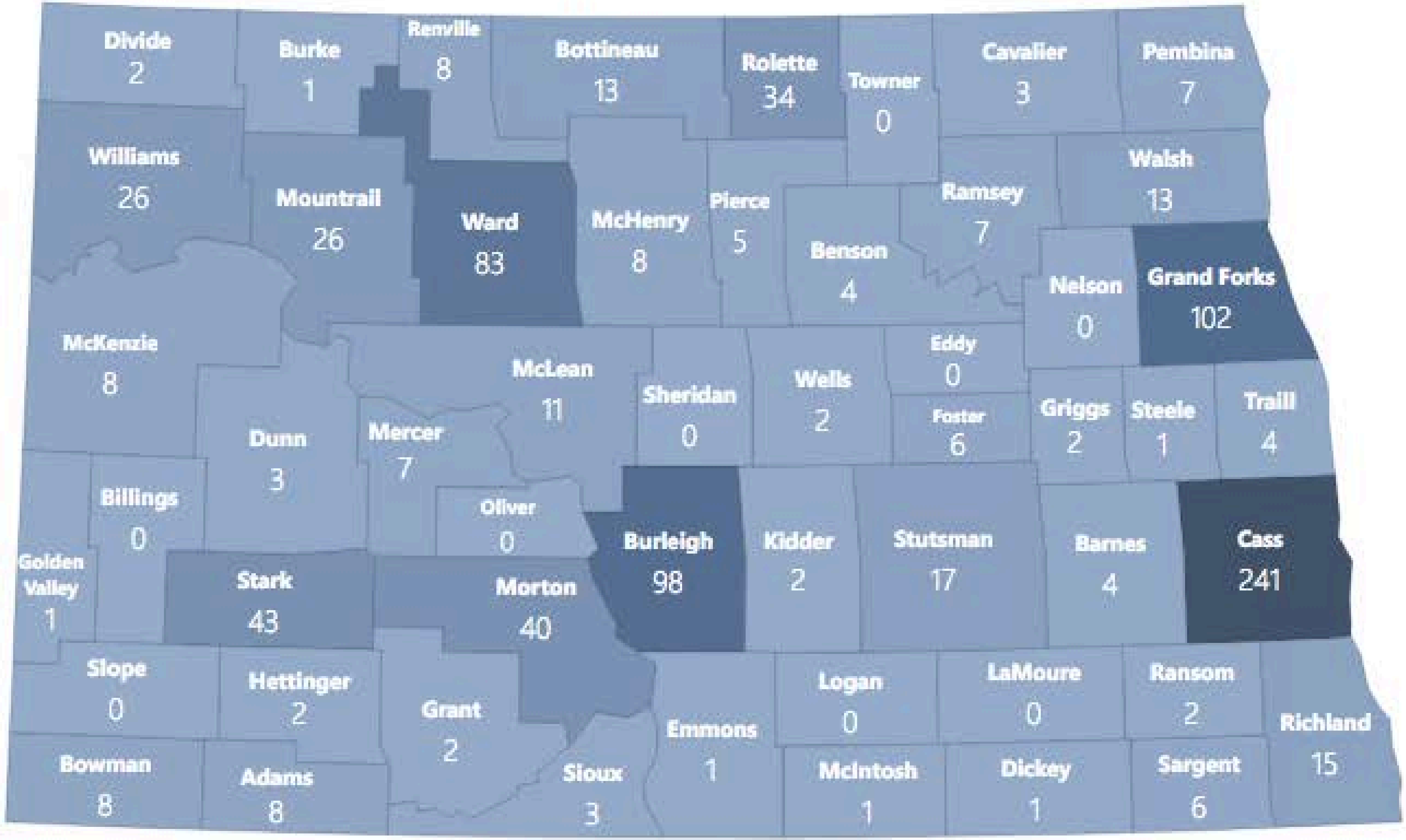

Current Status | Trends | Demographics | NV Health Response Homepage | Office of Analytics Homepage | Download Data

Total Cases | Hospitalizations | Deaths | Vaccinations

DASHBOARD UPDATED ON 6/8/2022

CURRENT STATUS - TOTAL CASES (MARCH 2020 - CURRENT)

DATA AS OF 6/6/2022

COVID-19 case data are summarized below and the key metrics are separated by confirmed and probable. Daily new cases are displayed as 14-day moving averages of all cases confirmed and probable. Cases per 100,000 are displayed for the most recent 30-day period and cumulatively. In the maps below, counties with higher rates are shaded darker. Results of at-home COVID-19 tests are not included in these data. **Learn more**

SELECT A COUNTY

Search

- North
  - Carson City
  - Washoe
- South
  - Clark
- Rural
  - Churchill
  - Douglas
  - Elko
  - Esmeralda
  - Eureka
  - Humboldt
  - Lander
  - Lincoln
  - Lyon
  - Mineral
  - Nye
  - Pershing
  - Storey
  - White Pine

KEY METRICS

773

NEW DAILY CONFIRMED CASES
14-day moving average

688 Confirmed Cases per 100K
30-day period

690,370 Cumulative Confirmed Cases

21,478 Confirmed Cases per 100K

74

NEW DAILY PROBABLE CASES
14-day moving average

67 Probable Cases per 100K
30-day period

TOTAL CASES PER 100K (30-day period)

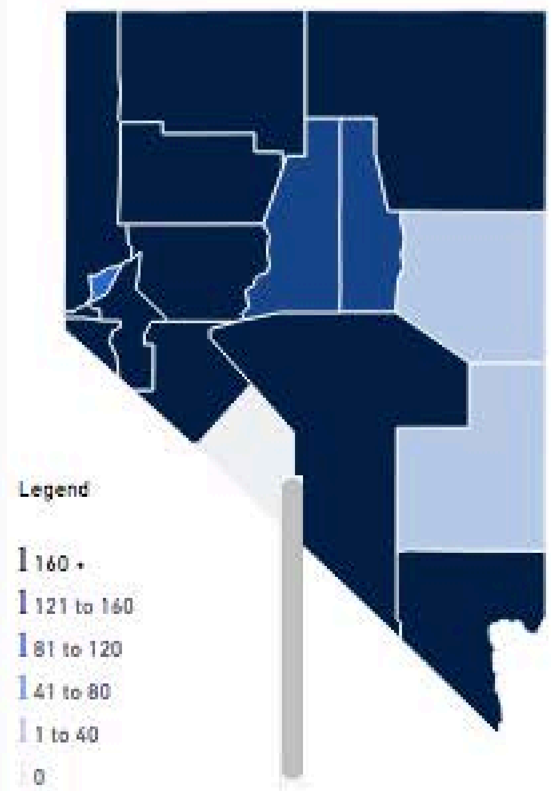

TOTAL DAILY NEW CASES (14-day moving avg.) STATEWIDE ONLY

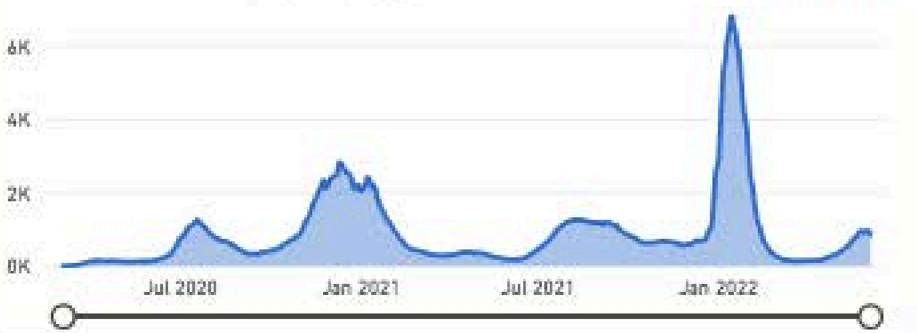

COUNTY	POPULATION	TOTAL CUMULATIVE CASES	TOTAL CUMULATIVE CASES PER 100K
Carson City	57,222	15,370	26,860
Churchill	26,242	6,691	25,497
Clark	2,378,903	563,205	23,675
Douglas	49,535	7,655	15,454
Elko	54,035	11,767	21,777
Esmeralda	971	108	11,123
Eureka	1,885	168	8,912
Humboldt	16,897	2,537	15,014
Lander	5,992	1,189	19,843
**Total**	**3,214,260**	**742,375**	**23,096**

This dashboard is updated Wednesdays at approximately 11:00 am. Please note that updates apply incrementally and may be in process for up to an hour. The Nevada DHHS also maintains a facility-specific COVID-19 dashboard, available **Here**

62%

Microsoft Power BI

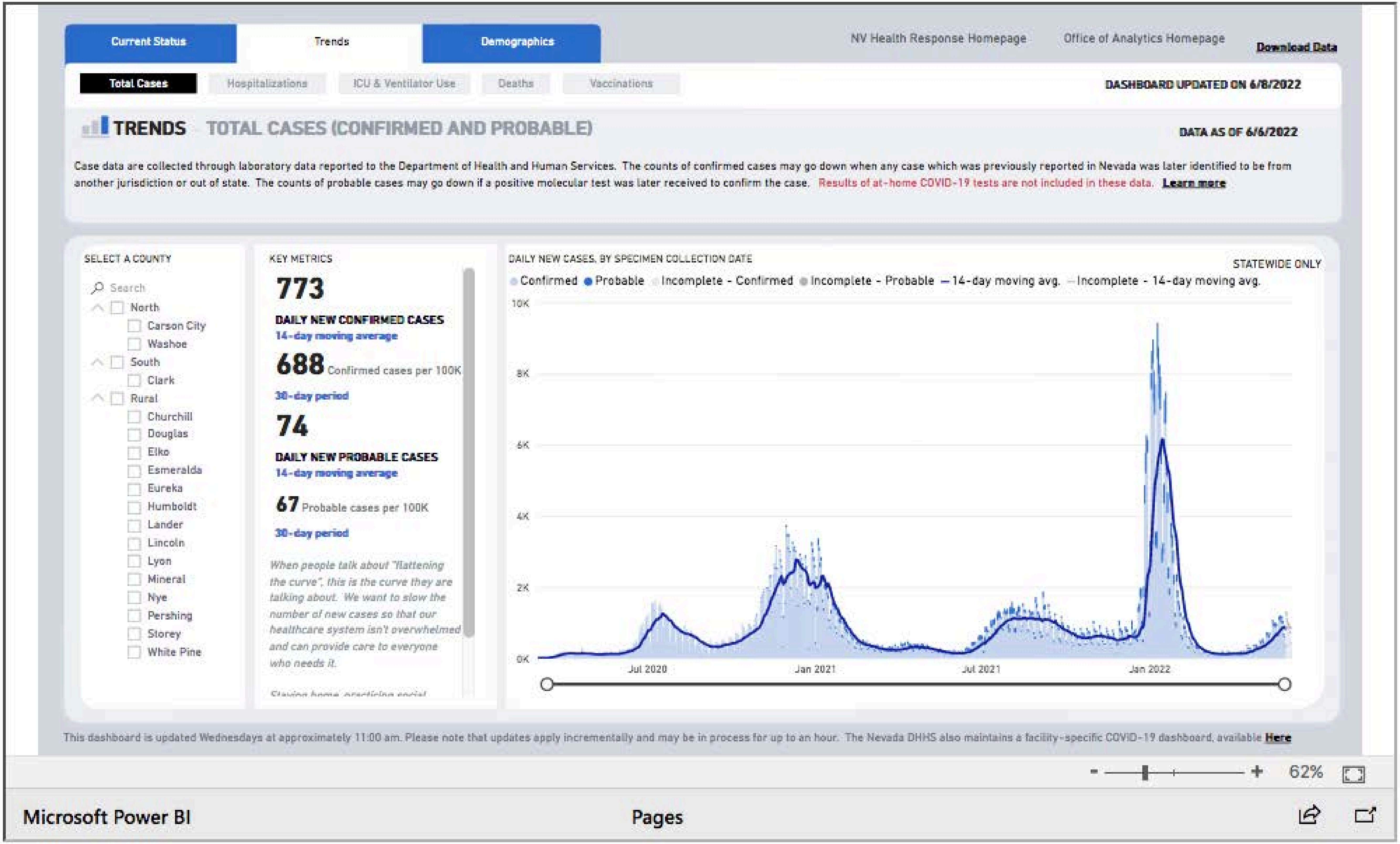
Current Status
Trends
Demographics
NV Health Response Homepage
Office of Analytics Homepage
Download Data
Total Cases
Hospitalizations
ICU & Ventilator Use
Deaths
Vaccinations
DASHBOARD UPDATED ON 6/8/2022
TRENDS
TOTAL CASES (CONFIRMED AND PROBABLE)
DATA AS OF 6/6/2022
Case data are collected through laboratory data reported to the Department of Health and Human Services. The counts of confirmed cases may go down when any case which was previously reported in Nevada was later identified to be from another jurisdiction or out of state. The counts of probable cases may go down if a positive molecular test was later received to confirm the case. Results of at-home COVID-19 tests are not included in these data. Learn more
SELECT A COUNTY
Search
North
Carson City
Washoe
South
Clark
Rural
Churchill
Douglas
Elko
Esmeralda
Eureka
Humboldt
Lander
Lincoln
Lyon
Mineral
Nye
Pershing
Storey
White Pine
KEY METRICS
773
DAILY NEW CONFIRMED CASES
14-day moving average
688 Confirmed cases per 100K
30-day period
74
DAILY NEW PROBABLE CASES
14-day moving average
67 Probable cases per 100K
30-day period
When people talk about "flattening the curve", this is the curve they are talking about. We want to slow the number of new cases so that our healthcare system isn't overwhelmed and can provide care to everyone who needs it.
DAILY NEW CASES, BY SPECIMEN COLLECTION DATE
STATEWIDE ONLY
Confirmed
Probable
Incomplete - Confirmed
Incomplete - Probable
14-day moving avg.
Incomplete - 14-day moving avg.
10K
8K
6K
4K
2K
0K
Jul 2020
Jan 2021
Jul 2021
Jan 2022
This dashboard is updated Wednesdays at approximately 11:00 am. Please note that updates apply incrementally and may be in process for up to an hour. The Nevada DHHS also maintains a facility-specific COVID-19 dashboard, available Here
62%
Microsoft Power BI
Pages

Current Status | Trends | Demographics | NV Health Response Homepage | Office of Analytics Homepage | Download Data

Total Cases | Deaths | Vaccinations

Demographics

DASHBOARD UPDATED ON 6/8/2022

DEMOGRAPHICS TOTAL CASES (CONFIR[illegible]BLE)

DATA AS OF 6/6/2022

COVID-19 can affect people of all age groups, including children. However, adults in Nevada are more frequently tested and diagnosed with COVID-19. Children can spread COVID-19 despite the absence of signs or symptoms. Adults aged 60 years or above are more vulnerable to COVID-19-associated mortality as opposed to other age groups. The total number of cases for each demographic group may not be equal due to missing data. Results of at-home COVID-19 tests are not included in these data. **Learn more**

Group	CUMULATIVE CASES	CASES %	POPULATION %	POPULATION
**AGE**				
<10	39,587	5.4%	12.4%	398,565
10-19	82,945	11.3%	13.5%	432,709
20-29	138,671	19.0%	13.9%	447,369
30-39	138,029	18.9%	13.6%	436,407
40-49	115,579	15.8%	13.0%	419,139
50-59	99,959	13.7%	12.7%	407,113
60-69	64,622	8.8%	11.0%	353,238
70+	52,252	7.1%	9.9%	319,719
**GENDER**				
Female	387,242	52.5%	50.0%	1,607,496
Male	349,938	47.5%	50.0%	1,606,764
**RACE/ETHNICITY**				
Hispanic	185,520	32.0%	30.7%	986,526
Non-Hispanic American Indian or Alaska Native	4,129	0.7%	1.1%	36,119
Non-Hispanic Asian/Pacific Islander	54,332	9.4%	10.1%	325,604
Non-Hispanic Black	56,673	9.8%	9.0%	290,120
Non-Hispanic Other Race	47,741	8.2%	0.0%	0

This dashboard is updated Wednesdays at approximately 11:00 am. Please note that updates apply incrementally and may be in process for up to an hour. The Nevada DHHS also maintains a facility-specific COVID-19 dashboard, available **Here**

62%

Microsoft Power BI | Pages

New Hampshire announced **329 cases** on June 10. There were an average of **302 cases per day** over the most recent 7-day period (June 4 to June 10). This is a **20% decrease** compared to the previous 7-day period.

328,318 Total Cases
329 New Cases

63.1% Fully Vaccinated
70.9% At Least 1 Dose

22 Hosp. Treated for COVID-19
2,562 Total Deaths

Total Deaths by Age

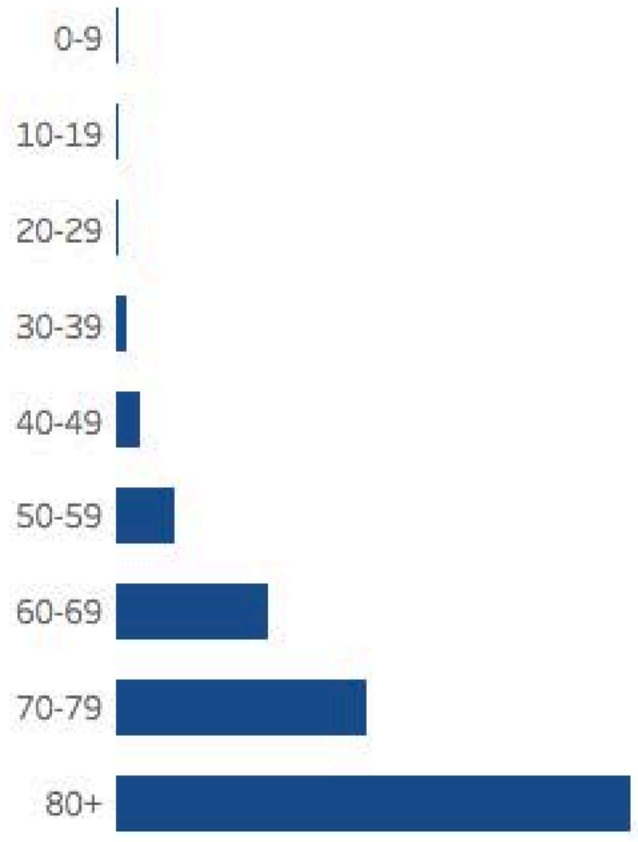

New Cases Trend

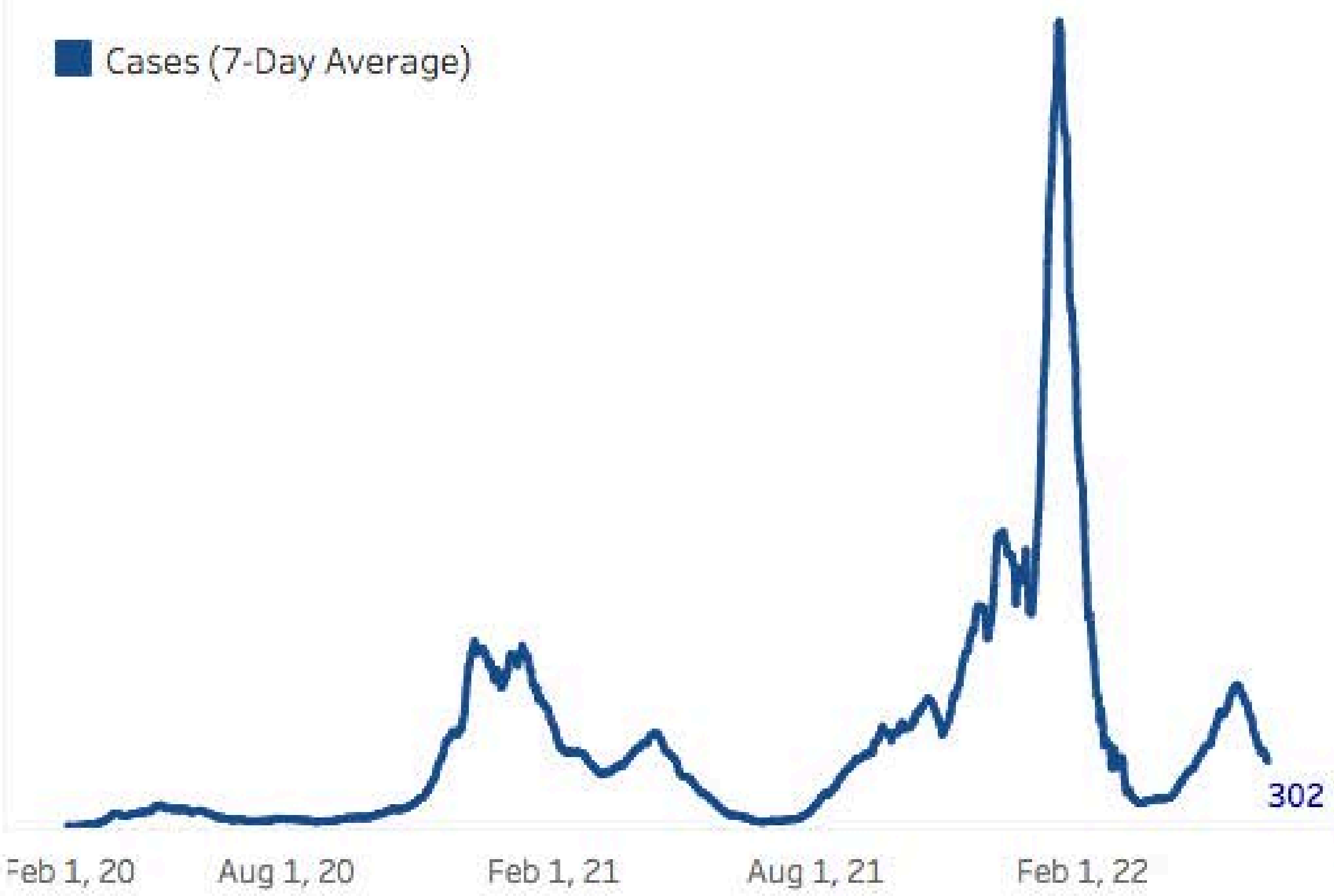

New Cases per 100k over 14 days

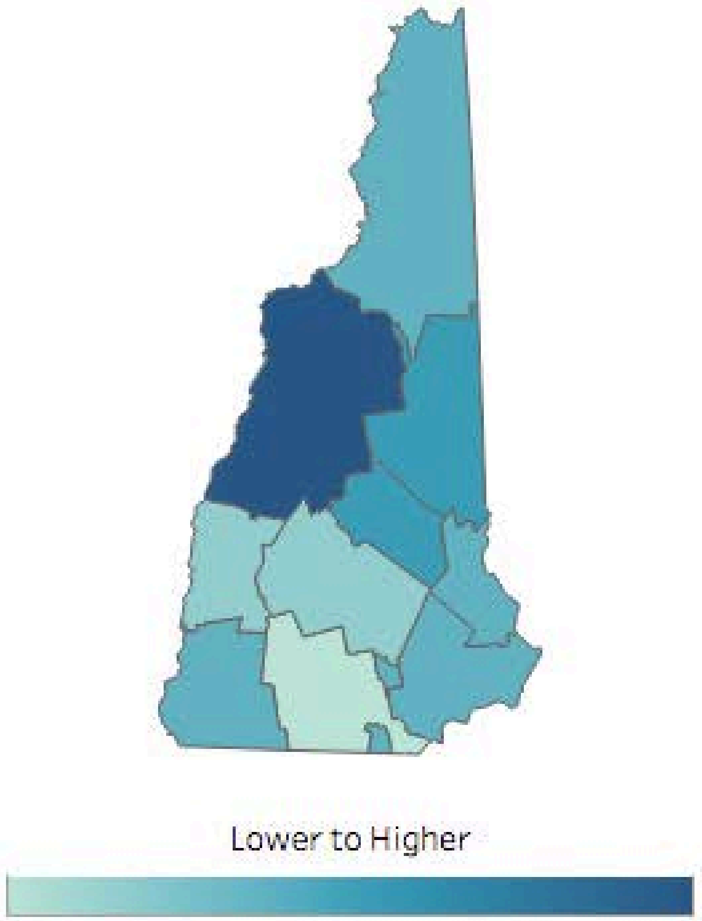

Data as of: 6/10/2022

Undo Redo Replay Revert Refresh Pause Share Download

COVID-19 Trends

Metrics

Select a Metric

- (•) New Cases by Report Date
- ( ) Long-Term Care Facility Cases
- ( ) Non Long-Term Care Facility Cases
- ( ) Deaths
- ( ) Long-Term Care Facility Deaths
- ( ) Non Long-Term Care Facility Deaths
- ( ) Positive PCR Tests - Test Based
- ( ) Positive Antigen Tests - Test Based
- ( ) Individuals Fully Vaccinated
- ( ) Individuals with At Least 1 Dose
- ( ) Individuals with a Booster Shot

Filters

Sex

(All)

Age

(All)

Daily Total

7-Day Average

Cumulative Total

There are 0 COVID-19 related cases not currently plotted due to unknown date of report to NH DHHS.

Data as of: 6/10/2022

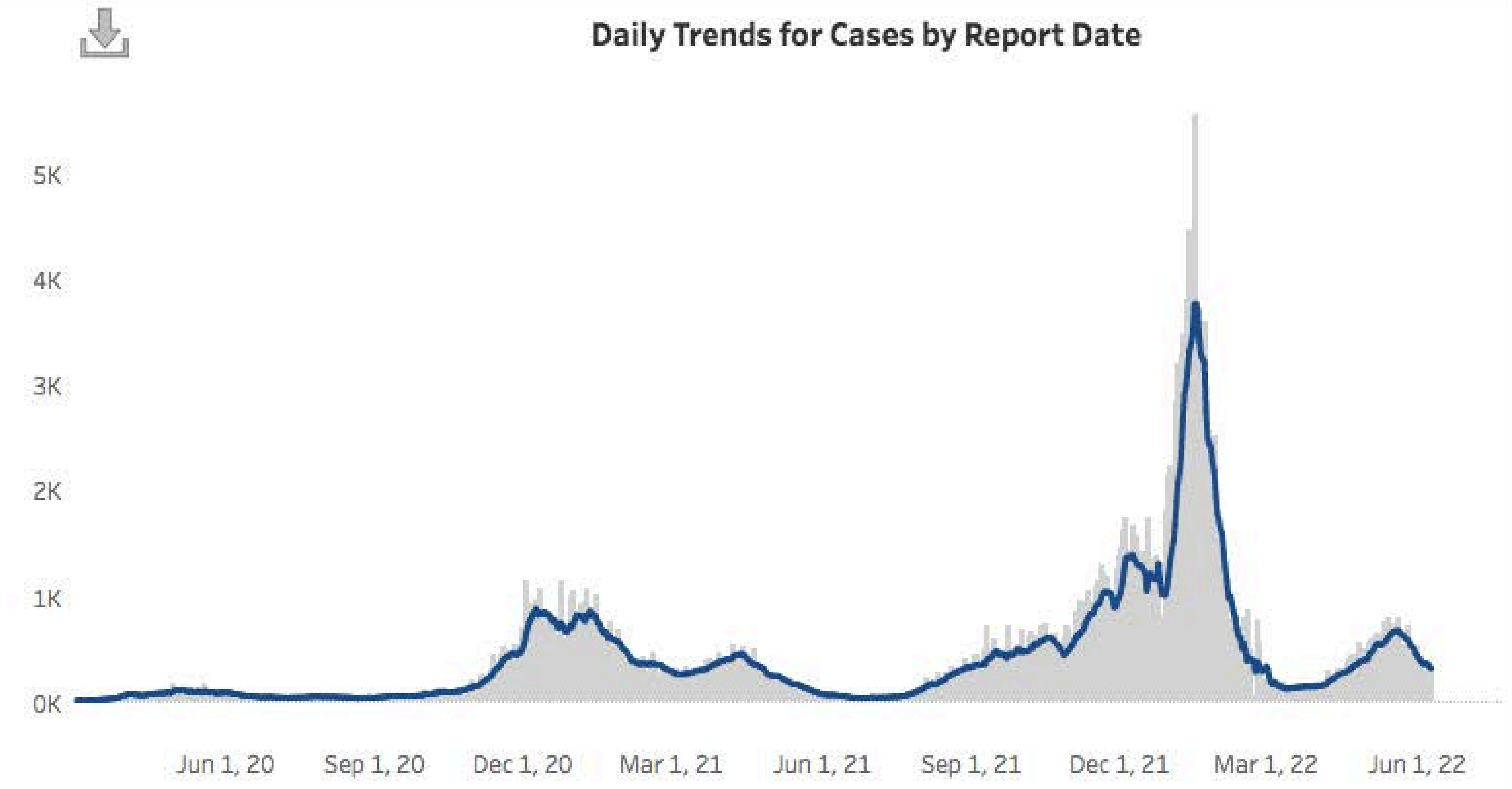

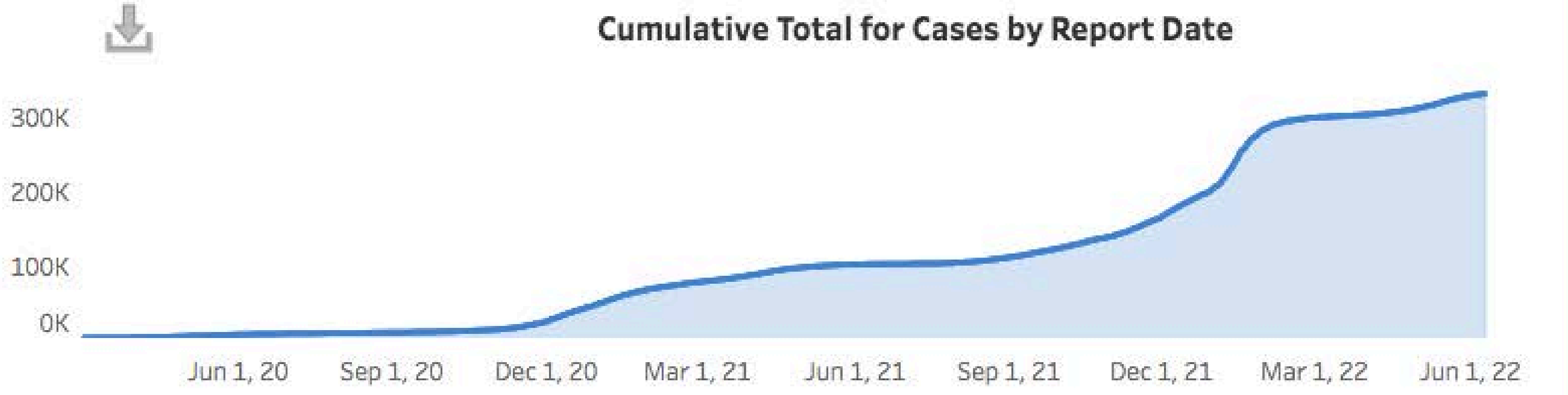

Undo Redo Replay Revert Refresh Pause Share Download

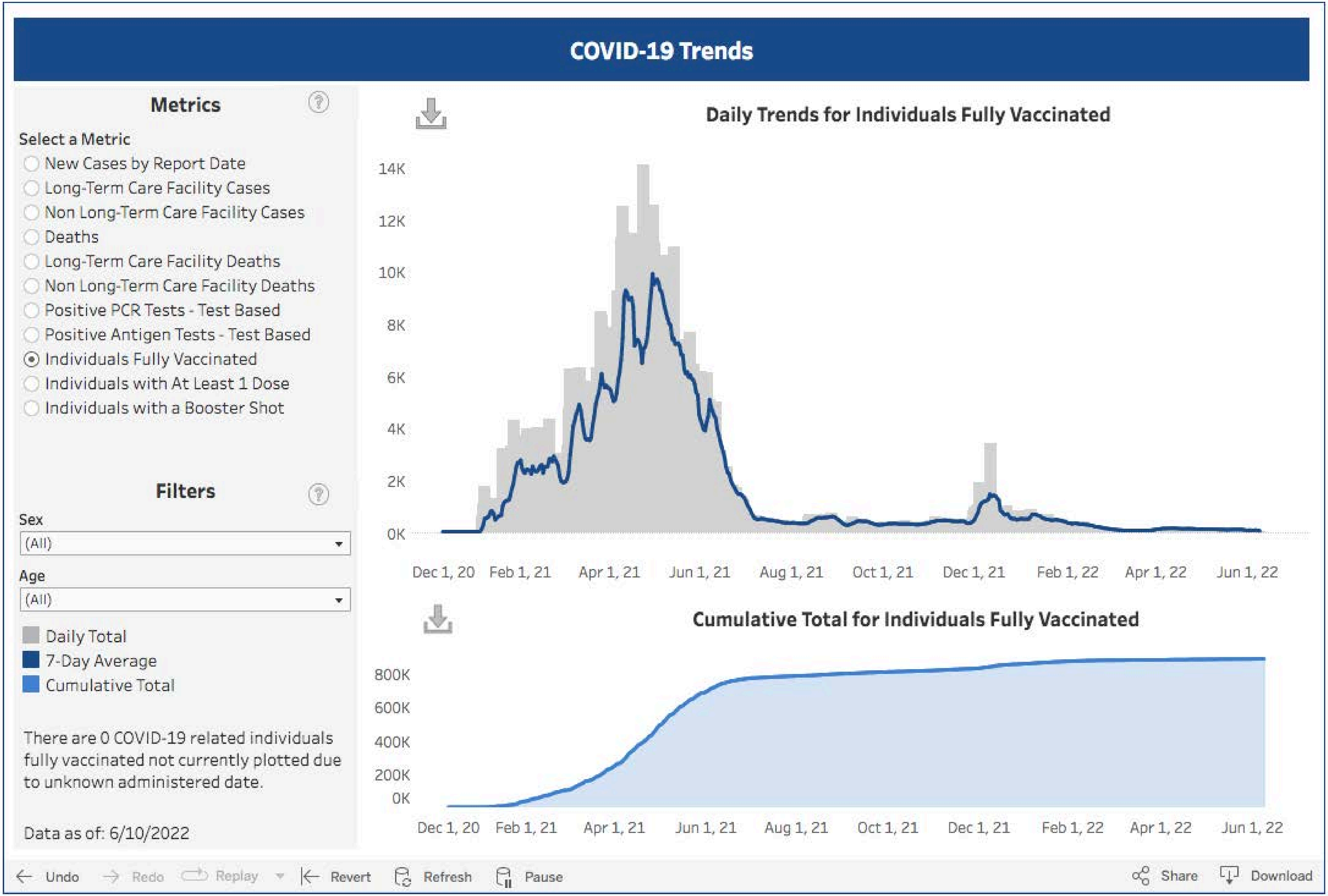
COVID-19 Trends
Metrics
Select a Metric
New Cases by Report Date
Long-Term Care Facility Cases
Non Long-Term Care Facility Cases
Deaths
Long-Term Care Facility Deaths
Non Long-Term Care Facility Deaths
Positive PCR Tests - Test Based
Positive Antigen Tests - Test Based
Individuals Fully Vaccinated
Individuals with At Least 1 Dose
Individuals with a Booster Shot
Filters
Sex
(All)
Age
(All)
Daily Total
7-Day Average
Cumulative Total
There are 0 COVID-19 related individuals fully vaccinated not currently plotted due to unknown administered date.
Data as of: 6/10/2022
Daily Trends for Individuals Fully Vaccinated
14K
12K
10K
8K
6K
4K
2K
0K
Dec 1, 20
Feb 1, 21
Apr 1, 21
Jun 1, 21
Aug 1, 21
Oct 1, 21
Dec 1, 21
Feb 1, 22
Apr 1, 22
Jun 1, 22
Cumulative Total for Individuals Fully Vaccinated
800K
600K
400K
200K
0K
Dec 1, 20
Feb 1, 21
Apr 1, 21
Jun 1, 21
Aug 1, 21
Oct 1, 21
Dec 1, 21
Feb 1, 22
Apr 1, 22
Jun 1, 22
Undo
Redo
Replay
Revert
Refresh
Pause
Share
Download

Just the basics: Total cases, hospitalizations, deaths and other key metrics for New Hampshire

The numbers below are pulled directly from New Hampshire's COVID-19 website. If you think there's an error, please email us at coronavirus@nhpr.org so that we can review and correct any mistakes as soon as possible.

328,064	People Have Been Diagnosed With COVID-19 Since March 2020
2,179	People Have Recovered From COVID-19 Since March 2020
2,555	People Have Died From COVID-19 Since March 2020
2,707	People Currently Have COVID-19
23	People Are Currently Hospitalized With COVID-19 and Being Treated With Remdesivir, Dexamethasone or both therapies

Data note: New Hampshire's recent COVID-19 hospitalization data offers only a limited window into coronavirus-related hospitalizations. State health officials no longer track patients who remain hospitalized while recovering from COVID-19. They also only include patients who are being treated with Remdesivir, Dexamethasone or both therapies. You can find more comprehensive COVID hospitalization data at this link.

Table: NHPR • Source: N.H. Department of Health and Human Services • Created with Datawrapper

How does New Hampshire's COVID-19 risk level compare to other states?

This graphic was produced by NPR. You can find more of their COVID-19 maps and charts here.

33 places are at the highest COVID-19 risk level

Data as of June 8

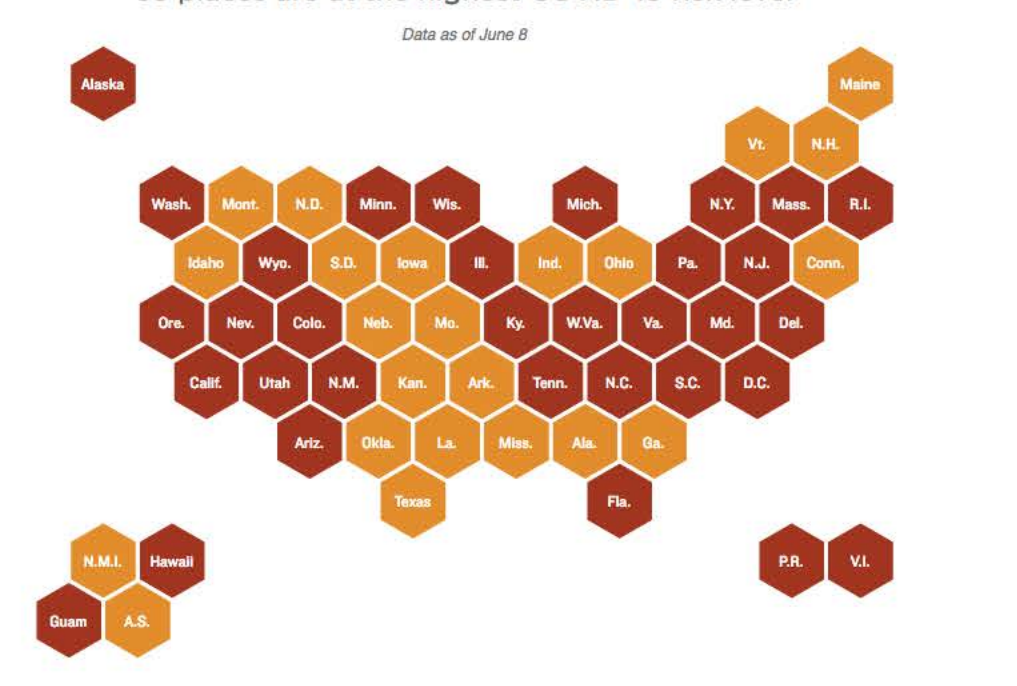

RED	ORANGE	YELLOW	GREEN
**Threshold:** 25+ daily new cases per 100,000 people	**Threshold:** 10-24 daily new cases per 100,000 people	**Threshold:** 1-9 daily new cases per 100,000 people	**Threshold:** <1 daily new case per 100,000 people
**Indicates:** unchecked community spread	**Indicates:** escalating community spread	**Indicates:** potential community spread	**Indicates:** close to containment

Notes

— Daily cases are a 7-day average to smooth out day-to-day variations in the data. Risk levels are based on a scale developed by the Harvard Global Health Institute and a collaboration of top scientists at institutions around the country.

Source: Center for Systems Science and Engineering at Johns Hopkins University, Census Bureau 2021 population estimates, 2020 Census (U.S. territories)

How many new cases are emerging each day in New Hampshire?

This shows how many new COVID-19 cases were announced each day and the seven-day average for the same metric. It doesn't reflect the number of new positive tests reported on each date or new infections by illness onset date, but more information on both of those data points can be found on the state's coronavirus dashboard.

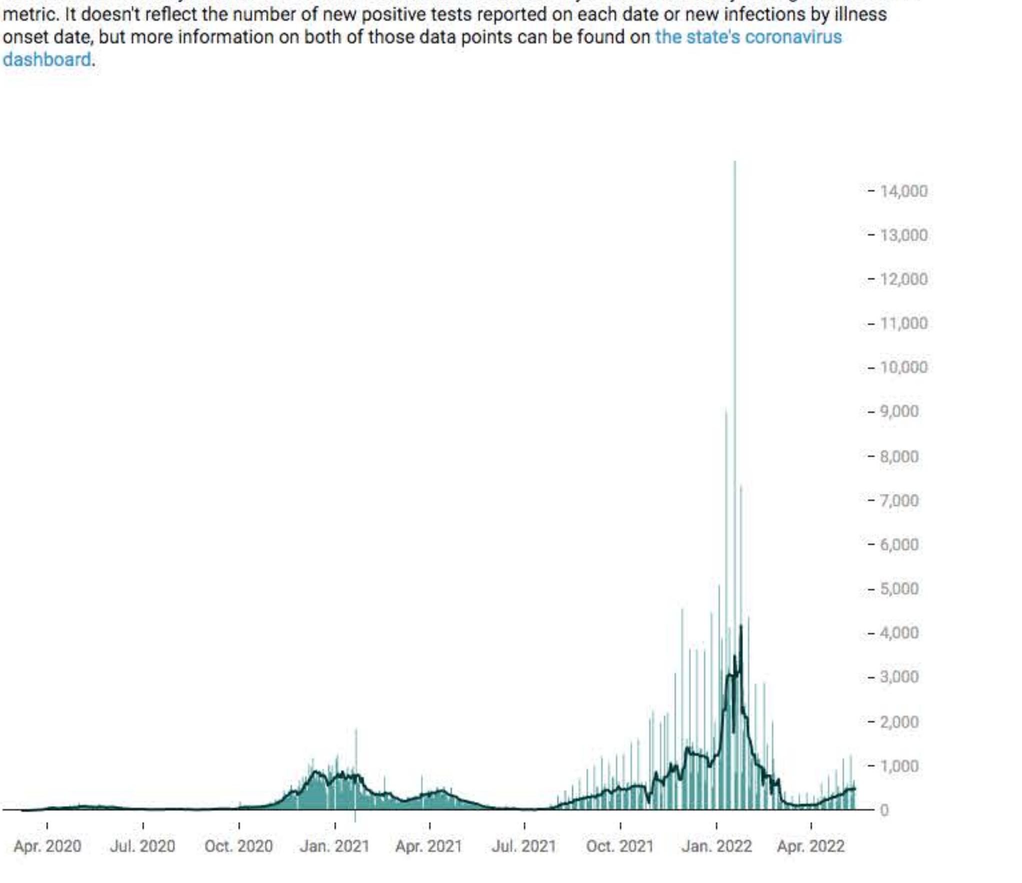

***Note:** We recently changed our method of calculating new cases reported by day, to account for changes in the state's approach to reporting this metric. We now use the raw difference in total cases reported each day, rather than the summary of new case counts provided by the Department of Health and Human Services. New Hampshire began including positive antigen tests in addition to positive PCR tests in its case totals in early October 2020. The state also skipped reporting on several holidays and stopped reporting new case counts on weekends in the summer of 2021, which could make it appear that cases decreased and then spiked more than they actually did after those dates.*

Chart: NHPR • Source: New Hampshire Department of Health and Human Services • Created with Datawrapper

How does New Hampshire's case count compare to other states?

This graphic was produced by NPR. You can find more of their COVID-19 maps and charts here.

Are new cases growing in your state?

7-day average of new cases, per 100,000 people, as of June 8. States are ordered from highest new daily cases to lowest.

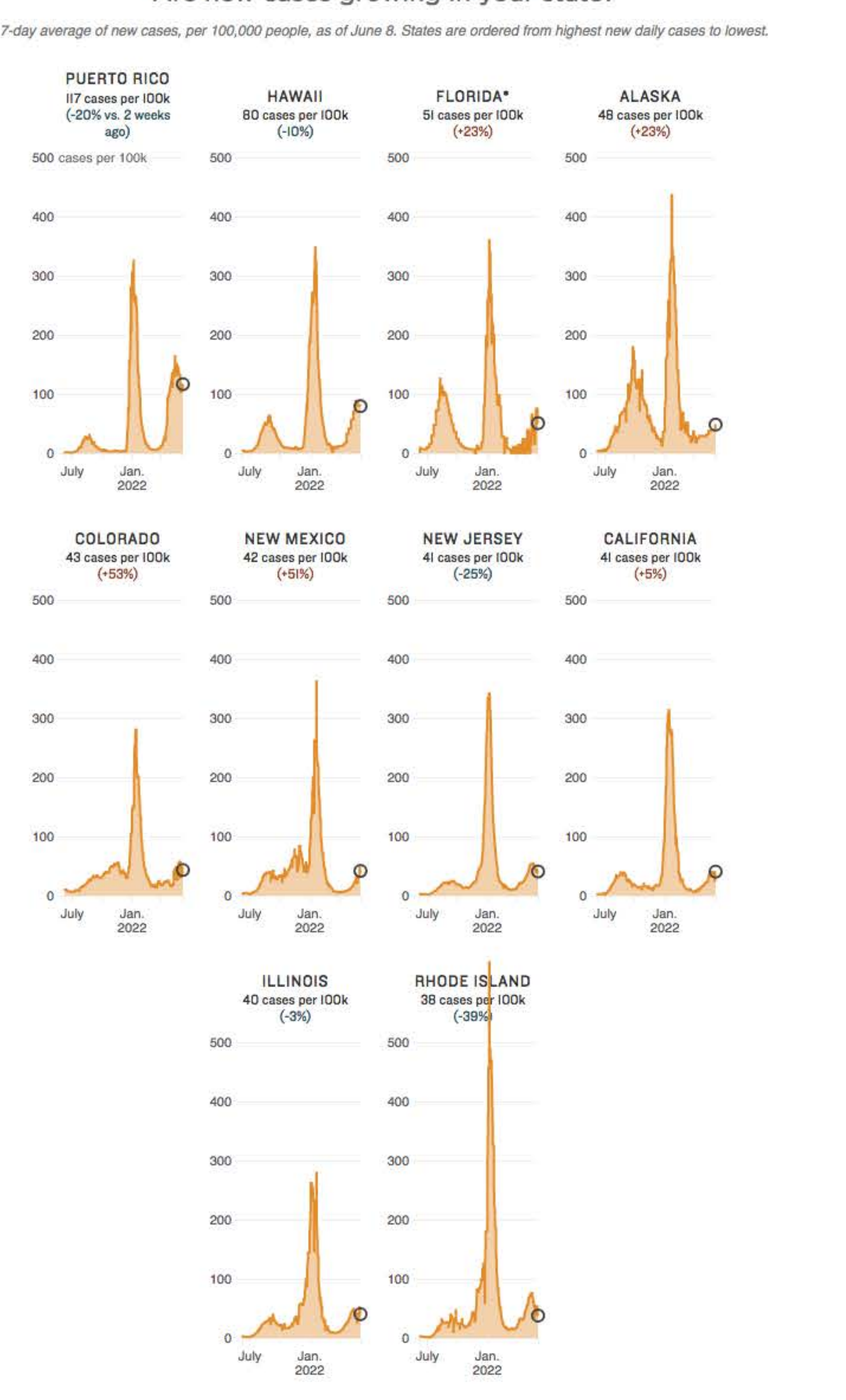

SHOW MORE ▼

Notes

*Data anomalies: In late March, Arizona and Georgia reported backlogged data with their new counts. Florida has shifted to reporting case and death numbers every other week. Johns Hopkins University relies largely on state dashboards for its data. This graphic excludes places where JHU reports no new data in the past week.

Source: Center for Systems Science and Engineering at Johns Hopkins University

How many people have COVID-19 right now in New Hampshire?

This reflects the number of people in New Hampshire confirmed to have COVID-19 on each date, as reported by state health officials, plus the seven-day rolling average for the same metric.

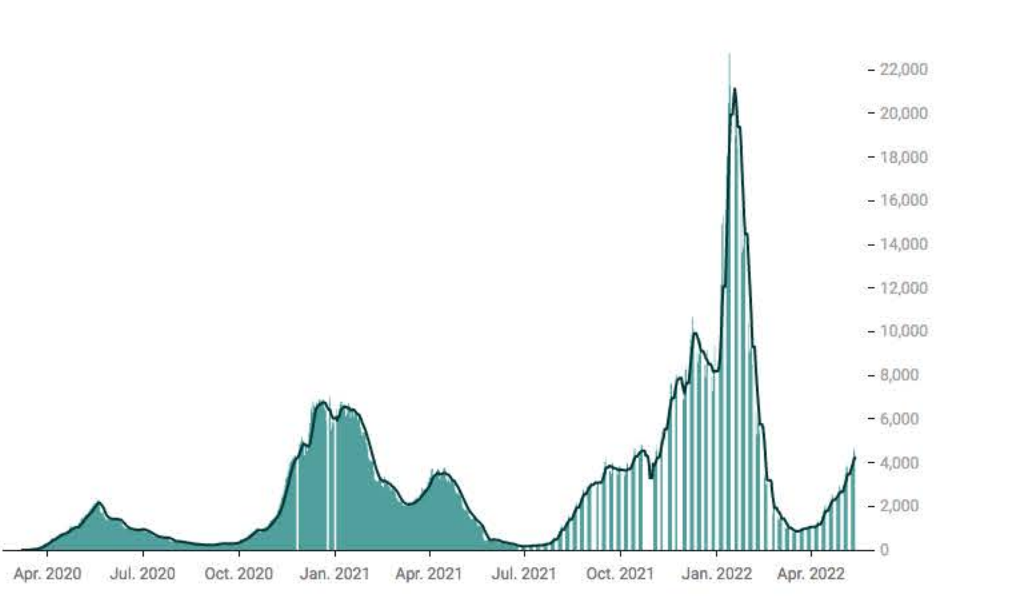

***Note:** The state skipped reporting on several holidays and, starting in June 2021, on weekends. This could make it appear that cases decreased and then spiked more than they actually did after those dates. We recently adjusted our formula for calculating weekly averages in response to the state's changes in its reporting schedule, which resulted in a slight shift in some of the numbers on this chart.*

Chart: NHPR • Source: New Hampshire Department of Health and Human Services • Created with Datawrapper

How many people are hospitalized in New Hampshire?

New Hampshire's recent COVID-19 hospitalization data offers only a limited window into coronavirus-related hospitalizations. The figures reported by state health officials don't include patients who remain hospitalized while recovering even after they're no longer considered to have active COVID-19 infections. And since March 29, 2022, the state has only counted COVID-19 patients being treated with Remdesivir, Dexamethasone or both therapies.

The New Hampshire Hospital Association has more comprehensive COVID-19 hospitalization statistics. You can find more of their hospitalization data at this link.

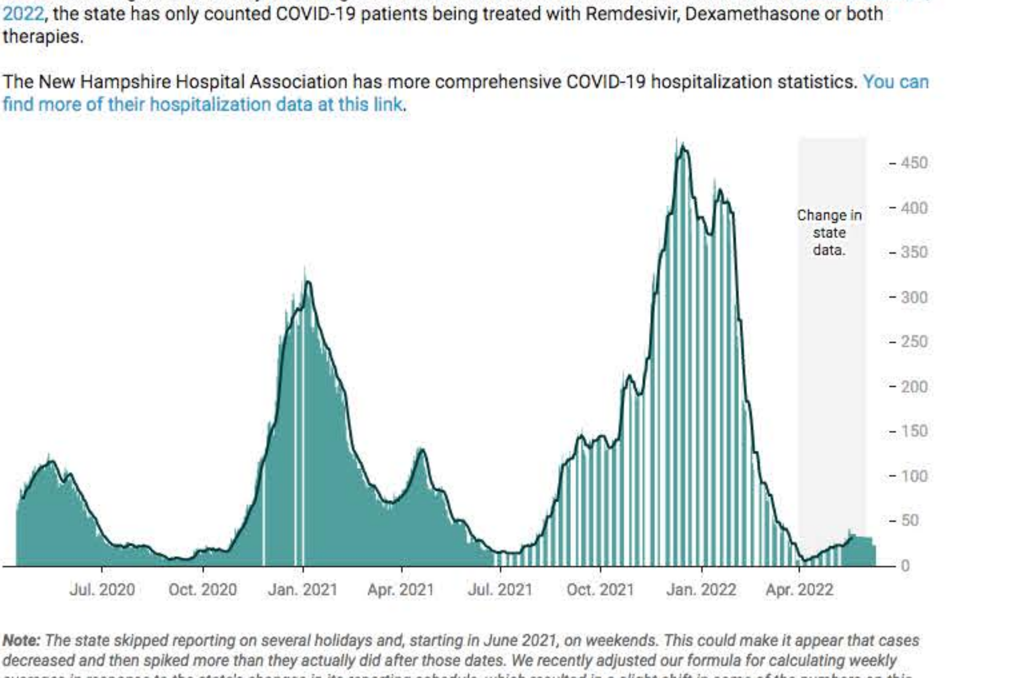

***Note:** The state skipped reporting on several holidays and, starting in June 2021, on weekends. This could make it appear that cases decreased and then spiked more than they actually did after those dates. We recently adjusted our formula for calculating weekly averages in response to the state's changes in its reporting schedule, which resulted in a slight shift in some of the numbers on this chart.*

Chart: NHPR • Source: New Hampshire Department of Health and Human Services • Created with Datawrapper

How full are local hospital beds, and how does that compare to other parts of the country?

These graphics were produced by NPR, and some of the numbers might differ from those reported by local agencies. For more context and details on NPR's methodology, we strongly encourage you to check out their full reporting here.

Where hospitals are filling up

Percent of hospital beds occupied by COVID-19 patients the week of May 27-June 2 (average by hospital)

No data | Low (0-5% COVID patients) | Moderate (5-9%) | High (10-20%) | Extreme (20+%)

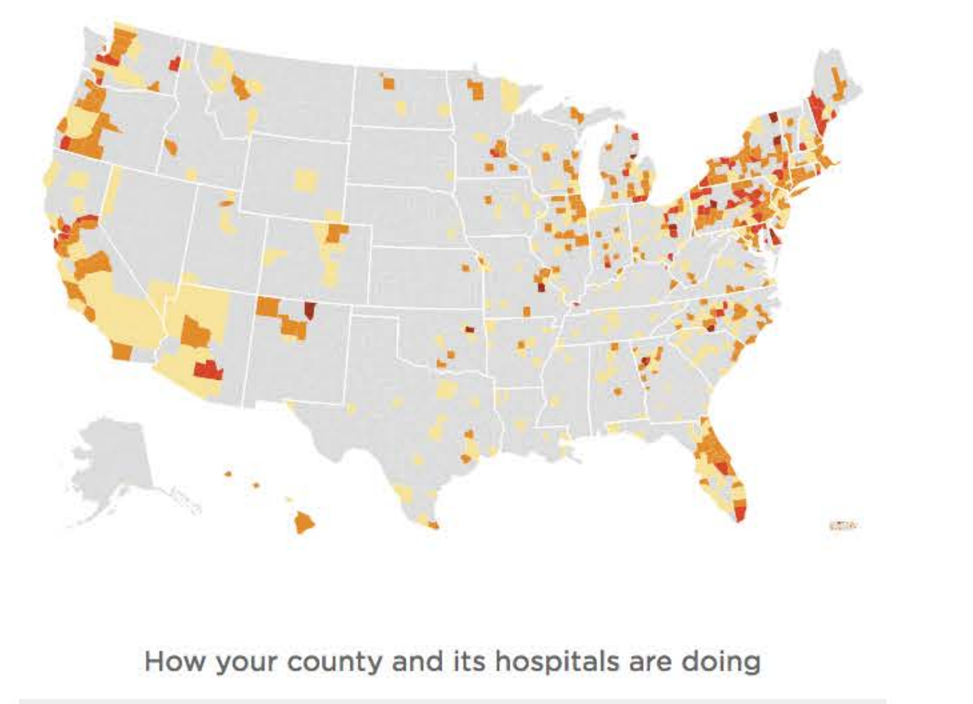

How your county and its hospitals are doing

SEARCH BY **COUNTY** TO SEE HOSPITALS

How many people are dying from COVID-19?

Please note that this only reflect the death totals *reported* on each date, not the number of deaths that occurred on each date. There can be a lag between when someone dies from COVID-19 and when their death is confirmed by state health officials. The state's COVID-19 dashboard has more information on cases by date of death.

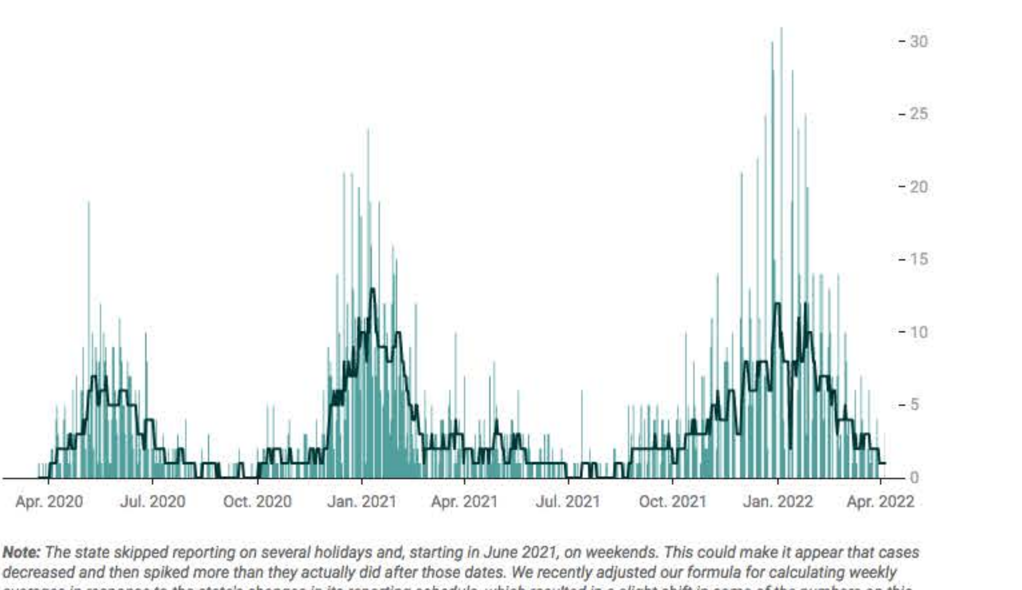

***Note:** The state skipped reporting on several holidays and, starting in June 2021, on weekends. This could make it appear that cases decreased and then spiked more than they actually did after those dates. We recently adjusted our formula for calculating weekly averages in response to the state's changes in its reporting schedule, which resulted in a slight shift in some of the numbers on this chart.*

Chart: NHPR • Source: New Hampshire Department of Health and Human Services • Created with Datawrapper

How many people have been vaccinated, and how is New Hampshire doing compared to other states?

A word of caution: In the fall of 2021, state officials disclosed that their vaccine data is likely inaccurate due to problems with New Hampshire's immunization tracking systems. The Centers for Disease Control and Prevention also disclosed that they were mistracking many of New Hampshire's booster shots and third shots as first doses of the COVID-19 vaccines. **That means that New Hampshire's rate of first doses, as reported by the CDC, is likely inaccurate.** Please keep this in mind as you're reading the charts below.

Read more: N.H.'s COVID-19 vaccination data hasn't been accurate since June. Why?

The first graphic below was produced by NPR. You can find more of their COVID-19 maps and charts here.

How is the vaccine rollout going in your state?

VACCINATIONS | DISTRIBUTION

STATE	% BOOSTED (OF FULLY VACCINATED POP.)	% FULLY VACCINATED (OF TOTAL POP.)	% ADULTS FULLY VACCINATED (18+)	% SENIORS FULLY VACCINATED (65+)	% WITH AT LEAST ONE DOSE (OF TOTAL POP.)
**United States**	47%	66.7%	76.7%	91.2%	78%
Rhode Island	54.4%	83.4%	91.7%	95%	95%
Vermont	61.4%	81.7%	88.3%	95%	94.2%
Maine	56.9%	80.2%	88.9%	95%	91.4%
Connecticut	52.3%	79.9%	88.8%	95%	95%
Massachusetts	52.1%	79.7%	87.6%	95%	95%
District of Columbia	41.5%	78.9%	88%	95%	95%
Hawaii	52.4%	78.7%	88%	93.2%	87.9%
New York	46.2%	77.5%	87.5%	93.1%	90.7%
Maryland	51.9%	76.2%	85.6%	95%	87.1%

SHOW MORE

Notes

— The vaccine is not currently authorized for use in children younger than 5 years old. Trials are underway on COVID-19 vaccines for younger age groups. Children under 18 represent 24% of the total U.S. population and can catch and spread the virus.

— "Fully vaccinated" means a person has received one shot of the Johnson & Johnson vaccine, or two shots of the Pfizer or Moderna vaccine.

— Doses distributed may underestimate the actual number of doses available (for example, if additional doses can be drawn out of the vials)

— Data on COVID-19 vaccines administered is pulled from the CDC, and may differ from state dashboard sites. According to CDC, the discrepancies occur because the data are reported by multiple sources, including pharmacies, states and federal entities, through various reporting systems.

Source: Centers for Disease Control and Prevention (as of June 8)

Who's Vaccinated in New Hampshire, By Age

Until late March, the COVID-19 vaccine was only open to the general public above age 65 in New Hampshire. State officials say this data might not reflect the full picture of vaccination rates, especially for older adults, because it doesn't include shots given through a federal long-term care vaccination program.

It's also likely that the numbers below are not fully accurate due to recently disclosed problems with New Hampshire's immunization tracking systems.

	Est. % of Population With 1+ Dose	Est. % of Population Fully Vaccinated
Statewide Totals	63%	56%

nhpr News from New Hampshire and npr
NHPR
Sound Opinions
NEXT UP

	Est. % of Population With 1+ Dose	Est. % of Population Fully Vaccinated
40-49	64%	59%
50-59	70%	64%
60-69	87%	81%
70-79	98%	89%
80+	94%	84%

These numbers reflect data reported on New Hampshire's COVID-19 Vaccination Dashboard as of Jan. 4, 2022.

Chart: NHPR • Source: N.H. Department of Health and Human Services • Created with Datawrapper

Who's Vaccinated in New Hampshire, By Race/Ethnicity

State data shows that Black and Latino residents have been falling behind in New Hampshire's COVID-19 vaccine rollout for months. For the most up-to-date data on vaccine equity, check out the state's dashboard.

It's also likely that the numbers below are not fully accurate due to recently disclosed problems with New Hampshire's immunization tracking systems.

	Est. % of Population With 1+ Dose	Est. % of Population Fully Vaccinated
Statewide Totals	64%	57%

	Est. % of Population With 1+ Dose	Est. % of Population Fully Vaccinated
Other Races	71%	65%
Asian	64%	56%
White	56%	50%
Hispanic/Latino	49%	41%
Black or African American	39%	33%

These numbers reflect data reported on New Hampshire's COVID-19 Vaccination Dashboard as of Feb. 16, 2022.

Chart: NHPR • Source: N.H. Department of Health and Human Services • Created with Datawrapper

New Jersey COVID-19

NJ COVID-19 Hub

Vaccination Overview | Cases and Trends | Hospital Census | Long Term Care | Case and Mortality Summaries

COVID-19 Dashboard

Filter by County:
STATEWIDE

Census data is updated every 10 years. When the pandemic began, the most recent Census data available was from 2010, and these numbers were used to calculate case, hospitalization, and death rates. Since 2020 Census data is now available (and should more accurately reflect current populations in NJ), the state Health Department updated the county populations upon which rates are calculated. Thus death (and other) rates on the dashboard will vary slightly from what had previously been posted. The total number of deaths has not been changed.

On April 26, 2021, New Jersey implemented an automated method to remove duplicate case reports, leading to a one-time drop of 10,442 confirmed cases from the total confirmed cumulative COVID-19 cases. Learn more.

Notes

More information about this data layer, including date/time

Click a county to view its metrics

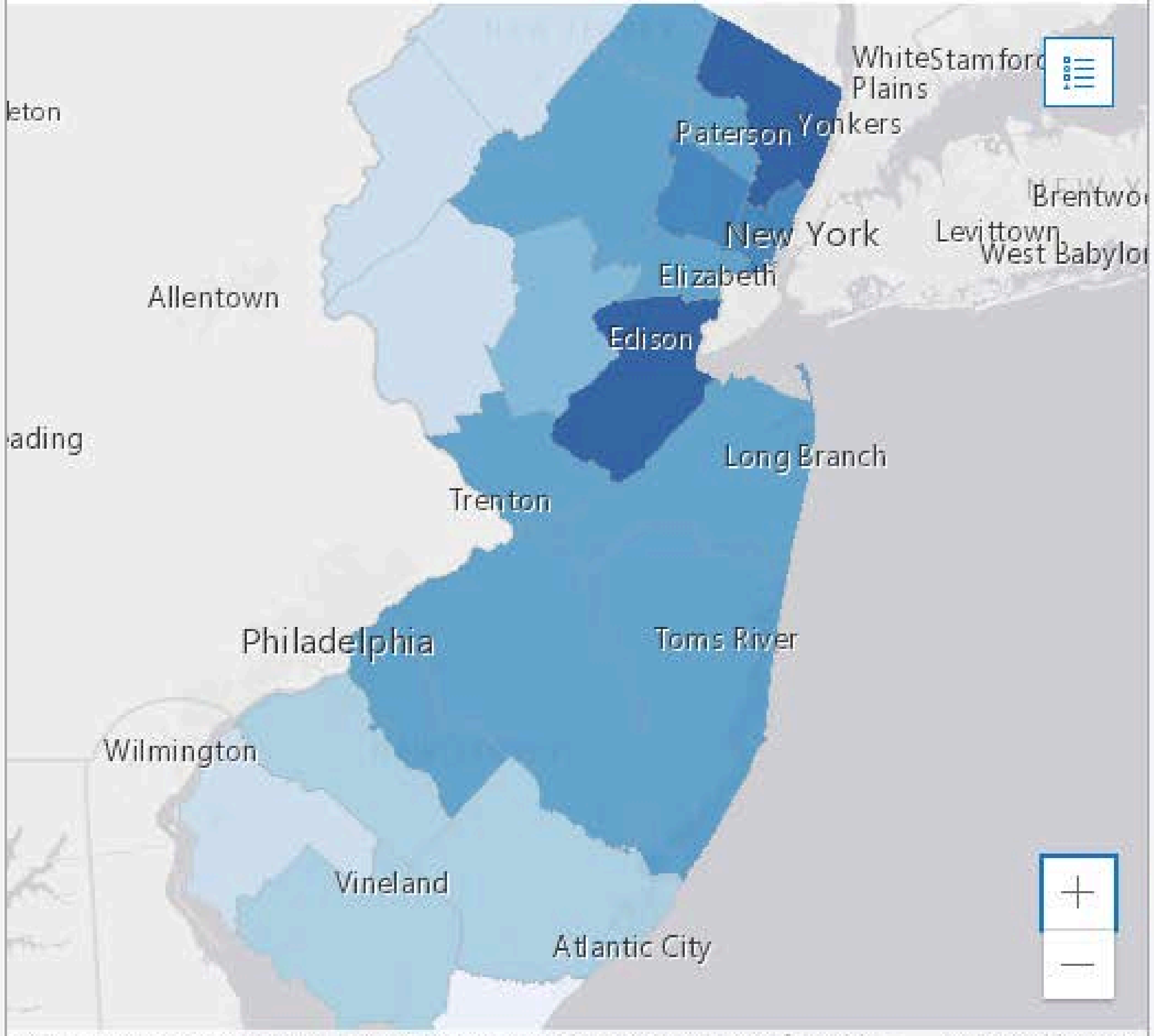

Esri, HERE, Garmin, USGS, EPA, NPS | New Jersey Department of Health -… Powered by Esri

2,461
Confirmed Cases - County Not Yet Assigned

Map | Case Counts | Variants

New Confirmed Cases[1]

1,918

Established via PCR testing

New Probable Cases[2]

371

Established via antigen testing

New Lab-Confirmed Deaths

1

Source: CDRSS

Hospital-reported Deaths (last 24h)[3]

7

Rate of Transmission (Rt)

0.89

Statewide

Daily Metrics

esri A Story Map

Go Back to Main COVID-19 page

New Jersey COVID-19

NJ COVID-19 Hub

Vaccination Overview | Cases and Trends | Hospital Census | Long Term Care | Case and Mortality Summaries

Vaccination Over...

Map Fi... All Ages | 5-11 | 5+ | 12-17 | 12+ | 18+ | 30+

Filter Demographics by C...
STATEWIDE

Due to a coding error, approximately 200,000 Pfizer (adult formulation) doses were not being classified as Pfizer doses. However, those doses were being included in the count of total vaccine doses administered in NJ. As of Feb 25, 2022, these doses are now included in the number of Pfizer doses administered. This change will contribute to statewide increases in the number of people with at least one dose, the number of fully vaccinated people, and the number of Pfizer additional/booster/3rd doses administered.

Due to a filtering error, the additional/3rd/booster doses were included in the counts for the People Vaccinated - Primary Series tab. These doses are now filtered out as of December 7th, 2021.

Notes

This dashboard is updated once daily, usually between 10:30-11:30 a.m.

Map of vaccination coverage refreshed weekly, current data as of 6/8/2022 and

Persons with at Least One Dose, % of population

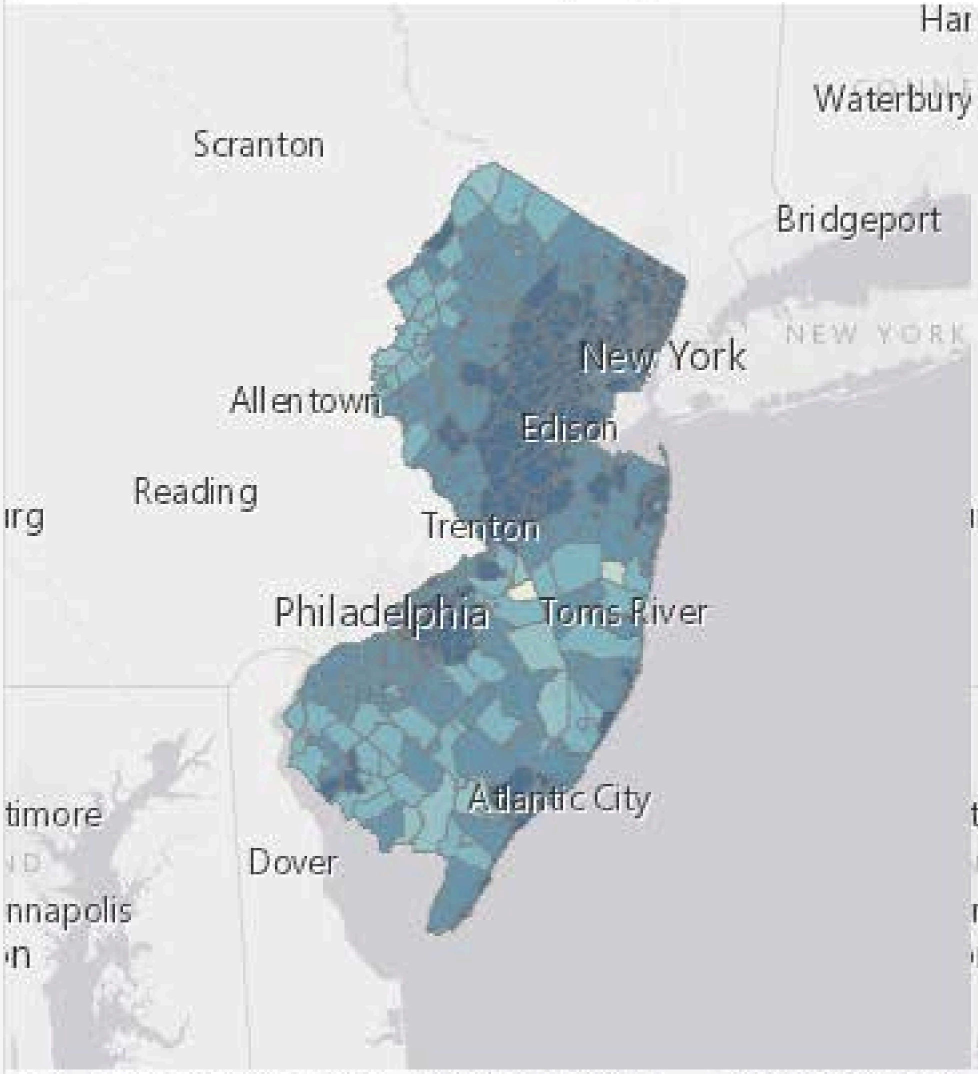

Esri, HERE, Garmin, USGS, EPA, NPS | Esri, H... Powered by Esri

Individuals who have received at least one dose of any vaccine (combines the population of those who have initiated courses or completed courses)

Persons with Vaccine Courses Complete, % of population

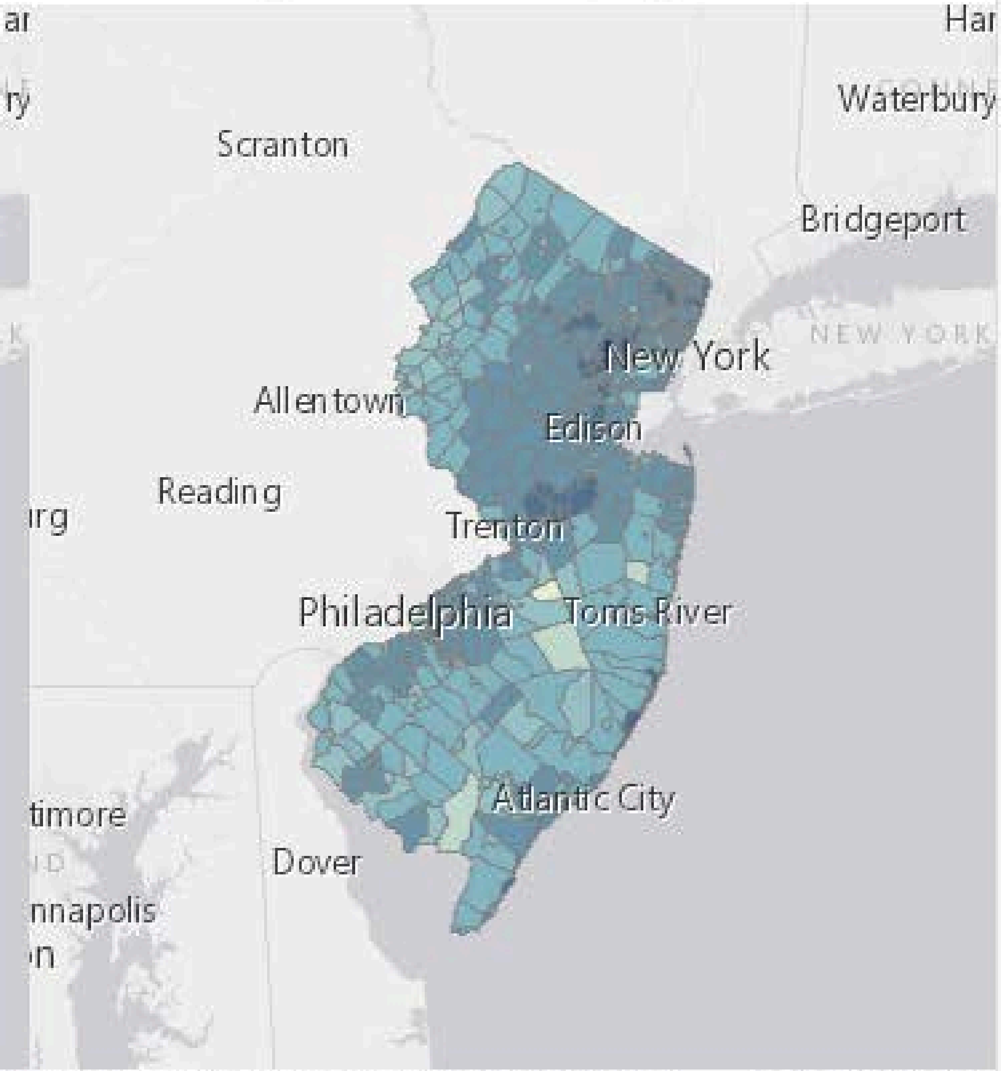

Esri, HERE, Garmin, USGS, EPA, NPS | Esri, H... Powered by Esri

Individuals who have received a single dose from a one-dose vaccine course, e.g., the J&J vaccine, or their second dose from a two-dose course

Map of Vaccination Coverage

esri A Story Map

NMDOH

New Mexico Department of Health

COVID-19 in New Mexico

Back to CV.NMHEALTH.ORG

NUMBER OF CASES

544,867

TOTAL # OF TESTS PERFORMED

7,293,689

TOTAL HOSPITALIZATIONS

27,132

CURRENT HOSPITALIZATIONS

130

TOTAL DEATHS

7,873

TOTAL RECOVERED

510,801

Show Historical Statewide Data

Current COVID case outlook 5/17/2022 - 5/30/2022

DATA IS UPDATED EVERY OTHER WEEK. NEXT UPDATE: 6/14/2022. DAILY CASE COUNT AND TEST NUMBERS ARE RAW DATA BASED ON INFORMATION THE STATE RECEIVES TODAY - MEANING DATA THAT HAS NOT YET BEEN SCRUTINIZED BY STATE EPIDEMIOLOGISTS TO IDENTIFY

Map Key: Fewer Cases — More Cases View Map Key

Choose a county

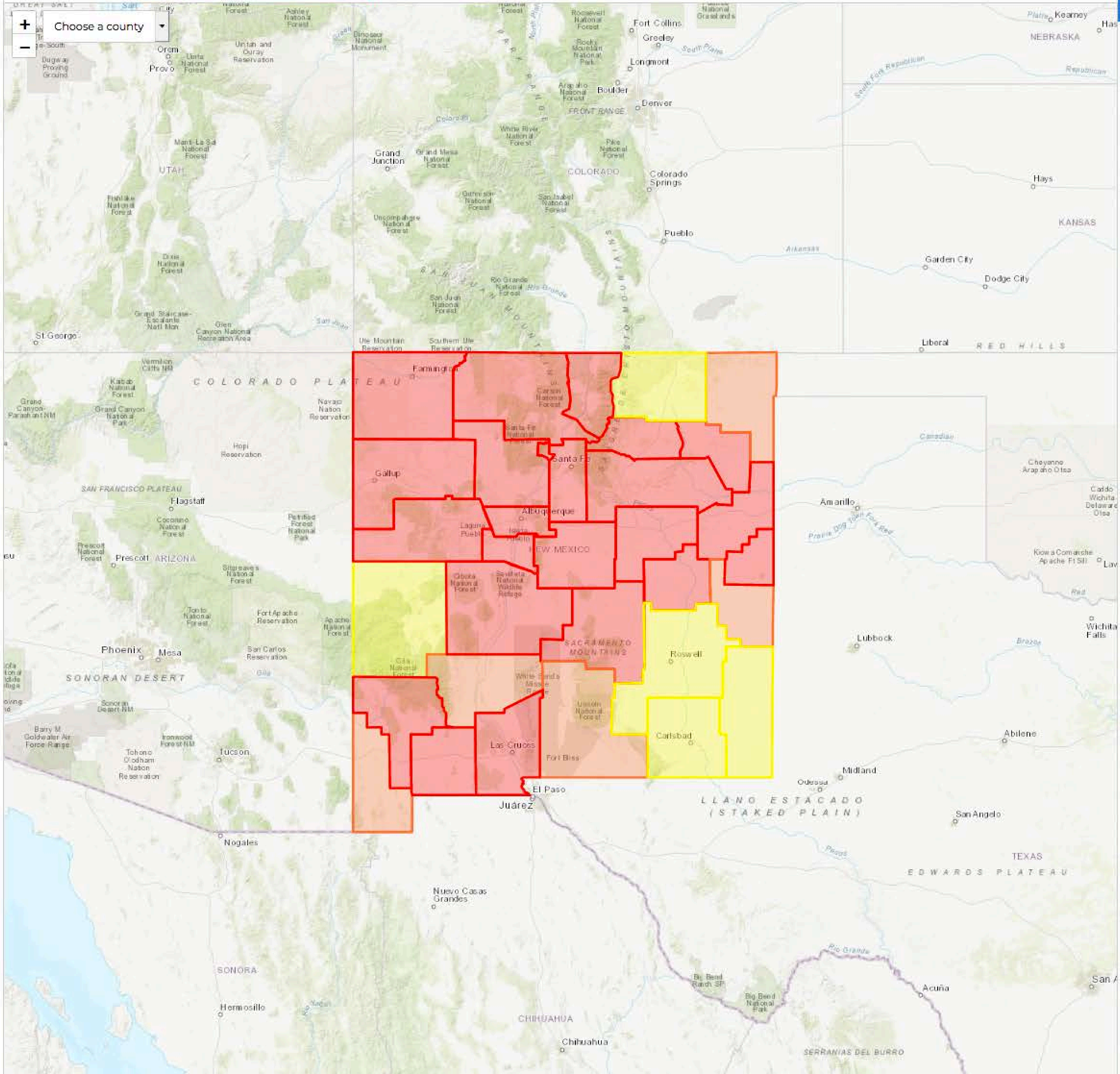

EXPAND DEMOGRAPHICS VIEW

Statewide Gender Breakdown

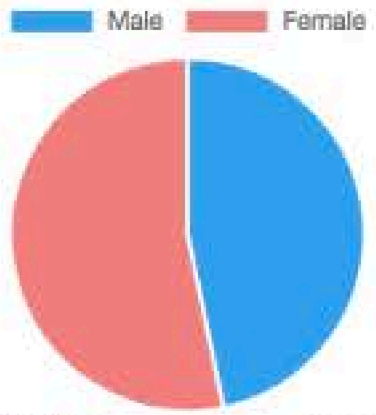

Gender	Cases
Male	46.74%
Female	53.26%

Statewide Age Breakdown

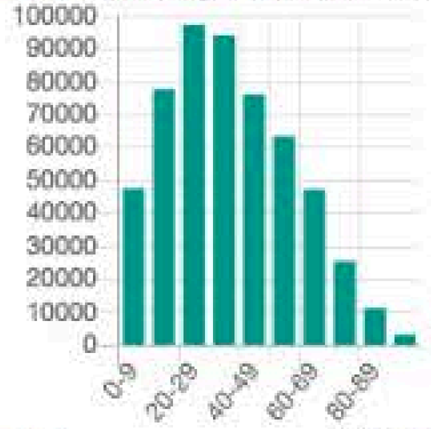

Age	Cases
0-9	47649
10-19	77898
20-29	97520
30-39	94283
40-49	76256
50-59	63532
60-69	47387
70-79	25623
80-89	11207
90+	3300

Statewide Race/Ethnicity Breakdown

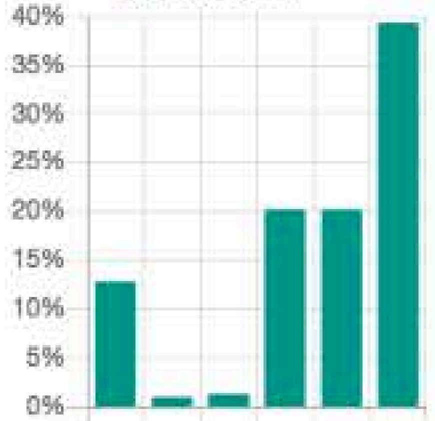

Race	Cases
American Indian/Alaska Native	12.82%
Asian	0.97%
Black/African American	1.24%
White	20.21%
Unknown	20.19%
Hispanic/Latino	39.35%

NEW YORK STATE | Department of Health

New York State Department of Health COVID-19 Tracker

Testing data as of: 6/11/2022 2 PM
Testing data last updated on: 6/12/2022

Statewide

Tested Positive (6/11)
4,278

Total Results Received (6/11)
83,995

Tested Positive (Cumulative)
5,478,263

Total Results Received (Cumulative)
112,277,448

Tested Positive by Sex (Cumulative)

Female	Male	Unknown
52.1%	46.2%	1.7%

Positive Tests by County

- ○ Cases per 100k, 7-day avg
- ◉ Test % Positive, 7-day avg*

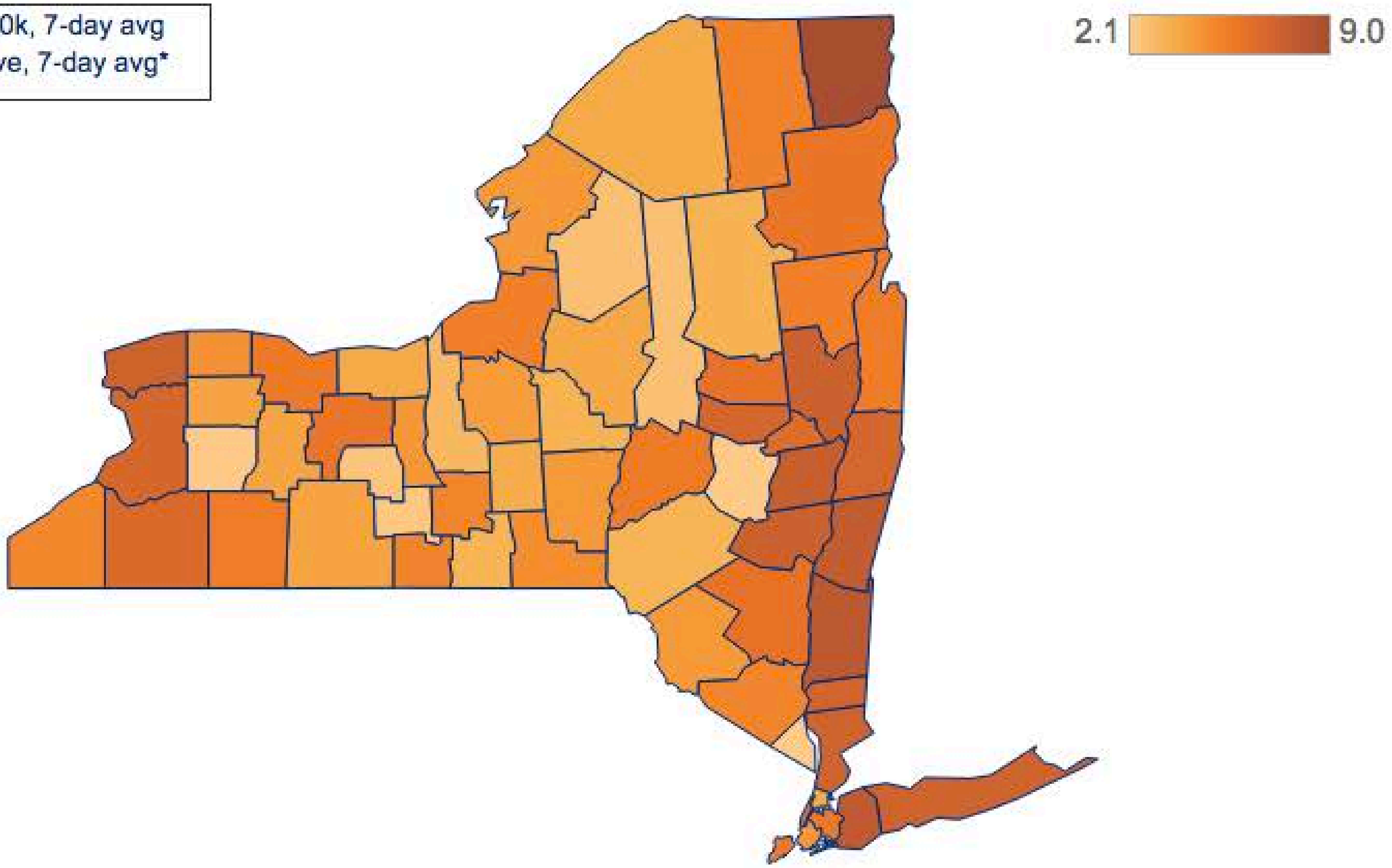

County Testing Details: **None**

Click County to See Detail
Click Again for Statewide

Albany
Allegany
Bronx
Broome
Cattaraugus
Cayuga
Chautauqua
Chemung
Chenango
Clinton
Columbia
Cortland
Delaware
Dutchess
Erie
Essex
Franklin
Fulton
Genesee
Greene
Hamilton
Herkimer
Jefferson
Kings
Lewis
Livingston
Madison
Monroe
Montgomery

State of Ohio | COVID-19 Dashboard

Last Updated: 06-09-22
Updated Thursdays

Other states do not send death certificates to ODH's Bureau of Vital Statistics on a regular schedule and therefore fluctuations will be reflected in reported mortality data. Deaths are assigned to their appropriate date of death.

Starting the week of June 6, there will be a two-week pause in the assignments of causes of death in the Ohio Department of Health's Office of Vital Statistics because the National Center for Health Statistics is implementing a new coding system.
While death records will continue to be added to our database, no specific causes of death will be assigned. This means there will be no additions to the COVID-19 death totals reported on June 9 and 16.
Once the new coding system is operational, all deaths that were entered during the pause will be updated with causes.

County Map | Cases

Select a County To Filter Dashboard

Case Classification Status
Filter all views by case classificati...
(All) Total Cases

View By
Pick an option to view in map
Cases

County Search
Search a county to highlight
Highlight County

County View:
View deaths by county
County of Residence

Ethnicity:
Select an Ethnicity to filter the vie...
(All)

Race
Select a race to filter the views
(All)

Start Date
1/1/2020

End Date
6/12/2022

Metrics | Cumulative and Daily Count

View Count By:
Select to view counts by daily or cumulative
Daily Count

Cases

* Preliminary

2,798,900

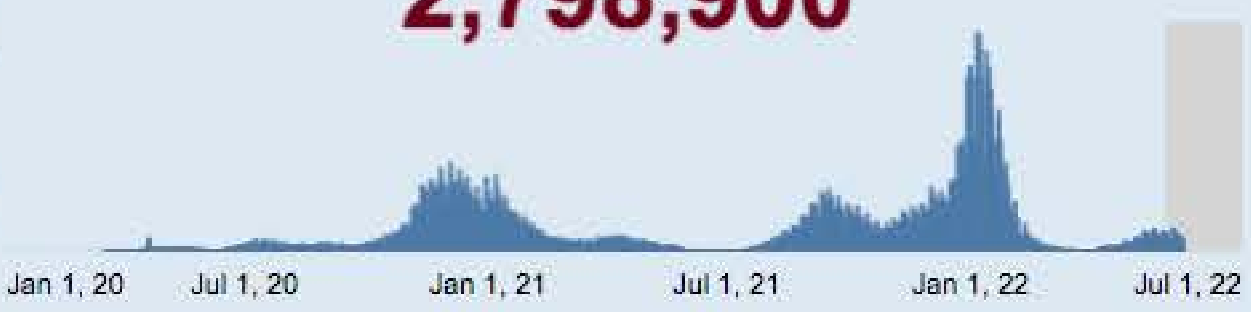

Hospitalizations

117,844

* Preliminary

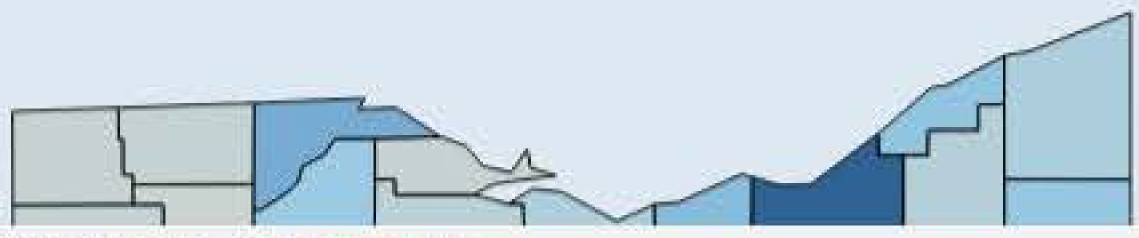

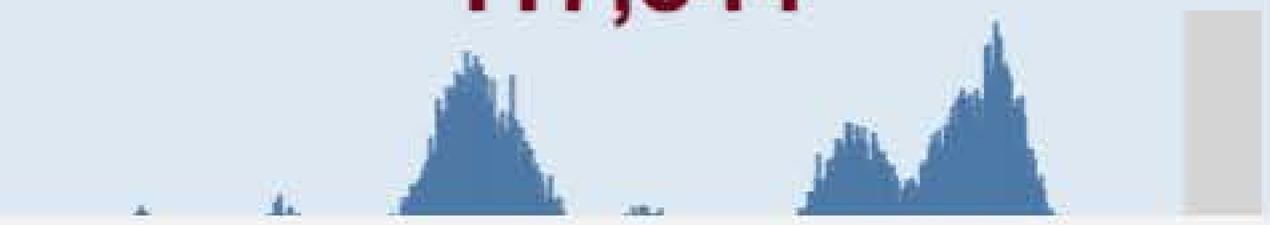

An official State of Ohio site. Here's how you know

Ohio Department of Health

Coronavirus (COVID-19)

COVID-19 Testing and Treatment
COVID-19 Vaccination Program
Equity and High-Risk Groups
Families and Individuals

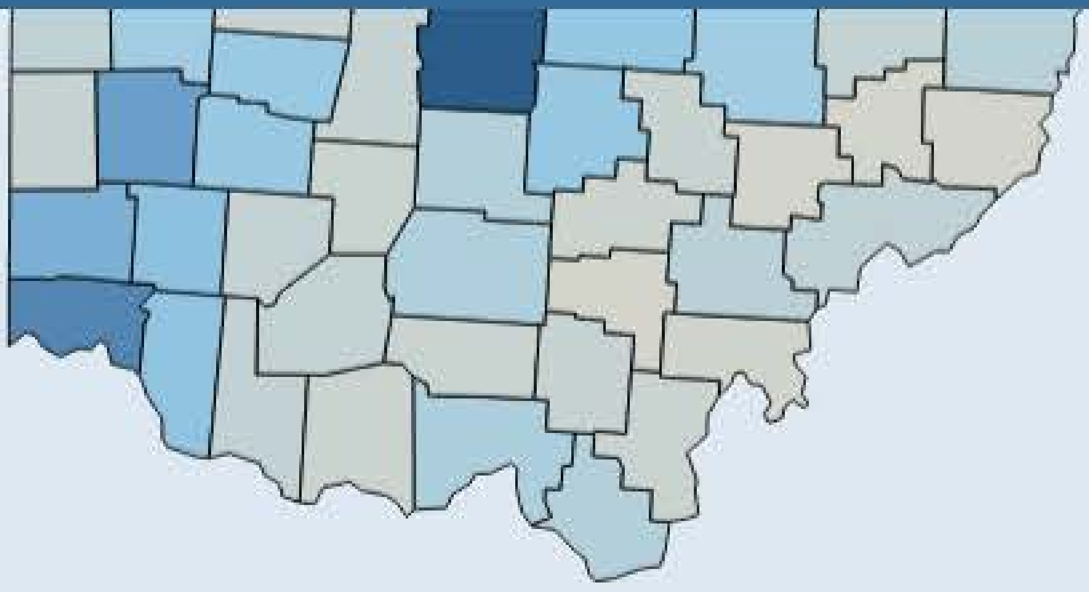

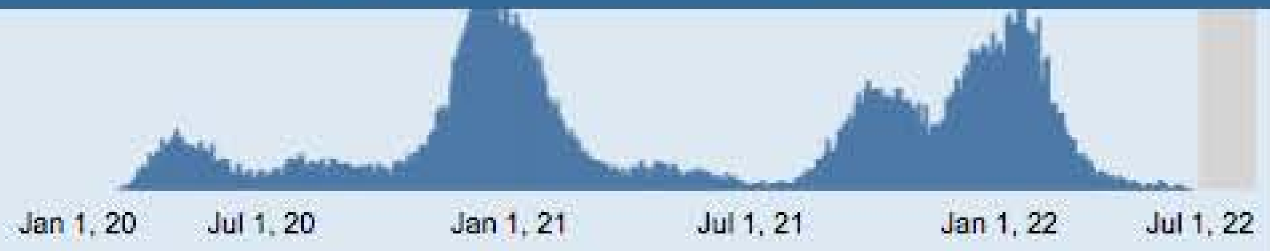

Presumed Recovered ***

2,704,859

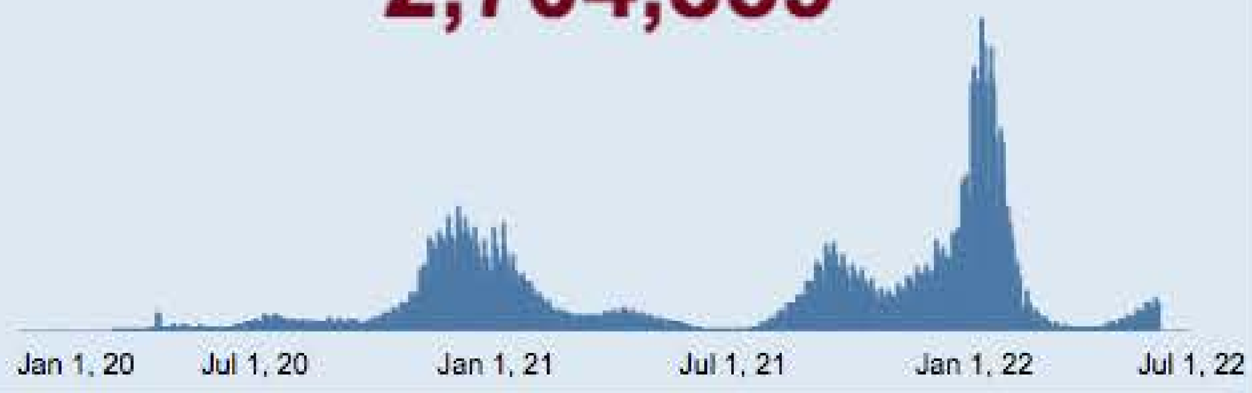

Key Metrics | By County

© OpenStreetMap

View By
Pick an option to change the view
County

View By	Case Count	Hospitalization Count	Death Count
Franklin	306,118	5,608	2,618
Cuyahoga	285,703	13,071	3,813
Hamilton	196,748	6,233	2,072
Montgomery	130,964	9,751	2,026
Summit	119,297	8,505	1,714
Lucas	106,736	3,973	1,423
Butler	96,063	3,277	1,160
Stark	82,906	4,253	1,720
Lorain	71,004	3,753	984
Warren	60,116	1,369	586
Mahoning	55,709	2,118	1,093
Clermont	53,444	1,506	562
Lake	51,329	1,503	738
Delaware	47,260	434	274

Note: Totals include Confirmed and CDC Expanded Case Definition (Probable)
All data displayed is preliminary and subject to change as more information is reported to ODH. Trends are based on the date of illness onset for cases, date of hospital admission for hospitalizations, and date of death for deaths. The date slider at the top of the dashboard will limit the graphical display of data using these date fields. The CSV download file will always show all dates, regardless of date slider settings. (For cases, if onset date is unknown, the earliest known date associated with the case is used as a substitute for the date of illness onset. For hospitalizations, if date of admission is not reported, these are omitted from the graphical display but are still included as counts in the CSV download file.)

* Preliminary case data in the shaded area is likely under-reported to ODH because of the delay between initial contact with a health care provider, testing, and diagnosis.
** Mortality data is verified by coded death certificate information received from the National Center for Health Statistics, and can take some time to receive. Deaths by county are presented based on the county recorded on the certificate of death. This information is not available daily, and is updated approximately once per week as data is received.
*** Presumed Recovered is defined as cases with a symptom onset date >21 days prior who are not deceased.

+ a b l e a u

Oklahoma COVID-19 Immunization Summary

6,012,281 Total Doses Administered

Source: CDC COVID Tracker - https://covid.cdc.gov/covid-data-tracker/#vaccinations_vacc-total-admin-rate-total

Immunization Updated As Of: **6/7/2022**

Goal: Vaccinate 70% of Oklahoma's eligible population.

Population (12+ yrs)

2,754,565 At Least 1 Dose

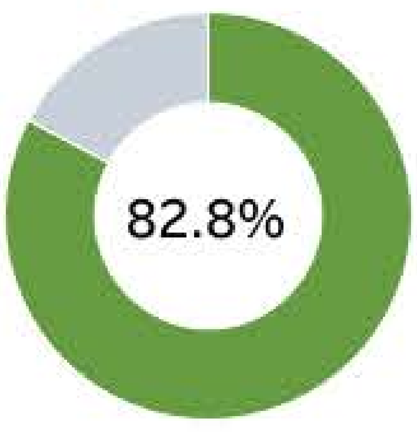

Total Population

2,831,900 At Least 1 Dose

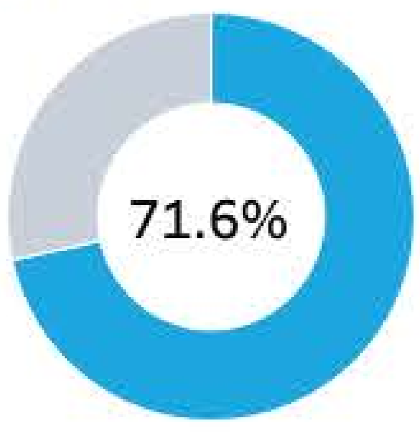

Population (12+ yrs)

2,223,148 Fully Vaccinated

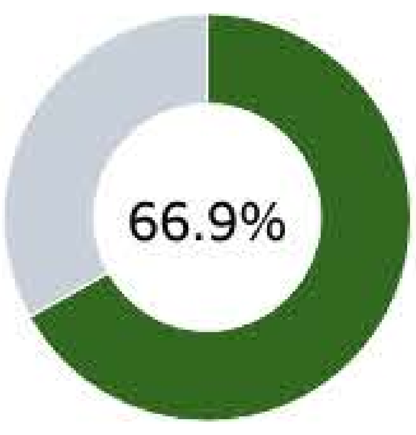

Total Population

2,284,445 Fully Vaccinated

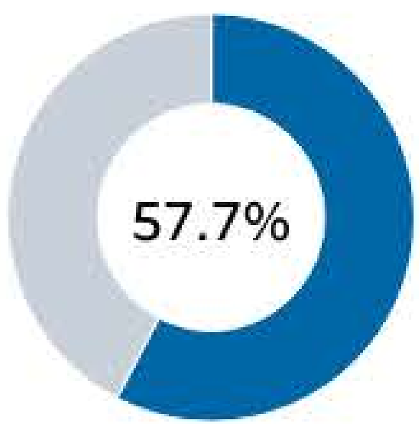

Vaccinations (Daily Doses Administered & Full Vaccinated People)

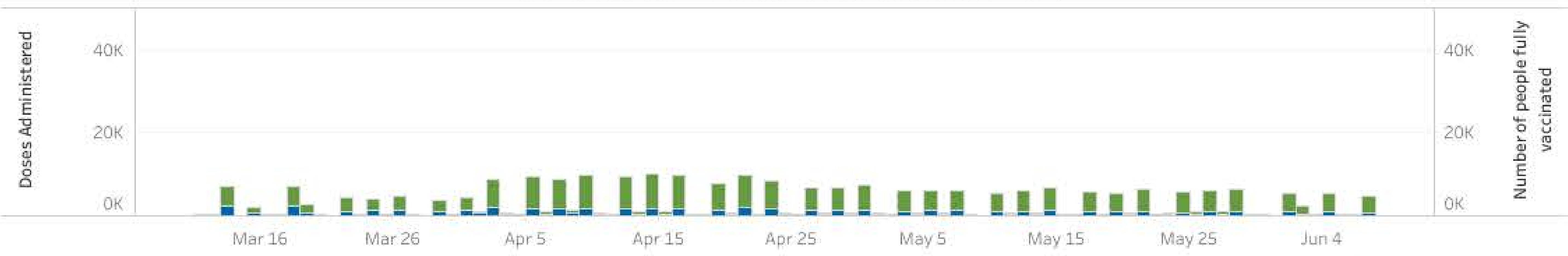

Vaccine Time Frame

Last 90 Days

Oklahoma Licensed Acute Care Hospitals

Source (unless otherwise noted): HHS Survey date **6/7/2022**

94 (88.7%) Facilities Reported

Response rate impact accuracy of data. Facilities may update previously reported information as necessary.

Hospitalizations by Vaccination Status,
Previous 30 days (as of April 12, 2022)
Source: OSDH Case Investigation Registry

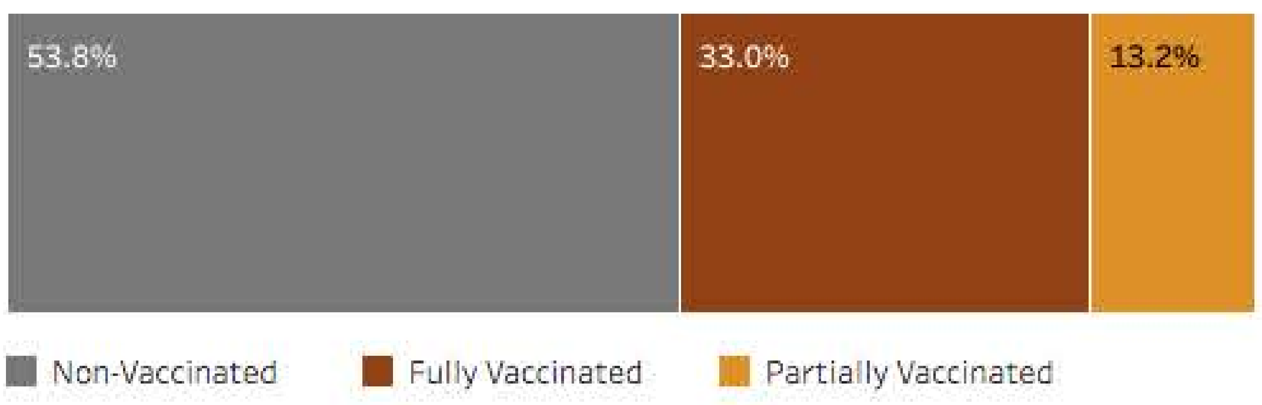

Most Recent Information Reported by Hosptials
(Response rates impact accuracy of regional totals)

Unoccupied Staffed Adult Inpatient Beds - 1,918
Unoccupied Staffed Adult ICU Beds - 192
Hospital Capacity Reports -
https://oklahoma.gov/covid19/newsroom/hhs-hospital-capacity-reports.html

COVID-19 Positive Adult Hospitalizations - 99
COVID-19 Positive Adults in the ICU - 23
COVID-19 Situation Updates and other News -
https://oklahoma.gov/covid19/newsroom.html

3 Day Average COIVD-19 Positive Adult **Hospitalizations** & **ICU**

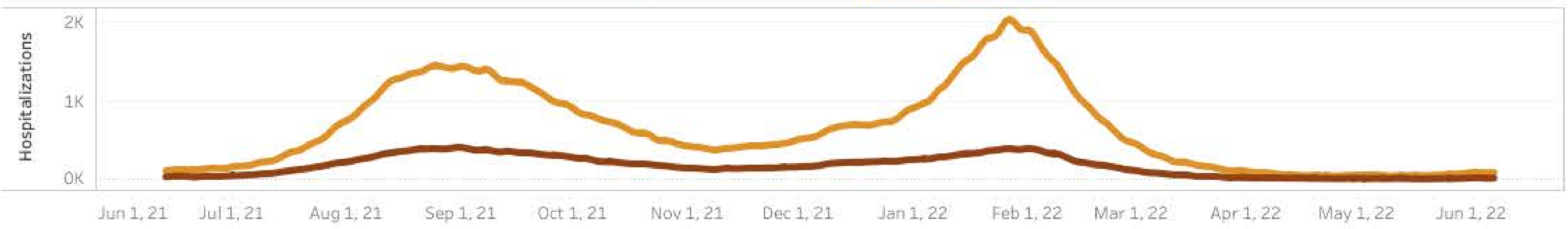

Case Information

Source (unless otherwise noted): OSDH Case Investigation Registry

Average Number of New Daily Cases 436.6

Weekly New Cases 3,056

Case Week May 29, 2022 - June 4, 2022

Cumulative Cases 1,054,953

CDC Provisional Deaths Mentioning COVID-19 as of **6/3/2022**

Oklahoma Occurances - **16,145**

https://www.cdc.gov/nchs/nvss/vsrr/COVID19/index.htm

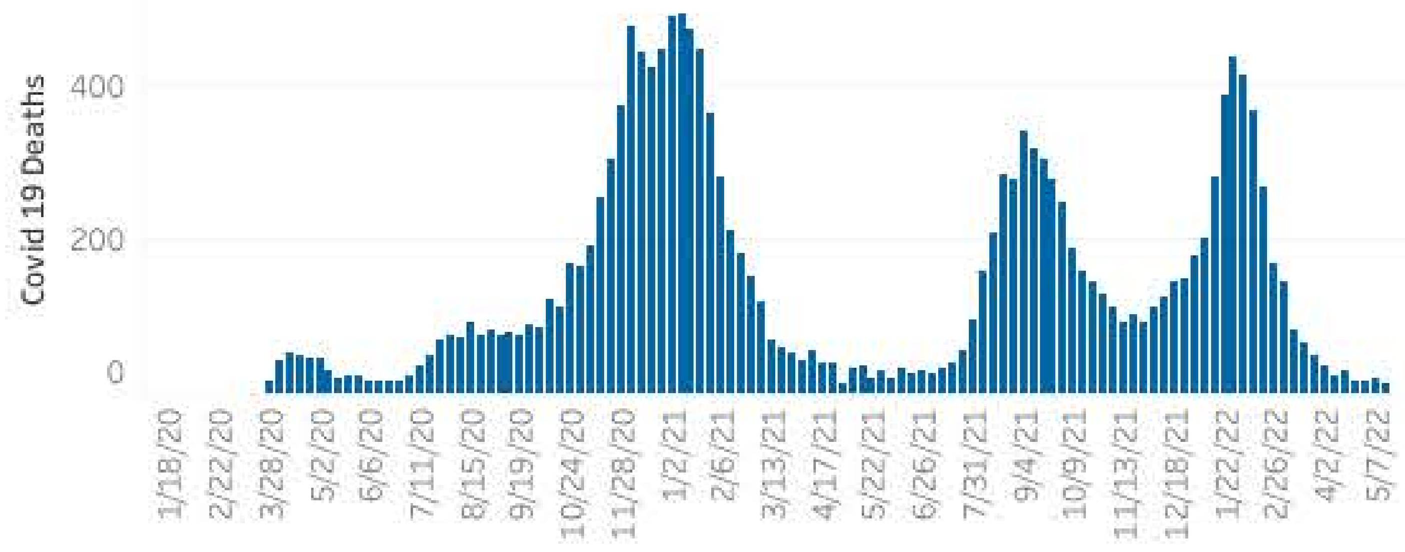

COVID-19 Cases per 100,000 Population (previous 4 weeks)

Click a County or select a Public Health District to see counts for that specific area.

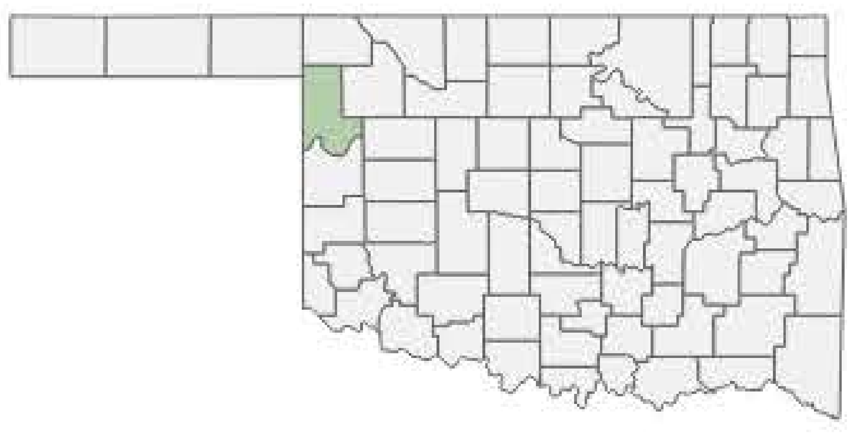

Age Distribution by Case Month

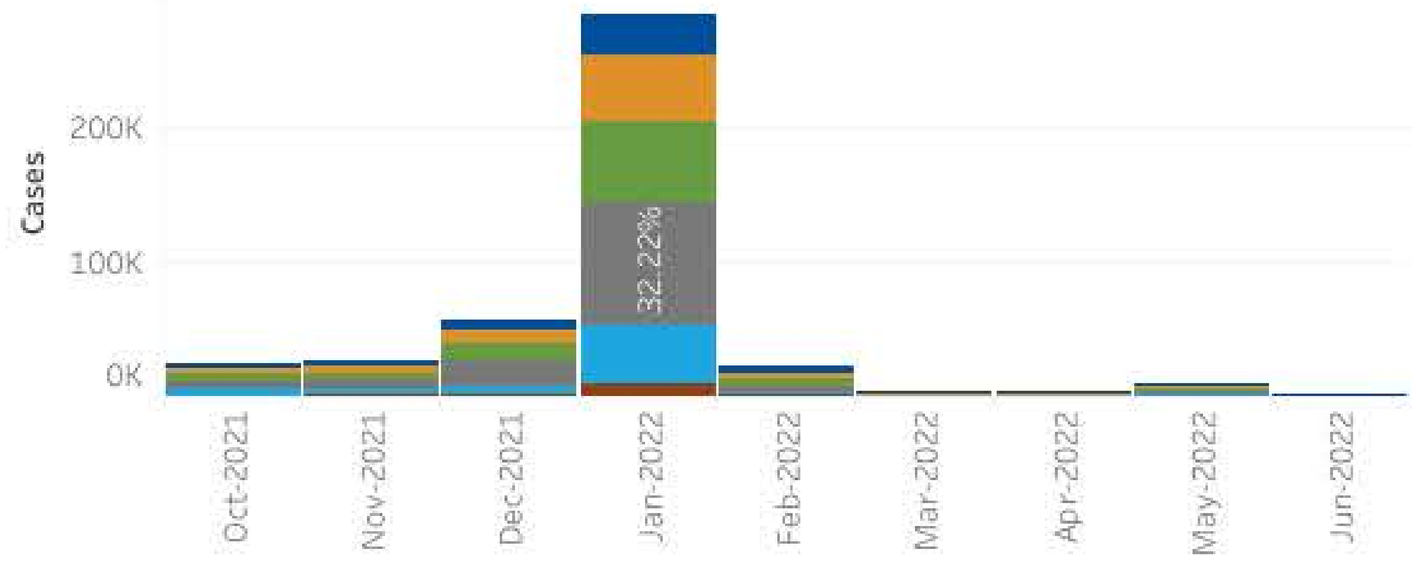

Public Health District

(All)

Age Group

65+ | 50-64 | 36-49 | 18-35 | 05-17 | 00-04 | UNK

Weekly Cases Last 90 Days

Public Health District: All

County: All

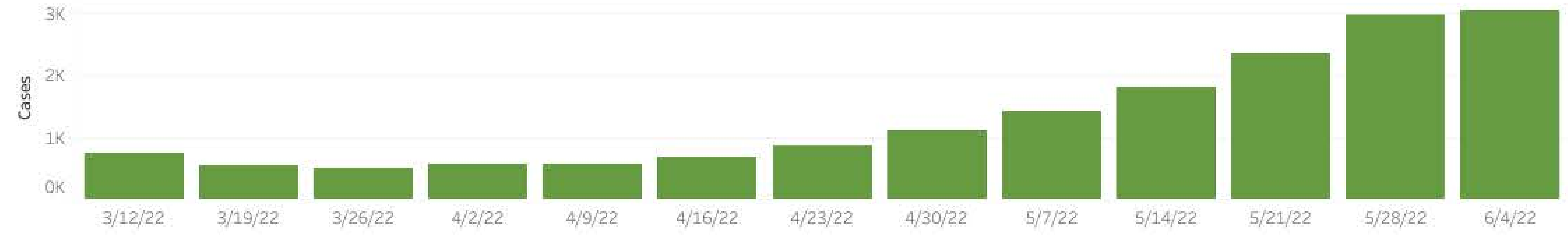

Case Time Frame

Last 90 Days

For additional COVID19 data please see the: **Weekly Epidemiology and Surveillance Report**-https://oklahoma.gov/covid19/newsroom/weekly-epidemiology-and-surveillance-report.html

Additional public health data, including a new COVID19 module, can be accessed through **OK2SHARE**-https://ok2share.health.ok.gov/

+ableau

Oregon Health Authority

The Oregon Health Authority (OHA) is part of the statewide response working to address and reduce the effects of COVID-19 on the lives of Oregonians. Data presented here are from the OHA Public Health Division.

OHA COVID-19 Website | View All COVID-19 Data Dashboards | Contact Us | County Table

Oregon's COVID-19 Update 6/9/2022

Data Current as of 12:01am 6/10/2022

CASES — Statewide

New cases	7-day average
1,146	1,570.0

Total cases

781,128

Source: Opera

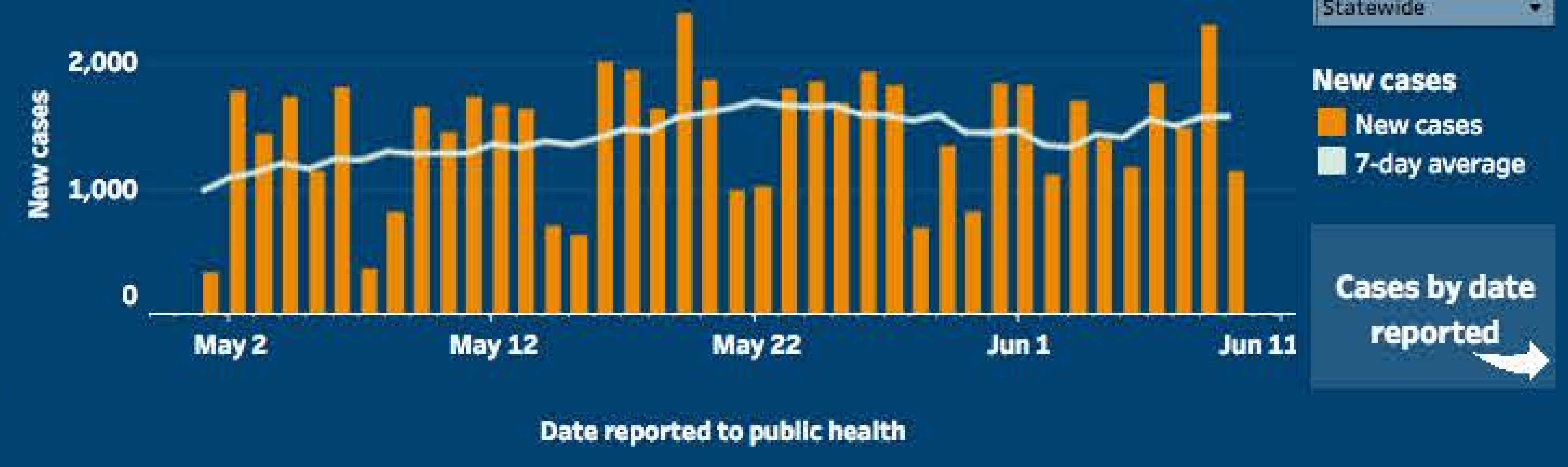

Newly reported deaths with COVID-19

6

Total deaths

7,693

Source: Opera

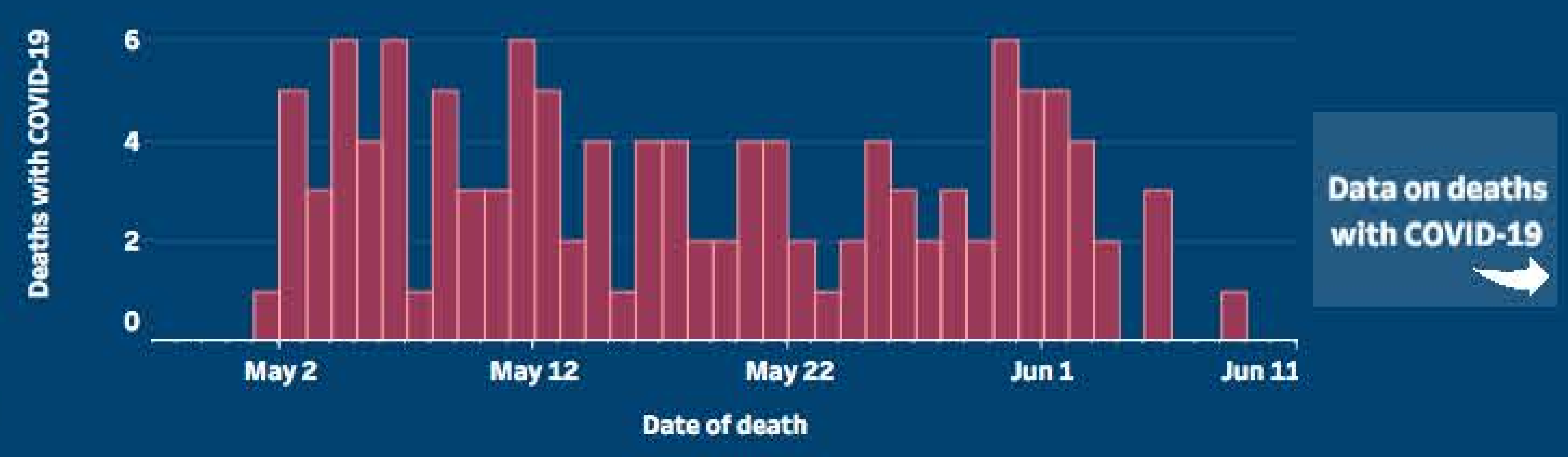

HEALTH CARE — Statewide

Current COVID-19 positive patients

Hospitalized	304
in ICU beds	29

Source: HOSCAP

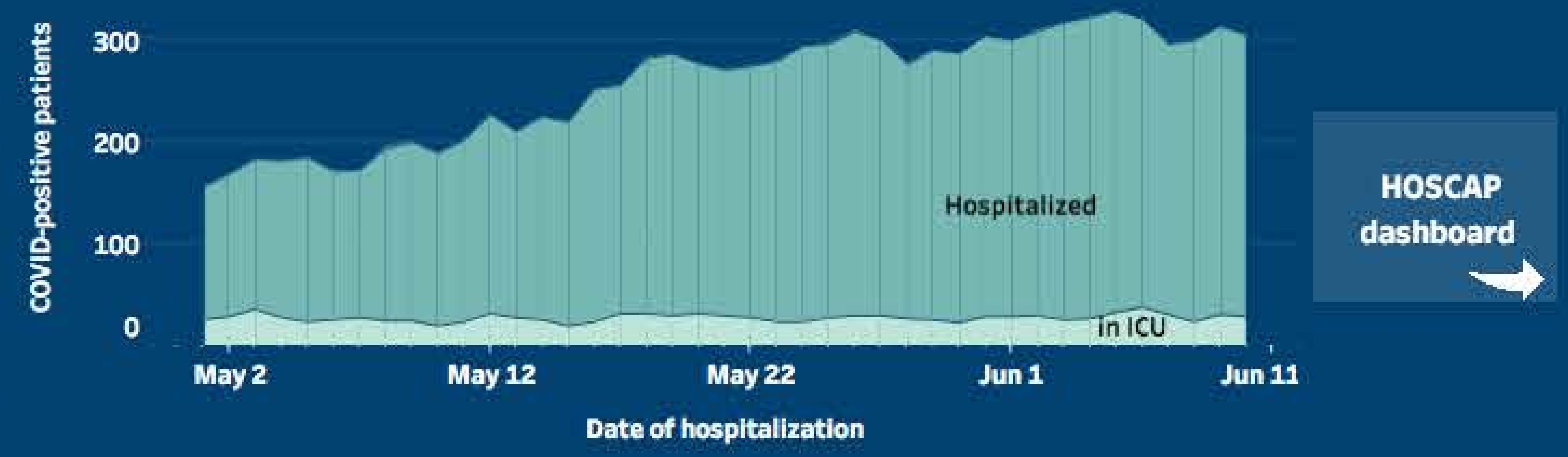

Emergency department visits for COVID-19-like illness (CLI)

3.9%

Source: Oregon ESSENCE

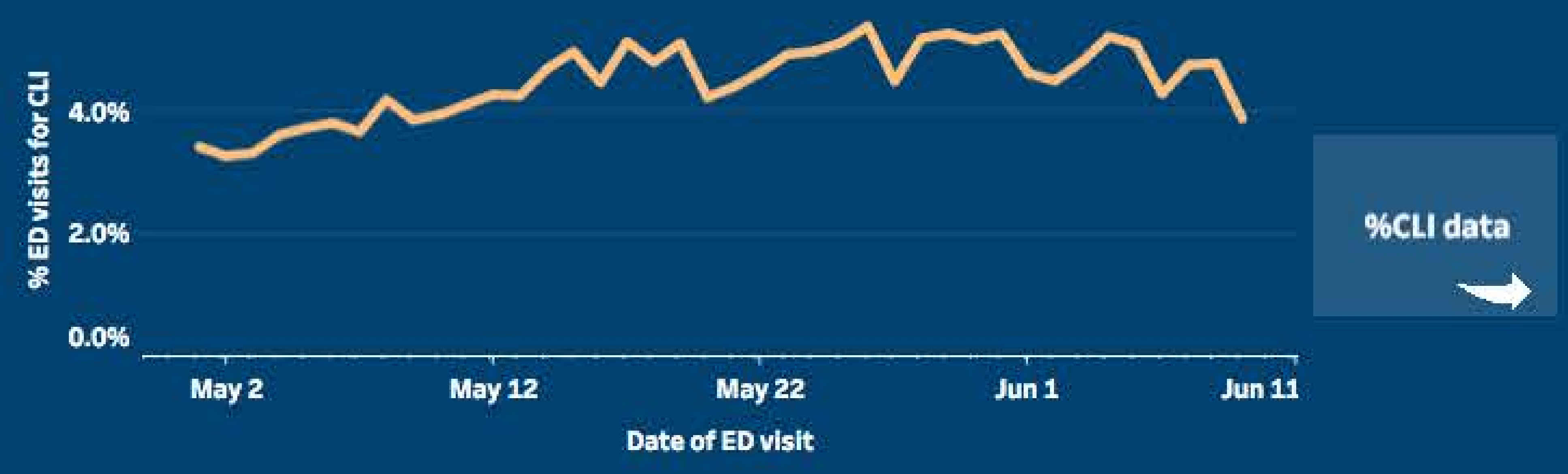

VACCINATION — Statewide

Vaccination coverage, statewide people ages 18+

One dose	84.0%
Boosted	46.1%

Updated weekly | Source: ALERT IIS

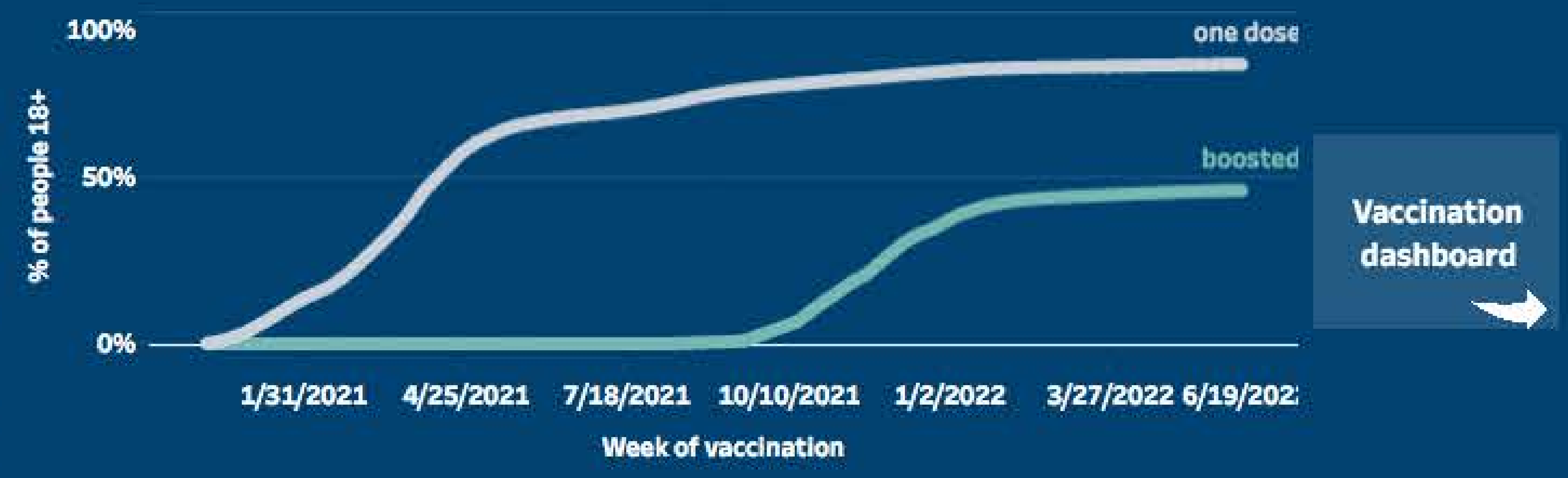

TESTING — Statewide | SEQUENCING — Statewide

Test positivity

11.2%

Tests reported

9,374

Source: ELR and OCRP

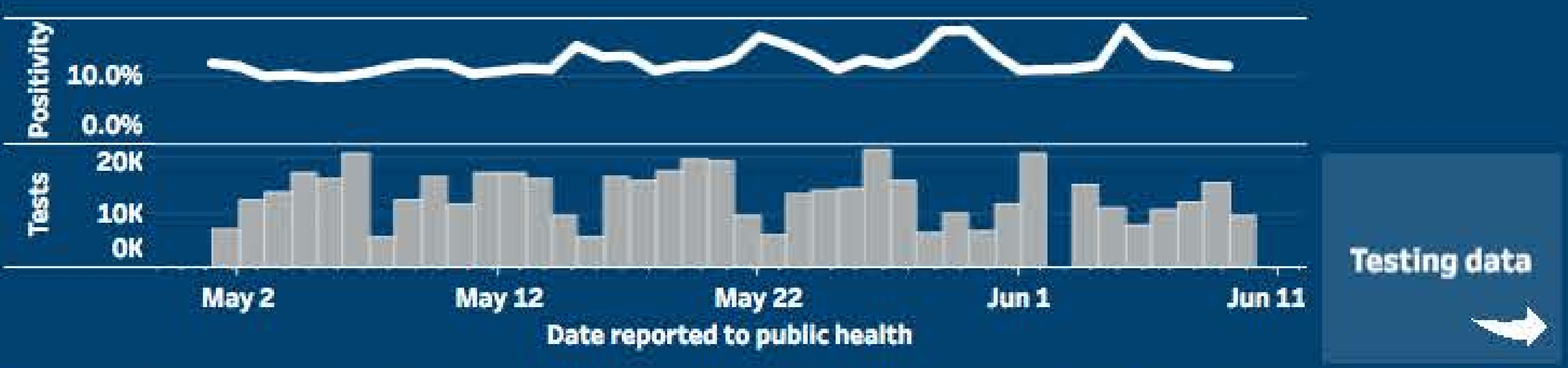

Due to a processing error, electronic laboratory report (ELR) counts for June 2, 2022 are inaccurate and will be excluded from data visualizations. Test counts and percent positivity for June 2 will not be available on this dashboard. Case counts for June 2, 2022 are accurate. OHA is working to find a solution to this processing error, which resulted in duplication, and will provide these data when available. The root cause of the issue has been addressed; OHA does not anticipate this error occurring again

Total Variants of Concern

Omicron (B.1.1.529, BA.1, BA.3)	12,142
Omicron (BA.2)	2,326
Omicron (BA.4)	15
Omicron (BA.5)	6

Updated weekly | Source: GISAID

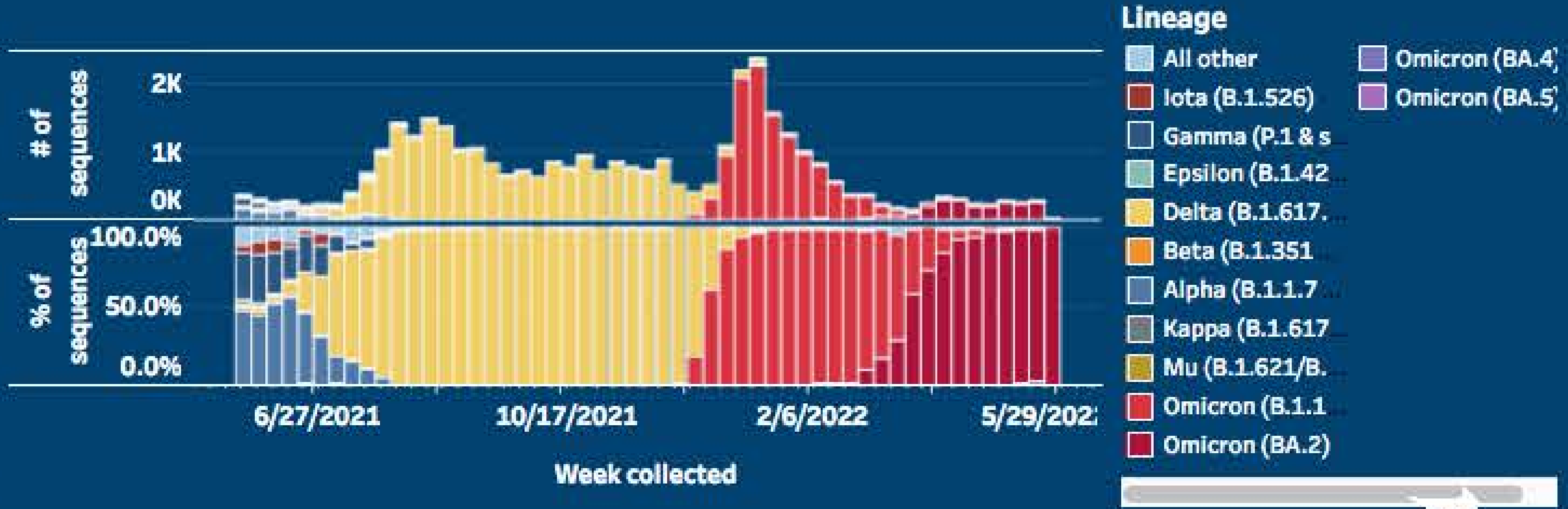

Data are provisional and subject to change.

Opera is Oregon's COVID-19 disease surveillance system. Total cases include both presumptive and confirmed cases. Presumptive cases are people without a positive diagnostic test who have COVID-19-like symptoms and had close contact with a laboratory confirmed case.

Oregon's Hospital Capacity Web System (HOSCAP) data are from the most recent report from the hospitals, as of 9:00am. Every hospital in Oregon is asked to submit data twice daily.

Oregon ESSENCE, Electronic Surveillance System for the Early Notification of Community-based Epidemics, allows for public health to monitor what is happening in emergency departments across the state. Oregon ESSENCE receives daily reports of emergency department visits from all 60 non-federal hospitals in Oregon.

Vaccination data are from the Oregon ALERT Immunization Information System (IIS). COVID-19 vaccination providers must report administration data to ALERT IIS no later than 72 hours after administration.

Testing data are from electronic laboratory reports (ELR) and the Oregon COVID-19 Reporting Portal (OCRP). Tests are counted by the date received by public health. Counts reflect individual tests and not individual persons tested. Non-antibody and non-'info only' COVID results are deduplicated based on medical record number (MRN), accession number, facility and result daily.

Variant data are from the open-source sequencing data platform GISAID and are updated weekly on Wednesdays. There is a lag of approximately two weeks between when a sample is collected, sequenced and then reported by GISAID; this chart reflects that time-lag.

tableau

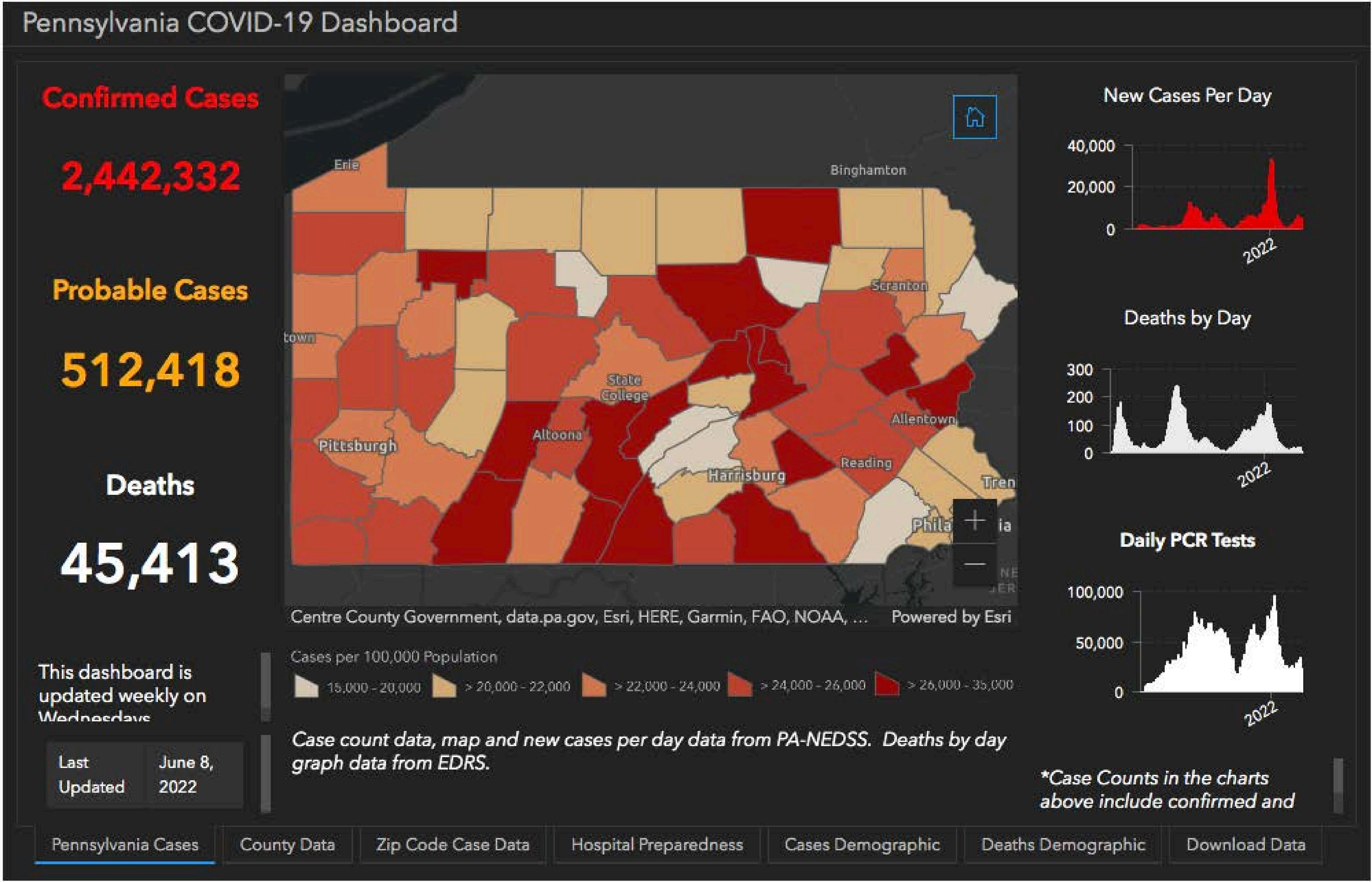

Pennsylvania COVID-19 Dashboard
Confirmed Cases
2,442,332
Probable Cases
512,418
Deaths
45,413
This dashboard is updated weekly on Wednesdays
Last Updated
June 8, 2022
Erie
Binghamton
Scranton
State College
Altoona
Pittsburgh
Allentown
Reading
Harrisburg
Centre County Government, data.pa.gov, Esri, HERE, Garmin, FAO, NOAA, ...
Powered by Esri
Cases per 100,000 Population
15,000 - 20,000
> 20,000 - 22,000
> 22,000 - 24,000
> 24,000 - 26,000
> 26,000 - 35,000
Case count data, map and new cases per day data from PA-NEDSS. Deaths by day graph data from EDRS.
New Cases Per Day
40,000
20,000
0
2022
Deaths by Day
300
200
100
0
2022
Daily PCR Tests
100,000
50,000
0
2022
*Case Counts in the charts above include confirmed and
Pennsylvania Cases
County Data
Zip Code Case Data
Hospital Preparedness
Cases Demographic
Deaths Demographic
Download Data

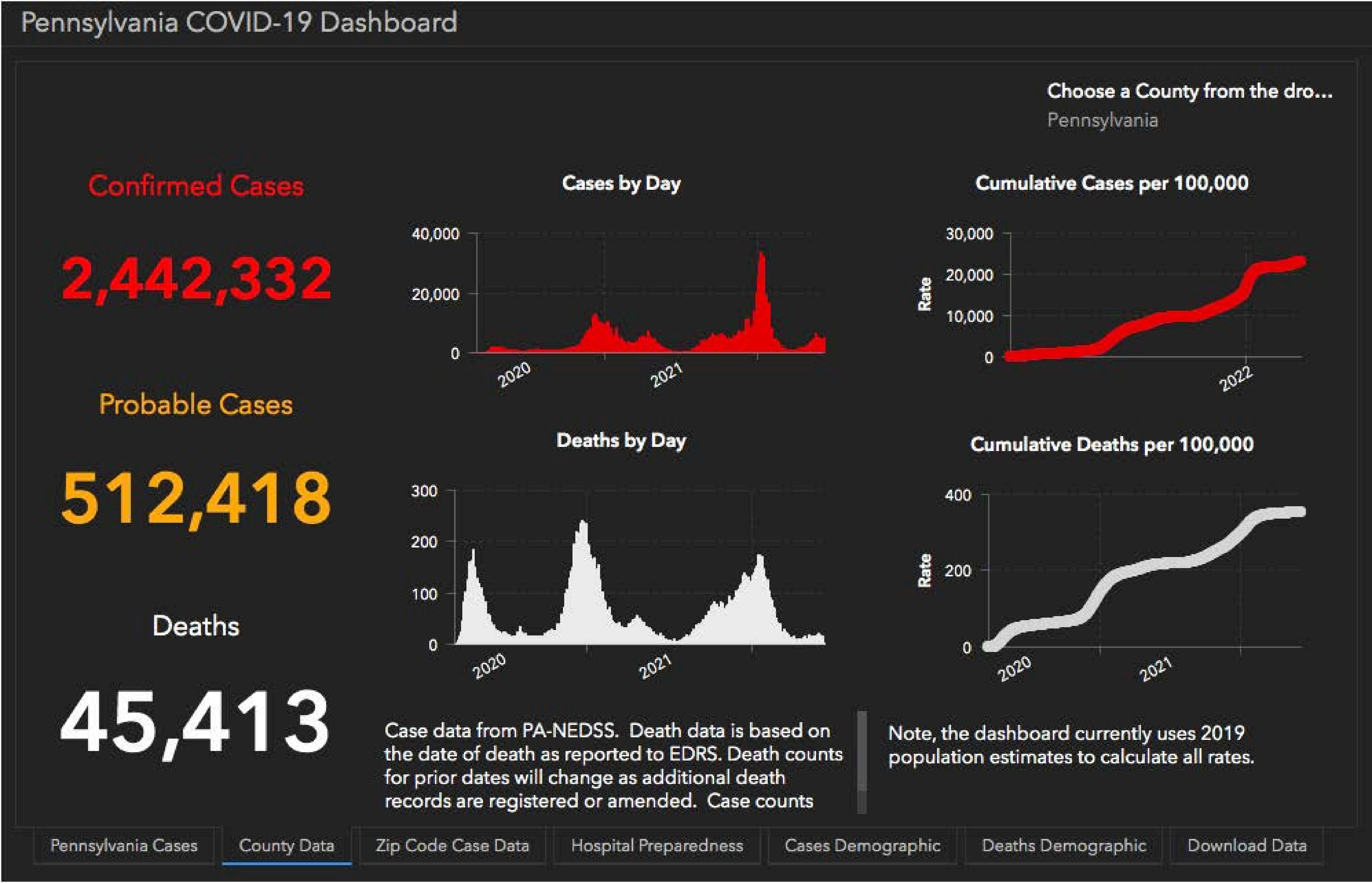
Pennsylvania COVID-19 Dashboard
Choose a County from the dro...
Pennsylvania
Confirmed Cases
2,442,332
Probable Cases
512,418
Deaths
45,413
Cases by Day
40,000
20,000
0
2020
2021
Cumulative Cases per 100,000
30,000
20,000
10,000
0
Rate
2022
Deaths by Day
300
200
100
0
2020
2021
Cumulative Deaths per 100,000
400
200
0
Rate
2020
2021
Case data from PA-NEDSS. Death data is based on the date of death as reported to EDRS. Death counts for prior dates will change as additional death records are registered or amended. Case counts
Note, the dashboard currently uses 2019 population estimates to calculate all rates.
Pennsylvania Cases
County Data
Zip Code Case Data
Hospital Preparedness
Cases Demographic
Deaths Demographic
Download Data

Pennsylvania COVID-19 Dashboard

Select a category
Pennsylvania

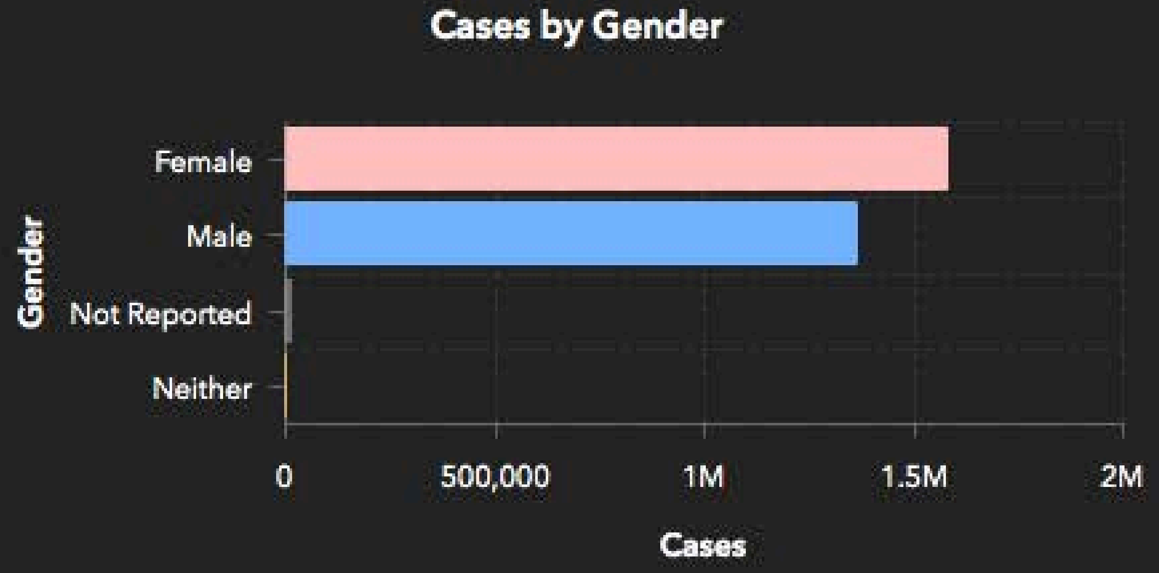

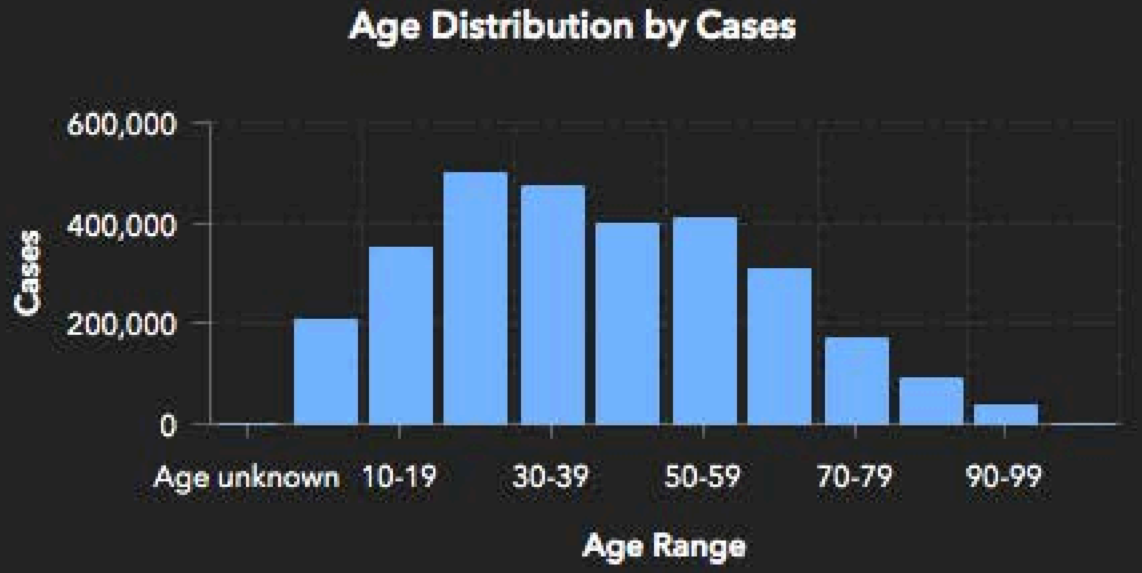

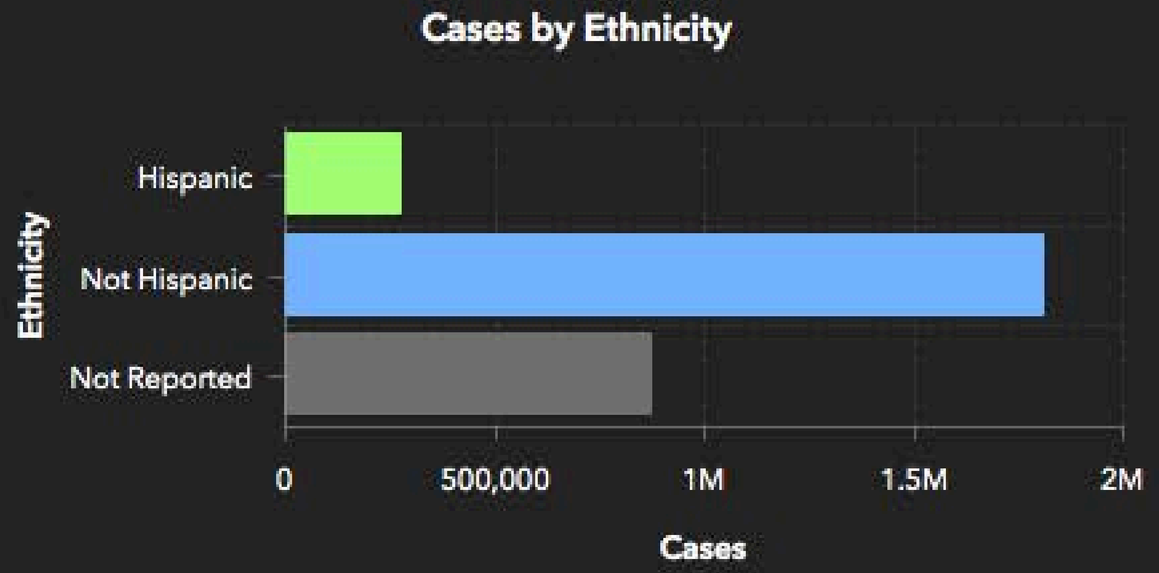

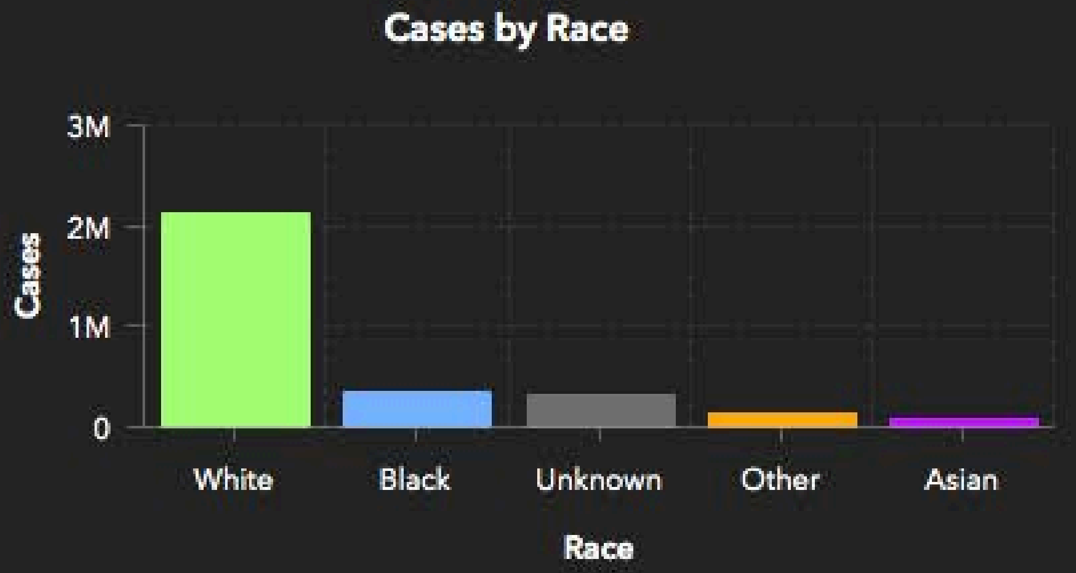

Daily data updated Monday through Friday by approximately 3 p.m.
Weekly charts updated on Tuesdays.
Daily data last updated: Jun 10, 2022

Rhode Island Department of Health COVID-19 Data Tracker

Total new cases per 100,000 persons in the past 7 days
219.8

Total Tests
8,116,032

Total Positive Cases
397,187

Tests Prior Day
5,001

New Positive Cases Prior Day
269

Total Fatalities
3,590

Fatalities Confirmed Prior Day
6

New Hospital Admissions by Week

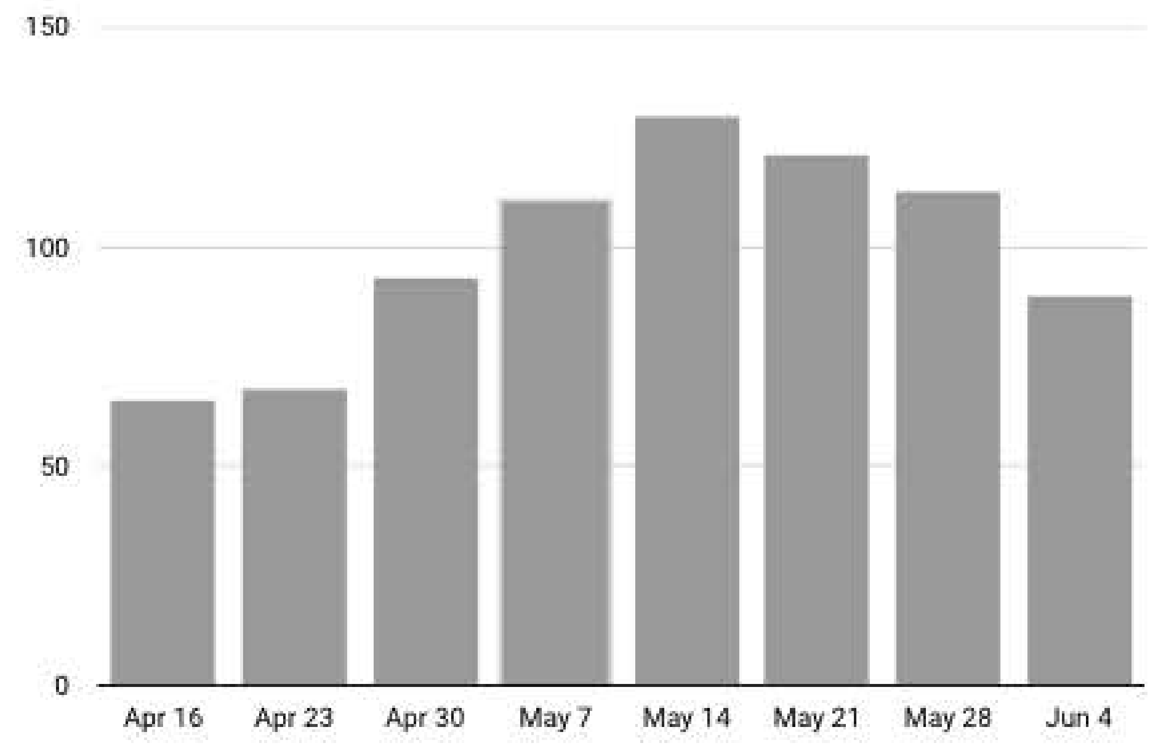

This week: 89
Last Week: 113

New Cases per 100,000 Population by Week

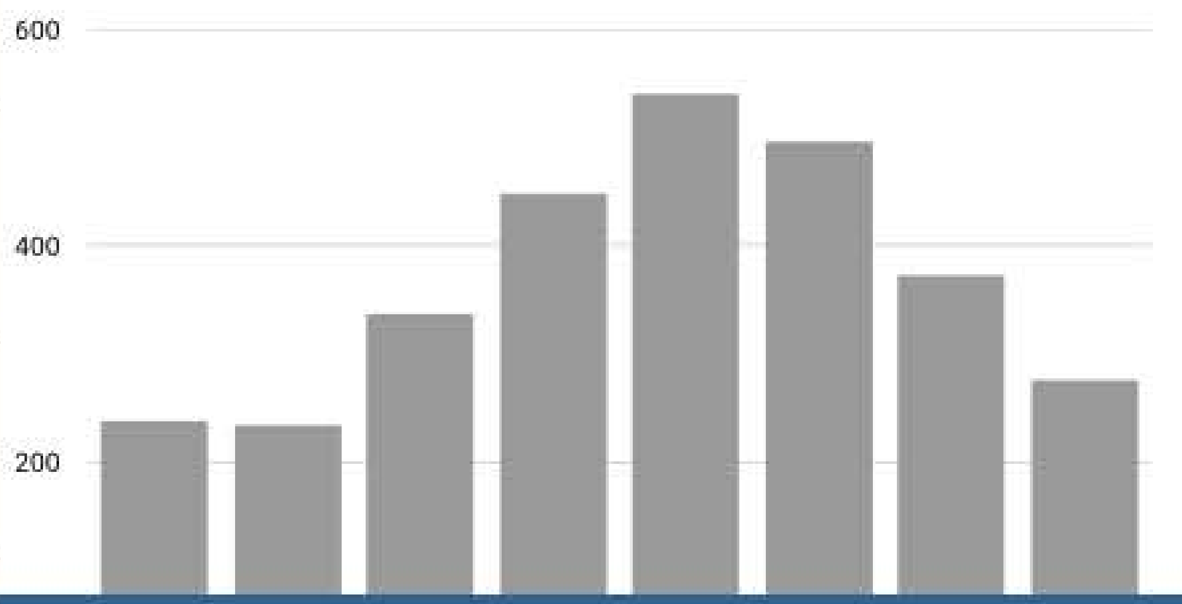

This week: 277
Last Week: 374

Sign In

Rhode Island COVID-19 Response Data [Click here] to return to the RIDOH COVID-19 Home Page

Weekly COVID-19 Community Level

Updated weekly on Fridays
Last updated: Jun 10, 2022

Bristol County	Kent County	Newport County	Providence County	Washington County
MEDIUM	MEDIUM	MEDIUM	MEDIUM	MEDIUM

Please note that county community levels reported above for the general population are determined by the CDC, taking into consideration case rate per 100,000 population, hospital admissions per 100,000 population, and percent of staffed inpatient beds occupied by COVID-19 patients. For more information about prevention guidance using this framework, and for details about the community level indicators and thresholds, please click on the "COVID-19 County Check" button below.

COVID-19 County Check

Healthcare and Congregate Care Settings
CDC's COVID-19 Community Level recommendations do not apply to healthcare settings such as hospitals or congregate care settings including corrections, nursing homes, assisted living facilities and shelters. Instead, these organizations should continue to use Community Transmission Rates.
Community Transmission Rates for Rhode Island can be found on the Congregate Care page.

Vaccine Administration Data

Data cumulative since 12/14/20
Updated daily, Monday-Friday

People At Least Partially Vaccinated in Rhode Island	People Who Have Completed Primary Vaccine Series in Rhode Island	Total Doses Administered in Rhode Island
955,625	835,560	2,196,960

Causes of COVID-19 Associated Hospitalizations

Data are for the most recent four weeks. Data are only for hospitalizations where the cause of admission was known.
Updated weekly on Wednesdays.

COVID-19 was the primary cause	COVID-19 was a contributing cause	COVID-19 was not a cause
35.3%	26.8%	37.9%

COVID-19 associated hospitalizations are hospitalizations among people who have tested positive for COVID-19. Federal and State health officials use this definition to measure the impact of COVID-19 on the hospital systen
When a patient is hospitalized with COVID-19, the same level of isolation resources is needed, regardless of the cause of the admission. See Hospitalization page for more detail.

Google Data Studio

COVID-19 in South Carolina

Data as of 11:59pm on Saturday, June 4, 2022

Currently Displaying 2/1/2020-6/4/2022

BUILD YOUR DASHBOARD VIEW

Select a Date Range

2/1/2020 — 6/4/2022

Select a Geography

South Carolina

Select a Map

Completed Vaccination Rate per 100k

Select a Trend

Cases by Day & 7-day Avg

Select Measures for Rates/Counts

(Multiple values)

Select a Demographic

by Age

CLICK TO BUILD A TABLE

CLICK TO BUILD A TREND

Download

AT A GLANCE

Percent Positivity	Total Tests	Cases	Deaths	Completed Vaccinations
11.8%	16,637,428	1,514,423	17,970	2,707,286
Fixed % Change Most Recent Week Compared to Previous	-5.1%	-1.4%	-77.8%	-22.0%

Completed Vaccination Rate per 100k in South Carolina

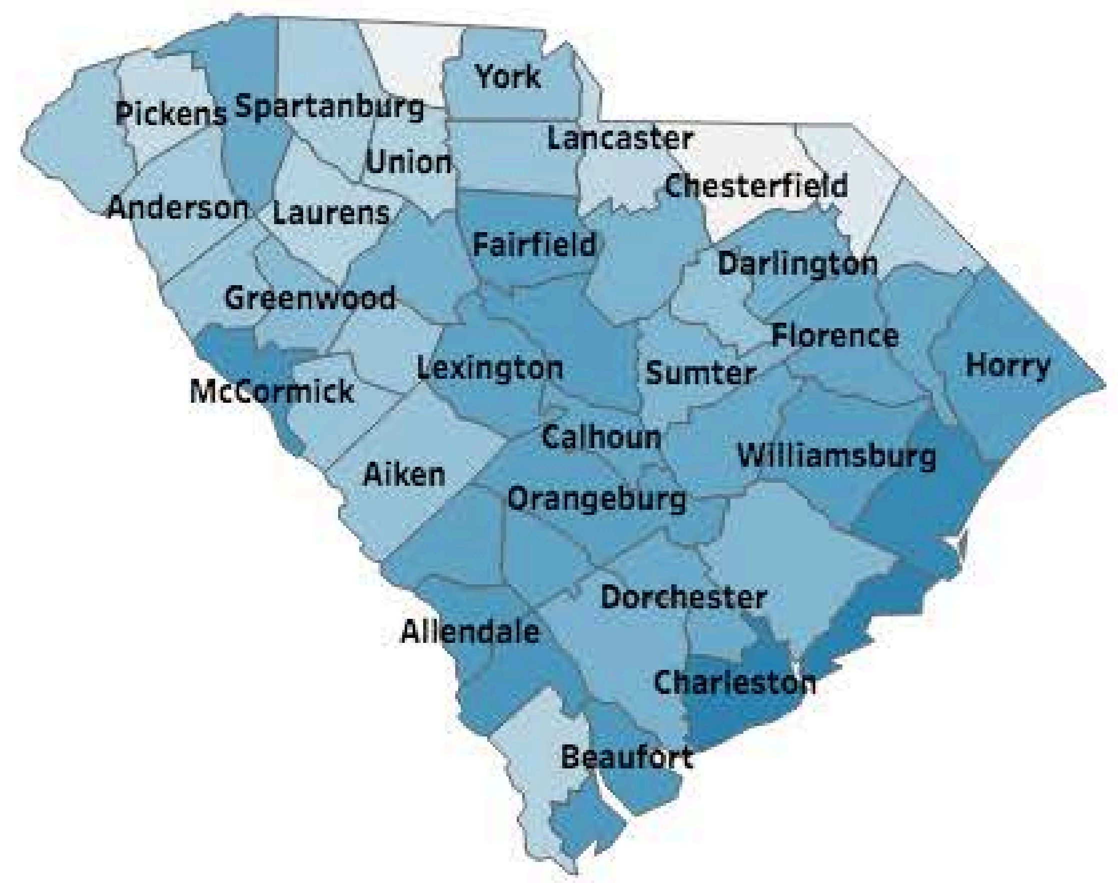

Rank	County
1	Charleston
2	Georgetown
3	McCormick
4	Hampton
5	Beaufort
6	Allendale
7	Richland
8	Williamsburg
9	Horry
10	Fairfield
11	Orangeburg
12	Barnwell
13	Lexington
14	Bamberg
15	Calhoun
16	Florence
17	Marion
18	Clarendon
19	Greenville
20	Dorchester
21	Darlington
22	Newberry
23	Colleton
24	Kershaw
25	Sumter
26	Berkeley
27	Greenwood
28	York
29	Lee
30	Aiken
31	Abbeville
32	Edgefield
33	Chester
34	Saluda
35	Oconee
36	Anderson
37	Spartanburg
38	Union
39	Jasper
40	Dillon
41	Laurens
42	Lancaster
43	Pickens
44	Marlboro
45	Cherokee
46	Chesterfield

Rate per 100k/Percent Population of Cases, Hospitalizations, Deaths and 1 more by Age in South Carolina

Key Population	Cases	Hospitalizations	Deaths	Completed Vaccinations
Under 5	18,180.8	107.5	1.7	0%
5 to 11	24,228.7	45.5	1.4	17.3%
12 to 17	31,383.9	64.6	3.6	41.8%
18 to 34	37,619.8	175.2	20.4	42.3%
35 to 49	34,214.1	444.3	105.4	55.1%
50 to 64	27,096.9	909.9	334.6	67.1%
65 to 84	19,751.8	1,982.4	1,060.9	80.%
85 & Older	22,929.3	4,319.5	3,935.6	72.9%
Overall	29,022.8	725.3	344.4	51.9%

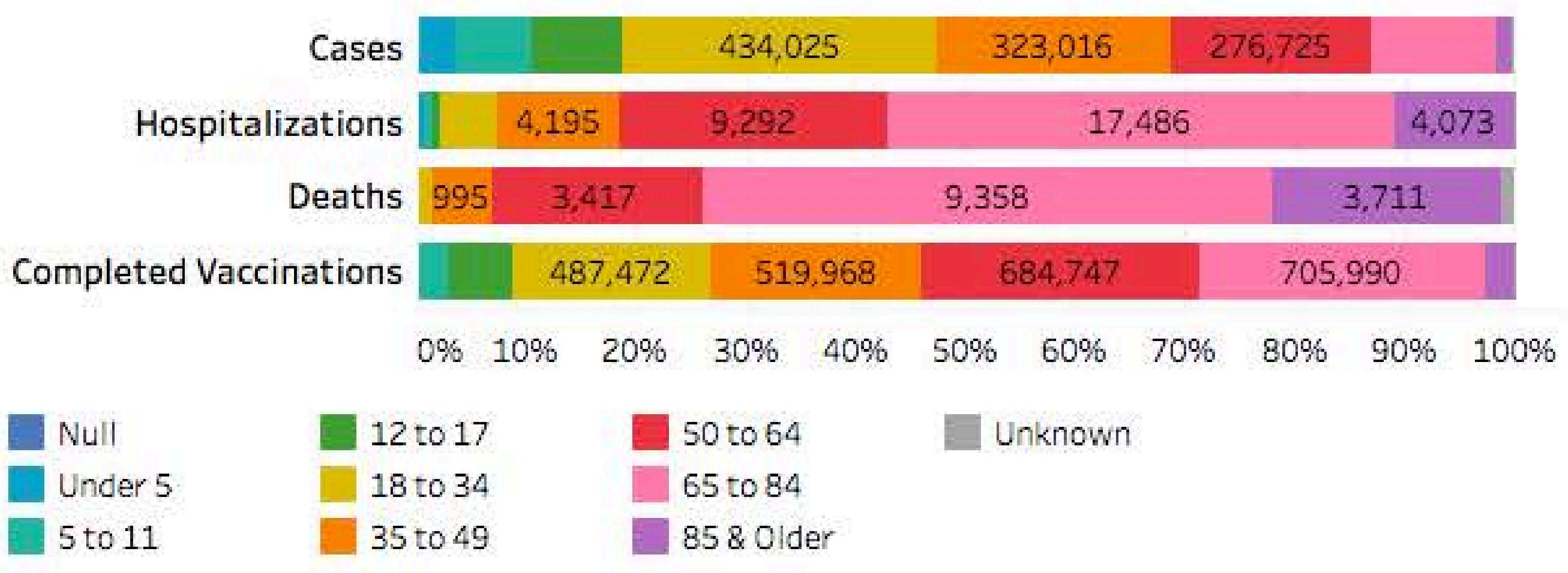

Trend of Cases by Day & 7-day Avg in South Carolina

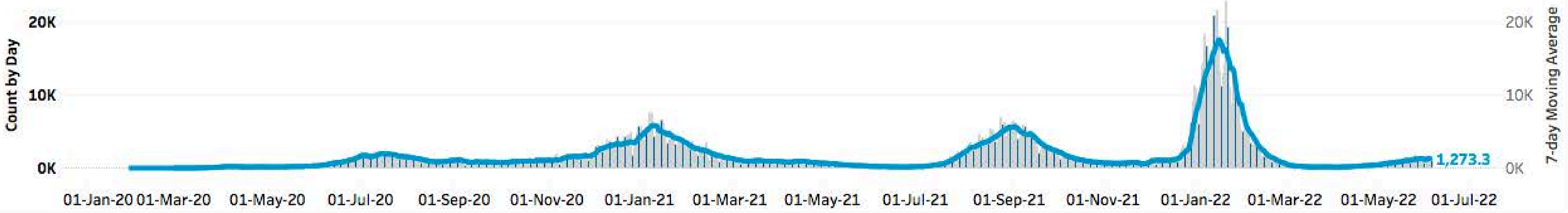

+ a b l e a u

COVID-19 Quick Numbers
Data as of 6/4/2022 at 11:59 pm

	Total to Date	Most Recent Week Count	Change over Previous Week	Change over Previous Month
**Tests**	16,637,428	63,361	-5.1%	9.5%
**Cases**	1,514,423	8,913	-1.4%	31.7%
**COVID+ hospitalized**		261	13.3%	53.3%
**Deaths**	17,970	4	-77.8%	-81.4%
**Completed Vaccinations**	2,707,286	2,356	-22.0%	-27.8%

Previous Month Trend
(7 day running average)

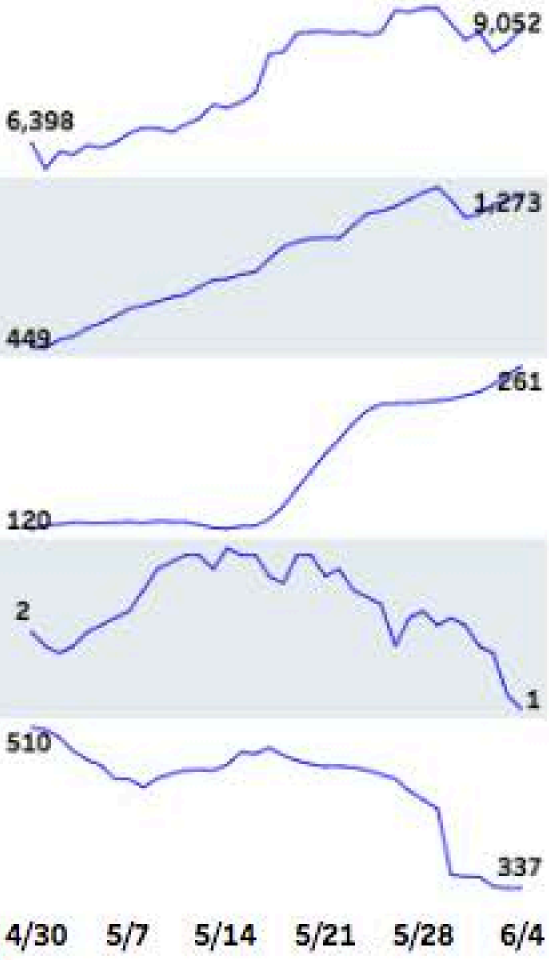

Click to Download PDF View

+ a b | e a u

COVID-19 VACCINATION
AMONG ELIGIBLE RESIDENTS OF SOUTH CAROLINA BY POPULATION

Data as of 11:59 PM on June 4, 2022

AT LEAST 1 DOSE

Kids aged 5-11 with at least 1 dose
population estimate: 439,755

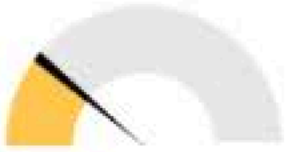

21.0%

Residents aged 12 & older with at least 1 dose
population estimate: 4,486,989

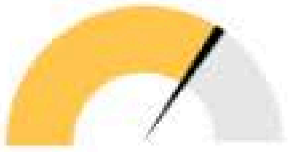

67.7%

All Eligible Residents with at least 1 dose
population estimate: 4,926,744

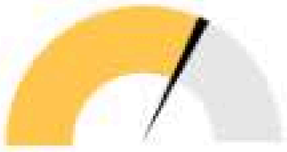

63.5%

COMPLETED VACCINATIONS

Kids aged 5-11 with Completed Vaccine* Series
population estimate: 439,755

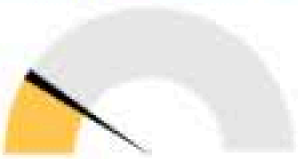

17.3%

Residents aged 12 & older with Completed Vaccine* Series
population estimate: 4,486,989

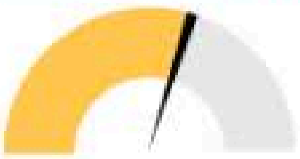

58.6%

All Eligible Residents with Completed Vaccine* Series
population estimate: 4,926,744

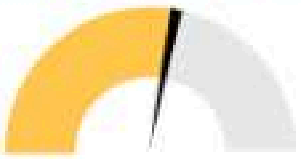

55.0%

* Data Notes: This page is updated daily using data from the Statewide Immunization Online Network (SIMON), used to track person-level vaccine doses. ***Completed Vaccination** is any resident who received 1 dose of Janssen or 2 doses of Pfizer/Moderna.

+ a b | e a u

SOUTH DAKOTA DEPARTMENT OF HEALTH doh.sd.gov/covid/

SOUTH DAKOTA COVID-19 DASHBOARD

SOUTH DAKOTA COVID-19 UPDATES

Please wait. The data dashboard below takes a moment to load and is best viewed on desktop or in landscape mode on mobile (i.e. holding the phone sideways).

NOTE: *This dashboard is updated weekly on Wednesdays.*

LAST UPDATED
6/8/2022 10:18:38 AM

SD Overview | Demographics | Trends | Hospital Capacity | Hospitalized & ILI | Tables | Vaccines | Deaths

New Confirmed and Probable Cases	Active Cases	Recovered Cases	Currently Hospitalized
970	2,173	236,225	49

Community Spread Map by County of Residence

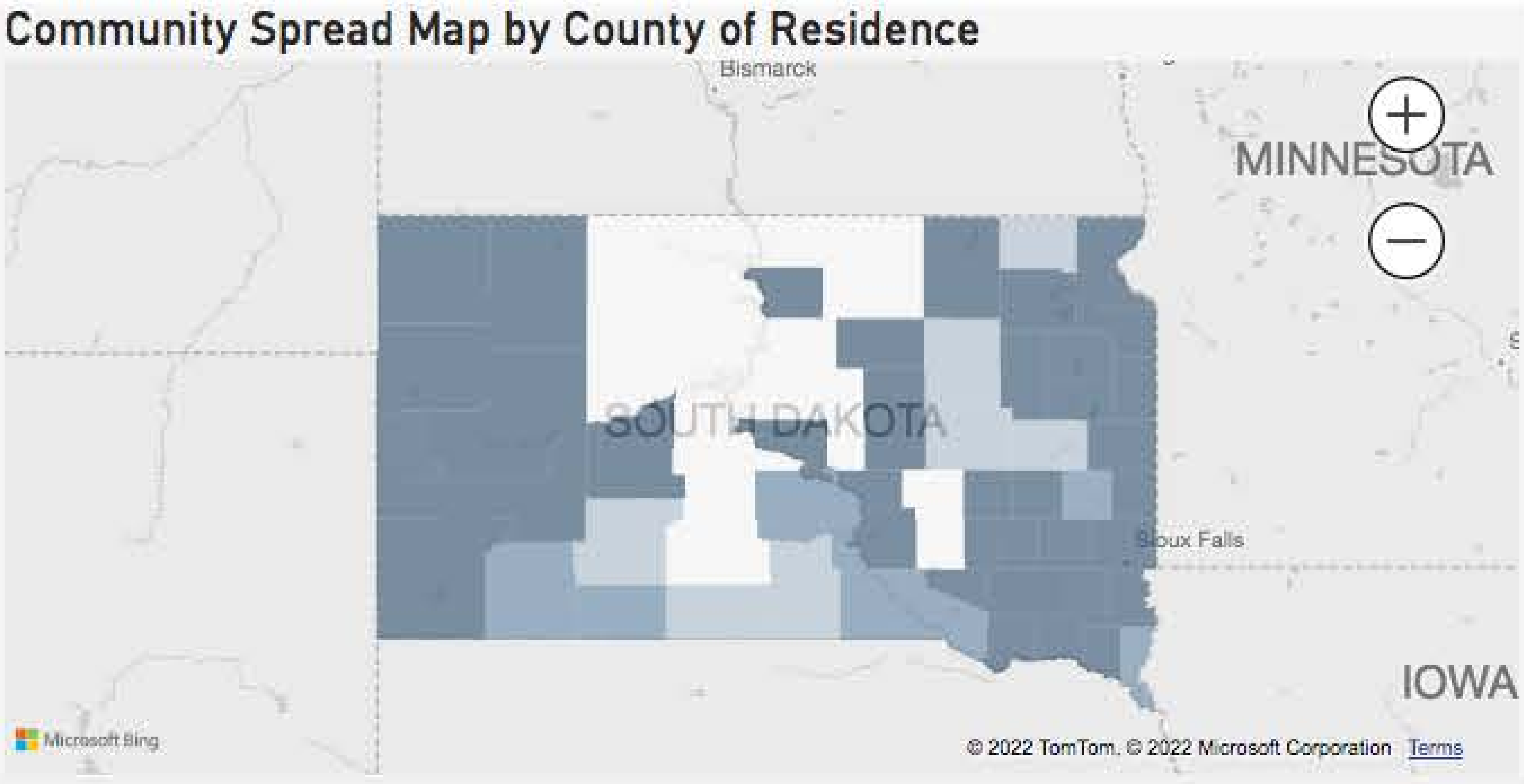

Community Spread Low Moderate Substantial High

Hover over a county to see its details, or click county to update the orange boxes.
More information on U.S COVID-19 Community levels can be found at: https://www.cdc.gov/coronavirus/2019-ncov/your-health/covid-by-county.html

Total Confirmed and Probable Cases	Total Tests	Deaths Among Cases	Ever Hospitalized	Percent of State Population with at least 1 Dose**
241,326	2,204,658	2,928	10,899	73%

***Includes aggregate federal data from IHS, VA, DoD, and BoP plus reports to SDIIS*

New Confirmed Cases by Residence

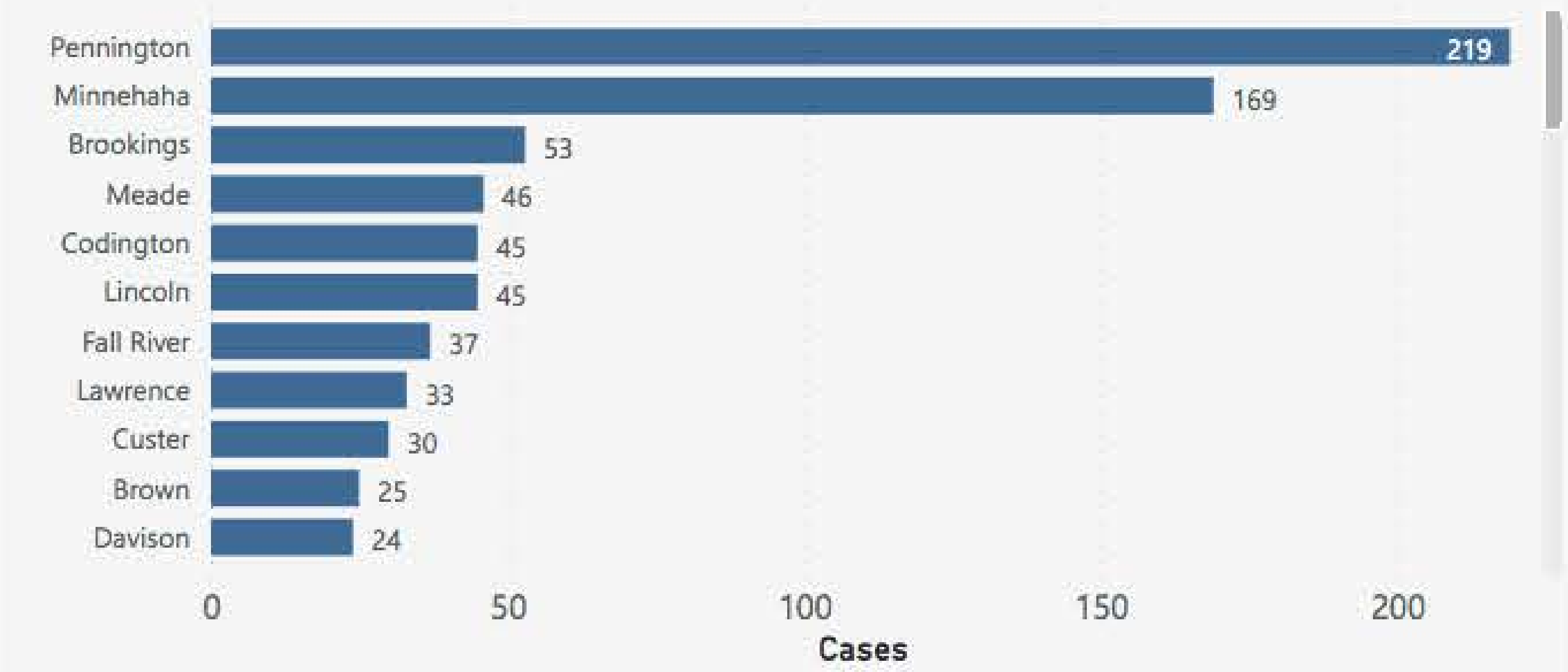

Cases: *Persons who meet the national surveillance case definition for COVID-19. According to the case definition, Confirmed Cases includes persons with a positive PCR test and Probable Cases includes persons with a positive antigen test for the SARS-CoV-2 virus that causes COVID-19. A positive case moves from Active to Recovered status based on the CDC guidelines for removal from isolation.*

Hospitalized: *Currently Hospitalized numbers include COVID-19 cases and suspects that are in the hospital under transmission-based precautions. The number may include out-of-state cases and suspects. SD-DOH reports on data directly entered by hospitals into the US Department of Health and Human Services TeleTracking system. Federal entities, such as the Veterans Affairs Medical Centers, are not subject to reporting. Ever Hospitalized include COVID-19 cases that were, at some point during their infection, hospitalized under transmission-based precautions. The number of persons ever hospitalized are cumulative and includes only South Dakota resident cases. Data are reported from the South Dakota Electronic Disease Surveillance System.*

Deaths among Cases: *This data includes deaths reported by death certificate or deaths within 20 days of a COVID-19 infection. SD-DOH does not report deaths until a certified death record has been filed with COVID-19 as a cause or contributing factor. By law, a death record must be filed within 5 days of the date of death. For more information see the Deaths Tab.*

Tests: *% Progress shows the percentage of PCR and antigen tests completed toward the monthly goal of 44,233. The monthly goal for South Dakota is equal to 5% of the state's population. The federal government established a 2% goal for SARS-CoV-2 testing for each state. Total tests are the number of tests performed, not the number of individuals tested, and include tests conducted by the South Dakota Public Health Laboratory and hospital, commercial, Indian Health Service, Veteran Affairs, and other medical laboratories.*

Community Spread *is updated every Wednesday based on two indicators: 1) the rate of new cases per week, and 2) the weekly % PCR test positivity rate - both calculated from the data reported during the previous calendar week (Sun-Sat). If the two indicators suggest different transmission levels for a county, the higher level is selected.*

Community spread is defined as:
Low: *Less than 10 cases per 100,000 AND weekly % PCR test positivity rate less than 5%*
Moderate: *10-49 cases per 100,000 OR weekly % PCR test positivity rate of 5-7.9%*
Substantial: *50-99 cases per 100,000 OR weekly % PCR test positivity rate of 8-9.9%*
High: *100 cases or greater per 100,000 OR weekly % PCR test positivity rate of 10% or greater*
**Small number of new cases (less than 10) reported during the previous calendar week*

% PCR Test Positivity Rate *is equal to the number of positive PCR tests divided by total PCR tests, within the specified period of time. Weekly % PCR Test Positivity Rate is updated every Wednesday based on the number of new PCR tests* [illegible]

COVID-19
Collapse All | Expand All
Dashboard
Your Health
Quarantine & Isolation Calculator
Community, Work, & School
Testing
COVID-19 Vaccine
Healthcare Providers
Resources

doh.sd.gov/covid/

SOUTH DAKOTA COVID-19 DASHBOARD

SOUTH DAKOTA COVID-19 UPDATES

Please wait. The data dashboard below takes a moment to load and is best viewed on desktop or in landscape mode on mobile (i.e. holding the phone sideways).

NOTE: *This dashboard is updated weekly on Wednesdays.*

LAST UPDATED
6/8/2022 10:18:38 AM

SD Overview | Demographics | Trends | Hospital Capacity | Hospitalized & ILI | Tables | Vaccines | Deaths

County
All

PCR Test Positivity Rate, Last 7 Days	PCR Test Positivity Rate, Last 14 Days	PCR Test Positivity Rate, Cumulative
16.4%	15.4%	15.0%
5/31/2022 - 6/6/2022	5/24/2022 - 6/6/2022	3/10/2020 - 6/6/2022

PCR Test Positivity Rate: *This rate is equal to the number of positive PCR tests divided by total PCR tests, within the specified period of time. Date range includes last full day (24 hours) captured in the data set.*

Confirmed Cases and 14-Day Trend Line by Date Reported to SD-DOH

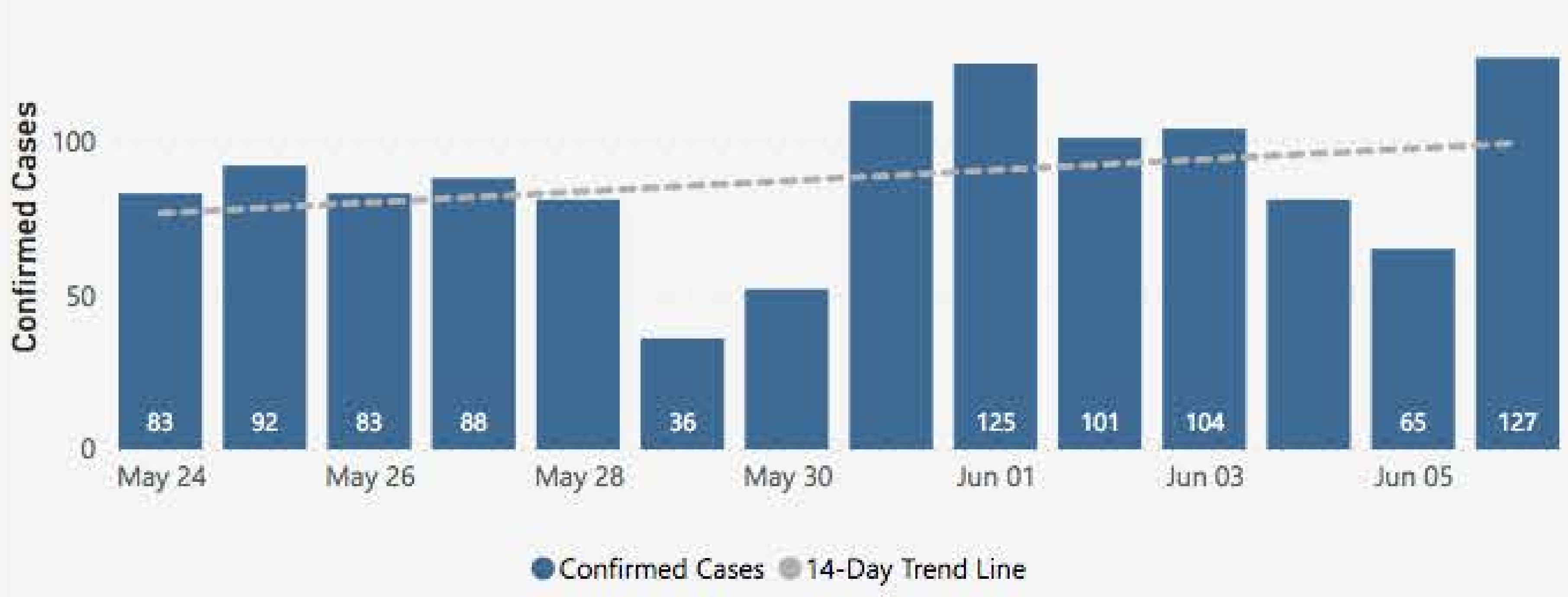

14-Day Trend of Confirmed Cases by Date Reported to SD-DOH: *This graph shows the trend (increasing or decreasing) of COVID-19 confirmed cases (persons who meet the national surveillance case definition case for COVID-19) at the state or county level.*

Daily Rate of Total Cases, Last 7 Days	Rate of Total Cases, Cumulative
5/31/2022 - 6/6/2022	3/10/2020 - 6/6/2022
15.29	27,346
Daily Case Rate per Capita	Case Rate per Capita

Rate of Cases: *Number of cases (persons who meet the national surveillance case definition for a confirmed or probable case of COVID-19) reported to SD-DOH in the last 7 days as a daily average (or cumulative) divided by the state or county population multiplied by 100,000. This is done to compare disease burden across geographic areas (eg. state or county).*

Date Reported to SD-DOH

3/10/2020 | 6/6/2022

Cases by Date Reported to SD-DOH

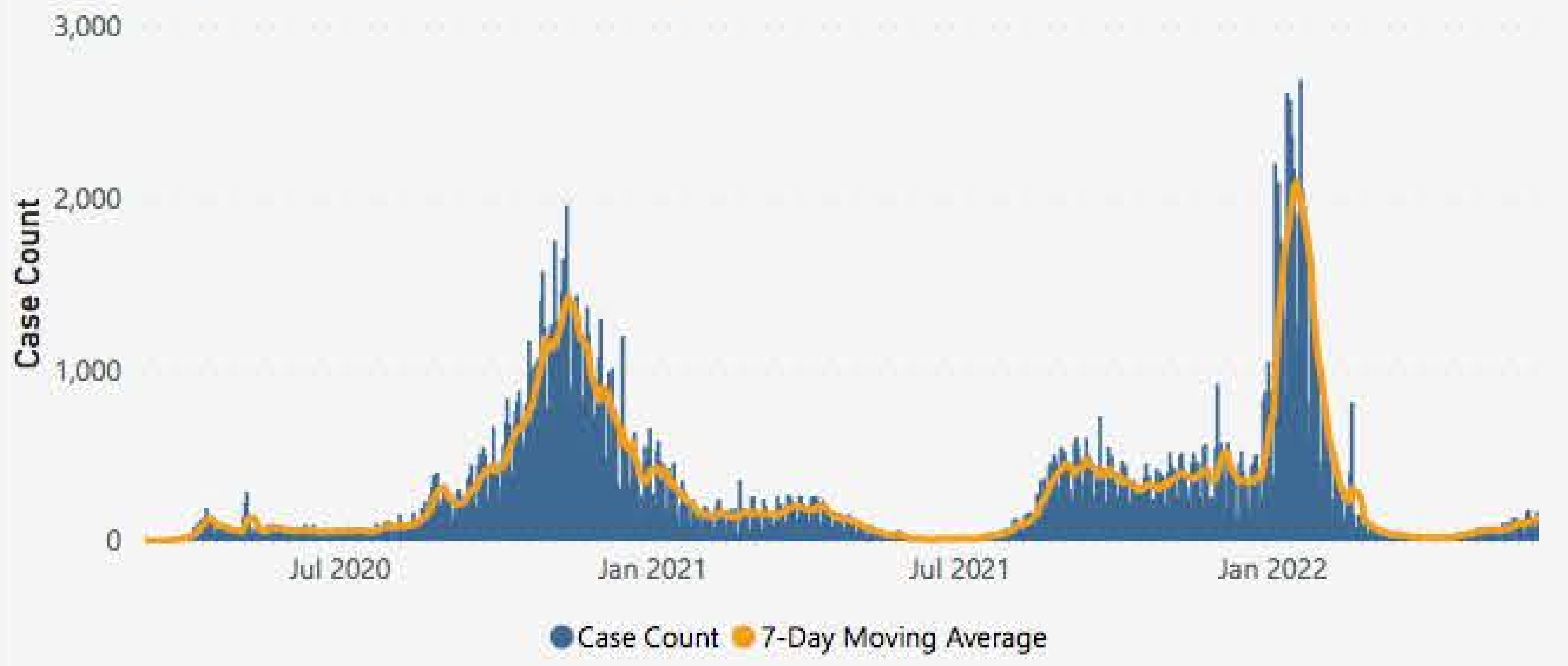

Confirmed | Probable

Cases by Date Reported to SD-DOH: *This graph shows cases (persons who meet the national surveillance case definition for a confirmed or probable case of COVID-19) over time includes results received by midnight and may not align with the daily data update.*

PCR Test Positivity Rate by Date Reported to SD-DOH

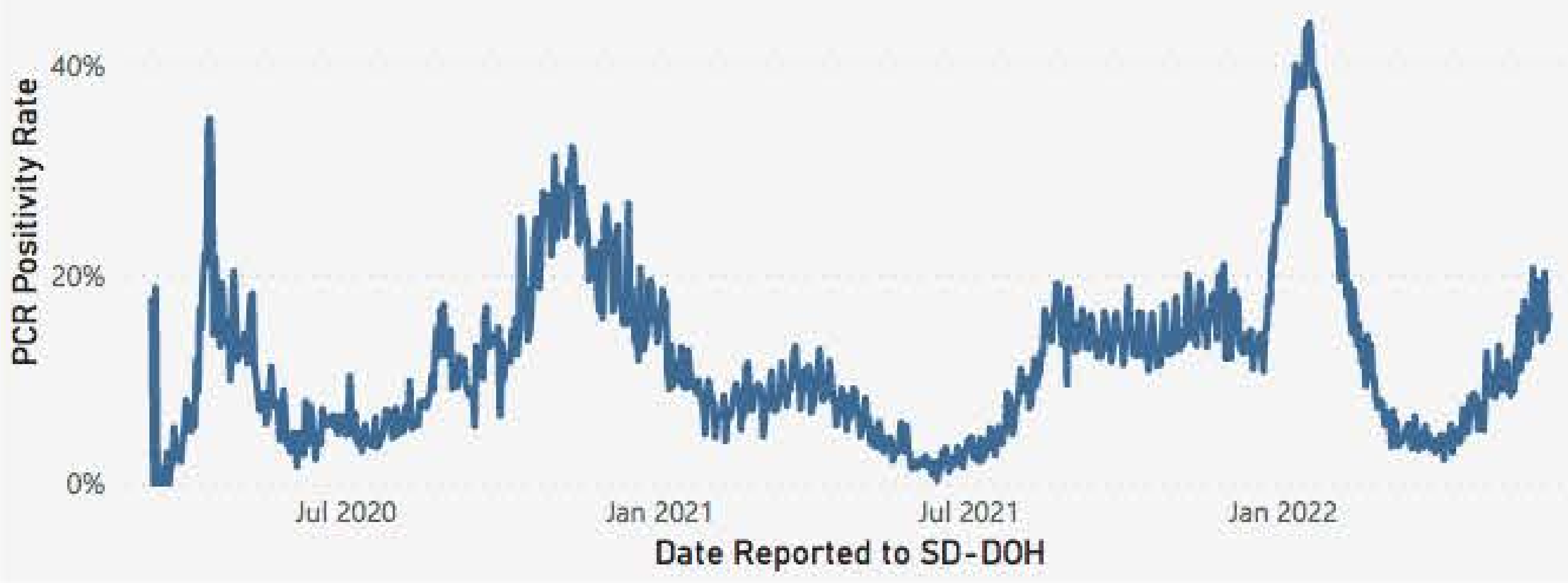

PCR Test Positive Rate by Date Reported to SD-DOH: *This graph shows % PCR Test Positivity Rate which is equal to the number of positive PCR tests divided by total PCR tests, within the specified period of time. Table view data also shows total positive tests and total tests which includes combined antigen and PCR test data..*

Note: *Data are provisional and subject to change. Data are updated weekly on Wednesday by about 12 p.m. Results include tests conducted by the South Dakota Public Health Laboratory and hospital, commercial, Indian Health Service, Veteran Affairs, and other medical laboratories.*

COVID-19

Collapse All | Expand All

- Dashboard
- Your Health
- Quarantine & Isolation Calculator
- Community, Work, & School
- + Testing
- + COVID-19 Vaccine
- + Healthcare Providers
- Resources

SOUTH DAKOTA DEPARTMENT OF HEALTH doh.sd.gov/covid/

SOUTH DAKOTA COVID-19 DASHBOARD

SOUTH DAKOTA COVID-19 UPDATES

Please wait. The data dashboard below takes a moment to load and is best viewed on desktop or in landscape mode on mobile (i.e. holding the phone sideways).

***NOTE:** This dashboard is updated weekly on Wednesdays.*

LAST UPDATED
6/8/2022 10:18:38 AM

SD Overview | Demographics | Trends | Hospital Capacity | Hospitalized & ILI | Tables | Vaccines | Deaths

Total Doses Administered
1,248,435

Pfizer Recipients
714,165

Percent of State Population with at least 1 Dose**
73%

Moderna Recipients
496,161

Percent of State Population with Series Complete**
60%

Total Persons Administered a Vaccine*
526,326

Janssen Recipients
38,109

Percent of State Population received Booster Dose**
32%

Based on 2019 Census Estimate for aged 5+ years for 1 dose and series complete (823,492) and 12+ years for Booster dose (738,101).

**Includes reports to SD Immunization Information System (SDIIS)*
***Includes aggregate federal data from IHS, VA, DoD, and BoP plus reports to SDIIS*

For COVID-19 Vaccine Coverage at 12+, 18+, or 65+ years click the link below: US Vaccine Coverage

CDC Covid Tracker

Doses & Persons Administered Vaccine by County of Residence

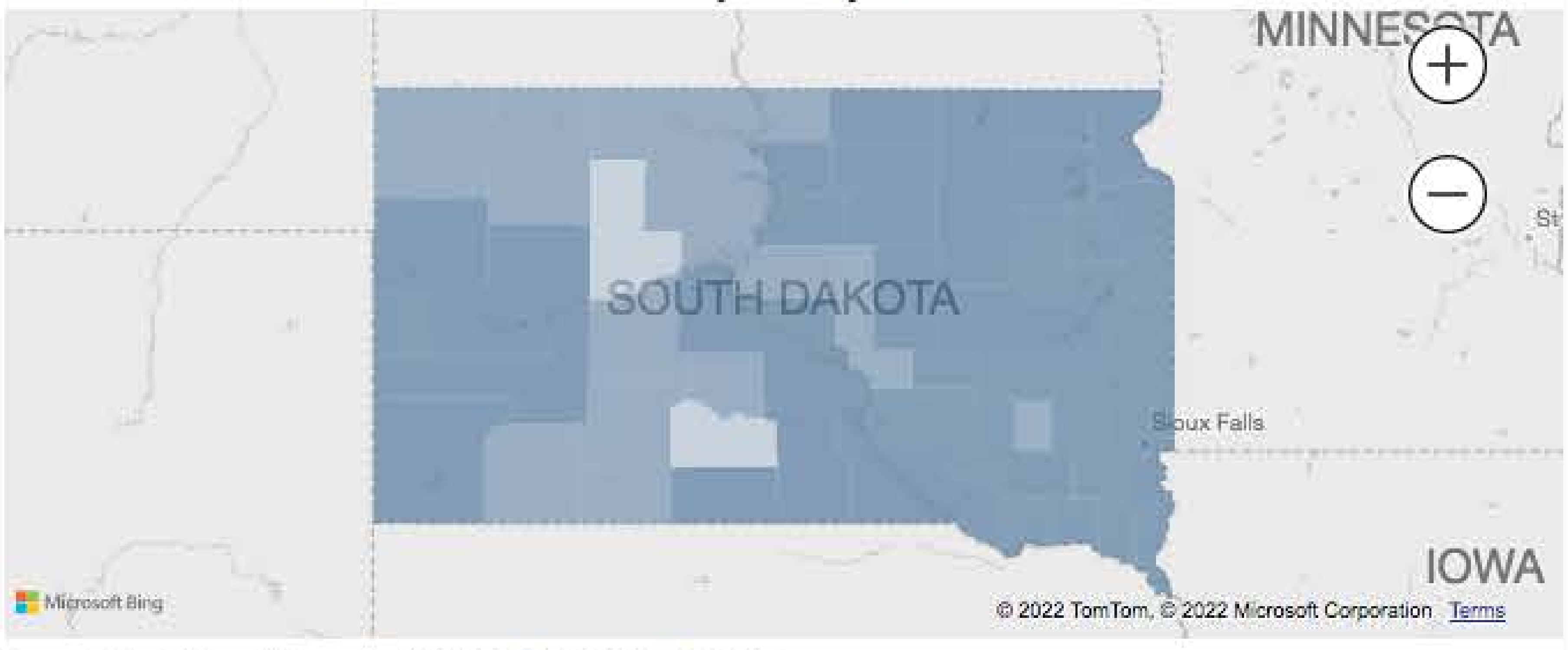

Doses Administered Range ● 100-499 ● 500-2,499 ● 2,500+

Hover over a county to see its details, or click county to update the orange boxes.

**Counties with tribes receiving vaccine through Indian Health Services (IHS). Doses administered by IHS, VA (Veteran's Affairs), DoD (Dept of Defense), and BoP (Bureau of Prisons) are not reported to SDIIS and are not shown, except for Percent of State Population with at least 1 Dose.*

County	# Doses	1-dose	2-series Complete	3rd/booster dose	Total # Persons
Minnehaha*	327,245	18,546	57,012	59,833	**134,703**
Pennington*	136,432	11,222	25,556	22,452	**58,646**
Lincoln	99,188	4,688	16,844	18,746	**40,122**
Other	50,570	12,834	11,793	4,324	**28,864**
Brown	59,470	2,684	10,086	11,291	**23,853**
Brookings	49,043	3,667	8,174	8,919	**20,549**
Codington*	37,385	1,749	6,411	7,091	**15,200**

Doses by Month (1 dose/Series Complete)

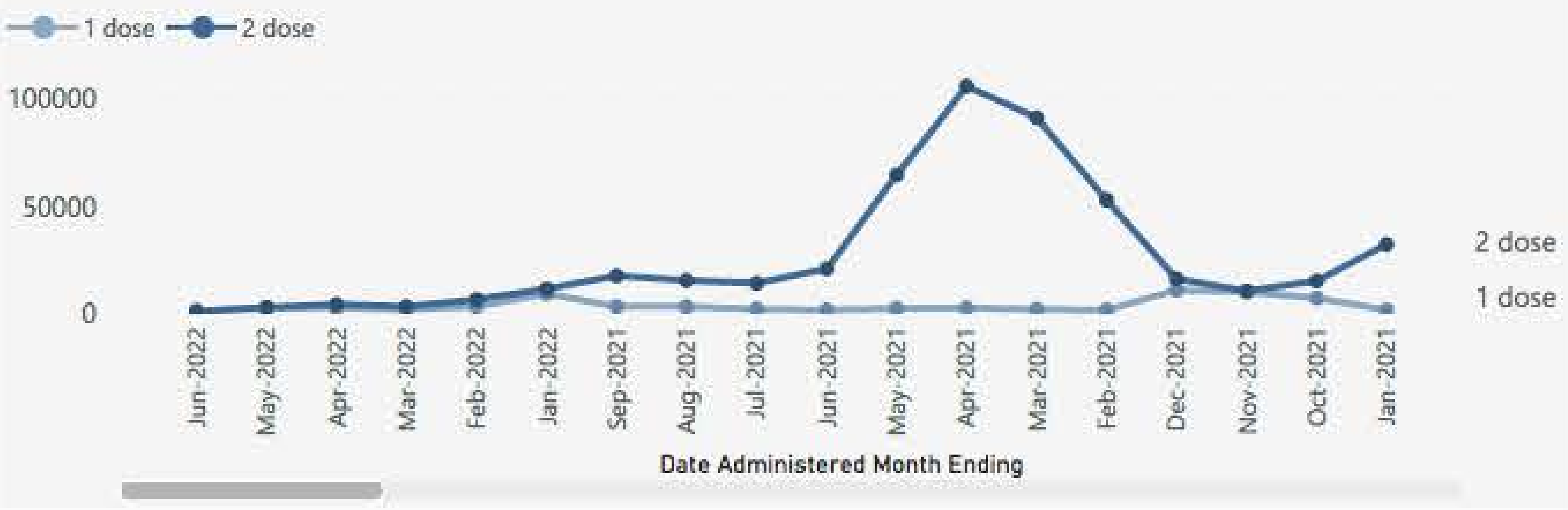

Age | Race/Ethnicity | Sex

Number of Persons Vaccinated by Age Group

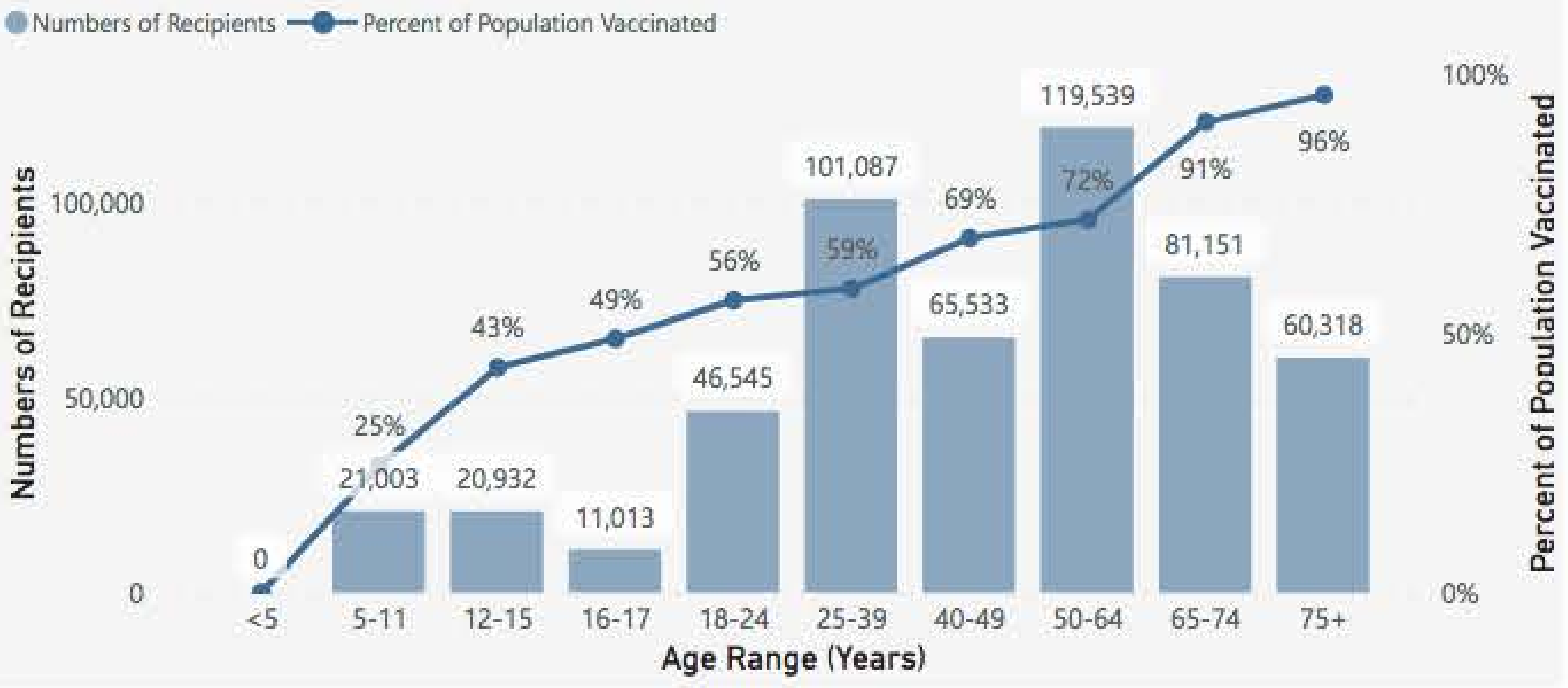

COVID-19 vaccine provided to veterans by the VA Medical Centers and American Indians by the Indian Health Service are not reported to SD-DOH and will lower the percentage of minority race/ethnicity represented when compared to the SD population. COVID-19 vaccine recipient race/ethnicity is a data element of the vaccination record reported to SD-DOH from hospitals, clinics, and other vaccination partners (e.g., federal pharmacy partners).

***Note:** Data are provisional and subject to change. Data are updated weekly on Wednesday by about 12 p.m. and include doses reported through 10 a.m. on Tuesday from SDIIS. Doses administered by IHS, VA, DoD, and BoP are not reported to*

100%

TN Department of Health

Tennessee COVID-19 - June 04, 2022
Epidemiology and Surveillance Data

PDF

Total Cases: **2,073,518**	Confirmed Cases: **1,571,822**	Probable Cases: **501,696**
Total Deaths: **26,491**	Confirmed Deaths: **19,355**	Probable Deaths: **7,136**

Cases by County

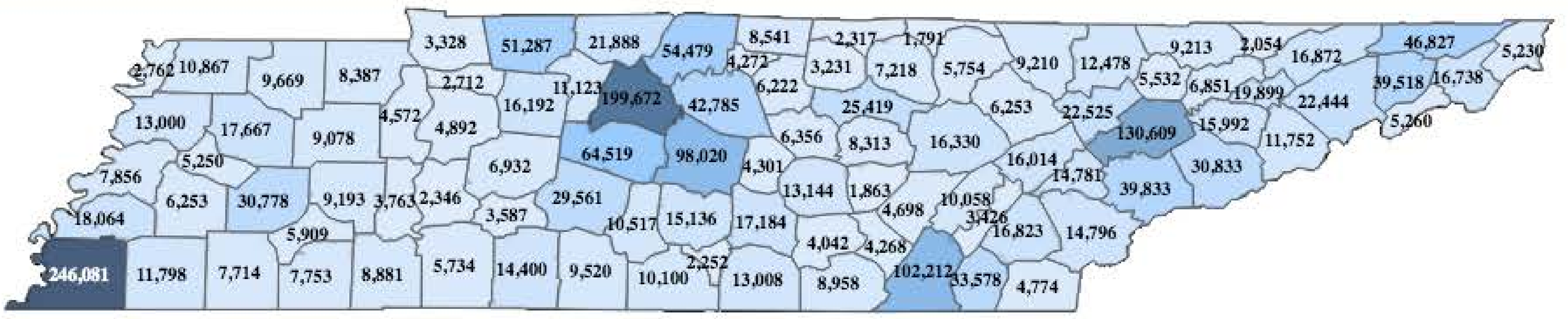

Daily Counts of Cases for Last 120 Days

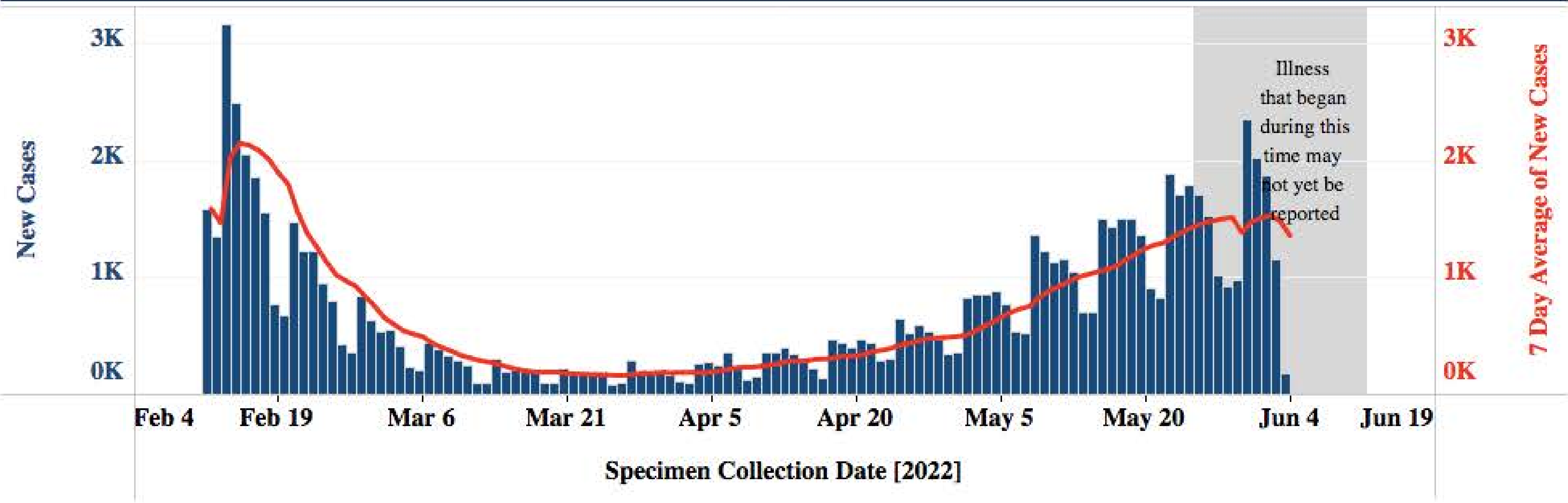

To see full epi curve visit: https://www.tn.gov/health/cedep/ncov/data/epi-curves.html

Demographics

Minimum Age	Average Age	Maximum Age
0	38	110

Age Range	Cases	% of Cases	Deaths	% of Deaths
0-10 years	187,012	9%	14	0%
11-20 years	289,046	14%	26	0%
21-30 years	363,112	18%	208	1%
[illegible]	[illegible]	[illegible]	[illegible]	[illegible]
41-50 years	294,497	14%	1,436	5%
51-60 years	268,101	13%	3,335	13%
61-70 years	186,881	9%	5,598	21%
71-80 years	105,461	5%	7,359	28%
81+ years	49,327	2%	7,946	30%
Pending	1,514	0%	0	0%

Patient Sex	Cases	% of Cases	Deaths	% of Deaths
Female	1,118,085	54%	12,153	46%
Male	942,254	45%	14,317	54%
Pending	13,179	1%	21	0%

Patient Race	Cases	% of Cases	Deaths	% of Deaths
American Indian or Alaska Native	3,395	0%	26	0%
Asian	20,472	1%	124	1%
Black or African American	290,167	14%	4,278	16%
Native Hawaiian or Other Pacific Islander	1,823	0%	12	0%
White	1,311,057	63%	20,135	76%
Other/Multiracial	146,337	7%	945	4%
Pending	300,267	15%	971	4%

Patient Ethnicity	Cases	% of Cases	Deaths	% of Deaths
Not Hispanic or Latino	1,390,730	66%	23,515	89%
Hispanic or Latino	92,449	3%	592	2%
Pending	590,339	30%	2,384	9%

Outcomes

Ever Hospitalized	Cases	% of Cases
Yes	48,481	2%
No	617,752	25%
Pending	1,407,285	73%

Please note for hospitalizations: Information about hospitalization status is gathered at the time [illegible] patients currently hospitalized.

TN Contact Us Program Areas News Health Professionals Parents Individuals Statistical Data Career Opportunities

Deceased	Cases	% of Cases
Yes	26,491	1%
No	2,047,027	99%

Please note for deaths: Some deaths may be reported by health care providers, hospitals, medical examiners, local health departments, or others before they are included in the statewide count.

Laboratory Tests

Test Type	Positive	Negative	Total
PCR	1,759,116	11,399,162	13,158,278
Antigen	560,778	4,838,632	5,399,410

Please note, these data are meant to provide a ***rough estimate*** *of testing volume. Due to different source data, numbers may differ slightly from day to day. Laboratory reports of positive cases are reported to metro and local health departments as soon as results are available. State numbers are updated weekdays before 5pm and there may be a lag in the reporting of cumulative numbers at the state level. Please note numbers within tables may not add up to total confirmed number of cases due to reporting delays and/or missing data. Percentages may not add up to 100.0% due to rounding.*

Cases and Labs by County

County	Cases (Confirmed and Probable)	PCR Positive Tests	PCR Negative Tests	Deaths
ANDERSON	22,525	15,689	96,361	342
BEDFORD	15,136	12,425	53,423	238
BENTON	4,572	3,066	14,807	94
BLEDSOE	4,698	3,778	24,808	60
BLOUNT	39,833	34,728	135,962	445
BRADLEY	33,578	27,722	115,294	419

Case Counts by Region or County

New Cases by Region | New Cases by County | New Cases by MMWR Week

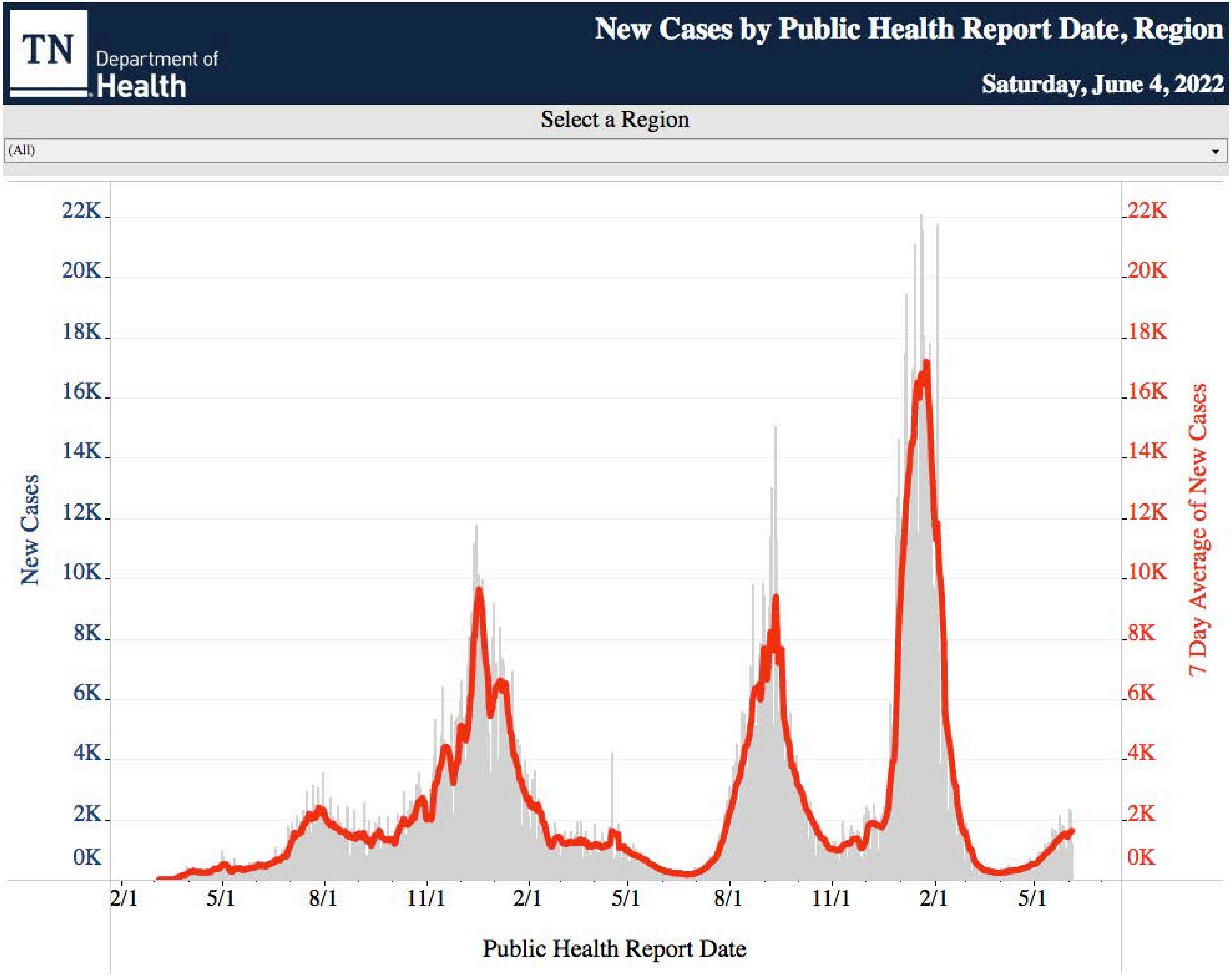

COVID

Tracking COVID: Interactive maps, charts show spread of virus across Houston, rest of Texas

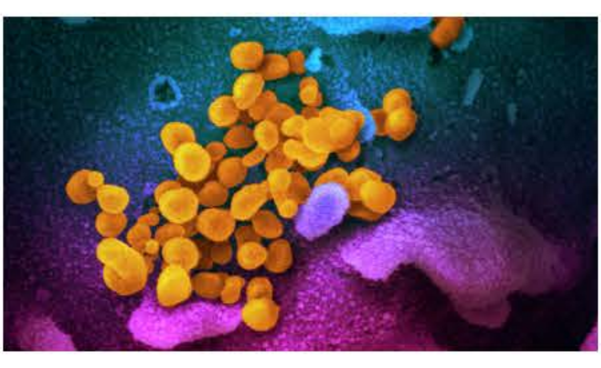

HANDOUT / National Institutes of Health / AFP via Getty Images

Jordan Ray-Hart, Stephanie Lamm, Daniel Simmons-Ritchie, Kirkland An
Feb. 17, 2022 | Updated: May 12, 2022 9:09 a.m.

To keep you up-to-date with COVID-19 cases in Houston, Texas and the rest of the world, the Houston Chronicle has come up with a few eye-catching, but easy-to-use interactives, based on our reporting and official data from the Texas Department of State Health Services.

To start, let's take a look at the test positivity rates in Texas.

Texas positive test rate

Percent of COVID-19 tests* that are positive for the virus

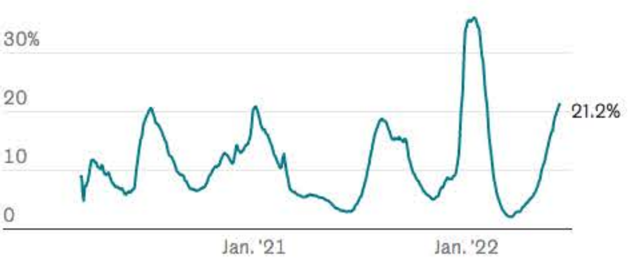

Chart: Texas Hearst Data Visualization Team • Source: Texas Department of State Health Services

*This chart uses molecular tests, which the state health department uses to count confirmed COVID-19 cases.

More locally, we've got test positivity rates in Harris County.

Harris County positive test rate

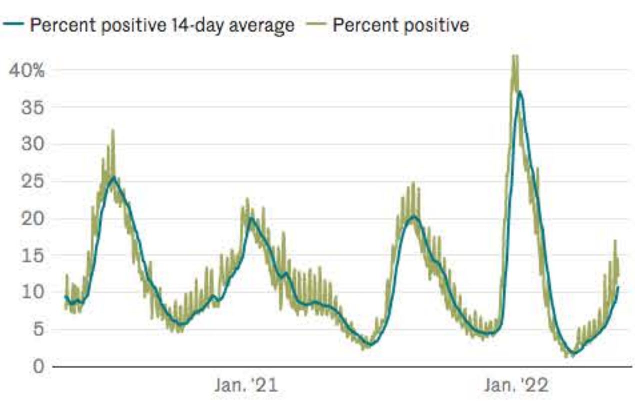

Chart: Texas Hearst Data Visualization Team • Source: Harris County

Positivity rates only tell one part of the story though, so here's the seven-day rolling average of new cases around the Lone Star State.

Daily new cases in Texas

7-day average

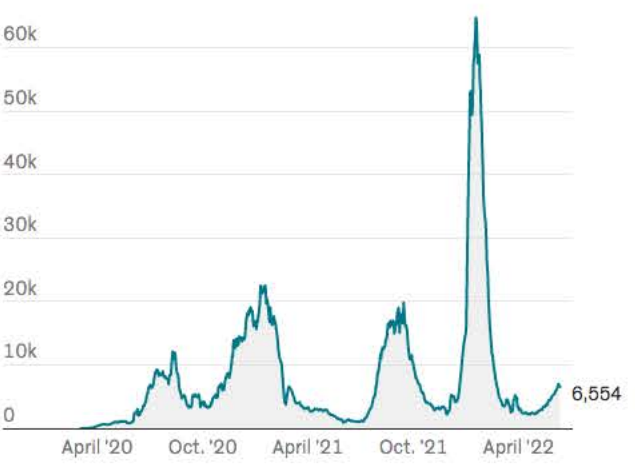

Chart: Texas Hearst Data Visualization Team • Source: Texas Department of State Health Services

Official positivity rates cases alone capture only a portion of the COVID activity in Texas. Decreased testing requirements and at-home testing make tracking the virus much more difficult.

As new COVID variants emerge, hospitalizations statewide and in Houston rise.

Current hospitalizations

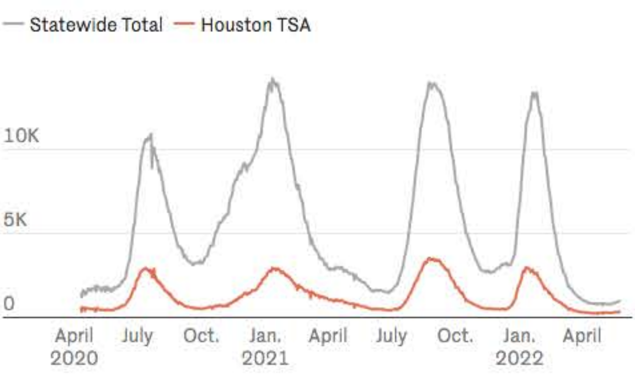

Chart: Texas Hearst Data Visualization Team • Source: Texas Department of State Health Services

Tens of thousands of Texans have died from COVID since the pandemic began. You can view state and local daily deaths and total deaths for the state below.

COVID daily deaths

7-day average

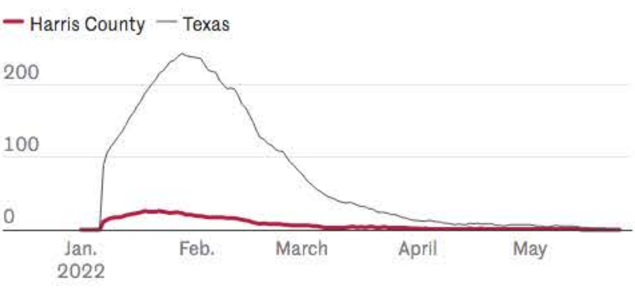

Chart: Texas Hearst Data Visualization Team • Source: Texas Department of State Health Services

Records may be updated by DHSH in subsequent data releases

Deaths due to the COVID-19 in Texas

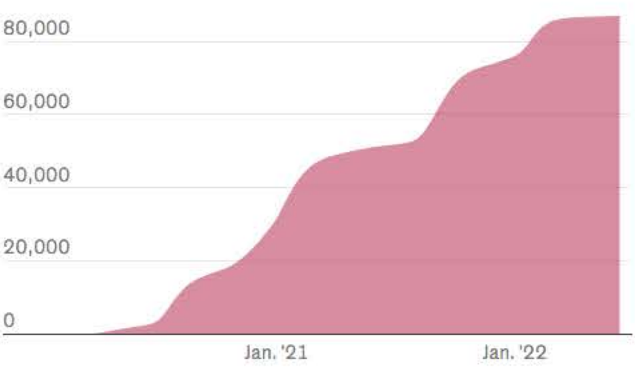

Chart: Texas Hearst Data Visualization Team • Source: Texas Department of State Health Services

Finally, let's dive into vaccines and the ongoing fight to reach herd immunity. Here's a chart showing what percentage of eligible Texans have received both vaccine doses in each county.

Percent of eligible people fully vaccinated by county

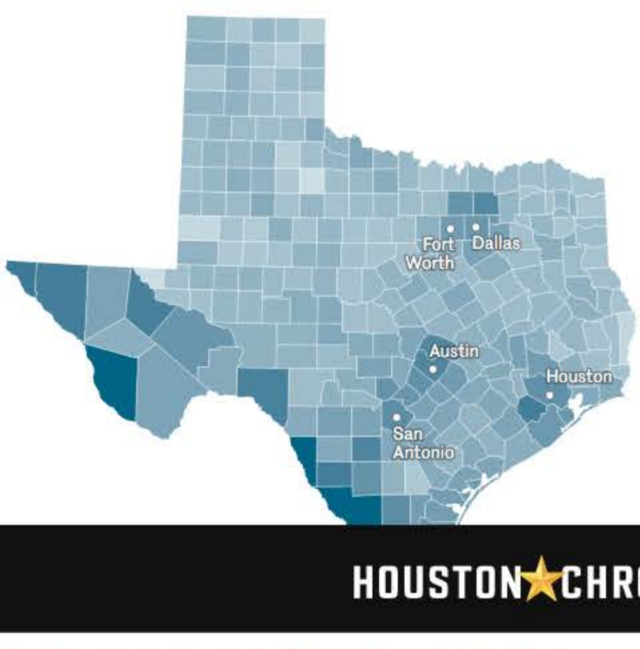

Map: Texas Hearst Data Visualization Team • Source: Texas Department of State Health Services

You can also view zip-code level vaccination rates for Harris County.

Fully vaccinated by zip code

Harris County

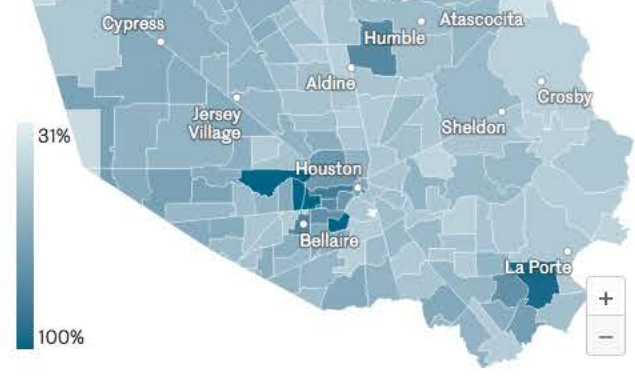

Map: Texas Hearst Data Visualization Team • Source: Texas Department of State Health Services

Greyed out zip codes either did not have data or had data suppression due to insufficient data. Some zip codes registered a vaccination rate of over 100%. Those areas have been set to max out at 100%.

As we create and tweak our interactives to best suit our audience, we'll be updating this list, so be sure to subscribe to HoustonChronicle.com for all the latest news and analysis. To stay on top of everything pandemic-related, you can also check out our COVID landing page.

Vaccination Status

COVID-19 vaccines have been proven to be safe and effective. However, no vaccines are 100% effective at preventing illness in vaccinated people. There will be a small percentage of fully vaccinated and boosted people who still get sick, are hospitalized, or die from COVID-19. These are called "breakthrough cases." Fortunately, there is evidence that vaccination may make breakthrough cases less severe and people who are fully vaccinated may be less likely to spread the disease to others.

Fully Vaccinated Breakthrough Case: A person who tests positive for COVID-19 14 days or more after they have completed the full series of an approved or authorized COVID-19 vaccine and who has not had a booster dose yet.

Boosted Breakthrough Case: A fully vaccinated person who tests positive for COVID-19 14 days or more after receiving an additional dose of approved or authorized COVID-19 vaccine after completing the full series on or after August 13, 2021. Booster data and rates are analyzed starting December 3, 2021, two weeks after the date boosters became available to those 18 years and older (November 19, 2021). Prior to this time boosters were only recommended for people 65+ and people with underlying medical conditions, making comparisons to fully vaccinated and unvaccinated individuals difficult before December 3, 2021.

Unvaccinated Case: A person who tests positive for COVID-19 and who is not fully vaccinated or boosted yet. This includes people who have not had any doses of a COVID-19 vaccine, those who have only had one dose of the Moderna or Pfizer vaccine, or those for whom 14 days have not passed between their positive COVID-19 test and their last dose in a primary vaccine series.

The Utah Department of Health determines vaccine status for cases using two methods: by automatically linking known cases to vaccination records reported into the Utah Statewide Immunization Information System (USIIS), and through self-report by asking interviewed cases if they have been fully vaccinated. Breakthrough cases may be under-represented if there is incomplete or incorrect data to link a person to their USIIS record, or if case investigators are unable to conduct an interview.

In the last 28 days, Unvaccinated Utahns had:

1.5x
Greater Risk of Testing Positive for COVID-19 than Fully Vaccinated

1.6x
Greater Risk of Hospitalization[3] than Fully Vaccinated

Death risk ratio has been suppressed at this time due to low numbers in the past 28 days

0.8x
Greater Risk of Testing Positive for COVID-19 than Boosted

1.8x
Greater Risk of Hospitalization[3] than Boosted

Death risk ratio has been suppressed at this time due to low numbers in the past 28 days

Age Adjusted Unvaccinated and Vaccinated Incidence Rates in People Eligible for the Vaccine in the last 28 days

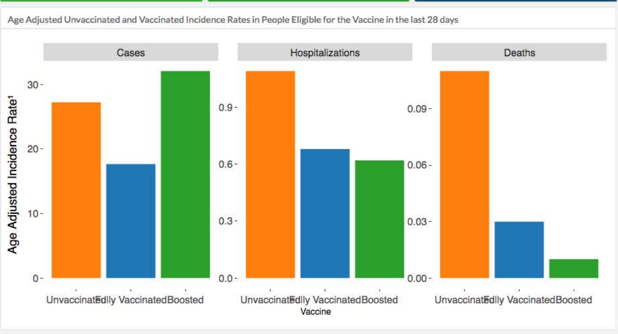

COVID-19 Case, Hospitalization, and Mortality Rates in Unvaccinated, Fully Vaccinated, and Boosted Utahns by Age Group Since December 21, 2021 (Omicron Period)

Cases

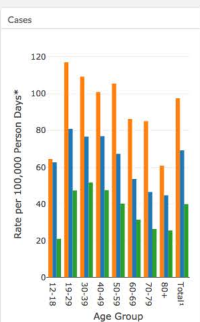

Hospitalizations

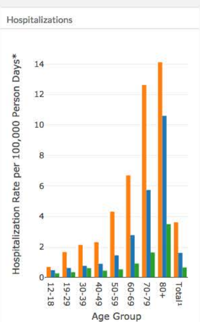

Deaths

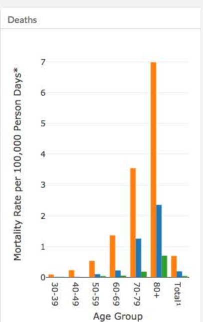

Weekly Age Adjusted Case, Hospitalization, and Mortality Rates[2] in Unvaccinated, Vaccinated, and Boosted Utahns Aged 12+

Case Rates

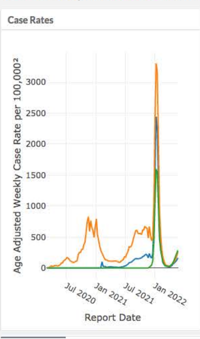

Hospitalization Rates

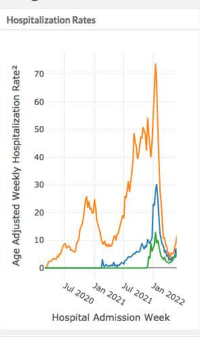

Mortality Rates

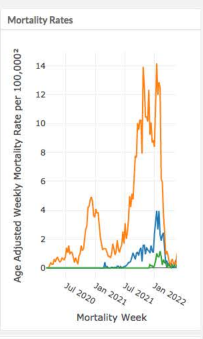

Last 28 days | Omicron Period (>12/21/2021) | Prior to Omicron Period (2/1/2021 - 12/21/2021)

Cases

Age Group/ Total	Unvaccinated		Fully Vaccinated				Boosted			
	Count	Rate	Count	Rate	Rate Ratio**	95% CI	Count	Rate	Rate Ratio**	95% CI
12-18 Years	389	9.4	408	8.0	1.18	1-1.4	311	19.8	0.48	0.4-0.6
19-29 Years	1588	27.5	1388	19.0	1.45	1.3-1.6	1236	35.1	0.78	0.7-0.8
30-39 Years	1113	26.9	927	17.8	1.51	1.4-1.6	1476	38.6	0.7	0.6-0.8
40-49 Years	797	26.0	755	17.2	1.51	1.4-1.7	1530	36.2	0.72	0.7-0.8
50-59 Years	598	32.2	600	19.2	1.68	1.5-1.9	1372	34.3	0.94	0.9-1
60-69 Years	433	33.0	411	19.4	1.7	1.5-1.9	1315	28.1	1.17	1-1.3
70-79 Years	282	46.2	249	25.2	1.83	1.5-2.2	825	24.9	1.86	1.6-2.1
80+ Years	143	37.6	130	27.7	1.36	1.1-1.7	447	28.8	1.31	1.1-1.6
**Total (Age Adjusted)**	**5343**	**27.3**	**4868**	**17.7**	**1.54**	**1.5-1.6**	**8512**	**32.1**	**0.85**	**0.8-0.9**

Hospitalizations[3]

Age Group/ Total	Unvaccinated		Fully Vaccinated				Boosted			
	Count	Rate	Count	Rate	Rate Ratio**	95% CI	Count	Rate	Rate Ratio**	95% CI
12-18 Years	9	0.2	6	0.1	1.86	0.7-5.2	<5	0.3	0.86	0.3-2.8
19-29 Years	26	0.5	17	0.2	1.94	1.1-3.6	9	0.3	1.76	0.8-3.8
30-39 Years	28	0.7	18	0.3	1.96	1.1-3.5	13	0.3	1.99	1-3.8
40-49 Years	11	0.4	<5	0.1	5.26	1.5-18.9	16	0.4	0.95	0.4-2
50-59 Years	15	0.8	20	0.6	1.26	0.6-2.5	19	0.5	1.7	0.9-3.3
60-69 Years	21	1.6	26	1.2	1.3	0.7-2.3	35	0.8	2.14	1.2-3.7
70-79 Years	27	4.4	26	2.6	1.68	1-2.9	64	1.9	2.29	1.5-3.6
80+ Years	30	7.9	26	5.5	1.43	0.8-2.4	79	5.1	1.55	1-2.4
**Total (Age Adjusted)**	**167**	**1.1**	**142**	**0.7**	**1.6**	**1.3-2**	**239**	**0.6**	**1.75**	**1.4-2.1**

Deaths[3] - Recent Deaths by Age have been Suppressed to Protect Patient Privacy

Age Group/ Total	Unvaccinated		Fully Vaccinated				Boosted			
	Count	Rate	Count	Rate	Rate Ratio**	95% CI	Count	Rate	Rate Ratio**	95% CI
**Total (Age Adjusted)**	**14**	**0.1**	**<5**	**0.0**	**4.23**	**1.4-12.9**	**<5**	**0.0**	**13.84**	**4.6-42**

Data Notes for Vaccinated and Unvaccinated Risk Analysis

To protect patient privacy, these analyses exclude the last seven days. Death counts and rates will be updated separately once per week and age groups with fewer than 20 deaths are suppressed.

Person-day population estimates: To calculate the eligible Utah population 12 years and older who are fully vaccinated, the UDOH takes the cumulative total of people 14 days after becoming fully vaccinated or boosted reported into USIIS each day and sums all the days. To calculate the eligible but unvaccinated population, the UDOH takes the cumulative total of people 14 days post-full vaccination or boosted minus the 2019 census population estimate for people 12 years and older each day and sums all the days.

[1]Age adjustment for total rates: As of 10/5/2021, the UDOH age-adjusts total rates to better show the risk of people who are fully vaccinated, boosted, and unvaccinated getting COVID-19, being hospitalized, or dying. Age adjusting is a way to control for the effects of age in multiple populations, in this case in vaccinated, boosted, and unvaccinated Utahns. We age-adjust using the direct age standardization method with the Utah 2019 census population from IBIS as the standard population. To calculate age-adjusted rates, the UDOH calculates each age groups' individual case rate for vaccinated and unvaccinated people and then multiplies that rate by the proportion of that age group in the 2019 Utah population. The UDOH then sums each weighted rate to get the total age-adjusted rate for the state.

*Rates are a cumulative incidence rate per 100,000 person-days and are calculated as the "sum of people observed with COVID-19 outcome each day" divided by the "sum of people vaccinated, boosted, or unvaccinated each day" times 100,000.

**The rate ratio is the "incidence rate of unvaccinated people" divided by "incidence rate of fully vaccinated or boosted people." The rate ratio is interpreted as how many times more the risk is for unvaccinated people than fully vaccinated or boosted people. If the lower bound of the 95% confidence interval (CI) is greater than 1, then the vaccines have a statistically significant effect on risk. Groups that don't have overlapping 95% confidence intervals have statistically significant differences in risk, while groups whose confidence intervals do overlap are not statistically different.

[2] Seven-day age-adjusted rates are calculated using the number of fully vaccinated, boosted, and unvaccinated cases per age group and dividing by the median vaccinated and unvaccinated population during that week. The total weekly rates are age-adjusted to the Utah 2019 census population estimates available on IBIS. Seven-day rates exclude the current calendar week as cases reported in this time may not yet be investigated. Hospitalizations and deaths will backfill as these events are reported to the UDOH.

[3]There may be large daily changes in recent case rates and age-specific hospitalizations and deaths because of small case counts. These changes do not indicate differences in vaccine efficacy but instead reflect the UDOH identifying additional hospitalizations and deaths in this time window, which often lag case reports.

This analysis relies on surveillance data and does not control for individual differences in risk, location, or testing practices so these data should be considered in conjunction with clinical trials and other vaccine efficacy studies that show the vaccine is very effective at preventing symptomatic illness and severe disease. Breakthrough cases may be under-represented if there is insufficient data to link a case to a USIIS record or reach a person for interview, or over-represented if vaccinated communities are more likely to have access to and receive testing.

Total Vaccine Breakthroughs

Total Vaccine Breakthroughs | Daily Case Counts by Vaccination Status

Type	Fully Vaccinated Breakthrough Cases	Boosted Breakthrough Cases	People 14 Days Post Fully Vaccination Without Booster	People 14 Days Post Booster	Rate per 100k Fully Vaccinated	% Fully Vaccinated to Get COVID-19	Rate per 100k Boosted
Cases	190,521	55,517	1,088,854	935,091	17497.4	17.497%	5937.1
Hospitalizations	4,892	1,449	1,088,854	935,091	449.3	0.449%	155
Deaths	600	135	1,088,854	935,091	55.1	0.055%	14.4

Pre-Existing Conditions

Data on pre-existing conditions are gathered from a variety of sources, including case interviews and medical records (when available). Case interviews are conducted by public health investigators across state and local health departments to determine what potential medical risk factors cases may have had prior to developing COVID-19. Data gathered through interviews is self-reported by cases and is dependent on their willingness to share this information with health departments. This is not a complete list of conditions and rates may change as we collect more data and performs additional analysis.

Data collection forms used by the Utah Department of Health and Utah's Local Health Departments have been updated to collect information on additional pre-existing conditions: autoimmune conditions, disabilities, hypertension, severe/morbid obesity, psychological/psychiatric conditions, and substance abuse. The previous neurologic pre-existing condition has been moved into the broader disability condition, which includes neurological, neurodevelopmental, intellectual, and physical disabilities. Additionally, investigators are now able to distinguish between type 1 and type 2 diabetes. All analyses of these new conditions are based on the subset of cases that use the new forms. These forms no longer collect information on specific conditions from cases without that condition or where the status is unknown; this information remains available for the overall 'Any Pre-Existing Condition' question.

Examples of persons with compromised immune systems include those with cancer and transplant patients who are taking certain immunosuppressive drugs, persons living with HIV/AIDS, and those with inherited diseases that affect the immune system. Chronic pulmonary conditions include uncontrolled asthma, emphysema, and COPD. Examples of persons with a disability include those with dementia, seizure disorders, cognitive impairment, and Alzheimer's disease. Psychological/psychiatric conditions include schizophrenia, major depressive disorder, and bipolar disorder.

Any Pre-Existing Condition in All Cases

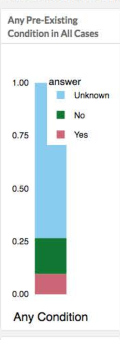

Pre-Existing Conditions of All Cases

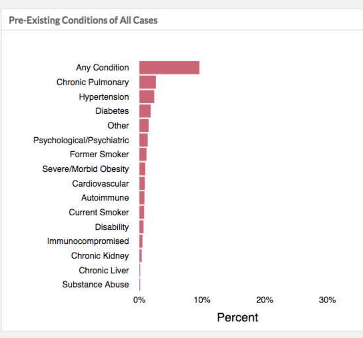

Diabetes Type in All Cases with Diabetes

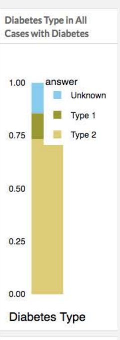

Any Pre-Existing Condition in Hospitalized Cases

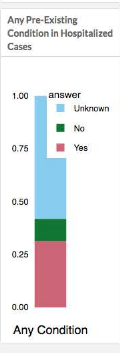

Pre-Existing Conditions of Hospitalized Cases

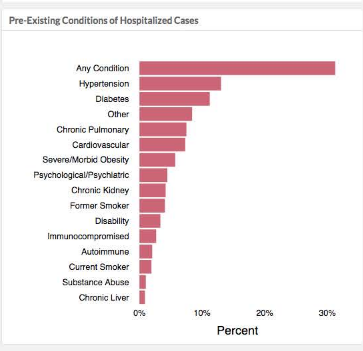

Diabetes Type in Hospitalized Cases with Diabetes

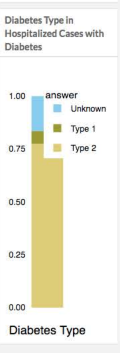

In the data below, counts below 5 are suppressed to protect privacy. Suppressed values in the chart are represented by the percent equivalent to a count of 5 in that category.

Pre-Existing Conditions of All Cases by Age

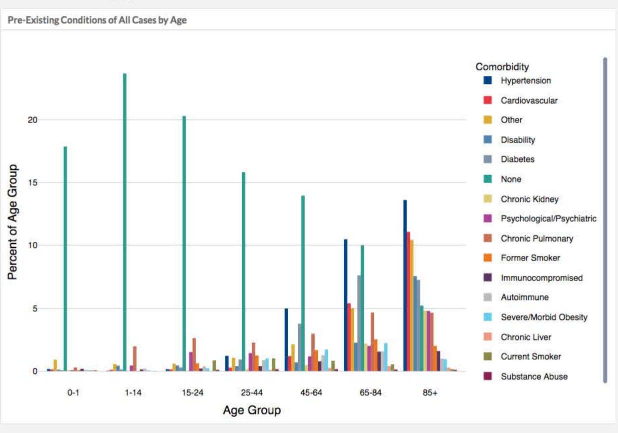

Potential Exposures

The charts below show if the public health investigator has determined a case to have potentially acquired COVID-19 through known contact to another confirmed case, or through "Community Spread", where a single source of infection cannot be identified. In this analysis, "Known Contact" represents any case with a known contact to a confirmed case in the 14 days before experiencing symptoms through contact tracing, self-report, or outbreak identification. "Community" is any case with a completed investigation, but no indication of a known contact. "Unknown/ Pending" are cases who either could not be contacted for an interview, would not provide contact information, are recently reported and not yet interviewed, or where incomplete information has been entered by the investigator. Data will be updated weekly on Wednesday for the past calendar week and may backfill significantly as investigations are completed and outbreaks are identified.

Potential exposures are identified through case investigations and are not mutually exclusive. "Household" exposures are defined as any case identified through contact tracing of household contacts, or who self-reports a confirmed case of COVID-19 in the household. "Social" exposures are defined as any case identified through contract tracing of social contacts (like church, group gathering, dating, friends, and coworkers outside of the worksite) or who self-report known social contact with a confirmed case. "Workplace" exposures are defined as any case who reports known contact to a confirmed case in the workplace, or is part of an identified worksite outbreak (two or more cases associated with a worksite within 14 days). Finally, Travel exposures are any case who reports known contact to a confirmed case while traveling outside of the state of Utah, or with known contact to a confirmed case through CDC contact tracing of flights. Often, cases have multiple exposures in the categories or, in some instances, no exposures in these categories.

Known Contact and Community Spread

Infection Source	Count	Percent
Community	130948	13.7
Known Contact	372556	38.9
Unknown/Pending	453642	47.4

Reported Exposures (categories are not mutually exclusive)

Potential Exposure	Count	Percent
Household	199263	20.8
Social	58952	6.2
Travel	126	0
Workplace	43678	4.6

Known Contact and Community Spread By Week - Counts | Known Contact and Community Spread By Week - Percent

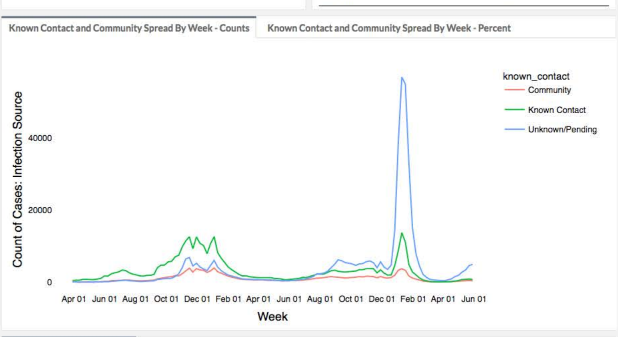

Potential Exposures - Counts | Potential Exposures - Percent

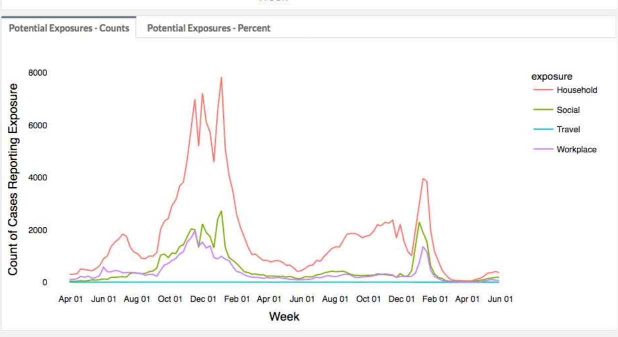

Mask Wearing Compliance

The Utah Department of Health collects information on mask compliance through the Behavioral Risk Factor Surveillance System (BRFSS) where Utah residents are asked to rank how often they wear a mask in public or when unable to socially distance. The response options are always, usually, sometimes, rarely, or never.

The BRFSS is a random-digit dial telephone survey of adults aged 18 and older. In 2020, about 25% of completed interviews came from landline phones and 75% from cellular phones in Utah. In order to have timely estimates, the data are downloaded weekly from the system and are not adjusted to reflect unequal sampling or population characteristics. However, sampling is done proportionally to population and the broad coverage of telephones makes this data sufficient for state and local estimates.

Note: The UDOH has transitioned all mask wearing surveillance data to the BRFSS methodology. The BRFSS is more robust and representative of Utah's population than the Physical Mask Wearing Survey data. Additionally, the data now show the three most recently completed months, plus the current month, rather than all available data. This is important for understanding current trends in mask wearing.

BRFSS: Mask Wearing Compliance – Past 3 Months

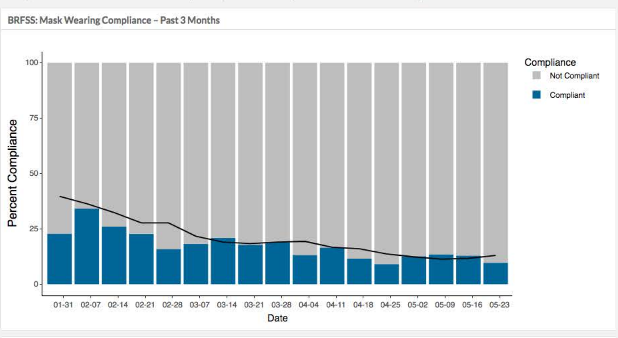

BRFSS Survey: Mask Wearing Compliance by Age and Sex – Past 3 Months

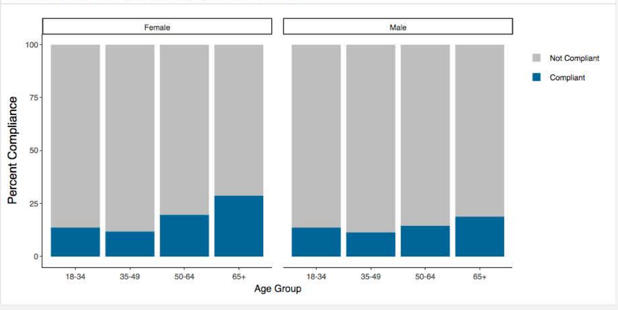

BRFSS Survey: Mask Wearing Compliance by County – Past 3 Months

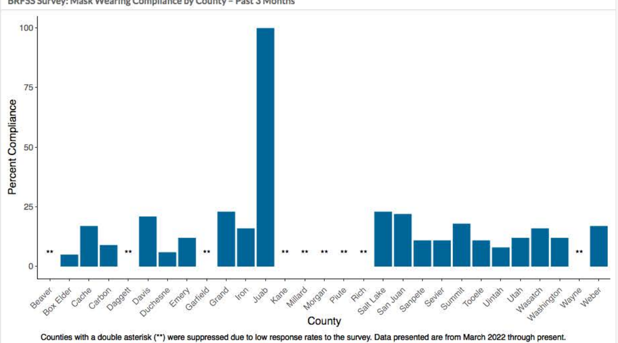

Counties with a double asterisk (**) were suppressed due to low response rates to the survey. Data presented are from March 2022 through present.

Healthcare Workers

These data reflect any individual who reports being an employee in the healthcare setting in any capacity such as cooks, environmental services, administrative roles, support staff, physicians, nurses, respiratory therapists, pharmacists, home health and personal care aids, etc. Healthcare workers also include those who commonly have patient interactions such as dentists, physical therapists, EMTS, firefighters, phlebotomists, and other technical occupations. These data are not intended to represent the amount of transmission occurring within the healthcare setting. Rather, the data can help us have a better understand the burdenour healthcare systems due to employees testing positive for COVID-19.

Total number of cases, hospitilizations, and deaths among healthcare workers

Jurisdiction	Cases	Hospitalizations	Deaths
State Total	28738	639	26

Number of Cases Among Healthcare Workers by Day

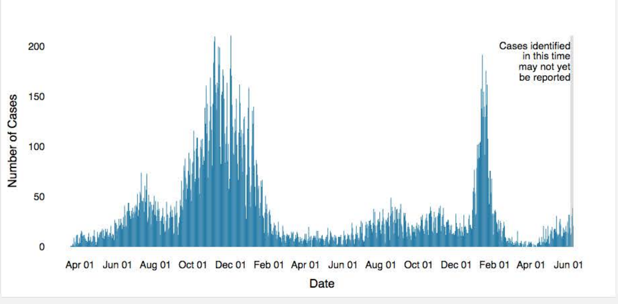

Data for this report were accessed on June 09, 2022 06:50 AM.

COVID-19 in Virginia: Vaccine Summary

VIRGINIA'S HEALTH IS IN OUR HANDS. Do your part, stop the spread.

VDH VIRGINIA DEPARTMENT OF HEALTH

Dashboard Updated: 6/10/2022

COVID-19 Vaccinations in Virginia

Total Doses Administered - 15,984,583

People Vaccinated with at Least One Dose*	% of the Population Vaccinated with at Least One Dose	People Fully Vaccinated^	% of the Population Fully Vaccinated	People Vaccinated with Booster/ Third Dose**
7,076,961	82.4%	6,341,888	73.8%	3,115,084

% of the Adult (18+) Population Vaccinated with at Least One Dose
93.2%

% of the Adult (18+) Population Fully Vaccinated
83.7%

* People vaccinated with one dose of a two-dose vaccine and one dose of a single dose vaccine, including doses administered through the Federal CDC Pharmacy Partnership. Doses on the Federal Doses Administered dashboard are included.
^ People vaccinated with two doses of a two-dose vaccine and one dose of a single dose vaccine, including doses administered through the Federal CDC Pharmacy Partnership. Doses on the Federal Doses Administered dashboard are included.
**People vaccinated with a booster or third dose after the primary vaccine series, including doses administered through the Federal CDC Pharmacy Partnership. Doses on the Federal Doses Administered dashboard are not included.

People Vaccinated by Locality of Residence and Vaccination Status - Percent of the Population

Percent of the Population with At Least One Dose

- 45.1% - 50.0%
- 50.1% - 55.0%
- 55.1% - 60.0%
- 60.1% - 65.0%
- 65.1% - 70.0%
- 70.1+%

Select Counts, Rates, or Percents
Percent of the Population

Select Vaccination Status
At Least One Dose

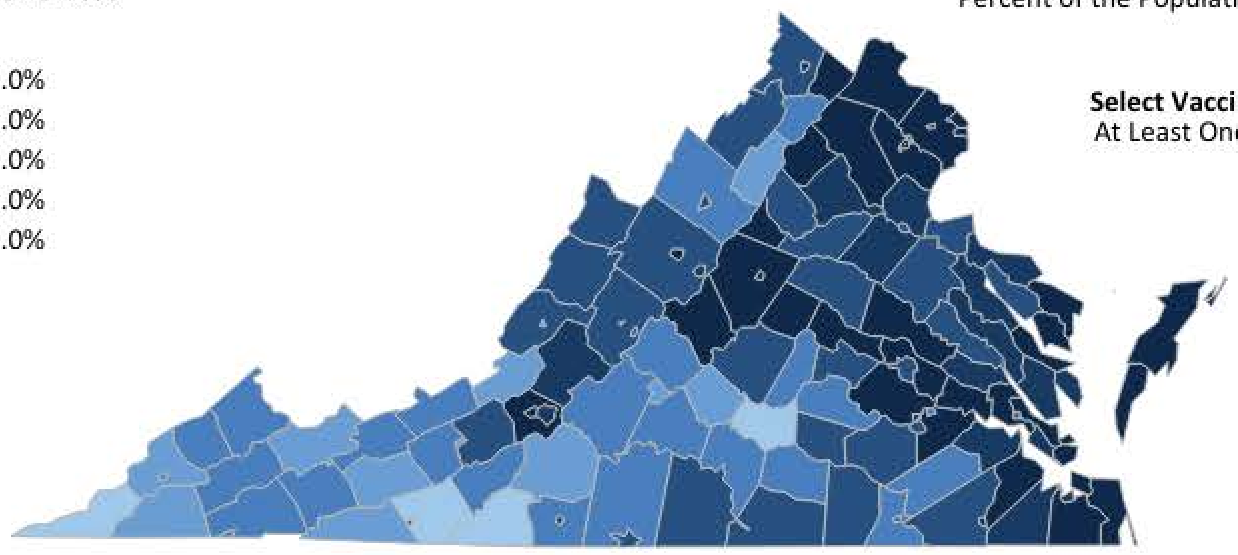

People Not Mapped : 1,097,270

COVID-19 Vaccine Doses Administered by Facility Type

Facility Type	Vaccine Doses Administered
Hospitals	1,362,217
Local Health Departments	2,694,208
Medical Practices	2,017,565
Other Community Health Provider	1,783,461
Pharmacies	7,321,588

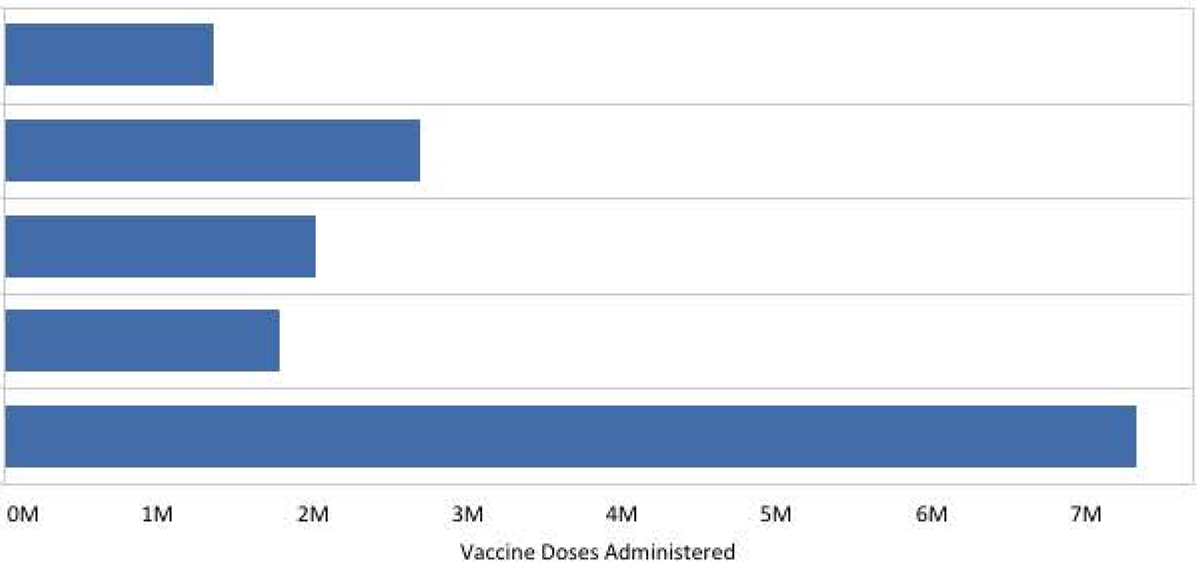

COVID-19 Vaccine Administration^ by Day

Average Doses Administered Each Day**

3,975

J&J Moderna Pfizer Average Doses Administered Each Day**

Select Date Range All

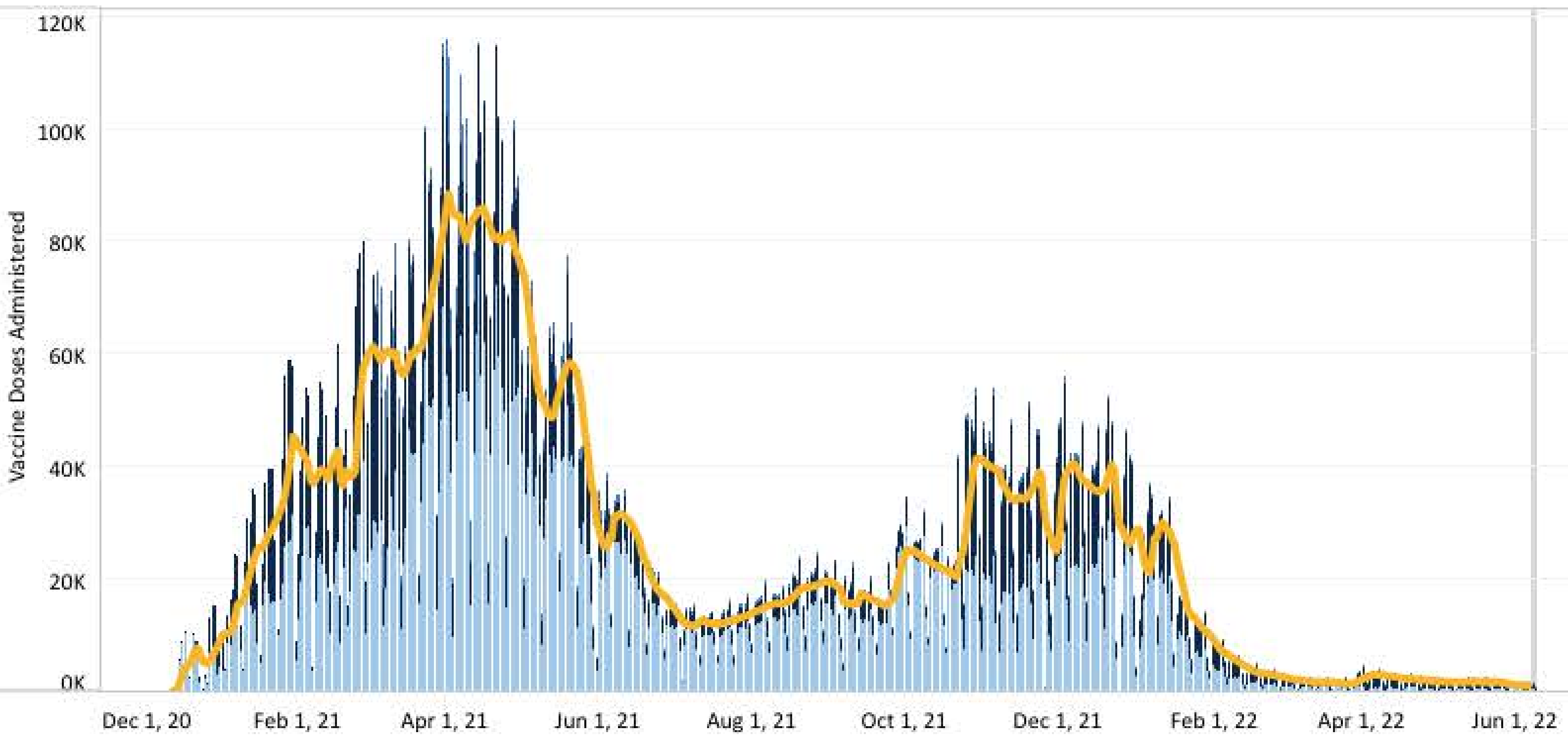

**The average doses administered each day takes the last 7 days of daily doses given, adds them up, and divides that number by 7. This is useful to get a clear picture of the data while taking into account reporting delays.
^Vaccine administrations may take up to 72 hours to be reported, shown by gray shaded area on graph.

VIRGINIA'S HEALTH IS IN OUR HANDS. Do your part, stop the spread.

COVID-19 in Virginia: Summary

VDH VIRGINIA DEPARTMENT OF HEALTH

Dashboard Updated: 6/10/2022
Data entered by 5:00 PM the prior day.

Cases, Hospitalizations and Deaths

Total Cases*		Total Hospital Admissions**		Total Deaths	
1,817,814 (New Cases: 3,149)^		52,212		20,441	
Confirmed†	Probable†	Confirmed†	Probable†	Confirmed†	Probable†
1,300,199	517,615	49,048	3,164	17,060	3,381

* Includes both people with a positive test (Confirmed), and symptomatic with a known exposure to COVID-19 (Probable).
** Hospitalization of a case is captured at the time VDH performs case investigation. This underrepresents the total number of hospitalizations in Virginia.
^New cases represent the number of confirmed and probable cases reported to VDH in the past 24 hours.
† VDH adopted the updated CDC COVID-19 confirmed and probable surveillance case definitions on August 27, 2020. Found here: https://wwwn.cdc.gov/nndss/conditions/coronavirus-disease-2019-covid-19/case-definition/2020/08/05/ ..

Outbreaks

Total Outbreaks*	Outbreak Associated Cases
8,694	141,185

* At least two (2) lab confirmed cases are required to classify an outbreak.

Testing (PCR Only)

Testing Encounters PCR Only*	Current 7-Day Positivity Rate PCR Only**
13,988,678	18.5%

* PCR" refers to "Reverse transcriptase polymerase chain reaction laboratory testing."
** Lab reports may not have been received yet. Percent positivity is not calculated for days with incomplete data.

Multisystem Inflammatory Syndrome in Children

Total Cases*	Total Deaths
177	1

*Cases defined by CDC HAN case definition: https://emergency.cdc.gov/han/2020/han00432.asp

VIRGINIA'S HEALTH IS IN OUR HANDS.
Do your part, stop the spread.

COVID-19 in Virginia: Demographics

VDH VIRGINIA DEPARTMENT OF HEALTH

Dashboard Updated: 6/10/2022
Data entered by 5:00 PM the prior day.

All Health Districts Cases*	All Health Districts Hospital Admissions**	All Health Districts Deaths
1,817,814	52,212	20,441

Health District
All

Past 13 Weeks | Past 26 Weeks | Past 52 Weeks | All Reporting
Cases | Case Rate | Deaths | Death Rate | Hospitalizations | Hospitalization Rate

Health District 2
Alexandria

Cases by Age Group - All Health Districts - Past 13 Weeks

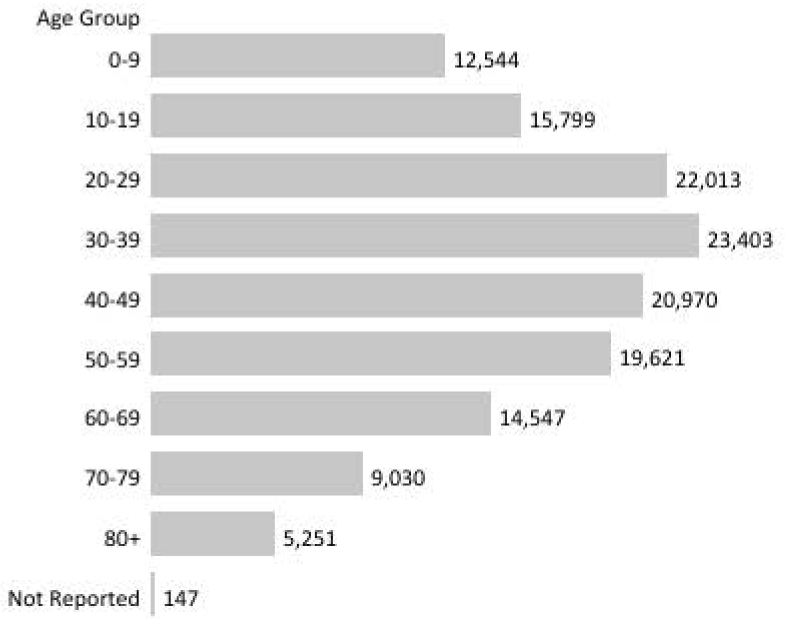

Cases by Age Group - Alexandria - Past 13 Weeks

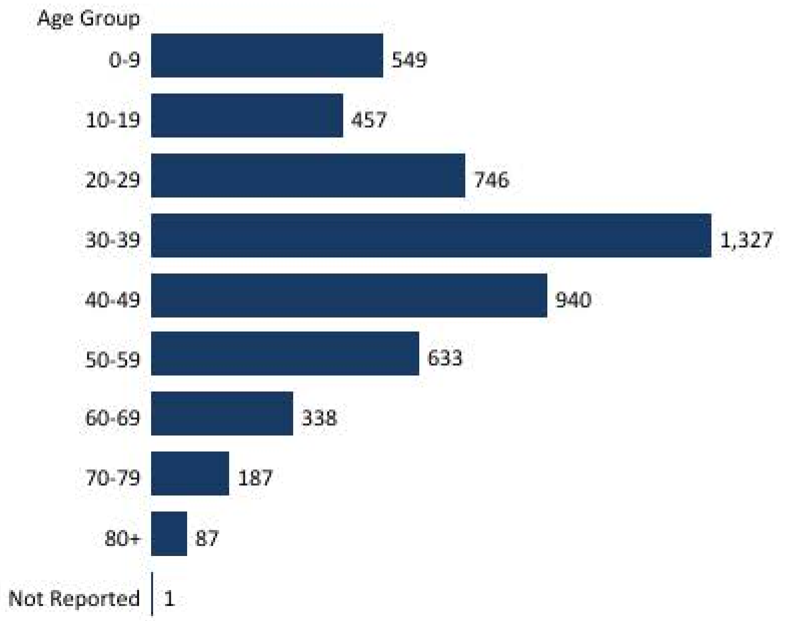

Cases by Race and Ethnicity^ - All Health Districts - Past 13 Weeks

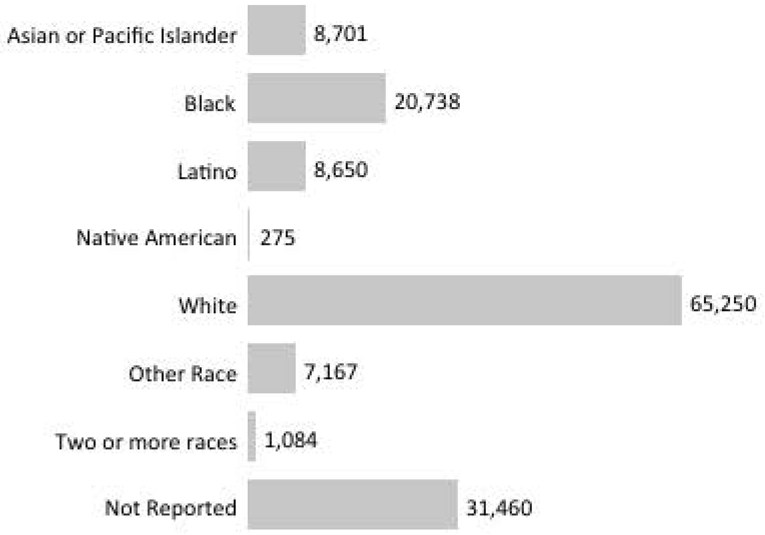

Cases by Race and Ethnicity^ - Alexandria - Past 13 Weeks

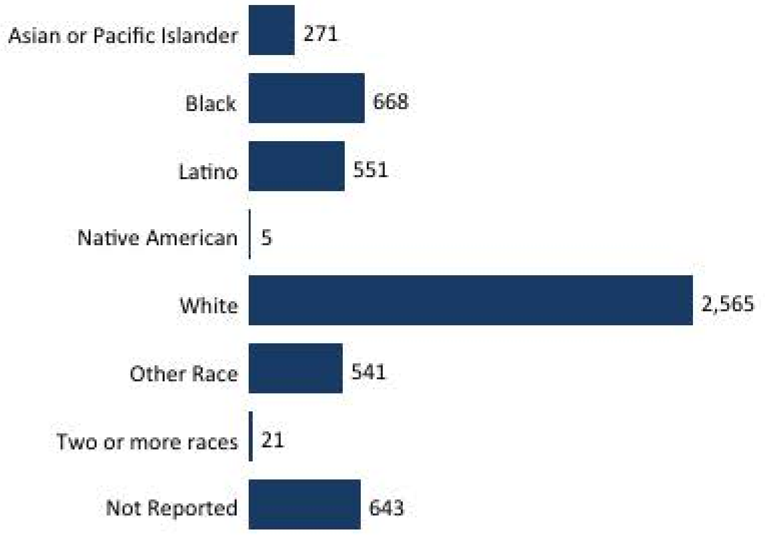

Cases by Sex - All Health Districts - Past 13 Weeks

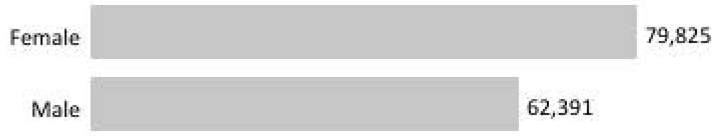

Cases by Sex - Alexandria - Past 13 Weeks

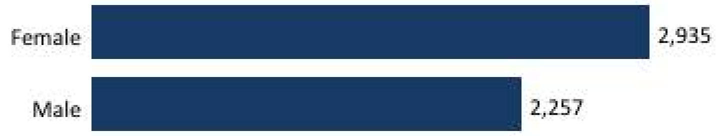

* Includes both people with a positive test (Confirmed), and symptomatic with a known exposure to COVID-19 (Probable).
** Hospitalization status at time case was investigated by VDH. This underrepresents the total number of hospitalizations in Virginia.
Source: Cases - Virginia Electronic Disease Surveillance System (VEDSS), data entered by 5:00 PM the prior day.
^ For more information about how VDH is presenting race and ethnicity data for COVID-19, please read our blog post:
https://www.vdh.virginia.gov/coronavirus/2020/06/16/race-and-ethnicity-reporting-update/

Coronavirus in Vermont

This page is updated every weekday, excluding holidays, when new data on Covid-19 in Vermont becomes available. Sources include the Vermont Department of Health and the U.S. Centers for Disease Control and Prevention. Read more of VTDigger's Covid-19 coverage.

Vermont Coronavirus report

Wednesday, June 8
141 new cases reported today, with 33 hospitalized (5 in ICU)
9.5% seven-day test positivity rate
Most recent death reported June 2, totaling 673 deaths statewide
88% of Vermonters age 5+ have received at least one vaccine dose
68% of vaccinated Vermonters have received a booster dose

Data retrieved from the Vermont Department of Health, updated every weekday representing the previous day's report. Vaccination percentage is based on the number of Vermonters age 5 and above, per Vermont Department of Health Population numbers (2019).

The Vermont Department of Health publishes daily Covid-19 case counts for the prior week every Wednesday. The daily counts include only positive PCR test results. As of January 2022, officials estimate that case counts far exceed this figure due to the increased use of at-home rapid antigen tests. The department publishes separate data on self-reported antigen test results, noting that that data is also partial.

Covid new cases per day, rolling average

Based on the latest Department of Health data, with a seven-day rolling average:

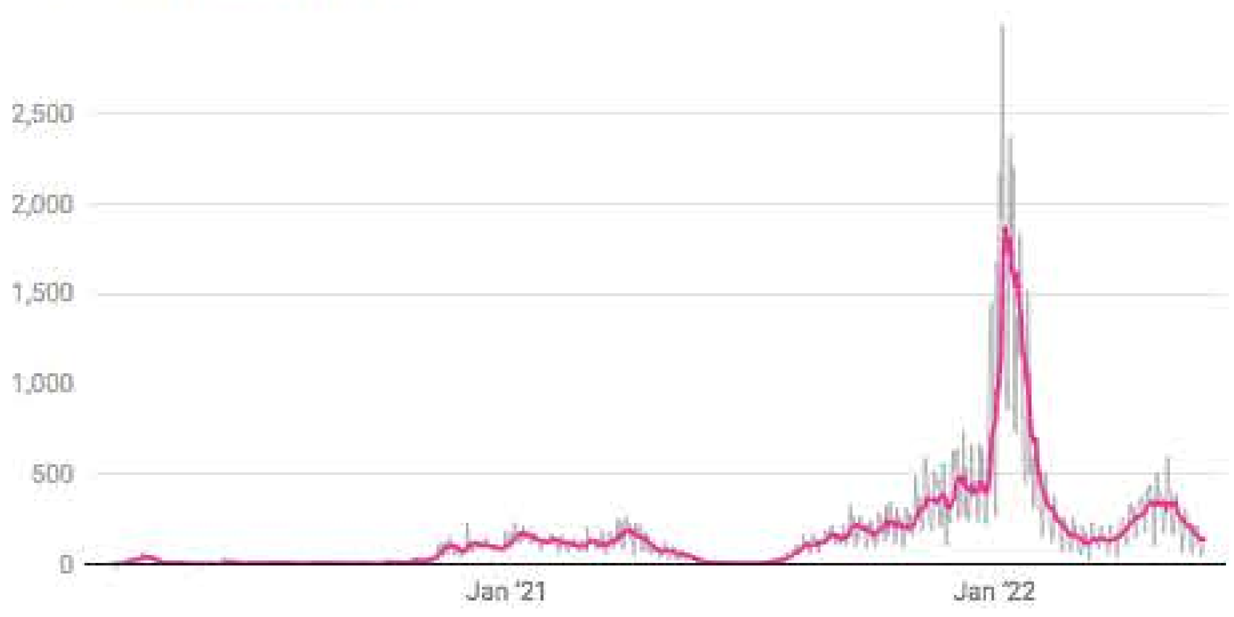

Chart: Erin Petenko • Source: VTDOH via Vermont Center for Geographic Information • Get the data • Download image • Created with Datawrapper

The Vermont Department of Health releases data every Monday, Wednesday and Friday on the daily number of patients in Vermont hospitals with Covid-19. That includes both patients who were admitted because of Covid symptoms, and patients who were admitted for other reasons and tested positive for Covid while hospitalized.

People hospitalized for Covid over time

Number of people currently hospitalized for Covid:

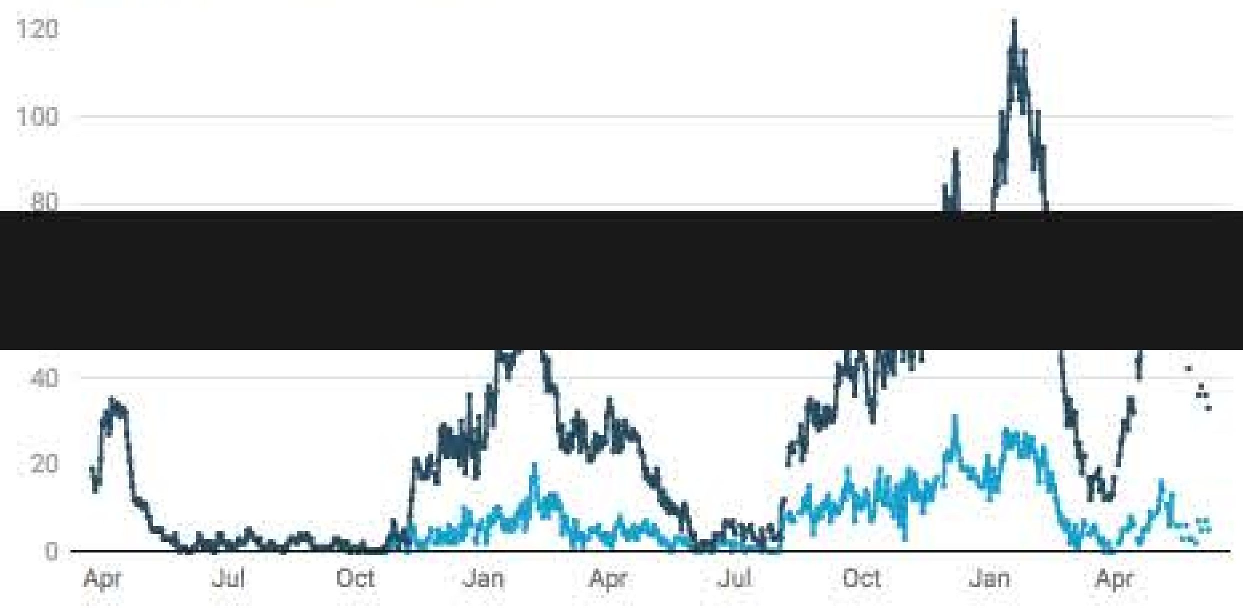

enter search term

Does not include people hospitalized under investigation for Covid. There are reporting gaps when the department did not report data over a holiday weekend.

Chart: Erin Petenko • Source: VTDOH via VCGI • Get the data • Created with Datawrapper

VTDigger is reader supported.
If you rely on our timely and rigorous news coverage to keep you informed, please support it Will you join us?

Covid-19 deaths include any deaths in Vermont that have Covid listed as a cause or probable cause on their death certificate. Because of the time it takes to investigate and report Covid deaths, these deaths can be added to the data days or weeks after they occur, which can raise previous months' count.

Monthly Covid-19 deaths

Most recent month is a partial total.

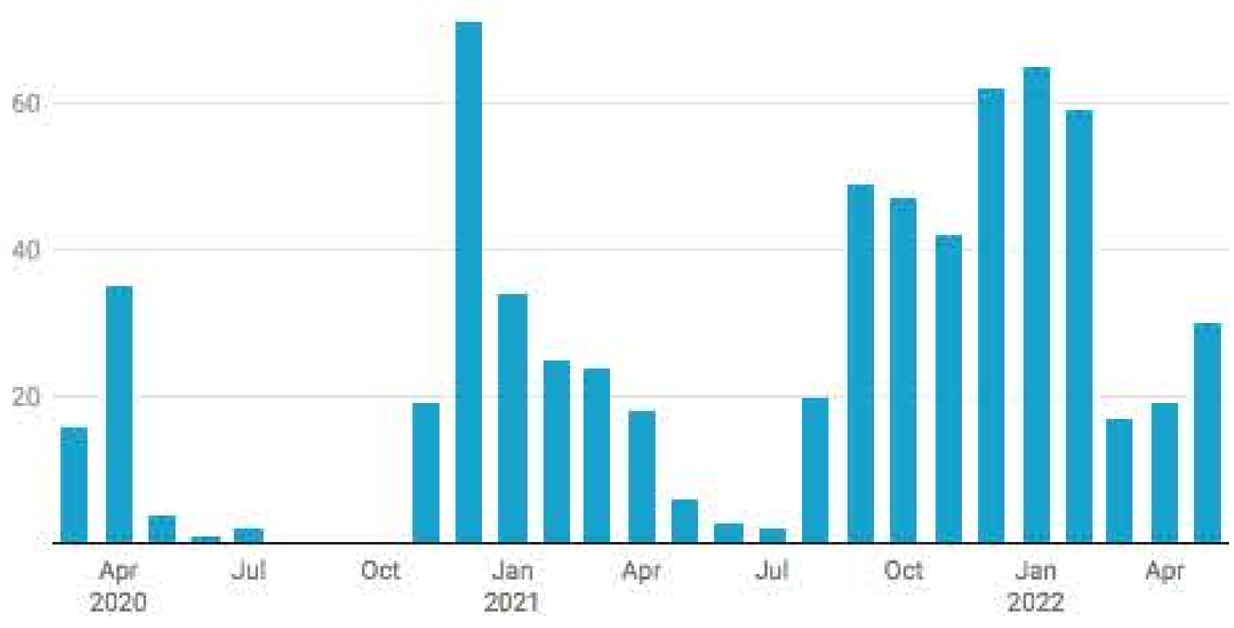

Chart: Erin Petenko • Source: VTDOH via VCGI • Get the data • Created with Datawrapper

Support VTDigger
DONATE
NEWSLETTER
SHOP

[illegible] Prevention has calculated county-by-county "community levels" of Covid-19. The agency's rubric is based on three metrics: recent Covid cases, hospital admissions and hospital beds taken up by Covid patients. The CDC recommends that people in high-level counties take precautions, including wearing masks, to prevent Covid spread. In medium-level counties, the CDC recommends that high-risk individuals take extra precautions to protect themselves. This map is updated on Friday mornings.

CDC Covid-19 Community Levels

High | Medium | Low

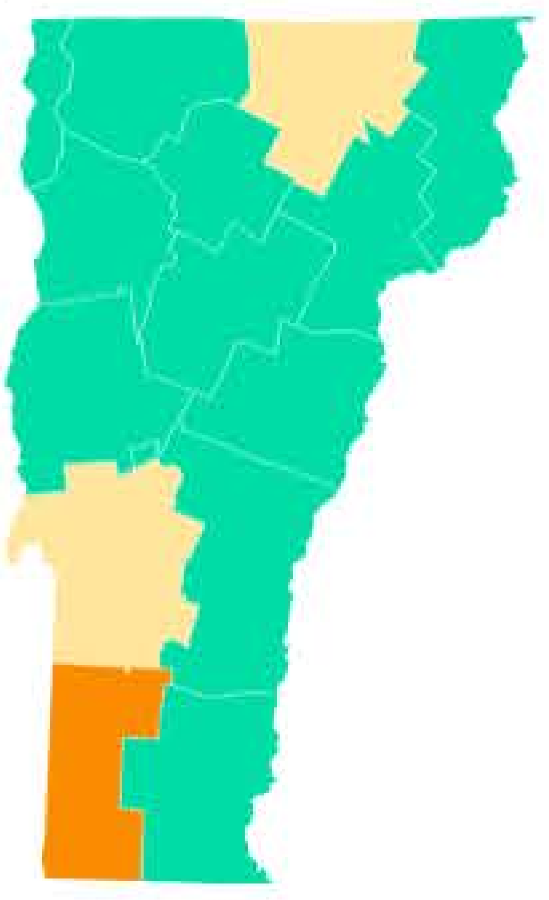

Map: Erin Petenko • Source: CDC • Get the data • Created with Datawrapper

CURRENT STATUS

COVID-19 IN WASHINGTON STATE

DATA AS OF 06/09/2022 11:59PM PT

The Current Status tab provides a snapshot of statewide and county-level COVID-19 information. The 7-day case rate and percent hospital occupancy measures are presented for the most recent 7-day period with complete data. The total population completing the primary vaccination series measure provides the most recent account of people in Washington who completed the recommended number of COVID-19 vaccinations. **Learn More**

State Summary

235 CASES PER 100,000 POPULATION

9% OF HOSPITAL BEDS OCCUPIED BY COVID-19 PATIE...

68% OF POPULATION COMPLETING PRIMARY SERIES

Low · Moderate · Substantial · High

TREND IN 7-DAY RATE OF NEW COVID-19 CASES PER 100,000 POPULATION

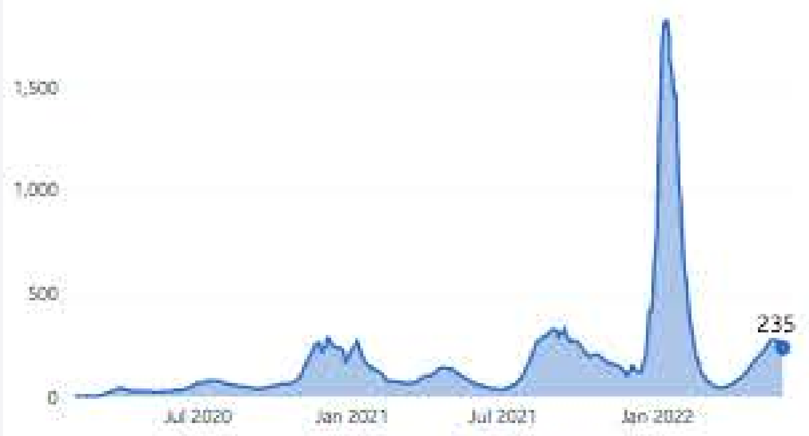

Cases from the last 8 days are not reported
Hospital bed occupancy is reported only at the state level

7-DAY CASE RATE PER 100,000 POPULATION

Measurement Period: 5/25/2022-6/1/2022

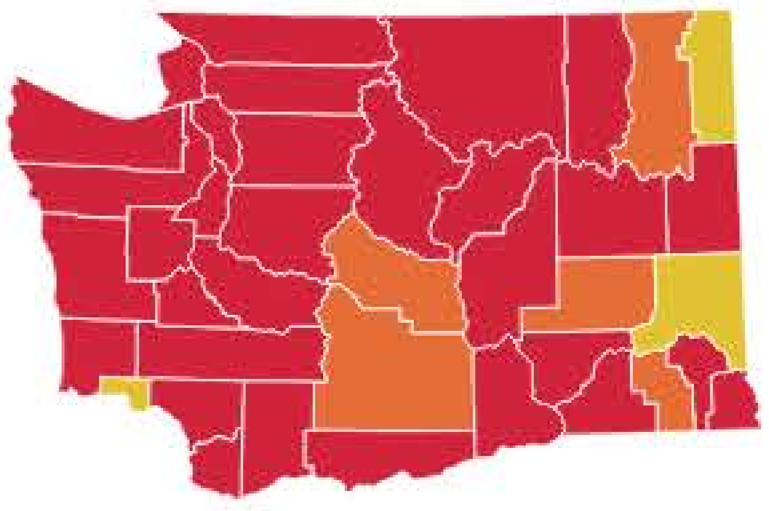

Click on the map for county data

0-9.99 · 10-49.99 · 50-99.99 · 100+

0 cases in the measurement period do not have an assigned county

TOTAL POPULATION COMPLETING PRIMARY VACCINATION SERIES

Measurement Period: 12/15/2020-06/08/2022

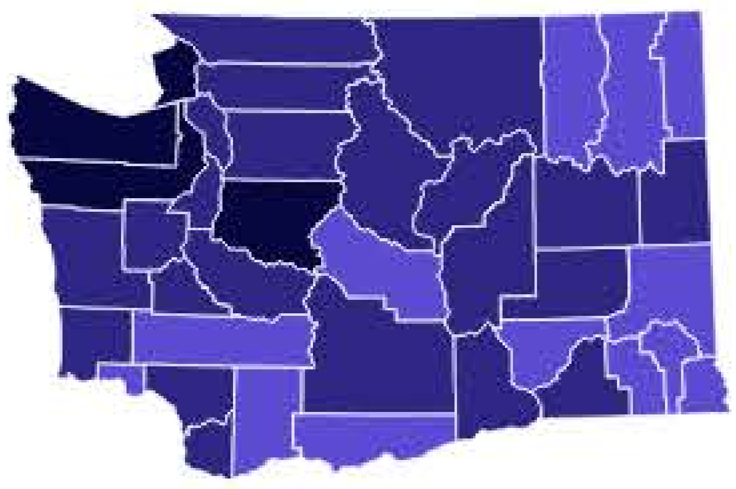

Click on the map for county data

Zero · 0-18% · 18%-35% · 35%-53% · 53%-70% · 70%+

67,326 people completing the primary vaccination series do not have a county reported

County-level information can be found on Local Health Jurisdiction (LHJ) websites

\- + 67%

Microsoft Power BI

DISEASE ACTIVITY AND TESTING

Disease Activity | Total Counts | Demographics | Testing

Rate / Count

COVID-19 IN WASHINGTON STATE — Cases and Deaths by Specimen Collection Date, and Hospitalizations by Admission Date

DATA AS OF 06/09/2022 11:59PM PT

Current Status | Disease Activity and Testing | Vaccination | Hospital Use

Summary Data Tables | Data Download | Reports | Technical Notes

Hospitalizations

Deaths

R-effective Estimates

SELECT COUNTIES

Search

- Select all
- Adams County
- Asotin County
- Benton County
- Chelan County
- Clallam County
- Clark County
- Columbia County
- Cowlitz County
- Douglas County

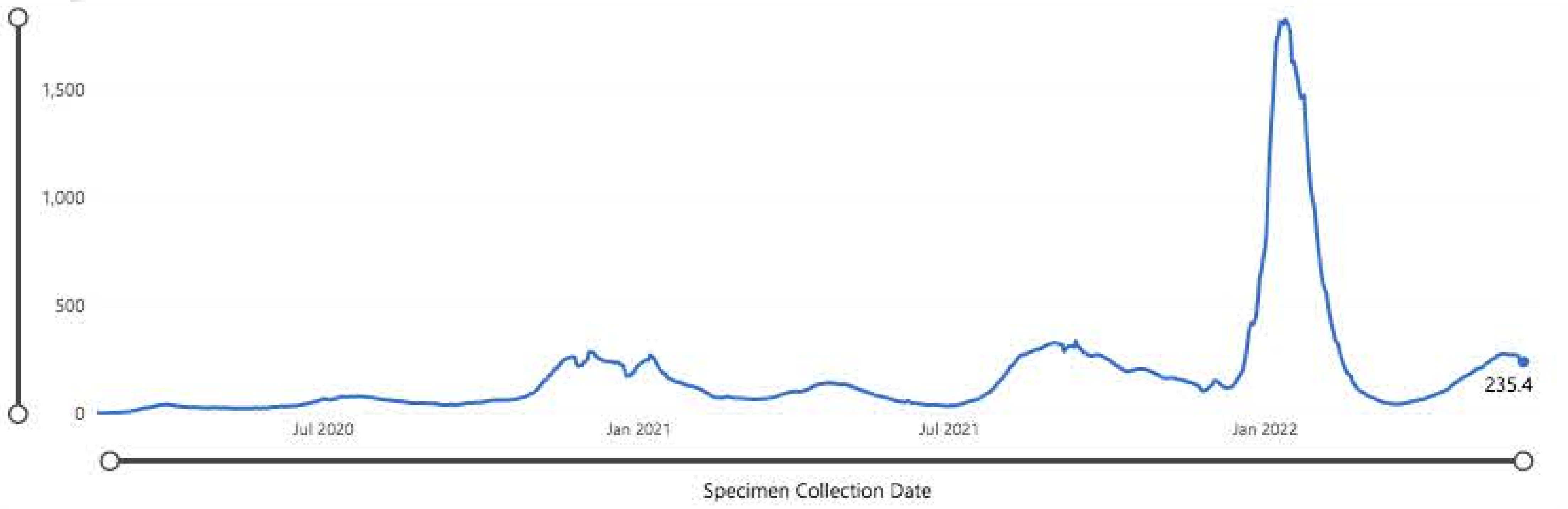

3,276 of 1,610,793 cases do not have an assigned county. Cases from the last 8 days may yet not be reported.

Download

County-level information can be found on Local Health Jurisdiction (LHJ) websites

\- + 67%

Microsoft Power BI

VACCINATIONS

Where and when are vaccinations being given? | How many people are getting vaccinated? | Who is getting vaccinated?

COVID-19 IN WASHINGTON STATE — Where and when are vaccinations being given?

DATA AS OF 6/8/2022 11:59:00 PM PT

This chart shows the number of COVID-19 vaccines given in Washington state facilities and reported to the state. Not all COVID-19 vaccines given in Washington are reported to the state. Vaccine doses given reflects the data reported to the state as of the latest update date. Please note, the measures "vaccines given" and "people vaccinated" are not comparable. People vaccinated counts the number of Washington residents initiating their primary series, completing their primary series, and receiving a booster. Numbers may not always align since people may live in a different county than where they

SELECT COUNTIES

Search

- Select all
- Adams County
- Asotin County
- Benton County
- Chelan County
- Clallam County
- Clark County
- Columbia County
- Cowlitz County
- Douglas County
- Ferry County
- Franklin County
- Garfield County
- Grant County
- Grays Harbor Co...
- Island County

By Location | By Date | Cumulative View

Vaccine Doses Given and Reported (statewide or selected counties)	14,002,539
**Statewide Measures Only**	
Vaccine Doses Given and Reported in Washington	14,002,539
Doses Delivered to Washington Providers	8,857,660
Doses Delivered for Federal Partnership Programs	6,942,690
Percentage of Delivered Doses Given	88.6%
Current Complete 7-Day Average	7,588

VACCINE DOSES GIVEN AND REPORTED IN WASHINGTON COUNTIES

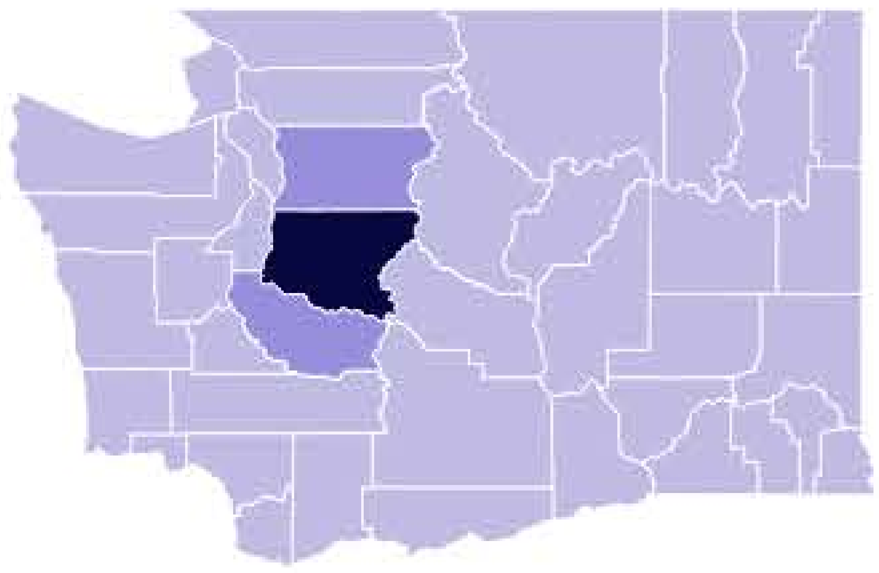

0 · 1 to 996,345 · 996,346 to 1,992... · 1,992,691 to 2,9... · 2,989,037 to 3,9... · 3,985,382 to 4,9...

County data for doses given are based on the location of the facility giving the vaccine. 91,631 vaccine doses given do not have a county reported.

County-level information can be found on Local Health Jurisdiction (LHJ) websites

\- + 67%

Microsoft Power BI

Coronavirus tracker in Washington State

This tracker is updated at least daily using data from the Washington State Department of Health. For a more detailed update on COVID-19 in Washington State, please review the DOH dashboard.

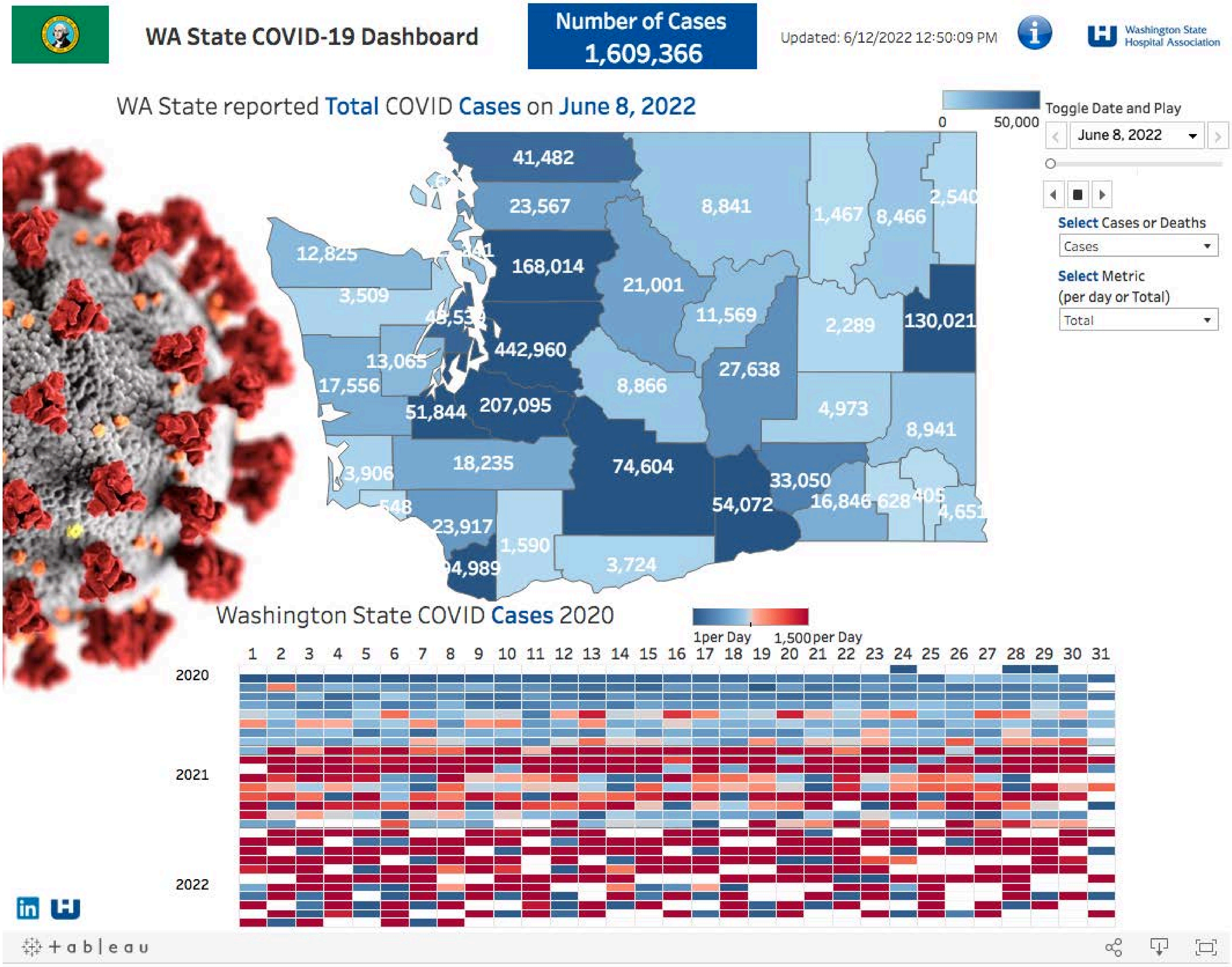

West Virginia COVID-19

West Virginia Department of Health & Human Resources
Bureau for Public Health

Overview | County Alert | 7-Day Trend | Cumulative Summary | Breakthrough Data | Outbreaks | Hospital | Other Trends ▾ | Vaccine Summary

Click here to view variant data

522,061
Total Number of Cases
Click here to view Breakthrough Cases and Total Cases

6,999
Deaths
Click here to view Breakthrough Deaths and Total Deaths

518
New Cases Received Since Last Update

1
Deaths Reported Since Last Update

2,198
Current Active Cases

6.39%
Daily Percent Positivity
Click here to view breakdown

8.14%
Cumulative Percent Positivity
Click here to view breakdown

Note: Boost Doses % is calculated with respect to the number of people fully

Vaccine Doses Administered Into Arms of West Virginia Residents

Key Metric	Doses	Population	%
At Least One Dose	1,133,615	1,792,147	63.3%
Fully Vaccinated	980,051	1,792,147	54.7%
Boost Doses	507,593	980,051	51.8%
5+ At Least One Dose	1,133,615	1,699,122	66.7%
5+ Fully Vaccinated	980,051	1,699,122	57.7%
5+ Boost Doses*	507,593	980,051	51.8%
18+ At Least One Dose	1,038,181	1,432,580	72.5%
18+ Fully Vaccinated	898,750	1,432,580	62.7%
18+ Boost Doses	495,297	898,750	55.1%
50+ At Least One Dose	617,528	736,823	83.8%
50+ Fully Vaccinated	551,213	736,823	74.8%
50+ Boost Doses	375,781	551,213	68.2%
65+ At Least One Dose	334,640	367,011	91.2%
65+ Fully Vaccinated	301,761	367,011	82.2%
[illegible]	[illegible]	[illegible]	[illegible]

Data Source: HHS, CDC and WV DHHR   Updated 10:00 AM 6/10/2022
*Booster doses include ages 12 and older

Individuals counted are West Virginia residents vaccinated in-state or out-of-state through State Allocations, Federal Pharmacy Program, Federal FQHC Program and Federal Entities

Note: Data posted on dashboard is from 12:00 midnight the previous day. All data are provisional and subject to change based on information obtained during public health investigations.

Updated 10:00 AM 6/10/2022

- + 45%

Microsoft Power BI

Wisconsin COVID-19 Summary Statistics

Click on the blue headers to see more data on a topic

Vaccines Updated: 6/10/22

64.5%

of residents have received at least one dose

61.3%

of residents have completed the vaccine series

34.7%

of residents have received an additional/booster dose

Testing Updated: 6/10/22

New Confirmed Cases
(7-day average)

1,639

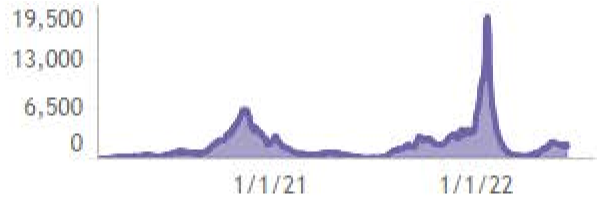

New Probable Cases
(7-day average)

219

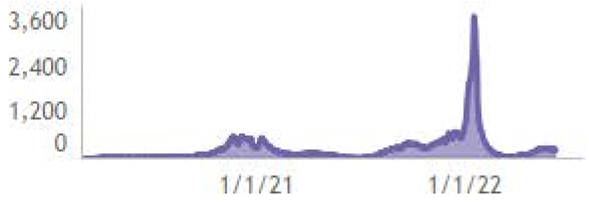

Percent Positive by Test
(7-day average)

12.0%

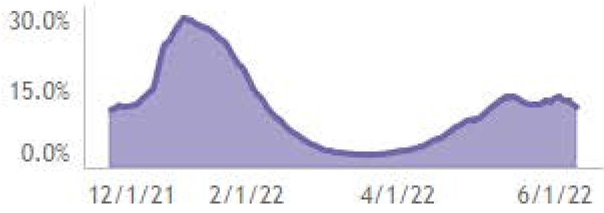

Deaths Updated: 6/10/22

Total Confirmed Deaths

13,058

Total Probable Deaths

1,626

New Deaths Reported
(7-day average)

4

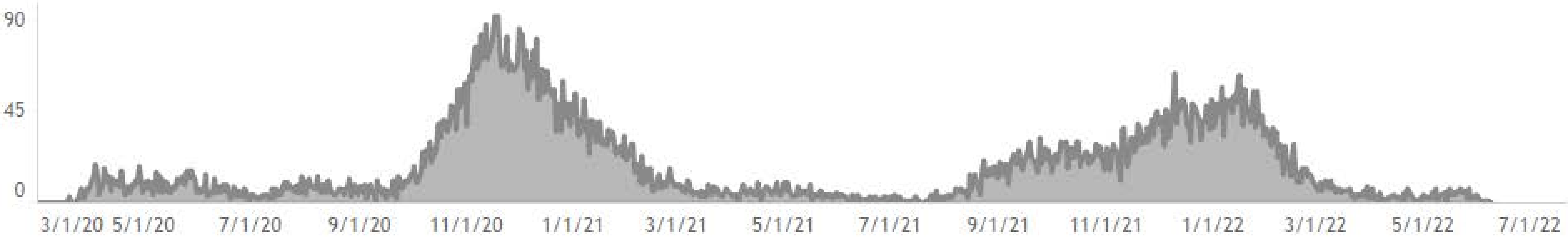

CDC COVID-19 Community Levels

Updated: 6/10/2022

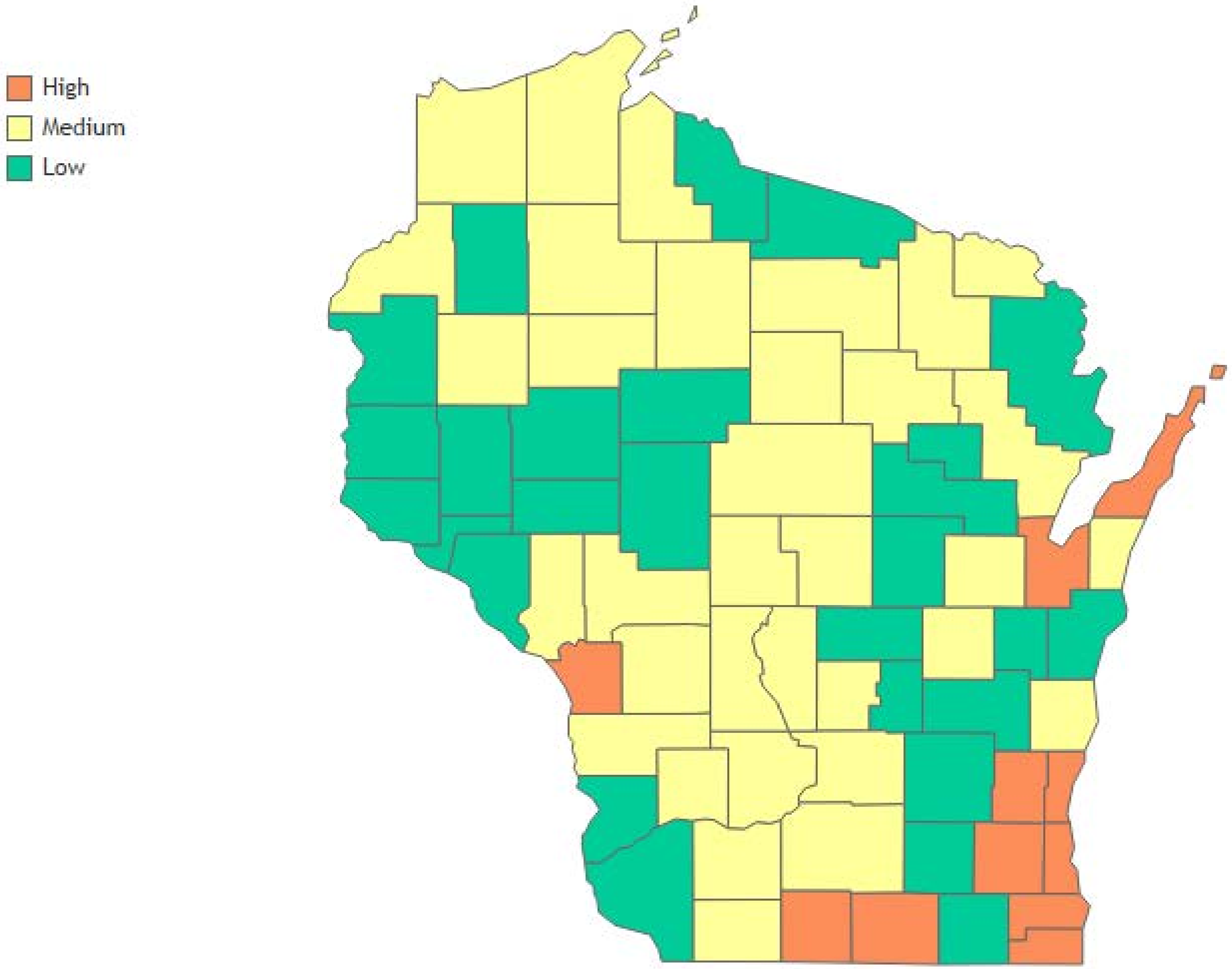

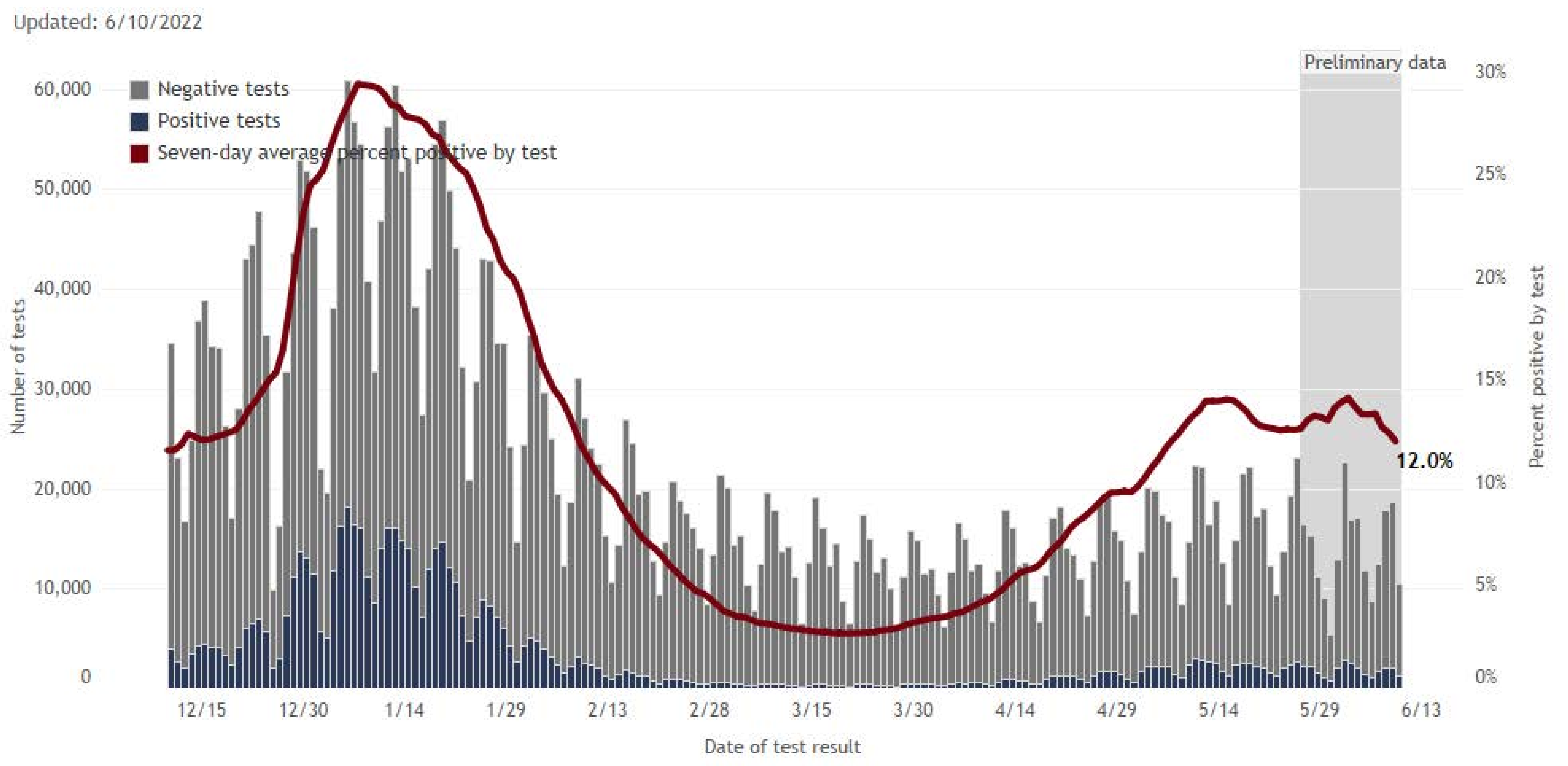

Updated: 6/10/2022
Negative tests
Positive tests
Seven-day average percent positive by test
Preliminary data
Number of tests
60,000
50,000
40,000
30,000
20,000
10,000
0
30%
25%
20%
15%
10%
5%
0%
Percent positive by test
12.0%
12/15
12/30
1/14
1/29
2/13
2/28
3/15
3/30
4/14
4/29
5/14
5/29
6/13
Date of test result

Tracking the COVID-19 curve in Wisconsin

COVID-19 Cases Total

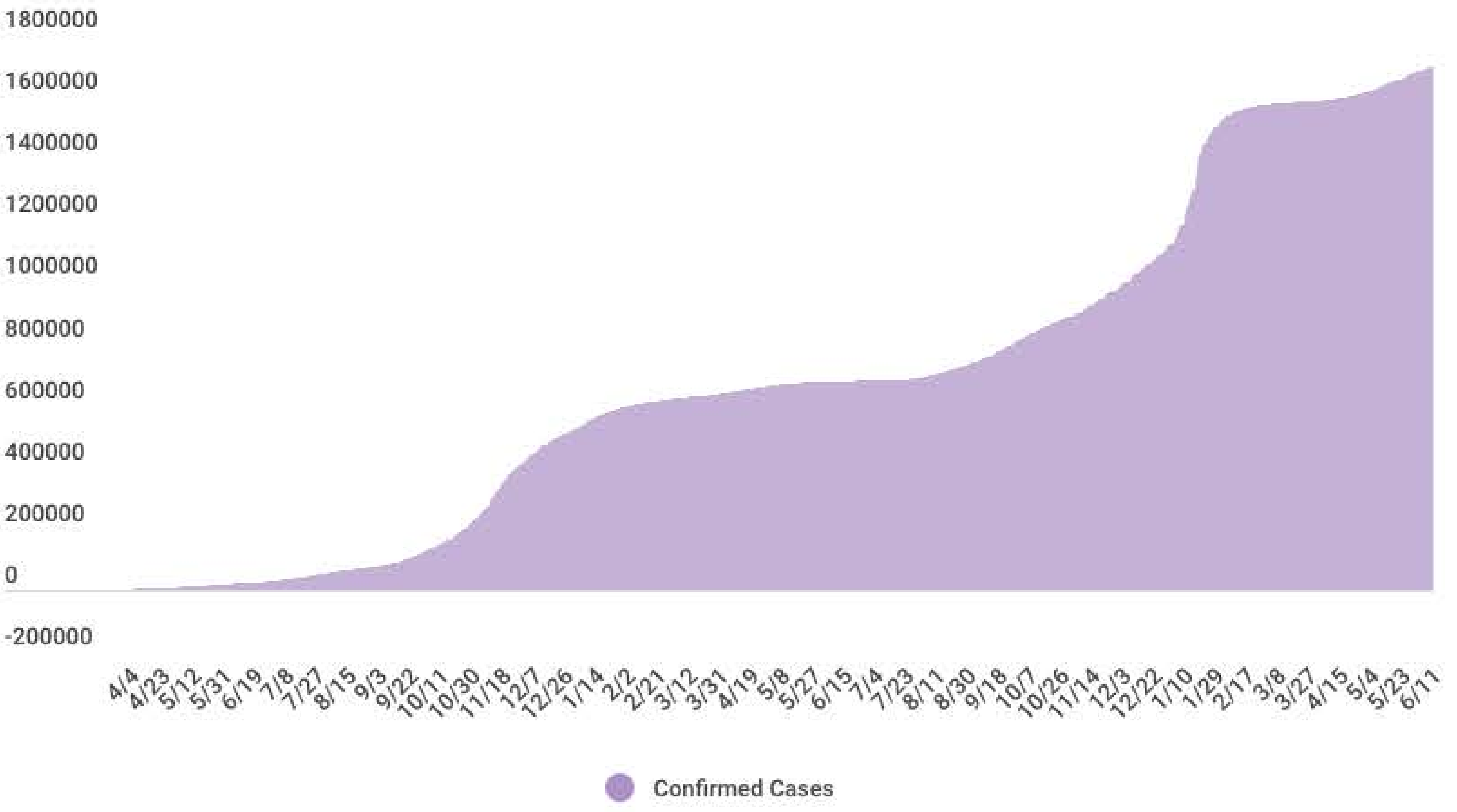

COVID-19 Cases Per Day

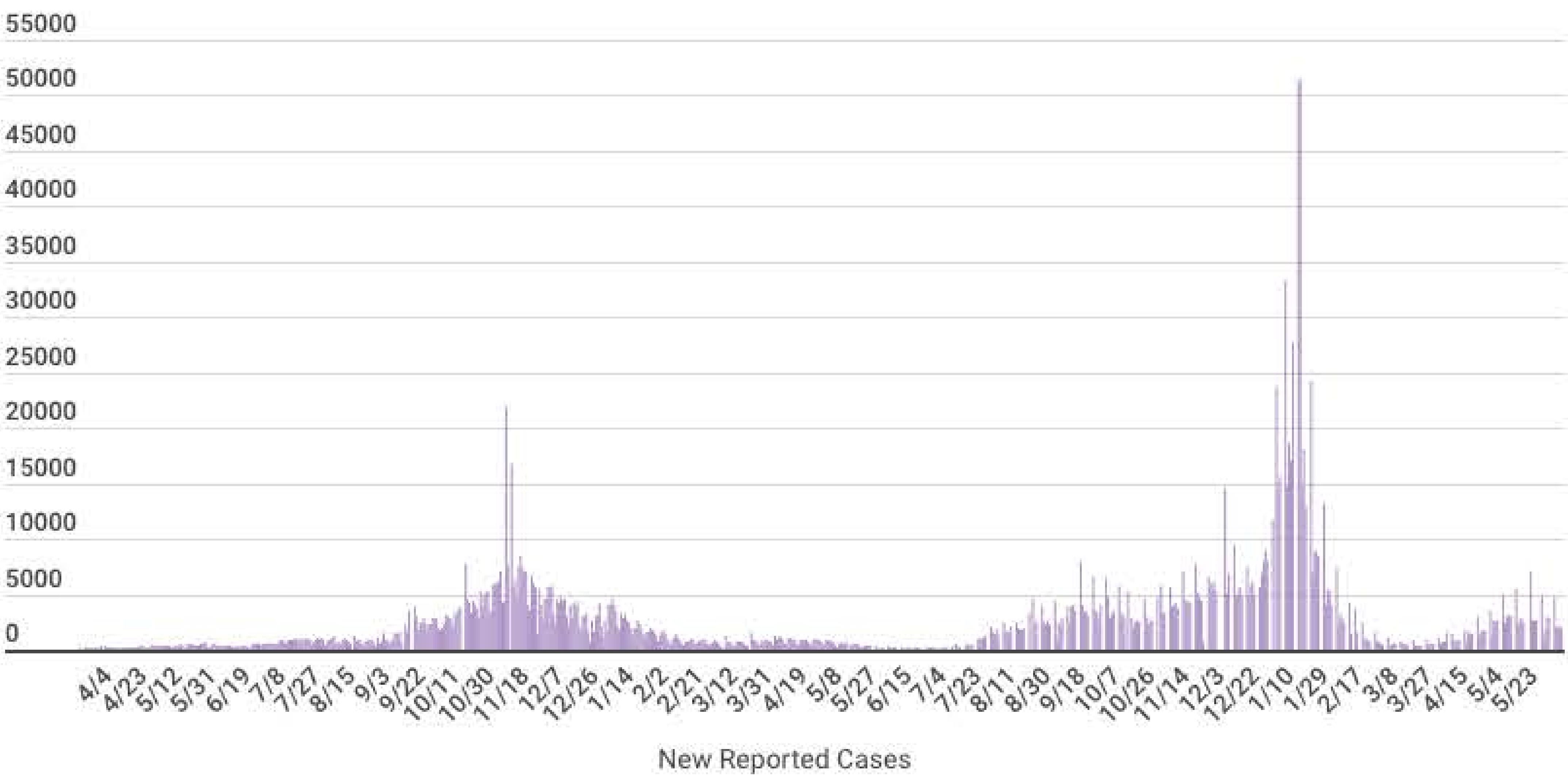

COVID-19 Deaths Total

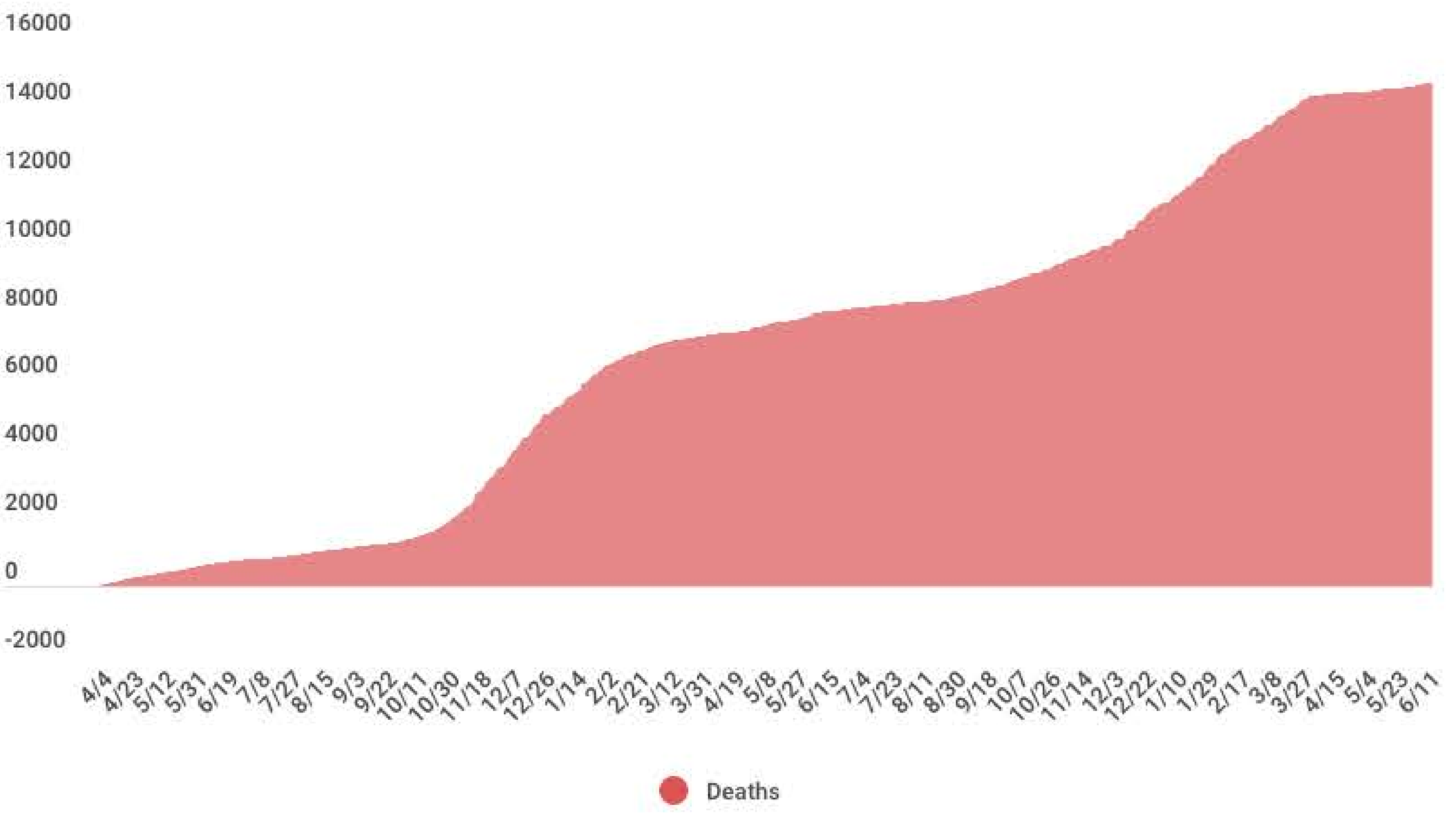

COVID-19 Deaths Per Day

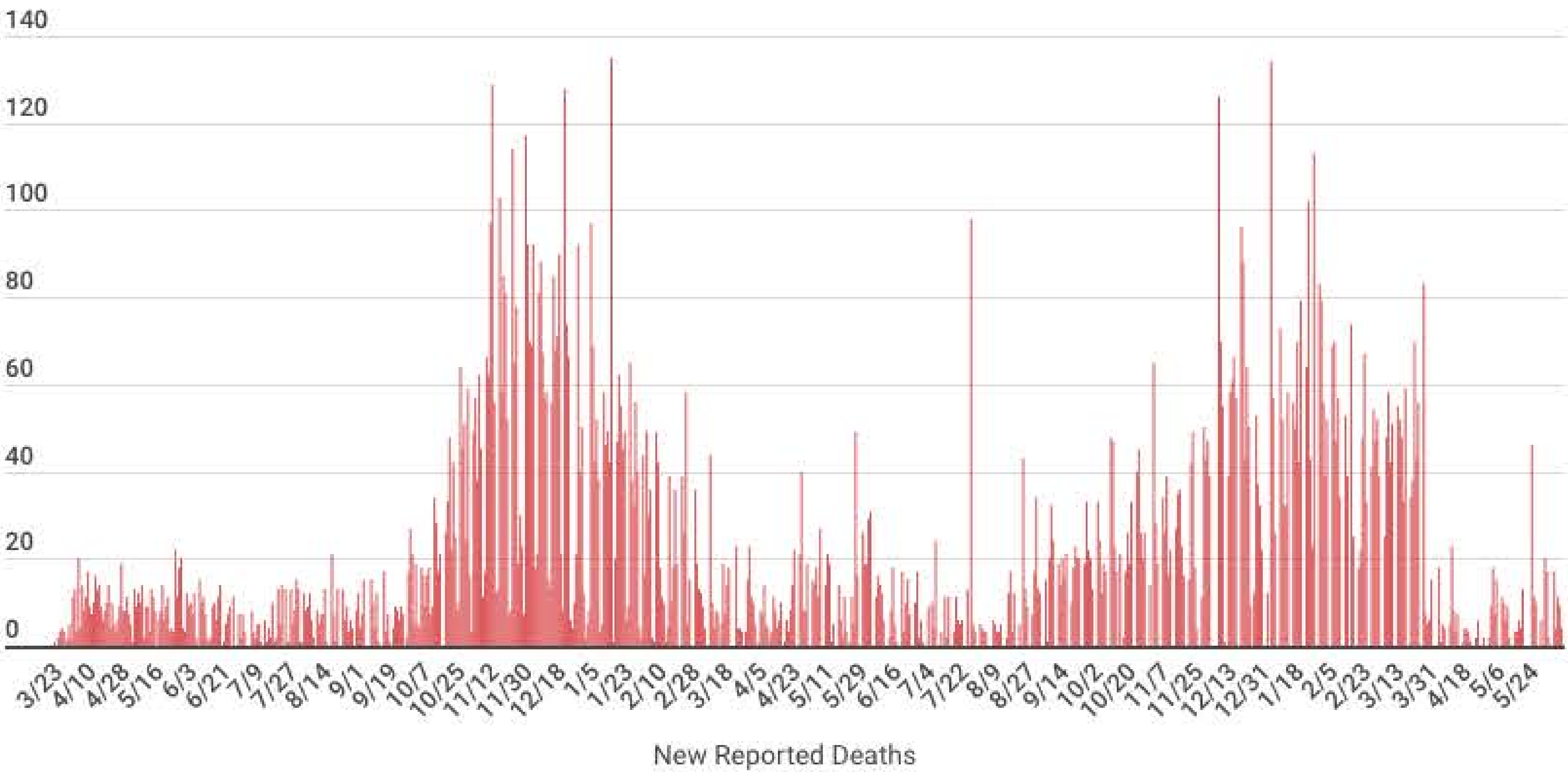

These charts are powered by data from Johns Hopkins University CSSE.
The coronavirus case numbers and death toll are updated daily.
This data may vary from state and local health department tallies.

COVID-19 County by County

HEARST television

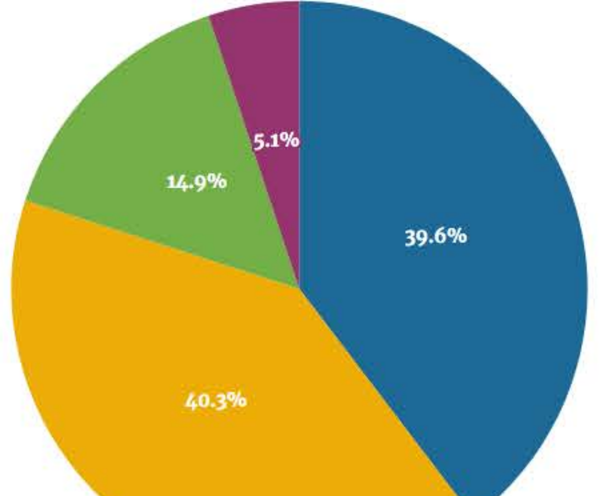

Sources: R.I. Department of Health, U.S. Census Bureau • Digital graphic created by Target 12
Fully and partially vaccinated includes Rhode Island residents who have been vaccinated anywhere in Rhode Island or out of state. Out-of-state residents are excluded. "Fully vaccinated" is defined as completing a one-dose or two-dose vaccine regimen. Due to a limitation of state data, "Boosted" excludes people who got extra doses at federal facilities or out of state.

A Flourish chart

COVID-19 First vs. Booster Doses in Rhode Island

7-day average

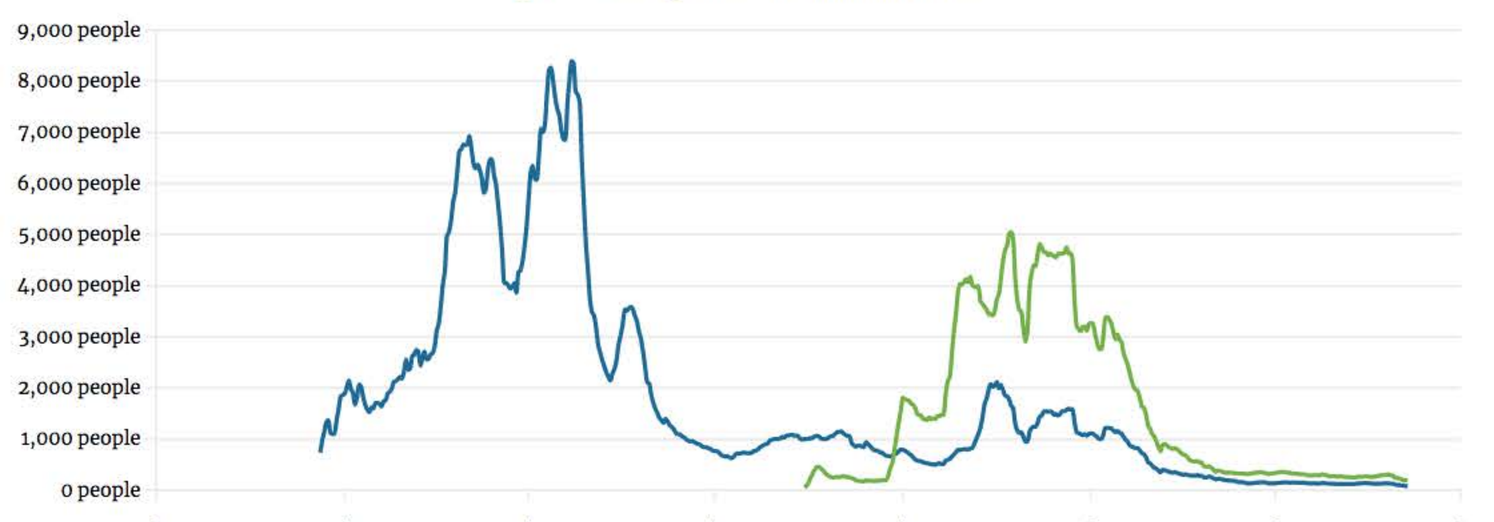

Source: R.I. Department of Health • Digital graphic created by Target 12
Data excludes doses administered at federal facilities in Rhode Island. Booster/additional doses includes extra doses recommended for some immunocompromised and other individuals.

A Flourish chart

Total COVID-19 Hospitalizations in Rhode Island

(RI data revised daily with a two-day lag in reporting)

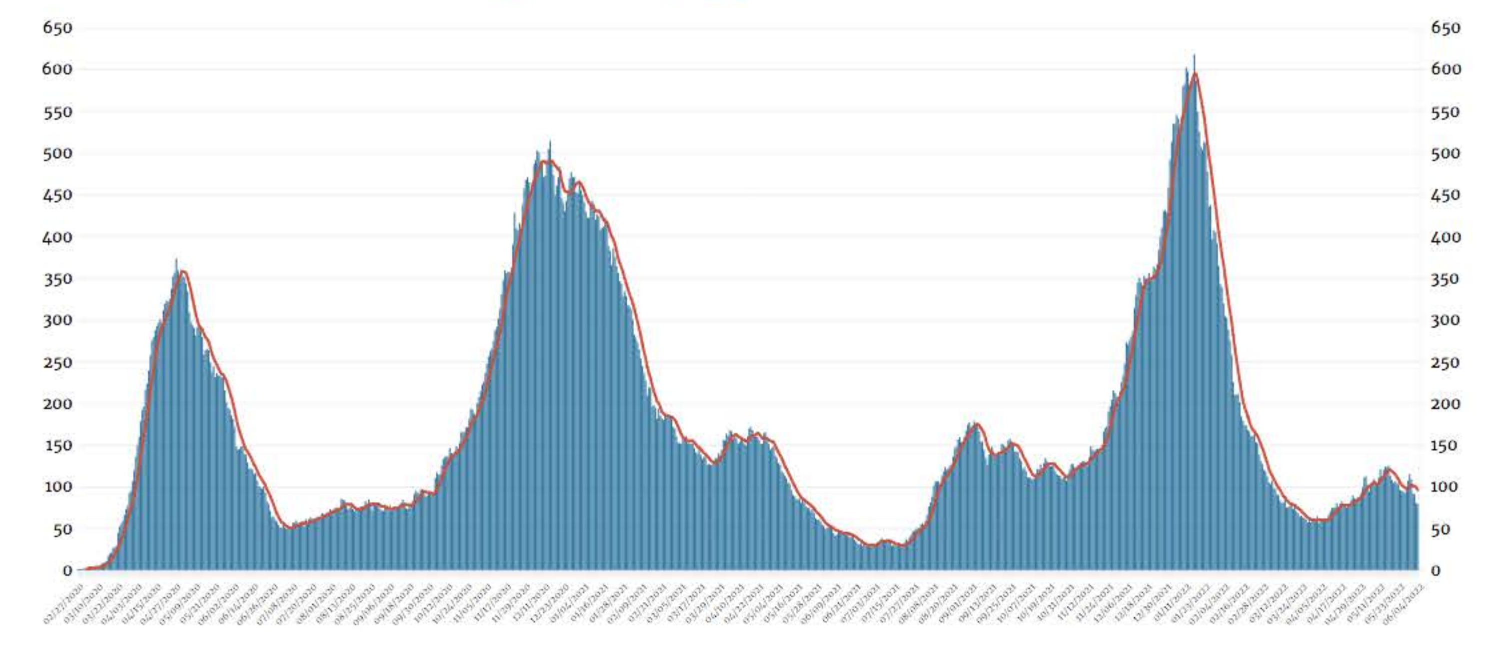

Source: R.I. Department of Health • Digital graphic created by Target 12

A Flourish chart

COVID-19 Hospitalizations (2020-2022)

7-day average

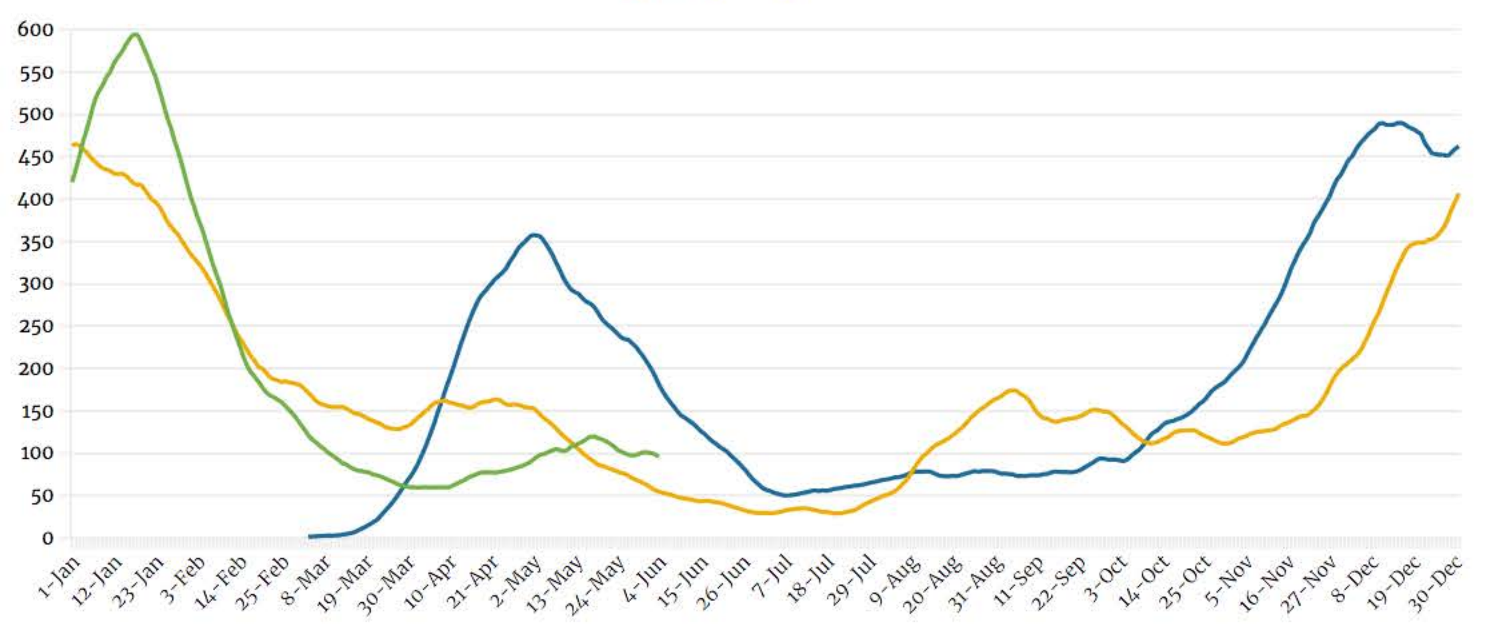

Source: R.I. Department of Health • Digital graphic created by Target 12

A Flourish chart

New Hospital Admissions in Rhode Island

(RI data revised daily with a two-day lag)

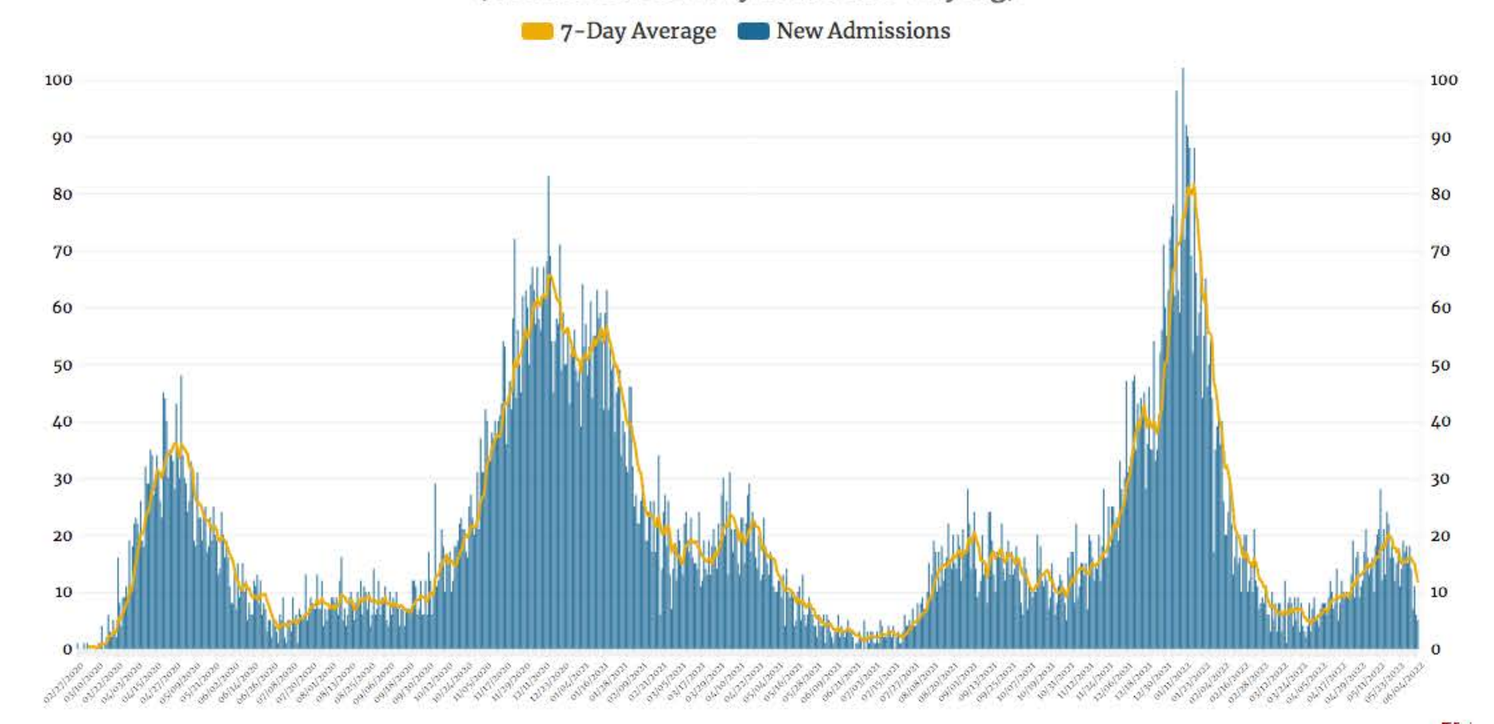

Source: R.I. Department of Health • Digital graphic created by Target 12

A Flourish chart

COVID-19 Deaths in Rhode Island

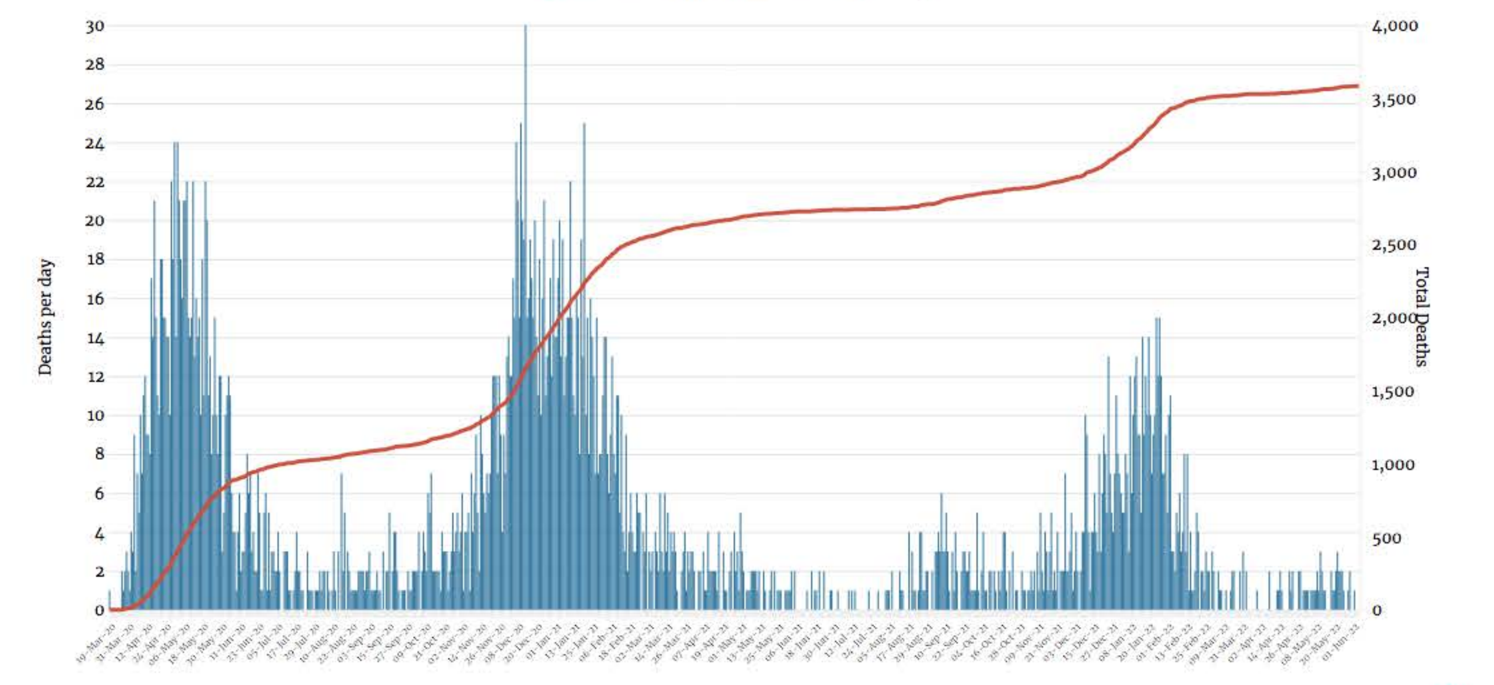

Source: R.I. Department of Health • Digital graphic created by Target 12

A Flourish chart

COVID-19 Deaths (2020-2022)

7-day average

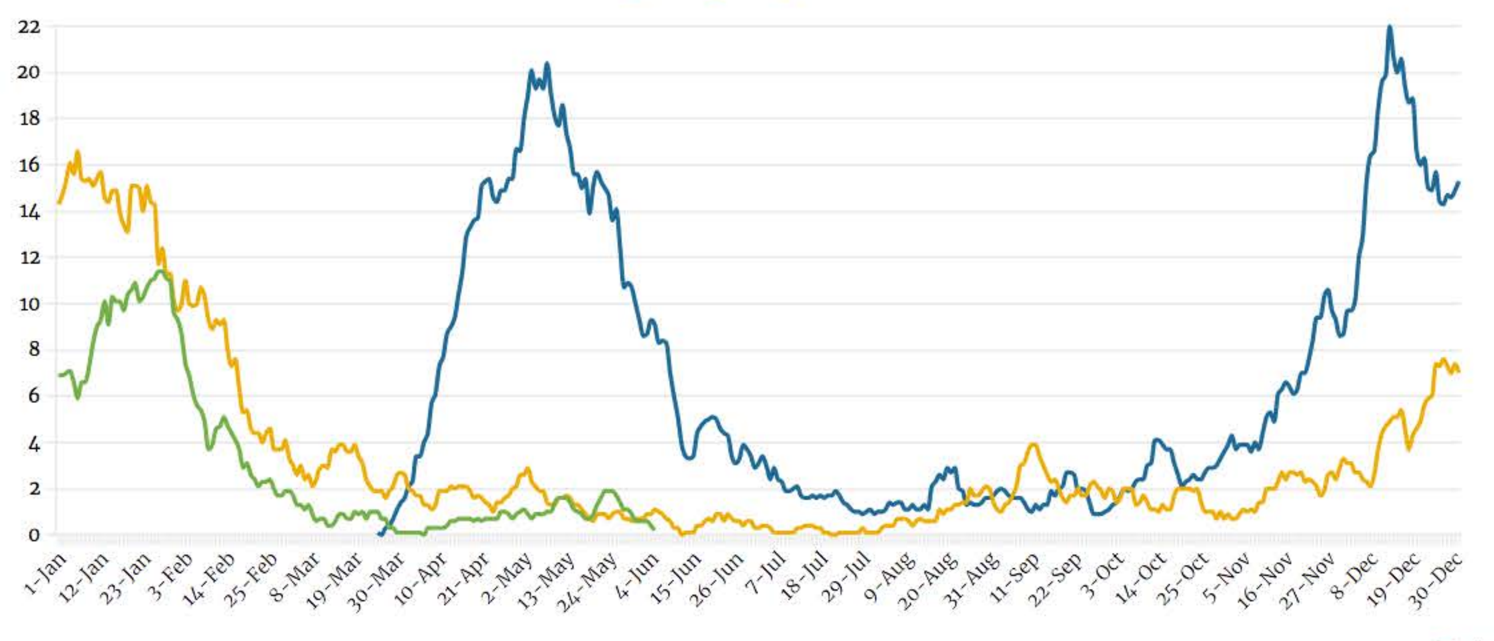

Source: R.I. Department of Health • Digital graphic created by Target 12

A Flourish chart

7-Day Average of New Positive Cases in Rhode Island

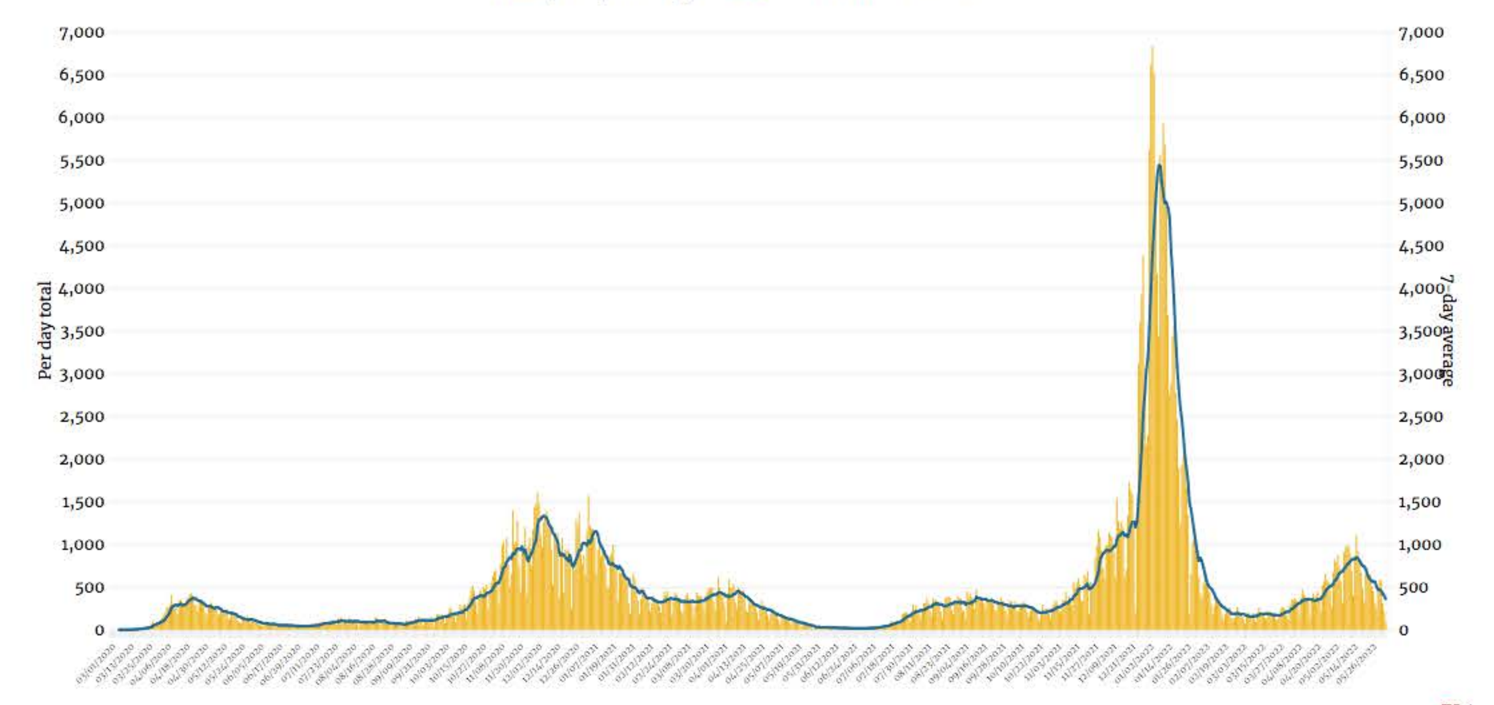

Source: R.I. Department of Health • Digital graphic created by Target 12

A Flourish chart

COVID-19 Cases (2020-2022)

7-day average

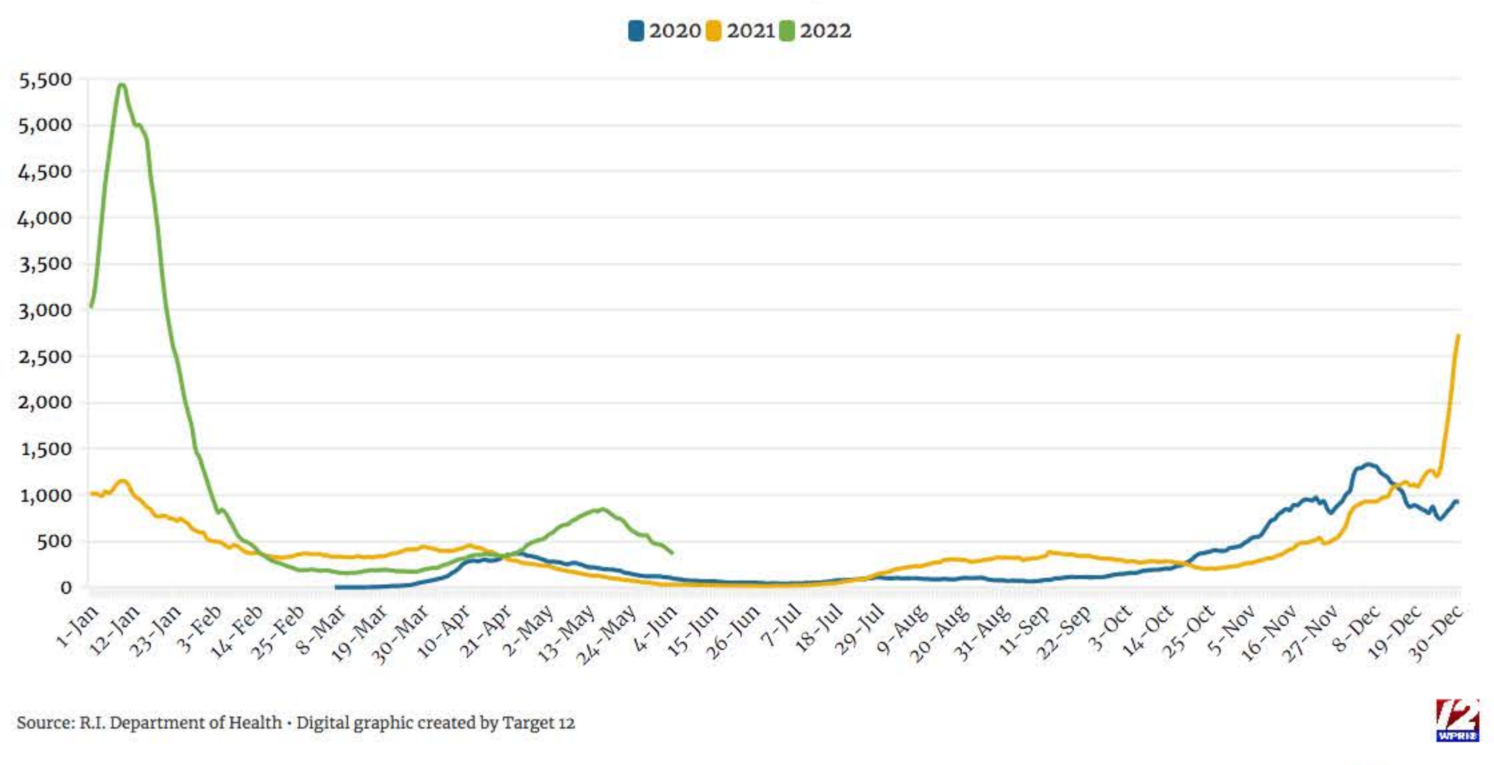

Source: R.I. Department of Health • Digital graphic created by Target 12

A Flourish chart

7-Day Average of Daily Testing in Rhode Island

Includes all negative and positive tests from each day

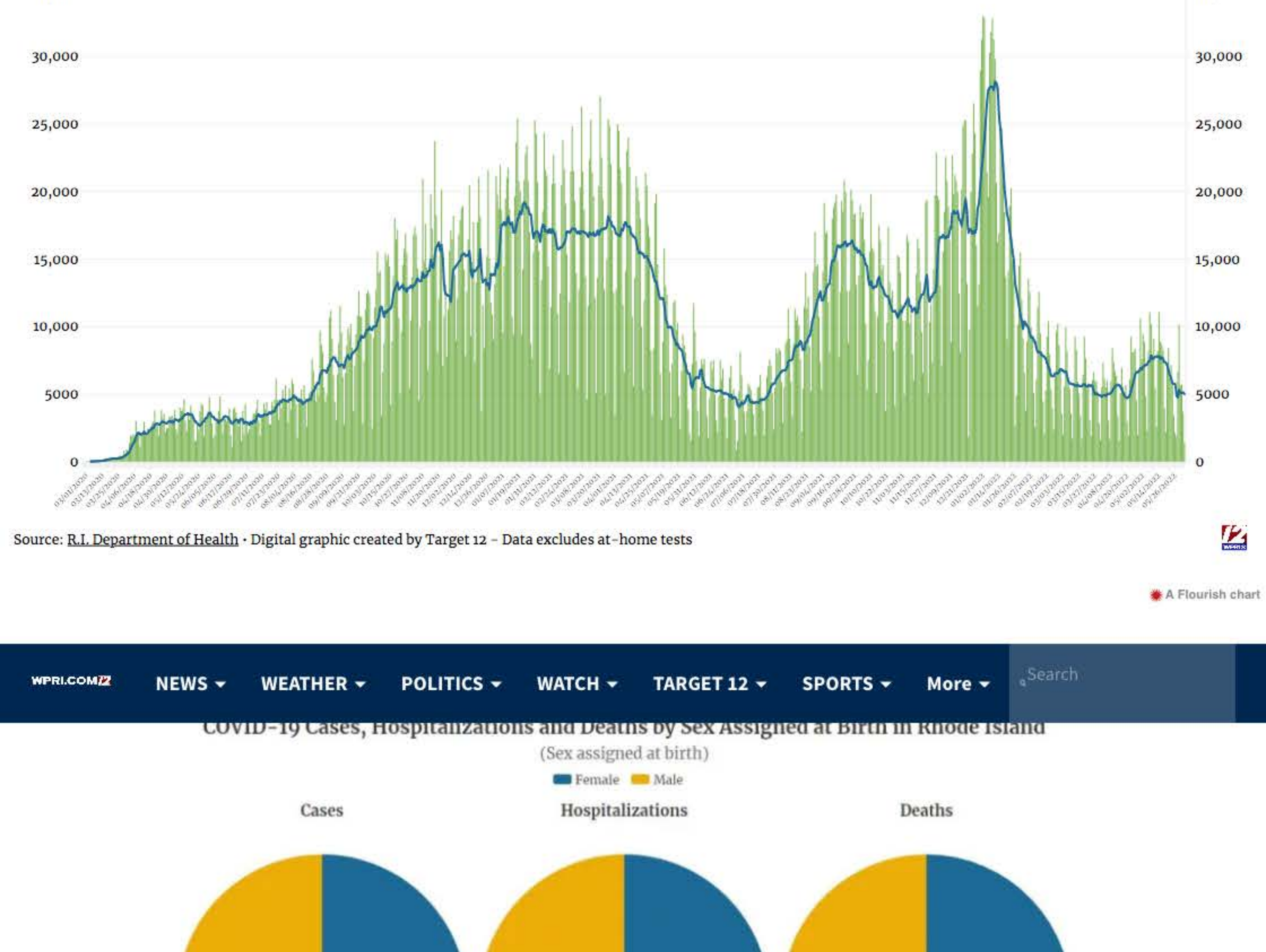

Source: R.I. Department of Health • Digital graphic created by Target 12 – Data excludes at-home tests

A Flourish chart

WPRI.COM 12 NEWS WEATHER POLITICS WATCH TARGET 12 SPORTS More Search

COVID-19 Cases, Hospitalizations and Deaths by Sex Assigned at Birth in Rhode Island

(Sex assigned at birth)

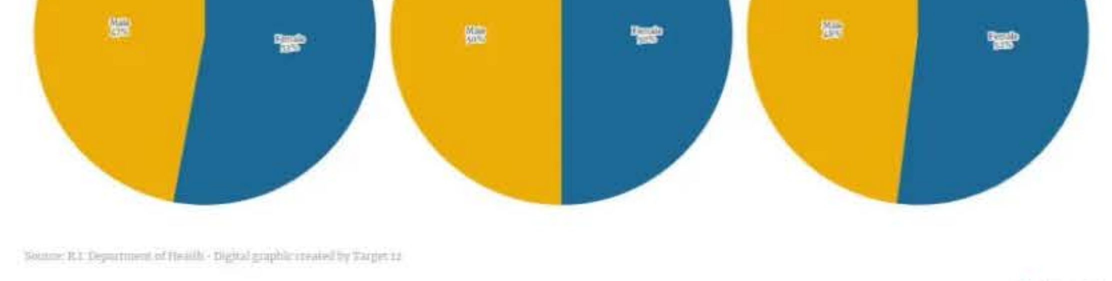

Source: R.I. Department of Health • Digital graphic created by Target 12

A Flourish chart

Bay Area		California		United States	
1,485,108 cases	8,501 deaths	9,771,861 cases	91,005 deaths	85,515,795 cases	1,011,275 deaths

Coronavirus Tracker

The Chronicle is mapping every reported coronavirus case in the Bay Area, California and the U.S. We are tallying the number of confirmed cases, deaths, hospitalizations and tests reported by the state on Tuesdays and Fridays.

By CHRONICLE DIGITAL TEAM | LAST UPDATED: June 12, 2022 4:38 p.m.

New reported cases and deaths in the Bay Area, seven-day average

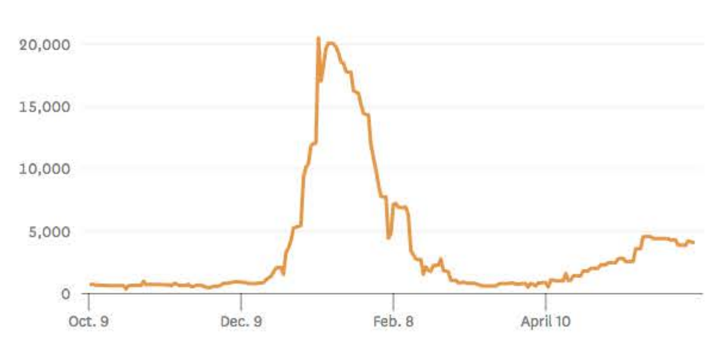

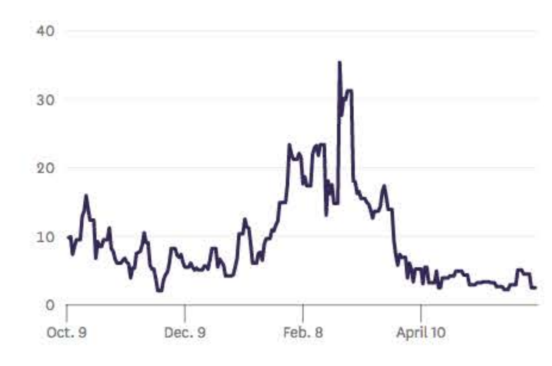

New reported cases and deaths in California, seven-day average

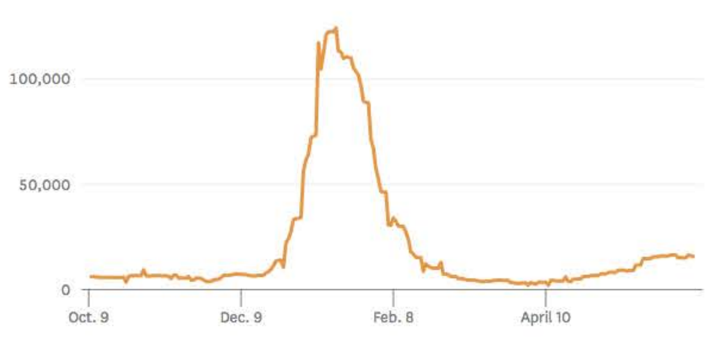

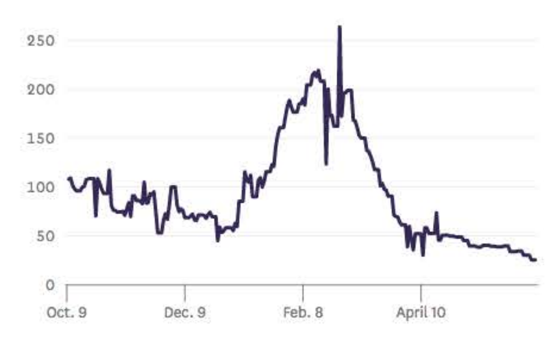

Sources: U.S. Centers for Disease Control and Prevention, California Department of Public Health and county public health departments, Chronicle reporting

The seven-day trailing average represents the average number of cases or deaths reported each day over the weeklong span ending on the given day. It is used to account for daily variances in reporting and large one-off changes in daily counts, such as April 20 when nearly 1,500 cases were reported in L.A. County as the result of a lab clearing a large backlog. Some counties do not provide daily updates which, combined with daily variances in the number of tests given, could result in randomly higher or lower counts for daily reported cases.

Update
Starting Sept. 3, the California Data Coalition switched its source for county virus data to counts released the California Department of Public Health. A one-time change in charts and totals occurred on the date of that change. Read more about this change below.

New cases | Total cases

New cases per 100,000 people over the past two weeks

349 426 541 704

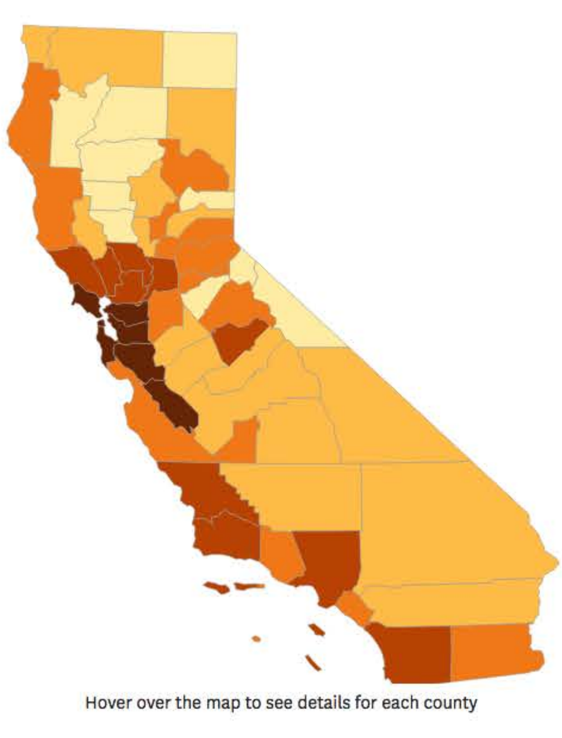

Hover over the map to see details for each county

California cases are organized by reporting county. Cases are based on reports from the U.S. Centers for Disease Control and Prevention, the California Department of Public Health and county public health departments, plus the Chronicle / Los Angeles Times data collaboration. For more information on the data, please read about our methodology.

Latest coronavirus news

COVID in California: Health officials in Florida undercounted COVID cases and deaths, audit says June 9, 2022

COVID in California: New variants likely to keep coronavirus levels high through the summer June 9, 2022

S.F.'s COVID positive test rate hits second-highest level ever June 8, 2022

How many people are hospitalized?

The number of people hospitalized with COVID-19 in California and in the Bay Area nearly doubled during the month of May. Experts said, however, that hospitalizations remain at a relatively low and manageable level compared to prior surges. The number of patients with COVID needing intensive care in the region also doubled in May, but ICU capacity is not tapped out. Hospitalizations and ICU availability were key numbers used to determine the level of restrictions during California's previous shutdown and continue to be monitored as the region deals with highly contagious variants.

Confirmed ICU and non-ICU COVID-19 patients in Bay Area hospitals

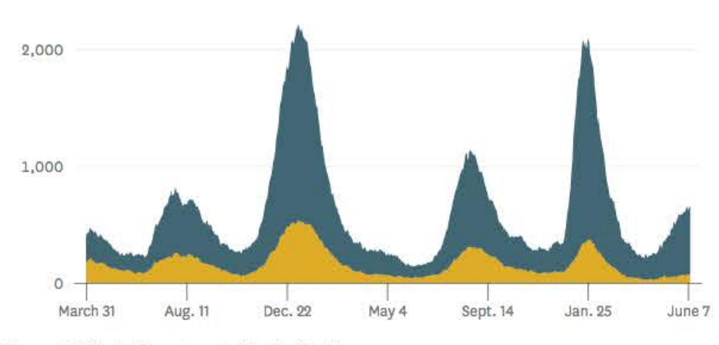

Source: California Department of Public Health

Confirmed ICU and non-ICU COVID-19 patients in California hospitals

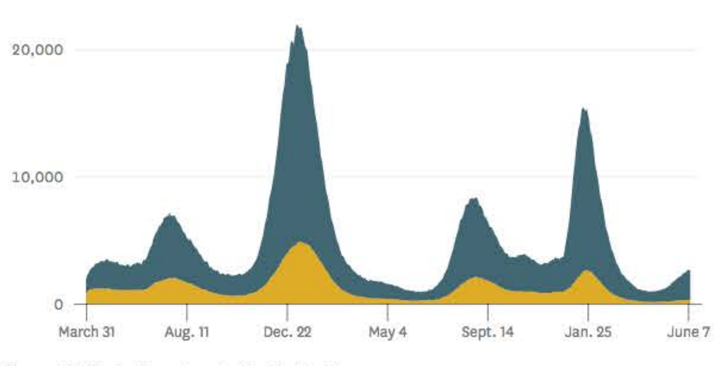

Source: California Department of Public Health

Due to limited testing capacity, not all hospitalized patients with COVID-19 symptoms have been tested. While not all suspected cases will ultimately test positive, including these patients may give a more comprehensive picture of the demands on hospitals.

Total confirmed and suspected COVID-19 patients in California hospitals

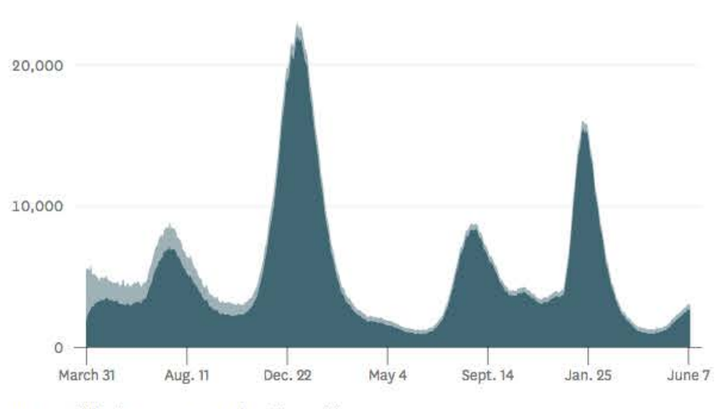

Source: California Department of Public Health

ADVERTISEMENT

What are the current trends in the Bay Area?

Reported case rates have climbed higher than during the first winter surge at the end of 2020 and experts say the total number infected could be nearing or surpassing the total during the peak of the omicron-driven surge this past winter. The Bay Area has been reporting some of the highest case rates in the state and those numbers do not include the growing number of people testing positive with home tests. One study suggested that cases in New York City could be thirtyfold higher than the numbers being reported. The Bay Area has relatively high vaccination rates but boosters are being recommended as variants continue to emerge.

Cases and deaths by Bay Area county

The new case curve represents the 7-day moving average which accounts for variances in daily reporting. The change in new cases compares the number of cases in the past seven days to the previous seven days.

County	Total deaths	Total cases	New cases curve	Weekly change in new cases	
Santa Clara	2,322	375,317	1132 (Oct. 11 – June 11)	▼409	-5%
Alameda	1,877	308,525	820	▼409	-7%
Contra Costa	1,315	226,595	548	▼314	-8%
San Francisco	890	159,180	515	▼92	-2%
San Mateo	715	150,508	448	▼206	-6%
Sonoma	492	97,025	219	▲19	+1%
Solano	453	95,675	195	▲58	+4%
Marin	294	42,896	125	▼127	-13%
Napa	143	29,387	61	▲66	+18%

Sources: U.S. Centers for Disease Control and Prevention, the California Department of Public Health and county public health departments, Chronicle / Los Angeles Times data collaboration

The Marin County figures include the San Quentin state prison outbreak that began June 20 and totaled more than 2,200 cases.

Bay Area cases by city or region

Some Bay Area county health departments are now providing details on where people who have tested positive reside, either by region, city or jurisdiction. Use the drop down to search counties (more will be added if data becomes available). Last updated June 1.

Alameda County

Place	Total cases	Cases last month
Alameda	10,286	1,020
Albany	1,776	185
Berkeley	16,316	1,699
Castro Valley MAC	9,983	845
Dublin	8,823	928
Eden Area MAC	15,797	800

Show more

What is the status of testing in California?

The volume of reported tests ramped up as the omicron variant took hold. Home tests, most of which are not included in these numbers, are being recommended in hopes that people who test positive will stay home and isolate. Free home tests are being offered on the White House website. Before vaccines were widely available, the World Health Organization had established a recommended positivity rate of 5% for reopening. California's positive test rate was up to nearly 20% during the peak of the winter surge. At one point it had declined to around 2%, but has been steadily climbing and is back above that WHO recommended benchmark.

Daily test results reported in California

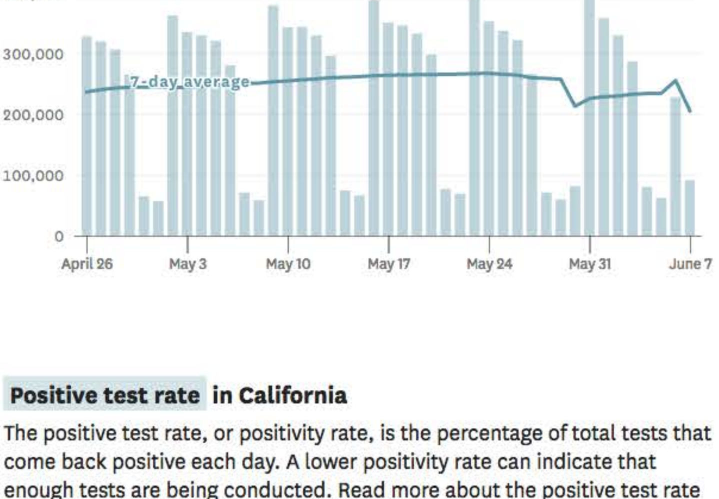

Positive test rate in California

The positive test rate, or positivity rate, is the percentage of total tests that come back positive each day. A lower positivity rate can indicate that enough tests are being conducted. Read more about the positive test rate here.

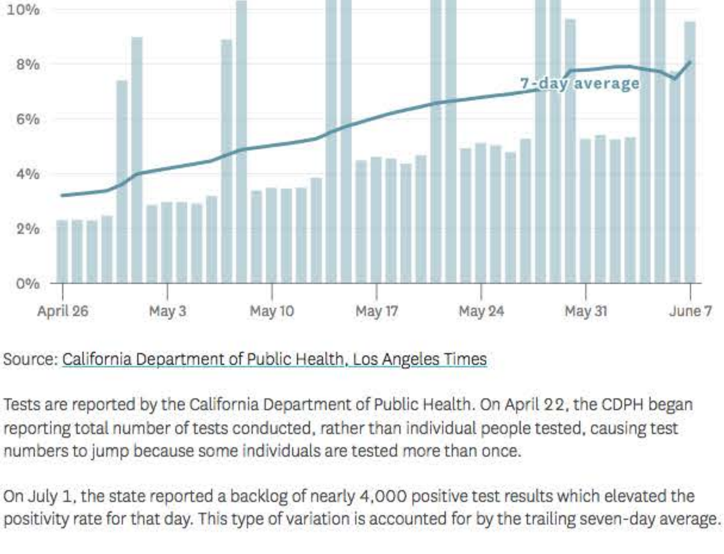

Source: California Department of Public Health, Los Angeles Times

Tests are reported by the California Department of Public Health. On April 22, the CDPH began reporting total number of tests conducted, rather than individual people tested, causing test numbers to jump because some individuals are tested more than once.

On July 1, the state reported a backlog of nearly 4,000 positive test results which elevated the positivity rate for that day. This type of variation is accounted for by the trailing seven-day average.

Total cases and deaths in the U.S.

The U.S. has the highest reported total COVID-19 cases of any country. California has the highest totals in the nation in terms of cases and deaths, but on a per-capita basis, the number of cases and deaths are below the national average.

85,515,795 confirmed cases in the U.S.
1,011,275 deaths

Cases per 100,000 people

0 10,000 20,000 30,000+

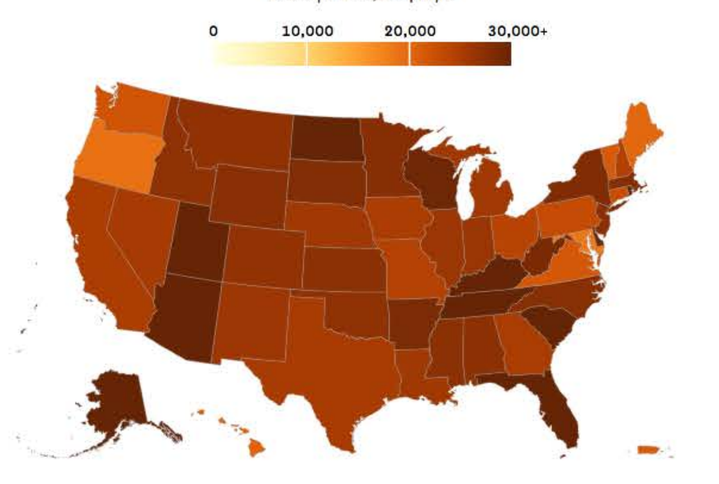

Figures include presumed COVID-19 deaths.

Sources: The Center for Systems Science and Engineering at Johns Hopkins University, U.S. Census Bureau
U.S. reports of COVID-19 are tabulated by Johns Hopkins, which is tracking cases using data collected from the Centers for Disease Control and Prevention and state and local public health departments. Numbers from Johns Hopkins for California may not match the numbers from the top map, which are being compiled by The Chronicle using county reports, county public health departments and Chronicle reporting.

Total cases

The Maine CDC breaks out cases at the county level by deaths, hospitalizations, recoveries and total confirmed cases. Each of the categories subtracted from total confirmed cases results in the other confirmed cases, shown below.

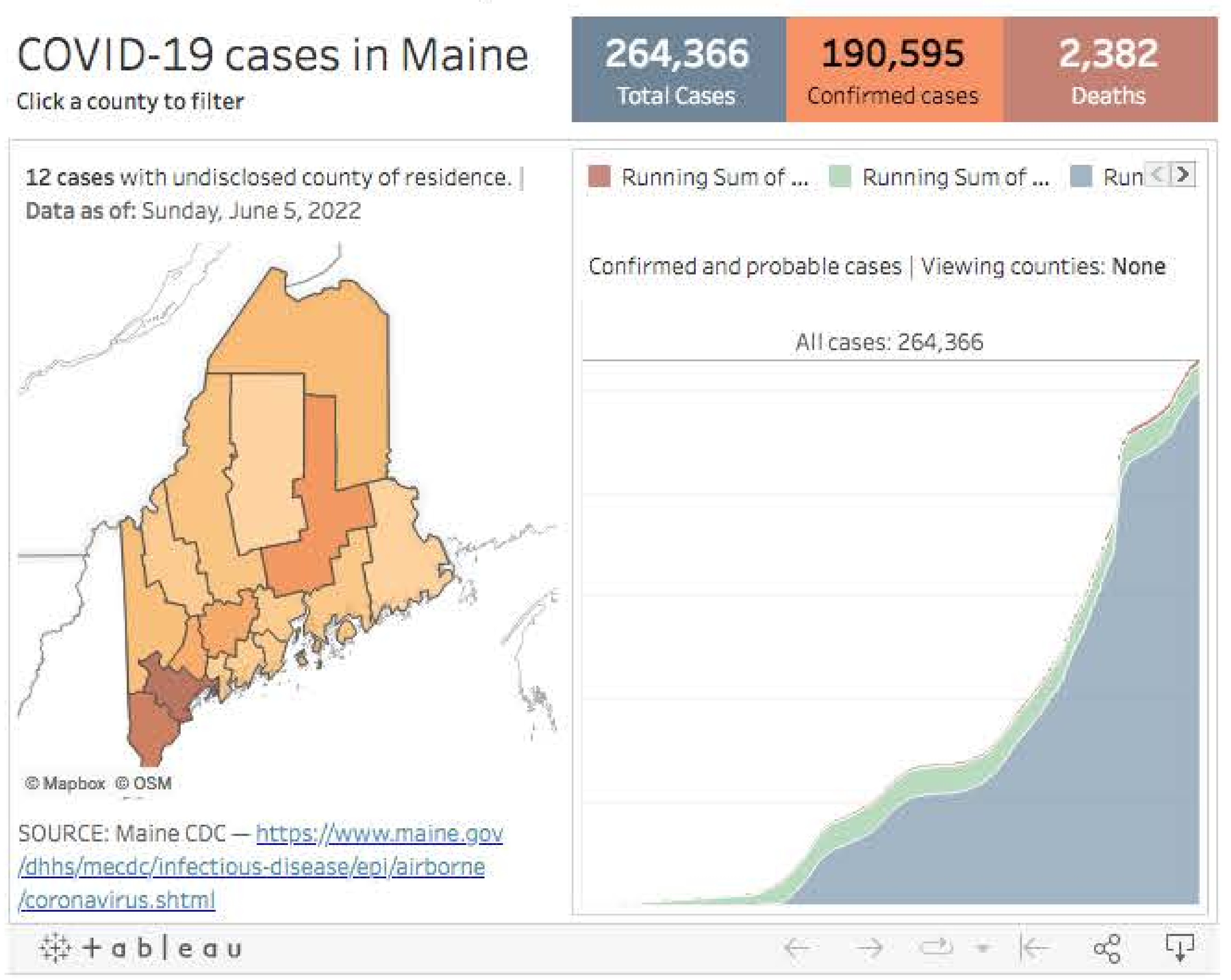

New cases

The rate of growth of cases is important to watch. Social distancing measures intend to slow the spread of the disease so that fewer people are sick all at once, which stands to overwhelm the healthcare system.

Cumberland County has seen the largest spikes in new cases, hitting a high for new cases each day. The early numbers could be impacted by delays in testing, as well.

The chart also illustrates some of the path the disease has taken through Maine counties, starting from March 18. Piscataquis, Aroostook and Washington counties had not reported a case as of April 1.

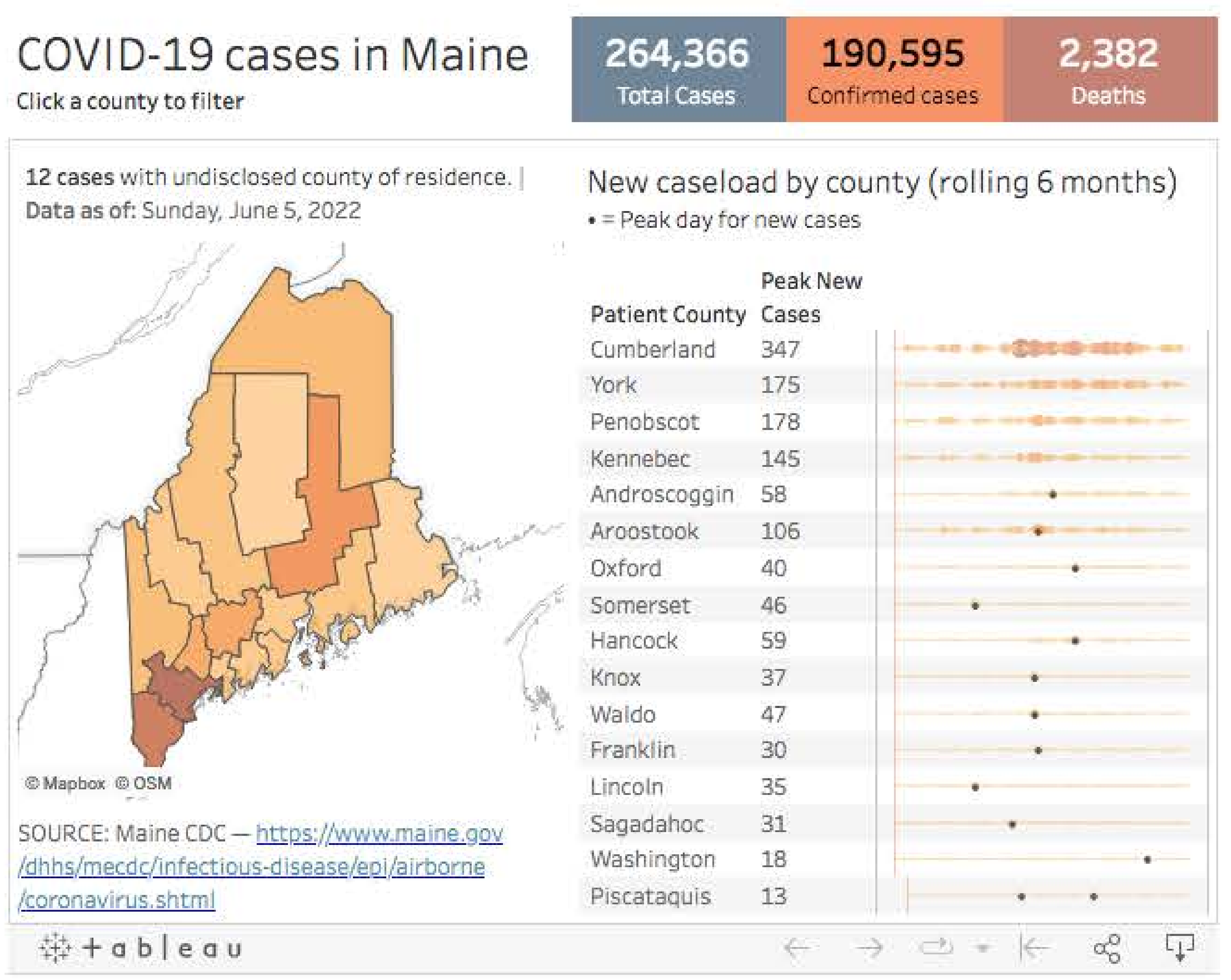

Case rates

For overall metrics, Cumberland and York will have the highest case counts because they have the most people.

The charts below adjust for population, showing the counties that have the highest current rates of confirmed infection.

These are shown alongside the latest calculations for the share of confirmed cases that have resulted in either recovery or hospitalization. These numbers only provide a small snapshot of the current status of testing and the disease in Maine.

In other words, those numbers reflect the situation on the ground in Maine and not necessarily a broader set of insights about the illness, COVID-19.

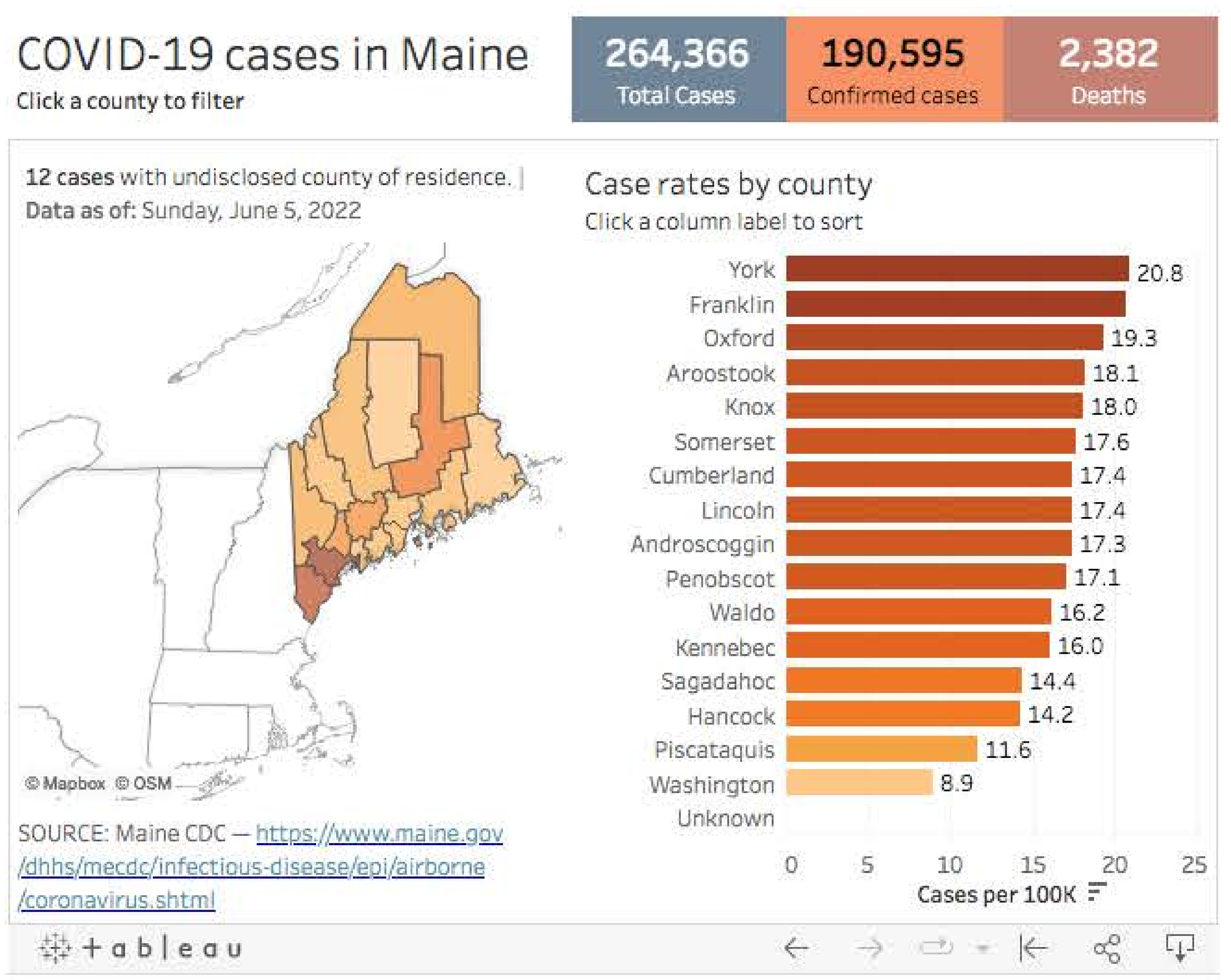

Age trends

The majority of Mainers infected so far have been in their 60s, followed by people in their 50s.

The latest studies of the disease have found that it is more deadly for older people, especially people over 80.

That has not meant that younger people are in the clear — the disease can still be fatal. The line chart shows how cases have trended among Mainers of different ages.

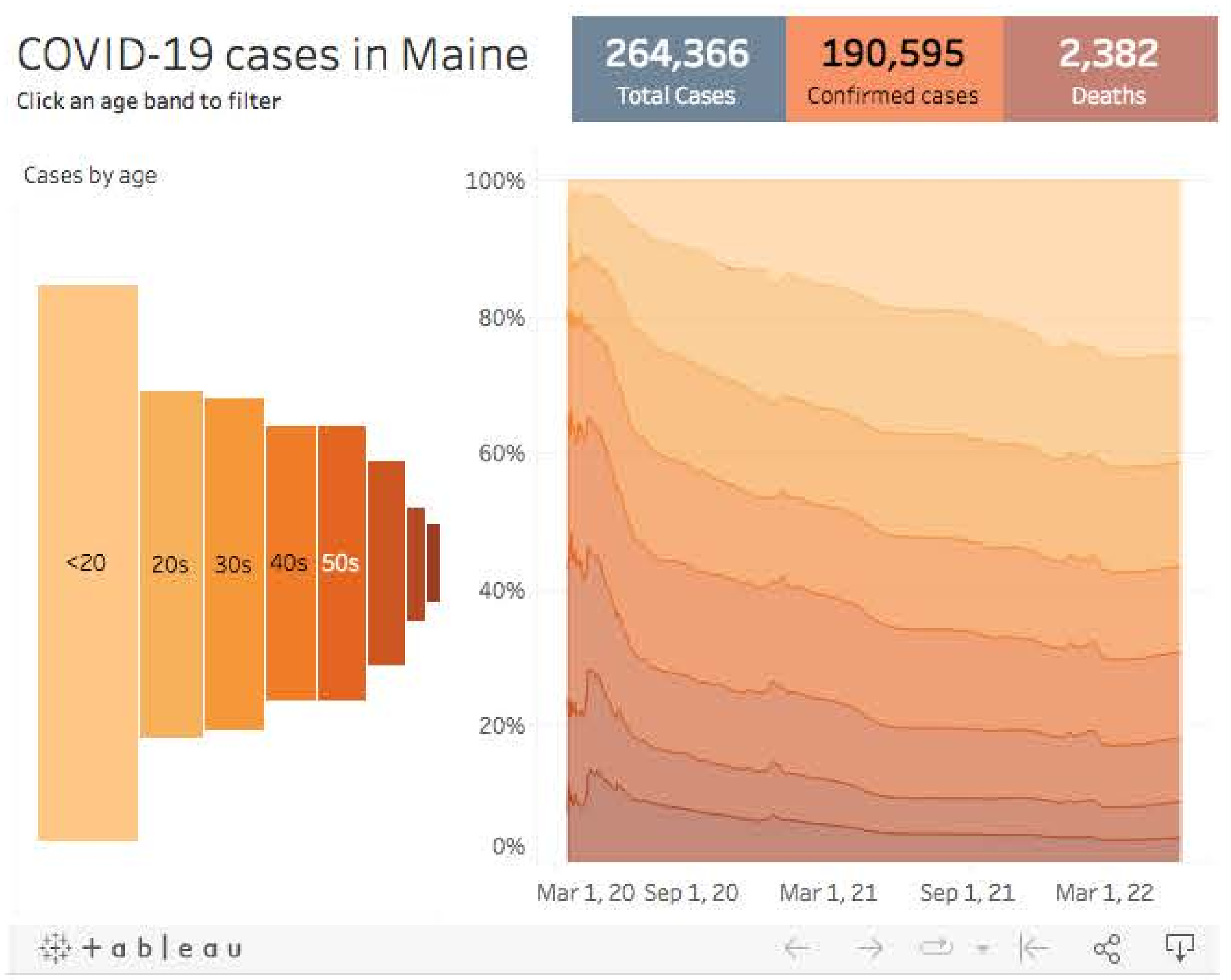

Comparing Maine with other states

The big question is: Can America stop the spread of this virus?

At the state level, this is reflected by looking at the rate of cases day by day. How quickly are they increasing? How many days is it taking for cases to double?

The goal is to slow that growth by: keeping six feet of distance from people you do not live with; washing your hands frequently and thoroughly; and disinfecting regularly touched surfaces.

The chart below uses a logarithmic y-axis, where the values double at every tick on the axis. As The New York Times explained, this makes it easier to tell when case growth begins to slow:

> Unconstrained, the coronavirus spreads exponentially, the caseload doubling at a steady rate. That curve, plotted linearly, is a skyrocketing curve. Plotted logarithmically, however, it transforms into a straight line — which means that deviations from the exponential spread of the virus become much easier to discern.

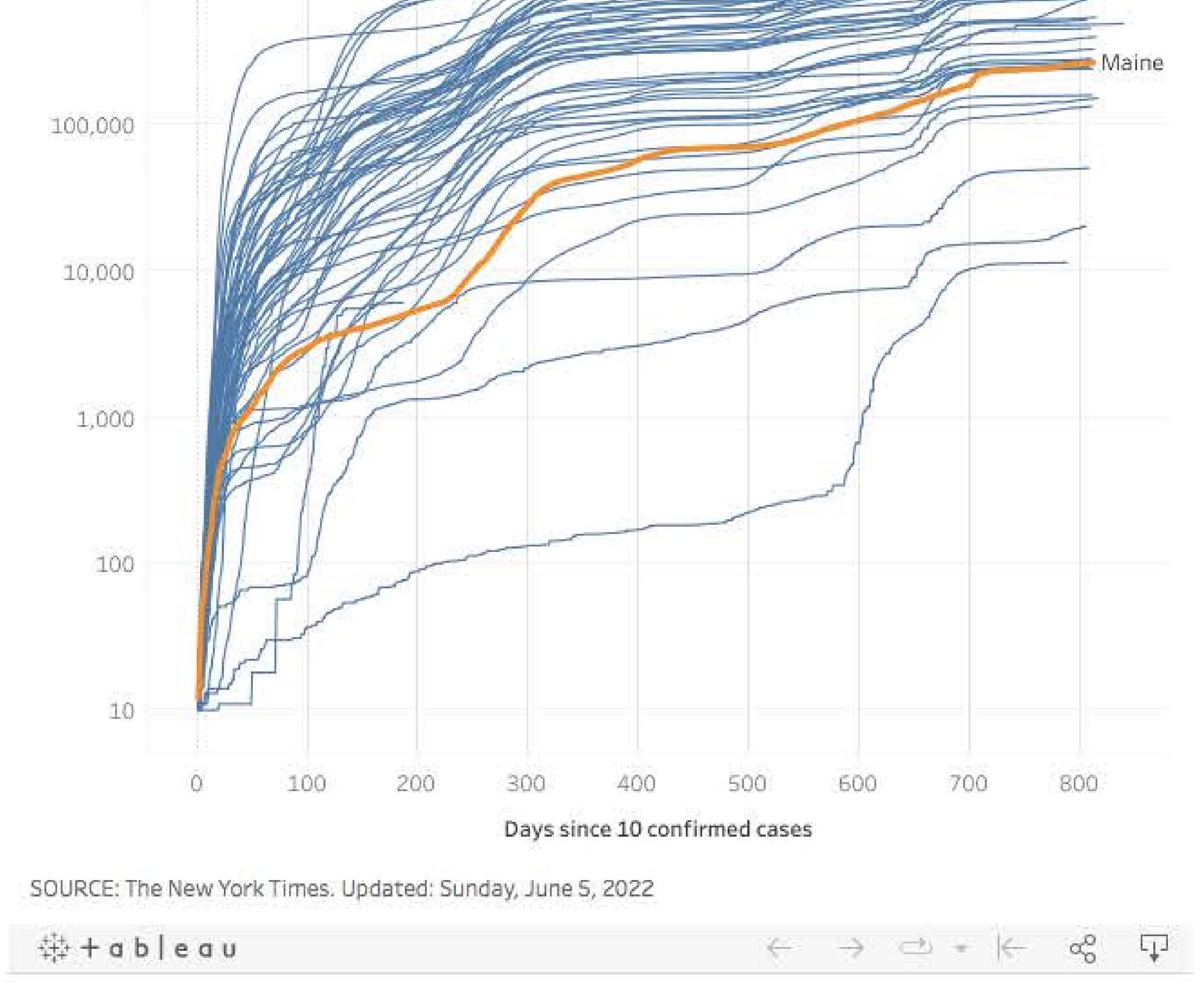

Coronavirus at a glance

The latest Oregon COVID-19 numbers, maps and charts to keep you up to date.

New cases by county

Seven-day case total per 10,000 residents

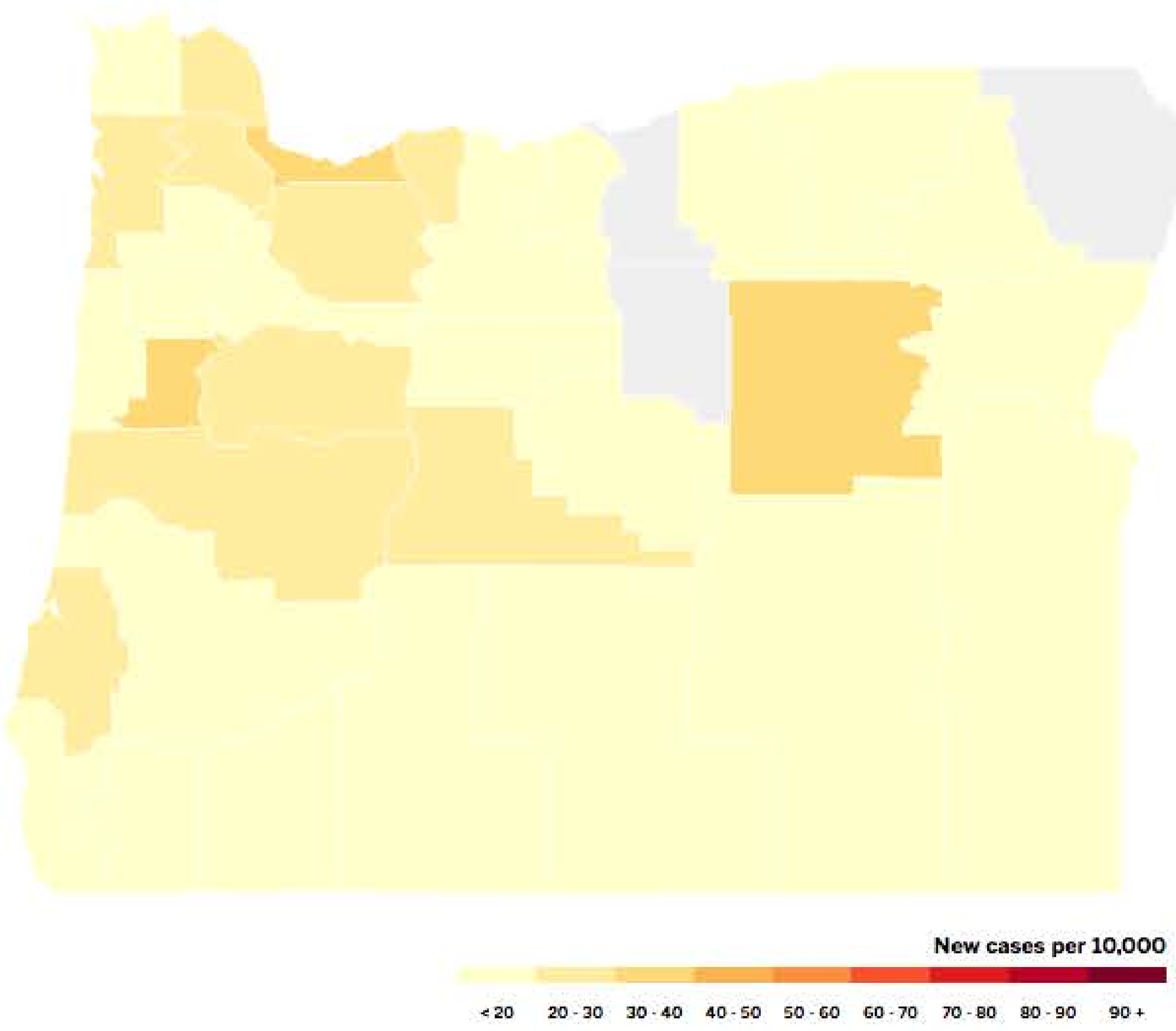

1,163
NEW CASES

2
NEW DEATHS

774,426
TOTAL CASES

1,400
AVG. CASES PER DAY

7,659
DEATHS

320
HOSPITALIZATIONS

Daily new cases

Daily confirmed and presumptive coronavirus cases reported in Oregon and the 7-day rolling average.

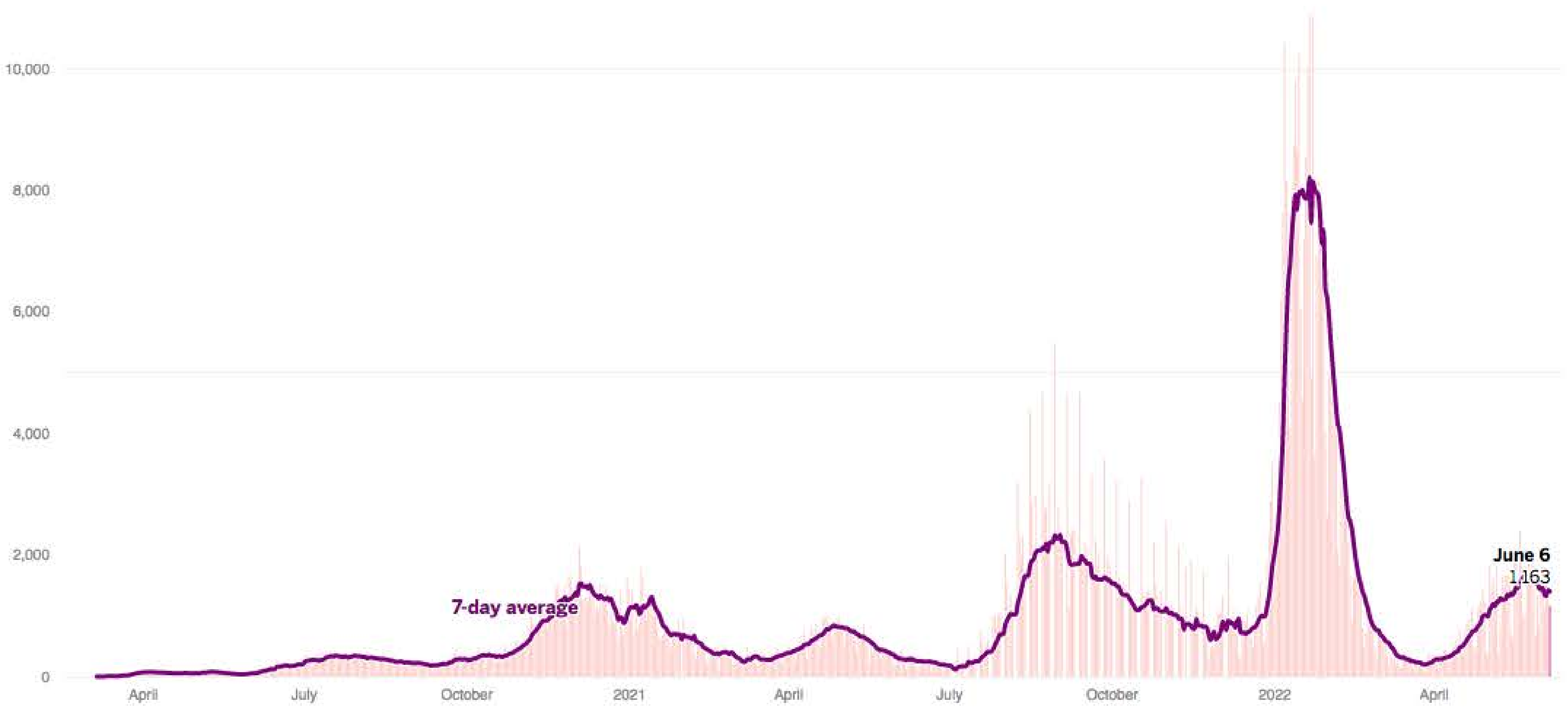

Daily hospitalization status

Number of confirmed coronavirus patients in Oregon who are **hospitalized,** in the **ICU** or on a **ventilator**

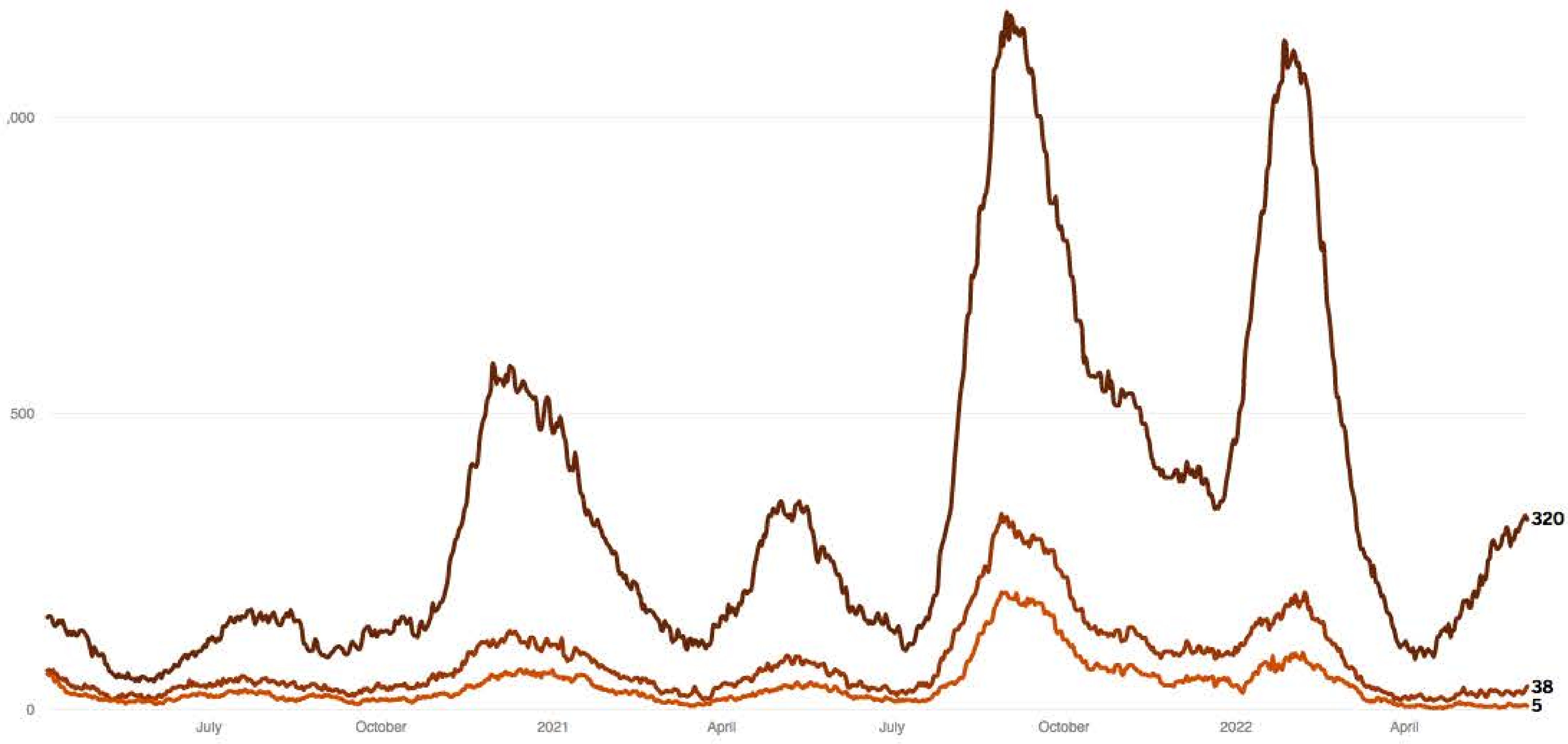

Daily new deaths

Daily coronavirus deaths reported in Oregon and the 7-day rolling average.

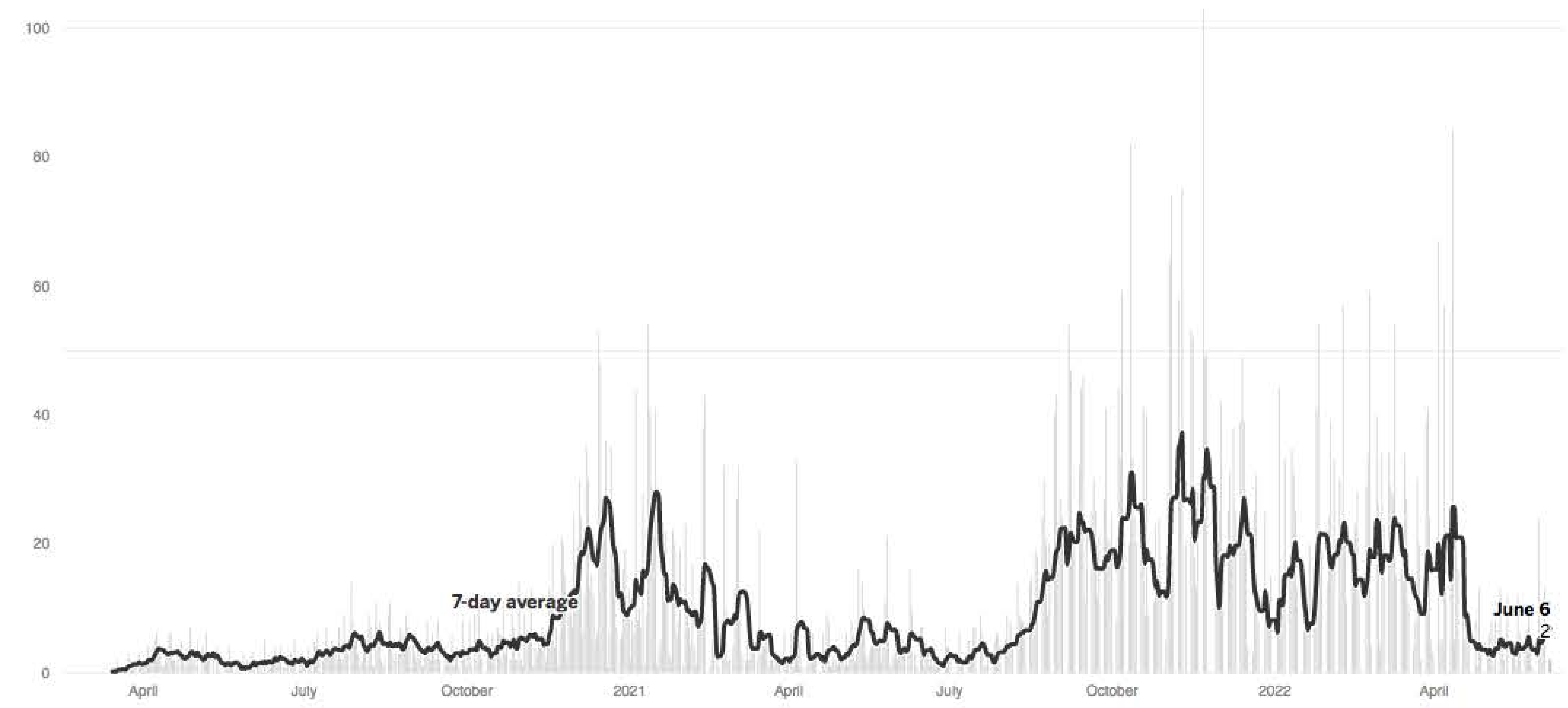

Deaths by month

Oregon coronavirus deaths each month by day of death.

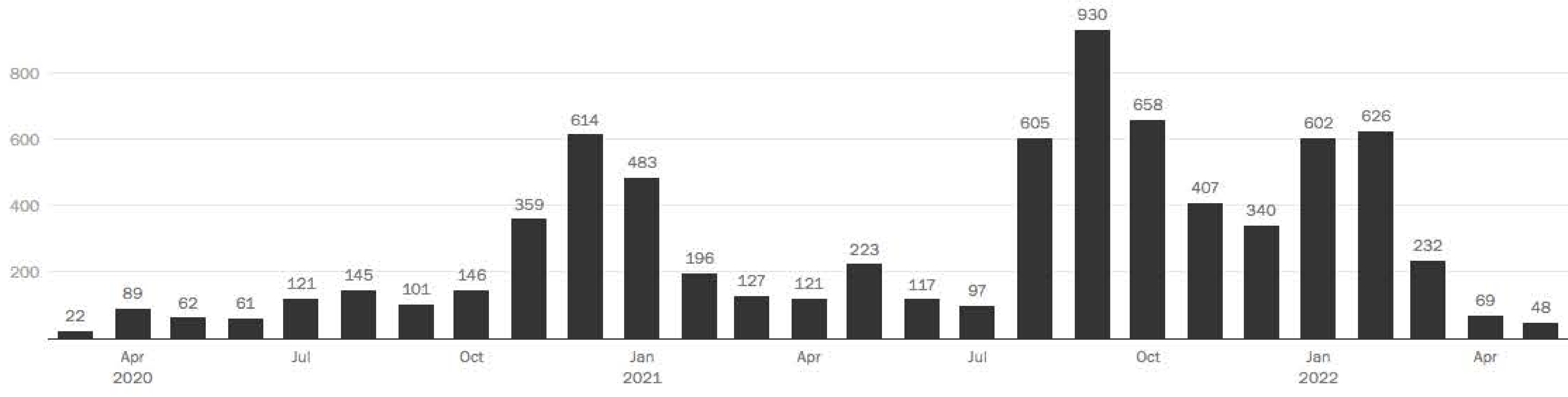

Interactives by Mark Friesen and David Cansler/staff

Sources: Oregon Health Authority, Centers for Disease Control and Prevention
